# Supplementary material for: A novel Drosophila injury model reveals severed axons are cleared through a Draper/MMP-1 signaling cascade
Source: eLife. 2017 Aug 21;6:e23611. doi: 10.7554/eLife.23611 (PMC5565368; doi:10.7554/eLife.23611)
Supplement: Table 1—source code 3. — DOI: http://dx.doi.org/10.7554/eLife.23611.015 [file elife-23611-table1-code3.zip › Table 1 - Source Data 9 - human_annokey_report.html]

 Hypoxia upregulates Rab11-family interacting protein 4 through HIF-1α to promote the metastasis of hepatocellular carcinoma. Hypoxic microenvironment is a powerful driving force for the invasion and metastasis of hepatocellular carcinoma (HCC). Hypoxia-inducible factor 1α (HIF-1α), as a crucial regulator of transcriptional responses to hypoxia, induces the expression of multiple target genes involved in different steps of HCC metastatic process. It is critical to find target genes associated with metastasis under hypoxia for shedding new light on molecular mechanism of HCC metastasis. In this study, we uncovered that hypoxia could induce the upregulation of Rab11-family interacting protein 4 (Rab11-FIP4) and activation of Rab11-FIP4 promoter by HIF-1α. The overexpression of Rab11-FIP4 significantly enhanced the mobility   and inva  siveness of HCC cells in vitro, also contributed to distant lung metastasis in vivo, whereas silencing of Rab11-FIP4 decreased the ability of migration and invasion in HCC cells in vitro and suppressed lung metastasis in vivo. Rab11-FIP4 facilitated HCC metastasis through the phosphorylation of PRAS40, which was regulated by mTOR. Furthermore, the expression level of Rab11-FIP4 was significantly increased in HCC tissues and high expression of Rab11-FIP4 was closely correlated with vascular invasion and poor prognosis in HCC patients. A markedly positive correlation between the expression of Rab11-FIP4 and HIF-1α was observed in HCC tissues and combination of Rab11-FIP4 and HIF-1α was a more valuable predictor of poor prognosis for HCC patients. In conclusion, Rab11-FIP4 is a target gene of HIF-1α and has a pro-metastatic role in HCC, suggesting that Rab11-FIP4 may be a promising candidate target for HCC treatment. 
  [Mm]etastasis  PubMed  
 Hypoxia upregulates Rab11-family interacting protein 4 through HIF-1α to promote the   metastasi  s of hepatocellular carcinoma. Hypoxic microenvironment is a powerful driving force for the invasion and   metastasi  s of hepatocellular carcinoma (HCC). Hypoxia-inducible factor 1α (HIF-1α), as a crucial regulator of transcriptional responses to hypoxia, induces the expression of multiple target genes involved in different steps of HCC metastatic process. It is critical to find target genes associated wi  th metasta  sis under hypoxia for shedding new light on molecular mechanism of H  CC metasta  sis. In this study, we uncovered that hypoxia could induce the upregulation of Rab11-family interacting protein 4 (Rab11-FIP4) and activation of Rab11-FIP4 promoter by HIF-1α. The overexpression of Rab11-FIP4 significantly enhanced the mobility and invasiveness of HCC cells in vitro, also contributed to distant l  ung metast  asis in vivo, whereas silencing of Rab11-FIP4 decreased the ability of migration and invasion in HCC cells in vitro and suppressed l  ung metast  asis in vivo. Rab11-FIP4 facilitated   HCC metast  asis through the phosphorylation of PRAS40, which was regulated by mTOR. Furthermore, the expression level of Rab11-FIP4 was significantly increased in HCC tissues and high expression of Rab11-FIP4 was closely correlated with vascular invasion and poor prognosis in HCC patients. A markedly positive correlation between the expression of Rab11-FIP4 and HIF-1α was observed in HCC tissues and combination of Rab11-FIP4 and HIF-1α was a more valuable predictor of poor prognosis for HCC patients. In conclusion, Rab11-FIP4 is a target gene of HIF-1α and has a pro-metastatic role in HCC, suggesting that Rab11-FIP4 may be a promising candidate target for HCC treatment. 
  GeneRIFs  
 Results show the expression of Rab11-FIP4 upregulated in hepatocellular carcinoma (HCC) and correlated with poor prognosis. Rab11-FIP4 dramatically contributed to HCC   metastasis   via phosphorylation of PRAS40. 
   
  
 RRAD 
 
  NCBI Gene  
  GeneRIF  
  Pubmed  
 
 
  Search Term  Rank  Fields  
  [Mm]igration  6  PubMed(4); GeneRIFs(1)  
  [Ii]nvasive  10  PubMed(1)  
  [Mm]etastasis  12  PubMed(4); GeneRIFs(1)  
  [Cc]ell [Ii]nvasion  13  PubMed(2); GeneRIFs(1)  
 
 
 hide/show details for RRAD 
  [Mm]igration  PubMed  
 Brahma-related gene 1 inhibits proliferation and   migration   of human aortic smooth muscle cells by directly up-regulating Ras-related associated with diabetes in the pathophysiologic processes of aortic dissection. To elucidate the mechanisms of Brahma-related gene 1 (Brg1) involvement in the pathophysiologic processes of aortic dissection.Seventeen dissecting, 4 dilated, and 10 healthy human aorta samples were collected. Expression of Brg1 in the medium of aorta was evaluated by quantitative real-time polymerase chain reaction, Western blot, and immunohistochemical staining, respectively. The regulation effect of Brg1 on proliferation and   migration   of human aortic smooth muscle cells (HASMCs) was analyzed in 3 ways: using cell counting, a   migration   chamber, and a wound scratch assay. A polymerase chain reaction array was used for screening potential target genes of Brg1. A chromatin immunoprecipitation assay was adopted for direct deoxyribonucleic acid-protein binding detection.Expression levels of Brg1 were increased in aortic dissection and aortic dilation patients. In vitro results indicated that overexpression of Brg1 inhibited proliferation and   migratio  n of HASMCs. The candidate proliferation- and   migratio  n-related Brg1 target gene found was Ras-related associated with diabetes (RRAD), expression levels of which were enhanced in dissecting aortic specimens. The direct regulation effect of Brg1 on RRAD was verified by chromatin immunoprecipitation assay results. Furthermore, down-regulating RRAD significantly alleviated the suppression effects of Brg1 on proliferation and   migratio  n of HASMCs.Our study illustrated that Brg1 inhibited the proliferation and   migratio  n capacity of HASMCs, via the mechanism of direct up-regulation of RRAD, thus playing an important role in the pathophysiologic processes of aortic dissection. 
 RRAD inhibits aerobic glycolysis, invasion, and   migration   and is associated with poor prognosis in hepatocellular carcinoma. Hepatocellular carcinoma (HCC) is one of the most prevalent and lethal cancer worldwide. However, the mechanism underlying the HCC development remains unclear. Ras-related associated with diabetes (RRAD) is a small Ras-related GTPase which has been implicated in metabolic disease and several types of cancer, yet its functions in HCC remain unknown. A tissue microarray constructed by 90 paired HCC tissues and adjacent non-cancerous liver tissues was used to examine the protein levels of RRAD, and the messenger RNA (mRNA) expression of RRAD was also detected in a subset of this cohort. The prognostic significance of RRAD was estimated by the Kaplan-Meier analysis and Cox regression. The glucose utilization assay and lactate production assay were performed to measure the role of RRAD in HCC glycolysis. The effect of RRAD in HCC invasion and metastasis was analyzed by transwell assays. Our results suggested that the expression of RRAD was downregulated in HCC tissues compared to the adjacent non-tumorous liver tissues both in mRNA and protein levels and lower RRAD expression served as an independent prognostic indicator for the survival of HCC patients. Moreover, RRAD inhibited hepatoma cell aerobic glycolysis by negatively regulating the expression of glucose transporter 1 (GLUT1) and hexokinase II (HK-II). In addition, RRAD inhibition dramatically increased hepatoma cell invasion and metastasis. In conclusion, our study revealed that RRAD expression was decreased in HCC tumor tissues and predicted poor clinical outcome for HCC patients and played an important role in regulating aerobic glycolysis and cell invasion and metastasis and may represent potential targets for improving the treatment of HCC. 
 Rad GTPase inhibits the NFκB pathway through interacting with RelA/p65 to impede its DNA binding and target gene transactivation. Rad is a Ras-related small GTPase shown to inhibit cancer cell   migratio  n, and its expression is frequently lost in lung cancer cells. Here we provide evidence that Rad can negatively regulate the NFκB pathway. Overexpressing Rad in cells lowered both the basal and TNFα-stimulated transcriptional activity of NFκB. Compared with control cells, Rad-overexpressing cells displayed more cytoplasmic distribution of the NFκB subunit RelA/p65, while Rad-knockdown cells had higher levels of nuclear RelA/p65. Depleting Rad did not affect the kinetics of TNFα-induced IκB degradation, suggesting that Rad-mediated regulation of NFκB was through an IκB-independent mechanism. Expression of a nucleus-localized mutant Rad was sufficient to inhibit the NFκB transcriptional activity, whereas expressing the scaffolding protein 14-3-3γ to retain Rad in the cytoplasm alleviated the suppressive effect of Rad on NFκB. GST pull-down assays showed that Rad could directly bind to RelA/p65, and co-immunoprecipitation demonstrated that the Rad-p65 interaction primarily occurred in the nucleus. Adding Rad-containing nuclear extracts or purified GST-Rad in the electrophoretic mobility shift assays dose-dependently decreased the binding of RelA/p65 to an oligonucleotide probe containing the NFκB response element, suggesting that Rad may directly impede the interaction between RelA/p65 and DNA. Rad depletion altered the expression of an array of NFκB target genes, including upregulating MMP9. Knockdown of Rad expression in cells increased both basal and TNFα-stimulated MMP9 activities and cell invasion. Collectively, our results disclose a novel role of nuclear Rad in inhibiting the NFκB pathway function. 
 Promoter hypermethylation of Ras-related GTPase gene RRAD inactivates a tumor suppressor function in nasopharyngeal carcinoma. Nasopharyngeal carcinoma (NPC) is endemic in southern China. In a genome-wide screen for genes inactivated by promoter hypermethylation, we identified Ras-related associated with diabetes (RRAD). Expression of RRAD was down-regulated in 83.3% (30/36) of the biopsies from NPC patients. RRAD was aberrantly methylated in 74.3% (26/35) of primary tumors, but not in normal nasopharyngeal epithelium. Ectopic RRAD expression in NPC cell lines inhibited the cell growth, colony formation, and cell   migration  . These results indicate that RRAD might act as a functional tumor suppressor and its epigenetic inactivation may play an important role in NPC development. 
  GeneRIFs  
 Brg1 inhibits proliferation and   migration   of human aortic smooth muscle cells by directly up-regulating RRAD in aortic dissection. 
  [Ii]nvasive  PubMed  
 Regulation of growth and tumorigenicity of breast cancer cells by the low molecular weight GTPase Rad and nm23. Rad is the prototypic member of a family of novel Ras-related GTPases that is normally expressed in heart, skeletal muscle, and lung and that has been shown to exhibit a novel form of bi-directional interaction with the nm23 metastasis suppressor. In the present study, we have investigated the expression of Rad in normal and neoplastic breast tissues by Western blot and immunohistochemistry and the functional effect of altered Rad expression in breast cancer cell lines. We found that, although Rad is frequently expressed in normal breast tissue (23/30 Rad+ve), expression is usually lost in adjacent   invasive   carcinoma (8/30 Rad+ve; P < 0.0001). However, where Rad expression persists in a small proportion of tumors, it is associated with higher grade, larger size, and extensive axillary nodal involvement (n = 48; P = 0.035, P = 0.016, P = 0.022, respectively). Furthermore, Rad is also highly expressed in a breast cancer cell line with high tumorigenic and metastatic potential (MDA-MB231). To further examine the role of Rad in breast cancer, we stably transfected a Rad-ve breast cancer cell line (MDA-MB435). We observed an increase in growth and marked increased colony formation in soft agar in vitro (P < 0.05) and an increase in tumor growth rate in nude mice (P < 0.05). Moreover, coexpression of nm23 with wild-type Rad inhibited the effect of Rad on growth of these cells in culture and markedly inhibited tumor growth in vivo. Additional transfection studies with mutated Rad cDNAs revealed that the growth-promoting effects of Rad appeared to be mediated through its NH2- and COOH-terminal regions, rather than its GTPase domain, and might involve acceleration of cell cycle transition. These findings suggest that Rad may act as an oncogenic protein in breast tissues and demonstrate a potential mechanism by which interaction between Rad and nm23 may regulate growth and tumorigenicity of breast cancer. 
  [Mm]etastasis  PubMed  
 Regulation of growth and tumorigenicity of breast cancer cells by the low molecular weight GTPase Rad and nm23. Rad is the prototypic member of a family of novel Ras-related GTPases that is normally expressed in heart, skeletal muscle, and lung and that has been shown to exhibit a novel form of bi-directional interaction with the nm23   metastasis   suppressor. In the present study, we have investigated the expression of Rad in normal and neoplastic breast tissues by Western blot and immunohistochemistry and the functional effect of altered Rad expression in breast cancer cell lines. We found that, although Rad is frequently expressed in normal breast tissue (23/30 Rad+ve), expression is usually lost in adjacent invasive carcinoma (8/30 Rad+ve; P < 0.0001). However, where Rad expression persists in a small proportion of tumors, it is associated with higher grade, larger size, and extensive axillary nodal involvement (n = 48; P = 0.035, P = 0.016, P = 0.022, respectively). Furthermore, Rad is also highly expressed in a breast cancer cell line with high tumorigenic and metastatic potential (MDA-MB231). To further examine the role of Rad in breast cancer, we stably transfected a Rad-ve breast cancer cell line (MDA-MB435). We observed an increase in growth and marked increased colony formation in soft agar in vitro (P < 0.05) and an increase in tumor growth rate in nude mice (P < 0.05). Moreover, coexpression of nm23 with wild-type Rad inhibited the effect of Rad on growth of these cells in culture and markedly inhibited tumor growth in vivo. Additional transfection studies with mutated Rad cDNAs revealed that the growth-promoting effects of Rad appeared to be mediated through its NH2- and COOH-terminal regions, rather than its GTPase domain, and might involve acceleration of cell cycle transition. These findings suggest that Rad may act as an oncogenic protein in breast tissues and demonstrate a potential mechanism by which interaction between Rad and nm23 may regulate growth and tumorigenicity of breast cancer. 
 Effects of phosphorylation on function of the Rad GTPase. Rad, Gem and Kir possess unique structural features in comparison with other Ras-like GTPases, including a C-terminal 31-residue extension that lacks typical prenylation motifs. We have recently shown that Rad and Gem bind calmodulin in a Ca2+-dependent manner via this C-terminal extension, involving residues 278-297 in human Rad. This domain also contains several consensus sites for serine phosphorylation, and Rad is complexed with calmodulin-dependent protein kinase II (CaMKII) in C2C12 cells. Here we show that Rad serves as a substrate for phosphorylation by CaMKII, cAMP-dependent protein kinase (PKA), protein kinase C (PKC) and casein kinase II (CKII) with stoichiometries in vitro of 0.2-1.3 mol of phosphate/mol of Rad. By deletion and point mutation analysis we show that phosphorylation by CaMKII and PKA occurs on a single serine residue at position 273, whereas PKC and CKII phosphorylate multiple C-terminal serine residues, including Ser214, Ser257, Ser273, Ser290 and Ser299. Incubation of Rad with PKA decreases GTP binding by 60-70%, but this effect seems to be independent of phosphorylation, as it is observed with the Ser273-->Ala mutant of Rad containing a mutation at the site of PKA phosphorylation. The remainder of the serine kinases have no effect on Rad GTP binding, intrinsic GTP hydrolysis or GTP hydrolysis stimulated by the putative tumour   metastasis   suppressor nm23. However, phosphorylation of Rad by PKC and CKII abolishes the interaction of Rad with calmodulin. These findings suggest that the binding of Rad to calmodulin, as well as its ability to bind GTP, might be regulated by the activation of several serine kinases. 
 RRAD inhibits aerobic glycolysis, invasion, and migration and is associated with poor prognosis in hepatocellular carcinoma. Hepatocellular carcinoma (HCC) is one of the most prevalent and lethal cancer worldwide. However, the mechanism underlying the HCC development remains unclear. Ras-related associated with diabetes (RRAD) is a small Ras-related GTPase which has been implicated in metabolic disease and several types of cancer, yet its functions in HCC remain unknown. A tissue microarray constructed by 90 paired HCC tissues and adjacent non-cancerous liver tissues was used to examine the protein levels of RRAD, and the messenger RNA (mRNA) expression of RRAD was also detected in a subset of this cohort. The prognostic significance of RRAD was estimated by the Kaplan-Meier analysis and Cox regression. The glucose utilization assay and lactate production assay were performed to measure the role of RRAD in HCC glycolysis. The effect of RRAD in HCC invasion and   metastasis   was analyzed by transwell assays. Our results suggested that the expression of RRAD was downregulated in HCC tissues compared to the adjacent non-tumorous liver tissues both in mRNA and protein levels and lower RRAD expression served as an independent prognostic indicator for the survival of HCC patients. Moreover, RRAD inhibited hepatoma cell aerobic glycolysis by negatively regulating the expression of glucose transporter 1 (GLUT1) and hexokinase II (HK-II). In addition, RRAD inhibition dramatically increased hepatoma cell invasion and   metastasis  . In conclusion, our study revealed that RRAD expression was decreased in HCC tumor tissues and predicted poor clinical outcome for HCC patients and played an important role in regulating aerobic glycolysis and cell invasion and   metastasis   and may represent potential targets for improving the treatment of HCC. 
 Interaction of the Ras-related protein associated with diabetes rad and the putative tumor   metastasis   suppressor NM23 provides a novel mechanism of GTPase regulation. Rad is the prototypic member of a new class of Ras-related GTPases. Purification of the GTPase-activating protein (GAP) for Rad revealed nm23, a putative tumor   metastasis   suppressor and a development gene in Drosophila. Antibodies against nm23 depleted Rad-GAP activity from human skeletal muscle cytosol, and bacterially expressed nm23 reconstituted the activity. The GAP activity of nm23 was specific for Rad, was absent with the S105N putative dominant negative mutant of Rad, and was reduced with mutations of nm23. In the presence of ATP, GDP.Rad was also reconverted to GTP.Rad by the nucleoside diphosphate (NDP) kinase activity of nm23. Simultaneously, Rad regulated nm23 by enhancing its NDP kinase activity and decreasing its autophosphorylation. Melanoma cells transfected with wild-type Rad, but not the S105N-Rad, showed enhanced DNA synthesis in response to serum; this effect was lost with coexpression of nm23. Thus, the interaction of nm23 and Rad provides a potential novel mechanism for bidirectional, bimolecular regulation in which nm23 stimulates both GTP hydrolysis and GTP loading of Rad whereas Rad regulates activity of nm23. This interaction may play important roles in the effects of Rad on glucose metabolism and the effects of nm23 on tumor   metastasis   and developmental regulation. 
  GeneRIFs  
 our study revealed that RRAD expression was decreased in Hepatocellular carcinoma (HCC) tumor tissues and predicted poor clinical outcome for HCC patients and played an important role in regulating aerobic glycolysis and cell invasion and   metastasis   and may represent potential targets for improving the treatment of HCC 
  [Cc]ell [Ii]nvasion  PubMed  
 RRAD inhibits aerobic glycolysis, invasion, and migration and is associated with poor prognosis in hepatocellular carcinoma. Hepatocellular carcinoma (HCC) is one of the most prevalent and lethal cancer worldwide. However, the mechanism underlying the HCC development remains unclear. Ras-related associated with diabetes (RRAD) is a small Ras-related GTPase which has been implicated in metabolic disease and several types of cancer, yet its functions in HCC remain unknown. A tissue microarray constructed by 90 paired HCC tissues and adjacent non-cancerous liver tissues was used to examine the protein levels of RRAD, and the messenger RNA (mRNA) expression of RRAD was also detected in a subset of this cohort. The prognostic significance of RRAD was estimated by the Kaplan-Meier analysis and Cox regression. The glucose utilization assay and lactate production assay were performed to measure the role of RRAD in HCC glycolysis. The effect of RRAD in HCC invasion and metastasis was analyzed by transwell assays. Our results suggested that the expression of RRAD was downregulated in HCC tissues compared to the adjacent non-tumorous liver tissues both in mRNA and protein levels and lower RRAD expression served as an independent prognostic indicator for the survival of HCC patients. Moreover, RRAD inhibited hepatoma cell aerobic glycolysis by negatively regulating the expression of glucose transporter 1 (GLUT1) and hexokinase II (HK-II). In addition, RRAD inhibition dramatically increased hepatoma   cell invasion   and metastasis. In conclusion, our study revealed that RRAD expression was decreased in HCC tumor tissues and predicted poor clinical outcome for HCC patients and played an important role in regulating aerobic glycolysis and   cell invasion   and metastasis and may represent potential targets for improving the treatment of HCC. 
 Rad GTPase inhibits the NFκB pathway through interacting with RelA/p65 to impede its DNA binding and target gene transactivation. Rad is a Ras-related small GTPase shown to inhibit cancer cell migration, and its expression is frequently lost in lung cancer cells. Here we provide evidence that Rad can negatively regulate the NFκB pathway. Overexpressing Rad in cells lowered both the basal and TNFα-stimulated transcriptional activity of NFκB. Compared with control cells, Rad-overexpressing cells displayed more cytoplasmic distribution of the NFκB subunit RelA/p65, while Rad-knockdown cells had higher levels of nuclear RelA/p65. Depleting Rad did not affect the kinetics of TNFα-induced IκB degradation, suggesting that Rad-mediated regulation of NFκB was through an IκB-independent mechanism. Expression of a nucleus-localized mutant Rad was sufficient to inhibit the NFκB transcriptional activity, whereas expressing the scaffolding protein 14-3-3γ to retain Rad in the cytoplasm alleviated the suppressive effect of Rad on NFκB. GST pull-down assays showed that Rad could directly bind to RelA/p65, and co-immunoprecipitation demonstrated that the Rad-p65 interaction primarily occurred in the nucleus. Adding Rad-containing nuclear extracts or purified GST-Rad in the electrophoretic mobility shift assays dose-dependently decreased the binding of RelA/p65 to an oligonucleotide probe containing the NFκB response element, suggesting that Rad may directly impede the interaction between RelA/p65 and DNA. Rad depletion altered the expression of an array of NFκB target genes, including upregulating MMP9. Knockdown of Rad expression in cells increased both basal and TNFα-stimulated MMP9   activities an  d cell invasion. Collectively, our results disclose a novel role of nuclear Rad in inhibiting the NFκB pathway function. 
  GeneRIFs  
 our study revealed that RRAD expression was decreased in Hepatocellular carcinoma (HCC) tumor tissues and predicted poor clinical outcome for HCC patients and played an important role in regulating aerobic glycolysis and   cell invasion   and metastasis and may represent potential targets for improving the treatment of HCC 
   
  
 ANKS1B 
 
  NCBI Gene  
  GeneRIF  
  Pubmed  
 
 
  Search Term  Rank  Fields  
  [Mm]igration  6  PubMed(1)  
 
 
 hide/show details for ANKS1B 
  [Mm]igration  PubMed  
 Identification of phosphotyrosine binding domain-containing proteins as novel downstream targets of the EphA8 signaling function. Eph receptors and ephrins have been implicated in a variety of cellular processes, including morphology and motility, because of their ability to modulate intricate signaling networks. Here we show that the phosphotyrosine binding (PTB) domain-containing proteins AIDA-1b and Odin are tightly associated with the EphA8 receptor in response to ligand stimulation. Both AIDA-1b and Odin belong to the ankyrin repeat and sterile alpha motif domain-containing (Anks) protein family. The PTB domain of Anks family proteins is crucial for their association with the juxtamembrane domain of EphA8, whereas EphA8 tyrosine kinase activity is not required for this protein-protein interaction. In addition, we found that Odin is a more physiologically relevant partner of EphA8 in mammalian cells. Interestingly, overexpression of the Odin PTB domain alone attenuated EphA8-mediated inhibition of cell   migration   in HEK293 cells, suggesting that it acts as a dominant-negative mutant of the endogenous Odin protein. More importantly, small interfering RNA-mediated Odin silencing significantly diminished ephrinA5-induced EphA8 signaling effects, which inhibit cell   migration   in HEK293 cells and retract growing neurites of Neuro2a cells. Taken together, our findings support a possible function for Anks family proteins as scaffolding proteins of the EphA8 signaling pathway. 
   
  
 ANKS1A 
 
  NCBI Gene  
  GeneRIF  
  Pubmed  
 
 
  Search Term  Rank  Fields  
  [Mm]igration  6  Process(1)  
 
 
 hide/show details for ANKS1A 
  [Mm]igration  Process  
 substrate-dependent cell   migration   
   
  
 NOVA1 
 
  NCBI Gene  
  GeneRIF  
  Pubmed  
 
 
  Search Term  Rank  Fields  
  [Mm]igration  6  PubMed(3)  
 
 
 hide/show details for NOVA1 
  [Mm]igration  PubMed  
 MicroRNA-339, an epigenetic modulating target is involved in human gastric carcinogenesis through targeting NOVA1. The role of miR-339 in human gastric cancer (GC) remains unclear. Here, we found that miR-339 is remarkably decreased in primary GC tissues. Overexpression of miR-339 in GC cells significantly suppressed proliferation,   migration  , invasion, and tumorigenicity. Furthermore, NOVA1 was confirmed as a target of miR-339. Restoration of NOVA1 in miR-339-overexpressing GC cells partially impaired the inhibitory effects of miR-339. More importantly, epigenetic modification may be involved in the modulation of miR-339 expression. These findings uncover a novel role for miR-339 in gastric carcinogenesis, and restoration of miR-339 could be considered as a potential therapeutic strategy for GC treatment. 
 High expression of neuro-oncological ventral antigen 1 correlates with poor prognosis in hepatocellular carcinoma. Neuro-oncological ventral antigen 1 (Nova1) is a neuron-specific RNA-binding protein in human paraneoplastic opsoclonus-myoclonus ataxia accompanying with malignant tumors, but its role in hepatocellular carcinoma (HCC) remains elusive. In this study, we found that overexpressed intratumoral Nova1 was associated with poor survival rate and increased recurrence rate of HCC, especially early recurrence, and was an independent prognostic factor for overall survival rate and tumor recurrence. HCC cell lines over-expressing Nova1 exhibited greater potentials in cell proliferation, invasion and   migration  , while knockdown of Nova1 had the opposite effects. All these findings indicate that Nova1 may act as a prognostic marker for poor outcome and high recurrence in HCC. 
 MiR-181b-5p downregulates NOVA1 to suppress proliferation,   migration   and invasion and promote apoptosis in astrocytoma. MicroRNAs (miRNAs) are small, short noncoding RNAs that modulate the expression of numerous genes by targeting their mRNA. Numerous abnormal miRNA expression patterns are observed in various human malignancies, and certain miRNAs can act as oncogenes or tumor suppressors. Astrocytoma, the most common neuroepithelial cancer, represents the majority of malignant brain tumors in humans. In our previous studies, we found that the downregulation of miR-181b-5p in astrocytomas is associated with a poor prognosis. The aim of the present study was to investigate the functional role of miR-181b-5p and its possible target genes. miR-181b-5p was significantly downregulated in astrocytoma specimens, and the reduced expression of miR-181b-5p was inversely correlated with the clinical stage. The ectopic expression of miR-181b-5p inhibited proliferation,   migration   and invasion and induced apoptosis in astrocytoma cancer cells in vitro. The NOVA1 (neuro-oncological ventral antigen 1) gene was further identified as a novel direct target of miR-181b-5p. Specifically, miR-181b-5p bound directly to the 3'-untranslated region (UTR) of NOVA1 and suppressed its expression. In clinical specimens, NOVA1 was overexpressed, and its protein levels were inversely correlated with miR-181b-5p expression. Furthermore, the changing level of NOVA1 was significantly associated with a poor survival outcome. Similar to restoring miR-181b-5p expression, downregulating NOVA1 inhibited cell growth,   migration   and invasion. Overexpression of NOVA1 reversed the inhibitory effects of miR-181b-5p. Our results indicate that miR-181b-5p is a tumor suppressor in astrocytoma that inhibits tumor progression by targeting NOVA1. These findings suggest that miR-181b-5p may serve as a novel therapeutic target for astrocytoma. 
   
  
 SIRT7 
 
  NCBI Gene  
  GeneRIF  
  Pubmed  
 
 
  Search Term  Rank  Fields  
  [Mm]igration  6  PubMed(1); GeneRIFs(1)  
  [Ii]nvasive  10  PubMed(1)  
  [Mm]etastasis  12  PubMed(2); GeneRIFs(1)  
 
 
 hide/show details for SIRT7 
  [Mm]igration  PubMed  
 SIRT7 Exhibits Oncogenic Potential in Human Ovarian Cancer Cells. Sirtuin7 (SIRT7) is a type of nicotinamide adenine dinucleotide oxidized form (NAD+)-dependent deacetylase and the least understood member of the sirtuins family; it is implicated in various processes, such as aging, DNA damage repair and cell signaling transduction. There is some evidence that SIRT7 may function as a tumor trigger for human malignancy. Here, we aimed to explore the biological function of SIRT7 in ovarian carcinoma cells and its potential mechanism.Expression of SIRT7 in ovarian cancer cell lines was detected by western blotting. Transduced cell lines with SIRT7 knockdown or overexpression were constructed. Cell viability, cologenic, apoptosis-associated and motility assays were performed to elucidate the biological function of SIRT7 in ovarian cancer cells.SIRT7 demonstrated a higher level in ovarian cancer cell lines compared with normal cells. On the one hand, down-regulation of SIRT7 significantly reduced ovarian cancer cell growth, repressed colony formation and increased cancer cell apoptosis; on the other hand, up-regulation promoted the   migration   of cancer cells. Additionally, repression of SIRT7 also induced change in apoptosis-related molecules and subunits of the NF-κB family.In the present study, our data indicated that SIRT7 might play a role of oncogene in ovarian malignancy and be a potential therapeutic target. 
  GeneRIFs  
 SIRT7 might play a role of oncogene in ovarian malignancy. Down-regulation of SIRT7 significantly reduced ovarian cancer cell growth, repressed colony formation and increased cancer cell apoptosis; up-regulation promoted the   migration   of cancer cells. 
  [Ii]nvasive  PubMed  
 Overexpression of sirt7 exhibits oncogenic property and serves as a prognostic factor in colorectal cancer. Sirtuins play an important role in cancer development. Sirt7, as a member of this family, is frequently overexpressed in certain carcinomas, but the oncogenic mechanism is seldom reported. In this study, Sirt7 was characterized for its role in colorectal cancer aggressiveness and underlying molecular mechanisms.Quantitative PCR, Western blotting, and immunohistochemistry were performed to study Sirt7 expression in a cohort of colorectal cancer tissues and non-tumor tissues and cells. A series of in vitro and in vivo assays was performed to elucidate the function of Sirt7 in colorectal cancer and its underlying mechanisms. Association between the Sirt7 signature and survival was examined using Kaplan-Meier analysis and log-rank tests.The Sirt7 protein level significantly correlated with tumor stage (P = 0.029), lymph node metastasis (P = 0.046), and poor patient survival (P < 0.05). Sirt7 knockdown significantly inhibited colorectal cancer cell proliferation, colony formation, and motility. Ectopic Sirt7 expression promoted colony formation, induced a more   invasive   phenotype, and accelerated cell growth both in vitro and in vivo. Moreover, Sirt7 enhanced MAPK pathway activity concomitantly with p-ERK and p-MEK upregulation. In Sirt7-overexpressing cells, the mesenchymal markers vimentin and fibronectin were upregulated, and the epithelial markers E-cadherin and β-catenin were downregulated, which was linked to enhanced invasion by colorectal cancer cells.Our findings suggest that Sirt7 plays an important role in the development and progression of human colorectal cancer and functions as a valuable marker of colorectal cancer prognosis. 
  [Mm]etastasis  PubMed  
 Sirt7 promotes gastric cancer growth and inhibits apoptosis by epigenetically inhibiting miR-34a. Gastric cancer is the fourth most common cancer worldwide, with a low 5-year survival rate. Epigenetic modification plays pivotal roles in gastric cancer development. However, the role of histone-modifying enzymes in gastric cancer remains largely unknown. Here we report that Sirt7, a NAD(+)-dependent class III histone deacetylase, is over-expressed in human gastric cancer tissues. Sirt7 level is significantly correlated with disease stage,   metastasis  , and survival. Knockdown of Sirt7 in gastric cancer cells inhibits cell proliferation and colony formation in vitro. In vivo subcutaneous xenograft results also show that Sirt7 knockdown can markedly repress gastric cancer cell growth. In addition, Sirt7 depletion induces apoptosis in gastric cancer cells via up-regulating expression of pro-apoptotic proteins and down-regulating anti-apoptotic proteins. Mechanically, Sirt7 binds to the promoter of miR-34a and deacetylases the H3K18ac, thus represses miR-34a expression. Reversely, depletion of miR-34a inhibits gastric cancer apoptosis induced by Sirt7 knockdown, and restores cellular capacity of proliferation and colony formation. miR-34a depletion reduces Sirt7-knockdown-induced arrest of gastric growth. Finally, miR-34a is tightly associated with survival of patients with gastric cancer. 
 Overexpression of sirt7 exhibits oncogenic property and serves as a prognostic factor in colorectal cancer. Sirtuins play an important role in cancer development. Sirt7, as a member of this family, is frequently overexpressed in certain carcinomas, but the oncogenic mechanism is seldom reported. In this study, Sirt7 was characterized for its role in colorectal cancer aggressiveness and underlying molecular mechanisms.Quantitative PCR, Western blotting, and immunohistochemistry were performed to study Sirt7 expression in a cohort of colorectal cancer tissues and non-tumor tissues and cells. A series of in vitro and in vivo assays was performed to elucidate the function of Sirt7 in colorectal cancer and its underlying mechanisms. Association between the Sirt7 signature and survival was examined using Kaplan-Meier analysis and log-rank tests.The Sirt7 protein level significantly correlated with tumor stage (P = 0.029), lymph node   metastasis   (P = 0.046), and poor patient survival (P < 0.05). Sirt7 knockdown significantly inhibited colorectal cancer cell proliferation, colony formation, and motility. Ectopic Sirt7 expression promoted colony formation, induced a more invasive phenotype, and accelerated cell growth both in vitro and in vivo. Moreover, Sirt7 enhanced MAPK pathway activity concomitantly with p-ERK and p-MEK upregulation. In Sirt7-overexpressing cells, the mesenchymal markers vimentin and fibronectin were upregulated, and the epithelial markers E-cadherin and β-catenin were downregulated, which was linked to enhanced invasion by colorectal cancer cells.Our findings suggest that Sirt7 plays an important role in the development and progression of human colorectal cancer and functions as a valuable marker of colorectal cancer prognosis. 
  GeneRIFs  
 Sirt7 is overexpressed in human gastric cancers. Expression of Sirt7 is markedly correlated with tumor size,   metastasis  , disease stage and prognosis. 
   
  
 PSD3 
 
  NCBI Gene  
  GeneRIF  
  Pubmed  
 
 
  Search Term  Rank  Fields  
  [Ii]nvasive  10  PubMed(1)  
  [Mm]etastasis  12  PubMed(1)  
 
 
 hide/show details for PSD3 
  [Ii]nvasive  PubMed  
 Systematic mapping and functional analysis of a family of human epididymal secretory sperm-located proteins. The mammalian spermatozoon has many cellular compartments, such as head and tail, permitting it to interact with the female reproductive tract and fertilize the egg. It acquires this fertilizing potential during transit through the epididymis, which secretes proteins that coat different sperm domains. Optimal levels of these proteins provide the spermatozoon with its ability to move to, bind to, fuse with, and penetrate the egg; otherwise male infertility results. As few human epididymal proteins have been characterized, this work was performed to generate a database of human epididymal sperm-located proteins involved in maturation. Two-dimensional gel electrophoresis of epididymal tissue and luminal fluid proteins, followed by identification using MALDI-TOF/MS or MALDI-TOF/TOF, revealed over a thousand spots in gels comprising 745 abundant nonstructural proteins, 408 in luminal fluids, of which 207 were present on spermatozoa. Antibodies raised to 619 recombinant or synthetic peptides, used in Western blots, histological sections, and washed sperm preparations to confirm antibody quality and protein expression, indicated their regional location in the epididymal epithelium and highly specific locations on washed functional spermatozoa. Sperm function tests suggested the role of some proteins in motility and protection against oxidative attack. A large database of these proteins, characterized by size, pI, chromosomal location, and function, was given a unified terminology reflecting their sperm domain location. These novel, secreted human epididymal proteins are potential targets for a posttesticular contraceptive acting to provide rapid, reversible, functional sterility in men and they are also biomarkers that could be used in non  invasive   assessments of male fertility. 
  [Mm]etastasis  PubMed  
 Large scale identification of human hepatocellular carcinoma-associated antigens by autoantibodies. Autoantibodies are often detected in hepatocellular carcinoma (HCC), and these responses may represent recognition of tumor Ags that are associated with transformation events. The identities of these Ags, however, are less well known. Using serological analysis of recombinant cDNA expression libraries (SEREX) from four HCC patients, we identified 55 independent cDNA sequences potentially encoding HCC tumor Ags. Of these genes, 15 are novel. Two such proteins, HCA587 and HCA661, were predominantly detected in testis, but not in other normal tissues, except for a weak expression in normal pancreas. In addition to HCC, these two Ags can be found in cancers of other histological types. Therefore, they can be categorized as cancer-testis (CT) Ags. Two other Ags (HCA519 and HCA90) were highly overexpressed in HCC and also expressed in cancer cell lines of lung, prostate, and pancreas, but not in the respective normal tissues. Four other Ags were identified to be expressed in particular types of cancer cell lines (HCA520 in an ovarian cancer cell line, HCA59 and HCA67 in a colon cancer cell line, HCA58 in colon and ovarian cancer cell lines), but not in the normal tissue counterpart(s). In addition, abundant expression of complement inactivation factors was found in HCC. These results indicate a broad range expression of autoantigens in HCC patients. Our findings open an avenue for the study of autoantigens in the transformation,   metastasis  , and immune evasion in HCC. 
   
  
 PSD 
 
  NCBI Gene  
  GeneRIF  
  Pubmed  
 
 
  Search Term  Rank  Fields  
  [Mm]igration  6  PubMed(3)  
  [Ii]nvasive  10  PubMed(1)  
  [Mm]etastasis  12  PubMed(1)  
  [Cc]ell [Ii]nvasion  13  PubMed(2)  
 
 
 hide/show details for PSD 
  [Mm]igration  PubMed  
 Aberrant methylation of PSD disturbs Rac1-mediated immune responses governing neutrophil chemotaxis and apoptosis in ulcerative colitis-associated carcinogenesis. We previously reported that the Pleckstrin and Sec7 domain-containing (PSD) gene is preferentially methylated in patients with ulcerative colitis (UC) who developed colorectal cancer (CRC), and is implicated in UC-associated carcinogenesis through its inhibition of apoptosis. This study aimed to determine the potential effect of PSD methylation on its downstream molecule, Ras-related C3 botulinum toxin substrate 1 (Rac1), which governs neutrophil chemotaxis and apoptosis signaling. PSD was knocked down in a normal human fibroblast cell line (HNDF) and a neutrophil-like cell line (HL-60). Both NHDF and HL-60 cells exhibited numerous filamentous-actin (F-actin) rich membrane extensions, resulting in the activation of Rac1; this activation was hampered by PSD silencing. Lipopolysaccharide, a reactive oxygen species (ROS) inducer, stimulated NHDF cells to release ROS and activated caspase‑3/7 in the presence of neutrophils, which was inhibited by PSD knockdown  . Migrati  on assays demonstrated that chemotaxis of HL-60 cells was affected by PSD silencing in NHDF cells. Tissue sections from 6 UC patients with CRC and 15 UC patients without CRC were examined. To verify Rac1-mediated chemotaxis in tissue sections, we evaluated the grade of neutrophil infiltration by histological assessment and assessed F-actin and PSD expression by immunohistochemistry. Neutrophil infiltration, F-actin and PSD expression were significantly decreased in specimens from UC patients with PSD methylation compared with those without. Decreased levels of F-actin expression were observed in colorectal mucosa, as well as in infiltrating cells with PSD methylation. PSD expression was preferentially inhibited in colorectal mucosa by PSD methylation, whereas PSD expression was rarely observed in infiltrating cells, regardless of PSD methylation status. These data indicate that aberrant methylation of PSD occurs in UC-associated colorectal mucosa, enabling circumvention of Rac1-mediated immune responses governing neutrophil chemotaxis and apoptosis, and thus plays a pivotal role in the mechanisms underlying UC-associated carcinogenesis. 
 Aberrant methylation of PSD disturbs Rac1-mediated immune responses governing neutrophil chemotaxis and apoptosis in ulcerative colitis-associated carcinogenesis. We previously reported that the Pleckstrin and Sec7 domain-containing (PSD) gene is preferentially methylated in patients with ulcerative colitis (UC) who developed colorectal cancer (CRC), and is implicated in UC-associated carcinogenesis through its inhibition of apoptosis. This study aimed to determine the potential effect of PSD methylation on its downstream molecule, Ras-related C3 botulinum toxin substrate 1 (Rac1), which governs neutrophil chemotaxis and apoptosis signaling. PSD was knocked down in a normal human fibroblast cell line (HNDF) and a neutrophil-like cell line (HL-60). Both NHDF and HL-60 cells exhibited numerous filamentous-actin (F-actin) rich membrane extensions, resulting in the activation of Rac1; this activation was hampered by PSD silencing. Lipopolysaccharide, a reactive oxygen species (ROS) inducer, stimulated NHDF cells to release ROS and activated caspase‑3/7 in the presence of neutrophils, which was inhibited by PSD knockdown  . Migrati  on assays demonstrated that chemotaxis of HL-60 cells was affected by PSD silencing in NHDF cells. Tissue sections from 6 UC patients with CRC and 15 UC patients without CRC were examined. To verify Rac1-mediated chemotaxis in tissue sections, we evaluated the grade of neutrophil infiltration by histological assessment and assessed F-actin and PSD expression by immunohistochemistry. Neutrophil infiltration, F-actin and PSD expression were significantly decreased in specimens from UC patients with PSD methylation compared with those without. Decreased levels of F-actin expression were observed in colorectal mucosa, as well as in infiltrating cells with PSD methylation. PSD expression was preferentially inhibited in colorectal mucosa by PSD methylation, whereas PSD expression was rarely observed in infiltrating cells, regardless of PSD methylation status. These data indicate that aberrant methylation of PSD occurs in UC-associated colorectal mucosa, enabling circumvention of Rac1-mediated immune responses governing neutrophil chemotaxis and apoptosis, and thus plays a pivotal role in the mechanisms underlying UC-associated carcinogenesis. 
 EFA6A enhances glioma cell invasion through ADP ribosylation factor 6/extracellular signal-regulated kinase signaling. EFA6A, or Pleckstrin and Sec7 domain protein, is a member of guanine nucleotide exchange factors for ADP ribosylation factor 6 (ARF6). Whereas EFA6A is specifically expressed in the brain, little is known about its function in glial cells or glioma. Here we show that elevated EFA6A expression is detectable in both low-grade and high-grade human glioma tissues samples. To investigate the role of EFA6A in glioma carcinogenesis, we generated a human glioblastoma cell line which conditionally overexpresses EFA6A (U373-EFA6A). We showed that overexpression of EFA6A had no effect on cell proliferation, apoptosis, or cell cycle control. However, as shown by wound healing and in vitro cell invasion assays, it significantly enhanced the cell motility and invasiveness whereas silencing EFA6A by its dominant negative mutant EFA6A(E242K) produced opposite effects. We further showed that ARF6/extracellular signal-regulated kinase (ERK) signaling is required for the EFA6A-mediated cell invasion because both EFA6A(E242K) and ARF6 dominant negative mutant ARF6(T27N) markedly reduced the phosphorylated ERK level and EFA6A-mediated invasive capacity. Consistently, mitogen-activated protein kinase/ERK kinase inhibitor U0126 could abolish the EFA6A-induced cell invasion. These results suggest for the first time a potential role of EFA6A/ARF6/ERK signal cascade in glioma cell   migration   and invasion. 
  [Ii]nvasive  PubMed  
 EFA6A enhances glioma cell invasion through ADP ribosylation factor 6/extracellular signal-regulated kinase signaling. EFA6A, or Pleckstrin and Sec7 domain protein, is a member of guanine nucleotide exchange factors for ADP ribosylation factor 6 (ARF6). Whereas EFA6A is specifically expressed in the brain, little is known about its function in glial cells or glioma. Here we show that elevated EFA6A expression is detectable in both low-grade and high-grade human glioma tissues samples. To investigate the role of EFA6A in glioma carcinogenesis, we generated a human glioblastoma cell line which conditionally overexpresses EFA6A (U373-EFA6A). We showed that overexpression of EFA6A had no effect on cell proliferation, apoptosis, or cell cycle control. However, as shown by wound healing and in vitro cell invasion assays, it significantly enhanced the cell motility and   invasive  ness whereas silencing EFA6A by its dominant negative mutant EFA6A(E242K) produced opposite effects. We further showed that ARF6/extracellular signal-regulated kinase (ERK) signaling is required for the EFA6A-mediated cell invasion because both EFA6A(E242K) and ARF6 dominant negative mutant ARF6(T27N) markedly reduced the phosphorylated ERK level and EFA6A-mediated   invasive   capacity. Consistently, mitogen-activated protein kinase/ERK kinase inhibitor U0126 could abolish the EFA6A-induced cell invasion. These results suggest for the first time a potential role of EFA6A/ARF6/ERK signal cascade in glioma cell migration and invasion. 
  [Mm]etastasis  PubMed  
 The Pleckstrin and Sec7 domain-containing gene as a novel epigenetic modification marker in human gastric cancer and its clinical significance. The Pleckstrin and Sec7 domain-containing (PSD) gene has been recently found to participate in the progression of several types of cancer. In the present study, we identified PSD as a candidate tumor suppressor gene silenced through epigenetic modification in gastric cancer (GC). PSD mRNA expression and DNA methylation were evaluated by real-time reverse-transcriptase polymerase chain reaction (RT-PCR) and methylation-specific PCR in GC cell lines and tissue samples. Involvement of histone modification in GC cell lines was examined by chromatin immunoprecipitation assay. We also used an siRNA-mediated approach to knock down the PSD gene in SGC7901 cells, which was utilized to detect the role of PSD in GC progression, followed by analysis of cell apoptosis and invasion. PSD gene expression was reduced in all GC cell lines compared with GES1 (an immortalized normal gastric cell line). In addition, PSD expression was markedly downregulated in gastric carcinoma tissues when compared to adjacent non-tumor tissues. Our data also indicated that PSD mRNA and protein levels were associated with tumor differentiation and lymph node   metastasis  . Aberrant DNA methylation status and histone modification were also found in GC cell lines. Enhanced gene expression was detected when the HGC27, AGS and BGC823 GC cell lines were treated with the DNA-demethylating agent 5-aza-2'-deoxycytidine. However, treatment with trichostatin A, a histone deacetylase inhibitor, had no effect on PSD expression in any of the GC cell lines. Suppression of PSD by siRNA led to enhanced SGC7901 cell invasion. The depletion of PSD expression inhibited cell proliferation and decreased apoptosis in SGC7901 cell lines. Knockdown of the PSD expression decreased caspase-3 and -7 protein levels in SGC7901 cells. PSD gene may function as a tumor suppressor in GC suggesting a vital role for DNA methylation and histone modification in PSD silencing. PSD expression might be a useful biomarker for epigenetic-based GC early diagnosis and may lead to the identification of new targets for pharmacological intervention. 
  [Cc]ell [Ii]nvasion  PubMed  
 EFA6A enhances glioma   cell invasion   through ADP ribosylation factor 6/extracellular signal-regulated kinase signaling. EFA6A, or Pleckstrin and Sec7 domain protein, is a member of guanine nucleotide exchange factors for ADP ribosylation factor 6 (ARF6). Whereas EFA6A is specifically expressed in the brain, little is known about its function in glial cells or glioma. Here we show that elevated EFA6A expression is detectable in both low-grade and high-grade human glioma tissues samples. To investigate the role of EFA6A in glioma carcinogenesis, we generated a human glioblastoma cell line which conditionally overexpresses EFA6A (U373-EFA6A). We showed that overexpression of EFA6A had no effect on cell proliferation, apoptosis, or cell cycle control. However, as shown by wound healing and in vitro   cell invasion   assays, it significantly enhanced the cell motility and invasiveness whereas silencing EFA6A by its dominant negative mutant EFA6A(E242K) produced opposite effects. We further showed that ARF6/extracellular signal-regulated kinase (ERK) signaling is required for the EFA6A-mediated   cell invasion   because both EFA6A(E242K) and ARF6 dominant negative mutant ARF6(T27N) markedly reduced the phosphorylated ERK level and EFA6A-mediated invasive capacity. Consistently, mitogen-activated protein kinase/ERK kinase inhibitor U0126 could abolish the EFA6A-induced   cell invasion  . These results suggest for the first time a potential role of EFA6A/ARF6/ERK signal cascade in glioma cell migration and invasion. 
 The Pleckstrin and Sec7 domain-containing gene as a novel epigenetic modification marker in human gastric cancer and its clinical significance. The Pleckstrin and Sec7 domain-containing (PSD) gene has been recently found to participate in the progression of several types of cancer. In the present study, we identified PSD as a candidate tumor suppressor gene silenced through epigenetic modification in gastric cancer (GC). PSD mRNA expression and DNA methylation were evaluated by real-time reverse-transcriptase polymerase chain reaction (RT-PCR) and methylation-specific PCR in GC cell lines and tissue samples. Involvement of histone modification in GC cell lines was examined by chromatin immunoprecipitation assay. We also used an siRNA-mediated approach to knock down the PSD gene in SGC7901 cells, which was utilized to detect the role of PSD in GC progression, followed by analysis of cell apoptosis and invasion. PSD gene expression was reduced in all GC cell lines compared with GES1 (an immortalized normal gastric cell line). In addition, PSD expression was markedly downregulated in gastric carcinoma tissues when compared to adjacent non-tumor tissues. Our data also indicated that PSD mRNA and protein levels were associated with tumor differentiation and lymph node metastasis. Aberrant DNA methylation status and histone modification were also found in GC cell lines. Enhanced gene expression was detected when the HGC27, AGS and BGC823 GC cell lines were treated with the DNA-demethylating agent 5-aza-2'-deoxycytidine. However, treatment with trichostatin A, a histone deacetylase inhibitor, had no effect on PSD expression in any of the GC cell lines. Suppression of PSD by siRNA led to enhanced SGC7901   cell invasion  . The depletion of PSD expression inhibited cell proliferation and decreased apoptosis in SGC7901 cell lines. Knockdown of the PSD expression decreased caspase-3 and -7 protein levels in SGC7901 cells. PSD gene may function as a tumor suppressor in GC suggesting a vital role for DNA methylation and histone modification in PSD silencing. PSD expression might be a useful biomarker for epigenetic-based GC early diagnosis and may lead to the identification of new targets for pharmacological intervention. 
   
  
 APC2 
 
  NCBI Gene  
  GeneRIF  
  Pubmed  
 
 
  Search Term  Rank  Fields  
  [Mm]igration  6  PubMed(1)  
 
 
 hide/show details for APC2 
  [Mm]igration  PubMed  
 Loss-of-Function Mutation in APC2 Causes Sotos Syndrome Features. Sotos syndrome, characterized by intellectual disability and characteristic facial features, is caused by haploinsufficiency in the NSD1 gene. We conducted an etiological study on two siblings with Sotos features without mutations in NSD1 and detected a homozygous frameshift mutation in the APC2 gene by whole-exome sequencing, which resulted in the loss of function of cytoskeletal regulation in neurons. Apc2-deficient (Apc2(-/-)) mice exhibited impaired learning and memory abilities along with an abnormal head shape. Endogenous Apc2 expression was downregulated by the knockdown of Nsd1, indicating that APC2 is a downstream effector of NSD1 in neurons. Nsd1 knockdown in embryonic mouse brains impaired the   migration   and laminar positioning of cortical neurons, as observed in Apc2(-/-) mice, and this defect was rescued by the forced expression of Apc2. Thus, APC2 is a crucial target of NSD1, which provides an explanation for the intellectual disability associated with Sotos syndrome. 
   
  
 APC 
 
  NCBI Gene  
  GeneRIF  
  Pubmed  
 
 
  Search Term  Rank  Fields  
  [Ff]ilopodia  1  PubMed(2)  
  [Mm]igration  6  Process(2); PubMed(35); GeneRIFs(6); Summary(1)  
  [Ii]nvasive  10  PubMed(23); GeneRIFs(5)  
  [Mm]etastasize  11  PubMed(1)  
  [Mm]etastasis  12  PubMed(25); GeneRIFs(4)  
  [Cc]ell [Ii]nvasion  13  PubMed(1)  
 
 
 hide/show details for APC 
  [Ff]ilopodia  PubMed  
 Identification and characterization of Asef2, a guanine-nucleotide exchange factor specific for Rac1 and Cdc42. The tumor suppressor adenomatous polyposis coli (APC) is mutated in sporadic and familial colorectal tumors. APC interacts with the Rac1-specific guanine-nucleotide exchange factor (GEF) Asef, which contains an APC-binding region (ABR) in addition to Dbl homology (DH), Pleckstrin (PH) and Src homology 3 (SH3) domains. APC stimulates the GEF activity of Asef, and thereby regulates cell adhesion and migration. Here, we have identified a second Asef, termed Asef2, that shows significant structural and functional similarities to Asef. We found that both the N-terminal ABR and SH3 domains of Asef2 are responsible for its interaction with APC. When expressed in HeLa cells, a mutant Asef2 lacking the ABR and SH3 domains, Asef2-DeltaABR/SH3, induced increases in the levels of the active forms of Rac1 and Cdc42. Full-length Asef2 also showed this activity when co-transfected with truncated mutant APC expressed in colorectal tumor cells. Consistent with this, either Asef2-DeltaABR/SH3 or Asef2 plus truncated mutant APC stimulated lamellipodia formation in MDCK cells and   filopodia   formation in HeLa cells. Furthermore, RNA interference experiments showed that Asef2 is required for migration of colorectal tumor cells expressing truncated APC. These results suggest that similar to Asef, Asef2 plays an important role in cell migration, and that Asef2 activated by truncated mutant APC is required for aberrant migration of colorectal tumor cells. 
 Asef2 and Neurabin2 cooperatively regulate actin cytoskeletal organization and are involved in HGF-induced cell migration. The tumor suppressor adenomatous polyposis coli (APC) is mutated in sporadic and familial colorectal tumors. APC interacts with the Rac1- and Cdc42-specific guanine-nucleotide exchange factors (GEF), Asef and Asef2, which contain an APC-binding region (ABR) in addition to Dbl homology, Pleckstrin homology (PH) and Src homology 3 (SH3) domains. APC stimulates the GEF activity of Asef and Asef2, and thereby regulates cell adhesion and migration. Here we show that Asef2, but not Asef, interacts with Neurabin2/Spinophilin, a scaffold protein that binds to Filamentous actin (F-actin). In response to hepatocyte growth factor (HGF) treatment of HeLa cells, Asef2, Neurabin2 and APC were induced to accumulate and colocalize in lamellipodia and membrane ruffles. Neurabin2 did not affect the GEF activity of Asef2. RNA interference experiments showed that Asef2, Neurabin2 and APC are involved in HGF-induced cell migration. Furthermore, knockdown of Neurabin2 resulted in the suppression of Asef2-induced   filopodia   formation. These results suggest that Asef2, Neurabin2 and APC cooperatively regulate actin cytoskeletal organization and are required for HGF-induced cell migration. 
  [Mm]igration  Process  
 cell   migration   
 positive regulation of cell   migration   
  PubMed  
 Binding of APC and dishevelled mediates Wnt5a-regulated focal adhesion dynamics in migrating cells. Wnt5a is a representative ligand that activates the Wnt/beta-catenin-independent pathway, resulting in the regulation of cell adhesion,   migration  , and polarity, but its molecular mechanism is poorly understood. This report shows that Dishevelled (Dvl) binds to adenomatous polyposis coli (APC) gene product, and this binding is enhanced by Wnt5a. Dvl was involved in the stabilization of the plus end dynamics of microtubules as well as APC. Frizzled2 (Fz2) was present with Wnt5a at the leading edge of migrating cells and formed a complex with APC through Dvl. Fz2 also interacted with integrins at the leading edge, and Dvl and APC associated with and activated focal adhesion kinase and paxillin. The binding of APC to Dvl enhanced the localization of paxillin to the leading edge and was involved in Wnt5a-dependent focal adhesion turnover. Furthermore, this new Wnt5a signalling pathway was important for the epithelial morphogenesis in the three-dimensional culture. These results suggest that the functional and physical interaction of Dvl and APC is involved in Wnt5a/Fz2-dependent focal adhesion dynamics during cell   migration   and epithelial morphogenesis. 
 Adenomatous polyposis coli (APC) plays multiple roles in the intestinal and colorectal epithelia. The adenomatous polyposis coli (APC) gene is mutated in familial adenomatous polyposis and in most sporadic colorectal tumors. During both embryonic and postnatal periods, APC is widely expressed in a variety of tissues, including the brain and gastrointestinal tract. The APC gene product (APC) is a large multidomain protein consisting of 2843 amino acids. APC downregulates the Wnt signaling pathway through its binding to beta-catenin and Axin. Most mutated APC proteins in colorectal tumors lack the beta-catenin-binding regions and fail to inhibit Wnt signaling, leading to the overproliferation of tumor cells. Several mouse models (APC580D, APCDelta716, APC1309, APCMin, APC1638T) have been established to investigate carcinogenesis caused by APC mutations. APC also binds to APC-stimulated guanine nucleotide exchange factor, the kinesin superfamily-associated protein 3, IQGAP1, microtubules, EB1, and discs large (DLG). APC has both nuclear localization signals and nuclear export signals in its molecule, suggesting its occasional nuclear localization and export of beta-catenin from the nucleus. APC is highly expressed in the intestinal and colorectal epithelia and may be involved in homeostasis of the enterocyte renewal phenomena, in which proliferation,   migration  , differentiation, and apoptosis are highly regulated both temporally and spatially. Through the many binding proteins mentioned, APC can exert multiple functions involved in epithelial homeostasis. 
 Adenomatous polyposis coli regulates endothelial cell   migration   independent of roles in beta-catenin signaling and cell-cell adhesion. Adenomatous polyposis coli (APC), a tumor suppressor commonly mutated in cancer, is a cytoskeletal organizer for cell   migration   and a scaffold for GSK3 beta/CKI-mediated phosphorylation and degradation of the Wnt effector beta-catenin. It remains unclear whether these different APC functions are coupled, or independently regulated and localized. In primary endothelial cells, we show that GSK3 beta/CKI-phosphorylated APC localizes to microtubule-dependent clusters at the tips of membrane extensions. Loss of GSK3 beta/CKI-phosphorylated APC from these clusters correlates with a decrease in cell   migration  . GSK3 beta/CKI-phosphorylated APC and beta-catenin at clusters is degraded rapidly by the proteasome, but inhibition of GSK3 beta/CKI does not increase beta-catenin-mediated transcription. GSK3 beta/CKI-phosphorylated and -nonphosphorylated APC also localize along adherens junctions, which requires actin and cell-cell adhesion. Significantly, inhibition of cell-cell adhesion results in loss of lateral membrane APC and a concomitant increase in GSK3 beta/CKI-phosphorylated APC in clusters. These results uncouple different APC functions and show that GSK3 beta/CKI phosphorylation regulates APC clusters and cell   migration   independently of cell-cell adhesion and beta-catenin transcriptional activity. 
 Adenomatous polyposis coli-mediated accumulation of abasic DNA lesions lead to cigarette smoke condensate-induced neoplastic transformation of normal breast epithelial cells. Adenomatous polyposis coli (APC) is a multifunctional protein having diverse cellular functions including cell   migration  , cell-cell adhesion, cell cycle control, chromosomal segregation, and apoptosis. Recently, we found a new role of APC in base excision repair (BER) and showed that it interacts with DNA polymerase β and 5'-flap endonuclease 1 and interferes in BER. Previously, we have also reported that cigarette smoke condensate (CSC) increases expression of APC and enhances the growth of normal human breast epithelial (MCF10A) cells in vitro. In the present study, using APC overexpression and knockdown systems, we have examined the molecular mechanisms by which CSC and its major component, Benzo[α]pyrene, enhances APC-mediated accumulation of abasic DNA lesions, which is cytotoxic and mutagenic in nature, leading to enhanced neoplastic transformation of MCF10A cells in an orthotopic xenograft model. 
 Adenomatous polyposis coli truncation alters cytoskeletal structure and microtubule stability in early intestinal tumorigenesis. Partial loss of function of adenomatous polyposis coli (APC) protein by truncation of its carboxy (C)-terminus is an early factor in the development of many sporadic colorectal cancers. In the C57BL/6J Min/+ (Min/+) mouse, an animal with a germline mutation of Apc, we found that APC truncation was associated with reduced enterocyte   migration   and loss of association and membrane expression of adherens junction proteins. We hypothesized that these defects were related to changes in cytoskeletal function resulting from truncation of the APC C-terminus, which contains microtubule binding regions, as well as putative sites for indirect actin binding. We investigated this further by determining whether APC truncation produced in vivo changes in actin cytoskeletal structure and microtubule stability. The actin cytoskeleton of histologically normal enterocytes from Min/+ mice was compared to that of Apc+/+ (wild-type) mice by confocal indirect immunofluorescence microscopy. We found a significant loss of actin localization at the apical plasma membrane in Min/+ enterocytes. In addition, immunoblotting revealed increased levels of both unstable Tyr-tubulin and alpha-tubulin turnover in Min/+ enterocytes, indicating an alteration in microtubule dynamics. These studies suggest that loss of actin localization and changes in microtubule dynamics may be dominant negative effects of truncated APC. These changes are consistent with the defects in enterocyte   migration   and junctional complex formation observed in the Min/+ model of early APC-associated colorectal tumorigenesis. 
 APC and Smad7 link TGFβ type I receptors to the microtubule system to promote cell   migratio  n. Cell   migratio  n occurs by activation of complex regulatory pathways that are spatially and temporally integrated in response to extracellular cues. Binding of adenomatous polyposis coli (APC) to the microtubule plus ends in polarized cells is regulated by glycogen synthase kinase 3β (GSK-3β). This event is crucial for establishment of cell polarity during direction  al migrat  ion. However, the role of APC for cellular extension in response to extracellular signals is less clear. Smad7 is a direct target gene for transforming growth factor-β (TGFβ) and is known to inhibit various TGFβ-induced responses. Here we report a new function for Smad7. We show that Smad7 and p38 mitogen-activated protein kinase together regulate the expression of APC and   cell mig  ration in prostate cancer cells in response to TGFβ stimulation. In addition, Smad7 forms a complex with APC and acts as an adaptor protein for p38 and GSK-3β kinases to facilitate local TGFβ/p38-dependent inactivation of GSK-3β, accumulation of β-catenin, and recruitment of APC to the microtubule plus end in the leading edge of migrating prostate cancer cells. Moreover, the Smad7-APC complex links the TGFβ type I receptor to the microtubule system to regulate directed cellular extension and migratory responses evoked by TGFβ. 
 Structural basis for the recognition of Asef by adenomatous polyposis coli. Adenomatous polyposis coli (APC) regulates cell-cell adhesion and cell   migration   through activating the APC-stimulated guanine nucleotide-exchange factor (GEF; Asef), which is usually autoinhibited through the binding between its Src homology 3 (SH3) and Dbl homology (DH) domains. The APC-activated Asef stimulates the small GTPase Cdc42, which leads to decreased cell-cell adherence and enhanced cell   migration  . In colorectal cancers, truncated APC constitutively activates Asef and promotes cancer cell   migration   and angiogenesis. Here, we report crystal structures of the human APC/Asef complex. We find that the armadillo repeat domain of APC uses a highly conserved surface groove to recognize the APC-binding region (ABR) of Asef, conformation of which changes dramatically upon binding to APC. Key residues on APC and Asef for the complex formation were mutated and their importance was demonstrated by binding and activity assays. Structural superimposition of the APC/Asef complex with autoinhibited Asef suggests that the binding between APC and Asef might create a steric clash between Asef-DH domain and APC, which possibly leads to a conformational change in Asef that stimulates its GEF activity. Our structures thus elucidate the molecular mechanism of Asef recognition by APC, as well as provide a potential target for pharmaceutical intervention against cancers. 
 Novel self-association of the APC molecule affects APC clusters and cell   migration  . Truncation mutations in the adenomatous polyposis coli (APC) gene are responsible for familial and sporadic colorectal cancer. APC is a multifunctional protein involved in cell   migration  , proliferation and differentiation. The APC protein forms specific clusters in the cell periphery that correlate with sites of active cell   migration  . Little is known about the molecular mechanisms that govern these clusters. Here, we identify a novel interaction of an N-terminal region of APC with the extreme C-terminal 300 amino acids of APC and also with itself. The latter interaction is phospho-sensitive and is enhanced by 14-3-3 (YWHA) protein. These interactions modulate the clustering of APC at the ends of membrane protrusions. Overexpressing this domain or inhibiting 14-3-3 proteins disperses APC clusters and leads to decreased cell   migration  . Moreover, deleting this domain from full-length APC results in less-dynamic clusters compared with wild-type APC. Our data indicate that this newly identified regions in the N-terminal third of APC contributes to the regulation of APC clusters, thus providing a molecular clue for how locally regulated phosphorylation events could mediate the dynamics of APC clusters and contribute to cell   migration  . 
 An EB1-binding motif acts as a microtubule tip localization signal. Microtubules are filamentous polymers essential for cell viability. Microtubule plus-end tracking proteins (+TIPs) associate with growing microtubule plus ends and control microtubule dynamics and interactions with different cellular structures during cell division,   migration  , and morphogenesis. EB1 and its homologs are highly conserved proteins that play an important role in the targeting of +TIPs to microtubule ends, but the underlying molecular mechanism remains elusive. By using live cell experiments and in vitro reconstitution assays, we demonstrate that a short polypeptide motif, Ser-x-Ile-Pro (SxIP), is used by numerous +TIPs, including the tumor suppressor APC, the transmembrane protein STIM1, and the kinesin MCAK, for localization to microtubule tips in an EB1-dependent manner. Structural and biochemical data reveal the molecular basis of the EB1-SxIP interaction and explain its negative regulation by phosphorylation. Our findings establish a general "microtubule tip localization signal" (MtLS) and delineate a unifying mechanism for this subcellular protein targeting process. 
 Restoration of full-length adenomatous polyposis coli (APC) protein in a colon cancer cell line enhances cell adhesion. The APC tumour suppressor gene is mutated in most colon cancers. A major role of APC is the downregulation of the beta-catenin/T-cell factor (Tcf)/lymphoid enhancer factor (LEF) signalling pathway; however, there are also suggestions that it plays a role in the organization of the cytoskeleton, and in cell adhesion and   migration  . For the first time, we have achieved stable expression of wild-type APC in SW480 colon cancer cells, which normally express a truncated form of APC. The ectopically expressed APC is functional, and results in the translocation of beta-catenin from the nucleus and cytoplasm to the cell periphery, and reduces beta-catenin/Tcf/LEF transcriptional signalling. E-cadherin is also translocated to the cell membrane, where it forms functional adherens junctions. Total cellular levels of E-cadherin are increased in the SW480APC cells and the altered charge distribution in the presence of full-length APC suggests that APC is involved in post-translational regulation of E-cadherin localization. Changes in the location of adherens junction proteins are associated with tighter cell-cell adhesion in SW480APC cells, with consequent changes in cell morphology, the actin cytoskeleton and cell   migration   in a wound assay. SW480APC cells have a reduced proliferation rate, a reduced ability to form colonies in soft agar and do not grow tumours in a xenograft mouse tumour model. By regulating the intracellular transport of junctional proteins, we propose that APC plays a role in cell adhesion in addition to its known role in beta-catenin transcriptional signalling. 
 IQ-domain GTPase-activating protein 1 regulates beta-catenin at membrane ruffles and its role in macropinocytosis of N-cadherin and adenomatous polyposis coli. Beta-catenin is an integral component of E-cadherin dependent cell-cell junctions. Here we show that beta-catenin co-localizes with IQ-domain GTPase-activating protein 1 (IQGAP1), adenomatous polyposis coli (APC), and N-cadherin at actin-positive membrane ruffles in NIH 3T3 fibroblasts. We used deletion mapping to identify the membrane ruffle-targeting region of beta-catenin, localizing it to amino acids 47-217, which overlap the IQGAP1 binding site. Knockdown by small interference RNA (siRNA) revealed IQGAP1-dependent membrane targeting of beta-catenin, APC, and N-cadherin. Transient overexpression of IQGAP1 or N-cadherin increased beta-catenin at membrane ruffles. IQGAP1/APC regulates cell   migration  , and using a wound healing assay we demonstrate that siRNA-mediated loss of beta-catenin also caused a modest reduction in the rate of cell   migration  . More significantly, we discovered that beta-catenin is internalized by Arf6-dependent macropinocytosis near sites of membrane ruffling. The beta-catenin macropinosomes co-stained for APC, N-cadherin, and to a lesser extent IQGAP1, and internalization of each binding partner was abrogated by siRNA-dependent knockdown of beta-catenin. In addition, beta-catenin macropinosomes co-localized with the lysosomal marker, lysosome associated membrane protein 1, consistent with their recycling by the late endosomal machinery. Our findings expand on current knowledge of beta-catenin function. We propose that in motile cells beta-catenin is recruited by IQGAP1 and N-cadherin to active membrane ruffles, wherein beta-catenin mediates the internalization and possible recycling of the membrane-associated proteins N-cadherin and APC. 
 Identification and characterization of Asef2, a guanine-nucleotide exchange factor specific for Rac1 and Cdc42. The tumor suppressor adenomatous polyposis coli (APC) is mutated in sporadic and familial colorectal tumors. APC interacts with the Rac1-specific guanine-nucleotide exchange factor (GEF) Asef, which contains an APC-binding region (ABR) in addition to Dbl homology (DH), Pleckstrin (PH) and Src homology 3 (SH3) domains. APC stimulates the GEF activity of Asef, and thereby regulates cell adhesion and   migration  . Here, we have identified a second Asef, termed Asef2, that shows significant structural and functional similarities to Asef. We found that both the N-terminal ABR and SH3 domains of Asef2 are responsible for its interaction with APC. When expressed in HeLa cells, a mutant Asef2 lacking the ABR and SH3 domains, Asef2-DeltaABR/SH3, induced increases in the levels of the active forms of Rac1 and Cdc42. Full-length Asef2 also showed this activity when co-transfected with truncated mutant APC expressed in colorectal tumor cells. Consistent with this, either Asef2-DeltaABR/SH3 or Asef2 plus truncated mutant APC stimulated lamellipodia formation in MDCK cells and filopodia formation in HeLa cells. Furthermore, RNA interference experiments showed that Asef2 is required for   migration   of colorectal tumor cells expressing truncated APC. These results suggest that similar to Asef, Asef2 plays an important role in cell   migration  , and that Asef2 activated by truncated mutant APC is required for aberrant   migration   of colorectal tumor cells. 
 Asef, a link between the tumor suppressor APC and G-protein signaling. The adenomatous polyposis coli gene (APC) is mutated in familial adenomatous polyposis and in sporadic colorectal tumors. Here the APC gene product is shown to bind through its armadillo repeat domain to a Rac-specific guanine nucleotide exchange factor (GEF), termed Asef. Endogenous APC colocalized with Asef in mouse colon epithelial cells and neuronal cells. Furthermore, APC enhanced the GEF activity of Asef and stimulated Asef-mediated cell flattening, membrane ruffling, and lamellipodia formation in MDCK cells. These results suggest that the APC-Asef complex may regulate the actin cytoskeletal network, cell morphology and   migration  , and neuronal function. 
 Regulation of APC and AXIN2 expression by intestinal tumor suppressor CDX2 in colon cancer cells. Wnt signaling is often constitutively active in colorectal cancer cells. The expression of the intestinal specific transcription factor CDX2 is found to be transiently decreased in invasive cells at the tumor/stroma interface. A recent ChIP-Seq study has indicated that several Wnt signaling-related genes are regulated by CDX2. The aim was to investigate the role of decreased CDX2 level on the expression of APC, AXIN2 and GSK3β in migrating colon cancer cells at the invasive front. CDX2-bound promoter and enhancer regions from APC, AXIN2 and GSK3β were analyzed for gene regulatory activity and the expression pattern of APC and GSK3β at the invasive front was evaluated by immunohistochemical procedures. Transfection of intestinal and non-intestinal cell lines demonstrated that CDX2 activated APC and AXIN2 promoter activities via intestinal cell-specific enhancer elements. Suppressed CDX2 expression was associated with endogenous downregulation of APC and AXIN2 expression in Caco-2 cells but did not affect GSK3β expression. Furthermore, elevated levels of nuclear β-catenin and reduced levels of cytoplasmic APC were correlated to a low CDX2 expression in migrating colon cancer cells in vivo. These results suggest that a low CDX2 level has influence on the Wnt signaling in invasive colon cancer cells possibly promoting cell  ular migr  ation. 
 APC binds intermediate filaments and is required for their reorganization during cell   migration  . Intermediate filaments (IFs) are components of the cytoskeleton involved in most cellular functions, including cell   migration  . Primary astrocytes mainly express glial fibrillary acidic protein, vimentin, and nestin, which are essential for   migration  . In a wound-induced   migration   assay, IFs reorganized to form a polarized network that was coextensive with microtubules in cell protrusions. We found that the tumor suppressor adenomatous polyposis coli (APC) was required for microtubule interaction with IFs and for microtubule-dependent rearrangements of IFs during astrocyte   migration  . We also show that loss or truncation of APC correlated with the disorganization of the IF network in glioma and carcinoma cells. In migrating astrocytes, vimentin-associated APC colocalized with microtubules. APC directly bound polymerized vimentin via its armadillo repeats. This binding domain promoted vimentin polymerization in vitro and contributed to the elongation of IFs along microtubules. These results point to APC as a crucial regulator of IF organization and confirm its fundamental role in the coordinated regulation of cytoskeletons. 
 Wnt signalling and the actin cytoskeleton. The tumour suppressor adenomatous polyposis coli (APC) is mutated in sporadic and familial colorectal tumours. APC binds to beta-catenin, a key component of the Wnt signalling pathway, and induces its degradation. In addition to this role, there is increasing evidence for additional roles of APC, including the organization of cytoskeletal networks. APC interacts with microtubules and accumulates at their plus ends in membrane protrusions. Also, it has been reported that APC is associated with the plasma membrane in an actin-dependent manner. Moreover, APC interacts with IQGAP1, an effector of Rac1 and Cdc42, and APC-stimulated guanine nucleotide exchange factor (Asef), a Rac1-specific guanine nucleotide exchange factor (GEF). IQGAP1 mediates association of APC with cortical actin in the leading edge of migrating cell and both proteins are required for cell polarization and directional   migration  . APC interacts with Asef and stimulates its activity, thereby regulating the actin cytoskeletal network, cell morphology, adhesion and   migration  . Truncated mutant APCs present in colorectal tumour cells activate Asef constitutively and contribute to their aberrant migratory properties, which may be important for adenoma formation as well as tumour progression to invasive malignancy. 
 Nup358 interacts with APC and plays a role in cell polarization. Asymmetric localization of adenomatous polyposis coli (APC) to the ends of a subset of microtubules located in the leading edges is essential for the establishment of front-rear polarity during cell   migration  . APC is known to associate with microtubules in three ways: through interaction with the plus-end tracking protein EB1, direct binding through a C-terminal basic region, and through interaction with the plus-end motor kinesin-2. Here we report that the middle region of APC has a previously unidentified microtubule plus-end-targeting function, suggesting an additional microtubule-binding mode for APC. Through the same region, APC interacts with Nup358 (also called RanBP2), a microtubule-binding nucleoporin. Ectopic expression of the middle region of APC is sufficient to recruit endogenous Nup358 to the plus ends of microtubules. Furthermore, our results indicate that Nup358 cooperates with kinesin-2 to regulate the localization of APC to the cell cortex through a nuclear-transport-independent mechanism. Using RNA interference and a scratch-induced wound-healing assay we demonstrate that Nup358 functions in polarized cell   migration  . These results reveal a more active role for structural nucleoporins in regulating fundamental cellular processes than previously anticipated. 
 Male-specific association of the APC rs383830 T allele with the risk of coronary heart disease. APC is a tumor suppressor gene that is involved in the processes of cell   migration   and adhesion, transcriptional activation, and apoptosis. The goal of this study was to evaluate the contribution of the APC rs383830 polymorphism to coronary heart disease (CHD) in Han Chinese. A total of 783 patients with CHD and 737 controls were tested in the current association study. Although our study did not identify an association between the APC rs383830 polymorphism and CHD, a breakdown analysis by gender indicated there was a significant contribution of the rs383830 T allele to the risk of CHD in males (P = 0.046, odds ratio = 1.267, 95% confidence interval = 1.004-1.598). In conclusion, our study suggested a male-specific association of the APC rs383830 polymorphism with CHD. 
 Identification of a link between the tumour suppressor APC and the kinesin superfamily. The tumour suppressor gene adenomatous polyposis coli (APC) is mutated in sporadic and familial colorectal tumours. APC is involved in the proteasome-mediated degradation of beta-catenin, through its interaction with beta-catenin, GSK-3 beta and Axin. APC also interacts with the microtubule cytoskeleton and has been localized to clusters near the distal ends of microtubules at the edges of migrating epithelial cells. Moreover, in Xenopus laevis epithelial cells, APC has been shown to move along microtubules and accumulate at their growing plus ends. However, the mechanism of APC accumulation and the nature of these APC clusters remain unknown. We show here that APC interacts with the kinesin superfamily (KIF) 3A-KIF3B proteins, microtubule plus-end-directed motor proteins, through an association with the kinesin superfamily-associated protein 3 (KAP3). The interaction of APC with KAP3 was required for its accumulation in clusters, and mutant APCs derived from cancer cells were unable to accumulate efficiently in clusters. These results suggest that APC and beta-catenin are transported along microtubules by KAP3-KIF3A-KIF3B, accumulate in the tips of membrane protrusions, and may thus regulate cell   migration  . 
 AMER1 regulates the distribution of the tumor suppressor APC between microtubules and the plasma membrane. APC is a multifunctional tumor suppressor protein that negatively controls Wnt signaling, but also regulates cell adhesion and   migration   by interacting with the plasma membrane and the microtubule cytoskeleton. Although the molecular basis for the microtubule association of APC is well understood, molecular mechanisms that underlie its plasma membrane localization have remained elusive. We show here that APC is recruited to the plasma membrane by binding to APC membrane recruitment 1 (AMER1), a novel membrane-associated protein that interacts with the ARM repeat domain of APC. The N-terminus of AMER1 contains two distinct phosphatidylinositol(4,5)-bisphosphate [PtdIns(4,5)P(2)]-binding domains, which mediate its localization to the plasma membrane. Overexpression of AMER1 increases APC levels and redirects APC from microtubule ends to the plasma membrane of epithelial cells. Conversely, siRNA-mediated knockdown of AMER1 reduces the overall levels of APC, promotes its association with microtubule ends in cellular protrusions and disturbs intercellular junctions. These data indicate that AMER1 controls the subcellular distribution of APC between membrane- and microtubule-associated pools, and might thereby regulate APC-dependent cellular morphogenesis, cell   migration   and cell-cell adhesion. 
 APC binds the Miro/Milton motor complex to stimulate transport of mitochondria to the plasma membrane. Mutations in adenomatous polyposis coli (APC) disrupt regulation of Wnt signaling, mitosis, and the cytoskeleton. We describe a new role for APC in the transport of mitochondria. Silencing of wild-type APC by small interfering RNA caused mitochondria to redistribute from the cell periphery to the perinuclear region. We identified novel APC interactions with the mitochondrial kinesin-motor complex Miro/Milton that were mediated by the APC C-terminus. Truncating mutations in APC abolished its ability to bind Miro/Milton and reduced formation of the Miro/Milton complex, correlating with disrupted mitochondrial distribution in colorectal cancer cells that could be recovered by reconstitution of wild-type APC. Using proximity ligation assays, we identified endogenous APC-Miro/Milton complexes at mitochondria, and live-cell imaging showed that loss of APC slowed the frequency of anterograde mitochondrial transport to the membrane. We propose that APC helps drive mitochondria to the membrane to supply energy for cellular processes such as directed cell   migration  , a process disrupted by cancer mutations. 
 Amer2 protein interacts with EB1 protein and adenomatous polyposis coli (APC) and controls microtubule stability and cell   migration  . EB1 is key factor in the organization of the microtubule cytoskeleton by binding to the plus-ends of microtubules and serving as a platform for a number of interacting proteins (termed +TIPs) that control microtubule dynamics. Together with its direct binding partner adenomatous polyposis coli (APC), EB1 can stabilize microtubules. Here, we show that Amer2 (APC membrane recruitment 2), a previously identified membrane-associated APC-binding protein, is a direct interaction partner of EB1 and acts as regulator of microtubule stability together with EB1. Amer2 binds to EB1 via specific (S/T)xIP motifs and recruits it to the plasma membrane. Coexpression of Amer2 and EB1 generates stabilized microtubules at the plasma membrane, whereas knockdown of Amer2 leads to destabilization of microtubules. Knockdown of Amer2, APC, or EB1 reduces cell   migration  , and morpholino-mediated down-regulation of Xenopus Amer2 blocks convergent extension cell movements, suggesting that the Amer2-EB1-APC complex regulates cell   migration   by altering microtubule stability. 
 Presence of c.3956delC mutation in familial adenomatous polyposis patients from Brazil. To characterize APC gene mutations and correlate them with patient phenotypes in individuals diagnosed with familial adenomatous polyposis (FAP) in northern Brazil.A total of 15 individuals diagnosed with FAP from 5 different families from the north of Brazil were analyzed in this study. In addition to patients with histopathological diagnosis of FAP, family members who had not developed the disease were also tested in order to identify mutations and for possible genetic counseling. All analyzed patients or their guardians signed a consent form approved by the Research Ethics Committee of the João de Barros Barreto University Hospital (Belem, Brazil). DNA extracted from the peripheral blood of a member of each of the affected families was subjected to direct sequencing. The proband of each family was sequenced to identify germline mutations using the Ion Torrent platform. To validate the detected mutations, Sanger sequencing was also performed. The samples from all patients were also tested for the identification of mutations by real-time quantitative polymerase chain reaction using the amplification refractory mutation system.Through interviews with relatives and a search of medical records, it was possible to construct genograms for three of the five families included in the study. All 15 patients from the five families with FAP exhibited mutations in the APC gene, and all mutations were detected in exon 15 of the APC gene. In addition to the patients with a histological diagnosis of FAP, family members without disease symptoms showed the mutation in the APC gene. In the present study, we detected two of the three most frequent germline mutations in the literature: the mutation at codon 1309 and the mutation at codon 1061. The presence of c.3956delC mutation was found in all families from this study, and suggests that this mutation was introduced in the population of the State of Pará through ancestor   immigrati  on (i.e., a de novo mutation that arose in one member belonging to this state from Brazil).Regardless of its origin, the c.3956delC mutation is a strong candidate biomarker of this hereditary cancer syndrome in families of northern Brazil. 
 A novel miR-193a-5p-YY1-APC regulatory axis in human endometrioid endometrial adenocarcinoma. Aberrant expression and altered function of transcription factors (TFs) have vital roles in many aspects of tumor development and progression. In this study, we investigated the functional significance of a TF, Yin Yang1 (YY1) in tumorigenesis of endometrioid endometrial carcinoma (EEC). We demonstrated that YY1 is upregulated in EEC cell lines and primary tumors; and its expression is associated with tumor stages. Depletion of YY1 inhibits EEC cell proliferation and   migration   both in vitro and in vivo, whereas overexpression of YY1 promotes EEC cell growth. These results suggest that YY1 functions as an oncogenic factor in EEC. Transcriptome analysis revealed a significant effect of YY1 on critical aspects of EEC tumorigenesis through inhibition of APC expression. Further mechanistic investigation uncovered a new epigenetic silencing mode of APC by YY1 through recruitment of EZH2 and trimethylation of histone 3 lysine 27 on its promoter region. Moreover, YY1 overexpression was found to be a consequence of miR-193a-5p downregulation through direct miR-193a-5p-YY1 interplay. Our results therefore establish a novel miR-193a-5p-YY1-APC axis, which contributes to EEC development, and may serve as future intervention target. 
 Adenomatous polyposis coli and Asef function downstream of hepatocyte growth factor and phosphatidylinositol 3-kinase. Mutations of the tumor suppressor adenomatous polyposis coli (APC) are responsible for sporadic and familial colorectal tumors. APC negatively regulates Wnt signaling by inducing beta-catenin degradation. It has also been shown that APC plays a role in the organization of cytoskeletal networks. APC interacts with Asef and Asef2, Rac1- and Cdc42-specific guanine nucleotide exchange factors (GEFs), and stimulates their GEF activity; thereby regulating cell morphology, adhesion, and   migration  . Truncated mutant APCs present in colorectal tumor cells activate Asef and Asef2 constitutively and contribute to their aberrant migratory properties. We show here that hepatocyte growth factor (HGF), as well as basic fibroblast growth factor (bFGF) and epidermal growth factor (EGF), induce the accumulation and colocalization of APC and Asef in membrane ruffles and lamellipodia of epithelial cells. Both APC and Asef were found to be required for HGF-induced cell   migration  . Furthermore, we show that the effects of HGF, bFGF, and EGF on APC and Asef are mediated by the activation of phosphatidylinositol 3-kinase (PI3-kinase) and require the PH domain of Asef. These results suggest that Asef and APC function downstream of HGF and PI3-kinase, and play critical roles in growth factor-mediated regulation of cell morphology and   migration  . 
 The armadillo repeat domain of the APC tumor suppressor protein interacts with Striatin family members. Adenomatous polyposis coli (APC) is a multifunctional tumor suppressor protein that negatively regulates the Wnt signaling pathway. The APC gene is ubiquitously expressed in various tissues, especially throughout the large intestine and central nervous system. Mutations in the gene encoding APC have been found in most colorectal cancers and in other types of cancer. The APC gene product is a large multidomain protein that interacts with a variety of proteins, many of which bind to the well conserved armadillo repeat domain of APC. Through its binding partners, APC affects a large number of important cellular processes, including cell-cell adhesion, cell   migration  , organization of the actin and microtubule cytoskeletons, spindle formation and chromosome segregation. The molecular mechanisms that control these diverse APC functions are only partly understood. Here we describe the identification of an additional APC armadillo repeat binding partner - the Striatin protein. The Striatin family members are multidomain molecules that are mainly neuronal and are thought to function as scaffolds. We have found that Striatin is expressed in epithelial cells and co-localizes with APC in the epithelial tight junction compartment and in neurite tips of PC12 cells. The junctional localization of APC and Striatin is actin-dependent. Depletion of APC or Striatin affected the localization of the tight junction protein ZO-1 and altered the organization of F-actin. These results raise the possibility that the contribution of APC to cell-cell adhesion may be through interaction with Striatin in the tight junction compartment of epithelial cells. 
 The adenomatous polyposis coli protein (APC) exists in two distinct soluble complexes with different functions. Mutations resulting in the truncation of the adenomatous polyposis coli (APC) protein are common to most colonic tumours. The APC protein has emerged as a multifunctional protein that contributes to cytoskeletal organisation and is involved in the regulation of beta-catenin. Both, changes in transcription due to increases in beta-catenin, as well as defects in directed cell   migration   and cell division contribute to cancer when APC is mutated. Little is known about how separate functions of APC are coordinated. In this study, we identified two distinct soluble protein pools containing APC. We found that one of these pools represents the fully assembled beta-catenin-targeting complex. The second pool contained at least two different forms of APC: APC that was bound to partially assembled beta-catenin-targeting complexes and APC that could bind microtubules. Consistent with the previously proposed role for glycogen synthase kinase 3beta (GSK3beta) in modulating the assembly and activity of the beta-catenin-targeting complex, formation of the fully assembled complex was reduced by inhibitors of GSK3beta. Similarly, tumour cells with truncated APC only contained the partially assembly beta-catenin-targeting complex. We also found that highly elevated levels of beta-catenin in tumour cells containing wild-type APC correlated with a decrease in the ability of the endogenous APC protein to bind microtubules. Additionally, APC lacking the direct microtubule binding site was more effective at downregulating beta-catenin. Together, our data suggest that the interaction of APC with microtubules and the beta-catenin-targeting complex are mutually exclusive, and indicate that the distribution of endogenous APC between different pools is dynamic, which allows cells to distribute it as required. 
 The frequency of founder mutations in the BRCA1, BRCA2, and APC genes in Australian Ashkenazi Jews: implications for the generality of U.S. population data. Several studies have shown that Ashkenazi Jews in the United States and Israel have a high prevalence of the founder mutations BRCA1 185delAG, BRCA1 5382insC, BRCA2 6174delT, and APC I1307K at frequencies of 1.0--1.1%, 0.2--0.3%, 0.6--1.4%, and 6.1--7.0%, respectively. The objective of this study was to compare the prevalence of these alleles in the Australian Jewish population with that of U.S. Jews. Australian Jews have a different history of   migration  , with less opportunity for changes in allele frequency due to conversion or intermarriage with non-Jewish Australians. The results obtained therefore can be used to assess whether U.S. data can be generalized to other Jewish populations. SUBJECTS AND METHODS. Subject samples were ascertained through a screening program for Tay-Sachs disease as part of a community-based screening program in New South Wales and Victoria. DNA extracted from 1200 deidentified blood samples was tested using amplification refractory mutation system polymerase chain reaction.The allele frequencies found were as follows: BRCA1 185delAG 1.25% (95% confidence interval [CI], 0.62--1.88%), BRCA1 5382insC 0.25% (95% CI, 0--0.53%), BRCA2 6174delT 1.08% (95% CI, 0.50--1.67%), and APC I1307K 8.67% (95% CI, 7.07--10.26%). The prevalence of breast carcinoma predisposition alleles therefore is greater than 2.5% in Australian Ashkenazim.There were no significant differences between the allele frequencies in Australian Ashkenazim and those identified in other studies with similar ascertainment strategies, despite the different   migration   patterns of Australian Jews. This suggests the broad applicability of the U.S. and Israeli data, not only to Australian Ashkenazim, but also to Ashkenazi communities throughout the world. 
 Lack of adenomatous polyposis coli protein correlates with a decrease in cell   migration   and overall changes in microtubule stability. Most sporadic colorectal tumors carry truncation mutations in the adenomatous polyposis coli (APC) gene. The APC protein is involved in many processes that govern gut tissue. In addition to its involvement in the regulation of beta-catenin, APC is a cytoskeletal regulator with direct and indirect effects on microtubules. Cancer-related truncation mutations lack direct and indirect binding sites for microtubules in APC, suggesting that loss of this function contributes to defects in APC-mutant cells. In this study, we show that loss of APC results in disappearance of cellular protrusions and decreased cell   migration  . These changes are accompanied by a decrease in overall microtubule stability and also by a decrease in posttranslationally modified microtubules in the cell periphery particularly the migrating edge. Consistent with the ability of APC to affect cell shape, the overexpression of APC in cells can induce cellular protrusions. These data demonstrate that cell   migration   and microtubule stability are linked to APC status, thereby revealing a weakness in APC-deficient cells with potential therapeutic implications. 
 The mitochondrial protein hTID-1 partners with the caspase-cleaved adenomatous polyposis cell tumor suppressor to facilitate apoptosis. The adenomatous polyposis cell (APC) tumor suppressor is a multifunctional protein involved in cell   migration  , proliferation, differentiation, and apoptosis. Cleavage of APC and the subsequent release of an amino-terminal segment are necessary for a transcription-independent mechanism of APC-mediated apoptosis. The aim of the current study is to elucidate the mechanism by which the amino-terminus of APC contributes to the enhancement of apoptosis.Previous yeast 2-hybrid screens, using the armadillo repeat domain of APC as bait, identified hTID-1 as a potential binding partner. Coimmunoprecipitations, coimmunofluorescence, and binding assays confirm a direct interaction between caspase-cleaved APC and hTID-1 in vivo at the mitochondria. Overexpression and small interfering RNA (siRNA) knockdown studies were designed to determine the significance of this interaction.These experiments have identified hTID-1 as a directly interacting protein partner of caspase-cleaved APC. hTID-1 is an apoptosis modulator: 2 of its known mitochondrial protein isoforms, 43-kilodaltons and 40-kilodaltons, have opposing effects in apoptosis. We demonstrate that the amino-terminal segment of APC interacts with both hTID-1 isoforms directly, although there is a stronger association with the apoptotic suppressor 40-kilodalton isoform in vitro. This interaction localizes to amino acids 202-512 of APC, a region including 2 of the 7 armadillo repeats. Overexpression of the 40-kilodalton hTID-1 isoform partially rescues cells from apoptosis mediated by APC 1-777, whereas siRNA knockdown of this hTID-1 isoform enhances apoptosis.These data suggest that the amino-terminal segment of APC promotes cell sensitivity to apoptosis modulated through its binding to 40- and 43-kilodalton hTID-1 isoforms. 
 Molecular basis for the recognition of adenomatous polyposis coli by the Discs Large 1 protein. The human Discs Large 1 (DLG1) protein uses two of its three PDZ domains to interact with the C-terminal peptide of the Adenomatous Polyposis Coli (APC) tumor suppressor protein. The DLG1/APC complex inhibits the cell cycle progression from the G0/G1 to the S phase, regulates epithelial cell   migration   and morphogenesis, and is required for polarization of the microtubule cytoskeleton. However, the molecular details of how DLG1 recognizes APC is not clear. In this study, we performed biochemical and biophysical assays to investigate the interactions between PDZ domains of DLG1 and the C-terminal peptide of APC. In addition, we determined the crystal structures of the PDZ1 and PDZ2 domains of DLG1 each in complex with the C-terminal 11-residue peptide of APC. Our biochemical, biophysical, and structural results revealed structural elements and residues on PDZ1 and PDZ2 domains of DLG1 and on APC crucial for their mutual interaction. In particular, our results show that the β2/β3 loops of PDZ1 and PDZ2 play important roles in contributing to the binding affinities between PDZ domains and APC, through interacting with the residues upstream of the canonical PDZ-binding S/T-X-V motif. The results provide new insights into the binding mode of a defined C-terminal segment of APC by the PDZ domains of DLG1. 
 Asef2 and Neurabin2 cooperatively regulate actin cytoskeletal organization and are involved in HGF-induced cell   migration  . The tumor suppressor adenomatous polyposis coli (APC) is mutated in sporadic and familial colorectal tumors. APC interacts with the Rac1- and Cdc42-specific guanine-nucleotide exchange factors (GEF), Asef and Asef2, which contain an APC-binding region (ABR) in addition to Dbl homology, Pleckstrin homology (PH) and Src homology 3 (SH3) domains. APC stimulates the GEF activity of Asef and Asef2, and thereby regulates cell adhesion and   migration  . Here we show that Asef2, but not Asef, interacts with Neurabin2/Spinophilin, a scaffold protein that binds to Filamentous actin (F-actin). In response to hepatocyte growth factor (HGF) treatment of HeLa cells, Asef2, Neurabin2 and APC were induced to accumulate and colocalize in lamellipodia and membrane ruffles. Neurabin2 did not affect the GEF activity of Asef2. RNA interference experiments showed that Asef2, Neurabin2 and APC are involved in HGF-induced cell   migration  . Furthermore, knockdown of Neurabin2 resulted in the suppression of Asef2-induced filopodia formation. These results suggest that Asef2, Neurabin2 and APC cooperatively regulate actin cytoskeletal organization and are required for HGF-induced cell   migration  . 
 The tumor suppressor adenomatous polyposis coli controls the direction in which a cell extrudes from an epithelium. Despite high rates of cell death, epithelia maintain intact barriers by squeezing dying cells out using a process termed cell extrusion. Cells can extrude apically into the lumen or basally into the tissue the epithelium encases, depending on whether actin and myosin contract at the cell base or apex, respectively. We previously found that microtubules in cells surrounding a dying cell target p115 RhoGEF to the actin cortex to control where contraction occurs. However, what controls microtubule targeting to the cortex and whether the dying cell also controls the extrusion direction were unclear. Here we find that the tumor suppressor adenomatous polyposis coli (APC) controls microtubule targeting to the cell base to drive apical extrusion. Whereas wild-type cells preferentially extrude apically, cells lacking APC or expressing an oncogenic APC mutation extrude predominantly basally in cultured monolayers and zebrafish epidermis. Thus APC is essential for driving extrusion apically. Surprisingly, although APC controls microtubule reorientation and attachment to the actin cortex in cells surrounding the dying cell, it does so by controlling actin and microtubules within the dying cell. APC disruptions that are common in colon and breast cancer may promote basal extrusion of tumor cells, which could enable their exit and subsequent   migration  . 
 APC/β-catenin-rich complexes at membrane protrusions regulate mammary tumor cell   migratio  n and mesenchymal morphology. The APC tumor suppressor is mutated or downregulated in many tumor types, and is prominently localized to punctate clusters at protrusion tips in migratory cells, such as in astrocytes where it has been implicated in directed cell motility. Although APC loss is considered an initiating event in colorectal cancer, for example, it is less clear what role APC plays in tumor cell motility and whether loss of APC might be an important promoter of tumor progression in addition to initiation.The localization of APC and β-catenin was analyzed in multiple cell lines, including non-transformed epithelial lines treated with a proteasome inhibitor or TGFβ to induce an epithelial-to-mesenchymal transition (EMT), as well as several breast cancer lines, by immunofluorescence. APC expression was knocked down in 4T07 mammary tumor cells using lentiviral-mediated delivery of APC-specific short-hairpin (sh) RNAs, and assessed using quantitative (q) reverse-transcriptase (RT)-PCR and western blotting. Tumor cell motility was analyzed by performing wound-filling assays, and morphology via immunofluorescence (IF) and phase-contrast microscopy. Additionally, proliferation was measured using BrdU incorporation, and TCF reporter assays were performed to determine β-catenin/TCF-mediated transcriptional activity.APC/β-catenin-rich complexes were observed at protrusion ends of migratory epithelial cells treated with a proteasome inhibitor or when EMT has been induced and in tumor cells with a mesenchymal, spindle-like morphology. 4T07 tumor cells with reduced APC levels were significantly less motile and had a more rounded morphology; yet, they did not differ significantly in proliferation or β-catenin/TCF transcriptional activity. Furthermore, we found that APC/β-catenin-rich complexes at protrusion ends were dependent upon an intact microtubule cytoskeleton.These findings indicate that membrane protrusions with APC/β-catenin-containing puncta control the migratory potential and mesenchymal morphology of mammary tumor cells and suggest that APC loss during later stages of tumor progression might impact tumor cell dissemination or colonization. 
 Cell type-specific expression of adenomatous polyposis coli in lung development, injury, and repair. Adenomatous polyposis coli (Apc) is critical for Wnt signaling and cell   migration  . The current study examined Apc expression during lung development, injury, and repair. Apc was first detectable in smooth muscle layers in early lung morphogenesis, and was highly expressed in ciliated and neuroendocrine cells in the advanced stages. No Apc immunoreactivity was detected in Clara or basal cells, which function as stem/progenitor cell in adult lung. In ciliated cells, Apc is associated mainly with apical cytoplasmic domain. In response to naphthalene-induced injury, Apc(positive) cells underwent squamous metaplasia, accompanied by changes in Apc subcellular distribution. In conclusion, both spatial and temporal expression of Apc is dynamically regulated during lung development and injury repair. Differential expression of Apc in progenitor vs. nonprogenitor cells suggests a functional role in cell-type specification. Subcellular localization changes of Apc in response to naphthalene injury suggest a role in cell shape and cell   migration  . 
  GeneRIFs  
 CDX2 activated APC and AXIN2 promoter activities via intestinal cell-specific enhancer elements; results suggest that a low CDX2 level has influence on the Wnt signaling in invasive colon cancer cells possibly promoting cellular   migration   
 the Amer2-EB1-APC complex regulates cell   migration   by altering microtubule stability. 
 These results uncouple different APC functions and show that GSK3 beta/CKI phosphorylation regulates APC clusters and cell   migration   independently of cell-cell adhesion and beta-catenin transcriptional activity. 
 Asef and APC function downstream of HGF and PI3-kinase, and play critical roles in growth factor-mediated regulation of cell morphology and   migration   
 APC is highly expressed in the intestinal and colorectal epithelia and it is involved in homeostasis of the enterocyte renewal phenomena, in which proliferation,   migration  , differentiation, and apoptosis are highly regulated both temporally and spatially 
 These data demonstrate that cell   migration   and microtubule stability are linked to APC status. 
  Summary  
 This gene encodes a tumor suppressor protein that acts as an antagonist of the Wnt signaling pathway. It is also involved in other processes including cell   migration   and adhesion, transcriptional activation, and apoptosis. Defects in this gene cause familial adenomatous polyposis (FAP), an autosomal dominant pre-malignant disease that usually progresses to malignancy. Disease-associated mutations tend to be clustered in a small region designated the mutation cluster region (MCR) and result in a truncated protein product. [provided by RefSeq, Jul 2008] 
  [Ii]nvasive  PubMed  
 Genetic susceptibility to distinct bladder cancer subphenotypes. Clinical, pathologic, and molecular evidence indicate that bladder cancer is heterogeneous with pathologic/molecular features that define distinct subphenotypes with different prognoses. It is conceivable that specific patterns of genetic susceptibility are associated with particular subphenotypes.To examine evidence for the contribution of germline genetic variation to bladder cancer heterogeneity.The Spanish Bladder Cancer/EPICURO Study is a case-control study based in 18 hospitals located in five areas in Spain. Cases were patients with a newly diagnosed, histologically confirmed, urothelial cell carcinoma of the bladder from 1998 to 2001. Case diagnoses were reviewed and uniformly classified by pathologists following the World Health Organisation/International Society of Urological Pathology 1999 criteria. Controls were hospital-matched patients (n=1149).A total of 1526 candidate variants in 423 candidate genes were analysed. Three distinct subphenotypes were defined according to stage and grade: low-grade nonmuscle   invasive   (n=586), high-grade nonmuscle   invasive   (n=219), and muscle   invasive   (n=246). The association between each variant and subphenotype was assessed by polytomous risk models adjusting for potential confounders. Heterogeneity in genetic susceptibility among subphenotypes was also tested.Two established bladder cancer susceptibility genotypes, NAT2 slow-acetylation and GSTM1-null, exhibited similar associations among the subphenotypes, as did VEGF-rs25648, which was previously identified in our study. Other variants conferred risks for specific tumour subphenotypes such as PMS2-rs6463524 and CD4-rs3213427 (respective heterogeneity p values of 0.006 and 0.004), which were associated with muscle-  invasive   tumours (per-allele odds ratios [95% confidence interval] of 0.56 [0.41-0.77] and 0.71 [0.57-0.88], respectively) but not with non-muscle-  invasive   tumours. Heterogeneity p values were not robust in multiple testing according to their false-discovery rate.These exploratory analyses suggest that genetic susceptibility loci might be related to the molecular/pathologic diversity of bladder cancer. Validation through large-scale replication studies and the study of additional genes and single nucleotide polymorphisms are required. 
 Genetic susceptibility to distinct bladder cancer subphenotypes. Clinical, pathologic, and molecular evidence indicate that bladder cancer is heterogeneous with pathologic/molecular features that define distinct subphenotypes with different prognoses. It is conceivable that specific patterns of genetic susceptibility are associated with particular subphenotypes.To examine evidence for the contribution of germline genetic variation to bladder cancer heterogeneity.The Spanish Bladder Cancer/EPICURO Study is a case-control study based in 18 hospitals located in five areas in Spain. Cases were patients with a newly diagnosed, histologically confirmed, urothelial cell carcinoma of the bladder from 1998 to 2001. Case diagnoses were reviewed and uniformly classified by pathologists following the World Health Organisation/International Society of Urological Pathology 1999 criteria. Controls were hospital-matched patients (n=1149).A total of 1526 candidate variants in 423 candidate genes were analysed. Three distinct subphenotypes were defined according to stage and grade: low-grade nonmuscle   invasive   (n=586), high-grade nonmuscle   invasive   (n=219), and muscle   invasive   (n=246). The association between each variant and subphenotype was assessed by polytomous risk models adjusting for potential confounders. Heterogeneity in genetic susceptibility among subphenotypes was also tested.Two established bladder cancer susceptibility genotypes, NAT2 slow-acetylation and GSTM1-null, exhibited similar associations among the subphenotypes, as did VEGF-rs25648, which was previously identified in our study. Other variants conferred risks for specific tumour subphenotypes such as PMS2-rs6463524 and CD4-rs3213427 (respective heterogeneity p values of 0.006 and 0.004), which were associated with muscle-  invasive   tumours (per-allele odds ratios [95% confidence interval] of 0.56 [0.41-0.77] and 0.71 [0.57-0.88], respectively) but not with non-muscle-  invasive   tumours. Heterogeneity p values were not robust in multiple testing according to their false-discovery rate.These exploratory analyses suggest that genetic susceptibility loci might be related to the molecular/pathologic diversity of bladder cancer. Validation through large-scale replication studies and the study of additional genes and single nucleotide polymorphisms are required. 
 Epigenetic alterations of CDH1 and APC genes: relationship with activation of Wnt/beta-catenin pathway in   invasive   ductal carcinoma of breast. Activation of canonical Wnt/beta-catenin pathway in   Invasive   Ductal Carcinoma of Breast (IDCs) was recently reported from our laboratory. Herein, we analyzed promoter methylation status of CDH1 and Adenomatous polyposis coli (APC) genes in 50 IDCs and correlated with expression of E-cadherin (E-CD) and APC proteins and with activation of oncogenic Wnt/beta-catenin signaling pathway components, Dvl, beta-catenin and CyclinD1. Further, Wnt/beta-catenin driven epithelial mesenchymal transition (EMT) was investigated by correlating the expression of Dvl, beta-catenin and CyclinD1 with vimentin expression in these IDCs. Promoter hypermethylation was observed in 25/50 (50%) IDCs for CDH1 and in 11/50 (22%) tumors for APC, associated with loss of expression of E-CD and APC proteins; concordant hypermethylation of these genes was observed in paired patients' sera. Further, 57% of tumors harboring CDH1 methylation and 50% tumors harboring the methylated APC gene showed nuclear localization of beta-catenin, suggesting activation of the canonical Wnt/beta-catenin pathway. Our study demonstrates significant association between vimentin expression and nuclear beta-catenin (p=0.001; Odds ratio (OR)=25.6) and Dvl (p=0.023; OR=8.0), suggesting that EMT may be driven by Wnt/beta-catenin activation in IDCs. In conclusion, we demonstrate correlation of CDH1 and APC promoter methylation with loss of E-CD and APC proteins and with activation of Wnt/beta-catenin signaling pathway. Association of nuclear Dvl and beta-catenin with vimentin expression suggests the importance of Wnt/beta-catenin pathway driven EMT in IDCs. The concordance between CDH1 and APC methylation in IDCs and paired circulating DNA underscores the utility of serum DNA as a non-  invasive   tool for methylation analysis in IDC patients. 
 Genetic and epigenetic alterations of the APC gene in malignant melanoma. High levels of beta-catenin and activating mutations in the beta-catenin gene (CTNNB1) have been demonstrated in malignant melanomas, implicating dysregulated Wnt signalling in the pathogenesis of this malignancy. We systematically examined melanoma cell lines for activating CTNNB1 mutations as well as genetic and epigenetic alterations of the adenomatous polyposis coli gene (APC), another key component of the Wnt signalling transduction pathway. Of 40 cell lines tested, one carried a truncating APC mutation and loss of the corresponding wild-type allele, and one carried a CTNNB1 missense mutation. Hypermethylation of APC promoter 1A was present in five of the cell lines (13%) and in nine of 54 melanoma biopsies (17%). Cells with truncating APC or activating CTNNB1 mutations showed increased transcription from endogenous and ectopic beta-catenin/T-cell factor (Tcf)-responsive target genes, consistent with the known effects of these alterations on beta-catenin stability and Tcf transactivation. In contrast, cell lines with APC promoter 1A hypermethylation did not show increased Wnt signalling, probably due to residual APC activity expressed from promoter 1B. Suppression of APC transcripts in melanoma cells by stable expression of short hairpin RNAs led to a Wnt signalling-independent increase in cell proliferation, but also reduced the   invasive   growth in collagen type I. Collectively, our data suggest that the tumour-suppressive function of APC in melanocytic cells is dose dependent. We propose that epigenetic silencing of promoter 1A may contribute to the development of malignant melanoma by reducing the expression of APC to a level that promotes cell proliferation without compromising the   invasive   capacity. 
 May the APC gene somatic mutations in tumor tissues influence the clinical features of Chinese sporadic colorectal cancers? The APC gene plays an important role in colorectal carcinogenesis. The impact of APC mutations on the clinical features in sporadic CRC remains to be uncovered. The APC gene was screened for mutations with systematic analysis techniques including DHPLC, PTT, MLPA and DNA sequencing in 43 Chinese sporadic CRC patients. Twenty nine somatic mutations (in 17 different types) in APC gene were found in 18 of 43 sporadic CRC patients. Of those, nine were novel mutations. Higher frequency of somatic APC mutations was found in younger CRC patients than that in elder ones. The biallelic somatic mutations of APC gene were identified in four CRC patients whose tumors had more   invasive   clinical features. The nonsense mutation Arg1114X in APC gene was found in five of 43 CRC tumor tissues. A higher cancer metastasis rate was uncovered in CRC patients with this mutation. The somatic mutations of APC gene may influence the clinical features of sporadic CRC. Arg1114X in APC gene, as a hot spot mutation in Chinese CRC, may predispose to the cancer metastasis of sporadic CRC. 
 Smad3 deficiency promotes tumorigenesis in the distal colon of ApcMin/+ mice. Colorectal cancer, one of the most common human malignancies in the Western world, is often subdivided based on tumor location in either the distal or proximal colon. Several mouse models have been developed to study human colorectal cancer, but few display this clear distinction between the two colonic locations. By crossing Apc(Min/+) and Smad3 mutant mice, we showed that combined activation of the Wnt pathway and attenuation of the transforming growth factor-beta (TGF-beta) pathway causes high multiplicity and rapid onset of   invasive   tumorigenesis almost exclusively in the distal colon, closely mimicking the familial adenomatous polyposis (FAP) disease and consisting with distinct colorectal cancer etiologies based on tumor location. Transcriptional profiling revealed higher expression of several TGF-beta activators in the normal distal mucosa than in proximal mucosa, suggesting a stronger reliance on TGF-beta-mediated growth control in the distal than in the proximal colon. Apc(Min/+)Smad3(-/-) mice provide an alternative model to Apc(Min/+) mice to study FAP and distal sporadic colorectal cancer. This model will be useful in dissecting mechanistic and etiologic differences between proximal and distal colonic cancer, whereas the confinement of tumorigenesis to the distal colon offers unique advantages in monitoring tumor progression by in vivo imaging. 
 APC mutations in sporadic medulloblastomas. The cerebellar medulloblastoma (WHO Grade IV) is a highly malignant,   invasive   embryonal tumor with preferential manifestation in children. Several molecular alterations appear to be involved, including isochromosome 17q and the p53, PTCH, and beta-catenin gene mutations. In this study, 46 sporadic medulloblastomas were screened for the presence of mutations in genes of the Wnt signaling pathway (APC and beta-catenin). Single-strand conformational polymorphism (SSCP) analysis followed by direct DNA sequencing revealed 3 miscoding APC mutations in 2 (4.3%) medulloblastomas. One case contained a GCA-->GTA mutation at codon 1296 (Ala-->Val), and another case had double point mutations at codons 1472 (GTA-->ATA, Val-->Ile) and 1495 (AGT-->GGT, Ser-->Gly). Miscoding beta-catenin mutations were detected in 4 tumors (8.7%). Three of these were located at codon 33 (TCT -->TTT, Ser-->Phe) and another at codon 37 (TCT-->GCT, Ser-->Ala). Adenomatous polyposis coli (APC) gene and beta-catenin mutations were mutually exclusive and occurred in a total of 6 of 46 cases (13%). Although germline APC mutations are a well established cause of familial colon and brain tumors (Turcot syndrome), this study provides the first evidence that APC mutations are also operative in a subset of sporadic medulloblastomas. 
 Somatic mutations of adenomatous polyposis coli gene and nuclear b-catenin accumulation have prognostic significance in   invasive   urothelial carcinomas: evidence for Wnt pathway implication. Wnt pathway signaling is crucial in many cancers and data indicate crosstalk with other key cancer pathways, however in urothelial carcinogenesis it has not been extensively studied. We searched for mutations in adenomatous polyposis coli (APC), a key regulator of the pathway, and studied b-catenin expression and interactions with the expression of other markers of apoptosis, angiogenesis, and proliferation in patients with   invasive   urothelial cancer. The mutation cluster region of APC was directly sequenced in 70 patients with muscle   invasive   disease who were treated with surgery and adjuvant chemotherapy. COX-2, p53, Ki67, and b-catenin were studied immunohistochemically and micro vessel density was quantified by CD105 expression. Single somatic amino-acid substitutions (missense) were found in 9 (13%) and frameshift deletions in 2 (3%) tumors, all located in regions adjacent to b-catenin binding sites. Patients having either APC missense mutations or b-catenin nuclear accumulation had less frequent COX-2 overexpression (24% vs. 76%, p = 0.043) and more frequent lymph node involvement (75% vs. 38%, p = 0.023). Patients with either APC mutations or b-catenin accumulation had shorter disease-free interval (13.4 vs. 28 months, p = 0.07), whereas in multivariate analysis they had shorter disease-specific survival (60.5 vs. 20.6 months, p = 0.048). Somatic APC missense mutations are not rare in advanced urothelial neoplasms. Either APC mutations and/or aberrant expression of b-catenin are associated with worse outcome. Further study of the role of the Wnt pathway, potential crosstalk with other pathways and potential candidate therapeutic targets in urothelial cancer is needed. 
 Epithelial to mesenchymal transition (EMT) biomarkers--E-cadherin, beta-catenin, APC and Vimentin--in oral squamous cell carcinogenesis and transformation. To investigate immunohistochemical (IHC) analysis of E-cadherin, β-catenin, APC and Vimentin for prediction of oral malignant transformation.Immunoreactivity for E-cadherin, β-catenin, APC and Vimentin were determined for 100 oral biopsies classified as normal, mild dysplasia, moderate-severe dysplasia or OSCC, using the IHC scoring or label index scoring systems. Co-expression of biomarkers and correlation with histopathological grading was analysed. Vimentin and E-cadherin results were confirmed by RT-PCR and further investigated in vitro using a novel organotypic cell invasion model based on human dermis.A trend for decreased E-cadherin expression but increased Vimentin expression that correlated with increased disease severity was observed. Epithelial β-catenin localisation shifted from being membranous to cytoplasmic/nuclear with increased histopathological grade severity. Relative to normal, APC expression was decreased for mild dysplasia but increased for OSCC. Co-expression of β-catenin, APC and Vimentin (Spearman rank correlation) suggests interdependence of these molecules and involvement of the Wnt pathway in oral malignant transformation. Relative mRNA expression of E-cadherin for dysplasia and OSCC were less than 1% of normal tissue values, and mRNA expression of Vimentin was 3.7 times higher for OSCC than normal. After 63 days of organotypic culture neoplastic oral keratinocytes (PE/CA-PJ15) lost expression of E-cadherin and gained expression of Vimentin relative to their   non-inva  sive counterparts in the epithelium.Trends in the expression of EMT markers - E-cadherin, β-catenin, APC and Vimentin - suggest their involvement in oral carcinogenesis via Wnt pathway dysregulation. Aberrant expression of β-catenin, APC and Vimentin are potential markers of malignant transformation. 
 Regulation of APC and AXIN2 expression by intestinal tumor suppressor CDX2 in colon cancer cells. Wnt signaling is often constitutively active in colorectal cancer cells. The expression of the intestinal specific transcription factor CDX2 is found to be transiently decreased in   invasive   cells at the tumor/stroma interface. A recent ChIP-Seq study has indicated that several Wnt signaling-related genes are regulated by CDX2. The aim was to investigate the role of decreased CDX2 level on the expression of APC, AXIN2 and GSK3β in migrating colon cancer cells at the   invasiv  e front. CDX2-bound promoter and enhancer regions from APC, AXIN2 and GSK3β were analyzed for gene regulatory activity and the expression pattern of APC and GSK3β at t  he invas  ive front was evaluated by immunohistochemical procedures. Transfection of intestinal and non-intestinal cell lines demonstrated that CDX2 activated APC and AXIN2 promoter activities via intestinal cell-specific enhancer elements. Suppressed CDX2 expression was associated with endogenous downregulation of APC and AXIN2 expression in Caco-2 cells but did not affect GSK3β expression. Furthermore, elevated levels of nuclear β-catenin and reduced levels of cytoplasmic APC were correlated to a low CDX2 expression in migrating colon cancer cells in vivo. These results suggest that a low CDX2 level has influence on the Wnt signalin  g in inv  asive colon cancer cells possibly promoting cellular migration. 
 Parafibromin and APC as screening markers for malignant potential in atypical parathyroid adenomas. The identification of parathyroid carcinomas is based upon histopathological criteria in which an   invasive   growth pattern or distant metastasis is demonstrated. A dilemma arises when tumours present with atypical histopathological features but lack direct evidence of malignancy. Recently, reduced expression or loss of the tumour suppressor proteins parafibromin and adenomatous polyposis coli (APC) has been associated with parathyroid malignancy. We report results from APC and parafibromin expression analyses by immunohistochemistry and Western blot in five cases of atypical adenoma, a single case of carcinoma and 54 adenomas without atypical features. Complete loss of APC immunoreactivity and reduced expression of parafibromin was evident in two of the atypical adenomas and in the parathyroid carcinoma. By contrast, all adenomas displayed APC expression, including two cases with hyperparathyroidism 2 gene (HRPT2) mutations and loss of parafibromin expression. We conclude that loss of APC is a frequent molecular event in atypical adenomas and carcinomas, but not in adenomas. Following verification in an independent material, APC could become a valuable tool when assessing parathyroid tumours in the clinical setting. Furthermore, the molecular resemblance of atypical adenomas with carcinoma concerning parafibromin and APC expression indicates that atypical adenomas should be subjects to watchful follow-up. 
 Methylation of the adenomatous polyposis coli (APC) gene in human placenta and hypermethylation in choriocarcinoma cells. Methylation of the human APC gene promoter is associated with several different types of cancers and has also been documented in some pre-cancerous tissues. We have examined the methylation of APC gene promoters in human placenta and choriocarcinoma cells. This revealed a general hypomethylation of the APC-1b promoter and a pattern with monoallelic methylation of the APC-1a promoter in full term placental tissue. However, there was no evidence of a parent-of-origin effect, suggesting random post zygotic origin of methylation. Increased methylation of this promoter was observed in all choriocarcinoma-derived trophoblast cell lines, suggesting a trophoblastic origin of placental APC methylation and implicating APC hypermethylation in the development of this group of gestational tumours. Our demonstration of placental methylation of the APC-1a promoter represents the first observation of monoallelic methylation of this gene in early development, and provides further support for a role of canonical Wnt signalling in placental trophoblast   invasive  ness. This also implicates tumour suppressor gene silencing as an integral part of normal human placental development. 
 Mutations of the APC gene in human sporadic colorectal cancers. Mutations of the APC gene are reported to occur frequently in sporadic colorectal adenomas and adenocarcinomas. We studied APC gene mutations in cases of human sporadic colorectal cancer in order to evaluate their correlation with pathologic characteristics and clinical prognosis.Most of the mutations of the APC gene (95%) are nonsense or frame shift mutations, encoding for truncated APC proteins. For this reason, mutation detection of the APC gene was performed using the in vitro synthesized protein (IVSP) assay, analysing the region between nucleotide 2058 and nucleotide 5079 of the gene, containing the mutation cluster region.Out of 58 cases of colorectal cancer, 29 presented a mutated form of APC (mutation frequency 50%). We did not find a statistically significant correlation between APC gene mutation and age, sex, localization of the primary tumour, grading, Crohn-like lymphoid reaction or presence of residual adenoma. Tumours with low invasivity (Dukes' stages A and B) were less frequently mutated (12/27, 44.5%) than tumours of Dukes' stage C (15 out of 21, 71.4%), which developed macroscopically secondary metastasis with variable latency after surgery. Highly   invasive   tumours with synchronous metastases (Dukes' stage D) had, instead, a low frequency of APC mutations (20%, 2/10) (P = 0.02, compared with Dukes' stages A, B and C).These data suggest that more aggressive Dukes' stage D tumours develop metastasis by means of an unknown mechanism, independent of APC mutation. 
 Wnt signalling and the actin cytoskeleton. The tumour suppressor adenomatous polyposis coli (APC) is mutated in sporadic and familial colorectal tumours. APC binds to beta-catenin, a key component of the Wnt signalling pathway, and induces its degradation. In addition to this role, there is increasing evidence for additional roles of APC, including the organization of cytoskeletal networks. APC interacts with microtubules and accumulates at their plus ends in membrane protrusions. Also, it has been reported that APC is associated with the plasma membrane in an actin-dependent manner. Moreover, APC interacts with IQGAP1, an effector of Rac1 and Cdc42, and APC-stimulated guanine nucleotide exchange factor (Asef), a Rac1-specific guanine nucleotide exchange factor (GEF). IQGAP1 mediates association of APC with cortical actin in the leading edge of migrating cell and both proteins are required for cell polarization and directional migration. APC interacts with Asef and stimulates its activity, thereby regulating the actin cytoskeletal network, cell morphology, adhesion and migration. Truncated mutant APCs present in colorectal tumour cells activate Asef constitutively and contribute to their aberrant migratory properties, which may be important for adenoma formation as well as tumour progression to   invasive   malignancy. 
 Physiological expression of the PI3K-activating mutation Pik3ca(H1047R) combines with Apc loss to promote development of   invasive   intestinal adenocarcinomas in mice. PIK3CA, the gene encoding the p110α catalytic subunit of PI3K (phosphoinositide 3-kinase), is mutated in approximately 20% of sporadic CRCs (colorectal cancers), but the role of these mutations in the pathogenesis of CRC remains unclear. In the present study we used a novel mouse model to investigate the role of the Pik3caH1047R mutation, the most common PIK3CA mutation in CRC, during the development and progression of intestinal cancer. Our results demonstrate that Pik3caH1047R, when expressed at physiological levels, is insufficient to initiate intestinal tumorigenesis; however, in the context of Apc (adenomatous polyposis coli) loss, which is observed in 80% of CRCs and by itself results in benign intestinal adenomas, the Pik3caH1047R mutation promotes the development of highly aggressive and   invasiv  e adenocarcinomas in both the small and large intestines. The results of the present study show that an activating Pik3ca mutation can act in tandem with Apc loss to drive the progression of gastrointestinal cancer and thus this disease may be susceptible to therapeutic targeting using PI3K pathway inhibitors. 
 Aberrant promoter methylation of the RASSF1A and APC genes in epithelial ovarian carcinoma development. Tumor suppressor gene (TSG) silencing through promoter hypermethylation plays an important role in cancer development. The aim of this study was to assess the extent of methylation of the RASSF1A and APC TSG promoters in ovarian epithelial adenomas, low malignant potential tumours and carcinomas in order to reveal a role for epigenetic TSG silencing in the development of these ovarian malignancies.The promoter methylation status of the RASSF1A and APC genes was assessed in 19 benign cystadenomas, 14 low malignant potential (LMP) tumours, and 86 carcinomas using methylation specific PCR (MSP).The methylation frequencies of the RASSF1A and APC gene promoters in benign cystadenomas were found to be 37 % and 16 %, respectively. The LMP tumours exhibited RASSF1A and APC gene promoter methylation frequencies of 50 % and 28 %, respectively, whereas the carcinomas exhibited methylation frequencies of 58 % and 29 %, respectively. Methylation of either the RASSF1A or the APC gene promoter was encountered in 58 %   of the i  nvasive carcinomas.The observed aberrant methylation frequencies of the RASSF1A and APC gene promoters indicate that an accumulation of epigenetic events at these specific TSG promoters may be associated with the malignant transformation of benign cystadenomas and LMP tumours to carcinomas. 
 Desmoid tumors complicating Familial Adenomatous Polyposis: a meta-analysis mutation spectrum of affected individuals. Desmoid tumors are a group of benign,   invasive  , solid tumors that are relatively rare in the general population, but can occur in up to 21 % of patients with Familial Adenomatous Polyposis (FAP). They can be difficult to treat and have high rates of recurrence even after resection. Our goal with this study was to identify the genetic mutations that put certain patients with FAP at high risk for desmoid tumors and could be future targets for research.We performed a search in Pubmed, Ovid Medline and Embase to identify subjects with desmoid tumors and FAP. As a reference group for APC mutations in the unselected FAP population, we used the UMD-APC database referenced in the Orphanet portal which includes APC mutation data on 2040 individuals with FAP.Mutations were able to be broken down into 7 regions based on previously published data. Mutations in the APC gene from codons 1310 to 2011 were the most common region encompassing 48 % of published desmoid cases and 40 % of the reference population. It had a slightly elevated odds ratio of 1.4 that was statistically significant along with codon region 543-713 that had an odds ratio of 2.0. Using a combination of p-value and CI, the remaining 5 regions did not meet statistical significance as either the p >0.05 or the CI included 1.0. The most common point mutation found was codon 1309 (13.1 %), but it was also the most commonly found mutation in our reference population (12.9 %) and had an odds ratio of 1.0.There is an increased risk for desmoid tumors in individuals with APC mutations between codons 543-713 and 1310-2011 when compared to a reference population. These patients may benefit from further study to develop surveillance protocols that could improve outcomes. 
 Association of genetic variation in genes implicated in the beta-catenin destruction complex with risk of breast cancer. Aberrant Wnt/beta-catenin signaling leading to nuclear accumulation of the oncogene product beta-catenin is observed in a wide spectrum of human malignancies. The destruction complex in the Wnt/beta-catenin pathway is critical for regulating the level of beta-catenin in the cytoplasm and in the nucleus. Here, we report a comprehensive study of the contribution of genetic variation in six genes encoding the beta-catenin destruction complex (APC, AXIN1, AXIN2, CSNK1D, CSNK1E, and GSK3B) to breast cancer using a Mayo Clinic Breast Cancer Case-Control Study. A total of 79 candidate functional and tagging single nucleotide polymorphisms (SNP) were genotyped in 798   invasive   cases and 843 unaffected controls. Of these, rs454886 in the APC tumor suppressor gene was associated with increased breast cancer risk (per allele odds ratio, 1.23; 95% confidence intervals, 1.05-1.43; P(trend) = 0.01). In addition, five SNPs in AXIN2 were associated with increased risk of breast cancer (P(trend) < 0.05). Haplotype-based tests identified significant associations between specific haplotypes in APC and AXIN2 (P < or = 0.03) and breast cancer risk. Further characterization of the APC and AXIN2 variants suggested that AXIN2 rs4791171 was significantly associated with risk in premenopausal (P(trend) = 0.0002) but not in postmenopausal women. The combination of our findings and numerous genetic and functional studies showing that APC and AXIN2 perform crucial tumor suppressor functions suggest that further investigation of the contribution of AXIN2 and APC SNPs to breast cancer risk are needed. 
 Ultrasensitive detection of unknown colon cancer-initiating mutations using the example of the Adenomatous polyposis coli gene. Detection of cancer precursors contributes to cancer prevention, for example, in the case of colorectal cancer. To record more patients early, ultrasensitive methods are required for the purpose of non  invasive   precursor detection in body fluids. Our aim was to develop a method for enrichment and detection of known as well as unknown driver mutations in the Adenomatous polyposis coli (APC) gene. By coupled wild-type blocking (WTB) PCR and high-resolution melting (HRM), referred to as WTB-HRM, a minimum detection limit of 0.01% mutant in excess wild-type was achieved according to as little as 1 pg mutated DNA in the assay. The technique was applied to 80 tissue samples from patients with colorectal cancer (n = 17), adenomas (n = 50), serrated lesions (n = 8), and normal mucosa (n = 5). Any kind of known and unknown APC mutations (deletions, insertions, and base exchanges) being situated inside the mutation cluster region was distinguishable from wild-type DNA. Furthermore, by WTB-HRM, nearly twice as many carcinomas and 1.5 times more precursor lesions were identified to be mutated in APC, as compared with direct sequencing. By analyzing 31 associated stool DNA specimens all but one of the APC mutations could be recovered. Transferability of the WTB-HRM method to other genes was proven using the example of KRAS mutation analysis. In summary, WTB-HRM is a new approach for ultrasensitive detection of cancer-initiating mutations. In this sense, it appears especially applicable for non  invasive   detection of colon cancer precursors in body fluids with excess wild-type DNA like stool. 
 Aberrant methylation of RARbeta2 and APC genes in voided urine as molecular markers for early detection of bilharzial and nonbilharzial bladder cancer. Bladder cancer cells illustrate major disruptions in their DNA methylation patterns as compared with normal ones. Authors aimed to identify epigenetic molecular markers in urine for early detection of bladder cancer.We retrospectively analyzed the methylation status of RARβ(2) and APC genes in urine samples from 210 bladder cancer patients, 61 patients with benign urological diseases, and 49 healthy volunteers by using methylation-specific PCR.Methylated RARβ(2) and APC were significantly higher in bladder cancer patients (62.8%, 59.5%) than benign (16.4%, 5%) but not detected in healthy volunteers (0%) at (P < 0.0001). Both methylated genes showed no significant difference among clinicopathologic factors; however, they were detected in all grades and stages. Among the 128 patients with bilharzial bladder cancer, 94 (73.4%) showed methylated RARβ(2) and 86 (67.2%) showed methylated APC. Homoplasmic methylation pattern of both genes were only detected in bilharzial bladder cancer cases. Both sensitivities and specificities of the methylated genes for bladder cancer detection were superior to urine cytology and when altogether combined, the sensitivities improved to (91.8%), (93.5%), (91.9%), and (80.9%) in detection of: bladder cancer, non-musc  le invas  ive bladder cancer, low-grade tumors, and bilharzial associated bladder cancer, respectively.Thus, methylated RARβ(2) and APC genes might be valuable urinary molecular markers for early detection of bilharzial and nonbilharzial bladder cancer. 
 Quantitative adenomatous polyposis coli promoter methylation analysis in tumor tissue, serum, and plasma DNA of patients with lung cancer. The serum of cancer patients often harbors increased free DNA levels, which can potentially be used for cancer detection. Because genetic and epigenetic alterations of the adenomatous polyposis coli (APC) gene are common events in gastrointestinal tumor development, we sought to investigate the frequency and level of aberrant APC promoter methylation in primary tumors and paired preoperative serum or plasma samples of lung cancer patients by semiquantitative methylation-specific fluorogenic real-time PCR. We detected methylation of APC in 95 of 99 (96%) primary lung cancer tissues. Forty-two of 89 (47%) available serum and/or plasma samples from these cases carried detectable amounts of methylated APC promoter DNA. In contrast, no methylated APC promoter DNA was detected in serum samples from 50 healthy controls. A highly elevated APC methylation level in lung cancer tissue was the only independent factor predicting inferior survival in this cohort (P = 0.015). APC methylation analysis appears to be promising as a prognostic factor in primary lung cancer and as a non  invasive   tumor marker in plasma and/or serum DNA. 
 Epigenetic and genetic alterations of APC and CDH1 genes in lobular breast cancer: relationships with abnormal E-cadherin and catenin expression and microsatellite instability. The causes and functional consequences of E-cadherin (E-CD) loss in breast cancer are poorly understood. E-CD loss might act in concert with alterations in the APC/beta-catenin pathway to permit oncogenic beta-catenin signaling. To test this hypothesis, we have analyzed the presence of genetic and epigenetic alterations affecting E-CD (CDH1), APC and beta-catenin (CTNNB1) genes and the immunohistochemical expression of E-CD, beta- and gamma-catenin in a series of 46 infiltrating lobular breast carcinomas (ILCs). Since 80% of ILCs featured complete loss of E-CD expression, we analyzed the molecular alterations responsible for E-CD inactivation in these tumors. We found that 10 of 46 (22%) cases harbored mutations in CDH1, including 1 case with 2 different mutations (1 of which was germline). CDH1 was also inactivated by loss of heterozygosity (LOH; 30/41, 73%) and promoter hypermethylation (19/46, 41%). Interestingly, LOH and mutations were also detected in the corresponding in situ lesions of the ILCs, implying that these alterations are early events in lobular cancer tumorogenesis. Additionally, the presence of a polymorphism in the CDH1 promoter was found to be inversely correlated with CDH1 mutations, but not with E-CD levels. We next examined whether alterations in the APC/beta-catenin pathway also occurred in the same series of ILCs. Although no CTNNB1 or APC mutations were detected, promoter methylation (25/46, 52%) and LOH (7/30, 23%) of APC were found. Moreover, methylation of APC and CDH1 occurred concordantly. However, beta- and gamma-catenin were severely reduced or absent in 90% of these tumors, implying that alterations in CDH1 and APC genes do not promote beta-catenin accumulation in ILC. These molecular alterations were not associated with microsatellite instability. In summary, several different mechanisms (mutations, LOH, methylation) are involved in the frequent CDH1 inactivation in   invasive   and in situ lobular breast cancer. The same tumors also show genetic and epigenetic alterations of APC gene. However, altered CDH1 and APC genes do not promote beta-catenin accumulation in this tumor type. 
 Analysis of Wnt/Beta catenin signalling in desmoid tumors. Desmoid tumors are fibromatous lesions occurring both sporadically and in patients with familial adenomatous polyposis (FAP). Because of the association of these tumors with the hereditary colorectal cancer syndrome FAP we set out to define the molecular events driving desmoid tumorigenesis, hypothezising these might be identical to events driving colorectal tumorigenesis. We found that whereas FAP-associated desmoid tumors are caused by germline APC mutations followed by somatic inactivation of the wild-type APC allele, sporadic desmoids are usually characterized by oncogenic mutations in the b-catenin gene, both identical molecular alterations to those found in the vast majority of colorectal cancers. Next we set out to investigate the cellular pathways activated by these mutations, and identified activation of the Wnt signaling pathway in desmoid tumors. Wnt signaling modulates expression of developmental genes and cell fate via beta-catenin, and has been implicated in many cancer types. Currently we are investigating tissue-specific downstream effectors of the Wnt pathway that might be responsible for the behaviour of these   invasive   fibrous tumors. Our findings also point to a role for this pathway in the regulation of normal myofibroblast proliferation and suggest novel treatments in desmoid tumors and other fibrous proliferative disorders. 
  GeneRIFs  
 CDX2 activated APC and AXIN2 promoter activities via intestinal cell-specific enhancer elements; results suggest that a low CDX2 level has influence on the Wnt signaling in   invasive   colon cancer cells possibly promoting cellular migration 
 adenomatous polyposis coli gene and nuclear b-catenin may have roles in wnt-related progression of   invasive   urothelial carcinomas 
 APC mutations have roles in adenoma formation as well as tumour progression to   invasive   malignancy [review] 
 APC inactivation due to mutations, LOH and methylation is associated with   invasive   and in situ lobular breast cancer. 
 The APC mutations associated wiht desmoid tumors and responsible for the behavior of the   invasive  ness of these neoplasms. 
  [Mm]etastasize  PubMed  
 Desmoid tumor: a disease opportune for molecular insights. Desmoid tumors are monoclonal proliferations that fall within a broad histologic spectrum of fibrous mesenchymal tumors that ranges from benign proliferations of scar tissue to high-grade fibrosarcomas. These low-grade tumors are extremely infiltrative locally, but lack the ability to   metastasize   systemically. While they are only rarely a direct cause of mortality, using current therapeutic modalities, these tumors have a high rate of local recurrence that can result in significant treatment related morbidity. Sporadic desmoids are usually associated with somatic mutations in codons 41 or 45 of exon 3 of beta-catenin (CTNNB1). Desmoid tumors occurring in the background of familial adenomatous polyposis (FAP) usually contain inactivating germline mutations in the adenomatous polyposis coli (APC) gene. CTNNB1 and APC are part of the Wnt signaling pathway and mutations in either gene result in stabilization of the beta-catenin protein and allow nuclear translocation and binding of beta-catenin to the T-cell factor/lymphoid enhancer factor (TCF/Lef) family of transcription factors, resulting in activation of target genes which may underlie desmoid tumor biology and clinical behavior. In an era of molecularly targeted therapeutics there is a real need to better grasp the molecular mechanisms behind desmoid tumorigenesis and progression. This knowledge will eventually result in the development of patient and tumor tailored therapies and assist in the control and eradication of this disease. 
  [Mm]etastasis  PubMed  
 Characterization of gene methylation in human papillomavirus associated-head and neck squamous cell carcinoma. Head and neck squamous cell carcinoma (HNSCC) has become one of the most common forms of cancer worldwide. Hypermethylation-induced silencing of tumor-associated gene has been proposed as an important cofactor in cancer pathology. This paper aimed to characterize the gene methylation patterns in human papillomavirus (HPV) associated-HNSCC. TIMP3 and APC methylation status in neoplastic (N = 92) and non-neoplastic tissues (N = 92) of HNSCC as well as their association with HPV infection were investigated via methylation-specific PCR assays. Results indicated that methylation level of TIMP3 was markedly higher in HPV-positive tumors as compared with HPV-negative tumors. Both TIMP3 and APC methylation were associated with lymph node   metastasis   and higher clinical stage of tumors. Patients with methylation at TIMP3 or APC had worse prognoses as compared to those without these alterations. This is the first study that shows a possible linkage between HPV infection and APC methylation. Methylation patterns of tumor-related genes may contribute to different disease prognosis in HNSCC according to the HPV infection status. 
 Characterization of gene methylation in human papillomavirus associated-head and neck squamous cell carcinoma. Head and neck squamous cell carcinoma (HNSCC) has become one of the most common forms of cancer worldwide. Hypermethylation-induced silencing of tumor-associated gene has been proposed as an important cofactor in cancer pathology. This paper aimed to characterize the gene methylation patterns in human papillomavirus (HPV) associated-HNSCC. TIMP3 and APC methylation status in neoplastic (N = 92) and non-neoplastic tissues (N = 92) of HNSCC as well as their association with HPV infection were investigated via methylation-specific PCR assays. Results indicated that methylation level of TIMP3 was markedly higher in HPV-positive tumors as compared with HPV-negative tumors. Both TIMP3 and APC methylation were associated with lymph node   metastasis   and higher clinical stage of tumors. Patients with methylation at TIMP3 or APC had worse prognoses as compared to those without these alterations. This is the first study that shows a possible linkage between HPV infection and APC methylation. Methylation patterns of tumor-related genes may contribute to different disease prognosis in HNSCC according to the HPV infection status. 
 Common genetic variants in Wnt signaling pathway genes as potential prognostic biomarkers for colorectal cancer. Compelling evidence has implicated the Wnt signaling pathway in the pathogenesis of colorectal cancer. We assessed the use of tag single nucleotide polymorphisms (tSNPs) in adenomatous polyposis coli (APC)/β-catenin (CTNNB1) genes to predict outcomes in patients with colorectal cancer. We selected and genotyped 10 tSNP to predict common variants across entire APC and CTNNB1 genes in 282 colorectal cancer patients. The associations of these tSNPs with distant   metastasi  s-free survival and overall survival were evaluated by Kaplan-Meier analysis, Cox regression model, and survival tree analysis. The 5-year overall survival rate was 68.3%. Survival tree analysis identified a higher-order genetic interaction profile consisting of the APC rs565453, CTNNB1 2293303, and APC rs1816769 that was significantly associated with overall survival. The 5-year survival overall rates were 89.2%, 66.1%, and 58.8% for the low-, medium-, and high-risk genetic profiles, respectively (log-rank P = 0.001). After adjusting for possible confounders, including age, gender, carcinoembryonic antigen levels, tumor differentiation, stage, lymphovascular invasion, perineural invasion, and lymph node involvement, the genetic interaction profile remained significant. None of the studied SNPs were individually associated with dis  tant metas  tasis-free survival and overall survival. Our results suggest that the genetic interaction profile among Wnt pathway SNPs might potentially increase the prognostic value in outcome prediction for colorectal cancer. 
 Expressions of two adenomatous polyposis coli and E-cadherin proteins on human colorectal cancers. Mutations in the adenomatous polyposis coli (APC) gene contribute to the progression of colorectal tumorigenesis. Despite the importance, few studies regarding the localization of this protein on surgically resected human colorectal cancer specimens using immunohistochemistry have been reported so far because of the unavailability of the antibodies for this use. The goal of this study has been to provide the APC protein expression and to validate the APC molecular studies. We took advantage of an immunohistochemistry procedure of applying the unique detergent-mediated antigen retrieval technique to frozen sections and examined the expressions of one amino (N)-terminal (AC4) and one carboxy (C)-terminal APC antibody (HG2). Further, we compared the stainings of APC antibodies with those of the E-cadherin antibody using a quantitative image analysis. E-cadherin is a critical morphogenetic regulator during embryogenesis and recent evidence strongly suggests that downregulation of E-cadherin expression in cancers is associated with a high rate of invasion and   metastasis  . The analysis indicated statistically that normal epithelia showed stronger staining than cancer cells ( P<0.05). Further, in normal epithelia, the amino (N)-terminal APC antibody (AC4) showed a positive correlation with another carboxy (C)-terminal APC antibody (HG2). E-cadherin showed no positive correlation with other APCs in either the normal epithelia or cancer cells. This study verified reduced expressions of APCs and E-cadherin proteins in colorectal cancer cells. This suggests that the normal APC and E-cadherin protein expressions in benign epithelium are progressively and independently lost in the sporadic colorectal cancers. 
 [The I1307K mutation and protein expression of APC gene in gastric cancer]. In order to explore the correlation of the abnormalities of tumor suppressor gene APC with the carcinogenesis and progression of gastric cancer. The I1307K mutation of APC gene in gastric cancer was analysed using Amplification Refractory Mutation System PCR(ARMS ,PCR),also the expression of APC protein in gastric cancer of different stages was detected by immunohistochemical method. We found that there wasn't I1307K mutation of APC gene in 62 cases of blood samples of susceptible population in high incidence areas of gastric cancer and 45 cases of gastric cancer tissues. The positive rates of APC protein in gastric cancer (both early and progressive gastric cancer) were significantly lower than that in normal mucosa,the positive rates of APC protein in progressive gastric cancer were significantly lower than that in early gastric cancer,the positive rates of APC protein in gastric cancer with lymph node   metastasis   were significantly lower than that in gastric cancer without lymph node   metastasis  . So it was thought that there might be no correlation between the I1307K mutation of APC gene and carcinogenesis of gastric cancer in China,but the decreased expression of APC protein was closely related to the carcinogenesis,progression and lymph node   metastasis   of gastric cancer. 
 [Expressions of APC and c-Myc and its implication on non-small cell lung cancer]. To investigate the expressions of adenomatous polyposis coli (APC) protein and c-Myc protein in non-small cell lung cancer (NSCLC) and their lymph node metastases.APC and c-Myc proteins were detected in 270 cases of primary NSCLC, 55 cases of lymph node metastatic tissues and 46 cases of adjacent normal lung tissues by EliVision and EnVision methods of immunohistochemical staining.Higher rates of the expressions of both APC and c-Myc proteins in NSCLC primary foci were found compared with those in lymph node metastases (P < 0.05). Furthermore, the expressions of APC and c-Myc proteins varied with histological types, TNM stagings and   metastasis   of the NSCLC (P < 0.05). The Spearman correlation analysis showed that the expressions of APC and c-Myc proteins were positively correlated (r(s) = 0.376, P = 0.000). The Kaplan-Meier survival analysis revealed that the survival rate was lower in patients with positive expressions of APC and c-Myc proteins than in patients with negative expressions (P < 0.05). Histological type, pathologic grading,   metastasis   and c-Myc were identified as independent risk factors with related to the prognosis of NSCLC patients in the multivariate Cox regression model (P < 0.05).APC and c-Myc may play an important role in the progression of NSCLC. 
 Genetic heterogeneity in synchronous colorectal cancers impacts genotyping approaches and therapeutic strategies. Synchronous colorectal carcinomas (sCRC) are clinically challenging neoplasms. Although the epidemiological characteristics are quite well established, their biological basis is still poorly understood. Hence, we performed comprehensive molecular profiling of 23 sCRC cases comprising 50 synchronous primary tumors, 5 metastases, and corresponding normal tissue by targeted deep sequencing of 30 CRC-related genes, microsatellite analysis and analysis for methylated MLH1. We identified a striking inter- and intratumoral genetic heterogeneity of sCRC. Twenty (87%) cases showed genetic heterogeneity leaving only three cases with tumors that had an identical genetic make-up. Intertumoral heterogeneity was frequently observed for clinically actionable genes, including KRAS. Specifically, 44% of the cases harbored tumors of which at least one was KRAS mutated and the other KRAS wildtype. Moreover, 48% of the cases had at least double, sometimes even triple or quadruple mutations in KRAS, APC, TP53, PIK3CA, and TGFBR2, most of them being subclonal events. Lastly, we detected four cases (17%) with microsatellite instable (MSI) tumors with one case harboring one MSI- and a distinct microsatellite stable carcinoma. Our data demonstrate a striking genetic heterogeneity not only between different sCRC of a single case but also within a single tumor. These results contribute to the biological understanding of sCRC and directly impact genotyping strategies and oncological decision making. Testing one tumor or a single   metastasis   may not suffice in the sCRC setting as clinically relevant and tumor-specific genetic information may be left undetected compromising optimal oncological therapy. © 2015 Wiley Periodicals, Inc. 
 May the APC gene somatic mutations in tumor tissues influence the clinical features of Chinese sporadic colorectal cancers? The APC gene plays an important role in colorectal carcinogenesis. The impact of APC mutations on the clinical features in sporadic CRC remains to be uncovered. The APC gene was screened for mutations with systematic analysis techniques including DHPLC, PTT, MLPA and DNA sequencing in 43 Chinese sporadic CRC patients. Twenty nine somatic mutations (in 17 different types) in APC gene were found in 18 of 43 sporadic CRC patients. Of those, nine were novel mutations. Higher frequency of somatic APC mutations was found in younger CRC patients than that in elder ones. The biallelic somatic mutations of APC gene were identified in four CRC patients whose tumors had more invasive clinical features. The nonsense mutation Arg1114X in APC gene was found in five of 43 CRC tumor tissues. A higher cancer   metastasis   rate was uncovered in CRC patients with this mutation. The somatic mutations of APC gene may influence the clinical features of sporadic CRC. Arg1114X in APC gene, as a hot spot mutation in Chinese CRC, may predispose to the cancer   metastasis   of sporadic CRC. 
 Microsatellite analysis of the APC gene and immunoexpression of E-cadherin, catenin, and tubulin in esophageal squamous cell carcinoma. The histological features that accompany the development and progression of solid tumors are known to be controlled by a distinct cascade of molecular events. One such event is the inactivation of tumor suppressor genes, such as the adenomatous polyposis coli (APC) gene. Disruption of the cadherin-catenin cell adhesion complex also plays a role in the initial steps of cancer invasion and   metastasis   whereas alterations in cell structural molecules, such as tubulin, may contribute to the cancer phenotype. The understanding of the status of these molecules in ESSC may provide novel markers that could impact on management of the disease. The present study examined alterations in the microsatellite sequence of the APC gene via fluorescent-based polymerase chain reaction in 100 cases of primary esophageal squamous cell carcinoma. In addition, the expression of E-cadherin, alpha- and beta-catenin, and alpha- and beta-tubulin was analyzed using immunohistochemistry. These data were then statistically compared with each other as well as the relevant clinicopathologic data. Although the APC markers (D5S210, D5S346, D5S299, and D5S82) tested did show an overall high frequency of allelic imbalance/loss of heterozygosity (62.48%) and microsatellite instability (41.27%), they did not show prognostic significance in the study cohort and were not correlated with the immunohistochemical data. The tubulin proteins showed no significant change in expression in the tumor tissue The decreased immunoreactivity of E-cadherin was statistically correlated with the presence of lymph node metastases (P = .0180). Although alpha- and beta-catenin as well as E-cadherin showed no direct prognostic value, E-cadherin may warrant further investigation as an indirect prognostic indicator by allowing more accurate prediction of lymph node metastases. 
 DNA alterations of microsatellite DNA, p53, APC and K-ras in Chinese colorectal cancer patients.   Colorectal cancer is one of the most rapidly increasing cancers in the world, and accumulation of alterations in oncogenes, tumour suppressor genes and mismatch repair (MMR) genes contributes to colorectal tumorigenesis. Thus, we investigated the alterations of 14 microsatellite loci adjacent to MMR genes, p53, adenomatous polyposis coli (APC) and K-ras in 52 Chinese patients with colorectal cancer.  We performed fluorescent polymerase chain reaction and capillary electrophoresis to analyse microsatellite instability (MSI) and loss of heterozygosity (LOH) in microsatellite loci, which included a panel of nine dinucleotide repeats and the Bethesda consensus panel. Additionally, we screened for mutations in exons 4-9 of p53 and the mutation cluster region (MCR) in APC by DHPLC. Codons 12, 13 and 61 in K-ras were analysed using direct sequencing. All variations were confirmed using clone sequencing.  The alteration frequency of microsatellite DNA was 55·8% (29/52). Among the microsatellites, five loci exhibited MSI and another nine loci exhibited LOH. The mutation rates of p53, APC and K-ras were 42·3%, 38·5% and 36·5%, respectively. All patients (n =   7) with li  ver metastasis had a mutation in p53, APC or K-ras. APC mutation was correlated with clinical stage and the presence   of lymph n  ode metastasis (P = 0·001 and P = 0·006, respectively). CONCLUSIONS>   A total of 80·8% of Chinese patients with colorectal cancer show variations in microsatellite DNA, p53, APC or K-ras. It appears that these microsatellite DNA alterations could be a new biomarker for colorectal cancer. 
 Aberrant promoter CpG island hypermethylation of the adenomatosis polyposis coli gene can serve as a good prognostic factor by affecting lymph node   metastasis   in squamous cell carcinoma of the esophagus. There has been no clear evidence demonstrating whether DNA hypermethylation can affect the prognosis of esophageal cancer. We collected tissue from 50 cases of squamous cell carcinoma of the esophagus and tested them for DNA hypermethylation using methylation-specific polymerase chain reaction. CpG island hypermethylations were observed in 10% for p16, 34% for RARbetaP2, 46% for adenomatosis polyposis coli (APC), 14% for RASSF1A, 84% for FHIT, and 8% for hMLH1. APC promoter hypermethylation was frequently found in patients without lymph node   metastasis   compared with those with lymph node   metastasis   (62.5% : 30.8%, P = 0.025). The number of metastatic lymph nodes were lower in patients with APC promoter hypermethylation (0.87 +/- 0.30 : 3.07 +/- 0.72, P = 0.008). Excluding operative mortalities and incomplete resections, 42 patients were analyzed for long-term outcome. During the mean follow-up period of 35 months, 17 developed recurrence and 14 died of cancer. Ten patients died of other causes. In univariable analysis, unmethylation of APC (P = 0.0015) and FHIT (P = 0.0044), as well as presence of lymph node   metastasis   (P = 0.0038), were risk factors for recurrence. In multivariable analysis, lymph nodes   metastasis   (P = 0.050) and unmethylation of APC promoter (P = 0.023) remained as significant risk factors. In conclusion, promoter hypermethylation of the APC gene is related to a lower number of metastatic lymph nodes and to superior prognosis in terms of recurrence, which suggests it might be involved in the process of lymph node   metastasis   in esophageal cancer. 
 Brain metastases exhibit gross deletions of the APC gene. Candidate genes involved in   metastasis   to the brain require investigation. In the present study, the adenomatous polyposis coli (APC) gene was analyzed in a set of human brain metastases. Gross deletions of the APC gene were tested by polymerase chain reaction/loss of heterozygosity (LOH) using the restriction fragment length polymorphism method performed by the use of MspI and RsaI genetic markers inside exon 15 and exon 11. Among 21 brain metastases analyzed, 58.8% of samples showed LOH of the APC gene. When assigning the genetic changes to a specific primary tumor type, 6 LOHs were found in metastases originated from lung and 4 LOHs in metastases from colon. The main effector of the wnt signaling, beta-catenin, was upregulated in 42.9% of cases and transferred to the nucleus in 28.6% of   metastasis   cases. Our findings suggest that genetic changes of the tumor suppressor gene APC, a component of the wnt pathway, represent a part of the brain   metastasis   genetic profile. 
 Mutation analysis of APC gene in gastric cancer with microsatellite instability. To evaluate the role of APC mutation in gastric carcinogenesis and to correlate APC mutation with microsatellite instability (MSI) in gastric carcinomas.APC mutation was measured with multiplex PCR, denaturing gradient gel electrophoresis and DNA sequencing; and MSI was analyzed by PCR-based methods.Sixty-eight cases of sporadic gastric carcinoma were studied for APC mutation at exon 15 and MSI. APC mutations were detected in 15(22.1 %) gastric cancers. Frequence of APC mutation (33.3 %) in intestinal type of gastric cancer was significantly higher than that in diffuse type (13.1 %, P<0.05). On the contrary, no association was observed between APC mutation and tumor size, differentiation, depth of invasion,   metastasis   or clinical stages. Using five microsatellite markers, MSI in at least one locus was detected in 17 of 68 (25 %) of the tumors analyzed. APC mutations were all detected in MSI-L (only one locus, n=9) or MSS(tumor lacking MSI or stable, n=51), but no mutation was found in MSI-H (> or =2 loci, n=8).APC mutation is involved in carcinogenesis of intestinal type of gastric cancer and is independent of MSI phenotype but related to the LOH pathway in gastric cancer. 
 Molecular genetic analysis of malignant melanomas for aberrations of the WNT signaling pathway genes CTNNB1, APC, ICAT and BTRC. Aberrant activation of the Wnt signaling pathway has been reported in different human tumor types, including malignant melanomas. We investigated 37 malignant melanomas (15 primary tumors and 22 metastases) for alterations of 4 genes encoding members of this pathway, i.e., CTNNB1 (beta-catenin gene, 3p22.1), APC (adenomatous polyposis coli gene, 5q22.2), BTRC (beta-transducin repeat-containing protein gene, 10q24.3) and ICAT (inhibitor of beta-catenin and Tcf-4, 1p36.2). Mutational analysis of CTNNB1 identified somatic mutations in 1 primary melanoma and 1 melanoma   metastasis   from 2 different patients (5%). Both mutations affected the N-terminal degradation box of beta-catenin, which is important for the regulation of beta-catenin homeostasis. Another primary melanoma carried a somatic APC missense mutation within the known mutation cluster region in exon 15. Fourteen tumors (40%) showed LOH at microsatellite markers on 1p36. None of the tumors had lost both copies of the ICAT gene, but 1 melanoma   metastasis   carried a somatic point mutation altering the translation start codon of ICAT. Real-time RT-PCR showed markedly reduced ICAT transcript levels (<or=20% relative to normal skin and benign melanocytic nevi) in 28/36 malignant melanomas (78%), including 13/14 tumors with LOH on 1p36. Allelic loss on 10q was detected in 15 tumors (44%). We found neither mutations nor complete loss of expression of the BTRC gene in our melanoma series. Taken together, our results indicate that the Wnt pathway may be altered in malignant melanomas by different mechanisms, including rare somatic mutations in CTNNB1, APC or ICAT, as well as low or absent expression of ICAT transcripts. 
 Parafibromin and APC as screening markers for malignant potential in atypical parathyroid adenomas. The identification of parathyroid carcinomas is based upon histopathological criteria in which an invasive growth pattern or distant   metastasis   is demonstrated. A dilemma arises when tumours present with atypical histopathological features but lack direct evidence of malignancy. Recently, reduced expression or loss of the tumour suppressor proteins parafibromin and adenomatous polyposis coli (APC) has been associated with parathyroid malignancy. We report results from APC and parafibromin expression analyses by immunohistochemistry and Western blot in five cases of atypical adenoma, a single case of carcinoma and 54 adenomas without atypical features. Complete loss of APC immunoreactivity and reduced expression of parafibromin was evident in two of the atypical adenomas and in the parathyroid carcinoma. By contrast, all adenomas displayed APC expression, including two cases with hyperparathyroidism 2 gene (HRPT2) mutations and loss of parafibromin expression. We conclude that loss of APC is a frequent molecular event in atypical adenomas and carcinomas, but not in adenomas. Following verification in an independent material, APC could become a valuable tool when assessing parathyroid tumours in the clinical setting. Furthermore, the molecular resemblance of atypical adenomas with carcinoma concerning parafibromin and APC expression indicates that atypical adenomas should be subjects to watchful follow-up. 
 APC, K-ras, and p53 gene mutations in colorectal cancer patients: correlation to clinicopathologic features and postoperative surveillance. Current researches have proposed a genetic model for colorectal cancer (CRC), in which the sequential accumulation of mutations in specific cancer-related genes, including adenomatous polyposis coli (APC), K-ras, and p53, drives the transition from normal epithelium through increasing adenomatous dysplasia to colorectal cancer. To identify patients with an increased risk of tumor recurrence or   metastasis   and evaluate the prognostic values of APC, K-ras, and p53 gene mutations, we investigated the frequency of these three mutated genes in tumors and sera of CRC patients. APC, K-ras, and p53 gene mutations in primary tumor tissues and their paired preoperative serum samples of 118 CRC patients were detected by using polymerase chain reaction-single strand conformation polymorphism (PCR-SSCP) analysis, followed by direct DNA sequencing of the PCR-amplified genomic DNA. Subsequently, serum molecular markers were analyzed for their correlation with patients' clinicopathologic features and presence of postoperative recurrence/  metastasis  . We did not observe any significant difference in the association of APC or K-ras or p53 gene mutations in primary tumors with patients' demographic data (all were P > 0.05). In contrast, both serum APC and p53 molecular markers were closely correlated with lymph node   metastasis   and TNM stage (both P < 0.05). Moreover, the serum overall molecular markers (at least one of the three markers) were prominently associated with depth of tumor invasion (P = 0.033), lymph node   metastasis   (P < 0.001), and TNM stage (P < 0.001). In addition, a significantly higher postoperative   metastasis  /recurrence rate in patients positive for overall molecular markers compared to those negative for these molecular markers were also demonstrated (P < 0.001). APC and K-ras molecular markers were more frequently observed in patients with locoregional   metastasis   (both P < 0.05), while p53 molecular marker was usually detected in the cases of peritoneal   metastasis   (P = 0.004). Our findings suggest that serum molecular markers are potentially useful in the determination of colorectal cancer patients harboring gene mutations at high risk of   metastasis  . Serial analysis is warranted in order to assess their long-term prognostic significance and the therapeutic implications. 
 Mutations of the APC gene in human sporadic colorectal cancers. Mutations of the APC gene are reported to occur frequently in sporadic colorectal adenomas and adenocarcinomas. We studied APC gene mutations in cases of human sporadic colorectal cancer in order to evaluate their correlation with pathologic characteristics and clinical prognosis.Most of the mutations of the APC gene (95%) are nonsense or frame shift mutations, encoding for truncated APC proteins. For this reason, mutation detection of the APC gene was performed using the in vitro synthesized protein (IVSP) assay, analysing the region between nucleotide 2058 and nucleotide 5079 of the gene, containing the mutation cluster region.Out of 58 cases of colorectal cancer, 29 presented a mutated form of APC (mutation frequency 50%). We did not find a statistically significant correlation between APC gene mutation and age, sex, localization of the primary tumour, grading, Crohn-like lymphoid reaction or presence of residual adenoma. Tumours with low invasivity (Dukes' stages A and B) were less frequently mutated (12/27, 44.5%) than tumours of Dukes' stage C (15 out of 21, 71.4%), which developed macroscopically secondary   metastasis   with variable latency after surgery. Highly invasive tumours with synchronous metastases (Dukes' stage D) had, instead, a low frequency of APC mutations (20%, 2/10) (P = 0.02, compared with Dukes' stages A, B and C).These data suggest that more aggressive Dukes' stage D tumours develop   metastasis   by means of an unknown mechanism, independent of APC mutation. 
 Association analysis of Wnt pathway genes on prostate-specific antigen recurrence after radical prostatectomy. Approximately one-third of prostate cancer (PCa) patients show biochemical failure after radical prostatectomy (RP) and are prone to develop   metastasis   with significant mortality. Although aberrant Wnt/beta-catenin (CTNNB1) signaling has been observed in numerous types of human cancers, including PCa, to our knowledge there is currently no information on the role of Wnt signaling gene polymorphisms in PCa.We comprehensively studied the contribution of genetic variations in CTNNB1 and adenomatous polyposis coli (APC), one of the key genes encoding the CTNNB1 destruction complex, to PCa risk and prognosis after RP using a hospital-based case-control study. We selected and genotyped 13 tagged single-nucleotide polymorphisms (tSNP) to predict common variants across entire APC and CTNNB1 genes in 307 patients with clinically localized PCa who received RP and 371 unaffected controls.Four tSNPs (rs3846716, rs2431238, rs41115, and rs565453) and a specific haplotype (GTAAGA) in the APC tumor suppressor gene were associated with a 0.57- to 0.71-fold lower risk of localized PCa. The association of tSNPs with prostate-specific antigen (PSA) recurrence in PCa patients was then analyzed by Kaplan-Meier analysis and Cox regression model. Interestingly, we found that the APC rs3846716 GA/AA genotypes were also significantly associated with poorer PSA-free survival (log-rank test, P = 0.037) compared with the GG genotype.This is the first report documenting the potential prognostic role of the APC rs3846716 GA/AA genotype on PSA recurrence after RP. 
 [Expression of Wnt5a, APC, β-catenin and their clinical significance in human colorectal adenocarcinoma]. To study the expression of Wnt5a gene mRNA and Wnt5a, APC, β-catenin proteins in human colorectal adenocarcinoma (CRC) and explore its clinical significance.Wnt5a mRNA level was measured in 30 patients with CRC and paired non-tumor tissues by real-time PCR. Immunohistochemical staining of Wnt5a, APC, β-catenin was performed in samples of 62 patients with CRC using SP system.The relative expression level of Wnt5a mRNA in fresh CRC is 0.1232 ± 0.0140, which is significantly higher than that in adjacent colorectal mucosa (0.0497 ± 0.0074, P = 0.02). A low expression of Wnt5a protein was observed in 38 of 62 CRC. Wnt5a protein expression was closely correlated with the tumor types and the degree of tumor differentiation (P < 0.05). There was no apparent relationship with lymph   node metas  tasis, depth of myometrial invasion and TNM stages (P > 0.05). APC protein was decreased in 38 of 62 CRC. The expression of APC was closely correlated with the tumor types (P < 0.05). There was no apparent relationship with the degree of tumor differentiation, lymph   node metas  tasis, depth of myometrial invasion and TNM stages (P > 0.05). The expression of β-catenin was observed in cytoplasm and/or cell nuclei in 50 of 62 CRC. The positive rate of β-catenin expression was closely correlated with the degree of tumor differentiation, lymp  h node met  astasis, depth of myometrial invasion and TNM stages (P < 0.05). There was no apparent relationship with the tumor types (P > 0.05). The expressions of Wnt5a (r = 0.271, P = 0.027) and APC (r = 0.343, P = 0.004) were correlated with that of β-catenin in CRC, respectively, but there was no correlation between the expressions of Wnt5a and APC protein (r = 0.218, P = 0.078) in the tumors.Wnt5a, APC and β-catenin genes might be involved in the carcinogenesis and development of CRC. It is hypothesized that down-regulation of APC and Wnt5a proteins may be one of causes of ectopic expression of β-catenin in CRC. 
 APC promoter methylation and protein expression in hepatocellular carcinoma. We investigated the impact of promoter methylation on APC protein expression in patients with hepatocellular carcinoma (HCC).50 patients [HCC (n=19), liver   metastasis   (n=19), cholangiocellular cancer (n=7), and benign liver tumors (n=5)] were studied for methylation using Methylight analysis. APC mutation was investigated by protein truncation test and direct sequencing of genomic DNA. The protein expression was evaluated by immunohistochemistry and Western blot analysis.The APC promoter was hypermethylated in 81.8% of non-cancerous liver tissue samples. All HCC samples and ten patients with liver   metastasis   (52.6%) exhibited APC promoter methylation. The degree of methylation was significantly higher in samples from HCC compared to the non-cancerous liver tissue samples (63.1% vs. 24.98%; p=0.001). The level of APC protein expression was significantly reduced in HCC samples compared to that of the corresponding non-tumor liver tissue (p<0.05).Promoter methylation of the APC gene seems to be of significance in hepatocarcinogenesis and results in reduced protein expression in HCC. Interestingly, APC promoter methylation is also present in the vast majority of non-cancerous liver tissue whose (patho)physiological function remains unresolved. 
 The COP9 signalosome mediates beta-catenin degradation by deneddylation and blocks adenomatous polyposis coli destruction via USP15. The Wnt/beta-catenin signalling pathway has important roles in normal cellular proliferation, development and angiogenesis. Many malignant transformations, including sporadic colorectal tumours, are caused by constitutive activation of the Wnt route due to mutations in the tumour suppressor protein adenomatous polyposis coli (APC) or the beta-catenin oncogene, ultimately resulting in reduced beta-catenin degradation by the ubiquitin (Ub) proteasome system (UPS). The COP9 signalosome (CSN) regulates the UPS by controlling cullin-RING Ub ligases (CRLs). We show here that the CSN and the beta-catenin destruction complex cooperate in targeting beta-catenin for degradation by the UPS. Together with the CRL that ubiquitinates beta-catenin, they form a supercomplex responsible for beta-catenin degradation. Wnt3A, glycogen synthase kinase 3beta inhibitors or mutation of CSN-mediated deneddylation induce the disassembly of the supercomplex and the accumulation of beta-catenin. Likewise, downregulation of the CSN in HeLa cells leads to retarded degradation of beta-catenin. Additionally, we found that the knockdown of the CSN causes accelerated proteolysis of APC, an essential component of the beta-catenin destruction complex, which is degraded by the UPS as beta-catenin. We show here that APC is stabilised by the Ub-specific protease 15 (USP15) associated with the CSN. This is demonstrated by over-expression of siRNA oligonucleotides against USP15 or by over-expression of an USP15 mutant, which is unable to degrade poly-Ub chains. Thus, the CSN controls the Wnt/beta-catenin signalling by assisting the assembly of beta-catenin-degrading supercomplexes by deneddylation and, simultaneously, by stabilising APC via CSN-associated USP15. The CSN regulates the balance between beta-catenin and APC. Disturbance of this balance can cause cancer by driving cell transformation, tumour angiogenesis and   metastasis  . A model is provided that proposes a role of CSN-mediated deneddylation in the formation of the beta-catenin-degrading supercomplex and the protection of complex-bound APC via CSN-associated USP15. 
 CpG methylation of APC promoter 1A in sporadic and familial breast cancer patients. Tumour suppressor gene (TSG) silencing through promoter hypermethylation plays an important role in cancer initiation. The aim of this study was to assess the extent of methylation of APC gene promoter in 91 sporadic and 44 familial cases of Tunisian patients with breast cancer (BC) in. The frequency of APC promoter methylation is somewhat similar for sporadic and familial breast cancer cases, (52.1%, and 54.5% respectively). For sporadic breast cancer patients, there was a significant correlation of APC promoter hypermethylation with TNM stage (p = 0.024) and 3-year survival (p = 0.025). Regarding the hormonal status (HR), we found significant association between negativity to PR and unmethylated APC (p= 0.005) while ER and Her2/neu are not correlated. Moreover, unmethylated APC promoter is more frequent in tumours expressing at least one out the 3 proteins compared to triple negative cases (p= 0.053). On the other hand, aberrant methylation of APC was associated with tumour size (p = 0.036), lymph node (p = 0.028), distant   metastasis   (p = 0.031), and 3-year survival (p = 0.046) in the group of patients with familial breast cancer. Moreover, patients with sporadic breast cancer displaying the unmethylated profile have a significant prolonged overall survival compared to those with the methylated pattern of APC promoter (p log rank = 0.008). Epigenetic change at the CpG islands in the APC promoter was associated with the silence of its transcript and the loss of protein expression suggesting that this event is the main mechanism regulating the APC expression in breast cancer. In conclusion, our data showed that the loss of APC through aberrant methylation is associated with the aggressive behavior of both sporadic and familial breast cancer in Tunisian patients. 
 Expression of APC, β-catenin and E-cadherin in Tunisian patients with gastric adenocarcinoma: clinical significance. Aberrant activation of the Wnt signalling pathway is a key feature of many cancers. β-Catenin, adenomatous polyposis coli (APC) and E-cadherin are major players in this pathway. The aim of this study is to examine the expression of β-catenin, APC and E-cadherin in tumour tissues of 80 Tunisian patients with gastric carcinoma and to determine the methylation status of the APC promoter in tumour tissues. Associations between protein expression and clinico-pathological parameters, including prognosis, were performed. Positive expression of β-catenin, APC and E-cadherin was observed in 77.5, 68.7 and 60% of cases, respectively. Tumours lacking membranous expression of β-catenin had greater extent of lymph   node metas  tasis, poor differentiation and advanced T-stage. The expression of E-cadherin correlated with poor differentiation (P = 0.05) and β-catenin expression (P = 0.004). With regards to prognosis, the overall survival time was significantly prolonged for patients showing normal β-catenin expression (exclusively or predominantly membranous staining) alone or combined with positive APC expression (P log rank = 0.008 and 0.003, respectively). The methylated pattern of APC promoter 1A was detected in 43.8% of cases and correlated with T-stage (P   = 0.046)   and distant metastasis (P = 0.037). No correlation was found between the methylated profile of APC promoter 1A and the expression of APC protein in tumour tissues. Our findings suggest that deregulation of the Wnt pathway via abnormal expression of β-catenin and E-cadherin occurred frequently in gastric carcinoma and correlated with worse clinical behaviour. 
 Expression and promoter methylation status of hMLH1, MGMT, APC, and CDH1 genes in patients with colon adenocarcinoma. Colorectal cancer (CRC) is the third most common cancer in men and the second in women worldwide. CRC development is the result of genetic and epigenetic alterations accumulation in the epithelial cells of colon mucosa. In the present study, DNA methylation, an epigenetic event, was evaluated in tumoral and matching normal epithelium in a cohort of 61 CRC patients. The results confirmed and expanded knowledge for the tumor suppressor genes hMLH1, MGMT, APC, and CDH1. Promoter methylation was observed for all the examined genes in different percentage. A total of 71% and 10% of the examined cases were found to be methylated in two or more and in all genes, respectively. mRNA and protein levels were also evaluated. Promoter methylation of hMLH1, MGMT, APC, and CDH1 genes was present at the early stages of tumor's formation and it could also be detected in the normal mucosa. Correlations of the methylated genes with patient's age and tumor's clinicopathological characteristics were also observed. Our findings suggest that DNA methylation is a useful marker for tumor progression monitoring and that promoter methylation in certain genes is associated with more advanced tumor stage, poor differentiation, and   metastasis  . 
 [Expression of DNA methylation of APC in peripheral blood and tumor tissue in patients with esophageal squamous cell carcinoma]. To investigate the relationship between methylation status of APC gene in both peripheral blood and tumor tissues and clinical-pathology characteristics in patients with esophageal squamous cell carcinoma(ESCC), and to study the dynamic change of APC methylation in peripheral blood in the perioperative period.Real-time MSP technique was used to detect methylation status of APC in tumor tissues, adjacent normal tissues and peripheral blood on the day before the surgery, intraoperative, postoperative day 7 in 76 cases with ESCC. Sixty healthy volunteers matched by age and gender were randomly selected as controls.The methylation rate of APC in tumor tissue and peripheral blood was 44.74%(34/76) and 42.11%(32/76), respectively, which were significantly higher than that in adjacent normal tissue and controls [6.58%(5/76) and 1.67%(1/60), P=0.000]. The methylation rates showed good agreement between tumor tissues and peripheral blood, which could be verified by ROC curve(A Zeta=0.849, P=0.000). APC methylation rate was significantly related to pathological staging, lymph node   metastasis  , depth of invasion, and invasion of nerve and vessel (P<0.05). The results demonstrated that family history of cancer was independently associated with APC methylation in peripheral blood(P<0.05). DNA methylation rates in peripheral blood showed an initial increase and then decreased in the preoperative period, intraoperative and postoperative.The methylation rates of APC among free DNA in peripheral blood in patients with ESCC reflect tumor progression, and decrease with the solid tumour resection. 
  GeneRIFs  
 Both TIMP3 and APC methylation were associated with lymph node   metastasis   and higher clinical stage of tumors. Patients with methylation at TIMP3 or APC had worse prognoses as compared to those without these alterations. 
 The methylated pattern of APC promoter 1A was detected in 43.8% of gastric adenocarcinoma cases and correlated with T-stage (P = 0.046) and distant   metastasis   
 Promoter hypermethylation of the APC gene is related to a lower number of metastatic lymph nodes and to superior prognosis in terms of recurrence, suggesting it might be involved in lymphatic   metastasis   in esophageal neoplasms. 
 A nonsense mutation in Arg1114X in the APC gene was found in 5 of 43 colorectal cancer patients. The patients also had higher rates of   metastasis  . 
  [Cc]ell [Ii]nvasion  PubMed  
 Epithelial to mesenchymal transition (EMT) biomarkers--E-cadherin, beta-catenin, APC and Vimentin--in oral squamous cell carcinogenesis and transformation. To investigate immunohistochemical (IHC) analysis of E-cadherin, β-catenin, APC and Vimentin for prediction of oral malignant transformation.Immunoreactivity for E-cadherin, β-catenin, APC and Vimentin were determined for 100 oral biopsies classified as normal, mild dysplasia, moderate-severe dysplasia or OSCC, using the IHC scoring or label index scoring systems. Co-expression of biomarkers and correlation with histopathological grading was analysed. Vimentin and E-cadherin results were confirmed by RT-PCR and further investigated in vitro using a novel organotypi  c cell invasi  on model based on human dermis.A trend for decreased E-cadherin expression but increased Vimentin expression that correlated with increased disease severity was observed. Epithelial β-catenin localisation shifted from being membranous to cytoplasmic/nuclear with increased histopathological grade severity. Relative to normal, APC expression was decreased for mild dysplasia but increased for OSCC. Co-expression of β-catenin, APC and Vimentin (Spearman rank correlation) suggests interdependence of these molecules and involvement of the Wnt pathway in oral malignant transformation. Relative mRNA expression of E-cadherin for dysplasia and OSCC were less than 1% of normal tissue values, and mRNA expression of Vimentin was 3.7 times higher for OSCC than normal. After 63 days of organotypic culture neoplastic oral keratinocytes (PE/CA-PJ15) lost expression of E-cadherin and gained expression of Vimentin relative to their non-invasive counterparts in the epithelium.Trends in the expression of EMT markers - E-cadherin, β-catenin, APC and Vimentin - suggest their involvement in oral carcinogenesis via Wnt pathway dysregulation. Aberrant expression of β-catenin, APC and Vimentin are potential markers of malignant transformation. 
   
  
 NINJ1 
 
  NCBI Gene  
  GeneRIF  
  Pubmed  
 
 
  Search Term  Rank  Fields  
  [Mm]igration  6  PubMed(2); GeneRIFs(1)  
  [Ii]nvasive  10  PubMed(2)  
  [Mm]etastasis  12  PubMed(1)  
 
 
 hide/show details for NINJ1 
  [Mm]igration  PubMed  
 Contribution of Ninjurin1 to Toll-like receptor 4 signaling and systemic inflammation. Nerve injury-induced protein (Ninjurin [Ninj]) 1 is an adhesion molecule originally identified in Schwann cells after nerve injury, whereas it is also expressed in leukocytes, epithelium, endothelium, and various organs, and is induced under inflammatory conditions. Its contribution to inflammation was so far restricted to the nervous system and exclusively attributed to its role during leukocyte   migration  . We hypothesized a proinflammatory role for Ninj1 also outside the nervous system. To elucidate its impact during inflammation, we analyzed expression levels and its contribution to inflammation in septic mice and studied its effect on inflammatory signaling in vitro. The effect on inflammation was analyzed by genetic (only in vitro) and pharmacologic repression in septic mice (cecal ligation and puncture) and cell culture, respectively. Repression of Ninj1 by an inhibitory peptide or small interfering RNA attenuated LPS-triggered inflammation in macrophages and endothelial cells by modulating p38 phosphorylation and activator protein-1 activation. Inhibition of Ninj1 in septic mice reduced systemic and pulmonary inflammation as well as organ damage, and ameliorated survival after 24 hours. Ninj1 is elevated under inflammatory conditions and contributes to inflammation not only by mediating leukocyte   migration  , but also by modulating Toll-like receptor 4-dependent expression of inflammatory mediators. We assume that, owing to both mechanisms, inhibition reduces systemic inflammation and organ damage in septic mice. Our data contribute to a better understanding of the complex inflammatory mechanisms and add a novel therapeutic target for inflammatory conditions such as sepsis. 
 Role of Ninjurin-1 in the   migration   of myeloid cells to central nervous system inflammatory lesions. Blood-derived myeloid antigen-presenting cells (APCs) account for a significant proportion of the leukocytes found within lesions of multiple sclerosis (MS) and experimental allergic encephalomyelitis (EAE). These APCs along with activated microglia are thought to be pivotal in the initiation of the central nervous system (CNS)-targeted immune response in MS and EAE. However, the exact molecules that direct the   migration   of myeloid cells from the periphery across the blood-brain barrier (BBB) remain largely unknown.We identified Ninjurin-1 in a proteomic screen of human BBB endothelial cells (ECs). We assessed the expression of Ninjurin-1 by BBB-ECs and immune cells, and we determined the role of Ninjurin-1 in immune cell   migration   to the CNS in vivo in EAE mice.Ninjurin-1 was found to be weakly expressed in the healthy human and mouse CNS but upregulated on BBB-ECs and on infiltrating APCs during the course of EAE and in active MS lesions. In human peripheral blood, Ninjurin-1 was predominantly expressed by monocytes, whereas it was barely detectable on T and B lymphocytes. Moreover, Ninjurin-1 neutralization specifically abrogated the adhesion and   migration   of human monocytes across BBB-ECs, without affecting lymphocyte recruitment. Finally, Ninjurin-1 blockade reduced clinical disease activity and histopathological indices of EAE and decreased infiltration of macrophages, dendritic cells, and APCs into the CNS.Our study uncovers an important cell-specific role for Ninjurin-1 in the trans  migration   of inflammatory APCs across the BBB and further emphasizes the importance of myeloid cell recruitment during the development of neuroinflammatory lesions. 
  GeneRIFs  
 Data show an important cell-specific role for Ninjurin-1 in the trans  migration   of inflammatory APCs across the BBB. 
  [Ii]nvasive  PubMed  
 Genetic susceptibility to distinct bladder cancer subphenotypes. Clinical, pathologic, and molecular evidence indicate that bladder cancer is heterogeneous with pathologic/molecular features that define distinct subphenotypes with different prognoses. It is conceivable that specific patterns of genetic susceptibility are associated with particular subphenotypes.To examine evidence for the contribution of germline genetic variation to bladder cancer heterogeneity.The Spanish Bladder Cancer/EPICURO Study is a case-control study based in 18 hospitals located in five areas in Spain. Cases were patients with a newly diagnosed, histologically confirmed, urothelial cell carcinoma of the bladder from 1998 to 2001. Case diagnoses were reviewed and uniformly classified by pathologists following the World Health Organisation/International Society of Urological Pathology 1999 criteria. Controls were hospital-matched patients (n=1149).A total of 1526 candidate variants in 423 candidate genes were analysed. Three distinct subphenotypes were defined according to stage and grade: low-grade nonmuscle   invasive   (n=586), high-grade nonmuscle   invasive   (n=219), and muscle   invasive   (n=246). The association between each variant and subphenotype was assessed by polytomous risk models adjusting for potential confounders. Heterogeneity in genetic susceptibility among subphenotypes was also tested.Two established bladder cancer susceptibility genotypes, NAT2 slow-acetylation and GSTM1-null, exhibited similar associations among the subphenotypes, as did VEGF-rs25648, which was previously identified in our study. Other variants conferred risks for specific tumour subphenotypes such as PMS2-rs6463524 and CD4-rs3213427 (respective heterogeneity p values of 0.006 and 0.004), which were associated with muscle-  invasive   tumours (per-allele odds ratios [95% confidence interval] of 0.56 [0.41-0.77] and 0.71 [0.57-0.88], respectively) but not with non-muscle-  invasive   tumours. Heterogeneity p values were not robust in multiple testing according to their false-discovery rate.These exploratory analyses suggest that genetic susceptibility loci might be related to the molecular/pathologic diversity of bladder cancer. Validation through large-scale replication studies and the study of additional genes and single nucleotide polymorphisms are required. 
 Genetic susceptibility to distinct bladder cancer subphenotypes. Clinical, pathologic, and molecular evidence indicate that bladder cancer is heterogeneous with pathologic/molecular features that define distinct subphenotypes with different prognoses. It is conceivable that specific patterns of genetic susceptibility are associated with particular subphenotypes.To examine evidence for the contribution of germline genetic variation to bladder cancer heterogeneity.The Spanish Bladder Cancer/EPICURO Study is a case-control study based in 18 hospitals located in five areas in Spain. Cases were patients with a newly diagnosed, histologically confirmed, urothelial cell carcinoma of the bladder from 1998 to 2001. Case diagnoses were reviewed and uniformly classified by pathologists following the World Health Organisation/International Society of Urological Pathology 1999 criteria. Controls were hospital-matched patients (n=1149).A total of 1526 candidate variants in 423 candidate genes were analysed. Three distinct subphenotypes were defined according to stage and grade: low-grade nonmuscle   invasive   (n=586), high-grade nonmuscle   invasive   (n=219), and muscle   invasive   (n=246). The association between each variant and subphenotype was assessed by polytomous risk models adjusting for potential confounders. Heterogeneity in genetic susceptibility among subphenotypes was also tested.Two established bladder cancer susceptibility genotypes, NAT2 slow-acetylation and GSTM1-null, exhibited similar associations among the subphenotypes, as did VEGF-rs25648, which was previously identified in our study. Other variants conferred risks for specific tumour subphenotypes such as PMS2-rs6463524 and CD4-rs3213427 (respective heterogeneity p values of 0.006 and 0.004), which were associated with muscle-  invasive   tumours (per-allele odds ratios [95% confidence interval] of 0.56 [0.41-0.77] and 0.71 [0.57-0.88], respectively) but not with non-muscle-  invasive   tumours. Heterogeneity p values were not robust in multiple testing according to their false-discovery rate.These exploratory analyses suggest that genetic susceptibility loci might be related to the molecular/pathologic diversity of bladder cancer. Validation through large-scale replication studies and the study of additional genes and single nucleotide polymorphisms are required. 
  [Mm]etastasis  PubMed  
 Ninjurin1 Is Up-regulated in Circulating Prostate Tumor Cells and Plays a Critical Role in Prostate Cancer Cell Motility. Ninjurin1 is a 17-kDa membrane protein that is highly expressed in circulating tumor cells (CTCs) obtained from locally-advanced prostate cancer patients. As CTCs are implicated in the initiation of distant   metastasis  , we examined the potential contribution of Ninjurin1 to the motility of prostate cancer cells.Ninjurin1 expression was evaluated in CTCs harvested from seven locally advanced patients with no metastatic hallmarks using real-time polymerase chain reaction (PCR). The role of Ninjurin1 in cell motility was investigated using small interfering RNA (siRNA), neutralizing antibodies against Ninjurin1 and Ninjurin1-overexpressing adenoviruses.Ninjurin1 was ranked as the most significantly up-regulated adhesion protein identified by RNA-Seq analysis. Both Ninjurin1 down-regulation by siRNA and neutralizing antibodies blocking Ninjurin1 homophilic interactions effectively inhibited cell motility. In contrast, cell motility was enhanced in prostate cancer cells infected with adenovirus enabling Ninjurin1 overexpression.Ninjurin1-neutralizing antibodies or Ninjurin1-targeting siRNA merit further development for patients with metastatic potential. 
   
  
 FLI1 
 
  NCBI Gene  
  GeneRIF  
  Pubmed  
 
 
  Search Term  Rank  Fields  
  [Mm]igration  6  PubMed(6); GeneRIFs(1)  
  ECM  7  PubMed(1)  
  [Ee]xtracellular [Mm]atrix  8  PubMed(6); GeneRIFs(1)  
  [Ii]nvasive  10  PubMed(3)  
  [Mm]etastasis  12  PubMed(6); GeneRIFs(1)  
  [Cc]ell [Ii]nvasion  13  PubMed(1)  
 
 
 hide/show details for FLI1 
  [Mm]igration  PubMed  
 Friend leukemia virus integration 1 activates the Rho GTPase pathway and is associated with metastasis in breast cancer. Breast cancer is the most prevalent malignant disease in women worldwide. In patients with breast cancer, metastasis to distant sites directly determines the survival outcome. However, the molecular mechanism underlying metastasis in breast cancer remains to be defined. In this report, we found that Friend leukemia virus integration 1 (FLI1) proto-oncogene was differentially expressed between the aggressive MDA-MB231 and the non-aggressive MCF-7 breast cancer cells. Congruently, immunohistochemical staining of clinical samples revealed that FLI1 was overexpressed in breast cancers as compared with the adjacent tissues. The abundance of FLI1 protein was strongly correlated with the advanced stage, poor differentiation, and lymph node metastasis in breast cancer patients. Knockdown of FLI1 with small interfering RNAs significantly attenuated the potential of   migration   and invasion in highly metastatic human breast cancer cells. FLI1 oncoprotein activated the Rho GTPase pathway that is known to play a role in tumor metastasis. This study for the first time identifies FLI1 as a clinically and functionally important target gene of metastasis, providing a rationale for developing FLI1 inhibitors in the treatment of breast cancer. 
 FLI1 expression is correlated with breast cancer cellular growth,   migration  , and invasion and altered gene expression. ETS factors have been shown to be dysregulated in breast cancer. ETS factors control the expression of genes involved in many biological processes, such as cellular proliferation, differentiation, and apoptosis. FLI1 is an ETS protein aberrantly expressed in retrovirus-induced hematological tumors, but limited attention has been directed towards elucidating the role of FLI1 in epithelial-derived cancers. Using data mining, we show that loss of FLI1 expression is associated with shorter survival and more aggressive phenotypes of breast cancer. Gain and loss of function cellular studies indicate the inhibitory effect of FLI1 expression on cellular growth,   migration  , and invasion. Using Fli1 mutant mice and both a transgenic murine breast cancer model and an orthotopic injection of syngeneic tumor cells indicates that reduced Fli1 contributes to accelerated tumor growth. Global expression analysis and RNA-Seq data from an invasive human breast cancer cell line with over expression of either FLI1 and another ETS gene, PDEF, shows changes in several cellular pathways associated with cancer, such as the cytokine-cytokine receptor interaction and PI3K-Akt signaling pathways. This study demonstrates a novel role for FLI1 in epithelial cells. In addition, these results reveal that FLI1 down-regulation in breast cancer may promote tumor progression. 
 EAPII interacts with ETS1 and modulates its transcriptional function. Ets proteins constitute a family of conserved sequence-specific DNA-binding proteins and function as transcription factors. ETS1 plays important roles in differentiation, lymphoid cell development, invasiveness and angiogenesis. Such diverse roles of ETS1 are likely to be dependent on its associated proteins. A yeast two-hybrid screen was conducted and here we describe a novel ETS1 interacting protein designated as ETS1-associated protein II (EAPII). EAPII protein interacts with ETS1 and other Ets proteins (ETS2 and FLI1) both in vitro and in vivo. Indirect immunofluorescence demonstrated that EAPII is predominately localized to the nucleus of mammalian cells. EAPII negatively modulates ETS1 transcriptional activity and attenuates synergistic transactivation by ETS1 and AP-1. Significantly, re-expression of EAPII inhibits the   migration   of epithelial cancer cells, but does not affect cell viability. Therefore, EAPII is a novel ETS1 modulator that regulates specific aspects of the ETS1 functions. 
 Oncogenic Fli-1 is a potential prognostic marker for the progression of epithelial ovarian cancer. Ovarian cancer is the most lethal gynecologic malignancy, but its etiology remains poorly understood. This study investigated the role of Fli-1 in ovarian carcinogenesis and disease survival.Fli-1 protein expression was evaluated by immunohistochemistry in 104 primary epithelial ovarian cancer (EOC) patients with known follow-up data and 20 controls. Correlation between Fli-1 expression and clinical characteristics was evaluated with the logistic regression. Kaplan Meier analysis was used to assess the impact of Fli-1 expression on overall survival (OS) and disease-free survival (DFS). Cell proliferation and   migration   assay were used to explore the function of Fli-1 in ovarian cancer cells.Fli-1 was expressed in 74% cases and up-regulated in EOC tissues compared with normal control tissues (p< 0.05). The high expression of Fli-1 was significantly associated with advanced tumor stage, positive lymph nodal involvement, and poor OS and DFS (p< 0.05). Further analysis showed Fli-1 is an independent prognostic factor for OS and DFS. Down-regulation of Fli-1 inhibited cell proliferation but did not affect cell   migration   in SKOV3 cells.This study revealed that Fli-1 played an essential role in the development and progression of ovarian cancers. Its overexpression is intimately related to malignant phenotypes and poor clinical outcome, suggesting that Fli-1 is a potential prognostic marker and therapeutic molecular target in ovarian cancer. 
 Overexpression of Fli-1 is associated with adverse prognosis of endometrial cancer. This study aimed to investigate the expression of Friend leukemia virus integration 1 (Fli-1) and its correlation with the prognosis of endometrial cancer (EC). Thirty-two EC tissue samples were evaluated for Fli-1 expression using immunohistochemistry. Fli-1 showed significantly high expression in EC cells, followed by hyperplasia cells, and was negative in adjacent normal tissues. The high expression of Fli-1 was significantly associated with a high differentiation grade, mutated P53 expression, and histological subtype (p < .05). Downregulation of Fli-1 in AN3CN cells using RNA interference inhibited cell clone formation and proliferation but did not affect apoptosis and   migration   of the cells. This study provides the first evidence that Fli-1 expression gradually increases in parallel with disease progression, and its overexpression might predict poor prognosis in EC. 
 14-3-3 proteins modulate the ETS transcription factor ETV1 in prostate cancer. Overexpression of the ETS-related transcription factor ETV1 can initiate neoplastic transformation of the prostate. ETV1 activity is highly regulated by phosphorylation, but the underlying mechanisms are unknown. Here we report that all 14-3-3 proteins, with the exception of the tumor suppressor 14-3-3σ, can bind to ETV1 in a condition manner dictated by its prominent phosphorylation site S216. Non-σ 14-3-3 proteins synergized with ETV1 to activate transcription of its target genes MMP-1 and MMP-7, which regulate extracellular matrix in the prostate tumor microenvironment. S216 mutation or 14-3-3τ downregulation was sufficient to reduce ETV1 protein levels in prostate cancer cells, indicating that non-σ 14-3-3 proteins protect ETV1 from degradation. Notably, S216 mutation also decreased ETV1-depend  ent migra  tion and invasion in benign prostate cells. Downregulation of 14-3-3τ reduced prostate cancer cell invasion and growth in the same manner as ETV1 attenuation. Finally, we showed that 14-3-3τ and 14-3-3ε were overexpressed in human prostate tumors. Taken together, our results showed that non-σ 14-3-3 proteins are important modulators of ETV1 function that promote prostate tumorigenesis. 
  GeneRIFs  
 FLI1 levels are reduced in human breast cancer samples and cell lines.FLI1 expression is correlated with breast cancer cellular growth,   migration  , and invasion and altered gene expression. 
  ECM  PubMed  
 Fli1 is a negative regulator of estrogen receptor α in dermal fibroblasts. Estrogen is an important regulator of dermal fibroblast functions, including extracellular matrix   (EC  M) synthesis. Estrogen mediates its effects through estrogen receptors (ERs), ERα and ERβ; however, regulation of ERs in dermal fibroblasts remains poorly understood. Friend leukemia integration factor 1 (Fli1), a member of the Ets transcription factor family, has been shown to play a pivotal role in regulation of t  he   ECM genes in dermal fibroblasts. The aim of this study was to examine a possible interaction between Fli1 and estrogen pathways, focusing on ERα. We show that treatment of human dermal fibroblasts with transforming growth factor-β (TGF-β) increases ERα protein and mRNA levels. Similarly, ERα expression was increased in response to small interfering RNA (siRNA)-mediated depletion of Fli1, suggesting that Fli1 is a mediator of the TGF-β effects on ERα expression. Accordingly, we showed that Fli1 binds to the most proximal region of the ERα promoter, and dissociates from the promoter upon TGF-β treatment. An inverse correlation between Fli1 and ERα expression levels was confirmed in cultured skin fibroblasts obtained from Fli1(+/-) mice and in the skin of Fli1(+/-) mice in vivo. This study supports a role of Fli1 as a negative regulator of the ERα gene in dermal fibroblasts. 
  [Ee]xtracellular [Mm]atrix  PubMed  
 Association between enhanced type I collagen expression and epigenetic repression of the FLI1 gene in scleroderma fibroblasts. Scleroderma (systemic sclerosis; SSc) is an autoimmune disease characterized by vasculopathy and widespread organ fibrosis. Altered fibroblast function, both in vivo and in vitro, is well documented and illustrated by augmented synthesis and deposition of   extracellular matrix   proteins. We undertook this study to investigate the possibility that epigenetic mechanisms mediate the emergence and persistence of the altered SSc fibroblast phenotype.The effects of DNA methyltransferase and histone deacetylase inhibitors on collagen expression and the level of epigenetic mediators in fibroblasts were examined. The effects of transient transfection of SSc fibroblasts with FLI1 gene and normal cells with FLI1 antisense construct on collagen expression were determined. The methylation status of the FLI1 promoter was tested in cultured cells and in SSc and normal skin biopsy specimens.Increased levels of epigenetic mediators in SSc fibroblasts were noted. The addition of epigenetic inhibitors to cell cultures normalized collagen expression in SSc fibroblasts. The augmented collagen synthesis by SSc fibroblasts was linked to epigenetic repression of the collagen suppressor gene FLI1. Heavy methylation of the CpG islands in the FLI1 promoter region was demonstrated in SSc fibroblasts and skin biopsy specimens.The results of this study indicate that epigenetic mechanisms may mediate the fibrotic manifestations of SSc. The signal transduction leading to the SSc fibrotic phenotype appears to converge on DNA methylation and histone deacetylation at the FLI1 gene. 
 Fli1 is a negative regulator of estrogen receptor α in dermal fibroblasts. Estrogen is an important regulator of dermal fibroblast functions, including   extracellular matri  x (ECM) synthesis. Estrogen mediates its effects through estrogen receptors (ERs), ERα and ERβ; however, regulation of ERs in dermal fibroblasts remains poorly understood. Friend leukemia integration factor 1 (Fli1), a member of the Ets transcription factor family, has been shown to play a pivotal role in regulation of the ECM genes in dermal fibroblasts. The aim of this study was to examine a possible interaction between Fli1 and estrogen pathways, focusing on ERα. We show that treatment of human dermal fibroblasts with transforming growth factor-β (TGF-β) increases ERα protein and mRNA levels. Similarly, ERα expression was increased in response to small interfering RNA (siRNA)-mediated depletion of Fli1, suggesting that Fli1 is a mediator of the TGF-β effects on ERα expression. Accordingly, we showed that Fli1 binds to the most proximal region of the ERα promoter, and dissociates from the promoter upon TGF-β treatment. An inverse correlation between Fli1 and ERα expression levels was confirmed in cultured skin fibroblasts obtained from Fli1(+/-) mice and in the skin of Fli1(+/-) mice in vivo. This study supports a role of Fli1 as a negative regulator of the ERα gene in dermal fibroblasts. 
 Molecular dissection of the mechanism by which EWS/FLI expression compromises actin cytoskeletal integrity and cell adhesion in Ewing sarcoma. Ewing sarcoma is the second-most-common bone cancer in children. Driven by an oncogenic chromosomal translocation that results in the expression of an aberrant transcription factor, EWS/FLI, the disease is typically aggressive and micrometastatic upon presentation. Silencing of EWS/FLI in patient-derived tumor cells results in the altered expression of hundreds to thousands of genes and is accompanied by dramatic morphological changes in cytoarchitecture and adhesion. Genes encoding focal adhesion,   extracellular matrix  , and actin regulatory proteins are dominant targets of EWS/FLI-mediated transcriptional repression. Reexpression of genes encoding just two of these proteins, zyxin and α5 integrin, is sufficient to restore cell adhesion and actin cytoskeletal integrity comparable to what is observed when the EWS/FLI oncogene expression is compromised. Using an orthotopic xenograft model, we show that EWS/FLI-induced repression of α5 integrin and zyxin expression promotes tumor progression by supporting anchorage-independent cell growth. This selective advantage is paired with a tradeoff in which metastatic lung colonization is compromised. 
 EWS/FLI-1 silencing and gene profiling of Ewing cells reveal downstream oncogenic pathways and a crucial role for repression of insulin-like growth factor binding protein 3. Ewing tumors are characterized by abnormal transcription factors resulting from the oncogenic fusion of EWS with members of the ETS family, most commonly FLI-1. RNA interference targeted to the junction between EWS and FLI-1 sequences was used to inactivate the EWS/FLI-1 fusion gene in Ewing cells and to explore the resulting phenotype and alteration of the gene expression profile. Loss of expression of EWS/FLI-1 resulted in the complete arrest of growth and was associated with a dramatic increase in the number of apoptotic cells. Gene profiling of Ewing cells in which the EWS/FLI-1 fusion gene had been inactivated identified downstream targets which could be grouped in two major functional clusters related to   extracellular matrix   structure or remodeling and regulation of signal transduction pathways. Among these targets, the insulin-like growth factor binding protein 3 gene (IGFBP-3), a major regulator of insulin-like growth factor 1 (IGF-1) proliferation and survival signaling, was strongly induced upon treating Ewing cells with EWS/FLI-1-specific small interfering RNAs. We show that EWS/FLI-1 can bind the IGFBP-3 promoter in vitro and in vivo and can repress its activity. Moreover, IGFBP-3 silencing can partially rescue the apoptotic phenotype caused by EWS/FLI-1 inactivation. Finally, IGFBP-3-induced Ewing cell apoptosis relies on both IGF-1-dependent and -independent pathways. These findings therefore identify the repression of IGFBP-3 as a key event in the development of Ewing's sarcoma. 
 Fli1 and Ets1 have distinct roles in connective tissue growth factor/CCN2 gene regulation and induction of the profibrotic gene program. CCN2 (connective tissue growth factor), an important regulator of angiogenesis, chondrogenesis, and wound healing, is overexpressed in a majority of fibrotic diseases and in various tumors. This study investigated regulation of CCN2 gene expression by Ets family of transcription factors, focusing on two members, Fli1 and Ets1, with deregulated expression during fibrosis and tumorigenesis. We show that Ets1 and Fli1 have opposite effects on CCN2 gene expression. Ets1 functions as an activator of CCN2 transcription, whereas Fli1 acts as a repressor. A functional Ets binding site was mapped at -114 within the CCN2 promoter. This site not only mediates stimulation by Ets factors, including Ets1, Ets2, and GABPalpha/beta, but is also required for the transforming growth factor (TGF)-beta response. The contrasting functions of Ets1 and Fli1 in regulation of the CCN2 gene were confirmed by suppressing their endogenous levels using adenoviral vectors expressing specific small interfering RNAs. Additional experiments using chromatin immunoprecipitation assays have revealed that in fibroblasts both Ets1 and Fli1 occupy the CCN2 promoter. TGF-beta stimulation resulted in displacement of Fli1 from the CCN2 promoter and a transient inhibition of Fli1 synthesis. Moreover, reduction of Fli1 expression resulted in up-regulation of COL1A1 and COL1A2 genes and down-regulation of the MMP1 gene. Thus, inhibition of Fli1 recapitulated some of the key effects of TGF-beta, suggesting that Fli1 suppression is involved in activation of the profibrotic gene program in fibroblasts. On the other hand, activation of the CCN2 gene downstream of Ets1 is consistent with its role in angiogenesis and   extracellular matrix   remodeling. This study strongly supports a critical role of Fli1 and Ets1 in the pathological   extracellular matrix   regulation during fibrosis and cancer. 
 14-3-3 proteins modulate the ETS transcription factor ETV1 in prostate cancer. Overexpression of the ETS-related transcription factor ETV1 can initiate neoplastic transformation of the prostate. ETV1 activity is highly regulated by phosphorylation, but the underlying mechanisms are unknown. Here we report that all 14-3-3 proteins, with the exception of the tumor suppressor 14-3-3σ, can bind to ETV1 in a condition manner dictated by its prominent phosphorylation site S216. Non-σ 14-3-3 proteins synergized with ETV1 to activate transcription of its target genes MMP-1 and MMP-7, which regulat  e extracellular matr  ix in the prostate tumor microenvironment. S216 mutation or 14-3-3τ downregulation was sufficient to reduce ETV1 protein levels in prostate cancer cells, indicating that non-σ 14-3-3 proteins protect ETV1 from degradation. Notably, S216 mutation also decreased ETV1-dependent migration and invasion in benign prostate cells. Downregulation of 14-3-3τ reduced prostate cancer cell invasion and growth in the same manner as ETV1 attenuation. Finally, we showed that 14-3-3τ and 14-3-3ε were overexpressed in human prostate tumors. Taken together, our results showed that non-σ 14-3-3 proteins are important modulators of ETV1 function that promote prostate tumorigenesis. 
  GeneRIFs  
 Fli1 and Ets1 have roles in the pathological   extracellular matrix   regulation during fibrosis and cancer 
  [Ii]nvasive  PubMed  
 FLI1 expression is correlated with breast cancer cellular growth, migration, and invasion and altered gene expression. ETS factors have been shown to be dysregulated in breast cancer. ETS factors control the expression of genes involved in many biological processes, such as cellular proliferation, differentiation, and apoptosis. FLI1 is an ETS protein aberrantly expressed in retrovirus-induced hematological tumors, but limited attention has been directed towards elucidating the role of FLI1 in epithelial-derived cancers. Using data mining, we show that loss of FLI1 expression is associated with shorter survival and more aggressive phenotypes of breast cancer. Gain and loss of function cellular studies indicate the inhibitory effect of FLI1 expression on cellular growth, migration, and invasion. Using Fli1 mutant mice and both a transgenic murine breast cancer model and an orthotopic injection of syngeneic tumor cells indicates that reduced Fli1 contributes to accelerated tumor growth. Global expression analysis and RNA-Seq data from an   invasive   human breast cancer cell line with over expression of either FLI1 and another ETS gene, PDEF, shows changes in several cellular pathways associated with cancer, such as the cytokine-cytokine receptor interaction and PI3K-Akt signaling pathways. This study demonstrates a novel role for FLI1 in epithelial cells. In addition, these results reveal that FLI1 down-regulation in breast cancer may promote tumor progression. 
 Ewing sarcoma cells secrete EWS/Fli-1 fusion mRNA via microvesicles. Tumours defined as Ewing sarcoma (ES) constitute a group of highly malignant neoplasms that most often affect children and young adults in the first 2 decades of life. The EWS/Fli-1 fusion gene, a product of the translocation t(11;22) (q24; 12), is detected in 95% of ES patients. Recently, it was validated that cells emit a heterogeneous mixture of vesicular, organelle-like structures (microvesicles, MVs) into their surroundings including blood and body fluids, and that these MVs contain a selected set of tumor-related proteins and high levels of mRNAs and miRNAs. In this present study, we detected the Ewing sarcoma-specific EWS/Fli-1 mRNA in MVs from the culture medium of ES cell lines carrying t(11;22) (q24; 12). Also, we detected this fusion gene in approximately 40% of the blood samples from mice inoculated with xenografts of TC135 or A673 cells. These findings indicate the EWS/Fli-1 mRNA in MVs might be a new non-  invasive   diagnostic marker for specific cases of Ewing sarcoma. 
 EAPII interacts with ETS1 and modulates its transcriptional function. Ets proteins constitute a family of conserved sequence-specific DNA-binding proteins and function as transcription factors. ETS1 plays important roles in differentiation, lymphoid cell development,   invasive  ness and angiogenesis. Such diverse roles of ETS1 are likely to be dependent on its associated proteins. A yeast two-hybrid screen was conducted and here we describe a novel ETS1 interacting protein designated as ETS1-associated protein II (EAPII). EAPII protein interacts with ETS1 and other Ets proteins (ETS2 and FLI1) both in vitro and in vivo. Indirect immunofluorescence demonstrated that EAPII is predominately localized to the nucleus of mammalian cells. EAPII negatively modulates ETS1 transcriptional activity and attenuates synergistic transactivation by ETS1 and AP-1. Significantly, re-expression of EAPII inhibits the migration of epithelial cancer cells, but does not affect cell viability. Therefore, EAPII is a novel ETS1 modulator that regulates specific aspects of the ETS1 functions. 
  [Mm]etastasis  PubMed  
 Clinical Relevance of Plasma DNA Methylation in Colorectal Cancer Patients Identified by Using a Genome-Wide High-Resolution Array. DNA methylation is a potential tumor marker for several cancers, including colorectal cancer (CRC), because of its heritable and stable characteristics.Using a high-resolution, genome-wide approach, we epigenotyped >450,000 CpG sites in tumor and adjacent non-tumor tissues from 23 microsatellite instability (MSI)/microsatellite stability (MSS) CRC cases. Using matrix-assisted laser desorption ionization-time of flight mass spectrometry, the methylation status of five frequently hypermethylated genes were confirmed in 75 independent CRC series and 353 CRC patients with available plasma.Compared with non-tumor tissues, 13 MSI tumors had 34,836 (7 %) aberrant methylation sites, 87 % of which were hypermethylated. In contrast, only 9,806 (2 %) differentially methylated sites were identified in ten MSS cases (62 % hypermethylated). In both MSI and MSS, 228 promoter-associated CpG islands were hypermethylated, with AGBL4, ZNF625, MDFI, TWIST1, and FLI1 being most frequently hypermethylated. In an independent set of 35 MSI and 40 MSS cases, the methylation status of these five genes significantly differed between tumor and adjacent non-tumor tissues. Of 353 CRC patients, 230 (65.2 %), 232 (65.7 %), and 247 (70.0 %) had AGBL4, FLI1, and TWIST1 promoter hypermethylation in circulating cell-free DNA, respectively. In patients without   metastasis  , the sensitivity of any two or three hypermethylation markers was 52.8-57.8 and 27.9-38.9 %, respectively. The sensitivity of any two or three markers was significantly high in patients with stage IV disease (73.0 and 55.6 %, respectively). The prognostic value of these epimarkers was inconclusive.DNA methylation patterns differed in CRC subtypes. The identified hypermethylation markers in CRC patients may have good sensitivity in different CRC stages. 
 Clinical Relevance of Plasma DNA Methylation in Colorectal Cancer Patients Identified by Using a Genome-Wide High-Resolution Array. DNA methylation is a potential tumor marker for several cancers, including colorectal cancer (CRC), because of its heritable and stable characteristics.Using a high-resolution, genome-wide approach, we epigenotyped >450,000 CpG sites in tumor and adjacent non-tumor tissues from 23 microsatellite instability (MSI)/microsatellite stability (MSS) CRC cases. Using matrix-assisted laser desorption ionization-time of flight mass spectrometry, the methylation status of five frequently hypermethylated genes were confirmed in 75 independent CRC series and 353 CRC patients with available plasma.Compared with non-tumor tissues, 13 MSI tumors had 34,836 (7 %) aberrant methylation sites, 87 % of which were hypermethylated. In contrast, only 9,806 (2 %) differentially methylated sites were identified in ten MSS cases (62 % hypermethylated). In both MSI and MSS, 228 promoter-associated CpG islands were hypermethylated, with AGBL4, ZNF625, MDFI, TWIST1, and FLI1 being most frequently hypermethylated. In an independent set of 35 MSI and 40 MSS cases, the methylation status of these five genes significantly differed between tumor and adjacent non-tumor tissues. Of 353 CRC patients, 230 (65.2 %), 232 (65.7 %), and 247 (70.0 %) had AGBL4, FLI1, and TWIST1 promoter hypermethylation in circulating cell-free DNA, respectively. In patients without   metastasis  , the sensitivity of any two or three hypermethylation markers was 52.8-57.8 and 27.9-38.9 %, respectively. The sensitivity of any two or three markers was significantly high in patients with stage IV disease (73.0 and 55.6 %, respectively). The prognostic value of these epimarkers was inconclusive.DNA methylation patterns differed in CRC subtypes. The identified hypermethylation markers in CRC patients may have good sensitivity in different CRC stages. 
 Friend leukemia virus integration 1 activates the Rho GTPase pathway and is associated with   metastasis   in breast cancer. Breast cancer is the most prevalent malignant disease in women worldwide. In patients with breast cancer,   metastasis   to distant sites directly determines the survival outcome. However, the molecular mechanism underlying   metastasis   in breast cancer remains to be defined. In this report, we found that Friend leukemia virus integration 1 (FLI1) proto-oncogene was differentially expressed between the aggressive MDA-MB231 and the non-aggressive MCF-7 breast cancer cells. Congruently, immunohistochemical staining of clinical samples revealed that FLI1 was overexpressed in breast cancers as compared with the adjacent tissues. The abundance of FLI1 protein was strongly correlated with the advanced stage, poor differentiation, and lymph node   metastasis   in breast cancer patients. Knockdown of FLI1 with small interfering RNAs significantly attenuated the potential of migration and invasion in highly metastatic human breast cancer cells. FLI1 oncoprotein activated the Rho GTPase pathway that is known to play a role in tumor   metastasis  . This study for the first time identifies FLI1 as a clinically and functionally important target gene of   metastasis  , providing a rationale for developing FLI1 inhibitors in the treatment of breast cancer. 
 GLI1 is a central mediator of EWS/FLI1 signaling in Ewing tumors. The Ewing Sarcoma Family Tumors (ESFT) consist of the classical pathologic entities of Ewing Sarcoma and peripheral Primitive Neuroectodermal Tumor. Occurring largely in the childhood through young adult years, these tumors have an unsurpassed propensity for   metastasis   and have no defined cell of origin. The biology of these aggressive malignancies centers around EWS/FLI1 and related EWS/ETS chimeric transcription factors, which are largely limited to this tumor class. Much progress has been made in the identification of a network of loci whose expression is modulated by EWS/FLI1 and its congeners. To date, little progress has been made in reconstructing the sequence of direct and indirect events that produce this network of modulated loci. The recent identification of GLI1 as an upregulated target of EWS/ETS transcription factors suggests a target which may be a more central mediator in the ESFT signaling network. In this paper, we further define the relationship of EWS/FLI1 expression and GLI1 upregulation in ESFT. This relationship is supported with data from primary tumor specimens. It is consistently observed across multiple ESFT cell lines and with multiple means of EWS/FLI1 inhibition. GLI1 inhibition affects tumor cell line phenotype whether shRNA or endogenous or pharmacologic inhibitors are employed. As is seen in model transformation systems, GLI1 upregulation by EWS/FLI1 appears to be independent of Hedgehog stimulation. Consistent with a more central role in ESFT pathogenesis, several known EWS/FLI1 targets appear to be targeted through GLI1. These findings further establish a central role for GLI1 in the pathogenesis of Ewing Tumors. 
 Functional roles of Fli-1, a member of the Ets family of transcription factors, in human breast malignancy. The Ets family of transcription factors is implicated in malignant transformation and tumor progression, including invasion,   metastasis   and neo-angiogenesis. In the present study, we found that the Fli-1 gene, a member of the Ets family, was highly expressed in several breast cancer cell lines (MDA-MB231, MDA-MB436, BT-549 and HCC1395). To investigate the functional roles of Fli-1 in breast cancer malignancy, we introduced an expression plasmid containing full-length Fli-1 cDNA into MCF7 breast cancer cells in which endogenous expression of Fli-1 was barely detectable.Overexpression of Fli-1 in MCF7 cells led to inhibition of apoptosis induced by serum depletion or ultraviolet irradiation, although it did not affect cell growth rate in liquid media, colony formation in soft agar or the in vitro invasion capacity of the cells. Expression of Fli-1 and antiapoptotic bcl-2 was coordinately upregulated by serum depletion in MCF7 cells, and the upregulation was inhibited by treatment of the cells with a c-Jun-NH(2)-terminal kinase-specific inhibitor. Furthermore, expression of the bcl-2 gene and protein was enhanced in Fli-1-overexpressing MCF7 cells compared with mock-transfected cells. In addition, human bcl-2 promoter activity was transactivated by Fli-1. These results suggest that Fli-1 contributes to the malignancy of human breast cancer by inhibiting apoptosis through upregulated expression of the bcl-2 gene. 
 Downregulation of Friend leukemia virus integration 1 as a feedback mechanism that restrains lipopolysaccharide induction of matrix metalloproteases and interleukin-10 in human macrophages. The E26 transformation-specific (Ets) proteins are a family of transcription factors with important roles in a variety of cellular processes ranging from proliferation and differentiation to transformation and   metastasis  . Tissue-specific expression of Ets proteins and their ability to interact with other families of transcription factors contribute to their versatility. In this study, we investigated the regulation of Ets factors in primary human monocytes and macrophages, and their role in matrix metalloprotease (MMP) and cytokine production. The macrophage-activating Toll-like receptor ligand, lipopolysaccharide (LPS), induced the expression of Ets family members epithelium-specific Ets factor 3 (ESE-3) and TEL-2 but rapidly suppressed Friend leukemia virus integration 1 (FLI-1) expression. Modulation of FLI-1 expression using either RNA interference or forced expression identified a positive role for FLI-1 in contributing to LPS-induced expression of MMP-1, MMP-3, MMP-10, and interleukin-10 (IL-10). Thus, the rapid downregulation of FLI-1 expression after LPS stimulation attenuates the induction of various MMPs and IL-10 under inflammatory conditions. In contrast, the expression of IL-6 and TNFα and the effects of interferon (IFN)γ on LPS responses were not dependent on FLI-1. Our results define a novel FLI-1-mediated self-regulatory feedback loop that limits MMP expression and thus may attenuate extent of tissue destruction associated with inflammatory responses. 
  GeneRIFs  
 This study for the first time identifies FLI1 as a clinically and functionally important target gene of   metastasis  , providing a rationale for developing FLI1 inhibitors in the treatment of breast cancer. 
  [Cc]ell [Ii]nvasion  PubMed  
 14-3-3 proteins modulate the ETS transcription factor ETV1 in prostate cancer. Overexpression of the ETS-related transcription factor ETV1 can initiate neoplastic transformation of the prostate. ETV1 activity is highly regulated by phosphorylation, but the underlying mechanisms are unknown. Here we report that all 14-3-3 proteins, with the exception of the tumor suppressor 14-3-3σ, can bind to ETV1 in a condition manner dictated by its prominent phosphorylation site S216. Non-σ 14-3-3 proteins synergized with ETV1 to activate transcription of its target genes MMP-1 and MMP-7, which regulate extracellular matrix in the prostate tumor microenvironment. S216 mutation or 14-3-3τ downregulation was sufficient to reduce ETV1 protein levels in prostate cancer cells, indicating that non-σ 14-3-3 proteins protect ETV1 from degradation. Notably, S216 mutation also decreased ETV1-dependent migration and invasion in benign prostate cells. Downregulation of 14-3-3τ reduced prostate ca  ncer cell inv  asion and growth in the same manner as ETV1 attenuation. Finally, we showed that 14-3-3τ and 14-3-3ε were overexpressed in human prostate tumors. Taken together, our results showed that non-σ 14-3-3 proteins are important modulators of ETV1 function that promote prostate tumorigenesis. 
   
  
 NFKB1 
 
  NCBI Gene  
  GeneRIF  
  Pubmed  
 
 
  Search Term  Rank  Fields  
  [Ff]ilopodia  1  PubMed(1)  
  [Mm]igration  6  PubMed(170); GeneRIFs(29)  
  ECM  7  PubMed(8); GeneRIFs(2)  
  [Ee]xtracellular [Mm]atrix  8  PubMed(33)  
  [Ii]nvasive  10  PubMed(92); GeneRIFs(16)  
  [Mm]etastasize  11  PubMed(1)  
  [Mm]etastasis  12  PubMed(147); GeneRIFs(25)  
  [Cc]ell [Ii]nvasion  13  PubMed(31); GeneRIFs(10)  
 
 
 hide/show details for NFKB1 
  [Ff]ilopodia  PubMed  
 TRAF6 activation of PI 3-kinase-dependent cytoskeletal changes is cooperative with Ras and is mediated by an interaction with cytoplasmic Src. Interleukin 1 (IL-1) has been implicated in the reorganization of the actin cytoskeleton. An expression vector encoding a PKB/Akt pleckstrin-homology domain fused to a fluorescent protein was used to detect phosphoinositide 3-kinase (PI 3-kinase) products. It was observed that PI 3-kinase was activated either by treatment with IL-1 or by expression of either TRAF6, Src, MyD88 or dominant-positive PI 3-kinase, and resulted in the formation of long   filopodia  -like cellular protrusions that appeared to branch at membrane sites consisting of clusters of phosphoinositide. This depended upon a TRAF6 polyproline motif and Src catalytic activity, and was blocked by inhibitors of PI 3-kinase, Src and Ras. Using both conventional and split fluorescent protein probes fused to expressed TRAF6 and Src in living cells, the polyproline sequence of TRAF6 and the Src-homology 3 (SH3) domain of Src were shown to be required for interaction between these two proteins. Interaction occurred within the cytoplasm, and not at either the cell membrane or cytoplasmic sequestosomes. In addition, co-transfection of vectors expressing fluorescent-protein-fused TRAF6 and non-fluorescent MyD88, IRAK1 and IRAK2 revealed an inverse correlation between increased sequestosome formation and activation of both PI 3-kinase and NF-kappaB. Although a key factor in TRAF6-dependent activation of PI 3-kinase, ectopic expression of Src was insufficient for NF-kappaB activation and, in contrast to NF-kappaB, was not inhibited by IRAK2. 
  [Mm]igration  PubMed  
 Stabilization of snail by NF-kappaB is required for inflammation-induced cell   migration   and invasion. The increased motility and invasiveness of tumor cells are reminiscent of epithelial-mesenchymal transition (EMT), which occurs during embryonic development, wound healing, and metastasis. In this study, we found that Snail is stabilized by the inflammatory cytokine TNFalpha through the activation of the NF-kappaB pathway. We demonstrated that NF-kappaB is required for the induction of COP9 signalosome 2 (CSN2), which, in turn, blocks the ubiquitination and degradation of Snail. Furthermore, we showed that the expression of Snail correlated with the activation of NF-kappaB in cancer cell lines and metastatic tumor samples. Knockdown of Snail expression inhibited cell   migration   and invasion induced by inflammatory cytokines and suppressed inflammation-mediated breast cancer metastasis. Our study provides a plausible mechanism for inflammation-induced metastasis. 
 Molecular basis of nuclear factor-kappaB activation by astrocyte elevated gene-1. Malignant glioma is a consistently fatal brain cancer. The tumor invades the surrounding tissue, limiting complete surgical removal and thereby initiating recurrence. Identifying molecules critical for glioma invasion is essential to develop targeted, effective therapies. The expression of astrocyte elevated gene-1 (AEG-1) increases in malignant glioma and AEG-1 regulates in vitro invasion and   migration   of malignant glioma cells by activating the nuclear factor-kappaB (NF-kappaB) signaling pathway. The present studies elucidate the domains of AEG-1 important for mediating its function. Serial NH(2)-terminal and COOH-terminal deletion mutants were constructed and functional analysis revealed that the NH(2)-terminal 71 amino acids were essential for invasion,   migration  , and NF-kappaB-activating properties of AEG-1. The p65-interaction domain was identified between amino acids 101 to 205, indicating that p65 interaction alone is not sufficient to mediate AEG-1 function. Coimmunoprecipitation assays revealed that AEG-1 interacts with cyclic AMP-responsive element binding protein-binding protein (CBP), indicating that it might act as a bridging factor between NF-kappaB, CBP, and the basal transcription machinery. Chromatin immunoprecipitation assays showed that AEG-1 is associated with the NF-kappaB binding element in the interleukin-8 promoter. Thus, AEG-1 might function as a coactivator for NF-kappaB, consequently augmenting expression of genes necessary for invasion of glioma cells. In these contexts, AEG-1 represents a viable potential target for the therapy of malignant glioma. 
 Aberrant IKKα and IKKβ cooperatively activate NF-κB and induce EGFR/AP1 signaling to promote survival a  nd migrat  ion of head and neck cancer. The inhibitor-κB kinase-nuclear factor-κB (IKK-NF-κB) and epidermal growth factor receptor-activator protein-1 (EGFR-AP1) pathways are often co-activated and promote malignant behavior, but the underlying basis for this relationship is unclear. Resistance to inhibitors of IKKβ or EGFR is observed in head and neck squamous cell carcinomas (HNSCC). Here, we reveal that both IKKα and β contribute to nuclear activation of canonical and alternate NF-κB/REL family transcription factors, and overexpression of signal components that enhance co-activation of the EGFR-AP1 pathway. We observed that IKKα and IKKβ exhibit increased protein expression, nuclear localization, and phosphorylation in HNSCC tissues and cell lines. Individually, IKK activity varied among different cell lines, but overexpression of both IKKs induced the strongest NF-κB activation. Conversely, siRNA knock down of both IKKs significantly decreased nuclear localization and phosphorylation of canonical RELA and IκBα and alternative p52 and RELB subunits. Knock down of both IKKs more effectively inhibited NF-κB activation, broadly modulated gene expression and suppressed cell pr  oliferati  on and migration. Global expression profiling revealed that NF-κB, cytokine, inflammatory response and growth factor signaling are among the top pathways and networks regulated by IKKs. Importantly, IKKα and IKKβ together promoted the expression and activity of transforming growth factor α, EGFR and AP1 transcription factors cJun, JunB and Fra1. Knock down of AP1 subunits individually decreased 8/15 (53%) of IKK-targeted genes sampled and similarly inhibited cel  l prolife  ration and migration. Mutations of NF-κB and AP1-binding sites abolished or decreased IKK-induced interleukin-8 (IL-8) promoter activity. Compounds such as wedelactone with dual IKK inhibitory activity and geldanomycins that block IKKα/β and EGFR pathways were more active than IKKβ-specific inhibitors in suppressing NF-κB activation and proliferation and inducing cell death. We conclude that IKKα and IKKβ cooperatively activate NF-κB and EGFR/AP1 networks of signaling pathways and contribute to the malignant phenotype and the intrinsic or acquired therapeutic resistance of HNSCC. 
 Differential effects of sulfated triterpene glycosides, holothurin A1, and 24-dehydroechinoside A, on antimetastasic activity via regulation of the MMP-9 signal pathway. Two sulfated triterpene glycosides, holothurin A(1) (HA(1)) and 24-dehydroechinoside A (DHEA), isolated from the sea cucumber Pearsonothuria graeffei, are of the holostane type with 18(20)-lactone and identical carbohydrate chains. DHEA has a side chain 23 (24)-double bond, while HA(1) has a hydroxyl group at C-21. In this study, we compared the effects of DHEA and HA(1) on metastasis in vitro and in vivo. The results show that HA(1) and DHEA treatment significantly suppressed adhesion of human hepatocellular liver carcinoma cells (HepG2) to both matrigel and human endothelial cells (ECV-304) and inhibited HepG2 cell   migration   and invasion in a dose-dependant manner. HA(1) and DHEA reduced tube formation of ECV-304 cells on the matrigel in vitro and attenuated neovascularization in the chick embryo using the chorioallantoic membrane (CAM) assay in vivo. Immunocytochemistry analyses revealed that both HA(1) and DHEA significantly decreased the expression of the matrix metallo-proteinase-9 (MMP-9) and increased the expression level of tissue inhibitor of metalloproteinase-1 (TIMP-1), an important regulator of MMP-9 activation. Western blot analyses demonstrated that HA(1) and DHEA remarkably abolished the expression of vascular endothelial growth factor (VEGF). The expression of nuclear factor-kappa B (NF-κB) was significantly decreased by HA(1), while DHEA treatment had no effect on the down regulation of NF-κB expression. These data suggest that both DHEA and HA(1) exert significant antimetastatic activities by inhibiting MMP-9 and VEGF expression. DHEA-induced antimetastasis was more potent than HA(1). In addition, only HA(1) treatment downregulated the expression level of NF-κB, suggesting that the antimetastatic activity of triterpene glycosides derived from P. graeffei can be either NF-κB-dependent or -independent, depending on their structure. 
 Lysophosphatidic acid upregulates vascular endothelial growth factor-C and tube formation in human endothelial cells through LPA(1/3), COX-2, and NF-kappaB activation- and EGFR transactivation-dependent mechanisms. Lysophosphatidic acid (LPA) is a lipid bioactive mediator which binds to G-protein-coupled receptors and activates a variety of cellular functions. LPA modulates multiple behaviors in endothelial cells, including cell proliferation and   migration  , capillary-like tube formation in vitro, activation of proteases, interactions with leukocytes, and expressions of inflammation-related genes, thereby regulating vessel formation. LPA has been reported to modulate the angiogenesis process. However, the role of LPA in the lymphangiogenesis process has not been studied. In this study, we showed that LPA upregulated vascular endothelial growth factor-C (VEGF-C) mRNA expression in human umbilical vein endothelial cells (HUVECs) and subsequent endothelial cell tube formation in vitro and in vivo. These enhancement effects were LPA(1)- and LPA(3)-dependent and required cyclooxygenase-2 (COX-2), endothelial growth factor receptor (EGFR) transactivation and activation of nuclear factor kappaB (NF-kappaB). Moreover, LPA induced the protein expressions of the lymphatic markers, Prox-1, LYVE-1, and podoplanin, in HUVECs, and these enhancement effects were dependent on LPA(1) and LPA(3) activation and EGFR transactivation. Our results demonstrated that LPA might regulate VEGF-C and lymphatic marker expression in endothelial cells, which contributes to endothelial cell tube formation in vitro and in vivo, thus facilitating endothelial cell participation in the lymphangiogenesis process. This study clarifies the signaling mechanism of LPA-regulated VEGF-C expression and lymphatic marker expressions in endothelial cells, which suggest that LPA may be a suitable target for generating therapeutics against lymphangiogenesis and tumor metastasis. 
 Association of variants in genes encoding for macrophage-related functions with clinical outcome in patients with locoregional gastric cancer. Nuclear factor-kappaB (NF-κB) and CCL2/CCR2 chemokine axis play a central role in tumor progression such as stimulation of angiogenesis, acceleration of tumor invasion and   migratio  n, and suppression of innate immunosurveillance in the macrophage-related functions. There have been few reports regarding association of the macrophage function-related genes with the clinical outcome in gastric cancer. We hypothesized that variants in genes encoding for NF-κB and CCL2/CCR2 axis may predict prognosis in gastric cancer and tested whether the functional single-nucleotide polymorphisms (SNPs) will be associated with clinical outcome in patients with gastric cancer across two independent groups.This study enrolled two cohorts which consisted of 160 Japanese patients and 104 US patients with locoregional gastric cancer. Genomic DNA was analyzed for association of 11 SNPs in NFKB1, RELA, CCL2, and CCR2 with clinical outcome using PCR-based direct DNA sequencing.The univariable analysis showed four SNPs had significant association with clinical outcome in the Japanese cohort, NFKB1 rs230510 remained significant upon multivariable analysis. The patients with the A allele of the NFKB1 rs230510 had significantly longer overall survival (OS) compared with those with the T/T genotype in both the Japanese and US cohort in the univariable analysis. In contrast, genotypes with the T allele of CCL2 rs4586 were significantly associated with shorter OS compared with the C/C genotype in the US cohort [hazard ratio (HR) 2.43; P = 0.015] but longer OS in the Japanese cohort (HR 0.58; P = 0.021), resulting in the statistically significant opposite impact on OS (P = 0.001).Our study provides the first evidence that the NFKB1 rs230510 and CCL2 rs4586 are significantly associated with the clinical outcome in patients with locoregional gastric cancer. These results also suggest that the genetic predisposition of the host may dictate the immune-related component of the tumor for progression in gastric cancer. 
 miR-508-3p concordantly silences NFKB1 and RELA to inactivate canonical NF-κB signaling in gastric carcinogenesis. NF-κB signaling pathway plays an important role in gastric carcinogenesis. The basic expression and functional role of NFKB1 and RELA (components of canonical NF-κB pathway) in gastric cancer (GC) have not been well elucidated. In this study, the role of NFKB1 and RELA in gastric tumorigenesis will be investigated and their regulation by microRNAs (miRNAs) will be deeply explored.The mRNA and protein expression of NFKB1 and RELA were investigated by qRT-PCR and Western blot in GC cell lines and primary tumors. The functional roles of NFKB1 and RELA in GC were demonstrated by MTT proliferation assay, monolayer colony formation, cell invasion a  nd migrat  ion, cell cycle analysis and in vivo study through siRNA mediated knockdown. Identification of NFKB1 as a direct target of tumor suppressor miRNA miR-508-3p was achieved by expression regulation assays together with dual luciferase activity experiments.NFKB1 and RELA were up-regulated in GC cell lines and primary tumors compared with normal gastric epithelium cells and their upregulation correlation with poor survival in GC. siRNA mediated knockdown of NFKB1 or RELA exhibited anti-oncogenic effect both in vitro and in vivo. NFKB1 was further revealed to be a direct target of miR-508-3p in gastric tumorigenesis and their expression showed negative correlation in primary GC samples. miR-508-3p was down-regulated in GC cells compared with normal gastric epithelium samples and its ectopic expression in GC cell lines also exerts tumor suppressor function. NFKB1 re-expression was found to partly abolish the tumor-suppressive effect of miR-508-3p in GC.All these findings supports that canonical NF-κB signaling pathway is activated in GC at least by the inactivation of miR-508-3p and this might have therapeutic potential in GC treatment. 
 ΔNp63 versatilely regulates a Broad NF-κB gene program and promotes squamous epithelial proliferation  , migrati  on, and inflammation. Head and neck squamous cell carcinoma (HNSCC) and many other epithelial malignancies exhibit increased proliferation, invasion, and inflammation, concomitant with aberrant nuclear activation of TP53 and NF-κB family members ΔNp63, cRel, and RelA. However, the mechanisms of cross-talk by which these transcription factors coordinate gene expression and the malignant phenotype remain elusive. In this study, we showed that ΔNp63 regulates a cohort of genes involved in cell growth, survival, adhesion, and inflammation, which substantially overlaps with the NF-κB transcriptome. ΔNp63 with cRel and/or RelA are recruited to form novel binding complexes on p63 or NF-κB/Rel sites of multitarget gene promoters. Overexpressed ΔNp63- or TNF-α-induced NF-κB and inflammatory cytokine interleukin-8 (IL-8) reporter activation depended on RelA/cRel regulatory binding sites. Depletion of RelA or ΔNp63 by small interfering RNA (siRNA) significantly inhibited NF-κB-specific, or TNF-α-induced IL-8 reporter activation. ΔNp63 siRNA significantly inhibited proliferation,   survival  , and migration by HNSCC cells in vitro. Consistent with these data, an increase in nuclear ΔNp63, accompanied by increased proliferation (Ki-67) and adhesion (β4 integrin) markers, and induced inflammatory cell infiltration was observed throughout HNSCC specimens, when compared with the basilar pattern of protein expression and minimal inflammation seen in nonmalignant mucosa. Furthermore, overexpression of ΔNp63α in squamous epithelial cells in transgenic mice leads to increased suprabasilar cRel, Ki-67, and cytokine expression, together with epidermal hyperplasia and diffuse inflammation, similar to HNSCC. Our study reveals ΔNp63 as a master transcription factor that, in coordination with NF-κB/Rels, orchestrates a broad gene program promoting epidermal hyperplasia, inflammation, and the malignant phenotype of HNSCC. 
 The RASSF8 candidate tumor suppressor inhibits cell growth and regulates the Wnt and NF-kappaB signaling pathways. The Ras-assocation domain family (RASSF) of tumor suppressor proteins until recently contained six proteins named RASSF1-6. Recently, four novel family members, RASSF7-10, have been identified by homology searches for RA-domain-containing proteins. These additional RASSF members are divergent and structurally distinct from RASSF1-6, containing an N-terminal RA domain and lacking the Sav/RASSF/Hpo (SARAH) domain. Here, we show that RASSF8 is ubiquitously expressed throughout the murine embryo and in normal human adult tissues. Functionally, RNAi-mediated knockdown of RASSF8 in non-small-cell lung cancer (NSCLC) cell lines, increased anchorage-independent growth in soft agar and enhanced tumor growth in severe combined immunodeficiency (SCID) mice. Furthermore, EdU staining of RASSF8-depleted cells showed growth suppression in a manner dependent on contact inhibition. We show that endogenous RASSF8 is not only found in the nucleus, but is also membrane associated at sites of cell-cell adhesion, co-localizing with the adherens junction (AJ) component beta-catenin and binding to E-cadherin. Following RASSF8 depletion in two different lung cancer cell lines using alternative small interfering RNA (siRNA) sequences, we show that AJs are destabilized and E-cadherin is lost from the cell membrane. The AJ components beta-catenin and p65 are also lost from sites of cell-cell contact and are relocalized to the nucleus with a concomitant increase in beta-catenin-dependent and nuclear factor-kappaB (NF-kappaB)-dependent signaling following RASSF8 depletion. RASSF8 may also be required to maintain actin -cytoskeletal organization since immunofluorescence analysis shows a striking disorganization of the actin- cytoskeleton following RASSF8 depletion. Accordingly, scratch wound healing studies show increased cellular   migration   in RASSF8-deficient cells. These results implicate RASSF8 as a tumor suppressor gene that is essential for maintaining AJs function in epithelial cells and have a role in epithelial cell   migration  . 
 Differential requirement of the epidermal growth factor receptor for G protein-mediated activation of transcription factors by lysophosphatidic acid. The role of the epidermal growth factor receptor (EGFR) and other receptor tyrosine kinases (RTKs) in provoking biological actions of G protein-coupled receptors (GPCRs) has been one of the most disputed subjects in the field of GPCR signal transduction. The purpose of the current study is to identify EGFR-mediated mechanisms involved in activation of G protein cascades and the downstream transcription factors by lysophosphatidic acid (LPA).In ovarian cancer cells highly responsive to LPA, activation of AP-1 by LPA was suppressed by inhibition of EGFR, an effect that could be reversed by co-stimulation of another receptor tyrosine kinase c-Met with hepatocyte growth factor, indicating that LPA-mediated activation of AP-1 requires activity of a RTK, not necessarily EGFR. Induction of AP-1 components by LPA lied downstream of Gi, G12/13, and Gq. Activation of the effectors of Gi, but not Gq or G12/13 was sensitive to inhibition of EGFR. In contrast, LPA stimulated another prominent transcription factor NF-kappaB via the Gq-PKC pathway in an EGFR-independent manner. Consistent with the importance of Gi-elicited signals in a plethora of biological processes, LPA-induced cytokine production, cell proliferation,   migration   and invasion require intact EGFR.An RTK activity is required for activation of the AP-1 transcription factor and other Gi-dependent cellular responses to LPA. In contrast, activation of G12/13, Gq and Gq-elicited NF-kappaB by LPA is independent of such an input. These results provide a novel insight into the role of RTK in GPCR signal transduction and biological functions. 
 Necrotic cells influence   migration   and invasion of glioblastoma via NF-κB/AP-1-mediated IL-8 regulation. Glioblastoma multiforme (GBM) is the most common primary intracranial tumor in adults and has poor prognosis. Diffuse infiltration into normal brain parenchyma, rapid growth, and the presence of necrosis are remarkable hallmarks of GBM. However, the effect of necrotic cells on GBM growth and metastasis is poorly understood at present. In this study, we examined the biological significance of necrotic tissues by exploring the molecular mechanisms underlying the signaling network between necrotic tissues and GBM cells. The   migratio  n and invasion of the GBM cell line CRT-MG was significantly enhanced by treatment with necrotic cells, as shown by assays for scratch wound healing and spheroid invasion. Incubation with necrotic cells induced IL-8 secretion in CRT-MG cells in a dose-dependent manner. In human GBM tissues, IL-8 positive cells were mainly distributed in the perinecrotic region, as seen in immunohistochemistry and immunofluorescence analysis. Necrotic cells induced NF-κB and AP-1 activation and their binding to the IL-8 promoter, leading to enhanced IL-8 production and secretion in GBM cells. Our data demonstrate that when GBM cells are exposed to and stimulated by necrotic cells, th  e migrati  on and invasion of GBM cells are enhanced and facilitated via NF-κB/AP-1 mediated IL-8 upregulation. 
 SPHK1 inhibitor suppresses cell proliferation and invasion associated with the inhibition of NF-κB pathway in hepatocellular carcinoma. Sphingosine kinase 1 (SphK1) is an oncogenic enzyme promoting transformation, proliferation, and angiogenesis of a number of human tumors. However, its effect on hepatocellular carcinoma (HCC) behavior has not been fully clarified. The purpose of this study was to determine the correlation between HCC and SphK1, and to evaluate the effect of SphK1 inhibitor N,N-dimethylsphingosine (DMS) in HCC. The expression of SphK1 was measured in tissue samples from 76 HCC and paired adjacent noncancerous liver tissues (NT) by immunohistochemistry, quantitative real-time PCR, and Western blotting analysis. The effect of DMS was tested on HCC cells by evaluating cell viability in vitro. Transwell cell   migratio  n and invasion assay were carried out for functional analysis. Furthermore, Western blotting analysis was performed to examine the impact of DMS on the PI3K/Akt/NF-kB signaling. High expression of Sphk1 was observed in 84.21% (64/76) of the HCC versus 15.79% (12/76) of the adjacent non-tumorous liver tissues; the difference of Sphk1 expression between HCC and the adjacent non-tumorous liver tissues was statistically significant (P < 0.001). The results were confirmed by Western blot analyses and quantitative real-time PCR. DMS inhibited the proliferation of SK-Hep1 and MHCCLM3 cells which have a relatively high level of SphK1 in a time- and concentration-dependent manner, and the invasion   and migr  ation of SK-Hep1 cells were distinctly suppressed after undergoing treatment with DMS. Furthermore, DMS markedly suppressed the expression of phosphorylations of Akt and NF-κB in HCC cells. Our data suggest that the pathogenesis of human HCC maybe mediated by Sphk1, and the specific Sphk1 inhibitor DMS can play a therapeutic role in the treatment of HCC and thus, Sphk1 could represent selective targets for the molecularly targeted treatments of HCC. 
 SASH1 is a scaffold molecule in endothelial TLR4 signaling. Recognition of microbial products by TLRs is critical for mediating innate immune responses to invading pathogens. In this study, we identify a novel scaffold protein in TLR4 signaling called SAM and SH3 domain containing protein 1 (SASH1). Sash1 is expressed across all microvascular beds and functions as a scaffold molecule to independently bind TRAF6, TAK1, IκB kinase α, and IκB kinase β. This interaction fosters ubiquitination of TRAF6 and TAK1 and promotes LPS-induced NF-κB, JNK, and p38 activation, culminating in increased production of proinflammatory cytokines and increased LPS-induced endothe  lial migr  ation. Our findings suggest that SASH1 acts to assemble a signaling complex downstream of TLR4 to activate early endothelial responses to receptor activation. 
 Constitutively active type I insulin-like growth factor receptor causes transformation and xenograft growth of immortalized mammary epithelial cells and is accompanied by an epithelial-to-mesenchymal transition mediated by NF-kappaB and snail. Type I insulin-like growth factor receptor (IGF-IR) can transform mouse fibroblasts; however, little is known about the transforming potential of IGF-IR in human fibroblasts or epithelial cells. We found that overexpression of a constitutively activated IGF-IR (CD8-IGF-IR) was sufficient to cause transformation of immortalized human mammary epithelial cells and growth in immunocompromised mice. Furthermore, CD8-IGF-IR caused cells to undergo an epithelial-to-mesenchymal transition (EMT) which was associated with dramatically increased   migration   and invasion. The EMT was mediated by the induction of the transcriptional repressor Snail and downregulation of E-cadherin. NF-kappaB was highly active in CD8-IGF-IR-MCF10A cells, and both increased levels of Snail and the EMT were partially reversed by blocking NF-kappaB or IGF-IR activity. This study places IGF-IR among a small group of oncogenes that, when overexpressed alone, can confer in vivo tumorigenic growth of MCF10A cells and indicates the hierarchy in the mechanism of IGF-IR-induced EMT. 
 Interleukin-1β enhances cell   migratio  n through AP-1 and NF-κB pathway-dependent FGF2 expression in human corneal endothelial cells. Interleukin (IL)-1β is a major pro-inflammatory cytokine that plays a crucial role in the regulation of inflammation and wound healing in the cornea. Elucidation of IL-1β signalling may help identify therapeutic targets for corneal wound healing; however, mechanisms such as c  ell migra  tion, a component of IL-1β-induced wound healing response in human corneal endothelial cells (CEC), have not been well characterised.Stimulation of human CEC with IL-1β activated expression of fibroblast growth factor 2 (FGF2) and resulted in enhanced   cell mig  ration. This, in turn, was abolished by treatment with either IL-1 receptor antagonist or SU-5402, a pan-fibroblast growth factor signalling inhibitor. Phosphatidyl inositol (PI) 3-kinase or IL receptor-associated kinase 1/4 antagonists demonstrated that IL receptor-associated kinase 1/4 activates PI 3-kinase, which in turn phosphorylates p38 and inhibitor κB kinase α/β, leading to FGF2 expression through activation of activator protein 1 (AP-1) and nuclear factor kappa-light-chain enhancer of activated B cells (NF-κB) in human CEC. Treatment of IL-1β-stimulated human CEC with either AP-1 or NF-κB antagonists decreased FGF2 expression and resulted in reduced IL-1β-e  nhanced c  ell migration. Co-treatment of IL-1β-stimulated human CEC with both inhibitors completely blocked FGF2 expression and IL-1β  -enhanced   cell migration. Chromatin immunoprecipitation assays demonstrated that AP-1 and NF-κB directly bind to the FGF2 promoter following IL-1β stimulation.The results show that binding of IL-1β to its receptor in human CEC leads to parallel activation of AP-1 and NF-κB pathways, leading, in turn, to FGF2 expression   and enha  nced cell migration. 
 Cell specific CD44 expression in breast cancer requires the interaction of AP-1 and NFκB with a novel cis-element. Breast cancers contain a heterogeneous population of cells with a small percentage that possess properties similar to those found in stem cells. One of the widely accepted markers of breast cancer stem cells (BCSCs) is the cell surface marker CD44. As a glycoprotein, CD44 is involved in many cellular processes including cell adhesion,   migratio  n and proliferation, making it pro-oncogenic by nature. CD44 expression is highly up-regulated in BCSCs, and has been implicated in tumorigenesis and metastasis. However, the genetic mechanism that leads to a high level of CD44 expression in breast cancer cells and BCSCs is not well understood. Here, we identify a novel cis-element of the CD44 directs gene expression in breast cancer cells in a cell type specific manner. We have further identified key trans-acting factor binding sites and nuclear factors AP-1 and NFκB that are involved in the regulation of cell-specific CD44 expression. These findings provide new insight into the complex regulatory mechanism of CD44 expression, which may help identify more effective therapeutic targets against the breast cancer stem cells and metastatic tumors. 
 Necrotic cells influence   migration   and invasion of glioblastoma via NF-κB/AP-1-mediated IL-8 regulation. Glioblastoma multiforme (GBM) is the most common primary intracranial tumor in adults and has poor prognosis. Diffuse infiltration into normal brain parenchyma, rapid growth, and the presence of necrosis are remarkable hallmarks of GBM. However, the effect of necrotic cells on GBM growth and metastasis is poorly understood at present. In this study, we examined the biological significance of necrotic tissues by exploring the molecular mechanisms underlying the signaling network between necrotic tissues and GBM cells. The   migratio  n and invasion of the GBM cell line CRT-MG was significantly enhanced by treatment with necrotic cells, as shown by assays for scratch wound healing and spheroid invasion. Incubation with necrotic cells induced IL-8 secretion in CRT-MG cells in a dose-dependent manner. In human GBM tissues, IL-8 positive cells were mainly distributed in the perinecrotic region, as seen in immunohistochemistry and immunofluorescence analysis. Necrotic cells induced NF-κB and AP-1 activation and their binding to the IL-8 promoter, leading to enhanced IL-8 production and secretion in GBM cells. Our data demonstrate that when GBM cells are exposed to and stimulated by necrotic cells, th  e migrati  on and invasion of GBM cells are enhanced and facilitated via NF-κB/AP-1 mediated IL-8 upregulation. 
 miR-508-3p concordantly silences NFKB1 and RELA to inactivate canonical NF-κB signaling in gastric carcinogenesis. NF-κB signaling pathway plays an important role in gastric carcinogenesis. The basic expression and functional role of NFKB1 and RELA (components of canonical NF-κB pathway) in gastric cancer (GC) have not been well elucidated. In this study, the role of NFKB1 and RELA in gastric tumorigenesis will be investigated and their regulation by microRNAs (miRNAs) will be deeply explored.The mRNA and protein expression of NFKB1 and RELA were investigated by qRT-PCR and Western blot in GC cell lines and primary tumors. The functional roles of NFKB1 and RELA in GC were demonstrated by MTT proliferation assay, monolayer colony formation, cell invasion a  nd migrat  ion, cell cycle analysis and in vivo study through siRNA mediated knockdown. Identification of NFKB1 as a direct target of tumor suppressor miRNA miR-508-3p was achieved by expression regulation assays together with dual luciferase activity experiments.NFKB1 and RELA were up-regulated in GC cell lines and primary tumors compared with normal gastric epithelium cells and their upregulation correlation with poor survival in GC. siRNA mediated knockdown of NFKB1 or RELA exhibited anti-oncogenic effect both in vitro and in vivo. NFKB1 was further revealed to be a direct target of miR-508-3p in gastric tumorigenesis and their expression showed negative correlation in primary GC samples. miR-508-3p was down-regulated in GC cells compared with normal gastric epithelium samples and its ectopic expression in GC cell lines also exerts tumor suppressor function. NFKB1 re-expression was found to partly abolish the tumor-suppressive effect of miR-508-3p in GC.All these findings supports that canonical NF-κB signaling pathway is activated in GC at least by the inactivation of miR-508-3p and this might have therapeutic potential in GC treatment. 
 Association of variants in genes encoding for macrophage-related functions with clinical outcome in patients with locoregional gastric cancer. Nuclear factor-kappaB (NF-κB) and CCL2/CCR2 chemokine axis play a central role in tumor progression such as stimulation of angiogenesis, acceleration of tumor invasion and   migratio  n, and suppression of innate immunosurveillance in the macrophage-related functions. There have been few reports regarding association of the macrophage function-related genes with the clinical outcome in gastric cancer. We hypothesized that variants in genes encoding for NF-κB and CCL2/CCR2 axis may predict prognosis in gastric cancer and tested whether the functional single-nucleotide polymorphisms (SNPs) will be associated with clinical outcome in patients with gastric cancer across two independent groups.This study enrolled two cohorts which consisted of 160 Japanese patients and 104 US patients with locoregional gastric cancer. Genomic DNA was analyzed for association of 11 SNPs in NFKB1, RELA, CCL2, and CCR2 with clinical outcome using PCR-based direct DNA sequencing.The univariable analysis showed four SNPs had significant association with clinical outcome in the Japanese cohort, NFKB1 rs230510 remained significant upon multivariable analysis. The patients with the A allele of the NFKB1 rs230510 had significantly longer overall survival (OS) compared with those with the T/T genotype in both the Japanese and US cohort in the univariable analysis. In contrast, genotypes with the T allele of CCL2 rs4586 were significantly associated with shorter OS compared with the C/C genotype in the US cohort [hazard ratio (HR) 2.43; P = 0.015] but longer OS in the Japanese cohort (HR 0.58; P = 0.021), resulting in the statistically significant opposite impact on OS (P = 0.001).Our study provides the first evidence that the NFKB1 rs230510 and CCL2 rs4586 are significantly associated with the clinical outcome in patients with locoregional gastric cancer. These results also suggest that the genetic predisposition of the host may dictate the immune-related component of the tumor for progression in gastric cancer. 
 SPHK1 inhibitor suppresses cell proliferation and invasion associated with the inhibition of NF-κB pathway in hepatocellular carcinoma. Sphingosine kinase 1 (SphK1) is an oncogenic enzyme promoting transformation, proliferation, and angiogenesis of a number of human tumors. However, its effect on hepatocellular carcinoma (HCC) behavior has not been fully clarified. The purpose of this study was to determine the correlation between HCC and SphK1, and to evaluate the effect of SphK1 inhibitor N,N-dimethylsphingosine (DMS) in HCC. The expression of SphK1 was measured in tissue samples from 76 HCC and paired adjacent noncancerous liver tissues (NT) by immunohistochemistry, quantitative real-time PCR, and Western blotting analysis. The effect of DMS was tested on HCC cells by evaluating cell viability in vitro. Transwell cell   migratio  n and invasion assay were carried out for functional analysis. Furthermore, Western blotting analysis was performed to examine the impact of DMS on the PI3K/Akt/NF-kB signaling. High expression of Sphk1 was observed in 84.21% (64/76) of the HCC versus 15.79% (12/76) of the adjacent non-tumorous liver tissues; the difference of Sphk1 expression between HCC and the adjacent non-tumorous liver tissues was statistically significant (P < 0.001). The results were confirmed by Western blot analyses and quantitative real-time PCR. DMS inhibited the proliferation of SK-Hep1 and MHCCLM3 cells which have a relatively high level of SphK1 in a time- and concentration-dependent manner, and the invasion   and migr  ation of SK-Hep1 cells were distinctly suppressed after undergoing treatment with DMS. Furthermore, DMS markedly suppressed the expression of phosphorylations of Akt and NF-κB in HCC cells. Our data suggest that the pathogenesis of human HCC maybe mediated by Sphk1, and the specific Sphk1 inhibitor DMS can play a therapeutic role in the treatment of HCC and thus, Sphk1 could represent selective targets for the molecularly targeted treatments of HCC. 
 Aberrant IKKα and IKKβ cooperatively activate NF-κB and induce EGFR/AP1 signaling to promote survival a  nd migrat  ion of head and neck cancer. The inhibitor-κB kinase-nuclear factor-κB (IKK-NF-κB) and epidermal growth factor receptor-activator protein-1 (EGFR-AP1) pathways are often co-activated and promote malignant behavior, but the underlying basis for this relationship is unclear. Resistance to inhibitors of IKKβ or EGFR is observed in head and neck squamous cell carcinomas (HNSCC). Here, we reveal that both IKKα and β contribute to nuclear activation of canonical and alternate NF-κB/REL family transcription factors, and overexpression of signal components that enhance co-activation of the EGFR-AP1 pathway. We observed that IKKα and IKKβ exhibit increased protein expression, nuclear localization, and phosphorylation in HNSCC tissues and cell lines. Individually, IKK activity varied among different cell lines, but overexpression of both IKKs induced the strongest NF-κB activation. Conversely, siRNA knock down of both IKKs significantly decreased nuclear localization and phosphorylation of canonical RELA and IκBα and alternative p52 and RELB subunits. Knock down of both IKKs more effectively inhibited NF-κB activation, broadly modulated gene expression and suppressed cell pr  oliferati  on and migration. Global expression profiling revealed that NF-κB, cytokine, inflammatory response and growth factor signaling are among the top pathways and networks regulated by IKKs. Importantly, IKKα and IKKβ together promoted the expression and activity of transforming growth factor α, EGFR and AP1 transcription factors cJun, JunB and Fra1. Knock down of AP1 subunits individually decreased 8/15 (53%) of IKK-targeted genes sampled and similarly inhibited cel  l prolife  ration and migration. Mutations of NF-κB and AP1-binding sites abolished or decreased IKK-induced interleukin-8 (IL-8) promoter activity. Compounds such as wedelactone with dual IKK inhibitory activity and geldanomycins that block IKKα/β and EGFR pathways were more active than IKKβ-specific inhibitors in suppressing NF-κB activation and proliferation and inducing cell death. We conclude that IKKα and IKKβ cooperatively activate NF-κB and EGFR/AP1 networks of signaling pathways and contribute to the malignant phenotype and the intrinsic or acquired therapeutic resistance of HNSCC. 
 Interleukin-1β enhances cell   migratio  n through AP-1 and NF-κB pathway-dependent FGF2 expression in human corneal endothelial cells. Interleukin (IL)-1β is a major pro-inflammatory cytokine that plays a crucial role in the regulation of inflammation and wound healing in the cornea. Elucidation of IL-1β signalling may help identify therapeutic targets for corneal wound healing; however, mechanisms such as c  ell migra  tion, a component of IL-1β-induced wound healing response in human corneal endothelial cells (CEC), have not been well characterised.Stimulation of human CEC with IL-1β activated expression of fibroblast growth factor 2 (FGF2) and resulted in enhanced   cell mig  ration. This, in turn, was abolished by treatment with either IL-1 receptor antagonist or SU-5402, a pan-fibroblast growth factor signalling inhibitor. Phosphatidyl inositol (PI) 3-kinase or IL receptor-associated kinase 1/4 antagonists demonstrated that IL receptor-associated kinase 1/4 activates PI 3-kinase, which in turn phosphorylates p38 and inhibitor κB kinase α/β, leading to FGF2 expression through activation of activator protein 1 (AP-1) and nuclear factor kappa-light-chain enhancer of activated B cells (NF-κB) in human CEC. Treatment of IL-1β-stimulated human CEC with either AP-1 or NF-κB antagonists decreased FGF2 expression and resulted in reduced IL-1β-e  nhanced c  ell migration. Co-treatment of IL-1β-stimulated human CEC with both inhibitors completely blocked FGF2 expression and IL-1β  -enhanced   cell migration. Chromatin immunoprecipitation assays demonstrated that AP-1 and NF-κB directly bind to the FGF2 promoter following IL-1β stimulation.The results show that binding of IL-1β to its receptor in human CEC leads to parallel activation of AP-1 and NF-κB pathways, leading, in turn, to FGF2 expression   and enha  nced cell migration. 
 Cell specific CD44 expression in breast cancer requires the interaction of AP-1 and NFκB with a novel cis-element. Breast cancers contain a heterogeneous population of cells with a small percentage that possess properties similar to those found in stem cells. One of the widely accepted markers of breast cancer stem cells (BCSCs) is the cell surface marker CD44. As a glycoprotein, CD44 is involved in many cellular processes including cell adhesion,   migratio  n and proliferation, making it pro-oncogenic by nature. CD44 expression is highly up-regulated in BCSCs, and has been implicated in tumorigenesis and metastasis. However, the genetic mechanism that leads to a high level of CD44 expression in breast cancer cells and BCSCs is not well understood. Here, we identify a novel cis-element of the CD44 directs gene expression in breast cancer cells in a cell type specific manner. We have further identified key trans-acting factor binding sites and nuclear factors AP-1 and NFκB that are involved in the regulation of cell-specific CD44 expression. These findings provide new insight into the complex regulatory mechanism of CD44 expression, which may help identify more effective therapeutic targets against the breast cancer stem cells and metastatic tumors. 
 Differential effects of sulfated triterpene glycosides, holothurin A1, and 24-dehydroechinoside A, on antimetastasic activity via regulation of the MMP-9 signal pathway. Two sulfated triterpene glycosides, holothurin A(1) (HA(1)) and 24-dehydroechinoside A (DHEA), isolated from the sea cucumber Pearsonothuria graeffei, are of the holostane type with 18(20)-lactone and identical carbohydrate chains. DHEA has a side chain 23 (24)-double bond, while HA(1) has a hydroxyl group at C-21. In this study, we compared the effects of DHEA and HA(1) on metastasis in vitro and in vivo. The results show that HA(1) and DHEA treatment significantly suppressed adhesion of human hepatocellular liver carcinoma cells (HepG2) to both matrigel and human endothelial cells (ECV-304) and inhibited HepG2 cell   migration   and invasion in a dose-dependant manner. HA(1) and DHEA reduced tube formation of ECV-304 cells on the matrigel in vitro and attenuated neovascularization in the chick embryo using the chorioallantoic membrane (CAM) assay in vivo. Immunocytochemistry analyses revealed that both HA(1) and DHEA significantly decreased the expression of the matrix metallo-proteinase-9 (MMP-9) and increased the expression level of tissue inhibitor of metalloproteinase-1 (TIMP-1), an important regulator of MMP-9 activation. Western blot analyses demonstrated that HA(1) and DHEA remarkably abolished the expression of vascular endothelial growth factor (VEGF). The expression of nuclear factor-kappa B (NF-κB) was significantly decreased by HA(1), while DHEA treatment had no effect on the down regulation of NF-κB expression. These data suggest that both DHEA and HA(1) exert significant antimetastatic activities by inhibiting MMP-9 and VEGF expression. DHEA-induced antimetastasis was more potent than HA(1). In addition, only HA(1) treatment downregulated the expression level of NF-κB, suggesting that the antimetastatic activity of triterpene glycosides derived from P. graeffei can be either NF-κB-dependent or -independent, depending on their structure. 
 ΔNp63 versatilely regulates a Broad NF-κB gene program and promotes squamous epithelial proliferation  , migrati  on, and inflammation. Head and neck squamous cell carcinoma (HNSCC) and many other epithelial malignancies exhibit increased proliferation, invasion, and inflammation, concomitant with aberrant nuclear activation of TP53 and NF-κB family members ΔNp63, cRel, and RelA. However, the mechanisms of cross-talk by which these transcription factors coordinate gene expression and the malignant phenotype remain elusive. In this study, we showed that ΔNp63 regulates a cohort of genes involved in cell growth, survival, adhesion, and inflammation, which substantially overlaps with the NF-κB transcriptome. ΔNp63 with cRel and/or RelA are recruited to form novel binding complexes on p63 or NF-κB/Rel sites of multitarget gene promoters. Overexpressed ΔNp63- or TNF-α-induced NF-κB and inflammatory cytokine interleukin-8 (IL-8) reporter activation depended on RelA/cRel regulatory binding sites. Depletion of RelA or ΔNp63 by small interfering RNA (siRNA) significantly inhibited NF-κB-specific, or TNF-α-induced IL-8 reporter activation. ΔNp63 siRNA significantly inhibited proliferation,   survival  , and migration by HNSCC cells in vitro. Consistent with these data, an increase in nuclear ΔNp63, accompanied by increased proliferation (Ki-67) and adhesion (β4 integrin) markers, and induced inflammatory cell infiltration was observed throughout HNSCC specimens, when compared with the basilar pattern of protein expression and minimal inflammation seen in nonmalignant mucosa. Furthermore, overexpression of ΔNp63α in squamous epithelial cells in transgenic mice leads to increased suprabasilar cRel, Ki-67, and cytokine expression, together with epidermal hyperplasia and diffuse inflammation, similar to HNSCC. Our study reveals ΔNp63 as a master transcription factor that, in coordination with NF-κB/Rels, orchestrates a broad gene program promoting epidermal hyperplasia, inflammation, and the malignant phenotype of HNSCC. 
 The RASSF8 candidate tumor suppressor inhibits cell growth and regulates the Wnt and NF-kappaB signaling pathways. The Ras-assocation domain family (RASSF) of tumor suppressor proteins until recently contained six proteins named RASSF1-6. Recently, four novel family members, RASSF7-10, have been identified by homology searches for RA-domain-containing proteins. These additional RASSF members are divergent and structurally distinct from RASSF1-6, containing an N-terminal RA domain and lacking the Sav/RASSF/Hpo (SARAH) domain. Here, we show that RASSF8 is ubiquitously expressed throughout the murine embryo and in normal human adult tissues. Functionally, RNAi-mediated knockdown of RASSF8 in non-small-cell lung cancer (NSCLC) cell lines, increased anchorage-independent growth in soft agar and enhanced tumor growth in severe combined immunodeficiency (SCID) mice. Furthermore, EdU staining of RASSF8-depleted cells showed growth suppression in a manner dependent on contact inhibition. We show that endogenous RASSF8 is not only found in the nucleus, but is also membrane associated at sites of cell-cell adhesion, co-localizing with the adherens junction (AJ) component beta-catenin and binding to E-cadherin. Following RASSF8 depletion in two different lung cancer cell lines using alternative small interfering RNA (siRNA) sequences, we show that AJs are destabilized and E-cadherin is lost from the cell membrane. The AJ components beta-catenin and p65 are also lost from sites of cell-cell contact and are relocalized to the nucleus with a concomitant increase in beta-catenin-dependent and nuclear factor-kappaB (NF-kappaB)-dependent signaling following RASSF8 depletion. RASSF8 may also be required to maintain actin -cytoskeletal organization since immunofluorescence analysis shows a striking disorganization of the actin- cytoskeleton following RASSF8 depletion. Accordingly, scratch wound healing studies show increased cellular   migration   in RASSF8-deficient cells. These results implicate RASSF8 as a tumor suppressor gene that is essential for maintaining AJs function in epithelial cells and have a role in epithelial cell   migration  . 
 Differential requirement of the epidermal growth factor receptor for G protein-mediated activation of transcription factors by lysophosphatidic acid. The role of the epidermal growth factor receptor (EGFR) and other receptor tyrosine kinases (RTKs) in provoking biological actions of G protein-coupled receptors (GPCRs) has been one of the most disputed subjects in the field of GPCR signal transduction. The purpose of the current study is to identify EGFR-mediated mechanisms involved in activation of G protein cascades and the downstream transcription factors by lysophosphatidic acid (LPA).In ovarian cancer cells highly responsive to LPA, activation of AP-1 by LPA was suppressed by inhibition of EGFR, an effect that could be reversed by co-stimulation of another receptor tyrosine kinase c-Met with hepatocyte growth factor, indicating that LPA-mediated activation of AP-1 requires activity of a RTK, not necessarily EGFR. Induction of AP-1 components by LPA lied downstream of Gi, G12/13, and Gq. Activation of the effectors of Gi, but not Gq or G12/13 was sensitive to inhibition of EGFR. In contrast, LPA stimulated another prominent transcription factor NF-kappaB via the Gq-PKC pathway in an EGFR-independent manner. Consistent with the importance of Gi-elicited signals in a plethora of biological processes, LPA-induced cytokine production, cell proliferation,   migration   and invasion require intact EGFR.An RTK activity is required for activation of the AP-1 transcription factor and other Gi-dependent cellular responses to LPA. In contrast, activation of G12/13, Gq and Gq-elicited NF-kappaB by LPA is independent of such an input. These results provide a novel insight into the role of RTK in GPCR signal transduction and biological functions. 
 Stabilization of snail by NF-kappaB is required for inflammation-induced cell   migration   and invasion. The increased motility and invasiveness of tumor cells are reminiscent of epithelial-mesenchymal transition (EMT), which occurs during embryonic development, wound healing, and metastasis. In this study, we found that Snail is stabilized by the inflammatory cytokine TNFalpha through the activation of the NF-kappaB pathway. We demonstrated that NF-kappaB is required for the induction of COP9 signalosome 2 (CSN2), which, in turn, blocks the ubiquitination and degradation of Snail. Furthermore, we showed that the expression of Snail correlated with the activation of NF-kappaB in cancer cell lines and metastatic tumor samples. Knockdown of Snail expression inhibited cell   migration   and invasion induced by inflammatory cytokines and suppressed inflammation-mediated breast cancer metastasis. Our study provides a plausible mechanism for inflammation-induced metastasis. 
 Lysophosphatidic acid upregulates vascular endothelial growth factor-C and tube formation in human endothelial cells through LPA(1/3), COX-2, and NF-kappaB activation- and EGFR transactivation-dependent mechanisms. Lysophosphatidic acid (LPA) is a lipid bioactive mediator which binds to G-protein-coupled receptors and activates a variety of cellular functions. LPA modulates multiple behaviors in endothelial cells, including cell proliferation and   migration  , capillary-like tube formation in vitro, activation of proteases, interactions with leukocytes, and expressions of inflammation-related genes, thereby regulating vessel formation. LPA has been reported to modulate the angiogenesis process. However, the role of LPA in the lymphangiogenesis process has not been studied. In this study, we showed that LPA upregulated vascular endothelial growth factor-C (VEGF-C) mRNA expression in human umbilical vein endothelial cells (HUVECs) and subsequent endothelial cell tube formation in vitro and in vivo. These enhancement effects were LPA(1)- and LPA(3)-dependent and required cyclooxygenase-2 (COX-2), endothelial growth factor receptor (EGFR) transactivation and activation of nuclear factor kappaB (NF-kappaB). Moreover, LPA induced the protein expressions of the lymphatic markers, Prox-1, LYVE-1, and podoplanin, in HUVECs, and these enhancement effects were dependent on LPA(1) and LPA(3) activation and EGFR transactivation. Our results demonstrated that LPA might regulate VEGF-C and lymphatic marker expression in endothelial cells, which contributes to endothelial cell tube formation in vitro and in vivo, thus facilitating endothelial cell participation in the lymphangiogenesis process. This study clarifies the signaling mechanism of LPA-regulated VEGF-C expression and lymphatic marker expressions in endothelial cells, which suggest that LPA may be a suitable target for generating therapeutics against lymphangiogenesis and tumor metastasis. 
 Molecular basis of nuclear factor-kappaB activation by astrocyte elevated gene-1. Malignant glioma is a consistently fatal brain cancer. The tumor invades the surrounding tissue, limiting complete surgical removal and thereby initiating recurrence. Identifying molecules critical for glioma invasion is essential to develop targeted, effective therapies. The expression of astrocyte elevated gene-1 (AEG-1) increases in malignant glioma and AEG-1 regulates in vitro invasion and   migration   of malignant glioma cells by activating the nuclear factor-kappaB (NF-kappaB) signaling pathway. The present studies elucidate the domains of AEG-1 important for mediating its function. Serial NH(2)-terminal and COOH-terminal deletion mutants were constructed and functional analysis revealed that the NH(2)-terminal 71 amino acids were essential for invasion,   migration  , and NF-kappaB-activating properties of AEG-1. The p65-interaction domain was identified between amino acids 101 to 205, indicating that p65 interaction alone is not sufficient to mediate AEG-1 function. Coimmunoprecipitation assays revealed that AEG-1 interacts with cyclic AMP-responsive element binding protein-binding protein (CBP), indicating that it might act as a bridging factor between NF-kappaB, CBP, and the basal transcription machinery. Chromatin immunoprecipitation assays showed that AEG-1 is associated with the NF-kappaB binding element in the interleukin-8 promoter. Thus, AEG-1 might function as a coactivator for NF-kappaB, consequently augmenting expression of genes necessary for invasion of glioma cells. In these contexts, AEG-1 represents a viable potential target for the therapy of malignant glioma. 
 Constitutively active type I insulin-like growth factor receptor causes transformation and xenograft growth of immortalized mammary epithelial cells and is accompanied by an epithelial-to-mesenchymal transition mediated by NF-kappaB and snail. Type I insulin-like growth factor receptor (IGF-IR) can transform mouse fibroblasts; however, little is known about the transforming potential of IGF-IR in human fibroblasts or epithelial cells. We found that overexpression of a constitutively activated IGF-IR (CD8-IGF-IR) was sufficient to cause transformation of immortalized human mammary epithelial cells and growth in immunocompromised mice. Furthermore, CD8-IGF-IR caused cells to undergo an epithelial-to-mesenchymal transition (EMT) which was associated with dramatically increased   migration   and invasion. The EMT was mediated by the induction of the transcriptional repressor Snail and downregulation of E-cadherin. NF-kappaB was highly active in CD8-IGF-IR-MCF10A cells, and both increased levels of Snail and the EMT were partially reversed by blocking NF-kappaB or IGF-IR activity. This study places IGF-IR among a small group of oncogenes that, when overexpressed alone, can confer in vivo tumorigenic growth of MCF10A cells and indicates the hierarchy in the mechanism of IGF-IR-induced EMT. 
 Aloe emodin inhibits colon cancer cell   migration  /angiogenesis by downregulating MMP-2/9, RhoB and VEGF via reduced DNA binding activity of NF-κB. Aloe emodin (AE), a natural anthraquinone, is reported to have antiproliferative activity in various cancer cell lines. In this study we analyzed molecular mechanisms involved in the antimigratory and antiangiogenic activity of this hydroxy anthraquinone in colon cancer cell, WiDr. Our results show that a relatively non toxic concentration of AE suppressed the phorbol-12-myristyl-13-acetate (PMA) induced   migratio  n and invasion of tumor cells. On analysis for the molecules involved in the   migratio  n/invasion, we found AE downregulated mRNA expression and promoter/gelatinolytic activity of Matrix Metalloproteinase (MMP)-2/9, as well as the RhoB expression at gene and protein level. It was also a strong inhibitor of Vascular Endothelial Growth Factor (VEGF) expression, promoter activity and endothelial cell   migratio  n/invasion and in vitro angiogenesis. AE suppressed the nuclear translocation and DNA binding of NF-κB, which is an important transcription factor for controlling MMP-2/9 and VEGF gene expression. Taken together these data indicate that AE target multiple molecules responsible for cellular invasion  , migrati  on and angiogenesis. Inhibitory effect on angiogenic and metastatic regulatory processes make AE a sensible candidate as a specific blocker of tumor associated events. 
 NF-κB signaling pathway confers neuroblastoma cells   migratio  n and invasion ability via the regulation of CXCR4. Accumulating evidence implicates the transcription factor NF-κB as a positive mediator of tumor metastasis, but the molecular mechanism(s) involved in this process remains largely unknown. In this study, we investigated the role of NF-κB signaling pathway in the regulation of CXC chemokine receptor-4 (CXCR4) in neuroblastoma metastasis.NF-κB, CXCR4 mRNA and protein expression were measured by RT-PCR, and Western blot. Tumor necrosis factor-α (TNF-α) was used to induce the upregulation of NF-κB and CXCR4. The knockdown of NF-κB and CXCR4 was achieved by PDTC. Transwell assay was used to investigate the role of NF-κB (P65) in neuroblast  oma cell   migration and invasion. An in vitro co-culture system was established to investigate the role of tumor microenvironment in regulation of the NF-κB signaling pathway.Over-expression of NF-κB (p65) prom  oted tumo  r migration and invasion through the upregulation of CXCR4; however, knockdown of NF-κB(P65) inhi  bited tum  or migration and invasion through blocking the expression of CXCR4. Consistently, in the co-culture system, the expression of CXCR4 was partly dependent on the expression of NF-κB (p65).Our studies reveal critical roles for the NF-κB signaling pathway in   neuroblas  toma migration and invasion. The mechanism may be through up-regulation of CXCR4, mediated by the NF-κB signaling pathways. Targeting NF-κB signalling pathways and ultimately CXCR4 could be a strategy in neuroblastoma therapy. 
 Antiviral signaling protein MITA acts as a tumor suppressor in breast cancer by regulating NF-κB induced cell death. Emerging evidences suggest that chronic inflammation is one of the major causes of tumorigenesis. The role of inflammation in regulation of breast cancer progression is not well established. Recently Mediator of IRF3 Activation (MITA) protein has been identified that regulates NF-κB and IFN pathways. Role of MITA in the context of inflammation and cancer progression has not been investigated. In the current report, we studied the role of MITA in the regulation of cross talk between cell death and inflammation in breast cancer cells. The expression of MITA was significantly lower on in estrogen receptor (ER) positive breast cancer cells than ER negative cells. Similarly, it was significantly down regulated in tumor tissue as compared to the normal tissue. The overexpression of MITA in MCF-7 and T47D decreases the cell proliferation and increases the cell death by activation of caspases. MITA positively regulates NF-κB transcription factor, which is essential for MITA induced cell death. The activation of NF-κB induces TNF-α production which further sensitizes MITA induced cell death by activation of death receptor pathway through capsase-8. MITA expression decreases the colony forming units   and migr  ation ability of MCF-7 cells. Thus, our finding suggests that MITA acts as a tumor suppressor which is down regulated during tumorigenesis providing survival advantage to tumor cell. 
 [C/EBPβ promotes NF-κB-mediated invasion an  d migrati  on of human renal carcinoma 786-O cells]. To explore the regulatory role of CCAAT/enhancer-binding protein beta (C/EBPβ) in invasion a  nd migrat  ion of 786-O human renal cancer cells.C/EBPβ adenoviral overexpression vector (pAd-C/EBPβ) and C/EBPβ siRNA were constructed and respectively transfered into 786-O cells. Wound healing and Transwell(TM) invasion methods were used to detect the invasio  n and mig  ration of the cells. Laser scanning confocal microscopy was applied to determine the translocation of nuclear factor κB (NF-κB), the central regulator of epithelial-mesenchymal transition (EMT); Western blotting was performed to examine the levels of EMT key markers E-cadherin, vimentin and tenascin.When C/EBPβ was overexpressed, inva  sion and   migration of 786-O cells were enhanced, translocation of NF-κB increased, E-cadherin level was reduced, and vimentin and tenascin levels were elevated. When C/EBPβ was knocked down, the results were completely opposite to those of C/EBPβ overexpression: i  nvasion a  nd migration of 786-O cells were inhibited, translocation of NF-κB decreased, E-cadherin level was elevated, and vimentin and tenascin expressions were suppressed. Moreover, the above changes were not found in the cells that co-treated with pAd-C/EBPβ and C/EBPβ siRNA.C/EBPβ could promote t  he invasi  on and migration of human renal carcinoma 786-O cells, and had an inducing effect on nuclear translocation of NF-κB in 786-O cells. 
 Perturbation of actin dynamics induces NF-kappaB activation in myelomonocytic cells through an NADPH oxidase-dependent pathway. Although several reports showed the effect of compounds disrupting microtubules on NF-kappaB (nuclear factor kappaB) activation, nothing is known about agents perturbing actin dynamics. In the present study, we have shown that actin cytoskeleton disruption induced by actin-depolymerizing agents such as cytochalasin D and latrunculin B and actin-polymerizing compounds such as jasplakinolide induced NF-kappaB activation in myelomonocytic cells. The transduction pathway involved the IkappaB (inhibitory kappaB) kinase complex and a degradation of IkappaBalpha. We have shown that NF-kappaB activation in response to the perturbation of actin dynamics required reactive oxygen species, as demonstrated by the effect of antioxidants. Actin cytoskeleton disruption by cytochalasin D induced O2- release from human monocytes, through the activation of the NADPH oxidase, as confirmed by the phosphorylation and by the membrane translocation of p47phox. NF-kappaB activation after actin cytoskeleton disruption could be physiologically relevant during monocyte activation and/or recruitment into injured tissues, where cellular attachment,   migration   and phagocytosis result in cyclic shifts in cytoskeletal organization and disorganization. 
 MCPIP1 Regulates Fibroblast   Migration   in 3-D Collagen Matrices Downstream of MAP Kinases and NF-κB. The fibroblast-populated three-dimensional (3-D) collagen matrix has been used to model matrix contraction, cell motility, and general fibroblast biology. MCPIP1 (monocyte chemotactic protein-induced protein 1) has been shown to regulate inflammation, angiogenesis, and cellular motility. In the present study, we demonstrated induction of MCPIP1 in human fibroblasts embedded in the stress-released 3-D collagen matrix, which occurred through activation of mitogen-activated protein kinases, phosphoinositide 3-kinase, and NF-κB. Furthermore, MCPIP1 induction was associated with inhibition of fibroblas  t migrati  on out of the nested collagen matrix. MCPIP1 induction or ectopic expression also upregulated p53. RNA interference of p53 prevented the inhibition o  f migrati  on produced by induction or ectopic expression of MCPIP1. Our findings suggest a new role for MCPIP1 as a molecular switch that regulates fibroblas  t migrati  on in the nested collagen matrix model. 
 12-O-tetradecanoylphorbol-13-acetate-induced invasion/  migration   of glioblastoma cells through activating PKCalpha/ERK/NF-kappaB-dependent MMP-9 expression. An increase in MMP-9 gene expression and enzyme activity with stimulating the   migration   of GBM8401 glioma cells via wound healing assay by 12-O-tetradecanoylphorbol-13-acetate (TPA) was detected in glioblastoma cells GBM8401. TPA-induced translocation of protein kinase C (PKC)alpha from the cytosol to membranes, and   migration   of GBM8401 elicited by TPA was suppressed by adding the PKCalpha inhibitors, GF109203X and H7. Activation of extracellular signal-regulated kinase (ERK) and c-Jun-N-terminal kinase (JNK) by TPA was identified, and TPA-induced   migration   and MMP-9 activity was significantly blocked by ERK inhibitor PD98059 and U0126, but not JNK inhibitor SP600125. Activation of NF-kappaB protein p65 nuclear translocation and IkappaBalpha protein phosphorylation with increased NF-kappaB-directed luciferase activity by TPA were observed, and these were blocked by the PD98059 and IkB inhibitor BAY117082 accompanied by reducing   migration   and MMP-9 activity induced by TPA in GBM8401 cells. Transfection of GBM8401 cells with PKCalpha siRNA specifically reduced PKCalpha protein expression with blocking TPA-induced MMP-9 activation and   migration  . Additionally, suppression of TPA-induced PKCalpha/ERK/NK-kappaB activation,   migration  , and MMP-9 activation by flavonoids including kaempferol (Kae; 3,5,7,4'-tetrahydroxyflavone), luteolin (Lut; 5,7,3'4'-tetrahydroxyflavone), and wogonin (Wog; 5,7-dihydroxy-8-methoxyflavone) was demonstrated, and structure-activity relationship (SAR) studies showed that hydroxyl (OH) groups at C4' and C8 are critical for flavonoids' action against MMP-9 enzyme activation and   migration  /invasion of glioblastoma cells elicited by TPA. Application of flavonoids to prevent the   migration  /invasion of glioblastoma cells through blocking PKCalpha/ERK/NF-kappaB activation is first demonstrated herein. 
 PF-3758309, p21-activated kinase 4 inhibitor, suppresses   migration   and invasion of A549 human lung cancer cells via regulation of CREB, NF-κB, and β-catenin signalings  . Migrati  on and invasion comprise key steps in cancer metastasis. Through th  e migrati  on and invasion into and out of lymphatic and/or blood vessels, cancer cells can be spread out into the tissues in remote site from the origin. Degradation of extracellular matrix (ECM) must be preceded prior to the metastasis of cancer cells. Matrix metalloproteinases (MMP) can degrade ECM, thus allow cells to migrate from the original site. Among MMPs, two gelatinase MMP-2 and MMP-9 play particularly important roles in ECM degradation. Here, we report that recently developed p21-activated kinase 4 inhibitor PF-3758309 shows anti-metastatic effect in A549 human lung cancer cell. PF-3758309 suppresses CREB, NF-κB, and β-catenin pathways, which are well known to be closely related with c  ell migra  tion. This leads to the downregulation of MMP-2/MMP-9 expressions and the inhibition of A549 lung cancer metastasis. 
 Knockdown of Radixin Suppresses Gastric Cancer Metastasis In Vitro by Up-Regulation of E-Cadherin via NF-κB/Snail Pathway. Radixin has recently been shown to correlate with the metastasis of gastric cancer, but the pathogenesis is elusive. Adhesion proteins contribute to the regulation of metastasis, and thus this study sought to investigate the role of radixin in the   migratio  n, invasion and adhesion of gastric cancer cells, as well as its interaction with adhesion proteins in vitro.Radixin stable knockdown human gastric carcinoma SGC-7901 cells were constructed. Alterations in the   migratio  n, invasion and adhesion ability were examined by matrigel-coated plate and transwell assays. The expression pattern of adhesion proteins, including E-cadherin, β-catenin and claudin-1, was determined by quantitative real-time PCR and western blot. Possible involvement of NF-κB/snail pathway was also evaluated.Stable knockdown of radixin significantly suppress  ed migrat  ion and invasion, but enhanced adhesion in SGC-7901 cells. The expression of E-cadherin was manifestly increased in radixin knockdown cells, whereas the expression of β-catenin and claudin-1 was unchanged. The nuclear exclusion of NF-κB followed by conspicuous reduction of snail expression was involved in the regulation of E-cadherin expression.Radixin knockdown suppresses the metastasis of SGC-7901 cells in vitro by up-regulation of E-cadherin. The NF-κB/snail pathway contributes to the regulation of E-cadherin in response to depletion of radixin. 
 CXCR2-driven ovarian cancer progression involves upregulation of proinflammatory chemokines by potentiating NF-κB activation via EGFR-transactivated Akt signaling. Ovarian cancer is an inflammation-associated malignancy with a high mortality rate. CXCR2 expressing ovarian cancers are aggressive with poorer outcomes. We therefore investigated molecular mechanisms involved in CXCR2-driven cancer progression by comparing CXCR2 positive and negative ovarian cancer cell lines. Stably CXCR2 transfected SKOV-3 cells had a faster growth rate as compared to control cells transfected with empty vector. Particularly, tumor necrosis factor (TNF), abundantly expressed in ovarian cancer, enhanced cell proliferation by decreasing the G0-G1 phase in CXCR2 transfected cells. TNF increased nuclear factor-κB (NF-κB) activity to a greater degree in CXCR2 transfected cells than control cells as well as provided a greater activation of IκB. CXCR2 transfected cells expressed higher levels of its proinflammatory ligands, CXCL1/2 and enhanced more proliferati  on, migra  tion, invasion and colony formation. CXCR2 positive cells also activated more EGFR, which led to higher Akt activation. Enhanced NF-κB activity in CXCR2 positive cells was reduced by a PI3K/Akt inhibitor rather than an Erk inhibitor. CXCL1 added to CXCR2 positive cells led to an increased activation of IκB. CXCL1 also led to a significantly greater number of invasive cells in CXCR2 transfected cells, which was blocked by the NF-κB inhibitor, Bay 11-7082. In addition, enhanced cell proliferation in CXCR2 positive cells was more sensitive to CXCL1 antibody or an NF-κB inhibitor. Finally, CXCR2 transfection of parental cells increased CXCL1 promoter activity via an NF-κB site. Thus augmentation of proinflammatory chemokines CXCL1/2, by potentiating NF-κB activation through EGFR-transactivated Akt, contributes to CXCR2-driven ovarian cancer progression. 
 Crebanine, an aporphine alkaloid, sensitizes TNF-α-induced apoptosis and suppressed invasion of human lung adenocarcinoma cells A549 by blocking NF-κB-regulated gene products. Crebanine is an alkaloid known to exhibit anticancer, but its mechanism is not well understood. Besides, the nuclear factor-kappa B (NF-κB) transcription factor has been correlated with inflammation, carcinogenesis, tumor cell survival, invasion, and angiogenesis. In this study, we investigated the effects of crebanine on tumor necrosis factor alpha (TNF-α)-induced NF-κB activation and the expression of NF-κB-regulated gene products. We found that crebanine reduced the cell proliferation of lung, ovarian, and breast cancer cells. Crebanine also potentiated TNF-α-induced apoptosis which correlated with the suppression of the gene products linked to cell survival, B cell lymphoma-extra large, and proliferation, cyclin D1. In addition, crebanine affected TNF-α-induced activation of caspase-8, caspase-3, and poly(ADP-ribose) polymerase cleavage, indicating that the apoptotic effects of TNF-α were enhanced by crebanine. Moreover, crebanine reduced TNF-α-induced A549 cell inv  asion and   migration. Furthermore, crebanine suppressed the TNF-α-mediated expression of proteins that involved cancer cell invasion (matrix metalloproteinase 9 urokinase-type plasminogen activator, urokinase-type plasminogen activator receptor and intercellular adhesion molecule 1) and angiogenesis (COX-2 and VEGF), all of which are known to be regulated by NF-κB. We also demonstrated that TNF-α induced NF-κB DNA-binding activity, which was inhibited by crebanine. Moreover, crebanine suppressed the TNF-α-induced degradation of inhibitor of NF-κB alpha (IκBa), which led to reduced NF-κB translocation to the nucleus. Taken together, our results demonstrated that crebanine reduced TNF-α-induced cancer cell proliferation, invasion, and survival by suppressing NF-κB activity and expression profile of its downstream genes. 
 NF-kappaB and epithelial to mesenchymal transition of cancer. During progression of an in situ to an invasive cancer, epithelial cells lose expression of proteins that promote cell-cell contact, and acquire mesenchymal markers, which promote cell   migration   and invasion. These events bear extensive similarities to the process of epithelial to mesenchymal transition (EMT), which has been recognized for several decades as critical feature of embryogenesis. The NF-kappaB family of transcription factors plays pivotal roles in both promoting and maintaining an invasive phenotype. After briefly describing the NF-kappaB family and its role in cancer, in this review we will first describe studies elucidating the functions of NF-kappaB in transcription of master regulator genes that repress an epithelial phenotype. In the second half, we discuss the roles of NF-kappaB in control of mesenchymal genes critical for promoting and maintaining an invasive phenotype. Overall, NF-kappaB is identified as a key target in prevention and in the treatment of invasive carcinomas. 
 Overexpression of RRM2 in gastric cancer cell promotes their invasiveness via AKT/NF-κB signaling pathway. The ribonucleotide reductase M2 subunit (RRM2) plays an active role in tumor progression and is frequently overexpressed in cancer. It plays a significant role in the regulation of cell invasiveness, cell   migratio  n and tumor metastasis. Elevated RRM2 expression has been reported to be associated with poor prognosis of gastric cancer. However, the molecular mechanisms of RRM2 in gastric cancer cells remain elusive. In our study, we found that RRM2 highly expressed in gastric cancer cells BGC823. RRM2 stimulation dose-dependently enhanced the invasion and   migratio  n of BGC823 cells. Furthermore, we found that the expressions of MMP-2 and MMP-9 in BGC823 cells were significantly increased after RRM2 stimulation. In addition, RRM2 time-dependently induced activation of AKT, IKBα, and NF-κB. These effects of RRM2 were prevented by AKT selective inhibitor GSK690693 as well as NF-κB selective inhibitor BAY117082. In conclusion, our findings establish a signaling role for RRM2 in gastric cancer cells and identify that the RRM2/AKT/NF-κB signaling pathway is essential for tumor invasiveness in gastric cancer cells. Thus, our data may provide knowledge for using RRM2 as a novel target for effective diagnosis and treatment of gastric cancer. 
 Hypoxia increases CX3CR1 expression via HIF-1 and NF‑κB in androgen-independent prostate cancer cells. The unique CX3C chemokine CX3CL1 and its cognate receptor CX3CR1 have been implicated in organ-specific metastasis of various types of tumors. Hypoxia, a common phenomenon in solid tumors, is associated with a malignant cancer phenotype. Previous studies have proved that hypoxia facilitates cancer cell metastasis through upregulation of specific chemokine receptors. We hypothesized that hypoxia could upregulate CX3CR1 expression and lead to an increased chemotactic response to CX3CL1 in prostate cancer cells. In the present study, we found that CX3CR1 expression was significantly increased in androgen-independent prostate cancer cells, including DU145, PC-3 and PC-3M, following exposure to hypoxia. This upregulation of CX3CR1 corresponded to a significant increase   in migrat  ion and invasion of prostate cancer cells under hypoxic conditions, which was attenuated after knocking down CX3CR1 expression. In addition, we examined the possible role of HIF-1 and NF-κB in the process of hypoxia-induced CX3CR1 expression and hypoxia-mediated metastasis. Attenuation of HIF-1 and NF-κB transcriptional activity by siRNAs or pharmacological inhibitors, abrogated hypoxia-induced upregulation of CX3CR1, and also prevented   the migr  ation and invasion of DU145 cells under a hypoxic environment. In summary, our study demonstrated that HIF-1 and NF-κB are essential for hypoxia-regulated CX3CR1 expression, which is associated with increased migratory and invasive potential of prostate cancer cells. CX3CR1 signaling is a potential therapeutic target in the adjuvant treatment of prostate cancer. 
 Deregulation of NF-kappaB and its upstream kinases in cancer. The transcription factor NF-kappaB is known for its function in regulating immune and inflammatory responses. However, recent evidence suggests that NF-kappaB also plays a pivotal role in controlling cell proliferation, apoptosis, and cell   migration  . Deregulated activation of NF-kappaB has been observed in various cancers. Over the past few years. significant progress has been made to elucidate the mechanisms of NF-kappaB activation in both normal and cancer cells. Notably, a large number of protein kinases have been shown to stimulate NF-kappaB activity under different conditions, and some of these kinases are aberrantly activated in cancer cells. In this review, we discuss our current knowledge of NF-kappaB activation, with a focus on the NF-kappaB-activating kinases and their roles in cancer formation. 
 Guggulsterone enhances antitumor activity of gemcitabine in gallbladder cancer cells through suppression of NF-κB. Patients with gallbladder cancer usually have a poor prognosis, and effective standard chemotherapeutic regimens have not been established. The anticancer activities of guggulsterone have been demonstrated in various cancer cells. The aims of the study were to determine the effect of guggulsterone on gallbladder cancer cells and to investigate whether treatment with guggulsterone influences the antitumor activities of gemcitabine.The Dojindo Cell Counting Kit-8 assay was used to determine the inhibition of proliferation by drugs in TGBC1 and TGBC2 cells. Cell   migratio  n and invasion were examined using 24-well inserts and Matrigel™-coated invasion chambers. The activities of NF-κB p65, VEGF-C, and MMP-2 were measured by ELISA.Guggulsterone inhibited the proliferation and suppres  sed migra  tion and invasion of gallbladder cancer cells in a dose-dependent manner. Guggulsterone significantly decreased NF-κB p65, VEGF-C, and MMP-2 activities in the gallbladder cancer cells examined. Gallbladder cancer cells treated with a combination of guggulsterone and gemcitabine demonstrated significant inhibition of cell proliferation and invasion when compared to treatment with gemcitabine alone. In addition, NF-κB p65 activation decreased significantly in cells treated with a combination of guggulsterone and gemcitabine when compared to treatment with gemcitabine alone.Guggulsterone exhibits anticancer activities and enhances the antitumor activities of gemcitabine through the suppression of NF-κB activation in gallbladder cancer cells. These results suggest that guggulsterone could be a potential therapeutic option for patients with gallbladder cancer. 
 Brain endothelial miR-146a negatively modulates T-cell adhesion through repressing multiple targets to inhibit NF-κB activation. Pro-inflammatory cytokine-induced activation of nuclear factor, NF-κB has an important role in leukocyte adhesion to, and subsequen  t migrati  on across, brain endothelial cells (BECs), which is crucial for the development of neuroinflammatory disorders such as multiple sclerosis (MS). In contrast, microRNA-146a (miR-146a) has emerged as an anti-inflammatory molecule by inhibiting NF-κB activity in various cell types, but its effect in BECs during neuroinflammation remains to be evaluated. Here, we show that miR-146a was upregulated in microvessels of MS-active lesions and the spinal cord of mice with experimental autoimmune encephalomyelitis. In vitro, TNFα and IFNγ treatment of human cerebral microvascular endothelial cells (hCMEC/D3) led to upregulation of miR-146a. Brain endothelial overexpression of miR-146a diminished, whereas knockdown of miR-146a augmented cytokine-stimulated adhesion of T cells to hCMEC/D3 cells, nuclear translocation of NF-κB, and expression of adhesion molecules in hCMEC/D3 cells. Furthermore, brain endothelial miR-146a modulates NF-κB activity upon cytokine activation through targeting two novel signaling transducers, RhoA and nuclear factor of activated T cells 5, as well as molecules previously identified, IL-1 receptor-associated kinase 1, and TNF receptor-associated factor 6. We propose brain endothelial miR-146a as an endogenous NF-κB inhibitor in BECs associated with decreased leukocyte adhesion during neuroinflammation. 
 Loss of BRMS1 promotes a mesenchymal phenotype through NF-κB-dependent regulation of Twist1. Breast cancer metastasis suppressor 1 (BRMS1) is downregulated in non-small cell lung cancer (NSCLC), and its reduction correlates with disease progression. Herein, we investigate the mechanisms through which loss of the BRMS1 gene contributes to epithelial-to-mesenchymal transition (EMT). Using a short hairpin RNA (shRNA) system, we show that loss of BRMS1 promotes basal and transforming growth factor beta-induced EMT in NSCLC cells. NSCLC cells expressing BRMS1 shRNAs (BRMS1 knockdown [BRMS1(KD)]) display mesenchymal characteristics, including enhanced cell   migratio  n and differential regulation of the EMT markers. Mesenchymal phenotypes observed in BRMS1(KD) cells are dependent on RelA/p65, the transcriptionally active subunit of nuclear factor kappa B (NF-κB). In addition, chromatin immunoprecipitation analysis demonstrates that loss of BRMS1 increases Twist1 promoter occupancy of RelA/p65 K310-a key histone modification associated with increased transcription. Knockdown of Twist1 results in reversal of BRMS1(KD)-mediated EMT phenotypic changes. Moreover, in our animal model, BRMS1(KD)/Twist1(KD) double knockdown cells were less efficient in establishing lung tumors than BRMS1(KD) cells. Collectively, this study demonstrates that loss of BRMS1 promotes malignant phenotypes that are dependent on NF-κB-dependent regulation of Twist1. These observations offer fresh insight into the mechanisms through which BRMS1 regulates the development of metastases in NSCLC. 
 NF-kappaB promotes epithelial-mesenchymal transition,   migration   and invasion of pancreatic carcinoma cells. The transcription factor NF-kappaB is constitutively active in pancreatic adenocarcinoma. Here we explore the contribution of NF-kappaB to the malignant phenotype of pancreatic cancer cells in addition to its anti-apoptotic role. Block of NF-kappaB signalling by non-destructible IkappaBalpha rendered cells resistant to TGF-beta-induced epithelial-mesenchymal transition (EMT). In contrast, NF-kappaB activation by TNF-alpha or expression of constitutively active IKK2 induced an EMT-phenotype with up-regulation of vimentin and ZEB1, and down-regulation of E-cadherin. EMT could also be induced in cells with defective TGF-beta signalling. Functional assays demonstrated reduced or strongly enhanced   migration   and invasion upon NF-kappaB inhibition or activation, respectively. 
 EGCG targeting efficacy of NF-κB downstream gene products is dictated by the monocytic/macrophagic differentiation status of promyelocytic leukemia cells. Central nervous system infiltration by circulating leukemic cells and enhanced in vitro transendothelial   migratio  n of promyelocytic leukemia HL-60-derived macrophages through a blood-brain barrier model was recently demonstrated. The intrinsic molecular and signaling mechanisms involved are, however, poorly documented. Drug targeting of such translocation event performed by circulating microbes and immune cells may prevent secondary cerebral infections and development of brain pathologies. In this study, we specifically investigated the in vitro targeting efficacy of the chemopreventive and dietary-derived epigallocatechin-3-gallate (EGCG) molecule on the NF-κB-mediated transcriptional regulation of a panel of 89 biomarkers associated with promyelocytic HL-60 differentiation into macrophages. NF-κB-mediated signaling during HL-60 macrophage differentiation was reversed by EGCG, in part through reduced IκB phosphorylation and led to the inhibition of moderately to highly expressed NF-κB gene targets among which the matrix metalloproteinase (MMP)-9 and the cyclooxygenase (COX)-2. In contrast, EGCG exhibited low efficacy in reversing NF-κB-regulated genes and showed selective antagonism toward COX-2 expression while that of MMP-9 remained high in terminally differentiated macrophages. Decreased expression of the 67-kDa non-integrin Laminin Receptor in terminally differentiated macrophages may explain such differential EGCG efficacy. Our results suggest that terminally differentiated macrophage transendoth  elial mig  ration associated with neuroinflammation may not be pharmacologically affected by such a specific class of flavonoid. The differentiation status of a given in vitro cell model must therefore be carefully considered for optimized assessment of therapeutic drugs. 
 Folate deprivation enhances invasiveness of human colon cancer cells mediated by activation of sonic hedgehog signaling through promoter hypomethylation and cross action with transcription nuclear factor-kappa B pathway. Low folate status is well recognized as one of the metabolic stressors for colorectal cancer carcinogenesis, but its role in colon cancer invasion remains unknown. Activation of the Sonic hedgehog (Shh) signal in interaction with the transcription nuclear factor-kappa B (NF-κB) pathway is crucial for cancer aggressiveness. The aims of this study were to investigate whether and how folate deprivation promotes invasion by colon cancer cells in relation to Shh signaling and NF-κB pathway activation. Cultivation of epithelial colon carcinoma-derived cells (HCT116) in folate-deficient (FD) medium enhanced cellula  r migrati  on and invasion, in correlation with epithelial-mesenchymal transition (EMT) associated with Snail expression and E-cadherin suppression, increased production of β1 integrin and increased proteolysis by matrix metalloproteinase 2. Blockade of Shh signaling by cyclopamine (CYC) or of NF-κB activation by BAY abolished FD-enhanced EMT and invasion by HCT116 cells. FD cells had 50-80% less intracellular folate, associated with aberrant hypomethylation of the Shh promoter, than control cells, and increased binding of nuclear NF-κB subunit p65 to the Shh promoter region, which coincided with increased Shh expression and protein production of Shh ligand; in addition, the FD-induced Shh signaling targeted Gli1 transcription activator as well as Ptch receptor. The FD-induced Shh induction and activated signaling were blocked by NF-κB inhibitor BAY. Blockade of Shh signaling abrogated FD-promoted NF-κB activation measured by IκBα degradation and by target gene TNFα expression. Taken together, these findings demonstrate that folate deprivation enhanced invasiveness of colon cancer cells mediated by activation of Shh signaling through promoter hypomethylation and cross actions with the NF-κB pathway. 
 Laryngeal squamous cell carcinoma progression is associated with the NFκB signaling pathway regulated by IκB kinase β. We aimed to analyze the association of NFκB signaling pathway with the carcinogenesis of laryngeal squamous cell carcinoma (LSCC).Protein array was used to identify the differentially expressed proteins involved in NFκB signaling pathway between LSCC cells and normal throat mucosa. Correlation analysis between significantly expressed proteins and clinical characteristics (differentiation, clinical stage, and node metastasis) was performed. The expression of IκB kinase-β (IKK-β) in Hep-2 cells transfected with IKK-β-siRNA or pcDNA3.1-IKK-β was detected both by real-time polymerase chain reaction and western blot. Besides, a tetrazolium-based colorimetric assay (MTT), flow cytometer, and transwell assay were used to examine the proliferation, apopt  osis, and   migration rate of Hep-2 cells, respectively.Three differentially expressed proteins were identified. Among them, tumor necrosis factor receptor 1 (TNFR1) and IKK-β were significantly up-regulated (p < 0.01) and Fas-associated via death domain (FADD) was significantly down-regulated (p < 0.01). The correlation analysis showed that IKK-β expression had a significant association with differentiation, clinical stage, and node metastasis (p < 0.05). Besides, high expressed IKK-β resulted in significantly increased proli  feration   and migration rate (p < 0.05). Reversely, Hep-2 cells transfected with IKK-β-siRNA showed significantly lower prol  iferation   and migration rate (p < 0.05) and significantly higher apoptosis rate (p < 0.05) than normal cells.The NFκB signaling pathway involved TNFR1, IKK-β, and FADD is significantly associated with the development of LSCC. Over-expressed IKK-β efficiently inhibits cell apoptosis and has positive effects on cell p  roliferat  ion and migration. 
 TGF-β and NF-κB signal pathway cross-talk is mediated through TAK1 and SMAD7 in a subset of head and neck cancers. Transforming growth factor-beta (TGF-β) has a dual role in epithelial malignancies, including head and neck squamous cell carcinoma (HNSCC). Attenuation of canonical TGF-β signaling enhances de novo tumor development, whereas TGF-β overexpression and signaling paradoxically promotes malignant progression. We recently observed that TGF-β-induced growth arrest response is attenuated, in association with aberrant activation of nuclear factor-κB (NF-κB), a transcription factor, which promotes malignant progression in HNSCC. However, what role cross-talk between components of the TGF-β and NF-κB pathways plays in altered activation of these pathways has not been established. Here, we show TGF-β receptor II and TGF-β-activated kinase 1 (TAK1) are predominantly expressed in a subset of HNSCC tumors with nuclear activation of NF-κB family member RELA (p65). Further, TGF-β1 treatment induced sequential phosphorylation of TAK1, IKK, IκBα and RELA in human HNSCC lines. TAK1 enhances TGF-β-induced NF-κB activation, as TAK1 siRNA knockdown decreased TGF-β1-induced phosphorylation of IKK, IκB and RELA, degradation of IκBα, RELA nuclear translocation and DNA binding, and NF-κB-induced reporter and target gene transcription. Functionally, TAK1 siRNA inhibit  ed cell p  roliferation, migration and invasion. Celastrol, a TAK1 inhibitor and anti-inflammatory compound used in traditional Chinese medicine, also decreased TGF-β1-induced phosphorylation of TAK1 and RELA, and suppressed basal, TGF-β1- and tumor necrosis factor-alpha (TNF-α)-induced NF-κB reporter gene activity. Celastrol also inhibited cell proliferation, while increasing sub-G0 DNA fragmentation and Annexin V markers of apoptosis. Furthermore, TGF-β and RELA activation promoted SMAD7 expression. In turn, SMAD7 preferentially suppressed TGF-β-induced SMAD and NF-κB reporters when compared with constitutive or TNF-α-induced NF-κB reporter gene activation. Thus, cross-talk by TGF-β via TAK1 and NF-κB promotes the malignant phenotype of HNSCC. Moreover, NF-κB may contribute to the downstream attenuation of canonical TGF-β signaling through increased SMAD7 expression. Celastrol highlights the therapeutic potential of agents targeting TAK1 as a key node in this pro-oncogenic TGF-β-NF-κB signal pathway. 
 Autocrine CCL5 signaling promotes invasion and   migration   of CD133+ ovarian cancer stem-like cells via NF-κB-mediated MMP-9 upregulation. The concept of cancer stem cells (CSCs) proposes that solely CSCs are capable of generating tumor metastases. However, how CSCs maintain their invasion and   migratio  n abilities, the most important properties of metastatic cells, still remains elusive. Here we used CD133 to mark a specific population from human ovarian cancer cell line and ovarian cancer tissue and determined its hyperactivity in   migratio  n and invasion. Therefore, we labeled this population as cancer stem-like cells (CSLCs). In comparison to CD133- non-CSLCs, chemokine CCL5 and its receptors, CCR1, CCR3, and CCR5, were consistently upregulated in CSLCs, and most importantly, blocking of CCL5, CCR1, or CCR3 effectively inhibits the invasive capacity of CSLCs. Mechanistically, we identified that this enhanced invasiveness is mediated through nuclear factor κB (NF-κB) activation and the consequently elevated MMP9 secretion. Our results suggested that the autocrine activation of CCR1 and CCR3 by CCL5 represents one of major mechanisms underlying the metastatic property of ovarian CSLCs. 
 Hepatitis B virus X protein upregulates expression of calpain small subunit 1 via nuclear factor-kappaB/p65 in hepatoma cells. Hepatitis B virus (HBV) infection is closely correlated with the development of hepatocellular carcinoma (HCC), in which hepatitis B virus X protein (HBx) plays crucial roles. HBx is believed to be a multifunctional oncoprotein. It has been reported that the calpain small subunit 1 (Capn4) is upregulated in the HCC tissues and involved in the metastasis of HCC. Therefore, we suppose that HBx may promote hepatoma cell   migration   through Capn4. In the present study, we investigated the effect of HBx on regulating Capn4 expression in human HCC cells. Our data showed that HBx could increase promoter activity of Capn4 and upregulate the expression of Capn4 at the levels of mRNA and protein in human hepatoma HepG2 (or H7402) cells using luciferase reporter gene assay, real-time quantitative RT-PCR assay and Western blot analysis. While, the RNA interference targeting HBx mRNA was able to abolish the upregulation. Interestingly, we found that the inhibition of nuclear factor-kappaB (NF-kappaB) mediated by siRNA targeting NF-kappaB/p65 mRNA or PDTC (an inhibitor of NF-kappaB) could attenuate the upregulation of Capn4. While, HBx failed to increase the promoter activity of Capn4 in hepatoma cells when the putative NF-kappaB binding site of the Capn4 promoter was mutant, suggesting that NF-kappaB is involved in the activation of Capn4 mediated by HBx. In function, wound healing assay showed that HBx could significantly enhance the   migration   ability of HepG2 cells through upregulating Capn4. Thus, we conclude that HBx upregulate Capn4 through NF-kappaB/p65 to promote   migration   of hepatoma cells. 
 Integrin-mediated transcriptional activation of inhibitor of apoptosis proteins protects smooth muscle cells against apoptosis induced by degraded collagen. Apoptosis of smooth muscle cells (SMC) and degradation of the extracellular matrix (ECM) have both been implicated in atherosclerotic plaque rupture. We have previously reported that degraded type I collagen fragments induce a rapid but transient apoptotic burst initiated by calpains in SMC. The aim of the current study was to identify the pathway responsible for consecutive SMC survival. We show that exposure of SMC to collagen fragments resulted in a sustained activation of nuclear factor (NF)-kappaB via phosphorylation and degradation of IkappaBalpha. Its prevention through retroviral expression of superrepressor IkappaBalpha or proteasome inhibition potently induced apoptosis. In the presence of blocking antibodies to alpha(v)beta(3) integrin and RGD peptides, collagen fragments no longer activated NF-kappaB and apoptosis was enhanced. The mechanism by which NF-kappaB was protecting SMC against collagen fragment-induced apoptosis was a transcriptional activation of several endogenous caspase inhibitors of the inhibitor of apoptosis protein (IAP) family as: (1) the expression of xIAP, c-IAP2, and survivin was potently induced by collagen fragments; (2) IAP expression was abrogated by superrepressor IkappaBalpha; and (3) knockdown of each of the 3 IAPs by small interfering RNA (siRNA) resulted in enhanced apoptosis after collagen fragment treatment. Our data suggest that SMC exposed to degraded collagen are protected against apoptosis by a mechanism involving alpha(v)beta(3)-dependent NF-kappaB activation with consequent activation of IAPs. This may constitute a novel antiapoptotic pathway ensuring SMC survival in settings of enhanced ECM degradation such as cell   migration  , vascular remodeling, and atherosclerotic plaque rupture. 
 Prognostic significance of nuclear factor-kappaB p105/p50 in human melanoma and its role in cell   migration  . Transcriptional factor nuclear factor-kappaB (NF-kappaB) family has been shown to play an important role in tumor pathogenesis and serve as a potential target in cancer therapy. However, it is necessary to clarify the specific functions of NF-kappaB members, which would provide the basis for the selective blockade and reduction of therapeutic side effects resulting from unspecific inhibition of NF-kappaB members. In this study, we explored the role of NF-kappaB p105/p50 in melanoma pathogenesis in vitro and in vivo. We found that the expression of NF-kappaB p105/p50 significantly increased in dysplastic nevi, primary melanoma, and metastatic melanoma compared with normal nevi (P = 0.0004, chi(2) test). Furthermore, NF-kappaB p105/p50 nuclear staining increased with melanoma progression and strong NF-kappaB p105/p50 nuclear staining was inversely correlated with disease-specific 5-year survival of patients with tumor thickness >2.0 mm (P = 0.014, log-rank test). Multivariate Cox regression analysis revealed that nuclear expression of NF-kappaB p105/p50 is an independent prognostic factor in this subgroup. Moreover, we found that up-regulation of NF-kappaB p50 enhanced melanoma cell   migration  , whereas small interfering RNA knockdown inhibited cell   migration  . In addition, overexpression of NF-kappaB p50 induced RhoA activity and Rock-mediated formation of stress fiber in melanoma cells. Taken together, our data indicate that NF-kappaB p105/p50 may be an important marker for human melanoma progression and prognosis as well as a potentially selective therapeutic target. 
 Survival advantage of EBV-associated gastric carcinoma: survivin up-regulation by viral latent membrane protein 2A. EBV-associated gastric carcinoma is a distinct subset of gastric carcinoma infected with EBV, which shows latency I type expression of EBV latent genes (EBNA1, EBER, BARF0, and LMP2A). To clarify the role of EBV in this type of gastric carcinoma, the cell biological characteristics (growth, apoptosis, and   migration  ) were evaluated in gastric carcinoma cell lines (MKN-1, TMK1, MKN-74 and MKN-7) with and without infection of recombinant EBV harboring the neomycin resistance gene. The infection reiterated the latency I type infection, and the only difference observed in EBV-infected gastric carcinoma cell lines was the resistance to serum deprivation-induced apoptosis. Comparative analyses of transcripts of apoptosis-associated genes in MKN-1 and EBV-MKN-1 and subsequent quantitative reverse transcription-PCR analysis showed up-regulation of the cellular survivin gene in EBV-infected gastric carcinoma cell lines. Small interfering RNA-mediated knockdown of survivin increased apoptosis in EBV-MKN-1 to the level of the original MKN-1 cells. Transfection of EBV-latent genes into MKN-1 showed that LMP2A, but not EBNA1, EBER, or BARF0, up-regulated survivin gene expression. LMP2A-mediated survivin up-regulation in gastric carcinoma cells was inhibited with a nuclear factor-kappaB (NF-kappaB) inhibitor, Bay 11-7082. In parallel with these findings in vitro, survivin expression was frequent in carcinoma tissues of gastric carcinoma by immunohistochemistry, and significantly more in EBV-associated gastric carcinoma (12 of 13) than in EBV-negative gastric carcinoma in the advanced stage (P = 0.0307). Thus, EBV uses its latent protein, LMP2A, to activate the NF-kappaB-survivin pathway to rescue EBV-infected epithelial cells from serum deprivation, and up-regulation of survivin may play a role in the progression of this specific type of gastric carcinoma infected with EBV. 
 TNFα and IFNγ synergistically enhance transcriptional activation of CXCL10 in human airway smooth muscle cells via STAT-1, NF-κB, and the transcriptional coactivator CREB-binding protein. Asthmatic airway smooth muscle (ASM) expresses interferon-γ-inducible protein-10 (CXCL10), a chemokine known to mediate mast c  ell migra  tion into ASM bundles that has been reported in the airways of asthmatic patients. CXCL10 is elevated in patients suffering from viral exacerbations of asthma and in patients with chronic obstructive pulmonary disease (COPD), diseases in which corticosteroids are largely ineffective. IFNγ and TNFα synergistically induce CXCL10 release from human ASM cells in a steroid-insensitive manner, via an as yet undefined mechanism. We report that TNFα activates the classical NF-κB (nuclear factor κB) pathway, whereas IFNγ activates JAK2/STAT-1α and that inhibition of the JAK/STAT pathway is more effective in abrogating CXCL10 release than the steroid fluticasone. The synergy observed with TNFα and IFNγ together, however, did not lie at the level of NF-κB activation, STAT-1α phosphorylation, or in vivo binding of these transcription factors to the CXCL10 promoter. Stimulation of human ASM cells with TNFα and IFNγ induced histone H4 but not histone H3 acetylation at the CXCL10 promoter, although no synergism was observed when both cytokines were combined. We show, however, that TNFα and IFNγ exert a synergistic effect on the recruitment of CREB-binding protein (CBP) to the CXCL10, which is accompanied by increased RNA polymerase II. Our results provide evidence that synergism between TNFα and IFNγ lies at the level of coactivator recruitment in human ASM and suggest that inhibition of JAK/STAT signaling may be of therapeutic benefit in steroid-resistant airway disease. 
 Suppression of LASP-1 attenuates the carcinogenesis of prostatic cancer cell lines: Key role of the NF-κB pathway. Prostate cancer (PCa) is one of the most frequently diagnosed cancers among males worldwide and causes a considerable number of deaths each year. One of the newly explored targets for the development of therapies against PCa is LIM and SH3 protein 1 (LASP-1). In the present study, the function of LASP-1 in the oncogenesis and metastasis of PCa was investigated using a series of in vitro experiments. Moreover, the mechanism through which LASP-1 exerted its effect on the carcinogenesis of PCa was also explored. The expression levels of LASP-1 in clinical PCa specimens were determined both at the mRNA and protein levels. Afterwards, the activity of LASP-1 in human PCa cell lines PC3 and DU145 was inhibited using a short hairpin RNA (shRNA) interfering method. The effects of LASP-1 knockdown on the cell growth, apoptosis, cell cycle distributi  on, migra  tion and invasion were assessed. It was demonstrated that the expression of LASP-1 was significantly higher in the clinical PCa tissues than the level in the corresponding para-carcinoma tissues. Following the knockdown of the LASP-1 gene in human PCa cell lines, the viabili  ty, migra  tion and invasion of the cancer cells were decreased. It was also demonstrated that the change in the cell viability and motile ability were associated with an induction of cell apoptosis and G1 phase cell cycle arrest. Based on the results of the detection of the expression of NF-κB-related factors, it was indicated that LASP-1 may affect the carcinogenesis of PCa through a NF-κB inhibition-dependent manner. Although the detailed explanation of the mechanism of LASP-1 in the carcinogenesis of PCa requires further elucidation, the present study highlights the potential of LASP-1 as a promising therapeutic target to ameliorate the oncogenesis and metastasis of PCa. 
 Activation of NF-kappa B signaling promotes growth of prostate cancer cells in bone. Patients with advanced prostate cancer almost invariably develop osseous metastasis. Although many studies indicate that the activation of NF-κB signaling appears to be correlated with advanced cancer and promotes tumor metastasis by influencing tumor cell   migratio  n and angiogenesis, the influence of altered NF-κB signaling in prostate cancer cells within boney metastatic lesions is not clearly understood. While C4-2B and PC3 prostate cancer cells grow well in the bone, LNCaP cells are difficult to grow in murine bone following intraskeletal injection. Our studies show that when compared to LNCaP, NF-κB activity is significantly higher in C4-2B and PC3, and that the activation of NF-κB signaling in prostate cancer cells resulted in the increased expression of the osteoclast inducing genes PTHrP and RANKL. Further, conditioned medium derived from NF-κB activated LNCaP cells induce osteoclast differentiation. In addition, inactivation of NF-κB signaling in prostate cancer cells inhibited tumor formation in the bone, both in the osteolytic PC3 and osteoblastic/osteoclastic mixed C4-2B cells; while the activation of NF-κB signaling in LNCaP cells promoted tumor establishment and proliferation in the bone. The activation of NF-κB in LNCaP cells resulted in the formation of an osteoblastic/osteoclastic mixed tumor with increased osteoclasts surrounding the new formed bone, similar to metastases commonly seen in patients with prostate cancer. These results indicate that osteoclastic reaction is required even in the osteoblastic cancer cells and the activation of NF-κB signaling in prostate cancer cells increases osteoclastogenesis by up-regulating osteoclastogenic genes, thereby contributing to bone metastatic formation. 
 Connective tissue growth factor enhances the   migration   of gastric cancer through downregulation of E-cadherin via the NF-κB pathway. Local invasion and distant metastasis are difficult problems for surgical intervention and treatment in gastric cancer. Connective tissue growth factor (CTGF/CCN2) was considered to have an important role in this process. In this study, we demonstrated that expression of CTGF was significantly upregulated in clinical tissue samples of gastric carcinoma (GC) samples. Forced expression of CTGF in AGS GC cells promoted their   migratio  n in culture and significantly increased tumor metastasis in nude mice, whereas RNA interference-mediated knockdown of CTGF in GC cells significantly inhibited cell   migratio  n in vitro. We disclose that CTGF downregulated the expression of E-cadherin through activation of the nuclear factor-κappa B (NF-κB) pathway. The effects of CTGF in GC cells were abolished by dominant negative IκappaB. Collectively, these data reported here demonstrate CTGF could modulate the NF-κappaB pathway and perhaps be a promising therapeutic target for gastric cancer invasion and metastasis. 
 Inhibition of adhesive interaction between multiple myeloma and bone marrow stromal cells by PPARgamma cross talk with NF-kappaB and C/EBP. Binding of multiple myeloma (MM) cells to bone marrow stromal cells (BMSCs) triggers expression of adhesive molecules and secretion of interleukin-6 (IL-6), promoting MM cell growth, survival, drug resistance, and   migration  , which highlights the possibility of developing and validating novel anti-MM therapeutic strategies targeting MM cells-host BMSC interactions and their sequelae. Recently, we have found that expression of the peroxisome proliferator-activated receptor gamma (PPARgamma) and its ligands can potently inhibit IL-6-regulated MM cell growth. Here we demonstrate that PPARgamma agonists 15-d-PGJ2 and troglitazone significantly suppress cell-cell adhesive events, including expression of adhesion molecules and IL-6 secretion from BMSCs triggered by adhesion of MM cells, as well as overcome drug resistance by a PPARgamma-dependent mechanism. The synthetic and natural PPARgamma agonists have diverging and overlapping mechanisms blocking transactivation of transcription factors NF-kappaB and 5'-CCAAT/enhancer-binding protein beta (C/EBPbeta). Both 15-d-PGJ2 and troglitazone blocked C/EBPbeta transcriptional activity by forming PPARgamma complexes with C/EBPbeta. 15-d-PGJ2 and troglitazone also blocked NF-kappaB activation by recruiting the coactivator PGC-1 from p65/p50 complexes. In addition, 15-d-PGJ2 had a non-PPARgamma-dependent effect by inactivation of phosphorylation of IKK and IkappaB. These studies provide the framework for PPARgamma-based pharmacological strategies targeting adhesive interactions of MM cells with the bone marrow microenvironment. 
 Ganoderic acids suppress growth and invasive behavior of breast cancer cells by modulating AP-1 and NF-kappaB signaling. Structurally related lanostane-type triterpenes, ganoderic acid A, F and H (GA-A, GA-F, GA-H), were identified in an oriental medicinal mushroom Ganoderma lucidum. In the present study we evaluated the effect of GA-A, GA-H and GA-F on highly invasive human breast cancer cells. We showed that GA-A and GA-H suppressed growth (cell proliferation and colony formation) and invasive behavior (adhesion,   migration   and invasion) of MDA-MB-231 cells. Our results suggest that GA-A and GA-H mediate their biological effects through the inhibition of transcription factors AP-1 and NF-kappaB, resulting in the down-regulation of expression of Cdk4 and the suppression of secretion of uPA, respectively. Furthermore, the activity of ganoderic acids is linked to the hydroxylation in the position 7 and 15 (GA-A) and 3 (GA-H) in their triterpene lanostane structure. In conclusion, hydroxylated triterpenes from G. lucidum could be promising natural agents for the therapy of invasive breast cancers. 
 14-3-3β Promotes   Migratio  n and Invasion of Human Hepatocellular Carcinoma Cells by Modulating Expression of MMP2 and MMP9 through PI3K/Akt/NF-κB Pathway. 14-3-3β has been demonstrated to possess the oncogenic potential, and its increased expression has been detected in multiple types of carcinomas. However, majority of previous studies focused on the role of 14-3-3β in tumor cell proliferation and apoptosis, leaving much to be elucidated about its function in tumor cell invasion and metastasis. Hence, the present study aimed to investigate the role of 14-3-3β in the invasion of hepatocellular carcinoma (HCC) cells and the implications in the prognosis of HCC patients. We first examined the expression of 14-3-3β in the primary tumors of HCC patients with or without portal vein tumor thrombus (PVTT), and found that 14-3-3β expression was higher in the primary tumors with PVTT, and the level was even higher in the PVTTs. Kaplan-Meier curves and multivariate analysis revealed that high expression of 14-3-3β was associated with overall survival (OS) and time to recurrence (TTR) of HCC patients. In addition, ectopic expression of 14-3-3β in HCC cell lines led to   enhanced   migration ability and invasiveness, as well as up-regulation of matrix metalloproteinase 2 and 9, which could be suppressed by inhibiting the activation of Akt and nuclear factor-κB (NF-κB) signaling. Furthermore, we identified a correlated elevation of 14-3-3β and p-Akt in the primary tumors of HCC patients, and showed that a combinatory detection of 14-3-3β and p-Akt could better predict post-surgical outcome of HCC patients. 
 Numbl inhibits glioma cell   migration   and invasion by suppressing TRAF5-mediated NF-κB activation. The Notch signaling regulator Numblike (Numbl) is expressed in the brain, but little is known regarding its role in the pathophysiology of glial cells. In this paper, we report that Numbl expression was down-regulated in high-grade human glioma tissue samples and glioblastoma cell lines. To investigate the role of Numbl in glioma   migratio  n and invasion, we generated human glioma cell lines in which Numbl was either overexpressed or depleted. Overexpression of Numbl suppressed, while elimination of Numbl promoted, the   migratio  n and invasion of glioma cells. Numbl inhibited glioma   migratio  n and invasion by dampening NF-κB activity. Furthermore, Numbl interacted directly with tumor necrosis factor receptor-associated factor 5 (TRAF5), which signals upstream and is required for the activation of NF-κB, and committed it to proteasomal degradation by promoting K48-linked polyubiquitination of TRAF5. In conclusion, our data suggest that Numbl negative regulates glioma ce  ll migrat  ion and invasion by abrogating TRAF5-induced activation of NF-κB. 
 HBXIP upregulates CD46, CD55 and CD59 through ERK1/2/NF-κB signaling to protect breast cancer cells from complement attack. Hepatitis B X-interacting protein (HBXIP) is able to enhance   migratio  n of breast cancer cells. However, the role of HBXIP in regulation of complement-dependent cytotoxicity (CDC) in breast cancer is not understood. Here, we report that HBXIP contributes to protecting breast cancer cells from CDC by upregulating membrane-bound complement regulatory protein (mCRPs), including CD46, CD55 and CD59. We found that HBXIP upregulated mCRPs through activating p-ERK1/2/NF-κB. Interestingly, the knockdown of CD59 was able to block the HBXIP-enhanced breast tumor growth in animal. Thus, we conclude that HBXIP upregulates CD46, CD55 and CD59 through p-ERK1/2/NF-κB signaling to protect breast cancer from CDC. 
 MicroRNA-26b suppresses the metastasis of non-small cell lung cancer by targeting MIEN1 via NF-κB/MMP-9/VEGF pathways. MicroRNAs (miRNAs) are involved in tumor initiation and progression. MiR-26b was reported to be significantly downregulated in non-small cell lung cancer (NSCLC). However, the underlying mechanisms of miR-26b involvement in the development and progression of NSCLC remains poorly understood. In the present study, we report that miR-26b suppresses cell metastasis in NSCLC through targeting   migratio  n and invasion enhancer 1 (MIEN1). We found that miR-26b was significantly downregulated and MIEN1 was significantly upregulated in both NSCLC tissues and cells lines. The expression levels of miR-26b were negatively related to those of MIEN1 mRNA in clinical NSCLC tissues. Furthermore, MIEN1 was confirmed to be a direct target of miR-26b by dual-luciferase reporter assay and MIEN1 expression was downregulated by miR-26b in NSCLC cells. In terms of function, transwell and wound healing assays demonstrated that the miR-26b remarkably inhibited invasion and   migratio  n of NSCLC cells, which was simulated by siRNA knockdown of MIEN1 and reversed by pcDNA/MIEN1 overexpression of MIEN1. Finally, we found that miR-26b could regulate NF-κB/MMP-9/VEGF pathway in NSCLC cells. In conclusion, this study revealed that miR-26b suppresses NSCLC metastasis by targeting MIEN1 via NF-κB/MMP-9/VEGF pathways, implicating a potential prognostic biomarker and therapeutic target for NSCLC treatment. 
 Antrodia salmonea inhibits TNF-α-induced angiogenesis and atherogenesis in human endothelial cells through the down-regulation of NF-κB and up-regulation of Nrf2 signaling pathways. Antrodia salmonea (AS) is known as a traditional Chinese medicine, but very few biological activities have been reported.The present study was aimed to investigate the anti-angiogenic and anti-atherosclerotic potential of the fermented culture broth of AS against tumor necrosis factor-α (TNF-α)-stimulated human endothelial (EA.hy 926) cells.The non-cytotoxic concentrations of AS significantly inhibited TNF-α-ind  uced migr  ation/invasion and capillary-like tube formation in EA.hy 926 cells. Furthermore, AS suppressed TNF-α-induced activity and expression of matrix metalloproteinase-9 (MMP-9), and cell-surface expression of intercellular adhesion molecule-1 (ICAM-1), which was associated with abridged adhesion of U937 leukocytes to endothelial cells. Moreover, AS significantly down-regulated TNF-α-induced nuclear translocation and transcriptional activation of nuclear factor κB (NF-κB) followed by suppression of I-κB degradation and phosphorylation of I-κB kinase-α (IKKα). Notably, the protective effect of AS was directly correlated with the increased expression of hemeoxygenase-1 (HO-1) and γ-glutamylcysteine synthetase (γ-GCLC), which was reasoned by nuclear translocation and transactivation of NF-E2 related factor-2 (Nrf2)/antioxidant response element (ARE). Furthermore, HO-1 knockdown by HO-1-specific shRNA diminished the protective effects of AS on TNF-α-stimulated invasion, tube formation, and U937 adhesion in EA.hy 926 cells.Taken together, these results suggest that Antrodia salmonea may be useful for the prevention of angiogenesis and atherosclerosis. 
 Tyrosine kinase p56lck regulates cell motility and nuclear factor kappaB-mediated secretion of urokinase type plasminogen activator through tyrosine phosphorylation of IkappaBalpha following hypoxia/reoxygenation. Nuclear factor kappaB (NFkappaB) plays major role in regulating cellular responses as a result of environmental injuries. The molecular mechanism(s) by which hypoxia/reoxygenation (H/R) regulates p56lck-dependent activation of NFkappaB through tyrosine phosphorylation of IkappaBalpha and modulates the expression of downstream genes that are involved in cell   migration   in human breast cancer cells are not well defined. In this paper, we investigated the involvement of protein-tyrosine kinase p56lck in the redox-regulated activation of NFkappaB following H/R in highly invasive (MDA-MB-231) and low invasive (MCF-7) breast cancer cells. We demonstrated that H/R induces tyrosine phosphorylation of p56lck, nuclear translocation of NFkappaB, NFkappaB-DNA binding, and transactivation of NFkappaB through tyrosine phosphorylation of IkappaBalpha. Transfection of these cells with wild type Lck but not with mutant Lck F394 followed by H/R induces the tyrosine phosphorylation of inhibitor of nuclear factor kappaB (IkappaBalpha) and transcriptional activation of NFkappaB, and these are inhibited by Lck inhibitors. In vitro kinase assay demonstrated that immunoprecipitated p56lck but not Lyn or Fyn directly phosphorylate IkappaBalpha in presence of H/R. Pervanadate, H2O2, and H/R induce the interaction between Lck and tyrosine-phosphorylated IkappaBalpha, and this interaction is inhibited by Src homology 2 domain inhibitory peptide, suggesting that tyrosine-phosphorylated IkappaBalpha interacts with Src homology 2 domain of Lck. Luciferase reporter gene assay indicated that Lck induces NFkappaB-dependent urokinase type plasminogen activator (uPA) promoter activity in presence of H/R. Furthermore, H/R stimulates the cell motility through secretion of uPA. To our knowledge, this is the first report that p56lck in presence of H/R regulates NFkappaB activation, uPA secretion, and cell motility through tyrosine phosphorylation of IkappaBalpha and further demonstrates an important redox-regulated pathway for NFkappaB activation following H/R injury that is independent of IkappaB kinase/IkappaBalpha-mediated signaling pathways. 
 Glial cell-derived neurotrophic factor increases   migration   of human chondrosarcoma cells via ERK and NF-kappaB pathways. Invasion of tumor cells is the primary cause of therapeutic failure in the treatment of malignant chondrosarcomas. Glial cell-derived neurotrophic factor (GDNF) plays a crucial role in   migration   and metastasis of human cancer cells. Integrins are the major adhesive molecules in mammalian cells. Here we found that GDNF directed the   migration   and increased cell surface expression of alphav and beta3 integrin in human chondrosarcoma cells. Pretreated of JJ012 cells with MAPK kinase (MEK) inhibitors PD98059 or U0126 inhibited the GDNF-mediated   migration   and integrin expression. Stimulation of cells with GDNF increased the phosphorylation of MEK and extracellular signal-regulating kinase (ERK). In addition, NF-kappaB inhibitor (PDTC) or IkappaB protease inhibitor (TPCK) also inhibited GDNF-mediated cells   migration   and integrin up-regulation. Stimulation of cells with GDNF induced IkappaB kinase (IKKalpha/beta) phosphorylation, IkappaB phosphorylation, p65 Ser(536) phosphorylation, and kappaB-luciferase activity. Furthermore, the GDNF-mediated increasing of kappaB-luciferase activity was inhibited by PD98059, U0126, PDTC and TPCK or MEK, ERK, IKKalpha, and IKKbeta mutants. Taken together, these results suggest that the GDNF acts through MEK/ERK, which in turn activates IKKalpha/beta and NF-kappaB, resulting in the activations of alphavbeta3 integrin and contributing the   migration   of human chondrosarcoma cells. 
 Regulation of HSP27 on NF-kappaB pathway activation may be involved in metastatic hepatocellular carcinoma cells apoptosis. During the process of metastasis, cells are subjected to various apoptotic stimuli. Aberrant expression of apoptotic regulators often contribute to cell metastasis. Heat shock protein 27(HSP27) is confirmed as an apoptosis regulator, but its antiapoptotic mechanism in metastatic hepatocellular carcinoma (HCC) cells remains unclear.Levels of HSP27 protein and its phosphorylation in Hep3B, MHCC97L to MHCC97H cells with different metastatic potentials were determined by western blot analysis. MHCC97H cells were transfected with specific small interference RNA (siRNA) against HSP27. The in vitro   migration   and invasion potentials of cells were evaluated by Transwell assay. The apoptosis ratio of MHCC97H cells was analyzed by TUNEL staining and Flow Cytometry. Alteration of signal transduction pathway after HSP27 knockdown in MHCC97H cells was evaluated through a Human Q Series Signal Transduction in Cancer Gene Array analysis. Nuclear NF-kappaB contentration and endogenous IKK activity were demonstrated by ELISA assay. The association of IKKalpha, IKKbeta, IkappaBalpha with HSP27 and the association between IKKbeta and IKKalpha in MHCC97H cells were determined by co-immunoprecipitation assay followed by western blot analysis.HSP27 protein and its phosphorylation increased in parallel with enhanced metastatic potentials of HCC cells. siRNA-mediated HSP27 knockdown in MHCC97H significantly suppressed cells   migration   and invasion in vitro and induced cell apoptosis; the prominently altered signal transduction pathway was NF-kappaB pathway after HSP27 knockdown in MHCC97H cells. Furthermore, inhibition of HSP27 expression led to a significant decrease of nuclear NF-kappaB contentration and endogenous IKK activity. In addition, HSP27 was associated with IKKalpha, IKKbeta, IkappaBalpha in three HCC cells above. ELISA assay and western blot analysis also showed a decrease of the association between IKKbeta and IKKalpha, the association between phosphor-HSP27 and IKK complex, and an increase of total IkappaBalpha but reducing tendency of phosphor-IkappaBalpha when HSP27 expression was efficiently knocked down in MHCC97H cells.Altogether, these findings revealed a possible effect of HSP27 on apoptosis in metastatic HCC cells, in which HSP27 may regulate NF-kB pathway activation. 
 Human β-defensin 3 promotes NF-κB-mediated CCR7 expression and anti-apoptotic signals in squamous cell carcinoma of the head and neck. The microenvironment of aerodigestive cancers contains tumor-promoting inflammatory signals often involved in innate immunity. The epithelial malignancy, squamous cell carcinoma of the head and neck (SCCHN), is characterized by secretion of inflammatory mediators that can promote tumorigenesis and lymph node metastasis. Human β-defensin (hBD) 3 is one such antimicrobial mediator of innate immunity produced by squamous epithelial cells in response to tissue damage and inflammation. Here, we hypothesized that the observed overexpression of hBD3 in SCCHN may have a tumor-promoting effect or contribute to nodal metastasis, which has previously been linked to chemokine receptor (CCR) 7 overexpression. Indeed, treatment of non-metastatic SCCHN cells with hBD3 induced surface CCR7 expression a  nd migrat  ion toward its ligand, CCL19. The hBD3-induced CCR7 upregulation in SCCHN cells was significantly reduced by inhibition of nuclear factor (NF)-κB, an inflammatory transcription factor known to influence CCR7 expression. Moreover, hBD3 stimulation provided anti-apoptotic signals to SCCHN cells, as evidenced by tumor resistance to cisplatin-induced cell death, which was regulated by phosphoinositide-3-kinase/Akt activation. Interestingly, the observed hBD3-mediated effects were not dependent on G-protein coupled receptors or toll-like receptors, as has been previously published, but hBD3 was internalized through endocytosis, allowing intracellular signal transduction. Our findings suggest that hBD3 represents a novel NF-κB-regulated mediator of CCR7 expression and anti-apoptotic pathways, which may be exploited by developing SCCHN tumors to enhance their survival and metastasis. 
 Resveratrol-induced apoptosis in MCF-7 human breast cancer cells involves a caspase-independent mechanism with downregulation of Bcl-2 and NF-kappaB. Resveratrol (RES), a chemopreventive molecule, inhibits the proliferation of tumor cells of different etiologies. We previously showed that RES alters the cell cycle and induces apoptosis in MCF-7 breast tumor cells by interfering with the estrogen receptor (ERaalpha)-dependent phosphoinositide 3-kinase (PI3K) pathway. Here, we analyzed signaling downstream of PI3K, to understand the mechanisms of RES-induced apoptosis. Apoptotic death by RES in MCF-7 was mediated by Bcl-2 downregulation since overexpression of this protein abolished apoptosis. Decreased Bcl-2 levels were not related to cytochrome c release, activation of caspases 3/8 or poly(ADP-ribose) polymerase proteolysis. However, RES decreased mitochondrial membrane potential and increased reactive oxygen species and nitric oxide production. NF-kappaB, a regulator of Bcl-2 expression, and calpain protease activity, a regulator of NF-kappaB, were both inhibited by RES. The patterns for NF-kappaB and calpain activities followed that of PI3K and were inhibited by LY294002. NF-kappaB inhibition coincided with diminished MMP-9 activity and cell   migration  . These data suggest that RES-induced apoptosis in MCF-7 could involve an oxidative, caspase-independent mechanism, whereby inhibition of PI3K signaling converges to Bcl-2 through NF-kappaB and calpain protease activity. Therefore, Bcl-2 and NF-kappaB could be considered potential targets for the chemopreventive activity of RES in estrogen-responsive tumor cells. 
 Keratinocyte growth factor/fibroblast growth factor-7-regulated cell   migration   and invasion through activation of NF-kappaB transcription factors. Keratinocyte growth factor (KGF)/fibroblast growth factor-7 (FGF-7) is a paracrine- and epithelium-specific growth factor produced by cells of mesenchymal origin. It acts exclusively through FGF-7 receptor (FGFR2/IIIb), which is expressed predominantly by epithelial cells, but not by fibroblasts, suggesting that it might function as a paracrine mediator of mesenchymal-epithelial interactions. KGF/FGF-7 plays an essential role in the growth of epithelial cells and is frequently overexpressed in cancers of epithelial origin such as pancreatic cancer, switching paracrine stimulation of KGF/FGF-7 to an autocrine loop. Less is known, however, about the signaling pathways by which KGF/FGF-7 regulates the response of epithelial cells. To delineate the signaling pathways activated by KGF/FGF-7 and examine cellular response to KGF/FGF-7 stimulation, we performed functional analysis of KGF/FGF-7 action. In this report, we show that KGF/FGF-7 activated nuclear factor kappaB (NF-kappaB), which in turn induced expression of VEGF, MMP-9, and urokinase-type plasminogen activator and increased   migration   and invasion of KGF/FGF-7-stimulated human pancreatic ductal epithelial cells. Expression of phosphorylation-defective IkappaBalpha (IkappaBalphaS32A,S36A), which blocked NF-kappaB activation, inhibited KGF/FGF-7-induced gene expression and cell   migration   and invasion. Our results demonstrate for the first time that KGF/FGF-7 induces NF-kappaB activation and that NF-kappaB plays an essential role in regulation of KGF/FGF-7-inducible gene expression and KGF/FGF-7-initiated cellular responses. Thus, these findings identify one signaling pathway for KGF/FGF-7-regulated cell   migration   and invasion and suggest that paracrine sources of KGF/FGF-7 are one of the malignancy-contributing factors from tumor stroma. 
 Role of osteopontin in induction of monocyte chemoattractant protein 1 and macrophage inflammatory protein 1beta through the NF-kappaB and MAPK pathways in rheumatoid arthritis. Osteopontin (OPN) is a proinflammatory protein with a critical role in leukocyte   migration  . Although OPN has been implicated in rheumatoid arthritis (RA), its underlying mechanism remains unknown. In this study, we investigated the role and molecular mechanism of OPN in the induction of 2 key chemokines, monocyte chemoattractant protein 1 (MCP-1) and macrophage inflammatory protein 1beta (MIP-1beta), in RA.Enzyme-linked immunosorbent assay and quantitative polymerase chain reaction were used to determine chemokine expression. Leukocyte   migration   in the presence of OPN was measured by chemotaxis assay. Signaling and molecular events were analyzed by immunoblotting and chromatin immunoprecipitation.The effect of OPN on inflammatory cell   migration   was mediated through its unique property of inducing the expression of MCP-1 and MIP-1beta in CD14+ monocytes. The concentration of OPN was significantly elevated in RA patients and appeared to correlate with the serum levels of inflammation markers and increased expression of MCP-1 or MIP-1beta in monocytes in RA patients. Endogenous production of OPN in RA synovial fluid was attributable to increased production of MCP-1 or MIP-1beta, and this effect could be blocked by an anti-OPN antibody. Furthermore, the structural motif responsible for this property resided within residues 50-83 of human OPN, sparing the known RGD or SVVYGLR sequences. It was evident that the effect of OPN on chemokine expression was mediated through both the NF-kappaB and MAPK pathways, involving the activation of IKKbeta, p38, and JNK.These results support a unique role of OPN in leukocyte   migration  , in the context of perpetuation of rheumatoid synovitis through the induction of MCP-1 and MIP-1beta. 
 Secreted heat shock protein 90α (HSP90α) induces nuclear factor-κB-mediated TCF12 protein expression to down-regulate E-cadherin and to enhance colorectal cancer ce  ll migrat  ion and invasion. Secreted levels of HSP90α and overexpression of TCF12 have been associated with the enhancement of colorectal cancer (CRC) c  ell migra  tion and invasion. In this study, we observed that CRC patients with tumor TCF12 overexpression exhibited both a higher rate of metastatic occurrence and a higher average serum HSP90α level compared with patients without TCF12 overexpression. Therefore, we studied the relationship between the actions of secreted HSP90α and TCF12. Like overexpressed TCF12, secreted HSP90α or recombinant HSP90α (rHSP90α) induced fibronectin expression and repressed E-cadherin, connexin-26, connexin-43, and gap junction levels in CRC cells. Consistently, rHSP90α stimulated invasive outgrowths of CRC cells from spherical structures during three-dimensional culture. rHSP90α also induced TCF12 expression in CRC cells. Its effects on CRC cell epithelial-mesenchymal t  ransition  , migration, and invasion were drastically prevented when TCF12 was knocked down. This suggests that TCF12 expression is required for secreted HSP90α to enhance CRC cell spreading. Through the cellular receptor CD91, rHSP90α facilitated the complex formation of CD91 with IκB kinases (IKKs) α and β and increased the levels of phosphorylated (active) IKKα/β and NF-κB. Use of an IKKα/β inhibitor or ectopic overexpression of dominant-negative IκBα efficiently repressed rHSP90α-induced TCF12 expression. Moreover, κB motifs were recognized in the gene sequence of the TCF12 promoter, and a physical association between NF-κB and the TCF12 promoter was detected in rHSP90α-treated CRC cells. Together, these results suggest that the CD91/IKK/NF-κB signaling cascade is involved in secreted HSP90α-induced TCF12 expression, leading to E-cadherin down-regu  lation an  d enhanced CRC cell migration/invasion. 
 Effect of weight loss on proinflammatory state of mononuclear cells in obese women. In order to investigate whether weight loss can lead to improvement of the mononuclear cell (MNC) proinflammatory state, 21 nondiabetic obese women with mean age 34+/-2 years (mean+/-s.e.m.) and BMI 32.5+/-1.2 kg/m2 were enrolled in a 12-week caloric restriction and light exercise-based weight loss program. Ten lean women served as controls. Reverse transcription-PCR of proinflammatory cytokines and adipocytokines as well as homeostasis model assessment of insulin resistance (HOMA-IR) were determined before and after weight reduction. Nuclear factor kappaB (NF-kappaB) binding to DNA and inhibitors of NF-kappaB (IkappaB-alpha and IkappaB-beta) obtained from peripheral MNCs were measured. Overall, subjects lost a mean of 4.0+/-0.4 kg (5.0+/-0.3% of their initial body weight) (P<0.01). In addition to significant reductions in BMI, fasting glucose and insulin concentrations, mean serum high-sensitivity C-reactive protein (hs-CRP),   migration   inhibitor factor (MIF), leptin and visfatin levels decreased by 49.0, 66.6, 17.2, and 50.2%, respectively (all P<0.05), while adiponectin concentrations rose by 33.9% (P<0.05). The DNA binding of the transcriptionally active NF-kappaB from (p65/p50) decreased by 38.1% (P<0.05). Elevated levels of mRNA of NF-kappaB related proinflammatory genes, tumor necrosis factor-alpha (TNF-alpha), interleukin-6 (IL-6), MIF, and matrix metalloproteinase-9 (MMP-9), decreased significantly after weight loss. Although mRNA expression of Rel-A, p105, IkappaB-alpha, IkappaB-beta decreased significantly, their protein levels did not change after weight loss. As a group, NF-kappaB binding activity correlated with HOMA-IR (r=0.332, P=0.049) and marginally with values of BMI (r=0.308, P=0.059). In conclusion, weight loss by 5% of initial weight in nondiabetic obese women led to significant improvement in activated intranuclear NF-kappaB binding as well as several transcriptions of proinflammatory genes regulated by NF-kappaB. 
 Alpha-mangostin suppresses phorbol 12-myristate 13-acetate-induced MMP-2/MMP-9 expressions via alphavbeta3 integrin/FAK/ERK and NF-kappaB signaling pathway in human lung adenocarcinoma A549 cells. The purpose of this study is to investigate the anti-metastatic effect of alpha-mangostin on phorbol 12-myristate 13-acetate (PMA)-induced matrix metalloproteinase-2 (MMP-2) and matrix metalloproteinase-9 (MMP-9) expressions in A549 human lung adenocarcinoma cells. Firstly, alpha-mangostin could inhibit PMA-induced abilities of the adhesion, invasion, and   migration  . Data also showed alpha-mangostin could inhibit the activation of alphavbeta3 integrin, focal adhesion kinase (FAK), and extracellular signal-regulated kinase1/2 (ERK1/2) involved in the downregulation the enzyme activities, protein and messenger RNA levels of MMP-2 and MMP-9 induced by PMA. Next, alpha-mangostin also strongly inhibited PMA-induced degradation of inhibitor of kappaBalpha (IkappaBalpha) and the nuclear levels of nuclear factor kappa B (NF-kappaB). Also, a dose-dependent inhibition on the binding abilities of NF-kappaB by alpha-mangostin treatment was further observed. Furthermore, reduction of FAK or ERK1/2 phosphorylation by FAK small interfering RNA (FAK siRNA) potentiated the effect of alpha-mangostin. Finally, the transient transfection of ERK siRNA significantly down-regulated the expressions of MMP-2 and MMP-9 concomitantly with a marked inhibition on cell invasion and   migration  . Presented results indicated alpha-mangostin is a novel, effect, anti-metastatic agent that functions by downregulating MMP-2 and MMP-9 gene expressions. 
 [Small interfering RNA targeting NF-kappaB Inhibits human umbilical venous endothelial cells   migration  ]. To investigate the effect of small interfering RNA(siRNA) targeting NF-kappaB on   migration   ability of human umbilical venous endothelial cells.EAhy926 cells (Human umbilical vein endothelial cell line) were cultured with stimulation of vascular endothelial growth factor (VEGF), and then were transfected with NF-kappaB -siRNA (40 pmol/L) by using catiotic liposome lipofectamine(TM); 2000 as the transfecting agent. Cells transfected with negative control-siRNA (40 pmol/L), empty Liposome and nothing as control. Forty-eight hours later, cells were collected and prepared for RT-PCR, Western blot analysis, and determination of the   migration   inhibition rate.Compared with normal control group, both mRNA and protein levels of NF-kappaB were decreased obviously and at the same time cell   migration   inhibition rate was increased obviously in NF-kappaB-siRNA group. There were no significant differences between empty liposome group, negative control siRNA group and normal control group, respectively (P>0.05).Synthetic siRNA can effectively inhibit endothelial cell   migration   ability by means of blocking the signaling pathway of NF-kappaB. And siRNA targeting NF-kappaB may be useful for antiangiogenesis treatment. 
 Ox-LDL induces dysfunction of endothelial progenitor cells via activation of NF-κB. Dyslipidemia increases the risks for atherosclerosis in part by impairing endothelial integrity. Endothelial progenitor cells (EPCs) are thought to contribute to endothelial recovery after arterial injury. Oxidized low-density lipoprotein (ox-LDL) can induce EPC dysfunction, but the underlying mechanism is not well understood. Human EPCs were cultured in endothelial growth medium supplemented with VEGF (10 ng/mL) and bFGF (10 ng/mL). The cells were treated with ox-LDL (50 µg/mL). EPC proliferation was assayed by using CCK8 kits. Expression and translocation of nuclear factor-kabba B (NF-κB) were evaluated. The level of reactive oxygen species (ROS) in cells was measured using H2DCF-DA as a fluorescence probe. The activity of NADPH oxidase activity was determined by colorimetric assay. Ox-LDL significantly decreased the prolif  eration,   migration, and adhesion capacity of EPCs, while significantly increased ROS production and NADPH oxidase expression. Ox-LDL induced NF-κB P65 mRNA expression and translocation in EPCs. Thus ox-LDL can induce EPC dysfunction at least by increasing expression and translocation of NF-κB P65 and NADPH oxidase activity, which represents a new mechanism of lipidemia-induced vascular injury. 
 YGLF motif in the Kaposi sarcoma herpes virus G-protein-coupled receptor adjusts NF-κB activation and paracrine actions. Kaposi sarcoma (KS) and primary effusion lymphoma (PEL) are two pathologies associated with KS herpes virus (KSHV/HHV-8) infection. KSHV genome contains several oncogenes, among which, the viral G-protein-coupled receptor (vGPCR open reading frame 74) has emerged as a major factor in KS pathogenicity. Indeed, vGPCR is a constitutively active receptor, whose expression is sufficient to drive cell transformation in vitro and tumour development in mice. However, neither the role of vGPCR in KSHV-infected B-lymphocytes nor the molecular basis for its constitutive activation is well understood. Here, we show that vGPCR expression contributes to nuclear factor-κB (NF-κB)-dependent cellular survival in both PEL cells and primary B cells from HIV-negative KS patients. We further identified within vGPCR an AP2 consensus binding motif, Y326GLF, that directs its localization between the plasma membrane and clathrin-coated vesicles. The introduction of a mutation in this site (Y326A) increased NF-κB activity and proinflammatory cytokines production. This correlated with exacerbated morphological rearrangeme  nt, migra  tion and proliferation of non-infected monocytes. Collectively, our work raises the possibility that KSHV-infected B-lymphocytes use vGPCR to impact ultimately the immune response and communication within the tumour microenvironment in KSHV-associated pathologies. 
 MicroRNA-9 up-regulates E-cadherin through inhibition of NF-κB1-Snail1 pathway in melanoma. MicroRNAs (miRNAs) are short non-coding RNAs that post-transcriptionally regulate gene expression. Hsa-miR-9 has been shown to have opposite functions in different tumour types; however, the underlying mechanism is unclear. Here we show that hsa-miR-9 is down-regulated in metastatic melanomas compared to primary melanomas. Overexpression of miR-9 in melanoma cells resulted in significantly decreased cell proliferation and migratory capacity with decreased F-actin polymerization and down-regulation of multiple GTPases involved in cytoskeleton remodelling. miR-9 overexpression induced significant down-regulation of Snail1 with a concomitant increase in E-cadherin expression. In contrast, knockdown of miR-9 increased Snail1 expression as well as melanoma cell proliferation and   migratio  n capacity. Mechanistically, miR-9 expression down-regulated NF-κB1 in melanoma and the effect was abolished by mutations in the putative miR-9 binding sites within the 3'-untranslated region (UTR) of NF-κB1. Anti-miR-9 miRNA inhibitor also increased the expression of NF-κB1. The effects of miR-9 on Snail1 expression and melanoma cell proliferation   and migra  tion were rescued by overexpression of NF-κB1 in these cells. Furthermore, miR-9 overexpression resulted in significantly decreased melanoma growth and metastasis in vivo. In summary, miR-9 inhibits melanoma proliferation and metastasis through down-regulation of the NF-κB1-Snail1 pathway. This study finds a new mechanism that miR-9 utilizes to decrease E-cadherin expression and inhibit melanoma progression. The results suggest that function of microRNAs is context and tumour type-specific. 
 Interleukin-20 promotes   migration   of bladder cancer cells through extracellular signal-regulated kinase (ERK)-mediated MMP-9 protein expression leading to nuclear factor (NF-κB) activation by inducing the up-regulation of p21(WAF1) protein expression. The role of inflammatory cytokine interleukin-20 (IL-20) has not yet been studied in cancer biology. Here, we demonstrated up-regulation of both IL-20 and IL-20R1 in muscle-invasive bladder cancer patients. The expressions of IL-20 and IL-20R1 were observed in bladder cancer 5637 and T-24 cells. We found that IL-20 significantly increased the expression of matrix metalloproteinase (MMP)-9 via binding activity of NF-κB and AP-1 in bladder cancer cells and stimulated the activation of ERK1/2, JNK, p38 MAPK, and JAK-STAT signaling. Among the pathways examined, only ERK1/2 inhibitor U0126 significantly inhibited IL-20-induce  d migrati  on and invasion. Moreover, siRNA knockdown of IL-20R1 suppresse  d migrati  on, invasion, ERK1/2 activation, and NF-κB-mediated MMP-9 expression induced by IL-20. Unexpectedly, the cell cycle inhibitor p21(WAF1) was induced by IL-20 treatment without altering cell cycle progression. Blockade of p21(WAF1) function by siRNA revers  ed migrat  ion, invasion, activation of ERK signaling, MMP-9 expression, and activation of NF-κB in IL-20-treated cells. In addition, IL-20 induced the activation of IκB kinase, the degradation and phosphorylation of IκBα, and NF-κB p65 nuclear translocation, which was regulated by ERK1/2. IL-20 stimulated the recruitment of p65 to the MMP-9 promoter region. Finally, the IL-20-  induced m  igration and invasion of cells was confirmed by IL-20 gene transfection and by addition of anti-IL-20 antibody. This is the first report that p21(WAF1) is involved in ERK1/2-mediated MMP-9 expression via increased binding activity of NF-κB, which resulted in the indu  ction of   migration in IL-20/IL-20R1 dyad-induced bladder cancer cells. These unexpected results might provide a critical new target for the treatment of bladder cancer. 
 CCL18/PITPNM3 enhances   migration  , invasion, and EMT through the NF-κB signaling pathway in hepatocellular carcinoma. Chemokine ligand 18 (CCL18) has been associated with hepatocellular carcinoma (HCC) metastasis. Here, we demonstrated a novel mechanism through which CCL18 enhances cell   migratio  n, invasion, and epithelial-mesenchymal transition (EMT) in HCC. (1) Using immunohistochemistry, we analyzed the expression of PITPNM3, a molecule that correlated with CCL18 signaling, in 149 HCC tissue specimens. The results showed that PITPNM3 expression is highly associated with tumor metastasis and differentiation; (2) in vitro experiments showed that CCL18 enhances cell   migratio  n, invasion, and EMT in PITPNM3((+)) HCC cells but not in PITPNM3((-)) cells. Silencing of PITPNM3 by short interfering RNA (siRNA) inhibited the induction of cell   migratio  n, invasion, and EMT by CCL18; (3) Cell   migratio  n, invasion, and EMT induced by CCL18 accompanied with the phosphorylation of IKK and IKBα as well as p65 nuclear translocation in PITPNM3((+)) HCC cells, but not in the cells that PITPNM3 is silenced with siRNA, implying that the activation of NF-κB signaling is involved in the action of CCL18/PITPNM3. These results suggest that CCL18 enhances HCC ce  ll migrat  ion, invasion, and EMT through the expression of PITPNM3 and the activation of the NF-κB signaling pathway. 
 Stromal cell-derived factor-1/CXCR4 enhanced motility of human osteosarcoma cells involves MEK1/2, ERK and NF-kappaB-dependent pathways. Osteosarcoma is characterized by a high malignant and metastatic potential. The chemokine stromal-derived factor-1alpha (SDF-1alpha) and its receptor, CXCR4, play a crucial role in adhesion and   migration   of human cancer cells. Integrins are the major adhesive molecules in mammalian cells, and has been associated with metastasis of cancer cells. Here, we found that human osteosarcoma cell lines had significant expression of SDF-1 and CXCR4 (SDF-1 receptor). Treatment of osteosarcoma cells with SDF-1alpha increased the   migration   and cell surface expression of alphavbeta3 integrin. CXCR4-neutralizing antibody, CXCR4 specific inhibitor (AMD3100) or small interfering RNA against CXCR4 inhibited the SDF-1alpha-induced increase the   migration   and integrin expression of osteosarcoma cells. Pretreated of osteosarcoma cells with MAPK kinase (MEK) inhibitor PD98059 inhibited the SDF-1alpha-mediated   migration   and integrin expression. Stimulation of cells with SDF-1alpha increased the phosphorylation of MEK and extracellular signal-regulating kinase (ERK). In addition, NF-kappaB inhibitor (PDTC) or IkappaB protease inhibitor (TPCK) also inhibited SDF-1alpha-mediated cell   migration   and integrin up-regulation. Stimulation of cells with SDF-1alpha induced IkappaB kinase (IKKalpha/beta) phosphorylation, IkappaB phosphorylation, p65 Ser(536) phosphorylation, and kappaB-luciferase activity. Furthermore, the SDF-1alpha-mediated increasing kappaB-luciferase activity was inhibited by AMD3100, PD98059, PDTC and TPCK or MEK1, ERK2, IKKalpha and IKKbeta mutants. Taken together, these results suggest that the SDF-1alpha acts through CXCR4 to activate MEK and ERK, which in turn activates IKKalpha/beta and NF-kappaB, resulting in the activations of alphavbeta3 integrins and contributing the   migration   of human osteosarcoma cells. 
 Plasmin induces intercellular adhesion molecule 1 expression in human endothelial cells via nuclear factor-κB/mitogen-activated protein kinases-dependent pathways. Activation of endothelial cells (ECs) by proinflammatory stimuli triggers expression of cellular adhesion molecules including intercellular adhesion molecule 1 (ICAM-1) on the cell surface. Such molecules mediate the transendothelial   migratio  n of inflammatory cells, which is an early key step of atherogenesis. We have previously demonstrated that plasmin activates human inflammatory cells via the annexin A2 heterotetramer (A2t). Here we show that human umbilical vein endothelial cells (HUVECs) and human microvascular endothelial cells express high amounts of A2t, as shown by Western blotting, fluorescence microscopy and flow cytometry. Activation of HUVEC by plasmin led to cleavage of the annexin A2 subunit of the receptor complex, followed by the activation of Akt/nuclear factor (NF)-κB signaling, and phosphorylation of MAP kinases p38 and ERK1/2. Further, plasmin stimulates the NF-κB/p38-dependent expression of ICAM-1 by HUVEC. The plasmin-induced activation of cells was abolished when annexin A2 was down-regulated by small-interfering RNA. In vivo, we show co-localization of the ECs marker CD31 with the plasmin receptor A2t and ICAM-1 in human atherosclerotic plaques of human femoral arteries, which also exhibit activated NF-κB signaling as revealed by immunofluorescence staining for phosphorylated IκBα. In addition, plasma of patients with advanced atherosclerosis exhibited enhanced plasmin activity and up-regulated levels of plasmin-α2-antiplasmin. These data point to a previously unrecognized functional role of plasmin in EC biology, which could be of particular relevance in the development of atherosclerosis. 
 CTGF enhances   migration   and MMP-13 up-regulation via alphavbeta3 integrin, FAK, ERK, and NF-kappaB-dependent pathway in human chondrosarcoma cells. Tumor malignancy is associated with several features such as proliferation ability and frequency of metastasis. Connective tissue growth factor (CTGF), a secreted protein that binds to integrins, modulates the invasive behavior of certain human cancer cells. However, the effect of CTGF on   migration   activity in human chondrosarcoma cells is mostly unknown. Here we found that CTGF increased the   migration   and expression of matrix metalloproteinase (MMP)-13 in human chondrosarcoma cells (JJ012 cells). RGD peptide, alphavbeta3 monoclonal antibody (mAb) and MAPK kinase (MEK) inhibitors (PD98059 and U0126) but not RAD peptide inhibited the CTGF-induced increase of the   migration   and MMP-13 up-regulation of chondrosarcoma cells. CTGF stimulation increased the phosphorylation of focal adhesion kinase (FAK) and extracellular signal-regulated kinase (ERK). In addition, treatment of JJ012 cells with NF-kappaB inhibitor (PDTC) or IkappaB protease inhibitor (TPCK) inhibited CTGF-induced cell   migration   and MMP-13 up-regulation. Stimulation of JJ012 cells with CTGF also induced IkappaB kinase alpha/beta (IKK alpha/beta) phosphorylation, IkappaBalpha phosphorylation, p65 Ser(536) phosphorylation, and kappaB-luciferase activity. The CTGF-mediated increases in kappaB-luciferase activities were inhibited by RGD, PD98059, U0126 or FAK, and ERK2 mutant. Taken together, our results indicated that CTGF enhances the   migration   of chondrosarcoma cells by increasing MMP-13 expression through the alphavbeta3 integrin, FAK, ERK, and NF-kappaB signal transduction pathway. 
 High expression of DDX20 enhances the proliferation and metastatic potential of prostate cancer cells through the NF-κB pathway. DEAD (Asp-Glu-Ala-Asp) box polypeptide 20 (DDX20), a member of the DEAD box protein family, encodes an RNA helicase. Previous research has revealed that DDX20 may act as a tumor suppressor in hepatocellular carcinoma and as a tumor promoter in breast cancer. These conflicting reports prompted us to explore the role of DDX20 in prostate cancer (PCa). To gain insight into the functions of DDX20 in PCa, we examined DDX20 expression patterns in a PCa tissue microarray with 99 PCa tissue samples. The results of immunohistochemical staining revealed that DDX20 expression is frequently upregulated in PCa tissues compared with that in the adjacent tissues and further clinicopathological analysis showed that the expression level of DDX20 closely correlates with tumor size, TNM stage (positive correlation) and patient prognosis (negative correlation). Both gain‑of- and loss‑of‑function assays were performed in vitro; the overexpression of DDX20 enhanced the proliferation and metastatic potential of cancer cells and this was examined by performing a cell counting kit-8 (CCK-8) assay, wound healing assa  y and Tra  nswell migration assay. Furthermore, we found that there is a positive correlation between the expression of matrix metallopeptidase 9 (MMP9) and DDX20 expression. These findings led us to examine whether DDX20 may exert effects through the NF‑κB pathway. Luciferase reporter assays suggested that DDX20 altered the activity of NF-κB. Taken together, these findings show that DDX20 may promote the progression of PCa through the NF-κB pathway. 
 DNA damage induces NF-κB-dependent microRNA-21 up-regulation and promotes breast cancer cell invasion. NF-κB activation induced by genotoxic treatment in cancer cells has been associated with therapeutic resistance in multiple human malignancies. Therapeutic resistance also correlates with high metastatic potential in human cancers, including breast cancer. Whether genotoxic treatment-activated NF-κB also contributes to cancer metastasis following radiation and chemotherapy is unclear. Here, we show that chemotherapeutic drug-induced NF-κB activation promotes breast cancer c  ell migra  tion and invasion. The increased metastatic potential is dependent on IL-6 induction mediated by genotoxic NF-κB activation. Moreover, genotoxic treatment also up-regulates oncogenic microRNA-21 (miR-21) expression through eliciting NF-κB recruitment to the miR-21 promoter region, where it cooperates with signal transducer and activator of transcription 3 (STAT3) to activate miR-21 transcription. DNA damage-induced histone H3 phosphorylation via activated MSK1 creates an open chromatin structure for NF-κB/STAT3-driven transactivation of miR-21. NF-κB-dependent IL-6 up-regulation is responsible for STAT3 activation and recruitment to the miR-21 promoter upon genotoxic stress. Induction of miR-21 may enable cancer cells to elude DNA damage-induced apoptosis and enhance the metastatic potential of breast cancer cells through repressing expression of PTEN and PDCD4. Our data support a critical role of DNA damage-induced NF-κB activation in promoting cancer metastasis following genotoxic treatment, and NF-κB-dependent miR-21 induction may contribute to both therapeutic resistance and metastasis in breast cancer. 
 Bradykinin enhances cell   migration   in human chondrosarcoma cells through BK receptor signaling pathways. Bradykinin (BK) is an inflammatory mediator, and shows elevated levels in regions of severe injury and inflammatory diseases. BK has recently been shown to be involved in carcinogenesis and cancer progression. In this study, we found that BK increased the   migration   and the expression of alpha2beta1 integrin in human chondrosarcoma cells. We also found that human chondrosarcoma tissues had significantly higher expression of the B1 and B2 receptors comparing to normal cartilage. BK-mediated   migration   and integrin up-regulation was attenuated by B1 and B2 BK receptor siRNA or antagonist. Activations of phospholipase C (PLC), protein kinase Cdelta (PKCdelta), and NF-kappaB pathways after BK treatment was demonstrated, and BK-induced integrin expression and   migration   activity was inhibited by the specific inhibitor of PLC, PKCdelta, and NF-kappaB cascades. Taken together, our results indicated that BK enhances the   migration   of chondrosarcoma cells by increasing alpha2beta1 integrin expression through the BK receptors/PLC/PKCdelta/NF-kappaB signal transduction pathway. 
 NF-κB mediates the 12(S)-HETE-induced endothelial to mesenchymal transition of lymphendothelial cells during the intravasation of breast carcinoma cells. The intravasation of breast cancer into the lymphendothelium is an early step of metastasis. Little is known about the mechanisms of bulky cancer invasion into lymph ducts.To particularly address this issue, we developed a 3-dimensional co-culture model involving MCF-7 breast cancer cell spheroids and telomerase-immortalised human lymphendothelial cell (LEC) monolayers, which resembles intravasation in vivo and correlated the malignant phenotype with specific protein expression of LECs.We show that tumour spheroids generate 'circular chemorepellent-induced defects' (CCID) in LEC monolayers through retraction of LECs, which was induced by 12(S)-hydroxyeicosatetraenoic acid (HETE) secreted by MCF-7 spheroids. This 12(S)-HETE-regulated retraction of LECs during intravasation particularly allowed us to investigate the key regulators involved in the motility and plasticity of LECs. In all, 12(S)-HETE induced pro-metastatic protein expression patterns and showed NF-κB-dependent up-regulation of the mesenchymal marker protein S100A4 and of transcriptional repressor ZEB1 concomittant with down-regulation of the endothelial adherence junction component VE-cadherin. This was in accordance with ∼50% attenuation of CCID formation by treatment of cells with 10 μM Bay11-7082. Notably, 12(S)-HETE-induced VE-cadherin repression was regulated by either NF-κB or by ZEB1 since ZEB1 siRNA knockdown abrogated not only 12(S)-HETE-mediated VE-cadherin repression but inhibited VE-cadherin expression in general.These data suggest an endothelial to mesenchymal transition-like process of LECs, which induces single cell motility during endotheli  al transm  igration of breast carcinoma cells. In conclusion, this study demonstrates that the 12(S)-HETE-induced intravasation of MCF-7 spheroids through LECs require an NF-κB-dependent process of LECs triggering the disintegration of cell-cell c  ontacts,   migration, and the generation of CCID. 
 Expression of microRNA-146 suppresses NF-kappaB activity with reduction of metastatic potential in breast cancer cells. Cancer cells often acquire a constitutively active nuclear factor-kappaB (NF-kappaB) program to promote survival, proliferation and metastatic potential by mechanisms that remain largely unknown. Extending observations from an immunologic setting, we demonstrate that microRNA-146a and microRNA-146b (miR-146a/b) when expressed in the highly metastatic human breast cancer cell line MDA-MB-231 function to negatively regulate NF-kappaB activity. Lentiviral-mediated expression of miR-146a/b significantly downregulated interleukin (IL)-1 receptor-associated kinase and TNF receptor-associated factor 6, two key adaptor/scaffold proteins in the IL-1 and Toll-like receptor signaling pathway, known to positively regulate NF-kappaB activity. Impaired NF-kappaB activity was evident from reduced phosphorylation of the NF-kappaB inhibitor IkappaBalpha, reduced NF-kappaB DNA-binding activity and suppressed expression of the NF-kappaB target genes IL-8, IL-6 and matrix metalloproteinase-9. Functionally, miR-146a/b-expressing MDA-MB-231 cells showed markedly impaired invasion and   migration   capacity relative to control cells. These findings implicate miR-146a/b as a negative regulator of constitutive NF-kappaB activity in a breast cancer setting and suggest that modulating miR-146a/b levels has therapeutic potential to suppress breast cancer metastases. 
 The secret second life of an innocent chaperone: the story of CD74 and B cell/chronic lymphocytic leukemia cell survival. This review deals with the cytokine macrophage   migration   inhibitory factor (MIF) and its receptor, CD74. MIF and CD74 have been shown to regulate peripheral B cell survival and were associated with tumor progression and metastasis. CD74 expression has been suggested to serve as a prognostic factor in many cancers, with higher relative expression of CD74 behaving as a marker of tumor progression. In chronic lymphocytic leukemia (CLL) cells, binding of MIF to CD74 induces nuclear factor-κB (NF-κB) activation and up-regulation of TAp63 expression, resulting in the secretion of interleukin 8 (IL-8), which in turn promotes cell survival. In addition, TAp63 expression elevates expression of the integrin VLA-4, particularly during the advanced stage of the disease. Blocking of CD74, TAp63, or VLA-4 inhibits the in vivo homing of CLL cells to the BM. Thus, CD74 and its target genes, TAp63 and VLA-4, facilitat  e migrati  on of CLL cells back to the BM, where they interact with the supportive BM environment that helps rescue them from apoptosis. These results are expected to pave the way toward novel therapeutic strategies aimed at interrupting this survival pathway. One such agent, the monocolonal antibody milatuzumab directed at CD74, is already being studied in early clinical trials. 
 TGF-β1 promoted MMP-2 mediated wound healing of anterior cruciate ligament fibroblasts through NF-κB. The adult human anterior cruciate ligament (ACL) has poor functional healing response. Transforming growth factor (TGF)-β1 enhances the wound repair by stimulating matrix proteins deposition as well as the proliferation a  nd migrat  ion of cells. However, the function of the TGF-β1-induced matrix metalloproteinases' (MMPs) activities in the wound healing process is poorly understood. In this study, exogenous MMP-2 is added to mimic the TGF-β1-induced MMP-2 expression. Role of NF-κB pathway is further examined. Our results show that TGF-β1 induces dramatic elevation of MMP-2 activities and the MMP-2/tissue inhibitors of metalloproteinases ratio. Furthermore, the exogenous MMP-2 significantly promoted in vitro wound healing abilities of ACL fibroblasts that are significantly blocked with the addition of its inhibitors. TGF-β1 also increases the proliferation of ACL fibroblasts whereas MMP-2 alone does not, indicating that MMP-2 activities are not involved in the proliferation. TGF-β1-induced MMP-2 activity is inhibited by Bay11-7082 and Bay11-7085 (NF-κB inhibitors). Our results demonstrate that increased TGF-β1 facilitates the ACL healing process by promoting the f  ibroblast  s migration and prolifer  ation. Th  e migration process is mediated by MMP-2 and NF-κB pathway is involved in TGF-β1-mediated MMP-2 release. 
 Cyclic AMP-responsive element binding protein- and nuclear factor-kappaB-regulated CXC chemokine gene expression in lung carcinogenesis. The recognition of the importance of angiogenesis in tumor progression has led to the development of antiangiogenesis as a new strategy for cancer treatment and prevention. By modulating tumor microenvironment and inducing angiogenesis, the proinflammatory cytokine interleukine (IL)-1beta has been reported to promote tumor development. However, the factors mediating IL-1beta-induced angiogenesis in non-small cell lung cancer (NSCLC) and the regulation of these angiogenic factors by IL-1beta are less clear. Here, we report that IL-1beta up-regulated an array of proangiogenic CXC chemokine genes in the NSCLC cell line A549 and in normal human tracheobronchial epithelium cells, as determined by microarray analysis. Further analysis revealed that IL-1beta induced much higher protein levels of CXC chemokines in NSCLC cells than in normal human tracheobronchial epithelium cells. Conditioned medium from IL-1beta-treated A549 cells markedly increased endothelial cell   migration  , which was suppressed by neutralizing antibodies against CXCL5 and CXCR2. We also found that IL-1beta-induced CXC chemokine gene overexpression in NSCLC cells was abrogated with the knockdown of cyclic AMP-responsive element binding protein (CREB) or nuclear factor kappaB (NF-kappaB). Moreover, the expression of the CXC chemokine genes as well as CREB and NF-kappaB activities was greatly increased in the tumorigenic NSCLC cell line compared with normal, premalignant immortalized or nontumorigenic cell lines. A disruptor of the interaction between CREB-binding protein and transcription factors such as CREB and NF-kappaB, 2-naphthol-AS-E-phosphate (KG-501), inhibited IL-1beta-induced CXC chemokine gene expression and angiogenic activity in NSCLC. We propose that targeting CREB or NF-kappaB using small-molecule inhibitors, such as KG-501, holds promise as a preventive and/or therapeutic approach for NSCLC. 
 Visfatin triggers the in vitro   migration   of osteosarcoma cells via activation of NF-κB/IL-6 signals. Pulmonary metastasis is the major challenge for clinical treatment of osteosarcoma patients. Recent studies indicated that visfatin, a 52kDa adipocytokine, can trigger the cell motility of various cancers, while its role in the progression of osteosarcoma remains not clear. Our present study revealed that visfatin can significantly promote the in vitro   migratio  n and invasion of osteosarcoma MG-63 and HOS cells and up regulate the expression of matrix metalloproteinase-2 (MMP-2) and fibronectin (FN). Furthermore, visfatin treatment also increased the expression of IL-6 in both MG-63 and HOS cells via a time dependent manner, while anti-IL-6 antibody can significantly attenuate visfatin induced cell invasion and up regulation of MMP-2 and FN. It suggested that up regulation of IL-6 mediated visfatin induced in vitro motility of osteosarcoma cells. Visfatin treatment can increase the phosphorylation of both p65 and ERK1/2 in MG-63 and HOS cells, while only the inhibitor of NF-κB, BAY 11-7082, can abolish visfatin induced up regulation of IL-6. BAY 11-7082 also attenuated visfatin induced upregulation of MMP-2 and FN in MG-63 cells. Western blot analysis revealed that visfatin treatment can significantly increase the phosphorylation of IκBα and IKKβ in MG-63 cells. ACHP, the inhibitor of IKK-β, blocked visfatin induced expression of IL-6 mRNA in both MG-63 and HOS cells. Collectively, our data suggested that visfatin can increase the motility of osteosarcoma cells via up regulation of NF-κB/IL-6 signals. It indicated that visfatin might be a potential therapeutic target of osteosarcoma treatment. 
 Unfractionated heparin suppresses lipopolysaccharide-induced monocyte chemoattractant protein-1 expression in human microvascular endothelial cells by blocking Krüppel-like factor 5 and nuclear factor-κB pathway. Unfractionated heparin (UFH) and low-molecular-weight heparins (LMWH), apart from anticoagulant activities, contain a variety of biological properties such as anti-inflammatory actions possibly affecting sepsis. Chemokines are vital for promoting the movement of circulating leukocytes to the site of infection and are involved in the pathogenesis of sepsis. The purpose of this study was to investigate the effects and potential mechanisms of UFH on lipopolysaccharide (LPS)-induced chemokine production in human pulmonary microvascular endothelial cells (HPMECs). HPMECs were pretreated with UFH (0.1 U/ml and 1 U/ml), 15 min prior to stimulation with LPS (10 μg/ml). Cells were cultured under various experimental conditions for 2 h and 6 h for analysis. UFH markedly decreased LPS-induced interleukin (IL)-8 and monocyte chemoattractant protein-1 (MCP-1) mRNA and protein expression in HPMECs. UFH also attenuated the secretion of these chemokines in culture supernatants. In addition, UFH blocked the chemotactic activities of LPS-stimulated HPMECs supernatants on monocyt  es migrat  ion as expected. UFH inhibited LPS-induced Krüppel-like factor 5 (KLF-5) mRNA and protein levels. Concurrently, UFH reduced nuclear factor (NF)-κB nuclear translocation. Importantly, transfection with siRNA targeting KLF-5 reduced NF-κB activation and chemokines expression. These results demonstrate that interfering with KLF-5 mediated NF-κB activation might contribute to the inhibitory effects of chemokines and mon  ocytes mi  gration by UFH in LPS-stimulated HPMECs. 
 TWEAK promotes ovarian cancer cell metastasis via NF-kappaB pathway activation and VEGF expression. The poor prognosis of human ovarian cancer is partly due to its metastasis and recurrence. It has been demonstrated that tumor necrosis factor (TNF)-like weak inducer of apoptosis (TWEAK)-fibroblast growth factor inducible-14 (Fn14) signaling system may be a potential regulator of human tumorigenesis. The objective of this study was to understand the effect of TWEAK on ovarian cancer metastasis. We recently showed that activation of Fn14 signaling by TWEAK promoted cell   migration   and invasion in human HO-8910PM cells. Treating HO-8910PM cells with TWEAK resulted in the activation of nuclear factor-kappa B (NF-kappaB) and subsequently the translocation of NF-kappaB from cytoplasm to nucleus. In addition, TWEAK promoted vascular endothelial growth factor (VEGF) protein expression, and this effect was dependent upon NF-kappaB transcriptional activity. Blocking the NF-kappaB pathway with PDTC suppressed TWEAK-induced up-regulation of VEGF protein expression and cell metastasis. Our results suggest that TWEAK-Fn14 functions, in part, through the NF-kappaB signaling pathway to up-regulate VEGF expression to foster ovarian cancer cell metastasis. Targeted therapy against TWEAK-Fn14 signaling system as an adjuvant to surgery may improve clinical management of invasive ovarian cancer cells and advance the outcome of this devastating cancer. 
 HBx protein induces expression of MIG and increases   migration   of leukocytes through activation of NF-kappaB. Elevated expression of monokine induced by the interferon-gamma (MIG) has been shown in HBV carriers, and it is involved in the infiltration of inflammatory cells and liver damage after HBV infection. However, the molecular mechanisms by which HBV-induced MIG expression have not been characterized. Our results indicated that HBx protein induced MIG expression in a dose-dependent manner. Such increase was due to the direct binding of NF-kappaB to the MIG promoter. By luciferase, chromatin immunoprecipitation and electrophoretic mobility shift assays, we demonstrated that the NF-kappaB binding site at positions -147 was essential for transcriptional activation of MIG promoter by HBx protein. Chemotaxis assay showed that the up-regulation of MIG protein levels enhanced the   migration   of peripheral blood lymphocytes (PBLs), and inhibition of NF-kappaB significantly decreased the chemotaxis activity. Our findings provide a new insight into how leukocytes migrate to liver, and disclose a new regulatory mechanism of MIG expression after HBV infection. 
 Tumor necrosis factor α up-regulates endometrial milk fat globule-epidermal growth factor 8 protein production via nuclear factor κB activation, resulting in cel  l migrati  on of epithelial cells. To explore the role of tumor necrosis factor (TNF) α, an early embryonic product, on endometrial epithelial ce  ll migrat  ion and endometrial milk fat globule-epidermal growth factor 8 protein (MFG-E8) production.In vitro study.Academic center.Ishikawa cells, used as surrogates for human epithelial cells, were treated with and without TNF-α.Effect of TNF-α on intracellular MFG-E8 protein was evaluated with the use of ELISA, Western blot, and subcellular fractionation. Specific inhibitors were used to study TNF-α mechanism of action. Effect of TNF-α o  n cell mi  gration was studied with the use of a wound healing assay and reorganization of E-cadherin.TNF-α induced: 1) significant up-regulation of MFG-E8 intracellular protein, which was attenuated by pretreatment with a specific inhibitor of nuclear factor κB; 2) increased transcription of MFG-E8 and other proinflammatory factors, such as interleukins 6 and 8, which were suppressed by cotreatment with hCG; and 3) signific  ant cell   migration with E-cadherin remodeling, changes associated with subcellular MFG-E8 relocalization.TNF-α up-regulates endometrial epithe  lial cell   migration and MFG-E8 production, which are critical steps required for the endometrial changes during menstrual cycle as well as during embryonic attachment and invasion. 
 Receptor-interacting protein kinase 2 promotes triple-negative breast cancer cell   migration   and invasion via activation of nuclear factor-kappaB and c-Jun N-terminal kinase pathways. Metastasis is the main cause of breast cancer morbidity and mortality. Processes that allow for tumor cell   migration   and invasion are important therapeutic targets. Here we demonstrate that receptor-interacting protein kinase 2 (RIP2), a kinase known to be involved in inflammatory processes, also has novel roles in cancer cell   migration   and invasion.A total of six breast cancer expression databases, including The Cancer Genome Atlas, were assessed for RIP2 expression among various clinical subtypes and its role as a prognostic biomarker. mRNA fluorescence in situ hybridization (FISH) for RIP2 was performed on 17 stage III breast cancers to determine if there was a correlation between RIP2 expression and lymph node involvement. RNA-interference was used to knock-down RIP2 expression in MDA-MB-231, Htb126, SUM149PT, MCF7, T47D, and HCC1428 cells. Cell   migration   and invasion were measured in vitro by scratch/wound healing and transwell   migration   assays. A xenograft mouse model was used to assess tumor growth and chemosensitivity to docetaxel in vivo in MDA-MB-231 cells with and without RIP2 small hairpin RNA knockdown. Western blot and immunofluorescence imaging were used to evaluate protein expressions.Interrogation of expression databases showed that RIP2 expression is significantly over-expressed in triple-negative breast cancers (TNBC: estrogen-receptor (ER) negative, progesterone-receptor (PR) negative, Her2/neu- (Her2) negative), compared to other clinical subtypes. High RIP2 expression correlates with worse progression-free survival using a combined breast cancer expression array dataset consisting of 946 patients. Multivariate analysis shows RIP2 as an independent prognostic biomarker. Knock-down of RIP2 significantly decreases   migration   in both scratch/wound healing and transwell   migration   assays in MDA-MB-231, Htb126, SUM149PT, MCF7, and T47D cells and is correlated with decreased Nuclear Factor-kappaB and c-Jun N-terminal kinase (JNK) activation. Finally, RIP2 knock-down leads to increased sensitivity to docetaxel and decreased tumor mass and lung metastases in a xenograft mouse model.These results highlight RIP2 as a pro-metastasis kinase in patients with advanced breast cancer. These results also illustrate a novel role for this kinase in addition to its known role in inflammation, and suggest that targeting RIP2 may improve outcomes in advanced breast cancer patients, in which it is overexpressed. 
 Vibrio vulnificus VvpE Stimulates IL-1β Production by the Hypomethylation of the IL-1β Promoter and NF-κB Activation via Lipid Raft-Dependent ANXA2 Recruitment and Reactive Oxygen Species Signaling in Intestinal Epithelial Cells. An inflammatory response is a hallmark of necrosis evoked by bacterial pathogens. Vibrio vulnificus, VvpE, is an elastase that is responsible for tissue necrosis and inflammation; however, the molecular mechanism by which it regulates host cell death has not been characterized. In the present study, we investigate the cellular mechanism of VvpE with regard to host cell death and the inflammatory response of human intestinal epithelial (INT-407) cells. The recombinant protein (r)VvpE (50 pg/ml) caused cytotoxicity mainly via necrosis coupled with IL-1β production. The necrotic cell death induced by rVvpE is highly susceptible to the knockdown of annexin A (ANXA)2 and the sequestration of membrane cholesterol. We found that rVvpE induces the recruitment of NADPH oxidase 2 and neutrophil cytosolic factor 1 into membrane lipid rafts coupled with ANXA2 to facilitate the production of reactive oxygen species (ROS). The bacterial signaling of rVvpE through ROS production is uniquely mediated by the phosphorylation of redox-sensitive transcription factor NF-κB. The silencing of NF-κB inhibited IL-1β production during necrosis. rVvpE induced hypomethylation and region-specific transcriptional occupancy by NF-κB in the IL-1β promoter and has the ability to induce pyroptosis via NOD-, LRR-, and pyrin domain-containing 3 inflammasome. In a mouse model of V. vulnificus infection, the mutation of the vvpE gene from V. vulnificus negated the proinflammatory responses and maintained the physiological levels of the prolifera  tion and   migration of enterocytes. These results demonstrate that VvpE induces the hypomethylation of the IL-1β promoter and the transcriptional regulation of NF-κB through lipid raft-dependent ANXA2 recruitment and ROS signaling to promote IL-1β production in intestinal epithelial cells. 
 Ethyl pyruvate, an anti-inflammatory agent, inhibits tumor angiogenesis through inhibition of the NF-κB signaling pathway. Ethyl pyruvate (EP), simple derivative of pyruvate, has been shown to have anti-inflammatory properties. Here, we demonstrate EP's strong anti-angiogenic activity. EP inhibited in vivo angiogenesis in the mouse Matrigel-plug assay and tumor growth in the mouse Lewis lung carcinoma model. EP also interfered with the angiogenic cascade, including growth, invasion,   migratio  n, and tube formation. Activation of NF-κB by vascular endothelial cell growth factor was reduced by EP. Taken together, we suggest that EP may have potential as a new multi-functional drug candidate for anti-angiogenesis and cancer therapy. 
 Endotoxin-induced vascular endothelial cell   migration   is dependent on TLR4/NF-κB pathway, NAD(P)H oxidase activation, and transient receptor potential melastatin 7 calcium channel activity. Endothelial dysfunction is decisive and leads to the development of several inflammatory diseases. Endotoxemia-derived sepsis syndrome exhibits a broad inflammation-induced endothelial dysfunction. We reported previously that the endotoxin, lipopolysaccharide (LPS), induces the conversion of endothelial cells (ECs) into activated fibroblasts, showing a myofibroblast-like protein expression profile. Enhanced   migratio  n is a hallmark of myofibroblast function. However, the mechanism involved in LPS-induced EC   migratio  n is no totally understood. Some studies have shown that the transient receptor potential melastatin 7 (TRPM7) ion channel is involved in fibroblast and tumor cell   migratio  n through the regulation of calcium influx. Furthermore, LPS modulates TRPM7 expression. However, whether TRPM7 is involved in LPS-induced EC   migratio  n remains unknown. Here, we study the participation of LPS as an inducer of EC   migratio  n and study the mechanism underlying evaluating the participation of the TRPM7 ion channel. Our results demonstrate that LPS induced EC   migratio  n in a dose-dependent manner. Furthermore, this migratory process was mediated by the TLR-4/NF-κB pathway and the generation of ROS through the PKC-activated NAD(P)H oxidase. In addition, LPS increased the intracellular calcium level and the number of focal adhesion kinase (FAK)-positive focal adhesions in EC. Finally, we demonstrate that using TRPM7 blockers or suppressing TRPM7 expression through siRNA successfully inhibits the calcium influx and the LPS-induced E  C migrati  on. These results point out TRPM7 as a new target in the drug design for several inflammatory diseases that impair vascular endothelium function. 
 Notch-1 signaling activates NF-κB in human breast carcinoma MDA-MB-231 cells via PP2A-dependent AKT pathway. Breast cancer has a high incidence in the world and is becoming a leading cause of death in female patients due to its high metastatic ability. High expression of Notch-1 and its ligand Jagged-1 correlates with poor prognosis in breast cancer. Our previous work has shown that Notch-1 signaling pathway upregulates NF-κB transcriptional activity and induces the adhesion  , migrati  on and invasion of human breast cancer cell line MDA-MB-231. However, the role of Notch-1 in NF-κB activation is still poorly understood. Here, we aim to understand the exact mechanism that Notch-1 regulates NF-κB activity. In MDA-MB-231 cells where Notch-1 is constitutively activated, the phosphorylation of p85 and AKT (Tyr308/Ser473) is upregulated, indicating PI3K/AKT pathway is activated. Notch-1 activation caused the increase of PP2A phosphorylation at Tyr307, indicating Notch-1 inhibits PP2A activity. NF-κB transcriptional activity was evaluated by dual-luciferase reporter assay, and the results showed that, while silencing of Notch-1, PP2A activity was upregulated and NF-κB activity was downregulated, whereas PP2A inhibitor okadaic acid (OA) restored NF-κB activity. Immunofluorescence and Western blots showed that OA treatment antagonized the decrease of p65 nuclear translocation caused by Notch-1 silencing. Moreover, OA treatment also upregulated MMP-2, MMP-9 and VEGF mRNA expression levels, indicating OA rescues Notch-1 silencing that caused low cell invasion. Taken together, our results suggest that Notch-1-activating PI3K/AKT/NF-κB pathway is PP2A dependent; PP2A may be a potential therapeutic target in breast cancer. 
 Bmi-1 promotes the aggressiveness of glioma via activating the NF-kappaB/MMP-9 signaling pathway. The prognosis of human glioma is poor, and the highly invasive nature of the disease represents a major impediment to current therapeutic modalities. The oncoprotein B-cell-specific Moloney murine leukemia virus integration site 1 protein (Bmi-1) has been linked to the development and progression of glioma; however, the biological role of Bmi-1 in the invasion of glioma remains unclear.A172 and LN229 glioma cells were engineered to overexpress Bmi-1 via stable transfection or to be silenced for Bmi-1 expression using RNA interfering method.   Migration   and invasiveness of the engineered cells were assessed using wound healing assay, Transwell   migration   assay, Transwell matrix penetration assay and 3-D spheroid invasion assay. MMP-9 expression and activity were measured using real-time PCR, ELISA and the gelatin zymography methods. Expression of NF-kappaB target genes was quantified using real-time PCR. NF-kappaB transcriptional activity was assessed using an NF-kappaB luciferase reporter system. Expression of Bmi-1 and MMP-9 in clinical specimens was analyzed using immunohistochemical assay.Ectopic overexpression of Bmi-1 dramatically increased, whereas knockdown of endogenous Bmi-1 reduced, the invasiveness and   migration   of glioma cells. NF-kappaB transcriptional activity and MMP-9 expression and activity were significantly increased in Bmi-1-overexpressing but reduced in Bmi-1-silenced cells. The reporter luciferase activity driven by MMP-9 promoter in Bmi-1-overexpressing cells was dependent on the presence of a functional NF-kappaB binding site, and blockade of NF-kappaB signaling inhibited the upregulation of MMP-9 in Bmi-1 overexpressing cells. Furthermore, expression of Bmi-1 correlated with NF-kappaB nuclear translocation as well as MMP-9 expression in clinical glioma samples.Bmi-1 may play an important role in the development of aggressive phenotype of glioma via activating the NF-kappaB/MMP-9 pathway and therefore might represent a novel therapeutic target for glioma. 
 The tumor marker Fascin is induced by the Epstein-Barr virus-encoded oncoprotein LMP1 via NF-κB in lymphocytes and contributes to their invasive   migratio  n. The actin-bundling protein Fascin (FSCN1) is a tumor marker that is highly expressed in numerous types of cancer including lymphomas and is important for   migratio  n and metastasis of tumor cells. Fascin has also been detected in B lymphocytes that are freshly-infected with Epstein-Barr virus (EBV), however, both the inducers and the mechanisms of Fascin upregulation are still unclear.Here we show that the EBV-encoded oncoprotein latent membrane protein 1 (LMP1), a potent regulator of cellular signaling and transformation, is sufficient to induce both Fascin mRNA and protein in lymphocytes. Fascin expression is mainly regulated by LMP1 via the C-terminal activation region 2 (CTAR2). Block of canonical NF-κB signaling using a chemical inhibitor of IκB kinase β (IKKβ) or cotransfection of a dominant-negative inhibitor of IκBα (NFKBIA) reduced not only expression of p100, a classical target of the canonical NF-κB-pathway, but also LMP1-induced Fascin expression. Furthermore, chemical inhibition of IKKβ reduced both Fascin mRNA and protein levels in EBV-transformed lymphoblastoid cell lines, indicating that canonical NF-κB signaling is required for LMP1-mediated regulation of Fascin both in transfected and transformed lymphocytes. Beyond that, chemical inhibition of IKKβ significantly reduce  d invasiv  e migration of EBV-transformed lymphoblastoid cells through extracellular matrix. Transient transfection experiments revealed that Fascin contributed to LMP1-mediated enhancement o  f invasiv  e migration through extracellular matrix. While LMP1 enhanced the number of invaded cells, functional knockdown of Fascin by two different small hairpin RNAs resulted in significant reduction of invaded, non-attached cells.Thus, our data show that LMP1-mediated upregulation of Fascin depends on NF-κB and both NF-κB and Fascin contribute   to invas  ive migration of LMP1-expressing lymphocytes. 
 An autocrine sphingosine-1-phosphate signaling loop enhances NF-kappaB-activation and survival. Sphingosine-1-phosphate (S1P) is a bioactive lipid that regulates a multitude of cellular functions, including cell proliferation, survival,   migration   and angiogenesis. S1P mediates its effects either by signaling through G protein-coupled receptors (GPCRs) or through an intracellular mode of action. In this study, we have investigated the mechanism behind S1P-induced survival signalling.We found that S1P protected cells from FasL-induced cell death in an NF-kappaB dependent manner. NF-kappaB was activated by extracellular S1P via S1P2 receptors and Gi protein signaling. Our study also demonstrates that extracellular S1P stimulates cells to rapidly produce and secrete additional S1P, which can further amplify the NF-kappaB activation.We propose a self-amplifying loop of autocrine S1P with capacity to enhance cell survival. The mechanism provides increased understanding of the multifaceted roles of S1P in regulating cell fate during normal development and carcinogenesis. 
 Celastrol inhibits interleukin-17A-stimulated rheumatoid fibroblast-like synoviocyte   migration   and invasion through suppression of NF-κB-mediated matrix metalloproteinase-9 expression. Interleukin-17A (IL-17A)-induced   migratio  n and invasion of fibroblast-like synoviocytes (FLSs) is critical for the pathogenesis of rheumatoid arthritis (RA). More than 30% of RA patients are resistant to available therapies, despite the introduction of novel biologic agents. Therefore, it is necessary to develop new anti-arthritic agents. Recent studies have demonstrated that celastrol has anti-arthritic activity in an adjuvant-induced arthritis (AIA) model. However, the effect and molecular mechanisms of celastrol on the   migratio  n and invasion of RA-FLSs are not yet understood. Results showed that treatment of RA-FLSs with celastrol suppressed the IL-17A-induced   migratio  n and invasion abilities of the cells. In addition, celastrol inhibited IL-17A-induced matrix metalloproteinase (MMP)-9 mRNA and protein expression, and the proteolytic activity of MMP-9 in RA-FLSs. Furthermore, our results revealed that celastrol inhibited the transcriptional activity of MMP-9 by suppression of the binding activity of nuclear factor-κB (NF-κB) in the MMP-9 promoter, and inhibited IκBα phosphorylation and nuclear translocation of NF-κB. In conclusion, celastrol can inhibit IL-17A-in  duced mig  ration and invasion by suppressing NF-κB-mediated MMP-9 expression in RA-FLSs. These results provide a strong rationale for further testing and validation of celastrol as an adjunct with conventional drugs for the treatment of RA in humans. 
 Suppression of NF-kappaB activity by NDRG2 expression attenuates the invasive potential of highly malignant tumor cells. Downregulation of the N-myc downstream-regulated gene 2 (NDRG2) gene is involved in the progression of aggressive forms of cancer, along with the poor prognosis of cancer patients. In the current study, we examined the effect of NDRG2 expression on the metastatic potential of HT1080 human fibrosarcoma and B16F10 murine melanoma cells in both in vitro and in vivo systems. In gelatin zymography, NDRG2 expression remarkably suppressed the matrix metalloproteinase (MMP)-9 activity and slightly inhibited MMP-2 activity of both cell lines. Tumor   migration   and invasion in vitro were significantly reduced by NDRG2 expression, and NDRG2 inhibited tumor cell proliferation in an anchorage-independent semisolid agar assay. Specifically, we found that NDRG2 affects invasion through suppression of nuclear factor kappa B (NF-kappaB) activity. In animal experiments, subcutaneously injected B16F10-NDRG2 cells showed delayed tumor growth compared with B16F10-mock cells. Furthermore, severe metastasis from primary tumor mass into the draining lymph nodes was observed after injection of B16F10-mock cells, but not with B16F10-NDRG2 cells. Pulmonary metastasis after intravenous injection of B16F10 cells was also reduced by NDRG2 expression. Intra- and peritumoral angiogenesis that is critical for the tumor growth and metastasis was clearly found in tumors after injection with B16F10-mock cells, whereas it was impaired in tumors after injection with B16F10-NDRG2 cells. Collectively, our data show that NDRG2 expression significantly suppresses tumor invasion by inhibiting MMP activities, which are regulated through the NF-kappaB signaling. Moreover, results from animal experiments provide evidence for the regulatory role of the NDRG2 gene in metastatic tumors. 
 The Ron receptor tyrosine kinase positively regulates angiogenic chemokine production in prostate cancer cells. Overexpression of the Ron receptor tyrosine kinase has recently been shown in a wide variety of human cancers. However, no studies have examined Ron receptor expression or function during prostate tumorigenesis. In this study we report that Ron is highly expressed in human prostate adenocarcinoma and metastatic lymph nodes when compared with normal prostate or benign prostate hyperplasia. Furthermore, we show that Ron is overexpressed in PC-3 and DU145 prostate cancer cell lines, and that the levels of angiogenic chemokines produced by prostate cancer cells positively correlate with Ron expression. The knockdown of Ron in PC-3 or DU145 cells results in a significant decrease in angiogenic chemokine production and is associated with a decreased activation of the transcription factor nuclear factor-kappaB (NF-kappaB). Moreover, exogenous overexpression of Ron in LNCaP cells is sufficient to induce a significant increase in angiogenic chemokines that can be abrogated by inhibition of NF-kappaB signaling. Given that the function of angiogenic chemokines is important in the development of new blood vessels, we also examined the ability of Ron to modulate endothelial cell   migration  . Our data show that knockdown of Ron in prostate cancer cells results in significantly less endothelial cell chemotaxis when compared with Ron-expressing cells in vitro as well as in reduced tumor growth and decreased microvessel density after orthotopic transplantation into the prostate in vivo. In total, our data suggest that the Ron receptor is important in modulating prostate tumor growth by modulating angiogenic chemokine production and subsequent endothelial cell recruitment. 
 Selective inhibition of prostaglandin E2 receptors EP2 and EP4 induces apoptosis of human endometriotic cells through suppression of ERK1/2, AKT, NFkappaB, and beta-catenin pathways and activation of intrinsic apoptotic mechanisms. Endometriosis is a benign chronic gynecological disease of reproductive-age women characterized by the presence of functional endometrial tissues outside the uterine cavity. It is an estrogen-dependent disease. Current treatment modalities to inhibit biosynthesis and actions of estrogen compromise menstruation, pregnancy, and the reproductive health of women and fail to prevent reoccurrence of disease. There is a critical need to identify new specific signaling modules for non-estrogen-targeted therapies for endometriosis. In our previous study, we reported that selective inhibition of cyclooxygenase-2 prevented survival,   migration  , and invasion of human endometriotic epithelial and stromal cells, which was due to decreased prostaglandin E(2) (PGE(2)) production. In this study, we determined mechanisms through which PGE(2) promoted survival of human endometriotic cells. Results of the present study indicate that 1) PGE(2) promotes survival of human endometriotic cells through EP2 and EP4 receptors by activating ERK1/2, AKT, nuclear factor-kappaB, and beta-catenin signaling pathways; 2) selective inhibition of EP2 and EP4 suppresses these cell survival pathways and augments interactions between proapoptotic proteins (Bax and Bad) and antiapoptotic proteins (Bcl-2/Bcl-XL), facilitates the release of cytochrome c, and thus activates caspase-3/poly (ADP-ribose) polymerase-mediated intrinsic apoptotic pathways; and 3) these PGE(2) signaling components are more abundantly expressed in ectopic endometriosis tissues compared with eutopic endometrial tissues during the menstrual cycle in women. These novel findings may provide an important molecular framework for further evaluation of selective inhibition of EP2 and EP4 as potential therapy, including nonestrogen target, to expand the spectrum of currently available treatment options for endometriosis in women. 
 The c-Rel subunit of NF-κB is a crucial regulator of phenotype and motility of HaCaT keratinocytes. The transcription factor NF-κB exerts key functions in epidermal homeostasis and carcinogenesis. Its c-Rel subunit is expressed in squamous cell carcinoma, and c-Rel down-regulation results in increased apoptosis, G2/M cell cycle delay with reduced proliferation and aberrant mitotic spindle formation. To further study the impact of c-Rel on essential keratinocyte features such a  s migrati  on and epithelial morphology, c-Rel was down-regulated in HaCaT keratinocytes by a siRNA approach. This inhibition of c-Rel impaired the keratinocyte-typical clustered growth leading to a more scattered appearance of the cultures. The cells were more spindle-shaped and elongated, albeit without expression changes of markers characteristic for epithelial mesenchymal transition. In addition, wound healing-relate  d migrati  on and adhesion to type I collagen, fibronectin, laminin and vitronectin were significantly impaired. On the sub-cellular level, these functional features were not associated with quantitatively altered adhesion receptor or Rho-GTPase expression, but rather with a significantly reduced length of cell-matrix adhesion complexes and altered appearance of filamentous actin. Thus, our studies support a role for c-Rel in processes crucial for keratinocyte integrity and malignant transformation such as adhesion an  d migrati  on. 
 An increase in integrin-linked kinase non-canonically confers NF-κB-mediated growth advantages to gastric cancer cells by activating ERK1/2. Increased activity or expression of integrin-linked kinase (ILK), which regulates cell adhesion,   migratio  n, and proliferation, leads to oncogenesis. We identified the molecular basis for the regulation of ILK and its alternative role in conferring ERK1/2/NF-κB-mediated growth advantages to gastric cancer cells.Inhibiting ILK with short hairpin RNA or T315, a putative ILK inhibitor, abolished NF-κB-mediated the growth in the human gastric cancer cells AGS, SNU-1, MKN45, and GES-1. ILK stimulated Ras activity to activate the c-Raf/MEK1/2/ERK1/2/ribosomal S6 kinase/inhibitor of κBα/NF-κB signaling by facilitating the formation of the IQ motif-containing GTPase-activating protein 1 (IQGAP1)-Ras complex. Forced enzymatic ILK expression promoted cell growth by facilitating ERK1/2/NF-κB signaling. PI3K activation or decreased PTEN expression prolonged ERK1/2 activation by protecting ILK from proteasome-mediated degradation. C-terminus of heat shock cognate 70 interacting protein, an HSP90-associated E3 ubiquitin ligase, mediated ILK ubiquitination to control PI3K- and HSP90-regulated ILK stabilization and signaling. In addition to cell growth, the identified pathway promote  d cell mi  gration and reduced the sensitivity of gastric cancer cells to the anticancer agents 5-fluorouracil and cisplatin. Additionally, exogenous administration of EGF as well as overexpression of EGFR triggered ILK- and IQGAP1-regulated ERK1/2/NF-κB activation, cell grow  th, and m  igration.An increase in ILK non-canonically promotes ERK1/2/NF-κB activation and leads to the growth of gastric cancer cells. 
 Activation of epidermal growth factor receptor is required for NTHi-induced NF-κB-dependent inflammation. Inflammation is a hallmark of many serious human diseases. Nontypeable Haemophilus influenzae (NTHi) is an important human pathogen causing respiratory tract infections in both adults and children. NTHi infections are characterized by inflammation, which is mainly mediated by nuclear transcription factor-kappa B (NF-κB)-dependent production of proinflammatory mediators. Epidermal growth factor receptor (EGFR) has been shown to play important roles in regulating diverse biological processes, including cell growth, differentiation, apoptosis, adhesion, an  d migrati  on. Its role in regulating NF-κB activation and inflammation, however, remains largely unknown.In the present study, we demonstrate that EGFR plays a vital role in NTHi-induced NF-κB activation and the subsequent induction of proinflammatory mediators in human middle ear epithelial cells and other cell types. Importantly, we found that AG1478, a specific tyrosine kinase inhibitor of EGFR potently inhibited NTHi-induced inflammatory responses in the middle ears and lungs of mice in vivo. Moreover, we found that MKK3/6-p38 and PI3K/Akt signaling pathways are required for mediating EGFR-dependent NF-κB activation and inflammatory responses by NTHi.Here, we provide direct evidence that EGFR plays a critical role in mediating NTHi-induced NF-κB activation and inflammation in vitro and in vivo. Given that EGFR inhibitors have been approved in clinical use for the treatment of cancers, current studies will not only provide novel insights into the molecular mechanisms underlying the regulation of inflammation, but may also lead to the development of novel therapeutic strategies for the treatment of respiratory inflammatory diseases and other inflammatory diseases. 
 Inflammation induces lymphangiogenesis through up-regulation of VEGFR-3 mediated by NF-kappaB and Prox1. The concept of inflammation-induced lymphangiogenesis (ie, formation of new lymphatic vessels) has long been recognized, but the molecular mechanisms remained largely unknown. The 2 primary mediators of lymphangiogenesis are vascular endothelial growth factor receptor-3 (VEGFR-3) and Prox1. The key factors that regulate inflammation-induced transcription are members of the nuclear factor-kappaB (NF-kappaB) family; however, the role of NF-kappaB in regulation of lymphatic-specific genes has not been defined. Here, we identified VEGFR-3 and Prox1 as downstream targets of the NF-kappaB pathway. In vivo time-course analysis of inflammation-induced lymphangiogenesis showed activation of NF-kappaB followed by sequential up-regulation of Prox1 and VEGFR-3 that preceded lymphangiogenesis by 4 and 2 days, respectively. Activation of NF-kappaB by inflammatory stimuli also elevated Prox1 and VEGFR-3 expression in cultured lymphatic endothelial cells, resulting in increased proliferation and   migration  . We also show that Prox1 synergizes with the p50 of NF-kappaB to control VEGFR-3 expression. Collectively, our findings suggest that induction of the NF-kappaB pathway by inflammatory stimuli activates Prox1, and both NF-kappaB and Prox1 activate the VEGFR-3 promoter leading to increased receptor expression in lymphatic endothelial cells. This, in turn, enhances the responsiveness of preexisting lymphatic endothelium to VEGFR-3 binding factors, VEGF-C and VEGF-D, ultimately resulting in robust lymphangiogenesis. 
 The Major Histocompatibility Complex Class II Transactivator CIITA Inhibits the Persistent Activation of NF-κB by the Human T Cell Lymphotropic Virus Type 1 Tax-1 Oncoprotein. Human T cell lymphotropic virus type 1 (HTLV-1) Tax-1, a key protein in HTLV-1-induced T cell transformation, deregulates diverse cell signaling pathways. Among them, the NF-κB pathway is constitutively activated by Tax-1, which binds to NF-κB proteins and activates the IκB kinase (IKK). Upon phosphorylation-dependent IκB degradation, NF-κB migrates into the nucleus, mediating Tax-1-stimulated gene expression. We show that the transcriptional regulator of major histocompatibility complex class II genes CIITA (class II transactivator), endogenously or ectopically expressed in different cells, inhibits the activation of the canonical NF-κB pathway by Tax-1 and map the region that mediates this effect. CIITA affects the subcellular localization of Tax-1, which is mostly retained in the cytoplasm, and this correlates with im  paired mi  gration of RelA into the nucleus. Cytoplasmic and nuclear mutant forms of CIITA reveal that CIITA exploits different strategies to suppress Tax-1-mediated NF-κB activation in both subcellular compartments. CIITA interacts with Tax-1 without preventing Tax-1 binding to both IKKγ and RelA. Nevertheless, CIITA affects Tax-1-induced IKK activity, causing retention of the inactive p50/RelA/IκB complex in the cytoplasm. Nuclear CIITA associates with Tax-1/RelA in nuclear bodies, blocking Tax-1-dependent activation of NF-κB-responsive genes. Thus, CIITA inhibits cytoplasmic and nuclear steps of Tax-1-mediated NF-κB activation. These results, together with our previous finding that CIITA acts as a restriction factor inhibiting Tax-1-promoted HTLV-1 gene expression and replication, indicate that CIITA is a versatile molecule that might also counteract Tax-1 transforming activity. Unveiling the molecular basis of CIITA-mediated inhibition of Tax-1 functions may be important in defining new strategies to control HTLV-1 spreading and oncogenic potential.HTLV-1 is the causative agent of human adult T cell leukemia-lymphoma (ATLL). The viral transactivator Tax-1 plays a central role in the onset of ATLL, mostly by deregulating the NF-κB pathway. We demonstrate that CIITA, a key regulator of adaptive immunity, suppresses Tax-1-dependent activation of NF-κB by acting at several levels: it retains most of Tax-1 and RelA in the cytoplasm and inhibits their residual functional activity in the nucleus. Importantly, this inhibition occurs in cells that are targets of HTLV-1 infection. These findings are of interest in the field of virology because they expand the current knowledge of the functional relationship between viral products and cellular interactors and provide the basis for a better understanding of the molecular countermeasures adopted by the host cell to antagonize HTLV-1 spreading and transforming properties. Within this framework, our results may contribute to the establishment of novel strategies against HTLV-1 infection and virus-dependent oncogenic transformation. 
 RANKL promotes   migration   and invasion of hepatocellular carcinoma cells via NF-κB-mediated epithelial-mesenchymal transition. Metastasis accounts for the most deaths in patients with hepatocellular carcinoma (HCC). Receptor activator of nuclear factor kappa B ligand (RANKL) is associated with cancer metastasis, while its role in HCC remains largely unknown.Immunohistochemistry was performed to determine the expression of RANK in HCC tissue (n = 398). Quantitative real-time polymerase chain reaction (qRT-PCR) and Western blot were used to examine the expression of RANK, E-cadherin, N-cadherin, vimentin, Snail, Slug, Twist and MMPs in HCC cells. Wound healing and Transwell assays were used to evaluate   cell migr  ation and invasion ability.We found that expression of RANK, the receptor of RANKL, was significantly higher in HCC tumor tissues than in peritumor liver tissues (p<0.001). Constitutive expression of RANK was detected in HCC cell lines, which can be up-regulated when HCC cells were stimulated with RANKL. Notably, in vitro experiments showed that activation of RANKL-RANK axis significantly prom  oted migr  ation and invasion ability of HCC cells. In addition, RANKL stimulation increased the expression levels of N-cadherin, Snail, and Twist, while decreased the expression of E-cadherin, with concomitant activation of NF-κB signaling pathway. Moreover, administration of the NF-κB inhibitor attenuated RANKL-i  nduced mi  gration, invasion and epithelial-mesenchymal transition of HCC cells.RANKL could pote  ntiate mi  gration and invasion ability of RANK-positive HCC cells through NF-κB pathway-mediated epithelial-mesenchymal transition, which means that RANKL-RANK axis could be a potential target for HCC therapy. 
 Up-regulation of human bradykinin B1 receptor by secreted components of Pseudomonas aeruginosa via a NF-κB pathway in epithelial cells. Pulmonary epithelial cells produce neutrophil chemotactic activity in response to pathogenic bacterial infections, resulting in neutrophil   migratio  n to infection sites. Elicited neutrophils in the inflamed tissues were found to be dependent on bradykinin B1 receptor (B1R), which shows high affinity for the active metabolites derived from bradykinin. Thus, the up-regulation of bradykinin and B1R expression represents an important host defense response against invading microbes such as Pseudomonas aeruginosa. However, the effect of P. aeruginosa on the expression of B1R remains unclear, while P. aeruginosa infection is known to stimulate the production of bradykinin. Here, we report that human B1R (hB1R) transcription is up-regulated in host cells co-cultured with P. aeruginosa. Components secreted from P. aeruginosa play a major role in the up-regulation, and the secretion of the components is not controlled by either type III secretion system or quorum sensing. Moreover, the B1R induction is mediated by a NF-κB signaling pathway in human lung epithelial cells. Taken together, this study demonstrates that P. aeruginosa is capable of up-regulating hB1R expression via the NF-κB signaling pathway. 
 GMCSF activates NF-kappaB via direct interaction of the GMCSF receptor with IkappaB kinase beta. Granulocyte-macrophage colony-stimulating factor (GMCSF) has a central role in proliferation and differentiation of hematopoetic cells. Furthermore, it influences the proliferation and   migration   of endothelial cells. GMCSF elicits these functions by activating a receptor consisting of a ligand-specific alpha-chain and a beta-chain, which is common for GMCSF, interleukin-3 (IL-3), and IL-5. It is known that various signaling molecules such as Janus kinase 2 or transcription factors of the signal transducer and activator of transcription (STAT) family bind to the common beta-chain and initiate signaling cascades. However, alpha-chain-specific signal transduction adapters have to be postulated given that IL-3, IL-5, and GMCSF induce partly distinct biologic responses. Using a yeast 2-hybrid system, we identified the alpha-chain of the GMCSF receptor (GMRalpha) as putative interaction partner of IkappaB kinase beta, one of the central signaling kinases activating the transcription factor nuclear factor-kappaB (NF-kappaB). Using endogenous protein levels of endothelial cell extracts, we could verify the interaction by coimmunoprecipitation experiments. Fluorescence resonance energy transfer (FRET) microscopy confirmed the direct interaction of CFP-IKKbeta and YFPGMRalpha in living cells. Functional studies demonstrated GMCSF-dependent activation of IkappaB kinase activity in endothelial cells, degradation of IkappaB, and activation of NF-kappaB. Further biologic studies using GMCSF-dependent TF-1 cells indicated that GMCSF-triggered activation of NF-kappaB is important for cell survival and proliferation. 
 EBP1 suppresses growth,   migration  , and invasion of thyroid cancer cells through upregulating RASAL expression. Ebp1, a protein identified by its interactions with the ErbB3 receptor, has been characterized as a negative regulator of cancers. RAS GTPase-activating protein (RasGAP), RASAL1, was recently identified as a major tumor suppressor in thyroid cancer. In this study, we examined EBP1 expression in papillary and follicular thyroid cancer cells. We found that compared with normal thyroid cells, TPC1, WRO, and FTC133 thyroid tumor cells exhibited lower EBP1 expression at messenger RNA (mRNA) and protein levels. We then investigated the effects of forced EBP1 expression on growth,   migration  , and invasiveness of thyroid tumor cells. By using MTT and Boyden chamber assays, we showed that EBP1 overexpression dramatically reduced growth rate,   migration  , and invasiveness of K1 and FTC133 thyroid tumor cells. Furthermore, we explored the molecular mechanism underlying the effects of EBP1 on the cells by disclosing the correlation of EBP1 and RASAL1 expression. RASAL expression was elevated in thyroid tumor cells overexpressing EBP1. Knockdown RASAL by transduction of RASAL1 shRNA lentiviral particles markedly reduced RASAL levels with restoration of EBP1, and RASAL1 knockdown abrogated the effects of forced EBP1 expression on cell growth,   migration  , and invasiveness of thyroid tumor cells. These findings suggest that Ebp1 suppressed thyroid cancer cell lines by upregulating RASRAL expression. 
 Role of beta(3)-endonexin in the regulation of NF-kappaB-dependent expression of urokinase-type plasminogen activator receptor. Endothelial   migration   on extracellular matrix is regulated by integrins and proteolysis. Previous studies showed that beta(3)-integrins regulate expression of the urokinase-type plasminogen activator receptor (uPAR) through outside-in signalling involving the cytoplasmic domain. Here we show that overexpression of the integrin-binding protein beta(3)-endonexin decreased uPAR promoter (-398 base-pair fragment) activity that is constitutively active in endothelial cells. Mutation of the NF-kappaB promoter binding site (-45 bp) impaired the ability of beta(3)-endonexin to downregulate uPAR promoter activity. Immunoprecipitation studies showed that beta(3)-endonexin interacts directly with the p50/p65 transactivation complex and thereby inhibits binding of kappaB oligonucleotides to the p50/p65 complex. Moreover, binding of beta(3)-endonexin to p50 was inhibited in the presence of kappaB but not mutated kappaB oligonucleotides, suggesting a sterical competition between beta(3)-endonexin and kappaB DNA for the p50/p65 complex. We therefore propose that beta(3)-endonexin acts as regulator of uPAR expression in beta(3)-integrin-mediated endothelial cell   migration   through direct interaction with p50/p65. Since NF-kappaB regulates the expression of matrix degrading enzymes, the present results define a role of beta(3)-endonexin in regulating beta(3)-integrin-mediated adhesion and pericellular proteolysis. 
 Trim44 facilitates the   migration   and invasion of human lung cancer cells via the NF-κB signaling pathway. Trim44 is an important member of the tripartite motif-containing protein (TRIM) family. Recent research reported that Trim44 might play an important role in tumorigenesis, although its role in non-small cell lung cancer (NSCLC) and the related mechanisms is not yet known.In this study we analyzed 30 pairs of NSCLC tumors and the matched adjacent normal tissue to define the relationship between Trim44 and NSCLC tumors. The function of Trim44 in cell   migratio  n and invasion was determined by overexpression of Trim44 in normal bronchial epithelial cell line 16HE or knockdown of Trim44 in A549 cells, respectively. Whether Trim44-mediated NF-κB signaling activation was involved in Trim44-mediated promotion of lung cancer was tested by q-PCR analysis and cel  l migrati  on and invasion assay using PDTC, an inhibitor of NF-κB.We found that Trim44 was upregulated in NSCLC tumors (14/30 cases; 46.7%). Furthermore, Trim44 was upregulated in many NSCLC cell lines, especially in A549 and H441. Moreover, Trim44 significantly enhanced ce  ll migrat  ion and invasion ability, which was related to increased CXCR6 and matrix metalloproteinase 9 (MMP9). Knockdown of Trim44 in A549 cells by siRNA showed a diminished effect in ce  ll migrat  ion and invasion. Further investigation revealed that blocking the NF-κB signaling pathway using PDTC, an inhibitor of NF-κB, reversed the expression of CXCR6 and MMP9, and alleviated the promotio  n of migr  ation and invasion mediated by Trim44.Our data suggest that Trim44 promotes NSCLC development through activation of NF-κB signaling via upregulating CXCL16 and MMP9 expression. 
 Hericium erinaceus Inhibits TNF-α-Induced Angiogenesis and ROS Generation through Suppression of MMP-9/NF-κB Signaling and Activation of Nrf2-Mediated Antioxidant Genes in Human EA.hy926 Endothelial Cells. Hericium erinaceus (HE) is an edible mushroom that has been shown to exhibit anticancer and anti-inflammatory activities. We investigated the antiangiogenic and antioxidant potentials of ethanol extracts of HE in human endothelial (EA.hy926) cells upon tumor necrosis factor-α- (TNF-α-) stimulation (10 ng/mL). The underlying molecular mechanisms behind the pharmacological efficacies were elucidated. We found that noncytotoxic concentrations of HE (50-200 μg/mL) significantly inhibited TNF-�  �-induced   migration/invasion and capillary-like tube formation of endothelial cells. HE treatment suppressed TNF-α-induced activity and/or overexpression of matrix metalloproteinase-9 (MMP-9) and intercellular adhesion molecule-1 (ICAM-1). Furthermore, HE downregulated TNF-α-induced nuclear translocation and transcriptional activation of nuclear factor-κB (NF-κB) followed by suppression of I-κB (inhibitor-κB) degradation. Data from fluorescence microscopy illustrated that increased intracellular ROS production upon TNF-α-stimulation was remarkably inhibited by HE pretreatment in a dose-dependent manner. Notably, HE triggered antioxidant gene expressions of heme oxygenase-1 (HO-1), γ-glutamylcysteine synthetase (γ-GCLC), and glutathione levels, which may contribute to inhibition of ROS. Increased antioxidant status was associated with upregulated nuclear translocation and transcriptional activation of NF-E2 related factor-2 (Nrf2) in HE treated cells. Our findings conclude that antiangiogenic and anti-inflammatory activities of H. erinaceus may contribute to its anticancer property through modulation of MMP-9/NF-κB and Nrf2-antioxidant signaling pathways. 
 Homeostatic NF-κB Signaling in Steady-State Migratory Dendritic Cells Regulates Immune Homeostasis and Tolerance. Migratory non-lymphoid tissue dendritic cells (NLT-DCs) transport antigens to lymph nodes (LNs) and are required for protective immune responses in the context of inflammation and to promote tolerance to self-antigens in steady-state. However, the molecular mechanisms that elicit steady-state NLT-DC maturation and   migratio  n are unknown. By comparing the transcriptome of NLT-DCs in the skin with their migratory counterparts in draining LNs, we have identified a novel NF-κB-regulated gene network specific to migratory DCs. We show that targeted deletion of IKKβ in DCs, a major activator of NF-κB, prevents NLT-DC accumulation in LNs and compromises regulatory T cell conversion in vivo. This was associated with impaired tolerance and autoimmunity. NF-κB is generally considered the prototypical pro-inflammatory transcription factor, but this study describes a role for NF-κB signaling in DCs for immune homeostasis and tolerance that could have implications in autoimmune diseases and immunity. 
 Type I collagen promotes epithelial-mesenchymal transition through ILK-dependent activation of NF-kappaB and LEF-1. Collagen I has been shown to promote epithelial-mesenchymal transition (EMT), a critical process of embryonic development and disease progression. However, little is known about the signaling mechanisms by which collagen I induces this cellular transformation. Here we show that collagen I causes ILK-dependent phosphorylation of IkappaB and subsequent nuclear translocation of active NF-kappaB, which in turn promotes increased expression of the Snail and LEF-1 transcription factors. ILK also causes inhibitory phosphorylation of GSK-3beta, a kinase that prevents functional activation of both Snail and LEF-1. These transcription factors alter expression of epithelial and mesenchymal markers to initiate EMT and stimulate cell   migration  . These data provide a foundation for understanding the mechanisms by which collagen I stimulates EMT and identify potential therapeutic targets for suppressing this transition in pathological conditions. 
 Sequestration of PDLIM2 in the cytoplasm of monocytic/macrophage cells is associated with adhesion and increased nuclear activity of NF-kappaB. PDLIM2 (Mystique/SLIM) is a postsynaptic density-95/discs large/zonula occludens-1-Lin-11, Isl-1, Mec-3 (PDZ-LIM) domain protein expressed in the nucleus of T lymphocytes, where it promotes degradation of the p65 subunit of NF-kappaB. It is also expressed at the cytoskeleton in epithelial cells, where it is essential for cell   migration  . It is not known whether PDLIM2 function at the nucleus and cytoskeleton is linked and whether PDLIM2 subcellular location is regulated in hematopoietic cells. To investigate this, we used the human monocytic leukemia cell line THP-1 that can differentiate into adherent macrophages and the adherent murine macrophage cell line RAW264.7. PMA-induced differentiation of THP-1 cells resulted in increased accumulation of PDLIM2. In differentiated cells, PDLIM2 exhibited retarded mobility indicative of serine phosphorylation, which could be reversed by phosphatases and by inhibition of protein kinase C or ERK kinases. In nondifferentiated THP-1 cells, PDLIM2 was located predominantly in the nucleus, whereas in differentiated cells, PDLIM2 was located predominantly in the cytoplasm. Suppression of PDLIM2 expression in THP-1 and RAW 264.7 cells resulted in decreased adhesion, increased NF-kappaB transcription reporter activity, and increased LPS-induced TNF-alpha production. Overexpression of PDLIM2 in THP-1 cells enhanced cell adhesion. Overall, these findings indicate that PDLIM2 sequestration in the cytoplasm is associated with cell adhesion and increased nuclear activity of NF-kappaB p65. The data suggest that sequestration of PDLIM2 at the cytoskeleton regulates its nuclear function. 
 S100A4 regulates   migration   and invasion in hepatocellular carcinoma HepG2 cells via NF-κB-dependent MMP-9 signal. We previously showed that the calcium-binding protein S100A4 is overexpressed and related to metastasis in hepatocellular carcinoma (HCC). However, whether S100A4 participates in the regulation of metastasis and its mechanisms in HCC is mostly unknown. Given the associations of S100A4, nuclear factor-kB (NF-kB/RelA) and MMP-9 with metastasis in a variety of malignancies, we explored a potential role of S100A4 in HCC metastasis and its mechanism.20 patients with HCC invasion (Lymph node metastasis, microvascular invasion, major portal vein invasion and intrahepatic metastasis) and 20 patients without HCC invasion were included. These tissues were detected for the expression of S100A4, NF-kB/RelA and MMP-9 by immunohistochemistry and quantitative real time polymerase chain reaction (Q-PCR). Correlation between the expressions of S100A4, NF-kB/RelA and MMP-9 with the invasion was analysed. The expressions of S100A4, nuclear factor-kB and MMP-9 was evaluated in HepG2 cells by western blot and immunohistochemistry. HepG2 cells were stably transfected with S100A4-specific small interfering RNA (S100A4 siRNA) to knockdown of S100A4, then transiently transfected with S100A4 cDNA to rescure the S100A4 level and evaluated for effects on invasion and expression analysis for molecules involved in invasion. After the HepG2 cells recurred the S100A4 levels, the HepG2 cells was treated with 5 µM Pyrrolidine Dithiocarbamate (PDTC) (a selective NF κ B inhibitor) to inhibit the NF-kB activity, or treated with Batimast (BB94: a MMPs inhibitor) to inhibit the MMP-9 activity. The expression analysis for molecules involved in invasion was analyzed.A significant increase of S100A4, NF-kB/RelA and MMP-9 expression in HCC tissues with invasion than that of without invasion. A positive correlation was observed between S100A4, NF-kB/RelA, MMP-9 and invasion, respectively. In addition, S100A4 was positively correlated with NF-kB and MMP-9. S100A4 siRNA mediated knockdown of S100A4 in HepG2 cells resulted in significant reduction in the NF-kB activity and MMP-9 expression, and dramatically decreased its invasion. Moreover, the HepG2 cell metastatic potential was rescued by overexpression of S100A4 completely, at the same time, the NF-kB activity and MMP-9 expression was also increased. Pretreatment with PDTC or BB94 was observed to significantly reduce NF-kB activity and MMP-9 expression and dramatically decreased S100A4 -induced invasion.Our findings indicate that S100A4 contributes to HCC metastasis by activation of NF-kB dependent MMP-9 expression, suggesting S100A4 as a novel diagnostic biomarker and therapeutic target in HCC. 
 Notch1 promotes glioma cell   migration   and invasion by stimulating β-catenin and NF-κB signaling via AKT activation. The Notch signaling pathway has been implicated in both developmental processes and tumorigenesis. Aberrant Notch signaling has been repeatedly demonstrated to facilitate the proliferation and survival of glioma cells by regulating downstream effectors or other signaling pathways. In glioblastoma multiforme specimens from 59 patients, Notch1 was highly expressed in tumor tissues compared with normal brain tissues, and this expression was correlated with elevated AKT phosphorylation and Snail expression. Increased nuclear localization of β-catenin and p50 as well as enhanced IKKα/AKT interaction were also observed in glioma tissues. In U87MG cells, the activation of Notch1 by DLL4 stimulation or by the overexpression of Notch intracellular domain (NICD) resulted in AKT activation and thereby promoted β-catenin activity and NF-κB signaling. Inhibition of EGFR partially blocked the β-catenin and NF-κB signaling stimulated by Notch1 activation. Furthermore, NICD overexpression in U87MG cells led to the upregulated expression of several metastasis-associated molecules, which could be abrogated by the knockdown of either β-catenin or p50. In U87MG and U251 cells, DLL4-induced   cellular   migration and invasion could be inhibited by either β-catenin or a p50 inhibitor. Collectively, these results indicate that Notch activation could stimulate β-catenin and NF-κB signaling through AKT activation in glioma cells. Thus, Notch activation-stimulated β-catenin and NF-κB signaling synergistically promote the migratory and invasive properties of glioma cells. 
 Knockdown of CypA inhibits interleukin-8 (IL-8) and IL-8-mediated proliferation and tumor growth of glioblastoma cells through down-regulated NF-κB. Although cyclophilin A (CypA) has been reported to be over-expressed in cancer cells and solid tumors, its expression and role in glioblastomas have not been studied. Herein, we show that expression of CypA in human glioblastoma cell lines and tissues is significantly higher than in normal human astrocytes and normal counterparts of brain tissue. To determine the role of over-expressed CypA in glioblastoma, stable RNA interference (RNAi)-mediated knockdown of CypA (CypA KD) was performed in gliobastoma cell line U87vIII (U87MG · ΔEGFR). CypA KD stable single clones decrease proliferation, infiltrat  ion, migr  ation, and anchorage-independent growth in vitro and with slower growth in vivo as xenografts in immunodeficient nude mice. We have also observed that knockdown of CypA inhibits expression of interleukin-8 (IL-8), a tumorigenic and proangiogenic cytokine. Conversely, enforced expression of CypA in the CypA KD cell line, Ud-12, markedly enhanced IL-8 transcripts and restored Ud-12 proliferation, suggesting that CypA-mediated IL-8 production provides a growth advantage to glioblastoma cells. CypA knockdown-mediated inhibition of IL-8 is due to reduced activity of NF-κB, which is one of the major transcription factors regulating IL-8 expression. These results not only establish the relevance of CypA to glioblastoma growth in vitro and in vivo, but also suggest that small interfering RNA-based CypA knockdown could be an effective therapeutic approach against glioblastomas. 
 The prolyl isomerase Pin1 regulates the NF-kappaB signaling pathway and interleukin-8 expression in glioblastoma. The brain tumor glioblastoma (GBM) remains one of the most aggressive and devastating tumors despite decades of effort to find more effective treatments. A hallmark of GBM is the constitutive activation of the nuclear factor kappa-light-chain-enhancer of activated B cells (NF-kappaB) signaling pathway, which regulates cell proliferation, inflammation,   migration   and apoptosis. The prolyl isomerase, Pin1, has been found to bind directly to the NF-kappaB protein, p65, and cause increases in NF-kappaB promoter activity in a breast cancer model. We now present evidence that this interaction occurs in GBM and that it has important consequences on NF-kappaB signaling. We demonstrate that Pin1 levels are enhanced in primary GBM tissues compared with controls, and that this difference in Pin1 expression affects the migratory capacity of GBM-derived cells. Pin1 knockdown decreases the amount of activated, phosphorylated p65 in the nucleus, resulting in inhibition of the transcriptional program of the IL-8 gene. Through the use of microarray, we also observed changes in the expression levels of other NF-kappaB regulated genes due to Pin1 knockdown. Taken together, these data suggest that Pin1 is an important regulator of NF-kappaB in GBM, and support the notion of using Pin1 as a therapeutic target in the future. 
 Involvement of ERK1/2/NF-κB signal transduction pathway in TF/FVIIa/PAR2-induced proliferation and   migratio  n of colon cancer cell SW620. Our previous study has demonstrated that TF/FVIIa and protease-activated receptor 2 (PAR2) are closely related to the proliferation and   migratio  n of colon cancer cell line SW620. However, the detailed signaling cascades and underlying molecular mechanisms remain unclear. This study has investigated whether extracellular signal-regulated kinase 1 and 2 (ERK1/2) and nuclear factor kappaB (NF-κB) signaling pathways are involved in the events. The results revealed that PAR2-activating peptide (PAR2-AP) or FVIIa elicited time-dependent upregulation of ERK1/2 phosphorylation in SW620 cells, and the effect of FVIIa was significantly attenuated by anti-TF antibody. PAR2-AP or FVIIa also increased NF-κB (p65/RelA) levels among cell nuclear proteins and simultaneously decreased IκB-α levels in the cytoplasmic proteins. Such effects of FVIIa can be inhibited with anti-PAR2 or anti-TF antibodies. While ERK1/2 inhibitor (U0126) intervened with the regulatory effects of PAR2-AP and FVIIa on IκB-α/NF-κB (p65/Rel) expression in the cells, NF-κB inhibitor (PDTC) partially blocked the enhancing effects of PAR2-AP and FVIIa on the proliferating and migratory ability of SW620 cells. Furthermore, the regulatory effects of PAR2-AP and FVIIa on expressions of certain proteins (IL-8, caspase-7, and TF) were also significantly abolished by PDTC. Collectively, the data in this study suggest that the interaction between FVIIa and TF induces PAR2 activation, thereby triggers the ERK1/2 and IκB-α/NF-κB signal transduction pathway to regulate the gene expression of IL-8, TF, and caspase-7, and ultimately promotes SW620 cell prolif  eration a  nd migration. 
 Antiproliferative and antimetastatic effects of emodin on human pancreatic cancer. Emodin (1, 3, 8-trihydroxy-6-methylanthraquinone) is an active constituent isolated from the root of Rheum palmatum L and is the main effective component of some Chinese herbs and plants. Pharmacological studies have demonstrated that emodin exhibits anti-cancer effects on several human cancers. However, the molecular mechanisms of emodin-mediated tumor regression have not been fully defined. This study was performed to investigate the antiproliferative and antimetastatic effects of emodin on pancreatic cancer in vitro and in vivo. Our results showed that emodin induced a higher percentage of growth inhibition and apoptosis in the pancreatic cancer cell line SW1990 compared to that of control, and emodin suppressed the   migration   and invasion of SW1990 cells in a dose-dependent manner. To investigate the possible mechanisms involved in these events, we performed electrophoretic mobility shift assay (EMSA) and Western blot analysis, and found that emodin significantly down-regulated NF-κB DNA-binding activity, survivin and MMP-9 in SW1990 cells. Moreover, the expression of cleaved caspase-3 was up-regulated in SW1990 cells after treatment with emodin. In addition, a metastatic model simulating human pancreatic cancer was established by orthotopic implantation of histologically intact human tumor tissue into the pancreatic wall of nude mice. Oral administration of emodin significantly decreased tumor weight and metastasis compared to control. Furthermore, the expression of NF-κB, survivin and MMP-9 were also suppressed in tumor tissues after treatment with emodin. Collectively, our results indicated that emodin exerts antiproliferative and antimetastatic activity on pancreatic cancer both in vitro and in vivo, which may be related to down-regulation of NF-κB and its regulated molecules such as survivin and MMP-9 proteins. Consequently, these results provide important insights into emodin as an anti-invasive agent for the therapy of human pancreatic cancer. 
 Factor VIIa regulates the expression of caspase-3, MMP-9, and CD44 in SW620 colon cancer cells involving PAR2/MAPKs/NF-κB signaling pathways. Our previous study has demonstrated that TF/FVIIa and PAR2 are closely related to the proliferation and   migratio  n of colon cancer cell line SW620. However, the detailed molecular mechanisms in the process remain unclear. This study further investigated whether some important molecules (caspase-3, MMP-9 and CD44) are involved in the events. The results showed that PAR2-AP or FVIIa elicited time-dependent downregulation of caspase-3, and up-regulation of MMP-9 and CD44 in SW620 cells. The effects of FVIIa were TF-dependent and involving PAR2/MAPKs/NF-κB signal transduction pathways. Our study suggests that the links among PAR2/MAPKs/NF-κB may be blocked for effective treatments of colorectal cancers. 
 Overexpression of SDF-1 activates the NF-κB pathway to induce epithelial to mesenchymal transition and cancer stem cell-like phenotypes of breast cancer cells. The formation of EMT and EMT-induced CSC-like phenotype is crucial for the metastasis of tumor cells. The stromal cell-derived factor-1 (SDF-1) is upregulated in various human carcinomas, which is closely associated with proliferation,   migratio  n, invasion and prognosis of malignancies. However, limited attention has been directed towards the effect of SDF-1 on epithelial to mesenchymal transition (EMT) or cancer stem cell (CSC)-like phenotype formation in breast cancer cells and the related mechanism. In the present study, we screened MCF-7 cells with low SDF-1 expression level for the purpose of evaluating whether SDF-1 is involved in EMT and CSC-like phenotype formation in MCF-7 cells. The pEGFP-N1-SDF-1 plasmid was transfected into MCF-7 cells, and the stably overexpressed SDF-1 in MCF-7 cells was confirmed by real-time PCR and western blot analysis. Colony formation assay, MTT, wound healing assay and Transwell invasion assay demonstrated that overexpression of SDF-1 significantly boosted the proliferation,   migratio  n and invasion of MCF-7 cells compared with parental (P<0.05). Flow cytometry analysis revealed a notable increase of CD44+/CD24- subpopulation in SDF-1 overexpressing MCF-7 cells (P<0.001), accompanied by the apparently elevated ALDH activity and the upregulation of the stem cell markers OCT-4, Nanog, and SOX2 compared with parental (P<0.01). Besides, western blot analysis and immunofluorescence assay observed the significant decreased expression of E-cadherin and enhanced expression of slug, fibronectin and vimentin in SDF-1 overexpressed MCF-7 cells in comparison with parental (P<0.01). Further study found that overexpression of SDF-1 induced the activation of NF-κB pathway in MCF-7 cells. Conversely, suppressing or silencing p65 expression by antagonist or RNA interference could remarkably increase the expression of E-cadherin in SDF-1 overexpressed MCF-7 cells (P<0.001). Overall, the above results indicated that overexpression of SDF-1 enhanced EMT by activating the NF-κB pathway of MCF-7 cells and further induced the formation of CSC-like phenotypes, ultimately promoting the proliferation and metastasis of MCF-7 cells. Therefore, SDF-1 may further be assessed as a potential target for gene therapy of breast cancer. 
 Poxviral regulation of the host NF-kappaB response: the vaccinia virus M2L protein inhibits induction of NF-kappaB activation via an ERK2 pathway in virus-infected human embryonic kidney cells. Exposure of eukaryotic cells to viruses will activate the host NF-kappaB transcription factor, resulting in proinflammatory and immune protein production. Vaccinia virus (VV), the prototypic orthopoxvirus, expresses products that inhibit this antiviral event. To identify novel mechanisms responsible for this effect, we made use of a VV deletion mutant (MVA) that stimulates NF-kappaBeta activation in infected 293T cells. In this virus-host system, the extents of NF-kappaBeta-regulated gene expression and nuclear translocation were reduced in the presence of either PD 98059 or U0126, two compounds capable of blocking ERK1 and ERK2 phosphorylation. A similar repression was also observed in cells that contained a dominant, nonactive form of ERK2 but not in cells where ERK1 phosphorylation was inhibited via overexpression of a dominant-negative mutant MEK1 protein. Presumably, proteins expressed from a wild-type VV that block ERK2 activity would also inhibit MVA-induced NF-kappaB activation. Indeed, the expression of one such open reading frame, M2L, supported this prediction. First, ectopic M2L expression hampered ERK2 phosphorylation induced by exposure to phorbol myristate acetate. Second, viral M2L expression via infection of cells with a recombinant MVA construct that stably expressed M2L decreased the phosphorylation of ERK2 compared to that in cells infected with the parental MVA strain. Finally, the recombinant M2L-expressing virus restored the "wild-type" NF-kappaB-inhibitory phenotype, as indicated by decreased NF-kappaB   migration   to infected cell nuclei and interference in transcription. Thus, in 293T cells, VV apparently utilizes its M2L protein to interfere with a step(s) that would otherwise enable ERK2 phosphorylation and the consequential activation of an NF-kappaBeta response. 
 Osteopontin increases   migration   and MMP-9 up-regulation via alphavbeta3 integrin, FAK, ERK, and NF-kappaB-dependent pathway in human chondrosarcoma cells. Tumor malignancy is associated with several features such as proliferation ability and frequency of metastasis. Osteopontin (OPN), which abundantly expressed in bone matrix, is involved in cell adhesion,   migration  , invasion and proliferation via interaction with its receptor, that is, alphavbeta3 integrin. However, the effect of OPN on   migration   activity in human chondrosarcoma cells is mostly unknown. Here we found that OPN increased the   migration   and expression of matrix metalloproteinase (MMP)-9 in human chondrosarcoma cells (JJ012 cells). RGD peptide, alphavbeta3 monoclonal antibody and MAPK kinase (MEK) inhibitors (PD98059 and U0126) but not RAD peptide inhibited the OPN-induced increase of the   migration   and MMP-9 up-regulation of chondrosarcoma cells. OPN stimulation increased the phosphorylation of focal adhesion kinase (FAK), MEK and extracellular signal-regulated kinase (ERK). In addition, treatment of JJ012 cells with NF-kappaB inhibitor (PDTC) or IkappaB protease inhibitor (TPCK) inhibited OPN-induced cell   migration   and MMP-9 up-regulation. Stimulation of JJ012 cells with OPN also induced IkappaB kinase alpha/beta (IKK alpha/beta) phosphorylation, IkappaBalpha phosphorylation, p65 Ser(536) phosphorylation, and kappaB-luciferase activity. The OPN-mediated increases in MMP-9 and kappaB-luciferase activities were inhibited by RGD peptide, PD98059 or FAK and ERK2 mutant. Taken together, our results indicated that OPN enhances the   migration   of chondrosarcoma cells by increasing MMP-9 expression through the alphavbeta3 integrin, FAK, MEK, ERK and NF-kappaB signal transduction pathway. 
 IL-1 beta induces urokinase-plasminogen activator expression and cell   migration   through PKC alpha, JNK1/2, and NF-kappaB in A549 cells. Breakdown of the extracellular matrix (ECM) is accomplished by the concerted action of several proteases, including the urokinase plasminogen-activator (uPA) system and matrix metalloproteinases (MMPs), which is crucial for cancer invasion and metastasis. Several reports have shown that the levels of IL-1 beta and MMPs in plasma of the patients with lung cancer are significantly elevated and link to the invasion of tumor cells. Therefore, we investigated whether IL-1 beta-induced expression of uPA participated in lung cancer progression. In this study, IL-1 beta significantly induced uPA expression and activity via PKC alpha-dependent JNK1/2 and NIK cascades, linking to IKK alpha/beta activation, p65 translocation and transcription activity, using pharmacological inhibitors and transfection with dominant negative mutants and siRNAs. IL-1 beta-induced uPA protein and mRNA expression in a time- and concentration-dependent manner, which was inhibited by pretreatment with the inhibitors of JNK1/2 (SP600125), PKC (Ro31-8220, Gö6976), or NF-kappaB (helenalin), and transfection with dominant negative mutants of PKC alpha, NIK, and IKK beta, and siRNAs of JNK1/2 and p65. IL-1 beta stimulated PKC alpha translocation to plasma membrane leading to phosphorylation of JNK1/2, which was attenuated by PKC inhibitors and transfection with shRNAs of JNK1/2, but not by helenalin. In addition, IL-1beta stimulated p65 phosphorylation and translocation into nucleus concomitant with I kappaB alpha phosphorylation and I kappaB alpha degradation, which was mediated via activation of PKC alpha-dependent JNK1/2-NIK/IKK beta cascade. These results demonstrated that in A549 cells, activation of p50/p65 heterodimer through sequential activation of PKC alpha-JNK-NIK-IKK beta-NF-kappaB was required for IL-1 beta-induced uPA expression associated with   migratio  n of tumor cells. 
 Essential role of cooperative NF-κB and Stat3 recruitment to ICAM-1 intronic consensus elements in the regulation of radiation-induced invasion and   migratio  n in glioma. Although radiotherapy improves survival in patients, glioblastoma multiformes (GBMs) tend to relapse with augmented tumor   migratio  n and invasion even after ionizing radiation (IR). Aberrant nuclear factor-κB (NF-κB) and signal transducer and activator of transcription factor 3 (Stat3) activation and interaction have been suggested in several human tumors. However, possible NF-κB/Stat3 interaction and the role of Stat3 in maintenance of NF-κB nuclear retention in GBM still remain unknown. Stat3 and NF-κB (p65) physically interact with one another in the nucleus in glioma tumors. Most importantly, glutathione S-transferase pull-down assays identified that Stat3 binds to the p65 transactivation domain and is present in the NF-κB DNA-binding complex. Irradiation significantly elevated nuclear phospho-p65/phospho-Stat3 interaction in correlation with increased intercellular adhesion molecule-1 (ICAM-1) and soluble-ICAM-1 l  evels, mi  gration and invasion in human glioma xenograft cell lines 4910 and 5310. Chromatin immunopreicipitation and promoter luciferase activity assays confirmed the critical role of adjacent NF-κB (+399) and Stat3 (+479) binding motifs in the proximal intron-1 in elevating IR-induced ICAM-1 expression. Specific inhibition of Stat3 or NF-κB with Stat3.siRNA or JSH-23 severely inhibited IR-induced p65 recruitment onto ICAM-1 intron-1 and suppressed migratory properties in both the cell lines. On the other hand, Stat3C- or IR-induced Stat3 promoter recruitment was significantly decreased in p65-knockdown cells, thereby suggesting the reciprocal regulation between p65 and Stat3. We also observed a significant increase in NF-κB enrichment on ICAM-1 intron-1 and ICAM-1 transactivation in Stat3C overexpressing cells. In in vivo orthotopic experiments, suppression of tumor growth in Stat3.si+IR-treated mice was associated with the inhibition of IR-induced p-p65/p-Stat3 nuclear colocalization and ICAM-1 levels. To our knowledge, this is the first study showing the crucial role of NF-κB/Stat3 nuclear association in IR-induced ICAM-1 regulation and implies that targeting NF-κB/Stat3 interaction may have future therapeutic significance in glioma treatment. 
 Host defence peptide LL-37 induces IL-6 expression in human bronchial epithelial cells by activation of the NF-kappaB signaling pathway. LL-37, the only member of the cathelicidin family of cationic host defence peptides in humans, has been shown to mediate multiple immunomodulatory effects and as such is thought to be an important component of innate immune responses. A growing body of evidence indicates that LL-37 affects lung mucosal responses to pathogens through altered regulation of cell   migration  , proliferation, wound healing and cell apoptosis. These functions are consistent with LL-37 playing a role in regulating lung epithelial inflammatory responses; however, that role has not been clearly defined. In this report we have demonstrated that host defence peptide LL-37 induced cytokine (IL-6) and chemokine (CXCL-1/GRO-alpha and CXCL-8/IL-8) release from human bronchial epithelial cells. It was demonstrated that LL-37-mediated IL-6 release was time and dose dependent and that LL-37 up-regulated this pleiotropic cytokine at the transcriptional level. Using specific inhibitors it was shown that NF-kappaB signaling led to the LL-37-stimulated production of IL-6. LL-37 stimulation of airway epithelial cells activated NF-kappaB signaling, as demonstrated by the phosphorylation and degradation of Ikappa-Balpha, and consequent nuclear translocation of p65 and p50 NF-kappaB subunits. Furthermore this host defence peptide augmented flagellin-mediated cytokine production, indicating that LL-37 likely modulates Toll-like receptor 5-mediated responses. 
 Annexin-A1 controls an ERK-RhoA-NFκB activation loop in breast cancer cells. Wound healing is critical for normal development and pathological processes including cancer cell metastasis. MAPK, Rho-GTPases and NFκB are important regulators of wound healing, but mechanisms for their integration are incompletely understood. Annexin-A1 (ANXA1) is upregulated in invasive breast cancer cells resulting in constitutive activation of NFκB. We show here that silencing ANXA1 increases the formation of stress fibers and focal adhesions, which may inhibit wound healing. ANXA1 regulated wound healing is dependent on the activation of ERK1/2. ANXA1 increases the activation of RhoA, which is dependent on ERK activation. Furthermore, active RhoA is important in NF-κB activation, where constitutively active RhoA potentiates NFκB activation, while dominant negative RhoA inhibits NFκB activation in response to CXCL12 stimulation and active MEKK plasmids. These findings establish a central role for ANXA1 in the   cell mig  ration through the activation of NFκB, ERK1/2 and RhoA. 
 Effects of NF-κB and hypoxia on the biological behavior of Y79 retinoblastoma cells. We aimed to investigate the influence of nuclear factor-κB (NF-κB) on the biological behavior of Y79 retinoblastoma cells exposed to hypoxia and its possible mechanism. The cells were administrated with hypoxia, and/or 5 μM pyrrolidine dithiocarbamate (PDTC) (a selective NF-κB inhibitor) to inhibit the NF-κB activity, expressions of NF-κB was measured by western blot, and the translocation of NF-κB was detected. To examine the proliferation of Y79 cells, MTT assay was applied. Transwell assay was used to detect the invas  ion and m  igration ability of cells. The expressions of molecules involved in invasion was analyzed including HIF-1α, MMP-2, 9, and VEGF. We found that hypoxia significantly activated NF-κB activity. While once the NF-κB was inhibited, the proliferation, in  vasion an  d migration ability of Y79 cells were also blocked. Interestingly, the expressions of invasion-involved molecules elevated by hypoxia induction were also decreased when NF-κB was inhibited. Hypoxia could significantly change the adhesive and invasive ability of Y79 retinoblastoma cells, NF-κB signal might be one of the main mediators for these hypoxia induced cell changes of biological behavior via downregulation of HIF-1α and the invasion related molecules, and the mechanism still needs further investigation. 
 Alchornea glandulosa ethyl acetate fraction exhibits antiangiogenic activity: preliminary findings from in vitro assays using human umbilical vein endothelial cells. Alchornea glandulosa has traditionally been used in Brazilian folk medicine for the treatment of immune-inflammatory diseases and as an antiulcer agent to heal gastric ulcer and gastritis. Angiogenesis is a complex multistep process that consists of proliferation,   migration  , and anastomosis of endothelial cells and has a major role in the development of pathologic conditions, such as inflammatory diseases. To investigate a possible link between the anti-inflammatory activities and antiangiogenic effects of A. glandulosa ethyl acetate fraction (AGF), this study examined which features of the angiogenic process could be disturbed by this fraction. The antiangiogenic activity of AGF was determined in vitro by using human umbilical vein endothelial cells (HUVEC), and cell viability, proliferation, apoptosis, invasion, and capillary-like structures formation were addressed. To elucidate the mechanism of action, nuclear factor κB (NFκB), a transcription factor implicated in these processes, was also evaluated in HUVEC incubated with AGF. A significant decrease in proliferation, a relevant increase in apoptosis, and a strong reduction in invasion capacity (as assessed by bromodeoxyuridine, terminal deoxynucleotidyl transferase-mediated deoxyuridine triphosphate nick-end labeling, and double-chamber assays, respectively) were observed. AGF also led to a drastic reduction in the number of capillary-like structures formed when HUVEC were cultured on growth factor-reduced Matrigel-coated plates. In addition, incubation of HUVEC with AGF resulted in reduced NFκB activity. These findings emphasize the antiangiogenic potential of AGF and support its therapeutic use for disorders that involve excessive angiogenesis, such as chronic inflammation and tumor growth. 
 Cooperative interactions between p53 and NFκB enhance cell plasticity. The p53 and NFκB sequence-specific transcription factors play crucial roles in cell proliferation and survival with critical, even if typically opposite, effects on cancer progression. To investigate a possible crosstalk between p53 and NFκB driven by chemotherapy-induced responses in the context of an inflammatory microenvironment, we performed a proof of concept study using MCF7 cells. Transcriptome analyses upon single or combined treatments with doxorubicin (Doxo, 1.5μM) and the NFκB inducer TNF-alpha (TNFα, 5ng/ml) revealed 432 up-regulated (log2 FC> 2), and 390 repressed genes (log2 FC< -2) for the Doxo+TNFα treatment. 239 up-regulated and 161 repressed genes were synergistically regulated by the double treatment. Annotation and pathway analyses of Doxo+TNFα selectively up-regulated genes indicated strong enrichment f  or cell m  igration terms. A panel of genes was examined by qPCR coupled to p53 activation by Doxo, 5-Fluoruracil and Nutlin-3a, or to p53 or NFκB inhibition. Transcriptome data were confirmed for 12 of 15 selected genes and seven (PLK3, LAMP3, ETV7, UNC5B, NTN1, DUSP5, SNAI1) were synergistically up-regulated after Doxo+TNFα and dependent both on p53   and NFκB  . Migration assays consistently showed an increase in motility for MCF7 cells upon Doxo+TNFα. A signature of 29 Doxo+TNFα highly synergistic genes exhibited prognostic value for luminal breast cancer patients, with adverse outcome correlating with higher relative expression. We propose that the crosstalk between p53 and NFκB can lead to the activation of specific gene expression programs that may impact on cancer phenotypes and potentially modify the efficacy of cancer therapy. 
 HIV-1 Tat disrupts CX3CL1-CX3CR1 axis in microglia via the NF-κBYY1 pathway. Microglia are critical for the pathogenesis of HIV-associated dementia not only by acting as conduits of viral entry but also as reservoirs for productive and latent virus infection, and as producers of neurotoxins. Interaction between CX3CL1 (fractalkine) and FKN receptor (CX3CR1) is highly functional in the brain, and is known to regulate a complex network of paracrine and autocrine interactions between neurons and microglia. The aim of the present study was to determine which extent of HIV-1 Tat protein causes the alteration of CX3CR1 expression and to investigate the regulatory mechanism for CX3CR1 expression. Here we showed that exposure of primary microglia and BV2 cells to exogenous Tat protein resulted in down-regulation of CX3CR1 mRNA and protein expression, with a concomitant induction of proinflammatory responses. Next, we further showed that NF-κB activation by Tat treatment negatively regulated CX3CR1 expression. Since a YY1 binding site ~10kb upstream of CX3CR1 promoter was predicted in rats, mice and humans, the classical NF-κB-YY1 regulatory pathway was considered. Our findings indicated that Tat repressed CX3CR1 expression via NF-κB-YY1 regulatory pathway. To gain insight into the effect of Tat on CX3CL1-CX3CR1 communication, calcium mobilization, MAPK activation and microgl  ial migra  tion, respectively, were tested in microglial cells after successive treatment with Tat and CX3CL1. The results suggested that Tat disrupted the responses of microglia to CX3CL1. Taken together, these results demonstrate that HIV-1 Tat protein suppresses CX3CR1 expression in microglia via NF-κB-YY1 pathway and attenuates CX3CL1-induced functional response of microglia. 
 Ghrelin induces gastric cancer cell proliferation,   migration  , and invasion through GHS-R/NF-κB signaling pathway. This study aims to investigate the roles of ghrelin signaling in human gastric carcinoma cell lines AGS and SGC7901. Effects of ghrelin signaling on CDK6, P53, NF-κB/P65 and MMP2 mRNA and/or protein expression were determined by real-time PCR and western blot. MTT method and flow cytometry were performed to assess the gastric cancer cell proliferation. The SGC7901 cells overexpressing ghrelin were inoculated into nude mice to produce tumors which were measured later. The wound-healing assay and cell invasion assay were used to test the cel  l migrati  on and invasive ability of gastric cancer. Ghrelin signaling promotes the oncogene CDK6 gene expression and represses the tumor suppressor gene P53 gene expression in gastric cancer. Ghrelin activates NF-κB/P65 signaling pathway through GHS-R in gastric cancer. Ghrelin upregulates the metastasis factor MMP2 expression via GHS-R/NF-κB signaling pathway in gastric cancer cells and promotes tumor ce  lls migra  tion and invasion, suggesting that ghrelin signaling is a critical pathway in cancer metastasis. Ghrelin induces cell proliferati  on, migra  tion and invasion via GHS-R/NF-κB signaling pathway in gastric cancer cells. Ghrelin treatment must be avoided for gastric cancer patients. 
 Exosomes derived from gastric cancer cells activate NF-κB pathway in macrophages to promote cancer progression. Exosomes are nano-sized membrane vesicles secreted by both normal and cancer cells. Emerging evidence indicates that cancer cells derived exosomes contribute to cancer progression through the modulation of tumor microenvironment. However, the effects of exosomes derived from gastric cancer cells on macrophages are not well understood. In this study, we investigated the biological role of gastric cancer cells derived exosomes in the activation of macrophages. We demonstrated that gastric cancer cells derived exosomes activated macrophages to express increased levels of proinflammatory factors, which in turn promoted tumor cell proliferation and   migratio  n. In addition, gastric cancer cells derived exosomes remarkably upregulated the phosphorylation of NF-κB in macrophages. Inhibiting the activation of NF-κB reversed the upregulation of proinflammatory factors in macrophages and blocked their promoting effects on gastric cancer cells. Moreover, we found that gastric cancer cells derived exosomes could also activate macrophages from human peripheral blood monocytes through the activation of NF-κB. In conclusion, our results suggest that gastric cancer cells derived exosomes stimulate the activation of NF-κB pathway in macrophages to promote cancer progression, which provides a potential therapeutic approach for gastric cancer by interfering with the interaction between exosomes and macrophages in tumor microenvironment. 
 CARMA3 is crucial for EGFR-Induced activation of NF-κB and tumor progression. EGF activates NF-κB, and constitutively activated NF-κB contributes to EGFR mutation-associated tumorigenesis, but it remains unclear precisely how EGFR signaling leads to NF-κB activation. Here we report that CARMA3, a caspase recruitment domain (CARD)-containing scaffold molecule, is required for EGF-induced NF-κB activation. CARMA3 deficiency impaired the activation of the IKK complex following EGF stimulation, resulting in a defect of EGF-induced IκBα phosphorylation and NF-κB activation. We found that CARMA3 and Bcl10 contributed to several characteristics of EGFR-associated malignancy, including proliferation, su  rvival, m  igration, and invasion. Most importantly, CARMA3 contributed to tumor growth in vivo. Our findings elucidate a crucial link between EGFR-proximal signaling components and the downstream IKK complex, and they suggest a new therapeutic target for treatment of EGFR-driven cancers. 
 Raf kinase inhibitor protein suppresses nuclear factor-κB-dependent cancer cell invasion through negative regulation of matrix metalloproteinase expression. Accumulating evidence suggests that Raf kinase inhibitor protein (RKIP), which negatively regulates multiple signaling cascades including the Raf and nuclear factor-κB (NF-κB) pathways, functions as a metastasis suppressor. However, the basis for this activity is not clear. We investigated this question in a panel of breast cancer, colon cancer and melanoma cell lines. We found that RKIP negatively regulated the invasion of the different cancer cells through three-dimensional extracellular matrix barriers by controlling the expression of matrix metalloproteinases (MMPs), particularly, MMP-1 and MMP-2. Silencing of RKIP expression resulted in a highly invasive phenotype and dramatically increased levels of MMP-1 and MMP-2 expression, while overexpression of RKIP decreased cancer cell invasion in vitro and metastasis in vivo of murine tumor allografts. Knockdown of MMP-1 or MMP-2 in RKIP-knockdown cells reverted their invasiveness to normal. In contrast, when examini  ng migrat  ion of the different cancer cells in a two-dimensional, barrier-less environment, we found that RKIP had either a positive regulatory activity or no activity, but in no case a negative one (as would be expected if RKIP suppressed metastasis at the level of ce  ll migrat  ion itself). Therefore, RKIP's function as a metastasis suppressor appears to arise from its ability to negatively regulate expression of specific MMPs, and thus invasion through barriers, and not from a direct effect on the raw capacity of cells to move. The NF-κB pathway, but not the Raf pathway, appeared to positively control the invasion of breast cancer cells. A regulatory loop involving an opposing relationship between RKIP and the NF-κB pathway may control the level of MMP expression and cell invasion. 
 Wogonin suppresses TNF-alpha-induced MMP-9 expression by blocking the NF-kappaB activation via MAPK signaling pathways in human aortic smooth muscle cells. Matrix metalloproteinase-9 (MMP-9) plays a major role in the pathogenesis of atherosclerosis and restenosis by regulating both   migration   and proliferation of vascular smooth muscle cells (VSMC) after an arterial injury. In this study, we examined the inhibitory effect of three major flavonoids in Scutellariae Radix, baicalin, baicalein, and wogonin, on TNF-alpha-induced MMP-9 expression in human aortic smooth muscle cells (HASMC). Wogonin, but not baicalin and baicalein, significantly and selectively suppressed TNF-alpha-induced MMP-9 expression in HASMC. Reporter gene, electrophoretic mobility shift, and Western blotting assays showed that wogonin inhibits MMP-9 gene transcriptional activity by blocking the activation of NF-kappaB via MAPK signaling pathways. Moreover, the Matrigel   migration   assay showed that wogonin reduced TNF-alpha-induced HASMC   migration  . These results suggest that wogonin effectively suppresses TNF-alpha-induced HASMC   migration   through the selective inhibition of MMP-9 expression and represents a potential agent for the prevention of vascular disorders related to the   migration   of VSMC. 
 Role of the adipocyte-specific NF-κB activity in the regulation of IP-10 and T cell   migratio  n. Infiltration of immune cells into adipose tissue plays a central role in the pathophysiology of obesity-associated low-grade inflammation. The aim of this study was to analyze the role of adipocyte NF-κB signaling in the regulation of the chemokine/adipokine interferon-γ-induced protein 10 kDa (IP-10) and adipocyte-mediated T ce  ll migrat  ion. Therefore, the regulation of IP-10 was investigated in adipose tissue of male C57BL/6J mice, primary human and 3T3-L1 preadipocytes/adipocytes. To specifically block the NF-κB pathway, 3T3-L1 cells stably overexpressing a transdominant mutant of IκBα were generated, and the chemical NF-κB inhibitor Bay117082 was used. Adipocyte-mediated   T cell mi  gration was assesse  d by a mi  gration assay. It could be shown that IP-10 expression was higher in mature adipocytes compared with preadipocytes. Induced IP-10 expression and secretion were completely blocked by an NF-κB inhibitor in 3T3-L1 and primary human adipocytes. Stable overexpression of a transdominant mutant of IκBα in 3T3-L1 adipocytes led to an inhibition of basal and stimulated IP-10 expression and secretio  n. T cell   migration was induced by 3T3-L1 adipocyte-conditioned medium, and both basal and induc  ed T cell   migration was strongly inhibited by stable overexpression of a transdominant IκBα mutant. In addition, with the use of an anti-IP-10 antibody, a significant decrease of adipocyte-ind  uced T ce  ll migration was shown. In conclusion, in this study, we could demonstrate that the NF-κB pathway is essential for the regulation of IP-10 in 3T3-L1 and primary human adipocytes. Adipocytes rather than preadipocytes contribute to NF-κB-dependent IP-10 expression and secretion. Furthermore, NF-κB-dependent factors and especially IP-10 represent novel signals from adipocytes to   induce T   cell migration. 
 Participation of the cell polarity protein PALS1 to T-cell receptor-mediated NF-κB activation. Beside their established function in shaping cell architecture, some cell polarity proteins were proposed to participate to lymphocyte   migratio  n, homing, scanning, as well as activation following antigen receptor stimulation. Although PALS1 is a central component of the cell polarity network, its expression and function in lymphocytes remains unknown. Here we investigated whether PALS1 is present in T cells and whether it contributes to T Cell-Receptor (TCR)-mediated activation.By combining RT-PCR and immunoblot assays, we found that PALS1 is constitutively expressed in human T lymphocytes as well as in Jurkat T cells. siRNA-based knockdown of PALS1 hampered TCR-induced activation and optimal proliferation of lymphocyte. We further provide evidence that PALS1 depletion selectively hindered TCR-driven activation of the transcription factor NF-κB.The cell polarity protein PALS1 is expressed in T lymphocytes and participates to the optimal activation of NF-κB following TCR stimulation. 
 Epidermal growth factor (EGF)-induced corneal epithelial wound healing through nuclear factor κB subtype-regulated CCCTC binding factor (CTCF) activation. Epidermal growth factor (EGF) plays an important role in corneal epithelial   migratio  n and proliferation to improve the wound healing process. This study aimed to understand the role of NFκB in EGF-induced corneal epithelial wound healing through regulation of CTCF activity, which plays important roles in cell motility an  d migrati  on to promote wound healing. The effect of NFκB p50 on corneal epithelial wound healing was investigated by comparing the eyes of wild-type and p50 knockout mice. We found that there was a significant retardation in corneal epithelial wound healing in the corneas of p50 knockout mice. Wound closure rates were measured in human corneal epithelial cells transfected with an NFκB activation-sensitive CTCF expression construct to demonstrate the effect of human CTCF expression under the control of EGF-induced NFκB activation on wound healing. EGF stimulation activated NFκB, which directly triggered the expression of the exogenous human CTCF in transfected cells and, subsequently, promoted human corneal epithelial cell moti  lity, mig  ration, and wound healing. Overexpression of CTCF in corneal epithelial cells and mouse corneas significantly enhanced the wound healing process. Furthermore, the effect of overexpressing NFκB p50 in corneal epithelial cells on the promotion of wound healing was abolished by knockdown of CTCF with CTCF-specific shRNA. Thus, a direct regulatory relationship between EGF-induced NFκB p50 and CTCF activation affecting corneal epithelial wound healing has been established, indicating that CTCF is, indeed, a NFκB p50-targeted and effective gene product in the core transcriptional network downstream from the growth factor-induced NFκB signaling pathway. 
 Signaling pathway for TNF-alpha-induced MMP-9 expression: mediation through p38 MAP kinase, and inhibition by anti-cancer molecule magnolol in human urinary bladder cancer 5637 cells. We investigated the molecular mechanisms involved in the expression of matrix metalloproteinase-9 (MMP-9) and the inhibition of MMP expression by magnolol in 5637 human urinary bladder cancer cells. Tumor necrosis factor-alpha (TNF-alpha) stimulated the secretion of MMP-9 in 5637 cells, as shown by zymography and promoter assay. The transcription factor nuclear factor kappaB (NF-kappaB) binding site was identified by gel-shift assay to be a cis-element for TNF-alpha activation of the MMP-9 promoter. Our results also demonstrated that TNF-alpha stimulates MMP-9 expression via the p38 MAP kinase signaling pathway in 5637 cells. Moreover, p38 MAP kinase-mediated MMP-9 gene regulation in response to TNF-alpha is involved in the NF-kappaB response element in 5637 cells. In addition, magnolol inhibited TNF-alpha-induced expression of the MMP-9, as determined by zymography and immunoblot, in 5637 cells. The TNF-alpha-induced invasion and   migration   of cells was inhibited by magnolol, as assessed by a modified boyden chamber and wound-healing assays, respectively. Finally, magnolol blocked MMP-9 expression, at least in part, by decreasing the binding of transcription factor NF-kappaB to DNA. In conclusion, TNF-alpha induced MMP-9 expression in 5637 cells by activating the transcription factor NF-kappaB, which is involved in the p38 MAP kinase-mediated control of MMP-9 regulation. Magnolol inhibited MMP-9 expression through the transcription factor NF-kappaB in TNF-alpha-induced 5637 cells. 
 TNF-α mediates mitochondrial uncoupling and enhances ROS-dependent cell   migratio  n via NF-κB activation in liver cells. Development of hepatocellular carcinoma (HCC) is accompanied by a continuous increase in reactive oxygen species (ROS) levels. To investigate the primary source of ROS in liver cells, we used tumor necrosis factor-alpha (TNF-α) as stimulus. Applying inhibitors against the respiratory chain complexes, we identified mitochondria as primary source of ROS production. TNF-α altered mitochondrial integrity by mimicking a mild uncoupling effect in liver cells, as indicated by a 40% reduction in membrane potential and ATP depletion (35%). TNF-α-induced ROS production activated NF-κB 3.5-fold and subsequently enh  anced mig  ration up to 12.7-fold. This study identifies complex I and complex III of the mitochondrial respiratory chain as point of release of ROS upon TNF-α stimulation of liver cells, which enhance  s cell mi  gration by activating NF-κB signalling. 
 KiSS1 suppresses TNFalpha-induced breast cancer cell invasion via an inhibition of RhoA-mediated NF-kappaB activation. Tumor necrosis factor-alpha (TNFalpha) induces cancer development and metastasis, which is prominently achieved by nuclear factor-kappa B (NF-kappaB) activation. TNFalpha-induced NF-kappaB activation enhances cellular mechanisms including proliferation,   migration  , and invasion. KiSS1, a key regulator of puberty, was initially discovered as a tumor metastasis suppressor. The expression of KiSS1 was lost or down-regulated in different metastatic tumors. However, it is unclear whether KiSS1 regulates TNFalpha-induced NF-kappaB activation and further tumor cell   migration  . In this study, we demonstrate that KiSS1 suppresses the   migration   of breast cancer cells by inhibiting TNFalpha-induced NF-kappaB pathway and RhoA activation. Both KiSS1 overexpression and KP10 (kisspeptin-10) stimulation inhibited TNFalpha-induced NF-kappaB activity, suppressed TNFalpha-induced cell   migration   and cell attachment to fibronectin in breast cancer cells while KP10 has little effect on cancer cell proliferation. Furthermore, KP10 inhibited TNFalpha-induced cell   migration   and RhoA GTPase activation. Therefore, our data demonstrate that KiSS1 inhibits TNFalpha-induced NF-kappaB activation via downregulation of RhoA activation and suppression of breast cancer cell   migration   and invasion. 
 Association of common polymorphisms in TNFA, NFkB1 and NFKBIA with risk and prognosis of esophageal squamous cell carcinoma. Tumour necrosis factor-alpha (TNF-α) and nuclear factor of kappa light chain gene enhancer in activated B cells (NF-κB) play critical role in carcinogenesis processes like tumour initiation, proliferation  , migrati  on and invasion. Single nucleotide polymorphisms in TNF-α, NF-κB and its inhibitor IκB genes were shown to be associated with susceptibility and prognosis of several cancers; however, their role in esophageal squamous cell carcinoma (ESCC) is not well recognised. Therefore, in present study, we aimed to investigate association of common polymorphisms in TNFA, NFkB1 and NFKBIA with risk and prognosis of ESCC in northern Indian population.We genotyped 290 ESCC patients (including 162 followed up cases) and 311 mean age, gender and ethnicity matched controls for TNFA -308G>A, NFkB1 -94ATTG ins/del and NFKBIA (-826C>T and 3'UTRA>G) polymorphisms using PCR alone or followed by RFLP and TaqMan assay.TNFA-308GA genotype was associated with increased risk of ESCC specifically in females and in patients with regional lymph node involvement, while, NFKBIA -826CT+TT genotype conferred decreased risk of ESCC in females. Haplotypes of NFKBIA -826C>T and 3'UTRA>G polymorphisms, C-826G3'UTR and T-826A3'UTR, were associated with reduced risk of ESCC. No independent role of NFkB1 -94ATTG ins/del polymorphism in susceptibility of ESCC was found. Multi-dimensionality reduction analysis showed three factor model TNFA-308, NFKBIA-826, NFKBIA 3'UTR as better predictor for risk of ESCC. Furthermore, combined risk genotype analysis of all studied polymorphisms showed increased risk of ESCC in patients with 1-3 risk genotype compared to '0' risk genotype. Survival analysis did not show any significant prognostic effect of studied polymorphisms. However, in stepwise multivariate analysis, metastasis was found to be independent prognostic predictor of ESCC patients.TNFA-308 and NFKBIA (-826C>T and 3'UTRA>G) polymorphisms may play role in susceptibility but not in prognosis of ESCC patients in northern Indian population. 
 NF-kappaB activation, dependent on acetylation/deacetylation, contributes to HIF-1 activity and   migration   of bone metastatic breast carcinoma cells. Here, we show that NF-kappaB-HIF-1 interaction contributed to breast cancer metastatic capacity by means of an incomplete epithelial/mesenchymal transition and influencing   migration  , as shown in 1833 (human) and 4T1 (mouse) metastatic cells after different stimuli. The 1833 and the transforming growth factor-beta1-exposed 4T1 cells showed both epithelial (E-cadherins) and mesenchymal (N-cadherins and vimentin) markers, and common mechanisms contributed to the retention of certain epithelial characteristics and the control of   migration  . The complex NF-kappaB-HIF-1 reciprocal regulation and the enhanced c-Jun expression played a functional role in exacerbating the invasiveness of 1833 cells after p50/p65 transfection and of 4T1 cells exposed to transforming growth factor-beta1. Twist expression seemed to exert a permissive role also regulating epithelial/mesenchymal transition markers. After c-Src wild-type (Srcwt) transfection, c-Src-signal transducer overexpression in 1833 cells increased HIF-1 transactivating activity and invasiveness, and changed E-cadherin/N-cadherin ratio versus mesenchymal phenotype. The transcription factor pattern and the motile phenotype of metastatic 1833 cells were influenced by p65-lysine acetylation and HDAC-dependent epigenetic mechanisms, which positively regulated basal NF-kappaB and HIF-1 activities. However, HDAC3 acted as a corepressor of NF-kappaB activity in parental MDA-MB231 cells, thus explaining many differences from the derived 1833 clone, including reduced HIF-1alpha and c-Jun expression. Invasiveness was differently affected by HDAC knockdown in 1833 and MDA-MB231 cells. We suggest that acetylation/deacetylation are critical in establishing the bone-metastatic gene signature of 1833 cells by regulating the activity of NF-kappaB and HIF-1, and further clarify the epigenetic control of transcription factor network in the motile phenotype of 1833 cells. 
 Activation of vascular adhesion protein-1 on liver endothelium results in an NF-kappaB-dependent increase in lymphocyte adhesion. Vascular adhesion protein-1 (VAP-1) is an adhesion molecule and amine oxidase that is expressed at high levels in the human liver. It promotes leukocyte adhesion to the liver in vivo and drives lymphocyte trans  migration   across hepatic sinusoidal endothelial cells in vitro. We report that in addition to supporting leukocyte adhesion, provision of specific substrate to VAP-1 results in hepatic endothelial cell activation, which can be abrogated by treatment with the enzyme inhibitor semicarbazide. VAP-1-mediated activation was rapid; dependent upon nuclear factor-kappaB, phosphatidylinositol-3 kinase, and mitogen-activated protein kinase pathways; and led to upregulation of the adhesion molecules E-selectin, intercellular adhesion molecule-1, and vascular cell adhesion molecule-1 and secretion of the chemokine CXCL8. This response resulted in enhanced lymphocyte adhesion, was restricted to hepatic endothelial cells that expressed VAP-1, and was not observed in human umbilical vein endothelial cells.We propose that as well as directly promoting adhesion via interactions with the as yet unknown ligand, binding of enzyme substrate to VAP-1 can indirectly promote inflammatory cell recruitment via upregulation of adhesion molecules and chemokines. This response is likely to be important for the recruitment of leukocytes to the liver and suggests that VAP-1 inhibitors have therapeutic potential for treating chronic inflammatory liver disease. 
 Down-regulation of TFF expression in gastrointestinal cell lines by cytokines and nuclear factors. Trefoil peptides (TFF1, TFF2 and TFF3) are acute phase proteins up-regulated in response to gastrointestinal mucosal damage. They promote cell   migration  , protect and heal the mucosa and may function as tumorsuppressors. We assumed them to be regulated by the proinflammatory cytokines interleukin-1beta (IL1beta) and interleukin-6 (IL6), which trigger the transcriptional factors NF-kappaB and C/EBPbeta.Following IL1beta and IL6 stimulation, expression of TFF genes was analyzed in gastrointestinal cell lines HT-29 and KATO III by reporter gene assays using TFF promoter constructs and by quantitative real-time PCR. NF-kappaB and C/EBPbeta were transiently co-expressed.We have functionally identified transcription factors NF-kappaB and C/EBPbeta to inhibit transcription of human TFF genes. Down-regulation of TFF transcription is also observed by IL1beta and IL6, suggesting crosstalk with or in response to the immune system. IL1beta and IL6 caused a 3- to 11-fold reduction in TFF mRNA expression, displayed in real-time PCR.Down-regulation of intestinal trefoil factor TFF3 due to transcriptional repression by IL1beta through NF-kappaB as well as by IL6 through C/EBPbeta activation in vitro may reflect the situation in vivo and may contribute to ulceration and decreased wound healing during inflammatory bowel disease. Additionally, IL1 and IL6 over-expression in chronic gastritis may lead to mucosal damage and gastric carcinogenesis through transcriptional repression of TFF1 and TFF2. 
 ESM-1 regulates cell growth and metastatic process through activation of NF-κB in colorectal cancer. In our previous study, we reported that endothelial cell specific molecule-1 (ESM-1) was increased in tissue and serum from colorectal cancer patients and suggested that ESM-1 can be used as a potential serum marker for early detection of colorectal cancer. The aim of this study was to evaluate the role of ESM-1 as an intracellular molecule in colorectal cancer. ESM-1 expression was knocked down by small interfering RNA (siRNA) in colorectal cancer cells. Expression of ESM-1 siRNA decreased cell survival through the Akt-dependent inhibition of NF-κB/IκB pathway and an interconnected reduction in phospho-Akt, -p38, -ERK1, -RSK1, -GSK-3α/β and -HSP27, as determined by a phospho-MAPK array. ESM-1 silencing induced G(1) phase cell cycle arrest by induction of PTEN, resulting in the inhibition of cyclin D1 and inhibited   cell migr  ation and invasion of COLO205 cells. Consistently, ESM-1 overexpression in HCT-116 cells enhanced cell proliferation through the Akt-dependent activation of NF-κB pathway. In addition, ESM-1 interacted with NF-κB and activated NF-κB promoter. This study demonstrates that ESM-1 is involved in cell survival, cell cycle progr  ession, m  igration, invasion and EMT during tumor invasion in colorectal cancer. Based on our results, ESM-1 may be a useful therapeutic target for colorectal cancer. 
 2-Methoxyestradiol inhibits the proliferation and   migration   and reduces the radioresistance of nasopharyngeal carcinoma CNE-2 stem cells via NF-κB/HIF-1 signaling pathway inactivation and EMT reversal. Accumulating evidence indicates that cancer stem cells (CSCs) are a source of resistance to radiation therapy (RT); however, the mechanism of this resistance remains unclear. 2-Methoxyestradiol (2-ME2) is a metabolic product of estrogen in the body. Recent studies have found that 2-ME2 regulates the activation of transcription factors, including nuclear factor (NF)-κB/hypoxia-inducible factor-1 (HIF-1), thus contributing to tumor cell apoptosis and chemosensitivity. Therefore, 2-ME2 is being studied as a potential anticancer drug. The purpose of this study was to determine the effect and mechanism by which 2-ME2 inhibits nasopharyngeal carcinoma CNE-2 stem-like cell (NPCSC) proliferatio  n and mig  ration and reduces NPCSC radioresistance. This study has important significance for reducing the radioresistance of these cells to improve the cure rate of NPC. First, the NPCSCs were collected in a serum-free culture system and then identified by relevant experiments. The NPCSCs were treated with 2-ME2 (0-8 µM) combined with X-ray exposure and Cell Counting Kit-8 (CCK-8), Transwell assay, colony formation assay, western blot analysis, RT-PCR, flow cytometry and RNA interference technology were used to explore the effect and mechanism of 2-ME2 on NPCSCs. The results showed that the microspheres collected in the serum‑free culture system possessed CSC traits and radioresistance. 2-ME2 obviously inhibited NPCSC   growth an  d migration and reduced NPCSC radioresistance. 2-ME2 decreased NF-κB p65 and HIF-1α protein expression, downregulated NF-κB p65 nuclear localization, and reversed epithelial-mesenchymal transition (EMT). NF-κB p65 knockdown reduced HIF-1α expression, reversed EMT, and enhanced the suppressive effect of 2-ME2 on NPCSCs. Collectively, these data indicate that 2-ME2 inhibits NPCSC p  roliferat  ion and migration and reduces the radioresistance of NPCSCs via NF-κB/HIF-1 signaling pathway inactivation and EMT reversal. 
 Nuclear factor-kappa B activation pathway in intestinal epithelial cells is a major regulator of chemokine gene expression and neutrophil   migration   induced by Bacteroides fragilis enterotoxin. Although intestinal epithelial cells are known to up-regulate the expression of several chemokine genes in response to the stimulation with B. fragilis enterotoxin (BFT), there has been little understanding on the cellular mechanisms of BFT-induced mucosal inflammation. To test whether nuclear transcriptional factor-kappa B (NF-kappaB) is involved in the process, we stimulated intestinal epithelial cells with BFT, and evaluated the signalling NF-kappaB pathways. BFT increased signals of NF-kappaB in HT-29 and T84 epithelial cell lines as well as primary human colon epithelial cells. NF-kappaB molecules activated by BFT stimulation were composed of p65 and p50 heterodimers. In contrast, BFT decreased the signals of IkappaBalpha and IkappaB epsilon, as assessed by immunoblot. Super-repressors of IkappaBalpha, IkappaB kinase (IKK)beta, and NF-kappaB inducing kinase (NIK) inhibited an up-regulated transcription of downstream target gene (CXCL8) of NF-kappaB. Moreover, blocking the activation of NF-kappaB by MG-132 or antisense p50 oligonucleotide transfection resulted in down-regulated expression of chemokines such as CXCL1, CXCL8, and CCL2 in BFT-stimulated HT-29 cells. In addition, NF-kappaB inhibition suppressed the BFT-induced neutrophil transepithelial   migration   in T84 cells. These results indicate that NF-kappaB can be a central regulator of chemokine gene expression in BFT-stimulated intestinal epithelial cells and may be an important regulator of neutrophil   migration  . 
 Interleukin 17A promotes hepatocellular carcinoma metastasis via NF-kB induced matrix metalloproteinases 2 and 9 expression.  IL-17A is a pro-inflammatory cytokine that plays important role in inflammatory disease pathology and tumor microenvironment. The aim of this study is to investigate the effect of IL-17A on the progression of hepatocellular carcinoma (HCC).Expression pattern of IL-17A in clinical HCC samples (n = 43) was determined by immunohistochemistry staining. Transcript levels of MMP2, MMP9 and IL-17A were measured in another 50 pairs (including tumor and related non-tumor tissues) HCC samples. Cell growth, focus formation,   cell mig  ration, invasion and western blot assays were used to characterize the functional and signaling mechanisms in IL-17A-treated HCC. Association study was used to identify clinical significance of IL-17A in HCC. Compared with paired non-tumor tissue, higher frequency of IL-17A-positive cells was detected in tumor tissues in HCCs with metastasis, and the frequency of IL-17A-positive cells was also significantly associated with poor prognosis of HCC (P = 0.01). Functional study found that IL-17A could promote   HCC cell   migration and invasion. Further molecular analysis also showed that IL-17A could upregulate MMP2 and MMP9 expression via NF-κB signaling activation. IL-17A could promote HCC metastasis by the upregulation of MMP2 and MMP9 expression via activating NF-κB signaling pathway. 
 Hepatitis C virus NS3 protein enhances cancer cell invasion by activating matrix metalloproteinase-9 and cyclooxygenase-2 through ERK/p38/NF-κB signal cascade. Hepatitis C virus (HCV) infection causes acute and chronic hepatitis, liver cirrhosis, and hepatocellular carcinoma (HCC). However, the mechanisms by which HCV causes the diseases are largely unknown. Here, we elucidated the effects of HCV on the invasion and   migratio  n of hepatoma cells, with the aim to reveal the mechanism by which HCV infection induces HCC. We initially showed that matrix metalloproteinase-9 (MMP-9) was elevated in the sera of HCV-infected patients, and demonstrated that HCV nonstructural protein 3 (NS3) activated MMP-9 transcription through nuclear factor-κB (NF-κB) by stimulating translocation of NF-κB from cytosol to the nucleus to enhance its binding to MMP-9 promoter. In addition, cyclooxygenase-2 (COX-2) and extracellular signal-regulated kinase (ERK1/2)/mitogen-activated protein kinase (p38) pathway were involved in HCV-activated MMP-9 expression. Moreover, NS3 enhanced hepatoma cell invasion   and migra  tion through MMP-9 and COX-2. Thus, this study provides new insights into the roles of HCV NS3, MMP-9 and COX-2 in regulating cancer cell invasion. 
 Down-regulation of HMGB1 expression by shRNA constructs inhibits the bioactivity of urothelial carcinoma cell lines via the NF-κB pathway. The high mobility group box 1 (HMGB1), which is a highly conserved and evolutionarily non-histone nuclear protein, has been shown to associate with a variety of biological important processes, such as transcription, DNA repair, differentiation, and extracellular signalling. High HMGB1 expression has been reported in many cancers, such as prostate, kidney, ovarian, and gastric cancer. However, there have been few studies of the function of HMGB1 in the malignant biological behaviour of bladder urothelial carcinoma (BUC), and the potential mechanism of HMGB1 in the pathogenesis of BUC remains unclear. Thus, in this study, we constructed plasmid vectors that are capable of synthesizing specific shRNAs targeting HMGB1 and transfected them into BUC cells to persistently suppress the endogenous gene expression of HMGB1. The expression of HMGB1, the bioactivity of BUC cells, including proliferation, apoptosis, cell cycle distribution,   migratio  n and invasion, and the effects of HMGB1 knockdown on downstream signalling pathways were investigated. Our data suggest that HMGB1 promotes the malignant biological behaviour of BUC, and that this effect may be partially mediated by the NF-κB signalling pathway. HMGB1 may serve as a potential therapeutic target for BUC in the future. 
 A synergistic interaction between transcription factors nuclear factor-κB and signal transducers and activators of transcription 3 promotes gastric cancer cell   migratio  n and invasion. The transcription factor nuclear factor-κB (NF-κB) has been implicated in gastric cancer metastasis, but the underlying molecular mechanisms remain unclear. We investigated the role of the interaction between NF-κB and signal transducers and activators of transcription 3 (STAT3) in controlling metastatic potential of gastric cancer cells.Immunohistochemistry for NF-κB p65 (RelA), phospho-Tyr705-STAT3 (pSTAT3), or matrix metalloproteinase 9 (MMP9) was performed on tissue array slides containing 255 gastric carcinoma specimens. NF-κB inhibition in SNU-638 and MKN1 gastric cancer cell lines were performed by transduction with a retroviral vector containing NF-κB repressor mutant of IκBα, and STAT3 was silenced by RNA interference. We also did luciferase reporter assay, double immunofluorescence staining and immunoblotti  ng. Cell   migration and invasion were determined by wound-healing assay and invasion assay, respectively.NF-κB and STAT3 were constitutively activated and were positively correlated (P=0.038) in gastric cancer tissue specimens. In cell culture experiments, NF-κB inhibition reduced STAT3 expression and activation, whereas STAT3 silencing did not affect NF-κB activation. Moreover, both NF-κB inhibition and STAT3 silencing decreased gastric   cancer c  ell migration and invasion in a synergistic manner. In addition, both NF-κB activation and STAT3 activation were positively correlated with MMP9 in gastric cancer tissues (P=0.001 and P=0.022, respectively), decreased E-cadherin expression and increased Snail and MMP9 expressions in cultured cells.NF-κB and STAT3 are positively associated and synergistically contribute to the metastatic potential of gastric cancer cells. Thus, dual use of NF-κB and STAT3 inhibitors may enhance the efficacy of the anti-metastatic treatment of gastric cancer. 
 BRAF activated non-coding RNA (BANCR) promoting gastric cancer cells proliferation via regulation of NF-κB1. Long non-coding RNA, BANCR, has been demonstrated to contribute to the proliferation and   migratio  n of tumors. However, its molecular mechanism underlying gastric cancer is still unknown. In present study, we investigated whether BANCR was involved in the development of gastric cancer cells via regulation of NF-κB1.Human gastric cancer tissues were isolated as well as human gastric cell lines MGC803 and BGC823 were cultured to investigate the role of BANCR in gastric cancer.BANCR expression was significantly up-regulated in gastric tumor tissues and gastric cell lines. Down-regulation of BANCR inhibited gastric cancer cell growth and promoted cell apoptosis, and it also contributed to a significant decrease of NF-κB1 (P50/105) expression and 3'UTR of NF-κB1 activity. Overexpression of NF-κB1 reversed the effect of BANCR on cancer cell growth and apoptosis. MiroRNA-9 (miR-9) targeted NF-κB1, and miR-9 inhibitor also reversed the effects of BANCR on gastric cancer cell growth and apoptosis.BANCR was highly expressed both in gastric tumor tissues and in cancer cells. NF-κB1 and miR-9 were involved in the role of BANCR in gastric cancer cell growth and apoptosis. 
  GeneRIFs  
 CCL18 enhances hepatocellular carcinoma (HCC) cell   migration  , invasion, an epithelial-mesenchymal transition (EMT) through the expression of PITPNM3 and the activation of the NF-kappaB signaling pathway. 
 C/EBPbeta could promote the invasion and   migration   of human renal carcinoma 786-O cells, and had an inducing effect on nuclear translocation of NF-kappaB in 786-O cells. 
 This study is the first to demonstrate that inhibition of NF-kappaB could significantly reduce hypoxia and retinoblastoma cell invasion and   migration   ability 
 Data show that lipopolysaccharide induced vascular endothelial cell   migration   is mediated by toll-like receptor TLR-4/NF-kappa B pathway and enzyme NAD(P)H oxidase in association with the transient receptor potential melastatin 7 (TRPM7) ion channel. 
 These findings establish a central role for ANXA1 in the cell   migration   through the activation of NFkappaB, ERK1/2 and RhoA. 
 RANKL could potentiate   migration   and invasion ability of RANK-positive hepatocellular carcinoma cells through NF-kappaB pathway mediated epithelial-mesenchymal transition. 
 LMP1-mediated upregulation of Fascin depends on NF-kappaB and both NF-kappaB and Fascin contribute to invasive   migration   of LMP1-expressing lymphocytes. 
 High NFKB expression enhanced hepatoma cell invasion and   migration  . 
 Ghrelin induces cell proliferation,   migration   and invasion via GHS-R/NF-kappaB signaling pathway in gastric cancer cells. 
 Data indicate that tumor necrosis factor-alpha (TNF-alpha)-induced reactive oxygen species (ROS) production activates NF-kappaB and enhances liver cell   migration  . 
 Il-1beta enhances cell   migration   through AP-1 and NF-kappaB pathway-dependent FGF2 expression in human corneal endothelial cells. 
 Knockdown of endogenous Bmi-1 reduced the invasiveness and   migration   of glioma cells. NF-kappaB transcriptional activity and MMP-9 expression and activity were significantly increased in Bmi-1-overexpressing but reduced in Bmi-1-silenced cells. 
 the CD91/IKK/NF-kappaB signaling cascade is involved in secreted HSP90alpha-induced TCF12 expression, leading to E-cadherin down-regulation and enhanced CRC cell   migration  /invasion 
 Interleukin-20 promotes   migration   of bladder cancer cells through extracellular signal-regulated kinase (ERK)-mediated MMP-9 protein expression leading to nuclear factor (NF-kappaB) activation by inducing the up-regulation of p21(WAF1) protein expression 
 data suggest that Numbl regulates glioma cell   migration   and invasion by abrogating TRAF5-induced activation of NF-KappaB 
 Data show that CARMA3 and Bcl10 contributed to several characteristics of EGFR-associated malignancy, including proliferation, survival,   migration  , and invasion. 
 The application of flavonoids to prevent the   migration  /invasion of glioblastoma cells through blocking PKCalpha/ERK/NF-kappaB activation is demonstrated. 
 NF-kappaB promotes epithelial-mesenchymal transition,   migration   and invasion of pancreatic carcinoma cells. 
 The authors conclude that hepatitis B virus X protein upregulate Capn4 through NF-kappaB/p65 to promote   migration   of hepatoma cells. 
 Bradykinin enhances the   migration   of chondrosarcoma cells by increasing alpha2beta1 integrin expression through the BK receptors/PLC/PKCdelta/NF-kappaB signal transduction pathway. 
 NF-kappaB-HIF-1 interaction contributed to breast cancer metastatic capacity by means of an incomplete epithelial/mesenchymal transition and influencing   migration  . 
 KiSS1 inhibits TNFalpha-induced NF-kappaB activation via downregulation of RhoA activation and suppression of breast cancer cell   migration   and invasion 
 SDF-1alpha acts through CXCR4 to activate MEK and ERK, which in turn activates IKKalpha/beta and NF-kappaB, resulting in the activations of alphavbeta3 integrins and contributing the   migration   of human osteosarcoma cells. 
 OPN enhances the   migration   of chondrosarcoma cells by increasing MMP-9 expression through the alphavbeta3 integrin, FAK, MEK, ERK and NF-kappaB signal transduction pathway. 
 Connective tissue growth factor enhances the   migration   of chondrosarcoma cells by increasing MMP-13 expression through the alphavbeta3 integrin, FAK, ERK, and NF-kappaB signal transduction pathway. 
 Results suggest that glial cell-derived neurotrophic factor acts through MEK/ERK, which in turn activates IKKalpha/beta and NF-kappaB, resulting in activation of alphavbeta3 integrin and   migration   of human chondrosarcoma cells. 
 These results demonstrated that in A549 cells, activation of p50/p65 heterodimer through sequential activation of PKC alpha-JNK-NIK-IKK beta-NF-kappaB was required for IL-1 beta-induced uPA expression associated with   migration   of tumor cells. 
 results suggest that wogonin effectively suppresses TNF-alpha-induced HASMC   migration   through the selective inhibition of MMP-9. 
 NF-kappaB can be a central regulator of chemokine gene expression in BFT-stimulated intestinal epithelial cells and may be an important regulator of neutrophil   migration  . 
  ECM  PubMed  
 PF-3758309, p21-activated kinase 4 inhibitor, suppresses migration and invasion of A549 human lung cancer cells via regulation of CREB, NF-κB, and β-catenin signalings. Migration and invasion comprise key steps in cancer metastasis. Through the migration and invasion into and out of lymphatic and/or blood vessels, cancer cells can be spread out into the tissues in remote site from the origin. Degradation of extracellular matrix   (E  CM) must be preceded prior to the metastasis of cancer cells. Matrix metalloproteinases (MMP) can degrad  e E  CM, thus allow cells to migrate from the original site. Among MMPs, two gelatinase MMP-2 and MMP-9 play particularly important roles i  n E  CM degradation. Here, we report that recently developed p21-activated kinase 4 inhibitor PF-3758309 shows anti-metastatic effect in A549 human lung cancer cell. PF-3758309 suppresses CREB, NF-κB, and β-catenin pathways, which are well known to be closely related with cell migration. This leads to the downregulation of MMP-2/MMP-9 expressions and the inhibition of A549 lung cancer metastasis. 
 Galectin-1 stimulates motility of human umbilical cord blood-derived mesenchymal stem cells by downregulation of smad2/3-dependent collagen 3/5 and upregulation of NF-κB-dependent fibronectin/laminin 5 expression. Galectin-1 (Gal-1) belongs to a family of endogenous lectins with conserved carbohydrate recognition domains binding β-galactosidase sugars and plays a vital role in regulating stem cell functions including determination of cell fate. However, our understanding of the functional roles of Gal-1 in human umbilical cord blood-derived mesenchymal stem cells (UCB-MSCs) is still fragmentary and incomplete. Gal-1 significantly increased motility after a 24-h incubation, and this effect was inhibited by β-lactose. We analyzed 17 extracellular matri  x (  ECM) genes in UCB-MSCs. Gal-1 decreased the expression of collagen genes COL3A1 (COL-3) and COL5A1 (COL-5) but increased the expression of fibronectin (FN) and laminin 5 (LM-5), that were reversed by β-lactose. Gal-1 increased protein kinase C (PKC), c-Src, and caveolin-1 (Cav-1) phosphorylation that was attenuated by β-lactose and the Src inhibitor PP2. In addition, pretreatment with the lipid raft disruptor Mβ-CD and the PKC inhibitors inhibited Gal-1-induced UCB-MSC motility. In addition, Gal-1 reduced smad2/3 phosphorylation and induced nuclear factor (NF)-κB phosphorylation. Pretreatment with Mβ-CD attenuated Gal-1-reduced smad2/3 phosphorylation, COL-3, and COL-5 expression but did not affect NF-κB phosphorylation, FN, or LM-5 expression. In contrast, PKC inhibitors only attenuated NF-κB phosphorylation, FN, and LM-5 expression. Reconstructing Gal-1-induced genetic changes by replacing it with siRNA specific for COL-3 or COL-5, or treatment of the cells with FN and LM-5 proteins, increased motility and its related proteins such as focal adhesion kinase, Akt, Erk, integrins, and matrix metalloproteinase-2. A combined treatment with COL-3/COL-5 siRNA or FN/LM-5 compared with that of single treatments was synergistic. However, a single Gal-1 treatment maximally stimulated motility and related protein phosphorylation/expression. These results demonstrate that Gal-1 stimulated human UCB-MSC motility by decreasing COL-3/COL-5 expression and increasing FN/LM-5 expression through a PKC-dependent NF-κB and c-Src/Cav-1-dependent smad2/3 pathway that was critical for governing the activation of FAK, Akt, Erk, integrins, and MMP2. 
 Bacterial endotoxin enhances colorectal cancer cell adhesion and invasion through TLR-4 and NF-kappaB-dependent activation of the urokinase plasminogen activator system. Perioperative exposure to lipopolysaccharide (LPS) is associated with accelerated metastatic colorectal tumour growth. LPS directly affects cells through Toll-like receptor 4 (TLR-4) and the transcription factor NF-kappaB. The urokinase plasminogen activator (u-PA) system is intimately implicated in tumour cell extracellular matrix (  ECM  ) interactions fundamental to tumour progression. Thus we sought to determine if LPS directly induces accelerated tumour cell   ECM   adhesion and invasion through activation of the u-PA system and to elucidate the cellular pathways involved. Human colorectal tumour cell lines were stimulated with LPS. u-PA concentration, u-PA activity, active u-PA, surface urokinase plasminogen activator receptor (u-PAR) and TLR-4 expression were assessed by ELISA, colorimetric assay, western blot analysis and flow cytometry respectively. In vitro tumour cell vitronectin adhesion and   ECM   invasion were analysed by vitronectin adhesion assay and   ECM   invasion chambers. u-PA and u-PAR function was inhibited with anti u-PA antibodies or the selective u-PA inhibitors amiloride or WXC-340, TLR-4 by TLR-4-blocking antibodies and NF-kappaB by the selective NF-kappaB inhibitor SN-50. LPS upregulates u-PA and u-PAR in a dose-dependent manner, enhancing in vitro tumour cell vitronectin adhesion and   ECM   invasion by >40% (P<0.01). These effects were ameliorated by u-PA and u-PAR inhibition. LPS activates NF-kappaB through TLR-4. TLR-4 and NF-kappaB inhibition ameliorated LPS-enhanced u-PA and u-PAR expression, tumour cell vitronectin adhesion and   ECM   invasion. LPS promotes tumour cell   ECM   adhesion and invasion through activation of the u-PA system in a TLR-4- and NF-kappaB-dependent manner. 
 Activation of Toll-like receptor 3 augments myofibroblast differentiation. Airway remodeling is observed in the airways of patients with asthma, and differentiation of fibroblasts to myofibroblasts plays a critical role in the progress of airway remodeling. Viral infection induces not only the disease development and exacerbations but also airway remodeling. The aim of this study was to evaluate whether the activation of Toll-like receptor 3 (TLR3) can affect the differentiation of fibroblasts to myofibroblasts and the extracellular matrix (  ECM  ) protein production. Human fetal lung fibroblasts (HFL-1) and adult lung fibroblasts were treated with a synthetic double-stranded RNA, polyinosine-polycytidylic acid (poly[I:C]) and the expression of alpha-smooth muscle actin (alpha-SMA), a marker of myofibroblast differentiation, was evaluated. The release of transforming growth factor-beta(1) (TGF-beta(1)) and   ECM   protein production were assessed. The effect of anti-TGF-beta antibody on the alpha-SMA and   ECM   production was also assessed. Poly(I:C) significantly augmented the alpha-SMA expression (P < 0.01) and release of TGF-beta(1) (P < 0.01) compared with control. Bafilomycin, an inhibitor of TLR3 signaling, diminished poly(I:C)-augmented TGF-beta(1) release. Anti-TGF-beta(1) antibody inhibited the poly(I:C)-augmented alpha-SMA expression. Poly(I:C) enhanced translocation of nuclear factor-kB (NF-kappaB) and interferon regulatory factor-3 (IRF-3) into the nucleus. Poly(I:C)-augmented TGF-beta(1) release was almost completely blocked by NF-kappaB inhibitors, but not by silencing IRF-3. The production of fibronectin and collagen I expression were significantly increased by poly(I:C) (P < 0.01) and they were inhibited by anti-TGF-beta antibody. These results suggest that activation of TLR3 can affect the differentiation to myofibroblasts and enhance   ECM   production via the NF-kappaB-TGF-beta(1)-dependent pathway. 
 Integrin-mediated transcriptional activation of inhibitor of apoptosis proteins protects smooth muscle cells against apoptosis induced by degraded collagen. Apoptosis of smooth muscle cells (SMC) and degradation of the extracellular matrix (  ECM  ) have both been implicated in atherosclerotic plaque rupture. We have previously reported that degraded type I collagen fragments induce a rapid but transient apoptotic burst initiated by calpains in SMC. The aim of the current study was to identify the pathway responsible for consecutive SMC survival. We show that exposure of SMC to collagen fragments resulted in a sustained activation of nuclear factor (NF)-kappaB via phosphorylation and degradation of IkappaBalpha. Its prevention through retroviral expression of superrepressor IkappaBalpha or proteasome inhibition potently induced apoptosis. In the presence of blocking antibodies to alpha(v)beta(3) integrin and RGD peptides, collagen fragments no longer activated NF-kappaB and apoptosis was enhanced. The mechanism by which NF-kappaB was protecting SMC against collagen fragment-induced apoptosis was a transcriptional activation of several endogenous caspase inhibitors of the inhibitor of apoptosis protein (IAP) family as: (1) the expression of xIAP, c-IAP2, and survivin was potently induced by collagen fragments; (2) IAP expression was abrogated by superrepressor IkappaBalpha; and (3) knockdown of each of the 3 IAPs by small interfering RNA (siRNA) resulted in enhanced apoptosis after collagen fragment treatment. Our data suggest that SMC exposed to degraded collagen are protected against apoptosis by a mechanism involving alpha(v)beta(3)-dependent NF-kappaB activation with consequent activation of IAPs. This may constitute a novel antiapoptotic pathway ensuring SMC survival in settings of enhanced   ECM   degradation such as cell migration, vascular remodeling, and atherosclerotic plaque rupture. 
 Classical macrophage activation up-regulates several matrix metalloproteinases through mitogen activated protein kinases and nuclear factor-κB. Remodelling of the extracellular matrix   (EC  M) and cell surface by matrix metalloproteinases (MMPs) is an important function of monocytes and macrophages. Recent work has emphasised the diverse roles of classically and alternatively activated macrophages but the consequent regulation of MMPs and their inhibitors has not been studied comprehensively. Classical activation of macrophages derived in vitro from un-fractionated CD16(+/-) or negatively-selected CD16(-) macrophages up-regulated MMP-1, -3, -7, -10, -12, -14 and -25 and decreased TIMP-3 steady-state mRNA levels. Bacterial lipopolysaccharide, IL-1 and TNFα were more effective than interferonγ except for the effects on MMP-25, and TIMP-3. By contrast, alternative activation decreased MMP-2, -8 and -19 but increased MMP -11, -12, -25 and TIMP-3 steady-state mRNA levels. Up-regulation of MMPs during classical activation depended on mitogen activated protein kinases, phosphoinositide-3-kinase and inhibitor of κB kinase-2. Effects of interferonγ depended on janus kinase-2. Where investigated, similar effects were seen on protein concentrations and collagenase activity. Moreover, activity of MMP-1 and -10 co-localised with markers of classical activation in human atherosclerotic plaques in vivo. In conclusion, classical macrophage activation selectively up-regulates several MMPs in vitro and in vivo and down-regulates TIMP-3, whereas alternative activation up-regulates a distinct group of MMPs and TIMP-3. The signalling pathways defined here suggest targets for selective modulation of MMP activity. 
 Bit-1 mediates integrin-dependent cell survival through activation of the NFkappaB pathway. Loss of properly regulated cell death and cell survival pathways can contribute to the development of cancer and cancer metastasis. Cell survival signals are modulated by many different receptors, including integrins. Bit-1 is an effector of anoikis (cell death due to loss of attachment) in suspended cells. The anoikis function of Bit-1 can be counteracted by integrin-mediated cell attachment. Here, we explored integrin regulation of Bit-1 in adherent cells. We show that knockdown of endogenous Bit-1 in adherent cells decreased cell survival and re-expression of Bit-1 abrogated this effect. Furthermore, reduction of Bit-1 promoted both staurosporine and serum-deprivation induced apoptosis. Indeed knockdown of Bit-1 in these cells led to increased apoptosis as determined by caspase-3 activation and positive TUNEL staining. Bit-1 expression protected cells from apoptosis by increasing phospho-IκB levels and subsequently bcl-2 gene transcription. Protection from apoptosis under serum-free conditions correlated with bcl-2 transcription and Bcl-2 protein expression. Finally, Bit-1-mediated regulation of bcl-2 was dependent on focal adhesion kinase, PI3K, and AKT. Thus, we have elucidated an integrin-controlled pathway in which Bit-1 is, in part, responsible for the survival effects of cell  -EC  M interactions. 
 IL-1 beta induces urokinase-plasminogen activator expression and cell migration through PKC alpha, JNK1/2, and NF-kappaB in A549 cells. Breakdown of the extracellular matrix (  ECM  ) is accomplished by the concerted action of several proteases, including the urokinase plasminogen-activator (uPA) system and matrix metalloproteinases (MMPs), which is crucial for cancer invasion and metastasis. Several reports have shown that the levels of IL-1 beta and MMPs in plasma of the patients with lung cancer are significantly elevated and link to the invasion of tumor cells. Therefore, we investigated whether IL-1 beta-induced expression of uPA participated in lung cancer progression. In this study, IL-1 beta significantly induced uPA expression and activity via PKC alpha-dependent JNK1/2 and NIK cascades, linking to IKK alpha/beta activation, p65 translocation and transcription activity, using pharmacological inhibitors and transfection with dominant negative mutants and siRNAs. IL-1 beta-induced uPA protein and mRNA expression in a time- and concentration-dependent manner, which was inhibited by pretreatment with the inhibitors of JNK1/2 (SP600125), PKC (Ro31-8220, Gö6976), or NF-kappaB (helenalin), and transfection with dominant negative mutants of PKC alpha, NIK, and IKK beta, and siRNAs of JNK1/2 and p65. IL-1 beta stimulated PKC alpha translocation to plasma membrane leading to phosphorylation of JNK1/2, which was attenuated by PKC inhibitors and transfection with shRNAs of JNK1/2, but not by helenalin. In addition, IL-1beta stimulated p65 phosphorylation and translocation into nucleus concomitant with I kappaB alpha phosphorylation and I kappaB alpha degradation, which was mediated via activation of PKC alpha-dependent JNK1/2-NIK/IKK beta cascade. These results demonstrated that in A549 cells, activation of p50/p65 heterodimer through sequential activation of PKC alpha-JNK-NIK-IKK beta-NF-kappaB was required for IL-1 beta-induced uPA expression associated with migration of tumor cells. 
  GeneRIFs  
 activation of TLR3 can affect the differentiation to myofibroblasts and enhance   ECM   production via the NF-kappaB-TGF-beta(1)-dependent pathway 
 LPS promotes tumour cell   ECM   adhesion and invasion through activation of the u-PA system in a TLR-4- and NF-kappaB-dependent manner. 
  [Ee]xtracellular [Mm]atrix  PubMed  
 Tissue inhibitor of metalloproteinases-2 growth-stimulatory activity is mediated by nuclear factor-kappa B in A549 lung epithelial cells. Tissue inhibitors of metalloproteinases (TIMPs) are pleiotropic factors that function as key regulators of   extracellular matrix   remodeling. They exhibit multifunctional roles including cell growth-stimulating activities and protection from apoptosis. In the present study, we showed that human recombinant TIMP-2 (hrTIMP-2) promotes growth of A549 lung cells. This effect was accompanied by increase in nuclear factor-kappa B (NF-kappaB) activity 24h after exposure as determined by electrophoretic mobility shift assay (EMSA) analysis. This effect was correlated with downregulation of IkappaBalpha and beta proteins and later increases in Bcl-3, IkappaB, and cyclin D1 proteins. Blocking induction of NF-kappaB activity using a dominant-negative mutated version of IkappaBalpha abrogated NF-kappaB activation and cell proliferation. 
 Identification of fetal and maternal single nucleotide polymorphisms in candidate genes that predispose to spontaneous preterm labor with intact membranes. The purpose of this study was to determine whether maternal/fetal single nucleotide polymorphisms (SNPs) in candidate genes are associated with spontaneous preterm labor/delivery.A genetic association study was conducted in 223 mothers and 179 fetuses (preterm labor with intact membranes who delivered <37 weeks of gestation [preterm birth (PTB)]), and 599 mothers and 628 fetuses (normal pregnancy); 190 candidate genes and 775 SNPs were studied. Single locus/haplotype association analyses were performed; the false discovery rate was used to correct for multiple testing.The strongest single locus associations with PTB were interleukin-6 receptor 1 (fetus; P=.000148) and tissue inhibitor of metalloproteinase 2 (mother; P=.000197), which remained significant after correction for multiple comparisons. Global haplotype analysis indicated an association between a fetal DNA variant in insulin-like growth factor F2 and maternal alpha 3 type IV collagen isoform 1 (global, P=.004 and .007, respectively).An SNP involved in controlling fetal inflammation (interleukin-6 receptor 1) and DNA variants in maternal genes encoding for proteins involved in   extracellular matrix   metabolism approximately doubled the risk of PTB. 
 A genetic association study of maternal and fetal candidate genes that predispose to preterm prelabor rupture of membranes (PROM). We sought to determine whether maternal/fetal single-nucleotide polymorphisms (SNPs) in candidate genes are associated with preterm prelabor rupture of membranes (pPROM).A case-control study was conducted in patients with pPROM (225 mothers and 155 fetuses) and 599 mothers and 628 fetuses with a normal pregnancy; 190 candidate genes and 775 SNPs were studied. Single locus/haplotype association analyses were performed; false discovery rate was used to correct for multiple testing (q* = 0.15).First, a SNP in tissue inhibitor of metalloproteinase 2 in mothers was significantly associated with pPROM (odds ratio, 2.12; 95% confidence interval, 1.47-3.07; P = .000068), and this association remained significant after correction for multiple comparisons. Second, haplotypes for Alpha 3 type IV collagen isoform precursor in the mother were associated with pPROM (global P = .003). Third, multilocus analysis identified a 3-locus model, which included maternal SNPs in collagen type I alpha 2, defensin alpha 5 gene, and endothelin 1.DNA variants in a maternal gene involved in   extracellular matrix   metabolism doubled the risk of pPROM. 
 A genetic association study of maternal and fetal candidate genes that predispose to preterm prelabor rupture of membranes (PROM). We sought to determine whether maternal/fetal single-nucleotide polymorphisms (SNPs) in candidate genes are associated with preterm prelabor rupture of membranes (pPROM).A case-control study was conducted in patients with pPROM (225 mothers and 155 fetuses) and 599 mothers and 628 fetuses with a normal pregnancy; 190 candidate genes and 775 SNPs were studied. Single locus/haplotype association analyses were performed; false discovery rate was used to correct for multiple testing (q* = 0.15).First, a SNP in tissue inhibitor of metalloproteinase 2 in mothers was significantly associated with pPROM (odds ratio, 2.12; 95% confidence interval, 1.47-3.07; P = .000068), and this association remained significant after correction for multiple comparisons. Second, haplotypes for Alpha 3 type IV collagen isoform precursor in the mother were associated with pPROM (global P = .003). Third, multilocus analysis identified a 3-locus model, which included maternal SNPs in collagen type I alpha 2, defensin alpha 5 gene, and endothelin 1.DNA variants in a maternal gene involved in   extracellular matrix   metabolism doubled the risk of pPROM. 
 Identification of fetal and maternal single nucleotide polymorphisms in candidate genes that predispose to spontaneous preterm labor with intact membranes. The purpose of this study was to determine whether maternal/fetal single nucleotide polymorphisms (SNPs) in candidate genes are associated with spontaneous preterm labor/delivery.A genetic association study was conducted in 223 mothers and 179 fetuses (preterm labor with intact membranes who delivered <37 weeks of gestation [preterm birth (PTB)]), and 599 mothers and 628 fetuses (normal pregnancy); 190 candidate genes and 775 SNPs were studied. Single locus/haplotype association analyses were performed; the false discovery rate was used to correct for multiple testing.The strongest single locus associations with PTB were interleukin-6 receptor 1 (fetus; P=.000148) and tissue inhibitor of metalloproteinase 2 (mother; P=.000197), which remained significant after correction for multiple comparisons. Global haplotype analysis indicated an association between a fetal DNA variant in insulin-like growth factor F2 and maternal alpha 3 type IV collagen isoform 1 (global, P=.004 and .007, respectively).An SNP involved in controlling fetal inflammation (interleukin-6 receptor 1) and DNA variants in maternal genes encoding for proteins involved in   extracellular matrix   metabolism approximately doubled the risk of PTB. 
 Tissue inhibitor of metalloproteinases-2 growth-stimulatory activity is mediated by nuclear factor-kappa B in A549 lung epithelial cells. Tissue inhibitors of metalloproteinases (TIMPs) are pleiotropic factors that function as key regulators of   extracellular matrix   remodeling. They exhibit multifunctional roles including cell growth-stimulating activities and protection from apoptosis. In the present study, we showed that human recombinant TIMP-2 (hrTIMP-2) promotes growth of A549 lung cells. This effect was accompanied by increase in nuclear factor-kappa B (NF-kappaB) activity 24h after exposure as determined by electrophoretic mobility shift assay (EMSA) analysis. This effect was correlated with downregulation of IkappaBalpha and beta proteins and later increases in Bcl-3, IkappaB, and cyclin D1 proteins. Blocking induction of NF-kappaB activity using a dominant-negative mutated version of IkappaBalpha abrogated NF-kappaB activation and cell proliferation. 
 Independent and cooperative roles of tumor necrosis factor-alpha, nuclear factor-kappaB, and bone morphogenetic protein-2 in regulation of metastasis and osteomimicry of prostate cancer cells and differentiation and mineralization of MC3T3-E1 osteoblast-like cells. The molecular mechanisms involved in prostate cancer (PC) metastasis and bone remodeling are poorly understood. We recently reported that phosphoinositide 3-kinase (PI3K)/protein kinase B (Akt) mediates transcriptional regulation and activation of bone morphogenetic protein (BMP)-2 signaling by nuclear factor (NF)-kappaB in bone metastatic prostate cancer cells. In the present study, we demonstrate that NF-kappaB, whether activated by recombinant human tumor necrosis factor (TNF)-alpha or by ectopic expression of the p65 subunit, is involved in   extracellular matrix   adhesion and invasion of osteotropic PC-3 and C4-2B, but not LNCaP, cells. The enhanced metastatic potential was associated with transcriptional upregulation of osteopontin, osteocalcin, and collagen IA1 in osteotropic PC cells, suggesting their role in osteomimicry of PC cells. Unlike BMP-4, BMP-2 protein enhanced the invasive properties of C4-2B cells, but not in LNCaP cells. Also, this effect was nullified by Noggin. In addition, BMP-2 mediates TNF-alpha-induced invasion of C4-2B cells in a NF-kappaB-dependent fashion. TNF-alpha or conditioned media (CM) of TNF-alpha-stimulated C4-2B cells upregulated BMP-2 and BMP-dependent Smad transcripts and inhibited receptor activator of NF-kappaB ligand transcripts in RAW 264.7 preosteoclast cells, respectively, implying that this factor may contribute to suppression of osteoclastogenesis via direct and paracrine mechanisms. In contrast, CM of TNF-alpha-stimulate or BMP2-stimulated C4-2B cells induced in vitro mineralization of MC3T3-E1 osteoblast cells in a BMP-2-dependent and NF-kappaB-dependent manner, respectively. Taken together, the results suggest that mutual interactions between these factors may be pivotal not only in enhancing the osteomimicry and metastatic potential of PC cells, but also in bone remodeling and in shifting the balance from osteoclastogenesis towards osteoblastogenesis. 
 PF-3758309, p21-activated kinase 4 inhibitor, suppresses migration and invasion of A549 human lung cancer cells via regulation of CREB, NF-κB, and β-catenin signalings. Migration and invasion comprise key steps in cancer metastasis. Through the migration and invasion into and out of lymphatic and/or blood vessels, cancer cells can be spread out into the tissues in remote site from the origin. Degradation o  f extracellular matr  ix (ECM) must be preceded prior to the metastasis of cancer cells. Matrix metalloproteinases (MMP) can degrade ECM, thus allow cells to migrate from the original site. Among MMPs, two gelatinase MMP-2 and MMP-9 play particularly important roles in ECM degradation. Here, we report that recently developed p21-activated kinase 4 inhibitor PF-3758309 shows anti-metastatic effect in A549 human lung cancer cell. PF-3758309 suppresses CREB, NF-κB, and β-catenin pathways, which are well known to be closely related with cell migration. This leads to the downregulation of MMP-2/MMP-9 expressions and the inhibition of A549 lung cancer metastasis. 
 Inflammation anergy in human intestinal macrophages is due to Smad-induced IkappaBalpha expression and NF-kappaB inactivation. Human intestinal macrophages contribute to tissue homeostasis in noninflamed mucosa through profound down-regulation of pro-inflammatory cytokine release. Here, we show that this down-regulation extends to Toll-like receptor (TLR)-induced cytokine release, as intestinal macrophages expressed TLR3-TLR9 but did not release cytokines in response to TLR-specific ligands. Likely contributing to this unique functional profile, intestinal macrophages expressed markedly down-regulated adapter proteins MyD88 and Toll interleukin receptor 1 domain-containing adapter-inducing interferon beta, which together mediate all TLR MyD88-dependent and -independent NF-kappaB signaling, did not phosphorylate NF-kappaB p65 or Smad-induced IkappaBalpha, and did not translocate NF-kappaB into the nucleus. Importantly, transforming growth factor-beta released from intestinal   extracellular matrix   (stroma) induced identical down-regulation in the NF-kappaB signaling and function of blood monocytes, the exclusive source of intestinal macrophages. Our findings implicate stromal transforming growth factor-beta-induced dysregulation of NF-kappaB proteins and Smad signaling in the differentiation of pro-inflammatory blood monocytes into noninflammatory intestinal macrophages. 
 Galectin-1 stimulates motility of human umbilical cord blood-derived mesenchymal stem cells by downregulation of smad2/3-dependent collagen 3/5 and upregulation of NF-κB-dependent fibronectin/laminin 5 expression. Galectin-1 (Gal-1) belongs to a family of endogenous lectins with conserved carbohydrate recognition domains binding β-galactosidase sugars and plays a vital role in regulating stem cell functions including determination of cell fate. However, our understanding of the functional roles of Gal-1 in human umbilical cord blood-derived mesenchymal stem cells (UCB-MSCs) is still fragmentary and incomplete. Gal-1 significantly increased motility after a 24-h incubation, and this effect was inhibited by β-lactose. We analyzed   17 extracellular mat  rix (ECM) genes in UCB-MSCs. Gal-1 decreased the expression of collagen genes COL3A1 (COL-3) and COL5A1 (COL-5) but increased the expression of fibronectin (FN) and laminin 5 (LM-5), that were reversed by β-lactose. Gal-1 increased protein kinase C (PKC), c-Src, and caveolin-1 (Cav-1) phosphorylation that was attenuated by β-lactose and the Src inhibitor PP2. In addition, pretreatment with the lipid raft disruptor Mβ-CD and the PKC inhibitors inhibited Gal-1-induced UCB-MSC motility. In addition, Gal-1 reduced smad2/3 phosphorylation and induced nuclear factor (NF)-κB phosphorylation. Pretreatment with Mβ-CD attenuated Gal-1-reduced smad2/3 phosphorylation, COL-3, and COL-5 expression but did not affect NF-κB phosphorylation, FN, or LM-5 expression. In contrast, PKC inhibitors only attenuated NF-κB phosphorylation, FN, and LM-5 expression. Reconstructing Gal-1-induced genetic changes by replacing it with siRNA specific for COL-3 or COL-5, or treatment of the cells with FN and LM-5 proteins, increased motility and its related proteins such as focal adhesion kinase, Akt, Erk, integrins, and matrix metalloproteinase-2. A combined treatment with COL-3/COL-5 siRNA or FN/LM-5 compared with that of single treatments was synergistic. However, a single Gal-1 treatment maximally stimulated motility and related protein phosphorylation/expression. These results demonstrate that Gal-1 stimulated human UCB-MSC motility by decreasing COL-3/COL-5 expression and increasing FN/LM-5 expression through a PKC-dependent NF-κB and c-Src/Cav-1-dependent smad2/3 pathway that was critical for governing the activation of FAK, Akt, Erk, integrins, and MMP2. 
 Bacterial endotoxin enhances colorectal cancer cell adhesion and invasion through TLR-4 and NF-kappaB-dependent activation of the urokinase plasminogen activator system. Perioperative exposure to lipopolysaccharide (LPS) is associated with accelerated metastatic colorectal tumour growth. LPS directly affects cells through Toll-like receptor 4 (TLR-4) and the transcription factor NF-kappaB. The urokinase plasminogen activator (u-PA) system is intimately implicated in tumour cell   extracellular matrix   (ECM) interactions fundamental to tumour progression. Thus we sought to determine if LPS directly induces accelerated tumour cell ECM adhesion and invasion through activation of the u-PA system and to elucidate the cellular pathways involved. Human colorectal tumour cell lines were stimulated with LPS. u-PA concentration, u-PA activity, active u-PA, surface urokinase plasminogen activator receptor (u-PAR) and TLR-4 expression were assessed by ELISA, colorimetric assay, western blot analysis and flow cytometry respectively. In vitro tumour cell vitronectin adhesion and ECM invasion were analysed by vitronectin adhesion assay and ECM invasion chambers. u-PA and u-PAR function was inhibited with anti u-PA antibodies or the selective u-PA inhibitors amiloride or WXC-340, TLR-4 by TLR-4-blocking antibodies and NF-kappaB by the selective NF-kappaB inhibitor SN-50. LPS upregulates u-PA and u-PAR in a dose-dependent manner, enhancing in vitro tumour cell vitronectin adhesion and ECM invasion by >40% (P<0.01). These effects were ameliorated by u-PA and u-PAR inhibition. LPS activates NF-kappaB through TLR-4. TLR-4 and NF-kappaB inhibition ameliorated LPS-enhanced u-PA and u-PAR expression, tumour cell vitronectin adhesion and ECM invasion. LPS promotes tumour cell ECM adhesion and invasion through activation of the u-PA system in a TLR-4- and NF-kappaB-dependent manner. 
 Resveratrol reduces TNF-α-induced U373MG human glioma cell invasion through regulating NF-κB activation and uPA/uPAR expression. High invasiveness of glioma cells is one of the reasons that patients with malignant glioma have a poor prognosis. Resveratrol, a plant compound abundant in the peel of grapes, has been suggested as a potential cancer chemopreventive agent. Therefore, we investigated the effect of resveratrol on glioma cell invasion.The effect of resveratrol on U373MG human glioma cell invasion was assessed by Matrigel assay and methylthiazoltetrazolium assay. Western blotting and reverse transcription-polymerase chain reaction were performed to elucidate the action mechanism of resveratrol.Resveratrol reduced tumor necrosis factor (TNF)-α-induced U373MG human glioma cell invasion. In addition, resveratrol repressed nuclear factor kappa B (NF-κB) activation and down-regulated mRNA expression of urokinase plasminogen activator (uPA) and its receptor in TNF-α-treated glioma cells.These findings suggest that resveratrol could prevent glioma cell invasion via inhibiting proteolysi  s of extracellular m  atrix. 
 Reverse signaling initiated from GITRL induces NF-kappaB activation through ERK in the inflammatory activation of macrophages. Glucocorticoid-induced TNF receptor family related protein ligand (GITRL) is known to interact with its cognate receptor GITR. In order to investigate the potential role of GITRL in the pro-inflammatory activation of macrophages and the signaling pathway induced by GITRL, we stimulated the macrophage cell line, THP-1, and primary macrophages with an anti-GITRL monoclonal antibody or a GITR:Fc fusion protein and analyzed the cellular responses. The stimulation of GITRL induced the expression of pro-inflammatory cytokines and matrix metalloproteinase (MMP)-9 and up-regulated ICAM-1 expression levels, which was responsible for enhanced cellular aggregation and adhesion to   extracellular matrix   proteins. The activation of these pro-inflammatory mediators required the activation of ERK1/2 mitogen-activated protein kinase (MAPK) and negatively regulated by p38 MAPK and JNK. Immunofluorescence analysis detected nuclear translocation of the NF-kappaB p50 subunit and this was blocked by ERK inhibitor, indicating that GITRL stimulation induced ERK1/2 phosphorylation and subsequent activation of NF-kappaB. Furthermore, the expression of GITRL and GITR was detected in macrophages in inflammatory disease specimens such as atherosclerotic plaques and synovial tissues of rheumatoid arthritis. These observations raise the possibility that the GITRL-mediated inflammatory activation of macrophages is involved in the pathogenesis of inflammatory diseases. 
 Activation of Toll-like receptor 3 augments myofibroblast differentiation. Airway remodeling is observed in the airways of patients with asthma, and differentiation of fibroblasts to myofibroblasts plays a critical role in the progress of airway remodeling. Viral infection induces not only the disease development and exacerbations but also airway remodeling. The aim of this study was to evaluate whether the activation of Toll-like receptor 3 (TLR3) can affect the differentiation of fibroblasts to myofibroblasts and the   extracellular matrix   (ECM) protein production. Human fetal lung fibroblasts (HFL-1) and adult lung fibroblasts were treated with a synthetic double-stranded RNA, polyinosine-polycytidylic acid (poly[I:C]) and the expression of alpha-smooth muscle actin (alpha-SMA), a marker of myofibroblast differentiation, was evaluated. The release of transforming growth factor-beta(1) (TGF-beta(1)) and ECM protein production were assessed. The effect of anti-TGF-beta antibody on the alpha-SMA and ECM production was also assessed. Poly(I:C) significantly augmented the alpha-SMA expression (P < 0.01) and release of TGF-beta(1) (P < 0.01) compared with control. Bafilomycin, an inhibitor of TLR3 signaling, diminished poly(I:C)-augmented TGF-beta(1) release. Anti-TGF-beta(1) antibody inhibited the poly(I:C)-augmented alpha-SMA expression. Poly(I:C) enhanced translocation of nuclear factor-kB (NF-kappaB) and interferon regulatory factor-3 (IRF-3) into the nucleus. Poly(I:C)-augmented TGF-beta(1) release was almost completely blocked by NF-kappaB inhibitors, but not by silencing IRF-3. The production of fibronectin and collagen I expression were significantly increased by poly(I:C) (P < 0.01) and they were inhibited by anti-TGF-beta antibody. These results suggest that activation of TLR3 can affect the differentiation to myofibroblasts and enhance ECM production via the NF-kappaB-TGF-beta(1)-dependent pathway. 
 Integrin-mediated transcriptional activation of inhibitor of apoptosis proteins protects smooth muscle cells against apoptosis induced by degraded collagen. Apoptosis of smooth muscle cells (SMC) and degradation of the   extracellular matrix   (ECM) have both been implicated in atherosclerotic plaque rupture. We have previously reported that degraded type I collagen fragments induce a rapid but transient apoptotic burst initiated by calpains in SMC. The aim of the current study was to identify the pathway responsible for consecutive SMC survival. We show that exposure of SMC to collagen fragments resulted in a sustained activation of nuclear factor (NF)-kappaB via phosphorylation and degradation of IkappaBalpha. Its prevention through retroviral expression of superrepressor IkappaBalpha or proteasome inhibition potently induced apoptosis. In the presence of blocking antibodies to alpha(v)beta(3) integrin and RGD peptides, collagen fragments no longer activated NF-kappaB and apoptosis was enhanced. The mechanism by which NF-kappaB was protecting SMC against collagen fragment-induced apoptosis was a transcriptional activation of several endogenous caspase inhibitors of the inhibitor of apoptosis protein (IAP) family as: (1) the expression of xIAP, c-IAP2, and survivin was potently induced by collagen fragments; (2) IAP expression was abrogated by superrepressor IkappaBalpha; and (3) knockdown of each of the 3 IAPs by small interfering RNA (siRNA) resulted in enhanced apoptosis after collagen fragment treatment. Our data suggest that SMC exposed to degraded collagen are protected against apoptosis by a mechanism involving alpha(v)beta(3)-dependent NF-kappaB activation with consequent activation of IAPs. This may constitute a novel antiapoptotic pathway ensuring SMC survival in settings of enhanced ECM degradation such as cell migration, vascular remodeling, and atherosclerotic plaque rupture. 
 Transcriptional regulation of the endogenous danger signal tenascin-C: a novel autocrine loop in inflammation. Inappropriate expression of proinflammatory mediators underpins the pathogenesis of autoimmune disease and tumor metastasis. The   extracellular matrix   glycoprotein tenascin-C is an endogenous activator of innate immunity that promotes the synthesis of inflammatory cytokines via activation of TLR4. Little tenascin-C is observed in most healthy adult tissues, but expression is specifically upregulated at sites of inflammation. Moreover, high levels of tenascin-C are associated with chronic inflammation and found in the tumor stroma. In this study, we show that the expression of tenascin-C is induced in immune myeloid cells activated by a variety of inflammatory stimuli, including specific TLR ligands. Its synthesis is transcriptionally regulated and requires the specific activation of AKT/PI3K and NF-kappaB signaling pathways. Using a bioinformatic approach, we identified a large number of conserved noncoding regions throughout the tenascin-C genomic locus that may contribute to its transcriptional regulation during inflammation. We also demonstrate that tenascin-C expression is transient during acute inflammation. In contrast, persistently high levels of expression occur in the inflamed synovium of joints from rheumatoid arthritis patients. Thus, misregulated expression of this endogenous danger signal may promote an autocrine loop of inflammation and contribute to the persistence of inflammation in autoimmune diseases or to tumor egress and invasion during metastasis. 
 NF-kappaB is a critical regulator of the survival of rodent and human hepatic myofibroblasts. Hepatic myofibroblast activation during injury causes deposition of   extracellular matrix   within the liver and promotes development of fibrosis. Hepatic myofibroblast apoptosis is associated with remodelling of fibrotic   extracellular matrix   and regression of fibrosis. Previous work showed that inhibition of constitutive NF-kappaB signaling promotes hepatic myofibroblast apoptosis and resolution of fibrosis in rodent models. However, to date agents used to target constitutive NF-kappaB transcriptional activity in hepatic myofibroblasts have been relatively non-specific with potential for off-target effects that may complicate data interpretation. Likewise, rat chronic liver disease models may not accurately recapitulate the activation of human hepatic myofibroblasts.We used a mutant recombinant IkappaBalpha super-repressor fused to the HIV-TAT domain to specifically target NF-kappaB signaling in hepatic myofibroblasts. Inhibition of NF-kappaB activity was measured using reporter assay. Apoptosis of hepatic myofibroblasts was assessed by morphological changes, cleavage of the PARP-1 protein and Caspase 3 activation.TAT-IkappaBalphaSR reduced NF-kappaB dependent transcription, Bcl-2 expression and promoted Jun-N-terminal kinase-dependent apoptosis in human and rat hepatic myofibroblasts.These data highlight the conserved role of NF-kappaB during fibrogenesis. Our data validate the use of rodent models for pre-clinical testing of NF-kappaB inhibitors as anti-fibrotics and stimulators of fibrotic   extracellular matrix   remodelling. 
 Classical macrophage activation up-regulates several matrix metalloproteinases through mitogen activated protein kinases and nuclear factor-κB. Remodelling of the   extracellular matri  x (ECM) and cell surface by matrix metalloproteinases (MMPs) is an important function of monocytes and macrophages. Recent work has emphasised the diverse roles of classically and alternatively activated macrophages but the consequent regulation of MMPs and their inhibitors has not been studied comprehensively. Classical activation of macrophages derived in vitro from un-fractionated CD16(+/-) or negatively-selected CD16(-) macrophages up-regulated MMP-1, -3, -7, -10, -12, -14 and -25 and decreased TIMP-3 steady-state mRNA levels. Bacterial lipopolysaccharide, IL-1 and TNFα were more effective than interferonγ except for the effects on MMP-25, and TIMP-3. By contrast, alternative activation decreased MMP-2, -8 and -19 but increased MMP -11, -12, -25 and TIMP-3 steady-state mRNA levels. Up-regulation of MMPs during classical activation depended on mitogen activated protein kinases, phosphoinositide-3-kinase and inhibitor of κB kinase-2. Effects of interferonγ depended on janus kinase-2. Where investigated, similar effects were seen on protein concentrations and collagenase activity. Moreover, activity of MMP-1 and -10 co-localised with markers of classical activation in human atherosclerotic plaques in vivo. In conclusion, classical macrophage activation selectively up-regulates several MMPs in vitro and in vivo and down-regulates TIMP-3, whereas alternative activation up-regulates a distinct group of MMPs and TIMP-3. The signalling pathways defined here suggest targets for selective modulation of MMP activity. 
 Matrix metalloproteinase-1-mediated up-regulation of vascular endothelial growth factor-2 in endothelial cells. Matrix metalloproteinase-1 (MMP-1) is a collagenase that is highly active in   extracellular matrix   and vascular remodeling, angiogenesis, and tumor progression. Vascular endothelial growth factor receptor-2 (VEGFR2), the main receptor for VEGF-A, is expressed on endothelial cells and promotes cell survival, proliferation, and other functions. Although MMP-1 and VEGFR2 co-exist in many normal and pathophysiological conditions, the effect of MMP-1 on cellular VEGFR2 that can promote the above processes is unknown. In this study we test the hypothesis that stimulation of endothelial cells with MMP-1 increases their levels of VEGFR2. The increased VEGFR2 is then available to bind VEGF-A, resulting in increased response. Indeed we found that endothelial cells incubated with active MMP-1 had higher mRNA and protein levels of VEGFR2. Furthermore, VEGF-A-dependent phosphorylation of intracellular signaling molecules and endothelial proliferation were elevated after MMP-1 treatment. MMP-1 caused activation of the nuclear factor-κB (NF-κB) pathway (p65/RelA) in endothelial cells, and this response was dependent upon activation of protease activated receptor-1 (PAR-1). Chromatin immunoprecipitation was used to confirm NF-κB-mediated active transcription of the VEGFR2 (KDR) gene. Elevation in VEGFR2 after MMP-1 stimulation was inhibited by PAR-1 knockdown and NF-κB specific inhibition. We conclude that MMP-1 promotes VEGFR2 expression and proliferation of endothelial cells through stimulation of PAR-1 and activation of NF-κB. These results suggest a mechanism by which MMP-1 may prime or sensitize endothelial cell functions. 
 The Paracrine Effect of Degenerated Disc Cells on Healthy Human Nucleus Pulposus Cells Is Mediated by MAPK and NF-κB Pathways and Can Be Reduced by TGF-β1. Inflammation is thought to have a major role in the pathogenesis of disc degeneration. Studies have shown that nucleus pulposus cells (NPCs) respond to one or two specific cytokines by regulating cell proliferation or matrix synthesis. However, the effects of a cocktail of factors secreted by degenerated disc cells on transplanted exogenous healthy NPCs remain unknown. Concentrations of multiple cytokines in degenerated disc tissue-conditioned medium (dCM) were measured using enzyme-linked immunosorbent assay (ELISA). 3-(4, 5-Dimethylthiazol-2-yl)-2, 5-diphenyltetrazolium bromide (MTT) assay and Ki67 immunofluorescence staining were used to evaluate the proliferation of cells in dCM. The function of exogenous NPCs cultured in dCM was evaluated by examining catabolic markers (ADAMTS-4, ADAMTS-5, MMP-1, MMP-3, and MMP-13), anabolic markers (TIMP-1, TIMP-2, and TIMP-3), and th  e extracellular matr  ix protein-aggrecan (ACAN) and collagen II (COL2)-expression with real time polymerase chain reaction (RT-PCR). Mitogen-activated protein kinase (MAPK) and nuclear factor-kappa B (NF-κB) pathway activation was observed using Western blotting. Finally, we examined the role of transforming growth factor (TGF)-β1 in reducing dCM-mediated exogenous NPC dysfunction. Levels of tumor necrosis factor (TNF)-α, interleukin (IL)-1β, IL-1α, IL-2, IL-4, IL-6, IL-8, IL-10, IL-17, interferon-γ (IFN-γ), and prostaglandin E2 (PGE2) were higher and TGF-β1 levels were lower in dCM compared with the control medium. Treatment with dCM increased the proliferation of healthy NPCs. NPCs exhibited significantly higher expression of ADAMTS-4, ADAMTS-5, MMP-1, MMP-3, and MMP-13 and decreased TIMP-2, ACAN, and COL2 expression in the dCM group in a dose- and time-dependent manner. Treatment with dCM moderately increased TIMP-1 expression and had no effect on TIMP-3 mRNA levels. The MAPK and NF-κB pathways were implicated in dCM-mediated responses of healthy NPCs. TGF-β1 partially reversed the dCM-mediated NPC dysfunction. Increased levels of inflammatory factors and decreased TGF-β1 levels in dCM suggest an inflammatory environment in degenerated disc tissue. The catabolic effect of dCM on human healthy NPCs is mediated by MAPK and NF-κB pathways and can be reduced by TGF-β1. 
 Carcinoma matrix controls resistance to cisplatin through talin regulation of NF-kB.   Extracellular matrix   factors within the tumor microenvironment that control resistance to chemotherapeutics are poorly understood. This study focused on understanding matrix adhesion pathways that control the oral carcinoma response to cisplatin. Our studies revealed that adhesion of HN12 and JHU012 oral carcinomas to carcinoma matrix supported tumor cell proliferation in response to treatment with cisplatin. Proliferation in response to 30 µM cisplatin was not observed in HN12 cells adherent to other purified extracellular matrices such as Matrigel, collagen I, fibronectin or laminin I. Integrin β₁ was important for adhesion to carcinoma matrix to trigger proliferation after treatment with cisplatin. Disruption of talin expression in HN12 cells adherent to carcinoma matrix increased cisplatin induced proliferation. Pharmacological inhibitors were used to determine signaling events required for talin deficiency to regulate cisplatin induced proliferation. Pharmacological inhibition of NF-kB reduced proliferation of talin-deficient HN12 cells treated with 30 µM cisplatin. Nuclear NF-kB activity was assayed in HN12 cells using a luciferase reporter of NF-kB transcriptional activity. Nuclear NF-kB activity was similar in HN12 cells adherent to carcinoma matrix and collagen I when treated with vehicle DMSO. Following treatment with 30 µM cisplatin, NF-kB activity is maintained in cells adherent to carcinoma matrix whereas NF-kB activity is reduced in collagen I adherent cells. Expression of talin was sufficient to trigger proliferation of HN12 cells adherent to collagen I following treatment with 1 and 30 µM cisplatin. Talin overexpression was sufficient to trigger NF-kB activity following treatment with cisplatin in carcinoma matrix adherent HN12 cells in a process disrupted by FAK siRNA. Thus, adhesions within the carcinoma matrix create a matrix environment in which exposure to cisplatin induces proliferation through the function of integrin β₁, talin and FAK pathways that regulate NF-kB nuclear activity. 
 Hypoxia activates heparanase expression in an NF-kappaB dependent manner. Hypoxia was shown to increase tumor cell invasion into the   extracellular matrix   in vitro. This result suggests that heparanase (Hpa), one of the key enzymes involved in tumor invasion and metastasis, may be regulated by hypoxia. RT-PCR, Western blot and Matrigel invasive assays were used to study the regulation of Hpa under hypoxia in human pancreatic MIA PaCa-2 cancer cells. Compared with those in normoxia (20% O2), Hpa mRNA, protein and enzymatic activity levels, were up-regulated by a reduction in the oxygen level (1% O2). Invasion by tumor cells into the   extracellular matrix   was found to be significantly enhanced. A reduction in Hpa protein levels was observed when nuclear factor kappaB (NF-kappaB) activation was blocked by pyrrolidine dithiocarbamate. The levels of Hpa were also reduced when Hpa was inhibited by an Hpa-specific antisense oligonucleotide. The MMP-9 mRNA, protein and gelatinase B activity levels in supernatants decreased significantly when Hpa was inhibited. We conclude that up-regulation of Hpa by hypoxia is NF-kappaB-dependent in MIA PaCa-2 cells and inhibition of Hpa reduces MMP-9 activity. This reduction in MMP-9 activity may be an important mechanism in tumor metastasis. 
 The tumor marker Fascin is induced by the Epstein-Barr virus-encoded oncoprotein LMP1 via NF-κB in lymphocytes and contributes to their invasive migration. The actin-bundling protein Fascin (FSCN1) is a tumor marker that is highly expressed in numerous types of cancer including lymphomas and is important for migration and metastasis of tumor cells. Fascin has also been detected in B lymphocytes that are freshly-infected with Epstein-Barr virus (EBV), however, both the inducers and the mechanisms of Fascin upregulation are still unclear.Here we show that the EBV-encoded oncoprotein latent membrane protein 1 (LMP1), a potent regulator of cellular signaling and transformation, is sufficient to induce both Fascin mRNA and protein in lymphocytes. Fascin expression is mainly regulated by LMP1 via the C-terminal activation region 2 (CTAR2). Block of canonical NF-κB signaling using a chemical inhibitor of IκB kinase β (IKKβ) or cotransfection of a dominant-negative inhibitor of IκBα (NFKBIA) reduced not only expression of p100, a classical target of the canonical NF-κB-pathway, but also LMP1-induced Fascin expression. Furthermore, chemical inhibition of IKKβ reduced both Fascin mRNA and protein levels in EBV-transformed lymphoblastoid cell lines, indicating that canonical NF-κB signaling is required for LMP1-mediated regulation of Fascin both in transfected and transformed lymphocytes. Beyond that, chemical inhibition of IKKβ significantly reduced invasive migration of EBV-transformed lymphoblastoid cel  ls through extracell  ular matrix. Transient transfection experiments revealed that Fascin contributed to LMP1-mediated enhancement of invasive migrati  on through extracell  ular matrix. While LMP1 enhanced the number of invaded cells, functional knockdown of Fascin by two different small hairpin RNAs resulted in significant reduction of invaded, non-attached cells.Thus, our data show that LMP1-mediated upregulation of Fascin depends on NF-κB and both NF-κB and Fascin contribute to invasive migration of LMP1-expressing lymphocytes. 
 Osteopontin stimulates cell motility and nuclear factor kappaB-mediated secretion of urokinase type plasminogen activator through phosphatidylinositol 3-kinase/Akt signaling pathways in breast cancer cells. We have recently reported that osteopontin (OPN) induces nuclear factor kappaB (NFkappaB)-mediated promatrix metalloproteinase-2 activation through IkappaBalpha/IKK signaling pathways and that curcumin (diferulolylmethane) down-regulates these pathways (Philip, S., and Kundu, G. C. (2003) J. Biol. Chem. 278, 14487-14497). However, the molecular mechanism by which upstream kinases regulate the OPN-induced NFkappaB activation and urokinase type plasminogen activator (uPA) secretion in human breast cancer cells is not well defined. Here we report that OPN induces the phosphatidylinositol 3'-kinase (PI 3'-kinase) activity and phosphorylation of Akt in highly invasive MDA-MB-231 and low invasive MCF-7 cells. The OPN-induced Akt phosphorylation was inhibited when cells were transfected with a dominant negative mutant of the p85 domain of PI 3-kinase (Deltap85) and enhanced when cells were transfected with an activated form of PI 3-kinase (p110CAAX), indicating that PI 3'-kinase is involved in Akt phosphorylation. OPN enhances the interaction between IkappaBalpha kinase (IKK) and phosphorylated Akt. OPN also induces NFkappaB activation through phosphorylation and degradation of IkappaBalpha by inducing the IKK activity. However, both pharmacological (wortmannin and LY294002) and genetic (Deltap85) inhibitors of PI 3'-kinase inhibited OPN-induced Akt phosphorylation, IKK activity, and NFkappaB activation through phosphorylation and degradation of IkappaBalpha. OPN also enhances uPA secretion, cell motility, and   extracellular matrix   invasion. Furthermore, cells transfected with Deltap85 or the super-repressor form of IkappaBalpha suppressed the OPN-induced uPA secretion and cell motility, whereas cells transfected with p110CAAX enhanced these effects. Pretreatment of cells with PI 3-kinase inhibitors or NFkappaB inhibitory peptide (SN-50) reduced the OPN-induced uPA secretion, cell motility, and invasion. To our knowledge, this is first report that OPN induces NFkappaB activity and uPA secretion by activating PI 3'-kinase/Akt/IKK-mediated signaling pathways and further demonstrates a functional molecular link between OPN-induced PI 3'-kinase-dependent Akt phosphorylation and NFkappaB-mediated uPA secretion, and all of these ultimately control the motility of breast cancer cells. 
 Role of beta(3)-endonexin in the regulation of NF-kappaB-dependent expression of urokinase-type plasminogen activator receptor. Endothelial migration on   extracellular matrix   is regulated by integrins and proteolysis. Previous studies showed that beta(3)-integrins regulate expression of the urokinase-type plasminogen activator receptor (uPAR) through outside-in signalling involving the cytoplasmic domain. Here we show that overexpression of the integrin-binding protein beta(3)-endonexin decreased uPAR promoter (-398 base-pair fragment) activity that is constitutively active in endothelial cells. Mutation of the NF-kappaB promoter binding site (-45 bp) impaired the ability of beta(3)-endonexin to downregulate uPAR promoter activity. Immunoprecipitation studies showed that beta(3)-endonexin interacts directly with the p50/p65 transactivation complex and thereby inhibits binding of kappaB oligonucleotides to the p50/p65 complex. Moreover, binding of beta(3)-endonexin to p50 was inhibited in the presence of kappaB but not mutated kappaB oligonucleotides, suggesting a sterical competition between beta(3)-endonexin and kappaB DNA for the p50/p65 complex. We therefore propose that beta(3)-endonexin acts as regulator of uPAR expression in beta(3)-integrin-mediated endothelial cell migration through direct interaction with p50/p65. Since NF-kappaB regulates the expression of matrix degrading enzymes, the present results define a role of beta(3)-endonexin in regulating beta(3)-integrin-mediated adhesion and pericellular proteolysis. 
 Significance of NF-kappaB/GATA axis in tumor necrosis factor-alpha-induced expression of 6-sulfated cell recognition glycans in human T-lymphocytes. Sulfated glycans play critical roles in various cell recognition events among leukocytes. The 6-sulfated lactosamine glycans in particular have been widely noted for their importance because they are involved in cell recognition events mediated by cell-adhesion molecules such as selectins and sialic acid-recognizing molecules such as siglecs and also in the activation of CD44 in binding to   extracellular matrix   hyaluronate. A pro-inflammatory cytokine, tumor necrosis factor-alpha, induces expression of 6-sulfated glycans on human leukocytes. Here we report that the transcription of the GlcNAc6ST-1 gene, the gene encoding a sulfotransferase for 6-sulfated glycan synthesis, is induced in human T-lymphoid cells through tandem NF-kappaB and GATA motifs in its 5'-regulatory region. Results of our reporter assays, immunoprecipitation, and chromatin immunoprecipitation analyses indicated that GATA-3 and/or GATA-2, but not GATA-1, associates with NF-kappaB in a transcription factor complex on the 5'-regulatory region of the gene and acts synergistically with NF-kappaB in triggering GlcNAc6ST-1 transcription. Recently, a skin-homing subset of helper memory T cells exhibiting the Th2 marker CCR4 was shown to specifically express 6-sulfated glycans. The transactivation mechanism described here suggested that GlcNAc6ST-1 transcription is coordinated with the NF-kappaB/GATA-3 axis, which is known to figure heavily in Th2 cell differentiation. In line with this, in vitro differentiation of human T cells to Th2 cells was found to significantly induce GlcNAc6ST-1 transcription and 6-sulfated glycan expression. 
 IL-1 beta induces urokinase-plasminogen activator expression and cell migration through PKC alpha, JNK1/2, and NF-kappaB in A549 cells. Breakdown of the   extracellular matrix   (ECM) is accomplished by the concerted action of several proteases, including the urokinase plasminogen-activator (uPA) system and matrix metalloproteinases (MMPs), which is crucial for cancer invasion and metastasis. Several reports have shown that the levels of IL-1 beta and MMPs in plasma of the patients with lung cancer are significantly elevated and link to the invasion of tumor cells. Therefore, we investigated whether IL-1 beta-induced expression of uPA participated in lung cancer progression. In this study, IL-1 beta significantly induced uPA expression and activity via PKC alpha-dependent JNK1/2 and NIK cascades, linking to IKK alpha/beta activation, p65 translocation and transcription activity, using pharmacological inhibitors and transfection with dominant negative mutants and siRNAs. IL-1 beta-induced uPA protein and mRNA expression in a time- and concentration-dependent manner, which was inhibited by pretreatment with the inhibitors of JNK1/2 (SP600125), PKC (Ro31-8220, Gö6976), or NF-kappaB (helenalin), and transfection with dominant negative mutants of PKC alpha, NIK, and IKK beta, and siRNAs of JNK1/2 and p65. IL-1 beta stimulated PKC alpha translocation to plasma membrane leading to phosphorylation of JNK1/2, which was attenuated by PKC inhibitors and transfection with shRNAs of JNK1/2, but not by helenalin. In addition, IL-1beta stimulated p65 phosphorylation and translocation into nucleus concomitant with I kappaB alpha phosphorylation and I kappaB alpha degradation, which was mediated via activation of PKC alpha-dependent JNK1/2-NIK/IKK beta cascade. These results demonstrated that in A549 cells, activation of p50/p65 heterodimer through sequential activation of PKC alpha-JNK-NIK-IKK beta-NF-kappaB was required for IL-1 beta-induced uPA expression associated with migration of tumor cells. 
 Aggregation of beta2 integrins activates human neutrophils through the IkappaB/NF-kappaB pathway. Neutrophils are now considered central to the pathogenesis of most forms of acute lung injury. Neutrophils do not cause damage while suspended in the bloodstream; however, a release of cytotoxic agents occurs when neutrophils are adherent to endothelium, epithelium, or   extracellular matrix   proteins in the interstitium. Such neutrophil adherence is mediated predominantly through beta(2) integrins (CD11/CD18) on its surface. This study was undertaken to investigate whether the IkappaB/nuclear factor (NF)-kappaB cascade is involved in this beta(2) integrin-mediated activation of human neutrophils. beta(2) Integrin Mac-1 (CD11b/CD18) aggregation was induced by antibody cross-linking of the integrins on the cell surface. beta(2) Integrin aggregation induced interleukin-1beta and tumor necrosis factor-alpha production, which suggests the activation of neutrophils by beta(2) integrin. IkappaBalpha was markedly degraded at 1 h, and NF-kappaB-DNA-binding activity markedly increased 2 h after beta(2) integrin aggregation, which activated IkappaB kinase activity at 1 h. beta(2) Integrin-induced cytokine production was suppressed by MG132 or SN50 pretreatment, which blocked the activation of NF-kappaB. These findings suggest that the activation of human neutrophils by beta(2) integrin aggregation is mediated through the activation of the IkappaB/NF-kappaB pathway. 
 Inhibition of matrix metalloproteinase-9 and nuclear factor kappa B contribute to melatonin prevention of motility and invasiveness in HepG2 liver cancer cells. Hepatocellular carcinoma (HCC) is one of the most lethal human cancers worldwide because of its high incidence and its metastatic potential.   Extracellular matrix   degradation by matrix metalloproteinases (MMPs) has been connected with cancer cell invasion, and it has been suggested that inhibition of MMPs by synthetic and natural inhibitors may be of great importance in the HCC therapies. Melatonin, the main product of the pineal gland, exerts antiproliferative, proapoptotic, and antiangiogenic properties in HepG2 human hepatocellular cells, and exhibits anti-invasive and antimetastatic activities by suppressing the enzymatic activity of MMP-9 in different tumor types. However, the underlying mechanism of anti-invasive activity in HCC models has not been fully elucidated. Here, we demonstrate that 1 mm melatonin dosage reduced in IL-1β-induced HepG2 cells MMP-9 gelatinase activity and inhibited cell invasion and motility through downregulation of MMP-9 gene expression and upregulation of the MMP-9-specific inhibitor tissue inhibitor of metalloproteinases (TIMP)-1. No significant changes were observed in the expression and activity of MMP-2, the other proteinase implicated in matrix collagen degradation, and its tissue inhibitor, TIMP-2. Also, melatonin significantly suppressed IL-1β-induced nuclear factor-kappaB (NF-κB) translocation and transcriptional activity. In summary, we demonstrate that melatonin modulates motility and invasiveness of HepG2 cell in vitro through a molecular mechanism that involves TIMP-1 upregulation and attenuation of MMP-9 expression and activity via NF-κB signal pathway inhibition. 
 Raf kinase inhibitor protein suppresses nuclear factor-κB-dependent cancer cell invasion through negative regulation of matrix metalloproteinase expression. Accumulating evidence suggests that Raf kinase inhibitor protein (RKIP), which negatively regulates multiple signaling cascades including the Raf and nuclear factor-κB (NF-κB) pathways, functions as a metastasis suppressor. However, the basis for this activity is not clear. We investigated this question in a panel of breast cancer, colon cancer and melanoma cell lines. We found that RKIP negatively regulated the invasion of the different cancer cells through three-dimension  al extracellular mat  rix barriers by controlling the expression of matrix metalloproteinases (MMPs), particularly, MMP-1 and MMP-2. Silencing of RKIP expression resulted in a highly invasive phenotype and dramatically increased levels of MMP-1 and MMP-2 expression, while overexpression of RKIP decreased cancer cell invasion in vitro and metastasis in vivo of murine tumor allografts. Knockdown of MMP-1 or MMP-2 in RKIP-knockdown cells reverted their invasiveness to normal. In contrast, when examining migration of the different cancer cells in a two-dimensional, barrier-less environment, we found that RKIP had either a positive regulatory activity or no activity, but in no case a negative one (as would be expected if RKIP suppressed metastasis at the level of cell migration itself). Therefore, RKIP's function as a metastasis suppressor appears to arise from its ability to negatively regulate expression of specific MMPs, and thus invasion through barriers, and not from a direct effect on the raw capacity of cells to move. The NF-κB pathway, but not the Raf pathway, appeared to positively control the invasion of breast cancer cells. A regulatory loop involving an opposing relationship between RKIP and the NF-κB pathway may control the level of MMP expression and cell invasion. 
 Interleukin-18 stimulates fibronectin expression in primary human cardiac fibroblasts via PI3K-Akt-dependent NF-kappaB activation. Fibronectin (FN), a key component of the   extracellular matrix  , is upregulated in cardiac tissue during myocardial hypertrophy and failure. Here we show that interleukin (IL)-18, a proinflammatory and pro-hypertrophic cytokine, stimulates FN expression in adult human cardiac fibroblasts (HCF), an effect blocked by either the IL-18BP:Fc chimera or IL-18 neutralizing antibodies. IL-18 stimulated FN promoter-reporter activity in HCF, a response attenuated by mutation of an NF-kappaB binding site in the FN promoter. Overexpression of p65 stimulated FN transcription. IL-18 stimulated in vitro (p65, p50) and in vivo NF-kappaB DNA binding activities, and induced kappaB-dependent reporter gene activity. These effects were inhibited by adenoviral transduction of dominant negative (dn) p65 (Ad.dnp65) and dnIKK2 (Ad.dnIKK2). Investigation of signaling intermediates revealed that IL-18 stimulated PI3 kinase activity (blocked by wortmannin, LY294002, or Ad.dnPI3Kp85), and Akt phosphorylation and kinase activity (blocked by SH-5 or Ad.dnAkt). Furthermore, targeting MyD88, IRAK1, TRAF6, PI3K, Akt, and NF-kappaB by RNA interference or dn expression vectors blunted IL-18 mediated FN transcription and mRNA expression. Conversely, FN stimulated IL-18 expression. These data provide the first evidence that IL-18 and FN stimulate each other's expression in HCF, and suggest a role for IL-18, FN and their crosstalk in myocardial hypertrophy and remodeling, disease states characterized by enhanced FN expression and fibrosis. 
 Stromal down-regulation of macrophage CD4/CCR5 expression and NF-κB activation mediates HIV-1 non-permissiveness in intestinal macrophages. Tissue macrophages are derived exclusively from blood monocytes, which as monocyte-derived macrophages support HIV-1 replication. However, among human tissue macrophages only intestinal macrophages are non-permissive to HIV-1, suggesting that the unique microenvironment in human intestinal mucosa renders lamina propria macrophages non-permissive to HIV-1. We investigated this hypothesis using blood monocytes and intestinal   extracellular matri  x (stroma)-conditioned media (S-CM) to model the exposure of newly recruited monocytes and resident macrophages to lamina propria stroma, where the cells take up residence in the intestinal mucosa. Exposure of monocytes to S-CM blocked up-regulation of CD4 and CCR5 expression during monocyte differentiation into macrophages and inhibited productive HIV-1 infection in differentiated macrophages. Importantly, exposure of monocyte-derived macrophages simultaneously to S-CM and HIV-1 also inhibited viral replication, and sorted CD4+ intestinal macrophages, a proportion of which expressed CCR5+, did not support HIV-1 replication, indicating that the non-permissiveness to HIV-1 was not due to reduced receptor expression alone. Consistent with this conclusion, S-CM also potently inhibited replication of HIV-1 pseudotyped with vesicular stomatitis virus glycoprotein, which provides CD4/CCR5-independent entry. Neutralization of TGF-β in S-CM and recombinant TGF-β studies showed that stromal TGF-β inhibited macrophage nuclear translocation of NF-κB and HIV-1 replication. Thus, the profound inability of intestinal macrophages to support productive HIV-1 infection is likely the consequence of microenvironmental down-regulation of macrophage HIV-1 receptor/coreceptor expression and NF-κB activation. 
 Relation between TLR4/NF-κB signaling pathway activation by 27-hydroxycholesterol and 4-hydroxynonenal, and atherosclerotic plaque instability. It is now thought that atherosclerosis, although due to increased plasma lipids, is mainly the consequence of a complicated inflammatory process, with immune responses at the different stages of plaque development. Increasing evidence points to a significant role of Toll-like receptor 4 (TLR4), a key player in innate immunity, in the pathogenesis of atherosclerosis. This study aimed to determine the effects on TLR4 activation of two reactive oxidized lipids carried by oxidized low-density lipoproteins, the oxysterol 27-hydroxycholesterol (27-OH) and the aldehyde 4-hydroxynonenal (HNE), both of which accumulate in atherosclerotic plaques and play a key role in the pathogenesis of atherosclerosis. Secondarily, it examined their potential involvement in mediating inflammation and   extracellular matri  x degradation, the hallmarks of high-risk atherosclerotic unstable plaques. In human promonocytic U937 cells, both 27-OH and HNE were found to enhance cell release of IL-8, IL-1β, and TNF-α and to upregulate matrix metalloproteinase-9 (MMP-9) via TLR4/NF-κB-dependent pathway; these actions may sustain the inflammatory response and matrix degradation that lead to atherosclerotic plaque instability and to their rupture. Using specific antibodies, it was also demonstrated that these inflammatory cytokines increase MMP-9 upregulation, thus enhancing the release of this matrix-degrading enzyme by macrophage cells and contributing to plaque instability. These innovative results suggest that, by accumulating in atherosclerotic plaques, the two oxidized lipids may contribute to plaque instability and rupture. They appear to do so by sustaining the release of inflammatory molecules and MMP-9 by inflammatory and immune cells, for example, macrophages, through activation of TLR4 and its NF-κB downstream signaling. 
  [Ii]nvasive  PubMed  
 Stabilization of snail by NF-kappaB is required for inflammation-induced cell migration and invasion. The increased motility and   invasive  ness of tumor cells are reminiscent of epithelial-mesenchymal transition (EMT), which occurs during embryonic development, wound healing, and metastasis. In this study, we found that Snail is stabilized by the inflammatory cytokine TNFalpha through the activation of the NF-kappaB pathway. We demonstrated that NF-kappaB is required for the induction of COP9 signalosome 2 (CSN2), which, in turn, blocks the ubiquitination and degradation of Snail. Furthermore, we showed that the expression of Snail correlated with the activation of NF-kappaB in cancer cell lines and metastatic tumor samples. Knockdown of Snail expression inhibited cell migration and invasion induced by inflammatory cytokines and suppressed inflammation-mediated breast cancer metastasis. Our study provides a plausible mechanism for inflammation-induced metastasis. 
 Molecular basis of anti-inflammatory action of platelet-rich plasma on human chondrocytes: mechanisms of NF-κB inhibition via HGF. Loss of articular cartilage through injury or disease presents major clinical challenges also because cartilage has very poor regenerative capacity, giving rise to the development of biological approaches. As autologous blood product, platelet-rich plasma (PRP) provides a promising alternative to surgery by promoting safe and natural healing. Here we tested the possibility that PRP might be effective as an anti-inflammatory agent, providing an attractive basis for regeneration of articular cartilage, and two principal observations were done. First, activated PRP in chondrocytes reduced the transactivating activity of NF-κB, critical regulator of the inflammatory process, and decreased the expression of COX-2 and CXCR4 target genes. By analyzing a panel of cytokines with different biological significance, in activated PRP we observed increases in hepatocyte growth factor (HGF), interleukin-4 and tumor necrosis factor-α (TNF-α). HGF and TNF-α, by disrupting NF-κB-transactivating activity, were important for the anti-inflammatory function of activated PRP. The key molecular mechanisms involved in PRP-inhibitory effects on NF-κB activity were for HGF the enhanced cellular IkBα expression, that contributed to NF-κB-p65 subunit retention in the cytosol and nucleo-cytoplasmic shuttling, and for TNF-α the p50/50 DNA-binding causing inhibition of target-gene expression. Second, activated PRP in U937-monocytic cells reduced chemotaxis by inhibiting chemokine transactivation and CXCR4-receptor expression, thus possibly controlling local inflammation in cartilage. In conclusion, activated PRP is a promising biological therapeutic agent, as a scaffold   in micr  o-invasive articular cartilage regeneration, not only for its content of proliferative/differentiative growth factors, but also for the presence of anti-inflammatory agents including HGF. 
 EGF stimulates uPAR expression and cell   invasive  ness through ERK, AP-1, and NF-kappaB signaling in human gastric carcinoma cells. Overexpression of epidermal growth factor (EGF) and urokinase plasminogen activator receptor (uPAR) have been observed in human gastric cancers. However, the interaction between EGF and uPAR in gastric cancer has not been well elucidated. In this study, we investigated the effect of EGF on uPAR expression and the underlying signal pathways in human gastric cancer AGS cells. EGF induced uPAR mRNA expression in a time- and concentration-dependent manner. EGF also induced uPAR promoter activity. In addition, EGF induced the activation of extracellular signal regulated kinase-1/2 (ERK-1/2) and P38 mitogen-activated protein kinase (MAPK) but not the activation of c-Jun amino terminal kinase. A specific inhibitor of MEK-1 (an upstream effector of ERK-1/2) and a dominant negative MEK-1 were able to suppress the EGF-induced uPAR promoter activity. Site-directed mutagenesis and electrophoretic mobility shift assays demonstrated that the binding sites of transcription factors, activator protein-1 (AP-1) and nuclear factor (NF)-kappaB, are involved in the EGF-induced uPAR transcription. Suppression of the EGF-induced uPAR promoter activity by the AP-1 decoy oligonuclotide, as well as expression vectors encoding mutated-type NF-kappaB-inducting kinase and I-kappaB, confirmed that the activation of AP-1 and NF-kappaB are essential for the EGF-induced uPAR upregulation. The AGS cells pretreated with EGF showed a remarkably enhanced   invasive  ness and this effect was partially abrogated by uPAR neutralizing antibodies and by the inhibitors of ERK-1/2, AP-1, and NF-kappaB. The above results suggest that EGF induces uPAR expression via ERK-1/2, AP-1, and NF-kappaB signaling pathways and, in turn, stimulates cell   invasive  ness in human gastric cancer AGS cells. 
 Degradation of the transcription factors NF-κB, STAT3, and STAT5 is involved in Entamoeba histolytica-induced cell death in Caco-2 colonic epithelial cells. Entamoeba histolytica is a tissue  -invasiv  e protozoan parasite causing dysentery in humans. During infection of colonic tissues, amoebic trophozoites are able to kill host cells via apoptosis or necrosis, both of which trigger IL-8-mediated acute inflammatory responses. However, the signaling pathways involved in host cell death induced by E. histolytica have not yet been fully defined. In this study, we examined whether calpain plays a role in the cleavage of pro-survival transcription factors during cell death of colonic epithelial cells, induced by live E. histolytica trophozoites. Incubation with amoebic trophozoites induced activation of m-calpain in a time- and dose-dependent manner. Moreover, incubation with amoebae resulted in marked degradation of STAT proteins (STAT3 and STAT5) and NF-κB (p65) in Caco-2 cells. However, IκB, an inhibitor of NF-κB, was not cleaved in Caco-2 cells following adherence of E. histolytica. Entamoeba-induced cleavage of STAT proteins and NF-κB was partially inhibited by pretreatment of cells with a cell-permeable calpain inhibitor, calpeptin. In contrast, E. histolytica did not induce cleavage of caspase-3 in Caco-2 cells. Furthermore, pretreatment of Caco-2 cells with a calpain inhibitor, calpeptin (but not the pan-caspase inhibitor, z-VAD-fmk) or m-calpain siRNA partially reduced Entamoeba-induced DNA fragmentation in Caco-2 cells. These results suggest that calpain plays an important role in E. histolytica-induced degradation of NF-κB and STATs in colonic epithelial cells, which ultimately accelerates cell death. 
 Genetic susceptibility to distinct bladder cancer subphenotypes. Clinical, pathologic, and molecular evidence indicate that bladder cancer is heterogeneous with pathologic/molecular features that define distinct subphenotypes with different prognoses. It is conceivable that specific patterns of genetic susceptibility are associated with particular subphenotypes.To examine evidence for the contribution of germline genetic variation to bladder cancer heterogeneity.The Spanish Bladder Cancer/EPICURO Study is a case-control study based in 18 hospitals located in five areas in Spain. Cases were patients with a newly diagnosed, histologically confirmed, urothelial cell carcinoma of the bladder from 1998 to 2001. Case diagnoses were reviewed and uniformly classified by pathologists following the World Health Organisation/International Society of Urological Pathology 1999 criteria. Controls were hospital-matched patients (n=1149).A total of 1526 candidate variants in 423 candidate genes were analysed. Three distinct subphenotypes were defined according to stage and grade: low-grade nonmuscle   invasive   (n=586), high-grade nonmuscle   invasive   (n=219), and muscle   invasive   (n=246). The association between each variant and subphenotype was assessed by polytomous risk models adjusting for potential confounders. Heterogeneity in genetic susceptibility among subphenotypes was also tested.Two established bladder cancer susceptibility genotypes, NAT2 slow-acetylation and GSTM1-null, exhibited similar associations among the subphenotypes, as did VEGF-rs25648, which was previously identified in our study. Other variants conferred risks for specific tumour subphenotypes such as PMS2-rs6463524 and CD4-rs3213427 (respective heterogeneity p values of 0.006 and 0.004), which were associated with muscle-  invasive   tumours (per-allele odds ratios [95% confidence interval] of 0.56 [0.41-0.77] and 0.71 [0.57-0.88], respectively) but not with non-muscle-  invasive   tumours. Heterogeneity p values were not robust in multiple testing according to their false-discovery rate.These exploratory analyses suggest that genetic susceptibility loci might be related to the molecular/pathologic diversity of bladder cancer. Validation through large-scale replication studies and the study of additional genes and single nucleotide polymorphisms are required. 
 MSP-induced RON activation upregulates uPAR expression and cell   invasive  ness via MAPK, AP-1 and NF-κB signals in gastric cancer cells. Overexpression of recepteur d'Origine nantais (RON) and urokinase plasminogen activator receptor (uPAR) have been observed in human gastric cancers. However, the interaction between RON and uPAR in gastric cancer is unclear. The present study investigated the effect of macrophage-stimulating protein (MSP, the RON ligand) on uPAR expression and the underlying signal pathways in human gastric cancer AGS cells. uPAR messenger RNA expression was induced by MSP in a time- and concentration-dependent manner. MSP also induced uPAR promoter activity. The introduction of RON-specific small interfering RNA (siRNA) significantly affected the MSP-induced uPAR transcription. Deleted and site-directed mutagenesis studies demonstrated the involvement of the binding sites of transcription factor nuclear factor-kappaB (NF-κB) and activator protein (AP)-1 in the MSP-induced uPAR expression. Studies with expression vectors encoding mutated-type NF-κB signaling molecules and AP-1 decoy confirmed that NF-κB and AP-1 were essential for the MSP-induced uPAR expression. In addition, MSP induced the activation of extracellular signal-regulated kinase-1/2 (Erk-1/2), c-Jun amino terminal kinase (JNK) and p38 mitogen-activated protein kinase (MAPK). Dominant-negative mutants (K97M and TAM67) and specific inhibitors of Erk-1/2 and JNK were able to suppress the MSP-induced uPAR expression. AGS cells pretreated with MSP showed a remarkably enhan  ced inva  siveness, which was partially abrogated by siRNA-targeted RON and uPAR-neutralizing antibodies. The above results suggest that MSP induces uPAR expression via MAPK, AP-1 and NF-κB signaling pathways and, in turn, stimulates   cell inv  asiveness in human gastric cancer AGS cells. 
 Dual knockdown of p65 and p50 subunits of NF-kappaB by siRNA inhibits the induction of inflammatory cytokines and significantly enhance apoptosis in human primary synoviocytes treated with tumor necrosis factor-alpha. In order to develop an anti-NF-kappaB siRNA as a novel class of anti-inflammatory drug, we have isolated a highly efficient siRNA targeting the p65 (RelA) subunit of NF-kappaB, hereafter named REL1096. To determine whether down-regulation of p65 by REL1096 can block the induction of inflammatory cytokines after treatment with tumor necrosis factor-alpha (TNF-alpha), human primary fibroblast-like synoviocytes were isolated from patients with rheumatoid arthritis. When treated with REL1096, the TNF-mediated induction of downstream target genes such as inflammatory cytokines, chemokines, and anti-apoptosis genes was drastically inhibited. To enhance the inhibitory effect of REL1096, cells were treated with siRNA targeting the p50 subunit of NF-kappaB together with REL1096. In addition to effective downregulation of inflammatory cytokines, knockdown of both p65 and p50 resulted in much more extensive apoptosis when compared to cells treated with either REL1096 or p50-siRNA alone. Thus, our results provide evidence for the potential use of siRNA targeting NF-kappaB as an effective means to treat rheumatoid arthritis. In addition to effective amelioration of synovial inflammation by downregulation of inflammatory cytokines, increased apoptosis by dual knockdown of p65 and p50 may prove advantageous in preventing   invasive  ness and destructiveness of hyperplastic synoviocytes. 
 Degradation of the transcription factors NF-κB, STAT3, and STAT5 is involved in Entamoeba histolytica-induced cell death in Caco-2 colonic epithelial cells. Entamoeba histolytica is a tissue  -invasiv  e protozoan parasite causing dysentery in humans. During infection of colonic tissues, amoebic trophozoites are able to kill host cells via apoptosis or necrosis, both of which trigger IL-8-mediated acute inflammatory responses. However, the signaling pathways involved in host cell death induced by E. histolytica have not yet been fully defined. In this study, we examined whether calpain plays a role in the cleavage of pro-survival transcription factors during cell death of colonic epithelial cells, induced by live E. histolytica trophozoites. Incubation with amoebic trophozoites induced activation of m-calpain in a time- and dose-dependent manner. Moreover, incubation with amoebae resulted in marked degradation of STAT proteins (STAT3 and STAT5) and NF-κB (p65) in Caco-2 cells. However, IκB, an inhibitor of NF-κB, was not cleaved in Caco-2 cells following adherence of E. histolytica. Entamoeba-induced cleavage of STAT proteins and NF-κB was partially inhibited by pretreatment of cells with a cell-permeable calpain inhibitor, calpeptin. In contrast, E. histolytica did not induce cleavage of caspase-3 in Caco-2 cells. Furthermore, pretreatment of Caco-2 cells with a calpain inhibitor, calpeptin (but not the pan-caspase inhibitor, z-VAD-fmk) or m-calpain siRNA partially reduced Entamoeba-induced DNA fragmentation in Caco-2 cells. These results suggest that calpain plays an important role in E. histolytica-induced degradation of NF-κB and STATs in colonic epithelial cells, which ultimately accelerates cell death. 
 MSP-induced RON activation upregulates uPAR expression and cell   invasive  ness via MAPK, AP-1 and NF-κB signals in gastric cancer cells. Overexpression of recepteur d'Origine nantais (RON) and urokinase plasminogen activator receptor (uPAR) have been observed in human gastric cancers. However, the interaction between RON and uPAR in gastric cancer is unclear. The present study investigated the effect of macrophage-stimulating protein (MSP, the RON ligand) on uPAR expression and the underlying signal pathways in human gastric cancer AGS cells. uPAR messenger RNA expression was induced by MSP in a time- and concentration-dependent manner. MSP also induced uPAR promoter activity. The introduction of RON-specific small interfering RNA (siRNA) significantly affected the MSP-induced uPAR transcription. Deleted and site-directed mutagenesis studies demonstrated the involvement of the binding sites of transcription factor nuclear factor-kappaB (NF-κB) and activator protein (AP)-1 in the MSP-induced uPAR expression. Studies with expression vectors encoding mutated-type NF-κB signaling molecules and AP-1 decoy confirmed that NF-κB and AP-1 were essential for the MSP-induced uPAR expression. In addition, MSP induced the activation of extracellular signal-regulated kinase-1/2 (Erk-1/2), c-Jun amino terminal kinase (JNK) and p38 mitogen-activated protein kinase (MAPK). Dominant-negative mutants (K97M and TAM67) and specific inhibitors of Erk-1/2 and JNK were able to suppress the MSP-induced uPAR expression. AGS cells pretreated with MSP showed a remarkably enhan  ced inva  siveness, which was partially abrogated by siRNA-targeted RON and uPAR-neutralizing antibodies. The above results suggest that MSP induces uPAR expression via MAPK, AP-1 and NF-κB signaling pathways and, in turn, stimulates   cell inv  asiveness in human gastric cancer AGS cells. 
 Molecular basis of anti-inflammatory action of platelet-rich plasma on human chondrocytes: mechanisms of NF-κB inhibition via HGF. Loss of articular cartilage through injury or disease presents major clinical challenges also because cartilage has very poor regenerative capacity, giving rise to the development of biological approaches. As autologous blood product, platelet-rich plasma (PRP) provides a promising alternative to surgery by promoting safe and natural healing. Here we tested the possibility that PRP might be effective as an anti-inflammatory agent, providing an attractive basis for regeneration of articular cartilage, and two principal observations were done. First, activated PRP in chondrocytes reduced the transactivating activity of NF-κB, critical regulator of the inflammatory process, and decreased the expression of COX-2 and CXCR4 target genes. By analyzing a panel of cytokines with different biological significance, in activated PRP we observed increases in hepatocyte growth factor (HGF), interleukin-4 and tumor necrosis factor-α (TNF-α). HGF and TNF-α, by disrupting NF-κB-transactivating activity, were important for the anti-inflammatory function of activated PRP. The key molecular mechanisms involved in PRP-inhibitory effects on NF-κB activity were for HGF the enhanced cellular IkBα expression, that contributed to NF-κB-p65 subunit retention in the cytosol and nucleo-cytoplasmic shuttling, and for TNF-α the p50/50 DNA-binding causing inhibition of target-gene expression. Second, activated PRP in U937-monocytic cells reduced chemotaxis by inhibiting chemokine transactivation and CXCR4-receptor expression, thus possibly controlling local inflammation in cartilage. In conclusion, activated PRP is a promising biological therapeutic agent, as a scaffold   in micr  o-invasive articular cartilage regeneration, not only for its content of proliferative/differentiative growth factors, but also for the presence of anti-inflammatory agents including HGF. 
 Stabilization of snail by NF-kappaB is required for inflammation-induced cell migration and invasion. The increased motility and   invasive  ness of tumor cells are reminiscent of epithelial-mesenchymal transition (EMT), which occurs during embryonic development, wound healing, and metastasis. In this study, we found that Snail is stabilized by the inflammatory cytokine TNFalpha through the activation of the NF-kappaB pathway. We demonstrated that NF-kappaB is required for the induction of COP9 signalosome 2 (CSN2), which, in turn, blocks the ubiquitination and degradation of Snail. Furthermore, we showed that the expression of Snail correlated with the activation of NF-kappaB in cancer cell lines and metastatic tumor samples. Knockdown of Snail expression inhibited cell migration and invasion induced by inflammatory cytokines and suppressed inflammation-mediated breast cancer metastasis. Our study provides a plausible mechanism for inflammation-induced metastasis. 
 EGF stimulates uPAR expression and cell   invasive  ness through ERK, AP-1, and NF-kappaB signaling in human gastric carcinoma cells. Overexpression of epidermal growth factor (EGF) and urokinase plasminogen activator receptor (uPAR) have been observed in human gastric cancers. However, the interaction between EGF and uPAR in gastric cancer has not been well elucidated. In this study, we investigated the effect of EGF on uPAR expression and the underlying signal pathways in human gastric cancer AGS cells. EGF induced uPAR mRNA expression in a time- and concentration-dependent manner. EGF also induced uPAR promoter activity. In addition, EGF induced the activation of extracellular signal regulated kinase-1/2 (ERK-1/2) and P38 mitogen-activated protein kinase (MAPK) but not the activation of c-Jun amino terminal kinase. A specific inhibitor of MEK-1 (an upstream effector of ERK-1/2) and a dominant negative MEK-1 were able to suppress the EGF-induced uPAR promoter activity. Site-directed mutagenesis and electrophoretic mobility shift assays demonstrated that the binding sites of transcription factors, activator protein-1 (AP-1) and nuclear factor (NF)-kappaB, are involved in the EGF-induced uPAR transcription. Suppression of the EGF-induced uPAR promoter activity by the AP-1 decoy oligonuclotide, as well as expression vectors encoding mutated-type NF-kappaB-inducting kinase and I-kappaB, confirmed that the activation of AP-1 and NF-kappaB are essential for the EGF-induced uPAR upregulation. The AGS cells pretreated with EGF showed a remarkably enhanced   invasive  ness and this effect was partially abrogated by uPAR neutralizing antibodies and by the inhibitors of ERK-1/2, AP-1, and NF-kappaB. The above results suggest that EGF induces uPAR expression via ERK-1/2, AP-1, and NF-kappaB signaling pathways and, in turn, stimulates cell   invasive  ness in human gastric cancer AGS cells. 
 Dual knockdown of p65 and p50 subunits of NF-kappaB by siRNA inhibits the induction of inflammatory cytokines and significantly enhance apoptosis in human primary synoviocytes treated with tumor necrosis factor-alpha. In order to develop an anti-NF-kappaB siRNA as a novel class of anti-inflammatory drug, we have isolated a highly efficient siRNA targeting the p65 (RelA) subunit of NF-kappaB, hereafter named REL1096. To determine whether down-regulation of p65 by REL1096 can block the induction of inflammatory cytokines after treatment with tumor necrosis factor-alpha (TNF-alpha), human primary fibroblast-like synoviocytes were isolated from patients with rheumatoid arthritis. When treated with REL1096, the TNF-mediated induction of downstream target genes such as inflammatory cytokines, chemokines, and anti-apoptosis genes was drastically inhibited. To enhance the inhibitory effect of REL1096, cells were treated with siRNA targeting the p50 subunit of NF-kappaB together with REL1096. In addition to effective downregulation of inflammatory cytokines, knockdown of both p65 and p50 resulted in much more extensive apoptosis when compared to cells treated with either REL1096 or p50-siRNA alone. Thus, our results provide evidence for the potential use of siRNA targeting NF-kappaB as an effective means to treat rheumatoid arthritis. In addition to effective amelioration of synovial inflammation by downregulation of inflammatory cytokines, increased apoptosis by dual knockdown of p65 and p50 may prove advantageous in preventing   invasive  ness and destructiveness of hyperplastic synoviocytes. 
 Genetic susceptibility to distinct bladder cancer subphenotypes. Clinical, pathologic, and molecular evidence indicate that bladder cancer is heterogeneous with pathologic/molecular features that define distinct subphenotypes with different prognoses. It is conceivable that specific patterns of genetic susceptibility are associated with particular subphenotypes.To examine evidence for the contribution of germline genetic variation to bladder cancer heterogeneity.The Spanish Bladder Cancer/EPICURO Study is a case-control study based in 18 hospitals located in five areas in Spain. Cases were patients with a newly diagnosed, histologically confirmed, urothelial cell carcinoma of the bladder from 1998 to 2001. Case diagnoses were reviewed and uniformly classified by pathologists following the World Health Organisation/International Society of Urological Pathology 1999 criteria. Controls were hospital-matched patients (n=1149).A total of 1526 candidate variants in 423 candidate genes were analysed. Three distinct subphenotypes were defined according to stage and grade: low-grade nonmuscle   invasive   (n=586), high-grade nonmuscle   invasive   (n=219), and muscle   invasive   (n=246). The association between each variant and subphenotype was assessed by polytomous risk models adjusting for potential confounders. Heterogeneity in genetic susceptibility among subphenotypes was also tested.Two established bladder cancer susceptibility genotypes, NAT2 slow-acetylation and GSTM1-null, exhibited similar associations among the subphenotypes, as did VEGF-rs25648, which was previously identified in our study. Other variants conferred risks for specific tumour subphenotypes such as PMS2-rs6463524 and CD4-rs3213427 (respective heterogeneity p values of 0.006 and 0.004), which were associated with muscle-  invasive   tumours (per-allele odds ratios [95% confidence interval] of 0.56 [0.41-0.77] and 0.71 [0.57-0.88], respectively) but not with non-muscle-  invasive   tumours. Heterogeneity p values were not robust in multiple testing according to their false-discovery rate.These exploratory analyses suggest that genetic susceptibility loci might be related to the molecular/pathologic diversity of bladder cancer. Validation through large-scale replication studies and the study of additional genes and single nucleotide polymorphisms are required. 
 Independent and cooperative roles of tumor necrosis factor-alpha, nuclear factor-kappaB, and bone morphogenetic protein-2 in regulation of metastasis and osteomimicry of prostate cancer cells and differentiation and mineralization of MC3T3-E1 osteoblast-like cells. The molecular mechanisms involved in prostate cancer (PC) metastasis and bone remodeling are poorly understood. We recently reported that phosphoinositide 3-kinase (PI3K)/protein kinase B (Akt) mediates transcriptional regulation and activation of bone morphogenetic protein (BMP)-2 signaling by nuclear factor (NF)-kappaB in bone metastatic prostate cancer cells. In the present study, we demonstrate that NF-kappaB, whether activated by recombinant human tumor necrosis factor (TNF)-alpha or by ectopic expression of the p65 subunit, is involved in extracellular matrix adhesion and invasion of osteotropic PC-3 and C4-2B, but not LNCaP, cells. The enhanced metastatic potential was associated with transcriptional upregulation of osteopontin, osteocalcin, and collagen IA1 in osteotropic PC cells, suggesting their role in osteomimicry of PC cells. Unlike BMP-4, BMP-2 protein enhanced the   invasive   properties of C4-2B cells, but not in LNCaP cells. Also, this effect was nullified by Noggin. In addition, BMP-2 mediates TNF-alpha-induced invasion of C4-2B cells in a NF-kappaB-dependent fashion. TNF-alpha or conditioned media (CM) of TNF-alpha-stimulated C4-2B cells upregulated BMP-2 and BMP-dependent Smad transcripts and inhibited receptor activator of NF-kappaB ligand transcripts in RAW 264.7 preosteoclast cells, respectively, implying that this factor may contribute to suppression of osteoclastogenesis via direct and paracrine mechanisms. In contrast, CM of TNF-alpha-stimulate or BMP2-stimulated C4-2B cells induced in vitro mineralization of MC3T3-E1 osteoblast cells in a BMP-2-dependent and NF-kappaB-dependent manner, respectively. Taken together, the results suggest that mutual interactions between these factors may be pivotal not only in enhancing the osteomimicry and metastatic potential of PC cells, but also in bone remodeling and in shifting the balance from osteoclastogenesis towards osteoblastogenesis. 
 53BP1 functions as a tumor suppressor in breast cancer via the inhibition of NF-κB through miR-146a. p53-binding protein-1 (53BP1) plays a critical role in cell cycle checkpoint and DNA repair activities. Recently, 53BP1 was recognized as a potential tumor suppressor gene. In this study, we investigated its tumor suppressor function in breast cancer. In clinical samples, we observed a lower level of 53BP1 expression in the cancer lesions than in the matched non-tumor tissues. Furthermore, the 53BP1 level showed a gradual decrease during the progression from precancerous to cancer lesion. Ectopic expression of 53BP1 can significantly inhibit cell proliferation and curb the   invasiv  eness in breast cancer cell lines, whereas knockdown of 53BP1 by RNA interference had the opposite effects. Additionally, 53BP1 markedly inhibited xenograft formation and metastasis of breast cancer cells in nude mice. Both in vitro and in vivo studies revealed that the 53BP1 expression level was inversely correlated to the function of nuclear factor-kappaB (NF-κB), which contributes to the invasion and metastasis of breast cancer. Importantly, the inhibitory effect of 53BP1 on NF-κB activity was shown to be mediated by the upregulation of miR-146a. Together, our findings demonstrated that 53BP1 has a potent tumor suppressor activity in breast cancer, and it may serve as a novel target for breast cancer prevention and treatment. 
 EMT-associated up-regulation of L1CAM provides insights into L1CAM-mediated integrin signalling and NF-κB activation. Expression of L1 cell adhesion molecule (L1CAM) is associated with poor prognosis in a variety of human carcinomas including breast, ovarian and pancreatic ductal adenocarcinoma (PDAC). Recently we reported that L1CAM induces sustained nuclear factor kappa B (NF-κB) activation by augmenting the autocrine production of interleukin 1 beta (IL-1β), a process dependent on interaction of L1CAM with integrins. In the present study, we demonstrate that transforming growth factor β1 (TGF-β1) treatment of breast carcinoma (MDA-MB231) and PDAC (BxPc3) cell lines induces an EMT (epithelial to mesenchymal transition)-like phenotype and leads to the expression of L1CAM. In MDA-MB231 cells, up-regulation of L1CAM augmented expression of IL-1β and NF-κB activation, which was reversed by depletion of L1CAM, L1CAM-binding membrane cytoskeleton linker protein ezrin, β1-integrin or focal adhesion kinase (FAK). Over-expression of L1CAM not only induced NF-κB activation but also mediated the phosphorylation of FAK and Src. Phosphorylation was not induced in cells expressing a mutant form of L1CAM (L1-RGE) devoid of the integrin-binding site. FAK- and Src-phosphorylation were inhibited by knock-down of various components of the integrin signalling pathway such as β1- and α5-integrins, integrin-linked kinase (ILK), FAK and the phosphoinositide 3-kinase (PI3K) subunit p110β. In summary, these results reveal that during EMT, L1CAM promotes IL-1β expression through a process dependent on integrin signalling and supports   a motile   and invasive tumour cell phenotype. We also identify important novel downstream effector molecules of the L1CAM-integrin signalling crosstalk that help to understand the molecular mechanisms underlying L1CAM-promoted tumour progression. 
 CXCR2-driven ovarian cancer progression involves upregulation of proinflammatory chemokines by potentiating NF-κB activation via EGFR-transactivated Akt signaling. Ovarian cancer is an inflammation-associated malignancy with a high mortality rate. CXCR2 expressing ovarian cancers are aggressive with poorer outcomes. We therefore investigated molecular mechanisms involved in CXCR2-driven cancer progression by comparing CXCR2 positive and negative ovarian cancer cell lines. Stably CXCR2 transfected SKOV-3 cells had a faster growth rate as compared to control cells transfected with empty vector. Particularly, tumor necrosis factor (TNF), abundantly expressed in ovarian cancer, enhanced cell proliferation by decreasing the G0-G1 phase in CXCR2 transfected cells. TNF increased nuclear factor-κB (NF-κB) activity to a greater degree in CXCR2 transfected cells than control cells as well as provided a greater activation of IκB. CXCR2 transfected cells expressed higher levels of its proinflammatory ligands, CXCL1/2 and enhanced more proliferation, migration, invasion and colony formation. CXCR2 positive cells also activated more EGFR, which led to higher Akt activation. Enhanced NF-κB activity in CXCR2 positive cells was reduced by a PI3K/Akt inhibitor rather than an Erk inhibitor. CXCL1 added to CXCR2 positive cells led to an increased activation of IκB. CXCL1 also led to a significantly greater numb  er of in  vasive cells in CXCR2 transfected cells, which was blocked by the NF-κB inhibitor, Bay 11-7082. In addition, enhanced cell proliferation in CXCR2 positive cells was more sensitive to CXCL1 antibody or an NF-κB inhibitor. Finally, CXCR2 transfection of parental cells increased CXCL1 promoter activity via an NF-κB site. Thus augmentation of proinflammatory chemokines CXCL1/2, by potentiating NF-κB activation through EGFR-transactivated Akt, contributes to CXCR2-driven ovarian cancer progression. 
 RRM2 induces NF-kappaB-dependent MMP-9 activation and enhances cellular   invasive  ness. Ribonucleotide reductase is a dimeric enzyme that catalyzes conversion of ribonucleotide 5'-diphosphates to their 2'-deoxynucleotide forms, a rate-limiting step in the production of 2'-deoxyribonucleoside 5'-triphosphates required for DNA synthesis. The ribonucleotide reductase M2 subunit (RRM2) is a determinant of malignant cellular behavior in a range of human cancers. We examined the effect of RRM2 overexpression on pancreatic adenocarcinoma cellular   invasive  ness and nuclear factor-kappaB (NF-kappaB) transcription factor activity. RRM2 overexpression increases pancreatic adenocarcinoma cellular   invasive  ness and MMP-9 expression in a NF-kappaB-dependent manner. RNA interference (RNAi)-mediated silencing of RRM2 expression attenuates cellular   invasive  ness and NF-kappaB activity. NF-kappaB is a key mediator of the   invasive   phenotypic changes induced by RRM2 overexpression. 
 NF-kappaB and epithelial to mesenchymal transition of cancer. During progression of an in situ to an   invasive   cancer, epithelial cells lose expression of proteins that promote cell-cell contact, and acquire mesenchymal markers, which promote cell migration and invasion. These events bear extensive similarities to the process of epithelial to mesenchymal transition (EMT), which has been recognized for several decades as critical feature of embryogenesis. The NF-kappaB family of transcription factors plays pivotal roles in both promoting and maintaining an   invasive   phenotype. After briefly describing the NF-kappaB family and its role in cancer, in this review we will first describe studies elucidating the functions of NF-kappaB in transcription of master regulator genes that repress an epithelial phenotype. In the second half, we discuss the roles of NF-kappaB in control of mesenchymal genes critical for promoting and maintaining an   invasive   phenotype. Overall, NF-kappaB is identified as a key target in prevention and in the treatment of   invasive   carcinomas. 
 Overexpression of RRM2 in gastric cancer cell promotes their   invasive  ness via AKT/NF-κB signaling pathway. The ribonucleotide reductase M2 subunit (RRM2) plays an active role in tumor progression and is frequently overexpressed in cancer. It plays a significant role in the regulation of cell   invasiv  eness, cell migration and tumor metastasis. Elevated RRM2 expression has been reported to be associated with poor prognosis of gastric cancer. However, the molecular mechanisms of RRM2 in gastric cancer cells remain elusive. In our study, we found that RRM2 highly expressed in gastric cancer cells BGC823. RRM2 stimulation dose-dependently enhanced the invasion and migration of BGC823 cells. Furthermore, we found that the expressions of MMP-2 and MMP-9 in BGC823 cells were significantly increased after RRM2 stimulation. In addition, RRM2 time-dependently induced activation of AKT, IKBα, and NF-κB. These effects of RRM2 were prevented by AKT selective inhibitor GSK690693 as well as NF-κB selective inhibitor BAY117082. In conclusion, our findings establish a signaling role for RRM2 in gastric cancer cells and identify that the RRM2/AKT/NF-κB signaling pathway is essential for t  umor inv  asiveness in gastric cancer cells. Thus, our data may provide knowledge for using RRM2 as a novel target for effective diagnosis and treatment of gastric cancer. 
 Hypoxia increases CX3CR1 expression via HIF-1 and NF‑κB in androgen-independent prostate cancer cells. The unique CX3C chemokine CX3CL1 and its cognate receptor CX3CR1 have been implicated in organ-specific metastasis of various types of tumors. Hypoxia, a common phenomenon in solid tumors, is associated with a malignant cancer phenotype. Previous studies have proved that hypoxia facilitates cancer cell metastasis through upregulation of specific chemokine receptors. We hypothesized that hypoxia could upregulate CX3CR1 expression and lead to an increased chemotactic response to CX3CL1 in prostate cancer cells. In the present study, we found that CX3CR1 expression was significantly increased in androgen-independent prostate cancer cells, including DU145, PC-3 and PC-3M, following exposure to hypoxia. This upregulation of CX3CR1 corresponded to a significant increase in migration and invasion of prostate cancer cells under hypoxic conditions, which was attenuated after knocking down CX3CR1 expression. In addition, we examined the possible role of HIF-1 and NF-κB in the process of hypoxia-induced CX3CR1 expression and hypoxia-mediated metastasis. Attenuation of HIF-1 and NF-κB transcriptional activity by siRNAs or pharmacological inhibitors, abrogated hypoxia-induced upregulation of CX3CR1, and also prevented the migration and invasion of DU145 cells under a hypoxic environment. In summary, our study demonstrated that HIF-1 and NF-κB are essential for hypoxia-regulated CX3CR1 expression, which is associated with increased migrator  y and in  vasive potential of prostate cancer cells. CX3CR1 signaling is a potential therapeutic target in the adjuvant treatment of prostate cancer. 
 ErbB2 and NFκB overexpression as predictors of chemoradiation resistance and putative targets to overcome resistance in muscle  -invasiv  e bladder cancer. Radical cystectomy for muscle  -invasiv  e bladder cancer (MIBC) patients frequently impairs their quality of life (QOL) due to urinary diversion. To improve their QOL, a bladder-sparing alternative strategy using chemoradiation has been developed. In bladder-sparing protocols, complete response (CR) to induction chemoradiation is a prerequisite for bladder preservation and favorable survival. Thus predicting chemoradiation resistance and overcoming it would increase individual MIBC patients' chances of bladder preservation. The aim of this study is to investigate putative molecular targets for treatment aimed at improving chemoradiation response. Expression levels of erbB2, NFκB, p53, and survivin were evaluated immunohistochemically in pretreatment biopsy samples from 35 MIBC patients in whom chemoradiation sensitivity had been pathologically evaluated in cystectomy specimens, and associations of these expression levels with chemoradiation sensitivity and cancer-specific survival (CSS) were investigated. Of the 35 patients, 11 (31%) achieved pathological CR, while tumors in the remaining 24 patients (69%) were chemoradiation-resistant. Multivariate analysis identified erbB2 and NFκB overexpression and hydronephrosis as significant and independent risk factors for chemoradiation resistance with respective relative risks of 11.8 (P = 0.014), 15.4 (P = 0.024) and 14.3 (P = 0.038). The chemoradiation resistance rate was 88.5% for tumors overexpressing erbB2 and/or NFκB, but only 11.1% for those negative for both (P <0.0001). The 5-year CSS rate was 74% overall. Through multivariate analysis, overexpression of erbB2 and/or NFκB was identified as an independent risk factor for bladder cancer death with marginal significance (hazard ratio 21.5, P = 0.056) along with chemoradiation resistance (P = 0.003) and hydronephrosis (P = 0.018). The 5-year CSS rate for the 11 patients achieving pathological CR was 100%, while that for the 24 with chemoradiation-resistant disease was 61% (P = 0.018). Thus, erbB2 and NFκB overexpression are relevant to chemoradiation resistance and are putative targets aimed at overcoming chemoradiation resistance in MIBC. 
 DDB2: a novel regulator of NF-κB and breast tumor invasion. The DNA repair protein damaged DNA-binding 2 (DDB2) has been implicated in promoting cell-cycle progression by regulating gene expression. DDB2 is selectively overexpressed in breast tumor cells that are no  ninvasiv  e, but not in those that are   invasiv  e. We found that its overexpression in   invasiv  e human breast tumor cells limited their motility and   invasiv  eness in vitro and blocked their ability to colonize lungs in vivo, defining a new function for DDB2 in malignant progression. DDB2 overexpression attenuated the activity of NF-κB and the expression of its target matrix metalloprotease 9 (MMP9). Mechanistic investigations indicated that DDB2 decreased NF-κB activity by upregulating expression of IκBα by binding the proximal promoter of this gene. This effect was causally linke  d to inv  asive capacity. Indeed, knockdown of DDB2-induced IκBα gene expression restored NF-κB activity and MMP9 expression, along w  ith the   invasive properties of breast tumor cells overexpressing DDB2. Taken together, our findings enlighten understanding of how breast cancer cells progres  s to an   invasive phenotype and underscore potential clinical interest in DDB2 as a prognostic marker or therapeutic target in this setting. 
 NF-κB regulates mesenchymal transition for the induction of non-small cell lung cancer initiating cells. The epithelial-to-mesenchymal transition (EMT) is a de-differentiation process that has been implicated in metastasis and the generation of cancer initiating cells (CICs) in solid tumors. To examine EMT in non-small cell lung cancer (NSCLC), we utilized a three dimensional (3D) cell culture system in which cells were co-stimulated with tumor necrosis factor alpha (TNF) and transforming growth factor beta (TGFβ). NSCLC spheroid cultures display elevated expression of EMT master-switch transcription factors, TWIST1, SNAI1/Snail1, SNAI2/Slug and ZEB2/Sip1, and are highl  y invasi  ve. Mesenchymal NSCLC cultures show CIC characteristics, displaying elevated expression of transcription factors KLF4, SOX2, POU5F1/Oct4, MYCN, and KIT. As a result, these putative CIC display a cancer "stem-like" phenotype by forming lung metastases under limiting cell dilution. The pleiotropic transcription factor, NF-κB, has been implicated in EMT and metastasis. Thus, we set out to develop a NSCLC model to further characterize the role of NF-κB activation in the development of CICs. Here, we demonstrate that induction of EMT in 3D cultures results in constitutive NF-κB activity. Furthermore, inhibition of NF-κB resulted in the loss of TWIST1, SNAI2, and ZEB2 induction, and a failure of cells to invade and metastasize. Our work indicates that NF-κB is required for NSCLC metastasis, in part, by transcriptionally upregulating master-switch transcription factors required for EMT. 
 The relationships between snail1 and estrogen receptor signaling in breast cancer cells. The loss of hormonal dependency of breast tumor cells is often accompanied with the appearance of epithelial-mesenchymal transition (EMT) features and increase in cell metastasis and   invasive  ness. The central role in the EMT belongs to transcription factors Snail responded for the decrease in E-cadherin expression and cell contacts, stimulation of cell mobility and   invasive  ness. Aim was to study the relationships between estrogen receptor machinery and Snail1 signaling, and mechanism of Snail1 regulation in hormone-resistant breast cancer cells. The experiments were performed on the estrogen-dependent MCF-7 breast cancer cells, estrogen-hyposensitive MCF-7/LS subline generated through long-term cultivation of the parental cells in steroid-free medium, and ER-negative estrogen-resistant HBL-100 cells. Snail1, estrogen receptor, p65 NF-κB, E-cadherin levels were analyzed by Western blot. We found that decrease in the estrogen dependency is correlated with increase in Snail1 expression and activity, we demonstrated the Snail1 involvement in the negative regulation of ER, and showed that Snail1 inhibition partially restores the sensitivity of the estrogen-hyposensitive cells to antiestrogen tamoxifen. Furthermore, NF-κB was found to serve as a positive regulator of Snail1 in breast cancer cells, and simultaneous inhibition of NF-κB and Snail1 resulted in additional increase in cell response to tamoxifen. In general, the results obtained demonstrate the phenomenon of Snail1 activation in the hormone-resistant breast cancer cells, and show that Snail1 and NF-κB may serve as an important targets in the treatment of breast cancer, both estrogen-dependent and estrogen-independent tumors. 
 Curcumin Inhibits Non-Small Cell Lung Cancer Cells Metastasis through the Adiponectin/NF-κb/MMPs Signaling Pathway. Adipose tissue is now considered as an endocrine organ involved in metabolic and inflammatory reactions. Adiponectin, a 244-amino acid peptide hormone, is associated with insulin resistance and carcinogenesis. Curcumin (diferuloylmethane) is the principal curcuminoid of the popular Indian spice, turmeric. Curcumin possesses antitumor effects, including the inhibition of neovascularization and regulation of cell cycle and apoptosis. However, the effects of adiponectin and curcumin on non-small cell lung cancer (NSCLC) remain unclear. In this study, we evaluated the expression of adiponectin in paired tumors and normal lung tissues from 77 patients with NSCLC using real-time polymerase chain reaction, western blotting, and immunohistochemistry. Kaplan-Meier survival analysis showed that patients with low adiponectin expression ratio (<1) had significantly longer survival time than those with high expression ratio (>1) (p = 0.015). Curcumin inhibited the migratory and   invasiv  e ability of A549 cells via the inhibition of adiponectin expression by blocking the adiponectin receptor 1. Curcumin treatment also inhibited the in vivo tumor growth of A549 cells and adiponectin expression. These results suggest that adiponectin can be a prognostic indicator of NSCLC. The effect of curcumin in decreasing the migratory and   invasiv  e ability of A549 cells by inhibiting adiponectin expression is probably mediated through NF-κB/MMP pathways. Curcumin could be an important potential adjuvant therapeutic agent for lung cancer in the future. 
 Resveratrol reduces TNF-α-induced U373MG human glioma cell invasion through regulating NF-κB activation and uPA/uPAR expression. Hig  h invasi  veness of glioma cells is one of the reasons that patients with malignant glioma have a poor prognosis. Resveratrol, a plant compound abundant in the peel of grapes, has been suggested as a potential cancer chemopreventive agent. Therefore, we investigated the effect of resveratrol on glioma cell invasion.The effect of resveratrol on U373MG human glioma cell invasion was assessed by Matrigel assay and methylthiazoltetrazolium assay. Western blotting and reverse transcription-polymerase chain reaction were performed to elucidate the action mechanism of resveratrol.Resveratrol reduced tumor necrosis factor (TNF)-α-induced U373MG human glioma cell invasion. In addition, resveratrol repressed nuclear factor kappa B (NF-κB) activation and down-regulated mRNA expression of urokinase plasminogen activator (uPA) and its receptor in TNF-α-treated glioma cells.These findings suggest that resveratrol could prevent glioma cell invasion via inhibiting proteolysis of extracellular matrix. 
 Folate deprivation enhances   invasive  ness of human colon cancer cells mediated by activation of sonic hedgehog signaling through promoter hypomethylation and cross action with transcription nuclear factor-kappa B pathway. Low folate status is well recognized as one of the metabolic stressors for colorectal cancer carcinogenesis, but its role in colon cancer invasion remains unknown. Activation of the Sonic hedgehog (Shh) signal in interaction with the transcription nuclear factor-kappa B (NF-κB) pathway is crucial for cancer aggressiveness. The aims of this study were to investigate whether and how folate deprivation promotes invasion by colon cancer cells in relation to Shh signaling and NF-κB pathway activation. Cultivation of epithelial colon carcinoma-derived cells (HCT116) in folate-deficient (FD) medium enhanced cellular migration and invasion, in correlation with epithelial-mesenchymal transition (EMT) associated with Snail expression and E-cadherin suppression, increased production of β1 integrin and increased proteolysis by matrix metalloproteinase 2. Blockade of Shh signaling by cyclopamine (CYC) or of NF-κB activation by BAY abolished FD-enhanced EMT and invasion by HCT116 cells. FD cells had 50-80% less intracellular folate, associated with aberrant hypomethylation of the Shh promoter, than control cells, and increased binding of nuclear NF-κB subunit p65 to the Shh promoter region, which coincided with increased Shh expression and protein production of Shh ligand; in addition, the FD-induced Shh signaling targeted Gli1 transcription activator as well as Ptch receptor. The FD-induced Shh induction and activated signaling were blocked by NF-κB inhibitor BAY. Blockade of Shh signaling abrogated FD-promoted NF-κB activation measured by IκBα degradation and by target gene TNFα expression. Taken together, these findings demonstrate that folate deprivation   enhance  d invasiveness of colon cancer cells mediated by activation of Shh signaling through promoter hypomethylation and cross actions with the NF-κB pathway. 
 Pathobiological implications of the expression of EGFR, pAkt, NF-κB and MIC-1 in prostate cancer stem cells and their progenies. The progression of prostate cancers (PCs) to locally   invasiv  e, androgen-independent and metastatic disease states is generally associated with treatment resistance and disease relapse. The present study was undertaken to establish the possibility of using a combination of specific oncogenic products, including epidermal growth factor receptor (EGFR), pAkt, nuclear factor-kappaB (NF-κB) and macrophage inhibitory cytokine-1 (MIC-1) as biomarkers and therapeutic targets for optimizing the management of patients with localized PC at earlier disease stages. The immunohistochemical and immunofluorescence data have revealed that the expression levels of EGFR, Ser(473)-pAkt, NF-κB p65 and MIC-1 proteins were significantly enhanced in the same subset of 76 cases of prostatic adenocarcinoma specimens during the disease progression and these biomarkers were expressed in a small subpopulation of CD133(+) PC cells and the bulk tumor mass of CD133(-) PC cells. Importantly, all of these biomarkers were also overexpressed in 80-100% of 30 PC metastasis bone tissue specimens. Moreover, the results have indicated that the EGF-EGFR signaling pathway can provide critical functions for the self-renewal of side population (SP) cells endowed with stem cell-like features from high  ly invas  ive WPE1-NB26 cells. Of therapeutic interest, the targeting of EGFR, pAkt, NF-κB or MIC-1 was also effective at suppressing the basal and EGF-promoted prostasphere formation by SP WPE1-NB26 cells, inducing disintegration of SP cell-derived prostaspheres and decreasing the viability of SP and non-SP WPE1-NB26 cell fractions. Also, the targeting of these oncogenic products induced the caspase-dependent apoptosis in chemoresistant SP WPE1-NB26 cells and enhanced their sensibility to the cytotoxic effects induced by docetaxel. These findings suggest that the combined use of EGFR, pAkt, NF-κB and/or MIC-1 may represent promising strategies for improving the accuracy of current diagnostic and prognostic methods and efficacy of treatments of PC patients in considering the disease heterogeneity, thereby preventing PC progression to metastatic and lethal disease states. 
 Predictive role of nuclear factor-kappaB activity in gastric cancer: a promising adjuvant approach with caffeic acid phenethyl ester. The biologic significance of nuclear factor-kappaB (NF-kappaB) activation in human gastric cancer is unclear. We clarify the clinical significance of NF-kappaB activation and its relationship to Helicobacter pylori infection, a well-known pathogenesis of gastric cancer. Moreover, we examine the effects and underlying mechanisms induced by caffeic acid phenethyl ester (CAPE), an inhibitor of NF-kappaB, for gastric carcinoma.NF-kappaB was located immunohistochemically in 90 human gastric cancer specimens and 50 nonmalignant gastric specimens. The correlations between NF-kappaB activation, pathologic staging, and H. pylori infection were analyzed. We also performed electrophoretic mobility gel shift assay, real-time reverse transcription polymerase chain reaction, and enzyme-linked immunosorbent assay to evaluate the responses of AGS (a gastric adenocarcinoma epithelial cell line) human gastric cancer cells subsequent to H. pylori infection or CAPE treatment.Nuclear expression of NF-kappaB was significantly more frequently observed in gastric cancer tissues than in nonmalignant gastric tissues (31% vs. 4%, P=0.0001). The activity of NF-kappaB and the expressions of MMP-9, IL-1beta, and IL-8 in AGS cells were activated by H. pylori infection. However, the augmented responses could be significantly reversed by CAPE treatment. Moreover, in vitro studies showed that CAPE inhibits tumor growth and capacity for invasion.NF-kappaB activation is related to carcinogenesis, tumor aggression, and H. pylori infection with the increased expression of MMP-9, IL-1beta, and IL-8. Moreover, NF-kappaB inhibitors or anti-inflammatory agents such as CAPE might be new adjuvant agent against   invasive   gastric carcinoma. 
 Autocrine CCL5 signaling promotes invasion and migration of CD133+ ovarian cancer stem-like cells via NF-κB-mediated MMP-9 upregulation. The concept of cancer stem cells (CSCs) proposes that solely CSCs are capable of generating tumor metastases. However, how CSCs maintain their invasion and migration abilities, the most important properties of metastatic cells, still remains elusive. Here we used CD133 to mark a specific population from human ovarian cancer cell line and ovarian cancer tissue and determined its hyperactivity in migration and invasion. Therefore, we labeled this population as cancer stem-like cells (CSLCs). In comparison to CD133- non-CSLCs, chemokine CCL5 and its receptors, CCR1, CCR3, and CCR5, were consistently upregulated in CSLCs, and most importantly, blocking of CCL5, CCR1, or CCR3 effectively inhibits the   invasiv  e capacity of CSLCs. Mechanistically, we identified that this enhanced   invasiv  eness is mediated through nuclear factor κB (NF-κB) activation and the consequently elevated MMP9 secretion. Our results suggested that the autocrine activation of CCR1 and CCR3 by CCL5 represents one of major mechanisms underlying the metastatic property of ovarian CSLCs. 
 Protease-activated Receptor-2 (PAR-2)-mediated Nf-κB Activation Suppresses Inflammation-associated Tumor Suppressor MicroRNAs in Oral Squamous Cell Carcinoma. Oral cancer is the sixth most common cause of death from cancer with an estimated 400,000 deaths worldwide and a low (50%) 5-year survival rate. The most common form of oral cancer is oral squamous cell carcinoma (OSCC). OSCC is highly inflammatory and   invasiv  e, and the degree of inflammation correlates with tumor aggressiveness. The G protein-coupled receptor protease-activated receptor-2 (PAR-2) plays a key role in inflammation. PAR-2 is activated via proteolytic cleavage by trypsin-like serine proteases, including kallikrein-5 (KLK5), or by treatment with activating peptides. PAR-2 activation induces G protein-α-mediated signaling, mobilizing intracellular calcium and Nf-κB signaling, leading to the increased expression of pro-inflammatory mRNAs. Little is known, however, about PAR-2 regulation of inflammation-related microRNAs. Here, we assess PAR-2 expression and function in OSCC cell lines and tissues. Stimulation of PAR-2 activates Nf-κB signaling, resulting in RelA nuclear translocation and enhanced expression of pro-inflammatory mRNAs. Concomitantly, suppression of the anti-inflammatory tumor suppressor microRNAs let-7d, miR-23b, and miR-200c was observed following PAR-2 stimulation. Analysis of orthotopic oral tumors generated by cells with reduced KLK5 expression showed smaller, less aggressive lesions with reduced inflammatory infiltrate relative to tumors generated by KLK5-expressing control cells. Together, these data support a model wherein KLK5-mediated PAR-2 activation regulates the expression of inflammation-associated mRNAs and microRNAs, thereby modulating progression of oral tumors. 
 Involvement of Akt/NF-κB pathway in antitumor effects of parthenolide on glioblastoma cells in vitro and in vivo. Glioblastoma is the most common and most aggressive form of malignant glioma and is very difficult to treat. Controlling tumour cell invasion and angiogenesis is essential to improve the prognosis of glioblastoma patients. Since constitutive activation of nuclear factor-κB (NF-κB) is necessary for tumour progression, NF-κB may be an important pharmacological target for this disease. Our study aimed to evaluate the antitumour effects of parthenolide, a NF-κB inhibitor, in two human glioblastoma cell lines (U87MG and U373) and in glioblastoma xenografts. Furthermore, we aimed to investigate the molecular mechanisms underlying these effects.The   anti-inv  asive and anti-angiogenic effects of parthenolide were analysed using in vitro invasion and angiogenesis assays. Parthenolide-induced growth inhibition of glioblastoma cells in vitro was determined using the MTT (methyl thiazolyl tetrazolium) assay. In addition, the effect of parthenolide on orthotropic implantation in vivo was evaluated using an intracerebral human glioblastoma xenograft model.We found that parthenolide suppresses proliferation, invasion, and tumour- induced angiogenesis of glioblastoma cells. Molecular studies demonstrated that parthenolide suppresses gene and protein expression of angiogenic factors. Furthermore, parthenolide reduced Akt phosphorylation and activated mitochondrial signalling, suggesting that the antitumour function of parthenolide may be mediated not only by the inhibition of NF-κB but also by the inhibition of Akt signalling and the activation of apoptotic proteins. Parthenolide suppressed neovascularity and tumour growth in glioblastoma xenografts.The present study identified parthenolide as a new therapeutic agent for glioblastomas. 
 The fibroblast growth factor-inducible 14 receptor is highly expressed in HER2-positive breast tumors and regulates breast cancer cell   invasive   capacity. Genomic characterization is beginning to define a molecular taxonomy for breast cancer; however, the molecular basis of invasion and metastasis remains poorly understood. We report a pivotal role for the fibroblast growth factor-inducible 14 (Fn14) receptor in this process. We examined whether Fn14 and its ligand tumor necrosis factor-like weak inducer of apoptosis (TWEAK) were expressed in breast tumors and whether deregulation of Fn14 levels affected malignant behavior of breast cancer cell lines. Analysis of TWEAK and Fn14 in publicly available gene expression data indicated that high Fn14 expression levels significantly correlated with several poor prognostic indicators (P < 0.05). Fn14 expression was highest in the HER2-positive/estrogen receptor-negative (HER2(+)/ER(-)) intrinsic subtype (P = 0.0008). An association between Fn14 and HER2 expression in breast tumors was confirmed by immunohistochemistry. Fn14 levels were elevated in   invasive  , ER(-) breast cancer cell lines. Overexpression of Fn14 in weakly   invasive   MCF7 and T47D cells resulted in a marked induction of invasion and activation of nuclear factor-kappaB (NF-kappaB) signaling. Ectopic expression of Fn14tCT, a Fn14 deletion mutant that cannot activate NF-kappaB signaling, was not able to induce invasion. Moreover, ectopic expression of Fn14tCT in highly   invasive   MDA-MB-231 cells reduced their   invasive   capability. RNA interference-mediated inhibition of Fn14 expression in both MDA-MB-231 and MDA-MB-436 cells reduced invasion. Expression profiling of the Fn14-depleted cells revealed deregulation of NF-kappaB activity. Our findings support a role for Fn14-mediated NF-kappaB pathway activation in breast tumor invasion and metastasis. 
 Acquisition of stable inducible up-regulation of nuclear factor-kappaB by tumor necrosis factor exposure confers increased radiation resistance without increased transformation in breast cancer cells. High-grade breast cancers are better adapted to hypoxia and more resistant to chemotherapy and radiotherapy. Constitutive activation of the transcription factor nuclear factor-kappaB (NF-kappaB) increases in breast tumors and in breast cancer cell lines, where it promotes chemoradiation resistance, in part by activation of antiapoptotic genes. The role for up-regulation of NF-kappaB in breast cancer progression is less clear. Here, we first show that whereas the constitutive activity of NF-kappaB is incrementally elevated from immortalized breast epithelial to frank transformed   invasive   ductal breast cancer cell lines (~3-fold, +/-0.1-fold, P < 0.05), inflammatory cytokine-inducible activity is further increased (up to 9-fold, +/-0.9-fold, P < 0.05). We then show that inhibition of NF-kappaB activity selectively sensitizes transformed but not immortalized cells to killing by ionizing radiation or low levels of tumor necrosis factor (TNF) by up to 10-fold (+/-1-fold, P < 0.05) but has little effect on hypoxia-mediated cell death. Prolonged cultivation of immortalized and partially transformed cells in TNF selected for cells displaying stable constitutive and strongly inducible overexpression of NF-kappaB even in the absence of TNF. Stable acquisition of increased NF-kappaB activity conferred resistance to ionizing radiation or inflammatory cytokines, which was dependent on elevated NF-kappaB activity, but had no effect on transformation potential measured by in vitro and in vivo parameters. Thus, TNF and possibly other inflammatory cytokines in the tumor-stroma matrix likely select for breast cancer cells that stably overexpress NF-kappaB, leading to greater cancer cell survival. Greater cell survival despite increased genomic injury may permit increased acquisition of malignant genetic alterations as well as resistance to chemoradiation therapy. 
 Ganoderic acids suppress growth and   invasive   behavior of breast cancer cells by modulating AP-1 and NF-kappaB signaling. Structurally related lanostane-type triterpenes, ganoderic acid A, F and H (GA-A, GA-F, GA-H), were identified in an oriental medicinal mushroom Ganoderma lucidum. In the present study we evaluated the effect of GA-A, GA-H and GA-F on highly   invasive   human breast cancer cells. We showed that GA-A and GA-H suppressed growth (cell proliferation and colony formation) and   invasive   behavior (adhesion, migration and invasion) of MDA-MB-231 cells. Our results suggest that GA-A and GA-H mediate their biological effects through the inhibition of transcription factors AP-1 and NF-kappaB, resulting in the down-regulation of expression of Cdk4 and the suppression of secretion of uPA, respectively. Furthermore, the activity of ganoderic acids is linked to the hydroxylation in the position 7 and 15 (GA-A) and 3 (GA-H) in their triterpene lanostane structure. In conclusion, hydroxylated triterpenes from G. lucidum could be promising natural agents for the therapy of   invasive   breast cancers. 
 14-3-3β Promotes Migration and Invasion of Human Hepatocellular Carcinoma Cells by Modulating Expression of MMP2 and MMP9 through PI3K/Akt/NF-κB Pathway. 14-3-3β has been demonstrated to possess the oncogenic potential, and its increased expression has been detected in multiple types of carcinomas. However, majority of previous studies focused on the role of 14-3-3β in tumor cell proliferation and apoptosis, leaving much to be elucidated about its function in tumor cell invasion and metastasis. Hence, the present study aimed to investigate the role of 14-3-3β in the invasion of hepatocellular carcinoma (HCC) cells and the implications in the prognosis of HCC patients. We first examined the expression of 14-3-3β in the primary tumors of HCC patients with or without portal vein tumor thrombus (PVTT), and found that 14-3-3β expression was higher in the primary tumors with PVTT, and the level was even higher in the PVTTs. Kaplan-Meier curves and multivariate analysis revealed that high expression of 14-3-3β was associated with overall survival (OS) and time to recurrence (TTR) of HCC patients. In addition, ectopic expression of 14-3-3β in HCC cell lines led to enhanced migration abi  lity and   invasiveness, as well as up-regulation of matrix metalloproteinase 2 and 9, which could be suppressed by inhibiting the activation of Akt and nuclear factor-κB (NF-κB) signaling. Furthermore, we identified a correlated elevation of 14-3-3β and p-Akt in the primary tumors of HCC patients, and showed that a combinatory detection of 14-3-3β and p-Akt could better predict post-surgical outcome of HCC patients. 
 Tyrosine kinase p56lck regulates cell motility and nuclear factor kappaB-mediated secretion of urokinase type plasminogen activator through tyrosine phosphorylation of IkappaBalpha following hypoxia/reoxygenation. Nuclear factor kappaB (NFkappaB) plays major role in regulating cellular responses as a result of environmental injuries. The molecular mechanism(s) by which hypoxia/reoxygenation (H/R) regulates p56lck-dependent activation of NFkappaB through tyrosine phosphorylation of IkappaBalpha and modulates the expression of downstream genes that are involved in cell migration in human breast cancer cells are not well defined. In this paper, we investigated the involvement of protein-tyrosine kinase p56lck in the redox-regulated activation of NFkappaB following H/R in highly   invasive   (MDA-MB-231) and low   invasive   (MCF-7) breast cancer cells. We demonstrated that H/R induces tyrosine phosphorylation of p56lck, nuclear translocation of NFkappaB, NFkappaB-DNA binding, and transactivation of NFkappaB through tyrosine phosphorylation of IkappaBalpha. Transfection of these cells with wild type Lck but not with mutant Lck F394 followed by H/R induces the tyrosine phosphorylation of inhibitor of nuclear factor kappaB (IkappaBalpha) and transcriptional activation of NFkappaB, and these are inhibited by Lck inhibitors. In vitro kinase assay demonstrated that immunoprecipitated p56lck but not Lyn or Fyn directly phosphorylate IkappaBalpha in presence of H/R. Pervanadate, H2O2, and H/R induce the interaction between Lck and tyrosine-phosphorylated IkappaBalpha, and this interaction is inhibited by Src homology 2 domain inhibitory peptide, suggesting that tyrosine-phosphorylated IkappaBalpha interacts with Src homology 2 domain of Lck. Luciferase reporter gene assay indicated that Lck induces NFkappaB-dependent urokinase type plasminogen activator (uPA) promoter activity in presence of H/R. Furthermore, H/R stimulates the cell motility through secretion of uPA. To our knowledge, this is the first report that p56lck in presence of H/R regulates NFkappaB activation, uPA secretion, and cell motility through tyrosine phosphorylation of IkappaBalpha and further demonstrates an important redox-regulated pathway for NFkappaB activation following H/R injury that is independent of IkappaB kinase/IkappaBalpha-mediated signaling pathways. 
 Expression of nuclear factor kappa B (NF-κB) as a predictor of poor pathologic response to chemotherapy in patients with locally advanced breast cancer. NF-κB inhibits apoptosis through induction of antiapoptotic proteins and suppression of proapoptotic genes. Various chemotherapy agents induce NF-κB translocation and target gene activation. We conducted the present study to assess the predictive value of NF-κB regarding pathologic responses after receiving neoadjuvant chemotherapy.We enrolled 131 patients with locally advan  ced inva  sive ductal breast carcinoma. Immunohistochemistry (IHC) was used to detect NF-κB expression. Evaluation of pathologic response was elaborated with the Ribero classification.Expression of NF-κB was significantly associated with poor pathological response (p=0.02). From the multivariate analysis, it was found that the positive expression of NF-κB yielded RR=1.74 (95%CI 0.77 to 3.94).NF-κB can be used as a predictor of poor pathological response after neoadjuvant chemotherapy. 
 Activated macrophages down-regulate expression of E-cadherin in hepatocellular carcinoma cells via NF-κB/Slug pathway. Hepatocellular carcinomas are an aggressive malignancy mainly due to metastasis or postsurgical recurrence. Expression of E-cadherin is strongly reduced in Hepatocellular carcinoma (HCC) tissues, and its downregulation is connected to   invasiv  eness and metastasis in hepatocellular carcinomas. The previous study showed that the supernatant from activated macrophages can downregulate the expression of E-cadherin in HCC cells. The partial known molecular mechanism is that tyrosine kinases c-Src- and EGFR phosphorylate β-catenin and E-cadherin leading to destabilization of E-cadherin/β-catenin complex. The aim of this study is to clarify other mechanism by which activated macrophages downregulate the expression of E-cadherin. We detect the expression of E-cadherin and macrophage infiltration in hepatocellular carcinoma tissues by double-staining immunohistochemistry and evaluate the relationship between macrophages and E-cadherin expression in hepatocellular carcinoma cells in vitro experiments. We found that reduced expression of E-cadherin was associated with macrophage infiltration along the border between the tumor nest and stroma in hepatocellular carcinoma tissues. Besides, protein expression of E-cadherin was significantly decreased in hepatocellular carcinoma cells co-cultured with macrophages derived from THP-1 cells. Consistently, mRNA expression of E-cadherin was also decreased in cancer cells co-cultured with THP-1-differentiated macrophages. Moreover, the downregulation of E-cadherin expression was companied by upregulation of Slug expression in cancer cells with conditional medium from THP-1-differentiated macrophage culture. The change in expression of E-cadherin and Slug was abrogated when NF-κB signaling pathway was blocked. All the findings suggested that macrophages contributed to the decreased expression of E-cadherin by NF-κB/Slug pathway in hepatocellular carcinomas. 
 [Inhibition of NF-kappaB through IkappaBalpha transfection affects invasion of human lung cancer cell line A549]. Recent studies have shown that activation of nuclear factor-kappaB(NF-kappaB) can regulate the invasion and metastasis of cancer cells. The present study was to investigate inhibition of NF-kappaB activity on invasion of human lung cancer cell line A549 and the possible mechanism.The recombinant plasmid pcDNA3.1(+)/IkappaBalpha, expressing the alpha isoform (IkappaBalpha) of the NF-kappaB inhibitor, was constructed. A549 cells were cultured in vitro and divided into non-transfection group, pcDNA3.1(+) transfected group and pcDNA3.1(+)/IkappaBalpha transfected group. The expression of IkappaBalpha was detected by reverse transcription-polymerase chain reaction (RT-PCR) and Western blotting. The activity of NF-kappaB was determined by electrophoretic mobility shift assay (EMSA). Invasion of A549 cells was assessed by transwell chamber assay. The expression of MMP-2 and MMP-9 was detected by RT-PCR and gelatin zymography.Plasmid pcDNA3.1(+)/IkappaBalpha was successfully constructed and expressed in A549 cells. The activity of NF-kappaB, the number of   invasive   cells, the activity of MMP-2 and MMP-9 of A549 cells in pcDNA3.1(+)/IkappaBalpha transfected group were significantly lower than those in non-transfection group and pcDNA3.1(+) transfected group (all P<0.05).Transfection of IkappaBalpha can inhibit NF-kappaB activity, thus inhibit cell invasion of A549, which may be through the down-regulation of MMP-2 and MMP-9 expressions. 
 Role of nuclear factor-κB in female breast cancer: a study in Indian patients. The nuclear factor κB (NF-κB) is a super family of transcription factors which plays important roles in development and progression of cancer. The present investigation concerns NF-κB /p65 activity in human breast cancers with overexpression of ER, PR, HER-2/neu, as well as the significance of p65 expression with regard to menopausal status, stage, grade, tumor size, nodal status, and NPI   of inva  sive ductal carcinomas in Eastern India.In this hospital based study 57 breast cancer patients attending a Breast Clinic of a reputed institute of Eastern India were assessed for p65 protein expression in breast tumor tissue samples by Western blotting. ER, PR and HER-2/neu expression was determined by immunohistochemistry.NF-κB/p65 was significantly associated with advanced stage, large tumor size (≥5 cm), high grade, negative ER, negative PR, and positive HER-2/neu. High NF-κB/p65 expression was more frequent in patients with a high NPI (NPI≥5.4, 84.6%) compared with low NPI (<5.4, 44.4%) and this association was statistically significant (p=0.002).NF-κB/p65 overexpression was associated with advanced stage, large tumor size, high grade, and high NPI which are poor prognostic factors linked to enhanced aggressiveness of the disease. NF-κB/p65 expression implies aggressive biological behavior of breast cancer and this study validates significant association of NF-κB /p65 overexpression with negative estrogen and progesterone receptor status and overexpression of HER-2/neu oncoprotein. In our good clinical practice, patients with NF-κB positive tumors need to be treated aggressively. 
 Secreted heat shock protein 90α (HSP90α) induces nuclear factor-κB-mediated TCF12 protein expression to down-regulate E-cadherin and to enhance colorectal cancer cell migration and invasion. Secreted levels of HSP90α and overexpression of TCF12 have been associated with the enhancement of colorectal cancer (CRC) cell migration and invasion. In this study, we observed that CRC patients with tumor TCF12 overexpression exhibited both a higher rate of metastatic occurrence and a higher average serum HSP90α level compared with patients without TCF12 overexpression. Therefore, we studied the relationship between the actions of secreted HSP90α and TCF12. Like overexpressed TCF12, secreted HSP90α or recombinant HSP90α (rHSP90α) induced fibronectin expression and repressed E-cadherin, connexin-26, connexin-43, and gap junction levels in CRC cells. Consistently, rHSP90α s  timulate  d invasive outgrowths of CRC cells from spherical structures during three-dimensional culture. rHSP90α also induced TCF12 expression in CRC cells. Its effects on CRC cell epithelial-mesenchymal transition, migration, and invasion were drastically prevented when TCF12 was knocked down. This suggests that TCF12 expression is required for secreted HSP90α to enhance CRC cell spreading. Through the cellular receptor CD91, rHSP90α facilitated the complex formation of CD91 with IκB kinases (IKKs) α and β and increased the levels of phosphorylated (active) IKKα/β and NF-κB. Use of an IKKα/β inhibitor or ectopic overexpression of dominant-negative IκBα efficiently repressed rHSP90α-induced TCF12 expression. Moreover, κB motifs were recognized in the gene sequence of the TCF12 promoter, and a physical association between NF-κB and the TCF12 promoter was detected in rHSP90α-treated CRC cells. Together, these results suggest that the CD91/IKK/NF-κB signaling cascade is involved in secreted HSP90α-induced TCF12 expression, leading to E-cadherin down-regulation and enhanced CRC cell migration/invasion. 
 Pterostilbene, a bioactive component of blueberries, suppresses the generation of breast cancer stem cells within tumor microenvironment and metastasis via modulating NF-κB/microRNA 448 circuit. Tumor-associated macrophages (TAMs) have been shown to promote metastasis and malignancy. Pterostilbene, a natural stilbene isolated from blueberries, has been suggested for anti-cancer effects. Here, we explored the potential cancer stem cells (CSCs)/TAM modulating effects of pterostilbene in breast cancer.Using flowcytometric and Boyden chamber assay, we showed MCF7 and MDA-MB-231 cells cocultured with M2 TAMs exhibited increased percentage of CD44(+) /CD24(-) CSC population and migratory  /invasiv  e abilities. RT-PCR results showed that CD44(+) /CD24(-) cells expressed an increased level of HIF-1α, β-catenin, Twist1, and NF-κB and enhanced tumor sphere forming ability. Additionally, pterostilbene treatment dose dependently overcame M2 TAM-induced enrichment of CSCs and metastatic potential of breast cancer cells. Mechanistically, pterostilbene suppressed NFκB, Twist1, vimentin, and increased E-cadherin expression. Using siRNA technique, we demonstrated that pterostilbene-mediated NFκB downregulation was correlated to an increased amount of microRNA 448. Finally, pterostilbene-mediated suppression in tumorigenesis and metastasis was validated   by nonin  vasive bioluminescence in mice bearing M2 TAM cocultured MDA-MB-231 tumor.Pterostilbene effectively suppresses the generation of CSCs and metastatic potential under the influence of M2 TAMs via modulating EMT associated signaling pathways, specifically NF-κB/miR488 circuit. Thus, pterostilbene could be an ideal anti-CSC agent in clinical settings. 
 1,25-Dihydroxyvitamin D3 alleviates salivary adenoid cystic carcinoma progression by suppressing GPX1 expression through the NF-κB pathway. 1,25-Dihydroxyvitamin D3 (1,25D3) is the active form of vitamin D with antineoplastic effects. The glutathione peroxidase-1 (GPX1) gene is associated with tumour progression. The present study aimed to explore the role of GPX1 in 1,25D3-mediated progression of salivary adenoid cystic carcinoma (SACC). Downregulating GPX1 expression inhibited SACC cell proliferation, chemoresistance, motility, and uPA secretion, but promoted apoptosis via the NF-κB pathway. Pre-processing 1,25D3 inhibited expression of NF-κB/GPX1/uPA, which subsequently suppressed cell motility and cisplatin-resistance in ACC-2 cells. In conclusion, 1,25D3 works as a modifier of NF-κB/GPX1/uPA expression, inhibiting cisplatin-resistance and c  ell inva  sive ability of SACC cells. The present study comprehensively elucidated the potential mechanism underlying the effects of vitamin D on chemoresistance   and inva  sive potential in SACC. 
 Interleukin-20 promotes migration of bladder cancer cells through extracellular signal-regulated kinase (ERK)-mediated MMP-9 protein expression leading to nuclear factor (NF-κB) activation by inducing the up-regulation of p21(WAF1) protein expression. The role of inflammatory cytokine interleukin-20 (IL-20) has not yet been studied in cancer biology. Here, we demonstrated up-regulation of both IL-20 and IL-20R1 in muscle  -invasiv  e bladder cancer patients. The expressions of IL-20 and IL-20R1 were observed in bladder cancer 5637 and T-24 cells. We found that IL-20 significantly increased the expression of matrix metalloproteinase (MMP)-9 via binding activity of NF-κB and AP-1 in bladder cancer cells and stimulated the activation of ERK1/2, JNK, p38 MAPK, and JAK-STAT signaling. Among the pathways examined, only ERK1/2 inhibitor U0126 significantly inhibited IL-20-induced migration and invasion. Moreover, siRNA knockdown of IL-20R1 suppressed migration, invasion, ERK1/2 activation, and NF-κB-mediated MMP-9 expression induced by IL-20. Unexpectedly, the cell cycle inhibitor p21(WAF1) was induced by IL-20 treatment without altering cell cycle progression. Blockade of p21(WAF1) function by siRNA reversed migration, invasion, activation of ERK signaling, MMP-9 expression, and activation of NF-κB in IL-20-treated cells. In addition, IL-20 induced the activation of IκB kinase, the degradation and phosphorylation of IκBα, and NF-κB p65 nuclear translocation, which was regulated by ERK1/2. IL-20 stimulated the recruitment of p65 to the MMP-9 promoter region. Finally, the IL-20-induced migration and invasion of cells was confirmed by IL-20 gene transfection and by addition of anti-IL-20 antibody. This is the first report that p21(WAF1) is involved in ERK1/2-mediated MMP-9 expression via increased binding activity of NF-κB, which resulted in the induction of migration in IL-20/IL-20R1 dyad-induced bladder cancer cells. These unexpected results might provide a critical new target for the treatment of bladder cancer. 
 CTGF enhances migration and MMP-13 up-regulation via alphavbeta3 integrin, FAK, ERK, and NF-kappaB-dependent pathway in human chondrosarcoma cells. Tumor malignancy is associated with several features such as proliferation ability and frequency of metastasis. Connective tissue growth factor (CTGF), a secreted protein that binds to integrins, modulates the   invasive   behavior of certain human cancer cells. However, the effect of CTGF on migration activity in human chondrosarcoma cells is mostly unknown. Here we found that CTGF increased the migration and expression of matrix metalloproteinase (MMP)-13 in human chondrosarcoma cells (JJ012 cells). RGD peptide, alphavbeta3 monoclonal antibody (mAb) and MAPK kinase (MEK) inhibitors (PD98059 and U0126) but not RAD peptide inhibited the CTGF-induced increase of the migration and MMP-13 up-regulation of chondrosarcoma cells. CTGF stimulation increased the phosphorylation of focal adhesion kinase (FAK) and extracellular signal-regulated kinase (ERK). In addition, treatment of JJ012 cells with NF-kappaB inhibitor (PDTC) or IkappaB protease inhibitor (TPCK) inhibited CTGF-induced cell migration and MMP-13 up-regulation. Stimulation of JJ012 cells with CTGF also induced IkappaB kinase alpha/beta (IKK alpha/beta) phosphorylation, IkappaBalpha phosphorylation, p65 Ser(536) phosphorylation, and kappaB-luciferase activity. The CTGF-mediated increases in kappaB-luciferase activities were inhibited by RGD, PD98059, U0126 or FAK, and ERK2 mutant. Taken together, our results indicated that CTGF enhances the migration of chondrosarcoma cells by increasing MMP-13 expression through the alphavbeta3 integrin, FAK, ERK, and NF-kappaB signal transduction pathway. 
 ALK+ALCLs induce cutaneous, HMGB-1-dependent IL-8/CXCL8 production by keratinocytes through NF-κB activation. Anaplastic large-cell lymphomas (ALCLs) bearing the t(2;5) translocation (ALK(+)ALCLs) are frequently characterized by skin colonization and associated with a poor prognosis. Using conditional transgenic models of anaplastic lymphoma kinase-positive (ALK(+)) lymphomas and human ALK(+)ALCL cell lines, in the present study, we show that high-mobility-group box-1 (HMGB-1), a proinflammatory cytokine, is released by ALK(+) cells, and demonstrate extracellular HMGB-1-stimulated secretion of the IL-8 chemokine by HaCaT keratinocytes through the involvement of MMP-9, PAR-2, and the NF-κB pathway. Furthermore, we demonstrate that, in vitro, IL-8 is able to induce th  e invasi  veness of ALK(+) cells, which express the IL-8 receptors CXCR1 and CXCR2. In vitro and in vivo, HMGB-1 inhibition achieved by glycyrrhizin treatment led to a drastic reduction in ALK(+) cel  l invasi  veness. The pathophysiological relevance of our observations was confirmed by demonstrating that the HMGB-1 and IL-8 receptors are expressed in ALK(+)ALCL biopsies. We have also shown that IL-8 secretion is correlated with leukemic dissemination of ALK(+) cells in a significant number of patients. The results of the present study demonstrate for the first time a relationship among the pro-inflammatory mediators HMGB-1, MMP-9, PAR-2, and IL-8. We propose that these mediators create a premetastatic niche within the skin, thereby participating in ALK(+) lymphoma epidermotropism. 
 Signaling mechanisms responsible for lysophosphatidic acid-induced urokinase plasminogen activator expression in ovarian cancer cells. Lysophosphatidic acid (LPA) enhances urokinase plasminogen activator (uPA) expression in ovarian cancer cells; however, the molecular mechanisms responsible for this event have not been investigated. In this study, we used the   invasive   ovarian cancer SK-OV-3 cell line to explore the signaling molecules and pathways essential for LPA-induced uPA up-regulation. With the aid of specific inhibitors and dominant negative forms of signaling molecules, we determined that the G(i)-associated pathway mediates this LPA-induced event. Moreover, constitutively active H-Ras and Raf-1-activating H-Ras mutant enhance uPA expression, whereas dominant negative H-Ras and Raf-1 block LPA-induced uPA up-regulation, suggesting that the Ras-Raf pathway works downstream of G(i) to mediate this LPA-induced process. Surprisingly, dominant negative MEK1 or Erk2 displays only marginal inhibitory effect on LPA-induced uPA up-regulation, suggesting that a signaling pathway distinct from Raf-MEK1/2-Erk is the prominent pathway responsible for this process. In this report, we demonstrate that LPA activates NF-kappaB in a Ras-Raf-dependent manner and that blocking NF-kappaB activation with either non-phosphorylable IkappaB or dominant negative IkappaB kinase abolished LPA-induced uPA up-regulation and uPA promoter activation. Furthermore, introducing mutations to knock out the NF-kappaB binding site of the uPA promoter results in over 80% reduction in LPA-induced uPA promoter activation, whereas this activity is largely intact with the promoter containing mutations in the AP1 binding sites. Thus these results suggest that the G(i)-Ras-Raf-NF-kappaB signaling cascade is responsible for LPA-induced uPA up-regulation in ovarian cancer cells. 
 TWEAK promotes ovarian cancer cell metastasis via NF-kappaB pathway activation and VEGF expression. The poor prognosis of human ovarian cancer is partly due to its metastasis and recurrence. It has been demonstrated that tumor necrosis factor (TNF)-like weak inducer of apoptosis (TWEAK)-fibroblast growth factor inducible-14 (Fn14) signaling system may be a potential regulator of human tumorigenesis. The objective of this study was to understand the effect of TWEAK on ovarian cancer metastasis. We recently showed that activation of Fn14 signaling by TWEAK promoted cell migration and invasion in human HO-8910PM cells. Treating HO-8910PM cells with TWEAK resulted in the activation of nuclear factor-kappa B (NF-kappaB) and subsequently the translocation of NF-kappaB from cytoplasm to nucleus. In addition, TWEAK promoted vascular endothelial growth factor (VEGF) protein expression, and this effect was dependent upon NF-kappaB transcriptional activity. Blocking the NF-kappaB pathway with PDTC suppressed TWEAK-induced up-regulation of VEGF protein expression and cell metastasis. Our results suggest that TWEAK-Fn14 functions, in part, through the NF-kappaB signaling pathway to up-regulate VEGF expression to foster ovarian cancer cell metastasis. Targeted therapy against TWEAK-Fn14 signaling system as an adjuvant to surgery may improve clinical management of   invasive   ovarian cancer cells and advance the outcome of this devastating cancer. 
 Hypoxia activates heparanase expression in an NF-kappaB dependent manner. Hypoxia was shown to increase tumor cell invasion into the extracellular matrix in vitro. This result suggests that heparanase (Hpa), one of the key enzymes involved in tumor invasion and metastasis, may be regulated by hypoxia. RT-PCR, Western blot and Matrigel   invasive   assays were used to study the regulation of Hpa under hypoxia in human pancreatic MIA PaCa-2 cancer cells. Compared with those in normoxia (20% O2), Hpa mRNA, protein and enzymatic activity levels, were up-regulated by a reduction in the oxygen level (1% O2). Invasion by tumor cells into the extracellular matrix was found to be significantly enhanced. A reduction in Hpa protein levels was observed when nuclear factor kappaB (NF-kappaB) activation was blocked by pyrrolidine dithiocarbamate. The levels of Hpa were also reduced when Hpa was inhibited by an Hpa-specific antisense oligonucleotide. The MMP-9 mRNA, protein and gelatinase B activity levels in supernatants decreased significantly when Hpa was inhibited. We conclude that up-regulation of Hpa by hypoxia is NF-kappaB-dependent in MIA PaCa-2 cells and inhibition of Hpa reduces MMP-9 activity. This reduction in MMP-9 activity may be an important mechanism in tumor metastasis. 
 Bmi-1 promotes the aggressiveness of glioma via activating the NF-kappaB/MMP-9 signaling pathway. The prognosis of human glioma is poor, and the highly   invasive   nature of the disease represents a major impediment to current therapeutic modalities. The oncoprotein B-cell-specific Moloney murine leukemia virus integration site 1 protein (Bmi-1) has been linked to the development and progression of glioma; however, the biological role of Bmi-1 in the invasion of glioma remains unclear.A172 and LN229 glioma cells were engineered to overexpress Bmi-1 via stable transfection or to be silenced for Bmi-1 expression using RNA interfering method. Migration and   invasive  ness of the engineered cells were assessed using wound healing assay, Transwell migration assay, Transwell matrix penetration assay and 3-D spheroid invasion assay. MMP-9 expression and activity were measured using real-time PCR, ELISA and the gelatin zymography methods. Expression of NF-kappaB target genes was quantified using real-time PCR. NF-kappaB transcriptional activity was assessed using an NF-kappaB luciferase reporter system. Expression of Bmi-1 and MMP-9 in clinical specimens was analyzed using immunohistochemical assay.Ectopic overexpression of Bmi-1 dramatically increased, whereas knockdown of endogenous Bmi-1 reduced, the   invasive  ness and migration of glioma cells. NF-kappaB transcriptional activity and MMP-9 expression and activity were significantly increased in Bmi-1-overexpressing but reduced in Bmi-1-silenced cells. The reporter luciferase activity driven by MMP-9 promoter in Bmi-1-overexpressing cells was dependent on the presence of a functional NF-kappaB binding site, and blockade of NF-kappaB signaling inhibited the upregulation of MMP-9 in Bmi-1 overexpressing cells. Furthermore, expression of Bmi-1 correlated with NF-kappaB nuclear translocation as well as MMP-9 expression in clinical glioma samples.Bmi-1 may play an important role in the development of aggressive phenotype of glioma via activating the NF-kappaB/MMP-9 pathway and therefore might represent a novel therapeutic target for glioma. 
 Relationship between NFKB1 -94 insertion/deletion ATTG polymorphism and susceptibility of cervical squamous cell carcinoma risk. A very high expression of nuclear factor-kappa B protein (nuclear p50, encoded by NFKB1) in high-grade squamous intraepithelial lesion and   invasive   cancers has been observed. The aim of this study was to determine whether the functional NFKB1 -94 insertion/deletion ATTG polymorphism (rs28362491) is associated with cervical squamous cell carcinoma (CSCC).PCR-polyacrylamide gel electrophoresis method was used to genotype the NFKB1 -94 insertion/deletion ATTG polymorphism in 233 women with CSCC and 365 ethnicity-matched healthy control women. The genotyping method was confirmed by the DNA sequencing analysis.The frequency of ATTG(2)/ATTG(2) genotype and ATTG(2) allele in the CSCC patients was significantly higher than that of controls, indicating that the -94 insertion/deletion ATTG polymorphism in NFKB1 promoter was associated with CSCC [P = 0.001, odds ratio (OR) = 2.560, 95% confidence interval (CI) 1.459-4.492 and P = 0.001, OR = 1.493, 95% CI 1.168-1.908, respectively]. Results of stratified analyses revealed that this polymorphism is associated with younger age (< or =35 years) and positive parametrial invasion but not with tumor differentiation, high clinical stage or lymph node status.Our results indicate that the functional NFKB1 -94 insertion/deletion ATTG polymorphism is associated with CSCC, especially with younger age (< or =35 years) and positive parametrial invasion of CSCC patients. 
 Reciprocal activation between ATPase inhibitory factor 1 and NF-κB drives hepatocellular carcinoma angiogenesis and metastasis. Hepatocellular carcinoma (HCC) is a highly vascularized tumor with frequent extrahepatic metastasis. Active angiogenesis and metastasis are responsible for rapid recurrence and poor survival of HCC. However, the mechanisms that contribute to tumor metastasis remain unclear. Here we evaluate the effects of ATPase inhibitory factor 1 (IF1), an inhibitor of the mitochondrial H(+)-adenosine triphosphate (ATP) synthase, on HCC angiogenesis and metastasis. We found that increased expression of IF1 in human HCC predicts poor survival and disease recurrence after surgery. Patients with HCC who have large tumors, with vascular invasion and metastasis, expressed high levels of IF1.   Invasiv  e tumors overexpressing IF1 were featured by active epithelial-mesenchymal transition (EMT) and increased angiogenesis, whereas silencing IF1 expression attenuated EMT and invasion of HCC cells. Mechanistically, IF1 promoted Snai1 and vascular endothelial growth factor (VEGF) expression by way of activating nuclear factor kappa B (NF-κB) signaling, which depended on the binding of tumor necrosis factor (TNF) receptor-associated factor 1 (TRAF1) to NF-κB-inducing kinase (NIK) and the disruption of NIK association with the TRAF2-cIAP2 complex. Suppression of the NF-κB pathway interfered with IF1-mediated EMT and invasion. Chromatin immunoprecipitation assay showed that NF-κB can bind to the Snai1 promoter and trigger its transcription. IF1 was directly transcribed by NF-κB, thus forming a positive feedback signaling loop. There was a significant correlation between IF1 expression and pp65 levels in a cohort of HCC biopsies, and the combination of these two parameters was a more powerful predictor of poor prognosis.IF1 promotes HCC angiogenesis and metastasis by up-regulation of Snai1 and VEGF transcription, thereby providing new insight into HCC progression and IF1 function. (Hepatology 2014;60:1659-1673). 
 The tumor marker Fascin is induced by the Epstein-Barr virus-encoded oncoprotein LMP1 via NF-κB in lymphocytes and contributes to their   invasiv  e migration. The actin-bundling protein Fascin (FSCN1) is a tumor marker that is highly expressed in numerous types of cancer including lymphomas and is important for migration and metastasis of tumor cells. Fascin has also been detected in B lymphocytes that are freshly-infected with Epstein-Barr virus (EBV), however, both the inducers and the mechanisms of Fascin upregulation are still unclear.Here we show that the EBV-encoded oncoprotein latent membrane protein 1 (LMP1), a potent regulator of cellular signaling and transformation, is sufficient to induce both Fascin mRNA and protein in lymphocytes. Fascin expression is mainly regulated by LMP1 via the C-terminal activation region 2 (CTAR2). Block of canonical NF-κB signaling using a chemical inhibitor of IκB kinase β (IKKβ) or cotransfection of a dominant-negative inhibitor of IκBα (NFKBIA) reduced not only expression of p100, a classical target of the canonical NF-κB-pathway, but also LMP1-induced Fascin expression. Furthermore, chemical inhibition of IKKβ reduced both Fascin mRNA and protein levels in EBV-transformed lymphoblastoid cell lines, indicating that canonical NF-κB signaling is required for LMP1-mediated regulation of Fascin both in transfected and transformed lymphocytes. Beyond that, chemical inhibition of IKKβ significant  ly reduc  ed invasive migration of EBV-transformed lymphoblastoid cells through extracellular matrix. Transient transfection experiments revealed that Fascin contributed to LMP1-mediated enha  ncement   of invasive migration through extracellular matrix. While LMP1 enhanced the number of invaded cells, functional knockdown of Fascin by two different small hairpin RNAs resulted in significant reduction of invaded, non-attached cells.Thus, our data show that LMP1-mediated upregulation of Fascin depends on NF-κB and both NF-κB and Fascin c  ontribut  e to invasive migration of LMP1-expressing lymphocytes. 
 High NF-κB and STAT3 activity in human urothelial carcinoma: a pilot study. Given that the tumor-promoting inflammation has been previously established in squamous cell carcinoma of the bladder but its contribution to development of urothelial carcinoma (UC) still remains elusive, our aim was to study changes in expression and activity of inflammation-mediating NF-κB and STAT3 transcription factors in human urothelial bladder carcinoma as well as expression of their target genes cyclin D1, VEGFA and TGFβ1.Gene expression of STAT3, NF-κB, TGFβ1, cyclin D1 and VEGFA was measured by quantitative real-time polymerase chain reaction in both tumor and healthy bladder tissue from 36 patients with UC of the bladder. Activation of STAT3 and NF-κB was assessed with immunohistochemistry and immunoblot.Urothelial bladder carcinoma displayed elevated expression as well as activation of NF-κB (P = 5.38e-10) and STAT3 (P = 0.002) transcription factors. Furthermore, elevated level of expression was observed for cyclin D1, VEGFA and TGFβ1 (P = 9.71e-09, P = 9.71e-09, P = 5.38e-10). Preliminary statistical analysis indicated that the level of upregulation of STAT3 or NF-κB was probably not dependent upon the grade (P = 0.984 and 0.803, respectiv  ely) and   invasiveness of the tumor (0.399 and 0.949), nor to the gender (0.780 and 0.536) and age (0.660 and 0.816) of the patients.NF-κB and STAT3 signaling pathways, as main inflammatory mediators, are found to be activated in urothelial bladder carcinoma indicating that chronic inflammatory processes are accompanying development of this tumor type. Future studies will have to determine possible causative role of inflammatory processes in development of urothelial bladder carcinomas. 
 Targeting Ribonucleotide Reductase M2 and NF-κB Activation with Didox to Circumvent Tamoxifen Resistance in Breast Cancer. Tamoxifen is widely used as an adjuvant therapy for patients with estrogen receptor (ERα)-positive tumors. However, the clinical benefit is often limited because of the emergence of drug resistance. In this study, overexpression of ribonucleotide reductase M2 (RRM2) in MCF-7 breast cancer cells resulted in a reduction in the effectiveness of tamoxifen, through downregulation of ERα66 and upregulation of the 36-kDa variant of ER (ERα36). We identified that NF-κB, HIF1α, and MAPK/JNK are the major pathways that are affected by RRM2 overexpression and result in increased NF-κB activity and increased protein levels of EGFR, HER2, IKKs, Bcl-2, RelB, and p50. RRM2-overexpressing cells also exhibited higher migrato  ry and i  nvasive properties. Through time-lapse microscopy and protein profiling studies of tamoxifen-treated MCF-7 and T-47D cells, we have identified that RRM2, along with other key proteins, is altered during the emergence of acquired tamoxifen resistance. Inhibition of RRM2 using siRRM2 or the ribonucleotide reductase (RR) inhibitor didox not only eradicated and effectively prevented the emergence of tamoxifen-resistant populations but also led to the reversal of many of the proteins altered during the process of acquired tamoxifen resistance. Because didox also appears to be a potent inhibitor of NF-κB activation, combining didox with tamoxifen treatment cooperatively reverses ER-α alterations and inhibits NF-κB activation. Finally, inhibition of RRM2 by didox reversed tamoxifen-resistant in vivo tumor growth and decreased in vitro migr  atory an  d invasive properties, revealing a beneficial effect of combination therapy that includes RRM2 inhibition to delay or abrogate tamoxifen resistance. 
 Suppression of NF-kappaB activity by NDRG2 expression attenuates the   invasive   potential of highly malignant tumor cells. Downregulation of the N-myc downstream-regulated gene 2 (NDRG2) gene is involved in the progression of aggressive forms of cancer, along with the poor prognosis of cancer patients. In the current study, we examined the effect of NDRG2 expression on the metastatic potential of HT1080 human fibrosarcoma and B16F10 murine melanoma cells in both in vitro and in vivo systems. In gelatin zymography, NDRG2 expression remarkably suppressed the matrix metalloproteinase (MMP)-9 activity and slightly inhibited MMP-2 activity of both cell lines. Tumor migration and invasion in vitro were significantly reduced by NDRG2 expression, and NDRG2 inhibited tumor cell proliferation in an anchorage-independent semisolid agar assay. Specifically, we found that NDRG2 affects invasion through suppression of nuclear factor kappa B (NF-kappaB) activity. In animal experiments, subcutaneously injected B16F10-NDRG2 cells showed delayed tumor growth compared with B16F10-mock cells. Furthermore, severe metastasis from primary tumor mass into the draining lymph nodes was observed after injection of B16F10-mock cells, but not with B16F10-NDRG2 cells. Pulmonary metastasis after intravenous injection of B16F10 cells was also reduced by NDRG2 expression. Intra- and peritumoral angiogenesis that is critical for the tumor growth and metastasis was clearly found in tumors after injection with B16F10-mock cells, whereas it was impaired in tumors after injection with B16F10-NDRG2 cells. Collectively, our data show that NDRG2 expression significantly suppresses tumor invasion by inhibiting MMP activities, which are regulated through the NF-kappaB signaling. Moreover, results from animal experiments provide evidence for the regulatory role of the NDRG2 gene in metastatic tumors. 
   Invasive   skin carcinoma--Ras and alpha6beta4 integrin lead the way. Although the genesis of   invasive   squamous cell carcinoma (SCC) from stratified epithelia of the skin is considered to be a complex, multistage process, recent work on human epidermis reveals that sustained Ras signaling coupled with suppression of Ras-induced growth arrest is sufficient to drive the entire process and that the alpha6beta4 integrin and its laminin 5 ligand are essential components of this process. 
 Identification of SNF1/AMP kinase-related kinase as an NF-kappaB-regulated anti-apoptotic kinase involved in CD95-induced motility and   invasive  ness. The death receptor CD95 (APO-1/Fas) induces apoptosis in many tissues. However, in apoptosis-resistant tumor cells, stimulation of CD95 induces up-regulation of a defined number of mostly anti-apoptotic genes, resulting in increased motility and   invasive  ness of tumor cells. The majority of these genes are known NF-kappaB target genes. We have identified one of the CD95-regulated genes as the serine/threonine kinase (SNF1/AMP kinase-related kinase (SNARK)), which is induced in response to various forms of metabolic stress. We demonstrate that up-regulation of SNARK in response to CD95 ligand and tumor necrosis factor alpha depends on activation of NF-kappaB. Overexpression of SNARK rendered tumor cells more resistant, whereas a kinase-inactive mutant of SNARK sensitized cells to CD95-mediated apoptosis. Furthermore, small interfering RNA-mediated knockdown of SNARK increased the sensitivity of tumor cells to CD95 ligand- and TRAIL-induced apoptosis. Importantly, cells with reduced expression of SNARK also showed reduced motility and   invasive  ness in response to CD95 engagement. SNARK therefore represents an NF-kappaB-regulated anti-apoptotic gene that contributes to the tumor-promoting activity of CD95 in apoptosis-resistant tumor cells. 
 GEF-H1 mediated control of NOD1 dependent NF-kappaB activation by Shigella effectors. Shigella flexneri has evolved the ability to modify host cell function with intracellular active effectors to overcome the intestinal barrier. The detection of these microbial effectors and the initiation of innate immune responses are critical for rapid mucosal defense activation. The guanine nucleotide exchange factor H1 (GEF-H1) mediates RhoA activation required for cell invasion by the entero  invasive   pathogen Shigella flexneri. Surprisingly, GEF-H1 is requisite for NF-kappaB activation in response to Shigella infection. GEF-H1 interacts with NOD1 and is required for RIP2 dependent NF-kappaB activation by H-Ala-D-gammaGlu-DAP (gammaTriDAP). GEF-H1 is essential for NF-kappaB activation by the Shigella effectors IpgB2 and OspB, which were found to signal in a NOD1 and RhoA Kinase (ROCK) dependent manner. Our results demonstrate that GEF-H1 is a critical component of cellular defenses forming an intracellular sensing system with NOD1 for the detection of microbial effectors during cell invasion by pathogens. 
 Osteopontin stimulates cell motility and nuclear factor kappaB-mediated secretion of urokinase type plasminogen activator through phosphatidylinositol 3-kinase/Akt signaling pathways in breast cancer cells. We have recently reported that osteopontin (OPN) induces nuclear factor kappaB (NFkappaB)-mediated promatrix metalloproteinase-2 activation through IkappaBalpha/IKK signaling pathways and that curcumin (diferulolylmethane) down-regulates these pathways (Philip, S., and Kundu, G. C. (2003) J. Biol. Chem. 278, 14487-14497). However, the molecular mechanism by which upstream kinases regulate the OPN-induced NFkappaB activation and urokinase type plasminogen activator (uPA) secretion in human breast cancer cells is not well defined. Here we report that OPN induces the phosphatidylinositol 3'-kinase (PI 3'-kinase) activity and phosphorylation of Akt in highly   invasive   MDA-MB-231 and low   invasive   MCF-7 cells. The OPN-induced Akt phosphorylation was inhibited when cells were transfected with a dominant negative mutant of the p85 domain of PI 3-kinase (Deltap85) and enhanced when cells were transfected with an activated form of PI 3-kinase (p110CAAX), indicating that PI 3'-kinase is involved in Akt phosphorylation. OPN enhances the interaction between IkappaBalpha kinase (IKK) and phosphorylated Akt. OPN also induces NFkappaB activation through phosphorylation and degradation of IkappaBalpha by inducing the IKK activity. However, both pharmacological (wortmannin and LY294002) and genetic (Deltap85) inhibitors of PI 3'-kinase inhibited OPN-induced Akt phosphorylation, IKK activity, and NFkappaB activation through phosphorylation and degradation of IkappaBalpha. OPN also enhances uPA secretion, cell motility, and extracellular matrix invasion. Furthermore, cells transfected with Deltap85 or the super-repressor form of IkappaBalpha suppressed the OPN-induced uPA secretion and cell motility, whereas cells transfected with p110CAAX enhanced these effects. Pretreatment of cells with PI 3-kinase inhibitors or NFkappaB inhibitory peptide (SN-50) reduced the OPN-induced uPA secretion, cell motility, and invasion. To our knowledge, this is first report that OPN induces NFkappaB activity and uPA secretion by activating PI 3'-kinase/Akt/IKK-mediated signaling pathways and further demonstrates a functional molecular link between OPN-induced PI 3'-kinase-dependent Akt phosphorylation and NFkappaB-mediated uPA secretion, and all of these ultimately control the motility of breast cancer cells. 
 EBP1 suppresses growth, migration, and invasion of thyroid cancer cells through upregulating RASAL expression. Ebp1, a protein identified by its interactions with the ErbB3 receptor, has been characterized as a negative regulator of cancers. RAS GTPase-activating protein (RasGAP), RASAL1, was recently identified as a major tumor suppressor in thyroid cancer. In this study, we examined EBP1 expression in papillary and follicular thyroid cancer cells. We found that compared with normal thyroid cells, TPC1, WRO, and FTC133 thyroid tumor cells exhibited lower EBP1 expression at messenger RNA (mRNA) and protein levels. We then investigated the effects of forced EBP1 expression on growth, migration, and   invasive  ness of thyroid tumor cells. By using MTT and Boyden chamber assays, we showed that EBP1 overexpression dramatically reduced growth rate, migration, and   invasive  ness of K1 and FTC133 thyroid tumor cells. Furthermore, we explored the molecular mechanism underlying the effects of EBP1 on the cells by disclosing the correlation of EBP1 and RASAL1 expression. RASAL expression was elevated in thyroid tumor cells overexpressing EBP1. Knockdown RASAL by transduction of RASAL1 shRNA lentiviral particles markedly reduced RASAL levels with restoration of EBP1, and RASAL1 knockdown abrogated the effects of forced EBP1 expression on cell growth, migration, and   invasive  ness of thyroid tumor cells. These findings suggest that Ebp1 suppressed thyroid cancer cell lines by upregulating RASRAL expression. 
 The involvement of nuclear factor-κappaB in the nuclear targeting and cyclin E1 upregulating activities of hepatoma upregulated protein. Hepatoma upregulated protein (HURP) is originally isolated during the search for the genes associated with hepatoma. HURP is upregulated in many human cancers. Culture cells exhibit transformed and   invasiv  e phenotype when ectopic HURP is introduced, revealing HURP as an oncogene candidate. Our previous studies demonstrated that Aurora-A regulated the cell transforming activities of HURP by phosphorylating HURP at four serines. To unravel how the Aurora-A/HURP cascade contributes to cell transformation, we firstly noticed that HURP shuttled between cytoplasm and nucleus. The nuclear localization activity of HURP was promoted or abolished by overexpression or knockdown of Aurora-A. Similarly, the HURP phosphorylation mimicking mutant 4E had higher nuclear targeting activity than the phosphorylation deficient mutant 4A. The HURP 4E accelerated G1 progression and upregulated cyclin E1, and the cyclin E1 upregulating and cell transforming activities of HURP were diminished when the nuclear localization signal (NLS) was removed from HURP. Furthermore, HURP employed p38/nuclear factor-κB (NF-κB) cascade to stimulate cell growth. Interestingly, NF-κB trapped HURP in nucleus by interacting with HURP 4E. At last, the HURP/NF-κB complex activated the cyclin E1 promoter. Collectively, Aurora-A/HURP relays cell transforming signal to NF-κB, and the HURP/NF-κB complex is engaged in the regulation of cyclin E1 expression. 
 Senescent cells develop a PARP-1 and nuclear factor-{kappa}B-associated secretome (PNAS). Melanoma cells can enter the process of senescence, but whether they express a secretory phenotype, as reported for other cells, is undetermined. This is of paramount importance, because this secretome can alter the tumor microenvironment and the response to chemotherapeutic drugs. More generally, the molecular events involved in formation of the senescent-associated secretome have yet to be determined. We reveal here that melanoma cells experiencing senescence in response to diverse stimuli, including anti-melanoma drugs, produce an inflammatory secretory profile, where the chemokine ligand-2 (CCL2) acts as a critical effector. Thus, we reveal how senescence induction might be involved in therapeutic failure in melanoma. We further provide a molecular relationship between senescence induction and secretome formation by revealing that the poly(ADP-ribose) polymerase-1 (PARP-1)/nuclear factor-κB (NF-κB) signaling cascade, activated during senescence, drives the formation of a secretome endowed with protumoral and prometastatic properties. Our findings also point to the existence of the PARP-1 and NF-κB-associated secretome, termed the PNAS, in nonmelanoma cells. Most importantly, inhibition of PARP-1 or NF-κB prevents the   proinva  sive properties of the secretome. Collectively, identification of the PARP-1/NF-κB axis in secretome formation opens new avenues for therapeutic intervention against cancers. 
 Notch1 promotes glioma cell migration and invasion by stimulating β-catenin and NF-κB signaling via AKT activation. The Notch signaling pathway has been implicated in both developmental processes and tumorigenesis. Aberrant Notch signaling has been repeatedly demonstrated to facilitate the proliferation and survival of glioma cells by regulating downstream effectors or other signaling pathways. In glioblastoma multiforme specimens from 59 patients, Notch1 was highly expressed in tumor tissues compared with normal brain tissues, and this expression was correlated with elevated AKT phosphorylation and Snail expression. Increased nuclear localization of β-catenin and p50 as well as enhanced IKKα/AKT interaction were also observed in glioma tissues. In U87MG cells, the activation of Notch1 by DLL4 stimulation or by the overexpression of Notch intracellular domain (NICD) resulted in AKT activation and thereby promoted β-catenin activity and NF-κB signaling. Inhibition of EGFR partially blocked the β-catenin and NF-κB signaling stimulated by Notch1 activation. Furthermore, NICD overexpression in U87MG cells led to the upregulated expression of several metastasis-associated molecules, which could be abrogated by the knockdown of either β-catenin or p50. In U87MG and U251 cells, DLL4-induced cellular migration and invasion could be inhibited by either β-catenin or a p50 inhibitor. Collectively, these results indicate that Notch activation could stimulate β-catenin and NF-κB signaling through AKT activation in glioma cells. Thus, Notch activation-stimulated β-catenin and NF-κB signaling synergistically promote the   migrator  y and invasive properties of glioma cells. 
 Antiproliferative and antimetastatic effects of emodin on human pancreatic cancer. Emodin (1, 3, 8-trihydroxy-6-methylanthraquinone) is an active constituent isolated from the root of Rheum palmatum L and is the main effective component of some Chinese herbs and plants. Pharmacological studies have demonstrated that emodin exhibits anti-cancer effects on several human cancers. However, the molecular mechanisms of emodin-mediated tumor regression have not been fully defined. This study was performed to investigate the antiproliferative and antimetastatic effects of emodin on pancreatic cancer in vitro and in vivo. Our results showed that emodin induced a higher percentage of growth inhibition and apoptosis in the pancreatic cancer cell line SW1990 compared to that of control, and emodin suppressed the migration and invasion of SW1990 cells in a dose-dependent manner. To investigate the possible mechanisms involved in these events, we performed electrophoretic mobility shift assay (EMSA) and Western blot analysis, and found that emodin significantly down-regulated NF-κB DNA-binding activity, survivin and MMP-9 in SW1990 cells. Moreover, the expression of cleaved caspase-3 was up-regulated in SW1990 cells after treatment with emodin. In addition, a metastatic model simulating human pancreatic cancer was established by orthotopic implantation of histologically intact human tumor tissue into the pancreatic wall of nude mice. Oral administration of emodin significantly decreased tumor weight and metastasis compared to control. Furthermore, the expression of NF-κB, survivin and MMP-9 were also suppressed in tumor tissues after treatment with emodin. Collectively, our results indicated that emodin exerts antiproliferative and antimetastatic activity on pancreatic cancer both in vitro and in vivo, which may be related to down-regulation of NF-κB and its regulated molecules such as survivin and MMP-9 proteins. Consequently, these results provide important insights into emodin as an   anti-inv  asive agent for the therapy of human pancreatic cancer. 
 Autotaxin stimulates urokinase-type plasminogen activator expression through phosphoinositide 3-kinase-Akt-nuclear [corrected] factor kappa B signaling cascade in human melanoma cells. Autotaxin, a lysophospholipase D producing lysophosphatidic acid, augments   invasive   and metastatic potential of tumor cells. Current investigations have focused on understanding the molecular mechanisms by which autotaxin regulates the expression of a major mediator of tumor invasion and metastasis, urokinase-type plasminogen activator (uPA) in human A2058 melanoma cells. Autotaxin induced uPA expression in a dose-dependent manner that was inhibited by pharmacological inhibitors for Gi (pertussis toxin), phosphoinositide 3-kinase (PI3K, LY294002), Akt inhibitor (AktI), proteosome activity and IkappaB phosphorylation (pyrrolidine dithiocarbamate), and by a dominant negative mutant (DN) of Akt. Autotaxin phosphorylated Akt and induced the translocation of nuclear [corrected] factor-kappaB (NF-kappaB) to the nucleus that were inhibited by AktI or by overexpressing DN-Akt. Consistently, green fluorescence protein-tagged p65 of NF-kappaB accumulated in the nucleus by autotaxin that was abrogated when the cells were transfected with DN-Akt. Moreover, autotaxin increased the DNA binding ability of NF-kappaB and promoter activity of uPA. Collectively, these data strongly suggest autotaxin induces uPA expression via the Gi-PI3K-Akt-NF-kappaB signaling pathway that might be critical for autotaxin-induced tumor cell invasion and metastasis. 
 NF-kappaB p50 promotes tumor cell invasion through negative regulation of invasion suppressor gene CRMP-1 in human lung adenocarcinoma cells. Lung adenocarcinoma Cl1-5 cells were selected from parental Cl1-0 cells based on their high metastatic potential. In a previous study, CRMP-1, an invasion suppressor gene, was shown to be suppressed in Cl1-5 cells. However, the regulation of CRMP-1 expression has not been explored. In this study, we showed nuclear factor-kappaB controls CRMP-1 expression. The electromobility shift assay showed that while Cl1-0 cells exhibited low NF-kappaB activity in response to TNF-alpha, an abundance of basal and TNF-alpha-induced NF-kappaB-DNA complex was detected in Cl1-5 cells. Supershift-coupled EMSA and Western blotting of nuclear proteins, however, revealed p50 protein, but not classic p65/p50 heterodimer in the complex. ChIP and EMSA demonstrated that p50 binds to a kappaB site residing between -1753 and -1743 of the CRMP-1 promoter region. Transfection of antisense p50 gene into Cl1-5 cells increased the CRMP-1 protein level and decreased the   invasive   activity of Cl1-5 cells. 
 Romo1 and the NF-κB pathway are involved in oxidative stress-induced tumor cell invasion. Reactive oxygen species (ROS) are important contributors to tumor cell invasion. ROS enhanced by reactive oxygen species modulator 1 (Romo1) expression has been reported to increase   invasiv  e potential and constitutive activation of nuclear factor-κB (NF-κB) in hepatocellular carcinoma (HCC). Therefore, we investigated whether constitutive NF-κB activation due to Romo1 expression is associated with breast cancer tumor cell invasion. In this study, we show that oxidative stress-induced invasion is mediated by Romo1 expression. The Romo1-induced increase   of inva  sive activity was blocked by an inhibitor of κB kinase (IKK). These results demonstrate that tumor cell invasion in response to oxidative stress is associated with Romo1 expression and the NF-κB signaling pathway. Romo1 is therefore a promising therapeutic target for diseases characterized by NF-κB deregulation. 
 NF-kappa B genes have a major role in inflammatory breast cancer. IBC (Inflammatory Breast cancer) is a rare form of breast cancer with a particular phenotype. New molecular targets are needed to improve the treatment of this rapidly fatal disease. Given the role of NF-kappaB-related genes in cell proliferation,   invasive  ness, angiogenesis and inflammation, we postulated that they might be deregulated in IBC.We measured the mRNA expression levels of 60 NF-kappaB-related genes by using real-time quantitative RT-PCR in a well-defined series of 35 IBCs, by comparison with 22 stage IIB and III non inflammatory breast cancers. Twenty-four distant metastases of breast cancer served as "poor prognosis" breast tumor controls.Thirty-five (58%) of the 60 NF-kappaB-related genes were significantly upregulated in IBC compared with non IBC. The upregulated genes were NF-kappaB genes (NFKB1, RELA, IKBKG, NFKBIB, NFKB2, REL, CHUK), apoptosis genes (MCL1L, TNFAIP3/A20, GADD45B, FASLG, MCL1S, IER3L, TNFRSF10B/TRAILR2), immune response genes (CD40, CD48, TNFSF11/RANKL, TNFRSF11A/RANK, CCL2/MCP-1, CD40LG, IL15, GBP1), proliferation genes (CCND2, CCND3, CSF1R, CSF1, SOD2), tumor-promoting genes (CXCL12, SELE, TNC, VCAM1, ICAM1, PLAU/UPA) or angiogenesis genes (PTGS2/COX2, CXCL1/GRO1). Only two of these 35 genes (PTGS2/COX2 and CXCL1/GRO1)were also upregulated in breast cancer metastases. We identified a five-gene molecular signature that matched patient outcomes, consisting of IL8 and VEGF plus three NF-kappaB-unrelated genes that we had previously identified as prognostic markers in the same series of IBC.The NF-kappaB pathway appears to play a major role in IBC, possibly contributing to the unusual phenotype and aggressiveness of this form of breast cancer. Some upregulated NF-kappaB-related genes might serve as novel therapeutic targets in IBC. 
 [NF-κB signaling pathways and the future perspectives of bone disease therapy using selective inhibitors of NF-κB]. The transcriptional factor nuclear factor κB(NF-κB)regulates the expression of a wide variety of genes that are involved in immune and inflammatory responses, proliferation, and tumorigenesis. NF-κB consists of five members, such as p65(RelA), RelB, c-Rel, p50/p105(NF-κB1), and p52/p100(NF-κB2). There are two distinct NF-κB activation pathways, termed the classical and alternative NF-κB signaling pathways. Since mice lacking both p50 and p52 subunits developed typical osteopetrosis, due to total lack of osteoclasts, NF-κB is also important osteoclast differentiation. A selective NF-κB inhibitor blocked receptor activator of NF-κB ligand(RANKL)-induced osteoclastogenesis both in vitro and in vivo. Recent findings have shown that inactivation of NF-κB enhances osteoblast differentiation in vitro and bone formation in vivo. NF-κB is constitutively activated in many cancers including oral squamous cell carcinoma(OSCC), and is in  volved i  n the invasive characteristics of OSCC. A selective NF-κB inhibitor also prevented jaw bone destruction by OSCC by reduced osteoclast numbers in animal model. Thus the inhibition of NF-κB might useful for the treatment of bone diseases, such as arthritis, osteoporosis, periodontitis, and bone invasion by OSCC by inhibiting bone resorption and by stimulating bone formation. 
 High EMT Signature Score of   Invasive   Non-Small Cell Lung Cancer (NSCLC) Cells Correlates with NFκB Driven Colony-Stimulating Factor 2 (CSF2/GM-CSF) Secretion by Neighboring Stromal Fibroblasts. We established co-cultures of   invasiv  e or non  -invasiv  e NSCLC cell lines and various types of fibroblasts (FBs) to more precisely characterize the molecular mechanism of tumor-stroma crosstalk in lung cancer. The HGF-MET-ERK1/2-CREB-axis was shown to contribute to the onset of the   invasiv  e phenotype of Calu-1 with HGF being secreted by FBs. Differential expression analysis of the respective mono- and co-cultures revealed an upregulation of NFκB-related genes exclusively in co-cultures with Calu-1. Cytokine Array- and ELISA-based characterization of the "cytokine fingerprints" identified CSF2 (GM-CSF), CXCL1, CXCL6, VEGF, IL6, RANTES and IL8 as being specifically upregulated in various co-cultures. Whilst CXCL6 exhibited a strictly FB-type-specific induction profile regardless of th  e invasi  veness of the tumor cell line, CSF2 was only induced in co-cultures o  f invasi  ve cell lines regardless of the partnered FB type. These cultures revealed a clear link between the induction of CSF2 and the EMT signature of the cancer cell line. The canonical NFκB signaling in FBs, but not in tumor cells, was shown to be responsible for the induced and constitutive CSF2 expression. In addition to CSF2, cytokine IL6, IL8 and IL1B, and chemokine CXCL1 and CXCL6 transcripts were also shown to be increased in co-cultured FBs. In contrast, their induction was not strictly dependent on t  he invas  iveness of the co-cultured tumor cell. In a multi-reporter assay, additional signaling pathways (AP-1, HIF1-α, KLF4, SP-1 and ELK-1) were found to be induced in FBs co-cultured with Calu-1. Most importantly, no difference was observed in the level of inducibility of these six signaling pathways with regard to the type of FBs used. Finally, upon tumor fibroblast interaction the massive induction of chemokines such as CXCL1 and CXCL6 in FBs might be responsible for increased recruitment of a monocytic cell line (THP-1) in a transwell assay. 
 Annexin-A1 controls an ERK-RhoA-NFκB activation loop in breast cancer cells. Wound healing is critical for normal development and pathological processes including cancer cell metastasis. MAPK, Rho-GTPases and NFκB are important regulators of wound healing, but mechanisms for their integration are incompletely understood. Annexin-A1 (ANXA1) is upregulated i  n invasi  ve breast cancer cells resulting in constitutive activation of NFκB. We show here that silencing ANXA1 increases the formation of stress fibers and focal adhesions, which may inhibit wound healing. ANXA1 regulated wound healing is dependent on the activation of ERK1/2. ANXA1 increases the activation of RhoA, which is dependent on ERK activation. Furthermore, active RhoA is important in NF-κB activation, where constitutively active RhoA potentiates NFκB activation, while dominant negative RhoA inhibits NFκB activation in response to CXCL12 stimulation and active MEKK plasmids. These findings establish a central role for ANXA1 in the cell migration through the activation of NFκB, ERK1/2 and RhoA. 
 Effects of NF-κB and hypoxia on the biological behavior of Y79 retinoblastoma cells. We aimed to investigate the influence of nuclear factor-κB (NF-κB) on the biological behavior of Y79 retinoblastoma cells exposed to hypoxia and its possible mechanism. The cells were administrated with hypoxia, and/or 5 μM pyrrolidine dithiocarbamate (PDTC) (a selective NF-κB inhibitor) to inhibit the NF-κB activity, expressions of NF-κB was measured by western blot, and the translocation of NF-κB was detected. To examine the proliferation of Y79 cells, MTT assay was applied. Transwell assay was used to detect the invasion and migration ability of cells. The expressions of molecules involved in invasion was analyzed including HIF-1α, MMP-2, 9, and VEGF. We found that hypoxia significantly activated NF-κB activity. While once the NF-κB was inhibited, the proliferation, invasion and migration ability of Y79 cells were also blocked. Interestingly, the expressions of invasion-involved molecules elevated by hypoxia induction were also decreased when NF-κB was inhibited. Hypoxia could significantly change the a  dhesive   and invasive ability of Y79 retinoblastoma cells, NF-κB signal might be one of the main mediators for these hypoxia induced cell changes of biological behavior via downregulation of HIF-1α and the invasion related molecules, and the mechanism still needs further investigation. 
 Simultaneous inactivation of Par-4 and PTEN in vivo leads to synergistic NF-kappaB activation and   invasive   prostate carcinoma. Prostate cancer is one of the most common neoplasias in men. The tumor suppressor Par-4 is an important negative regulator of the canonical NF-kappaB pathway and is highly expressed in prostate. Here we show that Par-4 expression is lost in a high percentage of human prostate carcinomas, and this occurs in association with phosphatase and tensin homolog deleted from chromosome 10 (PTEN) loss. Par-4 null mice, similar to PTEN-heterozygous mice, only develop benign prostate lesions, but, importantly, concomitant Par-4 ablation and PTEN-heterozygosity lead to   invasive   prostate carcinoma in mice. This strong tumorigenic cooperation is anticipated in the preneoplastic prostate epithelium by an additive increase in Akt activation and a synergistic stimulation of NF-kappaB. These results establish the cooperation between Par-4 and PTEN as relevant for the development of prostate cancer and implicate the NF-kappaB pathway as a critical event in prostate tumorigenesis. 
 The NF-κB RelB protein is an oncogenic driver of mesenchymal glioma. High-grade gliomas, such as glioblastomas (GBMs), are very aggressive,   invasiv  e brain tumors with low patient survival rates. The recent identification of distinct glioma tumor subtypes offers the potential for understanding disease pathogenesis, responses to treatment and identification of molecular targets for personalized cancer therapies. However, the key alterations that drive tumorigenesis within each subtype are still poorly understood. Although aberrant NF-κB activity has been implicated in glioma, the roles of specific members of this protein family in tumorigenesis and pathogenesis have not been elucidated. In this study, we show that the NF-κB protein RelB is expressed in a particularly aggressive mesenchymal subtype of glioma, and loss of RelB significantly attenuated glioma cell survival, motility and invasion. We find that RelB promotes the expression of mesenchymal genes including YKL-40, a marker of the MES glioma subtype. Additionally, RelB regulates expression of Olig2, a regulator of cancer stem cell proliferation and a candidate marker for the cell of origin in glioma. Furthermore, loss of RelB in glioma cells significantly diminished tumor growth in orthotopic mouse xenografts. The relevance of our studies for human disease was confirmed by analysis of a human GBM genome database, which revealed that high RelB expression strongly correlates with rapid tumor progression and poor patient survival rates. Thus, our findings demonstrate that RelB is an oncogenic driver of mesenchymal glioma tumor growth and invasion, highlighting the therapeutic potential of inhibiting the noncanonical NF-κB (RelB-mediated) pathway to treat these deadly tumors. 
 Ghrelin induces gastric cancer cell proliferation, migration, and invasion through GHS-R/NF-κB signaling pathway. This study aims to investigate the roles of ghrelin signaling in human gastric carcinoma cell lines AGS and SGC7901. Effects of ghrelin signaling on CDK6, P53, NF-κB/P65 and MMP2 mRNA and/or protein expression were determined by real-time PCR and western blot. MTT method and flow cytometry were performed to assess the gastric cancer cell proliferation. The SGC7901 cells overexpressing ghrelin were inoculated into nude mice to produce tumors which were measured later. The wound-healing assay and cell invasion assay were used to test the cell migration an  d invasi  ve ability of gastric cancer. Ghrelin signaling promotes the oncogene CDK6 gene expression and represses the tumor suppressor gene P53 gene expression in gastric cancer. Ghrelin activates NF-κB/P65 signaling pathway through GHS-R in gastric cancer. Ghrelin upregulates the metastasis factor MMP2 expression via GHS-R/NF-κB signaling pathway in gastric cancer cells and promotes tumor cells migration and invasion, suggesting that ghrelin signaling is a critical pathway in cancer metastasis. Ghrelin induces cell proliferation, migration and invasion via GHS-R/NF-κB signaling pathway in gastric cancer cells. Ghrelin treatment must be avoided for gastric cancer patients. 
 Inhibition of matrix metalloproteinase-9 and nuclear factor kappa B contribute to melatonin prevention of motility and   invasive  ness in HepG2 liver cancer cells. Hepatocellular carcinoma (HCC) is one of the most lethal human cancers worldwide because of its high incidence and its metastatic potential. Extracellular matrix degradation by matrix metalloproteinases (MMPs) has been connected with cancer cell invasion, and it has been suggested that inhibition of MMPs by synthetic and natural inhibitors may be of great importance in the HCC therapies. Melatonin, the main product of the pineal gland, exerts antiproliferative, proapoptotic, and antiangiogenic properties in HepG2 human hepatocellular cells, and exhibits anti-  invasive   and antimetastatic activities by suppressing the enzymatic activity of MMP-9 in different tumor types. However, the underlying mechanism of anti-  invasive   activity in HCC models has not been fully elucidated. Here, we demonstrate that 1 mm melatonin dosage reduced in IL-1β-induced HepG2 cells MMP-9 gelatinase activity and inhibited cell invasion and motility through downregulation of MMP-9 gene expression and upregulation of the MMP-9-specific inhibitor tissue inhibitor of metalloproteinases (TIMP)-1. No significant changes were observed in the expression and activity of MMP-2, the other proteinase implicated in matrix collagen degradation, and its tissue inhibitor, TIMP-2. Also, melatonin significantly suppressed IL-1β-induced nuclear factor-kappaB (NF-κB) translocation and transcriptional activity. In summary, we demonstrate that melatonin modulates motility a  nd invas  iveness of HepG2 cell in vitro through a molecular mechanism that involves TIMP-1 upregulation and attenuation of MMP-9 expression and activity via NF-κB signal pathway inhibition. 
 Raf kinase inhibitor protein suppresses nuclear factor-κB-dependent cancer cell invasion through negative regulation of matrix metalloproteinase expression. Accumulating evidence suggests that Raf kinase inhibitor protein (RKIP), which negatively regulates multiple signaling cascades including the Raf and nuclear factor-κB (NF-κB) pathways, functions as a metastasis suppressor. However, the basis for this activity is not clear. We investigated this question in a panel of breast cancer, colon cancer and melanoma cell lines. We found that RKIP negatively regulated the invasion of the different cancer cells through three-dimensional extracellular matrix barriers by controlling the expression of matrix metalloproteinases (MMPs), particularly, MMP-1 and MMP-2. Silencing of RKIP expression resulted in a high  ly invas  ive phenotype and dramatically increased levels of MMP-1 and MMP-2 expression, while overexpression of RKIP decreased cancer cell invasion in vitro and metastasis in vivo of murine tumor allografts. Knockdown of MMP-1 or MMP-2 in RKIP-knockdown cells reverted the  ir invas  iveness to normal. In contrast, when examining migration of the different cancer cells in a two-dimensional, barrier-less environment, we found that RKIP had either a positive regulatory activity or no activity, but in no case a negative one (as would be expected if RKIP suppressed metastasis at the level of cell migration itself). Therefore, RKIP's function as a metastasis suppressor appears to arise from its ability to negatively regulate expression of specific MMPs, and thus invasion through barriers, and not from a direct effect on the raw capacity of cells to move. The NF-κB pathway, but not the Raf pathway, appeared to positively control the invasion of breast cancer cells. A regulatory loop involving an opposing relationship between RKIP and the NF-κB pathway may control the level of MMP expression and cell invasion. 
 Nuclear factor-κB-dependent epithelial to mesenchymal transition induced by HIF-1α activation in pancreatic cancer cells under hypoxic conditions. Epithelial to mesenchymal transition (EMT) induced by hypoxia is one of the critical causes of treatment failure in different types of human cancers. NF-κB is closely involved in the progression of EMT. Compared with HIF-1α, the correlation between NF-κB and EMT during hypoxia has been less studied, and although the phenomenon was observed in the past, the molecular mechanisms involved remained unclear.Here, we report that hypoxia or overexpression of hypoxia-inducible factor-1α (HIF-1α) promotes EMT in pancreatic cancer cells. On molecular or pharmacologic inhibition of NF-κB, hypoxic cells regained expression of E-cadherin, lost expression of N-cadherin, and attenuated their   highly   invasive and drug-resistant phenotype. Introducing a pcDNA3.0/HIF-1α into pancreatic cancer cells under normoxic conditions heightened NF-κB activity, phenocopying EMT effects produced by hypoxia. Conversely, inhibiting the heightened NF-κB activity in this setting attenuated the EMT phenotype.These results suggest that hypoxia or overexpression of HIF-1α induces the EMT that is largely dependent on NF-κB in pancreatic cancer cells. 
 Correlated expression of Fas, NF-kappaB, and VEGF-C in infiltrating ductal carcinoma of the breast. To investigate the expressions of Fas, NF-kappaB, and vascular endothelial growth factor-C (VEGF-C) in infiltrating ductal carcinoma of the breast and provide scientific basis for early diagnosis and prognosis of breast cancer.The immunohistochemical technique (SP method) was used to detect expression of Fas, NF-kappaB, and VEGF-C in 137 cases of breast-infiltrating ductal carcinoma, 17 cases of intraductal carcinoma of the breast, and 20 cases of normal breast tissues, and analyze its relationship with clinicopathologic factors of breast cancer and patients' survival rate, as well as the correlation among their expression, clinicopathologic factors, and survival rate.Fas expression was less commonly detected in infiltrating ductal carcinoma than in intraductal carcinoma and normal tissue. In contrast, both NF-kappaB and VEGF-C were more commonly detected in infiltrating ductal carcinoma than in intraductal carcinoma and normal tissue. Fas expression was correlated with tumor size, histological grade, and clinicopathological stage; NF-kappaB expression was correlated with tumor size, histological grade, lymph node metastasis; VEGF-C expression was correlated with lymph node metastasis and clinical and pathological stages of breast cancer (p < or = 0.05). Spearman rank correlation analysis revealed a negative correlation between Fas expression and both NF-kappaB and VEGF-C expression in infiltrating breast cancer (p < 0.05) Additionally, Kaplan-Meier survival analysis demonstrated that five-year survival was higher for patients with Fas-positive samples but lower for those with VEGF-C-positive samples.The present results demonstrate that Fas and NF-B play a role in the initiation and development of breast cancer, while VEGF-C appears to promote lymph node metastasis. Thus, these proteins may serve as useful diagnostic and prognostic markers of   invasive   breast cancer. 
 Trastuzumab-Resistant Luminal B Breast Cancer Cells Show Basal-Like Cell Growth Features Through NF-κB-Activation. A major clinical problem in the treatment of breast cancer is mortality due to metastasis. Understanding the molecular mechanisms associated with metastasis should aid in designing new therapeutic approaches for breast cancer. Trastuzumab is the main therapeutic option for HER2+ breast cancer patients; however, the molecular basis for trastuzumab resistance (TZR) and subsequent metastasis is not known. Earlier, we found expression of basal-like molecular markers in TZR tissues from patients with   invasiv  e breast cancer.(( 1 )) The basal-like phenotype is a particularly aggressive form of breast cancer. This observation suggests that TZR might contribute to an aggressive phenotype. To understand if resistance to TZR can lead to basal-like phenotype, we generated a trastuzumab-resistant human breast cancer cell line (BT-474-R) that maintained human epidermal growth factor receptor 2 (HER2) overexpression and HER2 mediated signaling. Analysis showed that nuclear factor-kappa B (NF-κB) was constitutively activated in the BT-474-R cells, a feature similar to the basal-like tumor phenotype. Pharmacologic inhibition of NF-κB improved sensitivity of BT-474-R cells to trastuzumab. Interestingly, activation of HER2 independent NF-κB is not shown in luminal B breast cancer cells. Our study suggests that by activating the NF-κB pathway, luminal B cells may acquire a HER2+ basal-like phenotype in which NF-κB is constitutively activated; this notion is consistent with the recently proposed "progression through grade" or "evolution of resistance" hypothesis. Furthermore, we identified IKK-α/IKK-β and nuclear accumulation of RelA/p65 as the major determinants in the resistant cells. Thus our study additionally suggests that the nuclear accumulation of p65 may be a useful marker for identifying metastasis-initiating tumor cells and targeting RelA/p65 may limit metastasis of breast and other cancers associated with NF-κB activation. 
 Genetic variation and alterations of genes involved in NFκB/TNFAIP3- and NLRP3-inflammasome signaling affect susceptibility and outcome of colorectal cancer. Colorectal tumors are continuously exposed to an inflammatory environment, which together with mitogenic signals sustain several cancer hallmarks. Nuclear factor-kappa B (NFκB) is a major regulator of inflammation and variation in NFκB-associated genes could potentially be used as biomarkers to identify patients with increased risk of colorectal cancer (CRC) development, and/or a rapidly progressing disease. In this study, 348 CRC cases and 806 randomly selected healthy individuals from southeastern Sweden were examined with regard to seven polymorphisms in NFκB pathway-associated genes. Log-rank-tests and Cox proportional hazard regression analysis examined the association between the polymorphisms and CRC-specific survival, whereas chi-square tests and logistic regression analysis were used to test for associations between the polymorphisms and CRC susceptibility. Gene expression and loss of heterozygosity analyses of TNFAIP3 were carried out in a subset of tumors to assess its role as a tumor suppressor in CRC. Heterozygous and polymorphic TNFAIP3 (rs6920220), heterozygous NLRP3 (Q705K) and polymorphic NFκB -94 ATTG ins/del genotypes were found to be associated with poorer survival in patients diagnosed   with inv  asive CRC (aHR = 5.2, 95% CI: 2.5-10.9, P < 0.001). TNFAIP3 mRNA levels were significantly decreased in tumors compared with adjacent non-neoplastic mucosa (P < 0.0001) and loss of heterozygosity of 6q23.3 (TNFAIP3) was detected in 17% of cases, whereas only 2.5% of the investigated specimens displayed TNFAIP3 gene mutations. We propose that TNFAIP3 (rs6920220), NLRP3 (Q705K) and NFκB -94 ATTG ins/del polymorphisms are associated with poor survival in patients with advanced CRC and may be used as prognostic markers. Experimental results indicate that TNFAIP3 may act as a tumor suppressor in CRC. 
 NF-kappaB activation, dependent on acetylation/deacetylation, contributes to HIF-1 activity and migration of bone metastatic breast carcinoma cells. Here, we show that NF-kappaB-HIF-1 interaction contributed to breast cancer metastatic capacity by means of an incomplete epithelial/mesenchymal transition and influencing migration, as shown in 1833 (human) and 4T1 (mouse) metastatic cells after different stimuli. The 1833 and the transforming growth factor-beta1-exposed 4T1 cells showed both epithelial (E-cadherins) and mesenchymal (N-cadherins and vimentin) markers, and common mechanisms contributed to the retention of certain epithelial characteristics and the control of migration. The complex NF-kappaB-HIF-1 reciprocal regulation and the enhanced c-Jun expression played a functional role in exacerbating the   invasive  ness of 1833 cells after p50/p65 transfection and of 4T1 cells exposed to transforming growth factor-beta1. Twist expression seemed to exert a permissive role also regulating epithelial/mesenchymal transition markers. After c-Src wild-type (Srcwt) transfection, c-Src-signal transducer overexpression in 1833 cells increased HIF-1 transactivating activity and   invasive  ness, and changed E-cadherin/N-cadherin ratio versus mesenchymal phenotype. The transcription factor pattern and the motile phenotype of metastatic 1833 cells were influenced by p65-lysine acetylation and HDAC-dependent epigenetic mechanisms, which positively regulated basal NF-kappaB and HIF-1 activities. However, HDAC3 acted as a corepressor of NF-kappaB activity in parental MDA-MB231 cells, thus explaining many differences from the derived 1833 clone, including reduced HIF-1alpha and c-Jun expression.   Invasive  ness was differently affected by HDAC knockdown in 1833 and MDA-MB231 cells. We suggest that acetylation/deacetylation are critical in establishing the bone-metastatic gene signature of 1833 cells by regulating the activity of NF-kappaB and HIF-1, and further clarify the epigenetic control of transcription factor network in the motile phenotype of 1833 cells. 
 Nuclear factor-kappaB-mediated transforming growth factor-beta-induced expression of vimentin is an independent predictor of biochemical recurrence after radical prostatectomy. Transforming growth factor-beta (TGF-beta)-mediated epithelial-to-mesenchymal transition (EMT) has been shown to occur in some cancers; however, the pathway remains controversial and varies with different cancers. In addition, the mechanisms by which TGF-beta and the EMT contribute to prostate cancer recurrence are largely unknown. In this study, we elucidated TGF-beta-mediated EMT as a predictor of disease recurrence after therapy for prostate cancer, which has not been reported before.We analyzed TGF-beta-induced EMT using nuclear factor-kappaB (NF-kappaB) as an intermediate mediator in prostate cancer cell lines. A total of 287 radical prostatectomy specimens were evaluated using immunohistochemistry in a high-throughput tissue microarray analysis. Levels of TGF-beta signaling components and EMT-related factors were analyzed using specific antibodies. Results were expressed as the percentage of cancer cells that stained positive for a given antibody and were correlated with disease recurrence rates at a mean of 7 years following radical prostatectomy.In prostate cancer cell lines, TGF-beta-induced EMT was mediated by NF-kappaB signaling. Blockade of NF-kappaB or TGF-beta signaling resulted in abrogation of vimentin expression and inhibition of the   invasive   capability of these cells. There was high risk of biochemical recurrence associated with tumors that displayed high levels of expression of TGF-beta1, vimentin, and NF-kappaB and low level of cytokeratin 18. This was particularly true for vimentin, which is independent of patients' Gleason score.The detection of NF-kappaB-mediated TGF-beta-induced EMT in primary tumors predicts disease recurrence in prostate cancer patients following radical prostatectomy. The changes in TGF-beta signaling and EMT-related factors provide novel molecular markers that may predict prostate cancer outcomes following treatment. 
 Knockdown of the inflammatory factor pentraxin-3 suppresses growth and invasion of lung adenocarcinoma through the AKT and NF-kappa B pathways. Pentraxin-3 (PTX3), a modulator of tumor-associated inflammation, is known to be positively correlated with tumor grade and severity of malignancies, but the function and molecular underlying mechanisms of PTX3 remain unclear. In the present study, the expression of PTX3 in human lung adenocarcinoma (LAC) was examined by immunohistochemical assay using a tissue microarray procedure. A loss-of-function experiment was performed to explore the effects of lentiviral vector-mediated PTX3 shRNA (Lv-shPTX3) on cell growth and   invasive   potential in LAC cell lines (A549 and LETPα-2), assessed by MTT and Transwell assays, respectively. We found that the expression of PTX3 protein was significantly increased in LAC tissues compared with that in adjacent non-cancerous tissues (ANCT) (60.42% vs. 29.17%, P=0.004), and positively correlated with lymphatic invasion of the tumor (P=0.006). Furthermore, knockdown of PTX3 suppressed tumor proliferation and invasion of LAC cells, followed by decreased expression of p-AKT, p-NF-kappa B, PCNA, and MMP-9. Taken together, our findings demonstrate that upregulation of PTX3 expression is correlated with tumor metastasis of LAC patients, and knockdown of PTX3 blocks the development of LAC through inhibition of the AKT and NF-kappa B pathways, suggesting that PTX3 may serve as a potential therapeutic target for the treatment of cancer. 
 Functional promoter -94 ins/del ATTG polymorphism in NFKB1 gene is associated with bladder cancer risk in a Chinese population. A functional -94 insertion/deletion polymorphism (rs28362491) in the promoter of the NFKB1 gene was reported to influence NFKB1 expression and confer susceptibility to different types of cancer. This study aims to determine whether the polymorphism is associated with risk of bladder cancer.TaqMan assay was used to determine genotype among 609 cases and 640 controls in a Chinese population. Logistic regression was used to assess the association between the polymorphism and bladder cancer risk, and quantitative real-time polymerase chain reaction was used to determine NFKB1 mRNA expression.Compared with the ins/ins/ins/del genotypes, the del/del genotype was associated with a significantly increased risk of bladder cancer [adjusted odd ratio (OR) = 1.92, 95% confidence interval (CI) = 1.42-2.59]. The increased risk was more prominent among subjects over 65 years old (OR = 2.37, 95% CI= 1.52-3.70), male subjects (OR = 1.97, 95% CI = 1.40-2.79) and subjects with self-reported family history of cancer (OR = 3.59, 95% CI = 1.19-10.9). Furthermore, the polymorphism was associated with a higher   risk of   developing non-muscle invasive bladder cancer (OR= 2.07, 95% CI= 1.51-2.85), grade 1 bladder cancer (OR = 2.40, 95% CI = 1.68-3.43), single tumor bladder cancer (OR = 2.04, 95% CI = 1.48-2.82) and smaller tumor size bladder cancer (OR = 2.10, 95% CI= 1.51-2.92). The expression of NFKB1 mRNA in bladder cancer tissues with homozygous insertion genotype was higher than that with deletion allele.In conclusion, the -94 ins/del ATTG polymorphism in NFKB1 promoter may contribute to the etiology of bladder cancer in the Chinese population. 
 The breast cancer susceptibility gene product fibroblast growth factor receptor 2 serves as a scaffold for regulation of NF-κB signaling. Fibroblast growth factor (FGF) receptor 2 (FGFR2) has been identified in genome-wide association studies to be associated with increased breast cancer risk; however, its mechanism of action remains unclear. Here we show that the two major FGFR2 alternatively spliced isoforms, FGFR2-IIIb and FGFR2-IIIc, interact with IκB kinase β and its downstream target, NF-κB. FGFR2 inhibits nuclear RelA/p65 NF-κB translocation and activity and reduces expression of dependent transcripts, including interleukin-6. These interactions result in diminished STAT3 phosphorylation and reduced breast cancer cell growth, motility,   and inv  asiveness. FGFR2 also arrests the epithelial cell-to-mesenchymal cell transition (EMT), resulting in attenuated neoplastic growth in orthotopic xenografts of breast cancer cells. Our studies provide strong evidence for the protective effects of FGFR2 on tumor progression. We propose that FGFR2 serves as a scaffold for multiple components of the NF-κB signaling complex. Through these interactions, FGFR2 isoforms can respond to tissue-specific FGF signals to modulate epithelial cell-stromal cell communications in cancer progression. 
 Pachymic acid impairs breast cancer cell invasion by suppressing nuclear factor-κB-dependent matrix metalloproteinase-9 expression. Pachymic acid (PA), a lanostane-type triterpenoid derived from Poria cocos, possesses demonstrated anti-inflammatory and anti-cancer activities. Nonetheless, the biological properties and mechanism/s of action of PA remain largely undefined. In this study, the activity of PA against breast cancer cell invasion was evaluated.   Invasiv  eness of human-derived MDA-MB-231 and MCF-7 breast carcinoma cells was suppressed by PA at non-lethal concentrations, which was associated with a decrease in matrix metalloproteinase-9 (MMP-9) secretion as a result of PA-mediated down-regulation of MMP-9 mRNA expression. In order to elucidate the underlying anti  -invasiv  e mechanism, the effect of PA on transcription factors activator protein-1 (AP-1) and nuclear factor kappaB (NF-κB) was examined using luciferase-based reporter gene assays. PA was found to bring about a reduction in phorbol 12-myristate 13-acetate (PMA)-induced transcriptional activity of NF-κB, but not that of AP-1. In accord with the luciferase activity data, western blot analysis showed that PA inhibited NF-κB signaling pathway, but did not alter the phosphorylation states of mitogen-activated protein kinases including ERK, JNK, and p38 kinase. The inhibition of PA on NF-κB signaling pathway was further attributed to PA-mediated diminution in PMA-induced degradation of inhibitor of kappaBα (IκBα) through preventing phosphorylation of the upstream signal IκB kinase (IKK). A decrease in p65 nuclear translocation was achieved, which led to attenuation of NF-κB transactivation. Taken together, it was concluded that by targeting NF-κB signaling, PA inhibited breast cancer cell invasion through decreasing MMP-9 expression. PA may thus be potentially exploited for use in tumor metastasis intervention. 
 NFKB1/NFKBIa polymorphisms are associated with the progression of cervical carcinoma in HPV-infected postmenopausal women from rural area. Human papillomavirus (HPV) is considered as the major etiological agent for development of cervical cancer but alone is not sufficient enough. So, other environmental factors and host genetic background may play an important role in the development of cervical cancer. HPV carries a minimal amount of structural and regulatory proteins so it is apparently dependent on its host for survival. NF-κB/IkB system plays an important regulatory role in the apoptotic pathway. In the present study, a total of 575 consecutive subjects including 285 cases (45 cervical pre-cancerous and 240   invasiv  e cervical carcinoma) and 290 age- and ethnicity-matched controls recruited from Lok Nayak Jai Prakash Hospital and Safdarjung Hospital, New Delhi between July 2009 to July 2013 were genotyped for NFKB1 -94 insertion/deletion (rs28362491) and NFKBIa 3'-UTR2758A > G (rs696) polymorphism by PCR-RFLP followed by sequencing. We observed a positive association of NFKB1 -94 insertion/insertion (II) and NFKBIa 3'-UTR 2758 GG genotypes with the progression of cervical carcinoma. Cervical cancer patients were found more pronounce to be a carrier of II + GG genotype of both the SNPs. We also noticed that HPV-infected postmenopausal women having higher parity along with the history of tobacco consumption and who carries insertion allele of NFKB1 -94 polymorphism in association of GG genotype of NFKBIa 3'-UTR polymorphism, were more susceptible to develop cervical carcinoma. II + GG genotype together were found to have direct proportionality with the aggressiveness of cervical carcinoma. In conclusion, alteration in the gene map of NFKB1/NFKBIa helps in the progression of cervical cancer accompanied by HPV infection in postmenopausal women from rural residential setup who had higher parity along with history of tobacco consumption. 
  GeneRIFs  
 1,25D3 works as a modifier of NF-kappaB/GPX1/uPA expression, inhibiting cisplatin-resistance and cell   invasive   ability of salivary adenoid cystic carcinoma cells 
 Data show that 14-3-3beta protein augmented the expression of matrix metalloproteinasea MMP2 and MMP9 through PI3 kinase/Akt protein/NF-kappaappa B pathway, thereby enhancing the   invasive  ness of hepatocellular carcinoma (HCC) cells. 
 the RRM2/AKT/NF-kappaB signaling pathway is essential for tumor   invasive  ness in gastric cancer cells 
 Data show that the effect of curcumin in decreasing the migratory and   invasive   ability of lung adenocarcinoma A549 cells by inhibiting adiponectin expression is probably mediated through NF-kappaB/MMP pathways 
 LMP1-mediated upregulation of Fascin depends on NF-kappaB and both NF-kappaB and Fascin contribute to   invasive   migration of LMP1-expressing lymphocytes. 
 Knockdown of endogenous Bmi-1 reduced the   invasive  ness and migration of glioma cells. NF-kappaB transcriptional activity and MMP-9 expression and activity were significantly increased in Bmi-1-overexpressing but reduced in Bmi-1-silenced cells. 
 HIF-1 and NF-kappaB are essential for hypoxia-regulated CX3CR1 expression, which is associated with increased migratory and   invasive   potential of prostate cancer cells. 
 Enhanced ovarian neoplasm   invasive  ness is mediated through nuclear factor kappaB activation and the consequently elevated MMP9 secretion. 
 findings demonstrate that folate deprivation enhanced   invasive  ness of colon cancer cells mediated by activation of Shh signaling through promoter hypomethylation and cross actions with the NF-kappaB pathway. 
 NFkappaB overexpression is associated with chemoradiation resistance in muscle-  invasive   bladder cancer. 
 Notch activation-stimulated beta-catenin and NF-kappaB signaling synergistically promote the migratory and   invasive   properties of glioma cells 
 EGF induces uPAR expression via ERK-1/2, AP-1, and NF-kappaB signaling pathways and, in turn, stimulates cell   invasive  ness in human gastric cancer AGS cells 
 increased apoptosis by dual knockdown of p65 and p50 may prove advantageous in preventing   invasive  ness and destructiveness of hyperplastic synoviocytes 
 NF-kappaB has roles in control of mesenchymal genes critical for promoting and maintaining an   invasive   phenotype [review] 
 Circumvention of Ras-induced growth arrest by blockade of NFKB1 function by expression of IkBalpha frees the oncogenic functions of Ras and results in   invasive   carcinoma resembling squamous cell carcinoma. 
 NF-kappaB is a key mediator of the   invasive   phenotypic changes induced by RRM2 overexpression. 
  [Mm]etastasize  PubMed  
 NF-κB regulates mesenchymal transition for the induction of non-small cell lung cancer initiating cells. The epithelial-to-mesenchymal transition (EMT) is a de-differentiation process that has been implicated in metastasis and the generation of cancer initiating cells (CICs) in solid tumors. To examine EMT in non-small cell lung cancer (NSCLC), we utilized a three dimensional (3D) cell culture system in which cells were co-stimulated with tumor necrosis factor alpha (TNF) and transforming growth factor beta (TGFβ). NSCLC spheroid cultures display elevated expression of EMT master-switch transcription factors, TWIST1, SNAI1/Snail1, SNAI2/Slug and ZEB2/Sip1, and are highly invasive. Mesenchymal NSCLC cultures show CIC characteristics, displaying elevated expression of transcription factors KLF4, SOX2, POU5F1/Oct4, MYCN, and KIT. As a result, these putative CIC display a cancer "stem-like" phenotype by forming lung metastases under limiting cell dilution. The pleiotropic transcription factor, NF-κB, has been implicated in EMT and metastasis. Thus, we set out to develop a NSCLC model to further characterize the role of NF-κB activation in the development of CICs. Here, we demonstrate that induction of EMT in 3D cultures results in constitutive NF-κB activity. Furthermore, inhibition of NF-κB resulted in the loss of TWIST1, SNAI2, and ZEB2 induction, and a failure of cells to invad  e and metas  tasize. Our work indicates that NF-κB is required for NSCLC metastasis, in part, by transcriptionally upregulating master-switch transcription factors required for EMT. 
  [Mm]etastasis  PubMed  
 Stabilization of snail by NF-kappaB is required for inflammation-induced cell migration and invasion. The increased motility and invasiveness of tumor cells are reminiscent of epithelial-mesenchymal transition (EMT), which occurs during embryonic development, wound healing, and   metastasis  . In this study, we found that Snail is stabilized by the inflammatory cytokine TNFalpha through the activation of the NF-kappaB pathway. We demonstrated that NF-kappaB is required for the induction of COP9 signalosome 2 (CSN2), which, in turn, blocks the ubiquitination and degradation of Snail. Furthermore, we showed that the expression of Snail correlated with the activation of NF-kappaB in cancer cell lines and metastatic tumor samples. Knockdown of Snail expression inhibited cell migration and invasion induced by inflammatory cytokines and suppressed inflammation-mediated breast cancer   metastasis  . Our study provides a plausible mechanism for inflammation-induced   metastasis  . 
 EGF-R is expressed and AP-1 and NF-kappaB are activated in stromal myofibroblasts surrounding colon adenocarcinomas paralleling expression of COX-2 and VEGF. COX-2 and VEGF are important triggers of colon cancer growth,   metastasis   and angiogenesis. Cox-2 promoter contains transcriptional regulatory elements for AP-1 and NF-kappaB transcription factors whilst vegf is a known AP-1 downstream target gene. We investigated whether stromal myofibroblasts surrounding colon adenocarcinomas express COX-2 and VEGF and whether activation of AP-1 and NF-kappaB, as well as expression of EGF-R parallel expression of COX-2 and VEGF in these cells.Immunohistochemical methodology was performed on archival sections from 40 patients with colon adenocarcinomas. We evaluated c-FOS, p-c-JUN (phosphorylated c-JUN), p-IkappaB-alpha (phosphorylated IkappaB-alpha), EGF-R, COX-2, NF-kappaB and VEGF expression in stromal myofibroblasts surrounding colon adenocarcinomas. Double immunostaining with a-smooth muscle actin and each antibody was done to verify the expression of these molecules in stromal myofibroblasts.VEGF, p-IkappaB-alpha, NF-kappaB, c-FOS, p-c-JUN, EGF-R and COX-2 were expressed in stromal myofibroblasts surrounding colon adenocarcinomas in the majority of cases. EGF-R, p-IkappaB-alpha, NF-kappaB, c-FOS and p-c-JUN correlated positively with COX-2 and VEGF expression.Stromal myofibroblasts surrounding colon adenocarcinomas are an important source of VEGF and COX-2 production, while AP-1 and NF-kappaB transcription factors are activated and EGF-R is expressed in these cells and associated with COX-2 and VEGF production. 
 Differential effects of sulfated triterpene glycosides, holothurin A1, and 24-dehydroechinoside A, on antimetastasic activity via regulation of the MMP-9 signal pathway. Two sulfated triterpene glycosides, holothurin A(1) (HA(1)) and 24-dehydroechinoside A (DHEA), isolated from the sea cucumber Pearsonothuria graeffei, are of the holostane type with 18(20)-lactone and identical carbohydrate chains. DHEA has a side chain 23 (24)-double bond, while HA(1) has a hydroxyl group at C-21. In this study, we compared the effects of DHEA and HA(1) on   metastasis   in vitro and in vivo. The results show that HA(1) and DHEA treatment significantly suppressed adhesion of human hepatocellular liver carcinoma cells (HepG2) to both matrigel and human endothelial cells (ECV-304) and inhibited HepG2 cell migration and invasion in a dose-dependant manner. HA(1) and DHEA reduced tube formation of ECV-304 cells on the matrigel in vitro and attenuated neovascularization in the chick embryo using the chorioallantoic membrane (CAM) assay in vivo. Immunocytochemistry analyses revealed that both HA(1) and DHEA significantly decreased the expression of the matrix metallo-proteinase-9 (MMP-9) and increased the expression level of tissue inhibitor of metalloproteinase-1 (TIMP-1), an important regulator of MMP-9 activation. Western blot analyses demonstrated that HA(1) and DHEA remarkably abolished the expression of vascular endothelial growth factor (VEGF). The expression of nuclear factor-kappa B (NF-κB) was significantly decreased by HA(1), while DHEA treatment had no effect on the down regulation of NF-κB expression. These data suggest that both DHEA and HA(1) exert significant antimetastatic activities by inhibiting MMP-9 and VEGF expression. DHEA-induced an  timetastas  is was more potent than HA(1). In addition, only HA(1) treatment downregulated the expression level of NF-κB, suggesting that the antimetastatic activity of triterpene glycosides derived from P. graeffei can be either NF-κB-dependent or -independent, depending on their structure. 
 Lysophosphatidic acid upregulates vascular endothelial growth factor-C and tube formation in human endothelial cells through LPA(1/3), COX-2, and NF-kappaB activation- and EGFR transactivation-dependent mechanisms. Lysophosphatidic acid (LPA) is a lipid bioactive mediator which binds to G-protein-coupled receptors and activates a variety of cellular functions. LPA modulates multiple behaviors in endothelial cells, including cell proliferation and migration, capillary-like tube formation in vitro, activation of proteases, interactions with leukocytes, and expressions of inflammation-related genes, thereby regulating vessel formation. LPA has been reported to modulate the angiogenesis process. However, the role of LPA in the lymphangiogenesis process has not been studied. In this study, we showed that LPA upregulated vascular endothelial growth factor-C (VEGF-C) mRNA expression in human umbilical vein endothelial cells (HUVECs) and subsequent endothelial cell tube formation in vitro and in vivo. These enhancement effects were LPA(1)- and LPA(3)-dependent and required cyclooxygenase-2 (COX-2), endothelial growth factor receptor (EGFR) transactivation and activation of nuclear factor kappaB (NF-kappaB). Moreover, LPA induced the protein expressions of the lymphatic markers, Prox-1, LYVE-1, and podoplanin, in HUVECs, and these enhancement effects were dependent on LPA(1) and LPA(3) activation and EGFR transactivation. Our results demonstrated that LPA might regulate VEGF-C and lymphatic marker expression in endothelial cells, which contributes to endothelial cell tube formation in vitro and in vivo, thus facilitating endothelial cell participation in the lymphangiogenesis process. This study clarifies the signaling mechanism of LPA-regulated VEGF-C expression and lymphatic marker expressions in endothelial cells, which suggest that LPA may be a suitable target for generating therapeutics against lymphangiogenesis and tumor   metastasis  . 
 Comprehensive Evaluation of Nuclear Factor-κΒ Expression Patterns in Non-Small Cell Lung Cancer. Nuclear factor (NF)-κB signalling is required for lung adenocarcinoma development in mice, and both of its subunits RelA and RelB were independently reported to be highly expressed in human non-small cell lung cancer (NSCLC). To comprehensively examine NF-κB expression in NSCLC, we analyzed serial sections of primary tumor samples from 77 well-documented patients (36 adenocarcinomas, 40 squamous cell carcinomas and 3 large cell carcinomas) for immunoreactivity of RelA, RelB, P50, and P52/P100. Tumor and intratumoral stroma areas were discriminated based on proliferating cell nuclear antigen immunoreactivity and inflammatory infiltration was assessed in intratumoral stroma areas. NF-κB immunoreactivity was quantified by intensity, extent, and nuclear localization and was cross-examined with tumor cell proliferation, inflammatory infiltration, and clinical-pathologic data. We found that the expression of the different NF-κB subunits was not concordant, warranting our integral approach. Overall, RelA, RelB, and P50 were expressed at higher levels compared with P52/P100. However, RelA and P50 were predominantly expressed in intratumoral stroma, but RelB in tumor cells. Importantly, tumor area RelA expression was correlated with the intensity of inflammatory infiltration, whereas RelB expression was identified in proliferating tumor cells. Using multiple logistic regression, we identified that tumor RelB expression was an independent predictor of lymph   node meta  stasis, and tumor P50 was an independent predictor of TNM6 stage IIB or higher, whereas tumor RelA was an independent predictor of inflammatory infiltration. We conclude that pathologic studies of NF-κB expression in cancer should include multiple pathway components. Utilizing such an approach, we identified intriguing associations between distinct NF-κB subunits and clinical and pathologic features of NSCLC. 
 Transforming growth factor-beta1-mediated activation of NF-kappaB contributes to enhanced ADAM-12 expression in mammary carcinoma cells. A disintegrin and metalloproteinase-12 (ADAM-12), a member of multifunctional family of proteins, is upregulated in many cancers, including breast, lung, liver, prostate, gastric, and bladder. The multidomain structure, composed of a prodomain, a metalloproteinase, disintegrin-like, epidermal growth factor-like, cysteine-rich and transmembrane domains, and a cytoplasmic tail, allows ADAM-12 to promote matrix degradation, cell-cell adhesion, and intracellular signaling capacities and thereby to play a critical role in cancer growth and   metastasis  . Despite ample evidence linking increased ADAM-12 expression with cancer, the mechanisms controlling its upregulation are still unknown. In the present study, transforming growth factor-β1 (TGF-β1) is shown to increase ADAM-12 mRNA expression in MDA-MB-231 breast carcinoma cells. We have identified a promoter element responsible for TGF-β1-mediated ADAM-12 induction. We show interaction of NF-κB with ADAM-12 promoter and that high level of NF-κB activity in breast carcinoma cells results in the upregulation of ADAM-12 expression. Site-directed mutagenesis of the NF-κB element in ADAM-12 promoter and inhibition of NF-κB activity by Bay-11-7085 and MG-132 significantly reduced TGF-β1-mediated increase of ADAM-12 promoter-driven gene expression. Transfection of cells with a dominant-negative mutant form of IκBα (IκBαΔN), which inhibits activation of NF-κB, significantly reduced transcription from ADAM-12 promoter-reporter in TGF-β1-stimulated MDA-MB-231 cancer cells. In correlation, overexpression of NF-κB induced ADAM-12 expression in a dose-dependent manner. DNA-binding and ChIP assays indicated that p65 subunit of NF-κB binds to ADAM-12 promoter. Together, our study identified a cellular mechanism for induction of ADAM-12, which involves NF-κB and its activation by TGF-β1. 
 Dysregulation of the cell survival/anti-apoptotic NF-kappaB pathway by the novel humanized BM-ca anti-CD20 mAb: implication in chemosensitization. Treatment of patients with relapsed or refractory low grade follicular B-NHL lymphoma with rituximab (chimeric anti-CD20 mAb) has resulted in approximately 50% response rate. The mechanism underlying the failure of rituximab to affect the remaining 50% of the patients is not clear, though their tumors express CD20. The in vivo effector functions of rituximab include ADCC, CDC and seldom apoptosis. In addition, we have reported that rituximab signals the cells and inhibits several intracellular cell survival pathways that are responsible for the immuno and chemo-sensitizing effects of rituximab on resistant B-NHL cell lines. The objective of this study was to develop novel and fully humanized anti-CD20 monoclonal antibodies with enhanced effector functions and molecular signaling that may potentiate their therapeutic efficacy. Novel humanized anti-CD20 monoclonal antibodies were derived from a chimerized form of murine anti-CD20 1K11791, shown to exert a more potent ADCC, CDC and apoptotic activities compared to rituximab. A representative humanized monoclonal antibody, BM-ca was used to examine its biological effect and molecular signaling using Ramos B-NHL cell line as a model. The studies were also performed in parallel with rituximab treatment for comparison. Ramos cells were treated with various concentrations of BM-ca monoclonal antibody. Inhibition of cell proliferation was observed in a concentration-dependent manner, suggesting cell signal perturbations must have occurred. Compared to untreated cells, treatment with BM-ca inhibited both the constitutively activated NF-kappaB and p38 MAPK pathways, as assessed by inhibition of both phospho-p65 and phospho-IkappaBalpha and phospho-p38, respectively, but not the unphosphorylated forms. BM-ca significantly induced the expression of the   metastasis   suppressor and immune surveillance cancer gene product, Raf-1 kinase inhibitor protein (RKIP). These alterations resulted in inhibition of anti-apoptotic gene products and sensitized Ramos cells to apoptosis by CDDP. In comparison with rituximab, BM-ca showed qualitative and quantitative differences in the above analyses. These findings demonstrate that BM-ca triggers CD20 expressing B-NHL cells resulting in a significant alteration of several gene products that regulate cell growth and chemoresistance. 
 Necrotic cells influence migration and invasion of glioblastoma via NF-κB/AP-1-mediated IL-8 regulation. Glioblastoma multiforme (GBM) is the most common primary intracranial tumor in adults and has poor prognosis. Diffuse infiltration into normal brain parenchyma, rapid growth, and the presence of necrosis are remarkable hallmarks of GBM. However, the effect of necrotic cells on GBM growth and   metastasi  s is poorly understood at present. In this study, we examined the biological significance of necrotic tissues by exploring the molecular mechanisms underlying the signaling network between necrotic tissues and GBM cells. The migration and invasion of the GBM cell line CRT-MG was significantly enhanced by treatment with necrotic cells, as shown by assays for scratch wound healing and spheroid invasion. Incubation with necrotic cells induced IL-8 secretion in CRT-MG cells in a dose-dependent manner. In human GBM tissues, IL-8 positive cells were mainly distributed in the perinecrotic region, as seen in immunohistochemistry and immunofluorescence analysis. Necrotic cells induced NF-κB and AP-1 activation and their binding to the IL-8 promoter, leading to enhanced IL-8 production and secretion in GBM cells. Our data demonstrate that when GBM cells are exposed to and stimulated by necrotic cells, the migration and invasion of GBM cells are enhanced and facilitated via NF-κB/AP-1 mediated IL-8 upregulation. 
 Cell specific CD44 expression in breast cancer requires the interaction of AP-1 and NFκB with a novel cis-element. Breast cancers contain a heterogeneous population of cells with a small percentage that possess properties similar to those found in stem cells. One of the widely accepted markers of breast cancer stem cells (BCSCs) is the cell surface marker CD44. As a glycoprotein, CD44 is involved in many cellular processes including cell adhesion, migration and proliferation, making it pro-oncogenic by nature. CD44 expression is highly up-regulated in BCSCs, and has been implicated in tumorigenesis and   metastasi  s. However, the genetic mechanism that leads to a high level of CD44 expression in breast cancer cells and BCSCs is not well understood. Here, we identify a novel cis-element of the CD44 directs gene expression in breast cancer cells in a cell type specific manner. We have further identified key trans-acting factor binding sites and nuclear factors AP-1 and NFκB that are involved in the regulation of cell-specific CD44 expression. These findings provide new insight into the complex regulatory mechanism of CD44 expression, which may help identify more effective therapeutic targets against the breast cancer stem cells and metastatic tumors. 
 Necrotic cells influence migration and invasion of glioblastoma via NF-κB/AP-1-mediated IL-8 regulation. Glioblastoma multiforme (GBM) is the most common primary intracranial tumor in adults and has poor prognosis. Diffuse infiltration into normal brain parenchyma, rapid growth, and the presence of necrosis are remarkable hallmarks of GBM. However, the effect of necrotic cells on GBM growth and   metastasi  s is poorly understood at present. In this study, we examined the biological significance of necrotic tissues by exploring the molecular mechanisms underlying the signaling network between necrotic tissues and GBM cells. The migration and invasion of the GBM cell line CRT-MG was significantly enhanced by treatment with necrotic cells, as shown by assays for scratch wound healing and spheroid invasion. Incubation with necrotic cells induced IL-8 secretion in CRT-MG cells in a dose-dependent manner. In human GBM tissues, IL-8 positive cells were mainly distributed in the perinecrotic region, as seen in immunohistochemistry and immunofluorescence analysis. Necrotic cells induced NF-κB and AP-1 activation and their binding to the IL-8 promoter, leading to enhanced IL-8 production and secretion in GBM cells. Our data demonstrate that when GBM cells are exposed to and stimulated by necrotic cells, the migration and invasion of GBM cells are enhanced and facilitated via NF-κB/AP-1 mediated IL-8 upregulation. 
 Comprehensive Evaluation of Nuclear Factor-κΒ Expression Patterns in Non-Small Cell Lung Cancer. Nuclear factor (NF)-κB signalling is required for lung adenocarcinoma development in mice, and both of its subunits RelA and RelB were independently reported to be highly expressed in human non-small cell lung cancer (NSCLC). To comprehensively examine NF-κB expression in NSCLC, we analyzed serial sections of primary tumor samples from 77 well-documented patients (36 adenocarcinomas, 40 squamous cell carcinomas and 3 large cell carcinomas) for immunoreactivity of RelA, RelB, P50, and P52/P100. Tumor and intratumoral stroma areas were discriminated based on proliferating cell nuclear antigen immunoreactivity and inflammatory infiltration was assessed in intratumoral stroma areas. NF-κB immunoreactivity was quantified by intensity, extent, and nuclear localization and was cross-examined with tumor cell proliferation, inflammatory infiltration, and clinical-pathologic data. We found that the expression of the different NF-κB subunits was not concordant, warranting our integral approach. Overall, RelA, RelB, and P50 were expressed at higher levels compared with P52/P100. However, RelA and P50 were predominantly expressed in intratumoral stroma, but RelB in tumor cells. Importantly, tumor area RelA expression was correlated with the intensity of inflammatory infiltration, whereas RelB expression was identified in proliferating tumor cells. Using multiple logistic regression, we identified that tumor RelB expression was an independent predictor of lymph   node meta  stasis, and tumor P50 was an independent predictor of TNM6 stage IIB or higher, whereas tumor RelA was an independent predictor of inflammatory infiltration. We conclude that pathologic studies of NF-κB expression in cancer should include multiple pathway components. Utilizing such an approach, we identified intriguing associations between distinct NF-κB subunits and clinical and pathologic features of NSCLC. 
 Cell specific CD44 expression in breast cancer requires the interaction of AP-1 and NFκB with a novel cis-element. Breast cancers contain a heterogeneous population of cells with a small percentage that possess properties similar to those found in stem cells. One of the widely accepted markers of breast cancer stem cells (BCSCs) is the cell surface marker CD44. As a glycoprotein, CD44 is involved in many cellular processes including cell adhesion, migration and proliferation, making it pro-oncogenic by nature. CD44 expression is highly up-regulated in BCSCs, and has been implicated in tumorigenesis and   metastasi  s. However, the genetic mechanism that leads to a high level of CD44 expression in breast cancer cells and BCSCs is not well understood. Here, we identify a novel cis-element of the CD44 directs gene expression in breast cancer cells in a cell type specific manner. We have further identified key trans-acting factor binding sites and nuclear factors AP-1 and NFκB that are involved in the regulation of cell-specific CD44 expression. These findings provide new insight into the complex regulatory mechanism of CD44 expression, which may help identify more effective therapeutic targets against the breast cancer stem cells and metastatic tumors. 
 Differential effects of sulfated triterpene glycosides, holothurin A1, and 24-dehydroechinoside A, on antimetastasic activity via regulation of the MMP-9 signal pathway. Two sulfated triterpene glycosides, holothurin A(1) (HA(1)) and 24-dehydroechinoside A (DHEA), isolated from the sea cucumber Pearsonothuria graeffei, are of the holostane type with 18(20)-lactone and identical carbohydrate chains. DHEA has a side chain 23 (24)-double bond, while HA(1) has a hydroxyl group at C-21. In this study, we compared the effects of DHEA and HA(1) on   metastasis   in vitro and in vivo. The results show that HA(1) and DHEA treatment significantly suppressed adhesion of human hepatocellular liver carcinoma cells (HepG2) to both matrigel and human endothelial cells (ECV-304) and inhibited HepG2 cell migration and invasion in a dose-dependant manner. HA(1) and DHEA reduced tube formation of ECV-304 cells on the matrigel in vitro and attenuated neovascularization in the chick embryo using the chorioallantoic membrane (CAM) assay in vivo. Immunocytochemistry analyses revealed that both HA(1) and DHEA significantly decreased the expression of the matrix metallo-proteinase-9 (MMP-9) and increased the expression level of tissue inhibitor of metalloproteinase-1 (TIMP-1), an important regulator of MMP-9 activation. Western blot analyses demonstrated that HA(1) and DHEA remarkably abolished the expression of vascular endothelial growth factor (VEGF). The expression of nuclear factor-kappa B (NF-κB) was significantly decreased by HA(1), while DHEA treatment had no effect on the down regulation of NF-κB expression. These data suggest that both DHEA and HA(1) exert significant antimetastatic activities by inhibiting MMP-9 and VEGF expression. DHEA-induced an  timetastas  is was more potent than HA(1). In addition, only HA(1) treatment downregulated the expression level of NF-κB, suggesting that the antimetastatic activity of triterpene glycosides derived from P. graeffei can be either NF-κB-dependent or -independent, depending on their structure. 
 Transforming growth factor-beta1-mediated activation of NF-kappaB contributes to enhanced ADAM-12 expression in mammary carcinoma cells. A disintegrin and metalloproteinase-12 (ADAM-12), a member of multifunctional family of proteins, is upregulated in many cancers, including breast, lung, liver, prostate, gastric, and bladder. The multidomain structure, composed of a prodomain, a metalloproteinase, disintegrin-like, epidermal growth factor-like, cysteine-rich and transmembrane domains, and a cytoplasmic tail, allows ADAM-12 to promote matrix degradation, cell-cell adhesion, and intracellular signaling capacities and thereby to play a critical role in cancer growth and   metastasis  . Despite ample evidence linking increased ADAM-12 expression with cancer, the mechanisms controlling its upregulation are still unknown. In the present study, transforming growth factor-β1 (TGF-β1) is shown to increase ADAM-12 mRNA expression in MDA-MB-231 breast carcinoma cells. We have identified a promoter element responsible for TGF-β1-mediated ADAM-12 induction. We show interaction of NF-κB with ADAM-12 promoter and that high level of NF-κB activity in breast carcinoma cells results in the upregulation of ADAM-12 expression. Site-directed mutagenesis of the NF-κB element in ADAM-12 promoter and inhibition of NF-κB activity by Bay-11-7085 and MG-132 significantly reduced TGF-β1-mediated increase of ADAM-12 promoter-driven gene expression. Transfection of cells with a dominant-negative mutant form of IκBα (IκBαΔN), which inhibits activation of NF-κB, significantly reduced transcription from ADAM-12 promoter-reporter in TGF-β1-stimulated MDA-MB-231 cancer cells. In correlation, overexpression of NF-κB induced ADAM-12 expression in a dose-dependent manner. DNA-binding and ChIP assays indicated that p65 subunit of NF-κB binds to ADAM-12 promoter. Together, our study identified a cellular mechanism for induction of ADAM-12, which involves NF-κB and its activation by TGF-β1. 
 Dysregulation of the cell survival/anti-apoptotic NF-kappaB pathway by the novel humanized BM-ca anti-CD20 mAb: implication in chemosensitization. Treatment of patients with relapsed or refractory low grade follicular B-NHL lymphoma with rituximab (chimeric anti-CD20 mAb) has resulted in approximately 50% response rate. The mechanism underlying the failure of rituximab to affect the remaining 50% of the patients is not clear, though their tumors express CD20. The in vivo effector functions of rituximab include ADCC, CDC and seldom apoptosis. In addition, we have reported that rituximab signals the cells and inhibits several intracellular cell survival pathways that are responsible for the immuno and chemo-sensitizing effects of rituximab on resistant B-NHL cell lines. The objective of this study was to develop novel and fully humanized anti-CD20 monoclonal antibodies with enhanced effector functions and molecular signaling that may potentiate their therapeutic efficacy. Novel humanized anti-CD20 monoclonal antibodies were derived from a chimerized form of murine anti-CD20 1K11791, shown to exert a more potent ADCC, CDC and apoptotic activities compared to rituximab. A representative humanized monoclonal antibody, BM-ca was used to examine its biological effect and molecular signaling using Ramos B-NHL cell line as a model. The studies were also performed in parallel with rituximab treatment for comparison. Ramos cells were treated with various concentrations of BM-ca monoclonal antibody. Inhibition of cell proliferation was observed in a concentration-dependent manner, suggesting cell signal perturbations must have occurred. Compared to untreated cells, treatment with BM-ca inhibited both the constitutively activated NF-kappaB and p38 MAPK pathways, as assessed by inhibition of both phospho-p65 and phospho-IkappaBalpha and phospho-p38, respectively, but not the unphosphorylated forms. BM-ca significantly induced the expression of the   metastasis   suppressor and immune surveillance cancer gene product, Raf-1 kinase inhibitor protein (RKIP). These alterations resulted in inhibition of anti-apoptotic gene products and sensitized Ramos cells to apoptosis by CDDP. In comparison with rituximab, BM-ca showed qualitative and quantitative differences in the above analyses. These findings demonstrate that BM-ca triggers CD20 expressing B-NHL cells resulting in a significant alteration of several gene products that regulate cell growth and chemoresistance. 
 Stabilization of snail by NF-kappaB is required for inflammation-induced cell migration and invasion. The increased motility and invasiveness of tumor cells are reminiscent of epithelial-mesenchymal transition (EMT), which occurs during embryonic development, wound healing, and   metastasis  . In this study, we found that Snail is stabilized by the inflammatory cytokine TNFalpha through the activation of the NF-kappaB pathway. We demonstrated that NF-kappaB is required for the induction of COP9 signalosome 2 (CSN2), which, in turn, blocks the ubiquitination and degradation of Snail. Furthermore, we showed that the expression of Snail correlated with the activation of NF-kappaB in cancer cell lines and metastatic tumor samples. Knockdown of Snail expression inhibited cell migration and invasion induced by inflammatory cytokines and suppressed inflammation-mediated breast cancer   metastasis  . Our study provides a plausible mechanism for inflammation-induced   metastasis  . 
 Lysophosphatidic acid upregulates vascular endothelial growth factor-C and tube formation in human endothelial cells through LPA(1/3), COX-2, and NF-kappaB activation- and EGFR transactivation-dependent mechanisms. Lysophosphatidic acid (LPA) is a lipid bioactive mediator which binds to G-protein-coupled receptors and activates a variety of cellular functions. LPA modulates multiple behaviors in endothelial cells, including cell proliferation and migration, capillary-like tube formation in vitro, activation of proteases, interactions with leukocytes, and expressions of inflammation-related genes, thereby regulating vessel formation. LPA has been reported to modulate the angiogenesis process. However, the role of LPA in the lymphangiogenesis process has not been studied. In this study, we showed that LPA upregulated vascular endothelial growth factor-C (VEGF-C) mRNA expression in human umbilical vein endothelial cells (HUVECs) and subsequent endothelial cell tube formation in vitro and in vivo. These enhancement effects were LPA(1)- and LPA(3)-dependent and required cyclooxygenase-2 (COX-2), endothelial growth factor receptor (EGFR) transactivation and activation of nuclear factor kappaB (NF-kappaB). Moreover, LPA induced the protein expressions of the lymphatic markers, Prox-1, LYVE-1, and podoplanin, in HUVECs, and these enhancement effects were dependent on LPA(1) and LPA(3) activation and EGFR transactivation. Our results demonstrated that LPA might regulate VEGF-C and lymphatic marker expression in endothelial cells, which contributes to endothelial cell tube formation in vitro and in vivo, thus facilitating endothelial cell participation in the lymphangiogenesis process. This study clarifies the signaling mechanism of LPA-regulated VEGF-C expression and lymphatic marker expressions in endothelial cells, which suggest that LPA may be a suitable target for generating therapeutics against lymphangiogenesis and tumor   metastasis  . 
 EGF-R is expressed and AP-1 and NF-kappaB are activated in stromal myofibroblasts surrounding colon adenocarcinomas paralleling expression of COX-2 and VEGF. COX-2 and VEGF are important triggers of colon cancer growth,   metastasis   and angiogenesis. Cox-2 promoter contains transcriptional regulatory elements for AP-1 and NF-kappaB transcription factors whilst vegf is a known AP-1 downstream target gene. We investigated whether stromal myofibroblasts surrounding colon adenocarcinomas express COX-2 and VEGF and whether activation of AP-1 and NF-kappaB, as well as expression of EGF-R parallel expression of COX-2 and VEGF in these cells.Immunohistochemical methodology was performed on archival sections from 40 patients with colon adenocarcinomas. We evaluated c-FOS, p-c-JUN (phosphorylated c-JUN), p-IkappaB-alpha (phosphorylated IkappaB-alpha), EGF-R, COX-2, NF-kappaB and VEGF expression in stromal myofibroblasts surrounding colon adenocarcinomas. Double immunostaining with a-smooth muscle actin and each antibody was done to verify the expression of these molecules in stromal myofibroblasts.VEGF, p-IkappaB-alpha, NF-kappaB, c-FOS, p-c-JUN, EGF-R and COX-2 were expressed in stromal myofibroblasts surrounding colon adenocarcinomas in the majority of cases. EGF-R, p-IkappaB-alpha, NF-kappaB, c-FOS and p-c-JUN correlated positively with COX-2 and VEGF expression.Stromal myofibroblasts surrounding colon adenocarcinomas are an important source of VEGF and COX-2 production, while AP-1 and NF-kappaB transcription factors are activated and EGF-R is expressed in these cells and associated with COX-2 and VEGF production. 
 NF-κB signaling pathway confers neuroblastoma cells migration and invasion ability via the regulation of CXCR4. Accumulating evidence implicates the transcription factor NF-κB as a positive mediator of tumo  r metastas  is, but the molecular mechanism(s) involved in this process remains largely unknown. In this study, we investigated the role of NF-κB signaling pathway in the regulation of CXC chemokine receptor-4 (CXCR4) in neuroblasto  ma metasta  sis.NF-κB, CXCR4 mRNA and protein expression were measured by RT-PCR, and Western blot. Tumor necrosis factor-α (TNF-α) was used to induce the upregulation of NF-κB and CXCR4. The knockdown of NF-κB and CXCR4 was achieved by PDTC. Transwell assay was used to investigate the role of NF-κB (P65) in neuroblastoma cell migration and invasion. An in vitro co-culture system was established to investigate the role of tumor microenvironment in regulation of the NF-κB signaling pathway.Over-expression of NF-κB (p65) promoted tumor migration and invasion through the upregulation of CXCR4; however, knockdown of NF-κB(P65) inhibited tumor migration and invasion through blocking the expression of CXCR4. Consistently, in the co-culture system, the expression of CXCR4 was partly dependent on the expression of NF-κB (p65).Our studies reveal critical roles for the NF-κB signaling pathway in neuroblastoma migration and invasion. The mechanism may be through up-regulation of CXCR4, mediated by the NF-κB signaling pathways. Targeting NF-κB signalling pathways and ultimately CXCR4 could be a strategy in neuroblastoma therapy. 
 Independent and cooperative roles of tumor necrosis factor-alpha, nuclear factor-kappaB, and bone morphogenetic protein-2 in regulation of   metastasis   and osteomimicry of prostate cancer cells and differentiation and mineralization of MC3T3-E1 osteoblast-like cells. The molecular mechanisms involved in prostate cancer (PC)   metastasis   and bone remodeling are poorly understood. We recently reported that phosphoinositide 3-kinase (PI3K)/protein kinase B (Akt) mediates transcriptional regulation and activation of bone morphogenetic protein (BMP)-2 signaling by nuclear factor (NF)-kappaB in bone metastatic prostate cancer cells. In the present study, we demonstrate that NF-kappaB, whether activated by recombinant human tumor necrosis factor (TNF)-alpha or by ectopic expression of the p65 subunit, is involved in extracellular matrix adhesion and invasion of osteotropic PC-3 and C4-2B, but not LNCaP, cells. The enhanced metastatic potential was associated with transcriptional upregulation of osteopontin, osteocalcin, and collagen IA1 in osteotropic PC cells, suggesting their role in osteomimicry of PC cells. Unlike BMP-4, BMP-2 protein enhanced the invasive properties of C4-2B cells, but not in LNCaP cells. Also, this effect was nullified by Noggin. In addition, BMP-2 mediates TNF-alpha-induced invasion of C4-2B cells in a NF-kappaB-dependent fashion. TNF-alpha or conditioned media (CM) of TNF-alpha-stimulated C4-2B cells upregulated BMP-2 and BMP-dependent Smad transcripts and inhibited receptor activator of NF-kappaB ligand transcripts in RAW 264.7 preosteoclast cells, respectively, implying that this factor may contribute to suppression of osteoclastogenesis via direct and paracrine mechanisms. In contrast, CM of TNF-alpha-stimulate or BMP2-stimulated C4-2B cells induced in vitro mineralization of MC3T3-E1 osteoblast cells in a BMP-2-dependent and NF-kappaB-dependent manner, respectively. Taken together, the results suggest that mutual interactions between these factors may be pivotal not only in enhancing the osteomimicry and metastatic potential of PC cells, but also in bone remodeling and in shifting the balance from osteoclastogenesis towards osteoblastogenesis. 
 53BP1 functions as a tumor suppressor in breast cancer via the inhibition of NF-κB through miR-146a. p53-binding protein-1 (53BP1) plays a critical role in cell cycle checkpoint and DNA repair activities. Recently, 53BP1 was recognized as a potential tumor suppressor gene. In this study, we investigated its tumor suppressor function in breast cancer. In clinical samples, we observed a lower level of 53BP1 expression in the cancer lesions than in the matched non-tumor tissues. Furthermore, the 53BP1 level showed a gradual decrease during the progression from precancerous to cancer lesion. Ectopic expression of 53BP1 can significantly inhibit cell proliferation and curb the invasiveness in breast cancer cell lines, whereas knockdown of 53BP1 by RNA interference had the opposite effects. Additionally, 53BP1 markedly inhibited xenograft formation and   metastasi  s of breast cancer cells in nude mice. Both in vitro and in vivo studies revealed that the 53BP1 expression level was inversely correlated to the function of nuclear factor-kappaB (NF-κB), which contributes to the invasion an  d metastas  is of breast cancer. Importantly, the inhibitory effect of 53BP1 on NF-κB activity was shown to be mediated by the upregulation of miR-146a. Together, our findings demonstrated that 53BP1 has a potent tumor suppressor activity in breast cancer, and it may serve as a novel target for breast cancer prevention and treatment. 
 Resistance to irinotecan (CPT-11) activates epidermal growth factor receptor/nuclear factor kappa B and increases cellular   metastasis   and autophagy in LoVo colon cancer cells. Chemotherapy is usually applied to treat colon cancer but leads to chemoresistance, and increased   metastasis   and invasion. The main focus of this study is to observe effects of resistance to irinotecan (CPT-11) on   metastasis  , invasion and autophagy in CPT-11 resistant (CPT-11-R) LoVo colon cancer cells. CPT-11, a topoisomerase I inhibitor and a first-line chemotherapeutic drug, is used to treat colon cancer. CPT-11-R cells were constructed in a step-wise fashion with increasing CPT-11 doses. The CPT-11-R strain had a significantly lower expression of Wnt/β-catenin pathway, but induced an EGFR/IKKα/β/NF-κB pathway with elevated cell cyc  le, metast  asis and basal autophagy. 
 PF-3758309, p21-activated kinase 4 inhibitor, suppresses migration and invasion of A549 human lung cancer cells via regulation of CREB, NF-κB, and β-catenin signalings. Migration and invasion comprise key steps in cance  r metastas  is. Through the migration and invasion into and out of lymphatic and/or blood vessels, cancer cells can be spread out into the tissues in remote site from the origin. Degradation of extracellular matrix (ECM) must be preceded prior to th  e metastas  is of cancer cells. Matrix metalloproteinases (MMP) can degrade ECM, thus allow cells to migrate from the original site. Among MMPs, two gelatinase MMP-2 and MMP-9 play particularly important roles in ECM degradation. Here, we report that recently developed p21-activated kinase 4 inhibitor PF-3758309 shows anti-metastatic effect in A549 human lung cancer cell. PF-3758309 suppresses CREB, NF-κB, and β-catenin pathways, which are well known to be closely related with cell migration. This leads to the downregulation of MMP-2/MMP-9 expressions and the inhibition of A549 lung can  cer metast  asis. 
 Knockdown of Radixin Suppresses Gastric Cancer   Metastasis   In Vitro by Up-Regulation of E-Cadherin via NF-κB/Snail Pathway. Radixin has recently been shown to correlate with the   metastasi  s of gastric cancer, but the pathogenesis is elusive. Adhesion proteins contribute to the regulation of   metastasi  s, and thus this study sought to investigate the role of radixin in the migration, invasion and adhesion of gastric cancer cells, as well as its interaction with adhesion proteins in vitro.Radixin stable knockdown human gastric carcinoma SGC-7901 cells were constructed. Alterations in the migration, invasion and adhesion ability were examined by matrigel-coated plate and transwell assays. The expression pattern of adhesion proteins, including E-cadherin, β-catenin and claudin-1, was determined by quantitative real-time PCR and western blot. Possible involvement of NF-κB/snail pathway was also evaluated.Stable knockdown of radixin significantly suppressed migration and invasion, but enhanced adhesion in SGC-7901 cells. The expression of E-cadherin was manifestly increased in radixin knockdown cells, whereas the expression of β-catenin and claudin-1 was unchanged. The nuclear exclusion of NF-κB followed by conspicuous reduction of snail expression was involved in the regulation of E-cadherin expression.Radixin knockdown suppresses   the metas  tasis of SGC-7901 cells in vitro by up-regulation of E-cadherin. The NF-κB/snail pathway contributes to the regulation of E-cadherin in response to depletion of radixin. 
 HOXB13 downregulates intracellular zinc and increases NF-κB signaling to promote prostate cancer   metastasi  s. Characteristically, prostate cancer (PCa) cells exhibit marked decrease in intracellular zinc; however, the mechanism responsible is not clearly understood. HOXB13 is involved in PCa progression and is overexpressed in castration-resistant PCa. DNA microarray analysis of LNCaP Pca cells showed that ZnT zinc output transporters were strikingly upregulated among androgen-independent HOXB13 target genes. Furthermore, exogenous HOXB13 caused intracellular zinc concentrations to fall in PCa cells, stimulated NF-κB-mediated signaling by reducing inhibitor of NF-κB alpha (IκBα) and enhanced the nuclear translocation of RelA/p65. Human prostate tumors also exhibited strong inverse correlation between the protein expressions of HOXB13 and IκBα. Consequently, HOXB13 stimulated PCa cell invasion, and this was inhibited by the suppression of ZnT4. In addition, studies in a PC3 orthotopic mouse model   of PCa met  astasis showed that HOXB13 is a strong metastatic stimulator. Taken together, these results show that HOXB13 promotes PCa invasi  on and met  astasis by decreasing intracellular zinc levels, thus stimulating NF-κB signals, and suggest that HOXB13 acts as a modulator of intracellular zinc levels that promotes the malignant characteristics of PCa. 
 Overexpression of RRM2 in gastric cancer cell promotes their invasiveness via AKT/NF-κB signaling pathway. The ribonucleotide reductase M2 subunit (RRM2) plays an active role in tumor progression and is frequently overexpressed in cancer. It plays a significant role in the regulation of cell invasiveness, cell migration and tumor   metastasi  s. Elevated RRM2 expression has been reported to be associated with poor prognosis of gastric cancer. However, the molecular mechanisms of RRM2 in gastric cancer cells remain elusive. In our study, we found that RRM2 highly expressed in gastric cancer cells BGC823. RRM2 stimulation dose-dependently enhanced the invasion and migration of BGC823 cells. Furthermore, we found that the expressions of MMP-2 and MMP-9 in BGC823 cells were significantly increased after RRM2 stimulation. In addition, RRM2 time-dependently induced activation of AKT, IKBα, and NF-κB. These effects of RRM2 were prevented by AKT selective inhibitor GSK690693 as well as NF-κB selective inhibitor BAY117082. In conclusion, our findings establish a signaling role for RRM2 in gastric cancer cells and identify that the RRM2/AKT/NF-κB signaling pathway is essential for tumor invasiveness in gastric cancer cells. Thus, our data may provide knowledge for using RRM2 as a novel target for effective diagnosis and treatment of gastric cancer. 
 Hypoxia increases CX3CR1 expression via HIF-1 and NF‑κB in androgen-independent prostate cancer cells. The unique CX3C chemokine CX3CL1 and its cognate receptor CX3CR1 have been implicated in organ-specif  ic metasta  sis of various types of tumors. Hypoxia, a common phenomenon in solid tumors, is associated with a malignant cancer phenotype. Previous studies have proved that hypoxia facilitates cancer ce  ll metasta  sis through upregulation of specific chemokine receptors. We hypothesized that hypoxia could upregulate CX3CR1 expression and lead to an increased chemotactic response to CX3CL1 in prostate cancer cells. In the present study, we found that CX3CR1 expression was significantly increased in androgen-independent prostate cancer cells, including DU145, PC-3 and PC-3M, following exposure to hypoxia. This upregulation of CX3CR1 corresponded to a significant increase in migration and invasion of prostate cancer cells under hypoxic conditions, which was attenuated after knocking down CX3CR1 expression. In addition, we examined the possible role of HIF-1 and NF-κB in the process of hypoxia-induced CX3CR1 expression and hypoxia-media  ted metast  asis. Attenuation of HIF-1 and NF-κB transcriptional activity by siRNAs or pharmacological inhibitors, abrogated hypoxia-induced upregulation of CX3CR1, and also prevented the migration and invasion of DU145 cells under a hypoxic environment. In summary, our study demonstrated that HIF-1 and NF-κB are essential for hypoxia-regulated CX3CR1 expression, which is associated with increased migratory and invasive potential of prostate cancer cells. CX3CR1 signaling is a potential therapeutic target in the adjuvant treatment of prostate cancer. 
 Elevated expression patterns and tight correlation of the PLCE1 and NF-κB signaling in Kazakh patients with esophageal carcinoma. This study investigated the expression of the phospholipase C epsilon 1 (PLCE1) and nuclear factor-kappaB (NF-κB)-related proteins in Kazakh patients with esophageal squamous cell carcinoma (ESCC). Tissue microarrays of 90 ethnic Kazakh patients with ESCC and exhibiting clinical characteristics were analyzed for protein expression of PLCE1, IKKβ, IKBα, p50, and p65 by immunohistochemistry. Correlations between histoscores of PLCE1 and NF-κB-related proteins were determined using Spearman's rank correlation tests. Expression of PLCE1 and NF-κB-related proteins significantly increased in tumor tissues compared with normal esophageal tissues (P = 9.48 × 10(-7), 1.24 × 10(-5), 0.004, 0.003, and 2.83 × 10(-5), respectively). Upregulation of PLCE1 was significantly correlated with advanced tu  mor-node-m  etastasis stages (P = 0.018) and ly  mph node m  etastasis (P = 0.003). Overexpression of IKKβ and IKBα was associated with ESCC stages I/II (P = 3.36 × 10(-4) and 0.022, respectively). Increased expression of p50 was significantly higher in patients with   lymph nod  e metastasis than without   lymph nod  e metastasis (P = 0.048). Elevated expression of p65 protein was significantly correlated with poor and moderately differentiated ESCC and depth of tumor invasion (P = 0.026 and 0.010, respectively). Significant positive correlations were observed between the expression of PLCE1 and NF-κB-related proteins, especially IKKβ (r = 0.246 and P = 0.025) and p50 (r = 0.244 and P = 0.024). These results suggest, for the first time, that upregulation of PLCE1 is correlated with increased expression of NF-κB-related proteins in Kazakh patients with ESCC, suggesting that interaction between PLCE1 with the NF-κB signal pathway may be responsible for the carcinogenesis of ESCC, such as ESCC-related inflammation. 
 Prognostic significance of XIAP and NF-κB expression in esophageal carcinoma with postoperative radiotherapy. X-chromosome-linked IAP (XIAP) and nuclear factor-κB (NF-κB) are frequently overexpressed and correlate closely with chemoradiotherapy resistance and poor prognosis in many cancers. However, the significance of XIAP and NF-κB expression in radiotherapy sensitivity and its effect on the prognosis of esophageal squamous cell carcinoma (ESCC) are still unknown. The aim of this study was to examine XIAP and NF-κB status in ESCC patients undergoing postoperative radiotherapy after radical surgery, and to evaluate their clinical significance.A total of 78 ESCC patients treated with postoperative radiotherapy after radical surgery were enrolled in this study. We immunohistochemically investigated the expression of XIAP and NF-κB in tissues from enrolled patients with specific antibodies. Then, the correlations among XIAP, NF-κB expression, clinicopathological features and its prognostic relevance in ESCC were analyzed.The increased expression of XIAP and NF-κB in ESCC tissues were clearly correlated with the tumor differentiation and p-TNM stage. Significant positive correlations were found between the expression status of XIAP and NF-κB (r = 0.779, P = 0.000). Overexpression of XI  AP and NF-  κB and metastasis were significantly associated with shorter overall survival times in univariate analysis (P < 0.05). Multivariate analysis also confirmed that XIAP expression was an independent prognostic factor (P = 0.005).XIAP and NF-κB are intensively expressed in ESCC. The level of XIAP is positively correlated to progression and prognosis of ESCC. 
 NF-κB regulates mesenchymal transition for the induction of non-small cell lung cancer initiating cells. The epithelial-to-mesenchymal transition (EMT) is a de-differentiation process that has been implicated in   metastasi  s and the generation of cancer initiating cells (CICs) in solid tumors. To examine EMT in non-small cell lung cancer (NSCLC), we utilized a three dimensional (3D) cell culture system in which cells were co-stimulated with tumor necrosis factor alpha (TNF) and transforming growth factor beta (TGFβ). NSCLC spheroid cultures display elevated expression of EMT master-switch transcription factors, TWIST1, SNAI1/Snail1, SNAI2/Slug and ZEB2/Sip1, and are highly invasive. Mesenchymal NSCLC cultures show CIC characteristics, displaying elevated expression of transcription factors KLF4, SOX2, POU5F1/Oct4, MYCN, and KIT. As a result, these putative CIC display a cancer "stem-like" phenotype by forming lung metastases under limiting cell dilution. The pleiotropic transcription factor, NF-κB, has been implicated in EMT a  nd metasta  sis. Thus, we set out to develop a NSCLC model to further characterize the role of NF-κB activation in the development of CICs. Here, we demonstrate that induction of EMT in 3D cultures results in constitutive NF-κB activity. Furthermore, inhibition of NF-κB resulted in the loss of TWIST1, SNAI2, and ZEB2 induction, and a failure of cells to invade and metastasize. Our work indicates that NF-κB is required for   NSCLC met  astasis, in part, by transcriptionally upregulating master-switch transcription factors required for EMT. 
 Loss of BRMS1 promotes a mesenchymal phenotype through NF-κB-dependent regulation of Twist1. Breast cancer   metastasi  s suppressor 1 (BRMS1) is downregulated in non-small cell lung cancer (NSCLC), and its reduction correlates with disease progression. Herein, we investigate the mechanisms through which loss of the BRMS1 gene contributes to epithelial-to-mesenchymal transition (EMT). Using a short hairpin RNA (shRNA) system, we show that loss of BRMS1 promotes basal and transforming growth factor beta-induced EMT in NSCLC cells. NSCLC cells expressing BRMS1 shRNAs (BRMS1 knockdown [BRMS1(KD)]) display mesenchymal characteristics, including enhanced cell migration and differential regulation of the EMT markers. Mesenchymal phenotypes observed in BRMS1(KD) cells are dependent on RelA/p65, the transcriptionally active subunit of nuclear factor kappa B (NF-κB). In addition, chromatin immunoprecipitation analysis demonstrates that loss of BRMS1 increases Twist1 promoter occupancy of RelA/p65 K310-a key histone modification associated with increased transcription. Knockdown of Twist1 results in reversal of BRMS1(KD)-mediated EMT phenotypic changes. Moreover, in our animal model, BRMS1(KD)/Twist1(KD) double knockdown cells were less efficient in establishing lung tumors than BRMS1(KD) cells. Collectively, this study demonstrates that loss of BRMS1 promotes malignant phenotypes that are dependent on NF-κB-dependent regulation of Twist1. These observations offer fresh insight into the mechanisms through which BRMS1 regulates the development of metastases in NSCLC. 
 The relationships between snail1 and estrogen receptor signaling in breast cancer cells. The loss of hormonal dependency of breast tumor cells is often accompanied with the appearance of epithelial-mesenchymal transition (EMT) features and increase in cell   metastasis   and invasiveness. The central role in the EMT belongs to transcription factors Snail responded for the decrease in E-cadherin expression and cell contacts, stimulation of cell mobility and invasiveness. Aim was to study the relationships between estrogen receptor machinery and Snail1 signaling, and mechanism of Snail1 regulation in hormone-resistant breast cancer cells. The experiments were performed on the estrogen-dependent MCF-7 breast cancer cells, estrogen-hyposensitive MCF-7/LS subline generated through long-term cultivation of the parental cells in steroid-free medium, and ER-negative estrogen-resistant HBL-100 cells. Snail1, estrogen receptor, p65 NF-κB, E-cadherin levels were analyzed by Western blot. We found that decrease in the estrogen dependency is correlated with increase in Snail1 expression and activity, we demonstrated the Snail1 involvement in the negative regulation of ER, and showed that Snail1 inhibition partially restores the sensitivity of the estrogen-hyposensitive cells to antiestrogen tamoxifen. Furthermore, NF-κB was found to serve as a positive regulator of Snail1 in breast cancer cells, and simultaneous inhibition of NF-κB and Snail1 resulted in additional increase in cell response to tamoxifen. In general, the results obtained demonstrate the phenomenon of Snail1 activation in the hormone-resistant breast cancer cells, and show that Snail1 and NF-κB may serve as an important targets in the treatment of breast cancer, both estrogen-dependent and estrogen-independent tumors. 
 NSCLC and the alternative pathway of NF-κB: uncovering an unknown relation. Lung cancer is the leading cause of cancer-related deaths worldwide. Although our knowledge on the pathobiology of the disease has increased in the last decades, the prognosis of lung cancer patients has hardly changed. Many signaling pathways are implicated in lung carcinogenesis, but the role of the alternative pathway of nuclear factor kappa-light-chain-enhancer of activated B cells (NF-κB) in lung cancer pathogenesis and progression has not been investigated. The aim of our study was to investigate the role of this pathway in non-small cell lung cancer (NSCLC) patients. NF-κB2 and RelB protein expression was retrospectively assessed by immunohistochemistry in tissue samples from 109 NSCLC patients. RelB and NF-κB2 protein levels differed between tumors and adjacent nonneoplastic lung parenchyma. Cytoplasmic immunoreactivity of NF-κB2 and RelB was correlated with tumor stage (p = 0.03 and p = 0.016, respectively). In addition, cytoplasmic NF-κB2 levels were related to tumor grade (p = 0.046). Expression of RelB in the cytoplasm was tumor histologic type-specific, with squamous cell carcinomas having the highest protein levels. Nuclear expression of RelB and NF-κB2 differed between tumor and nonneoplastic tissues, possibly indicating activation of the alternative pathway of NF-κB in cancer cells. Moreover, lym  ph node me  tastasis was related to nuclear NF-κB2 expression in tumor cells. The deregulation of the alternative NF-κB pathway in NSCLC could play a role in the development and progression of the disease. 
 Curcumin Inhibits Non-Small Cell Lung Cancer Cells   Metastasis   through the Adiponectin/NF-κb/MMPs Signaling Pathway. Adipose tissue is now considered as an endocrine organ involved in metabolic and inflammatory reactions. Adiponectin, a 244-amino acid peptide hormone, is associated with insulin resistance and carcinogenesis. Curcumin (diferuloylmethane) is the principal curcuminoid of the popular Indian spice, turmeric. Curcumin possesses antitumor effects, including the inhibition of neovascularization and regulation of cell cycle and apoptosis. However, the effects of adiponectin and curcumin on non-small cell lung cancer (NSCLC) remain unclear. In this study, we evaluated the expression of adiponectin in paired tumors and normal lung tissues from 77 patients with NSCLC using real-time polymerase chain reaction, western blotting, and immunohistochemistry. Kaplan-Meier survival analysis showed that patients with low adiponectin expression ratio (<1) had significantly longer survival time than those with high expression ratio (>1) (p = 0.015). Curcumin inhibited the migratory and invasive ability of A549 cells via the inhibition of adiponectin expression by blocking the adiponectin receptor 1. Curcumin treatment also inhibited the in vivo tumor growth of A549 cells and adiponectin expression. These results suggest that adiponectin can be a prognostic indicator of NSCLC. The effect of curcumin in decreasing the migratory and invasive ability of A549 cells by inhibiting adiponectin expression is probably mediated through NF-κB/MMP pathways. Curcumin could be an important potential adjuvant therapeutic agent for lung cancer in the future. 
 Expression of NF-kappaB in prostate cancer lymph node metastases. Nuclear factor-kappaB (NF-kappaB) is a transcription factor that transactivates genes involved in the regulation of cell growth, apoptosis, angiogenesis, and   metastasis  . Our aim was to assess NF-kappaB expression in lymph node (LN) metastases of prostate cancer.Immunohistochemical staining was performed using the p65 anti-NF-kappaB antibody. Seventy-seven paraffin-embedded LN specimens obtained from 54 prostate cancer patients were analyzed. Of the 54 patients, 32 had positive LN metastases, while 22 showed no evidence of   metastasis   and were considered as controls. The overall percentage of NF-kappaB-nuclear localization was assessed, as well as the intensity of staining.Nuclear localization of NF-kappaB was significantly greater in the metastatic LN group compared to controls. In patients with positive-LN metastases, 84.4% showed >10% nuclear staining in tumor cells. Moreover, 64.4% of the malignant LN specimens had >10% nuclear staining in lymphocytes compared to 0% in controls. Intensity of cytoplasmic and nuclear staining was higher in the metastatic LN group than in controls (P < 0.01).Nuclear localization/activation of NF-kappaB is up-regulated in prostate cancer LN   metastasis  . Such up-regulation of NF-kappaB activity is observed in the tumor cells as well as in the surrounding lymphocytes. 
 Expression of vascular endothelial growth factor and transcription factors HIF-1, NF-kB expression in squamous cell carcinoma of head and neck; association with proteasome and calpain activities. The transcription factors NF-kB, HIF-1 and vascular endothelial growth factors (VEGF) are known to play an important role in pathogenesis of squamous cell carcinoma of head and neck (SCCHN).The aim of the study was to determine the NF-kB, HIF-1 and VEGF, expression their characteristics in squamous cell carcinoma of head and neck.Transcription factors and VEGF expression were measured by ELISA kits. Proteasome and calpain activity were determined using specific fluorogenic substrate. Proteasome subunits composition was measured by Western blot analysis.In the present study, we revealed the connection between SCCHN lymphogenous   metastasis   development and NF-kB p50 expression. An increase in total, 26S and 20S proteasome activities and calpain activity was observed in cancer tissues in comparison with agreed standard (non-transformed tissue). The dynamics of changes in proteasome activity and proteasome subunits content during lymph nodes   metastasis   development had a complex pattern. Nonparametric analysis of variance showed the connection between the extent of metastatic affection of regional lymph nodes, total proteasome activity and LMP2 expression. Proteasome and calpain systems corresponded and interacted with each other. We also revealed a positive correlation between the NF-kB p65 and p50 expression and proteasome activity.Taken together, our results suggest that above mentioned transcription factors and intracellular proteolytic systems are involved in SCCHN progression and   metastasis  . Moreover, the opportunity of transcription factors regulation by proteasome takes place in oncogenesis of SCCHN. The results provide a basis for new prognostic tests and development of novel targeted therapy. 
 Dexamethasone protection from TNF-alpha-induced cell death in MCF-7 cells requires NF-kappaB and is independent from AKT. The biochemical bases for hormone dependence in breast cancer have been recognized as an important element in tumor resistance, proliferation and   metastasis  . On this respect, dexamethasone (Dex) dependent protection against TNF-alpha-mediated cell death in the MCF-7 cell line has been demonstrated to be a useful model for the study of this type of cancer. Recently, cytoplasmic signaling induced by steroid receptors has been described, such as the activation of the PI3K/Akt and NF-kappaB pathways. We evaluated their possible participation in the Dex-dependent protection against TNF-alpha-mediated cell death.Cellular cultures of the MCF-7 cell line were exposed to either, TNF-alpha or TNF-alpha and Dex, and cell viability was evaluated. Next, negative dominants of PI3K and IkappaB-alpha, designed to block the PI3K/Akt and NF-kappaB pathways, respectively, were transfected and selection and evaluation of several clones overexpressing the mutants were examined. Also, correlation with inhibitor of apoptosis proteins (IAPs) expression was examined. Independent inhibition of these two pathways allowed us to test their participation in Dex-dependent protection against TNF-alpha-cytotoxicity in MCF-7 cells. Expression of the PI3K dominant negative mutant did not alter the protection conferred by Dex against TNF-alpha mediated cell death. Contrariwise, clones expressing the IkappaB-alpha dominant negative mutant lost the Dex-conferred protection against TNF-alpha. In these clones degradation of c-IAP was accelerated, while that of XIAP was remained unaffected.NF-kappaB, but not PI3K/Akt activation, is required for the Dex protective effect against TNF-alpha-mediated cell death, and correlates with lack of degradation of the anti-apoptotic protein c-IAP1. 
 Laryngeal squamous cell carcinoma progression is associated with the NFκB signaling pathway regulated by IκB kinase β. We aimed to analyze the association of NFκB signaling pathway with the carcinogenesis of laryngeal squamous cell carcinoma (LSCC).Protein array was used to identify the differentially expressed proteins involved in NFκB signaling pathway between LSCC cells and normal throat mucosa. Correlation analysis between significantly expressed proteins and clinical characteristics (differentiation, clinical stage, and   node metas  tasis) was performed. The expression of IκB kinase-β (IKK-β) in Hep-2 cells transfected with IKK-β-siRNA or pcDNA3.1-IKK-β was detected both by real-time polymerase chain reaction and western blot. Besides, a tetrazolium-based colorimetric assay (MTT), flow cytometer, and transwell assay were used to examine the proliferation, apoptosis, and migration rate of Hep-2 cells, respectively.Three differentially expressed proteins were identified. Among them, tumor necrosis factor receptor 1 (TNFR1) and IKK-β were significantly up-regulated (p < 0.01) and Fas-associated via death domain (FADD) was significantly down-regulated (p < 0.01). The correlation analysis showed that IKK-β expression had a significant association with differentiation, clinical stag  e, and nod  e metastasis (p < 0.05). Besides, high expressed IKK-β resulted in significantly increased proliferation and migration rate (p < 0.05). Reversely, Hep-2 cells transfected with IKK-β-siRNA showed significantly lower proliferation and migration rate (p < 0.05) and significantly higher apoptosis rate (p < 0.05) than normal cells.The NFκB signaling pathway involved TNFR1, IKK-β, and FADD is significantly associated with the development of LSCC. Over-expressed IKK-β efficiently inhibits cell apoptosis and has positive effects on cell proliferation and migration. 
 Effects of NFKB1 and NFKBIA gene polymorphisms on susceptibility to environmental factors and the clinicopathologic development of oral cancer. Oral cancer, which is the fourth most common cancer in Taiwanese men, is associated with environmental carcinogens. The possibility that genetic predisposition in nuclear factor-kappa B (NF-κB)-signaling pathways activation is linked to the development of oral squamous cell carcinoma (OSCC) requires investigation. The current study examines associations between polymorphisms within promoter regions of NFKB1 encoding NF-κB1 and NFKBIA encoding IkappaBalpha (IκBα) with both the susceptibility to develop OSCC and the clinicopathological characteristics of the tumors.Genetic polymorphisms of NFKB1 and NFKBIA were analyzed by a real-time polymerase chain reaction (real-time PCR) for 462 patients with oral cancer and 520 non-cancer controls. We found that NFKB1 -94 ATGG1/ATGG2, -94 ATGG2/ATGG2, and the combination of -94 ATGG1/ATGG2 and ATGG2/ATGG2 genotypes NFKBIA -826 T (CT+TT) and -881 G (AG+GG) allelic carriages, were more prevalent in OSCC patients than in non-cancer participants. Moreover, we found that NFKB1 or NFKBIA gene polymorphisms seem to be related to susceptibility to develop oral cancer linked to betel nut and tobacco consumption. Finally, patients with oral cancer who had at least one -519 T allele of the NFKBIA gene were at higher risk for developing dist  ant metast  asis (P<.05), compared with those patients CC homozygotes.Our results suggest that NFKB1 -94 ATTG2, NFKBIA -826 T, and -881 G alleles are associated with oral carcinogenesis. The combination of NFKB1 or NFKBIA gene polymorphisms and environmental carcinogens appears related to an increased risk of oral cancer. More importantly, the genetic polymorphism of NFKBIA -519 might be a predictive factor for the dis  tal metast  asis of OSCC in Taiwanese. 
 Pathobiological implications of the expression of EGFR, pAkt, NF-κB and MIC-1 in prostate cancer stem cells and their progenies. The progression of prostate cancers (PCs) to locally invasive, androgen-independent and metastatic disease states is generally associated with treatment resistance and disease relapse. The present study was undertaken to establish the possibility of using a combination of specific oncogenic products, including epidermal growth factor receptor (EGFR), pAkt, nuclear factor-kappaB (NF-κB) and macrophage inhibitory cytokine-1 (MIC-1) as biomarkers and therapeutic targets for optimizing the management of patients with localized PC at earlier disease stages. The immunohistochemical and immunofluorescence data have revealed that the expression levels of EGFR, Ser(473)-pAkt, NF-κB p65 and MIC-1 proteins were significantly enhanced in the same subset of 76 cases of prostatic adenocarcinoma specimens during the disease progression and these biomarkers were expressed in a small subpopulation of CD133(+) PC cells and the bulk tumor mass of CD133(-) PC cells. Importantly, all of these biomarkers were also overexpressed in 80-100% of 30   PC metasta  sis bone tissue specimens. Moreover, the results have indicated that the EGF-EGFR signaling pathway can provide critical functions for the self-renewal of side population (SP) cells endowed with stem cell-like features from highly invasive WPE1-NB26 cells. Of therapeutic interest, the targeting of EGFR, pAkt, NF-κB or MIC-1 was also effective at suppressing the basal and EGF-promoted prostasphere formation by SP WPE1-NB26 cells, inducing disintegration of SP cell-derived prostaspheres and decreasing the viability of SP and non-SP WPE1-NB26 cell fractions. Also, the targeting of these oncogenic products induced the caspase-dependent apoptosis in chemoresistant SP WPE1-NB26 cells and enhanced their sensibility to the cytotoxic effects induced by docetaxel. These findings suggest that the combined use of EGFR, pAkt, NF-κB and/or MIC-1 may represent promising strategies for improving the accuracy of current diagnostic and prognostic methods and efficacy of treatments of PC patients in considering the disease heterogeneity, thereby preventing PC progression to metastatic and lethal disease states. 
 Hepatitis B virus X protein upregulates expression of calpain small subunit 1 via nuclear factor-kappaB/p65 in hepatoma cells. Hepatitis B virus (HBV) infection is closely correlated with the development of hepatocellular carcinoma (HCC), in which hepatitis B virus X protein (HBx) plays crucial roles. HBx is believed to be a multifunctional oncoprotein. It has been reported that the calpain small subunit 1 (Capn4) is upregulated in the HCC tissues and involved in the   metastasis   of HCC. Therefore, we suppose that HBx may promote hepatoma cell migration through Capn4. In the present study, we investigated the effect of HBx on regulating Capn4 expression in human HCC cells. Our data showed that HBx could increase promoter activity of Capn4 and upregulate the expression of Capn4 at the levels of mRNA and protein in human hepatoma HepG2 (or H7402) cells using luciferase reporter gene assay, real-time quantitative RT-PCR assay and Western blot analysis. While, the RNA interference targeting HBx mRNA was able to abolish the upregulation. Interestingly, we found that the inhibition of nuclear factor-kappaB (NF-kappaB) mediated by siRNA targeting NF-kappaB/p65 mRNA or PDTC (an inhibitor of NF-kappaB) could attenuate the upregulation of Capn4. While, HBx failed to increase the promoter activity of Capn4 in hepatoma cells when the putative NF-kappaB binding site of the Capn4 promoter was mutant, suggesting that NF-kappaB is involved in the activation of Capn4 mediated by HBx. In function, wound healing assay showed that HBx could significantly enhance the migration ability of HepG2 cells through upregulating Capn4. Thus, we conclude that HBx upregulate Capn4 through NF-kappaB/p65 to promote migration of hepatoma cells. 
 Transcriptional regulation of the endogenous danger signal tenascin-C: a novel autocrine loop in inflammation. Inappropriate expression of proinflammatory mediators underpins the pathogenesis of autoimmune disease and tumor   metastasis  . The extracellular matrix glycoprotein tenascin-C is an endogenous activator of innate immunity that promotes the synthesis of inflammatory cytokines via activation of TLR4. Little tenascin-C is observed in most healthy adult tissues, but expression is specifically upregulated at sites of inflammation. Moreover, high levels of tenascin-C are associated with chronic inflammation and found in the tumor stroma. In this study, we show that the expression of tenascin-C is induced in immune myeloid cells activated by a variety of inflammatory stimuli, including specific TLR ligands. Its synthesis is transcriptionally regulated and requires the specific activation of AKT/PI3K and NF-kappaB signaling pathways. Using a bioinformatic approach, we identified a large number of conserved noncoding regions throughout the tenascin-C genomic locus that may contribute to its transcriptional regulation during inflammation. We also demonstrate that tenascin-C expression is transient during acute inflammation. In contrast, persistently high levels of expression occur in the inflamed synovium of joints from rheumatoid arthritis patients. Thus, misregulated expression of this endogenous danger signal may promote an autocrine loop of inflammation and contribute to the persistence of inflammation in autoimmune diseases or to tumor egress and invasion during   metastasis  . 
 Constitutive activation of nuclear factor-kappaB is preferentially involved in the proliferation of basal-like subtype breast cancer cell lines. Constitutive nuclear factor (NF)-kappaB activation is thought to be involved in survival, invasion, and   metastasis   in various types of cancers. However, neither the subtypes of breast cancer cells with constitutive NF-kappaB activation nor the molecular mechanisms leading to its constitutive activation have been clearly defined. Here, we quantitatively analyzed basal NF-kappaB activity in 35 human breast cancer cell lines and found that most of the cell lines with high constitutive NF-kappaB activation were categorized in the estrogen receptor negative, progesterone receptor negative, ERBB2 negative basal-like subtype, which is the most malignant form of breast cancer. Inhibition of constitutive NF-kappaB activation by expression of IkappaBalpha super-repressor reduced proliferation of the basal-like subtype cell lines. Expression levels of mRNA encoding NF-kappaB-inducing kinase (NIK) were elevated in several breast cancer cell lines, and RNA interference-mediated knockdown of NIK reduced NF-kappaB activation in a subset of the basal-like subtype cell lines with upregulated NIK expression. Taken together, these results suggest that constitutive NF-kappaB activation, partially dependent on NIK, is preferentially involved in proliferation of basal-like subtype breast cancer cells and may be a useful therapeutic target for this subtype of cancer. 
 Zerumbone inhibits angiogenesis by blocking NF-κB activity in pancreatic cancer. Because angiogenesis is essential for tumor growth and   metastasi  s, the development of antiangiogenic agents is an urgent issue in cancer treatment. Zerumbone, a component of subtropical ginger, has been shown to exhibit anticancer activities in various cancer cells; however, little is known about its biological mechanisms against angiogenesis in pancreatic cancer (PaCa). Here, we evaluated the effects of zerumbone on PaCa angiogenesis.The cytotoxicity of zerumbone in PaCa was measured using premix WST-1 cell proliferation assays. The influence of zerumbone on the angiogenic factors vascular endothelial growth factor and interleukin 8 was measured using the reverse transcription-polymerase chain reaction and enzyme-linked immunosorbent assays. Changes in nuclear factor-κB (NF-κB) activities were measured using NF-κB transcription factor assays. We also examined the effects of zerumbone on PaCa-induced angiogenesis using angiogenesis assays.Zerumbone inhibited mRNA expression and protein secretion of angiogenic factors and NF-κB activity. Tube formation in human umbilical vein endothelial cells was enhanced by coculture with PaCa cells, and these enhancements were significantly inhibited by zerumbone treatment.Zerumbone blocked the PaCa-associated angiogenesis through the inhibition of NF-κB and NF-κB-dependent proangiogenic gene products. 
 Suppression of LASP-1 attenuates the carcinogenesis of prostatic cancer cell lines: Key role of the NF-κB pathway. Prostate cancer (PCa) is one of the most frequently diagnosed cancers among males worldwide and causes a considerable number of deaths each year. One of the newly explored targets for the development of therapies against PCa is LIM and SH3 protein 1 (LASP-1). In the present study, the function of LASP-1 in the oncogenesis a  nd metasta  sis of PCa was investigated using a series of in vitro experiments. Moreover, the mechanism through which LASP-1 exerted its effect on the carcinogenesis of PCa was also explored. The expression levels of LASP-1 in clinical PCa specimens were determined both at the mRNA and protein levels. Afterwards, the activity of LASP-1 in human PCa cell lines PC3 and DU145 was inhibited using a short hairpin RNA (shRNA) interfering method. The effects of LASP-1 knockdown on the cell growth, apoptosis, cell cycle distribution, migration and invasion were assessed. It was demonstrated that the expression of LASP-1 was significantly higher in the clinical PCa tissues than the level in the corresponding para-carcinoma tissues. Following the knockdown of the LASP-1 gene in human PCa cell lines, the viability, migration and invasion of the cancer cells were decreased. It was also demonstrated that the change in the cell viability and motile ability were associated with an induction of cell apoptosis and G1 phase cell cycle arrest. Based on the results of the detection of the expression of NF-κB-related factors, it was indicated that LASP-1 may affect the carcinogenesis of PCa through a NF-κB inhibition-dependent manner. Although the detailed explanation of the mechanism of LASP-1 in the carcinogenesis of PCa requires further elucidation, the present study highlights the potential of LASP-1 as a promising therapeutic target to ameliorate the oncogenes  is and met  astasis of PCa. 
 Activation of NF-kappa B signaling promotes growth of prostate cancer cells in bone. Patients with advanced prostate cancer almost invariably develop osseous   metastasis  . Although many studies indicate that the activation of NF-κB signaling appears to be correlated with advanced cancer and promotes tumor   metastasi  s by influencing tumor cell migration and angiogenesis, the influence of altered NF-κB signaling in prostate cancer cells within boney metastatic lesions is not clearly understood. While C4-2B and PC3 prostate cancer cells grow well in the bone, LNCaP cells are difficult to grow in murine bone following intraskeletal injection. Our studies show that when compared to LNCaP, NF-κB activity is significantly higher in C4-2B and PC3, and that the activation of NF-κB signaling in prostate cancer cells resulted in the increased expression of the osteoclast inducing genes PTHrP and RANKL. Further, conditioned medium derived from NF-κB activated LNCaP cells induce osteoclast differentiation. In addition, inactivation of NF-κB signaling in prostate cancer cells inhibited tumor formation in the bone, both in the osteolytic PC3 and osteoblastic/osteoclastic mixed C4-2B cells; while the activation of NF-κB signaling in LNCaP cells promoted tumor establishment and proliferation in the bone. The activation of NF-κB in LNCaP cells resulted in the formation of an osteoblastic/osteoclastic mixed tumor with increased osteoclasts surrounding the new formed bone, similar to metastases commonly seen in patients with prostate cancer. These results indicate that osteoclastic reaction is required even in the osteoblastic cancer cells and the activation of NF-κB signaling in prostate cancer cells increases osteoclastogenesis by up-regulating osteoclastogenic genes, thereby contributing to bone metastatic formation. 
 The fibroblast growth factor-inducible 14 receptor is highly expressed in HER2-positive breast tumors and regulates breast cancer cell invasive capacity. Genomic characterization is beginning to define a molecular taxonomy for breast cancer; however, the molecular basis of invasion and   metastasis   remains poorly understood. We report a pivotal role for the fibroblast growth factor-inducible 14 (Fn14) receptor in this process. We examined whether Fn14 and its ligand tumor necrosis factor-like weak inducer of apoptosis (TWEAK) were expressed in breast tumors and whether deregulation of Fn14 levels affected malignant behavior of breast cancer cell lines. Analysis of TWEAK and Fn14 in publicly available gene expression data indicated that high Fn14 expression levels significantly correlated with several poor prognostic indicators (P < 0.05). Fn14 expression was highest in the HER2-positive/estrogen receptor-negative (HER2(+)/ER(-)) intrinsic subtype (P = 0.0008). An association between Fn14 and HER2 expression in breast tumors was confirmed by immunohistochemistry. Fn14 levels were elevated in invasive, ER(-) breast cancer cell lines. Overexpression of Fn14 in weakly invasive MCF7 and T47D cells resulted in a marked induction of invasion and activation of nuclear factor-kappaB (NF-kappaB) signaling. Ectopic expression of Fn14tCT, a Fn14 deletion mutant that cannot activate NF-kappaB signaling, was not able to induce invasion. Moreover, ectopic expression of Fn14tCT in highly invasive MDA-MB-231 cells reduced their invasive capability. RNA interference-mediated inhibition of Fn14 expression in both MDA-MB-231 and MDA-MB-436 cells reduced invasion. Expression profiling of the Fn14-depleted cells revealed deregulation of NF-kappaB activity. Our findings support a role for Fn14-mediated NF-kappaB pathway activation in breast tumor invasion and   metastasis  . 
 Deregulated Levels of the NF-κB1, NF-κB2, and Rel Genes in Ukrainian Patients with Leukemia and Lymphoma in the Post-Chernobyl Period. Nuclear factor kappa B (NF-κB) is an important transcription factor in cancer and NF-κB activation has been seen in angiogenesis, tumor progression,   and metast  asis. Relationships between specific NF-κB gene networks, leukemogenesis, and radiation exposure are still unknown. Our aim was to study the expression levels of the NF-κB1, NF-κB2, and Rel genes in hematological malignancies in the post-Chernobyl period.We analyzed gene expression levels of NF-κB1, NF-κB2, and Rel in 49 B-cell chronic lymphocytic leukemia, 8 B-cell non-Hodgkin's lymphoma, 3 acute myeloid leukemia, 3 chronic myeloid leukemia, 2 hairy cell leukemia, 2 myelodysplastic syndrome, and 2 T-cell large granular lymphocytic leukemia patients using real-time polymerase chain reaction.Expression levels of NF-κB1, NF-κB2, and Rel genes were found to be deregulated.These results could be accepted as specific gene traces to radiation-induced leukemia or as potential candidates for new diagnostic biomarker studies. Larger experiments and non-exposed control malignant cell populations are needed to clarify these suggestions. 
 Connective tissue growth factor enhances the migration of gastric cancer through downregulation of E-cadherin via the NF-κB pathway. Local invasion and distant   metastasi  s are difficult problems for surgical intervention and treatment in gastric cancer. Connective tissue growth factor (CTGF/CCN2) was considered to have an important role in this process. In this study, we demonstrated that expression of CTGF was significantly upregulated in clinical tissue samples of gastric carcinoma (GC) samples. Forced expression of CTGF in AGS GC cells promoted their migration in culture and significantly increased tumor   metastasi  s in nude mice, whereas RNA interference-mediated knockdown of CTGF in GC cells significantly inhibited cell migration in vitro. We disclose that CTGF downregulated the expression of E-cadherin through activation of the nuclear factor-κappa B (NF-κB) pathway. The effects of CTGF in GC cells were abolished by dominant negative IκappaB. Collectively, these data reported here demonstrate CTGF could modulate the NF-κappaB pathway and perhaps be a promising therapeutic target for gastric cancer invasion   and metas  tasis. 
 Targeting the NF-κB and mTOR pathways with a quinoxaline urea analog that inhibits IKKβ for pancreas cancer therapy. The presence of TNF-α in approximately 50% of surgically resected tumors suggests that the canonical NF-κB and the mTOR pathways are activated. Inhibitor of IκB kinase β (IKKβ) acts as the signaling node that regulates transcription via the p-IκBα/NF-κB axis and regulates translation via the mTOR/p-S6K/p-eIF4EBP axis. A kinome screen identified a quinoxaline urea analog 13-197 as an IKKβ inhibitor. We hypothesized that targeting the NF-κB and mTOR pathways with 13-197 will be effective in malignancies driven by these pathways.Retrospective clinical and preclinical studies in pancreas cancers have implicated NF-κB. We examined the effects of 13-197 on the downstream targets of the NF-κB and mTOR pathways in pancreatic cancer cells, pharmacokinetics, toxicity and tumor growth, and metastases in vivo.13-197 inhibited the kinase activity of IKKβ in vitro and TNF-α-mediated NF-κB transcription in cells with low-μmol/L potency. 13-197 inhibited the phosphorylation of IκBα, S6K, and eIF4EBP, induced G1 arrest, and downregulated the expression of antiapoptotic proteins in pancreatic cancer cells. Prolonged administration of 13-197 did not induce granulocytosis and protected mice from lipopolysaccharide (LPS)-induced death. Results also show that 13-197 is orally available with extensive distribution to peripheral tissues and inhibit  ed tumor g  rowth and metastasis in an orthotopic pancreatic cancer model without any detectable toxicity.These results suggest that 13-197 targets IKKβ and thereby inhibits mTOR and NF-κB pathways. Oral availability along with in vivo efficacy without obvious toxicities makes this quinoxaline urea chemotype a viable cancer therapeutic. 
 14-3-3β Promotes Migration and Invasion of Human Hepatocellular Carcinoma Cells by Modulating Expression of MMP2 and MMP9 through PI3K/Akt/NF-κB Pathway. 14-3-3β has been demonstrated to possess the oncogenic potential, and its increased expression has been detected in multiple types of carcinomas. However, majority of previous studies focused on the role of 14-3-3β in tumor cell proliferation and apoptosis, leaving much to be elucidated about its function in tumor cell invasion   and metast  asis. Hence, the present study aimed to investigate the role of 14-3-3β in the invasion of hepatocellular carcinoma (HCC) cells and the implications in the prognosis of HCC patients. We first examined the expression of 14-3-3β in the primary tumors of HCC patients with or without portal vein tumor thrombus (PVTT), and found that 14-3-3β expression was higher in the primary tumors with PVTT, and the level was even higher in the PVTTs. Kaplan-Meier curves and multivariate analysis revealed that high expression of 14-3-3β was associated with overall survival (OS) and time to recurrence (TTR) of HCC patients. In addition, ectopic expression of 14-3-3β in HCC cell lines led to enhanced migration ability and invasiveness, as well as up-regulation of matrix metalloproteinase 2 and 9, which could be suppressed by inhibiting the activation of Akt and nuclear factor-κB (NF-κB) signaling. Furthermore, we identified a correlated elevation of 14-3-3β and p-Akt in the primary tumors of HCC patients, and showed that a combinatory detection of 14-3-3β and p-Akt could better predict post-surgical outcome of HCC patients. 
 Stathmin, interacting with Nf-κB, promotes tumor growth and predicts poor prognosis of pancreatic cancer. Stathmin (STMN) has been known as a p53-regulated protein and has been shown to play an oncogenic role in a range of human malignancies. Paradoxically, most recent studies demonstrated that stathmin has a dual function as both an oncogene and a   metastasi  s suppressor. Stathmin is a member of microtubule dynamic destabilizing proteins and stathmin-regulated microtubule disruption could lead to a variety of cell dysfunctions such as enhanced chronic hypoxia in pancreatic cancer. In this study, we identified that stathmin promotes proliferation of pancreatic cancer cells by an underlying nuclear factor kappa B (Nf-κB) interacting mechanism. In human specimens, stathmin was significantly overexpressed in pancreatic cancer tissues and high expression of stathmin was correlated with vascular emboli (p=0.028), tumor size (p=0.019), and overall survival (p=0.031). Functional assays showed that knockdown of stathmin significantly reduced pancreatic cancer cell viability, colony formation, and arrested the cell cycle at the G2/M phase. Furthermore, silence of stathmin could reduce pancreatic tumor growth in nude mice. For the mechanism, Western blot analyses demonstrated that Nf-κB (p65) was significantly down-regulated when stathmin was silenced. In addition, co-immunoprecipitation (CoIP) assay suggested that stathmin was able to interact with Nf-κB (p65). Our findings indicate that stathmin might play its oncogenic role by an interaction with Nf-κB pathway, which may reveal a novel mechanism to uncover the role of microtubule-destabilizing stathmin in pancreatic cancer environment as well as provide a potential therapeutic strategy for pancreatic cancer. 
 MicroRNA-26b suppresses the   metastasis   of non-small cell lung cancer by targeting MIEN1 via NF-κB/MMP-9/VEGF pathways. MicroRNAs (miRNAs) are involved in tumor initiation and progression. MiR-26b was reported to be significantly downregulated in non-small cell lung cancer (NSCLC). However, the underlying mechanisms of miR-26b involvement in the development and progression of NSCLC remains poorly understood. In the present study, we report that miR-26b suppresses cell   metastasi  s in NSCLC through targeting migration and invasion enhancer 1 (MIEN1). We found that miR-26b was significantly downregulated and MIEN1 was significantly upregulated in both NSCLC tissues and cells lines. The expression levels of miR-26b were negatively related to those of MIEN1 mRNA in clinical NSCLC tissues. Furthermore, MIEN1 was confirmed to be a direct target of miR-26b by dual-luciferase reporter assay and MIEN1 expression was downregulated by miR-26b in NSCLC cells. In terms of function, transwell and wound healing assays demonstrated that the miR-26b remarkably inhibited invasion and migration of NSCLC cells, which was simulated by siRNA knockdown of MIEN1 and reversed by pcDNA/MIEN1 overexpression of MIEN1. Finally, we found that miR-26b could regulate NF-κB/MMP-9/VEGF pathway in NSCLC cells. In conclusion, this study revealed that miR-26b suppresses NSCL  C metastas  is by targeting MIEN1 via NF-κB/MMP-9/VEGF pathways, implicating a potential prognostic biomarker and therapeutic target for NSCLC treatment. 
 Activin upregulation by NF-κB is required to maintain mesenchymal features of cancer stem-like cells in non-small cell lung cancer. Soluble growth factors and cytokines within the tumor microenvironment aid in the induction of the epithelial-to-mesenchymal transition (EMT). Although EMT promotes the development of cancer-initiating cells (CIC), cellular mechanisms by which cancer cells maintain mesenchymal phenotypes remain poorly understood. Work presented here indicates that induction of EMT stimulates non-small cell lung cancer (NSCLC) to secrete soluble factors that function in an autocrine fashion. Using gene expression profiling of all annotated and predicted secreted gene products, we find that NF-κB activity is required to upregulate INHBA/Activin, a morphogen in the TGFβ superfamily. INHBA is capable of inducing and maintaining mesenchymal phenotypes, including the expression of EMT master-switch regulators and self-renewal factors that sustain CIC phenotypes and promote lu  ng metasta  sis. Our work demonstrates that INHBA mRNA and protein expression are commonly elevated in primary human NSCLC and provide evidence that INHBA is a critical autocrine factor that maintains mesenchymal properties of CICs to promo  te metasta  sis in NSCLC. 
 Glial cell-derived neurotrophic factor increases migration of human chondrosarcoma cells via ERK and NF-kappaB pathways. Invasion of tumor cells is the primary cause of therapeutic failure in the treatment of malignant chondrosarcomas. Glial cell-derived neurotrophic factor (GDNF) plays a crucial role in migration and   metastasis   of human cancer cells. Integrins are the major adhesive molecules in mammalian cells. Here we found that GDNF directed the migration and increased cell surface expression of alphav and beta3 integrin in human chondrosarcoma cells. Pretreated of JJ012 cells with MAPK kinase (MEK) inhibitors PD98059 or U0126 inhibited the GDNF-mediated migration and integrin expression. Stimulation of cells with GDNF increased the phosphorylation of MEK and extracellular signal-regulating kinase (ERK). In addition, NF-kappaB inhibitor (PDTC) or IkappaB protease inhibitor (TPCK) also inhibited GDNF-mediated cells migration and integrin up-regulation. Stimulation of cells with GDNF induced IkappaB kinase (IKKalpha/beta) phosphorylation, IkappaB phosphorylation, p65 Ser(536) phosphorylation, and kappaB-luciferase activity. Furthermore, the GDNF-mediated increasing of kappaB-luciferase activity was inhibited by PD98059, U0126, PDTC and TPCK or MEK, ERK, IKKalpha, and IKKbeta mutants. Taken together, these results suggest that the GDNF acts through MEK/ERK, which in turn activates IKKalpha/beta and NF-kappaB, resulting in the activations of alphavbeta3 integrin and contributing the migration of human chondrosarcoma cells. 
 Overexpression of Forkhead Box M1 transcription factor and nuclear factor-κB in laryngeal squamous cell carcinoma: a potential indicator for poor prognosis. The Forkhead Box M1 transcription factor and nuclear factor-κB have been shown to play important roles in the development and progression of human cancers. However, the functional significance of Forkhead Box M1 transcription factor in laryngeal squamous cell carcinoma and the correlation between Forkhead Box M1 transcription factor and nuclear factor-κB remain unclear. In the current study, we have shown that Forkhead Box M1 transcription factor and nuclear factor-κB were significantly overexpressed in laryngeal squamous cell carcinoma tissues and precancerous lesions, compared with adjacent normal tissues (both P < .001). The overexpression of Forkhead Box M1 transcription factor was significantly associated with histologic differentiation (rs = 0.321, P = .002), T stage (rs = 0.276, P = .009), lymph n  ode metast  asis (rs = 0.266, P = .012), and clinical stage (rs = 0.272, P = .010); overexpression of nuclear factor-κB was significantly associated with T stage (rs = 0.404, P < .001), lymph   node metas  tasis (rs = 0.293, P = .005), and clinical stage (rs = 0.425, P < .001). Overexpressions of both Forkhead Box M1 transcription factor and nuclear factor-κB were associated with worse overall survival (P = .041 and P < .001, respectively). Multivariate Cox regression analysis showed that T stage, lymph   node meta  stasis, and nuclear factor-κB were independent prognostic factors for laryngeal squamous cell carcinoma (P = .038, P = .014, and P = .005, respectively). Furthermore, a significant correlation was observed between Forkhead Box M1 transcription factor and nuclear factor-κB (rs = 0.683, P < .001), indicating the potential direct or indirect interaction between them. In conclusion, our results suggest that overexpressions of Forkhead Box M1 transcription factor and nuclear factor-κB and the possible interaction between them may play important roles in the development and progression of laryngeal squamous cell carcinoma, and Forkhead Box M1 transcription factor and nuclear factor-κB may serve as useful prognostic markers for laryngeal squamous cell carcinoma. 
 A signal transducer and activator of transcription 3·Nuclear Factor κB (Stat3·NFκB) complex is necessary for the expression of fascin in metastatic breast cancer cells in response to interleukin (IL)-6 and tumor necrosis factor (TNF)-α. IL-6 mediated activation of Stat3 is a major signaling pathway in the process of breast ca  ncer metas  tasis. One important mechanism by which the IL-6/Stat3 pathway prom  otes metas  tasis is through transcriptional regulation of the actin-bundling protein fascin. In this study, we further analyzed the transcriptional regulation of the fascin gene promoter. We show that in addition to IL-6, TNF-α increases Stat3 and NFκB binding to the fascin promoter to induce its expression. We also show that NFκB is required for Stat3 recruitment to the fascin promoter in response to IL-6. Furthermore, Stat3 and NFκB form a protein complex in response to cytokine stimulation. Finally, we demonstrate that an overlapping STAT/NFκB site in a highly conserved 160-bp region of the fascin promoter is sufficient and necessary to induce transcription in response to IL-6 and TNF-α. 
 Regulation of HSP27 on NF-kappaB pathway activation may be involved in metastatic hepatocellular carcinoma cells apoptosis. During the process of   metastasis  , cells are subjected to various apoptotic stimuli. Aberrant expression of apoptotic regulators often contribute to cell   metastasis  . Heat shock protein 27(HSP27) is confirmed as an apoptosis regulator, but its antiapoptotic mechanism in metastatic hepatocellular carcinoma (HCC) cells remains unclear.Levels of HSP27 protein and its phosphorylation in Hep3B, MHCC97L to MHCC97H cells with different metastatic potentials were determined by western blot analysis. MHCC97H cells were transfected with specific small interference RNA (siRNA) against HSP27. The in vitro migration and invasion potentials of cells were evaluated by Transwell assay. The apoptosis ratio of MHCC97H cells was analyzed by TUNEL staining and Flow Cytometry. Alteration of signal transduction pathway after HSP27 knockdown in MHCC97H cells was evaluated through a Human Q Series Signal Transduction in Cancer Gene Array analysis. Nuclear NF-kappaB contentration and endogenous IKK activity were demonstrated by ELISA assay. The association of IKKalpha, IKKbeta, IkappaBalpha with HSP27 and the association between IKKbeta and IKKalpha in MHCC97H cells were determined by co-immunoprecipitation assay followed by western blot analysis.HSP27 protein and its phosphorylation increased in parallel with enhanced metastatic potentials of HCC cells. siRNA-mediated HSP27 knockdown in MHCC97H significantly suppressed cells migration and invasion in vitro and induced cell apoptosis; the prominently altered signal transduction pathway was NF-kappaB pathway after HSP27 knockdown in MHCC97H cells. Furthermore, inhibition of HSP27 expression led to a significant decrease of nuclear NF-kappaB contentration and endogenous IKK activity. In addition, HSP27 was associated with IKKalpha, IKKbeta, IkappaBalpha in three HCC cells above. ELISA assay and western blot analysis also showed a decrease of the association between IKKbeta and IKKalpha, the association between phosphor-HSP27 and IKK complex, and an increase of total IkappaBalpha but reducing tendency of phosphor-IkappaBalpha when HSP27 expression was efficiently knocked down in MHCC97H cells.Altogether, these findings revealed a possible effect of HSP27 on apoptosis in metastatic HCC cells, in which HSP27 may regulate NF-kB pathway activation. 
 Human β-defensin 3 promotes NF-κB-mediated CCR7 expression and anti-apoptotic signals in squamous cell carcinoma of the head and neck. The microenvironment of aerodigestive cancers contains tumor-promoting inflammatory signals often involved in innate immunity. The epithelial malignancy, squamous cell carcinoma of the head and neck (SCCHN), is characterized by secretion of inflammatory mediators that can promote tumorigenesis and lymph nod  e metastas  is. Human β-defensin (hBD) 3 is one such antimicrobial mediator of innate immunity produced by squamous epithelial cells in response to tissue damage and inflammation. Here, we hypothesized that the observed overexpression of hBD3 in SCCHN may have a tumor-promoting effect or contribute to nod  al metasta  sis, which has previously been linked to chemokine receptor (CCR) 7 overexpression. Indeed, treatment of non-metastatic SCCHN cells with hBD3 induced surface CCR7 expression and migration toward its ligand, CCL19. The hBD3-induced CCR7 upregulation in SCCHN cells was significantly reduced by inhibition of nuclear factor (NF)-κB, an inflammatory transcription factor known to influence CCR7 expression. Moreover, hBD3 stimulation provided anti-apoptotic signals to SCCHN cells, as evidenced by tumor resistance to cisplatin-induced cell death, which was regulated by phosphoinositide-3-kinase/Akt activation. Interestingly, the observed hBD3-mediated effects were not dependent on G-protein coupled receptors or toll-like receptors, as has been previously published, but hBD3 was internalized through endocytosis, allowing intracellular signal transduction. Our findings suggest that hBD3 represents a novel NF-κB-regulated mediator of CCR7 expression and anti-apoptotic pathways, which may be exploited by developing SCCHN tumors to enhance their survival   and metas  tasis. 
 Targeting NF-kappaB in hematologic malignancies. The transcription factor nuclear factor kappa B (NF-kappaB) can intervene in oncogenesis by virtue of its capacity to regulate the expression of a plethora of genes that modulate apoptosis, and cell survival as well as proliferation, inflammation, tumor   metastasis   and angiogenesis. Different reports demonstrate the intrinsic activation of NF-kappaB in lymphoid and myeloid malignancies, including preneoplastic conditions such as myelodysplastic syndromes, underscoring its implication in malignant transformation. Targeting intrinsic NF-kappaB activation, as well as its upstream and downstream regulators, may hence constitute an additional approach to the oncologist's armamentarium. Several small inhibitors of the NF-kappaB-activatory kinase IkappaB kinase, of the proteasome, or of the DNA binding of NF-kappaB subunits are under intensive investigation. Currently used cytotoxic agents can induce NF-kappaB activation as an unwarranted side effect, which confers apoptosis suppression and hence resistance to these drugs. Thus, NF-kappaB inhibitory molecules may be clinically useful, either as single therapeutic agents or in combination with classical chemotherapeutic agents, for the treatment of hematological malignancies. 
 The relationship between BRAFV600E, NF-κB and TgAb expression in papillary thyroid carcinoma. The aim of this study was to investigate the potential link between thyroglobulin antibody (TgAb) titers and papillary thyroid carcinoma (PTC) aggressiveness.This retrospective study included 122 patients who had undergone total thyroidectomy and neck central area lymph dissection followed by radioactive iodine (RAI) ablation for PTC between July 2012 and July 2014. The parameters included clinical characteristics of PTC, TgAb levels and tumor histopathological characteristics. Then, BRAFV600E and NF-κB expression in 113 PTC patients in our hospital were examined by immunohistochemistry.The group of TgAb-positive PTC patients more frequently presented with lymph node metastases (80.0% vs 61.2%, p=0.000), distant metastases (10.0% vs 1.9%, p=0.000), more RAI ablation treatments in their treatment history (344.4±88.2 vs 222.5±91.1, p=0.003) and imaging-documented disease (20.0% vs 7.8%, p=0.000) after two years' follow-up. We also found that protein expression levels of BRAFV600E and NF-κB were greater in TgAb-positive patients compared with TgAb-negative PTC patients (p=0.005, p=0.001). Immunoreactivity scores of BRAFV600E and NF-κB in TgAb-positive PTC patients with bilateral tumor foci, lymph node metastases, HT and imaging-documented disease was highest. Multivariate analysis revealed that lymph node metastases significantly correlated with NF-κB levels and TgAb levels (p=0.004, p=0.014).The expression levels of BRAFV600E and NF-κB were significantly greater in TgAb-positive than in TgAb-negative PTC patients. In PTC, overexpression of NF-κB, as well as positive TgAb, may be a risk factor for cervical ly  mph node m  etastasis. 
 Activated macrophages down-regulate expression of E-cadherin in hepatocellular carcinoma cells via NF-κB/Slug pathway. Hepatocellular carcinomas are an aggressive malignancy mainly due to   metastasi  s or postsurgical recurrence. Expression of E-cadherin is strongly reduced in Hepatocellular carcinoma (HCC) tissues, and its downregulation is connected to invasiveness and   metastasi  s in hepatocellular carcinomas. The previous study showed that the supernatant from activated macrophages can downregulate the expression of E-cadherin in HCC cells. The partial known molecular mechanism is that tyrosine kinases c-Src- and EGFR phosphorylate β-catenin and E-cadherin leading to destabilization of E-cadherin/β-catenin complex. The aim of this study is to clarify other mechanism by which activated macrophages downregulate the expression of E-cadherin. We detect the expression of E-cadherin and macrophage infiltration in hepatocellular carcinoma tissues by double-staining immunohistochemistry and evaluate the relationship between macrophages and E-cadherin expression in hepatocellular carcinoma cells in vitro experiments. We found that reduced expression of E-cadherin was associated with macrophage infiltration along the border between the tumor nest and stroma in hepatocellular carcinoma tissues. Besides, protein expression of E-cadherin was significantly decreased in hepatocellular carcinoma cells co-cultured with macrophages derived from THP-1 cells. Consistently, mRNA expression of E-cadherin was also decreased in cancer cells co-cultured with THP-1-differentiated macrophages. Moreover, the downregulation of E-cadherin expression was companied by upregulation of Slug expression in cancer cells with conditional medium from THP-1-differentiated macrophage culture. The change in expression of E-cadherin and Slug was abrogated when NF-κB signaling pathway was blocked. All the findings suggested that macrophages contributed to the decreased expression of E-cadherin by NF-κB/Slug pathway in hepatocellular carcinomas. 
 Subcellular localization of apurinic endonuclease 1 promotes lung tumor aggressiveness via NF-kappaB activation. Apurinic endonuclease 1 (Ape1) is not only involved in base excision repair, but also activates some transcriptional factors through its redox activity. However, which subcellular localization of Ape1 is involved in the activation of transcriptional factor remains unclear. We first observed that Cox-2 expression was associated with cytoplasmic Ape1 expression in lung tumors and cancer cell lines. We thus hypothesize that nuclear factor (NF)-kappaB is activated by cytoplasmic Ape1 to cause Cox-2 expression. Herein, we generated cytoplasmic and nuclear Ape1 in Ape1-knockdown lung cancer cells by exogenous expression of Ape1 containing various deletions and/or mutations of the nuclear localization sequence. It was observed that cytoplasmic Ape1, but not nuclear Ape1, induced Cox-2 expression through NF-kappaB activation. NF-kappaB activation by cytoplasmic Ape1 was diminished by the Ape1 redox activity inhibitor resveratrol. Cells expressing cytoplasmic Ape1 exhibited tumor progression and   metastasis   in vitro and in vivo as xenografts, but cells expressing nuclear Ape1 did not. Patients with tumors containing elevated cytoplasmic Ape1 had a poor prognosis and a 3.722-fold risk of tumor recurrence and/or   metastasis  . Cytoplasmic Ape1 could therefore enhance lung tumor malignancy through NF-kappaB activation, suggesting that combination of cisplatin and specific redox inhibitor could improve chemotherapeutic response in patients with tumors containing elevated cytoplasmic Ape1. 
 BRAF-induced tumorigenesis is IKKα-dependent but NF-κB-independent. KRAS mutations contribute to cell proliferation and survival in numerous cancers, including colorectal cancers (CRC). One pathway through which mutant KRAS acts is an inflammatory pathway that involves the kinase IKK and activates the transcription factor NF-κB. BRAF, a kinase that is downstream of KRAS, is mutated in a subset of CRC and is predictive of poor prognosis and therapeutic resistance. We found that, in contrast to mutant KRAS, mutant BRAF (BRAF(V600E)) did not trigger NF-κB activation but instead triggered the phosphorylation of a proteolytic fragment of IKKα (p45-IKKα) in CRC cells. BRAF(V600E) CRC cells had a high abundance of phosphorylated p45-IKKα, which was decreased by a RAF inhibitor. However, the abundance and DNA binding of NF-κB in these cells were unaffected by the RAF inhibitor, and expression of BRAF(V600E) in human embryonic kidney-293T cells did not activate an NF-κB reporter. Moreover, BRAF-induced transformation of NIH-3T3 cells and BRAF-dependent transcription required phosphorylation of p45-IKKα. The kinase TAK1, which was associated with the endosomal compartment, phosphorylated p45-IKKα. Inhibition of endosomal vacuolar adenosine triphosphatase (V-ATPase) with chloroquine or bafilomycin A1 blocked p45-IKKα phosphorylation and induced apoptosis in BRAF-mutant CRC cells independent of autophagy. Treating mice with V-ATPase inhibitors reduced the   growth an  d metastasis of BRAF(V600E) xenograft tumors in the cecum of mice. 
 Polymorphic variants of NFKB1 and its inhibitory protein NFKBIA, and their involvement in sporadic breast cancer. Nuclear factor kappa-beta (NF-kappaB) is a transcription factor responsible for modulating the expression of many genes involved in cell proliferation, differentiation, apoptosis and   metastasis  . NF-kappaB interacts with IkappaB inhibitory proteins to regulate gene expression. This study investigated common variants within the genes coding for NF-kappaB and IkappaB, NFKB1 and NFKBIA, for involvement in sporadic breast cancer. Genotypes were determined in a population of breast cancer affected individuals and age-matched controls. Results do not support an involvement of the tested NFKB1 and NFKBIA polymorphisms in susceptibility to sporadic breast cancer, in the tested Caucasian population. 
 Tissue transglutaminase constitutively activates HIF-1α promoter and nuclear factor-κB via a non-canonical pathway. Constitutive activation of nuclear factor kappa B (NF-κB) has been linked with carcinogenesis and cancer progression, includi  ng metasta  sis, chemoresistance, and radiation resistance. However, the molecular mechanisms that result in constitutive activation of NF-κB are poorly understood. Here we show that chronic expression of the pro-inflammatory protein tissue transglutaminase (TG2) reprograms the transcription regulatory network in epithelial cells via constitutive activation of NF-κB. TG2-induced NF-κB binds the functional NF-κB binding site in hypoxia-inducible factor-1 (HIF-1α) promoter and results in its increased expression at transcription and protein levels even under normoxic conditions. TG2/NF-κB-induced HIF-1 was deemed essential for increased expression of some transcription repressors, like Zeb1, Zeb2, Snail, and Twist. Unlike tumor necrosis factor-alpha (TNFα), TG2 did not require IκB kinase (IKK) for NF-κB activation. Our data suggest that TG2 binds with IκBα and results in its rapid degradation via a non-proteasomal pathway. Importantly, the catalytically inactive (C277S) mutant form of TG2 was as effective as was wild-type TG2 in activating NF-κB and inducing HIF-1 expression. We also found that TG2 interacted with p65/RelA protein, both in the cytosolic and the nuclear compartment. The TG2/p65(NF-κB) complex binds to the HIF-1 promoter and induced its transcriptional regulation. Inhibition of TG2 or p65/RelA also inhibited the HIF-1α expression and attenuated Zeb1, Zeb2, and Twist expression. To our knowledge, these findings show for the first time a direct link between TG2, NF-κB, and HIF-1α, demonstrating TG2's important role in cancer progression. 
 [Inhibition of NF-kappaB through IkappaBalpha transfection affects invasion of human lung cancer cell line A549]. Recent studies have shown that activation of nuclear factor-kappaB(NF-kappaB) can regulate the invasion and   metastasis   of cancer cells. The present study was to investigate inhibition of NF-kappaB activity on invasion of human lung cancer cell line A549 and the possible mechanism.The recombinant plasmid pcDNA3.1(+)/IkappaBalpha, expressing the alpha isoform (IkappaBalpha) of the NF-kappaB inhibitor, was constructed. A549 cells were cultured in vitro and divided into non-transfection group, pcDNA3.1(+) transfected group and pcDNA3.1(+)/IkappaBalpha transfected group. The expression of IkappaBalpha was detected by reverse transcription-polymerase chain reaction (RT-PCR) and Western blotting. The activity of NF-kappaB was determined by electrophoretic mobility shift assay (EMSA). Invasion of A549 cells was assessed by transwell chamber assay. The expression of MMP-2 and MMP-9 was detected by RT-PCR and gelatin zymography.Plasmid pcDNA3.1(+)/IkappaBalpha was successfully constructed and expressed in A549 cells. The activity of NF-kappaB, the number of invasive cells, the activity of MMP-2 and MMP-9 of A549 cells in pcDNA3.1(+)/IkappaBalpha transfected group were significantly lower than those in non-transfection group and pcDNA3.1(+) transfected group (all P<0.05).Transfection of IkappaBalpha can inhibit NF-kappaB activity, thus inhibit cell invasion of A549, which may be through the down-regulation of MMP-2 and MMP-9 expressions. 
 Toll-like receptor 9 agonists up-regulates the expression of cyclooxygenase-2 via activation of NF-kappaB in prostate cancer cells. CpG-oligonucleotides (CpG-ODNs), mimicking bacterial DNA, have recently been shown to stimulate prostate cancer invasion in vitro via Toll-like receptor 9 (TLR9). Since cyclooxygenase 2 (COX-2), frequently overexpressed in multiple tumor types including prostate cancer, is a causal factor for tumor development, invasion and   metastasis  , an interesting question is raised whether TLR9 regulates COX-2 expression in prostate cancer cells. To address this question, herein we examined COX-2 expression in PC-3 cells stimulated with different doses and time courses of CpG-ODNs. The regulatory role of NF-kappaB in TLR9-mediated COX-2 expression was also investigated. CpG-ODN was found to up-regulate the expression of COX-2 in PC-3 cells in a dose- and time-dependent manner, but have little impact on COX-1 expression. Moreover, CpG-ODN also promoted nuclear translocation and activation of NF-kappaB, which appeared to be required for COX-2 induction by CpG-ODN. Overall, TLR9 up-regulates COX-2 expression in prostate cancer cells, at least partially through the activation of NF-kappaB, which may be implicated in tumor invasion and   metastasis  . 
 [Expression and significance of RhoA and NF-ΚB in human gastric carcinoma]. To investigate the expression of RhoA and NF-κB in gastric carcinoma and their correlation with clinicopathological fearures. To determine the effective prognostic factors of long-term suivival of gastric carcinoma patients.The role of RhoA and NF-κB in gastric carcinoma was assessed by tissue array technology and the levels of RhoA and NF-κB expression in paraffin-embedded tissues was quantified by immunohistochemistry from 189 cases of gastric carcinoma, 54 cases of their adjacent tissues, and 32 cases of normal gastric mucosa. The prognosis of gastric carcinoma was evaluated by Kaplan-Meier survival analysis and Cox multivariate regression analysis.The positive rates of RhoA expression were 84.7%, 68.5% and 65.6% in gastric carcinoma, adjacent tissues and normal mucosa, respectively. The expression of RhoA in gasric carcinoma was significantly higher than that in adjacent tissues and normal mucosa (P < 0.05). The positive rates of NF-κB expression were 75.1%, 42.6% and 15.6%% in gastric carcinoma, adjacent tissues and normal mucosa, respectively. The expression of NF-κB in gasric carcinoma was significantly higher than that in adjacent tissues and normal mucosa (P < 0.05). RhoA was positively linked with NF-κB (r = 0.203, P = 0.005). In gastric carcinoma, the expression of RhoA was related with depth of invasion (P < 0.05), and the expression of NF-κB was related with depth of invasion and lym  ph node me  tastasis (P < 0.05). The Kaplan-Meier survival analysis showed that the tumor size, lym  ph node me  tastasis, depth of invasion, expression of RhoA and NF-κB can shorten the cumulative survival rate. With these paramaters entering the Cox multivariate regression analysis mode, it was revealed that expression of NF-κB, l  ymph node   metastasis and depth of invasion are independent prognostic factors.The overexpression of RhoA and NF-κB is involved in the occurrence and development of gastric carcinoma. RhoA is positively linked with NF-κB. They are correlated with the i  nvasion an  d metastasis of gastric carcinoma. The expression of NF-κB  , lymph no  de metastasis, depth of invasion are independent prognostic factors playing an important role in prediction of the clinical outcome after radical resection of gastric carcinoma. 
 Evaluation of nuclear factor-kappaB, urokinase-type plasminogen activator, and HBx and their clinicopathological significance in hepatocellular carcinoma. Nuclear factor kappaB (NF-kappaB) signaling pathway is an important regulating pathway in human diseases and cancers. One of its downstream target genes is urokinase plasminogen activator (uPA), which is involved in cancer invasion and   metastasis  . The purpose of this study was to evaluate NF-kappaB activation, uPA up-regulation, and hepatitis B viral X protein (HBx) expression in human hepatocellular carcinoma (HCC) and to assess their clinicopathological significance.We evaluated NF-kappaB activation, expression of uPA, and presence of HBx in 32 human HCCs. Their clinicopathological significance was assessed by correlation with the clinicopathological features. Aberrant NF-kappaB signaling pathway and uPA up-regulation mediated by HBx were also analyzed in vitro.We found that NF-kappaB activation and uPA up-regulation were frequently (56% and 59%, respectively) observed in HCCs, and particularly in HBx-positive HCCs. NF-kappaB activation and uPA overexpression were closely associated with one another (P < 0.0001). Furthermore, both activation of NF-kappaB and up-regulation of uPA were significantly associated with a more aggressive tumor behavior in terms of venous invasion, direct liver invasion, and absence of tumor encapsulation. In vitro, NF-kappaB activation was induced by HBx transfection in HepG2 cells through inhibitor of nuclear factor-kappaB kinase beta (IKKbeta). HBx also up-regulated uPA and enhanced cell invasion synergistically with IKKbeta.The data indicate that NF-kappaB dysregulation and uPA overexpression may lead to a more aggressive tumor behavior in HCC. In addition, our data suggest that IKKbeta plays a critical role in the HBx-activated NF-kappaB signaling pathway. 
 The homeoprotein DLX4 stimulates NF-κB activation and CD44-mediated tumor-mesothelial cell interactions in ovarian cancer. Ovarian cancers often highly express inflammatory cytokines and form implants throughout the peritoneal cavity. However, the mechanisms that drive inflammatory signaling and peritoneal   metastasi  s of ovarian cancer are poorly understood. We previously identified that high expression of DLX4, a transcription factor encoded by a homeobox gene, is associated with reduced survival of ovarian cancer patients. In this study, we identified that DLX4 stimulates attachment of ovarian tumor cells to peritoneal mesothelial cells in vitro and increases the numbers of peritoneal implants in xenograft models. DLX4 induced expression of the cell surface molecule CD44 in ovarian tumor cells, and inhibition of CD44 abrogated the ability of DLX4 to stimulate tumor-mesothelial cell interactions. The induction of CD44 by DLX4 was attributed to increased activity of NF-κB that was stimulated by the inflammatory cytokine IL-1β, a transcriptional target of DLX4. The stimulatory effects of DLX4 on CD44 levels and tumor-mesothelial cell interactions were abrogated when IL-1β or NF-κB was inhibited in tumor cells. Furthermore, DLX4 expression levels strongly correlated with NF-κB activation and disease stage in clinical specimens of ovarian cancer. Collectively, these findings indicate that DLX4 induces CD44 by stimulating IL-1β-mediated NF-κB activity, thereby promoting pe  ritoneal m  etastasis of ovarian cancer. 
 Loss of E-cadherin in the vicinity of necrosis in colorectal carcinomas: association with NFkappaB expression. The transcription factor NFkappaB regulates the expression of several tumor-related molecules associated with tumor progression and   metastasis  . However, the precise mechanisms by which its activation mediates these processes in diverse tumors are unknown. In this study we determined the expression of NFkappaB in various colorectal carcinoma cell lines, in a series of 90 non-metastatic and metastatic colorectal tumors and in an in vitro 3D-spheroid model of HT-29 cells simulating morphological hallmark of these adenocarcinomas, namely neoplastic glandular nests around a necrotic center. We show that the inactive cytoplasmic NFkappaB form is evidently up-regulated in the tumor epithelium, especially in the metastatic cases, as compared to normal tissue. We found that in situ nuclear NFkappaB staining is characteristic for cells that are still viable but dissociated from the surrounding cohesive tumor tissue and destined to die. Evidence for a possible association between NFkappaB expression and loss of cell adhesion mediated by E-cadherin function has been provided in vivo and in vitro using the HT-29 3D-spheroid model. In both cases, we found a strong correlation between activation of NFkappaB and loss of E-cadherin expression. Considering the fact that cancer cell necrosis plays a crucial role in   metastasis  , NFkappaB activation mediated by loss of E-cadherin may represent an essential, even initial event in this process. Furthermore, we present in vitro data implicating LPS, the endotoxin of gram-negative bacteria, in the triggering of NFkappaB up-regulation. Thus, release of bacterial endotoxin may essentially contribute to the progression of colon cancer in vivo. 
 Livin mediates tumor cell invasion in the DU-145 cell line via NF-κB. Livin is a member of the family of inhibitors of apoptosis proteins (IAPs) and tumor cell invasion is a general property of multiple IAPs. Livin is highly expressed in prostate cancer (PCa) tissues. Livin overexpressing cells are more resistant to apoptotic stimuli than normal cells. Thus, aberrantly increased cell survival is an invariable requirement of   metastasi  s. In this study, we investigated whether livin signaling affects   metastasi  s by transfecting siRNA targeting livin into the DU-145 prostate cancer cell line to confirm the anti-invasion effect and blockade of the livin gene. We found that livin knockdown inhibited DU-145 prostate carcinoma cell invasion. We investigated how livin promotes tumor cell invasion, and found that livin induction of fibronectin contributed to tumor cell invasion. In addition, we found that livin induction of fibronectin regulates tumor cell invasion via nuclear factor κB (NF-κB) signaling. These data showed that livin, as a gene directly promoti  ng metasta  sis, can be useful for therapeutic intervention against advanced and disseminated PCa. 
 Pterostilbene, a bioactive component of blueberries, suppresses the generation of breast cancer stem cells within tumor microenvironment and   metastasis   via modulating NF-κB/microRNA 448 circuit. Tumor-associated macrophages (TAMs) have been shown to promote   metastasi  s and malignancy. Pterostilbene, a natural stilbene isolated from blueberries, has been suggested for anti-cancer effects. Here, we explored the potential cancer stem cells (CSCs)/TAM modulating effects of pterostilbene in breast cancer.Using flowcytometric and Boyden chamber assay, we showed MCF7 and MDA-MB-231 cells cocultured with M2 TAMs exhibited increased percentage of CD44(+) /CD24(-) CSC population and migratory/invasive abilities. RT-PCR results showed that CD44(+) /CD24(-) cells expressed an increased level of HIF-1α, β-catenin, Twist1, and NF-κB and enhanced tumor sphere forming ability. Additionally, pterostilbene treatment dose dependently overcame M2 TAM-induced enrichment of CSCs and metastatic potential of breast cancer cells. Mechanistically, pterostilbene suppressed NFκB, Twist1, vimentin, and increased E-cadherin expression. Using siRNA technique, we demonstrated that pterostilbene-mediated NFκB downregulation was correlated to an increased amount of microRNA 448. Finally, pterostilbene-mediated suppression in tumorigenesi  s and meta  stasis was validated by noninvasive bioluminescence in mice bearing M2 TAM cocultured MDA-MB-231 tumor.Pterostilbene effectively suppresses the generation of CSCs and metastatic potential under the influence of M2 TAMs via modulating EMT associated signaling pathways, specifically NF-κB/miR488 circuit. Thus, pterostilbene could be an ideal anti-CSC agent in clinical settings. 
 Monocytes mediate metastatic breast tumor cell adhesion to endothelium under flow. Endothelial adhesion is necessary for the hematogenous dissemination of tumor cells. However, the metastatic breast tumor cell MDA-MB-231 does not bind to the endothelium under physiological flow conditions, suggesting alternate mechanisms of adhesion. Since monocytes are highly represented in the tumor microenvironment, and also bind to endothelium during inflammation, we hypothesized that the monocytes assist in the arrest of MDA-MB-231 on the endothelium. Using in vitro models of the dynamic shear environment of the vasculature, we show that TNF-α-activated THP1/primary human monocytes and MDA-MB-231 cells form stable aggregates, and that the monocytes in these aggregates mediate the adhesion of otherwise nonadherent MDA-MB-231 cells to inflamed endothelium under flow (55±2.4 vs. 1.7±0.82 at a shear stress of 0.5 dyn/cm(2), P<0.01). We also show that the hydrodynamic forces determine the size and orientation of aggregates adhered to the endothelium, and strongly favor the attachment of small aggregates with tumor cells downstream of flow (74-86% doublets at 0.5-2 dyn/cm(2), P<0.01). The 5-fold up-regulation of ICAM-1 on TNF-α-activated MDA-MB-231 cells through the Nf-κB pathway was found to be critical in MDA-MB-231-monocyte aggregation and endothelial adhesion. Our results demonstrate that, under inflammatory conditions, monocytes may serve to disseminate tumor cells through circulation, and the tumor-monocyte-endothelial axis may represent a new therapeutic target to reduce ca  ncer metas  tasis. 
 Combined Proteomics and Transcriptomics Identifies Carboxypeptidase B1 and Nuclear Factor κB (NF-κB) Associated Proteins as Putative Biomarkers o  f Metastas  is in Low Grade Breast Cancer. Current prognostic factors are insufficient for precise risk-discrimination in breast cancer patients with low grade breast tumors, which, in disagreement with theoretical prognosis, occasionally form early lymph nod  e metastas  is. To identify markers for this group of patients, we employed iTRAQ-2DLC-MS/MS proteomics to 24 lymph node positive and 24 lymph node negative grade 1 luminal A primary breast tumors. Another group of 48 high-grade tumors (luminal B, triple negative, Her-2 subtypes) was also analyzed to investigate marker specificity for grade 1 luminal A tumors. From the total of 4405 proteins identified (FDR < 5%), the top 65 differentially expressed together with 30 previously identified and control markers were analyzed also at transcript level. Increased levels of carboxypeptidase B1 (CPB1), PDZ and LIM domain protein 2 (PDLIM2), and ring finger protein 25 (RNF25) were associated specifically with lymph node positive grade 1 tumors, whereas stathmin 1 (STMN1) and thymosin beta 10 (TMSB10) associated with aggressive tumor phenotype also in high grade tumors at both protein and transcript level. For CPB1, these differences were also observed by immunohistochemical analysis on tissue microarrays. Up-regulation of putative biomarkers in lymph node positive (versus negative) luminal A tumors was validated by gene expression analysis of an independent published data set (n = 343) for CPB1 (p = 0.00155), PDLIM2 (p = 0.02027) and RELA (p = 0.00015). Moreover, statistically significant connections with patient survival were identified in another public data set (n = 1678). Our findings indicate unique pro-metastatic mechanisms in grade 1 tumors that can include up-regulation of CPB1, activation of NF-κB pathway and changes in cell survival and cytoskeleton. These putative biomarkers have potential to identify the specific minor subpopulation of breast cancer patients with low grade tumors who are at higher than expected risk of recurrence and who would benefit from more intensive follow-up and may require more personalized therapy. 
 MicroRNA-9 up-regulates E-cadherin through inhibition of NF-κB1-Snail1 pathway in melanoma. MicroRNAs (miRNAs) are short non-coding RNAs that post-transcriptionally regulate gene expression. Hsa-miR-9 has been shown to have opposite functions in different tumour types; however, the underlying mechanism is unclear. Here we show that hsa-miR-9 is down-regulated in metastatic melanomas compared to primary melanomas. Overexpression of miR-9 in melanoma cells resulted in significantly decreased cell proliferation and migratory capacity with decreased F-actin polymerization and down-regulation of multiple GTPases involved in cytoskeleton remodelling. miR-9 overexpression induced significant down-regulation of Snail1 with a concomitant increase in E-cadherin expression. In contrast, knockdown of miR-9 increased Snail1 expression as well as melanoma cell proliferation and migration capacity. Mechanistically, miR-9 expression down-regulated NF-κB1 in melanoma and the effect was abolished by mutations in the putative miR-9 binding sites within the 3'-untranslated region (UTR) of NF-κB1. Anti-miR-9 miRNA inhibitor also increased the expression of NF-κB1. The effects of miR-9 on Snail1 expression and melanoma cell proliferation and migration were rescued by overexpression of NF-κB1 in these cells. Furthermore, miR-9 overexpression resulted in significantly decreased melanoma growth   and metas  tasis in vivo. In summary, miR-9 inhibits melanoma proliferation   and metas  tasis through down-regulation of the NF-κB1-Snail1 pathway. This study finds a new mechanism that miR-9 utilizes to decrease E-cadherin expression and inhibit melanoma progression. The results suggest that function of microRNAs is context and tumour type-specific. 
 CCL18/PITPNM3 enhances migration, invasion, and EMT through the NF-κB signaling pathway in hepatocellular carcinoma. Chemokine ligand 18 (CCL18) has been associated with hepatocellular carcinoma (HCC)   metastasi  s. Here, we demonstrated a novel mechanism through which CCL18 enhances cell migration, invasion, and epithelial-mesenchymal transition (EMT) in HCC. (1) Using immunohistochemistry, we analyzed the expression of PITPNM3, a molecule that correlated with CCL18 signaling, in 149 HCC tissue specimens. The results showed that PITPNM3 expression is highly associated with tumor   metastasi  s and differentiation; (2) in vitro experiments showed that CCL18 enhances cell migration, invasion, and EMT in PITPNM3((+)) HCC cells but not in PITPNM3((-)) cells. Silencing of PITPNM3 by short interfering RNA (siRNA) inhibited the induction of cell migration, invasion, and EMT by CCL18; (3) Cell migration, invasion, and EMT induced by CCL18 accompanied with the phosphorylation of IKK and IKBα as well as p65 nuclear translocation in PITPNM3((+)) HCC cells, but not in the cells that PITPNM3 is silenced with siRNA, implying that the activation of NF-κB signaling is involved in the action of CCL18/PITPNM3. These results suggest that CCL18 enhances HCC cell migration, invasion, and EMT through the expression of PITPNM3 and the activation of the NF-κB signaling pathway. 
 Stromal cell-derived factor-1/CXCR4 enhanced motility of human osteosarcoma cells involves MEK1/2, ERK and NF-kappaB-dependent pathways. Osteosarcoma is characterized by a high malignant and metastatic potential. The chemokine stromal-derived factor-1alpha (SDF-1alpha) and its receptor, CXCR4, play a crucial role in adhesion and migration of human cancer cells. Integrins are the major adhesive molecules in mammalian cells, and has been associated with   metastasis   of cancer cells. Here, we found that human osteosarcoma cell lines had significant expression of SDF-1 and CXCR4 (SDF-1 receptor). Treatment of osteosarcoma cells with SDF-1alpha increased the migration and cell surface expression of alphavbeta3 integrin. CXCR4-neutralizing antibody, CXCR4 specific inhibitor (AMD3100) or small interfering RNA against CXCR4 inhibited the SDF-1alpha-induced increase the migration and integrin expression of osteosarcoma cells. Pretreated of osteosarcoma cells with MAPK kinase (MEK) inhibitor PD98059 inhibited the SDF-1alpha-mediated migration and integrin expression. Stimulation of cells with SDF-1alpha increased the phosphorylation of MEK and extracellular signal-regulating kinase (ERK). In addition, NF-kappaB inhibitor (PDTC) or IkappaB protease inhibitor (TPCK) also inhibited SDF-1alpha-mediated cell migration and integrin up-regulation. Stimulation of cells with SDF-1alpha induced IkappaB kinase (IKKalpha/beta) phosphorylation, IkappaB phosphorylation, p65 Ser(536) phosphorylation, and kappaB-luciferase activity. Furthermore, the SDF-1alpha-mediated increasing kappaB-luciferase activity was inhibited by AMD3100, PD98059, PDTC and TPCK or MEK1, ERK2, IKKalpha and IKKbeta mutants. Taken together, these results suggest that the SDF-1alpha acts through CXCR4 to activate MEK and ERK, which in turn activates IKKalpha/beta and NF-kappaB, resulting in the activations of alphavbeta3 integrins and contributing the migration of human osteosarcoma cells. 
 IL-1beta-mediated up-regulation of HIF-1alpha via an NFkappaB/COX-2 pathway identifies HIF-1 as a critical link between inflammation and oncogenesis. Growing evidence indicates that inflammation is a contributing factor leading to cancer development. However, pathways involved in this progression are not well understood. To examine whether HIF-1alpha is a factor linking inflammation and tumorigenesis, we investigated whether the HIF-1 signaling pathway was stimulated by the pro-inflammatory cytokine interleukin-1beta (IL-1beta) in A549 cells. We find that IL-1beta up-regulated HIF-1alpha protein under normoxia and activated the HIF-1-responsive gene vascular endothelial growth factor (VEGF) via a pathway dependent on nuclear factor kappaB (NFkB). Interestingly, although this pathway is stimulated by upstream signaling via AKT and mTOR and requires new transcription, IL-1 mediated HIF-1alpha induction also utilizes a post-transcriptional mechanism that involves antagonism of VHL-dependent HIF-1alpha degradation, which results in increased HIF-1alpha protein stability. IL-1 mediated NFkB-dependent cyclooxygenases-2 (COX-2) expression served as a positive effector for HIF-1alpha induction. Although COX-2 inhibitors attenuated IL-1 mediated HIF-1alpha induction, prostaglandin E2 (PGE2), a physiological product of COX-2, induced HIF-1alpha protein in a dose-dependent manner. Our data, therefore, demonstrate that IL-1beta up-regulates functional HIF-1alpha protein through a classical inflammatory signaling pathway involving NFkB and COX-2, culminating in up-regulation of VEGF, a potent angiogenic factor required for tumor growth and   metastasis  . Thus, HIF-1 is identified as a pivotal transcription factor linking the inflammatory and oncogenic pathways. 
 CTGF enhances migration and MMP-13 up-regulation via alphavbeta3 integrin, FAK, ERK, and NF-kappaB-dependent pathway in human chondrosarcoma cells. Tumor malignancy is associated with several features such as proliferation ability and frequency of   metastasis  . Connective tissue growth factor (CTGF), a secreted protein that binds to integrins, modulates the invasive behavior of certain human cancer cells. However, the effect of CTGF on migration activity in human chondrosarcoma cells is mostly unknown. Here we found that CTGF increased the migration and expression of matrix metalloproteinase (MMP)-13 in human chondrosarcoma cells (JJ012 cells). RGD peptide, alphavbeta3 monoclonal antibody (mAb) and MAPK kinase (MEK) inhibitors (PD98059 and U0126) but not RAD peptide inhibited the CTGF-induced increase of the migration and MMP-13 up-regulation of chondrosarcoma cells. CTGF stimulation increased the phosphorylation of focal adhesion kinase (FAK) and extracellular signal-regulated kinase (ERK). In addition, treatment of JJ012 cells with NF-kappaB inhibitor (PDTC) or IkappaB protease inhibitor (TPCK) inhibited CTGF-induced cell migration and MMP-13 up-regulation. Stimulation of JJ012 cells with CTGF also induced IkappaB kinase alpha/beta (IKK alpha/beta) phosphorylation, IkappaBalpha phosphorylation, p65 Ser(536) phosphorylation, and kappaB-luciferase activity. The CTGF-mediated increases in kappaB-luciferase activities were inhibited by RGD, PD98059, U0126 or FAK, and ERK2 mutant. Taken together, our results indicated that CTGF enhances the migration of chondrosarcoma cells by increasing MMP-13 expression through the alphavbeta3 integrin, FAK, ERK, and NF-kappaB signal transduction pathway. 
 Expression levels and significance of nuclear factor-κB and epidermal growth factor receptor in hepatolithiasis associated with intrahepatic cholangiocarcinoma. An increasing incidence of cholangiocarcinoma (CCA) and CCA mortality rates has been observed around the world. Patients with intrahepatic biliary stones have a 10% risk of developing CCA, and up to 70% of patients with histologically confirmed CCA have hepatolithiasis. Few previous studies have addressed the associations between the expression of nuclear factor (NF)-κB and epidermal growth factor receptor (EGFR) and clinicopathological prognosis in patients with hepatolithiasis-associated intrahepatic CCA.This study was designed to investigate the clinicopathological and prognostic significance of NF-κB and EGFR expression in hepatolithiasis-associated intrahepatic CCA and hepatolithiasis.A total of 90 liver specimens were immunohistochemically stained for NF-κB and EGFR expression, and the characteristics of 90 individual patients were retrospectively reviewed.Differences in the positive rates of NF-κB and EGFR expression between the hepatolithiasis-associated intrahepatic CCA group, intrahepatic lithiasis group, and control group were found to be statistically significant. EGFR expression was closely associated with the degree of differentiation and depth of invasion (p < 0.05). The 1-, 3-, and 5-year overall survival rates were respectively 42.8, 21.0, and 10.3% in intrahepatic CCA groups. The survival rate of the EGFR-negative group was higher than in the EGFR-positive group (p < 0.01). Lymph   node metas  tasis (HR 1.24, 95% CI 1.02-1.51) and EGFR positivity (HR 1.74, 95% CI 1.30-2.23) were associated with decreases in the survival rate.The expression of NF-κB may be an early step in intrahepatic cholangiocarcinogenesis. Overexpression of EGFR is associated with the degree of malignancy and with poor prognosis. NF-κB and EGFR may cooperate during intrahepatic cholangiocarcinogenesis and progression. Lymp  h node met  astasis and EGFR positivity were associated with decreases in the survival rate. 
 Up-regulation of vascular endothelial growth factor C in breast cancer cells by heregulin-beta 1. A critical role of p38/nuclear factor-kappa B signaling pathway. Vascular endothelial growth factor C (VEGF-C) is a critical activator of tumor lymphangiogenesis that recently has been strongly implicated in the tumor   metastasis   process. In this study, we identified that HRG-beta 1 stimulated up-regulation of VEGF-C mRNA and protein of human breast cancer cells in a dosage- and time-dependent manner and that this up-regulation was de novo RNA synthesis-dependent. The HRG-beta 1-induced increase in VEGF-C expression was effectively reduced by treatment with Herceptin, an antibody specifically against HER2. Also, when HER2 was overexpressed in MCF-7 cells that resulted in an evident increase in the VEGF-C level, suggesting an essential role of HER2 in mediating VEGF-C up-regulation by HRG-beta 1. NF-kappa B has been shown to be probably involved in interleukin-1 beta- or tumor necrosis factor-alpha-induced VEGF-C mRNA expression in human fibroblasts. Here we found that HRG-beta 1 could stimulate NF-kappa B nuclear translocation and DNA-binding activity via the I kappa B alpha phosphorylation-degradation mechanism. Blockage of the NF-kappa B activation cascade caused a complete inhibition of the HRG-beta 1-induced elevation of VEGF-C. In promoter-reporter assay, the luciferase activities of the reporter constructs, including the putative NF-kappa B site deleted and mutated form were significantly reduced after HRG-beta 1 treatment as compared with the 1.5-kb VEGF-C promoter. Although investigating the upstream kinase pathway(s) involved in HRG-beta 1-elicited NF-kappa B activation and VEGF-C up-regulation, we found that HRG-beta1 could activate extracellular signal-regulated protein kinase 1/2, phosphatidylinositol 3'-kinase, and p38 mitogen-activated protein kinase (MAPK) in MCF-7. However, only SB203580 (a specific inhibitor of p38 MAPK), not PD98059 nor LY294002, blocked the up-regulation of VEGF-C by HRG-beta 1. A similar inhibition in VEGF-C expression was obtained by cell transfection with dominant-negative p38 (p38AF). Interestingly, the HRG-beta 1-induced NF-kappa B activation cascade was also effectively blocked by SB203580 treatment or p38AF transfection. Our data thus suggests that HRG-beta 1 stimulated a NF-kappa B-dependent up-regulation of VEGF-C through the p38 MAPK signaling pathway in human breast cancer cells. 
 DNA damage induces NF-κB-dependent microRNA-21 up-regulation and promotes breast cancer cell invasion. NF-κB activation induced by genotoxic treatment in cancer cells has been associated with therapeutic resistance in multiple human malignancies. Therapeutic resistance also correlates with high metastatic potential in human cancers, including breast cancer. Whether genotoxic treatment-activated NF-κB also contributes to canc  er metasta  sis following radiation and chemotherapy is unclear. Here, we show that chemotherapeutic drug-induced NF-κB activation promotes breast cancer cell migration and invasion. The increased metastatic potential is dependent on IL-6 induction mediated by genotoxic NF-κB activation. Moreover, genotoxic treatment also up-regulates oncogenic microRNA-21 (miR-21) expression through eliciting NF-κB recruitment to the miR-21 promoter region, where it cooperates with signal transducer and activator of transcription 3 (STAT3) to activate miR-21 transcription. DNA damage-induced histone H3 phosphorylation via activated MSK1 creates an open chromatin structure for NF-κB/STAT3-driven transactivation of miR-21. NF-κB-dependent IL-6 up-regulation is responsible for STAT3 activation and recruitment to the miR-21 promoter upon genotoxic stress. Induction of miR-21 may enable cancer cells to elude DNA damage-induced apoptosis and enhance the metastatic potential of breast cancer cells through repressing expression of PTEN and PDCD4. Our data support a critical role of DNA damage-induced NF-κB activation in promotin  g cancer m  etastasis following genotoxic treatment, and NF-κB-dependent miR-21 induction may contribute to both therapeutic resis  tance and   metastasis in breast cancer. 
 NF-κB mediates the 12(S)-HETE-induced endothelial to mesenchymal transition of lymphendothelial cells during the intravasation of breast carcinoma cells. The intravasation of breast cancer into the lymphendothelium is an early step of   metastasi  s. Little is known about the mechanisms of bulky cancer invasion into lymph ducts.To particularly address this issue, we developed a 3-dimensional co-culture model involving MCF-7 breast cancer cell spheroids and telomerase-immortalised human lymphendothelial cell (LEC) monolayers, which resembles intravasation in vivo and correlated the malignant phenotype with specific protein expression of LECs.We show that tumour spheroids generate 'circular chemorepellent-induced defects' (CCID) in LEC monolayers through retraction of LECs, which was induced by 12(S)-hydroxyeicosatetraenoic acid (HETE) secreted by MCF-7 spheroids. This 12(S)-HETE-regulated retraction of LECs during intravasation particularly allowed us to investigate the key regulators involved in the motility and plasticity of LECs. In all, 12(S)-HETE induced pro-metastatic protein expression patterns and showed NF-κB-dependent up-regulation of the mesenchymal marker protein S100A4 and of transcriptional repressor ZEB1 concomittant with down-regulation of the endothelial adherence junction component VE-cadherin. This was in accordance with ∼50% attenuation of CCID formation by treatment of cells with 10 μM Bay11-7082. Notably, 12(S)-HETE-induced VE-cadherin repression was regulated by either NF-κB or by ZEB1 since ZEB1 siRNA knockdown abrogated not only 12(S)-HETE-mediated VE-cadherin repression but inhibited VE-cadherin expression in general.These data suggest an endothelial to mesenchymal transition-like process of LECs, which induces single cell motility during endothelial transmigration of breast carcinoma cells. In conclusion, this study demonstrates that the 12(S)-HETE-induced intravasation of MCF-7 spheroids through LECs require an NF-κB-dependent process of LECs triggering the disintegration of cell-cell contacts, migration, and the generation of CCID. 
 Targeted knockdown of EGR-1 inhibits IL-8 production and IL-8-mediated invasion of prostate cancer cells through suppressing EGR-1/NF-kappaB synergy. IL-8 produced by prostate cancer cells may be responsible for the androgen-independent growth of advanced prostate cancers. Accumulating evidence from microarray analyses and animal genetic models highlights the central involvement of the transcription factor early growth response-1 (EGR-1) in prostate carcinoma progression. It is unknown, however, whether knockdown of EGR-1 inhibits IL-8 production and IL-8-mediated tumor   metastasis  . Here we show that EGR-1 knockdown by a specific shRNA-Egr1 inhibited gene transcription and production of IL-8 by the human prostate cancer cell line DU145. Conversely, enforced expression of EGR-1 in EGR-1-lacking PC3 prostate cancer cells markedly enhanced IL-8 transcription and secretion. By using wild type and a series of mutant IL-8 promoter luciferase constructs, we found that the NF-kappaB binding site is important for EGR-1 regulation of IL-8. Furthermore, silencing EGR-1 suppressed a synergistically functional interaction between EGR-1 and NF-kappaB. Consequently, knockdown of EGR-1 inhibited IL-8-mediated tumor colony formation and invasion. Thus, targeted knockdown of EGR-1 could be an effective therapeutic approach against prostate cancer. 
 [Activation and prognostic significance of AKT, NF-kappaB and STAT3 in breast cancer with lymph node   metastasis   and estrogen receptor expression]. Being downstream targets of the phosphatase and tensin homolog deleted on chromosome ten (PTEN) and epidermal growth factor receptor (EGFR) pathways, serine/threonine kinase AKT, nuclear factor-kappaB (NF-kappaB) and signal transducer and activator of transcription-3 (STAT3), play important roles in cell proliferation, apoptosis and oncogenesis. This study was to investigate the activation and prognostic values of AKT, NF-kappaB and STAT3 in breast cancer with lymph node   metastasis   and estrogen receptor (ER) expression.The expression of phosphatized AKT (p-AKT), NF-kappaB (p-NF-kappaB) and STAT3 (p-STAT3), and the expression of EGFR, PTEN, HER-2, and Ki67 in tissue samples from 130 breast cancer patients with lymph node   metastasis   and ER expression were detected by immunohistochemistry.The activity of AKT (p-AKT) and NF-kappaB (p-NF-kappaB) were correlated to HER-2 overexpression (P=0.023 and P=0.017) and histological grade of breast cancer (P=0.035 and P=0.004). The expression of p-AKT, p-NF-kappaB and p-STAT3 had no correlation to tumor size, EGFR overexpression, and proliferation index assessed by Ki67. The expression of p-AKT was positively correlated to that of p-NF-kappaB (r=0.43, P<0.001) and negatively correlated to that of p-PTEN (r=-0.20, P=0.002). The overexpression of p-AKT and p-NF-kappaB were significantly related to shorter survival (P=0.002 and P=0.003). The up-regulation of p-NF-kappaB expression was also related to enhanced risk of recurrence and   metastasis   (P=0.006). Cox multivariate analysis showed that the expression of p-AKT and p-NF-kappaB were correlated to shorter overall survival (OS) (P=0.017 and P=0.008) and disease-free survival (DFS) (P=0.005 and P=0.012), while the expression of p-STAT3 had no correlation to OS (P=0.332) and DFS (P=0.237).The activation of AKT and NF-kappaB, but not STAT3, significantly contributes to the progression of breast cancer. Activated AKT and NF-kappaB may indicate poor prognosis of breast cancer with lymph node   metastasis   and ER expression. 
 The secret second life of an innocent chaperone: the story of CD74 and B cell/chronic lymphocytic leukemia cell survival. This review deals with the cytokine macrophage migration inhibitory factor (MIF) and its receptor, CD74. MIF and CD74 have been shown to regulate peripheral B cell survival and were associated with tumor progression and   metastasis  . CD74 expression has been suggested to serve as a prognostic factor in many cancers, with higher relative expression of CD74 behaving as a marker of tumor progression. In chronic lymphocytic leukemia (CLL) cells, binding of MIF to CD74 induces nuclear factor-κB (NF-κB) activation and up-regulation of TAp63 expression, resulting in the secretion of interleukin 8 (IL-8), which in turn promotes cell survival. In addition, TAp63 expression elevates expression of the integrin VLA-4, particularly during the advanced stage of the disease. Blocking of CD74, TAp63, or VLA-4 inhibits the in vivo homing of CLL cells to the BM. Thus, CD74 and its target genes, TAp63 and VLA-4, facilitate migration of CLL cells back to the BM, where they interact with the supportive BM environment that helps rescue them from apoptosis. These results are expected to pave the way toward novel therapeutic strategies aimed at interrupting this survival pathway. One such agent, the monocolonal antibody milatuzumab directed at CD74, is already being studied in early clinical trials. 
 The expression of Survivin and NF-κB associated with prognostically worse clinicopathologic variables in hepatocellular carcinoma. Expressions of Survivin and nuclear factor of kappa light polypeptide gene enhancer in B cells (NF-κB) are associated with a poor prognosis in many malignancies. However, their relationship in hepatocellular carcinoma remains unclear. To investigate the protein expression of Survivin and NF-κB, determine their role in the pathogenesis of hepatocellular carcinoma, and correlate expression with patient survival outcome, immunohistochemistry was used to detect the protein expression of Survivin and NF-κB in 305 cases of hepatocellular carcinoma. Statistical analysis was performed to determine the relationship between the protein expression of Survivin and NF-κB and clinicopathological parameters, survival time, and prognosis. Survivin was expressed predominantly in the cytoplasm, and NF-κB was expressed mostly in the nucleolus. Survivin and NF-κB are expressed at significantly higher rates in hepatocellular carcinoma compared with benign tissue (75.7 vs 13.4 %, P < 0.01 and 79.0 vs 17.1 %, P < 0.01, respectively). Both Survivin and NF-κB expression levels are associated with poor prognostic factors, including tumor size, capsular invasion, tumor thrombus of t  he portal   vein, metastasis of the lymph node, and clinical staging. There was an obvious positive correlation between the expression of Survivin and NF-κB in hepatocellular carcinoma (r = 0.23, P < 0.01). Patients expressing Survivin and NF-κB had significantly shorter survival compared with patients negative for protein expression (P < 0.01). The overexpressions of both Survivin and NF-κB are associated with worse survival outcome in patients with hepatocellular carcinoma. Thus, these proteins could be used as negative prognostic indicators. 
 Visfatin triggers the in vitro migration of osteosarcoma cells via activation of NF-κB/IL-6 signals. Pulmonary   metastasi  s is the major challenge for clinical treatment of osteosarcoma patients. Recent studies indicated that visfatin, a 52kDa adipocytokine, can trigger the cell motility of various cancers, while its role in the progression of osteosarcoma remains not clear. Our present study revealed that visfatin can significantly promote the in vitro migration and invasion of osteosarcoma MG-63 and HOS cells and up regulate the expression of matrix metalloproteinase-2 (MMP-2) and fibronectin (FN). Furthermore, visfatin treatment also increased the expression of IL-6 in both MG-63 and HOS cells via a time dependent manner, while anti-IL-6 antibody can significantly attenuate visfatin induced cell invasion and up regulation of MMP-2 and FN. It suggested that up regulation of IL-6 mediated visfatin induced in vitro motility of osteosarcoma cells. Visfatin treatment can increase the phosphorylation of both p65 and ERK1/2 in MG-63 and HOS cells, while only the inhibitor of NF-κB, BAY 11-7082, can abolish visfatin induced up regulation of IL-6. BAY 11-7082 also attenuated visfatin induced upregulation of MMP-2 and FN in MG-63 cells. Western blot analysis revealed that visfatin treatment can significantly increase the phosphorylation of IκBα and IKKβ in MG-63 cells. ACHP, the inhibitor of IKK-β, blocked visfatin induced expression of IL-6 mRNA in both MG-63 and HOS cells. Collectively, our data suggested that visfatin can increase the motility of osteosarcoma cells via up regulation of NF-κB/IL-6 signals. It indicated that visfatin might be a potential therapeutic target of osteosarcoma treatment. 
 TWEAK promotes ovarian cancer cell   metastasis   via NF-kappaB pathway activation and VEGF expression. The poor prognosis of human ovarian cancer is partly due to its   metastasis   and recurrence. It has been demonstrated that tumor necrosis factor (TNF)-like weak inducer of apoptosis (TWEAK)-fibroblast growth factor inducible-14 (Fn14) signaling system may be a potential regulator of human tumorigenesis. The objective of this study was to understand the effect of TWEAK on ovarian cancer   metastasis  . We recently showed that activation of Fn14 signaling by TWEAK promoted cell migration and invasion in human HO-8910PM cells. Treating HO-8910PM cells with TWEAK resulted in the activation of nuclear factor-kappa B (NF-kappaB) and subsequently the translocation of NF-kappaB from cytoplasm to nucleus. In addition, TWEAK promoted vascular endothelial growth factor (VEGF) protein expression, and this effect was dependent upon NF-kappaB transcriptional activity. Blocking the NF-kappaB pathway with PDTC suppressed TWEAK-induced up-regulation of VEGF protein expression and cell   metastasis  . Our results suggest that TWEAK-Fn14 functions, in part, through the NF-kappaB signaling pathway to up-regulate VEGF expression to foster ovarian cancer cell   metastasis  . Targeted therapy against TWEAK-Fn14 signaling system as an adjuvant to surgery may improve clinical management of invasive ovarian cancer cells and advance the outcome of this devastating cancer. 
 Effect of PI3K/Akt Signaling Pathway on the Process of Prostate Cancer   Metastasis   to Bone. We sought to study the effects of PI3K/Akt pathway and its downstream substrate NF-κB on prostate cancer bone metastatic process. Expression level of active p-Akt in PC3 cells was upregulated by transient expression with constitutively active plasmid CA-Akt or, alternatively, suppressed by dominant negative construct DN-Akt. NF-κB activity was determined by luciferase reporter assays. mRNA and protein expressions of receptor activator of NF-κB ligand (RANKL), parathyroid hormone-related protein (PTHrP), and bone morphogenetic protein 2 (BMP-2) were evaluated using RT-PCR and Western blotting. The effect of cross-talk between PC3 and SaOS2 cells on cell proliferation was analyzed using a co-culture system. Stimulation of p-Akt promoted NF-κB activity, and led to an increase in mRNA and protein expressions of RANKL, PTHrP, and BMP-2 in PC3 PCa cells through NF-κB. Co-culturing PC3 and SaOS2 cells significantly increased the expression of p-Akt and the activity of NF-κB, and promoted proliferation of both PC3 and SaOS2 cells. Increasing expression levels of p-Akt by transfection with CA-Akt led to further increase in cells proliferation, whereas NF-κB inhibitor PDTC partially blocked this effect. PI3K/Akt pathway stimulates the expressions of RANKL, PTHrP, and BMP-2 partly through NF-κB, suggesting its importance f  or bone me  tastasis of prostate carcinoma. Interaction of prostate cancer cells with bone cells has a stimulatory effect on cell proliferation. 
 High-mobility group box 1 protein activating nuclear factor-κB to upregulate vascular endothelial growth factor C is involved in lymphangiogenesis and lymphatic node   metastasi  s in colon cancer. To investigate the roles of high-mobility group box 1 (HMGB1) protein in lymphangiogenesis and lymphatic node   metastasi  s in colon cancer.Archival tumour specimens from patients with colon cancer were analysed in this retrospective immunohistochemical study. HMGB1, vascular endothelial growth factor C (VEGF-C) and podoplanin protein levels were analysed immunohistochemically. In vitro studies using the colon cancer cell line HCT116 were also undertaken to investigate the relationship between HMGB1, VEGF-C and nuclear factor (NF)-κB.Specimens from 70 patients with colon cancer were reviewed. The presence of positive HMGB1 immunohistochemical staining significantly correlated with lymphatic microvessel density, lymph no  de metasta  sis and VEGF-C immunohistochemical staining in colon cancer specimens. The presence of positive VEGF-C immunohistochemical staining significantly correlated with lymph no  de metasta  sis. The in vitro studies demonstrated that HMGB1 upregulated VEGF-C mRNA and protein in a dose-dependent manner in HCT116 cells, and that this was mediated via NF-κB.HMGB1 immunohistochemical staining was significantly associated with lymphangiogenesis and lymphatic   node metas  tasis in colon cancer. There was evidence that HMGB1 upregulates VEGF-C by activating NF-κB in a colon cancer cell line. 
 β-Arrestin 1 is required for endothelin-1-induced NF-κB activation in ovarian cancer cells. In epithelial ovarian cancer (EOC), activation of endothelin-1 (ET-1)/endothelin A receptor (ETAR) signalling is linked to many tumor promoting effects, such as proliferation, angiogenesis, invasion an  d metastas  is. These effects are dependent by the activation of critical signalling pathways, such as MAPK, Akt, and β-catenin, through specific cytosolic and nuclear scaffolding functions of β-arrestin 1 (β-arr1). Here, we have assessed the potential role of ET-1/ETAR in promoting NF-κB signalling in EOC cells through β-arr-1 recruitment.We used cultured HEY EOC cells cultured in the presence or absence of ET-1 and the ETAR antagonist BQ123. The phosphorylation of p65 and Iκ-Bα was evaluated by immunoblotting analysis. The interaction between p65 and β-arr1 was evaluated by immunoprecipitation experiments in nuclear extracts. NF-κB promoter activity was evaluated by transfection with NF-κB-driven luciferase reporter construct. Assessment of the function of β-arr1 was achieved by β-arr1 silencing with shRNA and expression of β-arr1-FLAG expression vector.In EOC cells, ET-1 promotes the phosphorylation of p65 subunit and the cytoplasmic inhibitor IκB that in turn led to increased NF-κB transcriptional activity. These effects were inhibited by the use of BQ123, as well as by β-arr-1 silencing, suggesting that ET-1 through ETAR promotes the recruitment of β-arr1 to regulate NF-κB signalling. Moreover, the nuclear physical interaction between p65 and β-arr1 indicates a nuclear function of β-arr-1 in ETAR-driven NF-κB transcriptional activity.Altogether these findings reveal a previously unrecognized pathway that depends on β-arr1 to sustain NF-κB signalling in response to ETAR activation in ovarian cancer. 
 Hypoxia activates heparanase expression in an NF-kappaB dependent manner. Hypoxia was shown to increase tumor cell invasion into the extracellular matrix in vitro. This result suggests that heparanase (Hpa), one of the key enzymes involved in tumor invasion and   metastasis  , may be regulated by hypoxia. RT-PCR, Western blot and Matrigel invasive assays were used to study the regulation of Hpa under hypoxia in human pancreatic MIA PaCa-2 cancer cells. Compared with those in normoxia (20% O2), Hpa mRNA, protein and enzymatic activity levels, were up-regulated by a reduction in the oxygen level (1% O2). Invasion by tumor cells into the extracellular matrix was found to be significantly enhanced. A reduction in Hpa protein levels was observed when nuclear factor kappaB (NF-kappaB) activation was blocked by pyrrolidine dithiocarbamate. The levels of Hpa were also reduced when Hpa was inhibited by an Hpa-specific antisense oligonucleotide. The MMP-9 mRNA, protein and gelatinase B activity levels in supernatants decreased significantly when Hpa was inhibited. We conclude that up-regulation of Hpa by hypoxia is NF-kappaB-dependent in MIA PaCa-2 cells and inhibition of Hpa reduces MMP-9 activity. This reduction in MMP-9 activity may be an important mechanism in tumor   metastasis  . 
 Receptor-interacting protein kinase 2 promotes triple-negative breast cancer cell migration and invasion via activation of nuclear factor-kappaB and c-Jun N-terminal kinase pathways.   Metastasis   is the main cause of breast cancer morbidity and mortality. Processes that allow for tumor cell migration and invasion are important therapeutic targets. Here we demonstrate that receptor-interacting protein kinase 2 (RIP2), a kinase known to be involved in inflammatory processes, also has novel roles in cancer cell migration and invasion.A total of six breast cancer expression databases, including The Cancer Genome Atlas, were assessed for RIP2 expression among various clinical subtypes and its role as a prognostic biomarker. mRNA fluorescence in situ hybridization (FISH) for RIP2 was performed on 17 stage III breast cancers to determine if there was a correlation between RIP2 expression and lymph node involvement. RNA-interference was used to knock-down RIP2 expression in MDA-MB-231, Htb126, SUM149PT, MCF7, T47D, and HCC1428 cells. Cell migration and invasion were measured in vitro by scratch/wound healing and transwell migration assays. A xenograft mouse model was used to assess tumor growth and chemosensitivity to docetaxel in vivo in MDA-MB-231 cells with and without RIP2 small hairpin RNA knockdown. Western blot and immunofluorescence imaging were used to evaluate protein expressions.Interrogation of expression databases showed that RIP2 expression is significantly over-expressed in triple-negative breast cancers (TNBC: estrogen-receptor (ER) negative, progesterone-receptor (PR) negative, Her2/neu- (Her2) negative), compared to other clinical subtypes. High RIP2 expression correlates with worse progression-free survival using a combined breast cancer expression array dataset consisting of 946 patients. Multivariate analysis shows RIP2 as an independent prognostic biomarker. Knock-down of RIP2 significantly decreases migration in both scratch/wound healing and transwell migration assays in MDA-MB-231, Htb126, SUM149PT, MCF7, and T47D cells and is correlated with decreased Nuclear Factor-kappaB and c-Jun N-terminal kinase (JNK) activation. Finally, RIP2 knock-down leads to increased sensitivity to docetaxel and decreased tumor mass and lung metastases in a xenograft mouse model.These results highlight RIP2 as a pro-  metastasis   kinase in patients with advanced breast cancer. These results also illustrate a novel role for this kinase in addition to its known role in inflammation, and suggest that targeting RIP2 may improve outcomes in advanced breast cancer patients, in which it is overexpressed. 
 Reciprocal activation between ATPase inhibitory factor 1 and NF-κB drives hepatocellular carcinoma angiogenesis and   metastasi  s. Hepatocellular carcinoma (HCC) is a highly vascularized tumor with frequent extrahepatic   metastasi  s. Active angiogenesis and   metastasi  s are responsible for rapid recurrence and poor survival of HCC. However, the mechanisms that contribute to tumor   metastasi  s remain unclear. Here we evaluate the effects of ATPase inhibitory factor 1 (IF1), an inhibitor of the mitochondrial H(+)-adenosine triphosphate (ATP) synthase, on HCC angiogenesis and   metastasi  s. We found that increased expression of IF1 in human HCC predicts poor survival and disease recurrence after surgery. Patients with HCC who have large tumors, with vascular invasion and   metastasi  s, expressed high levels of IF1. Invasive tumors overexpressing IF1 were featured by active epithelial-mesenchymal transition (EMT) and increased angiogenesis, whereas silencing IF1 expression attenuated EMT and invasion of HCC cells. Mechanistically, IF1 promoted Snai1 and vascular endothelial growth factor (VEGF) expression by way of activating nuclear factor kappa B (NF-κB) signaling, which depended on the binding of tumor necrosis factor (TNF) receptor-associated factor 1 (TRAF1) to NF-κB-inducing kinase (NIK) and the disruption of NIK association with the TRAF2-cIAP2 complex. Suppression of the NF-κB pathway interfered with IF1-mediated EMT and invasion. Chromatin immunoprecipitation assay showed that NF-κB can bind to the Snai1 promoter and trigger its transcription. IF1 was directly transcribed by NF-κB, thus forming a positive feedback signaling loop. There was a significant correlation between IF1 expression and pp65 levels in a cohort of HCC biopsies, and the combination of these two parameters was a more powerful predictor of poor prognosis.IF1 promotes HCC angiogenesi  s and meta  stasis by up-regulation of Snai1 and VEGF transcription, thereby providing new insight into HCC progression and IF1 function. (Hepatology 2014;60:1659-1673). 
 The tumor marker Fascin is induced by the Epstein-Barr virus-encoded oncoprotein LMP1 via NF-κB in lymphocytes and contributes to their invasive migration. The actin-bundling protein Fascin (FSCN1) is a tumor marker that is highly expressed in numerous types of cancer including lymphomas and is important for migration and   metastasi  s of tumor cells. Fascin has also been detected in B lymphocytes that are freshly-infected with Epstein-Barr virus (EBV), however, both the inducers and the mechanisms of Fascin upregulation are still unclear.Here we show that the EBV-encoded oncoprotein latent membrane protein 1 (LMP1), a potent regulator of cellular signaling and transformation, is sufficient to induce both Fascin mRNA and protein in lymphocytes. Fascin expression is mainly regulated by LMP1 via the C-terminal activation region 2 (CTAR2). Block of canonical NF-κB signaling using a chemical inhibitor of IκB kinase β (IKKβ) or cotransfection of a dominant-negative inhibitor of IκBα (NFKBIA) reduced not only expression of p100, a classical target of the canonical NF-κB-pathway, but also LMP1-induced Fascin expression. Furthermore, chemical inhibition of IKKβ reduced both Fascin mRNA and protein levels in EBV-transformed lymphoblastoid cell lines, indicating that canonical NF-κB signaling is required for LMP1-mediated regulation of Fascin both in transfected and transformed lymphocytes. Beyond that, chemical inhibition of IKKβ significantly reduced invasive migration of EBV-transformed lymphoblastoid cells through extracellular matrix. Transient transfection experiments revealed that Fascin contributed to LMP1-mediated enhancement of invasive migration through extracellular matrix. While LMP1 enhanced the number of invaded cells, functional knockdown of Fascin by two different small hairpin RNAs resulted in significant reduction of invaded, non-attached cells.Thus, our data show that LMP1-mediated upregulation of Fascin depends on NF-κB and both NF-κB and Fascin contribute to invasive migration of LMP1-expressing lymphocytes. 
 Suppression of NF-kappaB activity by NDRG2 expression attenuates the invasive potential of highly malignant tumor cells. Downregulation of the N-myc downstream-regulated gene 2 (NDRG2) gene is involved in the progression of aggressive forms of cancer, along with the poor prognosis of cancer patients. In the current study, we examined the effect of NDRG2 expression on the metastatic potential of HT1080 human fibrosarcoma and B16F10 murine melanoma cells in both in vitro and in vivo systems. In gelatin zymography, NDRG2 expression remarkably suppressed the matrix metalloproteinase (MMP)-9 activity and slightly inhibited MMP-2 activity of both cell lines. Tumor migration and invasion in vitro were significantly reduced by NDRG2 expression, and NDRG2 inhibited tumor cell proliferation in an anchorage-independent semisolid agar assay. Specifically, we found that NDRG2 affects invasion through suppression of nuclear factor kappa B (NF-kappaB) activity. In animal experiments, subcutaneously injected B16F10-NDRG2 cells showed delayed tumor growth compared with B16F10-mock cells. Furthermore, severe   metastasis   from primary tumor mass into the draining lymph nodes was observed after injection of B16F10-mock cells, but not with B16F10-NDRG2 cells. Pulmonary   metastasis   after intravenous injection of B16F10 cells was also reduced by NDRG2 expression. Intra- and peritumoral angiogenesis that is critical for the tumor growth and   metastasis   was clearly found in tumors after injection with B16F10-mock cells, whereas it was impaired in tumors after injection with B16F10-NDRG2 cells. Collectively, our data show that NDRG2 expression significantly suppresses tumor invasion by inhibiting MMP activities, which are regulated through the NF-kappaB signaling. Moreover, results from animal experiments provide evidence for the regulatory role of the NDRG2 gene in metastatic tumors. 
 Microtubule disruption utilizes an NFkappa B-dependent pathway to stabilize HIF-1alpha protein. Hypoxia-inducible factor (HIF)-1alpha levels are elevated in normoxic cells undergoing physiological processes involving large scale microtubule reorganization, such as embryonic development, wound healing, and tumor cell   metastasis  . Although alterations in microtubules affect numerous cellular responses, no data have yet implicated microtubule dynamics in HIF-1alpha regulation. To investigate the effect of microtubule change upon HIF-1alpha regulation, we treated cells with the microtubule-depolymerizing agents (MDAs) colchicine, vinblastine or nocodazole. We demonstrate that these agents are able to induce transcriptionally active HIF-1. MDA-mediated induction of HIF-1alpha required microtubule depolymerization, since HIF-1alpha levels were unchanged in cells treated with either the microtubule-stabilizing agent paclitaxel, or an inactive form of colchicine, or in colchicine-resistant cells. HIF-1 induction was dependent upon cellular transcription, as transcription inhibitors abrogated HIF-1alpha protein up-regulation. The ability of transcriptional inhibitors to interfere with HIF-1alpha accumulation was specific to the MDA-initiated pathway, as they were ineffective in preventing hypoxia-mediated HIF-1 induction, which occurs by a distinct post-translational pathway. Moreover, we provide evidence implicating a requirement for NFkappaB transcription in the HIF-1 induction mediated by MDAs. The ability of MDAs to induce HIF-1alpha is dependent upon activation of NFkappaB, since inhibition of NFkappaB either pharmacologically or by transfection of an NFkappaB super-repressor plasmid abrogated this induction. Collectively, these data support a model in which NFkappaB is a focal point for the convergence of MDA-mediated signaling events leading to HIF-1 induction, thus revealing a novel aspect of HIF-1alpha regulation and function. 
 Apoptosis of human primary osteoclasts treated with molecules targeting nuclear factor-kappaB. Osteoclasts (OCs) are involved in several pathologies associated with bone loss, including rheumatoid arthritis, osteoporosis, bone   metastasis   of myeloma, osteosarcoma, and breast cancer. In this review we determined the effects of natural compounds, including extracts from medicinal plants, on differentiation and survival of human primary OCs obtained from peripheral blood. We found that OCs from umbilical cord blood and peripheral blood behave differently in response to molecules inducing apoptosis in this experimental system. Apoptosis induced by decoy oligonucleotides was reproducibly obtained in OCs from peripheral blood but not in OCs derived from cord blood. With respect to effects of medicinal plants, we found that crude extracts of Emblica officinalis are able to induce specifically programmed cell death of mature OCs without altering the process of osteoclastogenesis. E. officinalis specifically increased the expression levels of Fas, a critical member of the apoptotic pathway. Gel shift experiments BioPharmaNet demonstrate that E. officinalis extracts specifically compete with the binding of a transcription factor involved in osteoclastogenesis NF-kappaB to its specific target DNA sequences. This might explain the observed effects of E. officinalis on the expression levels of IL-6, an NF-kappaB-specific target gene. We suggest the application of natural products as an alternative tool for therapy applied to bone diseases. 
 RANKL promotes migration and invasion of hepatocellular carcinoma cells via NF-κB-mediated epithelial-mesenchymal transition.   Metastasi  s accounts for the most deaths in patients with hepatocellular carcinoma (HCC). Receptor activator of nuclear factor kappa B ligand (RANKL) is associated with cancer   metastasi  s, while its role in HCC remains largely unknown.Immunohistochemistry was performed to determine the expression of RANK in HCC tissue (n = 398). Quantitative real-time polymerase chain reaction (qRT-PCR) and Western blot were used to examine the expression of RANK, E-cadherin, N-cadherin, vimentin, Snail, Slug, Twist and MMPs in HCC cells. Wound healing and Transwell assays were used to evaluate cell migration and invasion ability.We found that expression of RANK, the receptor of RANKL, was significantly higher in HCC tumor tissues than in peritumor liver tissues (p<0.001). Constitutive expression of RANK was detected in HCC cell lines, which can be up-regulated when HCC cells were stimulated with RANKL. Notably, in vitro experiments showed that activation of RANKL-RANK axis significantly promoted migration and invasion ability of HCC cells. In addition, RANKL stimulation increased the expression levels of N-cadherin, Snail, and Twist, while decreased the expression of E-cadherin, with concomitant activation of NF-κB signaling pathway. Moreover, administration of the NF-κB inhibitor attenuated RANKL-induced migration, invasion and epithelial-mesenchymal transition of HCC cells.RANKL could potentiate migration and invasion ability of RANK-positive HCC cells through NF-κB pathway-mediated epithelial-mesenchymal transition, which means that RANKL-RANK axis could be a potential target for HCC therapy. 
 Identification of an autoregulatory feedback pathway involving interleukin-1alpha in induction of constitutive NF-kappaB activation in pancreatic cancer cells. We previously reported that NF-kappaB is constitutively activated in most human pancreatic cancer tissues and cell lines but not in normal pancreatic tissues and immortalized pancreatic ductal epithelial cells. IkappaBalphaM-mediated inhibition of constitutive NF-kappaB activity in human pancreatic cancer cells suppressed tumorigenesis and liver   metastasis   in an orthotopic nude mouse model, suggesting that constitutive NF-kappaB activation plays an important role in pancreatic tumor progression and   metastasis  . However, the underlying mechanism by which NF-kappaB is activated in pancreatic cancer remains to be elucidated. In this study, we found that an autocrine mechanism accounts for the constitutive activation of NF-kappaB in metastatic human pancreatic cancer cell lines. Further investigation showed that interleukin-1alpha was the primary cytokine secreted by these cells that activates NF-kappaB. Neutralization of interleukin-1alpha activity suppressed the constitutive activation of NF-kappaB and the expression of its downstream target gene, urokinase-type plasminogen activator, in metastatic pancreatic cancer cell lines. Our results demonstrate that regulation of interleukin-1alpha expression is primarily dependent on AP-1 activity, which is in part induced by signaling pathways that are epidermal growth factor receptor-dependent and -independent. In conclusion, our findings suggest a possible mechanism for the constitutive activation of NF-kappaB in metastatic human pancreatic cancer cells and a possible missing mechanistic link between inflammation and cancer. 
 Elevated TAK1 augments tumor growth and metastatic capacities of ovarian cancer cells through activation of NF-κB signaling. Transforming growth factor (TGF)-β-activating kinase 1 (TAK1) is a serine/threonine kinase which is frequently associated with human cancer progression. However, its functional role in tumorigenesis is still controversial. Here, we report that TAK1 enhances the oncogenic capacity of ovarian cancer cells through the activation of NF-κB signaling. We found that TAK1 is frequently upregulated and significantly associated with high-grade and metastatic ovarian cancers. Mechanistic studies showed that Ser412 phosphorylation is required for TAK1 in activating NF-κB signaling and promotes aggressiveness of ovarian cancer cells. Conversely, suppression of TAK1 activity by point mutation at Ser412, RNAi mediated gene knockdown or TAK1 specific inhibitor ((5Z) -7-Oxozeaenol) remarkably impairs tumor growth   and metast  asis in ovarian cancer in vitro and in vivo. Our study underscores the importance of targeting TAK1 as a promising therapeutic approach to counteract the ovarian cancer progression. 
 Clinicopathologic significance of nuclear factor-κB and vascular endothelial growth factor expression in advanced gastric cancer patients. Nuclear factor-κB (NF-κB) and vascular endothelial growth factor (VEGF) are involved in cell proliferation, invasion, angiogenesis, a  nd metasta  sis. The principal objective of this study was to assess the prognostic significance of NF-κB and VEGF expression in gastric cancer.Tumor tissues of 154 patients with gastric cancer, all of whom underwent potentially curative resection, were immunohistochemically evaluated using monoclonal antibodies against NF-κB and VEGF.The positivity rates of NF-κB and VEGF staining were 44.2% and 39.6%, respectively. NF-κB expression in tumor tissues was significantly correlated with VEGF expression (p < 0.001). VEGF expression was related to Lauren's classification (p = 0.002), differentiation (p = 0.043), depth of invasion (p = 0.005), carcinoembryonic antigen expression (p = 0.032), and stage (p = 0.026). Univariate analysis demonstrated that NF-κB expression was significantly related to both the 5-year disease-free survival (65.2% vs. 46.4%, p = 0.007) and the 5-year overall survival (60.0% vs. 42.5%, p = 0.014). Multivariate analysis verified that NF-κB was independently associated with disease-free survival (hazard ratio: 2.082, p = 0.005) and overall survival (hazard ratio: 1.841, p = 0.008).NF-κB expression in tumor tissue is associated with poor survival in gastric cancer patients. 
 Prolyl hydroxylase-3 is down-regulated in colorectal cancer cells and inhibits IKKbeta independent of hydroxylase activity. Prolyl hydroxylase (PHD) hydroxylates hypoxia inducible factor (HIF) alpha, leading to HIFalpha degradation. The PHD family comprises PHD1, PHD2, and PHD3. The enzymatic activity of PHDs is O(2)-dependent, so PHDs are believed to be oxygen sensors as well as tumor suppressors. However, the expression pattern of PHDs in colorectal cancer and the correlation between their expression level and tumorigenesis is unclear.We determined the expression of PHDs in 60 human primary colorectal carcinoma tissues, paired with normal colorectal tissues. PHD3 expression levels were knocked down using small interfering RNA (siRNA); cells were analyzed by immunoblotting, immunoprecipitation, and histochemical analyses. In vivo tumor growth was analyzed in nu/nu mice.Expression of PHD3 is decreased in colorectal cancer tissues. Decreased expression of PHD3 is associated with higher tumor grade and   metastasis  . PHD3 inhibits phosphorylation of inhibitor of kappaB (IkappaB) kinase (IKK) beta and activation of (NF) kappaB, independent of its hydroxylase activity. PHD3 associates with IKKbeta but does not target it for destruction; instead, PHD3 blocks the interaction between IKKbeta and Hsp90 that is required for phosphorylation of IKKbeta. Knockdown of PHD3 increased resistance of colorectal cancer cells to the effects of tumor necrosis factor-alpha and tumorigenesis.PHD3 appears to be a tumor suppressor in colorectal cancer cells that inhibits IKKbeta/NF-kappaB signaling, independent of its hydroxylase activity. Activation of NF-kappaB has been observed in colon cancer. Determination of PHD3 status could aid targeted therapy selection for patients with colorectal tumors that have increased NF-kappaB activity. 
 S100A4 regulates migration and invasion in hepatocellular carcinoma HepG2 cells via NF-κB-dependent MMP-9 signal. We previously showed that the calcium-binding protein S100A4 is overexpressed and related to   metastasi  s in hepatocellular carcinoma (HCC). However, whether S100A4 participates in the regulation of   metastasi  s and its mechanisms in HCC is mostly unknown. Given the associations of S100A4, nuclear factor-kB (NF-kB/RelA) and MMP-9 with   metastasi  s in a variety of malignancies, we explored a potential role of S100A4 in HCC   metastasi  s and its mechanism.20 patients with HCC invasion (Lymph node   metastasi  s, microvascular invasion, major portal vein invasion and intrahepatic   metastasi  s) and 20 patients without HCC invasion were included. These tissues were detected for the expression of S100A4, NF-kB/RelA and MMP-9 by immunohistochemistry and quantitative real time polymerase chain reaction (Q-PCR). Correlation between the expressions of S100A4, NF-kB/RelA and MMP-9 with the invasion was analysed. The expressions of S100A4, nuclear factor-kB and MMP-9 was evaluated in HepG2 cells by western blot and immunohistochemistry. HepG2 cells were stably transfected with S100A4-specific small interfering RNA (S100A4 siRNA) to knockdown of S100A4, then transiently transfected with S100A4 cDNA to rescure the S100A4 level and evaluated for effects on invasion and expression analysis for molecules involved in invasion. After the HepG2 cells recurred the S100A4 levels, the HepG2 cells was treated with 5 µM Pyrrolidine Dithiocarbamate (PDTC) (a selective NF κ B inhibitor) to inhibit the NF-kB activity, or treated with Batimast (BB94: a MMPs inhibitor) to inhibit the MMP-9 activity. The expression analysis for molecules involved in invasion was analyzed.A significant increase of S100A4, NF-kB/RelA and MMP-9 expression in HCC tissues with invasion than that of without invasion. A positive correlation was observed between S100A4, NF-kB/RelA, MMP-9 and invasion, respectively. In addition, S100A4 was positively correlated with NF-kB and MMP-9. S100A4 siRNA mediated knockdown of S100A4 in HepG2 cells resulted in significant reduction in the NF-kB activity and MMP-9 expression, and dramatically decreased its invasion. Moreover, the HepG2 cell metastatic potential was rescued by overexpression of S100A4 completely, at the same time, the NF-kB activity and MMP-9 expression was also increased. Pretreatment with PDTC or BB94 was observed to significantly reduce NF-kB activity and MMP-9 expression and dramatically decreased S100A4 -induced invasion.Our findings indicate that S100A4 contributes to H  CC metasta  sis by activation of NF-kB dependent MMP-9 expression, suggesting S100A4 as a novel diagnostic biomarker and therapeutic target in HCC. 
 Notch1 promotes glioma cell migration and invasion by stimulating β-catenin and NF-κB signaling via AKT activation. The Notch signaling pathway has been implicated in both developmental processes and tumorigenesis. Aberrant Notch signaling has been repeatedly demonstrated to facilitate the proliferation and survival of glioma cells by regulating downstream effectors or other signaling pathways. In glioblastoma multiforme specimens from 59 patients, Notch1 was highly expressed in tumor tissues compared with normal brain tissues, and this expression was correlated with elevated AKT phosphorylation and Snail expression. Increased nuclear localization of β-catenin and p50 as well as enhanced IKKα/AKT interaction were also observed in glioma tissues. In U87MG cells, the activation of Notch1 by DLL4 stimulation or by the overexpression of Notch intracellular domain (NICD) resulted in AKT activation and thereby promoted β-catenin activity and NF-κB signaling. Inhibition of EGFR partially blocked the β-catenin and NF-κB signaling stimulated by Notch1 activation. Furthermore, NICD overexpression in U87MG cells led to the upregulated expression of   several me  tastasis-associated molecules, which could be abrogated by the knockdown of either β-catenin or p50. In U87MG and U251 cells, DLL4-induced cellular migration and invasion could be inhibited by either β-catenin or a p50 inhibitor. Collectively, these results indicate that Notch activation could stimulate β-catenin and NF-κB signaling through AKT activation in glioma cells. Thus, Notch activation-stimulated β-catenin and NF-κB signaling synergistically promote the migratory and invasive properties of glioma cells. 
 High expression of ErbB3 binding protein 1 (EBP1) predicts poor prognosis of pancreatic ductal adenocarcinoma (PDAC). Recent studies have identified that ErbB3 binding protein 1 (EBP1) is broadly expressed in various cancer tissues and critically involved in plenty of biological processes in this regard. However, the functional role of EBP1 in pancreatic ductal adenocarcinoma (PDAC) has never been elucidated. In this study, we found that EBP1 could serve as a prognostic biomarker of PDAC. Western blot analysis revealed that EBP1 was remarkably upregulated in PDAC tissues and cell lines. Using immunohistochemical analysis, we showed that the expression of EBP1 was correlated with tumor size (P = 0.004), histological differentiation (P = 0.041), and tum  or node me  tastasis (TNM) stage (P = 0.000). Notably, Kaplan-Meier curve showed that high expression of EBP1 predicted significantly worsened prognosis of PDAC patients (P = 0.001). In addition, knockdown of EBP1 expression suppressed PDAC cell proliferation and retarded cell cycle progression. Furthermore, depletion of EBP1 induced the apoptosis of Panc-1 cells. Of great interest, we found that EBP1 interacted with anti-apoptotic protein, Bcl-xL, and promoted its accumulation. In summary, our results suggest that EBP1 is a novel prognostic indicator and potential therapeutic target of PDAC, shedding new insights into the important role of EBP1 in cancer development. 
 Functional promoter polymorphisms of NFKB1 influence susceptibility to the diffuse type of gastric cancer. In the present study, we report an association between gastric cancer and polymorphisms in NFKB1 (rs28362941 and rs78696119). We employed the PCR-SSCP method to detect gene polymorphisms in 479 gastric cancer cases and 880 controls. The rs28362941 del/del homozygote was significantly associated with gastric cancer development; in particular it was closely associated with diffuse type gastric cancer. The rs78696119 GG homozygote was also associated with the diffuse type of gastric cancer. In young subjects, both polymorphisms were significantly associated with the development of gastric cancer. In addition, both polymorphisms were related to tumor progression such as tumor invasion and lymph node   metastasis  . The inflammatory cell infiltration into non-cancerous gastric mucosa was greater in the subjects with the rs28362491 del/del or rs78696119 GG genotype when compared to those with the other genotypes. In conclusion, functional polymorphisms of NFKB1 are associated with an increased risk of gastric cancer; in particular they are closely associated with the development of diffuse type of gastric cancer via severe gastric inflammation. These polymorphisms also appear to be associated with gastric cancer progression. 
 Antiproliferative and antimetastatic effects of emodin on human pancreatic cancer. Emodin (1, 3, 8-trihydroxy-6-methylanthraquinone) is an active constituent isolated from the root of Rheum palmatum L and is the main effective component of some Chinese herbs and plants. Pharmacological studies have demonstrated that emodin exhibits anti-cancer effects on several human cancers. However, the molecular mechanisms of emodin-mediated tumor regression have not been fully defined. This study was performed to investigate the antiproliferative and antimetastatic effects of emodin on pancreatic cancer in vitro and in vivo. Our results showed that emodin induced a higher percentage of growth inhibition and apoptosis in the pancreatic cancer cell line SW1990 compared to that of control, and emodin suppressed the migration and invasion of SW1990 cells in a dose-dependent manner. To investigate the possible mechanisms involved in these events, we performed electrophoretic mobility shift assay (EMSA) and Western blot analysis, and found that emodin significantly down-regulated NF-κB DNA-binding activity, survivin and MMP-9 in SW1990 cells. Moreover, the expression of cleaved caspase-3 was up-regulated in SW1990 cells after treatment with emodin. In addition, a metastatic model simulating human pancreatic cancer was established by orthotopic implantation of histologically intact human tumor tissue into the pancreatic wall of nude mice. Oral administration of emodin significantly decreased tumor weight and   metastasi  s compared to control. Furthermore, the expression of NF-κB, survivin and MMP-9 were also suppressed in tumor tissues after treatment with emodin. Collectively, our results indicated that emodin exerts antiproliferative and antimetastatic activity on pancreatic cancer both in vitro and in vivo, which may be related to down-regulation of NF-κB and its regulated molecules such as survivin and MMP-9 proteins. Consequently, these results provide important insights into emodin as an anti-invasive agent for the therapy of human pancreatic cancer. 
 Betaglycan blocks metastatic behaviors in human granulosa cell tumors by suppressing NFκB-mediated induction of MMP2. Metastatic ovarian granulosa cell tumors (GCT) exhibit loss of betaglycan. Here we test the hypothesis that betaglycan blocks GCT   metastasi  s by suppressing NFκB/TGFβ2-induced matrix metalloprotinease-2 (MMP2). Human GCT and a human GCT cell model demonstrated prominent MMP2 expression, which was dependent on NFκB activity and stimulated by TGFβ2 in an NFκB-dependent manner. Betaglycan suppressed both basal and TGFβ2-induced MMP2 expression and countered metastatic behaviors of GCT cells in non-adherent spheroid culture and in vivo xenograft mod  els of met  astasis. These data suggest that NFκB/TGFβ2 promotes, and betaglycan impedes, the early stage  s of GCT m  etastasis, when tumor cells first invade the peritoneum. 
 Autotaxin stimulates urokinase-type plasminogen activator expression through phosphoinositide 3-kinase-Akt-nuclear [corrected] factor kappa B signaling cascade in human melanoma cells. Autotaxin, a lysophospholipase D producing lysophosphatidic acid, augments invasive and metastatic potential of tumor cells. Current investigations have focused on understanding the molecular mechanisms by which autotaxin regulates the expression of a major mediator of tumor invasion and   metastasis  , urokinase-type plasminogen activator (uPA) in human A2058 melanoma cells. Autotaxin induced uPA expression in a dose-dependent manner that was inhibited by pharmacological inhibitors for Gi (pertussis toxin), phosphoinositide 3-kinase (PI3K, LY294002), Akt inhibitor (AktI), proteosome activity and IkappaB phosphorylation (pyrrolidine dithiocarbamate), and by a dominant negative mutant (DN) of Akt. Autotaxin phosphorylated Akt and induced the translocation of nuclear [corrected] factor-kappaB (NF-kappaB) to the nucleus that were inhibited by AktI or by overexpressing DN-Akt. Consistently, green fluorescence protein-tagged p65 of NF-kappaB accumulated in the nucleus by autotaxin that was abrogated when the cells were transfected with DN-Akt. Moreover, autotaxin increased the DNA binding ability of NF-kappaB and promoter activity of uPA. Collectively, these data strongly suggest autotaxin induces uPA expression via the Gi-PI3K-Akt-NF-kappaB signaling pathway that might be critical for autotaxin-induced tumor cell invasion and   metastasis  . 
 Overexpression of SDF-1 activates the NF-κB pathway to induce epithelial to mesenchymal transition and cancer stem cell-like phenotypes of breast cancer cells. The formation of EMT and EMT-induced CSC-like phenotype is crucial for the   metastasi  s of tumor cells. The stromal cell-derived factor-1 (SDF-1) is upregulated in various human carcinomas, which is closely associated with proliferation, migration, invasion and prognosis of malignancies. However, limited attention has been directed towards the effect of SDF-1 on epithelial to mesenchymal transition (EMT) or cancer stem cell (CSC)-like phenotype formation in breast cancer cells and the related mechanism. In the present study, we screened MCF-7 cells with low SDF-1 expression level for the purpose of evaluating whether SDF-1 is involved in EMT and CSC-like phenotype formation in MCF-7 cells. The pEGFP-N1-SDF-1 plasmid was transfected into MCF-7 cells, and the stably overexpressed SDF-1 in MCF-7 cells was confirmed by real-time PCR and western blot analysis. Colony formation assay, MTT, wound healing assay and Transwell invasion assay demonstrated that overexpression of SDF-1 significantly boosted the proliferation, migration and invasion of MCF-7 cells compared with parental (P<0.05). Flow cytometry analysis revealed a notable increase of CD44+/CD24- subpopulation in SDF-1 overexpressing MCF-7 cells (P<0.001), accompanied by the apparently elevated ALDH activity and the upregulation of the stem cell markers OCT-4, Nanog, and SOX2 compared with parental (P<0.01). Besides, western blot analysis and immunofluorescence assay observed the significant decreased expression of E-cadherin and enhanced expression of slug, fibronectin and vimentin in SDF-1 overexpressed MCF-7 cells in comparison with parental (P<0.01). Further study found that overexpression of SDF-1 induced the activation of NF-κB pathway in MCF-7 cells. Conversely, suppressing or silencing p65 expression by antagonist or RNA interference could remarkably increase the expression of E-cadherin in SDF-1 overexpressed MCF-7 cells (P<0.001). Overall, the above results indicated that overexpression of SDF-1 enhanced EMT by activating the NF-κB pathway of MCF-7 cells and further induced the formation of CSC-like phenotypes, ultimately promoting the proliferation a  nd metasta  sis of MCF-7 cells. Therefore, SDF-1 may further be assessed as a potential target for gene therapy of breast cancer. 
 Function of nuclear factor kappaB in pancreatic cancer   metastasis  . We seek to elucidate the role of constitutive nuclear factor kappaB (NFkappaB) activity in human pancreatic cancer cells. We have demonstrated that the transcription factor NFkappaB is activated constitutively in human pancreatic adenocarcinoma and human pancreatic cancer cell lines but not in normal pancreatic tissues or in immortalized/nontumorigenic pancreatic epithelial cells, suggesting that NFkappaB plays a critical role in development of pancreatic adenocarcinoma.By pooling all of the puromycin resistant clones after inhibitor of nuclear factor-kappaB phosphorylation mutant (IkappaBalphaM) retroviral infection, we generated pancreatic tumor cell lines that express a IkappaBalphaM (S32, 36A) that blocks NFkappaB activity. Inhibition of metastatic phenotype was assayed in an orthotopic nude mouse model. NFkappaB activity was determined by electrophoretic mobility shift assay, and the expression of NFkappaB downstream target genes was analyzed by Northern, Western, and immunohistochemical analyses.We showed that inhibiting constitutive NFkappaB activity by expressing IkappaBalphaM suppresses liver   metastasis  , but not tumorigenesis, from the metastatic human pancreatic tumor cell line AsPc-1 in an orthotopic nude mouse model. Furthermore, inhibiting NFkappaB activation by expressing IkappaBalphaM significantly reduced in vivo expression of a major proangiogenic molecule, vascular endothelial growth factor, and, hence, decreased neoplastic angiogenesis. Inhibiting NFkappaB activation by expressing IkappaBalphaM and using pharmacologic NFkappaB inhibitor PS-341 also significantly reduced cytokine-induced vascular endothelial growth factor and interleukin-8 expression in AsPc-1 pancreatic cancer cells.These results demonstrated that the inhibition of NFkappaB signaling can suppress the angiogenic potential and   metastasis   of pancreatic cancer, and suggest that the NFkappaB signaling pathway is a potential target for anticancer agents. 
 Functional polymorphism in NFKB1 promoter is related to the risks of oral squamous cell carcinoma occurring on older male areca (betel) chewers. Areca (betel)-chewing is tightly associated with the high prevalence of oral squamous cell carcinoma (OSCC) in Asians. NFKB1 encodes a 105kDa protein that can be processed to produce p50 subunit of nuclear factor-kappaB protein complex. A insertion (ins)/deletion (del) polymorphism (-94ins/delATTG) in NFKB1 promoter, which may drive the ins allele two-fold increase in NFKB1 transcription relative to del allele, was recently found. This study identified that the odds ratio in OSCC carrying ins allelotype were 1.78 relative to controls (56.7 vs 41.8%) in subjects more than 50 years old. L allelotype of Heme oxygenase-1 (HO-1), accounting for a long (GT)(n) repeat in HO-1 promoter, is associated with the risks of areca-related OSCC. Subjects carried both NFKB1 ins and HO-1 L allelotypes had significant risks for various subsets of OSCC. OSCC with lymph node   metastasis   or advanced stage had significantly higher frequency of NFKB1 ins and HO-1 L allelotypes. This study suggested that the functional NFKB1promoter polymorphism could be valuable for assessment of cancer risk. 
 Association of constitutive nuclear factor-kappaB activation with aggressive aspects and poor prognosis in cervical cancer. Although nuclear factor-kappaB (NF-kappaB) is generally believed to be involved in carcinogenesis, the relationship between NF-kappaB activation and progression of cervical cancer in clinical settings has not been reported. In this study, we investigated the association of NF-kappaB activation with aggressive aspects and prognosis in cervical cancer.Nuclear factor-kappaB subunits p65 and p50 were detected in 159 paraffin tissues including normal cervical, precancerous (squamous intraepithelial lesions), and cervical carcinoma tissues by immunohistochemistry. Nuclear factor-kappaB nuclear translocation and DNA-binding activity in precancerous or carcinoma tissues were examined by Western blot and electrophoretic mobility shift assay, respectively.A gradual NF-kappaB activation from normal cervical epithelial cells to precancerous and carcinoma cells was detected by immunohistochemistry (nuclear expression of p65 and p50, P < 0.001), Western blot (NF-kappaB nuclear translocation), and electrophoretic mobility shift assay (enhanced DNA-binding activity). In 79 cancer tissues, increased nuclear p65, an active NF-kappaB form, was correlated with poor tumor grade, lymphatic   metastasis  , interstitial invasion, and larger tumor size (P < 0.05). Similarly, increased nuclear p50 was correlated with poor tumor grade, interstitial invasion, and larger tumor size (P < 0.05). Moreover, increased nuclear p65 was associated with lower survival rate in patients with cancer (P < 0.05).Constitutive NF-kappaB activation is correlated to tumor progression, aggressive behaviors, and poor prognosis in cervical cancer, suggesting that NF-kappaB is a tumor promoter, a prognostic indicator, and a possible therapeutic target for this malignant disease. 
 Osteopontin increases migration and MMP-9 up-regulation via alphavbeta3 integrin, FAK, ERK, and NF-kappaB-dependent pathway in human chondrosarcoma cells. Tumor malignancy is associated with several features such as proliferation ability and frequency of   metastasis  . Osteopontin (OPN), which abundantly expressed in bone matrix, is involved in cell adhesion, migration, invasion and proliferation via interaction with its receptor, that is, alphavbeta3 integrin. However, the effect of OPN on migration activity in human chondrosarcoma cells is mostly unknown. Here we found that OPN increased the migration and expression of matrix metalloproteinase (MMP)-9 in human chondrosarcoma cells (JJ012 cells). RGD peptide, alphavbeta3 monoclonal antibody and MAPK kinase (MEK) inhibitors (PD98059 and U0126) but not RAD peptide inhibited the OPN-induced increase of the migration and MMP-9 up-regulation of chondrosarcoma cells. OPN stimulation increased the phosphorylation of focal adhesion kinase (FAK), MEK and extracellular signal-regulated kinase (ERK). In addition, treatment of JJ012 cells with NF-kappaB inhibitor (PDTC) or IkappaB protease inhibitor (TPCK) inhibited OPN-induced cell migration and MMP-9 up-regulation. Stimulation of JJ012 cells with OPN also induced IkappaB kinase alpha/beta (IKK alpha/beta) phosphorylation, IkappaBalpha phosphorylation, p65 Ser(536) phosphorylation, and kappaB-luciferase activity. The OPN-mediated increases in MMP-9 and kappaB-luciferase activities were inhibited by RGD peptide, PD98059 or FAK and ERK2 mutant. Taken together, our results indicated that OPN enhances the migration of chondrosarcoma cells by increasing MMP-9 expression through the alphavbeta3 integrin, FAK, MEK, ERK and NF-kappaB signal transduction pathway. 
 Bit-1 mediates integrin-dependent cell survival through activation of the NFkappaB pathway. Loss of properly regulated cell death and cell survival pathways can contribute to the development of cancer and cancer   metastasis  . Cell survival signals are modulated by many different receptors, including integrins. Bit-1 is an effector of anoikis (cell death due to loss of attachment) in suspended cells. The anoikis function of Bit-1 can be counteracted by integrin-mediated cell attachment. Here, we explored integrin regulation of Bit-1 in adherent cells. We show that knockdown of endogenous Bit-1 in adherent cells decreased cell survival and re-expression of Bit-1 abrogated this effect. Furthermore, reduction of Bit-1 promoted both staurosporine and serum-deprivation induced apoptosis. Indeed knockdown of Bit-1 in these cells led to increased apoptosis as determined by caspase-3 activation and positive TUNEL staining. Bit-1 expression protected cells from apoptosis by increasing phospho-IκB levels and subsequently bcl-2 gene transcription. Protection from apoptosis under serum-free conditions correlated with bcl-2 transcription and Bcl-2 protein expression. Finally, Bit-1-mediated regulation of bcl-2 was dependent on focal adhesion kinase, PI3K, and AKT. Thus, we have elucidated an integrin-controlled pathway in which Bit-1 is, in part, responsible for the survival effects of cell-ECM interactions. 
 Chromatin remodeling protein SMAR1 regulates NF-κB dependent Interleukin-8 transcription in breast cancer. Interleukin-8 (IL-8) is a pleiotropic chemokine involved in   metastasi  s and angiogenesis of breast tumors. The expression of IL-8 is deregulated in metastatic breast carcinomas owing to aberrant NF-κB activity, which is known to positively regulate IL-8 transcription. Earlier, we have shown that tumor suppressor SMAR1 suppresses NF-κB transcriptional activity by modulating IκBα function. Here, we show that NF-κB target gene IL-8, is a direct transcriptional target of SMAR1. Using chromatin immunoprecipitation and reporter assays, we demonstrate that SMAR1 binds to IL-8 promoter MAR (matrix attachment region) and recruits HDAC1 dependent co-repressor complex. Further, we also show that SMAR1 antagonizes p300-mediated acetylation of RelA/p65, a post-translational modification indispensable for IL-8 transactivation. Thus, we decipher a new role of SMAR1 in NF-κB dependent transcriptional regulation of pro-angiogenic chemokine IL-8. 
 Over-expression of nuclear NF-κB1 and c-Rel correlates with chemoresistance and prognosis of serous epithelial ovarian cancer. This study aims to measure the expression and subcellular location of NF-κB1 and c-Rel protein in serous epithelial ovarian cancer (EOC), and to test the correlation between NF-κB1 and c-Rel expression and clinicopathological parameters, chemoresistance, and prognosis of serous EOC.A total of 63 specimens of serous EOC patients meeting the inclusion criteria with complete follow-up data were enrolled in our study. The specimens were divided into chemo-resistant group and sensitive group. The expression and subcellular location of NF-κB1 and c-Rel were assessed in paraffin sections using immunohistochemistry. The relationship between NF-κB1 and c-Rel protein expression and pathological characteristics of serous EOC, chemoresistance, prognosis and survival time was analyzed.The positive nuclear staining of NF-κB1 and c-Rel were significantly higher in the chemo-resistant serous EOC specimens than that in chemo-sensitive group. Lymph   node meta  stasis and the nuclear expression of NF-κB1 and c-Rel were independent risk factors associated with chemotherapy resistance of serous EOC. Nucleus NF-κB1 and c-Rel expression along with FIGO stage were independent risk factors that strongly correlated with prognosis of serous EOC. Western blot result showed the protein level of NF-κB1 and c-Rel was significantly higher in chemoresistant group compared with in sensitive group.Over-expression of nuclear NF-κB1 and c-Rel are strong risk factors associated with chemoresistance and the prognosis of serous epithelial ovarian cancer. 
 IL-1 beta induces urokinase-plasminogen activator expression and cell migration through PKC alpha, JNK1/2, and NF-kappaB in A549 cells. Breakdown of the extracellular matrix (ECM) is accomplished by the concerted action of several proteases, including the urokinase plasminogen-activator (uPA) system and matrix metalloproteinases (MMPs), which is crucial for cancer invasion and   metastasis  . Several reports have shown that the levels of IL-1 beta and MMPs in plasma of the patients with lung cancer are significantly elevated and link to the invasion of tumor cells. Therefore, we investigated whether IL-1 beta-induced expression of uPA participated in lung cancer progression. In this study, IL-1 beta significantly induced uPA expression and activity via PKC alpha-dependent JNK1/2 and NIK cascades, linking to IKK alpha/beta activation, p65 translocation and transcription activity, using pharmacological inhibitors and transfection with dominant negative mutants and siRNAs. IL-1 beta-induced uPA protein and mRNA expression in a time- and concentration-dependent manner, which was inhibited by pretreatment with the inhibitors of JNK1/2 (SP600125), PKC (Ro31-8220, Gö6976), or NF-kappaB (helenalin), and transfection with dominant negative mutants of PKC alpha, NIK, and IKK beta, and siRNAs of JNK1/2 and p65. IL-1 beta stimulated PKC alpha translocation to plasma membrane leading to phosphorylation of JNK1/2, which was attenuated by PKC inhibitors and transfection with shRNAs of JNK1/2, but not by helenalin. In addition, IL-1beta stimulated p65 phosphorylation and translocation into nucleus concomitant with I kappaB alpha phosphorylation and I kappaB alpha degradation, which was mediated via activation of PKC alpha-dependent JNK1/2-NIK/IKK beta cascade. These results demonstrated that in A549 cells, activation of p50/p65 heterodimer through sequential activation of PKC alpha-JNK-NIK-IKK beta-NF-kappaB was required for IL-1 beta-induced uPA expression associated with migration of tumor cells. 
 The prognostic impact of TGF-β1, fascin, NF-κB and PKC-ζ expression in soft tissue sarcomas. Transforming growth factor-β (TGF-β), fascin, nuclear factor-kappa B (NF-κB) p105, protein-kinase C-zeta (PKC-ζ), partioning-defective protein-6 (Par-6), E-cadherin and vimentin are tumor promoting molecules through mechanisms involved in cell dedifferentiation. In soft tissue sarcomas, their expression profile is poorly defined and their significance is uncertain. We aimed to investigate the prognostic impact of TGF-β1, NF-κB p105, PKC-ζ, Par-6α, E-cadherin and vimentin in non-gastrointestinal stromal tumor soft tissue sarcomas (non-GIST STSs).Tumor samples and clinical data from 249 patients with non-GIST STS were obtained, and tissue microarrays (TMAs) were constructed for each specimen. Immunohistochemistry (IHC) was used to evaluate marker expression in tumor cells.In univariate analysis, the expression levels of TGF-β1 (P = 0.016), fascin (P = 0.006), NF-κB p105 (P = 0.022) and PKC-ζ, (P = 0.042) were significant indicators for disease specific survival (DSS). In the multivariate analysis, high TGF-β1 expression was an independent negative prognostic factor for DSS (HR = 1.6, 95% CI = 1.1-2.4, P = 0.019) in   addition t  o tumor depth, malignancy grade, metastasis at diagnosis, surgery and positive resection margins.Expression of TGF-β1 was significantly associated with aggressive behavior and shorter DSS in non-GIST STSs. 
 The role of NF-kappaB in Hepatitis b virus X protein-mediated upregulation of VEGF and MMPs. Hepatitis B virus X protein (HBx) promotes hepatocellular carcinoma (HCC) invasion and   metastasis   by a poorly understood mechanism. This study investigated the role of NF-kappa B in HBx-mediated upregulation of vascular endothelial growth factor (VEGF) and matrix metalloproteinases (MMPs). In a stably expressing HBx cell line, NF-kappa B level was examined by laser scanning confocal microscopy before and after treatment with pyrrolidine dithiocarbamate (PDTC; an NF-kappa B inhibitor). VEGF, MMP2, MMP9, and MMP14 mRNA and protein levels were quantitated by real-time PCR and Western blotting, respectively. HBx stimulated NF-kappa B signaling and increased VEGF, MMP2, MMP9, and MMP14 mRNA and protein levels. PDTC treatment blocked HBx-mediated stimulation of NF-kappa B signaling and decreased VEGF, MMP9, and MMP14 (but not MMP2) mRNA and protein levels. In vivo studies, PDTC reduced angiogenesis in subcutaneous xenograft of nude mice which injected HepG2-HBx cells. This suggests that NF-kappa B is involved in upregulating these genes and in the HBx-mediated invasion and   metastasis   of HCC. 
 Annexin-A1 controls an ERK-RhoA-NFκB activation loop in breast cancer cells. Wound healing is critical for normal development and pathological processes including cancer cell   metastasi  s. MAPK, Rho-GTPases and NFκB are important regulators of wound healing, but mechanisms for their integration are incompletely understood. Annexin-A1 (ANXA1) is upregulated in invasive breast cancer cells resulting in constitutive activation of NFκB. We show here that silencing ANXA1 increases the formation of stress fibers and focal adhesions, which may inhibit wound healing. ANXA1 regulated wound healing is dependent on the activation of ERK1/2. ANXA1 increases the activation of RhoA, which is dependent on ERK activation. Furthermore, active RhoA is important in NF-κB activation, where constitutively active RhoA potentiates NFκB activation, while dominant negative RhoA inhibits NFκB activation in response to CXCL12 stimulation and active MEKK plasmids. These findings establish a central role for ANXA1 in the cell migration through the activation of NFκB, ERK1/2 and RhoA. 
 NF-κB signaling in cancer stem cells: a promising therapeutic target? Cancer stem cells (CSCs) are regulated by several signaling pathways that ultimately control their maintenance and expansion. NF-κB (nuclear factor kappa-light-chain-enhancer of activated B cells) forms a protein complex that controls DNA transcription and, as such, plays an important role in proliferation, inflammation, angiogenesis, invasion an  d metastas  is. The NF-κB signaling pathway, which has been found to be constitutively activated in CSCs from a variety of cancers, participates in the maintenance, expansion, proliferation and survival of CSCs. Targeted disruption of this pathway may profoundly impair the adverse phenotype of CSCs and may provide a therapeutic opportunity to remove the CSC fraction. In particular, it may be attractive to use specific NF-κB inhibitors in chronic therapeutic schemes to reduce disease progression. Exceptional low toxicity profiles of these inhibitors are a prerequisite for use in combined treatment regimens and to avoid resistance.Although still preliminary, recent evidence shows that such targeted strategies may be useful in adjuvant chemo-preventive settings. 
 CXCL12/CXCR4 protein signaling axis induces sonic hedgehog expression in pancreatic cancer cells via extracellular regulated kinase- and Akt kinase-mediated activation of nuclear factor κB: implications for bidirectional tumor-stromal interactions. Recent evidence suggests a major role of tumor-stromal interactions in pancreatic cancer pathobiology. The chemokine CXCL12 (stromal cell-derived factor 1 (SDF-1)), abundantly produced by stromal cells, promotes progression,   metastasi  s, and chemoresistance of pancreatic cancer cells. On the other hand, pancreatic tumor cell-derived sonic hedgehog (SHH) acts predominantly on stromal cells to induce desmoplasia and, thus, has a paracrine effect on tumorigenesis and therapeutic outcome. In this study, we examined the association between these two proteins of pathological significance in pancreatic cancer. Our data demonstrate that CXCL12 leads to a dose- and time-dependent up-regulation of SHH in pancreatic cancer cells. CXCL12-induced SHH up-regulation is specifically mediated through the receptor CXCR4 and is dependent on the activation of downstream Akt and ERK signaling pathways. Both Akt and ERK cooperatively promote nuclear accumulation of NF-κB by inducing the phosphorylation and destabilization of its inhibitory protein, IκB-α. Using dominant negative IκB-α, a SHH promoter (deletion mutant) reporter, and chromatin immunoprecipitation assays, we demonstrate that CXCL12 exposure enhances direct binding of NF-κB to the SHH promoter and that suppression of NF-κB activation abrogates CXCL12-induced SHH expression. Finally, our data demonstrate a strong correlative expression of CXCR4 and SHH in human pancreatic cancer tissues, whereas their expression is not observed in the normal pancreas. Altogether, our data reveal a novel mechanism underlying aberrant SHH expression in pancreatic cancer and identify a molecular link facilitating bidirectional tumor-stromal interactions. 
 Ghrelin induces gastric cancer cell proliferation, migration, and invasion through GHS-R/NF-κB signaling pathway. This study aims to investigate the roles of ghrelin signaling in human gastric carcinoma cell lines AGS and SGC7901. Effects of ghrelin signaling on CDK6, P53, NF-κB/P65 and MMP2 mRNA and/or protein expression were determined by real-time PCR and western blot. MTT method and flow cytometry were performed to assess the gastric cancer cell proliferation. The SGC7901 cells overexpressing ghrelin were inoculated into nude mice to produce tumors which were measured later. The wound-healing assay and cell invasion assay were used to test the cell migration and invasive ability of gastric cancer. Ghrelin signaling promotes the oncogene CDK6 gene expression and represses the tumor suppressor gene P53 gene expression in gastric cancer. Ghrelin activates NF-κB/P65 signaling pathway through GHS-R in gastric cancer. Ghrelin upregulates t  he metasta  sis factor MMP2 expression via GHS-R/NF-κB signaling pathway in gastric cancer cells and promotes tumor cells migration and invasion, suggesting that ghrelin signaling is a critical pathway in can  cer metast  asis. Ghrelin induces cell proliferation, migration and invasion via GHS-R/NF-κB signaling pathway in gastric cancer cells. Ghrelin treatment must be avoided for gastric cancer patients. 
 Raf kinase inhibitor protein suppresses nuclear factor-κB-dependent cancer cell invasion through negative regulation of matrix metalloproteinase expression. Accumulating evidence suggests that Raf kinase inhibitor protein (RKIP), which negatively regulates multiple signaling cascades including the Raf and nuclear factor-κB (NF-κB) pathways, functions as   a metasta  sis suppressor. However, the basis for this activity is not clear. We investigated this question in a panel of breast cancer, colon cancer and melanoma cell lines. We found that RKIP negatively regulated the invasion of the different cancer cells through three-dimensional extracellular matrix barriers by controlling the expression of matrix metalloproteinases (MMPs), particularly, MMP-1 and MMP-2. Silencing of RKIP expression resulted in a highly invasive phenotype and dramatically increased levels of MMP-1 and MMP-2 expression, while overexpression of RKIP decreased cancer cell invasion in vitro a  nd metasta  sis in vivo of murine tumor allografts. Knockdown of MMP-1 or MMP-2 in RKIP-knockdown cells reverted their invasiveness to normal. In contrast, when examining migration of the different cancer cells in a two-dimensional, barrier-less environment, we found that RKIP had either a positive regulatory activity or no activity, but in no case a negative one (as would be expected if RKIP suppress  ed metasta  sis at the level of cell migration itself). Therefore, RKIP's function as   a metasta  sis suppressor appears to arise from its ability to negatively regulate expression of specific MMPs, and thus invasion through barriers, and not from a direct effect on the raw capacity of cells to move. The NF-κB pathway, but not the Raf pathway, appeared to positively control the invasion of breast cancer cells. A regulatory loop involving an opposing relationship between RKIP and the NF-κB pathway may control the level of MMP expression and cell invasion. 
 NF-kappaB activation in human prostate cancer: important mediator or epiphenomenon? The NF-kappaB family of transcription factors has been shown to be constitutively activated in various human malignancies, including leukemias, lymphomas, and a number of solid tumors. NF-kappaB is hypothesized to contribute to development and/or progression of malignancy by regulating the expression of genes involved in cell growth and proliferation, anti-apoptosis, angiogenesis, and   metastasis  . Prostate cancer cells have been reported to have constitutive NF-kappaB activity due to increased activity of the IkappaB kinase complex. Furthermore, an inverse correlation between androgen receptor (AR) status and NF-kappaB activity was observed in prostate cancer cell lines. NF-kappaB may promote cell growth and proliferation in prostate cancer cells by regulating expression of genes such as c-myc, cyclin D1, and IL-6. NF-kappaB may also inhibit apoptosis in prostate cancer cells through activation of expression of anti-apoptotic genes, such as Bcl-2, although pro-apoptotic activity of NF-kappaB has also been reported. NF-kappaB-mediated expression of genes involved in angiogenesis (IL-8, VEGF), and invasion and   metastasis   (MMP9, uPA, uPA receptor) may further contribute to the progression of prostate cancer. Constitutive NF-kappaB activity has also been demonstrated in primary prostate cancer tissue samples and suggested to have prognostic importance for a subset of primary tumors. The limited number of samples analyzed in those studies and the relative lack of NF-kappaB target genes identified in RNA expression microarray analyses of prostate cancer cells suggest that further studies will be required in order to determine if NF-kappaB actually plays a role in human prostate cancer development, and/or progression, and to characterize its potential as a therapeutic target. 
 Interleukin-8 induces nuclear transcription factor-kappaB through a TRAF6-dependent pathway. Considering the potential role of interleukin-8 (IL-8) in inflammation, angiogenesis, tumorigenesis, and   metastasis  , we investigated the molecular mechanism involved in IL-8-mediated signaling. In this report we provide evidence that like TNF, an inducer of NF-kappaB and also a NF-kappaB-dependent gene product, IL-8 induces NF-kappaB in a unique pathway. IL-8 induces NF-kappaB activation in a dose-dependent manner in different cell types as detected by a DNA-protein binding assay. IL-8 induces NF-kappaB-dependent reporter gene expression as well as ICAM-1, VCAM-1, and Cox-2 expression. IL-8 also induces IkappaBalpha phosphorylation followed by degradation and p65 translocation. IL-8 induces c-Jun N-terminal kinase (JNK) and mitogen-activated protein kinase (MAPK) in a dose- and time-dependent manner. IL-8-induced NF-kappaB activation is for the most part unaltered when cells are transfected with dominant-negative TRADD, FADD, or TRAF2, but is inhibited with dominant-negative TRAF6-, NIK-, IKK-, or IkappaBalpha-transfected cells. The data suggest that IL-8-induced NF-kappaB activation proceeds through a TRAF2-independent but TRAF6-dependent pathway, followed by recruitment of IRAK and activation of IKK. IL-8-induced NF-kappaB activation is not observed in a cell-permeable peptide that has TRAF6 binding motif-treated cells or IRAK-deficient cells. IL-8-induced NF-kappaB activation proceeds mostly through interaction with TRAF6 and partially through the Rho-GTPase pathways. This is the first report that IL-8 induces NF-kappaB in a distinct pathway, and activation of NF-kappaB and its dependent genes may be one of the pathways of IL-8-induced inflammation and angiogenesis. 
 Correlated expression of Fas, NF-kappaB, and VEGF-C in infiltrating ductal carcinoma of the breast. To investigate the expressions of Fas, NF-kappaB, and vascular endothelial growth factor-C (VEGF-C) in infiltrating ductal carcinoma of the breast and provide scientific basis for early diagnosis and prognosis of breast cancer.The immunohistochemical technique (SP method) was used to detect expression of Fas, NF-kappaB, and VEGF-C in 137 cases of breast-infiltrating ductal carcinoma, 17 cases of intraductal carcinoma of the breast, and 20 cases of normal breast tissues, and analyze its relationship with clinicopathologic factors of breast cancer and patients' survival rate, as well as the correlation among their expression, clinicopathologic factors, and survival rate.Fas expression was less commonly detected in infiltrating ductal carcinoma than in intraductal carcinoma and normal tissue. In contrast, both NF-kappaB and VEGF-C were more commonly detected in infiltrating ductal carcinoma than in intraductal carcinoma and normal tissue. Fas expression was correlated with tumor size, histological grade, and clinicopathological stage; NF-kappaB expression was correlated with tumor size, histological grade, lymph node   metastasis  ; VEGF-C expression was correlated with lymph node   metastasis   and clinical and pathological stages of breast cancer (p < or = 0.05). Spearman rank correlation analysis revealed a negative correlation between Fas expression and both NF-kappaB and VEGF-C expression in infiltrating breast cancer (p < 0.05) Additionally, Kaplan-Meier survival analysis demonstrated that five-year survival was higher for patients with Fas-positive samples but lower for those with VEGF-C-positive samples.The present results demonstrate that Fas and NF-B play a role in the initiation and development of breast cancer, while VEGF-C appears to promote lymph node   metastasis  . Thus, these proteins may serve as useful diagnostic and prognostic markers of invasive breast cancer. 
 Hepatocyte nuclear factor 4α-nuclear factor-κB feedback circuit modulates liver cancer progression. Hepatocyte nuclear factor 4α (HNF4α) is a liver enriched transcription factor and is indispensable for liver development. However, the role of HNF4α in hepatocellular carcinoma (HCC) progression remains to be elucidated. We report that reduced HNF4α expression correlated well with the aggressive clinicopathological characteristics of HCC and predicted poor prognosis of patients. HNF4α levels were even lower in metastatic HCCs, and ectopic HNF4α expression suppres  sed the me  tastasis of hepatoma cells both in vitro and in vivo. Forced HNF4α expression attenuated the expression and nuclear translocation of RelA (p65) and impaired NF-κB activation through an IKK-independent mechanism. Blockage of RelA robustly attenuated the suppressive effect of HNF4α on hep  atoma cell   metastasis. MicroRNA (miR)-7 and miR-124 were transcriptionally up-regulated by HNF4α, which repressed RelA expression by way of interaction with RelA-3' untranslated region (UTR). In addition, nuclear factor kappa B (NF-κB) up-regulated the expression of miR-21 in hepatoma cells, resulting in decreased HNF4α levels through down-regulating HNF4α-3'UTR activity.Collectively, an HNF4α-NF-κB feedback circuit including miR-124, miR-7, and miR-21 was identified in HCC, and the combination of HNF4α and NF-κB exhibited more powerful predictive efficiency of patient prognosis. These findings broaden the knowledge of hepatic inflammation and cancer initiation/progression, and also provide novel prognostic biomarkers and therapeutic targets for HCC. 
 Trastuzumab-Resistant Luminal B Breast Cancer Cells Show Basal-Like Cell Growth Features Through NF-κB-Activation. A major clinical problem in the treatment of breast cancer is mortality due to   metastasi  s. Understanding the molecular mechanisms associated with   metastasi  s should aid in designing new therapeutic approaches for breast cancer. Trastuzumab is the main therapeutic option for HER2+ breast cancer patients; however, the molecular basis for trastuzumab resistance (TZR) and subsequent   metastasi  s is not known. Earlier, we found expression of basal-like molecular markers in TZR tissues from patients with invasive breast cancer.(( 1 )) The basal-like phenotype is a particularly aggressive form of breast cancer. This observation suggests that TZR might contribute to an aggressive phenotype. To understand if resistance to TZR can lead to basal-like phenotype, we generated a trastuzumab-resistant human breast cancer cell line (BT-474-R) that maintained human epidermal growth factor receptor 2 (HER2) overexpression and HER2 mediated signaling. Analysis showed that nuclear factor-kappa B (NF-κB) was constitutively activated in the BT-474-R cells, a feature similar to the basal-like tumor phenotype. Pharmacologic inhibition of NF-κB improved sensitivity of BT-474-R cells to trastuzumab. Interestingly, activation of HER2 independent NF-κB is not shown in luminal B breast cancer cells. Our study suggests that by activating the NF-κB pathway, luminal B cells may acquire a HER2+ basal-like phenotype in which NF-κB is constitutively activated; this notion is consistent with the recently proposed "progression through grade" or "evolution of resistance" hypothesis. Furthermore, we identified IKK-α/IKK-β and nuclear accumulation of RelA/p65 as the major determinants in the resistant cells. Thus our study additionally suggests that the nuclear accumulation of p65 may be a useful marker for iden  tifying me  tastasis-initiating tumor cells and targeting RelA/p65 ma  y limit me  tastasis of breast and other cancers associated with NF-κB activation. 
 [Proteasome inhibitor induces apoptosis and influences the expression of Notch1 and NF-kappaB in multiple myeloma RPMI8226 cells]. The occurrence and development of Multiple myeloma (MM) are closely related to bone marrow microenvironment in which many signal pathways were involved. Notch signal plays an important role in regulating hematopoietic cells proliferation and differentiation in hemopoietic microenvironment, especially Notch1 has close touch with the occurrence and development of neoplastic hematologic disorders. MM deeply depends on NF-kappaB, and the latter has something to do with oncogenesis,   metastasis  , proliferation, apoptosis and drug resistance of tumor cells. NF-kappaB2 family has been confirmed as one of target genes of Notch signal. Bortezomib as representative of proteasome inhibitors has been licensed by FDA in treating refractory and relapsing MM, but the mechanism of bortezomib inducing cells apoptosis is not yet clear. This study was purposed to investigate the effects of bortezomib inducing cell apoptosis and influencing the erpression of Notch1 and NF-kappaB in MM RPMI 8226 cells. The effect of bortezomib on apoptosis of MM RPMI8226 cells was assayed by transmission electron microscopy, flow cytometry and TUNEL; the expressions of Notch 1 and NF-kappaB in apoptotic RPMI8226 cells were detected by RT-PCR and immunofluorescence cytochemical staining respectively. The results showed that Notch1 and NF-kappaB highly expressed in RPMI 8226 cells. With the increase of bortezomib concentration, the typical apoptosis features of RPMI8226 cells were observed, the expressions of Notch1 and the expression of NF-kappaB decreased in nuclei and increased in cytoplasm. These changes had significant difference from control after concentration of bortezomib was raised to a certain degree. It is concluded that Notch1 and NF-kappaB signaling pathways participate in bortezomib-inducing RPMI8226 cell apoptosis and there may be some correlation between the Notch 1 and NF-kappaB signaling pathways, indicating that Notch 1 signal may be a latent target in treating MM. This study provides a certain experimental basis for research and development of new drugs. 
 KiSS1 suppresses TNFalpha-induced breast cancer cell invasion via an inhibition of RhoA-mediated NF-kappaB activation. Tumor necrosis factor-alpha (TNFalpha) induces cancer development and   metastasis  , which is prominently achieved by nuclear factor-kappa B (NF-kappaB) activation. TNFalpha-induced NF-kappaB activation enhances cellular mechanisms including proliferation, migration, and invasion. KiSS1, a key regulator of puberty, was initially discovered as a tumor   metastasis   suppressor. The expression of KiSS1 was lost or down-regulated in different metastatic tumors. However, it is unclear whether KiSS1 regulates TNFalpha-induced NF-kappaB activation and further tumor cell migration. In this study, we demonstrate that KiSS1 suppresses the migration of breast cancer cells by inhibiting TNFalpha-induced NF-kappaB pathway and RhoA activation. Both KiSS1 overexpression and KP10 (kisspeptin-10) stimulation inhibited TNFalpha-induced NF-kappaB activity, suppressed TNFalpha-induced cell migration and cell attachment to fibronectin in breast cancer cells while KP10 has little effect on cancer cell proliferation. Furthermore, KP10 inhibited TNFalpha-induced cell migration and RhoA GTPase activation. Therefore, our data demonstrate that KiSS1 inhibits TNFalpha-induced NF-kappaB activation via downregulation of RhoA activation and suppression of breast cancer cell migration and invasion. 
 Association of common polymorphisms in TNFA, NFkB1 and NFKBIA with risk and prognosis of esophageal squamous cell carcinoma. Tumour necrosis factor-alpha (TNF-α) and nuclear factor of kappa light chain gene enhancer in activated B cells (NF-κB) play critical role in carcinogenesis processes like tumour initiation, proliferation, migration and invasion. Single nucleotide polymorphisms in TNF-α, NF-κB and its inhibitor IκB genes were shown to be associated with susceptibility and prognosis of several cancers; however, their role in esophageal squamous cell carcinoma (ESCC) is not well recognised. Therefore, in present study, we aimed to investigate association of common polymorphisms in TNFA, NFkB1 and NFKBIA with risk and prognosis of ESCC in northern Indian population.We genotyped 290 ESCC patients (including 162 followed up cases) and 311 mean age, gender and ethnicity matched controls for TNFA -308G>A, NFkB1 -94ATTG ins/del and NFKBIA (-826C>T and 3'UTRA>G) polymorphisms using PCR alone or followed by RFLP and TaqMan assay.TNFA-308GA genotype was associated with increased risk of ESCC specifically in females and in patients with regional lymph node involvement, while, NFKBIA -826CT+TT genotype conferred decreased risk of ESCC in females. Haplotypes of NFKBIA -826C>T and 3'UTRA>G polymorphisms, C-826G3'UTR and T-826A3'UTR, were associated with reduced risk of ESCC. No independent role of NFkB1 -94ATTG ins/del polymorphism in susceptibility of ESCC was found. Multi-dimensionality reduction analysis showed three factor model TNFA-308, NFKBIA-826, NFKBIA 3'UTR as better predictor for risk of ESCC. Furthermore, combined risk genotype analysis of all studied polymorphisms showed increased risk of ESCC in patients with 1-3 risk genotype compared to '0' risk genotype. Survival analysis did not show any significant prognostic effect of studied polymorphisms. However, in stepwise multivariate analy  sis, metas  tasis was found to be independent prognostic predictor of ESCC patients.TNFA-308 and NFKBIA (-826C>T and 3'UTRA>G) polymorphisms may play role in susceptibility but not in prognosis of ESCC patients in northern Indian population. 
 Use of bioluminescent imaging to investigate the role of nuclear factor-kappaBeta in experimental non-small cell lung cancer   metastasis  . Nuclear factor (NF)-kappaB is frequently over-expressed in non-small cell lung cancer (NSCLC), but the exact role of this observation remains unclear. In this regard, activation of the transcription factor may govern distinct steps of NSCLC progression, such as carcinogenesis, angiogenesis, and   metastasis  . In these studies we attempted to dissect the effects of two proteins of the NF-kappaB pathway (p65/RelA and IkappaBetaalpha) on experimental   metastasis   of murine NSCLC, using a novel approach of bioluminescent detection of NF-kappaB activation in tumor cells. Stable integration of a NF-kappaBeta reporter confirmed high basal activation of the transcription factor in mouse NSCLC cells in vitro and during experimental   metastasis   to the lungs, like human NSCLC. In the mouse model of NSCLC   metastasis  , NF-kappaBeta-dependent luciferase expression served as a reliable indicator of tumor cell delivery to the lungs, establishment of metastatic tumors, and lung tumor burden. In vitro transient p65/RelA and IkappaBetaalpha gene transfer to mouse NSCLC cells resulted, respectively, in significant NF-kappaB activation and inhibition, without affecting cell growth. However, p65/RelA overexpression in NSCLC cells drastically reduced in vivo   metastasis   to the lungs, while overexpression of IkappaBetaalpha had no effect. In conclusion, using bioluminescent detection of NF-kappaB activation in mouse lug adenocarcinoma cells, we found a negative impact of p65/RelA on NSCLC   metastasis  . 
 NF-kappaB and IKK as therapeutic targets in cancer. The transcription factor NF-kappaB and associated regulatory factors (including IkappaB kinase subunits and the IkappaB family member Bcl-3) are strongly implicated in a variety of hematologic and solid tumor malignancies. A role for NF-kappaB in cancer cells appears to involve regulation of cell proliferation, control of apoptosis, promotion of angiogenesis, and stimulation of invasion/  metastasis  . Consistent with a role for NF-kappaB in oncogenesis are observations that inhibition of NF-kappaB alone or in combination with cancer therapies leads to tumor cell death or growth inhibition. However, other experimental data indicate that NF-kappaB can play a tumor suppressor role in certain settings and that it can be important in promoting an apoptotic signal downstream of certain cancer therapy regimens. In order to appropriately move NF-kappaB inhibitors in the clinic, thorough approaches must be initiated to determine the molecular mechanisms that dictate the complexity of oncologic and therapeutic outcomes that are controlled by NF-kappaB. 
 Knockdown of the inflammatory factor pentraxin-3 suppresses growth and invasion of lung adenocarcinoma through the AKT and NF-kappa B pathways. Pentraxin-3 (PTX3), a modulator of tumor-associated inflammation, is known to be positively correlated with tumor grade and severity of malignancies, but the function and molecular underlying mechanisms of PTX3 remain unclear. In the present study, the expression of PTX3 in human lung adenocarcinoma (LAC) was examined by immunohistochemical assay using a tissue microarray procedure. A loss-of-function experiment was performed to explore the effects of lentiviral vector-mediated PTX3 shRNA (Lv-shPTX3) on cell growth and invasive potential in LAC cell lines (A549 and LETPα-2), assessed by MTT and Transwell assays, respectively. We found that the expression of PTX3 protein was significantly increased in LAC tissues compared with that in adjacent non-cancerous tissues (ANCT) (60.42% vs. 29.17%, P=0.004), and positively correlated with lymphatic invasion of the tumor (P=0.006). Furthermore, knockdown of PTX3 suppressed tumor proliferation and invasion of LAC cells, followed by decreased expression of p-AKT, p-NF-kappa B, PCNA, and MMP-9. Taken together, our findings demonstrate that upregulation of PTX3 expression is correlated with tumor   metastasi  s of LAC patients, and knockdown of PTX3 blocks the development of LAC through inhibition of the AKT and NF-kappa B pathways, suggesting that PTX3 may serve as a potential therapeutic target for the treatment of cancer. 
 Induction of Mac-2BP by nerve growth factor is regulated by the PI3K/Akt/NF-kappaB-dependent pathway in the HEK293 cell line. Mac-2BP is a ligand of the galectin family that has been suggested to affect tumor proliferation and   metastasis   formation. We assessed Mac-2BP expression at the transcriptional and translational levels to evaluate nerve growth factor (NGF)-induced Mac-2BP expression. A time kinetic analysis using reverse transcription-polymerase chain reaction showed that NGF-induced Mac-2BP transcript levels were 4-5 times higher than in controls. Mac-2BP enzyme-linked immunosorbent assay and immunofluorescence staining showed a 2-3-fold increase in intracellular and secreted Mac-2BP as a result of NGF stimulation. This increase was regulated by Akt activation and NF-kappaB binding. p65 and p50-NF-kappaB are major transcriptional factors in the Mac-2BP promoter region, and were shown to be regulated in accordance with the Akt activation states. Collectively, these results suggest that NGF induces Mac-2BP expression via the PI3K/Akt/NF-kappaB pathway. 
 Nuclear factor-kappaB mediates angiogenesis and   metastasis   of human bladder cancer through the regulation of interleukin-8. Interleukin (IL)-8 is an important mediator of angiogenesis, tumorigenicity, and   metastasis   in transitional cell carcinoma (TCC) of the bladder. Nuclear factor kappaB (NF-kappaB)/relA regulates IL-8 expression in several neoplasms. The purpose of this study was to determine whether the organ microenvironment (hypoxia, acidosis) regulates the expression of IL-8 in TCC via NF-kappaB, and whether inhibition of NF-kappaB function by mutant IkappaB-alpha prevents induction of IL-8 expression.IL-8 mRNA expression and protein production by human TCC cell lines (UM-UC-14, HTB-9, RT-4, KU-7 and 253J B-V) were measured by Northern blot analysis and ELISA under acidic (pH 7.35-6.0) and hypoxic (1.0% O(2)) conditions. The involvement of NF-kappaB and activator protein 1 in the regulation of IL-8 production was evaluated by electrophoretic mobility shift assay. Furthermore, the tumorigenicity and metastatic potential of UM-UC-14 cells were determined after transfection with mutant IkappaB-alpha.We found that acidic and hypoxic conditions increased IL-8 mRNA expression and protein production by several, but not all, TCC cell lines evaluated. NF-kappaB, but not activator protein 1, was inducibly activated in UM-UC-14 under both acidic and hypoxic conditions, but not in UM-UC-14 mutant IkappaB-alpha transfectants. Tumor growth and lymph node   metastasis   were inhibited in UM-UC-14 mutant IkappaB-alpha transfectants compared with UM-UC-14 controls. This effect was associated with the inhibition of IL-8 production, cellular proliferation, and angiogenesis.These results suggest that TCCs of the bladder have heterogenic responses to physicochemical changes in the microenvironment and identify NF-kappaB as a potential molecular target for therapy. 
 Interleukin 17A promotes hepatocellular carcinoma   metastasis   via NF-kB induced matrix metalloproteinases 2 and 9 expression.  IL-17A is a pro-inflammatory cytokine that plays important role in inflammatory disease pathology and tumor microenvironment. The aim of this study is to investigate the effect of IL-17A on the progression of hepatocellular carcinoma (HCC).Expression pattern of IL-17A in clinical HCC samples (n = 43) was determined by immunohistochemistry staining. Transcript levels of MMP2, MMP9 and IL-17A were measured in another 50 pairs (including tumor and related non-tumor tissues) HCC samples. Cell growth, focus formation, cell migration, invasion and western blot assays were used to characterize the functional and signaling mechanisms in IL-17A-treated HCC. Association study was used to identify clinical significance of IL-17A in HCC. Compared with paired non-tumor tissue, higher frequency of IL-17A-positive cells was detected in tumor tissues in HCCs   with meta  stasis, and the frequency of IL-17A-positive cells was also significantly associated with poor prognosis of HCC (P = 0.01). Functional study found that IL-17A could promote HCC cell migration and invasion. Further molecular analysis also showed that IL-17A could upregulate MMP2 and MMP9 expression via NF-κB signaling activation. IL-17A could   promote H  CC metastasis by the upregulation of MMP2 and MMP9 expression via activating NF-κB signaling pathway. 
 Turmeric (Curcuma longa) inhibits inflammatory nuclear factor (NF)-κB and NF-κB-regulated gene products and induces death receptors leading to suppressed proliferation, induced chemosensitization, and suppressed osteoclastogenesis. The incidence of cancer is significantly lower in regions where turmeric is heavily consumed. Whether lower cancer incidence is due to turmeric was investigated by examining its effects on tumor cell proliferation, on pro-inflammatory transcription factors NF-κB and STAT3, and on associated gene products.Cell proliferation and cell cytotoxicity were measured by the MTT method, NF-κB activity by EMSA, protein expression by Western blot analysis, ROS generation by FACS analysis, and osteoclastogenesis by TRAP assay. Turmeric inhibited NF-κB activation and down-regulated NF-κB-regulated gene products linked to survival (Bcl-2, cFLIP, XIAP, and cIAP1), proliferation (cyclin D1 and c-Myc)  , and meta  stasis (CXCR4) of cancer cells. The spice suppressed the activation of STAT3, and induced the death receptors (DR)4 and DR5. Turmeric enhanced the production of ROS, and suppressed the growth of tumor cell lines. Furthermore, turmeric sensitized the tumor cells to chemotherapeutic agents capecitabine and taxol. Turmeric was found to be more potent than pure curcumin for cell growth inhibition. Turmeric also inhibited NF-κB activation induced by RANKL that correlated with the suppression of osteoclastogenesis.Our results indicate that turmeric can effectively block the proliferation of tumor cells through the suppression of NF-κB and STAT3 pathways. 
 A synergistic interaction between transcription factors nuclear factor-κB and signal transducers and activators of transcription 3 promotes gastric cancer cell migration and invasion. The transcription factor nuclear factor-κB (NF-κB) has been implicated in gastric canc  er metasta  sis, but the underlying molecular mechanisms remain unclear. We investigated the role of the interaction between NF-κB and signal transducers and activators of transcription 3 (STAT3) in controlling metastatic potential of gastric cancer cells.Immunohistochemistry for NF-κB p65 (RelA), phospho-Tyr705-STAT3 (pSTAT3), or matrix metalloproteinase 9 (MMP9) was performed on tissue array slides containing 255 gastric carcinoma specimens. NF-κB inhibition in SNU-638 and MKN1 gastric cancer cell lines were performed by transduction with a retroviral vector containing NF-κB repressor mutant of IκBα, and STAT3 was silenced by RNA interference. We also did luciferase reporter assay, double immunofluorescence staining and immunoblotting. Cell migration and invasion were determined by wound-healing assay and invasion assay, respectively.NF-κB and STAT3 were constitutively activated and were positively correlated (P=0.038) in gastric cancer tissue specimens. In cell culture experiments, NF-κB inhibition reduced STAT3 expression and activation, whereas STAT3 silencing did not affect NF-κB activation. Moreover, both NF-κB inhibition and STAT3 silencing decreased gastric cancer cell migration and invasion in a synergistic manner. In addition, both NF-κB activation and STAT3 activation were positively correlated with MMP9 in gastric cancer tissues (P=0.001 and P=0.022, respectively), decreased E-cadherin expression and increased Snail and MMP9 expressions in cultured cells.NF-κB and STAT3 are positively associated and synergistically contribute to the metastatic potential of gastric cancer cells. Thus, dual use of NF-κB and STAT3 inhibitors may enhance the efficacy of the anti-metastatic treatment of gastric cancer. 
 Global analysis of L1-transcriptomes identified IGFBP-2 as a target of ezrin and NF-κB signaling that promotes colon cancer progression. L1, a neuronal cell adhesion receptor of the immunoglobulin-like protein family is expressed in invading colorectal cancer (CRC) cells as a target gene of Wnt/β-catenin signaling. Overexpression of L1 in CRC cells enhances cell motility and proliferation, and confers live  r metastas  is. We recently identified ezrin and the IκB-NF-κB pathway as essential for the biological properties conferred by L1 in CRC cells. Here, we studied the underlying molecular mechanisms and found that L1 enhances ezrin phosphorylation, via Rho-associated protein kinase (ROCK), and is required for L1-ezrin co-localization at the juxtamembrane domain and for enhancing cell motility. Global transcriptomes from L1-expressing CRC cells were compared with transcriptomes from the same cells expressing small hairpin RNA (shRNA) to ezrin. Among the genes whose expression was elevated by L1 and ezrin we identified insulin-like growth factor-binding protein 2 (IGFBP-2) and showed that its increased expression is mediated by an NF-κB-mediated transactivation of the IGFBP-2 gene promoter. Expression of a constitutively activated mutant ezrin (Ezrin567D) could also increase IGFBP-2 levels in CRC cells. Overexpression of IGFBP-2 in CRC cells lacking L1-enhanced cell proliferation (in the absence of serum), cell motility, tumorigenesis and induced l  iver metas  tasis, similar to L1 overexpression. Suppression of endogenous IGFBP-2 in L1-transfected cells inhibited these properties conferred by L1. We detected IGFBP-2 in a unique organization at the bottom of human colonic crypts in normal mucosa and at increased levels throughout human CRC tissue samples co-localizing with the phosphorylated p65 subunit of NF-κB. Finally, we found that IGFBP-2 and L1 can form a molecular complex suggesting that L1-mediated signaling by the L1-ezrin-NF-κB pathway, that induces IGFBP-2 expression, has an important role in CRC progression. 
 Pachymic acid impairs breast cancer cell invasion by suppressing nuclear factor-κB-dependent matrix metalloproteinase-9 expression. Pachymic acid (PA), a lanostane-type triterpenoid derived from Poria cocos, possesses demonstrated anti-inflammatory and anti-cancer activities. Nonetheless, the biological properties and mechanism/s of action of PA remain largely undefined. In this study, the activity of PA against breast cancer cell invasion was evaluated. Invasiveness of human-derived MDA-MB-231 and MCF-7 breast carcinoma cells was suppressed by PA at non-lethal concentrations, which was associated with a decrease in matrix metalloproteinase-9 (MMP-9) secretion as a result of PA-mediated down-regulation of MMP-9 mRNA expression. In order to elucidate the underlying anti-invasive mechanism, the effect of PA on transcription factors activator protein-1 (AP-1) and nuclear factor kappaB (NF-κB) was examined using luciferase-based reporter gene assays. PA was found to bring about a reduction in phorbol 12-myristate 13-acetate (PMA)-induced transcriptional activity of NF-κB, but not that of AP-1. In accord with the luciferase activity data, western blot analysis showed that PA inhibited NF-κB signaling pathway, but did not alter the phosphorylation states of mitogen-activated protein kinases including ERK, JNK, and p38 kinase. The inhibition of PA on NF-κB signaling pathway was further attributed to PA-mediated diminution in PMA-induced degradation of inhibitor of kappaBα (IκBα) through preventing phosphorylation of the upstream signal IκB kinase (IKK). A decrease in p65 nuclear translocation was achieved, which led to attenuation of NF-κB transactivation. Taken together, it was concluded that by targeting NF-κB signaling, PA inhibited breast cancer cell invasion through decreasing MMP-9 expression. PA may thus be potentially exploited for us  e in tumor   metastasis intervention. 
  GeneRIFs  
 Radixin knockdown suppresses the   metastasis   of SGC-7901 cells in vitro by up-regulation of E-cadherin. The NF-kappaB/snail pathway contributes to the regulation of E-cadherin in response to depletion of radixin. 
 PI3K/Akt pathway stimulates the expressions of RANKL, PTHrP, and BMP-2 partly through NF-kappaB, suggesting its importance for bone   metastasis   of prostate carcinoma 
 overexpression of SDF-1 enhanced EMT by activating the NF-kappaB pathway of MCF-7 cells and further induced the formation of CSC-like phenotypes, ultimately promoting the proliferation and   metastasis   of MCF-7 cells 
 In conclusion, this study revealed that miR-26b suppresses NSCLC   metastasis   by targeting MIEN1 via NF-kappaB/MMP-9/VEGF pathways, implicating a potential prognostic biomarker and therapeutic target for NSCLC treatment. 
 DLX4 induces CD44 by stimulating IL-1beta-mediated NF-kappaB activity, thereby promoting peritoneal   metastasis   of ovarian cancer 
 The role of NF-kappaB signaling pathway in the regulation of CXC chemokine receptor-4 (CXCR4) in neuroblastoma   metastasis  , was investigated. 
 HOXB13 promotes PCa invasion and   metastasis   by decreasing intracellular zinc levels, thus stimulating NF-kappaB signals, and acts as a modulator of intracellular zinc levels that promotes the malignant characteristics of PCa 
 NFkappaB/TGFbeta2 promotes, and betaglycan impedes, the early stages of granulosa cell tumor   metastasis  , when tumor cells first invade the peritoneum 
 NF-kappaB and EGFR may cooperate during intrahepatic cholangiocarcinogenesis and progression. Lymph node   metastasis   and EGFR positivity were associated with decreases in the survival rate. 
 NF-kappaB is required for NSCLC   metastasis  , in part, by transcriptionally upregulating master-switch transcription factors required for EMT. 
 Our findings indicate that S100A4 contributes to HCC   metastasis   by activation of NF-kB dependent MMP-9 expression. 
 DNA damage-induced NF-kappaB activation has a role in promoting cancer   metastasis   following genotoxic treatment, and NF-kappaB-dependent miR-21 induction may contribute to both therapeutic resistance and   metastasis   in breast cancer 
 miR-9 inhibits melanoma proliferation and   metastasis   through down-regulation of the NF-kappaB1-Snail1 pathway. 
 TLR9 up-regulates COX-2 expression in prostate cancer cells, at least partially through the activation of NF-kappaB, which may be implicated in tumor invasion and   metastasis  . 
 NF-kappa B is involved in upregulating these genes and in the HBx-mediated invasion and   metastasis   of HCC. 
 TWEAK-Fn14 functions, in part, through the NF-kappaB signaling pathway to up-regulate VEGF expression to foster ovarian cancer cell   metastasis   
 Snail is stabilized by the inflammatory cytokine TNFalpha through the activation of the NF-kappaB pathway; a plausible mechanism for inflammation-induced   metastasis   
 Fn14-mediated NF-kappaB pathway activation has a role in in breast tumor invasion and   metastasis   
 using bioluminescent detection of NF-kappaB activation in mouse lug adenocarcinoma cells, we found a negative impact of p65/RelA on non-small cell lung cancer   metastasis   
 NFkappaB activation mediated by loss of E-cadherin may represent an essential, even initial event in colorectal neoplasm   metastasis   
 Nuclear localization/activation of NF-kappaB is up-regulated in prostate cancer lymph node   metastasis  . Such up-regulation of NF-kappaB activity is observed in the tumor cells as well as in the surrounding lymphocytes. 
 Nuclear factor-kappaB has a role in mediating angiogenesis and   metastasis   of human bladder cancer through the regulation of interleukin-8 
 autotaxin induces uPA expression via the Gi-PI3K-Akt-NF-kappaB signaling pathway that might be critical for autotaxin-induced tumor cell invasion and   metastasis   
 Oral squamous cell carcinoma with lymph node   metastasis   or advanced stage had significantly higher frequency of NFKB1 ins allele. 
 NFkappaB signaling can suppress the angiogenic potential and   metastasis   of pancreatic cancer 
  [Cc]ell [Ii]nvasion  PubMed  
 miR-508-3p concordantly silences NFKB1 and RELA to inactivate canonical NF-κB signaling in gastric carcinogenesis. NF-κB signaling pathway plays an important role in gastric carcinogenesis. The basic expression and functional role of NFKB1 and RELA (components of canonical NF-κB pathway) in gastric cancer (GC) have not been well elucidated. In this study, the role of NFKB1 and RELA in gastric tumorigenesis will be investigated and their regulation by microRNAs (miRNAs) will be deeply explored.The mRNA and protein expression of NFKB1 and RELA were investigated by qRT-PCR and Western blot in GC cell lines and primary tumors. The functional roles of NFKB1 and RELA in GC were demonstrated by MTT proliferation assay, monolayer colony formatio  n, cell invas  ion and migration, cell cycle analysis and in vivo study through siRNA mediated knockdown. Identification of NFKB1 as a direct target of tumor suppressor miRNA miR-508-3p was achieved by expression regulation assays together with dual luciferase activity experiments.NFKB1 and RELA were up-regulated in GC cell lines and primary tumors compared with normal gastric epithelium cells and their upregulation correlation with poor survival in GC. siRNA mediated knockdown of NFKB1 or RELA exhibited anti-oncogenic effect both in vitro and in vivo. NFKB1 was further revealed to be a direct target of miR-508-3p in gastric tumorigenesis and their expression showed negative correlation in primary GC samples. miR-508-3p was down-regulated in GC cells compared with normal gastric epithelium samples and its ectopic expression in GC cell lines also exerts tumor suppressor function. NFKB1 re-expression was found to partly abolish the tumor-suppressive effect of miR-508-3p in GC.All these findings supports that canonical NF-κB signaling pathway is activated in GC at least by the inactivation of miR-508-3p and this might have therapeutic potential in GC treatment. 
 miR-508-3p concordantly silences NFKB1 and RELA to inactivate canonical NF-κB signaling in gastric carcinogenesis. NF-κB signaling pathway plays an important role in gastric carcinogenesis. The basic expression and functional role of NFKB1 and RELA (components of canonical NF-κB pathway) in gastric cancer (GC) have not been well elucidated. In this study, the role of NFKB1 and RELA in gastric tumorigenesis will be investigated and their regulation by microRNAs (miRNAs) will be deeply explored.The mRNA and protein expression of NFKB1 and RELA were investigated by qRT-PCR and Western blot in GC cell lines and primary tumors. The functional roles of NFKB1 and RELA in GC were demonstrated by MTT proliferation assay, monolayer colony formatio  n, cell invas  ion and migration, cell cycle analysis and in vivo study through siRNA mediated knockdown. Identification of NFKB1 as a direct target of tumor suppressor miRNA miR-508-3p was achieved by expression regulation assays together with dual luciferase activity experiments.NFKB1 and RELA were up-regulated in GC cell lines and primary tumors compared with normal gastric epithelium cells and their upregulation correlation with poor survival in GC. siRNA mediated knockdown of NFKB1 or RELA exhibited anti-oncogenic effect both in vitro and in vivo. NFKB1 was further revealed to be a direct target of miR-508-3p in gastric tumorigenesis and their expression showed negative correlation in primary GC samples. miR-508-3p was down-regulated in GC cells compared with normal gastric epithelium samples and its ectopic expression in GC cell lines also exerts tumor suppressor function. NFKB1 re-expression was found to partly abolish the tumor-suppressive effect of miR-508-3p in GC.All these findings supports that canonical NF-κB signaling pathway is activated in GC at least by the inactivation of miR-508-3p and this might have therapeutic potential in GC treatment. 
 Sulfoglucuronosyl paragloboside is a ligand for T cell adhesion: regulation of sulfoglucuronosyl paragloboside expression via nuclear factor kappaB signaling. Inflammatory cytokines such as tumor necrosis factor (TNF)-alpha and interleukin (IL)-1beta stimulate glucuronosyltransferase genes (S and P) in endothelial cells (ECs) and up-regulate sulfoglucuronosyl paragloboside (SGPG) expression, which serves as a ligand for T cell adhesion. However, the mechanism of cytokine-mediated gene up-regulation has not been elucidated. To evaluate the precise mechanism of SGPG up-regulation, we have specifically inhibited the SGPG synthesis in the cerebromicrovascular EC line (SV-HCECs), a transformed brain ECs of human origin. SV-HCECs were transfected with small interfering RNA designed to mimic the human natural killer epitope-1 sulfotransferase (HNK-1ST), the ultimate enzyme that transfers the sulfate group to glucuronic acid for SGPG synthesis. An inhibition of SGPG expression along with a reduction of human CD4(+) cell adhesion was observed in siRNA HNK-1ST (siHNK-1)-transfected cells after TNFalpha stimulation. A thorough screening of the signaling system confirmed that TNFalpha/IL-1beta stimulation up-regulated nuclear factor kappaB (NFkappaB) signaling in SV-HCECs. siHNK-1 transfection interfered with the SGPG up-regulation after TNFalpha/IL-1beta stimulation in transfected cells and reduced the T cell adhesion. Hence, our study indicates that T cell-SGPG adhesion in SV-HCECs may proceed through NFkappaB activation. In addition, siHNK-1 transfection reduced the NFkappaB activity compared with cells that were transfected with scrambled siRNA, before and after TNFalpha/IL-1beta stimulation. This is the first report indicating that NFkappaB signaling is involved in SGPG gene expression in brain ECs by an unknown mechanism. Its down-regulation by inhibiting HNK-1ST expression may have a potential use in preventing the T   cell invasion   and consequently nerve damage during inflammation. 
 Crebanine, an aporphine alkaloid, sensitizes TNF-α-induced apoptosis and suppressed invasion of human lung adenocarcinoma cells A549 by blocking NF-κB-regulated gene products. Crebanine is an alkaloid known to exhibit anticancer, but its mechanism is not well understood. Besides, the nuclear factor-kappa B (NF-κB) transcription factor has been correlated with inflammation, carcinogenesis, tumor cell survival, invasion, and angiogenesis. In this study, we investigated the effects of crebanine on tumor necrosis factor alpha (TNF-α)-induced NF-κB activation and the expression of NF-κB-regulated gene products. We found that crebanine reduced the cell proliferation of lung, ovarian, and breast cancer cells. Crebanine also potentiated TNF-α-induced apoptosis which correlated with the suppression of the gene products linked to cell survival, B cell lymphoma-extra large, and proliferation, cyclin D1. In addition, crebanine affected TNF-α-induced activation of caspase-8, caspase-3, and poly(ADP-ribose) polymerase cleavage, indicating that the apoptotic effects of TNF-α were enhanced by crebanine. Moreover, crebanine reduced TNF-α-ind  uced A549 cel  l invasion and migration. Furthermore, crebanine suppressed the TNF-α-mediated expression of proteins that invol  ved cancer ce  ll invasion (matrix metalloproteinase 9 urokinase-type plasminogen activator, urokinase-type plasminogen activator receptor and intercellular adhesion molecule 1) and angiogenesis (COX-2 and VEGF), all of which are known to be regulated by NF-κB. We also demonstrated that TNF-α induced NF-κB DNA-binding activity, which was inhibited by crebanine. Moreover, crebanine suppressed the TNF-α-induced degradation of inhibitor of NF-κB alpha (IκBa), which led to reduced NF-κB translocation to the nucleus. Taken together, our results demonstrated that crebanine reduced TNF-α-induced cancer cell proliferation, invasion, and survival by suppressing NF-κB activity and expression profile of its downstream genes. 
 HOXB13 downregulates intracellular zinc and increases NF-κB signaling to promote prostate cancer metastasis. Characteristically, prostate cancer (PCa) cells exhibit marked decrease in intracellular zinc; however, the mechanism responsible is not clearly understood. HOXB13 is involved in PCa progression and is overexpressed in castration-resistant PCa. DNA microarray analysis of LNCaP Pca cells showed that ZnT zinc output transporters were strikingly upregulated among androgen-independent HOXB13 target genes. Furthermore, exogenous HOXB13 caused intracellular zinc concentrations to fall in PCa cells, stimulated NF-κB-mediated signaling by reducing inhibitor of NF-κB alpha (IκBα) and enhanced the nuclear translocation of RelA/p65. Human prostate tumors also exhibited strong inverse correlation between the protein expressions of HOXB13 and IκBα. Consequently, HOXB13 stimulat  ed PCa cell i  nvasion, and this was inhibited by the suppression of ZnT4. In addition, studies in a PC3 orthotopic mouse model of PCa metastasis showed that HOXB13 is a strong metastatic stimulator. Taken together, these results show that HOXB13 promotes PCa invasion and metastasis by decreasing intracellular zinc levels, thus stimulating NF-κB signals, and suggest that HOXB13 acts as a modulator of intracellular zinc levels that promotes the malignant characteristics of PCa. 
 Inhibition of nuclear factor-kappa B differentially affects thyroid cancer cell growth, apoptosis, and invasion. Nuclear factor-kappaB (NF-kappaB) is constitutively activated in many cancers and plays a key role in promoting cell proliferation, survival, and invasion. Our understanding of NF-kappaB signaling in thyroid cancer, however, is limited. In this study, we have investigated the role of NF-kappaB signaling in thyroid cancer cell proliferation, invasion, and apoptosis using selective genetic inhibition of NF-kappaB in advanced thyroid cancer cell lines.Three pharmacologic inhibitors of NF-kappaB differentially inhibited growth in a panel of advanced thyroid cancer cell lines, suggesting that these NF-kappaB inhibitors may have off-target effects. We therefore used a selective genetic approach to inhibit NF-kappaB signaling by overexpression of a dominant-negative IkappaBalpha (mIkappaBalpha). These studies revealed decreased cell growth in only one of five thyroid cancer cell lines (8505C), which occurred through a block in the S-G2/M transition. Resistance to TNFalpha-induced apoptosis was observed in all cell lines, likely through an NF-kappaB-dependent mechanism. Inhibition of NF-kappaB by mIkappaBalpha sensitized a subset of cell lines to TNFalpha-induced apoptosis. Sensitive cell lines displayed sustained activation of the stress-activated protein kinase/c-Jun NH2-terminal kinase (SAPK/JNK) pathway, defining a potential mechanism of response. Finally, NF-kappaB inhibition by mIkappaBalpha expression differentially reduced thyroid cancer   cell invasion   in these thyroid cancer cell lines. Sensitive cell lines demonstrated approximately a two-fold decrease in invasion, which was associated with differential expression of MMP-13. MMP-9 was reduced by mIkappaBalpha expression in all cell lines tested.These data indicate that selective inhibition of NF-kappaB represents an attractive therapeutic target for the treatment of advanced thyroid. However, it is apparent that global regulation of thyroid cancer cell growth and invasion is not achieved by NF-kappaB signaling alone. Instead, our findings suggest that other important molecular processes play a critical role in defining the extent of NF-kappaB function within cancer cells. 
 Plumbagin (5-hydroxy-2-methyl-1,4-naphthoquinone) suppresses NF-kappaB activation and NF-kappaB-regulated gene products through modulation of p65 and IkappaBalpha kinase activation, leading to potentiation of apoptosis induced by cytokine and chemotherapeutic agents. Plumbagin, derived from the medicinal plant Plumbago zeylanica, modulates cellular proliferation, carcinogenesis, and radioresistance, all known to be regulated by the activation of the transcription factor NF-kappaB, suggesting plumbagin might affect the NF-kappaB activation pathway. We found that plumbagin inhibited NF-kappaB activation induced by TNF, and other carcinogens and inflammatory stimuli (e.g. phorbol 12-myristate 13-acetate, H2O2, cigarette smoke condensate, interleukin-1beta, lipopolysaccharide, and okadaic acid). Plumbagin also suppressed the constitutive NF-kappaB activation in certain tumor cells. The suppression of NF-kappaB activation correlated with sequential inhibition of the tumor necrosis factor (TNF)-induced activation of IkappaBalpha kinase, IkappaBalpha phosphorylation, IkappaBalpha degradation, p65 phosphorylation, p65 nuclear translocation, and the NF-kappaB-dependent reporter gene expression activated by TNF, TNFR1, TRAF2, NIK, IKK-beta, and the p65 subunit of NF-kappaB. Plumbagin also suppressed the direct binding of nuclear p65 and recombinant p65 to the DNA, and this binding was reversed by dithiothreitol both in vitro and in vivo. However, plumbagin did not inhibit p65 binding to DNA when cells were transfected with the p65 plasmid containing cysteine 38 mutated to serine. Plumbagin down-regulated the expression of NF-kappaB-regulated anti-apoptotic (IAP1, IAP2, Bcl-2, Bcl-xL, cFLIP, Bfl-1/A1, and survivin), proliferative (cyclin D1 and COX-2), and angiogenic (matrix metalloproteinase-9 and vascular endothelial growth factor) gene products. This led to potentiation of apoptosis induced by TNF and paclitaxel and inhibited   cell invasion  . Overall, our results indicate that plumbagin is a potent inhibitor of the NF-kappaB activation pathway that leads to suppression of NF-kappaB-regulated gene products. This may explain its cell growth modulatory, anticarcinogenic, and radiosensitizing effects previously described. 
 Autotaxin expression and its connection with the TNF-alpha-NF-kappaB axis in human hepatocellular carcinoma. Autotaxin (ATX) is an extracellular lysophospholipase D that generates lysophosphatidic acid (LPA) from lysophosphatidylcholine (LPC). Both ATX and LPA have been shown to be involved in many cancers. However, the functional role of ATX and the regulation of ATX expression in human hepatocellular carcinoma (HCC) remain elusive.In this study, ATX expression was evaluated in tissues from 38 human HCC and 10 normal control subjects. ATX was detected mainly in tumor cells within tissue sections and its over-expression in HCC was specifically correlated with inflammation and liver cirrhosis. In addition, ATX expression was examined in normal human hepatocytes and liver cancer cell lines. Hepatoma Hep3B and Huh7 cells displayed stronger ATX expression than hepatoblastoma HepG2 cells and normal hepatocytes did. Proinflammtory cytokine tumor necrosis factor alpha (TNF-alpha) promoted ATX expression and secretion selectively in Hep3B and Huh7 cells, which led to a corresponding increase in lysophospholipase-D activity. Moreover, we explored the mechanism governing the expression of ATX in hepatoma cells and established a critical role of nuclear factor-kappa B (NF-kappaB) in basal and TNF-alpha induced ATX expression. Further study showed that secreted enzymatically active ATX stimulated Hep3B   cell invasion  .This report highlights for the first time the clinical and biological evidence for the involvement of ATX in human HCC. Our observation that links the TNF-alpha/NF-kappaB axis and the ATX-LPA signaling pathway suggests that ATX is likely playing an important role in inflammation related liver tumorigenesis. 
 Resveratrol reduces TNF-α-induced U373MG human glioma   cell invasio  n through regulating NF-κB activation and uPA/uPAR expression. High invasiveness of glioma cells is one of the reasons that patients with malignant glioma have a poor prognosis. Resveratrol, a plant compound abundant in the peel of grapes, has been suggested as a potential cancer chemopreventive agent. Therefore, we investigated the effect of resveratrol on gliom  a cell invasi  on.The effect of resveratrol on U373MG human gliom  a cell invasi  on was assessed by Matrigel assay and methylthiazoltetrazolium assay. Western blotting and reverse transcription-polymerase chain reaction were performed to elucidate the action mechanism of resveratrol.Resveratrol reduced tumor necrosis factor (TNF)-α-induced U373MG human glio  ma cell invas  ion. In addition, resveratrol repressed nuclear factor kappa B (NF-κB) activation and down-regulated mRNA expression of urokinase plasminogen activator (uPA) and its receptor in TNF-α-treated glioma cells.These findings suggest that resveratrol could prevent gl  ioma cell inv  asion via inhibiting proteolysis of extracellular matrix. 
 Involvement of Akt/NF-κB pathway in antitumor effects of parthenolide on glioblastoma cells in vitro and in vivo. Glioblastoma is the most common and most aggressive form of malignant glioma and is very difficult to treat. Controlling tumour   cell invasio  n and angiogenesis is essential to improve the prognosis of glioblastoma patients. Since constitutive activation of nuclear factor-κB (NF-κB) is necessary for tumour progression, NF-κB may be an important pharmacological target for this disease. Our study aimed to evaluate the antitumour effects of parthenolide, a NF-κB inhibitor, in two human glioblastoma cell lines (U87MG and U373) and in glioblastoma xenografts. Furthermore, we aimed to investigate the molecular mechanisms underlying these effects.The anti-invasive and anti-angiogenic effects of parthenolide were analysed using in vitro invasion and angiogenesis assays. Parthenolide-induced growth inhibition of glioblastoma cells in vitro was determined using the MTT (methyl thiazolyl tetrazolium) assay. In addition, the effect of parthenolide on orthotropic implantation in vivo was evaluated using an intracerebral human glioblastoma xenograft model.We found that parthenolide suppresses proliferation, invasion, and tumour- induced angiogenesis of glioblastoma cells. Molecular studies demonstrated that parthenolide suppresses gene and protein expression of angiogenic factors. Furthermore, parthenolide reduced Akt phosphorylation and activated mitochondrial signalling, suggesting that the antitumour function of parthenolide may be mediated not only by the inhibition of NF-κB but also by the inhibition of Akt signalling and the activation of apoptotic proteins. Parthenolide suppressed neovascularity and tumour growth in glioblastoma xenografts.The present study identified parthenolide as a new therapeutic agent for glioblastomas. 
 14-3-3β Promotes Migration and Invasion of Human Hepatocellular Carcinoma Cells by Modulating Expression of MMP2 and MMP9 through PI3K/Akt/NF-κB Pathway. 14-3-3β has been demonstrated to possess the oncogenic potential, and its increased expression has been detected in multiple types of carcinomas. However, majority of previous studies focused on the role of 14-3-3β in tumor cell proliferation and apoptosis, leaving much to be elucidated about its function in tu  mor cell inva  sion and metastasis. Hence, the present study aimed to investigate the role of 14-3-3β in the invasion of hepatocellular carcinoma (HCC) cells and the implications in the prognosis of HCC patients. We first examined the expression of 14-3-3β in the primary tumors of HCC patients with or without portal vein tumor thrombus (PVTT), and found that 14-3-3β expression was higher in the primary tumors with PVTT, and the level was even higher in the PVTTs. Kaplan-Meier curves and multivariate analysis revealed that high expression of 14-3-3β was associated with overall survival (OS) and time to recurrence (TTR) of HCC patients. In addition, ectopic expression of 14-3-3β in HCC cell lines led to enhanced migration ability and invasiveness, as well as up-regulation of matrix metalloproteinase 2 and 9, which could be suppressed by inhibiting the activation of Akt and nuclear factor-κB (NF-κB) signaling. Furthermore, we identified a correlated elevation of 14-3-3β and p-Akt in the primary tumors of HCC patients, and showed that a combinatory detection of 14-3-3β and p-Akt could better predict post-surgical outcome of HCC patients. 
 Regulation of HGF-mediated cell proliferation and invasion through NF-κB, JunB, and MMP-9 cascades in stomach cancer cells. Blocking of activator protein-1 activity leads to suppression of growth factor-induced transformation. However, the role of individual components of the Jun family in this process is unclear. Over-expression of JunB has been reported to be associated with neoplastic transformation and regulation of Matrix metalloproteinase-9 (MMP-9) expression. Using cDNA microarray, we found that JunB might be one of target genes up-regulated by hepatocyte growth factor (HGF). Therefore, we tried to investigate the role of JunB in HGF-medicated cell proliferation and   cell invasio  n in human gastric cancer cells, NUGC3 and MKN28. We verified that HGF increased JunB in time and dose-dependent manners. The JunB levels were decreased by the treatment with an NF-κB inhibitor, pyrrolidine dithiocarbamate. Down-regulation of JunB by transfecting cells with JunB-short hairpin RNAs (shRNA) inhibited MMP-9 up-regulation induced by HGF. Furthermore, transfection with JunB-shRNA repressed HGF-mediated increases in cell proliferation an  d cell invasi  on. These data suggest that JunB might be regulated through an NF-κB pathway and up-regulation of JunB induced by HGF might play an important role in the regulation of cell proliferation a  nd cell invas  ion through MMP-9 expression. In conclusion, these results may contribute to the JunB-associated malignant phenotype of gastric cancers by regulating MMP-9, and serve as a novel therapeutic target for stomach cancer therapy in the future. 
 [Inhibition of NF-kappaB through IkappaBalpha transfection affects invasion of human lung cancer cell line A549]. Recent studies have shown that activation of nuclear factor-kappaB(NF-kappaB) can regulate the invasion and metastasis of cancer cells. The present study was to investigate inhibition of NF-kappaB activity on invasion of human lung cancer cell line A549 and the possible mechanism.The recombinant plasmid pcDNA3.1(+)/IkappaBalpha, expressing the alpha isoform (IkappaBalpha) of the NF-kappaB inhibitor, was constructed. A549 cells were cultured in vitro and divided into non-transfection group, pcDNA3.1(+) transfected group and pcDNA3.1(+)/IkappaBalpha transfected group. The expression of IkappaBalpha was detected by reverse transcription-polymerase chain reaction (RT-PCR) and Western blotting. The activity of NF-kappaB was determined by electrophoretic mobility shift assay (EMSA). Invasion of A549 cells was assessed by transwell chamber assay. The expression of MMP-2 and MMP-9 was detected by RT-PCR and gelatin zymography.Plasmid pcDNA3.1(+)/IkappaBalpha was successfully constructed and expressed in A549 cells. The activity of NF-kappaB, the number of invasive cells, the activity of MMP-2 and MMP-9 of A549 cells in pcDNA3.1(+)/IkappaBalpha transfected group were significantly lower than those in non-transfection group and pcDNA3.1(+) transfected group (all P<0.05).Transfection of IkappaBalpha can inhibit NF-kappaB activity, thus inhibit   cell invasion   of A549, which may be through the down-regulation of MMP-2 and MMP-9 expressions. 
 Alpha-mangostin suppresses phorbol 12-myristate 13-acetate-induced MMP-2/MMP-9 expressions via alphavbeta3 integrin/FAK/ERK and NF-kappaB signaling pathway in human lung adenocarcinoma A549 cells. The purpose of this study is to investigate the anti-metastatic effect of alpha-mangostin on phorbol 12-myristate 13-acetate (PMA)-induced matrix metalloproteinase-2 (MMP-2) and matrix metalloproteinase-9 (MMP-9) expressions in A549 human lung adenocarcinoma cells. Firstly, alpha-mangostin could inhibit PMA-induced abilities of the adhesion, invasion, and migration. Data also showed alpha-mangostin could inhibit the activation of alphavbeta3 integrin, focal adhesion kinase (FAK), and extracellular signal-regulated kinase1/2 (ERK1/2) involved in the downregulation the enzyme activities, protein and messenger RNA levels of MMP-2 and MMP-9 induced by PMA. Next, alpha-mangostin also strongly inhibited PMA-induced degradation of inhibitor of kappaBalpha (IkappaBalpha) and the nuclear levels of nuclear factor kappa B (NF-kappaB). Also, a dose-dependent inhibition on the binding abilities of NF-kappaB by alpha-mangostin treatment was further observed. Furthermore, reduction of FAK or ERK1/2 phosphorylation by FAK small interfering RNA (FAK siRNA) potentiated the effect of alpha-mangostin. Finally, the transient transfection of ERK siRNA significantly down-regulated the expressions of MMP-2 and MMP-9 concomitantly with a marked inhibition on   cell invasion   and migration. Presented results indicated alpha-mangostin is a novel, effect, anti-metastatic agent that functions by downregulating MMP-2 and MMP-9 gene expressions. 
 Evaluation of nuclear factor-kappaB, urokinase-type plasminogen activator, and HBx and their clinicopathological significance in hepatocellular carcinoma. Nuclear factor kappaB (NF-kappaB) signaling pathway is an important regulating pathway in human diseases and cancers. One of its downstream target genes is urokinase plasminogen activator (uPA), which is involved in cancer invasion and metastasis. The purpose of this study was to evaluate NF-kappaB activation, uPA up-regulation, and hepatitis B viral X protein (HBx) expression in human hepatocellular carcinoma (HCC) and to assess their clinicopathological significance.We evaluated NF-kappaB activation, expression of uPA, and presence of HBx in 32 human HCCs. Their clinicopathological significance was assessed by correlation with the clinicopathological features. Aberrant NF-kappaB signaling pathway and uPA up-regulation mediated by HBx were also analyzed in vitro.We found that NF-kappaB activation and uPA up-regulation were frequently (56% and 59%, respectively) observed in HCCs, and particularly in HBx-positive HCCs. NF-kappaB activation and uPA overexpression were closely associated with one another (P < 0.0001). Furthermore, both activation of NF-kappaB and up-regulation of uPA were significantly associated with a more aggressive tumor behavior in terms of venous invasion, direct liver invasion, and absence of tumor encapsulation. In vitro, NF-kappaB activation was induced by HBx transfection in HepG2 cells through inhibitor of nuclear factor-kappaB kinase beta (IKKbeta). HBx also up-regulated uPA and enhanced   cell invasion   synergistically with IKKbeta.The data indicate that NF-kappaB dysregulation and uPA overexpression may lead to a more aggressive tumor behavior in HCC. In addition, our data suggest that IKKbeta plays a critical role in the HBx-activated NF-kappaB signaling pathway. 
 Induction of the NFκ-B signal transduction pathway in response to Corynebacterium diphtheriae infection. Corynebacterium diphtheriae, the causative agent of diphtheria, has been thoroughly studied with respect to toxin production and pili formation, while knowledge on host responses to C. diphtheriae infection is limited. In this study, we studied adhesion to and invasion of epithelial cells by different C. diphtheriae isolates. When NFκ-B reporter cell lines were used to monitor the effect of C. diphtheriae infection on human cells, strain-specific differences were observed. While adhesion to host cells had no effect, a correlation of invasion rate with NFκ-B induction was found, which indicates that internalization of bacteria is crucial for NFκ-B induction. Immunofluorescence microscopy experiments used to support the reporter assays showed that translocation of p65, as a hallmark of NFκ-B induction, was only observed in association   with cell inv  asion by C. diphtheriae. Our data indicate that the response of epithelial cells to C. diphtheriae infection is determined by internalization of bacteria and that invasion of these cells is an active process; tetracycline-treated C. diphtheriae was still able to attach to host cells, but lost its ability to invade the cytoplasm. Recognition of pathogen-associated molecular patterns such as pili subunits by membrane-bound receptors facing the outside of the cell is not sufficient for NFκ-B induction. 
 Livin mediates tumor   cell invasion   in the DU-145 cell line via NF-κB. Livin is a member of the family of inhibitors of apoptosis proteins (IAPs) and tumor   cell invasio  n is a general property of multiple IAPs. Livin is highly expressed in prostate cancer (PCa) tissues. Livin overexpressing cells are more resistant to apoptotic stimuli than normal cells. Thus, aberrantly increased cell survival is an invariable requirement of metastasis. In this study, we investigated whether livin signaling affects metastasis by transfecting siRNA targeting livin into the DU-145 prostate cancer cell line to confirm the anti-invasion effect and blockade of the livin gene. We found that livin knockdown inhibited DU-145 prostate carcinoma   cell invasio  n. We investigated how livin promotes tumor   cell invasio  n, and found that livin induction of fibronectin contributed to tumor   cell invasio  n. In addition, we found that livin induction of fibronectin regulates tumor   cell invasio  n via nuclear factor κB (NF-κB) signaling. These data showed that livin, as a gene directly promoting metastasis, can be useful for therapeutic intervention against advanced and disseminated PCa. 
 DNA damage induces NF-κB-dependent microRNA-21 up-regulation and promotes breast cancer   cell invasio  n. NF-κB activation induced by genotoxic treatment in cancer cells has been associated with therapeutic resistance in multiple human malignancies. Therapeutic resistance also correlates with high metastatic potential in human cancers, including breast cancer. Whether genotoxic treatment-activated NF-κB also contributes to cancer metastasis following radiation and chemotherapy is unclear. Here, we show that chemotherapeutic drug-induced NF-κB activation promotes breast cancer cell migration and invasion. The increased metastatic potential is dependent on IL-6 induction mediated by genotoxic NF-κB activation. Moreover, genotoxic treatment also up-regulates oncogenic microRNA-21 (miR-21) expression through eliciting NF-κB recruitment to the miR-21 promoter region, where it cooperates with signal transducer and activator of transcription 3 (STAT3) to activate miR-21 transcription. DNA damage-induced histone H3 phosphorylation via activated MSK1 creates an open chromatin structure for NF-κB/STAT3-driven transactivation of miR-21. NF-κB-dependent IL-6 up-regulation is responsible for STAT3 activation and recruitment to the miR-21 promoter upon genotoxic stress. Induction of miR-21 may enable cancer cells to elude DNA damage-induced apoptosis and enhance the metastatic potential of breast cancer cells through repressing expression of PTEN and PDCD4. Our data support a critical role of DNA damage-induced NF-κB activation in promoting cancer metastasis following genotoxic treatment, and NF-κB-dependent miR-21 induction may contribute to both therapeutic resistance and metastasis in breast cancer. 
 Visfatin triggers the in vitro migration of osteosarcoma cells via activation of NF-κB/IL-6 signals. Pulmonary metastasis is the major challenge for clinical treatment of osteosarcoma patients. Recent studies indicated that visfatin, a 52kDa adipocytokine, can trigger the cell motility of various cancers, while its role in the progression of osteosarcoma remains not clear. Our present study revealed that visfatin can significantly promote the in vitro migration and invasion of osteosarcoma MG-63 and HOS cells and up regulate the expression of matrix metalloproteinase-2 (MMP-2) and fibronectin (FN). Furthermore, visfatin treatment also increased the expression of IL-6 in both MG-63 and HOS cells via a time dependent manner, while anti-IL-6 antibody can significantly attenuate visfatin induced   cell invasio  n and up regulation of MMP-2 and FN. It suggested that up regulation of IL-6 mediated visfatin induced in vitro motility of osteosarcoma cells. Visfatin treatment can increase the phosphorylation of both p65 and ERK1/2 in MG-63 and HOS cells, while only the inhibitor of NF-κB, BAY 11-7082, can abolish visfatin induced up regulation of IL-6. BAY 11-7082 also attenuated visfatin induced upregulation of MMP-2 and FN in MG-63 cells. Western blot analysis revealed that visfatin treatment can significantly increase the phosphorylation of IκBα and IKKβ in MG-63 cells. ACHP, the inhibitor of IKK-β, blocked visfatin induced expression of IL-6 mRNA in both MG-63 and HOS cells. Collectively, our data suggested that visfatin can increase the motility of osteosarcoma cells via up regulation of NF-κB/IL-6 signals. It indicated that visfatin might be a potential therapeutic target of osteosarcoma treatment. 
 Hypoxia activates heparanase expression in an NF-kappaB dependent manner. Hypoxia was shown to increase tumor   cell invasion   into the extracellular matrix in vitro. This result suggests that heparanase (Hpa), one of the key enzymes involved in tumor invasion and metastasis, may be regulated by hypoxia. RT-PCR, Western blot and Matrigel invasive assays were used to study the regulation of Hpa under hypoxia in human pancreatic MIA PaCa-2 cancer cells. Compared with those in normoxia (20% O2), Hpa mRNA, protein and enzymatic activity levels, were up-regulated by a reduction in the oxygen level (1% O2). Invasion by tumor cells into the extracellular matrix was found to be significantly enhanced. A reduction in Hpa protein levels was observed when nuclear factor kappaB (NF-kappaB) activation was blocked by pyrrolidine dithiocarbamate. The levels of Hpa were also reduced when Hpa was inhibited by an Hpa-specific antisense oligonucleotide. The MMP-9 mRNA, protein and gelatinase B activity levels in supernatants decreased significantly when Hpa was inhibited. We conclude that up-regulation of Hpa by hypoxia is NF-kappaB-dependent in MIA PaCa-2 cells and inhibition of Hpa reduces MMP-9 activity. This reduction in MMP-9 activity may be an important mechanism in tumor metastasis. 
 Notch-1 signaling activates NF-κB in human breast carcinoma MDA-MB-231 cells via PP2A-dependent AKT pathway. Breast cancer has a high incidence in the world and is becoming a leading cause of death in female patients due to its high metastatic ability. High expression of Notch-1 and its ligand Jagged-1 correlates with poor prognosis in breast cancer. Our previous work has shown that Notch-1 signaling pathway upregulates NF-κB transcriptional activity and induces the adhesion, migration and invasion of human breast cancer cell line MDA-MB-231. However, the role of Notch-1 in NF-κB activation is still poorly understood. Here, we aim to understand the exact mechanism that Notch-1 regulates NF-κB activity. In MDA-MB-231 cells where Notch-1 is constitutively activated, the phosphorylation of p85 and AKT (Tyr308/Ser473) is upregulated, indicating PI3K/AKT pathway is activated. Notch-1 activation caused the increase of PP2A phosphorylation at Tyr307, indicating Notch-1 inhibits PP2A activity. NF-κB transcriptional activity was evaluated by dual-luciferase reporter assay, and the results showed that, while silencing of Notch-1, PP2A activity was upregulated and NF-κB activity was downregulated, whereas PP2A inhibitor okadaic acid (OA) restored NF-κB activity. Immunofluorescence and Western blots showed that OA treatment antagonized the decrease of p65 nuclear translocation caused by Notch-1 silencing. Moreover, OA treatment also upregulated MMP-2, MMP-9 and VEGF mRNA expression levels, indicating OA rescues Notch-1 silencing that caus  ed low cell i  nvasion. Taken together, our results suggest that Notch-1-activating PI3K/AKT/NF-κB pathway is PP2A dependent; PP2A may be a potential therapeutic target in breast cancer. 
 GEF-H1 mediated control of NOD1 dependent NF-kappaB activation by Shigella effectors. Shigella flexneri has evolved the ability to modify host cell function with intracellular active effectors to overcome the intestinal barrier. The detection of these microbial effectors and the initiation of innate immune responses are critical for rapid mucosal defense activation. The guanine nucleotide exchange factor H1 (GEF-H1) mediates RhoA activation required for   cell invasion   by the enteroinvasive pathogen Shigella flexneri. Surprisingly, GEF-H1 is requisite for NF-kappaB activation in response to Shigella infection. GEF-H1 interacts with NOD1 and is required for RIP2 dependent NF-kappaB activation by H-Ala-D-gammaGlu-DAP (gammaTriDAP). GEF-H1 is essential for NF-kappaB activation by the Shigella effectors IpgB2 and OspB, which were found to signal in a NOD1 and RhoA Kinase (ROCK) dependent manner. Our results demonstrate that GEF-H1 is a critical component of cellular defenses forming an intracellular sensing system with NOD1 for the detection of microbial effectors during   cell invasion   by pathogens. 
 Autotaxin stimulates urokinase-type plasminogen activator expression through phosphoinositide 3-kinase-Akt-nuclear [corrected] factor kappa B signaling cascade in human melanoma cells. Autotaxin, a lysophospholipase D producing lysophosphatidic acid, augments invasive and metastatic potential of tumor cells. Current investigations have focused on understanding the molecular mechanisms by which autotaxin regulates the expression of a major mediator of tumor invasion and metastasis, urokinase-type plasminogen activator (uPA) in human A2058 melanoma cells. Autotaxin induced uPA expression in a dose-dependent manner that was inhibited by pharmacological inhibitors for Gi (pertussis toxin), phosphoinositide 3-kinase (PI3K, LY294002), Akt inhibitor (AktI), proteosome activity and IkappaB phosphorylation (pyrrolidine dithiocarbamate), and by a dominant negative mutant (DN) of Akt. Autotaxin phosphorylated Akt and induced the translocation of nuclear [corrected] factor-kappaB (NF-kappaB) to the nucleus that were inhibited by AktI or by overexpressing DN-Akt. Consistently, green fluorescence protein-tagged p65 of NF-kappaB accumulated in the nucleus by autotaxin that was abrogated when the cells were transfected with DN-Akt. Moreover, autotaxin increased the DNA binding ability of NF-kappaB and promoter activity of uPA. Collectively, these data strongly suggest autotaxin induces uPA expression via the Gi-PI3K-Akt-NF-kappaB signaling pathway that might be critical for autotaxin-induced tumor   cell invasion   and metastasis. 
 NF-kappaB p50 promotes tumor   cell invasion   through negative regulation of invasion suppressor gene CRMP-1 in human lung adenocarcinoma cells. Lung adenocarcinoma Cl1-5 cells were selected from parental Cl1-0 cells based on their high metastatic potential. In a previous study, CRMP-1, an invasion suppressor gene, was shown to be suppressed in Cl1-5 cells. However, the regulation of CRMP-1 expression has not been explored. In this study, we showed nuclear factor-kappaB controls CRMP-1 expression. The electromobility shift assay showed that while Cl1-0 cells exhibited low NF-kappaB activity in response to TNF-alpha, an abundance of basal and TNF-alpha-induced NF-kappaB-DNA complex was detected in Cl1-5 cells. Supershift-coupled EMSA and Western blotting of nuclear proteins, however, revealed p50 protein, but not classic p65/p50 heterodimer in the complex. ChIP and EMSA demonstrated that p50 binds to a kappaB site residing between -1753 and -1743 of the CRMP-1 promoter region. Transfection of antisense p50 gene into Cl1-5 cells increased the CRMP-1 protein level and decreased the invasive activity of Cl1-5 cells. 
 Romo1 and the NF-κB pathway are involved in oxidative stress-induced tumor   cell invasio  n. Reactive oxygen species (ROS) are important contributors to tumor   cell invasio  n. ROS enhanced by reactive oxygen species modulator 1 (Romo1) expression has been reported to increase invasive potential and constitutive activation of nuclear factor-κB (NF-κB) in hepatocellular carcinoma (HCC). Therefore, we investigated whether constitutive NF-κB activation due to Romo1 expression is associated with breast cancer tu  mor cell inva  sion. In this study, we show that oxidative stress-induced invasion is mediated by Romo1 expression. The Romo1-induced increase of invasive activity was blocked by an inhibitor of κB kinase (IKK). These results demonstrate that t  umor cell inv  asion in response to oxidative stress is associated with Romo1 expression and the NF-κB signaling pathway. Romo1 is therefore a promising therapeutic target for diseases characterized by NF-κB deregulation. 
 Ghrelin induces gastric cancer cell proliferation, migration, and invasion through GHS-R/NF-κB signaling pathway. This study aims to investigate the roles of ghrelin signaling in human gastric carcinoma cell lines AGS and SGC7901. Effects of ghrelin signaling on CDK6, P53, NF-κB/P65 and MMP2 mRNA and/or protein expression were determined by real-time PCR and western blot. MTT method and flow cytometry were performed to assess the gastric cancer cell proliferation. The SGC7901 cells overexpressing ghrelin were inoculated into nude mice to produce tumors which were measured later. The wound-healing assay an  d cell invasi  on assay were used to test the cell migration and invasive ability of gastric cancer. Ghrelin signaling promotes the oncogene CDK6 gene expression and represses the tumor suppressor gene P53 gene expression in gastric cancer. Ghrelin activates NF-κB/P65 signaling pathway through GHS-R in gastric cancer. Ghrelin upregulates the metastasis factor MMP2 expression via GHS-R/NF-κB signaling pathway in gastric cancer cells and promotes tumor cells migration and invasion, suggesting that ghrelin signaling is a critical pathway in cancer metastasis. Ghrelin induces cell proliferation, migration and invasion via GHS-R/NF-κB signaling pathway in gastric cancer cells. Ghrelin treatment must be avoided for gastric cancer patients. 
 Inhibition of matrix metalloproteinase-9 and nuclear factor kappa B contribute to melatonin prevention of motility and invasiveness in HepG2 liver cancer cells. Hepatocellular carcinoma (HCC) is one of the most lethal human cancers worldwide because of its high incidence and its metastatic potential. Extracellular matrix degradation by matrix metalloproteinases (MMPs) has been connected with cancer   cell invasion  , and it has been suggested that inhibition of MMPs by synthetic and natural inhibitors may be of great importance in the HCC therapies. Melatonin, the main product of the pineal gland, exerts antiproliferative, proapoptotic, and antiangiogenic properties in HepG2 human hepatocellular cells, and exhibits anti-invasive and antimetastatic activities by suppressing the enzymatic activity of MMP-9 in different tumor types. However, the underlying mechanism of anti-invasive activity in HCC models has not been fully elucidated. Here, we demonstrate that 1 mm melatonin dosage reduced in IL-1β-induced HepG2 cells MMP-9 gelatinase activity and inhibited   cell invasio  n and motility through downregulation of MMP-9 gene expression and upregulation of the MMP-9-specific inhibitor tissue inhibitor of metalloproteinases (TIMP)-1. No significant changes were observed in the expression and activity of MMP-2, the other proteinase implicated in matrix collagen degradation, and its tissue inhibitor, TIMP-2. Also, melatonin significantly suppressed IL-1β-induced nuclear factor-kappaB (NF-κB) translocation and transcriptional activity. In summary, we demonstrate that melatonin modulates motility and invasiveness of HepG2 cell in vitro through a molecular mechanism that involves TIMP-1 upregulation and attenuation of MMP-9 expression and activity via NF-κB signal pathway inhibition. 
 Raf kinase inhibitor protein suppresses nuclear factor-κB-dependent cancer   cell invasio  n through negative regulation of matrix metalloproteinase expression. Accumulating evidence suggests that Raf kinase inhibitor protein (RKIP), which negatively regulates multiple signaling cascades including the Raf and nuclear factor-κB (NF-κB) pathways, functions as a metastasis suppressor. However, the basis for this activity is not clear. We investigated this question in a panel of breast cancer, colon cancer and melanoma cell lines. We found that RKIP negatively regulated the invasion of the different cancer cells through three-dimensional extracellular matrix barriers by controlling the expression of matrix metalloproteinases (MMPs), particularly, MMP-1 and MMP-2. Silencing of RKIP expression resulted in a highly invasive phenotype and dramatically increased levels of MMP-1 and MMP-2 expression, while overexpression of RKIP decreased canc  er cell invas  ion in vitro and metastasis in vivo of murine tumor allografts. Knockdown of MMP-1 or MMP-2 in RKIP-knockdown cells reverted their invasiveness to normal. In contrast, when examining migration of the different cancer cells in a two-dimensional, barrier-less environment, we found that RKIP had either a positive regulatory activity or no activity, but in no case a negative one (as would be expected if RKIP suppressed metastasis at the level of cell migration itself). Therefore, RKIP's function as a metastasis suppressor appears to arise from its ability to negatively regulate expression of specific MMPs, and thus invasion through barriers, and not from a direct effect on the raw capacity of cells to move. The NF-κB pathway, but not the Raf pathway, appeared to positively control the invasion of breast cancer cells. A regulatory loop involving an opposing relationship between RKIP and the NF-κB pathway may control the level of MMP expression   and cell inv  asion. 
 KiSS1 suppresses TNFalpha-induced breast cancer   cell invasion   via an inhibition of RhoA-mediated NF-kappaB activation. Tumor necrosis factor-alpha (TNFalpha) induces cancer development and metastasis, which is prominently achieved by nuclear factor-kappa B (NF-kappaB) activation. TNFalpha-induced NF-kappaB activation enhances cellular mechanisms including proliferation, migration, and invasion. KiSS1, a key regulator of puberty, was initially discovered as a tumor metastasis suppressor. The expression of KiSS1 was lost or down-regulated in different metastatic tumors. However, it is unclear whether KiSS1 regulates TNFalpha-induced NF-kappaB activation and further tumor cell migration. In this study, we demonstrate that KiSS1 suppresses the migration of breast cancer cells by inhibiting TNFalpha-induced NF-kappaB pathway and RhoA activation. Both KiSS1 overexpression and KP10 (kisspeptin-10) stimulation inhibited TNFalpha-induced NF-kappaB activity, suppressed TNFalpha-induced cell migration and cell attachment to fibronectin in breast cancer cells while KP10 has little effect on cancer cell proliferation. Furthermore, KP10 inhibited TNFalpha-induced cell migration and RhoA GTPase activation. Therefore, our data demonstrate that KiSS1 inhibits TNFalpha-induced NF-kappaB activation via downregulation of RhoA activation and suppression of breast cancer cell migration and invasion. 
 Hepatitis C virus NS3 protein enhances cancer   cell invasion   by activating matrix metalloproteinase-9 and cyclooxygenase-2 through ERK/p38/NF-κB signal cascade. Hepatitis C virus (HCV) infection causes acute and chronic hepatitis, liver cirrhosis, and hepatocellular carcinoma (HCC). However, the mechanisms by which HCV causes the diseases are largely unknown. Here, we elucidated the effects of HCV on the invasion and migration of hepatoma cells, with the aim to reveal the mechanism by which HCV infection induces HCC. We initially showed that matrix metalloproteinase-9 (MMP-9) was elevated in the sera of HCV-infected patients, and demonstrated that HCV nonstructural protein 3 (NS3) activated MMP-9 transcription through nuclear factor-κB (NF-κB) by stimulating translocation of NF-κB from cytosol to the nucleus to enhance its binding to MMP-9 promoter. In addition, cyclooxygenase-2 (COX-2) and extracellular signal-regulated kinase (ERK1/2)/mitogen-activated protein kinase (p38) pathway were involved in HCV-activated MMP-9 expression. Moreover, NS3 enhanced hepat  oma cell inva  sion and migration through MMP-9 and COX-2. Thus, this study provides new insights into the roles of HCV NS3, MMP-9 and COX-2 in regulating can  cer cell inva  sion. 
 Pachymic acid impairs breast cancer   cell invasion   by suppressing nuclear factor-κB-dependent matrix metalloproteinase-9 expression. Pachymic acid (PA), a lanostane-type triterpenoid derived from Poria cocos, possesses demonstrated anti-inflammatory and anti-cancer activities. Nonetheless, the biological properties and mechanism/s of action of PA remain largely undefined. In this study, the activity of PA against breast cancer   cell invasio  n was evaluated. Invasiveness of human-derived MDA-MB-231 and MCF-7 breast carcinoma cells was suppressed by PA at non-lethal concentrations, which was associated with a decrease in matrix metalloproteinase-9 (MMP-9) secretion as a result of PA-mediated down-regulation of MMP-9 mRNA expression. In order to elucidate the underlying anti-invasive mechanism, the effect of PA on transcription factors activator protein-1 (AP-1) and nuclear factor kappaB (NF-κB) was examined using luciferase-based reporter gene assays. PA was found to bring about a reduction in phorbol 12-myristate 13-acetate (PMA)-induced transcriptional activity of NF-κB, but not that of AP-1. In accord with the luciferase activity data, western blot analysis showed that PA inhibited NF-κB signaling pathway, but did not alter the phosphorylation states of mitogen-activated protein kinases including ERK, JNK, and p38 kinase. The inhibition of PA on NF-κB signaling pathway was further attributed to PA-mediated diminution in PMA-induced degradation of inhibitor of kappaBα (IκBα) through preventing phosphorylation of the upstream signal IκB kinase (IKK). A decrease in p65 nuclear translocation was achieved, which led to attenuation of NF-κB transactivation. Taken together, it was concluded that by targeting NF-κB signaling, PA inhibited bre  ast cancer ce  ll invasion through decreasing MMP-9 expression. PA may thus be potentially exploited for use in tumor metastasis intervention. 
  GeneRIFs  
 This study is the first to demonstrate that inhibition of NF-kappaB could significantly reduce hypoxia and retinoblastoma   cell invasion   and migration ability 
 These results demonstrate that tumor   cell invasion   in response to oxidative stress is associated with Romo1 expression and the NF-kappaB signaling pathway. 
 High NFKB expression enhanced hepatoma   cell invasion   and migration. 
 livin induction of fibronectin regulates tumor   cell invasion   via nuclear factor kappa-B signaling 
 Resveratrol reduces TNF-alpha-induced U373MG human glioma   cell invasion   through regulating NF-kappaB activation and uPA/uPAR expression 
 Pachymic acid impairs breast cancer   cell invasion   by suppressing nuclear factor-kappaB-dependent matrix metalloproteinase-9 expression 
 Data show that a regulatory loop involving an opposing relationship between RKIP and the NF-kappaB pathway may control the level of MMP expression and   cell invasion  . 
 GEF-H1 is a critical component of cellular defenses forming an intracellular sensing system with NOD1 for the detection of microbial effectors during   cell invasion   by pathogens. 
 Transfection of IkappaBalpha can inhibit NF-kappaB activity, thus inhibit   cell invasion   of A549, which may be through the down-regulation of MMP-2 and MMP-9 expressions. 
 autotaxin induces uPA expression via the Gi-PI3K-Akt-NF-kappaB signaling pathway that might be critical for autotaxin-induced tumor   cell invasion   and metastasis 
   
  
 SOCS4 
 
  NCBI Gene  
  GeneRIF  
  Pubmed  
 
 
  Search Term  Rank  Fields  
  [Mm]igration  6  PubMed(1); GeneRIFs(1)  
 
 
 hide/show details for SOCS4 
  [Mm]igration  PubMed  
 Interleukin-23 Facilitates Thyroid Cancer Cell   Migration   and Invasion by Inhibiting SOCS4 Expression via MicroRNA-25. Interleukin-23 (IL-23) is a conventional proinflammatory cytokine that plays a role in tumor progression by inducing inflammation in the tumor microenvironment. However, the role of IL-23 in thyroid cancer   migration   and invasion remains unclear. In the present study, we observed that the treatment with IL-23, induced   migration   and invasion in human thyroid cancer cells. Additional data demonstrate that SOCS4 negatively regulates IL-23-mediated   migration   and invasion. On investigating the mechanisms involved in IL-23 mediated   migration   and invasion, we observed that miR-25 promotes the   migration   and invasion of thyroid cancer cells by directly binding to the 3'-UTR of SOCS4 that leads to the inhibition of SOCS4. In addition, we also demonstrated that IL-23 increases miR-25 expression levels, and overexpressed miR-25 is involved in IL-23-associated SOCS4 inhibition and cell   migration   and invasion. Together, our data suggest that IL-23 induces   migration   and invasion in thyroid cancer cells by mediating the miR-25/SOCS4 signaling pathway. 
  GeneRIFs  
 miR-25 promotes the   migration   and invasion of thyroid cancer cells by directly binding to the 3'-UTR of SOCS4 that leads to the inhibition of SOCS4. 
   
  
 CREB3L2 
 
  NCBI Gene  
  GeneRIF  
  Pubmed  
 
 
  Search Term  Rank  Fields  
  [Mm]etastasize  11  PubMed(1)  
 
 
 hide/show details for CREB3L2 
  [Mm]etastasize  PubMed  
 Fusion of the FUS and CREB3L2 genes in a supernumerary ring chromosome in low-grade fibromyxoid sarcoma. Low-grade fibromyxoid sarcoma (LGFMS) is a rare, low-grade malignant soft tissue tumor that is often mistaken for either benign or more malignant tumor types. Commonly, this tumor affects young adults and typically arises in the deep proximal extremities or trunk with frequent recurrences and can   metastasize   to the lungs many years later. Most cases have a recurrent balanced translocation involving chromosomes 7 and 16, t(7;16)(q32-34;p11), which leads to the fusion of the FUS and CREB3L2 genes. However, supernumerary ring chromosomes have been identified in a subset of FUS/CREB3L2-positive LGFMS, but it has not yet been formally demonstrated that such ring chromosomes harbor the FUS/CREB3L2 fusion gene. Here, we report the genetic findings of a supernumerary ring chromosome from an LGFMS from a 77-year-old man. Chromosome banding analysis revealed a supernumerary ring chromosome, and further studies with fluorescence in situ hybridization and reverse transcriptase-polymerase chain reaction (RT-PCR) showed that the ring contained material from chromosomes 7 and 16, that the FUS gene was present in two rearranged copies, and that it expressed the FUS/CREB3L2 fusion gene. Moreover, an assessment of previously reported cases showed that tumors with ring chromosomes relapsed more often than tumors with a balanced t(7;16), suggesting that ring formation in LGFMS is correlated with tumor progression. 
   
  
 GADD45G 
 
  NCBI Gene  
  GeneRIF  
  Pubmed  
 
 
  Search Term  Rank  Fields  
  [Mm]igration  6  PubMed(1)  
  [Mm]etastasis  12  PubMed(1); GeneRIFs(1)  
 
 
 hide/show details for GADD45G 
  [Mm]igration  PubMed  
 Paired box 5 is a frequently methylated lung cancer tumour suppressor gene interfering β-catenin signalling and GADD45G expression. Recent studies suggest that paired box 5 (PAX5) is down-regulated in multiple tumours through its promoter methylation. However, the role of PAX5 in non-small cell lung cancer (NSCLC) pathogenesis remains unclear. The aim of this study is to examine PAX5 expression, its methylation status, biological functions and related molecular mechanism in NSCLC. We found that PAX5 was widely expressed in normal adult tissues but silenced or down-regulated in 88% (7/8) of NSCLC cell lines. PAX5 expression level was significantly lower in NSCLC than that in adjacent non-cancerous tissues (P = 0.0201). PAX5 down-regulation was closely associated with its promoter hypermethylation status and PAX5 expression could be restored by demethylation treatment. Frequent PAX5 promoter methylation in primary tumours (70%) was correlated with lung tumour histological types (P = 0.006). Ectopic expression of PAX5 in silenced lung cancer cell lines (A549 and H1975) inhibited their colony formation and cell viability, arrested cell cycle at G2 phase and suppressed cell   migratio  n/invasion as well as tumorigenicity in nude mice. Restoration of PAX5 expression resulted in the down-regulation of β-catenin and up-regulation of tissue inhibitors of metalloproteinase 2, GADD45G in lung tumour cells. In summary, PAX5 was found to be an epigenetically inactivated tumour suppressor that inhibits NSCLC cell proliferation and metastasis, through down-regulating the β-catenin pathway and up-regulating GADD45G expression. 
  [Mm]etastasis  PubMed  
 Paired box 5 is a frequently methylated lung cancer tumour suppressor gene interfering β-catenin signalling and GADD45G expression. Recent studies suggest that paired box 5 (PAX5) is down-regulated in multiple tumours through its promoter methylation. However, the role of PAX5 in non-small cell lung cancer (NSCLC) pathogenesis remains unclear. The aim of this study is to examine PAX5 expression, its methylation status, biological functions and related molecular mechanism in NSCLC. We found that PAX5 was widely expressed in normal adult tissues but silenced or down-regulated in 88% (7/8) of NSCLC cell lines. PAX5 expression level was significantly lower in NSCLC than that in adjacent non-cancerous tissues (P = 0.0201). PAX5 down-regulation was closely associated with its promoter hypermethylation status and PAX5 expression could be restored by demethylation treatment. Frequent PAX5 promoter methylation in primary tumours (70%) was correlated with lung tumour histological types (P = 0.006). Ectopic expression of PAX5 in silenced lung cancer cell lines (A549 and H1975) inhibited their colony formation and cell viability, arrested cell cycle at G2 phase and suppressed cell migration/invasion as well as tumorigenicity in nude mice. Restoration of PAX5 expression resulted in the down-regulation of β-catenin and up-regulation of tissue inhibitors of metalloproteinase 2, GADD45G in lung tumour cells. In summary, PAX5 was found to be an epigenetically inactivated tumour suppressor that inhibits NSCLC cell proliferation an  d metastas  is, through down-regulating the β-catenin pathway and up-regulating GADD45G expression. 
  GeneRIFs  
 PAX5 was found to be an epigenetically inactivated tumor suppressor that inhibited non-small-cell lung proliferation and   metastasis  , through down-regulating the beta-catenin pathway and up-regulating GADD45G expression. 
   
  
 PLPP1 
 
  NCBI Gene  
  GeneRIF  
  Pubmed  
 
 
  Search Term  Rank  Fields  
  [Mm]igration  6  Process(1); PubMed(1)  
  [Ii]nvasive  10  PubMed(1)  
 
 
 hide/show details for PLPP1 
  [Mm]igration  Process  
 germ cell   migration   
  PubMed  
 Cloning and characterization of two human isozymes of Mg2+-independent phosphatidic acid phosphatase. We obtained two human cDNA clones encoding phosphatidic acid phosphatase (PAP) isozymes named PAP-2a (Mr = 32,158) and -2b (Mr = 35, 119), both of which contained six putative transmembrane domains. Both enzymes were glycosylated and cleaved by N-glycanase and endo-beta-galactosidase, thus suggesting their post-Golgi localization. PAP-2a and -2b shared 47% identical sequence and were judged to be the human counterparts of the previously sequenced mouse 35-kDa PAP(83% identity) and rat Dri42 protein (94% identity), respectively. Furthermore, the sequences of both PAPs were 34-39% identical to that of Drosophila Wunen protein. In view of the functions ascribed to Wunen and Dri42 in germ cell   migration   and epithelial differentiation, respectively, these findings unexpectedly suggest critical roles of PAP isoforms in cell growth and differentiation. Although the two PAPs hydrolyzed lysophosphatidate and ceramide-1-phosphate in addition to phosphatidate, the hydrolysis of sphingosine-1-phosphate was detected only for PAP-2b. PAP-2b was expressed almost ubiquitously in all human tissues examined, whereas the expression of PAP-2a was relatively variable, being extremely low in the placenta and thymus. In HeLa cells, the transcription of PAP-2a was not affected by different stimuli, whereas PAP-2b was induced (up to 3-fold) by epidermal growth factor. These findings indicate that despite structural similarities, the two PAP isozymes may play distinct functions through their different patterns of substrate utilization and transcriptional regulation. 
  [Ii]nvasive  PubMed  
 Role of decreased levels of lipid phosphate phosphatase-1 in accumulation of lysophosphatidic acid in ovarian cancer. The levels of lysophosphatidic acid (LPA) are consistently elevated in the ascites of ovarian cancer patients, suggesting that ovarian cancer cells are exposed to an LPA replete environment. LPA stimulates cell proliferation, cell survival, resistance to cisplatin, production and activation of proteases,   invasive  ness and production of the neovascularizing factors, vascular endothelial growth factor, and interleukin 8. Although ovarian cancer cells can produce LPA, this may not be the major reason for altered LPA levels in ascites. We have demonstrated that the major mechanism of degradation of LPA by ovarian cancer cells is through a lipid phosphate phosphatase (LPP)-like activity. We demonstrate herein that LPP-1 mRNA is decreased in the majority of ovarian cancers. This is recapitulated in ovarian cancer cell lines, where LPP-1 RNA levels are lower than those in normal ovarian epithelium and immortalized ovarian epithelial cells. Introduction of LPP-1 into ovarian cancer cell lines results in increased LPA hydrolysis, which is associated with a marked inhibition of cell proliferation and colony-forming activity and a marked increase in apoptosis. Thus, the LPA-rich environment of the ovarian cancer cell in vivo and the subsequent effects of cellular pathophysiology may be a consequence of both increased LPA production and decreased LPA metabolism by ovarian cancer cells. 
   
  
 NFKBIA 
 
  NCBI Gene  
  GeneRIF  
  Pubmed  
 
 
  Search Term  Rank  Fields  
  [Mm]igration  6  PubMed(11)  
  [Ee]xtracellular [Mm]atrix  8  PubMed(6)  
  [Ii]nvasive  10  PubMed(10)  
  [Mm]etastasis  12  PubMed(19)  
  [Cc]ell [Ii]nvasion  13  PubMed(5); GeneRIFs(1)  
 
 
 hide/show details for NFKBIA 
  [Mm]igration  PubMed  
 Alpha-mangostin suppresses phorbol 12-myristate 13-acetate-induced MMP-2/MMP-9 expressions via alphavbeta3 integrin/FAK/ERK and NF-kappaB signaling pathway in human lung adenocarcinoma A549 cells. The purpose of this study is to investigate the anti-metastatic effect of alpha-mangostin on phorbol 12-myristate 13-acetate (PMA)-induced matrix metalloproteinase-2 (MMP-2) and matrix metalloproteinase-9 (MMP-9) expressions in A549 human lung adenocarcinoma cells. Firstly, alpha-mangostin could inhibit PMA-induced abilities of the adhesion, invasion, and   migration  . Data also showed alpha-mangostin could inhibit the activation of alphavbeta3 integrin, focal adhesion kinase (FAK), and extracellular signal-regulated kinase1/2 (ERK1/2) involved in the downregulation the enzyme activities, protein and messenger RNA levels of MMP-2 and MMP-9 induced by PMA. Next, alpha-mangostin also strongly inhibited PMA-induced degradation of inhibitor of kappaBalpha (IkappaBalpha) and the nuclear levels of nuclear factor kappa B (NF-kappaB). Also, a dose-dependent inhibition on the binding abilities of NF-kappaB by alpha-mangostin treatment was further observed. Furthermore, reduction of FAK or ERK1/2 phosphorylation by FAK small interfering RNA (FAK siRNA) potentiated the effect of alpha-mangostin. Finally, the transient transfection of ERK siRNA significantly down-regulated the expressions of MMP-2 and MMP-9 concomitantly with a marked inhibition on cell invasion and   migration  . Presented results indicated alpha-mangostin is a novel, effect, anti-metastatic agent that functions by downregulating MMP-2 and MMP-9 gene expressions. 
 Effect of weight loss on proinflammatory state of mononuclear cells in obese women. In order to investigate whether weight loss can lead to improvement of the mononuclear cell (MNC) proinflammatory state, 21 nondiabetic obese women with mean age 34+/-2 years (mean+/-s.e.m.) and BMI 32.5+/-1.2 kg/m2 were enrolled in a 12-week caloric restriction and light exercise-based weight loss program. Ten lean women served as controls. Reverse transcription-PCR of proinflammatory cytokines and adipocytokines as well as homeostasis model assessment of insulin resistance (HOMA-IR) were determined before and after weight reduction. Nuclear factor kappaB (NF-kappaB) binding to DNA and inhibitors of NF-kappaB (IkappaB-alpha and IkappaB-beta) obtained from peripheral MNCs were measured. Overall, subjects lost a mean of 4.0+/-0.4 kg (5.0+/-0.3% of their initial body weight) (P<0.01). In addition to significant reductions in BMI, fasting glucose and insulin concentrations, mean serum high-sensitivity C-reactive protein (hs-CRP),   migration   inhibitor factor (MIF), leptin and visfatin levels decreased by 49.0, 66.6, 17.2, and 50.2%, respectively (all P<0.05), while adiponectin concentrations rose by 33.9% (P<0.05). The DNA binding of the transcriptionally active NF-kappaB from (p65/p50) decreased by 38.1% (P<0.05). Elevated levels of mRNA of NF-kappaB related proinflammatory genes, tumor necrosis factor-alpha (TNF-alpha), interleukin-6 (IL-6), MIF, and matrix metalloproteinase-9 (MMP-9), decreased significantly after weight loss. Although mRNA expression of Rel-A, p105, IkappaB-alpha, IkappaB-beta decreased significantly, their protein levels did not change after weight loss. As a group, NF-kappaB binding activity correlated with HOMA-IR (r=0.332, P=0.049) and marginally with values of BMI (r=0.308, P=0.059). In conclusion, weight loss by 5% of initial weight in nondiabetic obese women led to significant improvement in activated intranuclear NF-kappaB binding as well as several transcriptions of proinflammatory genes regulated by NF-kappaB. 
 Association of common polymorphisms in TNFA, NFkB1 and NFKBIA with risk and prognosis of esophageal squamous cell carcinoma. Tumour necrosis factor-alpha (TNF-α) and nuclear factor of kappa light chain gene enhancer in activated B cells (NF-κB) play critical role in carcinogenesis processes like tumour initiation, proliferation  , migrati  on and invasion. Single nucleotide polymorphisms in TNF-α, NF-κB and its inhibitor IκB genes were shown to be associated with susceptibility and prognosis of several cancers; however, their role in esophageal squamous cell carcinoma (ESCC) is not well recognised. Therefore, in present study, we aimed to investigate association of common polymorphisms in TNFA, NFkB1 and NFKBIA with risk and prognosis of ESCC in northern Indian population.We genotyped 290 ESCC patients (including 162 followed up cases) and 311 mean age, gender and ethnicity matched controls for TNFA -308G>A, NFkB1 -94ATTG ins/del and NFKBIA (-826C>T and 3'UTRA>G) polymorphisms using PCR alone or followed by RFLP and TaqMan assay.TNFA-308GA genotype was associated with increased risk of ESCC specifically in females and in patients with regional lymph node involvement, while, NFKBIA -826CT+TT genotype conferred decreased risk of ESCC in females. Haplotypes of NFKBIA -826C>T and 3'UTRA>G polymorphisms, C-826G3'UTR and T-826A3'UTR, were associated with reduced risk of ESCC. No independent role of NFkB1 -94ATTG ins/del polymorphism in susceptibility of ESCC was found. Multi-dimensionality reduction analysis showed three factor model TNFA-308, NFKBIA-826, NFKBIA 3'UTR as better predictor for risk of ESCC. Furthermore, combined risk genotype analysis of all studied polymorphisms showed increased risk of ESCC in patients with 1-3 risk genotype compared to '0' risk genotype. Survival analysis did not show any significant prognostic effect of studied polymorphisms. However, in stepwise multivariate analysis, metastasis was found to be independent prognostic predictor of ESCC patients.TNFA-308 and NFKBIA (-826C>T and 3'UTRA>G) polymorphisms may play role in susceptibility but not in prognosis of ESCC patients in northern Indian population. 
 Down-regulation of the tumor suppressor PTEN by the tumor necrosis factor-alpha/nuclear factor-kappaB (NF-kappaB)-inducing kinase/NF-kappaB pathway is linked to a default IkappaB-alpha autoregulatory loop. The PTEN (phosphatase and tensin homolog deleted on chromosome ten) tumor suppressor gene affects multiple cellular processes including cell growth, proliferation, and cell   migration   by antagonizing phosphatidylinositol 3-kinase (PI3K). However, mechanisms by which PTEN expression is regulated have not been studied extensively. Similar to PTEN, tumor necrosis factor-alpha (TNF-alpha) affects a wide spectrum of diseases including inflammatory processes and cancer by acting as a mediator of apoptosis, inflammation, and immunity. In this study, we show that treatment of cancer cell lines with TNF-alpha decreases PTEN expression. In addition, overexpression of TNF-alpha downstream signaling targets, nuclear factor-kappaB (NF-kappaB)-inducing kinase (NIK) and p65 nuclear factor NF-kappaB, lowers PTEN expression, suggesting that TNF-alpha-induced down-regulation of PTEN is mediated through a TNF-alpha/NIK/NF-kappaB pathway. Down-regulation of PTEN by NIK/NF-kappaB results in activation of the PI3K/Akt pathway and augmentation of TNF-alpha-induced PI3K/Akt stimulation. Importantly, we demonstrate that this effect is associated with a lack of an inhibitor of kappaB (IkappaB)-alpha autoregulatory loop. Moreover, these findings suggest the interaction between PI3K/Akt and NF-kappaB via transcriptional regulation of PTEN and offer one possible explanation for increased tumorigenesis in systems in which NF-kappaB is chronically activated. In such a tumor system, these findings suggest a positive feedback loop whereby Akt activation of NF-kappaB further stimulates Akt via down-regulation of the PI3K inhibitor PTEN. 
 Association of common polymorphisms in TNFA, NFkB1 and NFKBIA with risk and prognosis of esophageal squamous cell carcinoma. Tumour necrosis factor-alpha (TNF-α) and nuclear factor of kappa light chain gene enhancer in activated B cells (NF-κB) play critical role in carcinogenesis processes like tumour initiation, proliferation  , migrati  on and invasion. Single nucleotide polymorphisms in TNF-α, NF-κB and its inhibitor IκB genes were shown to be associated with susceptibility and prognosis of several cancers; however, their role in esophageal squamous cell carcinoma (ESCC) is not well recognised. Therefore, in present study, we aimed to investigate association of common polymorphisms in TNFA, NFkB1 and NFKBIA with risk and prognosis of ESCC in northern Indian population.We genotyped 290 ESCC patients (including 162 followed up cases) and 311 mean age, gender and ethnicity matched controls for TNFA -308G>A, NFkB1 -94ATTG ins/del and NFKBIA (-826C>T and 3'UTRA>G) polymorphisms using PCR alone or followed by RFLP and TaqMan assay.TNFA-308GA genotype was associated with increased risk of ESCC specifically in females and in patients with regional lymph node involvement, while, NFKBIA -826CT+TT genotype conferred decreased risk of ESCC in females. Haplotypes of NFKBIA -826C>T and 3'UTRA>G polymorphisms, C-826G3'UTR and T-826A3'UTR, were associated with reduced risk of ESCC. No independent role of NFkB1 -94ATTG ins/del polymorphism in susceptibility of ESCC was found. Multi-dimensionality reduction analysis showed three factor model TNFA-308, NFKBIA-826, NFKBIA 3'UTR as better predictor for risk of ESCC. Furthermore, combined risk genotype analysis of all studied polymorphisms showed increased risk of ESCC in patients with 1-3 risk genotype compared to '0' risk genotype. Survival analysis did not show any significant prognostic effect of studied polymorphisms. However, in stepwise multivariate analysis, metastasis was found to be independent prognostic predictor of ESCC patients.TNFA-308 and NFKBIA (-826C>T and 3'UTRA>G) polymorphisms may play role in susceptibility but not in prognosis of ESCC patients in northern Indian population. 
 Alpha-mangostin suppresses phorbol 12-myristate 13-acetate-induced MMP-2/MMP-9 expressions via alphavbeta3 integrin/FAK/ERK and NF-kappaB signaling pathway in human lung adenocarcinoma A549 cells. The purpose of this study is to investigate the anti-metastatic effect of alpha-mangostin on phorbol 12-myristate 13-acetate (PMA)-induced matrix metalloproteinase-2 (MMP-2) and matrix metalloproteinase-9 (MMP-9) expressions in A549 human lung adenocarcinoma cells. Firstly, alpha-mangostin could inhibit PMA-induced abilities of the adhesion, invasion, and   migration  . Data also showed alpha-mangostin could inhibit the activation of alphavbeta3 integrin, focal adhesion kinase (FAK), and extracellular signal-regulated kinase1/2 (ERK1/2) involved in the downregulation the enzyme activities, protein and messenger RNA levels of MMP-2 and MMP-9 induced by PMA. Next, alpha-mangostin also strongly inhibited PMA-induced degradation of inhibitor of kappaBalpha (IkappaBalpha) and the nuclear levels of nuclear factor kappa B (NF-kappaB). Also, a dose-dependent inhibition on the binding abilities of NF-kappaB by alpha-mangostin treatment was further observed. Furthermore, reduction of FAK or ERK1/2 phosphorylation by FAK small interfering RNA (FAK siRNA) potentiated the effect of alpha-mangostin. Finally, the transient transfection of ERK siRNA significantly down-regulated the expressions of MMP-2 and MMP-9 concomitantly with a marked inhibition on cell invasion and   migration  . Presented results indicated alpha-mangostin is a novel, effect, anti-metastatic agent that functions by downregulating MMP-2 and MMP-9 gene expressions. 
 Effect of weight loss on proinflammatory state of mononuclear cells in obese women. In order to investigate whether weight loss can lead to improvement of the mononuclear cell (MNC) proinflammatory state, 21 nondiabetic obese women with mean age 34+/-2 years (mean+/-s.e.m.) and BMI 32.5+/-1.2 kg/m2 were enrolled in a 12-week caloric restriction and light exercise-based weight loss program. Ten lean women served as controls. Reverse transcription-PCR of proinflammatory cytokines and adipocytokines as well as homeostasis model assessment of insulin resistance (HOMA-IR) were determined before and after weight reduction. Nuclear factor kappaB (NF-kappaB) binding to DNA and inhibitors of NF-kappaB (IkappaB-alpha and IkappaB-beta) obtained from peripheral MNCs were measured. Overall, subjects lost a mean of 4.0+/-0.4 kg (5.0+/-0.3% of their initial body weight) (P<0.01). In addition to significant reductions in BMI, fasting glucose and insulin concentrations, mean serum high-sensitivity C-reactive protein (hs-CRP),   migration   inhibitor factor (MIF), leptin and visfatin levels decreased by 49.0, 66.6, 17.2, and 50.2%, respectively (all P<0.05), while adiponectin concentrations rose by 33.9% (P<0.05). The DNA binding of the transcriptionally active NF-kappaB from (p65/p50) decreased by 38.1% (P<0.05). Elevated levels of mRNA of NF-kappaB related proinflammatory genes, tumor necrosis factor-alpha (TNF-alpha), interleukin-6 (IL-6), MIF, and matrix metalloproteinase-9 (MMP-9), decreased significantly after weight loss. Although mRNA expression of Rel-A, p105, IkappaB-alpha, IkappaB-beta decreased significantly, their protein levels did not change after weight loss. As a group, NF-kappaB binding activity correlated with HOMA-IR (r=0.332, P=0.049) and marginally with values of BMI (r=0.308, P=0.059). In conclusion, weight loss by 5% of initial weight in nondiabetic obese women led to significant improvement in activated intranuclear NF-kappaB binding as well as several transcriptions of proinflammatory genes regulated by NF-kappaB. 
 Down-regulation of the tumor suppressor PTEN by the tumor necrosis factor-alpha/nuclear factor-kappaB (NF-kappaB)-inducing kinase/NF-kappaB pathway is linked to a default IkappaB-alpha autoregulatory loop. The PTEN (phosphatase and tensin homolog deleted on chromosome ten) tumor suppressor gene affects multiple cellular processes including cell growth, proliferation, and cell   migration   by antagonizing phosphatidylinositol 3-kinase (PI3K). However, mechanisms by which PTEN expression is regulated have not been studied extensively. Similar to PTEN, tumor necrosis factor-alpha (TNF-alpha) affects a wide spectrum of diseases including inflammatory processes and cancer by acting as a mediator of apoptosis, inflammation, and immunity. In this study, we show that treatment of cancer cell lines with TNF-alpha decreases PTEN expression. In addition, overexpression of TNF-alpha downstream signaling targets, nuclear factor-kappaB (NF-kappaB)-inducing kinase (NIK) and p65 nuclear factor NF-kappaB, lowers PTEN expression, suggesting that TNF-alpha-induced down-regulation of PTEN is mediated through a TNF-alpha/NIK/NF-kappaB pathway. Down-regulation of PTEN by NIK/NF-kappaB results in activation of the PI3K/Akt pathway and augmentation of TNF-alpha-induced PI3K/Akt stimulation. Importantly, we demonstrate that this effect is associated with a lack of an inhibitor of kappaB (IkappaB)-alpha autoregulatory loop. Moreover, these findings suggest the interaction between PI3K/Akt and NF-kappaB via transcriptional regulation of PTEN and offer one possible explanation for increased tumorigenesis in systems in which NF-kappaB is chronically activated. In such a tumor system, these findings suggest a positive feedback loop whereby Akt activation of NF-kappaB further stimulates Akt via down-regulation of the PI3K inhibitor PTEN. 
 Aurora-A down-regulates IkappaBalpha via Akt activation and interacts with insulin-like growth factor-1 induced phosphatidylinositol 3-kinase pathway for cancer cell survival. The mitotic Aurora-A kinase exerts crucial functions in maintaining mitotic fidelity. As a bona fide oncoprotein, Aurora-A aberrant overexpression leads to oncogenic transformation. Yet, the mechanisms by which Aurora-A enhances cancer cell survival remain to be elucidated.Here, we found that Aurora-A overexpression was closely correlated with clinic stage and lymph node metastasis in tongue carcinoma. Aurora-A inhibitory VX-680 suppressed proliferation, induced apoptosis and markedly reduced   migration   in cancer cells. We further showed that insulin-like growth factor-1, a PI3K physiological activator, reversed VX-680-decreased cell survival and motility. Conversely, wortmannin, a PI3K inhibitor, combined with VX-680 showed a synergistic effect on inducing apoptosis and suppressing   migration  . In addition, Aurora-A inhibition suppressed Akt activation, and VX-680-induced apoptosis was attenuated by Myr-Akt overexpression, revealing a cross-talk between Aurora-A and PI3K pathway interacting at Akt activation. Significantly, we showed that suppression of Aurora-A decreased phosphorylated Akt and was associated with increased IkappaBalpha expression. By contrast, Aurora-A overexpression upregulated Akt activity and downregulated IkappaBalpha, these changes were accompanied by nuclear translocation of nuclear factor-kappaB and increased expression of its target gene Bcl-xL. Lastly, Aurora-A overexpression induced IkappaBalpha reduction was abrogated by suppression of Akt either chemically or genetically.Taken together, our data established that Aurora-A, via activating Akt, stimulated nuclear factor-kappaB signaling pathway to promote cancer cell survival, and promised a novel combined chemotherapy targeting both Aurora-A and PI3K in cancer treatment. 
 Mechanisms of microvascular wound repair I. Role of mitosis, oxygen tension, and I-kappa B. To better understand the mechanisms of both normal reendothelialization and neointimal hyperplasia following injury, human dermal microvascular endothelial cells (HDMEC) were isolated from neonatal foreskin and studied in an in vitro model of the microvascular endothelium. In a standard 3-mm wound of nonproliferative HDMEC cultures, reendothelialization was complete at 32 h at a 20.8% (atmospheric) O(2) level. Inhibition of mitosis by mitomycin C did not reduce reendothelialization and both actinomycin D and cycloheximide inhibited repair by 80%. To determine if signals from injured cells communicated with noninjured cells, diffusion of the dye Lucifer Yellow was followed into injured and surrounding noninjured HDMEC. Diffusion was increased into both injured and noninjured cells, indicating a role for gap junctional intercellular communication (GJIC) in HDMEC wound repair. To determine if a more physiologic O(2) tension (5%) also increased vascular repair, reendothelialization at 5% O(2) was compared to 20.8% O(2) (atmospheric) levels and found to be increased by up to 50% at 5% O(2) at 12 and 24 h postinjury. I-kappa B alpha, the inhibitory subunit of NF-kappa B (a transcription factor activated by oxidative stress), was upregulated following wounding. Retroviral transfection of I-kappa B alpha into HDMEC increased the rate of reendothelialization by 35%, supporting an inhibitory role for NF-kappa B in the control of HDMEC   migration  . 
 MiR-196a promotes pancreatic cancer progression by targeting nuclear factor kappa-B-inhibitor alpha. Aberrant expression of miR-196a has been frequently reported in different cancers including pancreatic cancer. However, its function in pancreatic cancer has not been fully elucidated. Here, we investigated the expression pattern and the biological role of miR-196a in pancreatic cancer cell lines, as well as its interaction with a metastasis-related gene, nuclear factor-kappa-B-inhibitor alpha (NFKBIA). We demonstrated that miR-196a was up-regulated in human pancreatic cancer cell lines compared with immortalized pancreatic ductal epithelial cells by means of microRNAs microarray and qRT-PCR. Furthermore, down-regulation of miR-196a in PANC-1 suppressed its proliferation and   migration   with an increase in G0/G1 transition and decreased expression of Cyclin D1 and CDK4/6. Meanwhile, an increased expression in E-cadherin and decreased expression in N-cadherin and Vimentin were also observed. We identified a novel miR-196a target, NFKBIA, and down-regulation of miR-196a enhanced the expression of NFKBIA protein. Luciferase assay confirmed that NFKBIA was a direct and specific target of miR-196a. Silencing NFKBIA in PANC-1 cells enhanced its proliferation and   migration  . Taken together, our findings indicate that miR-196a is highly expressed in pancreatic cancer cell lines, and may play a crucial role in pancreatic cancer proliferation and   migration  , possibly through its downstream target, NFKBIA. Thus, miR-196a may serve as a potential therapeutic target for pancreatic cancer. 
  [Ee]xtracellular [Mm]atrix  PubMed  
 Aggregation of beta2 integrins activates human neutrophils through the IkappaB/NF-kappaB pathway. Neutrophils are now considered central to the pathogenesis of most forms of acute lung injury. Neutrophils do not cause damage while suspended in the bloodstream; however, a release of cytotoxic agents occurs when neutrophils are adherent to endothelium, epithelium, or   extracellular matrix   proteins in the interstitium. Such neutrophil adherence is mediated predominantly through beta(2) integrins (CD11/CD18) on its surface. This study was undertaken to investigate whether the IkappaB/nuclear factor (NF)-kappaB cascade is involved in this beta(2) integrin-mediated activation of human neutrophils. beta(2) Integrin Mac-1 (CD11b/CD18) aggregation was induced by antibody cross-linking of the integrins on the cell surface. beta(2) Integrin aggregation induced interleukin-1beta and tumor necrosis factor-alpha production, which suggests the activation of neutrophils by beta(2) integrin. IkappaBalpha was markedly degraded at 1 h, and NF-kappaB-DNA-binding activity markedly increased 2 h after beta(2) integrin aggregation, which activated IkappaB kinase activity at 1 h. beta(2) Integrin-induced cytokine production was suppressed by MG132 or SN50 pretreatment, which blocked the activation of NF-kappaB. These findings suggest that the activation of human neutrophils by beta(2) integrin aggregation is mediated through the activation of the IkappaB/NF-kappaB pathway. 
 Inflammation anergy in human intestinal macrophages is due to Smad-induced IkappaBalpha expression and NF-kappaB inactivation. Human intestinal macrophages contribute to tissue homeostasis in noninflamed mucosa through profound down-regulation of pro-inflammatory cytokine release. Here, we show that this down-regulation extends to Toll-like receptor (TLR)-induced cytokine release, as intestinal macrophages expressed TLR3-TLR9 but did not release cytokines in response to TLR-specific ligands. Likely contributing to this unique functional profile, intestinal macrophages expressed markedly down-regulated adapter proteins MyD88 and Toll interleukin receptor 1 domain-containing adapter-inducing interferon beta, which together mediate all TLR MyD88-dependent and -independent NF-kappaB signaling, did not phosphorylate NF-kappaB p65 or Smad-induced IkappaBalpha, and did not translocate NF-kappaB into the nucleus. Importantly, transforming growth factor-beta released from intestinal   extracellular matrix   (stroma) induced identical down-regulation in the NF-kappaB signaling and function of blood monocytes, the exclusive source of intestinal macrophages. Our findings implicate stromal transforming growth factor-beta-induced dysregulation of NF-kappaB proteins and Smad signaling in the differentiation of pro-inflammatory blood monocytes into noninflammatory intestinal macrophages. 
 The regulating role of mutant IkappaBalpha in expression of TIMP-2 and MMP-9 in human glioblastoma multiform. Our previous studies demonstrated that mutant IkappaBalpha (IkappaBalphaM) inhibited the occurrence, growth and angiogenesis of human glioblastoma multiform (GBM). However, the specific mechanism by which IkappaBalphaM regulates protein-degrading enzymes secreted from GBM to inhibit invasion and metastasis has remained unclear. The aim of the present study was to investigate the regulatory role and significance of IkappaBalphaM genes in the expression of tissue inhibitor of metalloproteinase (TIMP)-2 and matrix metalloproteinase (MMP)-9 in human GBM.We established the following four GBM cell lines stably expressing IkappaBalphaM by plasmid construction, gene transfection and screening for IkappaBalphaM protein expression: mutant IkappaBalpha-transfected cells (G36Delta-M), wild-type IkappaBalpha-transfected cells (G36Delta-W), empty plasmid transfected cells (G36Delta-P) and untransfected cells (G36Delta). The TIMP-2 and MMP-9 expression was detected by RT-PCR and Western blotting. Tumor cells were then implanted subcutaneously into nude mice to establish an animal model of ectopic tumor growth, and TIMP-2 and MMP-9 expression was determined by immunohistochemical methods.The results showed that there was a significant increase in TIMP-2 expression and a significant decrease in MMP-9 expression in the G36Delta-M group at both the RNA and protein levels compared with the G36Delta-W group, G36Delta-P group and G36Delta group. Similar results were observed in the immunohistochemical staining analysis of tumor tissues. In the G36Delta-M group, TIMP-2 expression was significantly higher while MMP-9 expression was significantly lower than in the other three groups.Our findings indicate that IkappaBalphaM inhibits the activation of NF-kappaB. It significantly up-regulates TIMP-2 expression in human malignant glioma cells and down-regulates the expression of MMP-9. Thus, IkappaBalphaM maintains the integrity of the   extracellular matrix   and further inhibits the growth and metastasis of tumor tissues. 
 Inflammation anergy in human intestinal macrophages is due to Smad-induced IkappaBalpha expression and NF-kappaB inactivation. Human intestinal macrophages contribute to tissue homeostasis in noninflamed mucosa through profound down-regulation of pro-inflammatory cytokine release. Here, we show that this down-regulation extends to Toll-like receptor (TLR)-induced cytokine release, as intestinal macrophages expressed TLR3-TLR9 but did not release cytokines in response to TLR-specific ligands. Likely contributing to this unique functional profile, intestinal macrophages expressed markedly down-regulated adapter proteins MyD88 and Toll interleukin receptor 1 domain-containing adapter-inducing interferon beta, which together mediate all TLR MyD88-dependent and -independent NF-kappaB signaling, did not phosphorylate NF-kappaB p65 or Smad-induced IkappaBalpha, and did not translocate NF-kappaB into the nucleus. Importantly, transforming growth factor-beta released from intestinal   extracellular matrix   (stroma) induced identical down-regulation in the NF-kappaB signaling and function of blood monocytes, the exclusive source of intestinal macrophages. Our findings implicate stromal transforming growth factor-beta-induced dysregulation of NF-kappaB proteins and Smad signaling in the differentiation of pro-inflammatory blood monocytes into noninflammatory intestinal macrophages. 
 The regulating role of mutant IkappaBalpha in expression of TIMP-2 and MMP-9 in human glioblastoma multiform. Our previous studies demonstrated that mutant IkappaBalpha (IkappaBalphaM) inhibited the occurrence, growth and angiogenesis of human glioblastoma multiform (GBM). However, the specific mechanism by which IkappaBalphaM regulates protein-degrading enzymes secreted from GBM to inhibit invasion and metastasis has remained unclear. The aim of the present study was to investigate the regulatory role and significance of IkappaBalphaM genes in the expression of tissue inhibitor of metalloproteinase (TIMP)-2 and matrix metalloproteinase (MMP)-9 in human GBM.We established the following four GBM cell lines stably expressing IkappaBalphaM by plasmid construction, gene transfection and screening for IkappaBalphaM protein expression: mutant IkappaBalpha-transfected cells (G36Delta-M), wild-type IkappaBalpha-transfected cells (G36Delta-W), empty plasmid transfected cells (G36Delta-P) and untransfected cells (G36Delta). The TIMP-2 and MMP-9 expression was detected by RT-PCR and Western blotting. Tumor cells were then implanted subcutaneously into nude mice to establish an animal model of ectopic tumor growth, and TIMP-2 and MMP-9 expression was determined by immunohistochemical methods.The results showed that there was a significant increase in TIMP-2 expression and a significant decrease in MMP-9 expression in the G36Delta-M group at both the RNA and protein levels compared with the G36Delta-W group, G36Delta-P group and G36Delta group. Similar results were observed in the immunohistochemical staining analysis of tumor tissues. In the G36Delta-M group, TIMP-2 expression was significantly higher while MMP-9 expression was significantly lower than in the other three groups.Our findings indicate that IkappaBalphaM inhibits the activation of NF-kappaB. It significantly up-regulates TIMP-2 expression in human malignant glioma cells and down-regulates the expression of MMP-9. Thus, IkappaBalphaM maintains the integrity of the   extracellular matrix   and further inhibits the growth and metastasis of tumor tissues. 
 Aggregation of beta2 integrins activates human neutrophils through the IkappaB/NF-kappaB pathway. Neutrophils are now considered central to the pathogenesis of most forms of acute lung injury. Neutrophils do not cause damage while suspended in the bloodstream; however, a release of cytotoxic agents occurs when neutrophils are adherent to endothelium, epithelium, or   extracellular matrix   proteins in the interstitium. Such neutrophil adherence is mediated predominantly through beta(2) integrins (CD11/CD18) on its surface. This study was undertaken to investigate whether the IkappaB/nuclear factor (NF)-kappaB cascade is involved in this beta(2) integrin-mediated activation of human neutrophils. beta(2) Integrin Mac-1 (CD11b/CD18) aggregation was induced by antibody cross-linking of the integrins on the cell surface. beta(2) Integrin aggregation induced interleukin-1beta and tumor necrosis factor-alpha production, which suggests the activation of neutrophils by beta(2) integrin. IkappaBalpha was markedly degraded at 1 h, and NF-kappaB-DNA-binding activity markedly increased 2 h after beta(2) integrin aggregation, which activated IkappaB kinase activity at 1 h. beta(2) Integrin-induced cytokine production was suppressed by MG132 or SN50 pretreatment, which blocked the activation of NF-kappaB. These findings suggest that the activation of human neutrophils by beta(2) integrin aggregation is mediated through the activation of the IkappaB/NF-kappaB pathway. 
  [Ii]nvasive  PubMed  
 Evaluation of candidate stromal epithelial cross-talk genes identifies association between risk of serous ovarian cancer and TERT, a cancer susceptibility "hot-spot". We hypothesized that variants in genes expressed as a consequence of interactions between ovarian cancer cells and the host micro-environment could contribute to cancer susceptibility. We therefore used a two-stage approach to evaluate common single nucleotide polymorphisms (SNPs) in 173 genes involved in stromal epithelial interactions in the Ovarian Cancer Association Consortium (OCAC). In the discovery stage, cases with epithelial ovarian cancer (n=675) and controls (n=1,162) were genotyped at 1,536 SNPs using an Illumina GoldenGate assay. Based on Positive Predictive Value estimates, three SNPs-PODXL rs1013368, ITGA6 rs13027811, and MMP3 rs522616-were selected for replication using TaqMan genotyping in up to 3,059 serous   invasive   cases and 8,905 controls from 16 OCAC case-control studies. An additional 18 SNPs with Pper-allele<0.05 in the discovery stage were selected for replication in a subset of five OCAC studies (n=1,233 serous   invasive   cases; n=3,364 controls). The discovery stage associations in PODXL, ITGA6, and MMP3 were attenuated in the larger replication set (adj. Pper-allele>or=0.5). However genotypes at TERT rs7726159 were associated with ovarian cancer risk in the smaller, five-study replication study (Pper-allele=0.03). Combined analysis of the discovery and replication sets for this TERT SNP showed an increased risk of serous ovarian cancer among non-Hispanic whites [adj. ORper-allele 1.14 (1.04-1.24) p=0.003]. Our study adds to the growing evidence that, like the 8q24 locus, the telomerase reverse transcriptase locus at 5p15.33, is a general cancer susceptibility locus. 
 [Inhibition of NF-kappaB through IkappaBalpha transfection affects invasion of human lung cancer cell line A549]. Recent studies have shown that activation of nuclear factor-kappaB(NF-kappaB) can regulate the invasion and metastasis of cancer cells. The present study was to investigate inhibition of NF-kappaB activity on invasion of human lung cancer cell line A549 and the possible mechanism.The recombinant plasmid pcDNA3.1(+)/IkappaBalpha, expressing the alpha isoform (IkappaBalpha) of the NF-kappaB inhibitor, was constructed. A549 cells were cultured in vitro and divided into non-transfection group, pcDNA3.1(+) transfected group and pcDNA3.1(+)/IkappaBalpha transfected group. The expression of IkappaBalpha was detected by reverse transcription-polymerase chain reaction (RT-PCR) and Western blotting. The activity of NF-kappaB was determined by electrophoretic mobility shift assay (EMSA). Invasion of A549 cells was assessed by transwell chamber assay. The expression of MMP-2 and MMP-9 was detected by RT-PCR and gelatin zymography.Plasmid pcDNA3.1(+)/IkappaBalpha was successfully constructed and expressed in A549 cells. The activity of NF-kappaB, the number of   invasive   cells, the activity of MMP-2 and MMP-9 of A549 cells in pcDNA3.1(+)/IkappaBalpha transfected group were significantly lower than those in non-transfection group and pcDNA3.1(+) transfected group (all P<0.05).Transfection of IkappaBalpha can inhibit NF-kappaB activity, thus inhibit cell invasion of A549, which may be through the down-regulation of MMP-2 and MMP-9 expressions. 
 [Expressions of NF-κBp65 and IκBα in gestational trophoblastic disease and clinical significance]. To investigate the roles of nuclear factor κB p65 (NF-κBp65) and inhibitor of nuclear factor κB α (IκBα) in the development and metastasis of gestational trophoblastic neoplasia (GTN) by analyzing the expressions of NF-κBp65 and IκBα in normal early pregnancy villi and gestational trophoblastic diseases, and to reveal the relationship of NF-κBp65 and IκBα with age and clinical stage of GTN patients.The expressions of NF-κBp65 and IκBα were detected by immunohistochemistry in normal pregnancy villi (20 cases), hydatidiform mole  s (HM, 3  0 cases), invasive hydatidiform moles (IHM, 13 cases) and chorionic carcinomas (CCA, 15 cases).NF-κBp65 expression was statistically different (P<0.05) between normal pregnancy villi and IHM (P=0.013), normal pregnancy villi and CCA(P=0.018), HM and IHM(P=0.026), HM and CCA (P=0.035). Differences in IκBα expression were statistically significant between normal pregnancy villi and IHM, normal pregnancy villi and CCA, HM and IHM, HM and CCA (P<0.01). The expressions of NF-κBp65 and IκBα were correlated to clinical stage (P=0.043, 0.042, P<0.05), but not to patients' ages. Spearman correlation analysis revealed that there was a negative association between the protein expressions of NF-κBp65 and IκBα in GTN (r=-0.403, P=0.034, P<0.05).Up-regulated expression of NF-κBp65 and down-regulated expression of IκBα may be related to the development, invasion and metastasis of GTN. The expressions of NF-κBp65 and IκBα are negatively correlated in gestational trophoblastic tumor tissues. 
 [Expressions of NF-κBp65 and IκBα in gestational trophoblastic disease and clinical significance]. To investigate the roles of nuclear factor κB p65 (NF-κBp65) and inhibitor of nuclear factor κB α (IκBα) in the development and metastasis of gestational trophoblastic neoplasia (GTN) by analyzing the expressions of NF-κBp65 and IκBα in normal early pregnancy villi and gestational trophoblastic diseases, and to reveal the relationship of NF-κBp65 and IκBα with age and clinical stage of GTN patients.The expressions of NF-κBp65 and IκBα were detected by immunohistochemistry in normal pregnancy villi (20 cases), hydatidiform mole  s (HM, 3  0 cases), invasive hydatidiform moles (IHM, 13 cases) and chorionic carcinomas (CCA, 15 cases).NF-κBp65 expression was statistically different (P<0.05) between normal pregnancy villi and IHM (P=0.013), normal pregnancy villi and CCA(P=0.018), HM and IHM(P=0.026), HM and CCA (P=0.035). Differences in IκBα expression were statistically significant between normal pregnancy villi and IHM, normal pregnancy villi and CCA, HM and IHM, HM and CCA (P<0.01). The expressions of NF-κBp65 and IκBα were correlated to clinical stage (P=0.043, 0.042, P<0.05), but not to patients' ages. Spearman correlation analysis revealed that there was a negative association between the protein expressions of NF-κBp65 and IκBα in GTN (r=-0.403, P=0.034, P<0.05).Up-regulated expression of NF-κBp65 and down-regulated expression of IκBα may be related to the development, invasion and metastasis of GTN. The expressions of NF-κBp65 and IκBα are negatively correlated in gestational trophoblastic tumor tissues. 
 Evaluation of candidate stromal epithelial cross-talk genes identifies association between risk of serous ovarian cancer and TERT, a cancer susceptibility "hot-spot". We hypothesized that variants in genes expressed as a consequence of interactions between ovarian cancer cells and the host micro-environment could contribute to cancer susceptibility. We therefore used a two-stage approach to evaluate common single nucleotide polymorphisms (SNPs) in 173 genes involved in stromal epithelial interactions in the Ovarian Cancer Association Consortium (OCAC). In the discovery stage, cases with epithelial ovarian cancer (n=675) and controls (n=1,162) were genotyped at 1,536 SNPs using an Illumina GoldenGate assay. Based on Positive Predictive Value estimates, three SNPs-PODXL rs1013368, ITGA6 rs13027811, and MMP3 rs522616-were selected for replication using TaqMan genotyping in up to 3,059 serous   invasive   cases and 8,905 controls from 16 OCAC case-control studies. An additional 18 SNPs with Pper-allele<0.05 in the discovery stage were selected for replication in a subset of five OCAC studies (n=1,233 serous   invasive   cases; n=3,364 controls). The discovery stage associations in PODXL, ITGA6, and MMP3 were attenuated in the larger replication set (adj. Pper-allele>or=0.5). However genotypes at TERT rs7726159 were associated with ovarian cancer risk in the smaller, five-study replication study (Pper-allele=0.03). Combined analysis of the discovery and replication sets for this TERT SNP showed an increased risk of serous ovarian cancer among non-Hispanic whites [adj. ORper-allele 1.14 (1.04-1.24) p=0.003]. Our study adds to the growing evidence that, like the 8q24 locus, the telomerase reverse transcriptase locus at 5p15.33, is a general cancer susceptibility locus. 
 [Inhibition of NF-kappaB through IkappaBalpha transfection affects invasion of human lung cancer cell line A549]. Recent studies have shown that activation of nuclear factor-kappaB(NF-kappaB) can regulate the invasion and metastasis of cancer cells. The present study was to investigate inhibition of NF-kappaB activity on invasion of human lung cancer cell line A549 and the possible mechanism.The recombinant plasmid pcDNA3.1(+)/IkappaBalpha, expressing the alpha isoform (IkappaBalpha) of the NF-kappaB inhibitor, was constructed. A549 cells were cultured in vitro and divided into non-transfection group, pcDNA3.1(+) transfected group and pcDNA3.1(+)/IkappaBalpha transfected group. The expression of IkappaBalpha was detected by reverse transcription-polymerase chain reaction (RT-PCR) and Western blotting. The activity of NF-kappaB was determined by electrophoretic mobility shift assay (EMSA). Invasion of A549 cells was assessed by transwell chamber assay. The expression of MMP-2 and MMP-9 was detected by RT-PCR and gelatin zymography.Plasmid pcDNA3.1(+)/IkappaBalpha was successfully constructed and expressed in A549 cells. The activity of NF-kappaB, the number of   invasive   cells, the activity of MMP-2 and MMP-9 of A549 cells in pcDNA3.1(+)/IkappaBalpha transfected group were significantly lower than those in non-transfection group and pcDNA3.1(+) transfected group (all P<0.05).Transfection of IkappaBalpha can inhibit NF-kappaB activity, thus inhibit cell invasion of A549, which may be through the down-regulation of MMP-2 and MMP-9 expressions. 
 [Expression of IκBα in bladder cancer cell lines is negatively correlated with epithelial-mesenchymal transition and cell invasion in vitro]. To investigate the expression of inhibitor of nuclear factor κBα (IκBα) in bladder cancer cell lines and its correlation with epithelial-mesenchymal transition (EMT  ) and in  vasive potential in vitro.The expressions of IκBα, epithelia cadherin (E-cadherin), neuronal cadherin (N-cadherin) and vimentin in human bladder cancer RT4, 5637, 235J, J82 and T24 cell lines were detected by Western bl  otting.   Invasive potential of these cell lines was evaluated by Transwell(TM) assay. In addition, morphological characteristics of those cells were carefully observed under a microscope.RT4 and 5637 cells exhibited epithelial cell morphological features, but 253J, J82 and T24 cells had mesenchymal cell morphological features. E-cadherin was over-expressed in RT4 and 5637 cell lines, but the expressions of vimentin and N-cadherin could not been detected in the two cells. E-cadherin could be detected in 253J cells with a lower expression level; J82 and T24 cells had no expression of E-cadherin; vimentin and N-cadherin were highly expressed in 253J, J82 and T24 cells. Transwell(TM) assay showed   higher   invasive potential of 253J, J82 and T24 cells than RT4 and 5637 cells in vitro. In addition, RT4 and 5637 cells expressed higher levels of IκBα than 253J, J82 and T24 cells.Expression of IκBα in human bladder cancer cells is negatively correlated with EMT and tumor invasion in vitro. 
 A NIK-IKKα module expands ErbB2-induced tumor-initiating cells by stimulating nuclear export of p27/Kip1. IκB kinase α (IKKα) activity is required for ErbB2-induced mammary tumorigenesis. Here, we show that IKKα and its activator, NF-κB-inducing kinase (NIK), support the expansion of tumor-initiating cells (TICs) that copurify with a CD24(med)CD49f(hi) population from premalignant ErbB2-expressing mammary glands. Upon activation, IKKα enters the nucleus, phosphorylates the cyclin-dependent kinase (CDK) inhibitor p27/Kip1, and stimulates its nuclear export or exclusion. Reduced p27 expression rescues mammary tumorigenesis in mice deficient in IKKα kinase activity and restores TIC self-renewal. IKKα is also likely to be involved in human breast cancer, where its expression shows an inverse correlation with metastasis-free survival, and its presence in the nu  cleus of   invasive ductal carcinomas (IDCs) is associated with decreased nuclear p27 abundance. 
 MicroRNA-30e* promotes human glioma cell   invasive  ness in an orthotopic xenotransplantation model by disrupting the NF-κB/IκBα negative feedback loop. Constitutive activation of NF-κB is a frequent event in human cancers, playing important roles in cancer development and progression. In nontransformed cells, NF-κB activation is tightly controlled by IκBs. IκBs bind NF-κB in the cytoplasm, preventing it from translocating to the nucleus to modulate gene expression. Stimuli that activate NF-κB signaling trigger IκB degradation, enabling nuclear translocation of NF-κB. Among the genes regulated by NF-κB are those encoding the IκBs, providing a negative feedback loop that limits NF-κB activity. How transformed cells override this NF-κB/IκB negative feedback loop remains unclear. Here, we report in human glioma cell lines that microRNA-30e* (miR-30e*) directly targets the IκBα 3ι-UTR and suppresses IκBα expression. Overexpression of miR-30e* in human glioma cell lines led to hyperactivation of NF-κB and enhanced expression of NF-κB-regulated genes, whic  h promot  ed glioma cell invasiveness in in vitro assays and in an orthotopic xenotransplantation model. These effects of miR-30e* were shown to be clinically relevant, as miR-30e* was found to be upregulated in primary human glioma cells and correlated with malignant progression and poor survival. Hence, miR-30e* provides an epigenetic mechanism that disrupts the NF-κB/IκBα loop and may represent a new therapeutic target and prognostic marker. 
 IkappaB genetic polymorphisms and   invasive   pneumococcal disease. Increasing evidence supports a key role for the transcription factor nuclear factor (NF)-kappaB in the host response to pneumococcal infection. Control of NF-kappaB activity is achieved through interactions with the IkappaB family of inhibitors, encoded by the genes NFKBIA, NFKBIB, and NFKBIE. Rare NFKBIA mutations cause immunodeficiency with severe bacterial infection, raising the possibility that common IkappaB gene polymorphisms confer susceptibility to common bacterial disease.To determine whether polymorphisms in NFKBIA, NFKBIB, and NFKBIE associate with susceptibility to   invasive   pneumococcal disease (IPD) and thoracic empyema.We studied the frequencies of 62 single-nucleotide polymorphisms (SNPs) across NFKBIA, NFKBIB, and NFKBIE in individuals with IPD and control subjects (n=1,060). Significantly associated SNPs were then studied in a group of individuals with thoracic empyema and a second control group (n=632).Two SNPs in the NFKBIA promoter region were associated with protection from IPD in both the initial study group and the pneumococcal empyema subgroup. Significant protection from IPD was observed for carriage of mutant alleles at these two loci on combining the groups (SNP rs3138053: Mantel-Haenszel 2x2 chi2=13.030, p=0.0003; odds ratio [OR], 0.60; 95% confidence interval [CI], 0.45-0.79; rs2233406: Mantel-Haenszel 2x2 chi2=18.927, p=0.00001; OR, 0.55; 95% CI, 0.42-0.72). An NFKBIE SNP associated with susceptibility to IPD but not pneumococcal empyema. None of the NFKBIB SNPs associated with IPD susceptibility.NFKBIA polymorphisms associate with susceptibility to IPD. Genetic variation in an inhibitor of NF-kappaB therefore not only causes a very rare immunodeficiency state but may also influence the development of common infectious disease. 
  [Mm]etastasis  PubMed  
 A cytoplasmic NF-κB interacting long noncoding RNA blocks IκB phosphorylation and suppresses breast cance  r metastas  is. NF-κB is a critical link between inflammation and cancer, but whether long non-coding RNAs (lncRNAs) regulate its activation remains unknown. Here, we identify an NF-KappaB Interacting LncRNA (NKILA), which is upregulated by NF-κB, binds to NF-κB/IκB, and directly masks phosphorylation motifs of IκB, thereby inhibiting IKK-induced IκB phosphorylation and NF-κB activation. Unlike DNA that is dissociated from NF-κB by IκB, NKILA interacts with NF-κB/IκB to form a stable complex. Importantly, NKILA is essential to prevent over-activation of NF-κB pathway in inflammation-stimulated breast epithelial cells. Furthermore, low NKILA expression is associated with   breast can  cer metastasis and poor patient prognosis. Therefore, lncRNAs can directly interact with functional domains of signaling proteins, serving as a class of NF-κB modulators to   suppress c  ancer metastasis. 
 Polymorphic variants of NFKB1 and its inhibitory protein NFKBIA, and their involvement in sporadic breast cancer. Nuclear factor kappa-beta (NF-kappaB) is a transcription factor responsible for modulating the expression of many genes involved in cell proliferation, differentiation, apoptosis and   metastasis  . NF-kappaB interacts with IkappaB inhibitory proteins to regulate gene expression. This study investigated common variants within the genes coding for NF-kappaB and IkappaB, NFKB1 and NFKBIA, for involvement in sporadic breast cancer. Genotypes were determined in a population of breast cancer affected individuals and age-matched controls. Results do not support an involvement of the tested NFKB1 and NFKBIA polymorphisms in susceptibility to sporadic breast cancer, in the tested Caucasian population. 
 [Inhibition of NF-kappaB through IkappaBalpha transfection affects invasion of human lung cancer cell line A549]. Recent studies have shown that activation of nuclear factor-kappaB(NF-kappaB) can regulate the invasion and   metastasis   of cancer cells. The present study was to investigate inhibition of NF-kappaB activity on invasion of human lung cancer cell line A549 and the possible mechanism.The recombinant plasmid pcDNA3.1(+)/IkappaBalpha, expressing the alpha isoform (IkappaBalpha) of the NF-kappaB inhibitor, was constructed. A549 cells were cultured in vitro and divided into non-transfection group, pcDNA3.1(+) transfected group and pcDNA3.1(+)/IkappaBalpha transfected group. The expression of IkappaBalpha was detected by reverse transcription-polymerase chain reaction (RT-PCR) and Western blotting. The activity of NF-kappaB was determined by electrophoretic mobility shift assay (EMSA). Invasion of A549 cells was assessed by transwell chamber assay. The expression of MMP-2 and MMP-9 was detected by RT-PCR and gelatin zymography.Plasmid pcDNA3.1(+)/IkappaBalpha was successfully constructed and expressed in A549 cells. The activity of NF-kappaB, the number of invasive cells, the activity of MMP-2 and MMP-9 of A549 cells in pcDNA3.1(+)/IkappaBalpha transfected group were significantly lower than those in non-transfection group and pcDNA3.1(+) transfected group (all P<0.05).Transfection of IkappaBalpha can inhibit NF-kappaB activity, thus inhibit cell invasion of A549, which may be through the down-regulation of MMP-2 and MMP-9 expressions. 
 Association of common polymorphisms in TNFA, NFkB1 and NFKBIA with risk and prognosis of esophageal squamous cell carcinoma. Tumour necrosis factor-alpha (TNF-α) and nuclear factor of kappa light chain gene enhancer in activated B cells (NF-κB) play critical role in carcinogenesis processes like tumour initiation, proliferation, migration and invasion. Single nucleotide polymorphisms in TNF-α, NF-κB and its inhibitor IκB genes were shown to be associated with susceptibility and prognosis of several cancers; however, their role in esophageal squamous cell carcinoma (ESCC) is not well recognised. Therefore, in present study, we aimed to investigate association of common polymorphisms in TNFA, NFkB1 and NFKBIA with risk and prognosis of ESCC in northern Indian population.We genotyped 290 ESCC patients (including 162 followed up cases) and 311 mean age, gender and ethnicity matched controls for TNFA -308G>A, NFkB1 -94ATTG ins/del and NFKBIA (-826C>T and 3'UTRA>G) polymorphisms using PCR alone or followed by RFLP and TaqMan assay.TNFA-308GA genotype was associated with increased risk of ESCC specifically in females and in patients with regional lymph node involvement, while, NFKBIA -826CT+TT genotype conferred decreased risk of ESCC in females. Haplotypes of NFKBIA -826C>T and 3'UTRA>G polymorphisms, C-826G3'UTR and T-826A3'UTR, were associated with reduced risk of ESCC. No independent role of NFkB1 -94ATTG ins/del polymorphism in susceptibility of ESCC was found. Multi-dimensionality reduction analysis showed three factor model TNFA-308, NFKBIA-826, NFKBIA 3'UTR as better predictor for risk of ESCC. Furthermore, combined risk genotype analysis of all studied polymorphisms showed increased risk of ESCC in patients with 1-3 risk genotype compared to '0' risk genotype. Survival analysis did not show any significant prognostic effect of studied polymorphisms. However, in stepwise multivariate analy  sis, metas  tasis was found to be independent prognostic predictor of ESCC patients.TNFA-308 and NFKBIA (-826C>T and 3'UTRA>G) polymorphisms may play role in susceptibility but not in prognosis of ESCC patients in northern Indian population. 
 The regulating role of mutant IkappaBalpha in expression of TIMP-2 and MMP-9 in human glioblastoma multiform. Our previous studies demonstrated that mutant IkappaBalpha (IkappaBalphaM) inhibited the occurrence, growth and angiogenesis of human glioblastoma multiform (GBM). However, the specific mechanism by which IkappaBalphaM regulates protein-degrading enzymes secreted from GBM to inhibit invasion and   metastasis   has remained unclear. The aim of the present study was to investigate the regulatory role and significance of IkappaBalphaM genes in the expression of tissue inhibitor of metalloproteinase (TIMP)-2 and matrix metalloproteinase (MMP)-9 in human GBM.We established the following four GBM cell lines stably expressing IkappaBalphaM by plasmid construction, gene transfection and screening for IkappaBalphaM protein expression: mutant IkappaBalpha-transfected cells (G36Delta-M), wild-type IkappaBalpha-transfected cells (G36Delta-W), empty plasmid transfected cells (G36Delta-P) and untransfected cells (G36Delta). The TIMP-2 and MMP-9 expression was detected by RT-PCR and Western blotting. Tumor cells were then implanted subcutaneously into nude mice to establish an animal model of ectopic tumor growth, and TIMP-2 and MMP-9 expression was determined by immunohistochemical methods.The results showed that there was a significant increase in TIMP-2 expression and a significant decrease in MMP-9 expression in the G36Delta-M group at both the RNA and protein levels compared with the G36Delta-W group, G36Delta-P group and G36Delta group. Similar results were observed in the immunohistochemical staining analysis of tumor tissues. In the G36Delta-M group, TIMP-2 expression was significantly higher while MMP-9 expression was significantly lower than in the other three groups.Our findings indicate that IkappaBalphaM inhibits the activation of NF-kappaB. It significantly up-regulates TIMP-2 expression in human malignant glioma cells and down-regulates the expression of MMP-9. Thus, IkappaBalphaM maintains the integrity of the extracellular matrix and further inhibits the growth and   metastasis   of tumor tissues. 
 Effects of NFKB1 and NFKBIA gene polymorphisms on susceptibility to environmental factors and the clinicopathologic development of oral cancer. Oral cancer, which is the fourth most common cancer in Taiwanese men, is associated with environmental carcinogens. The possibility that genetic predisposition in nuclear factor-kappa B (NF-κB)-signaling pathways activation is linked to the development of oral squamous cell carcinoma (OSCC) requires investigation. The current study examines associations between polymorphisms within promoter regions of NFKB1 encoding NF-κB1 and NFKBIA encoding IkappaBalpha (IκBα) with both the susceptibility to develop OSCC and the clinicopathological characteristics of the tumors.Genetic polymorphisms of NFKB1 and NFKBIA were analyzed by a real-time polymerase chain reaction (real-time PCR) for 462 patients with oral cancer and 520 non-cancer controls. We found that NFKB1 -94 ATGG1/ATGG2, -94 ATGG2/ATGG2, and the combination of -94 ATGG1/ATGG2 and ATGG2/ATGG2 genotypes NFKBIA -826 T (CT+TT) and -881 G (AG+GG) allelic carriages, were more prevalent in OSCC patients than in non-cancer participants. Moreover, we found that NFKB1 or NFKBIA gene polymorphisms seem to be related to susceptibility to develop oral cancer linked to betel nut and tobacco consumption. Finally, patients with oral cancer who had at least one -519 T allele of the NFKBIA gene were at higher risk for developing dist  ant metast  asis (P<.05), compared with those patients CC homozygotes.Our results suggest that NFKB1 -94 ATTG2, NFKBIA -826 T, and -881 G alleles are associated with oral carcinogenesis. The combination of NFKB1 or NFKBIA gene polymorphisms and environmental carcinogens appears related to an increased risk of oral cancer. More importantly, the genetic polymorphism of NFKBIA -519 might be a predictive factor for the dis  tal metast  asis of OSCC in Taiwanese. 
 [Expressions of NF-κBp65 and IκBα in gestational trophoblastic disease and clinical significance]. To investigate the roles of nuclear factor κB p65 (NF-κBp65) and inhibitor of nuclear factor κB α (IκBα) in the develop  ment and m  etastasis of gestational trophoblastic neoplasia (GTN) by analyzing the expressions of NF-κBp65 and IκBα in normal early pregnancy villi and gestational trophoblastic diseases, and to reveal the relationship of NF-κBp65 and IκBα with age and clinical stage of GTN patients.The expressions of NF-κBp65 and IκBα were detected by immunohistochemistry in normal pregnancy villi (20 cases), hydatidiform moles (HM, 30 cases), invasive hydatidiform moles (IHM, 13 cases) and chorionic carcinomas (CCA, 15 cases).NF-κBp65 expression was statistically different (P<0.05) between normal pregnancy villi and IHM (P=0.013), normal pregnancy villi and CCA(P=0.018), HM and IHM(P=0.026), HM and CCA (P=0.035). Differences in IκBα expression were statistically significant between normal pregnancy villi and IHM, normal pregnancy villi and CCA, HM and IHM, HM and CCA (P<0.01). The expressions of NF-κBp65 and IκBα were correlated to clinical stage (P=0.043, 0.042, P<0.05), but not to patients' ages. Spearman correlation analysis revealed that there was a negative association between the protein expressions of NF-κBp65 and IκBα in GTN (r=-0.403, P=0.034, P<0.05).Up-regulated expression of NF-κBp65 and down-regulated expression of IκBα may be related to   the develo  pment, invasion and metastasis of GTN. The expressions of NF-κBp65 and IκBα are negatively correlated in gestational trophoblastic tumor tissues. 
 Potential biomarkers involving IKK/RelA signal in early stage non-small cell lung cancer. The clinical relevance of nuclear factor kappaB (NF-kappaB) and its regulatory molecules on prognosis of patient with early stages of non-small cell lung cancer (NSCLC), remains unclear. Therefore, we conducted biomarker analyses with survival in patients with stages I and II NSCLC. Tumor samples were collected from 88 patients with early-stage NSCLC (stages I, II). A minimum follow-up period of 5 years was required. RelA, phosphorylated I kappaB (pI kappaB alpha), pIKK alpha/beta were detected by immunostaining. NF-kappaB DNA binding activity was assessed by electrophoretic mobility shift assay. Association of clinical and pathologic variables (e.g. sex, age, pathologic stage) with relevant molecules was determined by Pearson's chi(2) test or Fisher's exact test. Survival analysis based on single expression of RelA, pI kappaB alpha, pIKK alpha/beta as well as composite expressions were evaluated using Cox proportional hazards regression models, and log rank test followed Kaplan-Meier estimates. RelA, pI kappaB alpha, pIKK alpha/beta were observed as increased expression in NSCLC tissues compared with adjacent normal tissues and normal lung tissues. These molecules were associated with tumor-node-  metastasis   stages, T stages and histological status, respectively. Among the molecules analyzed, RelA and pI kappaB alpha-positive were statistically significant predictors of patient death in the entire patient population adjusted by age, gender and smoking status; furthermore both RelA and pI kappaB alpha-positive was the strongest prognostic indicators of poor prognosis by univariate and multivariate analyses. Borderline positive correlations were observed between RelA and pI kappaB alpha or pIKK alpha/beta expression. In this cohort of early-stage NSCLC patients, molecular markers, especially composite application of multiple biomarkers (both nuclear RelA and cytoplasmic pI kappaB-alpha expression) that independently predict overall survival have been identified. 
 Association of common polymorphisms in TNFA, NFkB1 and NFKBIA with risk and prognosis of esophageal squamous cell carcinoma. Tumour necrosis factor-alpha (TNF-α) and nuclear factor of kappa light chain gene enhancer in activated B cells (NF-κB) play critical role in carcinogenesis processes like tumour initiation, proliferation, migration and invasion. Single nucleotide polymorphisms in TNF-α, NF-κB and its inhibitor IκB genes were shown to be associated with susceptibility and prognosis of several cancers; however, their role in esophageal squamous cell carcinoma (ESCC) is not well recognised. Therefore, in present study, we aimed to investigate association of common polymorphisms in TNFA, NFkB1 and NFKBIA with risk and prognosis of ESCC in northern Indian population.We genotyped 290 ESCC patients (including 162 followed up cases) and 311 mean age, gender and ethnicity matched controls for TNFA -308G>A, NFkB1 -94ATTG ins/del and NFKBIA (-826C>T and 3'UTRA>G) polymorphisms using PCR alone or followed by RFLP and TaqMan assay.TNFA-308GA genotype was associated with increased risk of ESCC specifically in females and in patients with regional lymph node involvement, while, NFKBIA -826CT+TT genotype conferred decreased risk of ESCC in females. Haplotypes of NFKBIA -826C>T and 3'UTRA>G polymorphisms, C-826G3'UTR and T-826A3'UTR, were associated with reduced risk of ESCC. No independent role of NFkB1 -94ATTG ins/del polymorphism in susceptibility of ESCC was found. Multi-dimensionality reduction analysis showed three factor model TNFA-308, NFKBIA-826, NFKBIA 3'UTR as better predictor for risk of ESCC. Furthermore, combined risk genotype analysis of all studied polymorphisms showed increased risk of ESCC in patients with 1-3 risk genotype compared to '0' risk genotype. Survival analysis did not show any significant prognostic effect of studied polymorphisms. However, in stepwise multivariate analy  sis, metas  tasis was found to be independent prognostic predictor of ESCC patients.TNFA-308 and NFKBIA (-826C>T and 3'UTRA>G) polymorphisms may play role in susceptibility but not in prognosis of ESCC patients in northern Indian population. 
 [Expressions of NF-κBp65 and IκBα in gestational trophoblastic disease and clinical significance]. To investigate the roles of nuclear factor κB p65 (NF-κBp65) and inhibitor of nuclear factor κB α (IκBα) in the develop  ment and m  etastasis of gestational trophoblastic neoplasia (GTN) by analyzing the expressions of NF-κBp65 and IκBα in normal early pregnancy villi and gestational trophoblastic diseases, and to reveal the relationship of NF-κBp65 and IκBα with age and clinical stage of GTN patients.The expressions of NF-κBp65 and IκBα were detected by immunohistochemistry in normal pregnancy villi (20 cases), hydatidiform moles (HM, 30 cases), invasive hydatidiform moles (IHM, 13 cases) and chorionic carcinomas (CCA, 15 cases).NF-κBp65 expression was statistically different (P<0.05) between normal pregnancy villi and IHM (P=0.013), normal pregnancy villi and CCA(P=0.018), HM and IHM(P=0.026), HM and CCA (P=0.035). Differences in IκBα expression were statistically significant between normal pregnancy villi and IHM, normal pregnancy villi and CCA, HM and IHM, HM and CCA (P<0.01). The expressions of NF-κBp65 and IκBα were correlated to clinical stage (P=0.043, 0.042, P<0.05), but not to patients' ages. Spearman correlation analysis revealed that there was a negative association between the protein expressions of NF-κBp65 and IκBα in GTN (r=-0.403, P=0.034, P<0.05).Up-regulated expression of NF-κBp65 and down-regulated expression of IκBα may be related to   the develo  pment, invasion and metastasis of GTN. The expressions of NF-κBp65 and IκBα are negatively correlated in gestational trophoblastic tumor tissues. 
 Effects of NFKB1 and NFKBIA gene polymorphisms on susceptibility to environmental factors and the clinicopathologic development of oral cancer. Oral cancer, which is the fourth most common cancer in Taiwanese men, is associated with environmental carcinogens. The possibility that genetic predisposition in nuclear factor-kappa B (NF-κB)-signaling pathways activation is linked to the development of oral squamous cell carcinoma (OSCC) requires investigation. The current study examines associations between polymorphisms within promoter regions of NFKB1 encoding NF-κB1 and NFKBIA encoding IkappaBalpha (IκBα) with both the susceptibility to develop OSCC and the clinicopathological characteristics of the tumors.Genetic polymorphisms of NFKB1 and NFKBIA were analyzed by a real-time polymerase chain reaction (real-time PCR) for 462 patients with oral cancer and 520 non-cancer controls. We found that NFKB1 -94 ATGG1/ATGG2, -94 ATGG2/ATGG2, and the combination of -94 ATGG1/ATGG2 and ATGG2/ATGG2 genotypes NFKBIA -826 T (CT+TT) and -881 G (AG+GG) allelic carriages, were more prevalent in OSCC patients than in non-cancer participants. Moreover, we found that NFKB1 or NFKBIA gene polymorphisms seem to be related to susceptibility to develop oral cancer linked to betel nut and tobacco consumption. Finally, patients with oral cancer who had at least one -519 T allele of the NFKBIA gene were at higher risk for developing dist  ant metast  asis (P<.05), compared with those patients CC homozygotes.Our results suggest that NFKB1 -94 ATTG2, NFKBIA -826 T, and -881 G alleles are associated with oral carcinogenesis. The combination of NFKB1 or NFKBIA gene polymorphisms and environmental carcinogens appears related to an increased risk of oral cancer. More importantly, the genetic polymorphism of NFKBIA -519 might be a predictive factor for the dis  tal metast  asis of OSCC in Taiwanese. 
 The regulating role of mutant IkappaBalpha in expression of TIMP-2 and MMP-9 in human glioblastoma multiform. Our previous studies demonstrated that mutant IkappaBalpha (IkappaBalphaM) inhibited the occurrence, growth and angiogenesis of human glioblastoma multiform (GBM). However, the specific mechanism by which IkappaBalphaM regulates protein-degrading enzymes secreted from GBM to inhibit invasion and   metastasis   has remained unclear. The aim of the present study was to investigate the regulatory role and significance of IkappaBalphaM genes in the expression of tissue inhibitor of metalloproteinase (TIMP)-2 and matrix metalloproteinase (MMP)-9 in human GBM.We established the following four GBM cell lines stably expressing IkappaBalphaM by plasmid construction, gene transfection and screening for IkappaBalphaM protein expression: mutant IkappaBalpha-transfected cells (G36Delta-M), wild-type IkappaBalpha-transfected cells (G36Delta-W), empty plasmid transfected cells (G36Delta-P) and untransfected cells (G36Delta). The TIMP-2 and MMP-9 expression was detected by RT-PCR and Western blotting. Tumor cells were then implanted subcutaneously into nude mice to establish an animal model of ectopic tumor growth, and TIMP-2 and MMP-9 expression was determined by immunohistochemical methods.The results showed that there was a significant increase in TIMP-2 expression and a significant decrease in MMP-9 expression in the G36Delta-M group at both the RNA and protein levels compared with the G36Delta-W group, G36Delta-P group and G36Delta group. Similar results were observed in the immunohistochemical staining analysis of tumor tissues. In the G36Delta-M group, TIMP-2 expression was significantly higher while MMP-9 expression was significantly lower than in the other three groups.Our findings indicate that IkappaBalphaM inhibits the activation of NF-kappaB. It significantly up-regulates TIMP-2 expression in human malignant glioma cells and down-regulates the expression of MMP-9. Thus, IkappaBalphaM maintains the integrity of the extracellular matrix and further inhibits the growth and   metastasis   of tumor tissues. 
 [Inhibition of NF-kappaB through IkappaBalpha transfection affects invasion of human lung cancer cell line A549]. Recent studies have shown that activation of nuclear factor-kappaB(NF-kappaB) can regulate the invasion and   metastasis   of cancer cells. The present study was to investigate inhibition of NF-kappaB activity on invasion of human lung cancer cell line A549 and the possible mechanism.The recombinant plasmid pcDNA3.1(+)/IkappaBalpha, expressing the alpha isoform (IkappaBalpha) of the NF-kappaB inhibitor, was constructed. A549 cells were cultured in vitro and divided into non-transfection group, pcDNA3.1(+) transfected group and pcDNA3.1(+)/IkappaBalpha transfected group. The expression of IkappaBalpha was detected by reverse transcription-polymerase chain reaction (RT-PCR) and Western blotting. The activity of NF-kappaB was determined by electrophoretic mobility shift assay (EMSA). Invasion of A549 cells was assessed by transwell chamber assay. The expression of MMP-2 and MMP-9 was detected by RT-PCR and gelatin zymography.Plasmid pcDNA3.1(+)/IkappaBalpha was successfully constructed and expressed in A549 cells. The activity of NF-kappaB, the number of invasive cells, the activity of MMP-2 and MMP-9 of A549 cells in pcDNA3.1(+)/IkappaBalpha transfected group were significantly lower than those in non-transfection group and pcDNA3.1(+) transfected group (all P<0.05).Transfection of IkappaBalpha can inhibit NF-kappaB activity, thus inhibit cell invasion of A549, which may be through the down-regulation of MMP-2 and MMP-9 expressions. 
 Potential biomarkers involving IKK/RelA signal in early stage non-small cell lung cancer. The clinical relevance of nuclear factor kappaB (NF-kappaB) and its regulatory molecules on prognosis of patient with early stages of non-small cell lung cancer (NSCLC), remains unclear. Therefore, we conducted biomarker analyses with survival in patients with stages I and II NSCLC. Tumor samples were collected from 88 patients with early-stage NSCLC (stages I, II). A minimum follow-up period of 5 years was required. RelA, phosphorylated I kappaB (pI kappaB alpha), pIKK alpha/beta were detected by immunostaining. NF-kappaB DNA binding activity was assessed by electrophoretic mobility shift assay. Association of clinical and pathologic variables (e.g. sex, age, pathologic stage) with relevant molecules was determined by Pearson's chi(2) test or Fisher's exact test. Survival analysis based on single expression of RelA, pI kappaB alpha, pIKK alpha/beta as well as composite expressions were evaluated using Cox proportional hazards regression models, and log rank test followed Kaplan-Meier estimates. RelA, pI kappaB alpha, pIKK alpha/beta were observed as increased expression in NSCLC tissues compared with adjacent normal tissues and normal lung tissues. These molecules were associated with tumor-node-  metastasis   stages, T stages and histological status, respectively. Among the molecules analyzed, RelA and pI kappaB alpha-positive were statistically significant predictors of patient death in the entire patient population adjusted by age, gender and smoking status; furthermore both RelA and pI kappaB alpha-positive was the strongest prognostic indicators of poor prognosis by univariate and multivariate analyses. Borderline positive correlations were observed between RelA and pI kappaB alpha or pIKK alpha/beta expression. In this cohort of early-stage NSCLC patients, molecular markers, especially composite application of multiple biomarkers (both nuclear RelA and cytoplasmic pI kappaB-alpha expression) that independently predict overall survival have been identified. 
 Polymorphic variants of NFKB1 and its inhibitory protein NFKBIA, and their involvement in sporadic breast cancer. Nuclear factor kappa-beta (NF-kappaB) is a transcription factor responsible for modulating the expression of many genes involved in cell proliferation, differentiation, apoptosis and   metastasis  . NF-kappaB interacts with IkappaB inhibitory proteins to regulate gene expression. This study investigated common variants within the genes coding for NF-kappaB and IkappaB, NFKB1 and NFKBIA, for involvement in sporadic breast cancer. Genotypes were determined in a population of breast cancer affected individuals and age-matched controls. Results do not support an involvement of the tested NFKB1 and NFKBIA polymorphisms in susceptibility to sporadic breast cancer, in the tested Caucasian population. 
 Association of NFKBIA polymorphism with colorectal cancer risk and prognosis in Swedish and Chinese populations. The inhibitory proteins, IkappaBs, regulate the activity of nuclear factor kappa-beta (NF-kappaB), which is implicated in tumorigenesis by regulating expression of a variety of genes involved in cellular transformation, proliferation, invasion, angiogenesis and   metastasis  . Variants in the genes encoding IkappaBs may be involved in cancer development through the activation of NF-kappaB. The objective of this study was to investigate the susceptibility of an A to G variation (rs696) in the 3' UTR of NFKBIA (encoding IkappaBalpha) to colorectal cancer (CRC) and the association of this polymorphism with clinicopathologic variables in CRC patients.A case-control study was carried out on a Swedish (155 CRCs, 438 controls) and a Chinese population (199 CRCs, 577 controls). The genotype of NFKBIA was determined by PCR-restriction fragment length polymorphism.The frequency of the AG genotype was increased in the Chinese patients >or=50 years of age compared with the Chinese controls (odds ratio (OR)=3.06, 95% confidence interval (CI)=1.55-6.02, p=0.001), even when adjusted for age (OR=3.20, 95% CI=1.61-6.38, p=0.001). The GG genotype of NFKBIA was related to a poorer survival rate in the Swedish patients, independent of gender, age, tumour location, Dukes' stage and differentiation (hazard ratio = 3.10, 95% Cl=1.28-7.60, p=0.01).Chinese individuals >or=50 years of age carrying the AG genotype of NFKBIA may be at an increased risk of developing CRC, and the GG genotype of NFKBIA may be considered as a prognostic factor for Swedish CRC patients. 
 Aurora-A down-regulates IkappaBalpha via Akt activation and interacts with insulin-like growth factor-1 induced phosphatidylinositol 3-kinase pathway for cancer cell survival. The mitotic Aurora-A kinase exerts crucial functions in maintaining mitotic fidelity. As a bona fide oncoprotein, Aurora-A aberrant overexpression leads to oncogenic transformation. Yet, the mechanisms by which Aurora-A enhances cancer cell survival remain to be elucidated.Here, we found that Aurora-A overexpression was closely correlated with clinic stage and lymph node   metastasis   in tongue carcinoma. Aurora-A inhibitory VX-680 suppressed proliferation, induced apoptosis and markedly reduced migration in cancer cells. We further showed that insulin-like growth factor-1, a PI3K physiological activator, reversed VX-680-decreased cell survival and motility. Conversely, wortmannin, a PI3K inhibitor, combined with VX-680 showed a synergistic effect on inducing apoptosis and suppressing migration. In addition, Aurora-A inhibition suppressed Akt activation, and VX-680-induced apoptosis was attenuated by Myr-Akt overexpression, revealing a cross-talk between Aurora-A and PI3K pathway interacting at Akt activation. Significantly, we showed that suppression of Aurora-A decreased phosphorylated Akt and was associated with increased IkappaBalpha expression. By contrast, Aurora-A overexpression upregulated Akt activity and downregulated IkappaBalpha, these changes were accompanied by nuclear translocation of nuclear factor-kappaB and increased expression of its target gene Bcl-xL. Lastly, Aurora-A overexpression induced IkappaBalpha reduction was abrogated by suppression of Akt either chemically or genetically.Taken together, our data established that Aurora-A, via activating Akt, stimulated nuclear factor-kappaB signaling pathway to promote cancer cell survival, and promised a novel combined chemotherapy targeting both Aurora-A and PI3K in cancer treatment. 
 A NIK-IKKα module expands ErbB2-induced tumor-initiating cells by stimulating nuclear export of p27/Kip1. IκB kinase α (IKKα) activity is required for ErbB2-induced mammary tumorigenesis. Here, we show that IKKα and its activator, NF-κB-inducing kinase (NIK), support the expansion of tumor-initiating cells (TICs) that copurify with a CD24(med)CD49f(hi) population from premalignant ErbB2-expressing mammary glands. Upon activation, IKKα enters the nucleus, phosphorylates the cyclin-dependent kinase (CDK) inhibitor p27/Kip1, and stimulates its nuclear export or exclusion. Reduced p27 expression rescues mammary tumorigenesis in mice deficient in IKKα kinase activity and restores TIC self-renewal. IKKα is also likely to be involved in human breast cancer, where its expression shows an inverse correlat  ion with m  etastasis-free survival, and its presence in the nucleus of invasive ductal carcinomas (IDCs) is associated with decreased nuclear p27 abundance. 
 MiR-196a promotes pancreatic cancer progression by targeting nuclear factor kappa-B-inhibitor alpha. Aberrant expression of miR-196a has been frequently reported in different cancers including pancreatic cancer. However, its function in pancreatic cancer has not been fully elucidated. Here, we investigated the expression pattern and the biological role of miR-196a in pancreatic cancer cell lines, as well as its interaction with a   metastasis  -related gene, nuclear factor-kappa-B-inhibitor alpha (NFKBIA). We demonstrated that miR-196a was up-regulated in human pancreatic cancer cell lines compared with immortalized pancreatic ductal epithelial cells by means of microRNAs microarray and qRT-PCR. Furthermore, down-regulation of miR-196a in PANC-1 suppressed its proliferation and migration with an increase in G0/G1 transition and decreased expression of Cyclin D1 and CDK4/6. Meanwhile, an increased expression in E-cadherin and decreased expression in N-cadherin and Vimentin were also observed. We identified a novel miR-196a target, NFKBIA, and down-regulation of miR-196a enhanced the expression of NFKBIA protein. Luciferase assay confirmed that NFKBIA was a direct and specific target of miR-196a. Silencing NFKBIA in PANC-1 cells enhanced its proliferation and migration. Taken together, our findings indicate that miR-196a is highly expressed in pancreatic cancer cell lines, and may play a crucial role in pancreatic cancer proliferation and migration, possibly through its downstream target, NFKBIA. Thus, miR-196a may serve as a potential therapeutic target for pancreatic cancer. 
  [Cc]ell [Ii]nvasion  PubMed  
 [Inhibition of NF-kappaB through IkappaBalpha transfection affects invasion of human lung cancer cell line A549]. Recent studies have shown that activation of nuclear factor-kappaB(NF-kappaB) can regulate the invasion and metastasis of cancer cells. The present study was to investigate inhibition of NF-kappaB activity on invasion of human lung cancer cell line A549 and the possible mechanism.The recombinant plasmid pcDNA3.1(+)/IkappaBalpha, expressing the alpha isoform (IkappaBalpha) of the NF-kappaB inhibitor, was constructed. A549 cells were cultured in vitro and divided into non-transfection group, pcDNA3.1(+) transfected group and pcDNA3.1(+)/IkappaBalpha transfected group. The expression of IkappaBalpha was detected by reverse transcription-polymerase chain reaction (RT-PCR) and Western blotting. The activity of NF-kappaB was determined by electrophoretic mobility shift assay (EMSA). Invasion of A549 cells was assessed by transwell chamber assay. The expression of MMP-2 and MMP-9 was detected by RT-PCR and gelatin zymography.Plasmid pcDNA3.1(+)/IkappaBalpha was successfully constructed and expressed in A549 cells. The activity of NF-kappaB, the number of invasive cells, the activity of MMP-2 and MMP-9 of A549 cells in pcDNA3.1(+)/IkappaBalpha transfected group were significantly lower than those in non-transfection group and pcDNA3.1(+) transfected group (all P<0.05).Transfection of IkappaBalpha can inhibit NF-kappaB activity, thus inhibit   cell invasion   of A549, which may be through the down-regulation of MMP-2 and MMP-9 expressions. 
 Alpha-mangostin suppresses phorbol 12-myristate 13-acetate-induced MMP-2/MMP-9 expressions via alphavbeta3 integrin/FAK/ERK and NF-kappaB signaling pathway in human lung adenocarcinoma A549 cells. The purpose of this study is to investigate the anti-metastatic effect of alpha-mangostin on phorbol 12-myristate 13-acetate (PMA)-induced matrix metalloproteinase-2 (MMP-2) and matrix metalloproteinase-9 (MMP-9) expressions in A549 human lung adenocarcinoma cells. Firstly, alpha-mangostin could inhibit PMA-induced abilities of the adhesion, invasion, and migration. Data also showed alpha-mangostin could inhibit the activation of alphavbeta3 integrin, focal adhesion kinase (FAK), and extracellular signal-regulated kinase1/2 (ERK1/2) involved in the downregulation the enzyme activities, protein and messenger RNA levels of MMP-2 and MMP-9 induced by PMA. Next, alpha-mangostin also strongly inhibited PMA-induced degradation of inhibitor of kappaBalpha (IkappaBalpha) and the nuclear levels of nuclear factor kappa B (NF-kappaB). Also, a dose-dependent inhibition on the binding abilities of NF-kappaB by alpha-mangostin treatment was further observed. Furthermore, reduction of FAK or ERK1/2 phosphorylation by FAK small interfering RNA (FAK siRNA) potentiated the effect of alpha-mangostin. Finally, the transient transfection of ERK siRNA significantly down-regulated the expressions of MMP-2 and MMP-9 concomitantly with a marked inhibition on   cell invasion   and migration. Presented results indicated alpha-mangostin is a novel, effect, anti-metastatic agent that functions by downregulating MMP-2 and MMP-9 gene expressions. 
 Alpha-mangostin suppresses phorbol 12-myristate 13-acetate-induced MMP-2/MMP-9 expressions via alphavbeta3 integrin/FAK/ERK and NF-kappaB signaling pathway in human lung adenocarcinoma A549 cells. The purpose of this study is to investigate the anti-metastatic effect of alpha-mangostin on phorbol 12-myristate 13-acetate (PMA)-induced matrix metalloproteinase-2 (MMP-2) and matrix metalloproteinase-9 (MMP-9) expressions in A549 human lung adenocarcinoma cells. Firstly, alpha-mangostin could inhibit PMA-induced abilities of the adhesion, invasion, and migration. Data also showed alpha-mangostin could inhibit the activation of alphavbeta3 integrin, focal adhesion kinase (FAK), and extracellular signal-regulated kinase1/2 (ERK1/2) involved in the downregulation the enzyme activities, protein and messenger RNA levels of MMP-2 and MMP-9 induced by PMA. Next, alpha-mangostin also strongly inhibited PMA-induced degradation of inhibitor of kappaBalpha (IkappaBalpha) and the nuclear levels of nuclear factor kappa B (NF-kappaB). Also, a dose-dependent inhibition on the binding abilities of NF-kappaB by alpha-mangostin treatment was further observed. Furthermore, reduction of FAK or ERK1/2 phosphorylation by FAK small interfering RNA (FAK siRNA) potentiated the effect of alpha-mangostin. Finally, the transient transfection of ERK siRNA significantly down-regulated the expressions of MMP-2 and MMP-9 concomitantly with a marked inhibition on   cell invasion   and migration. Presented results indicated alpha-mangostin is a novel, effect, anti-metastatic agent that functions by downregulating MMP-2 and MMP-9 gene expressions. 
 [Inhibition of NF-kappaB through IkappaBalpha transfection affects invasion of human lung cancer cell line A549]. Recent studies have shown that activation of nuclear factor-kappaB(NF-kappaB) can regulate the invasion and metastasis of cancer cells. The present study was to investigate inhibition of NF-kappaB activity on invasion of human lung cancer cell line A549 and the possible mechanism.The recombinant plasmid pcDNA3.1(+)/IkappaBalpha, expressing the alpha isoform (IkappaBalpha) of the NF-kappaB inhibitor, was constructed. A549 cells were cultured in vitro and divided into non-transfection group, pcDNA3.1(+) transfected group and pcDNA3.1(+)/IkappaBalpha transfected group. The expression of IkappaBalpha was detected by reverse transcription-polymerase chain reaction (RT-PCR) and Western blotting. The activity of NF-kappaB was determined by electrophoretic mobility shift assay (EMSA). Invasion of A549 cells was assessed by transwell chamber assay. The expression of MMP-2 and MMP-9 was detected by RT-PCR and gelatin zymography.Plasmid pcDNA3.1(+)/IkappaBalpha was successfully constructed and expressed in A549 cells. The activity of NF-kappaB, the number of invasive cells, the activity of MMP-2 and MMP-9 of A549 cells in pcDNA3.1(+)/IkappaBalpha transfected group were significantly lower than those in non-transfection group and pcDNA3.1(+) transfected group (all P<0.05).Transfection of IkappaBalpha can inhibit NF-kappaB activity, thus inhibit   cell invasion   of A549, which may be through the down-regulation of MMP-2 and MMP-9 expressions. 
 [Expression of IκBα in bladder cancer cell lines is negatively correlated with epithelial-mesenchymal transition an  d cell invasi  on in vitro]. To investigate the expression of inhibitor of nuclear factor κBα (IκBα) in bladder cancer cell lines and its correlation with epithelial-mesenchymal transition (EMT) and invasive potential in vitro.The expressions of IκBα, epithelia cadherin (E-cadherin), neuronal cadherin (N-cadherin) and vimentin in human bladder cancer RT4, 5637, 235J, J82 and T24 cell lines were detected by Western blotting. Invasive potential of these cell lines was evaluated by Transwell(TM) assay. In addition, morphological characteristics of those cells were carefully observed under a microscope.RT4 and 5637 cells exhibited epithelial cell morphological features, but 253J, J82 and T24 cells had mesenchymal cell morphological features. E-cadherin was over-expressed in RT4 and 5637 cell lines, but the expressions of vimentin and N-cadherin could not been detected in the two cells. E-cadherin could be detected in 253J cells with a lower expression level; J82 and T24 cells had no expression of E-cadherin; vimentin and N-cadherin were highly expressed in 253J, J82 and T24 cells. Transwell(TM) assay showed higher invasive potential of 253J, J82 and T24 cells than RT4 and 5637 cells in vitro. In addition, RT4 and 5637 cells expressed higher levels of IκBα than 253J, J82 and T24 cells.Expression of IκBα in human bladder cancer cells is negatively correlated with EMT and tumor invasion in vitro. 
  GeneRIFs  
 Transfection of IkappaBalpha can inhibit NF-kappaB activity, thus inhibit   cell invasion   of A549, which may be through the down-regulation of MMP-2 and MMP-9 expressions. 
   
  
 FAM43A 
 
  NCBI Gene  
  GeneRIF  
  Pubmed  
 
 
  Search Term  Rank  Fields  
  [Ee]xtracellular [Mm]atrix  8  PubMed(1)  
 
 
 hide/show details for FAM43A 
  [Ee]xtracellular [Mm]atrix  PubMed  
 Isolation of novel and known genes from a human fetal cochlear cDNA library using subtractive hybridization and differential screening. We used a combination of subtractive hybridization and differential screening strategies to identify genes that may function normally in hearing and, when mutated, result in deafness. A human fetal cochlear (membranous labyrinth) cDNA library was subtracted against total human fetal brain RNAs by an avidin-biotin-based procedure to enrich for cochlear transcripts. Subtracted cochlear clones were differentially screened with 32P-labeled total cochlear and total brain cDNA probes. Sequence analysis of clones that hybridized more intensely with cochlear than with brain cDNA probes revealed some previously characterized genes, including mitochondrial sequences, collagen type I alpha-2 (COL1A2), collagen type II alpha-1 (COL2A1), collagen type III alpha-1 (COL3A1), spermidine/spermine N1-acetyltransferase (SAT), osteonectin (SPARC), and peripheral myelin protein 22 (PMP22). Also identified were clones that are potential novel cochlear genes. Northern blots of cochlear and brain RNAs probed with COL1A2, COL2A1, COL3A1, SAT, SPARC, PMP22, and a novel sequence, designated Coch-5B2, confirm results of the subtractive procedure by showing preferential cochlear expression. A number of these genes serve structural or regulatory functions in   extracellular matrix   or neural conduction; defects in some of these genes are associated with disorders involving hearing loss. Partial sequence analysis of Coch-5B2 reveals a von Willebrand factor type A-like domain in this cDNA. To assess the cochlear specificity of Coch-5B2, a Northern blot panel of 14 human fetal tissue RNAs was probed with Coch-5B2, showing differential expression of this novel gene in the cochlea. 
   
  
 GPR84 
 
  NCBI Gene  
  GeneRIF  
  Pubmed  
 
 
  Search Term  Rank  Fields  
  [Mm]igration  6  PubMed(1)  
 
 
 hide/show details for GPR84 
  [Mm]igration  PubMed  
 Cloning and expression analysis of a novel G-protein-coupled receptor selectively expressed on granulocytes. The   migration   of neutrophils into sites of acute and chronic inflammation is mediated by chemokines. We used degenerate-primer reverse transcriptase-polymerase chain reaction (RT-PCR) to analyze chemokine receptor expression in neutrophils and identify novel receptors. RNA was isolated from human peripheral blood neutrophils and from neutrophils that had been stimulated for 5 h with granulocyte-macrophage colony-stimulating factor or by coculturing with primary human bronchial epithelial cells. Amplification products were cloned, and clone redundancy was determined. Seven known G-protein-coupled receptors were identified among 38 clones-CCR1, CCR4, CXCR1, CXCR2, CXCR4, HM63, and FPR1-as well as a novel gene, EX33. The full-length EX33 clone was obtained, and an in silico approach was used to identify the putative murine homologue. The EX33 gene encodes a 396-amino-acid protein with limited sequence identity to known receptors. Expression studies of several known chemokine receptors and EX33 revealed that resting neutrophils expressed higher levels of CXCRs and EX33 compared with activated neutrophils. Northern blot experiments revealed that EX33 is expressed mainly in bone marrow, lung, and peripheral blood leukocytes. Using RT-PCR analysis, we showed more abundant expression of EX33 in neutrophils and eosinophils, in comparison with that in T- or B-lymphocytes, indicating cell-specific expression among leukocytes. 
   
  
 FOXG1 
 
  NCBI Gene  
  GeneRIF  
  Pubmed  
 
 
  Search Term  Rank  Fields  
  [Mm]igration  6  Process(1); PubMed(5)  
  [Mm]etastasis  12  PubMed(2)  
 
 
 hide/show details for FOXG1 
  [Mm]igration  Process  
 pyramidal neuron   migration   
  PubMed  
 MicroRNA-9 regulates neurogenesis in mouse telencephalon by targeting multiple transcription factors. microRNA-9-2 and microRNA-9-3 double-mutant mice demonstrate that microRNA-9 (miR-9) controls neural progenitor proliferation and differentiation in the developing telencephalon by regulating the expression of multiple transcription factors. As suggested by our previous study, the Foxg1 expression was elevated, and the production of Cajal-Retzius cells and early-born neurons was suppressed in the miR-9-2/3 double-mutant pallium. At embryonic day 16.5 (E16.5), however, the Foxg1 expression was no longer elevated. The expression of an AU-rich RNA-binding protein Elavl2 increased at E16.5, Elav2 associated with Foxg1 3' untranslated region (UTR), and it countered the Foxg1 suppression by miR-9. Later, progenitor proliferation was reduced in the miR-9-2/3 double-mutant pallium with the decrease in Nr2e1 and Pax6 expression and the increase in Meis2 expression. The analyses suggest that microRNA-9 indirectly inhibits Pax6 expression by suppressing Meis2 expression. In contrast, together with Elavl1 and Msi1, microRNA-9 targets Nr2e1 mRNA 3' UTR to enhance the expression. Concomitantly, cortical layers were reduced, each cortical projection was malformed, and the tangential   migration   of interneurons into the pallium was impaired in the miR-9-2/3 double mutants. miR-9 also targets Gsh2 3' UTR, and Gsh2, as well as Foxg1, expression was elevated in the miR-9-2/3 double-mutant subpallium. The subpallium progenitor proliferation was enhanced, and the development of basal ganglia including striatum and globus pallidus was suppressed. Pallial/subpallial boundary shifted dorsally, and the ventral pallium was lost. Corridor was malformed, and thalamocortical and corticofugal axons were misrouted in the miR-9-2/3 double mutants. 
 An AKT3-FOXG1-reelin network underlies defective   migration   in human focal malformations of cortical development. Focal malformations of cortical development (FMCDs) account for the majority of drug-resistant pediatric epilepsy. Postzygotic somatic mutations activating the phosphatidylinositol-4,5-bisphosphate-3-kinase (PI3K)-protein kinase B (AKT)-mammalian target of rapamycin (mTOR) pathway are found in a wide range of brain diseases, including FMCDs. It remains unclear how a mutation in a small fraction of cells disrupts the architecture of the entire hemisphere. Within human FMCD-affected brain, we found that cells showing activation of the PI3K-AKT-mTOR pathway were enriched for the AKT3(E17K) mutation. Introducing the FMCD-causing mutation into mouse brain resulted in electrographic seizures and impaired hemispheric architecture. Mutation-expressing neural progenitors showed misexpression of reelin, which led to a non-cell autonomous   migration   defect in neighboring cells, due at least in part to derepression of reelin transcription in a manner dependent on the forkhead box (FOX) transcription factor FOXG1. Treatments aimed at either blocking downstream AKT signaling or inactivating reelin restored   migration  . These findings suggest a central AKT-FOXG1-reelin signaling pathway in FMCD and support pathway inhibitors as potential treatments or therapies for some forms of focal epilepsy. 
 Double-negative feedback loop between microRNA-422a and forkhead box (FOX)G1/Q1/E1 regulates hepatocellular carcinoma tumor growth and metastasis. Growing evidence indicates that the aberrant expression of microRNAs (miRNAs) contributes to tumor development; however, the function of miRNAs in human hepatocellular carcinoma (HCC) remains largely undefined. In this study, we report that microRNA-422a (miR-422a) is significantly down-regulated in HCC tumor samples and cell lines compared with normal controls, and its expression level is negatively correlated with pathological grading, recurrence, and metastasis. The restoration of miR-422a expression in HCC tumor cells significantly inhibited cell proliferation and   migration   in vitro. At the same time, the overexpression of miR-422a in HCC tumor cells significantly inhibits tumor growth and liver metastasis in xenograft tumor models. A mechanistic study identified three genes, forkhead box G1 (FOXG1), FOXQ1, and FOXE1, as miR-422a targets in the regulation of HCC development. We also investigated the function of the three targets themselves in HCC tumorigenesis using RNAi manipulation and demonstrated that the knockdown of these targets led to significant inhibition of tumor cell proliferation and   migration   both in vitro and in vivo. More interestingly, a potential miR-422a promoter region was identified. Both the promoter activity and miR-422a expression were negatively regulated by the three targets, indicating that a double-negative feedback loop exists between miR-422a and its targets. Moreover, we explored the therapeutic potential of miR-422a in HCC treatment and found that the therapeutic delivery of miR-422a significantly inhibited tumor development in a xenograft tumor model and a diethylnitrosamine-induced primary HCC model.Our findings show the critical roles of miR-422a and its targets--FOXG1, FOXQ1, and FOXE1--in the regulation of HCC development and provide new potential candidates for HCC therapy. 
 MiR-200b promotes the cell proliferation and metastasis of cervical cancer by inhibiting FOXG1. Previous studies have revealed the important role of miR-200b in cancer biology, including its upregulation in cervical cancer. However, miR-200b function in cervical cancer progression remains unclear. Thus, this study aims to explore the functional role of miR-200b in cervical cancer development, involving its potential regulation on FoxG1, one transcriptional repressor.Thirty paired cervical cancer samples were used to analyze the expression of miR-200b and FoxG1 by real time PCR and western blot analysis. Further gain- and loss-of-function studies were performed to validate FoxG1 as one miR-200b target, in line with luciferase report assays. MiR-200b silence was also conducted to observe its regulation on cell viability,   migration   and invasion in vitro, while tumor growth in vivo was tracked through the delivery of miR-200b inhibitor.MiR-200b upregulation was confirmed in cancer tissues or cells as compared to normal controls, while FoxG1 downregulation was observed and then FoxG1 was definitely validated as one miR-200b target. Further in vitro studies showed that enforced miR-200b downregulation induced the decrease of cell ability, with increased cell apoptosis, and attenuated ability of cell   migration   and invasion in both HeLa and C33A cells, while further inhibition of FoxG1 expression could reverse all these changes. In addition, miR-200b silence in vivo strongly inhibited tumor growth.Upregulated miR-200b in cervical cancer was proven to show positive regulation on cervical cancer development by directly targeting FoxG1. Moreover, miR-200b silence was proposed to inhibit tumor growth in vivo, implying its therapeutic value in cervical cancer treatment. 
 Foxg1 has an essential role in postnatal development of the dentate gyrus. Foxg1, formerly BF-1, is expressed continuously in the postnatal and adult hippocampal dentate gyrus (DG). This transcription factor (TF) is thought to be involved in Rett syndrome, which is characterized by reduced hippocampus size, indicating its important role in hippocampal development. Due to the perinatal death of Foxg1(-/-) mice, the function of Foxg1 in postnatal DG neurogenesis remains to be explored. Here, we describe the generation of a Foxg1(fl/fl) mouse line. Foxg1 was conditionally ablated from the DG during prenatal and postnatal development by crossing this line with a Frizzled9-CreER(TM) line and inducing recombination with tamoxifen. In this study, we first show that disruption of Foxg1 results in the loss of the subgranular zone and a severely disrupted secondary radial glial scaffold, leading to the impaired   migration   of granule cells. Moreover, detailed analysis reveals that Foxg1 may be necessary for the maintenance of the DG progenitor pool and that the lack of Foxg1 promotes both gliogenesis and neurogenesis. We additionally show that Foxg1 may be required for the survival and maturation of postmitotic neurons and that Foxg1 may be involved in Reelin signaling in regulating postnatal DG development. Last, prenatal deletion of Foxg1 suggests that it is rarely involved in the   migration   of primordial granule cells. In summary, we report that Foxg1 is critical for DG formation, especially during early postnatal stage. 
  [Mm]etastasis  PubMed  
 Double-negative feedback loop between microRNA-422a and forkhead box (FOX)G1/Q1/E1 regulates hepatocellular carcinoma tumor growth and   metastasis  . Growing evidence indicates that the aberrant expression of microRNAs (miRNAs) contributes to tumor development; however, the function of miRNAs in human hepatocellular carcinoma (HCC) remains largely undefined. In this study, we report that microRNA-422a (miR-422a) is significantly down-regulated in HCC tumor samples and cell lines compared with normal controls, and its expression level is negatively correlated with pathological grading, recurrence, and   metastasis  . The restoration of miR-422a expression in HCC tumor cells significantly inhibited cell proliferation and migration in vitro. At the same time, the overexpression of miR-422a in HCC tumor cells significantly inhibits tumor growth and liver   metastasis   in xenograft tumor models. A mechanistic study identified three genes, forkhead box G1 (FOXG1), FOXQ1, and FOXE1, as miR-422a targets in the regulation of HCC development. We also investigated the function of the three targets themselves in HCC tumorigenesis using RNAi manipulation and demonstrated that the knockdown of these targets led to significant inhibition of tumor cell proliferation and migration both in vitro and in vivo. More interestingly, a potential miR-422a promoter region was identified. Both the promoter activity and miR-422a expression were negatively regulated by the three targets, indicating that a double-negative feedback loop exists between miR-422a and its targets. Moreover, we explored the therapeutic potential of miR-422a in HCC treatment and found that the therapeutic delivery of miR-422a significantly inhibited tumor development in a xenograft tumor model and a diethylnitrosamine-induced primary HCC model.Our findings show the critical roles of miR-422a and its targets--FOXG1, FOXQ1, and FOXE1--in the regulation of HCC development and provide new potential candidates for HCC therapy. 
 MiR-200b promotes the cell proliferation and   metastasis   of cervical cancer by inhibiting FOXG1. Previous studies have revealed the important role of miR-200b in cancer biology, including its upregulation in cervical cancer. However, miR-200b function in cervical cancer progression remains unclear. Thus, this study aims to explore the functional role of miR-200b in cervical cancer development, involving its potential regulation on FoxG1, one transcriptional repressor.Thirty paired cervical cancer samples were used to analyze the expression of miR-200b and FoxG1 by real time PCR and western blot analysis. Further gain- and loss-of-function studies were performed to validate FoxG1 as one miR-200b target, in line with luciferase report assays. MiR-200b silence was also conducted to observe its regulation on cell viability, migration and invasion in vitro, while tumor growth in vivo was tracked through the delivery of miR-200b inhibitor.MiR-200b upregulation was confirmed in cancer tissues or cells as compared to normal controls, while FoxG1 downregulation was observed and then FoxG1 was definitely validated as one miR-200b target. Further in vitro studies showed that enforced miR-200b downregulation induced the decrease of cell ability, with increased cell apoptosis, and attenuated ability of cell migration and invasion in both HeLa and C33A cells, while further inhibition of FoxG1 expression could reverse all these changes. In addition, miR-200b silence in vivo strongly inhibited tumor growth.Upregulated miR-200b in cervical cancer was proven to show positive regulation on cervical cancer development by directly targeting FoxG1. Moreover, miR-200b silence was proposed to inhibit tumor growth in vivo, implying its therapeutic value in cervical cancer treatment. 
   
  
 RELB 
 
  NCBI Gene  
  GeneRIF  
  Pubmed  
 
 
  Search Term  Rank  Fields  
  [Mm]igration  6  PubMed(6); GeneRIFs(2)  
  [Ii]nvasive  10  PubMed(4)  
  [Mm]etastasis  12  PubMed(2); GeneRIFs(1)  
 
 
 hide/show details for RELB 
  [Mm]igration  PubMed  
 RelB, together with RelA, sustains cell survival and confers proteasome inhibitor sensitivity of chronic lymphocytic leukemia cells from bone marrow. Although the biological factors that contribute to the pathogenesis of chronic lymphocytic leukemia (CLL) remain widely unresolved, it has been suggested that dysregulated cell survival and proliferation are fundamental to this process. Constitutive classical nuclear factor kappa-light-chain-enhancer of activated B cells (NF-κB) activation protects CLL B-cells from cell death and plays a critical role in the acquisition of chemoresistance. RelB, representing the alternative NF-κB activity, functions specifically in lymphoid organogenesis and B-cell maturation. RelB indeed plays a tumor-supportive role and confers radiation resistance in tumors. However, the involvement of RelB in CLL has not been addressed. Here, we analyzed the NF-κB activation in 67 of CLL bone marrow (BM). Both the RelA and RelB activity were detected in CLL B-cells from BM, in spite of inevitable variability. Low RelB activity was linked to a favorable prognosis of CLL. T  he migrat  ion and adhesion abilities of CLL B-cells were not affected by the RelB activity. High RelB activity, together with the RelA activity, maintained basal survival of cells. The induction of RelA and RelB expression in the nucleus was responsible for better survival of CLL B-cells supported by bone marrow stromal cells. In addition, the presence of high RelB activity in CLL B-cells was correlated with sensitivity to proteasome inhibitor but not fludarabine. Taken together, we provided evidences that not only RelA but also RelB, subunits of NF-κB family, played an important role in the cellular behaviors of CLL cells from BM. The strength of RelB activity influenced the prognosis of CLL patients.RelB, with RelA activity, maintained the basal survival of CLL cells from BM. RelB, with RelA, conferred the proteasome inhibitor sensitivity of CLL cells. Induction of RelA and RelB was responsible for the better survival of CLL B-cells. The strength of RelB activity influenced the prognosis of CLL patients. 
 The NF-κB subunit RelB regulates the   migratio  n and invasion abilities and the radio-sensitivity of prostate cancer cells. NF-κB subunits play important roles in carcinogenesis of a variety of human malignancies and response to cancer therapy; however, the contribution of an individual subunit has not been thoroughly defined. Constitutive activation of the canonical NF-κB subunit is a critical event in prostate carcinogenesis. Recent findings point out that RelB, which contributes to the non-canonical NF-κB activity, functions importantly in the prostate cancer progression. Here, we investigated systemically the functional roles of RelB in prostate cancer and examine its significance as a therapeutic target. Targeting RelB using short hairpin RNA approach in androgen-independent DU145 prostate cancer cells interfered with various biological behaviors of cells. We observed that RelB knockdown inhibited prostate cancer cell grow  th, migra  tion, and invasion, and enhanced proteasome inhibitor sensitivity. The altered expression of anti-apoptotic gene Bcl-2 played critical roles in regulating both spontaneous and radiation-induced apoptosis in the presence of RelB knockdown. For the first time, we showed that RelB knockdown significantly attenuated   the migra  tion and invasion of DU145 prostate cancer cells, due to the reduction of integrin β-1. Collectively, we provided evidence that RelB functioned as an oncogene in prostate cancer. Developing a RelB-targeted therapeutic intervention, is valuable in treating advanced, metastatic prostate cancer. 
 The NF-κB subunit RelB regulates the   migratio  n and invasion abilities and the radio-sensitivity of prostate cancer cells. NF-κB subunits play important roles in carcinogenesis of a variety of human malignancies and response to cancer therapy; however, the contribution of an individual subunit has not been thoroughly defined. Constitutive activation of the canonical NF-κB subunit is a critical event in prostate carcinogenesis. Recent findings point out that RelB, which contributes to the non-canonical NF-κB activity, functions importantly in the prostate cancer progression. Here, we investigated systemically the functional roles of RelB in prostate cancer and examine its significance as a therapeutic target. Targeting RelB using short hairpin RNA approach in androgen-independent DU145 prostate cancer cells interfered with various biological behaviors of cells. We observed that RelB knockdown inhibited prostate cancer cell grow  th, migra  tion, and invasion, and enhanced proteasome inhibitor sensitivity. The altered expression of anti-apoptotic gene Bcl-2 played critical roles in regulating both spontaneous and radiation-induced apoptosis in the presence of RelB knockdown. For the first time, we showed that RelB knockdown significantly attenuated   the migra  tion and invasion of DU145 prostate cancer cells, due to the reduction of integrin β-1. Collectively, we provided evidence that RelB functioned as an oncogene in prostate cancer. Developing a RelB-targeted therapeutic intervention, is valuable in treating advanced, metastatic prostate cancer. 
 RelB, together with RelA, sustains cell survival and confers proteasome inhibitor sensitivity of chronic lymphocytic leukemia cells from bone marrow. Although the biological factors that contribute to the pathogenesis of chronic lymphocytic leukemia (CLL) remain widely unresolved, it has been suggested that dysregulated cell survival and proliferation are fundamental to this process. Constitutive classical nuclear factor kappa-light-chain-enhancer of activated B cells (NF-κB) activation protects CLL B-cells from cell death and plays a critical role in the acquisition of chemoresistance. RelB, representing the alternative NF-κB activity, functions specifically in lymphoid organogenesis and B-cell maturation. RelB indeed plays a tumor-supportive role and confers radiation resistance in tumors. However, the involvement of RelB in CLL has not been addressed. Here, we analyzed the NF-κB activation in 67 of CLL bone marrow (BM). Both the RelA and RelB activity were detected in CLL B-cells from BM, in spite of inevitable variability. Low RelB activity was linked to a favorable prognosis of CLL. T  he migrat  ion and adhesion abilities of CLL B-cells were not affected by the RelB activity. High RelB activity, together with the RelA activity, maintained basal survival of cells. The induction of RelA and RelB expression in the nucleus was responsible for better survival of CLL B-cells supported by bone marrow stromal cells. In addition, the presence of high RelB activity in CLL B-cells was correlated with sensitivity to proteasome inhibitor but not fludarabine. Taken together, we provided evidences that not only RelA but also RelB, subunits of NF-κB family, played an important role in the cellular behaviors of CLL cells from BM. The strength of RelB activity influenced the prognosis of CLL patients.RelB, with RelA activity, maintained the basal survival of CLL cells from BM. RelB, with RelA, conferred the proteasome inhibitor sensitivity of CLL cells. Induction of RelA and RelB was responsible for the better survival of CLL B-cells. The strength of RelB activity influenced the prognosis of CLL patients. 
 Expression of the RelB transcription factor correlates with the activation of human dendritic cells. The RelB gene product is a member of the nuclear factor (NF)-kappaB family of transcription factors. It has been identified recently within mouse antigen-presenting cells and human monocyte-derived dendritic cells (DC). Disruption of the mouse RelB gene is accompanied, amongst other phenotypes, by abnormalities in the antigen-presenting cell lineages. In order to define RelB expression during human DC differentiation, we have analysed RelB mRNA by reverse transcriptase-polymerase chain reaction and RelB protein by intracellular staining in CD34+ precursors and different types of DC preparations. RelB mRNA was not detected in CD34+ precursor populations. Fresh blood DC (lineage-human leucocyte antigen-DR+ (lin-HLA-DR+)) lacked RelB mRNA and cytoplasmic RelB protein but a period of in vitro culture induced RelB expression in blood DC. Purified Langerhans' cells (LC) (CD1a+ HLA-DR+) failed to express RelB mRNA. Immunocytochemical staining identified RelB protein in human skin epithelium. RelB protein was expressed in a very few CD1a+, CD83+ or CMRF-44+ dermal DC but was not present in CD1a+ LC. Tonsil DC (lin-HLA-DR+ CMRF-44+) were positive for RelB mRNA and RelB protein. Intestinal DC (HLA-DR+) also lacked immunoreactive RelB protein. The majority of interdigitating CD83+, CMRF-44+, CMRF-56+ or p55+ DC located in paracortical T-lymphocyte areas of lymph node and tonsil contained RelB protein. The expression of RelB mRNA and RelB protein correlates with the activated phase of blood DC and the post  migration   cell (activated) stage of tissue DC development. 
 The adhesion G protein-coupled receptor G2 (ADGRG2/GPR64) constitutively activates SRE and NFκB and is involved in cell adhesion and   migratio  n. Adhesion G protein-coupled receptors (ADGRs) are believed to be activated by auto-proteolytic cleavage of their very large extracellular N-terminal domains normally acting as a negative regulator of the intrinsically constitutively active seven transmembrane domain. ADGRG2 (or GPR64) which originally was described to be expressed in the epididymis and studied for its potential role in male fertility, is highly up-regulated in a number of carcinomas, including breast cancer. Here, we demonstrate that ADGRG2 is a functional receptor, which in transfected HEK293 cells signals with constitutive activity through the adhesion- and   migratio  n-related transcription factors serum response element (SRE) and nuclear factor kappa-light-chain-enhancer of activated B cells (NFκB) presumably via coupling to Gα12/13 and Gαq. However, activation of these two pathways appears to occur through distinct molecular activation mechanisms as auto-proteolytic cleavage is essential for SRE activation but not required for NFκB signaling. The overall activation mechanism for ADGRG2 is clearly distinct from the established ADGR activation mechanism as it requires the large extracellular N-terminal domain for proper intracellular signal transduction. Knockdown of ADGRG2 by siRNA in the highly motile breast cancer cell lines Hs578T and MDA-MB-231 resulted in a strong reduction in cell adhesion and subsequent   cell migr  ation which was associated with a selective reduction in RelB, an NFκB family member. It is concluded that the adhesion GPCR ADGRG2 is critically involved in the adhesio  n and mig  ration of certain breast cancer cells through mechanisms including a non-canonical NFkB pathway and that ADGRG2 could be a target for treatment of certain types of cancer. 
  GeneRIFs  
 The altered expression of anti-apoptotic gene Bcl-2 played critical roles in regulating both spontaneous and radiation-induced apoptosis in the presence of RelB knockdown. For the first time, we showed that RelB knockdown significantly attenuated the   migration   and invasion of DU145 prostate cancer cells, due to the reduction of integrin b-1 
 Knockdown of ADGRG2 breast cancer cell lines resulted in a strong reduction in cell adhesion and subsequent cell   migration   which was associated with a selective reduction in RelB. 
  [Ii]nvasive  PubMed  
 The NF-κB RelB protein is an oncogenic driver of mesenchymal glioma. High-grade gliomas, such as glioblastomas (GBMs), are very aggressive,   invasiv  e brain tumors with low patient survival rates. The recent identification of distinct glioma tumor subtypes offers the potential for understanding disease pathogenesis, responses to treatment and identification of molecular targets for personalized cancer therapies. However, the key alterations that drive tumorigenesis within each subtype are still poorly understood. Although aberrant NF-κB activity has been implicated in glioma, the roles of specific members of this protein family in tumorigenesis and pathogenesis have not been elucidated. In this study, we show that the NF-κB protein RelB is expressed in a particularly aggressive mesenchymal subtype of glioma, and loss of RelB significantly attenuated glioma cell survival, motility and invasion. We find that RelB promotes the expression of mesenchymal genes including YKL-40, a marker of the MES glioma subtype. Additionally, RelB regulates expression of Olig2, a regulator of cancer stem cell proliferation and a candidate marker for the cell of origin in glioma. Furthermore, loss of RelB in glioma cells significantly diminished tumor growth in orthotopic mouse xenografts. The relevance of our studies for human disease was confirmed by analysis of a human GBM genome database, which revealed that high RelB expression strongly correlates with rapid tumor progression and poor patient survival rates. Thus, our findings demonstrate that RelB is an oncogenic driver of mesenchymal glioma tumor growth and invasion, highlighting the therapeutic potential of inhibiting the noncanonical NF-κB (RelB-mediated) pathway to treat these deadly tumors. 
 The NF-κB RelB protein is an oncogenic driver of mesenchymal glioma. High-grade gliomas, such as glioblastomas (GBMs), are very aggressive,   invasiv  e brain tumors with low patient survival rates. The recent identification of distinct glioma tumor subtypes offers the potential for understanding disease pathogenesis, responses to treatment and identification of molecular targets for personalized cancer therapies. However, the key alterations that drive tumorigenesis within each subtype are still poorly understood. Although aberrant NF-κB activity has been implicated in glioma, the roles of specific members of this protein family in tumorigenesis and pathogenesis have not been elucidated. In this study, we show that the NF-κB protein RelB is expressed in a particularly aggressive mesenchymal subtype of glioma, and loss of RelB significantly attenuated glioma cell survival, motility and invasion. We find that RelB promotes the expression of mesenchymal genes including YKL-40, a marker of the MES glioma subtype. Additionally, RelB regulates expression of Olig2, a regulator of cancer stem cell proliferation and a candidate marker for the cell of origin in glioma. Furthermore, loss of RelB in glioma cells significantly diminished tumor growth in orthotopic mouse xenografts. The relevance of our studies for human disease was confirmed by analysis of a human GBM genome database, which revealed that high RelB expression strongly correlates with rapid tumor progression and poor patient survival rates. Thus, our findings demonstrate that RelB is an oncogenic driver of mesenchymal glioma tumor growth and invasion, highlighting the therapeutic potential of inhibiting the noncanonical NF-κB (RelB-mediated) pathway to treat these deadly tumors. 
 RelB NF-kappaB represses estrogen receptor alpha expression via induction of the zinc finger protein Blimp1. Aberrant constitutive expression of NF-kappaB subunits, reported in more than 90% of breast cancers and multiple other malignancies, plays pivotal roles in tumorigenesis. Higher RelB subunit expression was demonstrated in estrogen receptor alpha (ERalpha)-negative breast cancers versus ERalpha-positive ones, due in part to repression of RelB synthesis by ERalpha signaling. Notably, RelB promoted a more   invasive   phenotype in ERalpha-negative cancers via induction of the BCL2 gene. We report here that RelB reciprocally inhibits ERalpha synthesis in breast cancer cells, which contributes to a more migratory phenotype. Specifically, RelB is shown for the first time to induce expression of the zinc finger repressor protein Blimp1 (B-lymphocyte-induced maturation protein), the critical mediator of B- and T-cell development, which is transcribed from the PRDM1 gene. Blimp1 protein repressed ERalpha (ESR1) gene transcription. Commensurately higher Blimp1/PRDM1 expression was detected in ERalpha-negative breast cancer cells and primary breast tumors. Induction of PRDM1 gene expression was mediated by interaction of Bcl-2, localized in the mitochondria, with Ras. Thus, the induction of Blimp1 represents a novel mechanism whereby the RelB NF-kappaB subunit mediates repression, specifically of ERalpha, thereby promoting a more migratory phenotype. 
 Inhibition of RelB by 1,25-dihydroxyvitamin D3 promotes sensitivity of breast cancer cells to radiation. Aberrant constitutive expression of the NF-kappaB c-Rel and RelA subunits in breast cancer cells was shown to promote their survival. Recently, we demonstrated that aggressive breast cancers constitutively express high levels of the RelB subunit, which promotes their more   invasive   phenotype via induction of the BCL2 gene. As these cancers are frequently resistant to therapy, here we tested the hypothesis that RelB promotes their survival. High RelB expressing Hs578T and MDA-MB-231 breast cancer cells were more resistant to gamma-radiation than MCF7 and ZR-75 cells, which express lower RelB levels. Knockdown of RelB in Hs578T led to decreased survival in response to gamma-irradiation, while conversely ectopic expression of RelB in MCF7 cells protected these cells from radiation. Similar data were obtained upon treatment of Hs578T or MCF7 cells with the chemotherapeutic agent doxorubicin. High serum levels of 25-hydroxyvitamin D are associated with decreased breast cancer risk and mortality, although, the mechanisms of its protective actions have not been fully elucidated. Treatment of Hs578T and Her-2/neu-driven NF639 cells with 1,25-dihydroxyvitamin D3 decreased RelB/RELB gene expression and levels of pro-survival targets Survivin, MnSOD and Bcl-2, while increasing their sensitivity to gamma-irradiation. Thus, RelB, which promotes survival and a more highly   invasive   phenotype of breast cancer cells, is a target of 1,25-dihydroxyvitamin D3, providing one mechanism for the observed protective role of 25-hydroxyvitamin D in patients with breast cancer. 
  [Mm]etastasis  PubMed  
 Comprehensive Evaluation of Nuclear Factor-κΒ Expression Patterns in Non-Small Cell Lung Cancer. Nuclear factor (NF)-κB signalling is required for lung adenocarcinoma development in mice, and both of its subunits RelA and RelB were independently reported to be highly expressed in human non-small cell lung cancer (NSCLC). To comprehensively examine NF-κB expression in NSCLC, we analyzed serial sections of primary tumor samples from 77 well-documented patients (36 adenocarcinomas, 40 squamous cell carcinomas and 3 large cell carcinomas) for immunoreactivity of RelA, RelB, P50, and P52/P100. Tumor and intratumoral stroma areas were discriminated based on proliferating cell nuclear antigen immunoreactivity and inflammatory infiltration was assessed in intratumoral stroma areas. NF-κB immunoreactivity was quantified by intensity, extent, and nuclear localization and was cross-examined with tumor cell proliferation, inflammatory infiltration, and clinical-pathologic data. We found that the expression of the different NF-κB subunits was not concordant, warranting our integral approach. Overall, RelA, RelB, and P50 were expressed at higher levels compared with P52/P100. However, RelA and P50 were predominantly expressed in intratumoral stroma, but RelB in tumor cells. Importantly, tumor area RelA expression was correlated with the intensity of inflammatory infiltration, whereas RelB expression was identified in proliferating tumor cells. Using multiple logistic regression, we identified that tumor RelB expression was an independent predictor of lymph   node meta  stasis, and tumor P50 was an independent predictor of TNM6 stage IIB or higher, whereas tumor RelA was an independent predictor of inflammatory infiltration. We conclude that pathologic studies of NF-κB expression in cancer should include multiple pathway components. Utilizing such an approach, we identified intriguing associations between distinct NF-κB subunits and clinical and pathologic features of NSCLC. 
 Comprehensive Evaluation of Nuclear Factor-κΒ Expression Patterns in Non-Small Cell Lung Cancer. Nuclear factor (NF)-κB signalling is required for lung adenocarcinoma development in mice, and both of its subunits RelA and RelB were independently reported to be highly expressed in human non-small cell lung cancer (NSCLC). To comprehensively examine NF-κB expression in NSCLC, we analyzed serial sections of primary tumor samples from 77 well-documented patients (36 adenocarcinomas, 40 squamous cell carcinomas and 3 large cell carcinomas) for immunoreactivity of RelA, RelB, P50, and P52/P100. Tumor and intratumoral stroma areas were discriminated based on proliferating cell nuclear antigen immunoreactivity and inflammatory infiltration was assessed in intratumoral stroma areas. NF-κB immunoreactivity was quantified by intensity, extent, and nuclear localization and was cross-examined with tumor cell proliferation, inflammatory infiltration, and clinical-pathologic data. We found that the expression of the different NF-κB subunits was not concordant, warranting our integral approach. Overall, RelA, RelB, and P50 were expressed at higher levels compared with P52/P100. However, RelA and P50 were predominantly expressed in intratumoral stroma, but RelB in tumor cells. Importantly, tumor area RelA expression was correlated with the intensity of inflammatory infiltration, whereas RelB expression was identified in proliferating tumor cells. Using multiple logistic regression, we identified that tumor RelB expression was an independent predictor of lymph   node meta  stasis, and tumor P50 was an independent predictor of TNM6 stage IIB or higher, whereas tumor RelA was an independent predictor of inflammatory infiltration. We conclude that pathologic studies of NF-κB expression in cancer should include multiple pathway components. Utilizing such an approach, we identified intriguing associations between distinct NF-κB subunits and clinical and pathologic features of NSCLC. 
  GeneRIFs  
 In non-small cell lung cancer, RelB expression was identified in proliferating tumor cells. tumor RelB expression was an independent predictor of lymph node   metastasis   
   
  
 PHLDB1 
 
  NCBI Gene  
  GeneRIF  
  Pubmed  
 
 
  Search Term  Rank  Fields  
  [Mm]igration  6  PubMed(1)  
  [Ee]xtracellular [Mm]atrix  8  PubMed(1)  
  [Ii]nvasive  10  PubMed(1)  
  [Cc]ell [Ii]nvasion  13  PubMed(1)  
 
 
 hide/show details for PHLDB1 
  [Mm]igration  PubMed  
 Liprin-α1, ERC1 and LL5 define polarized and dynamic structures that are implicated in cell   migratio  n. Cell   migratio  n during development and metastatic invasion requires the coordination of actin and adhesion dynamics to promote protrusive activity at the front of the cell. The knowledge of the molecular mechanisms required to achieve such coordination is fragmentary. Here, we identify a new functional complex that drives cell motility. ERC1a (an isoform of ERC1) and the LL5 proteins LL5α and LL5β (encoded by PHLDB1 and PHLDB2, respectively) are required, together with liprin-α1, for effect  ive migra  tion and tumor cell invasion, and do so by stabilizing the protrusive activity at the cell front. Depletion of either protein negatively affects invasi  on, migra  tion on extracellular matrix, lamellipodial persistence and the internalization of active integrin β1 receptors needed for adhesion turnover at the front of the cell. Liprin-α1, ERC1a and LL5 also define new highly polarized and dynamic cytoplasmic structures uniquely localized near the protruding cell edge. Our results indicate that the functional complex and the associated structures described here represent an important mechanism to drive tumor   cell mig  ration. 
  [Ee]xtracellular [Mm]atrix  PubMed  
 Liprin-α1, ERC1 and LL5 define polarized and dynamic structures that are implicated in cell migration. Cell migration during development and metastatic invasion requires the coordination of actin and adhesion dynamics to promote protrusive activity at the front of the cell. The knowledge of the molecular mechanisms required to achieve such coordination is fragmentary. Here, we identify a new functional complex that drives cell motility. ERC1a (an isoform of ERC1) and the LL5 proteins LL5α and LL5β (encoded by PHLDB1 and PHLDB2, respectively) are required, together with liprin-α1, for effective migration and tumor cell invasion, and do so by stabilizing the protrusive activity at the cell front. Depletion of either protein negatively affects invasion, migration   on extracellular ma  trix, lamellipodial persistence and the internalization of active integrin β1 receptors needed for adhesion turnover at the front of the cell. Liprin-α1, ERC1a and LL5 also define new highly polarized and dynamic cytoplasmic structures uniquely localized near the protruding cell edge. Our results indicate that the functional complex and the associated structures described here represent an important mechanism to drive tumor cell migration. 
  [Ii]nvasive  PubMed  
 Association between RTEL1, PHLDB1, and TREH Polymorphisms and Glioblastoma Risk: A Case-Control Study. Glioblastoma (GBM) is a highly   invasive  , aggressive, and incurable brain tumor. Genetic factors play important roles in GBM risk. The aim of this study was to elucidate the influence of gene polymorphism on GBM susceptibility.In this case-control study, we included 72 GBM patients and 320 healthy controls to analyze the association between 29 single-nucleotide polymorphisms and GBM cancer risk in the Chinese Han population. The single-nucleotide polymorphisms were determined by Sequenom MassARRAY RS1000 and statistical analysis was performed using SPSS software and SNPStats software.Using the χ(2) test, we found that rs2297440 and rs6010620 in RTEL1 increased risk of GBM. In the recessive model, we also found that the genotypes "CC" of rs2297440 and "GG" of rs6010620 in RTEL1 significantly increased GBM risk. The variant TT genotype of TREH rs17748 and the variant TT genotype of PHLDB1 rs498872 decreased GBM risk in the recessive model. We also found that the TREH rs17748 variant C allele showed an increased risk in males in the dominant model.Our results suggest a significant association between the RETL1, TREH, and PHLDB1 genes and GBM development in the Han Chinese population. 
  [Cc]ell [Ii]nvasion  PubMed  
 Liprin-α1, ERC1 and LL5 define polarized and dynamic structures that are implicated in cell migration. Cell migration during development and metastatic invasion requires the coordination of actin and adhesion dynamics to promote protrusive activity at the front of the cell. The knowledge of the molecular mechanisms required to achieve such coordination is fragmentary. Here, we identify a new functional complex that drives cell motility. ERC1a (an isoform of ERC1) and the LL5 proteins LL5α and LL5β (encoded by PHLDB1 and PHLDB2, respectively) are required, together with liprin-α1, for effective migration and tu  mor cell inva  sion, and do so by stabilizing the protrusive activity at the cell front. Depletion of either protein negatively affects invasion, migration on extracellular matrix, lamellipodial persistence and the internalization of active integrin β1 receptors needed for adhesion turnover at the front of the cell. Liprin-α1, ERC1a and LL5 also define new highly polarized and dynamic cytoplasmic structures uniquely localized near the protruding cell edge. Our results indicate that the functional complex and the associated structures described here represent an important mechanism to drive tumor cell migration. 
   
  
 EGFL7 
 
  NCBI Gene  
  GeneRIF  
  Pubmed  
 
 
  Search Term  Rank  Fields  
  [Mm]igration  6  PubMed(13); GeneRIFs(6)  
  ECM  7  PubMed(1)  
  [Ee]xtracellular [Mm]atrix  8  PubMed(3); Component(1)  
  [Ii]nvasive  10  PubMed(5)  
  [Mm]etastasize  11  PubMed(1)  
  [Mm]etastasis  12  PubMed(10); GeneRIFs(4)  
  [Cc]ell [Ii]nvasion  13  PubMed(2)  
 
 
 hide/show details for EGFL7 
  [Mm]igration  PubMed  
 EGFL7-overexpressing epidermal stem cells promotes fibroblast proliferation and   migration   via mediating cell adhesion and strengthening cytoskeleton. Epidermal growth factor (EGF)-like family members mediate a wide range of biological activities including cell proliferation and   migration  . Increasing evidence indicated that EGF plays an important role in the process of wound healing by stimulating fibroblast motility. The aim of this study was to see whether EGF-like domain 7 (EGFL7)-overexpressing epidermal stem cells (EGFL7-ESCs) would promote fibroblast proliferation and   migration  . We found that mRNA and protein levels of EGFL7 expression were significantly increased in EGFL7-ESCs. The protein expression of EGFL7 was significantly elevated in conditioned media (CM) of EGFL7-ESCs compared to ESCs CM or vector-ESCs CM. The cell count and cell viability of EGFL7-ESCs CM-treated fibroblasts were also significantly increased compared to control. In addition, EGFL7-ESCs CM-treated fibroblasts showed elevated   migration   compared with control. Moreover, the expressions of β1-integrin, β-tubulin, β-actin, and Vimentin were increased, while that of E-cadherin was decreased in EGFL7-ESCs CM-treated fibroblasts. These results indicate that EGFL7-ESCs contribute towards promoting fibrobla  st migrat  ion through enhancing cell adhesion, strengthening cytoskeleton, and reducing intercellular aggregation. These findings suggest that the stimulating effect of EGFL7-ESCs on fibroblast proliferation a  nd migrat  ion may provide a useful strategy for wound healing. 
 The endothelial-cell-derived secreted factor Egfl7 regulates vascular tube formation. Vascular development is a complex but orderly process that is tightly regulated. A number of secreted factors produced by surrounding cells regulate endothelial cell (EC) differentiation, proliferation,   migration   and coalescence into cord-like structures. Vascular cords then undergo tubulogenesis to form vessels with a central lumen. But little is known about how tubulogenesis is regulated in vivo. Here we report the identification and characterization of a new EC-derived secreted factor, EGF-like domain 7 (Egfl7). Egfl7 is expressed at high levels in the vasculature associated with tissue proliferation, and is downregulated in most of the mature vessels in normal adult tissues. Loss of Egfl7 function in zebrafish embryos specifically blocks vascular tubulogenesis. We uncover a dynamic process during which gradual separation and proper spatial arrangement of the angioblasts allow subsequent assembly of vascular tubes. This process fails to take place in Egfl7 knockdown embryos, leading to the failure of vascular tube formation. Our study defines a regulator that controls a specific and important step in vasculogenesis. 
 Targeting EGFL7 expression through RNA interference suppresses renal cell carcinoma growth by inhibiting angiogenesis. Renal cell carcinoma (RCC) is the most lethal of all urological cancers and tumor angiogenesis is closely related with its growth, invasion, and metastasis. Recent studies have suggested that epidermal growth factor-like domain multiple 7 (EGFL7) is overexpressed by many tumors, such as colorectal cancer and hepatocellular carcinoma; it is also correlated with progression, metastasis, and a poor prognosis. However, the role of EGFL7 in RCC is not clear. In this study, we examined how EGFL7 contributes to the growth of RCC using a co-culture system in vitro and a xenograft model in vivo. Downregulated EGFL7 expression in RCC cells affected the   migration   and tubule formation of HMEC-1 cells, but not their growth and apoptosis in vitro. The level of focal adhesion kinase (FAK) phosphorylation in HMEC-1 cells decreased significantly when co-cultured with 786-0/iEGFL7 cells compared with 786-0 cells. After adding rhEGFL7, the level of FAK phosphorylation in HMEC-1 cells was significantly elevated compared with phosphate-buffered saline (PBS) control. However, FAK phosphorylation was abrogated by EGFR inhibition. The average size of RCC local tumors in the 786-0/iEGFL7 group was noticeably smaller than those in the 786-0 cell group and their vascular density was also significantly decreased. These data suggest that EGFL7 has an important function in the growth of RCC by facilitating angiogenesis. 
 Epidermal growth factor-like domain 7 promotes   migration   and invasion of human trophoblast cells through activation of MAPK, PI3K and NOTCH signaling pathways. Epidermal growth factor-like domain 7 (Egfl7) is a gene that encodes a partially secreted protein and whose expression is largely restricted to the endothelia. We recently reported that EGFL7 is also expressed by trophoblast cells in mouse and human placentas. Here, we investigated the molecular pathways that are regulated by EGFL7 in trophoblast cells. Stable EGFL7 overexpression in a Jeg3 human choriocarcinoma cell line resulted in significantly increased cell   migration   and invasiveness, while cell proliferation was unaffected. Analysis of mitogen-activated protein kinase (MAPK) and phosphatidylinositol 3-kinase (PI3K) pathways showed that EGFL7 promotes Jeg3 cell motility by activating both pathways. We show that EGFL7 activates the epidermal growth factor receptor (EGFR) in Jeg3 cells, resulting in downstream activation of extracellular regulated kinases (ERKs). In addition, we provide evidence that EGFL7-triggered   migration   of Jeg3 cells involves activation of NOTCH signaling. EGFL7 and NOTCH1 are co-expressed in Jeg3 cells, and blocking of NOTCH activation abrogates enhanced   migration   of Jeg3 cells overexpressing EGFL7. We also demonstrate that signaling through EGFR and NOTCH converged to mediate EGFL7 effects. Reduction of endogenous EGFL7 expression in Jeg3 cells significantly decreased cell   migration  . We further confirmed that EGFL7 stimulates cell   migration   by using primary human first trimester trophoblast (PTB) cells overexpressing EGFL7. In conclusion, our data suggest that in trophoblast cells, EGFL7 regulates cell   migration   and invasion by activating multiple signaling pathways. Our results provide a possible explanation for the correlation between reduced expression of EGFL7 and inadequate trophoblast invasion observed in placentopathies. 
 Overexpressed MALAT1 promotes invasion and metastasis of gastric cancer cells via increasing EGFL7 expression. Long non-coding RNAs (lncRNAs) have been demonstrated to participate in various cancers. Here, the role and its potential mechanism of MALAT1 in invasion and   migration   of gastric cancer (GC) were investigated.Gastric adenocarcinoma tissues and matched normal adjacent tissues were isolated from 25 patients with GC. The expression of epidermal growth factor-like domain-containing protein 7 (EGFL7) was detected in the normal gastric mucosa epithelial GES-1 cell line and three different differentiation GC cell lines, including MKN28 (well-differentiated adenocarcinoma), SGC7901 (moderately differentiated adenocarcinoma) and BGC823 (poorly differentiated adenocarcinoma). Tumor xenotransplant mouse model was established with the injection of cell line pretreated with lentiviral vectors for si-MALAT1 or si-control.The expression of MALAT1 was up-regulated in GC tissues and three cell lines. Si-MALAT1/pcDNA-MALAT1 induced the decrease of cell invasion and   migration  , while the effects were reversed by the transfection of pcDNA-EGFL7/si-EGFL7. ChIP assay showed that MALAT1 regulated EGFL7 expression by altering the level of H3 histone acetylation in EGFL7 promoter. In tumor xenotransplant mice, down-regulated MALAT1 contributed to the inhibition of tumor metastasis.Up-regulated MALAT1 promoted the invasion and metastasis of GC, and the increase of EGFL7 expression was a potential mechanism via altering its H3 histone acetylation level. 
 miR-126 inhibits non-small cell lung cancer cells proliferation by targeting EGFL7. MicroRNAs (miRNAs) represent an abundant group of small non-coding RNAs that regulate gene expression, and have been demonstrated to play roles as tumor suppressor genes (oncogenes), and affect homeostatic processes such as development, cell proliferation, and cell death. Subsequently, epidermal growth factor-like domain 7 (EGFL7), which is confirmed to be involved in cellular responses such as cell   migration   and blood vessel formation, is identified as a potential miR-126 target by bioinformatics. However, there is still no evidence showing EGFL7's relationship with miR-126 and the proliferation of lung cancer cells. The aim of this work is to investigate whether miR-126, together with EGFL7, have an effect on non-small cell lung cancer (NSCLC) cells' proliferation. Therefore, we constructed overexpressed miR-126 plasmid to target EGFL7 and transfected them into NSCLC cell line A549 cells. Then, we used methods like quantitative RT-PCR, Western blot, flow cytometry assay, and immunohistochemistry staining to confirm our findings. The result was that overexpression of miR-126 in A549 cells could increase EGFL7 expression. Furthermore, the most notable finding by cell proliferation related assays is that miR-126 can inhibit A549 cells proliferation in vitro and inhibit tumor growth in vivo by targeting EGFL7. As a result, our study demonstrates that miR-126 can inhibit proliferation of non-small cell lung cancer cells through one of its targets, EGFL7. 
 Epidermal growth factor-like domain-containing protein 7 (EGFL7) enhances EGF receptor-AKT signaling, epithelial-mesenchymal transition, and metastasis of gastric cancer cells. Epidermal growth factor-like domain-containing protein 7 (EGFL7) is upregulated in human epithelial tumors and so is a potential biomarker for malignancy. Indeed, previous studies have shown that high EGFL7 expression promotes infiltration and metastasis of gastric carcinoma. The epithelial-mesenchymal transition (EMT) initiates the metastatic cascade and endows cancer cells with invasive and migratory capacity; however, it is not known if EGFL7 promotes metastasis by triggering EMT. We found that EGFL7 was overexpressed in multiple human gastric cancer (GC) cell lines and that overexpression promoted cell invasion and   migration   as revealed by scratch wound and transwell   migration   assays. Conversely, shRNA-mediated EGFL7 knockdown reduced invasion and   migration  . Furthermore, EGFL7-overexpressing cells grew into larger tumors and were more likely to metastasize to the liver compared to underexpressing CG cells following subcutaneous injection in mice. EGFL7 overexpression protected GC cell lines against anoikis, providing a plausible mechanism for this enhanced metastatic capacity. In excised human gastric tumors, expression of EGFL7 was positively correlated with expression levels of the mesenchymal marker vimentin and the EMT-associated transcription repressor Snail, and negatively correlated with expression of the epithelial cell marker E-cadherin. In GC cell lines, EGFL7 knockdown reversed morphological signs of EMT and decreased both vimentin and Snail expression. In addition, EGFL7 overexpression promoted EGF receptor (EGFR) and protein kinase B (AKT) phospho-activation, effects markedly suppressed by the EGFR tyrosine kinase inhibitor AG1478. Moreover, AG1478 also reduced the elevated invasive and migratory capacity of GC cell lines overexpressing EGFL7. Collectively, these results strongly suggest that EGFL7 promotes metastasis by activating EMT through an EGFR-AKT-Snail signaling pathway. Disruption of EGFL7-EGFR-AKT-Snail signaling may a promising therapeutic strategy for gastric cancer. 
 miR-126 and miR-126*: new players in cancer. Cancer progression is characterized by autarky in growth signals, insensitivity to growth-restrictive signals, evasion of apoptosis, a limitless potential to replicate, sustained angiogenesis, and tissue invasion, including metastasis. The regulation of these cellular processes relies on a fine-tuned control of molecular signal cascades. In recent years, short noncoding RNAs termed microRNAs (miRNAs) have been described as a novel class of molecular regulators. These affect various signaling cascades during the progression of neoplastic diseases by the regulation of gene expression on the post-transcriptional level. The novel endothelial cell-derived secreted protein epidermal growth factor-like domain 7 (EGFL7) has been suggested to control vascular tubulogenesis. Further, the two biologically active miRNAs miR-126 and its complement miR-126*, which are encoded by intron 7 of the egfl7 gene, have been described to mediate vascular functions. Knock-out studies in zebrafish and mice suggested a major role of miR-126 in angiogenesis and vascular integrity, which was mediated by the repression of inhibitors of VEGF-induced proliferation in endothelial cells. Recent studies revealed the distribution and function of miR-126 and miR-126* in various types of cancer, and assigned a role to both miRNAs as suppressors of tumor formation. Indeed, miR-126 and miR-126* have been reported to impair cancer progression through signaling pathways that control tumor cell proliferation,   migration  , invasion, and survival. Conversely, miR-126 and miR-126* may have a supportive role in the progression of cancer as well, which might be mediated by the promotion of blood vessel growth and inflammation. In this work, we will summarize the current knowledge on functions of miR-126/miR-126* that are relevant for cancer formation, and we will discuss their potential clinical use as predictive markers of survival and application as novel therapeutic targets for the treatment of neoplastic diseases. 
 VE-statin, an endothelial repressor of smooth muscle cell   migration  . The recruitment and proliferation of smooth muscle cells and pericytes are two key events for the stabilization of newly formed capillaries during angiogenesis and, when out of control in the adult, are the main causes of arteriosclerosis. We have identified a novel gene, named VE-statin for vascular endothelial-statin, which is expressed specifically by endothelial cells of the developing mouse embryo and in the adult, and in early endothelial progenitors. The mouse and human VE-statin genes have been located on chromosome 2 and 9, respectively, they span >10 kbp and are transcribed in two major variants arising from independent initiation sites. The VE-statin transcripts code for a unique protein of 30 kDa that contains a signal peptide and two epidermal growth factor (EGF)-like modules. VE-statin is found in the cellular endoplasmic reticulum and secreted in the cell supernatant. Secreted VE-statin inhibits platelet-derived growth factor (PDGF)-BB-induced smooth muscle cell   migration  , but has no effects on endothelial cell   migration  . VE-statin is the first identified inhibitor of mural cell   migration   specifically produced by endothelial cells. 
 Suppression of the Epidermal Growth Factor-like Domain 7 and Inhibition of   Migration   and Epithelial-Mesenchymal Transition in Human Pancreatic Cancer PANC-1 Cells. Epidermal growth factor-like domain multiple 7 (EGFL7), a secreted protein specifically expressed by endothelial cells during embryogenesis, recently was identified as a critical gene in tumor metastasis. Epithelial-mesenchymal transition (EMT) was found to be closely related with tumor progression. Accordingly, it is important to investigate the   migration   and EMT change after knock-down of EGFL7 gene expression in human pancreatic cancer cells.EGFL7 expression was firstly testified in 4 pancreatic cancer cell lines by real-time polymerase chain reaction (Real-time PCR) and western blot, and the highest expression of EGFL7 was found in PANC-1 cell line. Then, PANC-1 cells transfected with small interference RNA (siRNA) of EGFL7 using plasmid vector were named si-PANC-1, while transfected with negative control plasmid vector were called NC-PANC-1. Transwell assay was used to analyze the   migration   of PANC-1 cells. Real-time PCR and western blotting were used to detect the expression change of EGFL7 gene, EMT markers like E-Cadherin, N-Cadherin, Vimentin, Fibronectin and transcription factors like snail, slug in PANC-1, NC- PANC-1, and si-PANC-1 cells, respectively.After successful plasmid transfection, EGFL7 gene were dramatically knock-down by RNA interference in si-PANC-1 group. Meanwhile,   migration   ability decreased significantly, compared with PANC-1 and NC-PANC-1 group. Meanwhile, the expression of epithelial phenotype marker E-Cadherin increased and that of mesenchymal phenotype markers N-Cadherin, Vimentin, Fibronectin dramatically decreased in si-PANC-1 group, indicating a reversion of EMT. Also, transcription factors snail and slug decreased significantly after RNA interference.Current study suggested that highly-expressed EGFL7 promotes   migration   of PANC-1 cells and acts through transcription factors snail and slug to induce EMT, and further study is needed to confirm this issue. 
 Impaired angiogenesis and altered Notch signaling in mice overexpressing endothelial Egfl7. Epidermal growth factor-like domain 7 (Egfl7) is important for regulating tubulogenesis in zebrafish, but its role in mammals remains unresolved. We show here that endothelial overexpression of Egfl7 in transgenic mice leads to partial lethality, hemorrhaging, and altered cardiac morphogenesis. These defects are accompanied by abnormal vascular patterning and remodeling in both the embryonic and postnatal vasculature. Egfl7 overexpression in the neonatal retina results in a hyperangiogenic response, and EGFL7 knockdown in human primary endothelial cells suppresses endothelial cell proliferation, sprouting, and   migration  . These phenotypes are reminiscent of Notch inhibition. In addition, our results show that EGFL7 and endothelial-specific NOTCH physically interact in vivo and strongly suggest that Egfl7 antagonizes Notch in both the postnatal retina and in primary endothelial cells. Specifically, Egfl7 inhibits Notch reporter activity and down-regulates the level of Notch target genes when overexpressed. In conclusion, we have uncovered a critical role for Egfl7 in vascular development and have shown that some of these functions are mediated through modulation of Notch signaling. 
 Novel role for epidermal growth factor-like domain 7 in metastasis of human hepatocellular carcinoma. Epidermal growth factor-like domain 7 (Egfl7) is a recently identified secreted protein that is believed to be primarily expressed in endothelial cells (ECs). Although its expression was reported elevated during tumorigenesis, whether and how Egfl7 contributes to human malignancies remains unknown. In the present study overexpression of Egfl7 was found predominantly in hepatocellular carcinoma (HCC) cells in HCC tissues and closely correlated with poor prognosis of HCC. The expression of Egfl7 in cancer cells was further verified with HCC cell lines including HepG2, MHCC97-L, and HCCLM3, and the Egfl7 expression levels positively correlated with metastatic potential of HCC cell lines was tested. To functionally characterize Egfl7 in HCC, we depleted its expression in HCCLM3 cells by using small interfering RNA. Interestingly, reduction of Egfl7 expression resulted in significant inhibition of   migration   but not growth of HCCLM3 cells. Biochemical analysis indicated that Egfl7 could facilitate the phosphorylation of focal adhesion kinase (FAK) and therefore promote the   migration   of HCCLM3 cells. In addition, this effect was almost completely blocked by inhibition of epidermal growth factor receptor (EGFR), indicating that the activation of FAK by Egfl7 is mediated through EGFR. Finally, we used a mouse model to demonstrate that down-regulation of Egfl7 was associated with suppression of intrahepatic and pulmonary metastases of HCC. Collectively, our study shows for the first time that overexpression of Egfl7 in HCC and Egfl7 promotes metastasis of HCC by enhancing cell motility through EGFR-dependent FAK phosphorylation.Our study suggests Egfl7 as a novel prognostic marker for metastasis of HCC and a potential therapeutic target. 
 Ectopic expression of miR-126*, an intronic product of the vascular endothelial EGF-like 7 gene, regulates prostein translation and invasiveness of prostate cancer LNCaP cells. MicroRNAs (miRNAs) are endogenous noncoding RNAs that down-regulate gene expression by promoting cleavage or translational arrest of target mRNAs. While most miRNAs are transcribed from their own dedicated genes, some map to introns of 'host' transcripts, the biological significance of which remains unknown. Here, we show that prostate cells are naturally devoid of EGF-like domain 7 (Egfl7) transcripts and hence also deficient in a miRNA, miR-126*, generated from splicing and processing of its ninth intron. Use of recombinant and synthetic miRNAs or a specific antagomir established a role of miR-126* in silencing prostein in non-endothelial cells. We mapped two miR-126*-binding sites in the 3'UTR of the prostein mRNA required for translational repression. Transfection of synthetic miR-126* into prostate cancer LNCaP cells strongly reduced the translation of prostein. Interestingly, loss of prostein correlated with reduction of LNCaP cell   migration   and invasion. Thus, the robust expression of prostein protein in the prostate cells results from a combination of transcriptional activation of the prostein gene and absence of intronic miRNA-126* due to the prostate-specific repression of the Egfl7 gene. We conclude that intronic miRNAs from tissue-specific transcripts, or their natural absence, make cardinal contributions to cellular gene expression and phenotype. These findings also open the door to tissue-specific miRNA therapy. 
  GeneRIFs  
 These findings suggest that the stimulating effect of EGFL7-ESCs on fibroblast proliferation and   migration   may provide a useful strategy for wound healing. 
 High EGFL7 expression promotes   migration   and Epithelial-Mesenchymal Transition in pancreatic Cancer. 
 Data suggest that, in trophoblast cells, EGFL7 regulates cell   migration   and invasion/placentation by activating multiple signaling pathways via MAPK, PI3K (phosphatidylinositol 3-kinase), and NOTCH1 (translocation-associated notch protein 1). 
 EGFL7 promotes growth of Renal cell carcinoma by facilitating   migration   and tube formation of endothelial cells. These effects were produced by EGFL7-mediated focal adhesion kinase phosphorylation through combination with epidermal growth factor receptor. 
 Studies indicate that Egfl7 controls blood vessel development by promoting endothelial cell   migration   and proliferation. 
 is the first identified inhibitor of mural cell   migration   specifically produced by endothelial cells. 
  ECM  PubMed  
 Comparative proteomic analysis of supportive and unsupportive extracellular matrix substrates for human embryonic stem cell maintenance. Human embryonic stem cells (hESCs) are pluripotent cells that have indefinite replicative potential and the ability to differentiate into derivatives of all three germ layers. hESCs are conventionally grown on mitotically inactivated mouse embryonic fibroblasts (MEFs) or feeder cells of human origin. In addition, feeder-free culture systems can be used to support hESCs, in which the adhesive substrate plays a key role in the regulation of stem cell self-renewal or differentiation. Extracellular matrix (  ECM  ) components define the microenvironment of the niche for many types of stem cells, but their role in the maintenance of hESCs remains poorly understood. We used a proteomic approach to characterize in detail the composition and interaction networks of   ECM  s that support the growth of self-renewing hESCs. Whereas many   ECM   components were produced by supportive and unsupportive MEF and human placental stromal fibroblast feeder cells, some proteins were only expressed in supportive   ECM  , suggestive of a role in the maintenance of pluripotency. We show that identified candidate molecules can support attachment and self-renewal of hESCs alone (fibrillin-1) or in combination with fibronectin (perlecan, fibulin-2), in the absence of feeder cells. Together, these data highlight the importance of specific   ECM   interactions in the regulation of hESC phenotype and provide a resource for future studies of hESC self-renewal. 
  [Ee]xtracellular [Mm]atrix  PubMed  
 Comparative proteomic analysis of supportive and unsupportive   extracellular matrix   substrates for human embryonic stem cell maintenance. Human embryonic stem cells (hESCs) are pluripotent cells that have indefinite replicative potential and the ability to differentiate into derivatives of all three germ layers. hESCs are conventionally grown on mitotically inactivated mouse embryonic fibroblasts (MEFs) or feeder cells of human origin. In addition, feeder-free culture systems can be used to support hESCs, in which the adhesive substrate plays a key role in the regulation of stem cell self-renewal or differentiation.   Extracellular matrix   (ECM) components define the microenvironment of the niche for many types of stem cells, but their role in the maintenance of hESCs remains poorly understood. We used a proteomic approach to characterize in detail the composition and interaction networks of ECMs that support the growth of self-renewing hESCs. Whereas many ECM components were produced by supportive and unsupportive MEF and human placental stromal fibroblast feeder cells, some proteins were only expressed in supportive ECM, suggestive of a role in the maintenance of pluripotency. We show that identified candidate molecules can support attachment and self-renewal of hESCs alone (fibrillin-1) or in combination with fibronectin (perlecan, fibulin-2), in the absence of feeder cells. Together, these data highlight the importance of specific ECM interactions in the regulation of hESC phenotype and provide a resource for future studies of hESC self-renewal. 
 EGFL7 ligates αvβ3 integrin to enhance vessel formation. Angiogenesis, defined as blood vessel formation from a preexisting vasculature, is governed by multiple signal cascades including integrin receptors, in particular integrin αVβ3. Here we identify the endothelial cell (EC)-secreted factor epidermal growth factor-like protein 7 (EGFL7) as a novel specific ligand of integrin αVβ3, thus providing mechanistic insight into its proangiogenic actions in vitro and in vivo. Specifically, EGFL7 attaches t  o the extracellular   matrix and by its interaction with integrin αVβ3 increases the motility of EC, which allows EC to move on a sticky underground during vessel remodeling. We provide evidence that the deregulation of EGFL7 in zebrafish embryos leads to a severe integrin-dependent malformation of the caudal venous plexus, pointing toward the significance of EGFL7 in vessel development. In biopsy specimens of patients with neurologic diseases, vascular EGFL7 expression rose with increasing EC proliferation. Further, EGFL7 became upregulated in vessels of the stroke penumbra using a mouse model of reversible middle cerebral artery occlusion. Our data suggest that EGFL7 expression depends on the remodeling state of the existing vasculature rather than on the phenotype of neurologic disease analyzed. In sum, our work sheds a novel light on the molecular mechanism EGFL7 engages to govern physiological and pathological angiogenesis. 
 EGFL7: a unique angiogenic signaling factor in vascular development and disease. EGFL7 is a secreted angiogenic factor that is highly conserved in vertebrates. Most secreted angiogenic signaling molecules, including VEGF and fibroblast growth factor-2, are mainly expressed by non-endothelial cell types such as fibroblasts. In contrast, EGFL7 is unique because it is almost exclusively expressed by and acts on endothelial cells. Egfl7 expression is highest when the endothelium is in an active, proliferating state. This factor acts as a chemoattractant for endothelial cells and binds to components of the   extracellular matrix  . In vivo, Egfl7 is important for regulating tubulogenesis in zebrafish and for controlling vascular patterning and integrity in mice. Its function in blood vessel development is mediated, at least in part, through modulation of Notch signaling. In this review, we summarize the findings that support a role for Egfl7 in developmental and postnatal angiogenesis and describe the EGFL7-signaling pathways that underlie these processes. In addition, we discuss a potential role for EGFL7 in vascular repair and its possible use as a therapeutic target for treatment of hypoxia-induced injury. Finally, we consider EGFL7 action during tumorigenesis and its potential as an antiangiogenic agent. 
  Component  
   extracellular matrix   
  [Ii]nvasive  PubMed  
 MicroRNA-126 and epidermal growth factor-like domain 7-an angiogenic couple of importance in metastatic colorectal cancer. Results from the Nordic ACT trial. This study investigated the clinical importance of linked angiogenetic biomarkers to chemotherapy, combined with the anti-vascular endothelial growth factor A (anti-VEGF-A), as a first-line treatment in patients with metastatic colorectal cancer (mCRC).A total of 230 patients from a randomised phase III study were included. The primary microRNA-126 (pri-miRNA-126) A24G single-nucleotide polymorphism and the mature miRNA-126 were analysed by PCR using genomic DNA (full blood) and formalin-fixed paraffin-embedded tissue sections, respectively. The epidermal growth factor-like domain 7 (EGFL7) protein was visualised and quantified using immunohistochemistry.High tumour expression of miRNA-126 was significantly related to a longer progression-free survival. The independent prognostic value of miRNA-126 was confirmed using a Cox regression analysis (hazard ratio=0.49, 95% confidence interval=0.29-0.84, P=0.009). Although not significant, a relationship between EGFL7 expression and response rates is suggested, with EGFL7 expression at the   invasive   front being lower in responding patients than in the non-responders (P=0.063).The results validate the previous findings on the prognostic value of miRNA-126 in mCRC and may suggest a relationship between treatment efficacy and EGFL7 expression. As miRNA-126 may target VEGF-A as well as EGFL7, the results may provide predictive information in relation to next-generation anti-angiogenetics. 
 Epidermal growth factor-like domain 7 promotes migration and invasion of human trophoblast cells through activation of MAPK, PI3K and NOTCH signaling pathways. Epidermal growth factor-like domain 7 (Egfl7) is a gene that encodes a partially secreted protein and whose expression is largely restricted to the endothelia. We recently reported that EGFL7 is also expressed by trophoblast cells in mouse and human placentas. Here, we investigated the molecular pathways that are regulated by EGFL7 in trophoblast cells. Stable EGFL7 overexpression in a Jeg3 human choriocarcinoma cell line resulted in significantly increased cell migration and   invasive  ness, while cell proliferation was unaffected. Analysis of mitogen-activated protein kinase (MAPK) and phosphatidylinositol 3-kinase (PI3K) pathways showed that EGFL7 promotes Jeg3 cell motility by activating both pathways. We show that EGFL7 activates the epidermal growth factor receptor (EGFR) in Jeg3 cells, resulting in downstream activation of extracellular regulated kinases (ERKs). In addition, we provide evidence that EGFL7-triggered migration of Jeg3 cells involves activation of NOTCH signaling. EGFL7 and NOTCH1 are co-expressed in Jeg3 cells, and blocking of NOTCH activation abrogates enhanced migration of Jeg3 cells overexpressing EGFL7. We also demonstrate that signaling through EGFR and NOTCH converged to mediate EGFL7 effects. Reduction of endogenous EGFL7 expression in Jeg3 cells significantly decreased cell migration. We further confirmed that EGFL7 stimulates cell migration by using primary human first trimester trophoblast (PTB) cells overexpressing EGFL7. In conclusion, our data suggest that in trophoblast cells, EGFL7 regulates cell migration and invasion by activating multiple signaling pathways. Our results provide a possible explanation for the correlation between reduced expression of EGFL7 and inadequate trophoblast invasion observed in placentopathies. 
 Epidermal growth factor-like domain-containing protein 7 (EGFL7) enhances EGF receptor-AKT signaling, epithelial-mesenchymal transition, and metastasis of gastric cancer cells. Epidermal growth factor-like domain-containing protein 7 (EGFL7) is upregulated in human epithelial tumors and so is a potential biomarker for malignancy. Indeed, previous studies have shown that high EGFL7 expression promotes infiltration and metastasis of gastric carcinoma. The epithelial-mesenchymal transition (EMT) initiates the metastatic cascade and endows cancer cells with   invasive   and migratory capacity; however, it is not known if EGFL7 promotes metastasis by triggering EMT. We found that EGFL7 was overexpressed in multiple human gastric cancer (GC) cell lines and that overexpression promoted cell invasion and migration as revealed by scratch wound and transwell migration assays. Conversely, shRNA-mediated EGFL7 knockdown reduced invasion and migration. Furthermore, EGFL7-overexpressing cells grew into larger tumors and were more likely to metastasize to the liver compared to underexpressing CG cells following subcutaneous injection in mice. EGFL7 overexpression protected GC cell lines against anoikis, providing a plausible mechanism for this enhanced metastatic capacity. In excised human gastric tumors, expression of EGFL7 was positively correlated with expression levels of the mesenchymal marker vimentin and the EMT-associated transcription repressor Snail, and negatively correlated with expression of the epithelial cell marker E-cadherin. In GC cell lines, EGFL7 knockdown reversed morphological signs of EMT and decreased both vimentin and Snail expression. In addition, EGFL7 overexpression promoted EGF receptor (EGFR) and protein kinase B (AKT) phospho-activation, effects markedly suppressed by the EGFR tyrosine kinase inhibitor AG1478. Moreover, AG1478 also reduced the elevated   invasive   and migratory capacity of GC cell lines overexpressing EGFL7. Collectively, these results strongly suggest that EGFL7 promotes metastasis by activating EMT through an EGFR-AKT-Snail signaling pathway. Disruption of EGFL7-EGFR-AKT-Snail signaling may a promising therapeutic strategy for gastric cancer. 
 Expression of Egfl7 correlates with low-grade   invasive   lesions in human breast cancer. Egfl7 (VE-statin) is specifically expressed by endothelial cells of normal tissues but its expression is deregulated in human cancers. Analysis of expression of Egfl7 protein and transcripts in 211 human breast cancer samples shows that Egfl7 is strongly expressed by breast tumor cells. Egfl7 expression is significantly higher in   invasive   ductal than in   invasive   lobular carcinoma. Expression of Egfl7 transcripts is also higher in lower SBR grade lesions and in lesions which are not associated with lymph node invasion. Within the   invasive   ductal carcinoma sub-population, expression of Egfl7 transcripts is correlated with the SBR score and with the ER+ status. High transcript and Egfl7 protein levels significantly correlate with the absence of axillary lymph node invasion. In lymph nodes, the levels of Egfl7 are correlated with the histological type of the primary lesions; they are higher in ductal than in lobular carcinoma. Egfl7 expression is thus associated with better prognosis factors and with the absence of lymph node invasion in human breast cancer lesions. 
 Ectopic expression of miR-126*, an intronic product of the vascular endothelial EGF-like 7 gene, regulates prostein translation and   invasive  ness of prostate cancer LNCaP cells. MicroRNAs (miRNAs) are endogenous noncoding RNAs that down-regulate gene expression by promoting cleavage or translational arrest of target mRNAs. While most miRNAs are transcribed from their own dedicated genes, some map to introns of 'host' transcripts, the biological significance of which remains unknown. Here, we show that prostate cells are naturally devoid of EGF-like domain 7 (Egfl7) transcripts and hence also deficient in a miRNA, miR-126*, generated from splicing and processing of its ninth intron. Use of recombinant and synthetic miRNAs or a specific antagomir established a role of miR-126* in silencing prostein in non-endothelial cells. We mapped two miR-126*-binding sites in the 3'UTR of the prostein mRNA required for translational repression. Transfection of synthetic miR-126* into prostate cancer LNCaP cells strongly reduced the translation of prostein. Interestingly, loss of prostein correlated with reduction of LNCaP cell migration and invasion. Thus, the robust expression of prostein protein in the prostate cells results from a combination of transcriptional activation of the prostein gene and absence of intronic miRNA-126* due to the prostate-specific repression of the Egfl7 gene. We conclude that intronic miRNAs from tissue-specific transcripts, or their natural absence, make cardinal contributions to cellular gene expression and phenotype. These findings also open the door to tissue-specific miRNA therapy. 
  [Mm]etastasize  PubMed  
 Epidermal growth factor-like domain-containing protein 7 (EGFL7) enhances EGF receptor-AKT signaling, epithelial-mesenchymal transition, and metastasis of gastric cancer cells. Epidermal growth factor-like domain-containing protein 7 (EGFL7) is upregulated in human epithelial tumors and so is a potential biomarker for malignancy. Indeed, previous studies have shown that high EGFL7 expression promotes infiltration and metastasis of gastric carcinoma. The epithelial-mesenchymal transition (EMT) initiates the metastatic cascade and endows cancer cells with invasive and migratory capacity; however, it is not known if EGFL7 promotes metastasis by triggering EMT. We found that EGFL7 was overexpressed in multiple human gastric cancer (GC) cell lines and that overexpression promoted cell invasion and migration as revealed by scratch wound and transwell migration assays. Conversely, shRNA-mediated EGFL7 knockdown reduced invasion and migration. Furthermore, EGFL7-overexpressing cells grew into larger tumors and were more likely to   metastasize   to the liver compared to underexpressing CG cells following subcutaneous injection in mice. EGFL7 overexpression protected GC cell lines against anoikis, providing a plausible mechanism for this enhanced metastatic capacity. In excised human gastric tumors, expression of EGFL7 was positively correlated with expression levels of the mesenchymal marker vimentin and the EMT-associated transcription repressor Snail, and negatively correlated with expression of the epithelial cell marker E-cadherin. In GC cell lines, EGFL7 knockdown reversed morphological signs of EMT and decreased both vimentin and Snail expression. In addition, EGFL7 overexpression promoted EGF receptor (EGFR) and protein kinase B (AKT) phospho-activation, effects markedly suppressed by the EGFR tyrosine kinase inhibitor AG1478. Moreover, AG1478 also reduced the elevated invasive and migratory capacity of GC cell lines overexpressing EGFL7. Collectively, these results strongly suggest that EGFL7 promotes metastasis by activating EMT through an EGFR-AKT-Snail signaling pathway. Disruption of EGFL7-EGFR-AKT-Snail signaling may a promising therapeutic strategy for gastric cancer. 
  [Mm]etastasis  PubMed  
 Multivariate analysis of molecular markers in peripheral blood associated with recurrence and   metastasis   of hepatocellular carcinoma. Invasion,   metastasis  , and recurrence are the most common causes of death in patients with hepatocellular carcinoma (HCC) and are therefore critical factors for both therapy and prognosis. Current methods for diagnosis of HCC rely mainly on serological markers such as alpha-fetoprotein and liver enzymes, together with physical assessment and imaging techniques. The availability of more accurate serum markers may facilitate screening and early diagnosis, which will improve prognosis. This retrospective cohort analysis included 50 consecutive patients with cirrhosis and single or multifocal HCC and 40 control subjects with no liver disease or risk factors for viral hepatitis. Expression of epidermal growth factor-like domain 7 (EGFL7), osteopontin (OPN), and prostaglandin E2 (PGE2) were detected using an enzyme-linked immunosorbent assay. The mean serum levels of EGFL7, OPN, and PGE2 in the HCC group were 132.11 pg/mL, 11.77 ng/mL, and 179.37 pg/mL, respectively, which were all significantly higher than the levels in the control group (23.03 pg/mL, 2.31 ng/mL, and 47.36 pg/mL, respectively; P < 0.001). Serum levels of EGFL7, OPN, and PGE2 levels may thus be useful for screening and surveillance of HCC among high-risk populations, and have the potential to improve prognosis of these patients. 
 Targeting EGFL7 expression through RNA interference suppresses renal cell carcinoma growth by inhibiting angiogenesis. Renal cell carcinoma (RCC) is the most lethal of all urological cancers and tumor angiogenesis is closely related with its growth, invasion, and   metastasis  . Recent studies have suggested that epidermal growth factor-like domain multiple 7 (EGFL7) is overexpressed by many tumors, such as colorectal cancer and hepatocellular carcinoma; it is also correlated with progression,   metastasis  , and a poor prognosis. However, the role of EGFL7 in RCC is not clear. In this study, we examined how EGFL7 contributes to the growth of RCC using a co-culture system in vitro and a xenograft model in vivo. Downregulated EGFL7 expression in RCC cells affected the migration and tubule formation of HMEC-1 cells, but not their growth and apoptosis in vitro. The level of focal adhesion kinase (FAK) phosphorylation in HMEC-1 cells decreased significantly when co-cultured with 786-0/iEGFL7 cells compared with 786-0 cells. After adding rhEGFL7, the level of FAK phosphorylation in HMEC-1 cells was significantly elevated compared with phosphate-buffered saline (PBS) control. However, FAK phosphorylation was abrogated by EGFR inhibition. The average size of RCC local tumors in the 786-0/iEGFL7 group was noticeably smaller than those in the 786-0 cell group and their vascular density was also significantly decreased. These data suggest that EGFL7 has an important function in the growth of RCC by facilitating angiogenesis. 
 Overexpressed MALAT1 promotes invasion and   metastasis   of gastric cancer cells via increasing EGFL7 expression. Long non-coding RNAs (lncRNAs) have been demonstrated to participate in various cancers. Here, the role and its potential mechanism of MALAT1 in invasion and migration of gastric cancer (GC) were investigated.Gastric adenocarcinoma tissues and matched normal adjacent tissues were isolated from 25 patients with GC. The expression of epidermal growth factor-like domain-containing protein 7 (EGFL7) was detected in the normal gastric mucosa epithelial GES-1 cell line and three different differentiation GC cell lines, including MKN28 (well-differentiated adenocarcinoma), SGC7901 (moderately differentiated adenocarcinoma) and BGC823 (poorly differentiated adenocarcinoma). Tumor xenotransplant mouse model was established with the injection of cell line pretreated with lentiviral vectors for si-MALAT1 or si-control.The expression of MALAT1 was up-regulated in GC tissues and three cell lines. Si-MALAT1/pcDNA-MALAT1 induced the decrease of cell invasion and migration, while the effects were reversed by the transfection of pcDNA-EGFL7/si-EGFL7. ChIP assay showed that MALAT1 regulated EGFL7 expression by altering the level of H3 histone acetylation in EGFL7 promoter. In tumor xenotransplant mice, down-regulated MALAT1 contributed to the inhibition of tumor   metastasis  .Up-regulated MALAT1 promoted the invasion and   metastasis   of GC, and the increase of EGFL7 expression was a potential mechanism via altering its H3 histone acetylation level. 
 Different expression of VEGF and EGFL7 in human hepatocellular carcinoma. Vascular endothelial growth factor (VEGF) is one of several angiogenic factors expressed in cirrhosis and during progression to malignancy, that seem to play a major role in hepatocellular carcinoma development. Lately, another angiogenic factor, epidermal growth factor-like domain multiple 7 (EGFL7), has attracted interest due to its possible relationship with hepatocellular carcinoma   metastasis  .To evaluate expression of VEGF and EGFL7 in human hepatocellular carcinoma, compared to corresponding cirrhotic surrounding tissue.Tumoural and cirrhotic tissue was harvested from 12 consecutive patients undergoing surgical resection. VEGF and EGFL7 were assessed by immunofluorescence and quantitative reverse transcriptase-polymerase chain reaction, compared with normal controls.Both angiogenic factors were over-expressed in cirrhotic livers compared to normal controls. VEGF and EGFL7 expressions did not differ according to disease aetiology, nodule size or other clinical variables. While VEGF expression was constant, regardless of tumour differentiation stage and unchanged compared to surrounding cirrhotic tissue, EGFL7 expression increased in less differentiated hepatocellular carcinoma.The preferential expression of EGFL7 in less differentiated hepatocellular carcinoma compared to VEGF, suggests a possible important role of this angiogenic factor in a later oncogenic and infiltrative/metastatic phase. 
 The expression of Egfl7 in human normal tissues and epithelial tumors. To investigate the expression of Egfl7 in normal adult human tissues and human epithelial tumors. RT-PCR and Western blot were employed to detect Egfl7 expression in normal adult human tissues and 10 human epithelial tumors including hepatocellular carcinoma (HCC), lung cancer, breast cancer, prostate cancer, colorectal cancer, gastric cancer, esophageal cancer, malignant glioma, ovarian cancer and renal cancer. Immunohistochemistry and cytoimmunofluorescence were subsequently used to determine the localization of Egfl7 in human epithelial tumor tissues and cell lines. ELISA was also carried out to examine the serum Egfl7 levels in cancer patients. In addition, correlations between Egfl7 expression and clinicopathological features as well as prognosis of HCC and breast cancer were also analyzed on the basis of immunohistochemistry results. Egfl7 was differentially expressed in 19 adult human normal tissues and was overexpressed in all 10 human epithelial tumor tissues. The serum Egfl7 level was also significantly elevated in cancer patients. The increased Egfl7 expression in HCC correlated with vein invasion, absence of capsule formation, multiple tumor nodes and poor prognosis. Similarly, upregulation of Egfl7 in breast cancer correlated strongly with TNM stage, lympha  tic metast  asis, estrogen receptor positivity, Her2 positivity and poor prognosis.  Egfl7 is significantly upregulated in human epithelial tumor tissues, suggesting Egfl7 to be a potential biomarker for human epithelial tumors, especially HCC and breast cancer. 
 Epidermal growth factor-like domain-containing protein 7 (EGFL7) enhances EGF receptor-AKT signaling, epithelial-mesenchymal transition, and   metastasis   of gastric cancer cells. Epidermal growth factor-like domain-containing protein 7 (EGFL7) is upregulated in human epithelial tumors and so is a potential biomarker for malignancy. Indeed, previous studies have shown that high EGFL7 expression promotes infiltration and   metastasis   of gastric carcinoma. The epithelial-mesenchymal transition (EMT) initiates the metastatic cascade and endows cancer cells with invasive and migratory capacity; however, it is not known if EGFL7 promotes   metastasis   by triggering EMT. We found that EGFL7 was overexpressed in multiple human gastric cancer (GC) cell lines and that overexpression promoted cell invasion and migration as revealed by scratch wound and transwell migration assays. Conversely, shRNA-mediated EGFL7 knockdown reduced invasion and migration. Furthermore, EGFL7-overexpressing cells grew into larger tumors and were more likely to metastasize to the liver compared to underexpressing CG cells following subcutaneous injection in mice. EGFL7 overexpression protected GC cell lines against anoikis, providing a plausible mechanism for this enhanced metastatic capacity. In excised human gastric tumors, expression of EGFL7 was positively correlated with expression levels of the mesenchymal marker vimentin and the EMT-associated transcription repressor Snail, and negatively correlated with expression of the epithelial cell marker E-cadherin. In GC cell lines, EGFL7 knockdown reversed morphological signs of EMT and decreased both vimentin and Snail expression. In addition, EGFL7 overexpression promoted EGF receptor (EGFR) and protein kinase B (AKT) phospho-activation, effects markedly suppressed by the EGFR tyrosine kinase inhibitor AG1478. Moreover, AG1478 also reduced the elevated invasive and migratory capacity of GC cell lines overexpressing EGFL7. Collectively, these results strongly suggest that EGFL7 promotes   metastasis   by activating EMT through an EGFR-AKT-Snail signaling pathway. Disruption of EGFL7-EGFR-AKT-Snail signaling may a promising therapeutic strategy for gastric cancer. 
 miR-126 and miR-126*: new players in cancer. Cancer progression is characterized by autarky in growth signals, insensitivity to growth-restrictive signals, evasion of apoptosis, a limitless potential to replicate, sustained angiogenesis, and tissue invasion, including   metastasis  . The regulation of these cellular processes relies on a fine-tuned control of molecular signal cascades. In recent years, short noncoding RNAs termed microRNAs (miRNAs) have been described as a novel class of molecular regulators. These affect various signaling cascades during the progression of neoplastic diseases by the regulation of gene expression on the post-transcriptional level. The novel endothelial cell-derived secreted protein epidermal growth factor-like domain 7 (EGFL7) has been suggested to control vascular tubulogenesis. Further, the two biologically active miRNAs miR-126 and its complement miR-126*, which are encoded by intron 7 of the egfl7 gene, have been described to mediate vascular functions. Knock-out studies in zebrafish and mice suggested a major role of miR-126 in angiogenesis and vascular integrity, which was mediated by the repression of inhibitors of VEGF-induced proliferation in endothelial cells. Recent studies revealed the distribution and function of miR-126 and miR-126* in various types of cancer, and assigned a role to both miRNAs as suppressors of tumor formation. Indeed, miR-126 and miR-126* have been reported to impair cancer progression through signaling pathways that control tumor cell proliferation, migration, invasion, and survival. Conversely, miR-126 and miR-126* may have a supportive role in the progression of cancer as well, which might be mediated by the promotion of blood vessel growth and inflammation. In this work, we will summarize the current knowledge on functions of miR-126/miR-126* that are relevant for cancer formation, and we will discuss their potential clinical use as predictive markers of survival and application as novel therapeutic targets for the treatment of neoplastic diseases. 
 Suppression of the Epidermal Growth Factor-like Domain 7 and Inhibition of Migration and Epithelial-Mesenchymal Transition in Human Pancreatic Cancer PANC-1 Cells. Epidermal growth factor-like domain multiple 7 (EGFL7), a secreted protein specifically expressed by endothelial cells during embryogenesis, recently was identified as a critical gene in tumor   metastasis  . Epithelial-mesenchymal transition (EMT) was found to be closely related with tumor progression. Accordingly, it is important to investigate the migration and EMT change after knock-down of EGFL7 gene expression in human pancreatic cancer cells.EGFL7 expression was firstly testified in 4 pancreatic cancer cell lines by real-time polymerase chain reaction (Real-time PCR) and western blot, and the highest expression of EGFL7 was found in PANC-1 cell line. Then, PANC-1 cells transfected with small interference RNA (siRNA) of EGFL7 using plasmid vector were named si-PANC-1, while transfected with negative control plasmid vector were called NC-PANC-1. Transwell assay was used to analyze the migration of PANC-1 cells. Real-time PCR and western blotting were used to detect the expression change of EGFL7 gene, EMT markers like E-Cadherin, N-Cadherin, Vimentin, Fibronectin and transcription factors like snail, slug in PANC-1, NC- PANC-1, and si-PANC-1 cells, respectively.After successful plasmid transfection, EGFL7 gene were dramatically knock-down by RNA interference in si-PANC-1 group. Meanwhile, migration ability decreased significantly, compared with PANC-1 and NC-PANC-1 group. Meanwhile, the expression of epithelial phenotype marker E-Cadherin increased and that of mesenchymal phenotype markers N-Cadherin, Vimentin, Fibronectin dramatically decreased in si-PANC-1 group, indicating a reversion of EMT. Also, transcription factors snail and slug decreased significantly after RNA interference.Current study suggested that highly-expressed EGFL7 promotes migration of PANC-1 cells and acts through transcription factors snail and slug to induce EMT, and further study is needed to confirm this issue. 
 Egfl7 promotes tumor escape from immunity by repressing endothelial cell activation. Downregulating the leukocyte adhesion molecules expressed by endothelial cells that line tumor blood vessels can limit the entry of immune effector cells into the tumor mass, thereby contributing to tumoral immune escape. Egfl7 (also known as VE-statin) is a secreted protein specifically expressed by endothelial cells in normal tissues and by cancer cells in various human tumors. High levels of Egfl7 correlate with higher tumor grade and poorer prognosis. Here we show that expression of Egfl7 in breast and lung carcinoma cells accelerates tumor growth and   metastasis   in immunocompetent mice but not in immunodeficient mice. Tumors expressing Egfl7 were infiltrated relatively poorly by immune cells and were characterized by reduced levels of immunostimulatory cytokines [IFN-γ, interleukin-12 (IL-12)] and fewer endothelial adhesion molecules [intercellular adhesion molecule 1 (ICAM-1) and vascular cell adhesion molecule 1 (VCAM-1)]. In vitro studies revealed that Egfl7 inhibited the expression of leukocyte adhesion molecules by endothelial cells, preventing lymphocyte adhesion. In contrast, Egfl7 did not exert any effects on immune cell activation. Human breast cancer lesions expressing high levels of Egfl7 also expressed less ICAM-1 and VCAM-1 in their blood vessels, also indicating an inverse correlation between expression levels of Egfl7 and IFN-γ. Thus, Egfl7 expression in tumors promotes tumor progression by reducing the expression of endothelial molecules that mediate immune cell infiltration. Our findings highlight a novel mechanism through which tumors escape immune control. 
 Novel role for epidermal growth factor-like domain 7 in   metastasis   of human hepatocellular carcinoma. Epidermal growth factor-like domain 7 (Egfl7) is a recently identified secreted protein that is believed to be primarily expressed in endothelial cells (ECs). Although its expression was reported elevated during tumorigenesis, whether and how Egfl7 contributes to human malignancies remains unknown. In the present study overexpression of Egfl7 was found predominantly in hepatocellular carcinoma (HCC) cells in HCC tissues and closely correlated with poor prognosis of HCC. The expression of Egfl7 in cancer cells was further verified with HCC cell lines including HepG2, MHCC97-L, and HCCLM3, and the Egfl7 expression levels positively correlated with metastatic potential of HCC cell lines was tested. To functionally characterize Egfl7 in HCC, we depleted its expression in HCCLM3 cells by using small interfering RNA. Interestingly, reduction of Egfl7 expression resulted in significant inhibition of migration but not growth of HCCLM3 cells. Biochemical analysis indicated that Egfl7 could facilitate the phosphorylation of focal adhesion kinase (FAK) and therefore promote the migration of HCCLM3 cells. In addition, this effect was almost completely blocked by inhibition of epidermal growth factor receptor (EGFR), indicating that the activation of FAK by Egfl7 is mediated through EGFR. Finally, we used a mouse model to demonstrate that down-regulation of Egfl7 was associated with suppression of intrahepatic and pulmonary metastases of HCC. Collectively, our study shows for the first time that overexpression of Egfl7 in HCC and Egfl7 promotes   metastasis   of HCC by enhancing cell motility through EGFR-dependent FAK phosphorylation.Our study suggests Egfl7 as a novel prognostic marker for   metastasis   of HCC and a potential therapeutic target. 
  GeneRIFs  
 Up-regulated MALAT1 promoted the invasion and   metastasis   of GC, and the increase of EGFL7 expression was a potential mechanism via altering its H3 histone acetylation level 
 EGFL7, OPN, and PGE2 may have a role in recurrence and   metastasis   of hepatocellular carcinoma 
 EGFL7 enhances EGFR-AKT signaling, epithelial-mesenchymal transition, and   metastasis   of gastric cancer cells. 
 Egfl7 promotes   metastasis   of hepatocellular carcinoma (HCC) by enhancing cell motility through EGFR-dependent FAK phosphorylation. 
  [Cc]ell [Ii]nvasion  PubMed  
 Overexpressed MALAT1 promotes invasion and metastasis of gastric cancer cells via increasing EGFL7 expression. Long non-coding RNAs (lncRNAs) have been demonstrated to participate in various cancers. Here, the role and its potential mechanism of MALAT1 in invasion and migration of gastric cancer (GC) were investigated.Gastric adenocarcinoma tissues and matched normal adjacent tissues were isolated from 25 patients with GC. The expression of epidermal growth factor-like domain-containing protein 7 (EGFL7) was detected in the normal gastric mucosa epithelial GES-1 cell line and three different differentiation GC cell lines, including MKN28 (well-differentiated adenocarcinoma), SGC7901 (moderately differentiated adenocarcinoma) and BGC823 (poorly differentiated adenocarcinoma). Tumor xenotransplant mouse model was established with the injection of cell line pretreated with lentiviral vectors for si-MALAT1 or si-control.The expression of MALAT1 was up-regulated in GC tissues and three cell lines. Si-MALAT1/pcDNA-MALAT1 induced the decrease of   cell invasion   and migration, while the effects were reversed by the transfection of pcDNA-EGFL7/si-EGFL7. ChIP assay showed that MALAT1 regulated EGFL7 expression by altering the level of H3 histone acetylation in EGFL7 promoter. In tumor xenotransplant mice, down-regulated MALAT1 contributed to the inhibition of tumor metastasis.Up-regulated MALAT1 promoted the invasion and metastasis of GC, and the increase of EGFL7 expression was a potential mechanism via altering its H3 histone acetylation level. 
 Epidermal growth factor-like domain-containing protein 7 (EGFL7) enhances EGF receptor-AKT signaling, epithelial-mesenchymal transition, and metastasis of gastric cancer cells. Epidermal growth factor-like domain-containing protein 7 (EGFL7) is upregulated in human epithelial tumors and so is a potential biomarker for malignancy. Indeed, previous studies have shown that high EGFL7 expression promotes infiltration and metastasis of gastric carcinoma. The epithelial-mesenchymal transition (EMT) initiates the metastatic cascade and endows cancer cells with invasive and migratory capacity; however, it is not known if EGFL7 promotes metastasis by triggering EMT. We found that EGFL7 was overexpressed in multiple human gastric cancer (GC) cell lines and that overexpression promoted   cell invasion   and migration as revealed by scratch wound and transwell migration assays. Conversely, shRNA-mediated EGFL7 knockdown reduced invasion and migration. Furthermore, EGFL7-overexpressing cells grew into larger tumors and were more likely to metastasize to the liver compared to underexpressing CG cells following subcutaneous injection in mice. EGFL7 overexpression protected GC cell lines against anoikis, providing a plausible mechanism for this enhanced metastatic capacity. In excised human gastric tumors, expression of EGFL7 was positively correlated with expression levels of the mesenchymal marker vimentin and the EMT-associated transcription repressor Snail, and negatively correlated with expression of the epithelial cell marker E-cadherin. In GC cell lines, EGFL7 knockdown reversed morphological signs of EMT and decreased both vimentin and Snail expression. In addition, EGFL7 overexpression promoted EGF receptor (EGFR) and protein kinase B (AKT) phospho-activation, effects markedly suppressed by the EGFR tyrosine kinase inhibitor AG1478. Moreover, AG1478 also reduced the elevated invasive and migratory capacity of GC cell lines overexpressing EGFL7. Collectively, these results strongly suggest that EGFL7 promotes metastasis by activating EMT through an EGFR-AKT-Snail signaling pathway. Disruption of EGFL7-EGFR-AKT-Snail signaling may a promising therapeutic strategy for gastric cancer. 
   
  
 XIAP 
 
  NCBI Gene  
  GeneRIF  
  Pubmed  
 
 
  Search Term  Rank  Fields  
  [Mm]igration  6  PubMed(9); GeneRIFs(1)  
  [Ee]xtracellular [Mm]atrix  8  PubMed(2)  
  [Ii]nvasive  10  PubMed(6); GeneRIFs(1)  
  [Mm]etastasize  11  PubMed(1)  
  [Mm]etastasis  12  PubMed(23); GeneRIFs(5)  
  [Cc]ell [Ii]nvasion  13  PubMed(4); GeneRIFs(1)  
 
 
 hide/show details for XIAP 
  [Mm]igration  PubMed  
 IAPs regulate the plasticity of cell   migration   by directly targeting Rac1 for degradation. Inhibitors of apoptosis proteins (IAPs) are a highly conserved class of multifunctional proteins. Rac1 is a well-studied Rho GTPase that controls numerous basic cellular processes. While the regulation of nucleotide binding to Rac1 is well understood, the molecular mechanisms controlling Rac1 degradation are not known. Here, we demonstrate X-linked IAP (XIAP) and cellular IAP1 (c-IAP1) directly bind to Rac1 in a nucleotide-independent manner to promote its polyubiquitination at Lys147 and proteasomal degradation. These IAPs are also required for degradation of Rac1 upon CNF1 toxin treatment or RhoGDI depletion. Consistently, downregulation of XIAP or c-IAP1 by various strategies led to an increase in Rac1 protein levels in primary and tumour cells, leading to an elongated morphology and enhanced cell   migration  . Further, XIAP counteracts Rac1-dependent cellular polarization in the developing zebrafish hindbrain and promotes the delamination of neurons from the normal tissue architecture. These observations unveil an evolutionarily conserved role of IAPs in controlling Rac1 stability thereby regulating the plasticity of cell   migration   and morphogenesis. 
 FOXM1 targets XIAP and Survivin to modulate breast cancer survival and chemoresistance. Drug resistance is a major hurdle for successful treatment of breast cancer, the leading cause of deaths in women throughout the world. The FOXM1 transcription factor is a potent oncogene that transcriptionally regulates a wide range of target genes involved in DNA repair, metastasis, cell invasion, and   migration  . However, little is known about the role of FOXM1 in cell survival and the gene targets involved. Here, we show that FOXM1-overexpressing breast cancer cells display an apoptosis-resistant phenotype, which associates with the upregulation of expression of XIAP and Survivin antiapoptotic genes. Conversely, FOXM1 knockdown results in XIAP and Survivin downregulation as well as decreased binding of FOXM1 to the promoter regions of XIAP and Survivin. Consistently, FOXM1, XIAP, and Survivin expression levels were higher in taxane and anthracycline-resistant cell lines when compared to their sensitive counterparts and could not be downregulated in response to drug treatment. In agreement with our in vitro findings, we found that FOXM1 expression is significantly associated with Survivin and XIAP expression in samples from patients with IIIa stage breast invasive ductal carcinoma. Importantly, patients co-expressing FOXM1, Survivin, and nuclear XIAP had significantly worst overall survival, further confirming the physiological relevance of the regulation of Survivin and XIAP by FOXM1. Together, these findings suggest that the overexpression of FOXM1, XIAP, and Survivin contributes to the development of drug-resistance and is associated with poor clinical outcome in breast cancer patients. 
 Simultaneous silencing of XIAP and survivin causes partial mesenchymal-epithelial transition of human pancreatic cancer cells via the PTEN/PI3K/Akt pathway. Pancreatic cancer has one of the highest mortality rates among malignant tumors and is characterized by rapid invasion, early metastasis and chemoresistance. X-linked inhibitor of apoptosis (XIAP) and survivin are two of the most important members of the IAP family. Previous studies have shown that XIAP and survivin were overexpressed in pancreatic cancer and were closely associated with cell proliferation and chemoresistance to gemcitabine. In the present study, stable inhibition of XIAP and survivin in Panc-1 cells was performed using lentivirus-carried short hairpin RNAs. The expression of XIAP, survivin, E-cadherin, Slug, phosphatase and tensin homolog (PTEN) and phosphorylated Akt was then measured. In addition, cell proliferation, apoptosis, invasion and   migration   were assessed. The results showed that stable inhibition of XIAP and survivin expression in Panc-1 cells significantly reduced cell proliferation, increased apoptosis and partially reversed the epithelial-mesenchymal transition (EMT). Furthermore, the results of the present study demonstrated that the partial reversal of the EMT was accompanied by inhibited cell invasion and   migration   as well as increased chemosensitivity to gemcitabine in pancreatic cancer cells; this was indicated to be mediated via the PTEN/phosphatidylinositol 3-kinase/Akt signaling pathway. In conclusion, these results suggested that simultaneous inhibition of XIAP and survivin may be a promising strategy for the treatment of pancreatic cancer. 
 X-linked and cellular IAPs modulate the stability of C-RAF kinase and cell motility. Inhibitor of apoptosis proteins (IAP) are evolutionarily conserved anti-apoptotic regulators. C-RAF protein kinase is a direct RAS effector protein, which initiates the classical mitogen-activated protein kinase (MAPK) cascade. This signalling cascade mediates diverse biological functions, such as cell growth, proliferation,   migration  , differentiation and survival. Here we demonstrate that XIAP and c-IAPs bind directly to C-RAF kinase and that siRNA-mediated silencing of XIAP and c-IAPs leads to stabilization of C-RAF in human cells. XIAP binds strongly to C-RAF and promotes the ubiquitylation of C-RAF in vivo through the Hsp90-mediated quality control system, independently of its E3 ligase activity. In addition, XIAP or c-IAP-1/2 knockdown cells showed enhanced cell   migration   in a C-RAF-dependent manner. XIAP promotes binding of CHIP (carboxy terminal Hsc70-interacting protein), a chaperone-associated ubiquitin ligase, to the C-RAF-Hsp90 complex in vivo. Interfering with CHIP expression resulted in stabilization of C-RAF and enhanced cell   migration  , as observed in XIAP knockdown cells. Our data show an unexpected role of XIAP and c-IAPs in the turnover of C-RAF protein, thereby modulating the MAPK signalling pathway and cell   migration  . 
 uPAR and cathepsin B shRNA impedes TGF-β1-driven proliferation and invasion of meningioma cells in a XIAP-dependent pathway. Overexpression of transforming growth factor β1 (TGF-β1) has been linked to immune suppression, tumor angiogenesis, tumor ce  ll migrat  ion, tumor cell survival, and tumor cell invasion in many cancers. In the present study, we found abundant expression of TGF-β1 in the microenvironment of four different pathological types of meningioma tumors. TGF-β1 induced invasion in malignant meningioma cells with an associated upregulation of urokinase-type plasminogen activator (uPA), uPAR, cathepsin B, and MMP-9, and this increase in proliferation was coupled with the expression of anti-apoptotic and pro-survival signaling molecules. In addition to the intense immunoreactivity of meningioma tumors to X-linked inhibitor to apoptosis (XIAP), its knockdown abolished the TGF-β1-induced proliferation of these cells. The stimulation of XIAP expression and the activation of pSMAD-2 is mediated by phosphatidylinositol 3-kinase (PI3K)- and MEK-dependent pathways, and the addition of anti-TGF-β1 antibodies prevented their expression with a consequent decrease in invasion. Bicistronic shRNA constructs targeting uPAR and cathepsin B (pUC) quenched TGF-β1-driven invasion and survival of meningioma cells by downregulation of XIAP and pSMAD-2 expression. Animal models with intracranial tumors showed elevated levels of TGF-β1, XIAP and pSMAD-2, and pUC treatment prevented this increased expression. Thus, targeted silencing of TGF-β1-induced signaling by pUC in meningioma would provide new treatment approaches for management of meningioma. 
 MicroRNA-509-3p Inhibits Cancer Cell Proliferation and   Migration   via Upregulation of XIAP in Gastric Cancer Cells. Gastric cancer (GC) is the fourth most common cancer globally. Recently, microRNAs (miRNAs) have been suggested to be closely associated with tumorigenesis. Aberrant expression of miR-509-3p has been reported in cancer studies. However, the expression and mechanism of its function in GC remain unclear. Here we showed that miR-509-3p was downregulated in GC specimens, which was associated with overall survival. Functional investigations demonstrated that the overexpression of miR-509-3p inhibited the   migration   and proliferation of the GC cells. Additionally, we identified X-linked inhibitor of apoptosis protein (XIAP) as a direct target of miR-509-3p. Knockdown of XIAP significantly attenuated the ability of proliferation,   migration  , and invasion of GC cells. The data therefore suggest that miR-509-3p plays an important role in the development and progression of GC, implicating possible applications in the clinic as a biomarker and a potential new target. 
 RhoGDI SUMOylation at Lys-138 increases its binding activity to Rho GTPase and its inhibiting cancer cell motility. The Rho GDP dissociation inhibitor (RhoGDI) can bind to small GTPases and keep them in a biologically inactive state in cytoplasm, through which it affects actin polymerization and cell motility. However, mechanisms underlying how RhoGDI regulates Rho GTPase complex formation/membrane extraction/GTPase dissociation remain largely unexplored. Our previous studies reported that X-linked inhibitor of apoptosis protein (XIAP) interacted with RhoGDI via its RING domain and negatively modulated RhoGDI SUMOylation and HCT116 cancer cell   migration  . Here, we identified that RhoGDI SUMOylation specifically occurred at Lys-138, which was inhibited by XIAP domain. We further demonstrated that RhoGDI SUMOylation at Lys-138 was crucial for inhibiting actin polymerization and cytoskeleton formation as well as cancer cell motility. Moreover, SUMO-RhoGDI had a much higher binding affinity to small Rho GTPase compared with the un-SUMOylated form of RhoGDI. Taken together, our study demonstrated a novel modification of RhoGDI, SUMOylation at Lys-138, which played a key role in regulating Rho GTPase activation in cancer cells. The physiological regulation of RhoGDI SUMOylation by the RING domain of XIAP may account for modulation of cancer cell invasion and metastasis by XIAP. 
 X-linked inhibitor of apoptosis protein (XIAP) mediates cancer cell motility via Rho GDP dissociation inhibitor (RhoGDI)-dependent regulation of the cytoskeleton. X-linked inhibitor of apoptosis protein (XIAP) overexpression has been found to be associated with malignant cancer progression and aggression in individuals with many types of cancers. However, the molecular basis of XIAP in the regulation of cancer cell biological behavior remains largely unknown. In this study, we found that a deficiency of XIAP expression in human cancer cells by either knock-out or knockdown leads to a marked reduction in β-actin polymerization and cytoskeleton formation. Consistently, cell   migratio  n and invasion were also decreased in XIAP-deficient cells compared with parental wild-type cells. Subsequent studies demonstrated that the regulation of cell motility by XIAP depends on its interaction with the Rho GDP dissociation inhibitor (RhoGDI) via the XIAP RING domain. Furthermore, XIAP was found to negatively regulate RhoGDI SUMOylation, which might affect its activity in controlling cell motility. Collectively, our studies provide novel insights into the molecular mechanisms by which XIAP regulates cancer invasion and offer a further theoretical basis for setting XIAP as a potential prognostic marker and specific target for treatment of cancers with metastatic properties. 
 E3 ligase activity of XIAP RING domain is required for XIAP-mediated cancer cell   migration  , but not for its RhoGDI binding activity. Although an increased expression level of XIAP is associated with cancer cell metastasis, the underlying molecular mechanisms remain largely unexplored. To verify the specific structural basis of XIAP for regulation of cancer cell   migration  , we introduced different XIAP domains into XIAP(-/-) HCT116 cells, and found that reconstitutive expression of full length HA-XIAP and HA-XIAP ΔBIR, both of which have intact RING domain, restored β-Actin expression, actin polymerization and cancer cell motility. Whereas introduction of HA-XIAP ΔRING or H467A mutant, which abolished its E3 ligase function, did not show obvious restoration, demonstrating that E3 ligase activity of XIAP RING domain played a crucial role of XIAP in regulation of cancer cell motility. Moreover, RING domain rather than BIR domain was required for interaction with RhoGDI independent on its E3 ligase activity. To sum up, our present studies found that role of XIAP in regulating cellular motility was uncoupled from its caspase-inhibitory properties, but related to physical interaction between RhoGDI and its RING domain. Although E3 ligase activity of RING domain contributed to ce  ll migrat  ion, it was not involved in RhoGDI binding nor its ubiquitinational modification. 
  GeneRIFs  
 These data show an unexpected role of XIAP and cellular-inhibitor of apoptosis proteins in the turnover of C-RAF protein, thereby modulating the MAPK signalling pathway and cell   migration  . 
  [Ee]xtracellular [Mm]atrix  PubMed  
 Role for X-linked Inhibitor of apoptosis protein upstream of mitochondrial permeabilization. Apoptosis is controlled by a signaling equilibrium between prosurvival and proapoptotic pathways, such that unwanted apoptosis is avoided, but when required it occurs rapidly and efficiently. Many apoptosis regulators display dual roles, depending upon whether a cell has received an apoptotic stimulus or not. Here, we identify a novel and unexpected function for X-linked inhibitor of apoptosis (XIAP) that occurs when apoptosis is triggered under physiological conditions. We show that in response to loss of survival signals provided by cell adhesion, endogenous XIAP translocates from the cytosol into a mitochondrial 400-kDa complex and that this occurs very early in the apoptosis process. Membrane-associated XIAP induces mitochondrial outer membrane permeabilization leading to cytochrome c and Smac release, which is dependent on Bax and Bak. Thus, although XIAP suppresses apoptosis in healthy cells, our data indicate that XIAP may contribute to it in response to a proapoptotic signal such as loss of   extracellular matrix  -dependent survival signaling. We suggest that, as with Bcl-2 family proteins, more diverse functions for XIAP exist than previously identified. Moreover, switching the function of proteins from anti- to proapoptotic forms may be a common theme in the efficient execution of cell death. 
 Detachment-induced upregulation of XIAP and cIAP2 delays anoikis of intestinal epithelial cells. Detachment of normal epithelial cells from the   extracellular matrix   triggers apoptosis, a phenomenon called anoikis. Conversely, carcinoma cells tend to be relatively more anoikis-resistant than their normal counterparts, and this increased resistance represents a critical feature of the malignant phenotype. Mechanisms that control susceptibility and resistance to anoikis are not fully understood. It is now known that detachment of non-malignant epithelial cells triggers both pro- and antiapoptotic signals, and it is the balance between these signals and the duration of detachment that determine further fate of the cells. Detachment-induced antiapoptotic events delay anoikis and if cells reattach relatively soon after detachment they survive. Direct regulators of apoptosis responsible for this delay of anoikis are unknown. We found that detachment of non-malignant intestinal epithelial cells triggers upregulation of inhibitors of apoptosis protein (IAP) family, such as X-chromosome-linked inhibitor of apoptosis protein and cellular inhibitor of apoptosis-2 (cIAP2). We demonstrated that this upregulation requires detachment-dependent activation of the transcription factor nuclear factor-kappaB. We further observed that various IAP antagonists accelerate anoikis, indicating that upregulation of the IAPs delays detachment-triggered apoptosis. We conclude that the IAPs are important regulators of the balance between detachment-triggered life and death signals. Perhaps, not by coincidence, these proteins are often upregulated in carcinomas, tumors composed of cells that tend to be anoikis-resistant. 
  [Ii]nvasive  PubMed  
 FOXM1 targets XIAP and Survivin to modulate breast cancer survival and chemoresistance. Drug resistance is a major hurdle for successful treatment of breast cancer, the leading cause of deaths in women throughout the world. The FOXM1 transcription factor is a potent oncogene that transcriptionally regulates a wide range of target genes involved in DNA repair, metastasis, cell invasion, and migration. However, little is known about the role of FOXM1 in cell survival and the gene targets involved. Here, we show that FOXM1-overexpressing breast cancer cells display an apoptosis-resistant phenotype, which associates with the upregulation of expression of XIAP and Survivin antiapoptotic genes. Conversely, FOXM1 knockdown results in XIAP and Survivin downregulation as well as decreased binding of FOXM1 to the promoter regions of XIAP and Survivin. Consistently, FOXM1, XIAP, and Survivin expression levels were higher in taxane and anthracycline-resistant cell lines when compared to their sensitive counterparts and could not be downregulated in response to drug treatment. In agreement with our in vitro findings, we found that FOXM1 expression is significantly associated with Survivin and XIAP expression in samples from patients with IIIa stage breast   invasive   ductal carcinoma. Importantly, patients co-expressing FOXM1, Survivin, and nuclear XIAP had significantly worst overall survival, further confirming the physiological relevance of the regulation of Survivin and XIAP by FOXM1. Together, these findings suggest that the overexpression of FOXM1, XIAP, and Survivin contributes to the development of drug-resistance and is associated with poor clinical outcome in breast cancer patients. 
 Prognostic value of the X-linked inhibitor of apoptosis protein for   invasive   ductal breast cancer with triple-negative phenotype. Molecular classification has raised new hopes of improving our understanding of breast cancer. Discovery of novel tumor markers that allow the identification of patients at higher risk for   invasive   ductal breast cancer with triple-negative phenotype remains a research and clinical priority. To evaluate the prognostic value of the X-linked inhibitor of apoptosis protein for   invasive   ductal breast cancer with triple-negative phenotype by correlating the expression of X-linked inhibitor of apoptosis protein with clinicopathologic parameters, thus determining its role in predicting tumor outcomes, 200 cases of patients with   invasive   ductal breast cancer, including their complete information, were obtained. Tissue microarrays were constructed; and immunohistochemical staining was performed to detect the expression of the estrogen receptor, progesterone receptor, HER2/neu, Ki-67, and X-linked inhibitor of apoptosis protein. We identified 42 cases of   invasive   ductal breast cancer with triple-negative phenotype. Of these, X-linked inhibitor of apoptosis protein expression was detected in 32 patients (80%). Significant correlations were found between X-linked inhibitor of apoptosis protein expression and primary tumor size (P = .027), and between X-linked inhibitor of apoptosis protein expression and Ki-67 index (P = .038). Kaplan-Meier survival analysis revealed a pattern of X-linked inhibitor of apoptosis protein expression with impaired overall and disease-free survival in patients with the disease. Most importantly, multivariate analysis also showed statistically significant worse outcomes for patients with tumors exhibiting X-linked inhibitor of apoptosis protein expression of at least 50% compared with those with X-linked inhibitor of apoptosis protein expression less than 50%. In conclusion, our results suggest that X-linked inhibitor of apoptosis protein is a novel biomarker and viable prognostic factor for   invasive   ductal breast cancer with triple-negative phenotype. Furthermore, the expression of X-linked inhibitor of apoptosis protein is significantly correlated with a more aggressive tumor phenotype and decreased overall and disease-free survival. 
 Expression of X-linked inhibitor-of-apoptosis protein in hepatocellular carcinoma promotes metastasis and tumor recurrence. Hepatocellular carcinoma (HCC) is one of the most common malignant tumors worldwide. Despite significantly improved diagnosis and treatment in recent years, the long-term therapeutic effect is compromised by the frequent recurrence and metastasis, of which the molecular mechanisms are not fully understood. Our initial studies in established HCC cell lines with different metastatic capabilities indicated a correlation of metastasis with the resistance to apoptosis and therefore the ability to survive in stressed conditions. Subsequent investigation revealed that increased expression of X-linked inhibitor-of-apoptosis protein (XIAP) was correlated with the resistance to apoptosis and enhanced   invasive  ness in vitro, which could contribute to increased metastatic foci in vivo. Furthermore, we found that nearly 90% of clinical samples from advanced HCC patients expressed high levels of XIAP. Patients with XIAP-positive tumors had a significantly increased risk of relapse, which resulted from metastasis after total liver resection and orthotopic liver transplantation. Indeed, XIAP expression could be an independent prognostic factor for predicting disease-free survival rate and overall survival rate of these patients. XIAP expression was also highly correlated with advanced cases that exceeded the Milan criteria and could be a prognostic factor for disease-free survival in these patients as well.Our studies have shown an important molecule in controlling HCC metastasis, defined a biomarker that can be used to predict HCC recurrence and patient survival after treatment, and suggest that XIAP can be a molecular target subject to intervention to reduce metastasis and recurrence. 
 Immunohistochemical detection of p63 and XIAP in thymic hyperplasia and thymomas. We subjected 23 formalin-fixed, paraffin-embedded tissue blocks (11 cases of thymic hyperplasia and 12 thymomas [3 encapsulated, 8 with capsular invasion, and 1 atypical]) to incubation with monoclonal anti-X-linked inhibitor of apoptosis protein (XIAP) (BD Biosciences, San Jose, CA) and monoclonal anti-p63 (4A4, Santa Cruz, Santa Cruz, CA). Granular or heterogeneous cytoplasmic XIAP staining and nuclear p63 staining were considered positive. We compared thymic hyperplasia with thymoma and capsulated thymoma with thymoma with capsular invasion or atypia. p63 was positive in virtually all thymic epithelial cells in hyperplasia and thymoma. XIAP was negative in all hyperplasia cases except one. Of 12 thymomas, 9 were XIAP+ with focal/weak to diffuse/strong positivity: 2 of 3 encapsulated and 7 of 8 thymomas with capsular invasion were XIAP+. One atypical thymoma was XIAP-. XIAP expression differed significantly between hyperplasia and thymoma (P = .0007) but not between capsulated and   invasive   thymomas (P = .3797). p63 is consistently positive in nonneoplastic and neoplastic thymic epithelium. XIAP expression in thymoma suggests a possible role in the pathogenesis of thymoma and may be helpful in differentiating thymic hyperplasia from thymoma, especially in small biopsy specimens. However, the level of expression does not correlate with capsular invasion or atypia. 
 XIAP associated factor 1 (XAF1) represses expression of X-linked inhibitor of apoptosis protein (XIAP) and regulates invasion, cell cycle, apoptosis, and cisplatin sensitivity of ovarian carcinoma cells. X-linked inhibitor of apoptosis protein (XIAP) associated factor 1 (XAF1) exhibits aberrantly low or absent expression in various human malignancies, closely associated with anti-apoptosis and overgrowth of cancer cells. However, limited attention has been directed towards the contribution of XAF1 to invasion, apoptosis, and cisplatin (DDP)-resistance of epithelial ovarian cancer (EOC) cells. This study aimed to evaluate the potential effects of XAF1 on invasion, cell cycle, apoptosis, and cisplatin-resistance by overexpressing XAF1 in SKOV-3 and SKOV-3/DDP cells.The pEGFP-C1-XAF1 plasmid was transfected into SKOV-3 and SKOV-3/DDP cells, and the expression of XAF1 at both mRNA and protein levels was analyzed by reverse transcription-PCR and Western blotting. Overexpression of XAF1 suppressed XIAP expression in both SKOV-3 and SKOV-3/DDP cells. Transwell invasion assays demonstrated that XAF1 exerted a strong anti-  invasive   effect in XAF1-overexpressing cells. Moreover, flow cytometry analysis revealed that XAF1 overexpression arrested the cell cycle at G0/G1 phase, and cell apoptosis analysis showed that overexpression of XAF1 enhanced apoptosis of SKOV-3 and SKOV-3/DDP cells apparently by activating caspase-9 and caspase-3. Furthermore, MTT assay confirmed a dose-dependent inhibitory effect of cisplatin in the tested tumor cells, and overexpression of XAF1 increased the sensitivity of SKOV-3 and SKOV-3/DDP cells to cisplatin-mediated anti- proliferative effects.In summary, our data indicated that overexpression of XAF1 could suppress XIAP expression, inhibit invasion, arrest cell cycle, promote apoptosis, and confer cisplatin-sensitivity in SKOV-3 and SKOV-3/DDP cells. Therefore, XAF1 may be further assessed as a potential target for the treatment of both cisplatin-resistant and non-resistant EOCs. 
 XIAP gene expression and function is regulated by autocrine and paracrine TGF-beta signaling. X-linked inhibitor of apoptosis protein (XIAP) is often overexpressed in cancer cells, where it plays a key role in survival and also promotes   invasive  ness. To date however, the extracellular signals and intracellular pathways regulating its expression and activity remain incompletely understood. We have previously showed that exposure to each of the three TGF-beta (transforming growth factor beta) isoforms upregulates XIAP protein content in endometrial carcinoma cells in vitro. In the present study, we have investigated the clinical relevance of TGF-beta isoforms in endometrial tumours and the mechanisms through which TGF-beta isoforms regulate XIAP content in uterine cancer cells.TGF-beta isoforms immunoreactivity in clinical samples from endometrial tumours was assessed using immunofluorescence. Two model cancer cell lines (KLE endometrial carcinoma cells and HeLa cervical cancer cells) and pharmacological inhibitors were used to investigate the signalling pathways regulating XIAP expression and activity in response to autocrine and paracrine TGF-beta in cancer cell.We have found immunoreactivity for each TGF-beta isoform in clinical samples from endometrial tumours, localizing to both stromal and epithelial/cancer cells. Blockade of autocrine TGF-beta signaling in KLE endometrial carcinoma cells and HeLa cervical cancer cells reduced endogenous XIAP mRNA and protein levels. In addition, each TGF-beta isoform upregulated XIAP gene expression when given exogenously, in a Smad/NF-kappaB dependent manner. This resulted in increased polyubiquitination of PTEN (phosphatase and tensin homolog on chromosome ten), a newly identified substrate for XIAP E3 ligase activity, and in a XIAP-dependent decrease of PTEN protein levels. Although each TGF-beta isoform decreased PTEN content in a XIAP- and a Smad-dependent manner, decrease of PTEN levels in response to only one isoform, TGF-beta3, was blocked by PI3-K inhibitor LY294002.XIAP gene expression and function is positively regulated by exposure to the three TGF-beta isoforms in a Smad-dependent manner, similar to constitutive XIAP gene expression which depends on autocrine TGF-beta/Smad signalling. 
  GeneRIFs  
 Kaplan-Meier survival analysis revealed a pattern of X-linked inhibitor of apoptosis protein expression with impaired overall and disease-free survival in patients with   invasive   ductal breast cancer. 
  [Mm]etastasize  PubMed  
 Immunohistochemical detection of XIAP in melanoma. The X-linked inhibitor of apoptosis protein (XIAP) is the most potent of the inhibitor of apoptosis family of eight proteins. High levels of XIAP have been found in melanoma cell lines and are believed to play a role in therapeutic resistance in a number of malignancies. XIAP expression has not been investigated in clinically obtained melanoma tissue samples, nor have studies attempted to correlate XIAP expression with prognostic variables or clinical aggressiveness of melanomas.Sixty-seven patients with primary cutaneous malignant melanoma for whom clinical follow up was available were identified from the records of the Mount Sinai Hospital, comprising 37 thin melanomas (Breslow thickness < 1.0 mm) and 30 thick melanomas (Breslow thickness > 1.0 mm). Archival paraffin sections from primary lesions and corresponding metastases were stained with monoclonal anti-XIAP antibody using routine immunohistochemical methods.Six benign intradermal nevi and four in situ melanomas were XIAP negative. 9 of 37 thin melanomas (24%) were XIAP positive. In contrast, 21 of 30 (73%) thick melanomas were XIAP positive, including 3 of 4 ulcerated melanomas that were strongly positive. Over a follow-up period ranging from 6 months to 6 years, 23 melanomas   metastasize  d (22 thick, 1 thin). In total, XIAP was immunohistochemically detected in 17 of 23 metastases (74%). Metastasis occurred in 1 of 9 XIAP-positive thin melanomas; 0 of 28 XIAP-negative thin melanomas; 17 of 22 XIAP-positive thick melanomas, and 5 of 8 XIAP-negative thick melanomas (63%).XIAP is immunohistochemically detectable nearly three times more frequently in thick compared with thin melanomas. These results suggest that XIAP elevation may be correlated with increasing melanoma thickness and tumor progression. 
  [Mm]etastasis  PubMed  
 Prognostic significance of XIAP and NF-κB expression in esophageal carcinoma with postoperative radiotherapy. X-chromosome-linked IAP (XIAP) and nuclear factor-κB (NF-κB) are frequently overexpressed and correlate closely with chemoradiotherapy resistance and poor prognosis in many cancers. However, the significance of XIAP and NF-κB expression in radiotherapy sensitivity and its effect on the prognosis of esophageal squamous cell carcinoma (ESCC) are still unknown. The aim of this study was to examine XIAP and NF-κB status in ESCC patients undergoing postoperative radiotherapy after radical surgery, and to evaluate their clinical significance.A total of 78 ESCC patients treated with postoperative radiotherapy after radical surgery were enrolled in this study. We immunohistochemically investigated the expression of XIAP and NF-κB in tissues from enrolled patients with specific antibodies. Then, the correlations among XIAP, NF-κB expression, clinicopathological features and its prognostic relevance in ESCC were analyzed.The increased expression of XIAP and NF-κB in ESCC tissues were clearly correlated with the tumor differentiation and p-TNM stage. Significant positive correlations were found between the expression status of XIAP and NF-κB (r = 0.779, P = 0.000). Overexpression of XI  AP and NF-  κB and metastasis were significantly associated with shorter overall survival times in univariate analysis (P < 0.05). Multivariate analysis also confirmed that XIAP expression was an independent prognostic factor (P = 0.005).XIAP and NF-κB are intensively expressed in ESCC. The level of XIAP is positively correlated to progression and prognosis of ESCC. 
 Prognostic significance of XIAP and NF-κB expression in esophageal carcinoma with postoperative radiotherapy. X-chromosome-linked IAP (XIAP) and nuclear factor-κB (NF-κB) are frequently overexpressed and correlate closely with chemoradiotherapy resistance and poor prognosis in many cancers. However, the significance of XIAP and NF-κB expression in radiotherapy sensitivity and its effect on the prognosis of esophageal squamous cell carcinoma (ESCC) are still unknown. The aim of this study was to examine XIAP and NF-κB status in ESCC patients undergoing postoperative radiotherapy after radical surgery, and to evaluate their clinical significance.A total of 78 ESCC patients treated with postoperative radiotherapy after radical surgery were enrolled in this study. We immunohistochemically investigated the expression of XIAP and NF-κB in tissues from enrolled patients with specific antibodies. Then, the correlations among XIAP, NF-κB expression, clinicopathological features and its prognostic relevance in ESCC were analyzed.The increased expression of XIAP and NF-κB in ESCC tissues were clearly correlated with the tumor differentiation and p-TNM stage. Significant positive correlations were found between the expression status of XIAP and NF-κB (r = 0.779, P = 0.000). Overexpression of XI  AP and NF-  κB and metastasis were significantly associated with shorter overall survival times in univariate analysis (P < 0.05). Multivariate analysis also confirmed that XIAP expression was an independent prognostic factor (P = 0.005).XIAP and NF-κB are intensively expressed in ESCC. The level of XIAP is positively correlated to progression and prognosis of ESCC. 
 Caspase-3 cleaves XIAP in a positive feedback loop to sensitize melanoma cells to TRAIL-induced apoptosis. Successful treatment of melanoma is still challenging, because   metastasis   remain chemoresistant and radioresistant. Accordingly, combinational treatments involving death ligands are mandatory. In a recent study from our lab, the majority out of 18 melanoma cell lines remained resistant against treatment with the death ligand TRAIL (tumor necrosis factor related apoptosis inducing ligand). Resistance was shown to be mainly due to incomplete processing of caspase-3 into catalytically inactive p21 by binding of the anti-apoptotic protein X-linked inhibitor of apoptosis protein (XIAP). Co-irradiation with sub-lethal ultraviolet (UV) B caused depletion of XIAP resulting in synergistic sensitization of all but two melanoma cell lines to TRAIL. We show here the XIAP depletion to essentially require initial caspase-mediated cleavage, which promotes proteasomal degradation of XIAP. Utilizing specific caspase inhibitors and small interfering RNA-mediated knockdown, we further identified caspase-3 to be responsible for performing the initial cleavage of XIAP after UVB treatment. Additional evidence suggests an accelerated mitochondrial outer membrane permeabilization in response to co-treatment with TRAIL and UVB, which directs the release of XIAP antagonizing factors including Smac. Distraction of XIAP consequently liberates caspase-3 to autocatalytically process into active p17. Activated caspase-3 cleaves XIAP and further enhances its activation in a positive regulatory feedback loop. The molecular mechanism discovered here appears to have broader implications, because cleavage of XIAP was also shown to accompany cisplatin-induced sensitization of melanoma cells to TRAIL. 
 Silencing XIAP suppresses osteosarcoma cell growth, and enhances the sensitivity of osteosarcoma cells to doxorubicin and cisplatin. X-chromosome-linked inhibitor of apoptosis protein (XIAP) is an important member of the inhibitors of apoptosis (IAP) family. It has been shown that XIAP promotes the invasion,   metastasis  , growth and survival of malignant cells, and confers resistance to some chemotherapeutic drugs in various types of cancer. However, little is known regarding its detailed role in osteosarcoma (OS). In the present study, we first investigated the expression of XIAP in OS tissues, and an increased expression of XIAP in OS tissues compared to adjacent non-tumor tissue was identified. Additionally, its expression level correlated with key pathological characteristics including clinical stage, tumor size and   metastasis  . Subsequently, small interfering RNA (siRNA) was used to block XIAP expression to evaluate the effect of XIAP siRNA on cell proliferation, colony formation, cell cycle, apoptosis, tumorigenicity, and the combined effects of doxorubicin or cisplatin in OS cell lines (MG63 cells). Downregulation of XIAP expression using the RNA silencing approach efficiently decreased cell proliferation and colony formation, and induced cell apoptosis and cell cycle in the G0/G1 stage. In addition, this downregulation inhibited tumor growth in a nude murine model. The resuls also showed that treatment with XIAP-shRNA in combination with doxorubicin or cisplatin enhanced chemosensitivity. These results suggested that XIAP may aid the diagnosis of OS and may be an effective strategy for gene therapy of this disease. 
 FOXM1 targets XIAP and Survivin to modulate breast cancer survival and chemoresistance. Drug resistance is a major hurdle for successful treatment of breast cancer, the leading cause of deaths in women throughout the world. The FOXM1 transcription factor is a potent oncogene that transcriptionally regulates a wide range of target genes involved in DNA repair,   metastasis  , cell invasion, and migration. However, little is known about the role of FOXM1 in cell survival and the gene targets involved. Here, we show that FOXM1-overexpressing breast cancer cells display an apoptosis-resistant phenotype, which associates with the upregulation of expression of XIAP and Survivin antiapoptotic genes. Conversely, FOXM1 knockdown results in XIAP and Survivin downregulation as well as decreased binding of FOXM1 to the promoter regions of XIAP and Survivin. Consistently, FOXM1, XIAP, and Survivin expression levels were higher in taxane and anthracycline-resistant cell lines when compared to their sensitive counterparts and could not be downregulated in response to drug treatment. In agreement with our in vitro findings, we found that FOXM1 expression is significantly associated with Survivin and XIAP expression in samples from patients with IIIa stage breast invasive ductal carcinoma. Importantly, patients co-expressing FOXM1, Survivin, and nuclear XIAP had significantly worst overall survival, further confirming the physiological relevance of the regulation of Survivin and XIAP by FOXM1. Together, these findings suggest that the overexpression of FOXM1, XIAP, and Survivin contributes to the development of drug-resistance and is associated with poor clinical outcome in breast cancer patients. 
 XIAP is a predictor of cisplatin-based chemotherapy response and prognosis for patients with advanced head and neck cancer. Approximately 60-80% of patients with advanced head and neck squamous cell carcinoma (HNSCC) die within five years after diagnosis. Cisplatin-based chemotherapy is the most commonly used palliative treatment for these patients. To evaluate the prognostic value of X-linked inhibitor of apoptosis (XIAP) level as a potential biomarker in these patients, we investigated the relationship between XIAP expression and cisplatin response of these patients and their prognosis.Sixty patients with advanced HNSCC were recruited in this study. Expression of XIAP was examined both before and after chemotherapy and was correlated with chemotherapy response, clinicopathology parameters and clinical outcomes of the patients. We found that XIAP was expressed in 17 (20.83%) of the 60 advanced HNSCC samples and the expression was significantly associated with cisplatin resistance (P = 0.036) and poor clinical outcome (P = 0.025). Cisplatin-based chemotherapy induced XIAP expression in those post-chemotherapy samples (P = 0.011), was further associated with poorer clinical outcome (P = 0.029). Multivariate analysis demonstrated that only alcohol consumption, lymph node   metastasis   and XIAP level were independently associated with the prognosis of advanced HNSCC patients. Inhibiting XIAP expression with siRNA in XIAP overexpressed HNSCC cells remarkably increased their sensitivity to cisplatin treatment to nearly a 3 fold difference.Our results demonstrate that XIAP overexpression plays an important role in the disease course and cisplatin-resistance of advanced HNSCC. XIAP is a valuable predictor of cisplatin-response and prognosis for patients with advanced head and neck cancer. Down-regulation of XIAP might be a promising adjuvant therapy for those patients of advanced HNSCC. 
 [Expression of X-linked inhibitor of apoptosis protein in colorectal cancer tissues]. To investigate the expression of X-linked inhibitor of apoptosis protein (XIAP) in colorectal cancer tissues and investigate its correlation with the clinicopathological factors of the malignancy.Immunohistochemistry and RT-PCR was employed to detect the expression of XIAP in 87 colorectal cancer tissues.XIAP mRNA expression was detected in 64.4% (56/87) of the colorectal cancer tissues, 49.4% (43/87) of the adjacent tissues, and in 11.4% (10/87) of the normal tissues, respectively. The cancer tissues showed significant greater positivity rate and higher expression level of XIAP mRNA than the adjacent and normal tissues. Immunohistochemistry also identified a significantly greater positivity rate for XIAP [70.1% (61/87)] in the colorectal cancer tissues. The pathological grade of the tumors was associated with the expression level of XIAP, whereas this association was not established between XIAP expression and the clinical stages, tumor position or lymph node   metastasis  .XIAP may play an important role in the development of colorectal cancer, which might serve as a potentially useful tumor marker. 
 Simultaneous silencing of XIAP and survivin causes partial mesenchymal-epithelial transition of human pancreatic cancer cells via the PTEN/PI3K/Akt pathway. Pancreatic cancer has one of the highest mortality rates among malignant tumors and is characterized by rapid invasion, early   metastasis   and chemoresistance. X-linked inhibitor of apoptosis (XIAP) and survivin are two of the most important members of the IAP family. Previous studies have shown that XIAP and survivin were overexpressed in pancreatic cancer and were closely associated with cell proliferation and chemoresistance to gemcitabine. In the present study, stable inhibition of XIAP and survivin in Panc-1 cells was performed using lentivirus-carried short hairpin RNAs. The expression of XIAP, survivin, E-cadherin, Slug, phosphatase and tensin homolog (PTEN) and phosphorylated Akt was then measured. In addition, cell proliferation, apoptosis, invasion and migration were assessed. The results showed that stable inhibition of XIAP and survivin expression in Panc-1 cells significantly reduced cell proliferation, increased apoptosis and partially reversed the epithelial-mesenchymal transition (EMT). Furthermore, the results of the present study demonstrated that the partial reversal of the EMT was accompanied by inhibited cell invasion and migration as well as increased chemosensitivity to gemcitabine in pancreatic cancer cells; this was indicated to be mediated via the PTEN/phosphatidylinositol 3-kinase/Akt signaling pathway. In conclusion, these results suggested that simultaneous inhibition of XIAP and survivin may be a promising strategy for the treatment of pancreatic cancer. 
 Association between expression of X-linked inhibitor of apoptosis protein and the clinical outcome in a BRAF V600E-prevalent papillary thyroid cancer population. The X-linked inhibitor of apoptosis protein (XIAP) is associated with carcinogenesis, cancer progression, and   metastasis   through inhibition of the caspase-mediated apoptotic pathway. The BRAF(V600E) mutation is the most common genetic alteration and an established prognostic marker in papillary thyroid cancer (PTC). The prevalence of the BRAF mutation is very high and is up to 80% in Korean PTC patients. In the present study, we evaluated the potential role of XIAP expression as a novel prognostic marker to predict recurrence, in combination with the BRAF(V600E) mutational status.The study enrolled 164 patients with conventional PTC who underwent bilateral thyroidectomy followed by immediate (131)I ablation. The presence of the BRAF(V600E) mutation was evaluated by direct sequencing. The degree of XIAP expression was evaluated by immunohistochemical (IHC) staining using a monoclonal antibody.The BRAF(V600E) mutation was found in 123 of 164 patients (75%) with classical PTC. XIAP expression was positive in 128 of 164 patients (75%), and positive XIAP expression was significantly associated with the presence of lateral cervical lymph node metastases (p=0.01). XIAP expression was more frequent in BRAF(V600E) mutated PTCs than in BRAF wild type PTCs (p=0.048). The BRAF(V600E) mutation was significantly associated with cancer recurrence in study subjects (hazard ratio=2.98, p=0.039). PTCs positive for the BRAF(V600E) mutation but negative for XIAP expression had a significantly higher rate of recurrent PTC (hazard ratio=4.53, p=0.012).The evaluation of XIAP expression and BRAF mutational analysis was more useful for the prediction of cancer recurrence in patients with PTC than BRAF genotype alone. 
 Expression of X-linked inhibitor-of-apoptosis protein in hepatocellular carcinoma promotes   metastasis   and tumor recurrence. Hepatocellular carcinoma (HCC) is one of the most common malignant tumors worldwide. Despite significantly improved diagnosis and treatment in recent years, the long-term therapeutic effect is compromised by the frequent recurrence and   metastasis  , of which the molecular mechanisms are not fully understood. Our initial studies in established HCC cell lines with different metastatic capabilities indicated a correlation of   metastasis   with the resistance to apoptosis and therefore the ability to survive in stressed conditions. Subsequent investigation revealed that increased expression of X-linked inhibitor-of-apoptosis protein (XIAP) was correlated with the resistance to apoptosis and enhanced invasiveness in vitro, which could contribute to increased metastatic foci in vivo. Furthermore, we found that nearly 90% of clinical samples from advanced HCC patients expressed high levels of XIAP. Patients with XIAP-positive tumors had a significantly increased risk of relapse, which resulted from   metastasis   after total liver resection and orthotopic liver transplantation. Indeed, XIAP expression could be an independent prognostic factor for predicting disease-free survival rate and overall survival rate of these patients. XIAP expression was also highly correlated with advanced cases that exceeded the Milan criteria and could be a prognostic factor for disease-free survival in these patients as well.Our studies have shown an important molecule in controlling HCC   metastasis  , defined a biomarker that can be used to predict HCC recurrence and patient survival after treatment, and suggest that XIAP can be a molecular target subject to intervention to reduce   metastasis   and recurrence. 
 Impact of X-linked inhibitor of apoptosis protein on survival of nasopharyngeal carcinoma patients following radiotherapy. This study aims to investigate CNE1 and CNE2 cell proliferation and apoptosis of nasopharyngeal cancer (NPC) and X-linked inhibitor of apoptosis protein (XIAP) expression in NPC patients after radiotherapy. Quantitative real-time quantitative polymerase chain reaction (qRT-PCR) and Western Blot detected XIAP and XIAP-associated factor1 (XAF1) messenger RNA (mRNA) and protein expression of CNE1 and CNE2 in NPC cells irradiated by γ-ray; MTT and flow cytometry assays detected CNE2 cells proliferation and apoptotic rate, respectively. With a retrospective analysis of 109 NPC patients in Xinxiang Central Hospital, immunohistochemistry (IHC) method detected XIAP expression, followed by a 5-year clinical analysis of the prognosis relevance after radiotherapy. In vitro, the inhibition and apoptotic rates of cells increased with the growth of radiation dose. qRT-PCR and Western blot detection declared that XIAP mRNA and protein expression increased, whereas XAF1 mRNA and protein expression decreased with the growth of radiation dose and exposure time. And XIAP mRNA and protein expression were negatively correlated with proliferation and apoptotic rates of the cells. In vivo, positive XIAP expression rate was negatively correlated with pathological tumor-node  -metastasi  s (p-TNM) staging and tumor differentiation. Further, high XIAP expression, high p-TNM staging, and lower degree of differentiation were significantly correlated with the decrease of NPC patients' survival rate. Additionally, XIAP expression, p-TNM staging, and degrees of differentiation were independent risk factors for the survival of the NPC patients after radiotherapy. Increased XIAP expression and decreased XAF1 expression may be one reason for the apoptosis delays of CNE1 and CNE2 cells after irradiation, and the XIAP expression or the p-TNM staging and degree of differentiation are independent risk factors for NPC patients' survival after radiotherapy, providing a molecular rationale for radiotherapy and prognosis of NPC. 
 [Expression and clinical significance of anti-apoptotic factors PED/PEA-15 and XIAP in hepatocellular carcinoma]. To investigate the expression of apoptosis arrestin PED/PEA-15 and XIAP in hepatocellular carcinoma (HCC) and explore their relationship with clinicopathological factors of HCC.The mRNA expressions of PED/PEA-15 and XIAP genes were detected by RT-PCR (reverse transcription-polymerase chain reaction) in resected liver tumor tissues and corresponding adjacent noncancerous tissues from 40 HCC patients and normal liver tissues from 12 patients with benign lesions. Their protein levels were detected by Western blot.The mRNA expressions of PED/PEA-15 and XIAP genes and their proteins were significantly higher than those in adjacent tissues and normal tissues (0.636 +/- 0.061, 0.352 +/- 0.068, 0.179 +/- 0.036; 0.579 +/- 0.090, 0.344 +/- 0.084, 0.184 +/- 0.038) (P < 0.01). The mRNA levels of PED/PEA-15 and XIAP genes and their protein levels were significantly associated with the pathological grade and clinical stage of HCC (P < 0.05). However there was not a significant correlation with such clinicopathological features as age, gender, size of tumor, tumor load,   metastasis   and recurrence (P > 0.05).The expressions of apoptosis arrestin PED/PEA-15 and XIAP are elevated in HCC as compared with adjacent tissues and normal tissues. Thus it may serve as both a reference indicator for biological behaviors of HCC and a new target for gene therapy. 
 RhoGDI SUMOylation at Lys-138 increases its binding activity to Rho GTPase and its inhibiting cancer cell motility. The Rho GDP dissociation inhibitor (RhoGDI) can bind to small GTPases and keep them in a biologically inactive state in cytoplasm, through which it affects actin polymerization and cell motility. However, mechanisms underlying how RhoGDI regulates Rho GTPase complex formation/membrane extraction/GTPase dissociation remain largely unexplored. Our previous studies reported that X-linked inhibitor of apoptosis protein (XIAP) interacted with RhoGDI via its RING domain and negatively modulated RhoGDI SUMOylation and HCT116 cancer cell migration. Here, we identified that RhoGDI SUMOylation specifically occurred at Lys-138, which was inhibited by XIAP domain. We further demonstrated that RhoGDI SUMOylation at Lys-138 was crucial for inhibiting actin polymerization and cytoskeleton formation as well as cancer cell motility. Moreover, SUMO-RhoGDI had a much higher binding affinity to small Rho GTPase compared with the un-SUMOylated form of RhoGDI. Taken together, our study demonstrated a novel modification of RhoGDI, SUMOylation at Lys-138, which played a key role in regulating Rho GTPase activation in cancer cells. The physiological regulation of RhoGDI SUMOylation by the RING domain of XIAP may account for modulation of cancer cell invasion and   metastasis   by XIAP. 
 Identification of novel biomarkers in neuroblastoma associated with the risk for bone marrow   metastasis  : a pilot study. Neuroblastoma is a common pediatric neoplasm with variable histopathological features that carry an inherent risk of developing distant metastases, in particular bone marrow   metastasis  . Nestin, X-linked inhibitor of apoptosis (XIAP) and vascular growth factors (VEGF) are biomarkers that are implicated in the tumorigenesis of various cancers. We studied the expression of these biomarkers in neuroblastoma, in relation to bone marrow (BM)   metastasis   and other histologic parameters.Patients with neuroblastoma included seven with BM   metastasis   and 12 with non-metastatic tumors. Slides from the primary tumors were immunostained with antibodies against nestin, XIAP, VEGF-A, VEGF-B, VEGF-D, VEGF-R1, and VEGF-R2. Immunostaining results were evaluated by two pathologists, graded and statistically correlated with the risk of developing BM   metastasis  .Nestin was expressed in 16/19 cases with no significant difference between patients with BM   metastasis   and those without BM   metastasis  . XIAP was identified in 18/19 tumor cases; the staining density was significantly lower in patients with bone marrow   metastasis   and those with unfavorable histology. VEGF-R1, VEGF-R2, and VEGF-B were expressed while VEGF-A and VEGF-D were not. Significantly, higher expression of VEGF-B was noted in patients with BM   metastasis  .Expression of VEGF-B and XIAP in neuroblastoma may play a role in the development of bone marrow   metastasis  . Given the limited number of patients in this study, a larger cohort is needed to validate these findings. 
 MicroRNA-874 inhibits growth, induces apoptosis and reverses chemoresistance in colorectal cancer by targeting X-linked inhibitor of apoptosis protein. MicroRNA-874 (miR-874) is downregulated and acts as a tumor suppressor in several types of cancers, whereas the biological function of miR-874 in colorectal cancer (CRC) remains unclear. The aims of the present study were to investigate the clinical significance, biological effects, and the underlying mechanisms of miR-874 in CRC. Reverse transcription-quantitative PCR (RT-qPCR) was used to detect miR-874 expression in CRC cell lines and tissue samples. MTT and colony formation assays and flow cytometry were performed to analyze the effects of miR-874 expression on growth, apoptosis and the chemoresistance of CRC cells. Regulation of putative miR-874 targets was determined by dual-luciferase reporter assays. RT-qPCR and western blot assays were performed to detected the levels of X-linked inhibitor of apoptosis protein (XIAP) mRNA and protein expression. It was found that expression of miR-874 was downregulated in CRC tissues and cell lines, and its expression was significantly negatively correlated with TNM stage and lymph node   metastasis   of the CRC patients. Functional assays revealed that restoration of miR-874 inhibited proliferation, reduced colony formation, enhanced apoptosis, as well as decreased the 5-fluorouracil (5-FU) resistance of the CRC cells. Through luciferase activity assay, RT-qPCR and western blot analysis, XIAP was shown to be a direct target of miR-874. In addition, XIAP expression was significantly increased in the CRC tissues and cell lines, and was inversely correlated with miR-874 expression. Importantly, downregulation of XIAP in CRC cells had an effect similar to that of miR-874 overexpression. Taken together, these data showed that miR-874 inhibits growth, increases apoptosis and enhances chemosensitivity in CRC cells by targeting XIAP, suggesting that miR-874 may be a potential molecular target for the treatment of human CRC. 
 A mitochondrial block and expression of XIAP lead to resistance to TRAIL-induced apoptosis during progression to   metastasis   of a colon carcinoma. A pair of isogenic colon carcinoma cells, SW480 and 620, was used to investigate the mechanisms of acquired tumour necrosis factor-related apoptosis-inducing ligand (TRAIL)-resistance during tumour progression. Whereas primary tumour SW480 cells are sensitive to TRAIL-induced apoptosis, metastatic SW620 cells are resistant. The apoptotic signalling activated by TRAIL in SW480 cells is a type II pathway. We show that in SW620 cells, although caspase-8 is recruited and activated at the death-inducing-signalling complex and Bid is cleaved, this does not lead to caspase-9 activation. Comparison of Bcl-2, Bcl-xL and Mcl-1 levels in both cell lines showed no difference. In SW620 cells transfected with a tBid-GFP construct, tBid-GFP was correctly localized to the mitochondria. Thus, the resistance of SW620 cells is at the level of the mitochondria that can withstand large amounts of tBid. Although caspase-3 was directly cleaved by caspase-8 in SW620 cells to yield the p20 fragment, no further autocatalytic maturation into the p17 fragment was observed. We show that, in contrast to SW480 cells, the SW620 cell line expresses high amounts of X-linked inhibitor of apoptosis (XIAP). Downregulation of XIAP with bortezomib or small-interfering RNA was sufficient to restore the sensitivity of SW620 cells to TRAIL-induced apoptosis in the absence of SMAC/Diablo or cytochrome c release from the mitochondria. Thus, SW620 cells have developed a dual resistance to TRAIL-induced apoptosis: a block at the level of the mitochondria and, after a conversion to a type I pathway, an increased expression of XIAP which inhibits this pathway. 
 Expression of X-linked inhibitor of apoptosis protein in neoplastic thyroid disorder. X-linked inhibitor of apoptosis protein (XIAP) is associated with tumor genesis, growth, progression and   metastasis  , and acts by blocking caspase-mediated apoptosis. In the present study, we sought to evaluate the expression patterns of XIAP in various neoplastic thyroid disorders and determine the association between XIAP expression and clinicopathologic factors. Expression of XIAP was evaluated with immunohistochemical staining using monoclonal anti-XIAP in 164 specimens of conventional papillary thyroid carcinoma (PTC) and 53 specimens of other malignant or benign thyroid tumors. XIAP positivity was observed in 128 (78%) of the 164 conventional PTC specimens. Positive rates of XIAP expression in follicular variant PTC, follicular, medullary, poorly differentiated, and anaplastic thyroid carcinoma specimens were 20%, 25%, 38%, 67%, and 38%, respectively. Six nodular hyperplasia specimens were negative and 1 of 7 follicular adenomas (8%) was positive for XIAP. Lateral neck lymph node metastases were more frequent in patients negative for XIAP expression (P = 0.01). Immunohistochemical staining for XIAP as a novel molecular marker may thus be helpful in the differential diagnosis of thyroid cancer. Moreover, high XIAP expression in conventional PTC is strongly associated with reduced risk of lateral neck lymph node   metastasis  . 
 Cord blood stem cell-mediated induction of apoptosis in glioma downregulates X-linked inhibitor of apoptosis protein (XIAP). XIAP (X-linked inhibitor of apoptosis protein) is one of the most important members of the apoptosis inhibitor family. XIAP is upregulated in various malignancies, including human glioblastoma. It promotes invasion,   metastasis  , growth and survival of malignant cells. We hypothesized that downregulation of XIAP by human umbilical cord blood mesenchymal stem cells (hUCBSC) in glioma cells would cause them to undergo apoptotic death.We observed the effect of hUCBSC on two malignant glioma cell lines (SNB19 and U251) and two glioma xenograft cell lines (4910 and 5310). In co-cultures of glioma cells with hUCBSC, proliferation of glioma cells was significantly inhibited. This is associated with increased cytotoxicity of glioma cells, which led to glioma cell death. Stem cells induced apoptosis in glioma cells, which was evaluated by TUNEL assay, FACS analyses and immunoblotting. The induction of apoptosis is associated with inhibition of XIAP in co-cultures of hUCBSC. Similar results were obtained by the treatment of glioma cells with shRNA to downregulate XIAP (siXIAP). Downregulation of XIAP resulted in activation of caspase-3 and caspase-9 to trigger apoptosis in glioma cells. Apoptosis is characterized by the loss of mitochondrial membrane potential and upregulation of mitochondrial apoptotic proteins Bax and Bad. Cell death of glioma cells was marked by downregulation of Akt and phospho-Akt molecules. We observed similar results under in vivo conditions in U251- and 5310-injected nude mice brains, which were treated with hUCBSC. Under in vivo conditions, Smac/DIABLO was found to be colocalized in the nucleus, showing that hUCBSC induced apoptosis is mediated by inhibition of XIAP and activation of Smac/DIABLO.Our results indicate that downregulation of XIAP by hUCBSC treatment induces apoptosis, which led to the death of the glioma cells and xenograft cells. This study demonstrates the therapeutic potential of XIAP and hUCBSC to treat malignant gliomas. 
 Immunohistochemical detection of XIAP in melanoma. The X-linked inhibitor of apoptosis protein (XIAP) is the most potent of the inhibitor of apoptosis family of eight proteins. High levels of XIAP have been found in melanoma cell lines and are believed to play a role in therapeutic resistance in a number of malignancies. XIAP expression has not been investigated in clinically obtained melanoma tissue samples, nor have studies attempted to correlate XIAP expression with prognostic variables or clinical aggressiveness of melanomas.Sixty-seven patients with primary cutaneous malignant melanoma for whom clinical follow up was available were identified from the records of the Mount Sinai Hospital, comprising 37 thin melanomas (Breslow thickness < 1.0 mm) and 30 thick melanomas (Breslow thickness > 1.0 mm). Archival paraffin sections from primary lesions and corresponding metastases were stained with monoclonal anti-XIAP antibody using routine immunohistochemical methods.Six benign intradermal nevi and four in situ melanomas were XIAP negative. 9 of 37 thin melanomas (24%) were XIAP positive. In contrast, 21 of 30 (73%) thick melanomas were XIAP positive, including 3 of 4 ulcerated melanomas that were strongly positive. Over a follow-up period ranging from 6 months to 6 years, 23 melanomas metastasized (22 thick, 1 thin). In total, XIAP was immunohistochemically detected in 17 of 23 metastases (74%).   Metastasis   occurred in 1 of 9 XIAP-positive thin melanomas; 0 of 28 XIAP-negative thin melanomas; 17 of 22 XIAP-positive thick melanomas, and 5 of 8 XIAP-negative thick melanomas (63%).XIAP is immunohistochemically detectable nearly three times more frequently in thick compared with thin melanomas. These results suggest that XIAP elevation may be correlated with increasing melanoma thickness and tumor progression. 
 XIAP gene downregulation by small interfering RNA inhibits proliferation, induces apoptosis, and reverses the cisplatin resistance of ovarian carcinoma. XIAP is one of the most important members of the inhibitors of apoptosis family. It is upregulated in various malignancies, including human ovarian carcinomas, it promotes invasion,   metastasis  , growth, and survival of malignant cells, and it also confers resistance to some chemotherapeutic drugs. We observed the effect of XIAP gene RNA interference (RNAi) on the proliferation, apoptosis, tumorigenicity, and chemosensitivity of the human ovarian carcinoma cells A2780/cp70.We used a human U6 promoter-driven DNA template approach to induce short hairpin RNA-triggered RNAi to block XIAP gene expression in the human ovarian cancer cell line A2780/cp70. A corresponding site-mutated vector was constructed as a negative control (pSilencer 2.1-NC). The expression of XIAP mRNA and protein among the stable transfected cells and the untransfected ones was detected by RT-PCR and Western blotting. The cell growth was examined and drug sensitivity was assayed using the MTT assay. Cell apoptosis was measured by flow cytometry and the tumorigenicity by using nude mice.Three stable transfected cell lines: A2780/cp70-s2, A2780/cp70-NC, and A2780/cp70-neo were established. The expression levels of XIAP gene mRNA and protein in A2780/cp70-s2 were 63.3% and 60.8%, lower than in A2780/cp70-NC, A2780/cp70-neo, and untransfected A2780/cp70 cells. The cell proliferation of A2780/cp70-s2 was reduced by up to 62.2+/-1.9%. Compared with the other cell lines, the changes in apoptotic rate in A2780/cp70-s2 was 31.1+/-1.3%, obviously increasing (P<0.05). The knockdown of XIAP retards tumorigenicity in nude mice and after 21 days the average tumor weight of the A2780/cp70-s2 group was lower by 1.62+/-0.11g (P<0.05). And the survival index in A2780/cp70-s2 cells was markedly reduced with the addition of 1 microM and 10 microM cisplatinum, respectively (P<0.05).XIAP gene RNAi inhibited the proliferation of ovarian carcinoma cells and caused cells to be more sensitive to cisplatin through the reduction of its mRNA and protein. These results suggest that XIAP is an ovarian cancer-related gene and that XIAP is a potential target for therapeutic anti-cancer drugs. 
 Ceramide targets xIAP and cIAP1 to sensitize metastatic colon and breast cancer cells to apoptosis induction to suppress tumor progression. Ceramide is a bioeffector that mediates various cellular processes, including apoptosis. However, the mechanism underlying ceramide function in apoptosis is apparently cell type-dependent and is not well-understood. We aimed at identifying molecular targets of ceramide in metastatic human colon and breast cancer cells, and determining the efficacy of ceramide analog in suppression of colon and breast cancer   metastasis  .The activity of and mechanism underlying ceramide as a cytotoxic agent, and as a sensitizer for Fas-mediated apoptosis was analyzed in human cell lines established from primary or metastatic colon and breast cancers. The efficacy of ceramide analog LCL85 in suppression of   metastasis   was examined in preclinical mouse tumor models.Exposure of human colon carcinoma cells to ceramide analog LCL85 results in apoptosis in a dose-dependent manner. Interestingly, a sublethal dose of LCL85 increased C16 ceramide content and overcame tumor cell resistance to Fas-mediated apoptosis. Subsequently, treatment of tumor cells with exogenous C16 ceramide resulted in increased tumor cell sensitivity to Fas-mediated apoptosis. LCL85 resembles Smac mimetic BV6 in sensitization of colon carcinoma cells to Fas-mediated apoptosis by inducing proteasomal degradation of cIAP1 and xIAP proteins. LCL85 also decreased xIAP1 and cIAP1 protein levels and sensitized metastatic human breast cancer cells to Fas-mediated apoptosis. Silencing xIAP and cIAP1 with specific siRNAs significantly increased the metastatic human colon carcinoma cell sensitivity to Fas-mediated apoptosis, suggesting that IAP proteins mediate apoptosis resistance in metastatic human colon carcinoma cells and ceramide induces IAP protein degradation to sensitize the tumor cells to apoptosis induction. Consistent with its apoptosis sensitization activity, subtoxic doses of LCL85 suppressed colon carcinoma cell metastatic potential in an experimental lung   metastasis   mouse model, as well as breast cancer growth and spontaneous lung   metastasis   in an orthotopic breast cancer mouse model.We have identified xIAP and cIAP1 as molecular targets of ceramide and determined that ceramide analog LCL85 is an effective sensitizer in overcoming resistance of human cell lines established from metastatic colon and breast cancers to apoptosis induction to suppress   metastasis   in vivo. 
 Tissue microarray analysis of X-linked inhibitor of apoptosis (XIAP) expression in breast cancer patients. The goal of this study was to determine the diagnostic and prognostic potential of X-linked inhibitor of apoptosis (XIAP) expression in breast cancer. We analyzed a tissue microarray comprised of 100 breast cancer cases and 70 matched normal samples. Analysis of an online database, which included 2,977 patients, was also performed. There was a significant difference in cytoplasmic expression of XIAP (XIAP-C) between breast cancer tissue and matched normal (p<0.001). Staining of XIAP-C was defined as negative (breast cancer 8.42% vs. normal 30.91%), slight (40.0 vs. 45.45%), moderate (43.16 vs. 23.64%), or high (8.42 vs. 0%). High XIAP-C protein expression correlated with human epidermal growth factor receptor 2 (HER-2) status (p=0.010) and with human p53 mutant-type (P53) status (p=0.039). We found that XIAP expression did not correlate with disease-free survival (p=0.706) and overall survival (p=0.496) of breast cancer patients. An Internet-based system analysis confirmed our results. In the subgroup analysis, basal-like breast cancer patients with high XIAP levels in the tumor had a significantly increased risk of relapse; thus, the up-regulation of XIAP appeared to be predictive of poor relapse-free survival (p=0.013). Kaplan-Meier curves also identified a significant correlation between distant   metastasis  -free survival and XIAP expression in patients with lymph-node-negative disease (p=0.030). In summary, expression of XIAP-C was significantly higher in breast cancer compared to normal tissue. XIAP-C expression correlated with HER-2 status and may be considered a prognostic biomarker for basal-like breast cancer patients. 
 E3 ligase activity of XIAP RING domain is required for XIAP-mediated cancer cell migration, but not for its RhoGDI binding activity. Although an increased expression level of XIAP is associated with cancer cell   metastasis  , the underlying molecular mechanisms remain largely unexplored. To verify the specific structural basis of XIAP for regulation of cancer cell migration, we introduced different XIAP domains into XIAP(-/-) HCT116 cells, and found that reconstitutive expression of full length HA-XIAP and HA-XIAP ΔBIR, both of which have intact RING domain, restored β-Actin expression, actin polymerization and cancer cell motility. Whereas introduction of HA-XIAP ΔRING or H467A mutant, which abolished its E3 ligase function, did not show obvious restoration, demonstrating that E3 ligase activity of XIAP RING domain played a crucial role of XIAP in regulation of cancer cell motility. Moreover, RING domain rather than BIR domain was required for interaction with RhoGDI independent on its E3 ligase activity. To sum up, our present studies found that role of XIAP in regulating cellular motility was uncoupled from its caspase-inhibitory properties, but related to physical interaction between RhoGDI and its RING domain. Although E3 ligase activity of RING domain contributed to cell migration, it was not involved in RhoGDI binding nor its ubiquitinational modification. 
  GeneRIFs  
 Identify xIAP and cIAP1 as molecular targets of ceramide and show ceramide analog LCL85 is an effective sensitizer in overcoming resistance of metastatic colon and breast cancers to apoptosis induction to suppress   metastasis   in vivo. 
 Low XIAP expression is associated with bone marrow   metastasis   in neuroblastoma. 
 The physiological regulation of RhoGDI SUMOylation by the RING domain of XIAP may account for modulation of cancer cell invasion and   metastasis   by XIAP 
 A mitochondrial block and expression of XIAP lead to resistance to TRAIL-induced apoptosis during progression to   metastasis   of a colon carcinoma. 
 Important molecule in controlling hepatocellular carcinoma   metastasis  . XIAP can be a molecular target subject to intervention to reduce   metastasis   and recurrence. 
  [Cc]ell [Ii]nvasion  PubMed  
 FOXM1 targets XIAP and Survivin to modulate breast cancer survival and chemoresistance. Drug resistance is a major hurdle for successful treatment of breast cancer, the leading cause of deaths in women throughout the world. The FOXM1 transcription factor is a potent oncogene that transcriptionally regulates a wide range of target genes involved in DNA repair, metastasis,   cell invasion  , and migration. However, little is known about the role of FOXM1 in cell survival and the gene targets involved. Here, we show that FOXM1-overexpressing breast cancer cells display an apoptosis-resistant phenotype, which associates with the upregulation of expression of XIAP and Survivin antiapoptotic genes. Conversely, FOXM1 knockdown results in XIAP and Survivin downregulation as well as decreased binding of FOXM1 to the promoter regions of XIAP and Survivin. Consistently, FOXM1, XIAP, and Survivin expression levels were higher in taxane and anthracycline-resistant cell lines when compared to their sensitive counterparts and could not be downregulated in response to drug treatment. In agreement with our in vitro findings, we found that FOXM1 expression is significantly associated with Survivin and XIAP expression in samples from patients with IIIa stage breast invasive ductal carcinoma. Importantly, patients co-expressing FOXM1, Survivin, and nuclear XIAP had significantly worst overall survival, further confirming the physiological relevance of the regulation of Survivin and XIAP by FOXM1. Together, these findings suggest that the overexpression of FOXM1, XIAP, and Survivin contributes to the development of drug-resistance and is associated with poor clinical outcome in breast cancer patients. 
 Simultaneous silencing of XIAP and survivin causes partial mesenchymal-epithelial transition of human pancreatic cancer cells via the PTEN/PI3K/Akt pathway. Pancreatic cancer has one of the highest mortality rates among malignant tumors and is characterized by rapid invasion, early metastasis and chemoresistance. X-linked inhibitor of apoptosis (XIAP) and survivin are two of the most important members of the IAP family. Previous studies have shown that XIAP and survivin were overexpressed in pancreatic cancer and were closely associated with cell proliferation and chemoresistance to gemcitabine. In the present study, stable inhibition of XIAP and survivin in Panc-1 cells was performed using lentivirus-carried short hairpin RNAs. The expression of XIAP, survivin, E-cadherin, Slug, phosphatase and tensin homolog (PTEN) and phosphorylated Akt was then measured. In addition, cell proliferation, apoptosis, invasion and migration were assessed. The results showed that stable inhibition of XIAP and survivin expression in Panc-1 cells significantly reduced cell proliferation, increased apoptosis and partially reversed the epithelial-mesenchymal transition (EMT). Furthermore, the results of the present study demonstrated that the partial reversal of the EMT was accompanied by inhibited   cell invasion   and migration as well as increased chemosensitivity to gemcitabine in pancreatic cancer cells; this was indicated to be mediated via the PTEN/phosphatidylinositol 3-kinase/Akt signaling pathway. In conclusion, these results suggested that simultaneous inhibition of XIAP and survivin may be a promising strategy for the treatment of pancreatic cancer. 
 uPAR and cathepsin B shRNA impedes TGF-β1-driven proliferation and invasion of meningioma cells in a XIAP-dependent pathway. Overexpression of transforming growth factor β1 (TGF-β1) has been linked to immune suppression, tumor angiogenesis, tumor cell migration, tumor cell survival, and tum  or cell invas  ion in many cancers. In the present study, we found abundant expression of TGF-β1 in the microenvironment of four different pathological types of meningioma tumors. TGF-β1 induced invasion in malignant meningioma cells with an associated upregulation of urokinase-type plasminogen activator (uPA), uPAR, cathepsin B, and MMP-9, and this increase in proliferation was coupled with the expression of anti-apoptotic and pro-survival signaling molecules. In addition to the intense immunoreactivity of meningioma tumors to X-linked inhibitor to apoptosis (XIAP), its knockdown abolished the TGF-β1-induced proliferation of these cells. The stimulation of XIAP expression and the activation of pSMAD-2 is mediated by phosphatidylinositol 3-kinase (PI3K)- and MEK-dependent pathways, and the addition of anti-TGF-β1 antibodies prevented their expression with a consequent decrease in invasion. Bicistronic shRNA constructs targeting uPAR and cathepsin B (pUC) quenched TGF-β1-driven invasion and survival of meningioma cells by downregulation of XIAP and pSMAD-2 expression. Animal models with intracranial tumors showed elevated levels of TGF-β1, XIAP and pSMAD-2, and pUC treatment prevented this increased expression. Thus, targeted silencing of TGF-β1-induced signaling by pUC in meningioma would provide new treatment approaches for management of meningioma. 
 RhoGDI SUMOylation at Lys-138 increases its binding activity to Rho GTPase and its inhibiting cancer cell motility. The Rho GDP dissociation inhibitor (RhoGDI) can bind to small GTPases and keep them in a biologically inactive state in cytoplasm, through which it affects actin polymerization and cell motility. However, mechanisms underlying how RhoGDI regulates Rho GTPase complex formation/membrane extraction/GTPase dissociation remain largely unexplored. Our previous studies reported that X-linked inhibitor of apoptosis protein (XIAP) interacted with RhoGDI via its RING domain and negatively modulated RhoGDI SUMOylation and HCT116 cancer cell migration. Here, we identified that RhoGDI SUMOylation specifically occurred at Lys-138, which was inhibited by XIAP domain. We further demonstrated that RhoGDI SUMOylation at Lys-138 was crucial for inhibiting actin polymerization and cytoskeleton formation as well as cancer cell motility. Moreover, SUMO-RhoGDI had a much higher binding affinity to small Rho GTPase compared with the un-SUMOylated form of RhoGDI. Taken together, our study demonstrated a novel modification of RhoGDI, SUMOylation at Lys-138, which played a key role in regulating Rho GTPase activation in cancer cells. The physiological regulation of RhoGDI SUMOylation by the RING domain of XIAP may account for modulation of cancer   cell invasion   and metastasis by XIAP. 
  GeneRIFs  
 The physiological regulation of RhoGDI SUMOylation by the RING domain of XIAP may account for modulation of cancer   cell invasion   and metastasis by XIAP 
   
  
 BIRC2 
 
  NCBI Gene  
  GeneRIF  
  Pubmed  
 
 
  Search Term  Rank  Fields  
  [Ff]ilopodia  1  PubMed(1)  
  [Mm]igration  6  PubMed(6)  
  [Ii]nvasive  10  PubMed(4)  
  [Mm]etastasis  12  PubMed(8); GeneRIFs(3)  
  [Cc]ell [Ii]nvasion  13  PubMed(1)  
 
 
 hide/show details for BIRC2 
  [Ff]ilopodia  PubMed  
 cIAP1 regulates TNF-mediated cdc42 activation and   filopodia   formation. Tumour necrosis factor-α (TNF) is a cytokine endowed with multiple functions, depending on the cellular and environmental context. TNF receptor engagement induces the formation of a multimolecular complex including the TNFR-associated factor TRAF2, the receptor-interaction protein kinase RIP1 and the cellular inhibitor of apoptosis cIAP1, the latter being essential for NF-κB activation. Here, we show that cIAP1 also regulates TNF-induced actin cytoskeleton reorganization through a cdc42-dependent, NF-κB-independent pathway. Deletion of cIAP1 prevents TNF-induc  ed filopo  dia and cdc42 activation. The expression of cIAP1 or its E3-ubiquitin ligase-defective mutant restores the ability of cIAP1(-/-) MEFs to produ  ce filopo  dia, whereas a cIAP1 mutant unable to bind TRAF2 does not. Accordingly, the silencing of TRAF2 inhibits TNF-mediat  ed filopo  dia formation, whereas silencing of RIP1 does not. cIAP1 directly binds cdc42 and promotes its RhoGDIα-mediated stabilization. TNF decreases cIAP1-cdc42 interaction, suggesting that TNF-induced recruitment of cIAP1/TRAF2 to the receptor releases cdc42, which in turn triggers actin remodeling. cIAP1 also regulates cdc42 activation in response to EGF and HRas-V12 expression. A downregulation of cIAP1 altered the cell polarization, the cell adhesion to endothelial cells and cell intercalation, which are cdc42-dependent processes. Finally, we demonstrated that the deletion of cIAP1 regulated the HRas-V12-mediated transformation process, including anchorage-dependent cell growth, tumour growth in a xenograft model and the development of experimental metastasis in the lung. 
  [Mm]igration  PubMed  
 IAPs regulate the plasticity of cell   migration   by directly targeting Rac1 for degradation. Inhibitors of apoptosis proteins (IAPs) are a highly conserved class of multifunctional proteins. Rac1 is a well-studied Rho GTPase that controls numerous basic cellular processes. While the regulation of nucleotide binding to Rac1 is well understood, the molecular mechanisms controlling Rac1 degradation are not known. Here, we demonstrate X-linked IAP (XIAP) and cellular IAP1 (c-IAP1) directly bind to Rac1 in a nucleotide-independent manner to promote its polyubiquitination at Lys147 and proteasomal degradation. These IAPs are also required for degradation of Rac1 upon CNF1 toxin treatment or RhoGDI depletion. Consistently, downregulation of XIAP or c-IAP1 by various strategies led to an increase in Rac1 protein levels in primary and tumour cells, leading to an elongated morphology and enhanced cell   migration  . Further, XIAP counteracts Rac1-dependent cellular polarization in the developing zebrafish hindbrain and promotes the delamination of neurons from the normal tissue architecture. These observations unveil an evolutionarily conserved role of IAPs in controlling Rac1 stability thereby regulating the plasticity of cell   migration   and morphogenesis. 
 CARD-mediated autoinhibition of cIAP1's E3 ligase activity suppresses cell proliferation and   migration  . E3 ligases mediate the covalent attachment of ubiquitin to target proteins thereby enabling ubiquitin-dependent signaling. Unraveling how E3 ligases are regulated is important because miscontrolled ubiquitylation can lead to disease. Cellular inhibitor of apoptosis (cIAP) proteins are E3 ligases that modulate diverse biological processes such as cell survival, proliferation, and   migration  . Here, we have solved the structure of the caspase recruitment domain (CARD) of cIAP1 and identified that it is required for cIAP1 autoregulation. We demonstrate that the CARD inhibits activation of cIAP1's E3 activity by preventing RING dimerization, E2 binding, and E2 activation. Moreover, we show that the CARD is required to suppress cell proliferation and   migration  . Further, CARD-mediated autoregulation is also necessary to maximally suppress caspase-8-dependent apoptosis and vascular tree degeneration in vivo. Taken together, our data reveal mechanisms by which the E3 ligase activity of cIAP1 is controlled, and how its deregulation impacts on cell proliferation,   migration   and cell survival. 
 cIAP1-dependent TRAF2 degradation regulates the differentiation of monocytes into macrophages and their response to CD40 ligand. Peripheral blood monocytes are plastic cells that migrate to tissues and differentiate into various cell types, including macrophages, dendritic cells, and osteoclasts. We have described the   migration   of cellular inhibitor of apoptosis protein 1 (cIAP1), a member of the IAP family of proteins, from the nucleus to the Golgi apparatus in monocytes undergoing differentiation into macrophages. Here we show that, once in the cytoplasm, cIAP1 is involved in the degradation of the adaptor protein tumor necrosis factor receptor-associated factor 2 (TRAF2) by the proteosomal machinery. Inhibition of cIAP1 prevents the decrease in TRAF2 expression that characterizes macrophage formation. We demonstrate that TRAF2 is initially required for macrophage differentiation as its silencing prevents Ikappa-Balpha degradation, nuclear factor-kappaB (NF-kappaB) p65 nuclear translocation, and the differentiation process. Then, we show that cIAP1-mediated degradation of TRAF2 allows the differentiation process to progress. This degradation is required for the macrophages to be fully functional as TRAF2 overexpression in differentiated cells decreases the c-Jun N-terminal kinase-mediated synthesis and the secretion of proinflammatory cytokines, such as interleukin-8 and monocyte chemoattractant protein 1 (MCP-1) in response to CD40 ligand. We conclude that TRAF2 expression and subsequent degradation are required for the differentiation of monocytes into fully functional macrophages. 
 Identification of ARIA regulating endothelial apoptosis and angiogenesis by modulating proteasomal degradation of cIAP-1 and cIAP-2. Endothelial apoptosis is a pivotal process for angiogenesis during embryogenesis as well as postnatal life. By using a retrovirus-mediated signal sequence trap method, we identified a previously undescribed gene, termed ARIA (apoptosis regulator through modulating IAP expression), which regulates endothelial apoptosis and angiogenesis. ARIA was expressed in blood vessels during mouse embryogenesis, as well as in endothelial cells both in vitro and in vivo. ARIA is a unique protein with no homology to previously reported conserved domain structures. Knockdown of ARIA in HUVECs by using small interfering RNA significantly reduced endothelial apoptosis without affecting either cell   migration   or proliferation. ARIA knockdown significantly increased inhibitor of apoptosis (cIAP)-1 and cIAP-2 protein expression, although their mRNA expression was not changed. Simultaneous knockdown of cIAP-1 and cIAP-2 abolished the antiapoptotic effect of ARIA knockdown. Using yeast 2-hybrid screening, we identified the interaction of ARIA with 20S proteasome subunit alpha-7. Thereafter, we found that cIAP-1 and cIAP-2 were degraded by proteasomes in endothelial cells under normal condition. Overexpression of ARIA significantly reduced cIAP-1 expression, and this reduction was abolished by proteasomal inhibition in BAECs. Also, knockdown of ARIA demonstrated an effect similar to proteasomal inhibition with respect to not only expression but also subcellular localization of cIAP-1 and cIAP-2. In vivo angiogenesis studied by Matrigel-plug assay, mouse ischemic retinopathy model, and tumor xenograft model was significantly enhanced by ARIA knockdown. Together, our data indicate that ARIA is a unique factor regulating endothelial apoptosis, as well as angiogenesis, presumably through modulating proteasomal degradation of cIAP-1 and cIAP-2 in endothelial cells. 
 Interaction of heat-shock protein 90 beta isoform (HSP90 beta) with cellular inhibitor of apoptosis 1 (c-IAP1) is required for cell differentiation. Members of the inhibitor of apoptosis protein (IAP) family have demonstrated functions in cell death, cell signalling, cell   migration   and mitosis. Several of them are E3 enzymes in the ubiquitination of proteins that leads to their degradation by the proteosomal machinery. We previously reported that one of them, cellular inhibitor of apoptosis protein-1 (c-IAP1), migrated from the nucleus to the surface of the Golgi apparatus in cells undergoing differentiation. Here, we show that c-IAP1 is a client protein of the stress protein HSP90 beta. In three distinct cellular models, the two proteins interact and migrate from the nucleus to the cytoplasm along the differentiation process through a leptomycin B-sensitive pathway. Inhibition of HSP90 proteins by small chemical molecules and specific depletion of HSP90 beta isoform by siRNA both lead to auto-ubiquitination of c-IAP1 and its degradation by the proteasome machinery. This chaperone function of HSP90 towards c-IAP1 is specific of its beta isoform as specific depletion of HSP90alpha does not affect c-IAP1 content. Chemical inhibition of HSP90 or siRNA-mediated depletion of HSP90 beta both inhibit cell differentiation, which can be reproduced by siRNA-mediated depletion of c-IAP1. Altogether, these results suggest that HSP90 beta prevents auto-ubiquitination and degradation of its client protein c-IAP1, whose depletion would be sufficient to inhibit cell differentiation. 
 Inhibitor of apoptosis proteins as novel targets in inflammatory processes. Inhibitor of apoptosis proteins (IAPs), such as X-linked or cellular IAP 1/2 (XIAP, cIAP1/2), are important regulators of apoptosis. IAP antagonists are currently under clinical investigation as anticancer agents. Interestingly, IAPs participate in the inflammation-associated TNF receptor signaling complex and regulate NFκB signaling. This raises the question about the role of IAPs in inflammation. Here, we investigated the anti-inflammatory potential of IAP inhibitors and the role of IAPs in inflammatory processes of endothelial cells.In mice, the small molecule IAP antagonist A-4.10099.1 (ABT) suppressed antigen-induced arthritis, leukocyte infiltration in concanavalin A-evoked liver injury, and leukocyte tran  smigratio  n in the TNFα-activated cremaster muscle. In vitro, we observed an attenuation of leukocyte-endothelial cell interaction by downregulation of the intercellular adhesion molecule-1. ABT did not impair NFκB signaling but decreased the TNFα-induced activation of the TGF-β-activated kinase 1, p38, and c-Jun N-terminal kinase. These effects are based on the proteasomal degradation of cIAP1/2 accompanied by an altered ratio of the levels of membrane-localized TNF receptor-associated factors 2 and 5.Our results reveal IAP antagonism as a profound anti-inflammatory principle in vivo and highlight IAPs as important regulators of inflammatory processes in endothelial cells. 
  [Ii]nvasive  PubMed  
 Genetic susceptibility to distinct bladder cancer subphenotypes. Clinical, pathologic, and molecular evidence indicate that bladder cancer is heterogeneous with pathologic/molecular features that define distinct subphenotypes with different prognoses. It is conceivable that specific patterns of genetic susceptibility are associated with particular subphenotypes.To examine evidence for the contribution of germline genetic variation to bladder cancer heterogeneity.The Spanish Bladder Cancer/EPICURO Study is a case-control study based in 18 hospitals located in five areas in Spain. Cases were patients with a newly diagnosed, histologically confirmed, urothelial cell carcinoma of the bladder from 1998 to 2001. Case diagnoses were reviewed and uniformly classified by pathologists following the World Health Organisation/International Society of Urological Pathology 1999 criteria. Controls were hospital-matched patients (n=1149).A total of 1526 candidate variants in 423 candidate genes were analysed. Three distinct subphenotypes were defined according to stage and grade: low-grade nonmuscle   invasive   (n=586), high-grade nonmuscle   invasive   (n=219), and muscle   invasive   (n=246). The association between each variant and subphenotype was assessed by polytomous risk models adjusting for potential confounders. Heterogeneity in genetic susceptibility among subphenotypes was also tested.Two established bladder cancer susceptibility genotypes, NAT2 slow-acetylation and GSTM1-null, exhibited similar associations among the subphenotypes, as did VEGF-rs25648, which was previously identified in our study. Other variants conferred risks for specific tumour subphenotypes such as PMS2-rs6463524 and CD4-rs3213427 (respective heterogeneity p values of 0.006 and 0.004), which were associated with muscle-  invasive   tumours (per-allele odds ratios [95% confidence interval] of 0.56 [0.41-0.77] and 0.71 [0.57-0.88], respectively) but not with non-muscle-  invasive   tumours. Heterogeneity p values were not robust in multiple testing according to their false-discovery rate.These exploratory analyses suggest that genetic susceptibility loci might be related to the molecular/pathologic diversity of bladder cancer. Validation through large-scale replication studies and the study of additional genes and single nucleotide polymorphisms are required. 
 Genetic susceptibility to distinct bladder cancer subphenotypes. Clinical, pathologic, and molecular evidence indicate that bladder cancer is heterogeneous with pathologic/molecular features that define distinct subphenotypes with different prognoses. It is conceivable that specific patterns of genetic susceptibility are associated with particular subphenotypes.To examine evidence for the contribution of germline genetic variation to bladder cancer heterogeneity.The Spanish Bladder Cancer/EPICURO Study is a case-control study based in 18 hospitals located in five areas in Spain. Cases were patients with a newly diagnosed, histologically confirmed, urothelial cell carcinoma of the bladder from 1998 to 2001. Case diagnoses were reviewed and uniformly classified by pathologists following the World Health Organisation/International Society of Urological Pathology 1999 criteria. Controls were hospital-matched patients (n=1149).A total of 1526 candidate variants in 423 candidate genes were analysed. Three distinct subphenotypes were defined according to stage and grade: low-grade nonmuscle   invasive   (n=586), high-grade nonmuscle   invasive   (n=219), and muscle   invasive   (n=246). The association between each variant and subphenotype was assessed by polytomous risk models adjusting for potential confounders. Heterogeneity in genetic susceptibility among subphenotypes was also tested.Two established bladder cancer susceptibility genotypes, NAT2 slow-acetylation and GSTM1-null, exhibited similar associations among the subphenotypes, as did VEGF-rs25648, which was previously identified in our study. Other variants conferred risks for specific tumour subphenotypes such as PMS2-rs6463524 and CD4-rs3213427 (respective heterogeneity p values of 0.006 and 0.004), which were associated with muscle-  invasive   tumours (per-allele odds ratios [95% confidence interval] of 0.56 [0.41-0.77] and 0.71 [0.57-0.88], respectively) but not with non-muscle-  invasive   tumours. Heterogeneity p values were not robust in multiple testing according to their false-discovery rate.These exploratory analyses suggest that genetic susceptibility loci might be related to the molecular/pathologic diversity of bladder cancer. Validation through large-scale replication studies and the study of additional genes and single nucleotide polymorphisms are required. 
 Overexpression of hSNF2H in glioma promotes cell proliferation, invasion, and chemoresistance through its interaction with Rsf-1. hSNF2H partners with Rsf-1 to compose the Rsf complex to regulate gene expression. Recent studies indicated that hSNF2H was overexpressed in several human cancers. However, its expression pattern and biological mechanism in glioma remain unexplored. In this study, we found that hSNF2H was overexpressed in 32 % of glioma specimens. hSNF2H overexpression correlated with advanced tumor grade (p = 0.0338) and Rsf-1 positivity in glioma tissues (p = 0.016). Small interfering RNA (siRNA) knockdown was performed in A172 and U87 cell lines. MTT, colony formation assay, and cell cycle analysis showed that knockdown of hSNF2H inhibited cell proliferation, colony formation ability, and cell cycle transition. Matrigel invasion assay showed that hSNF2H depletion i  nhibited   invasive ability of glioma cells. In addition, we demonstrated that hSNF2H depletion decreased temozolomide resistance of A172 and U87 cell lines and increased temozolomide induced apoptosis. Furthermore, hSNF2H depletion decreased cyclin D1, cyclin E, p-Rb, MMP2, cIAP1, Bcl-2 expression, and phosphorylation of IκBα and p65, suggesting hSNF2H regulates apoptosis through NF-κB pathway. Immunoprecipitation showed that hSNF2H could interact with Rsf-1 in both cell lines. To validate the involvement of Rsf-1, we checked the change of its downstream targets in Rsf-1 depleted cells. In Rsf-1 depleted cells, changes of cyclin E, Bcl-2, and p-IκBα were not significant using hSNF2H siRNA treatment. In conclusion, our study demonstrated that hSNF2H was overexpressed in human gliomas and contributed to glioma proliferation, invasion, and chemoresistance through regulation of cyclin E and NF-κB pathway, which is dependent on its interaction with Rsf-1. 
 Nuclear cIAP1 overexpression is a tumor stage- and grade-independent predictor of poor prognosis in human bladder cancer patients. To evaluate the tumor-related expression profile of cellular inhibitor of apoptosis protein 1 (cIAP1) and cellular inhibitor of apoptosis protein (cIAP2) in patients with bladder cell carcinoma (BCC) and to investigate its potential prognostic value.The expression of cIAP1 and cIAP2 was examined immunohistochemically in archival bladder specimens from 32 normal controls and 102 consecutive patients who underwent surgical operations at our department from January 2004 through December 2005. Cytoplasm cIAP1 and cIAP2 expression was scored as 0 (negative), +1 (weak), +2 (medium), and +3 (strong). Nuclear cIAP1 expression was scored as 0 (0%), +1 (1%-25%), +2 (26%-50%), and +3 (>50%). Proliferation was determined by Ki67 staining as percentage of positive cells.cIAP1 and cIAP2 expression were significantly increased in bladder cancer compared with normal bladder urothelium (cIAP1-C: P < 0.01, cIAP2-C: P = 0.017, cIAP1-N: P < 0.01). Nuclear staining of cIAP1 (cIAP1-N) was significantly associated with tumor stage (muscle   invasive   vs. non-muscle   invasive  , P = 0.03) and tumor grade (low vs. high, P = 0.01). Both the mean overall survival and mean recurrence-free survival were significantly decreased in the high cIAP1-N group compared to the low cIAP1-N group (low cIAP1-N: mean overall survival 62.7 months, high cIAP1-N: mean overall survival 45.6 months, P < 0.01; low cIAP1-N: mean recurrence-free survival 44.2 months, high cIAP1-N: mean recurrence-free survival 30.1 months, P < 0.01). cIAP1-N expression correlated strongly with KI67 expression (r = 0.744, P < 0.01).Nuclear cIAP-1 expression strongly correlated to bladder cancer stage, tumor grade, tumor recurrence and tumor related death. This marker expression was also appears to be a marker in bladder cancer prognosis. 
  [Mm]etastasis  PubMed  
 Ceramide targets xIAP and cIAP1 to sensitize metastatic colon and breast cancer cells to apoptosis induction to suppress tumor progression. Ceramide is a bioeffector that mediates various cellular processes, including apoptosis. However, the mechanism underlying ceramide function in apoptosis is apparently cell type-dependent and is not well-understood. We aimed at identifying molecular targets of ceramide in metastatic human colon and breast cancer cells, and determining the efficacy of ceramide analog in suppression of colon and breast cancer   metastasis  .The activity of and mechanism underlying ceramide as a cytotoxic agent, and as a sensitizer for Fas-mediated apoptosis was analyzed in human cell lines established from primary or metastatic colon and breast cancers. The efficacy of ceramide analog LCL85 in suppression of   metastasis   was examined in preclinical mouse tumor models.Exposure of human colon carcinoma cells to ceramide analog LCL85 results in apoptosis in a dose-dependent manner. Interestingly, a sublethal dose of LCL85 increased C16 ceramide content and overcame tumor cell resistance to Fas-mediated apoptosis. Subsequently, treatment of tumor cells with exogenous C16 ceramide resulted in increased tumor cell sensitivity to Fas-mediated apoptosis. LCL85 resembles Smac mimetic BV6 in sensitization of colon carcinoma cells to Fas-mediated apoptosis by inducing proteasomal degradation of cIAP1 and xIAP proteins. LCL85 also decreased xIAP1 and cIAP1 protein levels and sensitized metastatic human breast cancer cells to Fas-mediated apoptosis. Silencing xIAP and cIAP1 with specific siRNAs significantly increased the metastatic human colon carcinoma cell sensitivity to Fas-mediated apoptosis, suggesting that IAP proteins mediate apoptosis resistance in metastatic human colon carcinoma cells and ceramide induces IAP protein degradation to sensitize the tumor cells to apoptosis induction. Consistent with its apoptosis sensitization activity, subtoxic doses of LCL85 suppressed colon carcinoma cell metastatic potential in an experimental lung   metastasis   mouse model, as well as breast cancer growth and spontaneous lung   metastasis   in an orthotopic breast cancer mouse model.We have identified xIAP and cIAP1 as molecular targets of ceramide and determined that ceramide analog LCL85 is an effective sensitizer in overcoming resistance of human cell lines established from metastatic colon and breast cancers to apoptosis induction to suppress   metastasis   in vivo. 
 Ceramide targets xIAP and cIAP1 to sensitize metastatic colon and breast cancer cells to apoptosis induction to suppress tumor progression. Ceramide is a bioeffector that mediates various cellular processes, including apoptosis. However, the mechanism underlying ceramide function in apoptosis is apparently cell type-dependent and is not well-understood. We aimed at identifying molecular targets of ceramide in metastatic human colon and breast cancer cells, and determining the efficacy of ceramide analog in suppression of colon and breast cancer   metastasis  .The activity of and mechanism underlying ceramide as a cytotoxic agent, and as a sensitizer for Fas-mediated apoptosis was analyzed in human cell lines established from primary or metastatic colon and breast cancers. The efficacy of ceramide analog LCL85 in suppression of   metastasis   was examined in preclinical mouse tumor models.Exposure of human colon carcinoma cells to ceramide analog LCL85 results in apoptosis in a dose-dependent manner. Interestingly, a sublethal dose of LCL85 increased C16 ceramide content and overcame tumor cell resistance to Fas-mediated apoptosis. Subsequently, treatment of tumor cells with exogenous C16 ceramide resulted in increased tumor cell sensitivity to Fas-mediated apoptosis. LCL85 resembles Smac mimetic BV6 in sensitization of colon carcinoma cells to Fas-mediated apoptosis by inducing proteasomal degradation of cIAP1 and xIAP proteins. LCL85 also decreased xIAP1 and cIAP1 protein levels and sensitized metastatic human breast cancer cells to Fas-mediated apoptosis. Silencing xIAP and cIAP1 with specific siRNAs significantly increased the metastatic human colon carcinoma cell sensitivity to Fas-mediated apoptosis, suggesting that IAP proteins mediate apoptosis resistance in metastatic human colon carcinoma cells and ceramide induces IAP protein degradation to sensitize the tumor cells to apoptosis induction. Consistent with its apoptosis sensitization activity, subtoxic doses of LCL85 suppressed colon carcinoma cell metastatic potential in an experimental lung   metastasis   mouse model, as well as breast cancer growth and spontaneous lung   metastasis   in an orthotopic breast cancer mouse model.We have identified xIAP and cIAP1 as molecular targets of ceramide and determined that ceramide analog LCL85 is an effective sensitizer in overcoming resistance of human cell lines established from metastatic colon and breast cancers to apoptosis induction to suppress   metastasis   in vivo. 
 Antagonism of inhibitor of apoptosis proteins increases bone   metastasis   via unexpected osteoclast activation. Inhibitor of apoptosis (IAP) proteins play a central role in many types of cancer, and IAP antagonists are in development as anticancer agents. IAP antagonists cause apoptosis in many cells, but they also activate alternative NF-κB signaling through NF-κB-inducing kinase (NIK), which regulates osteoclasts. In bon  e metastas  is, a positive feedback loop between tumors and osteoclasts promotes tumor growth and osteolysis. We therefore tested the effect of IAP antagonists on the bone microenvironment fo  r metastas  is. In both drug-sensitive and drug-resistant tumors, growth in bone was favored, as compared with other sites during IAP antagonist treatment. These drugs also caused osteoporosis and increased osteoclastogenesis, mediated by NIK, and enhanced tumor-associated osteolysis. Cotreatment with zoledronic acid, a potent osteoclast inhibitor, reduced IAP antagonist-enhanced tumor growth in bone and osteolysis. Thus, IAP antagonist-based cancer treatment may be compromised by osteoporosis and enhanced skeleta  l metastas  is, which may be prevented by antiresorptive agents.Although IAP antagonists are a class of anticancer agents with proven efficacy in multiple cancers, we show that these agents can paradoxically increase tumor growth an  d metastas  is in the bone by stabilizing NIK and activating the alternative NF-κB pathway in osteoclasts. Future clinical trials of IAP antagonist-based therapy may require detailed examination of this potential for enhanced bo  ne metasta  sis and osteoporosis, as well as possible combination with antiresorptive agents. 
 cIAP1 regulates TNF-mediated cdc42 activation and filopodia formation. Tumour necrosis factor-α (TNF) is a cytokine endowed with multiple functions, depending on the cellular and environmental context. TNF receptor engagement induces the formation of a multimolecular complex including the TNFR-associated factor TRAF2, the receptor-interaction protein kinase RIP1 and the cellular inhibitor of apoptosis cIAP1, the latter being essential for NF-κB activation. Here, we show that cIAP1 also regulates TNF-induced actin cytoskeleton reorganization through a cdc42-dependent, NF-κB-independent pathway. Deletion of cIAP1 prevents TNF-induced filopodia and cdc42 activation. The expression of cIAP1 or its E3-ubiquitin ligase-defective mutant restores the ability of cIAP1(-/-) MEFs to produce filopodia, whereas a cIAP1 mutant unable to bind TRAF2 does not. Accordingly, the silencing of TRAF2 inhibits TNF-mediated filopodia formation, whereas silencing of RIP1 does not. cIAP1 directly binds cdc42 and promotes its RhoGDIα-mediated stabilization. TNF decreases cIAP1-cdc42 interaction, suggesting that TNF-induced recruitment of cIAP1/TRAF2 to the receptor releases cdc42, which in turn triggers actin remodeling. cIAP1 also regulates cdc42 activation in response to EGF and HRas-V12 expression. A downregulation of cIAP1 altered the cell polarization, the cell adhesion to endothelial cells and cell intercalation, which are cdc42-dependent processes. Finally, we demonstrated that the deletion of cIAP1 regulated the HRas-V12-mediated transformation process, including anchorage-dependent cell growth, tumour growth in a xenograft model and the development of experimen  tal metast  asis in the lung. 
 Nuclear expression of cIAP-1, an apoptosis inhibiting protein, predicts lymph node   metastasis   and poor patient prognosis in head and neck squamous cell carcinomas. cIAP-1, an apoptosis inhibiting protein, has been suggested to play important roles in the development of cervical and esophageal squamous cell carcinomas (SCCs). In order to clarify the subcellular localization of cIAP-1 and to investigate its clinicopathological significance in head and neck SCCs (HNSCCs), we examined cIAP-1 expression in four oral SCC cell lines by immunocytochemistry and Western blot. Expressions of nuclear and cytoplasmic cIAP-1, caspase-3, and Smac/DIABLO were also examined immunohistochemically in 57 cases of the HNSCCs. cIAP-1 expression was detected in HSC-2, HSC-3, and HSC-4 cells by immunohistochemistry and Western blot. In HSC-2 and HSC-4 cells, cIAP-1 was detected in both the nuclear and cytoplasmic fractions. Nuclear cIAP-1 expression was positive in 17 (30%) of HNSCCs, was correlated with lymph node   metastasis   (P=0.020) and advanced disease stage (P=0.032), and tended to be correlated with poor patient prognosis (P=0.059). Cytoplasmic cIAP-1 expression showed similar but weaker clinicopathological correlations. Nuclear cIAP-1 expression was inversely correlated with caspase-3 expression, but was correlated with Smac/DIABLO expression. Nuclear cIAP-1 expression appears to be a useful marker for predicting poor patient prognosis in HNSCCs, and may play roles in HNSCCs through the signaling pathway mediated by Smac/DIABLO and caspase-3. 
 Human papillomavirus 16 E6/E7-immortalized human gingival keratinocytes with epithelial mesenchymal transition acquire increased expression of cIAP-1, Bclx and p27(Kip1). Epithelial mesenchymal transition (EMT) has been implicated in neoplastic invasion and   metastasis  . We have previously generated six cell lines from human gingival mucosal keratinocytes immortalized by E6/E7 of human papillomavirus type 16. Ldk and NuB1 lines represented EMT phenotype and other four lines represented cobblestone non-EMT phenotype. These cell lines were utilized as model cells to determine whether inhibitors of apoptosis proteins and cell-cycle regulators were molecular players during EMT. EMT cells exhibited increased expression of vimentin, vascular endothelial growth factor (VEGF) receptor1 and the ability to form tubules on Matrigel as well as to grow anchorage independently. We found that EMT cells expressed significantly elevated levels of cIAP-1, Bclx and p27(kip) higher than non-EMT cells. These proteins could therefore be used as intrinsic indicators of EMT of human gingival keratinocytes. 
 Expression of cIAP-1 correlates with nodal   metastasis   in squamous cell carcinoma of the tongue. Cellular inhibitor-of-apoptosis protein 1 (cIAP-1) is a member of the inhibitor-of- apoptosis protein family, which predominantly regulates apoptosis. It has been suggested that expression of cIAP-1 correlates with certain clinicopathological features. The possible significance of cIAP-1 expression in cervical lymph node   metastasis   of tongue squamous cell carcinoma (SCC) is investigated. Seventy-five tongue SCCs were analyzed by immunohistochemistry. cIAP-1 immunoreactivity patterns were nuclear in 38 (51%), cytoplasmic in 47 (63%), and concurrent in 37 (49%) cases. Nuclear, cytoplasmic and concurrent cIAP-1 immunoreactions were significantly correlated with lymph node   metastasis   in tongue SCCs (P=0.0011, 0.0012, and 0.0006, respectively). The cleaved caspase-3, which is a marker of tumor apoptosis, and Ki-67 index, which is a marker of tumor proliferation, were immunohistochemically examined in 21 tongue SCCs with concurrent nuclear and cytoplasmic cIAP-1 expression and with   metastasis  , and in 23 tongue SCCs without concurrent nuclear and cytoplasmic cIAP-1 expression and without   metastasis  . Concurrent cIAP-1 expression was inversely correlated with caspase-3 (P=0.0066), but was positively correlated with Ki-67 expression (P=0.0028). The mode of invasion was associated with lymph node   metastasis   (P=0.014) and differentiation (P=0.013), but was not correlated with cIAP-1 expression. There was no statistically significant correlation between nuclear or cytoplasmic cIAP-1 expression and the clinicopathological factors of gender, age, clinical stage or differentiation. These results suggest that both patterns of cIAP-1 are useful markers for predicting cervical lymph node   metastasis   in tongue SCC. 
 IAPs: from caspase inhibitors to modulators of NF-kappaB, inflammation and cancer. The realization that alterations in inhibitor of apoptosis (IAP) proteins are found in many types of human cancer and are associated with chemoresistance, disease progression and poor prognosis, has sparked a worldwide frenzy in the development of small pharmacological inhibitors of IAPs. The development of such inhibitors has radically changed our knowledge of the signalling processes that are regulated by IAPs. Recent studies indicate that IAPs not only regulate caspases and apoptosis, but also modulate inflammatory signalling and immunity, mitogenic kinase signalling, proliferation and mitosis, as well as cell invasion and   metastasis  . 
  GeneRIFs  
 Identify xIAP and cIAP1 as molecular targets of ceramide and show ceramide analog LCL85 is an effective sensitizer in overcoming resistance of metastatic colon and breast cancers to apoptosis induction to suppress   metastasis   in vivo. 
 Data suggest that inhibitor of apoptosis (IAP) antagonist-based cancer treatment may be compromised by osteoporosis and enhanced skeletal   metastasis  , which may be prevented by antiresorptive agents. 
 Nuclear, cytoplasmic and concurrent cIAP-1 immunoreactions were significantly correlated with lymph node   metastasis   in tongue squamous cell carcinomas. 
  [Cc]ell [Ii]nvasion  PubMed  
 IAPs: from caspase inhibitors to modulators of NF-kappaB, inflammation and cancer. The realization that alterations in inhibitor of apoptosis (IAP) proteins are found in many types of human cancer and are associated with chemoresistance, disease progression and poor prognosis, has sparked a worldwide frenzy in the development of small pharmacological inhibitors of IAPs. The development of such inhibitors has radically changed our knowledge of the signalling processes that are regulated by IAPs. Recent studies indicate that IAPs not only regulate caspases and apoptosis, but also modulate inflammatory signalling and immunity, mitogenic kinase signalling, proliferation and mitosis, as well as   cell invasion   and metastasis. 
   
  
 FMN2 
 
  NCBI Gene  
  GeneRIF  
  Pubmed  
 
 
  Search Term  Rank  Fields  
  [Ff]ilopodia  1  PubMed(1)  
  [Mm]igration  6  PubMed(1)  
  [Ii]nvasive  10  PubMed(1)  
  [Mm]etastasis  12  PubMed(1)  
 
 
 hide/show details for FMN2 
  [Ff]ilopodia  PubMed  
 Metastasis suppressor microRNA-335 targets the formin family of actin nucleators. MiRNAs can have pleiotropic effects by targeting multiple genes belonging to diverse signalling networks. Alternatively, miRNAs can enhance the potency of their cellular effects by targeting multiple genes within the same genetic pathway. Previously, we and others have demonstrated that miR-335 is a potent suppressor of tumour cell migration, invasion and metastasis, in part by targeting several genes involved in these cellular processes, including ROCK1, MAPK1, LRG1, SP1 and SOX4. Here, we demonstrate that direct targeting of multiple members of the formin family of actin nucleators contributes to the inhibitory effects of miR-335 in neuroblastoma cells. We demonstrate that miR-335 regulates the expression of at least five formin family members and validate three family members, FMNL3, FMN2 and DAAM2, as direct targets of miR-335. The contribution of the formin family genes to cancer progression and metastasis has recently begun to emerge and here we demonstrate for the first time the ability of FMN2 and DAAM2 to regulate tumour cell migration and invasion, using siRNA-mediated inhibition of each of these formin genes. Finally, we demonstrate that the formin genes, in particular FMNL3, are responsible for the protrusion of actin-rich   filopodia   structures that contribute to the enhanced migratory and invasive potential associated with reduced expression of miR-335. Thus, direct targeting of the formin family contributes to the metastasis suppressing abilities of miR-335 by providing a direct regulatory link to the actin assembly machinery of the cell. We conclude that miR-335 is a master regulator of tumour cell migration and invasion by directly targeting a plethora of genes that effectively control cell migratory processes. 
  [Mm]igration  PubMed  
 Metastasis suppressor microRNA-335 targets the formin family of actin nucleators. MiRNAs can have pleiotropic effects by targeting multiple genes belonging to diverse signalling networks. Alternatively, miRNAs can enhance the potency of their cellular effects by targeting multiple genes within the same genetic pathway. Previously, we and others have demonstrated that miR-335 is a potent suppressor of tumour cell   migration  , invasion and metastasis, in part by targeting several genes involved in these cellular processes, including ROCK1, MAPK1, LRG1, SP1 and SOX4. Here, we demonstrate that direct targeting of multiple members of the formin family of actin nucleators contributes to the inhibitory effects of miR-335 in neuroblastoma cells. We demonstrate that miR-335 regulates the expression of at least five formin family members and validate three family members, FMNL3, FMN2 and DAAM2, as direct targets of miR-335. The contribution of the formin family genes to cancer progression and metastasis has recently begun to emerge and here we demonstrate for the first time the ability of FMN2 and DAAM2 to regulate tumour cell   migration   and invasion, using siRNA-mediated inhibition of each of these formin genes. Finally, we demonstrate that the formin genes, in particular FMNL3, are responsible for the protrusion of actin-rich filopodia structures that contribute to the enhanced migratory and invasive potential associated with reduced expression of miR-335. Thus, direct targeting of the formin family contributes to the metastasis suppressing abilities of miR-335 by providing a direct regulatory link to the actin assembly machinery of the cell. We conclude that miR-335 is a master regulator of tumour cell   migration   and invasion by directly targeting a plethora of genes that effectively control cell migratory processes. 
  [Ii]nvasive  PubMed  
 Metastasis suppressor microRNA-335 targets the formin family of actin nucleators. MiRNAs can have pleiotropic effects by targeting multiple genes belonging to diverse signalling networks. Alternatively, miRNAs can enhance the potency of their cellular effects by targeting multiple genes within the same genetic pathway. Previously, we and others have demonstrated that miR-335 is a potent suppressor of tumour cell migration, invasion and metastasis, in part by targeting several genes involved in these cellular processes, including ROCK1, MAPK1, LRG1, SP1 and SOX4. Here, we demonstrate that direct targeting of multiple members of the formin family of actin nucleators contributes to the inhibitory effects of miR-335 in neuroblastoma cells. We demonstrate that miR-335 regulates the expression of at least five formin family members and validate three family members, FMNL3, FMN2 and DAAM2, as direct targets of miR-335. The contribution of the formin family genes to cancer progression and metastasis has recently begun to emerge and here we demonstrate for the first time the ability of FMN2 and DAAM2 to regulate tumour cell migration and invasion, using siRNA-mediated inhibition of each of these formin genes. Finally, we demonstrate that the formin genes, in particular FMNL3, are responsible for the protrusion of actin-rich filopodia structures that contribute to the enhanced migratory and   invasive   potential associated with reduced expression of miR-335. Thus, direct targeting of the formin family contributes to the metastasis suppressing abilities of miR-335 by providing a direct regulatory link to the actin assembly machinery of the cell. We conclude that miR-335 is a master regulator of tumour cell migration and invasion by directly targeting a plethora of genes that effectively control cell migratory processes. 
  [Mm]etastasis  PubMed  
   Metastasis   suppressor microRNA-335 targets the formin family of actin nucleators. MiRNAs can have pleiotropic effects by targeting multiple genes belonging to diverse signalling networks. Alternatively, miRNAs can enhance the potency of their cellular effects by targeting multiple genes within the same genetic pathway. Previously, we and others have demonstrated that miR-335 is a potent suppressor of tumour cell migration, invasion and   metastasis  , in part by targeting several genes involved in these cellular processes, including ROCK1, MAPK1, LRG1, SP1 and SOX4. Here, we demonstrate that direct targeting of multiple members of the formin family of actin nucleators contributes to the inhibitory effects of miR-335 in neuroblastoma cells. We demonstrate that miR-335 regulates the expression of at least five formin family members and validate three family members, FMNL3, FMN2 and DAAM2, as direct targets of miR-335. The contribution of the formin family genes to cancer progression and   metastasis   has recently begun to emerge and here we demonstrate for the first time the ability of FMN2 and DAAM2 to regulate tumour cell migration and invasion, using siRNA-mediated inhibition of each of these formin genes. Finally, we demonstrate that the formin genes, in particular FMNL3, are responsible for the protrusion of actin-rich filopodia structures that contribute to the enhanced migratory and invasive potential associated with reduced expression of miR-335. Thus, direct targeting of the formin family contributes to the   metastasis   suppressing abilities of miR-335 by providing a direct regulatory link to the actin assembly machinery of the cell. We conclude that miR-335 is a master regulator of tumour cell migration and invasion by directly targeting a plethora of genes that effectively control cell migratory processes. 
   
  
 HSPD1 
 
  NCBI Gene  
  GeneRIF  
  Pubmed  
 
 
  Search Term  Rank  Fields  
  [Mm]igration  6  PubMed(4); GeneRIFs(1)  
  [Ee]xtracellular [Mm]atrix  8  PubMed(2); Component(1)  
  [Ii]nvasive  10  PubMed(5)  
  [Mm]etastasis  12  PubMed(5); GeneRIFs(2)  
  [Cc]ell [Ii]nvasion  13  PubMed(2)  
 
 
 hide/show details for HSPD1 
  [Mm]igration  PubMed  
 Molecular chaperones as a common set of proteins that regulate the invasion phenotype of head and neck cancer. The goal of this study was to establish a common set of molecules that regulate cell invasion in head and neck cancer (HNC).Five invasive sublines derived from HNC cell lines were established using the Matrigel selection method. Proteomic technology, MetaCore algorithm, and reverse transcriptase-PCR methods were used to search for molecules that contribute to the invasion phenotype. Cellular functional analyses and clinical association studies were applied to examine the significance of the molecules.Fifty-two proteins were identified in more than two of the four independent proteomic experiments, including 10 (19%) molecular chaperones. Seven chaperones were confirmed to be differentially expressed in five sublines, Hsp90α, Hsp90β, Hsp90-B1/Gp96, Hsp70-A5/Grp78, and HYOU1, that upregulate, whereas Hsp60 and glucosidase-α neutral AB (GANAB) downregulate. Four molecules were further investigated. In all cell lines, knockdown of Hsp60 or GANAB and silencing of Gp96 or Grp78 considerably enhanced or reduced ce  ll migrat  ion and invasion, respectively. Clinical association studies consistently revealed that low levels of Hsp60 or GANAB and high levels of Gp96 or Grp78 are significantly associated with advanced cancer (P < 0.001 to P = 0.047, respectively, for the four molecules) and poor survival (P < 0.001 to P = 0.025, respectively, for the four molecules).Our study defined molecular chaperones as a common set of proteins that regulate the invasion phenotype of HNC. Loss of the tumor suppression function of Hsp60 or GANAB and acquisition of the oncogenic function of Gp96 or Grp78 contribute to aggressive cancers. These molecules may serve as prognostic markers and targets for cancer drug development. 
 Molecular chaperones as a common set of proteins that regulate the invasion phenotype of head and neck cancer. The goal of this study was to establish a common set of molecules that regulate cell invasion in head and neck cancer (HNC).Five invasive sublines derived from HNC cell lines were established using the Matrigel selection method. Proteomic technology, MetaCore algorithm, and reverse transcriptase-PCR methods were used to search for molecules that contribute to the invasion phenotype. Cellular functional analyses and clinical association studies were applied to examine the significance of the molecules.Fifty-two proteins were identified in more than two of the four independent proteomic experiments, including 10 (19%) molecular chaperones. Seven chaperones were confirmed to be differentially expressed in five sublines, Hsp90α, Hsp90β, Hsp90-B1/Gp96, Hsp70-A5/Grp78, and HYOU1, that upregulate, whereas Hsp60 and glucosidase-α neutral AB (GANAB) downregulate. Four molecules were further investigated. In all cell lines, knockdown of Hsp60 or GANAB and silencing of Gp96 or Grp78 considerably enhanced or reduced ce  ll migrat  ion and invasion, respectively. Clinical association studies consistently revealed that low levels of Hsp60 or GANAB and high levels of Gp96 or Grp78 are significantly associated with advanced cancer (P < 0.001 to P = 0.047, respectively, for the four molecules) and poor survival (P < 0.001 to P = 0.025, respectively, for the four molecules).Our study defined molecular chaperones as a common set of proteins that regulate the invasion phenotype of HNC. Loss of the tumor suppression function of Hsp60 or GANAB and acquisition of the oncogenic function of Gp96 or Grp78 contribute to aggressive cancers. These molecules may serve as prognostic markers and targets for cancer drug development. 
 Plasminogen and angiostatin interact with heat shock proteins. Previous studies from this laboratory have demonstrated that plasminogen and angiostatin bind to endothelial cell (EC) surface-associated actin via their kringles in a specific manner. Heat shock proteins (hsps) like hsp 27 are constitutively expressed by vascular ECs and regulate actin polymerization, cell growth, and   migration  . Since many hsps have also been found to be highly abundant on cell surfaces and there is evidence that bacterial surface hsps may interact with human plasminogen, the purpose of this study was to determine whether human plasminogen and angiostatin would interact with human hsps. ELISAs were developed in our laboratory to assess these interactions. It was observed that plasminogen bound to hsps 27, 60, and 70. In all cases, binding was inhibited (85-90%) by excess (50 mM) lysine indicating kringle involvement. Angiostatin predominantly bound to hsp 27 and to hsp 70 in a concentration- and kringle-dependent manner. As observed previously for actin, there was concentration-dependent inhibition of angiostatin's interaction with hsp 27 by plasminogen. In addition, 30-fold molar excess actin inhibited (up to 50%), the interaction of plasminogen with all hsps. However, 30-fold molar excess actin could only inhibit the interaction of angiostatin with hsp 27 by 15-20%. Collectively, these data indicate that (i) while plasminogen interacts specifically with hsp 27, 60, and 70, angiostatin interacts predominantly with hsp 27 and to some extent with hsp 70; (ii) plasminogen only partially displaces angiostatin's binding to hsp 27 and (iii) actin only partially displaces plasminogen/angiostatin binding to hsps. It is conceivable therefore that surface-associated hsps could mediate the binding of these ligands to cells like ECs. 
 Heat shock protein 60 stimulates the   migration   of vascular smooth muscle cells via Toll-like receptor 4 and ERK MAPK activation. Accumulating evidence indicates that heat shock protein (HSP) 60 is strongly associated with the pathology of atherosclerosis (AS). However, the precise mechanisms by which HSP60 promotes atherosclerosis remain unclear. In the present study, we found that HSP60 mRNA and protein expression levels in the thoracic aorta are enhanced not only in a mouse model of AS but also in high-fat diet (HFD) mice. HSP60 expression and secretion was activated by platelet-derived growth factor-BB (PDGF-BB) and interleukin (IL)-8 in both human umbilical vein endothelial cells (HUVECs) and vascular smooth muscle cells (VSMCs). HSP60 was found to induce VSMC   migration  , and exposure to HSP60 activated ERK MAPK signaling. U0126, an inhibitor of ERK, reduced VSMC   migration  . The HSP60-stimulated VSMCs were found to express TLR4 mRNA but not TLR2 mRNA. Knockdown of TLR4 by siRNA reduced HSP60-induced VSMC   migration   and HSP60-induced ERK activation. Finally, HSP60 induced IL-8 secretion in VSMCs. Together these results suggest that HSP60 is involved in the stimulation of VSMC   migration  , via TLR4 and ERK MAPK activation. Meanwhile, activation of HSP60 is one of the most powerful methods of sending a 'danger signal' to the immune system to generate IL-8, which assists in the management of an infection or disease. 
  GeneRIFs  
 Heat shock protein 60 stimulates the   migration   of vascular smooth muscle cells via Toll-like receptor 4 and ERK MAPK activation 
  [Ee]xtracellular [Mm]atrix  PubMed  
 SILAC-based proteomics of human primary endothelial cell morphogenesis unveils tumor angiogenic markers. Proteomics has been successfully used for cell culture on dishes, but more complex cellular systems have proven to be challenging and so far poorly approached with proteomics. Because of the complexity of the angiogenic program, we still do not have a complete understanding of the molecular mechanisms involved in this process, and there have been no in depth quantitative proteomic studies. Plating endothelial cells on matrigel recapitulates aspects of vessel growth, and here we investigate this mechanism by using a spike-in SILAC quantitative proteomic approach. By comparing proteomic changes in primary human endothelial cells morphogenesis on matrigel to general adhesion mechanisms in cells spreading on culture dish, we pinpoint pathways and proteins modulated by endothelial cells. The cell-  extracellular matrix   adhesion proteome depends on the adhesion substrate, and a detailed proteomic profile of the   extracellular matrix   secreted by endothelial cells identified CLEC14A as a matrix component, which binds to MMRN2. We verify deregulated levels of these proteins during tumor angiogenesis in models of multistage carcinogenesis. This is the most in depth quantitative proteomic study of endothelial cell morphogenesis, which shows the potential of applying high accuracy quantitative proteomics to in vitro models of vessel growth to shed new light on mechanisms that accompany pathological angiogenesis. The mass spectrometry proteomics data have been deposited to the ProteomeXchange Consortium with the data set identifier PXD000359. 
 Quantitative proteome analysis of multidrug resistance in human ovarian cancer cell line. In order to understand the molecular mechanisms of multidrug resistance (MDR) in ovarian cancer, we employed the proteomic approach of isobaric tags for relative and absolute quantification (iTRAQ), followed by LC-MS/MS, using the cisplatin-resistant COC1/DDP cell line and its parental COC1 cell line as a model. A total number of 28 proteins differentially expressed were identified, and then the differential expression levels of partially identified proteins were confirmed by Western blot analysis and/or real-time RT-PCR. Furthermore, the association of PKM2 and HSPD1, two differentially expressed proteins, with MDR were analyzed, and the results showed that they could contribute considerably to the cisplatin resistance in ovarian cancer cell. The differential expression proteins could be classified into eight categories based on their functions, that is, calcium binding proteins, chaperones,   extracellular matrix  , proteins involved in drug detoxification or repair of DNA damage, metabolic enzymes, transcription factor, proteins related to cellular structure and proteins relative to signal transduction. These data will be valuable for further study of the mechanisms of MDR in the ovarian cancer. 
  Component  
   extracellular matrix   
  [Ii]nvasive  PubMed  
 Molecular chaperones as a common set of proteins that regulate the invasion phenotype of head and neck cancer. The goal of this study was to establish a common set of molecules that regulate cell invasion in head and neck cancer (HNC).Five   invasive   sublines derived from HNC cell lines were established using the Matrigel selection method. Proteomic technology, MetaCore algorithm, and reverse transcriptase-PCR methods were used to search for molecules that contribute to the invasion phenotype. Cellular functional analyses and clinical association studies were applied to examine the significance of the molecules.Fifty-two proteins were identified in more than two of the four independent proteomic experiments, including 10 (19%) molecular chaperones. Seven chaperones were confirmed to be differentially expressed in five sublines, Hsp90α, Hsp90β, Hsp90-B1/Gp96, Hsp70-A5/Grp78, and HYOU1, that upregulate, whereas Hsp60 and glucosidase-α neutral AB (GANAB) downregulate. Four molecules were further investigated. In all cell lines, knockdown of Hsp60 or GANAB and silencing of Gp96 or Grp78 considerably enhanced or reduced cell migration and invasion, respectively. Clinical association studies consistently revealed that low levels of Hsp60 or GANAB and high levels of Gp96 or Grp78 are significantly associated with advanced cancer (P < 0.001 to P = 0.047, respectively, for the four molecules) and poor survival (P < 0.001 to P = 0.025, respectively, for the four molecules).Our study defined molecular chaperones as a common set of proteins that regulate the invasion phenotype of HNC. Loss of the tumor suppression function of Hsp60 or GANAB and acquisition of the oncogenic function of Gp96 or Grp78 contribute to aggressive cancers. These molecules may serve as prognostic markers and targets for cancer drug development. 
 Molecular chaperones as a common set of proteins that regulate the invasion phenotype of head and neck cancer. The goal of this study was to establish a common set of molecules that regulate cell invasion in head and neck cancer (HNC).Five   invasive   sublines derived from HNC cell lines were established using the Matrigel selection method. Proteomic technology, MetaCore algorithm, and reverse transcriptase-PCR methods were used to search for molecules that contribute to the invasion phenotype. Cellular functional analyses and clinical association studies were applied to examine the significance of the molecules.Fifty-two proteins were identified in more than two of the four independent proteomic experiments, including 10 (19%) molecular chaperones. Seven chaperones were confirmed to be differentially expressed in five sublines, Hsp90α, Hsp90β, Hsp90-B1/Gp96, Hsp70-A5/Grp78, and HYOU1, that upregulate, whereas Hsp60 and glucosidase-α neutral AB (GANAB) downregulate. Four molecules were further investigated. In all cell lines, knockdown of Hsp60 or GANAB and silencing of Gp96 or Grp78 considerably enhanced or reduced cell migration and invasion, respectively. Clinical association studies consistently revealed that low levels of Hsp60 or GANAB and high levels of Gp96 or Grp78 are significantly associated with advanced cancer (P < 0.001 to P = 0.047, respectively, for the four molecules) and poor survival (P < 0.001 to P = 0.025, respectively, for the four molecules).Our study defined molecular chaperones as a common set of proteins that regulate the invasion phenotype of HNC. Loss of the tumor suppression function of Hsp60 or GANAB and acquisition of the oncogenic function of Gp96 or Grp78 contribute to aggressive cancers. These molecules may serve as prognostic markers and targets for cancer drug development. 
 Serum antibody response to the heat shock protein 60 of Chlamydia trachomatis in women with developing cervical cancer. The purpose of this study was to determine whether serum antibody response to the three versions of chlamydial heat shock protein 60 is associated with an increased risk for cervical cancer.Women with cervical carcinoma were identified by linking the data files of three Nordic serum banks with cancer registries. Overall, 178 women with   invasive   cervical carcinoma were identified. For each case, the earliest prediagnostic serum sample was chosen, and three matched control subjects who were free of cancer at the time of the case diagnosis were selected randomly. Serum antibodies to the chlamydial heat shock protein 60 were measured by enzyme-linked immunosorbent assay and correlated with the risk of the subsequent development of cervical cancer.Antibodies to chlamydial heat shock protein 60-1 were associated with cervical squamous cell carcinoma among cases with long lag time (>3.5 years; odds ratio, 2.4; 95% CI, 1.1-5.1). Antibodies to chlamydial heat shock protein 60-2 or chlamydial heat shock protein 60-3 were not associated with cervical cancer risk.The finding that chlamydial heat shock protein 60-1 antibodies are associated with an increased cervical cancer risk suggests that persistent Chlamydia trachomatis infection may contribute to cervical neoplasia. 
 Detection of Hsp60 in saliva and serum from type 2 diabetic and non-diabetic control subjects. There is increasing evidence that mitochondrial dysfunction and oxidative stress may be integral to the pathogenesis of type 2 diabetes mellitus. Heat shock protein (Hsp60) is a mitochondrial stress protein known to be induced under conditions of mitochondrial impairment. Although this intracellular protein is normally found in the mitochondrion, several studies have shown that this protein is also present in systemic circulation. In this study, we report the presence of elevated levels of Hsp60 in both saliva and serum of type 2 diabetic patients compared to non-diabetic controls. Hsp60 was detectable in the saliva of 10% of control and 93% of type 2 diabetic patients. Levels detected were in the range of 3-7 ng/ml in control and 3-75 ng/ml in type 2 diabetic patients. Serum Hsp60 levels in the range of 3-88 ng/ml were detected in 33% of control subjects, and levels in the range of 28-1,043 ng/ml were detected in 100% of type 2 diabetic patients. This is the first reporting of the presence of mitochondrial stress protein in salivary secretions. The serum Hsp60 levels were 16-fold higher compared to those in saliva, and there was a good positive correlation between salivary and serum Hsp60 levels (r = 0.55). While the exact mechanisms responsible for the secretion of Hsp60 into biological fluids such as saliva and blood are not yet known. The presence of this molecular marker of mitochondrial stress in saliva offer  s a non-  invasive route to further investigate the biological functions of extracellular Hsp60 in type 2 diabetes mellitus and other conditions. 
 Heat shock protein 60 overexpression is associated with the progression and prognosis in gastric cancer. Heat shock protein 60 (HSP60) is a chaperonin with essential functions for cell physiology and survival, and its expression correlates with prognosis in a number of malignancies. The aim of this study is to determine the relationship of HSP60 status with clinicopathological parameters and prognosis in gastric cancer.The levels of HSP60 and matrix metallopeptidase 9 (MMP-9) antigen was evaluated by immunohistochemistry in 223 gastric carcinoma samples. The association between HSP60 and MMP-9, clinicopathological parameters, and prognosis of gastric cancer was examined.The level of HSP60 protein was significantly associated with depth invasion, lymph node metastasis and stage of disease (all P<0.05). Both univariate and multivariate analyses revealed that HSP60 was an independent prognostic factor for both overall survival (OS) and recurrence-free survival (RFS) (both P<0.05). Furthermore, HSP60 overexpression was associated with a poor prognosis in patients with advanced gastric cancer in different risk groups. Moreover, HSP60 was significantly correlated with MMP-9 among 223 gastric cancer tissues (P<0.001). Patients who had HSP60 overexpression, in which tumor cells displayed high   invasive  ness, had poor OS and shorter RFS.HSP60 plays an important role on tumor aggressiveness and prognosis, and may act as a promising target for prognostic prediction. 
  [Mm]etastasis  PubMed  
 Differential protein expression of lymph node metastases of papillary thyroid carcinoma harboring the BRAF mutation. The prognostic role of the T1799A BRAF mutation is controversial. We investigated the protein expression in papillary thyroid carcinoma (PTCs) samples harboring the specific mutation using proteomic tools.We performed two-dimensional gel electrophoresis to identify differential protein expression regarding lymph node   metastasis   (LNM). Proteins were identified by matrix-assisted laser desorption/ionization time-of-flight mass spectrometry. Immunohistochemical staining was performed for 38 PTCs harboring the mutation. We validated the association between these proteins and clinicopathological factors in a test set of 121 PTCs.The expression of vimentin was increased in PTCs with LNM, but the one for HSP60, SOD2 and PEBP1 was increased in samples without LNM. HSP60 protein was up-regulated in PTCs without LNM (84.2% vs. 36.8%. p=0.003) and in PTCs without LNM harboring the mutation (58% vs. 41.8%. p=0.003) in the test set as shown by immunohistochemical staining.HSP60 protein expression may inhibit LNM in PTCs harboring the BRAF mutation and may be a useful prognostic marker. 
 The expression of HSP60 and HSP10 in large bowel carcinomas with lymph node metastase. The involvement of Heat Shock Proteins (HSP) in cancer development and progression is a widely debated topic. The objective of the present study was to evaluate the presence and expression of HSP60 and HSP10 in a series of large bowel carcinomas and locoregional lymph nodes with and without metastases.82 Astler and Coller's stage C2 colorectal cancers, of which 48 well-differentiated and 34 poorly-differentiated, were selected along with 661 lymph nodes, including 372 with metastases and 289 with reactive hyperplasia only, from the same tumours. Primitive tumours and both metastatic and reactive lymph nodes were studied; specifically, three different compartments of the lymph nodes, secondary follicle, paracortex and medullary sinus, were also analysed. An immunohistochemical research for HSP60 and HSP10 was performed and the semiquantitative results were analysed by statistical analysis to determine the correlation between HSPs expression and 1) tumour grading; 2) degree of inflammation; 3) number of lymph nodes involved; 4) lymph node compartment hyperplasia. Moreover, western blotting was performed on a smaller group of samples to confirm the immunohistochemical results.Our data show that the expression of HSP60, in both primary tumour and lymph node   metastasis  , is correlated with the tumoral grade, while the HSP10 expression is not. Nevertheless, the levels of HSP10 are commonly higher than the levels of HSP60. In addition, statistical analyses do not show any correlation between the degree of inflammation and the immunopositivity for both HSP60 and HSP10. Moreover, we find a significant correlation between the presence of lymph node metastases and the positivity for both HSP60 and HSP10. In particular, metastatic lymph nodes show a higher percentage of cells positive for both HSP60 and HSP10 in the secondary follicles, and for HSP10 in the medullary sinuses, when compared with hyperplastic lymph nodes.HSP60 and HSP10 may have diagnostic and prognostic significance in the management of this tumour and their overexpression in tumoral cells may be functionally related to tumoral progression. We hypothesise that their expression in follicular and medullary cells of lymph nodes may be induced by formation of metastases. Further studies based on these observations could lead to a better understanding of the HSPs involvement in colorectal cancer progression, as well as other neoplasms. 
 Immunohistochemical expression of Hsp60 correlates with tumor progression and hormone resistance in prostate cancer. To investigate the expression of Hsp60 protein in prostate cancer biopsy samples, and its association with prognostic clinical parameters and hormone resistance and survival. Molecular chaperones are involved in protein folding, protein degradation, and protein trafficking among subcellular compartments.We selected 107 patients with localized and locally advanced prostate cancer at our hospital from 1999 through 2004. We performed an analysis by western blot and immunohistochemistry on paraffin-embedded tissue sections. Clinical data were used to determine associations between immunohistochemical expression of Hsp60 and tumor behavior.The expression level of Hsp60 was significantly increased in tumors with high Gleason score (P < .001). Hsp60 expression was also significantly associated with initial serum PSA levels (P < .01) and with the presence of lymph node   metastasis   (P < .003). In 50 locally advanced cancers treated by androgen ablation we found an association between high Hsp60-expressing tumors and an early onset of hormone refractory disease (P < .02) and reduced cancer-specific survival (P < .05).Hsp60 protein is overexpressed in poorly differentiated prostate cancers. Hsp60 expression is strongly associated with prognostic clinical parameters, such as Gleason score, initial serum PSA levels, and lymph node   metastasis   and with the onset of hormone-refractory disease and reduced cancer-specific survival. Identification of such markers could be of relevance in the clinical management of prostate cancer. 
 Heat shock protein 60 overexpression is associated with the progression and prognosis in gastric cancer. Heat shock protein 60 (HSP60) is a chaperonin with essential functions for cell physiology and survival, and its expression correlates with prognosis in a number of malignancies. The aim of this study is to determine the relationship of HSP60 status with clinicopathological parameters and prognosis in gastric cancer.The levels of HSP60 and matrix metallopeptidase 9 (MMP-9) antigen was evaluated by immunohistochemistry in 223 gastric carcinoma samples. The association between HSP60 and MMP-9, clinicopathological parameters, and prognosis of gastric cancer was examined.The level of HSP60 protein was significantly associated with depth invasion, lymph node   metastasis   and stage of disease (all P<0.05). Both univariate and multivariate analyses revealed that HSP60 was an independent prognostic factor for both overall survival (OS) and recurrence-free survival (RFS) (both P<0.05). Furthermore, HSP60 overexpression was associated with a poor prognosis in patients with advanced gastric cancer in different risk groups. Moreover, HSP60 was significantly correlated with MMP-9 among 223 gastric cancer tissues (P<0.001). Patients who had HSP60 overexpression, in which tumor cells displayed high invasiveness, had poor OS and shorter RFS.HSP60 plays an important role on tumor aggressiveness and prognosis, and may act as a promising target for prognostic prediction. 
 Interaction between HSP60 and beta-catenin promotes   metastasis  . Heat shock protein 60 (HSP60) plays an essential role in assisting many newly synthesized proteins to reach their native forms. Increased HSP60 expression is observed in different types of human cancers with   metastasis   (e.g. pancreatic cancer and large bowel carcinoma). However, the role of HSP60 in   metastasis   remains little known. Aberrant activation of beta-catenin plays a key role in tumorigenesis and   metastasis  . Here, we show that overexpression of HSP60 induces metastatic phenotypes in vitro and in vivo. HSP60 interacts with beta-catenin, increases beta-catenin protein levels through the apical domain and enhances its transcriptional activity. Short-interference RNA-mediated repression of beta-catenin reverts metastatic activity caused by HSP60 overexpression. Proteosomal activity is not required for the induction of beta-catenin by HSP60. Coexpression of HSP60 and nuclear beta-catenin predicts a worse prognosis of metastatic head and neck cancer patients. These results implicate a novel role of HSP60 in   metastasis  . 
  GeneRIFs  
 HSP60 protein expression may inhibit lymph node   metastasis   in papillary thyroid carcinomas harboring the BRAF mutation. 
 Hsp60 is overexpressed in poorly differentiated prostate cancers & is associated with prognostic clinical parameters, like Gleason score, initial serum PSA, lymphatic   metastasis  , hormone-refractory disease & reduced cancer-specific survival. 
  [Cc]ell [Ii]nvasion  PubMed  
 Molecular chaperones as a common set of proteins that regulate the invasion phenotype of head and neck cancer. The goal of this study was to establish a common set of molecules that regulate   cell invasion   in head and neck cancer (HNC).Five invasive sublines derived from HNC cell lines were established using the Matrigel selection method. Proteomic technology, MetaCore algorithm, and reverse transcriptase-PCR methods were used to search for molecules that contribute to the invasion phenotype. Cellular functional analyses and clinical association studies were applied to examine the significance of the molecules.Fifty-two proteins were identified in more than two of the four independent proteomic experiments, including 10 (19%) molecular chaperones. Seven chaperones were confirmed to be differentially expressed in five sublines, Hsp90α, Hsp90β, Hsp90-B1/Gp96, Hsp70-A5/Grp78, and HYOU1, that upregulate, whereas Hsp60 and glucosidase-α neutral AB (GANAB) downregulate. Four molecules were further investigated. In all cell lines, knockdown of Hsp60 or GANAB and silencing of Gp96 or Grp78 considerably enhanced or reduced cell migration and invasion, respectively. Clinical association studies consistently revealed that low levels of Hsp60 or GANAB and high levels of Gp96 or Grp78 are significantly associated with advanced cancer (P < 0.001 to P = 0.047, respectively, for the four molecules) and poor survival (P < 0.001 to P = 0.025, respectively, for the four molecules).Our study defined molecular chaperones as a common set of proteins that regulate the invasion phenotype of HNC. Loss of the tumor suppression function of Hsp60 or GANAB and acquisition of the oncogenic function of Gp96 or Grp78 contribute to aggressive cancers. These molecules may serve as prognostic markers and targets for cancer drug development. 
 Molecular chaperones as a common set of proteins that regulate the invasion phenotype of head and neck cancer. The goal of this study was to establish a common set of molecules that regulate   cell invasion   in head and neck cancer (HNC).Five invasive sublines derived from HNC cell lines were established using the Matrigel selection method. Proteomic technology, MetaCore algorithm, and reverse transcriptase-PCR methods were used to search for molecules that contribute to the invasion phenotype. Cellular functional analyses and clinical association studies were applied to examine the significance of the molecules.Fifty-two proteins were identified in more than two of the four independent proteomic experiments, including 10 (19%) molecular chaperones. Seven chaperones were confirmed to be differentially expressed in five sublines, Hsp90α, Hsp90β, Hsp90-B1/Gp96, Hsp70-A5/Grp78, and HYOU1, that upregulate, whereas Hsp60 and glucosidase-α neutral AB (GANAB) downregulate. Four molecules were further investigated. In all cell lines, knockdown of Hsp60 or GANAB and silencing of Gp96 or Grp78 considerably enhanced or reduced cell migration and invasion, respectively. Clinical association studies consistently revealed that low levels of Hsp60 or GANAB and high levels of Gp96 or Grp78 are significantly associated with advanced cancer (P < 0.001 to P = 0.047, respectively, for the four molecules) and poor survival (P < 0.001 to P = 0.025, respectively, for the four molecules).Our study defined molecular chaperones as a common set of proteins that regulate the invasion phenotype of HNC. Loss of the tumor suppression function of Hsp60 or GANAB and acquisition of the oncogenic function of Gp96 or Grp78 contribute to aggressive cancers. These molecules may serve as prognostic markers and targets for cancer drug development. 
   
  
 SDHA 
 
  NCBI Gene  
  GeneRIF  
  Pubmed  
 
 
  Search Term  Rank  Fields  
  [Mm]igration  6  PubMed(2); GeneRIFs(2)  
  [Ii]nvasive  10  PubMed(2)  
  [Mm]etastasis  12  PubMed(2)  
  [Cc]ell [Ii]nvasion  13  PubMed(1)  
 
 
 hide/show details for SDHA 
  [Mm]igration  PubMed  
 Cancer-associated fibroblasts enhance pancreatic cancer cell invasion by remodeling the metabolic conversion mechanism. We investigated the mechanism of cancer-associated fibroblasts (CAFs) in promoting the invasion and metastasis of pancreatic cancer cells in a non-vascular manner. We verified the original generation of isolated cultured CAFs and normal fibroblasts (NFs) based on the expression of α-SMA and vimentin, and we examined the cell glycolysis level through glucose consumption and lactate production experiments. The mRNA and protein expression of CAF glycolytic enzymes, lactate dehydrogenase and pyruvate kinase m2, were examined by RT-PCR and western blotting, respectively. In vitro culture first-generation pancreatic CAFs were collected and cultured together with pancreas cancer BxPc-3 and Panc-1 cells. Cell invasion a  nd migrat  ion were assessed using a Transwell assay and scratch test, respectively. Mitochondrial activity was assessed by experimentally determining oxidative phosphorylation (OP) activity. The aerobic oxidation index of cancer cells was also examined. Succinate dehydrogenase, fumarate hydratase (FH), and monocarboxylate transporter 1 (MCT1) expression were examined using an MCT1-specific inhibitor to remove 'tumor-stromal' metabolic coupling to observe the influence of cell interstices on pancreas cancer progression. First-generation isolated cultured CAFs and NFs both grew well, and showed active proliferation. Glucose absorption and lactate production were significantly enhanced in CAFs compared with that in NFs. PCR and western blotting showed that the lactate dehydrogenase and pyruvate kinase m2 mRNA and protein expression levels were increased in the CAFs. After indirect co-culture, OP was increased in the BxPc-3 and Panc-1 cells; correspondingly, succinate dehydrogenase, FH and MCT expression were increased. After the MCT1-specific inhibitor removed 'tumor-stromal' metabolic coupling,   the migra  tion and invasion abilities of the pancreatic cancer cells were decreased. Pancreatic CAFs can alter metabolism as well as communicate with and respond to cancer c  ell migra  tion and invasion. This may be an important mechanism for promoting tumor progression in a non-vascular manner in the tumor microenvironment. The mechanism by which CAFs reshape metabolic transition requires further analysis. 
 Inhibition of succinate dehydrogenase sensitizes cyclin E-driven ovarian cancer to CDK inhibition. High-grade serous ovarian cancer (HGS-OvCa) is characterized by widespread CCNE1 amplification. Current treatments lack specificity to target Cyclin E-driven OvCa.By in silico analysis of the TCGA OvCa dataset we searched association between genes involved in glucose metabolism and cell cycle control. Metabolic shift was studied in Cyclin E-driven OvCa cells treated with CDK inhibition (CDKi). Genetic and pharmaceutical inhibition of succinate dehydrogenase (SDH) was tested in combination with CDKi.OvCa patients with CCNE1 amplification could be divided by concomitant SDHA amplification. A2780 OvCa cells were similar to the Cyclin E-driven and SDHA neutral genotype. CDKi in A2780 cells using Dinaciclib resulted in compensatory enhancement of tricarboxylicacid cycle (TCA) cycle activity. Combined blockade of CDK and SDH, both genetically and pharmaceutically, showed synergy and resulted in inhibited proliferation,   migration  , invasion and   migration   in A2780 cells. The combined inhibition did not further alter cell cycle population, but induced apoptosis of A2780 cells.Cyclin E-driven OvCa cells appeared addicted to glucose metabolism via TCA. Combined CDKi with modalities targeting TCA, like SDHA inhibition showed promising effects for this genotype. 
  GeneRIFs  
 After indirect co-culture, OP was increased in the BxPc-3 and Panc-1 cells; correspondingly, succinate dehydrogenase, FH and MCT expression were increased. After the MCT1-specific inhibitor removed 'tumor-stromal' metabolic coupling, the   migration   and invasion abilities of the pancreatic cancer cells were decreased. 
 Combined blockade of CDK and SDH, both genetically and pharmaceutically, showed synergy and resulted in inhibited proliferation,   migration  , invasion and   migration   in A2780 cells Cyclin E-driven OvCa cells appeared addicted to glucose metabolism via TCA. Combined CDKi with modalities targeting TCA, like SDHA inhibition showed promising effects for this genotype. 
  [Ii]nvasive  PubMed  
 An abnormality in glucocorticoid receptor expression differentiates steroid responders from nonresponders in keloid disease. Glucocorticoids (GCs) are first-line treatment for keloid disease (KD) but are limited by high incidence of resistance, recurrence and undesirable side-effects. Identifying patient responsiveness early could guide therapy.Nineteen patients with KD were recruited at week 0 (before treatment) and received intralesional steroids. At weeks 0, 2 and 4, non  invasive   imaging and biopsies were performed. Responsiveness was determined by clinical response and a significant reduction in vascular perfusion following steroid treatment, using full-field laser perfusion imaging (FLPI). Responsiveness was also evaluated using (i) spectrophotometric intracutaneous analysis to quantify changes in collagen and melanin and (ii) histology to identify changes in epidermal thickness and glycosaminoglycan (GAG) expression. Biopsies were used to quantify changes in glucocorticoid receptor (GR) expression using quantitative reverse transcriptase polymerase chain reaction, immunoblotting and immunohistochemistry.At week 2, the FLPI was used to separate patients into steroid responsive (n = 12) and nonresponsive groups (n = 7). All patients demonstrated a significant decrease in GAG at week 2 (P < 0.05). At week 4, responsive patients exhibited significant reduction in melanin, GAG, epidermal thickness (all P < 0.05) and a continued reduction in perfusion (P < 0.001) compared with nonresponders. Steroid-responsive patients had increased GR expression at baseline and showed autoregulation of GR compared with nonresponders, who showed no change in GR transcription or protein.This is the first demonstration that keloid response to steroids can be measured objectively using non  invasive   imaging. FLPI is a potentially reliable tool to stratify KD responsiveness. Altered GR expression may be the mechanism gating therapeutic response. 
 In Vivo Detection of Succinate by Magnetic Resonance Spectroscopy as a Hallmark of SDHx Mutations in Paraganglioma. Germline mutations in genes encoding mitochondrial succinate dehydrogenase (SDH) are found in patients with paragangliomas, pheochromocytomas, gastrointestinal stromal tumors, and renal cancers. SDH inactivation leads to a massive accumulation of succinate, acting as an oncometabolite and which levels, assessed on surgically resected tissue are a highly specific biomarker of SDHx-mutated tumors. The aim of this study was to address the feasibility of detecting succinate in vivo by magnetic resonance spectroscopy.A pulsed proton magnetic resonance spectroscopy ((1)H-MRS) sequence was developed, optimized, and applied to image nude mice grafted with Sdhb(-/-) or wild-type chromaffin cells. The method was then applied to patients with paraganglioma carrying (n = 5) or not (n = 4) an SDHx gene mutation. Following surgery, succinate was measured using gas chromatography/mass spectrometry, and SDH protein expression was assessed by immunohistochemistry in resected tumors.A succinate peak was observed at 2.44 ppm by (1)H-MRS in all Sdhb(-/-)-derived tumors in mice and in all paragangliomas of patients carrying an SDHx gene mutation, but neither in wild-type mouse tumors nor in patients exempt of SDHx mutation. In one patient, (1)H-MRS results led to the identification of an unsuspected SDHA gene mutation. In another case, it helped define the pathogenicity of a variant of unknown significance in the SDHB gene.Detection of succinate by (1)H-MRS is a highly specific and sensitive hallmark of SDHx mutations. This non  invasive   approach is a simple and robust method allowing in vivo detection of the major biomarker of SDHx-mutated tumors. 
  [Mm]etastasis  PubMed  
 Loss of SDHA expression identifies SDHA mutations in succinate dehydrogenase-deficient gastrointestinal stromal tumors. Succinate dehydrogenase-deficient gastrointestinal stromal tumors (SDH-deficient GISTs) are a unique class of GIST defined by negative immunohistochemical staining for succinate dehydrogenase B (SDHB). SDH-deficient GISTs show distinctive clinical and pathologic features including absence of KIT and PDGFRA mutations, exclusive gastric location, common lymph node   metastasis  , a prognosis not predicted by size and mitotic rate, and indolent behavior of metastases. They may be syndromal with some being associated with the Carney Triad or germline SDHA, SDHB, SDHC, or SDHD mutations (Carney-Stratakis syndrome). It is normally recommended that genetic testing for SDHA, SDHB, SDHC, and SDHD be offered whenever an SDH-deficient GIST is encountered. However, testing for all 4 genes is burdensome and beyond the means of most centers. In this study we performed SDHA mutation and immunohistochemical analyses for SDHA on 10 SDH-deficient GISTs. Three showed negative staining for SDHA, and all of these were associated with germline SDHA mutations. In 2 tumors, 3 novel mutations were identified (p.Gln54X, p.Thr267Met, and c.1663+3G>C), none of which have previously been reported in GISTs or other SDH-associated tumors. Seven showed positive staining for SDHA and were not associated with SDHA mutation. In conclusion, 30% of SDH-deficient GISTs in this study were associated with germline SDHA mutation. Negative staining for SDHA can be used to triage formal genetic testing for SDHA when an SDH-deficient GIST is encountered. 
 Cancer-associated fibroblasts enhance pancreatic cancer cell invasion by remodeling the metabolic conversion mechanism. We investigated the mechanism of cancer-associated fibroblasts (CAFs) in promoting the invasion and   metastasis   of pancreatic cancer cells in a non-vascular manner. We verified the original generation of isolated cultured CAFs and normal fibroblasts (NFs) based on the expression of α-SMA and vimentin, and we examined the cell glycolysis level through glucose consumption and lactate production experiments. The mRNA and protein expression of CAF glycolytic enzymes, lactate dehydrogenase and pyruvate kinase m2, were examined by RT-PCR and western blotting, respectively. In vitro culture first-generation pancreatic CAFs were collected and cultured together with pancreas cancer BxPc-3 and Panc-1 cells. Cell invasion and migration were assessed using a Transwell assay and scratch test, respectively. Mitochondrial activity was assessed by experimentally determining oxidative phosphorylation (OP) activity. The aerobic oxidation index of cancer cells was also examined. Succinate dehydrogenase, fumarate hydratase (FH), and monocarboxylate transporter 1 (MCT1) expression were examined using an MCT1-specific inhibitor to remove 'tumor-stromal' metabolic coupling to observe the influence of cell interstices on pancreas cancer progression. First-generation isolated cultured CAFs and NFs both grew well, and showed active proliferation. Glucose absorption and lactate production were significantly enhanced in CAFs compared with that in NFs. PCR and western blotting showed that the lactate dehydrogenase and pyruvate kinase m2 mRNA and protein expression levels were increased in the CAFs. After indirect co-culture, OP was increased in the BxPc-3 and Panc-1 cells; correspondingly, succinate dehydrogenase, FH and MCT expression were increased. After the MCT1-specific inhibitor removed 'tumor-stromal' metabolic coupling, the migration and invasion abilities of the pancreatic cancer cells were decreased. Pancreatic CAFs can alter metabolism as well as communicate with and respond to cancer cell migration and invasion. This may be an important mechanism for promoting tumor progression in a non-vascular manner in the tumor microenvironment. The mechanism by which CAFs reshape metabolic transition requires further analysis. 
  [Cc]ell [Ii]nvasion  PubMed  
 Cancer-associated fibroblasts enhance pancreatic cancer   cell invasion   by remodeling the metabolic conversion mechanism. We investigated the mechanism of cancer-associated fibroblasts (CAFs) in promoting the invasion and metastasis of pancreatic cancer cells in a non-vascular manner. We verified the original generation of isolated cultured CAFs and normal fibroblasts (NFs) based on the expression of α-SMA and vimentin, and we examined the cell glycolysis level through glucose consumption and lactate production experiments. The mRNA and protein expression of CAF glycolytic enzymes, lactate dehydrogenase and pyruvate kinase m2, were examined by RT-PCR and western blotting, respectively. In vitro culture first-generation pancreatic CAFs were collected and cultured together with pancreas cancer BxPc-3 and Panc-1 cell  s. Cell invas  ion and migration were assessed using a Transwell assay and scratch test, respectively. Mitochondrial activity was assessed by experimentally determining oxidative phosphorylation (OP) activity. The aerobic oxidation index of cancer cells was also examined. Succinate dehydrogenase, fumarate hydratase (FH), and monocarboxylate transporter 1 (MCT1) expression were examined using an MCT1-specific inhibitor to remove 'tumor-stromal' metabolic coupling to observe the influence of cell interstices on pancreas cancer progression. First-generation isolated cultured CAFs and NFs both grew well, and showed active proliferation. Glucose absorption and lactate production were significantly enhanced in CAFs compared with that in NFs. PCR and western blotting showed that the lactate dehydrogenase and pyruvate kinase m2 mRNA and protein expression levels were increased in the CAFs. After indirect co-culture, OP was increased in the BxPc-3 and Panc-1 cells; correspondingly, succinate dehydrogenase, FH and MCT expression were increased. After the MCT1-specific inhibitor removed 'tumor-stromal' metabolic coupling, the migration and invasion abilities of the pancreatic cancer cells were decreased. Pancreatic CAFs can alter metabolism as well as communicate with and respond to cancer cell migration and invasion. This may be an important mechanism for promoting tumor progression in a non-vascular manner in the tumor microenvironment. The mechanism by which CAFs reshape metabolic transition requires further analysis. 
   
  
 SLC9A3R2 
 
  NCBI Gene  
  GeneRIF  
  Pubmed  
 
 
  Search Term  Rank  Fields  
  [Ff]ilopodia  1  PubMed(1)  
  [Mm]igration  6  PubMed(5); GeneRIFs(1)  
  [Ii]nvasive  10  PubMed(1)  
  [Mm]etastasis  12  PubMed(1)  
 
 
 hide/show details for SLC9A3R2 
  [Ff]ilopodia  PubMed  
 Binding to Na(+) /H(+) exchanger regulatory factor 2 (NHERF2) affects trafficking and function of the enteropathogenic Escherichia coli type III secretion system effectors Map, EspI and NleH. Enteropathogenic Escherichia coli (EPEC) strains are diarrhoeal pathogens that use a type III secretion system to translocate effector proteins into host cells in order to colonize and multiply in the human gut. Map, EspI and NleH1 are conserved EPEC effectors that possess a C-terminal class I PSD-95/Disc Large/ZO-1 (PDZ)-binding motif. Using a PDZ array screen we identified Na(+)/H(+) exchanger regulatory factor 2 (NHERF2), a scaffold protein involved in tethering and recycling ion channels in polarized epithelia that contains two PDZ domains, as a common target of Map, EspI and NleH1. Using recombinant proteins and co-immunoprecipitation we confirmed that NHERF2 binds each of the effectors. We generated a HeLa cell line stably expressing HA-tagged NHERF2 and found that Map, EspI and NleH1 colocalize and interact with intracellular NHERF2 via their C-terminal PDZ-binding motif. Overexpression of NHERF2 enhanced the formation and persistence of Map-induced   filopodia  , accelerated the trafficking of EspI to the Golgi and diminished the anti-apoptotic activity of NleH1. The binding of multiple T3SS effectors to a single scaffold protein is unique. Our data suggest that NHERF2 may act as a plasma membrane sorting site, providing a novel regulatory mechanism to control the intracellular spatial and temporal effector protein activity. 
  [Mm]igration  PubMed  
 MCC, a new interacting protein for Scrib, is required for cell   migration   in epithelial cells. To further characterize the molecular events supporting the tumor suppressor activity of Scrib in mammals, we aim to identify new binding partners. We isolated MCC, a recently identified binding partner for beta-catenin, as a new interacting protein for Scrib. MCC interacts with both Scrib and the NHERF1/NHERF2/Ezrin complex in a PDZ-dependent manner. In T47D cells, MCC and Scrib proteins colocalize at the cell membrane and reduced expression of MCC results in impaired cell   migration  . By contrast to Scrib, MCC inhibits cell directed   migration   independently of Rac1, Cdc42 and PAK activation. Altogether, these results identify MCC as a potential scaffold protein regulating cell movement and able to bind Scrib, beta-catenin and NHERF1/2. 
 Analysis of the myosin-II-responsive focal adhesion proteome reveals a role for β-Pix in negative regulation of focal adhesion maturation. Focal adhesions undergo myosin-II-mediated maturation wherein they grow and change composition to modulate integrin signalling for cell   migratio  n, growth and differentiation. To determine how focal adhesion composition is affected by myosin II activity, we performed proteomic analysis of isolated focal adhesions and compared protein abundance in focal adhesions from cells with and without myosin II inhibition. We identified 905 focal adhesion proteins, 459 of which changed in abundance with myosin II inhibition, defining the myosin-II-responsive focal adhesion proteome. The abundance of 73% of the proteins in the myosin-II-responsive focal adhesion proteome was enhanced by contractility, including proteins involved in Rho-mediated focal adhesion maturation and endocytosis- and calpain-dependent focal adhesion disassembly. During myosin II inhibition, 27% of proteins in the myosin-II-responsive focal adhesion proteome, including proteins involved in Rac-mediated lamellipodial protrusion, were enriched in focal adhesions, establishing that focal adhesion protein recruitment is also negatively regulated by contractility. We focused on the Rac guanine nucleotide exchange factor β-Pix, documenting its role in the negative regulation of focal adhesion maturation and the promotion of lamellipodial protrusion and focal adhesion turnover to drive cel  l migrati  on. 
 MAGI-3 competes with NHERF-2 to negatively regulate LPA2 receptor signaling in colon cancer cells. Lysophosphatidic acid (LPA) is a potent inducer of colon cancer and LPA receptor type 2 (LPA(2)) is overexpressed in colon tumors. LPA(2) interacts with membrane-associated guanylate kinase with inverted orientation-3 (MAGI-3) and the Na+/H+ exchanger regulatory factor 2 (NHERF-2), but the biological effects of these interactions are unknown. We investigated the roles of MAGI-3 and NHERF-2 in LPA(2)-mediated signaling in human colon cancer cells.We overexpressed or knocked down MAGI-3 in HCT116 and SW480 cells. The effects of MAGI-3 and NHERF-2 in LPA-induced cell   migration  , invasion, inositol phosphate generation, and nuclear factor-κB activation were determined. Expression of MAGI-3 and NHERF-2 in human colon tumor tissues was analyzed using tissue microarray analysis.NHERF-2 promoted   migratio  n and invasion of colon cancer cells, whereas MAGI-3 inhibited these processes. MAGI-3 competed with NHERF-2 for binding to LPA(2) and phospholipase C-β3. However, NHERF-2 and MAGI-3 reciprocally regulated LPA(2)-induced phospholipase C activity. MAGI-3 increased the interaction of LPA(2) with Gα(12), whereas NHERF-2 preferentially promoted interaction between LPA(2) and Gα(q). MAGI-3 decreased the tumorigenic capacity of LPA(2) by attenuating the activities of nuclear factor-κB and c-Jun N-terminal kinase. MAGI-3 and NHERF-2 were expressed differentially in colon adenocarcinomas, consistent with their opposing effects.LPA(2) is dynamically regulated by 2 distinct PDZ proteins via modulation of G-protein coupling and receptor signaling. MAGI-3 is a negative regulator of LPA(2) signaling. 
 PTEN tumor suppressor associates with NHERF proteins to attenuate PDGF receptor signaling. PTEN, a tumor suppressor frequently inactivated in many human cancers, directly antagonizes the activity of phosphatidylinositol-3-OH kinase (PI3K) by dephosphorylating phosphoinositides. We show here that PTEN interacts directly with the NHERF1 and NHERF2 (Na+/H+ exchanger regulatory factor) homologous adaptor proteins through the PDZ motif of PTEN and the PDZ1 domain of NHERF1 or both PDZ domains of NHERF2. NHERFs were shown to interact directly with platelet-derived growth factor receptor (PDGFR), and we demonstrate the assembly of a ternary complex between PTEN, NHERFs and PDGFR. The activation of the PI3K pathway after PDGFR stimulation was prolonged in NHERF1(-/-) mouse embryonic fibroblasts as compared to wild-type cells, consistent with defective PTEN recruitment to PDGFR in the absence of NHERF1. Depletion of NHERF2 by small interfering RNA similarly increased PI3K signaling. Phenotypically, the loss of NHERF1 enhanced the PDGF-induced cytoskeletal rearrangements and chemotactic   migration   of the cells. These data indicate that, in normal cells, NHERF proteins recruit PTEN to PDGFR to restrict the activation of the PI3K. 
 NHERF links the N-cadherin/catenin complex to the platelet-derived growth factor receptor to modulate the actin cytoskeleton and regulate cell motility. Using phage display, we identified Na+/H+ exchanger regulatory factor (NHERF)-2 as a novel binding partner for the cadherin-associated protein, beta-catenin. We showed that the second of two PSD-95/Dlg/ZO-1 (PDZ) domains of NHERF interacts with a PDZ-binding motif at the very carboxy terminus of beta-catenin. N-cadherin expression has been shown to induce motility in a number of cell types. The first PDZ domain of NHERF is known to bind platelet-derived growth factor-receptor beta (PDGF-Rbeta), and the interaction of PDGF-Rbeta with NHERF leads to enhanced cell spreading and motility. Here we show that beta-catenin and N-cadherin are in a complex with NHERF and PDGF-Rbeta at membrane ruffles in the highly invasive fibrosarcoma cell line HT1080. Using a stable short hairpin RNA system, we showed that HT1080 cells knocked down for either N-cadherin or NHERF had impaired ability to migrate into the wounded area in a scratch assay, similar to cells treated with a PDGF-R kinase inhibitor. Cells expressing a mutant NHERF that is unable to associate with beta-catenin had increased stress fibers, reduced lamellipodia, and impaired cell   migration  . Using HeLa cells, which express little to no PDGF-R, we introduced PDGF-Rbeta and showed that it coimmunoprecipitates with N-cadherin and that PDGF-dependent cell   migration   was reduced in these cells when we knocked-down expression of N-cadherin or NHERF. These studies implicate N-cadherin and beta-catenin in cell   migration   via PDGF-R-mediated signaling through the scaffolding molecule NHERF. 
  GeneRIFs  
 N-cadherin and beta-catenin play role in cell   migration   via PDGF-Rbeta-mediated signaling through the scaffolding molecule NHERF2 
  [Ii]nvasive  PubMed  
 NHERF links the N-cadherin/catenin complex to the platelet-derived growth factor receptor to modulate the actin cytoskeleton and regulate cell motility. Using phage display, we identified Na+/H+ exchanger regulatory factor (NHERF)-2 as a novel binding partner for the cadherin-associated protein, beta-catenin. We showed that the second of two PSD-95/Dlg/ZO-1 (PDZ) domains of NHERF interacts with a PDZ-binding motif at the very carboxy terminus of beta-catenin. N-cadherin expression has been shown to induce motility in a number of cell types. The first PDZ domain of NHERF is known to bind platelet-derived growth factor-receptor beta (PDGF-Rbeta), and the interaction of PDGF-Rbeta with NHERF leads to enhanced cell spreading and motility. Here we show that beta-catenin and N-cadherin are in a complex with NHERF and PDGF-Rbeta at membrane ruffles in the highly   invasive   fibrosarcoma cell line HT1080. Using a stable short hairpin RNA system, we showed that HT1080 cells knocked down for either N-cadherin or NHERF had impaired ability to migrate into the wounded area in a scratch assay, similar to cells treated with a PDGF-R kinase inhibitor. Cells expressing a mutant NHERF that is unable to associate with beta-catenin had increased stress fibers, reduced lamellipodia, and impaired cell migration. Using HeLa cells, which express little to no PDGF-R, we introduced PDGF-Rbeta and showed that it coimmunoprecipitates with N-cadherin and that PDGF-dependent cell migration was reduced in these cells when we knocked-down expression of N-cadherin or NHERF. These studies implicate N-cadherin and beta-catenin in cell migration via PDGF-R-mediated signaling through the scaffolding molecule NHERF. 
  [Mm]etastasis  PubMed  
 Overexpression of SIP1 and downregulation of E-cadherin predict delayed neck   metastasis   in stage I/II oral tongue squamous cell carcinoma after partial glossectomy. Patients with clinical stage I/II (T1-2N0M0) oral tongue squamous cell carcinoma (TSCC) usually undergo partial glossectomy alone. However, 14-48% of them develop delayed neck   metastasis   (DNM), which may lead to an unfavorable course. Recently epithelial-to-mesenchymal transition (EMT) has been thought to play a crucial role in cancer   metastasis  . The present study aimed to examine the associations of EMT-involved molecular factors and clinicopathological factors with DNM in stage I/II TSCC.mRNA expression levels of E-cadherin and its transcriptional repressors (snail, SIP1, and twist) in 7 head and neck squamous cell carcinoma (HNSCC) cell lines were evaluated by quantitative real-time PCR. Clinicopathological parameters and immunohistochemical expressions of E-cadherin and its repressors were examined in surgical specimens of 37 stage I/II TSCC patients who underwent partial glossectomy alone.In HNSCC cells, E-cadherin expression was inversely correlated with SIP1 expression (P = 0.023). Univariate analysis of immunohistochemistry showed that overexpression of SIP1 and loss of E-cadherin were significantly correlated with DNM, although no inverse correlation was found between E-cadherin and its repressors. Multiple logistic regression analysis including clinicopathological and molecular factors revealed that overexpression of SIP1 (P = 0.005), loss of E-cadherin (P = 0.046), and vascular invasion (P = 0.024) were independently correlated with DNM.These results suggest that development of DNM in stage I/II TSCC is closely related to induction of EMT in primary tumor cells. Especially, SIP1 and E-cadherin are considered to be the possible markers for selecting patients at high risk of DNM. 
   
  
 KLF7 
 
  NCBI Gene  
  GeneRIF  
  Pubmed  
 
 
  Search Term  Rank  Fields  
  [Ee]xtracellular [Mm]atrix  8  PubMed(1)  
 
 
 hide/show details for KLF7 
  [Ee]xtracellular [Mm]atrix  PubMed  
 Cell and tissue specific expression of human Krüppel-like transcription factors in human ocular surface. The ocular surface, composed of the conjunctiva and the cornea, is essential for vision. Its integrity depends on numerous molecular and cellular processes such as proliferation, differentiation, apoptosis, adhesion, and   extracellular matri  x homeostasis, whose deregulation can induce ophthalmological pathologies. The Krüppel-like transcription factors (KLFs) family is made up of 15 C2H2 zinc-finger proteins involved in vertebrate development and able to control cell proliferation, growth, and differentiation. In order to better define their respective roles in the human ocular surface, we decided to determine their pattern of expression in ocular tissues. We then focused on the expression of KLF4 and some of its target genes to establish KLF4's biological activities in human ocular surface.Firstly, total mRNA was extracted from human total cornea, conjunctiva, corneal epithelial cells (primary culture and established cell line), corneal keratocytes (primary culture), corneal endothelial cells (established cell line), and conjunctival epithelial cells (established cell line) and submitted to RT-PCR experiments in order to determine the expression patterns of the different KLFs. Secondly, KLF4 protein localization was visualized by immunofluorescence assays at tissue and cell levels. Finally, KLF4 target genes (endoglin, ornithine decarboxylase) mRNA expression levels were determined by semi-quantitative RT-PCR, after KLF4 transient transfection in human corneal epithelium (HCE) cells.We detected the presence of twelve transcripts of KLFs in the cornea (KLF2, KLF3, KLF4, KLF5, KLF6, KLF7, KLF8, KLF10, KLF11, KLF12, KLF13, and KLF16) and eight in the conjunctiva (KLF2, KLF3, KLF4, KLF6, KLF7, KLF10, KLF11, and KLF12). Under our conditions, the transcripts encoding KLF1 and KLF9 were never detected. Specific expression patterns of each KLF were also determined for the major cellular components of the human cornea and conjunctiva. KLF4 immunolocalization assays indicated its presence in both the cytoplasmic and nuclear compartments of conjunctival and corneal cells. KLF4 transient overexpression in HCE cells down regulated both endoglin and ODC mRNA levels.For the first time, we established the presence of a KLF network in the human ocular surface and illustrated the conservation of KLF4's biological properties in a corneal derived epithelial cell line. 
   
  
 KLF6 
 
  NCBI Gene  
  GeneRIF  
  Pubmed  
 
 
  Search Term  Rank  Fields  
  [Mm]igration  6  PubMed(6); GeneRIFs(3)  
  [Ee]xtracellular [Mm]atrix  8  PubMed(2); GeneRIFs(1)  
  [Ii]nvasive  10  PubMed(7); GeneRIFs(2)  
  [Mm]etastasize  11  PubMed(1)  
  [Mm]etastasis  12  PubMed(11); GeneRIFs(2)  
  [Cc]ell [Ii]nvasion  13  PubMed(1)  
 
 
 hide/show details for KLF6 
  [Mm]igration  PubMed  
 Disruption of an SP2/KLF6 repression complex by SHP is required for farnesoid X receptor-induced endothelial cell   migration  . The farnesoid X receptor (FXR) signaling pathway regulates bile acid and cholesterol homeostasis. Here, we demonstrate, using a variety of gain- and loss-of-function approaches, a role of FXR in the process of cell motility, which involves the small heterodimeric partner (SHP)-dependent up-regulation of matrix metalloproteinase-9. We use this observation to reveal a transcriptional regulatory mechanism involving the SP/KLF transcription factors, SP2 and KLF6. Small interference RNA-based silencing studies in combination with promoter, gel shift, and chromatin immunoprecipitation assays indicate that SP2 and KLF6 bind to the matrix metalloproteinase-9 promoter and together function to maintain this gene in a silenced state. However, upon activation of FXR, SHP interacts with SP2 and KLF6, disrupting the SP2/KLF6 repressor complex. Thus, together, these studies identify a mechanism for antagonizing Sp/KLF protein repression function via SHP, with this process regulating endothelial cell motility. 
 A small interfering RNA targeting the KLF6 splice variant, KLF6-SV1, as gene therapy for gastric cancer. Accumulating evidence suggests that the tumor suppressor gene Kruppel-like factor 6 (KLF6) and its dominant-negative splice form KLF6-SV1 play important roles in both the development and progression of cancer. However, the role of KLF6-SV1 in gastric cancer remains largely unknown.KLF6-SV1 expression was detected in various human gastric cancer cell lines and gastric cancer patient samples by reverse transcriptase polymerase chain reaction (RT-PCR) and Western blotting. Small interfering RNA (siRNA) was used to inhibit KLF6-SV1 expression in BGC-823 and SGC-7901 cell lines. The effects of downregulation of KLF6-SV1 by siRNA on cell proliferation,   migration  , invasion, and tumor growth were examined in vitro and in vivo.Overexpression of KLF6-SV1 was detected in tumor samples from gastric cancer patients, and in various differentiated gastric cancer cell lines. In vitro downregulation of KLF6-SV1 by siRNA inhibited BGC-823 and SGC-7901 cell proliferation, anchorage-independent growth,   migration  , and invasion through the altered expression of Ki-67, vascular endothelial growth factor (VEGF), E-cadherin, and matrix metalloproteinase (MMP)-9. Also, KLF6-SV1 silencing promoted caspase-dependent apoptosis of BGC-823 and SGC-7901 cells via the regulation of phosphatidylinositol 3-OH kinase (PI3K)/Akt activity and Bcl-2-related protein expression. In vivo animal studies showed that KLF6-SV1 siRNA significantly inhibited the tumorigenicity of BGC-823 and SGC-7901 cells. Gene therapy with polyethylenimine/si-SV1 intratumoral injection also resulted in the suppression of tumor growth and prolonged animal survival in an established xenograft tumor model.These data demonstrate that KLF6-SV1 is an important regulator of the growth,   migration  , invasion, and survival of gastric cancer cells, and downregulation of KLF6-SV1 by siRNA may offer a new potential gene therapy approach for gastric cancer. 
 Increased H3K9me3 drives dedifferentiated phenotype via KLF6 repression in liposarcoma. Liposarcoma (LPS) can be divided into 4 different subtypes, of which well-differentiated LPS (WDLPS) and dedifferentiated LPS (DDLPS) are the most common. WDLPS is typically low grade, whereas DDLPS is high grade, aggressive, and carries a worse prognosis. WDLPS and DDLPS frequently co-occur in patients. However, it is not clear whether DDLPS arises independently from WDLPS, or whether epigenomic alterations underly the histopathological differences of these subtypes. Here, we profiled 9 epigenetic marks in tumor samples from 151 patients with LPS and showed elevated trimethylation of histone H3 at Lys9 (H3K9me3) levels in DDLPS tumors. Integrated ChIP-seq and gene expression analyses of patient-derived cell lines revealed that H3K9me3 mediates differential regulation of genes involved in cellular differentiation and   migration  . Among these, Kruppel-like factor 6 (KLF6) was reduced in DDLPS, with increased H3K9me3 at associated regulatory regions. Pharmacologic inhibition of H3K9me3 with chaetocin decreased DDLPS proliferation and increased expression of the adipogenesis-associated factors PPARγ, CEBPα, and CEBPβ, suggesting that increased H3K9me3 may mediate DDLPS-associated aggressiveness and dedifferentiation properties. KLF6 overexpression partially phenocopied chaetocin treatment in DDLPS cells and induced phenotypic changes that were consistent with adipocytic differentiation, suggesting that the effects of increased H3K9me3 may be mediated through KLF6. In conclusion, we provide evidence of an epigenetic basis for the transition between WDLPS and DDLPS. 
 miR-101 acts as a tumor suppressor by targeting Kruppel-like factor 6 in glioblastoma stem cells. Great interest persists in useful therapeutic targets in glioblastoma (GBM). Deregulation of microRNAs (miRNAs) expression has been associated with cancer formation through alterations in gene targets. In this study, we reported the role of miR-101 in human glioblastoma stem cells (GSCs) and the potential mechanisms.Quantitative real-time PCR showed that miR-101 expression was decreased in GSCs. Overexpression of miR-101 reduced the proliferation,   migration  , invasion, and promoted apoptosis of GSCs. One direct target of miR-101, the transcription factor Kruppel-like factor 6 (KLF6), was identified using the Dual-Luciferase Reporter Assay System, which mediated the tumor suppressor activity of miR-101. This process was coincided with the reduced expression of Chitinase-3-like protein 1 (CHI3L1) whose promoter could be bound with and be promoted by KLF6 demonstrated by luciferase assays and chromatin immunoprecipitation assays. The downregulation of CHI3L1 led to the inactivation of MEK1/2 and PI3K signal pathways. Furthermore, nude mice carrying the tumors of overexpressed miR-101 combined with knockdown of KLF6 produced the smallest tumors and showed the highest survival rate.Our findings provided a comprehensive analysis of miR-101 and further defining it as a potential therapeutic candidate for GBM. 
 MicroRNA-181a promotes gastric cancer by negatively regulating tumor suppressor KLF6. MicroRNAs have emerged as crucial regulators of tumorigenesis. However, it remains unknown whether miR-181a is involved in the pathogenesis of gastric cancer. In this study, we found that miR-181a is overexpressed in human gastric cancer tissues. Ectopic expression of miR-181a mimic promoted the proliferation, colony formation,   migration  , and invasion and inhibited the apoptosis of SGC-7901 gastric cancer cells, whereas ectopic expression of miR-181a inhibitor inhibited the malignant phenotypes of SGC-7901 cells. Site-directed mutagenesis and luciferase reporter assay demonstrated that miR-181a repressed KLF6 expression by targeting its 3'-UTR. Western blot analysis further showed that KLF6 protein was significantly decreased or increased when miR-181a mimic or inhibitor was transfected into SGC-7901 cells, respectively. In summary, these data suggest that KLF6 gene is a direct target of miR-181a and miR-181a functions as an oncomir in gastric cancer by repressing the expression of tumor suppressor KLF6. 
 Identification of miRNAs that specifically target tumor suppressive KLF6-FL rather than oncogenic KLF6-SV1 isoform. The Krüppel like factor 6 (KLF6) gene encodes multiple protein isoforms derived from alternative mRNA splicing, most of which are intimately involved in hepatocarcinogenesis and tumor progression. Recent bioinformatics analysis shows that alternative mRNA splicing of the KLF6 gene produces around 16 alternatively spliced variants with divergent or even opposing functions. Intriguingly, the full-length KLF6 (KLF6-FL) is a tumor suppressor gene frequently inactivated in liver cancer, whereas KLF6 splice variant 1 (KLF6-SV1) is an oncogenic isoform with antagonistic function against KLF6-FL. Compelling evidence indicates that miRNA, the small endogenous non-coding RNA (ncRNA), acts as a vital player in modulating a variety of cellular biological processes through targeting different mRNA regions of protein-coding genes. To identify the potential miRNAs specifically targeting KLF6-FL, we utilized bioinformatics analysis in combination with the luciferase reporter assays and screened out two miRNAs, namely miR-210 and miR-1301, specifically targeted the tumor suppressive KLF6-FL rather than the oncogenic KLF6-SV1. Our in vitro experiments demonstrated that stable expression of KLF6-FL inhibited cell proliferation,   migratio  n and angiogenesis while overexpression of miR-1301 promoted cell   migratio  n and angiogenesis. Further experiments demonstrated that miR-1301 was highly expressed in liver cancer cell lines as well as clinical specimens and we also identified the potential methylation and histone acetylation for miR-1301 gene. To sum up, our findings unveiled a novel molecular mechanism that specific miRNAs promoted tumorigenesis by targeting the tumor suppressive isoform KLF6-FL rather than its oncogenic isoform KLF6-SV1. 
  GeneRIFs  
 Study revealed that overexpression of miR-101 inhibited the proliferation, cell   migration  , invasion, and promoted apoptosis of glioblastoma stem cells by directly targeting KLF6 
 dominant-negative splice form KLF6-SV1 is an important regulator of the growth,   migration  , invasion, and survival of gastric cancer cells. 
 an SP2/KLF6 repression complex by SHP is required for farnesoid X receptor-induced endothelial cell   migration   
  [Ee]xtracellular [Mm]atrix  PubMed  
 Genetic susceptibility to dupuytren disease: association of Zf9 transcription factor gene. Transforming growth factor beta 1 (TGF-beta 1) is a key fibrogenic cytokine that has been shown to stimulate fibroblast proliferation and   extracellular matrix   deposition and has been implicated in the pathogenesis of Dupuytren disease. Zf9 is a transcription factor that increases TGF-beta 1 expression in tissue. The authors previously demonstrated a lack of association between common TGF-beta 1 and TGF-beta 2 polymorphisms and Dupuytren disease. Therefore, Zf9 transcription factor represents an additional candidate susceptibility gene for investigating hereditary predisposition to Dupuytren disease. Dupuytren disease, or Dupuytren contracture, is a progressive and recurrent fibroproliferative disease of unknown etiology that affects the human hands. It is often a familial disorder affecting Northern European Caucasians. Genes implicated in the development of Dupuytren disease have not yet been found. Using a polymerase chain reaction-restriction fragment polymorphism method, the authors genotyped a novel single nucleotide polymorphism in the 3' untranslated region of the Zf9 gene. A case-control association study of 138 patients with Dupuytren disease versus 255 controls demonstrated a statistically significant difference in the genotype and allele frequencies between patients and controls for Zf9 gene polymorphism. Presence of the G allele versus the A allele is associated with an increased risk of developing Dupuytren disease (odds ratio, 1.9; 95 percent confidence interval, 1.2 to 2.9). The authors believe this to be the first report of a positive genetic association study in Dupuytren disease using single nucleotide polymorphisms. 
 KLF6 Gene and early melanoma development in a collagen I-rich extracellular environment. A putative tumor suppressor gene at chromosome 10p15, which contains KLF6 and other genes, is predicted to be lost during melanoma development, and its identity is unknown. In this study, we investigated the biological roles and identity of this tumor suppressor gene.The human UACC 903 melanoma cell line containing introduced DNA fragments from the 10p15 region with (10E6/3, 10E6/11, and 10E6/18) and without (10ER4S.2/1) the tumor suppressor gene was used. Xenograft tumors were generated in a total of 40 mice with melanoma cell lines, and tumor size was measured. Cells were cultured on plastic or a gel of type I collagen. Viability, proliferation, and apoptosis were assessed. Expression of KLF6 protein was assessed by immunohistochemistry and immunoblot analysis. Expression of phosphorylated Erk1/2 and cyclin D1 was assessed by immunoblot analysis. Protein expression of KLF6 was inhibited with small interfering RNA (siRNA). KLF6 protein expression was assessed in 17 human nevi and human melanoma specimens from 29 patients. Statistical analyses were adjusted for multiple comparisons by use of Dunnett method. All statistical tests were two-sided.Melanoma cells containing KLF6 generated smaller subcutaneous xenograft tumors with fewer proliferating cells than control cells. When grown on collagen 1, viability of cells with ectopic KLF6 expression (72%) was lower than that of control cells (100%) (group difference = -28%, 95% confidence interval = -31.3% to -25.2%, P < .001). Viability of melanoma cells with or without the KLF6 tumor suppressor gene on plastic dishes was similar. When KLF6 expression was inhibited with KLF6 siRNA, viability of cells with the tumor suppressor gene on collagen I gel increased compared with that of control cells carrying scrambled siRNA. KLF6 protein was detected in all nevi examined but not in human metastatic melanoma tissue examined. Ectopic expression of KLF6 protein in melanoma cells grown on collagen I decreased levels of phosphorylated Erk1/2 and cyclin D1 in the mitogen-activated protein kinase signaling pathway.In melanoma cells, the tumor suppressor gene at 10p15 appears to be KLF6. Signaling from the collagen I-rich   extracellular matrix   appears to be involved in the tumor suppressive activity of KLF6 protein. 
  GeneRIFs  
 In melanoma cells, the tumor suppressor gene at 10p15 appears to be KLF6. Signaling from the collagen I-rich   extracellular matrix   appears to be involved in the tumor suppressive activity of KLF6 protein. 
  [Ii]nvasive  PubMed  
 Effects of Kruppel-like factor 6 on osteosarcoma cell biological behavior. Kruppel-like factor 6 (KLF6) is a tumor suppressor gene frequently downregulated in a number of human cancers, including osteosarcoma. However, the role of KLF6 in osteosarcoma remains unclear. This study was aimed at investigating the effects of KLF6 on osteosarcoma cell biological behavior. First, the expression of KLF6 in osteosarcoma cell lines (MG63, SaOS-2, U2OS, and HOS) and a human osteoblastic cell line (hFOB1.19) was detected by Western blotting. Results showed that KLF6 displayed a significant downregulation in osteosarcoma cell lines (MG63, SaOS-2, U2OS, and HOS) compared with human osteoblastic cell line (hFOB1.19). To investigate the role of KLF6 in osteosarcoma cell proliferation, apoptosis, and invasion, we generated human osteosarcoma MG63 cells in which KLF6 was either overexpressed or depleted. The MG63 cell viability, cycle, apoptosis, and   invasive   ability were analyzed by 3-(4,5-dimethylthiazol-2-yl)-2,5-diphenyltetrazolium bromide staining, propidium iodide (PI) staining, Annexin-V-FITC/PI double staining, and Transwell invasion experiment, respectively. Results showed that the viability, proliferation, and   invasive   abilities were suppressed, and the apoptosis was enhanced in MG63 cells with overexpression of KLF6. The viability, proliferation, and   invasive   abilities were improved, and the apoptosis was inhibited in MG63 cells with knockdown of KLF6. At the same time, these molecules, including p21, bcl-2, and MMP-9, associated with the events about cell cycle, apoptosis, and invasion, were detected. Results showed that the expressions of bcl-2 and MMP-9 were downregulated, and the expressions of p21 were upregulated in the MG-63 cells with overexpression of KLF6. Taken together, our results suggested that KLF6 could inhibit proliferation and invasion, and facilitate apoptosis of osteosarcoma cells, which might be a potential target for the treatment of osteosarcoma. 
 Kruppel-like factor 6 suppresses growth and invasion of hepatocellular carcinoma cells in vitro and in vivo. Kruppel-like factor 6 (KLF6) as a novel tumor suppressive gene participates in multiple biological behaviors and plays an important role in regulating tumor cell growth and invasion. However, the functions of KLF6 in hepatocellular carcinoma (HCC) remain poorly understood. The expression level of KLF6 was examined by immunohistochemical assay in human HCC tissues, and KLF6-overexpressed HCC cells (SMCC-7721 and HepG2) were used for evaluating cell proliferation and invasion by MTT and Transwell assays. A subcutaneous HCC tumor model was established for assessing tumor growth in vivo. Our results showed that the expression of KLF6 was significantly downregulated in HCC tissues compared with the adjacent non-cancerous tissues (50.0% vs. 72.0%, P = 0.034) and negatively associated with the lymph-vascular space invasion (LVSI) in HCC patients (P = 0.003). Furthermore, overexpression of KLF6 reduced cell proliferation and weakened the cell   invasive   potential followed with the decreased expression of PCNA and MMP-9 in HCC cells. The in vivo experiment indicated that KLF6 overexpression suppressed the xenograft tumor growth. Therefore, our findings show that KLF6 suppresses growth and invasion of HCC cells in vitro and in vivo, suggesting a tumor suppressive function in HCC and provides the potential therapeutic target for the treatment of HCC. 
 [Transcriptional activation of TMSG-1 by complex of KLF6 and Sp1]. To investigate the regulatory mechanism of the transcription of tumor metastasis suppressor gene TMSG-1.Luciferase reporter assay and site-directed mutagenesis were used to analyze the regulatory region of TMSG-1. Electrophoretic mobility shift assay (EMSA) and chromatin immunoprecipitation (ChIP) were carried out to verify the interaction of KLF6 and Sp1 with the regulatory region of TMSG-1. Co-immunoprecipitation (CoIP) was performed to analyze the interaction between KLF6 and Sp1. TMSG-1 and wt-KLF6 mRNA expressions in cells with different metastatic capacities were quantitated by real-time PCR. Cell   invasive   capability was determined by Matrigel invasion assay.A 63 bp inducible regulatory region (+59 bp - +123 bp) in exon 1 was identified by luciferase assay using reporter plasmids with a series of TMSG-1 regulatory region deletions. Mutations in KLF6/Sp1 binding sites of this region resulted in a decrease of luciferase activity, while cotransfection with KLF6 or Sp1 expressing plasmids led to a remarkable increase of luciferase activity. EMSA and ChIP demonstrated that KLF6 as well as Sp1 interacted with this region. CoIP also indicated a possible interaction between KLF6 and Sp1 proteins. In the highly metastatic cell sublines, a low level of wild type KLF6 was associated synchronously with a low TMSG-1 level. Prostate carcinoma cells overexpressing KLF6 exhibited a higher TMSG-1 level and a lower   invasive   capability.Transcription factor complex of KLF6 and Sp1 may participate in the inducible transcriptional regulation of TMSG-1, and a decreased wild type KLF6 expression is likely associated with a low TMSG-1 level in the highly metastatic cell sublines. 
 Tissue factor pathway inhibitor-2 was repressed by CpG hypermethylation through inhibition of KLF6 binding in highly   invasive   breast cancer cells. Tissue factor pathway inhibitor-2 (TFPI-2) is a matrix-associated Kunitz inhibitor that inhibits plasmin and trypsin-mediated activation of zymogen matrix metalloproteinases involved in tumor progression, invasion and metastasis. Here, we have investigated the mechanism of DNA methylation on the repression of TFPI-2 in breast cancer cell lines.We found that both protein and mRNA of TFPI-2 could not be detected in highly   invasive   breast cancer cell line MDA-MB-435. To further investigate the mechanism of TFPI-2 repression in breast cancer cells, 1.5 Kb TFPI-2 promoter was cloned, and several genetic variations were detected, but the promoter luciferase activities were not affected by the point mutation in the promoter region and the phenomena was further supported by deleted mutation. Scan mutation and informatics analysis identified a potential KLF6 binding site in TFPI-2 promoter. It was revealed, by bisulfite modified sequence, that the CpG island in TFPI-2 promoter region was hypermethylated in MDA-MB-435. Finally, using EMSA and ChIP assay, we demonstrated that the CpG methylation in the binding site of KLF-6 diminished the binding of KLF6 to TFPI-2 promoter.In this study, we found that the CpG islands in TFPI-2 promoter was hypermethylated in highly   invasive   breast cancer cell line, and DNA methylation in the entire promoter region caused TFPI-2 repression by inducing inactive chromatin structure and decreasing KLF6 binding to its DNA binding sequence. 
 KLF6/Sp1 initiates transcription of the tmsg-1 gene in human prostate carcinoma cells: an exon involved mechanism. The tumor metastasis suppressor gene-1 (tmsg-1) was first cloned as a new tumor suppressor gene in our laboratory several years ago. Since then, however, despite the substantial progression that has been made in investigation of the biologic roles played by this gene, the manner in which it exerts its regulatory influence is still unknown. With transfection of various deletion or mutation constructs, we identified a potential enhancer and three potential silencers in the 5'-flanking region. However, it was particularly interesting to find that a region (+59 to +123 bp) of exon 1 exhibited a strong role in initiation of tmsg-1 gene transcription. Deletion of this region led to essentially complete loss of driving activity of exon-1 sequence on luciferase. Further analysis showed that transcription factors KLF6 and Sp1 are able to interact with each other and bind to their elements in this region. Co-transfection of pGL3-114/+123 with KLF6- and/or Sp1-expressing plasmids resulted in an elevation of luciferase activity and transcription level of tmsg-1, which was abolished by knockdown of KLF6 or Sp1. Analysis of metastatic capacity showed that cells with high metastatic capability exhibited a lower level of KLF6/TMSG-1 proteins with highe  r invasi  ve capability and vice versa. Thus, we concluded that interaction of KLF6 and Sp1, together with their binding of elements in exon 1 are critical events in initiation of transcription of the tmsg-1 gene. These results reveal a hitherto unreported mechanism for initiation of transcription of the tmsg-1 gene. 
 Difference in the role of loss of heterozygosity at 10p15 (KLF6 locus) in colorectal carcinogenesis between sporadic and familial adenomatous polyposis and hereditary nonpolyposis colorectal cancer patients. To clarify the role of the KLF6 (Kruppel-like factor 6) locus in multistep colorectal carcinogenesis, we analyzed loss of heterozygosity (LOH) at 10p15 (KLF6 locus) and mutations of the KLF6gene in 298 colorectal tumors at various pathological stages of sporadic and familial adenomatous polyposis (FAP) and hereditary nonpolyposis colorectal cancer (HNPCC) patients.10p15 LOH was analyzed using KLF6M1 and KLF6M2, and KLF6 gene mutation was analyzed using PCR-SSCP and sequencing.It was found that the frequencies of LOH (sum of M1 and M2) were 4% in adenomas, 0% in intramucosal carcinomas, 35% in   invasive   carcinomas, and 33% in liver metastases in sporadic cases.   Invasive   carcinomas from FAP patients showed only 6% LOH, and   invasive   carcinomas from HNPCC patients exhibited 0% LOH. Mutation analysis of the KLF6 gene in 298 colorectal tumors detected no somatic mutations.The present data suggest that LOH of the KLF6 locus at chromosome 10p15 contributes to the invasion step from an intramucosal carcinoma to an   invasive   carcinoma specifically in sporadic colorectal carcinogenesis, but is rarely involved in the carcinogenesis of FAP and HNPCC cases. Moreover, the absence of somatic mutations suggests the uncertainty of the KLF6 gene as a classical tumor suppressor gene at the lost 10p15 region in colorectal carcinogenesis. 
 KLF6-SV1 drives breast cancer metastasis and is associated with poor survival. Metastasis is the major cause of cancer mortality. A more thorough understanding of the mechanisms driving this complex multistep process will aid in the identification and characterization of therapeutically targetable genetic drivers of disease progression. We demonstrate that KLF6-SV1, an oncogenic splice variant of the KLF6 tumor suppressor gene, is associated with increased metastatic potential and poor survival in a cohort of 671 lymph node-negative breast cancer patients. KLF6-SV1 overexpression in mammary epithelial cell lines resulted in an epithelial-to-mesenchymal-like transition and drove aggressive multiorgan metastatic disease in multiple in vivo models. Additionally, KLF6-SV1 loss-of-function studies demonstrated reversion to an epithelial and less   invasive   phenotype. Combined, these findings implicate KLF6-SV1 as a key driver of breast cancer metastasis that distinguishes between indolent and lethal early-stage disease and provides a potential therapeutic target for   invasive   breast cancer. 
  GeneRIFs  
 The CpG islands in TFPI-2 promoter was hypermethylated in highly   invasive   breast cancer cell line, and DNA methylation in the entire promoter region caused TFPI-2 repression by inducing inactive chromatin structure and decreasing KLF6 binding. 
 Loss of heterozygosity of the KLF6 at chromosome 10p15 contributes to the invasion step from an intramucosal carcinoma to an   invasive   carcinoma specifically in sporadic colorectal carcinogenesis 
  [Mm]etastasize  PubMed  
 KLF6-SV1 overexpression accelerates human and mouse prostate cancer progression and metastasis. Metastatic prostate cancer (PCa) is one of the leading causes of death from cancer in men. The molecular mechanisms underlying the transition from localized tumor to hormone-refractory metastatic PCa remain largely unknown, and their identification is key for predicting prognosis and targeted therapy. Here we demonstrated that increased expression of a splice variant of the Kruppel-like factor 6 (KLF6) tumor suppressor gene, known as KLF6-SV1, in tumors from men after prostatectomy predicted markedly poorer survival and disease recurrence profiles. Analysis of tumor samples revealed that KLF6-SV1 levels were specifically upregulated in hormone-refractory metastatic PCa. In 2 complementary mouse models of metastatic PCa, KLF6-SV1-overexpressing PCa cells were shown by in vivo and ex vivo bioluminescent imaging to   metastasize   more rapidly and to disseminate to lymph nodes, bone, and brain more often. Interestingly, while KLF6-SV1 overexpression increased metastasis, it did not affect localized tumor growth. KLF6-SV1 inhibition using RNAi induced spontaneous apoptosis in cultured PCa cell lines and suppressed tumor growth in mice. Together, these findings demonstrate that KLF6-SV1 expression levels in PCa tumors at the time of diagnosis can predict the metastatic behavior of the tumor; thus, KLF-SV1 may represent a novel therapeutic target. 
  [Mm]etastasis  PubMed  
 Kruppel-like factor 6 in the progression and prognosis of malignant melanoma. The aims of this study were to investigate the incidence of Krüppel-like factor 6 (KLF6) protein staining in patients with cutaneous malignant melanoma and examine its potential relevance to clinicopathological characteristics and tumour cell proliferation.Clinicopathological data from patients with cutaneous malignant melanoma were analysed retrospectively. Presence of KLF6 and the antigen Ki-67 in malignant melanoma and healthy tissue samples from each patient was detected by immunohistochemistry. The proliferation index was calculated on the basis of Ki-67 expression. The relationship between KLF6 and clinicopathological characteristics was also analysed.KLF6 was detected more frequently in normal healthy skin tissue compared with cutaneous malignant melanoma lesions (n = 40). There was a negative correlation between the presence of KLF6 and the proliferation index. The presence of KLF6 was also significantly correlated with tumour diameter, lymph   node metas  tasis, tumour-  node-metas  tasis stage and 3-year survival rate.KLF6 protein is downregulated in human cutaneous malignant melanoma lesions compared with healthy skin tissue. KLF6 may be involved in tumour progression and may be a tumour suppressor and prognostic marker for cutaneous malignant melanoma. 
 [Transcriptional activation of TMSG-1 by complex of KLF6 and Sp1]. To investigate the regulatory mechanism of the transcription of tumor   metastasis   suppressor gene TMSG-1.Luciferase reporter assay and site-directed mutagenesis were used to analyze the regulatory region of TMSG-1. Electrophoretic mobility shift assay (EMSA) and chromatin immunoprecipitation (ChIP) were carried out to verify the interaction of KLF6 and Sp1 with the regulatory region of TMSG-1. Co-immunoprecipitation (CoIP) was performed to analyze the interaction between KLF6 and Sp1. TMSG-1 and wt-KLF6 mRNA expressions in cells with different metastatic capacities were quantitated by real-time PCR. Cell invasive capability was determined by Matrigel invasion assay.A 63 bp inducible regulatory region (+59 bp - +123 bp) in exon 1 was identified by luciferase assay using reporter plasmids with a series of TMSG-1 regulatory region deletions. Mutations in KLF6/Sp1 binding sites of this region resulted in a decrease of luciferase activity, while cotransfection with KLF6 or Sp1 expressing plasmids led to a remarkable increase of luciferase activity. EMSA and ChIP demonstrated that KLF6 as well as Sp1 interacted with this region. CoIP also indicated a possible interaction between KLF6 and Sp1 proteins. In the highly metastatic cell sublines, a low level of wild type KLF6 was associated synchronously with a low TMSG-1 level. Prostate carcinoma cells overexpressing KLF6 exhibited a higher TMSG-1 level and a lower invasive capability.Transcription factor complex of KLF6 and Sp1 may participate in the inducible transcriptional regulation of TMSG-1, and a decreased wild type KLF6 expression is likely associated with a low TMSG-1 level in the highly metastatic cell sublines. 
 Tissue factor pathway inhibitor-2 was repressed by CpG hypermethylation through inhibition of KLF6 binding in highly invasive breast cancer cells. Tissue factor pathway inhibitor-2 (TFPI-2) is a matrix-associated Kunitz inhibitor that inhibits plasmin and trypsin-mediated activation of zymogen matrix metalloproteinases involved in tumor progression, invasion and   metastasis  . Here, we have investigated the mechanism of DNA methylation on the repression of TFPI-2 in breast cancer cell lines.We found that both protein and mRNA of TFPI-2 could not be detected in highly invasive breast cancer cell line MDA-MB-435. To further investigate the mechanism of TFPI-2 repression in breast cancer cells, 1.5 Kb TFPI-2 promoter was cloned, and several genetic variations were detected, but the promoter luciferase activities were not affected by the point mutation in the promoter region and the phenomena was further supported by deleted mutation. Scan mutation and informatics analysis identified a potential KLF6 binding site in TFPI-2 promoter. It was revealed, by bisulfite modified sequence, that the CpG island in TFPI-2 promoter region was hypermethylated in MDA-MB-435. Finally, using EMSA and ChIP assay, we demonstrated that the CpG methylation in the binding site of KLF-6 diminished the binding of KLF6 to TFPI-2 promoter.In this study, we found that the CpG islands in TFPI-2 promoter was hypermethylated in highly invasive breast cancer cell line, and DNA methylation in the entire promoter region caused TFPI-2 repression by inducing inactive chromatin structure and decreasing KLF6 binding to its DNA binding sequence. 
 KLF6/Sp1 initiates transcription of the tmsg-1 gene in human prostate carcinoma cells: an exon involved mechanism. The tumor   metastasis   suppressor gene-1 (tmsg-1) was first cloned as a new tumor suppressor gene in our laboratory several years ago. Since then, however, despite the substantial progression that has been made in investigation of the biologic roles played by this gene, the manner in which it exerts its regulatory influence is still unknown. With transfection of various deletion or mutation constructs, we identified a potential enhancer and three potential silencers in the 5'-flanking region. However, it was particularly interesting to find that a region (+59 to +123 bp) of exon 1 exhibited a strong role in initiation of tmsg-1 gene transcription. Deletion of this region led to essentially complete loss of driving activity of exon-1 sequence on luciferase. Further analysis showed that transcription factors KLF6 and Sp1 are able to interact with each other and bind to their elements in this region. Co-transfection of pGL3-114/+123 with KLF6- and/or Sp1-expressing plasmids resulted in an elevation of luciferase activity and transcription level of tmsg-1, which was abolished by knockdown of KLF6 or Sp1. Analysis of metastatic capacity showed that cells with high metastatic capability exhibited a lower level of KLF6/TMSG-1 proteins with higher invasive capability and vice versa. Thus, we concluded that interaction of KLF6 and Sp1, together with their binding of elements in exon 1 are critical events in initiation of transcription of the tmsg-1 gene. These results reveal a hitherto unreported mechanism for initiation of transcription of the tmsg-1 gene. 
 E-cadherin is a novel transcriptional target of the KLF6 tumor suppressor. The tumor suppressor KLF6 is a member of the Krüppel-like family of transcription factors, which has been implicated in the pathogenesis of several human carcinomas. Uncovering the transcriptional targets relevant for its tumorigenic properties, including cellular proliferation and invasion, will be essential to understanding possible mechanisms by which KLF6 and its antagonistic splice form, KLF6-SV1, regulate this development. To begin defining possible metastatic-related pathways, we analysed the effect of KLF6 dysregulation on a recognized suppressor of cellular invasion, E-cadherin. Targeted KLF6 reduction in an ovarian cancer cell line, SKOV-3, resulted in a 50% reduction of E-cadherin expression (P<0.01) and conversely, KLF6-SV1 silencing upregulated E-cadherin approximately fivefold (P<0.0001). These changes resulted from KLF6 directly transactivating the E-cadherin promoter as demonstrated by luciferase promoter assay and chromatin immunoprecipitation (ChIP). KLF6-mediated changes in E-cadherin levels were accompanied by downstream changes in both the subcellular localization of beta-catenin and c-myc expression levels. Moreover, and consistent with these experimental findings, patient-derived epithelial ovarian tumors with low KLF6 and high KLF6-SV1 expression ratios had significantly decreased E-cadherin expression (P<0.0001). These combined findings highlight the E-cadherin pathway as a novel and functionally important mediator by which changes in KLF6 and KLF6-SV1 can directly alter ovarian tumor invasion and   metastasi  s. 
 Decreased expression of KLF6 and its significance in gastric carcinoma. To study the expression of the Krüppel-like transcription factor 6 (KLF6) in human gastric carcinoma and normal gastric mucosa tissues, and to explore the role of KLF6 in the carcinogenesis and tumor progression and its clinical significance. Expression of KLF6, P21WAF1 and PCNA was investigated by immunohistochemistry for 69 surgically resected gastric carcinoma tissues and corresponding normal gastric mucosa tissues, respectively. The correlations of KLF6 expression with clinicopathological characteristics, P21WAF1 and PCNA were examined. Positive-expression of KLF6 was 64 out of 69 cases (92.8%) in normal gastric mucosa and only 23 cases (33.3%) in gastric carcinoma. Expression of KLF6 in the gastric carcinoma was remarkably lower than normal gastric mucosa. Decreased expression of KLF6 in gastric carcinoma was significantly associated with histological differentiation (P<0.01), TNM stage (P<0.05), lymph node   metastasi  s (P<0.01) and distant   metastasi  s (P<0.05). There was no significant correlation between KLF6 expression and sex, age. Meanwhile, expression of KLF6 was associated with expression of P21WAF1 in both normal gastric mucosa and gastric carcinoma (P<0.05). In addition, decreased expression of KLF6 in gastric carcinoma was positively associated with PCNA level (r=0.719, P<0.01) by association analysis. Down-regulation of KLF6 might play an important role in the carcinogenesis and development of human gastric carcinoma and have significant clinical value. 
 KLF6-SV1 drives breast cancer   metastasis   and is associated with poor survival.   Metastasis   is the major cause of cancer mortality. A more thorough understanding of the mechanisms driving this complex multistep process will aid in the identification and characterization of therapeutically targetable genetic drivers of disease progression. We demonstrate that KLF6-SV1, an oncogenic splice variant of the KLF6 tumor suppressor gene, is associated with increased metastatic potential and poor survival in a cohort of 671 lymph node-negative breast cancer patients. KLF6-SV1 overexpression in mammary epithelial cell lines resulted in an epithelial-to-mesenchymal-like transition and drove aggressive multiorgan metastatic disease in multiple in vivo models. Additionally, KLF6-SV1 loss-of-function studies demonstrated reversion to an epithelial and less invasive phenotype. Combined, these findings implicate KLF6-SV1 as a key driver of breast cancer   metastasis   that distinguishes between indolent and lethal early-stage disease and provides a potential therapeutic target for invasive breast cancer. 
 New candidate tumor-suppressor gene KLF6 and its splice variant KLF6 SV2 counterbalancing expression in primary hepatocarcinoma. This study aimed to detect the expression of newly discovered zinc finger transcriptional factor KLF6 and its splice variant KLF6 SV2 in primary hepatocarcinoma (PHC) tissues and hepatoma cell strains, and to evaluate their clinicopathologic relationship with PHC.Wild-type KLF6 and KLF6 SV2 mRNA expression was determined by RTPCR in 27 cases of PHC tissues and cell strains of HepG2, SMMC7721 and LO2. Western blotting and immunohistochemical staining were adopted to detect KLF6 protein expression. Positive area ratio of wild-type KLF6 protein expression and its relationship with clinicopathological parameters of PHC was analyzed.Wild-type KLF6 expression in PHC tissues was lower than that in paracancerous tissues. In contrast, KLF6 SV2 mRNA expression was higher in PHC tissues and hepatoma cell strains (p<0.05). Positive area ratio of wild-type KLF6 protein expression was positively correlated with cellular differentiation degree of PHC (p<0.01), but negatively correlated not only with liver cirrhosis, tumor size and extrahepatic metastases (p<0.01), but also with portal vein thrombus and the number of lymph nodes with   metastasis   (p<0.05).Wild-type KLF6 deletion and inactivation was involved in the growth, cell differentiation and other physiological processes of PHC. The upregulation of KLF6 splice variant might counterbalance the wildtype KLF6 and contribute to the occurrence and development of PHC. 
 Genetic alterations of the KLF6 gene in gastric cancer. The KLF6 is a zinc-finger tumor suppressor that is frequently mutated in several human cancers and broadly involved in differentiation and development, growth-related signal transduction, cell proliferation, apoptosis, and angiogenesis. To determine whether genetic alterations of KLF6 gene are involved in the development and/or progression of gastric cancer, we have screened a set of 80 sporadic gastric cancers for mutations and allele loss of the KLF6 gene. Four missense mutations, S155R, P172 T, S180L, and R198 K, were detected in transactivation domain of the KLF6 gene and one of them had biallelic mutations with somatic mutation of one allele and loss of the remaining allele. All of the cases with mutation were of advanced intestinal-type gastric cancer with lymph node   metastasis  . In addition, 16 (43.2%) of 37 informative cases showed allelic loss at KLF6 locus. Interestingly, allelic loss was also frequent in intestinal-type gastric cancer. Therefore, our data suggest that genetic alterations of KLF6 gene might play an important role in the development or progression of sporadic gastric cancers. 
 KLF6-SV1 overexpression accelerates human and mouse prostate cancer progression and   metastasis  . Metastatic prostate cancer (PCa) is one of the leading causes of death from cancer in men. The molecular mechanisms underlying the transition from localized tumor to hormone-refractory metastatic PCa remain largely unknown, and their identification is key for predicting prognosis and targeted therapy. Here we demonstrated that increased expression of a splice variant of the Kruppel-like factor 6 (KLF6) tumor suppressor gene, known as KLF6-SV1, in tumors from men after prostatectomy predicted markedly poorer survival and disease recurrence profiles. Analysis of tumor samples revealed that KLF6-SV1 levels were specifically upregulated in hormone-refractory metastatic PCa. In 2 complementary mouse models of metastatic PCa, KLF6-SV1-overexpressing PCa cells were shown by in vivo and ex vivo bioluminescent imaging to metastasize more rapidly and to disseminate to lymph nodes, bone, and brain more often. Interestingly, while KLF6-SV1 overexpression increased   metastasis  , it did not affect localized tumor growth. KLF6-SV1 inhibition using RNAi induced spontaneous apoptosis in cultured PCa cell lines and suppressed tumor growth in mice. Together, these findings demonstrate that KLF6-SV1 expression levels in PCa tumors at the time of diagnosis can predict the metastatic behavior of the tumor; thus, KLF-SV1 may represent a novel therapeutic target. 
 Hepatocyte growth factor enhances alternative splicing of the Kruppel-like factor 6 (KLF6) tumor suppressor to promote growth through SRSF1. Alternative splicing of the Krüppel-like factor 6 (KLF6) tumor suppressor into an antagonistic splice variant 1 (SV1) is a pathogenic event in several cancers including hepatocellular carcinoma (HCC) because elevated SV1 is associated with increased tumor   metastasi  s and mortality. Ras activation is one factor that can enhance KLF6 splicing in cancer cells, however pathways driving KLF6 splicing are unknown. Splice site selection is regulated by splice factors that include serine/arginine-rich (SR) proteins such as SRSF1 (ASF-SF2), which in turn is controlled by phosphoinositide 3-kinase (PI3K)/Akt and the mitogen-activated protein kinase (MAPK) signaling pathway. Because signaling pathways downstream of the liver mitogen hepatocyte growth factor (HGF) include Akt, we explored whether HGF induces KLF6 alternative splicing. In HepG2 cells, HGF (25 ng/mL) significantly increases the ratio of SV1/KLF6 full by 40% through phosphorylation of Akt and subsequent downregulation of two splicing regulators, SRSF3 (SRp20) and SRSF1. Decreased SRSF3 levels regulate SRSF1 levels by alternative splicing associated with the nonsense-mediated mRNA decay pathway (AS-NMD), which stimulates cell growth by decreasing p21 levels. Enhanced cell replication through increased KLF6 alternative splicing is a novel growth-promoting pathway of HGF that could contribute to the molecule's mitogenic activity in physiologic liver growth and hepatocellular carcinoma. 
  GeneRIFs  
 These findings implicate KLF6-SV1 as a key driver of breast cancer   metastasis  . 
 KLF6 overexpression accelerates human prostate cancer progression and   metastasis  . 
  [Cc]ell [Ii]nvasion  PubMed  
 miR-4262 Promotes Proliferation and Invasion of Human Breast Cancer Cells Through Directly Targeting KLF6 and KLF15. miRNAs have been shown to be involved in breast cancer growth and progression. miR-4262 is a potential tumor promoter in human cancers. In this study, we first investigated the role of miR-4262 in the proliferation and invasion of human breast cancer cells. Our results showed that, compared with the adjacent tissues and MCF-10A normal breast epithelial cells, miR-4262 was markedly increased in the breast cancer tissues and five cell lines, including MDA-MB-231, MDA-MB-468, MDA-MB-435, SKBR3, and MCF-7. Then the miR-4262 mimic or oligo anta-miR-4262 was transfected into MDA-MB-231 and MCF-7 breast cancer cell lines. The results showed that the miR-4262 mimic greatly increased the miR-4262 level and the proliferation and invasion of MDA-MB-231 and MCF-7 cells. In contrast, the anta-miR-4262 had a completely opposite effect on miR-4262 expression, cell proliferation, and   cell invasion   in MDA-MB-231 and MCF-7 cells. Moreover, bioinformatics and luciferase reporter gene assays confirmed that miR-4262 targeted the mRNA 3'-UTR region of KLF6 and KLF15, two characterized tumor suppressor genes. miR-4262 suppressed protein levels of KLF6 and KLF15 in MDA-MB-231 cells, and the suppression could be rescued by the transfection of pcDNA-KLF6 and -KLF15. In conclusion, miR-4262 positively regulates proliferation and invasion of human breast cancer cells via suppression of KLF6 and KLF15. 
   
  
 DAB2IP 
 
  NCBI Gene  
  GeneRIF  
  Pubmed  
 
 
  Search Term  Rank  Fields  
  [Mm]igration  6  Process(5); PubMed(5); GeneRIFs(1)  
  [Ii]nvasive  10  PubMed(7)  
  [Mm]etastasis  12  PubMed(9); GeneRIFs(2)  
  [Cc]ell [Ii]nvasion  13  PubMed(1)  
 
 
 hide/show details for DAB2IP 
  [Mm]igration  Process  
 cell motility involved in cerebral cortex radial glia guided   migration   
 negative regulation of endothelial cell   migration   
 negative regulation of epithelial cell   migration   
 negative regulation of epithelial cell   migration   
 positive regulation of neuron   migration   
  PubMed  
 miR-92b targets DAB2IP to promote EMT in bladder cancer   migration   and invasion. Muscle-invasive or metastatic bladder cancer (BCa) has a very poor prognosis; however, its mechanisms remain largely unknown. Previous studies have discovered multiple microRNAs (miRs) that are involved in BCa progression and regarded as potential biomarkers or therapeutic targets. In this study, we demonstrated that miR-92b could uniquely promote cell   migratio  n and invasion of BCa cells, but had no effect on cell proliferation. Mechanistically, our data provided evidence to verify that miR-92b was able to directly target DAB2IP, a well-known tumor suppressor, and inhibit epithelial‑mesenchymal transition of BCa cells. Moreover, the increased expression levels of miR-92b were negatively correlated with DAB2IP, and predicted poor prognosis of patients with BCa. Overall, this study reveals a new promising biomarker and its mechanisms contributing to BCa invasion or metastasis. 
 Downregulation of DAB2IP results in cell proliferation and invasion and contributes to unfavorable outcomes in bladder cancer. The DOC-2/DAB2 interactive protein (DAB2IP) is a member of the Ras GTPase-activating protein family. It has been shown to be often downregulated and a poor prognostic factor in several human malignancies. In this study, we analyzed the clinicopathological features and outcomes of DAB2IP expression in 135 patients with urothelial carcinoma of the bladder (UCB) treated by radical cystectomy plus bilateral lymph node dissection, and evaluated the effect of DAB2IP knockdown in vitro using the MTT method, colony formation assay, cell cycle assay, and cell   migration   and invasive assay. We found low expression of DAB2IP was significantly associated with high pathological stage (P = 0.002), high pathological grade (P = 0.02), tumor size more than 3 cm (P = 0.04), and presence of histological variants (P = 0.01). DAB2IP was an independent prognostic factor of disease recurrence (hazard ratio, 2.67; P = 0.034) and cancer-specific survival (hazard ratio, 2.79; P = 0.038). Knockdown of DAB2IP could promote cell proliferation,   migration  , and invasion. Downregulation of DAB2IP could activate the ERK and Akt pathways and was correlated with the expression of epithelial-mesenchymal transition markers, such as E-cadherin and vimentin. In conclusion, downregulation of DAB2IP is associated with features of biologically aggressive UCB and results in cell proliferation,   migration  , and invasion of bladder cancer. DAB2IP may serve as a promising biomarker in patients with UCB treated by radical cystectomy and bilateral lymph node dissection. 
 High glucose and/or high insulin affects HIF-1 signaling by regulating AIP1 in human umbilical vein endothelial cells. The objective of this study was to explore the effects of high glucose/high insulin on AIP1 expression in HUVECs and the possible regulation of HIF-1α signaling by AIP1.We investigated the expression of AIP1 and HIF-1α signaling in HUVECs at the levels of mRNA and protein following exposure to 30 mmol/L glucose (high glucose), 1 nmol/L insulin (high insulin), and the combination of the two (high glucose/high insulin). We detected changes in HIF-1α and VEGF expression with AIP1 siRNA interference by real-time PCR and western blotting. The CCK8 cell proliferation assay, the scratch/wound-healing assay, and flow cytometry were used to assess cell proliferatio  n, migrat  ion and apoptosis, respectively. Matrigel was used to perform a tubule formation assay.Compared with 5.5 mmol/L glucose alone (control), high glucose, high insulin, and the combination of high glucose+high insulin increased AIP1 expression at 24 h at the mRNA and protein levels. High glucose, high insulin, and high glucose+high insulin decreased HIF-1α expression at the mRNA and protein levels. AIP1 knockdown significantly increased HIF-1α and VEGF expression at both the mRNA and protein levels in HUVECs under high glucose conditions. In the presence of high insulin, the effect of high glucose on target gene expression was altered. The downregulation of AIP1 promoted cell proliferat  ion, migr  ation, and tubule formation, and it decreased apoptosis.High glucose increases AIP1 expression and decreases the expression of HIF-1α and downstream molecules. Decreased HIF-1α signaling may be regulated by increased AIP1 under high glucose. 
 Expression of DAB2IP in human trophoblast and its role in trophoblast invasion. DAB2IP is a growth inhibitor present in many types of cancer cells and is associated with epigenetic regulations controlling tumor development. The primary objective of this study is to determine whether DAB2IP participates in the invasion and   migration   of trophoblasts during placental development.The expressions of DAB2IP in human placentas (10 villi, 18 term placentas and 20 pre-eclampsia placentas) were determined by immunohistochemistry, Western blotting and quantitative RT-PCR. HTR8/SVneo cells were treated with hypoxia-reoxygenation (H/R) to test how DAB2IP expression would affect the invasion and   migration   of trophoblasts. JEG-3 andHTR8/SVneo cells were treated with 5-aza-2-deoxycytidine (5-aza-dC) to study the role of DAB2IP promoter methylation in trophoblasts.DAB2IP was strongly expressed in human villi and extravillous trophoblasts as well as in HTR8/SVneo cells, but not in pre-eclampsia placentas. DAB2IP expression increased after H/R treatment, but the invasive and migratory abilities of trophoblasts were reduced. DAB2IP expression in JEG-3 cells also increased after treatment with 5-aza-dC.These findings strongly suggest that DAB2IP is an important negative regulator at the maternal-fetal interface during early pregnancy. Excessive oxidative stress can increase DAB2IP expression in trophoblasts. The mechanism of DNA methylation may involve in its function during the development of pathologic pregnancy. 
 AIP1 functions as an endogenous inhibitor of VEGFR2-mediated signaling and inflammatory angiogenesis in mice. ASK1-interacting protein-1 (AIP1), a recently identified member of the Ras GTPase-activating protein family, is highly expressed in vascular ECs and regulates EC apoptosis in vitro. However, its function in vivo has not been established. To study this, we generated AIP1-deficient mice (KO mice). Although these mice showed no obvious defects in vascular development, they exhibited dramatically enhanced angiogenesis in 2 models of inflammatory angiogenesis. In one of these models, the enhanced angiogenesis observed in the KO mice was associated with increased VEGF-VEGFR2 signaling. Consistent with this, VEGF-induced ear, cornea, and retina neovascularization were greatly augmented in KO mice and the enhanced retinal angiogenesis was markedly diminished by overexpression of AIP1. In vitro, VEGF-induced EC   migration   was inhibited by AIP1 overexpression, whereas it was augmented by both AIP1 knockout and knockdown, with the enhanced EC   migration   caused by AIP1 knockdown being associated with increased VEGFR2 signaling. We present mechanistic data that suggest AIP1 is recruited to the VEGFR2-PI3K complex, binding to both VEGFR2 and PI3K p85, at a late phase of the VEGF response, and that this leads to inhibition of VEGFR2 signaling. Taken together, our data demonstrate that AIP1 functions as an endogenous inhibitor in VEGFR2-mediated adaptive angiogenesis in mice. 
  GeneRIFs  
 downregulation of DAB2IP is associated with features of biologically aggressive urothelial carcinoma of the bladder and results in cell proliferation,   migration  , and invasion of bladder cancer. 
  [Ii]nvasive  PubMed  
 miR-92b targets DAB2IP to promote EMT in bladder cancer migration and invasion. Muscle-  invasive   or metastatic bladder cancer (BCa) has a very poor prognosis; however, its mechanisms remain largely unknown. Previous studies have discovered multiple microRNAs (miRs) that are involved in BCa progression and regarded as potential biomarkers or therapeutic targets. In this study, we demonstrated that miR-92b could uniquely promote cell migration and invasion of BCa cells, but had no effect on cell proliferation. Mechanistically, our data provided evidence to verify that miR-92b was able to directly target DAB2IP, a well-known tumor suppressor, and inhibit epithelial‑mesenchymal transition of BCa cells. Moreover, the increased expression levels of miR-92b were negatively correlated with DAB2IP, and predicted poor prognosis of patients with BCa. Overall, this study reveals a new promising biomarker and its mechanisms contributing to BCa invasion or metastasis. 
 DAB2IP regulates the chemoresistance to pirarubicin and tumor recurrence of non-muscle   invasive   bladder cancer through STAT3/Twist1/P-glycoprotein signaling. There is a high frequency of tumor recurrence in non-muscle   invasive   bladder cancer (NMIBC) after transurethral resection and postoperative intravesical chemotherapy, however, the molecular mechanisms leading to the chemoresistance and tumor re-growth remain largely unknown. In this study, we observed a significant decrease of DAB2IP expression in high-grade and recurrent NMIBC specimens, which was negatively correlated with Twist1 expression and predicted a lower recurrence-free survival of patients. Mechanistically, DAB2IP could inhibit the phosphorylation and transactivation of STAT3, and then subsequently suppress the expression of Twist1 and its target gene P-glycoprotein, both of which were crucial for the pirarubicin chemoresistance and tumor re-growth of bladder cancer cells. Overall, this study reveals a new promising biomarker modulating the chemoresistance and tumor recurrence of NMIBC after bladder preservation surgery. 
 Low expression of DAB2IP contributes to malignant development and poor prognosis in hepatocellular carcinoma. DOC-2/DAB2 interactive protein gene (DAB2IP) is a novel member of the Ras GTPase-activating protein family and plays a tumor suppressive role in cancer progression, but its function in hepatocellular carcinoma (HCC) remains unclear. This aims of this study were to analyze the clinicopathological features of DAB2IP expression in HCC, and to determine the effect of DAB2IP on HCC cell behaviors in vitro.The expression of DAB2IP was detected in hepatocyte cell line and HCC cell lines by real-time reverse transcription-polymerase chain reaction and western blot. DAB2IP expression was then examined in 120 cases of clinical paraffin-embedded HCC tissue by immunohistochemistry (IHC). 3-(4,5)-dimethylthiahiazo (-z-y1)-3,5-di- phenytetrazoliumromide (MTT) method and in vitro   invasive   assay were finally performed to evaluate the effect of DAB2IP depletion on cell proliferation or invasion of HCC cells.DAB2IP expression was lower in HCC cell lines or tissues than in hepatocyte cell lines, adjacent cirrhotic livers or normal livers (P < 0.05). Its expression was positively correlated with tumor size (P = 0.01). Patients with lower DAB2IP expression had shorter overall survival time (P = 0.013). DAB2IP suppresses proliferation and invasion of HCC cells in vitro.DAB2IP is a valuable marker for progression of HCC patients. Downregulation of DAB2IP is associated with poor prognosis in HCC patients. DAB2IP silence alone is sufficient to promote HCC cell proliferation and invasion in vitro. 
 Mutant p53 reprograms TNF signaling in cancer cells through interaction with the tumor suppressor DAB2IP. Inflammation is a significant factor in cancer development, and a molecular understanding of the parameters dictating the impact of inflammation on cancers could significantly improve treatment. The tumor suppressor p53 is frequently mutated in cancer, and p53 missense mutants (mutp53) can acquire oncogenic properties. We report that cancer cells with mutp53 respond to inflammatory cytokines increasing their   invasive   behavior. Notably, this action is coupled to expression of chemokines that can expose the tumor to host immunity, potentially affecting response to therapy. Mechanistically, mutp53 fuels NF-κB activation while it dampens activation of ASK1/JNK by TNFα, and this action depends on mutp53 binding and inhibiting the tumor suppressor DAB2IP in the cytoplasm. Interfering with such interaction reduced aggressiveness of cancer cells in xenografts. This interaction is an unexplored mechanism by which mutant p53 can influence tumor evolution, with implications for our understanding of the complex role of inflammation in cancer. 
 Downregulation of DAB2IP results in cell proliferation and invasion and contributes to unfavorable outcomes in bladder cancer. The DOC-2/DAB2 interactive protein (DAB2IP) is a member of the Ras GTPase-activating protein family. It has been shown to be often downregulated and a poor prognostic factor in several human malignancies. In this study, we analyzed the clinicopathological features and outcomes of DAB2IP expression in 135 patients with urothelial carcinoma of the bladder (UCB) treated by radical cystectomy plus bilateral lymph node dissection, and evaluated the effect of DAB2IP knockdown in vitro using the MTT method, colony formation assay, cell cycle assay, and cell migration and   invasive   assay. We found low expression of DAB2IP was significantly associated with high pathological stage (P = 0.002), high pathological grade (P = 0.02), tumor size more than 3 cm (P = 0.04), and presence of histological variants (P = 0.01). DAB2IP was an independent prognostic factor of disease recurrence (hazard ratio, 2.67; P = 0.034) and cancer-specific survival (hazard ratio, 2.79; P = 0.038). Knockdown of DAB2IP could promote cell proliferation, migration, and invasion. Downregulation of DAB2IP could activate the ERK and Akt pathways and was correlated with the expression of epithelial-mesenchymal transition markers, such as E-cadherin and vimentin. In conclusion, downregulation of DAB2IP is associated with features of biologically aggressive UCB and results in cell proliferation, migration, and invasion of bladder cancer. DAB2IP may serve as a promising biomarker in patients with UCB treated by radical cystectomy and bilateral lymph node dissection. 
 Transitional cell carcinomas and nonurothelial carcinomas of the urinary bladder differ in the promoter methylation status of the caveolin-1, hDAB2IP and p53 genes, but not in the global methylation of Alu elements. Tumor suppressor genes play a prominent role in the modification and progression of urinary bladder carcinogenesis as a result of classic genetic alterations. Little is known about the potential significance of epigenetic events, mediated by DNA hypermethylation. This prompted our investigation to explore the global Alu methylation and the promoter methylation of the novel putative tumor suppressor genes caveolin-1 and hDAB2IP, and of p53 in transitional cell carcinomas (TCC), squamous cell carcinomas and undifferentiated small cell carcinomas of the urinary bladder. Quantitative GeneScan analysis revealed that the various histopathological tumor entities showed considerable interindividual variations in the global methylation, but the overall rate did not significantly differ between the various cancer subtypes. With methylation-specific PCR, a high frequency of methylation of the promoter region of the caveolin-1 gene was detected in undifferentiated small cell carcinomas (50%) and in squamous cell carcinomas (25.9%), while TCC were found not to be methylated. By immunohistochemistry, all squamous cell carcinomas showed a strong diffuse overexpression of caveolin-1, whereas undifferentiated small cell cancers lacked any expression. High-grade, high-stage TCC disclosed a higher incidence (60%) and a substantially stronger expression than low-grade, low-stage TCC (42.9%). Our findings suggest that hypermethylation of the caveolin-1 gene and an abnormal protein expression play a crucial role in cell differentiation, and in the phenotypical conversion of TCC into nonurothelial carcinomas. Promoter methylation of the hDAB2IP gene occurred more frequently in advanced muscle   invasive   (72.7%) than in superficial non  invasive   (50%) TCC. DNA hypermethylation of p53 was detected in a quarter of the low-grade, low-stage TCC and undifferentiated small cell carcinomas, but only sporadically in squamous cell carcinomas, and was absent in high-grade, high-stage TCC. In conclusion, aberrant methylation and abnormal protein expression of the caveolin-1-gene is involved in the formation of nonurothelial carcinomas of the urinary bladder and promoter methylation of the hDAB2IP gene in the progression of TCC from a low to a high malignant potential. 
 Expression of DAB2IP in human trophoblast and its role in trophoblast invasion. DAB2IP is a growth inhibitor present in many types of cancer cells and is associated with epigenetic regulations controlling tumor development. The primary objective of this study is to determine whether DAB2IP participates in the invasion and migration of trophoblasts during placental development.The expressions of DAB2IP in human placentas (10 villi, 18 term placentas and 20 pre-eclampsia placentas) were determined by immunohistochemistry, Western blotting and quantitative RT-PCR. HTR8/SVneo cells were treated with hypoxia-reoxygenation (H/R) to test how DAB2IP expression would affect the invasion and migration of trophoblasts. JEG-3 andHTR8/SVneo cells were treated with 5-aza-2-deoxycytidine (5-aza-dC) to study the role of DAB2IP promoter methylation in trophoblasts.DAB2IP was strongly expressed in human villi and extravillous trophoblasts as well as in HTR8/SVneo cells, but not in pre-eclampsia placentas. DAB2IP expression increased after H/R treatment, but the   invasive   and migratory abilities of trophoblasts were reduced. DAB2IP expression in JEG-3 cells also increased after treatment with 5-aza-dC.These findings strongly suggest that DAB2IP is an important negative regulator at the maternal-fetal interface during early pregnancy. Excessive oxidative stress can increase DAB2IP expression in trophoblasts. The mechanism of DNA methylation may involve in its function during the development of pathologic pregnancy. 
  [Mm]etastasis  PubMed  
 miR-92b targets DAB2IP to promote EMT in bladder cancer migration and invasion. Muscle-invasive or metastatic bladder cancer (BCa) has a very poor prognosis; however, its mechanisms remain largely unknown. Previous studies have discovered multiple microRNAs (miRs) that are involved in BCa progression and regarded as potential biomarkers or therapeutic targets. In this study, we demonstrated that miR-92b could uniquely promote cell migration and invasion of BCa cells, but had no effect on cell proliferation. Mechanistically, our data provided evidence to verify that miR-92b was able to directly target DAB2IP, a well-known tumor suppressor, and inhibit epithelial‑mesenchymal transition of BCa cells. Moreover, the increased expression levels of miR-92b were negatively correlated with DAB2IP, and predicted poor prognosis of patients with BCa. Overall, this study reveals a new promising biomarker and its mechanisms contributing to BCa invasion   or metasta  sis. 
 Negative regulation of DAB2IP by Akt and SCFFbw7 pathways. Deletion of ovarian carcinoma 2/disabled homolog 2 (DOC-2/DAB2) interacting protein (DAB2IP), is a tumor suppressor that serves as a scaffold protein involved in coordinately regulating cell proliferation, survival and apoptotic pathways. DAB2IP is epigenetically down-regulated in a variety of tumors through the action of the histone methyltransferase EZH2. Although DAB2IP is transcriptionally down-regulated in a variety of tumors, it remains unclear if other mechanisms contribute to functional inactivation of DAB2IP. Here we demonstrate that DAB2IP can be functionally down-regulated by two independent mechanisms. First, we identified that Akt1 can phosphorylate DAB2IP on S847, which regulates the interaction between DAB2IP and its effector molecules H-Ras and TRAF2. Second, we demonstrated that DAB2IP can be degraded in part through ubiquitin-proteasome pathway by SCF(Fbw7). DAB2IP harbors two Fbw7 phosho-degron motifs, which can be regulated by the kinase, CK1δ. Our data hence indicate that in addition to epigenetic down-regulation, two additional pathways can functional inactivate DAB2IP. Given that DAB2IP has previously been identified to possess direct causal role in tumorigenesis and   metastasi  s, our data indicate that a variety of pathways may pass through DAB2IP to govern cancer development, and therefore highlight DAB2IP agonists as potential therapeutic approaches for future anti-cancer drug development. 
 Pretreatment biopsy analysis of DAB2IP identifies subpopulation of high-risk prostate cancer patients with worse survival following radiation therapy. Decreased expression of tumor suppressor DAB2IP is linked to aggressive cancer and radiation resistance in several malignancies, but clinical survival data is largely unknown. We hypothesized that pretreatment DAB2IP reduction would predict worse prostate cancer-specific survival (PCSS). Immunohistochemistry of pretreatment biopsies was scored by an expert genitourinary pathologist. Other endpoints analyzed include freedom from biochemical failure (FFBF), castration resistance-free survival (CRFS), and distant   metastasis  -free survival (DMFS). Seventy-nine patients with NCCN-defined high-risk prostate cancer treated with radiotherapy from 2005 to 2012 at our institution were evaluated. Twenty-eight percent (22/79) of pretreatment biopsies revealed DAB2IP-reduction. The median follow up times were 4.8 years and 5.3 years for patients in the DAB2IP-reduced group and DAB2IP-retained group, respectively. Patients with reduced DAB2IP demonstrated worse outcome compared to patients retaining DAB2IP, including FFBF (4-year: 34 vs. 92%; P < 0.0001), CRFS (4-year: 58 vs. 96%; P = 0.0039), DMFS (4-year: 58 vs. 100%; P = 0.0006), and PCSS (5-year: 83 vs. 100%; P = 0.0102). Univariate analysis showed T stage, N stage, and Gleason score were statistically significant variables. Pretreatment tumor DAB2IP status remained significant in multivariable analyses. This study suggests that about one-fourth of men with high-risk prostate cancer have decreased tumor expression of DAB2IP. This subpopulation with reduced DAB2IP has a suboptimal response and worse malignancy-specific survival following radiation therapy and androgen deprivation. DAB2IP loss may be a genetic explanation for the observed differences in aggressive tumor characteristics and radiation resistance. Further study into improving treatment response and survival in this subpopulation is warranted. 
 Role of DAB2IP in modulating epithelial-to-mesenchymal transition and prostate cancer   metastasis  . A single nucleotide polymorphism in the DAB2IP gene is associated with risk of aggressive prostate cancer (PCa), and loss of DAB2IP expression is frequently detected in metastatic PCa. However, the functional role of DAB2IP in PCa remains unknown. Here, we show that the loss of DAB2IP expression initiates epithelial-to-mesenchymal transition (EMT), which is visualized by repression of E-cadherin and up-regulation of vimentin in both human normal prostate epithelial and prostate carcinoma cells as well as in clinical prostate-cancer specimens. Conversely, restoring DAB2IP in metastatic PCa cells reversed EMT. In DAB2IP knockout mice, prostate epithelial cells exhibited elevated mesenchymal markers, which is characteristic of EMT. Using a human prostate xenograft-mouse model, we observed that knocking down endogenous DAB2IP in human carcinoma cells led to the development of multiple lymph node and distant organ metastases. Moreover, we showed that DAB2IP functions as a scaffold protein in regulating EMT by modulating nuclear beta-catenin/T-cell factor activity. These results show the mechanism of DAB2IP in EMT and suggest that assessment of DAB2IP may provide a prognostic biomarker and potential therapeutic target for PCa   metastasis  . 
 The positive feedback between Snail and DAB2IP regulates EMT, invasion and   metastasis   in colorectal cancer. DAB2IP has been identified as a tumor suppressor in several cancers but its oncogenic role and transcriptionally regulatory mechanisms in the progression of colorectal carcinoma (CRC) remain unknown. In this study, DAB2IP was down-regulated in CRC tissues and a valuable prognostic marker for survival of CRC patients, especially in the late stage. Moreover, DAB2IP was sufficient to suppress proliferation, epithelial-mesenchymal transition (EMT), invasion and   metastasis   in CRC. Mechanically, the linear complex of EZH2/HDAC1/Snail contributed to DAB2IP silencing in CRC cells. The study further proved that the positive feedback loop between Snail and DAB2IP existed in CRC cells and DAB2IP was required for Snail-induced aggressive cell behaviors. Finally, DAB2IP correlated negatively with Snail and EZH2 expressions in CRC tissues. Our findings reveal the suppressive role and a novel regulatory mechanism of DAB2IP expression in the progression of CRC. DAB2IP may be a potential, novel therapeutic and prognostic target for clinical CRC patients. 
 Individual and cumulative association of prostate cancer susceptibility variants with clinicopathologic characteristics of the disease. Recent genome-wide association studies have identified several independent single nucleotide polymorphisms (SNPs) strongly associated with prostate cancer (PCa) risk. However, their individual and cumulative associations with clinicopathologic characteristics of the disease remain inconclusive.We systematically evaluated 20 PCa risk SNPs in a cohort of 320 localized PCa patients receiving radical prostatectomy, and the associations of these variants with age at diagnosis, preoperative prostate-specific antigen concentration, Gleason score, pathologic stage, surgical margin, and lymph node   metastasis   were evaluated.Eight SNPs, rs10486567 at 7p15, rs6465657 at 7q21, rs6983267, rs1447295, and rs4242382 at 8q24, 10993994 at 10q11, rs4430796 at 17q12, and rs266849 at 19q13, were significantly (P< or =0.048) associated with some specific clinicopathologic features. In combination of these 8 SNPs, men who carried 4 or 5, or more than 5 unfavorable alleles had an increasing likelihood of adverse clinicopathologic features, as compared with men who carried fewer than 4 unfavorable alleles (P for trend, 0.031, <0.001, 0.006, and 0.003, for Gleason scores 8-10, advanced pathologic stage, positive surgical margin, and lymph node   metastasis  , respectively).Our results suggested that loci associated with PCa risk might also have a cumulative and significant association with disease aggressiveness. 
 Absence of DAB2IP promotes cancer stem cell like signatures and indicates poor survival outcome in colorectal cancer.   Metastasis   is a critical factor for the high mortality of colorectal cancer (CRC), but its mechanism is not completely understood. Epithelial-mesenchymal transition (EMT) is thought to play a key role in   metastasis   and also increases the cancer stem cell (CSC) feature that facilitates metastatic colonization. In this study, we investigated the biological roles of DAB2IP regulating EMT and stem cell-like features in human CRC. We demonstrate that DAB2IP suppresses NF-κB-mediated EMT and CSC features in CRC cells. In DAB2IP knockout mice, we discovered the hyperplasia in colonic epithelium which aberrantly represents the mesenchymal feature and NF-κB pathway activation. In clinic CRC tissue, we also reveal that reduced DAB2IP can enrich the CD133(+) subpopulation. DAB2IP expression was inversely correlated with tumor differentiation an  d metastas  is, and patients with lower DAB2IP expression had shorter overall survival time. Taken together, our study demonstrates that DAB2IP inhibits NF-κB-inducing EMT and CSC to suppress the CRC progression, and also suggests that DAB2IP is a beneficial prediction factor for CRC patient prognosis. 
 Aberrant promoter methylation in human DAB2 interactive protein (hDAB2IP) gene in breast cancer. Human DOC-2/DAB2 interactive protein (hDAB2IP) gene is a novel member of the Ras GTPase-activating family and has been demonstrated to be a tumor suppressor gene inactivated by methylation in prostate cancer. We analyzed methylation and expression status of hDAB2IP in breast cancer.The promoter region of hDAB2IP was divided into two regions (m2a and m2b) following our previous report on prostate cancer, and methylation status was determined in breast cancer cell lines with bisulfited DNA sequencing. Expression was semiquantified with real-time reverse transcription-PCR to find that aberrant methylation showed the inverse relationship with expression. On the basis of sequence data, we developed methylation-specific PCR for m2a and m2b regions and applied to samples.Aberrant methylation was detected in 11 of 25 breast cancer cell lines (44%) and 15 of 39 primary tumors (38%) at the m2a region and in 12 of 25 cell lines (48%) and 13 of 39 tumors (33%) at the m2b region. In addition, gene expression was restored in methylated cell lines with 5-aza-2'-deoxycytidine, confirming that methylation caused gene down-regulation. We also examined the relationship between hDAB2IP methylation and clinicopathologic features in primary tumors and found that methylation in the m2b region was associated with progressive nodal status of tumors.We developed methylation-specific PCR for hDAB2IP and examined its methylation status in breast cancer. Our results demonstrate that hDAB2IP methylation frequently is present in breast cancer and plays a key role in hDAB2IP inactivation, suggesting the relationship between hDAB2IP methylation and lymph node   metastasis   of breast cancer. 
 An oncogene-tumor suppressor cascade drives metastatic prostate cancer by coordinately activating Ras and nuclear factor-kappaB.   Metastasis   is responsible for the majority of prostate cancer-related deaths; however, little is known about the molecular mechanisms that underlie this process. Here we identify an oncogene-tumor suppressor cascade that promotes prostate cancer growth and   metastasis   by coordinately activating the small GTPase Ras and nuclear factor-kappaB (NF-kappaB). Specifically, we show that loss of the Ras GTPase-activating protein (RasGAP) gene DAB2IP induces metastatic prostate cancer in an orthotopic mouse tumor model. Notably, DAB2IP functions as a signaling scaffold that coordinately regulates Ras and NF-kappaB through distinct domains to promote tumor growth and   metastasis  , respectively. DAB2IP is suppressed in human prostate cancer, where its expression inversely correlates with tumor grade and predicts prognosis. Moreover, we report that epigenetic silencing of DAB2IP is a key mechanism by which the polycomb-group protein histone-lysine N-methyltransferase EZH2 activates Ras and NF-kappaB and triggers   metastasis  . These studies define the mechanism by which two major pathways can be simultaneously activated in metastatic prostate cancer and establish EZH2 as a driver of   metastasis  . 
  GeneRIFs  
 Snail and DAB2IP interact to regulate EMT, invasion and   metastasis   in colorectal cancer 
 functions as a signaling scaffold that coordinately regulates Ras and NF-kappaB through distinct domains to promote prostate cancer growth and   metastasis   
  [Cc]ell [Ii]nvasion  PubMed  
 Infiltrating T cells promote renal cell carcinoma (RCC) progression via altering the estrogen receptor β-DAB2IP signals. Previous studies indicated the T cells, one of the most common types of immune cells existing in the microenvironment of renal cell carcinoma (RCC), may influence the progression of RCC. The potential linkage of T cells and the estrogen receptor beta (ERβ), a key player to impact RCC progression, however, remains unclear. Our results demonstrate that RCC cells can recruit more T cells than non-malignant kidney cells. Using an in vitro matrigel invasion system, we found infiltrating T cells could promote RCC cells invasion via increasing ERβ expression and transcriptional activity. Mechanism dissection suggested that co-culturing T cells with RCC cells released more T cell attraction factors, including IFN-γ, CCL3 and CCL5, suggesting a positive regulatory feed-back mechanism. Meanwhile, infiltrating T cells may also promote   RCC cell inva  sion via increased ERβ and decreased DAB2IP expressions, and knocking down DAB2IP can then reverse the T cells-promoted   RCC cell inv  asion. Together, our results suggest that infiltrating T cells may promote   RCC cell inv  asion via increasing the RCC cell ERβ expression to inhibit the tumor suppressor DAB2IP signals. Further mechanism dissection showed that co-culturing T cells with RCC cells could produce more IGF-1 and FGF-7, which may enhance the ERβ transcriptional activity. The newly identified relationship between infiltrating T cells/ERβ/DAB2IP signals may provide a novel therapeutic target in the development of agents against RCC. 
   
  
 RASAL2 
 
  NCBI Gene  
  GeneRIF  
  Pubmed  
 
 
  Search Term  Rank  Fields  
  [Mm]igration  6  PubMed(5)  
  [Ii]nvasive  10  PubMed(6); GeneRIFs(1)  
  [Mm]etastasis  12  PubMed(7); GeneRIFs(1)  
 
 
 hide/show details for RASAL2 
  [Mm]igration  PubMed  
 ECT2 and RASAL2 mediate mesenchymal-amoeboid transition in human astrocytoma cells. Malignant astrocytomas are highly invasive brain tumors. The Rho family of cytoskeletal GTPases are key regulators of astrocytoma   migration   and invasion; expression of the guanine nucleotide exchange factor ECT2 is elevated in primary astrocytomas and predicts both survival and malignancy. Mice bearing orthotopically implanted astrocytoma cells with diminished ECT2 levels following ECT2 knockdown exhibit longer survival. Although ECT2 is normally expressed in the nucleus, we show that ECT2 is aberrantly localized to the cytoplasm in both astrocytoma cell lines and primary human astrocytomas, and colocalizes with RAC1 and CDC42 at the leading edge of migrating astrocytoma cells. Inhibition of ECT2 expression by RNA interference resulted in decreased RAC1 and CDC42 activity, but no change in RHO activity, suggesting that ECT2 is capable of activating these pro-migratory Rho family members. ECT2 overexpression in astrocytoma cells resulted in a transition to an amoeboid phenotype that was abolished with the ROCK inhibitor, Y-27632. Cytoplasmic fractionation of astrocytoma cells followed by ECT2 immunoprecipitation and mass spectrometry were used to identify protein-binding partners that modulate the activity of ECT2 toward RAC1 and RHO/ROCK. We identified RASAL2 as an ECT2-interacting protein that regulates RHO activity in astrocytoma cells. RASAL2 knockdown leads to a conversion to an amoeboid phenotype. Our studies reveal that ECT2 has a novel role in mesenchymal-amoeboid transition in human astrocytoma cells. 
 ECT2 and RASAL2 mediate mesenchymal-amoeboid transition in human astrocytoma cells. Malignant astrocytomas are highly invasive brain tumors. The Rho family of cytoskeletal GTPases are key regulators of astrocytoma   migration   and invasion; expression of the guanine nucleotide exchange factor ECT2 is elevated in primary astrocytomas and predicts both survival and malignancy. Mice bearing orthotopically implanted astrocytoma cells with diminished ECT2 levels following ECT2 knockdown exhibit longer survival. Although ECT2 is normally expressed in the nucleus, we show that ECT2 is aberrantly localized to the cytoplasm in both astrocytoma cell lines and primary human astrocytomas, and colocalizes with RAC1 and CDC42 at the leading edge of migrating astrocytoma cells. Inhibition of ECT2 expression by RNA interference resulted in decreased RAC1 and CDC42 activity, but no change in RHO activity, suggesting that ECT2 is capable of activating these pro-migratory Rho family members. ECT2 overexpression in astrocytoma cells resulted in a transition to an amoeboid phenotype that was abolished with the ROCK inhibitor, Y-27632. Cytoplasmic fractionation of astrocytoma cells followed by ECT2 immunoprecipitation and mass spectrometry were used to identify protein-binding partners that modulate the activity of ECT2 toward RAC1 and RHO/ROCK. We identified RASAL2 as an ECT2-interacting protein that regulates RHO activity in astrocytoma cells. RASAL2 knockdown leads to a conversion to an amoeboid phenotype. Our studies reveal that ECT2 has a novel role in mesenchymal-amoeboid transition in human astrocytoma cells. 
 RASAL2 down-regulation in ovarian cancer promotes epithelial-mesenchymal transition and metastasis. Ovarian cancer is the most lethal gynecologic malignancy, and transcoelomic metastasis is responsible for the greatest disease mortality. Although intensive efforts have been made, the mechanism behind this process remains unclear. RASAL2 is a GTPase activating proteins (GAPs) which was recently reported as a tumor suppressor in breast cancer. In this study, we identified RASAL2 as a regulator of epithelial-mesenchymal transition (EMT) and metastasis in ovarian cancer. RASAL2 was down-regulated in ovarian cancer samples compared with normal tissue samples, especially in advanced stages and grades. RASAL2 knockdown in ovarian cancer cell lines promoted in vitro anchorage-independent growth, cell   migration   and invasion and in vivo tumor formation. Moreover, we observed EMT in RASAL2-depleted cells. E-cadherin-mediated cell-cell adhesion was attenuated, and mesenchymal markers were up-regulated. Further investigation revealed that the oncogenic role of RASAL2 down-regulation was mediated by the Ras-ERK pathway. RASAL2 knockdown activated the Ras-ERK pathway, and inhibition of the pathway reversed the functional effects of RASAL2 depletion. Together, our results implicate RASAL2 as an EMT regulator and tumor suppressor in ovarian cancer, and down-regulation of RASAL2 promotes ovarian cancer progression. 
 RASAL2 promotes lung cancer metastasis through epithelial-mesenchymal transition. It was reported that genetically-engineered RASAL2 knockout mice are prone to development of several sporadic tumor, including lung adenocarcinoma. However, a causative relationship between RASAL2 deficiency and lung adenocarcinoma development still remains unknown. In the present study, RASAL2 level was determined in patients with lung adenocarcinoma and control subjects in an attempt to explore its potential clinical diagnostic and prognostic value. Low RASAL2 expression levels were found in 71% (37 of 52) of lung adenocarcinoma, which were correlated with lymph node metastasis in lung adenocarcinoma. Moreover, Low RASAL2 expression levels were correlated with reduced overall survival (OS) in lung adenocarcinoma. We find that inactivation of RASAL2 promotes lung cancer cell   migration   through the induction of epithelial mesenchymal transition (EMT) and promoted lung metastasis in nude mice. Our results suggest that the down-regulation of RASAL2 promotes metastatic progression of lung adenocarcinoma, hence it could serve as a potential target for the development of lung cancer therapies. 
 miR-136 suppresses tumor invasion and metastasis by targeting RASAL2 in triple-negative breast cancer. MicroRNAs play an important role in the regulation of cancer   migration  , invasion and metastasis. Patients with triple-negative breast cancer (TNBC) have a high incidence of early relapse and metastasis; however, the molecular basis for metastasis and recurrence in these individuals remains largely unknown. Herein, we demonstrate that miR-136 is an anti-invasive microRNA in TNBC and suppresses mesenchymal invasion and metastasis. Our results demonstrated that miR-136 was downregulated in TNBC and negative correlated with the WHO grades. However, RASAL2 was identified as a functional target of miR-136, and was overexpressed in TNBC and correlates with pathological grades. Moreover, overexpression of RASAL2 in a breast cancer cell line rescued miR-136-mediated cell   migration   and invasion. In conclusion, these results indicate that the miR-136/RASAL2/MET axis act as a suppressor of TNBC metastasis. 
  [Ii]nvasive  PubMed  
 RASAL2 activates RAC1 to promote triple-negative breast cancer progression. Patients with triple-negative breast cancer (TNBC) have a high incidence of early relapse and metastasis; however, the molecular basis for recurrence in these individuals remains poorly understood. Here, we demonstrate that RASAL2, which encodes a RAS-GTPase-activating protein (RAS-GAP), is a functional target of anti-  invasive   microRNA-203 and is overexpressed in a subset of triple-negative or estrogen receptor-negative (ER-negative) breast tumors. As opposed to luminal B ER-positive breast cancers, in which RASAL2 has been shown to act as a RAS-GAP tumor suppressor, we found that RASAL2 is oncogenic in TNBC and drives mesenchymal invasion and metastasis. Moreover, high RASAL2 expression was predictive of poor disease outcomes in patients with TNBC. RASAL2 acted independently of its RAS-GAP catalytic activity in TNBC; however, RASAL2 promoted small GTPase RAC1 signaling, which promotes mesenchymal invasion, through binding and antagonizing the RAC1-GAP protein ARHGAP24. Together, these results indicate that activation of a RASAL2/ARHGAP24/RAC1 module contributes to TNBC tumorigenesis and identify a context-dependent role of RASAL2 in breast cancer. 
 ECT2 and RASAL2 mediate mesenchymal-amoeboid transition in human astrocytoma cells. Malignant astrocytomas are highly   invasive   brain tumors. The Rho family of cytoskeletal GTPases are key regulators of astrocytoma migration and invasion; expression of the guanine nucleotide exchange factor ECT2 is elevated in primary astrocytomas and predicts both survival and malignancy. Mice bearing orthotopically implanted astrocytoma cells with diminished ECT2 levels following ECT2 knockdown exhibit longer survival. Although ECT2 is normally expressed in the nucleus, we show that ECT2 is aberrantly localized to the cytoplasm in both astrocytoma cell lines and primary human astrocytomas, and colocalizes with RAC1 and CDC42 at the leading edge of migrating astrocytoma cells. Inhibition of ECT2 expression by RNA interference resulted in decreased RAC1 and CDC42 activity, but no change in RHO activity, suggesting that ECT2 is capable of activating these pro-migratory Rho family members. ECT2 overexpression in astrocytoma cells resulted in a transition to an amoeboid phenotype that was abolished with the ROCK inhibitor, Y-27632. Cytoplasmic fractionation of astrocytoma cells followed by ECT2 immunoprecipitation and mass spectrometry were used to identify protein-binding partners that modulate the activity of ECT2 toward RAC1 and RHO/ROCK. We identified RASAL2 as an ECT2-interacting protein that regulates RHO activity in astrocytoma cells. RASAL2 knockdown leads to a conversion to an amoeboid phenotype. Our studies reveal that ECT2 has a novel role in mesenchymal-amoeboid transition in human astrocytoma cells. 
 RASAL2 activates RAC1 to promote triple-negative breast cancer progression. Patients with triple-negative breast cancer (TNBC) have a high incidence of early relapse and metastasis; however, the molecular basis for recurrence in these individuals remains poorly understood. Here, we demonstrate that RASAL2, which encodes a RAS-GTPase-activating protein (RAS-GAP), is a functional target of anti-  invasive   microRNA-203 and is overexpressed in a subset of triple-negative or estrogen receptor-negative (ER-negative) breast tumors. As opposed to luminal B ER-positive breast cancers, in which RASAL2 has been shown to act as a RAS-GAP tumor suppressor, we found that RASAL2 is oncogenic in TNBC and drives mesenchymal invasion and metastasis. Moreover, high RASAL2 expression was predictive of poor disease outcomes in patients with TNBC. RASAL2 acted independently of its RAS-GAP catalytic activity in TNBC; however, RASAL2 promoted small GTPase RAC1 signaling, which promotes mesenchymal invasion, through binding and antagonizing the RAC1-GAP protein ARHGAP24. Together, these results indicate that activation of a RASAL2/ARHGAP24/RAC1 module contributes to TNBC tumorigenesis and identify a context-dependent role of RASAL2 in breast cancer. 
 ECT2 and RASAL2 mediate mesenchymal-amoeboid transition in human astrocytoma cells. Malignant astrocytomas are highly   invasive   brain tumors. The Rho family of cytoskeletal GTPases are key regulators of astrocytoma migration and invasion; expression of the guanine nucleotide exchange factor ECT2 is elevated in primary astrocytomas and predicts both survival and malignancy. Mice bearing orthotopically implanted astrocytoma cells with diminished ECT2 levels following ECT2 knockdown exhibit longer survival. Although ECT2 is normally expressed in the nucleus, we show that ECT2 is aberrantly localized to the cytoplasm in both astrocytoma cell lines and primary human astrocytomas, and colocalizes with RAC1 and CDC42 at the leading edge of migrating astrocytoma cells. Inhibition of ECT2 expression by RNA interference resulted in decreased RAC1 and CDC42 activity, but no change in RHO activity, suggesting that ECT2 is capable of activating these pro-migratory Rho family members. ECT2 overexpression in astrocytoma cells resulted in a transition to an amoeboid phenotype that was abolished with the ROCK inhibitor, Y-27632. Cytoplasmic fractionation of astrocytoma cells followed by ECT2 immunoprecipitation and mass spectrometry were used to identify protein-binding partners that modulate the activity of ECT2 toward RAC1 and RHO/ROCK. We identified RASAL2 as an ECT2-interacting protein that regulates RHO activity in astrocytoma cells. RASAL2 knockdown leads to a conversion to an amoeboid phenotype. Our studies reveal that ECT2 has a novel role in mesenchymal-amoeboid transition in human astrocytoma cells. 
 miR-136 suppresses tumor invasion and metastasis by targeting RASAL2 in triple-negative breast cancer. MicroRNAs play an important role in the regulation of cancer migration, invasion and metastasis. Patients with triple-negative breast cancer (TNBC) have a high incidence of early relapse and metastasis; however, the molecular basis for metastasis and recurrence in these individuals remains largely unknown. Herein, we demonstrate that miR-136 is an anti-  invasive   microRNA in TNBC and suppresses mesenchymal invasion and metastasis. Our results demonstrated that miR-136 was downregulated in TNBC and negative correlated with the WHO grades. However, RASAL2 was identified as a functional target of miR-136, and was overexpressed in TNBC and correlates with pathological grades. Moreover, overexpression of RASAL2 in a breast cancer cell line rescued miR-136-mediated cell migration and invasion. In conclusion, these results indicate that the miR-136/RASAL2/MET axis act as a suppressor of TNBC metastasis. 
 Genome-wide study of hypomethylated and induced genes in patients with liver cancer unravels novel anticancer targets. We utilized whole-genome mapping of promoters that are activated by DNA hypomethylation in hepatocellular carcinoma (HCC) clinical samples to shortlist novel targets for anticancer therapeutics. We provide a proof of principle of this approach by testing six genes short-listed in our screen for their essential role in cancer growth and   invasive  ness.We used siRNA- or shRNA-mediated depletion to determine whether inhibition of these genes would reduce human tumor xenograft growth in mice as well as cell viability, anchorage-independent growth,   invasive   capacities, and state of activity of nodal signaling pathways in liver, breast, and bladder cancer cell lines.Depletion of EXOSC4, RNMT, SENP6, WBSCR22, RASAL2, and NENF effectively and specifically inhibits cancer cell growth and cell   invasive   capacities in different types of cancer, but, remarkably, there is no effect on normal cell growth, suggesting a ubiquitous causal role for these genes in driving cancer growth and metastasis. Depletion of RASAL2 and NENF in vitro reduces their growth as explants in vivo in mice. RASAL2 and NENF depletion interferes with AKT, WNT, and MAPK signaling pathways as well as regulation of epigenetic proteins that were previously demonstrated to drive cancer growth and metastasis.Our results prove that genes that are hypomethylated and induced in tumors are candidate targets for anticancer therapeutics in multiple cancer cell types. Because these genes are particularly activated in cancer, they constitute a group of targets for specific pharmacologic inhibitors of cancer and cancer metastasis. Clin Cancer Res; 20(12); 3118-32. ©2014 AACR. 
  GeneRIFs  
 these results demonstrated that miRNA-136 was a key anti-  invasive   miRNA and further confirmed the oncogenic role of RASAL2 in triple-negative breast cancer. 
  [Mm]etastasis  PubMed  
 RASAL2 activates RAC1 to promote triple-negative breast cancer progression. Patients with triple-negative breast cancer (TNBC) have a high incidence of early relapse and   metastasis  ; however, the molecular basis for recurrence in these individuals remains poorly understood. Here, we demonstrate that RASAL2, which encodes a RAS-GTPase-activating protein (RAS-GAP), is a functional target of anti-invasive microRNA-203 and is overexpressed in a subset of triple-negative or estrogen receptor-negative (ER-negative) breast tumors. As opposed to luminal B ER-positive breast cancers, in which RASAL2 has been shown to act as a RAS-GAP tumor suppressor, we found that RASAL2 is oncogenic in TNBC and drives mesenchymal invasion and   metastasis  . Moreover, high RASAL2 expression was predictive of poor disease outcomes in patients with TNBC. RASAL2 acted independently of its RAS-GAP catalytic activity in TNBC; however, RASAL2 promoted small GTPase RAC1 signaling, which promotes mesenchymal invasion, through binding and antagonizing the RAC1-GAP protein ARHGAP24. Together, these results indicate that activation of a RASAL2/ARHGAP24/RAC1 module contributes to TNBC tumorigenesis and identify a context-dependent role of RASAL2 in breast cancer. 
 RASAL2 activates RAC1 to promote triple-negative breast cancer progression. Patients with triple-negative breast cancer (TNBC) have a high incidence of early relapse and   metastasis  ; however, the molecular basis for recurrence in these individuals remains poorly understood. Here, we demonstrate that RASAL2, which encodes a RAS-GTPase-activating protein (RAS-GAP), is a functional target of anti-invasive microRNA-203 and is overexpressed in a subset of triple-negative or estrogen receptor-negative (ER-negative) breast tumors. As opposed to luminal B ER-positive breast cancers, in which RASAL2 has been shown to act as a RAS-GAP tumor suppressor, we found that RASAL2 is oncogenic in TNBC and drives mesenchymal invasion and   metastasis  . Moreover, high RASAL2 expression was predictive of poor disease outcomes in patients with TNBC. RASAL2 acted independently of its RAS-GAP catalytic activity in TNBC; however, RASAL2 promoted small GTPase RAC1 signaling, which promotes mesenchymal invasion, through binding and antagonizing the RAC1-GAP protein ARHGAP24. Together, these results indicate that activation of a RASAL2/ARHGAP24/RAC1 module contributes to TNBC tumorigenesis and identify a context-dependent role of RASAL2 in breast cancer. 
 RASAL2 down-regulation in ovarian cancer promotes epithelial-mesenchymal transition and   metastasis  . Ovarian cancer is the most lethal gynecologic malignancy, and transcoelomic   metastasis   is responsible for the greatest disease mortality. Although intensive efforts have been made, the mechanism behind this process remains unclear. RASAL2 is a GTPase activating proteins (GAPs) which was recently reported as a tumor suppressor in breast cancer. In this study, we identified RASAL2 as a regulator of epithelial-mesenchymal transition (EMT) and   metastasis   in ovarian cancer. RASAL2 was down-regulated in ovarian cancer samples compared with normal tissue samples, especially in advanced stages and grades. RASAL2 knockdown in ovarian cancer cell lines promoted in vitro anchorage-independent growth, cell migration and invasion and in vivo tumor formation. Moreover, we observed EMT in RASAL2-depleted cells. E-cadherin-mediated cell-cell adhesion was attenuated, and mesenchymal markers were up-regulated. Further investigation revealed that the oncogenic role of RASAL2 down-regulation was mediated by the Ras-ERK pathway. RASAL2 knockdown activated the Ras-ERK pathway, and inhibition of the pathway reversed the functional effects of RASAL2 depletion. Together, our results implicate RASAL2 as an EMT regulator and tumor suppressor in ovarian cancer, and down-regulation of RASAL2 promotes ovarian cancer progression. 
 The RasGAP gene, RASAL2, is a tumor and   metastasis   suppressor. RAS genes are commonly mutated in cancer; however, RAS mutations are rare in breast cancer, despite frequent hyperactivation of Ras and ERK. Here, we report that the RasGAP gene, RASAL2, functions as a tumor and   metastasis   suppressor. RASAL2 is mutated or suppressed in human breast cancer, and RASAL2 ablation promotes tumor growth, progression, and   metastasis   in mouse models. In human breast cancer, RASAL2 loss is associated with metastatic disease; low RASAL2 levels correlate with recurrence of luminal B tumors; and RASAL2 ablation promotes   metastasis   of luminal mouse tumors. Additional data reveal a broader role for RASAL2 inactivation in other tumor types. These studies highlight the expanding role of RasGAPs and reveal an alternative mechanism of activating Ras in cancer. 
 RASAL2 promotes lung cancer   metastasis   through epithelial-mesenchymal transition. It was reported that genetically-engineered RASAL2 knockout mice are prone to development of several sporadic tumor, including lung adenocarcinoma. However, a causative relationship between RASAL2 deficiency and lung adenocarcinoma development still remains unknown. In the present study, RASAL2 level was determined in patients with lung adenocarcinoma and control subjects in an attempt to explore its potential clinical diagnostic and prognostic value. Low RASAL2 expression levels were found in 71% (37 of 52) of lung adenocarcinoma, which were correlated with lymph node   metastasis   in lung adenocarcinoma. Moreover, Low RASAL2 expression levels were correlated with reduced overall survival (OS) in lung adenocarcinoma. We find that inactivation of RASAL2 promotes lung cancer cell migration through the induction of epithelial mesenchymal transition (EMT) and promoted lung   metastasis   in nude mice. Our results suggest that the down-regulation of RASAL2 promotes metastatic progression of lung adenocarcinoma, hence it could serve as a potential target for the development of lung cancer therapies. 
 miR-136 suppresses tumor invasion and   metastasis   by targeting RASAL2 in triple-negative breast cancer. MicroRNAs play an important role in the regulation of cancer migration, invasion and   metastasis  . Patients with triple-negative breast cancer (TNBC) have a high incidence of early relapse and   metastasis  ; however, the molecular basis for   metastasis   and recurrence in these individuals remains largely unknown. Herein, we demonstrate that miR-136 is an anti-invasive microRNA in TNBC and suppresses mesenchymal invasion and   metastasis  . Our results demonstrated that miR-136 was downregulated in TNBC and negative correlated with the WHO grades. However, RASAL2 was identified as a functional target of miR-136, and was overexpressed in TNBC and correlates with pathological grades. Moreover, overexpression of RASAL2 in a breast cancer cell line rescued miR-136-mediated cell migration and invasion. In conclusion, these results indicate that the miR-136/RASAL2/MET axis act as a suppressor of TNBC   metastasis  . 
 Genome-wide study of hypomethylated and induced genes in patients with liver cancer unravels novel anticancer targets. We utilized whole-genome mapping of promoters that are activated by DNA hypomethylation in hepatocellular carcinoma (HCC) clinical samples to shortlist novel targets for anticancer therapeutics. We provide a proof of principle of this approach by testing six genes short-listed in our screen for their essential role in cancer growth and invasiveness.We used siRNA- or shRNA-mediated depletion to determine whether inhibition of these genes would reduce human tumor xenograft growth in mice as well as cell viability, anchorage-independent growth, invasive capacities, and state of activity of nodal signaling pathways in liver, breast, and bladder cancer cell lines.Depletion of EXOSC4, RNMT, SENP6, WBSCR22, RASAL2, and NENF effectively and specifically inhibits cancer cell growth and cell invasive capacities in different types of cancer, but, remarkably, there is no effect on normal cell growth, suggesting a ubiquitous causal role for these genes in driving cancer growth and   metastasis  . Depletion of RASAL2 and NENF in vitro reduces their growth as explants in vivo in mice. RASAL2 and NENF depletion interferes with AKT, WNT, and MAPK signaling pathways as well as regulation of epigenetic proteins that were previously demonstrated to drive cancer growth and   metastasis  .Our results prove that genes that are hypomethylated and induced in tumors are candidate targets for anticancer therapeutics in multiple cancer cell types. Because these genes are particularly activated in cancer, they constitute a group of targets for specific pharmacologic inhibitors of cancer and cancer   metastasis  . Clin Cancer Res; 20(12); 3118-32. ©2014 AACR. 
  GeneRIFs  
 The RasGAP gene, RASAL2, is a tumor and   metastasis   suppressor. 
   
  
 DLC1 
 
  NCBI Gene  
  GeneRIF  
  Pubmed  
 
 
  Search Term  Rank  Fields  
  [Mm]igration  6  Process(2); PubMed(32); GeneRIFs(10)  
  [Ee]xtracellular [Mm]atrix  8  PubMed(1)  
  [Ii]nvasive  10  PubMed(4)  
  [Mm]etastasis  12  PubMed(22); GeneRIFs(5)  
  [Cc]ell [Ii]nvasion  13  PubMed(3); GeneRIFs(1)  
 
 
 hide/show details for DLC1 
  [Mm]igration  Process  
 negative regulation of cell   migration   
 negative regulation of cell   migration   
  PubMed  
 Mutation of ARHGAP9 in patients with coronary spastic angina. Coronary artery spasm has an important function in the etiology of variant angina and other acute coronary syndromes. Abnormal activation of Rho-family GTPases has been observed in cardiovascular disorders, but the function of genetic variability in Rho-family GTPases remains to be evaluated in cardiovascular disorders. We examined the genetic variability of Rho-family GTPases and their regulators in coronary artery spasm. We performed a comprehensive candidate gene analysis of 67 single nucleotide polymorphisms with amino-acid substitution in Rho-family GTPases and their regulators in 103 unrelated Japanese patients with acetylcholine-induced coronary artery spasm and 102 control Japanese subjects without acetylcholine-induced coronary artery spasm. We noted an association of the single nucleotide polymorphism of ARHGAP9 (rs11544238, Ala370Ser) with coronary artery spasm (odds ratio =2.67). We found that ARHGAP9 inactivated Rac as RacGAP and that the mRNA level of ARHGAP9 was strongly detected in hematopoietic cells. ARHGAP9 negatively regulated cell   migration  . The Ala370Ser polymorphism counteracted ARHGAP9-reduced cell   migration  , spreading and adhesion. The Ala370Ser polymorphism in the ARHGAP9 gene is associated with coronary artery spasm. These data suggest that the polymorphism of ARHGAP9 has a critical function in the infiltration of hematopoietic cells into the endothelium and inflammation leading to endothelial dysfunction. 
 Mutation of ARHGAP9 in patients with coronary spastic angina. Coronary artery spasm has an important function in the etiology of variant angina and other acute coronary syndromes. Abnormal activation of Rho-family GTPases has been observed in cardiovascular disorders, but the function of genetic variability in Rho-family GTPases remains to be evaluated in cardiovascular disorders. We examined the genetic variability of Rho-family GTPases and their regulators in coronary artery spasm. We performed a comprehensive candidate gene analysis of 67 single nucleotide polymorphisms with amino-acid substitution in Rho-family GTPases and their regulators in 103 unrelated Japanese patients with acetylcholine-induced coronary artery spasm and 102 control Japanese subjects without acetylcholine-induced coronary artery spasm. We noted an association of the single nucleotide polymorphism of ARHGAP9 (rs11544238, Ala370Ser) with coronary artery spasm (odds ratio =2.67). We found that ARHGAP9 inactivated Rac as RacGAP and that the mRNA level of ARHGAP9 was strongly detected in hematopoietic cells. ARHGAP9 negatively regulated cell   migration  . The Ala370Ser polymorphism counteracted ARHGAP9-reduced cell   migration  , spreading and adhesion. The Ala370Ser polymorphism in the ARHGAP9 gene is associated with coronary artery spasm. These data suggest that the polymorphism of ARHGAP9 has a critical function in the infiltration of hematopoietic cells into the endothelium and inflammation leading to endothelial dysfunction. 
 Restoration of DLC1 gene inhibits proliferation and   migration   of human colon cancer HT29 cells. DLC1 (deleted in liver cancer-1) is a new candidate tumor suppressor gene, which is inactive in various types of human cancers including colon cancer. To study the function of DLC1, we constructed a pcDNA3.1 vector containing the DLC1 gene and transfected it into HT29 colon cancer cells that were deficient in DLC1 expression. The restoration of DLC1 expression in HT29 cells significantly inhibited cell proliferation and   migration  . Flow cytometry showed that DLC1 transfection into HT29 cells induced apoptosis and that the cell cycle was arrested at S-phase. Additionally, cyclinD1 mRNA and protein expression were down-regulated while p21 expression was increased in pcDNA3.1-DLC1-HT29 cells compared to wild HT29 cells. These results confirm the role of DLC1 gene as a tumor suppressor, which may be manifested by regulation of p21 and cyclinDl. The DLC1 gene has a potential therapeutic role in inhibiting the development of colon cancer. 
 DLC-1, a GTPase-activating protein for Rho, is associated with cell proliferation, morphology, and   migration   in human hepatocellular carcinoma. DLC-1 (deleted in liver cancer-1) is a tumor suppressor gene for hepatocellular carcinoma and other cancers. To characterize its functions, we constructed recombinant adenovirus encoding the wild-type DLC-1 and examined its effects on behaviors of a hepatocellular carcinoma cell line (SNU-368), which does not express DLC-1. Here, we found that restoration of DLC-1 expression in the SNU-368 cells caused an inhibition of cell proliferation with an increase of a subG1 population. Furthermore, DLC-1 overexpression induced disassembly of stress fibers and extensive membrane protrusions around cells on laminin-1. DLC-1 overexpression also inhibited cell   migration   and dephosphorylated focal adhesion proteins such as focal adhesion kinase (FAK), Cas (p130Cas; Crk-associated substrate), and paxillin. These observations suggest that DLC-1 plays important roles in signal transduction pathway regulating cell proliferation, cell morphology, and cell   migration   by affecting Rho family GTPases and focal adhesion proteins. 
 DLC1 interaction with S100A10 mediates inhibition of in vitro cell invasion and tumorigenicity of lung cancer cells through a RhoGAP-independent mechanism. The DLC1 gene encodes a Rho GTPase-activating protein (RhoGAP) that functions as a tumor suppressor in several common human cancers. The multidomain structure of DLC1 enables interaction with a number of other proteins. Here we report that the proinflammatory protein S100A10 (also known as p11), a key cell surface receptor for plasminogen which regulates pericellular proteolysis and tumor cell invasion, is a new binding partner of DLC1 in human cells. We determined that the 2 proteins colocalize in the cell cytoplasm and that their binding is mediated by central sequences in the central domain of DLC1 and the C-terminus of S100A10. Because the same S100A10 sequence also mediates binding to Annexin 2, we found that DLC1 competed with Annexin 2 for interaction with S100A10. DLC1 binding to S100A10 did not affect DLC1's RhoGAP activity, but it decreased the steady-state level of S100A10 expression in a dose-dependent manner by displacing it from Annexin 2 and making it accessible to ubiquitin-dependent degradation. This process attenuated plasminogen activation and resulted in inhibition of in vitro cell   migration  , invasion, colony formation, and anchorage-independent growth of aggressive lung cancer cells. These results suggest that a novel GAP-independent mechanism contributes to the tumor suppressive activity of DLC1, and highlight the importance and complexity of protein-protein interactions involving DLC1 in certain cancers. 
 Tensin1 requires protein phosphatase-1alpha in addition to RhoGAP DLC-1 to control cell polarization,   migration  , and invasion. Tensin is a family of multidomain scaffold proteins that bind the cytoplasmic tail of beta-integrins and localize to adhesions that anchor stress fibers in cells. Tensin expression is suppressed in cancer, especially metastatic cancer. The N-terminal domain of tensin1 associates with protein phosphatase-1alpha (PP1alpha) and mediates PP1alpha localization to adhesions. Here, we show F302A mutation in a KVXF motif of tensin1 abrogates binding to PP1alpha. The SH2 domain in tensin family member c-ten requires R474 to bind a RhoGAP called DLC-1 (deleted in liver cancer). We mutated the corresponding residue in tensin1, R1488A, and showed this reduces association with DLC-1. Unexpectedly, tensin1 F302A also had reduced association with DLC-1. Expression of tensin1 F302A or R1488A showed similar dominant phenotypes, with reduced cell polarization, lowered MLC20 phosphorylation and reduced levels of RhoA(GTP) compared with cells expressing tensin1 WT. However,   migration   and invasion of metastatic MDA MB 231 breast cancer cells were differentially affected by tensin1 mutated at F302A or R1488A. Cancer cells stably expressing F302A tensin1 showed increased   migration   and invasion compared with cells stably expressing either R1488A tensin1 or WT tensin1. This suggests that PP1alpha bound to tensin1 has additional effects in reducing   migration   and invasion that are not mediated through DLC-1. Our results show the importance of PP1alpha binding to tensin1 for the regulation of cell polarization,   migration  , and invasion. 
 Uncovering the rare variants of DLC1 isoform 1 and their functional effects in a Chinese sporadic congenital heart disease cohort. Congenital heart disease (CHD) is the most common birth defect affecting the structure and function of fetal hearts. Despite decades of extensive studies, the genetic mechanism of sporadic CHD remains obscure. Deleted in liver cancer 1 (DLC1) gene, encoding a GTPase-activating protein, is highly expressed in heart and essential for heart development according to the knowledge of Dlc1-deficient mice. To determine whether DLC1 is a susceptibility gene for sporadic CHD, we sequenced the coding region of DLC1 isoform 1 in 151 sporadic CHD patients and identified 13 non-synonymous rare variants (including 6 private variants) in the case cohort. Importantly, these rare variants (8/13) were enriched in the N-terminal region of the DLC1 isoform 1 protein. Seven of eight amino acids at the N-terminal variant positions were conserved among the primates. Among the 9 rare variants that were predicted as "damaging", five were located at the N-terminal region. Ensuing in vitro functional assays showed that three private variants (Met360Lys, Glu418Lys and Asp554Val) impaired the ability of DLC1 to inhibit cell   migration   or altered the subcellular location of the protein compared to wild-type DLC1 isoform 1. These data suggest that DLC1 might act as a CHD-associated gene in addition to its role as a tumor suppressor in cancer. 
 DLC-1 as a modulator of proliferation, apoptosis and   migration   in Burkitt's lymphoma cells. Deleted in liver cancer-1(DLC-1) gene expression is frequently down-regulated or deleted in many types of human cancer. To evaluate whether DLC-1 could be a therapeutic target for non-Hodgkin lymphoma (NHL), we examined the expressions of DLC-1 in Burkitt's lymphoma (BL) cell lines and tested the effects of DLC-1 on cellular growth and   migration   in BL cells. DLC-1 expression was not detectable in two human BL cell lines, Raji and Daudi, by reverse transcription-PCR. The transfer of DLC-1 into Raji and Daudi cell lines caused a significant inhibition in cell proliferation. This inhibitory effect on cell proliferation in BL cell lines was accompanied by induction of apoptosis. Furthermore, restoration of DLC-1 expression in BL cells had a significant inhibitory effect on   migration  . Our findings suggest that DLC-1 may play an important role in lymphoma by acting as a bona fide new tumor suppressor gene. 
 The transcriptional coactivators megakaryoblastic leukemia 1/2 mediate the effects of loss of the tumor suppressor deleted in liver cancer 1. Deleted in Liver Cancer 1 (DLC1) is a tumor suppressor whose allele is lost in 50% of liver, breast, lung and 70% of colon cancers. Here, we show that the transcriptional coactivators Megakaryoblastic Leukemia 1 and 2 (MKL1/2) are constitutively localized to the nucleus in hepatocellular and mammary carcinoma cells that lack DLC1. Moreover, DLC1 loss and MKL1 nuclear localization correlate in primary human hepatocellular carcinoma. Nuclear accumulation of MKL1 in DLC1-deficient cancer cells is accomplished by activation of the RhoA/actin signaling pathway and concomitant impairment of MKL1 phosphorylation, which results in constitutive activation of MKL1/2 target genes. We provide evidence that MKL1/2 mediates cancerous transformation in DLC1-deficient hepatocellular and mammary carcinoma cells. Depletion of MKL1/2 suppresses cell   migration  , cell proliferation and anchorage-independent cell growth induced by DLC1 loss. 
 DLC-1, a candidate tumor suppressor gene, inhibits the proliferation,   migration   and tumorigenicity of human nasopharyngeal carcinoma cells. In our previous study we demonstrated the downregulation or loss of deleted in liver cancer‑1 (DLC-1) gene expression in nasopharyngeal carcinoma (NPC). In this study, we report the effects of the DLC-1 gene on NPC cells and its mechanisms of action. DLC-1 expression was restored in the 5-8F NPC cell line, which lacks DLC-1 expression, and the biological characteristics of 5-8F-DLC‑1 cells were analyzed by MTT assay, colony formation assay, flow cytometry (FCM), tumorigenesis analysis in nude mice, as well as invasion   and migra  tion assay. Differentially expressed genes in response to DLC-1 expression were screened using microarray analysis and identified by RT-PCR. The re-expression of DLC-1 in the NPC cells attenuated the proliferation and colony formation ability of the cells in vitro, blocked NPC cells at the G0/G1 phase, reduced tumorigenicity potential in vivo, inhibited the invasion   and migra  tion ability of NPC cells and resulted in the reorganization of the actin cytoskeleton. DLC-1 altered the gene expression profile in 5-8F cells. Some tumor suppressor genes (TSGs) were upregulated and some oncogenes were downregulated. These results demonstrate that DLC-1 gene can partially reverse the malignant phenotype of NPC cells by changing the tumor-related gene expression profile, and may be a candidate tumor suppressor gene and a promising diagnostic and therapeutic target in NPC. 
 Effects of structure of Rho GTPase-activating protein DLC-1 on cell morphology and   migration  . DLC-1 encodes a Rho GTPase-activating protein (RhoGAP) and negative regulator of specific Rho family proteins (RhoA-C and Cdc42). DLC-1 is a multi-domain protein, with the RhoGAP catalytic domain flanked by an amino-terminal sterile alpha motif (SAM) and a carboxyl-terminal START domain. The roles of these domains in the regulation of DLC-1 function remain to be determined. We undertook a structure-function analysis involving truncation and missense mutants of DLC-1. We determined that the amino-terminal SAM domain functions as an autoinhibitory domain of intrinsic RhoGAP activity. Additionally, we determined that the SAM and START domains are dispensable for DLC-1 association with focal adhesions. We then characterized several mutants for their ability to regulate cell   migration   and identified constitutively activated and dominant negative mutants of DLC-1. We report that DLC-1 activation profoundly alters cell morphology, enhances protrusive activity, and can increase the velocity but reduce directionality of cell   migration  . Conversely, the expression of the amino-terminal domain of DLC-1 acts as a dominant negative and profoundly inhibits cell   migration   by displacing endogenous DLC-1 from focal adhesions. 
 The Tensin-3 protein, including its SH2 domain, is phosphorylated by Src and contributes to tumorigenesis and metastasis. In cell lines from advanced lung cancer, breast cancer, and melanoma, endogenous tensin-3 contributes to cell   migration  , anchorage-independent growth, and tumorigenesis. Although SH2 domains have not been reported previously to be phosphorylated, the tensin-3 SH2 domain is a physiologic substrate for Src. Tyrosines in the SH2 domain contribute to the biological activity of tensin-3, and phosphorylation of these tyrosines can regulate ligand binding. In a mouse breast cancer model, tensin-3 tyrosines are phosphorylated in a Src-associated manner in primary tumors, and experimental metastases induced by tumor-derived cell lines depend on endogenous tensin-3. Thus, tensin-3 is implicated as an oncoprotein regulated by Src and possessing an SH2 domain with a previously undescribed mechanism for the regulation of ligand binding. 
 GAP-independent functions of DLC1 in metastasis. Metastases are responsible for most cancer-related deaths. One of the hallmarks of metastatic cells is increased motility and   migration   through extracellular matrixes. These processes rely on specific small GTPases, in particular those of the Rho family. Deleted in liver cancer-1 (DLC1) is a tumor suppressor that bears a RhoGAP activity. This protein is lost in most cancers, allowing malignant cells to proliferate and disseminate in a Rho-dependent manner. However, DLC1 is also a scaffold protein involved in alternative pathways leading to tumor and metastasis suppressor activities. Recently, substantial information has been gathered on these mechanisms and this review is aiming at describing the potential and known alternative GAP-independent mechanisms allowing DLC1 to impair   migration  , invasion, and metastasis formation. 
 DLC1 activation requires lipid interaction through a polybasic region preceding the RhoGAP domain. Deleted in Liver Cancer 1 (DLC1) is a GTPase-activating protein (GAP) with specificity for RhoA, RhoB, and RhoC that is frequently deleted in various tumor types. By inactivating these small GTPases, DLC1 controls actin cytoskeletal remodeling and biological processes such as cell   migration   and proliferation. Here we provide evidence that DLC1 binds to phosphatidylinositol-4,5-bisphosphate (PI(4,5)P(2)) through a previously unrecognized polybasic region (PBR) adjacent to its RhoGAP domain. Importantly, PI(4,5)P(2)-containing membranes are shown to stimulate DLC1 GAP activity in vitro. In living cells, a DLC1 mutant lacking an intact PBR inactivated Rho signaling less efficiently and was severely compromised in suppressing cell spreading, directed   migration  , and proliferation. We therefore propose that PI(4,5)P(2) is an important cofactor in DLC1 regulation in vivo and that the PBR is essential for the cellular functions of the protein. 
 Inhibitory effects of deleted in liver cancer 1 gene on gallbladder cancer growth through induction of cell cycle arrest and apoptosis. The biological function of tumor suppressor deleted in liver cancer 1 (DLC1) has been investigated in several types of human cancer, but its role in gallbladder cancer (GBC) is yet to be determined. In this research, we conducted in vitro and in vivo analysis to evaluate the inhibitory activities of DLC1 gene against GBC growth.DLC1 expression in GBC tissues and cell lines was examined by immunohistochemical staining, reverse transcription polymerase chain reaction, and Western blot assay. The in vitro and in vivo effects of ectopic DLC1 expression on cell growth were evaluated. In addition, the effects of ectopic DLC1 expression on cell cycle, apoptosis, and   migration   were also evaluated. The expressions of cell cycle-related and apoptosis-related proteins were examined.The downregulation of DLC1 expression was a common event in GBC tissues and cell lines. Restoration of DLC1 expression in GBC-SD and NOZ cells significantly reduced cell proliferation,   migration   in vitro, and the ability of these cells to form tumors in vivo. Restoration of DLC1 expression arrested GBC-SD and NOZ cells in G0/G1 phase through inducing p21 in a p53-independent manner. In addition, restoration of DLC1 expression induced extrinsic and intrinsic apoptotic pathway through promoting the expressions of Fas L/FADD, Bax, cytochrome c, cleaved caspase-8, -9, -3, and cleaved poly-(ADP-ribose) polymerase and suppressing bcl-2 expression in GBC-SD and NOZ cells.Our findings suggested that dysregulated expression of DLC1 is involved in proliferation and invasion of GBC cells and may serve as a potential therapeutic target. 
 H2O2 inhibits proliferation and mediates suppression of   migration   via DLC1/RhoA signaling in cancer cells. RhoGTPase-activating proteins (RhoGAPs) regulate RhoGTPases in cells, but whether individual reactive oxygen species (ROS) regulate RhoGAPs is unknown. Our previous published papers have shown that deleted in liver cancer 1 (DLC1) inhibits cancer cell   migration   by its RhoGAP activity. The present study was designed to explore the role of H2O2 in regulation of DLC1.We treated cells with H2O2 for 24h and phenotypic changes were analyzed by MTT, RT-PCR, Western blotting, immunofluorescence staining and wound healing assays.H2O2 downregulated cyclin D1 and cyclin E to inhibit proliferation, and upregulated BAX to induce apoptosis in MCF-7 cells. Compared with non-tumorigenic cells, H2O2 increased expression of DLC1 and reduced activity of RhoA in cancer cells. Stress fiber production and   migration   were also suppressed by H2O2 in MDA-MB-231 cells.Our study suggests that H2O2 inhibits proliferation through modulation of cell cycle and apoptosis-related genes, and inhibits   migration   by decreasing stress fibers via DLC1/RhoA signaling. 
 Morphological changes and nuclear translocation of DLC1 tumor suppressor protein precede apoptosis in human non-small cell lung carcinoma cells. We have previously shown that reactivation of DLC1, a RhoGAP containing tumor suppressor gene, inhibits tumorigenicity of human non-small cell lung carcinoma cells (NSCLC). After transfection of NSCLC cells with wild type (WT) DLC1, changes in cell morphology were observed. To determine whether such changes have functional implications, we generated several DLC1 mutants and examined their effects on cell morphology, proliferation,   migration   and apoptosis in a DLC1 deficient NSCLC cell line. We show that WT DLC1 caused actin cytoskeleton-based morphological alterations manifested as cytoplasmic extensions and membrane blebbings in most cells. Subsequently, a fraction of cells exhibiting DLC1 protein nuclear translocation (PNT) underwent caspase 3-dependent apoptosis. We also show that the RhoGAP domain is essential for the occurrence of morphological alterations, PNT and apoptosis, and the inhibition of cell   migration  . DLC1 PNT is dependent on a bipartite nuclear localizing sequence and most likely is regulated by a serine-rich domain at N-terminal part of the DLC1 protein. Also, we found that DLC1 functions in the cytoplasm as an inhibitor of tumor cell proliferation and   migration  , but in the nucleus as an inducer of apoptosis. Our analyses provide evidence for a possible link between morphological alterations, PNT and proapoptotic and anti-oncogenic activities of DLC1 in lung cancer. 
 The SAM domain of the RhoGAP DLC1 binds EF1A1 to regulate cell   migration  . Deleted in liver cancer 1 (DLC1) is a multi-modular Rho-GTPase-activating protein (RhoGAP) and a tumor suppressor. Besides its RhoGAP domain, functions of other domains in DLC1 remain largely unknown. By protein precipitation and mass spectrometry, we identified eukaryotic elongation factor 1A1 (EF1A1) as a novel partner for the sterile alpha motif (SAM) domain of DLC1 but not the SAM domain of DLC2. The solution structure of DLC1 SAM revealed a new monomeric fold with four parallel helices, similar to that of DLC2 SAM but distinct from other SAM domains. Mutating F38, L39 and F40 within a hydrophobic patch retained its overall structure but abolished its interaction with EF1A1 with F38 and L39 forming an indispensable interacting motif. DLC1 SAM did not localize to and was not required for DLC1 to suppress the turnover of focal adhesions. Instead, DLC1 SAM facilitated EF1A1 distribution to the membrane periphery and ruffles upon growth factor stimulation. Compared with wild-type DLC1, the non-interactive DLC1 mutant is less potent in suppressing cell   migration  , whereas overexpression of the DLC1 SAM domain alone, but not the non-interactive mutant SAM or DLC2 SAM, greatly enhanced cell   migration  . This finding reveals a novel contribution of the SAM-EF1A1 interaction as a potentially important GAP-independent modulation of cell   migration   by DLC1. 
 Synergistic antineoplastic effect of DLC1 tumor suppressor protein and histone deacetylase inhibitor, suberoylanilide hydroxamic acid (SAHA), on prostate and liver cancer cells: perspectives for therapeutics. Inactivation of tumor suppressor genes is a major contributing alteration in the initiation or progression of cancer. The human tumor suppressor gene DLC1 (deleted in liver cancer 1) is frequently downregulated or silenced in multiple cancers, predominantly by epigenetic mechanisms. With the current considerable interest and progress in epigenetic therapy, a number of promising antineoplastic agents, particularly histone deacetylase (HDAC) inhibitors, have been developed and used successfully in clinical trials. Both DLC1 and HDAC inhibitors exert antineoplastic functions, and their combined action could be exploited for a more effective cancer therapy. To evaluate the potential benefits of this approach, we examined the antineoplastic effects of adenoviral (Ad)-DLC1-mediated transduction and exposure to suberoylanilide hydroxamic acid (SAHA), a powerful HDAC inhibitor, in two human cancer cell lines that lack intrinsic DLC1 expression, 22Rv1 prostate cancer cells and 7703K human hepatocellular carcinoma cells. Consistent with the oncosuppressive function of DLC1 in several cancers, including prostate and liver cancer, transduction of 22Rv1 and 7703K cells with an Ad-DLC1 expression vector resulted in alterations of cell morphology, induction of apoptosis, and inhibition of cell proliferation,   migration  , and anchorage-independent growth. A low concentration of SAHA (5 microM) efficiently restored the expression of DLC1 in 22Rv1 cells that lack DLC1 expression due to histone deacetylation but had a minimal effect in 7703K cells in which silencing of the DLC1 gene is due mainly to promoter hypermethylation. Regardless of the epigenetic mechanism of DLC1 inactivation, SAHA treatment of DLC1-transduced cells had a synergistic inhibitory effect on tumor cell proliferation and tumorigenesis in both cell lines. In 22Rv1 cells, this combination regimen nearly abolished the formation of colonies in semisolid media as a measure of tumorigenicity in vitro. Current in vitro results validate this protocol as a potentially new therapeutic option in certain cancers. 
 Concerted modulation of paxillin dynamics at focal adhesions by Deleted in Liver Cancer-1 and focal adhesion kinase during early cell spreading. Deleted in Liver Cancer-1 (DLC1) is a RhoGTPase-activating protein (GAP) and a tumor suppressor often downregulated in cancers. It is localized to the focal adhesions (FAs) and its absence leads to enhanced cell   migration  , invasion, and metastasis. Although DLC1 interacts with focal adhesion kinase (FAK), talin, and tensin, its role in focal adhesions dynamics remains unclear. We examined the effect of DLC1 in Human Foreskin Fibroblasts and determined its localization, dynamics and impact on paxillin by Fluorescence Recovery After Photobleaching at both nascent and mature focal adhesions. During early cell spreading, DLC1 is preferentially localized at the inner/mature adhesions whereas phosphorylated paxillin occupies the outer/nascent FAs. In addition, DLC1 downregulates paxillin turnover in a process, that does not require its GAP activity. Instead, it requires the presence of FAK. Acting in concert, both DLC1 and FAK could provide a unique spatio-temporal mechanism to regulate paxillin function in tissue homeostasis. 
 Inhibition of DLC-1 gene expression by RNA interference in the colon cancer LoVo cell line. DLC-1 (deleted in liver cancer-1) is a potential tumor suppressor gene, which is inactive in liver carcinogenesis. To observe the effects of DLC-1 gene expression on cell proliferation and   migration   in the human colon cancer cell line, RNAi Lipo-recombinant of the DLC-1 gene (pGCsil-DLC-1) was constructed and transduced into LoVo cells which are positive for DLC-1 gene expression. Results showed that the RNAi recombinant effectively inhibited the expression of the DLC-1 gene in LoVo cells. Additionally, our data showed decreased DLC-1 gene expression which resulted in the promotion of LoVo cell proliferation. Flow cytometry in cell cycle detection further indicated that the DLC-1 gene induced cell cycle arrest at G2/M and a cell   migration   assay confirmed that the knocking down of DLC-1 gene expression promotes LoVo cell   migration  . Our observations suggest that the DLC-1 gene is associated with LoVo cell proliferation,   migration   and cell cycle distribution. DLC-1 is a potential suppressor gene in the colon cancer LoVo cell line and may play an important role in colon cancer mechanisms. 
 Focal adhesion-localization of START-GAP1/DLC1 is essential for cell motility and morphology. There is a class of GTPase activating proteins for the Rho family GTPases (RhoGAPs) that contain the steroidogenic acute regulatory protein (STAR)-related lipid transfer (START) domain. In mammals three genes encode such proteins and they are designated START-GAP1-3 or deleted in liver cancer 1-3 (DLC1-3). In this study, we examined the intracellular localization and roles of START-GAP1/DLC1 in cell motility. Immunofluorescence microscopic analysis of NRK cells and HeLa cells revealed that START-GAP1 was localized in focal adhesions. Amino acid residues 265-459 of START-GAP1 were found to be necessary for focal adhesion targeting and we name the region "the focal adhesion-targeting (FAT) domain." It was previously known that ectopic expression of START-GAP1 induced cell rounding. We demonstrated that the FAT domain of START-GAP1 was partially required for this morphological change. Furthermore, expression of this domain in HeLa cells resulted in dissociation of endogenous START-GAP1 from focal adhesions as a dominant negative modulator, reducing cell   migration   and spreading. Taken together, START-GAP1 is targeted to focal adhesions via the FAT domain and regulates actin rearrangement through down-regulation of active RhoA and Cdc42. Its absence from focal adhesions could, therefore, cause abnormal cell motility and spreading. 
 DLC1 tumor suppressor gene inhibits   migration   and invasion of multiple myeloma cells through RhoA GTPase pathway. DLC1 (deleted in liver cancer 1), a tumor suppressor gene that encodes a RhoGTPase-activating protein, is recurrently downregulated or silenced in various solid tumors and hematological malignancies because of epigenetic modifications or genomic deletion. Here, we identified DLC1 promoter hypermethylation in 43 out of 44 multiple myeloma (MM) cell lines, which resulted in downregulation or silencing of DLC1 in 41 samples. High frequency of tumor-specific methylation and attenuation or silencing of DLC1 expression could serve as an independent diagnostic marker for MM. Combined treatment with demethylating and acetylating agents significantly elevated the expression of DLC1 and suppressed MM cell proliferation. Two cell lines exhibiting complete promoter methylation and the absence of DLC1 expression were transduced by an adenoviral vector containing DLC1 cDNA. In both cell lines, the reexpression of DLC1 inhibited myeloma cell invasion and   migration  , reduced RhoA activity and resulted in the reorganization of actin cytoskeleton. These results provide the first evidence for the antiproliferative effect of DLC1 in a hematological cancer and implicate RhoA pathway in suppression of MM   migration   and invasion. Given the myeloma cells sensitivity to the reactivation of DLC1 function, the potential for molecular targeted therapy of DLC1-mediated pathways as well as epigenetic therapies hold prospects. 
 Tumor suppressor DLC-1 induces apoptosis and inhibits the growth and invasion of colon cancer cells through the Wnt/β-catenin signaling pathway. The aim of the present study was to investigate the biological role and molecular mechanism of the deleted in liver cancer-1 (DLC-1) gene in human colon cancer growth and invasion. Recombinant lentiviral vectors encoding the DLC-1 gene were constructed for transfection into the human colon cancer cell line SW480. Real-time quantitative polymerase chain reaction (real-time qPCR) and western blot analysis were employed to evaluate the expression of DLC-1, β-catenin, GSK-3β and c-myc in DLC-1-transfected cells. Moreover, cell proliferation assay, cell colony formation assay, cell cycle analysis, apoptosis analysis and ce  ll migrat  ion and invasion assays were performed in order to elucidate the role of DLC-1 in colorectal cancer development and progression. Both real-time qPCR and western blot analyses showed that the DLC-1 gene and protein were overexpressed in the DLC-1-transfected SW480 cells. In addition, the expression of β-catenin and GSK-3β was upregulated and the expression of the c-myc gene was downregulated in the DLC-1-transfected SW480 cells. Furthermore, DLC-1 overexpression inhibited cell proliferation, colony format  ion, migr  ation and invasion, and induced cell cycle arrest at the G1 phase with subsequent apoptosis. DLC-1 inhibits cell growth and invasion in human colon cancer, functioning as a tumor-suppressor gene, possibly through the regulation of the Wnt/β-catenin signaling pathway. 
 Simultaneous loss of the DLC1 and PTEN tumor suppressors enhances breast cancer cell   migration  . The phosphatase and tensin homolog (PTEN) gene is a tumor suppressor frequently deleted or mutated in sporadic tumors of the breast, prostate, endometrium and brain. The protein acts as a dual specificity phosphatase for lipids and proteins. PTEN loss confers a growth advantage to cells, protects from apoptosis and favors cell   migration  . The deleted in liver cancer 1 (DLC1) gene has emerged as a novel tumor suppressor downregulated in a variety of tumor types including those of the breast. DLC1 contains a Rho GTPase activating domain that is involved in the inhibition of cell proliferation,   migration   and invasion. To investigate how simultaneous loss of PTEN and DLC1 contributes to cell transformation, we downregulated both proteins by RNA interference in the non-invasive MCF7 breast carcinoma cell line. Joint depletion of PTEN and DLC1 resulted in enhanced cell   migration   in wounding and chemotactic transwell assays. Interestingly, both proteins were found to colocalize at the plasma membrane and interacted physically in biochemical pulldowns and coimmunoprecipitations. We therefore postulate that the concerted local inactivation of signaling pathways downstream of PTEN and DLC1, respectively, is required for the tight control of cell   migration  . 
 Silencing of DLC1 upregulates PAI-1 expression and reduces   migration   in normal prostate cells. Deleted in liver cancer 1 (DLC1) is a GTPase-activating protein (GAP) domain containing tumor suppressor that localizes to focal adhesions. In cancer cells, loss of DLC1 is known to enhance cancer cell   migration  . However, the role of DLC1 in normal cell   migration   has not been well studied. Here, we show that silencing of DLC1 (shDLC1) in normal prostate epithelial cells reduces cell   migration   in both Transwell and wound-healing assays. This   migration   defect is mainly due to upregulation of plasminogen activator inhibitor 1 (PAI-1). Silencing of PAI-1 rescues the shDLC1-reduced   migration   phenotype. Reexpression of DLC1 suppresses PAI-1 and restores the   migration   defect as well. In contrast, DLC1-K714E (GAP inactive) mutant neither decreases the PAI-1 level nor rescues the shDLC1   migration   defect. Interestingly, DLC1-Y442F (tensin-binding and focal adhesion-localizing defective) mutant is able to suppress PAI-1 expression but does not restore the   migration   defect. Furthermore, PAI-1 upregulation in shDLC1 cells is EGFR-MEK pathway dependent and is able to promote in vitro angiogenesis. Together, our results show that at least the following two new mechanisms are involved in DLC1-mediated normal cell   migration  : (i) DLC1 modulates the expression of PAI-1, which is a negative regulator for cell   migration  , in a GAP domain and EGFR-MEK-dependent manner and (ii) Independent of PAI-1, the interaction of DLC1 with tensin members positively regulates cell   migration  . 
 Overexpression of DLC-1 induces cell apoptosis and proliferation inhibition in the renal cell carcinoma. The lack of effective anti-tumor therapy for renal cell carcinoma (RCC) has stimulated the search for novel target whose inhibition could block tumorigenesis. Recently, reduced DLC-1 has been shown to be associated with aggressive and highly metastatic renal cell carcinoma. In this study, the biological role of DLC-1 on cell growth,   migration   and cell cycle progression in RCC cells was investigated. Over-expression of DLC-1 was associated with a marked inhibition of cell growth (P<0.01). The inhibitory effect was partly due to the induction of apoptosis and cell cycle arrest in G(0)/G(1) accompanied by up-regulation of the intracellular signal proteins of p27 and down-regulation of cyclin D1 and cyclin E. Furthermore, DLC-1 induced FAK dephosphorylation of focal adhesion proteins inhibited cell   migration   (P<0.05). Decreased DLC-1 expression strongly correlated with proliferative activity, as indicated by the elevated levels of Ki67. Restoration of DLC-1 expression in RCC cells led to Bcl-2 and caspase-3 mediated apoptosis as well as attenuated the ability of the cells to form RCC tumors in athymic nude mice (P<0.05). Taken together, these results suggest that DLC-1 plays a crucial role in signal transduction pathway regulating the cell proliferation,   migration  , and carcinogenesis of human RCC. 
 A phosphorylation switch controls the spatiotemporal activation of Rho GTPases in directional cell   migration  . Although cell   migration   plays a central role in development and disease, the underlying molecular mechanism is not fully understood. Here we report that a phosphorylation-mediated molecular switch comprising deleted in liver cancer 1 (DLC1), tensin-3 (TNS3), phosphatase and tensin homologue (PTEN) and phosphoinositide-3-kinase (PI3K) controls the spatiotemporal activation of the small GTPases, Rac1 and RhoA, thereby initiating directional cell   migration   induced by growth factors. On epidermal growth factor (EGF) or platelet-derived growth factor (PDGF) stimulation, TNS3 and PTEN are phosphorylated at specific Thr residues, which trigger the rearrangement of the TNS3-DLC1 and PTEN-PI3K complexes into the TNS3-PI3K and PTEN-DLC1 complexes. Subsequently, the TNS3-PI3K complex translocates to the leading edge of a migrating cell to promote Rac1 activation, whereas PTEN-DLC1 translocates to the posterior for localized RhoA activation. Our work identifies a core signalling mechanism by which an external motility stimulus is coupled to the spatiotemporal activation of Rac1 and RhoA to drive directional cell   migration  . 
 Differential regulation of the activity of deleted in liver cancer 1 (DLC1) by tensins controls cell   migration   and transformation. The epithelial growth factor receptor plays an important role in cell   migration   and cancer metastasis, but the underlying molecular mechanism is not fully understood. We show here that differential regulation of the Ras-homology-GTPase-activating protein [corrected] (Rho-GAP) activity of deleted in liver cancer 1 (DLC1) by tensin3 and COOH-terminal tensin-like protein (cten) controls EGF-driven cell   migration   and transformation. Tensin3 binds DLC1 through its actin-binding domain, a region that is missing in cten, and thereby releases an autoinhibitory interaction between the sterile alpha motif and Rho-GAP domains of DLC1. Consequently, tensin3, but not cten, promotes the activation of DLC1, which, in turn, leads to inactivation of RhoA and decreased cell   migration  . Depletion of endogenous tensin3, but not cten, augmented the formation of actin stress fibers and focal adhesions and enhanced cell motility. These effects were, however, ablated by an inhibitor of the Rho-associated protein kinase. Importantly, activation of DLC1 by tensin3 or its actin-binding domain drastically reduced the anchorage-independent growth of transformed cells. Our study therefore links dynamic regulation of tensin family members by EGF to Rho-GAP through DLC1 and suggests that the tensin-DLC1-RhoA signaling axis plays an important role in tumorigenesis and cancer metastasis, and may be explored for cancer intervention. 
 Role of DLC-1, a tumor suppressor protein with RhoGAP activity, in regulation of the cytoskeleton and cell motility. DLC-1 was originally identified as a potential tumor suppressor. One of the key biochemical functions of DLC-1 is to serve as a GTPase activating protein (GAP) for members of the Rho family of GTPases, particularly Rho A-C and Cdc 42. Since these GTPases are critically involved in regulation of the cytoskeleton and cell   migration  , it seems clear that DLC-1 will also influence these processes. In this review we examine basic aspects of the actin cyoskeleton and how it relates to cell motility. We then delineate the characteristics of DLC-1 and other members of its family, and describe how they may have multiple effects on the regulation of cell polarity, actin organization, and cell   migration  . 
 Genome-wide analysis of chromosomal alterations in patients with esophageal squamous cell carcinoma exposed to tobacco and betel quid from high-risk area in India. Genomic alterations such as chromosomal amplifications, deletions and loss of heterozygosity play an important role in the pathogenesis and progression of cancer. Environmental risk factors contribute to the development and progression of tumors by facilitating the loss of tumor suppressor genes and amplification of oncogenes. In this current study, Affymetrix 10K single nucleotide polymorphism (SNP) arrays were used to evaluate genomic alterations in 20 pairs of matched germ-line and tumor DNA obtained from patients with esophageal squamous cell carcinoma (ESCC) from high-risk area of India where tobacco, betel quid and alcohol use are widespread. Twenty-two amplified regions and 16 deleted regions identified across chromosomal arms were biologically relevant. The candidate genes located at amplified regions of chromosomes or low-level gain regions such as PLA2G5 (1p36-p34), COL11A1 (1p21), KCNK2 (1q41), S100A3 (1q21), ENAH (1q42.12), RGS1 (1q31), KCNH1 (1q32-q41), INSIG2 (2q14.1), FGF12 (3q28), TRIO (5p15.2), RNASEN (5p15.2), FGF10 (5p13-p12), EDN1(6p24.1-p22.3), SULF1 (8q13.2-13.3), TLR4 (9q32-q33), TNC (9q33), NTRK2 (9q22.1), CD44 (11p13), NCAM1 (11q23.1), TRIM29 (11q22-q23), PAK1 (11q13-q14) and RAB27A (15q15-q21.1), are found to be associated with cellular   migration   and proliferation, tumor cell metastasis and invasion, anchorage independent growth and inhibition of apoptosis. The candidate genes located at deleted regions of chromosomes, such as FBLN2 (3p25.1), WNT7A (3p25), DLC1 (8p22), LZTS1 (8p22), CDKN2A (9p21), COL4A1 (13q34), CDK8 (13q12) and DCC (18q21.3), are found to be associated with the suppression of tumor. The suggested candidate genes were mostly involved in potential signaling pathways such as focal adhesion (COL4A1), tight junction (CLDN10), MAPK signaling pathway (FGF12) and neuroactive ligand receptor interaction pathway (CCKAR). Expression of FGF12 and COL4A1 was validated by tissue microarray. These unique copy number alteration profiles should be taken into consideration when developing biomarkers for the early detection of ESCC in high-risk areas of India in association with tobacco and betel quid use. 
 Deleted in liver cancer 1 controls cell   migration   through a Dia1-dependent signaling pathway. Deleted in liver cancer (DLC) 1 and 2 are Rho GTPase-activating proteins that are frequently down-regulated in various types of cancer. Ectopic expression in carcinoma cell lines lacking these proteins has been shown to inhibit cell   migration   and invasion. However, whether the loss of DLC1 or DLC2 is the cause of aberrant Rho signaling in transformed cells has not been investigated. Here, we have down-regulated DLC1 and DLC2 expression in breast cancer cells using a RNA interference approach. Silencing of DLC1 led to the stabilization of stress fibers and focal adhesions and enhanced cell motility in wound-healing as well as chemotactic Transwell assays. We provide evidence that enhanced   migration   of cells lacking DLC1 is dependent on the Rho effector protein Dia1 but does not require the activity of Rho kinase. By contrast, DLC2 knockdown failed to affect the migratory behavior of cells, suggesting that the two proteins have distinct functions. This is most likely due to their differential subcellular localizations, with DLC1 found in focal adhesions and DLC2 being mainly cytosolic. Collectively, our data show that DLC1 is critically involved in the control of Rho signaling and actin cytoskeleton remodeling and that its cellular loss is sufficient for the acquisition of a more migratory phenotype of breast cancer cells. 
  GeneRIFs  
 A phosphorylation-mediated molecular switch comprising DLC), TNS3, PTEN and PI3K controls the spatiotemporal activation of Rac1 and RhoA, thereby initiating directional cell   migration   induced by growth factors. 
 DLC-1 inhibits the proliferation,   migration   and tumorigenicity of human nasopharyngeal carcinoma cells. 
 DLC1 modulates the expression of PAI-1, which is a negative regulator for cell   migration  , in a GAP domain and EGFR-MEK-dependent manner and independent of PAI-1, the interaction of DLC1 with tensin members positively regulates cell   migration  . 
 Results suggest that PP1alpha bound to tensin1 has effects in reducing   migration   and invasion that are not mediated through DLC-1, and show the importance of PP1alpha binding to tensin1 for the regulation of cell polarization,   migration  , and invasion. 
 Results suggest that the concerted local inactivation of signaling pathways downstream of PTEN and DLC1, respectively, is required for the tight control of cell   migration  . 
 DLC-1 plays a crucial role in signal transduction pathway regulating the cell proliferation,   migration  , and carcinogenesis of human renal cell carcinoma 
 This finding reveals a novel contribution of the SAM-EF1A1 interaction as a potentially important GAP-independent modulation of cell   migration   by DLC1. 
 expression of the amino-terminal domain of DLC-1 acts as a dominant negative and profoundly inhibits cell   migration   by displacing endogenous DLC-1 from focal adhesions 
 Results suggest that the DLC-1 gene is associated with cell proliferation,   migration   and cell cycle distribution in a colon cancer cell line. 
 Involved in signal transduction pathway regulating cell proliferation, cell morphology, and cell   migration   in liver cancers. 
  [Ee]xtracellular [Mm]atrix  PubMed  
 GAP-independent functions of DLC1 in metastasis. Metastases are responsible for most cancer-related deaths. One of the hallmarks of metastatic cells is increased motility and migration through   extracellular matrix  es. These processes rely on specific small GTPases, in particular those of the Rho family. Deleted in liver cancer-1 (DLC1) is a tumor suppressor that bears a RhoGAP activity. This protein is lost in most cancers, allowing malignant cells to proliferate and disseminate in a Rho-dependent manner. However, DLC1 is also a scaffold protein involved in alternative pathways leading to tumor and metastasis suppressor activities. Recently, substantial information has been gathered on these mechanisms and this review is aiming at describing the potential and known alternative GAP-independent mechanisms allowing DLC1 to impair migration, invasion, and metastasis formation. 
  [Ii]nvasive  PubMed  
 Correlation of DLC1 gene methylation with oncogenic PIK3CA mutations in extramammary Paget's disease. Extramammary Paget's disease is a rare cutaneous malignant neoplasm. The genetic and epigenetic mechanisms underlying its pathology remain unknown. In this study, we investigated the expression levels, and mutation and methylation status of a common tumor suppressor gene, deleted in liver cancer 1 (DLC1), and an oncogene, PIK3CA, in tumor (n=132) and normal tissues (n=20) from unrelated patients. The presence of epigenetic and genetic lesions was then correlated to the patient pathology data to determine the potential role of these genes in extramammary Paget's disease etiology and progression. The DLC1 gene was found to be downregulated in 43 (33%) tumors, as compared with immunohistochemistry results from normal tissues. Methylation-sensitive, high-resolution melting analysis indicated that the DLC1 promoter was hypermethylated in 51 (39%) extramammary Paget's disease tumors. This hypermethylation was associated with significantly decreased DLC1 levels (P=0.011), and had a strong positive correlation with advanced age (P=0.002). PIK3CA mutations were detected by direct sequencing in 32 (24%) tumors, the majority of which were   invasive  . Furthermore, PIK3CA mutations significantly correlated with DLC1 hypermethylation. Thus, aberrant DLC1 methylation and PIK3CA mutations may have important roles in extramammary Paget's disease pathogenesis, and may represent potential molecular targets for therapy. 
 Simultaneous loss of the DLC1 and PTEN tumor suppressors enhances breast cancer cell migration. The phosphatase and tensin homolog (PTEN) gene is a tumor suppressor frequently deleted or mutated in sporadic tumors of the breast, prostate, endometrium and brain. The protein acts as a dual specificity phosphatase for lipids and proteins. PTEN loss confers a growth advantage to cells, protects from apoptosis and favors cell migration. The deleted in liver cancer 1 (DLC1) gene has emerged as a novel tumor suppressor downregulated in a variety of tumor types including those of the breast. DLC1 contains a Rho GTPase activating domain that is involved in the inhibition of cell proliferation, migration and invasion. To investigate how simultaneous loss of PTEN and DLC1 contributes to cell transformation, we downregulated both proteins by RNA interference in the non-  invasive   MCF7 breast carcinoma cell line. Joint depletion of PTEN and DLC1 resulted in enhanced cell migration in wounding and chemotactic transwell assays. Interestingly, both proteins were found to colocalize at the plasma membrane and interacted physically in biochemical pulldowns and coimmunoprecipitations. We therefore postulate that the concerted local inactivation of signaling pathways downstream of PTEN and DLC1, respectively, is required for the tight control of cell migration. 
 Restoration of DLC-1 gene expression induces apoptosis and inhibits both cell growth and tumorigenicity in human hepatocellular carcinoma cells. The gene deleted in liver cancer-1 (DLC-1) is located on human chromosome 8p21-22, a region thought to harbor tumor suppressor genes on the basis of its frequent deletion or loss of heterozygosity in a variety of human cancers, including hepatocellular carcinoma (HCC). Deletion or altered expression of DLC-1 is common in HCC. In the current study, the subcellular localization of Dlc-1 protein was determined by immunostaining with antibody to DLC-1 and the possible tumor growth suppressor activity of DLC-1 was investigated by examining the effects of of DLC-1 cDNA transfection in two human HCC cell lines lacking expression of the endogenous gene. The results show that Dlc-1protein is localized in the cell cytoplasm, and the restoration of DLC-1 expression in HCC cells resulted in caspase-3-mediated apoptosis, inhibition of cell growth and   invasive  ness in vitro as well as in reduction of the ability of the cells to form tumors in athymic nude mice. These observations thus support the notion that Dlc-1 protein is involved in hepatocarcinogenesis and has oncosuppressive activity in HCC. 
 DLC-1 expression levels in breast cancer assessed by qRT- PCR are negatively associated with malignancy. The aim of this study was to explore the expression of DLC-l in breast carcinoma and any association with tumor metastasis.51 surgical specimens of human breast carcinoma, divided into high   invasive   and low   invasive   groups according to their clinicopathological features, 30 cases of adjacent normal tissue and 28 benign breast lesions were examined by qRT-PCR for expression of DLC-1.Expression level of DLC-1 in adjacent normal tissue and benign breast lesion specimens was higher than that in breast carcinoma (P<0.0001); the values in the high   invasive   group with synchronous metastases were also lower than in the low   invasive   group (P=0.0275). The correlation between DLC-1 expression level and tumor progression and metastasis of breast cancer was negative.As an anti-oncogene, DLC-1 could play an important part in breast carcinoma occurrence, progression,   invasive  ness and metastasis. Detecting the changes of the expression of DLC-1 in the breast carcinoma may contribute to earlier auxiliary diagnosis of   invasive  ness, metastasis and recrudescence. 
  [Mm]etastasis  PubMed  
 DLC-1:a Rho GTPase-activating protein and tumour suppressor. The deleted in liver cancer 1 (DLC-1) gene encodes a GTPase activating protein that acts as a negative regulator of the Rho family of small GTPases. Rho proteins transduce signals that influence cell morphology and physiology, and their aberrant up-regulation is a key factor in the neoplastic process, including   metastasis  . Since its discovery, compelling evidence has accumulated that demonstrates a role for DLC-1 as a bona fide tumour suppressor gene in different types of human cancer. Loss of DLC-1 expression mediated by genetic and epigenetic mechanisms has been associated with the development of many human cancers, and restoration of DLC-1 expression inhibited the growth of tumour cells in vivo and in vitro. Two closely related genes, DLC-2 and DLC-3, may also be tumour suppressors. This review presents the current status of progress in understanding the biological functions of DLC-1 and its relatives and their roles in neoplasia. 
 Deleted in liver cancer 1 (DLC1) negatively regulates Rho/ROCK/MLC pathway in hepatocellular carcinoma. Deleted in liver cancer 1 (DLC1), a member of RhoGTPase activating protein (GAP) family, is known to have suppressive activities in tumorigenicity and cancer   metastasis  . However, the underlying molecular mechanisms of how DLC1 suppresses cell motility have not been fully elucidated. Rho-kinase (ROCK) is an immediate down-stream effector of RhoA in mediating cellular cytoskeletal events and cell motility. In the present study, we aimed to investigate the effects of DLC1 on Rho/ROCK signaling pathway in hepatocellular carcinoma (HCC).We demonstrated that DLC1 negatively regulated ROCK-dependent actomyosin contractility. From immunofluorescence study, we found that ectopic expression of DLC1 abrogated Rho/ROCK-mediated cytoskeletal reorganization including formation of stress fibers and focal adhesions. It also downregulated cortical phosphorylation of myosin light chain 2 (MLC2). These inhibitory events by DLC1 were RhoGAP-dependent, as RhoGAP-deficient mutant of DLC1 (DLC1 K714E) abolished these inhibitory events. In addition, from western study, DLC1 inhibited ROCK-related myosin light chain phosphatase targeting unit 1 (MYPT1) phosphorylation at Threonine 853. By examining cell morphology under microscope, we found that ectopic expression of dominant-active ROCK released cells from DLC1-induced cytoskeletal collapse and cell shrinkage.Our data suggest that DLC1 negatively regulates Rho/ROCK/MLC2. This implicates a ROCK-mediated pathway of DLC1 in suppressing   metastasis   of HCC cells and enriches our understanding in the molecular mechanisms involved in the progression of hepatocellular carcinoma. 
 Expression of deleted in liver cancer 1 and plasminogen activator inhibitor 1 protein in ovarian carcinoma and their clinical significance. The deleted in liver cancer 1 (DLC1) and plasminogen activator inhibitor 1 (PAI-1) are known to be closely associated with tumor growth and   metastasis   in several kinds of human tumors. The aim of this study was to investigate the expression of DLC1 and PAI-1 in ovarian carcinoma, and evaluate their relations with the prognosis of ovarian carcinoma.Immunohistochemical staining and Western blot were used to examine the expressions of DLC1 and PAI-1 protein in 25 specimens normal ovarian tissues, 52 specimens of serous cystadenocarcinoma tissues and 23 specimens of mucinous cystadenocarcinoma tissues. Chi-square test, Logistic regression and Partial Correlate analysis were performed to evaluate the association between DLC1 and PAI-1 with clinicopathological characteristics. Overall survival was estimated by Kaplan-Meier curves and multivariate Cox analysis. The relationships between DLC1 and PAI-1 protein expression were analyzed by Pearson's correlation coefficient.The expression of DLC1 protein in ovarian carcinoma tissues was significantly lower than that in normal ovarian tissues, but it was converse for PAI-1. In ovarian carcinoma, the expression of DLC1 was significantly associated with advanced FIGO stage, ascites and positive lymph node   metastasis  , whereas PAI-1 protein was closely related with advanced FIGO stage, poor histological differentiation and lymph node   metastasis  . The expression of DLC1 was negatively correlated with PAI-1 in ovarian carcinoma. Ovarian cancer patients with negative expression of DLC1 and positive expression of PAI-1 had the worst overall survival time compared to other patients.The expression of DLC1 and PAI-1 were closely related with the   metastasis   and invasion of ovarian carcinoma, only the combination of DLC1 and PAI-1 could serve as an independent prognostic factor of ovarian carcinoma. 
 DLC1 as a regulator of proliferation, invasion, cell cycle, and apoptosis in cutaneous squamous cell carcinoma. Increasing evidence has demonstrated that the tumor suppressor gene deleted in liver cancer-1 (DLC1) is tightly implicated in the development and progression of tumors and is verified to be downregulated in a variety of tumors. However, the roles and precise molecular mechanisms of DLC1 in cutaneous squamous cell carcinoma (cutaneous SCC) remain to be elucidated. In the present study, we confirmed the reduced level in cutaneous SCC tissues and cells, and DLC1 mRNA relative level in cutaneous SCC tissues with lymph node   metastasis   (0.801 ± 0.079) was markedly lower than those without lymph node   metastasi  s (1.245 ± 0.071) (P < 0.0001). Importantly, the survival rates of patients with low DLC1 level were lower than those with high DLC1 level (P = 0.0051). Further investigation revealed that DLC1 overexpression inhibited proliferation and arrested cell cycle at G0/G1 phase in A431 cells, which may be tightly associated with upregulation of p21 protein and downregulation of cyclin D1 and cdk2 proteins. Moreover, the decreases of FAK and p-FAK as well as the increase of E-cadherin level mediated by elevated DLC1 level suppressed invasion in A431 cells. Additionally, DLC1 overexpression induced apoptosis coupled with elevations of Bax level and caspase-3 activity and decrease of Bcl-2 level in A431 cells. Taken altogether, our data presented herein suggest that DLC1 plays a pivotal role in the development and progression of cutaneous SCC, which may be in part achieved by regulating the signaling pathway related to proliferation, invasion, cell cycle, and apoptosis in cutaneous SCC cells. 
 Aberrant deleted in liver cancer-1 expression is associated with tumor   metastasis   and poor prognosis in urothelial carcinoma. This study explored the potential role of deleted in liver cancer-1 (DLC-1) as a prognostic indicator of cancer   metastasis   and survival in urothelial carcinoma (UC). Tissue microarrays were constructed from paraffin-embedded specimens from 88 UC patients, and immunohistochemical staining was performed to investigate the association of DLC-1 with clinicopathologic characteristics and clinical outcome. The DLC-1 expression showed a significant positive correlation with tumor location (p = 0.041) and a significant negative correlation with advanced histological grade (p = 0.013). In tumors with low DLC-1 expression, Bcl-2 positivity was observed in 24.4% of cases. The DLC-1 expression had significant negative associations with Bcl-2 expression (p = 0.032) and with highly metastatic UC (p = 0.032). Kaplan-Meier analysis showed that DLC-1 protein expression was negatively associated with both overall survival (OS) (p = 0.035) and with distant   metastasis  -free survival (DMFS) (p = 0.041), but not with disease-free survival. Multivariate analyses indicated that tumor size was the significant independent predictors of OS (p = 0.048); however, only DLC-1 expression was a significant independent predictor of DMFS (p = 0.019). In conclusion, reduced DLC-1 protein expression may be an important factor in tumor progression and a useful prognostic molecular marker in UC. 
 Low expression of DLC1 is predictive of poor therapeutic efficiency of fluoropyrimidine and oxaliplatin as adjuvant chemotherapy in gastric cancer. The Rho‑GTPase‑activating protein, deleted in liver cancer‑1 (DLC1), has been reported to be a tumor suppressor. However, the prognostic value of DLC1 in gastric cancer (GC) remains to be fully elucidated. Fluoropyrimidine‑oxaliplatin (FP‑LOHP) combination therapy has been widely used for the adjuvant chemotherapy of GC, however, no reliable marker has been identified to determine its efficiency. Thus, the present study performed a retrospective investigation involving 251 patients with stage IB‑III GC, who received adjuvant chemotherapy following radical resection and 37 patients with stage IV GC, who underwent palliative resection. The expression of DLC1 was found to be reduced in the majority of GC samples (212/288 pairs of samples), compared with normal mucosa, in immunohistochemical analyses. Lower expression levels of DLC1 indicated a more advanced   tumor‑n  ode‑metastasis stage, incre  ased lymph   node metastasis, deeper tumor invasion, increased tumor size and a higher   rate of di  stant metastasis. By contrast, relatively increased expression levels of DLC1 indicated a longer time to recurrence (TTR) [hazard ratio (HR), 2.232; P=0.004] and overall survival (OS) rate (HR, 2.910; P=0.001). Patients receiving FP‑LOHP adjuvant chemotherapy were significantly less likely to suffer GC recurrence (P=0.001) and succumb to mortality (P=0.004), compared with those who received alternative chemotherapies. However, only the patients with DLC1‑positive GC receiving FP‑LOHP [DLC1 (+)/FP‑LOHP (+)] exhibited a more favorable TTR and OS, compared with the patients with DLC1 (+)/FP‑LOHP (‑) (TTR, P=0.001; OS, P=0.020). No significant improvement in clinical outcome was observed in GC patients with low DLC1 receiving FP‑LOHP treatment (TTR, P=0.270; OS, P=0.197). In conclusion, low expression of DLC1 correlated with GC progression and is predictive of higher rates of recurrence and mortality. Only patients with DLC1‑positive GC may have an improved treatment outcome from the use of FP‑LOHP as adjuvant chemotherapy. 
 Deleted in liver cancer-1 (DLC1): an emerging   metastasis   suppressor gene. While significant progress continues to be made in the early detection and therapeutic management of primary tumors, the incidence of metastatic disease remains the major cause of mortality. Accordingly, the development of novel effective therapies that can ameliorate dissemination and secondary tumor growth are a clinical priority. The identification of genetic and functional alterations in cancer cells that affect factors implicated in the metastatic process is critical for designing preventive and therapeutic strategies. Evidence implicating the protein deleted in liver cancer-1 (DLC1), a Rho GTPase activator, in   metastasis   has accumulated to a point where DLC1 may be considered as a   metastasis   suppressor gene. This review presents evidence supporting an anti-metastatic role for DLC1 in several human cancers and discusses the mechanisms contributing to its inhibitory effects. In addition, promising opportunities for therapeutic interventions based on DLC1 function and downstream pathways involved in the metastatic process are considered. 
 The Tensin-3 protein, including its SH2 domain, is phosphorylated by Src and contributes to tumorigenesis and   metastasis  . In cell lines from advanced lung cancer, breast cancer, and melanoma, endogenous tensin-3 contributes to cell migration, anchorage-independent growth, and tumorigenesis. Although SH2 domains have not been reported previously to be phosphorylated, the tensin-3 SH2 domain is a physiologic substrate for Src. Tyrosines in the SH2 domain contribute to the biological activity of tensin-3, and phosphorylation of these tyrosines can regulate ligand binding. In a mouse breast cancer model, tensin-3 tyrosines are phosphorylated in a Src-associated manner in primary tumors, and experimental metastases induced by tumor-derived cell lines depend on endogenous tensin-3. Thus, tensin-3 is implicated as an oncoprotein regulated by Src and possessing an SH2 domain with a previously undescribed mechanism for the regulation of ligand binding. 
 GAP-independent functions of DLC1 in   metastasis  . Metastases are responsible for most cancer-related deaths. One of the hallmarks of metastatic cells is increased motility and migration through extracellular matrixes. These processes rely on specific small GTPases, in particular those of the Rho family. Deleted in liver cancer-1 (DLC1) is a tumor suppressor that bears a RhoGAP activity. This protein is lost in most cancers, allowing malignant cells to proliferate and disseminate in a Rho-dependent manner. However, DLC1 is also a scaffold protein involved in alternative pathways leading to tumor and   metastasis   suppressor activities. Recently, substantial information has been gathered on these mechanisms and this review is aiming at describing the potential and known alternative GAP-independent mechanisms allowing DLC1 to impair migration, invasion, and   metastasis   formation. 
 DLC1 interaction with α-catenin stabilizes adherens junctions and enhances DLC1 antioncogenic activity. The DLC1 (for deleted in liver cancer 1) tumor suppressor gene encodes a RhoGAP protein that inactivates Rho GTPases, which are implicated in regulation of the cytoskeleton and adherens junctions (AJs), a cell-cell adhesion protein complex associated with the actin cytoskeleton. Malignant transformation and tumor progression to   metastasi  s are often associated with changes in cytoskeletal organization and cell-cell adhesion. Here we have established in human cells that the AJ-associated protein α-catenin is a new binding partner of DLC1. Their binding was mediated by the N-terminal amino acids 340 to 435 of DLC1 and the N-terminal amino acids 117 to 161 of α-catenin. These proteins colocalized in the cytosol and in the plasma membrane, where together they associated with E-cadherin and β-catenin, constitutive AJ proteins. Binding of DLC1 to α-catenin led to their accumulation at the plasma membrane and required DLC1 GAP activity. Knocking down α-catenin in DLC1-positive cells diminished DLC1 localization at the membrane. The DLC1-α-catenin complex reduced the Rho GTP level at the plasma membrane, increased E-cadherin's mobility, affected actin organization, and stabilized AJs. This process eventually contributed to a robust oncosuppressive effect of DLC1 in metastatic prostate carcinoma cells. Together, these results unravel a new mechanism through which DLC1 exerts its strong oncosuppressive function by positively influencing AJ stability. 
 DLC-1 is an independent prognostic marker and potential therapeutic target in hepatocellular cancer. The 5-year survival rate of patients with hepatocellular cancer (HCC) was very low because of invasion and   metastasi  s in the early stage. Biomarkers might help predict early occurrence of invasion and   metastasi  s. Accumulating evidence has shown that deleted in liver cancer-1 (DLC1) may be considered as a   metastasi  s suppressor gene in numerous solid and hematological cancers. However, its prognostic role and mechanisms that regulate and coordinate these activities remain poorly understood.With the method of immunohistochemistry, the expression of DLC-1 as well as Rho A, ROCK2, moesin had been characterized in 80 HCC tissues and adjacent noncancerous tissues. The correlation between their expression and their relationships with clinicopathological characteristics of HCC were also investigated. In addition, the prognostic value of DLC1 expression within the tumor tissues was assessed by Cox regression and Kaplan-Meier analysis.DLC1 expression was significantly lower in HCC tissues than in adjacent noncancerous tissues, and DLC-1 expression was found to be negatively correlated with tumor differentiation, TNM stage and lymph nod  e metastas  is. Furthermore, DLC-1 expression was found to inversely correlate with Rho A, ROCK2 and moesin which were all highly expressed in HCC tissues. Kaplan-Meier analysis showed that significantly longer 5-year survival rate was seen in HCC patients with higher DLC1 expression, compared to those with lower expression of DLC1. Multivariate Cox proportional hazard analyses revealed that DLC1 was an independent factor affecting the overall survival probability.DLC1 could be served as a tumor suppressor gene in the progression especially in the invasion an  d metastas  is of HCC. DLC1 perhaps played its role by regulating the expression of Rho A, ROCK2 and moesin. Evaluation of the expression of DLC-1 might be a good prognostic marker for patients with HCC. 
 [Expression of deleted in liver cancer 1 and phosphorelated focal adhesion kinase in breast cancer]. To analyze the expression of deleted in liver cancer 1 (DLC1) and phosphorelated focal adhesion kinase (p-FAK) in breast cancer tissue to further understand the molecular mechanisms of the carcinogenesis and   metastasis   of breast cancer.Immunohistochemistry was employed to determine the protein level of DLC1 and p-FAK in 61 breast cancer, 30 benign breast disease and the adjacent normal breast tissues.The positivity rates of DLC1 differed significantly between breast cancer, benign and normal tissues (34.43%, 80.00% and 76.67%, respectively, P<0.001). The positivity rates of p-FAK in the 3 tissues were 77.05%, 33.33% and 26.67%, also showing significant differences (P<0.001). The aberrant expression of DLC1 showed an inverse correlation to p-FAK (κ=-0.4591). Both DLC1 and p-FAK were closely correlated to the carcinogenesis, clinical stage, PR and lymphatic   metastasi  s of breast cancer (P<0.05), but not to the patients age, pathological subtype, familial history, ER or CerbB-2 (P>0.05).The abnormal expression of DLC1 and p-FAK might participate in the carcinogenesis, progression, and   metastasi  s of breast cancer. The role of DLC1 and p-FAK might be related to the regulation of progestone. DLC1 and p-FAK may serve as candidate markers for early diagnosis, prognostic evaluation and target treatment of breast cancer. 
 PKA-induced dimerization of the RhoGAP DLC1 promotes its inhibition of tumorigenesis and   metastasis  . Deleted in Liver Cancer 1 (DLC1) is a tumour suppressor that encodes a RhoGTPase-activating protein (RhoGAP) and is frequently inactivated in many human cancers. The RhoGAP activity of DLC1 against Rho signalling is well documented and is strongly associated with the tumour suppressor functions of DLC1. However, the mechanism by which the RhoGAP activity of DLC1 is regulated remains obscure. Here, we report that phosphorylation of DLC1 at Ser549 by cyclic AMP-dependent protein kinase A contributes to enhanced RhoGAP activity and promotes the activation of DLC1, which suppresses hepatoma cell growth, motility and   metastasis   in both in vitro and in vivo models. Intriguingly, we found that Ser549 phosphorylation induces the dimerization of DLC1 and that inducible dimerization of DLC1 can rescue the tumour suppressive and RhoGAP activities of DLC1 containing a Ser549 deletion. Our study establishes a novel regulatory mechanism for DLC1 RhoGAP activity via dimerization induced by protein kinase A signalling. 
 rs621554 single nucleotide polymorphism of DLC1 is associated with breast cancer susceptibility and prognosis. Deleted in liver cancer 1 (DLC1) on chromosome 8p22, is an important tumor suppressor gene originally identified to be deleted in hepatocellular carcinoma. It can regulate the structure of the actin cytoskeleton and inhibit cell proliferation, motility and angiogenesis, which predominantly depends on its homology to rat RhoGAP. There are many genetic variants in DLC1, which may influence its antitumor efficacy. The rs621554 (IVS19+108C>T) polymorphism is a synonymous single nucleotide polymorphism (SNP) previously found to be associated with hepatocellular carcinoma. In the present study, 453 patients with breast cancer and 330 healthy females were analyzed using a cycling probe method. It was determined that the rs621554 polymorphism of DLC1 was associated with breast cancer susceptibility, with the CC and CT genotypes resulting in a higher risk of developing breast cancer. In regard to clinicopathological variables, it was demonstrated that the CT and CC genotype were associated with tumor size, lymph node   metastasis   and progesterone receptor status. Patients with the CT and CC genotype had shorter disease-free survival and overall survival rates compared with those with the TT genotype. Additionally, it was demonstrated that the rs621554 polymorphism was correlated with DLC1 expression at the mRNA level. These results suggested that the rs621554 polymorphism is associated with breast cancer susceptibility and prognosis, and may serve as a biomarker for breast cancer development and progression. 
 Concerted modulation of paxillin dynamics at focal adhesions by Deleted in Liver Cancer-1 and focal adhesion kinase during early cell spreading. Deleted in Liver Cancer-1 (DLC1) is a RhoGTPase-activating protein (GAP) and a tumor suppressor often downregulated in cancers. It is localized to the focal adhesions (FAs) and its absence leads to enhanced cell migration, invasion, and   metastasis  . Although DLC1 interacts with focal adhesion kinase (FAK), talin, and tensin, its role in focal adhesions dynamics remains unclear. We examined the effect of DLC1 in Human Foreskin Fibroblasts and determined its localization, dynamics and impact on paxillin by Fluorescence Recovery After Photobleaching at both nascent and mature focal adhesions. During early cell spreading, DLC1 is preferentially localized at the inner/mature adhesions whereas phosphorylated paxillin occupies the outer/nascent FAs. In addition, DLC1 downregulates paxillin turnover in a process, that does not require its GAP activity. Instead, it requires the presence of FAK. Acting in concert, both DLC1 and FAK could provide a unique spatio-temporal mechanism to regulate paxillin function in tissue homeostasis. 
 Clinicopathological Significance of DLC-1 Expression in Cancer: a Meta-Analysis. Recent reports have shown that DLC-1 is widely expressed in normal tissues and is down- regulated in a wide range of human tumors, suggesting it may act as a tumor suppressor gene. We conducted a meta-analysis to determine the correlation between DLC-1 expression and clinicopathological characteristics in cancers.A detailed literature search was made for relevant publications from PubMed, EMBASE, Cochrane library databases, Web of Science, CNKI. The methodological quality of the studies was also evaluated. Analyses of pooled data were performed and odds ratios (ORs) were calculated and summarized.Final analysis was performed of 1,815 cancer patients from 19 eligible studies. We observed that DLC- 1 expression was significantly lower in cancers than in normal tissues. DLC-1 expression was not found to be associated with tumor differentiation status. However, DLC-1 expression was obviously lower in advance stage than in early-stage cancers and was more down-regulated in metastatic than non-metastatic cancers.The results of our meta-analysis suggested that DLC-1 expression is significantly lower in cancers than in normal tissues. Aberrant DLC-1 expression may play an important role in cancer genesis and   metastasis  . 
 DLC-1 is a candidate biomarker methylated and down-regulated in pancreatic ductal adenocarcinoma. Pancreatic ductal adenocarcinoma (PDA) is one of the most aggressive malignancies in humans, and its prognosis is generally poor even after surgery. Many advances have been made to understand the pathogenesis of PDA; however, the molecular mechanisms that lead to pancreatic carcinogenesis are still not clearly understood. The aims of this study were to investigate the relationship between DLC-1 methylation status and clinicopathological characteristics of PDA patients and evaluate the role of DLC-1 methylation status in PDA. The expression of DLC-1 mRNA in PDA tissues was analyzed by real-time PCR. The methylation status of DLC-1 was analyzed by methylation-specific polymerase chain reaction (MSP). Furthermore, we determined the prognostic importance of DLC-1 methylation status in PDA patients. Our results showed that the expression level of DLC-1 mRNA in PDA tissues was lower than that in non-cancerous tissues. The rate of DLC-1 promoter methylation was significantly higher in PDA tissues than in adjacent non-cancerous tissues (p < 0.001). Downregulation of DLC-1 was strongly correlated with promoter methylation (P = 0.003). The presence of DLC-1 methylation in PDA tissue samples was significantly correlated with clinical stage (P = 0.005), histological differentiation (P = 0.05), and lymph node   metastasis   (P = 0.006). Kaplan-Meier survival analysis showed that DLC-1 methylation status was inversely correlated with overall survival of the PDA patients. Further, Cox multivariate analysis indicated that DLC-1 methylation status was an independent prognostic factor for the overall survival rate of PDA patients. In conclusion, our data suggest that downregulation of DLC-1 may be explained by DNA methylation; DLC-1 may be a biomarker for PDA. 
 Differential regulation of the activity of deleted in liver cancer 1 (DLC1) by tensins controls cell migration and transformation. The epithelial growth factor receptor plays an important role in cell migration and cancer   metastasis  , but the underlying molecular mechanism is not fully understood. We show here that differential regulation of the Ras-homology-GTPase-activating protein [corrected] (Rho-GAP) activity of deleted in liver cancer 1 (DLC1) by tensin3 and COOH-terminal tensin-like protein (cten) controls EGF-driven cell migration and transformation. Tensin3 binds DLC1 through its actin-binding domain, a region that is missing in cten, and thereby releases an autoinhibitory interaction between the sterile alpha motif and Rho-GAP domains of DLC1. Consequently, tensin3, but not cten, promotes the activation of DLC1, which, in turn, leads to inactivation of RhoA and decreased cell migration. Depletion of endogenous tensin3, but not cten, augmented the formation of actin stress fibers and focal adhesions and enhanced cell motility. These effects were, however, ablated by an inhibitor of the Rho-associated protein kinase. Importantly, activation of DLC1 by tensin3 or its actin-binding domain drastically reduced the anchorage-independent growth of transformed cells. Our study therefore links dynamic regulation of tensin family members by EGF to Rho-GAP through DLC1 and suggests that the tensin-DLC1-RhoA signaling axis plays an important role in tumorigenesis and cancer   metastasis  , and may be explored for cancer intervention. 
 DLC-1 expression levels in breast cancer assessed by qRT- PCR are negatively associated with malignancy. The aim of this study was to explore the expression of DLC-l in breast carcinoma and any association with tumor   metastasis  .51 surgical specimens of human breast carcinoma, divided into high invasive and low invasive groups according to their clinicopathological features, 30 cases of adjacent normal tissue and 28 benign breast lesions were examined by qRT-PCR for expression of DLC-1.Expression level of DLC-1 in adjacent normal tissue and benign breast lesion specimens was higher than that in breast carcinoma (P<0.0001); the values in the high invasive group with synchronous metastases were also lower than in the low invasive group (P=0.0275). The correlation between DLC-1 expression level and tumor progression and   metastasis   of breast cancer was negative.As an anti-oncogene, DLC-1 could play an important part in breast carcinoma occurrence, progression, invasiveness and   metastasis  . Detecting the changes of the expression of DLC-1 in the breast carcinoma may contribute to earlier auxiliary diagnosis of invasiveness,   metastasis   and recrudescence. 
 DLC1-dependent parathyroid hormone-like hormone inhibition suppresses breast cancer bone   metastasis  . Bone   metastasis   is a frequent complication of breast cancer that is often accelerated by TGF-β signaling; however, little is known about how the TGF-β pathway is regulated during bon  e metastas  is. Here we report that deleted in liver cancer 1 (DLC1) is an important regulator of TGF-β responses and osteolyt  ic metasta  sis of breast cancer cells. In murine models, breast cancer cells lacking DLC1 expression exhibited enhanced capabilities of bo  ne metasta  sis. Knockdown of DLC1 in cancer cells promoted bo  ne metasta  sis, leading to manifested osteolysis and accelerated death in mice, while DLC1 overexpression suppressed bo  ne metasta  sis. Activation of Rho-ROCK signaling in the absence of DLC1 mediated SMAD3 linker region phosphorylation and TGF-β-induced expression of parathyroid hormone-like hormone (PTHLH), leading to osteoclast maturation for osteolytic colonization. Furthermore, pharmacological inhibition of Rho-ROCK effectively reduced PTHLH production and breast cancer b  one metast  asis in vitro and in vivo. Evaluation of clinical breast tumor samples revealed that reduced DLC1 expression was linked to elevated PTHLH expression and organ-speci  fic metast  asis to bone. Overall, our findings define a stroma-dependent paradigm of Rho signaling in cancer and implicate Rho-TGF-β crosstalk in osteolytic   bone metas  tasis. 
 Genome-wide analysis of chromosomal alterations in patients with esophageal squamous cell carcinoma exposed to tobacco and betel quid from high-risk area in India. Genomic alterations such as chromosomal amplifications, deletions and loss of heterozygosity play an important role in the pathogenesis and progression of cancer. Environmental risk factors contribute to the development and progression of tumors by facilitating the loss of tumor suppressor genes and amplification of oncogenes. In this current study, Affymetrix 10K single nucleotide polymorphism (SNP) arrays were used to evaluate genomic alterations in 20 pairs of matched germ-line and tumor DNA obtained from patients with esophageal squamous cell carcinoma (ESCC) from high-risk area of India where tobacco, betel quid and alcohol use are widespread. Twenty-two amplified regions and 16 deleted regions identified across chromosomal arms were biologically relevant. The candidate genes located at amplified regions of chromosomes or low-level gain regions such as PLA2G5 (1p36-p34), COL11A1 (1p21), KCNK2 (1q41), S100A3 (1q21), ENAH (1q42.12), RGS1 (1q31), KCNH1 (1q32-q41), INSIG2 (2q14.1), FGF12 (3q28), TRIO (5p15.2), RNASEN (5p15.2), FGF10 (5p13-p12), EDN1(6p24.1-p22.3), SULF1 (8q13.2-13.3), TLR4 (9q32-q33), TNC (9q33), NTRK2 (9q22.1), CD44 (11p13), NCAM1 (11q23.1), TRIM29 (11q22-q23), PAK1 (11q13-q14) and RAB27A (15q15-q21.1), are found to be associated with cellular migration and proliferation, tumor cell   metastasis   and invasion, anchorage independent growth and inhibition of apoptosis. The candidate genes located at deleted regions of chromosomes, such as FBLN2 (3p25.1), WNT7A (3p25), DLC1 (8p22), LZTS1 (8p22), CDKN2A (9p21), COL4A1 (13q34), CDK8 (13q12) and DCC (18q21.3), are found to be associated with the suppression of tumor. The suggested candidate genes were mostly involved in potential signaling pathways such as focal adhesion (COL4A1), tight junction (CLDN10), MAPK signaling pathway (FGF12) and neuroactive ligand receptor interaction pathway (CCKAR). Expression of FGF12 and COL4A1 was validated by tissue microarray. These unique copy number alteration profiles should be taken into consideration when developing biomarkers for the early detection of ESCC in high-risk areas of India in association with tobacco and betel quid use. 
 Akt phosphorylation of deleted in liver cancer 1 abrogates its suppression of liver cancer tumorigenesis and   metastasis  . Deleted in liver cancer 1 (DLC1), which encodes a Rho GTPase activating protein, is a bona fide tumor suppressor in hepatocellular carcinoma. Underexpression of DLC1 in cancer has been attributed to genomic deletion and epigenetic silencing. However, the regulatory mechanism of the tumor suppressive activity of DLC1 remains elusive. In this study, we elucidated a novel post-translational modification by which the activity of DLC1 is functionally regulated.Molecular and biochemical approaches were employed to study Akt phosphorylation of DLC1. In vitro and in vivo functional assays were performed to elucidate the functional significance of Akt phosphorylation of DLC1.Phosphorylation of ectopically expressed and endogenous DLC1 was enhanced upon insulin induction or with Akt expression in liver cancer cell lines. Conversely, addition of a phosphatidylinositol 3-kinase/Akt pathway inhibitor or silencing of Akt attenuated the phosphorylation level of DLC1. Site-directed mutagenesis was employed to replace the serine residue of the consensus Akt substrate motifs of DLC1 with alanine. S567 of DLC1 was identified as the only target of Akt phosphorylation. S567 is well conserved in all DLC family members. DLC2 was phosphorylated by Akt at the corresponding residue. Functional assays demonstrated that the S567D phosphomimetic DLC1 mutant lost its inhibitory activities in tumorigenesis and   metastasis   of oncogenically transformed hepatoblasts in a mouse model.This study has revealed a novel post-translational modification that functionally deregulates the biologic activities of DLC1. Phosphorylation of DLC1 and DLC2 by Akt at the conserved residue points to a common regulatory mechanism of the DLC tumor suppressor family. 
  GeneRIFs  
 DLC-1 protein expression was negatively associated with both overall survival and with distant   metastasis  -free survival, but not with disease-free survival. 
 The expression of DLC1 and PAI-1 were closely related with the   metastasis   and invasion of ovarian carcinoma, only the combination of DLC1 and PAI-1 could serve as an independent prognostic factor of ovarian carcinoma. 
 Evaluation of clinical breast tumor samples revealed that reduced DLC1 expression was linked to elevated PTHLH expression and organ-specific   metastasis   to bone. 
 The abnormal expression of DLC1 and p-FAK might participate in the carcinogenesis, progression, and   metastasis   of breast cancer. 
 study links dynamic regulation of tensin family members by EGF to Rho-GAP through DLC1 and suggests that the tensin-DLC1-RhoA signaling axis plays an important role in tumorigenesis and cancer   metastasis  , and may be explored for cancer intervention 
  [Cc]ell [Ii]nvasion  PubMed  
 DLC1 interaction with S100A10 mediates inhibition of in vitro   cell invasion   and tumorigenicity of lung cancer cells through a RhoGAP-independent mechanism. The DLC1 gene encodes a Rho GTPase-activating protein (RhoGAP) that functions as a tumor suppressor in several common human cancers. The multidomain structure of DLC1 enables interaction with a number of other proteins. Here we report that the proinflammatory protein S100A10 (also known as p11), a key cell surface receptor for plasminogen which regulates pericellular proteolysis and tumor   cell invasion  , is a new binding partner of DLC1 in human cells. We determined that the 2 proteins colocalize in the cell cytoplasm and that their binding is mediated by central sequences in the central domain of DLC1 and the C-terminus of S100A10. Because the same S100A10 sequence also mediates binding to Annexin 2, we found that DLC1 competed with Annexin 2 for interaction with S100A10. DLC1 binding to S100A10 did not affect DLC1's RhoGAP activity, but it decreased the steady-state level of S100A10 expression in a dose-dependent manner by displacing it from Annexin 2 and making it accessible to ubiquitin-dependent degradation. This process attenuated plasminogen activation and resulted in inhibition of in vitro cell migration, invasion, colony formation, and anchorage-independent growth of aggressive lung cancer cells. These results suggest that a novel GAP-independent mechanism contributes to the tumor suppressive activity of DLC1, and highlight the importance and complexity of protein-protein interactions involving DLC1 in certain cancers. 
 DLC1 induces expression of E-cadherin in prostate cancer cells through Rho pathway and suppresses invasion. E-cadherin is a cell-cell adhesion molecule that acts as a suppressor of cancer   cell invasion   and its expression is downregulated in many advanced, poorly differentiated, human cancers. In this study, we found that the expression of DLC1 (deleted in liver cancer 1) tumor-suppressor gene in metastatic prostate carcinoma (PCA) cells increased the expression of E-cadherin and resulted in an elevated rate of cell-cell aggregation as measured by aggregation assay. DLC1-mediated increase in E-cadherin expression was not dependent on α-catenin, a DLC1-binding protein associated with E-cadherin, and/or cellular density. The increase of E-cadherin expression occurred at mRNA level and relied on DLC1 RhoGAP function, leading to suppression of high level of RhoA-GTP and RhoC-GTP activity in metastatic PCA cells. Application of Rho/ROCK inhibitors produced the same effect as introduction of DLC1. Knocking down of RhoA produced a moderate increase in E-cadherin whereas knocking down of RhoC resulted in a significant increase of E-cadherin. Downregulation of E-cadherin caused by constitutively active RhoA(V14) and RhoC(V14) could not be reversed by expression of DLC1 in DLC1-negative cell line. DLC1-mediated suppression of metastatic PCA cells invasion was comparable with the one associated with ectopic E-cadherin expression, or caused by suppression of Rho pathway either by Rho/ROCK inhibitors, or by shRNA repression. This study demonstrates that DLC1 expression positively regulates E-cadherin and suppresses highly metastatic PCA   cell invasio  n by modulating Rho pathway, which appears as a feasible therapeutic target in cancers with high activity of RhoGTPases. 
 DLC1 tumor suppressor gene inhibits migration and invasion of multiple myeloma cells through RhoA GTPase pathway. DLC1 (deleted in liver cancer 1), a tumor suppressor gene that encodes a RhoGTPase-activating protein, is recurrently downregulated or silenced in various solid tumors and hematological malignancies because of epigenetic modifications or genomic deletion. Here, we identified DLC1 promoter hypermethylation in 43 out of 44 multiple myeloma (MM) cell lines, which resulted in downregulation or silencing of DLC1 in 41 samples. High frequency of tumor-specific methylation and attenuation or silencing of DLC1 expression could serve as an independent diagnostic marker for MM. Combined treatment with demethylating and acetylating agents significantly elevated the expression of DLC1 and suppressed MM cell proliferation. Two cell lines exhibiting complete promoter methylation and the absence of DLC1 expression were transduced by an adenoviral vector containing DLC1 cDNA. In both cell lines, the reexpression of DLC1 inhibited myeloma   cell invasion   and migration, reduced RhoA activity and resulted in the reorganization of actin cytoskeleton. These results provide the first evidence for the antiproliferative effect of DLC1 in a hematological cancer and implicate RhoA pathway in suppression of MM migration and invasion. Given the myeloma cells sensitivity to the reactivation of DLC1 function, the potential for molecular targeted therapy of DLC1-mediated pathways as well as epigenetic therapies hold prospects. 
  GeneRIFs  
 Study demonstrates that DLC1 expression positively regulates E-cadherin and suppresses highly metastatic PCA   cell invasion   by modulating Rho pathway. 
   
  
 USO1 
 
  NCBI Gene  
  GeneRIF  
  Pubmed  
 
 
  Search Term  Rank  Fields  
  [Mm]igration  6  PubMed(3); GeneRIFs(1)  
 
 
 hide/show details for USO1 
  [Mm]igration  PubMed  
 P115 promotes growth of gastric cancer through interaction with macrophage   migration   inhibitory factor. To investigate the role of P115 in the proliferation of gastric cancer cells and the mechanism involved.The RNA and protein level of P115 and macrophage   migration   inhibitory factor (MIF) in gastric cancer and normal gastric tissue/cells were measured and the effect of P115 on cell proliferation was assessed. The role of P115 in cell cycle checkpoints was investigated and the related proteins and signaling pathways, such as cyclin D1, Mcm2, p53, PCNA as well as the MAPK signaling pathway were determined. The interaction between P115 and MIF and the effect of P115 on MIF secretion were examined. The data were analyzed via one-way ANOVA comparisons between groups and P < 0.05 was considered significant.P115 and MIF were both specifically expressed in gastric cancer tissues compared with normal gastric mucosa (both P < 0.01). The mRNA and protein levels of P115 and MIF in gastric cancer cell lines MKN-28 and BGC-823 were higher than in the human gastric epithelial cell line GES-1 (both P < 0.01). In MKN-28 and BGC-823 cell lines, P115 promoted cell proliferation and G0-G1 to S phase transition. In addition, several cell cycle-related regulators, including cyclin D1, Mcm2, PCNA, pERK1/2 and p53 were up-regulated by P115. Furthermore, the interaction between P115 and MIF was confirmed by co-immunoprecipitation assay. ELISA showed that P115 stimulated the secretion of MIF into the culture supernatant (P < 0.01) and the compensative expression of MIF in cells was observed by Western blotting.P115 promotes proliferation of gastric cancer cells through an interaction with MIF. 
 Lentivirus-mediated silencing of USO1 inhibits cell proliferation and   migration   of human colon cancer cells. Belonging to the tether factors family, USO1 (vesicle transport factor) plays a critical role in endoplasmic reticulum-Golgi trafficking and vesicular transport which is important to tumorigenesis. However, the mechanism of USO1 in colon cancer was still unknown. In our research, the expression of USO1 was knockdown in colon cancer cells (HCT116 and HT-29) by using a special lentivirus shRNA approach. A series of experiments were carried out to evaluate the effect of deregulation of USO1, including cell proliferation, apoptosis, cell   migration   and cell cycle. Knockdown of USO1 inhibits the ability of cell proliferation and   migration  . Furthermore, the deregulation of USO1 induces early apoptosis and decreased cells in G2-M phase. We demonstrate for the first time that USO1 gene has a critical role in human colon cancer. Our finding represents that USO1 gene may be a promising target for therapy and diagnosis in treatment of human colon cancer. 
 The Golgi-associated protein p115 mediates the secretion of macrophage   migration   inhibitory factor. Macrophage   migration   inhibitory factor (MIF) is a leaderless protein that is secreted from cells by a specialized, nonclassical export pathway. The release of MIF nevertheless is regulated and its production in response to different inflammatory, mitogenic, and hormonal stimuli plays an important role in diverse physiologic and pathologic processes. We report herein the identification of the Golgi complex-associated protein p115 as an intracellular binding partner for MIF. MIF interacts with p115 in the cytoplasm and the stimulated secretion of MIF results in the accumulation of both proteins in supernatants, which is consistent with MIF release from cells in conjunction with p115. The depletion of p115 from monocytes/macrophages decreases the release of MIF but not other cytokines following inflammatory stimulation or intracellular bacterial infection. Notably, the small molecule MIF inhibitor 4-iodo-6-phenylpyrimidine inhibits MIF secretion by targeting the interaction between MIF and p115. These data reveal p115 to be a critical intermediary component in the regulated secretion of MIF from monocytes/macrophages. 
  GeneRIFs  
 USO1 gene has a critical role in human colon cancer: USO1 is involved in cell proliferation and cell   migration   of colon cancer cells. 
   
  
 MAPRE1 
 
  NCBI Gene  
  GeneRIF  
  Pubmed  
 
 
  Search Term  Rank  Fields  
  [Mm]igration  6  Process(1); PubMed(7); GeneRIFs(2)  
  [Ii]nvasive  10  PubMed(2)  
 
 
 hide/show details for MAPRE1 
  [Mm]igration  Process  
 positive regulation of cell   migration   
  PubMed  
 Amer2 protein interacts with EB1 protein and adenomatous polyposis coli (APC) and controls microtubule stability and cell   migration  . EB1 is key factor in the organization of the microtubule cytoskeleton by binding to the plus-ends of microtubules and serving as a platform for a number of interacting proteins (termed +TIPs) that control microtubule dynamics. Together with its direct binding partner adenomatous polyposis coli (APC), EB1 can stabilize microtubules. Here, we show that Amer2 (APC membrane recruitment 2), a previously identified membrane-associated APC-binding protein, is a direct interaction partner of EB1 and acts as regulator of microtubule stability together with EB1. Amer2 binds to EB1 via specific (S/T)xIP motifs and recruits it to the plasma membrane. Coexpression of Amer2 and EB1 generates stabilized microtubules at the plasma membrane, whereas knockdown of Amer2 leads to destabilization of microtubules. Knockdown of Amer2, APC, or EB1 reduces cell   migration  , and morpholino-mediated down-regulation of Xenopus Amer2 blocks convergent extension cell movements, suggesting that the Amer2-EB1-APC complex regulates cell   migration   by altering microtubule stability. 
 An EB1-binding motif acts as a microtubule tip localization signal. Microtubules are filamentous polymers essential for cell viability. Microtubule plus-end tracking proteins (+TIPs) associate with growing microtubule plus ends and control microtubule dynamics and interactions with different cellular structures during cell division,   migration  , and morphogenesis. EB1 and its homologs are highly conserved proteins that play an important role in the targeting of +TIPs to microtubule ends, but the underlying molecular mechanism remains elusive. By using live cell experiments and in vitro reconstitution assays, we demonstrate that a short polypeptide motif, Ser-x-Ile-Pro (SxIP), is used by numerous +TIPs, including the tumor suppressor APC, the transmembrane protein STIM1, and the kinesin MCAK, for localization to microtubule tips in an EB1-dependent manner. Structural and biochemical data reveal the molecular basis of the EB1-SxIP interaction and explain its negative regulation by phosphorylation. Our findings establish a general "microtubule tip localization signal" (MtLS) and delineate a unifying mechanism for this subcellular protein targeting process. 
 Amer2 protein interacts with EB1 protein and adenomatous polyposis coli (APC) and controls microtubule stability and cell   migration  . EB1 is key factor in the organization of the microtubule cytoskeleton by binding to the plus-ends of microtubules and serving as a platform for a number of interacting proteins (termed +TIPs) that control microtubule dynamics. Together with its direct binding partner adenomatous polyposis coli (APC), EB1 can stabilize microtubules. Here, we show that Amer2 (APC membrane recruitment 2), a previously identified membrane-associated APC-binding protein, is a direct interaction partner of EB1 and acts as regulator of microtubule stability together with EB1. Amer2 binds to EB1 via specific (S/T)xIP motifs and recruits it to the plasma membrane. Coexpression of Amer2 and EB1 generates stabilized microtubules at the plasma membrane, whereas knockdown of Amer2 leads to destabilization of microtubules. Knockdown of Amer2, APC, or EB1 reduces cell   migration  , and morpholino-mediated down-regulation of Xenopus Amer2 blocks convergent extension cell movements, suggesting that the Amer2-EB1-APC complex regulates cell   migration   by altering microtubule stability. 
 ROS-mediated EB1 phosphorylation through Akt/GSK3β pathway: implication in cancer cell response to microtubule-targeting agents. Microtubule-targeting agents (MTAs) are largely administered in adults and children cancers. Better deciphering their mechanism of action is of prime importance to develop more convenient therapy strategies. Here, we addressed the question of how reactive oxygen species (ROS) generation by mitochondria can be necessary for MTA efficacy. We showed for the first time that EB1 associates with microtubules in a phosphorylation-dependent manner, under control of ROS. By using phospho-defective mutants, we further characterized the Serine 155 residue as critical for EB1 accumulation at microtubule plus-ends, and both cancer cell   migratio  n and proliferation. Phosphorylation of EB1 on the Threonine 166 residue triggered opposite effects, and was identified as a requisite molecular switch in MTA activities. We then showed that GSK3β activation was responsible for MTA-triggered EB1 phosphorylation, resulting from ROS-mediated inhibition of upstream Akt. We thus disclosed here a novel pathway by which generation of mitochondrial ROS modulates microtubule dynamics through phosphorylation of EB1, improving our fundamental knowledge about this oncogenic protein, and pointing out the need to re-examine the current dogma of microtubule targeting by MTAs. The present work also provides a strong mechanistic rational to the promising therapeutic strategies that currently combine MTAs with anti-Akt targeted therapies. 
 CYLD coordinates with EB1 to regulate microtubule dynamics and cell   migration  . Cylindromatosis (CYLD), a deubiquitinase involved in inflammation and tumorigenesis via the modulation of cell signaling, has recently been identified as a critical regulator of microtubule dynamics. CYLD has also been shown to stimulate cell   migration   and thereby contribute to normal physiological processes. However, it remains elusive how the regulation of microtubule dynamic properties by CYLD is connected to its role in mediating cell   migration  . In this study, we performed yeast 2-hybrid screening with CYLD as bait and identified 7 CYLD-interacting proteins, including end-binding protein 1 (EB1). The CYLD-EB1 interaction was confirmed both in cells and in vitro, and these 2 proteins colocalized at the plus ends of microtubules. Interestingly, the association of CYLD with EB1 was significantly increased upon the stimulation of cell   migration  . CYLD coordinated with EB1 to orchestrate tail retraction, centrosome reorientation, and leading-edge microtubule stabilization in migratory cells. In addition, CYLD acted in concert with EB1 to regulate microtubule assembly in vitro, microtubule nucleation at the centrosome, and microtubule growth at the cell periphery. These data provide mechanistic insights into the actions of CYLD in the regulation of microtubule dynamics and cell   migration  . These findings also support the notion that coordinated actions of microtubule-binding proteins are critical for microtubule-mediated cellular events. 
 Interactions between the Microtubule Binding Protein EB1 and F-Actin. Many cellular processes including cell division and cell   migration   require coordination between the actin and microtubule (MT) cytoskeletons. This coordination is as-yet poorly understood, but proteins such as formins and IQGAP1 are known to be involved. We show that the MT binding protein EB1 (end-binding protein 1), a key regulator of MT dynamics, can bind directly to filamentous actin (F-actin) F-actin. We determined that the EB1:F-actin interaction is salt sensitive and weak under physiological salt concentrations but might be relevant in contexts where the local concentration of actin is high. Using bioinformatics and mutagenesis, we found that the EB1:F-actin binding site partially overlaps the well-characterized EB1:MT binding interface. Congruently, competition experiments indicate that EB1 can bind to F-actin or MTs but not both simultaneously. These observations suggest that EB1:F-actin interactions may negatively regulate EB1:MT interactions, and we speculate that this interaction may assist cells in differentially regulating MT stability in the actin-rich cortex as opposed to the cell interior. 
 TIP150 interacts with and targets MCAK at the microtubule plus ends. The microtubule (MT) cytoskeleton orchestrates the cellular plasticity and dynamics that underlie morphogenesis and cell division. Growing MT plus ends have emerged as dynamic regulatory machineries in which specialized proteins-called plus-end tracking proteins (+TIPs)-bind to and control the plus-end dynamics that are essential for cell division and   migration  . However, the molecular mechanisms underlying the plus-end regulation by +TIPs at spindle and astral MTs have remained elusive. Here, we show that TIP150 is a new +TIP that binds to end-binding protein 1 (EB1) in vitro and co-localizes with EB1 at the MT plus ends in vivo. Suppression of EB1 eliminates the plus-end localization of TIP150. Interestingly, TIP150 also binds to mitotic centromere-associated kinesin (MCAK), an MT depolymerase that localizes to the plus end of MTs. Suppression of TIP150 diminishes the plus-end localization of MCAK. Importantly, aurora B-mediated phosphorylation disrupts the TIP150-MCAK association in vitro. We reason that TIP150 facilitates the EB1-dependent loading of MCAK onto MT plus ends and orchestrates the dynamics at the plus end of MTs. 
  GeneRIFs  
 The CYLD-EB1 interaction was confirmed both in cells and in vitro, and these 2 proteins colocalized at the plus ends of microtubules. The association of CYLD with EB1 was significantly increased upon the stimulation of cell   migration  . 
 the Amer2-EB1-APC complex regulates cell   migration   by altering microtubule stability. 
  [Ii]nvasive  PubMed  
 Centrosome-related genes, genetic variation, and risk of breast cancer. Centrosome amplification has been detected in premalignant lesions and in situ tumors in the breast and in over 70% of   invasive   breast tumors, and has been associated with aneuploidy and tumor development. Based on these observations, the contribution of commonly inherited genetic variation in candidate genes related to centrosome structure and function to breast cancer risk was evaluated in an association study. Seven-hundred and 82 single nucleotide polymorphisms (SNPs) from 101 centrosomal genes were analyzed in 798 breast cancer cases and 843 controls from the Mayo Clinic Breast Cancer Study to assess the association between these SNPs (both individually and combined) and risk of breast cancer in this population. Eleven SNPs out of 782 from six genes displayed associations with breast cancer risk (P < 0.01). Haplotypes in five genes also displayed significant associations with risk. A two SNP combination of rs10145182 in NIN and rs2134808 in the TUBG1 locus (P-interaction = 0.00001), suggested SNPs in mediators of microtubule nucleation from the centrosome contribute to breast cancer. Evaluation of the simultaneous significance of all SNPs in the centrosome pathway suggested that the centrosome pathway is highly enriched (P = 4.76 × 10(-50)) for SNPs that are associated with breast cancer risk. Collections of weakly associated genetic variants in the centrosome pathway, rather than individual highly significantly associated SNPs, may account for a putative role for the centrosome pathway in predisposition to breast cancer. 
 Centrosome-related genes, genetic variation, and risk of breast cancer. Centrosome amplification has been detected in premalignant lesions and in situ tumors in the breast and in over 70% of   invasive   breast tumors, and has been associated with aneuploidy and tumor development. Based on these observations, the contribution of commonly inherited genetic variation in candidate genes related to centrosome structure and function to breast cancer risk was evaluated in an association study. Seven-hundred and 82 single nucleotide polymorphisms (SNPs) from 101 centrosomal genes were analyzed in 798 breast cancer cases and 843 controls from the Mayo Clinic Breast Cancer Study to assess the association between these SNPs (both individually and combined) and risk of breast cancer in this population. Eleven SNPs out of 782 from six genes displayed associations with breast cancer risk (P < 0.01). Haplotypes in five genes also displayed significant associations with risk. A two SNP combination of rs10145182 in NIN and rs2134808 in the TUBG1 locus (P-interaction = 0.00001), suggested SNPs in mediators of microtubule nucleation from the centrosome contribute to breast cancer. Evaluation of the simultaneous significance of all SNPs in the centrosome pathway suggested that the centrosome pathway is highly enriched (P = 4.76 × 10(-50)) for SNPs that are associated with breast cancer risk. Collections of weakly associated genetic variants in the centrosome pathway, rather than individual highly significantly associated SNPs, may account for a putative role for the centrosome pathway in predisposition to breast cancer. 
   
  
 PTPRG 
 
  NCBI Gene  
  GeneRIF  
  Pubmed  
 
 
  Search Term  Rank  Fields  
  [Mm]igration  6  Process(1); PubMed(1)  
  [Ee]xtracellular [Mm]atrix  8  PubMed(2)  
  [Ii]nvasive  10  PubMed(1)  
  [Cc]ell [Ii]nvasion  13  PubMed(1)  
 
 
 hide/show details for PTPRG 
  [Mm]igration  Process  
 negative regulation of epithelial cell   migration   
  PubMed  
 Receptor tyrosine phosphatase PTPγ is a regulator of spinal cord neurogenesis. During spinal cord development the proliferation,   migratio  n and survival of neural progenitors and precursors is tightly controlled, generating the fine spatial organisation of the cord. In order to understand better the control of these processes, we have examined the function of an orphan receptor protein tyrosine phosphatase (RPTP) PTPγ, in the developing chick spinal cord. Widespread expression of PTPγ occurs post-embryonic day 3 in the early cord and is consistent with a potential role in either neurogenesis or neuronal maturation. Using gain-of-function and loss-of-function approaches in ovo, we show that PTPγ perturbation significantly reduces progenitor proliferation rates and neuronal precursor numbers, resulting in hypoplasia of the neuroepithelium. PTPγ gain-of-function causes widespread suppression of Wnt/β-catenin-driven TCF signalling. One potential target of PTPγ may therefore be β-catenin itself, since PTPγ can dephosphorylate it in vitro, but alternative targets are also likely. PTPγ loss-of-function is not sufficient to alter TCF signalling. Instead, loss-of-function leads to increased apoptosis and defective cell-cell adhesion in progenitors and precursors. Furthermore, motor neuron   precursor   migration is specifically defective. PTPγ therefore regulates neurogenesis during a window of spinal cord development, with molecular targets most likely related to Wnt/β-catenin signalling, cell survival and cell adhesion. 
  [Ee]xtracellular [Mm]atrix  PubMed  
 E2-2 protein and Fuchs's corneal dystrophy. Fuchs's corneal dystrophy (FCD) is a leading cause of corneal transplantation and affects 5% of persons in the United States who are over the age of 40 years. Clinically visible deposits called guttae develop under the corneal endothelium in patients with FCD. A loss of endothelial cells and deposition of an abnormal   extracellular matrix   are observed microscopically. In advanced disease, the cornea swells and becomes cloudy because the remaining endothelial cells are not sufficient to keep the cornea dehydrated and clear. Although rare genetic variation that contributes to both early-onset and typical late-onset forms of FCD has been identified, to our knowledge, no common variants have been reported.We performed a genomewide association study and replicated the most significant observations in a second, independent group of subjects.Alleles in the transcription factor 4 gene (TCF4), encoding a member of the E-protein family (E2-2), were associated with typical FCD (P=2.3x10(-26)). The association increased the odds of having FCD by a factor of 30 for persons with two copies of the disease variants (homozygotes) and discriminated between case subjects and control subjects with about 76% accuracy. At least two regions of the TCF4 locus were associated independently with FCD. Alleles in the gene encoding protein tyrosine phosphatase receptor type G (PTPRG) were associated with FCD (P=4.0x10(-7)), but the association did not reach genomewide significance.Genetic variation in TCF4 contributes to the development of FCD. (Funded by the National Eye Institute and others.) 
 Functional analysis of a cell cycle-associated, tumor-suppressive gene, protein tyrosine phosphatase receptor type G, in nasopharyngeal carcinoma. Functional studies to identify the potential role of a chromosome 3p14-21 gene, protein tyrosine phosphatase receptor type G (PTPRG), were performed. PTPRG was identified as a candidate tumor suppressor gene (TSG) in nasopharyngeal carcinoma (NPC) by differential gene profiling of tumorigenic and nontumorigenic NPC chromosome 3 microcell hybrids (MCH). Down-regulation of this gene was found in tumor segregants when compared with their corresponding tumor-suppressive MCHs, as well as in NPC cell lines and tumor biopsies. Promoter hypermethylation and loss of heterozygosity were found to be important mechanisms contributing to PTPRG silencing. PTPRG overexpression in NPC cell lines induces growth suppression and reduced anchorage-independent growth in vitro. This is the first study to use a tetracycline-responsive vector expression system to study PTPRG stable transfectants. Results indicate its ability to induce significant tumor growth suppression in nude mice under conditions activating transgene expression. These studies now provide functional evidence indicating critical interactions of PTPRG in the   extracellular matrix   milieu induce cell arrest and changes in cell cycle status. This is associated with inhibition of pRB phosphorylation through down-regulation of cyclin D1. These novel findings enhance our current understanding of how PTPRG may contribute to tumorigenesis. 
  [Ii]nvasive  PubMed  
 Identification of PTPN23 as a novel regulator of cell invasion in mammary epithelial cells from a loss-of-function screen of the 'PTP-ome'. We used an RNAi-mediated loss-of-function screen to study systematically the role of the protein tyrosine phosphatase (PTP) superfamily of enzymes in mammary epithelial cell motility in the absence or presence of the oncoprotein tyrosine kinase ERBB2. We report that although shRNAs directed against most of the PTP family were without effect, suppression of three PTPs-PRPN23, PTPRG, and PTPRR-enhanced cell motility. Furthermore, we found that suppression of PTPN23, but not PTPRG or PTPRR, induced cell invasion. Suppression of PTPN23 increased E-cadherin internalization, impaired early endosome trafficking of E-cadherin, induced the expression of mesenchymal proteins, and caused cell scattering. The activity of SRC and β-catenin was elevated when PTPN23 was suppressed. Moreover, we identified SRC, E-cadherin, and β-catenin as direct substrates of PTPN23. Inhibition of SRC with the small molecular inhibitor SU6656 blocked the effects of PTPN23 depletion. These findings suggest that loss of PTPN23 may increase the activity of SRC and the phosphorylation status of the E-cadherin/β-catenin signaling complex to promote tumor growth a  nd invas  ive behavior in breast cancer. In addition, our studies highlight functional specificity among PTPs and reveal new roles for PTPs in mammary epithelial cell biology. 
  [Cc]ell [Ii]nvasion  PubMed  
 Identification of PTPN23 as a novel regulator of   cell invasion   in mammary epithelial cells from a loss-of-function screen of the 'PTP-ome'. We used an RNAi-mediated loss-of-function screen to study systematically the role of the protein tyrosine phosphatase (PTP) superfamily of enzymes in mammary epithelial cell motility in the absence or presence of the oncoprotein tyrosine kinase ERBB2. We report that although shRNAs directed against most of the PTP family were without effect, suppression of three PTPs-PRPN23, PTPRG, and PTPRR-enhanced cell motility. Furthermore, we found that suppression of PTPN23, but not PTPRG or PTPRR, induced   cell invasion  . Suppression of PTPN23 increased E-cadherin internalization, impaired early endosome trafficking of E-cadherin, induced the expression of mesenchymal proteins, and caused cell scattering. The activity of SRC and β-catenin was elevated when PTPN23 was suppressed. Moreover, we identified SRC, E-cadherin, and β-catenin as direct substrates of PTPN23. Inhibition of SRC with the small molecular inhibitor SU6656 blocked the effects of PTPN23 depletion. These findings suggest that loss of PTPN23 may increase the activity of SRC and the phosphorylation status of the E-cadherin/β-catenin signaling complex to promote tumor growth and invasive behavior in breast cancer. In addition, our studies highlight functional specificity among PTPs and reveal new roles for PTPs in mammary epithelial cell biology. 
   
  
 GNAL 
 
  NCBI Gene  
  GeneRIF  
  Pubmed  
 
 
  Search Term  Rank  Fields  
  [Ii]nvasive  10  PubMed(1)  
  [Mm]etastasis  12  PubMed(1)  
 
 
 hide/show details for GNAL 
  [Ii]nvasive  PubMed  
 G-protein alpha(olf) subunit promotes cellular invasion, survival, and neuroendocrine differentiation in digestive and urogenital epithelial cells. The heterotrimeric G-protein subunits Galpha and Gbetagamma are involved in cellular transformation and tumor development. Here, we report the expression of Galpha(olf) in human digestive and urogenital epithelial cells using RT-PCR and Western blot. When the constitutively activated form of Galpha(olf)Q214L (AGalpha(olf)) was stably transfected in canine kidney MDCKts.src and human colonic HCT-8/S11 epithelial cells, it induced cellular invasion in collagen gels. AGalpha(olf)-mediated invasion was abrogated by agonists of platelet activating factor receptors (PAF-R) and protease-activated receptors -1 (PAR-1), pharmacological inhibitors of PI3'-Kinase (wortmannin), protein kinase C (Gö6976 and GF109203X), Rho GTPase (C3T exoenzyme), but was independent of protein kinase A. Accordingly, the   invasiv  e phenotype induced by AGalpha(olf) in HCT-8/S11 cells was reversed by the RhoA antagonist RhoD (G26V). Although AGalpha(olf) protected MDCKts.src cells against serum starvation-mediated apoptosis via a Rho-independent pathway, both AGalpha(olf) and Rho inhibition by C3T induced neuroendocrine-like differentiation linked to extensive neurite outgrowth and parathyroid hormone-related protein expression in human prostatic LNCaP-AGalpha(olf) cells. Since prostate tumors with a larger neuroendocrine cell population display increased   invasiv  eness, persistent activation of the G-protein alpha(olf) may exert convergent adverse effects on cellular invasion and survival in solid tumors during the neoplastic progression towards metastasis. doi:10.1038/sj.onc.1205498 
  [Mm]etastasis  PubMed  
 G-protein alpha(olf) subunit promotes cellular invasion, survival, and neuroendocrine differentiation in digestive and urogenital epithelial cells. The heterotrimeric G-protein subunits Galpha and Gbetagamma are involved in cellular transformation and tumor development. Here, we report the expression of Galpha(olf) in human digestive and urogenital epithelial cells using RT-PCR and Western blot. When the constitutively activated form of Galpha(olf)Q214L (AGalpha(olf)) was stably transfected in canine kidney MDCKts.src and human colonic HCT-8/S11 epithelial cells, it induced cellular invasion in collagen gels. AGalpha(olf)-mediated invasion was abrogated by agonists of platelet activating factor receptors (PAF-R) and protease-activated receptors -1 (PAR-1), pharmacological inhibitors of PI3'-Kinase (wortmannin), protein kinase C (Gö6976 and GF109203X), Rho GTPase (C3T exoenzyme), but was independent of protein kinase A. Accordingly, the invasive phenotype induced by AGalpha(olf) in HCT-8/S11 cells was reversed by the RhoA antagonist RhoD (G26V). Although AGalpha(olf) protected MDCKts.src cells against serum starvation-mediated apoptosis via a Rho-independent pathway, both AGalpha(olf) and Rho inhibition by C3T induced neuroendocrine-like differentiation linked to extensive neurite outgrowth and parathyroid hormone-related protein expression in human prostatic LNCaP-AGalpha(olf) cells. Since prostate tumors with a larger neuroendocrine cell population display increased invasiveness, persistent activation of the G-protein alpha(olf) may exert convergent adverse effects on cellular invasion and survival in solid tumors during the neoplastic progression towards   metastasi  s. doi:10.1038/sj.onc.1205498 
   
  
 HSPA1A 
 
  NCBI Gene  
  GeneRIF  
  Pubmed  
 
 
  Search Term  Rank  Fields  
  [Mm]igration  6  PubMed(5); GeneRIFs(1)  
  [Ee]xtracellular [Mm]atrix  8  PubMed(2)  
  [Mm]etastasis  12  PubMed(12)  
 
 
 hide/show details for HSPA1A 
  [Mm]igration  PubMed  
 Analysis of the myosin-II-responsive focal adhesion proteome reveals a role for β-Pix in negative regulation of focal adhesion maturation. Focal adhesions undergo myosin-II-mediated maturation wherein they grow and change composition to modulate integrin signalling for cell   migratio  n, growth and differentiation. To determine how focal adhesion composition is affected by myosin II activity, we performed proteomic analysis of isolated focal adhesions and compared protein abundance in focal adhesions from cells with and without myosin II inhibition. We identified 905 focal adhesion proteins, 459 of which changed in abundance with myosin II inhibition, defining the myosin-II-responsive focal adhesion proteome. The abundance of 73% of the proteins in the myosin-II-responsive focal adhesion proteome was enhanced by contractility, including proteins involved in Rho-mediated focal adhesion maturation and endocytosis- and calpain-dependent focal adhesion disassembly. During myosin II inhibition, 27% of proteins in the myosin-II-responsive focal adhesion proteome, including proteins involved in Rac-mediated lamellipodial protrusion, were enriched in focal adhesions, establishing that focal adhesion protein recruitment is also negatively regulated by contractility. We focused on the Rac guanine nucleotide exchange factor β-Pix, documenting its role in the negative regulation of focal adhesion maturation and the promotion of lamellipodial protrusion and focal adhesion turnover to drive cel  l migrati  on. 
 Quercetin suppresses heat shock-induced nuclear translocation of Hsp72. The effect of quercetin and heat shock on the Hsp72 level and distribution in HeLa cells was studied by Western blotting, indirect immunofluorescence and immunogold electron microscopy. In control cells and after quercetin treatment, Hsp72 was located both in the cytoplasm and in the nucleus in comparable amounts. After hyperthermia, the level of nuclear Hsp72 raised dramatically. Expression of Hsp72 in cytoplasm was also higher but not to such extent as that observed in the nucleus. Preincubation of heated cells with quercetin inhibited strong Hsp72 expression observed after hyperthermia and changed the intracellular Hsp72 distribution. The cytoplasmic level of protein exceeded the nuclear one, especially around the nucleus, where the coat of Hsp72 was noticed. Observations indicating that quercetin was present around and in the nuclear envelope suggested an involvement of this drug in the inhibition of nuclear translocation. Our results indicate that pro-apoptotic activity of quercetin may be correlated not only with the inhibition of Hsp72 expression but also with suppression of its   migration   to the nucleus. 
 Heat shock protein 70-1A is a novel angiogenic regulator. Heat shock protein 70-1A (HSP70-1A) is a stress-inducible protein that provides an essential intracellular molecular chaperone function; however, the mechanism of HSP70-1A in angiogenesis has not been clarified. Herein, HSP70-1A gene silencing implicated this protein in angiogenesis. Additionally, recombinant human HSP70-1A (rhHSP70-1A) was able to stimulate human umbilical vein endothelial cell (HUVEC)   migration   and tube formation in vitro and microvessel formation in vivo similarly to recombinant human vascular endothelial growth factor (rhVEGF). Furthermore, rhHSP70-1A was tightly bound to the surface of HUVECs and participated in extracellular signal-related kinase (ERK)-dependent angiogenesis. Together, these results implicate HSP70-1A as a novel angiogenic regulator. 
 Stress-induced localization of HSPA6 (HSP70B') and HSPA1A (HSP70-1) proteins to centrioles in human neuronal cells. The localization of yellow fluorescent protein (YFP)-tagged HSP70 proteins was employed to identify stress-sensitive sites in human neurons following temperature elevation. Stable lines of human SH-SY5Y neuronal cells were established that expressed YFP-tagged protein products of the human inducible HSP70 genes HSPA6 (HSP70B') and HSPA1A (HSP70-1). Following a brief period of thermal stress, YFP-tagged HSPA6 and HSPA1A rapidly appeared at centrioles in the cytoplasm of human neuronal cells, with HSPA6 demonstrating a more prolonged signal compared to HSPA1A. Each centriole is composed of a distal end and a proximal end, the latter linking the centriole doublet. The YFP-tagged HSP70 proteins targeted the proximal end of centrioles (identified by γ-tubulin marker) rather than the distal end (centrin marker). Centrioles play key roles in cellular polarity and   migratio  n during neuronal differentiation. The proximal end of the centriole, which is involved in centriole stabilization, may be stress-sensitive in post-mitotic, differentiating human neurons. 
 Bmx is a downstream Rap1 effector in VEGF-induced endothelial cell activation. We had previously shown that Rap1 mediates certain of the signaling pathways involved in VEGF-induced endothelial cell   migration  , although the downstream Rap1 effectors are not known. Towards the goal of identifying those effectors, we utilized a commercially available antibody array filter to identify proteins that either directly interact with Rap1 or interact indirectly through a multi-protein complex. The protocol identified 10 possible Rap1-interacting proteins, including the Bmx non-receptor tyrosine kinase. The conclusion that VEGF treatment leads to a Rap1/Bmx complex was confirmed by an experiment in which cell lysates from VEGF and control cells were immunoprecipitated with Bmx antibodies and Western blotting was done using anti-Rap1 antibodies. VEGF treatment led to the recruitment of Bmx to the CAS scaffolding protein, and inhibition of the Bmx kinase blocked VEGF-induced cell   migration  . Formation of a Rap1/Bmx complex was not observed in cells transfected with an expression vector for a dominant-negative Rap1, indicating that Bmx is a downstream Rap1 effector in VEGF-induced endothelial cell activation. 
  GeneRIFs  
 Our results indicate that pro-apoptotic activity of quercetin may be correlated not only with the inhibition of Hsp72 expression but also with suppression of its   migration   to the nucleus. 
  [Ee]xtracellular [Mm]atrix  PubMed  
 Proteomic analysis of human parotid gland exosomes by multidimensional protein identification technology (MudPIT). Human ductal saliva contributes over a thousand unique proteins to whole oral fluids. The mechanism by which most of these proteins are secreted by salivary glands remains to be determined. The present study used a mass spectrometry-based, shotgun proteomics approach to explore the possibility that a subset of the proteins found in saliva are derived from exosomes, membrane-bound vesicles of endosomal origin within multivesicular endosomes. Using MudPIT (multidimensional protein identification technology) mass spectrometry, we catalogued 491 proteins in the exosome fraction of human parotid saliva. Many of these proteins were previously observed in ductal saliva from parotid glands (265 proteins). Furthermore, 72 of the proteins in parotid exosomes overlap with those previously identified as urinary exosome proteins, proteins which are also frequently associated with exosomes from other tissues and cell types. Gene Ontology (GO) and KEGG pathway analyses found that cytosolic proteins comprise the largest category of proteins in parotid exosomes (43%), involved in such processes as phosphatidylinositol signaling system, calcium signaling pathway, inositol metabolism, protein export, and signal transduction, among others; whereas the integral plasma membrane proteins and associated/peripheral plasma membrane proteins (26%) were associated with   extracellular matrix  -receptor interaction, epithelial cell signaling, T-cell and B-cell receptor signaling, cytokine receptor interaction, and antigen processing and presentation, among other biological functions. In addition, these putative saliva exosomal proteins were linked to specific diseases (e.g., neurodegenerative disorders, prion disease, cancers, type I and II diabetes). Consequently, parotid glands secrete exosomes that reflect the metabolic and functional status of the gland and may also carry informative protein markers useful in the diagnosis and treatment of systemic diseases. 
 GRP-induced up-regulation of Hsp72 promotes CD16+/94+ natural killer cell binding to colon cancer cells causing tumor cell cytolysis. Gastrin-releasing peptide (GRP) and its receptor (GRPR) are not normally expressed by epithelial cells lining the adult human colon. However post malignant transformation both GRP and its receptor are aberrantly expressed in the colon where we have previously shown they act to retard metastasis by enhancing tumor cell attachment to the   extracellular matrix  . In the present study, we show that GRP signaling via its cognate receptor when both are aberrantly expressed in human colon cancer cells causes heat shock protein 72 (Hsp72) to be expressed. We show that GRP/GRPR induces expression of Hsp72 by signaling via focal adhesion kinase. When expressed, Hsp72 promotes the binding of CD16+ and CD94+ natural killer cells, resulting in tumor cell cytolysis. These findings demonstrate the presence of a novel mechanism whereby aberrantly expressed GRP/GRPR in human colorectal cancer attenuates tumor progression and may promote a favorable outcome. 
  [Mm]etastasis  PubMed  
 Opposite roles of metastasin (S100A4) in two potentially tumoricidal mechanisms involving human lymphocyte protein Tag7 and Hsp70. We compare the physical and functional interactions between three widespread multifunctional proteins [metastasin (Mts1/S100A4), innate immunity-related Tag7/PGRP-S, and Hsp70] in two experimental models relevant to host-tumor relationships on humoral and cellular levels. (i) Tag7 and Hsp70 in solution or in a lymphocyte make a stable binary complex that is highly cytotoxic for some tumor cells. Here, we show that Mts1 prevents Tag7.Hsp70 assembly in solution, and an excess of Mts1 disrupts the existing Tag7.Hsp70 complex; accordingly, Tag7.Hsp70 cytotoxicity (exemplified with L929 cells) is diminished in the presence of excess Mts1. (ii) Tag7 exposed on a specialized subset of lymphokine-activated killer cells makes specific contact with Hsp70 exposed on some HLA-negative tumor cells, thus enabling FasL/Fas-mediated induction of apoptosis. Here, we show that some CD4(+)CD25(+) cells coexpose Mts1 with Tag7 and FasL, that Mts1 and Tag7 closely contact the same Hsp70 molecule on the target K562 cell (as evidenced by cross-linking), and that killing of such targets is abolished by Mts1-specific antibodies (or selective removal of Mts1-exposing lymphocytes). Thus, this phenotype active against immunoevasive cancerous cells is defined as CD4(+)CD25(+), FasL(+), Tag7(+)Mts1(+) (approximately 0.5% of total lymphocytes in culture). Remarkably, similar effectors with at least the same activity are often found in fresh donor blood samples (approximately 10(4) effectors/mL). Thus, our models suggest that interactions between the three proteins in different situations may have opposite functional outcomes as regards antitumor defense, immune escape, and   metastasis  . 
 Immunolocalisation of heat shock protein 72 and glycoprotein 96 in colonic adenocarcinoma. Heat shock protein 72 (HSP72) and glycoprotein 96 (gp96) are highly expressed in cancer tissues. Recent studies indicate the possible roles of HSP72 and gp96 in the development and progression of colonic carcinomas, but detailed information is still ambiguous. In this study, we investigated the correlation between clinical pathology and immunolocalisation of HSP72 and gp96 in human colonic carcinoma. The distribution of HSP72 and gp96 was studied in 160 human colonic carcinomas, with or without   metastasis  , as well as in mucous membranes adjacent to cancers by means of immunohistochemistry. HSP72 immunoreactivity was detected in 145 of 160 primary tumours (90.6%) and in 44 of 160 mucous membranes adjacent to cancers (27.5%). Gp96 was detected in 81.3% colonic carcinomas and in 13.8% mucous membranes adjacent to cancer. Immunolocalisation of HSP72 and gp96 was mainly cytoplasmic. HSP72 and gp96 immunolabelling was significantly higher in colonic carcinomas with   metastasis   than in those without   metastasis   (P<0.05). The results indicate a significant correlation between the immunopositivity of HSP72 and gp96 and the progression of colonic carcinomas. Immunolabelling of HSP72 and gp96 may be useful as diagnostic or prognostic markers in colonic carcinoma. 
 HSP72 and gp96 in gastroenterological cancers. Heat shock protein 72 (HSP72) and glycoprotein 96 (gp96) are highly expressed in cancer tissues. Recent studies indicate the possible roles of HSP72 and gp96 in the development and progression of gastrointestinal carcinomas but detailed mechanisms are still ambiguous. Human esophageal cancer, gastric cancer, colon cancer and liver cancer are common gastrointestinal malignant carcinomas in the world. The studies indicated that there existed a significant correlation between the expression of HSP72, gp96 and the development and progression of digestive carcinomas. HSP72 and gp96 expression were significantly associated with the presence of tumor infiltration, lymph node and remote   metastasis  . Interestingly, studies have found that HSP72 chaperoned alpha-fetoprotein (AFP), HBx in hepatocellular carcinoma, and CD44 in colonic carcinomas. The further researches demonstrated that HSP72-AFP or gp96-AFP recombined vaccine could elicit specific anti-tumor immunity. The high-level expression of HSP72 and gp96 may be not only used as diagnostic or prognostic markers for gastrointestinal carcinomas but also as better immunotherapeutic vaccines in the cancers. 
 Correlation between clinicopathology and expression of heat shock protein 72 and glycoprotein 96 in human esophageal squamous cell carcinoma. Heat shock protein 72 (HSP72) and glycoprotein 96 (gp96) are highly expressed in cancer tissues. Recent studies indicate the possible roles of HSP72 and gp96 in the development and progression of gastrointestinal carcinomas but detailed information is still ambiguous. We investigated the correlation between clinicopathology and expression of HSP72 and gp96 in human esophageal squamous cell carcinoma. The expression of HSP72 and gp96 was studied in 120 human esophageal squamous cell carcinomas with or without   metastasis   as well as in mucous membrane adjacent to cancers by way of immunohistochemistry. HSP72 immunoreactivities were detected in 112 of 120 primary tumors (93.3%) and in 30 of 120 mucous membranes adjacent to cancers (25.0%). Gp96 detected in esophageal squamous cell carcinoma and inmucous membrane adjacent to cancer was 85.0% and 20.0%, respectively. Both HSP72 and gp96 stained in cytoplasm. HSP72 and gp96 expression in esophageal squamous cell carcinomas with   metastasis   was significantly higher than those with non  metastasis   (P < .05). The results indicate that there exists a significant correlation between the expression of HSP72 and gp96 and the progression of esophageal squamous cell carcinomas. HSP72 and gp96 expression were significantly associated with the presence of tumor infiltration, lymph node, and remote   metastasis  . 
 Proteomic profiling of hepatocellular carcinoma in Chinese cohort reveals heat-shock proteins (Hsp27, Hsp70, GRP78) up-regulation and their associated prognostic values. To facilitate the identification of candidate molecular biomarkers that are linked to the pathogenesis of hepatocellular carcinoma (HCC), we investigated protein-expression profiles of 146 tissue specimens including 67 pairs of tumors and adjacent non-tumors resected from HCC patients as well as 12 normal livers by 2-DE. Among the 1800 spots displayed in the liver proteome, a total of 90 protein species were found to be significantly different between the three groups (P < 0.05). Three of the top candidate markers up-regulated in HCC, with high receiver operating characteristic (ROC) curves, were identified by MS/MS analysis and belonged to the chaperone members: heat-shock protein (Hsp)27, Hsp70 and glucose-regulated protein (GRP)78. Over-expression of these chaperone proteins in HCC tissues was confirmed by Western blotting and immunohistochemistry. In correlation with clinico-pathological parameters, expression of Hsp27 was linked to alpha-fetoprotein level (P = 0.007) whereas up-regulation of GRP78 was associated with tumor venous infiltration (P = 0.035). No significant association of Hsp70 with any pathologic features was observed. The present HCC proteome analysis revealed that in response to the stressful cancerous microenvironment, tumor cells strived to increase the expression of chaperone proteins for cyto-protective function and to enhance tumor growth and   metastasis  . 
 HSP72 and gp96 in gastroenterological cancers. Heat shock protein 72 (HSP72) and glycoprotein 96 (gp96) are highly expressed in cancer tissues. Recent studies indicate the possible roles of HSP72 and gp96 in the development and progression of gastrointestinal carcinomas but detailed mechanisms are still ambiguous. Human esophageal cancer, gastric cancer, colon cancer and liver cancer are common gastrointestinal malignant carcinomas in the world. The studies indicated that there existed a significant correlation between the expression of HSP72, gp96 and the development and progression of digestive carcinomas. HSP72 and gp96 expression were significantly associated with the presence of tumor infiltration, lymph node and remote   metastasis  . Interestingly, studies have found that HSP72 chaperoned alpha-fetoprotein (AFP), HBx in hepatocellular carcinoma, and CD44 in colonic carcinomas. The further researches demonstrated that HSP72-AFP or gp96-AFP recombined vaccine could elicit specific anti-tumor immunity. The high-level expression of HSP72 and gp96 may be not only used as diagnostic or prognostic markers for gastrointestinal carcinomas but also as better immunotherapeutic vaccines in the cancers. 
 Correlation between clinicopathology and expression of heat shock protein 72 and glycoprotein 96 in human esophageal squamous cell carcinoma. Heat shock protein 72 (HSP72) and glycoprotein 96 (gp96) are highly expressed in cancer tissues. Recent studies indicate the possible roles of HSP72 and gp96 in the development and progression of gastrointestinal carcinomas but detailed information is still ambiguous. We investigated the correlation between clinicopathology and expression of HSP72 and gp96 in human esophageal squamous cell carcinoma. The expression of HSP72 and gp96 was studied in 120 human esophageal squamous cell carcinomas with or without   metastasis   as well as in mucous membrane adjacent to cancers by way of immunohistochemistry. HSP72 immunoreactivities were detected in 112 of 120 primary tumors (93.3%) and in 30 of 120 mucous membranes adjacent to cancers (25.0%). Gp96 detected in esophageal squamous cell carcinoma and inmucous membrane adjacent to cancer was 85.0% and 20.0%, respectively. Both HSP72 and gp96 stained in cytoplasm. HSP72 and gp96 expression in esophageal squamous cell carcinomas with   metastasis   was significantly higher than those with non  metastasis   (P < .05). The results indicate that there exists a significant correlation between the expression of HSP72 and gp96 and the progression of esophageal squamous cell carcinomas. HSP72 and gp96 expression were significantly associated with the presence of tumor infiltration, lymph node, and remote   metastasis  . 
 Opposite roles of metastasin (S100A4) in two potentially tumoricidal mechanisms involving human lymphocyte protein Tag7 and Hsp70. We compare the physical and functional interactions between three widespread multifunctional proteins [metastasin (Mts1/S100A4), innate immunity-related Tag7/PGRP-S, and Hsp70] in two experimental models relevant to host-tumor relationships on humoral and cellular levels. (i) Tag7 and Hsp70 in solution or in a lymphocyte make a stable binary complex that is highly cytotoxic for some tumor cells. Here, we show that Mts1 prevents Tag7.Hsp70 assembly in solution, and an excess of Mts1 disrupts the existing Tag7.Hsp70 complex; accordingly, Tag7.Hsp70 cytotoxicity (exemplified with L929 cells) is diminished in the presence of excess Mts1. (ii) Tag7 exposed on a specialized subset of lymphokine-activated killer cells makes specific contact with Hsp70 exposed on some HLA-negative tumor cells, thus enabling FasL/Fas-mediated induction of apoptosis. Here, we show that some CD4(+)CD25(+) cells coexpose Mts1 with Tag7 and FasL, that Mts1 and Tag7 closely contact the same Hsp70 molecule on the target K562 cell (as evidenced by cross-linking), and that killing of such targets is abolished by Mts1-specific antibodies (or selective removal of Mts1-exposing lymphocytes). Thus, this phenotype active against immunoevasive cancerous cells is defined as CD4(+)CD25(+), FasL(+), Tag7(+)Mts1(+) (approximately 0.5% of total lymphocytes in culture). Remarkably, similar effectors with at least the same activity are often found in fresh donor blood samples (approximately 10(4) effectors/mL). Thus, our models suggest that interactions between the three proteins in different situations may have opposite functional outcomes as regards antitumor defense, immune escape, and   metastasis  . 
 Immunolocalisation of heat shock protein 72 and glycoprotein 96 in colonic adenocarcinoma. Heat shock protein 72 (HSP72) and glycoprotein 96 (gp96) are highly expressed in cancer tissues. Recent studies indicate the possible roles of HSP72 and gp96 in the development and progression of colonic carcinomas, but detailed information is still ambiguous. In this study, we investigated the correlation between clinical pathology and immunolocalisation of HSP72 and gp96 in human colonic carcinoma. The distribution of HSP72 and gp96 was studied in 160 human colonic carcinomas, with or without   metastasis  , as well as in mucous membranes adjacent to cancers by means of immunohistochemistry. HSP72 immunoreactivity was detected in 145 of 160 primary tumours (90.6%) and in 44 of 160 mucous membranes adjacent to cancers (27.5%). Gp96 was detected in 81.3% colonic carcinomas and in 13.8% mucous membranes adjacent to cancer. Immunolocalisation of HSP72 and gp96 was mainly cytoplasmic. HSP72 and gp96 immunolabelling was significantly higher in colonic carcinomas with   metastasis   than in those without   metastasis   (P<0.05). The results indicate a significant correlation between the immunopositivity of HSP72 and gp96 and the progression of colonic carcinomas. Immunolabelling of HSP72 and gp96 may be useful as diagnostic or prognostic markers in colonic carcinoma. 
 Proteomic profiling of hepatocellular carcinoma in Chinese cohort reveals heat-shock proteins (Hsp27, Hsp70, GRP78) up-regulation and their associated prognostic values. To facilitate the identification of candidate molecular biomarkers that are linked to the pathogenesis of hepatocellular carcinoma (HCC), we investigated protein-expression profiles of 146 tissue specimens including 67 pairs of tumors and adjacent non-tumors resected from HCC patients as well as 12 normal livers by 2-DE. Among the 1800 spots displayed in the liver proteome, a total of 90 protein species were found to be significantly different between the three groups (P < 0.05). Three of the top candidate markers up-regulated in HCC, with high receiver operating characteristic (ROC) curves, were identified by MS/MS analysis and belonged to the chaperone members: heat-shock protein (Hsp)27, Hsp70 and glucose-regulated protein (GRP)78. Over-expression of these chaperone proteins in HCC tissues was confirmed by Western blotting and immunohistochemistry. In correlation with clinico-pathological parameters, expression of Hsp27 was linked to alpha-fetoprotein level (P = 0.007) whereas up-regulation of GRP78 was associated with tumor venous infiltration (P = 0.035). No significant association of Hsp70 with any pathologic features was observed. The present HCC proteome analysis revealed that in response to the stressful cancerous microenvironment, tumor cells strived to increase the expression of chaperone proteins for cyto-protective function and to enhance tumor growth and   metastasis  . 
 GRP-induced up-regulation of Hsp72 promotes CD16+/94+ natural killer cell binding to colon cancer cells causing tumor cell cytolysis. Gastrin-releasing peptide (GRP) and its receptor (GRPR) are not normally expressed by epithelial cells lining the adult human colon. However post malignant transformation both GRP and its receptor are aberrantly expressed in the colon where we have previously shown they act to retard   metastasis   by enhancing tumor cell attachment to the extracellular matrix. In the present study, we show that GRP signaling via its cognate receptor when both are aberrantly expressed in human colon cancer cells causes heat shock protein 72 (Hsp72) to be expressed. We show that GRP/GRPR induces expression of Hsp72 by signaling via focal adhesion kinase. When expressed, Hsp72 promotes the binding of CD16+ and CD94+ natural killer cells, resulting in tumor cell cytolysis. These findings demonstrate the presence of a novel mechanism whereby aberrantly expressed GRP/GRPR in human colorectal cancer attenuates tumor progression and may promote a favorable outcome. 
 Heat shock protein 72 associated with CD44v6 in human colonic adenocarcinoma. CD44v6 is a splice variant of CD44 (CD44v), probably promoting cancer cell adherence to vascular endothelium and base membranes and enhancing the invasion and   metastasis   of colonic carcinomas. Heat shock protein 72 (HSP72) as a molecular chaperone has been confirmed to be overexpressed in epithelial carcinoma cells. There may be a possible association between the expression of HSP72 and CD44v6 during the growth and progression of colonic carcinoma cells. The aim of the study was to investigate the interaction between heat shock protein 72 and CD44v6 in human colonic carcinomas. The localization of HSP72 and CD44v6 in human colonic carcinomas was determined by immunohistochemistry and confocal laser microscopy. The interaction between HSP72 and CD44v6 in colonic carcinoma cells was analyzed by immunoprecipitation and Western immunoblots. Our results revealed that colonic carcinoma synchronously co-expressed higher levels of HSP72 and CD44v6 than that in adjacent normal colonic tissues. HSP72 and CD44v6 were mainly immunolocalized in the cytoplasm, and also immunolabelled on the cell membrane. Based on immunoprecipitation and Western immunoblots, we found that HSP72 was associated with CD44v6 precursor fragments in human colonic carcinoma cells. The interaction between HSP72 and CD44v6 in human colonic carcinoma cells may contribute to study the pathogenesis and immunotherapy of colonic carcinoma. 
   
  
 HSPA1B 
 
  NCBI Gene  
  GeneRIF  
  Pubmed  
 
 
  Search Term  Rank  Fields  
  [Mm]igration  6  PubMed(3)  
  [Ee]xtracellular [Mm]atrix  8  PubMed(1)  
  [Ii]nvasive  10  PubMed(1); GeneRIFs(1)  
  [Mm]etastasis  12  PubMed(1)  
 
 
 hide/show details for HSPA1B 
  [Mm]igration  PubMed  
 Analysis of the myosin-II-responsive focal adhesion proteome reveals a role for β-Pix in negative regulation of focal adhesion maturation. Focal adhesions undergo myosin-II-mediated maturation wherein they grow and change composition to modulate integrin signalling for cell   migratio  n, growth and differentiation. To determine how focal adhesion composition is affected by myosin II activity, we performed proteomic analysis of isolated focal adhesions and compared protein abundance in focal adhesions from cells with and without myosin II inhibition. We identified 905 focal adhesion proteins, 459 of which changed in abundance with myosin II inhibition, defining the myosin-II-responsive focal adhesion proteome. The abundance of 73% of the proteins in the myosin-II-responsive focal adhesion proteome was enhanced by contractility, including proteins involved in Rho-mediated focal adhesion maturation and endocytosis- and calpain-dependent focal adhesion disassembly. During myosin II inhibition, 27% of proteins in the myosin-II-responsive focal adhesion proteome, including proteins involved in Rac-mediated lamellipodial protrusion, were enriched in focal adhesions, establishing that focal adhesion protein recruitment is also negatively regulated by contractility. We focused on the Rac guanine nucleotide exchange factor β-Pix, documenting its role in the negative regulation of focal adhesion maturation and the promotion of lamellipodial protrusion and focal adhesion turnover to drive cel  l migrati  on. 
 Stress-induced localization of HSPA6 (HSP70B') and HSPA1A (HSP70-1) proteins to centrioles in human neuronal cells. The localization of yellow fluorescent protein (YFP)-tagged HSP70 proteins was employed to identify stress-sensitive sites in human neurons following temperature elevation. Stable lines of human SH-SY5Y neuronal cells were established that expressed YFP-tagged protein products of the human inducible HSP70 genes HSPA6 (HSP70B') and HSPA1A (HSP70-1). Following a brief period of thermal stress, YFP-tagged HSPA6 and HSPA1A rapidly appeared at centrioles in the cytoplasm of human neuronal cells, with HSPA6 demonstrating a more prolonged signal compared to HSPA1A. Each centriole is composed of a distal end and a proximal end, the latter linking the centriole doublet. The YFP-tagged HSP70 proteins targeted the proximal end of centrioles (identified by γ-tubulin marker) rather than the distal end (centrin marker). Centrioles play key roles in cellular polarity and   migratio  n during neuronal differentiation. The proximal end of the centriole, which is involved in centriole stabilization, may be stress-sensitive in post-mitotic, differentiating human neurons. 
 Targeting the testis-specific heat-shock protein 70-2 (HSP70-2) reduces cellular growth,   migration  , and invasion in renal cell carcinoma cells. Renal cell carcinoma (RCC) represents one of the most resistant tumors to radiotherapy and chemotherapy. Current therapies for the RCC patients are limited owing to lack of diagnosis and therapeutic treatments. Testis-specific heat-shock protein 70-2 (HSP70-2), a member of HSP70 chaperone family, has been shown to be associated with various cancers. In the present study, we investigated the putative role of HSP70-2 in various malignant properties of the RCC cells. HSP70-2 messenger RNA (mRNA) and protein expression was investigated in A704, ACHN, and Caki-1 cells derived from the RCC patients. We assessed the expression of HSP70-2 gene and protein by reverse transcription-polymerase chain reaction (RT-PCR) and Western blotting, respectively. The expression of HSP70-2 protein was further validated by performing indirect immunofluorescence (IIF) and flow cytometry. The malignant properties of high-grade invasive A704 and Caki-1 cells, such as cellular proliferation, colony formation,   migration  , invasion, and wound healing, were evaluated by silencing the expression of HSP70-2 gene in these cells. Statistical significance was defined using Student's t test. Our RT-PCR and Western blotting data showed the expression of HSP70-2 in all RCC cells. Our results showed that HSP70-2 was predominantly expressed in cytoplasm and found to be colocalized with endoplasmic reticulum, mitochondria, Golgi body, and plasma membrane but not the nuclear envelope. Knockdown of HSP70-2 expression with specific short hairpin RNA (shRNA) demonstrated significant reduction in cell growth and colony formation. Further, a marked reduction in cell   migration   and invasion was also observed, indicating the potential role of HSP70-2 in metastasis. Collectively, our data suggest that HSP70-2 plays a key role in cancerous growth and invasive potential of RCC cells. Thus, HSP70-2 could serve as a novel potential therapeutic target for the RCC. 
  [Ee]xtracellular [Mm]atrix  PubMed  
 Proteomic analysis of human parotid gland exosomes by multidimensional protein identification technology (MudPIT). Human ductal saliva contributes over a thousand unique proteins to whole oral fluids. The mechanism by which most of these proteins are secreted by salivary glands remains to be determined. The present study used a mass spectrometry-based, shotgun proteomics approach to explore the possibility that a subset of the proteins found in saliva are derived from exosomes, membrane-bound vesicles of endosomal origin within multivesicular endosomes. Using MudPIT (multidimensional protein identification technology) mass spectrometry, we catalogued 491 proteins in the exosome fraction of human parotid saliva. Many of these proteins were previously observed in ductal saliva from parotid glands (265 proteins). Furthermore, 72 of the proteins in parotid exosomes overlap with those previously identified as urinary exosome proteins, proteins which are also frequently associated with exosomes from other tissues and cell types. Gene Ontology (GO) and KEGG pathway analyses found that cytosolic proteins comprise the largest category of proteins in parotid exosomes (43%), involved in such processes as phosphatidylinositol signaling system, calcium signaling pathway, inositol metabolism, protein export, and signal transduction, among others; whereas the integral plasma membrane proteins and associated/peripheral plasma membrane proteins (26%) were associated with   extracellular matrix  -receptor interaction, epithelial cell signaling, T-cell and B-cell receptor signaling, cytokine receptor interaction, and antigen processing and presentation, among other biological functions. In addition, these putative saliva exosomal proteins were linked to specific diseases (e.g., neurodegenerative disorders, prion disease, cancers, type I and II diabetes). Consequently, parotid glands secrete exosomes that reflect the metabolic and functional status of the gland and may also carry informative protein markers useful in the diagnosis and treatment of systemic diseases. 
  [Ii]nvasive  PubMed  
 Targeting the testis-specific heat-shock protein 70-2 (HSP70-2) reduces cellular growth, migration, and invasion in renal cell carcinoma cells. Renal cell carcinoma (RCC) represents one of the most resistant tumors to radiotherapy and chemotherapy. Current therapies for the RCC patients are limited owing to lack of diagnosis and therapeutic treatments. Testis-specific heat-shock protein 70-2 (HSP70-2), a member of HSP70 chaperone family, has been shown to be associated with various cancers. In the present study, we investigated the putative role of HSP70-2 in various malignant properties of the RCC cells. HSP70-2 messenger RNA (mRNA) and protein expression was investigated in A704, ACHN, and Caki-1 cells derived from the RCC patients. We assessed the expression of HSP70-2 gene and protein by reverse transcription-polymerase chain reaction (RT-PCR) and Western blotting, respectively. The expression of HSP70-2 protein was further validated by performing indirect immunofluorescence (IIF) and flow cytometry. The malignant properties of high-grade   invasive   A704 and Caki-1 cells, such as cellular proliferation, colony formation, migration, invasion, and wound healing, were evaluated by silencing the expression of HSP70-2 gene in these cells. Statistical significance was defined using Student's t test. Our RT-PCR and Western blotting data showed the expression of HSP70-2 in all RCC cells. Our results showed that HSP70-2 was predominantly expressed in cytoplasm and found to be colocalized with endoplasmic reticulum, mitochondria, Golgi body, and plasma membrane but not the nuclear envelope. Knockdown of HSP70-2 expression with specific short hairpin RNA (shRNA) demonstrated significant reduction in cell growth and colony formation. Further, a marked reduction in cell migration and invasion was also observed, indicating the potential role of HSP70-2 in metastasis. Collectively, our data suggest that HSP70-2 plays a key role in cancerous growth and   invasive   potential of RCC cells. Thus, HSP70-2 could serve as a novel potential therapeutic target for the RCC. 
  GeneRIFs  
 HSP70-2 plays a key role in cancerous growth and   invasive   potential of RCC cells. 
  [Mm]etastasis  PubMed  
 Targeting the testis-specific heat-shock protein 70-2 (HSP70-2) reduces cellular growth, migration, and invasion in renal cell carcinoma cells. Renal cell carcinoma (RCC) represents one of the most resistant tumors to radiotherapy and chemotherapy. Current therapies for the RCC patients are limited owing to lack of diagnosis and therapeutic treatments. Testis-specific heat-shock protein 70-2 (HSP70-2), a member of HSP70 chaperone family, has been shown to be associated with various cancers. In the present study, we investigated the putative role of HSP70-2 in various malignant properties of the RCC cells. HSP70-2 messenger RNA (mRNA) and protein expression was investigated in A704, ACHN, and Caki-1 cells derived from the RCC patients. We assessed the expression of HSP70-2 gene and protein by reverse transcription-polymerase chain reaction (RT-PCR) and Western blotting, respectively. The expression of HSP70-2 protein was further validated by performing indirect immunofluorescence (IIF) and flow cytometry. The malignant properties of high-grade invasive A704 and Caki-1 cells, such as cellular proliferation, colony formation, migration, invasion, and wound healing, were evaluated by silencing the expression of HSP70-2 gene in these cells. Statistical significance was defined using Student's t test. Our RT-PCR and Western blotting data showed the expression of HSP70-2 in all RCC cells. Our results showed that HSP70-2 was predominantly expressed in cytoplasm and found to be colocalized with endoplasmic reticulum, mitochondria, Golgi body, and plasma membrane but not the nuclear envelope. Knockdown of HSP70-2 expression with specific short hairpin RNA (shRNA) demonstrated significant reduction in cell growth and colony formation. Further, a marked reduction in cell migration and invasion was also observed, indicating the potential role of HSP70-2 in   metastasis  . Collectively, our data suggest that HSP70-2 plays a key role in cancerous growth and invasive potential of RCC cells. Thus, HSP70-2 could serve as a novel potential therapeutic target for the RCC. 
   
  
 PER3 
 
  NCBI Gene  
  GeneRIF  
  Pubmed  
 
 
  Search Term  Rank  Fields  
  [Mm]etastasis  12  PubMed(1)  
 
 
 hide/show details for PER3 
  [Mm]etastasis  PubMed  
 Aberrant expression of Per1, Per2 and Per3 and their prognostic relevance in non-small cell lung cancer. The objective of the current study was to investigate the expression pattern and clinicopathological significance of Period1 (Per1), Period2 (Per2) and Period3 (Per3) in patients with non-small cell lung cancer (NSCLC). In 130 archived NSCLC tissues, the positive rate of Per1 (86/130, 66.2%), Per2 (77/130, 59.2%) and Per3 (82/130, 63.1%) were reduced in human lung cancer samples compared with adjacent normal lung tissues (Per1, 119/130, 91.5%; Per2, 115/130, 88.5%; Per3, 121/130, 93.1%), as measured by immunohistochemical staining. Loss of Per1 was correlated with poor differentiation (P < 0.001), tumor status (P=0.04), high p-TNM stage (P < 0.001) and lymph node   metastasis   (P=0.045). The similar tendencies were also found in the correlation of the expression of Per2 and Per3 with clinicopathological factors. In addition, a significant correlation was found between Per1 and Per2 (P < 0.001) in 130 cases of NSCLC. Similarly, a significant correlation was found between Per2 and Per3 (P=0.045). Patients with lower expression of Per1, Per2 and Per3 had a shorter survival time than those with higher expression. These results indicate that loss of Per may promote tumor progression in NSCLC, and may serve as a novel prognostic biomarker of NSCLC. 
   
  
 PER2 
 
  NCBI Gene  
  GeneRIF  
  Pubmed  
 
 
  Search Term  Rank  Fields  
  [Mm]igration  6  PubMed(1)  
  [Ii]nvasive  10  PubMed(1)  
  [Mm]etastasis  12  PubMed(7); GeneRIFs(1)  
 
 
 hide/show details for PER2 
  [Mm]igration  PubMed  
 Modulation of E-cadherin expression promotes   migration   ability of esophageal cancer cells. Losing the E-cadherin plays an important role in the metastasis of cancer. The regulation of the expression of E-cadherin is unclear. Circadian rhythm alteration is associated with the pathogenesis of a number of cancers. This study aims to investigate the role of one of the circadian proteins, period-2 (Per2) in repressing the expression of E-cadherin in esophageal cancer (esophageal cancer). We observed that the levels of circadian protein Per2 were significantly increased and E-cadherin was significantly decreased in the tissue of human esophageal cancer with metastasis as compared with non-metastatic esophageal cancer. Overexpression of Per2 in the esophageal cancer cells markedly repressed the expression of E-cadherin. The pHDAC1 was detected in human esophageal cancer with metastasis, which was much less in the esophageal cancer tissue without metastasis. Overexpression of Per2 increased the levels of pHDAC1 as well as the E-cadherin repressors at the E-cadherin promoter locus. Overexpression of Per2 markedly increased the migratory capacity of esophageal cancer cells, which was abolished by the inhibition of HDAC1. We conclude that Per-2 plays an important role in the esophageal cancer cell metastasis, which may be a novel therapeutic target for the treatment of esophageal cancer. 
  [Ii]nvasive  PubMed  
 Evening exposure to blue light stimulates the expression of the clock gene PER2 in humans. We developed a non-  invasive   method to measure and quantify human circadian PER2 gene expression in oral mucosa samples and show that this gene oscillates in a circadian (= about a day) fashion. We also have the first evidence that induction of human PER2 expression is stimulated by exposing subjects to 2 h of light in the evening. This increase in PER2 expression was statistically significant in comparison to a non-light control condition only after light at 460 nm (blue) but not after light exposure at 550 nm (green). Our results indicate that the non-image-forming visual system is involved in human circadian gene expression. The demonstration of a functional circadian machinery in human buccal samples and its response to light opens the door for investigation of human circadian rhythms at the gene level and their associated disorders. 
  [Mm]etastasis  PubMed  
 Aberrant expression of Per1, Per2 and Per3 and their prognostic relevance in non-small cell lung cancer. The objective of the current study was to investigate the expression pattern and clinicopathological significance of Period1 (Per1), Period2 (Per2) and Period3 (Per3) in patients with non-small cell lung cancer (NSCLC). In 130 archived NSCLC tissues, the positive rate of Per1 (86/130, 66.2%), Per2 (77/130, 59.2%) and Per3 (82/130, 63.1%) were reduced in human lung cancer samples compared with adjacent normal lung tissues (Per1, 119/130, 91.5%; Per2, 115/130, 88.5%; Per3, 121/130, 93.1%), as measured by immunohistochemical staining. Loss of Per1 was correlated with poor differentiation (P < 0.001), tumor status (P=0.04), high p-TNM stage (P < 0.001) and lymph node   metastasis   (P=0.045). The similar tendencies were also found in the correlation of the expression of Per2 and Per3 with clinicopathological factors. In addition, a significant correlation was found between Per1 and Per2 (P < 0.001) in 130 cases of NSCLC. Similarly, a significant correlation was found between Per2 and Per3 (P=0.045). Patients with lower expression of Per1, Per2 and Per3 had a shorter survival time than those with higher expression. These results indicate that loss of Per may promote tumor progression in NSCLC, and may serve as a novel prognostic biomarker of NSCLC. 
 Aberrant expression of Per1, Per2 and Per3 and their prognostic relevance in non-small cell lung cancer. The objective of the current study was to investigate the expression pattern and clinicopathological significance of Period1 (Per1), Period2 (Per2) and Period3 (Per3) in patients with non-small cell lung cancer (NSCLC). In 130 archived NSCLC tissues, the positive rate of Per1 (86/130, 66.2%), Per2 (77/130, 59.2%) and Per3 (82/130, 63.1%) were reduced in human lung cancer samples compared with adjacent normal lung tissues (Per1, 119/130, 91.5%; Per2, 115/130, 88.5%; Per3, 121/130, 93.1%), as measured by immunohistochemical staining. Loss of Per1 was correlated with poor differentiation (P < 0.001), tumor status (P=0.04), high p-TNM stage (P < 0.001) and lymph node   metastasis   (P=0.045). The similar tendencies were also found in the correlation of the expression of Per2 and Per3 with clinicopathological factors. In addition, a significant correlation was found between Per1 and Per2 (P < 0.001) in 130 cases of NSCLC. Similarly, a significant correlation was found between Per2 and Per3 (P=0.045). Patients with lower expression of Per1, Per2 and Per3 had a shorter survival time than those with higher expression. These results indicate that loss of Per may promote tumor progression in NSCLC, and may serve as a novel prognostic biomarker of NSCLC. 
 Effects of Per2 overexpression on growth inhibition and   metastasis  , and on MTA1, nm23-H1 and the autophagy-associated PI3K/PKB signaling pathway in nude mice xenograft models of ovarian cancer. The aim of the present study was to evaluate the association between Period2 (Per2) and the occurrence and development of ovarian cancer, in addition to evaluating the effect of this gene on the growth and   metastasis   of ovarian cancer in nude mice xenograft models. The detection of Per2 by reverse transcription‑quantitative polymerase chain reaction (RT‑qPCR) and western blotting methods at various stages of ovarian cancer in tumor tissue samples was conducted. Nude mice xenograft models of ovarian cancer were constructed using an ovarian cancer cell line and, using a gene transfection technique, exogenous infusion of the recombinant gene, Per2, was performed. To assess for the successful and stable expression of Per2 in the tumor tissue, levels of Per2 expression in the nude mice xenograft models were detected by RT‑qPCR. During the experimental period, the tumor volumes were measured every three days. Two weeks following treatment cessation, the nude mice were sacrificed and the tumor weight and volume were measured. Furthermore, detection of the changes in expression leve  ls of meta  stasis‑associated gene 1 (MTA‑1)   and tumor   metastasis suppressor   gene, non  ‑metastasis protein 23‑H1 (nm23‑H1), and the expression change of autophagy‑associated signal transduction pathway, phosphatidylinositol 3‑kinase (PI3K)/protein kinase B (PKB) kinase were analyzed. The findings demonstrated that with ovarian cancer stage development, the expression of Per2 gradually reduced or ceased. In addition, exogenous Per2 was successfully and stably expressed in nude mice tumor tissue samples. Furthermore, in the Per2 overexpression group, MTA‑1 protein expression was significantly reduced when compared with the phosphate‑buffered saline (PBS) control and empty plasmid groups, while nm23‑H1 protein expression was significantly higher when compared with those two groups. The expression levels of PI3K and PKB kinase, which are marker proteins of the autophagy associated signaling pathway PI3K/PKB, were significantly downregulated, when compared with the PBS control and empty plasmid groups (P<0.001). Thus, it was demonstrated that Per2 is closely associated with the development of ovarian cancer, and late‑stage ovarian cancer is associated with Per2 mutation or deletion. Per2 overexpression, via exogenous infusion reduced the ovarian cancer growth rate, which was demonstrated by a significant increase in the tumor inhibition rate. In addition, Per2 may inhibit the expression of MTA‑1 and promote the expression of nm23‑H1 to   restrict   ovarian tumor growth and metastasis. Finally, it is hypothesized that Per2 affects autophagy by interfering with the PI3K/PKB signaling pathway, causing inhibition of tumor angiogenesis in order to inhibit tumor growth. 
 Expression of circadian clock gene human Period2 (hPer2) in human colorectal carcinoma. Recent studies have shown that disruption of circadian rhythms is one of the tumor promoting factors which contribute to mammalian cancer development and progression, but very little is known about the molecular changes of circadian genes in colorectal carcinoma (CRC). Thus, in this study, changes in the expression of human Period2 (hPer2), one of the key circadian clock regulators, in CRC and its correlation with prognosis were investigated.Immunohistochemical (IHC) staining and real-time PCR for hPer2 were performed for 38 CRC cases.IHC analysis detected positive staining for hPer2 in 81.6% (31/38) of CRC tissues and 97.4% (37/38) of surrounding non-cancerous tissues (P < 0.05). Most colorectal cells in non-cancerous tissues were homogeneously stained. In contrast, in the paired cancerous tissues, a heterogeneous pattern was found with a significant portion of cancer cells displaying negative or weak hPer2 staining. In over 60% cases (24/38), the staining for hPer2 was much stronger in non-cancerous cells than in the paired cancerous cells. Well-differentiated cancer cells are more likely to maintain hPer2 expression than poorly-differentiated ones. Furthermore, associations of decreased hPer2 levels with patients' age, histological grade, TNM stage and expression of nucleus proliferation related antigen: Ki67 were also detected (P < 0.05). Expression of hPer2 did not correlate with that of either p53 or C-erB-2. Similar to hPer2 protein expression, quantitative RT-PCR for hPer2 also showed decreased mRNA expression in CRC.These results suggest a role for hPer2 in normal colorectal cell function and the potential deregulation of hPer2 expression in the development, invasion, and   metastasis   of CRC. 
 Modulation of E-cadherin expression promotes migration ability of esophageal cancer cells. Losing the E-cadherin plays an important role in the   metastasis   of cancer. The regulation of the expression of E-cadherin is unclear. Circadian rhythm alteration is associated with the pathogenesis of a number of cancers. This study aims to investigate the role of one of the circadian proteins, period-2 (Per2) in repressing the expression of E-cadherin in esophageal cancer (esophageal cancer). We observed that the levels of circadian protein Per2 were significantly increased and E-cadherin was significantly decreased in the tissue of human esophageal cancer with   metastasis   as compared with non-metastatic esophageal cancer. Overexpression of Per2 in the esophageal cancer cells markedly repressed the expression of E-cadherin. The pHDAC1 was detected in human esophageal cancer with   metastasis  , which was much less in the esophageal cancer tissue without   metastasis  . Overexpression of Per2 increased the levels of pHDAC1 as well as the E-cadherin repressors at the E-cadherin promoter locus. Overexpression of Per2 markedly increased the migratory capacity of esophageal cancer cells, which was abolished by the inhibition of HDAC1. We conclude that Per-2 plays an important role in the esophageal cancer cell   metastasis  , which may be a novel therapeutic target for the treatment of esophageal cancer. 
 Prognostic relevance of Period1 (Per1) and Period2 (Per2) expression in human gastric cancer. Period1 (Per1) and Period2 (Per2) are members of the circadian genes. Mounting evidence suggests that the deregulation of the circadian clock plays an important role in the development of mammalian cancer. However, the expression and clinical significance of Per1 and Per2 in gastric cancer is still unexplored. Here, we evaluated the expression pattern of Per1 and Per2 in 246 gastric cancer specimens and their adjacent, non-tumorous tissues using immunohistochemical assays. Per1 expression was significantly associated with clinical stage (p < 0.001), depth invasion (p < 0.001), lymph node   metastasis   (p < 0.001) and pathologic differentiation (p < 0.001). On the other hand, Per2 was associated with clinical stage (p = 0.021) and depth invasion (p = 0.007). Per1 expression was positively correlated with Per2 expression in the 246 gastric cancer patients (r = 0.378, p < 0.001), and the expression levels of Per1 and Per2 were down-regulated in gastric cancer tissues when compared with adjacent, non-tumorous tissues in 45 gastric cancer samples (p < 0.001, p = 0.003). Patients with lower Per1 and Per2 tumor expression had a shorter survival time than those with higher expression. Univariate and Multivariate analyses indicated that Per2 expression is an independent prognostic factor (p = 0.023). Our results demonstrate that Per1 and Per2 may play important roles in tumor development, invasion and prognosis, and Per2 may serve as a novel prognostic biomarker of human gastric cancer. 
 Expression of circadian genes correlates with liver   metastasis   and outcomes in colorectal cancer. Circadian rhythms are daily oscillations in various biological processes, generated by the feedback loops of eight core circadian genes: Period1 (Per1), Period2 (Per2), Period3 (Per3), Cryptochrome1 (Cry1), Cryptochrome2 (Cry2), Clock, Bmal1 and Casein Kinase I ε (CKIε). Recent studies have suggested that circadian genes participate in the growth and development of various cancers. This study examined the relations of circadian gene expression to clinicopathological factors and outcomes in patients with colorectal cancer. We studied surgical specimens of cancer tissue and adjacent normal mucosa obtained from 202 patients with untreated colorectal cancer. The relative expression levels of the circadian genes in the specimens were measured by quantitative real-time, reverse-transcription polymerase chain reaction. Expression of the Clock gene and the CKIε gene in cancer tissue were significantly higher compared to that in adjacent normal mucosa. Expression of the Per1 and Per3 genes in cancer tissue was significantly lower compared to that in adjacent normal mucosa. Analysis of the relations between clinicopathological features and expression of the eight circadian genes in cancer tissue showed that high expression of the Bmal1 gene and low expression of the Per1 gene correlated with liv  er metasta  sis. On analysis of the relations between outcomes and gene expression, high expression of the Per2 gene was associated with significantly better outcomes than low expression of the Per2 gene. Overexpression of the Bmal1 gene and reduced expression of the Per1 gene may thus be useful predictors of liv  er metasta  sis. Moreover, reduced expression of the Per2 gene may be a predictor of outcomes in patients with colorectal cancer. 
  GeneRIFs  
 The levels of circadian protein Per2 were significantly increased and E-cadherin was significantly decreased in the tissue of human esophageal cancer with   metastasis   as compared with non-metastatic esophageal cancer. 
   
  
 MYC 
 
  NCBI Gene  
  GeneRIF  
  Pubmed  
 
 
  Search Term  Rank  Fields  
  [Ff]ilopodia  1  PubMed(1)  
  [Mm]igration  6  PubMed(42); GeneRIFs(6)  
  ECM  7  PubMed(1)  
  [Ee]xtracellular [Mm]atrix  8  PubMed(5); GeneRIFs(1)  
  [Ii]nvasive  10  PubMed(60); GeneRIFs(13)  
  [Mm]etastasize  11  PubMed(1)  
  [Mm]etastasis  12  PubMed(67); GeneRIFs(13)  
  [Cc]ell [Ii]nvasion  13  PubMed(17); GeneRIFs(2)  
 
 
 hide/show details for MYC 
  [Ff]ilopodia  PubMed  
 The enforced expression of c-Myc in pig fibroblasts triggers mesenchymal-epithelial transition (MET) via F-actin reorganization and RhoA/Rock pathway inactivation. In previous studies from other labs it has been well demonstrated that the ectopic expression of c-Myc in mammary epithelial cells can induce epithelial-mesenchymal transition (EMT), whereas in our pilot experiment, epithelial-like morphological changes were unexpectedly observed in c-Myc-expressing pig fibroblasts [i.e., porcine embryonic fibroblasts (PEFs) and porcine dermal fibroblasts (PDFs)] and pig mesenchymal stem cells, suggesting that the same c-Myc gene is entitled to trigger EMT in epithelial cells and mesenchymal-epithelial transition (MET) in fibroblasts. This prompted us to characterize the existence of a MET in c-Myc-expressing PEFs and PDFs at the molecular level. qRT-PCR, immunofluorescence and western blot analysis illustrated that epithelial-like morphological changes were accompanied by the increased expression of epithelial markers [such as cell adhesion proteins (E-cadherin, α-catenin and Bves), tight junction protein occludin and cytokeratins (Krt8 and Krt18)], the reduced expression of mesenchymal markers [vimentin, fibronectin 1 (FN1), snail1, collagen family of proteins (COL1A1, COL5A2) and matrix metalloproteinase (MMP) family (MMP12 and MMP14)] and the decreased cell motility and increased cell adhesion in c-Myc-expressing PEFs and PDFs. Furthermore, the ectopic expression of c-Myc in pig fibroblasts disrupted the stress fiber network, suppressed the formation of   filopodi  a and lamellipodia, and resulted in RhoA/Rock pathway inactivation, which finally participates in epithelial-like morphological conversion. Taken together, these findings demonstrate, for the first time, that the enforced expression of c-Myc in fibroblasts can trigger MET, to which cytoskeleton depolymerization and RhoA/Rock pathway inactivation contribute. 
  [Mm]igration  PubMed  
 HMGCR is necessary for the tumorigenecity of esophageal squamous cell carcinoma and is regulated by Myc. Hydroxymethylglutaryl coenzyme A reductase (HMGCR), the rate-limiting enzyme of mevalonate pathway, has been involved in the tumorigenesis of several tumor types. Our previous study has showed that statin, the inhibitor of HMGCR, inhibited the tumorigenecity of esophageal squamous cell carcinoma (ESCC) in vitro and in vivo. However, the function of HMGCR in the carcinogenesis of ESCC cells remains unknown. In this study, we have observed the up-regulation of HMGCR in ESCC tissues compared with the paired normal tissues. Over-expression of HMGCR in ESCC cells promoted cell growth and   migration  , while knockdown of the expression of HMGCR inhibited the growth,   migration   and colony formation of ESCC cells in vitro and in vivo. Furthermore, we found that oncogene Myc positively regulated the expression of HMGCR. Taken together, our study revealed the pivotal function of HMGCR and mevalonate pathway in the progression of ESCC and supported the clinical application of statin. 
 HMGCR is necessary for the tumorigenecity of esophageal squamous cell carcinoma and is regulated by Myc. Hydroxymethylglutaryl coenzyme A reductase (HMGCR), the rate-limiting enzyme of mevalonate pathway, has been involved in the tumorigenesis of several tumor types. Our previous study has showed that statin, the inhibitor of HMGCR, inhibited the tumorigenecity of esophageal squamous cell carcinoma (ESCC) in vitro and in vivo. However, the function of HMGCR in the carcinogenesis of ESCC cells remains unknown. In this study, we have observed the up-regulation of HMGCR in ESCC tissues compared with the paired normal tissues. Over-expression of HMGCR in ESCC cells promoted cell growth and   migration  , while knockdown of the expression of HMGCR inhibited the growth,   migration   and colony formation of ESCC cells in vitro and in vivo. Furthermore, we found that oncogene Myc positively regulated the expression of HMGCR. Taken together, our study revealed the pivotal function of HMGCR and mevalonate pathway in the progression of ESCC and supported the clinical application of statin. 
 p63-Mediated activation of the β-catenin/c-Myc signaling pathway stimulates esophageal squamous carcinoma cell invasion and metastasis. The development of esophageal squamous carcinomas (ESC) results from numerous genetic alterations. Our previous study demonstrated that p63 is highly expressed in human ESC cells and stimulates their growth; however, the mechanism by which p63 regulates ESC cell adhesion and invasion remains unclear. In the present study, we further elucidated the underlying molecular mechanisms by which p63 regulates metastasis in ESC cells. Knockdown of p63 significantly diminished the invasion of ESC cell lines TE-8 and TE-12, whereas overexpression of p63 significantly increased the   migratio  n rates of BE3 and OE33 cells. The mRNA and protein levels of vimentin, twist, SUSD2, and uPA were significantly decreased in p63-knockdown ESC cells, while overexpression of p63 induced an increase in vimentin, SUSD2, and uPA. In addition, knockdown of p63 in ESC cells significantly reduced levels of β-catenin and c-Myc, while overexpression of p63 increased β-catenin, but reduced p-β-catenin level. Therefore, p63 regulates   the migra  tion and invasion of ESC cells through activation of the β-catenin/c-Myc pathway. Our results suggest that targeting p63 may constitute a potential therapeutic strategy for ESC. 
 Molecular pathogenesis of chronic wounds: the role of beta-catenin and c-myc in the inhibition of epithelialization and wound healing. Lack of understanding of the molecular mechanisms and pathogenesis of impaired healing in chronic ulcers is a serious health issue that contributes to excessive limb amputations and mortality. Here we show that beta-catenin and its downstream targets in keratinocytes, c-myc, and keratins K6 and K16, play important roles in the development of chronic wounds. In contrast to normal epidermis, we observed a significant nuclear presence of beta-catenin and elevated c-myc expression at the nonhealing wound edge of chronic ulcers from 10 patients. In vitro studies indicated that stabilization of nuclear beta-catenin inhibited wound healing and keratinocyte   migration   by blocking epidermal growth factor response, inducing c-myc and repressing the K6/K16 keratins (cytoskeletal components important for   migration  ). The molecular mechanism of K6/K16 repression involved beta-catenin and arginine methyltransferase (CARM-1) acting as co-repressors of glucocorticoid receptor monomers. We conclude that activation of the beta-catenin/c-myc pathway(s) contributes to impaired healing by inhibiting keratinocyte   migration   and altering their differentiation. The presence of activated beta-catenin and c-myc in the epidermis of chronic wounds may serve as a molecular marker of impaired healing and may provide future targets for therapeutic intervention. 
 Decorin antagonizes Met receptor activity and down-regulates {beta}-catenin and Myc levels. A theme emerging during the past few years is that members of the small leucine-rich proteoglycan gene family affect cell growth by interacting with multiple receptor tyrosine kinases (RTKs), mostly by a physical down-regulation of the receptors, thereby depriving tumor cells of pro-survival signals. Decorin binds and down-regulates several RTKs, including Met, the receptor for hepatocyte growth factor. Here we demonstrate that decorin blocks several biological activities mediated by the Met signaling axis, including cell scatter, evasion, and   migration  . These effects were mediated by a profound down-regulation of noncanonical β-catenin levels. In addition, Myc, a downstream target of β-catenin, was markedly down-regulated by decorin, whereas phosphorylation of Myc at threonine 58 was markedly induced. The latter is known to destabilize Myc and target it for proteasomal degradation. We also discovered that systemic delivery of decorin using three distinct tumor xenograft models caused down-regulation of Met and a concurrent suppression of β-catenin and Myc levels. We found that decorin protein core labeled with the near infrared dye IR800 specifically targeted the tumor cells expressing Met. Even 68-h post-injection, decorin was found to reside within the tumor xenografts with little or no binding to other tissues. Collectively, our results indicate a role for a secreted proteoglycan in suppressing the expression of key oncogenic factors required for tumor progression. 
 Novel c-MYC target genes mediate differential effects on cell proliferation and   migration  . The developmental and oncogenic roles of MYC proteins are well established, but the transcriptional targets mediating their functions remain elusive. Using small interfering RNA-mediated knockdown in breast and cervix carcinoma cell lines, which overexpress c-MYC, we show that c-MYC independently controls metabolism and cell proliferation, and can, depending on the cells, promote or inhibit   migration  . We identified new c-MYC target genes in these cell lines, and show that selective regulation of some targets correlates with the phenotypic responses of these different cell lines to c-MYC depletion. Notably, we show that a positive regulation of the WNT signalling pathway contributes to c-MYC pro-mitogenic effects in breast and cervix carcinoma cells. We also show that repression of CCL5/RANTES accounts for c-MYC anti-migratory effects in specific breast cancer cells. Our combined genomic and phenotypic analysis indicates that c-MYC functions are cellular-context-dependent and that selectively regulated genes are responsible for its differential properties. 
 MYCN downregulates integrin alpha1 to promote invasion of human neuroblastoma cells. Neuroblastoma is a childhood tumor thought to arise through improper differentiation of neural crest cells. MYCN amplification is a prognostic factor that indicates a highly malignant disease and poor patient prognosis. Integrins are important regulators of neuroblastoma attachment and   migration   and participate in many aspects of metastasis. However, the role of integrins in neuroblastoma metastasis, the leading cause of death from this disease, remains less well understood. Screening of neuroblastoma cell lines for integrin mRNA expression showed that integrin alpha1 expression was higher in lines such as SK-N-SH and NB69 that do not have MYCN amplification than in cell lines such as IMR32, NB1, NB9 and NB19 that have MYCN amplification. A knockdown of MYCN in NB1 and NB19 cells resulted in increased expression of integrin alpha1, which correlated with enhanced attachment to the extracellular matrix and reduced migratory activity. In contrast, the overexpression of MYCN in SK-N-SH and NB69 cells resulted in decreased expression of integrin alpha1, which correlated with reduced attachment to the extracellular matrix and enhanced migratory activity. These results show that MYCN may limit cell adhesion to the extracellular matrix and promote cell   migration   by downregulating integrin alpha1. 
 Repression of SRF target genes is critical for Myc-dependent apoptosis of epithelial cells. Oncogenic levels of Myc expression sensitize cells to multiple apoptotic stimuli, and this protects long-lived organisms from cancer development. How cells discriminate physiological from supraphysiological levels of Myc is largely unknown. Here, we show that induction of apoptosis by Myc in breast epithelial cells requires association of Myc with Miz1. Gene expression and ChIP-Sequencing experiments show that high levels of Myc invade target sites that lack consensus E-boxes in a complex with Miz1 and repress transcription. Myc/Miz1-repressed genes encode proteins involved in cell adhesion and   migration   and include several integrins. Promoters of repressed genes are enriched for binding sites of the serum-response factor (SRF). Restoring SRF activity antagonizes Myc repression of SRF target genes, attenuates Myc-induced apoptosis, and reverts a Myc-dependent decrease in Akt phosphorylation and activity, a well-characterized suppressor of Myc-induced apoptosis. We propose that high levels of Myc engage Miz1 in repressive DNA binding complexes and suppress an SRF-dependent transcriptional program that supports survival of epithelial cells. 
 A dihydroindolizino indole derivative selectively stabilizes G-quadruplex DNA and down-regulates c-MYC expression in human cancer cells. Telomeric and NHE III1, a c-MYC promoter region is abundant in guanine content and readily form G-quadruplex structures. Small molecules that stabilize G-quadruplex DNA were shown to reduce oncoprotein expression, initiate apoptosis and they may function as anticancer molecules.Electrospray ionization mass spectrometry, spectroscopy, isothermal titration calorimetry, Taq DNA polymerase stop assay, real time PCR and luciferase reporter assay. Cell   migration   assay to find out the effect of derivatives on normal as well as cancer cell proliferation.Among three different dihydroindolizino indole derivatives, 4-cyanophenyl group attached derivative has shown maximum affinity, selective interaction and higher stability towards G-quadruplex DNA over dsDNA. Further, as a potential G-quadruplex DNA stabilizer, 4-cyanophenyl linked dihydroindolizino indole derivative was found to be more efficient in inhibiting in vitro DNA synthesis, c-MYC expression and cancer cell proliferation among human cancer cells.The present study reveals that dihydroindolizino indole derivative having 4-cyanophenyl group has potential to stabilize G-quadruplex DNA and exhibit anticancer activity.These studies are useful in the identification and synthesis of lead derivatives that will selectively stabilize G-quadruplex DNA and function as anticancer agents. 
 c-MYC-regulated miR-23a/24-2/27a cluster promotes mammary carcinoma cell invasion and hepatic metastasis by targeting Sprouty2. Emerging evidence indicates that the miR-23a/24-2/27a cluster may possess a causal role in mammary tumorigenesis and function as a novel class of oncogenes. However, the regulatory mechanism of the miR-23a/24-2/27a cluster in mammary carcinoma cell invasion and   migration   is still largely unknown. We observed that the expression levels of miR-23a, miR-24-2 and miR-27a were significantly higher in breast cancer with lymph node metastasis, compared with that from patients without lymph node metastasis or normal tissue. Forced expression of the miR-23a/24-2/27a cluster promoted mammary carcinoma cell   migration  , invasion, and hepatic metastasis, through targeting Sprouty2 (SPRY2) and consequent activation of p44/42 MAPK. Epidermal growth factor induced the expression of the transcription factor c-MYC, which promoted the expression of mature miR-23a, miR-24-2, and miR-27a and subsequently decreased expression of SPRY2 and activated p44/42 MAPK to promote mammary carcinoma cell   migration   and invasion. We therefore suggest a novel link between epidermal growth factor and the miR-23a/24-2/27a cluster via the regulation of c-MYC, providing the potential for the miR-23a/24-2/27a cluster to be used as biomarker in the diagnosis and/or treatment of breast cancer. 
 The MZF1/c-MYC axis mediates lung adenocarcinoma progression caused by wild-type lkb1 loss. Liver kinase B1 (LKB1) loss in lung adenocarcinoma is commonly caused by genetic mutations, but these mutations rarely occur in Asian patients. We recently reported wild-type LKB1 loss via the alteration of NKX2-1/p53-axis-promoted tumor aggressiveness and predicted poor outcomes in cases of lung adenocarcinoma. The mechanistic action of wild-type LKB1 loss within tumor progression remains unknown. The suppression of MYC by LKB1 controls epithelial organization; therefore, we hypothesize that MYC expression can be increased via wild-type LKB1 loss and promotes tumor progression. Here, MYC transcription is upregulated by LKB1-loss-mediated MZF1 expression. The wild-type LKB1-loss-mediated MZF1/MYC axis is responsible for soft-agar growth,   migration   and invasion in lung adenocarcinoma cells. Moreover, wild-type LKB1 loss-induced cell invasiveness was markedly suppressed by MYC inhibitors (10058-F4 and JQ1). Patients with low-LKB1/high-MZF1 or low-LKB1/high-MYC tumors have shorter overall survival and relapse-free-survival periods than patients with high-LKB1/low-MZF1 or high-LKB1/low-MYC tumors. In summary, MZF1-mediated MYC expression may promote tumor progression, resulting in poor outcomes in cases of lung adenocarcinoma with low-wild-type-LKB1 tumors. 
 Overexpression of GYS1, MIF, and MYC is associated with adverse outcome and poor response to azacitidine in myelodysplastic syndromes and acute myeloid leukemia. The prognosis of myelodysplastic syndromes (MDS) and acute myeloid leukaemia (AML) is very heterogeneous.We analyzed the prognostic value of several genes in a cohort of 85 MDS and AML patients.Overexpression of glycogen synthase 1 and macrophage   migration   inhibitory factor genes had an adverse outcome in multivariate analysis (P = .003 and P < .001, respectively). Furthermore, the higher expression of myelocytomatosis oncogene was associated with a lower response to azacitidine (P = .03).In the current study we identified a specific gene expression profile as prognostic factors for response to azacitidine and survival in MDS and AML. 
 Tumor suppressor miR-375 regulates MYC expression via repression of CIP2A coding sequence through multiple miRNA-mRNA interactions. MicroRNAs (miRNAs) are small, noncoding RNAs involved in posttranscriptional regulation of protein-coding genes in various biological processes. In our preliminary miRNA microarray analysis, miR-375 was identified as the most underexpressed in human oral tumor versus controls. The purpose of the present study is to examine the function of miR-375 as a candidate tumor suppressor miRNA in oral cancer. Cancerous inhibitor of PP2A (CIP2A), a guardian of oncoprotein MYC, is identified as a candidate miR-375 target based on bioinformatics. Luciferase assay accompanied by target sequence mutagenesis elucidates five functional miR-375-binding sites clustered in the CIP2A coding sequence close to the C-terminal domain. Overexpression of CIP2A is clearly demonstrated in oral cancers, and inverse correlation between miR-375 and CIP2A is observed in the tumors, as well as in NCI-60 cell lines, indicating the potential generalized involvement of the miR-375-CIP2A relationship in many other cancers. Transient transfection of miR-375 in oral cancer cells reduces the expression of CIP2A, resulting in decrease of MYC protein levels and leading to reduced proliferation, colony formation,   migration  , and invasion. Therefore this study shows that underexpression of tumor suppressor miR-375 could lead to uncontrolled CIP2A expression and extended stability of MYC, which contributes to promoting cancerous phenotypes. 
 Integrin α1β1 expression is controlled by c-MYC in colorectal cancer cells. The α1β1 collagen receptor is only present in a few epithelial cell types. In the intestine, it is specifically expressed in proliferating crypt cells. This integrin has been reported to be involved in various cancers where it mediates the downstream activation of the Ras/ERK proliferative pathway. We have recently shown that integrin α1β1 is present in two-thirds of colon adenocarcinomas, but the mechanism by which ITGA1 expression is regulated is not known. DNA methylation, involved in ITGA1 repression during megakaryocyte differentiation, is not the mechanism of ITGA1 regulation in colorectal cancer cells. Our in silico analysis of the ITGA1 promoter revealed two response elements for MYC, an oncogenic factor known to regulate cancer cell proliferation, invasio  n and mig  ration. In situ, the expressions of both MYC and ITGA1 are localized in the lower crypt of the normal colon and correlate in 72% of the 65 analyzed colorectal cancers. MYC pharmacological inhibition or downregulation of expression with short hairpin RNA in HT29, T84 and SW480 cells resulted in reduced ITGA1 expression at both the transcript and protein levels. Chromatin immunoprecipitation assays revealed that MYC was bound to the chromatin region of the ITGA1 proximal promoter, whereas MYC overexpression enhanced ITGA1 promoter activity that was reduced with MAD co-transfection or by the disruption of the response elements. We concluded that MYC is a key regulating factor for the control of ITGA1 expression. 
 Myc coordinates transcription and translation to enhance transformation and suppress invasiveness. c-Myc is one of the major human proto-oncogenes and is often associated with tumor aggression and poor clinical outcome. Paradoxically, Myc was also reported as a suppressor of cell motility, invasiveness, and metastasis. Among the direct targets of Myc are many components of the protein synthesis machinery whose induction results in an overall increase in protein synthesis that empowers tumor cell growth. At present, it is largely unknown whether beyond the global enhancement of protein synthesis, Myc activation results in translation modulation of specific genes. Here, we measured Myc-induced global changes in gene expression at the transcription, translation, and protein levels and uncovered extensive transcript-specific regulation of protein translation. Particularly, we detected a broad coordination between regulation of transcription and translation upon modulation of Myc activity and showed the connection of these responses to mTOR signaling to enhance oncogenic transformation and to the TGFβ pathway to modulate cell   migratio  n and invasiveness. Our results elucidate novel facets of Myc-induced cellular responses and provide a more comprehensive view of the consequences of its activation in cancer cells. 
 MYC suppresses cancer metastasis by direct transcriptional silencing of αv and β3 integrin subunits. Overexpression of MYC transforms cells in culture, elicits malignant tumours in experimental animals and is found in many human tumours. We now report the paradoxical finding that this powerful oncogene can also act as a suppressor of cell motility, invasiveness and metastasis. Overexpression of MYC stimulated proliferation of breast cancer cells both in culture and in vivo as expected, but inhibited motility and invasiveness in culture, and lung and liver metastases in xenografted tumours. We show further that MYC represses transcription of both subunits of αvβ3 integrin, and that exogenous expression of β3 integrin in human breast cancer cells that do not express this integrin rescues invasiveness   and migr  ation when MYC is downregulated. These data uncover an unexpected function of MYC, provide an explanation for the hitherto puzzling literature on the relationship between MYC and metastasis, and reveal a variable that could influence the development of therapies that target MYC. 
 Overexpression of the LKB1 gene inhibits lung carcinoma cell proliferation partly through degradation of c-myc protein. LKB1 encodes a serine/threonine kinase generally inactivated in human lung cancers, which mediates cancer cell proliferation,   migration   and differentiation, but its biological function has not been completely elucidated. In this study, we demonstrated that LKB1 was associated with a substantial reduction of c-myc expression by using an inducible LKB1 expression system in the LKB1-null lung cell line A549. Nevertheless, the reduction of the c-Myc gene expression was not accompanied by corresponding reduction of mRNAs but protein, which can be abrogated by a proteosome inhibitor (MG132), suggesting that the reduction was associated with their increased degradation rather than transcriptional controls. Our results implied that the expression of c-Myc protein decreased by LKB1 in transfected cells may be a contributory factor in the process of cell proliferation. Overexpression of the LKB1 gene could inhibit the activation of ERK1/2 and STAT3 signaling pathways involved in the cell proliferation. Thus, LKB1-induced functional operation on c-Myc in promoting cell proliferation may occur in a novel mechanism, which may be regulated by ERK1/2 and/or STAT3 signal pathways in human lung carcinoma cells. Furthermore, our results give some insights into the understanding of how LKB1 inactivation contributes to lung carcinogenesis and emphasizes the central role played by LKB1 in lung cancer development. 
 Down-Regulation of MiR-1294 is Related to Dismal Prognosis of Patients with Esophageal Squamous Cell Carcinoma through Elevating C-MYC Expression. Changes in the expression of microRNAs (miRNAs) have been found in many cancers. This study aimed to investigate the expression of miR-1294 in patients with esophageal squamous cell carcinoma (ESCC) and its effect on prognosis. The underlying mechanism was explored as well.We examined the expression of miRNA in human ESCC cancer tissues and adjacent non-tumor controls using quantitative reverse transcription polymerase chain reaction (qRT-PCR). And the relationship between expressions of miR-1294 and ESCC prognosis was analyzed in this study. Over-expression and knock-down methods were used to investigate the biological functions of miRNA-1294. The effect of miRNA-1294 on cell proliferation was evaluated by MTT. Besides, the function of miR-1294 on cell   migration   and invasion were evaluated by transwell assays.MiR-1294 was significantly down-regulated in human ESCC tissues compared with the non-tumor controls tissues (P=0.014). And patients with low miR-1294 expression had a significantly poorer prognosis than those with a high miR-1294 expression (P=0.040). Negative association was defined between the expression of miR-1294 and the c-MYC expression in ESCC patients (Pearson correlation, r=-0.299, P=0.0079). Additionally, it was found that miR-1294 suppress esophageal cancer cells proliferation,   migration   and invasion capacity through targeting c-MYC in vitro.Down-regulation of miR-1294 correlates with poor prognosis of ESCC. It's partially due to the reduced function of c-MYC. This study may give insight into the understanding of pathogenesis of esophageal cancer and provide evidence for diagnosis and treatment of esophageal cancer. 
 p63 regulates human keratinocyte proliferation via MYC-regulated gene network and differentiation commitment through cell adhesion-related gene network. Although p63 and MYC are important in the control of epidermal homeostasis, the underlying molecular mechanisms governing keratinocyte proliferation or differentiation downstream of these two genes are not completely understood. By analyzing the transcriptional changes and phenotypic consequences of the loss of either p63 or MYC in human developmentally mature keratinocytes, we have characterized the networks acting downstream of these two genes to control epidermal homeostasis. We show that p63 is required to maintain growth and to commit to differentiation by two distinct mechanisms. Knockdown of p63 led to down-regulation of MYC via the Wnt/β-catenin and Notch signaling pathways and in turn reduced keratinocyte proliferation. We demonstrate that a p63-controlled keratinocyte cell fate network is essential to induce the onset of keratinocyte differentiation. This network contains several secreted proteins involved in cell   migratio  n/adhesion, including fibronectin 1 (FN1), interleukin-1β (IL1B), cysteine-rich protein 61 (CYR61), and jagged-1 (JAG1), that act downstream of p63 as key effectors to trigger differentiation. Our results characterized for the first time a connection between p63 and MYC and a cell adhesion-related network that controls differentiation. Furthermore, we show that the balance between the MYC-controlled cell cycle progression network and the p63-controlled cell adhesion-related network could dictate skin cell fate. 
 The impact of C-MYC gene expression on gastric cancer cell. The upregulation or mutation of C-MYC has been observed in gastric, colon, breast, and lung tumors and in Burkitt's lymphoma. However, little is known about the role C-MYC plays in gastric adenocarcinoma. In the present study, we intended to investigate the influence of C-MYC on the growth, proliferation, apoptosis, invasion, and cell cycle of the gastric cancer cell line SGC7901 and the gastric cell line HFE145. C-MYC cDNA was subcloned into a constitutive vector PCDNA3.1 followed by transfection in normal gastric cell line HFE145 by using liposome. Then stable transfectants were selected and appraised. Specific inhibition of C-MYC was achieved using a vector-based siRNA system which was transfected in gastric cancer cell line SGC7901. The apoptosis and cell cycles of these clones were analyzed by using flow cytometric assay. The growth and proliferation were analyzed by cell growth curves and colony-forming assay, respectively. The invasion of these clones was analyzed by using cell   migration   assay. The C-MYC stable expression clones (HFE-Myc) and C-MYC RNAi cells (SGC-MR) were detected and compared with their control groups, respectively. HFE-Myc grew faster than HFE145 and HFE-PC (HFE145 transfected with PCDNA3.1 vector). SGC-MR1, 2 grew slower than SGC7901 and SGC-MS1, 2 (SGC7901 transfected with scrambled control duplexes). The cell counts of HFE-Myc in the third, fourth, fifth, sixth, and seventh days were significantly more than those of control groups (P < 0.05). Those of SGC-MR1, 2 in the fourth, fifth, sixth, and seventh days were significantly fewer than those of control groups (P < 0.05). Cell cycle analysis showed that proportions of HFE-Myc and SGC-MR cells in G0-G1 and G2-M were different significantly with their control groups, respectively (P < 0.05). The apoptosis rate of HFE-Myc was significantly higher than those of control groups (P < 0.05). Results of colony-forming assay showed that the colony formation rate of HFE-Myc was higher than those of control groups; otherwise, the rate of SGC-MR was lower than those of their control groups (P < 0.05). The results of cell   migration   assay showed that there were no significant differences between experimental groups and control groups (P > 0.05). In conclusion, C-MYC can promote the growth and proliferation of normal gastric cells, and knockdown of C-MYC can restrain the growth and proliferation of gastric cancer cells. It can induce cell apoptosis and help tumor cell maintain malignant phenotype. But it can have not a detectable influence on the ability of invasion of gastric cancer cells. 
 Death-associated protein kinase 3 controls the tumor progression of A549 cells through ERK MAPK/c-Myc signaling. Death-associated protein kinases (DAPKs) are members of the serine/threonine protein kinase family, which regulate cell death. Although DAPK3 has been implicated as a tumor suppressor, a recent study revealed an oncogenic role of DAPK3. However, the role of DAPK3 in non-small cell lung cancer (NSCLC) remains unclear. Therefore, we examined whether DAPK3 controls the progression of NSCLC using the NSCLC cell line, A549. We generated A549 cells stably expressing small hairpin RNA (shRNA) targeting DAPK3. In the A549 cells, the protein level of DAPK3 was decreased and the cell proliferation was inhibited. DAPK3 knockdown caused G1/G0 cell cycle arrest as assessed by flow cytometric assay and reduced cyclin D1 expression in A549 cells. Phosphorylation of ERK and c-Myc, but not Akt and JNK, was inhibited by DAPK3 knockdown. Cell   migratio  n and invasion were also inhibited by DAPK3 knockdown as determined by a Boyden chamber assay and an invasion assay, respectively. Moreover, DAPK3 knockdown inhibited anchorage-independent cell growth as determined by soft-agar colony formation assay. In a mouse xenograft model, tumors derived from DAPK3-knockdown cells exhibited reduced tumor growth. The present results demonstrated for the first time that DAPK3 controls proliferation,   migratio  n, invasion, soft‑agar colony formation and tumor growth through activation of ERK/c-Myc signaling in A549 cells. These findings indicate that DAPK3 may be a novel target for the treatment of NSCLC. 
 CIP2A mediates prostate cancer progression via the c-MYC signaling pathway. Recent evidence suggests that cancerous inhibitor of protein phosphatase 2A (CIP2A) is an oncoprotein that acts as a novel therapeutic target in a variety of tumors. In this study, we investigated the clinical significance of CIP2A and its function in our large collection of prostate samples. Between August 2000 and December 2013, 126 patients with histologically confirmed PCa and 92 with benign prostate hyperplasia (BPH) were recruited into the study. Quantitative RT-PCR, Western blot, and immunohistochemistry analyses were used to quantify CIP2A expression in PCa clinical samples and cell lines. The relationships between CIP2A expression and clinicopathological features were analyzed. The functional role of CIP2A in PCa cells was evaluated by small interfering RNA-mediated depletion of the protein followed by analyses of cell proliferation and invasion. High expression of CIP2A staining was 86.51 % (109/126) in 126 cases of PCa and 17.39 % (16/92) in 92 cases of BPH, and the difference of CIP2A expression between PCa and BPH was statistically significant. CIP2A was significantly elevated in all five PCa cell lines when compared to the RWPE-1 cells at both the messenger RNA (mRNA) and protein levels. Silencing of CIP2A inhibited the proliferation of DU-145 cells which have a relatively high level of CIP2A in a time- and concentration-dependent manner, and the invasion and   migration   of DU-145 cells were distinctly suppressed. Furthermore, CIP2A knockdown led to substantial reductions in c-Myc levels in PCa cell lines, but no significant change in phosphorylated Akt expression after CIP2A knockdown in DU-145 cells. Our data suggest that the pathogenesis of human PCa maybe mediated by CIP2A, and CIP2A inhibition treatment may provide a promising strategy for the antitumor therapy of PCa, and thus CIP2A could represent selective targets for the molecularly targeted treatments of PCa. 
 Regulation of adrenomedullin gene transcription and degradation by the c-myc gene. Adrenomedullin, a vasodilatory peptide originally isolated from pheochromocytoma, is known to regulate cell growth, apoptosis, and   migration  . Overexpression of the c-myc oncogene has been shown to suppress the mouse adrenomedullin gene via the initiator element. We investigated whether c-myc regulates rat and human adrenomedullin genes because there appears to be no initiator elements in their promoter regions. Transactivation of the human adrenomedullin gene by c-myc was demonstrated using a luciferase reporter construct containing an upstream sequence. Using a panel of isogenic rat fibroblast cell lines with differential c-myc expression obtained by targeted homologous recombination, we found markedly elevated adrenomedullin transcript levels in cells stably overexpressing c-myc but a minimal decrease in two independent cell lines containing a homozygous null deletion of c-myc. Degradation of adrenomedullin mRNA was enhanced by a c-myc transgene, resulting in a relatively reserved increase in cellular secretion of adrenomedullin-like immunoreactivity. These results indicate that c-myc transactivates rat and human adrenomedullin genes and accelerates the degradation rate of adrenomedullin mRNA. However, c-myc is not essential for basal expression of the adrenomedullin gene. 
 HepaCAM induces G1 phase arrest and promotes c-Myc degradation in human renal cell carcinoma. Hepatocyte cell adhesion molecule (hepaCAM) encodes a generally inactive phosphorylated glycoprotein which mediates cancer cell proliferation,   migration  , and differentiation. We have reported that hepaCAM is down-regulated in renal cell carcinoma (RCC) and takes responsibility of cell growth inhibition. However, the precise mechanisms of hepaCAM inhibits cell growth is still unknown. In this study, we demonstrated that re-expression of hepaCAM can cause an accumulation in G0/G1 phase in 786-0 cells. This reaction was accompanied by a substantial reduction of c-Myc expression through using an ectopic hepaCAM expression system. Furthermore, we found a comparable decrease in proliferation and G0/G1 accumulation of 786-0 and RC-2 cells after treatment with a small molecule c-Myc inhibitor, 10058-F4. This indicated that the down regulation of c-Myc was an essential process in controlling growth inhibitory actions of hepaCAM. Nevertheless, re-expression of hepaCAM results in apparent reduction of c-Myc protein with no corresponding reduction of c-Myc mRNA. This suggests that this reaction might take place at a post-transcriptional level rather than transcriptional one. Consistent with these findings, hepaCAM decreased c-Myc stability by increasing the proportion of c-Myc phosphorylation on T58 which can be abrogated by a proteasomal inhibitor (MG132). Thus, our research implies that the decrease in c-Myc protein expression, resulting from ectopic expression of hepaCAM, may contribute to the inhibition of proliferation in these cells. 
 Integration of proteomic and transcriptomic profiles identifies a novel PDGF-MYC network in human smooth muscle cells. Platelet-derived growth factor-BB (PDGF-BB) has been implicated in the proliferation,   migration   and synthetic activities of smooth muscle cells that characterize physiologic and pathologic tissue remodeling in hollow organs. However, neither the molecular basis of PDGFR-regulated signaling webs, nor the extent to which specific components within these networks could be exploited for therapeutic benefit has been fully elucidated.Expression profiling and quantitative proteomics analysis of PDGF-treated primary human bladder smooth muscle cells identified 1,695 genes and 241 proteins as differentially expressed versus non-treated cells. Analysis of gene expression data revealed MYC, JUN, EGR1, MYB, RUNX1, as the transcription factors most significantly networked with up-regulated genes. Forty targets were significantly altered at both the mRNA and protein levels. Proliferation,   migration   and angiogenesis were the biological processes most significantly associated with this signature, and MYC was the most highly networked master regulator. Alterations in master regulators and gene targets were validated in PDGF-stimulated smooth muscle cells in vitro and in a model of bladder injury in vivo. Pharmacologic inhibition of MYC and JUN confirmed their role in SMC proliferation and   migration  . Network analysis identified the diaphanous-related formin 3 as a novel PDGF target regulated by MYC and JUN, which was necessary for PDGF-stimulated lamellipodium formation.These findings provide the first systems-level analysis of the PDGF-regulated transcriptome and proteome in normal smooth muscle cells. The analyses revealed an extensive cohort of PDGF-dependent biological processes and connected key transcriptional effectors to their regulation, significantly expanding current knowledge of PDGF-stimulated signaling cascades. These observations also implicate MYC as a novel target for pharmacological intervention in fibroproliferative expansion of smooth muscle, and potentially in cancers in which PDGFR-dependent signaling or MYC activation promote tumor progression. 
 miR-451 suppresses bladder cancer cell   migration   and invasion via directly targeting c-Myc. MicroRNA (miRNA) expression is shown dysregulated in tumors. It has been reported that miR-451 alters gene expression and regulates tumorigenesis in various cancer tissues. However, its underlying biological significance in bladder cancer remains to be clarified. In the present study, we investigated the function and molecular mechanism of miR-451 involved in bladder cancer cell   migration   and invasion. Our results showed that miR-451 was downregulated in clinical bladder carcinoma tissues compared with adjacent bladder tissues. Overexpression of miR-451 significantly retarded the proliferation,   migration   and invasion of bladder cancer T24 and 5637 cells in vitro. Moreover, the attenuated cell   migratio  n and invasion by miR-451 was correlated with increased apoptosis. However, our dual-luciferase reporter assay validated that c-Myc, an oncogene in many tumors, was a direct target gene of miR-451 in bladder cancer. The expression of c-Myc was repressed by miR-451 in bladder cancer cells, and knockdown of c-Myc mimicked the effects of miR-451 overexpression. This discovery suggested that miR-451 is a tumor suppressor modulating bladder cancer cell   migratio  n and invasion by directly targeting c-Myc. In addition, apoptosis promoted by miR-451 may participates in this biological behavior. Therefore, target miR-451 may be a novel therapeutic intervention for bladder cancer. 
 B7-H1, which represses EBV-immortalized B cell killing by autologous T and NK cells, is oppositely regulated by c-Myc and EBV latency III program at both mRNA and secretory lysosome levels. EBV-immortalized B cells induce a complex immune response such that the virus persists as a clinically silent infection for the lifetime of the infected host. B7-H1, also called PD-L1, is a cosignaling molecule of the B7 family that can inhibit activated T cell effectors by interaction with its receptor PD-1. In this work, we have studied the dependence of B7-H1 on NF-κB and c-Myc, the two main transcription factors in EBV latency III proliferating B cells, on various lymphoblastoid and Burkitt lymphoma cell lines, some of them being inducible or not for the EBV latency III program and/or for c-Myc. We found that B7-H1 repressed killing of EBV-immortalized B cells by their autologous T and NK cells. At the mRNA level, NF-κB was a weak inducer whereas c-Myc was a strong repressor of B7-H1 expression, an effect mediated by STAT1 inhibition. At the protein level, B7-H1 molecules were stored in both degradative and unconventional secretory lysosomes. Surface membrane B7-H1 molecules were constitutively internalized and proteolyzed in lysosomes. The EBV latency III program increased the amounts of B7-H1-containing secretory lysosomes and their export to the surface membrane. By repressing actin polymerization, c-Myc blocked secretory lysosom  e migrati  on and B7-H1 surface membrane export. In addition to B7-H1, various immunoregulatory molecules participating in the immunological synapse are stored in secretory lysosomes. By playing on actin polymerization, c-Myc could thus globally regulate the immunogenicity of transformed B cells, acting on export of secretory lysosomes to plasma membrane. 
 Overexpression of SOX18 promotes prostate cancer progression via the regulation of TCF1, c-Myc, cyclin D1 and MMP-7. Sex determining region Y (SRY)-box 18 (SOX18) gene encodes transcription factors that have been recently confirmed to be overexpressed in various human types of cancer and maintain the malignant behavior of cancer cells. However, the role and its potential function in prostate cancer (PCa) has not been demonstrated and the mechanisms of SOX18 involved in tumor progression remain largely unclear. In the present study, the expression of SOX18 was analyzed in 98 PCa and 81 adjacent non-tumor tissues using immunohistochemistry. The data showed that SOX18 was overexpressed in 72 of 98 (73.5%) PCa tissues compared with that in 28 of 81 (34.6%) non-tumor tissues. In addition, the expression of SOX18 was related with the clinical features of patients with PCa. To explore the potential role of SOX18 in PCa cells, Cell Counting Kit-8 (CCK-8  ), migrat  ion, invasion and xenograft assays were performed. Our data showed that knockdown of SOX18 decreased the proliferatio  n, migrat  ion and invasion of PCa cells in vitro, in addition to the tumor growth in vivo. Markedly, SOX18 knockdown caused the decreased expression of TCF1, c-Myc, cyclin D1 and MMP-7. In conclusion, SOX18 was overexpressed in PCa and may regulate the malignant capacity of cells via the upregulation of TCF1, c-Myc, cyclin D1 and MMP-7. 
 Repression of miR-17-5p with elevated expression of E2F-1 and c-MYC in non-metastatic hepatocellular carcinoma and enhancement of cell growth upon reversing this expression pattern. E2F-1, c-MYC, and miR-17-5p is a triad of two regulatory loops: a negative and a positive loop, where c-MYC induces the expression of E2F-1 that induces the expression of miR-17-5p which in turn reverses the expression of E2F-1 to close the loop. In this study, we investigated this triad for the first time in hepatocellular carcinoma (HCC), where miR-17-5p showed a significant down-regulation in 23 non-metastatic HCC biopsies compared to 10 healthy tissues; however, E2F-1 and c-MYC transcripts were markedly elevated. Forced over-expression of miR-17-5p in HuH-7 cells resulted in enhanced cell proliferation, growth,   migration   and clonogenicity with concomitant inhibition of E2F-1 and c-MYC transcripts expressions, while antagomirs of miR-17-5p reversed these events. In conclusion, this study revealed a unique pattern of expression for miR-17-5p in non-metastatic HCC patients in contrast to metastatic HCC patients. In addition we show that miR-17-5p is the key player among the triad that tumor growth and spread. 
 MUC16-mediated activation of mTOR and c-Myc reprograms pancreatic cancer metabolism. MUC16, a transmembrane mucin, facilitates pancreatic adenocarcinoma progression and metastasis. In the current studies, we observed that MUC16 knockdown pancreatic cancer cells exhibit reduced glucose uptake and lactate secretion along with reduced   migration   and invasion potential, which can be restored by supplementing the culture media with lactate, an end product of aerobic glycolysis. MUC16 knockdown leads to inhibition of mTOR activity and reduced expression of its downstream target c-MYC, a key player in cellular growth, proliferation and metabolism. Ectopic expression of c-MYC in MUC16 knockdown pancreatic cancer cells restores the altered cellular physiology. Our LC-MS/MS based metabolomics studies indicate global metabolic alterations in MUC16 knockdown pancreatic cancer cells, as compared to the controls. Specifically, glycolytic and nucleotide metabolite pools were significantly decreased. We observed similar metabolic alterations that correlated with MUC16 expression in primary tumor tissue specimens from human pancreatic adenocarcinoma cancer patients. Overall, our results demonstrate that MUC16 plays an important role in metabolic reprogramming of pancreatic cancer cells by increasing glycolysis and enhancing motility and invasiveness. 
 MiR-494 Inhibits Epithelial Ovarian Cancer Growth by Targeting c-Myc. BACKGROUND Epithelial ovarian cancer (EOC) is the most lethal malignant gynecological cancer. MicroRNAs (miRNAs) play important roles in the pathogenesis of ovarian cancer. The role of miR-494 in EOC has not been fully investigated. MATERIAL AND METHODS MiR-494 levels in ovarian cancer tissues and cells were tested by qRT-PCR. Cells were transfected with miR-494 mimics or miR-494 ASO by Lipofectamine. Bioinformatics algorithms from TargetScanHuman were used to predict the target genes of miR-494. The c-Myc protein level was assayed by Western blot. The interaction between miR-494 and c-Myc was confirmed by dual luciferase assays. RESULTS MiR-494 showed low levels in EOC tissues and cells. Overexpression of miR-494 inhibited cell growth and   migration   of EOC cells and vice versa. c-Myc is the targeted gene of miR-494. CONCLUSIONS MiR-494 has an anti-tumor role in EOC via c-Myc. 
 Long noncoding ribonucleic acids maternally expressed gene 3 inhibits lung cancer tumor progression through downregulation of MYC. Long noncoding ribonucleic acids (RNAs) nowadays emerge as important biomarkers or potential therapeutic targets discussed in human cancers. Among them, maternally expressed gene 3 (MEG3) is known to be decreased in a variety of malignancies.Quantitative reverse transcription-polymerase chain reaction (qRT-PCR) was performed to detect the expression of MEG3 in forty pairs of lung cancer (LC) tissues. Overexpression of MEG3 was carried out, and we determined its effect on cell proliferation, apoptosis, and   migration   evaluated by cell counting kit-8, flow cytometric, and transwell analysis. Messenger RNA and protein expression of MYC were determined by qRT-PCR and western blot, respectively.The expression of MEG3 was downregulated in LC tissues. Forced expression of MEG3 led to reduced abilities of cell proliferation and elevated apoptosis rate. It also slightly inhibited cell   migration   capacity in vitro. In addition, MYC was inhibited by MEG3 overexpression at both transcriptional and translational levels.Our findings revealed MEG3 could regulate LC progression and serve as an important target for LC treatment. 
 CIP2A mediates prostate cancer progression via the c-Myc signaling pathway. Recent evidence suggests that cancerous inhibitor of protein phosphatase 2A (CIP2A) is an oncoprotein that acts as a novel therapeutic target in a variety of tumors. In this study, we investigated the clinical significance of CIP2A and its function in our large collection of prostate samples. Between August 2000 and December 2013, 126 patients with histologically confirmed prostate cancer (PCa) and 92 with benign prostate hyperplasia (BPH) were recruited into the study. Quantitative real-time PCR (RT-PCR), Western blot, and immunohistochemistry analyses were used to quantify CIP2A expression in PCa clinical samples and cell lines. The relationships between CIP2A expression and clinicopathological features were analyzed. The functional role of CIP2A in PCa cells was evaluated by small interfering RNA-mediated depletion of the protein followed by analyses of cell proliferation and invasion. High expression of CIP2A staining was 86.51 % (109/126) in 126 cases of PCa and 17.39 % (16/92) in 92 cases of BPH; the difference of CIP2A expression between PCa and BPH was statistically significant. CIP2A was significantly elevated in all five PCa cell lines when compared to the RWPE-1 cells at both the messenger RNA (mRNA) and protein levels. Silencing of CIP2A inhibited the proliferation of DU-145 cells which have a relatively high level of CIP2A in a time- and concentration-dependent manner, and the invasion and   migration   of DU-145 cells were distinctly suppressed. Furthermore, CIP2A knockdown led to substantial reductions in c-Myc levels in DU-145 cells, but no significant change in phosphorylated Akt expression after CIP2A knockdown in DU-145 cells. Our data suggest that the pathogenesis of human PCa maybe mediated by CIP2A, and CIP2A inhibition treatment may provide a promising strategy for the antitumor therapy of PCa, and thus, CIP2A could represent selective targets for the molecularly targeted treatments of PCa. 
 MYC and metastasis. Aggressive primary tumors express transcriptional signatures that correlate with their metastatic propensity. A number of these signatures have been deployed in the clinic as risk stratification tools. However, the molecular basis of these clinically useful prognostic signatures has remained a largely unresolved area of controversy. We recently found that many prognostic signatures reflect the activity of the MYC oncogene, which in turn regulates tumor metastasis through specific effects on cancer cell invasion and   migration  . These findings offer a general framework for understanding the molecular basis of clinically prognostic transcriptional signatures and suggest potentially new avenues for studying metastasis. 
 MicroRNA-34a modulates c-Myc transcriptional complexes to suppress malignancy in human prostate cancer cells. MicroRNA-34a (miR-34a), a potent mediator of tumor suppressor p53, has been reported to function as a tumor suppressor and miR-34a was found to be downregulated in prostate cancer tissues. We studied the functional effects of miR-34a on c-Myc transcriptional complexes in PC-3 prostate cancer cells. Transfection of miR-34a into PC-3 cells strongly inhibited in vitro cell proliferation, cell invasion and promoted apoptosis. Transfection of miR-34a into PC-3 cells also significantly inhibited in vivo xenograft tumor growth in nude mice. miR-34a downregulated expression of c-Myc oncogene by targeting its 3' UTR as shown by luciferase reporter assays. miR-34a was found to repress RhoA, a regulator of cell   migration   and invasion, by suppressing c-Myc-Skp2-Miz1 transcriptional complex that activates RhoA. Overexpression of c-Myc reversed miR-34a suppression of RhoA expression, suggesting that miR-34a inhibits invasion by suppressing RhoA through c-Myc. miR-34a was also found to repress c-Myc-pTEFB transcription elongation complex, indicating one of the mechanisms by which miR-34a has profound effects on cellular function. This is the first report to document that miR-34a suppresses assembly and function of the c-Myc-Skp2-Miz1 complex that activates RhoA and the c-Myc-pTEFB complex that elongates transcription of various genes, suggesting a novel role of miR-34a in the regulation of transcription by c-Myc complex. 
 Long noncoding RNA CCAT1, which could be activated by c-Myc, promotes the progression of gastric carcinoma. Long noncoding RNAs (lncRNAs) have been shown to have functional roles in cancer biology and are deregulated in many tumors. The specific aim of this study was to determine the role of a long noncoding RNA CCAT1 in the progression of gastric carcinoma and discover which factors contribute to the deregulation of CCAT1.A computational screen of CCAT1 promoter was conducted to search for transcription-factor-binding sites. The association of c-Myc with the CCAT1 promoter in vivo was tested by chromatin immunoprecipitation assay. CCAT1 promoter activities were examined by luciferase reporter assay. The function of the c-Myc binding site in the CCAT1 promoter region was tested by a promoter assay with nucleotide substitutions in the putative E-box. The effect of CCAT1 on gastric carcinoma cell proliferation and   migration   was tested using in vitro cell proliferation and   migration   assays.CCAT1 levels were markedly increased in gastric carcinoma tissues compared with normal tissues. c-Myc directly binds to the E-box element in the promoter region of CCAT1, and when ectopically expressed increased promoter activity and expression of CCAT1. Nucleotide substitutions in the E-box element in the promoter region abrogated c-Myc-dependent promoter activation. The expression of CCAT1 and c-Myc shows strong association in gastric carcinomas. Moreover, abnormally expressed CCAT1 promotes cell proliferation and   migration  .These data suggest that c-Myc induction of CCAT1 holds an important role in gastric carcinoma and implicate the potential application of CCAT1 in the treatment of gastric carcinoma. 
 Novel interaction of the Z-DNA binding domain of human ADAR1 with the oncogenic c-Myc promoter G-quadruplex. Both G-quadruplex and Z-DNA can be formed in G-rich and repetitive sequences on genome, and their formation and biological functions are controlled by specific proteins. Z-DNA binding proteins, such as human ADAR1, have a highly conserved Z-DNA binding domain having selective affinity to Z-DNA. Here, our study identifies the Z-DNA binding domain of human ADAR1 (hZαADAR1) as a novel G-quadruplex binding protein that recognizes c-myc promoter G-quadruplex formed in NHEIII1 region and represses the gene expression. An electrophoretic   migratio  n shift assay shows the binding of hZαADAR1 to the intramolecular c-myc promoter G-quadruplex-forming DNA oligomer. To corroborate the binding of hZαADAR1 to the G-quadruplex, we conducted CD and NMR chemical shift perturbation analyses. CD results indicate that hZαADAR1 stabilizes the parallel-stranded conformation of the c-myc G-quadruplex. The NMR chemical shift perturbation data reveal that the G-quadruplex binding region in hZαADAR1 was almost identical with the Z-DNA binding region. Finally, promoter assay and Western blot analysis show that hZαADAR1 suppresses the c-myc expression promoted by NHEIII1 region containing the G-quadruplex-forming sequence. This finding suggests a novel function of Z-DNA binding protein as a regulator of G-quadruplex-mediated gene expression. 
 CIP2A signature reveals the MYC dependency of CIP2A-regulated phenotypes and its clinical association with breast cancer subtypes. Protein phosphatase 2A (PP2A) is a critical human tumor-suppressor complex. A recently characterized PP2A inhibitor protein, namely cancerous inhibitor of PP2A (CIP2A), has been found to be overexpressed at a high frequency in most of the human cancer types. However, our understanding of gene expression programs regulated by CIP2A is almost absent. Moreover, clinical relevance of the CIP2A-regulated transcriptome has not been addressed thus far. Here, we report a high-confidence transcriptional signature regulated by CIP2A. Bioinformatic pathway analysis of the CIP2A signature revealed that CIP2A regulates several MYC-dependent and MYC-independent gene programs. With regard to MYC-independent signaling, JNK2 expression and transwell   migration   were inhibited by CIP2A depletion, whereas MYC depletion did not affect either of these phenotypes. Instead, depletion of either CIP2A or MYC inhibited cancer cell colony growth with statistically indistinguishable efficiency. Moreover, CIP2A depletion was shown to regulate the expression of several established MYC target genes, out of which most were MYC-repressed genes. CIP2A small-interfering RNA-elicited inhibition of colony growth or activation of MYC-repressed genes was reversed at large by concomitant PP2A inhibition. Finally, the CIP2A signature was shown to cluster with basal-type and human epidermal growth factor receptor (HER)2-positive (HER2+) breast cancer signatures. Accordingly, CIP2A protein expression was significantly associated with basal-like (P=0.0014) and HER2+ (P<0.0001) breast cancers. CIP2A expression also associated with MYC gene amplification (P<0.001). Taken together, identification of CIP2A-driven transcriptional signature, and especially novel MYC-independent signaling programs regulated by CIP2A, provides important resource for understanding CIP2A's role as a clinically relevant human oncoprotein. With regard to MYC, these results both validate CIP2A's role in regulating MYC-mediated gene expression and provide a plausible novel explanation for the high MYC activity in basal-like and HER2+ breast cancers. 
 Analysis of secretome changes uncovers an autocrine/paracrine component in the modulation of cell proliferation and motility by c-Myc. Proteins secreted by cancer cells are a major component of tumor microenvironment. However, little is known on the impact of single oncogenic lesions on the expression of secreted proteins at early stages of tumor development. Because c-Myc overexpression is among the most frequent alterations in cancer, here we investigated the effect of sustained c-Myc expression on the secretome of a nontransformed human epithelial cell line (hT-RPE). By using a quantitative proteomic approach, we have identified 125 proteins in conditioned media of hT-RPE/MycER cells upon c-Myc induction. Analysis of the 49 proteins significantly down-regulated by c-Myc revealed a marked enrichment of factors associated with growth inhibition and cellular senescence. Accordingly, media conditioned by hT-RPE cells expressing c-Myc show an increased ability to sustain hT-RPE cellular proliferation/viability. We also find a marked down-regulation of several structural and regulatory components of the extracellular matrix (ECM), which correlates with an increased chemotactic potency of the conditioned media toward fibroblasts, a major cellular component of tumor stroma. In accordance with these data, the expression of the majority of the genes encoding proteins down-regulated in hT-RPE was significantly reduced also in colorectal adenomatous polyps, early tumors in which c-Myc is invariably overexpressed. These findings help to elucidate the significance of c-Myc overexpression at early stages of tumor development and uncover a remarkable autocrine/paracrine component in the ability of c-Myc to stimulate proliferation, sustain tumor maintenance, and modulate cell   migration  . 
 MicroRNA-34a inhibits human trophoblast cell invasion by targeting MYC. Preeclampsia, one of the major disorders of pregnancy, is characterized by inadequate trophoblast invasion and defective trophoblast-mediated remodeling of placental vasculature. MicroRNA-34a (miR-34a) has been found to be aberrantly expressed in the placentas of preeclamptic patients, yet its role in placental development and in the pathogenesis of preeclampsia remains elusive.The levels of miR-34a in the placentas of 20 preeclamptic patients and 20 healthy subjects were determined by real time-PCR, and miR-34a was found significantly elevated in the preeclamptic placentas. Further, the function of miR-34a in trophoblast cells was investigated by overexpressing miR-34a in JEG-3 trophoblast cell line. Overexpression of miR-34a in JEG-3 cells inhibited cell proliferation,   migration   and invasion. In addition, elevated expression of miR-34a reduced the expression of both endogenous and ectopic MYC. Moreover, we identified that MYC mRNA was a direct target of miR-34a in JEG-3 cells by dual luciferase reporter assay, and found that downregulation of MYC expression by miR-34a targeting significantly reduced the invasiveness of JEG-3 cells.Our findings provide preliminary evidence for the diverse functions of miR-34a in trophoblast biology, and suggest that miR-34a suppresses trophoblast invasion by directly targeting MYC. 
 The tumor-suppressive microRNA-135b targets c-myc in osteoscarcoma. Osteosarcoma is the most common primary tumor of the bone. It leads to many deaths because of its rapid proliferation and metastasis. Recent studies have shown that microRNAs are important gene regulators that are involved in various cancer-related processes. In this study, we found that miR-135b was down-regulated in both osteoscarcoma patient tumor tissues and osteoscarcoma cell lines in comparison to paired adjacent non-tumor bone tissue. We observed that a lower level of miR-135b was associated with metastasis. The ectopic expression of miR-135b markedly suppressed osteoscarcoma cell proliferation,   migration  , and invasion. Conversely, the inhibition of miR-135b expression dramatically accelerated cell proliferation,   migration  , and invasion. The forced expression of miR-135b in osteosarcoma cells resulted in a significant reduction in the protein level of c-Myc and repressed the activity of a luciferase reporter that contained the 3'-untranslated region of the c-Myc mRNA. These effects were abolished by the mutation of the predicted miR-135b-binding site, which indicates that c-Myc may be a miR-135b target gene. Moreover, the ectopic expression of c-Myc partially reversed the inhibition of cell proliferation and invasion that was caused by miR-135b. These data therefore suggest that miR-135b may function as a tumor suppressor to regulate osteosarcoma cell proliferation and invasion through a mechanism that targets the c-Myc oncogene. These findings indicate that miR-135b may play a role in the pathogenesis of osteosarcoma. 
 Tumor suppressor PDCD4 modulates miR-184-mediated direct suppression of C-MYC and BCL2 blocking cell growth and survival in nasopharyngeal carcinoma. Programmed cell death 4 (PDCD4), a novel tumor suppressor, inhibits cell proliferation,   migration   and invasion as well as promotes cell apoptosis in tumors. However, the molecular mechanism of its tumor-suppressive function remains largely unknown in tumors including nasopharyngeal carcinoma (NPC). In this study, downregulated PDCD4 expression was significantly associated with the status of NPC progression and poor prognosis. PDCD4 markedly suppressed the ability of cell proliferation and cell survival by modulating C-MYC-controlled cell cycle and BCL-2-mediated mitochondrion apoptosis resistance signals, and oncogenic transcription factor C-JUN in NPC. Furthermore, miR-184, a tumor-suppressive miRNA modulated by PDCD4 directly targeting BCL2 and C-MYC, participated in PDCD4-mediated suppression of cell proliferation and survival in NPC. Further, we found that PDCD4 decreased the binding of C-Jun to the AP-1 element on the miR-184 promoter regions by PI3K/AKT/JNK/C-Jun pathway and stimulated miR-184 expression. In clinical fresh specimens, reduced PDCD4 mRNA level was positively correlated with miR-184 expression in NPC. Our studies are the first to demonstrate that PDCD4 as tumor suppressor regulated miR-184-mediated direct targeting of BCL2 and C-MYC via PI3K/AKT and JNK/C-Jun pathway attenuating cell proliferation and survival in NPC. 
  GeneRIFs  
 This discovery suggested that miR-451 is a tumor suppressor modulating bladder cancer cell   migration   and invasion by directly targeting c-Myc 
 detected coordination between regulation of transcription and translation upon modulation of Myc activity and showed connection of these responses to mTOR signaling to enhance oncogenic transformation and to the TGFbeta pathway to modulate cell   migration   and invasiveness 
 p63 Regulates the   migration   and invasion of esophageal squamous carcinoma cells through activation of the beta-catenin/c-Myc pathway. 
 Over-expression of HMGCR in esophageal squamous cell carcinoma (ESCC) cells promoted cell growth and   migration  . 
 Studies indicate that many prognostic signatures reflect the activity of the MYC oncogene, which in turn regulates tumor metastasis through specific effects on cancer cell invasion and   migration  . 
 MYCN may limit cell adhesion to the extracellular matrix and promote cell   migration   by downregulating integrin alpha1 
  ECM  PubMed  
 Analysis of secretome changes uncovers an autocrine/paracrine component in the modulation of cell proliferation and motility by c-Myc. Proteins secreted by cancer cells are a major component of tumor microenvironment. However, little is known on the impact of single oncogenic lesions on the expression of secreted proteins at early stages of tumor development. Because c-Myc overexpression is among the most frequent alterations in cancer, here we investigated the effect of sustained c-Myc expression on the secretome of a nontransformed human epithelial cell line (hT-RPE). By using a quantitative proteomic approach, we have identified 125 proteins in conditioned media of hT-RPE/MycER cells upon c-Myc induction. Analysis of the 49 proteins significantly down-regulated by c-Myc revealed a marked enrichment of factors associated with growth inhibition and cellular senescence. Accordingly, media conditioned by hT-RPE cells expressing c-Myc show an increased ability to sustain hT-RPE cellular proliferation/viability. We also find a marked down-regulation of several structural and regulatory components of the extracellular matrix (  ECM  ), which correlates with an increased chemotactic potency of the conditioned media toward fibroblasts, a major cellular component of tumor stroma. In accordance with these data, the expression of the majority of the genes encoding proteins down-regulated in hT-RPE was significantly reduced also in colorectal adenomatous polyps, early tumors in which c-Myc is invariably overexpressed. These findings help to elucidate the significance of c-Myc overexpression at early stages of tumor development and uncover a remarkable autocrine/paracrine component in the ability of c-Myc to stimulate proliferation, sustain tumor maintenance, and modulate cell migration. 
  [Ee]xtracellular [Mm]atrix  PubMed  
 MYCN downregulates integrin alpha1 to promote invasion of human neuroblastoma cells. Neuroblastoma is a childhood tumor thought to arise through improper differentiation of neural crest cells. MYCN amplification is a prognostic factor that indicates a highly malignant disease and poor patient prognosis. Integrins are important regulators of neuroblastoma attachment and migration and participate in many aspects of metastasis. However, the role of integrins in neuroblastoma metastasis, the leading cause of death from this disease, remains less well understood. Screening of neuroblastoma cell lines for integrin mRNA expression showed that integrin alpha1 expression was higher in lines such as SK-N-SH and NB69 that do not have MYCN amplification than in cell lines such as IMR32, NB1, NB9 and NB19 that have MYCN amplification. A knockdown of MYCN in NB1 and NB19 cells resulted in increased expression of integrin alpha1, which correlated with enhanced attachment to the   extracellular matrix   and reduced migratory activity. In contrast, the overexpression of MYCN in SK-N-SH and NB69 cells resulted in decreased expression of integrin alpha1, which correlated with reduced attachment to the   extracellular matrix   and enhanced migratory activity. These results show that MYCN may limit cell adhesion to the   extracellular matrix   and promote cell migration by downregulating integrin alpha1. 
 MYC/BCL2 protein coexpression contributes to the inferior survival of activated B-cell subtype of diffuse large B-cell lymphoma and demonstrates high-risk gene expression signatures: a report from The International DLBCL Rituximab-CHOP Consortium Program. Diffuse large B-cell lymphoma (DLBCL) is stratified into prognostically favorable germinal center B-cell (GCB)-like and unfavorable activated B-cell (ABC)-like subtypes based on gene expression signatures. In this study, we analyzed 893 de novo DLBCL patients treated with R-CHOP (rituximab, cyclophosphamide, doxorubicin, vincristine, and prednisone). We show that MYC/BCL2 protein coexpression occurred significantly more commonly in the ABC subtype. Patients with the ABC or GCB subtype of DLBCL had similar prognoses with MYC/BCL2 coexpression and without MYC/BCL2 coexpression. Consistent with the notion that the prognostic difference between the 2 subtypes is attributable to MYC/BCL2 coexpression, there is no difference in gene expression signatures between the 2 subtypes in the absence of MYC/BCL2 coexpression. DLBCL with MYC/BCL2 coexpression demonstrated a signature of marked downregulation of genes encoding   extracellular matrix   proteins, those involving matrix deposition/remodeling and cell adhesion, and upregulation of proliferation-associated genes. We conclude that MYC/BCL2 coexpression in DLBCL is associated with an aggressive clinical course, is more common in the ABC subtype, and contributes to the overall inferior prognosis of patients with ABC-DLBCL. In conclusion, the data suggest that MYC/BCL2 coexpression, rather than cell-of-origin classification, is a better predictor of prognosis in patients with DLBCL treated with R-CHOP. 
 Amplification of   extracellular matrix   and oncogenes in tat-transfected human salivary gland cell lines with expression of laminin, fibronectin, collagens I, III, IV, c-myc and p53. Considerable progress has been made in the transfer of foreign genes into salivary glands in vivo using adenovirus vectors in rats. In an attempt to avoid the transient expression inherent, when using these vectors, retroviral vectors and human cell lines where used here in attempt to develop an in vitro model of HIV-associated salivary gland disease. The HIV-1-tat protein is increasingly implicated in the pathogenesis of the AIDS through altering the expression of strategic cellular genes. The purpose of this study was to transfect human salivary gland (HSG) cell lines in vitro, with the pHIV-1/LTR-tat plasmid, and examine the effect of tat on expression of matrix and basement membrane genes known to be important in the pathogenesis of salivary gland disease. HSG cells were transfected with HIV-1-tat plasmid by the lipofection method. Transfection was confirmed by polymerase chain reaction (PCR) and Southern blot, which verified that tat-specific DNA was present. Tat-mRNA was analysed by Northern blotting and quantified by reverse transcriptase polymerase chain reaction (RT-PCR) to demonstrate its expression. Numerous clones were found to contain integrated tat DNA sequences and analysis of mRNA showed stable expression of tat-specific RNA. Further analysis of mRNA expression for various marker proteins important in HIV pathogenesis showed that the HSG cell line transfected with HIV-1-tat, was associated with significant induction of mRNA expression for   extracellular matrix   protein. Tat-amplified transcription of the major basement membrane protein laminin, as well as of fibronectin, collagen I and III, and c-myc oncogene was demonstrated. Conversely, expression of p53 suppressor gene mRNA was reduced. Post-transfection expression of collagen IV was erratic and inconclusive. It was concluded that the presence of HIV-tat in this in vitro model of salivary ductal epithelial cell model alters the mRNA expression of several matrix, basement membrane and oncoproteins known to be involved in HIV pathogenesis. These cell lines provide a useful system for studying the role of tat in the immunopathogenesis of HIV-associated salivary gland disease. 
 K-Ras and B-Raf oncogenes inhibit colon epithelial polarity establishment through up-regulation of c-myc. KRAS, BRAF, and PI3KCA are the most frequently mutated oncogenes in human colon cancer. To explore their effects on morphogenesis, we used the colon cancer-derived cell line Caco-2. When seeded in   extracellular matrix  , individual cells proliferate and generate hollow, polarized cysts. The expression of oncogenic phosphatidylinositol 3-kinase (PI3KCA H1047R) in Caco-2 has no effect, but K-Ras V12 or B-Raf V600E disrupts polarity and tight junctions and promotes hyperproliferation, resulting in large, filled structures. Inhibition of mitogen-activated protein/extracellular signal-regulated kinase (ERK) kinase blocks the disruption of morphology, as well as the increased levels of c-myc protein induced by K-Ras V12 and B-Raf V600E. Apical polarity is already established after the first cell division (two-cell stage) in Caco-2 three-dimensional cultures. This is disrupted by expression of K-Ras V12 or B-Raf V600E but can be rescued by ribonucleic acid interference-mediated depletion of c-myc. We conclude that ERK-mediated up-regulation of c-myc by K-Ras or B-Raf oncogenes disrupts the establishment of apical/basolateral polarity in colon epithelial cells independently of its effect on proliferation. 
 Analysis of secretome changes uncovers an autocrine/paracrine component in the modulation of cell proliferation and motility by c-Myc. Proteins secreted by cancer cells are a major component of tumor microenvironment. However, little is known on the impact of single oncogenic lesions on the expression of secreted proteins at early stages of tumor development. Because c-Myc overexpression is among the most frequent alterations in cancer, here we investigated the effect of sustained c-Myc expression on the secretome of a nontransformed human epithelial cell line (hT-RPE). By using a quantitative proteomic approach, we have identified 125 proteins in conditioned media of hT-RPE/MycER cells upon c-Myc induction. Analysis of the 49 proteins significantly down-regulated by c-Myc revealed a marked enrichment of factors associated with growth inhibition and cellular senescence. Accordingly, media conditioned by hT-RPE cells expressing c-Myc show an increased ability to sustain hT-RPE cellular proliferation/viability. We also find a marked down-regulation of several structural and regulatory components of the   extracellular matrix   (ECM), which correlates with an increased chemotactic potency of the conditioned media toward fibroblasts, a major cellular component of tumor stroma. In accordance with these data, the expression of the majority of the genes encoding proteins down-regulated in hT-RPE was significantly reduced also in colorectal adenomatous polyps, early tumors in which c-Myc is invariably overexpressed. These findings help to elucidate the significance of c-Myc overexpression at early stages of tumor development and uncover a remarkable autocrine/paracrine component in the ability of c-Myc to stimulate proliferation, sustain tumor maintenance, and modulate cell migration. 
  GeneRIFs  
 MYCN may limit cell adhesion to the   extracellular matrix   and promote cell migration by downregulating integrin alpha1 
  [Ii]nvasive  PubMed  
 Genetic susceptibility to distinct bladder cancer subphenotypes. Clinical, pathologic, and molecular evidence indicate that bladder cancer is heterogeneous with pathologic/molecular features that define distinct subphenotypes with different prognoses. It is conceivable that specific patterns of genetic susceptibility are associated with particular subphenotypes.To examine evidence for the contribution of germline genetic variation to bladder cancer heterogeneity.The Spanish Bladder Cancer/EPICURO Study is a case-control study based in 18 hospitals located in five areas in Spain. Cases were patients with a newly diagnosed, histologically confirmed, urothelial cell carcinoma of the bladder from 1998 to 2001. Case diagnoses were reviewed and uniformly classified by pathologists following the World Health Organisation/International Society of Urological Pathology 1999 criteria. Controls were hospital-matched patients (n=1149).A total of 1526 candidate variants in 423 candidate genes were analysed. Three distinct subphenotypes were defined according to stage and grade: low-grade nonmuscle   invasive   (n=586), high-grade nonmuscle   invasive   (n=219), and muscle   invasive   (n=246). The association between each variant and subphenotype was assessed by polytomous risk models adjusting for potential confounders. Heterogeneity in genetic susceptibility among subphenotypes was also tested.Two established bladder cancer susceptibility genotypes, NAT2 slow-acetylation and GSTM1-null, exhibited similar associations among the subphenotypes, as did VEGF-rs25648, which was previously identified in our study. Other variants conferred risks for specific tumour subphenotypes such as PMS2-rs6463524 and CD4-rs3213427 (respective heterogeneity p values of 0.006 and 0.004), which were associated with muscle-  invasive   tumours (per-allele odds ratios [95% confidence interval] of 0.56 [0.41-0.77] and 0.71 [0.57-0.88], respectively) but not with non-muscle-  invasive   tumours. Heterogeneity p values were not robust in multiple testing according to their false-discovery rate.These exploratory analyses suggest that genetic susceptibility loci might be related to the molecular/pathologic diversity of bladder cancer. Validation through large-scale replication studies and the study of additional genes and single nucleotide polymorphisms are required. 
 DYRK2 priming phosphorylation of c-Jun and c-Myc modulates cell cycle progression in human cancer cells. Dysregulation of the G(1)/S transition in the cell cycle contributes to tumor development. The oncogenic transcription factors c-Jun and c-Myc are indispensable regulators at this transition, and their aberrant expression is associated with many malignancies. Degradation of c-Jun/c-Myc is a critical process for the G(1)/S transition, which is initiated upon phosphorylation by glycogen synthase kinase 3 β (GSK3β). However, a specific kinase or kinases responsible for priming phosphorylation events that precede this GSK3β modification has not been definitively identified. Here, we found that the dual-specificity tyrosine phosphorylation-regulated kinase DYRK2 functions as a priming kinase of c-Jun and c-Myc. Knockdown of DYRK2 in human cancer cells shortened the G(1) phase and accelerated cell proliferation due to escape of c-Jun and c-Myc from ubiquitination-mediated degradation. In concert with these results, silencing DYRK2 increased cell proliferation in human cancer cells, and this promotion was completely impeded by codeprivation of c-Jun or c-Myc in vivo. We also found marked attenuation of DYRK2 expression in multiple human tumor samples. Downregulation of DYRK2 correlated with high levels of unphosphorylated c-Jun and c-Myc and, importantly, wi  th invas  iveness of human breast cancers. These results reveal that DYRK2 regulates tumor progression through modulation of c-Jun and c-Myc. 
 DYRK2 priming phosphorylation of c-Jun and c-Myc modulates cell cycle progression in human cancer cells. Dysregulation of the G(1)/S transition in the cell cycle contributes to tumor development. The oncogenic transcription factors c-Jun and c-Myc are indispensable regulators at this transition, and their aberrant expression is associated with many malignancies. Degradation of c-Jun/c-Myc is a critical process for the G(1)/S transition, which is initiated upon phosphorylation by glycogen synthase kinase 3 β (GSK3β). However, a specific kinase or kinases responsible for priming phosphorylation events that precede this GSK3β modification has not been definitively identified. Here, we found that the dual-specificity tyrosine phosphorylation-regulated kinase DYRK2 functions as a priming kinase of c-Jun and c-Myc. Knockdown of DYRK2 in human cancer cells shortened the G(1) phase and accelerated cell proliferation due to escape of c-Jun and c-Myc from ubiquitination-mediated degradation. In concert with these results, silencing DYRK2 increased cell proliferation in human cancer cells, and this promotion was completely impeded by codeprivation of c-Jun or c-Myc in vivo. We also found marked attenuation of DYRK2 expression in multiple human tumor samples. Downregulation of DYRK2 correlated with high levels of unphosphorylated c-Jun and c-Myc and, importantly, wi  th invas  iveness of human breast cancers. These results reveal that DYRK2 regulates tumor progression through modulation of c-Jun and c-Myc. 
 Genetic susceptibility to distinct bladder cancer subphenotypes. Clinical, pathologic, and molecular evidence indicate that bladder cancer is heterogeneous with pathologic/molecular features that define distinct subphenotypes with different prognoses. It is conceivable that specific patterns of genetic susceptibility are associated with particular subphenotypes.To examine evidence for the contribution of germline genetic variation to bladder cancer heterogeneity.The Spanish Bladder Cancer/EPICURO Study is a case-control study based in 18 hospitals located in five areas in Spain. Cases were patients with a newly diagnosed, histologically confirmed, urothelial cell carcinoma of the bladder from 1998 to 2001. Case diagnoses were reviewed and uniformly classified by pathologists following the World Health Organisation/International Society of Urological Pathology 1999 criteria. Controls were hospital-matched patients (n=1149).A total of 1526 candidate variants in 423 candidate genes were analysed. Three distinct subphenotypes were defined according to stage and grade: low-grade nonmuscle   invasive   (n=586), high-grade nonmuscle   invasive   (n=219), and muscle   invasive   (n=246). The association between each variant and subphenotype was assessed by polytomous risk models adjusting for potential confounders. Heterogeneity in genetic susceptibility among subphenotypes was also tested.Two established bladder cancer susceptibility genotypes, NAT2 slow-acetylation and GSTM1-null, exhibited similar associations among the subphenotypes, as did VEGF-rs25648, which was previously identified in our study. Other variants conferred risks for specific tumour subphenotypes such as PMS2-rs6463524 and CD4-rs3213427 (respective heterogeneity p values of 0.006 and 0.004), which were associated with muscle-  invasive   tumours (per-allele odds ratios [95% confidence interval] of 0.56 [0.41-0.77] and 0.71 [0.57-0.88], respectively) but not with non-muscle-  invasive   tumours. Heterogeneity p values were not robust in multiple testing according to their false-discovery rate.These exploratory analyses suggest that genetic susceptibility loci might be related to the molecular/pathologic diversity of bladder cancer. Validation through large-scale replication studies and the study of additional genes and single nucleotide polymorphisms are required. 
 c-Myc expression and MEK1-induced Erk2 nuclear localization are required for TGF-beta induced epithelial-mesenchymal transition and invasion in prostate cancer. Understanding the initial mechanisms by which epithelial cells transform to an   invasive   phenotype is critical to the development of diagnostics that can identify the metastatic potential of cancers as well as therapeutic agents that can prevent metastases. Changes in cellular response to the transforming growth factor-beta (TGF-β) cytokine are known to promote epithelial cell invasion and metastasis in part through induction of epithelial-mesenchymal transitions (EMTs). In this report, we demonstrate that non-metastatic human prostate cancer cell lines of increasing Gleason score can be induced to undergo EMT when treated with TGF-β in combination with epidermal growth factor. Mechanistic studies revealed that in cells stably transfected with activated Ras, TGF-β alone induced EMT and that a Ras-Raf-MEK1, but not MEK2, signaling cascade is necessary and sufficient for Erk2 nuclear localization that works in concert with TGF-β to promote EMT. Furthermore, we show for the first time that expression of the transcription factor c-myc, which is phosphorlyated by Erk2, is required for EMT. Characteristically, EMT involved adoption of a spindle-shaped morphology, loss of E-cadherin and increased expression of Vimentin, Fibronectin and Fibroblast Specific Protein-1 (S100A4). Prostate cells undergoing EMT bec  ame inva  sive and expressed several genes associated with metastasis, including MT-MMP1, MMP-2/9, the MMP-9 homodimer, Slug and Twist2. In sum, we demonstrate a novel mechanism by which   non-inva  sive primary prostate tumor cells transition to   an inva  sive phenotype characteristic of malignant tumor cells in response to TGF-β signaling. 
 Proto-oncogene c-myc in uterine cervix carcinogenesis. The biological behaviour of precancerous and early stages of uterine cervix carcinoma is not always easily predictable. It is important therefore to identify new biological markers which could more reliably predict the evolution of the disease or provide important therapeutic targets. To establish the role of the proto-oncogene c-myc in uterine cervix tumorigenesis, we examined 96 tissue samples of different degrees of cervical intraepithelial neoplasia (CIN1-CIN 3), in situ (CIS) or   invasive   squamous cell carcinoma (ISCC) and control cases. Indirect immunohistochemical techniques were used to detect the c-myc expression. Significantly higher levels of Myc protein were found in keratinocytes of high-grade dysplasias in comparison to low-grade dysplasias and control cases. There was no difference between low-grade CIN and a control group of patients. The same significant changes between above mentioned groups were seen in surrounding stromal cells (fibrocytes, fibroblasts, some endothelial cells and lymphocytes). We confirm that expression of c-Myc protein is increased not only in uterine cervix cancer but also in the premalignant lesions. Problem for discussion seems there for whether increased Myc expression in stromal cells might create a more tumor promoting microenvironment which may support the growth and proliferation of transformed cells. 
 Stem cell-like micro-RNA signature driven by Myc in aggressive liver cancer. Myc activation has been implicated in the pathogenesis of hepatoblastoma (HB), a rare embryonal neoplasm derived from liver progenitor cells. Here, microRNA (miR) expression profiling of 65 HBs evidenced differential patterns related to developmental stage and Myc activity. Undifferentiated aggressive HBs overexpressed the miR-371-3 cluster with concomitant down-regulation of the miR-100/let-7a-2/miR-125b-1 cluster, evoking an ES cell expression profile. ChIP and Myc inhibition assays in hepatoma cells demonstrated that both miR clusters are regulated by Myc in an opposite manner. We show that the two miR clusters exert antagonistic effects on cell proliferation and tumorigenicity. Moreover, their combined deregulation cooperated in modulating the hepatic tumor phenotype, implicating stem cell-like regulation of Myc-dependent miRs in poorly differentiated HBs. Importantly, a four-miR signature representative of these clusters efficiently stratified HB patients, and when applied to 241 hepatocellular carcinomas (HCCs), it identified   invasive   tumors with a poor prognosis. Our data argue that Myc-driven reprogramming of miR expression patterns contributes to the aggressive phenotype of liver tumors originating from hepatic progenitor cells. 
 Expression of frizzled-related protein and Wnt-signalling molecules in   invasive   human breast tumours. Frizzled-related protein (Frp) is a new family of secreted proteins that contain a region homologous to the extracellular cysteine-rich domain (CRD) of the frizzled family proteins. The role of Frp protein is far from clear. To explore the role of Frp and its relationship to the Wnt-signalling pathway in breast cancer, in situ hybridization and immunohistochemical analyses of Frp, Wnt-1, APC, beta-catenin, and its target genes c-myc and cyclin D1 were conducted in 70 specimens of   invasive   ductal carcinomas of the human breast. Frp mRNA was down-regulated in 62 and elevated in eight tumour specimens, compared with adjacent normal tissues. In the course of tumour progression, however, Frp mRNA steadily increased in both tumour and the adjacent tissues. Interestingly, the number of cases with axillary lymph node metastasis was significantly lower in the group with elevated Frp than in the group with decreased Frp, suggesting that Frp may contribute as a prognostic factor in   invasive   breast cancer. Wnt-1, a gene implicated in human breast cancer, was markedly elevated in grade 1 tumours, but declined as tumour grade declined. The level of Wnt-1 was linearly correlated with its downstream target beta-catenin (p<0.05), but was inversely correlated with Frp (p<0.05), suggesting a possible negative regulatory role of Frp with regard to Wnt-1. APC was inversely correlated with beta-catenin (p<0.05). Beta-catenin, a key transcriptional activator responsible for the activation of both c-myc and cyclin D1 in colorectal tumours, was detected at high levels in the plasma membranes of cells in normal tissue. In tumour masses, however, beta-catenin lost its tight association with the membrane and diffused into the cytoplasm. Surprisingly, it clearly did not penetrate the nuclei, despite the fact that both c-myc and cyclin D1 were markedly elevated in all tumour tissues. As revealed in this study, Wnt-1/beta-catenin plays very different roles in the oncogenesis of breast and colon cancers. This first systemic analysis of the Frp and the Wnt-signalling pathway in human breast cancer provides a springboard for further work on the role of Frp in the development of breast cancer. 
 MicroRNA-184 Deregulated by the MicroRNA-21 Promotes Tumor Malignancy and Poor Outcomes in Non-small Cell Lung Cancer via Targeting CDC25A and c-Myc. MicroRNA (miR)-184 has been reported to have a dual role in human cancers. However, the role of miR-184 in non-small cell lung cancer (NSCLC) remains unclear.Wild-type or mutant CDC25A promoters were constructed by PCR and site-directed mutagenesis to verify whether miR-184 could inhibit CDC25A expression at post-transcription level. Boyden chamber assay was used to assess whether miR-184 could modulate cell   invasive  ness via targeting CDC25A and c-Myc. We utilized 124 tumors from NSCLC patients to determine miR-184, miR-21, PDCD4 mRNA, c-Myc mRNA, and CDC25A mRNA expression levels by means of real-time PCR analysis. The prognostic value of CDC25A, c-Myc, and miR-184 on overall survival (OS) and relapse-free survival (RFS) was evaluated by Kaplan-Meier and Cox regression analysis.MiR-184 suppressed CDC25A expression by enhancing the instability of its mRNA as a result of miR-184 binding to its coding region. An increase in CDC25A expression by means of a reduction in miR-184 promotes cell   invasive  ness. Moreover, a concomitant increase in CDC25A and c-Myc expression as a result of decreased miR-184 via the miR-21-mediated PDCD4 reduction is responsible for cell   invasive  ness. Among patients, miR-184 expression in lung tumors was found to correlate negatively with CDC25A mRNA, c-Myc mRNA, and miR-21 expression, but was positively related to PDCD4 mRNA expression. High-miR-184, High-CDC25A, or high-c-Myc mRNA tumors exhibited shorter OS and RFS periods than their counterparts. The worst OS and RFS were observed in low-miR-184/high-CDC25A/high-c-Myc tumors, followed by low-miR-184 /high-CDC25A, low-miR-184/high-c-Myc, high-c-Myc, and high-CDC25A tumors.MiR-184 as a tumor suppressor miR inhibits cell proliferation and invasion capability via targeting CDC25A and c-Myc. Low miR-184 level may predict worse prognosis in NSCLC patients. 
 MIR106B and MIR93 prevent removal of bacteria from epithelial cells by disrupting ATG16L1-mediated autophagy. Variants in genes that regulate autophagy have been associated with Crohn's disease (CD). Defects in autophagy-mediated removal of pathogenic microbes could contribute to the pathogenesis of CD. We investigated the role of the microRNAs (miRs) MIR106B and MIR93 in induction of autophagy and bacterial clearance in human cell lines and the correlation between MIR106B and autophagy-related gene 16L1 (ATG16L1) expression in tissues from patients with CD.We studied the ability of MIR106B and MIR93 to regulate ATG transcripts in human cancer cell lines (HCT116, SW480, HeLa, and U2OS) using luciferase report assays and bioinformatics analyses; MIR106B and MIR93 mimics and antagonists were transfected into cells to modify levels of miRs. Cells were infected with LF82, a CD-associated adherent-  invasive   strain of Escherichia coli, and monitored by confocal microscopy and for colony-forming units. Colon tissues from 41 healthy subjects (controls), 22 patients with active CD, 16 patients with inactive CD, and 7 patients with chronic inflammation were assessed for levels of MIR106B and ATG16L1 by in situ hybridization and immunohistochemistry.Silencing Dicer1, an essential processor of miRs, increased levels of ATG protein and formation of autophagosomes in cells, indicating that miRs regulate autophagy. Luciferase reporter assays indicated that MIR106B and MIR93 targeted ATG16L1 messenger RNA. MIR106B and MIR93 reduced levels of ATG16L1 and autophagy; these increased after expression of ectopic ATG16L1. In contrast, MIR106B and MIR93 antagonists increased formation of autophagosomes. Levels of MIR106B were increased in intestinal epithelia from patients with active CD, whereas levels of ATG16L1 were reduced compared with controls. Levels of c-Myc were also increased in intestinal epithelia of patients with active CD compared with controls. These alterations could impair removal of CD-associated bacteria by autophagy.In human cell lines, MIR106B and MIR93 reduce levels of ATG16L1 and autophagy and prevent autophagy-dependent eradication of intracellular bacteria. This process also appears to be altered in colon tissues from patients with active CD. 
 Pisosterol induces interphase arrest in HL60 cells with c-MYC amplification. The leukaemia cell line HL60 is widely used in studies of the cell cycle, apoptosis and adhesion mechanisms in cancer cells. One marked characteristic of HL60 cells is the c-MYC proto-oncogene amplification, resulting in the formation of homogeneously staining regions (HSRs) at 8p24. We conducted a fluorescence in situ hybridization study in an HL60 cell line, using a locus-specific probe for c-MYC, before and after treatment with pisosterol (at 0.5, 1.0 and 1.8 microg/mL), a triterpene isolated from the fungus Pisolithus tinctorius. Before treatment, 87.5% of the cells showed HSRs. After treatment, no effects were detected at lower concentrations of pisosterol (0.5 and 1.0 microg/mL). However, at 1.8 microg/mL only 15% of the cells presented HSRs, and 39.5% presented few fluorescent signals (3 or 4 alleles), suggesting that pisosterol probably blocks the cells with HSRs at interphase. This result is particularly interesting because cells that do not show a high degree of c-MYC gene amplification have a less aggressive and   invasive   behaviour and are easy targets for chemotherapy. Therefore, further studies are needed to examine the use of pisosterol in combination with conventional anti-cancer therapy. 
 Premalignant cervical lesions are characterized by dihydrofolate reductase gene amplification and c-Myc overexpression: possible biomarkers. The c-Myc oncoprotein deregulation is associated with overall genomic instability and locus-specific genomic instability involving the dihydrofolate reductase (DHFR) locus. This study analyzes c-Myc protein levels and the stability of the DHFR gene in cervical tissue biopsies.The stability of the DHFR gene was examined by fluorescence in situ hybridization (FISH). c-Myc protein levels were evaluated using quantitative fluorescent immunohistochemistry. Forty-four cervical tissue biopsies were analyzed and included 33 pre  invasive   cervical lesions identified by histology, 14 samples were cervical intraepithelial neoplasia (CIN) 1; 7 were CIN 2; and 12 were CIN 3. Eleven biopsies had negative histology.c-Myc protein levels were elevated in CIN 1, 2, and 3 (p = .02) biopsies. Concomitantly, DHFR gene amplification was detected in CIN 1, 2, and 3 (p = .0001). The degrees of DHFR gene amplification and of c-Myc protein levels were a measure of the progressive degree of the lesion. 
 WIF1, a Wnt pathway inhibitor, regulates SKP2 and c-myc expression leading to G1 arrest and growth inhibition of human   invasive   urinary bladder cancer cells. Epigenetic silencing of secreted wingless-type (Wnt) antagonists through hypermethylation is associated with tobacco smoking and with   invasive   bladder cancer. The secreted Wnt inhibitory factor-1 (WIF1) has shown consistent growth-inhibitory effect on various cancer cell lines. Therefore, we assessed the mechanisms of action of WIF1 by either restoring WIF1 expression in   invasive   bladder cancer cell lines (T24 and TSU-PR1) or using a recombinant protein containing functional WIF1 domain. Both ectopic expression of WIF1 and treatment with WIF1 domain protein resulted in cell growth inhibition via G(1) arrest. The G(1) arrest induced by WIF1 is associated with down-regulation of SKP2 and c-myc and up-regulation of p21/WAF1 and p27/Kip1. Conversely, reexpression of SKP2 in WIF1-overexpressing TSU-PR1 cells attenuated the WIF1-induced G(1) arrest. Furthermore, inhibition of nuclear Wnt signaling by either dominant-negative LEF1 or short hairpin RNA of TCF4 also reduced SKP2 expression. The human SKP2 gene contains two TCF/LEF1 consensus binding sites within the promoter. Chromatin immunoprecipitation/real-time PCR analysis revealed that both WIF1 and dominant-negative LEF1 expression decreased the in vivo binding of TCF4 and beta-catenin to the SKP2 promoter. Together, our results suggest that mechanisms of WIF1-induced G(1) arrest include (a) SKP2 down-regulation leading to p27/Kip1 accumulation and (b) c-myc down-regulation releasing p21/WAF1 transcription. Additionally, we show that WIF1 inhibits in vivo bladder tumor growth in nude mice. These observations suggest a mechanism for transformation of bladder epithelium on loss of WIF1 function and provide new targets such as SKP2 for intervention in WIF1-deficient bladder cancer. 
 Human immunodeficiency virus activates c-myc and Epstein-Barr virus in human B lymphocytes. We have established a line of malignantly transformed human B cells by infecting purified primary B lymphocytes with human immunodeficiency virus type 1 (HIV-1). This line, termed B-HIV1, may serve as a model system for a subset of AIDS-related B-cell lymphomas in which the transformed phenotype may be initiated and/or maintained through an HIV-1 gene product. The B-HIV1 line contains both Epstein-Barr virus (EBV) and HIV-1 genomes. In addition, the c-myc gene is expressed at levels 10 to 20 times those in normal B cells. Similarly, EBV sequences, including those for the latent membrane protein (LMP), are expressed at greatly enhanced levels relative to expression in normal, EBV-immortalized B cells. The upregulation of c-myc and of EBV gene expression can both be produced by infection of susceptible B cells (not already harboring HIV) with exogenous HIV-1. The B-HIV1 line exhibits properties of malignantly transformed cells, in that it grows logarithmically in 1% serum, clones in soft agar, and produces   invasive  , malignant B-cell lymphomas in severe combined immunodeficient (SCID) mice. We have shown that HIV-1 has the ability to infect primary human B cells and to activate expression of EBV and c-myc. HIV activation of EBV has been documented previously in certain cell lines, here we note that such activation can occur in primary B cells and, under certain conditions, can result in outgrowth of immortalized cell lines. This phenomenon may contribute to the clinical manifestation of lymphadenopathy early after infection with HIV. In addition, we have demonstrated that HIV infection of primary B cells in vitro can result in appearance of a fully malignant phenotype. This phenotype is likely to be due, at least in part, to the activation of c-myc by HIV. Preliminary experiments indicate that Tat, the gene product of the transactivator of viral gene transcription tat, can upregulate c-myc transcription after addition to the culture media of certain B-cell lines. This raises the possibility that Tat can bind to target sequences in cellular RNA and enhance transcription as it does for HIV.(ABSTRACT TRUNCATED AT 400 WORDS) 
 Focal amplifications are associated with high grade and recurrences in stage Ta bladder carcinoma. Urinary bladder cancer is a heterogeneous disease with tumors ranging from papillary non  invasive   (stage Ta) to solid muscle infiltrating tumors (stage T2+). The risk of progression and death for the most frequent diagnosed type, Ta, is low, but the high incidence of recurrences has a significant effect on the patients' quality of life and poses substantial costs for health care systems. Consequently, the purpose of this study was to search for predictive factors of recurrence on the basis of genetic profiling. A clinically well characterized cohort of Ta bladder carcinomas, selected by the presence or absence of recurrences, was evaluated by an integrated analysis of DNA copy number changes and gene expression (clone-based 32K, respectively, U133Plus2.0 arrays). Only a few chromosomal aberrations have previously been defined in superficial bladder cancer. Surprisingly, the profiling of Ta tumors with a high-resolution array showed that DNA copy alterations are relatively common in this tumor type. Furthermore, we observed an overrepresentation of focal amplifications within high-grade and recurrent cases. Known (FGFR3, CCND1, MYC, MDM2) and novel candidate genes were identified within the loci. For example, MYBL2, a nuclear transcription factor involved in cell-cycle progression; YWHAB, an antiapoptotic protein; and SDC4, an important component of focal adhesions represent interesting candidates detected within two amplicons on chromosome 20, for which DNA amplification correlated with transcript up-regulation. The observed overrepresentation of amplicons within high-grade and recurrent cases may be clinically useful for the identification of patients who will benefit from a more aggressive therapy. 
 Tumour angiogenesis and C-myc expression in breast carcinomas. Angiogenesis in tumors has been widely studied and has been considered to be a prognostic factor particularly in breast carcinomas. The myc family of proto oncogenes has shown an amplification in breast carcinomas. The aim of this study was to assess MVD (micro vessel density) and the expression of c-myc in   invasive   breast carcinomas. Formalin fixed, routinely processed, paraffin embedded tissue was stained with H&E for classification and grading according to the Nottingham modification of Bloom and Richardson criteria. Immunohistochemical staining using primary antibody against F VIII antigen and anti C-myc were carried out. LSAB method was employed with AEC as chromogen and Gill's Haematoxylin as counter stain. Micro vessels were counted and positivity indicated by a cherry red staining in the cytoplasm of the tumour cells. Grades I, II and III showed 12.22 %, 20.9% and 24.5 % positivity of micro vessel density (MVD). Fifty percent of Grade 1, 69.2 % of Grade II and 24.5 % of Grade III tumours expressed c-myc proteins. In our study we found a positive association between tumour grade and micro vessel density, signifying a poor prognosis for tumours with high MVD. C-myc expression also showed a positive association with increasing grade. 
 Myc inhibition is effective against glioma and reveals a role for Myc in proficient mitosis. Gliomas are the most common primary tumours affecting the adult central nervous system and respond poorly to standard therapy. Myc is causally implicated in most human tumours and the majority of glioblastomas have elevated Myc levels. Using the Myc dominant negative Omomyc, we previously showed that Myc inhibition is a promising strategy for cancer therapy. Here, we preclinically validate Myc inhibition as a therapeutic strategy in mouse and human glioma, using a mouse model of spontaneous multifocal   invasive   astrocytoma and its derived neuroprogenitors, human glioblastoma cell lines, and patient-derived tumours both in vitro and in orthotopic xenografts. Across all these experimental models we find that Myc inhibition reduces proliferation, increases apoptosis and remarkably, elicits the formation of multinucleated cells that then arrest or die by mitotic catastrophe, revealing a new role for Myc in the proficient division of glioma cells. 
 Multiple genetic copy number alterations in oral squamous cell carcinoma: study of MYC, TP53, CCDN1, EGFR and ERBB2 status in primary and metastatic tumours. Oncogenesis in the oral cavity is believed to result from genetic alterations that cause a stepwise transformation of the mucosa to   invasive   carcinoma. In oral squamous cell carcinoma (OSCC) multiple cytogenetic abnormalities have been reported, but their practical significance remains uncertain.To evaluate the usefulness of the assessment of CCND1, MYC, EGFR, ERBB2 and TP53 in OSCC and lymph node metastases.Fifty-one consecutive samples of OSCC, nine lymph node biopsies showing metastatic spread from OSCC, 16 biopsies diagnosed as oral leucoplakia (OLK), 13 samples corresponding to oral lichen planus (OLP) and 14 samples from normal oral mucosa were included in the study. Clinical and histopathological characteristics were reviewed. The genetic and protein status of the CCND1, MYC, EGFR, ERBB2 oncogenes and the TP53 tumour suppressor gene were assessed by fluorescence in situ hybridization (FISH) and immunohistochemistry (IHC). The obtained results were compared with the clinical characteristics and the outcome of the OSCCs.TP53 gene losses and MYC, ERBB2, CCND1 and EGFR copy number gains and amplifications were detected in a higher proportion in OSCC and lymph node samples than in OLK and OLP samples (P < 0·005). Overexpression of p53, Myc, Cyclin D1, c-erbB-2 and epidermal growth factor receptor (EGFR) was more prevalent in malignant samples than benign samples (P < 0·05). Correlation between FISH and IHC results was demonstrated in MYC, EGFR and CCND1 studies. The presence of two or more genetic abnormalities in the studied loci was exclusively detected in primary and metastatic OSCC.In our series, genetic abnormalities in TP53, MYC, CCND1, ERBB2 and EGFR detected by FISH were absent in inflammatory lesions, infrequent in precursor lesions and common in tumoral lesions. Evaluation of the genetic status of TP53, MYC, CCND1, ERBB2 and EGFR may be an additional diagnostic tool in distinguishing benign from malignant oral lesions in histopathologically challenging cases. 
 Correlation of amplification and overexpression of the c-myc oncogene in high-grade breast cancer: FISH, in situ hybridisation and immunohistochemical analyses. In this study, we analysed gene amplification, RNA expression and protein expression of the c-myc gene on archival tissue specimens of high-grade human breast cancer, using fluorescent in situ hybridisation (FISH), nonradioactive in situ hybridisation and immunohistochemistry. The specific question that we addressed was whether expression of c-Myc mRNA and protein were correlated with its gene copy amplification, as determined by FISH. Although c-Myc is one of the most commonly amplified oncogenes in human breast cancer, few studies have utilised in situ approaches to directly analyse the gene copy amplification, RNA transcription and protein expression on human breast tumour tissue sections. We now report that by using the sensitive FISH technique, a high proportion (70%) of high-grade breast carcinoma were amplified for the c-myc gene, irrespective of status of the oestrogen receptor. However, the level of amplification was low, ranging between one and four copies of gene gains, and the majority (84%) of the cases with this gene amplification gained only one to two copies. Approximately 92% of the cases were positive for c-myc RNA transcription, and essentially all demonstrated c-myc protein expression. In fact, a wide range of expression levels were detected. Statistically significant correlations were identified among the gene amplification indices, the RNA expression scores and protein expression scores. c-myc gene amplification, as detected by FISH, was significantly associated with expression of its mRNA, as measured by the intensity of in situ hybridisation in   invasive   cells (P=0.0067), and by the percentage of   invasive   cells positive for mRNA expression (P=0.0006). c-myc gene amplification was also correlated with the percentage of tumour cells which expressed high levels of its protein, as detected by immunohistochemistry in   invasive   cells (P=0.0016). Thus, although multiple mechanisms are known to regulate normal and aberrent expression of c-myc, in this study, where in situ methodologies were used to evaluate high-grade human breast cancers, gene amplification of c-myc appears to play a key role in regulating expression of its mRNA and protein. 
 c-myc proto-oncogene expression in hemophilic synovitis: in vitro studies of the effects of iron and ceramide. Hemophilia is a rare congenital bleeding disorder that is due to the deficiency of blood coagulation factor VIII or IX. Recurrent musculoskeletal bleeding is common and bleeding into joints results in a chronic inflammatory condition termed hemophilic synovitis. This destructive process is characterized by hemosiderin deposition in the superficial and deeper layers of the synovial membrane as well as a proliferation of synovial fibroblasts and vascular cells. The hyperplastic synovium and neovascular changes are reminiscent of the histopathologic appearance observed in malignant tissues. Indeed, the benign hyperplastic synovium in patients with hemophilia displays similar   invasive   and destructive behaviors suggesting the possibility of analogous disturbances in growth control and locally   invasive   mechanisms. Iron plays a role in malignant cell growth, local invasion, and tumor progression, possibly due to changes in the expression of the proto-oncogene, c-myc. We hypothesized that iron plays a similar role in hemophilic synovitis. To explore this hypothesis, we investigated the in vitro effects of iron on the proliferation of a primary, human synovial fibroblast cell (HSFC) line and the involvement of c-myc in this process. We also examined the role of ceramide, a sphingolipid capable of inducing apoptosis in this model system. HSFC proliferation was increased in a dose-dependent fashion and c-myc expression was enhanced by ferric citrate compared to sodium citrate control. Ceramide prevented both the iron-induced increases in HSFC proliferation and c-myc expression. These results indicate that iron probably plays a role in the proliferative changes observed in hemophilic joint disease and that aberrant expression of c-myc may underlie the iron effects. Furthermore, these results suggest that there may be a therapeutic role for ceramide in reversing these changes. 
 The MZF1/c-MYC axis mediates lung adenocarcinoma progression caused by wild-type lkb1 loss. Liver kinase B1 (LKB1) loss in lung adenocarcinoma is commonly caused by genetic mutations, but these mutations rarely occur in Asian patients. We recently reported wild-type LKB1 loss via the alteration of NKX2-1/p53-axis-promoted tumor aggressiveness and predicted poor outcomes in cases of lung adenocarcinoma. The mechanistic action of wild-type LKB1 loss within tumor progression remains unknown. The suppression of MYC by LKB1 controls epithelial organization; therefore, we hypothesize that MYC expression can be increased via wild-type LKB1 loss and promotes tumor progression. Here, MYC transcription is upregulated by LKB1-loss-mediated MZF1 expression. The wild-type LKB1-loss-mediated MZF1/MYC axis is responsible for soft-agar growth, migration and invasion in lung adenocarcinoma cells. Moreover, wild-type LKB1 loss-induced cell   invasiv  eness was markedly suppressed by MYC inhibitors (10058-F4 and JQ1). Patients with low-LKB1/high-MZF1 or low-LKB1/high-MYC tumors have shorter overall survival and relapse-free-survival periods than patients with high-LKB1/low-MZF1 or high-LKB1/low-MYC tumors. In summary, MZF1-mediated MYC expression may promote tumor progression, resulting in poor outcomes in cases of lung adenocarcinoma with low-wild-type-LKB1 tumors. 
 Inhibition of 5-lipoxygenase selectively triggers disruption of c-Myc signaling in prostate cancer cells. Myc is up-regulated in almost all cancer types and is the subject of intense investigation because of its pleiotropic effects controlling a broad spectrum of cell functions. However, despite its recognition as a stand-alone molecular target, development of suitable strategies to block its function is hindered because of its nonenzymatic nature. We reported earlier that arachidonate 5-lipoxygenase (5-Lox) plays an important role in the survival and growth of prostate cancer cells, although details of the underlying mechanisms have yet to be characterized. By whole genome gene expression array, we observed that inhibition of 5-Lox severely down-regulates the expression of c-Myc oncogene in prostate cancer cells. Moreover, inhibition of 5-Lox dramatically decreases the protein level, nuclear accumulation, DNA binding, and transcriptional activities of c-Myc. Both the 5-Lox inhibition-induced down-regulation of c-Myc and induction of apoptosis are mitigated when the cells are treated with 5-oxoeicosatetraenoic acid, a metabolite of 5-Lox, confirming a role of 5-Lox in these processes. c-Myc is a transforming oncogene widely expressed in prostate cancer cells and maintains their transformed phenotype. Interestingly, MK591, a specific 5-Lox inhibitor, strongly affects the viability of Myc-overactivated prostate cancer cells and completely blocks their   invasive   and soft agar colony-forming abilities, but it spares nontransformed cells where expression of 5-Lox is undetectable. These findings indicate that the oncogenic function of c-Myc in prostate cancer cells is regulated by 5-Lox activity, revealing a novel mechanism of 5-Lox action and suggesting that the oncogenic function of c-Myc can be suppressed by suitable inhibitors of 5-Lox. 
 Myc coordinates transcription and translation to enhance transformation and suppress   invasive  ness. c-Myc is one of the major human proto-oncogenes and is often associated with tumor aggression and poor clinical outcome. Paradoxically, Myc was also reported as a suppressor of cell motility,   invasive  ness, and metastasis. Among the direct targets of Myc are many components of the protein synthesis machinery whose induction results in an overall increase in protein synthesis that empowers tumor cell growth. At present, it is largely unknown whether beyond the global enhancement of protein synthesis, Myc activation results in translation modulation of specific genes. Here, we measured Myc-induced global changes in gene expression at the transcription, translation, and protein levels and uncovered extensive transcript-specific regulation of protein translation. Particularly, we detected a broad coordination between regulation of transcription and translation upon modulation of Myc activity and showed the connection of these responses to mTOR signaling to enhance oncogenic transformation and to the TGFβ pathway to modulate cell migration and   invasiv  eness. Our results elucidate novel facets of Myc-induced cellular responses and provide a more comprehensive view of the consequences of its activation in cancer cells. 
 MYC suppresses cancer metastasis by direct transcriptional silencing of αv and β3 integrin subunits. Overexpression of MYC transforms cells in culture, elicits malignant tumours in experimental animals and is found in many human tumours. We now report the paradoxical finding that this powerful oncogene can also act as a suppressor of cell motility  , invasi  veness and metastasis. Overexpression of MYC stimulated proliferation of breast cancer cells both in culture and in vivo as expected, but inhibited motility an  d invasi  veness in culture, and lung and liver metastases in xenografted tumours. We show further that MYC represses transcription of both subunits of αvβ3 integrin, and that exogenous expression of β3 integrin in human breast cancer cells that do not express this integrin res  cues inv  asiveness and migration when MYC is downregulated. These data uncover an unexpected function of MYC, provide an explanation for the hitherto puzzling literature on the relationship between MYC and metastasis, and reveal a variable that could influence the development of therapies that target MYC. 
 MYC cooperates with AKT in prostate tumorigenesis and alters sensitivity to mTOR inhibitors. MYC and phosphoinositide 3-kinase (PI3K)-pathway deregulation are common in human prostate cancer. Through examination of 194 human prostate tumors, we observed statistically significant co-occurrence of MYC amplification and PI3K-pathway alteration, raising the possibility that these two lesions cooperate in prostate cancer progression. To investigate this, we generated bigenic mice in which both activated human AKT1 and human MYC are expressed in the prostate (MPAKT/Hi-MYC model). In contrast to mice expressing AKT1 alone (MPAKT model) or MYC alone (Hi-MYC model), the bigenic phenotype demonstrates accelerated progression of mouse prostate intraepithelial neoplasia (mPIN) to micro  invasive   disease with disruption of basement membrane, significant stromal remodeling and infiltration of macrophages, B- and T-lymphocytes, similar to inflammation observed in human prostate tumors. In contrast to the reversibility of mPIN lesions in young MPAKT mice after treatment with mTOR inhibitors, Hi-MYC and bigenic MPAKT/Hi-MYC mice were resistant. Additionally, older MPAKT mice showed reduced sensitivity to mTOR inhibition, suggesting that additional genetic events may dampen mTOR dependence. Since increased MYC expression is an early feature of many human prostate cancers, these data have implications for treatment of human prostate cancers with PI3K-pathway alterations using mTOR inhibitors. 
 MYC activation associated with the integration of HPV DNA at the MYC locus in genital tumors. To determine whether integration of human papillomavirus (HPV) DNA sequences could lead to the deregulation of genes implied in oncogenesis, we analysed the HPV integration sites in a series of nine cell lines derived from   invasive   genital carcinomas. Using in situ hybridization, HPV16 or 18 sequences were found at chromosome band 8q24, the localization of MYC, in IC1, IC2, IC3, IC6 and CAC-1 cells and at other sites in IC4, IC5, IC7 and IC8 cells. We then localized viral sequences at the molecular level and searched for alterations of MYC structure and expression in these cells. MYC genomic status and viral integration sites were also analysed in primary tumors from which IC1, IC2, IC3 and IC6 cells were derived. In IC1, IC2 and CAC-1 cells, HPV DNA was located within 58 kb of MYC, downstream, upstream, or within MYC. In IC3 and IC6 cells, HPV DNA was located 400-500 kb upstream of MYC. Amplification studies showed that, in IC1, IC2 and IC3, viral and MYC sequences were co-amplified in an amplicon between less than 50 and 800 kb in size. MYC amplification was also observed in primary tumors, indicating that this genetic alteration, together with viral insertion at the MYC locus, had already taken place in vivo. MYC was not amplified in the other cell lines. MYC mRNA and protein overexpression was observed in the five cell lines in which the HPV DNA was inserted close to the MYC locus, but in none of the lines where the insertion had occurred at other sites. MYC activation, triggered by the insertion of HPV DNA sequences, can be an important genetic event in cervical oncogenesis. 
 PEG10 is a c-MYC target gene in cancer cells. The product of the imprinted gene paternally expressed gene-10 (PEG10) has been reported to support proliferation in hepatocellular carcinomas, but how this gene is regulated has been an open question. We find that MYC knockdown by RNA interference suppresses PEG10 expression in Panc1 pancreatic carcinoma and HepG2 hepatocellular carcinoma cells and that knockdown of PEG10 inhibits the proliferation of Panc1, HepG2, and Hep3B cells. Conversely, PEG10 was up-regulated by inducing c-MYC expression in a B-lymphocyte cell line. Chromatin immunoprecipitation from Panc1 cells showed c-MYC bound to an E-box-containing region in the PEG10 first intron and site-directed mutagenesis showed that the most proximal E-box is essential for promoter activity. In a mouse mammary tumor virus (MMTV)-MYC transgenic mouse model of breast cancer, most but not all of the mammary carcinomas had strongly increased Peg10 mRNA compared with normal mammary gland. By immunohistochemistry, normal human breast and prostate epithelium was negative for the major isoform [reading frame-1 (RF1)] of PEG10 protein, but this cytoplasmic protein was strongly expressed in a subset of breast carcinomas in situ and   invasive   ductal carcinomas ( approximately 30%) and in a similar percentage of prostate cancers. As in the mouse model, we found positive, but not absolute, correlations between PEG10 and c-MYC in tissue arrays containing 161 human breast cancers (P < 0.002) and 30 prostate cancers (P = 0.014). Immunostaining of human placenta showed PEG10 and c-MYC proteins coexpressed in proliferating cytotrophoblast and coordinately lost in postmitotic syncytiotrophoblast. These findings link cancer genetics and epigenetics by showing that a classic proto-oncogene, MYC, acts directly upstream of a proliferation-positive imprinted gene, PEG10. 
 Hepatic stem-like phenotype and interplay of Wnt/beta-catenin and Myc signaling in aggressive childhood liver cancer. Hepatoblastoma, the most common pediatric liver cancer, is tightly linked to excessive Wnt/beta-catenin signaling. Here, we used microarray analysis to identify two tumor subclasses resembling distinct phases of liver development and a discriminating 16-gene signature. beta-catenin activated different transcriptional programs in the two tumor types, with distinctive expression of hepatic stem/progenitor markers in immature tumors. This highly proliferating subclass was typified by gains of chromosomes 8q and 2p and upregulated Myc signaling. Myc-induced hepatoblastoma-like tumors in mice strikingly resembled the human immature subtype, and Myc downregulation in hepatoblastoma cells impaired tumorigenesis in vivo. Remarkably, the 16-gene signature discriminated   invasive   and metastatic hepatoblastomas and predicted prognosis with high accuracy. 
 Clinical significance of hTERC and C-Myc genes amplification in a group of Egyptian patients with cancer cervix. Cervical cancer is the second most common cancer in women worldwide after breast cancer. Cervical cancer is a preventable disease. The implementation of cervical cancer screening programs has greatly decreased the morbidity and mortality, as precancerous lesions and early   invasive   cervical cancer could be detected and treated effectively. The detection of hTERC gene amplification was suggested as a possible diagnostic marker for use in routine cytological screening.The present study was designed to detect genomic gains of the hTERC and C-MYC genes using FISH technique and to investigate the relationship between genes amplification and the clinical data of the patients.The current study was carried out on twelve cases with cervical cancer at different grades (three cases were grade I, six cases were grade II and three cases were grade III). Interphase FISH analysis using LSI probe, Cervical Cancer probe hTERC (3q26) & C-MYC (8q24), was successfully performed on 12 patients with cancer cervix.Interphase FISH analysis revealed positive hTERC gene amplification in all cases of cancer cervix (100%). However C-MYC gene amplification was detected in four cases only (33.3%). Statistical analysis of the data revealed significant correlation between hTERC amplification and grading. Also, there was significant correlation between C-MYC amplification and grading and highly significant correlation between C-MYC amplification and hTERC amplification. On the other hand hTERC and C-MYC genes amplification showed an inverse correlation with the ages of the patients.The present study highlights the importance of using hTERC and C-MYC genes FISH probes for cases with cancer cervix or pre-malignant lesions as a sensitive technique. This method provides an easy and effective applicable approach which helps in the diagnosis and prognosis, as an increased copy number is associated with a more advanced grade that could be detected in the early stages of the disease. 
 Down-regulation of c-Myc expression inhibits the invasion of bile duct carcinoma cells. Cholangiocarcinoma is the second most common primary hepatic tumour originating from biliary tract epithelial cells with poor prognosis. Enhanced c-Myc protein expression contributes to many aspects of tumour cell biology. Although the ability of c-Myc to drive unrestricted cell proliferation and to inhibit cell differentiation had been well recognized, whether down-regulated c-Myc expression can inhibit tumour cell invasion still remains to be explored. The c-Myc ASODN (antisense oligodeoxyribonucleotide) and NSODN (nonsense oligodeoxyribonucleotide) were designed, synthesized and transfected into human QBC939 bile duct carcinoma cells using the Lipofectamine 2000 reagent. The protein expression of c-Myc was detected by Western blot. A transwell experiment was applied to evaluate the   invasive   capacity of the QBC939 cells. c-Myc ASODN could significantly suppress the c-Myc protein expression (P<0.05) and the invasion (P<0.01) of QBC939 cells transfected with c-Myc ASODN compared with that in the control and c-Myc NSODN-transfected group. Thus in the present study we show that down-regulation of c-Myc expression can inhibit the invasion of QBC939 cells in vitro. 
 Co-overexpression of bcl-2 and c-myc in uterine cervix carcinomas and premalignant lesions. To establish the role of co-overexpression of bcl-2 and c-myc protooncogenes in uterine cervix carcinogenesis, we examined 138 tissue samples of low grade cervical squamous intraepithelial lesions (SIL), high grade SIL, portio vaginalis uteri (PVU) carcinoma in situ and PVU carcinoma   invasive  , stage IA-IIA (study group) and 36 samples without SIL or malignancy (control group). The expression of bcl-2 and c-myc was detected immunohistochemically using a monoclonal antibody. Fisher&rsquo;s exact test (P&lt;0.05) was used to assess statistical significance. Overexpression of bcl-2 was found to increase in direct relation to the grade of the cervical lesions. High sensitivity was of great diagnostic significance for the detection of these types of changes in the uterine cervix. On the basis of high predictive values it can be said that in patients with bcl-2 overexpression there is a great possibility that they have premalignant or malignant changes in the uterine cervix. Co-overexpression of bcl-2 and c-myc oncogenes was found only in patients with PVU   invasive   carcinoma (6/26-23.0%). Statistically significant difference was not found in the frequency of co-overexpression in patients with PVU   invasive   carcinoma in relation to the control group (Fisher&rsquo;s test; P=0.064). The method's sensitivity of determining these oncogenes with the aim of detecting PVU   invasive   carcinoma was 23%, while specificity was 72.2%. On the basis of high predictive values (100%), speaking in statistical terms, it can be concluded that all patients with co-overexpression of bcl-2 and c-myc oncogenes will have PVU   invasive   carcinoma. We confirmed in our research that co-overexpression of bcl-2 and c-myc oncogenes was increased only in PVU   invasive   carcinoma. However, a more extensive series of samples and additional tests are required to establish the prognostic significance of bcl-2 and c-myc co-overexpression in cervical carcinogenesis. 
 Inversed relationship between CD44 variant and c-Myc due to oxidative stress-induced canonical Wnt activation. Cancer stem-like cells express high amount of CD44 variant8-10 which protects cancer cells from redox stress. We have demonstrated by immunohistochemical analysis and Western blotting, and reverse-transcription polymerase chain reaction, that CD44 variant8-10 and c-Myc tend to show the inversed expression manner in gastric cancer cells. That is attributable to the oxidative stress-induced canonical Wnt activation, and furthermore, the up-regulation of the downstream molecules, one of which is oncogenic c-Myc, is not easily to occur in CD44 variant-positive cancer cells. We have also found out that CD44v8-10 expression is associated with the turn-over of the c-Myc with the experiments using gastric cancer cell lines. This cannot be simply explained by the model of oxidative stress-induced Wnt activation. CD44v8-10-positive cancer cells are enriched at the   invasive   front. Tumor tissue at the   invasive   area is considered to be composed of heterogeneous cellular population; dormant cancer stem-like cells with CD44v8-10 (high)/ Fbw7 (high)/ c-Myc (low) and proliferative cancer stem-like cells with CD44v8-10 (high)/ Fbw7 (low)/ c-Myc (high). 
 miR-9, a MYC/MYCN-activated microRNA, regulates E-cadherin and cancer metastasis. MicroRNAs (miRNAs) are increasingly implicated in regulating the malignant progression of cancer. Here we show that miR-9, which is upregulated in breast cancer cells, directly targets CDH1, the E-cadherin-encoding messenger RNA, leading to increased cell motility and   invasive  ness. miR-9-mediated E-cadherin downregulation results in the activation of beta-catenin signalling, which contributes to upregulated expression of the gene encoding vascular endothelial growth factor (VEGF); this leads, in turn, to increased tumour angiogenesis. Overexpression of miR-9 in otherwise non-metastatic breast tumour cells enables these cells to form pulmonary micrometastases in mice. Conversely, inhibiting miR-9 by using a 'miRNA sponge' in highly malignant cells inhibits metastasis formation. Expression of miR-9 is activated by MYC and MYCN, both of which directly bind to the mir-9-3 locus. Significantly, in human cancers, miR-9 levels correlate with MYCN amplification, tumour grade and metastatic status. These findings uncover a regulatory and signalling pathway involving a metastasis-promoting miRNA that is predicted to directly target expression of the key metastasis-suppressing protein E-cadherin. 
 Deregulation of MYC and TP53 through genetic and epigenetic alterations in gallbladder carcinomas. Gallbladder cancer is a rare malignancy and presents a poor prognosis. MYC and p53 have been implicated in gallbladder carcinogenesis. However, little is known about the molecular mechanisms involved in their regulation in this neoplasia. Here, we evaluated the MYC and TP53 copy numbers in gallbladder tumors and their possible association with protein expression. We also investigated whether MYC may be controlled by mutations and DNA promoter methylation. In the present study, 15 samples of   invasive   gallbladder carcinomas and six control samples were analyzed. On the other hand, the expression of MYC and p53 was more frequent in gallbladder carcinomas than in control samples (p = 0.002, p = 0.046, respectively). Gain of copies of the MYC and TP53 genes was detected in 86.7 and 50 % of gallbladder carcinomas, respectively. MYC and TP53 amplifications were associated with immunoreactivity of their protein (p = 0.029, p = 0.001, respectively). MYC hypomethylation was only detected in tumoral samples and was associated with its protein expression (p = 0.029). MYC mutations were detected in 80 % of tumor samples. The G allele at rs117856857 was associated with the presence of gallbladder tumors (p = 0.019) and with MYC expression (p = 0.044). Moreover, two tumors presented a pathogenic mutation in MYC exon 2 (rs28933407). Our study highlights that the gain of MYC and TP53 copies seems to be a frequent finding in gallbladder cancer. In addition, gain of copies, hypomethylation and point mutations at MYC may contribute to overexpression of its protein in this type of cancer. 
 Ezrin mediates c-Myc actions in prostate cancer cell invasion. The forced overexpression of c-Myc in mouse prostate and in normal human prostate epithelial cells results in tumor transformation with an   invasive   phenotype. How c-Myc regulates cell invasion is poorly understood. In this study, we have investigated the interplay of c-Myc and androgens in the regulation of prostate cancer cell invasion. We found that c-Myc induces cell invasion and anchorage-independent growth by regulating ezrin protein expression in the presence of androgens. The activity of the ezrin promoter is controlled by androgens through c-Myc, which binds to a phylogenetically conserved E-Box located in the proximal promoter region. Besides, we also show that ezrin is an important regulator of c-Myc protein levels. These effects are achieved through androgen-induced changes in ezrin phosphorylation, which results in the regulation of downstream signals. These downstream signals involve the modulation of Akt and GSK-3beta activity resulting in increased c-Myc protein synthesis and inhibition of its degradation. In summary, we have shown a key role for ezrin as a mediator of c-Myc-induced tumorigenesis in prostate cancer cells. 
 Induction of c-Myc-dependent cell proliferation through toll-like receptor 3 in head and neck cancer. Protein expression of human toll-like receptors (TLR) 1-10 was measured in cell lines and solid tumors of head and neck squamous cell carcinoma (HNSCC). All HNSCC cell lines and 80% of solid tumors were found to express TLR3 as a predominantly intracellular protein, while no other TLR proteins were expressed. TLR3 has previously been shown to contribute to the activation of nuclear factor-kappaB (NF-kappaB), a transcription factor which promotes several types of human cancers. Significantly, NF-kappaB expression was strongest in protein extracts from carcinoma tissue in which TLR3 was overexpressed. Inhibition of TLR3 expression in permanent HNSCC cell lines resulted in decreased expression of the oncoprotein c-Myc resulting in decreased cell proliferation. Correspondingly, overexpression of human TLR3 in mouse fibroblasts resulted in an upregulation of c-Myc and increased sensitivity for PolyI:C-induced cell proliferation. Our data suggest that TLR3 contributes to the malignant phenotype leading to   invasive   carcinoma in HNSCC. 
 A sequence variant at 4p16.3 confers susceptibility to urinary bladder cancer. Previously, we reported germline DNA variants associated with risk of urinary bladder cancer (UBC) in Dutch and Icelandic subjects. Here we expanded the Icelandic sample set and tested the top 20 markers from the combined analysis in several European case-control sample sets, with a total of 4,739 cases and 45,549 controls. The T allele of rs798766 on 4p16.3 was found to associate with UBC (odds ratio = 1.24, P = 9.9 x 10(-12)). rs798766 is located in an intron of TACC3, 70 kb from FGFR3, which often harbors activating somatic mutations in low-grade, non  invasive   UBC. Notably, rs798766[T] shows stronger association with low-grade and low-stage UBC than with more aggressive forms of the disease and is associated with higher risk of recurrence in low-grade stage Ta tumors. The frequency of rs798766[T] is higher in Ta tumors that carry an activating mutation in FGFR3 than in Ta tumors with wild-type FGFR3. Our results show a link between germline variants, somatic mutations of FGFR3 and risk of UBC. 
 Prostaglandin E2 promotes the cell growth and   invasive   ability of hepatocellular carcinoma cells by upregulating c-Myc expression via EP4 receptor and the PKA signaling pathway. Hepatocellular carcinoma (HCC) represents a major health problem worldwide. Prostaglandin E2 (PGE2), the predominant product of cyclooxygenase-2, has been implicated in hepatocarcinogenesis. However, the underlying molecular mechanisms remain to be further elucidated. c-myc, a cellular proto-oncogene, is activated or overexpressed in many types of human cancer, including HCC. The present study was designed to investigate the internal relationship and molecular mechanisms between PGE2 and c-Myc in HCC, and to define its role in HCC cell growth and invasion. Our results showed that PGE2 significantly upregulated c-Myc expression at both the mRNA and protein levels, and knockdown of c-Myc blocked PGE2-induced HCC cell growth and   invasive   ability in human HCC Huh-7 cells. The effect of PGE2 on c-Myc expression was mainly through the EP4 receptor, and EP4 receptor-mediated c-Myc protein upregulation largely depended on de novo biosynthesis of c-Myc mRNA and its protein. EP4 receptor signaling activated GS/AC and increased the intracellular cAMP level in Huh-7 cells. The adenylate cyclase (AC) activator forskolin mimicked the effects of the EP4 receptor agonist on c-Myc expression, while the AC inhibitor SQ22536 reduced EP4 receptor-mediated c-Myc upregulation. These data confirm the involvement of the GS/AC/cAMP pathway in EP4 receptor-mediated c-Myc upregulation. Moreover, the phosphorylation levels of CREB protein were markedly elevated by EP4 receptor signaling, and by using specific inhibitor and siRNA interference, we demonstrated that PKA/CREB was also involved in the EP4 receptor-mediated c-Myc upregulation. In summary, the present study revealed that PGE2 significantly upregulates c-Myc expression at both mRNA and protein levels through the EP4R/GS/AC/cAMP/PKA/CREB signaling pathway, thus promoting cell growth and invasion in HCC cells. Targeting of the PGE2/EP4R/c-Myc pathway may be a new therapeutic strategy to prevent and cure human HCC. 
 c-MYC amplification in mucinous gastric carcinoma: a possible genetic alteration leading to deeply   invasive   tumors. Patients with mucinous gastric carcinoma (MGC) usually have a poor prognosis, largely due to the advanced stage of disease. In this study, we evaluated the effects of c-MYC amplification on tumor stage and disease-specific survival of 128 patients with MGC and compared the results with those of 302 patients with non-mucinous gastric carcinoma (non-MGC).Two-color fluorescence in situ hybridization (FISH) for c-MYC was performed on 430 GC samples. Real-time quantitative polymerase chain reaction (q-PCR) analysis for c-MYC was also performed after tumor microdissection.c-MYC amplification was found in 10.2% of MGCs and 6.0% of non-MGCs. c-MYC amplification was more frequently found in MGCs of higher tumor stage than in MGCs of lower stage (p=0.038). c-MYC amplification in MGC was correlated with greater invasion depth (p=0.007). The mean survival time of patients with c-MYC amplification was shorter than that of patients without c-MYC amplification in MGC. Real-time q-PCR results showed that the calculated c-MYC/GAPDH ratios were higher in c-MYC-amplified MGC than in c-MYC-non-amplified MGC.This study showed that c-MYC amplification in MGC is highly correlated with advanced stage and deeply   invasive   MGC. This suggests that c-MYC amplification in MGC could be a possible genetic alteration contributing to the frequent presentation of advanced-stage MGC. 
 1Alpha,25-dihydroxyvitamin D3 reduces c-Myc expression, inhibiting proliferation and causing G1 accumulation in C4-2 prostate cancer cells. There is an inverse correlation between exposure to sunlight (the major source of vitamin D) and the risk for prostate cancer, the most common noncutaneous cancer and second most common cause of death from cancer in American men. The active metabolite of vitamin D, 1alpha,25-dihydroxyvitamin D(3) [1,25(OH)(2)D(3)] acting through the vitamin D receptor decreases prostate cancer cell growth and   invasive  ness. The precise mechanisms by which 1,25(OH)(2)D(3) inhibits growth in prostate cancer have not been fully elucidated. Treatment with 1,25(OH)(2)D(3) causes an accumulation in the G(0)/G(1) phase of the cell cycle in several prostate cancer cell lines. One potential target known to regulate the G(0)/G(1) to S phase transition is c-Myc, a transcription factor whose overexpression is associated with a number of cancers including prostate cancer. We find that 1,25(OH)(2)D(3) reduces c-Myc expression in multiple prostate epithelial cell lines, including C4-2 cells, an androgen-independent prostate cancer cell line. Reducing c-Myc expression to the levels observed after 1,25(OH)(2)D(3) treatment resulted in a comparable decrease in proliferation and G(1) accumulation demonstrating that down-regulation of c-Myc is a major component in the growth-inhibitory actions of 1,25(OH)2D(3). Treatment with 1,25(OH)(2)D(3) resulted in a 50% decrease in c-Myc mRNA but a much more extensive reduction in c-Myc protein. Treatment with 1,25(OH)(2)D(3) decreased c-Myc stability by increasing the proportion of c-Myc phosphorylated on T58, a glycogen synthase kinase-3beta site that serves as a signal for ubiquitin-mediated proteolysis. Thus, 1,25(OH)(2)D(3) reduces both c-Myc mRNA levels and c-Myc protein stability to inhibit growth of prostate cancer cells. 
 MYC regulation of a "poor-prognosis" metastatic cancer cell state. Gene expression signatures are used in the clinic as prognostic tools to determine the risk of individual patients with localized breast tumors developing distant metastasis. We lack a clear understanding, however, of whether these correlative biomarkers link to a common biological network that regulates metastasis. We find that the c-MYC oncoprotein coordinately regulates the expression of 13 different "poor-outcome" cancer signatures. In addition, functional inactivation of MYC in human breast cancer cells specifically inhibits distant metastasis in vivo and   invasive   behavior in vitro of these cells. These results suggest that MYC oncogene activity (as marked by "poor-prognosis" signature expression) may be necessary for the translocation of poor-outcome human breast tumors to distant sites. 
 Enhanced epithelial to mesenchymal transition (EMT) and upregulated MYC in ectopic lesions contribute independently to endometriosis. Epithelial to mesenchymal transition (EMT) is a process in which epithelial cells lose polarity and cell-to-cell contacts and acquire the migratory and   invasive   abilities of mesenchymal cells. These abilities are thought to be prerequisites for the establishment of endometriotic lesions. A hallmark of EMT is the functional loss of E-cadherin (CDH1) expression in epithelial cells. TWIST1, a transcription factor that represses E-cadherin transcription, is among the EMT inducers. SNAIL, a zinc-finger transcription factor, and its close relative SLUG have similar properties to TWIST1 and are thus also EMT inducers. MYC, which is upregulated by estrogens in the uterus by an estrogen response cis-acting element (ERE) in its promoter, is associated with proliferation in endometriosis. The role of EMT and proliferation in the pathogenesis of endometriosis was evaluated by analyzing TWIST1, CDH1 and MYC expression.CDH1, TWIST1, SNAIL and SLUG mRNA expression was analyzed by qRT-PCR from 47 controls and 74 patients with endometriosis. Approximately 42 ectopic and 62 eutopic endometrial tissues, of which 30 were matched samples, were collected during the same surgical procedure. We evaluated TWIST1 and MYC protein expression by immunohistochemistry (IHC) in the epithelial and stromal tissue of 69 eutopic and 90 ectopic endometrium samples, of which 49 matched samples were analyzed from the same patient. Concordant expression of TWIST1/SNAIL/SLUG and CDH1 but also of TWIST1 and MYC was analyzed.We found that TWIST1, SNAIL and SLUG are overexpressed (p < 0.001, p = 0.016 and p < 0.001) in endometriosis, while CDH1 expression was concordantly reduced in these samples (p < 0.001). Similar to TWIST1, the epithelial expression of MYC was also significantly enhanced in ectopic endometrium compared to eutopic tissues (p = 0.008). We found exclusive expression of either TWIST1 or MYC in the same samples (p = 0.003).Epithelial TWIST1 is overexpressed in endometriosis and may contribute to the formation of endometriotic lesions by inducing epithelial to mesenchymal transition, as CDH1 was reduced in ectopic lesions. We found exclusive expression of either TWIST1 or MYC in the same samples, indicating that EMT and proliferation contribute independently of each other to the formation of endometriotic lesions. 
 c-Myc represses FOXO3a-mediated transcription of the gene encoding the p27(Kip1) cyclin dependent kinase inhibitor. The p27(Kip1) (p27) cyclin-dependent kinase inhibitor and c-Myc oncoprotein play essential roles in control of cell cycle progression and apoptosis. Induction of p27 (CDKN1B) gene transcription by Forkhead box O proteins such as FOXO3a leads to growth arrest and apoptosis. Previously, we observed that B cell receptor (surface IgM) engagement of WEHI 231 immature B lymphoma cells with an anti-IgM antibody results in activation of FOXO3a, growth arrest and apoptosis. As ectopic c-Myc expression in these cells prevented anti-IgM induction of p27 and cell death, we hypothesized that c-Myc represses FOXO3a-mediated transcription. Here we show that c-Myc inhibits FOXO3a-mediated activation of the p27 promoter in multiple cell lines. The mechanism of this repression was explored using a combination of co-immunoprecipitation, oligonucleotide precipitation, and chromatin immunoprecipitation experiments. The studies demonstrate a functional association of FOXO3a and c-Myc on a proximal Forkhead binding element in the p27 promoter. This association involves the Myc box II domain of c-Myc and the N-terminal DNA-binding portion of FOXO3a. Analysis of publicly available microarray datasets showed an inverse pattern of c-MYC and p27 RNA expression in primary acute myeloid leukemia, prostate cancer and tongue squamous cell carcinoma samples. The inhibition of FOXO3a-mediated activation of the p27 gene by the high aberrant expression of c-Myc in many tumor cells likely contributes to their uncontrolled proliferation and   invasive   phenotype. 
 MUC16-mediated activation of mTOR and c-Myc reprograms pancreatic cancer metabolism. MUC16, a transmembrane mucin, facilitates pancreatic adenocarcinoma progression and metastasis. In the current studies, we observed that MUC16 knockdown pancreatic cancer cells exhibit reduced glucose uptake and lactate secretion along with reduced migration and invasion potential, which can be restored by supplementing the culture media with lactate, an end product of aerobic glycolysis. MUC16 knockdown leads to inhibition of mTOR activity and reduced expression of its downstream target c-MYC, a key player in cellular growth, proliferation and metabolism. Ectopic expression of c-MYC in MUC16 knockdown pancreatic cancer cells restores the altered cellular physiology. Our LC-MS/MS based metabolomics studies indicate global metabolic alterations in MUC16 knockdown pancreatic cancer cells, as compared to the controls. Specifically, glycolytic and nucleotide metabolite pools were significantly decreased. We observed similar metabolic alterations that correlated with MUC16 expression in primary tumor tissue specimens from human pancreatic adenocarcinoma cancer patients. Overall, our results demonstrate that MUC16 plays an important role in metabolic reprogramming of pancreatic cancer cells by increasing glycolysis and enhancing motility and   invasive  ness. 
 c-Myc suppresses microRNA-29b to promote tumor aggressiveness and poor outcomes in non-small cell lung cancer by targeting FHIT. The dual role of the microRNA-29 (miR-29) family in tumor progression and metastasis in solid tumors has been reported. Evidence for the role of miR-29 in tumor malignancy and its prognostic value in overall survival (OS) and relapse-free survival (RFS) in non-small cell lung cancer (NSCLC) remains conflicting. Mechanistic studies presented herein demonstrated that c-Myc suppressed the expression of miR-29b, promoting soft agar growth and invasion capability in lung cancer cells. Interestingly, the decrease in the expression of miR-29b by c-Myc is responsible for soft agar growth and   invasive  ness mediated by FHIT loss due to promoter methylation. Among patients, low expression of miR-29b and FHIT was more common in tumors with high c-Myc expression than in tumors with low c-Myc expression. Kaplan-Meier and Cox regression analysis showed that tumors with high c-Myc, low miR-29b and low FHIT expression had shorter OS and RFS periods than their counterparts. In conclusion, the decrease in the expression of miR-29b by c-Myc may be responsible for FHIT loss-mediated tumor aggressiveness and for poor outcome in NSCLC. Therefore, we suggest that restoration of the miR-29b expression using the c-Myc inhibitor might be helpful in suppressing tumor aggressiveness mediated by FHIT loss and consequently improving outcomes in NSCLC patients with tumors with low expression of FHIT. 
 Alterations in nucleolar structure and gene expression programs in prostatic neoplasia are driven by the MYC oncogene. Increased nucleolar size and number are hallmark features of many cancers. In prostate cancer, nucleolar enlargement and increased numbers are some of the earliest morphological changes associated with development of premalignant prostate intraepithelial neoplasia (PIN) lesions and   invasive   adenocarcinomas. However, the molecular mechanisms that induce nucleolar alterations in PIN and prostate cancer remain largely unknown. We verify that activation of the MYC oncogene, which is overexpressed in most human PIN and prostatic adenocarcinomas, leads to formation of enlarged nucleoli and increased nucleolar number in prostate luminal epithelial cells in vivo. In prostate cancer cells in vitro, MYC expression is needed for maintenance of nucleolar number, and a nucleolar program of gene expression. To begin to decipher the functional relevance of this transcriptional program in prostate cancer, we examined FBL (encoding fibrillarin), a MYC target gene, and report that fibrillarin is required for proliferation, clonogenic survival, and proper ribosomal RNA accumulation/processing in human prostate cancer cells. Further, fibrillarin is overexpressed in PIN lesions induced by MYC overexpression in the mouse prostate, and in human clinical prostate adenocarcinoma and PIN lesions, where its expression correlates with MYC levels. These studies demonstrate that overexpression of the MYC oncogene increases nucleolar number and size and a nucleolar program of gene expression in prostate epithelial cells, thus providing a molecular mechanism responsible for hallmark nucleolar alterations in prostatic neoplasia. 
 A c-Myc/miR-17-5p feedback loop regulates metastasis and invasion of hepatocellular carcinoma. The molecular mechanisms that control metastasis of hepatocellular cancer (HCC) are still poorly understood. It has been determined that microRNA (miRNA) expression has tissue and cell specific, and decreased expression of specific miRNA could induce tumor genesis or metastasis. In this study, we identified that miR-17-5p was expressed lower in high metastatic capability HCC cell lines HCCLM3 and MHCC97H than low metastatic HCC cell line HepG2 by real-time (RT)-PCR. Restoration of miR-17-5p could significantly repress the   invasive  ness and metastasis of MHCC97H cell line. Furthermore, we validated c-Myc as a downstream and functional target of miR-17-5p using luciferase reporter assay. Immunohistochemical assay revealed that the expression of c-Myc protein levels was significantly increased in cancerous tissues compared with para-tumor tissues. After clinical data analysis, we observed that the higher level of c-Myc was significantly associated with a reduced overall survival (p = 0.0209). Consistent with previous research, we also demonstrated that c-Myc could upregulate the expression of miR-17-5p. Taken together, our data indicated that there is a regulatory feedback loop between miR-17-5p and c-Myc, in which miR-17-5p could suppress some of the distinguishing features, invasion, and metastasis, of oncogenic c-Myc in HCC cells, and meanwhile, miR-17-5p is upregulated by c-Myc role as a transcription factor, although further studies are still needed. 
 MYC amplification in breast cancer: a chromogenic in situ hybridisation study. To analyse the correlation between MYC amplification and various clinicopathological features and outcome in a cohort of 245 patients with   invasive   breast carcinoma treated with surgery followed by anthracycline-based chemotherapy. Given the high prevalence of MYC amplification in tumours of BRCA1 mutation carriers and the similarities between these and sporadic "basal-like" carcinomas, the prevalence of MYC amplification in "basal-like" breast carcinomas was investigated.MYC gene copy number was assessed on tissue microarrays containing duplicate cores of 245   invasive   breast carcinomas by means of chromogenic in situ hybridisation using SpotLight C-MYC amplification probe and chromosome 8 centromeric probe (CEP8). Signals were evaluated at 400x magnification; 30 morphologically unequivocal neoplastic cells in each core were counted for the presence of the gene and CEP8 probes.Amplification was defined as a MYC:CEP8 ratio >2. Signals for both MYC and CEP8 were assessable in 196/245 (80%) tumours. MYC amplification was found in 19/196 cases (9.7%) and was not associated with tumour size, histological grade, positivity for oestrogen receptor, progesterone receptor, HER2, epidermal growth factor, cytokeratins 14, 5/6 and 17, MIB1 or p53. Only 4% of basal-like carcinomas showed MYC amplification, compared to 8.75% and 10.7% of luminal and HER2 tumours respectively. On univariate analysis, MYC amplification displayed a significant association with shorter metastasis-free and overall survival and proved to be an independent prognostic factor on multivariate survival analysis.MYC amplification is not associated with "basal-like" phenotype and proved to be an independent prognostic factor for breast cancer patients treated with anthracycline-based chemotherapy. 
 SALL4 as an Epithelial-Mesenchymal Transition and Drug Resistance Inducer through the Regulation of c-Myc in Endometrial Cancer. SALL4 plays important roles in the development and progression of many cancers. However, the role and molecular mechanism of SALL4 in endometrial cancer remain elusive. In the present research, we have demonstrated that the expression of SALL4 was upregulated in endometrial cancer and correlated positively with tumor stage, metastases and poor survival of patients. The overexpression of SALL4 promoted the   invasive  ness in endometrial cancer cells, as indicated by the upregulation of mesenchymal cell marker N-cadherin and downregulation of the epithelial marker E-cadherin, and invasion assays in vitro. Additionally, there was also an increase in drug resistance in these cell models due to the upregulation of ATP-binding cassette multidrug transporter ABCB1 expression. Moreover, we also found that ABCB1 was critical for SALL4-induced drug resistance. In contrast, SALL4 knockdown restored drug sensitivity, reversed EMT, diminished cell metastasis and suppressed the downregulation of E-cadherin and the upregulation of N-cadherin and ABCB1. Furthermore, we showed that SALL4 upregulated c-Myc expression and c-Myc was a direct target for SALL4 by ChIP assay, depletion of c-Myc with siRNA abolished the SALL4-induced downregulation of E-cadherin, upregulation of N-cadherin and ABCB1, suggesting that c-Myc was a downstream target for SALL4 and required for SALL4-induced EMT, invasion and drugs resistance in endometrial cancer cells. These results indicated that SALL4 could induce EMT and resistance to antineoplastic drugs through the regulation of c-Myc. SALL4 and c-Myc may be novel therapeutic targets for endometrial cancer. 
 Global levels of H3K27me3 track with differentiation in vivo and are deregulated by MYC in prostate cancer. Cancer cells and stem cells share a number of biological characteristics including abundant amounts of decondensed chromatin. However, the molecular correlates and the factors involved in altering chromatin structure in cancer cells are not well known. Here, we report that less differentiated stem-like cells in the basal compartment of human and mouse prostate contain lower levels of the polycomb heterochromatin marker H3K27me3 than more differentiated luminal cells. This link to differentiated normal cells is also found in a number of other human and rodent tissues characterized by hierarchical differentiation. In addition to MYC's traditional role as a gene-specific transcription factor, recent studies indicate that MYC also affects global chromatin structure where it is required to maintain "open" or active chromatin. We now demonstrate that in both MYC-driven prostate cancers in mice and human prostate cancers, global levels of H3K27me3 are reduced in prostatic intraepithelial neoplasia and   invasive   adenocarcinoma lesions. Moreover, decreased levels of H3K27me3 correlate with increased markers of disease aggressiveness (eg, Gleason score and pathological stage). In vitro, experimentally forced reductions in MYC levels result in increased global levels of H3K27me3. These findings suggest that increased levels of decondensed chromatin in both normal progenitor cells and cancer cells are associated with global loss of H3K27me3, which is linked to MYC overexpression. 
 Cooperation of Ras- and c-Myc-dependent pathways in regulating the growth and   invasive  ness of synovial fibroblasts in rheumatoid arthritis. To study the specific contribution of MAP kinase activator c-Raf-1 and one of its downstream transcription factors, c-Myc, to the growth and   invasive   behavior of rheumatoid arthritis synovial fibroblasts (RASFs).RASFs were transduced with retroviral constructs expressing dominant-negative mutants of c-Raf-1 or c-Myc (DN c-Raf-1 or DN c-Myc, respectively) or with the mock vector. The expression of wild-type and mutant proteins was confirmed by Western blotting. Growth curves of RASFs were recorded, and apoptosis was measured by flow cytometry.   Invasive  ness of RASFs was assessed in the SCID mouse model of RA. Immunohistochemistry was used to study the effects of DN c-Raf-1 on phosphorylated c-Jun and matrix metalloproteinase 1 (MMP-1) in RASFs implanted into SCID mice. The phosphorylation of ERK and JNK in DN c-Raf-1- and mock-transduced RASFs was determined in vitro by Western blotting. The levels of MMPs in these cells were measured by quantitative polymerase chain reaction (PCR).Neither DN c-Raf-1 alone nor DN c-Myc alone significantly altered proliferation or apoptosis of RASFs, but both mutants together rapidly induced apoptosis. Inhibition of c-Raf-1 or c-Myc significantly reduced the   invasive  ness of RASFs in the SCID mouse model. DN c-Raf-1 decreased the phosphorylation of ERK and JNK in vitro and reduced the in vivo expression of phosphorylated c-Jun as well as the expression of disease-relevant MMPs. As determined by quantitative PCR, the inhibition was most pronounced for MMP-1 and MMP-3.The data demonstrate that Ras- and c-Myc-dependent signaling events cooperate to regulate the growth and   invasive  ness of RASFs. Targeting of both c-Raf-1 and c-Myc may constitute an interesting therapeutic approach in RA. 
 High-throughput tissue microarray analysis of CMYC amplificationin urinary bladder cancer. Alterations of chromosome 8, preferentially deletions of 8p and gains of 8q, belong to the most frequent cytogenetic changes in bladder cancer. CMYC on 8q24 is a candidate oncogene in this region. Little is known about the clinical significance of CMYC copy number changes in urinary bladder cancer because its frequency is low and a limited numbers of tumors were analyzed so far. To investigate the impact of CMYC alterations on tumor progression and patient prognosis in bladder cancer, we applied FISH to a tissue microarray containing 2317 bladder cancer samples. Presence of CMYC copy number increase was associated with advanced stage and high grade. CMYC amplifications were seen in 3 of 467 pTa (0.6%), 10 of 247 pT1 (4%) and 11 of 201 pT2-4 urothelial carcinomas (5.5%; p < 0.0001), as well as in 1 of 123 G1 (0.8%), 8 of 470 G2 (1.7%) and 17 of 365 G3 urothelial carcinomas (4.7%; p < 0.0001). CMYC gains were present in 49 of 467 pTa (10.5%), 39 of 247 pT1 (15.8%) and 43 of 201 pT2-4 urothelial carcinoma (21.4%; p < 0.0001), as well as in 7 of 123 G1 (5.7%), 56 of 470 G2 (11.9%) and 72 of 365 G3 urothelial carcinomas (19.7%; p < 0.0001). CMYC copy number changes were unrelated to prognosis of bladder cancer patients. We conclude that alterations of the CMYC gene, including copy number gains and amplifications, are linked to genetically unstable bladder cancers that are characterized by a high histologic grade and/or   invasive   growth. Patient prognosis was not affected by CMYC gene copy number changes. 
 MicroRNA-34a inhibits human trophoblast cell invasion by targeting MYC. Preeclampsia, one of the major disorders of pregnancy, is characterized by inadequate trophoblast invasion and defective trophoblast-mediated remodeling of placental vasculature. MicroRNA-34a (miR-34a) has been found to be aberrantly expressed in the placentas of preeclamptic patients, yet its role in placental development and in the pathogenesis of preeclampsia remains elusive.The levels of miR-34a in the placentas of 20 preeclamptic patients and 20 healthy subjects were determined by real time-PCR, and miR-34a was found significantly elevated in the preeclamptic placentas. Further, the function of miR-34a in trophoblast cells was investigated by overexpressing miR-34a in JEG-3 trophoblast cell line. Overexpression of miR-34a in JEG-3 cells inhibited cell proliferation, migration and invasion. In addition, elevated expression of miR-34a reduced the expression of both endogenous and ectopic MYC. Moreover, we identified that MYC mRNA was a direct target of miR-34a in JEG-3 cells by dual luciferase reporter assay, and found that downregulation of MYC expression by miR-34a targeting significantly reduced the   invasive  ness of JEG-3 cells.Our findings provide preliminary evidence for the diverse functions of miR-34a in trophoblast biology, and suggest that miR-34a suppresses trophoblast invasion by directly targeting MYC. 
 MYC, FBXW7 and TP53 copy number variation and expression in gastric cancer. MYC deregulation is a common event in gastric carcinogenesis, usually as a consequence of gene amplification, chromosomal translocations, or posttranslational mechanisms. FBXW7 is a p53-controlled tumor-suppressor that plays a role in the regulation of cell cycle exit and reentry via MYC degradation.We evaluated MYC, FBXW7, and TP53 copy number, mRNA levels, and protein expression in gastric cancer and paired non-neoplastic specimens from 33 patients and also in gastric adenocarcinoma cell lines. We also determined the invasion potential of the gastric cancer cell lines.MYC amplification was observed in 51.5% of gastric tumor samples. Deletion of one copy of FBXW7 and TP53 was observed in 45.5% and 21.2% of gastric tumors, respectively. MYC mRNA expression was significantly higher in tumors than in non-neoplastic samples. FBXW7 and TP53 mRNA expression was markedly lower in tumors than in paired non-neoplastic specimens. Moreover, deregulated MYC and FBXW7 mRNA expression was associated with the presence of lymph node metastasis and tumor stage III-IV. Additionally, MYC immunostaining was more frequently observed in intestinal-type than diffuse-type gastric cancers and was associated with MYC mRNA expression. In vitro studies showed that increased MYC and reduced FBXW7 expression is associated with a more   invasive   phenotype in gastric cancer cell lines. This result encouraged us to investigate the activity of the gelatinases MMP-2 and MMP-9 in both cell lines. Both gelatinases are synthesized predominantly by stromal cells rather than cancer cells, and it has been proposed that both contribute to cancer progression. We observed a significant increase in MMP-9 activity in ACP02 compared with ACP03 cells. These results confirmed that ACP02 cells have greater invasion capability than ACP03 cells.In conclusion, FBXW7 and MYC mRNA may play a role in aggressive biologic behavior of gastric cancer cells and may be a useful indicator of poor prognosis. Furthermore, MYC is a candidate target for new therapies against gastric cancer. 
 Single-cell genetic analysis of ductal carcinoma in situ and   invasive   breast cancer reveals enormous tumor heterogeneity yet conserved genomic imbalances and gain of MYC during progression. Ductal carcinoma in situ (DCIS) is a precursor lesion of   invasive   ductal carcinoma (IDC) of the breast. To understand the dynamics of genomic alterations in this progression, we used four multicolor fluorescence in situ hybridization probe panels consisting of the oncogenes COX2, MYC, HER2, CCND1, and ZNF217 and the tumor suppressor genes DBC2, CDH1, and TP53 to visualize copy number changes in 13 cases of synchronous DCIS and IDC based on single-cell analyses. The DCIS had a lower degree of chromosomal instability than the IDC. Despite enormous intercellular heterogeneity in DCIS and IDC, we observed signal patterns consistent with a nonrandom distribution of genomic imbalances. CDH1 was most commonly lost, and gain of MYC emerged during progression from DCIS to IDC. Four of 13 DCISs showed identical clonal imbalances in the IDCs. Six cases revealed a switch, and in four of those, the IDC had acquired a gain of MYC. In one case, the major clone in the IDC was one of several clones in the DCIS, and in another case, the major clone in the DCIS became one of the two major clones in the IDC. Despite considerable chromosomal instability, in most cases the evolution from DCIS to IDC is determined by recurrent patterns of genomic imbalances, consistent with a biological continuum. 
 Genetic aberrations of c-myc and CCND1 in the development of   invasive   bladder cancer. Detrusor muscle   invasive   transitional cell carcinoma is associated with poor prognosis and is responsible for the majority of bladder cancer related deaths. Amplifications of c-myc and CCND1 are associated with detrusor-muscle-  invasive   transitional cell carcinoma, however, their precise role in driving disease progression is unclear. Fluorescence in situ hybridisation on archival tissue from 16 patients with primary diagnosis of > or = pT2 transitional cell carcinoma and 15 cases with primary pTa/pT1 disease subsequently progressing to detrusor-muscle-invasion was performed, in the latter group both pre and post muscle   invasive   events were studied. No patients presenting with >/=pT2 had amplification of c-myc, two out of 16 (12.5%) had CCND1 amplification. Of patients who developed > or = pT2, two out of 15 (13.3%) had amplification of c-myc, both in > or = pT2, five out of 15 (33.3%) had CCND1 amplification, two in pTa/pT1 tumours, three in > or = pT2 transitional cell carcinomas. In total, two out of 31 (6.5%) of patients' > or = pT2 TCCs were amplified for c-myc and six out of 31 (19%) were amplified for CCND1. Eighty-seven per cent (40 out of 46) of tumours were polysomic for chromosome 8 and 80% (37 out of 46) were polysomic for chromosome 11 and this reflected the high copy numbers of c-myc and CCND1 observed. In almost all cases an increase in c-myc/CCND1 copy number occurred prior to invasion and persisted in advanced disease. Amplification of CCND1 or alterations in c-myc/CCND1 early in bladder cancer may have clinical relevance in promoting and predicting progression to detrusor-muscle-  invasive   transitional cell carcinoma. 
 A regulatory loop involving miR-22, Sp1, and c-Myc modulates CD147 expression in breast cancer invasion and metastasis. Breast cancer is the most common cancer in women for which the metastatic process is still poorly understood. CD147 is upregulated in breast cancer and has been associated with tumor progression, but little is known about its regulatory mechanisms. In this study, we demonstrated that CD147 was overexpressed in breast cancer tissues and cell lines, and the high expression correlated with tumor invasion and metastasis. We also found that the transcription factors Sp1 and c-Myc could bind to the CD147 promoter and enhance its expression. The CD147 mRNA has a 748-bp 3'-untranslated region (UTR) with many miRNA target sites, suggesting possible regulation by miRNAs. We discovered that miR-22 repressed CD147 expression by directly targeting the CD147 3'UTR. We also determined that miR-22 could indirectly participate in CD147 modulation by downregulating Sp1 expression. miR-22 could form an autoregulatory loop with Sp1, which repressed miR-22 transcription by binding to the miR-22 promoter. Together with the c-Myc-mediated inhibition of miR-22 expression, our investigation identified a miR-22/Sp1/c-Myc network that regulates CD147 gene transcription. In addition, miR-22 overexpression suppressed breast cancer cell invasion, metastasis, and proliferation by targeting CD147 in vitro and in vivo. Furthermore, we found that miR-22 was significantly downregulated in breast cancer tissues and that its expression was inversely correlated with the tumor-node-metastasis stage and lymphatic metastasis in patients. Our study provides the first evidence that an miR-22/Sp1/c-Myc network regulates CD147 upregulation in breast cancer and that miR-22 represses breast cancer   invasive   and metastatic capacities. 
 MYC copy number gains are associated with poor outcome in penile squamous cell carcinoma. We determined MYC gene numerical aberrations and protein expression at different stages of penile squamous cell carcinoma carcinogenesis. We correlated these findings with clinicopathological parameters and HPV infection.We evaluated 79 cases of penile squamous cell carcinoma, including 11 in situ and 68   invasive   carcinomas. The MYC cytogenetic profile was evaluated by fluorescence in situ hybridization. HPV was detected by polymerase chain reaction amplification.MYC gains were identified in 4 of 11 in situ carcinomas (36%) and 50 of 68   invasive   penile squamous cell carcinomas (73%). A significant association between MYC gains, and tumor progression and poor outcome was demonstrated (p <0.05). HPV DNA was detected in 32 of 79 penile squamous cell carcinomas (39%). High risk type 16 was the most prevalent type. MYC numerical aberrations did not correlate with HPV status. A significant association between HPV and MYC protein over expression was noted. In HPV negative cases MYC gains correlated with MYC over expression.MYC gains progressively increased during penile squamous cell carcinoma progression from in situ samples to metastases. MYC gains were an independent factor for poor prognosis. These findings were independent of HPV infection. MYC expression was increased in samples with HPV infection, probably reflecting direct activation of MYC. 
 IκB kinases increase Myc protein stability and enhance progression of breast cancer cells. Both IκB kinase (IKK) complex and oncgenic protein Myc play important roles in cancer progression, including cancer cel  l invasi  veness and metastasis. The levels of Myc is regulated by the phosphorylation of Myc at Thr58 and Ser62.In this study, we show that the expression of Myc is associated with IKKα and IKKβ in breast cancers and that Myc is an IKKs substrate. Suppression of IKK activity by either chemical inhibitor or transfection of kinase-dead mutants decreases the phosphorylation of Myc at Ser62 and enhances the degradation of Myc. Consequently, these treatments decrease the tumorigenic   and inva  sive ability of breast cancer cells. Furthermore, doxorubicin, a frequently used anticancer drug in breast cancer, activates IKKs and Myc, thereby increas  ing inva  siveness and tumorigenesis of breast carcinoma MCF7 cells. Inhibition of IKKs prevents these doxorubicin-induced effects.Our study indicates that IKKs tightly regulate Myc expression through prolonging protein stability, and suggests that IKKs are potentially therapeutic targets and that suppression of IKKs may be used following chemotherapy to reduce the risk of treatment-induced tumor progression. 
 HER2 overexpression and amplification in urothelial carcinoma of the bladder is associated with MYC coamplification in a subset of cases. We examined 53   invasive   high-grade urothelial carcinomas (UCCs) and 42 paired lymph node metastases to determine frequency of HER2 overexpression, HER2/MYC coamplification, and association between HER2 and MYC status and clinicopathologic features. HER2 overexpression occurred in 19 UCCs (36%) and 14 metastases (30%), with an 88% concordance rate between UCCs and matched metastases. HER2 amplification occurred in 5 (10%) of 50 UCCs and 4 (11%) of 36 metastases, with a 100% concordance rate; MYC amplification occurred in 7 (18%) of 40 UCCs and 4 (13%) of 32 metastases, with a concordance rate of 50% between UCCs and metastases. Of 7 cases demonstrating HER2 amplification, MYC was coamplified in 4 (57%; P = .01), and coamplification was associated in all cases with metastasis and advanced local disease (pT4). Coamplification of HER2 and MYC occurs in a subset of patients with metastatic UCC. HER2 overexpression and amplification in metastatic lesions suggest that HER2-targeted therapy may be valuable for patients undergoing treatment for metastatic UCC, for which current therapy is limited. Further studies into the role of MYC coamplification in this population are needed to determine impacts on treatment with HER2-targeted therapy. 
  GeneRIFs  
 Increased c-myc is associated with   invasive  ness in Non-small Cell Lung Cancer. 
 detected coordination between regulation of transcription and translation upon modulation of Myc activity and showed connection of these responses to mTOR signaling to enhance oncogenic transformation and to the TGFbeta pathway to modulate cell migration and   invasive  ness 
 C-MYC amplification in gastric cancer is correlated with survival in advanced stages and deeply   invasive   stomach cancer. 
 Our study provides the first evidence that an miR-22/Sp1/c-Myc network regulates CD147 upregulation in breast cancer and that miR-22 represses breast cancer   invasive   and metastatic capacities. 
 CDH1 was most commonly lost, and gain of MYC emerged during progression from ductal carcinoma in situ to   invasive   ductal carcinoma. 
 c-MYC amplification is highly correlated with advanced stage and deeply   invasive   mucinous gastric carcinoma 
 ll patients with co-overexpression of bcl-2 and c-myc oncogenes will have PVU   invasive   carcinoma 
 MYC is necessary to specifically maintain an   invasive   and migratory state of tumors. 
 The inhibition of FOXO3a-mediated activation of the p27 gene by the high aberrant expression of c-Myc in many tumor cells likely contributes to their uncontrolled proliferation and   invasive   phenotype. 
 These data suggest that TLR3 contributes to the malignant phenotype leading to   invasive   carcinoma in head and neck squamous cell carcinoma. 
 Inhibition of c-Myc (and c-Raf-1) significantly reduced the growth and   invasive  ness of rheumatoid arthritis synovial fibroblasts in the SCID mouse model. 
 conclude that alterations of the CMYC gene, including copy number gains and amplifications, are linked to genetically unstable bladder cancers that are characterized by a high histologic grade and/or   invasive   growth 
 Amplifications of c-myc and CCND1 are associated with detrusor-muscle-  invasive   transitional cell carcinoma 
  [Mm]etastasize  PubMed  
 Combined MYC Activation and Pten Loss Are Sufficient to Create Genomic Instability and Lethal Metastatic Prostate Cancer. Genetic instability, a hallmark feature of human cancers including prostatic adenocarcinomas, is considered a driver of metastasis. Somatic copy number alterations (CNA) are found in most aggressive primary human prostate cancers, and the overall number of such changes is increased in metastases. Chromosome 10q23 deletions, encompassing PTEN, and amplification of 8q24, harboring MYC, are frequently observed, and the presence of both together portends a high risk of prostate cancer-specific mortality. In extant genetically engineered mouse prostate cancer models (GEMM), isolated MYC overexpression or targeted Pten loss can each produce early prostate adenocarcinomas, but are not sufficient to induce genetic instability or metastases with high penetrance. Although a previous study showed that combining Pten loss with focal MYC overexpression in a small fraction of prostatic epithelial cells exhibits cooperativity in GEMMs, additional targeted Tp53 disruption was required for formation of metastases. We hypothesized that driving combined MYC overexpression and Pten loss using recently characterized Hoxb13 transcriptional control elements that are active in prostate luminal epithelial cells would induce the development of genomic instability and aggressive disease with metastatic potential. Neoplastic lesions that developed with either MYC activation alone (Hoxb13-MYC) or Pten loss alone (Hoxb13-Cre∣Pten(Fl/Fl)) failed to progress beyond prostatic intraepithelial neoplasia and did not harbor genomic CNAs. By contrast, mice with both alterations (Hoxb13-MYC∣Hoxb13-Cre∣Pten(Fl/Fl), hereafter, BMPC mice) developed lethal adenocarcinoma with distant metastases and widespread genome CNAs that were independent of forced disruption of Tp53 and telomere shortening. BMPC cancers lacked neuroendocrine or sarcomatoid differentiation, features uncommon in human disease but common in other models of prostate cancer   that metas  tasize. These data show that combined MYC activation and Pten loss driven by the Hoxb13 regulatory locus synergize to induce genomic instability and aggressive prostate cancer that phenocopies the human disease at the histologic and genomic levels. 
  [Mm]etastasis  PubMed  
 [Expressions of APC and c-Myc and its implication on non-small cell lung cancer]. To investigate the expressions of adenomatous polyposis coli (APC) protein and c-Myc protein in non-small cell lung cancer (NSCLC) and their lymph node metastases.APC and c-Myc proteins were detected in 270 cases of primary NSCLC, 55 cases of lymph node metastatic tissues and 46 cases of adjacent normal lung tissues by EliVision and EnVision methods of immunohistochemical staining.Higher rates of the expressions of both APC and c-Myc proteins in NSCLC primary foci were found compared with those in lymph node metastases (P < 0.05). Furthermore, the expressions of APC and c-Myc proteins varied with histological types, TNM stagings and   metastasis   of the NSCLC (P < 0.05). The Spearman correlation analysis showed that the expressions of APC and c-Myc proteins were positively correlated (r(s) = 0.376, P = 0.000). The Kaplan-Meier survival analysis revealed that the survival rate was lower in patients with positive expressions of APC and c-Myc proteins than in patients with negative expressions (P < 0.05). Histological type, pathologic grading,   metastasis   and c-Myc were identified as independent risk factors with related to the prognosis of NSCLC patients in the multivariate Cox regression model (P < 0.05).APC and c-Myc may play an important role in the progression of NSCLC. 
 Molecular risk stratification of medulloblastoma patients based on immunohistochemical analysis of MYC, LDHB, and CCNB1 expression. Medulloblastoma is the most common malignant embryonal brain tumor in children. The current clinical risk stratification to select treatment modalities is not optimal because it does not identify the standard-risk patients with resistant disease or the unknown number of high-risk patients who might be overtreated with current protocols. The aim of this study is to improve the risk stratification of medulloblastoma patients by using the expression of multiple prognostic markers in combination with current clinical parameters.Candidate prognostic markers were selected from literature or from medulloblastoma expression data. Selected genes were immunohistochemically analyzed for their prognostic value using medulloblastoma tissue arrays containing 124 well-characterized patient samples.Protein expression analyses showed that the combined expression of three genes was able to predict survival in medulloblastoma patients. Low MYC expression identified medulloblastoma patients with a very good outcome. In contrast, concomitant expression of LDHB and CCNB1 characterized patients with a very poor outcome. Multivariate analyses showed that both expression of MYC and the LDHB/CCNB1 gene signature were strong prognostic markers independent of the clinical parameters   metastasis   and residual disease. Combined analysis of clinical and molecular markers enabled greater resolution of disease risk than clinical factors alone.A molecular risk stratification model for medulloblastoma patients is proposed based on the signature of MYC, LDHB, and CCNB1 expression. Combined with clinical variables, the model may provide a more accurate basis for targeting therapy in children with this disease. 
 [Expressions of APC and c-Myc and its implication on non-small cell lung cancer]. To investigate the expressions of adenomatous polyposis coli (APC) protein and c-Myc protein in non-small cell lung cancer (NSCLC) and their lymph node metastases.APC and c-Myc proteins were detected in 270 cases of primary NSCLC, 55 cases of lymph node metastatic tissues and 46 cases of adjacent normal lung tissues by EliVision and EnVision methods of immunohistochemical staining.Higher rates of the expressions of both APC and c-Myc proteins in NSCLC primary foci were found compared with those in lymph node metastases (P < 0.05). Furthermore, the expressions of APC and c-Myc proteins varied with histological types, TNM stagings and   metastasis   of the NSCLC (P < 0.05). The Spearman correlation analysis showed that the expressions of APC and c-Myc proteins were positively correlated (r(s) = 0.376, P = 0.000). The Kaplan-Meier survival analysis revealed that the survival rate was lower in patients with positive expressions of APC and c-Myc proteins than in patients with negative expressions (P < 0.05). Histological type, pathologic grading,   metastasis   and c-Myc were identified as independent risk factors with related to the prognosis of NSCLC patients in the multivariate Cox regression model (P < 0.05).APC and c-Myc may play an important role in the progression of NSCLC. 
 Molecular risk stratification of medulloblastoma patients based on immunohistochemical analysis of MYC, LDHB, and CCNB1 expression. Medulloblastoma is the most common malignant embryonal brain tumor in children. The current clinical risk stratification to select treatment modalities is not optimal because it does not identify the standard-risk patients with resistant disease or the unknown number of high-risk patients who might be overtreated with current protocols. The aim of this study is to improve the risk stratification of medulloblastoma patients by using the expression of multiple prognostic markers in combination with current clinical parameters.Candidate prognostic markers were selected from literature or from medulloblastoma expression data. Selected genes were immunohistochemically analyzed for their prognostic value using medulloblastoma tissue arrays containing 124 well-characterized patient samples.Protein expression analyses showed that the combined expression of three genes was able to predict survival in medulloblastoma patients. Low MYC expression identified medulloblastoma patients with a very good outcome. In contrast, concomitant expression of LDHB and CCNB1 characterized patients with a very poor outcome. Multivariate analyses showed that both expression of MYC and the LDHB/CCNB1 gene signature were strong prognostic markers independent of the clinical parameters   metastasis   and residual disease. Combined analysis of clinical and molecular markers enabled greater resolution of disease risk than clinical factors alone.A molecular risk stratification model for medulloblastoma patients is proposed based on the signature of MYC, LDHB, and CCNB1 expression. Combined with clinical variables, the model may provide a more accurate basis for targeting therapy in children with this disease. 
 p63-Mediated activation of the β-catenin/c-Myc signaling pathway stimulates esophageal squamous carcinoma cell invasion and   metastasi  s. The development of esophageal squamous carcinomas (ESC) results from numerous genetic alterations. Our previous study demonstrated that p63 is highly expressed in human ESC cells and stimulates their growth; however, the mechanism by which p63 regulates ESC cell adhesion and invasion remains unclear. In the present study, we further elucidated the underlying molecular mechanisms by which p63 regulates   metastasi  s in ESC cells. Knockdown of p63 significantly diminished the invasion of ESC cell lines TE-8 and TE-12, whereas overexpression of p63 significantly increased the migration rates of BE3 and OE33 cells. The mRNA and protein levels of vimentin, twist, SUSD2, and uPA were significantly decreased in p63-knockdown ESC cells, while overexpression of p63 induced an increase in vimentin, SUSD2, and uPA. In addition, knockdown of p63 in ESC cells significantly reduced levels of β-catenin and c-Myc, while overexpression of p63 increased β-catenin, but reduced p-β-catenin level. Therefore, p63 regulates the migration and invasion of ESC cells through activation of the β-catenin/c-Myc pathway. Our results suggest that targeting p63 may constitute a potential therapeutic strategy for ESC. 
 c-Myc expression and MEK1-induced Erk2 nuclear localization are required for TGF-beta induced epithelial-mesenchymal transition and invasion in prostate cancer. Understanding the initial mechanisms by which epithelial cells transform to an invasive phenotype is critical to the development of diagnostics that can identify the metastatic potential of cancers as well as therapeutic agents that can prevent metastases. Changes in cellular response to the transforming growth factor-beta (TGF-β) cytokine are known to promote epithelial cell invasion and   metastasi  s in part through induction of epithelial-mesenchymal transitions (EMTs). In this report, we demonstrate that non-metastatic human prostate cancer cell lines of increasing Gleason score can be induced to undergo EMT when treated with TGF-β in combination with epidermal growth factor. Mechanistic studies revealed that in cells stably transfected with activated Ras, TGF-β alone induced EMT and that a Ras-Raf-MEK1, but not MEK2, signaling cascade is necessary and sufficient for Erk2 nuclear localization that works in concert with TGF-β to promote EMT. Furthermore, we show for the first time that expression of the transcription factor c-myc, which is phosphorlyated by Erk2, is required for EMT. Characteristically, EMT involved adoption of a spindle-shaped morphology, loss of E-cadherin and increased expression of Vimentin, Fibronectin and Fibroblast Specific Protein-1 (S100A4). Prostate cells undergoing EMT became invasive and expressed several genes associated w  ith metast  asis, including MT-MMP1, MMP-2/9, the MMP-9 homodimer, Slug and Twist2. In sum, we demonstrate a novel mechanism by which non-invasive primary prostate tumor cells transition to an invasive phenotype characteristic of malignant tumor cells in response to TGF-β signaling. 
 A SUMOylation-dependent transcriptional subprogram is required for Myc-driven tumorigenesis. Myc is an oncogenic transcription factor frequently dysregulated in human cancer. To identify pathways supporting the Myc oncogenic program, we used a genome-wide RNA interference screen to search for Myc-synthetic lethal genes and uncovered a role for the SUMO-activating enzyme (SAE1/2). Loss of SAE1/2 enzymatic activity drives synthetic lethality with Myc. Inactivation of SAE2 leads to mitotic catastrophe and cell death upon Myc hyperactivation. Mechanistically, SAE2 inhibition switches a transcriptional subprogram of Myc from activated to repressed. A subset of these SUMOylation-dependent Myc switchers (SMS genes) is required for mitotic spindle function and to support the Myc oncogenic program. SAE2 is required for growth of Myc-dependent tumors in mice, and gene expression analyses of Myc-high human breast cancers suggest that low SAE1 and SAE2 abundance in the tumors correlates with longer   metastasis  -free survival of the patients. Thus, inhibition of SUMOylation may merit investigation as a possible therapy for Myc-driven human cancers. 
 Expression of c-Myc and Fas correlates with perineural invasion of pancreatic cancer. Lymph node   metastasis   (LNM) and perineural invasion (PNI) are regarded as two important prognostic factors in pancreatic cancer. The aim of this study was to identify the LNM-associated and PNI-associated markers in pancreatic cancer. We have constructed a formalin-fixed paraffin embedded pancreatic tissue microarrays containing 324 cylindrical tissue cores of human pancreatic cancer and its paracancerous nonmalignant pancreatic specimens (NMPs) from 162 patients. Among those patients, there were 83 of 162 with PNI, and 65 of 162 with LNM. The protein products of 2 genes encompassing a variety of functional classes, such as oncogenes (c-Myc), apoptosis (Fas), were analyzed by immunohistochemistry on the tissue microarray. There was marked increase in cancer tissues cytoplasmic c-Myc expression level in pancreatic cancer compared with the NMPs (P < 0.0001). Additionally, the expression level of c-Myc was also significant increase in pancreatic cancer with PNI compared with cancer without PNI (P < 0.01). In contrast, cytoplasmic Fas, low expressed in pancreatic cancer (P < 0.0001) was negatively correlated with PNI (P < 0.05). Logistic regression analysis showed that the c-Myc expression in the cancer tissues cytoplasm acted as a potential and independently predictive factor for PNI. However, there were no significant association between the expression of these two genes and LNM (P > 0.05). This study for the first time described expression levels of c-Myc and Fas played an important role in PNI of pancreatic cancer. Combined detection can be used as predictive factors, especially c-Myc. 
 Expression of frizzled-related protein and Wnt-signalling molecules in invasive human breast tumours. Frizzled-related protein (Frp) is a new family of secreted proteins that contain a region homologous to the extracellular cysteine-rich domain (CRD) of the frizzled family proteins. The role of Frp protein is far from clear. To explore the role of Frp and its relationship to the Wnt-signalling pathway in breast cancer, in situ hybridization and immunohistochemical analyses of Frp, Wnt-1, APC, beta-catenin, and its target genes c-myc and cyclin D1 were conducted in 70 specimens of invasive ductal carcinomas of the human breast. Frp mRNA was down-regulated in 62 and elevated in eight tumour specimens, compared with adjacent normal tissues. In the course of tumour progression, however, Frp mRNA steadily increased in both tumour and the adjacent tissues. Interestingly, the number of cases with axillary lymph node   metastasis   was significantly lower in the group with elevated Frp than in the group with decreased Frp, suggesting that Frp may contribute as a prognostic factor in invasive breast cancer. Wnt-1, a gene implicated in human breast cancer, was markedly elevated in grade 1 tumours, but declined as tumour grade declined. The level of Wnt-1 was linearly correlated with its downstream target beta-catenin (p<0.05), but was inversely correlated with Frp (p<0.05), suggesting a possible negative regulatory role of Frp with regard to Wnt-1. APC was inversely correlated with beta-catenin (p<0.05). Beta-catenin, a key transcriptional activator responsible for the activation of both c-myc and cyclin D1 in colorectal tumours, was detected at high levels in the plasma membranes of cells in normal tissue. In tumour masses, however, beta-catenin lost its tight association with the membrane and diffused into the cytoplasm. Surprisingly, it clearly did not penetrate the nuclei, despite the fact that both c-myc and cyclin D1 were markedly elevated in all tumour tissues. As revealed in this study, Wnt-1/beta-catenin plays very different roles in the oncogenesis of breast and colon cancers. This first systemic analysis of the Frp and the Wnt-signalling pathway in human breast cancer provides a springboard for further work on the role of Frp in the development of breast cancer. 
 [The expressions of STAT3, WWOX and c-myc in human non small cell lung cancer tissue and correlativity analysis]. To investigate the expression of STAT3, WWOX and c-myc in non small cell lung cancer(NSCLC) and their clinical significance, and study their relation ship.Forty resected tissue samples from patients with NSCLC and twenty normal lung tissues were subjected to immunohistochemical staining(SP) for STAT3, WWOX and c-myc.(1)The positive expression rate of STAT3 and c-myc in NSCLC were 92.5% and 90% respectively, obviously higher than normal lung tissues (20% and 15%, P<0.01); The positive expression rate of WWOX was 30%, apparently lower than normal lung tissues(75%, P<0.01). (2) The expression levels of STAT3 and c-myc were related to pathologic types in NSCLC, which in adenocarcinomas were higher than squmous cell carcinomas (P<0.01), which were regardless with tumor size, histodifferentiation level, clinical stage and lymphatic   metastasis  ; The expression of WWOX was ralated to whether lymph node   metastasis   in NSCLC(P<0.01).(3) There was a negativie correlation between the expressions of STAT3 and WWOX (r=-0.443, P<0.01); But no relationship was found between STAT3 and c-myc overexpression(r=-0.084) and between WWOX and c-myc overexpression(r=0.115).STAT3 and c-myc were overexpressed in human NSCLC. It indicated these proteins may make an important role in occurrence and development of lung cancer.The expressions of WWOX in human NSCLC was lower, which non expression was may related to the invision and   metastasis   of tumor. In the tumorigenesis of NSCLC, there is no relationship between expression of STAT3 and c-myc, They played independent roles in oncogenesis of NSCLC. There was a negativie correlation between the expressions of STAT3 and WWOX, but no relationship was found between WWOX and c-myc. 
 Association between c-myc amplification and pathological complete response to neoadjuvant chemotherapy in breast cancer. The aim of this study was to investigate whether c-myc amplification in human breast cancer is associated with response to neoadjuvant chemotherapy comprising paclitaxel followed by 5-FU/epirubicin/cyclophosphamide (P-FEC).Tumour tissue samples were obtained before neoadjuvant chemotherapy (P-FEC) from 100 primary breast cancer patients (stage II/III). C-myc and HER2 amplification were examined by FISH, and oestrogen receptor (ER), progesterone receptor (PR), Ki67, and topoisomerase 2α (TOP2A) expression were examined immunohistochemically. Pathological complete response (pCR) was defined by a complete loss of tumour cells in the breast without any lymph node   metastasi  s.C-myc amplification was observed in 40% (40/100) of breast tumours, and was significantly associated with ER-negative tumours (23/40 for ER(-) versus 17/60 for ER(+), P=0.004), high histological grade tumours (11/18 for grade 3 versus 29/82 for grades 1+2, P=0.043) and TOP2A-positive tumours (28/51 for TOP2A(+) versus 12/49 for TOP2A(-), P=0.002). pCR rate was 20% for total patients (10.0% for ER(+) and 35.0% for ER(-)). Further, breast tumours with c-myc amplification (c-myc(+)) showed a significantly (P=0.041) higher pCR rate (12/40) than those without such amplification (c-myc(-)) (8/60). This association between pCR and c-myc amplification was observed in ER-positive tumours (4/17 for c-myc(+) versus 2/43 for c-myc(-), P=0.048) but not in ER-negative tumours (8/23 for c-myc(+) versus 6/17 for c-myc(-), P=0.973).Our results suggest that c-myc amplification is significantly associated with a high pCR rate to P-FEC in breast tumours, especially in ER-positive tumours. 
 PIWIL2 induces c-Myc expression by interacting with NME2 and regulates c-Myc-mediated tumor cell proliferation. c-Myc serves as a crucial regulator in multiple cellular events. Cumulative evidences demonstrate that anomalous c-Myc overexpression correlates with proliferation, invasion and   metastasis   in various human tumors. However, the transcriptionally activating mechanisms responsible for c-Myc overexpression are complex and continue to be intangible. Here we showed that Piwi-Like RNA-Mediated Gene Silencing 2 (PIWIL2) can upregulate c-Myc via binding with NME/NM23 nucleoside diphosphate kinase 2 (NME2). PIWIL2 promotes c-Myc transcription by interacting with and facilitating NME2 to bind to G4-motif region within c-Myc promoter. Interestingly, in a c-Myc-mediated manner, PIWIL2 upregulates RhoA, which in turn induces filamentary F-actin. Deficiency of PIWIL2 results in obstacle for c-Myc expression, cell cycle progress and cell proliferation. Taken together, our present work demonstrates that PIWIL2 modulates tumor cell proliferation and F-actin filaments via promoting c-Myc expression. 
 Bmi-1, c-myc, and Snail expression in primary breast cancers and their metastases--elevated Bmi-1 expression in late breast cancer relapses. Breast cancer is known for its propensity to recur decades after treatment. The biology behind the phenomenon of tumor dormancy is still poorly understood. Bmi-1, c-myc, and Snail are transcription factors that have prognostic roles in several malignancies. In order to reveal whether any of these markers has impact on late relapses, we used immunohistochemistry to study the expression of Bmi-1, c-myc, Snail, and estrogen receptor in 73 primary breast cancers and in their metastatic relapses detected within 2 years, or 5 or 10 years after primary surgery. The expression of Bmi-1 was higher in the metastases than in their corresponding primary tumors in both early and late relapses. The highest expression of Bmi-1 was seen in the very late relapsing tumors (first tumor relapse after 10 years). Previously, Bmi-1 has been reported to function as a marker of tumor stem cells in breast cancer. Our results indicate that metastases, when compared to primary tumors, arise from tumor cells that have retained stem cell properties. We also analyzed the relationship between the expression of these markers and clinical parameters. A significant association between the expression of Bmi-1 and estrogen receptor was found. Nuclear expression of c-myc in primary tumors correlated with an increased risk for axillary lymph no  de metasta  sis. 
 c-Myc-mediated repression of miR-15-16 in hypoxia is induced by increased HIF-2α and promotes tumor angiogenesis and   metastasi  s by upregulating FGF2. Previous studies have established the link between aberrant microRNA (miRNA) expression and hypoxia in various neoplasms. However, how these hypoxia-related miRNAs modulate tumor progression is still unclear. Therefore, the patterns of miRNA in colorectal carcinoma cell lines in response to hypoxia or not were first screened and the hypoxia-induced repression of the miR-15-16 cluster was confirmed. Then, this repression was found to be associated with high tumor stage and poor prognosis in colorectal carcinoma and is shown to promote tumor angiogenesis a  nd metasta  sis by the loss of restriction of its target gene, fibroblast growth factor-2 (FGF2). Moreover, the general and alterative promoters of the miR-15-16 host (deleted in lymphocytic leukemia 2, DLEU2) were mapped, and three c-Myc/Max binding sites in response to the hypoxia-induced repression of miR-15-16 were further identified. Finally, an enhanced stability of c-Myc/Max heterodimer promoted by increased hypoxia-inducible factor-2α (HIF-2α) was validated, and we also verified that the enhancement contributed to the hypoxia-induced repression of miR-15-16. In brief, the c-Myc-mediated transcriptional repression of miR-15-16 in hypoxia is induced by increased HIF-2α and promoted tumor angiogenesis and hemat  ogenous me  tastasis by the further loss of post-transcriptional inhibition of FGF2. Our study provides a better understanding of the coping mechanisms in response to tumor hypoxia and may be helpful in developing an effective prognostic marker or treatment target against solid tumors. 
 MYCN downregulates integrin alpha1 to promote invasion of human neuroblastoma cells. Neuroblastoma is a childhood tumor thought to arise through improper differentiation of neural crest cells. MYCN amplification is a prognostic factor that indicates a highly malignant disease and poor patient prognosis. Integrins are important regulators of neuroblastoma attachment and migration and participate in many aspects of   metastasis  . However, the role of integrins in neuroblastoma   metastasis  , the leading cause of death from this disease, remains less well understood. Screening of neuroblastoma cell lines for integrin mRNA expression showed that integrin alpha1 expression was higher in lines such as SK-N-SH and NB69 that do not have MYCN amplification than in cell lines such as IMR32, NB1, NB9 and NB19 that have MYCN amplification. A knockdown of MYCN in NB1 and NB19 cells resulted in increased expression of integrin alpha1, which correlated with enhanced attachment to the extracellular matrix and reduced migratory activity. In contrast, the overexpression of MYCN in SK-N-SH and NB69 cells resulted in decreased expression of integrin alpha1, which correlated with reduced attachment to the extracellular matrix and enhanced migratory activity. These results show that MYCN may limit cell adhesion to the extracellular matrix and promote cell migration by downregulating integrin alpha1. 
 Tumor suppressor NDRG2 inhibits glycolysis and glutaminolysis in colorectal cancer cells by repressing c-Myc expression. Cancer cells use glucose and glutamine as the major sources of energy and precursor intermediates, and enhanced glycolysis and glutamimolysis are the major hallmarks of metabolic reprogramming in cancer. Oncogene activation and tumor suppressor gene inactivation alter multiple intracellular signaling pathways that affect glycolysis and glutaminolysis. N-Myc downstream regulated gene 2 (NDRG2) is a tumor suppressor gene inhibiting cancer growth,   metastasis   and invasion. However, the role and molecular mechanism of NDRG2 in cancer metabolism remains unclear. In this study, we discovered the role of the tumor suppressor gene NDRG2 in aerobic glycolysis and glutaminolysis of cancer cells. NDRG2 inhibited glucose consumption and lactate production, glutamine consumption and glutamate production in colorectal cancer cells. Analysis of glucose transporters and the catalytic enzymes involved in glycolysis revealed that glucose transporter 1 (GLUT1), hexokinase 2 (HK2), pyruvate kinase M2 isoform (PKM2) and lactate dehydrogenase A (LDHA) was significantly suppressed by NDRG2. Analysis of glutamine transporter and the catalytic enzymes involved in glutaminolysis revealed that glutamine transporter ASC amino-acid transporter 2 (ASCT2) and glutaminase 1 (GLS1) was also significantly suppressed by NDRG2. Transcription factor c-Myc mediated inhibition of glycolysis and glutaminolysis by NDRG2. More importantly, NDRG2 inhibited the expression of c-Myc by suppressing the expression of β-catenin, which can transcriptionally activate C-MYC gene in nucleus. In addition, the growth and proliferation of colorectal cancer cells were suppressed significantly by NDRG2 through inhibition of glycolysis and glutaminolysis. Taken together, these findings indicate that NDRG2 functions as an essential regulator in glycolysis and glutaminolysis via repression of c-Myc, and acts as a suppressor of carcinogenesis through coordinately targeting glucose and glutamine transporter, multiple catalytic enzymes involved in glycolysis and glutaminolysis, which fuels the bioenergy and biomaterials needed for cancer proliferation and progress. 
   Metastasis  -associated protein 1 (MTA1) is an essential downstream effector of the c-MYC oncoprotein. The c-myc oncogene is among the most commonly overexpressed genes in human cancer. c-myc encodes a basic helix-loop-helix/leucine zipper (bHLH/LZ) transcription factor (c-MYC) that activates a cascade of downstream targets that ultimately mediate cellular transformation. Although a large number of genes are regulated by c-MYC, only a few have been functionally linked to c-MYC-mediated transformation. By expression profiling, the   metastasis  -associated protein 1 (MTA1) gene was identified here as a target of the c-MYC oncoprotein in primary human cells, a result confirmed in human cancer cells. MTA1 itself has been previously implicated in cellular transformation, in part through its ability to regulate the epithelial-to-mesenchymal transition and   metastasis  . MTA1 is a component of the Mi-2/nucleosome remodeling and deacetylating (NURD) complex that contains both histone deacetylase and nucleosome remodeling activity. The data reported here demonstrate that endogenous c-MYC binds to the genomic MTA1 locus and recruits transcriptional coactivators. Most importantly, short hairpin RNA (shRNA)-mediated knockdown of MTA1 blocks the ability of c-MYC to transform mammalian cells. These data implicate MTA1 and the Mi-2/NURD complex as one of the first downstream targets of c-MYC function that are essential for the transformation potential of c-MYC. 
 A signal transduction pathway from TGF-β1 to SKP2 via Akt1 and c-Myc and its correlation with progression in human melanoma. Both SKP2 (S-phase kinase-associated protein 2) and transforming growth factor-β1 (TGF-β1) play important roles in canc  er metasta  sis through different mechanisms: TGF-β1 via induction of epithelial-mesenchymal transition (EMT) and SKP2 via downregulating p27(kip1). Recent studies indicated that c-Myc and Akt1 were active players   in metast  asis. In this study we demonstrated a crosstalk between these pathways. Specifically, we found that TGF-β1 treatment increased SKP2 expression accompanied with increased phosphorylation of Akt1 and c-Myc protein accumulation during EMT. We demonstrated that Akt1 was required for TGF-β1-mediated SKP2 upregulation and that c-Myc transcription factor specifically bound to the promoter of SKP2 for its enhanced transcription. Analysis of 25 samples of normal human skin, nevi, and melanomas revealed a positive correlation between c-Myc and SKP2 accumulation. Furthermore, accumulation of SKP2 and c-Myc proteins was significantly higher in metastatic melanoma samples as compared with that in primary melanomas, which again was higher than that in normal skin or nevi. In summary, our results integrated TGF-β1 signals to SKP2 via Akt1 and c-Myc during EMT, and provided, to our knowledge, a previously unreported mechanistic molecular event for TGF-β1-  induced me  tastasis in human melanoma. 
 MYC Drives Pten/Trp53-Deficient Proliferation and   Metastasis   due to IL6 Secretion and AKT Suppression via PHLPP2. We have recently recapitulated   metastasis   of human PTEN/TP53-mutant prostate cancer in the mouse using the RapidCaP system. Surprisingly, we found that this   metastasis   is driven by MYC, and not AKT, activation. Here, we show that cell-cell communication by IL6 drives the AKT-MYC switch through activation of the AKT-suppressing phosphatase PHLPP2, when PTEN and p53 are lost together, but not separately. IL6 then communicates a downstream program of STAT3-mediated MYC activation, which drives cell proliferation. Similarly, in tissues, peak proliferation in Pten/Trp53-mutant primary and metastatic prostate cancer does not correlate with activated AKT, but with STAT3/MYC activation instead. Mechanistically, MYC strongly activates the AKT phosphatase PHLPP2 in primary cells and prostate cancer   metastasis  . We show genetically that Phlpp2 is essential for dictating the proliferation of MYC-mediated AKT suppression. Collectively, our data reveal competition between two proto-oncogenes, MYC and AKT, which ensnarls the Phlpp2 gene to facilitate MYC-driven prostate cancer   metastasis   after loss of Pten and Trp53.Our data identify IL6 detection as a potential causal biomarker for MYC-driven   metastasis   after loss of PTEN and p53. Second, our finding that MYC then must supersede AKT to drive cell proliferation points to MYC inhibition as a critical part of PI3K pathway therapy in lethal prostate cancer. 
 ELL targets c-Myc for proteasomal degradation and suppresses tumour growth. Increasing evidence supports that ELL (eleven-nineteen lysine-rich leukaemia) is a key regulator of transcriptional elongation, but the physiological function of Ell in mammals remains elusive. Here we show that ELL functions as an E3 ubiquitin ligase and targets c-Myc for proteasomal degradation. In addition, we identify that UbcH8 serves as a ubiquitin-conjugating enzyme in this pathway. Cysteine 595 of ELL is an active site of the enzyme; its mutation to alanine (C595A) renders the protein unable to promote the ubiquitination and degradation of c-Myc. ELL-mediated c-Myc degradation inhibits c-Myc-dependent transcriptional activity and cell proliferation, and also suppresses c-Myc-dependent xenograft tumour growth. In contrast, the ELL(C595A) mutant not only loses the ability to inhibit cell proliferation and xenograft tumour growth, but also promotes tumour   metastasis  . Thus, our work reveals a previously unrecognized function for ELL as an E3 ubiquitin ligase for c-Myc and a potential tumour suppressor. 
 Prognostic value of β-catenin, c-myc, and cyclin D1 expressions in patients with esophageal squamous cell carcinoma. Esophageal squamous cell carcinoma (ESCC) is one of the most frequently diagnosed malignant tumors in North China. We have identified that Wnt2/β-catenin pathway is activated in ESCC cells and sodium nitroprusside (SNP) and siRNA against β-catenin not only inhibit the expressions of β-catenin and its major downstream effectors including c-myc and cyclin D1 but induce cell cycle arrest and apoptosis. The purpose of the present study was to analyze the relationship between pathological parameters including invasion depth and lymph n  ode metast  asis and the expressions of β-catenin, c-myc, and cyclin D1 in order to evaluate their values of prognosis in patients with ESCC. The expressions of β-catenin, c-myc, and cyclin D1 were detected immunohistochemically in the resected cancer tissues from 40 patients with ESCC. The β-catenin expression was reduced in 22 (55.0%) patients, which was closely correlated with invasion depth (P = 0.023) and lymp  h node met  astasis (P = 0.003). There was the positive c-myc expression in 21 (52.5%), which was significantly correlated with invasion depth (P = 0.009) and lymp  h node met  astasis (P = 0.001). Furthermore, the results of survival rates analyzed by Kaplan-Meier curve revealed that patients with the reduced expression of β-catenin had a poorer prognosis than those with the preserved expression (P = 0.031), and patients with the positive expression of c-myc also had a significantly poorer prognosis than those with the negative expression (P = 0.008). These findings demonstrate that β-catenin pathway plays a crucial role in the progression of ESCC, suggesting that both β-catenin and c-myc may be used as markers for predicting the prognosis of patients with ESCC. 
 Combined MYC Activation and Pten Loss Are Sufficient to Create Genomic Instability and Lethal Metastatic Prostate Cancer. Genetic instability, a hallmark feature of human cancers including prostatic adenocarcinomas, is considered a driver of   metastasis  . Somatic copy number alterations (CNA) are found in most aggressive primary human prostate cancers, and the overall number of such changes is increased in metastases. Chromosome 10q23 deletions, encompassing PTEN, and amplification of 8q24, harboring MYC, are frequently observed, and the presence of both together portends a high risk of prostate cancer-specific mortality. In extant genetically engineered mouse prostate cancer models (GEMM), isolated MYC overexpression or targeted Pten loss can each produce early prostate adenocarcinomas, but are not sufficient to induce genetic instability or metastases with high penetrance. Although a previous study showed that combining Pten loss with focal MYC overexpression in a small fraction of prostatic epithelial cells exhibits cooperativity in GEMMs, additional targeted Tp53 disruption was required for formation of metastases. We hypothesized that driving combined MYC overexpression and Pten loss using recently characterized Hoxb13 transcriptional control elements that are active in prostate luminal epithelial cells would induce the development of genomic instability and aggressive disease with metastatic potential. Neoplastic lesions that developed with either MYC activation alone (Hoxb13-MYC) or Pten loss alone (Hoxb13-Cre∣Pten(Fl/Fl)) failed to progress beyond prostatic intraepithelial neoplasia and did not harbor genomic CNAs. By contrast, mice with both alterations (Hoxb13-MYC∣Hoxb13-Cre∣Pten(Fl/Fl), hereafter, BMPC mice) developed lethal adenocarcinoma with distant metastases and widespread genome CNAs that were independent of forced disruption of Tp53 and telomere shortening. BMPC cancers lacked neuroendocrine or sarcomatoid differentiation, features uncommon in human disease but common in other models of prostate cancer that metastasize. These data show that combined MYC activation and Pten loss driven by the Hoxb13 regulatory locus synergize to induce genomic instability and aggressive prostate cancer that phenocopies the human disease at the histologic and genomic levels. 
 c-MYC-regulated miR-23a/24-2/27a cluster promotes mammary carcinoma cell invasion and hepatic   metastasis   by targeting Sprouty2. Emerging evidence indicates that the miR-23a/24-2/27a cluster may possess a causal role in mammary tumorigenesis and function as a novel class of oncogenes. However, the regulatory mechanism of the miR-23a/24-2/27a cluster in mammary carcinoma cell invasion and migration is still largely unknown. We observed that the expression levels of miR-23a, miR-24-2 and miR-27a were significantly higher in breast cancer with lymph node   metastasis  , compared with that from patients without lymph node   metastasis   or normal tissue. Forced expression of the miR-23a/24-2/27a cluster promoted mammary carcinoma cell migration, invasion, and hepatic   metastasis  , through targeting Sprouty2 (SPRY2) and consequent activation of p44/42 MAPK. Epidermal growth factor induced the expression of the transcription factor c-MYC, which promoted the expression of mature miR-23a, miR-24-2, and miR-27a and subsequently decreased expression of SPRY2 and activated p44/42 MAPK to promote mammary carcinoma cell migration and invasion. We therefore suggest a novel link between epidermal growth factor and the miR-23a/24-2/27a cluster via the regulation of c-MYC, providing the potential for the miR-23a/24-2/27a cluster to be used as biomarker in the diagnosis and/or treatment of breast cancer. 
 MYC-induced epigenetic activation of GATA4 in lung adenocarcinoma. Human lung cancer is a disease with high incidence and accounts for most cancer-related deaths in both men and women.   Metastasis   is a common event in non-small cell lung carcinoma (NSCLC), diminishing the survival chance of the patients with this type of tumor. It has been shown that MYC is involved in the development of   metastasis   from NSCLC, but the mechanism underlying this switch remained to be identified. Here, we focus on GATA4 as a MYC target in the development of   metastasis   with origin in lung adenocarcinoma, the most common type of NSCLC. Epigenetic alterations at the GATA4 promoter level were observed after MYC expression in lung adenocarcinoma in vivo and in vitro. Such alterations include site-specific demethylation that accompanies the displacement of the MYC-associated zinc finger protein (MAZ) from the GATA4 promoter, which leads to GATA4 expression. Histone modification analysis of the GATA4 promoter revealed a switch from repressive histone marks to active histone marks after MYC binding, which corresponds to active GATA4 expression. Our results thus identify a novel epigenetic mechanism by which MYC activates GATA4 leading to   metastasis   in lung adenocarcinoma, suggesting novel potential targets for the development of antimetastatic therapy. 
 Metabolic reprogramming in triple-negative breast cancer through Myc suppression of TXNIP. Triple-negative breast cancers (TNBCs) are aggressive and lack targeted therapies. Understanding how nutrients are used in TNBCs may provide new targets for therapeutic intervention. We demonstrate that the transcription factor c-Myc drives glucose metabolism in TNBC cells but does so by a previously unappreciated mechanism that involves direct repression of thioredoxin-interacting protein (TXNIP). TXNIP is a potent negative regulator of glucose uptake, aerobic glycolysis, and glycolytic gene expression; thus its repression by c-Myc provides an alternate route to c-Myc-driven glucose metabolism. c-Myc reduces TXNIP gene expression by binding to an E-box-containing region in the TXNIP promoter, possibly competing with the related transcription factor MondoA. TXNIP suppression increases glucose uptake and drives a dependence on glycolysis. Ectopic TXNIP expression decreases glucose uptake, reduces cell proliferation, and increases apoptosis. Supporting the biological significance of the reciprocal relationship between c-Myc and TXNIP, a Mychigh/TXNIPlow gene signature correlates with decreased overall survival and decreased   metastasis  -free survival in breast cancer. The correlation between the Mychigh/TXNIPlow gene signature and poor clinical outcome is evident only in TNBC, not in other breast cancer subclasses. Mutation of TP53, which is a defining molecular feature of TNBC, enhances the correlation between the Mychigh/TXNIPlow gene signature and death from breast cancer. Because Myc drives nutrient utilization and TXNIP restricts glucose availability, we propose that the Mychigh/TXNIPlow gene signature coordinates nutrient utilization with nutrient availability. Further, our data suggest that loss of the p53 tumor suppressor cooperates with Mychigh/TXNIPlow-driven metabolic dysregulation to drive the aggressive clinical behavior of TNBC. 
 Myc coordinates transcription and translation to enhance transformation and suppress invasiveness. c-Myc is one of the major human proto-oncogenes and is often associated with tumor aggression and poor clinical outcome. Paradoxically, Myc was also reported as a suppressor of cell motility, invasiveness, and   metastasis  . Among the direct targets of Myc are many components of the protein synthesis machinery whose induction results in an overall increase in protein synthesis that empowers tumor cell growth. At present, it is largely unknown whether beyond the global enhancement of protein synthesis, Myc activation results in translation modulation of specific genes. Here, we measured Myc-induced global changes in gene expression at the transcription, translation, and protein levels and uncovered extensive transcript-specific regulation of protein translation. Particularly, we detected a broad coordination between regulation of transcription and translation upon modulation of Myc activity and showed the connection of these responses to mTOR signaling to enhance oncogenic transformation and to the TGFβ pathway to modulate cell migration and invasiveness. Our results elucidate novel facets of Myc-induced cellular responses and provide a more comprehensive view of the consequences of its activation in cancer cells. 
 DOT1L cooperates with the c-Myc-p300 complex to epigenetically derepress CDH1 transcription factors in breast cancer progression. DOT1L has emerged as an anticancer target for MLL-associated leukaemias; however, its functional role in solid tumours is largely unknown. Here we identify that DOT1L cooperates with c-Myc and p300 acetyltransferase to epigenetically activate epithelial-mesenchymal transition (EMT) regulators in breast cancer progression. DOT1L recognizes SNAIL, ZEB1 and ZEB2 promoters via interacting with the c-Myc-p300 complex and facilitates lysine-79 methylation and acetylation towards histone H3, leading to the dissociation of HDAC1 and DNMT1 in the regions. The upregulation of these EMT regulators by the DOT1L-c-Myc-p300 complex enhances EMT-induced breast cancer stem cell (CSC)-like properties. Furthermore, in vivo orthotopic xenograft models show that DOT1L is required for malignant transformation of breast epithelial cells and breast tumour initiation and   metastasis  . Clinically, DOT1L expression is associated with poorer survival and aggressiveness of breast cancers. Collectively, we suggest that cooperative effect of DOT1L and c-Myc-p300 is critical for acquisition of aggressive phenotype of breast cancer by promoting EMT/CSC. 
 SATB1 Promotes Pancreatic Cancer Growth and Invasion Depending on MYC Activation. SATB1 plays an important role in human malignant progression, inducing cancer cell proliferation and   metastasis   by regulating downstream gene expressions. However, little is known about the underlying mechanisms in which SATB1 promotes pancreatic cancer tumorigenesis.To investigate SATB1 expression levels and its biological functions in promoting pancreatic cancer growth and invasion.SATB1 expression levels were detected in seven human pancreatic cancer cell lines and 16 pairs of normal pancreatic/pancreatic cancer tissues using RT-PCR and western blot. SW1990 or Capan-1 cells stably knockdown (shRNA) or transiently knockdown (siRNA) SATB1 cells, and PANC-1 stably overexpressing SATB1 cells were investigated with MTT, EdU assay, flow cytometry, and transwell invasion assay for cell proliferation and invasion activity. The binding of SATB1 to MYC promoter region was examined using reporter assay. Expression of SATB1 in 68 pancreatic cancer samples was studied by immunohistochemical staining and scoring.SATB1 was overexpressed in pancreatic cancer tissues samples, showing strong correlation with pancreatic cancer invasion depth and tumor staging. SATB1 induced MYC mRNA and protein expression; promoted pancreatic cancer cell growth; increased cell population in S phase; and enhanced pancreatic cancer cell invasion in vitro. On the other hand, SATB1 knockdown showed opposite effects. Furthermore, MYC blocking in SATB1-overexpressing cells attenuated the promotion of pancreatic cancer cell growth and invasion. Our data also indicated that SATB1 bound to specific promoter region of MYC.SATB1 is overexpressed in pancreatic cancer, promoting cancer cell proliferation and invasion through the activation of MYC. 
 MYC suppresses cancer   metastasis   by direct transcriptional silencing of αv and β3 integrin subunits. Overexpression of MYC transforms cells in culture, elicits malignant tumours in experimental animals and is found in many human tumours. We now report the paradoxical finding that this powerful oncogene can also act as a suppressor of cell motility, invasiveness an  d metastas  is. Overexpression of MYC stimulated proliferation of breast cancer cells both in culture and in vivo as expected, but inhibited motility and invasiveness in culture, and lung and liver metastases in xenografted tumours. We show further that MYC represses transcription of both subunits of αvβ3 integrin, and that exogenous expression of β3 integrin in human breast cancer cells that do not express this integrin rescues invasiveness and migration when MYC is downregulated. These data uncover an unexpected function of MYC, provide an explanation for the hitherto puzzling literature on the relationship between MYC   and metas  tasis, and reveal a variable that could influence the development of therapies that target MYC. 
 Overexpression of Suprabasin is Associated with Proliferation and Tumorigenicity of Esophageal Squamous Cell Carcinoma. Suprabasin is a recently identified oncoprotein that is upregulated in multiple cancers. However, the clinical significance and biological role of suprabasin in human esophageal squamous cell carcinoma (ESCC) remains unclear. In the current study, we reported that suprabasin was markedly overexpressed in ESCC cell lines and tissues at both mRNA and protein levels, and this was associated with advanced clinical stage, tumor-nodes-  metastasis   (TNM) classification, histological differentiation, tumor size and poorer survival. Furthermore, we found that both proliferation and tumorigenicity of ESCC cells were significantly induced by suprabasin overexpression, but inhibited by suprabasin knock-down. Moreover, we demonstrated that upregulation of suprabasin activated the Wnt/β-catenin signaling pathway and led to nuclear localization of β-catenin and upregulation of Cyclin D1 and c-Myc. Together, our results suggest that suprabasin plays an important oncogenic role in promoting proliferation and tumorigenesis of ESCC. 
 [Expression and significance of hTERT, c-myc and Ki-67 in hepatocellular carcinoma]. To study the correlation of expression of hTERT, c-myc and Ki-67 with the clinical pathological feature of hepatocellular carcinoma and explore the relationship among the three factors.The expressions of hTERT, c-myc and Ki-67 in 37 cancer tissues and 5 normal tissues were assessed by immunohistochemical method.The positive rates of hTERT, c-myc and Ki-67 in the cancer tissue were higher than those in normal tissues (P<0.05). The expressions of hTERT, c-myc and Ki-67 had nothing to do with age, sex,   metastasis   and the volume of tumor(P>0.05). With histology stage reducing, the expression of hTERT, c-myc and Ki-67 increased obviously. There is no correlation between hTERT and c-myc(P>0.05), but it's positive correlation between hTERT and Ki-67(P<0.05). With the expression of Ki-67 increasing, hTERT and c-myc increased.The over-expression of hTERT, c-myc and Ki-67 may play a vital role in the progress of hepatocellular carcinoma developing. The expressions of three factors in hepatocellular carcinoma are closely associated. 
 RanGTPase: a candidate for Myc-mediated cancer progression. Ras-related nuclear protein (Ran) is required for cancer cell survival in vitro and human cancer progression, but the molecular mechanisms are largely unknown.We investigated the effect of the v-myc myelocytomatosis viral oncogene homolog (Myc) on Ran expression by Western blot, chromatin immunoprecipitation, and luciferase reporter assays and the effects of Myc and Ran expression in cancer cells by soft-agar, cell adhesion, and invasion assays. The correlation between Myc and Ran and the association with patient survival were investigated in 14 independent patient cohorts (n = 2430) and analyzed with Spearman's rank correlation and Kaplan-Meier plots coupled with Wilcoxon-Gehan tests, respectively. All statistical tests were two-sided.Myc binds to the upstream sequence of Ran and transactivates Ran promoter activity. Overexpression of Myc upregulates Ran expression, whereas knockdown of Myc downregulates Ran expression. Myc or Ran overexpression in breast cancer cells is associated with cancer progression and   metastasis  . Knockdown of Ran reverses the effect induced by Myc overexpression in breast cancer cells. In clinical data, a positive association between Myc and Ran expression was revealed in 288 breast cancer and 102 lung cancer specimens. Moreover, Ran expression levels differentiate better or poorer survival in Myc overexpressing breast (χ2 = 24.1; relative risk [RR] = 9.1, 95% confidence interval [CI] = 3.3 to 24.7, P < .001) and lung (χ2 = 6.04; RR = 2.8, 95% CI = 1.2 to 6.3; P = .01) cancer cohorts.Our results suggest that Ran is required for and is a potential therapeutic target of Myc-driven cancer progression in both breast and lung cancers. 
 Prognostic significance of c-Myc expression in soft tissue leiomyosarcoma. The biological potential of soft tissue leiomyosarcoma is difficult to predict using current standard prognostic parameters, and control of systemic disease is challenging with current chemotherapeutic protocols. Additional prognostic markers and alternative treatment options are very much required. Previous studies implicate upregulation of the oncogenic nuclear transcription factor c-Myc with aggressive behavior of many solid tumors. Therefore, this oncoprotein was evaluated as a prognostic marker for overall and   metastasis  -free survival in leiomyosarcoma. Immunohistochemical stains for c-Myc were performed on 28 cases of leiomyosarcoma occurring in the deep somatic soft tissues. Comparisons of Kaplan-Meier survival curves stratified by c-Myc status and conventional prognostic factors (histological grade, tumor size, and tumor stage) were evaluated using standard univariate statistical methods. A subsequent multivariate survival analysis was carried out according to the Cox proportional hazards regression model adjusting for potential confounding prognostic factors. A total of 15 cases (54%) were positive for nuclear c-Myc expression. Patients with c-Myc-positive tumors had significantly shorter   metastasis  -free survival intervals compared with those with c-Myc-negative tumors (median, 9 months vs. >94 months; P=0.014). c-Myc positivity also correlated with decreased overall survival (median, 23 months vs. >94 months; P=0.017). Histological grade was the only other prognostic marker predictive of poor outcome in the univariate analyses. In the multivariate survival analysis, only c-Myc status reached statistical significance, suggesting that it is an important and independent predictor of prognosis in leiomyosarcoma. Detection of nuclear c-Myc in leiomyosarcoma predicts decreased overall and   metastasis  -free survival, independent of standard prognostic variables, tumor size, histological grade, and TNM stage. The expression of this oncoprotein may represent a useful prognostic marker and potential therapeutic target in leiomyosarcoma. 
 miR-9, a MYC/MYCN-activated microRNA, regulates E-cadherin and cancer   metastasis  . MicroRNAs (miRNAs) are increasingly implicated in regulating the malignant progression of cancer. Here we show that miR-9, which is upregulated in breast cancer cells, directly targets CDH1, the E-cadherin-encoding messenger RNA, leading to increased cell motility and invasiveness. miR-9-mediated E-cadherin downregulation results in the activation of beta-catenin signalling, which contributes to upregulated expression of the gene encoding vascular endothelial growth factor (VEGF); this leads, in turn, to increased tumour angiogenesis. Overexpression of miR-9 in otherwise non-metastatic breast tumour cells enables these cells to form pulmonary micrometastases in mice. Conversely, inhibiting miR-9 by using a 'miRNA sponge' in highly malignant cells inhibits   metastasis   formation. Expression of miR-9 is activated by MYC and MYCN, both of which directly bind to the mir-9-3 locus. Significantly, in human cancers, miR-9 levels correlate with MYCN amplification, tumour grade and metastatic status. These findings uncover a regulatory and signalling pathway involving a   metastasis  -promoting miRNA that is predicted to directly target expression of the key   metastasis  -suppressing protein E-cadherin. 
 NFATC1 promotes cell growth and tumorigenesis in ovarian cancer up-regulating c-Myc through ERK1/2/p38 MAPK signal pathway. It has been reported that nuclear factor of activated T cells (NFATC1) was up-regulated in cancers mediating malignant behaviors. However, the role of NFATC1 in ovarian cancer has not been elucidated. In the present study, we undertook to explore the clinicopathological significance of NFATC1 expression and the mechanism by which NFATC1 works in ovarian cancer. Expression status of NFATC1 was examined using immunohistochemistry. Both knockdown and re-expression of NFATC1 on ovarian cancer cells were employed to observe the effect overgrowth. It was found that NFATC1 was significantly overexpressed in ovarian cancer tissues in comparison with paired normal control tissues and that overexpression of NFATC1 was significantly associated with   metastasis   and poor prognosis on clinical tissue level. In in vitro ovarian cancer cell lines, we found that NFATC1 can promote proliferation up-regulating c-myc through activation of ERK1/2/p38/MAPK signal pathway. Together, the results we obtained demonstrated that NFATC1 played oncogenic role in ovarian cancer. Mechanistically, NFATC1 promoted growth of ovarian cancer cells up-regulating c-myc through activation of ERK1/2/p38/MAPK signal pathway, suggesting that NFATC1 might be used as a therapeutic target for ovarian cancer. 
 ROCK has a crucial role in regulating prostate tumor growth through interaction with c-Myc. Rho-associated kinase (ROCK) has an essential role in governing cell morphology and motility, and increased ROCK activity contributes to cancer cell invasion and   metastasis  . Burgeoning data suggest that ROCK is also involved in the growth regulation of tumor cells. However, thus far, the molecular mechanisms responsible for ROCK-governed tumor cell growth have not been clearly elucidated. Here we showed that inhibition of ROCK kinase activity, either by a selective ROCK inhibitor Y27632 or by specific ROCK small interfering RNA (siRNA) molecules, attenuated not only motility but also the proliferation of PC3 prostate cancer cells in vitro and in vivo. Importantly, mechanistic investigation revealed that ROCK endowed cancer cells with tumorigenic capability, mainly by targeting c-Myc. ROCK could increase the transcriptional activity of c-Myc by promoting c-Myc protein stability, and ROCK inhibition reduced c-Myc-mediated expression of mRNA targets (such as HSPC111) and microRNA targets (such as miR-17-92 cluster). We provided evidence demonstrating that ROCK1 directly interacted with and phosphorylated c-Myc, resulting in stabilization of the protein and activation of its transcriptional activity. Suppression of ROCK-c-Myc downstream molecules, such as c-Myc-regulated miR-17, also impaired tumor cell growth in vitro and in vivo. In addition, c-Myc was shown to exert a positive feedback regulation on ROCK by increasing RhoA mRNA expression. Therefore, inhibition of ROCK and its stimulated signaling might prove to be a promising strategy for restraining tumor progression in prostate cancer. 
 PTTG regulates the metabolic switch of ovarian cancer cells via the c-myc pathway. Human pituitary tumor-transforming gene (PTTG) is a proto-oncogene involved in the development, invasion, and   metastasis   of many types of cancer, including ovarian cancer. However, little is known about the role of PTTG in the metabolic shift of ovarian cancer cells. In our study, we show that PTTG expression was positively correlated with the differentiation degree of ovarian cancer tissue. In addition, PTTG suppression by specific shRNA could inhibit the proliferation of ovarian cancer cells A2780 and SKOV-3. Furthermore, aerobic glycolysis was suppressed and oxidative phosphorylation was increased in ovarian cancer cells after PTTG suppression. We further found that the expression of c-myc and several crucial enzymes involved in aerobic glycolysis (e.g., PKM2, LDHA, and glucose transporter 1 (GLUT-1)) were downregulated by PTTG knockwown. Overexpression of c-myc could prevent the metabolic shift induced by PTTG knockwown. Together, our findings suggest that the oncogene PTTG promotes the progression of ovarian cancer cells, and its loss resists tumor development, in part, by regulating cellular metabolic reprogramming that supports cell growth and proliferation via c-myc pathway. 
 c-myc but not Hif-1α-dependent downregulation of VEGF influences the proliferation and differentiation of HL-60 cells induced by ATRA. Vascular endothelial growth factor (VEGF) plays an important role in solid tumor growth, progression and   metastasi  s as well as in the proliferation and differentiation of hematological malignancies. However, the molecular mechanism that modulates VEGF expression and secretion in leukemia cells has not yet to be elucidated. The purpose of the present study was to investigate the role of the signal pathway in modulating the expression of VEGF in HL-60 cells. Specific siRNAs targeting VEGF were transfected into HL-60 cells and the VEGF expression was measured with reverse transcription-polymerase chain reaction (RT-PCR) and western blot assay. The cell proliferation of HL-60 cells was detected by the cell counting kit-8 (CCK-8) assay and the differentiation of HL-60 cells induced by all-trans-retinoic acid (ATRA) was detected by the RT-PCR assay and flow cytometry assay for CD11b. The upstream transcription factors that were related to VEGF expression such as P53, SP-1, c-jun, VHL, cox-2, c-myc and stat3 were detected by RT-PCR assay. In addition, the chromatin immunoprecipitation (ChIP) assay was used to reveal the role of c-myc by binding the target gene VEGF. The results demonstrated the hypoxia-inducible factor 1α-related signaling pathway, not the same as in solid tumors, might not play a key role in modulating VEGF expression. c-myc contributes to the modulation of VEGF expression by targeting the promoter of VEGF, which was indicated by the ChIP assay. In conclusion, our data demonstrate that VEGF plays an important role in the differentiation and proliferation of HL-60 cells; c-myc-dependent downregulation of VEGF induced by ATRA contributes to the differentiation of HL-60 cells. 
 c-MYC functions as a molecular switch to alter the response of human mammary epithelial cells to oncostatin M. Cytokines play an important role in creating an inflammatory microenvironment, which is now considered a hallmark of cancer. Although tumor cells can exploit cytokine signaling to promote growth, invasion, and   metastasis  , the response of normal and premalignant epithelial cells to cytokines present in a developing tumor microenvironment remains unclear. Oncostatin M (OSM), an IL-6 family cytokine responsible for STAT3 activation, has been implicated in cancer development, progression, invasion, and   metastasis  . Paradoxically, OSM can also suppress the growth of normal cells and certain tumor-derived cell lines. Using isogenic human mammary epithelial cells (HMEC) at different stages of neoplastic transformation, we found that OSM signaling suppressed c-MYC expression and engaged a p16- and p53-independent growth arrest that required STAT3 activity. Inhibition of STAT3 activation by expressing a dominant-negative STAT3 protein or a STAT3-shRNA prevented the OSM-mediated arrest. In addition, expression of c-MYC from a constitutive promoter also abrogated the STAT3-mediated arrest, and strikingly, cooperated with OSM to promote anchorage-independent growth (AIG), a property associated with malignant transformation. Cooperative transformation by c-MYC and OSM required PI3K and AKT signaling, showing the importance of multiple signaling pathways downstream of the OSM receptor in defining the cellular response to cytokines. These findings identify c-MYC as an important molecular switch that alters the cellular response to OSM-mediated signaling from tumor suppressive to tumor promoting. 
 MYC overexpression and poor prognosis in sporadic breast cancer with BRCA1 deficiency. Breast cancer is a complex disease; the molecular mechanisms involved in sporadic breast carcinogenesis remain to be elucidated. The present study aimed to explore the deficiency of breast cancer susceptibility gene 1 (BRCA1), including protein loss expression, promoter hypermethylation and gene copy deletion, its correlationship with other tumor markers expression (TP53, MYC, etc.), and clinical significance in sporadic breast cancer. BRCA1 protein expression was negative in 226 of 374 (60.4%) cases of this study. Cases negative for BRCA1 protein were more often with pathological tumor-node-  metastasis   stage III, positive for lymph node   metastasis   and MYC overexpression than BRCA1-positive tumors. BRCA1 hypermethylation was detected in 16.4% (31 of 189) breast cancers, which correlated with BRCA1 negative, ER negative, MYC overexpression, and triple-negative phenotype. In addition, the percentage of cells with BRCA1 gene copy deletion was significantly increased in BRCA1-methylated tumors. Kaplan-Meier survival analysis showed that patients with BRCA1-negative expression showed a worse overall survival (OS) than those with BRCA1-positive expression, and patients with BRCA1-methylated tumors had a significantly worse disease-free survival than did patients with unmethylated tumors. Furthermore, BRCA1 hypermethylation showed an inverse association with OS in LN-positive or p53-negative subgroup patients. Importantly, uni- and multivariate Cox regression analyses revealed that BRCA1 was an independent prognostic indicator of OS in sporadic breast cancer. Thus, we found MYC overexpression and poor prognosis in sporadic breast cancer with BRCA1 deficiency. The targeting of BRCA1 deficiency in combination with MYC-pathways inhibitors may provide a promising strategy for sporadic breast cancer care, the triple-negative subtype in particular. 
 Characterization and significance of MUC1 and c-myc expression in elderly patients with papillary thyroid carcinoma. The aim of this study was to examine the expression of mucin 1 (MUC1) and c-myc and the significance thereof in elderly patients with papillary thyroid carcinoma. The expression levels of MUC1 and c-myc were examined by immunohistochemical methods in 58 patients with papillary thyroid carcinoma, 35 with nodular goiter, and 30 subjects with normal thyroid tissue. The positive rate of MUC1 detection in papillary thyroid carcinoma was 77.6% (45/58), while it was 90.0% (9/10) in those with infiltration and 88.2% (15/17) in those with lymphatic   metastasis  . The positive rate of c-myc was 81.0% (47/58) in those with papillary thyroid carcinoma and 100.0% (17/17) in those with lymphatic   metastasis  . These results demonstrated that there were differences in MUC1 and c-myc expression in benign and papillary thyroid carcinoma and that these differences were related to thyroid cancer lymphatic   metastasis  . 
 Caspase-mediated cleavage of RNA-binding protein HuR regulates c-Myc protein expression after hypoxic stress. Altered expression of RNA-binding proteins modulates gene expression in association with mRNAs encoding many proto-oncogenes, cytokines, chemokines, and proinflammatory factors. Hu antigen R (HuR), a ubiquitously expressed protein, controls a range of cellular functions such as tumor progression, apoptosis, invasion, and   metastasis   by stabilizing the AU-rich element located at the 3'-untranslated region (UTR) of target mRNAs. Although significant progress has been made in understanding HuR regulation in gene expression, little is known about how HuR undergoes post-translational modifications and recruits target mRNAs during hypoxic stress. Here, we report that during CoCl(2)-induced hypoxic stress, HuR is significantly overexpressed and undergoes caspase-dependent cleavage in head and neck squamous cell carcinoma cells. Unexpectedly, the HuR-cleavage product 1 (HuR-CP1) was found to strongly associate with the 3'-UTR of c-myc mRNA and block mRNA translation. The binding efficiency of HuR to the 3'-UTR of c-myc mRNA was confirmed using ribonucleoprotein immunoprecipitation and site-directed mutagenesis at the AU-rich element sequences of the c-myc mRNA. Overexpression of a non-cleavable isoform, HuR-D226A, revealed a potent dominant-negative effect, repressing cleavage of endogenous HuR and promoting cell viability. Surprisingly, under hypoxia, siRNA knockdown of HuR elevated c-Myc protein expression. These findings suggest an important role for HuR in hypoxia, and we may have revealed a novel post-transcriptional mechanism that controls c-Myc expression in oral cancer progression. 
 MYC regulation of a "poor-prognosis" metastatic cancer cell state. Gene expression signatures are used in the clinic as prognostic tools to determine the risk of individual patients with localized breast tumors developing distant   metastasis  . We lack a clear understanding, however, of whether these correlative biomarkers link to a common biological network that regulates   metastasis  . We find that the c-MYC oncoprotein coordinately regulates the expression of 13 different "poor-outcome" cancer signatures. In addition, functional inactivation of MYC in human breast cancer cells specifically inhibits distant   metastasis   in vivo and invasive behavior in vitro of these cells. These results suggest that MYC oncogene activity (as marked by "poor-prognosis" signature expression) may be necessary for the translocation of poor-outcome human breast tumors to distant sites. 
 Hepatitis B virus X protein upregulates HSP90alpha expression via activation of c-Myc in human hepatocarcinoma cell line, HepG2. The Hepatitis B Virus X protein (HBx) plays a major role in hepatocellular carcinoma (HCC) development, however, its contribution to tumor invasion and   metastasis   has not been established so far. Heat shock protein 90 alpha (HSP90alpha) isoform is an ATP-dependent molecular chaperone that maintains the active conformation of client oncoproteins in cancer cells, which is abundantly expressed in HCC, especially in hepatitis B virus (HBV)-related tumors, might be involved in tumor progression.The levels of HSP90alpha, extracellular signal-regulated kinase 1/2 (ERK1/2), phosphorylated ERK1/2 (p-ERK1/2) and c-Myc in HBx-transfected HepG2 cells were determined by western blots analysis. The endogenous ERKs activity was demonstrated by ELISA assay. The regulation of c-Myc-mediated HSP90 alpha promoter transactivation by HBx was evaluated through electrophoretic mobility shift analysis (EMSA). The c-Myc-mediated HSP90alpha transcription was analysed by promoter assay. The HBx-expressing cells were transfected with specific small interference RNA (siRNA) against c-Myc. The in vitro invasion potentials of cells were evaluated by Transwell cell invasion assay.HBx induces HSP90alpha expression at the transcription level. The induction effect of HBx was inhibited after treatment with c-Myc inhibitor, 10058-F4. In addition, the luciferase activity of the HSP90alpha promoter analysis revealed that the HBx is directly involved in the c-Myc-mediated transcriptional activation of HSP90alpha. Furthermore, HBx induces c-Myc expression by activation of Ras/Raf/ERK1/2 cascades, which in turn results in activation of the c-Myc-mediated HSP90alpha promoter and subsequently up-regulation of the HSP90alpha expression. Overexpression of HSP90alpha in HBx-transfected cells enhances tumor cells invasion. siRNA-mediated c-Myc knockdown in HBx-transfected cells significantly suppressed HSP90alpha expression and cells invasion in vitro.These results demonstrate the ability of HBx to promote tumor cells invasion by a mechanism involving the up-regulation of HSP90alpha and provide new insights into the mechanism of action of HBx and its involvement in tumor   metastasis   and recurrence of HCC. 
 MUC16-mediated activation of mTOR and c-Myc reprograms pancreatic cancer metabolism. MUC16, a transmembrane mucin, facilitates pancreatic adenocarcinoma progression and   metastasis  . In the current studies, we observed that MUC16 knockdown pancreatic cancer cells exhibit reduced glucose uptake and lactate secretion along with reduced migration and invasion potential, which can be restored by supplementing the culture media with lactate, an end product of aerobic glycolysis. MUC16 knockdown leads to inhibition of mTOR activity and reduced expression of its downstream target c-MYC, a key player in cellular growth, proliferation and metabolism. Ectopic expression of c-MYC in MUC16 knockdown pancreatic cancer cells restores the altered cellular physiology. Our LC-MS/MS based metabolomics studies indicate global metabolic alterations in MUC16 knockdown pancreatic cancer cells, as compared to the controls. Specifically, glycolytic and nucleotide metabolite pools were significantly decreased. We observed similar metabolic alterations that correlated with MUC16 expression in primary tumor tissue specimens from human pancreatic adenocarcinoma cancer patients. Overall, our results demonstrate that MUC16 plays an important role in metabolic reprogramming of pancreatic cancer cells by increasing glycolysis and enhancing motility and invasiveness. 
 c-Myc suppresses microRNA-29b to promote tumor aggressiveness and poor outcomes in non-small cell lung cancer by targeting FHIT. The dual role of the microRNA-29 (miR-29) family in tumor progression and   metastasis   in solid tumors has been reported. Evidence for the role of miR-29 in tumor malignancy and its prognostic value in overall survival (OS) and relapse-free survival (RFS) in non-small cell lung cancer (NSCLC) remains conflicting. Mechanistic studies presented herein demonstrated that c-Myc suppressed the expression of miR-29b, promoting soft agar growth and invasion capability in lung cancer cells. Interestingly, the decrease in the expression of miR-29b by c-Myc is responsible for soft agar growth and invasiveness mediated by FHIT loss due to promoter methylation. Among patients, low expression of miR-29b and FHIT was more common in tumors with high c-Myc expression than in tumors with low c-Myc expression. Kaplan-Meier and Cox regression analysis showed that tumors with high c-Myc, low miR-29b and low FHIT expression had shorter OS and RFS periods than their counterparts. In conclusion, the decrease in the expression of miR-29b by c-Myc may be responsible for FHIT loss-mediated tumor aggressiveness and for poor outcome in NSCLC. Therefore, we suggest that restoration of the miR-29b expression using the c-Myc inhibitor might be helpful in suppressing tumor aggressiveness mediated by FHIT loss and consequently improving outcomes in NSCLC patients with tumors with low expression of FHIT. 
 Differential expression of Pim-3, c-Myc, and p-p27 proteins in adenocarcinomas of the gastric cardia and distal stomach. Gastric cardia adenocarcinoma (GCA) is distinct from adenocarcinoma of the distal stomach because of its different etiological factors, tumor characteristics, and biological behavior. However, its pathogenesis is not fully understood. The purpose of this study is to characterize the role of Pim-3, c-Myc, and p-p27 in the tumorigenesis and progression of different sites of gastric adenocarcinoma by determining its pathogenetic significance. The expression of Pim-3, c-Myc, and p-p27 proteins was evaluated by immunohistochemistry in 140 resection specimens of gastric adenocarcinomas (78 GCAs , 62 DGAs and 20 normal gastric tissues). The level of expression of Pim-3, c-Myc, and p-p27 and the co-expression of all three markers (Pim-3+/c-Myc+/p-p27+) in GCA were significantly lower than that in DGA tumors (P < 0.05). Detailed analysis of the immunoreactivity patterns showed that in DGA, Pim-3 immunoreactivity was associated significantly with poor tumor differentiation, advanced tumor stage, and presence of lymph n  ode metast  asis. In addition, c-Myc overexpression correlated with tumor stage and lymph n  ode metast  asis, and positive p-p27 expression correlated with poor differentiation and tumor stage. The phenotype of Pim-3(+)/c-Myc(+)/p-p27(+) co-expression was closely correlated with tumor stage and lymph n  ode metast  asis (P < 0.05). In contrast, GCA only demonstrated a close correlation of Pim-3 overexpression with poor tumor differentiation and tumor stage (P < 0.05). Our results demonstrate the presence of different expression patterns of Pim-3, c-Myc, p-p27, and Pim-3(+)/c-Myc(+)/p-p27(+) and their clinicopathologic significance in GCA and DGA tumors. Our results add support to the notion that distinct molecular mechanisms may be involved in the development and progression of adenocarcinomas from the gastric cardia and distal portion of stomach. 
 A c-Myc/miR-17-5p feedback loop regulates   metastasis   and invasion of hepatocellular carcinoma. The molecular mechanisms that control   metastasis   of hepatocellular cancer (HCC) are still poorly understood. It has been determined that microRNA (miRNA) expression has tissue and cell specific, and decreased expression of specific miRNA could induce tumor genesis or   metastasis  . In this study, we identified that miR-17-5p was expressed lower in high metastatic capability HCC cell lines HCCLM3 and MHCC97H than low metastatic HCC cell line HepG2 by real-time (RT)-PCR. Restoration of miR-17-5p could significantly repress the invasiveness and   metastasis   of MHCC97H cell line. Furthermore, we validated c-Myc as a downstream and functional target of miR-17-5p using luciferase reporter assay. Immunohistochemical assay revealed that the expression of c-Myc protein levels was significantly increased in cancerous tissues compared with para-tumor tissues. After clinical data analysis, we observed that the higher level of c-Myc was significantly associated with a reduced overall survival (p = 0.0209). Consistent with previous research, we also demonstrated that c-Myc could upregulate the expression of miR-17-5p. Taken together, our data indicated that there is a regulatory feedback loop between miR-17-5p and c-Myc, in which miR-17-5p could suppress some of the distinguishing features, invasion,   and metast  asis, of oncogenic c-Myc in HCC cells, and meanwhile, miR-17-5p is upregulated by c-Myc role as a transcription factor, although further studies are still needed. 
 MYC amplification in breast cancer: a chromogenic in situ hybridisation study. To analyse the correlation between MYC amplification and various clinicopathological features and outcome in a cohort of 245 patients with invasive breast carcinoma treated with surgery followed by anthracycline-based chemotherapy. Given the high prevalence of MYC amplification in tumours of BRCA1 mutation carriers and the similarities between these and sporadic "basal-like" carcinomas, the prevalence of MYC amplification in "basal-like" breast carcinomas was investigated.MYC gene copy number was assessed on tissue microarrays containing duplicate cores of 245 invasive breast carcinomas by means of chromogenic in situ hybridisation using SpotLight C-MYC amplification probe and chromosome 8 centromeric probe (CEP8). Signals were evaluated at 400x magnification; 30 morphologically unequivocal neoplastic cells in each core were counted for the presence of the gene and CEP8 probes.Amplification was defined as a MYC:CEP8 ratio >2. Signals for both MYC and CEP8 were assessable in 196/245 (80%) tumours. MYC amplification was found in 19/196 cases (9.7%) and was not associated with tumour size, histological grade, positivity for oestrogen receptor, progesterone receptor, HER2, epidermal growth factor, cytokeratins 14, 5/6 and 17, MIB1 or p53. Only 4% of basal-like carcinomas showed MYC amplification, compared to 8.75% and 10.7% of luminal and HER2 tumours respectively. On univariate analysis, MYC amplification displayed a significant association with shorter   metastasis  -free and overall survival and proved to be an independent prognostic factor on multivariate survival analysis.MYC amplification is not associated with "basal-like" phenotype and proved to be an independent prognostic factor for breast cancer patients treated with anthracycline-based chemotherapy. 
 SALL4 as an Epithelial-Mesenchymal Transition and Drug Resistance Inducer through the Regulation of c-Myc in Endometrial Cancer. SALL4 plays important roles in the development and progression of many cancers. However, the role and molecular mechanism of SALL4 in endometrial cancer remain elusive. In the present research, we have demonstrated that the expression of SALL4 was upregulated in endometrial cancer and correlated positively with tumor stage, metastases and poor survival of patients. The overexpression of SALL4 promoted the invasiveness in endometrial cancer cells, as indicated by the upregulation of mesenchymal cell marker N-cadherin and downregulation of the epithelial marker E-cadherin, and invasion assays in vitro. Additionally, there was also an increase in drug resistance in these cell models due to the upregulation of ATP-binding cassette multidrug transporter ABCB1 expression. Moreover, we also found that ABCB1 was critical for SALL4-induced drug resistance. In contrast, SALL4 knockdown restored drug sensitivity, reversed EMT, diminished cell   metastasis   and suppressed the downregulation of E-cadherin and the upregulation of N-cadherin and ABCB1. Furthermore, we showed that SALL4 upregulated c-Myc expression and c-Myc was a direct target for SALL4 by ChIP assay, depletion of c-Myc with siRNA abolished the SALL4-induced downregulation of E-cadherin, upregulation of N-cadherin and ABCB1, suggesting that c-Myc was a downstream target for SALL4 and required for SALL4-induced EMT, invasion and drugs resistance in endometrial cancer cells. These results indicated that SALL4 could induce EMT and resistance to antineoplastic drugs through the regulation of c-Myc. SALL4 and c-Myc may be novel therapeutic targets for endometrial cancer. 
 Low expression levels of p27 correlate with loco-regional recurrence in nasopharyngeal carcinoma. To clarify the roles of p27 and c-myc in nasopharyngeal carcinoma (NPC), we used immunohistochemical studies to examine the expression of p27 and c-myc proteins in 69 patients with NPC. The results show that a low level of expression was observed in 47 and 41 cases, respectively. A low level of p27 expression was significantly correlated with loco-regional recurrence, and reduction in the c-myc protein was also correlated with neck   metastasis   in NPC. Our study indicates that a reduction in p27 expression is frequently seen in NPC. Furthermore, low expression levels of p27 and c-myc may contribute to the aggressive behavior of NPC. 
 8q24 amplification is associated with Myc expression and prostate cancer progression and is an independent predictor of recurrence after radical prostatectomy. Genomic alterations affecting the 8q24 region are frequent in prostate cancer. Together with the oncogene MYC, other genes located in the surrounding of the amplified region could also be candidate targets. Tissue microarrays were constructed with prostate cancer tissues from (1) a case-control population of patients treated by radical prostatectomy (n = 242; 121 cases with biochemical relapse matched with 121 cancers with identical clinicopathologic features but without relapse), (2) castration-resistant disease (n = 55), and (3) metastatic cancers (n = 28). Fluorescence in situ hybridization and immunohistochemistry were used on tissue microarrays and slides to analyze, respectively, the amplification status of 8q24 and protein expression of genes located at 8q24. Amplification at the MYC locus was observed in 29% of cases and was closely associated with both disease progression (from 15% in pT2 tumors to 53% in   metastasis  ; P = .001), and Gleason score (from <3% in Gleason 6 tumors to 66% in Gleason 8 and more tumors; P < .0001). The expression of genes located at 8q24 did not correlated with the amplification status, except for the Myc protein (P = .002). MYC amplification status but not Myc protein expression was significantly predictive of biochemical recurrence after prostatectomy, together with the proliferation marker Ki-67 and independently from known prognostic factors, including TNM stage and Gleason score. The MYC amplification status could constitute a useful prognostic tool for patients treated by radical prostatectomy, particularly for those with d'Amico intermediate risk, whose clinical behavior is currently difficult to predict. 
 The epigenetic modifier JMJD6 is amplified in mammary tumors and cooperates with c-Myc to enhance cellular transformation, tumor progression, and   metastasis  . Oncogene overexpression in primary cells often triggers the induction of a cellular safeguard response promoting senescence or apoptosis. Secondary cooperating genetic events are generally required for oncogene-induced tumorigenesis to overcome these biologic obstacles. We employed comparative genomic hybridization for eight genetically engineered mouse models of mammary cancer to identify loci that might harbor genes that enhance oncogene-induced tumorigenesis.Unlike many other mammary tumor models, the MMTV-Myc tumors displayed few copy number variants except for amplification of distal mouse chromosome 11 in 80 % of the tumors (syntenic to human 17q23-qter often amplified in human breast cancer). Analyses of candidate genes located in this region identified JMJD6 as an epigenetic regulatory gene that cooperates with Myc to enhance tumorigenesis. It suppresses Myc-induced apoptosis under varying stress conditions through inhibition of p19ARF messenger RNA (mRNA) and protein, leading to reduced levels of p53. JMJD6 binds to the p19ARF promoter and exerts its inhibitory function through demethylation of H4R3me2a. JMJD6 overexpression in MMTV-Myc cell lines increases tumor burden, induces EMT, and greatly enhances tumor   metastasi  s. Importantly, we demonstrate that co-expression of high levels of JMJD6 and Myc is associated with poor prognosis for human ER+ breast cancer patients.A novel epigenetic mechanism has been identified for how JMJD6 cooperates with Myc during oncogenic transformation. Combined high expression of Myc and JMJD6 confers a more aggressive phenotype in mouse and human tumors. Given the pleiotropic pro-tumorigenic activities of JMJD6, it may be useful as a prognostic factor and a therapeutic target for Myc-driven mammary tumorigenesis. 
 MicroRNAs as regulators and mediators of c-MYC function. In the past ten years microRNAs (miRNAs) have been widely implicated as components of tumor suppressive and oncogenic pathways. Also the proto-typic oncogene c-MYC has been connected to miRNAs. The c-MYC gene is activated in approximately half of all tumors, and its product, the c-MYC transcription factor, regulates numerous processes e.g. cell cycle progression, metabolism, epithelial-mesenchymal transition (EMT),   metastasis  , stemness, and angiogenesis, thereby facilitating tumor initiation and progression. c-MYC target-genes, which mediate these functions of c-MYC, represent a complex network of protein- and non-coding RNAs, including numerous miRNAs. For example, c-MYC directly regulates expression of the miR-17-92 cluster, miR-34a, miR-15a/16-1 and miR-9. Moreover, the expression and activity of c-MYC itself are under the control of miRNAs. Here, we survey how these networks mediate and regulate c-MYC functions. In the future, miRNAs connected to c-MYC may be used for diagnostic and therapeutic approaches. This article is part of a Special Issue entitled: Myc proteins in cell biology and pathology. 
 c-MYC Copy-Number Gain Is an Independent Prognostic Factor in Patients with Colorectal Cancer. The aim of this study was to determine the incidence and clinicopathological significance of c-MYC gene copy-number (GCN) gain in patients with primary colorectal cancer (CRC).The c-MYC GCN was investigated in 367 consecutive CRC patients (cohort 1) by using dual-color silver in situ hybridization. Additionally, to evaluate regional heterogeneity, we examined CRC tissue from 3 sites including the primary cancer, distant   metastasis  , and lymph-node   metastasis   in 152 advanced CRC patients (cohort 2). KRAS exons 2 and 3 were investigated for mutations.In cohort 1, c-MYC gene amplification, defined by a c-MYC:centromere of chromosome 8 ratio ≥ 2.0, was detected in 31 (8.4%) of 367 patients. A c-MYC GCN gain, defined by ≥ 4.0 c-MYC copies/nucleus, was found in 63 (17.2%) patients and was associated with poor prognosis (P = 0.015). Multivariate Cox regression analysis showed that the hazard ratio for c-MYC GCN gain was 2.35 (95% confidence interval, 1.453-3.802; P < 0.001). In a subgroup of stage II-III CRC patients, c-MYC GCN gain was significantly associated with poor prognosis by univariate (P = 0.034) and multivariate (P = 0.040) analyses. c-MYC protein overexpression was observed in 201 (54.8%) out of 367 patients and weakly correlated with c-MYC GCN gain (ρ, 0.211). In cohort 2, the c-MYC genetic status was heterogenous in advanced CRC patients. Discordance between GCN gain in the primary tumor and either distant or lymph-  node metas  tasis was 25.7% and 30.4%, respectively. A similar frequency for c-MYC GCN gain and amplification was observed in CRC patients with both wild-type and mutated KRAS.c-MYC GCN gain was an independent factor for poor prognosis in consecutive CRC patients and in the stage II-III subgroup. Our findings indicate that the status of c-MYC may be helpful in predicting the patients' outcome and for managing CRC patients. 
 MYC and   metastasis  . Aggressive primary tumors express transcriptional signatures that correlate with their metastatic propensity. A number of these signatures have been deployed in the clinic as risk stratification tools. However, the molecular basis of these clinically useful prognostic signatures has remained a largely unresolved area of controversy. We recently found that many prognostic signatures reflect the activity of the MYC oncogene, which in turn regulates tumor   metastasis   through specific effects on cancer cell invasion and migration. These findings offer a general framework for understanding the molecular basis of clinically prognostic transcriptional signatures and suggest potentially new avenues for studying   metastasis  . 
 Study on the different expression of molecular markers between cardiac cancer and distal gastric cancer and their correlations with clinicopathological features. Currently, few studies have reported the different expression of molecular markers between distal gastric cancer and cardiac cancer. Here, we sought to make an investigation about it by a retrospective analysis.The expression of 8 proteins, including epidermal growth factor receptor, glutathione S-transferase pi (GST-pi), cyclin D1, Neu/Her-2, C-myc, p53, p27 and p21, were evaluated in 110 cases with cardiac cancer and 101 cases with distal gastric cancer who underwent curative surgery at the Cancer Hospital, Fudan University, in 2005 by immunohistochemistry method.The TNM stage, differentiation grade, invasion depth and lymph node   metastasis   were significantly different between cardiac cancer and distal gastric cancer. p21 (p = 0.034), Neu (p = 0.017), and GST-pi (p = 0.003) were expressed in relatively higher levels in cardiac cancer than in distal gastric cancer. Furthermore, the clinical pathological parameters were significantly correlated with the expression of molecules mentioned above.Different molecular mechanisms may be involved in the tumorigenesis and development of cardiac cancer and distal gastric cancer. 
 Transcriptional regulation of the c-Myc promoter by NFAT1 involves negative and positive NFAT-responsive elements. A number of physiological processes in both normal and cancer cells are regulated by the proto-oncogene c-Myc. Among them, processes such as cell cycle regulation, apoptosis, angiogenesis and   metastasis   are also controlled by the nuclear factor of activated T cells (NFAT) family of transcription factors. It is already known that NFAT upregulates c-Myc expression by binding to an element located in the minimal c-Myc promoter. However, the importance of other NFAT sites in the context of the full promoter has not been evaluated. In this work, we demonstrate that the regulation of c-Myc by NFAT1 is more complex than previously conceived. In addition to the proximal site, NFAT1 directly binds to distal sites in the c-Myc promoter with different affinities. Promoter deletions and site-directed mutagenesis of NFAT binding sites in HEK293T cells suggest that in NFAT1-mediated transactivation, some NFAT elements are negative and dominant and others are positive and recessive. Furthermore, we demonstrate that cooperation with partner proteins, such as p300, enhances NFAT1-mediated transactivation of the c-Myc promoter. At last, the newly identified sites are also responsive to NFAT2 in HEK293T cells. However, in NIH3T3 cells, the regulation mediated by NFAT proteins is not dependent on the known NFAT sites, including the site previously described. Thus, our data suggest that the contribution of NFAT to the regulation of c-Myc expression may depend on a balance between the binding to positive and negative NFAT-responsive elements and cooperation with transcriptional cofactors, which may differ according to the context and/or cell type. 
 Genetic dissimilarity between primary colorectal carcinomas and their lymph node metastases: ploidy, p53, bcl-2, and c-myc expression--a pilot study. The current paradigm of   metastasis   proposes that rare cells within primary tumors acquire metastatic capability via sequential mutations, suggesting that metastases are genetically dissimilar from their primary tumors. This study investigated the changes in the level of expression of a well-defined panel of cell proliferation, differentiation, and apoptosis markers between the primary colorectal cancer (CRC) and the corresponding synchronous lymph node (LN)   metastasis   from the same patients. DNA flow cytometry and immunostaining of p53, bcl-2, and c-myc were carried out on 36 cases of CRC radical resection specimens with their corresponding LN metastases. There was very low probability that the histological patterns of primary tumors and LN metastases are independent (p < 0.001). Metastatic tumors were significantly more diffusely positive for p53 than the primary tumors (p < 0.001). Conversely, primary tumors were significantly more diffusely positive for c-myc than metastatic tumors (p = 0.011). No significant difference was found between the LNs and the primary tumors in bcl-2 positivity (p = 0.538) and DNA aneuploidy (p = 0.35), with a tendency towards negative bcl-2 and less aneuploidy in LN metastases than primary tumors. In conclusion, LN metastatic colorectal carcinomas have a tendency of being less differentiated, with a higher incidence of diffuse p53 staining, lower incidence of bcl-2 staining, and less aneuploidy in comparison to their primary counterparts suggesting a more aggressive biological behavior, which could indicate the necessity for more aggressive adjuvant therapy. 
 Nuclear C-MYC expression level is associated with disease progression and potentially predictive of two year overall survival in prostate cancer. Upregulation of nuclear C-MYC protein has been reported to be an early event in prostate cancer (PCa); however, its clinicopathological and prognostic significance remain controversial. We determined the association of nuclear C-MYC protein expression with clinicopathological parameters, prognosis, ETS-related gene (ERG) expression, and TMPRSS2-ERG status in PCa.Nuclear C-MYC and ERG expression by immunohistochemistry and TMPRSS2-ERG status by triple-color probe fluorescence in situ hybridization assay were determined in 50 hormone-naïve PCa patients and 31 radical prostatectomy specimens.Nuclear C-MYC immunostaining was negative, positive, and strong positive in 27.5%, 32.5%, and 40.0% of cases, respectively. C-MYC immunostaining was significantly associated with clinical T stage (P < 0.001), distant   metastasi  s at the time of diagnosis (P < 0.001) and TMPRSS2-ERG status (P = 0.001) but not with ERG immunostaining (P = 0.818). In the Kaplan-Meier analysis, C-MYC positive cases were found to have worse 2-year OS compared with C-MYC negative cases (P = 0.027). However, in the univariate Cox analysis, only TMPRSS2-ERG status (hazard ratio [HR] 0.189, 95% CI 0.057-0.629; P = 0.007) and distant   metastasi  s (HR 3.545, 95% CI 1.056-11.894; P = 0.040) were significantly associated with 2-year OS. After adjusting for these two factors, TMPRSS2-ERG status still impacted 2-year OS (HR 0.196, 95% CI 0.049-0.778; P = 0.020).Nuclear C-MYC overexpression may be associated with disease progression and potentially predictive of 2-year OS in PCa. This is the first study to demonstrate an association between nuclear C-MYC immunostaining and TMPRSS2-ERG status in PCa. 
 The long non-coding RNA PCAT-1 promotes prostate cancer cell proliferation through cMyc. Long non-coding RNAs (lncRNAs) represent an emerging layer of cancer biology, contributing to tumor proliferation, invasion, and   metastasis  . Here, we describe a role for the oncogenic lncRNA PCAT-1 in prostate cancer proliferation through cMyc. We find that PCAT-1-mediated proliferation is dependent on cMyc protein stabilization, and using expression profiling, we observed that cMyc is required for a subset of PCAT-1-induced expression changes. The PCAT-1-cMyc relationship is mediated through the post-transcriptional activity of the MYC 3' untranslated region, and we characterize a role for PCAT-1 in the disruption of MYC-targeting microRNAs. To further elucidate a role for post-transcriptional regulation, we demonstrate that targeting PCAT-1 with miR-3667-3p, which does not target MYC, is able to reverse the stabilization of cMyc by PCAT-1. This work establishes a basis for the oncogenic role of PCAT-1 in cancer cell proliferation and is the first study to implicate lncRNAs in the regulation of cMyc in prostate cancer. 
 RapidCaP, a novel GEM model for metastatic prostate cancer analysis and therapy, reveals myc as a driver of Pten-mutant   metastasis  . Genetically engineered mouse (GEM) models are a pillar of functional cancer research. Here, we developed RapidCaP, a GEM modeling system that uses surgical injection for viral gene delivery to the prostate. We show that in Pten deficiency, loss of p53 suffices to trigger   metastasis   to distant sites at greater than 50% penetrance by four months, consistent with results from human prostate cancer genome analysis. Live bioluminescence tracking showed that endogenous primary and metastatic disease responds to castration before developing lethal castration resistance. To our surprise, the resulting lesions showed no activation of Akt but activation of the Myc oncogene. Using RapidCaP, we find that Myc drives local prostate   metastasis   and is critical for maintenance of   metastasis  , as shown by using the Brd4 inhibitor JQ1. Taken together, our data suggest that a "MYC-switch" away from AKT forms a critical and druggable event in PTEN-mutant prostate cancer   metastasis   and castration resistance. 
 MYC, FBXW7 and TP53 copy number variation and expression in gastric cancer. MYC deregulation is a common event in gastric carcinogenesis, usually as a consequence of gene amplification, chromosomal translocations, or posttranslational mechanisms. FBXW7 is a p53-controlled tumor-suppressor that plays a role in the regulation of cell cycle exit and reentry via MYC degradation.We evaluated MYC, FBXW7, and TP53 copy number, mRNA levels, and protein expression in gastric cancer and paired non-neoplastic specimens from 33 patients and also in gastric adenocarcinoma cell lines. We also determined the invasion potential of the gastric cancer cell lines.MYC amplification was observed in 51.5% of gastric tumor samples. Deletion of one copy of FBXW7 and TP53 was observed in 45.5% and 21.2% of gastric tumors, respectively. MYC mRNA expression was significantly higher in tumors than in non-neoplastic samples. FBXW7 and TP53 mRNA expression was markedly lower in tumors than in paired non-neoplastic specimens. Moreover, deregulated MYC and FBXW7 mRNA expression was associated with the presence of lymph node   metastasis   and tumor stage III-IV. Additionally, MYC immunostaining was more frequently observed in intestinal-type than diffuse-type gastric cancers and was associated with MYC mRNA expression. In vitro studies showed that increased MYC and reduced FBXW7 expression is associated with a more invasive phenotype in gastric cancer cell lines. This result encouraged us to investigate the activity of the gelatinases MMP-2 and MMP-9 in both cell lines. Both gelatinases are synthesized predominantly by stromal cells rather than cancer cells, and it has been proposed that both contribute to cancer progression. We observed a significant increase in MMP-9 activity in ACP02 compared with ACP03 cells. These results confirmed that ACP02 cells have greater invasion capability than ACP03 cells.In conclusion, FBXW7 and MYC mRNA may play a role in aggressive biologic behavior of gastric cancer cells and may be a useful indicator of poor prognosis. Furthermore, MYC is a candidate target for new therapies against gastric cancer. 
 A regulatory loop involving miR-22, Sp1, and c-Myc modulates CD147 expression in breast cancer invasion and   metastasis  . Breast cancer is the most common cancer in women for which the metastatic process is still poorly understood. CD147 is upregulated in breast cancer and has been associated with tumor progression, but little is known about its regulatory mechanisms. In this study, we demonstrated that CD147 was overexpressed in breast cancer tissues and cell lines, and the high expression correlated with tumor invasion and   metastasis  . We also found that the transcription factors Sp1 and c-Myc could bind to the CD147 promoter and enhance its expression. The CD147 mRNA has a 748-bp 3'-untranslated region (UTR) with many miRNA target sites, suggesting possible regulation by miRNAs. We discovered that miR-22 repressed CD147 expression by directly targeting the CD147 3'UTR. We also determined that miR-22 could indirectly participate in CD147 modulation by downregulating Sp1 expression. miR-22 could form an autoregulatory loop with Sp1, which repressed miR-22 transcription by binding to the miR-22 promoter. Together with the c-Myc-mediated inhibition of miR-22 expression, our investigation identified a miR-22/Sp1/c-Myc network that regulates CD147 gene transcription. In addition, miR-22 overexpression suppressed breast cancer cell invasion,   metastasis  , and proliferation by targeting CD147 in vitro and in vivo. Furthermore, we found that miR-22 was significantly downregulated in breast cancer tissues and that its expression was inversely correlated with the tumor-node-  metastasis   stage and lymphatic   metastasis   in patients. Our study provides the first evidence that an miR-22/Sp1/c-Myc network regulates CD147 upregulation in breast cancer and that miR-22 represses breast cancer invasive and metastatic capacities. 
 The tumor-suppressive microRNA-135b targets c-myc in osteoscarcoma. Osteosarcoma is the most common primary tumor of the bone. It leads to many deaths because of its rapid proliferation and   metastasis  . Recent studies have shown that microRNAs are important gene regulators that are involved in various cancer-related processes. In this study, we found that miR-135b was down-regulated in both osteoscarcoma patient tumor tissues and osteoscarcoma cell lines in comparison to paired adjacent non-tumor bone tissue. We observed that a lower level of miR-135b was associated with   metastasis  . The ectopic expression of miR-135b markedly suppressed osteoscarcoma cell proliferation, migration, and invasion. Conversely, the inhibition of miR-135b expression dramatically accelerated cell proliferation, migration, and invasion. The forced expression of miR-135b in osteosarcoma cells resulted in a significant reduction in the protein level of c-Myc and repressed the activity of a luciferase reporter that contained the 3'-untranslated region of the c-Myc mRNA. These effects were abolished by the mutation of the predicted miR-135b-binding site, which indicates that c-Myc may be a miR-135b target gene. Moreover, the ectopic expression of c-Myc partially reversed the inhibition of cell proliferation and invasion that was caused by miR-135b. These data therefore suggest that miR-135b may function as a tumor suppressor to regulate osteosarcoma cell proliferation and invasion through a mechanism that targets the c-Myc oncogene. These findings indicate that miR-135b may play a role in the pathogenesis of osteosarcoma. 
 IκB kinases increase Myc protein stability and enhance progression of breast cancer cells. Both IκB kinase (IKK) complex and oncgenic protein Myc play important roles in cancer progression, including cancer cell invasiveness an  d metastas  is. The levels of Myc is regulated by the phosphorylation of Myc at Thr58 and Ser62.In this study, we show that the expression of Myc is associated with IKKα and IKKβ in breast cancers and that Myc is an IKKs substrate. Suppression of IKK activity by either chemical inhibitor or transfection of kinase-dead mutants decreases the phosphorylation of Myc at Ser62 and enhances the degradation of Myc. Consequently, these treatments decrease the tumorigenic and invasive ability of breast cancer cells. Furthermore, doxorubicin, a frequently used anticancer drug in breast cancer, activates IKKs and Myc, thereby increasing invasiveness and tumorigenesis of breast carcinoma MCF7 cells. Inhibition of IKKs prevents these doxorubicin-induced effects.Our study indicates that IKKs tightly regulate Myc expression through prolonging protein stability, and suggests that IKKs are potentially therapeutic targets and that suppression of IKKs may be used following chemotherapy to reduce the risk of treatment-induced tumor progression. 
 HER2 overexpression and amplification in urothelial carcinoma of the bladder is associated with MYC coamplification in a subset of cases. We examined 53 invasive high-grade urothelial carcinomas (UCCs) and 42 paired lymph node metastases to determine frequency of HER2 overexpression, HER2/MYC coamplification, and association between HER2 and MYC status and clinicopathologic features. HER2 overexpression occurred in 19 UCCs (36%) and 14 metastases (30%), with an 88% concordance rate between UCCs and matched metastases. HER2 amplification occurred in 5 (10%) of 50 UCCs and 4 (11%) of 36 metastases, with a 100% concordance rate; MYC amplification occurred in 7 (18%) of 40 UCCs and 4 (13%) of 32 metastases, with a concordance rate of 50% between UCCs and metastases. Of 7 cases demonstrating HER2 amplification, MYC was coamplified in 4 (57%; P = .01), and coamplification was associated in all cases with   metastasis   and advanced local disease (pT4). Coamplification of HER2 and MYC occurs in a subset of patients with metastatic UCC. HER2 overexpression and amplification in metastatic lesions suggest that HER2-targeted therapy may be valuable for patients undergoing treatment for metastatic UCC, for which current therapy is limited. Further studies into the role of MYC coamplification in this population are needed to determine impacts on treatment with HER2-targeted therapy. 
  GeneRIFs  
 our data indicated that there is a regulatory feedback loop between miR-17-5p and c-Myc, in which miR-17-5p could suppress some of the distinguishing features, invasion, and   metastasis  , of oncogenic c-Myc in HCC cells, and meanwhile, miR-17-5p is upregulated by c-Myc role as a transcription factor, although further studies are still needed 
 Our data identify IL6 detection as a potential causal biomarker for MYC-driven   metastasis   after loss of PTEN and p53. 
 the c-Myc-mediated transcriptional repression of miR-15-16 in hypoxia is induced by increased HIF-2alpha and promoted tumor angiogenesis and hematogenous   metastasis   by the further loss of post-transcriptional inhibition of FGF2. 
 Results show that Myc drives local prostate   metastasis   and is critical for maintenance of   metastasis   prostate cancer. 
 Data indicate that patients negative for BRCA1 protein were more often with pathological tumor-node-  metastasis   stage III, positive for lymph node   metastasis   and MYC overexpression than BRCA1-positive tumors. 
 Results thus identify a novel epigenetic mechanism by which MYC activates GATA4 leading to   metastasis   in lung adenocarcinoma. 
 Overexpression of Myc upregulates Ran expression, whereas knockdown of Myc downregulates Ran expression. Myc or Ran overexpression in breast cancer cells is associated with cancer progression and   metastasis  . 
 loss of SAE1/2 activity drives synthetic lethality with Myc; inactivation of SAE2 leads to mitotic catastrophe and cell death upon Myc hyperactivation; findings in Myc-high breast cancers suggest low tumor SAE1 and SAE2 correlates   metastasis  -free survival 
 Nuclear expression of c-myc in primary breast tumors correlated with an increased risk for axillary lymph node   metastasis  . 
 Studies indicate that many prognostic signatures reflect the activity of the MYC oncogene, which in turn regulates tumor   metastasis   through specific effects on cancer cell invasion and migration. 
 miR-9 miRNA downregulates E-cadherin.  Expression of miR-9 is activated by MYC and MYCN, which bind to the mir-9-3 locus. Elevated expression of miR-9 may contribute to epithelial-mesenchymal transition and   metastasis   in some and possibly many tumours. 
 Detection of nuclear c-Myc in leiomyosarcoma predicts decreased overall and   metastasis  -free survival, independent of standard prognostic variables, tumor size, histological grade, and TNM stage. 
 Reduction in the c-myc protein was correlated with neck   metastasis   in nasopharyngeal carcinoma. 
  [Cc]ell [Ii]nvasion  PubMed  
 Pdcd4 knockdown up-regulates MAP4K1 expression and activation of AP-1 dependent transcription through c-Myc. Programmed cell death 4 (Pdcd4) is a novel tumor suppressor, whose expression is frequently down-regulated in several types of cancers. In the present study, we demonstrated that Pdcd4 knockdown up-regulates MAP kinase kinase kinase kinase 1 (MAP4K1) expression and increases phosphorylation of c-Jun. Over-expression of c-Myc in HEK293 cells increases the levels of MAP4K1, MAP4K1 promoter activity, and phospho-c-Jun. Mutation analysis showed that the c-Myc binding site at -536bp (relative to the initiation ATG) of map4k1 promoter responds to c-Myc regulation. In addition, chromatin immunoprecipitation demonstrated that c-Myc directly binds to map4k1 promoter at this site. Down-regulation of c-Myc reverses MAP4K1 expression and AP-1 activation in Pdcd4 knockdown cells. Moreover, over-expression of dominant negative Tcf4 decreases expression of c-Myc and MAP4K1, JNK activation, and AP-1 dependent transcription. Thus, activation of β-catenin/Tcf dependent transcription in Pdcd4 knockdown cells up-regulates MAP4K1 expression and AP-1 activity via c-Myc. The study presented here further reveals in detail the mechanism of how Pdcd4 inhibits tumor   cell invasio  n and provides a functional connection between β-catenin/Tcf and AP-1 dependent transcription. 
 Pdcd4 knockdown up-regulates MAP4K1 expression and activation of AP-1 dependent transcription through c-Myc. Programmed cell death 4 (Pdcd4) is a novel tumor suppressor, whose expression is frequently down-regulated in several types of cancers. In the present study, we demonstrated that Pdcd4 knockdown up-regulates MAP kinase kinase kinase kinase 1 (MAP4K1) expression and increases phosphorylation of c-Jun. Over-expression of c-Myc in HEK293 cells increases the levels of MAP4K1, MAP4K1 promoter activity, and phospho-c-Jun. Mutation analysis showed that the c-Myc binding site at -536bp (relative to the initiation ATG) of map4k1 promoter responds to c-Myc regulation. In addition, chromatin immunoprecipitation demonstrated that c-Myc directly binds to map4k1 promoter at this site. Down-regulation of c-Myc reverses MAP4K1 expression and AP-1 activation in Pdcd4 knockdown cells. Moreover, over-expression of dominant negative Tcf4 decreases expression of c-Myc and MAP4K1, JNK activation, and AP-1 dependent transcription. Thus, activation of β-catenin/Tcf dependent transcription in Pdcd4 knockdown cells up-regulates MAP4K1 expression and AP-1 activity via c-Myc. The study presented here further reveals in detail the mechanism of how Pdcd4 inhibits tumor   cell invasio  n and provides a functional connection between β-catenin/Tcf and AP-1 dependent transcription. 
 p63-Mediated activation of the β-catenin/c-Myc signaling pathway stimulates esophageal squamous carcinoma   cell invasio  n and metastasis. The development of esophageal squamous carcinomas (ESC) results from numerous genetic alterations. Our previous study demonstrated that p63 is highly expressed in human ESC cells and stimulates their growth; however, the mechanism by which p63 regulates ESC cell adhesion and invasion remains unclear. In the present study, we further elucidated the underlying molecular mechanisms by which p63 regulates metastasis in ESC cells. Knockdown of p63 significantly diminished the invasion of ESC cell lines TE-8 and TE-12, whereas overexpression of p63 significantly increased the migration rates of BE3 and OE33 cells. The mRNA and protein levels of vimentin, twist, SUSD2, and uPA were significantly decreased in p63-knockdown ESC cells, while overexpression of p63 induced an increase in vimentin, SUSD2, and uPA. In addition, knockdown of p63 in ESC cells significantly reduced levels of β-catenin and c-Myc, while overexpression of p63 increased β-catenin, but reduced p-β-catenin level. Therefore, p63 regulates the migration and invasion of ESC cells through activation of the β-catenin/c-Myc pathway. Our results suggest that targeting p63 may constitute a potential therapeutic strategy for ESC. 
 c-Myc expression and MEK1-induced Erk2 nuclear localization are required for TGF-beta induced epithelial-mesenchymal transition and invasion in prostate cancer. Understanding the initial mechanisms by which epithelial cells transform to an invasive phenotype is critical to the development of diagnostics that can identify the metastatic potential of cancers as well as therapeutic agents that can prevent metastases. Changes in cellular response to the transforming growth factor-beta (TGF-β) cytokine are known to promote epithelial   cell invasio  n and metastasis in part through induction of epithelial-mesenchymal transitions (EMTs). In this report, we demonstrate that non-metastatic human prostate cancer cell lines of increasing Gleason score can be induced to undergo EMT when treated with TGF-β in combination with epidermal growth factor. Mechanistic studies revealed that in cells stably transfected with activated Ras, TGF-β alone induced EMT and that a Ras-Raf-MEK1, but not MEK2, signaling cascade is necessary and sufficient for Erk2 nuclear localization that works in concert with TGF-β to promote EMT. Furthermore, we show for the first time that expression of the transcription factor c-myc, which is phosphorlyated by Erk2, is required for EMT. Characteristically, EMT involved adoption of a spindle-shaped morphology, loss of E-cadherin and increased expression of Vimentin, Fibronectin and Fibroblast Specific Protein-1 (S100A4). Prostate cells undergoing EMT became invasive and expressed several genes associated with metastasis, including MT-MMP1, MMP-2/9, the MMP-9 homodimer, Slug and Twist2. In sum, we demonstrate a novel mechanism by which non-invasive primary prostate tumor cells transition to an invasive phenotype characteristic of malignant tumor cells in response to TGF-β signaling. 
 c-MYC-regulated miR-23a/24-2/27a cluster promotes mammary carcinoma   cell invasion   and hepatic metastasis by targeting Sprouty2. Emerging evidence indicates that the miR-23a/24-2/27a cluster may possess a causal role in mammary tumorigenesis and function as a novel class of oncogenes. However, the regulatory mechanism of the miR-23a/24-2/27a cluster in mammary carcinoma   cell invasion   and migration is still largely unknown. We observed that the expression levels of miR-23a, miR-24-2 and miR-27a were significantly higher in breast cancer with lymph node metastasis, compared with that from patients without lymph node metastasis or normal tissue. Forced expression of the miR-23a/24-2/27a cluster promoted mammary carcinoma cell migration, invasion, and hepatic metastasis, through targeting Sprouty2 (SPRY2) and consequent activation of p44/42 MAPK. Epidermal growth factor induced the expression of the transcription factor c-MYC, which promoted the expression of mature miR-23a, miR-24-2, and miR-27a and subsequently decreased expression of SPRY2 and activated p44/42 MAPK to promote mammary carcinoma cell migration and invasion. We therefore suggest a novel link between epidermal growth factor and the miR-23a/24-2/27a cluster via the regulation of c-MYC, providing the potential for the miR-23a/24-2/27a cluster to be used as biomarker in the diagnosis and/or treatment of breast cancer. 
 SATB1 Promotes Pancreatic Cancer Growth and Invasion Depending on MYC Activation. SATB1 plays an important role in human malignant progression, inducing cancer cell proliferation and metastasis by regulating downstream gene expressions. However, little is known about the underlying mechanisms in which SATB1 promotes pancreatic cancer tumorigenesis.To investigate SATB1 expression levels and its biological functions in promoting pancreatic cancer growth and invasion.SATB1 expression levels were detected in seven human pancreatic cancer cell lines and 16 pairs of normal pancreatic/pancreatic cancer tissues using RT-PCR and western blot. SW1990 or Capan-1 cells stably knockdown (shRNA) or transiently knockdown (siRNA) SATB1 cells, and PANC-1 stably overexpressing SATB1 cells were investigated with MTT, EdU assay, flow cytometry, and transwell invasion assay for cell proliferation and invasion activity. The binding of SATB1 to MYC promoter region was examined using reporter assay. Expression of SATB1 in 68 pancreatic cancer samples was studied by immunohistochemical staining and scoring.SATB1 was overexpressed in pancreatic cancer tissues samples, showing strong correlation with pancreatic cancer invasion depth and tumor staging. SATB1 induced MYC mRNA and protein expression; promoted pancreatic cancer cell growth; increased cell population in S phase; and enhanced pancreatic cancer   cell invasion   in vitro. On the other hand, SATB1 knockdown showed opposite effects. Furthermore, MYC blocking in SATB1-overexpressing cells attenuated the promotion of pancreatic cancer cell growth and invasion. Our data also indicated that SATB1 bound to specific promoter region of MYC.SATB1 is overexpressed in pancreatic cancer, promoting cancer cell proliferation and invasion through the activation of MYC. 
 Down-regulation of c-Myc expression inhibits the invasion of bile duct carcinoma cells. Cholangiocarcinoma is the second most common primary hepatic tumour originating from biliary tract epithelial cells with poor prognosis. Enhanced c-Myc protein expression contributes to many aspects of tumour cell biology. Although the ability of c-Myc to drive unrestricted cell proliferation and to inhibit cell differentiation had been well recognized, whether down-regulated c-Myc expression can inhibit tumour   cell invasion   still remains to be explored. The c-Myc ASODN (antisense oligodeoxyribonucleotide) and NSODN (nonsense oligodeoxyribonucleotide) were designed, synthesized and transfected into human QBC939 bile duct carcinoma cells using the Lipofectamine 2000 reagent. The protein expression of c-Myc was detected by Western blot. A transwell experiment was applied to evaluate the invasive capacity of the QBC939 cells. c-Myc ASODN could significantly suppress the c-Myc protein expression (P<0.05) and the invasion (P<0.01) of QBC939 cells transfected with c-Myc ASODN compared with that in the control and c-Myc NSODN-transfected group. Thus in the present study we show that down-regulation of c-Myc expression can inhibit the invasion of QBC939 cells in vitro. 
 Ezrin mediates c-Myc actions in prostate cancer   cell invasion  . The forced overexpression of c-Myc in mouse prostate and in normal human prostate epithelial cells results in tumor transformation with an invasive phenotype. How c-Myc regulates   cell invasion   is poorly understood. In this study, we have investigated the interplay of c-Myc and androgens in the regulation of prostate cancer   cell invasion  . We found that c-Myc induces   cell invasion   and anchorage-independent growth by regulating ezrin protein expression in the presence of androgens. The activity of the ezrin promoter is controlled by androgens through c-Myc, which binds to a phylogenetically conserved E-Box located in the proximal promoter region. Besides, we also show that ezrin is an important regulator of c-Myc protein levels. These effects are achieved through androgen-induced changes in ezrin phosphorylation, which results in the regulation of downstream signals. These downstream signals involve the modulation of Akt and GSK-3beta activity resulting in increased c-Myc protein synthesis and inhibition of its degradation. In summary, we have shown a key role for ezrin as a mediator of c-Myc-induced tumorigenesis in prostate cancer cells. 
 ROCK has a crucial role in regulating prostate tumor growth through interaction with c-Myc. Rho-associated kinase (ROCK) has an essential role in governing cell morphology and motility, and increased ROCK activity contributes to cancer   cell invasion   and metastasis. Burgeoning data suggest that ROCK is also involved in the growth regulation of tumor cells. However, thus far, the molecular mechanisms responsible for ROCK-governed tumor cell growth have not been clearly elucidated. Here we showed that inhibition of ROCK kinase activity, either by a selective ROCK inhibitor Y27632 or by specific ROCK small interfering RNA (siRNA) molecules, attenuated not only motility but also the proliferation of PC3 prostate cancer cells in vitro and in vivo. Importantly, mechanistic investigation revealed that ROCK endowed cancer cells with tumorigenic capability, mainly by targeting c-Myc. ROCK could increase the transcriptional activity of c-Myc by promoting c-Myc protein stability, and ROCK inhibition reduced c-Myc-mediated expression of mRNA targets (such as HSPC111) and microRNA targets (such as miR-17-92 cluster). We provided evidence demonstrating that ROCK1 directly interacted with and phosphorylated c-Myc, resulting in stabilization of the protein and activation of its transcriptional activity. Suppression of ROCK-c-Myc downstream molecules, such as c-Myc-regulated miR-17, also impaired tumor cell growth in vitro and in vivo. In addition, c-Myc was shown to exert a positive feedback regulation on ROCK by increasing RhoA mRNA expression. Therefore, inhibition of ROCK and its stimulated signaling might prove to be a promising strategy for restraining tumor progression in prostate cancer. 
 Hepatitis B virus X protein upregulates HSP90alpha expression via activation of c-Myc in human hepatocarcinoma cell line, HepG2. The Hepatitis B Virus X protein (HBx) plays a major role in hepatocellular carcinoma (HCC) development, however, its contribution to tumor invasion and metastasis has not been established so far. Heat shock protein 90 alpha (HSP90alpha) isoform is an ATP-dependent molecular chaperone that maintains the active conformation of client oncoproteins in cancer cells, which is abundantly expressed in HCC, especially in hepatitis B virus (HBV)-related tumors, might be involved in tumor progression.The levels of HSP90alpha, extracellular signal-regulated kinase 1/2 (ERK1/2), phosphorylated ERK1/2 (p-ERK1/2) and c-Myc in HBx-transfected HepG2 cells were determined by western blots analysis. The endogenous ERKs activity was demonstrated by ELISA assay. The regulation of c-Myc-mediated HSP90 alpha promoter transactivation by HBx was evaluated through electrophoretic mobility shift analysis (EMSA). The c-Myc-mediated HSP90alpha transcription was analysed by promoter assay. The HBx-expressing cells were transfected with specific small interference RNA (siRNA) against c-Myc. The in vitro invasion potentials of cells were evaluated by Transwell   cell invasion   assay.HBx induces HSP90alpha expression at the transcription level. The induction effect of HBx was inhibited after treatment with c-Myc inhibitor, 10058-F4. In addition, the luciferase activity of the HSP90alpha promoter analysis revealed that the HBx is directly involved in the c-Myc-mediated transcriptional activation of HSP90alpha. Furthermore, HBx induces c-Myc expression by activation of Ras/Raf/ERK1/2 cascades, which in turn results in activation of the c-Myc-mediated HSP90alpha promoter and subsequently up-regulation of the HSP90alpha expression. Overexpression of HSP90alpha in HBx-transfected cells enhances tumor cells invasion. siRNA-mediated c-Myc knockdown in HBx-transfected cells significantly suppressed HSP90alpha expression and cells invasion in vitro.These results demonstrate the ability of HBx to promote tumor cells invasion by a mechanism involving the up-regulation of HSP90alpha and provide new insights into the mechanism of action of HBx and its involvement in tumor metastasis and recurrence of HCC. 
 Long non-coding RNA HOTAIR, a c-Myc activated driver of malignancy, negatively regulates miRNA-130a in gallbladder cancer. Protein coding genes account for only about 2% of the human genome, whereas the vast majority of transcripts are non-coding RNAs including long non-coding RNAs. A growing volume of literature has proposed that lncRNAs are important players in cancer. HOTAIR was previously shown to be an oncogene and negative prognostic factor in a variety of cancers. However, the factors that contribute to its upregulation and the interaction between HOTAIR and miRNAs are largely unknown.A computational screen of HOTAIR promoter was conducted to search for transcription-factor-binding sites. HOTAIR promoter activities were examined by luciferase reporter assay. The function of the c-Myc binding site in the HOTAIR promoter region was tested by a promoter assay with nucleotide substitutions in the putative E-box. The association of c-Myc with the HOTAIR promoter in vivo was confirmed by chromatin immunoprecipitation assay and Electrophoretic mobility shift assay. A search for miRNAs with complementary base paring with HOTAIR was performed utilizing online software program. Gain and loss of function approaches were employed to investigate the expression changes of HOTAIR or miRNA-130a. The expression levels of HOTAIR, c-Myc and miRNA-130a were examined in 65 matched pairs of gallbladder cancer tissues. The effects of HOTAIR and miRNA-130a on gallbladder cancer   cell invasion   and proliferation was tested using in vitro   cell invasion   and flow cytometric assays.We demonstrate that HOTAIR is a direct target of c-Myc through interaction with putative c-Myc target response element (RE) in the upstream region of HOTAIR in gallbladder cancer cells. A positive correlation between c-Myc and HOTAIR mRNA levels was observed in gallbladder cancer tissues. We predicted that HOTAIR harbors a miRNA-130a binding site. Our data showed that this binding site is vital for the regulation of miRNA-130a by HOTAIR. Moreover, a negative correlation between HOTAIR and miRNA-130a was observed in gallbladder cancer tissues. Finally, we demonstrate that the oncogenic activity of HOTAIR is in part through its negative regulation of miRNA-130a.Together, these results suggest that HOTAIR is a c-Myc-activated driver of malignancy, which acts in part through repression of miRNA-130a. 
 SATB1 and SATB2 play opposing roles in c-Myc expression and progression of colorectal cancer. Special AT-rich sequence-binding protein 1 and 2 (SATB1/2) are nuclear matrix-associated proteins involved in chromatin remodeling and regulation of gene expression. SATB2 acts as a tumor suppressor in laryngeal squamous cell carcinoma and colon cancer, whereas SATB1 promotes the progression of numerous types of cancers. In this study, we examined the effects of SATB1 and SATB2 on the malignant characteristics of colorectal cancer cells. SATB1 and SATB2 expression were negatively correlated in colorectal cancer specimens. SATB1 expression was increased, whereas SATB2 expression was reduced, in colorectal cancer tissues compared to control tissues. Exogenous expression of SATB2 in colorectal cancer cells suppressed cell proliferation, colony formation and tumor proliferation in mice. c-Myc was reduced by SATB2 expression, and exogenous expression of c-Myc in SATB2-expressing cells restored proliferation, colony formation and in vivo tumor growth of colorectal cancer cells. We also showed that c-Myc reduction by SATB2 was mediated by the inactivation of ERK5. In contrast, SATB1 promoted c-Myc expression. The expression of SATB1 in colorectal cancer tissues was positively correlated with c-Myc expression, and SATB1 knockdown reduced c-Myc expression in colorectal cancer cells. Finally, we showed that SATB1 knockdown in colorectal cancer cells suppressed cell proliferation, colony formation and   cell invasion  . Our results reveal interesting features of how the structural homologs SATB1 and SATB2 exert opposing functions in colorectal tumorigenesis. 
 MYC and metastasis. Aggressive primary tumors express transcriptional signatures that correlate with their metastatic propensity. A number of these signatures have been deployed in the clinic as risk stratification tools. However, the molecular basis of these clinically useful prognostic signatures has remained a largely unresolved area of controversy. We recently found that many prognostic signatures reflect the activity of the MYC oncogene, which in turn regulates tumor metastasis through specific effects on cancer   cell invasion   and migration. These findings offer a general framework for understanding the molecular basis of clinically prognostic transcriptional signatures and suggest potentially new avenues for studying metastasis. 
 MicroRNA-34a suppresses malignant transformation by targeting c-Myc transcriptional complexes in human renal cell carcinoma. We investigated the functional effects of microRNA-34a (miR-34a) on c-Myc transcriptional complexes in renal cell carcinoma. miR-34a down-regulated expression of multiple oncogenes including c-Myc by targeting its 3' untranslated region, which was revealed by luciferase reporter assays. miR-34a was also found to repress RhoA expression by suppressing the c-Myc-Skp2-Miz1 transcriptional complex that activates RhoA. Overexpression of c-Myc reversed miR-34a suppression of RhoA expression and inhibition of   cell invasion  , suggesting that miR-34a inhibits invasion by suppressing RhoA through c-Myc. miR-34a was also found to repress the c-Myc-P-TEFb transcription elongation complex, indicating one of the mechanisms by which miR-34a has profound effects on cellular functions. Our results demonstrate that miR-34a suppresses assembly and function of the c-Myc complex that activates or elongates transcription, indicating a novel role of miR-34a in the regulation of transcription by c-Myc. 
 MicroRNA-34a modulates c-Myc transcriptional complexes to suppress malignancy in human prostate cancer cells. MicroRNA-34a (miR-34a), a potent mediator of tumor suppressor p53, has been reported to function as a tumor suppressor and miR-34a was found to be downregulated in prostate cancer tissues. We studied the functional effects of miR-34a on c-Myc transcriptional complexes in PC-3 prostate cancer cells. Transfection of miR-34a into PC-3 cells strongly inhibited in vitro cell proliferation,   cell invasion   and promoted apoptosis. Transfection of miR-34a into PC-3 cells also significantly inhibited in vivo xenograft tumor growth in nude mice. miR-34a downregulated expression of c-Myc oncogene by targeting its 3' UTR as shown by luciferase reporter assays. miR-34a was found to repress RhoA, a regulator of cell migration and invasion, by suppressing c-Myc-Skp2-Miz1 transcriptional complex that activates RhoA. Overexpression of c-Myc reversed miR-34a suppression of RhoA expression, suggesting that miR-34a inhibits invasion by suppressing RhoA through c-Myc. miR-34a was also found to repress c-Myc-pTEFB transcription elongation complex, indicating one of the mechanisms by which miR-34a has profound effects on cellular function. This is the first report to document that miR-34a suppresses assembly and function of the c-Myc-Skp2-Miz1 complex that activates RhoA and the c-Myc-pTEFB complex that elongates transcription of various genes, suggesting a novel role of miR-34a in the regulation of transcription by c-Myc complex. 
 MicroRNA-34a inhibits human trophoblast   cell invasion   by targeting MYC. Preeclampsia, one of the major disorders of pregnancy, is characterized by inadequate trophoblast invasion and defective trophoblast-mediated remodeling of placental vasculature. MicroRNA-34a (miR-34a) has been found to be aberrantly expressed in the placentas of preeclamptic patients, yet its role in placental development and in the pathogenesis of preeclampsia remains elusive.The levels of miR-34a in the placentas of 20 preeclamptic patients and 20 healthy subjects were determined by real time-PCR, and miR-34a was found significantly elevated in the preeclamptic placentas. Further, the function of miR-34a in trophoblast cells was investigated by overexpressing miR-34a in JEG-3 trophoblast cell line. Overexpression of miR-34a in JEG-3 cells inhibited cell proliferation, migration and invasion. In addition, elevated expression of miR-34a reduced the expression of both endogenous and ectopic MYC. Moreover, we identified that MYC mRNA was a direct target of miR-34a in JEG-3 cells by dual luciferase reporter assay, and found that downregulation of MYC expression by miR-34a targeting significantly reduced the invasiveness of JEG-3 cells.Our findings provide preliminary evidence for the diverse functions of miR-34a in trophoblast biology, and suggest that miR-34a suppresses trophoblast invasion by directly targeting MYC. 
 A regulatory loop involving miR-22, Sp1, and c-Myc modulates CD147 expression in breast cancer invasion and metastasis. Breast cancer is the most common cancer in women for which the metastatic process is still poorly understood. CD147 is upregulated in breast cancer and has been associated with tumor progression, but little is known about its regulatory mechanisms. In this study, we demonstrated that CD147 was overexpressed in breast cancer tissues and cell lines, and the high expression correlated with tumor invasion and metastasis. We also found that the transcription factors Sp1 and c-Myc could bind to the CD147 promoter and enhance its expression. The CD147 mRNA has a 748-bp 3'-untranslated region (UTR) with many miRNA target sites, suggesting possible regulation by miRNAs. We discovered that miR-22 repressed CD147 expression by directly targeting the CD147 3'UTR. We also determined that miR-22 could indirectly participate in CD147 modulation by downregulating Sp1 expression. miR-22 could form an autoregulatory loop with Sp1, which repressed miR-22 transcription by binding to the miR-22 promoter. Together with the c-Myc-mediated inhibition of miR-22 expression, our investigation identified a miR-22/Sp1/c-Myc network that regulates CD147 gene transcription. In addition, miR-22 overexpression suppressed breast cancer   cell invasion  , metastasis, and proliferation by targeting CD147 in vitro and in vivo. Furthermore, we found that miR-22 was significantly downregulated in breast cancer tissues and that its expression was inversely correlated with the tumor-node-metastasis stage and lymphatic metastasis in patients. Our study provides the first evidence that an miR-22/Sp1/c-Myc network regulates CD147 upregulation in breast cancer and that miR-22 represses breast cancer invasive and metastatic capacities. 
  GeneRIFs  
 Overexpression of c-Myc reversed miR-34a suppression of RhoA expression and inhibition of   cell invasion  . 
 Studies indicate that many prognostic signatures reflect the activity of the MYC oncogene, which in turn regulates tumor metastasis through specific effects on cancer   cell invasion   and migration. 
   
  
 SOX12 
 
  NCBI Gene  
  GeneRIF  
  Pubmed  
 
 
  Search Term  Rank  Fields  
  [Mm]igration  6  PubMed(1)  
  [Mm]etastasis  12  PubMed(1); GeneRIFs(1)  
 
 
 hide/show details for SOX12 
  [Mm]igration  PubMed  
 Sox12, a direct target of FoxQ1, promotes hepatocellular carcinoma metastasis through up-regulating Twist1 and FGFBP1. Metastasis is the main reason for high recurrence and poor survival of hepatocellular carcinoma (HCC) after curative resection. However, the molecular mechanism underlying HCC metastasis remains unclear. Here, we report on a novel function of SRY (sex determining region Y)-box 12 (Sox12), a member of the SYR-related high mobility group box family proteins, in promoting HCC metastasis. Overexpression of Sox12 was significantly correlated with loss of tumor encapsulation, microvascular invasion, and a higher tumor-nodule-metastasis (TNM) stage and indicated poor prognosis in human HCC patients. Sox12 expression was an independent and significant risk factor for recurrence and reduced survival after curative resection. Overexpression of Sox12 induced epithelial-mesenchymal transition by transactivating Twist1 expression. Down-regulation of Twist1 decreased Sox12-enhanced HCC   migration  , invasion, and metastasis, whereas up-regulation of Twist1 rescued the decreased   migration  , invasion, and metastasis induced by Sox12 knockdown. Additionally, serial deletion, site-directed mutagenesis, and chromatin immunoprecipitation assays showed that fibroblast growth factor binding protein 1 (FGFBP1) was a direct transcriptional target of Sox12. Knockdown of FGFBP1 decreased Sox12-mediated HCC invasion and metastasis, whereas overexpression of FGFBP1 rescued the decreased invasion and metastasis induced by Sox12 knockdown. Furthermore, forkhead box Q1 (FoxQ1) directly bound to the Sox12 promoter and transactivated its expression, which contributed to Sox12 overexpression in human HCC. Knockdown of Sox12 dramatically decreased FoxQ1-mediated HCC metastasis. In two independent cohorts of human HCC tissues, Sox12 expression was positively correlated with Twist1, FGFBP1, and FoxQ1 expression, and patients with positive coexpression of Sox12/Twist1, Sox12/FGFBP1, or FoxQ1/Sox12 were associated with poorer prognosis.Up-regulated Sox12 induced by FoxQ1 promotes HCC invasion and metastasis by transactivating Twist1 and FGFBP1 expression. Thus, our study implicates Sox12 as a potential prognostic biomarker and a novel therapeutic target for HCC. 
  [Mm]etastasis  PubMed  
 Sox12, a direct target of FoxQ1, promotes hepatocellular carcinoma   metastasis   through up-regulating Twist1 and FGFBP1.   Metastasis   is the main reason for high recurrence and poor survival of hepatocellular carcinoma (HCC) after curative resection. However, the molecular mechanism underlying HCC   metastasis   remains unclear. Here, we report on a novel function of SRY (sex determining region Y)-box 12 (Sox12), a member of the SYR-related high mobility group box family proteins, in promoting HCC   metastasis  . Overexpression of Sox12 was significantly correlated with loss of tumor encapsulation, microvascular invasion, and a higher tumor-nodule-  metastasis   (TNM) stage and indicated poor prognosis in human HCC patients. Sox12 expression was an independent and significant risk factor for recurrence and reduced survival after curative resection. Overexpression of Sox12 induced epithelial-mesenchymal transition by transactivating Twist1 expression. Down-regulation of Twist1 decreased Sox12-enhanced HCC migration, invasion, and   metastasis  , whereas up-regulation of Twist1 rescued the decreased migration, invasion, and   metastasis   induced by Sox12 knockdown. Additionally, serial deletion, site-directed mutagenesis, and chromatin immunoprecipitation assays showed that fibroblast growth factor binding protein 1 (FGFBP1) was a direct transcriptional target of Sox12. Knockdown of FGFBP1 decreased Sox12-mediated HCC invasion and   metastasis  , whereas overexpression of FGFBP1 rescued the decreased invasion and   metastasis   induced by Sox12 knockdown. Furthermore, forkhead box Q1 (FoxQ1) directly bound to the Sox12 promoter and transactivated its expression, which contributed to Sox12 overexpression in human HCC. Knockdown of Sox12 dramatically decreased FoxQ1-mediated HCC   metastasis  . In two independent cohorts of human HCC tissues, Sox12 expression was positively correlated with Twist1, FGFBP1, and FoxQ1 expression, and patients with positive coexpression of Sox12/Twist1, Sox12/FGFBP1, or FoxQ1/Sox12 were associated with poorer prognosis.Up-regulated Sox12 induced by FoxQ1 promotes HCC invasion and   metastasis   by transactivating Twist1 and FGFBP1 expression. Thus, our study implicates Sox12 as a potential prognostic biomarker and a novel therapeutic target for HCC. 
  GeneRIFs  
 Up-regulated Sox12 induced by FoxQ1 promotes hepatocellular carcinoma invasion and   metastasis   by transactivating Twist1 and FGFBP1 expression. 
   
  
 FOSL2 
 
  NCBI Gene  
  GeneRIF  
  Pubmed  
 
 
  Search Term  Rank  Fields  
  [Mm]igration  6  PubMed(3); GeneRIFs(2)  
  [Ee]xtracellular [Mm]atrix  8  PubMed(1)  
  [Ii]nvasive  10  PubMed(1)  
  [Mm]etastasis  12  PubMed(3)  
  [Cc]ell [Ii]nvasion  13  PubMed(1)  
 
 
 hide/show details for FOSL2 
  [Mm]igration  PubMed  
 Transcription factor fos-related antigen-2 induces progressive peripheral vasculopathy in mice closely resembling human systemic sclerosis. Microvascular damage is one of the first pathological changes in systemic sclerosis. In this study, we investigated the role of Fos-related antigen-2 (Fra-2), a transcription factor of the activator protein-1 family, in the peripheral vasculopathy of systemic sclerosis and examined the underlying mechanisms.Expression of Fra-2 protein was significantly increased in skin biopsies of systemic sclerosis patients compared with healthy controls, especially in endothelial and vascular smooth muscle cells. Fra-2 transgenic mice developed a severe loss of small blood vessels in the skin that was paralleled by progressive skin fibrosis at 12 weeks of age. The reduction in capillary density was preceded by a significant increase in apoptosis in endothelial cells at week 9 as detected by immunohistochemistry. Similarly, suppression of Fra-2 by small interfering RNA prevented human microvascular endothelial cells from staurosporine-induced apoptosis and improved both the number of tubes and the cumulative tube lengths in the tube formation assay. In addition, cell   migration   in the scratch assay and vascular endothelial growth factor-dependent chemotaxis in a modified Boyden chamber assay were increased after transfection of human microvascular endothelial cells with Fra-2 small interfering RNA, whereas proliferation was not affected.Fra-2 is present in human systemic sclerosis and may contribute to the development of microvasculopathy by inducing endothelial cell apoptosis and by reducing endothelial cell   migration   and chemotaxis. Fra-2 transgenic mice are a promising preclinical model to study the mechanisms and therapeutic approaches of the peripheral vasculopathy in systemic sclerosis. 
 FOSL2 positively regulates TGF-β1 signalling in non-small cell lung cancer. Fos-related antigen 2 (FRA-2/FOSL2) belongs to the AP-1 transcription factor family. Although FOSL2 has been shown to be involved in diverse physiological and pathological processes, very little is known about the signalling pathways that regulate FOSL2 expression and the mechanisms of FOSL2 function. Here, we show that FOSL2 expression is regulated by TGF-β1 and that FOSL2 is required for TGF-β1-induc  ed migrat  ion. We demonstrate that FOSL2 interacts with Smad3 in vitro and in vivo and thus up-regulates TGF-β1-induced signalling responses. Mechanistically, FOSL2 promotes P300 binding to Smad3 and the acetylation of Smad3 by P300. Furthermore, we show that the expression of FOSL2 correlates with activated Smad3 expression in clinical non-small cell lung cancer (NSCLC) samples. In summary, the present study indicates that FOSL2 facilitates TGF-β1-ind  uced migr  ation by interaction with Smad3 in NSCLC and suggests FOSL2 as a potential therapeutic target for NSCLC. 
 Role of Fra-2 in breast cancer: influence on tumor cell invasion and motility. Fra-2 (Fos-related antigen 2) is a member of the Fos family of AP-1 transcription factors which is often up-regulated in mammary carcinomas. Previous results suggested that it might be involved in the regulation of breast cancer invasion and metastasis. In order to analyze the role of Fra-2 in breast cancer cells, it was silenced in the highly invasive MDA-MB231 cells using RNA interference. On the other hand, stable transfectants of the weakly invasive MCF7 cell line were established in order to analyze the effects of Fra-2 overexpression. In both approaches, cell proliferation was not or only weakly influenced by Fra-2. In contrast, the invasive potential of the cells was increased, and a weaker effect on motility was observed. By cDNA microarray analysis of the MCF7 transfectants followed by validation on a protein level, we identified several Fra-2 target genes which might be involved in cell invasion and   migration  , i.e., ALCAM and connexin 43. Additionally, mRNA expression levels of various genes which are associated with a more malignant behavior of the tumors in vivo were up- or downregulated, i.e., members of the MAGE family, S100P, TIMP2, IL24 etc. These results show that Fra-2 overexpression is associated with a more aggressive tumor phenotype and is probably involved in breast cancer progression in vivo. 
  GeneRIFs  
 FOSL2 facilitates TGF-beta1-induced   migration   by interaction with Smad3 in non-small cell lung cancer.FOSL2 positively regulates TGF-beta1 signalling . 
 Fra-2 is present in human systemic sclerosis and may contribute to the development of microvasculopathy by inducing endothelial cell apoptosis and by reducing endothelial cell   migration   and chemotaxis. 
  [Ee]xtracellular [Mm]atrix  PubMed  
 The transcription factor Fra-2 regulates the production of   extracellular matrix   in systemic sclerosis. Fra-2 belongs to the activator protein 1 family of transcription factors. Mice transgenic for Fra-2 develop a systemic fibrotic disease with vascular manifestations similar to those of systemic sclerosis (SSc). The aim of the present study was to investigate whether Fra-2 plays a role in the pathogenesis of SSc and to identify the molecular mechanisms by which Fra-2 induces fibrosis.Dermal thickness and the number of myofibroblasts were determined in skin sections from Fra-2-transgenic and wild-type mice. The expression of Fra-2 in SSc patients and in animal models of SSc was analyzed by real-time polymerase chain reaction and immunohistochemistry. Fra-2, transforming growth factor beta (TGFbeta), and ERK signaling in SSc fibroblasts were inhibited using small interfering RNA, neutralizing antibodies, and small-molecule inhibitors.Fra-2-transgenic mice developed a skin fibrosis with increases in dermal thickness and increased myofibroblast differentiation starting at age 12 weeks. The expression of Fra-2 was up-regulated in SSc patients and in different mouse models of SSc. Stimulation with TGFbeta and platelet-derived growth factor (PDGF) significantly increased the expression of Fra-2 in SSc fibroblasts and induced DNA binding of Fra-2 in an ERK-dependent manner. Knockdown of Fra-2 potently reduced the stimulatory effects of TGFbeta and PDGF and decreased the release of collagen from SSc fibroblasts.We demonstrate that Fra-2 is overexpressed in SSc and acts as a novel downstream mediator of the profibrotic effects of TGFbeta and PDGF. Since transgenic overexpression of Fra-2 causes not only fibrosis but also vascular disease, Fra-2 might be an interesting novel candidate for molecular-targeted therapies for SSc. 
  [Ii]nvasive  PubMed  
 Role of Fra-2 in breast cancer: influence on tumor cell invasion and motility. Fra-2 (Fos-related antigen 2) is a member of the Fos family of AP-1 transcription factors which is often up-regulated in mammary carcinomas. Previous results suggested that it might be involved in the regulation of breast cancer invasion and metastasis. In order to analyze the role of Fra-2 in breast cancer cells, it was silenced in the highly   invasive   MDA-MB231 cells using RNA interference. On the other hand, stable transfectants of the weakly   invasive   MCF7 cell line were established in order to analyze the effects of Fra-2 overexpression. In both approaches, cell proliferation was not or only weakly influenced by Fra-2. In contrast, the   invasive   potential of the cells was increased, and a weaker effect on motility was observed. By cDNA microarray analysis of the MCF7 transfectants followed by validation on a protein level, we identified several Fra-2 target genes which might be involved in cell invasion and migration, i.e., ALCAM and connexin 43. Additionally, mRNA expression levels of various genes which are associated with a more malignant behavior of the tumors in vivo were up- or downregulated, i.e., members of the MAGE family, S100P, TIMP2, IL24 etc. These results show that Fra-2 overexpression is associated with a more aggressive tumor phenotype and is probably involved in breast cancer progression in vivo. 
  [Mm]etastasis  PubMed  
 Microenvironmental regulation of BRCA1 gene expression by c-Jun and Fra2 in premalignant human ovarian surface epithelial cells. Reduced BRCA1 gene expression is common in the sporadic form of ovarian carcinoma. The spread of this highly lethal cancer often begins when tumor cell clusters are shed into the fluid of the abdominopelvic cavity such that they can float freely before seeding distant sites on the peritoneal walls and organs. Thus, the microenvironment that tumor cells find themselves in changes dramatically during these early shedding and floating stages of transperitoneal   metastasis  . To mimic this microenvironmental change in vitro, we released premalignant human ovarian surface epithelial cells from the substratum and forced them to cluster in suspension. Under these conditions, steady state levels of BRCA1 mRNA and protein fell significantly and the transcriptional activation state of the BRCA1 promoter was suppressed. Analysis of the promoter indicated that the previously identified "CRE" element located within the "positive regulatory region" (PRR) contributed to this suppression. More specifically, we show that the suppression was mediated, at least in part, by a suspension culture-driven decrease in the levels of two members of the AP1 transcription factor complex, c-Jun and Fra2, that bind to the CRE element. Therefore, a microenvironmental change that is manifested during the initial stages of ovarian carcinoma dissemination may, potentially, help suppress BRCA1 expression in sporadic tumors and thus promote their progression. 
 Microenvironmental regulation of BRCA1 gene expression by c-Jun and Fra2 in premalignant human ovarian surface epithelial cells. Reduced BRCA1 gene expression is common in the sporadic form of ovarian carcinoma. The spread of this highly lethal cancer often begins when tumor cell clusters are shed into the fluid of the abdominopelvic cavity such that they can float freely before seeding distant sites on the peritoneal walls and organs. Thus, the microenvironment that tumor cells find themselves in changes dramatically during these early shedding and floating stages of transperitoneal   metastasis  . To mimic this microenvironmental change in vitro, we released premalignant human ovarian surface epithelial cells from the substratum and forced them to cluster in suspension. Under these conditions, steady state levels of BRCA1 mRNA and protein fell significantly and the transcriptional activation state of the BRCA1 promoter was suppressed. Analysis of the promoter indicated that the previously identified "CRE" element located within the "positive regulatory region" (PRR) contributed to this suppression. More specifically, we show that the suppression was mediated, at least in part, by a suspension culture-driven decrease in the levels of two members of the AP1 transcription factor complex, c-Jun and Fra2, that bind to the CRE element. Therefore, a microenvironmental change that is manifested during the initial stages of ovarian carcinoma dissemination may, potentially, help suppress BRCA1 expression in sporadic tumors and thus promote their progression. 
 Role of Fra-2 in breast cancer: influence on tumor cell invasion and motility. Fra-2 (Fos-related antigen 2) is a member of the Fos family of AP-1 transcription factors which is often up-regulated in mammary carcinomas. Previous results suggested that it might be involved in the regulation of breast cancer invasion and   metastasis  . In order to analyze the role of Fra-2 in breast cancer cells, it was silenced in the highly invasive MDA-MB231 cells using RNA interference. On the other hand, stable transfectants of the weakly invasive MCF7 cell line were established in order to analyze the effects of Fra-2 overexpression. In both approaches, cell proliferation was not or only weakly influenced by Fra-2. In contrast, the invasive potential of the cells was increased, and a weaker effect on motility was observed. By cDNA microarray analysis of the MCF7 transfectants followed by validation on a protein level, we identified several Fra-2 target genes which might be involved in cell invasion and migration, i.e., ALCAM and connexin 43. Additionally, mRNA expression levels of various genes which are associated with a more malignant behavior of the tumors in vivo were up- or downregulated, i.e., members of the MAGE family, S100P, TIMP2, IL24 etc. These results show that Fra-2 overexpression is associated with a more aggressive tumor phenotype and is probably involved in breast cancer progression in vivo. 
  [Cc]ell [Ii]nvasion  PubMed  
 Role of Fra-2 in breast cancer: influence on tumor   cell invasion   and motility. Fra-2 (Fos-related antigen 2) is a member of the Fos family of AP-1 transcription factors which is often up-regulated in mammary carcinomas. Previous results suggested that it might be involved in the regulation of breast cancer invasion and metastasis. In order to analyze the role of Fra-2 in breast cancer cells, it was silenced in the highly invasive MDA-MB231 cells using RNA interference. On the other hand, stable transfectants of the weakly invasive MCF7 cell line were established in order to analyze the effects of Fra-2 overexpression. In both approaches, cell proliferation was not or only weakly influenced by Fra-2. In contrast, the invasive potential of the cells was increased, and a weaker effect on motility was observed. By cDNA microarray analysis of the MCF7 transfectants followed by validation on a protein level, we identified several Fra-2 target genes which might be involved in   cell invasion   and migration, i.e., ALCAM and connexin 43. Additionally, mRNA expression levels of various genes which are associated with a more malignant behavior of the tumors in vivo were up- or downregulated, i.e., members of the MAGE family, S100P, TIMP2, IL24 etc. These results show that Fra-2 overexpression is associated with a more aggressive tumor phenotype and is probably involved in breast cancer progression in vivo. 
   
  
 FOSL1 
 
  NCBI Gene  
  GeneRIF  
  Pubmed  
 
 
  Search Term  Rank  Fields  
  [Ff]ilopodia  1  PubMed(1)  
  [Mm]igration  6  PubMed(18); GeneRIFs(2)  
  [Ee]xtracellular [Mm]atrix  8  PubMed(1)  
  [Ii]nvasive  10  PubMed(15); GeneRIFs(1)  
  [Mm]etastasize  11  PubMed(2)  
  [Mm]etastasis  12  PubMed(20); GeneRIFs(3)  
  [Cc]ell [Ii]nvasion  13  PubMed(5)  
 
 
 hide/show details for FOSL1 
  [Ff]ilopodia  PubMed  
 Identification of a pharmacologically tractable Fra-1/ADORA2B axis promoting breast cancer metastasis. Metastasis confronts clinicians with two major challenges: estimating the patient's risk of metastasis and identifying therapeutic targets. Because they are key signal integrators connecting cellular processes to clinical outcome, we aimed to identify transcriptional nodes regulating cancer cell metastasis. Using rodent xenograft models that we previously developed, we identified the transcription factor Fos-related antigen-1 (Fra-1) as a key coordinator of metastasis. Because Fra-1 often is overexpressed in human metastatic breast cancers and has been shown to control their invasive potential in vitro, we aimed to assess the implication and prognostic significance of the Fra-1-dependent genetic program in breast cancer metastasis and to identify potential Fra-1-dependent therapeutic targets. In several in vivo assays in mice, we demonstrate that stable RNAi depletion of Fra-1 from human breast cancer cells strongly suppresses their ability to metastasize. These results support a clinically important role for Fra-1 and the genetic program it controls. We show that a Fra-1-dependent gene-expression signature accurately predicts recurrence of breast cancer. Furthermore, a synthetic lethal drug screen revealed that antagonists of the adenosine receptor A2B (ADORA2B) are preferentially toxic to breast tumor cells expressing Fra-1. Both RNAi silencing and pharmacologic blockade of ADORA2B inhibited   filopodia   formation and invasive activity of breast cancer cells and correspondingly reduced tumor outgrowth in the lungs. These data show that Fra-1 activity is causally involved in and is a prognostic indicator of breast cancer metastasis. They suggest that Fra-1 activity predicts responsiveness to inhibition of pharmacologically tractable targets, such as ADORA2B, which may be used for clinical interference of metastatic breast cancer. 
  [Mm]igration  PubMed  
 Fra-1/AP-1 induces EMT in mammary epithelial cells by modulating Zeb1/2 and TGFβ expression. Epithelial-to-mesenchymal transition (EMT) is essential for embryonic morphogenesis and wound healing and critical for tumour cell invasion and dissemination. The AP-1 transcription factor Fra-1 has been implicated in tumorigenesis and in tumour-associated EMT in human breast cancer. We observed a significant inverse correlation between Fra-1 mRNA expression and distant-metastasis-free survival in a large cohort of breast cancer patients derived from multiple array data sets. This unique correlation among Fos genes prompted us to assess the evolutionary conservation between Fra-1 functions in EMT of human and mouse cells. Ectopic expression of Fra-1 in fully polarized, non-tumourigenic, mouse mammary epithelial EpH4 cells induced a mesenchymal phenotype, characterized by a loss of epithelial and gain of mesenchymal markers. Proliferation, motility and invasiveness were also increased in the resulting EpFra1 cells, and the cells were tumourigenic and efficiently colonized the lung upon transplantation. Molecular analyses revealed increased expression of Tgfβ1 and the EMT-inducing transcription factors Zeb1, Zeb2 and Slug. Mechanistically, Fra-1 binds to the tgfb1 and zeb2 promoters and to an evolutionarily conserved region in the first intron of zeb1. Furthermore, increased activity of a zeb2 promoter reporter was detected in EpFra1 cells and shown to depend on AP-1-binding sites. Inhibiting TGFβ signalling in EpFra1 cells moderately increased the expression of epithelial markers, whereas silencing of zeb1 or zeb2 restored the epithelial phenotype and decreas  ed migrat  ion in vitro and tumorigenesis in vivo. Thus Fra-1 induces changes in the expression of genes encoding EMT-related transcription factors leading to the acquisition of mesenchymal, invasive and tumorigenic capacities by epithelial cells. This study defines a novel function of Fra-1/AP-1 in modulating tgfb1, zeb1 and zeb2 expression through direct binding to genomic regulatory regions, which establishes a basis for future in vivo genetic manipulations and preclinical studies using mouse models. 
 AP-1 (Fra-1/c-Jun)-mediated induction of expression of matrix metalloproteinase-2 is required for 15S-hydroxyeicosatetraenoic acid-induced angiogenesis. To understand the involvement of matrix metalloproteinases (MMPs) in 15(S)-hydroxyeicosatetraenoic acid (15(S)-HETE)-induced angiogenesis, we have studied the role of MMP-2. 15(S)-HETE induced MMP-2 expression and activity in a time-dependent manner in human dermal microvascular endothelial cells (HDMVECs). Inhibition of MMP-2 activity or depletion of its levels attenuated 15(S)-HETE-induced HDMVEC   migration  , tube formation, and Matrigel plug angiogenesis. 15(S)-HETE also induced Fra-1 and c-Jun expression in a Rac1-MEK1-JNK1-dependent manner. In addition, 15(S)-HETE-induced MMP-2 expression and activity were mediated by Rac1-MEK1-JNK1-dependent activation of AP-1 (Fra-1/c-Jun). Cloning and site-directed mutagenesis of MMP-2 promoter revealed that AP-1 site proximal to the transcriptional start site is required for 15(S)-HETE-induced MMP-2 expression, and Fra-1 and c-Jun are the essential components of AP-1 that bind to MMP-2 promoter in response to 15(S)-HETE. Hind limb ischemia led to an increase in MEK1 and JNK1 activation and Fra-1, c-Jun, and MMP-2 expression resulting in enhanced neovascularization and recovery of blood perfusion in wild-type mice as compared with 12/15-Lox(-/-) mice. Together, these results provide the first direct evidence for a role of 12/15-Lox-12/15(S)-HETE axis in the regulation of ischemia-induced angiogenesis. 
 Fra-1/AP-1 induces EMT in mammary epithelial cells by modulating Zeb1/2 and TGFβ expression. Epithelial-to-mesenchymal transition (EMT) is essential for embryonic morphogenesis and wound healing and critical for tumour cell invasion and dissemination. The AP-1 transcription factor Fra-1 has been implicated in tumorigenesis and in tumour-associated EMT in human breast cancer. We observed a significant inverse correlation between Fra-1 mRNA expression and distant-metastasis-free survival in a large cohort of breast cancer patients derived from multiple array data sets. This unique correlation among Fos genes prompted us to assess the evolutionary conservation between Fra-1 functions in EMT of human and mouse cells. Ectopic expression of Fra-1 in fully polarized, non-tumourigenic, mouse mammary epithelial EpH4 cells induced a mesenchymal phenotype, characterized by a loss of epithelial and gain of mesenchymal markers. Proliferation, motility and invasiveness were also increased in the resulting EpFra1 cells, and the cells were tumourigenic and efficiently colonized the lung upon transplantation. Molecular analyses revealed increased expression of Tgfβ1 and the EMT-inducing transcription factors Zeb1, Zeb2 and Slug. Mechanistically, Fra-1 binds to the tgfb1 and zeb2 promoters and to an evolutionarily conserved region in the first intron of zeb1. Furthermore, increased activity of a zeb2 promoter reporter was detected in EpFra1 cells and shown to depend on AP-1-binding sites. Inhibiting TGFβ signalling in EpFra1 cells moderately increased the expression of epithelial markers, whereas silencing of zeb1 or zeb2 restored the epithelial phenotype and decreas  ed migrat  ion in vitro and tumorigenesis in vivo. Thus Fra-1 induces changes in the expression of genes encoding EMT-related transcription factors leading to the acquisition of mesenchymal, invasive and tumorigenic capacities by epithelial cells. This study defines a novel function of Fra-1/AP-1 in modulating tgfb1, zeb1 and zeb2 expression through direct binding to genomic regulatory regions, which establishes a basis for future in vivo genetic manipulations and preclinical studies using mouse models. 
 AP-1 (Fra-1/c-Jun)-mediated induction of expression of matrix metalloproteinase-2 is required for 15S-hydroxyeicosatetraenoic acid-induced angiogenesis. To understand the involvement of matrix metalloproteinases (MMPs) in 15(S)-hydroxyeicosatetraenoic acid (15(S)-HETE)-induced angiogenesis, we have studied the role of MMP-2. 15(S)-HETE induced MMP-2 expression and activity in a time-dependent manner in human dermal microvascular endothelial cells (HDMVECs). Inhibition of MMP-2 activity or depletion of its levels attenuated 15(S)-HETE-induced HDMVEC   migration  , tube formation, and Matrigel plug angiogenesis. 15(S)-HETE also induced Fra-1 and c-Jun expression in a Rac1-MEK1-JNK1-dependent manner. In addition, 15(S)-HETE-induced MMP-2 expression and activity were mediated by Rac1-MEK1-JNK1-dependent activation of AP-1 (Fra-1/c-Jun). Cloning and site-directed mutagenesis of MMP-2 promoter revealed that AP-1 site proximal to the transcriptional start site is required for 15(S)-HETE-induced MMP-2 expression, and Fra-1 and c-Jun are the essential components of AP-1 that bind to MMP-2 promoter in response to 15(S)-HETE. Hind limb ischemia led to an increase in MEK1 and JNK1 activation and Fra-1, c-Jun, and MMP-2 expression resulting in enhanced neovascularization and recovery of blood perfusion in wild-type mice as compared with 12/15-Lox(-/-) mice. Together, these results provide the first direct evidence for a role of 12/15-Lox-12/15(S)-HETE axis in the regulation of ischemia-induced angiogenesis. 
 Fos-related antigen 1 (Fra-1) pairing with and transactivation of JunB in GBM cells. Fos-related antigen 1 (Fra-1) plays an important role in maintenance/progression of various cancers, including glioblastoma multiforme (GBM). In this study, we used both shRNA and siRNA to examine the effect of fra-1 knockdown in GBM cells over-expressing Fra-1. Furthermore, we analyzed both the expression of JunB and its knockdown, a previously identified target for Fra-1, and also examined its potential association with Fra-1. When using fra-1 shRNA and siRNA, we found that GBM cells has Fra-1 levels diminished together with the levels of JunB, but Fra-1 remains unchanged in cells with junB knockdown. This is accompanied by dramatic changes in cell morphology and significant alteration in their   migration  . We next uncovered that the expression of JunB increased in response to ectopic Fra-1 and also to EGF-induced signaling, similarly to Fra-1. This was associated with an avid pairing between phosphorylated Fra-1 and JunB. Importantly, we found that Fra-1 paired with JunB binds to an AP-1 site in the junB gene promoter. JunB knockdown did not affect Fra-1 and the changes in cell morphology did not fully replicate that seen with Fra-1 knockdown. Thus, Fra-1 takes part in a control of architecture and migratory nature of GBM cells. Moreover, Fra-1 is a phosphorylated factor that transactivates JunB with which it makes effectively AP-1 pairs in GBM cells. 
 TRPS1 targeting by miR-221/222 promotes the epithelial-to-mesenchymal transition in breast cancer. The basal-like subtype of breast cancer has an aggressive clinical behavior compared to that of the luminal subtype. We identified the microRNAs (miRNAs) miR-221 and miR-222 (miR-221/222) as basal-like subtype-specific miRNAs and showed that expression of miR-221/222 decreased expression of epithelial-specific genes and increased expression of mesenchymal-specific genes, and increased cell   migration   and invasion in a manner characteristic of the epithelial-to-mesenchymal transition (EMT). The transcription factor FOSL1 (also known as Fra-1), which is found in basal-like breast cancers but not in the luminal subtype, stimulated the transcription of miR-221/222, and the abundance of these miRNAs decreased with inhibition of the epidermal growth factor receptor (EGFR) or MEK (mitogen-activated or extracellular signal-regulated protein kinase kinase), placing miR-221/222 downstream of the RAS pathway. Furthermore, miR-221/222-mediated reduction in E-cadherin abundance depended on their targeting the 3' untranslated region of the GATA family transcriptional repressor TRPS1 (tricho-rhino-phalangeal syndrome type 1), which inhibited EMT by decreasing ZEB2 (zinc finger E-box-binding homeobox2) expression. We conclude that by promoting EMT, miR-221/222 may contribute to the more aggressive clinical behavior of basal-like breast cancers. 
 MicroRNA-34a inhibits   migration   and invasion of colon cancer cells via targeting to Fra-1. MicroRNA-34a (miR-34a), a transcriptional target of p53, is a well-known tumor suppressor gene. Here, we identified Fra-1 as a new target of miR-34a and demonstrated that miR-34a inhibits Fra-1 expression at both protein and messenger RNA levels. In addition, we found that p53 indirectly regulates Fra-1 expression via a miR-34a-dependant manner in colon cancer cells. Overexpression of miR-34a strongly inhibited colon cancer cell   migration   and invasion, which can be partially rescued by forced expression of the Fra-1 transcript lacking the 3'-untranslated region. The expression of matrix metalloproteinase (MMP)-1 and MMP-9, two enzymes involved in cell   migration   and invasion, was decreased in miR-34a-transfected cells, and this can be rescued by Fra-1 overexpression. Moreover, we found that miR-34a was downregulated in 25 of 40 (62.5%) colon cancer tissues, as compared with the adjacent normal colon tissues and that the expression of miR-34a was correlated with the DNA-binding activity of p53. Unexpectedly, the DNA-binding activity of p53 was not inversely correlated with Fra-1 expression, and a significant statistical inverse correlation between miR-34a and Fra-1 expression was only observed in 14 of 40 (35%) colon cancer tissues. Taken together, our in vitro data suggest that p53 regulates Fra-1 expression, and eventually cell   migration  /invasion, via a miR-34a-dependent manner. However, in vivo data indicate that the p53-miR-34a pathway is not the major regulator of Fra-1 expression in human colon cancer tissues. 
 Role of the AP-1 transcription factor FOSL1 in endothelial cells adhesion and   migration  . Vasculogenesis and angiogenesis, the fundamental processes by which new blood vessels are formed, involve the proliferation,   migration  , and remodeling of endothelial cells. Dynamic adhesion of endothelial cells to extracellular matrix plays a fundamental role in all these events. Key regulators of endothelial cells adhesion and   migration   are the αvβ3 and uPA-uPAR complexes. The αvβ3 integrin heterodimer is the receptor for extracellular matrix components such as vitronectin and is overexpressed on the cell surface of angiogenic endothelial cells, but not quiescent cells lining normal vessels. The uPA-uPAR complex contributes to extracellular matrix remodeling by mediating proteolytic activity at the leading edge of migrating cells. We recently reported that the FOSL1 transcription factor of the AP-1 family plays a pivotal role in the regulation of the level of the αvβ3 and uPA-uPAR complexes on the surface of endothelial cells. In this commentary, we review the current knowledge of αv and β3 transcriptional regulation in endothelial cells and discuss the role of FOSL1 in angiogenesis. 
 Fra-1 regulates vimentin during Ha-RAS-induced epithelial mesenchymal transition in human colon carcinoma cells. The process of epithelial mesenchymal transition, whereby cells acquire molecular alterations and fibroblastic features, is a fundamental process of embryogenesis and cancer invasion/metastasis. The mechanisms responsible for epithelial mesenchymal transition remain elusive. Human tumors frequently establish constitutively activated RAS signaling, which contributes to the malignant phenotype. In an effort to dissect distinct RAS isoform specific functions, we previously established human colon cell lines stably overexpressing activated Harvey-RAS (Ha-RAS) and Kirsten-RAS (Ki-RAS). Using these, we observed that only oncogenic Ha-RAS overexpression resulted in morphologic and molecular changes suggestive of epithelial to mesenchymal transition. We showed that vimentin, a key molecule of epithelial mesenchymal transition, was differentially regulated between Ha-RAS and Ki-RAS leading to a Ha-RAS specific induction of a migratory phenotype and eventually epithelial to mesenchymal transition. We demonstrated that the AP-1 sites in vimentin promoter could be involved in this regulation. A potential role of FRA-1 was suggested in the regulation of vimentin during the Ha-RAS-induced epithelial to mesenchymal transition, in association with colon cell   migration  . Our results therefore propose that in colon cells, the induction of epithelial mesenchymal transition by oncogenic Ha-RAS could occur through the overexpression of proteins like FRA-1 and vimentin. 
 The PKCθ pathway participates in the aberrant accumulation of Fra-1 protein in invasive ER-negative breast cancer cells. Fra-1 is aberrantly expressed in a large number of cancer cells and tissues, and emerging evidence suggests an important role for this Fos family protein in both oncogenesis and the progression or maintenance of many tumour types. Here, we show that the concentration of Fra-1 is high in invasive oestrogen receptor (ER)-negative (ER-) breast cancer cell lines, regardless of their Ras pathway status. All of the ER- cells express high levels of activated PKCθ, and the inhibition of PKCθ activity using RNA interference or the expression of a dominant-negative mutant results in a dramatic reduction in Fra-1 abundance. Conversely, the ectopic expression of constitutively active PKCθ leads to Fra-1 phosphorylation and accumulation in poorly invasive ER+ cells. This accumulation is due to the stabilisation of the Fra-1 protein through PKCθ signalling, whereas other members of the PKC family are ineffective. Both Ste20-related proline-alanine-rich kinase (SPAK) and ERK1/2, whose activities are upregulated by PKCθ, participate in PKCθ-driven Fra-1 stabilisation. Interestingly, their relative contributions appear to be different depending on the cell line studied. ERK1/2 signalling has a major role in ER- MDA-MB-231 cells, whereas Fra-1 accumulation occurs mainly through SPAK signalling in ER- BT549 cells. Fra-1 mutational analysis shows that the phosphorylation of S265, T223 and T230 is critical for PKCθ-driven Fra-1 stabilisation. Phosphorylation of the protein was confirmed using specific antisera against Fra-1 phosphorylated on T223 or S265. In addition, Fra-1 participates in PKCθ-induced cell invasion and is necessary for PKCθ-ind  uced cell   migration. In summary, we identified PKCθ signalling as an important regulator of Fra-1 accumulation in ER- breast cancer cells. Moreover, our results suggest that PKCθ could participate in progression of some breast cancers and could be a new therapeutic target. 
 Aberrantly expressed Fra-1 by IL-6/STAT3 transactivation promotes colorectal cancer aggressiveness through epithelial-mesenchymal transition. The pro-inflammatory cytokine interleukin-6 (IL-6) in tumor microenvironment has been suggested to promote development and progression of colorectal cancer (CRC). However, the underlying molecular mechanisms remain elusive. In this study, we demonstrate that fos-related antigen-1 (Fra-1) plays a critical role in IL-6 induced CRC aggressiveness and epithelial-mesenchymal transition (EMT). In CRC cell lines, the expression of Fra-1 gene was found significantly upregulated during IL-6-driven EMT process. The Fra-1 induction occurred at transcriptional level in a manner dependent on signal transducer and activator of transcription 3 (STAT3), during which both phosphorylated and acetylated post-translational modifications were required for STAT3 activation to directly bind to the Fra-1 promoter. Importantly, RNA interference-based attenuation of either STAT3 or Fra-1 prevented IL-6-induced EMT, cell   migration   and invasion, whereas ectopic expression of Fra-1 markedly reversed the STAT3-knockdown effect and enhanced CRC cell aggressiveness by regulating the expression of EMT-promoting factors (ZEB1, Snail, Slug, MMP-2 and MMP-9). Furthermore, Fra-1 levels were positively correlated with the local invasion depth as well as lymph node and liver metastasis in a total of 229 CRC patients. Intense immunohistochemical staining of Fra-1 was observed at the tumor marginal area adjacent to inflammatory cells and in parallel with IL-6 secretion and STAT3 activation in CRC tissues. Together, this study proposes the existence of an aberrant IL-6/STAT3/Fra-1 signaling axis leading to CRC aggressiveness through EMT induction, which suggests novel therapeutic opportunities for the malignant disease. 
 MicroRNA-34 suppresses breast cancer invasion and metastasis by directly targeting Fra-1. MicroRNAs have key roles in tumor metastasis. Here, we describe the regulation and function of miR-34a and miR-34c (miR-34a/c) in breast cancer metastasis. Expression analysis verified that miR-34a/c expression is significantly decreased in metastatic breast cancer cells and human primary breast tumors with lymph node metastases. Overexpression of miR-34a/c could inhibit breast cancer cell   migration   and invasion in vitro and distal pulmonary metastasis in vivo. Further studies revealed that Fos-related antigen 1 (Fra-1 or Fosl1) is a downstream target of miR-34a/c as miR-34a/c bound directly to the 3'untranslated region of Fra-1, subsequently reducing both the mRNA and protein levels of Fra-1. Silencing of Fra-1 recapitulated the effects of miR-34a/c overexpression, whereas enforced expression of Fra-1 reverses the suppressive effects of miR-34a/c. Moreover, significant downregulation of miR-34a in metastatic breast cancer tissues was found to be inversely correlated with Fra-1 expression. Our results demonstrate that miR-34a/c functions as a metastasis suppressor to regulate breast cancer   migration   and invasion through targeting Fra-1 oncogene and suggest a therapeutic application of miR-34 in breast cancer. 
 CD44 affects the expression level of FOS‑like antigen 1 in cervical cancer tissues. Cervical carcinoma is the second most prevalent type of malignancy in females worldwide. The crucial etiological factors involved in the development of cervical carcinoma include infection with the papillomavirus, and the structural or functional mutation of oncogenes and tumor suppressor genes. CD44 refers to a multifunctional family of type I transmembrane proteins. These proteins have been implicated in numerous biological processes, including cell adhesion, cel  l migrati  on and metastasis. The present study examined the differences in the expression levels of ATP-binding cassette sub-family G member 2, CD24, CD44, CD133, cytokeratin (CK) 14 and CK19 between cervical cancer tissues and corresponding normal non-tumor tissues by flow cytometry. Then, the CD44+ or CD44‑ cells from cervical cancer tissues were sorted for identification and confirmation of differential expression by flow cytometry. The results demonstrated that the expression level of CD44 in cervical cancer tissues was higher than in the corresponding non-tumor normal tissues (t=3.12; P=0.0102). Compared with the CD44‑ cells, the FOS-like antigen 1 (Fra-1), nestin, nuclear receptor subfamily 4, group A, member 2, OCT4 and p63 genes were highly expressed in CD44+ cells. The fold changes were 3.55, 3.55, 2.46, 2.87 and 2.56, respectively (P<0.05). However, BMI1 polycomb ring finger oncogene, ck5, tumor protein p53 and lactotransferrin genes exhibited low expression levels in CD44+ cells. It was verified by western blot analysis and flow cytometry that Fra-1 was highly expressed in CD44+ cells. Fra-1 was a potential target of miR-19a and miR-19b. The expression of miR-19a and miR-19b was downregulated by ~50% in CD44+ cells compared with CD44‑ cells. These findings suggested that CD44 dysregulated the activation of the Fra‑1 gene. The interaction of Fra-1 and CD44 may therefore be important in cervical carcinoma. 
 MicroRNA-195-5p, a new regulator of Fra-1, suppresses the   migration   and invasion of prostate cancer cells. An increasing number of studies have demonstrated that deregulation of microRNAs (miRNAs) was a common event in tumor tissues and miRNAs would be treated as ideal tumor biomarkers or therapeutic targets. miR-195-5p (termed as miR-195 for briefly in the following part) was suggested to function as a tumor suppressor in cancer development and progression. However, the roles of miR-195 in human prostate cancer are still elusive. Thus, this study was performed to investigate the biological functions and its molecular mechanisms of miR-195 in human prostate cancer cell lines, discussing whether it has a potential to be a therapeutic way of prostate cancer.Two human prostate cancer cell lines were analyzed for the expression of miR-195 by quantitative real-time reverse transcription-polymerase chain reaction (RT-PCR). A gain-of-function study of miR-195 was conducted by transfecting mimics into DU145 and PC3 cells and cell motility and invasion ability were evaluated by wound healing assay and transwell assay. Tissue microarray, and immunohistochemistry with antibodies against Fra-1 was performed using the peroxidase and DAB methods. The target gene of miR-195 was determined by luciferase assay, quantitative RT-PCR and western blot. The regulation of motility by miR-195 was analyzed by western blot.miR-195 was frequently down-regulated in both prostate cancer cell lines, DU145 and PC3. Overexpression of miR-195 significantly repressed the capability of   migration   and invasion of prostate cancer cells. In addition, we identified Fra-1, a cell motility regulator, as a novel target of miR-195. Fra-1 was up-regulated in prostate cancer tissues. We also observed that inhibition of miR-195 or restoration of Fra-1 in miR-195-over-expressed prostate cancer cells partially reversed the suppressive effects of miR-195. Furthermore, we demonstrated miR-195 could inhibit prostate cancer cell motility by regulated the expression of c-Met, MMP1, MMP9.miR-195 can repress the   migration   and invasion of prostate cancer cells via regulating Fra-1. Our results indicate that miR-195 could be a tumor suppressor and may have a potential to be a diagnostics or therapeutic target in prostate cancer. 
 Fra-1 governs cell   migration   via modulation of CD44 expression in human mesotheliomas. Silencing of Fra-1, a component of the dimeric transcription factor, activator protein-1 (AP-1), inhibits mRNA expression of c-met and cd44 in rat mesothelioma cells and is causally linked to maintenance of the transformed phenotype. However, the mechanisms of Fra-1 regulation and Fra-1 regulated gene expression in human malignant mesothelioma (MM) are unclear. We first show in a panel of human MM cells that Fra-1 mRNA expression in MM is complex and regulated by extracellular signal-regulated kinase (ERK1, ERK2), Src, and phosphatidyl-inositol-3-kinase (PI3K) pathways in a tumor-specific fashion. Cell lines with PI3K-dependent Fra-1 expression were SV40 positive and expressed the lowest basal Fra-1 levels. Levels of Fra-1 expression correlated with amounts of CD44 expression that were greater in simian virus 40 negative (SV40-) MM cells. Using dominant negative (dn), short hairpin (sh) and small interference (si) RNA constructs, we next demonstrate that expression of CD44, the principal hyaluronic receptor in MMs, correlates with Fra-expression in both simian virus 40 positive (SV40+) and SV40- MMs. Moreover, both Fra-1 and CD44 expression are linked to cell   migration   in SV40- MM cells. Lastly, in contrast to normal lung tissue, tissue microarrays revealed that Fra-1 was expressed in 33 of 34 human MMs, and that all CD44+ tumors were SV40-. These results suggest that Fra-1 is associated with cell   migration   in human MMs and that Fra-1 modulation of CD44 may govern   migration   of selected MMs. 
 SIRT1 promotes epithelial-mesenchymal transition and metastasis in colorectal cancer by regulating Fra-1 expression. Understanding molecular mechanisms of colorectal cancer (CRC) metastasis is urgently required for targeted therapy and prognosis of metastatic CRC. In this study, we explored potential effects of silent mating type information regulation 2 homolog 1 (SIRT1) on CRC metastasis. Our data showed that ectopic expression of SIRT1 markedly increased the   migration   and invasion of CRC cells. In contrast, silencing SIRT1 repressed this behavior in aggressive CRC cells. Tumor xenograft experiments revealed that knockdown of SIRT1 impaired CRC metastasis in vivo. Silencing SIRT1 in CRC cells induced mesenchymal-epithelial transition (MET), which is the reverse process of epithelial-mesenchymal transition (EMT) and characterized by a gain of epithelial and loss of mesenchymal markers. We provided a mechanistic insight toward regulation of Fra-1 by SIRT1 and demonstrated a direct link between the SIRT1-Fra-1 axis and EMT. Moreover, SIRT1 expression correlated positively with Fra-1 expression, metastasis and overall survival in patients with CRC. Taken together, our data provide a novel mechanistic role of SIRT1 in CRC metastasis, suggesting that SIRT1 may serve as a potential therapeutic target for metastatic CRC. 
 Gene expression analysis after receptor tyrosine kinase activation reveals new potential melanoma proteins. Melanoma is an aggressive tumor with increasing incidence. To develop accurate prognostic markers and targeted therapies, changes leading to malignant transformation of melanocytes need to be understood. In the Xiphophorus melanoma model system, a mutated version of the EGF receptor Xmrk (Xiphophorus melanoma receptor kinase) triggers melanomagenesis. Cellular events downstream of Xmrk, such as the activation of Akt, Ras, B-Raf or Stat5, were also shown to play a role in human melanomagenesis. This makes the elucidation of Xmrk downstream targets a useful method for identifying processes involved in melanoma formation.Here, we analyzed Xmrk-induced gene expression using a microarray approach. Several highly expressed genes were confirmed by realtime PCR, and pathways responsible for their induction were revealed using small molecule inhibitors. The expression of these genes was also monitored in human melanoma cell lines, and the target gene FOSL1 was knocked down by siRNA. Proliferation and   migration   of siRNA-treated melanoma cell lines were then investigated.Genes with the strongest upregulation after receptor activation were FOS-like antigen 1 (Fosl1), early growth response 1 (Egr1), osteopontin (Opn), insulin-like growth factor binding protein 3 (Igfbp3), dual-specificity phosphatase 4 (Dusp4), and tumor-associated antigen L6 (Taal6). Interestingly, most genes were blocked in presence of a SRC kinase inhibitor. Importantly, we found that FOSL1, OPN, IGFBP3, DUSP4, and TAAL6 also exhibited increased expression levels in human melanoma cell lines compared to human melanocytes. Knockdown of FOSL1 in human melanoma cell lines reduced their proliferation and   migration  .Altogether, the data show that the receptor tyrosine kinase Xmrk is a useful tool in the identification of target genes that are commonly expressed in Xmrk-transgenic melanocytes and melanoma cell lines. The identified molecules constitute new possible molecular players in melanoma development. Specifically, a role of FOSL1 in melanomagenic processes is demonstrated. These data are the basis for future detailed analyses of the investigated target genes. 
 Widespread FRA1-dependent control of mesenchymal transdifferentiation programs in colorectal cancer cells. Tumor invasion and metastasis involves complex remodeling of gene expression programs governing epithelial homeostasis. Mutational activation of the RAS-ERK is a frequent occurrence in many cancers and has been shown to drive overexpression of the AP-1 family transcription factor FRA1, a potent regulator of   migration   and invasion in a variety of tumor cell types. However, the nature of FRA1 transcriptional targets and the molecular pathways through which they promote tumor progression remain poorly understood. We found that FRA1 was strongly expressed in tumor cells at the invasive front of human colorectal cancers (CRCs), and that its depletion suppressed mesenchymal-like features in CRC cells in vitro. Genome-wide analysis of FRA1 chromatin occupancy and transcriptional regulation identified epithelial-mesenchymal transition (EMT)-related genes as a major class of direct FRA1 targets in CRC cells. Expression of the pro-mesenchymal subset of these genes predicted adverse outcomes in CRC patients, and involved FRA-1-dependent regulation and cooperation with TGFβ signaling pathway. Our findings reveal an unexpectedly widespread and direct role for FRA1 in control of epithelial-mesenchymal plasticity in CRC cells, and suggest that FRA1 plays an important role in mediating cross talk between oncogenic RAS-ERK and TGFβ signaling networks during tumor progression. 
  GeneRIFs  
 miR-195 can repress the   migration   and invasion of prostate cancer cells via regulating Fra-1 
 Fra-1 is associated with cell   migration   in human MMs and that Fra-1 modulation of CD44 may govern   migration   of selected MMs. 
  [Ee]xtracellular [Mm]atrix  PubMed  
 Role of the AP-1 transcription factor FOSL1 in endothelial cells adhesion and migration. Vasculogenesis and angiogenesis, the fundamental processes by which new blood vessels are formed, involve the proliferation, migration, and remodeling of endothelial cells. Dynamic adhesion of endothelial cells to   extracellular matrix   plays a fundamental role in all these events. Key regulators of endothelial cells adhesion and migration are the αvβ3 and uPA-uPAR complexes. The αvβ3 integrin heterodimer is the receptor   for extracellular ma  trix components such as vitronectin and is overexpressed on the cell surface of angiogenic endothelial cells, but not quiescent cells lining normal vessels. The uPA-uPAR complex contributes   to extracellular ma  trix remodeling by mediating proteolytic activity at the leading edge of migrating cells. We recently reported that the FOSL1 transcription factor of the AP-1 family plays a pivotal role in the regulation of the level of the αvβ3 and uPA-uPAR complexes on the surface of endothelial cells. In this commentary, we review the current knowledge of αv and β3 transcriptional regulation in endothelial cells and discuss the role of FOSL1 in angiogenesis. 
  [Ii]nvasive  PubMed  
 Fra-1/AP-1 induces EMT in mammary epithelial cells by modulating Zeb1/2 and TGFβ expression. Epithelial-to-mesenchymal transition (EMT) is essential for embryonic morphogenesis and wound healing and critical for tumour cell invasion and dissemination. The AP-1 transcription factor Fra-1 has been implicated in tumorigenesis and in tumour-associated EMT in human breast cancer. We observed a significant inverse correlation between Fra-1 mRNA expression and distant-metastasis-free survival in a large cohort of breast cancer patients derived from multiple array data sets. This unique correlation among Fos genes prompted us to assess the evolutionary conservation between Fra-1 functions in EMT of human and mouse cells. Ectopic expression of Fra-1 in fully polarized, non-tumourigenic, mouse mammary epithelial EpH4 cells induced a mesenchymal phenotype, characterized by a loss of epithelial and gain of mesenchymal markers. Proliferation, motility and   invasiv  eness were also increased in the resulting EpFra1 cells, and the cells were tumourigenic and efficiently colonized the lung upon transplantation. Molecular analyses revealed increased expression of Tgfβ1 and the EMT-inducing transcription factors Zeb1, Zeb2 and Slug. Mechanistically, Fra-1 binds to the tgfb1 and zeb2 promoters and to an evolutionarily conserved region in the first intron of zeb1. Furthermore, increased activity of a zeb2 promoter reporter was detected in EpFra1 cells and shown to depend on AP-1-binding sites. Inhibiting TGFβ signalling in EpFra1 cells moderately increased the expression of epithelial markers, whereas silencing of zeb1 or zeb2 restored the epithelial phenotype and decreased migration in vitro and tumorigenesis in vivo. Thus Fra-1 induces changes in the expression of genes encoding EMT-related transcription factors leading to the acquisition of mesenchyma  l, invas  ive and tumorigenic capacities by epithelial cells. This study defines a novel function of Fra-1/AP-1 in modulating tgfb1, zeb1 and zeb2 expression through direct binding to genomic regulatory regions, which establishes a basis for future in vivo genetic manipulations and preclinical studies using mouse models. 
 In situ proximity ligation detection of c-Jun/AP-1 dimers reveals increased levels of c-Jun/Fra1 complexes in aggressive breast cancer cell lines in vitro and in vivo. Genetic and biochemical studies have shown that selective interactions between the Jun, Fos, and activating transcription factor (ATF) components of transcription factor activating protein 1 (AP-1) exhibit specific and critical functions in the regulation of cell proliferation, differentiation, and survival. For instance, the ratio between c-Jun/c-Fos and c-Jun/ATF2 dimers in the cell can be a determining factor in the cellular response to oncogenic or apoptotic stimuli. Until recently, no methods were available to detect endogenous AP-1 complexes in cells and tissues in situ. Here, we validated the proximity ligation assay (PLA) for its ability to specifically visualize and quantify changes in endogenous c-Jun/c-Fos, c-Jun/ATF2, and c-Jun/Fra1 complexes by using, among others, partner-selective c-Jun mutants. Furthermore, we examined the levels of c-Jun/AP-1 dimers in cell lines representing different types of human breast cancer and found that aggressive basal-like breast cancer cells can be discriminated from much less   invasive   luminal-like cells by PLA detection of c-Jun/Fra1 rather than of c-Jun/ATF2 and c-Jun/c-Fos. Also in tumor tissue derived from highly metastatic basal-like MDA-MB231 cells, high levels of c-Jun/Fra1 complexes were detected. Together, these results demonstrate that in situ PLA is a powerful diagnostic tool to analyze and quantify the amounts of biologically critical AP-1 dimers in fixed cells and tissue material. 
 Phosphorylated c-Jun and Fra-1 induce matrix metalloproteinase-1 and thereby regulate invasion activity of 143B osteosarcoma cells. Osteosarcoma is the most common primary malignancy of bone and patients often develop pulmonary metastases. Despite the advances in surgical and medical management, the mechanisms underlying human osteosarcoma progression and metastasis remain to be elucidated. Gene expression profiles were compared by the cDNA microarray technique between two different human osteosarcoma sublines, MNNG/HOS and 143B, which differ greatly in spontaneous pulmonary metastatic potential. Here we report an enhanced expression of matrix metalloproteinase (MMP)-1 in the highly metastatic human osteosarcoma cell line 143B. Moreover, the in vitro invasion activity of 143B cells was MMP-1-dependent. The activator protein (AP)-1 binding site in the MMP-1 gene promoter was required for the constitutive expression of MMP-1 in 143B cells. Two AP-1 components, c-Jun and Fra-1, were phosphorylated, and bound to the AP-1 binding site of the MMP-1 promoter in 143B cells. Activated c-Jun and Fra-1 were essential for MMP-1 gene expression in 143B cells. Mitogen-activated protein kinase pathways including the c-Jun NH(2)-terminal kinase and the extracellular signal-regulated kinase activate c-Jun and Fra-1 and thereby regulate c-Jun/Fra-1 mediated events, establishing the mitogen-activated protein kinase/AP-1/MMP-1 axis as important in 143B cells. These data suggest that MMP-1 plays a central role in osteosarcoma invasion. Accordingly, MMP-1 might be a biomarker and therapeutic target for   invasive   osteosarcomas and pulmonary metastases. 
 Fra-1/AP-1 induces EMT in mammary epithelial cells by modulating Zeb1/2 and TGFβ expression. Epithelial-to-mesenchymal transition (EMT) is essential for embryonic morphogenesis and wound healing and critical for tumour cell invasion and dissemination. The AP-1 transcription factor Fra-1 has been implicated in tumorigenesis and in tumour-associated EMT in human breast cancer. We observed a significant inverse correlation between Fra-1 mRNA expression and distant-metastasis-free survival in a large cohort of breast cancer patients derived from multiple array data sets. This unique correlation among Fos genes prompted us to assess the evolutionary conservation between Fra-1 functions in EMT of human and mouse cells. Ectopic expression of Fra-1 in fully polarized, non-tumourigenic, mouse mammary epithelial EpH4 cells induced a mesenchymal phenotype, characterized by a loss of epithelial and gain of mesenchymal markers. Proliferation, motility and   invasiv  eness were also increased in the resulting EpFra1 cells, and the cells were tumourigenic and efficiently colonized the lung upon transplantation. Molecular analyses revealed increased expression of Tgfβ1 and the EMT-inducing transcription factors Zeb1, Zeb2 and Slug. Mechanistically, Fra-1 binds to the tgfb1 and zeb2 promoters and to an evolutionarily conserved region in the first intron of zeb1. Furthermore, increased activity of a zeb2 promoter reporter was detected in EpFra1 cells and shown to depend on AP-1-binding sites. Inhibiting TGFβ signalling in EpFra1 cells moderately increased the expression of epithelial markers, whereas silencing of zeb1 or zeb2 restored the epithelial phenotype and decreased migration in vitro and tumorigenesis in vivo. Thus Fra-1 induces changes in the expression of genes encoding EMT-related transcription factors leading to the acquisition of mesenchyma  l, invas  ive and tumorigenic capacities by epithelial cells. This study defines a novel function of Fra-1/AP-1 in modulating tgfb1, zeb1 and zeb2 expression through direct binding to genomic regulatory regions, which establishes a basis for future in vivo genetic manipulations and preclinical studies using mouse models. 
 Phosphorylated c-Jun and Fra-1 induce matrix metalloproteinase-1 and thereby regulate invasion activity of 143B osteosarcoma cells. Osteosarcoma is the most common primary malignancy of bone and patients often develop pulmonary metastases. Despite the advances in surgical and medical management, the mechanisms underlying human osteosarcoma progression and metastasis remain to be elucidated. Gene expression profiles were compared by the cDNA microarray technique between two different human osteosarcoma sublines, MNNG/HOS and 143B, which differ greatly in spontaneous pulmonary metastatic potential. Here we report an enhanced expression of matrix metalloproteinase (MMP)-1 in the highly metastatic human osteosarcoma cell line 143B. Moreover, the in vitro invasion activity of 143B cells was MMP-1-dependent. The activator protein (AP)-1 binding site in the MMP-1 gene promoter was required for the constitutive expression of MMP-1 in 143B cells. Two AP-1 components, c-Jun and Fra-1, were phosphorylated, and bound to the AP-1 binding site of the MMP-1 promoter in 143B cells. Activated c-Jun and Fra-1 were essential for MMP-1 gene expression in 143B cells. Mitogen-activated protein kinase pathways including the c-Jun NH(2)-terminal kinase and the extracellular signal-regulated kinase activate c-Jun and Fra-1 and thereby regulate c-Jun/Fra-1 mediated events, establishing the mitogen-activated protein kinase/AP-1/MMP-1 axis as important in 143B cells. These data suggest that MMP-1 plays a central role in osteosarcoma invasion. Accordingly, MMP-1 might be a biomarker and therapeutic target for   invasive   osteosarcomas and pulmonary metastases. 
 In situ proximity ligation detection of c-Jun/AP-1 dimers reveals increased levels of c-Jun/Fra1 complexes in aggressive breast cancer cell lines in vitro and in vivo. Genetic and biochemical studies have shown that selective interactions between the Jun, Fos, and activating transcription factor (ATF) components of transcription factor activating protein 1 (AP-1) exhibit specific and critical functions in the regulation of cell proliferation, differentiation, and survival. For instance, the ratio between c-Jun/c-Fos and c-Jun/ATF2 dimers in the cell can be a determining factor in the cellular response to oncogenic or apoptotic stimuli. Until recently, no methods were available to detect endogenous AP-1 complexes in cells and tissues in situ. Here, we validated the proximity ligation assay (PLA) for its ability to specifically visualize and quantify changes in endogenous c-Jun/c-Fos, c-Jun/ATF2, and c-Jun/Fra1 complexes by using, among others, partner-selective c-Jun mutants. Furthermore, we examined the levels of c-Jun/AP-1 dimers in cell lines representing different types of human breast cancer and found that aggressive basal-like breast cancer cells can be discriminated from much less   invasive   luminal-like cells by PLA detection of c-Jun/Fra1 rather than of c-Jun/ATF2 and c-Jun/c-Fos. Also in tumor tissue derived from highly metastatic basal-like MDA-MB231 cells, high levels of c-Jun/Fra1 complexes were detected. Together, these results demonstrate that in situ PLA is a powerful diagnostic tool to analyze and quantify the amounts of biologically critical AP-1 dimers in fixed cells and tissue material. 
 The molecular role of Fra-1 and its prognostic significance in human esophageal squamous cell carcinoma. The expression of Fra-1 (Fos related antigen 1) involves tumor progression and invasion, and its gene ablation could suppress the   invasive   phenotypes of human tumor cells. The authors investigated the significance of Fra-1 expression in esophageal squamous cell carcinoma (ESCC) and studied the effect of its down-regulation on cell proliferation, motility, and invasion.Surgical specimens from 164 patients with ESCC were evaluated. Fra-1 expression in the primary tumor along with metastatic lymph nodes was compared among various clinicopathological characteristics, and overall survival was analyzed. The rate and intensity of Fra-1 immunoreactivity were also investigated. The molecular role of Fra-1 was assessed by its down-regulation in human ESCC cell lines.Fra-1 expression was positive in 127 (77.4%) ESCC patients. Immunoreactivity was localized to the marginal areas of the ESCC tumors. Positive Fra-1 expression correlated with depth of tumor, lymph node metastasis, stage, and infiltrative growth pattern. A significant difference was seen in the survival between tumors with and without Fra-1, and positive Fra-1 expression was revealed to be an independent factor related to poor prognosis. Patients with metastatic lymph nodes with positive Fra-1 expression presented decreased survival compared with negative Fra-1 expression. After the down-regulation of Fra-1 expression, a significant decrease in cell proliferation, motility, and invasion was observed.This study demonstrated ESCC patients positive for Fra-1 to be associated with poor prognosis. The findings also suggest that Fra-1 regulation may play an important role in the progression of ESCC. 
 Identification of a pharmacologically tractable Fra-1/ADORA2B axis promoting breast cancer metastasis. Metastasis confronts clinicians with two major challenges: estimating the patient's risk of metastasis and identifying therapeutic targets. Because they are key signal integrators connecting cellular processes to clinical outcome, we aimed to identify transcriptional nodes regulating cancer cell metastasis. Using rodent xenograft models that we previously developed, we identified the transcription factor Fos-related antigen-1 (Fra-1) as a key coordinator of metastasis. Because Fra-1 often is overexpressed in human metastatic breast cancers and has been shown to control their   invasive   potential in vitro, we aimed to assess the implication and prognostic significance of the Fra-1-dependent genetic program in breast cancer metastasis and to identify potential Fra-1-dependent therapeutic targets. In several in vivo assays in mice, we demonstrate that stable RNAi depletion of Fra-1 from human breast cancer cells strongly suppresses their ability to metastasize. These results support a clinically important role for Fra-1 and the genetic program it controls. We show that a Fra-1-dependent gene-expression signature accurately predicts recurrence of breast cancer. Furthermore, a synthetic lethal drug screen revealed that antagonists of the adenosine receptor A2B (ADORA2B) are preferentially toxic to breast tumor cells expressing Fra-1. Both RNAi silencing and pharmacologic blockade of ADORA2B inhibited filopodia formation and   invasive   activity of breast cancer cells and correspondingly reduced tumor outgrowth in the lungs. These data show that Fra-1 activity is causally involved in and is a prognostic indicator of breast cancer metastasis. They suggest that Fra-1 activity predicts responsiveness to inhibition of pharmacologically tractable targets, such as ADORA2B, which may be used for clinical interference of metastatic breast cancer. 
 Role of Fos-related antigen 1 in the progression and prognosis of ductal breast carcinoma. Fos-related antigen 1 (Fra-1) is a member of the activator protein 1 (AP-1) transcription factor family. Our objective was to evaluate the role of Fra-1 expression in breast carcinoma progression and prognosis.Fra-1 expression was investigated by immunohistochemistry in two tissue microarrays containing, respectively, 85 ductal carcinoma in situ (DCIS) and 771   invasive   ductal carcinoma (IDC) samples. Staining was observed in the nucleus and cytoplasm of the carcinomas, but only nuclear staining was considered to be positive. Fibroblasts associated with IDC were also Fra-1-positive. The frequency of Fra-1 positivity in IDC (22.8%) was lower than that in DCIS (42.2%). No association was found between Fra-1 and clinico-pathological variables in DCIS. In IDC, Fra-1 expression correlated with aggressive phenotype markers, including: high grade, oestrogen receptor negativity and human epidermal growth factor receptor 2 (HER-2) positivity (P=0.001, 0.015 and 0.004, respectively), and marginally with the presence of metastasis (P=0.07). Fra-1 was more frequently positive in basal-like (34%) and in HER-2-positive (38.5%) subtypes than in luminal subtypes. Fra-1 presence did not correlate with survival.A high frequency of Fra-1 in DCIS tumours may be associated with early events in breast carcinogenesis. Although Fra-1 expression correlated with features of a more aggressive phenotype in IDC, no relationship with overall survival was found. 
 Epstein-Barr virus latent membrane protein 2A promotes invasion of nasopharyngeal carcinoma cells through ERK/Fra-1-mediated induction of matrix metalloproteinase 9. Epstein-Barr virus (EBV)-associated nasopharyngeal carcinoma (NPC) is highly metastatic, and this malignant feature may be promoted by an EBV oncoprotein, latent membrane protein 2A (LMP2A). Acting as a signal regulator, LMP2A can enhance   invasive  ness and motility of epithelial cells. Downstream from the LMP2A-triggered signaling events, it is largely unknown what key effector proteins are induced and essentially promote cell invasion. In the present study, we found that in NPC cells, LMP2A upregulated matrix metalloproteinase 9 (MMP9), a metastasis-associated protease. LMP2A increased MMP9 expression at both the mRNA and protein levels. It also activated the MMP9 promoter, in which two AP-1 elements were required for the promoter activation. Among AP-1 transcription factors, Fra-1 was induced by LMP2A and is essential for LMP2A-triggered MMP9 expression. Induction of Fra-1 was dependent on the LMP2A-activated ERK1/2 pathway, and induction of the ERK1/2-Fra-1-MMP9 axis required PY motifs in the amino-terminal domain of LMP2A. Notably, LMP2A-promoted invasion of NPC cells was blocked when MMP9 expression, Fra-1 induction, or ERK1/2 activation was inhibited. In addition, we found an association of LMP2A with MMP9 expression in NPC tumor biopsy specimens, where Fra-1 was a major mediation factor. This study reveals an underlying mechanism of LMP2A-induced cell invasion, from signal transduction to upregulation of a critical protease. Considering that MMP9 can also be upregulated by another EBV oncoprotein, LMP1, this protease may be a pivotal effector at which the EBV-induced, invasion-promoting mechanisms converge, serving as an attractive therapeutic target for NPC treatment. 
 The PKCθ pathway participates in the aberrant accumulation of Fra-1 protein in   invasiv  e ER-negative breast cancer cells. Fra-1 is aberrantly expressed in a large number of cancer cells and tissues, and emerging evidence suggests an important role for this Fos family protein in both oncogenesis and the progression or maintenance of many tumour types. Here, we show that the concentration of Fra-1 is high in   invasiv  e oestrogen receptor (ER)-negative (ER-) breast cancer cell lines, regardless of their Ras pathway status. All of the ER- cells express high levels of activated PKCθ, and the inhibition of PKCθ activity using RNA interference or the expression of a dominant-negative mutant results in a dramatic reduction in Fra-1 abundance. Conversely, the ectopic expression of constitutively active PKCθ leads to Fra-1 phosphorylation and accumulation in poo  rly inva  sive ER+ cells. This accumulation is due to the stabilisation of the Fra-1 protein through PKCθ signalling, whereas other members of the PKC family are ineffective. Both Ste20-related proline-alanine-rich kinase (SPAK) and ERK1/2, whose activities are upregulated by PKCθ, participate in PKCθ-driven Fra-1 stabilisation. Interestingly, their relative contributions appear to be different depending on the cell line studied. ERK1/2 signalling has a major role in ER- MDA-MB-231 cells, whereas Fra-1 accumulation occurs mainly through SPAK signalling in ER- BT549 cells. Fra-1 mutational analysis shows that the phosphorylation of S265, T223 and T230 is critical for PKCθ-driven Fra-1 stabilisation. Phosphorylation of the protein was confirmed using specific antisera against Fra-1 phosphorylated on T223 or S265. In addition, Fra-1 participates in PKCθ-induced cell invasion and is necessary for PKCθ-induced cell migration. In summary, we identified PKCθ signalling as an important regulator of Fra-1 accumulation in ER- breast cancer cells. Moreover, our results suggest that PKCθ could participate in progression of some breast cancers and could be a new therapeutic target. 
 Fra-1 controls motility of bladder cancer cells via transcriptional upregulation of the receptor tyrosine kinase AXL. Fos-related antigen 1 (Fra-1) is a Fos family member overexpressed in several types of human cancers. Here, we report that Fra-1 is highly expressed in the muscle-  invasive   form of the carcinoma of the bladder (80%) and to a lesser extent in superficial bladder cancer (42%). We demonstrate that in this type of cancer Fra-1 is regulated via a C-terminal instability signal and C-terminal phosphorylation. We show that manipulation of Fra-1 expression levels in bladder cancer cell lines affects cell morphology, motility and proliferation. The gene coding for AXL tyrosine kinase is directly upregulated by Fra-1 in bladder cancer and in other cell lines. Importantly, our data demonstrate that AXL mediates the effect of Fra-1 on tumour cell motility but not on cell proliferation. We suggest that AXL may represent an attractive therapeutic target in cancers expressing high Fra-1 levels. 
 Prognostic impact of transcription factor Fra-1 in ER-positive breast cancer: contribution to a metastatic phenotype through modulation of tumor cell adhesive properties. The transcription factor Fos-related antigen-1 (Fra-1) has been described to affect the morphology, motility and   invasive   potential of breast cancer cells. Since tumor cell adhesion plays an essential role in the metastatic process, especially for extravasation from blood vessels, we investigated the influence of Fra-1 on breast cancer cell interactions with the endothelium.Using Fra-1-overexpressing MCF7 [weakly   invasive  , estrogen receptor (ER)-positive] and MDA MB231 (strongly   invasive  , ER-negative) cells, we performed dynamic cell flow adhesion assays on surfaces coated with E-selectin or with human pulmonary microvascular endothelial cells.We found a significant increased adhesion of Fra-1-overexpressing MCF7 cells to E-selectin but also to activate endothelial cells, whereas the MDA MB231 cell line showed moderate enhanced cell rolling and tethering on both coated surfaces. These different adhesion behaviors corresponded to an up-regulation of various adhesion-related proteins such as CD44 and integrin α5 in Fra-1-overexpressing MCF7 cells measured by microarray analysis and flow cytometry in comparison with no deregulation of key adhesion molecules observed in Fra-1-overexpressing MDA MB231 cells. In line with these results and based on cDNA microarray data of breast cancer patients (n = 197), high Fra-1 expression significantly correlates with shorter overall survival and higher rate of lung metastasis in ER-positive breast cancer patients (n = 130), but has no impact on the prognosis of patients with ER-negative tumors.Thus, in addition to its pro  -invasiv  e and pro-migratory effect, Fra-1 might influence the metastatic potential of breast cancer cells by changing the expression of adhesion molecules, resulting in increased adherence to endothelial cells under flow conditions. 
 FRA-1 expression level regulates proliferation and   invasive  ness of breast cancer cells. Breast cancer progression is likely a multistep process involving the activation and inactivation of a number of genes. Previously, we showed that the mRNA coding for Fra-1, a FOS family member and an AP-1 transcription factor component, was highly expressed in the more   invasive   estrogen receptor negative (ER-) breast cancer cell lines. We used a tet-off system to stably overexpress Fra-1 in MCF7 ER+ cells and evaluate the impact of Fra-1 on this aggressive phenotype. Conversely, Fra-1 was silenced in highly   invasive   ER-MDA-MB231 cells using RNA interference. We report that in both systems the Fra-1 expression level was positively associated with cell proliferation, cell motility and   invasive  ness assessed in vitro. In addition, Fra-1 inhibition in fibroblastoid ER- cells, which formed colonies with large stellate projections in Matrigel, resulted in morphological changes. Cells acquired an epithelioid shape and had a spherical appearance in Matrigel. Fra-1 regulated several genes, implicated in invasion, angiogenesis and cell proliferation independently of beta1-integrin activation, and directly induced MMP-1 and MMP-9 promoter activity. These overall results show that high Fra-1 expression is associated with a more malignant cell phenotype and suggest that Fra-1 could have a pivotal role in breast cancer progression. 
 Widespread FRA1-dependent control of mesenchymal transdifferentiation programs in colorectal cancer cells. Tumor invasion and metastasis involves complex remodeling of gene expression programs governing epithelial homeostasis. Mutational activation of the RAS-ERK is a frequent occurrence in many cancers and has been shown to drive overexpression of the AP-1 family transcription factor FRA1, a potent regulator of migration and invasion in a variety of tumor cell types. However, the nature of FRA1 transcriptional targets and the molecular pathways through which they promote tumor progression remain poorly understood. We found that FRA1 was strongly expressed in tumor cells at the   invasive   front of human colorectal cancers (CRCs), and that its depletion suppressed mesenchymal-like features in CRC cells in vitro. Genome-wide analysis of FRA1 chromatin occupancy and transcriptional regulation identified epithelial-mesenchymal transition (EMT)-related genes as a major class of direct FRA1 targets in CRC cells. Expression of the pro-mesenchymal subset of these genes predicted adverse outcomes in CRC patients, and involved FRA-1-dependent regulation and cooperation with TGFβ signaling pathway. Our findings reveal an unexpectedly widespread and direct role for FRA1 in control of epithelial-mesenchymal plasticity in CRC cells, and suggest that FRA1 plays an important role in mediating cross talk between oncogenic RAS-ERK and TGFβ signaling networks during tumor progression. 
  GeneRIFs  
 study reports Fra-1 is highly expressed in the muscle-  invasive   form of bladder cancer and to a lesser extent in superficial bladder cancer; gene coding for AXL tyrosine kinase is directly upregulated by Fra-1 in bladder cancer and other cell lines; data demonstrate that AXL mediates the effect of Fra-1 on tumour cell motility but not on cell proliferation 
  [Mm]etastasize  PubMed  
 Fra-1 is a key driver of colon cancer metastasis and a Fra-1 classifier predicts disease-free survival. Fra-1 (Fos-related antigen-1) is a member of the AP-1 (activator protein-1) family of transcription factors. We previously showed that Fra-1 is necessary for breast cancer cells to   metastasize   in vivo, and that a classifier comprising genes that are expressed in a Fra-1-dependent fashion can predict breast cancer outcome. Here, we show that Fra-1 plays an important role also in colon cancer progression. Whereas Fra-1 depletion does not affect 2D proliferation of human colon cancer cells, it impairs growth in soft agar and in suspension. Consistently, subcutaneous tumors formed by Fra-1-depleted colon cancer cells are three times smaller than those produced by control cells. Most remarkably, when injected intravenously, Fra-1 depletion causes a 200-fold reduction in tumor burden. Moreover, a Fra-1 classifier generated by comparing RNA profiles of parental and Fra-1-depleted colon cancer cells can predict the prognosis of colon cancer patients. Functional pathway analysis revealed Wnt as one of the central pathways in the classifier, suggesting a possible mechanism of Fra-1 function in colon cancer metastasis. Our results demonstrate that Fra-1 is an important determinant of the metastatic potential of human colon cancer cells, and that the Fra-1 classifier can be used as a prognostic predictor in colon cancer patients. 
 Identification of a pharmacologically tractable Fra-1/ADORA2B axis promoting breast cancer metastasis. Metastasis confronts clinicians with two major challenges: estimating the patient's risk of metastasis and identifying therapeutic targets. Because they are key signal integrators connecting cellular processes to clinical outcome, we aimed to identify transcriptional nodes regulating cancer cell metastasis. Using rodent xenograft models that we previously developed, we identified the transcription factor Fos-related antigen-1 (Fra-1) as a key coordinator of metastasis. Because Fra-1 often is overexpressed in human metastatic breast cancers and has been shown to control their invasive potential in vitro, we aimed to assess the implication and prognostic significance of the Fra-1-dependent genetic program in breast cancer metastasis and to identify potential Fra-1-dependent therapeutic targets. In several in vivo assays in mice, we demonstrate that stable RNAi depletion of Fra-1 from human breast cancer cells strongly suppresses their ability to   metastasize  . These results support a clinically important role for Fra-1 and the genetic program it controls. We show that a Fra-1-dependent gene-expression signature accurately predicts recurrence of breast cancer. Furthermore, a synthetic lethal drug screen revealed that antagonists of the adenosine receptor A2B (ADORA2B) are preferentially toxic to breast tumor cells expressing Fra-1. Both RNAi silencing and pharmacologic blockade of ADORA2B inhibited filopodia formation and invasive activity of breast cancer cells and correspondingly reduced tumor outgrowth in the lungs. These data show that Fra-1 activity is causally involved in and is a prognostic indicator of breast cancer metastasis. They suggest that Fra-1 activity predicts responsiveness to inhibition of pharmacologically tractable targets, such as ADORA2B, which may be used for clinical interference of metastatic breast cancer. 
  [Mm]etastasis  PubMed  
 ERK signaling regulates tumor promoter induced c-Jun recruitment at the Fra-1 promoter. Fra-1 as an integral part of AP-1 (Jun/Fos) drives transcriptional programs involved in several physiologic and pathologic processes. It is also critical for tumor cell motility and   metastasis  . We have previously shown that two critical elements of Fra-1 promoter, the upstream TPA response element (TRE) and the serum response element (SRE), are necessary for its induction in response to phorbol esters in human pulmonary epithelial cell lines. Here, we have investigated the roles of various MAP kinases in regulating Fra-1 expression in response to TPA. Using pharmacologic and genetic tools, we demonstrate a prominent role for ERK1/2, but not JNK1/2 and p38, signaling in the TPA-induced activation of specific transcription factors that bind to the AP1 site and the SRE. Inhibition of ERK1/2 pathway suppresses Elk1 activation, and c-Jun and Fra-2 recruitment to the promoter. 
 Fra-1/AP-1 induces EMT in mammary epithelial cells by modulating Zeb1/2 and TGFβ expression. Epithelial-to-mesenchymal transition (EMT) is essential for embryonic morphogenesis and wound healing and critical for tumour cell invasion and dissemination. The AP-1 transcription factor Fra-1 has been implicated in tumorigenesis and in tumour-associated EMT in human breast cancer. We observed a significant inverse correlation between Fra-1 mRNA expression and distant  -metastasi  s-free survival in a large cohort of breast cancer patients derived from multiple array data sets. This unique correlation among Fos genes prompted us to assess the evolutionary conservation between Fra-1 functions in EMT of human and mouse cells. Ectopic expression of Fra-1 in fully polarized, non-tumourigenic, mouse mammary epithelial EpH4 cells induced a mesenchymal phenotype, characterized by a loss of epithelial and gain of mesenchymal markers. Proliferation, motility and invasiveness were also increased in the resulting EpFra1 cells, and the cells were tumourigenic and efficiently colonized the lung upon transplantation. Molecular analyses revealed increased expression of Tgfβ1 and the EMT-inducing transcription factors Zeb1, Zeb2 and Slug. Mechanistically, Fra-1 binds to the tgfb1 and zeb2 promoters and to an evolutionarily conserved region in the first intron of zeb1. Furthermore, increased activity of a zeb2 promoter reporter was detected in EpFra1 cells and shown to depend on AP-1-binding sites. Inhibiting TGFβ signalling in EpFra1 cells moderately increased the expression of epithelial markers, whereas silencing of zeb1 or zeb2 restored the epithelial phenotype and decreased migration in vitro and tumorigenesis in vivo. Thus Fra-1 induces changes in the expression of genes encoding EMT-related transcription factors leading to the acquisition of mesenchymal, invasive and tumorigenic capacities by epithelial cells. This study defines a novel function of Fra-1/AP-1 in modulating tgfb1, zeb1 and zeb2 expression through direct binding to genomic regulatory regions, which establishes a basis for future in vivo genetic manipulations and preclinical studies using mouse models. 
 Phosphorylated c-Jun and Fra-1 induce matrix metalloproteinase-1 and thereby regulate invasion activity of 143B osteosarcoma cells. Osteosarcoma is the most common primary malignancy of bone and patients often develop pulmonary metastases. Despite the advances in surgical and medical management, the mechanisms underlying human osteosarcoma progression and   metastasis   remain to be elucidated. Gene expression profiles were compared by the cDNA microarray technique between two different human osteosarcoma sublines, MNNG/HOS and 143B, which differ greatly in spontaneous pulmonary metastatic potential. Here we report an enhanced expression of matrix metalloproteinase (MMP)-1 in the highly metastatic human osteosarcoma cell line 143B. Moreover, the in vitro invasion activity of 143B cells was MMP-1-dependent. The activator protein (AP)-1 binding site in the MMP-1 gene promoter was required for the constitutive expression of MMP-1 in 143B cells. Two AP-1 components, c-Jun and Fra-1, were phosphorylated, and bound to the AP-1 binding site of the MMP-1 promoter in 143B cells. Activated c-Jun and Fra-1 were essential for MMP-1 gene expression in 143B cells. Mitogen-activated protein kinase pathways including the c-Jun NH(2)-terminal kinase and the extracellular signal-regulated kinase activate c-Jun and Fra-1 and thereby regulate c-Jun/Fra-1 mediated events, establishing the mitogen-activated protein kinase/AP-1/MMP-1 axis as important in 143B cells. These data suggest that MMP-1 plays a central role in osteosarcoma invasion. Accordingly, MMP-1 might be a biomarker and therapeutic target for invasive osteosarcomas and pulmonary metastases. 
 Fra-1/AP-1 induces EMT in mammary epithelial cells by modulating Zeb1/2 and TGFβ expression. Epithelial-to-mesenchymal transition (EMT) is essential for embryonic morphogenesis and wound healing and critical for tumour cell invasion and dissemination. The AP-1 transcription factor Fra-1 has been implicated in tumorigenesis and in tumour-associated EMT in human breast cancer. We observed a significant inverse correlation between Fra-1 mRNA expression and distant  -metastasi  s-free survival in a large cohort of breast cancer patients derived from multiple array data sets. This unique correlation among Fos genes prompted us to assess the evolutionary conservation between Fra-1 functions in EMT of human and mouse cells. Ectopic expression of Fra-1 in fully polarized, non-tumourigenic, mouse mammary epithelial EpH4 cells induced a mesenchymal phenotype, characterized by a loss of epithelial and gain of mesenchymal markers. Proliferation, motility and invasiveness were also increased in the resulting EpFra1 cells, and the cells were tumourigenic and efficiently colonized the lung upon transplantation. Molecular analyses revealed increased expression of Tgfβ1 and the EMT-inducing transcription factors Zeb1, Zeb2 and Slug. Mechanistically, Fra-1 binds to the tgfb1 and zeb2 promoters and to an evolutionarily conserved region in the first intron of zeb1. Furthermore, increased activity of a zeb2 promoter reporter was detected in EpFra1 cells and shown to depend on AP-1-binding sites. Inhibiting TGFβ signalling in EpFra1 cells moderately increased the expression of epithelial markers, whereas silencing of zeb1 or zeb2 restored the epithelial phenotype and decreased migration in vitro and tumorigenesis in vivo. Thus Fra-1 induces changes in the expression of genes encoding EMT-related transcription factors leading to the acquisition of mesenchymal, invasive and tumorigenic capacities by epithelial cells. This study defines a novel function of Fra-1/AP-1 in modulating tgfb1, zeb1 and zeb2 expression through direct binding to genomic regulatory regions, which establishes a basis for future in vivo genetic manipulations and preclinical studies using mouse models. 
 Phosphorylated c-Jun and Fra-1 induce matrix metalloproteinase-1 and thereby regulate invasion activity of 143B osteosarcoma cells. Osteosarcoma is the most common primary malignancy of bone and patients often develop pulmonary metastases. Despite the advances in surgical and medical management, the mechanisms underlying human osteosarcoma progression and   metastasis   remain to be elucidated. Gene expression profiles were compared by the cDNA microarray technique between two different human osteosarcoma sublines, MNNG/HOS and 143B, which differ greatly in spontaneous pulmonary metastatic potential. Here we report an enhanced expression of matrix metalloproteinase (MMP)-1 in the highly metastatic human osteosarcoma cell line 143B. Moreover, the in vitro invasion activity of 143B cells was MMP-1-dependent. The activator protein (AP)-1 binding site in the MMP-1 gene promoter was required for the constitutive expression of MMP-1 in 143B cells. Two AP-1 components, c-Jun and Fra-1, were phosphorylated, and bound to the AP-1 binding site of the MMP-1 promoter in 143B cells. Activated c-Jun and Fra-1 were essential for MMP-1 gene expression in 143B cells. Mitogen-activated protein kinase pathways including the c-Jun NH(2)-terminal kinase and the extracellular signal-regulated kinase activate c-Jun and Fra-1 and thereby regulate c-Jun/Fra-1 mediated events, establishing the mitogen-activated protein kinase/AP-1/MMP-1 axis as important in 143B cells. These data suggest that MMP-1 plays a central role in osteosarcoma invasion. Accordingly, MMP-1 might be a biomarker and therapeutic target for invasive osteosarcomas and pulmonary metastases. 
 ERK signaling regulates tumor promoter induced c-Jun recruitment at the Fra-1 promoter. Fra-1 as an integral part of AP-1 (Jun/Fos) drives transcriptional programs involved in several physiologic and pathologic processes. It is also critical for tumor cell motility and   metastasis  . We have previously shown that two critical elements of Fra-1 promoter, the upstream TPA response element (TRE) and the serum response element (SRE), are necessary for its induction in response to phorbol esters in human pulmonary epithelial cell lines. Here, we have investigated the roles of various MAP kinases in regulating Fra-1 expression in response to TPA. Using pharmacologic and genetic tools, we demonstrate a prominent role for ERK1/2, but not JNK1/2 and p38, signaling in the TPA-induced activation of specific transcription factors that bind to the AP1 site and the SRE. Inhibition of ERK1/2 pathway suppresses Elk1 activation, and c-Jun and Fra-2 recruitment to the promoter. 
 Fra-1 is a key driver of colon cancer   metastasis   and a Fra-1 classifier predicts disease-free survival. Fra-1 (Fos-related antigen-1) is a member of the AP-1 (activator protein-1) family of transcription factors. We previously showed that Fra-1 is necessary for breast cancer cells to metastasize in vivo, and that a classifier comprising genes that are expressed in a Fra-1-dependent fashion can predict breast cancer outcome. Here, we show that Fra-1 plays an important role also in colon cancer progression. Whereas Fra-1 depletion does not affect 2D proliferation of human colon cancer cells, it impairs growth in soft agar and in suspension. Consistently, subcutaneous tumors formed by Fra-1-depleted colon cancer cells are three times smaller than those produced by control cells. Most remarkably, when injected intravenously, Fra-1 depletion causes a 200-fold reduction in tumor burden. Moreover, a Fra-1 classifier generated by comparing RNA profiles of parental and Fra-1-depleted colon cancer cells can predict the prognosis of colon cancer patients. Functional pathway analysis revealed Wnt as one of the central pathways in the classifier, suggesting a possible mechanism of Fra-1 function in colon cancer   metastasis  . Our results demonstrate that Fra-1 is an important determinant of the metastatic potential of human colon cancer cells, and that the Fra-1 classifier can be used as a prognostic predictor in colon cancer patients. 
 The molecular role of Fra-1 and its prognostic significance in human esophageal squamous cell carcinoma. The expression of Fra-1 (Fos related antigen 1) involves tumor progression and invasion, and its gene ablation could suppress the invasive phenotypes of human tumor cells. The authors investigated the significance of Fra-1 expression in esophageal squamous cell carcinoma (ESCC) and studied the effect of its down-regulation on cell proliferation, motility, and invasion.Surgical specimens from 164 patients with ESCC were evaluated. Fra-1 expression in the primary tumor along with metastatic lymph nodes was compared among various clinicopathological characteristics, and overall survival was analyzed. The rate and intensity of Fra-1 immunoreactivity were also investigated. The molecular role of Fra-1 was assessed by its down-regulation in human ESCC cell lines.Fra-1 expression was positive in 127 (77.4%) ESCC patients. Immunoreactivity was localized to the marginal areas of the ESCC tumors. Positive Fra-1 expression correlated with depth of tumor, lymph node   metastasis  , stage, and infiltrative growth pattern. A significant difference was seen in the survival between tumors with and without Fra-1, and positive Fra-1 expression was revealed to be an independent factor related to poor prognosis. Patients with metastatic lymph nodes with positive Fra-1 expression presented decreased survival compared with negative Fra-1 expression. After the down-regulation of Fra-1 expression, a significant decrease in cell proliferation, motility, and invasion was observed.This study demonstrated ESCC patients positive for Fra-1 to be associated with poor prognosis. The findings also suggest that Fra-1 regulation may play an important role in the progression of ESCC. 
 Identification of a pharmacologically tractable Fra-1/ADORA2B axis promoting breast cancer   metastasis  .   Metastasis   confronts clinicians with two major challenges: estimating the patient's risk of   metastasis   and identifying therapeutic targets. Because they are key signal integrators connecting cellular processes to clinical outcome, we aimed to identify transcriptional nodes regulating cancer cell   metastasis  . Using rodent xenograft models that we previously developed, we identified the transcription factor Fos-related antigen-1 (Fra-1) as a key coordinator of   metastasis  . Because Fra-1 often is overexpressed in human metastatic breast cancers and has been shown to control their invasive potential in vitro, we aimed to assess the implication and prognostic significance of the Fra-1-dependent genetic program in breast cancer   metastasis   and to identify potential Fra-1-dependent therapeutic targets. In several in vivo assays in mice, we demonstrate that stable RNAi depletion of Fra-1 from human breast cancer cells strongly suppresses their ability to metastasize. These results support a clinically important role for Fra-1 and the genetic program it controls. We show that a Fra-1-dependent gene-expression signature accurately predicts recurrence of breast cancer. Furthermore, a synthetic lethal drug screen revealed that antagonists of the adenosine receptor A2B (ADORA2B) are preferentially toxic to breast tumor cells expressing Fra-1. Both RNAi silencing and pharmacologic blockade of ADORA2B inhibited filopodia formation and invasive activity of breast cancer cells and correspondingly reduced tumor outgrowth in the lungs. These data show that Fra-1 activity is causally involved in and is a prognostic indicator of breast cancer   metastasis  . They suggest that Fra-1 activity predicts responsiveness to inhibition of pharmacologically tractable targets, such as ADORA2B, which may be used for clinical interference of metastatic breast cancer. 
 Role of Fos-related antigen 1 in the progression and prognosis of ductal breast carcinoma. Fos-related antigen 1 (Fra-1) is a member of the activator protein 1 (AP-1) transcription factor family. Our objective was to evaluate the role of Fra-1 expression in breast carcinoma progression and prognosis.Fra-1 expression was investigated by immunohistochemistry in two tissue microarrays containing, respectively, 85 ductal carcinoma in situ (DCIS) and 771 invasive ductal carcinoma (IDC) samples. Staining was observed in the nucleus and cytoplasm of the carcinomas, but only nuclear staining was considered to be positive. Fibroblasts associated with IDC were also Fra-1-positive. The frequency of Fra-1 positivity in IDC (22.8%) was lower than that in DCIS (42.2%). No association was found between Fra-1 and clinico-pathological variables in DCIS. In IDC, Fra-1 expression correlated with aggressive phenotype markers, including: high grade, oestrogen receptor negativity and human epidermal growth factor receptor 2 (HER-2) positivity (P=0.001, 0.015 and 0.004, respectively), and marginally with the presence of   metastasis   (P=0.07). Fra-1 was more frequently positive in basal-like (34%) and in HER-2-positive (38.5%) subtypes than in luminal subtypes. Fra-1 presence did not correlate with survival.A high frequency of Fra-1 in DCIS tumours may be associated with early events in breast carcinogenesis. Although Fra-1 expression correlated with features of a more aggressive phenotype in IDC, no relationship with overall survival was found. 
 Fra-1 regulates vimentin during Ha-RAS-induced epithelial mesenchymal transition in human colon carcinoma cells. The process of epithelial mesenchymal transition, whereby cells acquire molecular alterations and fibroblastic features, is a fundamental process of embryogenesis and cancer invasion/  metastasis  . The mechanisms responsible for epithelial mesenchymal transition remain elusive. Human tumors frequently establish constitutively activated RAS signaling, which contributes to the malignant phenotype. In an effort to dissect distinct RAS isoform specific functions, we previously established human colon cell lines stably overexpressing activated Harvey-RAS (Ha-RAS) and Kirsten-RAS (Ki-RAS). Using these, we observed that only oncogenic Ha-RAS overexpression resulted in morphologic and molecular changes suggestive of epithelial to mesenchymal transition. We showed that vimentin, a key molecule of epithelial mesenchymal transition, was differentially regulated between Ha-RAS and Ki-RAS leading to a Ha-RAS specific induction of a migratory phenotype and eventually epithelial to mesenchymal transition. We demonstrated that the AP-1 sites in vimentin promoter could be involved in this regulation. A potential role of FRA-1 was suggested in the regulation of vimentin during the Ha-RAS-induced epithelial to mesenchymal transition, in association with colon cell migration. Our results therefore propose that in colon cells, the induction of epithelial mesenchymal transition by oncogenic Ha-RAS could occur through the overexpression of proteins like FRA-1 and vimentin. 
 Epstein-Barr virus latent membrane protein 2A promotes invasion of nasopharyngeal carcinoma cells through ERK/Fra-1-mediated induction of matrix metalloproteinase 9. Epstein-Barr virus (EBV)-associated nasopharyngeal carcinoma (NPC) is highly metastatic, and this malignant feature may be promoted by an EBV oncoprotein, latent membrane protein 2A (LMP2A). Acting as a signal regulator, LMP2A can enhance invasiveness and motility of epithelial cells. Downstream from the LMP2A-triggered signaling events, it is largely unknown what key effector proteins are induced and essentially promote cell invasion. In the present study, we found that in NPC cells, LMP2A upregulated matrix metalloproteinase 9 (MMP9), a   metastasis  -associated protease. LMP2A increased MMP9 expression at both the mRNA and protein levels. It also activated the MMP9 promoter, in which two AP-1 elements were required for the promoter activation. Among AP-1 transcription factors, Fra-1 was induced by LMP2A and is essential for LMP2A-triggered MMP9 expression. Induction of Fra-1 was dependent on the LMP2A-activated ERK1/2 pathway, and induction of the ERK1/2-Fra-1-MMP9 axis required PY motifs in the amino-terminal domain of LMP2A. Notably, LMP2A-promoted invasion of NPC cells was blocked when MMP9 expression, Fra-1 induction, or ERK1/2 activation was inhibited. In addition, we found an association of LMP2A with MMP9 expression in NPC tumor biopsy specimens, where Fra-1 was a major mediation factor. This study reveals an underlying mechanism of LMP2A-induced cell invasion, from signal transduction to upregulation of a critical protease. Considering that MMP9 can also be upregulated by another EBV oncoprotein, LMP1, this protease may be a pivotal effector at which the EBV-induced, invasion-promoting mechanisms converge, serving as an attractive therapeutic target for NPC treatment. 
 Aberrantly expressed Fra-1 by IL-6/STAT3 transactivation promotes colorectal cancer aggressiveness through epithelial-mesenchymal transition. The pro-inflammatory cytokine interleukin-6 (IL-6) in tumor microenvironment has been suggested to promote development and progression of colorectal cancer (CRC). However, the underlying molecular mechanisms remain elusive. In this study, we demonstrate that fos-related antigen-1 (Fra-1) plays a critical role in IL-6 induced CRC aggressiveness and epithelial-mesenchymal transition (EMT). In CRC cell lines, the expression of Fra-1 gene was found significantly upregulated during IL-6-driven EMT process. The Fra-1 induction occurred at transcriptional level in a manner dependent on signal transducer and activator of transcription 3 (STAT3), during which both phosphorylated and acetylated post-translational modifications were required for STAT3 activation to directly bind to the Fra-1 promoter. Importantly, RNA interference-based attenuation of either STAT3 or Fra-1 prevented IL-6-induced EMT, cell migration and invasion, whereas ectopic expression of Fra-1 markedly reversed the STAT3-knockdown effect and enhanced CRC cell aggressiveness by regulating the expression of EMT-promoting factors (ZEB1, Snail, Slug, MMP-2 and MMP-9). Furthermore, Fra-1 levels were positively correlated with the local invasion depth as well as lymph node and liver   metastasis   in a total of 229 CRC patients. Intense immunohistochemical staining of Fra-1 was observed at the tumor marginal area adjacent to inflammatory cells and in parallel with IL-6 secretion and STAT3 activation in CRC tissues. Together, this study proposes the existence of an aberrant IL-6/STAT3/Fra-1 signaling axis leading to CRC aggressiveness through EMT induction, which suggests novel therapeutic opportunities for the malignant disease. 
 A Fra-1-dependent, matrix metalloproteinase driven EGFR activation promotes human lung epithelial cell motility and invasion. We and others have shown a persistently high induction of Fra-1 transcription factor (a dimeric partner of AP-1) levels by respiratory carcinogens in pulmonary epithelial cells. Fra-1 is frequently overexpressed in various human tumors and cancer cells. We have recently shown that Fra-1 significantly promotes growth, motility, and invasion of human pulmonary epithelial cells, the precise molecular mechanisms by which this enhancement occurs are unclear. Because matrix metalloproteinases (MMPs) play key roles in wound healing and lung tumor   metastasis  , we tested the hypothesis that Fra-1 promotes lung epithelial cell motility and invasion via MMP activation. We show here that MMP-9 and MMP-2 activated signaling plays a critical role in regulating Fra-1-induced lung epithelial cell growth and invasion. Ectopic Fra-1 markedly stimulates MMP-2 and MMP-9 mRNA expression. Inhibition of MMP-2 and MMP-9 activity significantly attenuated Fra-1-driven cell motility and invasion. Furthermore, Fra-1 induced EGFR phosphorylation in an MMP-dependent manner, and an EGFR-specific inhibitor was able to block Fra-1-enhanced cell motility and invasion. Taken together, our data suggest that Fra-1 enhances lung cancer epithelial cell motility and invasion by inducing the activity of MMPs, in particular MMP-2 and MMP-9, and EGFR-activated signaling. 
 Prognostic impact of transcription factor Fra-1 in ER-positive breast cancer: contribution to a metastatic phenotype through modulation of tumor cell adhesive properties. The transcription factor Fos-related antigen-1 (Fra-1) has been described to affect the morphology, motility and invasive potential of breast cancer cells. Since tumor cell adhesion plays an essential role in the metastatic process, especially for extravasation from blood vessels, we investigated the influence of Fra-1 on breast cancer cell interactions with the endothelium.Using Fra-1-overexpressing MCF7 [weakly invasive, estrogen receptor (ER)-positive] and MDA MB231 (strongly invasive, ER-negative) cells, we performed dynamic cell flow adhesion assays on surfaces coated with E-selectin or with human pulmonary microvascular endothelial cells.We found a significant increased adhesion of Fra-1-overexpressing MCF7 cells to E-selectin but also to activate endothelial cells, whereas the MDA MB231 cell line showed moderate enhanced cell rolling and tethering on both coated surfaces. These different adhesion behaviors corresponded to an up-regulation of various adhesion-related proteins such as CD44 and integrin α5 in Fra-1-overexpressing MCF7 cells measured by microarray analysis and flow cytometry in comparison with no deregulation of key adhesion molecules observed in Fra-1-overexpressing MDA MB231 cells. In line with these results and based on cDNA microarray data of breast cancer patients (n = 197), high Fra-1 expression significantly correlates with shorter overall survival and higher rate of lung   metastasi  s in ER-positive breast cancer patients (n = 130), but has no impact on the prognosis of patients with ER-negative tumors.Thus, in addition to its pro-invasive and pro-migratory effect, Fra-1 might influence the metastatic potential of breast cancer cells by changing the expression of adhesion molecules, resulting in increased adherence to endothelial cells under flow conditions. 
 FRA-1 proto-oncogene induces lung epithelial cell invasion and anchorage-independent growth in vitro, but is insufficient to promote tumor growth in vivo. FRA-1 forms activator protein-1 complexes in association with members of the JUN family and drives gene transcription. FRA-1 has been implicated in the development of airway squamous metaplasia and is frequently overexpressed in squamous cell carcinomas of the esophagus and stomach. We and others have shown a high level of persistent induction of FRA-1 by lung carcinogens, such as cigarette smoke and asbestos, in pulmonary epithelial cells. However, the exact roles of FRA-1 in regulating lung epithelial cell growth and invasion are poorly understood. To examine this aspect, we have stably overexpressed FRA-1 in human type-II-like alveolar malignant cell line (A549) and a nonmalignant bronchial epithelial cell line (BEAS-2B). FRA-1 greatly enhanced the rate of proliferation, motility, and invasion of A549 and BEAS-2B cells. In athymic nude mice, FRA-1, but not the control vector, rapidly enhanced tumor formation and   metastasis   by A549 cells. In contrast, FRA-1 failed to promote tumor formation by BEAS-2B. We suggest that FRA-1 can promote motility, invasion, and anchorage-independent growth of lung epithelial cells in vitro, but is insufficient for tumor formation. 
 MicroRNA-34 suppresses breast cancer invasion and   metastasis   by directly targeting Fra-1. MicroRNAs have key roles in tumor   metastasis  . Here, we describe the regulation and function of miR-34a and miR-34c (miR-34a/c) in breast cancer   metastasis  . Expression analysis verified that miR-34a/c expression is significantly decreased in metastatic breast cancer cells and human primary breast tumors with lymph node metastases. Overexpression of miR-34a/c could inhibit breast cancer cell migration and invasion in vitro and distal pulmonary   metastasis   in vivo. Further studies revealed that Fos-related antigen 1 (Fra-1 or Fosl1) is a downstream target of miR-34a/c as miR-34a/c bound directly to the 3'untranslated region of Fra-1, subsequently reducing both the mRNA and protein levels of Fra-1. Silencing of Fra-1 recapitulated the effects of miR-34a/c overexpression, whereas enforced expression of Fra-1 reverses the suppressive effects of miR-34a/c. Moreover, significant downregulation of miR-34a in metastatic breast cancer tissues was found to be inversely correlated with Fra-1 expression. Our results demonstrate that miR-34a/c functions as a   metastasis   suppressor to regulate breast cancer migration and invasion through targeting Fra-1 oncogene and suggest a therapeutic application of miR-34 in breast cancer. 
 CD44 affects the expression level of FOS‑like antigen 1 in cervical cancer tissues. Cervical carcinoma is the second most prevalent type of malignancy in females worldwide. The crucial etiological factors involved in the development of cervical carcinoma include infection with the papillomavirus, and the structural or functional mutation of oncogenes and tumor suppressor genes. CD44 refers to a multifunctional family of type I transmembrane proteins. These proteins have been implicated in numerous biological processes, including cell adhesion, cell migration an  d metastas  is. The present study examined the differences in the expression levels of ATP-binding cassette sub-family G member 2, CD24, CD44, CD133, cytokeratin (CK) 14 and CK19 between cervical cancer tissues and corresponding normal non-tumor tissues by flow cytometry. Then, the CD44+ or CD44‑ cells from cervical cancer tissues were sorted for identification and confirmation of differential expression by flow cytometry. The results demonstrated that the expression level of CD44 in cervical cancer tissues was higher than in the corresponding non-tumor normal tissues (t=3.12; P=0.0102). Compared with the CD44‑ cells, the FOS-like antigen 1 (Fra-1), nestin, nuclear receptor subfamily 4, group A, member 2, OCT4 and p63 genes were highly expressed in CD44+ cells. The fold changes were 3.55, 3.55, 2.46, 2.87 and 2.56, respectively (P<0.05). However, BMI1 polycomb ring finger oncogene, ck5, tumor protein p53 and lactotransferrin genes exhibited low expression levels in CD44+ cells. It was verified by western blot analysis and flow cytometry that Fra-1 was highly expressed in CD44+ cells. Fra-1 was a potential target of miR-19a and miR-19b. The expression of miR-19a and miR-19b was downregulated by ~50% in CD44+ cells compared with CD44‑ cells. These findings suggested that CD44 dysregulated the activation of the Fra‑1 gene. The interaction of Fra-1 and CD44 may therefore be important in cervical carcinoma. 
 SIRT1 promotes epithelial-mesenchymal transition and   metastasis   in colorectal cancer by regulating Fra-1 expression. Understanding molecular mechanisms of colorectal cancer (CRC)   metastasis   is urgently required for targeted therapy and prognosis of metastatic CRC. In this study, we explored potential effects of silent mating type information regulation 2 homolog 1 (SIRT1) on CRC   metastasis  . Our data showed that ectopic expression of SIRT1 markedly increased the migration and invasion of CRC cells. In contrast, silencing SIRT1 repressed this behavior in aggressive CRC cells. Tumor xenograft experiments revealed that knockdown of SIRT1 impaired CRC   metastasis   in vivo. Silencing SIRT1 in CRC cells induced mesenchymal-epithelial transition (MET), which is the reverse process of epithelial-mesenchymal transition (EMT) and characterized by a gain of epithelial and loss of mesenchymal markers. We provided a mechanistic insight toward regulation of Fra-1 by SIRT1 and demonstrated a direct link between the SIRT1-Fra-1 axis and EMT. Moreover, SIRT1 expression correlated positively with Fra-1 expression,   metastasis   and overall survival in patients with CRC. Taken together, our data provide a novel mechanistic role of SIRT1 in CRC   metastasis  , suggesting that SIRT1 may serve as a potential therapeutic target for metastatic CRC. 
 Widespread FRA1-dependent control of mesenchymal transdifferentiation programs in colorectal cancer cells. Tumor invasion and   metastasis   involves complex remodeling of gene expression programs governing epithelial homeostasis. Mutational activation of the RAS-ERK is a frequent occurrence in many cancers and has been shown to drive overexpression of the AP-1 family transcription factor FRA1, a potent regulator of migration and invasion in a variety of tumor cell types. However, the nature of FRA1 transcriptional targets and the molecular pathways through which they promote tumor progression remain poorly understood. We found that FRA1 was strongly expressed in tumor cells at the invasive front of human colorectal cancers (CRCs), and that its depletion suppressed mesenchymal-like features in CRC cells in vitro. Genome-wide analysis of FRA1 chromatin occupancy and transcriptional regulation identified epithelial-mesenchymal transition (EMT)-related genes as a major class of direct FRA1 targets in CRC cells. Expression of the pro-mesenchymal subset of these genes predicted adverse outcomes in CRC patients, and involved FRA-1-dependent regulation and cooperation with TGFβ signaling pathway. Our findings reveal an unexpectedly widespread and direct role for FRA1 in control of epithelial-mesenchymal plasticity in CRC cells, and suggest that FRA1 plays an important role in mediating cross talk between oncogenic RAS-ERK and TGFβ signaling networks during tumor progression. 
  GeneRIFs  
 SIRT1 expression correlated positively with Fra-1 expression,   metastasis   and overall survival in patients with colorectal cancer 
 MicroRNA-34 suppresses breast cancer invasion and   metastasis   by directly targeting Fra-1. 
 Identification of a pharmacologically tractable Fra-1/ADORA2B axis promoting breast cancer   metastasis  . 
  [Cc]ell [Ii]nvasion  PubMed  
 Fra-1/AP-1 induces EMT in mammary epithelial cells by modulating Zeb1/2 and TGFβ expression. Epithelial-to-mesenchymal transition (EMT) is essential for embryonic morphogenesis and wound healing and critical for tumour   cell invasio  n and dissemination. The AP-1 transcription factor Fra-1 has been implicated in tumorigenesis and in tumour-associated EMT in human breast cancer. We observed a significant inverse correlation between Fra-1 mRNA expression and distant-metastasis-free survival in a large cohort of breast cancer patients derived from multiple array data sets. This unique correlation among Fos genes prompted us to assess the evolutionary conservation between Fra-1 functions in EMT of human and mouse cells. Ectopic expression of Fra-1 in fully polarized, non-tumourigenic, mouse mammary epithelial EpH4 cells induced a mesenchymal phenotype, characterized by a loss of epithelial and gain of mesenchymal markers. Proliferation, motility and invasiveness were also increased in the resulting EpFra1 cells, and the cells were tumourigenic and efficiently colonized the lung upon transplantation. Molecular analyses revealed increased expression of Tgfβ1 and the EMT-inducing transcription factors Zeb1, Zeb2 and Slug. Mechanistically, Fra-1 binds to the tgfb1 and zeb2 promoters and to an evolutionarily conserved region in the first intron of zeb1. Furthermore, increased activity of a zeb2 promoter reporter was detected in EpFra1 cells and shown to depend on AP-1-binding sites. Inhibiting TGFβ signalling in EpFra1 cells moderately increased the expression of epithelial markers, whereas silencing of zeb1 or zeb2 restored the epithelial phenotype and decreased migration in vitro and tumorigenesis in vivo. Thus Fra-1 induces changes in the expression of genes encoding EMT-related transcription factors leading to the acquisition of mesenchymal, invasive and tumorigenic capacities by epithelial cells. This study defines a novel function of Fra-1/AP-1 in modulating tgfb1, zeb1 and zeb2 expression through direct binding to genomic regulatory regions, which establishes a basis for future in vivo genetic manipulations and preclinical studies using mouse models. 
 Fra-1/AP-1 induces EMT in mammary epithelial cells by modulating Zeb1/2 and TGFβ expression. Epithelial-to-mesenchymal transition (EMT) is essential for embryonic morphogenesis and wound healing and critical for tumour   cell invasio  n and dissemination. The AP-1 transcription factor Fra-1 has been implicated in tumorigenesis and in tumour-associated EMT in human breast cancer. We observed a significant inverse correlation between Fra-1 mRNA expression and distant-metastasis-free survival in a large cohort of breast cancer patients derived from multiple array data sets. This unique correlation among Fos genes prompted us to assess the evolutionary conservation between Fra-1 functions in EMT of human and mouse cells. Ectopic expression of Fra-1 in fully polarized, non-tumourigenic, mouse mammary epithelial EpH4 cells induced a mesenchymal phenotype, characterized by a loss of epithelial and gain of mesenchymal markers. Proliferation, motility and invasiveness were also increased in the resulting EpFra1 cells, and the cells were tumourigenic and efficiently colonized the lung upon transplantation. Molecular analyses revealed increased expression of Tgfβ1 and the EMT-inducing transcription factors Zeb1, Zeb2 and Slug. Mechanistically, Fra-1 binds to the tgfb1 and zeb2 promoters and to an evolutionarily conserved region in the first intron of zeb1. Furthermore, increased activity of a zeb2 promoter reporter was detected in EpFra1 cells and shown to depend on AP-1-binding sites. Inhibiting TGFβ signalling in EpFra1 cells moderately increased the expression of epithelial markers, whereas silencing of zeb1 or zeb2 restored the epithelial phenotype and decreased migration in vitro and tumorigenesis in vivo. Thus Fra-1 induces changes in the expression of genes encoding EMT-related transcription factors leading to the acquisition of mesenchymal, invasive and tumorigenic capacities by epithelial cells. This study defines a novel function of Fra-1/AP-1 in modulating tgfb1, zeb1 and zeb2 expression through direct binding to genomic regulatory regions, which establishes a basis for future in vivo genetic manipulations and preclinical studies using mouse models. 
 Epstein-Barr virus latent membrane protein 2A promotes invasion of nasopharyngeal carcinoma cells through ERK/Fra-1-mediated induction of matrix metalloproteinase 9. Epstein-Barr virus (EBV)-associated nasopharyngeal carcinoma (NPC) is highly metastatic, and this malignant feature may be promoted by an EBV oncoprotein, latent membrane protein 2A (LMP2A). Acting as a signal regulator, LMP2A can enhance invasiveness and motility of epithelial cells. Downstream from the LMP2A-triggered signaling events, it is largely unknown what key effector proteins are induced and essentially promote   cell invasion  . In the present study, we found that in NPC cells, LMP2A upregulated matrix metalloproteinase 9 (MMP9), a metastasis-associated protease. LMP2A increased MMP9 expression at both the mRNA and protein levels. It also activated the MMP9 promoter, in which two AP-1 elements were required for the promoter activation. Among AP-1 transcription factors, Fra-1 was induced by LMP2A and is essential for LMP2A-triggered MMP9 expression. Induction of Fra-1 was dependent on the LMP2A-activated ERK1/2 pathway, and induction of the ERK1/2-Fra-1-MMP9 axis required PY motifs in the amino-terminal domain of LMP2A. Notably, LMP2A-promoted invasion of NPC cells was blocked when MMP9 expression, Fra-1 induction, or ERK1/2 activation was inhibited. In addition, we found an association of LMP2A with MMP9 expression in NPC tumor biopsy specimens, where Fra-1 was a major mediation factor. This study reveals an underlying mechanism of LMP2A-induced   cell invasion  , from signal transduction to upregulation of a critical protease. Considering that MMP9 can also be upregulated by another EBV oncoprotein, LMP1, this protease may be a pivotal effector at which the EBV-induced, invasion-promoting mechanisms converge, serving as an attractive therapeutic target for NPC treatment. 
 The PKCθ pathway participates in the aberrant accumulation of Fra-1 protein in invasive ER-negative breast cancer cells. Fra-1 is aberrantly expressed in a large number of cancer cells and tissues, and emerging evidence suggests an important role for this Fos family protein in both oncogenesis and the progression or maintenance of many tumour types. Here, we show that the concentration of Fra-1 is high in invasive oestrogen receptor (ER)-negative (ER-) breast cancer cell lines, regardless of their Ras pathway status. All of the ER- cells express high levels of activated PKCθ, and the inhibition of PKCθ activity using RNA interference or the expression of a dominant-negative mutant results in a dramatic reduction in Fra-1 abundance. Conversely, the ectopic expression of constitutively active PKCθ leads to Fra-1 phosphorylation and accumulation in poorly invasive ER+ cells. This accumulation is due to the stabilisation of the Fra-1 protein through PKCθ signalling, whereas other members of the PKC family are ineffective. Both Ste20-related proline-alanine-rich kinase (SPAK) and ERK1/2, whose activities are upregulated by PKCθ, participate in PKCθ-driven Fra-1 stabilisation. Interestingly, their relative contributions appear to be different depending on the cell line studied. ERK1/2 signalling has a major role in ER- MDA-MB-231 cells, whereas Fra-1 accumulation occurs mainly through SPAK signalling in ER- BT549 cells. Fra-1 mutational analysis shows that the phosphorylation of S265, T223 and T230 is critical for PKCθ-driven Fra-1 stabilisation. Phosphorylation of the protein was confirmed using specific antisera against Fra-1 phosphorylated on T223 or S265. In addition, Fra-1 participates in PKCθ  -induced cell   invasion and is necessary for PKCθ-induced cell migration. In summary, we identified PKCθ signalling as an important regulator of Fra-1 accumulation in ER- breast cancer cells. Moreover, our results suggest that PKCθ could participate in progression of some breast cancers and could be a new therapeutic target. 
 FRA-1 proto-oncogene induces lung epithelial   cell invasion   and anchorage-independent growth in vitro, but is insufficient to promote tumor growth in vivo. FRA-1 forms activator protein-1 complexes in association with members of the JUN family and drives gene transcription. FRA-1 has been implicated in the development of airway squamous metaplasia and is frequently overexpressed in squamous cell carcinomas of the esophagus and stomach. We and others have shown a high level of persistent induction of FRA-1 by lung carcinogens, such as cigarette smoke and asbestos, in pulmonary epithelial cells. However, the exact roles of FRA-1 in regulating lung epithelial cell growth and invasion are poorly understood. To examine this aspect, we have stably overexpressed FRA-1 in human type-II-like alveolar malignant cell line (A549) and a nonmalignant bronchial epithelial cell line (BEAS-2B). FRA-1 greatly enhanced the rate of proliferation, motility, and invasion of A549 and BEAS-2B cells. In athymic nude mice, FRA-1, but not the control vector, rapidly enhanced tumor formation and metastasis by A549 cells. In contrast, FRA-1 failed to promote tumor formation by BEAS-2B. We suggest that FRA-1 can promote motility, invasion, and anchorage-independent growth of lung epithelial cells in vitro, but is insufficient for tumor formation. 
   
  
 FOS 
 
  NCBI Gene  
  GeneRIF  
  Pubmed  
 
 
  Search Term  Rank  Fields  
  [Mm]igration  6  PubMed(23); GeneRIFs(4)  
  [Ee]xtracellular [Mm]atrix  8  PubMed(5)  
  [Ii]nvasive  10  PubMed(15); GeneRIFs(3)  
  [Mm]etastasis  12  PubMed(16); GeneRIFs(5)  
  [Cc]ell [Ii]nvasion  13  PubMed(5); GeneRIFs(1)  
 
 
 hide/show details for FOS 
  [Mm]igration  PubMed  
 Tissue factor/activated factor VIIa induces matrix metalloproteinase-7 expression through activation of c-Fos via ERK1/2 and p38 MAPK signaling pathways in human colon cancer cell. Increased expression of tissue factor (TF) is associated with tumor invasion and metastasis in human colorectal cancer. We have previously observed that TF/FVIIa upregulates matrix metalloproteinase-7 (MMP-7) expression at the transcriptional level in colon cancer cells. MMP-7 overexpression is believed to play an important role in tumor invasion and metastasis. The aim of this study is to elucidate the molecular mechanisms by which TF/FVIIa induced MMP-7 expression and cell invasion in vitro.Reverse transcription polymerase chain reaction, Western blot, luciferase assay, and chromatin immunoprecipitation (ChIP) were used to determine the potential mechanism and signaling pathways by which TF/FVIIa induced MMP-7 expression and cell invasion in LoVo cells. Small interfering RNA (siRNA) and cell invasion assay was used to examine whether blocking c-Fos expression could abolish FVIIa-mediated upregulation of MMP-7 and cell invasion in vitro.The results showed that FVIIa induced the upregulation of MMP-7 both at the mRNA and protein levels in a time- and dose-dependent manner and increased the invasive behavior of LoVo cells. FVIIa enhanced the promoter activity of MMP-7, and the activator protein-1 (AP-1) binding site was responsible for the activation. Site mutation of the AP-1 binding site in the promoter almost completely abolished FVIIa-mediated response. Furthermore, ChIP assay confirmed that FVIIa promoted the direct binding of c-Fos with the MMP-7 promoter in vivo. FVIIa also induced the expression and nuclear accumulation of the AP-1 subunit c-Fos. siRNA-mediated knockdown of c-Fos eliminated FVIIa-stimulated MMP-7 expression and cell   migration   in vitro. In addition, selective mitogen-activated protein kinase (MAPK) kinase (MEK1/2) inhibitor (PD98059) and p38 MAPK inhibitor SB203580 suppressed MMP-7 upregulation induced by FVIIa.Our data suggest that a novel TF/FVIIa/MAPK/c-Fos/MMP-7 axis plays an important role in modulating the invasion of colon cancer cells and blockage of this pathway holds promise to treat colon cancer metastasis. 
 Ganoderic acids suppress growth and invasive behavior of breast cancer cells by modulating AP-1 and NF-kappaB signaling. Structurally related lanostane-type triterpenes, ganoderic acid A, F and H (GA-A, GA-F, GA-H), were identified in an oriental medicinal mushroom Ganoderma lucidum. In the present study we evaluated the effect of GA-A, GA-H and GA-F on highly invasive human breast cancer cells. We showed that GA-A and GA-H suppressed growth (cell proliferation and colony formation) and invasive behavior (adhesion,   migration   and invasion) of MDA-MB-231 cells. Our results suggest that GA-A and GA-H mediate their biological effects through the inhibition of transcription factors AP-1 and NF-kappaB, resulting in the down-regulation of expression of Cdk4 and the suppression of secretion of uPA, respectively. Furthermore, the activity of ganoderic acids is linked to the hydroxylation in the position 7 and 15 (GA-A) and 3 (GA-H) in their triterpene lanostane structure. In conclusion, hydroxylated triterpenes from G. lucidum could be promising natural agents for the therapy of invasive breast cancers. 
 Expression of Jun and Fos proteins in ovarian tumors of different malignant potential and in ovarian cancer cell lines. Jun and Fos proteins form the transcription factor activating protein 1 (AP-1). They play a role in cell proliferation, malignant transformation and invasion in various tumors. The aim of the current study was to characterize the role of AP-1 in ovarian cancer. Fifty-six ovarian tumors of different invasive potential including 13 metastases as well as 5 ovarian cancer cell lines were analyzed by Western blot analysis regarding their expression of pc-Jun, Jun B, Jun D, c-Fos, Fos B, Fra-1 and Fra-2. In addition, invasion, cell proliferation and   migration   assays were performed. The expression of pc-Jun, Jun B, Jun D and Fra-2 was higher in invasive cancer compared to benign tumors. In metastases, c-Fos and Fos B expression was significantly lower than in the respective primary ovarian carcinomas. The invasive and proliferative potential of the cells was not associated with AP-1 protein expression. These results suggest that AP-1 proteins are differentially expressed in benign ovarian tumors, tumors with low malignant potential and epithelial ovarian carcinomas and metastases. No correlation with the proliferative and invasive potential of ovarian cancer cell lines could be found. 
 Interleukin-1β enhances cell   migratio  n through AP-1 and NF-κB pathway-dependent FGF2 expression in human corneal endothelial cells. Interleukin (IL)-1β is a major pro-inflammatory cytokine that plays a crucial role in the regulation of inflammation and wound healing in the cornea. Elucidation of IL-1β signalling may help identify therapeutic targets for corneal wound healing; however, mechanisms such as c  ell migra  tion, a component of IL-1β-induced wound healing response in human corneal endothelial cells (CEC), have not been well characterised.Stimulation of human CEC with IL-1β activated expression of fibroblast growth factor 2 (FGF2) and resulted in enhanced   cell mig  ration. This, in turn, was abolished by treatment with either IL-1 receptor antagonist or SU-5402, a pan-fibroblast growth factor signalling inhibitor. Phosphatidyl inositol (PI) 3-kinase or IL receptor-associated kinase 1/4 antagonists demonstrated that IL receptor-associated kinase 1/4 activates PI 3-kinase, which in turn phosphorylates p38 and inhibitor κB kinase α/β, leading to FGF2 expression through activation of activator protein 1 (AP-1) and nuclear factor kappa-light-chain enhancer of activated B cells (NF-κB) in human CEC. Treatment of IL-1β-stimulated human CEC with either AP-1 or NF-κB antagonists decreased FGF2 expression and resulted in reduced IL-1β-e  nhanced c  ell migration. Co-treatment of IL-1β-stimulated human CEC with both inhibitors completely blocked FGF2 expression and IL-1β  -enhanced   cell migration. Chromatin immunoprecipitation assays demonstrated that AP-1 and NF-κB directly bind to the FGF2 promoter following IL-1β stimulation.The results show that binding of IL-1β to its receptor in human CEC leads to parallel activation of AP-1 and NF-κB pathways, leading, in turn, to FGF2 expression   and enha  nced cell migration. 
 IL-1β-induced activation of p38 promotes metastasis in gastric adenocarcinoma via upregulation of AP-1/c-fos, MMP2 and MMP9. Interleukin-1β (IL-1β) has been implicated in the progression of gastric adenocarcinoma (GA); however, the molecular mechanisms of action of IL-1β in GA are poorly characterized. P38 and JNK are the major MAPK family members that regulate IL-1β signaling pathways. Here, we investigated the role of both p38 and JNK in IL-1β-induced GA   cell mig  ration, invasion and metastatic potential.The effects of IL-1β-induced p38 and JNK activation in GA cells were determined using in vitro Tra  nswell mi  gration and invasion assays of MKN-45 and AGS cells, or an in vivo metastasis assay in nude mice. The IL-1β-induced p38 signaling pathway was further characterized in GA cells. Activation of the IL-1β/p38 signaling pathway was also assessed in human primary GA tissues by immunohistochemistry.IL-1β-induced activation of p38 increase  d GA cell   migration and invasion in vitro and promoted the metastatic potential of GA cells in vivo; these effects were attenuated by p38 siRNA or the p38 inhibitor SB202190. MMP2 or MMP9 siRNAs and the MMP2/9 inhibitor BiPS also inhibited IL-1β-induc  ed GA cel  l migration and invasion in vitro. IL-1β-induced p38 activation significantly increased MMP2 and MMP9 mRNA and protein expression and activity. Luciferase reporter assays demonstrated that the activator protein-1 (AP-1) and the AP-1 binding sites of the MMP9 promoter (-670/MMP9) were activated by IL-1β-induced p38 activation. Phospho-p38 was significantly upregulated in human GA tissues (compared to matched non-neoplastic tissues), and significantly associated with lymph node metastasis, and invasion beyond the serosa. Expression of phospho-p38 significantly correlated with IL-1β, MMP2, MMP9, and c-fos expression in both human GA tissues and GA cell metastases in the lungs of nude mice. IL-1β was also capable of activating JNK in GA cells, but activation of JNK was not associate  d with GA   cell migration and invasion. Therefore, IL-  1β-induc  ed the migration and invasion in GA cells were regulated by p38, but not by JNK.IL-1β-induced p38 activation and the IL-1β/p38/AP-1(c-fos)/MMP2 & MMP9 pathway play an important role in metastasis in GA; this pathway may provide a novel therapeutic target for GA. 
 Involvement of connexin 43 in angiotensin II-induced   migration   and proliferation of saphenous vein smooth muscle cells via the MAPK-AP-1 signaling pathway. Proliferation and   migration   of vascular smooth muscle cells (VSMCs) lead to intimal thickening and influence the long-term patency of venous graft post coronary arterial bypass graft. There is increasing evidence that connexins are involved in the development of intimal hyperplasia and restenosis. We assessed connexin 43 (Cx43) expression and its role in angiotensin II-induced proliferation and   migration   of smooth muscle cells and the signal pathways involved in human saphenous vein bypass conduits. Angiotensin II significantly increased gap junctional intercellular communication and induced the expression of Cx43 in human saphenous vein SMCs in a dose- and time-dependent manner through angiotensin II type 1 receptor. The effect of angiotensin II was blocked by siRNA of ERK 1/2, p38 MAPK and JNK, respectively. Overexpression of Cx43 markedly increased the proliferation of saphenous vein SMCs. However, siRNA for Cx43 inhibited angiotensin II-induced proliferation, cyclin E expression and   migration   of human saphenous vein SMCs. In dual-luciferase reporter assay, angiotensin II markedly activated AP-1 transcription factor, which was significantly attenuated by a dominant-negative AP-1 (A-Fos) with subsequent inhibition of angiotensin II-induced transcriptional expression of Cx43. These data demonstrate the role of Cx43 in the proliferation and   migration   of human saphenous vein SMCs and angiotensin II-induced Cx43 expression via mitogen-activated protein kinases (MAPK)-AP-1 signaling pathway. 
 Inhibition of AP-1 by SARI negatively regulates transformation progression mediated by CCN1. Enhanced expression of the CCN family of secretory integrin-binding proteins correlates with many essential components of the cancerous state, including tumor cell adhesion, proliferation, invasion and   migration  . Consequently, CCN1 expression is elevated in various cancers, including breast cancer, and its expression directly correlates with poor patient prognosis. Using subtraction-hybridization, combined with induction of cancer cell terminal differentiation, we cloned SARI (suppressor of activator protein (AP)-1, regulated by interferon (IFN)), an IFN-beta-inducible, potent tumor suppressor gene that exerts cancer-selective growth inhibitory effects. Forced expression of SARI using an adenovirus (Ad.SARI) inhibits AP-1 function and downregulates CCN1 expression in multiple cancer lineages, resulting in a profound inhibition in anchorage-independent cell growth and tumor cell invasion. Overexpression of SARI reduces CCN1-promoter activity through inhibition of AP-1 binding. Accordingly, SARI selectively blocks expression of the transformed state in rat embryo fibroblast cells that stably overexpress c-Jun. These results illustrate that SARI inhibits AP-1 transactivating factor binding to the cis-element of the CCN1 promoter, possibly through its interaction with c-Jun. Overall, SARI can directly inhibit CCN1-induced transformation by inhibiting the transcription of CCN1, as well as indirectly by inhibiting the expression of c-Jun (and hence blocking AP-1 activity). In these contexts, transformed cells 'addicted' to AP-1 activity are rendered susceptible to SARI-mediated inhibition of expression of the transformed phenotype. 
 IL-1β-induced activation of p38 promotes metastasis in gastric adenocarcinoma via upregulation of AP-1/c-fos, MMP2 and MMP9. Interleukin-1β (IL-1β) has been implicated in the progression of gastric adenocarcinoma (GA); however, the molecular mechanisms of action of IL-1β in GA are poorly characterized. P38 and JNK are the major MAPK family members that regulate IL-1β signaling pathways. Here, we investigated the role of both p38 and JNK in IL-1β-induced GA   cell mig  ration, invasion and metastatic potential.The effects of IL-1β-induced p38 and JNK activation in GA cells were determined using in vitro Tra  nswell mi  gration and invasion assays of MKN-45 and AGS cells, or an in vivo metastasis assay in nude mice. The IL-1β-induced p38 signaling pathway was further characterized in GA cells. Activation of the IL-1β/p38 signaling pathway was also assessed in human primary GA tissues by immunohistochemistry.IL-1β-induced activation of p38 increase  d GA cell   migration and invasion in vitro and promoted the metastatic potential of GA cells in vivo; these effects were attenuated by p38 siRNA or the p38 inhibitor SB202190. MMP2 or MMP9 siRNAs and the MMP2/9 inhibitor BiPS also inhibited IL-1β-induc  ed GA cel  l migration and invasion in vitro. IL-1β-induced p38 activation significantly increased MMP2 and MMP9 mRNA and protein expression and activity. Luciferase reporter assays demonstrated that the activator protein-1 (AP-1) and the AP-1 binding sites of the MMP9 promoter (-670/MMP9) were activated by IL-1β-induced p38 activation. Phospho-p38 was significantly upregulated in human GA tissues (compared to matched non-neoplastic tissues), and significantly associated with lymph node metastasis, and invasion beyond the serosa. Expression of phospho-p38 significantly correlated with IL-1β, MMP2, MMP9, and c-fos expression in both human GA tissues and GA cell metastases in the lungs of nude mice. IL-1β was also capable of activating JNK in GA cells, but activation of JNK was not associate  d with GA   cell migration and invasion. Therefore, IL-  1β-induc  ed the migration and invasion in GA cells were regulated by p38, but not by JNK.IL-1β-induced p38 activation and the IL-1β/p38/AP-1(c-fos)/MMP2 & MMP9 pathway play an important role in metastasis in GA; this pathway may provide a novel therapeutic target for GA. 
 Interleukin-1β enhances cell   migratio  n through AP-1 and NF-κB pathway-dependent FGF2 expression in human corneal endothelial cells. Interleukin (IL)-1β is a major pro-inflammatory cytokine that plays a crucial role in the regulation of inflammation and wound healing in the cornea. Elucidation of IL-1β signalling may help identify therapeutic targets for corneal wound healing; however, mechanisms such as c  ell migra  tion, a component of IL-1β-induced wound healing response in human corneal endothelial cells (CEC), have not been well characterised.Stimulation of human CEC with IL-1β activated expression of fibroblast growth factor 2 (FGF2) and resulted in enhanced   cell mig  ration. This, in turn, was abolished by treatment with either IL-1 receptor antagonist or SU-5402, a pan-fibroblast growth factor signalling inhibitor. Phosphatidyl inositol (PI) 3-kinase or IL receptor-associated kinase 1/4 antagonists demonstrated that IL receptor-associated kinase 1/4 activates PI 3-kinase, which in turn phosphorylates p38 and inhibitor κB kinase α/β, leading to FGF2 expression through activation of activator protein 1 (AP-1) and nuclear factor kappa-light-chain enhancer of activated B cells (NF-κB) in human CEC. Treatment of IL-1β-stimulated human CEC with either AP-1 or NF-κB antagonists decreased FGF2 expression and resulted in reduced IL-1β-e  nhanced c  ell migration. Co-treatment of IL-1β-stimulated human CEC with both inhibitors completely blocked FGF2 expression and IL-1β  -enhanced   cell migration. Chromatin immunoprecipitation assays demonstrated that AP-1 and NF-κB directly bind to the FGF2 promoter following IL-1β stimulation.The results show that binding of IL-1β to its receptor in human CEC leads to parallel activation of AP-1 and NF-κB pathways, leading, in turn, to FGF2 expression   and enha  nced cell migration. 
 Tissue factor/activated factor VIIa induces matrix metalloproteinase-7 expression through activation of c-Fos via ERK1/2 and p38 MAPK signaling pathways in human colon cancer cell. Increased expression of tissue factor (TF) is associated with tumor invasion and metastasis in human colorectal cancer. We have previously observed that TF/FVIIa upregulates matrix metalloproteinase-7 (MMP-7) expression at the transcriptional level in colon cancer cells. MMP-7 overexpression is believed to play an important role in tumor invasion and metastasis. The aim of this study is to elucidate the molecular mechanisms by which TF/FVIIa induced MMP-7 expression and cell invasion in vitro.Reverse transcription polymerase chain reaction, Western blot, luciferase assay, and chromatin immunoprecipitation (ChIP) were used to determine the potential mechanism and signaling pathways by which TF/FVIIa induced MMP-7 expression and cell invasion in LoVo cells. Small interfering RNA (siRNA) and cell invasion assay was used to examine whether blocking c-Fos expression could abolish FVIIa-mediated upregulation of MMP-7 and cell invasion in vitro.The results showed that FVIIa induced the upregulation of MMP-7 both at the mRNA and protein levels in a time- and dose-dependent manner and increased the invasive behavior of LoVo cells. FVIIa enhanced the promoter activity of MMP-7, and the activator protein-1 (AP-1) binding site was responsible for the activation. Site mutation of the AP-1 binding site in the promoter almost completely abolished FVIIa-mediated response. Furthermore, ChIP assay confirmed that FVIIa promoted the direct binding of c-Fos with the MMP-7 promoter in vivo. FVIIa also induced the expression and nuclear accumulation of the AP-1 subunit c-Fos. siRNA-mediated knockdown of c-Fos eliminated FVIIa-stimulated MMP-7 expression and cell   migration   in vitro. In addition, selective mitogen-activated protein kinase (MAPK) kinase (MEK1/2) inhibitor (PD98059) and p38 MAPK inhibitor SB203580 suppressed MMP-7 upregulation induced by FVIIa.Our data suggest that a novel TF/FVIIa/MAPK/c-Fos/MMP-7 axis plays an important role in modulating the invasion of colon cancer cells and blockage of this pathway holds promise to treat colon cancer metastasis. 
 Inhibition of AP-1 by SARI negatively regulates transformation progression mediated by CCN1. Enhanced expression of the CCN family of secretory integrin-binding proteins correlates with many essential components of the cancerous state, including tumor cell adhesion, proliferation, invasion and   migration  . Consequently, CCN1 expression is elevated in various cancers, including breast cancer, and its expression directly correlates with poor patient prognosis. Using subtraction-hybridization, combined with induction of cancer cell terminal differentiation, we cloned SARI (suppressor of activator protein (AP)-1, regulated by interferon (IFN)), an IFN-beta-inducible, potent tumor suppressor gene that exerts cancer-selective growth inhibitory effects. Forced expression of SARI using an adenovirus (Ad.SARI) inhibits AP-1 function and downregulates CCN1 expression in multiple cancer lineages, resulting in a profound inhibition in anchorage-independent cell growth and tumor cell invasion. Overexpression of SARI reduces CCN1-promoter activity through inhibition of AP-1 binding. Accordingly, SARI selectively blocks expression of the transformed state in rat embryo fibroblast cells that stably overexpress c-Jun. These results illustrate that SARI inhibits AP-1 transactivating factor binding to the cis-element of the CCN1 promoter, possibly through its interaction with c-Jun. Overall, SARI can directly inhibit CCN1-induced transformation by inhibiting the transcription of CCN1, as well as indirectly by inhibiting the expression of c-Jun (and hence blocking AP-1 activity). In these contexts, transformed cells 'addicted' to AP-1 activity are rendered susceptible to SARI-mediated inhibition of expression of the transformed phenotype. 
 Expression of Jun and Fos proteins in ovarian tumors of different malignant potential and in ovarian cancer cell lines. Jun and Fos proteins form the transcription factor activating protein 1 (AP-1). They play a role in cell proliferation, malignant transformation and invasion in various tumors. The aim of the current study was to characterize the role of AP-1 in ovarian cancer. Fifty-six ovarian tumors of different invasive potential including 13 metastases as well as 5 ovarian cancer cell lines were analyzed by Western blot analysis regarding their expression of pc-Jun, Jun B, Jun D, c-Fos, Fos B, Fra-1 and Fra-2. In addition, invasion, cell proliferation and   migration   assays were performed. The expression of pc-Jun, Jun B, Jun D and Fra-2 was higher in invasive cancer compared to benign tumors. In metastases, c-Fos and Fos B expression was significantly lower than in the respective primary ovarian carcinomas. The invasive and proliferative potential of the cells was not associated with AP-1 protein expression. These results suggest that AP-1 proteins are differentially expressed in benign ovarian tumors, tumors with low malignant potential and epithelial ovarian carcinomas and metastases. No correlation with the proliferative and invasive potential of ovarian cancer cell lines could be found. 
 Ganoderic acids suppress growth and invasive behavior of breast cancer cells by modulating AP-1 and NF-kappaB signaling. Structurally related lanostane-type triterpenes, ganoderic acid A, F and H (GA-A, GA-F, GA-H), were identified in an oriental medicinal mushroom Ganoderma lucidum. In the present study we evaluated the effect of GA-A, GA-H and GA-F on highly invasive human breast cancer cells. We showed that GA-A and GA-H suppressed growth (cell proliferation and colony formation) and invasive behavior (adhesion,   migration   and invasion) of MDA-MB-231 cells. Our results suggest that GA-A and GA-H mediate their biological effects through the inhibition of transcription factors AP-1 and NF-kappaB, resulting in the down-regulation of expression of Cdk4 and the suppression of secretion of uPA, respectively. Furthermore, the activity of ganoderic acids is linked to the hydroxylation in the position 7 and 15 (GA-A) and 3 (GA-H) in their triterpene lanostane structure. In conclusion, hydroxylated triterpenes from G. lucidum could be promising natural agents for the therapy of invasive breast cancers. 
 Involvement of connexin 43 in angiotensin II-induced   migration   and proliferation of saphenous vein smooth muscle cells via the MAPK-AP-1 signaling pathway. Proliferation and   migration   of vascular smooth muscle cells (VSMCs) lead to intimal thickening and influence the long-term patency of venous graft post coronary arterial bypass graft. There is increasing evidence that connexins are involved in the development of intimal hyperplasia and restenosis. We assessed connexin 43 (Cx43) expression and its role in angiotensin II-induced proliferation and   migration   of smooth muscle cells and the signal pathways involved in human saphenous vein bypass conduits. Angiotensin II significantly increased gap junctional intercellular communication and induced the expression of Cx43 in human saphenous vein SMCs in a dose- and time-dependent manner through angiotensin II type 1 receptor. The effect of angiotensin II was blocked by siRNA of ERK 1/2, p38 MAPK and JNK, respectively. Overexpression of Cx43 markedly increased the proliferation of saphenous vein SMCs. However, siRNA for Cx43 inhibited angiotensin II-induced proliferation, cyclin E expression and   migration   of human saphenous vein SMCs. In dual-luciferase reporter assay, angiotensin II markedly activated AP-1 transcription factor, which was significantly attenuated by a dominant-negative AP-1 (A-Fos) with subsequent inhibition of angiotensin II-induced transcriptional expression of Cx43. These data demonstrate the role of Cx43 in the proliferation and   migration   of human saphenous vein SMCs and angiotensin II-induced Cx43 expression via mitogen-activated protein kinases (MAPK)-AP-1 signaling pathway. 
 Interleukin-1 Receptor Type 2 Acts with c-Fos to Enhance the Expression of Interleukin-6 and Vascular Endothelial Growth Factor A in Colon Cancer Cells and Induce Angiogenesis. Interleukin-1 receptor type 2 (IL1R2) acts as a decoy receptor of exogenous IL-1; however, its intracellular activity is poorly understood. We previously demonstrated that IL1R2 intracellularly activates the expression of several proinflammatory cytokines and affects cell   migration  . In this study, we found that intracellular IL1R2 expression was increased in human colorectal cancer cells (CRCs) compared with normal colon cells. We also observed that the mRNA levels of IL1R2 were highly correlated with IL-6 in tumor tissues of CRC patients. By modulating its expression in CRC cells, we verified that enhanced IL1R2 expression transcriptionally activated the expression of IL-6 and VEGF-A. Conditioned medium harvested from IL1R2-overexpressing CRC cells contained higher levels of IL-6 and VEGF-A than that from vector control cells and significantly enhanced the proliferation,   migration  , and tube formation of cultured endothelial cells. We further demonstrated a positive association of intracellular IL1R2 levels with tumor growth and microvessel density in xenograft mouse models. These results revealed that IL1R2 activates the expression of angiogenic factors. Mechanistically, we revealed that IL1R2 complexes with c-Fos and binds to the AP-1 site at the IL-6 and VEGF-A promoters. Together, these results reveal a novel function of intracellular IL1R2 that acts with c-Fos to enhance the transcription of IL-6 and VEGF-A, which promotes angiogenesis in CRC. 
 AP-1 transcription factor mediates VEGF-induced endothelial cell   migration   and proliferation. VEGF, upon binding to its endothelial cell specific receptors VEGF-R1 and VEGF-R2, can induce endothelial cell   migration  , proliferation and angiogenesis. However, the molecular mechanism of these effects still remains unclear. In this study, we investigated whether VEGF promotes human umbilical vascular endothelial cell (HUVEC)   migration   and proliferation through activator protein-1 transcription factor (AP-1) family. We first showed that VEGF induces immediate-early genes AP-1 family gene expression differentially with the profound induction of JunB (both mRNA and protein) under various conditions (PBS, DMSO or control adenoviruses). The increase in AP-1 mRNA expression occurs primarily at the transcriptional level. Inhibition of AP-1 DNA binding activity by adenovirus expressing a potent dominant negative form of c-Fos (Afos) significantly attenuated VEGF-induced HUVEC   migration   and proliferation and cyclin D1 expression. Knockdown of JunB with adenovirus expressing JunB shRNA reduces VEGF-induced JunB expression and attenuated HUVEC   migration  . However the shJunB-expressing virus has no effect on VEGF-induced cyclin D1 protein expression and proliferation. These results suggest that VEGF-induced endothelial   migration   is mediated primarily by induction of JunB whereas the promotion of endothelial proliferation by VEGF is mediated by JunB-independent AP-1 family members. 
 The nuclear import of oncoprotein hepatitis B X-interacting protein depends on interacting with c-Fos and phosphorylation of both proteins in breast cancer cells. Aberrant nuclear localization of oncogenic transcription factors and coactivators always leads to the development of cancer. We have reported that the oncoprotein hepatitis B X-interacting protein (HBXIP) acts as a novel transcriptional coactivator to promote proliferation and   migration   of breast cancer cells. However, the mechanism of regulating the nuclear import of HBXIP remains unclear. In the present study, we found that HBXIP interacted with c-Fos through their leucine zipper domains in vitro and in vivo. Interestingly, the leucine zipper mutant of HBXIP (or c-Fos) was unavailable to bind to c-Fos (or HBXIP), resulting in the disappearance of nuclear localization of HBXIP. Moreover, we revealed that the nuclear import of HBXIP was required for phosphorylation of c-Fos at Thr(232), Thr(325), Thr(331), and Ser(374) by ERK1/2. In addition, the mutant of HBXIP at the Ser(108) phosphorylation site failed to import into the nucleus. Strikingly, we found that the kinase ataxia telangiectasia mutated (ATM) phosphorylated HBXIP at Ser(108). The knockdown of ATM by siRNA remarkably decreased the levels of serine phosphorylation and blocked the nuclear import of HBXIP. Then, we identified that ATM could bind to HBXIP. Moreover, we validated that the nuclear import of HBXIP contributed to its nuclear function. Therefore, we conclude that the nuclear import of the oncoprotein HBXIP requires interaction with c-Fos through their leucine zipper domains and phosphorylation of both proteins in breast cancer cells. Thus, our findings provide new insights into the mechanism of the nuclear import of HBXIP. Therapeutically, the block of the nuclear import of HBXIP is significant in breast cancer. 
 PRL-3 suppresses c-Fos and integrin α2 expression in ovarian cancer cells. Phosphatase of regenerating liver-3 (PRL-3), a protein tyrosine phosphatase, is highly expressed in multiple human cancers and strongly implicated in tumor progression and cancer metastasis. However, the mechanisms by which PRL-3 promotes cancer cell   migratio  n, invasion, and metastasis are not very well understood. In this study, we investigated the contribution and molecular mechanisms of PRL-3 in ovarian cancer progression.PRL-3 protein expression was detected on ovarian cancer tissue microarrays using immunohistochemistry. Stable PRL-3 depleted cell lines were generated using short hairpin RNA (shRNA) constructs. The   migratio  n and invasion potential of these cells were analyzed using Transwell and Matrigel assays, respectively. Immunoblotting and immunofluorescence were used to detect protein levels and distribution in PRL-3-ablated cells and the control cells. Cell morphology was observed with hematoxylin-eosin staining and transmission electron microscopy. Finally, PRL-3-ablated and control cells were injected into nude mice for xenograft tumorigenicity assays.Elevated PRL-3 expression was detected in 19% (26 out of 135) of human ovarian cancer patient samples, but not in normal ovary tissues (0 out of 14). Stable depletion of PRL-3 in A2780 ovarian cancer cells resulted in decreased   migratio  n ability and invasion activity compared with control parental A2780 cells. In addition, PRL-3-ablated cells also exhibited flattened morphology and extended lamellipodia. To address the possible molecular basis for the altered phenotypes associated with PRL-3 down-regulation, we assessed the expression profiles of various proteins involved in cell-matrix adhesion. Depletion of PRL-3 dramatically enhanced both RNA and protein levels of the cell surface receptor integrin α2, but not its heterologous binding partner integrin β1. Inhibition of PRL-3 also correlated with elevated expression and phosphorylation of paxillin. A pronounced increase in the expression and activation of c-fos, a transcriptional activator of integrin α2, was observed in these PRL-3 knock-down cells. Moreover, forced expression of EGFP-PRL-3 resulted in the suppression of both integrin α2 and c-fos expression in A2780 cells. Significantly, using a xenograft tumor model, we observed a greatly reduced tumorigenicity of A2780 PRL-3 knock-down cells in vivo.These results suggest that PRL-3 plays a critical role in ovarian cancer tumorigenicity and maintaining the malignant phenotype. PRL-3 may inhibit c-fos transcriptional regulation of integrin α2 signaling. Our results strongly support a role for PRL-3 as a promising therapeutic target and potential early biomarker in ovarian cancer progression. 
 Derepression of c-Fos caused by microRNA-139 down-regulation contributes to the metastasis of human hepatocellular carcinoma. This study investigates whether the anti-metastasis effect of microRNA-139 (miR-139) on hepatocellular carcinoma (HCC) is mediated through regulating c-fos expression. The expression levels of miR-139 and c-fos in human HCC cell sublines with high (MHCC97H) and low (MHCC97L) spontaneous metastatic potentials were quantified using QPCR or Western blot. miR-139 mimics was transfected into MHCC97H cells to overexpress miR-139, and miR-139 inhibitor was transfected into MHCC97L cells to down-express miR-139. The effect of overexpression or down-expression of miR-139 on c-fos expression of MHCC97H and MHCC97L cells was evaluated using QPCR and Western blot. The 3' untranslated region segments of FOS containing the miR-139 binding sites were amplified by PCR, and the luciferase activity in the transfected cells was assayed. In comparison with the expression level of miR-139 in MHCC97L cells, the expression level in MHCC97H cells was significantly decreased, whereas c-Fos was significantly up-regulated in MHCC97H. The overexpression of miR-139 significantly inhibited the expression of c-fos in MHCC97H cells, and the down-expression of miR-139 significantly promoted the expression of c-fos in MHCC97L cells. miR-139 suppressed the luciferase activity of the pGL-FOS by approximately 40% compared with the negative control. In vitro cell   migration   analysis demonstrated that depletion of c-fos or overexpression of miR-139 in MHCC97H cells reduced cell   migration  , whereas overexpression of c-fos or depletion of miR-139 in MHCC97L cells increased cell   migration  . Thus, we got the conclusion that miR-139 expression is down-regulated in human HCC cell sublines with high spontaneous metastatic potentials (MHCC97H). Derepression of c-Fos caused by miR-139 down-regulation contributes to the metastasis of HCC. 
 c-FOS suppresses ovarian cancer progression by changing adhesion. C-Fos was initially described as oncogene, but was associated with favourable prognosis in ovarian cancer (OvCa) patients. The molecular and functional aspects underlying this effect are still unknown.Using stable transfectants of SKOV3 and OVCAR8 cells, proliferation,   migration  , invasion and apoptotic potential of c-FOS-overexpressing clones and controls were compared. Adherence to components of the extracellular matrix was analysed in static assays, and adhesion to E-selectin, endothelial and mesothelial cells in dynamic flow assays. The effect of c-FOS in vivo was studied after intraperitoneal injection of SKOV3 clones into SCID mice, and changes in gene expression were determined by microarray analysis.Tumour growth after injection into SCID mice was strongly delayed by c-FOS overexpression, with reduction of lung metastases and circulating tumour cells. In vitro, c-FOS had only weak influence on proliferation and   migration  , but was strongly pro-apoptotic. Adhesion to components of the extracellular matrix (collagen I, IV) and to E-selectin, endothelial and mesothelial cells was significantly reduced in c-FOS-overexpressing OvCa cells. This corresponds to deregulation of adhesion proteins and glycosylation enzymes in microarray analysis.In addition to its known pro-apoptotic effect, c-FOS might influence OvCa progression by changing the adhesion of OvCa cells to peritoneal surfaces. 
 Angiopoietin-1 promotes endothelial cell proliferation and   migration   through AP-1-dependent autocrine production of interleukin-8. Angiopoietin-1 (Ang-1), ligand for the endothelial cell-specific Tie-2 receptors, promotes   migration   and proliferation of endothelial cells, however, whether these effects are promoted through the release of a secondary mediator remains unclear. In this study, we assessed whether Ang-1 promotes endothelial cell   migration   and proliferation through the release of interleukin-8 (IL-8). Ang-1 elicited in human umbilical vein endothelial cells (HUVECs) a dose- and time-dependent increase in IL-8 production as a result of induction of mRNA and enhanced mRNA stability of IL-8 transcripts. IL-8 production is also elevated in HUVECs transduced with retroviruses expressing Ang-1. Neutralization of IL-8 in these cells with a specific antibody significantly attenuated proliferation and   migration   and induced caspase-3 activation. Exposure to Ang-1 triggered a significant increase in DNA binding of activator protein-1 (AP-1) to a relatively short fragment of IL-8 promoter. Upstream from the AP-1 complex, up-regulation of IL-8 transcription by Ang-1 was mediated through the Erk1/2, SAPK/JNK, and PI-3 kinase pathways, which triggered c-Jun phosphorylation on Ser63 and Ser73. These results suggest that promotion of endothelial   migration   and proliferation by Ang-1 is mediated, in part, through the production of IL-8, which acts in an autocrine fashion to suppress apoptosis and facilitate cell proliferation and   migration  . 
 PRIP promotes tumor formation through enhancing serum-responsive factor-mediated FOS expression. PRIP (peroxisome proliferator-activator receptor interacting protein) is a nuclear receptor coactivator required for mammary gland development. To understand the function of PRIP in breast tumorigenesis, we established a mammary tumor cell line with the PRIP(Loxp/Loxp) genotype. By knocking out the PRIP gene in the tumor cell line, we demonstrated that PRIP deficiency led to inhibited tumor formation without affecting tumor cell proliferation. The PRIP deficiency was associated with decreased cell invasion and   migration   capabilities. We found that PRIP deficiency substantially reduced FOS gene expression. A chromatin immunoprecipitation assay revealed that PRIP was recruited to the FOS promoter. In addition, we demonstrated that PRIP also directly up-regulated the FOS gene expression in human breast cancer cells. Promoter analysis showed that PRIP acted through serum-responsive factor to regulate FOS gene expression. Finally, by re-expressing the FOS gene, we confirmed that the inhibited tumor formation of PRIP-deficient tumor cells was due to reduced expression of the FOS gene. 
 c-Fos is required for TGFbeta1 production and the associated paracrine migratory effects of human colon carcinoma cells. In tumor cells that have lost responsiveness to the growth inhibitory effects of transforming growth factor beta (TGFbeta), increased TGFbeta production by the tumor cells often contributes to cancer progression, primarily through paracrine mechanisms. Here we investigated the major components of the activator protein-1 (AP-1) complex in the TGFbeta1 promoter of human colon carcinoma cells (HCCCs). In contrast to untransformed epithelial cells (UECs), HCCCs displayed constitutive activation of AP-1 at the proximal AP-1 site in the human TGFbeta1 promoter. Further, in contrast to the JunD and Fra-2 components present in the AP-1 complex at this AP-1 site in UECs, c-Fos was the major detectable AP-1 component in HCCCs. Thus, transcriptional factor switching had occurred in HCCCs relative to the UECs, with regard to the proximal AP-1 site of the human TGFbeta1 promoter. Small interfering RNAs (siRNAs) against c-Fos significantly suppressed AP-1 activity at the relevant AP-1 site, and led to a decrease in TGFbeta1 secretion by the HCCCs. Our results indicate for the first time that c-Fos binding at the TGFbeta1 promoter proximal AP-1 site in HCCCs is required for TGFbeta1 production by the tumor cells. Further, we demonstrated that blockade of TGFbeta1 secretion by c-Fos siRNA led to a suppression of the cellular   migration   and mitogenesis of NIH 3T3 fibroblasts in a paracrine fashion. Thus, c-Fos may have utility as a target for blocking tumor cell-secreted TGFbeta1, thereby suppressing the migratory behavior associated with the malignant phenotype of HCCCs. 
  GeneRIFs  
 VEGF-induced endothelial   migration   is mediated primarily by induction of JunB whereas the promotion of endothelial proliferation by VEGF is mediated by JunB-independent AP-1 family members. 
 Il-1beta enhances cell   migration   through AP-1 and NF-kappaB pathway-dependent FGF2 expression in human corneal endothelial cells. 
 These data demonstrate the role of Cx43 in the proliferation and   migration   of human saphenous vein smooth muscle cells and angiotensin II-induced Cx43 expression via mitogen-activated protein kinases (MAPK)-AP-1 signaling pathway. 
 These results suggest that promotion of endothelial   migration   and proliferation by Ang-1 is mediated, in part, through the production of IL-8, which acts in an autocrine fashion to suppress apoptosis and facilitate cell proliferation and   migration  . 
  [Ee]xtracellular [Mm]atrix  PubMed  
 Synergistic transcriptional activation of the tissue inhibitor of metalloproteinases-1 promoter via functional interaction of AP-1 and Ets-1 transcription factors. The tissue inhibitor of metalloproteinases-1 (TIMP-1) is an inhibitor of the   extracellular matrix  -degrading metalloproteinases. We characterized response elements that control TIMP-1 gene expression. One contains a binding site that selectively binds c-Fos and c-Jun in vitro and confers a response to multiple AP-1 family members in vivo. Adjacent to this is a binding site for Ets domain proteins. Although c-Ets-1 alone did not activate transcription from this element, it enhanced transcription synergistically with AP-1 either in the context of the natural promoter or when the sequence was linked upstream of a heterologous promoter. Furthermore, a complex of c-Jun and c-Fos interacted with c-Ets-1 in vitro. These results suggest that AP-1 tethers c-Ets-1 to the TIMP-1 promoter via protein-protein interaction to achieve Ets-dependent transcriptional regulation. Collectively, our results indicate that TIMP-1 expression is controlled by several DNA response elements that respond to variations in the level and activity of AP-1 and Ets transcriptional regulatory proteins. 
 Phosphorylation of c-Fos by members of the p38 MAPK family. Role in the AP-1 response to UV light. Exposure to sources of UV radiation, such as sunlight, induces a number of cellular alterations that are highly dependent on its ability to affect gene expression. Among them, the rapid activation of genes coding for two subfamilies of proto-oncoproteins, Fos and Jun, which constitute the AP-1 transcription factor, plays a key role in the subsequent regulation of expression of genes involved in DNA repair, cell proliferation, cell cycle arrest, death by apoptosis, and tissue and   extracellular matrix   remodeling proteases. Besides being regulated at the transcriptional level, Jun and Fos transcriptional activities are also regulated by phosphorylation as a result of the activation of intracellular signaling cascades. In this regard, the phosphorylation of c-Jun by UV-induced JNK has been readily documented, whereas a role for Fos proteins in UV-mediated responses and the identification of Fos-activating kinases has remained elusive. Here we identify p38 MAPKs as proteins that can associate with c-Fos and phosphorylate its transactivation domain both in vitro and in vivo. This phosphorylation is transduced into changes in its transcriptional ability as p38-activated c-Fos enhances AP1-driven gene expression. Our findings indicate that as a consequence of the activation of stress pathways induced by UV light, endogenous c-Fos becomes a substrate of p38 MAPKs and, for the first time, provide evidence that support a critical role for p38 MAPKs in mediating stress-induced c-Fos phosphorylation and gene transcription activation. Using a specific pharmacological inhibitor for p38alpha and -beta, we found that most likely these two isoforms mediate UV-induced c-Fos phosphorylation in vivo. Thus, these newly described pathways act concomitantly with the activation of c-Jun by JNK/MAPKs, thereby contributing to the complexity of AP1-driven gene transcription regulation. 
 Phosphorylation of c-Fos by members of the p38 MAPK family. Role in the AP-1 response to UV light. Exposure to sources of UV radiation, such as sunlight, induces a number of cellular alterations that are highly dependent on its ability to affect gene expression. Among them, the rapid activation of genes coding for two subfamilies of proto-oncoproteins, Fos and Jun, which constitute the AP-1 transcription factor, plays a key role in the subsequent regulation of expression of genes involved in DNA repair, cell proliferation, cell cycle arrest, death by apoptosis, and tissue and   extracellular matrix   remodeling proteases. Besides being regulated at the transcriptional level, Jun and Fos transcriptional activities are also regulated by phosphorylation as a result of the activation of intracellular signaling cascades. In this regard, the phosphorylation of c-Jun by UV-induced JNK has been readily documented, whereas a role for Fos proteins in UV-mediated responses and the identification of Fos-activating kinases has remained elusive. Here we identify p38 MAPKs as proteins that can associate with c-Fos and phosphorylate its transactivation domain both in vitro and in vivo. This phosphorylation is transduced into changes in its transcriptional ability as p38-activated c-Fos enhances AP1-driven gene expression. Our findings indicate that as a consequence of the activation of stress pathways induced by UV light, endogenous c-Fos becomes a substrate of p38 MAPKs and, for the first time, provide evidence that support a critical role for p38 MAPKs in mediating stress-induced c-Fos phosphorylation and gene transcription activation. Using a specific pharmacological inhibitor for p38alpha and -beta, we found that most likely these two isoforms mediate UV-induced c-Fos phosphorylation in vivo. Thus, these newly described pathways act concomitantly with the activation of c-Jun by JNK/MAPKs, thereby contributing to the complexity of AP1-driven gene transcription regulation. 
 Protein kinase C isoforms zeta and iota mediate collagenase expression and cartilage destruction via STAT3- and ERK-dependent c-fos induction. The protein kinase C (PKC) signaling pathway is a major regulator of cellular functions and is implicated in pathologies involving   extracellular matrix   remodeling. Inflammatory joint disease is characterized by excessive   extracellular matrix   catabolism, and here we assess the role of PKC in the induction of the collagenases, matrix metalloproteinase (MMP)-1 and MMP-13, in human chondrocytes by the potent cytokine stimulus interleukin-1 (IL-1) in combination with oncostatin M (OSM). IL-1 + OSM-stimulated collagenolysis and gelatinase activity were ameliorated by pharmacological PKC inhibition in bovine cartilage, as was collagenase gene induction in human chondrocytes. Small interfering RNA-mediated silencing of PKC gene expression showed that both novel (nPKC delta, nPKC eta) and atypical (aPKC zeta, aPKC iota) isoforms were involved in collagenase induction by IL-1. However, MMP1 and MMP13 induction by IL-1 + OSM was inhibited only by aPKC silencing, suggesting that only atypical isoforms play a significant role in complex inflammatory milieus. Silencing of either aPKC led to diminished IL-1 + OSM-dependent extracellular signal-regulated kinase (ERK) and signal transducer and activator of transcription (STAT) 3 phosphorylation, and c-fos expression. STAT3 gene silencing or ERK pathway inhibition also resulted in loss of IL-1 + OSM-stimulated c-fos and collagenase expression. Silencing of c-fos and c-jun expression was sufficient to abrogate IL-1 + OSM-stimulated collagenase gene induction, and overexpression of both c-fos and c-jun was sufficient to drive transcription from the MMP1 promoter in the absence of a stimulus. Our data identify atypical PKC isozymes as STAT and ERK activators that mediate c-fos and collagenase expression during IL-1 + OSM synergy in human chondrocytes. aPKCs may constitute potential therapeutic targets for inflammatory joint diseases involving increased collagenase expression. 
 c-FOS suppresses ovarian cancer progression by changing adhesion. C-Fos was initially described as oncogene, but was associated with favourable prognosis in ovarian cancer (OvCa) patients. The molecular and functional aspects underlying this effect are still unknown.Using stable transfectants of SKOV3 and OVCAR8 cells, proliferation, migration, invasion and apoptotic potential of c-FOS-overexpressing clones and controls were compared. Adherence to components of the   extracellular matrix   was analysed in static assays, and adhesion to E-selectin, endothelial and mesothelial cells in dynamic flow assays. The effect of c-FOS in vivo was studied after intraperitoneal injection of SKOV3 clones into SCID mice, and changes in gene expression were determined by microarray analysis.Tumour growth after injection into SCID mice was strongly delayed by c-FOS overexpression, with reduction of lung metastases and circulating tumour cells. In vitro, c-FOS had only weak influence on proliferation and migration, but was strongly pro-apoptotic. Adhesion to components of the   extracellular matrix   (collagen I, IV) and to E-selectin, endothelial and mesothelial cells was significantly reduced in c-FOS-overexpressing OvCa cells. This corresponds to deregulation of adhesion proteins and glycosylation enzymes in microarray analysis.In addition to its known pro-apoptotic effect, c-FOS might influence OvCa progression by changing the adhesion of OvCa cells to peritoneal surfaces. 
  [Ii]nvasive  PubMed  
 Cadmium induces matrix metalloproteinase-9 expression via ROS-dependent EGFR, NF-кB, and AP-1 pathways in human endothelial cells. Cadmium (Cd), a widespread cumulative pollutant, is a known human carcinogen, associated with inflammation and tumors. Matrix metalloproteinase-9 (MMP-9) plays a pivotal role in tumor metastasis; however, the mechanisms underlying the MMP-9 expression induced by Cd remain obscure in human endothelial cells. Here, Cd elevated MMP-9 expression in dose- and time-dependent manners in human endothelial cells. Cd increased ROS production and the ROS-producing NADPH oxidase. Cd translocates p47(phox), a key subunit of NADPH oxidase, to the cell membrane. Cd also activated the phosphorylation of EGFR, Akt, Erk1/2, and JNK1/2 in addition to promoting NF-кB and AP-1 binding activities. Specific inhibitor and mutagenesis studies showed that EGFR, Akt, Erk1/2, JNK1/2 and transcription factors NF-κB and AP-1 were related to Cd-induced MMP-9 expression in endothelial cells. Akt, Erk1/2, and JNK1/2 functioned as upstream signals in the activation of NF-κB and AP-1, respectively. In addition, N-acetyl-l-cystein (NAC), diphenyleneiodonium chloride (DPI) and apocynin (APO) inhibited the Cd-induced activation of EGFR, Akt, Erk1/2, JNK1/2, and p38 MAPK, indicating that ROS production by NADPH oxidase is the furthest upstream signal in MMP-9 expression. At present, it states that Cd displayed mar  ked inva  siveness in ECV304 cells, which was partially abrogated by MMP-9 neutralizing antibodies. These results demonstrated that Cd induces MMP-9 expression via ROS-dependent EGFR->Erk1/2, JNK1/2->AP-1 and EGFR->Akt->NF-κB signaling pathways and, in turn, stimul  ates inv  asiveness in human endothelial cells. 
 Tissue factor/activated factor VIIa induces matrix metalloproteinase-7 expression through activation of c-Fos via ERK1/2 and p38 MAPK signaling pathways in human colon cancer cell. Increased expression of tissue factor (TF) is associated with tumor invasion and metastasis in human colorectal cancer. We have previously observed that TF/FVIIa upregulates matrix metalloproteinase-7 (MMP-7) expression at the transcriptional level in colon cancer cells. MMP-7 overexpression is believed to play an important role in tumor invasion and metastasis. The aim of this study is to elucidate the molecular mechanisms by which TF/FVIIa induced MMP-7 expression and cell invasion in vitro.Reverse transcription polymerase chain reaction, Western blot, luciferase assay, and chromatin immunoprecipitation (ChIP) were used to determine the potential mechanism and signaling pathways by which TF/FVIIa induced MMP-7 expression and cell invasion in LoVo cells. Small interfering RNA (siRNA) and cell invasion assay was used to examine whether blocking c-Fos expression could abolish FVIIa-mediated upregulation of MMP-7 and cell invasion in vitro.The results showed that FVIIa induced the upregulation of MMP-7 both at the mRNA and protein levels in a time- and dose-dependent manner and increased the   invasive   behavior of LoVo cells. FVIIa enhanced the promoter activity of MMP-7, and the activator protein-1 (AP-1) binding site was responsible for the activation. Site mutation of the AP-1 binding site in the promoter almost completely abolished FVIIa-mediated response. Furthermore, ChIP assay confirmed that FVIIa promoted the direct binding of c-Fos with the MMP-7 promoter in vivo. FVIIa also induced the expression and nuclear accumulation of the AP-1 subunit c-Fos. siRNA-mediated knockdown of c-Fos eliminated FVIIa-stimulated MMP-7 expression and cell migration in vitro. In addition, selective mitogen-activated protein kinase (MAPK) kinase (MEK1/2) inhibitor (PD98059) and p38 MAPK inhibitor SB203580 suppressed MMP-7 upregulation induced by FVIIa.Our data suggest that a novel TF/FVIIa/MAPK/c-Fos/MMP-7 axis plays an important role in modulating the invasion of colon cancer cells and blockage of this pathway holds promise to treat colon cancer metastasis. 
 Ganoderic acids suppress growth and   invasive   behavior of breast cancer cells by modulating AP-1 and NF-kappaB signaling. Structurally related lanostane-type triterpenes, ganoderic acid A, F and H (GA-A, GA-F, GA-H), were identified in an oriental medicinal mushroom Ganoderma lucidum. In the present study we evaluated the effect of GA-A, GA-H and GA-F on highly   invasive   human breast cancer cells. We showed that GA-A and GA-H suppressed growth (cell proliferation and colony formation) and   invasive   behavior (adhesion, migration and invasion) of MDA-MB-231 cells. Our results suggest that GA-A and GA-H mediate their biological effects through the inhibition of transcription factors AP-1 and NF-kappaB, resulting in the down-regulation of expression of Cdk4 and the suppression of secretion of uPA, respectively. Furthermore, the activity of ganoderic acids is linked to the hydroxylation in the position 7 and 15 (GA-A) and 3 (GA-H) in their triterpene lanostane structure. In conclusion, hydroxylated triterpenes from G. lucidum could be promising natural agents for the therapy of   invasive   breast cancers. 
 Evaluation of candidate stromal epithelial cross-talk genes identifies association between risk of serous ovarian cancer and TERT, a cancer susceptibility "hot-spot". We hypothesized that variants in genes expressed as a consequence of interactions between ovarian cancer cells and the host micro-environment could contribute to cancer susceptibility. We therefore used a two-stage approach to evaluate common single nucleotide polymorphisms (SNPs) in 173 genes involved in stromal epithelial interactions in the Ovarian Cancer Association Consortium (OCAC). In the discovery stage, cases with epithelial ovarian cancer (n=675) and controls (n=1,162) were genotyped at 1,536 SNPs using an Illumina GoldenGate assay. Based on Positive Predictive Value estimates, three SNPs-PODXL rs1013368, ITGA6 rs13027811, and MMP3 rs522616-were selected for replication using TaqMan genotyping in up to 3,059 serous   invasive   cases and 8,905 controls from 16 OCAC case-control studies. An additional 18 SNPs with Pper-allele<0.05 in the discovery stage were selected for replication in a subset of five OCAC studies (n=1,233 serous   invasive   cases; n=3,364 controls). The discovery stage associations in PODXL, ITGA6, and MMP3 were attenuated in the larger replication set (adj. Pper-allele>or=0.5). However genotypes at TERT rs7726159 were associated with ovarian cancer risk in the smaller, five-study replication study (Pper-allele=0.03). Combined analysis of the discovery and replication sets for this TERT SNP showed an increased risk of serous ovarian cancer among non-Hispanic whites [adj. ORper-allele 1.14 (1.04-1.24) p=0.003]. Our study adds to the growing evidence that, like the 8q24 locus, the telomerase reverse transcriptase locus at 5p15.33, is a general cancer susceptibility locus. 
 Expression of Jun and Fos proteins in ovarian tumors of different malignant potential and in ovarian cancer cell lines. Jun and Fos proteins form the transcription factor activating protein 1 (AP-1). They play a role in cell proliferation, malignant transformation and invasion in various tumors. The aim of the current study was to characterize the role of AP-1 in ovarian cancer. Fifty-six ovarian tumors of different   invasive   potential including 13 metastases as well as 5 ovarian cancer cell lines were analyzed by Western blot analysis regarding their expression of pc-Jun, Jun B, Jun D, c-Fos, Fos B, Fra-1 and Fra-2. In addition, invasion, cell proliferation and migration assays were performed. The expression of pc-Jun, Jun B, Jun D and Fra-2 was higher in   invasive   cancer compared to benign tumors. In metastases, c-Fos and Fos B expression was significantly lower than in the respective primary ovarian carcinomas. The   invasive   and proliferative potential of the cells was not associated with AP-1 protein expression. These results suggest that AP-1 proteins are differentially expressed in benign ovarian tumors, tumors with low malignant potential and epithelial ovarian carcinomas and metastases. No correlation with the proliferative and   invasive   potential of ovarian cancer cell lines could be found. 
 Genetic susceptibility to distinct bladder cancer subphenotypes. Clinical, pathologic, and molecular evidence indicate that bladder cancer is heterogeneous with pathologic/molecular features that define distinct subphenotypes with different prognoses. It is conceivable that specific patterns of genetic susceptibility are associated with particular subphenotypes.To examine evidence for the contribution of germline genetic variation to bladder cancer heterogeneity.The Spanish Bladder Cancer/EPICURO Study is a case-control study based in 18 hospitals located in five areas in Spain. Cases were patients with a newly diagnosed, histologically confirmed, urothelial cell carcinoma of the bladder from 1998 to 2001. Case diagnoses were reviewed and uniformly classified by pathologists following the World Health Organisation/International Society of Urological Pathology 1999 criteria. Controls were hospital-matched patients (n=1149).A total of 1526 candidate variants in 423 candidate genes were analysed. Three distinct subphenotypes were defined according to stage and grade: low-grade nonmuscle   invasive   (n=586), high-grade nonmuscle   invasive   (n=219), and muscle   invasive   (n=246). The association between each variant and subphenotype was assessed by polytomous risk models adjusting for potential confounders. Heterogeneity in genetic susceptibility among subphenotypes was also tested.Two established bladder cancer susceptibility genotypes, NAT2 slow-acetylation and GSTM1-null, exhibited similar associations among the subphenotypes, as did VEGF-rs25648, which was previously identified in our study. Other variants conferred risks for specific tumour subphenotypes such as PMS2-rs6463524 and CD4-rs3213427 (respective heterogeneity p values of 0.006 and 0.004), which were associated with muscle-  invasive   tumours (per-allele odds ratios [95% confidence interval] of 0.56 [0.41-0.77] and 0.71 [0.57-0.88], respectively) but not with non-muscle-  invasive   tumours. Heterogeneity p values were not robust in multiple testing according to their false-discovery rate.These exploratory analyses suggest that genetic susceptibility loci might be related to the molecular/pathologic diversity of bladder cancer. Validation through large-scale replication studies and the study of additional genes and single nucleotide polymorphisms are required. 
 In situ proximity ligation detection of c-Jun/AP-1 dimers reveals increased levels of c-Jun/Fra1 complexes in aggressive breast cancer cell lines in vitro and in vivo. Genetic and biochemical studies have shown that selective interactions between the Jun, Fos, and activating transcription factor (ATF) components of transcription factor activating protein 1 (AP-1) exhibit specific and critical functions in the regulation of cell proliferation, differentiation, and survival. For instance, the ratio between c-Jun/c-Fos and c-Jun/ATF2 dimers in the cell can be a determining factor in the cellular response to oncogenic or apoptotic stimuli. Until recently, no methods were available to detect endogenous AP-1 complexes in cells and tissues in situ. Here, we validated the proximity ligation assay (PLA) for its ability to specifically visualize and quantify changes in endogenous c-Jun/c-Fos, c-Jun/ATF2, and c-Jun/Fra1 complexes by using, among others, partner-selective c-Jun mutants. Furthermore, we examined the levels of c-Jun/AP-1 dimers in cell lines representing different types of human breast cancer and found that aggressive basal-like breast cancer cells can be discriminated from much less   invasive   luminal-like cells by PLA detection of c-Jun/Fra1 rather than of c-Jun/ATF2 and c-Jun/c-Fos. Also in tumor tissue derived from highly metastatic basal-like MDA-MB231 cells, high levels of c-Jun/Fra1 complexes were detected. Together, these results demonstrate that in situ PLA is a powerful diagnostic tool to analyze and quantify the amounts of biologically critical AP-1 dimers in fixed cells and tissue material. 
 Tissue factor/activated factor VIIa induces matrix metalloproteinase-7 expression through activation of c-Fos via ERK1/2 and p38 MAPK signaling pathways in human colon cancer cell. Increased expression of tissue factor (TF) is associated with tumor invasion and metastasis in human colorectal cancer. We have previously observed that TF/FVIIa upregulates matrix metalloproteinase-7 (MMP-7) expression at the transcriptional level in colon cancer cells. MMP-7 overexpression is believed to play an important role in tumor invasion and metastasis. The aim of this study is to elucidate the molecular mechanisms by which TF/FVIIa induced MMP-7 expression and cell invasion in vitro.Reverse transcription polymerase chain reaction, Western blot, luciferase assay, and chromatin immunoprecipitation (ChIP) were used to determine the potential mechanism and signaling pathways by which TF/FVIIa induced MMP-7 expression and cell invasion in LoVo cells. Small interfering RNA (siRNA) and cell invasion assay was used to examine whether blocking c-Fos expression could abolish FVIIa-mediated upregulation of MMP-7 and cell invasion in vitro.The results showed that FVIIa induced the upregulation of MMP-7 both at the mRNA and protein levels in a time- and dose-dependent manner and increased the   invasive   behavior of LoVo cells. FVIIa enhanced the promoter activity of MMP-7, and the activator protein-1 (AP-1) binding site was responsible for the activation. Site mutation of the AP-1 binding site in the promoter almost completely abolished FVIIa-mediated response. Furthermore, ChIP assay confirmed that FVIIa promoted the direct binding of c-Fos with the MMP-7 promoter in vivo. FVIIa also induced the expression and nuclear accumulation of the AP-1 subunit c-Fos. siRNA-mediated knockdown of c-Fos eliminated FVIIa-stimulated MMP-7 expression and cell migration in vitro. In addition, selective mitogen-activated protein kinase (MAPK) kinase (MEK1/2) inhibitor (PD98059) and p38 MAPK inhibitor SB203580 suppressed MMP-7 upregulation induced by FVIIa.Our data suggest that a novel TF/FVIIa/MAPK/c-Fos/MMP-7 axis plays an important role in modulating the invasion of colon cancer cells and blockage of this pathway holds promise to treat colon cancer metastasis. 
 Evaluation of candidate stromal epithelial cross-talk genes identifies association between risk of serous ovarian cancer and TERT, a cancer susceptibility "hot-spot". We hypothesized that variants in genes expressed as a consequence of interactions between ovarian cancer cells and the host micro-environment could contribute to cancer susceptibility. We therefore used a two-stage approach to evaluate common single nucleotide polymorphisms (SNPs) in 173 genes involved in stromal epithelial interactions in the Ovarian Cancer Association Consortium (OCAC). In the discovery stage, cases with epithelial ovarian cancer (n=675) and controls (n=1,162) were genotyped at 1,536 SNPs using an Illumina GoldenGate assay. Based on Positive Predictive Value estimates, three SNPs-PODXL rs1013368, ITGA6 rs13027811, and MMP3 rs522616-were selected for replication using TaqMan genotyping in up to 3,059 serous   invasive   cases and 8,905 controls from 16 OCAC case-control studies. An additional 18 SNPs with Pper-allele<0.05 in the discovery stage were selected for replication in a subset of five OCAC studies (n=1,233 serous   invasive   cases; n=3,364 controls). The discovery stage associations in PODXL, ITGA6, and MMP3 were attenuated in the larger replication set (adj. Pper-allele>or=0.5). However genotypes at TERT rs7726159 were associated with ovarian cancer risk in the smaller, five-study replication study (Pper-allele=0.03). Combined analysis of the discovery and replication sets for this TERT SNP showed an increased risk of serous ovarian cancer among non-Hispanic whites [adj. ORper-allele 1.14 (1.04-1.24) p=0.003]. Our study adds to the growing evidence that, like the 8q24 locus, the telomerase reverse transcriptase locus at 5p15.33, is a general cancer susceptibility locus. 
 Expression of Jun and Fos proteins in ovarian tumors of different malignant potential and in ovarian cancer cell lines. Jun and Fos proteins form the transcription factor activating protein 1 (AP-1). They play a role in cell proliferation, malignant transformation and invasion in various tumors. The aim of the current study was to characterize the role of AP-1 in ovarian cancer. Fifty-six ovarian tumors of different   invasive   potential including 13 metastases as well as 5 ovarian cancer cell lines were analyzed by Western blot analysis regarding their expression of pc-Jun, Jun B, Jun D, c-Fos, Fos B, Fra-1 and Fra-2. In addition, invasion, cell proliferation and migration assays were performed. The expression of pc-Jun, Jun B, Jun D and Fra-2 was higher in   invasive   cancer compared to benign tumors. In metastases, c-Fos and Fos B expression was significantly lower than in the respective primary ovarian carcinomas. The   invasive   and proliferative potential of the cells was not associated with AP-1 protein expression. These results suggest that AP-1 proteins are differentially expressed in benign ovarian tumors, tumors with low malignant potential and epithelial ovarian carcinomas and metastases. No correlation with the proliferative and   invasive   potential of ovarian cancer cell lines could be found. 
 Ganoderic acids suppress growth and   invasive   behavior of breast cancer cells by modulating AP-1 and NF-kappaB signaling. Structurally related lanostane-type triterpenes, ganoderic acid A, F and H (GA-A, GA-F, GA-H), were identified in an oriental medicinal mushroom Ganoderma lucidum. In the present study we evaluated the effect of GA-A, GA-H and GA-F on highly   invasive   human breast cancer cells. We showed that GA-A and GA-H suppressed growth (cell proliferation and colony formation) and   invasive   behavior (adhesion, migration and invasion) of MDA-MB-231 cells. Our results suggest that GA-A and GA-H mediate their biological effects through the inhibition of transcription factors AP-1 and NF-kappaB, resulting in the down-regulation of expression of Cdk4 and the suppression of secretion of uPA, respectively. Furthermore, the activity of ganoderic acids is linked to the hydroxylation in the position 7 and 15 (GA-A) and 3 (GA-H) in their triterpene lanostane structure. In conclusion, hydroxylated triterpenes from G. lucidum could be promising natural agents for the therapy of   invasive   breast cancers. 
 Genetic susceptibility to distinct bladder cancer subphenotypes. Clinical, pathologic, and molecular evidence indicate that bladder cancer is heterogeneous with pathologic/molecular features that define distinct subphenotypes with different prognoses. It is conceivable that specific patterns of genetic susceptibility are associated with particular subphenotypes.To examine evidence for the contribution of germline genetic variation to bladder cancer heterogeneity.The Spanish Bladder Cancer/EPICURO Study is a case-control study based in 18 hospitals located in five areas in Spain. Cases were patients with a newly diagnosed, histologically confirmed, urothelial cell carcinoma of the bladder from 1998 to 2001. Case diagnoses were reviewed and uniformly classified by pathologists following the World Health Organisation/International Society of Urological Pathology 1999 criteria. Controls were hospital-matched patients (n=1149).A total of 1526 candidate variants in 423 candidate genes were analysed. Three distinct subphenotypes were defined according to stage and grade: low-grade nonmuscle   invasive   (n=586), high-grade nonmuscle   invasive   (n=219), and muscle   invasive   (n=246). The association between each variant and subphenotype was assessed by polytomous risk models adjusting for potential confounders. Heterogeneity in genetic susceptibility among subphenotypes was also tested.Two established bladder cancer susceptibility genotypes, NAT2 slow-acetylation and GSTM1-null, exhibited similar associations among the subphenotypes, as did VEGF-rs25648, which was previously identified in our study. Other variants conferred risks for specific tumour subphenotypes such as PMS2-rs6463524 and CD4-rs3213427 (respective heterogeneity p values of 0.006 and 0.004), which were associated with muscle-  invasive   tumours (per-allele odds ratios [95% confidence interval] of 0.56 [0.41-0.77] and 0.71 [0.57-0.88], respectively) but not with non-muscle-  invasive   tumours. Heterogeneity p values were not robust in multiple testing according to their false-discovery rate.These exploratory analyses suggest that genetic susceptibility loci might be related to the molecular/pathologic diversity of bladder cancer. Validation through large-scale replication studies and the study of additional genes and single nucleotide polymorphisms are required. 
 Ecotopic viral integration site 1 (EVI1) regulates multiple cellular processes important for cancer and is a synergistic partner for FOS protein in   invasive   tumors. Ecotropic viral integration site 1 (EVI1) is an oncogenic dual domain zinc finger transcription factor that plays an essential role in the regulation of hematopoietic stem cell renewal, and its overexpression in myeloid leukemia and epithelial cancers is associated with poor patient survival. Despite the discovery of EVI1 in 1988 and its emerging role as a dominant oncogene in various types of cancer, few EVI1 target genes are known. This lack of knowledge has precluded a clear understanding of exactly how EVI1 contributes to cancer. Using a combination of ChIP-Seq and microarray studies in human ovarian carcinoma cells, we show that the two zinc finger domains of EVI1 bind to DNA independently and regulate different sets of target genes. Strikingly, an enriched fraction of EVI1 target genes are cancer genes or genes associated with cancer. We also show that more than 25% of EVI1-occupied genes contain linked EVI1 and activator protein (AP)1 DNA binding sites, and this finding provides evidence for a synergistic cooperative interaction between EVI1 and the AP1 family member FOS in the regulation of cell adhesion, proliferation, and colony formation. An increased number of dual EVI1/AP1 target genes are also differentially regulated in late-stage ovarian carcinomas, further confirming the importance of the functional cooperation between EVI1 and FOS. Collectively, our data indicate that EVI1 is a multipurpose transcription factor that synergizes with FOS in   invasive   tumors. 
 Increased expression of c-fos protein associated with increased matrix metalloproteinase-9 protein expression in the endometrium of endometriotic patients. To investigate c-fos and matrix metalloproteinase-9 (MMP-9) expression in the endometrium from women with or without endometriosis throughout the menstrual cycle, and to explore the correlation of c-fos expression with MMP-9 expression and 17beta-E(2) levels in serum.Molecular studies in human tissue.A women's hospital in China.Fifty-five premenopausal women (25 with endometriosis and 30 without endometriosis) undergoing laparoscopic surgery or hysterectomy.Eutopic and ectopic endometrium tissue were obtained at the time of surgery. Peripheral sera were also collected on the same day.Immunohistochemical localization of c-fos in the endometrium, c-fos and MMP-9 protein levels in the endometrium, and 17beta-E(2) levels in the serum.c-fos protein was predominantly located in the nuclei of glandular epithelial cells and stromal cells. c-fos and MMP-9 protein levels in paired eutopic and ectopic endometria from women with endometriosis were significantly higher than those in the endometrium from women without endometriosis. No significant difference in c-fos or MMP-9 protein levels was observed between paired eutopic and ectopic endometria. c-fos protein levels in endometrium positively correlated with endometrial MMP-9 levels and serum 17beta-E(2) levels.Expression of c-fos in the human endometrium may be regulated by 17beta-E(2), and c-fos may be involved in development of endometriosis by promoting MMP-9 gene expression and subsequently the   invasive   potential of endometrial explants. 
 MicroRNA-101 inhibits the proliferation and invasion of bladder cancer cells via targeting c-FOS. MicroRNAs (miRs) have important roles in the parthenogenesis of malignancies. While it has been suggested that deregulation of miR‑101 is involved in bladder cancer, the underlying mechanisms have remained largely elusive. The present study aimed to investigate the roles of miR‑101 in the regulation of bladder cancer cell proliferation and invasion. Reverse‑transcription quantitative polymerase chain reaction analysis revealed that the expression of miR‑101 was significantly reduced in the HT‑1376, BIU87, T24 and 5637 several human bladder cancer cell lines compared to that in the SV‑HUC‑1 normal bladder epithelial cell line. Furthermore, a Targetscan search and a luciferase assay were used to identify c‑FOS as a novel target of miR‑101, and western blot analysis indicated that the protein expression of c‑FOS was shown to be negatively regulated by miR‑101 in bladder cancer T24 cells; however, c‑FOS mRNA expression was not affected. In addition, plasmid‑mediated overexpression of miR‑101 and small hairpin RNA‑mediated inhibition of c‑FOS significantly   inhibite  d the proliferation and invasive capacity of T24 cells, as indicated by an MTT and a Transwell assay, respectively. However, plasmid‑mediated overexpression of c‑FOS reversed the inhibitory effects of miR‑101 overexpression on T24‑cell proliferation and invasion. In conclusion, the present study demonstrated that miR‑101 inhibits the proliferation and invasion of bladder cancer cells, at least partly via targeting c‑FOS, suggesting that miR-101/c‑FOS signaling may represent a potential therapeutic target for bladder cancer. 
  GeneRIFs  
 EVI1 is a multipurpose transcription factor that synergizes with FOS in   invasive   tumors. 
 AP-1 proteins (jun/fos) are differentially expressed in ovarian tumors of differing malignant potential. No correlation with the proliferative and   invasive   potential of ovarian cancer cell lines could be found. 
 Expression of c-fos in the human endometrium may be regulated by 17beta-E(2), and c-fos may be involved in development of endometriosis by promoting MMP-9 gene expression and subsequently the   invasive   potential of endometrial explants. 
  [Mm]etastasis  PubMed  
 Cadmium induces matrix metalloproteinase-9 expression via ROS-dependent EGFR, NF-кB, and AP-1 pathways in human endothelial cells. Cadmium (Cd), a widespread cumulative pollutant, is a known human carcinogen, associated with inflammation and tumors. Matrix metalloproteinase-9 (MMP-9) plays a pivotal role in tumor   metastasi  s; however, the mechanisms underlying the MMP-9 expression induced by Cd remain obscure in human endothelial cells. Here, Cd elevated MMP-9 expression in dose- and time-dependent manners in human endothelial cells. Cd increased ROS production and the ROS-producing NADPH oxidase. Cd translocates p47(phox), a key subunit of NADPH oxidase, to the cell membrane. Cd also activated the phosphorylation of EGFR, Akt, Erk1/2, and JNK1/2 in addition to promoting NF-кB and AP-1 binding activities. Specific inhibitor and mutagenesis studies showed that EGFR, Akt, Erk1/2, JNK1/2 and transcription factors NF-κB and AP-1 were related to Cd-induced MMP-9 expression in endothelial cells. Akt, Erk1/2, and JNK1/2 functioned as upstream signals in the activation of NF-κB and AP-1, respectively. In addition, N-acetyl-l-cystein (NAC), diphenyleneiodonium chloride (DPI) and apocynin (APO) inhibited the Cd-induced activation of EGFR, Akt, Erk1/2, JNK1/2, and p38 MAPK, indicating that ROS production by NADPH oxidase is the furthest upstream signal in MMP-9 expression. At present, it states that Cd displayed marked invasiveness in ECV304 cells, which was partially abrogated by MMP-9 neutralizing antibodies. These results demonstrated that Cd induces MMP-9 expression via ROS-dependent EGFR->Erk1/2, JNK1/2->AP-1 and EGFR->Akt->NF-κB signaling pathways and, in turn, stimulates invasiveness in human endothelial cells. 
 Tissue factor/activated factor VIIa induces matrix metalloproteinase-7 expression through activation of c-Fos via ERK1/2 and p38 MAPK signaling pathways in human colon cancer cell. Increased expression of tissue factor (TF) is associated with tumor invasion and   metastasis   in human colorectal cancer. We have previously observed that TF/FVIIa upregulates matrix metalloproteinase-7 (MMP-7) expression at the transcriptional level in colon cancer cells. MMP-7 overexpression is believed to play an important role in tumor invasion and   metastasis  . The aim of this study is to elucidate the molecular mechanisms by which TF/FVIIa induced MMP-7 expression and cell invasion in vitro.Reverse transcription polymerase chain reaction, Western blot, luciferase assay, and chromatin immunoprecipitation (ChIP) were used to determine the potential mechanism and signaling pathways by which TF/FVIIa induced MMP-7 expression and cell invasion in LoVo cells. Small interfering RNA (siRNA) and cell invasion assay was used to examine whether blocking c-Fos expression could abolish FVIIa-mediated upregulation of MMP-7 and cell invasion in vitro.The results showed that FVIIa induced the upregulation of MMP-7 both at the mRNA and protein levels in a time- and dose-dependent manner and increased the invasive behavior of LoVo cells. FVIIa enhanced the promoter activity of MMP-7, and the activator protein-1 (AP-1) binding site was responsible for the activation. Site mutation of the AP-1 binding site in the promoter almost completely abolished FVIIa-mediated response. Furthermore, ChIP assay confirmed that FVIIa promoted the direct binding of c-Fos with the MMP-7 promoter in vivo. FVIIa also induced the expression and nuclear accumulation of the AP-1 subunit c-Fos. siRNA-mediated knockdown of c-Fos eliminated FVIIa-stimulated MMP-7 expression and cell migration in vitro. In addition, selective mitogen-activated protein kinase (MAPK) kinase (MEK1/2) inhibitor (PD98059) and p38 MAPK inhibitor SB203580 suppressed MMP-7 upregulation induced by FVIIa.Our data suggest that a novel TF/FVIIa/MAPK/c-Fos/MMP-7 axis plays an important role in modulating the invasion of colon cancer cells and blockage of this pathway holds promise to treat colon cancer   metastasis  . 
 Inhibitor of Differentiation/DNA Binding 1 (ID1) Inhibits Etoposide-induced Apoptosis in a c-Jun/c-Fos-dependent Manner. ID1 (inhibitor of differentiation/DNA binding 1) acts an important role in   metastasis  , tumorigenesis, and maintenance of cell viability. It has been shown that the up-regulation of ID1 is correlated with poor prognosis and the resistance to chemotherapy of human cancers. However, the underlying molecular mechanism remains elusive. Here, we determined for the first time that up-regulating ID1 upon etoposide activation was mediated through AP-1 binding sites within theID1promoter and confirmed that ID1 enhanced cell resistance to DNA damage-induced apoptosis in esophageal squamous cell carcinoma cells. Ablation of c-Jun/c-Fos or ID1 expression enhanced etoposide-mediated apoptosis through increasing activity of caspase 3 and PARP cleavage. Moreover, c-Jun/c-Fos and ID1 were positively correlated in human cancers. More importantly, simultaneous high expression of ID1 and c-Jun or c-Fos was correlated with poor survival in cancer patients. Collectively, we demonstrate the importance of c-Jun/c-Fos-ID1 signaling pathway in chemoresistance of esophageal cancer cells and provide considerable insight into understanding the underlying molecular mechanisms in esophageal squamous cell carcinoma cell biology. 
 Combined Expression of c-jun, c-fos, and p53 Improves Estimation of Prognosis in Oral Squamous Cell Carcinoma. To identify the prognostic value of c-jun, c-fos, and p53 in oral cancer, we examined the impact of immunohistochemical expression of these markers on tumor progression in 157 oral squamous cell carcinoma (OSCC). We found that c-jun or c-fos was significantly associated with lymph node   metastasis  , and coexpression of c-jun/c-fos, or c-jun/c-fos/p53 were significantly associated with lymph node   metastasis  , poor differentiation and clinical stage. The coexpression of c-jun/c-fos/p53 was identified as independent prognostic factors for overall survival. Simultaneous coexpression of these markers in OSCCs might prove to be a useful indicator for differentiation of low and high-risk patients. 
 IL-1β-induced activation of p38 promotes   metastasi  s in gastric adenocarcinoma via upregulation of AP-1/c-fos, MMP2 and MMP9. Interleukin-1β (IL-1β) has been implicated in the progression of gastric adenocarcinoma (GA); however, the molecular mechanisms of action of IL-1β in GA are poorly characterized. P38 and JNK are the major MAPK family members that regulate IL-1β signaling pathways. Here, we investigated the role of both p38 and JNK in IL-1β-induced GA cell migration, invasion and metastatic potential.The effects of IL-1β-induced p38 and JNK activation in GA cells were determined using in vitro Transwell migration and invasion assays of MKN-45 and AGS cells, or an i  n vivo met  astasis assay in nude mice. The IL-1β-induced p38 signaling pathway was further characterized in GA cells. Activation of the IL-1β/p38 signaling pathway was also assessed in human primary GA tissues by immunohistochemistry.IL-1β-induced activation of p38 increased GA cell migration and invasion in vitro and promoted the metastatic potential of GA cells in vivo; these effects were attenuated by p38 siRNA or the p38 inhibitor SB202190. MMP2 or MMP9 siRNAs and the MMP2/9 inhibitor BiPS also inhibited IL-1β-induced GA cell migration and invasion in vitro. IL-1β-induced p38 activation significantly increased MMP2 and MMP9 mRNA and protein expression and activity. Luciferase reporter assays demonstrated that the activator protein-1 (AP-1) and the AP-1 binding sites of the MMP9 promoter (-670/MMP9) were activated by IL-1β-induced p38 activation. Phospho-p38 was significantly upregulated in human GA tissues (compared to matched non-neoplastic tissues), and significantly associated wit  h lymph no  de metastasis, and invasion beyond the serosa. Expression of phospho-p38 significantly correlated with IL-1β, MMP2, MMP9, and c-fos expression in both human GA tissues and GA cell metastases in the lungs of nude mice. IL-1β was also capable of activating JNK in GA cells, but activation of JNK was not associated with GA cell migration and invasion. Therefore, IL-1β-induced the migration and invasion in GA cells were regulated by p38, but not by JNK.IL-1β-induced p38 activation and the IL-1β/p38/AP-1(c-fos)/MMP2 & MMP9 pathway play an   important   role in metastasis in GA; this pathway may provide a novel therapeutic target for GA. 
 EGF-R is expressed and AP-1 and NF-kappaB are activated in stromal myofibroblasts surrounding colon adenocarcinomas paralleling expression of COX-2 and VEGF. COX-2 and VEGF are important triggers of colon cancer growth,   metastasis   and angiogenesis. Cox-2 promoter contains transcriptional regulatory elements for AP-1 and NF-kappaB transcription factors whilst vegf is a known AP-1 downstream target gene. We investigated whether stromal myofibroblasts surrounding colon adenocarcinomas express COX-2 and VEGF and whether activation of AP-1 and NF-kappaB, as well as expression of EGF-R parallel expression of COX-2 and VEGF in these cells.Immunohistochemical methodology was performed on archival sections from 40 patients with colon adenocarcinomas. We evaluated c-FOS, p-c-JUN (phosphorylated c-JUN), p-IkappaB-alpha (phosphorylated IkappaB-alpha), EGF-R, COX-2, NF-kappaB and VEGF expression in stromal myofibroblasts surrounding colon adenocarcinomas. Double immunostaining with a-smooth muscle actin and each antibody was done to verify the expression of these molecules in stromal myofibroblasts.VEGF, p-IkappaB-alpha, NF-kappaB, c-FOS, p-c-JUN, EGF-R and COX-2 were expressed in stromal myofibroblasts surrounding colon adenocarcinomas in the majority of cases. EGF-R, p-IkappaB-alpha, NF-kappaB, c-FOS and p-c-JUN correlated positively with COX-2 and VEGF expression.Stromal myofibroblasts surrounding colon adenocarcinomas are an important source of VEGF and COX-2 production, while AP-1 and NF-kappaB transcription factors are activated and EGF-R is expressed in these cells and associated with COX-2 and VEGF production. 
 Combined Expression of c-jun, c-fos, and p53 Improves Estimation of Prognosis in Oral Squamous Cell Carcinoma. To identify the prognostic value of c-jun, c-fos, and p53 in oral cancer, we examined the impact of immunohistochemical expression of these markers on tumor progression in 157 oral squamous cell carcinoma (OSCC). We found that c-jun or c-fos was significantly associated with lymph node   metastasis  , and coexpression of c-jun/c-fos, or c-jun/c-fos/p53 were significantly associated with lymph node   metastasis  , poor differentiation and clinical stage. The coexpression of c-jun/c-fos/p53 was identified as independent prognostic factors for overall survival. Simultaneous coexpression of these markers in OSCCs might prove to be a useful indicator for differentiation of low and high-risk patients. 
 Inhibitor of Differentiation/DNA Binding 1 (ID1) Inhibits Etoposide-induced Apoptosis in a c-Jun/c-Fos-dependent Manner. ID1 (inhibitor of differentiation/DNA binding 1) acts an important role in   metastasis  , tumorigenesis, and maintenance of cell viability. It has been shown that the up-regulation of ID1 is correlated with poor prognosis and the resistance to chemotherapy of human cancers. However, the underlying molecular mechanism remains elusive. Here, we determined for the first time that up-regulating ID1 upon etoposide activation was mediated through AP-1 binding sites within theID1promoter and confirmed that ID1 enhanced cell resistance to DNA damage-induced apoptosis in esophageal squamous cell carcinoma cells. Ablation of c-Jun/c-Fos or ID1 expression enhanced etoposide-mediated apoptosis through increasing activity of caspase 3 and PARP cleavage. Moreover, c-Jun/c-Fos and ID1 were positively correlated in human cancers. More importantly, simultaneous high expression of ID1 and c-Jun or c-Fos was correlated with poor survival in cancer patients. Collectively, we demonstrate the importance of c-Jun/c-Fos-ID1 signaling pathway in chemoresistance of esophageal cancer cells and provide considerable insight into understanding the underlying molecular mechanisms in esophageal squamous cell carcinoma cell biology. 
 IL-1β-induced activation of p38 promotes   metastasi  s in gastric adenocarcinoma via upregulation of AP-1/c-fos, MMP2 and MMP9. Interleukin-1β (IL-1β) has been implicated in the progression of gastric adenocarcinoma (GA); however, the molecular mechanisms of action of IL-1β in GA are poorly characterized. P38 and JNK are the major MAPK family members that regulate IL-1β signaling pathways. Here, we investigated the role of both p38 and JNK in IL-1β-induced GA cell migration, invasion and metastatic potential.The effects of IL-1β-induced p38 and JNK activation in GA cells were determined using in vitro Transwell migration and invasion assays of MKN-45 and AGS cells, or an i  n vivo met  astasis assay in nude mice. The IL-1β-induced p38 signaling pathway was further characterized in GA cells. Activation of the IL-1β/p38 signaling pathway was also assessed in human primary GA tissues by immunohistochemistry.IL-1β-induced activation of p38 increased GA cell migration and invasion in vitro and promoted the metastatic potential of GA cells in vivo; these effects were attenuated by p38 siRNA or the p38 inhibitor SB202190. MMP2 or MMP9 siRNAs and the MMP2/9 inhibitor BiPS also inhibited IL-1β-induced GA cell migration and invasion in vitro. IL-1β-induced p38 activation significantly increased MMP2 and MMP9 mRNA and protein expression and activity. Luciferase reporter assays demonstrated that the activator protein-1 (AP-1) and the AP-1 binding sites of the MMP9 promoter (-670/MMP9) were activated by IL-1β-induced p38 activation. Phospho-p38 was significantly upregulated in human GA tissues (compared to matched non-neoplastic tissues), and significantly associated wit  h lymph no  de metastasis, and invasion beyond the serosa. Expression of phospho-p38 significantly correlated with IL-1β, MMP2, MMP9, and c-fos expression in both human GA tissues and GA cell metastases in the lungs of nude mice. IL-1β was also capable of activating JNK in GA cells, but activation of JNK was not associated with GA cell migration and invasion. Therefore, IL-1β-induced the migration and invasion in GA cells were regulated by p38, but not by JNK.IL-1β-induced p38 activation and the IL-1β/p38/AP-1(c-fos)/MMP2 & MMP9 pathway play an   important   role in metastasis in GA; this pathway may provide a novel therapeutic target for GA. 
 Tissue factor/activated factor VIIa induces matrix metalloproteinase-7 expression through activation of c-Fos via ERK1/2 and p38 MAPK signaling pathways in human colon cancer cell. Increased expression of tissue factor (TF) is associated with tumor invasion and   metastasis   in human colorectal cancer. We have previously observed that TF/FVIIa upregulates matrix metalloproteinase-7 (MMP-7) expression at the transcriptional level in colon cancer cells. MMP-7 overexpression is believed to play an important role in tumor invasion and   metastasis  . The aim of this study is to elucidate the molecular mechanisms by which TF/FVIIa induced MMP-7 expression and cell invasion in vitro.Reverse transcription polymerase chain reaction, Western blot, luciferase assay, and chromatin immunoprecipitation (ChIP) were used to determine the potential mechanism and signaling pathways by which TF/FVIIa induced MMP-7 expression and cell invasion in LoVo cells. Small interfering RNA (siRNA) and cell invasion assay was used to examine whether blocking c-Fos expression could abolish FVIIa-mediated upregulation of MMP-7 and cell invasion in vitro.The results showed that FVIIa induced the upregulation of MMP-7 both at the mRNA and protein levels in a time- and dose-dependent manner and increased the invasive behavior of LoVo cells. FVIIa enhanced the promoter activity of MMP-7, and the activator protein-1 (AP-1) binding site was responsible for the activation. Site mutation of the AP-1 binding site in the promoter almost completely abolished FVIIa-mediated response. Furthermore, ChIP assay confirmed that FVIIa promoted the direct binding of c-Fos with the MMP-7 promoter in vivo. FVIIa also induced the expression and nuclear accumulation of the AP-1 subunit c-Fos. siRNA-mediated knockdown of c-Fos eliminated FVIIa-stimulated MMP-7 expression and cell migration in vitro. In addition, selective mitogen-activated protein kinase (MAPK) kinase (MEK1/2) inhibitor (PD98059) and p38 MAPK inhibitor SB203580 suppressed MMP-7 upregulation induced by FVIIa.Our data suggest that a novel TF/FVIIa/MAPK/c-Fos/MMP-7 axis plays an important role in modulating the invasion of colon cancer cells and blockage of this pathway holds promise to treat colon cancer   metastasis  . 
 EGF-R is expressed and AP-1 and NF-kappaB are activated in stromal myofibroblasts surrounding colon adenocarcinomas paralleling expression of COX-2 and VEGF. COX-2 and VEGF are important triggers of colon cancer growth,   metastasis   and angiogenesis. Cox-2 promoter contains transcriptional regulatory elements for AP-1 and NF-kappaB transcription factors whilst vegf is a known AP-1 downstream target gene. We investigated whether stromal myofibroblasts surrounding colon adenocarcinomas express COX-2 and VEGF and whether activation of AP-1 and NF-kappaB, as well as expression of EGF-R parallel expression of COX-2 and VEGF in these cells.Immunohistochemical methodology was performed on archival sections from 40 patients with colon adenocarcinomas. We evaluated c-FOS, p-c-JUN (phosphorylated c-JUN), p-IkappaB-alpha (phosphorylated IkappaB-alpha), EGF-R, COX-2, NF-kappaB and VEGF expression in stromal myofibroblasts surrounding colon adenocarcinomas. Double immunostaining with a-smooth muscle actin and each antibody was done to verify the expression of these molecules in stromal myofibroblasts.VEGF, p-IkappaB-alpha, NF-kappaB, c-FOS, p-c-JUN, EGF-R and COX-2 were expressed in stromal myofibroblasts surrounding colon adenocarcinomas in the majority of cases. EGF-R, p-IkappaB-alpha, NF-kappaB, c-FOS and p-c-JUN correlated positively with COX-2 and VEGF expression.Stromal myofibroblasts surrounding colon adenocarcinomas are an important source of VEGF and COX-2 production, while AP-1 and NF-kappaB transcription factors are activated and EGF-R is expressed in these cells and associated with COX-2 and VEGF production. 
 PRL-3 suppresses c-Fos and integrin α2 expression in ovarian cancer cells. Phosphatase of regenerating liver-3 (PRL-3), a protein tyrosine phosphatase, is highly expressed in multiple human cancers and strongly implicated in tumor progression and cancer   metastasi  s. However, the mechanisms by which PRL-3 promotes cancer cell migration, invasion, and   metastasi  s are not very well understood. In this study, we investigated the contribution and molecular mechanisms of PRL-3 in ovarian cancer progression.PRL-3 protein expression was detected on ovarian cancer tissue microarrays using immunohistochemistry. Stable PRL-3 depleted cell lines were generated using short hairpin RNA (shRNA) constructs. The migration and invasion potential of these cells were analyzed using Transwell and Matrigel assays, respectively. Immunoblotting and immunofluorescence were used to detect protein levels and distribution in PRL-3-ablated cells and the control cells. Cell morphology was observed with hematoxylin-eosin staining and transmission electron microscopy. Finally, PRL-3-ablated and control cells were injected into nude mice for xenograft tumorigenicity assays.Elevated PRL-3 expression was detected in 19% (26 out of 135) of human ovarian cancer patient samples, but not in normal ovary tissues (0 out of 14). Stable depletion of PRL-3 in A2780 ovarian cancer cells resulted in decreased migration ability and invasion activity compared with control parental A2780 cells. In addition, PRL-3-ablated cells also exhibited flattened morphology and extended lamellipodia. To address the possible molecular basis for the altered phenotypes associated with PRL-3 down-regulation, we assessed the expression profiles of various proteins involved in cell-matrix adhesion. Depletion of PRL-3 dramatically enhanced both RNA and protein levels of the cell surface receptor integrin α2, but not its heterologous binding partner integrin β1. Inhibition of PRL-3 also correlated with elevated expression and phosphorylation of paxillin. A pronounced increase in the expression and activation of c-fos, a transcriptional activator of integrin α2, was observed in these PRL-3 knock-down cells. Moreover, forced expression of EGFP-PRL-3 resulted in the suppression of both integrin α2 and c-fos expression in A2780 cells. Significantly, using a xenograft tumor model, we observed a greatly reduced tumorigenicity of A2780 PRL-3 knock-down cells in vivo.These results suggest that PRL-3 plays a critical role in ovarian cancer tumorigenicity and maintaining the malignant phenotype. PRL-3 may inhibit c-fos transcriptional regulation of integrin α2 signaling. Our results strongly support a role for PRL-3 as a promising therapeutic target and potential early biomarker in ovarian cancer progression. 
 AP1-dependent repression of TGFα-mediated MMP9 upregulation by PPARδ agonists in keratinocytes. Peroxisome proliferator-activated receptors (PPARs) are ligand-activated transcription factors that function mainly in the regulation of glucose and lipid homeostasis. PPAR agonists have been shown to control inflammation by inhibition of distinct proinflammatory genes. Aberrant activation of the epidermal growth factor receptor and/or overexpression of its ligand, transforming growth factor-α (TGFα), are key features of both neoplastic and inflammatory hyperproliferative epithelia. Matrix metalloproteinase 9 (MMP9) belongs to the set of genes that are effectively induced by TGFα in keratinocytes. Induction of MMP9 expression is strongly linked to regenerative skin repair mechanisms, inflammatory skin diseases and t  umor metas  tasis. We explored whether the known anti-inflammatory effects of PPARδ ligands involve inhibiting the TGFα-mediated upregulation of MMP9. The PPARδ agonists potently inhibited TGFα-induced MMP9 expression in human keratinocytes. This inhibition was observed at both the protein and mRNA levels. Transcriptional activation studies with deletion constructs of a reporter gene revealed that PPARδ agonists mediate their inhibitory effects via an AP1-binding site. Electromobility shift assay analysis indicated that MMP9 gene expression is inhibited by repressing site-dependent DNA binding and transactivation by c-fos. In conclusion, our data provide the first evidence that MMP9 expression induced by TGFα is a valid target of PPARδ ligands in keratinocytes. 
 Derepression of c-Fos caused by microRNA-139 down-regulation contributes to the   metastasis   of human hepatocellular carcinoma. This study investigates whether the anti-  metastasis   effect of microRNA-139 (miR-139) on hepatocellular carcinoma (HCC) is mediated through regulating c-fos expression. The expression levels of miR-139 and c-fos in human HCC cell sublines with high (MHCC97H) and low (MHCC97L) spontaneous metastatic potentials were quantified using QPCR or Western blot. miR-139 mimics was transfected into MHCC97H cells to overexpress miR-139, and miR-139 inhibitor was transfected into MHCC97L cells to down-express miR-139. The effect of overexpression or down-expression of miR-139 on c-fos expression of MHCC97H and MHCC97L cells was evaluated using QPCR and Western blot. The 3' untranslated region segments of FOS containing the miR-139 binding sites were amplified by PCR, and the luciferase activity in the transfected cells was assayed. In comparison with the expression level of miR-139 in MHCC97L cells, the expression level in MHCC97H cells was significantly decreased, whereas c-Fos was significantly up-regulated in MHCC97H. The overexpression of miR-139 significantly inhibited the expression of c-fos in MHCC97H cells, and the down-expression of miR-139 significantly promoted the expression of c-fos in MHCC97L cells. miR-139 suppressed the luciferase activity of the pGL-FOS by approximately 40% compared with the negative control. In vitro cell migration analysis demonstrated that depletion of c-fos or overexpression of miR-139 in MHCC97H cells reduced cell migration, whereas overexpression of c-fos or depletion of miR-139 in MHCC97L cells increased cell migration. Thus, we got the conclusion that miR-139 expression is down-regulated in human HCC cell sublines with high spontaneous metastatic potentials (MHCC97H). Derepression of c-Fos caused by miR-139 down-regulation contributes to the   metastasis   of HCC. 
 Prognostic significance of loss of c-fos protein in gastric carcinoma. c-fos was first identified as a viral oncoprotein, and has been studied in terms of its oncogenic function in tumorigenesis. Many experimental and clinical data indicated that c-fos expression plays a role in the progression of several types of carcinomas. However, some recent studies challenge this view as they indicate that c-fos has tumor suppressor activity. In the present study, we assessed c-fos protein expression in 625 consecutive gastric cancers immunohistochemically, and analyzed its relationship with clinicopathologic factors and survival. We found that a loss of c-fos expression is correlated with a more advanced stage, lymph node   metastasis  , lymphatic invasion and shorter survival, indicating that c-fos expression in gastric cancer cells is lost during progression and that this loss is associated with a poor prognosis. The above findings suggest that loss of c-fos expression has tumor suppressor activity in gastric cancer and we suspect that this suppressor activity might be related to the pro-apoptotic function of c-fos. 
 P53 and C-FOS overexpression in patients with thyroid cancer: an immunohistochemical study. A sequence of genetic events characterized by deletion and expression of several oncogenes may lead progressively to tumorgenesis. The expression of certain oncogenes is believed to be related with thyroid carcinogenesis and tumor progression. We investigated immunohistochemically p53 tumor suppressor gene and c-fos oncogene expression in forty patients with thyroid cancer. Thyroid biopsies from twenty patients with benign thyroid diseases were also examined. The forty patients with thyroid cancer varied histologically; 24 with papillary carcinoma (60%), 12 with follicular carcinoma (30%), 3 with anaplastic carcinoma (7.5%) and one with medullary carcinoma (2.5%). The patients with benign thyroid diseases consisted of 10 with adenomatous goiter (50%), 7 with goiter (35%) and three with Hashimoto thyroiditis (15%). Individual p53 and c-fos expression was more prevalent in thyroid carcinomas compared to benign tumors (p=0.001 and p=0.04, respectively). A marked increase of p53 and c-fos coexpression was found (p=0.02) in patients with thyroid cancer and   metastasis   to the regional lymph nodes. Furthermore c-fos was overexpressed in only female thyroid cancer patients. In conclusion, p53 and c-fos are significantly overexpressed in thyroid cancer patients, indicating their role in the genetic mechanisms leading to thyroid tumorigenesis. This hypothesis is further supported by the observation that p53/c-fos coexpression was related with more advanced disease status. 
  GeneRIFs  
 We found that c-jun or c-fos was significantly associated with lymph node   metastasis  , and coexpression of c-jun/c-fos, or c-jun/c-fos/p53 were significantly associated with lymph node   metastasis  , poor differentiation and clinical stage. 
 S100A4, FOS and CXCR4, playing a major role in tumor progression and   metastasis  , are downregulated by sorafenib. 
 the IL-1beta/p38/AP-1(c-fos)/MMP2 & MMP9 pathway play an important role in   metastasis   in gastric adenocarcinoma 
 Derepression of c-Fos caused by miR-139 down-regulation contributes to the   metastasis   of hepatocellular carcinoma. 
 A loss of c-fos expression is correlated with a more advanced stage, lymph node   metastasis  , lymphatic invasion and shorter survival in gastric carcinoma. 
  [Cc]ell [Ii]nvasion  PubMed  
 Tissue factor/activated factor VIIa induces matrix metalloproteinase-7 expression through activation of c-Fos via ERK1/2 and p38 MAPK signaling pathways in human colon cancer cell. Increased expression of tissue factor (TF) is associated with tumor invasion and metastasis in human colorectal cancer. We have previously observed that TF/FVIIa upregulates matrix metalloproteinase-7 (MMP-7) expression at the transcriptional level in colon cancer cells. MMP-7 overexpression is believed to play an important role in tumor invasion and metastasis. The aim of this study is to elucidate the molecular mechanisms by which TF/FVIIa induced MMP-7 expression and   cell invasion   in vitro.Reverse transcription polymerase chain reaction, Western blot, luciferase assay, and chromatin immunoprecipitation (ChIP) were used to determine the potential mechanism and signaling pathways by which TF/FVIIa induced MMP-7 expression and   cell invasion   in LoVo cells. Small interfering RNA (siRNA) and   cell invasion   assay was used to examine whether blocking c-Fos expression could abolish FVIIa-mediated upregulation of MMP-7 and   cell invasion   in vitro.The results showed that FVIIa induced the upregulation of MMP-7 both at the mRNA and protein levels in a time- and dose-dependent manner and increased the invasive behavior of LoVo cells. FVIIa enhanced the promoter activity of MMP-7, and the activator protein-1 (AP-1) binding site was responsible for the activation. Site mutation of the AP-1 binding site in the promoter almost completely abolished FVIIa-mediated response. Furthermore, ChIP assay confirmed that FVIIa promoted the direct binding of c-Fos with the MMP-7 promoter in vivo. FVIIa also induced the expression and nuclear accumulation of the AP-1 subunit c-Fos. siRNA-mediated knockdown of c-Fos eliminated FVIIa-stimulated MMP-7 expression and cell migration in vitro. In addition, selective mitogen-activated protein kinase (MAPK) kinase (MEK1/2) inhibitor (PD98059) and p38 MAPK inhibitor SB203580 suppressed MMP-7 upregulation induced by FVIIa.Our data suggest that a novel TF/FVIIa/MAPK/c-Fos/MMP-7 axis plays an important role in modulating the invasion of colon cancer cells and blockage of this pathway holds promise to treat colon cancer metastasis. 
 Inhibition of AP-1 by SARI negatively regulates transformation progression mediated by CCN1. Enhanced expression of the CCN family of secretory integrin-binding proteins correlates with many essential components of the cancerous state, including tumor cell adhesion, proliferation, invasion and migration. Consequently, CCN1 expression is elevated in various cancers, including breast cancer, and its expression directly correlates with poor patient prognosis. Using subtraction-hybridization, combined with induction of cancer cell terminal differentiation, we cloned SARI (suppressor of activator protein (AP)-1, regulated by interferon (IFN)), an IFN-beta-inducible, potent tumor suppressor gene that exerts cancer-selective growth inhibitory effects. Forced expression of SARI using an adenovirus (Ad.SARI) inhibits AP-1 function and downregulates CCN1 expression in multiple cancer lineages, resulting in a profound inhibition in anchorage-independent cell growth and tumor   cell invasion  . Overexpression of SARI reduces CCN1-promoter activity through inhibition of AP-1 binding. Accordingly, SARI selectively blocks expression of the transformed state in rat embryo fibroblast cells that stably overexpress c-Jun. These results illustrate that SARI inhibits AP-1 transactivating factor binding to the cis-element of the CCN1 promoter, possibly through its interaction with c-Jun. Overall, SARI can directly inhibit CCN1-induced transformation by inhibiting the transcription of CCN1, as well as indirectly by inhibiting the expression of c-Jun (and hence blocking AP-1 activity). In these contexts, transformed cells 'addicted' to AP-1 activity are rendered susceptible to SARI-mediated inhibition of expression of the transformed phenotype. 
 Tissue factor/activated factor VIIa induces matrix metalloproteinase-7 expression through activation of c-Fos via ERK1/2 and p38 MAPK signaling pathways in human colon cancer cell. Increased expression of tissue factor (TF) is associated with tumor invasion and metastasis in human colorectal cancer. We have previously observed that TF/FVIIa upregulates matrix metalloproteinase-7 (MMP-7) expression at the transcriptional level in colon cancer cells. MMP-7 overexpression is believed to play an important role in tumor invasion and metastasis. The aim of this study is to elucidate the molecular mechanisms by which TF/FVIIa induced MMP-7 expression and   cell invasion   in vitro.Reverse transcription polymerase chain reaction, Western blot, luciferase assay, and chromatin immunoprecipitation (ChIP) were used to determine the potential mechanism and signaling pathways by which TF/FVIIa induced MMP-7 expression and   cell invasion   in LoVo cells. Small interfering RNA (siRNA) and   cell invasion   assay was used to examine whether blocking c-Fos expression could abolish FVIIa-mediated upregulation of MMP-7 and   cell invasion   in vitro.The results showed that FVIIa induced the upregulation of MMP-7 both at the mRNA and protein levels in a time- and dose-dependent manner and increased the invasive behavior of LoVo cells. FVIIa enhanced the promoter activity of MMP-7, and the activator protein-1 (AP-1) binding site was responsible for the activation. Site mutation of the AP-1 binding site in the promoter almost completely abolished FVIIa-mediated response. Furthermore, ChIP assay confirmed that FVIIa promoted the direct binding of c-Fos with the MMP-7 promoter in vivo. FVIIa also induced the expression and nuclear accumulation of the AP-1 subunit c-Fos. siRNA-mediated knockdown of c-Fos eliminated FVIIa-stimulated MMP-7 expression and cell migration in vitro. In addition, selective mitogen-activated protein kinase (MAPK) kinase (MEK1/2) inhibitor (PD98059) and p38 MAPK inhibitor SB203580 suppressed MMP-7 upregulation induced by FVIIa.Our data suggest that a novel TF/FVIIa/MAPK/c-Fos/MMP-7 axis plays an important role in modulating the invasion of colon cancer cells and blockage of this pathway holds promise to treat colon cancer metastasis. 
 Inhibition of AP-1 by SARI negatively regulates transformation progression mediated by CCN1. Enhanced expression of the CCN family of secretory integrin-binding proteins correlates with many essential components of the cancerous state, including tumor cell adhesion, proliferation, invasion and migration. Consequently, CCN1 expression is elevated in various cancers, including breast cancer, and its expression directly correlates with poor patient prognosis. Using subtraction-hybridization, combined with induction of cancer cell terminal differentiation, we cloned SARI (suppressor of activator protein (AP)-1, regulated by interferon (IFN)), an IFN-beta-inducible, potent tumor suppressor gene that exerts cancer-selective growth inhibitory effects. Forced expression of SARI using an adenovirus (Ad.SARI) inhibits AP-1 function and downregulates CCN1 expression in multiple cancer lineages, resulting in a profound inhibition in anchorage-independent cell growth and tumor   cell invasion  . Overexpression of SARI reduces CCN1-promoter activity through inhibition of AP-1 binding. Accordingly, SARI selectively blocks expression of the transformed state in rat embryo fibroblast cells that stably overexpress c-Jun. These results illustrate that SARI inhibits AP-1 transactivating factor binding to the cis-element of the CCN1 promoter, possibly through its interaction with c-Jun. Overall, SARI can directly inhibit CCN1-induced transformation by inhibiting the transcription of CCN1, as well as indirectly by inhibiting the expression of c-Jun (and hence blocking AP-1 activity). In these contexts, transformed cells 'addicted' to AP-1 activity are rendered susceptible to SARI-mediated inhibition of expression of the transformed phenotype. 
 PRIP promotes tumor formation through enhancing serum-responsive factor-mediated FOS expression. PRIP (peroxisome proliferator-activator receptor interacting protein) is a nuclear receptor coactivator required for mammary gland development. To understand the function of PRIP in breast tumorigenesis, we established a mammary tumor cell line with the PRIP(Loxp/Loxp) genotype. By knocking out the PRIP gene in the tumor cell line, we demonstrated that PRIP deficiency led to inhibited tumor formation without affecting tumor cell proliferation. The PRIP deficiency was associated with decreased   cell invasion   and migration capabilities. We found that PRIP deficiency substantially reduced FOS gene expression. A chromatin immunoprecipitation assay revealed that PRIP was recruited to the FOS promoter. In addition, we demonstrated that PRIP also directly up-regulated the FOS gene expression in human breast cancer cells. Promoter analysis showed that PRIP acted through serum-responsive factor to regulate FOS gene expression. Finally, by re-expressing the FOS gene, we confirmed that the inhibited tumor formation of PRIP-deficient tumor cells was due to reduced expression of the FOS gene. 
  GeneRIFs  
 The results indicate that 17beta-estradiol-induced endometrial stromal   cell invasion   is dependent on c-fos-mediated MMP-9 expression. 
   
  
 LVRN 
 
  NCBI Gene  
  GeneRIF  
  Pubmed  
 
 
  Search Term  Rank  Fields  
  [Mm]igration  6  PubMed(2); GeneRIFs(2)  
  [Ii]nvasive  10  PubMed(1)  
 
 
 hide/show details for LVRN 
  [Mm]igration  PubMed  
 Placental expression of aminopeptidase-Q (laeverin) and its role in the pathophysiology of preeclampsia. The purpose of this study was to investigate the expression and subcellular localization of laeverin, a placenta-specific membrane-bound aminopeptidase, in preeclamptic placentas and its role in trophoblast cell   migration   and invasion.Expression of laeverin was investigated in 6 normal and 6 preeclamptic placentas with the use of immunofluorescence, sodium dodecylsulfate-polyacrylamide gel electrophoresis with Western blot analysis and immunoelectron microscopy. The role of laeverin in trophoblast   migration   and invasion was studied with the use of the xCelligence system and Boyden chambers with Matrigel in HTR-8/SVneo cells. The effect of laeverin gene-silencing on selected genes that are involved in cell transformation and tumorigenesis was evaluated by polymerase chain reaction array. The Student t test, Mann-Whitney U test, χ(2) test, or F-test was used to compare groups as appropriate.Laeverin was expressed in the cell membrane of villous trophoblasts in third-trimester healthy placentas; in preeclamptic placentas, it was expressed ectopically in the cytoplasm, especially in microvesicles. Immunoelectron microscopy showed laeverin leakage into the fetal capillaries and abundant expression in microvesicles in preeclamptic placentas.   Migratio  n and invasion of HTR-8/SVneo cells were reduced by 11.5% (P = .023) and 56.7% (P = .001), respectively, by laeverin gene-silencing. Analysis of downstream pathways affected by laeverin-silencing demonstrated significant down-regulation of integrin A2 (39-fold), integrin B3 (5-fold), and matrix metalloprotease 1 (36-fold).Expression of laeverin protein is altered in preeclamptic placentas. Its ectopic expression in the cytoplasm and microvesicles, rather than the cell membrane and leakage into the fetal capillaries, may have a role in the pathophysiologic condition of preeclampsia. Laeverin gene appears to be involved in trophoblast cell   migratio  n and invasion through interaction with integrins and matrix metalloprotease 1. 
 Laeverin/aminopeptidase Q induces trophoblast invasion during human early placentation. In primate placenta, extravillous trophoblast (EVT) invades maternal tissue in temporally- and spatially-regulated fashions. We previously identified a novel placenta-specific cell-surface aminopeptidase, laeverin/aminopeptidase Q, which is expressed on EVT-lineage cells in the fetal membrane. Laeverin possesses a peptide-binding site that is evolutionally unique to primates, suggesting possible involvement of laeverin in a primate-specific phenomenon during placentation. Thus, this study was designed to elucidate the molecular characteristics and physiological roles of laeverin in human EVT.Placental tissues of various developmental stages were subjected to immunostaining and western blotting. Effects of siRNA and a soluble form of recombinant laeverin on EVT cells isolated from primary villous explant cultures were examined using Matrigel invasion assays and cell proliferation assays.Laeverin was specifically immunolocalized to HLA-G-positive EVT in placentas from early and term pregnancy. In primary villous explant cultures, laeverin expression was induced on the cell surface of the outgrowing EVT. In western blotting, laeverin protein was detected as two distinct bands at 130 and 160 kDa along with a broad band ranging from 200 to 270 kDa. De-glycosylation treatment showed that these native laeverin isotypes are N-linked glycoproteins sharing a common 115-kDa core protein. In invasion assays, the reduction of laeverin expression by siRNA suppressed   migration   of the isolated EVT, while the soluble form of recombinant laeverin enhanced its   migration  .Laeverin is a specific cell-surface marker for human EVT and plays a regulatory role in EVT   migration  . 
  GeneRIFs  
 In preeclamptic placentas, laeverin is experssed in the cytoplasm and microvesicles, rather than the cell membrane. Laeverin appears to be involved in trophoblast cell   migration   and invasion through interaction with integrins and matrix metalloprotease 1. 
 Laeverin is a specific cell-surface marker for human extravillous trophoblast (EVT) and plays a regulatory role in EVT   migration  . 
  [Ii]nvasive  PubMed  
 Regulation of human extravillous trophoblast function by membrane-bound peptidases. During human placentation, the invasion of extravillous trophoblasts (EVTs) into maternal decidual tissues, especially toward maternal spiral arteries, is considered an essential process for subsequent normal fetal development. However, the precise regulatory mechanisms to induce EVT invasion toward arteries and/or to protect EVTs from further invasion have not been well understood. Recently, we found that two cell surface peptidases, dipeptidyl peptidase IV (DPPIV) and carboxypeptidase-M (CP-M,) are differentially expressed on EVTs. DPPIV expression was mainly observed on EVTs that had already ceased invasion. CP-M was detected on migrating EVTs including endovascular trophoblasts in the maternal arteries. The enzymatic inhibition of these peptidases affected the   invasive   property of choriocarcinoma-derived cell lines, BeWo and JEG3 cells. In addition, a chemokine, RANTES, that is one of the substrates for DPPIV, enhanced invasion of EVTs isolated from primary villous explant culture and its receptor, CCR1, was specifically expressed on migrating EVTs toward maternal arteries. Furthermore, a novel membrane-bound cell surface peptidase, named laeverin, was found to be specifically expressed on EVTs that had almost ceased invasion. These findings suggest that membrane-bound peptidases are important factors regulating EVT invasion during early placentation in humans. 
   
  
 ANPEP 
 
  NCBI Gene  
  GeneRIF  
  Pubmed  
 
 
  Search Term  Rank  Fields  
  [Ff]ilopodia  1  PubMed(2)  
  [Mm]igration  6  PubMed(9); GeneRIFs(1)  
  ECM  7  PubMed(2); GeneRIFs(1)  
  [Ee]xtracellular [Mm]atrix  8  PubMed(5)  
  [Ii]nvasive  10  PubMed(12); GeneRIFs(4)  
  [Mm]etastasis  12  PubMed(9)  
  [Cc]ell [Ii]nvasion  13  PubMed(5)  
 
 
 hide/show details for ANPEP 
  [Ff]ilopodia  PubMed  
 CD13/APN regulates endothelial invasion and   filopodia   formation. CD13/aminopeptidase N is a transmembrane peptidase that is induced in the vasculature of solid tumors and is a potent angiogenic regulator. Here, we demonstrate that CD13 controls endothelial cell invasion in response to the serum peptide bradykinin by facilitating signal transduction at the level of the plasma membrane. Inhibition of CD13 abrogates bradykinin B(2) receptor internalization, leading to the attenuation of downstream events such as bradykinin-induced activation of Cdc42 and   filopodia   formation, and thus affects endothelial cell motility. Investigation into mechanisms underlying this block led us to focus on B(2)R internalization via membrane-dependent mechanisms. Membrane disruption by depletion of cholesterol or trypsinization halts B(2)R internalization, invasion, and   filopodia   formation, which can be recovered with addition of cholesterol. However, this functional recovery is severely impaired in the presence of CD13 antagonists, and the distribution of membrane proteins is disordered in treated cells, suggesting a role for CD13 in plasma membrane protein organization. Finally, exogenous expression of wild-type but not mutant CD13 further alters protein distribution, suggesting peptidase activity is required for CD13's regulatory activity. Therefore, CD13 functions as a novel modulator of signal transduction and cell motility via its influence on specific plasma membrane organization, thus regulating angiogenesis. 
 CD13 is a novel mediator of monocytic/endothelial cell adhesion. During inflammation, cell surface adhesion molecules guide the adhesion and migration of circulating leukocytes across the endothelial cells lining the blood vessels to access the site of injury. The transmembrane molecule CD13 is expressed on monocytes and endothelial cells and has been shown to mediate homotypic cell adhesion, which may imply a role for CD13 in inflammatory monocyte trafficking. Here, we show that ligation and clustering of CD13 by mAb or viral ligands potently induce myeloid cell/endothelial adhesion in a signal transduction-dependent manner involving monocytic cytoskeletal rearrangement and   filopodia   formation. Treatment with soluble recombinant (r)CD13 blocks this CD13-dependent adhesion, and CD13 molecules from monocytic and endothelial cells are present in the same immunocomplex, suggesting a direct participation of CD13 in the adhesive interaction. This concept is strengthened by the fact that activated monocytic cells adhere to immobilized recombinant CD13. Furthermore, treatment with anti-CD13 antibodies in a murine model of peritonitis results in a decrease in leukocyte infiltration into the peritoneum, suggesting a potential role for CD13 in leukocyte trafficking in vivo. Therefore, this work supports a new direction for CD13 biology, where these cell surface molecules act as true molecular interfaces that induce and participate in critical inflammatory cell interactions. 
  [Mm]igration  PubMed  
 Promoter methylation of aminopeptidase N/CD13 in malignant melanoma. Aminopeptidase N (APN)/CD13 as ubiquitously expressed membrane peptidase exerts important functions in diverse cellular processes, such as proliferation,   migration   and differentiation. Previously, a role of APN in the invasiveness of melanoma cells has been demonstrated, but the underlying molecular mechanisms controlling APN expression are not understood. The present study demonstrates that lack of APN expression in primary and established melanoma cells was directly associated with a high-grade DNA methylation status of the myeloid APN promoter. Demethylation by 5-aza-2'-desoxycytidine not only induced constitutive and cytokine-regulated APN protein expression but also resulted in an increased APN-dependent   migration   of melanoma cells. Furthermore, its heterogeneous expression was inversely correlated to the expression of melanocytic marker proteins in established as well as in short-term cultured human melanoma cells. Staining of tissue microarrays generated from a large series of melanoma samples and control tissues demonstrated a higher APN expression in primary melanoma lesions when compared with nevi and metastases, which was neither associated with clinico-pathological parameters nor with the patients' outcome. Thus, the heterogeneous APN expression pattern in melanoma cells is epigenetically controlled and directly associated with an altered   migration   capacity but not of clinical significance in our study group. 
 CD13/Aminopeptidase N overexpression by basic fibroblast growth factor mediates enhanced invasiveness of 1F6 human melanoma cells. CD13/Aminopeptidase N (CD13) is known to play an important role in tumour cell invasion. We examined whether basic fibroblast growth factor (bFGF) is involved in the regulation of CD13 expression in human melanoma cells. 1F6 human melanoma cells were stably transfected with constructs encoding either the 18 kDa (18 kD) or all (ALL) bFGF isoform proteins. We observed highly increased CD13 mRNA and protein expression in the 1F6 clones regardless of the overexpression of either the 18 kD or all isoform proteins. Neutral aminopeptidase activity was increased five-fold and could be inhibited by bestatin and the CD13-neutralising antibody WM15. The enhanced invasion through Matrigel, but not   migration   in a wound assay, was efficiently abrogated by both bestatin and WM15. Upregulation of CD13 expression was the result of increased epithelial and myeloid promoter activity up to 4.5-fold in 1F6-18 kD and 1F6-ALL clones. Interestingly, in a panel of human melanoma cell lines, a significant correlation (r(2)=0.883, P<0.05) between bFGF and CD13 mRNA and protein expression was detected. High bFGF and CD13 expression were clearly related with an aggressive phenotype. Taken together, our data indicate that high bFGF expression upregulates CD13 expression in human melanoma cells by activating both the myeloid and the epithelial CD13 promoter. In addition, we show that high bFGF and CD13 expression results in enhanced invasive capacity and metastatic behaviour of human melanoma cells. 
 The angiogenic regulator CD13/APN is a transcriptional target of Ras signaling pathways in endothelial morphogenesis. Angiogenesis, the formation of new blood vessels, is a critical step for tumor growth and metastasis and an integral component of the pathologic inflammatory response in arthritis and the proliferative retinopathies. The CD13/aminopeptidase N (CD13/APN) metalloprotease is an important regulator of angiogenesis where its expression on activated blood vessels is induced by angiogenic signals. Here, we show that cytokine induction of CD13/APN in endothelial cells is regulated by distinct Ras effector pathways involving Ras/mitogen-activated protein kinase (MAPK) or PI-3K. Signals transduced by activated Ras, Raf, and mitogen-induced extracellular kinase (MEK) stimulate transcription from the CD13/APN proximal promoter. Inhibition of these pathways and extracellular signal-regulated serine/threonine kinase (ERK-2) and PI-3K by expression of dominant-negative proteins or chemical inhibitors prevented induction of CD13/APN transcription in response to basic fibroblast growth factor (bFGF). We show that Ras-induced signal transduction is required for growth factor-induced angiogenesis, because inhibition of downstream mediators of Ras signaling (MEK or PI-3K) abrogated endothelial cell   migration  , invasion, and morphogenesis in vitro. Reintroduction of CD13/APN, a shared downstream target of these pathways, overrode the suppressive effect of these inhibitors and restored the function of endothelial cells in   migration  /invasion and capillary morphogenesis assays. Similarly, inhibition of MEK abrogated cell invasion and the formation of endothelial-lined capillaries in vivo, which was effectively rescued by addition of exogenous CD13/APN protein. These studies provide strong evidence that CD13/APN is an important target of Ras signaling in angiogenesis and is a limiting factor in angiogenic progression. 
 Targeting CD13 (aminopeptidase-N) in turn downregulates ADAM17 by internalization in acute myeloid leukaemia cells. Secreted matrix metalloproteinases (MMP)-2 and MMP-9 and membrane-anchored aminopeptidase-N/CD13 are abnormally expressed in human acute myeloid leukaemia (AML). We previously showed that CD13 ligation by anti-CD13 monoclonal antibodies can induce apoptosis in AML cells. Here, we assessed ADAM17 expression in primary blood blasts CD13+CD33+ from patients with AML. Primary AML cells expressed ADAM17 transcript and its surface expression was higher in subtype M4 (myelomonocytic) and M5 (monocytic) AML specimens than in M0 and M1/M2 (early and granulocytic) specimens. In AML cell lines defining distinct AML subfamilies (HL-60/M2, NB4/M3, THP-1/M5, U937/M5) and primary AML cells cultured ex vivo, anti-CD13 antibodies downregulated surface CD13 and ADAM17 without affecting MMP-2/-9 release. Knockdown of CD13 by siRNA prevented anti-CD13-mediated ADAM17 downregulation, indicating that CD13 is required for ADAM17 downregulation. Soluble ADAM17 was not detected in the medium of anti-CD13 treated cells, suggesting that ADAM17 was not shed. After ligation by anti-CD13, CD13 and ADAM17 were internalized. Subsequently, we found that ADAM17 interacts with CD13. We postulate that the interaction of ADAM17 with CD13 and its downregulation following CD13 engagement has important implications in AML for the known roles of ADAM17 in tumour-associated cell growth,   migration   and invasion. 
 Proteolytic processing of CXCL11 by CD13/aminopeptidase N impairs CXCR3 and CXCR7 binding and signaling and reduces lymphocyte and endothelial cell   migration  . CXCR3 ligands were secreted by tissue fibroblasts and peripheral blood-derived mononuclear leukocytes in response to interferon-gamma (IFN-gamma) and Toll-like receptor (TLR) ligands. Subsequent purification and identification revealed the presence of truncated CXCL11 variants missing up to 6 amino acids. In combination with CD26/dipeptidyl peptidase IV, the metalloprotease aminopeptidase N (APN), identical to the myeloid cell marker CD13, rapidly processed CXCL11, but not CXCL8, to generate truncated CXCL11 forms. Truncated CXCL11 had reduced binding, signaling, and chemotactic properties for lymphocytes and CXCR3- or CXCR7-transfected cells. CD13/APN-truncated CXCL11 failed to induce an intracellular calcium increase but was still able to bind and desensitize CXCR3 for intact CXCL11 signaling. CXCL11 efficiently bound to CXCR7, but CXCL11 was not able to induce calcium signaling or ERK1/2 or Akt phosphorylation through CXCR7. CD26-truncated CXCL11 failed to attract lymphocytes but still inhibited microvascular endothelial cell (HMVEC)   migration  . However, further processing of CXCL11 by CD13 resulted in significant reduction of inhibition of HMVEC   migration  . Taken together, during inflammation or cancer, CXCL11 processing by CD13 may lead to a reduced number of tumor-infiltrating lymphocytes and in a more angiogenic environment. 
 CD13 is a novel mediator of monocytic/endothelial cell adhesion. During inflammation, cell surface adhesion molecules guide the adhesion and   migration   of circulating leukocytes across the endothelial cells lining the blood vessels to access the site of injury. The transmembrane molecule CD13 is expressed on monocytes and endothelial cells and has been shown to mediate homotypic cell adhesion, which may imply a role for CD13 in inflammatory monocyte trafficking. Here, we show that ligation and clustering of CD13 by mAb or viral ligands potently induce myeloid cell/endothelial adhesion in a signal transduction-dependent manner involving monocytic cytoskeletal rearrangement and filopodia formation. Treatment with soluble recombinant (r)CD13 blocks this CD13-dependent adhesion, and CD13 molecules from monocytic and endothelial cells are present in the same immunocomplex, suggesting a direct participation of CD13 in the adhesive interaction. This concept is strengthened by the fact that activated monocytic cells adhere to immobilized recombinant CD13. Furthermore, treatment with anti-CD13 antibodies in a murine model of peritonitis results in a decrease in leukocyte infiltration into the peritoneum, suggesting a potential role for CD13 in leukocyte trafficking in vivo. Therefore, this work supports a new direction for CD13 biology, where these cell surface molecules act as true molecular interfaces that induce and participate in critical inflammatory cell interactions. 
 CD13 (aminopeptidase N) can associate with tumor-associated antigen L6 and enhance the motility of human lung cancer cells. Cancer metastasis is a multiple-step process that involves the regulated interaction of diverse cellular proteins. We recently reported that the expression of tumor-associated antigen L6 (TAL6) promoted the invasiveness of lung cancer cells and was inversely correlated with disease-free survival of squamous lung carcinoma patients. We now report that CD13 (aminopeptidase N) can associate with TAL6 and can enhance cancer cell   migration  . CD13 was shown by coimmunoprecipitation to associate in vitro with TAL6 on several cancer cell lines and to associate in vivo by antibody-mediated copatching immunofluorescence. CD13 was selectively expressed on highly invasive CL1-5 lung cancer cells as compared to poorly invasive CL1-0 lung cancer cells. The role of CD13 aminopeptidase activity in regulating cell motility was investigated with chemical inhibitors, specific antibodies and a catalytically inactive CD13 protein. Inhibition of CD13 aminopeptidase activity by nontoxic concentrations of leuhistin modestly decreased the   migration   of CL1-5 cells. In contrast, binding of CD13 by specific antibodies significantly reduced both the   migration   and the invasion of CL1-5 cells. Poorly invasive CL1-0 cells that stably expressed CD13 displayed significantly (p < or = 0.0005) enhanced cell   migration   (300% of control). Expression of an enzymatically inactive CD13 mutant on CL1-0 cells also significantly (p < or = 0.0005) enhanced cell   migration   (200% of control). Our results show that TAL6 and CD13 can form a complex on lung cancer cells, that these molecules can modulate cell   migration   and invasion and that the influence of CD13 on cell motility did not strictly depend on its aminopeptidase activity. 
 Aminopeptidase N (APN/CD13) is selectively expressed in vascular endothelial cells and plays multiple roles in angiogenesis. Proteolytic enzyme-mediated degradation of the extracellular matrix (ECM) is crucial for the formation of both tumor metastasis and angiogenesis. Recently, several reports have suggested that aminopeptidases are involved in this process, but precisely how is largely unknown. We found here that aminopeptidase N (APN/CD13) was selectively expressed in vascular endothelial cells including human umbilical vein endothelial cells (HUVEC) and human aortic endothelial cells (HAEC), and was not detectable in a majority of normal cells and tumor cell lines we examined. RNA interference (RNAi) of APN resulted in the inhibition of capillary tube formation of HUVEC on Matrigel. APN siRNA suppressed the   migration   of HUVEC through a fibronectin-coated Transwell membrane, and reduced the cellular adhesion to Matrigel and various adhesion molecules including type IV collagen, type I collagen and fibronectin. These findings suggest that APN is a multifunctional protein with important roles in vascular endothelial morphogenesis during angiogenesis. 
 Possible contribution of aminopeptidase N (APN/CD13) to   migratio  n and invasion of human osteosarcoma cell lines. Osteosarcoma is the most common primary malignancy of the bone. Aminopeptidase N (APN/CD13), a Zn+2-dependent ectopeptidase localized on the cell surface, is widely considered to influence the invasion mechanism. This study explores the potential involvement of APN in   migratio  n and invasion of human osteosarcoma cells in vitro using inhi-bitors and activators of APN. Cells treated with APN inhibitor bestatin displayed decreased   migratio  n and invasion in a Boyden chamber Transwell assay. Western blotting revealed reduced levels of mitogen-activated protein kinase (MAPK) and phosphatidylinositol 3-kinase (PI3K) pathway proteins, reduced phosphorylation of p38, ERK1/2 and JNK and decreased levels of NF-κB. Bestatin treatment also lowered APN, matrix metalloproteinase (MMP)-2 and -9 enzymatic activity and their mRNA expression. Reduced MMP-2 and -9 protein levels were also observed. By comparison, cells treated with cytokine interleukin-6 (IL-6), a stimulator of APN, displayed increase  d migrati  on and invasion. Western blotting revealed increased levels of MAPK and PI3K pathway proteins, phosphorylated p38, ERK1/2 and JNK, and NF-κB. IL-6 treatment also increased APN and MMP-2 and -9 enzymatic activity. An increase of APN, MMP-2 and -9 mRNA levels, and MMP-2 and -9 protein levels was also observed. Together these experiments reveal potential enzymatic and signalling roles for APN in osteosarcoma and establish a starting point for an in-depth analysis of the role of APN in regulating invasiveness. A deeper knowledge about the regulatory mechanisms of APN may contribute to the development of anti-metastatic therapies. 
  GeneRIFs  
 TAL6 and CD13 can form a complex on lung cancer cells; these molecules can modulate cell   migration   and invasion, and the influence of CD13 on cell motility does not strictly depend on its aminopeptidase activity 
  ECM  PubMed  
 Microarray-based identification of aminopeptidase N target genes in keratinocyte conditioned medium-stimulated dermal fibroblasts. Of the many processes that affect the outcome of wound repair, epidermal-dermal interactions are essential to extracellular matrix (  ECM  ) remodeling and in particular, soluble factors released by keratinocytes are known to have a direct impact on the production of   ECM   by dermal fibroblasts. Aminopeptidase N (APN) has recently been proposed as a cell-surface receptor for stratifin and is responsible for the stratifin-mediated matrix metalloproteinase-1 (MMP-1) upregulation in fibroblasts. The present study examines whether modulation of APN gene expression has any impact on the fibroblast   ECM   gene expression profile. The result reveals that in the presence of keratinocyte-derived soluble factors, transient knockdown of APN in dermal fibroblasts affects the expression of key   ECM   components such as fibronectin, tenascin-C, MMP-1, MMP-3, and MMP-12. The regulatory effects of APN on fibronectin and selective MMPs appear to be associated with receptor-mediated signal transduction independently of its peptidase activity. On the contrary, inhibition of the APN enzymatic activity by bestatin significantly reduces the tenascin-C expression and enhances the contraction of fibroblast-populated collagen gel, suggesting an activity-dependent regulation of fibroblast contractility by APN. The overall effects of APN on the expression of fibronectin, tenascin-C, and MMPs in fibroblasts propose an important role for APN in the regulation of keratinocyte-mediated   ECM   remodeling and fibroblast contractile activity. 
 Aminopeptidase N (APN/CD13) is selectively expressed in vascular endothelial cells and plays multiple roles in angiogenesis. Proteolytic enzyme-mediated degradation of the extracellular matrix (  ECM  ) is crucial for the formation of both tumor metastasis and angiogenesis. Recently, several reports have suggested that aminopeptidases are involved in this process, but precisely how is largely unknown. We found here that aminopeptidase N (APN/CD13) was selectively expressed in vascular endothelial cells including human umbilical vein endothelial cells (HUVEC) and human aortic endothelial cells (HAEC), and was not detectable in a majority of normal cells and tumor cell lines we examined. RNA interference (RNAi) of APN resulted in the inhibition of capillary tube formation of HUVEC on Matrigel. APN siRNA suppressed the migration of HUVEC through a fibronectin-coated Transwell membrane, and reduced the cellular adhesion to Matrigel and various adhesion molecules including type IV collagen, type I collagen and fibronectin. These findings suggest that APN is a multifunctional protein with important roles in vascular endothelial morphogenesis during angiogenesis. 
  GeneRIFs  
 The overall effects of APN on the expression of fibronectin, tenascin-C, and MMPs in fibroblasts propose an important role for APN in the regulation of keratinocyte-mediated   ECM   remodeling and fibroblast contractile activity. 
  [Ee]xtracellular [Mm]atrix  PubMed  
 Proteomic analysis of human parotid gland exosomes by multidimensional protein identification technology (MudPIT). Human ductal saliva contributes over a thousand unique proteins to whole oral fluids. The mechanism by which most of these proteins are secreted by salivary glands remains to be determined. The present study used a mass spectrometry-based, shotgun proteomics approach to explore the possibility that a subset of the proteins found in saliva are derived from exosomes, membrane-bound vesicles of endosomal origin within multivesicular endosomes. Using MudPIT (multidimensional protein identification technology) mass spectrometry, we catalogued 491 proteins in the exosome fraction of human parotid saliva. Many of these proteins were previously observed in ductal saliva from parotid glands (265 proteins). Furthermore, 72 of the proteins in parotid exosomes overlap with those previously identified as urinary exosome proteins, proteins which are also frequently associated with exosomes from other tissues and cell types. Gene Ontology (GO) and KEGG pathway analyses found that cytosolic proteins comprise the largest category of proteins in parotid exosomes (43%), involved in such processes as phosphatidylinositol signaling system, calcium signaling pathway, inositol metabolism, protein export, and signal transduction, among others; whereas the integral plasma membrane proteins and associated/peripheral plasma membrane proteins (26%) were associated with   extracellular matrix  -receptor interaction, epithelial cell signaling, T-cell and B-cell receptor signaling, cytokine receptor interaction, and antigen processing and presentation, among other biological functions. In addition, these putative saliva exosomal proteins were linked to specific diseases (e.g., neurodegenerative disorders, prion disease, cancers, type I and II diabetes). Consequently, parotid glands secrete exosomes that reflect the metabolic and functional status of the gland and may also carry informative protein markers useful in the diagnosis and treatment of systemic diseases. 
 Clinical significance of aminopeptidase N in non-small cell lung cancer. The aim of our study is to investigate the mechanism of metastasis, to detect novel metastasis-associated molecules, and to evaluate the molecules from the point of view of clinical application. A monoclonal antibody MH8-11, which we established, recognizes a glycoprotein that is identical to aminopeptidase N (APN/CD13). APN/CD13 degrades the   extracellular matrix  , while it is also involved in cell motility and improves angiogenesis.We investigated the expression of APN/CD13 in 194 cases of non-small cell lung cancer (NSCLC) by immunohistochemical analyses and reverse transcription-PCR assay to determine the significance of this prognostic factor; 95 tumors were stage I, 36 were stage II, 39 were stage IIIA, and 24 were stage IIIB. Moreover, we investigated that the relationship between the expression of APN/CD13 and angiogenesis and prognosis for patients with NSCLC.We found a correlation between the expression of APN/CD13 and angiogenesis (r = 0.659; P < 0.0001). In the 194 patients with NSCLC, we found 68 patients to be APN/CD13+ and 126 patients to be APN/CD13-. The 5-year survival rate in patients with APN/CD13+ tumors was significantly lower than in those whose tumors had negative APN/CD13 (48.3% versus 67.1%; P = 0.0001).Our data suggest the expression of APN/CD13 for patients with NSCLC to be associated with a poor prognosis and angiogenesis. This is the first study to show the relationship between the expression of APN/CD13 and the prognosis of patients with NSCLC. The inhibition of APN/CD13 may be an effective new molecular target therapy for patients with NSCLC. 
 Reduced activity of CD13/aminopeptidase N (APN) in aggressive meningiomas is associated with increased levels of SPARC. Meningiomas are the second most common brain tumors in adults, and meningiomas exhibit a tendency to invade adjacent structures. Compared with high-grade gliomas, little is known about the molecular changes that potentially underlie the invasive behavior of meningiomas. In this study, we examined the expression and function of the membrane alanyl-aminopeptidase [mAAP, aminopeptidase N (APN), CD13, EC3.4.11.2] zinc-dependent ectopeptidase in meningiomas and meningioma cell lines, based on its prior association with tumor invasion in colorectal and renal carcinomas. We found a significant reduction of APNmRNA and protein expression, as well as enzymatic activity, in high-grade meningiomas. While meningioma tumor cell proliferation was not affected by either pharmacologic APN inhibition or siRNA-mediated APN silencing, APN pharmacologic and siRNA knockdown significantly reduced meningioma cell invasion in vitro. Next, we employed pathway-specific cDNA microarray analyses to identify   extracellular matrix   and adhesion molecules regulated by APN, and found that APN-siRNA knockdown substantially increased the expression of secreted protein, acidic and rich in cysteine (SPARC)/osteonectin. Finally, we demonstrated that SPARC, which has been previously associated with meningioma invasiveness, was increased in aggressive meningiomas. Collectively, these results suggest that APN expression and enzymatic function is reduced in aggressive meningiomas, and that alterations in the balance between APN and SPARC might favor meningioma invasion. 
 Microarray-based identification of aminopeptidase N target genes in keratinocyte conditioned medium-stimulated dermal fibroblasts. Of the many processes that affect the outcome of wound repair, epidermal-dermal interactions are essential to   extracellular matrix   (ECM) remodeling and in particular, soluble factors released by keratinocytes are known to have a direct impact on the production of ECM by dermal fibroblasts. Aminopeptidase N (APN) has recently been proposed as a cell-surface receptor for stratifin and is responsible for the stratifin-mediated matrix metalloproteinase-1 (MMP-1) upregulation in fibroblasts. The present study examines whether modulation of APN gene expression has any impact on the fibroblast ECM gene expression profile. The result reveals that in the presence of keratinocyte-derived soluble factors, transient knockdown of APN in dermal fibroblasts affects the expression of key ECM components such as fibronectin, tenascin-C, MMP-1, MMP-3, and MMP-12. The regulatory effects of APN on fibronectin and selective MMPs appear to be associated with receptor-mediated signal transduction independently of its peptidase activity. On the contrary, inhibition of the APN enzymatic activity by bestatin significantly reduces the tenascin-C expression and enhances the contraction of fibroblast-populated collagen gel, suggesting an activity-dependent regulation of fibroblast contractility by APN. The overall effects of APN on the expression of fibronectin, tenascin-C, and MMPs in fibroblasts propose an important role for APN in the regulation of keratinocyte-mediated ECM remodeling and fibroblast contractile activity. 
 Aminopeptidase N (APN/CD13) is selectively expressed in vascular endothelial cells and plays multiple roles in angiogenesis. Proteolytic enzyme-mediated degradation of the   extracellular matrix   (ECM) is crucial for the formation of both tumor metastasis and angiogenesis. Recently, several reports have suggested that aminopeptidases are involved in this process, but precisely how is largely unknown. We found here that aminopeptidase N (APN/CD13) was selectively expressed in vascular endothelial cells including human umbilical vein endothelial cells (HUVEC) and human aortic endothelial cells (HAEC), and was not detectable in a majority of normal cells and tumor cell lines we examined. RNA interference (RNAi) of APN resulted in the inhibition of capillary tube formation of HUVEC on Matrigel. APN siRNA suppressed the migration of HUVEC through a fibronectin-coated Transwell membrane, and reduced the cellular adhesion to Matrigel and various adhesion molecules including type IV collagen, type I collagen and fibronectin. These findings suggest that APN is a multifunctional protein with important roles in vascular endothelial morphogenesis during angiogenesis. 
  [Ii]nvasive  PubMed  
 Promoter methylation of aminopeptidase N/CD13 in malignant melanoma. Aminopeptidase N (APN)/CD13 as ubiquitously expressed membrane peptidase exerts important functions in diverse cellular processes, such as proliferation, migration and differentiation. Previously, a role of APN in the   invasive  ness of melanoma cells has been demonstrated, but the underlying molecular mechanisms controlling APN expression are not understood. The present study demonstrates that lack of APN expression in primary and established melanoma cells was directly associated with a high-grade DNA methylation status of the myeloid APN promoter. Demethylation by 5-aza-2'-desoxycytidine not only induced constitutive and cytokine-regulated APN protein expression but also resulted in an increased APN-dependent migration of melanoma cells. Furthermore, its heterogeneous expression was inversely correlated to the expression of melanocytic marker proteins in established as well as in short-term cultured human melanoma cells. Staining of tissue microarrays generated from a large series of melanoma samples and control tissues demonstrated a higher APN expression in primary melanoma lesions when compared with nevi and metastases, which was neither associated with clinico-pathological parameters nor with the patients' outcome. Thus, the heterogeneous APN expression pattern in melanoma cells is epigenetically controlled and directly associated with an altered migration capacity but not of clinical significance in our study group. 
 Genetic variants and susceptibility to neurological complications following West Nile virus infection. To determine genetic factors predisposing to neurological complications following West Nile virus infection, we analyzed a cohort of 560 neuro  invasive   case patients and 950 control patients for 13 371 mostly nonsynonymous single-nucleotide polymorphisms (SNPs). The top 3 SNPs on the basis of statistical significance were also in genes of biological plausibility: rs2066786 in RFC1 (replication factor C1) (P = 1.88 × 10(-5); odds ratio [OR], 0.68 [95% confidence interval {CI}, .56-.81]); rs2298771 in SCN1A (sodium channel, neuronal type I α subunit) (P = 5.87 × 10(-5); OR, 1.47 [95% CI, 1.21-1.77]); and rs25651 in ANPEP (ananyl aminopeptidase) (P = 1.44 × 10(-4); OR, 0.69 [95% CI, .56-.83]). Additional genotyping of these SNPs in a separate sample of 264 case patients and 296 control patients resulted in a lack of significance in the replication cohort; joint significance was as follows: rs2066786, P = .0022; rs2298771, P = .005; rs25651, P = .042. Using mostly nonsynonymous variants, we therefore did not identify genetic variants associated with n  euroinva  sive disease. 
 Plasma renin-angiotensin system-regulating aminopeptidase activities are modified in early stage Alzheimer's disease and show gender differences but are not related to apolipoprotein E genotype. Alterations in blood pressure and components of the renin-angiotensin system (RAS) contribute to the development and progression of Alzheimer's disease (AD), resulting in changes that can lead or contribute to cognitive decline. Aspartyl aminopeptidase (ASAP), aminopeptidase A (APA), aminopeptidase N (APN) and aminopeptidase B (APB) catabolise circulating angiotensins, whereas insulin-regulated aminopeptidase (IRAP) has been described as the AT4 receptor. We have found in AD patients a significant decrease of APA activity in men but not in women, and of APN, APB and IRAP in both genders, when compared with control subjects. No changes were found in ASAP activity. Also, APN, APB and IRAP but not APA correlated with the Mini-Mental test, but no relationship with APOE genotype was found. We conclude that several components of the RAS are modified in AD patients, with gender differences. Furthermore, ROC analysis indicates that APN, APB and IRAP activities could be useful non-  invasive   biomarkers of AD from the earliest stages. 
 Reduced activity of CD13/aminopeptidase N (APN) in aggressive meningiomas is associated with increased levels of SPARC. Meningiomas are the second most common brain tumors in adults, and meningiomas exhibit a tendency to invade adjacent structures. Compared with high-grade gliomas, little is known about the molecular changes that potentially underlie the   invasive   behavior of meningiomas. In this study, we examined the expression and function of the membrane alanyl-aminopeptidase [mAAP, aminopeptidase N (APN), CD13, EC3.4.11.2] zinc-dependent ectopeptidase in meningiomas and meningioma cell lines, based on its prior association with tumor invasion in colorectal and renal carcinomas. We found a significant reduction of APNmRNA and protein expression, as well as enzymatic activity, in high-grade meningiomas. While meningioma tumor cell proliferation was not affected by either pharmacologic APN inhibition or siRNA-mediated APN silencing, APN pharmacologic and siRNA knockdown significantly reduced meningioma cell invasion in vitro. Next, we employed pathway-specific cDNA microarray analyses to identify extracellular matrix and adhesion molecules regulated by APN, and found that APN-siRNA knockdown substantially increased the expression of secreted protein, acidic and rich in cysteine (SPARC)/osteonectin. Finally, we demonstrated that SPARC, which has been previously associated with meningioma   invasive  ness, was increased in aggressive meningiomas. Collectively, these results suggest that APN expression and enzymatic function is reduced in aggressive meningiomas, and that alterations in the balance between APN and SPARC might favor meningioma invasion. 
 CD13/Aminopeptidase N overexpression by basic fibroblast growth factor mediates enhanced   invasive  ness of 1F6 human melanoma cells. CD13/Aminopeptidase N (CD13) is known to play an important role in tumour cell invasion. We examined whether basic fibroblast growth factor (bFGF) is involved in the regulation of CD13 expression in human melanoma cells. 1F6 human melanoma cells were stably transfected with constructs encoding either the 18 kDa (18 kD) or all (ALL) bFGF isoform proteins. We observed highly increased CD13 mRNA and protein expression in the 1F6 clones regardless of the overexpression of either the 18 kD or all isoform proteins. Neutral aminopeptidase activity was increased five-fold and could be inhibited by bestatin and the CD13-neutralising antibody WM15. The enhanced invasion through Matrigel, but not migration in a wound assay, was efficiently abrogated by both bestatin and WM15. Upregulation of CD13 expression was the result of increased epithelial and myeloid promoter activity up to 4.5-fold in 1F6-18 kD and 1F6-ALL clones. Interestingly, in a panel of human melanoma cell lines, a significant correlation (r(2)=0.883, P<0.05) between bFGF and CD13 mRNA and protein expression was detected. High bFGF and CD13 expression were clearly related with an aggressive phenotype. Taken together, our data indicate that high bFGF expression upregulates CD13 expression in human melanoma cells by activating both the myeloid and the epithelial CD13 promoter. In addition, we show that high bFGF and CD13 expression results in enhanced   invasive   capacity and metastatic behaviour of human melanoma cells. 
 Identification of APN/CD13 as the target antigen of FU3, a human monoclonal antibody that recognizes malignant fibrous histiocytoma. Malignant fibrous histiocytoma (MFH), a high-grade, undifferentiated sarcoma, is highly aggressive, resistant to radiochemotherapy and associated with poor prognosis. There are no specific immunohistochemical markers for its diagnosis. The MFH cell line SFT7913 served as and immunogen for the generation of the FU3 monoclonal antibody in our laboratory. FU3 reacted strongly with MFH cells and with perivascular mesenchymal cells. In this study, we demonstrated that the antigen recognized by FU3 was identical to aminopeptidase N (APN/CD13) using FU3 immunoaffinity chromatography and N-terminal amino acid sequencing. Frequent (80%) and high-grade (>50% of cells) expression of APN/CD13 was observed in MFH, although low-grade expression was seen in some other sarcomas. Moreover, small interfering RNA (siRNA) that specifically targets APN/CD13 significantly suppressed MFH cell invasion in vitro. The newly developed monoclonal antibody FU3 specifically recognizes CD13 on MFH cells. Decreased expression of CD13, mediated by siRNA-mediated knockdown, attenuated the   invasive   capacity of MFH cells. Thus, results indicate that APN/CD13 could be an important diagnostic biomarker and therapeutic target for MFH. 
 Prognostic significance of aminopeptidase-N (CD13) in hepatoblastoma. Hepatoblastoma is a rare childhood malignant tumor that originates from immature hepatic cells. Aminopeptidase-N(CD13), an ectopeptidase that promotes tumor invasion and metastasis, is expressed in fetal stage hepatic progenitor cells, although its role in hepatoblastoma remains unclear.The expression pattern of CD13 was investigated on immunohistochemistry in 30 tissue samples from 27 hepatoblastoma patients (16 with predominantly embryonal [pE] histology and 14 with predominantly fetal [pF] histology). Immunoreactive score (IRS) was used to quantify staining data, and the relationship between CD13 expression, clinicopathological factors, and clinical outcome was investigated. The biological function of CD13 was also examined in the hepatoblastoma cell lines Huh6 and HepG2.All specimens stained positive for CD13, with higher CD13 expression in pE than in pF hepatoblastoma samples (median IRS, 4; range, 2-9 vs 2; range, 1-4). Strong CD13 expression was correlated with vascular invasion. Five year event-free survival and overall survival were better in patients with CD13(low) than in those with CD13(high) tumors (100% vs 51.0%, P = 0.026; and 100% vs 74.0%, P = 0.114, respectively). A CD13-neutralizing antibody and the potent CD13 inhibitor, Ubenimex, suppressed   invasive   activity in HepG2 cells in vitro.CD13 expression is associated with hepatoblastoma   invasive  ness and could be a novel prognostic marker for hepatoblastoma. 
 CD13 (aminopeptidase N) can associate with tumor-associated antigen L6 and enhance the motility of human lung cancer cells. Cancer metastasis is a multiple-step process that involves the regulated interaction of diverse cellular proteins. We recently reported that the expression of tumor-associated antigen L6 (TAL6) promoted the   invasive  ness of lung cancer cells and was inversely correlated with disease-free survival of squamous lung carcinoma patients. We now report that CD13 (aminopeptidase N) can associate with TAL6 and can enhance cancer cell migration. CD13 was shown by coimmunoprecipitation to associate in vitro with TAL6 on several cancer cell lines and to associate in vivo by antibody-mediated copatching immunofluorescence. CD13 was selectively expressed on highly   invasive   CL1-5 lung cancer cells as compared to poorly   invasive   CL1-0 lung cancer cells. The role of CD13 aminopeptidase activity in regulating cell motility was investigated with chemical inhibitors, specific antibodies and a catalytically inactive CD13 protein. Inhibition of CD13 aminopeptidase activity by nontoxic concentrations of leuhistin modestly decreased the migration of CL1-5 cells. In contrast, binding of CD13 by specific antibodies significantly reduced both the migration and the invasion of CL1-5 cells. Poorly   invasive   CL1-0 cells that stably expressed CD13 displayed significantly (p < or = 0.0005) enhanced cell migration (300% of control). Expression of an enzymatically inactive CD13 mutant on CL1-0 cells also significantly (p < or = 0.0005) enhanced cell migration (200% of control). Our results show that TAL6 and CD13 can form a complex on lung cancer cells, that these molecules can modulate cell migration and invasion and that the influence of CD13 on cell motility did not strictly depend on its aminopeptidase activity. 
 Aminopeptidase N activity predicts 5-year survival in colorectal cancer patients. Aminopeptidase N (APN; EC 3.4.11.2) is a membrane dimeric metallopeptidase involved in differentiation, development, and proliferative processes of several tissues. Recent studies have demonstrated the increased expression and activity of this enzyme in several cancers. However, there are no available data about the impact of this peptidase in the biological aggressiveness and the survival of colorectal cancer (CRC) patients.The activity and mRNA expression of APN in tumor tissue (n = 81) and plasma (n = 40) of patients with CRC of low and high grades and stages were prospectively analyzed by fluorimetric and quantitative reverse transcriptase-polymerase chain reaction methods. Data obtained in adenoma and CRC were compared with those from the surrounding normal mucosa. Classic clinical and pathological parameters were stratified following APN data and analyzed for 5-year survival.mRNA levels of APN (ANPEP) were lower in colorectal adenomas and adenocarcinomas than in the surrounding uninvolved mucosa (Kruskal-Wallis, P < 0.001). Aminopeptidase N activity in CRC tissue was higher in patients with better overall survival (log-rank P < 0.05, Cox analysis P < 0.05). By contrast, higher plasmatic APN activity correlated with worse overall survival (log-rank P < 0.01, Cox analysis P < 0.05).Aminopeptidase N activity in tissue and plasma from CRC patients is an independent prognostic factor of 5-year survival. The determination of APN activity levels in the plasma may be a safe, minimally   invasive  , and inexpensive way to define the aggressiveness of CRC in daily practice. 
 Possible contribution of aminopeptidase N (APN/CD13) to migration and invasion of human osteosarcoma cell lines. Osteosarcoma is the most common primary malignancy of the bone. Aminopeptidase N (APN/CD13), a Zn+2-dependent ectopeptidase localized on the cell surface, is widely considered to influence the invasion mechanism. This study explores the potential involvement of APN in migration and invasion of human osteosarcoma cells in vitro using inhi-bitors and activators of APN. Cells treated with APN inhibitor bestatin displayed decreased migration and invasion in a Boyden chamber Transwell assay. Western blotting revealed reduced levels of mitogen-activated protein kinase (MAPK) and phosphatidylinositol 3-kinase (PI3K) pathway proteins, reduced phosphorylation of p38, ERK1/2 and JNK and decreased levels of NF-κB. Bestatin treatment also lowered APN, matrix metalloproteinase (MMP)-2 and -9 enzymatic activity and their mRNA expression. Reduced MMP-2 and -9 protein levels were also observed. By comparison, cells treated with cytokine interleukin-6 (IL-6), a stimulator of APN, displayed increased migration and invasion. Western blotting revealed increased levels of MAPK and PI3K pathway proteins, phosphorylated p38, ERK1/2 and JNK, and NF-κB. IL-6 treatment also increased APN and MMP-2 and -9 enzymatic activity. An increase of APN, MMP-2 and -9 mRNA levels, and MMP-2 and -9 protein levels was also observed. Together these experiments reveal potential enzymatic and signalling roles for APN in osteosarcoma and establish a starting point for an in-depth analysis of the role of APN in regulati  ng invas  iveness. A deeper knowledge about the regulatory mechanisms of APN may contribute to the development of anti-metastatic therapies. 
 Inhibition of APN/CD13 leads to suppressed progressive potential in ovarian carcinoma cells. Aminopeptidase N (APN/CD13), a 150-kDa metalloprotease, is a multifunctional cell surface aminopeptidase with ubiquitous expression. Recent studies have suggested that APN/CD13 plays an important role in tumor progression of several human malignancies. In the current study, we investigated the role of APN/CD13 in ovarian carcinoma (OVCA) progression.We first examined the expression of APN/CD13 at the protein level in a variety of OVCA cell lines and tissues. We subsequently investigated whether there was a correlation between APN/CD13 expression and   invasive   potential of various OVCA cell lines. Moreover, we investigated the function of APN/CD13 in OVCA cells using bestatin, an APN/CD13 inhibitor, or transfection of siRNA for APN/CD13.We confirmed that APN/CD13 was expressed in OVCA tissues and cell lines to various extents. There was a positive correlation between APN/CD13 expression and migratory potential in various OVCA cell lines with accordingly enhanced secretion of endogenous MMP-2. Subsequently, we found a significant decrease in the proliferative and migratory abilities of OVCA cells after the addition of bestatin or the inhibition of APN/CD13 expression by siRNA. Furthermore, in an animal model, daily intraperitoneal administration of bestatin after inoculation of OVCA cells resulted in a decrease of peritoneal dissemination and in prolonged survival of nude mice.The current data indicate the possible involvement of APN/CD13 in the development of OVCA, and suggest that clinical use of bestatin may contribute to better prognosis for ovarian carcinoma patients. 
 Comprehensive analysis of low-abundance proteins in human urinary exosomes using peptide ligand library technology, peptide OFFGEL fractionation and nanoHPLC-chip-MS/MS. Human urinary exosomes are 30-100 nm vesicles that originate as the internal vesicles in multivesicular bodies from every renal epithelial cell type facing the urinary track and may serve as a suitable non  invasive   starting material for biomarker discovery relevant to a variety of renal disease. To comprehensively explore the low-abundance proteome, combinatorial peptide ligand libraries, combined with peptide OFFGEL electrophoresis were employed for the enrichment and separation of relatively low-abundant proteins in urinary exosomes. After analysis by nanoHPLC-chip-MS/MS, 512 proteins were identified, including a large number of proteins with extreme molecular weight or extreme pI value, which could not be well mapped by using traditional 2-D-gel-based separation methods. This in-depth analysis of low-abundant proteins in urinary exosomes led to an increased understanding of molecular composition of these little vesicles and may be helpful for the discovery of novel biomarker. Our work also provides an effective strategy of concentration and identification of low-abundance proteome from complex bio-samples. 
  GeneRIFs  
 CD13 expression is associated with hepatoblastoma   invasive  ness and could be a novel prognostic marker for hepatoblastoma. 
 Together these experiments reveal potential enzymatic and signalling roles for APN in osteosarcoma and establish a starting point for an in-depth analysis of the role of APN in regulating   invasive  ness. 
 Activities of aminopeptidases N and B and insulin-regulated aminopeptidase could be useful non-  invasive   biomarkers of Alzheimer's disease from the earliest stages. 
 bFGF expression upregulates CD13 expression in human melanoma cells and results in enhanced   invasive   capacity and metastatic behaviour of human melanoma cells. 
  [Mm]etastasis  PubMed  
 Clinical significance of aminopeptidase N in non-small cell lung cancer. The aim of our study is to investigate the mechanism of   metastasis  , to detect novel   metastasis  -associated molecules, and to evaluate the molecules from the point of view of clinical application. A monoclonal antibody MH8-11, which we established, recognizes a glycoprotein that is identical to aminopeptidase N (APN/CD13). APN/CD13 degrades the extracellular matrix, while it is also involved in cell motility and improves angiogenesis.We investigated the expression of APN/CD13 in 194 cases of non-small cell lung cancer (NSCLC) by immunohistochemical analyses and reverse transcription-PCR assay to determine the significance of this prognostic factor; 95 tumors were stage I, 36 were stage II, 39 were stage IIIA, and 24 were stage IIIB. Moreover, we investigated that the relationship between the expression of APN/CD13 and angiogenesis and prognosis for patients with NSCLC.We found a correlation between the expression of APN/CD13 and angiogenesis (r = 0.659; P < 0.0001). In the 194 patients with NSCLC, we found 68 patients to be APN/CD13+ and 126 patients to be APN/CD13-. The 5-year survival rate in patients with APN/CD13+ tumors was significantly lower than in those whose tumors had negative APN/CD13 (48.3% versus 67.1%; P = 0.0001).Our data suggest the expression of APN/CD13 for patients with NSCLC to be associated with a poor prognosis and angiogenesis. This is the first study to show the relationship between the expression of APN/CD13 and the prognosis of patients with NSCLC. The inhibition of APN/CD13 may be an effective new molecular target therapy for patients with NSCLC. 
 Increased CD13 expression reduces reactive oxygen species, promoting survival of liver cancer stem cells via an epithelial-mesenchymal transition-like phenomenon. Recently, it has been reported that a small population of cancer stem cells (CSCs) play a role in resistance to chemotherapy and radiation therapy. We reported that CD13(+) liver CSCs survive in hypoxic lesions after chemotherapy, presumably through increased expression of CD13/Aminopeptidase N, which is a scavenger enzyme in the reactive oxygen species (ROS) metabolic pathway. On the other hand, the concept of epithelial-mesenchymal transition (EMT) was indicated by a recent study showing an increased plasticity linked to the cellular "stemness" of CSCs.To study the relationship between CSCs and EMT, we examined biological characteristics of liver cancer cell lines with EMT by exposing transforming growth factor-β (TGF-β).We showed that a TGF-β-induced EMT-like phenomenon is associated with increased CD13 expression in liver cancer cells. This phenomenon prevents further increases in the ROS level as well as the induction of apoptosis, promoting the survival of CD13(+) CSCs, whereas inhibition of CD13 stimulates apoptosis. Immunohistochemical analysis also indicated that after chemotherapy, CD13 was coexpressed with N-cadherin in surviving cancer cells within fibrous capsules. We have demonstrated that CD13 expression plays a role in supporting the survival of CSCs and that there is an EMT-associated reduction in ROS elevation.This novel and consistent linkage between functional CSC markers and the EMT phenomenon suggests a bona fide candidate for targeted therapy for EMT-mediated invasion a  nd metasta  sis of liver cancer. 
 The angiogenic regulator CD13/APN is a transcriptional target of Ras signaling pathways in endothelial morphogenesis. Angiogenesis, the formation of new blood vessels, is a critical step for tumor growth and   metastasis   and an integral component of the pathologic inflammatory response in arthritis and the proliferative retinopathies. The CD13/aminopeptidase N (CD13/APN) metalloprotease is an important regulator of angiogenesis where its expression on activated blood vessels is induced by angiogenic signals. Here, we show that cytokine induction of CD13/APN in endothelial cells is regulated by distinct Ras effector pathways involving Ras/mitogen-activated protein kinase (MAPK) or PI-3K. Signals transduced by activated Ras, Raf, and mitogen-induced extracellular kinase (MEK) stimulate transcription from the CD13/APN proximal promoter. Inhibition of these pathways and extracellular signal-regulated serine/threonine kinase (ERK-2) and PI-3K by expression of dominant-negative proteins or chemical inhibitors prevented induction of CD13/APN transcription in response to basic fibroblast growth factor (bFGF). We show that Ras-induced signal transduction is required for growth factor-induced angiogenesis, because inhibition of downstream mediators of Ras signaling (MEK or PI-3K) abrogated endothelial cell migration, invasion, and morphogenesis in vitro. Reintroduction of CD13/APN, a shared downstream target of these pathways, overrode the suppressive effect of these inhibitors and restored the function of endothelial cells in migration/invasion and capillary morphogenesis assays. Similarly, inhibition of MEK abrogated cell invasion and the formation of endothelial-lined capillaries in vivo, which was effectively rescued by addition of exogenous CD13/APN protein. These studies provide strong evidence that CD13/APN is an important target of Ras signaling in angiogenesis and is a limiting factor in angiogenic progression. 
 Prognostic significance of aminopeptidase-N (CD13) in hepatoblastoma. Hepatoblastoma is a rare childhood malignant tumor that originates from immature hepatic cells. Aminopeptidase-N(CD13), an ectopeptidase that promotes tumor invasion and   metastasis  , is expressed in fetal stage hepatic progenitor cells, although its role in hepatoblastoma remains unclear.The expression pattern of CD13 was investigated on immunohistochemistry in 30 tissue samples from 27 hepatoblastoma patients (16 with predominantly embryonal [pE] histology and 14 with predominantly fetal [pF] histology). Immunoreactive score (IRS) was used to quantify staining data, and the relationship between CD13 expression, clinicopathological factors, and clinical outcome was investigated. The biological function of CD13 was also examined in the hepatoblastoma cell lines Huh6 and HepG2.All specimens stained positive for CD13, with higher CD13 expression in pE than in pF hepatoblastoma samples (median IRS, 4; range, 2-9 vs 2; range, 1-4). Strong CD13 expression was correlated with vascular invasion. Five year event-free survival and overall survival were better in patients with CD13(low) than in those with CD13(high) tumors (100% vs 51.0%, P = 0.026; and 100% vs 74.0%, P = 0.114, respectively). A CD13-neutralizing antibody and the potent CD13 inhibitor, Ubenimex, suppressed invasive activity in HepG2 cells in vitro.CD13 expression is associated with hepatoblastoma invasiveness and could be a novel prognostic marker for hepatoblastoma. 
 Stromal aminopeptidase N expression: correlation with angiogenesis in non-small-cell lung cancer. Aminopeptidase-N (APN) is a membranebound protein that acts as a zinc-binding protease and participates in extracellular proteolysis. APN plays important roles in tumor progression through promoting invasion and   metastasis  , prolonging survival of tumor cells, and tumor angiogenesis.We evaluated APN expression in non-small-cell lung cancer patients by immunohistochemistry.Of the 95 patients reviewed in the present study, 9 (9.5%), all with adenocarcinoma (Ad), showed positive APN expression on the tumors' cells. In all, 31 (32.6%) and 19 (20.0%) patients showed positive APN expression on the tumors' stromal cells (fibroblasts) and microvessels, respectively. APN expression on the tumors' stromal cells was more frequently observed in squamous cell carcinoma patients than in adenocarcinoma patients (P = 0.005). The mean microvessel density (MVD) for APNpositive tumor stromal cells was 59.9, which was significantly higher than that for APN-negative tumor stromal cells (mean MVD 27.4; P = 0.001). The 5-year survival rates for APN-positive and APN-negative tumor stromal cells were 66.0% and 69.8%, respectively, showing no difference in patient survival according to APN status on the tumors' stromal cells.That APN expression on stromal cells was observed predominantly in squamous cell carcinoma may account for the efficacy of ubenimex in the postoperative adjuvant setting for squamous cell carcinoma. 
 CD13 (aminopeptidase N) can associate with tumor-associated antigen L6 and enhance the motility of human lung cancer cells. Cancer   metastasis   is a multiple-step process that involves the regulated interaction of diverse cellular proteins. We recently reported that the expression of tumor-associated antigen L6 (TAL6) promoted the invasiveness of lung cancer cells and was inversely correlated with disease-free survival of squamous lung carcinoma patients. We now report that CD13 (aminopeptidase N) can associate with TAL6 and can enhance cancer cell migration. CD13 was shown by coimmunoprecipitation to associate in vitro with TAL6 on several cancer cell lines and to associate in vivo by antibody-mediated copatching immunofluorescence. CD13 was selectively expressed on highly invasive CL1-5 lung cancer cells as compared to poorly invasive CL1-0 lung cancer cells. The role of CD13 aminopeptidase activity in regulating cell motility was investigated with chemical inhibitors, specific antibodies and a catalytically inactive CD13 protein. Inhibition of CD13 aminopeptidase activity by nontoxic concentrations of leuhistin modestly decreased the migration of CL1-5 cells. In contrast, binding of CD13 by specific antibodies significantly reduced both the migration and the invasion of CL1-5 cells. Poorly invasive CL1-0 cells that stably expressed CD13 displayed significantly (p < or = 0.0005) enhanced cell migration (300% of control). Expression of an enzymatically inactive CD13 mutant on CL1-0 cells also significantly (p < or = 0.0005) enhanced cell migration (200% of control). Our results show that TAL6 and CD13 can form a complex on lung cancer cells, that these molecules can modulate cell migration and invasion and that the influence of CD13 on cell motility did not strictly depend on its aminopeptidase activity. 
 Aminopeptidase N (APN/CD13) is selectively expressed in vascular endothelial cells and plays multiple roles in angiogenesis. Proteolytic enzyme-mediated degradation of the extracellular matrix (ECM) is crucial for the formation of both tumor   metastasis   and angiogenesis. Recently, several reports have suggested that aminopeptidases are involved in this process, but precisely how is largely unknown. We found here that aminopeptidase N (APN/CD13) was selectively expressed in vascular endothelial cells including human umbilical vein endothelial cells (HUVEC) and human aortic endothelial cells (HAEC), and was not detectable in a majority of normal cells and tumor cell lines we examined. RNA interference (RNAi) of APN resulted in the inhibition of capillary tube formation of HUVEC on Matrigel. APN siRNA suppressed the migration of HUVEC through a fibronectin-coated Transwell membrane, and reduced the cellular adhesion to Matrigel and various adhesion molecules including type IV collagen, type I collagen and fibronectin. These findings suggest that APN is a multifunctional protein with important roles in vascular endothelial morphogenesis during angiogenesis. 
 Expression and clinical significance of aminopeptidase N/CD13 in non-small cell lung cancer. The objective of this study was to analyze the expression of aminopeptidase N/CD13 (APN/CD13) in non-small cell lung cancer (NSCLC) and investigate its correlation with various clinical factors, including prognosis and efficacy of adjuvant chemotherapy.Using immunohistochemistry analysis, we analyzed the expression of CD13 in clinicopathologically characterized 127 NSCLC cases. The relationship between the expression levels of CD13 and clinical features was analyzed and presented.The data were analyzed using statistical package for the social sciences software (Ver 13.0, IBM, USA). Those conforming to Gauss distribution criteria was represented as Mean ΁ SD and those not conforming to Gauss distribution criteria was represented as median (M). The overall survival was recorded from the date of surgery to the date of cancer-specific death. APN/CD13 expression levels and clinicopathological factors were analyzed by Chi-square test or by Fisher's exact test. The Kaplan-Meier method was used to evaluate the probability of survival data and analyzed by Log rank test. Multivariate analysis was performed by Cox regression model. P < 0.05 was considered to be statistically significant.APN/CD13 was mainly expressed in the cellular membrane of cancer cells in pulmonary adenocarcinoma and in the cellular membrane of interstitial cells in squamous carcinoma. Positive APN/CD13 was detected in 62.3% (43 of 69) squamous carcinoma patients and in 50% (29/58) adenocarcinoma patients. Expression of APN/CD13 was not correlated with age, gender, tumor, node,   metastasi  s (TNM) stage, histological type and tumor size, but with TNM stage (P = 0.041) and lymph node   metastasi  s status (P = 0.009). As indicated by Kaplan-Meier survival curve, over-expression of APN/CD13 was significantly correlated with the low survival rate. Cox regression analysis showed that APN/CD13 expression was an independent impact factor for the survival of lung adenocarcinoma patients receiving adjuvant chemotherapy (P = 0.006).Expression of APN/CD13 is a potential unfavorable factor to predict the efficacy and prognosis of post-operative chemotherapy in NSCLC patients, especially in lung adenocarcinoma patients. 
 Aminopeptidase N (CD13) is involved in phagocytic processes in human dendritic cells and macrophages. Aminopeptidase N (APN or CD13) is a membrane ectopeptidase expressed by many cell types, including myelomonocytic lineage cells: monocytes, macrophages, and dendritic cells. CD13 is known to regulate the biological activity of various peptides by proteolysis, and it has been proposed that CD13 also participates in several functions such as angiogenesis, cell adhesion,   metastasis  , and tumor invasion. We had previously reported that, in human monocytes and macrophages, CD13 modulates the phagocytosis mediated by receptors for the Fc portion of IgG antibodies (FcγRs). In this work, we analyzed the possible interaction of CD13 with other phagocytic receptors. We found out that the cross-linking of CD13 positively modulates the phagocytosis mediated by receptors of the innate immune system, since a significant increase in the phagocytosis of zymosan particles or heat-killed E. coli was observed when CD13 was cross-linked using anti-CD13 antibodies, in both macrophages and dendritic cells. Also, we observed that, during the phagocytosis of zymosan, CD13 redistributes and is internalized into the phagosome. These findings suggest that, besides its known functions, CD13 participates in phagocytic processes in dendritic cells and macrophages. 
  [Cc]ell [Ii]nvasion  PubMed  
 Reduced activity of CD13/aminopeptidase N (APN) in aggressive meningiomas is associated with increased levels of SPARC. Meningiomas are the second most common brain tumors in adults, and meningiomas exhibit a tendency to invade adjacent structures. Compared with high-grade gliomas, little is known about the molecular changes that potentially underlie the invasive behavior of meningiomas. In this study, we examined the expression and function of the membrane alanyl-aminopeptidase [mAAP, aminopeptidase N (APN), CD13, EC3.4.11.2] zinc-dependent ectopeptidase in meningiomas and meningioma cell lines, based on its prior association with tumor invasion in colorectal and renal carcinomas. We found a significant reduction of APNmRNA and protein expression, as well as enzymatic activity, in high-grade meningiomas. While meningioma tumor cell proliferation was not affected by either pharmacologic APN inhibition or siRNA-mediated APN silencing, APN pharmacologic and siRNA knockdown significantly reduced meningioma   cell invasion   in vitro. Next, we employed pathway-specific cDNA microarray analyses to identify extracellular matrix and adhesion molecules regulated by APN, and found that APN-siRNA knockdown substantially increased the expression of secreted protein, acidic and rich in cysteine (SPARC)/osteonectin. Finally, we demonstrated that SPARC, which has been previously associated with meningioma invasiveness, was increased in aggressive meningiomas. Collectively, these results suggest that APN expression and enzymatic function is reduced in aggressive meningiomas, and that alterations in the balance between APN and SPARC might favor meningioma invasion. 
 CD13/Aminopeptidase N overexpression by basic fibroblast growth factor mediates enhanced invasiveness of 1F6 human melanoma cells. CD13/Aminopeptidase N (CD13) is known to play an important role in tumour   cell invasion  . We examined whether basic fibroblast growth factor (bFGF) is involved in the regulation of CD13 expression in human melanoma cells. 1F6 human melanoma cells were stably transfected with constructs encoding either the 18 kDa (18 kD) or all (ALL) bFGF isoform proteins. We observed highly increased CD13 mRNA and protein expression in the 1F6 clones regardless of the overexpression of either the 18 kD or all isoform proteins. Neutral aminopeptidase activity was increased five-fold and could be inhibited by bestatin and the CD13-neutralising antibody WM15. The enhanced invasion through Matrigel, but not migration in a wound assay, was efficiently abrogated by both bestatin and WM15. Upregulation of CD13 expression was the result of increased epithelial and myeloid promoter activity up to 4.5-fold in 1F6-18 kD and 1F6-ALL clones. Interestingly, in a panel of human melanoma cell lines, a significant correlation (r(2)=0.883, P<0.05) between bFGF and CD13 mRNA and protein expression was detected. High bFGF and CD13 expression were clearly related with an aggressive phenotype. Taken together, our data indicate that high bFGF expression upregulates CD13 expression in human melanoma cells by activating both the myeloid and the epithelial CD13 promoter. In addition, we show that high bFGF and CD13 expression results in enhanced invasive capacity and metastatic behaviour of human melanoma cells. 
 The angiogenic regulator CD13/APN is a transcriptional target of Ras signaling pathways in endothelial morphogenesis. Angiogenesis, the formation of new blood vessels, is a critical step for tumor growth and metastasis and an integral component of the pathologic inflammatory response in arthritis and the proliferative retinopathies. The CD13/aminopeptidase N (CD13/APN) metalloprotease is an important regulator of angiogenesis where its expression on activated blood vessels is induced by angiogenic signals. Here, we show that cytokine induction of CD13/APN in endothelial cells is regulated by distinct Ras effector pathways involving Ras/mitogen-activated protein kinase (MAPK) or PI-3K. Signals transduced by activated Ras, Raf, and mitogen-induced extracellular kinase (MEK) stimulate transcription from the CD13/APN proximal promoter. Inhibition of these pathways and extracellular signal-regulated serine/threonine kinase (ERK-2) and PI-3K by expression of dominant-negative proteins or chemical inhibitors prevented induction of CD13/APN transcription in response to basic fibroblast growth factor (bFGF). We show that Ras-induced signal transduction is required for growth factor-induced angiogenesis, because inhibition of downstream mediators of Ras signaling (MEK or PI-3K) abrogated endothelial cell migration, invasion, and morphogenesis in vitro. Reintroduction of CD13/APN, a shared downstream target of these pathways, overrode the suppressive effect of these inhibitors and restored the function of endothelial cells in migration/invasion and capillary morphogenesis assays. Similarly, inhibition of MEK abrogated   cell invasion   and the formation of endothelial-lined capillaries in vivo, which was effectively rescued by addition of exogenous CD13/APN protein. These studies provide strong evidence that CD13/APN is an important target of Ras signaling in angiogenesis and is a limiting factor in angiogenic progression. 
 Identification of APN/CD13 as the target antigen of FU3, a human monoclonal antibody that recognizes malignant fibrous histiocytoma. Malignant fibrous histiocytoma (MFH), a high-grade, undifferentiated sarcoma, is highly aggressive, resistant to radiochemotherapy and associated with poor prognosis. There are no specific immunohistochemical markers for its diagnosis. The MFH cell line SFT7913 served as and immunogen for the generation of the FU3 monoclonal antibody in our laboratory. FU3 reacted strongly with MFH cells and with perivascular mesenchymal cells. In this study, we demonstrated that the antigen recognized by FU3 was identical to aminopeptidase N (APN/CD13) using FU3 immunoaffinity chromatography and N-terminal amino acid sequencing. Frequent (80%) and high-grade (>50% of cells) expression of APN/CD13 was observed in MFH, although low-grade expression was seen in some other sarcomas. Moreover, small interfering RNA (siRNA) that specifically targets APN/CD13 significantly suppressed MFH   cell invasion   in vitro. The newly developed monoclonal antibody FU3 specifically recognizes CD13 on MFH cells. Decreased expression of CD13, mediated by siRNA-mediated knockdown, attenuated the invasive capacity of MFH cells. Thus, results indicate that APN/CD13 could be an important diagnostic biomarker and therapeutic target for MFH. 
 CD13/APN regulates endothelial invasion and filopodia formation. CD13/aminopeptidase N is a transmembrane peptidase that is induced in the vasculature of solid tumors and is a potent angiogenic regulator. Here, we demonstrate that CD13 controls endothelial   cell invasion   in response to the serum peptide bradykinin by facilitating signal transduction at the level of the plasma membrane. Inhibition of CD13 abrogates bradykinin B(2) receptor internalization, leading to the attenuation of downstream events such as bradykinin-induced activation of Cdc42 and filopodia formation, and thus affects endothelial cell motility. Investigation into mechanisms underlying this block led us to focus on B(2)R internalization via membrane-dependent mechanisms. Membrane disruption by depletion of cholesterol or trypsinization halts B(2)R internalization, invasion, and filopodia formation, which can be recovered with addition of cholesterol. However, this functional recovery is severely impaired in the presence of CD13 antagonists, and the distribution of membrane proteins is disordered in treated cells, suggesting a role for CD13 in plasma membrane protein organization. Finally, exogenous expression of wild-type but not mutant CD13 further alters protein distribution, suggesting peptidase activity is required for CD13's regulatory activity. Therefore, CD13 functions as a novel modulator of signal transduction and cell motility via its influence on specific plasma membrane organization, thus regulating angiogenesis. 
   
  
 PRKG2 
 
  NCBI Gene  
  GeneRIF  
  Pubmed  
 
 
  Search Term  Rank  Fields  
  [Mm]igration  6  PubMed(3); GeneRIFs(1)  
 
 
 hide/show details for PRKG2 
  [Mm]igration  PubMed  
 Type II cGMP‑dependent protein kinase inhibits RhoA activation in gastric cancer cells. Small GTPase RhoA is a key signaling component regulating cel  l migrati  on and stress fiber formation. Previous studies have shown that RhoA activity is regulated by protein kinases, such as cAMP‑dependent protein kinase (PKA) and type I cGMP‑dependent protein kinase (PKGI), which phosphorylate the protein. This study was designed to investigate the effect of type II cGMP‑dependent protein kinase (PKGII) on RhoA activity. Cells of the human gastric cancer line AGS were infected with adenoviral constructs bearing the PKGII cDNA in order to increase its endogenous expression, and were treated with 8‑pCPT‑cGMP to activate the PKGII enzyme. A transwell assay was performed to measure the migratory activity of the treated cells, and immunofluorescent microscopy was used to observe the formation of stress fibers. The phosphorylation of RhoA was detected by western blotting, and the activity of RhoA was measured by a pull‑down assay. Co‑immunoprecipitation (co‑IP) was performed to detect binding of PKGII to RhoA. Glutathione S‑transferase (GST)‑fused fragments of RhoA and PKGII were expressed in Escherichia coli and used to investigate the domains required for the binding. The results showed that lysophosphatidic acid (LPA) tr  eatment i  ncreased the migration and the formation of stress fibers in AGS cells and that this effect was RhoA‑dependent. An increase in PKGII activity, not onl  y inhibit  ed LPA‑induced migration and stress fiber formation, but also suppressed LPA‑induced activation of RhoA. PKGII caused serine 188 (Ser188) phosphorylation of RhoA, but not the phosphorylation of the mutant RhoA Ser188A, and therefore had no inhibitory effect on the activity of the mutant protein. Co‑IP results showed that there is direct binding of PKGII to RhoA. The GST pull‑down assay showed that the fragment containing RhoA amino acid residues 1‑44 and the N‑terminal fragment of PKGII containing amino acid residues 1‑176 are required for the binding between the two proteins. These results suggested that PKGII inhibits RhoA activity by binding to this small GTPase and causing phosphorylation at its Ser188 site. 
 Actin binding of human LIM and SH3 protein is regulated by cGMP- and cAMP-dependent protein kinase phosphorylation on serine 146. Various drugs that elevate cGMP levels and activate cGMP-dependent protein kinase (cGK) inhibit agonist-induced platelet activation. In the present study we identified the LIM and SH3 domain protein (LASP) that was recently cloned from human breast cancer cells (Tomasetto, C., Regnier, C., Moog-Lutz, C., Mattei, M. G., Chenard, M. P., Liderau, R., Basset, P., and Rio, M. C. (1995) Genomics 28, 367-376) as a novel substrate of cGK in human platelets. Recombinant human LASP was phosphorylated by cGMP- and cAMP-dependent protein kinase (cAK) in vitro. Cotransfection of PtK-2 cells with LASP and cGK confirmed phosphorylation of LASP in vivo. Studies with human LASP mutants identified serine 146 as a specific phosphorylation site for cGK and cAK in vivo. LASP is an actin-binding protein, and the phospho-LASP-mimicking mutant S146D showed reduced binding affinity for F-actin in cosedimentation experiments. Immunofluorescence of transfected PtK2 cells demonstrated the localization of LASP in the tips of cell membrane extensions and at cell-cell contacts. Expression of the human LASP mutant S146D resulted in nearly complete relocalization to the cytosol and reduced   migration   of the cells. Taken together, these data suggest that phosphorylation of LASP by cGK and cAK may be involved in cytoskeletal organization and cell motility. 
 Type II, but not type I, cGMP-dependent protein kinase reverses bFGF-induced proliferation and   migration   of U251 human glioma cells. Previous data have shown that the type II cGMP‑dependent protein kinase (PKG II) inhibits the EGF‑induced MAPK signaling pathway. In order to thoroughly investigate PKG, it is necessary to elucidate the function of another type of PKG, PKG I. The aim of this study was to investigate the possible inhibitory effect of PKG II and PKG I activity on the basic fibroblast growth factor (bFGF)‑induced proliferatio  n and mig  ration of U251 human glioma cells and the possible underlying mechanisms. U251 cells were infected with adenoviral constructs encoding cDNA of PKG I (Ad‑PKG I) or PKG II (Ad‑PKG II) to increase the expression levels of PKG I or PKG II and then treated with 8‑Br‑cGMP and 8‑pCPT‑cGMP, respectively, to activate the enzyme. An MTT assay was used to detect the proliferation of th  e U251 ce  lls. The migration of the U251 cells was analyzed   using a T  ranswell migration assay. Western blot analysis was used to detect the phosphorylation/activation of the fibroblast growth factor receptor (FGFR), MEK and ERK and the nuclear distribution of p-ERK. The results showed that bFGF treatment increased the   prolifera  tion and migration of U251 cells, accompanied by increased phosphorylation of FGFR, MEK and ERK. Furthermore, the nuclear distribution of p-ERK increased following bFGF treatment. Increasing the activity of PKG II through infection with Ad-PKG II and stimulation with 8-pCPT-cGMP significantly attenuated the aforementioned effects of the bFGF treatment, while increased PKG I activity did not inhibit the effects of bFGF treatment. These data suggest that increased PKG II activity attenuates bFGF‑induce  d prolife  ration and migration by inhibiting the MAPK/ERK signaling pathway, whereas PKG I does not. 
  GeneRIFs  
 These data suggest that increased PKG II activity attenuates bFGFinduced proliferation and   migration   by inhibiting the MAPK/ERK signaling pathway, whereas PKG I does not 
   
  
 ARHGEF16 
 
  NCBI Gene  
  GeneRIF  
  Pubmed  
 
 
  Search Term  Rank  Fields  
  [Mm]igration  6  PubMed(4); GeneRIFs(1)  
  [Ii]nvasive  10  PubMed(2)  
 
 
 hide/show details for ARHGEF16 
  [Mm]igration  PubMed  
 Mutation of ARHGAP9 in patients with coronary spastic angina. Coronary artery spasm has an important function in the etiology of variant angina and other acute coronary syndromes. Abnormal activation of Rho-family GTPases has been observed in cardiovascular disorders, but the function of genetic variability in Rho-family GTPases remains to be evaluated in cardiovascular disorders. We examined the genetic variability of Rho-family GTPases and their regulators in coronary artery spasm. We performed a comprehensive candidate gene analysis of 67 single nucleotide polymorphisms with amino-acid substitution in Rho-family GTPases and their regulators in 103 unrelated Japanese patients with acetylcholine-induced coronary artery spasm and 102 control Japanese subjects without acetylcholine-induced coronary artery spasm. We noted an association of the single nucleotide polymorphism of ARHGAP9 (rs11544238, Ala370Ser) with coronary artery spasm (odds ratio =2.67). We found that ARHGAP9 inactivated Rac as RacGAP and that the mRNA level of ARHGAP9 was strongly detected in hematopoietic cells. ARHGAP9 negatively regulated cell   migration  . The Ala370Ser polymorphism counteracted ARHGAP9-reduced cell   migration  , spreading and adhesion. The Ala370Ser polymorphism in the ARHGAP9 gene is associated with coronary artery spasm. These data suggest that the polymorphism of ARHGAP9 has a critical function in the infiltration of hematopoietic cells into the endothelium and inflammation leading to endothelial dysfunction. 
 Ephexin4 and EphA2 mediate cell   migration   through a RhoG-dependent mechanism. EphA2, a member of the Eph receptor family, is frequently overexpressed in a variety of human cancers, including breast cancers, and promotes cancer cell motility and invasion independently of its ligand ephrin stimulation. In this study, we identify Ephexin4 as a guanine nucleotide exchange factor (GEF) for RhoG that interacts with EphA2 in breast cancer cells, and knockdown and rescue experiments show that Ephexin4 acts downstream of EphA2 to promote ligand-independent breast cancer cell   migration   and invasion toward epidermal growth factor through activation of RhoG. The activation of RhoG recruits its effector ELMO2 and a Rac GEF Dock4 to form a complex with EphA2 at the tips of cortactin-rich protrusions in migrating breast cancer cells. In addition, the Dock4-mediated Rac activation is required for breast cancer cell   migration  . Our findings reveal a novel link between EphA2 and Rac activation that contributes to the cell motility and invasiveness of breast cancer cells. 
 Ephexin4 and EphA2 mediate cell   migration   through a RhoG-dependent mechanism. EphA2, a member of the Eph receptor family, is frequently overexpressed in a variety of human cancers, including breast cancers, and promotes cancer cell motility and invasion independently of its ligand ephrin stimulation. In this study, we identify Ephexin4 as a guanine nucleotide exchange factor (GEF) for RhoG that interacts with EphA2 in breast cancer cells, and knockdown and rescue experiments show that Ephexin4 acts downstream of EphA2 to promote ligand-independent breast cancer cell   migration   and invasion toward epidermal growth factor through activation of RhoG. The activation of RhoG recruits its effector ELMO2 and a Rac GEF Dock4 to form a complex with EphA2 at the tips of cortactin-rich protrusions in migrating breast cancer cells. In addition, the Dock4-mediated Rac activation is required for breast cancer cell   migration  . Our findings reveal a novel link between EphA2 and Rac activation that contributes to the cell motility and invasiveness of breast cancer cells. 
 Mutation of ARHGAP9 in patients with coronary spastic angina. Coronary artery spasm has an important function in the etiology of variant angina and other acute coronary syndromes. Abnormal activation of Rho-family GTPases has been observed in cardiovascular disorders, but the function of genetic variability in Rho-family GTPases remains to be evaluated in cardiovascular disorders. We examined the genetic variability of Rho-family GTPases and their regulators in coronary artery spasm. We performed a comprehensive candidate gene analysis of 67 single nucleotide polymorphisms with amino-acid substitution in Rho-family GTPases and their regulators in 103 unrelated Japanese patients with acetylcholine-induced coronary artery spasm and 102 control Japanese subjects without acetylcholine-induced coronary artery spasm. We noted an association of the single nucleotide polymorphism of ARHGAP9 (rs11544238, Ala370Ser) with coronary artery spasm (odds ratio =2.67). We found that ARHGAP9 inactivated Rac as RacGAP and that the mRNA level of ARHGAP9 was strongly detected in hematopoietic cells. ARHGAP9 negatively regulated cell   migration  . The Ala370Ser polymorphism counteracted ARHGAP9-reduced cell   migration  , spreading and adhesion. The Ala370Ser polymorphism in the ARHGAP9 gene is associated with coronary artery spasm. These data suggest that the polymorphism of ARHGAP9 has a critical function in the infiltration of hematopoietic cells into the endothelium and inflammation leading to endothelial dysfunction. 
  GeneRIFs  
 The ephexin4 protein mediates cell   migration   through a RhoG-dependent mechanism. 
  [Ii]nvasive  PubMed  
 Ephexin4 and EphA2 mediate cell migration through a RhoG-dependent mechanism. EphA2, a member of the Eph receptor family, is frequently overexpressed in a variety of human cancers, including breast cancers, and promotes cancer cell motility and invasion independently of its ligand ephrin stimulation. In this study, we identify Ephexin4 as a guanine nucleotide exchange factor (GEF) for RhoG that interacts with EphA2 in breast cancer cells, and knockdown and rescue experiments show that Ephexin4 acts downstream of EphA2 to promote ligand-independent breast cancer cell migration and invasion toward epidermal growth factor through activation of RhoG. The activation of RhoG recruits its effector ELMO2 and a Rac GEF Dock4 to form a complex with EphA2 at the tips of cortactin-rich protrusions in migrating breast cancer cells. In addition, the Dock4-mediated Rac activation is required for breast cancer cell migration. Our findings reveal a novel link between EphA2 and Rac activation that contributes to the cell motility and   invasive  ness of breast cancer cells. 
 Ephexin4 and EphA2 mediate cell migration through a RhoG-dependent mechanism. EphA2, a member of the Eph receptor family, is frequently overexpressed in a variety of human cancers, including breast cancers, and promotes cancer cell motility and invasion independently of its ligand ephrin stimulation. In this study, we identify Ephexin4 as a guanine nucleotide exchange factor (GEF) for RhoG that interacts with EphA2 in breast cancer cells, and knockdown and rescue experiments show that Ephexin4 acts downstream of EphA2 to promote ligand-independent breast cancer cell migration and invasion toward epidermal growth factor through activation of RhoG. The activation of RhoG recruits its effector ELMO2 and a Rac GEF Dock4 to form a complex with EphA2 at the tips of cortactin-rich protrusions in migrating breast cancer cells. In addition, the Dock4-mediated Rac activation is required for breast cancer cell migration. Our findings reveal a novel link between EphA2 and Rac activation that contributes to the cell motility and   invasive  ness of breast cancer cells. 
   
  
 MOV10 
 
  NCBI Gene  
  GeneRIF  
  Pubmed  
 
 
  Search Term  Rank  Fields  
  [Ii]nvasive  10  PubMed(1)  
 
 
 hide/show details for MOV10 
  [Ii]nvasive  PubMed  
 Comparison of tear protein levels in breast cancer patients and healthy controls using a de novo proteomic approach. Non  invasive   biomarkers are urgently needed for early detection of breast cancer since the risk of recurrence, morbidity and mortality are closely related to disease stage at the time of primary surgery. In the past decade, many proteomics-based approaches were developed that utilize the protein profiling of human body fluids or identification of putative biomarkers to obtain more knowledge on the effects of cancer emergence and progression. Herein, we report on an analysis of proteins in the tear fluid from breast carcinoma patients and healthy women using a de novo proteomic approach and 25 mixed samples from each group. This study included 25 patients with primary   invasiv  e breast carcinoma and 25 age-matched healthy controls. We performed a MALDI-TOF-TOF-driven semi-quantitative comparison of tear protein levels in cancer (CA) and control (CTRL) using a de novo approach in pooled samples. Over 150 proteins in the tear fluid of CTRL and CA were identified. Using an in-house-developed algorithm we found more than 20 proteins distinctly upregulated or downregulated in the CTRL and CA groups. We identified several proteins that had modified expression in breast cancer patients. These proteins are involved in host immune system pathways (e.g., C1Q1 or S100A8) and different metabolic cascades (ALDH3A or TPI). Further validation of the results in an independent population combined with individual protein profiling of participants is needed to confirm the specificity of our findings and may lead to a better understanding of the pathological mechanism of breast cancer. 
   
  
 ABCC4 
 
  NCBI Gene  
  GeneRIF  
  Pubmed  
 
 
  Search Term  Rank  Fields  
  [Mm]igration  6  PubMed(4); GeneRIFs(1)  
  [Ii]nvasive  10  PubMed(3)  
  [Mm]etastasize  11  PubMed(1)  
 
 
 hide/show details for ABCC4 
  [Mm]igration  PubMed  
 ABCC multidrug transporters in childhood neuroblastoma: clinical and biological effects independent of cytotoxic drug efflux. Although the prognostic value of the ATP-binding cassette, subfamily C (ABCC) transporters in childhood neuroblastoma is usually attributed to their role in cytotoxic drug efflux, certain observations have suggested that these multidrug transporters might contribute to the malignant phenotype independent of cytotoxic drug efflux.A v-myc myelocytomatosis viral related oncogene, neuroblastoma derived (MYCN)-driven transgenic mouse neuroblastoma model was crossed with an Abcc1-deficient mouse strain (658 hMYCN(1/-), 205 hMYCN(+/1) mice) or, alternatively, treated with the ABCC1 inhibitor, Reversan (n = 20). ABCC genes were suppressed using short interfering RNA or overexpressed by stable transfection in neuroblastoma cell lines BE(2)-C, SH-EP, and SH-SY5Y, which were then assessed for wound closure ability, clonogenic capacity, morphological differentiation, and cell growth. Real-time quantitative polymerase chain reaction was used to examine the clinical significance of ABCC family gene expression in a large prospectively accrued cohort of patients (n = 209) with primary neuroblastomas. Kaplan-Meier survival analysis and Cox regression were used to test for associations with event-free and overall survival. Except where noted, all statistical tests were two-sided.Inhibition of ABCC1 statistically significantly inhibited neuroblastoma development in hMYCN transgenic mice (mean age for palpable tumor: treated mice, 47.2 days; control mice, 41.9 days; hazard ratio [HR] = 9.3, 95% confidence interval [CI] = 2.65 to 32; P < .001). Suppression of ABCC1 in vitro inhibited wound closure (P < .001) and clonogenicity (P = .006); suppression of ABCC4 enhanced morphological differentiation (P < .001) and inhibited cell growth (P < .001). Analysis of 209 neuroblastoma patient tumors revealed that, in contrast with ABCC1 and ABCC4, low rather than high ABCC3 expression was associated with reduced event-free survival (HR of recurrence or death = 2.4, 95% CI = 1.4 to 4.2; P = .001), with 23 of 53 patients with low ABCC3 expression experiencing recurrence or death compared with 31 of 155 patients with high ABCC3. Moreover, overexpression of ABCC3 in vitro inhibited neuroblastoma cell   migration   (P < .001) and clonogenicity (P = .03). The combined expression of ABCC1, ABCC3, and ABCC4 was associated with patients having an adverse event, such that of the 12 patients with the "poor prognosis" expression pattern, 10 experienced recurrence or death (HR of recurrence or death = 12.3, 95% CI = 6 to 27; P < .001).ABCC transporters can affect neuroblastoma biology independently of their role in chemotherapeutic drug efflux, enhancing their potential as targets for therapeutic intervention. 
 ABCC multidrug transporters in childhood neuroblastoma: clinical and biological effects independent of cytotoxic drug efflux. Although the prognostic value of the ATP-binding cassette, subfamily C (ABCC) transporters in childhood neuroblastoma is usually attributed to their role in cytotoxic drug efflux, certain observations have suggested that these multidrug transporters might contribute to the malignant phenotype independent of cytotoxic drug efflux.A v-myc myelocytomatosis viral related oncogene, neuroblastoma derived (MYCN)-driven transgenic mouse neuroblastoma model was crossed with an Abcc1-deficient mouse strain (658 hMYCN(1/-), 205 hMYCN(+/1) mice) or, alternatively, treated with the ABCC1 inhibitor, Reversan (n = 20). ABCC genes were suppressed using short interfering RNA or overexpressed by stable transfection in neuroblastoma cell lines BE(2)-C, SH-EP, and SH-SY5Y, which were then assessed for wound closure ability, clonogenic capacity, morphological differentiation, and cell growth. Real-time quantitative polymerase chain reaction was used to examine the clinical significance of ABCC family gene expression in a large prospectively accrued cohort of patients (n = 209) with primary neuroblastomas. Kaplan-Meier survival analysis and Cox regression were used to test for associations with event-free and overall survival. Except where noted, all statistical tests were two-sided.Inhibition of ABCC1 statistically significantly inhibited neuroblastoma development in hMYCN transgenic mice (mean age for palpable tumor: treated mice, 47.2 days; control mice, 41.9 days; hazard ratio [HR] = 9.3, 95% confidence interval [CI] = 2.65 to 32; P < .001). Suppression of ABCC1 in vitro inhibited wound closure (P < .001) and clonogenicity (P = .006); suppression of ABCC4 enhanced morphological differentiation (P < .001) and inhibited cell growth (P < .001). Analysis of 209 neuroblastoma patient tumors revealed that, in contrast with ABCC1 and ABCC4, low rather than high ABCC3 expression was associated with reduced event-free survival (HR of recurrence or death = 2.4, 95% CI = 1.4 to 4.2; P = .001), with 23 of 53 patients with low ABCC3 expression experiencing recurrence or death compared with 31 of 155 patients with high ABCC3. Moreover, overexpression of ABCC3 in vitro inhibited neuroblastoma cell   migration   (P < .001) and clonogenicity (P = .03). The combined expression of ABCC1, ABCC3, and ABCC4 was associated with patients having an adverse event, such that of the 12 patients with the "poor prognosis" expression pattern, 10 experienced recurrence or death (HR of recurrence or death = 12.3, 95% CI = 6 to 27; P < .001).ABCC transporters can affect neuroblastoma biology independently of their role in chemotherapeutic drug efflux, enhancing their potential as targets for therapeutic intervention. 
 A role for multidrug resistance protein 4 (MRP4; ABCC4) in human dendritic cell   migration  . The capacity of dendritic cells (DCs) to migrate from peripheral organs to lymph nodes (LNs) is important in the initiation of a T cell-mediated immune response. The ATP-binding cassette (ABC) transporters P-glycoprotein (P-gp; ABCB1) and the multidrug resistance protein 1 (MRP1; ABCC1) have been shown to play a role in both human and murine DC   migration  . Here we show that a more recently discovered family member, MRP4 (ABCC4), is expressed on both epidermal and dermal human skin DCs and contributes to the migratory capacity of DCs. Pharmacological inhibition of MRP4 activity or down-regulation through RNAi in DCs resulted in reduced   migration   of DCs from human skin explants and of in vitro generated Langerhans cells. The responsible MRP4 substrate remains to be identified as exogenous addition of MRP4's known substrates prostaglandin E(2), leukotriene B(4) and D(4), or cyclic nucleotides (all previously implicated in DC   migration  ) could not restore   migration  . This notwithstanding, our data show that MRP4 is an important protein, significantly contributing to human DC   migration   toward the draining lymph nodes, and therefore relevant for the initiation of an immune response and a possible target for immunotherapy. 
 MRP4 knockdown enhances   migration  , suppresses apoptosis, and produces aggregated morphology in human retinal vascular endothelial cells. The multidrug resistance protein (MRP) MRP4/ABCC4 is an ATP-binding cassette transporter that actively effluxes endogenous and xenobiotic substrates out of cells. In the rodent retina, Mrp4 mRNA and protein are exclusively expressed in vascular endothelial cells, but the angiogenic properties of Mrp4 are poorly understood so far. This study aims to explore the angiogenic properties of MRP4 in human retinal microvascular endothelial cells (HRECs) utilizing the RNA interference (RNAi) technique. MRP4 expression was decreased at the mRNA and protein levels after stimulation with exogenous vascular endothelial growth factor in a dose-dependent manner. RNAi-mediated MRP4 knockdown in HRECs do not affect cell proliferation but enhances cell   migration  . Moreover, cell apoptosis induced by serum starvation was less prominent in MRP4 siRNA-treated HRECs as compared to control siRNA-treated HRECs. In a Matrigel-based tube-formation assay, although MRP4 knockdown did not lead to a significant change in the total tube length, MRP4 siRNA-treated HRECs assembled and aggregated into a massive tube-like structure, which was not observed in control siRNA-treated HRECs. These results suggest that MRP4 is uniquely involved in retinal angiogenesis. 
  GeneRIFs  
 contributes to human DC   migration   toward the draining lymph nodes 
  [Ii]nvasive  PubMed  
 Genetic susceptibility to distinct bladder cancer subphenotypes. Clinical, pathologic, and molecular evidence indicate that bladder cancer is heterogeneous with pathologic/molecular features that define distinct subphenotypes with different prognoses. It is conceivable that specific patterns of genetic susceptibility are associated with particular subphenotypes.To examine evidence for the contribution of germline genetic variation to bladder cancer heterogeneity.The Spanish Bladder Cancer/EPICURO Study is a case-control study based in 18 hospitals located in five areas in Spain. Cases were patients with a newly diagnosed, histologically confirmed, urothelial cell carcinoma of the bladder from 1998 to 2001. Case diagnoses were reviewed and uniformly classified by pathologists following the World Health Organisation/International Society of Urological Pathology 1999 criteria. Controls were hospital-matched patients (n=1149).A total of 1526 candidate variants in 423 candidate genes were analysed. Three distinct subphenotypes were defined according to stage and grade: low-grade nonmuscle   invasive   (n=586), high-grade nonmuscle   invasive   (n=219), and muscle   invasive   (n=246). The association between each variant and subphenotype was assessed by polytomous risk models adjusting for potential confounders. Heterogeneity in genetic susceptibility among subphenotypes was also tested.Two established bladder cancer susceptibility genotypes, NAT2 slow-acetylation and GSTM1-null, exhibited similar associations among the subphenotypes, as did VEGF-rs25648, which was previously identified in our study. Other variants conferred risks for specific tumour subphenotypes such as PMS2-rs6463524 and CD4-rs3213427 (respective heterogeneity p values of 0.006 and 0.004), which were associated with muscle-  invasive   tumours (per-allele odds ratios [95% confidence interval] of 0.56 [0.41-0.77] and 0.71 [0.57-0.88], respectively) but not with non-muscle-  invasive   tumours. Heterogeneity p values were not robust in multiple testing according to their false-discovery rate.These exploratory analyses suggest that genetic susceptibility loci might be related to the molecular/pathologic diversity of bladder cancer. Validation through large-scale replication studies and the study of additional genes and single nucleotide polymorphisms are required. 
 Genetic susceptibility to distinct bladder cancer subphenotypes. Clinical, pathologic, and molecular evidence indicate that bladder cancer is heterogeneous with pathologic/molecular features that define distinct subphenotypes with different prognoses. It is conceivable that specific patterns of genetic susceptibility are associated with particular subphenotypes.To examine evidence for the contribution of germline genetic variation to bladder cancer heterogeneity.The Spanish Bladder Cancer/EPICURO Study is a case-control study based in 18 hospitals located in five areas in Spain. Cases were patients with a newly diagnosed, histologically confirmed, urothelial cell carcinoma of the bladder from 1998 to 2001. Case diagnoses were reviewed and uniformly classified by pathologists following the World Health Organisation/International Society of Urological Pathology 1999 criteria. Controls were hospital-matched patients (n=1149).A total of 1526 candidate variants in 423 candidate genes were analysed. Three distinct subphenotypes were defined according to stage and grade: low-grade nonmuscle   invasive   (n=586), high-grade nonmuscle   invasive   (n=219), and muscle   invasive   (n=246). The association between each variant and subphenotype was assessed by polytomous risk models adjusting for potential confounders. Heterogeneity in genetic susceptibility among subphenotypes was also tested.Two established bladder cancer susceptibility genotypes, NAT2 slow-acetylation and GSTM1-null, exhibited similar associations among the subphenotypes, as did VEGF-rs25648, which was previously identified in our study. Other variants conferred risks for specific tumour subphenotypes such as PMS2-rs6463524 and CD4-rs3213427 (respective heterogeneity p values of 0.006 and 0.004), which were associated with muscle-  invasive   tumours (per-allele odds ratios [95% confidence interval] of 0.56 [0.41-0.77] and 0.71 [0.57-0.88], respectively) but not with non-muscle-  invasive   tumours. Heterogeneity p values were not robust in multiple testing according to their false-discovery rate.These exploratory analyses suggest that genetic susceptibility loci might be related to the molecular/pathologic diversity of bladder cancer. Validation through large-scale replication studies and the study of additional genes and single nucleotide polymorphisms are required. 
 Effect of GSTP1 and ABCC4 gene polymorphisms on response and toxicity of cyclophosphamide-epirubicin-5-fluorouracil-based chemotherapy in Bangladeshi breast cancer patients. The most important cytotoxic drug namely, cyclophosphamide used in breast cancer along with epirubicin and 5-fluorouracil, is transported by ABCC transporters and detoxified by glutathione S-transferases (GSTs). The activities of these enzymes and transporters may vary in different population due to the presence of genetic polymorphisms. This study was aimed to evaluate the effects of GSTP1rs1695 and ABCC4rs9561778 polymorphisms on the response and toxicities produced by chemotherapy used in the treatment of Bangladeshi breast cancer patients. A total of 200 and 56 patients with   invasive   breast cancers were recruited from different public and private hospitals of Bangladesh of which 117 patients received neoadjuvant chemotherapy to examine the response as well as the toxicity, and another 139 patients received adjuvant chemotherapy to evaluate only the toxicity. Genetic polymorphisms of the mentioned genes were detected by using Polymerase Chain Reaction Restriction Fragment Length Polymorphism (PCR RFLP). Patients carrying AG and AG plus GG genotype of GSTP1rs1695 were more likely to have a good response, whereas no association of ABCC4rs9561778 was found with the chemotherapy response. Patients carrying GT and GT plus TT genotypes of ABCC4rs9561778 were found to be associated with anemia, neutropenia, leukopenia, and gastrointestinal toxicities when compared with GG genotype whereas no association was found with thrombocytopenia. GSTP1rs1695 was not associated with any type of toxicities investigated. Our result indicates that GSTP1rs1695 polymorphism was strongly associated with the response of chemotherapy, whereas ABCC4rs9561778 polymorphism was significantly related with chemotherapy-induced toxicities. 
  [Mm]etastasize  PubMed  
 Upregulation of COX-2 in the lung cancer promotes overexpression of multidrug resistance protein 4 (MRP4) via PGE2-dependent pathway. It is apparent that lung cancer is associated with inflammation, with accompanying hallmark elevations of cyclooxygenase 2 (COX-2) and prostaglandin E2 (PGE2) levels. However, the effects of these changes on MRP efflux transporters have not been thoroughly investigated before. Here, we report that upregulation of COX-2 can induce overexpression of MRP4 in both A549 non-small-cell lung cancer cell lines and mouse lung cancer models. In A549 cells, phorbol 12-myristate 13-acetate (PMA) treatment induced upregulation of COX-2 and MRP4 together, but not other MRP transporters. Transient overexpression of human COX-2 cDNA also specifically increased COX-2 and MRP4. Moreover, COX inhibitor treatment and COX-2-specific siRNA significantly inhibited the upregulation of MRP4. Additionally, PMA-treatment increased extracellular PGE2 levels, likely due to increased MRP4 function. Likewise, COX-2-specific siRNA reduced extracellular PGE2 levels. Furthermore, COX-2 upregulation resulted in an increase in mPGES-1, an enzyme responsible for PGE2 production. Finally,   metastasize  d lung cancer model mice exhibited increased expression levels of COX-2 and MRP4, as well as mPGES-1. In conclusion, the present study suggests that overexpression of MRP4 in lung cancer may be attributable to COX-2 upregulation via a PGE2-dependent pathway. 
   
  
 AGPAT2 
 
  NCBI Gene  
  GeneRIF  
  Pubmed  
 
 
  Search Term  Rank  Fields  
  [Mm]igration  6  PubMed(1)  
 
 
 hide/show details for AGPAT2 
  [Mm]igration  PubMed  
 Lysophosphatidic acid acyltransferase β (LPAATβ) promotes the tumor growth of human osteosarcoma. Osteosarcoma is the most common primary malignancy of bone with poorly characterized molecular pathways important in its pathogenesis. Increasing evidence indicates that elevated lipid biosynthesis is a characteristic feature of cancer. We sought to investigate the role of lysophosphatidic acid acyltransferase β (LPAATβ, aka, AGPAT2) in regulating the proliferation and growth of human osteosarcoma cells. LPAATβ can generate phosphatidic acid, which plays a key role in lipid biosynthesis as well as in cell proliferation and survival. Although elevated expression of LPAATβ has been reported in several types of human tumors, the role of LPAATβ in osteosarcoma progression has yet to be elucidated.Endogenous expression of LPAATβ in osteosarcoma cell lines is analyzed by using semi-quantitative PCR and immunohistochemical staining. Adenovirus-mediated overexpression of LPAATβ and silencing LPAATβ expression is employed to determine the effect of LPAATβ on osteosarcoma cell prolife  ration an  d migration in vitro and osteosarcoma tumor growth in vivo. We have found that expression of LPAATβ is readily detected in 8 of the 10 analyzed human osteosarcoma lines. Exogenous expression of LPAATβ promotes osteosarcoma cell proli  feration   and migration, while silencing LPAATβ expression inhibits these cellular characteristics. We further demonstrate that exogenous expression of LPAATβ effectively promotes tumor growth, while knockdown of LPAATβ expression inhibits tumor growth in an orthotopic xenograft model of human osteosarcoma.Our results strongly suggest that LPAATβ expression may be associated with the aggressive phenotypes of human osteosarcoma and that LPAATβ may play an important role in regulating osteosarcoma cell proliferation and tumor growth. Thus, targeting LPAATβ may be exploited as a novel therapeutic strategy for the clinical management of osteosarcoma. This is especially attractive given the availability of selective pharmacological inhibitors. 
   
  
 HES6 
 
  NCBI Gene  
  GeneRIF  
  Pubmed  
 
 
  Search Term  Rank  Fields  
  [Mm]igration  6  PubMed(2)  
  [Cc]ell [Ii]nvasion  13  PubMed(1)  
 
 
 hide/show details for HES6 
  [Mm]igration  PubMed  
 HES6 promotes prostate cancer aggressiveness independently of Notch signalling. Notch signalling is implicated in the pathogenesis of a variety of cancers, but its role in prostate cancer is poorly understood. However, selected Notch pathway members are overrepresented in high-grade prostate cancers. We comprehensively profiled Notch pathway components in prostate cells and found prostate cancer-specific up-regulation of NOTCH3 and HES6. Their expression was particularly high in androgen responsive lines. Up- and down-regulating Notch in these cells modulated expression of canonical Notch targets, HES1 and HEY1, which could also be induced by androgen. Surprisingly, androgen treatment also suppressed Notch receptor expression, suggesting that androgens can activate Notch target genes in a receptor-independent manner. Using a Notch-sensitive Recombination signal binding protein for immunoglobulin kappa J region (RBPJ) reporter assay, we found that basal levels of Notch signalling were significantly lower in prostate cancer cells compared to benign cells. Accordingly pharmacological Notch pathway blockade did not inhibit cancer cell growth or viability. In contrast to canonical Notch targets, HES6, a HES family member known to antagonize Notch signalling, was not regulated by Notch signalling, but relied instead on androgen levels, both in cultured cells and in human cancer tissues. When engineered into prostate cancer cells, reduced levels of HES6 resulted in reduced cancer cell invasion and clonogenic growth. By molecular profiling, we identified potential roles for HES6 in regulating hedgehog signalling, apoptosis and cell   migration  . Our results did not reveal any cell-autonomous roles for canonical Notch signalling in prostate cancer. However, the results do implicate HES6 as a promoter of prostate cancer progression. 
 HES6 gene is selectively overexpressed in glioma and represents an important transcriptional regulator of glioma proliferation. Malignant glioma is the most common brain tumor with 16,000 new cases diagnosed annually in the United States. We performed a systematic large-scale transcriptomics data mining study of 9783 tissue samples from the GeneSapiens database to systematically identify genes that are most glioma-specific. We searched for genes that were highly expressed in 322 glioblastoma multiforme tissue samples and 66 anaplastic astrocytomas as compared with 425 samples from histologically normal central nervous system. Transcription cofactor HES6 (hairy and enhancer of split 6) emerged as the most glioma-specific gene. Immunostaining of a tissue microarray showed HES6 expression in 335 (98.8%) out of the 339 glioma samples. HES6 was expressed in endothelial cells of the normal brain and glioma tissue. Recurrent grade 2 astrocytomas and grade 2 or 3 oligodendrogliomas showed higher levels of HES6 immunoreactivity than the corresponding primary tumors. High HES6 mRNA expression correlated with the proneural subtype that generally has a favorable outcome but is prone to recur. Functional studies suggested an important role for HES6 in supporting survival of glioma cells, as evidenced by reduction of cancer cell proliferation and   migration   after HES6 silencing. The biological role and consequences of HES6 silencing and overexpression was explored with genome-wide analyses, which implicated a role for HES6 in p53, c-myc and nuclear factor-κB transcriptional networks. We conclude that HES6 is important for glioma cell proliferation and   migratio  n, and may have a role in angiogenesis. 
  [Cc]ell [Ii]nvasion  PubMed  
 HES6 promotes prostate cancer aggressiveness independently of Notch signalling. Notch signalling is implicated in the pathogenesis of a variety of cancers, but its role in prostate cancer is poorly understood. However, selected Notch pathway members are overrepresented in high-grade prostate cancers. We comprehensively profiled Notch pathway components in prostate cells and found prostate cancer-specific up-regulation of NOTCH3 and HES6. Their expression was particularly high in androgen responsive lines. Up- and down-regulating Notch in these cells modulated expression of canonical Notch targets, HES1 and HEY1, which could also be induced by androgen. Surprisingly, androgen treatment also suppressed Notch receptor expression, suggesting that androgens can activate Notch target genes in a receptor-independent manner. Using a Notch-sensitive Recombination signal binding protein for immunoglobulin kappa J region (RBPJ) reporter assay, we found that basal levels of Notch signalling were significantly lower in prostate cancer cells compared to benign cells. Accordingly pharmacological Notch pathway blockade did not inhibit cancer cell growth or viability. In contrast to canonical Notch targets, HES6, a HES family member known to antagonize Notch signalling, was not regulated by Notch signalling, but relied instead on androgen levels, both in cultured cells and in human cancer tissues. When engineered into prostate cancer cells, reduced levels of HES6 resulted in reduced cancer   cell invasion   and clonogenic growth. By molecular profiling, we identified potential roles for HES6 in regulating hedgehog signalling, apoptosis and cell migration. Our results did not reveal any cell-autonomous roles for canonical Notch signalling in prostate cancer. However, the results do implicate HES6 as a promoter of prostate cancer progression. 
   
  
 ZFPM1 
 
  NCBI Gene  
  GeneRIF  
  Pubmed  
 
 
  Search Term  Rank  Fields  
  [Ii]nvasive  10  PubMed(2)  
 
 
 hide/show details for ZFPM1 
  [Ii]nvasive  PubMed  
 Genetic susceptibility to distinct bladder cancer subphenotypes. Clinical, pathologic, and molecular evidence indicate that bladder cancer is heterogeneous with pathologic/molecular features that define distinct subphenotypes with different prognoses. It is conceivable that specific patterns of genetic susceptibility are associated with particular subphenotypes.To examine evidence for the contribution of germline genetic variation to bladder cancer heterogeneity.The Spanish Bladder Cancer/EPICURO Study is a case-control study based in 18 hospitals located in five areas in Spain. Cases were patients with a newly diagnosed, histologically confirmed, urothelial cell carcinoma of the bladder from 1998 to 2001. Case diagnoses were reviewed and uniformly classified by pathologists following the World Health Organisation/International Society of Urological Pathology 1999 criteria. Controls were hospital-matched patients (n=1149).A total of 1526 candidate variants in 423 candidate genes were analysed. Three distinct subphenotypes were defined according to stage and grade: low-grade nonmuscle   invasive   (n=586), high-grade nonmuscle   invasive   (n=219), and muscle   invasive   (n=246). The association between each variant and subphenotype was assessed by polytomous risk models adjusting for potential confounders. Heterogeneity in genetic susceptibility among subphenotypes was also tested.Two established bladder cancer susceptibility genotypes, NAT2 slow-acetylation and GSTM1-null, exhibited similar associations among the subphenotypes, as did VEGF-rs25648, which was previously identified in our study. Other variants conferred risks for specific tumour subphenotypes such as PMS2-rs6463524 and CD4-rs3213427 (respective heterogeneity p values of 0.006 and 0.004), which were associated with muscle-  invasive   tumours (per-allele odds ratios [95% confidence interval] of 0.56 [0.41-0.77] and 0.71 [0.57-0.88], respectively) but not with non-muscle-  invasive   tumours. Heterogeneity p values were not robust in multiple testing according to their false-discovery rate.These exploratory analyses suggest that genetic susceptibility loci might be related to the molecular/pathologic diversity of bladder cancer. Validation through large-scale replication studies and the study of additional genes and single nucleotide polymorphisms are required. 
 Genetic susceptibility to distinct bladder cancer subphenotypes. Clinical, pathologic, and molecular evidence indicate that bladder cancer is heterogeneous with pathologic/molecular features that define distinct subphenotypes with different prognoses. It is conceivable that specific patterns of genetic susceptibility are associated with particular subphenotypes.To examine evidence for the contribution of germline genetic variation to bladder cancer heterogeneity.The Spanish Bladder Cancer/EPICURO Study is a case-control study based in 18 hospitals located in five areas in Spain. Cases were patients with a newly diagnosed, histologically confirmed, urothelial cell carcinoma of the bladder from 1998 to 2001. Case diagnoses were reviewed and uniformly classified by pathologists following the World Health Organisation/International Society of Urological Pathology 1999 criteria. Controls were hospital-matched patients (n=1149).A total of 1526 candidate variants in 423 candidate genes were analysed. Three distinct subphenotypes were defined according to stage and grade: low-grade nonmuscle   invasive   (n=586), high-grade nonmuscle   invasive   (n=219), and muscle   invasive   (n=246). The association between each variant and subphenotype was assessed by polytomous risk models adjusting for potential confounders. Heterogeneity in genetic susceptibility among subphenotypes was also tested.Two established bladder cancer susceptibility genotypes, NAT2 slow-acetylation and GSTM1-null, exhibited similar associations among the subphenotypes, as did VEGF-rs25648, which was previously identified in our study. Other variants conferred risks for specific tumour subphenotypes such as PMS2-rs6463524 and CD4-rs3213427 (respective heterogeneity p values of 0.006 and 0.004), which were associated with muscle-  invasive   tumours (per-allele odds ratios [95% confidence interval] of 0.56 [0.41-0.77] and 0.71 [0.57-0.88], respectively) but not with non-muscle-  invasive   tumours. Heterogeneity p values were not robust in multiple testing according to their false-discovery rate.These exploratory analyses suggest that genetic susceptibility loci might be related to the molecular/pathologic diversity of bladder cancer. Validation through large-scale replication studies and the study of additional genes and single nucleotide polymorphisms are required. 
   
  
 GAB2 
 
  NCBI Gene  
  GeneRIF  
  Pubmed  
 
 
  Search Term  Rank  Fields  
  [Mm]igration  6  PubMed(17); GeneRIFs(4)  
  [Ee]xtracellular [Mm]atrix  8  PubMed(1)  
  [Ii]nvasive  10  PubMed(7)  
  [Mm]etastasis  12  PubMed(6); GeneRIFs(2)  
  [Cc]ell [Ii]nvasion  13  PubMed(2)  
 
 
 hide/show details for GAB2 
  [Mm]igration  PubMed  
 Gab2 phosphorylation by RSK inhibits Shp2 recruitment and cell motility. The scaffolding adapter protein Gab2 (Grb2-associated binder) participates in the signaling response evoked by various growth factors and cytokines. Gab2 is overexpressed in several human malignancies, including breast cancer, and was shown to promote mammary epithelial cell   migration  . The role of Gab2 in the activation of different signaling pathways is well documented, but less is known regarding the feedback mechanisms responsible for its inactivation. We now demonstrate that activation of the Ras/mitogen-activated protein kinase (MAPK) pathway promotes Gab2 phosphorylation on basic consensus motifs. More specifically, we show that RSK (p90 ribosomal S6 kinase) phosphorylates Gab2 on three conserved residues, both in vivo and in vitro. Mutation of these phosphorylation sites does not alter Gab2 binding to Grb2, but instead, we show that Gab2 phosphorylation inhibits the recruitment of the tyrosine phosphatase Shp2 in response to growth factors. Expression of an unphosphorylatable Gab2 mutant in mammary epithelial cells promotes an invasion-like phenotype and increases cell motility. Taken together, these results suggest that RSK is part of a negative-feedback loop that restricts Gab2-dependent epithelial cell motility. On the basis of the widespread role of Gab2 in receptor signaling, these findings also suggest that RSK plays a regulatory function in diverse receptor systems. 
 Non-redundant roles of the Gab1 and Gab2 scaffolding adapters in VEGF-mediated signalling,   migration  , and survival of endothelial cells. Gab1 was previously described as a positive modulator of Akt, Src, ERK1/2, endothelial cell   migration  , and capillary formation in response to vascular endothelial growth factor (VEGF). However, its involvement in endothelial cell survival, as well as the potential contribution of the other family member Gab2 to signalling and biological responses remained unknown. Here, we show that Gab2 is tyrosine phosphorylated in a Grb2-dependent manner downstream of activated VEGF receptor-2 (VEGFR2), and that it associates with signalling proteins including PI3K and SHP2, but apparently not with the receptor. Similarly to Gab1, over-expression of Gab2 induces endothelial cell   migration   in response to VEGF, whereas its depletion using siRNAs results in its reduction. Importantly, depletion of both Gab1 and Gab2 leads to an even greater inhibition of VEGF-induced cell   migration  . However, contrary to what has been reported for Gab1, the silencing of Gab2 results in increased Src, Akt and ERK1/2 activation, slightly reduced p38 phosphorylation, and up-regulation of Gab1 protein levels. Accordingly, re-expression of Gab2 in Gab2-/- fibroblasts leads to opposite results, suggesting that the modulation of both Gab2 and Gab1 expression in these conditions might contribute to the impaired signalling observed. Consistent with their opposite roles on Akt, the depletion of Gab1, but not of Gab2, results in reduced FOXO1 phosphorylation and VEGF-mediated endothelial cell survival. Mutation of VEGFR2 Y801 and Y1214, which abrogates the phosphorylation of Gab1, also correlates with inhibition of Akt. Altogether, these results underscore the non-redundant and essential roles of Gab1 and Gab2 in endothelial cells, and suggest major contributions of these proteins during in vivo angiogenesis. 
 MicroRNA-302a targets GAB2 to suppress cell proliferation,   migration   and invasion of glioma. Glioma is the most frequent and aggressive primary tumor of the brain in humans. Over the last few decades, significant progress has been made in early detection and multi-mode treatments, but the prognosis of gliomas is still extremely poor. MicroRNAs are endogenously expressed non-coding, single strand and short RNA molecules. Increasing number of studies demonstrated that microRNAs are dysregulated in a variety of human cancers, and play significant roles in tumorigenesis and tumor development, including glioma. In the present study, we for the first time found that microRNA-302a (miR-302a) was significantly downregulated in both glioma tissues and cell lines. In glioma patients, low miR-302a expression was correlated with KPS score and WHO grade. Restoration of miR-302a expression inhibited cell proliferation,   migration   and invasion of glioma in vitro. In addition, GAB2 was identified as a direct target of miR-320a. In clinical glioma tissues, GAB2 was upregulated and in-versely correlated with miR-302a expression. GAB2 underexpression had similar biological roles with miR-302a overexpression in glio-ma cells, further confirming that GAB2 was a functional downstream target of miR-302a. Moreover, rescue experiments showed that upregulation of GAB2 effectively reversed the inhibition effects of miR-302a on glioma cells proliferation,   migratio  n and invasion. These findings suggested that miR-302a is an important tumor suppressor of glioma progression by directly targeting GAB2, thus providing new insight into the molecular mechanisms underlying glioma occurrence, development and evolution of glioma. 
 Gab2 regulates cytoskeletal organization and   migration   of mammary epithelial cells by modulating RhoA activation. The docking protein Gab2 is overexpressed in several human malignancies, including breast cancer, and is associated with increased metastatic potential. Here we report that Gab2 overexpression in MCF-10A mammary epithelial cells led to delayed cell spreading, a decrease in stress fibers and mature focal adhesions, and enhanced cell   migration  . Expression of a Gab2 mutant uncoupled from 14-3-3-mediated negative feedback (Gab2(2xA)) led to a more mesenchymal morphology and acquisition of invasive potential. Expression of either Gab2 or Gab2(2xA) led to decreased activation of RhoA, but only the latter increased levels of Rac-GTP. Expression of constitutively active RhoA in MCF-10A/Gab2 cells restored stress fibers and focal adhesions, indicating that Gab2 signals upstream of RhoA to suppress these structures. Mutation of the two Shp2-binding sites to phenylalanine (Gab2(ΔShp2)) markedly reduced the effects of Gab2 on cellular phenotype and RhoA activation. Expression of Gab2 or Gab2(2xA), but not Gab2(ΔShp2), promoted Vav2 phosphorylation and plasma membrane recruitment of p190A RhoGAP. Knockdown of p190A RhoGAP reversed Gab2-mediated effects on stress fibers and focal adhesions. The identification of a novel pathway downstream of Gab2 involving negative regulation of RhoA by p190A RhoGAP sheds new light on the role of Gab2 in cancer progression. 
 Gab2 and Src co-operate in human mammary epithelial cells to promote growth factor independence and disruption of acinar morphogenesis. The Gab2 docking protein is a target of several oncogenic protein tyrosine kinases and potentiates activation of the Ras/extracellular signal regulated kinase and phosphatidylinositol 3-kinase (PI3-kinase) pathways. Since Gab2 is phosphorylated by c-Src, and both proteins are overexpressed in breast cancers, we have determined the biological consequences of their co-expression in the immortalized human mammary epithelial cell line MCF-10A. While overexpression of c-Src did not affect acinar morphogenesis or growth factor dependence in three-dimensional culture, c-Src co-operated with Gab2 to promote epidermal growth factor (EGF)-independent acinar growth. In contrast, expression of v-Src or the activated mutant c-SrcY527F led to a spectrum of aberrant phenotypes ranging from spheroids with incomplete luminal clearance to highly disrupted, dispersed structures. Gab2 co-expression shifted the phenotypic distribution towards the dispersed phenotype, an effect not observed with a Gab2 mutant unable to bind the p85 subunit of PI3-kinase (Gab2Deltap85). In v-Src-expressing cells, Gab2, but not Gab2Deltap85, significantly decreased E-cadherin adhesive strength without altering its surface expression. Gab2 associated with E-cadherin in the presence and absence of v-Src, indicating that the ability of Gab2 to weaken the strength of cell-cell contacts may reflect enhanced activation of PI3-kinase at adherens junctions. Gab2 also increased   migration   and invasion of these cells in transwell assays, but these effects were p85-independent. Overall, these findings demonstrate a novel mechanism whereby Gab2 may promote metastatic spread and indicate that Gab2 may play several roles during breast cancer progression. 
 Participation of both Gab1 and Gab2 in the activation of the ERK/MAPK pathway by epidermal growth factor. Three members of Gab family docking proteins, Gab1, Gab2 and Gab3, have been identified in humans. Previous studies have found that the hepatocyte growth factor preferentially utilizes Gab1 for signalling, whereas Bcr-Abl selectively signals through Gab2. Gab1-SHP2 interaction has been shown to mediate ERK (extracellular-signal-regulated kinase) activation by EGF (epidermal growth factor). However, it was unclear whether EGF selectively utilizes Gab1 for signalling to ERK and whether Gab2 is dispensable in cells where Gab1 and Gab2 are co-expressed. Using T47D and MCF-7 human breast carcinoma cells that express endogenous Gab1 and Gab2, we examined the role of these docking proteins in EGF-induced ERK activation. It was found that EGF induced a similar amount of SHP2-Gab1 and SHP2-Gab2 complexes. Expression of either SHP2-binding defective Gab1 or Gab2 mutant blocked EGF-induced ERK activation. Down-regulation of either Gab1 or Gab2 by siRNAs (small interfering RNAs) effectively inhibited the EGF-stimulated ERK activation pathway and cell   migration  . Interestingly, the inhibitory effect of Gab1 siRNA could be rescued not only by expression of an exogenous mouse Gab1 but also by an exogenous human Gab2 and vice versa, but not by IRS1 (insulin receptor substrate 1). These results reveal that Gab2 plays a pivotal role in the EGF-induced ERK activation pathway and that it can complement the function of Gab1 in the EGF signalling pathway. Furthermore, Gab1 and Gab2 are critical signalling threshold proteins for ERK activation by EGF. 
 Role of the docking protein Gab2 in beta(1)-integrin signaling pathway-mediated hematopoietic cell adhesion and   migration  . Gab2, a newly identified pleckstrin homology domain-containing docking protein, is a major binding protein of SHP-2 tyrosine phosphatase in interleukin (IL)-3-stimulated hematopoietic cells. Its signaling mechanism remains largely unknown. We report here an important regulatory role for Gab2 in beta(1) integrin signaling pathway that mediates hematopoietic cell adhesion and   migration  . Cross-linking of the beta(1) integrin on Ba/F3 cells induced rapid tyrosine phosphorylation of Gab2 and its association with Syk kinase, SHP-2 phosphatase, and the p85 subunit of phosphatidylinositol (PI)-3 kinase. In addition, Gab2 was also constitutively associated with SHP-1 phosphatase via its C-terminal Src homology 2 domain. Overexpression of the pleckstrin homology domain or a mutant Gab2 molecule lacking SHP-2 binding sites resulted in significant reductions in Ba/F3 cell adhesion and   migration  . Biochemical analyses revealed that enforced expression of Gab2 mutant molecules dramatically reduced beta(1)-integrin ligation-triggered PI3 kinase activation, whereas Erk kinase activation remained unaltered. Furthermore, transduction of primary hematopoietic progenitor cells from viable motheaten mice with these mutant Gab2 molecules also significantly ameliorated their enhanced   migration   capacity associated with the SHP1 gene mutation. Taken together, these results suggest an important signaling role for Gab2 in regulating hematopoietic cell adhesion and   migration  . 
 MiR125a-5p acting as a novel Gab2 suppressor inhibits invasion of glioma. Poor prognosis of glioma is due to the characteristics of high invasiveness. Recently, it was demonstrated that Gab2 was over-expressed and related to cellular   migration   and invasion in glioma, however, the mechanisms of regulation are still unknown. A better understanding of molecular events key to the carcinogenesis and tumor progression may facilitate development of new therapeutic targets and anti-glioma strategies. This study is the first to focus on miR125a-5p, which was predicted to regulate Gab2 with directly targeting the 3' un-translated region (3'UTR) of Gab2 and could inhibit   migration   and invasion of glioma cells by mediating Gab2 to affect cytoskeleton rearrangement and matrix metalloproteinases expression. Interestingly, further evaluation revealed that the miR125a-5p promoter was hypermethylated and that attenuating promoter methylation was sufficient to up-regulate miR125a-5p expression in glioma cells. Additionally, we reported that miR125a-5p was down-regulated in glioma as well as statistical analysis suggested that its expression level correlated with the World Health Organization grades of glioma (P < 0.05) and that patients with a low miR125a-5p level exhibited shorter survival time (P < 0.05). Taken together, these results reveal that miR125a-5p represents potential therapeutic targets in glioma by modulating Gab2. 
 Gab2 regulates the migratory behaviors and E-cadherin expression via activation of the PI3K pathway in ovarian cancer cells. Ovarian cancer, the most deadly gynecologic malignancy, is often diagnosed late and at the advanced stage when the cancer cells have already migrated and invaded into other tissues and organs. Better understanding of the mechanism of metastasis in ovarian cancer cells is essential to the design of effective therapy. In this study, we investigated the function of scaffolding adaptor protein Gab2 in ovarian cancer cells. Gab2 is found to be overexpressed in a subset of ovarian tumors and cancer cell lines. Gab2 expression mainly regulates the migratory behaviors of ovarian cancer cells. Overexpression of Gab2 promotes the   migration   and invasion, and downregulates E-cadherin expression in ovarian cancer cells with low-Gab2 expression. Conversely, knockdown of Gab2 expression inhibits the   migration   and invasion, and promotes E-cadherin expression in ovarian cancer cells with high-Gab2 expression. By expressing Gab2 wild-type and Gab2 mutants that are defective in activation of the PI3K and Shp2-Erk pathways, we find that Gab2 inhibits E-cadherin expression and enhances the expression of Zeb1, a transcription factor involved in epithelial-to-mesenchymal transition (EMT), and cell   migration   and invasion through the activation of the PI3K pathway. Knockdown of Zeb1 expression blocks Gab2-induced suppression of E-cadherin expression and increase in cell invasion. LY294002 and GDC-0941, inhibitors of PI3K, or Rapamycin, an inhibitor of PI3K downstream target mTOR, can reverse the effects of Gab2 on   migration   and invasion. Overall, our studies reveal that Gab2 overexpression, via activation of the PI3K-Zeb1 pathway, promotes characteristics of EMT in ovarian cancer cells. 
 Gab2 expression in glioma and its implications for tumor invasion. Gliomas are characterized by high invasiveness and poor prognosis. Better understanding of the mechanism of invasion in glioma cells is essential to the design of effective therapy. Recently Grb2-associated binder 2 (Gab2), a member of the DOS/Gab family of scaffolding adapters, has been reported to play important roles in the development and progression of human cancers. However, it is not known whether Gab2 has any role in the   migration   and invasion of gliomas. This study attempts to investigate the association between Gab2 expression and progression of gliomas and the molecular mechanism of Gab2 in the glioma cell invasion. Methods. The expression of Gab2 in pairs of matched glioma tissues and their normal brain tissues was detected by Western blot. Immunohistochemistry was applied to evaluate the expression of Gab2 in 163 cases of histologically diagnosed gliomas. The invasive character of Gab2 decreased glioma cells and control glioma cells were investigated in vitro and in vivo in SCID mice brain. Results. Gab2 is found to be high expressed in gliomas and a subset of cancer cell lines. Statistical analysis suggested that the up-regulation of Gab2 correlated with the WHO grade of gliomas (p < 0.01) and that patients with high Gab2 expression levels exhibited shorter survival time (p < 0.01). In an animal experiment, knockdown of Gab2 through siRNA inhibited invasive ability of glioma cells into the brain of SCID mice. In cell research, reduction of Gab2 by siRNA inhibits the   migration   and invasion of glioma cells by mediating cytoskeleton rearrangement and MMPs expression. Additionally, IGF-1-induced pAkt and pmTOR phosphorylation was suppressed by the knockdown of Gab2. Conclusion. Gab2 may be a useful prognostic marker for gliomas and a novel therapeutic target for glioma invasion intervention. 
 The GAB2 signaling scaffold promotes anchorage independence and drives a transcriptional response associated with metastatic progression of breast cancer. Acquisition of independence from anchorage to the extracellular matrix is a critical event for onset and progression of solid cancers. To identify and characterize new genes conferring anchorage independence, we transduced MCF10A human normal breast cells with a retroviral cDNA expression library and selected them by growth in suspension. Microarray analysis targeted on library-derived transcripts revealed robust and reproducible enrichment, after selection, of cDNAs encoding the scaffolding adaptor Gab2. Gab2 was confirmed to strongly promote anchorage-independent growth when overexpressed. Interestingly, downregulation by RNA interference of endogenous Gab2 in neoplastic cells did not affect their adherent growth, but abrogated their growth in soft agar. Gab2-driven anchorage independence was found to specifically involve activation of the Src-Stat3 signaling axis. A transcriptional 'signature' of 205 genes was obtained from GAB2-transduced, anchorage-independent MCF10A cells, and found to contain two main functional modules, controlling proliferation and cell adhesion/  migration  /invasion, respectively. Extensive validation on breast cancer data sets showed that the GAB2 signature provides a robust prognostic classifier for breast cancer metastatic relapse, largely independent from existing clinical and genomic indicators and from estrogen receptor status. This work highlights a pivotal role for GAB2 and its transcriptional targets in anchorage-independent growth and breast cancer metastatic progression. 
 Participation of Gab1 and Gab2 in IL-22-mediated keratinocyte proliferation,   migration  , and differentiation. Interleukin-22 (IL-22) is one of the key mediators of keratinocyte alterations in psoriasis. IL-22 inhibits keratinocyte differentiation and induces the   migration   of human keratinocytes. Grb2-associated binder 1 (Gab1) has been shown to mediate epidermal growth factor-induced epidermal growth and differentiation via interaction with the Src homology-2-containing protein-tyrosine phosphatase (Shp2). In this investigation, we explore the role of Gab1 and Gab2 in IL-22-mediated keratinocyte activities. We show that both Gab1 and Gab2 were tyrosine phosphorylated in IL-22-stimulated HaCaT cells and human primary epidermal keratinocytes and contributed to the activation of Extracellular signal regulated kinase 1/2 (Erk1/2) through interaction with Shp2. We further demonstrate that HaCaT cells infected with adenoviruses expressing Shp2-binding-defective Gab1/2 mutants exhibited decreased cell proliferation and   migration  , as well as increased differentiation. Moreover, similar results were observed in HaCaT cells infected with adenovirus-based small interfering RNAs targeting Gab1 and/or Gab2. Altogether, these data underscore the critical roles of Gab1 and Gab2 in IL-22-mediated HaCaT cell proliferation,   migration  , and differentiation. 
 Gab2 facilitates epithelial-to-mesenchymal transition via the MEK/ERK/MMP signaling in colorectal cancer. Grb2-associated binder 2 (Gab2), a scaffolding adaptor protein, has recently been implicated in cancer progression. However, the role of Gab2 in the progression and metastasis of colorectal cancer (CRC) remains unclear.Gab2 expression was assessed in CRC patient specimens as well as in CRC cell lines. Recombinant lentivirus vector containing Gab2 gene and its small interfering RNAs were constructed and introduced into CRC cells. Cell   migration   and invasion ability were evaluated by transwell assays in vitro, and in vivo metastasis was performed on nude mice model. Moreover, the expression of Gab2 and epithelial-to-mesenchymal transition (EMT)-associated proteins (E-cadherin and vimentin) were assessed by western blot and qRT-PCR in CRC cells to evaluate the correlation between Gab2 and EMT. Finally, we evaluated the impact of Gab2 on the activation of its downstream signaling effectors, and furthermore the effects of these pathways on Gab2 induced-EMT were also detected.We confirmed that increased Gab2 expression correlated with higher tumor node metastasis stage and highly invasive CRC cell lines. Ectopic expression of Gab2 promoted metastasis of CRC cells, whereas silencing of Gab2 resulted in inhibited metastasis both in vitro and in vivo. Overexpression of Gab2 in CRC cells induced EMT, whereas knockdown of Gab2 had the opposite effect. Furthermore, upregulation of Gab2 expression obviously stimulated the activation of extracellular signal-regulated kinase-1/2 (ERK1/2), and increased the expression of matrix metalloproteinase-7 (MMP7) and matrix metalloproteinase-9 (MMP9) in CRC cells. Conversely, downregulation of Gab2 expression significantly decreased the activation of ERK1/2, and inhibited MMP7 and MMP9 expression. U0126, an inhibitor of mitogen-activated protein kinase (MEK), can reverse the effects of Gab2 on EMT.Our work highlights that Gab2 induces EMT through the MEK/ERK/MMP pathway, which in turn promotes intestinal tumor metastasis. 
 Phosphorylation of Grb2-associated binder 2 on serine 623 by ERK MAPK regulates its association with the phosphatase SHP-2 and decreases STAT5 activation. IL-2 stimulation of T lymphocytes induces the tyrosine phosphorylation and adaptor function of the insulin receptor substrate/Grb2-associated binder (Gab) family member, Gab2. In addition, Gab2 undergoes a marked decrease in its mobility in SDS-PAGE, characteristic of   migration   shifts induced by serine/threonine phosphorylations in many proteins. This   migration   shift was strongly diminished by treating cells with the MEK inhibitor U0126, indicating a possible role for ERK in Gab2 phosphorylation. Indeed, ERK phosphorylated Gab2 on a consensus phosphorylation site at serine 623, a residue located between tyrosine 614 and tyrosine 643 that are responsible for Gab2/Src homology 2 domain-containing tyrosine phosphatase (SHP)-2 interaction. We report that pretreatment of Kit 225 cells with U0126 increased Gab2/SHP-2 association and tyrosine phosphorylation of SHP-2 in response to IL-2, suggesting that ERK phosphorylation of serine 623 regulates the interaction between Gab2 and SHP-2, and consequently the activity of SHP-2. This hypothesis was confirmed by biochemical analysis of cells expressing Gab2 WT, Gab2 serine 623A or Gab2 tyrosine 614F, a mutant that cannot interact with SHP-2 in response to IL-2. Activation of the ERK pathway was indeed blocked by Gab2 tyrosine 614F and slightly increased by Gab2 serine 623A. In contrast, STAT5 activation was strongly enhanced by Gab2 tyrosine 614F, slightly reduced by Gab2 WT and strongly inhibited by Gab2 serine 623A. Analysis of the rate of proliferation of cells expressing these mutants of Gab2 demonstrated that tyrosine 614F mutation enhanced proliferation whereas serine 623A diminished it. These results demonstrate that ERK-mediated phosphorylation of Gab2 serine 623 is involved in fine tuning the proliferative response of T lymphocytes to IL-2. 
 Gab2-mediated signaling promotes melanoma metastasis. Metastatic melanoma is a disease with a poor prognosis that currently lacks effective treatments. Critical biological features of metastasis include acquisition of migratory competence, growth factor independence, and invasive potential. In an attempt to identify genes that contribute to melanoma pathogenesis, a genome-wide search using bacterial artificial chromosome array comparative genomic hybridization and single nucleotide polymorphism arrays in a series of 64 metastatic melanoma samples and 20 melanoma cell lines identified increased copy numbers of Gab2 located on 11q14.1. Gab2 is an adaptor protein that potentiates the activation of the Ras-Erk and PI3K-Akt pathways and has recently been implicated in human cancer; however, its role in melanoma has not been explored. In this study, we found that Gab2 was either amplified (approximately 11%) and/or overexpressed (approximately 50%) in melanoma. Gab2 protein expression correlated with clinical melanoma progression, and higher levels of expression were seen in metastatic melanomas compared with primary melanoma and melanocytic nevi. We found that overexpression of Gab2 potentiates, whereas silencing of Gab2 reduces,   migration   and invasion of melanoma cells. Gab2 mediated the hyperactivation of Akt signaling in the absence of growth factors, whereas inhibition of the PI3K-Akt pathway decreased Gab2-mediated tumor cell   migration   and invasive potential. Gab2 overexpression resulted in enhanced tumor growth and metastatic potential in vivo. These studies demonstrate a previously undefined role for Gab2 in melanoma tumor progression and metastasis. 
 Proteinase-activated receptor 2 promotes cancer cell   migration   through RNA methylation-mediated repression of miR-125b. Proteinase activated-receptor 2 (PAR2) participates in cancer metastasis promoted by serine proteinases. The current study aimed to test the molecular mechanism by which PAR2 promotes cancer cell   migration  . In different cancer cells, activation of PAR2 by activating peptide (PAR2-AP) dramatically increased cell   migration  , whereas knock down of PAR2 inhibited cellular motility. The PAR2 activation also repressed miR-125b expression while miR-125b mimic successfully blocked PAR2-induced cell   migration  . Moreover, Grb associated-binding protein 2 (Gab2) was identified as a novel target gene of miR-125b and it mediated PAR2-induced cell   migration  . The correlation of PAR2 with miR-125b and Gab2 was further supported by the findings obtained from human colorectal carcinoma specimens. Remarkably, knock down of NOP2/Sun domain family, member 2 (NSun2), a RNA methyltransferase, blocked the reduction in miR-125b induced by PAR2. Furthermore, PAR2 activation increased the level of N(6)-methyladenosine (m(6)A)-containing pre-miR-125b in NSun2-dependent manner. Taken together, our results demonstrated that miR-125b mediates PAR2-induced cancer cell   migration   by targeting Gab2 and that NSun2-dependent RNA methylation contributes to the down-regulation of miR-125b by PAR2 signaling. These findings suggest a novel epigenetic mechanism by which microenvironment regulates cancer cell   migration   by altering miRNA expression. 
 Grb2-associated binder 2 silencing impairs growth and   migration   of H1975 cells via modulation of PI3K-Akt signaling. Non-small cell lung cancer (NSCLC) is a leading cause of cancer-related death and often has a poor prognosis. Investigation of NSCLC cancer cell   migration  , invasion and development of strategies to block this process is essential to improve the disease prognosis. In this study, we tested our hypothesis that Grb2-associated binder 2 (Gab2) regulate NSCLC cancer cell H1975 malignant biological behaviors, and silencing Gab2 reduced H1975 cellular colony forming ability,   migration   and invasion. Moreover, silenced cells present defects in phosphatidylinositol 3-kinase (PI3K)-serine/threonine kinase (Akt) signaling, and reduced expression/activity of matrix metallopeptidase (MMP)-2/9. Furthermore, in Gab2 siRNA-transfected cells, we detected a decrease in signal transducer and activator of transcription 3 (STAT3) phosphorylation and nuclear translocation. In vivo, Gab2 siRNA cells inoculated subcutaneously in nude mice demonstrated decreased tumor growth and PI3K-Akt signaling inhibition. These results indicate that Gab2 is a key factor in H1975 tumor   migration  , invasion, suggesting that Gab2 can be a novel therapeutic target in NSCLC. 
  GeneRIFs  
 GAB2 is a functional downstream target of miR-302a in glioma. GAB2 role in cell proliferation,   migration   and invasion of glioma. 
 Grb2-associated binder 2 silencing impairs growth and   migration   of non-small cell lung cancer cells via PI3K-Akt signaling. 
 In colorectal cancer, miR-125b mediates PAR2-induced cancer cell   migration   by targeting Gab2. 
 these data underscore the critical roles of Gab1 and Gab2 in IL-22-mediated HaCaT cell proliferation,   migration  , and differentiation. 
  [Ee]xtracellular [Mm]atrix  PubMed  
 The GAB2 signaling scaffold promotes anchorage independence and drives a transcriptional response associated with metastatic progression of breast cancer. Acquisition of independence from anchorage to the   extracellular matrix   is a critical event for onset and progression of solid cancers. To identify and characterize new genes conferring anchorage independence, we transduced MCF10A human normal breast cells with a retroviral cDNA expression library and selected them by growth in suspension. Microarray analysis targeted on library-derived transcripts revealed robust and reproducible enrichment, after selection, of cDNAs encoding the scaffolding adaptor Gab2. Gab2 was confirmed to strongly promote anchorage-independent growth when overexpressed. Interestingly, downregulation by RNA interference of endogenous Gab2 in neoplastic cells did not affect their adherent growth, but abrogated their growth in soft agar. Gab2-driven anchorage independence was found to specifically involve activation of the Src-Stat3 signaling axis. A transcriptional 'signature' of 205 genes was obtained from GAB2-transduced, anchorage-independent MCF10A cells, and found to contain two main functional modules, controlling proliferation and cell adhesion/migration/invasion, respectively. Extensive validation on breast cancer data sets showed that the GAB2 signature provides a robust prognostic classifier for breast cancer metastatic relapse, largely independent from existing clinical and genomic indicators and from estrogen receptor status. This work highlights a pivotal role for GAB2 and its transcriptional targets in anchorage-independent growth and breast cancer metastatic progression. 
  [Ii]nvasive  PubMed  
 Gab2 regulates cytoskeletal organization and migration of mammary epithelial cells by modulating RhoA activation. The docking protein Gab2 is overexpressed in several human malignancies, including breast cancer, and is associated with increased metastatic potential. Here we report that Gab2 overexpression in MCF-10A mammary epithelial cells led to delayed cell spreading, a decrease in stress fibers and mature focal adhesions, and enhanced cell migration. Expression of a Gab2 mutant uncoupled from 14-3-3-mediated negative feedback (Gab2(2xA)) led to a more mesenchymal morphology and acquisition of   invasive   potential. Expression of either Gab2 or Gab2(2xA) led to decreased activation of RhoA, but only the latter increased levels of Rac-GTP. Expression of constitutively active RhoA in MCF-10A/Gab2 cells restored stress fibers and focal adhesions, indicating that Gab2 signals upstream of RhoA to suppress these structures. Mutation of the two Shp2-binding sites to phenylalanine (Gab2(ΔShp2)) markedly reduced the effects of Gab2 on cellular phenotype and RhoA activation. Expression of Gab2 or Gab2(2xA), but not Gab2(ΔShp2), promoted Vav2 phosphorylation and plasma membrane recruitment of p190A RhoGAP. Knockdown of p190A RhoGAP reversed Gab2-mediated effects on stress fibers and focal adhesions. The identification of a novel pathway downstream of Gab2 involving negative regulation of RhoA by p190A RhoGAP sheds new light on the role of Gab2 in cancer progression. 
 Overexpression of the oncogenic signal transducer Gab2 occurs early in breast cancer development. Gab2, a docking-type signaling protein with demonstrated oncogenic potential, is overexpressed in breast cancer, but its prognostic significance and role in disease evolution remain unclear. Immunohistochemical detection of Gab2 in a large cohort of primary human breast cancers of known outcome revealed that while Gab2 expression was positively correlated with increased tumor grade, it did not correlate with disease recurrence or breast cancer-related death in the total cohort or in patients stratified according to lymph node, estrogen receptor (ER) or HER2 status. Interestingly, analysis of a "progression series" that included premalignant and pre  invasive   breast lesions as well as samples of metastatic disease revealed that Gab2 expression was significantly enhanced in the earliest lesion examined, usual ductal hyperplasia, with a further increase detected in ductal carcinoma in situ (DCIS). Furthermore, expression was less in   invasive   cancers and lymph node metastases than in DCIS, but still higher than in normal breast. These findings indicate that while Gab2 expression is not prognostic in breast cancer, its role in early disease evolution warrants further analysis, as Gab2 and its effectors may provide targets for novel strategies aimed at preventing breast cancer development. 
 MiR125a-5p acting as a novel Gab2 suppressor inhibits invasion of glioma. Poor prognosis of glioma is due to the characteristics of high   invasive  ness. Recently, it was demonstrated that Gab2 was over-expressed and related to cellular migration and invasion in glioma, however, the mechanisms of regulation are still unknown. A better understanding of molecular events key to the carcinogenesis and tumor progression may facilitate development of new therapeutic targets and anti-glioma strategies. This study is the first to focus on miR125a-5p, which was predicted to regulate Gab2 with directly targeting the 3' un-translated region (3'UTR) of Gab2 and could inhibit migration and invasion of glioma cells by mediating Gab2 to affect cytoskeleton rearrangement and matrix metalloproteinases expression. Interestingly, further evaluation revealed that the miR125a-5p promoter was hypermethylated and that attenuating promoter methylation was sufficient to up-regulate miR125a-5p expression in glioma cells. Additionally, we reported that miR125a-5p was down-regulated in glioma as well as statistical analysis suggested that its expression level correlated with the World Health Organization grades of glioma (P < 0.05) and that patients with a low miR125a-5p level exhibited shorter survival time (P < 0.05). Taken together, these results reveal that miR125a-5p represents potential therapeutic targets in glioma by modulating Gab2. 
 A role for the scaffolding adapter GAB2 in breast cancer. The scaffolding adapter GAB2 maps to a region (11q13-14) commonly amplified in human breast cancer, and is overexpressed in breast cancer cell lines and primary tumors, but its functional role in mammary carcinogenesis has remained unexplored. We found that overexpression of GAB2 (Grb2-associated binding protein 2) increases proliferation of MCF10A mammary cells in three-dimensional culture. Coexpression of GAB2 with antiapoptotic oncogenes causes lumenal filling, whereas coexpression with Neu (also known as ErbB2 and HER2) results in an   invasive   phenotype. These effects of GAB2 are mediated by hyperactivation of the Shp2-Erk pathway. Furthermore, overexpression of Gab2 potentiates, whereas deficiency of Gab2 ameliorates, Neu-evoked breast carcinogenesis in mice. Finally, GAB2 is amplified in some GAB2-overexpressing human breast tumors. Our data suggest that GAB2 may be a key gene within an 11q13 amplicon in human breast cancer and propose a role for overexpression of GAB2 in mammary carcinogenesis. Agents that target GAB2 or GAB2-dependent pathways may be useful for treating breast tumors that overexpress GAB2 or HER2 or both. 
 Gab2 expression in glioma and its implications for tumor invasion. Gliomas are characterized by high   invasive  ness and poor prognosis. Better understanding of the mechanism of invasion in glioma cells is essential to the design of effective therapy. Recently Grb2-associated binder 2 (Gab2), a member of the DOS/Gab family of scaffolding adapters, has been reported to play important roles in the development and progression of human cancers. However, it is not known whether Gab2 has any role in the migration and invasion of gliomas. This study attempts to investigate the association between Gab2 expression and progression of gliomas and the molecular mechanism of Gab2 in the glioma cell invasion. Methods. The expression of Gab2 in pairs of matched glioma tissues and their normal brain tissues was detected by Western blot. Immunohistochemistry was applied to evaluate the expression of Gab2 in 163 cases of histologically diagnosed gliomas. The   invasive   character of Gab2 decreased glioma cells and control glioma cells were investigated in vitro and in vivo in SCID mice brain. Results. Gab2 is found to be high expressed in gliomas and a subset of cancer cell lines. Statistical analysis suggested that the up-regulation of Gab2 correlated with the WHO grade of gliomas (p < 0.01) and that patients with high Gab2 expression levels exhibited shorter survival time (p < 0.01). In an animal experiment, knockdown of Gab2 through siRNA inhibited   invasive   ability of glioma cells into the brain of SCID mice. In cell research, reduction of Gab2 by siRNA inhibits the migration and invasion of glioma cells by mediating cytoskeleton rearrangement and MMPs expression. Additionally, IGF-1-induced pAkt and pmTOR phosphorylation was suppressed by the knockdown of Gab2. Conclusion. Gab2 may be a useful prognostic marker for gliomas and a novel therapeutic target for glioma invasion intervention. 
 Gab2 facilitates epithelial-to-mesenchymal transition via the MEK/ERK/MMP signaling in colorectal cancer. Grb2-associated binder 2 (Gab2), a scaffolding adaptor protein, has recently been implicated in cancer progression. However, the role of Gab2 in the progression and metastasis of colorectal cancer (CRC) remains unclear.Gab2 expression was assessed in CRC patient specimens as well as in CRC cell lines. Recombinant lentivirus vector containing Gab2 gene and its small interfering RNAs were constructed and introduced into CRC cells. Cell migration and invasion ability were evaluated by transwell assays in vitro, and in vivo metastasis was performed on nude mice model. Moreover, the expression of Gab2 and epithelial-to-mesenchymal transition (EMT)-associated proteins (E-cadherin and vimentin) were assessed by western blot and qRT-PCR in CRC cells to evaluate the correlation between Gab2 and EMT. Finally, we evaluated the impact of Gab2 on the activation of its downstream signaling effectors, and furthermore the effects of these pathways on Gab2 induced-EMT were also detected.We confirmed that increased Gab2 expression correlated with higher tumor node metastasis stage and highly   invasive   CRC cell lines. Ectopic expression of Gab2 promoted metastasis of CRC cells, whereas silencing of Gab2 resulted in inhibited metastasis both in vitro and in vivo. Overexpression of Gab2 in CRC cells induced EMT, whereas knockdown of Gab2 had the opposite effect. Furthermore, upregulation of Gab2 expression obviously stimulated the activation of extracellular signal-regulated kinase-1/2 (ERK1/2), and increased the expression of matrix metalloproteinase-7 (MMP7) and matrix metalloproteinase-9 (MMP9) in CRC cells. Conversely, downregulation of Gab2 expression significantly decreased the activation of ERK1/2, and inhibited MMP7 and MMP9 expression. U0126, an inhibitor of mitogen-activated protein kinase (MEK), can reverse the effects of Gab2 on EMT.Our work highlights that Gab2 induces EMT through the MEK/ERK/MMP pathway, which in turn promotes intestinal tumor metastasis. 
 Gab2-mediated signaling promotes melanoma metastasis. Metastatic melanoma is a disease with a poor prognosis that currently lacks effective treatments. Critical biological features of metastasis include acquisition of migratory competence, growth factor independence, and   invasive   potential. In an attempt to identify genes that contribute to melanoma pathogenesis, a genome-wide search using bacterial artificial chromosome array comparative genomic hybridization and single nucleotide polymorphism arrays in a series of 64 metastatic melanoma samples and 20 melanoma cell lines identified increased copy numbers of Gab2 located on 11q14.1. Gab2 is an adaptor protein that potentiates the activation of the Ras-Erk and PI3K-Akt pathways and has recently been implicated in human cancer; however, its role in melanoma has not been explored. In this study, we found that Gab2 was either amplified (approximately 11%) and/or overexpressed (approximately 50%) in melanoma. Gab2 protein expression correlated with clinical melanoma progression, and higher levels of expression were seen in metastatic melanomas compared with primary melanoma and melanocytic nevi. We found that overexpression of Gab2 potentiates, whereas silencing of Gab2 reduces, migration and invasion of melanoma cells. Gab2 mediated the hyperactivation of Akt signaling in the absence of growth factors, whereas inhibition of the PI3K-Akt pathway decreased Gab2-mediated tumor cell migration and   invasive   potential. Gab2 overexpression resulted in enhanced tumor growth and metastatic potential in vivo. These studies demonstrate a previously undefined role for Gab2 in melanoma tumor progression and metastasis. 
  [Mm]etastasis  PubMed  
 Gab2 regulates the migratory behaviors and E-cadherin expression via activation of the PI3K pathway in ovarian cancer cells. Ovarian cancer, the most deadly gynecologic malignancy, is often diagnosed late and at the advanced stage when the cancer cells have already migrated and invaded into other tissues and organs. Better understanding of the mechanism of   metastasis   in ovarian cancer cells is essential to the design of effective therapy. In this study, we investigated the function of scaffolding adaptor protein Gab2 in ovarian cancer cells. Gab2 is found to be overexpressed in a subset of ovarian tumors and cancer cell lines. Gab2 expression mainly regulates the migratory behaviors of ovarian cancer cells. Overexpression of Gab2 promotes the migration and invasion, and downregulates E-cadherin expression in ovarian cancer cells with low-Gab2 expression. Conversely, knockdown of Gab2 expression inhibits the migration and invasion, and promotes E-cadherin expression in ovarian cancer cells with high-Gab2 expression. By expressing Gab2 wild-type and Gab2 mutants that are defective in activation of the PI3K and Shp2-Erk pathways, we find that Gab2 inhibits E-cadherin expression and enhances the expression of Zeb1, a transcription factor involved in epithelial-to-mesenchymal transition (EMT), and cell migration and invasion through the activation of the PI3K pathway. Knockdown of Zeb1 expression blocks Gab2-induced suppression of E-cadherin expression and increase in cell invasion. LY294002 and GDC-0941, inhibitors of PI3K, or Rapamycin, an inhibitor of PI3K downstream target mTOR, can reverse the effects of Gab2 on migration and invasion. Overall, our studies reveal that Gab2 overexpression, via activation of the PI3K-Zeb1 pathway, promotes characteristics of EMT in ovarian cancer cells. 
 Focal amplification and oncogene dependency of GAB2 in breast cancer. DNA amplifications in breast cancer are frequent on chromosome 11q, in which multiple driver oncogenes likely reside in addition to cyclin D1 (CCND1). One such candidate, the scaffolding adapter protein, GRB2-associated binding protein 2 (GAB2), functions in ErbB signaling and was recently shown to enhance mammary epithelial cell proliferation, and   metastasis   of ERBB2 (HER2/neu)-driven murine breast cancer. However, the amplification status and function of GAB2 in the context of amplification remain undefined. In this study, by genomic profiling of 172 breast tumors, and fluorescence in situ hybridization validation in an independent set of 210 scorable cases, we observed focal amplification spanning GAB2 (11q14.1) independent of CCND1 (11q13.2) amplification, consistent with a driver role. Further, small interfering RNA (siRNA)-mediated knockdown of GAB2 in breast cancer lines (SUM52, SUM44PE and MDA468) with GAB2 amplification revealed a dependency on GAB2 for cell proliferation, cell-cycle progression, survival and invasion, likely mediated through altered phosphatidylinositol 3-kinase (PI3K) and mitogen-activated protein kinase (MAPK) signaling. GAB2 knockdown also reduced proliferation and survival in a cell line (BT474) with ERBB2 amplification, consistent with the possibility that GAB2 can function downstream of ERBB2. Our studies implicate focal amplification of GAB2 in breast carcinogenesis, and underscore an oncogenic role of scaffolding adapter proteins, and a potential new point of therapeutic intervention. 
 Gab2 facilitates epithelial-to-mesenchymal transition via the MEK/ERK/MMP signaling in colorectal cancer. Grb2-associated binder 2 (Gab2), a scaffolding adaptor protein, has recently been implicated in cancer progression. However, the role of Gab2 in the progression and   metastasis   of colorectal cancer (CRC) remains unclear.Gab2 expression was assessed in CRC patient specimens as well as in CRC cell lines. Recombinant lentivirus vector containing Gab2 gene and its small interfering RNAs were constructed and introduced into CRC cells. Cell migration and invasion ability were evaluated by transwell assays in vitro, and in vivo   metastasis   was performed on nude mice model. Moreover, the expression of Gab2 and epithelial-to-mesenchymal transition (EMT)-associated proteins (E-cadherin and vimentin) were assessed by western blot and qRT-PCR in CRC cells to evaluate the correlation between Gab2 and EMT. Finally, we evaluated the impact of Gab2 on the activation of its downstream signaling effectors, and furthermore the effects of these pathways on Gab2 induced-EMT were also detected.We confirmed that increased Gab2 expression correlated with higher tumor node   metastasis   stage and highly invasive CRC cell lines. Ectopic expression of Gab2 promoted   metastasis   of CRC cells, whereas silencing of Gab2 resulted in inhibited   metastasis   both in vitro and in vivo. Overexpression of Gab2 in CRC cells induced EMT, whereas knockdown of Gab2 had the opposite effect. Furthermore, upregulation of Gab2 expression obviously stimulated the activation of extracellular signal-regulated kinase-1/2 (ERK1/2), and increased the expression of matrix metalloproteinase-7 (MMP7) and matrix metalloproteinase-9 (MMP9) in CRC cells. Conversely, downregulation of Gab2 expression significantly decreased the activation of ERK1/2, and inhibited MMP7 and MMP9 expression. U0126, an inhibitor of mitogen-activated protein kinase (MEK), can reverse the effects of Gab2 on EMT.Our work highlights that Gab2 induces EMT through the MEK/ERK/MMP pathway, which in turn promotes intestinal tumor   metastasis  . 
 Gab2-mediated signaling promotes melanoma   metastasis  . Metastatic melanoma is a disease with a poor prognosis that currently lacks effective treatments. Critical biological features of   metastasis   include acquisition of migratory competence, growth factor independence, and invasive potential. In an attempt to identify genes that contribute to melanoma pathogenesis, a genome-wide search using bacterial artificial chromosome array comparative genomic hybridization and single nucleotide polymorphism arrays in a series of 64 metastatic melanoma samples and 20 melanoma cell lines identified increased copy numbers of Gab2 located on 11q14.1. Gab2 is an adaptor protein that potentiates the activation of the Ras-Erk and PI3K-Akt pathways and has recently been implicated in human cancer; however, its role in melanoma has not been explored. In this study, we found that Gab2 was either amplified (approximately 11%) and/or overexpressed (approximately 50%) in melanoma. Gab2 protein expression correlated with clinical melanoma progression, and higher levels of expression were seen in metastatic melanomas compared with primary melanoma and melanocytic nevi. We found that overexpression of Gab2 potentiates, whereas silencing of Gab2 reduces, migration and invasion of melanoma cells. Gab2 mediated the hyperactivation of Akt signaling in the absence of growth factors, whereas inhibition of the PI3K-Akt pathway decreased Gab2-mediated tumor cell migration and invasive potential. Gab2 overexpression resulted in enhanced tumor growth and metastatic potential in vivo. These studies demonstrate a previously undefined role for Gab2 in melanoma tumor progression and   metastasis  . 
 Proteinase-activated receptor 2 promotes cancer cell migration through RNA methylation-mediated repression of miR-125b. Proteinase activated-receptor 2 (PAR2) participates in cancer   metastasis   promoted by serine proteinases. The current study aimed to test the molecular mechanism by which PAR2 promotes cancer cell migration. In different cancer cells, activation of PAR2 by activating peptide (PAR2-AP) dramatically increased cell migration, whereas knock down of PAR2 inhibited cellular motility. The PAR2 activation also repressed miR-125b expression while miR-125b mimic successfully blocked PAR2-induced cell migration. Moreover, Grb associated-binding protein 2 (Gab2) was identified as a novel target gene of miR-125b and it mediated PAR2-induced cell migration. The correlation of PAR2 with miR-125b and Gab2 was further supported by the findings obtained from human colorectal carcinoma specimens. Remarkably, knock down of NOP2/Sun domain family, member 2 (NSun2), a RNA methyltransferase, blocked the reduction in miR-125b induced by PAR2. Furthermore, PAR2 activation increased the level of N(6)-methyladenosine (m(6)A)-containing pre-miR-125b in NSun2-dependent manner. Taken together, our results demonstrated that miR-125b mediates PAR2-induced cancer cell migration by targeting Gab2 and that NSun2-dependent RNA methylation contributes to the down-regulation of miR-125b by PAR2 signaling. These findings suggest a novel epigenetic mechanism by which microenvironment regulates cancer cell migration by altering miRNA expression. 
 Gab2 is a novel prognostic factor for colorectal cancer patients. Gab2 (Grb2-associated binder 2), a member of the DOS/Gab family of scaffolding adapters, serves as a critical signal amplifier downstream of various growth factor receptors. Recent studies have identified that Gab2 is overexpressed in several cancer types and that increased Gab2 expression promotes cell proliferation, cell transformation, and tumor progression. Here, we show for the first time that Gab2 protein is overexpressed in clinical colorectal cancer (CRC) specimens. Elevated mRNA (P=0.014) expression and protein (P=0.003) expression of Gab2 were found in most CRC tissues compared with the matched adjacent non-tumor tissues using real-time quantitative reverse transcription PCR (qRT-PCR) and western blotting, respectively. Immunohistochemical analyses showed that Gab2 protein was upregulated in CRC tissues relative to adjacent normal tissues (P<0.001), and this overexpression was significantly correlated with lymph node   metastasis   (P=0.007), distant   metastasis   (P<0.001) and TNM stage (P=0.002). According to Kaplan-Meier model, CRC patients with Gab2-positive had a significantly poorer prognosis compared to those with Gab2-negative (P=0.007). Multivariate analysis suggested that the positive expression of Gab2 protein was an independent prognostic factor for CRC patients. In conclusion, our data demonstrated that Gab2 expression may play an important role in the progression of CRC, and underscored that Gab2 has the potential value as a prognostic predictor for CRC patients. 
  GeneRIFs  
 Study shows that Gab2 is significantly upregulated in   metastasis  -positive colorectal cancer (CRC) tissues and suggest that it plays a vital role in regulating CRC   metastasis   and a potential target for diagnosis. 
 These studies demonstrate a previously undefined role for Gab2 in melanoma tumor progression and   metastasis   
  [Cc]ell [Ii]nvasion  PubMed  
 Gab2 regulates the migratory behaviors and E-cadherin expression via activation of the PI3K pathway in ovarian cancer cells. Ovarian cancer, the most deadly gynecologic malignancy, is often diagnosed late and at the advanced stage when the cancer cells have already migrated and invaded into other tissues and organs. Better understanding of the mechanism of metastasis in ovarian cancer cells is essential to the design of effective therapy. In this study, we investigated the function of scaffolding adaptor protein Gab2 in ovarian cancer cells. Gab2 is found to be overexpressed in a subset of ovarian tumors and cancer cell lines. Gab2 expression mainly regulates the migratory behaviors of ovarian cancer cells. Overexpression of Gab2 promotes the migration and invasion, and downregulates E-cadherin expression in ovarian cancer cells with low-Gab2 expression. Conversely, knockdown of Gab2 expression inhibits the migration and invasion, and promotes E-cadherin expression in ovarian cancer cells with high-Gab2 expression. By expressing Gab2 wild-type and Gab2 mutants that are defective in activation of the PI3K and Shp2-Erk pathways, we find that Gab2 inhibits E-cadherin expression and enhances the expression of Zeb1, a transcription factor involved in epithelial-to-mesenchymal transition (EMT), and cell migration and invasion through the activation of the PI3K pathway. Knockdown of Zeb1 expression blocks Gab2-induced suppression of E-cadherin expression and increase in   cell invasion  . LY294002 and GDC-0941, inhibitors of PI3K, or Rapamycin, an inhibitor of PI3K downstream target mTOR, can reverse the effects of Gab2 on migration and invasion. Overall, our studies reveal that Gab2 overexpression, via activation of the PI3K-Zeb1 pathway, promotes characteristics of EMT in ovarian cancer cells. 
 Gab2 expression in glioma and its implications for tumor invasion. Gliomas are characterized by high invasiveness and poor prognosis. Better understanding of the mechanism of invasion in glioma cells is essential to the design of effective therapy. Recently Grb2-associated binder 2 (Gab2), a member of the DOS/Gab family of scaffolding adapters, has been reported to play important roles in the development and progression of human cancers. However, it is not known whether Gab2 has any role in the migration and invasion of gliomas. This study attempts to investigate the association between Gab2 expression and progression of gliomas and the molecular mechanism of Gab2 in the glioma   cell invasion  . Methods. The expression of Gab2 in pairs of matched glioma tissues and their normal brain tissues was detected by Western blot. Immunohistochemistry was applied to evaluate the expression of Gab2 in 163 cases of histologically diagnosed gliomas. The invasive character of Gab2 decreased glioma cells and control glioma cells were investigated in vitro and in vivo in SCID mice brain. Results. Gab2 is found to be high expressed in gliomas and a subset of cancer cell lines. Statistical analysis suggested that the up-regulation of Gab2 correlated with the WHO grade of gliomas (p < 0.01) and that patients with high Gab2 expression levels exhibited shorter survival time (p < 0.01). In an animal experiment, knockdown of Gab2 through siRNA inhibited invasive ability of glioma cells into the brain of SCID mice. In cell research, reduction of Gab2 by siRNA inhibits the migration and invasion of glioma cells by mediating cytoskeleton rearrangement and MMPs expression. Additionally, IGF-1-induced pAkt and pmTOR phosphorylation was suppressed by the knockdown of Gab2. Conclusion. Gab2 may be a useful prognostic marker for gliomas and a novel therapeutic target for glioma invasion intervention. 
   
  
 GAB1 
 
  NCBI Gene  
  GeneRIF  
  Pubmed  
 
 
  Search Term  Rank  Fields  
  [Ff]ilopodia  1  PubMed(1)  
  [Ii]nvadopodia  3  PubMed(1); GeneRIFs(1)  
  [Pp]odosome(s)?  5  PubMed(1)  
  [Mm]igration  6  Process(1); PubMed(17); GeneRIFs(2)  
  [Ee]xtracellular [Mm]atrix  8  PubMed(1)  
  [Ii]nvasive  10  PubMed(4); GeneRIFs(1)  
  [Mm]etastasize  11  PubMed(1)  
  [Mm]etastasis  12  PubMed(8); GeneRIFs(1)  
  [Cc]ell [Ii]nvasion  13  PubMed(4)  
 
 
 hide/show details for GAB1 
  [Ff]ilopodia  PubMed  
 Adaptor molecule Crk is required for sustained phosphorylation of Grb2-associated binder 1 and hepatocyte growth factor-induced cell motility of human synovial sarcoma cell lines. Activation of the c-Met receptor tyrosine kinase through its ligand, hepatocyte growth factor (HGF), promotes mitogenic, motogenic, and morphogenic cellular responses. Aberrant HGF/c-Met signaling has been strongly implicated in tumor cell invasion and metastasis. Both HGF and its receptor c-Met have been shown to be overexpressed in human synovial sarcoma, which often metastasizes to the lung; however, little is known about HGF-mediated biological effects in this sarcoma. Here, we provide evidence that Crk adaptor protein is required for the sustained phosphorylation of c-Met-docking protein Grb2-associated binder 1 (Gab1) in response to HGF, leading to the enhanced cell motility of human synovial sarcoma cell lines SYO-1, HS-SY-II, and Fuji. HGF stimulation induced the sustained phosphorylation on Y307 of Gab1 where Crk was recruited. Crk knockdown by RNA interference disturbed this HGF-induced tyrosine phosphorylation of Gab1. By mutational analysis, we identified that Src homology 2 domain of Crk is indispensable for the induction of the phosphorylation on multiple Tyr-X-X-Pro motifs containing Y307 in Gab1. HGF remarkably stimulated cell motility and scattering of synovial sarcoma cell lines, consistent with the prominent activation of Rac1, extreme   filopodia   formation, and membrane ruffling. Importantly, the elimination of Crk in these cells induced the disorganization of actin cytoskeleton and complete abolishment of HGF-mediated Rac1 activation and cell motility. Time-lapse microscopic analysis revealed the significant attenuation in scattering of Crk knockdown cells following HGF treatment. Furthermore, the depletion of Crk remarkably inhibited the tumor formation and its invasive growth in vivo. These results suggest that the sustained phosphorylation of Gab1 through Crk in response to HGF contributes to the prominent activation of Rac1 leading to enhanced cell motility, scattering, and cell invasion, which may support the crucial role of Crk in the aggressiveness of human synovial sarcoma. 
  [Ii]nvadopodia  PubMed  
 Met receptor tyrosine kinase signals through a cortactin-Gab1 scaffold complex, to mediate   invadopodia  . Invasive carcinoma cells form actin-rich matrix-degrading protrusions called   invadopodia  . These structures resemble podosomes produced by some normal cells and play a crucial role in extracellular matrix remodeling. In cancer, formation of   invadopodia   is strongly associated with invasive potential. Although deregulated signals from the receptor tyrosine kinase Met (also known as hepatocyte growth factor are linked to cancer metastasis and poor prognosis, its role in   invadopodia   formation is not known. Here we show that stimulation of breast cancer cells with the ligand for Met, hepatocyte growth factor, promotes   invadopodia   formation, and in aggressive gastric tumor cells where Met is amplified,   invadopodia   formation is dependent on Met activity. Using both GRB2-associated-binding protein 1 (Gab1)-null fibroblasts and specific knockdown of Gab1 in tumor cells we show that Met-mediated   invadopodia   formation and cell invasion requires the scaffold protein Gab1. By a structure-function approach, we demonstrate that two proline-rich motifs (P4/5) within Gab1 are essential for   invadopodia   formation. We identify the actin regulatory protein, cortactin, as a direct interaction partner for Gab1 and show that a Gab1-cortactin interaction is dependent on the SH3 domain of cortactin and the integrity of the P4/5 region of Gab1. Both cortactin and Gab1 localize to   invadopodia   rosettes in Met-transformed cells and the specific uncoupling of cortactin from Gab1 abrogates   invadopodia   biogenesis and cell invasion downstream from the Met receptor tyrosine kinase. Met localizes to   invadopodia   along with cortactin and promotes phosphorylation of cortactin. These findings provide insights into the molecular mechanisms of   invadopodia   formation and identify Gab1 as a scaffold protein involved in this process. 
  GeneRIFs  
 Met signals through a cortactin-Gab1 scaffold complex, to mediate   invadopodia  . 
  [Pp]odosome(s)?  PubMed  
 Met receptor tyrosine kinase signals through a cortactin-Gab1 scaffold complex, to mediate invadopodia. Invasive carcinoma cells form actin-rich matrix-degrading protrusions called invadopodia. These structures resemble   podosomes   produced by some normal cells and play a crucial role in extracellular matrix remodeling. In cancer, formation of invadopodia is strongly associated with invasive potential. Although deregulated signals from the receptor tyrosine kinase Met (also known as hepatocyte growth factor are linked to cancer metastasis and poor prognosis, its role in invadopodia formation is not known. Here we show that stimulation of breast cancer cells with the ligand for Met, hepatocyte growth factor, promotes invadopodia formation, and in aggressive gastric tumor cells where Met is amplified, invadopodia formation is dependent on Met activity. Using both GRB2-associated-binding protein 1 (Gab1)-null fibroblasts and specific knockdown of Gab1 in tumor cells we show that Met-mediated invadopodia formation and cell invasion requires the scaffold protein Gab1. By a structure-function approach, we demonstrate that two proline-rich motifs (P4/5) within Gab1 are essential for invadopodia formation. We identify the actin regulatory protein, cortactin, as a direct interaction partner for Gab1 and show that a Gab1-cortactin interaction is dependent on the SH3 domain of cortactin and the integrity of the P4/5 region of Gab1. Both cortactin and Gab1 localize to invadopodia rosettes in Met-transformed cells and the specific uncoupling of cortactin from Gab1 abrogates invadopodia biogenesis and cell invasion downstream from the Met receptor tyrosine kinase. Met localizes to invadopodia along with cortactin and promotes phosphorylation of cortactin. These findings provide insights into the molecular mechanisms of invadopodia formation and identify Gab1 as a scaffold protein involved in this process. 
  [Mm]igration  Process  
 regulation of cell   migration   
  PubMed  
 Non-redundant roles of the Gab1 and Gab2 scaffolding adapters in VEGF-mediated signalling,   migration  , and survival of endothelial cells. Gab1 was previously described as a positive modulator of Akt, Src, ERK1/2, endothelial cell   migration  , and capillary formation in response to vascular endothelial growth factor (VEGF). However, its involvement in endothelial cell survival, as well as the potential contribution of the other family member Gab2 to signalling and biological responses remained unknown. Here, we show that Gab2 is tyrosine phosphorylated in a Grb2-dependent manner downstream of activated VEGF receptor-2 (VEGFR2), and that it associates with signalling proteins including PI3K and SHP2, but apparently not with the receptor. Similarly to Gab1, over-expression of Gab2 induces endothelial cell   migration   in response to VEGF, whereas its depletion using siRNAs results in its reduction. Importantly, depletion of both Gab1 and Gab2 leads to an even greater inhibition of VEGF-induced cell   migration  . However, contrary to what has been reported for Gab1, the silencing of Gab2 results in increased Src, Akt and ERK1/2 activation, slightly reduced p38 phosphorylation, and up-regulation of Gab1 protein levels. Accordingly, re-expression of Gab2 in Gab2-/- fibroblasts leads to opposite results, suggesting that the modulation of both Gab2 and Gab1 expression in these conditions might contribute to the impaired signalling observed. Consistent with their opposite roles on Akt, the depletion of Gab1, but not of Gab2, results in reduced FOXO1 phosphorylation and VEGF-mediated endothelial cell survival. Mutation of VEGFR2 Y801 and Y1214, which abrogates the phosphorylation of Gab1, also correlates with inhibition of Akt. Altogether, these results underscore the non-redundant and essential roles of Gab1 and Gab2 in endothelial cells, and suggest major contributions of these proteins during in vivo angiogenesis. 
 Participation of both Gab1 and Gab2 in the activation of the ERK/MAPK pathway by epidermal growth factor. Three members of Gab family docking proteins, Gab1, Gab2 and Gab3, have been identified in humans. Previous studies have found that the hepatocyte growth factor preferentially utilizes Gab1 for signalling, whereas Bcr-Abl selectively signals through Gab2. Gab1-SHP2 interaction has been shown to mediate ERK (extracellular-signal-regulated kinase) activation by EGF (epidermal growth factor). However, it was unclear whether EGF selectively utilizes Gab1 for signalling to ERK and whether Gab2 is dispensable in cells where Gab1 and Gab2 are co-expressed. Using T47D and MCF-7 human breast carcinoma cells that express endogenous Gab1 and Gab2, we examined the role of these docking proteins in EGF-induced ERK activation. It was found that EGF induced a similar amount of SHP2-Gab1 and SHP2-Gab2 complexes. Expression of either SHP2-binding defective Gab1 or Gab2 mutant blocked EGF-induced ERK activation. Down-regulation of either Gab1 or Gab2 by siRNAs (small interfering RNAs) effectively inhibited the EGF-stimulated ERK activation pathway and cell   migration  . Interestingly, the inhibitory effect of Gab1 siRNA could be rescued not only by expression of an exogenous mouse Gab1 but also by an exogenous human Gab2 and vice versa, but not by IRS1 (insulin receptor substrate 1). These results reveal that Gab2 plays a pivotal role in the EGF-induced ERK activation pathway and that it can complement the function of Gab1 in the EGF signalling pathway. Furthermore, Gab1 and Gab2 are critical signalling threshold proteins for ERK activation by EGF. 
 Participation of Gab1 and Gab2 in IL-22-mediated keratinocyte proliferation,   migration  , and differentiation. Interleukin-22 (IL-22) is one of the key mediators of keratinocyte alterations in psoriasis. IL-22 inhibits keratinocyte differentiation and induces the   migration   of human keratinocytes. Grb2-associated binder 1 (Gab1) has been shown to mediate epidermal growth factor-induced epidermal growth and differentiation via interaction with the Src homology-2-containing protein-tyrosine phosphatase (Shp2). In this investigation, we explore the role of Gab1 and Gab2 in IL-22-mediated keratinocyte activities. We show that both Gab1 and Gab2 were tyrosine phosphorylated in IL-22-stimulated HaCaT cells and human primary epidermal keratinocytes and contributed to the activation of Extracellular signal regulated kinase 1/2 (Erk1/2) through interaction with Shp2. We further demonstrate that HaCaT cells infected with adenoviruses expressing Shp2-binding-defective Gab1/2 mutants exhibited decreased cell proliferation and   migration  , as well as increased differentiation. Moreover, similar results were observed in HaCaT cells infected with adenovirus-based small interfering RNAs targeting Gab1 and/or Gab2. Altogether, these data underscore the critical roles of Gab1 and Gab2 in IL-22-mediated HaCaT cell proliferation,   migration  , and differentiation. 
 Analysis of protein-protein interactions in cross-talk pathways reveals CRKL protein as a novel prognostic marker in hepatocellular carcinoma. Deciphering the network of signaling pathways in cancer via protein-protein interactions (PPIs) at the cellular level is a promising approach but remains incomplete. We used an in situ proximity ligation assay to identify and quantify 67 endogenous PPIs among 21 interlinked pathways in two hepatocellular carcinoma (HCC) cells, Huh7 (minimally migratory cells) and Mahlavu (highly migratory cells). We then applied a differential network biology analysis and determined that the novel interaction, CRKL-FLT1, has a high centrality ranking, and the expression of this interaction is strongly correlated with the migratory ability of HCC and other cancer cell lines. Knockdown of CRKL and FLT1 in HCC cells leads to a decrease in cell   migration   via ERK signaling and the epithelial-mesenchymal transition process. Our immunohistochemical analysis shows high expression levels of the CRKL and CRKL-FLT1 pair that strongly correlate with reduced disease-free and overall survival in HCC patient samples, and a multivariate analysis further established CRKL and the CRKL-FLT1 as novel prognosis markers. This study demonstrated that functional exploration of a disease network with interlinked pathways via PPIs can be used to discover novel biomarkers. 
 Participation of Gab1 and Gab2 in IL-22-mediated keratinocyte proliferation,   migration  , and differentiation. Interleukin-22 (IL-22) is one of the key mediators of keratinocyte alterations in psoriasis. IL-22 inhibits keratinocyte differentiation and induces the   migration   of human keratinocytes. Grb2-associated binder 1 (Gab1) has been shown to mediate epidermal growth factor-induced epidermal growth and differentiation via interaction with the Src homology-2-containing protein-tyrosine phosphatase (Shp2). In this investigation, we explore the role of Gab1 and Gab2 in IL-22-mediated keratinocyte activities. We show that both Gab1 and Gab2 were tyrosine phosphorylated in IL-22-stimulated HaCaT cells and human primary epidermal keratinocytes and contributed to the activation of Extracellular signal regulated kinase 1/2 (Erk1/2) through interaction with Shp2. We further demonstrate that HaCaT cells infected with adenoviruses expressing Shp2-binding-defective Gab1/2 mutants exhibited decreased cell proliferation and   migration  , as well as increased differentiation. Moreover, similar results were observed in HaCaT cells infected with adenovirus-based small interfering RNAs targeting Gab1 and/or Gab2. Altogether, these data underscore the critical roles of Gab1 and Gab2 in IL-22-mediated HaCaT cell proliferation,   migration  , and differentiation. 
 Non-redundant roles of the Gab1 and Gab2 scaffolding adapters in VEGF-mediated signalling,   migration  , and survival of endothelial cells. Gab1 was previously described as a positive modulator of Akt, Src, ERK1/2, endothelial cell   migration  , and capillary formation in response to vascular endothelial growth factor (VEGF). However, its involvement in endothelial cell survival, as well as the potential contribution of the other family member Gab2 to signalling and biological responses remained unknown. Here, we show that Gab2 is tyrosine phosphorylated in a Grb2-dependent manner downstream of activated VEGF receptor-2 (VEGFR2), and that it associates with signalling proteins including PI3K and SHP2, but apparently not with the receptor. Similarly to Gab1, over-expression of Gab2 induces endothelial cell   migration   in response to VEGF, whereas its depletion using siRNAs results in its reduction. Importantly, depletion of both Gab1 and Gab2 leads to an even greater inhibition of VEGF-induced cell   migration  . However, contrary to what has been reported for Gab1, the silencing of Gab2 results in increased Src, Akt and ERK1/2 activation, slightly reduced p38 phosphorylation, and up-regulation of Gab1 protein levels. Accordingly, re-expression of Gab2 in Gab2-/- fibroblasts leads to opposite results, suggesting that the modulation of both Gab2 and Gab1 expression in these conditions might contribute to the impaired signalling observed. Consistent with their opposite roles on Akt, the depletion of Gab1, but not of Gab2, results in reduced FOXO1 phosphorylation and VEGF-mediated endothelial cell survival. Mutation of VEGFR2 Y801 and Y1214, which abrogates the phosphorylation of Gab1, also correlates with inhibition of Akt. Altogether, these results underscore the non-redundant and essential roles of Gab1 and Gab2 in endothelial cells, and suggest major contributions of these proteins during in vivo angiogenesis. 
 Participation of both Gab1 and Gab2 in the activation of the ERK/MAPK pathway by epidermal growth factor. Three members of Gab family docking proteins, Gab1, Gab2 and Gab3, have been identified in humans. Previous studies have found that the hepatocyte growth factor preferentially utilizes Gab1 for signalling, whereas Bcr-Abl selectively signals through Gab2. Gab1-SHP2 interaction has been shown to mediate ERK (extracellular-signal-regulated kinase) activation by EGF (epidermal growth factor). However, it was unclear whether EGF selectively utilizes Gab1 for signalling to ERK and whether Gab2 is dispensable in cells where Gab1 and Gab2 are co-expressed. Using T47D and MCF-7 human breast carcinoma cells that express endogenous Gab1 and Gab2, we examined the role of these docking proteins in EGF-induced ERK activation. It was found that EGF induced a similar amount of SHP2-Gab1 and SHP2-Gab2 complexes. Expression of either SHP2-binding defective Gab1 or Gab2 mutant blocked EGF-induced ERK activation. Down-regulation of either Gab1 or Gab2 by siRNAs (small interfering RNAs) effectively inhibited the EGF-stimulated ERK activation pathway and cell   migration  . Interestingly, the inhibitory effect of Gab1 siRNA could be rescued not only by expression of an exogenous mouse Gab1 but also by an exogenous human Gab2 and vice versa, but not by IRS1 (insulin receptor substrate 1). These results reveal that Gab2 plays a pivotal role in the EGF-induced ERK activation pathway and that it can complement the function of Gab1 in the EGF signalling pathway. Furthermore, Gab1 and Gab2 are critical signalling threshold proteins for ERK activation by EGF. 
 The scaffolding adapter Gab1 mediates vascular endothelial growth factor signaling and is required for endothelial cell   migration   and capillary formation. Vascular endothelial growth factor (VEGF) is involved in the promotion of endothelial cell proliferation,   migration  , and capillary formation. These activities are mainly mediated by the VEGFR2 receptor tyrosine kinase that upon stimulation, promotes the activation of numerous proteins including phospholipase Cgamma (PLCgamma), phosphatidylinositol 3-kinase (PI3K), Akt, Src, and ERK1/2. However, the VEGFR2-proximal signaling events leading to the activation of these targets remain ill defined. We have identified the Gab1 adapter as a novel tyrosine-phosphorylated protein in VEGF-stimulated cells. In bovine aortic endothelial cells, Gab1 associates with VEGFR2, Grb2, PI3K, SHP2, Shc, and PLCgamma, and its overexpression enhances VEGF-dependent cell   migration  . Importantly, silencing of Gab1 using small interfering RNAs leads to the impaired activation of PLCgamma, ERK1/2, Src, and Akt; blocks VEGF-induced endothelial cell   migration  ; and perturbs actin reorganization and capillary formation. In addition, co-expression of VEGFR2 with Gab1 mutants unable to bind SHP2 or PI3K in human embryonic kidney 293 cells and bovine aortic endothelial cells mimics the defects observed in Gab1-depleted cells. Our work thus identifies Gab1 as a novel critical regulatory component of endothelial cell   migration   and capillary formation and reveals its key role in the activation of VEGF-evoked signaling pathways required for angiogenesis. 
 MicroRNA-200a Suppresses Cell Invasion and   Migration   by Directly Targeting GAB1 in Hepatocellular Carcinoma. MicroRNA-200a (miR-200a) is frequently downregulated in most cancer types and plays an important role in carcinogenesis and cancer progression. In this study, we determined that miR-200a was downregulated in hepatocellular carcinoma (HCC) tissues and cell lines, consistent with the results of our previous study. Because a previous study suggested that downregulation of miR-200a is correlated with HCC metastasis, we aimed to elucidate the mechanism underlying the role of miR-200a in metastasis in HCC. Here we observed that overexpression of miR-200a resulted in suppression of HCC metastatic ability, including HCC cell   migration  , invasion, and metastasis, in vitro and in vivo. Furthermore, bioinformatics and luciferase reporter assays indicated that GAB1 is a direct target of miR-200a. Inhibition of GAB1 resulted in substantially decreased cell invasion and   migration   similar to that observed with overexpression of miR-200a in HCC cell lines, whereas restoration of GAB1 partially rescued the inhibitory effects of miR-200a. Taken together, these data provide novel information for comprehending the tumor-suppressive role of miR-200a in HCC pathogenesis through inhibition of GAB1 translation. 
 Gab1 regulates proliferation and   migration   through the PI3K/Akt signaling pathway in intrahepatic cholangiocarcinoma. Intrahepatic cholangiocarcinoma is the second most common primary malignant tumor of the liver, and it originates from the intrahepatic biliary duct epithelium. Prognosis is poor due to lack of effective comprehensive treatments. In this study, we assessed the expression of Gab1, VEGFR-2, and MMP-9 in intrahepatic cholangiocarcinoma solid tumors by immunohistochemistry and determined whether their expression was associated with clinical and pathological features. We found that expression of Gab1, VEGFR-2, and MMP-9 was highly and positively correlated with each other and with lymph node metastasis and TNM stage in intrahepatic cholangiocarcinoma tissues. Interference of Gab1 and VEGFR-2 expression via siRNA in the intrahepatic cholangiocarcinoma cell line RBE resulted in decreased PI3K/Akt pathway activity. Inhibition of Gab1 and VEGFR-2 expression also caused decreased cell proliferation, cell cycle arrested in G1 phase, increased apoptosis, and decreased invasion in RBE cells. These results suggest that Gab1, VEGFR-2, and MMP-9 contribute significantly to the highly malignant behavior of intrahepatic cholangiocarcinoma. The regulation of growth, apoptosis, and invasion by Gab1 through the VEGFR-2/Gab1/PI3K/Akt signaling pathway may represent potential targets for improving the treatment of intrahepatic cholangiocarcinoma. 
 The adaptor protein Gab1 couples the stimulation of vascular endothelial growth factor receptor-2 to the activation of phosphoinositide 3-kinase. Phosphoinositide 3-kinase (PI3K) mediates essential functions of vascular endothelial growth factor (VEGF), including the stimulation of endothelial cell proliferation and   migration  . Nevertheless, the mechanisms coupling the receptor VEGFR-2 to PI3K remain obscure. We observed that the Grb2-bound adapter Gab1 is tyrosine-phosphorylated and relocated to membrane fractions upon VEGF stimulation of endothelial cells. We could detect the PI3K regulatory subunit p85 in immunoprecipitates of endogenous Gab1, and vice versa, and measure a Gab1-associated lipid kinase activity upon VEGF stimulation. Furthermore, transfection of the Gab1-YF3 mutant lacking all p85-binding sites strongly repressed PI3K activation measured in vitro. Moreover, Gab1-YF3 severely decreased the cellular amount of phosphatidylinositol 3,4,5-trisphosphate (PtdIns(3,4,5)P3) generated in response to VEGF. Furthermore, adenoviral expression of Gab1-YF3 suppressed both Akt phosphorylation and recovery of wounded human umbilical vein endothelial cell monolayers, a VEGF-dependent process involving cell   migration   and proliferation under PI3K control. Transfection of other Gab1 mutants, lacking Grb2-binding sites or the pleckstrin homology (PH) domain, also prevented Akt activation, further demonstrating Gab1 involvement in PI3K activation. These mutants were also used to show that interactions with both Grb2 and PtdIns(3,4,5)P3 mediate Gab1 recruitment by VEGFR-2. Importantly, Gab1 mobilization was impaired by (i) PI3K inhibitors, (ii) deletion of Gab1 PH domain, (iii) PTEN (phosphatase and tensin homolog deleted on chromosome 10) overexpression to repress PtdIns(3,4,5)P3 production, and (iv) overexpression of a competitor PH domain for PtdIns(3,4,5)P3 binding, which altogether demonstrated that PI3K is also an upstream regulator of Gab1. Gab1 thus appears as a primary actor in coupling VEGFR-2 to PI3K/Akt, recruited through an amplification loop involving PtdIns(3,4,5)P3 and its PH domain. 
 Essential role of Gab1 for signaling by the c-Met receptor in vivo. The docking protein Gab1 binds phosphorylated c-Met receptor tyrosine kinase directly and mediates signals of c-Met in cell culture. Gab1 is phosphorylated by c-Met and by other receptor and nonreceptor tyrosine kinases. Here, we report the functional analysis of Gab1 by targeted mutagenesis in the mouse, and compare the phenotypes of the Gab1 and c-Met mutations. Gab1 is essential for several steps in development:   migration   of myogenic precursor cells into the limb anlage is impaired in Gab1-/- embryos. As a consequence, extensor muscle groups of the forelimbs are virtually absent, and the flexor muscles reach less far. Fewer hindlimb muscles exist, which are smaller and disorganized. Muscles in the diaphragm, which also originate from migratory precursors, are missing. Moreover, Gab1-/- embryos die in a broad time window between E13.5 and E18.5, and display reduced liver size and placental defects. The labyrinth layer, but not the spongiotrophoblast layer, of the placenta is severely reduced, resulting in impaired communication between maternal and fetal circulation. Thus, extensive similarities between the phenotypes of c-Met and HGF/SF mutant mice exist, and the muscle   migration   phenotype is even more pronounced in Gab1-/-:c-Met+/- embryos. This is genetic evidence that Gab1 is essential for c-Met signaling in vivo. Analogy exists to signal transmission by insulin receptors, which require IRS1 and IRS2 as specific docking proteins. 
 Requirement of Gαi1/3-Gab1 signaling complex for keratinocyte growth factor-induced PI3K-AKT-mTORC1 activation. Keratinocyte growth factor (KGF), also termed as fibroblast growth factor-7, promotes proliferation,   migratio  n, and adhesion of skin keratinocytes via binding to keratinocyte growth factor receptor (KGFR) and subsequent activation of downstream signaling including the PI3K-AKT-mTORC1 pathway. Here, we found that the α-subunits of the G proteins (Gαi1/3) and growth factor receptor binding 2-associated binding protein 1 (Gab1) are required for this activation process. With KGF stimulation, Gαi1/3 formed a complex with KGFR and was required for subsequent Gab1 recruitment, phosphorylation, and following PI3K-p85 activation. In addition, Gαi1/3 short hairpin RNA knockdown largely inhibited KGF-induced cell proliferat  ion, migr  ation, and the accumulation of cyclin D1/fibronectin in cultured skin keratinocytes. Furthermore, we observed increased expression of Gαi1/3 in wounded human skin and keloid skin tissues, suggesting the possible involvement of Gαi1/3 in wound healing and keloid formation. Overall, we suggest that Gαi1/3 proteins lie downstream of KGFR, but upstream of Gab1-mediated activation of PI3K-AKT-mTORC1 signaling, thus revealing a role for Gαi proteins in mediating KGFR signali  ng, cell   migration, and possible wound healing. 
 Signaling of hepatocyte growth factor/scatter factor (HGF) to the small GTPase Rap1 via the large docking protein Gab1 and the adapter protein CRKL. Hepatocyte growth factor (HGF; scatter factor) is a multipotent protein with mitogenic, motogenic, and developmental functions. Upon activation, the HGF-receptor c-Met binds and phosphorylates the multisite docking protein Gab1. Besides binding motifs for phosphatidylinositol 3-kinase and Grb2, Gab 1 contains multiple Tyr-X-X-Pro (YXXP) motifs which, when phosphorylated, are potential binding sites for the adapter proteins c-Crk and Crk-like (CRKL). Stimulation of human embryonic kidney cells (HEK293) with HGF leads to Gab1 association with CRKL. The Gab1-CRKL interaction requires both, the SH2 domain of CRKL and the region containing the YXXP motifs in Gab1. CRKL binds via its first SH3 domain to several downstream signal transducers, including C3G an activator of the small GTPase Rap1. Indeed, Rap1 was rapidly activated after HGF stimulation of HEK293 cells. Rap1 activation through HGF was suppressed through transfection of a truncated C3G protein which only contains the SH3-binding motifs of C3G. Transfection of nonmutated Gab1 led to a strong increase of Rap1.GTP in the absence of HGF. In contrast, transfection of the GabDeltaYXXP mutant abolished the elevation of Rap1.GTP by HGF. A replating assay indicated that HGF decreases the adhesion of HEK293 cells. The results presented here delineate a novel signaling pathway from HGF to the GTPase Rap1 which depends on the interaction of the adapter protein CRKL with the exchange factor C3G and could be linked to cell   migration  . 
 Roles of Gab1 and SHP2 in paxillin tyrosine dephosphorylation and Src activation in response to epidermal growth factor. Epidermal growth factor (EGF) induces paxillin tyrosine dephosphorylation and Src activation, but the signaling pathways that mediate these responses were largely undefined. We found that Gab1, a docking protein for the SHP2 protein-tyrosine phosphatase in EGF-stimulated cells, was associated with paxillin. SHP2 dephosphorylated paxillin and caused dissociation of Csk, a negative regulator of Src, from paxillin but had no effect on paxillin-Src association. A lower level of Src Tyr-530 phosphorylation was detected in paxillin-associated Src in EGF-stimulated cells. Expression of an SHP2 binding defective mutant of Gab1 (Gab1FF) or a catalytically inactive mutant of SHP2 (SHP2DN) prevented paxillin tyrosine dephosphorylation and Src activation induced by EGF. Importantly, Gab1FF blocked paxillin-SHP2 complex formation, Src Tyr-530 dephosphorylation, Erk activation, and cell   migration   induced by EGF. Inhibition of Src tyrosine kinase activity abrogated EGF-stimulated Erk activation and cell   migration  . Together, these results reveal that Gab1 recruits SHP2 to dephosphorylate paxillin, leading to dissociation of Csk from the paxillin-Src complex and Src activation and that Src is an SHP2 effector involved in EGF-stimulated Erk activation and cell   migration  . 
 MicroRNA-409-3p suppresses colorectal cancer invasion and metastasis partly by targeting GAB1 expression. Colorectal cancer (CRC) is one of the most common cancers worldwide and its metastasis accounts for the majority of deaths. However, the molecular mechanisms underlying CRC progression are not well characterized. In this study, we identified miR-409-3p as a tumor suppressor of CRC. MiR-409-3p expression was significantly downregulated in CRC tissue compared to adjacent non-tumor tissue, and reduced miR-409-3p expression was correlated with CRC metastasis. In vitro and in vivo studies revealed that miR-409-3p negatively regulated CRC metastatic capacities, including suppressing cancer cell   migration  , invasion and metastasis. To explore the mechanism of action of miR-409-3p, we adopted a pathway and pathophysiological event-based target screening and validation approach, and found nine known metastasis-related genes as potential targets. The 3'-UTR binding assays between the candidates and miR-409-3p suggested that only GAB1, NR4A2 and LMO4 were directly regulated by the miRNA. However, endogenous expression analysis revealed that only GAB1 was modulated by miR-409-3p in CRC cells at both the mRNA and protein levels. Furthermore, we provided evidence to conclude that GAB1 was partially responsible for miR-409-3p-mediated metastasis. Taken together, our data demonstrate that miR-409-3p is a metastatic suppressor, and post-transcriptional inhibition of the oncoprotein GAB1 is one of the mechanisms of action of this miRNA. Our finding suggests miR-409-3p might be a novel target for CRC metastasis treatment. 
 Down-regulation of Gab1 inhibits cell proliferation and   migration   in hilar cholangiocarcinoma. Hilar cholangiocarcinoma is a highly aggressive malignancy originating from the hilar biliary duct epithelium. Due to few effective comprehensive treatments, the prognosis of hilar cholangiocarcinoma is poor. In this study, immunohistochemistry was first used to detect and analyze the expression of Gab1, VEGFR-2, and MMP-9 in hilar cholangiocarcinoma solid tumors and the relationships to the clinical pathological features. Furthermore, Gab1 and VEGFR-2 siRNA were used to interfere the hilar cholangiocarcinoma cell line ICBD-1 and then detect the PI3K/Akt signaling pathway, MMP-9 levels and malignant biological behaviors of tumor cells. The data showed that 1. Gab1, VEGFR-2, and MMP-9 were highly expressed and positively correlated with each other in hilar cholangiocarcinoma tissues, which were related to lymph node metastasis and differentiation. 2. After Gab1 or VEGFR-2 siRNA interference, PI3K/Akt pathway activity and MMP-9 levels were decreased in ICBD-1 cells. At the same time, cell proliferation decreased, cell cycle arrested in G1 phase, apoptosis increased and invasion decreased. These results suggest that the expression of Gab1, VEGFR-2, and MMP-9 are significantly related to the malignant biological behavior of hilar cholangiocarcinoma. Gab1 regulates growth, apoptosis and invasion through the VEGFR-2/Gab1/PI3K/Akt signaling pathway in hilar cholangiocarcinoma cells and influences the invasion of tumor cells via MMP-9. 
  GeneRIFs  
 these data underscore the critical roles of Gab1 and Gab2 in IL-22-mediated HaCaT cell proliferation,   migration  , and differentiation. 
 Gab1 is a novel critical regulatory component of endothelial cell   migration   and capillary formation with a key role in the activation of VEGF-evoked signaling pathways required for angiogenesis 
  [Ee]xtracellular [Mm]atrix  PubMed  
 Met receptor tyrosine kinase signals through a cortactin-Gab1 scaffold complex, to mediate invadopodia. Invasive carcinoma cells form actin-rich matrix-degrading protrusions called invadopodia. These structures resemble podosomes produced by some normal cells and play a crucial role in   extracellular matrix   remodeling. In cancer, formation of invadopodia is strongly associated with invasive potential. Although deregulated signals from the receptor tyrosine kinase Met (also known as hepatocyte growth factor are linked to cancer metastasis and poor prognosis, its role in invadopodia formation is not known. Here we show that stimulation of breast cancer cells with the ligand for Met, hepatocyte growth factor, promotes invadopodia formation, and in aggressive gastric tumor cells where Met is amplified, invadopodia formation is dependent on Met activity. Using both GRB2-associated-binding protein 1 (Gab1)-null fibroblasts and specific knockdown of Gab1 in tumor cells we show that Met-mediated invadopodia formation and cell invasion requires the scaffold protein Gab1. By a structure-function approach, we demonstrate that two proline-rich motifs (P4/5) within Gab1 are essential for invadopodia formation. We identify the actin regulatory protein, cortactin, as a direct interaction partner for Gab1 and show that a Gab1-cortactin interaction is dependent on the SH3 domain of cortactin and the integrity of the P4/5 region of Gab1. Both cortactin and Gab1 localize to invadopodia rosettes in Met-transformed cells and the specific uncoupling of cortactin from Gab1 abrogates invadopodia biogenesis and cell invasion downstream from the Met receptor tyrosine kinase. Met localizes to invadopodia along with cortactin and promotes phosphorylation of cortactin. These findings provide insights into the molecular mechanisms of invadopodia formation and identify Gab1 as a scaffold protein involved in this process. 
  [Ii]nvasive  PubMed  
 Met receptor tyrosine kinase signals through a cortactin-Gab1 scaffold complex, to mediate invadopodia.   Invasive   carcinoma cells form actin-rich matrix-degrading protrusions called invadopodia. These structures resemble podosomes produced by some normal cells and play a crucial role in extracellular matrix remodeling. In cancer, formation of invadopodia is strongly associated with   invasive   potential. Although deregulated signals from the receptor tyrosine kinase Met (also known as hepatocyte growth factor are linked to cancer metastasis and poor prognosis, its role in invadopodia formation is not known. Here we show that stimulation of breast cancer cells with the ligand for Met, hepatocyte growth factor, promotes invadopodia formation, and in aggressive gastric tumor cells where Met is amplified, invadopodia formation is dependent on Met activity. Using both GRB2-associated-binding protein 1 (Gab1)-null fibroblasts and specific knockdown of Gab1 in tumor cells we show that Met-mediated invadopodia formation and cell invasion requires the scaffold protein Gab1. By a structure-function approach, we demonstrate that two proline-rich motifs (P4/5) within Gab1 are essential for invadopodia formation. We identify the actin regulatory protein, cortactin, as a direct interaction partner for Gab1 and show that a Gab1-cortactin interaction is dependent on the SH3 domain of cortactin and the integrity of the P4/5 region of Gab1. Both cortactin and Gab1 localize to invadopodia rosettes in Met-transformed cells and the specific uncoupling of cortactin from Gab1 abrogates invadopodia biogenesis and cell invasion downstream from the Met receptor tyrosine kinase. Met localizes to invadopodia along with cortactin and promotes phosphorylation of cortactin. These findings provide insights into the molecular mechanisms of invadopodia formation and identify Gab1 as a scaffold protein involved in this process. 
 Crk synergizes with epidermal growth factor for epithelial invasion and morphogenesis and is required for the met morphogenic program. Activation of the Met receptor tyrosine kinase through its ligand, hepatocyte growth factor, stimulates cell spreading, cell dispersal, and the inherent morphogenic program of various epithelial cell lines. Although both hepatocyte growth factor and epidermal growth factor (EGF) can activate downstream signaling pathways in Madin-Darby canine kidney epithelial cells, EGF fails to promote the breakdown of cell-cell junctional complexes and initiate an   invasive   morphogenic program. We have undertaken a strategy to identify signals that synergize with EGF in this process. We provide evidence that the overexpression of the CrkII adapter protein complements EGF-stimulated pathways to induce cell dispersal in two-dimensional cultures and cell invasion and branching morphogenesis in three-dimensional collagen gels. This finding correlates with the ability of CrkII to promote the breakdown of adherens junctions in stable cell lines and the ability of EGF to stimulate enhanced Rac activity in cells overexpressing CrkII. We have previously shown that the Gab1-docking protein is required for branching morphogenesis downstream of the Met receptor. Consistent with a role for CrkII in promoting EGF-dependent branching morphogenesis, the binding of Gab1 to CrkII is required for the branching morphogenic program downstream of Met. Together, our data support a role for the CrkII adapter protein in epithelial invasion and morphogenesis and underscores the importance of considering the synergistic actions of signaling pathways in cancer progression. 
 Protein kinase C-betaII represses hepatocyte growth factor-induced invasion by preventing the association of adapter protein Gab1 and phosphatidylinositol 3-kinase in melanoma cells. The hepatocyte growth factor (HGF) signaling pathway was examined in human normal melanocytes and three malignant melanoma cell lines. HGF-induced activation of c-Met, its receptor-tyrosine kinase, was observed in both melanocytes and melanoma cells, whereas phosphatidylinositol 3-kinase (PI3K), a downstream target of c-Met, was not activated in the melanocytes but enhanced in the melanoma cell lines. The electrophoretic mobility of Gab1, the scaffolding adapter protein that couples activated c-Met and PI3K, was slower in the melanocytes than that in the melanoma cells, and the mobility shifted to that of the melanoma cells after treatment with alkaline phosphatase, indicating that Gab1 is highly phosphorylated on serine and threonine in the melanocytes. Introduction of protein kinase C (PKC)-betaII into the melanoma cells, which is expressed in melanocytes but absent in melanoma cells, resulted in serine and threonine phosphorylation of Gab1 and also prevented tyrosine phosphorylation of Gab1 and its association with PI3K. Furthermore, the introduction of PKC-betaII suppressed HGF-induced activation of PI3K, and attenuated the in vitro invasion activity of the melanoma cells. These results indicate that the HGF signaling process from Gab1 to PI3K is negatively regulated by PKC-betaII, and its loss is critical for melanoma cells to gain   invasive   potential. 
 Adaptor molecule Crk is required for sustained phosphorylation of Grb2-associated binder 1 and hepatocyte growth factor-induced cell motility of human synovial sarcoma cell lines. Activation of the c-Met receptor tyrosine kinase through its ligand, hepatocyte growth factor (HGF), promotes mitogenic, motogenic, and morphogenic cellular responses. Aberrant HGF/c-Met signaling has been strongly implicated in tumor cell invasion and metastasis. Both HGF and its receptor c-Met have been shown to be overexpressed in human synovial sarcoma, which often metastasizes to the lung; however, little is known about HGF-mediated biological effects in this sarcoma. Here, we provide evidence that Crk adaptor protein is required for the sustained phosphorylation of c-Met-docking protein Grb2-associated binder 1 (Gab1) in response to HGF, leading to the enhanced cell motility of human synovial sarcoma cell lines SYO-1, HS-SY-II, and Fuji. HGF stimulation induced the sustained phosphorylation on Y307 of Gab1 where Crk was recruited. Crk knockdown by RNA interference disturbed this HGF-induced tyrosine phosphorylation of Gab1. By mutational analysis, we identified that Src homology 2 domain of Crk is indispensable for the induction of the phosphorylation on multiple Tyr-X-X-Pro motifs containing Y307 in Gab1. HGF remarkably stimulated cell motility and scattering of synovial sarcoma cell lines, consistent with the prominent activation of Rac1, extreme filopodia formation, and membrane ruffling. Importantly, the elimination of Crk in these cells induced the disorganization of actin cytoskeleton and complete abolishment of HGF-mediated Rac1 activation and cell motility. Time-lapse microscopic analysis revealed the significant attenuation in scattering of Crk knockdown cells following HGF treatment. Furthermore, the depletion of Crk remarkably inhibited the tumor formation and its   invasive   growth in vivo. These results suggest that the sustained phosphorylation of Gab1 through Crk in response to HGF contributes to the prominent activation of Rac1 leading to enhanced cell motility, scattering, and cell invasion, which may support the crucial role of Crk in the aggressiveness of human synovial sarcoma. 
  GeneRIFs  
 HGF signaling process from Gab1 to PI3K is negatively regulated by PKC-betaII, and its loss is critical for melanoma cells to gain   invasive   potential. 
  [Mm]etastasize  PubMed  
 Adaptor molecule Crk is required for sustained phosphorylation of Grb2-associated binder 1 and hepatocyte growth factor-induced cell motility of human synovial sarcoma cell lines. Activation of the c-Met receptor tyrosine kinase through its ligand, hepatocyte growth factor (HGF), promotes mitogenic, motogenic, and morphogenic cellular responses. Aberrant HGF/c-Met signaling has been strongly implicated in tumor cell invasion and metastasis. Both HGF and its receptor c-Met have been shown to be overexpressed in human synovial sarcoma, which often   metastasize  s to the lung; however, little is known about HGF-mediated biological effects in this sarcoma. Here, we provide evidence that Crk adaptor protein is required for the sustained phosphorylation of c-Met-docking protein Grb2-associated binder 1 (Gab1) in response to HGF, leading to the enhanced cell motility of human synovial sarcoma cell lines SYO-1, HS-SY-II, and Fuji. HGF stimulation induced the sustained phosphorylation on Y307 of Gab1 where Crk was recruited. Crk knockdown by RNA interference disturbed this HGF-induced tyrosine phosphorylation of Gab1. By mutational analysis, we identified that Src homology 2 domain of Crk is indispensable for the induction of the phosphorylation on multiple Tyr-X-X-Pro motifs containing Y307 in Gab1. HGF remarkably stimulated cell motility and scattering of synovial sarcoma cell lines, consistent with the prominent activation of Rac1, extreme filopodia formation, and membrane ruffling. Importantly, the elimination of Crk in these cells induced the disorganization of actin cytoskeleton and complete abolishment of HGF-mediated Rac1 activation and cell motility. Time-lapse microscopic analysis revealed the significant attenuation in scattering of Crk knockdown cells following HGF treatment. Furthermore, the depletion of Crk remarkably inhibited the tumor formation and its invasive growth in vivo. These results suggest that the sustained phosphorylation of Gab1 through Crk in response to HGF contributes to the prominent activation of Rac1 leading to enhanced cell motility, scattering, and cell invasion, which may support the crucial role of Crk in the aggressiveness of human synovial sarcoma. 
  [Mm]etastasis  PubMed  
 MicroRNA-200a Suppresses Cell Invasion and Migration by Directly Targeting GAB1 in Hepatocellular Carcinoma. MicroRNA-200a (miR-200a) is frequently downregulated in most cancer types and plays an important role in carcinogenesis and cancer progression. In this study, we determined that miR-200a was downregulated in hepatocellular carcinoma (HCC) tissues and cell lines, consistent with the results of our previous study. Because a previous study suggested that downregulation of miR-200a is correlated with HCC   metastasis  , we aimed to elucidate the mechanism underlying the role of miR-200a in   metastasis   in HCC. Here we observed that overexpression of miR-200a resulted in suppression of HCC metastatic ability, including HCC cell migration, invasion, and   metastasis  , in vitro and in vivo. Furthermore, bioinformatics and luciferase reporter assays indicated that GAB1 is a direct target of miR-200a. Inhibition of GAB1 resulted in substantially decreased cell invasion and migration similar to that observed with overexpression of miR-200a in HCC cell lines, whereas restoration of GAB1 partially rescued the inhibitory effects of miR-200a. Taken together, these data provide novel information for comprehending the tumor-suppressive role of miR-200a in HCC pathogenesis through inhibition of GAB1 translation. 
 Met receptor tyrosine kinase signals through a cortactin-Gab1 scaffold complex, to mediate invadopodia. Invasive carcinoma cells form actin-rich matrix-degrading protrusions called invadopodia. These structures resemble podosomes produced by some normal cells and play a crucial role in extracellular matrix remodeling. In cancer, formation of invadopodia is strongly associated with invasive potential. Although deregulated signals from the receptor tyrosine kinase Met (also known as hepatocyte growth factor are linked to cancer   metastasis   and poor prognosis, its role in invadopodia formation is not known. Here we show that stimulation of breast cancer cells with the ligand for Met, hepatocyte growth factor, promotes invadopodia formation, and in aggressive gastric tumor cells where Met is amplified, invadopodia formation is dependent on Met activity. Using both GRB2-associated-binding protein 1 (Gab1)-null fibroblasts and specific knockdown of Gab1 in tumor cells we show that Met-mediated invadopodia formation and cell invasion requires the scaffold protein Gab1. By a structure-function approach, we demonstrate that two proline-rich motifs (P4/5) within Gab1 are essential for invadopodia formation. We identify the actin regulatory protein, cortactin, as a direct interaction partner for Gab1 and show that a Gab1-cortactin interaction is dependent on the SH3 domain of cortactin and the integrity of the P4/5 region of Gab1. Both cortactin and Gab1 localize to invadopodia rosettes in Met-transformed cells and the specific uncoupling of cortactin from Gab1 abrogates invadopodia biogenesis and cell invasion downstream from the Met receptor tyrosine kinase. Met localizes to invadopodia along with cortactin and promotes phosphorylation of cortactin. These findings provide insights into the molecular mechanisms of invadopodia formation and identify Gab1 as a scaffold protein involved in this process. 
 Gab1 regulates proliferation and migration through the PI3K/Akt signaling pathway in intrahepatic cholangiocarcinoma. Intrahepatic cholangiocarcinoma is the second most common primary malignant tumor of the liver, and it originates from the intrahepatic biliary duct epithelium. Prognosis is poor due to lack of effective comprehensive treatments. In this study, we assessed the expression of Gab1, VEGFR-2, and MMP-9 in intrahepatic cholangiocarcinoma solid tumors by immunohistochemistry and determined whether their expression was associated with clinical and pathological features. We found that expression of Gab1, VEGFR-2, and MMP-9 was highly and positively correlated with each other and with lymph node   metastasis   and TNM stage in intrahepatic cholangiocarcinoma tissues. Interference of Gab1 and VEGFR-2 expression via siRNA in the intrahepatic cholangiocarcinoma cell line RBE resulted in decreased PI3K/Akt pathway activity. Inhibition of Gab1 and VEGFR-2 expression also caused decreased cell proliferation, cell cycle arrested in G1 phase, increased apoptosis, and decreased invasion in RBE cells. These results suggest that Gab1, VEGFR-2, and MMP-9 contribute significantly to the highly malignant behavior of intrahepatic cholangiocarcinoma. The regulation of growth, apoptosis, and invasion by Gab1 through the VEGFR-2/Gab1/PI3K/Akt signaling pathway may represent potential targets for improving the treatment of intrahepatic cholangiocarcinoma. 
 Expression of Gab1 Is Associated with Poor Prognosis of Patients with Epithelial Ovarian Cancer. Growth factor receptor-bound protein-2 (Grb2) can act as the scaffold protein recruiting other molecules to the stimulated receptors. Grb2-associated binding protein 1 (Gab1) is involved in cell proliferation, and its expression may enhance the carcinogenesis and cancer progression. However, the function of Gab1 remains to be investigated. Epithelial ovarian cancer (EOC) is the most lethal malignancy in the female reproductive system with increasing incidence and unsatisfied overall survival (OS). We investigated the expression of Gab1 in EOC tissues and the correlations between Gab1 expression and the clinicopathological characteristics of patients with EOC using Spearman rank test. The staining results were evaluated based on both the percentage of Gab1-positive tumor cells and the staining intensity for Gab1 expression. Kaplan-Meier survival analysis and Cox proportional hazards analysis were used to compare the postoperative OS between EOC patients with high Gab1 expression and those with low Gab1 expression. The high expression of Gab1 was positively correlated with advanced FIGO stage and lymph node   metastasis   of EOC. Univariate analysis showed that advanced FIGO stage, pathological grade, lymph node   metastasis   or Gab1 expression were associated with poor OS. Moreover, multivariate analysis revealed that Gab1 expression could be an independent prognostic factor for the poor OS of EOC patients (P = 0.042). We propose that Gab1 expression is correlated with poor prognosis of EOC patients and may act as an independent prognostic indicator. 
 Gab1 regulates SDF-1-induced progression via inhibition of apoptosis pathway induced by PI3K/AKT/Bcl-2/BAX pathway in human chondrosarcoma. In recent decades, the stromal cell-derived factor-l (SDF-1) and Gab1 have been investigated to be involved in oncogenesis. However, it is scarcely reported that SDF-1-Gab1 pathway mediates proliferation and apoptosis in human chondrosarcoma (CS). In this study, we assessed the expression of Gab1 in 90 CS solid tumors by immunohistochemistry, immunoblotting, and qRT-PCR, and then, some in vitro assays were also applied to CS cells treated with SDF-1. We observed that the overexpression of Gab1 was positively correlated with lung   metastasis   and recurrence, and acts as an independent prognostic factor for CS patients. Gab1 expression was up-regulated in response to SDF-1 stimulation in CS cell line JJ012, SW1353, L3252. Overexpression of Gab1 increased Bcl-2/BAX ratio to promote cell growth via PI3K/AKT. On the other hand, silencing of Gab1 accelerated apoptosis and repressed the growth of CS cells, which further caused the inhibition of G1/S phase transition and decreased invasion capacity in CS cell lines. In vivo assay identified that the knockdown of Gab1 interfered with the tumor mass formation. In conclusion, our data identified overexpression of Gab1 in CS tissues, and Gab1 can be recommended as a novel biomarker for diagnosis and prognosis in patients with CS. Additionally, PI3K/AKT/Bcl-2/BAX axis was involved in Gab1-induced CS progression, indicating Gab1 might act as a new target for the treatment of CS patients. 
 MicroRNA-409-3p suppresses colorectal cancer invasion and   metastasis   partly by targeting GAB1 expression. Colorectal cancer (CRC) is one of the most common cancers worldwide and its   metastasis   accounts for the majority of deaths. However, the molecular mechanisms underlying CRC progression are not well characterized. In this study, we identified miR-409-3p as a tumor suppressor of CRC. MiR-409-3p expression was significantly downregulated in CRC tissue compared to adjacent non-tumor tissue, and reduced miR-409-3p expression was correlated with CRC   metastasis  . In vitro and in vivo studies revealed that miR-409-3p negatively regulated CRC metastatic capacities, including suppressing cancer cell migration, invasion and   metastasis  . To explore the mechanism of action of miR-409-3p, we adopted a pathway and pathophysiological event-based target screening and validation approach, and found nine known   metastasis  -related genes as potential targets. The 3'-UTR binding assays between the candidates and miR-409-3p suggested that only GAB1, NR4A2 and LMO4 were directly regulated by the miRNA. However, endogenous expression analysis revealed that only GAB1 was modulated by miR-409-3p in CRC cells at both the mRNA and protein levels. Furthermore, we provided evidence to conclude that GAB1 was partially responsible for miR-409-3p-mediated   metastasis  . Taken together, our data demonstrate that miR-409-3p is a metastatic suppressor, and post-transcriptional inhibition of the oncoprotein GAB1 is one of the mechanisms of action of this miRNA. Our finding suggests miR-409-3p might be a novel target for CRC   metastasis   treatment. 
 Down-regulation of Gab1 inhibits cell proliferation and migration in hilar cholangiocarcinoma. Hilar cholangiocarcinoma is a highly aggressive malignancy originating from the hilar biliary duct epithelium. Due to few effective comprehensive treatments, the prognosis of hilar cholangiocarcinoma is poor. In this study, immunohistochemistry was first used to detect and analyze the expression of Gab1, VEGFR-2, and MMP-9 in hilar cholangiocarcinoma solid tumors and the relationships to the clinical pathological features. Furthermore, Gab1 and VEGFR-2 siRNA were used to interfere the hilar cholangiocarcinoma cell line ICBD-1 and then detect the PI3K/Akt signaling pathway, MMP-9 levels and malignant biological behaviors of tumor cells. The data showed that 1. Gab1, VEGFR-2, and MMP-9 were highly expressed and positively correlated with each other in hilar cholangiocarcinoma tissues, which were related to lymph node   metastasis   and differentiation. 2. After Gab1 or VEGFR-2 siRNA interference, PI3K/Akt pathway activity and MMP-9 levels were decreased in ICBD-1 cells. At the same time, cell proliferation decreased, cell cycle arrested in G1 phase, apoptosis increased and invasion decreased. These results suggest that the expression of Gab1, VEGFR-2, and MMP-9 are significantly related to the malignant biological behavior of hilar cholangiocarcinoma. Gab1 regulates growth, apoptosis and invasion through the VEGFR-2/Gab1/PI3K/Akt signaling pathway in hilar cholangiocarcinoma cells and influences the invasion of tumor cells via MMP-9. 
 Adaptor molecule Crk is required for sustained phosphorylation of Grb2-associated binder 1 and hepatocyte growth factor-induced cell motility of human synovial sarcoma cell lines. Activation of the c-Met receptor tyrosine kinase through its ligand, hepatocyte growth factor (HGF), promotes mitogenic, motogenic, and morphogenic cellular responses. Aberrant HGF/c-Met signaling has been strongly implicated in tumor cell invasion and   metastasis  . Both HGF and its receptor c-Met have been shown to be overexpressed in human synovial sarcoma, which often metastasizes to the lung; however, little is known about HGF-mediated biological effects in this sarcoma. Here, we provide evidence that Crk adaptor protein is required for the sustained phosphorylation of c-Met-docking protein Grb2-associated binder 1 (Gab1) in response to HGF, leading to the enhanced cell motility of human synovial sarcoma cell lines SYO-1, HS-SY-II, and Fuji. HGF stimulation induced the sustained phosphorylation on Y307 of Gab1 where Crk was recruited. Crk knockdown by RNA interference disturbed this HGF-induced tyrosine phosphorylation of Gab1. By mutational analysis, we identified that Src homology 2 domain of Crk is indispensable for the induction of the phosphorylation on multiple Tyr-X-X-Pro motifs containing Y307 in Gab1. HGF remarkably stimulated cell motility and scattering of synovial sarcoma cell lines, consistent with the prominent activation of Rac1, extreme filopodia formation, and membrane ruffling. Importantly, the elimination of Crk in these cells induced the disorganization of actin cytoskeleton and complete abolishment of HGF-mediated Rac1 activation and cell motility. Time-lapse microscopic analysis revealed the significant attenuation in scattering of Crk knockdown cells following HGF treatment. Furthermore, the depletion of Crk remarkably inhibited the tumor formation and its invasive growth in vivo. These results suggest that the sustained phosphorylation of Gab1 through Crk in response to HGF contributes to the prominent activation of Rac1 leading to enhanced cell motility, scattering, and cell invasion, which may support the crucial role of Crk in the aggressiveness of human synovial sarcoma. 
  GeneRIFs  
 We found that expression of Gab1, VEGFR-2, and MMP-9 was highly and positively correlated with each other and with lymph node   metastasis   and TNM stage in intrahepatic cholangiocarcinoma tissues 
  [Cc]ell [Ii]nvasion  PubMed  
 MicroRNA-200a Suppresses   Cell Invasion   and Migration by Directly Targeting GAB1 in Hepatocellular Carcinoma. MicroRNA-200a (miR-200a) is frequently downregulated in most cancer types and plays an important role in carcinogenesis and cancer progression. In this study, we determined that miR-200a was downregulated in hepatocellular carcinoma (HCC) tissues and cell lines, consistent with the results of our previous study. Because a previous study suggested that downregulation of miR-200a is correlated with HCC metastasis, we aimed to elucidate the mechanism underlying the role of miR-200a in metastasis in HCC. Here we observed that overexpression of miR-200a resulted in suppression of HCC metastatic ability, including HCC cell migration, invasion, and metastasis, in vitro and in vivo. Furthermore, bioinformatics and luciferase reporter assays indicated that GAB1 is a direct target of miR-200a. Inhibition of GAB1 resulted in substantially decreased   cell invasion   and migration similar to that observed with overexpression of miR-200a in HCC cell lines, whereas restoration of GAB1 partially rescued the inhibitory effects of miR-200a. Taken together, these data provide novel information for comprehending the tumor-suppressive role of miR-200a in HCC pathogenesis through inhibition of GAB1 translation. 
 Met receptor tyrosine kinase signals through a cortactin-Gab1 scaffold complex, to mediate invadopodia. Invasive carcinoma cells form actin-rich matrix-degrading protrusions called invadopodia. These structures resemble podosomes produced by some normal cells and play a crucial role in extracellular matrix remodeling. In cancer, formation of invadopodia is strongly associated with invasive potential. Although deregulated signals from the receptor tyrosine kinase Met (also known as hepatocyte growth factor are linked to cancer metastasis and poor prognosis, its role in invadopodia formation is not known. Here we show that stimulation of breast cancer cells with the ligand for Met, hepatocyte growth factor, promotes invadopodia formation, and in aggressive gastric tumor cells where Met is amplified, invadopodia formation is dependent on Met activity. Using both GRB2-associated-binding protein 1 (Gab1)-null fibroblasts and specific knockdown of Gab1 in tumor cells we show that Met-mediated invadopodia formation and   cell invasion   requires the scaffold protein Gab1. By a structure-function approach, we demonstrate that two proline-rich motifs (P4/5) within Gab1 are essential for invadopodia formation. We identify the actin regulatory protein, cortactin, as a direct interaction partner for Gab1 and show that a Gab1-cortactin interaction is dependent on the SH3 domain of cortactin and the integrity of the P4/5 region of Gab1. Both cortactin and Gab1 localize to invadopodia rosettes in Met-transformed cells and the specific uncoupling of cortactin from Gab1 abrogates invadopodia biogenesis and   cell invasion   downstream from the Met receptor tyrosine kinase. Met localizes to invadopodia along with cortactin and promotes phosphorylation of cortactin. These findings provide insights into the molecular mechanisms of invadopodia formation and identify Gab1 as a scaffold protein involved in this process. 
 Crk synergizes with epidermal growth factor for epithelial invasion and morphogenesis and is required for the met morphogenic program. Activation of the Met receptor tyrosine kinase through its ligand, hepatocyte growth factor, stimulates cell spreading, cell dispersal, and the inherent morphogenic program of various epithelial cell lines. Although both hepatocyte growth factor and epidermal growth factor (EGF) can activate downstream signaling pathways in Madin-Darby canine kidney epithelial cells, EGF fails to promote the breakdown of cell-cell junctional complexes and initiate an invasive morphogenic program. We have undertaken a strategy to identify signals that synergize with EGF in this process. We provide evidence that the overexpression of the CrkII adapter protein complements EGF-stimulated pathways to induce cell dispersal in two-dimensional cultures and   cell invasion   and branching morphogenesis in three-dimensional collagen gels. This finding correlates with the ability of CrkII to promote the breakdown of adherens junctions in stable cell lines and the ability of EGF to stimulate enhanced Rac activity in cells overexpressing CrkII. We have previously shown that the Gab1-docking protein is required for branching morphogenesis downstream of the Met receptor. Consistent with a role for CrkII in promoting EGF-dependent branching morphogenesis, the binding of Gab1 to CrkII is required for the branching morphogenic program downstream of Met. Together, our data support a role for the CrkII adapter protein in epithelial invasion and morphogenesis and underscores the importance of considering the synergistic actions of signaling pathways in cancer progression. 
 Adaptor molecule Crk is required for sustained phosphorylation of Grb2-associated binder 1 and hepatocyte growth factor-induced cell motility of human synovial sarcoma cell lines. Activation of the c-Met receptor tyrosine kinase through its ligand, hepatocyte growth factor (HGF), promotes mitogenic, motogenic, and morphogenic cellular responses. Aberrant HGF/c-Met signaling has been strongly implicated in tumor   cell invasion   and metastasis. Both HGF and its receptor c-Met have been shown to be overexpressed in human synovial sarcoma, which often metastasizes to the lung; however, little is known about HGF-mediated biological effects in this sarcoma. Here, we provide evidence that Crk adaptor protein is required for the sustained phosphorylation of c-Met-docking protein Grb2-associated binder 1 (Gab1) in response to HGF, leading to the enhanced cell motility of human synovial sarcoma cell lines SYO-1, HS-SY-II, and Fuji. HGF stimulation induced the sustained phosphorylation on Y307 of Gab1 where Crk was recruited. Crk knockdown by RNA interference disturbed this HGF-induced tyrosine phosphorylation of Gab1. By mutational analysis, we identified that Src homology 2 domain of Crk is indispensable for the induction of the phosphorylation on multiple Tyr-X-X-Pro motifs containing Y307 in Gab1. HGF remarkably stimulated cell motility and scattering of synovial sarcoma cell lines, consistent with the prominent activation of Rac1, extreme filopodia formation, and membrane ruffling. Importantly, the elimination of Crk in these cells induced the disorganization of actin cytoskeleton and complete abolishment of HGF-mediated Rac1 activation and cell motility. Time-lapse microscopic analysis revealed the significant attenuation in scattering of Crk knockdown cells following HGF treatment. Furthermore, the depletion of Crk remarkably inhibited the tumor formation and its invasive growth in vivo. These results suggest that the sustained phosphorylation of Gab1 through Crk in response to HGF contributes to the prominent activation of Rac1 leading to enhanced cell motility, scattering, and   cell invasion  , which may support the crucial role of Crk in the aggressiveness of human synovial sarcoma. 
   
  
 MID1IP1 
 
  NCBI Gene  
  GeneRIF  
  Pubmed  
 
 
  Search Term  Rank  Fields  
  [Mm]igration  6  PubMed(1)  
 
 
 hide/show details for MID1IP1 
  [Mm]igration  PubMed  
 Mig12, a novel Opitz syndrome gene product partner, is expressed in the embryonic ventral midline and co-operates with Mid1 to bundle and stabilize microtubules. Opitz G/BBB syndrome is a genetic disorder characterized by developmental midline abnormalities, such as hypertelorism, cleft palate, and hypospadias. The gene responsible for the X-linked form of this disease, MID1, encodes a TRIM/RBCC protein that is anchored to the microtubules. The association of Mid1 with the cytoskeleton is regulated by dynamic phosphorylation, through the interaction with the alpha4 subunit of phosphatase 2A (PP2A). Mid1 acts as an E3 ubiquitin ligase, regulating PP2A degradation on microtubules.In spite of these findings, the biological role exerted by the Opitz syndrome gene product is still unclear and the presence of other potential interacting moieties in the Mid1 structure prompted us to search for additional cellular partners. Through a yeast two-hybrid screening approach, we identified a novel gene, MIG12, whose protein product interacts with Mid1. We confirmed by immunoprecipitation that this interaction occurs in vivo and that it is mediated by the Mid1 coiled-coil domain. We found that Mig12 is mainly expressed in the neuroepithelial midline, urogenital apparatus, and digits during embryonic development. Transiently expressed Mig12 is found diffusely in both nucleus and cytoplasm, although it is enriched in the microtubule-organizing center region. Consistently with this, endogenous Mig12 protein is partially detected in the polymerized tubulin fraction after microtubule stabilization. When co-transfected with Mid1, Mig12 is massively recruited to thick filamentous structures composed of tubulin. These microtubule bundles are resistant to high doses of depolymerizing agents and are composed of acetylated tubulin, thus representing stabilized microtubule arrays.Our findings suggest that Mig12 co-operates with Mid1 to stabilize microtubules. Mid1-Mig12 complexes might be implicated in cellular processes that require microtubule stabilization, such as cell division and   migration  . Impairment in Mig12/Mid1-mediated microtubule dynamic regulation, during the development of embryonic midline, may cause the pathological signs observed in Opitz syndrome patients. 
   
  
 PTPA 
 
  NCBI Gene  
  GeneRIF  
  Pubmed  
 
 
  Search Term  Rank  Fields  
  [Mm]igration  6  PubMed(1)  
  [Ee]xtracellular [Mm]atrix  8  PubMed(3)  
  [Ii]nvasive  10  PubMed(1); GeneRIFs(1)  
  [Cc]ell [Ii]nvasion  13  PubMed(2)  
 
 
 hide/show details for PTPA 
  [Mm]igration  PubMed  
 Focal adhesion kinase regulates the phosphorylation protein tyrosine phosphatase-α at Tyr789 in breast cancer cells. Protein tyrosine phosphatase (PTP)‑α regulates the phosphorylation of focal adhesion kinase (FAK), which is important in cellular signal transduction and integration of proteins. It has been demonstrated that a FAK‑Del33 mutation (deletion of exon 33; KF437463) in breast cancer tissues regul  ates cell   migration through FAK/Src signaling activation. However, the detailed pathway for Src activation with FAK‑Del33 remains to be elucidated. The present study used a retroviral expression system to examine changes in PTPα phosphorylation affected by the FAK‑Del33 protein in breast cancer cells. Small interfering (si)RNA targeting PTPα interfered with the phosphorylation of Src. Wo  und‑hea  ling and migration assays were performed to identify cell morphology and quantitative analysis was performed by examining band color depth in western blot analysis. Significant differences were observed in the phosphorylation level of PTPα at Tyr789 between the FAK‑Del33 and the wild‑type breast cancer cells, suggesting that FAK regulated the phosphorylation level of PTPα at Tyr789 in breast cancer mutant FAK‑Del33 cells. The gene expression profile with FAK siRNA did not alter the levels of phosphorylation in other mutants, including autophosphorylation disability (Y397F), ATP kinase dominant negative (K454R) and protein 4.1, ezrin, radixin, moesin domain attenuate (Δ375). FAK RNAi inhibited the activity of the FAK‑Del33 at the Src si  te and re  scued the elevated cell migration and invasion. The present study demonstrated for the first time, to the best of our knowledge, an increase in the phosphorylation level of PTPα‑Tyr789 by its upstream activator, FAK‑Del33, leading to Src activation in certain breast cancer cells, which has significant implications for metastatic potential. 
  [Ee]xtracellular [Mm]atrix  PubMed  
 Eukaryotic translation initiation factor 4E binding protein 1 (4EBP-1) function is suppressed by Src and protein phosphatase 2A (PP2A) on   extracellular matrix  . Human lung fibroblasts utilize integrins to attach and proliferate on type I collagen. β1 integrin is the major integrin subunit for this attachment. Integrins coordinate cellular responses to cell-cell and cell  -extracellular matri  x interactions that regulate a variety of biological processes. Although β1 integrin-mediated signaling pathways in lung fibroblasts have been studied, a detailed molecular mechanism regulating translational control of gene expression by 4EBP-1 is not understood. 4EBP-1 inhibits cap-dependent translation by binding to the eIF4E translation initiation factor. We found that when lung fibroblasts attach to collagen via β1 integrin, high Src activity suppresses 4EBP-1 expression via PP2A, and the decrease of 4EBP-1 is due to protein degradation. The inhibition of Src activity dramatically increases PP2A and 4EBP-1 expression. Furthermore ectopic expression of PP2A, or PP2A silencing using PP2A siRNA confirmed that 4EBP-1 is regulated by PP2A. In addition, we found that 4EBP-1 inhibition by fibroblast attachment to collagen increases cap-dependent translation. Our study showed that when lung fibroblasts are attached to collagen matrix, the β1 integrin/Src/PP2A-mediated 4EBP-1 regulatory pathway is activated. We suggest that β1 integrin-mediated signaling pathway may be a crucial event in regulating fibroblast translational control machinery on collagen matrix. 
 Eukaryotic translation initiation factor 4E binding protein 1 (4EBP-1) function is suppressed by Src and protein phosphatase 2A (PP2A) on   extracellular matrix  . Human lung fibroblasts utilize integrins to attach and proliferate on type I collagen. β1 integrin is the major integrin subunit for this attachment. Integrins coordinate cellular responses to cell-cell and cell  -extracellular matri  x interactions that regulate a variety of biological processes. Although β1 integrin-mediated signaling pathways in lung fibroblasts have been studied, a detailed molecular mechanism regulating translational control of gene expression by 4EBP-1 is not understood. 4EBP-1 inhibits cap-dependent translation by binding to the eIF4E translation initiation factor. We found that when lung fibroblasts attach to collagen via β1 integrin, high Src activity suppresses 4EBP-1 expression via PP2A, and the decrease of 4EBP-1 is due to protein degradation. The inhibition of Src activity dramatically increases PP2A and 4EBP-1 expression. Furthermore ectopic expression of PP2A, or PP2A silencing using PP2A siRNA confirmed that 4EBP-1 is regulated by PP2A. In addition, we found that 4EBP-1 inhibition by fibroblast attachment to collagen increases cap-dependent translation. Our study showed that when lung fibroblasts are attached to collagen matrix, the β1 integrin/Src/PP2A-mediated 4EBP-1 regulatory pathway is activated. We suggest that β1 integrin-mediated signaling pathway may be a crucial event in regulating fibroblast translational control machinery on collagen matrix. 
 RhoB loss induces Rac1-dependent mesenchymal cell invasion in lung cells through PP2A inhibition. Non-small-cell lung cancer (NSCLC) is the leading cause of cancer-related death worldwide, which is mainly due to its high risk of metastatic dissemination. One critical point of this process is the ability of cancer cells to detach from the primary tumor and migrate through the   extracellular matrix  ; however, the underlying molecular mechanisms are not yet fully understood. In the present study, we identified the small GTPase RhoB as a key regulator of bronchial cell morphology in a three-dimensional (3D) matrix. RhoB loss, which is frequently observed during lung cancer progression, induced an epithelial-mesenchymal transition (EMT) characterized by an increased proportion of invasive elongated cells in 3D. The process was mediated by Slug induction and E-cadherin repression. In addition, downregulation of RhoB induced Akt1 activation, which in turn activated Rac1 through the guanine-exchange factor Trio to control cell shape rearrangement. Further, we provide evidence that RhoB interacted with and positively regulates phosphatase PP2A through the recruitment of its regulatory subunit B55, which was found to be crucial for Akt dephosphorylation. B55 inhibition completely suppressed RhoB-mediated PP2A regulation. Finally, we show that PP2A inactivation, by targeting either its catalytic or its regulatory B55 subunit, completely reversed RhoB-dependent morphological changes and also fully prevented the ability of RhoB to decrease the invasiveness of bronchial cells. Altogether, these results highlight a novel signaling axis and describe new molecular mechanisms that could explain the tumor suppressor role of RhoB in lung cancer. Therefore, we propose that RhoB could be responsible for early metastatic prevention by inhibiting the EMT-derived invasiveness of lung cells through the control of PP2A activity. 
  [Ii]nvasive  PubMed  
 RhoB loss induces Rac1-dependent mesenchymal cell invasion in lung cells through PP2A inhibition. Non-small-cell lung cancer (NSCLC) is the leading cause of cancer-related death worldwide, which is mainly due to its high risk of metastatic dissemination. One critical point of this process is the ability of cancer cells to detach from the primary tumor and migrate through the extracellular matrix; however, the underlying molecular mechanisms are not yet fully understood. In the present study, we identified the small GTPase RhoB as a key regulator of bronchial cell morphology in a three-dimensional (3D) matrix. RhoB loss, which is frequently observed during lung cancer progression, induced an epithelial-mesenchymal transition (EMT) characterized by an increased proportion of   invasive   elongated cells in 3D. The process was mediated by Slug induction and E-cadherin repression. In addition, downregulation of RhoB induced Akt1 activation, which in turn activated Rac1 through the guanine-exchange factor Trio to control cell shape rearrangement. Further, we provide evidence that RhoB interacted with and positively regulates phosphatase PP2A through the recruitment of its regulatory subunit B55, which was found to be crucial for Akt dephosphorylation. B55 inhibition completely suppressed RhoB-mediated PP2A regulation. Finally, we show that PP2A inactivation, by targeting either its catalytic or its regulatory B55 subunit, completely reversed RhoB-dependent morphological changes and also fully prevented the ability of RhoB to decrease the   invasive  ness of bronchial cells. Altogether, these results highlight a novel signaling axis and describe new molecular mechanisms that could explain the tumor suppressor role of RhoB in lung cancer. Therefore, we propose that RhoB could be responsible for early metastatic prevention by inhibiting the EMT-derived   invasive  ness of lung cells through the control of PP2A activity. 
  GeneRIFs  
 we propose that RhoB could be responsible for early metastatic prevention by inhibiting the EMT-derived   invasive  ness of lung cells through the control of PP2A activity. 
  [Cc]ell [Ii]nvasion  PubMed  
 PP2A phosphatase suppresses function of the mesenchymal invasion regulator NEDD9. The mesenchymal mode of cancer   cell invasion   characterized by active adhesion turnover and a polarized actin cytoskeleton, is critically regulated by the adaptor protein NEDD9/HEF1/Cas-L. While it is known that NEDD9 is subject to extensive phosphorylation modification, the molecules that determine NEDD9 phosphorylation to stimulate adhesion turnover and mesenchymal cell morphologies are currently unknown. Earlier studies have suggested that the serine/threonine phosphatase PP2A regulates interconversion between a low molecular mass NEDD9 phosphoform and higher molecular mass phosphoforms. However, previous studies have used chemical inhibitors to block PP2A activity. In the present study we therefore aimed to specifically inhibit PP2A activity via siRNA and dominant negative approaches to investigate the effect of PP2A on interconversion between 115 kDa and 105 kDa NEDD9 and determine the functional consequence of PP2A activity for NEDD9 function. Strikingly, we find that while the phosphatase inhibitor Calyculin A indeed abrogates detachment-induced dephosphorylation of the 115 kDa NEDD9 phosphoform, PP2A depletion does not inhibit 115 kDa to 105 kDa interconversion. Our data suggest instead that PP2A targets discrete NEDD9 phosphorylation modifications separate to the events that mediate interconversion between the two forms. Functionally, PP2A depletion increases NEDD9 mediated cell spreading and mutation of S369 in the serine-rich region of NEDD9 to aspartate mimics this effect. Importantly, mutation of S369 to alanine abrogates the ability of dominant negative PP2A to increase NEDD9-mediated cell spreading. Collectively, our data reveal that the tumour suppressor PP2A may act via S369 to regulated NEDD9-mediated cell spreading. 
 RhoB loss induces Rac1-dependent mesenchymal   cell invasion   in lung cells through PP2A inhibition. Non-small-cell lung cancer (NSCLC) is the leading cause of cancer-related death worldwide, which is mainly due to its high risk of metastatic dissemination. One critical point of this process is the ability of cancer cells to detach from the primary tumor and migrate through the extracellular matrix; however, the underlying molecular mechanisms are not yet fully understood. In the present study, we identified the small GTPase RhoB as a key regulator of bronchial cell morphology in a three-dimensional (3D) matrix. RhoB loss, which is frequently observed during lung cancer progression, induced an epithelial-mesenchymal transition (EMT) characterized by an increased proportion of invasive elongated cells in 3D. The process was mediated by Slug induction and E-cadherin repression. In addition, downregulation of RhoB induced Akt1 activation, which in turn activated Rac1 through the guanine-exchange factor Trio to control cell shape rearrangement. Further, we provide evidence that RhoB interacted with and positively regulates phosphatase PP2A through the recruitment of its regulatory subunit B55, which was found to be crucial for Akt dephosphorylation. B55 inhibition completely suppressed RhoB-mediated PP2A regulation. Finally, we show that PP2A inactivation, by targeting either its catalytic or its regulatory B55 subunit, completely reversed RhoB-dependent morphological changes and also fully prevented the ability of RhoB to decrease the invasiveness of bronchial cells. Altogether, these results highlight a novel signaling axis and describe new molecular mechanisms that could explain the tumor suppressor role of RhoB in lung cancer. Therefore, we propose that RhoB could be responsible for early metastatic prevention by inhibiting the EMT-derived invasiveness of lung cells through the control of PP2A activity. 
   
  
 SRSF3 
 
  NCBI Gene  
  GeneRIF  
  Pubmed  
 
 
  Search Term  Rank  Fields  
  [Ii]nvasive  10  PubMed(2); GeneRIFs(1)  
  [Mm]etastasis  12  PubMed(1)  
 
 
 hide/show details for SRSF3 
  [Ii]nvasive  PubMed  
 Knockdown of splicing factor SRp20 causes apoptosis in ovarian cancer cells and its expression is associated with malignancy of epithelial ovarian cancer. Our previous study revealed that two splicing factors, polypyrimidine tract-binding protein (PTB) and SRp20, were upregulated in epithelial ovarian cancer (EOC) and knockdown of PTB expression inhibited ovarian tumor cell growth and transformation properties. In this report, we show that knockdown of SRp20 expression in ovarian cancer cells also causes substantial inhibition of tumor cell growth and colony formation in soft agar and the extent of such inhibition appeared to correlate with the extent of suppression of SRp20. Massive knockdown of SRp20 expression triggered remarkable apoptosis in these cells. These results suggest that overexpression of SRp20 is required for ovarian tumor cell growth and survival. Immunohistochemical staining for PTB and SRp20 of two specialized tissue microarrays, one containing benign ovarian tumors, borderline/low malignant potential (LMP) ovarian tumors as well as   invasive   EOC and the other containing   invasive   EOC ranging from stage I to stage IV disease, reveals that PTB and SRp20 are both expressed differentially between benign tumors and   invasive   EOC, and between borderline/LMP tumors and   invasive   EOC. There were more all-negative or mixed staining cases (at least two evaluable section cores per case) in benign tumors than in   invasive   EOC, whereas there were more all-positive staining cases in   invasive   EOC than in the other two disease classifications. Among   invasive   EOC, the majority of cases were stained all positive for both PTB and SRp20, and there were no significant differences in average staining or frequency of positive cancer cells between any of the tumor stages. Therefore, the expression of PTB and SRp20 is associated with malignancy of ovarian tumors but not with stage of   invasive   EOC. 
 SRSF3 and hnRNP H1 regulate a splicing hotspot of HER2 in breast cancer cells. Overexpression of the oncogene HER2 occurs in 20-30% of   invasive   breast cancer and is associated with poor prognosis. A number of different splice variants of HER2 have been identified which produce functionally different proteins. Previously these splice variants have been investigated separately, but in the present study we collectively look at the expression and regulation of a group of HER2 splice variants produced by a splicing hotspot. Initial investigation in a cohort of tumor samples showed large variations in HER2 variant expression between patient samples. RNA interference studies identified 2 splicing factors involved in the regulation of splicing within this region, hnRNP H1 and SRSF3. siRNA targeting hnRNP H1 increases levels of X5 and the oncogenic variant Δ16HER2. Furthermore RNA chromatography assays demonstrated binding of hnRNP H1 to RNA in this region. Additionally the proto-oncogene SRSF3 was also identified as an important regulator of splicing with SRSF3 knockdown resulting in changes in all the splice variants located at the hotspot. Most notably knockdown of SRSF3 resulted in a switch from the oncogenic Δ16HER2 to p100 which inhibits cell proliferation. Binding of SRSF3 to RNA within this region was also demonstrated by RNA chromatography and more specifically 2 SRSF3 binding sites were identified within exon 15. SRSF3 and hnRNP H1 are the first splicing factors identified which regulate the production of these functionally distinct HER2 splice variants and therefore maybe important for the regulation of HER2 signaling. 
  GeneRIFs  
 expression of PTB and SRp20 is associated with malignancy of ovarian tumors but not with stage of   invasive   epithelial ovarian cancer 
  [Mm]etastasis  PubMed  
 Expression of SRSF3 is Correlated with Carcinogenesis and Progression of Oral Squamous Cell Carcinoma. Oral squamous cell carcinoma (OSCC) is the most common malignancy of head and neck with high mortality rates. The mechanisms of initiation and development of OSCC remain largely unknown. Dysregulated alternative splicing of pre-mRNA has been associated with OSCC. Splicing factor SRSF3 is a proto-oncogene and overexpressed in multiple cancers. The aim of this study was to uncover the relationship between SRSF3 and carcinogenesis and progression of oral squamous cell carcinoma.The expression of SRSF3 in oral normal, dysplasia, or carcinoma tissues was analyzed by immunohistochemistry. The expression levels of EMT-related genes were quantified by real-time quantitative RT-PCR. The expression of SRSF3 in DMBA treated primary cultured oral epithelial cells were analyzed by western blot.SRSF3 is overexpressed in oral cancer and moderate or severe dysplasia tissues. Patients with high grade cancer or lymphatic   metastasis   showed up-regulated expression of SRSF3. Knockdown of SRSF3 repressed the expression of Snail and N-cadherin in vitro. Carcinogen DMBA treated primary cultured oral epithelial cells showed significantly increased SRSF3 level than in control cells.Our results suggested that SRSF3 is associated with the initiation and development of OSCC and may be a biomarker and therapeutic target of OSCC. 
   
  
 RNF111 
 
  NCBI Gene  
  GeneRIF  
  Pubmed  
 
 
  Search Term  Rank  Fields  
  [Mm]igration  6  PubMed(1)  
  [Ee]xtracellular [Mm]atrix  8  PubMed(1)  
  [Mm]etastasis  12  PubMed(2); GeneRIFs(1)  
 
 
 hide/show details for RNF111 
  [Mm]igration  PubMed  
 RNF111/Arkadia is regulated by DNA methylation and affects TGF-β/Smad signaling associated invasion in NSCLC cells. RNF111/Arkadia is a critical regulator of TGF-β signaling, being required for SMAD3-mediated responses such as TGF-β-induced repression of E-cadherin. Previous studies show that mutations in RNF111 in human cancers are rare and RNF111 promotes lung tumor metastasis. However, the epigenetic mechanisms underlying the role of RNF111 in non-small cell lung cancer (NSCLC) metastasis remain unknown. Here, we mainly focused on low- (95C) and high-metastatic (95D) NSCLC cell lines, which share a similar genetic background, and investigated the methylation-based regulation of RNF111 expression.Clonal bisulfite sequencing, real-time qRT-PCR, western blot analysis, luciferase reporter assays, RNA interference, chromatin immunoprecipitation (ChIP) assay and transwe  ll migrat  ion and invasion assays were performed on human NSCLC cell lines 95C and 95D.RNF111 was high-expressed in 95D cells, which showed low-level methylation at -459CpG site in RNF111 promoter. The opposite results were obtained in 95C cells. Cell-based and biochemical assays revealed that -459CpG methylation can inhibit RNF111 transcriptional expression by interfering with the recruitment of Sp1 to RNF111 promoter. On TGF-β stimulation, siRNA-mediated RNF111 knockdown inhibited TGF-β/Smad signaling activity and Snail (an inducer of metastasis) expression, and enhanced E-cadherin (an epithelial-to-mesenchymal transition marker) expression in 95C and 95D cells. Furthermore, demethylation-induced upregulation of RNF111 enhanced phosphorylation of SMAD3 and Snail expression, and repressed E-cadherin expression in 95C cells expressing low RNF111.Our results suggest that -459CpG methylation in Sp1-binding site of RNF111 promoter transcriptionally decreases RNF111 expression, which inhibits TGF-β/Smad signaling associated invasion in NSCLC cells. 
  [Ee]xtracellular [Mm]atrix  PubMed  
 c-Ski, Smurf2, and Arkadia as regulators of TGF-beta signaling: new targets for managing myofibroblast function and cardiac fibrosis. Recent studies demonstrate the critical role of the   extracellular matrix   in the organization of parenchymal cells in the heart. Thus, an understanding of the modes of regulation of matrix production by cardiac myofibroblasts is essential. Transforming growth factor beta (TGF-beta) signaling is transduced through the canonical Smad pathway, and the involvement of this pathway in matrix synthesis and other processes requires precise control. Inhibition of Smad signaling may be achieved at the receptor level through the targeting of the TGF-beta type I receptors with an inhibitory Smad7/Smurf2 complex, or at the transcriptional level through c-Ski/receptor-Smad/co-mediator Smad4 interactions. Conversely, Arkadia protein intensifies TGF-beta-induced effects by marking c-Ski and inhibitory Smad7 for destruction. The study of these TGF-beta mediators is essential for future treatment of fibrotic disease, and this review highlights recent relevant findings that may impact our understanding of cardiac fibrosis. 
  [Mm]etastasis  PubMed  
 Arkadia regulates tumor   metastasis   by modulation of the TGF-β pathway. TGF-β can act as a tumor suppressor at early stages of cancer progression and as a tumor promoter at later stages. The E3 ubiquitin ligase Arkadia (RNF111) is a critical component of the TGF-β signaling pathway, being required for a subset of responses, those mediated by Smad3-Smad4 complexes. It acts by mediating ligand-induced degradation of Ski and SnoN (SKIL), which are 2 potent transcriptional repressors. Here, we investigate the role of Arkadia in cancer using model systems to address both potential tumor-suppressive and tumor-promoting roles. Stable reexpression of Arkadia in lung carcinoma NCI-H460 cells, which we show contain a hemizygous nonsense mutation in the Arkadia/RNF111 gene, efficiently restored TGF-β-induced Smad3-dependent transcription, and substantially decreased the ability of these cells to grow in soft agar in vitro. However, it had no effect on tumor growth in vivo in mouse models. Moreover, loss of Arkadia in cancer cell lines and human tumors is rare, arguing against a prominent tumor-suppressive role. In contrast, we have uncovered a potent tumor-promoting function for Arkadia. Using 3 different cancer cell lines whose tumorigenic properties are driven by TGF-β signaling, we show that loss of Arkadia function, either by overexpression of dominant negative Arkadia or by siRNA-induced knockdown, substantially inhibited lung colonization in tail vein injection experiments in immunodeficient mice. Our findings indicate that Arkadia is not critical for regulating tumor growth per se, but is required for the early stages of cancer cell colonization at the site  s of metas  tasis. 
 RNF111/Arkadia is regulated by DNA methylation and affects TGF-β/Smad signaling associated invasion in NSCLC cells. RNF111/Arkadia is a critical regulator of TGF-β signaling, being required for SMAD3-mediated responses such as TGF-β-induced repression of E-cadherin. Previous studies show that mutations in RNF111 in human cancers are rare and RNF111 promotes lung tum  or metasta  sis. However, the epigenetic mechanisms underlying the role of RNF111 in non-small cell lung cancer (NSCL  C) metasta  sis remain unknown. Here, we mainly focused on low- (95C) and high-metastatic (95D) NSCLC cell lines, which share a similar genetic background, and investigated the methylation-based regulation of RNF111 expression.Clonal bisulfite sequencing, real-time qRT-PCR, western blot analysis, luciferase reporter assays, RNA interference, chromatin immunoprecipitation (ChIP) assay and transwell migration and invasion assays were performed on human NSCLC cell lines 95C and 95D.RNF111 was high-expressed in 95D cells, which showed low-level methylation at -459CpG site in RNF111 promoter. The opposite results were obtained in 95C cells. Cell-based and biochemical assays revealed that -459CpG methylation can inhibit RNF111 transcriptional expression by interfering with the recruitment of Sp1 to RNF111 promoter. On TGF-β stimulation, siRNA-mediated RNF111 knockdown inhibited TGF-β/Smad signaling activity and Snail (an induce  r of metas  tasis) expression, and enhanced E-cadherin (an epithelial-to-mesenchymal transition marker) expression in 95C and 95D cells. Furthermore, demethylation-induced upregulation of RNF111 enhanced phosphorylation of SMAD3 and Snail expression, and repressed E-cadherin expression in 95C cells expressing low RNF111.Our results suggest that -459CpG methylation in Sp1-binding site of RNF111 promoter transcriptionally decreases RNF111 expression, which inhibits TGF-β/Smad signaling associated invasion in NSCLC cells. 
  GeneRIFs  
 findings indicate that Arkadia is not critical for regulating tumor growth per se, but is required for the early stages of cancer cell colonization at the sites of   metastasis   
   
  
 B3GAT3 
 
  NCBI Gene  
  GeneRIF  
  Pubmed  
 
 
  Search Term  Rank  Fields  
  [Ee]xtracellular [Mm]atrix  8  PubMed(2)  
 
 
 hide/show details for B3GAT3 
  [Ee]xtracellular [Mm]atrix  PubMed  
 Proteomic analysis of human parotid gland exosomes by multidimensional protein identification technology (MudPIT). Human ductal saliva contributes over a thousand unique proteins to whole oral fluids. The mechanism by which most of these proteins are secreted by salivary glands remains to be determined. The present study used a mass spectrometry-based, shotgun proteomics approach to explore the possibility that a subset of the proteins found in saliva are derived from exosomes, membrane-bound vesicles of endosomal origin within multivesicular endosomes. Using MudPIT (multidimensional protein identification technology) mass spectrometry, we catalogued 491 proteins in the exosome fraction of human parotid saliva. Many of these proteins were previously observed in ductal saliva from parotid glands (265 proteins). Furthermore, 72 of the proteins in parotid exosomes overlap with those previously identified as urinary exosome proteins, proteins which are also frequently associated with exosomes from other tissues and cell types. Gene Ontology (GO) and KEGG pathway analyses found that cytosolic proteins comprise the largest category of proteins in parotid exosomes (43%), involved in such processes as phosphatidylinositol signaling system, calcium signaling pathway, inositol metabolism, protein export, and signal transduction, among others; whereas the integral plasma membrane proteins and associated/peripheral plasma membrane proteins (26%) were associated with   extracellular matrix  -receptor interaction, epithelial cell signaling, T-cell and B-cell receptor signaling, cytokine receptor interaction, and antigen processing and presentation, among other biological functions. In addition, these putative saliva exosomal proteins were linked to specific diseases (e.g., neurodegenerative disorders, prion disease, cancers, type I and II diabetes). Consequently, parotid glands secrete exosomes that reflect the metabolic and functional status of the gland and may also carry informative protein markers useful in the diagnosis and treatment of systemic diseases. 
 Faulty initiation of proteoglycan synthesis causes cardiac and joint defects. Proteoglycans are a major component of   extracellular matrix   and contribute to normal embryonic and postnatal development by ensuring tissue stability and signaling functions. We studied five patients with recessive joint dislocations and congenital heart defects, including bicuspid aortic valve (BAV) and aortic root dilatation. We identified linkage to chromosome 11 and detected a mutation (c.830G>A, p.Arg277Gln) in B3GAT3, the gene coding for glucuronosyltransferase-I (GlcAT-I). The enzyme catalyzes an initial step in the synthesis of glycosaminoglycan side chains of proteoglycans. Patients' cells as well as recombinant mutant protein showed reduced glucuronyltransferase activity. Patient fibroblasts demonstrated decreased levels of dermatan sulfate, chondroitin sulfate, and heparan sulfate proteoglycans, indicating that the defect in linker synthesis affected all three lines of O-glycanated proteoglycans. Further studies demonstrated that GlcAT-I resides in the cis and cis-medial Golgi apparatus and is expressed in the affected tissues, i.e., heart, aorta, and bone. The study shows that reduced GlcAT-I activity impairs skeletal as well as heart development and results in variable combinations of heart malformations, including mitral valve prolapse, ventricular septal defect, and bicuspid aortic valve. The described family constitutes a syndrome characterized by heart defects and joint dislocations resulting from altered initiation of proteoglycan synthesis (Larsen-like syndrome, B3GAT3 type). 
   
  
 LGALS9 
 
  NCBI Gene  
  GeneRIF  
  Pubmed  
 
 
  Search Term  Rank  Fields  
  [Mm]igration  6  PubMed(9); GeneRIFs(2)  
  ECM  7  PubMed(1)  
  [Ee]xtracellular [Mm]atrix  8  PubMed(6); GeneRIFs(1)  
  [Mm]etastasis  12  PubMed(12); GeneRIFs(3)  
  [Cc]ell [Ii]nvasion  13  PubMed(1); GeneRIFs(1)  
 
 
 hide/show details for LGALS9 
  [Mm]igration  PubMed  
 Galectin-9 binding to cell surface protein disulfide isomerase regulates the redox environment to enhance T-cell   migration   and HIV entry. Interaction of cell surface glycoproteins with endogenous lectins on the cell surface regulates formation and maintenance of plasma membrane domains, clusters signaling complexes, and controls the residency time of glycoproteins on the plasma membrane. Galectin-9 is a soluble, secreted lectin that binds to glycoprotein receptors to form galectin-glycoprotein lattices on the cell surface. Whereas galectin-9 binding to specific glycoprotein receptors induces death of CD4 Th1 cells, CD4 Th2 cells are resistant to galectin-9 death due to alternative glycosylation. On Th2 cells, galectin-9 binds cell surface protein disulfide isomerase (PDI), increasing retention of PDI on the cell surface and altering the redox status at the plasma membrane. Cell surface PDI regulates integrin function on platelets and also enhances susceptibility of T cells to infection with HIV. We find that galectin-9 binding to PDI on Th2 cells results in increased cell   migration   through extracellular matrix via β3 integrins, identifying a unique mechanism to regulate T-cell   migratio  n. In addition, galectin-9 binding to PDI on T cells potentiates infection with HIV. We identify a mechanism for regulating cell surface redox status via a galectin-glycoprotein lattice, to regulate distinct T-cell functions. 
 Up-regulation of galectin-9 induces cell   migration   in human dendritic cells infected with dengue virus. Galectin-9 (Gal-9) exerts immunosuppressive effects by inducing apoptosis in T cells that produce interferon-γ and interleukin (IL)-17. However, Gal-9 can be pro-inflammatory in lipopolysaccharide-stimulated monocytes. Using microarray analysis, we observed that Gal-9 was up-regulated in human dendritic cells (DCs) after dengue virus (DV) infection. The investigation into the immunomodulatory effects and mechanisms of Gal-9 in DCs exposed to DV revealed that DV infection specifically increased mRNA and protein levels of Gal-9 but not those of Gal-1 or Gal-3. Blocking p38, but not c-Jun N-terminal kinase or extracellular signal-regulated kinase (ERK), inhibited DV-induced expression of Gal-9. Reduction in Gal-9 by small interference RNA treatment suppressed DV-stimulated   migratio  n of DCs towards the chemoattractants CCL19 and CCL21. In addition, DV-induced IL-12p40 production was reduced after knockdown of Gal-9 in DCs. Furthermore, Gal-9 deficiency suppressed DV-induced activation of nuclear factor-κB. Inhibition of DV-induced D  C migrati  on under conditions of Gal-9 deficiency was mediated through suppressing ERK activation but not by regulating the expression of CCR7, the receptor for CCL19 and CCL21. Both the reduction in IL-12 production and the suppression of ERK activity might account for the inhibition of DV-induced D  C migrati  on after knockdown of Gal-9. In summary, this study reveals the roles of Gal-9 in DV-induce  d migrati  on of DCs. The findings indicate that Gal-9 might be a therapeutic target for preventing immunopathogenesis induced by DV infection. 
 Up-regulation of galectin-9 induces cell   migration   in human dendritic cells infected with dengue virus. Galectin-9 (Gal-9) exerts immunosuppressive effects by inducing apoptosis in T cells that produce interferon-γ and interleukin (IL)-17. However, Gal-9 can be pro-inflammatory in lipopolysaccharide-stimulated monocytes. Using microarray analysis, we observed that Gal-9 was up-regulated in human dendritic cells (DCs) after dengue virus (DV) infection. The investigation into the immunomodulatory effects and mechanisms of Gal-9 in DCs exposed to DV revealed that DV infection specifically increased mRNA and protein levels of Gal-9 but not those of Gal-1 or Gal-3. Blocking p38, but not c-Jun N-terminal kinase or extracellular signal-regulated kinase (ERK), inhibited DV-induced expression of Gal-9. Reduction in Gal-9 by small interference RNA treatment suppressed DV-stimulated   migratio  n of DCs towards the chemoattractants CCL19 and CCL21. In addition, DV-induced IL-12p40 production was reduced after knockdown of Gal-9 in DCs. Furthermore, Gal-9 deficiency suppressed DV-induced activation of nuclear factor-κB. Inhibition of DV-induced D  C migrati  on under conditions of Gal-9 deficiency was mediated through suppressing ERK activation but not by regulating the expression of CCR7, the receptor for CCL19 and CCL21. Both the reduction in IL-12 production and the suppression of ERK activity might account for the inhibition of DV-induced D  C migrati  on after knockdown of Gal-9. In summary, this study reveals the roles of Gal-9 in DV-induce  d migrati  on of DCs. The findings indicate that Gal-9 might be a therapeutic target for preventing immunopathogenesis induced by DV infection. 
 Galectin-9 binding to cell surface protein disulfide isomerase regulates the redox environment to enhance T-cell   migration   and HIV entry. Interaction of cell surface glycoproteins with endogenous lectins on the cell surface regulates formation and maintenance of plasma membrane domains, clusters signaling complexes, and controls the residency time of glycoproteins on the plasma membrane. Galectin-9 is a soluble, secreted lectin that binds to glycoprotein receptors to form galectin-glycoprotein lattices on the cell surface. Whereas galectin-9 binding to specific glycoprotein receptors induces death of CD4 Th1 cells, CD4 Th2 cells are resistant to galectin-9 death due to alternative glycosylation. On Th2 cells, galectin-9 binds cell surface protein disulfide isomerase (PDI), increasing retention of PDI on the cell surface and altering the redox status at the plasma membrane. Cell surface PDI regulates integrin function on platelets and also enhances susceptibility of T cells to infection with HIV. We find that galectin-9 binding to PDI on Th2 cells results in increased cell   migration   through extracellular matrix via β3 integrins, identifying a unique mechanism to regulate T-cell   migratio  n. In addition, galectin-9 binding to PDI on T cells potentiates infection with HIV. We identify a mechanism for regulating cell surface redox status via a galectin-glycoprotein lattice, to regulate distinct T-cell functions. 
 Endothelial LGALS9 splice variant expression in endothelial cell biology and angiogenesis. Galectins are carbohydrate binding proteins with versatile functions in tumor progression. Galectin-9, encoded by LGALS9, has been associated with metastasis and immunosuppression. We previously reported on regulation of LGALS9 expression during endothelial cell activation. Here, we show increased galectin-9 protein levels in the endothelium of different tumors, including carcinomas of the lung, liver, breast and kidney. Endothelial cells were found to express five LGALS9 splice variants, two of which have not been reported before. Splicing was found to be confined to exons 5, 6 and 10. Transfection of human microvascular endothelial cells (HMEC) with galectin-9∆5, a specific LGALS9 splice variant, induced a small but significant increase of proliferation, whil  e migrati  on was not affected by any LGALS9 splice variant. Application of recombinant galectin-9∆5 protein dose-dependently reduced proliferation   and migra  tion of HMEC as well as human umbilical vein endothelial cells in vitro. Enhanced sprouting   and migra  tion of human umbilical vein endothelial cell (HUVEC) towards a galectin-9∆5 gradient were observed. Interestingly, galectin-9∆5 was found to induce a small inhibitory effect on angiogenesis in vivo. Collectively, these data show that endothelial cells regulate the expression and splicing of LGALS9 during angiogenesis. The function of the dominant splice variant, i.e. galectin-9∆5, in endothelial cell biology depends on the concentration and environmental context in which it is presented to the cells. 
 Peroxisome proliferator-activated receptor γ upregulates galectin-9 and predicts prognosis in intestinal-type gastric cancer. The importance of PPARγ (peroxisome proliferator-activated receptor γ) in gastric cancer (GC) is unclear. We investigated the role of PPARγ in GC cell lines and an animal model, and its prognostic significance of PPARγ in GC patients. We controlled PPARγ and galectin-9 expression by using siRNAs and lentiviral constructs. Interaction between PPARγ and galectin-9 was evaluated using luciferase and chromatin immunoprecipitation assays. PPARγ expression in GCs was determined by immunohistochemical staining of tissue microarrays and survival analysis was done. Overexpression of PPARγ was accompanied by increased galectin-9. Enhanced PPARγ or galectin-9 expression increased E-cadherin expression; decreased expression of N-cadherin, fibronectin, snail, twist and slug and reduced cell inv  asion and   migration. PPARγ bound to the galectin-9 promoter region. Galectin-9 activity increased in PPARγ-overexpressing cells but decreased in PPARγ siRNA-treated cells. In a zebrafish xenograft model, the number of migrated cancer cells and number of fish with AGS cells in the tail vein were reduced in PPARγ-overexpressing GC cells. PPARγ was expressed in 462 of the 688 patients (69.2%) with GC. In 306 patients with intestinal-type GC, those with PPARγ-positive tumors had lower overall and cancer-specific mortalities than those with PPARγ-negative tumors. PPARγ expression was an independent prognostic factor for overall and GC-specific mortality in patients with intestinal-type GC (adjusted hazard ratio, 0.42; 95% CI, 0.22-0.81). PPARγ inhib  its cell   invasion, migration and epithelial-mesenchymal transition through upregulation of galectin-9 in vitro and in vivo. 
 Participation of T cell immunoglobulin and mucin domain-3 (TIM-3) and its ligand (galectin-9) in the pathogenesis of active generalized vitiligo. Vitiligo is a depigmentary disease where melanocytes of the basal layer of epidermis are selectively destroyed by immune-cell-mediated cytotoxicity. The T cell immunoglobulin- and mucin-domain-containing molecules (TIMs) are involved in immune regulation, and their participation is not known in vitiligo. The present study revealed significant increase in the percentage of CD3+CD4+TIM3+ T cells (P < 0.05) in peripheral blood and was positively correlated with percentage body surface area involvement in aGV group. Further, increased expression of TIM-3 and its ligand galectin-9 (Gal-9) mRNA was found in peripheral blood and lesional/perilesional skin of active generalized vitiligo (aGV) compared with controls. Characteristic   migration   pattern of TIM-3-positive immune cells in lesional (near/in the epidermis) and perilesional (towards epidermis) skin section suggested that TIM-3+ immune cells may be involved in melanocyte destruction. Further, investigation is required to understand the role of TIM-3/Gal-9 signalling pathways in aGV and it can be targeted in the management of vitiligo. 
 Reduced Expression of Galectin-9 Contributes to a Poor Outcome in Colon Cancer by Inhibiting NK Cell Chemotaxis Partially through the Rho/ROCK1 Signaling Pathway. Galectin-9 is a widely expressed protein that is involved in immune regulation and tumorpathogenesis and serves as a marker of a poor prognosis in various types of cancers. However, the clinical impact and the precise mechanism by which this protein contributes to colon tumor progression are unclear. In the present study, we detected the expression of galectin-9 and CD56 cells using immunohistochemistry. Spearman's rank correlation was used to clarify the association between galectin-9 expression and natural killer (NK) cell infiltration. The influence of galectin-9 on NK-92 cell   migration   was evaluated in vitro using transwell chemotaxis assays. The role of rh-galectin-9 in F-actin polarization in NK-92 cells was investigated using laser scanning confocal microscopy. We showed that galectin-9 was expressed in 101 (78.91%) colon tumor tissues and that was expressed at lower levels in these tissues than in para-tumor tissues. Low levels of galectin-9 expression were positively correlated with a poor histological grade and lymph node metastasis (P<0.05). A Kaplan-Meier method and Cox proportional hazards regression analysis showed that overall survival was longer in patients with high galectin-9 expression in an 8-year follow-up (P<0.05). Spearman's rank correlation indicated that there was a linear correlation between galectin-9 expression and CD56+ NK cell infiltration (R(2) = 0.658; P<0.0001). Galectin-9 stimulated   migration   in human NK-92 cells by affecting F-actin polarization through the Rho/ROCK1 signaling pathway. These results suggest that galectin-9 expression potentially represents a novel mechanism for tumors to escape immune surveillance in colon tumors. 
 Galectin-9 inhibits CD44-hyaluronan interaction and suppresses a murine model of allergic asthma. Galectin-9 (Gal-9) belongs to the galectin family, which exhibits affinity for beta-galactosides. Gal-9 has a variety of biological activities; however, its role in allergic inflammation is unknown.We evaluated the effect of a stable form of the human protein on allergic airway inflammation in a mite allergen-induced asthma model.Human stable Gal-9 was given by intravenous injection to mice during antigen challenge. The effect of Gal-9 on airway inflammation and airway hyperresponsiveness (AHR) was then evaluated.Gal-9 reduced AHR as well as Th2-associated airway inflammation. Furthermore, administration of Gal-9 as well as anti-CD44 monoclonal antibody inhibited the infiltration of peripheral blood Th2 cells into the airway. Interestingly, Gal-9 directly bound the CD44 adhesion molecule and inhibited interactions with hyaluronan (HA). Consistent with the concept that CD44-HA interactions mediate the   migration   of T cells into the lung, Gal-9 blocked CD44-dependent adhesion of BW5147 mouse T cells to HA.We conclude that Gal-9 inhibits allergic inflammation of the airway and AHR by modulating CD44-dependent leukocyte recognition of the extracellular matrix. 
  GeneRIFs  
 Galectin-9 stimulated   migration   in human NK-92 cells by affecting F-actin polarization through the Rho/ROCK1 signaling pathway. 
 PPARG inhibits cell invasion,   migration   and epithelial-mesenchymal transition through upregulation of galectin-9 in vitro and in vivo. 
  ECM  PubMed  
 Galectin-9 acts as a prognostic factor with antimetastatic potential in hepatocellular carcinoma. Considerable research has been conducted concerning galectin-9 and carcinomas, but little information is available about any relation with the hepatocellular carcinoma. In this study, we employed a small interfering RNA (siRNA) targeting galectin-9 to down-regulate the expression in HepG2 cells. As a result, after galectin-9 expression was reduced, cell aggregation was suppressed, while other behaviour such as the proliferation, adhesion and invasion to   ECM  , cell-endothelial adhesion and transendothelial invasion of the cells were markedly enhanced. When tumors of 200 patients with hepatocellular carcinoma were tested for galectin-9 expression by immunohistochemistry, binding levels demonstrated intimate correlations with the histopathologic grade, lymph node metastasis, vascular invasion and intrahepatic metastasis (P<0.05). Moreover, survival analysis indicated that patients with galectin-9 expression had much longer survival time than those with negative lesions, and the Log-rank test indicated that this difference was statistical significant (P<0.0001). The Cox proportional hazards model suggested that negative galectin-9 expression in hepatocellular carcinoma represented a significant risk factor for patient survival. We propose that galectin-9 might be a new prognostic factor with antimetastatic potential in patients with hepatocellular carcinoma. 
  [Ee]xtracellular [Mm]atrix  PubMed  
 Galectin-9 binding to cell surface protein disulfide isomerase regulates the redox environment to enhance T-cell migration and HIV entry. Interaction of cell surface glycoproteins with endogenous lectins on the cell surface regulates formation and maintenance of plasma membrane domains, clusters signaling complexes, and controls the residency time of glycoproteins on the plasma membrane. Galectin-9 is a soluble, secreted lectin that binds to glycoprotein receptors to form galectin-glycoprotein lattices on the cell surface. Whereas galectin-9 binding to specific glycoprotein receptors induces death of CD4 Th1 cells, CD4 Th2 cells are resistant to galectin-9 death due to alternative glycosylation. On Th2 cells, galectin-9 binds cell surface protein disulfide isomerase (PDI), increasing retention of PDI on the cell surface and altering the redox status at the plasma membrane. Cell surface PDI regulates integrin function on platelets and also enhances susceptibility of T cells to infection with HIV. We find that galectin-9 binding to PDI on Th2 cells results in increased cell migration through   extracellular matrix   via β3 integrins, identifying a unique mechanism to regulate T-cell migration. In addition, galectin-9 binding to PDI on T cells potentiates infection with HIV. We identify a mechanism for regulating cell surface redox status via a galectin-glycoprotein lattice, to regulate distinct T-cell functions. 
 Galectin-9 binding to cell surface protein disulfide isomerase regulates the redox environment to enhance T-cell migration and HIV entry. Interaction of cell surface glycoproteins with endogenous lectins on the cell surface regulates formation and maintenance of plasma membrane domains, clusters signaling complexes, and controls the residency time of glycoproteins on the plasma membrane. Galectin-9 is a soluble, secreted lectin that binds to glycoprotein receptors to form galectin-glycoprotein lattices on the cell surface. Whereas galectin-9 binding to specific glycoprotein receptors induces death of CD4 Th1 cells, CD4 Th2 cells are resistant to galectin-9 death due to alternative glycosylation. On Th2 cells, galectin-9 binds cell surface protein disulfide isomerase (PDI), increasing retention of PDI on the cell surface and altering the redox status at the plasma membrane. Cell surface PDI regulates integrin function on platelets and also enhances susceptibility of T cells to infection with HIV. We find that galectin-9 binding to PDI on Th2 cells results in increased cell migration through   extracellular matrix   via β3 integrins, identifying a unique mechanism to regulate T-cell migration. In addition, galectin-9 binding to PDI on T cells potentiates infection with HIV. We identify a mechanism for regulating cell surface redox status via a galectin-glycoprotein lattice, to regulate distinct T-cell functions. 
 Galectin-9 as a prognostic factor with antimetastatic potential in breast cancer. Galectin-9, a member of the beta-galactoside-binding galectin family, induces aggregation of certain cell types. We assessed the contribution of galectin-9 to the aggregation of breast cancer cells as well as the relation between galectin-9 expression in tumor tissue and distant metastasis in patients with breast cancer.Subclones of MCF-7 breast cancer cells with high or low levels of galectin-9 expression were established and either cultured on plastic dishes or transplanted into nude mice. The tumors of 84 patients with breast cancer were tested for galectin-9 expression by immunohistochemistry. The patients were followed up for 14 years.MCF-7 subclones with a high level of galectin-9 expression formed tight clusters during proliferation in vitro, whereas a subclone (K10) with the lowest level of galectin-9 expression did not. However, K10 cells stably transfected with a galectin-9 expression vector aggregated in culture and in nude mice. Ectopic expression of galectin-9 also reduced MCF-7 cell adhesion to   extracellular matrix   proteins. Tumors of 42 of the 84 patients were galectin-9 positive, and those of 19 of the 21 patients with distant metastasis were galectin-9 negative. None of the 13 patients with galectin-9-positive tumors and lymph node metastasis up to level II manifested distant metastasis. The cumulative disease-free survival ratio for galectin-9-positive patients was more favorable than that for the galectin-9-negative group (P < 0.0001). Multivariate analysis revealed that galectin-9 status influenced distant metastasis independently of and to a greater extent than lymph node metastasis.Galectin-9 is a possible prognostic factor with antimetastatic potential in breast cancer. 
 Hypothesis: Tim-3/galectin-9, a new pathway for leukemia stem cells survival by promoting expansion of myeloid-derived suppressor cells and differentiating into tumor-associated macrophages. Despite the improvements in chemotherapy, about 60 % of acute myeloid leukemia (AML) remission patients still relapse. Leukemic stem cells (LSCs) are the main causes for the relapse and refractory. T cell immunoglobulin mucin-3 (TIM-3), a specific surface molecule expressed on LSCs in most types of AML, is a candidate for AML LSC-targeted therapies. It is important to know how this molecule functions in the maintenance of LSCs and suppression of anti-tumor immunity. Recent data have shown that Tim-3 which expresses on T cells can suppress immune responses indirectly by inducing expansion of myeloid-derived suppressor cells (MDSCs). MDSCs at the leukemia site can also differentiate into tumor-associated macrophages (TAMs). TAMs can promote proliferation and survival of LSCs by the diversion of adaptive immunity and the facilitation of   extracellular matrix   remodeling, angiogenesis, and lymphangiogenesis. Our previous study in AML patient bone marrow samples showed CD68(+) macrophages around AML clone. Based on the known evidence and our experimental findings, we hypothesize that Tim-3, which specifically expresses on LSCs, is beneficial for LSCs survival and AML progression by promoting expansion of MDSCs and differentiating into TAMs at the leukemia site. 
 Galectin-9 inhibits CD44-hyaluronan interaction and suppresses a murine model of allergic asthma. Galectin-9 (Gal-9) belongs to the galectin family, which exhibits affinity for beta-galactosides. Gal-9 has a variety of biological activities; however, its role in allergic inflammation is unknown.We evaluated the effect of a stable form of the human protein on allergic airway inflammation in a mite allergen-induced asthma model.Human stable Gal-9 was given by intravenous injection to mice during antigen challenge. The effect of Gal-9 on airway inflammation and airway hyperresponsiveness (AHR) was then evaluated.Gal-9 reduced AHR as well as Th2-associated airway inflammation. Furthermore, administration of Gal-9 as well as anti-CD44 monoclonal antibody inhibited the infiltration of peripheral blood Th2 cells into the airway. Interestingly, Gal-9 directly bound the CD44 adhesion molecule and inhibited interactions with hyaluronan (HA). Consistent with the concept that CD44-HA interactions mediate the migration of T cells into the lung, Gal-9 blocked CD44-dependent adhesion of BW5147 mouse T cells to HA.We conclude that Gal-9 inhibits allergic inflammation of the airway and AHR by modulating CD44-dependent leukocyte recognition of the   extracellular matrix  . 
 Different roles of galectin-9 isoforms in modulating E-selectin expression and adhesion function in LoVo colon carcinoma cells. Galectin-9, a member of galectin family, plays multiple roles in a variety of cellular functions, including cell adhesion, aggregation, and apoptosis. Galectin-9 also has three isoforms (named galectin-9L, galectin-9M, and galectin-9S), but whether these isoforms differ in their functions remains poorly understood. In this study, we showed that transient expression of galectin-9L decreased E-selectin levels, while transient expression of galectin-9M or galectin-9S increased E-selectin levels in LoVo cells, which do not express endogenous galectin-9. We also found that over-expression of three galectin-9 isoforms led to increased attachment of LoVo cells to   extracellular matrix   proteins respectively, while over-expression of galectin-9M or galectin-9S increased the adhesion of LoVo cells to human umbilical vein endothelial cells in vitro. In summary, these findings indicate that different isoforms of galectin-9 exhibit distinct biological functions. 
  GeneRIFs  
 Gal-9 inhibits allergic inflammation of the airway and airway hyperresponsiveness by modulating CD44-dependent leukocyte recognition of the   extracellular matrix   
  [Mm]etastasis  PubMed  
 Galectin-9 as a prognostic factor with antimetastatic potential in breast cancer. Galectin-9, a member of the beta-galactoside-binding galectin family, induces aggregation of certain cell types. We assessed the contribution of galectin-9 to the aggregation of breast cancer cells as well as the relation between galectin-9 expression in tumor tissue and distant   metastasis   in patients with breast cancer.Subclones of MCF-7 breast cancer cells with high or low levels of galectin-9 expression were established and either cultured on plastic dishes or transplanted into nude mice. The tumors of 84 patients with breast cancer were tested for galectin-9 expression by immunohistochemistry. The patients were followed up for 14 years.MCF-7 subclones with a high level of galectin-9 expression formed tight clusters during proliferation in vitro, whereas a subclone (K10) with the lowest level of galectin-9 expression did not. However, K10 cells stably transfected with a galectin-9 expression vector aggregated in culture and in nude mice. Ectopic expression of galectin-9 also reduced MCF-7 cell adhesion to extracellular matrix proteins. Tumors of 42 of the 84 patients were galectin-9 positive, and those of 19 of the 21 patients with distant   metastasis   were galectin-9 negative. None of the 13 patients with galectin-9-positive tumors and lymph node   metastasis   up to level II manifested distant   metastasis  . The cumulative disease-free survival ratio for galectin-9-positive patients was more favorable than that for the galectin-9-negative group (P < 0.0001). Multivariate analysis revealed that galectin-9 status influenced distant   metastasis   independently of and to a greater extent than lymph node   metastasis  .Galectin-9 is a possible prognostic factor with antimetastatic potential in breast cancer. 
 Galectin-9 acts as a prognostic factor with antimetastatic potential in hepatocellular carcinoma. Considerable research has been conducted concerning galectin-9 and carcinomas, but little information is available about any relation with the hepatocellular carcinoma. In this study, we employed a small interfering RNA (siRNA) targeting galectin-9 to down-regulate the expression in HepG2 cells. As a result, after galectin-9 expression was reduced, cell aggregation was suppressed, while other behaviour such as the proliferation, adhesion and invasion to ECM, cell-endothelial adhesion and transendothelial invasion of the cells were markedly enhanced. When tumors of 200 patients with hepatocellular carcinoma were tested for galectin-9 expression by immunohistochemistry, binding levels demonstrated intimate correlations with the histopathologic grade, lymph node   metastasis  , vascular invasion and intrahepatic   metastasis   (P<0.05). Moreover, survival analysis indicated that patients with galectin-9 expression had much longer survival time than those with negative lesions, and the Log-rank test indicated that this difference was statistical significant (P<0.0001). The Cox proportional hazards model suggested that negative galectin-9 expression in hepatocellular carcinoma represented a significant risk factor for patient survival. We propose that galectin-9 might be a new prognostic factor with antimetastatic potential in patients with hepatocellular carcinoma. 
 [Galectin-9 isoforms influence the adhesion between colon carcinoma LoVo cells and human umbilical vein endothelial cells in vitro by regulating the expression of E-selectin in LoVo cells]. To study the regulatory effect of galectin-9 isoforms on some molecules involved in cell adhesion/invasion, and the influence of this regulation action on the adhesion between colon carcinoma LoVo cells and human umbilical vein endothelial cells (HUVECs) in vitro.Various expression vectors of galectin-9 isoforms were transfected into LoVo cells. 24 h after transfection, the expression of integrin-beta1, E-cadherin, E-selectin, ICAM-1, CD44 and MMP-9 was detected by RT-PCR and Western blot analysis. LoVo cell-HUVEC adhesion assay was performed under conditions of galectin-9 transfection, galectin-9 transfection + galectin-9 antibody, galectin-9 transfection + E-selectin antibody and galectin-9 transfection + beta-lactose, respectively.Galectin-9L down-regulates the mRNA and protein levels of E-selectin while galectin-9M and galectin-9S up-regulate the expression of E-selectin. In LoVo cell-HUVEC adhesion assay, the average fluorescence intensity of vector transfection group, galectin-9L transfection group, galectin-9M transfection group and galectin-9S transfection group was 0.90 +/- 0.20, 0.94 +/- 0.24, 1.60 +/- 0.11 and 1.45 +/- 0.13, respectively, indicating that galectin-9M and galectin-9S facilitated the adherence of LoVo cells to HUVECs (P < 0.05). E-selectin antibody, galectin-9 antibody or beta-lactose inhibited that effect.Galectin-9 isoforms regulate the E-selectin expression in LoVo cells differently and thus influence the adhesion between LoVo cells and HUVECs in vitro in different modes. The mechanisms through which galectin-9 isoforms participate in the   metastasis   process of colon cancer may not be the same. 
 Galectin-9 as a regulator of cellular adhesion in human oral squamous cell carcinoma cell lines. Oral squamous cell carcinomas (OSCCs), a major public health problem worldwide, are the most common neoplasms of the head and neck. The most important prognostic indicator for patients with OSCC is   metastasis   to cervical lymph nodes or distant organs. Galectin-9 is correlated with cellular adhesion and aggregation in melanoma cells. To investigate expression levels of galectin-9 mRNA and protein, we performed qRT-PCR and Western blot analyses on OSCC cell lines (Ca9-22, HSC-2, and HSC-3) and normal oral keratinocytes (NOKs). Galectin-9 mRNA and protein were commonly down-regulated in OSCC cell lines compared with NOKs. We further analyzed Ca9-22, which had the lowest expression of galectin-9. We then transfected the galectin-9 cDNA into Ca9-22 cells to examine whether overexpression of galectin-9 increases cellular adhesion in vitro. An adhesion assay using a fibronectin and collagen I coating plate revealed an increased cellular adherence ratio in overexpressed galectin-9 cells compared with nontransfected cells (p < 0.05). The data suggest that galectin-9 is correlated with oral cancer cell-matrix interactions and may therefore play an important role in the   metastasis   of OSCCs. 
 Galectin-9: From cell biology to complex disease dynamics. Galectins is a family of non-classically secreted, beta-galactoside-binding proteins that has recently received considerable attention in the spatio-temporal regulation of surface 'signal lattice' organization, membrane dynamics, cell-adhesion and disease therapeutics. Galectin-9 is a unique member of this family, with two non-homologous carbohydrate recognition domains joined by a linker peptide sequence of variable lengths, generating isoforms with distinct properties and functions in both physiological and pathological settings, such as during development, immune reaction, neoplastic transformations and   metastasis  . In this review, we summarize the latest knowledge on the structure, receptors, cellular targets, trafficking pathways and functional properties of galectin-9 and discuss how galectin-9-mediated signalling cascades can be exploited in cancers and immunotherapies. 
 Endothelial LGALS9 splice variant expression in endothelial cell biology and angiogenesis. Galectins are carbohydrate binding proteins with versatile functions in tumor progression. Galectin-9, encoded by LGALS9, has been associated with   metastasis   and immunosuppression. We previously reported on regulation of LGALS9 expression during endothelial cell activation. Here, we show increased galectin-9 protein levels in the endothelium of different tumors, including carcinomas of the lung, liver, breast and kidney. Endothelial cells were found to express five LGALS9 splice variants, two of which have not been reported before. Splicing was found to be confined to exons 5, 6 and 10. Transfection of human microvascular endothelial cells (HMEC) with galectin-9∆5, a specific LGALS9 splice variant, induced a small but significant increase of proliferation, while migration was not affected by any LGALS9 splice variant. Application of recombinant galectin-9∆5 protein dose-dependently reduced proliferation and migration of HMEC as well as human umbilical vein endothelial cells in vitro. Enhanced sprouting and migration of human umbilical vein endothelial cell (HUVEC) towards a galectin-9∆5 gradient were observed. Interestingly, galectin-9∆5 was found to induce a small inhibitory effect on angiogenesis in vivo. Collectively, these data show that endothelial cells regulate the expression and splicing of LGALS9 during angiogenesis. The function of the dominant splice variant, i.e. galectin-9∆5, in endothelial cell biology depends on the concentration and environmental context in which it is presented to the cells. 
 Galectin-9 predicts postoperative recurrence and survival of patients with clear-cell renal cell carcinoma. Galectin-9 (Gal-9), a member of animal lectin family with evolutionary conserved carbohydrate recognition domains, has been reported to exert a large variety of functional roles in tumorigenesis due to its β-galactoside-binding affinity. The aim of this study is to evaluate the expression and prognostic significance of Gal-9 in patients with clear-cell renal cell carcinoma (ccRCC). The expression of Gal-9 was assessed by immunohistochemistry in 196 patients with ccRCC who underwent nephrectomy. In the cohort, 48 patients died and 61 patients suffered recurrence. Kaplan-Meier method with log-rank test was applied to compare survival curves. The authors employed univariate and multivariate Cox regression models to evaluate the prognostic value of Gal-9 expression in overall survival (OS) and recurrence-free survival (RFS). In patients with ccRCC, Gal-9 expression, which was positively associated with tumor size (P = 0.014), Fuhrman grade (P = 0.010), and necrosis (P = 0.025), was determined to be an independent prognostic indicator for OS (hazard ratio [HR] 2.394; P = 0.005) and RFS (HR 2.096; P = 0.006). High expression of Gal-9 was associated with poor survival (P = 0.001) and early recurrence (P = 0.006). Furthermore, Gal-9 expression could significantly stratify the patients in early (gra  des I +�  ��II) tumor, node, and metastasis (TNM) stage (OS: P = 0.005; RFS: P = 0.041) and low (grades 1 + 2) Fuhrman grade (OS: P = 0.004; RFS: P = 0.006). The prognostic accuracy of TNM, SSIGN, and UISS prognostic models was improved when Gal-9 expression was added. Gal-9 expression is a potential independent prognostic factor for OS and RFS in patients with ccRCC. 
 Expression of galectin-9 mRNA in hepatocellular carcinoma. Liver cancer, with a very high prevalence, is one of the most common causes of death worldwide. Galectin-9, a semi-galactoside-binding protein, was demonstrated to be involved in the formation and   metastasis   processes of tumors such as breast cancer, and has significant impact on the development and prognosis of tumor. In this study, 90 cases of liver cancer patients who had liver cancer resection surgery treatment, were selected. Samples of liver cancer tissues and cancer-adjacent tissues from the surgery resection of liver cancer patients, which also confirmed by pathology after operation as specimens, were obtained to detect the expression level of Galectin-9 mRNA. The comparing results showed that there were significant differences between the expression of Galectin-9 mRNA in cancer-adjacent tissues and that in cancer tissues (P < 0.05), in terms of pathology differentiation, TNM, and recurrence transfer aspects. However, there were no obvious correlations with gender, age, the size of tumor, and HBsAg. The expression of Galectin-9 mRNA has a close relationship with pathological differentiation, TNM, and recurrence   metastasis  . Our data presented here provide theoretical basis for new target of liver cancer diagnosis as well as potential prognosis. 
 Role of downregulation of galectin-9 in the tumorigenesis of gastric cancer. Galectin-9 (Gal-9), a member of the β-galactoside-binding galectin family, plays a role in immune response, apoptosis, cell proliferation and cell death. Recent studies have shown that abnormal expression of Gal-9 is involved in certain primary cancers. The present study is the first investigation of the role of Gal-9 gene expression in clinically diagnosed primary gastric cancer tissues. Gal-9 mRNA expression was assessed in 44 clinically diagnosed frozen primary cancer tissue samples using quantitative PCR (qPCR). Analysis of the qPCR data revealed a significant reduction (>2-fold decreased) of Gal-9 gene expression in gastric cancer tissues in 77% (34/44) of patients. In patients with gastric cancer, although no statistically significant difference was found between adjacent (<2 cm away from the cancer tissue) and normal tissues (>5 cm away from the cancer tissue), a >2-fold reduction in Gal-9 expression was observed in the adjacent tissues of 34% of the patients. Compared to matched normal or adjacent tissues, the gene expression of Gal-9 was significantly decreased in tumor tissues (p<0.001). The correlation of Gal-9 expression and clinicopathological features in gastric cancer was analyzed according to the TNM classification system using AJCC stage grouping. In patients with gastric cancer, clinical staging, tumor pathological stage (pT stage), tumor cell differentiation, lymph node   metastasi  s and survival rate were found to be associated with Gal-9 expression. However, no significant association was found between Gal-9 expression and distant   metastasi  s (p>0.05). No significant difference was found between patients of different genders, levels of cell differentiation, distant   metastasi  s status or different survival time of patients. Compared to normal tissues, >2-fold reduction of Tim-3 expression in gastric cancer tissues occurred in 59% of patients, but no correlation was found between Gal-9 and Tim-3 in gastric cancer. These results strongly suggest that Gal-9 is involved in tumorigenesis of gastric cancer. 
 Reduced Expression of Galectin-9 Contributes to a Poor Outcome in Colon Cancer by Inhibiting NK Cell Chemotaxis Partially through the Rho/ROCK1 Signaling Pathway. Galectin-9 is a widely expressed protein that is involved in immune regulation and tumorpathogenesis and serves as a marker of a poor prognosis in various types of cancers. However, the clinical impact and the precise mechanism by which this protein contributes to colon tumor progression are unclear. In the present study, we detected the expression of galectin-9 and CD56 cells using immunohistochemistry. Spearman's rank correlation was used to clarify the association between galectin-9 expression and natural killer (NK) cell infiltration. The influence of galectin-9 on NK-92 cell migration was evaluated in vitro using transwell chemotaxis assays. The role of rh-galectin-9 in F-actin polarization in NK-92 cells was investigated using laser scanning confocal microscopy. We showed that galectin-9 was expressed in 101 (78.91%) colon tumor tissues and that was expressed at lower levels in these tissues than in para-tumor tissues. Low levels of galectin-9 expression were positively correlated with a poor histological grade and lymph node   metastasis   (P<0.05). A Kaplan-Meier method and Cox proportional hazards regression analysis showed that overall survival was longer in patients with high galectin-9 expression in an 8-year follow-up (P<0.05). Spearman's rank correlation indicated that there was a linear correlation between galectin-9 expression and CD56+ NK cell infiltration (R(2) = 0.658; P<0.0001). Galectin-9 stimulated migration in human NK-92 cells by affecting F-actin polarization through the Rho/ROCK1 signaling pathway. These results suggest that galectin-9 expression potentially represents a novel mechanism for tumors to escape immune surveillance in colon tumors. 
 Decreased galectin-9 and increased Tim-3 expression are related to poor prognosis in gastric cancer. Galectin-9 (Gal-9) induces adhesion and aggregation of certain cell types and inhibits the   metastasis   of tumor cells. T-cell immunoglobulin-and mucin domain-3-containing molecule 3 (TIM-3) plays a pivotal role in immune regulation. The aim of this study is to investigate Gal-9 and TIM-3 alterations in gastric cancer and their prognostic values.Gal-9 and Tim-3 expression was evaluated using a tissue microarray immunohistochemistry method in 305 gastric cancers, of which 84 had paired adjacent normal samples. Cell lines SGC-7901, BGC-823, MGC-803, MKN45 and GES-1 were also stained. Correlations were analyzed between expression levels of Gal-9 and Tim-3 protein and tumor parameters or clinical outcomes.Gal-9 and Tim-3 stained positive on tumor cells in 86.2% (263/305), and 60.0% (183/305) patients with gastric cancer, respectively. Gal-9 expression was significantly higher in cancer than in normal mucosa (P<0.001). Reduced Gal-9 expression was associated with lymph-vascular invasion, lymph node   metastasis  , distant   metastasis   and worse TNM staging (P = 0.034, P = 0.009, P = 0.002 and P = 0.043, respectively). In contrast, Tim-3 expression was significantly lower in cancer than in control mucosa (P<0.001). Patients with lymph-vascular invasion had higher expression levels of Tim-3 (P<0.001). Moreover, multivariate analysis shows that both high Gal-9 expression and low Tim-3 expression were significantly associated with long overall survival (P = 0.002, P = 0.010, respectively); the combination of Gal-9 and Tim-3 expression was an independent prognostic predictor for patients with gastric cancer (RR: 0.43; 95%CI: 0.20-0.93). H.pylori infection status was not associated with Gal-9 and Tim-3 expression (P = 0.102, P = 0.565).The results suggest that expression of Gal-9 and Tim-3 in tumor cells may be a potential, independent prognostic factor for patients with gastric cancer. Gal-9 and TIM-3 may play an important part in the gastric carcinogenesis. 
 Changed expression of E-cadherin and galectin-9 in oral squamous cell carcinomas but lack of potential as prognostic markers. The survival rate for oral squamous cell carcinoma (OSCC) has remained generally unchanged in the past three decades, underlining the need for more biomarkers to be developed to aid prognostication and effective management. The prognostic potential of E-cadherin expression in OSCCs has been variable in previous studies while galectin-9 expression has been correlated with improved prognosis in other cancers. The aim of the present study was to investigate the expression of galectin-9 and E-cadherin in OSCC and their potential as prognostic biomarkers.E-cadherin and Galectin-9 expression was examined by immunohistochemistry in 32 cases of OSCC of the buccal mucosa (13 with and 19 without lymph node   metastasis  ), as well as 6 samples of reactive lesions and 5 of normal buccal mucosa.The expression of E-cadherin in OSCC was significantly lower than the control tissues but galectin-9 expression was conversely higher. Median E-cadherin HSCOREs between OSCCs positive and negative for nodal   metastasis   were not significantly different. Mean HSCOREs for galectin-9 in OSCC without lymph node   metastasis   (127.7 ± 81.8) was higher than OSCC with lymph node   metastasi  s (97.9 ± 62.9) but this difference was not statistically significant.E-cadherin expression is reduced whilst galectin-9 expression is increased in OSCC. However, the present results suggest that E-cadherin and galectin-9 expression may not be useful as prognostic markers for OSCC. 
  GeneRIFs  
 The expression of Galectin-9 mRNA has a close relationship with pathological differentiation, tumor staging, and recurrence   metastasis   in hepatocellular carcinoma 
 galectin-9 suppressed   metastasis   of hepatocelllular carcinoma cells in multiple steps; propose that galectin-9 might be a new prognostic factor with antimetastatic potential in patients with hepatocellular carcinoma 
 Data suggest that galectin-9 is correlated with oral cancer cell-matrix interactions and may therefore play an important role in the   metastasis   of oral squamous cell carcinomas. 
  [Cc]ell [Ii]nvasion  PubMed  
 Peroxisome proliferator-activated receptor γ upregulates galectin-9 and predicts prognosis in intestinal-type gastric cancer. The importance of PPARγ (peroxisome proliferator-activated receptor γ) in gastric cancer (GC) is unclear. We investigated the role of PPARγ in GC cell lines and an animal model, and its prognostic significance of PPARγ in GC patients. We controlled PPARγ and galectin-9 expression by using siRNAs and lentiviral constructs. Interaction between PPARγ and galectin-9 was evaluated using luciferase and chromatin immunoprecipitation assays. PPARγ expression in GCs was determined by immunohistochemical staining of tissue microarrays and survival analysis was done. Overexpression of PPARγ was accompanied by increased galectin-9. Enhanced PPARγ or galectin-9 expression increased E-cadherin expression; decreased expression of N-cadherin, fibronectin, snail, twist and slug an  d reduced cel  l invasion and migration. PPARγ bound to the galectin-9 promoter region. Galectin-9 activity increased in PPARγ-overexpressing cells but decreased in PPARγ siRNA-treated cells. In a zebrafish xenograft model, the number of migrated cancer cells and number of fish with AGS cells in the tail vein were reduced in PPARγ-overexpressing GC cells. PPARγ was expressed in 462 of the 688 patients (69.2%) with GC. In 306 patients with intestinal-type GC, those with PPARγ-positive tumors had lower overall and cancer-specific mortalities than those with PPARγ-negative tumors. PPARγ expression was an independent prognostic factor for overall and GC-specific mortality in patients with intestinal-type GC (adjusted hazard ratio, 0.42; 95% CI, 0.22-0.81  ). PPARγ inh  ibits cell invasion, migration and epithelial-mesenchymal transition through upregulation of galectin-9 in vitro and in vivo. 
  GeneRIFs  
 PPARG inhibits   cell invasion  , migration and epithelial-mesenchymal transition through upregulation of galectin-9 in vitro and in vivo. 
   
  
 PLEKHG5 
 
  NCBI Gene  
  GeneRIF  
  Pubmed  
 
 
  Search Term  Rank  Fields  
  [Mm]igration  6  PubMed(1); GeneRIFs(1)  
 
 
 hide/show details for PLEKHG5 
  [Mm]igration  PubMed  
 The Rho guanine nucleotide exchange factor Syx regulates the balance of dia and ROCK activities to promote polarized-cancer-cell   migration  . The role of RhoA in promoting directed cell   migration   has been complicated by studies showing that it is activated both in the front and the rear of migrating cells. We report here that the RhoA-specific guanine nucleotide exchange factor Syx is required for the polarity of actively migrating brain and breast tumor cells. This function of Syx is mediated by the selective activation of the RhoA downstream effector Dia1, the subsequent reorganization of microtubules, and the downregulation of focal adhesions and actin stress fibers. The data argue that directed cell   migration   requires the precise spatiotemporal regulation of Dia1 and ROCK activities in the cell. The recruitment of Syx to the cell membrane and the subsequent selective activation of Dia1 signaling, coupled with the suppression of ROCK and activation of cofilin-mediated actin reorganization, plays a key role in establishing cell polarity during directed cell   migration  . 
  GeneRIFs  
 Recruitment of Syx to the cell membrane, the selective activation of Dia1 signaling, coupled with the suppression of ROCK and actin reorganization, plays a key role in establishing cell polarity during directed cell   migration  . 
   
  
 ME1 
 
  NCBI Gene  
  GeneRIF  
  Pubmed  
 
 
  Search Term  Rank  Fields  
  [Mm]igration  6  PubMed(1)  
  [Ii]nvasive  10  PubMed(2); GeneRIFs(1)  
  [Mm]etastasis  12  PubMed(1)  
 
 
 hide/show details for ME1 
  [Mm]igration  PubMed  
 Repressing malic enzyme 1 redirects glucose metabolism, unbalances the redox state, and attenuates migratory and invasive abilities in nasopharyngeal carcinoma cell lines. A large amount of nicotinamide adenine dinucleotide phosphate (NADPH) is required for fatty acid synthesis and maintenance of the redox state in cancer cells. Malic enzyme 1(ME1)-dependent NADPH production is one of the three pathways that contribute to the formation of the cytosolic NADPH pool. ME1 is generally considered to be overexpressed in cancer cells to meet the high demand for increased de novo fatty acid synthesis. In the present study, we found that glucose induced higher ME1 activity and that repressing ME1 had a profound impact on glucose metabolism of nasopharyngeal carcinoma(NPC) cells. High incorporation of glucose and an enhancement of the pentose phosphate pathway were observed in ME1-repressed cells. However, there were no obvious changes in the other two pathways for glucose metabolism: glycolysis and oxidative phosphorylation. Interestingly, NADPH was decreased under low-glucose condition in ME1-repressed cells relative to wild-type cells, whereas no significant difference was observed under high-glucose condition. ME1-repressed cells had significantly decreased tolerance to low-glucose condition. Moreover, NADPH produced by ME1 was not only important for fatty acid synthesis but also essential for maintenance of the intracellular redox state and the protection of cells from oxidative stress. Furthermore, diminished   migration   and invasion were observed in ME1-repressed cells due to a reduced level of Snail protein. Collectively, these results suggest an essential role for ME1 in the production of cytosolic NADPH and maintenance of migratory and invasive abilities of NPC cells. 
  [Ii]nvasive  PubMed  
 Repressing malic enzyme 1 redirects glucose metabolism, unbalances the redox state, and attenuates migratory and   invasive   abilities in nasopharyngeal carcinoma cell lines. A large amount of nicotinamide adenine dinucleotide phosphate (NADPH) is required for fatty acid synthesis and maintenance of the redox state in cancer cells. Malic enzyme 1(ME1)-dependent NADPH production is one of the three pathways that contribute to the formation of the cytosolic NADPH pool. ME1 is generally considered to be overexpressed in cancer cells to meet the high demand for increased de novo fatty acid synthesis. In the present study, we found that glucose induced higher ME1 activity and that repressing ME1 had a profound impact on glucose metabolism of nasopharyngeal carcinoma(NPC) cells. High incorporation of glucose and an enhancement of the pentose phosphate pathway were observed in ME1-repressed cells. However, there were no obvious changes in the other two pathways for glucose metabolism: glycolysis and oxidative phosphorylation. Interestingly, NADPH was decreased under low-glucose condition in ME1-repressed cells relative to wild-type cells, whereas no significant difference was observed under high-glucose condition. ME1-repressed cells had significantly decreased tolerance to low-glucose condition. Moreover, NADPH produced by ME1 was not only important for fatty acid synthesis but also essential for maintenance of the intracellular redox state and the protection of cells from oxidative stress. Furthermore, diminished migration and invasion were observed in ME1-repressed cells due to a reduced level of Snail protein. Collectively, these results suggest an essential role for ME1 in the production of cytosolic NADPH and maintenance of migratory and   invasive   abilities of NPC cells. 
 Malic enzyme 1 induces epithelial-mesenchymal transition and indicates poor prognosis in hepatocellular carcinoma. Malic enzyme 1 (ME1) links the glycolytic and citric acid cycles and is important for NADPH production, glutamine metabolism, and lipogenesis. Recently, its deregulation has been implicated in the progression of various cancers. However, the role of ME1 in the progression of hepatocellular carcinoma (HCC) remains unclear. In this study, we utilized short hairpin RNA-mediated gene silencing to investigate the biological effects of ME1 depletion in HCC and determined its prognostic significance in HCC. ME1 expression was examined by real-time (RT)-PCR and Western blot using five HCC cell lines and one normal liver cell line. We used polyethylenimine nanoparticles to deliver a short hairpin RNA to induce cessation of ME1 expression in HCC cells. Changes in NADPH production and reactive oxygen species (ROS) production were studied. Metastatic potentials of HCC cells were evaluated in vitro. Furthermore, we evaluated the protein level of ME1 in para-tumor and cancerous tissues of 65 HCC patients with detailed clinical, pathological, and clinical follow-up data. Patients' survivals were further assessed as well. Upregulated ME1 expression was observed in HCC cell lines. Downregulation of ME1 attenuated NADPH production and stimulated ROS production. Silencing ME1 was noted to inhibit migratory and   invasive   properties of HCC cells by inducing the E-cadherin expression and decreasing of N-cadherin and vimentin expression in a ROS-dependent pathway. Overexpression of ME1 was observed in a major fraction of HCC samples. Higher level of ME1 in tumors was significantly associated with reduced overall survival (Kaplan-Meier analysis, P = 0.024) and reduced progression-free survival (Kaplan-Meier analysis, P = 0.011). Inhibition of ME1 expression decreases HCC metastasis via suppression of epithelial-mesenchymal transition (EMT) processes in ROS-induced pathways. ME1 overexpression associates with unfavorable prognoses in patients with HCC, suggesting that ME1 is a poor prognostic predictor of hepatocellular carcinoma. 
  GeneRIFs  
 essential role for ME1 in the production of cytosolic NADPH and maintenance of migratory and   invasive   abilities of nasopharyngeal carcinoma cells 
  [Mm]etastasis  PubMed  
 Malic enzyme 1 induces epithelial-mesenchymal transition and indicates poor prognosis in hepatocellular carcinoma. Malic enzyme 1 (ME1) links the glycolytic and citric acid cycles and is important for NADPH production, glutamine metabolism, and lipogenesis. Recently, its deregulation has been implicated in the progression of various cancers. However, the role of ME1 in the progression of hepatocellular carcinoma (HCC) remains unclear. In this study, we utilized short hairpin RNA-mediated gene silencing to investigate the biological effects of ME1 depletion in HCC and determined its prognostic significance in HCC. ME1 expression was examined by real-time (RT)-PCR and Western blot using five HCC cell lines and one normal liver cell line. We used polyethylenimine nanoparticles to deliver a short hairpin RNA to induce cessation of ME1 expression in HCC cells. Changes in NADPH production and reactive oxygen species (ROS) production were studied. Metastatic potentials of HCC cells were evaluated in vitro. Furthermore, we evaluated the protein level of ME1 in para-tumor and cancerous tissues of 65 HCC patients with detailed clinical, pathological, and clinical follow-up data. Patients' survivals were further assessed as well. Upregulated ME1 expression was observed in HCC cell lines. Downregulation of ME1 attenuated NADPH production and stimulated ROS production. Silencing ME1 was noted to inhibit migratory and invasive properties of HCC cells by inducing the E-cadherin expression and decreasing of N-cadherin and vimentin expression in a ROS-dependent pathway. Overexpression of ME1 was observed in a major fraction of HCC samples. Higher level of ME1 in tumors was significantly associated with reduced overall survival (Kaplan-Meier analysis, P = 0.024) and reduced progression-free survival (Kaplan-Meier analysis, P = 0.011). Inhibition of ME1 expression decrea  ses HCC me  tastasis via suppression of epithelial-mesenchymal transition (EMT) processes in ROS-induced pathways. ME1 overexpression associates with unfavorable prognoses in patients with HCC, suggesting that ME1 is a poor prognostic predictor of hepatocellular carcinoma. 
   
  
 ME2 
 
  NCBI Gene  
  GeneRIF  
  Pubmed  
 
 
  Search Term  Rank  Fields  
  [Mm]igration  6  PubMed(1)  
 
 
 hide/show details for ME2 
  [Mm]igration  PubMed  
 Human mitochondrial NAD(P)(+)-dependent malic enzyme participates in cutaneous melanoma progression and invasion. Cutaneous melanoma is the most life-threatening neoplasm of the skin, accounting for most of the skin cancer deaths. Accumulating evidence suggests that targeting metabolism is an appealing strategy for melanoma therapy. Mitochondrial NAD(P)(+)-dependent malic enzyme (ME2), an oxidative decarboxylase, was evaluated for its biological significance in cutaneous melanoma progression. ME2 mRNA and protein expression significantly increased during melanoma progression, as evidenced by Gene Expression Omnibus analysis and immunohistochemistry on clinically annotated tissue microarrays, respectively. In addition, ME2 knockdown attenuated melanoma cell proliferation in vitro. ME2 ablation resulted in reduced cellular ATP levels and elevated cellular reactive oxygen species production, which activated the AMP-activated protein kinase pathway and inhibited acetyl-CoA carboxylase. Furthermore, ME2 expression was associated with cell   migration   and invasion. ME2 knockdown decreased anchorage-independent growth in vitro and tumor cell growth in vivo. These results suggested that ME2 might be an important factor in melanoma progression and a novel biomarker of invasion. 
   
  
 PAK1 
 
  NCBI Gene  
  GeneRIF  
  Pubmed  
 
 
  Search Term  Rank  Fields  
  [Ff]ilopodia  1  PubMed(15); GeneRIFs(2)  
  [Ff]ilopodium  2  PubMed(1)  
  [Ii]nvadopodia  3  PubMed(3); GeneRIFs(1)  
  [Ii]nvadopodium  4  Component(1)  
  [Mm]igration  6  Process(3); PubMed(73); GeneRIFs(19)  
  ECM  7  PubMed(5)  
  [Ee]xtracellular [Mm]atrix  8  PubMed(10); GeneRIFs(1)  
  [Ii]nvasive  10  PubMed(30); GeneRIFs(5)  
  [Mm]etastasize  11  PubMed(2)  
  [Mm]etastasis  12  PubMed(49); GeneRIFs(10)  
  [Cc]ell [Ii]nvasion  13  PubMed(8); GeneRIFs(2)  
 
 
 hide/show details for PAK1 
  [Ff]ilopodia  PubMed  
 Cdc42/Rac1-dependent activation of the p21-activated kinase (PAK) regulates human platelet lamellipodia spreading: implication of the cortical-actin binding protein cortactin. Platelet activation by thrombin or thrombin receptor-activating peptide (TRAP) results in extensive actin reorganization that leads to   filopodia   emission and lamellae spreading concomitantly with activation of the Rho family small G proteins, Cdc42 and Rac1. Evidence has been provided that direct binding of Cdc42-guanosine triphosphate (GTP) and Rac1-GTP to the N-terminal regulatory domain of the p21-activated kinase (PAK) stimulates PAK activation and actin reorganization. In the present study, we have investigated the relationship between shape change and PAK activation. We show that thrombin, TRAP, or monoclonal antibody (MoAb) anti-Fc(gamma)RIIA IV.3 induces an activation of Cdc42 and Rac1. The GpVI ligand, convulxin (CVX), that forces platelets to lamellae spreading efficiently activates Rac1. Thrombin, TRAP, MoAb IV.3, and CVX stimulate autophosphorylation and kinase activity of PAK. Inhibition of Cdc42 and Rac1 with clostridial toxin B inhibits PAK activation and lamellae spreading. The cortical-actin binding protein, p80/85 cortactin, is constitutively associated with PAK in resting platelets and dissociates from PAK after thrombin stimulation. Inhibition of PAK autophosphorylation by toxin B prevents the dissociation of cortactin. These results suggest that Cdc42/Rac1-dependent activation of PAK may trigger early platelet shape change, at least in part through the regulation of cortactin binding to PAK. 
 Cdc42/Rac1-dependent activation of the p21-activated kinase (PAK) regulates human platelet lamellipodia spreading: implication of the cortical-actin binding protein cortactin. Platelet activation by thrombin or thrombin receptor-activating peptide (TRAP) results in extensive actin reorganization that leads to   filopodia   emission and lamellae spreading concomitantly with activation of the Rho family small G proteins, Cdc42 and Rac1. Evidence has been provided that direct binding of Cdc42-guanosine triphosphate (GTP) and Rac1-GTP to the N-terminal regulatory domain of the p21-activated kinase (PAK) stimulates PAK activation and actin reorganization. In the present study, we have investigated the relationship between shape change and PAK activation. We show that thrombin, TRAP, or monoclonal antibody (MoAb) anti-Fc(gamma)RIIA IV.3 induces an activation of Cdc42 and Rac1. The GpVI ligand, convulxin (CVX), that forces platelets to lamellae spreading efficiently activates Rac1. Thrombin, TRAP, MoAb IV.3, and CVX stimulate autophosphorylation and kinase activity of PAK. Inhibition of Cdc42 and Rac1 with clostridial toxin B inhibits PAK activation and lamellae spreading. The cortical-actin binding protein, p80/85 cortactin, is constitutively associated with PAK in resting platelets and dissociates from PAK after thrombin stimulation. Inhibition of PAK autophosphorylation by toxin B prevents the dissociation of cortactin. These results suggest that Cdc42/Rac1-dependent activation of PAK may trigger early platelet shape change, at least in part through the regulation of cortactin binding to PAK. 
 Cx43 increases serum induced   filopodia   formation via activation of p21-activated protein kinase 1. In a previous study we could show that connexin 43 (Cx43) expression increased the migration of cells in a channel-independent manner involving the MAPK p38. We analyzed here the mechanism by which Cx43 enhanced p38 activation and migration related changes of the actin cytoskeleton. HeLa cells were used as a model system for the controlled expression of Cx43 and truncated Cx43 proteins. The expression of Cx43 altered the actin cytoskeleton organization in response to serum stimulation. Cx43 expressing HeLa cells had significantly more   filopodia  l protrusions per cell than empty-vector transfected control cells. The expression of the channel incompetent carboxyl tail of Cx43 was sufficient to enhance the   filopodia   formation whereas the N-terminal, channel-building part, had no such effect. The enhanced   filopodia   formation was p38 dependent since the p38 blocker SB203580 significantly diminished it. Immunoprecipitation revealed an interaction of the upstream regulator of p38, p21-activated protein kinase 1 (PAK1), with Cx43 resulting in an enhanced phosphorylation of PAK1. Moreover, p38 activation,   filopodia   formation and cell migration were significantly reduced by blocking the PAK1 activity with its pharmacological inhibitor, IPA-3. The p38 target Hsp27, which favors the actin polymerization in its phosphorylated form, was significantly more phosphorylated characterizing it as a potential candidate molecule to enhance the serum-induced actin polymerization in Cx43 expressing cells. Our results provide a novel mechanism by which Cx43 can modify actin cytoskeletal dynamics and may thereby enhance cell migration. 
 Trihydrophobin 1 Interacts with PAK1 and Regulates ERK/MAPK Activation and Cell Migration. The Rac1/Cdc42 effector, p21-activated kinase (PAK), is activated by various signaling cascades, including receptor-tyrosine kinases and integrins, and regulates a number of processes such as cell proliferation and motility. PAK activity has been shown to be required for maximal activation of the canonical RAF-MEK-MAPK signaling cascade, possibly because of PAK co-activation of RAF and MEK. Here we have shown that trihydrophobin 1 (TH1), originally identified as a negative regulator of A-RAF kinase, also interacted with PAK1 in cultured cells. Confocal microscopy assay indicated that TH1 colocalized with PAK1 in both the cytoplasm and nucleus, which is consistent with our previous results. GST pulldown and coimmunoprecipitation experiments demonstrated that TH1 interacted directly with PAK1 and bound selectively to the carboxyl-terminal kinase domain of PAK1, and the ability of the binding was enhanced along with activation of PAK1. The binding pattern of PAK1 implies that this interaction was mediated in part by PAK1 kinase activity. As indicated by in vitro kinase activity assays and Western blot detections, TH1 inhibited PAK1 kinase activity and negatively regulated MAPK signal transduction. Interestingly, TH1 bound with MEK1/ERK in cells and in vitro without directly suppressing their kinase activity. Furthermore, we observed that TH1 localized to focal adhesions and   filopodia   in the leading edge of cells, where TH1 reduced cell migration through affecting actin and adhesion dynamics. Based on these observations, we propose a model in which TH1 interacts with PAK1 and specifically restricts the activation of MAPK modules through the upstream region of the MAPK pathway, thereby influencing cell migration. 
 Heregulin regulates cytoskeletal reorganization and cell migration through the p21-activated kinase-1 via phosphatidylinositol-3 kinase. The mechanisms through which heregulin (HRG) regulates the activities of breast cancer cells are currently unknown. We demonstrate that HRG stimulation of noninvasive breast cancer cells enhanced the conversion of globular to filamentous actin and the formation of membrane ruffles, stress fibers,   filopodia  , and lamellipodia and accompanied by increased cell migration. In addition, HRG triggered a rapid stimulation of p21-activated kinase1 (PAK1) activity and its redistribution into the leading edges of motile cells. The HRG-induced stimulation of PAK1 kinase activity followed phosphatidylinositol-3 kinase (PI-3 kinase) activation. Inhibition of PI-3 kinase activity blocked the activation of PAK1 kinase and also blocked cell migration in response to HRG. Furthermore, direct inhibition of PAK1 functions by the dominant-negative mutant suppressed the capacity of HRG to reorganize actin cytoskeleon structures. We also demonstrated that HRG stimulation promoted physical interactions between PAK1, actin, and human epidermal growth factor receptor 2 (HER2) receptors, and these interactions were dependent on the activation of PI-3 kinase. The blockade of HER2 receptor by an anti-HER2 monoclonal antibody resulted in the inhibition of HRG-mediated stimulation of PI-3 kinase/PAK pathway and also the formation of motile actin cytoskeleton structures but not extracellular signal-regulated kinases. These findings suggest a role of PI-3 kinase/PAK1-dependent reorganization of the cortical actin cytoskeleton in HRG-mediated increased cell migration, and these changes may have significant consequences leading to enhanced invasion by breast cancer cells. 
 Atypical mechanism of regulation of the Wrch-1 Rho family small GTPase. Rho family GTPases are GDP/GTP-regulated molecular switches that regulate signaling pathways controlling diverse cellular processes. Wrch-1 was identified as a Wnt-1 regulated Cdc42 homolog, upregulated by Wnt1 signaling in Wnt1-transformed mouse mammary cells, and was able to promote formation of   filopodia   and activate the PAK serine/threonine kinase. Wrch-1 shares significant sequence and functional similarity with the Cdc42 small GTPase. However, Wrch-1 possesses a unique N-terminal 46 amino acid sequence extension that contains putative Src homology 3 (SH3) domain-interacting motifs. We determined the contribution of the N terminus to Wrch-1 regulation and activity. We observed that Wrch-1 possesses properties that distinguish it from Cdc42 and other Rho family GTPases. Unlike Cdc42, Wrch-1 possesses an extremely rapid, intrinsic guanine nucleotide exchange activity. Although the N terminus did not influence GTPase or GDP/GTP cycling activity in vitro, N-terminal truncation of Wrch-1 enhanced its ability to interact with and activate PAK and to cause growth transformation. The N terminus associated with the Grb2 SH3 domain-containing adaptor protein, and this association increased the levels of active Wrch-1 in cells. We propose that Grb2 overcomes N-terminal negative regulation to promote Wrch-1 effector interaction. Thus, Wrch-1 exhibits an atypical model of regulation not seen in other Rho family GTPases. 
 Wrch1 is a GTPase-deficient Cdc42-like protein with unusual binding characteristics and cellular effects. The Rho family of small GTPases controls many biological processes, including cytoskeletal regulation, membrane trafficking, cell adhesion, cell polarization, transcriptional activity, apoptosis, and cell proliferation. Wrch1, which belongs to the Cdc42 subfamily, is one of the least characterized family member. Despite its homology to other Cdc42-like proteins, we found that Wrch1 has unique characteristics. Biochemical experiments showed that Wrch1 has no detectable GTPase activity in vitro and that its intrinsic nucleotide exchange rate is very high in comparison to Cdc42. Furthermore, NIH3T3 cells transiently transfected with Wrch1 showed an up-rounded, retracted phenotype. In addition, Wrch1 was shown to be more efficient than Cdc42 in triggering the formation of   filopodia  . Serum stimulation of cells expressing Wrch1 induces vigorous membrane blebbing, a phenomenon dependent on the activity of ROCK. In a search for proteins interacting with Wrch1, PAK1 and NCKbeta were identified as binding partners. Interestingly, the interaction to NCKbeta was shown to be mediated via PxxP motifs present in an N-terminal extension of Wrch1 to the second and third SH3 domains of NCKbeta. 
 The atypical Rho GTPase Wrch1 collaborates with the nonreceptor tyrosine kinases Pyk2 and Src in regulating cytoskeletal dynamics. The Cdc42-like GTPase Wnt responsive Cdc42 homolog 1 (Wrch1) has several atypical features; it has an N-terminal proline-rich extension that confers binding to SH3 domains, and it harbors an extremely high intrinsic nucleotide exchange activity, which overrides the normal GTPase activity. As a result, Wrch1 resides mainly in the active, GTP-loaded conformation under normal cellular conditions. We have previously shown that ectopic expression of Wrch1 in fibroblasts resulted in an altered cell morphology visible as a formation of   filopodia  , a loss of stress fibers, and a reduction in focal adhesions. Here, we show that Wrch1 binds to the nonreceptor tyrosine kinase Pyk2. The interaction required Wrch1 to be in a GTP conformation and also required an intact N-terminal proline-rich extension as well as an intact effector loop. Wrch1 requires Pyk2 in imposing the cytoskeletal effects, seen as the formation of   filopodia  , since treatment of cells with a Pyk2-specific small interfering RNA abrogated this response. Interestingly, we found that the presence and activity of Src were needed for the formation of a Wrch1-Pyk2 complex as well as for the Wrch1-induced formation of   filopodia  . We propose a model in which Pyk2 and Src function to coordinate the Wrch1-dependent effects on cytoskeletal dynamics. 
 Contra-regulation of calcium- and integrin-binding protein 1-induced cell migration on fibronectin by PAK1 and MAP kinase signaling. Calcium- and integrin-binding protein 1 (CIB1) has been shown to be involved in cell spreading and migration. The signaling events regulated by CIB1 during cell migration are poorly understood. Here we found that accumulation of CIB1 at the tip of the   filopodia   requires an intact cytoskeleton. Depletion of CIB1 using shRNA affects formation of FAK- and phosphotyrosine-rich focal adhesions without affecting stress fiber formation. Overexpression of CIB1 results in cell migration on fibronectin and Erk1/2 MAP kinase activation. CIB1-induced cell migration is dependent upon Erk1/2 activation, since it is inhibited by the MEK-specific inhibitor PD98059. Furthermore, CIB1-induced cell migration, as well as Erk1/2 activation, is dependent on PKC, Src family kinases as well as PI-3 kinase as it is inhibited by bisindolylmaleimide 1, PP2, and wortmannin, respectively, in a dose-dependent manner. Co-expression of dominant-negative Cdc42 completely abolished CIB1-induced cell migration. Additionally, co-expression of constitutively active, but not dominant negative PAK1, a CIB1 binding protein, inhibited CIB1-induced cell migration. These results suggest that CIB1 positively regulates cell migration and is necessary for the recruitment of FAK to the focal adhesions. Furthermore, CIB1-induced cell migration is dependent on MAP kinase signaling and its function is attenuated by PAK1. 
 Neuronal filopodium formation induced by the membrane glycoprotein M6a (Gpm6a) is facilitated by coronin-1a, Rac1, and p21-activated kinase 1 (Pak1). Stress-responsive neuronal membrane glycoprotein M6a (Gpm6a) functions in neurite extension, filopodium and spine formation and synaptogenesis. The mechanisms of Gpm6a action in these processes are incompletely understood. Previously, we identified the actin regulator coronin-1a (Coro1a) as a putative Gpm6a interacting partner. Here, we used co-immunoprecipitation assays with the anti-Coro1a antibody to show that Coro1a associates with Gpm6a in rat hippocampal neurons. By immunofluorescence microscopy, we demonstrated that in hippocampal neurons Coro1a localizes in F-actin-enriched regions and some of Coro1a spots co-localize with Gpm6a labeling. Notably, the over-expression of a dominant-negative form of Coro1a as well as its down-regulation by siRNA interfered with Gpm6a-induced filopodium formation. Coro1a is known to regulate the plasma membrane translocation and activation of small GTPase Rac1. We show that Coro1a co-immunoprecipitates with Rac1 together with Gpm6a. Pharmacological inhibition of Rac1 resulted in a significant decrease in filopodium formation by Gpm6a. The same was observed upon the co-expression of Gpm6a with the inactive GDP-bound form of Rac1. In this case, the elevated membrane recruitment of GDP-bound Rac1 was detected as well. Moreover, the kinase activity of the p21-activated kinase 1 (Pak1), a main downstream effector of Rac1 that acts downstream of Coro1a, was required for Gpm6a-induced filopodium formation. Taken together, our results provide evidence that a signaling pathway including Coro1a, Rac1, and Pak1 facilitates Gpm6a-induced filopodium formation. Formation of   filopodia   by membrane glycoprotein M6a (Gpm6a) requires actin regulator coronin-1a (Coro1a), known to regulate plasma membrane localization and activation of Rac1 and its downstream effector Pak1. Coro1a associates with Gpm6a. Blockage of Coro1a, Rac1, or Pak1 interferes with Gpm6a-induced filopodium formation. Moreover, Gpm6a facilitates Rac1 membrane recruitment. Altogether, a mechanistic insight into the process of Gpm6a-induced neuronal filopodium formation is provided. 
 Rho GTPases have diverse effects on the organization of the actin filament system. The Rho GTPases are related to the Ras proto-oncogenes and consist of 22 family members. These proteins have important roles in regulating the organization of the actin filament system, and thereby the morphogenesis of vertebrate cells as well as their ability to migrate. In an effort to compare the effects of all members of the Rho GTPase family, active Rho GTPases were transfected into porcine aortic endothelial cells and the effects on the actin filament system were monitored. Cdc42, TCL (TC10-like), Rac1-Rac3 and RhoG induced the formation of lamellipodia, whereas Cdc42, Rac1 and Rac2 also induced the formation of thick bundles of actin filaments. In contrast, transfection with TC10 or Chp resulted in the formation of focal adhesion-like structures, whereas Wrch-1 induced long and thin   filopodia  . Transfection with RhoA, RhoB or RhoC induced the assembly of stress fibres, whereas Rnd1-Rnd3 resulted in the loss of stress fibres, but this effect was associated with the formation of actin- and ezrin-containing dorsal microvilli. Cells expressing RhoD and Rif had extremely long and flexible   filopodia  . None of the RhoBTB or Miro GTPases had any major influence on the organization of the actin filament system; instead, RhoBTB1 and RhoBTB2 were present in vesicular structures, and Miro-1 and Miro-2 were present in mitochondria. Collectively, the data obtained in this study to some extent confirm earlier observations, but also allow the identification of previously undetected roles of the different members of the Rho GTPases. 
 PAK-PIX interactions regulate adhesion dynamics and membrane protrusion to control neurite outgrowth. The roles of P21-activated kinase (PAK) in the regulation of axon outgrowth downstream of extracellular matrix (ECM) proteins are poorly understood. Here we show that PAK1-3 and PIX are expressed in the developing spinal cord and differentially localize to point contacts and   filopodia  l tips within motile growth cones. Using a specific interfering peptide called PAK18, we found that axon outgrowth is robustly stimulated on laminin by partial inhibition of PAK-PIX interactions and PAK function, whereas complete inhibition of PAK function stalls axon outgrowth. Furthermore, modest inhibition of PAK-PIX stimulates the assembly and turnover of growth cone point contacts, whereas strong inhibition over-stabilizes adhesions. Point mutations within PAK confirm the importance of PIX binding. Together our data suggest that regulation of PAK-PIX interactions in growth cones controls neurite outgrowth by influencing the activity of several important mediators of actin filament polymerization and retrograde flow, as well as integrin-dependent adhesion to laminin. 
 Human p21-activated kinase (Pak1) regulates actin organization in mammalian cells. The Rho family GTPases Cdc42, Rac1 and RhoA regulate the reorganization of the actin cytoskeleton induced by extracellular signals such as growth factors. In mammalian cells, Cdc42 regulates the formation of   filopodia  , whereas Rac regulates lamellipodia formation and membrane ruffling, and RhoA regulates the formation of stress fibers. Recently, the serine/threonine protein kinase p65(pak) autophosphorylates, thereby increasing its catalytic activity towards exogenous substrates. This kinase is therefore a candidate effector for the changes in cell shape induced by growth factors.Here, we report that the microinjection of activated Pak1 protein into quiescent Swiss 3T3 cells induces the rapid formation of polarized   filopodia   and membrane ruffles. The prolonged overexpression of Pak1 amino-terminal mutants that are unable to bind Cdc42 or Rac1 results in the accumulation of filamentous actin in large, polarized membrane ruffles and the formation of vinculin-containing focal complexes within these structures. This phenotype resembles that seen in motile fibroblasts. The amino-terminal Pak1 mutant displays enhanced binding to the adaptor protein Nck, which contains three Src-homology 3 (SH3) domains. Mutation of a proline residue within a conserved SH3-binding region at the amino terminus of Pak1 interferes with SH3-protein binding and alters the effects of Pak1 on the cytoskeleton.These results indicate that Pak1, acting through a protein that contains an SH3 domain, regulates the structure of the actin cytoskeleton in mammalian cells, and may serve as an effector for Cdc42 and/or Rac1 in promoting cell motility. 
 Localization of p21-activated kinase 1 (PAK1) to pinocytic vesicles and cortical actin structures in stimulated cells. The mechanisms through which the small GTPases Rac1 and Cdc42 regulate the formation of membrane ruffles, lamellipodia, and   filopodia   are currently unknown. The p21-activated kinases (PAKs) are direct targets of active Rac and Cdc42 which can induce the assembly of polarized cytoskeletal structures when expressed in fibroblasts, suggesting that they may play a role in mediating the effects of these GTPases on cytoskeletal dynamics. We have examined the subcellular localization of endogenous PAK1 in fibroblast cell lines using specific PAK1 antibodies. PAK1 is detected in submembranous vesicles in both unstimulated and stimulated fibroblasts that colocalize with a marker for fluid-phase uptake. In cells stimulated with PDGF, in v-Src-transformed fibroblasts, and in wounded cells, PAK1 redistributed into dorsal and membrane ruffles and into the edges of lamellipodia, where it colocalizes with polymerized actin. PAK1 was also colocalized with F-actin in membrane ruffles extended as a response to constitutive activation of Rac1. PAK1 appears to precede F-actin in translocating to cytoskeletal structures formed at the cell periphery. The association of PAK1 with the actin cytoskeleton is prevented by the actin filament-disrupting agent cytochalasin D and by the phosphatidylinositol 3-kinase inhibitor wortmannin. Co-immunoprecipitation experiments demonstrate an in vivo interaction of PAK1 with filamentous (F)-actin in stimulated cells. Microinjection of a constitutively active PAK1 mutant into Rat-1 fibroblasts overexpressing the insulin receptor (HIRcB cells) induced the formation of F-actin- and PAK1-containing structures reminiscent of dorsal ruffles. These data indicate a close correlation between the subcellular distribution of endogenous PAK1 and the formation of Rac/Cdc42-dependent cytoskeletal structures and support an active role for PAK1 in regulating cortical actin rearrangements. 
 Activation of PAK1/2 during the shedding of platelet microvesicles. Simultaneously to phospholipid flip-flop that supports the procoagulant activity of activated platelets, blebs, supported by actin reorganization, are formed at the plasma membrane and generate microvesicles. The molecular mechanism of microvesicle shedding from activated platelets implicates Ca influx and Ca-dependent protease, calpain. We previously demonstrated that the formation of lamellipodias and   filopodia  s associated with platelet shape change involved the reorganization of actin filaments through a Cdc42/Rac1/p21-activated kinase (PAK)-dependent pathway. Here, we investigated whether platelet blebbing also depends on the Cdc42/Rac1/PAK pathway. Exposure of platelets in vitro to either a mixture of thrombin receptor-activating peptide (TRAP) and collagen or the Ca ionophore A23187 in the presence of Ca generates microvesicles that can be identified by flow cytometry. The calpain inhibitor, calpeptin, diminished microvesicle formation induced by the Ca ionophore A23187, confirming the role of calpain in this process. PAK1/2 is cleaved in a calpain-dependent manner, and calpeptin prevents this cleavage and allows a transient activation of the kinase. Inhibition of Cdc42 and Rac1 by toxin B from Clostridium difficile, that suppresses PAK1/2 activation induced by TRAP and collagen or by A23187 in the presence of calpeptin, decreases polymerization of actin, lamellipodia and   filopodia   formation and interferes with the shedding of microvesicles. We conclude that the Rac1/Cdc42/PAK pathway controls actin reorganization that is necessary for microvesicle shedding. 
  GeneRIFs  
 Formation of   filopodia   by membrane glycoprotein M6a (Gpm6a) requires actin regulator coronin-1a (Coro1a), known to regulate plasma membrane localization and activation of Rac1 and its downstream effector Pak1. 
 Cx43 increases serum induced   filopodia   formation via activation of p21-activated protein kinase 1. 
  [Ff]ilopodium  PubMed  
 Neuronal   filopodium   formation induced by the membrane glycoprotein M6a (Gpm6a) is facilitated by coronin-1a, Rac1, and p21-activated kinase 1 (Pak1). Stress-responsive neuronal membrane glycoprotein M6a (Gpm6a) functions in neurite extension,   filopodium   and spine formation and synaptogenesis. The mechanisms of Gpm6a action in these processes are incompletely understood. Previously, we identified the actin regulator coronin-1a (Coro1a) as a putative Gpm6a interacting partner. Here, we used co-immunoprecipitation assays with the anti-Coro1a antibody to show that Coro1a associates with Gpm6a in rat hippocampal neurons. By immunofluorescence microscopy, we demonstrated that in hippocampal neurons Coro1a localizes in F-actin-enriched regions and some of Coro1a spots co-localize with Gpm6a labeling. Notably, the over-expression of a dominant-negative form of Coro1a as well as its down-regulation by siRNA interfered with Gpm6a-induced   filopodium   formation. Coro1a is known to regulate the plasma membrane translocation and activation of small GTPase Rac1. We show that Coro1a co-immunoprecipitates with Rac1 together with Gpm6a. Pharmacological inhibition of Rac1 resulted in a significant decrease in   filopodium   formation by Gpm6a. The same was observed upon the co-expression of Gpm6a with the inactive GDP-bound form of Rac1. In this case, the elevated membrane recruitment of GDP-bound Rac1 was detected as well. Moreover, the kinase activity of the p21-activated kinase 1 (Pak1), a main downstream effector of Rac1 that acts downstream of Coro1a, was required for Gpm6a-induced   filopodium   formation. Taken together, our results provide evidence that a signaling pathway including Coro1a, Rac1, and Pak1 facilitates Gpm6a-induced   filopodium   formation. Formation of filopodia by membrane glycoprotein M6a (Gpm6a) requires actin regulator coronin-1a (Coro1a), known to regulate plasma membrane localization and activation of Rac1 and its downstream effector Pak1. Coro1a associates with Gpm6a. Blockage of Coro1a, Rac1, or Pak1 interferes with Gpm6a-induced   filopodium   formation. Moreover, Gpm6a facilitates Rac1 membrane recruitment. Altogether, a mechanistic insight into the process of Gpm6a-induced neuronal   filopodium   formation is provided. 
  [Ii]nvadopodia  PubMed  
 A Trio-Rac1-Pak1 signalling axis drives   invadopodia   disassembly. Rho family GTPases control cell migration and participate in the regulation of cancer metastasis.   Invadopodia  , associated with invasive tumour cells, are crucial for cellular invasion and metastasis. To study Rac1 GTPase in   invadopodia   dynamics, we developed a genetically encoded, single-chain Rac1 fluorescence resonance energy (FRET) transfer biosensor. The biosensor shows Rac1 activity exclusion from the core of   invadopodia  , and higher activity when   invadopodia   disappear, suggesting that reduced Rac1 activity is necessary for their stability, and Rac1 activation is involved in disassembly. Photoactivating Rac1 at   invadopodia   confirmed this previously unknown Rac1 function. We describe here an   invadopodia   disassembly model, where a signalling axis involving TrioGEF, Rac1, Pak1, and phosphorylation of cortactin, causes   invadopodia   dissolution. This mechanism is critical for the proper turnover of invasive structures during tumour cell invasion, where a balance of proteolytic activity and locomotory protrusions must be carefully coordinated to achieve a maximally invasive phenotype. 
 A Trio-Rac1-Pak1 signalling axis drives   invadopodia   disassembly. Rho family GTPases control cell migration and participate in the regulation of cancer metastasis.   Invadopodia  , associated with invasive tumour cells, are crucial for cellular invasion and metastasis. To study Rac1 GTPase in   invadopodia   dynamics, we developed a genetically encoded, single-chain Rac1 fluorescence resonance energy (FRET) transfer biosensor. The biosensor shows Rac1 activity exclusion from the core of   invadopodia  , and higher activity when   invadopodia   disappear, suggesting that reduced Rac1 activity is necessary for their stability, and Rac1 activation is involved in disassembly. Photoactivating Rac1 at   invadopodia   confirmed this previously unknown Rac1 function. We describe here an   invadopodia   disassembly model, where a signalling axis involving TrioGEF, Rac1, Pak1, and phosphorylation of cortactin, causes   invadopodia   dissolution. This mechanism is critical for the proper turnover of invasive structures during tumour cell invasion, where a balance of proteolytic activity and locomotory protrusions must be carefully coordinated to achieve a maximally invasive phenotype. 
 PAK1 regulates RUFY3-mediated gastric cancer cell migration and invasion. Actin protrusion at the cell periphery is central to the formation of   invadopodia   during tumor cell migration and invasion. Although RUFY3 (RUN and FYVE domain containing 3)/SINGAR1 (single axon-related1)/RIPX (Rap2 interacting protein X) has an important role in neuronal development, its pathophysiologic role and relevance to cancer are still largely unknown. The purpose of this study was to elucidate the molecular mechanisms by which RUFY3 involves in gastric cancer cell migration and invasion. Here, our data show that overexpression of RUFY3 leads to the formation of F-actin-enriched protrusive structures at the cell periphery and induces gastric cancer cell migration. Furthermore, P21-activated kinase-1 (PAK1) interacts with RUFY3, and promotes RUFY3 expression and RUFY3-induced gastric cancer cell migration; inhibition of PAK1 attenuates RUFY3-induced SGC-7901 cell migration and invasion. Importantly, we found that the inhibitory effect of cell migration and invasion is significantly enhanced by knockdown of both PAK1 and RUFY3 compared with knockdown of RUFY3 alone or PAK1 alone. Strikingly, we found significant upregulation of RUFY3 in gastric cancer samples with invasive carcinoma at pathologic TNM III and TNM IV stages, compared with their non-tumor counterparts. Moreover, an obvious positive correlation was observed between the protein expression of RUFY3 and PAK1 in 40 pairs of gastric cancer samples. Therefore, these findings provide important evidence that PAK1 can positively regulate RUFY3 expression, which contribute to the metastatic potential of gastric cancer cells, maybe blocking PAK1-RUFY3 signaling would become a potential metastasis therapeutic strategy for gastric cancer. 
  GeneRIFs  
 Describe here an   invadopodia   disassembly model, where a signalling axis involving TrioGEF, Rac1, Pak1, and phosphorylation of cortactin, causes   invadopodia   dissolution. 
  [Ii]nvadopodium  Component  
   invadopodium   
  [Mm]igration  Process  
 positive regulation of cell   migration   
 positive regulation of fibroblast   migration   
 positive regulation of vascular associated smooth muscle cell   migration   
  PubMed  
 A small GTPase‑like protein fragment of Mycoplasma promotes tumor cel  l migrati  on and proliferation in vitro via interaction with Rac1 and Stat3. The Mycoplasma genus comprises a group of microbes that cause persistent infection in humans and its role in promoting tumor development has long been a concern. Although mixtures of components isolated from Mycoplasma have been shown to activate host Rho family small GTPases and Stat3, no individual factor with this activity has been reported. In the current study, a conserved small GTPase-like protein fragment (SGLP) from Mycoplasma pulmonis chromosome partition protein, Smc, was identified as a virulence factor. SGLP was observed to interact with Rac1 and Stat3. The wild‑type (wt) SGLP, which contains a WxxxE motif, induced activation of Rac1 and phosphorylation of Stat3 at the tyrosine‑705 residue, while the SGLP mutant containing a mutation from WxxxE to AxxxA did not exert the same effects. Moreover, SGLP‑induced Stat3 phosphorylation was observed to be dependent upon Rac1 activity. Furthermore, wt SGLP was observed to prom  ote cell   migration and increase bromodeoxyuridine incorporation in HeLa cells and the SGLP mutant did not elicit these effects in HeLa cells. In conclusion, the current observations suggest that SGLP is an important virulence factor of Mycoplasma, which contributes to tu  mor cell   migration and proliferation in vitro via interaction with Rac1 and Stat3. 
 RhoG activates Rac1 by direct interaction with the Dock180-binding protein Elmo. The small GTPase Rac has a central role in regulating the actin cytoskeleton during cell   migration   and axon guidance. Elmo has been identified as an upstream regulator of Rac1 that binds to and functionally cooperates with Dock180 (refs 2-4). Dock180 does not contain a conventional catalytic domain for guanine nucleotide exchange on Rac, but possesses a domain that directly binds to and specifically activates Rac1 (refs 5, 6). The small GTPase RhoG mediates several cellular morphological processes, such as neurite outgrowth in neuronal cells, through a signalling cascade that activates Rac1 (refs 7-12); however, the downstream target of RhoG and the mechanism by which RhoG regulates Rac1 activity remain unclear. Here we show that RhoG interacts directly with Elmo in a GTP-dependent manner and forms a ternary complex with Dock180 to induce activation of Rac1. The RhoG-Elmo-Dock180 pathway is required for activation of Rac1 and cell spreading mediated by integrin, as well as for neurite outgrowth induced by nerve growth factor. We conclude that RhoG activates Rac1 through Elmo and Dock180 to control cell morphology. 
 16-kDa prolactin inhibits endothelial cell   migration   by down-regulating the Ras-Tiam1-Rac1-Pak1 signaling pathway. Angiogenesis plays a key role in promoting tumorigenesis and metastasis. The 16-kDa fragment of prolactin (16k PRL) is an NH(2)-terminal natural breakdown fragment of the intact 23-kDa prolactin and has been shown to have potent antiangiogenic and antitumor activities. The mechanism(s) involved in the action of 16k PRL in endothelial cells remains unclear. In this study, we showed that 16k PRL reduced rat aortic endothelial cell (RAEC)   migration   in a wound-healing assay and in a Matrigel tube formation assay, suggesting that 16k PRL inhibits endothelial cell   migration  , an important activity involved in angiogenesis and tumorigenesis. We further investigated how 16k PRL attenuates endothelial cell   migration  . We first showed that RAEC   migration   is mediated through the Rho GTPase Rac1, as Rac1 inhibition by the Rac1-specific inhibitor NSC27366 or Rac1 knockdown by small interfering RNA both blocked RAEC   migration  . We next showed that 16k PRL reduced the activation of Rac1 in a concentration-dependent manner. Furthermore, 16k PRL inhibition of Rac1 is mediated through the suppression of T lymphoma invasion and metastasis 1 (Tiam1) and its upstream activator Ras in a phosphoinositide-3-kinase-independent manner. 16k PRL also down-regulated the phosphorylation of the downstream effector of Rac1, p21-activating kinase 1 (Pak1), and inhibited its translocation to the leading edge of migrating cells. Thus, 16k PRL inhibits cell   migration   by blocking the Ras-Tiam1-Rac1-Pak1 signaling pathway in endothelial cells. 
 β-arrestin-dependent activation of the cofilin pathway is required for the scavenging activity of the atypical chemokine receptor D6. Chemokines promote the recruitment of leukocytes to sites of infection and inflammation by activating conventional heterotrimeric guanine nucleotide-binding protein (G protein)-coupled receptors (GPCRs). Chemokines are also recognized by a set of atypical chemokine receptors (ACRs), which cannot induce directional cell   migratio  n but are required for the generation of chemokine gradients in tissues. ACRs are presently considered "silent receptors" because no G protein-dependent signaling activity is observed after their engagement by cognate ligands. We report that engagement of the ACR D6 by its ligands activates a β-arrestin1-dependent, G protein-independent signaling pathway that results in the phosphorylation of the actin-binding protein cofilin through the Rac1-p21-activated kinase 1 (PAK1)-LIM kinase 1 (LIMK1) cascade. This signaling pathway is required for the increased abundance of D6 protein at the cell surface and for its chemokine-scavenging activity. We conclude that D6 is a signaling receptor that exerts its regulatory function on chemokine-mediated responses in inflammation and immunity through a distinct signaling pathway. 
 Rac1-induced cell   migration   requires membrane recruitment of the nuclear oncogene SET. The Rho GTPase Rac1 controls cell adhesion and motility. The effector loop of Rac1 mediates interactions with downstream effectors, whereas its C-terminus binds the exchange factor beta-Pix, which mediates Rac1 targeting and activation. Here, we report that Rac1, through its C-terminus, also binds the nuclear oncogene SET/I2PP2A, an inhibitor of the serine/threonine phosphatase PP2A. We found that SET translocates to the plasma membrane in cells that express active Rac1 as well as in migrating cells. Membrane targeting of SET stimulates cell   migration   in a Rac1-dependent manner. Conversely, reduction of SET expression inhibits Rac1-induced   migration  , indicating that efficient Rac1 signalling requires membrane recruitment of SET. The recruitment of the SET oncogene to the plasma membrane represents a new feature of Rac1 signalling. Our results suggest a model in which Rac1-stimulated cell motility requires both effector loop-based downstream signalling and recruitment of a signalling amplifier, that is, SET, through the hypervariable C-terminus. 
 Paxillin LD4 motif binds PAK and PIX through a novel 95-kD ankyrin repeat, ARF-GAP protein: A role in cytoskeletal remodeling. Paxillin is a focal adhesion adaptor protein involved in the integration of growth factor- and adhesion-mediated signal transduction pathways. Repeats of a leucine-rich sequence named paxillin LD motifs (Brown M.C., M.S. Curtis, and C.E. Turner. 1998. Nature Struct. Biol. 5:677-678) have been implicated in paxillin binding to focal adhesion kinase (FAK) and vinculin. Here we demonstrate that the individual paxillin LD motifs function as discrete and selective protein binding interfaces. A novel scaffolding function is described for paxillin LD4 in the binding of a complex of proteins containing active p21 GTPase-activated kinase (PAK), Nck, and the guanine nucleotide exchange factor, PIX. The association of this complex with paxillin is mediated by a new 95-kD protein, p95PKL (paxillin-kinase linker), which binds directly to paxillin LD4 and PIX. This protein complex also binds to Hic-5, suggesting a conservation of LD function across the paxillin superfamily. Cloning of p95PKL revealed a multidomain protein containing an NH2-terminal ARF-GAP domain, three ankyrin-like repeats, a potential calcium-binding EF hand, calmodulin-binding IQ motifs, a myosin homology domain, and two paxillin-binding subdomains (PBS). Green fluorescent protein- (GFP-) tagged p95PKL localized to focal adhesions/complexes in CHO.K1 cells. Overexpression in neuroblastoma cells of a paxillin LD4 deletion mutant inhibited lamellipodia formation in response to insulin-like growth fac- tor-1. Microinjection of GST-LD4 into NIH3T3 cells significantly decreased cell   migration   into a wound. These data implicate paxillin as a mediator of p21 GTPase-regulated actin cytoskeletal reorganization through the recruitment to nascent focal adhesion structures of an active PAK/PIX complex potentially via interactions with p95PKL. 
 A Trio-Rac1-Pak1 signalling axis drives invadopodia disassembly. Rho family GTPases control cell   migration   and participate in the regulation of cancer metastasis. Invadopodia, associated with invasive tumour cells, are crucial for cellular invasion and metastasis. To study Rac1 GTPase in invadopodia dynamics, we developed a genetically encoded, single-chain Rac1 fluorescence resonance energy (FRET) transfer biosensor. The biosensor shows Rac1 activity exclusion from the core of invadopodia, and higher activity when invadopodia disappear, suggesting that reduced Rac1 activity is necessary for their stability, and Rac1 activation is involved in disassembly. Photoactivating Rac1 at invadopodia confirmed this previously unknown Rac1 function. We describe here an invadopodia disassembly model, where a signalling axis involving TrioGEF, Rac1, Pak1, and phosphorylation of cortactin, causes invadopodia dissolution. This mechanism is critical for the proper turnover of invasive structures during tumour cell invasion, where a balance of proteolytic activity and locomotory protrusions must be carefully coordinated to achieve a maximally invasive phenotype. 
 Scrib regulates PAK activity during the cell   migration   process. Genetic studies have highlighted the key role of Scrib in the development of Metazoans. Deficiency in Scrib impairs many aspects of cell polarity and cell movement although the mechanisms involved remain unclear. In mammals, Scrib belongs to a protein complex containing betaPIX, an exchange factor for Rac/Cdc42, and GIT1, a GTPase activating protein for ARF6 implicated in receptor recycling and exocytosis. Here we show that the Scrib complex associates with PAK, a serine-threonine kinase family crucial for cell   migration  . PAK colocalizes with members of the Scrib complex at the leading edge of heregulin-treated T47D breast cancer cells. We demonstrate that the Scrib complex is required for epithelial cells and primary mouse embryonic fibroblasts to efficiently respond to chemoattractant cues. In Scrib-deficient cells, the pool of cortical PAK is decreased, thereby precluding its proper activation by Rac. Loss of Scrib also impairs the polarized distribution of active Rac at the leading edge and compromises the regulated activation of the GTPase in T47D cells and mouse embryonic fibroblasts. These data underscore the role of Scrib in cell   migration   and show the strong impact of Scrib in the function of PAK and Rac, two key molecules implicated in this process. 
 IAPs regulate the plasticity of cell   migration   by directly targeting Rac1 for degradation. Inhibitors of apoptosis proteins (IAPs) are a highly conserved class of multifunctional proteins. Rac1 is a well-studied Rho GTPase that controls numerous basic cellular processes. While the regulation of nucleotide binding to Rac1 is well understood, the molecular mechanisms controlling Rac1 degradation are not known. Here, we demonstrate X-linked IAP (XIAP) and cellular IAP1 (c-IAP1) directly bind to Rac1 in a nucleotide-independent manner to promote its polyubiquitination at Lys147 and proteasomal degradation. These IAPs are also required for degradation of Rac1 upon CNF1 toxin treatment or RhoGDI depletion. Consistently, downregulation of XIAP or c-IAP1 by various strategies led to an increase in Rac1 protein levels in primary and tumour cells, leading to an elongated morphology and enhanced cell   migration  . Further, XIAP counteracts Rac1-dependent cellular polarization in the developing zebrafish hindbrain and promotes the delamination of neurons from the normal tissue architecture. These observations unveil an evolutionarily conserved role of IAPs in controlling Rac1 stability thereby regulating the plasticity of cell   migration   and morphogenesis. 
 Invasion of breast cancer cells into collagen matrix requires TGF-α and Cdc42 signaling. Invasion of MDA-MB-231 breast cancer cells into three-dimensional (3-D) type I-collagen matrices depends on TGF-α. We characterized the steps of invasion mediated by TGF-α. Ce  ll migrat  ion, as observed by videomicroscopy, was effectively stimulated by collagen, suggesting that TGF-α may specifically participate in the invasion of a 3-D collagen matrix. We assessed the role of small GTPases of the Rho family in the invasion. Cdc42 was found to be necessary for invasion but dispensable for c  ell migra  tion. These results suggest that TGF-α mediates invasion into 3-D collagen matrices by initiating the formation of protrusions into collagen, likely through activation of Cdc42. 
 Adiponectin promotes   migration   activities of endothelial progenitor cells via Cdc42/Rac1. Adiponectin has anti-atherosclerotic effects through its direct actions on vascular cells. The present study investigates the molecular mechanisms of adiponectin in the   migration   of endothelial progenitor cells (EPCs) which play an important role in neovascularization and re-endothelization. The phosphorylation of Akt and the activations of Cdc42 and Rac1 were significantly increased by adiponectin. Adiponectin increased the   migration   activity of EPCs, which was completely inhibited by a PI3-kinase inhibitor. siRNA of Cdc42 or Rac1 completely inhibited the adiponectin-induced   migration  , but siRNA of Akt had no effects, indicating that adiponectin promotes the   migration   activities of EPCs mainly through PI3-kinase/Cdc42/Rac1. 
 Reciprocal regulation of Rac1 and PAK-1 by HIF-1alpha: a positive-feedback loop promoting pulmonary vascular remodeling. Pulmonary vascular remodeling associated with pulmonary hypertension is characterized by media thickening, disordered proliferation, and in situ thrombosis. The p21-activated kinase-1 (PAK-1) can control growth,   migration  , and prothrombotic activity, and the hypoxia-inducible transcription factor HIF-1alpha was associated with pulmonary vascular remodeling. Here we studied whether PAK-1 and HIF-1alpha are linked in pulmonary vascular remodeling. PAK-1 was expressed in the media of remodeled pulmonary vessels from patients with pulmonary vasculopathy and was upregulated, together with its upstream regulator Rac1 and HIF-1alpha in lung tissue from lambs with pulmonary vascular remodeling. PAK-1 and Rac1 were activated by thrombin involving calcium, thus resulting in enhanced generation of reactive oxygen species (ROS) in human pulmonary artery smooth muscle cells (PASMCs). Activation of PAK-1 stimulated HIF activity and HIF-1alpha expression involving ROS and NF-kappaB, enhanced the expression of the HIF-1 target gene plasminogen activator inhibitor-1, and stimulated PASMC proliferation. Importantly, HIF-1 itself bound to the Rac1 promoter and enhanced Rac1 and PAK-1 transcription. Thus, PAK-1 and its activator Rac1 are novel HIF-1 targets that may constitute a positive-feedback loop for induction of HIF-1alpha by thrombin and ROS, thus explaining elevated levels of PAK-1, Rac1, and HIF-1alpha in remodeled pulmonary vessels. 
 Opposing roles for Akt1 and Akt2 in Rac/Pak signaling and cell   migration  . The Akt/PKB isoforms have different roles in animals, with Akt2 primarily regulating metabolic signaling and Akt1 regulating growth and survival. Here we show distinct roles for Akt1 and Akt2 in mouse embryo fibroblast cell   migration   and regulation of the cytoskeleton. Akt1-deficient cells responded poorly to platelet-derived growth factor while Akt2-deficient cells had a dramatically enhanced response, resulting in a substantial increase in dorsal ruffling. Swapping domains between Akt1 and Akt2 demonstrated that the N-terminal region containing the pleckstrin homology domain and a linker region distinguishes the two isoforms, while the catalytic domains are interchangeable. Akt2 knock-out cells also migrated faster than wild-type cells, especially through extracellular matrix (ECM), while Akt1 knock-out cells migrated more slowly than wild-type cells. Consistently, Akt2 knock-out cells had elevated Pak1 and Rac activities, suggesting that Akt2 inhibits Rac and Pak1. Both Akt2 and Akt1 associated in complexes with Pak1, but only Akt2 inhibited Pak1 in kinase assays, suggesting an underlying molecular basis for the different cellular phenotypes. Together these data provide evidence for an unexpected functional link between Akt2 and Pak1 that opposes the actions of Akt1 on cell   migration  . 
 Adhesion molecule L1 stimulates neuronal   migration   through Vav2-Pak1 signaling. Organized neuronal   migration   and guided axon outgrowth are key determinants of the development of the functional nervous system. L1, a member of the Ig superfamily of cell surface receptors, stimulates cell   migration   and neurite outgrowth through the MAP kinases ERK1, 2. The signaling molecules participating in this signaling cascade have only partly been identified. Here it is shown that L1 clustering activates the guanine nucleotide exchange factor (GEF) Vav2 and the Rac1 effector p21 associated kinase 1 (Pak1). Also, we found that Pak1 kinase activity contributes to ERK activation by L1, and is necessary for L1-potentiated haptotactic cell   migration  . A signaling pathway is proposed from L1 through Vav2, Rac1, Pak1 and ERK that may be important for L1 mediated neuronal cell   migration  . 
 srGAP2 arginine methylation regulates cell   migration   and cell spreading through promoting dimerization. The Slit-Robo GTPase-activating proteins (srGAPs) are critical for neuronal   migration   through inactivation of Rho GTPases Cdc42, Rac1, and RhoA. Here we report that srGAP2 physically interacts with protein arginine methyltransferase 5 (PRMT5). srGAP2 localizes to the cytoplasm and plasma membrane protrusion. srGAP2 knockdown reduces cell adhesion spreading and increases cell   migration  , but has no effect on cell proliferation. PRMT5 binds to the N terminus of srGAP2 (225-538 aa) and methylates its C-terminal arginine residue Arg-927. The methylation mutant srGAP2-R927A fails to rescue the cell spreading rate, is unable to localize to the plasma membrane leading edge, and perturbs srGAP2 homodimer formation mediated by the F-BAR domain. These results suggest that srGAP2 arginine methylation plays important roles in cell spreading and cell   migration   through influencing membrane protrusion. 
 Tyrosyl phosphorylated PAK1 regulates breast cancer cell motility in response to prolactin through filamin A. The p21-activated serine-threonine kinase (PAK1) is activated by small GTPase-dependent and -independent mechanisms and regulates cell motility. Both PAK1 and the hormone prolactin (PRL) have been implicated in breast cancer by numerous studies. We have previously shown that the PRL-activated tyrosine kinase JAK2 (Janus tyrosine kinase 2) phosphorylates PAK1 in vivo and identified tyrosines (Tyr) 153, 201, and 285 in the PAK1 molecule as sites of JAK2 tyrosyl phosphorylation. Here, we have used human breast cancer T47D cells stably overexpressing PAK1 wild type or PAK1 Y3F mutant in which Tyr(s) 153, 201, and 285 were mutated to phenylalanines to demonstrate that phosphorylation of these three tyrosines are required for maximal PRL-dependent ruffling. In addition, phosphorylation of these three tyrosines is required for increased   migration   of T47D cells in response to PRL as assessed by two independent motility assays. Finally, we show that PAK1 phosphorylates serine (Ser) 2152 of the actin-binding protein filamin A to a greater extent when PAK1 is tyrosyl phosphorylated by JAK2. Down-regulation of PAK1 or filamin A abolishes the effect of PRL on cell   migration  . Thus, our data presented here bring some insight into the mechanism of PRL-stimulated motility of breast cancer cells. 
 14-3-3zeta Cooperates with ErbB2 to promote ductal carcinoma in situ progression to invasive breast cancer by inducing epithelial-mesenchymal transition. ErbB2, a metastasis-promoting oncoprotein, is overexpressed in approximately 25% of invasive/metastatic breast cancers, but in 50%-60% of noninvasive ductal carcinomas in situ (DCIS). It has been puzzling how a subset of ErbB2-overexpressing DCIS develops into invasive breast cancer (IBC). We found that co-overexpression of 14-3-3zeta in ErbB2-overexpressing DCIS conferred a higher risk of progression to IBC. ErbB2 and 14-3-3zeta overexpression, respectively, increased cell   migration   and decreased cell adhesion, two prerequisites of tumor cell invasion. 14-3-3zeta overexpression reduced cell adhesion by activating the TGF-beta/Smads pathway that led to ZFHX1B/SIP-1 upregulation, E-cadherin loss, and epithelial-mesenchymal transition. Importantly, patients whose breast tumors overexpressed both ErbB2 and 14-3-3zeta had higher rates of metastatic recurrence and death than those whose tumors overexpressed only one. 
 GIT2 represses Crk- and Rac1-regulated cell spreading and Cdc42-mediated focal adhesion turnover. G protein-coupled receptor kinase interactors (GITs) regulate focal adhesion (FA) turnover, cell spreading, and motility through direct interaction with paxillin and the Rac-exchange factor Pak-interacting exchange factor beta (betaPIX). However, it is not clear whether GITs function to activate or repress motility or if the predominant GIT forms, GIT1 and GIT2, serve distinct or redundant roles. Here we demonstrate an obligatory role for endogenous GIT2 in repression of lamellipodial extension and FA turnover by Rac1- and Cdc42-dependent signaling pathways, respectively. Moreover, we show that the SH2-SH3 adaptor protein Crk is an essential target of GIT2 inhibition. Unexpectedly, we find that betaPIX is dispensable for the effects elicited by knockdown of GIT2. Finally, we show that loss of GIT2 is sufficient to induce   migration   of the nontransformed epithelial cell line MCF10A. These results suggest that inactivation of GIT2 function is a required step for induction of cell motility and that GIT2 may be a target of oncogenic signaling pathways that regulate cell   migration  . 
 Targeting and activation of Rac1 are mediated by the exchange factor beta-Pix. Rho guanosine triphosphatases (GTPases) are critical regulators of cytoskeletal dynamics and control complex functions such as cell adhesion, spreading,   migration  , and cell division. It is generally accepted that localized GTPase activation is required for the proper initiation of downstream signaling events, although the molecular mechanisms that control targeting of Rho GTPases are unknown. In this study, we show that the Rho GTPase Rac1, via a proline stretch in its COOH terminus, binds directly to the SH3 domain of the Cdc42/Rac activator beta-Pix (p21-activated kinase [Pak]-interacting exchange factor). The interaction with beta-Pix is nucleotide independent and is necessary and sufficient for Rac1 recruitment to membrane ruffles and to focal adhesions. In addition, the Rac1-beta-Pix interaction is required for Rac1 activation by beta-Pix as well as for Rac1-mediated spreading. Finally, using cells deficient for the beta-Pix-binding kinase Pak1, we show that Pak1 regulates the Rac1-beta-Pix interaction and controls cell spreading and adhesion-induced Rac1 activation. These data provide a model for the intracellular targeting and localized activation of Rac1 through its exchange factor beta-Pix. 
 Cancer cell-autonomous TRAIL-R signaling promotes KRAS-driven cancer progression, invasion, and metastasis. Many cancers harbor oncogenic mutations of KRAS. Effectors mediating cancer progression, invasion, and metastasis in KRAS-mutated cancers are only incompletely understood. Here we identify cancer cell-expressed murine TRAIL-R, whose main function ascribed so far has been the induction of apoptosis as a crucial mediator of KRAS-driven cancer progression, invasion, and metastasis and in vivo Rac-1 activation. Cancer cell-restricted genetic ablation of murine TRAIL-R in autochthonous KRAS-driven models of non-small-cell lung cancer (NSCLC) and pancreatic ductal adenocarcinoma (PDAC) reduces tumor growth, blunts metastasis, and prolongs survival by inhibiting cancer cell-autonomous   migratio  n, proliferation, and invasion. Consistent with this, high TRAIL-R2 expression correlates with invasion of human PDAC into lymph vessels and with shortened metastasis-free survival of KRAS-mutated colorectal cancer patients. 
 Genome-wide analysis of chromosomal alterations in patients with esophageal squamous cell carcinoma exposed to tobacco and betel quid from high-risk area in India. Genomic alterations such as chromosomal amplifications, deletions and loss of heterozygosity play an important role in the pathogenesis and progression of cancer. Environmental risk factors contribute to the development and progression of tumors by facilitating the loss of tumor suppressor genes and amplification of oncogenes. In this current study, Affymetrix 10K single nucleotide polymorphism (SNP) arrays were used to evaluate genomic alterations in 20 pairs of matched germ-line and tumor DNA obtained from patients with esophageal squamous cell carcinoma (ESCC) from high-risk area of India where tobacco, betel quid and alcohol use are widespread. Twenty-two amplified regions and 16 deleted regions identified across chromosomal arms were biologically relevant. The candidate genes located at amplified regions of chromosomes or low-level gain regions such as PLA2G5 (1p36-p34), COL11A1 (1p21), KCNK2 (1q41), S100A3 (1q21), ENAH (1q42.12), RGS1 (1q31), KCNH1 (1q32-q41), INSIG2 (2q14.1), FGF12 (3q28), TRIO (5p15.2), RNASEN (5p15.2), FGF10 (5p13-p12), EDN1(6p24.1-p22.3), SULF1 (8q13.2-13.3), TLR4 (9q32-q33), TNC (9q33), NTRK2 (9q22.1), CD44 (11p13), NCAM1 (11q23.1), TRIM29 (11q22-q23), PAK1 (11q13-q14) and RAB27A (15q15-q21.1), are found to be associated with cellular   migration   and proliferation, tumor cell metastasis and invasion, anchorage independent growth and inhibition of apoptosis. The candidate genes located at deleted regions of chromosomes, such as FBLN2 (3p25.1), WNT7A (3p25), DLC1 (8p22), LZTS1 (8p22), CDKN2A (9p21), COL4A1 (13q34), CDK8 (13q12) and DCC (18q21.3), are found to be associated with the suppression of tumor. The suggested candidate genes were mostly involved in potential signaling pathways such as focal adhesion (COL4A1), tight junction (CLDN10), MAPK signaling pathway (FGF12) and neuroactive ligand receptor interaction pathway (CCKAR). Expression of FGF12 and COL4A1 was validated by tissue microarray. These unique copy number alteration profiles should be taken into consideration when developing biomarkers for the early detection of ESCC in high-risk areas of India in association with tobacco and betel quid use. 
 A Trio-Rac1-Pak1 signalling axis drives invadopodia disassembly. Rho family GTPases control cell   migration   and participate in the regulation of cancer metastasis. Invadopodia, associated with invasive tumour cells, are crucial for cellular invasion and metastasis. To study Rac1 GTPase in invadopodia dynamics, we developed a genetically encoded, single-chain Rac1 fluorescence resonance energy (FRET) transfer biosensor. The biosensor shows Rac1 activity exclusion from the core of invadopodia, and higher activity when invadopodia disappear, suggesting that reduced Rac1 activity is necessary for their stability, and Rac1 activation is involved in disassembly. Photoactivating Rac1 at invadopodia confirmed this previously unknown Rac1 function. We describe here an invadopodia disassembly model, where a signalling axis involving TrioGEF, Rac1, Pak1, and phosphorylation of cortactin, causes invadopodia dissolution. This mechanism is critical for the proper turnover of invasive structures during tumour cell invasion, where a balance of proteolytic activity and locomotory protrusions must be carefully coordinated to achieve a maximally invasive phenotype. 
 Tyrosyl phosphorylated PAK1 regulates breast cancer cell motility in response to prolactin through filamin A. The p21-activated serine-threonine kinase (PAK1) is activated by small GTPase-dependent and -independent mechanisms and regulates cell motility. Both PAK1 and the hormone prolactin (PRL) have been implicated in breast cancer by numerous studies. We have previously shown that the PRL-activated tyrosine kinase JAK2 (Janus tyrosine kinase 2) phosphorylates PAK1 in vivo and identified tyrosines (Tyr) 153, 201, and 285 in the PAK1 molecule as sites of JAK2 tyrosyl phosphorylation. Here, we have used human breast cancer T47D cells stably overexpressing PAK1 wild type or PAK1 Y3F mutant in which Tyr(s) 153, 201, and 285 were mutated to phenylalanines to demonstrate that phosphorylation of these three tyrosines are required for maximal PRL-dependent ruffling. In addition, phosphorylation of these three tyrosines is required for increased   migration   of T47D cells in response to PRL as assessed by two independent motility assays. Finally, we show that PAK1 phosphorylates serine (Ser) 2152 of the actin-binding protein filamin A to a greater extent when PAK1 is tyrosyl phosphorylated by JAK2. Down-regulation of PAK1 or filamin A abolishes the effect of PRL on cell   migration  . Thus, our data presented here bring some insight into the mechanism of PRL-stimulated motility of breast cancer cells. 
 Reciprocal regulation of Rac1 and PAK-1 by HIF-1alpha: a positive-feedback loop promoting pulmonary vascular remodeling. Pulmonary vascular remodeling associated with pulmonary hypertension is characterized by media thickening, disordered proliferation, and in situ thrombosis. The p21-activated kinase-1 (PAK-1) can control growth,   migration  , and prothrombotic activity, and the hypoxia-inducible transcription factor HIF-1alpha was associated with pulmonary vascular remodeling. Here we studied whether PAK-1 and HIF-1alpha are linked in pulmonary vascular remodeling. PAK-1 was expressed in the media of remodeled pulmonary vessels from patients with pulmonary vasculopathy and was upregulated, together with its upstream regulator Rac1 and HIF-1alpha in lung tissue from lambs with pulmonary vascular remodeling. PAK-1 and Rac1 were activated by thrombin involving calcium, thus resulting in enhanced generation of reactive oxygen species (ROS) in human pulmonary artery smooth muscle cells (PASMCs). Activation of PAK-1 stimulated HIF activity and HIF-1alpha expression involving ROS and NF-kappaB, enhanced the expression of the HIF-1 target gene plasminogen activator inhibitor-1, and stimulated PASMC proliferation. Importantly, HIF-1 itself bound to the Rac1 promoter and enhanced Rac1 and PAK-1 transcription. Thus, PAK-1 and its activator Rac1 are novel HIF-1 targets that may constitute a positive-feedback loop for induction of HIF-1alpha by thrombin and ROS, thus explaining elevated levels of PAK-1, Rac1, and HIF-1alpha in remodeled pulmonary vessels. 
 Genome-wide analysis of chromosomal alterations in patients with esophageal squamous cell carcinoma exposed to tobacco and betel quid from high-risk area in India. Genomic alterations such as chromosomal amplifications, deletions and loss of heterozygosity play an important role in the pathogenesis and progression of cancer. Environmental risk factors contribute to the development and progression of tumors by facilitating the loss of tumor suppressor genes and amplification of oncogenes. In this current study, Affymetrix 10K single nucleotide polymorphism (SNP) arrays were used to evaluate genomic alterations in 20 pairs of matched germ-line and tumor DNA obtained from patients with esophageal squamous cell carcinoma (ESCC) from high-risk area of India where tobacco, betel quid and alcohol use are widespread. Twenty-two amplified regions and 16 deleted regions identified across chromosomal arms were biologically relevant. The candidate genes located at amplified regions of chromosomes or low-level gain regions such as PLA2G5 (1p36-p34), COL11A1 (1p21), KCNK2 (1q41), S100A3 (1q21), ENAH (1q42.12), RGS1 (1q31), KCNH1 (1q32-q41), INSIG2 (2q14.1), FGF12 (3q28), TRIO (5p15.2), RNASEN (5p15.2), FGF10 (5p13-p12), EDN1(6p24.1-p22.3), SULF1 (8q13.2-13.3), TLR4 (9q32-q33), TNC (9q33), NTRK2 (9q22.1), CD44 (11p13), NCAM1 (11q23.1), TRIM29 (11q22-q23), PAK1 (11q13-q14) and RAB27A (15q15-q21.1), are found to be associated with cellular   migration   and proliferation, tumor cell metastasis and invasion, anchorage independent growth and inhibition of apoptosis. The candidate genes located at deleted regions of chromosomes, such as FBLN2 (3p25.1), WNT7A (3p25), DLC1 (8p22), LZTS1 (8p22), CDKN2A (9p21), COL4A1 (13q34), CDK8 (13q12) and DCC (18q21.3), are found to be associated with the suppression of tumor. The suggested candidate genes were mostly involved in potential signaling pathways such as focal adhesion (COL4A1), tight junction (CLDN10), MAPK signaling pathway (FGF12) and neuroactive ligand receptor interaction pathway (CCKAR). Expression of FGF12 and COL4A1 was validated by tissue microarray. These unique copy number alteration profiles should be taken into consideration when developing biomarkers for the early detection of ESCC in high-risk areas of India in association with tobacco and betel quid use. 
 16-kDa prolactin inhibits endothelial cell   migration   by down-regulating the Ras-Tiam1-Rac1-Pak1 signaling pathway. Angiogenesis plays a key role in promoting tumorigenesis and metastasis. The 16-kDa fragment of prolactin (16k PRL) is an NH(2)-terminal natural breakdown fragment of the intact 23-kDa prolactin and has been shown to have potent antiangiogenic and antitumor activities. The mechanism(s) involved in the action of 16k PRL in endothelial cells remains unclear. In this study, we showed that 16k PRL reduced rat aortic endothelial cell (RAEC)   migration   in a wound-healing assay and in a Matrigel tube formation assay, suggesting that 16k PRL inhibits endothelial cell   migration  , an important activity involved in angiogenesis and tumorigenesis. We further investigated how 16k PRL attenuates endothelial cell   migration  . We first showed that RAEC   migration   is mediated through the Rho GTPase Rac1, as Rac1 inhibition by the Rac1-specific inhibitor NSC27366 or Rac1 knockdown by small interfering RNA both blocked RAEC   migration  . We next showed that 16k PRL reduced the activation of Rac1 in a concentration-dependent manner. Furthermore, 16k PRL inhibition of Rac1 is mediated through the suppression of T lymphoma invasion and metastasis 1 (Tiam1) and its upstream activator Ras in a phosphoinositide-3-kinase-independent manner. 16k PRL also down-regulated the phosphorylation of the downstream effector of Rac1, p21-activating kinase 1 (Pak1), and inhibited its translocation to the leading edge of migrating cells. Thus, 16k PRL inhibits cell   migration   by blocking the Ras-Tiam1-Rac1-Pak1 signaling pathway in endothelial cells. 
 Opposing roles for Akt1 and Akt2 in Rac/Pak signaling and cell   migration  . The Akt/PKB isoforms have different roles in animals, with Akt2 primarily regulating metabolic signaling and Akt1 regulating growth and survival. Here we show distinct roles for Akt1 and Akt2 in mouse embryo fibroblast cell   migration   and regulation of the cytoskeleton. Akt1-deficient cells responded poorly to platelet-derived growth factor while Akt2-deficient cells had a dramatically enhanced response, resulting in a substantial increase in dorsal ruffling. Swapping domains between Akt1 and Akt2 demonstrated that the N-terminal region containing the pleckstrin homology domain and a linker region distinguishes the two isoforms, while the catalytic domains are interchangeable. Akt2 knock-out cells also migrated faster than wild-type cells, especially through extracellular matrix (ECM), while Akt1 knock-out cells migrated more slowly than wild-type cells. Consistently, Akt2 knock-out cells had elevated Pak1 and Rac activities, suggesting that Akt2 inhibits Rac and Pak1. Both Akt2 and Akt1 associated in complexes with Pak1, but only Akt2 inhibited Pak1 in kinase assays, suggesting an underlying molecular basis for the different cellular phenotypes. Together these data provide evidence for an unexpected functional link between Akt2 and Pak1 that opposes the actions of Akt1 on cell   migration  . 
 p21-Activated kinase-1 promotes aggressive phenotype, cell proliferation, and invasion in gestational trophoblastic disease. Gestational trophoblastic disease (GTD) includes hydatidiform mole (HM), which can develop persistent gestational trophoblastic neoplasia requiring chemotherapy; choriocarcinoma, which is a frankly malignant tumor; placental site trophoblastic tumor; and epithelioid trophoblastic tumor. p21-Activated kinases (PAKs) promote malignant tumor progression. Therefore, this study investigated PAK1, PAK2, and p-PAK2 Ser(20) in the pathogenesis of GTD. By real-time PCR, PAK1 mRNA was significantly higher in HMs, particularly metastatic HMs (P = 0.046) and HMs that developed persistent disease (P = 0.011), when compared with normal placentas. By immunohistochemistry, significantly increased cytoplasmic PAK1 immunoreactivity in cytotrophoblasts was also detected in HMs (P = 0.042) and choriocarcinomas (P = 0.003). In addition, HMs that developed persistent disease displayed higher PAK1 immunoreactivity than those that regressed (P = 0.016), and elevated PAK1 immunoreactivity was observed in placental site trophoblastic tumors. Indeed, there was significant positive correlation between PAK1 expression and the proliferative indices Ki-67 (P = 0.016) and MCM7 (P = 0.026). Moreover, higher PAK1 mRNA and protein expression was confirmed in the choriocarcinoma cell-lines JEG-3 and JAR; however, PAK2 mRNA and p-PAK2 immunoreactivity showed a similar expression pattern in normal first trimester placentas and GTD. Knockdown of PAK1 in JEG-3 and JAR reduced cell proliferation,   migration  , and invasion ability, up-regulated p16, and down-regulated vascular endothelial growth factor and MT1-MMP expression. This is the first report revealing the involvement of PAK1 in the pathogenesis and clinical progress of GTD. 
 Cx43 increases serum induced filopodia formation via activation of p21-activated protein kinase 1. In a previous study we could show that connexin 43 (Cx43) expression increased the   migration   of cells in a channel-independent manner involving the MAPK p38. We analyzed here the mechanism by which Cx43 enhanced p38 activation and   migration   related changes of the actin cytoskeleton. HeLa cells were used as a model system for the controlled expression of Cx43 and truncated Cx43 proteins. The expression of Cx43 altered the actin cytoskeleton organization in response to serum stimulation. Cx43 expressing HeLa cells had significantly more filopodial protrusions per cell than empty-vector transfected control cells. The expression of the channel incompetent carboxyl tail of Cx43 was sufficient to enhance the filopodia formation whereas the N-terminal, channel-building part, had no such effect. The enhanced filopodia formation was p38 dependent since the p38 blocker SB203580 significantly diminished it. Immunoprecipitation revealed an interaction of the upstream regulator of p38, p21-activated protein kinase 1 (PAK1), with Cx43 resulting in an enhanced phosphorylation of PAK1. Moreover, p38 activation, filopodia formation and cell   migration   were significantly reduced by blocking the PAK1 activity with its pharmacological inhibitor, IPA-3. The p38 target Hsp27, which favors the actin polymerization in its phosphorylated form, was significantly more phosphorylated characterizing it as a potential candidate molecule to enhance the serum-induced actin polymerization in Cx43 expressing cells. Our results provide a novel mechanism by which Cx43 can modify actin cytoskeletal dynamics and may thereby enhance cell   migration  . 
 Trihydrophobin 1 Interacts with PAK1 and Regulates ERK/MAPK Activation and Cell   Migration  . The Rac1/Cdc42 effector, p21-activated kinase (PAK), is activated by various signaling cascades, including receptor-tyrosine kinases and integrins, and regulates a number of processes such as cell proliferation and motility. PAK activity has been shown to be required for maximal activation of the canonical RAF-MEK-MAPK signaling cascade, possibly because of PAK co-activation of RAF and MEK. Here we have shown that trihydrophobin 1 (TH1), originally identified as a negative regulator of A-RAF kinase, also interacted with PAK1 in cultured cells. Confocal microscopy assay indicated that TH1 colocalized with PAK1 in both the cytoplasm and nucleus, which is consistent with our previous results. GST pulldown and coimmunoprecipitation experiments demonstrated that TH1 interacted directly with PAK1 and bound selectively to the carboxyl-terminal kinase domain of PAK1, and the ability of the binding was enhanced along with activation of PAK1. The binding pattern of PAK1 implies that this interaction was mediated in part by PAK1 kinase activity. As indicated by in vitro kinase activity assays and Western blot detections, TH1 inhibited PAK1 kinase activity and negatively regulated MAPK signal transduction. Interestingly, TH1 bound with MEK1/ERK in cells and in vitro without directly suppressing their kinase activity. Furthermore, we observed that TH1 localized to focal adhesions and filopodia in the leading edge of cells, where TH1 reduced cell   migration   through affecting actin and adhesion dynamics. Based on these observations, we propose a model in which TH1 interacts with PAK1 and specifically restricts the activation of MAPK modules through the upstream region of the MAPK pathway, thereby influencing cell   migration  . 
 Autophosphorylation-dependent degradation of Pak1, triggered by the Rho-family GTPase, Chp. The Paks (p21-activated kinases) Pak1, Pak2 and Pak3 are among the most studied effectors of the Rho-family GTPases, Rac, Cdc42 (cell division cycle 42) and Chp (Cdc42 homologous protein). Pak kinases influence a variety of cellular functions, but the process of Pak down-regulation, following activation, is poorly understood. In the present study, we describe for the first time a negative-inhibitory loop generated by the small Rho-GTPases Cdc42 and Chp, resulting in Pak1 inhibition. Upon overexpression of Chp, we unexpectedly observed a T-cell   migration   phenotype consistent with Paks inhibition. In line with this observation, overexpression of either Chp or Cdc42 caused a marked reduction in the level of Pak1 protein in a number of different cell lines. Chp-induced degradation was accompanied by ubiquitination of Pak1, and was dependent on the proteasome. The susceptibility of Pak1 to Chp-induced degradation depended on its p21-binding domain, kinase activity and a number of Pak1 autophosphorylation sites, whereas the PIX- (Pak-interacting exchange factor) and Nck-binding sites were not required. Together, these results implicate Chp-induced kinase autophosphorylation in the degradation of Pak1. The N-terminal domain of Chp was found to be required for Chp-induced degradation, although not for Pak1 activation, suggesting that Chp provides a second function, distinct from kinase activation, to trigger Pak degradation. Collectively, our results demonstrate a novel mechanism of signal termination mediated by the Rho-family GTPases Chp and Cdc42, which results in ubiquitin-mediated degradation of one of their direct effectors, Pak1. 
 FRAX597, a PAK1 inhibitor, synergistically reduces pancreatic cancer growth when combined with gemcitabine. Pancreatic ductal adenocarcinoma remains one of the most lethal of all solid tumours. Treatment options are limited and gemcitabine-based chemotherapy remains the standard of care. Although growing evidence shows that p21-activated kinase 1 (PAK1) plays a crucial role in pancreatic cancer, its role has not been fully elucidated. This study aimed to characterise the expression and functional relevance of PAK1 in pancreatic cancer.PAK1 expression was measured in pancreatic cancer specimens by immunohistochemistry and in pancreatic cancer cell lines by western blotting. The effect of inhibition of PAK1 by either shRNA knock-down (KD), or by a selective inhibitor, FRAX597, alone or in combination with gemcitabine, on cell proliferation and   migration  /invasion was measured by thymidine uptake and Boyden chamber assays, respectively. The effect on tumour growth and survival was assessed in orthotopic murine models.PAK1 was expressed in all human pancreatic cancer samples tested, an7d was upregulated in all pancreatic cancer cell lines tested. PAK1 KD inhibited pancreatic cancer cell growth and survival, and increased sensitivity to gemcitabine treatment. AKT activity and HIF1α expression were also inhibited. FRAX597 inhibited pancreatic cancer cell proliferation, survival, and   migratio  n/invasion. When combined with gemcitabine, FRAX597 synergistically inhibited pancreatic cancer proliferation in vitro and inhibited tumour growth in vivo.These results implicate PAK1 as a regulator of pancreatic cancer cell growth and survival. Combination of a PAK1 inhibitor such as FRAX597 with cytotoxic chemotherapy deserves further study as a novel therapeutic approach to pancreatic cancer treatment. 
 Heregulin regulates cytoskeletal reorganization and cell   migration   through the p21-activated kinase-1 via phosphatidylinositol-3 kinase. The mechanisms through which heregulin (HRG) regulates the activities of breast cancer cells are currently unknown. We demonstrate that HRG stimulation of noninvasive breast cancer cells enhanced the conversion of globular to filamentous actin and the formation of membrane ruffles, stress fibers, filopodia, and lamellipodia and accompanied by increased cell   migration  . In addition, HRG triggered a rapid stimulation of p21-activated kinase1 (PAK1) activity and its redistribution into the leading edges of motile cells. The HRG-induced stimulation of PAK1 kinase activity followed phosphatidylinositol-3 kinase (PI-3 kinase) activation. Inhibition of PI-3 kinase activity blocked the activation of PAK1 kinase and also blocked cell   migration   in response to HRG. Furthermore, direct inhibition of PAK1 functions by the dominant-negative mutant suppressed the capacity of HRG to reorganize actin cytoskeleon structures. We also demonstrated that HRG stimulation promoted physical interactions between PAK1, actin, and human epidermal growth factor receptor 2 (HER2) receptors, and these interactions were dependent on the activation of PI-3 kinase. The blockade of HER2 receptor by an anti-HER2 monoclonal antibody resulted in the inhibition of HRG-mediated stimulation of PI-3 kinase/PAK pathway and also the formation of motile actin cytoskeleton structures but not extracellular signal-regulated kinases. These findings suggest a role of PI-3 kinase/PAK1-dependent reorganization of the cortical actin cytoskeleton in HRG-mediated increased cell   migration  , and these changes may have significant consequences leading to enhanced invasion by breast cancer cells. 
 Lysophosphatidic acid induces MDA-MB-231 breast cancer cells   migration   through activation of PI3K/PAK1/ERK signaling. Enhanced motility of cancer cells is a critical step in promoting tumor metastasis. Lysophosphatidic acid (LPA), representing the major mitogenic activity in serum, stimulates   migration   in various types of cancer cells. However, the underlying signaling mechanisms for LPA-induced motility of cancer cells remain to be elucidated.In this study, we found that LPA dose-dependently stimulated   migration   of MDA-MB-231 breast cancer cells, with 10 µM being the most effective. LPA also increased ERK activity and the MEK inhibitor U0126 could block LPA-induced ERK activity and cell   migratio  n. In addition, LPA induced PAK1 activation while ERK activation and cell   migratio  n were inhibited by ectopic expression of an inactive mutant form of PAK1 in MDA-MB-231 cells. Furthermore, LPA increased PI3K activity, and the PI3K inhibitor LY294002 inhibited both LPA-induced PAK1/ERK activation and cell   migratio  n. Moreover, in the breast cancer cell, LPA treatment resulted in remarkable production of reactive oxygen species (ROS), while LPA-induced ROS generation, PI3K/PAK1/ERK activation and cell   migratio  n could be inhibited by N-acetyl-L-Cysteine, a scavenger of ROS.Taken together, this study identifies a PI3K/PAK1/ERK signaling pathway for LPA-stimulated breast cancer cell   migratio  n. These data also suggest that ROS generation plays an essential role in the activation of LPA-stimulated PI3K/PAK1/ERK signaling and breast cancer cell   migratio  n. These findings may provide a basis for designing future therapeutic strategy for blocking breast cancer metastasis. 
 Phorbol ester-induced   migration   of HepG2 cells is accompanied by intensive stress fibre formation, enhanced integrin expression and transient down-regulation of p21-activated kinase 1. Previously, we observed that phorbol ester induced more intensive scattering of HepG2 human hepatoma cells than hepatocyte growth factor (HGF). Regulatory components accounting for this intensive   migration   were studied. Phorbol ester-activated protein kinase C induced the early appearance of a great number of actin stress fibres. Whereas in response to HGF, the activation of phosphatidylinositol 3-kinase initiates the rearrangements of the actin cytoskeleton, in phorbol ester-treated cells, the activation of this enzyme was not required to the actin polymerisation. Activation of Erk1/Erk2 MAP kinases that was essential to the   migration   had a key role in enhancing the adherence of cells to the extracellular matrix via the increased expression of integrins alpha2, alpha6 and beta1. Protein kinase C stimulated the activation of p21-activated kinase (PAK), as well. However, it also stimulated the selective and transient down-regulation of PAK1, which coincided with the formation of stress fibres. 
 p21-Activated kinase 1 coordinates aberrant cell survival and pericellular proteolysis in a three-dimensional culture model for premalignant progression of human breast cancer. Overexpression of p21-activated kinase 1 (PAK1) occurs during the progression of human breast cancer. We have investigated the role of PAK1 in the premalignant progression of the MCF10 series of human breast epithelial cell lines. Levels of PAK1 expression and activation increased with premalignant progression, and expression of dominant-negative (DN) PAK1 reduced both cell proliferation and   migration  /invasion. In three-dimensional (3D) overlay cultures in reconstituted basement membrane, the MCF10 series produced an in vitro model for premalignant progression. MCF10AneoT cells formed a hyperplastic morphology in which some spheroids developed abnormal lumens. The MCF10.AT1 line exhibited an atypical hyperplastic morphology of abnormal spheroid clusters that did not form lumens. The MCF10.DCIS cells exhibited dysplastic growth. Expression of DN-PAK1 promoted lumen formation in 3D-cultured MCF10A, NeoT, and AT1 structures, suggesting partial reversion of the premalignant phenotype, but did not affect the atypical budding of AT1 structures or the dysplastic growth of ductal carcinoma in situ structures. Aberrant proteolysis is another important characteristic of breast cancer progression and invasion. DN-PAK1 or knock-down of PAK1 reduced pericellular proteolysis of DQ-collagen IV in the 3D cultures. Treatment of cells with an inhibitor of Rac1 also reduced pericellular proteolysis, and this reduction was reversed by the expression of activated PAK1. Our conclusion is that overexpressed and activated PAK1 may be a key coordinator of aberrant cell survival and proteolysis in breast cancer progression. 
 P21-activated kinase 1 (PAK1) as a therapeutic target in BRAF wild-type melanoma. Although remarkable clinical response rates in melanoma have been observed using vemurafenib or dabrafenib in patients with tumors carrying oncogenic mutations in BRAF, a substantial unmet medical need remains for the subset of patients with wild-type BRAF tumors.To investigate the role of p21-activated kinases (PAKs) in melanoma, we determined PAK1 genomic copy number and protein expression for a panel of human melanoma tissues. PAK1 was inhibited in vitro and in vivo using RNA interference or PF-3758309 inhibitor treatment in a panel of melanoma cell lines with known BRAF and RAS (rat sarcoma) genotype to better understand its role in melanoma cell proliferation and   migration  . Tumorigenesis was assessed in vivo in female NCR nude mice and analyzed with cubic spline regression and area under the curve analyses. All statistical tests were two-sided.Strong cytoplasmic PAK1 protein expression was prevalent in melanomas (27%) and negatively associated with activating mutation of the BRAF oncogene (P < .001). Focal copy number gain of PAK1 at 11q13 was also observed in 9% of melanomas (n = 87; copy number ≥ 2.5) and was mutually exclusive with BRAF mutation (P < .005). Selective PAK1 inhibition attenuated signaling through mitogen-activated protein kinase (MAPK) as well as cytoskeleton-regulating pathways to modulate the proliferation an  d migrati  on of BRAF wild-type melanoma cells. Treatment of BRAF wild-type melanomas with PF-3758309 PAK inhibitor decreased tumor growth for SK-MEL23 and 537MEL xenografts (91% and 63% inhibition, respectively; P < .001) and MAPK pathway activation in vivo.Taken together, our results provide evidence for a functional role of PAK1 in BRAF wild-type melanoma and therapeutic use of PAK inhibitors in this indication. 
 Directional sensing requires G beta gamma-mediated PAK1 and PIX alpha-dependent activation of Cdc42. Efficient chemotaxis requires directional sensing and cell polarization. We describe a signaling mechanism involving G beta gamma, PAK-associated guanine nucleotide exchange factor (PIX alpha), Cdc42, and p21-activated kinase (PAK) 1. This pathway is utilized by chemoattractants to regulate directional sensing and directional   migration   of myeloid cells. Our results suggest that G beta gamma binds PAK1 and, via PAK-associated PIX alpha, activates Cdc42, which in turn activates PAK1. Thus, in this pathway, PAK1 is not only an effector for Cdc42, but it also functions as a scaffold protein required for Cdc42 activation. This G beta gamma-PAK1/PIX alpha/Cdc42 pathway is essential for the localization of F-actin formation to the leading edge, the exclusion of PTEN from the leading edge, directional sensing, and the persistent directional   migration   of chemotactic leukocytes. Although ligand-induced production of PIP(3) is not required for activation of this pathway, PIP(3) appears to localize the activation of Cdc42 by the pathway. 
 Pak1 and PIX regulate contact inhibition during epithelial wound healing. Wound healing in epithelia requires coordinated cell   migration   and proliferation regulated by signaling mechanisms that are poorly understood. Here we show that epithelial cells expressing constitutively active or kinase-dead mutants of the Rac/Cdc42 effector Pak1 fail to undergo growth arrest upon wound closure. Strikingly, this phenotype is only observed when the Pak1 kinase mutants are expressed in cells possessing a free lateral surface, i.e. one that is not engaged in contact with neighboring cells. The Pak1 kinase mutants perturb contact inhibition by a mechanism that depends on the Pak-interacting Rac-GEF PIX. In control cells, endogenous activated Pak and PIX translocate from focal complexes to cell-cell contacts during wound closure. This process is abrogated in cells expressing Pak1 kinase mutants. In contrast, Pak1 mutants rendered defective in PIX binding do not impede translocation of activated Pak and PIX, and exhibit normal wound healing. Thus, recruitment of activated Pak and PIX to cell-cell contacts is pivotal to transduction of growth-inhibitory signals from neighboring cells in epithelial wound healing. 
 Integrin engagement differentially modulates epithelial cell motility by RhoA/ROCK and PAK1. Integrin-ligand binding regulates tumor cell motility and invasion. Cell   migration   also involves the Rho GTPases that control the interplay between adhesion receptors and the cytoskeleton. We evaluated how specific extracellular matrix ligands modulate Rho GTPases and control motility of human squamous cell carcinoma cells. On laminin-5 substrates, the epithelial cells rapidly spread and migrated, but on type I collagen the cells spread slowly and showed reduced motility. We found that RhoA activity was suppressed in cells attached to laminin-5 through the alpha3 integrin receptor. In contrast, RhoA was strongly activated in cells bound to type I collagen and this was mediated by the alpha2 integrin. Inhibiting the RhoA pathway by expression of a dominant-negative RhoA mutant or by directly inhibiting ROCK, reduced focal adhesion formation and enhanced cell   migration   on type I collagen. Cdc42 and Rac and their downstream target PAK1 were activated following adhesion to laminin-5. PAK1 activation induced by laminin-5 was suppressed by expression of a dominant-negative Cdc42. Moreover, constitutively active PAK1 stimulated   migration   on collagen I substrates. Our results indicate that in squamous epithelial cells, collagen-alpha2beta1 integrin binding activates RhoA, slowing cell locomotion, whereas laminin-5-alpha3beta1 integrin interaction inhibits RhoA and activates PAK1, stimulating cell   migration  . The data demonstrate that specific ligand-integrin pairs regulate cell motility differentially by selectively modulating activities of Rho GTPases and their effectors. 
 Requirement of reversible caldesmon phosphorylation at P21-activated kinase-responsive sites for lamellipodia extensions during cell   migration  . Caldesmon is believed to be one of the key regulators for actin dynamics and thereby cell polarity, membrane extension, and cell motility. We have shown previously that stress fiber formation and cell movement are severely impaired in the cells expressing human fibroblast caldesmon fragment defective in Ca2+/CaM binding sites. Both Ser458 and Ser489, adjacent to the Ca2+/CaM-binding sites, are phosphorylated by p21-activated kinase (PAK) in vitro. Here we report that Ser458 is phosphorylated in response to cell movement. We substituted Ser458 and Ser489 on C-terminal caldesmon (CaD39) with alanine or glutamic acid to mimic under-phosphorylated (CaD39-PAKA) or constitutively phosphorylated (CaD39-PAKE) caldesmon. In vitro, CaD39-PAKE, but not CaD39-PAKA, fails to inhibit myosin ATPase activity and exhibits reduced binding to Ca2+/CaM. When stably expressed in Chinese Hamster Ovary cells, both CaD39-PAKA and CaD39-PAKE incorporate into stress fibers and localize to the leading edge of the migrating cell. Expression of CaD39-PAKE, but not CaD39-PAKA, fails to protect stress fibers from cytochalasin depolymerization. However, both mutations inhibit cell polarization and lead to defects in membrane extension and cell   migration  . We conclude that phosphorylation of caldesmon by PAK is a dynamic process required to regulate actin dynamics and membrane protrusions in wound-induced cell   migration  . 
 PAK1 mediates pancreatic cancer cell   migration   and resistance to MET inhibition. Pancreatic adenocarcinoma (PDAC) is a major unmet medical need and a deeper understanding of molecular drivers is needed to advance therapeutic options for patients. We report here that p21-activated kinase 1 (PAK1) is a central node in PDAC cells downstream of multiple growth factor signalling pathways, including hepatocyte growth factor (HGF) and MET receptor tyrosine kinase. PAK1 inhibition blocks signalling to cytoskeletal effectors and tumour cell motility driven by HGF/MET. MET antagonists, such as onartuzumab and crizotinib, are currently in clinical development. Given that even highly effective therapies have resistance mechanisms, we show that combination with PAK1 inhibition overcomes potential resistance mechanisms mediated either by activation of parallel growth factor pathways or by direct amplification of PAK1. Inhibition of PAK1 attenuated in vivo tumour growth and metastasis in a model of pancreatic adenocarcinoma. In human tissues, PAK1 is highly expressed in a proportion of PDACs (33% IHC score 2 or 3; n = 304) and its expression is significantly associated with MET positivity (p < 0.0001) and linked to a widespread metastatic pattern in patients (p = 0.067). Taken together, our results provide evidence for a functional role of MET/PAK1 signalling in pancreatic adenocarcinoma and support further characterization of therapeutic inhibitors in this indication. 
 LKB1 suppresses p21-activated kinase-1 (PAK1) by phosphorylation of Thr109 in the p21-binding domain. The serine/threonine protein kinase LKB1 is a tumor suppressor gene mutated in Peutz-Jeghers syndrome patients. The mutations are found also in several types of sporadic cancer. Although LKB1 is implicated in suppression of cell growth and metastasis, the detailed mechanisms have not yet been elucidated. In this study, we investigated the effect of LKB1 on cell motility, whose acquisition occurs in early metastasis. The knockdown of LKB1 enhanced cell   migration   and PAK1 activity in human colon cancer HCT116 cells, whereas forced expression of LKB1 in Lkb1-null mouse embryonic fibroblasts suppressed PAK1 activity and PAK1-mediated cell   migration   simultaneously. Notably, LKB1 directly phosphorylated PAK1 at Thr(109) in the p21-binding domain in vitro. The phosphomimetic T109E mutant showed significantly lower protein kinase activity than wild-type PAK1, suggesting that the phosphorylation at Thr(109) by LKB1 was responsible for suppression of PAK1. Consistently, the nonphosphorylatable T109A mutant was resistant to suppression by LKB1. Furthermore, we found that PAK1 was activated in the hepatocellular carcinomas and the precancerous liver lesions of Lkb1(+/-) mice. Taken together, these results suggest that PAK1 is a direct downstream target of LKB1 and plays an essential role in LKB1-induced suppression of cell   migration  . 
 P21-activated protein kinase 1 is overexpressed in gastric cancer and induces cancer metastasis. P21-activated protein kinase (Pak1), a main downstream effector of small Rho GTPases, plays an important role in the regulation of cell morphogenesis, motility, mitosis and angiogenesis. However, the role of Pak1 in gastric cancer metastasis remains unclear. Here, we showed that Pak1 is overexpressed in gastric cancer tissues from 74 patients by immunohistochemistry. Overexpression of Pak1 was associated with metastasis and prognosis of gastric cancer. In addition, overexpression of Pak1 increased gastric cancer cell motility and invasion, whereas downregulation of Pak1 expression reduced gastric cancer cell   migration   and invasion. In further study, data showed that activated Pak1 inhibited stress fiber and focal adhesion complex formation in gastric cancer cells and led to the formation of motile phenotypes. Importantly, activated Pak1 elicited phosphorylation of the ERK and JNK-dependent pathway in gastric cancer cell lines. In conclusion, our results suggest that Pak1 is overexpressed in gastric cancer and plays an important role in the metastasis of gastric cancer. The mechanism by which Pak1 induces cancer metastasis may involve activation of ERK and JNK. 
 Association of p21-activated kinase-1 activity with aggressive tumor behavior and poor prognosis of head and neck cancer. This study investigated the role of p21-activated kinase (PAK)-1 in progression of head and neck squamous cell carcinoma (HNSCC).We examined PAK isoforms and explored whether PAK activation enhanced in vitro invasion of the HNSCC cell line. We analyzed the relationship between PAK1 expression and various clinicopathological features and investigated the effect of PAK1 overexpression on survival in 119 patients with HNSCC.PAK1 and PAK2 are predominantly expressed in HNSCC cells and patient tissues. Particularly, PAK1 makes the dominant contribution to increase in cell   migration   and invasion. There was a statistically significant correlation between PAK1 overexpression and aggressive cancer behavior. Moreover, PAK1 seemed to be a prognostic factor for overall and disease-specific survival in patients. Interestingly, enhancement of PAK1 expression was found in the invasive front of cancer.PAK1 is associated with the aggressive tumor behavior and poor prognosis of head and neck cancer. 
 PAK1 regulates RUFY3-mediated gastric cancer cell   migration   and invasion. Actin protrusion at the cell periphery is central to the formation of invadopodia during tumor cell   migration   and invasion. Although RUFY3 (RUN and FYVE domain containing 3)/SINGAR1 (single axon-related1)/RIPX (Rap2 interacting protein X) has an important role in neuronal development, its pathophysiologic role and relevance to cancer are still largely unknown. The purpose of this study was to elucidate the molecular mechanisms by which RUFY3 involves in gastric cancer cell   migration   and invasion. Here, our data show that overexpression of RUFY3 leads to the formation of F-actin-enriched protrusive structures at the cell periphery and induces gastric cancer cell   migration  . Furthermore, P21-activated kinase-1 (PAK1) interacts with RUFY3, and promotes RUFY3 expression and RUFY3-induced gastric cancer cell   migration  ; inhibition of PAK1 attenuates RUFY3-induced SGC-7901 cell   migration   and invasion. Importantly, we found that the inhibitory effect of cell   migration   and invasion is significantly enhanced by knockdown of both PAK1 and RUFY3 compared with knockdown of RUFY3 alone or PAK1 alone. Strikingly, we found significant upregulation of RUFY3 in gastric cancer samples with invasive carcinoma at pathologic TNM III and TNM IV stages, compared with their non-tumor counterparts. Moreover, an obvious positive correlation was observed between the protein expression of RUFY3 and PAK1 in 40 pairs of gastric cancer samples. Therefore, these findings provide important evidence that PAK1 can positively regulate RUFY3 expression, which contribute to the metastatic potential of gastric cancer cells, maybe blocking PAK1-RUFY3 signaling would become a potential metastasis therapeutic strategy for gastric cancer. 
 Klotho endows hepatoma cells with resistance to anoikis via VEGFR2/PAK1 activation in hepatocellular carcinoma. Klotho was originally characterized as an aging suppressor gene that predisposed Klotho-deficient mice to premature aging-like syndrome. Although Klotho was recently reported to exhibit tumor suppressive properties during various malignant transformations, the functional role and molecular mechanism of Klotho in hepatocarcinogenesis remains poorly understood. In our present study, immunohistochemical Klotho staining levels in a clinical follow-up of 52 hepatoma patients were significantly associated with liver cirrhosis, tumor multiplicity and venous invasion. The overall survival rate of hepatoma patients with high Klotho expression was significantly lower than those patients with low Klotho expression. Moreover, Klotho overexpression increased cellular   migration  , anchorage-independent growth, and anoikis resistance in hepatoma cells. Klotho overexpression elevated p21-activated kinase 1 (PAK1) expression and shRNA-mediated PAK1 knockdown and kinase activity inhibition with kinase dead mutant PAK1 K299R coexpression or allosteric inhibitor IPA3 treatment reversed anoikis resistance in Klotho-overexpressed hepatoma cells. More importantly, the pivotal significance of upregulated VEGFR2 protein levels mediated by Klotho expression was confirmed by VEGFR2 inhibitor Axitinib and blocking antibody treatment in hepatoma cells. Axitinib treatment sensitized anoikis was reversed by constitutive active mutant PAK1 T423E coexpression in Klotho-overexpressed hepatoma cells. Conversely, knockdown of Klotho reduced VEGFR2/PAK1 dependent anoikis resistance, which could be reversed by PAK1 T423E. These results revealed a novel oncogenic function of Klotho in promoting anoikis resistance via activating VEGFR2/PAK1 signaling, thus facilitating tumor   migration   and invasion during hepatoma progression, which could provide a putative molecular mechanism for tumor metastasis. 
 Contra-regulation of calcium- and integrin-binding protein 1-induced cell   migration   on fibronectin by PAK1 and MAP kinase signaling. Calcium- and integrin-binding protein 1 (CIB1) has been shown to be involved in cell spreading and   migration  . The signaling events regulated by CIB1 during cell   migration   are poorly understood. Here we found that accumulation of CIB1 at the tip of the filopodia requires an intact cytoskeleton. Depletion of CIB1 using shRNA affects formation of FAK- and phosphotyrosine-rich focal adhesions without affecting stress fiber formation. Overexpression of CIB1 results in cell   migration   on fibronectin and Erk1/2 MAP kinase activation. CIB1-induced cell   migration   is dependent upon Erk1/2 activation, since it is inhibited by the MEK-specific inhibitor PD98059. Furthermore, CIB1-induced cell   migration  , as well as Erk1/2 activation, is dependent on PKC, Src family kinases as well as PI-3 kinase as it is inhibited by bisindolylmaleimide 1, PP2, and wortmannin, respectively, in a dose-dependent manner. Co-expression of dominant-negative Cdc42 completely abolished CIB1-induced cell   migration  . Additionally, co-expression of constitutively active, but not dominant negative PAK1, a CIB1 binding protein, inhibited CIB1-induced cell   migration  . These results suggest that CIB1 positively regulates cell   migration   and is necessary for the recruitment of FAK to the focal adhesions. Furthermore, CIB1-induced cell   migration   is dependent on MAP kinase signaling and its function is attenuated by PAK1. 
 ACF7 regulates cytoskeletal-focal adhesion dynamics and   migration   and has ATPase activity. Coordinated interactions between microtubule (MT) and actin cytoskeletons are involved in many polarized cellular processes. Spectraplakins are enormous (>500 kDa) proteins able to bind both MTs and actin filaments (F-actin) directly. To elucidate the physiological significance and functions of mammalian spectraplakin ACF7, we've conditionally targeted it in skin epidermis. Intriguingly, ACF7 deficiency compromises the targeting of microtubules along F-actin to focal adhesions (FAs), stabilizes FA-actin networks, and impairs epidermal   migration  . Exploring underlying mechanisms, we show that ACF7's binding domains for F-actin, MTs, and MT plus-end proteins are not sufficient to rescue the defects in FA-cytoskeletal dynamics and   migration   functions of ACF7 null keratinocytes. We've uncovered an intrinsic actin-regulated ATPase domain in ACF7 and demonstrate that it is both functional and essential for these roles. Our findings provide insight into the functions of this important cytoskeletal crosslinking protein in regulating dynamic interactions between MTs and F-actin to sustain directional cell movement. 
 PAK1 and PAK2 have different roles in HGF-induced morphological responses. Hepatocyte growth factor (HGF) stimulates dissociation of epithelial cells (scattering) and cell   migration  . Several Rho GTPases are required for HGF-induced scattering. PAK1 and PAK2 are members of the p21-activated kinase (PAK) family of serine/threonine kinases, and are activated by the Rho GTPases Rac and Cdc42. Here we investigate the contributions of PAK1 and PAK2 to HGF-induced motile response. HGF stimulates phosphorylation of PAK1 and PAK2. Knockdown of PAK1 inhibits HGF-stimulated   migration   and loss of cell-cell junctions in DU145 prostate carcinoma cells, whereas knockdown of PAK2 enhances loss of cell-cell junctions and increases lamellipodium extension but does not affect   migration   speed. On the other hand, in PC3 prostate carcinoma cells, which lack cell-cell junctions, knockdown of PAK1 or PAK2 reduces HGF-stimulated   migration  . PAK2 knockdown increases phosphorylation of PAK1, indicating that PAK2 provides a negative feedback on PAK1. We hypothesise that PAK2 acts in part via PAK1 to regulate HGF-induced scattering. 
 The Role of the Pleckstrin Homology Domain-containing Protein CKIP-1 in Activation of p21-activated Kinase 1 (PAK1). Upon growth factor stimulation, PAK1 is recruited to the plasma membrane and activated by a mechanism that requires its phosphorylation at Ser-223 by the protein kinase CK2. However, the upstream signaling molecules that regulate this phosphorylation event are not clearly defined. Here, we demonstrate a major role of the CK2α-interacting protein CKIP-1 in activation of PAK1. CK2α, CKIP-1, and PAK1 are translocated to membrane ruffles in response to the epidermal growth factor (EGF), where CKIP-1 mediates the interaction between CK2α and PAK1 in a PI3K-dependent manner. Consistently, PAK1 mediates phosphorylation and modulation of the activity of p41-Arc, one of its plasma membrane substrate, in a fashion that requires PI3K and CKIP-1. Moreover, CKIP-1 knockdown or PI3K inhibition suppresses PAK1-mediated ce  ll migrat  ion and invasion, demonstrating the physiological significance of the PI3K-CKIP-1-CK2-PAK1 signaling pathway. Taken together, these findings identify a novel mechanism for the activation of PAK1 at the plasma membrane, which is critical for ce  ll migrat  ion and invasion. 
 P21 activated kinase-1 (Pak1) promotes prostate tumor growth and microinvasion via inhibition of transforming growth factor β expression and enhanced matrix metalloproteinase 9 secretion. P21-activated kinases (Paks) are major effectors downstream of the small Rho family of GTPases. Among the six isoforms, Pak1 is the most ubiquitous and the best characterized member. Previous studies have shown that inhibition of Pak6, which is predominantly present in the prostate compared with other tissues, inhibits prostate tumor growth in vivo. Even though Pak1 has been identified in normal prostatic epithelial cells and cancer cells, its specific role in the development of prostate cancer remains unclear. We report here that highly invasive prostate cancer cells express significantly higher levels of Pak1 protein compared with non-invasive prostate cancer cells. Furthermore, prostate tumor tissues and prostate cancer metastasized to lungs showed a higher expression of Pak1 compared with normal tissues. Interestingly, Pak6 protein expression levels did not change with the invasive/metastatic potential of the cancer cells or tumors. Although inhibition of Pak1, and not Pak6, resulted in impaired PC3 cell   migratio  n, the effects of Pak1 knockdown on transendothelial   migratio  n (microinvasion), tumor growth, and tumor angiogenesis was higher compared with Pak6 knockdown. Finally, gene array data revealed reduced expression of matrix metalloproteinase 9 with the ablation of either Pak1 or Pak6 gene expression in PC3 cells, whereas protein levels of TGFβ was elevated significantly with specific modulation of Pak1 activity or ablation of the Pak1 gene. Our observations suggest that although some level of functional redundancy exists between Pak1 and Pak6 in prostate cancer cells, targeting Pak1 is a potential option for the management of prostate tumor growth, microinvasion, and metastasis. 
 Forced expression of NESH suppresses motility and metastatic dissemination of malignant cells. To characterize the function of a novel Src homology 3 (SH3) adapter protein termed NESH, we have established transfectants stably expressing NESH. We observed that every clone of NESH transfectants caused a marked reduction in motility, although the clones exhibited no significant differences in intrinsic cell growth compared with the control cells in vitro. The NESH transfectants also exhibited significant reduction in tumor metastatic potential in vivo. We found that NESH expression is frequently lost in invasive cancer cell lines despite its ubiquitous expression in normal tissues. The SH3 domain of NESH seems to interact with p21-activated kinase (PAK), which is involved in regulation of cell motility. Furthermore, a significant decrease in PAK phosphorylation at (402)Thr was found in NESH transfectants. Taken together, these results suggest that NESH inhibits ectopic metastasis of tumor cells as well as cell   migration   through interaction with intracellular molecules such as PAK that are essential for cell motility. 
 Adhesion-related kinase repression of gonadotropin-releasing hormone gene expression requires Rac activation of the extracellular signal-regulated kinase pathway. Recent studies suggest that adhesion-related kinase (Ark) plays a role in gonadotropin-releasing hormone (GnRH) neuronal physiology. Ark promotes   migration   of GnRH neurons via Rac GTPase and concomitantly suppresses GnRH gene expression via homeodomain and myocyte enhancer factor-2 (MEF2) transcription factors. Here, we investigated the signaling cascade required for Ark inhibition of the GnRH promoter in GT1-7 GnRH neuronal cells. Ark repression was blocked by the MEK/ERK pathway inhibitor, PD98059, and dominant negative MEK1 but was unaffected by dominant negative Ras. Inhibitors of the Rho family GTPases, Clostridium difficile toxin B (Rho/Rac/Cdc42 inhibitor) and Clostridium sordellii lethal toxin (Rac/Cdc42 inhibitor), blocked Ark inhibition of GnRH transcription. Moreover, dominant negative Rac blunted both Ark activation of ERK and repression of the GnRH promoter, demonstrating an essential role for Rac in coupling Ark to ERK activation. Like Ark, a constitutively active mutant of Rac suppressed GnRH transcription in an ERK-dependent manner. Finally, Ark-mediated repression was significantly attenuated by a dominant negative MEF2C, whereas repression induced by constitutively active Rac was unaffected, indicating that MEF2 proteins are not targets of the Ark --> Rac --> MEK --> ERK cascade. The data suggest that Ark suppresses GnRH gene expression via the coordinated activation of a Rac --> ERK signaling pathway and a distinct MEF2- dependent mechanism. 
 p21-activated kinase 1 determines stem-like phenotype and sunitinib resistance via NF-κB/IL-6 activation in renal cell carcinoma. The p21-activated kinase 1 (PAK1), a serine/threonine kinase that orchestrates cytoskeletal remodeling and cell motility, has been shown to function as downstream node for various oncogenic signaling pathways to promote cell proliferation, regulate apoptosis and accelerate mitotic abnormalities, resulting in tumor formation and invasiveness. Although alterations in PAK1 expression and activity have been detected in various human malignancies, its potential biological and clinical significance in renal cell carcinoma (RCC) remains obscure. In this study, we found increased PAK1 and phosphorylated PAK1 levels in tumor tissues according to TNM stage progression. Elevated phosphorylated PAK1 levels associated with progressive features and indicated unfavorable overall survival (OS) as an independent adverse prognosticator for patients with RCC. Moreover, PAK1 kinase activation with constitutive active PAK1 mutant T423E promoted growth, colony formation,   migratio  n, invasion and stem-like phenotype of RCC cells, and vice versa, in PAK1 inhibition by PAK1 kinase inactivation with specific PAK1 shRNA, dead kinase PAK1 mutant K299R or allosteric inhibitor IPA3. Stem-like phenotype due to sunitinib administration via increased PAK1 kinase activation could be ameliorated by PAK1 shRNA, PAK1 mutant K299R and IPA3. Furthermore, nuclear factor-κB (NF-κB)/interleukin-6 (IL-6) activation was found to be responsible for PAK1-mediated stem-like phenotype following sunitinib treatment. Both IL-6 neutralizing antibody and IPA3 administration enhanced tumor growth inhibition effect of sunitinib treatment on RCC cells in vitro and in vivo. Our results unraveled that oncogenic activation of PAK1 defines an important mechanism for maintaining stem-like phenotype and sunitinib resistance through NF-κB/IL-6 activation in RCC, lending PAK1-mediated NF-κB/IL-6 activation considerable appeal as novel pharmacological therapeutic targets against sunitinib resistance. 
 P21-activated protein kinase 1 induces colorectal cancer metastasis involving ERK activation and phosphorylation of FAK at Ser-910. Pak1 has been reported to be overexpressed in colorectal cancer, but the role of Pak1 in colorectal cancer remains unclear. In this study, Pak1 expression and activity were associated with aggressive behavior of colorectal cancer. Overexpression of Pak1 increased colorectal cancer cell motility and invasion, whereas down-regulation of Pak1 expression or activity reduced colorectal cancer cell   migration   and invasion. In addition, activated Pak1 inhibited stress fiber and focal adhesion complex formation in colorectal cancer cells and led to formation of motile phenotypes. Importantly, activated Pak1 elicited phosphorylation of FAK at Ser-910 via an ERK-dependent pathway in colorectal cancer cell lines and clinical samples. In conclusion, our results suggest that activated Pak1 regulates colorectal cancer metastasis requiring an ERK-dependent phosphorylation of FAK at Ser-910. 
 ERK activation of p21 activated kinase-1 (Pak1) is critical for medulloblastoma cell   migration  . We previously identified that overexpression of the platelet-derived growth factor receptor (PDGFR) is associated with metastatic medulloblastoma (MB) and showed that PDGF treatment increases ERK activity and promotes MB cell   migration  . In this study, we investigated whether ERK regulates Rac1/Pak1 signaling and is critically linked to MB cell   migration  . Herein we demonstrate that PDGF-BB treatment of MB cells induces concomitant activation of PDGFRβ, MEK1/ERK, Rac1 and Pak1, but suppresses Rho activity, which together significantly promotes cell   migratio  n. Conversely, cells transfected with either PDGFRβ or Pak1 siRNA or treated with an inhibitor of Rac1 (NSC23766) or N-myristoyltransferase-1 (Tris-dipalladium) are unable to activate Rac1 or Pak1 in response to PDGF, and consequently, are unable to undergo PDGF-mediated cel  l migrati  on. Furthermore, we also demonstrate that either chemical inhibition of MEK/ERK (U0126) or stable downregulation of PDGFRβ by shRNA similarly results in the loss of PDGF-induced ERK phosphorylation and abolishes Rac1/Pak1 activation and ce  ll migrat  ion in response to PDGF. However, specific depletion of Pak1 by siRNA has no effect on PDGF-induced ERK phosphorylation, indicating that in MB cells ERK signaling is Pak1-independent, but PDGF-induc  ed migrat  ion is dependent on ERK-mediated activation of Pak1. Finally, using tissue microarrays, we detect phosphorylated Pak1 in 53% of medulloblastomas and show that immunopositivity is associated with unfavorable outcome. We conclude that Rac1/Pak1 signaling is critical to MB ce  ll migrat  ion and is functionally dependent on PDGFRβ/ERK activity. 
 Role of p21-activated kinase 1 in regulating the   migration   and invasion of fibroblast-like synoviocytes from rheumatoid arthritis patients. To investigate the role of p21-activated kinase 1 (PAK1) in regulating   migration  , invasion and MMP expression in RA fibroblast-like synoviocytes (FLS).RA FLS   migration   and invasion in vitro were measured by the Boyden chamber method. Invasion of RA FLS into cartilage was detected in the severe combined immunodeficiency (SCID) mouse co-implantation model of RA in vivo. PAK1 and MT1-MMP expression were examined by western blotting. ELISA was used to measure the production and activity of MMPs.Phosphorylated PAK1 (p-PAK1) protein expression was increased in ex vivo synovial membrane cells from RA patients. Stimulation with IL-1β or TNF-α up-regulated p-PAK1 expression. Inhibition of PAK1 by transfection with dominant negative PAK1 mutant (dnPAK1) reduced in vitr  o migrati  on and invasion of RA FLS. In the SCID mouse model, RA FLS invasion into cartilage was attenuated by transfection with dnPAK1 in vivo. PAK1 regulated IL-1β-induced production and activity of MMP-13 and MT1-MMP. Inhibition of MMP-13 or MT1-MMP activity also reduced RA FLS invasion. Furthermore, dnPAK1 transfection inhibited c-Jun N-terminal kinase (JNK) activation, but did not affect the activities of extracellular signal-regulated kinases and p38. Inhibition of the JNK activity by chemical inhibitor significantly reduced t  he migrat  ion, invasion and production of MMP-13 and MT1-MMP.PAK1 plays an important role in regulating t  he migrat  ion, invasion and production and activity of MMPs in RA FLS, which is mediated by the JNK pathway. This suggests a novel strategy targeting PAK1 to prevent joint destruction of RA. 
 microRNA-7 regulates cell growth,   migration   and invasion via direct targeting of PAK1 in thyroid cancer. The expression and function of microRNA-7 (miR-7) has been studied in a variety of different cancer types. However, to date, no studies have investigated the expression of miR‑7 in human thyroid cancer. In the present study, the expression levels and biological function of miR‑7 were investigated in human thyroid cancer, with the aim of evaluating whether it may serve as a therapeutic biomarker. The expression levels of miR‑7 in human thyroid cancer tissues, matched, adjacent normal tissues, normal thyroid tissues and human thyroid cancer cell lines were determined using RT‑qPCR and western blot analysis. To explore the functional role of miR‑7 in human thyroid cancer cell lines, MTT ass  ays, cell   migration and invasion assays were employed. TargetScan software identified p21 activated kinase‑1 (PAK1) as a putative interacting partner of miR‑7. Therefore, functional assays were performed to explore the effects of endogenous PAK1 in thyroid cancer. In the present study, miR‑7 was significantly downregulated in thyroid cancer tissues and cells compared with normal thyroid tissue samples. A correlation between miR‑7 expression and thyroid tumor stage was also observed. Ectopic expression of miR‑7 was found to suppress the proliferation, migra-tion and invasion of thyroid cancer cells in vitro. Dual-luciferase reporter assays demonstrated that PAK1 was a direct target of miR-7 in vitro. RT-qPCR and western blot analysis demonstrated that miR‑7 negatively regulates PAK1 protein expression but has no effect on PAK1 mRNA expression. Knockdown of PAK1 expression markedly suppressed thyroid can  cer cell   proliferation, migration and invasion. These results suggest that miR‑7 functions as a tumor suppressor by targeting PAK1 directly and may therefore present a novel therapeutic target for the treatment of thyroid cancer. 
 P21-activated kinase 1 stimulates colon cancer cell growth and   migration  /invasion via ERK- and AKT-dependent pathways. The p21-activated kinase (PAK) family of serine/threonine kinases plays an important role in cell proliferation, survival and motility, as well as in cell transformation and tumor progression. PAK1 promotes transformation through facilitating the ERK/MAPK pathway and enhances cell   migration   and survival by stimulating AKT. PAK1 expression increases with the progression of colorectal cancer (CRC). In this study, we have investigated the importance of PAK1 in the biology of colon cancer cells. Reduction of PAK1 expression decreased the activities of ERK and AKT leading to decreased cell proliferation,   migration  /invasion, and survival. Dual inhibition of ERK and AKT suppressed these cellular processes to levels comparable to those achieved by reduction of PAK1 expression, whereas inactivation of either the ERK or AKT pathway alone partially inhibited cell   migration  /invasion and survival and had no effect on proliferation. We conclude that PAK1 stimulates colon cancer cell proliferation,   migration  /invasion, and survival via ERK- and AKT-dependent pathways. These findings establish the central importance of PAK1 in CRC signal transduction and clarify the mechanism by which PAK1 regulates CRC growth and   migration  . Instead of simultaneously inhibiting both ERK and AKT, the PAK1 convergence point could be an alternative target for CRC therapy. 
 Nuclear localization and chromatin targets of p21-activated kinase 1. Pak1 (p21-activated kinase 1), a conserved, mammalian signaling kinase, is a downstream effector of small GTPases Rac1 and Cdc42 and of growth factor signaling. Until now, a major focus of study has been on the cytosolic functions of Pak1, where it is an important modulator of cytoskeletal reorganization, consequently playing a major role in cell survival,   migration  , and invasion. In this report, we demonstrate the nuclear localization of Pak1 upon stimulation by epidermal growth factor. Three nuclear localization signals (NLSs) were identified in the N-terminal domain of Pak1. With mutational analysis, the importance of each NLS was elucidated. Mutation of all three NLSs eliminated the nuclear localization of Pak1. Expression of Pak1 as a fusion protein with Gal4-DNA binding domain and Gal4-luciferase activity showed that Pak1 might increase transcription. To identify the potential targets of nuclear Pak1, we used a Pak1-specific chromatin immunoprecipitation-based screening assay and identified a series of Pak1-interacting target chromatins, including phosphofructokinase-muscle isoform (PFK-M) and nuclear factor of activated T-cell (NFAT1) genes. Pak1 associated with the upstream enhancer sequence and promoter of PFK-M and was involved in the stimulation of the PFK-M expression. It also associated with a portion of the NFAT1 gene and its upstream region, leading to the repression of NFAT1 expression. These investigations provide proof-of-principle evidence that Pak1 could influence the expression of its putative chromatin targets in both a positive and a negative manner. Together, for the first time, these findings defined the NLSs of the Pak1, its association with chromatin, and the resulting modulation of transcription, thus opening new avenues to further the search for nuclear Pak1 functions and identify putative Pak1-interacting nuclear proteins. 
 BRAF activates and physically interacts with PAK to regulate cell motility. Increased p21-activated kinase (PAK) signaling and expression have been identified in the invasive fronts of aggressive papillary thyroid cancers (PTCs), including those with RET/PTC, BRAFV600E, and mutant RAS expression. Functionally, thyroid cancer cell motility in vitro is dependent on group 1 PAKs, particularly PAK1. In this study, we hypothesize that BRAF, a central kinase in PTC tumorigenesis and invasion, regulates thyroid cancer cell motility in part through PAK activation. Using three well-characterized human thyroid cancer cell lines, we demonstrated in all cell lines that BRAF knockdown reduced PAK phosphorylation of direct downstream targets. In contrast, inhibition of MEK activity either pharmacologically or with siRNA did not reduce PAK activity, indicating MEK is dispensable for PAK activity. Inhibition of cell   migration   through BRAF loss is rescued by overexpression of either constitutive active MEK1 or PAK1, demonstrating that both signaling pathways are involved in BRAF-regulated cell motility. To further characterize BRAF-PAK signaling, immunofluorescence and immunoprecipitation demonstrated that both exogenously overexpressed and endogenous PAK1 and BRAF co-localize and physically interact, and that this interaction was enhanced in mitosis. Finally, we demonstrated that acute induction of BRAFV600E expression in vivo in murine thyroid glands results in increased PAK expression and activity confirming a positive signaling relationship in vivo. In conclusion, we have identified a signaling pathway in thyroid cancer cells which BRAF activates and physically interacts with PAK and regulates cell motility. 
 Phosphoinositide-dependent kinase 1 and p21-activated protein kinase mediate reactive oxygen species-dependent regulation of platelet-derived growth factor-induced smooth muscle cell   migration  . Smooth muscle cell   migration   in response to platelet-derived growth factor (PDGF) is a key event in several vascular pathologies, including atherosclerosis and restenosis. PDGF increases intracellular levels of reactive oxygen species (ROS) in vascular smooth muscle cells (VSMCs), but the ROS sensitivity of   migration   and of the signaling pathways leading to   migration   are largely unknown. In VSMCs, PDGF dose-dependently increased   migration   compared with nonstimulated cells, with a maximum increase at 10 ng/mL. Pretreatment with the antioxidant N-acetyl-cysteine, the flavin-containing enzyme inhibitor diphenylene iodonium, or the glutathione peroxidase mimetic ebselen significantly attenuated   migration   (PDGF alone, 5.0+/-1.1-fold; NAC, 1.8+/-0.2-fold; diphenylene iodonium, 1.4+/-0.3-fold   migration  ; and ebselen, 2.0+/-0.5-fold   migration  ), as did overexpression of catalase. Pretreatment of VSMCs with the Src inhibitor PP1 or dominant-negative Rac adenovirus significantly inhibited   migration  , but only Src activation was attenuated by ROS inhibitors. Phosphorylation of the Src- and Rac-effector p21-activated protein kinase (PAK) 1 on Thr423 (the phosphoinositide-dependent kinase-1 [PDK1] site) was attenuated by ROS inhibition, and infection of VSMCs with dominant-negative PAK1 adenovirus attenuated   migration  . Moreover, kinase-inactive K111N-PDK1 inhibited PAK1 phosphorylation on Thr423, and both K111N-PDK1 and Y9F-PDK1 significantly inhibited VSMC   migration  . PDK1 tyrosine phosphorylation was also ROS dependent. These data indicate that PDGF-induced VSMC   migration   is ROS dependent and identify the Src/PDK1/PAK1 signaling pathway as an important ROS-sensitive mediator of   migration  . Such information is critical to understanding the role of ROS in vascular diseases in which   migration   of VSMCs is an important component. 
 Pak1 and Pak2 mediate tumor cell invasion through distinct signaling mechanisms. Pak kinases are thought to play critical roles in cell   migration   and invasion. Here, we analyze the roles of Pak1 and Pak2 in breast carcinoma cell invasion using the transient transfection of small interfering RNA. We find that although both Pak1 and Pak2 contribute to breast carcinoma invasion stimulated by heregulin, these roles are mediated by distinct signaling mechanisms. Thus, whereas the depletion of Pak1 interferes with the heregulin-mediated dephosphorylation of cofilin, the depletion of Pak2 does not. The depletion of Pak1 also has a stronger inhibitory effect on lamellipodial protrusion than does the depletion of Pak2. Interestingly, Pak1 and Pak2 play opposite roles in regulating the phosphorylation of the myosin light chain (MLC). Whereas the depletion of Pak1 decreases phospho-MLC levels in heregulin-stimulated cells, the depletion of Pak2 enhances MLC phosphorylation. Consistent with their opposite effects on MLC phosphorylation, Pak1 and Pak2 differentially modulate focal adhesions. Pak2-depleted cells display an increase in focal adhesion size, whereas in Pak1-depleted cells, focal adhesions fail to mature. We also found that the depletion of Pak2, but not Pak1, enhances RhoA activity and that the inhibition of RhoA signaling in Pak2-depleted cells decreases MLC phosphorylation and restores cell invasion. In summary, this work presents the first comprehensive analysis of functional differences between the Pak1 and Pak2 isoforms. 
 PAK1-cofilin phosphorylation mediates human lung adenocarcinoma cells   migration   induced by apelin-13. Adipocytokines apelin peptide, the ligand of APJ (putative receptor related to the angiotensin receptor AT1), plays key roles in the pathogenesis and deterioration of cancer. In lung cancer, apelin elevating microvessel densities has been reported. Our previous research has characterized that apelin-13 promoted lung adenocarcinoma cell proliferation. However, the effect of apelin on metastasis in lung adenocarcinoma and the underlying mechanisms remain unclear. This study shows that apelin-13 induced human adenocarcinoma cell   migration   via the APJ receptor. Apelin-13 phosphorylated PAK1 and cofilin increase the   migration   of lung adenocarcinoma cells. Moreover, the results verify that over-expression of apelin and APJ contributed to reducing the effect of doxorubicin and razoxane on inhibiting lung adenocarcinoma cells metastasis. Hypoxia activated APJ expression and apelin release in lung adenocarcinoma cells. The results demonstrate a PAK1-cofilin phosphorylation mechanism to mediate lung adenocarcinoma cells   migration   promoted by apelin-13. This discovery further suggests that APJ and its downstream signalling is a potential target for anti-metastatic therapies in lung adenocarcinoma patients. 
 Nuclear factor of activated T cells c1 mediates p21-activated kinase 1 activation in the modulation of chemokine-induced human aortic smooth muscle cell F-actin stress fiber formation,   migration  , and proliferation and injury-induced vascular wall remodeling. Recent literature suggests that cyclin-dependent kinases (CDKs) mediate cell   migration  . However, the mechanisms were not known. Therefore, the objective of this study is to test whether cyclin/CDKs activate Pak1, an effector of Rac1, whose involvement in the modulation of cell   migration   and proliferation is well established. Monocyte chemotactic protein 1 (MCP1) induced Pak1 phosphorylation/activation in human aortic smooth muscle cells (HASMCs) in a delayed time-dependent manner. MCP1 also stimulated F-actin stress fiber formation in a delayed manner in HASMCs, as well as the   migration   and proliferation of these cells. Inhibition of Pak1 suppressed MCP1-induced HASMC F-actin stress fiber formation,   migration  , and proliferation. MCP1 induced cyclin D1 expression as well as CDK6 and CDK4 activities, and these effects were dependent on activation of NFATc1. Depletion of NFATc1, cyclin D1, CDK6, or CDK4 levels attenuated MCP1-induced Pak1 phosphorylation/activation and resulted in decreased HASMC F-actin stress fiber formation,   migration  , and proliferation. CDK4, which appeared to be activated downstream of CDK6, formed a complex with Pak1 in response to MCP1. MCP1 also activated Rac1 in a time-dependent manner, and depletion/inhibition of its levels/activation abrogated MCP1-induced NFATc1-cyclin D1-CDK6-CDK4-Pak1 signaling and, thereby, decreased HASMC F-actin stress fiber formation,   migration  , and proliferation. In addition, smooth muscle-specific deletion of NFATc1 led to decreased cyclin D1 expression and CDK6, CDK4, and Pak1 activities, resulting in reduced neointima formation in response to injury. Thus, these observations reveal that Pak1 is a downstream effector of CDK4 and Rac1-dependent, NFATc1-mediated cyclin D1 expression and CDK6 activity mediate this effect. In addition, smooth muscle-specific deletion of NFATc1 prevented the capacity of vascular smooth muscle cells for MCP-1-induced activation of the cyclin D1-CDK6-CDK4-Pak1 signaling axis, affecting their   migration   and proliferation in vitro and injury-induced neointima formation in vivo. 
 Angiotensin II-induced activation of p21-activated kinase 1 requires Ca2+ and protein kinase C{delta} in vascular smooth muscle cells. ANG II promotes remodeling of vascular smooth muscle cells (VSMCs) in cardiovascular diseases. It has been shown to activate p21-activated kinase (PAK)1, a critical component of signaling pathways implicated in growth and   migration  . However, the detailed signaling mechanism by which ANG II induces PAK1 activation in VSMCs remains unclear. Therefore, we have examined the mechanism required for activation of PAK1 by ANG II in VSMCs. ANG II, through activation of the ANG II type 1 receptor, rapidly promotes phosphorylation of PAK1 in VSMCs via a pathway independent of transactivation of the epidermal growth factor receptor. Using selective agonists and inhibitors, we demonstrated that mobilization of intracellular Ca(2+) and PKCdelta activation are required for ANG II-induced PAK1 phosphorylation. Rottlerin, a PKCdelta inhibitor, significantly blocked ANG II-induced PAK1 phosphorylation. Further support for this notion was provided through infection of VSMCs with adenovirus encoding a dominant-negative (dn)PKCdelta, which also markedly reduced phosphorylation of PAK1 by ANG II. In this pathway, Ca(2+) acts upstream of PKCdelta because a Ca(2+) ionophore rapidly induced PKCdelta phosphorylation at Tyr311 and Ca(2+)-dependent PAK1 phosphorylation was blocked by rottlerin. In addition, dnPYK-2, dnRac, and antioxidants inhibited ANG II-induced PAK1 phosphorylation, suggesting that PYK-2, Rac, and reactive oxygen species are involved in the upstream signaling. Finally, dnPAK1 markedly inhibited ANG II-induced protein synthesis in VSMCs. These data provide a novel signaling pathway by which ANG II may contribute to vascular remodeling. 
 Tyrosyl phosphorylated serine-threonine kinase PAK1 is a novel regulator of prolactin-dependent breast cancer cell motility and invasion. Despite efforts to discover the cellular pathways regulating breast cancer metastasis, little is known as to how prolactin (PRL) cooperates with extracellular environment and cytoskeletal proteins to regulate breast cancer cell motility and invasion. We implicated serine-threonine kinase p21-activated kinase 1 (PAK1) as a novel target for PRL-activated Janus-kinase 2 (JAK2). JAK2-dependent PAK1 tyrosyl phosphorylation plays a critical role in regulation of both PAK1 kinase activity and scaffolding properties of PAK1. Tyrosyl phosphorylated PAK1 facilitates PRL-dependent motility via at least two mechanisms: formation of paxillin/GIT1/βPIX/pTyr-PAK1 complexes resulting in increased adhesion turnover and phosphorylation of actin-binding protein filamin A. Increased adhesion turnover is the basis for cell   migratio  n and phosphorylated filamin A stimulates the kinase activity of PAK1 and increases actin-regulating activity to facilitate cell motility. Tyrosyl phosphorylated PAK1 also stimulates invasion of breast cancer cells in response to PRL and three-dimensional (3D) collagen IV via transcription and secretion of MMP-1 and MMP-3 in a MAPK-dependent manner. These data illustrate the complex interaction between PRL and the cell microenvironment in breast cancer cells and suggest a pivotal role for PRL/PAK1 signaling in breast cancer metastasis. 
 HGF and IGF-1 synergize with SDF-1α in promoting   migratio  n of myeloma cells by cooperative activation of p21-activated kinase. Stromal-derived factor (SDF)-1α, insulin-like growth factor (IGF)-1 and hepatocyte growth factor (HGF) are potent mediators of cel  l migrati  on. We studied the effect of combinations of these cytokines on th  e migrati  on of myeloma cells. When SDF-1α was combined with either HGF or IGF-1, we found a striking synergy in the cytokines' ability to guide cells across a transwell membrane. Between HGF and IGF-1 there was no cooperativity. However, the effects of HGF and IGF-1 were not redundant. HGF and SDF-1 caused concentration gradient-direct  ed migrat  ion, as opposed to IGF-1, which apparently caused randomly directed cell movement. The SDF-1α-dri  ven migra  tion of JJN-3 cells, a myeloma cell line secreting large amounts of HGF, was reduced when JJN-3 cells were given an inhibitor of the HGF receptor, demonstrating a cooperative activity between autocrine HGF and exogenous SDF-1α. There was a clear positive correlation between the degree of cytokine-ind  uced migr  ation and phosphorylation of p21-activated kinase (PAK) both in primary myeloma cells and in cell lines including INA-6 and IH-1. Downregulation of PAK with small interfering RNA in INA-6 cells resulted in decreased cytokine-dr  iven migr  ation. This study shows synergy between SDF-1α and HGF/IGF-1 in ind  ucing mig  ration of myeloma cells, yet each cytokine has distinct properties in the way it regulates   cell mig  ration. These findings are likely to be of clinical relevance because multiple myeloma cells are located in an environment containing HGF and IGF-1 and are exposed to an SDF-1α gradient between the bone marrow and peripheral blood. 
 Erk5 inhibits endothelial   migration   via KLF2-dependent down-regulation of PAK1. The MEK5/Erk5 pathway mediates beneficial effects of laminar flow, a major physiological factor preventing vascular dysfunction. Forced Erk5 activation induces a protective phenotype in endothelial cell (EC) that is associated with a dramatically decreased   migration   capacity of those cells. Transcriptional profiling identified the Krüppel-like transcription factors KLF2 and KLF4 as central mediators of Erk5-dependent gene expression. However, their downstream role regarding   migratio  n is unclear and relevant secondary effectors remain elusive. Here, we further investigated the mechanism underlying Erk5-dependent   migratio  n arrest in ECs.Our experiments reveal KLF2-dependent loss of the pro-migratory Rac/Cdc42 mediator, p21-activated kinase 1 (PAK1), as an important mechanism of Erk5-induced   migratio  n inhibition. We show that endothelial Erk5 activation by expression of a constitutively active MEK5 mutant, by statin treatment, or by application of laminar shear stress strongly decreased PAK1 mRNA and protein expression. Knockdown of KLF2 but not of KLF4 prevented Erk5-mediated PAK1 mRNA inhibition, revealing KLF2 as a novel PAK1 repressor in ECs. Importantly, both PAK1 re-expression and KLF2 knockdown restored the   migratio  n capacity of Erk5-activated ECs underscoring their functional relevance downstream of Erk5.Our data provide first evidence for existence of a previously unknown Erk5/KLF2/PAK1 axis, which may limit undesired cell   migratio  n in unperturbed endothelium and lower its sensitivity for migratory cues that promote vascular diseases including atherosclerosis. 
 Clinicopathological and cellular signature of PAK1 in human bladder cancer. Bladder cancer (BC) is the ninth most common cancer and the 13th most common cause of cancer death. Although p21 protein-activated kinase (PAK) regulates cell growth, motility, and morphology, the expression and function of PAK1 associated with the clinicopathological and cellular signature of human BC are not clear. This study was to examine the expression of PAK1 in human BC, the association of PAK1 with clinicopathological features, and the effect of PAK1 on cell proliferation,   migration  , and invasion in BC cells. A total of 54 BC and 12 normal bladder tissue specimens were retrieved. Among 54 BC patients, 39 cases were superficial BC and 15 cases were invasive BC. Histological examination revealed 29 patients with low-grade and 25 patients with high-grade papillary urothelial carcinomas. Immunohistochemical staining showed that PAK1 was overexpressed in BC tissue compared with normal bladder tissue. The overexpression of PAK1 was significantly associated with tumor size, histological grade, and lymph node metastasis, but not with gender, age, clinical stage, tumor number, and recurrence. Furthermore, the cytoplasmic distribution of PAK1 was observed in BC cells. Knocking down of PAK1 using lentiviral transduction decreased BC cell proliferation,   migration  , and invasion. In conclusion, we demonstrated that the overexpression of PAK1 is closely associated with the clinicopathological features of BC, suggesting that PAK1 may play an important role in the development and progression of BC and may be a potential therapeutic target for the treatment of BC. 
 Overexpression of p21-activated kinase 1 promotes endometrial cancer progression. Endometrial cancer (EC) is the most common gynecologic malignancy, but the molecular events involved in the development and progression of EC remain unclear. P21-activated kinase 1 (Pak1) plays important roles in cell motility and survival. This study investigated the clinical significance of Pak1 expression and its functional roles in EC. The expression of Pak1 in clinical samples and EC cell lines was evaluated. The effects of Pak1 on EC cell functions were determined by either overexpressing it via plasmid transfection or depleting its expression using short hairpin RNA (shRNA) in human EC cell lines. Pak1 was overexpressed in clinical samples of EC compared with normal endometrium. High Pak1 expression in EC was positively correlated with lymph node metastasis, advanced disease stage and poor histological differentiation. Pak1 over-expression was also observed in multiple human EC cell lines. In EC cell lines, Pak1 overexpression promoted cell proliferation,   migratio  n, invasion and anchorage-independent growth in vitro. Conversely, shRNA-mediated stable knockdown of Pak1 reduced cell proliferation  , migrati  on, invasion and anchorage-independent growth. In addition, ectopic Pak1 overexpression protected EC cells from apoptosis, along with decreased caspase-3 activation. These results suggest that Pak1 plays important roles at multiple stages of EC progression. 
 BET Bromodomain Suppression Inhibits VEGF-induced Angiogenesis and Vascular Permeability by Blocking VEGFR2-mediated Activation of PAK1 and eNOS. The tyrosine kinase receptor vascular endothelial growth factor receptor 2 (VEGFR2) is a critical modulator of angiogenesis. Increasing evidence indicate the important role of bromodomain and extra-terminal domain (BET) of chromatin adaptors in regulating tumor growth and inflammatory response. However, whether BET proteins have a role in angiogenesis and endothelial permeability is unclear. In this study, we observed that treatment with JQ1, a specific BET inhibitor, suppressed in vitro tube formation of human umbilical vein endothelial cells (HUVECs) and in vivo angiogenesis in a Matrigel plug and oxygen-induced retinopathy neovascularization. JQ1 attenuated the VEGF-induced decrease in TEER in HUVECs and prevented Evans blue dye leakage in the VEGF-induced Miles assay in athymic Balb/c nude mice. BET inhibition with JQ1 or shRNA for Brd2 or Brd4 suppressed VEGF-induced   migration  , proliferation, and stress fiber formation of HUVECs. Furthermore, BET inhibition suppressed phosphorylation of VEGFR2 and PAK1, as well as eNOS activation in VEGF-stimulated HUVECs. Inhibition with VEGFR2 and PAK1 also reduced   migration   and proliferation, and attenuated the VEGF-induced decrease in TEER. Thus, our observations suggest the important role of BET bromodomain in regulating VEGF-induced angiogenesis. Strategies that target the BET bromodomain may provide a new therapeutic approach for angiogenesis-related diseases. 
  GeneRIFs  
 miR7 negatively regulates PAK1 protein expression but has no effect on PAK1 mRNA expression. Knockdown of PAK1 expression markedly suppressed thyroid cancer cell proliferation,   migration   and invasion. 
 PAK1-cofilin phosphorylation mechanism to mediate lung adenocarcinoma cells   migration   promoted by apelin-13 
 Provide first evidence for existence of a previously unknown Erk5/KLF2/PAK1 axis, which may limit undesired cell   migration   in unperturbed endothelium and lower its sensitivity for migratory cues that promote vascular diseases including atherosclerosis. 
 Data indicate that depletion of NFATc1, cyclin D1, CDK6, or CDK4 levels attenuated MCP1-induced Pak1 phosphorylation/activation and resulted in decreased aortic smooth muscle cells (HASMCs) F-actin stress fiber formation,   migration  , and proliferation. 
 Cytokine-induced   migration   correlated with phosphorylation of PAK1 in primary myeloma cells & cell lines. Downregulation of PAK1 with siRNA in INA-6 cells resulted in decreased cytokine-driven   migration  . 
 PAK1 plays an important role in regulating the   migration  , invasion and production and activity of MMPs in RA FLS, which is mediated by the JNK pathway. 
 In colorectal neoplasms p-21 activated kinase 1 knockdown inhibits beta-catenin signalling and blocks cell proliferation and   migration  . 
 results suggest that CIB1 positively regulates cell   migration   and is necessary for the recruitment of FAK to the focal adhesions. Furthermore, CIB1-induced cell   migration   is dependent on MAP kinase signaling and its function is attenuated by PAK1 
 the PI3K/PAK1/ERK signaling pathway has a role in LPA-stimulated breast cancer cell   migration   
 P21-activated kinase 1 stimulates colon cancer cell growth and   migration  /invasion via ERK- and AKT-dependent pathways. 
 Rac1/Pak1 signaling is critical to MB cell   migration   and is functionally dependent on PDGFRbeta/ERK activity 
 PAK1 is a direct downstream target of LKB1 and plays an essential role in LKB1-induced suppression of cell   migration   
 Knockdown of PAK1 inhibits Hepatocyte Growth Factor (HGF) stimulated   migration   and loss of cell-cell junctions in DU145 prostate carcinoma cells. In PC3 prostate carcinoma cells, knockdown of PAK1 reduces HGF-stimulated   migration  . 
 TH1 interacts with PAK1 and specifically restricts the activation of MAPK modules through the upstream region of the MAPK pathway, thereby influencing cell   migration  . 
 16k PRL inhibits cell   migration   by blocking the Ras-Tiam1-Rac1-Pak1 signaling pathway in endothelial cells 
 Phosphorylation of caldesmon by PAK is a dynamic process required to regulate actin dynamics and membrane protrusions in wound-induced cell   migration  . 
 Src- & ROS-dependent PDK1 activation leads to site-specific PAK1 phosphorylation. This is critically important for PDGF-induced VSMC   migration  , a process integral to the vascular response to injury that leads to vessel occlusion & plaque formation. 
 Akt1 and Akt2 have opposing roles in Rac/Pak signaling and cell   migration   
 phorbol ester induced cell   migration   is accompanied by selective and transient down-regulation of PAK1, which coincided with the formation of stress fibres. 
  ECM  PubMed  
 Opposing roles for Akt1 and Akt2 in Rac/Pak signaling and cell migration. The Akt/PKB isoforms have different roles in animals, with Akt2 primarily regulating metabolic signaling and Akt1 regulating growth and survival. Here we show distinct roles for Akt1 and Akt2 in mouse embryo fibroblast cell migration and regulation of the cytoskeleton. Akt1-deficient cells responded poorly to platelet-derived growth factor while Akt2-deficient cells had a dramatically enhanced response, resulting in a substantial increase in dorsal ruffling. Swapping domains between Akt1 and Akt2 demonstrated that the N-terminal region containing the pleckstrin homology domain and a linker region distinguishes the two isoforms, while the catalytic domains are interchangeable. Akt2 knock-out cells also migrated faster than wild-type cells, especially through extracellular matrix (  ECM  ), while Akt1 knock-out cells migrated more slowly than wild-type cells. Consistently, Akt2 knock-out cells had elevated Pak1 and Rac activities, suggesting that Akt2 inhibits Rac and Pak1. Both Akt2 and Akt1 associated in complexes with Pak1, but only Akt2 inhibited Pak1 in kinase assays, suggesting an underlying molecular basis for the different cellular phenotypes. Together these data provide evidence for an unexpected functional link between Akt2 and Pak1 that opposes the actions of Akt1 on cell migration. 
 Opposing roles for Akt1 and Akt2 in Rac/Pak signaling and cell migration. The Akt/PKB isoforms have different roles in animals, with Akt2 primarily regulating metabolic signaling and Akt1 regulating growth and survival. Here we show distinct roles for Akt1 and Akt2 in mouse embryo fibroblast cell migration and regulation of the cytoskeleton. Akt1-deficient cells responded poorly to platelet-derived growth factor while Akt2-deficient cells had a dramatically enhanced response, resulting in a substantial increase in dorsal ruffling. Swapping domains between Akt1 and Akt2 demonstrated that the N-terminal region containing the pleckstrin homology domain and a linker region distinguishes the two isoforms, while the catalytic domains are interchangeable. Akt2 knock-out cells also migrated faster than wild-type cells, especially through extracellular matrix (  ECM  ), while Akt1 knock-out cells migrated more slowly than wild-type cells. Consistently, Akt2 knock-out cells had elevated Pak1 and Rac activities, suggesting that Akt2 inhibits Rac and Pak1. Both Akt2 and Akt1 associated in complexes with Pak1, but only Akt2 inhibited Pak1 in kinase assays, suggesting an underlying molecular basis for the different cellular phenotypes. Together these data provide evidence for an unexpected functional link between Akt2 and Pak1 that opposes the actions of Akt1 on cell migration. 
 PAK-PIX interactions regulate adhesion dynamics and membrane protrusion to control neurite outgrowth. The roles of P21-activated kinase (PAK) in the regulation of axon outgrowth downstream of extracellular matrix (  ECM  ) proteins are poorly understood. Here we show that PAK1-3 and PIX are expressed in the developing spinal cord and differentially localize to point contacts and filopodial tips within motile growth cones. Using a specific interfering peptide called PAK18, we found that axon outgrowth is robustly stimulated on laminin by partial inhibition of PAK-PIX interactions and PAK function, whereas complete inhibition of PAK function stalls axon outgrowth. Furthermore, modest inhibition of PAK-PIX stimulates the assembly and turnover of growth cone point contacts, whereas strong inhibition over-stabilizes adhesions. Point mutations within PAK confirm the importance of PIX binding. Together our data suggest that regulation of PAK-PIX interactions in growth cones controls neurite outgrowth by influencing the activity of several important mediators of actin filament polymerization and retrograde flow, as well as integrin-dependent adhesion to laminin. 
 Tumor necrosis factor-alpha induced expression of matrix metalloproteinase-9 through p21-activated kinase-1. Expressed in embryonic development, matrix metalloprotein-9 (MMP-9) is absent in most of developed adult tissues, but recurs in inflammation during tissue injury, wound healing, tumor formation and metastasis. Expression of MMP-9 is tightly controlled by extracellular cues including pro-inflammatory cytokines and extracellular matrix (  ECM  ). While the pathologic functions of MMP-9 are evident, the intracellular signaling pathways to control its expression are not fully understood. In this study we investigated mechanism of cytokine induced MMP-9 with particular emphasis on the role of p21-activated-kinase-1 (PAK1) and the down stream signaling.In response to TNF-alpha or IL-1alpha, PAK1 was promptly activated, as characterized by a sequential phosphorylation, initiated at threonine-212 followed by at threonine-423 in the activation loop of the kinase, in human skin keratinocytes, dermal fibroblasts, and rat hepatic stellate cells. Ectopic expression of PAK1 variants, but not p38 MAP kinase, impaired the TNF-alpha-induced MMP-9 expression, while other MMPs such as MMP-2, -3 and -14 were not affected. Activation of Jun N-terminal kinase (JNK) and NF-kappaB has been demonstrated to be essential for MMP-9 expression. Expression of inactive PAK1 variants impaired JNK but not NF-kappaB activation, which consequently suppressed the 5'-promoter activities of the MMP-9 gene. After the cytokine-induced phosphorylation, both ectopically expressed and endogenous PAK1 proteins were promptly accumulated even in the condition of suppressing protein synthesis, suggesting the PAK1 protein is stabilized upon TNF-alpha stimulation. Stabilization of PAK1 protein by TNF-alpha treatment is independent of the kinase catalytic activity and p21 GTPase binding capacities. In contrast to epithelial cells, mesenchymal cells require 3-dimensional type-I collagen in response to TNF-alpha to massively express MMP-9. The collagen effect is mediated, in part, by boost JNK activation in a way to cooperate the cytokine signaling.We identified a novel mechanism for MMP-9 expression in response to injury signals, which is mediated by PAK1 activation and stabilization leading JNK activation. 
 Cell adhesion regulates the interaction between Nck and p21-activated kinase. The p21-activated kinases (PAKs) are important mediators of cytoskeletal reorganization, cell motility and transcriptional events regulated by the Rho family GTPases Rac and Cdc42. PAK activation by serum components is strongly dependent on cell adhesion to the extracellular matrix (  ECM  ). PAK binds directly to the Nck adapter protein, an interaction thought to play an important role in regulation and localization of PAK activity. This report demonstrates that the interaction of PAK with Nck is regulated dynamically by cell adhesion. PAK-Nck binding is rapidly lost after cell detachment and rapidly restored after re-adhesion to the   ECM   protein fibronectin, suggesting a rapidly reversible mode of regulation. Furthermore, the loss of Nck binding correlates with changes in the phosphorylation state of PAK in nonadherent cells, as evidenced by electrophoretic mobility shift and phosphorylation within a sequence known to mediate interaction with Nck. The ability of cell adhesion to regulate PAK phosphorylation and interaction with Nck may contribute to the anchorage-dependence of PAK activation as well as to the localization of activated PAK within a cell. 
  [Ee]xtracellular [Mm]atrix  PubMed  
 alpha6beta4 integrin activates Rac-dependent p21-activated kinase 1 to drive NF-kappaB-dependent resistance to apoptosis in 3D mammary acini. Malignant transformation and multidrug resistance are linked to resistance to apoptosis, yet the molecular mechanisms that mediate tumor survival remain poorly understood. Because the stroma can influence tumor behavior by regulating the tissue phenotype, we explored the role of   extracellular matrix   signaling and tissue organization in epithelial survival. We report that elevated (alpha6)beta4 integrin-dependent Rac-Pak1 signaling supports resistance to apoptosis in mammary acini by permitting stress-dependent activation of the p65 subunit of NF-kappaB through Pak1. We found that inhibiting Pak1 through expression of N17Rac or PID compromises NF-kappaB activation and renders mammary acini sensitive to death, but that resistance to apoptosis could be restored to these structures by overexpressing wild-type NF-kappaB p65. We also observed that acini expressing elevated levels of Pak1 can activate p65 and survive death treatments, even in the absence of activated Rac, yet will die if activation of NF-kappaB is simultaneously inhibited through expression of IkappaBalphaM. Thus, mammary tissues can resist apoptotic stimuli by activating NF-kappaB through alpha6beta4 integrin-dependent Rac-Pak1 signaling. Our data emphasize the importance of the   extracellular matrix   stroma in tissue survival and suggest that alpha6beta4 integrin-dependent Rac stimulation of Pak1 could be an important mechanism mediating apoptosis-resistance in some breast tumors. 
 Opposing roles for Akt1 and Akt2 in Rac/Pak signaling and cell migration. The Akt/PKB isoforms have different roles in animals, with Akt2 primarily regulating metabolic signaling and Akt1 regulating growth and survival. Here we show distinct roles for Akt1 and Akt2 in mouse embryo fibroblast cell migration and regulation of the cytoskeleton. Akt1-deficient cells responded poorly to platelet-derived growth factor while Akt2-deficient cells had a dramatically enhanced response, resulting in a substantial increase in dorsal ruffling. Swapping domains between Akt1 and Akt2 demonstrated that the N-terminal region containing the pleckstrin homology domain and a linker region distinguishes the two isoforms, while the catalytic domains are interchangeable. Akt2 knock-out cells also migrated faster than wild-type cells, especially through   extracellular matrix   (ECM), while Akt1 knock-out cells migrated more slowly than wild-type cells. Consistently, Akt2 knock-out cells had elevated Pak1 and Rac activities, suggesting that Akt2 inhibits Rac and Pak1. Both Akt2 and Akt1 associated in complexes with Pak1, but only Akt2 inhibited Pak1 in kinase assays, suggesting an underlying molecular basis for the different cellular phenotypes. Together these data provide evidence for an unexpected functional link between Akt2 and Pak1 that opposes the actions of Akt1 on cell migration. 
 alpha6beta4 integrin activates Rac-dependent p21-activated kinase 1 to drive NF-kappaB-dependent resistance to apoptosis in 3D mammary acini. Malignant transformation and multidrug resistance are linked to resistance to apoptosis, yet the molecular mechanisms that mediate tumor survival remain poorly understood. Because the stroma can influence tumor behavior by regulating the tissue phenotype, we explored the role of   extracellular matrix   signaling and tissue organization in epithelial survival. We report that elevated (alpha6)beta4 integrin-dependent Rac-Pak1 signaling supports resistance to apoptosis in mammary acini by permitting stress-dependent activation of the p65 subunit of NF-kappaB through Pak1. We found that inhibiting Pak1 through expression of N17Rac or PID compromises NF-kappaB activation and renders mammary acini sensitive to death, but that resistance to apoptosis could be restored to these structures by overexpressing wild-type NF-kappaB p65. We also observed that acini expressing elevated levels of Pak1 can activate p65 and survive death treatments, even in the absence of activated Rac, yet will die if activation of NF-kappaB is simultaneously inhibited through expression of IkappaBalphaM. Thus, mammary tissues can resist apoptotic stimuli by activating NF-kappaB through alpha6beta4 integrin-dependent Rac-Pak1 signaling. Our data emphasize the importance of the   extracellular matrix   stroma in tissue survival and suggest that alpha6beta4 integrin-dependent Rac stimulation of Pak1 could be an important mechanism mediating apoptosis-resistance in some breast tumors. 
 Opposing roles for Akt1 and Akt2 in Rac/Pak signaling and cell migration. The Akt/PKB isoforms have different roles in animals, with Akt2 primarily regulating metabolic signaling and Akt1 regulating growth and survival. Here we show distinct roles for Akt1 and Akt2 in mouse embryo fibroblast cell migration and regulation of the cytoskeleton. Akt1-deficient cells responded poorly to platelet-derived growth factor while Akt2-deficient cells had a dramatically enhanced response, resulting in a substantial increase in dorsal ruffling. Swapping domains between Akt1 and Akt2 demonstrated that the N-terminal region containing the pleckstrin homology domain and a linker region distinguishes the two isoforms, while the catalytic domains are interchangeable. Akt2 knock-out cells also migrated faster than wild-type cells, especially through   extracellular matrix   (ECM), while Akt1 knock-out cells migrated more slowly than wild-type cells. Consistently, Akt2 knock-out cells had elevated Pak1 and Rac activities, suggesting that Akt2 inhibits Rac and Pak1. Both Akt2 and Akt1 associated in complexes with Pak1, but only Akt2 inhibited Pak1 in kinase assays, suggesting an underlying molecular basis for the different cellular phenotypes. Together these data provide evidence for an unexpected functional link between Akt2 and Pak1 that opposes the actions of Akt1 on cell migration. 
 Phorbol ester-induced migration of HepG2 cells is accompanied by intensive stress fibre formation, enhanced integrin expression and transient down-regulation of p21-activated kinase 1. Previously, we observed that phorbol ester induced more intensive scattering of HepG2 human hepatoma cells than hepatocyte growth factor (HGF). Regulatory components accounting for this intensive migration were studied. Phorbol ester-activated protein kinase C induced the early appearance of a great number of actin stress fibres. Whereas in response to HGF, the activation of phosphatidylinositol 3-kinase initiates the rearrangements of the actin cytoskeleton, in phorbol ester-treated cells, the activation of this enzyme was not required to the actin polymerisation. Activation of Erk1/Erk2 MAP kinases that was essential to the migration had a key role in enhancing the adherence of cells to the   extracellular matrix   via the increased expression of integrins alpha2, alpha6 and beta1. Protein kinase C stimulated the activation of p21-activated kinase (PAK), as well. However, it also stimulated the selective and transient down-regulation of PAK1, which coincided with the formation of stress fibres. 
 Integrin engagement differentially modulates epithelial cell motility by RhoA/ROCK and PAK1. Integrin-ligand binding regulates tumor cell motility and invasion. Cell migration also involves the Rho GTPases that control the interplay between adhesion receptors and the cytoskeleton. We evaluated how specific   extracellular matrix   ligands modulate Rho GTPases and control motility of human squamous cell carcinoma cells. On laminin-5 substrates, the epithelial cells rapidly spread and migrated, but on type I collagen the cells spread slowly and showed reduced motility. We found that RhoA activity was suppressed in cells attached to laminin-5 through the alpha3 integrin receptor. In contrast, RhoA was strongly activated in cells bound to type I collagen and this was mediated by the alpha2 integrin. Inhibiting the RhoA pathway by expression of a dominant-negative RhoA mutant or by directly inhibiting ROCK, reduced focal adhesion formation and enhanced cell migration on type I collagen. Cdc42 and Rac and their downstream target PAK1 were activated following adhesion to laminin-5. PAK1 activation induced by laminin-5 was suppressed by expression of a dominant-negative Cdc42. Moreover, constitutively active PAK1 stimulated migration on collagen I substrates. Our results indicate that in squamous epithelial cells, collagen-alpha2beta1 integrin binding activates RhoA, slowing cell locomotion, whereas laminin-5-alpha3beta1 integrin interaction inhibits RhoA and activates PAK1, stimulating cell migration. The data demonstrate that specific ligand-integrin pairs regulate cell motility differentially by selectively modulating activities of Rho GTPases and their effectors. 
 PAK-PIX interactions regulate adhesion dynamics and membrane protrusion to control neurite outgrowth. The roles of P21-activated kinase (PAK) in the regulation of axon outgrowth downstream of   extracellular matrix   (ECM) proteins are poorly understood. Here we show that PAK1-3 and PIX are expressed in the developing spinal cord and differentially localize to point contacts and filopodial tips within motile growth cones. Using a specific interfering peptide called PAK18, we found that axon outgrowth is robustly stimulated on laminin by partial inhibition of PAK-PIX interactions and PAK function, whereas complete inhibition of PAK function stalls axon outgrowth. Furthermore, modest inhibition of PAK-PIX stimulates the assembly and turnover of growth cone point contacts, whereas strong inhibition over-stabilizes adhesions. Point mutations within PAK confirm the importance of PIX binding. Together our data suggest that regulation of PAK-PIX interactions in growth cones controls neurite outgrowth by influencing the activity of several important mediators of actin filament polymerization and retrograde flow, as well as integrin-dependent adhesion to laminin. 
 Tumor necrosis factor-alpha induced expression of matrix metalloproteinase-9 through p21-activated kinase-1. Expressed in embryonic development, matrix metalloprotein-9 (MMP-9) is absent in most of developed adult tissues, but recurs in inflammation during tissue injury, wound healing, tumor formation and metastasis. Expression of MMP-9 is tightly controlled by extracellular cues including pro-inflammatory cytokines and   extracellular matrix   (ECM). While the pathologic functions of MMP-9 are evident, the intracellular signaling pathways to control its expression are not fully understood. In this study we investigated mechanism of cytokine induced MMP-9 with particular emphasis on the role of p21-activated-kinase-1 (PAK1) and the down stream signaling.In response to TNF-alpha or IL-1alpha, PAK1 was promptly activated, as characterized by a sequential phosphorylation, initiated at threonine-212 followed by at threonine-423 in the activation loop of the kinase, in human skin keratinocytes, dermal fibroblasts, and rat hepatic stellate cells. Ectopic expression of PAK1 variants, but not p38 MAP kinase, impaired the TNF-alpha-induced MMP-9 expression, while other MMPs such as MMP-2, -3 and -14 were not affected. Activation of Jun N-terminal kinase (JNK) and NF-kappaB has been demonstrated to be essential for MMP-9 expression. Expression of inactive PAK1 variants impaired JNK but not NF-kappaB activation, which consequently suppressed the 5'-promoter activities of the MMP-9 gene. After the cytokine-induced phosphorylation, both ectopically expressed and endogenous PAK1 proteins were promptly accumulated even in the condition of suppressing protein synthesis, suggesting the PAK1 protein is stabilized upon TNF-alpha stimulation. Stabilization of PAK1 protein by TNF-alpha treatment is independent of the kinase catalytic activity and p21 GTPase binding capacities. In contrast to epithelial cells, mesenchymal cells require 3-dimensional type-I collagen in response to TNF-alpha to massively express MMP-9. The collagen effect is mediated, in part, by boost JNK activation in a way to cooperate the cytokine signaling.We identified a novel mechanism for MMP-9 expression in response to injury signals, which is mediated by PAK1 activation and stabilization leading JNK activation. 
 Transcriptional regulation of fibronectin by p21-activated kinase-1 modulates pancreatic tumorigenesis. Pancreatic ductal adenocarcinoma (PDAC) is the eighth largest cause of cancer-related mortality across the world, with a median 5-year survival rate of less than 3.5%. This is partly because the molecules and the molecular mechanisms that contribute to PDAC are not well understood. Our goal is to understand the role of p21-activated kinase 1 (Pak1) signaling axis in the progression of PDAC. Pak1, a serine/threonine kinase, is a well-known regulator of cytoskeletal remodeling, cell motility, cell proliferation and cell survival. Recent reports suggest that Pak1 by itself can have an oncogenic role in a wide variety of cancers. In this study, we analyzed the expression of Pak1 in human pancreatic cancer tissues and found that Pak1 levels are significantly upregulated in PDAC samples as compared with adjacent normals. Further, to study the functional role of Pak1 in pancreatic cancer model systems, we developed stable overexpression and lentiviral short hairpin RNA-mediated knockdown (KD) clones of Pak1 and studied the changes in transforming properties of the cells. We also observed that Pak1 KD clones failed to form tumors in nude mice. By adopting a quantitative PCR array-based approach, we identified fibronectin, a component of the   extracellular matrix   and a mesenchymal marker, as a transcriptional target of Pak1 signaling. The underlying molecular mechanism of Pak1-mediated transformation includes its nuclear import and recruitment to the fibronectin promoter via interaction with nuclear factor-κB (NF-κB)-p65 complex. To our knowledge, this is the first study illustrating Pak1-NF-κB-p65-mediated fibronectin regulation as a potent tumor-promoting mechanism in KRAS intact model. 
 Cell adhesion regulates the interaction between Nck and p21-activated kinase. The p21-activated kinases (PAKs) are important mediators of cytoskeletal reorganization, cell motility and transcriptional events regulated by the Rho family GTPases Rac and Cdc42. PAK activation by serum components is strongly dependent on cell adhesion to the   extracellular matrix   (ECM). PAK binds directly to the Nck adapter protein, an interaction thought to play an important role in regulation and localization of PAK activity. This report demonstrates that the interaction of PAK with Nck is regulated dynamically by cell adhesion. PAK-Nck binding is rapidly lost after cell detachment and rapidly restored after re-adhesion to the ECM protein fibronectin, suggesting a rapidly reversible mode of regulation. Furthermore, the loss of Nck binding correlates with changes in the phosphorylation state of PAK in nonadherent cells, as evidenced by electrophoretic mobility shift and phosphorylation within a sequence known to mediate interaction with Nck. The ability of cell adhesion to regulate PAK phosphorylation and interaction with Nck may contribute to the anchorage-dependence of PAK activation as well as to the localization of activated PAK within a cell. 
  GeneRIFs  
 identified fibronectin, a component of the   extracellular matrix   and a mesenchymal marker, as a transcriptional target of Pak1 signaling 
  [Ii]nvasive  PubMed  
 HER2/Neu (ErbB2) signaling to Rac1-Pak1 is temporally and spatially modulated by transforming growth factor beta. In HER2 (ErbB2)-overexpressing cells, transforming growth factor beta (TGF-beta), via activation of phosphoinositide-3 kinase (PI3K), recruits actin and actinin to HER2, which then colocalizes with Vav2, activated Rac1, and Pak1 at cell protrusions. This results in prolonged Rac1 activation, enhanced motility and   invasive  ness, Bad phosphorylation, uncoupling of Bad/Bcl-2, and enhanced cell survival. The recruitment of the HER2/Vav2/Rac1/Pak1/actin/actinin complex to lamellipodia was abrogated by actinin siRNAs, dominant-negative (dn) p85, gefitinib, and dn-Rac1 or dn-Pak1, suggesting that the reciprocal interplay of PI3K, HER2 kinase, and Rac GTPases with the actin cytoskeleton is necessary for TGF-beta action in oncogene-overexpressing cells. Thus, by recruiting the actin skeleton, TGF-beta "cross-links" this signaling complex at cell lamellipodia; this prolongs Rac1 activation and increases metastatic properties and survival of HER2-overexpressing cells. 
 A Trio-Rac1-Pak1 signalling axis drives invadopodia disassembly. Rho family GTPases control cell migration and participate in the regulation of cancer metastasis. Invadopodia, associated with   invasive   tumour cells, are crucial for cellular invasion and metastasis. To study Rac1 GTPase in invadopodia dynamics, we developed a genetically encoded, single-chain Rac1 fluorescence resonance energy (FRET) transfer biosensor. The biosensor shows Rac1 activity exclusion from the core of invadopodia, and higher activity when invadopodia disappear, suggesting that reduced Rac1 activity is necessary for their stability, and Rac1 activation is involved in disassembly. Photoactivating Rac1 at invadopodia confirmed this previously unknown Rac1 function. We describe here an invadopodia disassembly model, where a signalling axis involving TrioGEF, Rac1, Pak1, and phosphorylation of cortactin, causes invadopodia dissolution. This mechanism is critical for the proper turnover of   invasive   structures during tumour cell invasion, where a balance of proteolytic activity and locomotory protrusions must be carefully coordinated to achieve a maximally   invasive   phenotype. 
 PI3K, Rac1 and pPAK1 are overexpressed in extramammary Paget's disease. Phosphatidylinositol 3-kinase (PI3K), Ras-related C3 botulinum toxin substrate 1 (Rac1) and P21-activated protein kinase 1 (PAK1) appear to play important roles in the pathogenesis of several tumors, but their expressions in extramammary Paget's disease (EMPD) have not been investigated yet.To investigate the potential contribution of the PI3K, Rac1 and PAK1 to the development of EMPD.Thirty-five paraffin-embedded EMPD specimens were subjected to immunohistochemical staining for PI3K (85α), Rac1 and pPAK1.All the 35 primary EMPD specimens, including 20 non  -invasiv  e EMPD, 13   invasiv  e EMPD and 2 metastatic lymph nodes, showed cytoplasm overexpression of PI3K (85α), Rac1 and pPAK1. The expression (% positive cells) of PI3K(85α), Rac1 and pPAK1 (90.1 ± 8.6, 91.4 ± 9.5 and 89.6 ± 10.8% ) in EMPD were significantly higher than in apocrine glands of normal skin ( 20.1 ± 11.9, 29.8 ± 8.9, 41.1 ± 13.4%), and the expre  ssion in   invasive EMPD with lymph node metastasis (98.2 ± 1.7, 98.8 ± 0.7 and 98.4 ± 0.9%) are significantly hig  her than   in invasive EMPD without lymph node metastasis (94.1 ± 2.6, 96.5 ± 1.7 and 95.3 ±   1.1%) a  nd non-invasive EMPD (85.2 ± 8.4, 87.1 ± 9.9 and 83.1 ± 10.6%). There were significant positive correlations of the expression levels between PI3K (85α) and Rac1, as well as between Rac1 and pPAK1 in EMPD.These results indicate that PI3K, Rac1 and PAK1 may play important roles in the pathogenesis of EMPD. 
 Centrosome-related genes, genetic variation, and risk of breast cancer. Centrosome amplification has been detected in premalignant lesions and in situ tumors in the breast and in over 70% of   invasive   breast tumors, and has been associated with aneuploidy and tumor development. Based on these observations, the contribution of commonly inherited genetic variation in candidate genes related to centrosome structure and function to breast cancer risk was evaluated in an association study. Seven-hundred and 82 single nucleotide polymorphisms (SNPs) from 101 centrosomal genes were analyzed in 798 breast cancer cases and 843 controls from the Mayo Clinic Breast Cancer Study to assess the association between these SNPs (both individually and combined) and risk of breast cancer in this population. Eleven SNPs out of 782 from six genes displayed associations with breast cancer risk (P < 0.01). Haplotypes in five genes also displayed significant associations with risk. A two SNP combination of rs10145182 in NIN and rs2134808 in the TUBG1 locus (P-interaction = 0.00001), suggested SNPs in mediators of microtubule nucleation from the centrosome contribute to breast cancer. Evaluation of the simultaneous significance of all SNPs in the centrosome pathway suggested that the centrosome pathway is highly enriched (P = 4.76 × 10(-50)) for SNPs that are associated with breast cancer risk. Collections of weakly associated genetic variants in the centrosome pathway, rather than individual highly significantly associated SNPs, may account for a putative role for the centrosome pathway in predisposition to breast cancer. 
 14-3-3zeta Cooperates with ErbB2 to promote ductal carcinoma in situ progression to   invasive   breast cancer by inducing epithelial-mesenchymal transition. ErbB2, a metastasis-promoting oncoprotein, is overexpressed in approximately 25% of   invasive  /metastatic breast cancers, but in 50%-60% of non  invasive   ductal carcinomas in situ (DCIS). It has been puzzling how a subset of ErbB2-overexpressing DCIS develops into   invasive   breast cancer (IBC). We found that co-overexpression of 14-3-3zeta in ErbB2-overexpressing DCIS conferred a higher risk of progression to IBC. ErbB2 and 14-3-3zeta overexpression, respectively, increased cell migration and decreased cell adhesion, two prerequisites of tumor cell invasion. 14-3-3zeta overexpression reduced cell adhesion by activating the TGF-beta/Smads pathway that led to ZFHX1B/SIP-1 upregulation, E-cadherin loss, and epithelial-mesenchymal transition. Importantly, patients whose breast tumors overexpressed both ErbB2 and 14-3-3zeta had higher rates of metastatic recurrence and death than those whose tumors overexpressed only one. 
 A Trio-Rac1-Pak1 signalling axis drives invadopodia disassembly. Rho family GTPases control cell migration and participate in the regulation of cancer metastasis. Invadopodia, associated with   invasive   tumour cells, are crucial for cellular invasion and metastasis. To study Rac1 GTPase in invadopodia dynamics, we developed a genetically encoded, single-chain Rac1 fluorescence resonance energy (FRET) transfer biosensor. The biosensor shows Rac1 activity exclusion from the core of invadopodia, and higher activity when invadopodia disappear, suggesting that reduced Rac1 activity is necessary for their stability, and Rac1 activation is involved in disassembly. Photoactivating Rac1 at invadopodia confirmed this previously unknown Rac1 function. We describe here an invadopodia disassembly model, where a signalling axis involving TrioGEF, Rac1, Pak1, and phosphorylation of cortactin, causes invadopodia dissolution. This mechanism is critical for the proper turnover of   invasive   structures during tumour cell invasion, where a balance of proteolytic activity and locomotory protrusions must be carefully coordinated to achieve a maximally   invasive   phenotype. 
 PI3K, Rac1 and pPAK1 are overexpressed in extramammary Paget's disease. Phosphatidylinositol 3-kinase (PI3K), Ras-related C3 botulinum toxin substrate 1 (Rac1) and P21-activated protein kinase 1 (PAK1) appear to play important roles in the pathogenesis of several tumors, but their expressions in extramammary Paget's disease (EMPD) have not been investigated yet.To investigate the potential contribution of the PI3K, Rac1 and PAK1 to the development of EMPD.Thirty-five paraffin-embedded EMPD specimens were subjected to immunohistochemical staining for PI3K (85α), Rac1 and pPAK1.All the 35 primary EMPD specimens, including 20 non  -invasiv  e EMPD, 13   invasiv  e EMPD and 2 metastatic lymph nodes, showed cytoplasm overexpression of PI3K (85α), Rac1 and pPAK1. The expression (% positive cells) of PI3K(85α), Rac1 and pPAK1 (90.1 ± 8.6, 91.4 ± 9.5 and 89.6 ± 10.8% ) in EMPD were significantly higher than in apocrine glands of normal skin ( 20.1 ± 11.9, 29.8 ± 8.9, 41.1 ± 13.4%), and the expre  ssion in   invasive EMPD with lymph node metastasis (98.2 ± 1.7, 98.8 ± 0.7 and 98.4 ± 0.9%) are significantly hig  her than   in invasive EMPD without lymph node metastasis (94.1 ± 2.6, 96.5 ± 1.7 and 95.3 ±   1.1%) a  nd non-invasive EMPD (85.2 ± 8.4, 87.1 ± 9.9 and 83.1 ± 10.6%). There were significant positive correlations of the expression levels between PI3K (85α) and Rac1, as well as between Rac1 and pPAK1 in EMPD.These results indicate that PI3K, Rac1 and PAK1 may play important roles in the pathogenesis of EMPD. 
 Centrosome-related genes, genetic variation, and risk of breast cancer. Centrosome amplification has been detected in premalignant lesions and in situ tumors in the breast and in over 70% of   invasive   breast tumors, and has been associated with aneuploidy and tumor development. Based on these observations, the contribution of commonly inherited genetic variation in candidate genes related to centrosome structure and function to breast cancer risk was evaluated in an association study. Seven-hundred and 82 single nucleotide polymorphisms (SNPs) from 101 centrosomal genes were analyzed in 798 breast cancer cases and 843 controls from the Mayo Clinic Breast Cancer Study to assess the association between these SNPs (both individually and combined) and risk of breast cancer in this population. Eleven SNPs out of 782 from six genes displayed associations with breast cancer risk (P < 0.01). Haplotypes in five genes also displayed significant associations with risk. A two SNP combination of rs10145182 in NIN and rs2134808 in the TUBG1 locus (P-interaction = 0.00001), suggested SNPs in mediators of microtubule nucleation from the centrosome contribute to breast cancer. Evaluation of the simultaneous significance of all SNPs in the centrosome pathway suggested that the centrosome pathway is highly enriched (P = 4.76 × 10(-50)) for SNPs that are associated with breast cancer risk. Collections of weakly associated genetic variants in the centrosome pathway, rather than individual highly significantly associated SNPs, may account for a putative role for the centrosome pathway in predisposition to breast cancer. 
 HER2/Neu (ErbB2) signaling to Rac1-Pak1 is temporally and spatially modulated by transforming growth factor beta. In HER2 (ErbB2)-overexpressing cells, transforming growth factor beta (TGF-beta), via activation of phosphoinositide-3 kinase (PI3K), recruits actin and actinin to HER2, which then colocalizes with Vav2, activated Rac1, and Pak1 at cell protrusions. This results in prolonged Rac1 activation, enhanced motility and   invasive  ness, Bad phosphorylation, uncoupling of Bad/Bcl-2, and enhanced cell survival. The recruitment of the HER2/Vav2/Rac1/Pak1/actin/actinin complex to lamellipodia was abrogated by actinin siRNAs, dominant-negative (dn) p85, gefitinib, and dn-Rac1 or dn-Pak1, suggesting that the reciprocal interplay of PI3K, HER2 kinase, and Rac GTPases with the actin cytoskeleton is necessary for TGF-beta action in oncogene-overexpressing cells. Thus, by recruiting the actin skeleton, TGF-beta "cross-links" this signaling complex at cell lamellipodia; this prolongs Rac1 activation and increases metastatic properties and survival of HER2-overexpressing cells. 
 Heregulin regulates cytoskeletal reorganization and cell migration through the p21-activated kinase-1 via phosphatidylinositol-3 kinase. The mechanisms through which heregulin (HRG) regulates the activities of breast cancer cells are currently unknown. We demonstrate that HRG stimulation of non  invasive   breast cancer cells enhanced the conversion of globular to filamentous actin and the formation of membrane ruffles, stress fibers, filopodia, and lamellipodia and accompanied by increased cell migration. In addition, HRG triggered a rapid stimulation of p21-activated kinase1 (PAK1) activity and its redistribution into the leading edges of motile cells. The HRG-induced stimulation of PAK1 kinase activity followed phosphatidylinositol-3 kinase (PI-3 kinase) activation. Inhibition of PI-3 kinase activity blocked the activation of PAK1 kinase and also blocked cell migration in response to HRG. Furthermore, direct inhibition of PAK1 functions by the dominant-negative mutant suppressed the capacity of HRG to reorganize actin cytoskeleon structures. We also demonstrated that HRG stimulation promoted physical interactions between PAK1, actin, and human epidermal growth factor receptor 2 (HER2) receptors, and these interactions were dependent on the activation of PI-3 kinase. The blockade of HER2 receptor by an anti-HER2 monoclonal antibody resulted in the inhibition of HRG-mediated stimulation of PI-3 kinase/PAK pathway and also the formation of motile actin cytoskeleton structures but not extracellular signal-regulated kinases. These findings suggest a role of PI-3 kinase/PAK1-dependent reorganization of the cortical actin cytoskeleton in HRG-mediated increased cell migration, and these changes may have significant consequences leading to enhanced invasion by breast cancer cells. 
 Etk/Bmx tyrosine kinase activates Pak1 and regulates tumorigenicity of breast cancer cells. Etk/Bmx, a member of the Tec family of nonreceptor protein-tyrosine kinases, is characterized by an N-terminal pleckstrin homology domain and has been shown to be a downstream effector of phosphatidylinositol 3-kinase. P21-activated kinase 1 (Pak1), another well characterized effector of phosphatidylinositol 3-kinase, has been implicated in the progression of breast cancer cells. In this study, we characterized the role of Etk in mammary development and tumorigenesis and explored the functional interactions between Etk and Pak1. We report that Etk expression is developmentally regulated in the mammary gland. Using transient transfection, coimmunoprecipitation and glutathione S-transferase-pull down assays, we showed that Etk directly associates with Pak1 via its N-terminal pleckstrin homology domain and also phosphorylates Pak1 on tyrosine residues. The expression of wild-type Etk in a non-  invasive   human breast cancer MCF-7 cells significantly increased proliferation and anchorage-independent growth of epithelial cancer cells. Conversely, expression of kinase-inactive mutant Etk-KQ suppressed the proliferation, anchorage-independent growth, and tumorigenicity of human breast cancer MDA-MB435 cells. These results indicate that Pak1 is a target of Etk and that Etk controls the proliferation as well as the anchorage-independent and tumorigenic growth of mammary epithelial cancer cells. 
 Association of p21-activated kinase-1 activity with aggressive tumor behavior and poor prognosis of head and neck cancer. This study investigated the role of p21-activated kinase (PAK)-1 in progression of head and neck squamous cell carcinoma (HNSCC).We examined PAK isoforms and explored whether PAK activation enhanced in vitro invasion of the HNSCC cell line. We analyzed the relationship between PAK1 expression and various clinicopathological features and investigated the effect of PAK1 overexpression on survival in 119 patients with HNSCC.PAK1 and PAK2 are predominantly expressed in HNSCC cells and patient tissues. Particularly, PAK1 makes the dominant contribution to increase in cell migration and invasion. There was a statistically significant correlation between PAK1 overexpression and aggressive cancer behavior. Moreover, PAK1 seemed to be a prognostic factor for overall and disease-specific survival in patients. Interestingly, enhancement of PAK1 expression was found in the   invasive   front of cancer.PAK1 is associated with the aggressive tumor behavior and poor prognosis of head and neck cancer. 
 PAK1 regulates RUFY3-mediated gastric cancer cell migration and invasion. Actin protrusion at the cell periphery is central to the formation of invadopodia during tumor cell migration and invasion. Although RUFY3 (RUN and FYVE domain containing 3)/SINGAR1 (single axon-related1)/RIPX (Rap2 interacting protein X) has an important role in neuronal development, its pathophysiologic role and relevance to cancer are still largely unknown. The purpose of this study was to elucidate the molecular mechanisms by which RUFY3 involves in gastric cancer cell migration and invasion. Here, our data show that overexpression of RUFY3 leads to the formation of F-actin-enriched protrusive structures at the cell periphery and induces gastric cancer cell migration. Furthermore, P21-activated kinase-1 (PAK1) interacts with RUFY3, and promotes RUFY3 expression and RUFY3-induced gastric cancer cell migration; inhibition of PAK1 attenuates RUFY3-induced SGC-7901 cell migration and invasion. Importantly, we found that the inhibitory effect of cell migration and invasion is significantly enhanced by knockdown of both PAK1 and RUFY3 compared with knockdown of RUFY3 alone or PAK1 alone. Strikingly, we found significant upregulation of RUFY3 in gastric cancer samples with   invasive   carcinoma at pathologic TNM III and TNM IV stages, compared with their non-tumor counterparts. Moreover, an obvious positive correlation was observed between the protein expression of RUFY3 and PAK1 in 40 pairs of gastric cancer samples. Therefore, these findings provide important evidence that PAK1 can positively regulate RUFY3 expression, which contribute to the metastatic potential of gastric cancer cells, maybe blocking PAK1-RUFY3 signaling would become a potential metastasis therapeutic strategy for gastric cancer. 
 Correlations of P21-activated kinase 1 expression to clinicopathological features of gastric carcinoma and patients' prognosis. Recent studies proved that P21-activated kinase 1 (PAK1) is highly expressed in many kinds of tumor and plays an important role in genesis, development, and metastasis of tumor. We aimed to detect the expression of PAK1 in gastric carcinoma and to analyze its relationship with clinicopathological features and prognosis of gastric carcinoma.Tissue microarray and immunohistochemical staining were performed to detect PAK1 in paraffin specimens of 189 gastric carcinomas, 54 paracancer tissues, 40 lymph nodes and 30 healthy tissues. Clinicopathologic features and follow-up data of the patients were analyzed by the Chi2 test and the Kaplan-Meier method.Positive rate of PAK1 was 73.0% in gastric carcinoma, 57.4% in paracancer tissues and 23.3% in healthy controls (Chi2 = 29.364, P < 0.05). Expression of PAK1 was significantly correlated with tumor size, tumor differentiation, lymph node metastasis, Lauren classification and   invasive   depth (all P < 0.05). The positive rate of PAK1 was significantly higher in primary gastric carcinomas than in metastatic lymph nodes (75.0% vs. 52.5%, Chi2 = 4.381, P < 0.05). Survival analysis using the Kaplan-Meier method showed that the expression of PAK1 was a predictor for poor prognosis of the patients with gastric carcinoma (Chi2 = 6.857, P < 0.01).Expression of PAK1 is an early molecular event in the tumorigenesis of gastric carcinoma. It is also closely correlated the development of gastric carcinoma and the patients' prognosis. 
 The investigation of miR-221-3p and PAK1 gene expressions in breast cancer cell lines. The most common malignancy in women is breast cancer. Drug resistance in the treatment of cancer still remains a major clinical concern. Resistance to tamoxifen is seen in half of the recurrences in breast cancer. The anti-estrogen tamoxifen gains agonistic property by transactivating ERα. PAK1-mediated phosphorylation of serine 305 (S305) of ERα leads to resistance to tamoxifen. In our study, PAK1-induced suggestive tamoxifen resistance was designed. According to our hypothesis, phosphorylation of ERα-S305 by PAK1 may be reversed by PAK1 transcriptional inhibition by miR-221-3p due to miR-221-3p targeting the 3' UTR of PAK1. For this purpose, we used Real-time PCR (qRT-PCR) to measure the expression level of miR-221-3p in ER-positive breast cancer cell lines (ZR-75-1, MCF7) and breast epithelial cell line, hTERT-HME1, as control in the laboratory in our department. The increase in the expression of PAK1 depending on miR-221-3p may be related to ZR-75-1 cell line which h  as invas  ive characteristic but other two ER+ cancer cell lines, MCF7 and HCC1500, have milder cancer severity. miR-221-3p may have a role on regulation of PAK1 expression because miR-221-3p expression level decreases while PAK1 expression level increases in SKBR3 cell line. miR-221-3p and PAK1 expressions in MDA-MB-231 cell line are higher than that of hTERT-HME1 cell line. This may mean that miR-221-3p has no regulatory effect on of PAK1 expression in this cell line. According to these results, miR-221-3p may give crucial information about molecular mechanism of the disease upon PAK1 activity or different mechanisms with respect to histopathology and severity of breast cancer. 
 P21 activated kinase-1 (Pak1) promotes prostate tumor growth and microinvasion via inhibition of transforming growth factor β expression and enhanced matrix metalloproteinase 9 secretion. P21-activated kinases (Paks) are major effectors downstream of the small Rho family of GTPases. Among the six isoforms, Pak1 is the most ubiquitous and the best characterized member. Previous studies have shown that inhibition of Pak6, which is predominantly present in the prostate compared with other tissues, inhibits prostate tumor growth in vivo. Even though Pak1 has been identified in normal prostatic epithelial cells and cancer cells, its specific role in the development of prostate cancer remains unclear. We report here that highly   invasiv  e prostate cancer cells express significantly higher levels of Pak1 protein compared with non  -invasiv  e prostate cancer cells. Furthermore, prostate tumor tissues and prostate cancer metastasized to lungs showed a higher expression of Pak1 compared with normal tissues. Interestingly, Pak6 protein expression levels did not change with the   invasiv  e/metastatic potential of the cancer cells or tumors. Although inhibition of Pak1, and not Pak6, resulted in impaired PC3 cell migration, the effects of Pak1 knockdown on transendothelial migration (microinvasion), tumor growth, and tumor angiogenesis was higher compared with Pak6 knockdown. Finally, gene array data revealed reduced expression of matrix metalloproteinase 9 with the ablation of either Pak1 or Pak6 gene expression in PC3 cells, whereas protein levels of TGFβ was elevated significantly with specific modulation of Pak1 activity or ablation of the Pak1 gene. Our observations suggest that although some level of functional redundancy exists between Pak1 and Pak6 in prostate cancer cells, targeting Pak1 is a potential option for the management of prostate tumor growth, microinvasion, and metastasis. 
 Axl as a downstream effector of TGF-β1 via PI3K/Akt-PAK1 signaling pathway promotes tumor invasion and chemoresistance in breast carcinoma. The invasion and chemoresistance are crucial causes of morbidity and death for cancer patients. Axl is closely associated with malignant phenotype of breast tumor cells, including   invasiv  eness and metastasis. Both breast cancer cell line and tissue displayed increased expression of Axl, especially in highly metastatic breast cancer. On the contrary, experimental inhibition of Axl or transforming growth factor beta 1 (TGF-β1) by RNAi assay could suppress cell invasion ability and chemoresistance. Moreover, the up-regulation of Axl was induced by TGF-β1, further activated phosphatidylinositol 3-kinase (PI3K)/Akt and PAK1 translocation, and resulted in greater cell motility, invasion, and chemoresistance in vitro and in vivo. After the detection and statistics in human breast cancer specimens, we found that the Axl expression was closely correlated with TGF-β1 level, tumor differentiation, lymph node metastasis, and clinical stage (p < 0.01). Our findings support the possibility that Axl is a significant regulator of invasion and chemosensitivity, and it means by targeting Axl or its related signaling pathways, we can reduce the invasion and chemosensitivity of breast tumor. 
 Forced expression of NESH suppresses motility and metastatic dissemination of malignant cells. To characterize the function of a novel Src homology 3 (SH3) adapter protein termed NESH, we have established transfectants stably expressing NESH. We observed that every clone of NESH transfectants caused a marked reduction in motility, although the clones exhibited no significant differences in intrinsic cell growth compared with the control cells in vitro. The NESH transfectants also exhibited significant reduction in tumor metastatic potential in vivo. We found that NESH expression is frequently lost in   invasive   cancer cell lines despite its ubiquitous expression in normal tissues. The SH3 domain of NESH seems to interact with p21-activated kinase (PAK), which is involved in regulation of cell motility. Furthermore, a significant decrease in PAK phosphorylation at (402)Thr was found in NESH transfectants. Taken together, these results suggest that NESH inhibits ectopic metastasis of tumor cells as well as cell migration through interaction with intracellular molecules such as PAK that are essential for cell motility. 
 p21-activated kinase 1 determines stem-like phenotype and sunitinib resistance via NF-κB/IL-6 activation in renal cell carcinoma. The p21-activated kinase 1 (PAK1), a serine/threonine kinase that orchestrates cytoskeletal remodeling and cell motility, has been shown to function as downstream node for various oncogenic signaling pathways to promote cell proliferation, regulate apoptosis and accelerate mitotic abnormalities, resulting in tumor formation and   invasiv  eness. Although alterations in PAK1 expression and activity have been detected in various human malignancies, its potential biological and clinical significance in renal cell carcinoma (RCC) remains obscure. In this study, we found increased PAK1 and phosphorylated PAK1 levels in tumor tissues according to TNM stage progression. Elevated phosphorylated PAK1 levels associated with progressive features and indicated unfavorable overall survival (OS) as an independent adverse prognosticator for patients with RCC. Moreover, PAK1 kinase activation with constitutive active PAK1 mutant T423E promoted growth, colony formation, migration, invasion and stem-like phenotype of RCC cells, and vice versa, in PAK1 inhibition by PAK1 kinase inactivation with specific PAK1 shRNA, dead kinase PAK1 mutant K299R or allosteric inhibitor IPA3. Stem-like phenotype due to sunitinib administration via increased PAK1 kinase activation could be ameliorated by PAK1 shRNA, PAK1 mutant K299R and IPA3. Furthermore, nuclear factor-κB (NF-κB)/interleukin-6 (IL-6) activation was found to be responsible for PAK1-mediated stem-like phenotype following sunitinib treatment. Both IL-6 neutralizing antibody and IPA3 administration enhanced tumor growth inhibition effect of sunitinib treatment on RCC cells in vitro and in vivo. Our results unraveled that oncogenic activation of PAK1 defines an important mechanism for maintaining stem-like phenotype and sunitinib resistance through NF-κB/IL-6 activation in RCC, lending PAK1-mediated NF-κB/IL-6 activation considerable appeal as novel pharmacological therapeutic targets against sunitinib resistance. 
 PAK1 kinase promotes cell motility and   invasive  ness through CRK-II serine phosphorylation in non-small cell lung cancer cells. The role of c-Crk (CRK) in promoting metastasis is well described however the role of CRK phosphorylation and the corresponding signaling events are not well explained. We have observed CRK-II serine 41 phosphorylation is inversely correlated with p120-catenin and E-cadherin expressions in non-small cell lung cancer (NSCLC) cells. Therefore, we investigated the role of CRK-II serine 41 phosphorylation in the down-regulation of p120-catenin, cell motility and cell   invasive  ness in NSCLC cells. For this purpose, we expressed phosphomimetic and phosphodeficient CRK-II serine 41 mutants in NSCLC cells. NSCLC cells expressing phosphomimetic CRK-II seine 41 mutant showed lower p120-catenin level while CRK-II seine 41 phosphodeficient mutant expression resulted in higher p120-catenin. In addition, A549 cells expressing CRK-II serine 41 phosphomimetic mutant demonstrated more aggressive behavior in wound healing and invasion assays and, on the contrary, expression of phosphodeficient CRK-II serine 41 mutant in A549 cells resulted in reduced cell motility and   invasive  ness. We also provide evidence that PAK1 mediates CRK-II serine 41 phosphorylation. RNAi mediated silencing of PAK1 increased p120-catenin level in A549 and H157 cells. Furthermore, PAK1 silencing decreased cell motility and   invasive  ness in A549 cells. These effects were abrogated in A549 cells expressing phosphomimetic CRK-II serine 41. In summary, these data provide evidence for the role of PAK1 in the promotion of cell motility, cell   invasive  ness and the down regulation of p120-catenin through CRK serine 41 phosphorylation in NSCLC cells. 
 Increased p21-activated kinase-1 expression is associated with   invasive   potential in uveal melanoma. Prognosis in patients with uveal melanoma is poor as approximately half of all tumors metastasize and currently there are no effective treatments for disseminated disease. Differences in   invasive  ness between uveal melanomas could therefore be of major significance regarding clinical outcome. To identify genes associated with   invasive   potential, we have used microarray expression profiling combined with phenotypic characterization of uveal melanoma and melanocyte cell lines to define a gene signature associated with cellular invasion. A panel of 14 uveal cell cultures was assessed using three assays of   invasive  ness: matrigel invasion chamber system, scratch wound closure and cell motility. We identified a set of 853 differentially expressed transcripts (Wilcoxon-Mann-Whitney test, P<0.01) that discriminated between samples with high or low   invasive   capacity based on a composite phenotype that takes into account behavior across all three assays. Of particular interest, expression of two members of the p21-activated kinase (PAK) family, PAK1 and PAK7, was elevated in the more   invasive   cultures. PAK1 has previously been shown to play a central role in regulating cell motility and   invasive  ness in other cell types, and increased expression has been observed in breast and colorectal carcinomas. Using small interfering RNA-mediated PAK1 knockdown, we showed up to a five-fold decrease in invasion through matrigel, indicating that elevated levels of PAK1 are associated with   invasive   potential in uveal melanoma. These data implicate PAK1 as a potential new target for therapy of these tumors. 
 BRAF activates and physically interacts with PAK to regulate cell motility. Increased p21-activated kinase (PAK) signaling and expression have been identified in the   invasive   fronts of aggressive papillary thyroid cancers (PTCs), including those with RET/PTC, BRAFV600E, and mutant RAS expression. Functionally, thyroid cancer cell motility in vitro is dependent on group 1 PAKs, particularly PAK1. In this study, we hypothesize that BRAF, a central kinase in PTC tumorigenesis and invasion, regulates thyroid cancer cell motility in part through PAK activation. Using three well-characterized human thyroid cancer cell lines, we demonstrated in all cell lines that BRAF knockdown reduced PAK phosphorylation of direct downstream targets. In contrast, inhibition of MEK activity either pharmacologically or with siRNA did not reduce PAK activity, indicating MEK is dispensable for PAK activity. Inhibition of cell migration through BRAF loss is rescued by overexpression of either constitutive active MEK1 or PAK1, demonstrating that both signaling pathways are involved in BRAF-regulated cell motility. To further characterize BRAF-PAK signaling, immunofluorescence and immunoprecipitation demonstrated that both exogenously overexpressed and endogenous PAK1 and BRAF co-localize and physically interact, and that this interaction was enhanced in mitosis. Finally, we demonstrated that acute induction of BRAFV600E expression in vivo in murine thyroid glands results in increased PAK expression and activity confirming a positive signaling relationship in vivo. In conclusion, we have identified a signaling pathway in thyroid cancer cells which BRAF activates and physically interacts with PAK and regulates cell motility. 
 Active p21-activated kinase 1 rescues MCF10A breast epithelial cells from undergoing anoikis. The protein kinase, PAK1, is overexpressed in human breast cancer and may contribute to malignancy through induction of proliferation and   invasive  ness. In this study, we examined the role of PAK1 in the survival of detached MCF10A breast epithelial cells to test whether it may also regulate the early stages of neoplasia. MCF10A cells undergo anoikis, as measured by the cleavage of caspase 3 and poly(ADP-ribose) polymerase (PARP), after more than 8 hours of detachment. Endogenous Akt, PAK1, and BAD are phosphorylated in attached MCF10A cells, but these phosphorylation events are all lost during the first 8 hours of detachment. Expression of constitutively active PAK1 or Akt suppresses the cleavage of caspase 3 and PARP in detached MCF10A cells. Co-overexpression of active PAK1 with dominant-negative Akt, or of active Akt with dominant-negative PAK1, still suppresses anoikis. Thus, Akt and PAK1 enhance survival through pathways that are at least partially independent. PAK1-dependent regulation of anoikis is likely to occur early in the apoptotic cascade as expression of dominant-negative PAK1 increased the cleavage of the upstream caspase 9, while constitutively active PAK1 inhibited caspase 9 activation. These results support a role for activated PAK1 in the suppression of anoikis in MCF10A epithelial cells. 
 MicroRNA-7, a homeobox D10 target, inhibits p21-activated kinase 1 and regulates its functions. MicroRNAs are noncoding RNAs that inhibit the expression of their targets in a sequence-specific manner and play crucial roles during oncogenesis. Here we show that microRNA-7 (miR-7) inhibits p21-activated kinase 1 (Pak1) expression, a widely up-regulated signaling kinase in multiple human cancers, by targeting the 3'-untranslated region (UTR) of Pak1 mRNA. We noticed an inverse correlation between the levels of endogenous miR-7 and Pak1 expression in human cancer cells. We discovered that endogenous miR-7 expression is positively regulated by a homeodomain transcription factor, HoxD10, the loss of which leads to an increased   invasive  ness. HoxD10 directly interacts with the miR-7 chromatin. Accordingly, the levels of Pak1 protein are progressively up-regulated whereas those of miR-7 and its upstream activator HoxD10 are progressively down-regulated in a cellular model of breast cancer progression from low to highly   invasive   phenotypes. Furthermore, HoxD10 expression in highly   invasive   breast cancer cells resulted in an increased miR-7 expression but reduced Pak1 3'-UTR-luciferase activity and reduced Pak1 protein. Finally, we show that miR-7 introduction inhibits the motility,   invasive  ness, anchorage-independent growth, and tumorigenic potential of highly   invasive   breast cancer cells. Collectively, these findings establish for the first time that Pak1 is a target of miR-7 and that HoxD10 plays a regulatory role in modifying the expression of miR-7 and, consequently, the functions of the miR-7-Pak1 pathway in human cancer cells. 
 Pak-1 expression increases with progression of colorectal carcinomas to metastasis. The p21-activated kinase-1 (Pak-1) promotes cell motility and   invasive  ness. Pak-1 is activated by the Rac, Rho, and Cdc42 small GTPases in response to a variety of stimuli including ras and phosphatidylinositol 3'-kinase/AKT pathway activation. Because Pak-1 plays a central role in regulating cell motility and   invasive  ness, we sought to determine whether Pak-1 may be involved in the malignant progression of colorectal carcinoma.Pak-1 expression was examined by immunohistochemistry in archived tissues from normal human colons, tubular and tubulovillous adenomas,   invasive   adenocarcinomas (stages I-III/IV), and lymph node metastases (184 total specimens from 38 patients). Specific cytoplasmic immunostaining was evaluated for overall intensity and uniformity to derive a combined histoscore (stain intensity x percentage of epithelium stained).Pak-1 expression was increased significantly with colorectal cancer progression from normal tissue to lymph node metastases (P < 0.0001). Furthermore, Pak-1 expression was increased significantly in adenomas,   invasive   carcinomas, and lymph node metastases compared with normal colon (P < 0.0001). Strikingly, Pak-1 expression was significantly higher in lymph node metastases than in   invasive   cancers, adenomas, or normal colon (P < 0.0001). Moreover, in patients with multiple lesions representing different stages of disease, Pak-1 expression was increased specifically in the most advanced lesions.This study demonstrates that Pak-1 expression is increased significantly with malignant progression of human colorectal carcinoma. These data, along with numerous functional studies demonstrating a central role for Pak-1 activity in tumor   invasive  ness and motility, implicate Pak-1 as an exciting target for therapy of colorectal carcinoma. 
 p21-activated kinase 1 interacts with and phosphorylates histone H3 in breast cancer cells. Stimulation of p21-activated kinase-1 (Pak1) signaling promotes motility,   invasive  ness, anchorage-independent growth and abnormal mitotic assembly in human breast cancer cells. Here, we provide new evidence that, before the onset of mitosis, activated Pak1 is specifically localized with the chromosomes during prophase and on the centrosomes in metaphase and moves to the contraction ring during cytokinesis. To identify mitosis-specific substrates of Pak1, we screened a synchronized G2-M expression library by using a glutathione transferase Pak1 solid-phase-based kinase reaction. This analysis identified histone H3 as a substrate of Pak1 both in vitro and in vivo, and it specifically interacted with Pak1 but not Pak2 or Pak3. Site-directed mutagenesis indicated that Pak1 phosphorylates histone H3 on Ser10. Expressions of the wild-type, or catalytically active, Pak1 caused it to appear at the poles corresponding to mitotic centrosomes in a variety of mammalian cells. Together, these results suggest for the first time that Pak1 interacts with and phosphorylates histone H3 and may thus influence the Pak1-histone H3 pathway, which in turn may influence mitotic events in breast cancer cells. 
 The adenomatous polyposis coli-associated exchange factors Asef and Asef2 are required for adenoma formation in Apc(Min/+)mice. Sporadic and familial colorectal tumours usually harbour biallelic adenomatous polyposis coli (APC)-associated mutations that result in constitutive activation of Wnt signalling. Furthermore, APC activates Asef and Asef2, which are guanine-nucleotide exchange factors specific for Rac1 and Cdc42. Here, we show that Asef and Asef2 expression is aberrantly enhanced in intestinal adenomas and tumours. We also show that deficiency of either Asef or Asef2 significantly reduces the number and size of adenomas in Apc(Min/+) mice, which are heterozygous for an APC mutation and spontaneously develop adenomas in the intestine. We observed that the APC-Asef/Asef2 complex induces c-Jun amino-terminal kinase-mediated transactivation of matrix metalloproteinase 9, and is required for the   invasive   activity of colorectal tumour cells. Furthermore, we show that Asef and Asef2 are required for tumour angiogenesis. These results suggest that Asef and Asef2 have a crucial role in intestinal adenoma formation and tumour progression, and might be promising molecular targets for the treatment of colorectal tumours. 
 [Clinicopathological significance of expression and amplification of P21-activated kinase 1 gene in colorectal carcinoma]. To investigate the clinicopathological value of the expression and amplification of P21-activated kinase 1 gene (PAK1) in colorectal carcinoma(CRC).Immunohistochemistry (IHC), fluorescence in situ hybridization (FISH) and terminal deoxynucleotidyl transferase-mediated dUTP nick end-labeling(TUNEL) methods were used to examine the protein expression, amplification of PAK1 and cell apoptosis in 80 cases of CRC and 30 cases of colorectal adenoma by tissue microarray.IHC showed an overexpression of PAK1 protein in 26% of colorectal adenomas and 62% of CRCs. Significant association was found between expression of PAK1 and tumor histological grade as well as tumor clinical stage(P<0.05). In poor-differentiated(G(3)) CRCs, PAK1 expression in 90% carcinoma was up-regulated, which was significantly higher than that in tumors of G(1/2)(51%). Overexpression of PAK1 was detected in 78% of CRCs in later clinical stages (Dukes C, D), which was significantly higher than that in early clinical stages (Dukes A,B, 53%). In addition, negative correlation between PAK1 overexpression and cell apoptosis was observed in these CRC cohorts(P<0.05). FISH revealed that amplification of PAK1 gene was examined in only 3% CRCs.Overexpression of PAK1 protein may play an important role in development and progression of colorectal neoplasms and it is closely associated with the malignant histological and   invasive   phenotype of CRCs. The expression of PAK1 in CRC may be used as one of the new molecular markers in predicting tumors malignant potential and progression. 
 Clinicopathological and cellular signature of PAK1 in human bladder cancer. Bladder cancer (BC) is the ninth most common cancer and the 13th most common cause of cancer death. Although p21 protein-activated kinase (PAK) regulates cell growth, motility, and morphology, the expression and function of PAK1 associated with the clinicopathological and cellular signature of human BC are not clear. This study was to examine the expression of PAK1 in human BC, the association of PAK1 with clinicopathological features, and the effect of PAK1 on cell proliferation, migration, and invasion in BC cells. A total of 54 BC and 12 normal bladder tissue specimens were retrieved. Among 54 BC patients, 39 cases were superficial BC and 15 cases were   invasive   BC. Histological examination revealed 29 patients with low-grade and 25 patients with high-grade papillary urothelial carcinomas. Immunohistochemical staining showed that PAK1 was overexpressed in BC tissue compared with normal bladder tissue. The overexpression of PAK1 was significantly associated with tumor size, histological grade, and lymph node metastasis, but not with gender, age, clinical stage, tumor number, and recurrence. Furthermore, the cytoplasmic distribution of PAK1 was observed in BC cells. Knocking down of PAK1 using lentiviral transduction decreased BC cell proliferation, migration, and invasion. In conclusion, we demonstrated that the overexpression of PAK1 is closely associated with the clinicopathological features of BC, suggesting that PAK1 may play an important role in the development and progression of BC and may be a potential therapeutic target for the treatment of BC. 
 Pak1 phosphorylation of snail, a master regulator of epithelial-to-mesenchyme transition, modulates snail's subcellular localization and functions. The process of epithelial-mesenchymal transition plays a pivotal role in the conversion of early stage tumors into   invasive   malignancies, and has been shown to be regulated by the zinc finger phosphoprotein, Snail; however, no upstream signaling kinases have been shown to modulate Snail functions. Since the   invasive  ness of breast cancer cells is also influenced by p21-activated kinase 1 (Pak1) signaling, we investigated Pak1's potential mechanistic role in the regulation of Snail functions. We found for the first time that Pak1 promotes transcription repression activity of Snail from E-cadherin, occludin, and aromatase promoters. Pak1 regulates the repressor activity of Snail by phosphorylating on Ser(246). Pak1 phosphorylation of Snail supports Snail's accumulation in the nucleus as well as its repressor functions. A Ser(246)Ala substitution in Snail or Pak1 knockdown by short interference RNA blocked Pak1-mediated Snail phosphorylation, leading to increased cytoplasmic accumulation of Snail and attenuation of Snail repressor activity in breast cancer cells. The regulation of phosphorylation and function of Snail by Pak1 represents a novel mechanism by which a signaling kinase might contribute to the process of epithelial-mesenchymal transition. 
  GeneRIFs  
 Increased p21-activated kinase is associated with   invasive   papillary thyroid cancers. 
 the role of PAK1 in the promotion of cell motility, cell   invasive  ness and the down regulation of p120-catenin through CRK serine 41 phosphorylation in NSCLC cells 
 Overexpression of PAK1 protein is closely associated with the malignant histological and   invasive   phenotype of colorectal carcinoma. 
 Increased p21-activated kinase-1 expression is associated with   invasive   potential in uveal melanoma 
 Pak-1 has a role in human colorectal tumor   invasive  ness and motility 
  [Mm]etastasize  PubMed  
 P21 activated kinase-1 (Pak1) promotes prostate tumor growth and microinvasion via inhibition of transforming growth factor β expression and enhanced matrix metalloproteinase 9 secretion. P21-activated kinases (Paks) are major effectors downstream of the small Rho family of GTPases. Among the six isoforms, Pak1 is the most ubiquitous and the best characterized member. Previous studies have shown that inhibition of Pak6, which is predominantly present in the prostate compared with other tissues, inhibits prostate tumor growth in vivo. Even though Pak1 has been identified in normal prostatic epithelial cells and cancer cells, its specific role in the development of prostate cancer remains unclear. We report here that highly invasive prostate cancer cells express significantly higher levels of Pak1 protein compared with non-invasive prostate cancer cells. Furthermore, prostate tumor tissues and prostate cancer   metastasiz  ed to lungs showed a higher expression of Pak1 compared with normal tissues. Interestingly, Pak6 protein expression levels did not change with the invasive/metastatic potential of the cancer cells or tumors. Although inhibition of Pak1, and not Pak6, resulted in impaired PC3 cell migration, the effects of Pak1 knockdown on transendothelial migration (microinvasion), tumor growth, and tumor angiogenesis was higher compared with Pak6 knockdown. Finally, gene array data revealed reduced expression of matrix metalloproteinase 9 with the ablation of either Pak1 or Pak6 gene expression in PC3 cells, whereas protein levels of TGFβ was elevated significantly with specific modulation of Pak1 activity or ablation of the Pak1 gene. Our observations suggest that although some level of functional redundancy exists between Pak1 and Pak6 in prostate cancer cells, targeting Pak1 is a potential option for the management of prostate tumor growth, microinvasion, and metastasis. 
 Increased p21-activated kinase-1 expression is associated with invasive potential in uveal melanoma. Prognosis in patients with uveal melanoma is poor as approximately half of all tumors   metastasize   and currently there are no effective treatments for disseminated disease. Differences in invasiveness between uveal melanomas could therefore be of major significance regarding clinical outcome. To identify genes associated with invasive potential, we have used microarray expression profiling combined with phenotypic characterization of uveal melanoma and melanocyte cell lines to define a gene signature associated with cellular invasion. A panel of 14 uveal cell cultures was assessed using three assays of invasiveness: matrigel invasion chamber system, scratch wound closure and cell motility. We identified a set of 853 differentially expressed transcripts (Wilcoxon-Mann-Whitney test, P<0.01) that discriminated between samples with high or low invasive capacity based on a composite phenotype that takes into account behavior across all three assays. Of particular interest, expression of two members of the p21-activated kinase (PAK) family, PAK1 and PAK7, was elevated in the more invasive cultures. PAK1 has previously been shown to play a central role in regulating cell motility and invasiveness in other cell types, and increased expression has been observed in breast and colorectal carcinomas. Using small interfering RNA-mediated PAK1 knockdown, we showed up to a five-fold decrease in invasion through matrigel, indicating that elevated levels of PAK1 are associated with invasive potential in uveal melanoma. These data implicate PAK1 as a potential new target for therapy of these tumors. 
  [Mm]etastasis  PubMed  
 16-kDa prolactin inhibits endothelial cell migration by down-regulating the Ras-Tiam1-Rac1-Pak1 signaling pathway. Angiogenesis plays a key role in promoting tumorigenesis and   metastasis  . The 16-kDa fragment of prolactin (16k PRL) is an NH(2)-terminal natural breakdown fragment of the intact 23-kDa prolactin and has been shown to have potent antiangiogenic and antitumor activities. The mechanism(s) involved in the action of 16k PRL in endothelial cells remains unclear. In this study, we showed that 16k PRL reduced rat aortic endothelial cell (RAEC) migration in a wound-healing assay and in a Matrigel tube formation assay, suggesting that 16k PRL inhibits endothelial cell migration, an important activity involved in angiogenesis and tumorigenesis. We further investigated how 16k PRL attenuates endothelial cell migration. We first showed that RAEC migration is mediated through the Rho GTPase Rac1, as Rac1 inhibition by the Rac1-specific inhibitor NSC27366 or Rac1 knockdown by small interfering RNA both blocked RAEC migration. We next showed that 16k PRL reduced the activation of Rac1 in a concentration-dependent manner. Furthermore, 16k PRL inhibition of Rac1 is mediated through the suppression of T lymphoma invasion and   metastasis   1 (Tiam1) and its upstream activator Ras in a phosphoinositide-3-kinase-independent manner. 16k PRL also down-regulated the phosphorylation of the downstream effector of Rac1, p21-activating kinase 1 (Pak1), and inhibited its translocation to the leading edge of migrating cells. Thus, 16k PRL inhibits cell migration by blocking the Ras-Tiam1-Rac1-Pak1 signaling pathway in endothelial cells. 
 A Trio-Rac1-Pak1 signalling axis drives invadopodia disassembly. Rho family GTPases control cell migration and participate in the regulation of cancer   metastasis  . Invadopodia, associated with invasive tumour cells, are crucial for cellular invasion and   metastasis  . To study Rac1 GTPase in invadopodia dynamics, we developed a genetically encoded, single-chain Rac1 fluorescence resonance energy (FRET) transfer biosensor. The biosensor shows Rac1 activity exclusion from the core of invadopodia, and higher activity when invadopodia disappear, suggesting that reduced Rac1 activity is necessary for their stability, and Rac1 activation is involved in disassembly. Photoactivating Rac1 at invadopodia confirmed this previously unknown Rac1 function. We describe here an invadopodia disassembly model, where a signalling axis involving TrioGEF, Rac1, Pak1, and phosphorylation of cortactin, causes invadopodia dissolution. This mechanism is critical for the proper turnover of invasive structures during tumour cell invasion, where a balance of proteolytic activity and locomotory protrusions must be carefully coordinated to achieve a maximally invasive phenotype. 
 PI3K, Rac1 and pPAK1 are overexpressed in extramammary Paget's disease. Phosphatidylinositol 3-kinase (PI3K), Ras-related C3 botulinum toxin substrate 1 (Rac1) and P21-activated protein kinase 1 (PAK1) appear to play important roles in the pathogenesis of several tumors, but their expressions in extramammary Paget's disease (EMPD) have not been investigated yet.To investigate the potential contribution of the PI3K, Rac1 and PAK1 to the development of EMPD.Thirty-five paraffin-embedded EMPD specimens were subjected to immunohistochemical staining for PI3K (85α), Rac1 and pPAK1.All the 35 primary EMPD specimens, including 20 non-invasive EMPD, 13 invasive EMPD and 2 metastatic lymph nodes, showed cytoplasm overexpression of PI3K (85α), Rac1 and pPAK1. The expression (% positive cells) of PI3K(85α), Rac1 and pPAK1 (90.1 ± 8.6, 91.4 ± 9.5 and 89.6 ± 10.8% ) in EMPD were significantly higher than in apocrine glands of normal skin ( 20.1 ± 11.9, 29.8 ± 8.9, 41.1 ± 13.4%), and the expression in invasive EMPD with ly  mph node m  etastasis (98.2 ± 1.7, 98.8 ± 0.7 and 98.4 ± 0.9%) are significantly higher than in invasive EMPD without   lymph nod  e metastasis (94.1 ± 2.6, 96.5 ± 1.7 and 95.3 ± 1.1%) and non-invasive EMPD (85.2 ± 8.4, 87.1 ± 9.9 and 83.1 ± 10.6%). There were significant positive correlations of the expression levels between PI3K (85α) and Rac1, as well as between Rac1 and pPAK1 in EMPD.These results indicate that PI3K, Rac1 and PAK1 may play important roles in the pathogenesis of EMPD. 
 A Rac1/PAK1 cascade controls β-catenin activation in colon cancer cells. P21-activated kinase 1 (PAK1) is associated with colon cancer progression and   metastasi  s, whereas the molecular mechanism remains elusive. Here, we show that downregulation of PAK1 in colon cancer cells reduces total β-catenin level, as well as cell proliferation. Mechanistically, PAK1 directly phosphorylates β-catenin proteins at Ser675 site and this leads to more stable and transcriptional active β-catenin. Corroborating these results, PAK1 is required for full Wnt signaling, and superactivation of β-catenin is achieved by simultaneous knockdown of adenomatous polyposis coli protein and activation of PAK1. Moreover, we show that Rac1 functions upstream of PAK1 in colon cancer cells and contributes to β-catenin phosphorylation and accumulation. We conclude that a Rac1/PAK1 cascade controls β-catenin S675 phosphorylation and full activation in colon cancer cells. Supporting this conclusion, overexpression of PAK1 is observed in 70% of colon cancer samples and is correlated with massive β-catenin accumulation. 
 14-3-3zeta Cooperates with ErbB2 to promote ductal carcinoma in situ progression to invasive breast cancer by inducing epithelial-mesenchymal transition. ErbB2, a   metastasis  -promoting oncoprotein, is overexpressed in approximately 25% of invasive/metastatic breast cancers, but in 50%-60% of noninvasive ductal carcinomas in situ (DCIS). It has been puzzling how a subset of ErbB2-overexpressing DCIS develops into invasive breast cancer (IBC). We found that co-overexpression of 14-3-3zeta in ErbB2-overexpressing DCIS conferred a higher risk of progression to IBC. ErbB2 and 14-3-3zeta overexpression, respectively, increased cell migration and decreased cell adhesion, two prerequisites of tumor cell invasion. 14-3-3zeta overexpression reduced cell adhesion by activating the TGF-beta/Smads pathway that led to ZFHX1B/SIP-1 upregulation, E-cadherin loss, and epithelial-mesenchymal transition. Importantly, patients whose breast tumors overexpressed both ErbB2 and 14-3-3zeta had higher rates of metastatic recurrence and death than those whose tumors overexpressed only one. 
 Expression and significance of Rac1, Pak1 and Rock1 in gastric carcinoma. Rac1, Pak1 and Rock1 are indicators related to gastric cancer invasion and   metastasis  , but few reports discuss all three kinds of protein in research on gastric cancer invasion and   metastasis  . The aim of this study was to investigate the expression and clinical significance of Rac1, Pak1 and Rock1 in gastric carcinoma.Rac1, Pak1 and Rock1 expression in 158 cases of gastric carcinoma were investigated via immunohistochemical staining and clinical analysis.The positive expression rates of Rac1, Pak1 and Rock1 in normal tissue, intraepithelial neoplastic tissues and gastric carcinoma showed an increasing trend (P < 0.05). Their expression in lymph n  ode metast  asis was significantly higher than in patients with lymph-n  ode metast  asis than in those without lymph no  des metast  asis (P < 0.05). Their expression in tumor (TNM stages III and IV) were significantly higher than that in stages I and II (P < 0.05). Rac1, Pak1 and Rock1 expression did not differ significantly with patients' sex (P > 0.05).Positive rates of Rac1, Pak1 and Rock1 expression in normal tissue, dysplasia and gastric carcinoma show an increasing trend and are correlated with t  umor lymph   node metastasis and TNM stage. Rac1, Pak1 and Rock1 may be important biomarkers of gastric carcino  ma invasio  n and metastasis. 
 Increased Rac1 activity and Pak1 overexpression are associated with lymphovascular invasion and lymph node   metastasis   of upper urinary tract cancer. Lymphovascular invasion (LVI) and lymph node   metastasis   are conventional pathological factors associated with an unfavorable prognosis of urothelial carcinoma of the upper urinary tract (UC-UUT), but little is known about the molecular mechanisms underlying LVI and nodal   metastasis   in this disease. Rac1 small GTPase (Rac1) is essential for tumor   metastasis  . Activated GTP-bound Rac1 (Rac1 activity) plays a key role in activating downstream effectors known as Pak (21-activated kinase), which are key regulators of cytoskeletal remolding, cell motility, and cell proliferation, and thus have a role in both carcinogenesis and tumor invasion.We analyzed Rac1 activity and Pak1 protein expression in matched sets of tumor tissue, non-tumor tissue, and metastatic lymph node tissue obtained from the surgical specimens of 108 Japanese patients with UC-UUT.Rac1 activity and Pak1 protein levels were higher in tumor tissue and metastatic lymph node tissue than in non-tumor tissue (both P < 0.0001). A high level of Rac1 activity and Pak1 protein expression in the primary tumor was related to poor differentiation (P < 0.05), muscle invasion (P < 0.01), LVI (P < 0.0001), and lymph node   metastasis   (P < 0.0001). Kaplan-Meier survival analysis showed that an increase of Rac1 activity and Pak1 protein was associated with a shorter disease-free survival time (P < 0.01) and shorter overall survival (P < 0.001). Cox proportional hazards analysis revealed that high Rac1 activity, Pak1 protein expression and LVI were independent prognostic factors for shorter overall and disease-free survival times (P < 0.01) on univariate analysis, although only Pak1 and LVI had an influence (P < 0.05) according to multivariate analysis.These findings suggest that Rac1 activity and Pak1 are involved in LVI and lymph node   metastasis   of UC-UUT, and may be prognostic markers for this disease. 
 Cancer cell-autonomous TRAIL-R signaling promotes KRAS-driven cancer progression, invasion, and   metastasis  . Many cancers harbor oncogenic mutations of KRAS. Effectors mediating cancer progression, invasion, and   metastasis   in KRAS-mutated cancers are only incompletely understood. Here we identify cancer cell-expressed murine TRAIL-R, whose main function ascribed so far has been the induction of apoptosis as a crucial mediator of KRAS-driven cancer progression, invasion, and   metastasis   and in vivo Rac-1 activation. Cancer cell-restricted genetic ablation of murine TRAIL-R in autochthonous KRAS-driven models of non-small-cell lung cancer (NSCLC) and pancreatic ductal adenocarcinoma (PDAC) reduces tumor growth, blunts   metastasi  s, and prolongs survival by inhibiting cancer cell-autonomous migration, proliferation, and invasion. Consistent with this, high TRAIL-R2 expression correlates with invasion of human PDAC into lymph vessels and with shortened   metastasi  s-free survival of KRAS-mutated colorectal cancer patients. 
 Genome-wide analysis of chromosomal alterations in patients with esophageal squamous cell carcinoma exposed to tobacco and betel quid from high-risk area in India. Genomic alterations such as chromosomal amplifications, deletions and loss of heterozygosity play an important role in the pathogenesis and progression of cancer. Environmental risk factors contribute to the development and progression of tumors by facilitating the loss of tumor suppressor genes and amplification of oncogenes. In this current study, Affymetrix 10K single nucleotide polymorphism (SNP) arrays were used to evaluate genomic alterations in 20 pairs of matched germ-line and tumor DNA obtained from patients with esophageal squamous cell carcinoma (ESCC) from high-risk area of India where tobacco, betel quid and alcohol use are widespread. Twenty-two amplified regions and 16 deleted regions identified across chromosomal arms were biologically relevant. The candidate genes located at amplified regions of chromosomes or low-level gain regions such as PLA2G5 (1p36-p34), COL11A1 (1p21), KCNK2 (1q41), S100A3 (1q21), ENAH (1q42.12), RGS1 (1q31), KCNH1 (1q32-q41), INSIG2 (2q14.1), FGF12 (3q28), TRIO (5p15.2), RNASEN (5p15.2), FGF10 (5p13-p12), EDN1(6p24.1-p22.3), SULF1 (8q13.2-13.3), TLR4 (9q32-q33), TNC (9q33), NTRK2 (9q22.1), CD44 (11p13), NCAM1 (11q23.1), TRIM29 (11q22-q23), PAK1 (11q13-q14) and RAB27A (15q15-q21.1), are found to be associated with cellular migration and proliferation, tumor cell   metastasis   and invasion, anchorage independent growth and inhibition of apoptosis. The candidate genes located at deleted regions of chromosomes, such as FBLN2 (3p25.1), WNT7A (3p25), DLC1 (8p22), LZTS1 (8p22), CDKN2A (9p21), COL4A1 (13q34), CDK8 (13q12) and DCC (18q21.3), are found to be associated with the suppression of tumor. The suggested candidate genes were mostly involved in potential signaling pathways such as focal adhesion (COL4A1), tight junction (CLDN10), MAPK signaling pathway (FGF12) and neuroactive ligand receptor interaction pathway (CCKAR). Expression of FGF12 and COL4A1 was validated by tissue microarray. These unique copy number alteration profiles should be taken into consideration when developing biomarkers for the early detection of ESCC in high-risk areas of India in association with tobacco and betel quid use. 
 Expression and significance of Rac1, Pak1 and Rock1 in gastric carcinoma. Rac1, Pak1 and Rock1 are indicators related to gastric cancer invasion and   metastasis  , but few reports discuss all three kinds of protein in research on gastric cancer invasion and   metastasis  . The aim of this study was to investigate the expression and clinical significance of Rac1, Pak1 and Rock1 in gastric carcinoma.Rac1, Pak1 and Rock1 expression in 158 cases of gastric carcinoma were investigated via immunohistochemical staining and clinical analysis.The positive expression rates of Rac1, Pak1 and Rock1 in normal tissue, intraepithelial neoplastic tissues and gastric carcinoma showed an increasing trend (P < 0.05). Their expression in lymph n  ode metast  asis was significantly higher than in patients with lymph-n  ode metast  asis than in those without lymph no  des metast  asis (P < 0.05). Their expression in tumor (TNM stages III and IV) were significantly higher than that in stages I and II (P < 0.05). Rac1, Pak1 and Rock1 expression did not differ significantly with patients' sex (P > 0.05).Positive rates of Rac1, Pak1 and Rock1 expression in normal tissue, dysplasia and gastric carcinoma show an increasing trend and are correlated with t  umor lymph   node metastasis and TNM stage. Rac1, Pak1 and Rock1 may be important biomarkers of gastric carcino  ma invasio  n and metastasis. 
 A Trio-Rac1-Pak1 signalling axis drives invadopodia disassembly. Rho family GTPases control cell migration and participate in the regulation of cancer   metastasis  . Invadopodia, associated with invasive tumour cells, are crucial for cellular invasion and   metastasis  . To study Rac1 GTPase in invadopodia dynamics, we developed a genetically encoded, single-chain Rac1 fluorescence resonance energy (FRET) transfer biosensor. The biosensor shows Rac1 activity exclusion from the core of invadopodia, and higher activity when invadopodia disappear, suggesting that reduced Rac1 activity is necessary for their stability, and Rac1 activation is involved in disassembly. Photoactivating Rac1 at invadopodia confirmed this previously unknown Rac1 function. We describe here an invadopodia disassembly model, where a signalling axis involving TrioGEF, Rac1, Pak1, and phosphorylation of cortactin, causes invadopodia dissolution. This mechanism is critical for the proper turnover of invasive structures during tumour cell invasion, where a balance of proteolytic activity and locomotory protrusions must be carefully coordinated to achieve a maximally invasive phenotype. 
 PI3K, Rac1 and pPAK1 are overexpressed in extramammary Paget's disease. Phosphatidylinositol 3-kinase (PI3K), Ras-related C3 botulinum toxin substrate 1 (Rac1) and P21-activated protein kinase 1 (PAK1) appear to play important roles in the pathogenesis of several tumors, but their expressions in extramammary Paget's disease (EMPD) have not been investigated yet.To investigate the potential contribution of the PI3K, Rac1 and PAK1 to the development of EMPD.Thirty-five paraffin-embedded EMPD specimens were subjected to immunohistochemical staining for PI3K (85α), Rac1 and pPAK1.All the 35 primary EMPD specimens, including 20 non-invasive EMPD, 13 invasive EMPD and 2 metastatic lymph nodes, showed cytoplasm overexpression of PI3K (85α), Rac1 and pPAK1. The expression (% positive cells) of PI3K(85α), Rac1 and pPAK1 (90.1 ± 8.6, 91.4 ± 9.5 and 89.6 ± 10.8% ) in EMPD were significantly higher than in apocrine glands of normal skin ( 20.1 ± 11.9, 29.8 ± 8.9, 41.1 ± 13.4%), and the expression in invasive EMPD with ly  mph node m  etastasis (98.2 ± 1.7, 98.8 ± 0.7 and 98.4 ± 0.9%) are significantly higher than in invasive EMPD without   lymph nod  e metastasis (94.1 ± 2.6, 96.5 ± 1.7 and 95.3 ± 1.1%) and non-invasive EMPD (85.2 ± 8.4, 87.1 ± 9.9 and 83.1 ± 10.6%). There were significant positive correlations of the expression levels between PI3K (85α) and Rac1, as well as between Rac1 and pPAK1 in EMPD.These results indicate that PI3K, Rac1 and PAK1 may play important roles in the pathogenesis of EMPD. 
 A Rac1/PAK1 cascade controls β-catenin activation in colon cancer cells. P21-activated kinase 1 (PAK1) is associated with colon cancer progression and   metastasi  s, whereas the molecular mechanism remains elusive. Here, we show that downregulation of PAK1 in colon cancer cells reduces total β-catenin level, as well as cell proliferation. Mechanistically, PAK1 directly phosphorylates β-catenin proteins at Ser675 site and this leads to more stable and transcriptional active β-catenin. Corroborating these results, PAK1 is required for full Wnt signaling, and superactivation of β-catenin is achieved by simultaneous knockdown of adenomatous polyposis coli protein and activation of PAK1. Moreover, we show that Rac1 functions upstream of PAK1 in colon cancer cells and contributes to β-catenin phosphorylation and accumulation. We conclude that a Rac1/PAK1 cascade controls β-catenin S675 phosphorylation and full activation in colon cancer cells. Supporting this conclusion, overexpression of PAK1 is observed in 70% of colon cancer samples and is correlated with massive β-catenin accumulation. 
 Increased Rac1 activity and Pak1 overexpression are associated with lymphovascular invasion and lymph node   metastasis   of upper urinary tract cancer. Lymphovascular invasion (LVI) and lymph node   metastasis   are conventional pathological factors associated with an unfavorable prognosis of urothelial carcinoma of the upper urinary tract (UC-UUT), but little is known about the molecular mechanisms underlying LVI and nodal   metastasis   in this disease. Rac1 small GTPase (Rac1) is essential for tumor   metastasis  . Activated GTP-bound Rac1 (Rac1 activity) plays a key role in activating downstream effectors known as Pak (21-activated kinase), which are key regulators of cytoskeletal remolding, cell motility, and cell proliferation, and thus have a role in both carcinogenesis and tumor invasion.We analyzed Rac1 activity and Pak1 protein expression in matched sets of tumor tissue, non-tumor tissue, and metastatic lymph node tissue obtained from the surgical specimens of 108 Japanese patients with UC-UUT.Rac1 activity and Pak1 protein levels were higher in tumor tissue and metastatic lymph node tissue than in non-tumor tissue (both P < 0.0001). A high level of Rac1 activity and Pak1 protein expression in the primary tumor was related to poor differentiation (P < 0.05), muscle invasion (P < 0.01), LVI (P < 0.0001), and lymph node   metastasis   (P < 0.0001). Kaplan-Meier survival analysis showed that an increase of Rac1 activity and Pak1 protein was associated with a shorter disease-free survival time (P < 0.01) and shorter overall survival (P < 0.001). Cox proportional hazards analysis revealed that high Rac1 activity, Pak1 protein expression and LVI were independent prognostic factors for shorter overall and disease-free survival times (P < 0.01) on univariate analysis, although only Pak1 and LVI had an influence (P < 0.05) according to multivariate analysis.These findings suggest that Rac1 activity and Pak1 are involved in LVI and lymph node   metastasis   of UC-UUT, and may be prognostic markers for this disease. 
 Genome-wide analysis of chromosomal alterations in patients with esophageal squamous cell carcinoma exposed to tobacco and betel quid from high-risk area in India. Genomic alterations such as chromosomal amplifications, deletions and loss of heterozygosity play an important role in the pathogenesis and progression of cancer. Environmental risk factors contribute to the development and progression of tumors by facilitating the loss of tumor suppressor genes and amplification of oncogenes. In this current study, Affymetrix 10K single nucleotide polymorphism (SNP) arrays were used to evaluate genomic alterations in 20 pairs of matched germ-line and tumor DNA obtained from patients with esophageal squamous cell carcinoma (ESCC) from high-risk area of India where tobacco, betel quid and alcohol use are widespread. Twenty-two amplified regions and 16 deleted regions identified across chromosomal arms were biologically relevant. The candidate genes located at amplified regions of chromosomes or low-level gain regions such as PLA2G5 (1p36-p34), COL11A1 (1p21), KCNK2 (1q41), S100A3 (1q21), ENAH (1q42.12), RGS1 (1q31), KCNH1 (1q32-q41), INSIG2 (2q14.1), FGF12 (3q28), TRIO (5p15.2), RNASEN (5p15.2), FGF10 (5p13-p12), EDN1(6p24.1-p22.3), SULF1 (8q13.2-13.3), TLR4 (9q32-q33), TNC (9q33), NTRK2 (9q22.1), CD44 (11p13), NCAM1 (11q23.1), TRIM29 (11q22-q23), PAK1 (11q13-q14) and RAB27A (15q15-q21.1), are found to be associated with cellular migration and proliferation, tumor cell   metastasis   and invasion, anchorage independent growth and inhibition of apoptosis. The candidate genes located at deleted regions of chromosomes, such as FBLN2 (3p25.1), WNT7A (3p25), DLC1 (8p22), LZTS1 (8p22), CDKN2A (9p21), COL4A1 (13q34), CDK8 (13q12) and DCC (18q21.3), are found to be associated with the suppression of tumor. The suggested candidate genes were mostly involved in potential signaling pathways such as focal adhesion (COL4A1), tight junction (CLDN10), MAPK signaling pathway (FGF12) and neuroactive ligand receptor interaction pathway (CCKAR). Expression of FGF12 and COL4A1 was validated by tissue microarray. These unique copy number alteration profiles should be taken into consideration when developing biomarkers for the early detection of ESCC in high-risk areas of India in association with tobacco and betel quid use. 
 16-kDa prolactin inhibits endothelial cell migration by down-regulating the Ras-Tiam1-Rac1-Pak1 signaling pathway. Angiogenesis plays a key role in promoting tumorigenesis and   metastasis  . The 16-kDa fragment of prolactin (16k PRL) is an NH(2)-terminal natural breakdown fragment of the intact 23-kDa prolactin and has been shown to have potent antiangiogenic and antitumor activities. The mechanism(s) involved in the action of 16k PRL in endothelial cells remains unclear. In this study, we showed that 16k PRL reduced rat aortic endothelial cell (RAEC) migration in a wound-healing assay and in a Matrigel tube formation assay, suggesting that 16k PRL inhibits endothelial cell migration, an important activity involved in angiogenesis and tumorigenesis. We further investigated how 16k PRL attenuates endothelial cell migration. We first showed that RAEC migration is mediated through the Rho GTPase Rac1, as Rac1 inhibition by the Rac1-specific inhibitor NSC27366 or Rac1 knockdown by small interfering RNA both blocked RAEC migration. We next showed that 16k PRL reduced the activation of Rac1 in a concentration-dependent manner. Furthermore, 16k PRL inhibition of Rac1 is mediated through the suppression of T lymphoma invasion and   metastasis   1 (Tiam1) and its upstream activator Ras in a phosphoinositide-3-kinase-independent manner. 16k PRL also down-regulated the phosphorylation of the downstream effector of Rac1, p21-activating kinase 1 (Pak1), and inhibited its translocation to the leading edge of migrating cells. Thus, 16k PRL inhibits cell migration by blocking the Ras-Tiam1-Rac1-Pak1 signaling pathway in endothelial cells. 
 Downregulation of p21-activated kinase-1 inhibits the growth of gastric cancer cells involving cyclin B1. p21-Activated kinase 1 (Pak1), a serine/threonine kinase, has been implicated in cytoskeletal remodelling, cell motility, apoptosis and transformation. However, the role of Pak1 in gastric cancer remains unclear. In this study, we detected Pak1 expression in gastric cancer tissues from 40 patients by western blot. Overexpression of Pak1 was associated with progression,   metastasis   and prognosis of gastric cancer. In addition, we found that knockdown of Pak1 expression significantly inhibited anchorage-dependent and anchorage-independent growth in gastric cancer cells, and markedly inhibited gastric cancer cell xenograft tumor growth. In further study, data showed that Pak1 could regulate the expression of cyclin B1 at the mRNA and protein levels, and impact the subcellular distribution and the promoter activity of cyclin B1. Results from deletion and mutant analysis supplied a new NF-kappaB binding sites at position -321 of cyclin B1 promoter, and indicated that Pak1 regulated the transcription of cyclin B1 in gastric cancer through NF-kappaB. In conclusion, Pak1 may be a potential prognostic marker and therapeutic target in gastric cancer. 
 Lysophosphatidic acid induces MDA-MB-231 breast cancer cells migration through activation of PI3K/PAK1/ERK signaling. Enhanced motility of cancer cells is a critical step in promoting tumor   metastasis  . Lysophosphatidic acid (LPA), representing the major mitogenic activity in serum, stimulates migration in various types of cancer cells. However, the underlying signaling mechanisms for LPA-induced motility of cancer cells remain to be elucidated.In this study, we found that LPA dose-dependently stimulated migration of MDA-MB-231 breast cancer cells, with 10 µM being the most effective. LPA also increased ERK activity and the MEK inhibitor U0126 could block LPA-induced ERK activity and cell migration. In addition, LPA induced PAK1 activation while ERK activation and cell migration were inhibited by ectopic expression of an inactive mutant form of PAK1 in MDA-MB-231 cells. Furthermore, LPA increased PI3K activity, and the PI3K inhibitor LY294002 inhibited both LPA-induced PAK1/ERK activation and cell migration. Moreover, in the breast cancer cell, LPA treatment resulted in remarkable production of reactive oxygen species (ROS), while LPA-induced ROS generation, PI3K/PAK1/ERK activation and cell migration could be inhibited by N-acetyl-L-Cysteine, a scavenger of ROS.Taken together, this study identifies a PI3K/PAK1/ERK signaling pathway for LPA-stimulated breast cancer cell migration. These data also suggest that ROS generation plays an essential role in the activation of LPA-stimulated PI3K/PAK1/ERK signaling and breast cancer cell migration. These findings may provide a basis for designing future therapeutic strategy for blocking breast cancer   metastasi  s. 
 Expression of P21-activated kinase 1 and cell division control protein 42 homolog correlates with clinicopathological features and prognosis in cervical carcinoma. This study investigated P21-activated kinase 1 (PAK1) and cell division control protein 42 homolog (CDC42) expression and their correlation with clinicopathological features and prognosis in cervical carcinoma.Cervical carcinoma (n = 55), cervical intraepithelial neoplasias (CIN, n = 93), and normal cervix (n = 26) tissues were sampled. PAK1 and CDC42 expressions were determined using real-time quantitative polymerase chain reaction, Western blot and immunohistochemistry. Correlation analysis of PAK1 and CDC42 expression was performed using a Pearson correlation test. Survival rate was calculated using the Kaplan-Meier method. Independent prognostic factors were analyzed by Cox proportional hazard model.PAK1 and CDC42 expression were positively correlated in all tissues. PAK1 and CDC42 expression in cervical carcinoma tissues were higher than those in normal tissues. PAK1 and CDC42 protein expression in normal cervix, CINI, CINII, CINIII and cervical carcinoma tissues showed a gradually increasing trend. PAK1 and CDC42 expression were correlated with lymph node   metastasis  , the depth of tumor invasion, the degree of tumor differentiation, histological type and lymphovascular space invasion (LVSI; all P < 0.05). Cox regression analysis showed that the degree of tumor differentiation, PAK1 protein expression, histological type and LVSI are independent risk factors for prognosis of cervical carcinoma.High PAK1 and CDC42 expressions are closely related to the clinicopathological features and poor prognosis of cervical carcinoma, serving as unfavorable prognostic factors. 
 P21-activated kinase 1 and 4 were associated with colorectal cancer   metastasis   and infiltration. P21-activated kinases (PAKs) are small guanosine triphosphate effectors that play critical roles in many fundamental cellular functions, including cytoskeletal reorganization and cell motility. PAKs are widely expressed in a variety of tissues and are often overexpressed in multiple cancer types. The aim of this study was to investigate the relationship between PAK1 and PAK4 and clinicopathologic features of colorectal cancer.PAK1 and PAK4 expression in colorectal cancer patients were investigated via TaqMan real-time polymerase chain reaction and immunohistochemistry and clinical analysis.The relative expression levels of PAK1 and PAK4 gene in colorectal carcinoma tissues were significantly higher than those in normal tissues (P < 0.01). PAK4 expression was higher than PAK1 in the same cancer tissue. The expression of PAK1 and PAK4 increased gradually with the clinical stages in carcinoma tissues (P < 0.01). PAK1 expression was higher in lymph node positive patients, and PAK4 expression was higher in infiltration into serous layer patients (P < 0.05). PAK1 overexpression group has a higher recurrence/  metastasis   rate compared with that of the PAK1 low expression group. Follow-up analysis showed that the median progression-free survival time of the PAK1 high expression group was significantly shorter than that of the PAK1 low expression group.PAK1 and PAK4 expression were associated with colorectal cancer   metastasis   and infiltration, PAK1 high expression may indicate poor prognosis of colorectal cancer. 
 Detailed map of a region commonly amplified at 11q13-->q14 in human breast carcinoma. Amplification of loci present on band q13 of human chromosome 11 is a feature of a subset of estrogen receptor positive breast carcinomas prone to   metastasis  . As many as five distinct amplification units have been described on 11q13. They include particularly a genomic area encompassing the GARP gene at 11q13.5-->q14.1. We have reassessed our current knowledge of this region, located telomeric to CCND1 and EMS1, which is amplified in 7-10% of mammary tumors. The loose definition of the driving forces of these amplification events led us to map accurately the boundaries of the amplifiable region, and thus to contribute a physical and transcriptional map of a 3-Mb region of chromosome 11. Four new genes were placed on the regional map, namely CBP2, CLNS1A, UVRAG, and PAK1. We have narrowed the core of the 11q13-->q14 amplicon to a 350-kb area encompassing D11S533, mostly on its telomeric side. The map reported here represents an indispensable step toward sequencing the entire region, and thus toward uncovering gene(s) which play(s) a critical role in breast cancer progression. 
 P-21 activated kinase 1 knockdown inhibits β-catenin signalling and blocks colorectal cancer growth. The p21-activated kinase 1 (PAK1) plays important roles in cell growth, motility, and transformation. The aims of this study were to delineate the signalling mechanisms downstream of PAK1, and to investigate the importance of PAK1 for colorectal cancer (CRC) growth and   metastasi  s in vivo. PAK1 knockdown in human CRC cell lines inhibited β-catenin expression, β-catenin/TCF4 transcriptional activity, and the expression of c-Myc. In mouse models PAK1 knockdown suppressed the growth a  nd metasta  sis of human CRC cells by decreasing proliferation and increasing apoptosis. Our findings demonstrate for the first time the crucial role of PAK1 in CRC progression in vivo. 
 PAK1 mediates pancreatic cancer cell migration and resistance to MET inhibition. Pancreatic adenocarcinoma (PDAC) is a major unmet medical need and a deeper understanding of molecular drivers is needed to advance therapeutic options for patients. We report here that p21-activated kinase 1 (PAK1) is a central node in PDAC cells downstream of multiple growth factor signalling pathways, including hepatocyte growth factor (HGF) and MET receptor tyrosine kinase. PAK1 inhibition blocks signalling to cytoskeletal effectors and tumour cell motility driven by HGF/MET. MET antagonists, such as onartuzumab and crizotinib, are currently in clinical development. Given that even highly effective therapies have resistance mechanisms, we show that combination with PAK1 inhibition overcomes potential resistance mechanisms mediated either by activation of parallel growth factor pathways or by direct amplification of PAK1. Inhibition of PAK1 attenuated in vivo tumour growth and   metastasis   in a model of pancreatic adenocarcinoma. In human tissues, PAK1 is highly expressed in a proportion of PDACs (33% IHC score 2 or 3; n = 304) and its expression is significantly associated with MET positivity (p < 0.0001) and linked to a widespread metastatic pattern in patients (p = 0.067). Taken together, our results provide evidence for a functional role of MET/PAK1 signalling in pancreatic adenocarcinoma and support further characterization of therapeutic inhibitors in this indication. 
 Axl gene knockdown inhibits the   metastasis   properties of hepatocellular carcinoma via PI3K/Akt-PAK1 signal pathway. The objective of this study is to clarify the possible role and mechanism of Axl in the tumorigenicity and   metastasis   process of hepatocellular carcinoma. The mRNA and protein expression levels of Axl in MHCC97-H and MHCC97-L cell lines were evaluated by real-time PCR and Western blot analysis. The key factor of phosphatidylinositol-3-kinase (PI3K)/Akt-p21-activated kinases-1 (PAK1) signaling pathway was studied after Axl expression was downregulated by shRNA. Finally, we analyzed the expression status of Axl protein expression in hepatocellular carcinoma tissues and its relationship with the prognosis of hepatocellular carcinoma. Axl was observed to be higher expressed in MHCC97-H cell lines compared to MHCC97-L cell lines. The downregulation of Axl in MHCC97-H cell lines resulted in the inhibition of the invasion ability of MHCC97-H cells both in vitro and in vivo. Interestingly, blocking PI3K/Akt signaling pathway by LY294002 or Akt siRNA could remarkably inhibit the PAK1 activation and cell invasion. Finally, the Axl protein expression was positively correlated with differentiation, lymph node   metastasis  , and clinical stage in patients with hepatocellular carcinoma patients (all P < 0.01). These findings suggest that Axl can also regulate the   metastasis   process of hepatocellular carcinoma and may serve as a new prognostic marker and therapeutic target for treating hepatocellular carcinoma   metastasis  . 
 LKB1 suppresses p21-activated kinase-1 (PAK1) by phosphorylation of Thr109 in the p21-binding domain. The serine/threonine protein kinase LKB1 is a tumor suppressor gene mutated in Peutz-Jeghers syndrome patients. The mutations are found also in several types of sporadic cancer. Although LKB1 is implicated in suppression of cell growth and   metastasis  , the detailed mechanisms have not yet been elucidated. In this study, we investigated the effect of LKB1 on cell motility, whose acquisition occurs in early   metastasis  . The knockdown of LKB1 enhanced cell migration and PAK1 activity in human colon cancer HCT116 cells, whereas forced expression of LKB1 in Lkb1-null mouse embryonic fibroblasts suppressed PAK1 activity and PAK1-mediated cell migration simultaneously. Notably, LKB1 directly phosphorylated PAK1 at Thr(109) in the p21-binding domain in vitro. The phosphomimetic T109E mutant showed significantly lower protein kinase activity than wild-type PAK1, suggesting that the phosphorylation at Thr(109) by LKB1 was responsible for suppression of PAK1. Consistently, the nonphosphorylatable T109A mutant was resistant to suppression by LKB1. Furthermore, we found that PAK1 was activated in the hepatocellular carcinomas and the precancerous liver lesions of Lkb1(+/-) mice. Taken together, these results suggest that PAK1 is a direct downstream target of LKB1 and plays an essential role in LKB1-induced suppression of cell migration. 
 P21-activated protein kinase 1 is overexpressed in gastric cancer and induces cancer   metastasis  . P21-activated protein kinase (Pak1), a main downstream effector of small Rho GTPases, plays an important role in the regulation of cell morphogenesis, motility, mitosis and angiogenesis. However, the role of Pak1 in gastric cancer   metastasis   remains unclear. Here, we showed that Pak1 is overexpressed in gastric cancer tissues from 74 patients by immunohistochemistry. Overexpression of Pak1 was associated with   metastasis   and prognosis of gastric cancer. In addition, overexpression of Pak1 increased gastric cancer cell motility and invasion, whereas downregulation of Pak1 expression reduced gastric cancer cell migration and invasion. In further study, data showed that activated Pak1 inhibited stress fiber and focal adhesion complex formation in gastric cancer cells and led to the formation of motile phenotypes. Importantly, activated Pak1 elicited phosphorylation of the ERK and JNK-dependent pathway in gastric cancer cell lines. In conclusion, our results suggest that Pak1 is overexpressed in gastric cancer and plays an important role in the   metastasis   of gastric cancer. The mechanism by which Pak1 induces cancer   metastasis   may involve activation of ERK and JNK. 
 PAK1 regulates RUFY3-mediated gastric cancer cell migration and invasion. Actin protrusion at the cell periphery is central to the formation of invadopodia during tumor cell migration and invasion. Although RUFY3 (RUN and FYVE domain containing 3)/SINGAR1 (single axon-related1)/RIPX (Rap2 interacting protein X) has an important role in neuronal development, its pathophysiologic role and relevance to cancer are still largely unknown. The purpose of this study was to elucidate the molecular mechanisms by which RUFY3 involves in gastric cancer cell migration and invasion. Here, our data show that overexpression of RUFY3 leads to the formation of F-actin-enriched protrusive structures at the cell periphery and induces gastric cancer cell migration. Furthermore, P21-activated kinase-1 (PAK1) interacts with RUFY3, and promotes RUFY3 expression and RUFY3-induced gastric cancer cell migration; inhibition of PAK1 attenuates RUFY3-induced SGC-7901 cell migration and invasion. Importantly, we found that the inhibitory effect of cell migration and invasion is significantly enhanced by knockdown of both PAK1 and RUFY3 compared with knockdown of RUFY3 alone or PAK1 alone. Strikingly, we found significant upregulation of RUFY3 in gastric cancer samples with invasive carcinoma at pathologic TNM III and TNM IV stages, compared with their non-tumor counterparts. Moreover, an obvious positive correlation was observed between the protein expression of RUFY3 and PAK1 in 40 pairs of gastric cancer samples. Therefore, these findings provide important evidence that PAK1 can positively regulate RUFY3 expression, which contribute to the metastatic potential of gastric cancer cells, maybe blocking PAK1-RUFY3 signaling would become a potential   metastasis   therapeutic strategy for gastric cancer. 
 Correlations of P21-activated kinase 1 expression to clinicopathological features of gastric carcinoma and patients' prognosis. Recent studies proved that P21-activated kinase 1 (PAK1) is highly expressed in many kinds of tumor and plays an important role in genesis, development, and   metastasis   of tumor. We aimed to detect the expression of PAK1 in gastric carcinoma and to analyze its relationship with clinicopathological features and prognosis of gastric carcinoma.Tissue microarray and immunohistochemical staining were performed to detect PAK1 in paraffin specimens of 189 gastric carcinomas, 54 paracancer tissues, 40 lymph nodes and 30 healthy tissues. Clinicopathologic features and follow-up data of the patients were analyzed by the Chi2 test and the Kaplan-Meier method.Positive rate of PAK1 was 73.0% in gastric carcinoma, 57.4% in paracancer tissues and 23.3% in healthy controls (Chi2 = 29.364, P < 0.05). Expression of PAK1 was significantly correlated with tumor size, tumor differentiation, lymph node   metastasis  , Lauren classification and invasive depth (all P < 0.05). The positive rate of PAK1 was significantly higher in primary gastric carcinomas than in metastatic lymph nodes (75.0% vs. 52.5%, Chi2 = 4.381, P < 0.05). Survival analysis using the Kaplan-Meier method showed that the expression of PAK1 was a predictor for poor prognosis of the patients with gastric carcinoma (Chi2 = 6.857, P < 0.01).Expression of PAK1 is an early molecular event in the tumorigenesis of gastric carcinoma. It is also closely correlated the development of gastric carcinoma and the patients' prognosis. 
 N-acetylglucosaminyltransferase V confers hepatoma cells with resistance to anoikis through EGFR/PAK1 activation. Elevated expression and activity of N-acetylglucosaminyltransferase V (Mgat5) in hepatocellular carcinoma (HCC) is a common early event involved in tumor invasion during hepatocarcinogenesis. A better understanding of the functional role and the molecular mechanism for Mgat5-targeted protein and downstream signaling pathway behind hepatoma invasion and   metastasis   is urgently needed. Here, we show that Mgat5 overexpression promoted anchorage-independent growth and inhibited anoikis in hepatoma cells. This effect was reversed by glycosyltransferase inactive mutant Mgat5 L188R transfection, α-mannosidase II inhibitor swainsonine treatment and N-acetyl glucosamine (GlcNAc) phosphotransferase (GPT) inhibitor tunicamycin administration. Mgat5 overexpression increased p21-activated kinase 1 (PAK1) expression and shRNA-mediated PAK1 knockdown and kinase inactivation with kinase dead mutant PAK1 K299R coexpression or allosteric inhibitor P21-activated kinase inhibitor III (IPA3) treatment reversed anoikis resistance in Mgat5-overexpressed hepatoma cells. Furthermore, Mgat5 overexpression upregulated β-1-6-GlcNAc branched N-glycosylation and following phosphorylation of epidermal growth factor receptor (EGFR) in hepatoma cells. EGFR tyrosine kinase inhibitors AG1478 and Iressa treatment declined anchorage-independent growth and anoikis resistance, which could be rescued by constitutive active mutant PAK1 T423E coexpression in Mgat5-overexpressed hepatoma cells. Conversely, knockdown of Mgat5 reduced EGFR/PAK1-dependent anoikis resistance, which could be reversed by PAK1 T423E. These results identified Mgat5-mediated β-1-6-GlcNAc branched N-glycosylation and following activation of EGFR as a potential novel upstream molecular event for PAK1-induced anoikis resistance in hepatoma cells, implicating that molecular targeted therapeutics against Mgat5/EGFR/PAK1 might open a new avenue for personalized medicine in advanced-stage HCC patients. 
 Klotho endows hepatoma cells with resistance to anoikis via VEGFR2/PAK1 activation in hepatocellular carcinoma. Klotho was originally characterized as an aging suppressor gene that predisposed Klotho-deficient mice to premature aging-like syndrome. Although Klotho was recently reported to exhibit tumor suppressive properties during various malignant transformations, the functional role and molecular mechanism of Klotho in hepatocarcinogenesis remains poorly understood. In our present study, immunohistochemical Klotho staining levels in a clinical follow-up of 52 hepatoma patients were significantly associated with liver cirrhosis, tumor multiplicity and venous invasion. The overall survival rate of hepatoma patients with high Klotho expression was significantly lower than those patients with low Klotho expression. Moreover, Klotho overexpression increased cellular migration, anchorage-independent growth, and anoikis resistance in hepatoma cells. Klotho overexpression elevated p21-activated kinase 1 (PAK1) expression and shRNA-mediated PAK1 knockdown and kinase activity inhibition with kinase dead mutant PAK1 K299R coexpression or allosteric inhibitor IPA3 treatment reversed anoikis resistance in Klotho-overexpressed hepatoma cells. More importantly, the pivotal significance of upregulated VEGFR2 protein levels mediated by Klotho expression was confirmed by VEGFR2 inhibitor Axitinib and blocking antibody treatment in hepatoma cells. Axitinib treatment sensitized anoikis was reversed by constitutive active mutant PAK1 T423E coexpression in Klotho-overexpressed hepatoma cells. Conversely, knockdown of Klotho reduced VEGFR2/PAK1 dependent anoikis resistance, which could be reversed by PAK1 T423E. These results revealed a novel oncogenic function of Klotho in promoting anoikis resistance via activating VEGFR2/PAK1 signaling, thus facilitating tumor migration and invasion during hepatoma progression, which could provide a putative molecular mechanism for tumor   metastasis  . 
 P21 activated kinase-1 (Pak1) promotes prostate tumor growth and microinvasion via inhibition of transforming growth factor β expression and enhanced matrix metalloproteinase 9 secretion. P21-activated kinases (Paks) are major effectors downstream of the small Rho family of GTPases. Among the six isoforms, Pak1 is the most ubiquitous and the best characterized member. Previous studies have shown that inhibition of Pak6, which is predominantly present in the prostate compared with other tissues, inhibits prostate tumor growth in vivo. Even though Pak1 has been identified in normal prostatic epithelial cells and cancer cells, its specific role in the development of prostate cancer remains unclear. We report here that highly invasive prostate cancer cells express significantly higher levels of Pak1 protein compared with non-invasive prostate cancer cells. Furthermore, prostate tumor tissues and prostate cancer metastasized to lungs showed a higher expression of Pak1 compared with normal tissues. Interestingly, Pak6 protein expression levels did not change with the invasive/metastatic potential of the cancer cells or tumors. Although inhibition of Pak1, and not Pak6, resulted in impaired PC3 cell migration, the effects of Pak1 knockdown on transendothelial migration (microinvasion), tumor growth, and tumor angiogenesis was higher compared with Pak6 knockdown. Finally, gene array data revealed reduced expression of matrix metalloproteinase 9 with the ablation of either Pak1 or Pak6 gene expression in PC3 cells, whereas protein levels of TGFβ was elevated significantly with specific modulation of Pak1 activity or ablation of the Pak1 gene. Our observations suggest that although some level of functional redundancy exists between Pak1 and Pak6 in prostate cancer cells, targeting Pak1 is a potential option for the management of prostate tumor growth, microinvasion, an  d metastas  is. 
 Axl as a downstream effector of TGF-β1 via PI3K/Akt-PAK1 signaling pathway promotes tumor invasion and chemoresistance in breast carcinoma. The invasion and chemoresistance are crucial causes of morbidity and death for cancer patients. Axl is closely associated with malignant phenotype of breast tumor cells, including invasiveness and   metastasi  s. Both breast cancer cell line and tissue displayed increased expression of Axl, especially in highly metastatic breast cancer. On the contrary, experimental inhibition of Axl or transforming growth factor beta 1 (TGF-β1) by RNAi assay could suppress cell invasion ability and chemoresistance. Moreover, the up-regulation of Axl was induced by TGF-β1, further activated phosphatidylinositol 3-kinase (PI3K)/Akt and PAK1 translocation, and resulted in greater cell motility, invasion, and chemoresistance in vitro and in vivo. After the detection and statistics in human breast cancer specimens, we found that the Axl expression was closely correlated with TGF-β1 level, tumor differentiation, lymph n  ode metast  asis, and clinical stage (p < 0.01). Our findings support the possibility that Axl is a significant regulator of invasion and chemosensitivity, and it means by targeting Axl or its related signaling pathways, we can reduce the invasion and chemosensitivity of breast tumor. 
 Forced expression of NESH suppresses motility and metastatic dissemination of malignant cells. To characterize the function of a novel Src homology 3 (SH3) adapter protein termed NESH, we have established transfectants stably expressing NESH. We observed that every clone of NESH transfectants caused a marked reduction in motility, although the clones exhibited no significant differences in intrinsic cell growth compared with the control cells in vitro. The NESH transfectants also exhibited significant reduction in tumor metastatic potential in vivo. We found that NESH expression is frequently lost in invasive cancer cell lines despite its ubiquitous expression in normal tissues. The SH3 domain of NESH seems to interact with p21-activated kinase (PAK), which is involved in regulation of cell motility. Furthermore, a significant decrease in PAK phosphorylation at (402)Thr was found in NESH transfectants. Taken together, these results suggest that NESH inhibits ectopic   metastasis   of tumor cells as well as cell migration through interaction with intracellular molecules such as PAK that are essential for cell motility. 
 Significance of KRAS/PAK1/Crk pathway in non-small cell lung cancer oncogenesis. Key effector(s) of mutated KRAS in lung cancer progression and   metastasis   are unknown. Here we investigated the role of PAK1/Crk axis in transduction of the oncogenic KRAS signal in non-small cell lung cancer (NSCLC).We used NSCLC clinical specimens to examine the correlation among KRAS mutations (codon 12, 13 and 61); PAK1/Crk axis activation [p-PAK1(Thr423), p-Crk(Ser41)]; and adhesion molecules expression by immunohistochemistry. For assessing the role of proto-oncogene c-Crk as a KRAS effector, we inhibited KRAS in NSCLC cells by a combination of farnesyltransferase inhibitor (FTI) and geranylgeranyltransferase inhibitor (GGTI) and measured p-Crk-II(Ser41) by western blotting. Finally, we disrupted the signaling network downstream of KRAS by blocking KRAS/PAK1/Crk axis with PAK1 inhibitors (i.e., IPA-3, FRAX597 or FRAX1036) along with partial inhibition of all other KRAS effectors by prenylation inhibitors (FTI + GGTI) and examined the motility, morphology and proliferation of the NSCLC cells.Immunohistochemical analysis demonstrated an inverse correlation between PAK1/Crk phosphorylation and E-cadherin/p120-catenin expression. Furthermore, KRAS mutant tumors expressed higher p-PAK1(Thr423) compared to KRAS wild type. KRAS prenylation inhibition by (FTI + GGTI) completely dephosphorylated proto-oncogene c-Crk on Serine 41 while Crk phosphorylation did not change by individual prenylation inhibitors or diluent. Combination of PAK1 inhibition and partial inhibition of all other KRAS effectors by (FTI + GGTI) dramatically altered morphology, motility and proliferation of H157 and A549 cells.Our data provide evidence that proto-oncogene c-Crk is operative downstream of KRAS in NSCLC. Previously we demonstrated that Crk receives oncogenic signals from PAK1. These data in conjunction with the work of others that have specified the role of PAK1 in transduction of KRAS signal bring forward the importance of KRAS/PAK1/Crk axis as a prominent pathway in the oncogenesis of KRAS mutant lung cancer. 
 Prognostic importance and therapeutic implications of PAK1, a drugable protein kinase, in gastroesophageal junction adenocarcinoma. Gastroesophageal junction (GEJ) adenocarcinoma is a lethal cancer with rising incidence, yet the molecular biomarkers that have strong prognostic impact and also hold great therapeutic promise remain elusive. We used a data mining approach and identified the p21 protein-activated kinase 1 (PAK1), an oncogene and drugable protein kinase, to be among the most promising targets for GEJ adenocarcinoma. Immunoblot analysis and data mining demonstrated that PAK1 protein and mRNA were upregulated in cancer tissues compared to the noncancerous tissues. Immunohistochemistry revealed PAK1 overexpression in 72.6% of primary GEJ adenocarcinomas (n = 113). A step-wise increase in PAK1 levels was noted from paired normal epithelium, to atypical hyperplasia and adenocarcinoma. PAK1 overexpression in tumor was associated with lymph node (LN)   metastasis   (P<0.001), advanced tumor stage (P<0.001), large tumor size (P = 0.006), residual surgical margin (P = 0.033), and unfavorable overall survival (P<0.001). Multivariate analysis showed PAK1 overexpression is an independent high-risk prognostic predictor (P<0.001). Collectively, PAK1 is overexpressed during tumorigenic progression and its upregulation correlates with malignant properties mainly relevant to invasion and   metastasis  . PAK1 expression could serve as a prognostic predictor that holds therapeutic promise for GEJ adenocarcinoma. 
 P21-activated protein kinase 1 induces colorectal cancer   metastasis   involving ERK activation and phosphorylation of FAK at Ser-910. Pak1 has been reported to be overexpressed in colorectal cancer, but the role of Pak1 in colorectal cancer remains unclear. In this study, Pak1 expression and activity were associated with aggressive behavior of colorectal cancer. Overexpression of Pak1 increased colorectal cancer cell motility and invasion, whereas down-regulation of Pak1 expression or activity reduced colorectal cancer cell migration and invasion. In addition, activated Pak1 inhibited stress fiber and focal adhesion complex formation in colorectal cancer cells and led to formation of motile phenotypes. Importantly, activated Pak1 elicited phosphorylation of FAK at Ser-910 via an ERK-dependent pathway in colorectal cancer cell lines and clinical samples. In conclusion, our results suggest that activated Pak1 regulates colorectal cancer   metastasis   requiring an ERK-dependent phosphorylation of FAK at Ser-910. 
 Dysregulation of PAK1 Is Associated with DNA Damage and Is of Prognostic Importance in Primary Esophageal Small Cell Carcinoma. Primary esophageal small cell carcinoma (PESCC) is a rare, but fatal subtype of esophageal carcinoma. No effective therapeutic regimen for it. P21-activated kinase 1 (PAK1) is known to function as an integrator and an indispensable node of major growth factor signaling and the molecular therapy targeting PAK1 has been clinical in pipeline. We thus set to examine the expression and clinical impact of PAK1 in PESCC. The expression of PAK1 was detected in a semi-quantitative manner by performing immunohistochemistry. PAK1 was overexpressed in 22 of 34 PESCC tumors, but in only 2 of 18 adjacent non-cancerous tissues. Overexpression of PAK1 was significantly associated with tumor location (p = 0.011), lymph node   metastasis   (p = 0.026) and patient survival (p = 0.032). We also investigated the association of PAK1 with DNA damage, a driven cause for malignancy progression. γH2AX, a DNA damage marker, was detectable in 18 of 24 (75.0%) cases, and PAK1 expression was associated with γH2AX (p = 0.027). Together, PAK1 is important i  n metastas  is and progression of PESCC. The contribution of PAK1 to clinical outcomes may be involved in its regulating DNA damage pathway. Further studies are worth determining the potentials of PAK1 as prognostic indicator and therapeutic target for PESCC. 
 Modulation of p21-activated kinase 1 alters the behavior of renal cell carcinoma. The p21-activated kinase 1 (Pak1) is a serine/threonine kinase whose activity is regulated by both Rho GTPases and AGC kinase family members. It plays a role in cytoskeletal remodeling and cell motility as well as cell proliferation, angiogenesis, tumorigenesis and   metastasis  . An involvement of Pak1 in renal cell carcinoma (RCC), which remains highly refractory to chemotherapy and radiotherapy, remains to be investigated. Pak1 expression, phosphorylation and kinase activity were examined in RCC cell lines and human tissue from normal and renal carcinoma. We report increased Pak1 expression and constitutive activity in the membrane and nucleus but not the cytoplasm of resected human RCC. To study a role for Pak1 in RCC, we developed 786-0 clones that expressed either a kinase-active Pak1L83,L86 2 different Pak1 dominant negative mutants, Pak1R299 and Pak1L83,L86,R299 or Pak1 siRNA. The expression of Pak1L83,L86 increased 786-0 proliferation, motility and anchorage independent growth, while the dominant negative mutants and Pak1 siRNA abrogated these effects. In addition, Pak1L83,L86 conferred resistance to 5-fluorouracil with a 40%+/-10% increase in cell viability. Conversely, Pak1L83,L86,R299, Pak1R299 and Pak1 siRNA conferred sensitivity with a 65.2%+/-5.5%, 69.2%+/-3.3% and 73.0%+/-8.4% loss in viability, respectively. Finally, Pak1 plays a role in renal tumor growth in vivo. Only 33% of mice developed tumors in the Pak1L83,L86,R299 group and no tumors developed from Pak1R299 cell challenge. Together these findings point to Pak1 as an exciting target for therapy of renal cancer, which remains highly refractory to existing treatments. 
 Pak protein kinases and their role in cancer. Some of the characteristics of cancer cells are high rates of cell proliferation, cell survival, and the ability to invade surrounding tissue. The cytoskeleton has an essential role in these processes. Dynamic changes in the cytoskeleton are necessary for cell motility and cancer cells are dependent on motility for invasion and   metastasis  . The signaling pathways behind the reshaping and migrating properties of the cytoskeleton in cancer cells involve a group of Ras-related small GTPases and their effectors, including the p21-activated kinases (Paks). Paks are a family of serine/threonine protein kinases comprised of six isoforms (Pak 1-6), all of which are direct targets of the small GTPases Rac and Cdc42. Besides their role in cytoskeletal dynamics, Paks have recently been shown to regulate various other cellular activities, including cell survival, mitosis, and transcription. Paks are overexpressed and/or hyperactivated in several human tumors and their role in cell transformation makes them attractive therapeutic targets. Pak-targeted therapeutics may efficiently inhibit certain types of tumors and efforts to identify selective Pak-inhibitors are underway. 
 PAK1 kinase promotes cell motility and invasiveness through CRK-II serine phosphorylation in non-small cell lung cancer cells. The role of c-Crk (CRK) in promoting   metastasis   is well described however the role of CRK phosphorylation and the corresponding signaling events are not well explained. We have observed CRK-II serine 41 phosphorylation is inversely correlated with p120-catenin and E-cadherin expressions in non-small cell lung cancer (NSCLC) cells. Therefore, we investigated the role of CRK-II serine 41 phosphorylation in the down-regulation of p120-catenin, cell motility and cell invasiveness in NSCLC cells. For this purpose, we expressed phosphomimetic and phosphodeficient CRK-II serine 41 mutants in NSCLC cells. NSCLC cells expressing phosphomimetic CRK-II seine 41 mutant showed lower p120-catenin level while CRK-II seine 41 phosphodeficient mutant expression resulted in higher p120-catenin. In addition, A549 cells expressing CRK-II serine 41 phosphomimetic mutant demonstrated more aggressive behavior in wound healing and invasion assays and, on the contrary, expression of phosphodeficient CRK-II serine 41 mutant in A549 cells resulted in reduced cell motility and invasiveness. We also provide evidence that PAK1 mediates CRK-II serine 41 phosphorylation. RNAi mediated silencing of PAK1 increased p120-catenin level in A549 and H157 cells. Furthermore, PAK1 silencing decreased cell motility and invasiveness in A549 cells. These effects were abrogated in A549 cells expressing phosphomimetic CRK-II serine 41. In summary, these data provide evidence for the role of PAK1 in the promotion of cell motility, cell invasiveness and the down regulation of p120-catenin through CRK serine 41 phosphorylation in NSCLC cells. 
 Overexpression of PAK-1 is an independent predictor of disease recurrence in colorectal carcinoma. Colorectal carcinoma (CRC) is a significant cause of major morbidity and mortality. PAK-1 is a protein that regulates cytoskeletal dynamics and cell motility. The purpose of the present study is to investigate the relationship between PAK-1 immunoexpression and CRC progression and its validity as an independent prognostic factor.Paraffin blocks of 103 primary CRCs and 37 nodal metastases were retrieved and tissue microarrays were constructed. Immunohistochemistry was performed using anti-PAK-1 antibody. Immunostaining was scored and results were analysed in relation to clinicopathological parameters.PAK-1 was overexpressed in primary CRC (P<0.001). No difference between low and high expression in nodal   metastasis   (P=0.139). There was no difference between PAK-1 immunoexpression in primary and nodal   metastasis   (P=0.275). High PAK-1 immunoexpression was associated with disease recurrence (P=0.03). However, there was no association with most clinicopathological parameters. PAK-1 overexpression was detected as an independent predictor of disease recurrence (P=0.05). No association was found between PAK-1 immunoexpression and disease free survival (log-rank =1.287, P=0.257).PAK-1 overexpression may be involved in CRC progression and could be considered an independent predictor of disease recurrence. Further in vivo and in vitro molecular studies are needed to investigate the role of PAK-1 in colorectal carcinogenesis. 
 Personalizing risk stratification by addition of PAK1 expression to TNM staging: improving the accuracy of clinical decision for gastroesophageal junction adenocarcinoma. Gastroesophageal junction adenocarcinoma (GEJA) is an aggressive malignancy with an alarmingly rising incidence. TNM staging is widely used by oncologists to stratify prognosis as well as direct therapeutic strategies. However, inadequate lymphadenectomy is frequently encountered for GEJA and largely confounds prognosis resulting from TNM staging. Thus, a molecular biomarker, which can accurately forecast the risk of nodal   metastasis   in patients with inadequate lymphadenectomy, is required to guide precisely clinical decision. In this study, bioinformatics and pathological analysis identified that p21 protein-activated kinase 1 (PAK1) is associated with lymph nodal   metastasis   of GEJA. The PAK1 H-score was lower in the patients with negative lymph nodes than that in patients with positive (metastatic) lymph nodes (6.865 ± 3.376, 9.370 ± 2.530, respectively; p < 0.001). The PAK1 H-score in lymph nodes was positively correlated with that in primary tumors (PTs; p < 0.001; r = 0.475). PAK1 H-scores in PTs had the best performance based on its area under the receiver-operating characteristic (ROC) curve compared with PAK1 H-scores in lymph nodes, histologic  al grade,   lymph nodal metastasis status, tumor size, depth of tumor, TNM stage and number of resected lymph nodes. Multivariate Cox proportional hazard and Fine and Gray models showed that histological grade 3, Charlson comorbidity index > 7 and high PAK1 expression in PTs were associated with significantly increased risk of recurrence and cancer-related death. In conclusion, high PAK1 expression in   PTs is pre  dictive of node metastasis and can be easily integrated in the clinical decision process for personalized therapeutics of GEJA. 
 Tumor necrosis factor-alpha induced expression of matrix metalloproteinase-9 through p21-activated kinase-1. Expressed in embryonic development, matrix metalloprotein-9 (MMP-9) is absent in most of developed adult tissues, but recurs in inflammation during tissue injury, wound healing, tumor formation and   metastasis  . Expression of MMP-9 is tightly controlled by extracellular cues including pro-inflammatory cytokines and extracellular matrix (ECM). While the pathologic functions of MMP-9 are evident, the intracellular signaling pathways to control its expression are not fully understood. In this study we investigated mechanism of cytokine induced MMP-9 with particular emphasis on the role of p21-activated-kinase-1 (PAK1) and the down stream signaling.In response to TNF-alpha or IL-1alpha, PAK1 was promptly activated, as characterized by a sequential phosphorylation, initiated at threonine-212 followed by at threonine-423 in the activation loop of the kinase, in human skin keratinocytes, dermal fibroblasts, and rat hepatic stellate cells. Ectopic expression of PAK1 variants, but not p38 MAP kinase, impaired the TNF-alpha-induced MMP-9 expression, while other MMPs such as MMP-2, -3 and -14 were not affected. Activation of Jun N-terminal kinase (JNK) and NF-kappaB has been demonstrated to be essential for MMP-9 expression. Expression of inactive PAK1 variants impaired JNK but not NF-kappaB activation, which consequently suppressed the 5'-promoter activities of the MMP-9 gene. After the cytokine-induced phosphorylation, both ectopically expressed and endogenous PAK1 proteins were promptly accumulated even in the condition of suppressing protein synthesis, suggesting the PAK1 protein is stabilized upon TNF-alpha stimulation. Stabilization of PAK1 protein by TNF-alpha treatment is independent of the kinase catalytic activity and p21 GTPase binding capacities. In contrast to epithelial cells, mesenchymal cells require 3-dimensional type-I collagen in response to TNF-alpha to massively express MMP-9. The collagen effect is mediated, in part, by boost JNK activation in a way to cooperate the cytokine signaling.We identified a novel mechanism for MMP-9 expression in response to injury signals, which is mediated by PAK1 activation and stabilization leading JNK activation. 
 PAK1-cofilin phosphorylation mediates human lung adenocarcinoma cells migration induced by apelin-13. Adipocytokines apelin peptide, the ligand of APJ (putative receptor related to the angiotensin receptor AT1), plays key roles in the pathogenesis and deterioration of cancer. In lung cancer, apelin elevating microvessel densities has been reported. Our previous research has characterized that apelin-13 promoted lung adenocarcinoma cell proliferation. However, the effect of apelin on   metastasis   in lung adenocarcinoma and the underlying mechanisms remain unclear. This study shows that apelin-13 induced human adenocarcinoma cell migration via the APJ receptor. Apelin-13 phosphorylated PAK1 and cofilin increase the migration of lung adenocarcinoma cells. Moreover, the results verify that over-expression of apelin and APJ contributed to reducing the effect of doxorubicin and razoxane on inhibiting lung adenocarcinoma cells   metastasis  . Hypoxia activated APJ expression and apelin release in lung adenocarcinoma cells. The results demonstrate a PAK1-cofilin phosphorylation mechanism to mediate lung adenocarcinoma cells migration promoted by apelin-13. This discovery further suggests that APJ and its downstream signalling is a potential target for anti-metastatic therapies in lung adenocarcinoma patients. 
 Tyrosyl phosphorylated serine-threonine kinase PAK1 is a novel regulator of prolactin-dependent breast cancer cell motility and invasion. Despite efforts to discover the cellular pathways regulating breast cancer   metastasis  , little is known as to how prolactin (PRL) cooperates with extracellular environment and cytoskeletal proteins to regulate breast cancer cell motility and invasion. We implicated serine-threonine kinase p21-activated kinase 1 (PAK1) as a novel target for PRL-activated Janus-kinase 2 (JAK2). JAK2-dependent PAK1 tyrosyl phosphorylation plays a critical role in regulation of both PAK1 kinase activity and scaffolding properties of PAK1. Tyrosyl phosphorylated PAK1 facilitates PRL-dependent motility via at least two mechanisms: formation of paxillin/GIT1/βPIX/pTyr-PAK1 complexes resulting in increased adhesion turnover and phosphorylation of actin-binding protein filamin A. Increased adhesion turnover is the basis for cell migration and phosphorylated filamin A stimulates the kinase activity of PAK1 and increases actin-regulating activity to facilitate cell motility. Tyrosyl phosphorylated PAK1 also stimulates invasion of breast cancer cells in response to PRL and three-dimensional (3D) collagen IV via transcription and secretion of MMP-1 and MMP-3 in a MAPK-dependent manner. These data illustrate the complex interaction between PRL and the cell microenvironment in breast cancer cells and suggest a pivotal role for PRL/PAK1 signaling in breast cancer   metastasi  s. 
 Pak-1 expression increases with progression of colorectal carcinomas to   metastasis  . The p21-activated kinase-1 (Pak-1) promotes cell motility and invasiveness. Pak-1 is activated by the Rac, Rho, and Cdc42 small GTPases in response to a variety of stimuli including ras and phosphatidylinositol 3'-kinase/AKT pathway activation. Because Pak-1 plays a central role in regulating cell motility and invasiveness, we sought to determine whether Pak-1 may be involved in the malignant progression of colorectal carcinoma.Pak-1 expression was examined by immunohistochemistry in archived tissues from normal human colons, tubular and tubulovillous adenomas, invasive adenocarcinomas (stages I-III/IV), and lymph node metastases (184 total specimens from 38 patients). Specific cytoplasmic immunostaining was evaluated for overall intensity and uniformity to derive a combined histoscore (stain intensity x percentage of epithelium stained).Pak-1 expression was increased significantly with colorectal cancer progression from normal tissue to lymph node metastases (P < 0.0001). Furthermore, Pak-1 expression was increased significantly in adenomas, invasive carcinomas, and lymph node metastases compared with normal colon (P < 0.0001). Strikingly, Pak-1 expression was significantly higher in lymph node metastases than in invasive cancers, adenomas, or normal colon (P < 0.0001). Moreover, in patients with multiple lesions representing different stages of disease, Pak-1 expression was increased specifically in the most advanced lesions.This study demonstrates that Pak-1 expression is increased significantly with malignant progression of human colorectal carcinoma. These data, along with numerous functional studies demonstrating a central role for Pak-1 activity in tumor invasiveness and motility, implicate Pak-1 as an exciting target for therapy of colorectal carcinoma. 
 STE20-related kinase adaptor protein α (STRADα) regulates cell polarity and invasion through PAK1 signaling in LKB1-null cells. LKB1 is a Ser/Thr kinase, and its activity is regulated by the pseudokinase, STE20-related adaptor α (STRADα). The STRADα-LKB1 pathway plays critical roles in epithelial cell polarity, neuronal polarity, and ca  ncer metas  tasis. Though much attention is given to the STRADα-LKB1 pathway, the function of STRADα itself, including a role outside of the LKB1 pathway, has not been well-studied. Data in Caenorhabditis elegans suggest that STRADα has an LKB1-independent role in regulating cell polarity, and therefore we tested the hypothesis that STRADα regulates cancer cell polarity and motility when wild-type LKB1 is absent. These results show that STRADα protein is reduced in LKB1-null cell lines (mutation or homozygous deletion) and this partial degradation occurs through the Hsp90-dependent proteasome pathway. The remaining STRADα participates in cell polarity and invasion, such that STRADα depletion results in misaligned lamellipodia, improper Golgi positioning, and reduced invasion. To probe the molecular basis of this defect, we show that STRADα associates in a complex with PAK1, and STRADα loss disrupts PAK1 activity via Thr(423) PAK1 phosphorylation. When STRADα is depleted, PAK1-induced invasion could not occur, suggesting that STRADα is necessary for PAK1 to drive motility. Furthermore, STRADα overexpression caused increased activity of the PAK1-activating protein, rac1, and a constitutively active rac1 mutant (Q61L) rescued pPAK(Thr423) and STRADα invasion defects. Taken together, these results show that a STRADα-rac1-PAK1 pathway regulates cell polarity and invasion in LKB1-null cells. It also suggests that while the function of LKB1 and STRADα undoubtedly overlap, they may also have mutually exclusive roles. 
 Clinicopathological and cellular signature of PAK1 in human bladder cancer. Bladder cancer (BC) is the ninth most common cancer and the 13th most common cause of cancer death. Although p21 protein-activated kinase (PAK) regulates cell growth, motility, and morphology, the expression and function of PAK1 associated with the clinicopathological and cellular signature of human BC are not clear. This study was to examine the expression of PAK1 in human BC, the association of PAK1 with clinicopathological features, and the effect of PAK1 on cell proliferation, migration, and invasion in BC cells. A total of 54 BC and 12 normal bladder tissue specimens were retrieved. Among 54 BC patients, 39 cases were superficial BC and 15 cases were invasive BC. Histological examination revealed 29 patients with low-grade and 25 patients with high-grade papillary urothelial carcinomas. Immunohistochemical staining showed that PAK1 was overexpressed in BC tissue compared with normal bladder tissue. The overexpression of PAK1 was significantly associated with tumor size, histological grade, and lymph node   metastasis  , but not with gender, age, clinical stage, tumor number, and recurrence. Furthermore, the cytoplasmic distribution of PAK1 was observed in BC cells. Knocking down of PAK1 using lentiviral transduction decreased BC cell proliferation, migration, and invasion. In conclusion, we demonstrated that the overexpression of PAK1 is closely associated with the clinicopathological features of BC, suggesting that PAK1 may play an important role in the development and progression of BC and may be a potential therapeutic target for the treatment of BC. 
 Overexpression of p21-activated kinase 1 promotes endometrial cancer progression. Endometrial cancer (EC) is the most common gynecologic malignancy, but the molecular events involved in the development and progression of EC remain unclear. P21-activated kinase 1 (Pak1) plays important roles in cell motility and survival. This study investigated the clinical significance of Pak1 expression and its functional roles in EC. The expression of Pak1 in clinical samples and EC cell lines was evaluated. The effects of Pak1 on EC cell functions were determined by either overexpressing it via plasmid transfection or depleting its expression using short hairpin RNA (shRNA) in human EC cell lines. Pak1 was overexpressed in clinical samples of EC compared with normal endometrium. High Pak1 expression in EC was positively correlated with lymph node   metastasi  s, advanced disease stage and poor histological differentiation. Pak1 over-expression was also observed in multiple human EC cell lines. In EC cell lines, Pak1 overexpression promoted cell proliferation, migration, invasion and anchorage-independent growth in vitro. Conversely, shRNA-mediated stable knockdown of Pak1 reduced cell proliferation, migration, invasion and anchorage-independent growth. In addition, ectopic Pak1 overexpression protected EC cells from apoptosis, along with decreased caspase-3 activation. These results suggest that Pak1 plays important roles at multiple stages of EC progression. 
  GeneRIFs  
 Study indicates that PAK1 is important in   metastasis   and progression of primary esophageal small cell carcinoma. The contribution of PAK1 to clinical outcomes may be involved in regulating DNA damage pathway. 
 PAK1 and PAK4 expression were associated with colorectal cancer   metastasis   and infiltration 
 high PAK1 expression in PTs is predictive of node   metastasis   and can be easily integrated in the clinical decision process for personalized therapeutics of GEJA. 
 Positive rates of Rac1, Pak1 and Rock1 expression in normal tissue, dysplasia and gastric carcinoma show an increasing trend and are correlated with tumor lymph node   metastasis   and TNM stage. 
 A pivotal role for PRL/PAK1 signaling in breast cancer   metastasis  .[review] 
 PAK1 is overexpressed during tumorigenic progression and its upregulation correlates with malignant properties mainly relevant to invasion and   metastasis  . 
 Pak1 is overexpressed in gastric cancer and plays an important role in the   metastasis   of gastric cancer. The mechanism by which Pak1 induces cancer   metastasis   may involve activation of ERK and JNK. 
 Results suggest that activated Pak1 regulates colorectal cancer   metastasis   requiring an ERK-dependent phosphorylation of FAK at Ser-910. 
 Suggest that Rac1 activity and Pak1 are involved in lymphovascular invasion and lymph node   metastasis   of urothelial carcinoma of the upper urinary tract , and may be prognostic markers for this disease. 
 Overexpression of Pak1 was associated with progression,   metastasis   and prognosis of gastric cancer. 
  [Cc]ell [Ii]nvasion  PubMed  
 A Trio-Rac1-Pak1 signalling axis drives invadopodia disassembly. Rho family GTPases control cell migration and participate in the regulation of cancer metastasis. Invadopodia, associated with invasive tumour cells, are crucial for cellular invasion and metastasis. To study Rac1 GTPase in invadopodia dynamics, we developed a genetically encoded, single-chain Rac1 fluorescence resonance energy (FRET) transfer biosensor. The biosensor shows Rac1 activity exclusion from the core of invadopodia, and higher activity when invadopodia disappear, suggesting that reduced Rac1 activity is necessary for their stability, and Rac1 activation is involved in disassembly. Photoactivating Rac1 at invadopodia confirmed this previously unknown Rac1 function. We describe here an invadopodia disassembly model, where a signalling axis involving TrioGEF, Rac1, Pak1, and phosphorylation of cortactin, causes invadopodia dissolution. This mechanism is critical for the proper turnover of invasive structures during tumour   cell invasion  , where a balance of proteolytic activity and locomotory protrusions must be carefully coordinated to achieve a maximally invasive phenotype. 
 14-3-3zeta Cooperates with ErbB2 to promote ductal carcinoma in situ progression to invasive breast cancer by inducing epithelial-mesenchymal transition. ErbB2, a metastasis-promoting oncoprotein, is overexpressed in approximately 25% of invasive/metastatic breast cancers, but in 50%-60% of noninvasive ductal carcinomas in situ (DCIS). It has been puzzling how a subset of ErbB2-overexpressing DCIS develops into invasive breast cancer (IBC). We found that co-overexpression of 14-3-3zeta in ErbB2-overexpressing DCIS conferred a higher risk of progression to IBC. ErbB2 and 14-3-3zeta overexpression, respectively, increased cell migration and decreased cell adhesion, two prerequisites of tumor   cell invasion  . 14-3-3zeta overexpression reduced cell adhesion by activating the TGF-beta/Smads pathway that led to ZFHX1B/SIP-1 upregulation, E-cadherin loss, and epithelial-mesenchymal transition. Importantly, patients whose breast tumors overexpressed both ErbB2 and 14-3-3zeta had higher rates of metastatic recurrence and death than those whose tumors overexpressed only one. 
 A Trio-Rac1-Pak1 signalling axis drives invadopodia disassembly. Rho family GTPases control cell migration and participate in the regulation of cancer metastasis. Invadopodia, associated with invasive tumour cells, are crucial for cellular invasion and metastasis. To study Rac1 GTPase in invadopodia dynamics, we developed a genetically encoded, single-chain Rac1 fluorescence resonance energy (FRET) transfer biosensor. The biosensor shows Rac1 activity exclusion from the core of invadopodia, and higher activity when invadopodia disappear, suggesting that reduced Rac1 activity is necessary for their stability, and Rac1 activation is involved in disassembly. Photoactivating Rac1 at invadopodia confirmed this previously unknown Rac1 function. We describe here an invadopodia disassembly model, where a signalling axis involving TrioGEF, Rac1, Pak1, and phosphorylation of cortactin, causes invadopodia dissolution. This mechanism is critical for the proper turnover of invasive structures during tumour   cell invasion  , where a balance of proteolytic activity and locomotory protrusions must be carefully coordinated to achieve a maximally invasive phenotype. 
 Axl gene knockdown inhibits the metastasis properties of hepatocellular carcinoma via PI3K/Akt-PAK1 signal pathway. The objective of this study is to clarify the possible role and mechanism of Axl in the tumorigenicity and metastasis process of hepatocellular carcinoma. The mRNA and protein expression levels of Axl in MHCC97-H and MHCC97-L cell lines were evaluated by real-time PCR and Western blot analysis. The key factor of phosphatidylinositol-3-kinase (PI3K)/Akt-p21-activated kinases-1 (PAK1) signaling pathway was studied after Axl expression was downregulated by shRNA. Finally, we analyzed the expression status of Axl protein expression in hepatocellular carcinoma tissues and its relationship with the prognosis of hepatocellular carcinoma. Axl was observed to be higher expressed in MHCC97-H cell lines compared to MHCC97-L cell lines. The downregulation of Axl in MHCC97-H cell lines resulted in the inhibition of the invasion ability of MHCC97-H cells both in vitro and in vivo. Interestingly, blocking PI3K/Akt signaling pathway by LY294002 or Akt siRNA could remarkably inhibit the PAK1 activation and   cell invasion  . Finally, the Axl protein expression was positively correlated with differentiation, lymph node metastasis, and clinical stage in patients with hepatocellular carcinoma patients (all P < 0.01). These findings suggest that Axl can also regulate the metastasis process of hepatocellular carcinoma and may serve as a new prognostic marker and therapeutic target for treating hepatocellular carcinoma metastasis. 
 Axl as a downstream effector of TGF-β1 via PI3K/Akt-PAK1 signaling pathway promotes tumor invasion and chemoresistance in breast carcinoma. The invasion and chemoresistance are crucial causes of morbidity and death for cancer patients. Axl is closely associated with malignant phenotype of breast tumor cells, including invasiveness and metastasis. Both breast cancer cell line and tissue displayed increased expression of Axl, especially in highly metastatic breast cancer. On the contrary, experimental inhibition of Axl or transforming growth factor beta 1 (TGF-β1) by RNAi assay could suppres  s cell invasi  on ability and chemoresistance. Moreover, the up-regulation of Axl was induced by TGF-β1, further activated phosphatidylinositol 3-kinase (PI3K)/Akt and PAK1 translocation, and resulted in greater cell motility, invasion, and chemoresistance in vitro and in vivo. After the detection and statistics in human breast cancer specimens, we found that the Axl expression was closely correlated with TGF-β1 level, tumor differentiation, lymph node metastasis, and clinical stage (p < 0.01). Our findings support the possibility that Axl is a significant regulator of invasion and chemosensitivity, and it means by targeting Axl or its related signaling pathways, we can reduce the invasion and chemosensitivity of breast tumor. 
 PAK1 regulates breast cancer   cell invasion   through secretion of matrix metalloproteinases in response to prolactin and three-dimensional collagen IV. p21-Activated serine-threonine kinase (PAK1) is implicated in breast cancer. We have shown previously that PAK1 is tyrosyl phosphorylated by prolactin (PRL)-activated Janus tyrosine kinase (JAK2). Although a role for both PRL and PAK1 in breast cancer is widely acknowledged, the mechanism remains poorly understood. In the present study, PRL-activated PAK1 stimulates the invasion of TMX2-28 human breast cancer cells through Matrigel. Three-dimensional (3D) collagen IV stimulates the secretion of the matrix proteases, metalloproteinase (MMP)-1 and -3 that is further enhanced by the PRL-dependent tyrosyl phosphorylation of PAK1. 3D collagen IV also stimulates the expression and secretion of MMP-2, but in contrast to MMP-1 and -3, PRL/PAK1 signaling down-regulates MMP-2 expression and secretion. In contrast, MMP-9 expression and secretion are stimulated by 3D collagen I, not collagen IV, and are not affected by PRL but are down-regulated by PAK1. MMP-1 and -3 are required and MMP-2 contributes to PRL-dependent invasion. ERK1/2 signaling appears to be required for the enhanced expression and secretion of MMP-1 and -3 and enhanced PRL-dependent invasion. p38 MAPK and c-Jun N-terminal kinase 1/2 pathways participate in production of MMP-1 and -3 as well as in PRL/PAK1-dependent   cell invasion  . Together, these data illustrate the complex interaction between the substratum and PRL/PAK1 signaling in human breast cancer cells and suggest a pivotal role for PRL-dependent PAK1 tyrosyl phosphorylation in MMP secretion. 
 miR-145 inhibits invasion of bladder cancer cells by targeting PAK1. MicroRNAs play important roles in cancer. In many cancers, miR-145 acts as a tumor suppressor, and it is down-regulated in bladder cancer. In the present study, we explored the modulation of oncogenic gene PAK1 by miR-145 in bladder cancer.Expression of miR-145 was detected in bladder cancer tissues and cell lines by quantitative real-time polymerase chain reaction. Through the bioinformatics approach, PAK1 has been predicted to be a direct target of miR-145 and was confirmed by the PAK1 messenger RNA 3'-untranslated region luciferase activity assay. To investigate whether miR-145 regulates PAK1 expression, it was overexpressed in J82 and T24 bladder cancer cells. In 10 paired bladder normal and tumor tissues, we determined the relationship between miR-145 and PAK1 through quantitative real-time polymerase chain reaction and western blot. By using transwell invasion assay and western blotting analysis, we investigated the effects of miR-145 and PAK1 on bladder cancer   cell invasion   and expression of invasion marker genes.The level of miR-145 decreases and PAK1 protein expression up-regulates in bladder cancer tissue, as compared with the paired normal bladder tissue. Moreover, miR-145 directly targets PAK1 in bladder cancer cells. The level of miR-145 negatively correlates with PAK1 protein expression in bladder cancer. In addition, PAK1 promotes invasion and enhances the expression and activity of MMP-9, whereas miR-145 inhibits bladder cancer   cell invasion   and expressions of PAK1 and MMP-9.Our results indicate that miR-145 inhibits bladder cancer   cell invasion  , at least partly through targeting PAK1. Restoration or replacement of miR-145 could be an efficient approach to inhibit PAK1 and bladder cancer development in the tumor therapy. 
 Pak1 and Pak2 mediate tumor   cell invasion   through distinct signaling mechanisms. Pak kinases are thought to play critical roles in cell migration and invasion. Here, we analyze the roles of Pak1 and Pak2 in breast carcinoma   cell invasion   using the transient transfection of small interfering RNA. We find that although both Pak1 and Pak2 contribute to breast carcinoma invasion stimulated by heregulin, these roles are mediated by distinct signaling mechanisms. Thus, whereas the depletion of Pak1 interferes with the heregulin-mediated dephosphorylation of cofilin, the depletion of Pak2 does not. The depletion of Pak1 also has a stronger inhibitory effect on lamellipodial protrusion than does the depletion of Pak2. Interestingly, Pak1 and Pak2 play opposite roles in regulating the phosphorylation of the myosin light chain (MLC). Whereas the depletion of Pak1 decreases phospho-MLC levels in heregulin-stimulated cells, the depletion of Pak2 enhances MLC phosphorylation. Consistent with their opposite effects on MLC phosphorylation, Pak1 and Pak2 differentially modulate focal adhesions. Pak2-depleted cells display an increase in focal adhesion size, whereas in Pak1-depleted cells, focal adhesions fail to mature. We also found that the depletion of Pak2, but not Pak1, enhances RhoA activity and that the inhibition of RhoA signaling in Pak2-depleted cells decreases MLC phosphorylation and restores   cell invasion  . In summary, this work presents the first comprehensive analysis of functional differences between the Pak1 and Pak2 isoforms. 
  GeneRIFs  
 blocking PI3K/Akt signaling pathway by LY294002 or Akt siRNA could remarkably inhibit the PAK1 activation and   cell invasion   
 Pak1 and Pak2 mediate tumor   cell invasion   through distinct signaling mechanisms 
   
  
 PAK3 
 
  NCBI Gene  
  GeneRIF  
  Pubmed  
 
 
  Search Term  Rank  Fields  
  [Ff]ilopodia  1  PubMed(1)  
  [Mm]igration  6  Process(1); PubMed(7)  
  [Mm]etastasis  12  PubMed(1)  
 
 
 hide/show details for PAK3 
  [Ff]ilopodia  PubMed  
 The mental retardation protein PAK3 contributes to synapse formation and plasticity in hippocampus. Mutations of the gene coding for PAK3 (p21-activated kinase 3) are associated with X-linked, nonsyndromic forms of mental retardation (MRX) in which the only distinctive clinical feature is the cognitive deficit. The mechanisms through which PAK3 mutation produces the mental handicap remain unclear, although an involvement in the mechanisms that regulate the formation or plasticity of synaptic networks has been proposed. Here we show, using a transient transfection approach, that antisense and small interfering RNA-mediated suppression of PAK3 or expression of a dominant-negative PAK3 carrying the human MRX30 mutation in rat hippocampal organotypic slice cultures results in the formation of abnormally elongated dendritic spines and   filopodia  -like protrusions and a decrease in mature spine synapses. Ultrastructural analysis of the changes induced by expression of PAK3 carrying the MRX30 mutation reveals that many elongated spines fail to express postsynaptic densities or contact presynaptic terminals. These defects are associated with a reduced spontaneous activity, altered expression of AMPA-type glutamate receptors, and defective long-term potentiation. Together, these data identify PAK3 as a key regulator of synapse formation and plasticity in the hippocampus and support interpretations that these defects might contribute to the cognitive deficits underlying this form of mental retardation. 
  [Mm]igration  Process  
 positive regulation of fibroblast   migration   
  PubMed  
 p21-activated kinase 3 (PAK3) is an AP-1 regulated gene contributing to actin organisation and   migration   of transformed fibroblasts. Activating Protein 1 (AP-1) plays a vital role in cell proliferation, differentiation and apoptosis. While de-regulation of AP-1 has been linked to many cancers, little is known regarding its downstream transcriptional targets that associate with cellular transformation. Previous studies identified PAK3, a serine/threonine kinase, as a potential AP-1 target gene. PAK3 has been implicated in a variety of pathological disorders and over-expression of other PAK-family members has been linked to cancer. In this study, we investigate AP-1 regulation of PAK3 expression and the role of PAK3 in cJun/AP-1-associated cellular transformation. Our results showed elevated PAK3 expression at both the mRNA and protein level in cJun-over-expressing Rat1a fibroblasts, as well as in transformed human fibroblasts. Elevated PAK3 expression in cJun/AP-1 over-expressing cells associated with a significant increase in PAK3 promoter activation. This increased promoter activity was lost when a single putative Jun binding site, which can bind AP-1 directly both in vitro and in vivo, was mutated. Further, inhibition of PAK3 using siRNA showed a regression in the cell morphology, migratory potential and actin organisation associated with AP-1 transformed cells. Our study is a first to describe a role for AP-1 in regulating PAK3 expression and suggest that PAK3 is an AP-1 target required for actin organization and   migration   observed in transformed cells. 
 p21-activated kinase 3 (PAK3) is an AP-1 regulated gene contributing to actin organisation and   migration   of transformed fibroblasts. Activating Protein 1 (AP-1) plays a vital role in cell proliferation, differentiation and apoptosis. While de-regulation of AP-1 has been linked to many cancers, little is known regarding its downstream transcriptional targets that associate with cellular transformation. Previous studies identified PAK3, a serine/threonine kinase, as a potential AP-1 target gene. PAK3 has been implicated in a variety of pathological disorders and over-expression of other PAK-family members has been linked to cancer. In this study, we investigate AP-1 regulation of PAK3 expression and the role of PAK3 in cJun/AP-1-associated cellular transformation. Our results showed elevated PAK3 expression at both the mRNA and protein level in cJun-over-expressing Rat1a fibroblasts, as well as in transformed human fibroblasts. Elevated PAK3 expression in cJun/AP-1 over-expressing cells associated with a significant increase in PAK3 promoter activation. This increased promoter activity was lost when a single putative Jun binding site, which can bind AP-1 directly both in vitro and in vivo, was mutated. Further, inhibition of PAK3 using siRNA showed a regression in the cell morphology, migratory potential and actin organisation associated with AP-1 transformed cells. Our study is a first to describe a role for AP-1 in regulating PAK3 expression and suggest that PAK3 is an AP-1 target required for actin organization and   migration   observed in transformed cells. 
 NCK and PAK participate in the signaling pathway by which vascular endothelial growth factor stimulates the assembly of focal adhesions. Vascular endothelial growth factor (VEGF)-induced endothelial cell   migration   is a key step in the angiogenic response and is mediated, in part, by an accelerated rate of focal adhesion complex assembly and disassembly. We investigated the signaling pathway by which VEGF regulates focal adhesion complex assembly by examining the signaling proteins involved. VEGF stimulated the tyrosine phosphorylation of the SH2 domain-containing signaling proteins NCK and CRK in human umbilical vein endothelial cells. The signaling pathways that couple the kinase insert domain-containing receptor to NCK and CRK is most likely mediated by another cellular protein, as NCK and CRK were tyrosine-phosphorylated in response to VEGF in cells expressing receptors mutated at each of several candidate SH2 domain-interacting cytosolic tyrosines. In the absence of VEGF treatment, NCK (but not CRK) associated with the p21 GTPase-activated kinase PAK. PAK catalytic activity was augmented after VEGF treatment; an association of PAK with 60- and 90-kDa tyrosine-phosphorylated proteins accompanied this. VEGF stimulated the recruitment of PAK to focal adhesions, and FAK immunoprecipitated with both NCK and PAK in VEGF-treated (but not untreated) human umbilical vein endothelial cells. Inhibition of NCK protein expression using antisense oligonucleotides led to the inhibition of both VEGF-induced focal adhesion assembly and VEGF-induced cell   migration  , demonstrating a necessary role of NCK in these cellular responses. 
 The four mammalian splice variants encoded by the p21-activated kinase 3 gene have different biological properties. The p21-activated kinases (PAK1), PAK2, and PAK3 are members of the PAK group I and share high sequence identity and common biochemical properties. PAK3 is specifically implicated in neuronal plasticity and also regulates cell cycle progression, neuronal   migration  , and apoptosis. Loss of function of PAK3 is responsible for X-linked non-syndromic mental retardation whereas gain of PAK3 function is associated with cancer. To understand the functional specificities of PAK3, we analyzed the structure of PAK3 gene products. We report here the characterization of a new alternatively spliced exon called c located upstream of the previously identified exon b. Exon b is detected in all tetrapods and not in fish, exon c is only present in mammals. Mammalian PAK3 genes encode four splice variants and the corresponding proteins were detected with specific antibodies in brain extracts. All PAK3 transcripts are specifically expressed in brain and in particular in neurons. The presence of the exons b and c renders the kinase constitutively active and decreases interaction with GTPases. The expression of the new splice variants in COS7 cells alters cell morphology and modifies the structure of focal adhesions. We propose that the appearance of new alternatively spliced exons during evolution and the resulting increase of complexity of PAK3 gene products may confer new functions to this kinase and contribute to its specific roles in neuronal signaling. 
 PAK signalling in neuronal physiology. Group I p21-activated kinases are a family of key effectors of Rac1 and Cdc42 and they regulate many aspects of cellular function, such as cytoskeleton dynamics, cell movement and cell   migration  , cell proliferation and differentiation, and gene expression. The three genes PAK1/2/3 are expressed in brain and recent evidence indicates their crucial roles in neuronal cell fate, in axonal guidance and neuronal polarisation, and in neuronal   migration  . Moreover they are implicated in neurodegenerative diseases and play an important role in synaptic plasticity, with PAK3 being specifically involved in mental retardation. The main goal of this review is to describe the molecular mechanisms that govern the different functions of group I PAK in neuronal signalling and to discuss the specific functions of each isoform. 
 p21-activated kinase 3 is overexpressed in thymic neuroendocrine tumors (carcinoids) with ectopic ACTH syndrome and participates in cell   migration  . Thymic carcinoid is an important component of the tumor spectrum causing Ectopic ACTH Syndrome (EAS) and usually carries a poor prognosis. Efforts have been focused on exploring the mechanism of the excessive ACTH production in non-pituitary tumors, whereas few studies have reported the molecular events underlying the tumor progression. In this study, seven patients with ACTH producing thymic carcinoids were enrolled. Of note is that five of them showed either lymph node metastasis, local invasion or distant metastasis. By using cDNA profiling approach, we evaluated the expression of cell adhesion pathway genes and found a remarkable overexpression of p21-activated kinase 3 (PAK3) in all thymic carcinoids which was further confirmed at both transcriptional and translational level. RAC1, an upstream activator of PAK3, was also overexpressed in thymic carcinoids. Overexpression of PAK3 in NIH3T3 cell enhanced cell   migration   and invasion. Importantly, we observed c-Jun NH(2)-terminal kinase (JNK) was activated in PAK3 transfected cells, and inhibition of JNK activity by SP600125, a JNK pathway inhibitor, abolished PAK3 mediated cell   migration  . Activation of JNK pathway was also detected in thymic carcinoid with high level of PAK3 expression. Our findings suggested a potential role of PAK3 in the progression of ACTH-producing thymic carcinoid. 
 A novel long non-coding RNA-ARA: adriamycin resistance-associated. Long non-coding RNAs (lncRNAs) are emerging as an integral functional component of human genome and have been investigated as critical regulators in molecular biology of cancer. A recent study reported that lncRNA-UCA1 induced drug resistance in adriamycin chemotherapy. However, the contributions of lncRNAs to adriamycin resistance in cancers remain largely unknown. To address this issue, we performed a genome-wide lncRNA microarray analysis in adriamycin resistant MCF-7/ADR and parental MCF-7 cells, and revealed differential expression of lncRNAs in distinct category and chromosome distribution patterns. A specific differentially expressed lncRNA (Adriamycin Resistance Associated, termed ARA) was validated in MCF-7/ADR and HepG2/ADR cells. ARA is derived from an intron of PAK3 gene, predicted to contain several stable hairpins in secondary structure and has conservative sequences in primates. ARA expression is significantly associated with adriamycin sensitivity in a panel of breast and liver cancer cell lines and is markedly up-regulated in parental sensitive MCF-7 and HepG2 cell lines after receiving adriamycin treatment. The functions of ARA were assessed by silencing this lncRNA in vitro, and we found that ARA knockdown reduced the proliferation, induced cell death, G2/M arrest and   migration   defects. Furthermore, microarray transcriptomic analysis was carried out to comprehensively depict the ARA-regulated genes. We showed that ARA can modulate multiple signalling pathways, including MAPK signalling pathway, metabolism pathways, cell cycle and cell adhesion-related biological pathways, and regulate cellular processes, including transcriptional processes and protein binding function. Overall, our results indicate novel insights of adriamycin resistance in lncRNA level. 
  [Mm]etastasis  PubMed  
 p21-activated kinase 3 is overexpressed in thymic neuroendocrine tumors (carcinoids) with ectopic ACTH syndrome and participates in cell migration. Thymic carcinoid is an important component of the tumor spectrum causing Ectopic ACTH Syndrome (EAS) and usually carries a poor prognosis. Efforts have been focused on exploring the mechanism of the excessive ACTH production in non-pituitary tumors, whereas few studies have reported the molecular events underlying the tumor progression. In this study, seven patients with ACTH producing thymic carcinoids were enrolled. Of note is that five of them showed either lymph node   metastasis  , local invasion or distant   metastasis  . By using cDNA profiling approach, we evaluated the expression of cell adhesion pathway genes and found a remarkable overexpression of p21-activated kinase 3 (PAK3) in all thymic carcinoids which was further confirmed at both transcriptional and translational level. RAC1, an upstream activator of PAK3, was also overexpressed in thymic carcinoids. Overexpression of PAK3 in NIH3T3 cell enhanced cell migration and invasion. Importantly, we observed c-Jun NH(2)-terminal kinase (JNK) was activated in PAK3 transfected cells, and inhibition of JNK activity by SP600125, a JNK pathway inhibitor, abolished PAK3 mediated cell migration. Activation of JNK pathway was also detected in thymic carcinoid with high level of PAK3 expression. Our findings suggested a potential role of PAK3 in the progression of ACTH-producing thymic carcinoid. 
   
  
 ACPP 
 
  NCBI Gene  
  GeneRIF  
  Pubmed  
 
 
  Search Term  Rank  Fields  
  [Ff]ilopodium  2  Component(1)  
 
 
 hide/show details for ACPP 
  [Ff]ilopodium  Component  
   filopodium   
   
  
 SLC46A1 
 
  NCBI Gene  
  GeneRIF  
  Pubmed  
 
 
  Search Term  Rank  Fields  
  [Mm]igration  6  PubMed(1)  
 
 
 hide/show details for SLC46A1 
  [Mm]igration  PubMed  
 Quantitative proteomics identifies a Dab2/integrin module regulating cell   migration  . Clathrin-associated endocytic adapters recruit cargoes to coated pits as a first step in endocytosis. We developed an unbiased quantitative proteomics approach to identify and quantify glycoprotein cargoes for an endocytic adapter, Dab2. Surface levels of integrins beta1, alpha1, alpha2, and alpha3 but not alpha5 or alphav chains were specifically increased on Dab2-deficient HeLa cells. Dab2 colocalizes with integrin beta1 in coated pits that are dispersed over the cell surface, suggesting that it regulates bulk endocytosis of inactive integrins. Depletion of Dab2 inhibits cell   migration   and polarized movement of integrin beta1 and vinculin to the leading edge. By manipulating intracellular and surface integrin beta1 levels, we show that   migration   speed correlates with the intracellular integrin pool but not the surface level. Together, these results suggest that Dab2 internalizes integrins freely diffusing on the cell surface and that Dab2 regulates   migration  , perhaps by maintaining an internal pool of integrins that can be recycled to create new adhesions at the leading edge. 
   
  
 ITGA4 
 
  NCBI Gene  
  GeneRIF  
  Pubmed  
 
 
  Search Term  Rank  Fields  
  [Ff]ilopodia  1  PubMed(1)  
  [Mm]igration  6  Process(3); PubMed(58); GeneRIFs(14); Summary(1)  
  ECM  7  PubMed(5)  
  [Ee]xtracellular [Mm]atrix  8  Process(1); PubMed(25)  
  [Ii]nvasive  10  PubMed(4)  
  [Mm]etastasize  11  PubMed(1)  
  [Mm]etastasis  12  PubMed(15); GeneRIFs(3)  
  [Cc]ell [Ii]nvasion  13  PubMed(1)  
 
 
 hide/show details for ITGA4 
  [Ff]ilopodia  PubMed  
 Alpha 4 integrin signaling activates phosphatidylinositol 3-kinase and stimulates T cell adhesion to intercellular adhesion molecule-1 to a similar extent as CD3, but induces a distinct rearrangement of the actin cytoskeleton. Dynamic regulation of beta(2) integrin-dependent adhesion is critical for a wide array of T cell functions. We previously showed that binding of high-affinity alpha(4)beta(1) integrins to VCAM-1 strengthens alpha(L)beta(2) integrin-mediated adhesion to ICAM-1. In this study, we compared beta(2) integrin-mediated adhesion of T cells to ICAM-1 under two different functional contexts: alpha(4) integrin signaling during emigration from blood into tissues and CD3 signaling during adhesion to APCs and target cells. Cross-linking either alpha(4) integrin or CD3 on Jurkat T cells induced adhesion to ICAM-1 of comparable strength. Adhesion was dependent on phosphatidylinositol (PI) 3-kinase but not p44/42 mitogen-activated protein kinase (extracellular regulated kinase 1/2), because it was inhibited by wortmannin and LY294002 but not U0126. These data suggest that PI 3-kinase is a ubiquitous regulator of beta(2) integrin-mediated adhesion. A distinct morphological change consisting of Jurkat cell spreading and extension of   filopodia   was induced by alpha(4) integrin signaling. In contrast, CD3 induced radial rings of cortical actin polymerization. Inhibitors of PI 3-kinase and extracellular regulated kinase 1/2 did not affect alpha(4) integrin-induced rearrangement of the actin cytoskeleton, but treatment with ionomycin, a Ca(2+) ionophore, modulated cell morphology by reducing   filopodia   and promoting lamellipodia formation. Qualitatively similar morphological and adhesive changes to those observed with Jurkat cells were observed following alpha(4) integrin or CD3 stimulation of human peripheral blood T cells. 
  [Mm]igration  Process  
 cell-matrix adhesion involved in ameboidal cell   migration   
 leukocyte   migration   
 positive regulation of T cell   migration   
  PubMed  
 Binding of paxillin to alpha4 integrins modifies integrin-dependent biological responses. The alpha4 integrins are indispensable for embryogenesis, haematopoiesis and immune responses, possibly because alpha4 regulates cellular functions differently from other integrins through its cytoplasmic tail. We used novel mimics of the alpha4 tail to identify molecules that could account for alpha4-specific signalling. Here we report that the alpha4 tail, but not several other alpha-subunit tails, binds tightly to the signalling adaptor paxillin. Paxillin physically associated with alpha4 integrins in Jurkat T cells at high stoichiometry, and joining the alpha4 tail to alphaIIb resulted in a complex of integrin alphaIIbbeta3 with paxillin. This association markedly enhanced the rates of alphaIIbbeta3-dependent phosphorylation of focal adhesion kinase and cell   migration  . It also reduced cell spreading, focal adhesion and stress fibre formation. A point mutation within the alpha4 tail that disrupts paxillin binding reversed all of these effects. Furthermore, alpha4beta1-dependent adhesion to VCAM-1 led to spreading of mouse embryonic fibroblasts derived from paxillin-null but not from wild-type mice. Thus, the tight association of paxillin with the alpha4 tail leads to distinct biochemical and biological responses to integrin-mediated cell adhesion. 
 High affinity interaction of integrin alpha4beta1 (VLA-4) and vascular cell adhesion molecule 1 (VCAM-1) enhances   migration   of human melanoma cells across activated endothelial cell layers. The capacity of tumor cells to form metastatic foci correlates with their ability to interact with and migrate through endothelial cell layers. This process involves multiple adhesive interactions between tumor cells and the endothelium. Only little is known about the molecular nature of these interactions during extravasation of tumor cells. In human melanoma cells, the integrin alphavbeta3 is involved in transendothelial   migration   and its expression correlates with metastasis. However, many human melanoma cells do not express beta3 integrins. Therefore, it remained unclear how these cells undergo transendothelial   migration  . In this study we show that human melanoma cells with different metastatic potency, which do not express beta2 or beta3 integrins, express the VCAM-1 receptor alpha4beta1. VCAM-1 is up-regulated on activated endothelial cells and is known to promote transendothelial   migration   of leukocytes. Interestingly, despite comparable cell surface levels of alpha4beta1, only the highly metastatic melanoma cell lines MV3 and BLM, but not the low metastatic cell lines IF6 and 530, bind VCAM-1 with high affinity without further stimulation, and are therefore able to adhere to and migrate on isolated VCAM-1. Moreover, we demonstrate that function-blocking antibodies against the integrin alpha4beta1, as well as siRNA-mediated knock-down of the alpha4 subunit in these highly metastatic human melanoma cells reduce their transendothelial   migration  . These data imply that only high affinity interactions between the integrin alpha4beta1 on melanoma cells and VCAM-1 on activated endothelial cells may enhance the metastatic capacity of human beta2/beta3-negative melanoma cells. 
 CCL25 and CCL28 promote alpha4 beta7-integrin-dependent adhesion of lymphocytes to MAdCAM-1 under shear flow. Inflammatory bowel disease is characterized by the recruitment of lymphocytes to the gut via mucosal vessels. Chemokines are believed to trigger alpha(4)beta(1)- and alpha(4)beta(7)-integrin-mediated adhesion to vascular cell adhesion molecule-1 (VCAM-1) and mucosal addressin cell adhesion molecule-1 (MAdCAM-1) on mucosal vessels, although the contribution of each pathway and the chemokines involved are not well characterized. These interactions occur under conditions of hemodynamic shear, which is critical in determining how lymphocytes integrate chemokine signals to promote trans  migration  . To define the role of specific chemokines in mediating lymphocyte adhesion to VCAM-1 and MAdCAM-1, we studied the ability of immobilized chemokines to activate adhesion of human lymphocytes in a flow-based adhesion assay. Adhesion to immobilized MAdCAM-1 was alpha(4)beta(7) dependent, with no contribution from alpha(4)beta(1), whereas alpha(4)beta(1) mediated rolling and static adhesion on VCAM-1. Immobilized CC-chemokine ligand (CCL) 25 and CCL28 were both able to trigger alpha(4)beta(7)-dependent lymphocyte arrest on MAdCAM-1 under shear, highlighting a potential role for these chemokines in the arrest of lymphocytes on postcapillary venules in the gut. Neither had any effect on adhesion to VCAM-1, suggesting that they selectively trigger alpha(4)beta(7)-mediated adhesion. Immobilized CCL21, CCL25, CCL28, and CXC-chemokine ligand (CXCL) 12 all converted rolling adhesion to static arrest on MAdCAM-1 by activating lymphocyte integrins, but only CCL21 and CXCL12 also triggered a motile phenotype characterized by lamelipodia and uropod formation. Thus alpha(4)beta(1)/VCAM-1 and alpha(4)beta(7)/MAdCAM-1 operate independently to support lymphocyte adhesion from flow, and chemokines may act in concert with one chemokine triggering integrin-mediated arrest and a second chemokine promoting motility and transendothelial   migration  . 
 JAML, a novel protein with characteristics of a junctional adhesion molecule, is induced during differentiation of myeloid leukemia cells. Retinoic acid induces clinical remission in acute promyelocytic leukemia (APL) by triggering differentiation of leukemia promyelocytes. Here, we have characterized a gene encoding a member of the immunoglobulin superfamily, among novel retinoic acid-induced genes identified in APL cells. This protein, which was named JAML (junctional adhesion molecule-like), contains 2 extracellular immunoglobulin-like domains, a transmembrane segment, and a cytoplasmic tail. JAML mRNA is expressed in hematopoietic tissues and is prominently expressed in granulocytes. The fact that JAML protein is localized at the cell plasma membrane in the areas of cell-cell contacts, whereas it is not detected at free cell borders, suggests that JAML is engaged in homophilic interactions. Furthermore, a conserved dimerization motif among JAM members was shown to be important for JAML localization at the cell membrane. Finally, exogenous expression of JAML in myeloid leukemia cells resulted in enhanced cell adhesion to endothelial cells. Altogether, our results point to JAML as a novel member of the JAM family expressed on leukocytes with a possible role in leukocyte trans  migration  . 
 Integrins mediate adherence and   migration   of T lymphocytes on human peritoneal mesothelial cells. We previously showed that a local immune response largely composed of type 1 T cells correlated with a favorable outcome of the peritonitis associated with peritoneal dialysis. To clarify how these subsets are recruited to the peritoneal cavity during inflammation, we measured integrin-mediated interactions between the T cells and human peritoneal mesothelial cells. Direct microscopy showed that lipopolysaccharide or peritoneal dialysis effluent stimulated the adherence of T cells to mesothelial cells, a process mediated by the integrins alpha6beta1 and alpha4beta1. Further, the   migration   of Th1 cell across human mesothelial cell monolayers grown on transwell surfaces was reduced by anti-alpha6beta1 integrin antibody while that of Th2 cell was inhibited by an anti-alpha4 integrin antibody. Pretreatment with either lipopolysaccharide or rapid response peritoneal dialysis effluent stimulated T cell   migration   and this was significantly decreased by the alpha6beta1 compared to the alpha4 antibody. These results suggest that integrins may play an important role in mediating selective T cell subset adhesion and   migration   across human peritoneal mesothelial cell monolayers and differential integrin expression and selective T cell subset recruitment during peritonitis may affect outcome. 
 Analysis of the myosin-II-responsive focal adhesion proteome reveals a role for β-Pix in negative regulation of focal adhesion maturation. Focal adhesions undergo myosin-II-mediated maturation wherein they grow and change composition to modulate integrin signalling for cell   migratio  n, growth and differentiation. To determine how focal adhesion composition is affected by myosin II activity, we performed proteomic analysis of isolated focal adhesions and compared protein abundance in focal adhesions from cells with and without myosin II inhibition. We identified 905 focal adhesion proteins, 459 of which changed in abundance with myosin II inhibition, defining the myosin-II-responsive focal adhesion proteome. The abundance of 73% of the proteins in the myosin-II-responsive focal adhesion proteome was enhanced by contractility, including proteins involved in Rho-mediated focal adhesion maturation and endocytosis- and calpain-dependent focal adhesion disassembly. During myosin II inhibition, 27% of proteins in the myosin-II-responsive focal adhesion proteome, including proteins involved in Rac-mediated lamellipodial protrusion, were enriched in focal adhesions, establishing that focal adhesion protein recruitment is also negatively regulated by contractility. We focused on the Rac guanine nucleotide exchange factor β-Pix, documenting its role in the negative regulation of focal adhesion maturation and the promotion of lamellipodial protrusion and focal adhesion turnover to drive cel  l migrati  on. 
 Real-time analysis of very late antigen-4 affinity modulation by shear. Shear promotes endothelial recruitment of leukocytes, cell activation, and trans  migration  . Mechanical stress on cells caused by shear can induce a rapid integrin conformational change and activation, followed by an increase in binding to the extracellular matrix. The molecular mechanism of increased avidity is unknown. We have shown previously that the affinity of the alpha(4)beta(1) integrin, very late antigen-4 (VLA-4), measured with an LDV-containing small molecule, varies with cellular avidity, measured from cell disaggregation rates. In this study, we measured in real time affinity changes of VLA-4 in response to shear. The resulting affinity was comparable with the state mediated by receptor signaling and corresponded in time with intracellular Ca(2+) responses. Ca(2+) ionophores and N,N'-[1,2-ethanediyl-bis(oxy-2,1-phenylene)]bis[N-[2-[(acetyloxy)methoxy]-2-oxoethyl]]-, bis[(acetyloxy)methyl]ester demonstrate that the affinity regulation of VLA-4 in the presence of shear was related to Ca(2+) signaling. Pertussis toxin treatment implicates G(i) in an unknown pathway that connects shear, Ca(2+) elevation, VLA-4 affinity, and cell avidity. 
 Alpha4beta1 integrin and 190-kDa CD44v constitute a cell surface docking complex for gelatinase B/MMP-9 in chronic leukemic but not in normal B cells. As B-cell chronic lymphocytic leukemia (B-CLL) progresses, malignant cells extravasate and infiltrate lymphoid tissues. Several molecules, including gelatinase B/MMP-9, contribute to these processes. Although mainly a secreted protease, some MMP-9 is present at the B-CLL cell surface and the function, mode of anchoring, and interactions of this MMP-9 are unknown. Here we show that anti-MMP-9 antibodies immunoprecipitated a 190-kDa CD44v isoform and alpha4beta1 integrin from B-CLL cells, but not from normal B cells. Function-blocking antibodies to alpha4beta1 or CD44, or transfection with specific siRNAs, decreased cell-associated proMMP-9 and increased the secreted form. B-CLL cells attached to and bound proMMP-9 and active MMP-9, and this was inhibited by blocking the expression or function of alpha4beta1 or CD44. The MMP-9 hemopexin domain was critical in these interactions. alpha4beta1 and 190-kDa CD44v (but not CD44H) formed a complex at the cell surface, since they both coimmunoprecipitated with anti-alpha4, anti-beta1, or anti-CD44 antibodies. Immunofluorescence analyses confirmed that alpha4beta1 and CD44v colocalized with MMP-9. Binding of proMMP-9 inhibited B-CLL cell   migration  , and this required MMP-9 proteolytic activity. Thus, we have identified alpha4beta1 and CD44v as a novel proMMP-9 cell surface docking complex and show that cell-associated MMP-9 may regulate B-CLL cell   migration   and arrest. 
 VLA-4 blockade promotes differential routes into human CNS involving PSGL-1 rolling of T cells and MCAM-adhesion of TH17 cells. The focus of this study is the characterization of human T cell blood-brain barrier   migration   and corresponding molecular trafficking signatures. We examined peripheral blood and cerebrospinal fluid immune cells from patients under long-term anti-very late antigen-4 (VLA-4)/natalizumab therapy (LTNT) and from CNS specimens. LTNT patients' cerebrospinal fluid T cells exhibited healthy central-/effector-memory ratios, but lacked CD49d and showed enhanced myeloma cell adhesion molecule (MCAM) expression. LTNT led to an increase of PSGL-1 expression on peripheral T cells. Although vascular cell adhesion molecule-1 (VLA-4 receptor) was expressed at all CNS barriers, P-selectin (PSGL-1-receptor) was mainly detected at the choroid plexus. Accordingly, in vitro experiments under physiological flow conditions using primary human endothelial cells and LTNT patients' T cells showed increased PSGL-1-mediated rolling and residual adhesion, even under VLA-4 blockade. Adhesion of MCAM(+)/TH17 cells was not affected by VLA-4 blocking alone, but was abrogated when both VLA-4 and MCAM were inhibited. Consistent with these data, MCAM(+) cells were detected in white matter lesions, and in gray matter of multiple sclerosis patients. Our data indicate that lymphocyte trafficking into the CNS under VLA-4 blockade can occur by using the alternative adhesion molecules, PSGL-1 and MCAM, the latter representing an exclusive pathway for TH17 cells to migrate over the blood-brain barrier. 
 Vav1 and Rac control chemokine-promoted T lymphocyte adhesion mediated by the integrin alpha4beta1. The chemokine CXCL12 promotes T lymphocyte adhesion mediated by the integrin alpha4beta1. CXCL12 activates the GTPase Rac, as well as Vav1, a guanine-nucleotide exchange factor for Rac, concomitant with up-regulation of alpha4beta1-dependent adhesion. Inhibition of CXCL12-promoted Rac and Vav1 activation by transfection of dominant negative Rac or Vav1 forms, or by transfection of their siRNA, remarkably impaired the increase in T lymphocyte attachment to alpha4beta1 ligands in response to this chemokine. Importantly, inhibition of Vav1 expression by RNA interference resulted in a blockade of Rac activation in response to CXCL12. Adhesions in flow chambers and soluble binding assays using these transfectants indicated that initial ligand binding and adhesion strengthening mediated by alpha4beta1 were dependent on Vav1 and Rac activation by CXCL12. Finally, CXCL12-promoted T-cell transendothelial   migration   involving alpha4beta1-mediated adhesion was notably inhibited by expression of dominant negative Vav1 and Rac. These results indicate that activation of Vav1-Rac signaling pathway by CXCL12 represents an important inside-out event controlling efficient up-regulation of alpha4beta1-dependent T lymphocyte adhesion. 
 Proteomic analysis of α4β1 integrin adhesion complexes reveals α-subunit-dependent protein recruitment. Integrin adhesion receptors mediate cell-cell and cell-extracellular matrix interactions, which control cell morphology a  nd migrat  ion, differentiation, and tissue integrity. Integrins recruit multimolecular adhesion complexes to their cytoplasmic domains, which provide structural and mechanosensitive signaling connections between the extracellular and intracellular milieux. The different functions of specific integrin heterodimers, such as α4β1 and α5β1, have been attributed to distinct signal transduction mechanisms that are initiated by selective recruitment of adhesion complex components to integrin cytoplasmic tails. Here, we report the isolation of ligand-induced adhesion complexes associated with wild-type α4β1 integrin, an activated α4β1 variant in the absence of the α cytoplasmic domain (X4C0), and a chimeric α4β1 variant with α5 leg and cytoplasmic domains (α4Pα5L), and the cataloguing of their proteomes by MS. Using hierarchical clustering and interaction network analyses, we detail the differential recruitment of proteins and highlight enrichment patterns of proteins to distinct adhesion complexes. We identify previously unreported components of integrin adhesion complexes and observe receptor-specific enrichment of molecules with previously report  ed links   to cell migration and cell signaling processes. Furthermore, we demonstrate colocalization of MYO18A with active integrin in migrating cells. These datasets provide a resource for future studies of integrin receptor-specific signaling events. 
 Integrin alpha4beta1 signaling is required for lymphangiogenesis and tumor metastasis. Recent studies have shown that lymphangiogenesis or the growth of lymphatic vessels at the periphery of tumors promotes tumor metastasis to lymph nodes. We show here that the fibronectin-binding integrin alpha4beta1 and its ligand fibronectin are novel functional markers of proliferative lymphatic endothelium. Tumors and lymphangiogenic growth factors, such as vascular endothelial growth factor-C (VEGF-C) and VEGF-A, induce lymphatic vessel expression of integrin alpha4beta1. Integrin alpha4beta1 then promotes growth factor and tumor-induced lymphangiogenesis, as genetic loss of integrin alpha4beta1 expression in Tie2Cre+ alpha4(loxp/loxp) mice or genetic loss of alpha4 signaling in alpha4Y991A knock-in mice blocks growth factor and tumor-induced lymphangiogenesis, as well as tumor metastasis to lymph nodes. In addition, antagonists of integrin alpha4beta1 suppress lymphangiogenesis and tumor metastasis. Our studies show that integrin alpha4beta1 and the signals it transduces regulate the adhesion,   migration  , invasion, and survival of proliferating lymphatic endothelial cells. As suppression of alpha4beta1 expression, signal transduction, or function in tumor lymphatic endothelium not only inhibits tumor lymphangiogenesis but also prevents metastatic disease, these results show that integrin alpha4beta1-mediated tumor lymphangiogenesis promotes metastasis and is a useful target for the suppression of metastatic disease. 
 CD47 plays a critical role in T-cell recruitment by regulation of LFA-1 and VLA-4 integrin adhesive functions. CD47 plays an important but incompletely understood role in the innate and adaptive immune responses. CD47, also called integrin-associated protein, has been demonstrated to associate in cis with β1 and β3 integrins. Here we test the hypothesis that CD47 regulates adhesive functions of T-cell α4β1 (VLA-4) and αLβ2 (LFA-1) in in vivo and in vitro models of inflammation. Intravital microscopy studies reveal that CD47(-/-) Th1 cells exhibit reduced interactions with wild-type (WT) inflamed cremaster muscle microvessels. Similarly, murine CD47(-/-) Th1 cells, as compared with WT, showed defects in adhesion and   transmig  ration across tumor necrosis factor-α (TNF-α)-activated murine endothelium and in adhesion to immobilized intercellular adhesion molecule 1 (ICAM-1) and vascular cell adhesion protein 1 (VCAM-1) under flow conditions. Human Jurkat T-cells lacking CD47 also showed reduced adhesion to TNF-α-activated endothelium and ICAM-1 and VCAM-1. In cis interactions between Jurkat T-cell β2 integrins and CD47 were detected by fluorescence lifetime imaging microscopy. Unexpectedly, Jurkat CD47 null cells exhibited a striking defect in β1 and β2 integrin activation in response to Mn(2+) or Mg(2+)/ethylene glycol tetraacetic acid treatment. Our results demonstrate that CD47 associates with β2 integrins and is necessary to induce high-affinity conformations of LFA-1 and VLA-4 that recognize their endothelial cell ligands and support leukocyte adhesion and tran  sendothel  ial migration. 
 Sphingosine-1-phosphate activates chemokine-promoted myeloma cell adhesion and   migration   involving α4β1 integrin function. Myeloma cell adhesion dependent on α4β1 integrin is crucial for the progression of multiple myeloma (MM). The α4β1-dependent myeloma cell adhesion is up-regulated by the chemokine CXCL12, and pharmacological blockade of the CXCL12 receptor CXCR4 leads to defective myeloma cell homing to bone marrow (BM). Sphingosine-1-phosphate (S1P) regulates immune cell trafficking upon binding to G-protein-coupled receptors. Here we show that myeloma cells express S1P1, a receptor for S1P. We found that S1P up-regulated the α4β1-mediated myeloma cell adhesion and transendo  thelial m  igration stimulated by CXCL12. S1P promoted generation of high-affinity α4β1 that efficiently bound the α4β1 ligand VCAM-1, a finding that was associated with S1P-triggered increase in talin-β1 integrin association. Furthermore, S1P cooperated with CXCL12 for enhancement of α4β1-dependent adhesion strengthening and spreading. CXCL12 and S1P activated the DOCK2-Rac1 pathway, which was required for stimulation of myeloma cell adhesion involving α4β1. Moreover, in vivo analyses indicated that S1P contributes to optimizing the interactions of MM cells with the BM microvasculture and for their lodging inside the bone marrow. The regulation of α4β1-depe  ndent adh  esion and migration of myeloma cells by CXCL12-S1P combined activities might have important consequences for myeloma disease progression. 
 VLA-4 blockade promotes differential routes into human CNS involving PSGL-1 rolling of T cells and MCAM-adhesion of TH17 cells. The focus of this study is the characterization of human T cell blood-brain barrier   migration   and corresponding molecular trafficking signatures. We examined peripheral blood and cerebrospinal fluid immune cells from patients under long-term anti-very late antigen-4 (VLA-4)/natalizumab therapy (LTNT) and from CNS specimens. LTNT patients' cerebrospinal fluid T cells exhibited healthy central-/effector-memory ratios, but lacked CD49d and showed enhanced myeloma cell adhesion molecule (MCAM) expression. LTNT led to an increase of PSGL-1 expression on peripheral T cells. Although vascular cell adhesion molecule-1 (VLA-4 receptor) was expressed at all CNS barriers, P-selectin (PSGL-1-receptor) was mainly detected at the choroid plexus. Accordingly, in vitro experiments under physiological flow conditions using primary human endothelial cells and LTNT patients' T cells showed increased PSGL-1-mediated rolling and residual adhesion, even under VLA-4 blockade. Adhesion of MCAM(+)/TH17 cells was not affected by VLA-4 blocking alone, but was abrogated when both VLA-4 and MCAM were inhibited. Consistent with these data, MCAM(+) cells were detected in white matter lesions, and in gray matter of multiple sclerosis patients. Our data indicate that lymphocyte trafficking into the CNS under VLA-4 blockade can occur by using the alternative adhesion molecules, PSGL-1 and MCAM, the latter representing an exclusive pathway for TH17 cells to migrate over the blood-brain barrier. 
 CD47 plays a critical role in T-cell recruitment by regulation of LFA-1 and VLA-4 integrin adhesive functions. CD47 plays an important but incompletely understood role in the innate and adaptive immune responses. CD47, also called integrin-associated protein, has been demonstrated to associate in cis with β1 and β3 integrins. Here we test the hypothesis that CD47 regulates adhesive functions of T-cell α4β1 (VLA-4) and αLβ2 (LFA-1) in in vivo and in vitro models of inflammation. Intravital microscopy studies reveal that CD47(-/-) Th1 cells exhibit reduced interactions with wild-type (WT) inflamed cremaster muscle microvessels. Similarly, murine CD47(-/-) Th1 cells, as compared with WT, showed defects in adhesion and   transmig  ration across tumor necrosis factor-α (TNF-α)-activated murine endothelium and in adhesion to immobilized intercellular adhesion molecule 1 (ICAM-1) and vascular cell adhesion protein 1 (VCAM-1) under flow conditions. Human Jurkat T-cells lacking CD47 also showed reduced adhesion to TNF-α-activated endothelium and ICAM-1 and VCAM-1. In cis interactions between Jurkat T-cell β2 integrins and CD47 were detected by fluorescence lifetime imaging microscopy. Unexpectedly, Jurkat CD47 null cells exhibited a striking defect in β1 and β2 integrin activation in response to Mn(2+) or Mg(2+)/ethylene glycol tetraacetic acid treatment. Our results demonstrate that CD47 associates with β2 integrins and is necessary to induce high-affinity conformations of LFA-1 and VLA-4 that recognize their endothelial cell ligands and support leukocyte adhesion and tran  sendothel  ial migration. 
 Sphingosine-1-phosphate activates chemokine-promoted myeloma cell adhesion and   migration   involving α4β1 integrin function. Myeloma cell adhesion dependent on α4β1 integrin is crucial for the progression of multiple myeloma (MM). The α4β1-dependent myeloma cell adhesion is up-regulated by the chemokine CXCL12, and pharmacological blockade of the CXCL12 receptor CXCR4 leads to defective myeloma cell homing to bone marrow (BM). Sphingosine-1-phosphate (S1P) regulates immune cell trafficking upon binding to G-protein-coupled receptors. Here we show that myeloma cells express S1P1, a receptor for S1P. We found that S1P up-regulated the α4β1-mediated myeloma cell adhesion and transendo  thelial m  igration stimulated by CXCL12. S1P promoted generation of high-affinity α4β1 that efficiently bound the α4β1 ligand VCAM-1, a finding that was associated with S1P-triggered increase in talin-β1 integrin association. Furthermore, S1P cooperated with CXCL12 for enhancement of α4β1-dependent adhesion strengthening and spreading. CXCL12 and S1P activated the DOCK2-Rac1 pathway, which was required for stimulation of myeloma cell adhesion involving α4β1. Moreover, in vivo analyses indicated that S1P contributes to optimizing the interactions of MM cells with the BM microvasculture and for their lodging inside the bone marrow. The regulation of α4β1-depe  ndent adh  esion and migration of myeloma cells by CXCL12-S1P combined activities might have important consequences for myeloma disease progression. 
 Proteomic analysis of α4β1 integrin adhesion complexes reveals α-subunit-dependent protein recruitment. Integrin adhesion receptors mediate cell-cell and cell-extracellular matrix interactions, which control cell morphology a  nd migrat  ion, differentiation, and tissue integrity. Integrins recruit multimolecular adhesion complexes to their cytoplasmic domains, which provide structural and mechanosensitive signaling connections between the extracellular and intracellular milieux. The different functions of specific integrin heterodimers, such as α4β1 and α5β1, have been attributed to distinct signal transduction mechanisms that are initiated by selective recruitment of adhesion complex components to integrin cytoplasmic tails. Here, we report the isolation of ligand-induced adhesion complexes associated with wild-type α4β1 integrin, an activated α4β1 variant in the absence of the α cytoplasmic domain (X4C0), and a chimeric α4β1 variant with α5 leg and cytoplasmic domains (α4Pα5L), and the cataloguing of their proteomes by MS. Using hierarchical clustering and interaction network analyses, we detail the differential recruitment of proteins and highlight enrichment patterns of proteins to distinct adhesion complexes. We identify previously unreported components of integrin adhesion complexes and observe receptor-specific enrichment of molecules with previously report  ed links   to cell migration and cell signaling processes. Furthermore, we demonstrate colocalization of MYO18A with active integrin in migrating cells. These datasets provide a resource for future studies of integrin receptor-specific signaling events. 
 Integrin alpha4beta1 signaling is required for lymphangiogenesis and tumor metastasis. Recent studies have shown that lymphangiogenesis or the growth of lymphatic vessels at the periphery of tumors promotes tumor metastasis to lymph nodes. We show here that the fibronectin-binding integrin alpha4beta1 and its ligand fibronectin are novel functional markers of proliferative lymphatic endothelium. Tumors and lymphangiogenic growth factors, such as vascular endothelial growth factor-C (VEGF-C) and VEGF-A, induce lymphatic vessel expression of integrin alpha4beta1. Integrin alpha4beta1 then promotes growth factor and tumor-induced lymphangiogenesis, as genetic loss of integrin alpha4beta1 expression in Tie2Cre+ alpha4(loxp/loxp) mice or genetic loss of alpha4 signaling in alpha4Y991A knock-in mice blocks growth factor and tumor-induced lymphangiogenesis, as well as tumor metastasis to lymph nodes. In addition, antagonists of integrin alpha4beta1 suppress lymphangiogenesis and tumor metastasis. Our studies show that integrin alpha4beta1 and the signals it transduces regulate the adhesion,   migration  , invasion, and survival of proliferating lymphatic endothelial cells. As suppression of alpha4beta1 expression, signal transduction, or function in tumor lymphatic endothelium not only inhibits tumor lymphangiogenesis but also prevents metastatic disease, these results show that integrin alpha4beta1-mediated tumor lymphangiogenesis promotes metastasis and is a useful target for the suppression of metastatic disease. 
 Integrins mediate adherence and   migration   of T lymphocytes on human peritoneal mesothelial cells. We previously showed that a local immune response largely composed of type 1 T cells correlated with a favorable outcome of the peritonitis associated with peritoneal dialysis. To clarify how these subsets are recruited to the peritoneal cavity during inflammation, we measured integrin-mediated interactions between the T cells and human peritoneal mesothelial cells. Direct microscopy showed that lipopolysaccharide or peritoneal dialysis effluent stimulated the adherence of T cells to mesothelial cells, a process mediated by the integrins alpha6beta1 and alpha4beta1. Further, the   migration   of Th1 cell across human mesothelial cell monolayers grown on transwell surfaces was reduced by anti-alpha6beta1 integrin antibody while that of Th2 cell was inhibited by an anti-alpha4 integrin antibody. Pretreatment with either lipopolysaccharide or rapid response peritoneal dialysis effluent stimulated T cell   migration   and this was significantly decreased by the alpha6beta1 compared to the alpha4 antibody. These results suggest that integrins may play an important role in mediating selective T cell subset adhesion and   migration   across human peritoneal mesothelial cell monolayers and differential integrin expression and selective T cell subset recruitment during peritonitis may affect outcome. 
 Alpha4beta1 integrin and 190-kDa CD44v constitute a cell surface docking complex for gelatinase B/MMP-9 in chronic leukemic but not in normal B cells. As B-cell chronic lymphocytic leukemia (B-CLL) progresses, malignant cells extravasate and infiltrate lymphoid tissues. Several molecules, including gelatinase B/MMP-9, contribute to these processes. Although mainly a secreted protease, some MMP-9 is present at the B-CLL cell surface and the function, mode of anchoring, and interactions of this MMP-9 are unknown. Here we show that anti-MMP-9 antibodies immunoprecipitated a 190-kDa CD44v isoform and alpha4beta1 integrin from B-CLL cells, but not from normal B cells. Function-blocking antibodies to alpha4beta1 or CD44, or transfection with specific siRNAs, decreased cell-associated proMMP-9 and increased the secreted form. B-CLL cells attached to and bound proMMP-9 and active MMP-9, and this was inhibited by blocking the expression or function of alpha4beta1 or CD44. The MMP-9 hemopexin domain was critical in these interactions. alpha4beta1 and 190-kDa CD44v (but not CD44H) formed a complex at the cell surface, since they both coimmunoprecipitated with anti-alpha4, anti-beta1, or anti-CD44 antibodies. Immunofluorescence analyses confirmed that alpha4beta1 and CD44v colocalized with MMP-9. Binding of proMMP-9 inhibited B-CLL cell   migration  , and this required MMP-9 proteolytic activity. Thus, we have identified alpha4beta1 and CD44v as a novel proMMP-9 cell surface docking complex and show that cell-associated MMP-9 may regulate B-CLL cell   migration   and arrest. 
 High affinity interaction of integrin alpha4beta1 (VLA-4) and vascular cell adhesion molecule 1 (VCAM-1) enhances   migration   of human melanoma cells across activated endothelial cell layers. The capacity of tumor cells to form metastatic foci correlates with their ability to interact with and migrate through endothelial cell layers. This process involves multiple adhesive interactions between tumor cells and the endothelium. Only little is known about the molecular nature of these interactions during extravasation of tumor cells. In human melanoma cells, the integrin alphavbeta3 is involved in transendothelial   migration   and its expression correlates with metastasis. However, many human melanoma cells do not express beta3 integrins. Therefore, it remained unclear how these cells undergo transendothelial   migration  . In this study we show that human melanoma cells with different metastatic potency, which do not express beta2 or beta3 integrins, express the VCAM-1 receptor alpha4beta1. VCAM-1 is up-regulated on activated endothelial cells and is known to promote transendothelial   migration   of leukocytes. Interestingly, despite comparable cell surface levels of alpha4beta1, only the highly metastatic melanoma cell lines MV3 and BLM, but not the low metastatic cell lines IF6 and 530, bind VCAM-1 with high affinity without further stimulation, and are therefore able to adhere to and migrate on isolated VCAM-1. Moreover, we demonstrate that function-blocking antibodies against the integrin alpha4beta1, as well as siRNA-mediated knock-down of the alpha4 subunit in these highly metastatic human melanoma cells reduce their transendothelial   migration  . These data imply that only high affinity interactions between the integrin alpha4beta1 on melanoma cells and VCAM-1 on activated endothelial cells may enhance the metastatic capacity of human beta2/beta3-negative melanoma cells. 
 Vav1 and Rac control chemokine-promoted T lymphocyte adhesion mediated by the integrin alpha4beta1. The chemokine CXCL12 promotes T lymphocyte adhesion mediated by the integrin alpha4beta1. CXCL12 activates the GTPase Rac, as well as Vav1, a guanine-nucleotide exchange factor for Rac, concomitant with up-regulation of alpha4beta1-dependent adhesion. Inhibition of CXCL12-promoted Rac and Vav1 activation by transfection of dominant negative Rac or Vav1 forms, or by transfection of their siRNA, remarkably impaired the increase in T lymphocyte attachment to alpha4beta1 ligands in response to this chemokine. Importantly, inhibition of Vav1 expression by RNA interference resulted in a blockade of Rac activation in response to CXCL12. Adhesions in flow chambers and soluble binding assays using these transfectants indicated that initial ligand binding and adhesion strengthening mediated by alpha4beta1 were dependent on Vav1 and Rac activation by CXCL12. Finally, CXCL12-promoted T-cell transendothelial   migration   involving alpha4beta1-mediated adhesion was notably inhibited by expression of dominant negative Vav1 and Rac. These results indicate that activation of Vav1-Rac signaling pathway by CXCL12 represents an important inside-out event controlling efficient up-regulation of alpha4beta1-dependent T lymphocyte adhesion. 
 Real-time analysis of very late antigen-4 affinity modulation by shear. Shear promotes endothelial recruitment of leukocytes, cell activation, and trans  migration  . Mechanical stress on cells caused by shear can induce a rapid integrin conformational change and activation, followed by an increase in binding to the extracellular matrix. The molecular mechanism of increased avidity is unknown. We have shown previously that the affinity of the alpha(4)beta(1) integrin, very late antigen-4 (VLA-4), measured with an LDV-containing small molecule, varies with cellular avidity, measured from cell disaggregation rates. In this study, we measured in real time affinity changes of VLA-4 in response to shear. The resulting affinity was comparable with the state mediated by receptor signaling and corresponded in time with intracellular Ca(2+) responses. Ca(2+) ionophores and N,N'-[1,2-ethanediyl-bis(oxy-2,1-phenylene)]bis[N-[2-[(acetyloxy)methoxy]-2-oxoethyl]]-, bis[(acetyloxy)methyl]ester demonstrate that the affinity regulation of VLA-4 in the presence of shear was related to Ca(2+) signaling. Pertussis toxin treatment implicates G(i) in an unknown pathway that connects shear, Ca(2+) elevation, VLA-4 affinity, and cell avidity. 
 Association between α4 integrin cytoplasmic tail and non-muscle myosin IIA regulates cell   migratio  n. α4β1 integrin regulates ce  ll migrat  ion via cytoplasmic interactions. Here, we report an association between the cytoplasmic tail of α4 integrin (α4 tail) and non-muscle myosin IIA (MIIA), demonstrated by co-immunoprecipitation of the MIIA heavy chain (HC) with anti-α4-integrin antibodies and pull-down of MIIA-HC with recombinant α4 tail from cell lysates. The association between the α4 tail and MIIA does not require paxillin binding or phosphorylation at Ser988 in the α4 tail. We found that substituting Glu982 in the α4 tail with alanine (E982A) disrupts the α4-MIIA association without interfering with the paxillin binding or Ser988 phosphorylation. By comparing stably transfected CHO cells, we show that the E982A mutation reduces the ability of α4β1 integrin to mediate cell spreading and to promote front-back polarization. In addition, we show that E982A impairs shear-  flow-indu  ced migration of the α4-integrin-expressing CHO cells by r  educing t  heir migration speed and directional persistence. The E982A mutation also leads to defects in the organization of MIIA filament bundles. Furthermore, when cells are plated on fibronectin and simulated with shear flow, α4β1 integrin forms filament-like patterns that co-align with MIIA filament bundles. These results provide a new mechanism for linking integrins to the actomyosin cytoskeleton and for   regulatin  g cell migration by integrins and non-muscle myosin II. 
 Paxillin binding to the alpha 4 integrin subunit stimulates LFA-1 (integrin alpha L beta 2)-dependent T cell   migration   by augmenting the activation of focal adhesion kinase/proline-rich tyrosine kinase-2. Engagement of very late Ag-4 (integrin alpha(4)beta(1)) by ligands such as VCAM-1 markedly stimulates leukocyte   migration   mediated by LFA-1 (integrin alpha(L)beta(2)). This form of integrin trans-regulation in T cells requires the binding of paxillin to the alpha(4) integrin cytoplasmic domain. This conclusion is based on the abolition of trans-regulation in Jurkat T cells by an alpha(4) mutation (alpha(4)(Y991A)) that disrupts paxillin binding. Furthermore, cellular expression of an alpha(4)-binding fragment of paxillin that blocks the alpha(4)-paxillin interaction, selectively blocked VCAM-1 stimulation of alpha(L)beta(2)-dependent cell   migration  . The alpha(4)-paxillin association mediates trans-regulation by enhancing the activation of tyrosine kinases, focal adhesion kinase (FAK) and/or proline-rich tyrosine kinase-2 (Pyk2), based on two lines of evidence. First, disruption of the paxillin-binding site in the alpha(4) tail resulted in much less alpha(4)beta(1)-mediated phosphorylation of Pyk2 and FAK. Second, transfection with cDNAs encoding C-terminal fragments of Pyk2 and FAK, which block the function of the intact kinases, blocked alpha(4)beta(1) stimulation of alpha(L)beta(2)-dependent   migration  . These results define a proximal protein-protein interaction of an integrin cytoplasmic domain required for trans-regulation between integrins, and establish that augmented activation of Pyk2 and/or FAK is an immediate signaling event required for the trans-regulation of integrin alpha(L)beta(2) by alpha(4)beta(1). 
 Integrin alpha 4 beta 1-dependent T cell   migration   requires both phosphorylation and dephosphorylation of the alpha 4 cytoplasmic domain to regulate the reversible binding of paxillin. alpha 4 integrins mediate increased cell   migration   and decreased cell spreading because the alpha 4 cytoplasmic domain (tail) binds tightly to paxillin, a signaling adaptor protein. Paxillin binding to the alpha 4 tail is blocked by alpha 4 phosphorylation at Ser988. To establish the biological role of alpha 4 phosphorylation, we reconstituted alpha 4-deficient Jurkat T cells with phosphorylation-mimicking (alpha 4(S988D)) or non-phosphorylatable (alpha 4(S988A)) mutants. alpha 4(S988D) disrupted paxillin binding and also inhibited cell   migration   and promoted cell spreading. In contrast, the non-phosphorylatable alpha 4(S988A) resulted in a further reduction in cell spreading; however, this mutation led to an unexpected suppression of cell   migration  . The suppression of cell   migration   by alpha 4(S988A) was ascribable to enhanced alpha 4-paxillin association, because enforced association by an alpha 4-paxillin fusion led to a phenotype similar to that of the non-phosphorylatable alpha 4(S988A) mutant. These data establish that optimal alpha 4-mediated cell   migration   requires both phosphorylation and dephosphorylation of the alpha 4 cytoplasmic domain to regulate the reversible binding of paxillin. 
 Microenvironmental Interaction Between Hypoxia and Endothelial Cells Controls the   Migration   Ability of Placenta-Derived Mesenchymal Stem Cells via α4 Integrin and Rho Signaling. Mesenchymal stem cells (MSCs) are a powerful source for cell therapy in degenerative diseases. The   migratio  n ability of MSCs is an important factor that enhances the therapeutic effect of the cells when they are transplanted into target tissues or organs. Hypoxia and the endothelial barrier, which are representative   migratio  n microenvironmental factors, are known to be regulated by the integrin-mediated pathway in several cancers. However, their regulatory mechanisms in MSCs remain unclear. Here, the objectives of the study were to compare the expression of markers related to integrin-mediated signaling in placenta-derived MSCs (PDMSCs) dependent on hypoxia and co-cultured with human umbilical vein endothelial cells (HUVECs) and to evaluate their correlations between   migratio  n ability and microenvironmetal factors including hypoxia and endothelial cells. The   migratio  n abilities of PDMSCs exposed to hypoxic conditions were significantly increased compared with normal fibroblasts (WI-38) and control (P < 0.05). Interestingly, decreased integrin α4 in PDMSCs under hypoxia induce to inc  rease mig  ration abilities of PDMSCs. Also, Rho family-related markers were significantly increased in PDMSCs under hypoxic conditions compared with normoxia (P < 0.05). Further  more, the   migration ability of PDMSCs was decreased by Rho kinase inhibitor treatment (Y-27632) and co-culturing with HUVECs in an ex vivo system. ROCK activity was increased by inhibiting integrin α4 with HUVECs and hypoxia compared with the absence of HUVECs and under normoxia. The findings suggest microenvironment event by hypoxia and the interaction with endothelial cells may be useful as a regula  tor of MS  C migration and provide insight into the migratory mechanism of MSCs in stem cell-based therapy. 
 Ligation of lymphocyte function-associated antigen-1 on monocytes decreases very late antigen-4-mediated adhesion through a reactive oxygen species-dependent pathway. Monocyte-endothelial adhesion plays an important role in monocyte trafficking and hence is important for immune responses and pathogenesis of inflammatory diseases including atherosclerosis. The cross-talk between different integrins on monocytes may be crucial for a coordinated regulation of the cellular adhesion during the complex process of transendothelial   migration  . By using monoclonal antibodies and recombinant intercellular adhesion molecule 1 (ICAM-1) to engage lymphocyte function-associated antigen 1 (LFA-1) on monocytic cells, we found that the cellular adhesion to vascular cell adhesion molecule 1 (VCAM-1) mediated by very late antigen 4 (VLA-4) was suppressed after this treatment and the suppression depended on the presence of reactive oxygen species (ROSs). Inhibition of production of ROSs through the use of inhibitor of the nicotinamide adenine dinucleotide phosphate (NADPH) oxidase, but not inhibitors of mitochondrial electron transport chain or xanthine oxidase, revealed that this suppression on VLA-4-mediated cellular binding was mediated by ROSs produced by phagocyte NADPH oxidase. Activation of phosphoinositol-3 kinase and Akt appears to mediate this NADPH oxidase activation through p47phox phosphorylation and Rac-1 activation. Our results provide a novel pathway in which ROSs play a critical role in integrin cross-talk in monocytes. This signaling pathway may be important for cellular transition from firm arrest to diapedesis during monocyte trafficking. 
 SDF-1/CXCR4 and VLA-4 interaction regulates homing in Waldenstrom macroglobulinemia. Waldenstrom macroglobulinemia (WM) is characterized by widespread involvement of the bone marrow at the time of diagnosis, implying continuous homing of WM cells into the marrow. The mechanisms by which trafficking of the malignant cells into the bone marrow has not been previously elucidated. In this study, we show that WM cells express high levels of chemokine and adhesion receptors, including CXCR4 and VLA-4. We showed that CXCR4 was essential for the   migration   and trans-endothelial   migration   of WM cells under static and dynamic shear flow conditions, with significant inhibition of   migration   using CXCR4 knockdown or the CXCR4 inhibitor AMD3100. Similarly, CXCR4 or VLA-4 inhibition led to significant inhibition of adhesion to fibronectin, stromal cells, and endothelial cells. Decreased adhesion of WM cells to stromal cells by AMD3100 led to increased sensitivity of these cells to cytotoxicity by bortezomib. To further investigate the mechanisms of CXCR4-dependent adhesion, we showed that CXCR4 and VLA-4 directly interact in response to SDF-1, we further investigated downstream signaling pathways regulating   migration   and adhesion in WM. Together, these studies demonstrate that the CXCR4/SDF-1 axis interacts with VLA-4 in regulating   migration   and adhesion of WM cells in the bone marrow microenvironment. 
 Alpha 4 integrin signaling activates phosphatidylinositol 3-kinase and stimulates T cell adhesion to intercellular adhesion molecule-1 to a similar extent as CD3, but induces a distinct rearrangement of the actin cytoskeleton. Dynamic regulation of beta(2) integrin-dependent adhesion is critical for a wide array of T cell functions. We previously showed that binding of high-affinity alpha(4)beta(1) integrins to VCAM-1 strengthens alpha(L)beta(2) integrin-mediated adhesion to ICAM-1. In this study, we compared beta(2) integrin-mediated adhesion of T cells to ICAM-1 under two different functional contexts: alpha(4) integrin signaling during e  migration   from blood into tissues and CD3 signaling during adhesion to APCs and target cells. Cross-linking either alpha(4) integrin or CD3 on Jurkat T cells induced adhesion to ICAM-1 of comparable strength. Adhesion was dependent on phosphatidylinositol (PI) 3-kinase but not p44/42 mitogen-activated protein kinase (extracellular regulated kinase 1/2), because it was inhibited by wortmannin and LY294002 but not U0126. These data suggest that PI 3-kinase is a ubiquitous regulator of beta(2) integrin-mediated adhesion. A distinct morphological change consisting of Jurkat cell spreading and extension of filopodia was induced by alpha(4) integrin signaling. In contrast, CD3 induced radial rings of cortical actin polymerization. Inhibitors of PI 3-kinase and extracellular regulated kinase 1/2 did not affect alpha(4) integrin-induced rearrangement of the actin cytoskeleton, but treatment with ionomycin, a Ca(2+) ionophore, modulated cell morphology by reducing filopodia and promoting lamellipodia formation. Qualitatively similar morphological and adhesive changes to those observed with Jurkat cells were observed following alpha(4) integrin or CD3 stimulation of human peripheral blood T cells. 
 The chemokine receptor CCR7 and alpha4 integrin are important for   migration   of chronic lymphocytic leukemia cells into lymph nodes. Malignant lymphocyte   migration   into lymph nodes is an important aspect of chronic lymphocytic leukemia (CLL), yet little is known about the processes involved. Here we demonstrate that CLL cells migrate across vascular endothelium in response to at least 3 chemokines, namely, CCL21, CCL19, and CXCL12. Moreover, transendothelial cell   migration   (TEM) in response to CCL21 and CCL19 was significantly higher for the malignant B cells of patients who had clinical lymph node involvement as compared with those of patients lacking such organomegaly. Furthermore, the expression of CCR7, the receptor for both CCL21 and CCL19, correlated with clinical lymphadenopathy, and blocking of CCR7 inhibited CLL cell TEM. By using immunohistochemistry we demonstrated that CCL21 and CCL19, but not CXCL12, are located in high endothelial venules and are, therefore, in an appropriate location to induce TEM. Regarding the adhesion receptors involved in TEM, alpha4 (most likely in association with beta1) and alphaLbeta2 were shown to be important in CLL cell TEM in vitro, but only the level of alpha4 expression correlated with the presence of clinical lymphadenopathy. The present studies are the first to shed light on the factors determining CLL cell entry into nodes and define the phenotype of circulating malignant cells likely to determine the pattern of lymph node enlargement in the disease. 
 VLA-4-mediated interactions between normal human hematopoietic progenitors and stromal cells. The alpha 4 beta 1 integrin very late activation antigen-4 (VLA-4) has been implicated to play a role in the adhesive interactions between hematopoietic progenitor cells (HPC) and bone marrow stromal cells which express the vascular cell adhesion molecule-1 (VCAM-1) or produce fibronectin (FN). Here, we summarize some of the recent advances made in the elucidation of the role of these particular adhesive interactions for the regulation of normal hematopoiesis. HPC bind to stroma mainly through VLA-4/VCAM-1 interactions. There is evidence which suggests that more primitive HPC constitutively express VLA-4 in a high-affinity state. In vitro studies in the mouse have shown that monoclonal antibodies (mAb) against VLA-4 partly block the development of lymphocytes, myelopoietic cells, and erythropoiesis, whereas in the human system outgrowth of TdT+ B cells is severely retarded by such mAb. In vivo studies revealed that VLA-4 is involved in erythropoietic development, and is particularly important for homing and lodgement of HPC in the bone marrow. Hematopoiesis in mice with deficient expression of alpha 4 integrin or VLA-4's ligand VCAM-1 appears to develop normally. However, chimeras developed from wild-type blastocysts and beta 1 -/- embryonic stem cells do not contain beta 1 -/- hematopoietic cells, although these are present as blood islands in the yolk sac. These beta 1 -/- hematopoietic cells are capable of forming colonies, indicating that beta 1-integrin is not involved in hematopoietic differentiation, but is primarily important for   migration   of hematopoietic cells into the fetal hematopoietic organs. In addition to the role of VLA-4 in   migration  , it may also have other regulatory functions. It has been demonstrated that ligation of VLA-4 induces phosphorylation of the protein tyrosine kinase (PTK) pp125FAK as well as other proteins which may be involved in the regulation of ligand affinity. Indeed, it has been shown that tyrosine kinase-dependent stimulation of CD34+ hematopoietic cell lines with c-kit ligand (KL), IL-3 or GM-CSF transiently activates the ability of VLA-4 to bind to VCAM-1 or FN. These events are most probably involved in the induction of quiescence in HPC which adhere to stromal cells. This claim was recently substantiated: when HPC were treated with Fab fragments of an anti-VLA-4 mAb, entry into S-phase of the cell cycle was prevented. Taken together, the present data point to a role for VLA-4 in HPC   migration  , cell cycle regulation, erythropoiesis and B-lymphopoiesis. Moreover, these insights may explain how defects in adhesive behavior of leukemic HPC through VLA-4 contribute to their dysregulated growth and provide a rationale for therapeutically correcting those defects. 
 Phosphorylation of the integrin alpha 4 cytoplasmic domain regulates paxillin binding. alpha4 integrins are essential for embryogenesis, hematopoiesis, inflammation, and immune response possibly because alpha4 integrins have distinct signaling properties from other integrins. Specifically, the alpha4 cytoplasmic domain binds tightly to paxillin, a signaling adaptor protein, leading to increased cell   migration   and an altered cytoskeletal organization that results in reduced cell spreading. The alpha4 tail contains potential phosphorylation sites clustered in its core paxillin binding region. We now report that the alpha4 tail is phosphorylated in vitro and in vivo. Furthermore, Ser(988) is a major phosphorylation site. Using antibodies specific for Ser(988)-phosphorylated alpha4, we found the stoichiometry of alpha4 phosphorylation varied in different cells. However, >60% of alpha4 was phosphorylated in Jurkat T cells. Phosphorylation at Ser(988) blocked paxillin binding to the alpha4 tail. A phosphorylation-mimicking mutant of alpha4 (alpha4S988D) blocked paxillin binding and reversed the inhibitory effect of alpha4 on cell spreading. Consequently, alpha4 phosphorylation is a biochemical mechanism to modulate paxillin binding to alpha4 integrins with consequent regulation of alpha4 integrin-dependent cellular functions. 
 Integrin α4 Enhances Metastasis and May Be Associated with Poor Prognosis in MYCN-low Neuroblastoma. High-risk neuroblastoma is associated with an overall survival rate of 30-50%. Neuroblastoma-expressed cell adhesion receptors of the integrin family impact cell adhesion,   migratio  n, proliferation and survival. Integrin α4 is essential for neural crest cell motility during development, is highly expressed on leukocytes, and is critical for transendothelia  l migrati  on. Thus, cancer cells that express this receptor may exhibit increased metastatic potential. We show that α4 expression in human and murine neuroblastoma cell lines selectively enhances in vitro interaction with the alternatively spliced connecting segment 1 of fibronectin, as well as vascular cell adhesion molecule-1 and increas  es migrat  ion. Integrin α4 expression enhanced experimental metastasis in a syngeneic tumor model, reconstituting a pattern of organ involvement similar to that seen in patients. Accordingly, antagonism of integrin α4 blocked metastasis, suggesting adhesive function of the integrin is required. However, adhesive function was not sufficient, as mutants of integrin α4 that conserved the matrix-adhesive and promigratory function in vitro were compromised in their metastatic capacity in vivo. Clinically, integrin α4 is more frequently expressed in non-MYNC amplified tumors, and is selectively associated with poor prognosis in this subset of disease. These results reveal an unexpected role for integrin α4 in neuroblastoma dissemination and identify α4 as a potential prognostic indicator and therapeutic target. 
 B-cell very late antigen-4 deficiency reduces leukocyte recruitment and susceptibility to central nervous system autoimmunity. Natalizumab, which binds very late antigen-4 (VLA-4), is a potent therapy for multiple sclerosis (MS). Studies have focused primarily upon its capacity to interfere with T-cell   migration   into the central nervous system (CNS). B cells are important in MS pathogenesis and express high levels of VLA-4. Here, we report that the selective inhibition of VLA-4 expression on B cells impedes CNS accumulation of B cells, and recruitment of Th17 cells and macrophages, and reduces susceptibility to experimental autoimmune encephalomyelitis. These results underscore the importance of B-cell VLA-4 expression in the pathogenesis of CNS autoimmunity and provide insight regarding mechanisms that may contribute to the benefit of natalizumab in MS, as well as candidate therapeutics that selectively target B cells. 
 Guanylate binding protein-1 inhibits spreading and   migration   of endothelial cells through induction of integrin alpha4 expression. Human guanylate binding protein-1 (GBP-1) is a large GTPase that is induced by inflammatory cytokines and acts antiangiogenically through the inhibition of endothelial cell proliferation and   migration  . In this study, we detected that GBP-1-expressing cells show a significantly reduced spreading and   migration   on fibronectin matrices. Investigating possible mechanisms of these effects, we found that integrin alpha(4) (ITGA4) was consistently up-regulated at both the RNA and protein level in GBP-1-expressing cell cultures. Inhibition of cell spreading and   migration   by GBP-1 was dependent on the binding of ITGA4 to fibronectin. The inflammatory cytokines IL-1beta and TNF-alpha induced ITGA4 expression in HUVECs and inhibited spreading and   migration  . Knockdown of GBP-1 by shRNA abrogated inflammatory cytokine induced ITGA4 expression and restored spreading and   migration   capabilities of the cells. These results show that inhibition of endothelial cell spreading and   migration   by inflammatory cytokines is mediated by GBP-1 through induction of ITGA4 expression. Endothelial cell   migration   is a key process during angiogenesis. Therefore, ITGA4 may be a novel molecular target to modulate angiogenesis in human disease. 
 CD4(+)CD25(High) Treg cells in HIV/HTLV co-infected patients with neuropathy: high expression of Alpha4 integrin and lower expression of Foxp3 transcription factor. Regulatory CD4 T cells (Tregs) are critical in maintaining the homeostasis of the immune system. Quantitative or phenotypic alterations and functional impairment of Tregs have been associated with the development of pathologies including those of the central nervous system. Individuals with HIV-1/HTLV-1 co-infection are more prone to develop neurological complications. The aim of this study was to characterize phenotypically Treg cells in HIV-1/HTLV-1 co-infected Mozambican individuals presenting neurological symptoms.A cross-sectional study was conducted among HIV-infected individuals presentingneurological symptoms, with and without HTLV co-infection, and blood donors. Peripheral bloodmononuclear cells were stained with monoclonal antibodies conjugated with fluorochromes to quantifyTregs and activated T cells by four colors flow cytometry.Higher Treg cell frequency (10.6%) was noted in HIV-1/HTLV-1 co-infected group with neurological symptoms when compared to HIV-1 mono-infected group with neurological symptoms (0.38%, p = 0.003) and control group (0.9%, p = 0.0105). An inverse correlation between Foxp3 and CD49d expression was observed in all study groups (rh = -0.71, p = 0.001). In addition, increased levels of Treg cells in co-infected patients were strongly associated with total activated CD4 T cells (rh = 0.8, p = 0.01).Treg cells in co-infected patients present phenotypic alterations and might have dysfunction marked by low expression of Foxp3 and increased expression of molecules not frequently seen on Treg cells, such as CD49d. These alterations may be related to (1) changes in Tr  eg cell t  rafficking and migration, possibly making those cells susceptible to HIV infection, and (2) inability to control the activation and proliferation of effector T lymphocytes. 
 Aberrant DNA methylation of integrin alpha4: a potential novel role for metastasis of cholangiocarcinoma. Although the altered expression of integrin alpha4 is known to be associated with transformation or metastasis in several human cancers, the information on cholangiocarcinoma (CC) is still poor. In this study, we investigated the promoter methylation status of integrin alpha4 gene in CC.A total of 29 CC, 19 adjacent non-tumor-containing tissue and 15 normal liver specimens were used for identification of gene methylation status by methylation-specific polymerase chain reaction.The frequency of DNA methylation was 55.17% (16 of 29) in the CC specimens (P < 0.001). Also, transcripts of the integrin alpha4 gene were decreased in all CC tissues in which there was DNA methylation of the integrin alpha4 gene. In addition, the downregulated expression of integrin alpha4 in CC cells with hypermethylation of the integrin alpha4 gene was restored by treatment with 5-aza-2'-deoxycytidine, a DNA methyltransferase inhibitor. Moreover, we found that DNA methylation of integrin alpha4 was detected in all CC tissues obtained from patients with LN metastasis (7/7). Furthermore, phosphorylation of paxillin, cell   migration  -related molecule, was regulated by silencing of integrin alpha4.Taken together, these results suggest that loss of the integrin alpha4 gene is caused by aberrant DNA methylation of the 5'-CpG island site of the gene, and methylation of the integrin alpha4 gene can be a useful marker of metastasis of CC. 
 Fibulin-5 binds human smooth-muscle cells through alpha5beta1 and alpha4beta1 integrins, but does not support receptor activation. Fibulin-5, an extracellular matrix glycoprotein expressed in elastin-rich tissues, regulates vascular cell behaviour and elastic fibre deposition. Recombinant full-length human fibulin-5 supported primary human aortic SMC (smooth-muscle cell) attachment through alpha5beta1 and alpha4beta1 integrins. Cells on fibulin-5 spread poorly and displayed prominent membrane ruffles but no stress fibres or focal adhesions, unlike cells on fibronectin that also binds these integrins. Cell   migration   and proliferation were significantly lower on fibulin-5 than on fibronectin. Treatment of cells on fibulin-5 with a beta1 integrin-activating antibody induced stress fibres, increased attachment,   migration   and proliferation, and stimulated signalling of epidermal growth factor receptor and platelet-derived growth factor receptors alpha and beta. Fibulin-5 also modulated fibronectin-mediated cell spreading and morphology. We have thus identified the beta1 integrins on primary SMCs that fibulin-5 interacts with, and have shown that failure of fibulin-5 to activate these receptors limits cell spreading,   migration   and proliferation. 
 An integrin alpha4beta7•IgG heterodimeric chimera binds to MAdCAM-1 on high endothelial venules in gut-associated lymphoid tissue. Lymphocyte homing is regulated by a multistep process mediated by sequential adhesive interactions between circulating lymphocytes and high endothelial venules (HEVs). In gut-associated lymphoid tissue (GALT), the initial interactive step, "tethering and rolling," is partly mediated by integrin α4β7 expressed on GALT-homing lymphocytes and its ligand MAdCAM-1, which is exclusively expressed on HEVs in GALT. To probe functional MAdCAM-1 in tissue sections, we developed a soluble integrin α4β7 heterodimeric IgG chimera by joining the extracellular region of mouse integrin α4 and β7 subunits to a human IgG Fc domain. Western blot analysis revealed that co-transfection of HEK 293T cells with expression vectors encoding integrin α4•IgG and β7•IgG results in the formation of α4β7•IgG heterodimeric chimeras. This complex preferentially binds to CHO cells expressing MAdCAM-1 and, to a lesser extent, to cells expressing VCAM-1, but not to cells expressing ICAM-1. Moreover, α4β7•IgG specifically binds to HEVs in GALT in situ in a divalent cation-dependent fashion and inhibits lymphocyte binding to HEVs in GALT. These findings indicate that α4β7•IgG can be used as a probe for functional MAdCAM-1 expressed on HEVs in GALT and could potentially serve as an anti-inflammatory drug inhibiting   GALT-spe  cific lymphocyte migration. 
 TAp63 regulates VLA-4 expression and chronic lymphocytic leukemia cell   migration   to the bone marrow in a CD74-dependent manner. The hallmark of chronic lymphocytic leukemia (CLL) is the relentless accumulation of mature lymphocytes, mostly due to their decreased apoptosis. CD74 was recently shown to serve as a survival receptor on CLL cells. In this study, we show that stimulation of CD74 with its natural ligand,   migration   inhibitory factor, initiates a signaling cascade that results in upregulation of TAp63, which directly regulates CLL survival. In addition, TAp63 expression elevates the expression of the integrin VLA-4, particularly during the advanced stage of the disease. Blocking of CD74, TAp63, or VLA-4 inhibits the in vivo homing of CLL cells to the bone marrow (BM). Thus, CD74 and its target genes TAp63 and VLA-4 facilitate   migration   of CLL cells back to the BM, where they interact with the supportive BM environment that rescues them from apoptosis. These results could form the basis of novel therapeutic strategies aimed at blocking homing of CLL cells in their return to the BM and attenuating their survival. 
 Activated integrin VLA-4 localizes to the lamellipodia and mediates T cell   migration   on VCAM-1. Lymphocyte   migration   from blood into lymphoid tissues or to sites of inflammation occurs through interactions between cell surface integrins and their ligands expressed on the vascular endothelium and the extracellular matrix. VLA-4 (alpha(4)beta(1)) is a key integrin in the effective trafficking of lymphocytes. Although it has been well established that integrins undergo functionally significant conformational changes to mediate cell adhesion, there is no mechanistic information that explains how these are dynamically and spatially regulated during lymphocyte polarization and   migration  . Using dynamic fluorescence resonance energy transfer analysis of a novel VLA-4 FRET sensor under total internal reflection fluorescence microscopy, we show that VLA-4 activation localizes to the lamellipodium in living cells. During T cell   migration   on VCAM-1, VLA-4 activation concurs with spatial redistribution of chemokine receptor and active Rap1 at the leading edge. Selective inhibition of the activated VLA-4 at the leading edge with a small molecule inhibitor is sufficient to block T cell   migration  . These data suggest that a subpopulation of activated VLA-4 is mainly localized to the leading edge of polarized human T cells and is critical for T cell   migration   on VCAM-1. 
 Integrin alpha4beta1 promotes focal adhesion kinase-independent cell motility via alpha4 cytoplasmic domain-specific activation of c-Src. The fibronectin binding integrins alpha5beta1 and alpha4beta1 generate signals pivotal for cell   migration   through distinct yet undefined mechanisms. For alpha5beta1, beta1-mediated activation of focal adhesion kinase (FAK) promotes c-Src recruitment to FAK and the formation of a FAK-Src signaling complex. Herein, we show that FAK expression is essential for alpha5beta1-stimulated cell motility and that exogenous expression of human alpha4 in FAK-null fibroblasts forms a functional alpha4beta1 receptor that promotes robust cell motility equal to the alpha5beta1 stimulation of wild-type and FAK-reconstituted fibroblasts. alpha4beta1-stimulated FAK-null cell spreading and motility were dependent on the integrity of the alpha4 cytoplasmic domain, independent of direct paxillin binding to alpha4, and were not affected by PRNK expression, a dominant-negative inhibitor of Pyk2. alpha4 cytoplasmic domain-initiated signaling led to a approximately 4-fold activation of c-Src which did not require paxillin binding to alpha4. Notably, alpha4-stimulated cell motility was inhibited by catalytically inactive receptor protein-tyrosine phosphatase alpha overexpression and blocked by the p50Csk phosphorylation of c-Src at Tyr-529. alpha4beta1-stimulated cell motility of triple-null Src(-/-), c-Yes(-/-), and Fyn(-/-) fibroblasts was dependent on c-Src reexpression that resulted in p130Cas tyrosine phosphorylation and Rac GTPase loading. As p130Cas phosphorylation and Rac activation are common downstream targets for alpha5beta1-stimulated FAK activation, our results support the existence of a novel alpha4 cytoplasmic domain connection leading to c-Src activation which functions as a FAK-independent linkage to a common motility-promoting signaling pathway. 
 Disruption of disulfide restriction at integrin knees induces activation and ligand-independent signaling of α₄β₇. Control of integrin activation and signaling plays crucial roles in cell adhesion, spreadin  g and mig  ration. Here, we report that selective breakage of two conserved disulfide bonds located at the knees of integrin α4C589-C594 and β7C494-C526 activated α4β7. This activated integrin had a unique structure that was different from the typical extended conformation of active integrin. In addition, these activated α4β7 integrins spontaneously clustered on the cell membrane and triggered integrin downstream signaling independent of ligand binding. Although these disulfide bonds were not broken during α4β7 activation by inside-out signaling or Mn(2+), they could be specifically reduced by 0.1 mM dithiothreitol, a reducing strength that could be produced in vivo under certain conditions. Our findings reveal a novel mechanism of integrin activation under specific reducing conditions by which integrin can signal and promote cell spreading in the absence of ligand. 
 Integrin α4 is involved in the regulation of glioma-induced motility of bone marrow mesenchymal stem cells. Bone marrow mesenchymal stem cells (BMSCs) have the ability of migrating towards glioma tissue. However, this migratory behavior remains to be elucidated. The aim of this study was to define the role of integrin α4 in the motility of BMSCs towards glioma. The role of integrin α4 in t  he migrat  ion of BMSCs towards glioma was evaluated using an in vit  ro migrat  ion assay with the application of a specific integrin α4‑blocking antibody. The effect of glioma conditioned medium (CM) on the integrin α4 expression level of BMSCs was assessed by RT-PCR, immunocytochemistry and western blot analysis. BAY11-7082, LY294002, SB203580, PD98059 and SP600125 were used to investigate the role of NF-κB, PI3K, p38 MAPK, MEK and JNK in the above process. In addition, the role of NF-κB in the tropism of BMSCs towards glioma was also evaluated using the in vitro mo  del. The   migration of BMSCs towards glioma CM was attenuated by blocking integrin α4. The stimulation of glioma CM increased integrin α4 expression of BMSCs. Furthermore, the inhibition of NF-κB and PI3K decreased the glioma-induced integrin α4 upregulation on BMSCs. Inhibition of NF-κB decreased the number of migrating BMSCs towards gliomas. Glioma cell  s induced   the migration of BMSCs by promoting the expression of integrin α4. NF-κB and PI3K contributed to the signal transduction of this process. Similar to PI3K, NF-κB is associated with the reg  ulation o  f BMSCs migration toward glioma. Thus, these results may be useful to elucidate the mechanism involved in th  e glioma-  induced migration of BMSCs. 
 The very late antigen family of heterodimers is part of a superfamily of molecules involved in adhesion and embryogenesis. The very late antigen (VLA) protein family contains at least five related heterodimers, including a fibronectin receptor structure, and probably other cell substrate adhesion receptors. These cell-surface VLA proteins were immunopurified from human placenta (VLA-1, VLA-3, and VLA-5), platelets (VLA-2), and Molt-4 cells (VLA-4) using a series of monoclonal antibody-Sepharose immunoaffinity columns. After further purification by gel electrophoresis, the N-terminal amino acid sequence for each of the five VLA alpha subunits was determined. In the first 14 positions, the five VLA alpha subunits showed an average of 42% homology to each other, rising to 59% including conservative amino acid substitutions. In addition, the alpha subunits from the LFA-1, Mac-1 (CR-3), and p150,95 family of heterodimers, the vitronectin receptor-platelet GPIIb/IIIa family, and a position-specific (PS) antigen important in Drosophila embryogenesis each showed average homologies of 31-40% to individual VLA alpha sequences and 46-52% homology to VLA alpha subunits including conservative substitutions. Taken together, these results suggest that the VLA proteins, the LFA-1, Mac-1, and p150,95 family, the GPIIb/IIIa, vitronectin receptor family, and the Drosophila PS antigens have evolved as four subgroups in a highly conserved supergene family of receptors involved in fundamentally important functions, such as cell adhesion,   migration  , and embryogenesis. 
 Communication between integrin receptors facilitates epicardial cell adhesion and matrix organization. Formation of the epicardium requires interactions between alpha(4)beta(1) integrin, and the extracellular matrix. We investigated the role of other integrins expressed by epicardial cells. We detected transcripts for alpha(5), alpha(8), alpha(v), beta(1), beta(3), and beta(5) integrins in the chick proepicardial organ (PE). We demonstrate that alpha(5)beta(1), alpha(8)beta(1), and alpha(v)beta(3) integrins are expressed by chick epicardial mesothelial cells (EMCs).   Migration   of EMCs in vitro was reduced by RGD-containing peptides. Using adenoviruses expressing an antisense to chick alpha(4) (AdGFPalpha4AS), full-length (Adhalpha4V5), and C-terminal deleted alpha(4) (Adhalpha4DeltaCV5), we found that EMCs were less able to adhere to vitronectin and fibronectin(120) indicating that alpha(4)beta(1) plays a role in regulating EMC adhesion to ligands of alpha(5)beta(1), alpha(8)beta(1), and alpha(v)beta(3). In Adhalpha4DeltaCV5-infected EMCs, alpha(5)beta(1) was diminished in fibrillar adhesions and new FN matrix assembly was abnormal. We propose that cooperation between alpha(4)beta(1) and RGD integrins is important for EMC adhesion and subepicardial matrix formation. 
 Functional differences between integrin alpha4 and integrins alpha5/alphav in modulating the motility of human oral squamous carcinoma cells in response to the V region and heparin-binding domain of fibronectin. The high-affinity heparin-binding domain and the V region of fibronectin (FN) mediate invasion and   migration   of human oral squamous cell carcinoma (SCC) cells. We investigated the role of the alpha4, alpha5, and alphav integrin receptors--which are central to mediating interactions with these domains of FN--in regulating SCC cell   migration  . SCC cells expressed the alpha4, alpha5, and alphav integrin subunits on their surface, although alpha4 expression was low. Treatment with recombinant FN proteins containing an alternatively spliced V region (V+) and either an unmutated (H+) or a mutated, nonfunctional high-affinity heparin-binding domain (H-) increased expression of alpha5 and alphav and cell motility. Antisense alpha5 or alphav oligonucleotides inhibited cell motility stimulated by FN proteins, as did blocking antibodies to alpha5 or alphav. Blocking antibodies to alpha5 increased alphav and alpha4 levels, and blocking antibodies to alphav increased the levels of alpha5 and alpha4, without increasing cell motility. In contrast, an antisense alpha4 oligonucleotide and alpha4-blocking antibodies increased cell motility, especially   migration   stimulated by V+H+ and V+H- FN proteins. alpha4-Blocking antibodies alone increased motility, probably by inducing alpha5 and alphav expression. Transfection with alpha4 cDNA decreased cell motility and alpha5 and alphav expression. Thus, the increased motility induced by the FN protein is probably mediated by alphav and alpha5, whereas alpha4 downregulates this process in a transdominant fashion. 
 ITGA4 polymorphisms and susceptibility to multiple sclerosis. In multiple sclerosis (MS), alpha(4)beta(1) integrin, also known as Very Late Antigen 4 (VLA4), facilitates   migration   of leukocytes across the blood brain barrier. Several studies suggest that expression of alpha(4) integrin may be increased in MS patients compared to controls, and down-regulation or antagonism of alpha(4) integrin may be associated with immunomodulatory treatment success. We analysed association of 13 single nucleotide polymorphisms (SNPs) in the gene encoding alpha(4) integrin (ITGA4) with susceptibility to MS in two distinct populations comprising cases and controls from the Basque Country in northern Spain (352 patients; 235 controls) and Nordic countries (1119 patients; 1235 controls). Carriage of the C allele of the ITGA4 promoter SNP rs1449263 was independently and weakly increased in MS patients from each population compared to respective controls (P = 0.037 in Basque; and P = 0.042 in Nordic cohorts), though these associations were lost upon application of permutation correction. Meta-analysis of rs1449263*C carriage revealed a Mantel-Haenszel common OR of 1.26 (95% CI 1.06-1.49; P = 0.0069). Though our data only modestly argue for a role of ITGA4 in determining susceptibility to MS, we suggest that further examination of this gene, particularly the promoter region, is warranted. 
 The differential amino acid requirement within osteopontin in alpha4 and alpha9 integrin-mediated cell binding and   migration  . Osteopontin (OPN) contains at least two major integrin recognition domains, Arg159-Gly-Asp161 (RGD) and Ser162-Val-Val-Tyr-Gly-Leu-Arg168 (SVVYGLR), recognized by alphavbeta3 and alpha5beta1 and alpha4 and alpha9 integrins, respectively. OPN is specifically cleaved by thrombin and matrix metalloproteinase (MMP)-3 or MMP-7 at a position of Arg168/Ser169 (R/S) and Gly166/Leu167 (G/L), respectively. We in this study examined the requirement of residues within SVVYGLR for the alpha4 and alpha9 integrin recognition and how MMP-cleavage influences the integrin recognition. The residues, Val164, Tyr165, and Leu167 are critical for alpha4 and alpha9 integrin recognition in both cell adhesion and cell   migration  . The residue Arg168 is additionally required for alpha9 integrin recognition in cell adhesion and this explains why alpha9 integrin binds to only thrombin cleaved form of OPN. alpha4 integrin is able to bind to SVVYG (MMP-cleaved form of RAA OPN-N half), while alpha9 integrin is not, supporting the above notion that Arg168 is additionally required for alpha9 integrin-mediated cell adhesion. The residue Val163 is important for alpha4, but not for alpha9 integrin recognition in cell   migration  . Importantly, we found that the replacement of Arg168 by Ala (R168A mutant) induces the augmentation of cell   migration   via alpha4 and alpha9 integrins. 
 The alpha4beta1 and alpha5beta1 integrins mediate engraftment of granulocyte-colony-stimulating factor-mobilized human hematopoietic progenitor cells. The alpha4beta1 and alpha5beta1 integrins are major adhesion molecules of murine and human hematopoietic progenitor cells. Granulocyte-colony-stimulating factor (G-CSF)-mobilized peripheral blood progenitor cells (PBPCs) are the most important source for clinical hematopoietic cell transplantation today. The contribution of alpha4beta1 and alpha5beta1 integrins to homing of PBPCs has not been studied yet.The expression of alpha4beta1 and alpha5beta1 integrins on purified human PBPCs was analyzed. Integrin function in adhesion to recombinant fibronectin and   migration   on fibronectin-coated transwells was assessed with fragments combining different adhesion domains and function-blocking antibodies. Finally, the function of those integrins in a transplantation model was investigated with repopulating cells of nonobese diabetic/severe combined immune-deficient (NOD/SCID) mice.More than 90 percent of all purified peripheral blood CD34+ cells express alpha4beta1 integrins, whereas only 10 to 15 percent express alpha5beta1. The alpha4beta1 integrin alone influences adhesion whereas alpha4beta1 and alpha5beta1 both mediate chemotaxis of clonogenic CD34+ progenitor cells on recombinant fibronectin. Importantly, antibodies against the integrins alpha4beta1 and alpha5beta1 independently reduce the repopulation of NOD/SCID mouse marrow after transplantation of human peripheral blood CD34+ cells.Alpha4beta1 and alpha5beta1 integrins are functional and critical adhesion receptors expressed on G-CSF-mobilized CD34+ hematopoietic blood progenitor cells with repopulating capacity mediating engraftment after transplantation. 
 Increased binding and defective   migration   across fibronectin of cycling hematopoietic progenitor cells. Engraftment of hematopoietic progenitor cells has been shown to decrease during cell cycle transit. We studied cell cycle-associated changes in adhesion and   migration   of mitotically activated cord blood CD34+ cells.   Migration   toward medium conditioned by the stromal-derived factor-1-producing cell line MS-5 was studied in bovine serum albumin- and fibronectin (Fn)-coated transwells.   Migration   was reduced in cycling CD34+ cells and long-term culture-initiating cells (LTC-ICs) compared with their noncycling counterparts across Fn but not across bovine serum albumin. Conversely, Fn binding was higher in cycling CD34+ cells and LTC-ICs compared with noncycling progenitor cells, while adhesion of both subsets to bovine serum albumin was undetectable. The contribution of alpha4 and alpha5 integrins in mediating adhesion and   migration   of activated CD34+ cells onto Fn was analyzed by neutralization experiments. While alpha4-mediated Fn binding decreased during G(2)/M, alpha5 integrin-mediated adhesion increased during transit from G(0)/G(1) to S and G(2)/M phases. As for   migration  , the contribution of alpha4 integrin was similar in all phases, whereas alpha5-directed   migration   was lower in G(2)/M compared with G(0)/G(1) and S phases. Defective   migration   of cycling CD34+ cells was not due to differences in alpha5 integrin expression. In conclusion, chemotaxis across Fn is less efficient in cycling progenitor cells in correlation with an increased Fn binding capacity. In addition, alpha4 and alpha5 integrin functions are independently modulated during cell cycle transit. 
 Fundamentally different roles for LFA-1, Mac-1 and alpha4-integrin in neutrophil chemotaxis. Although the LFA-1, Mac-1 and alpha(4) integrins are required for chemotaxis, it is unknown how they are regulated or what specific role they play. Previously we demonstrated that fMLP and IL-8 induce chemotaxis via the p38 MAPK and phosphoinositide 3-kinase (PI3K) pathways, respectively. Here we show that these chemoattractants also activate and use Mac-1 and LFA-1 in a differential manner during chemotaxis. Using integrin-specific substrata, we demonstrate that cell movement in response to IL-8 is mediated by Mac-1, whereas LFA-1 is required for directional   migration  . By contrast, chemotaxis to fMLP requires Mac-1 for cell movement, whereas LFA-1 and alpha(4)-integrin are required for directional   migration  . On serum protein, which contains ligands for LFA-1, Mac-1 and alpha(4)-integrin, chemotaxis to fMLP is dependent on Mac-1, whereas chemotaxis to IL-8 is dependent on LFA-1. These results suggest that Mac-1 is the dominant integrin involved in chemotaxis to fMLP, and LFA-1 is the dominant integrin involved in chemotaxis to IL-8. Consistent with these observations, higher quantities of high-affinity Mac-1 are found on cells chemotaxing to fMLP then on cells chemotaxing to IL-8. Moreover, a much larger quantity of clustered LFA-1 was found on cells migrating to IL-8 compared to cells moving towards fMLP. When cells are presented with competing gradients of fMLP and IL-8, they preferentially migrate towards fMLP and activate/utilize integrins in a manner identical to fMLP alone. Under the same conditions, p38 MAPK inhibition abolishes the preferential   migration   to fMLP; instead, the cells migrate preferentially towards IL-8. The activation and utilization of integrins under these conditions are consistent with patterns observed with IL-8 alone. Together, these data suggest that fMLP and IL-8 differentially activate integrins for use during chemotaxis, that p38 MAPK is a major mediator in the activation and utilization of integrins, and selective integrin activation occurs during chemotaxis between opposing gradients. 
 VLA-4 integrin concentrates at the peripheral supramolecular activation complex of the immune synapse and drives T helper 1 responses. The integrin alpha 4 beta 1 (VLA-4) not only mediates the adhesion and transendothelial   migration   of leukocytes, but also provides costimulatory signals that contribute to the activation of T lymphocytes. However, the behavior of alpha 4 beta 1 during the formation of the immune synapse is currently unknown. Here, we show that alpha 4 beta 1 is recruited to both human and murine antigen-dependent immune synapses, when the antigen-presenting cell is a B lymphocyte or a dendritic cell, colocalizing with LFA-1 at the peripheral supramolecular activation complex. However, when conjugates are formed in the presence of anti-alpha 4 antibodies, VLA-4 colocalizes with the CD3-zeta chain at the center of the synapse. In addition, antibody engagement of alpha 4 integrin promotes polarization toward a T helper 1 (Th1) response in human in vitro models of CD4(+) T cell differentiation and naïve T cell priming by dendritic cells. The in vivo administration of anti-alpha 4 integrin antibodies also induces an immune deviation to Th1 response that dampens a Th2-driven autoimmune nephritis in Brown Norway rats. These data reveal a regulatory role of alpha 4 integrins on T lymphocyte-antigen presenting cell cognate immune interactions. 
 A small molecule that inhibits the interaction of paxillin and alpha 4 integrin inhibits accumulation of mononuclear leukocytes at a site of inflammation. Extracellular antagonists of alpha 4 integrin are an effective therapy for several autoimmune and inflammatory diseases; however, these agents that directly block ligand binding may exhibit mechanism-based toxicities. Inhibition of alpha 4 integrin signaling by mutations of alpha 4 that block paxillin binding inhibits inflammation while limiting mechanism-based toxicities. Here, we test a pharmacological approach by identifying small molecules that inhibit the alpha 4 integrin-paxillin interaction. By screening a large (approximately 40,000-compound) chemical library, we identified a noncytotoxic inhibitor of this interaction that impaired integrin alpha 4-mediated but not alpha L beta 2-mediated Jurkat T cell   migration  . The identified compound had no effect on alpha 4-mediated   migration   in cells bearing the alpha 4(Y991A) mutation that disrupts the alpha 4-paxillin interaction, establishing the specificity of its action. Administration of this compound to mice led to impaired recruitment of mononuclear leukocytes to a site of inflammation in vivo, whereas an isomer that does not inhibit the alpha 4-paxillin interaction had no effect on alpha 4-mediated cell   migration  , cell spreading, or recruitment of leukocytes to an inflammatory site. Thus, a small molecule inhibitor that interferes with alpha 4 integrin signaling reduces alpha 4-mediated T cell   migration   in vivo, thus providing proof of principle for inhibition of alpha 4 integrin signaling as a target for the pharmacological reduction of inflammation. 
 Contribution of very late antigen-4 (VLA-4) integrin to cancer progression and metastasis. The integrin "very late antigen-4" (VLA-4) is expressed by numerous cells of hematopoietic origin and possesses a key function in the cellular immune response, e.g., by mediating leukocyte tethering, rolling, binding, and finally trans  migration   of the vascular wall at inflammatory sites. Thus, VLA-4 is a valuable target in medical sciences to interfere with pathological inflammations. In addition, leukemic cells and different solid tumors, which express VLA-4, make use of these adhesive functions and confer VLA-4 a progressive role in the metastatic spread. With a growing insight into the molecular mechanisms for creating a tumor-friendly microenvironment at metastatic sites and various tumor host interactions, the multiple functions of VLA-4 became evident recently, e.g., in leukocyte recruitment to micrometastases, the protection of tumors from immune surveillance, or contribution to a chemoresistance. Nevertheless, despite accumulating evidence for several functions of VLA-4 in tumorigenicity, a therapeutic interference with VLA-4 in cancer sciences has not been developed yet to the clinical level, undoubtedly by a marked impact on the physiological immune response. This review gives an up to date insight into the multiple functional role of VLA-4 in cancer and introduces this integrin as a promising target worthwhile to attract attention in biomedical cancer research. 
 Selective integrin endocytosis is driven by interactions between the integrin α-chain and AP2. Integrins are heterodimeric cell-surface adhesion molecules comprising one of 18 possible α-chains and one of eight possible β-chains. They control a range of cell functions in a matrix- and ligand-specific manner. Integrins can be internalized by clathrin-mediated endocytosis (CME) through β subunit-based motifs found in all integrin heterodimers. However, whether specific integrin heterodimers can be selectively endocytosed was unknown. Here, we found that a subset of α subunits contain an evolutionarily conserved and functional YxxΦ motif directing integrins to selective internalization by the most abundant endocytic clathrin adaptor, AP2. We determined the structure of the human integrin α4-tail motif in complex with the AP2 C-μ2 subunit and confirmed the interaction by isothermal titration calorimetry. Mutagenesis of the motif impaired selective heterodimer endocytosis and attenuated integrin-mediat  ed cell m  igration. We propose that integrins evolved to enable selective integrin-receptor turnover in response to changing matrix conditions. 
  GeneRIFs  
 Glioma cells induced the   migration   of BMSCs by promoting the expression of integrin a4. 
 The regulation of alpha4beta1-dependent adhesion and   migration   of myeloma cells by CXCL12-S1P combined activities might have important consequences for myeloma disease progression. 
 findings indicate that alpha4beta7*IgG can be used as a probe for functional MAdCAM-1 expressed on HEVs in GALT and could potentially serve as an anti-inflammatory drug inhibiting GALT-specific lymphocyte   migration   
 CD74 and its target gene transcripts (TA)p63 and very late antigen (VLA)-4 facilitate   migration   of chronic lymphocytic leukemia cells back to the bone marrow (BM), where they interact with the supportive BM environment that rescues them from apoptosis. 
 Activation of VLA-4 subunit of integrin alpha4beta1 localizes to the lamellipodium of polarized human T cells and is critical for T cell   migration   on VCAM-1. 
 residue Val163 is important for alpha4, but not for alpha9 integrin recognition in cell   migration   
 Inhibition of endothelial cell spreading and   migration   by inflammatory cytokines is mediated by GBP-1 through induction of ITGA4 expression. 
 the CXCR4/SDF-1 axis interacts with VLA-4 in regulating   migration   and adhesion of Waldenstrom macroglobulinemia cells in the bone marrow microenvironment 
 role in mediating adhesion and   migration   of activated cycling CD34+ hematopoietic progenitor cells onto fibronectin 
 important for   migration   of chronic lymphocytic leukemia cells into lymph nodes 
 We demonstrate that cell movement in response to IL-8 is mediated by Mac-1, whereas LFA-1 is required for directional   migration  . By contrast, chemotaxis to fMLP requires Mac-1 for cell movement. 
 This study identified the beta1 integrins on primary smooth muscle cells that fibulin-5 interacts with, and showed that failure of fibulin-5 to activate these receptors limits cell spreading,   migration   and proliferation. 
 Requires integrin alpha4beta1 to control T cell   migration   and requires both phosphorylation and dephosphorylation of the alpha 4 cytoplasmic domain to regulate the reversible binding of paxillin. 
 Role of alpha4, alpha5, and alphav integrin receptors--which are central to mediating interactions with these domains of FN--in regulating ssquamous cell carcinoma   migration  . 
  Summary  
 The gene encodes a member of the integrin alpha chain family of proteins. Integrins are heterodimeric integral membrane proteins composed of an alpha chain and a beta chain that function in cell surface adhesion and signaling. The encoded preproprotein is proteolytically processed to generate light and heavy chains that comprise the alpha 4 subunit. This subunit associates with a beta 1 or beta 7 subunit to form an integrin that may play a role in cell motility and   migration  . This integrin is a therapeutic target for the treatment of multiple sclerosis, Crohn's disease and inflammatory bowel disease. Alternative splicing results in multiple transcript variants. [provided by RefSeq, Oct 2015] 
  ECM  PubMed  
 Regulation of endodermal differentiation of human embryonic stem cells through integrin-  ECM   interactions. Many cellular responses during development are regulated by interactions between integrin receptors and extracellular matrix proteins (  ECM  Ps). Although the majority of recent studies in human embryonic stem cell (hESC) differentiation have focused on the role of growth factors, such as FGF, TGFβ, and WNT, relatively little is known about the role of   EC  MP-integrin signaling in this process. Moreover, current strategies to direct hESC differentiation into various lineages are inefficient and have yet to produce functionally mature cells in vitro. This suggests that additional factors, such as   EC  MPs, are required for the efficient differentiation of hESCs. Using a high-throughput multifactorial cellular array technology, we investigated the effect of hundreds of   EC  MP combinations and concentrations on differentiation of several hPSC lines to definitive endoderm (DE), an early embryonic cell population fated to give rise to internal organs such as the lung, liver, pancreas, stomach, and intestine. From this screen we identified fibronectin (FN) and vitronectin (VTN) as   EC  MP components that promoted DE differentiation. Analysis of integrin expression revealed that differentiation toward DE led to an increase in FN-binding integrin α5 (ITGA5) and VTN-binding integrin αV (ITGAV). Conditional short hairpin RNA-mediated knockdown of ITGA5 and ITGAV disrupted hESC differentiation toward DE. Finally, fluorescence-based cell sorting for ITGA5 and ITGAV significantly enriched cells with gene expression signatures associated with DE, demonstrating that these cell surface proteins permit isolation and enrichment of DE from hESCs. These data provide evidence that FN and VTN promote endoderm differentiation of hESCs through interaction with ITGA5 and ITGAV, and th  at   ECMP-integrin interactions are required for hESC differentiation into functionally mature cells. 
 Additive effect of recombinant Mycobacterium tuberculosis ESAT-6 protein and ESAT-6/CFP-10 fusion protein in adhesion of macrophages through fibronectin receptors. Tuberculous granulomas are the sites of interaction between the T cells, macrophages, and extracellular matrix (  ECM  ) to control the infection caused by Mycobacterium tuberculosis (M. tuberculosis). A predominant role of RD-1-encoded secretory proteins, early secreted antigenic target-6 (ESAT-6), and culture filtrate protein-10 (CFP-10) in the formation of granulomas has recently been emphasized. However, the precise molecular events that induce the formation of these granulomatous structures are yet to be elucidated. Macrophages use integrins to adhere to fibronectin (FN) as a major component of the   ECM  . The major goal of this study was to investigate whether recombinant M. tuberculosis antigens can modulate integrin-mediated macrophage adhesion.Differentiated THP-1 cell line was stimulated with recombinant ESAT-6, CFP-10, and ESAT-6/CFP-10 proteins and evaluated for alterations in the expression levels of α5β1 and α4β1 by semiquantitative real-time polymerase chain reaction. The role of these recombinant antigens in the cytoskeleton rearrangement was determined by adhesion assay and immunofluorescent microscopy.Our data showed that ESAT-6 and ESAT-6/CFP-10 fusion proteins could induce adhesion of macrophages to FN through α4β1 integrin. An increased expression level of α4β1 integrin in comparison with α5β1 integrin in differentiated THP-1 cells was also observed. Results of immunofluorescence studies showed that recombinant proteins-treated THP-1 cells form well-organized stress fibers and focal contacts containing vinculin compared with untreated THP-1 cells.Increased expression level of α4β1 in differentiated THP-1 cells could suggest the important role of α4β1 integrin in adhesion and focal contact formation of macrophages exposed to M. tuberculosis antigens. 
 Additive effect of recombinant Mycobacterium tuberculosis ESAT-6 protein and ESAT-6/CFP-10 fusion protein in adhesion of macrophages through fibronectin receptors. Tuberculous granulomas are the sites of interaction between the T cells, macrophages, and extracellular matrix (  ECM  ) to control the infection caused by Mycobacterium tuberculosis (M. tuberculosis). A predominant role of RD-1-encoded secretory proteins, early secreted antigenic target-6 (ESAT-6), and culture filtrate protein-10 (CFP-10) in the formation of granulomas has recently been emphasized. However, the precise molecular events that induce the formation of these granulomatous structures are yet to be elucidated. Macrophages use integrins to adhere to fibronectin (FN) as a major component of the   ECM  . The major goal of this study was to investigate whether recombinant M. tuberculosis antigens can modulate integrin-mediated macrophage adhesion.Differentiated THP-1 cell line was stimulated with recombinant ESAT-6, CFP-10, and ESAT-6/CFP-10 proteins and evaluated for alterations in the expression levels of α5β1 and α4β1 by semiquantitative real-time polymerase chain reaction. The role of these recombinant antigens in the cytoskeleton rearrangement was determined by adhesion assay and immunofluorescent microscopy.Our data showed that ESAT-6 and ESAT-6/CFP-10 fusion proteins could induce adhesion of macrophages to FN through α4β1 integrin. An increased expression level of α4β1 integrin in comparison with α5β1 integrin in differentiated THP-1 cells was also observed. Results of immunofluorescence studies showed that recombinant proteins-treated THP-1 cells form well-organized stress fibers and focal contacts containing vinculin compared with untreated THP-1 cells.Increased expression level of α4β1 in differentiated THP-1 cells could suggest the important role of α4β1 integrin in adhesion and focal contact formation of macrophages exposed to M. tuberculosis antigens. 
 Dynamic alterations in integrin α4 expression by hypoxia are involved in trophoblast invasion during early implantation. Implantation of the blastocyst into the maternal endometrium is mediated by a population of well-differentiated primary cells of the placenta known as trophoblasts, which grow in an invasive and destructive fashion similar to tumor cells. Interactions between the endometrium and trophoblasts are regulated by a coordinated interplay of extracellular matrix   (EC  M) proteins secreted by the invading extravillous trophoblasts. Integrins act as adhesion receptors and mediate both cell  -EC  M and cell-cell interactions. However, the correlation between integrin expression and trophoblast invasion under hypoxia is unclear. Here, we analyzed the expression of integrins in HTR-8/SVneo trophoblast cells exposed to hypoxic conditions in order to demonstrate an association between invasion activity and integrin expression in trophoblasts. Trophoblasts were examined by microarray analysis, RT-PCR, western blotting, and zymography after 1% hypoxic treatment, and cell invasion was estimated. The dynamic expression of integrins and human matrix metalloproteinases (MMPs) was observed under hypoxic conditions. The invasiveness of trophoblasts cultured under 1% hypoxic conditions was significantly greater than that of trophoblasts cultured under normoxic conditions through alterations in MMP-2 and -9 (P < 0.05). Notably, integrin α4 expression during early hypoxia was negatively regulated by hypoxia-inducible factor-1alpha (HIF-1alpha) expression in trophoblasts. The downregulation of integrin α4 expression by siRNA treatment controlled trophoblast invasion activity (P < 0.05). Taken together, we suggest that dynamic changes in integrins, including those in integrin α4 expression by hypoxia, play a regulatory role in trophoblast invasion. These findings expand our understanding of the potential roles of integrin α4 in implantation. 
 Integrins α4 and αM, collagen1A1, and matrix metalloproteinase 7 are upregulated in acute Kawasaki disease vasculopathy. Kawasaki disease (KD) can result in fatal coronary artery (CA) aneurysms, especially if left untreated. Our recent studies of its vascular pathology revealed subacute/chronic vasculitis that begins early in the illness with the proliferation of smooth muscle cell-derived myofibroblasts in a complex extracellular matrix   (E  CM). We hypothesized that a dysregulation of specifi  c E  CM and adhesion molecules occurs in KD CAs.Gene expression profiling fo  r E  CM and adhesion molecules was performed on six acute KD and eight control CAs using a targeted real-time PCR array approach.Integrins α4 and αM (ITGA4, ITGAM), collagen type I, α1 (COL1A1), and matrix metalloproteinase 7 (MMP7) were significantly upregulated in KD CAs as compared with controls. Immunohistochemistry with anti-ITGAM antibodies revealed expression on inflammatory cells within the CA wall in patients with KD but not in controls.Integrins ITGA4 and ITGAM are upregulated in KD vasculopathy, probably promoting inflammatory recruitment that stimulates smooth muscle cell transition to myofibroblasts and their proliferation. MMP7 probably enhances myofibroblast proliferation and luminal lesion expansion, and overexpression of COL1A1 may lead to CA stenosis. Identification of the molecular pathogenesis of KD vasculopathy may lead to the development of circulating biomarkers and to directed therapeutic interventions. 
  [Ee]xtracellular [Mm]atrix  Process  
   extracellular matrix   organization 
  PubMed  
 Real-time analysis of very late antigen-4 affinity modulation by shear. Shear promotes endothelial recruitment of leukocytes, cell activation, and transmigration. Mechanical stress on cells caused by shear can induce a rapid integrin conformational change and activation, followed by an increase in binding to the   extracellular matrix  . The molecular mechanism of increased avidity is unknown. We have shown previously that the affinity of the alpha(4)beta(1) integrin, very late antigen-4 (VLA-4), measured with an LDV-containing small molecule, varies with cellular avidity, measured from cell disaggregation rates. In this study, we measured in real time affinity changes of VLA-4 in response to shear. The resulting affinity was comparable with the state mediated by receptor signaling and corresponded in time with intracellular Ca(2+) responses. Ca(2+) ionophores and N,N'-[1,2-ethanediyl-bis(oxy-2,1-phenylene)]bis[N-[2-[(acetyloxy)methoxy]-2-oxoethyl]]-, bis[(acetyloxy)methyl]ester demonstrate that the affinity regulation of VLA-4 in the presence of shear was related to Ca(2+) signaling. Pertussis toxin treatment implicates G(i) in an unknown pathway that connects shear, Ca(2+) elevation, VLA-4 affinity, and cell avidity. 
 Proteomic analysis of α4β1 integrin adhesion complexes reveals α-subunit-dependent protein recruitment. Integrin adhesion receptors mediate cell-cell and ce  ll-extracellular mat  rix interactions, which control cell morphology and migration, differentiation, and tissue integrity. Integrins recruit multimolecular adhesion complexes to their cytoplasmic domains, which provide structural and mechanosensitive signaling connections between the extracellular and intracellular milieux. The different functions of specific integrin heterodimers, such as α4β1 and α5β1, have been attributed to distinct signal transduction mechanisms that are initiated by selective recruitment of adhesion complex components to integrin cytoplasmic tails. Here, we report the isolation of ligand-induced adhesion complexes associated with wild-type α4β1 integrin, an activated α4β1 variant in the absence of the α cytoplasmic domain (X4C0), and a chimeric α4β1 variant with α5 leg and cytoplasmic domains (α4Pα5L), and the cataloguing of their proteomes by MS. Using hierarchical clustering and interaction network analyses, we detail the differential recruitment of proteins and highlight enrichment patterns of proteins to distinct adhesion complexes. We identify previously unreported components of integrin adhesion complexes and observe receptor-specific enrichment of molecules with previously reported links to cell migration and cell signaling processes. Furthermore, we demonstrate colocalization of MYO18A with active integrin in migrating cells. These datasets provide a resource for future studies of integrin receptor-specific signaling events. 
 Characterization of plasminogen as an adhesive ligand for integrins alphaMbeta2 (Mac-1) and alpha5beta1 (VLA-5). Plasminogen (Pg) has been implicated in many biologic processes involving extracellular proteolysis. We investigated whether Pg, by virtue of its capacity to be deposited within the   extracellular matrix  , can serve as a ligand for cell surface integrins. We report here that Pg supports cell adhesion by engaging integrins alphaMbeta2 and alpha5beta1. The immobilized Glu-Pg, but not its derivatives with the N-terminal peptide lacking, plasmin and Lys-Pg, supported efficient adhesion that was abolished by anti-alphaMbeta2 and anti-alpha5beta1 integrin-specific monoclonal antibodies (mAbs). In addition, lysine binding sites of Glu-Pg contributed to cell adhesion inasmuch as tranexamic acid and epsilon-aminocaproic acid inhibited cell adhesion. The involvement of alphaMbeta2 and alpha5)beta1 in adhesion to Glu-Pg was demonstrable with blood neutrophils, U937 monocytoid cells, and genetically engineered alphaMbeta2-transfected human embryonic kidney (HEK) 293 cells. In alphaMbeta2, the alphaMI-domain is the binding site for Glu-Pg because the "I-less" form of alphaMbeta2 did not support cell adhesion and the recombinant alphaMI-domain bound Glu-Pg directly. In comparison with cell adhesion, the binding of soluble Glu-Pg to cells and the concomitant generation of plasmin activity was inhibited by anti-alpha5beta1 but not by anti-alphaMbeta2. These findings identify Glu-Pg as an adhesive ligand for integrins alphaMbeta2 and alpha5beta1 and suggest that alpha5beta1 may participate in the binding of soluble Glu-Pg and assist in its activation. 
 Regulation of endodermal differentiation of human embryonic stem cells through integrin-ECM interactions. Many cellular responses during development are regulated by interactions between integrin receptors and   extracellular matrix   proteins (ECMPs). Although the majority of recent studies in human embryonic stem cell (hESC) differentiation have focused on the role of growth factors, such as FGF, TGFβ, and WNT, relatively little is known about the role of ECMP-integrin signaling in this process. Moreover, current strategies to direct hESC differentiation into various lineages are inefficient and have yet to produce functionally mature cells in vitro. This suggests that additional factors, such as ECMPs, are required for the efficient differentiation of hESCs. Using a high-throughput multifactorial cellular array technology, we investigated the effect of hundreds of ECMP combinations and concentrations on differentiation of several hPSC lines to definitive endoderm (DE), an early embryonic cell population fated to give rise to internal organs such as the lung, liver, pancreas, stomach, and intestine. From this screen we identified fibronectin (FN) and vitronectin (VTN) as ECMP components that promoted DE differentiation. Analysis of integrin expression revealed that differentiation toward DE led to an increase in FN-binding integrin α5 (ITGA5) and VTN-binding integrin αV (ITGAV). Conditional short hairpin RNA-mediated knockdown of ITGA5 and ITGAV disrupted hESC differentiation toward DE. Finally, fluorescence-based cell sorting for ITGA5 and ITGAV significantly enriched cells with gene expression signatures associated with DE, demonstrating that these cell surface proteins permit isolation and enrichment of DE from hESCs. These data provide evidence that FN and VTN promote endoderm differentiation of hESCs through interaction with ITGA5 and ITGAV, and that ECMP-integrin interactions are required for hESC differentiation into functionally mature cells. 
 Additive effect of recombinant Mycobacterium tuberculosis ESAT-6 protein and ESAT-6/CFP-10 fusion protein in adhesion of macrophages through fibronectin receptors. Tuberculous granulomas are the sites of interaction between the T cells, macrophages, and   extracellular matrix   (ECM) to control the infection caused by Mycobacterium tuberculosis (M. tuberculosis). A predominant role of RD-1-encoded secretory proteins, early secreted antigenic target-6 (ESAT-6), and culture filtrate protein-10 (CFP-10) in the formation of granulomas has recently been emphasized. However, the precise molecular events that induce the formation of these granulomatous structures are yet to be elucidated. Macrophages use integrins to adhere to fibronectin (FN) as a major component of the ECM. The major goal of this study was to investigate whether recombinant M. tuberculosis antigens can modulate integrin-mediated macrophage adhesion.Differentiated THP-1 cell line was stimulated with recombinant ESAT-6, CFP-10, and ESAT-6/CFP-10 proteins and evaluated for alterations in the expression levels of α5β1 and α4β1 by semiquantitative real-time polymerase chain reaction. The role of these recombinant antigens in the cytoskeleton rearrangement was determined by adhesion assay and immunofluorescent microscopy.Our data showed that ESAT-6 and ESAT-6/CFP-10 fusion proteins could induce adhesion of macrophages to FN through α4β1 integrin. An increased expression level of α4β1 integrin in comparison with α5β1 integrin in differentiated THP-1 cells was also observed. Results of immunofluorescence studies showed that recombinant proteins-treated THP-1 cells form well-organized stress fibers and focal contacts containing vinculin compared with untreated THP-1 cells.Increased expression level of α4β1 in differentiated THP-1 cells could suggest the important role of α4β1 integrin in adhesion and focal contact formation of macrophages exposed to M. tuberculosis antigens. 
 Tetraspanins CD81 and CD82 facilitate α4β1-mediated adhesion of human erythroblasts to vascular cell adhesion molecule-1. The proliferation and terminal differentiation of erythroid progenitors occurs in human bone marrow within erythroblastic islands, specialised structures consisting of a central macrophage surrounded by developing erythroid cells. Many cell-cell and cell-matrix adhesive interactions maintain and regulate the co-ordinated daily production of reticulocytes. Erythroid cells express only one integrin, α4β1, throughout differentiation, and its interactions with both macrophage Vascular Cell Adhesion Molecule-1 and w  ith extracellular ma  trix fibronectin are critical for erythropoiesis. We observed that proerythroblasts expressed a broad tetraspanin phenotype, and investigated whether any tetraspanin could modulate integrin function. A specific association between α4β1 and CD81, CD82 and CD151 was demonstrated by confocal microscopy and co-immune precipitation. We observed that antibodies to CD81 and CD82 augmented adhesion of proerythroblasts to Vascular Cell Adhesion Molecule-1 but not to the fibronectin spliceoforms FnIII12-IIICS-15 and FnIII12-15. In contrast, different anti-CD151 antibodies augmented or inhibited adhesion of proerythroblasts to Vascular Cell Adhesion Molecule-1 and the fibronectin spliceoform FnIII12-IIICS-15 but not to FnIII12-15. These results strongly suggest that tetraspanins have a functional role in terminal erythropoiesis by modulating interactions of erythroblast α4β1 with both macropha  ges and extracellula  r matrix. 
 Conformational regulation of alpha 4 beta 1-integrin affinity by reducing agents. "Inside-out" signaling is independent of and additive to reduction-regulated integrin activation. The alpha(4)beta(1)-integrin (very late antigen-4 (VLA-4), CD49d/CD29) is an adhesion receptor involved in the interaction of lymphocytes, dendritic cells, and stem cells with the   extracellular matrix   and endothelial cells. This and other integrins have the ability to regulate their affinity for ligands through a process termed "inside-out" signaling that affects cell adhesion avidity. Several mechanisms are known to regulate integrin affinity and conformation: conformational changes induced by separation of the C-terminal tails, divalent ions, and reducing agents. Recently, we described a fluorescent LDV-containing small molecule that was used to monitor VLA-4 affinity changes in live cells (Chigaev, A., Blenc, A. M., Braaten, J. V., Kumaraswamy, N., Kepley, C. L., Andrews, R. P., Oliver, J. M., Edwards, B. S., Prossnitz, E. R., Larson, R. S., and Sklar, L. A. (2001) J. Biol. Chem. 276, 48670-48678). Using the same molecule, we also developed a fluorescence resonance energy transfer-based assay to probe the "switchblade-like" opening of VLA-4 upon activation. Here, we investigated the effect of reducing agents on the affinity and conformational state of the VLA-4 integrin simultaneously with cell activation initiated by inside-out signaling through G protein-coupled receptors or Mn(2+) in live cells in real time. We found that reducing agents (dithiothreitol and 2,3-dimercapto-1-propanesulfonic acid) induced multiple states of high affinity of VLA-4, where the affinity change was accompanied by an extension of the integrin molecule. Bacitracin, an inhibitor of the reductive function of the plasma membrane, diminished the effect of dithiothreitol, but had no effect on inside-out signaling. Based on this result and differences in the kinetics of integrin activation, we conclude that conformational activation of VLA-4 by inside-out signaling is independent of and additive to reduction-regulated integrin activation. 
 Additive effect of recombinant Mycobacterium tuberculosis ESAT-6 protein and ESAT-6/CFP-10 fusion protein in adhesion of macrophages through fibronectin receptors. Tuberculous granulomas are the sites of interaction between the T cells, macrophages, and   extracellular matrix   (ECM) to control the infection caused by Mycobacterium tuberculosis (M. tuberculosis). A predominant role of RD-1-encoded secretory proteins, early secreted antigenic target-6 (ESAT-6), and culture filtrate protein-10 (CFP-10) in the formation of granulomas has recently been emphasized. However, the precise molecular events that induce the formation of these granulomatous structures are yet to be elucidated. Macrophages use integrins to adhere to fibronectin (FN) as a major component of the ECM. The major goal of this study was to investigate whether recombinant M. tuberculosis antigens can modulate integrin-mediated macrophage adhesion.Differentiated THP-1 cell line was stimulated with recombinant ESAT-6, CFP-10, and ESAT-6/CFP-10 proteins and evaluated for alterations in the expression levels of α5β1 and α4β1 by semiquantitative real-time polymerase chain reaction. The role of these recombinant antigens in the cytoskeleton rearrangement was determined by adhesion assay and immunofluorescent microscopy.Our data showed that ESAT-6 and ESAT-6/CFP-10 fusion proteins could induce adhesion of macrophages to FN through α4β1 integrin. An increased expression level of α4β1 integrin in comparison with α5β1 integrin in differentiated THP-1 cells was also observed. Results of immunofluorescence studies showed that recombinant proteins-treated THP-1 cells form well-organized stress fibers and focal contacts containing vinculin compared with untreated THP-1 cells.Increased expression level of α4β1 in differentiated THP-1 cells could suggest the important role of α4β1 integrin in adhesion and focal contact formation of macrophages exposed to M. tuberculosis antigens. 
 Tetraspanins CD81 and CD82 facilitate α4β1-mediated adhesion of human erythroblasts to vascular cell adhesion molecule-1. The proliferation and terminal differentiation of erythroid progenitors occurs in human bone marrow within erythroblastic islands, specialised structures consisting of a central macrophage surrounded by developing erythroid cells. Many cell-cell and cell-matrix adhesive interactions maintain and regulate the co-ordinated daily production of reticulocytes. Erythroid cells express only one integrin, α4β1, throughout differentiation, and its interactions with both macrophage Vascular Cell Adhesion Molecule-1 and w  ith extracellular ma  trix fibronectin are critical for erythropoiesis. We observed that proerythroblasts expressed a broad tetraspanin phenotype, and investigated whether any tetraspanin could modulate integrin function. A specific association between α4β1 and CD81, CD82 and CD151 was demonstrated by confocal microscopy and co-immune precipitation. We observed that antibodies to CD81 and CD82 augmented adhesion of proerythroblasts to Vascular Cell Adhesion Molecule-1 but not to the fibronectin spliceoforms FnIII12-IIICS-15 and FnIII12-15. In contrast, different anti-CD151 antibodies augmented or inhibited adhesion of proerythroblasts to Vascular Cell Adhesion Molecule-1 and the fibronectin spliceoform FnIII12-IIICS-15 but not to FnIII12-15. These results strongly suggest that tetraspanins have a functional role in terminal erythropoiesis by modulating interactions of erythroblast α4β1 with both macropha  ges and extracellula  r matrix. 
 Proteomic analysis of α4β1 integrin adhesion complexes reveals α-subunit-dependent protein recruitment. Integrin adhesion receptors mediate cell-cell and ce  ll-extracellular mat  rix interactions, which control cell morphology and migration, differentiation, and tissue integrity. Integrins recruit multimolecular adhesion complexes to their cytoplasmic domains, which provide structural and mechanosensitive signaling connections between the extracellular and intracellular milieux. The different functions of specific integrin heterodimers, such as α4β1 and α5β1, have been attributed to distinct signal transduction mechanisms that are initiated by selective recruitment of adhesion complex components to integrin cytoplasmic tails. Here, we report the isolation of ligand-induced adhesion complexes associated with wild-type α4β1 integrin, an activated α4β1 variant in the absence of the α cytoplasmic domain (X4C0), and a chimeric α4β1 variant with α5 leg and cytoplasmic domains (α4Pα5L), and the cataloguing of their proteomes by MS. Using hierarchical clustering and interaction network analyses, we detail the differential recruitment of proteins and highlight enrichment patterns of proteins to distinct adhesion complexes. We identify previously unreported components of integrin adhesion complexes and observe receptor-specific enrichment of molecules with previously reported links to cell migration and cell signaling processes. Furthermore, we demonstrate colocalization of MYO18A with active integrin in migrating cells. These datasets provide a resource for future studies of integrin receptor-specific signaling events. 
 Conformational regulation of alpha 4 beta 1-integrin affinity by reducing agents. "Inside-out" signaling is independent of and additive to reduction-regulated integrin activation. The alpha(4)beta(1)-integrin (very late antigen-4 (VLA-4), CD49d/CD29) is an adhesion receptor involved in the interaction of lymphocytes, dendritic cells, and stem cells with the   extracellular matrix   and endothelial cells. This and other integrins have the ability to regulate their affinity for ligands through a process termed "inside-out" signaling that affects cell adhesion avidity. Several mechanisms are known to regulate integrin affinity and conformation: conformational changes induced by separation of the C-terminal tails, divalent ions, and reducing agents. Recently, we described a fluorescent LDV-containing small molecule that was used to monitor VLA-4 affinity changes in live cells (Chigaev, A., Blenc, A. M., Braaten, J. V., Kumaraswamy, N., Kepley, C. L., Andrews, R. P., Oliver, J. M., Edwards, B. S., Prossnitz, E. R., Larson, R. S., and Sklar, L. A. (2001) J. Biol. Chem. 276, 48670-48678). Using the same molecule, we also developed a fluorescence resonance energy transfer-based assay to probe the "switchblade-like" opening of VLA-4 upon activation. Here, we investigated the effect of reducing agents on the affinity and conformational state of the VLA-4 integrin simultaneously with cell activation initiated by inside-out signaling through G protein-coupled receptors or Mn(2+) in live cells in real time. We found that reducing agents (dithiothreitol and 2,3-dimercapto-1-propanesulfonic acid) induced multiple states of high affinity of VLA-4, where the affinity change was accompanied by an extension of the integrin molecule. Bacitracin, an inhibitor of the reductive function of the plasma membrane, diminished the effect of dithiothreitol, but had no effect on inside-out signaling. Based on this result and differences in the kinetics of integrin activation, we conclude that conformational activation of VLA-4 by inside-out signaling is independent of and additive to reduction-regulated integrin activation. 
 Real-time analysis of very late antigen-4 affinity modulation by shear. Shear promotes endothelial recruitment of leukocytes, cell activation, and transmigration. Mechanical stress on cells caused by shear can induce a rapid integrin conformational change and activation, followed by an increase in binding to the   extracellular matrix  . The molecular mechanism of increased avidity is unknown. We have shown previously that the affinity of the alpha(4)beta(1) integrin, very late antigen-4 (VLA-4), measured with an LDV-containing small molecule, varies with cellular avidity, measured from cell disaggregation rates. In this study, we measured in real time affinity changes of VLA-4 in response to shear. The resulting affinity was comparable with the state mediated by receptor signaling and corresponded in time with intracellular Ca(2+) responses. Ca(2+) ionophores and N,N'-[1,2-ethanediyl-bis(oxy-2,1-phenylene)]bis[N-[2-[(acetyloxy)methoxy]-2-oxoethyl]]-, bis[(acetyloxy)methyl]ester demonstrate that the affinity regulation of VLA-4 in the presence of shear was related to Ca(2+) signaling. Pertussis toxin treatment implicates G(i) in an unknown pathway that connects shear, Ca(2+) elevation, VLA-4 affinity, and cell avidity. 
 Characterization of plasminogen as an adhesive ligand for integrins alphaMbeta2 (Mac-1) and alpha5beta1 (VLA-5). Plasminogen (Pg) has been implicated in many biologic processes involving extracellular proteolysis. We investigated whether Pg, by virtue of its capacity to be deposited within the   extracellular matrix  , can serve as a ligand for cell surface integrins. We report here that Pg supports cell adhesion by engaging integrins alphaMbeta2 and alpha5beta1. The immobilized Glu-Pg, but not its derivatives with the N-terminal peptide lacking, plasmin and Lys-Pg, supported efficient adhesion that was abolished by anti-alphaMbeta2 and anti-alpha5beta1 integrin-specific monoclonal antibodies (mAbs). In addition, lysine binding sites of Glu-Pg contributed to cell adhesion inasmuch as tranexamic acid and epsilon-aminocaproic acid inhibited cell adhesion. The involvement of alphaMbeta2 and alpha5)beta1 in adhesion to Glu-Pg was demonstrable with blood neutrophils, U937 monocytoid cells, and genetically engineered alphaMbeta2-transfected human embryonic kidney (HEK) 293 cells. In alphaMbeta2, the alphaMI-domain is the binding site for Glu-Pg because the "I-less" form of alphaMbeta2 did not support cell adhesion and the recombinant alphaMI-domain bound Glu-Pg directly. In comparison with cell adhesion, the binding of soluble Glu-Pg to cells and the concomitant generation of plasmin activity was inhibited by anti-alpha5beta1 but not by anti-alphaMbeta2. These findings identify Glu-Pg as an adhesive ligand for integrins alphaMbeta2 and alpha5beta1 and suggest that alpha5beta1 may participate in the binding of soluble Glu-Pg and assist in its activation. 
 Toll-like receptor-9 triggering modulates expression of α-4 integrin on human B lymphocytes and their adhesion to   extracellular matri  x proteins. The interaction of human B lymphocytes as recirculating cells with their microenvironment components including fibronectin is an instrumental process that directs their further responses in an inflammatory milieu or during their development in secondary lymphoid organs. Factors derived from extracellular environment, including those of pathogens, termed pathogen-associated molecular patterns, may have effects on this interaction, yet no study to date has addressed these effects. In this study, we explored the effect of Toll-like receptor 9 (TLR9) triggering on the interaction of normal B cells with fibronectin and collagen.The synthetic analog of TLR9 ligand, CpG-C, was used for stimulating the cells. The expression pattern of very late antigen-4 integrin was studied by fluorescence-activated cell sorting and Western blotting experiments, and cell adhesion was analyzed by fluorometric adhesion assay.CpG at 0.5 μM upregulated fibronectin receptor (very late antigen-4) expression and cell adhesion, and increasing the CpG concentration did not have further effect. Blocking experiments with TLR9 signaling inhibitor, TTAGGG, anti-α4 antibody, and IκBα phosphorylation inhibitor, Bay 11-7082, confirmed that the CpG-induced induction level was TLR9 (partly), very late antigen-4, and nuclear factor-κB-mediated, respectively.This study indicates that TLR9 triggering on B cells influences their interaction   with extracellular   matrix, which will be critical in modulating activation of these cells in conditions, such as infections, and gives a basic insight into the contribution of innate immunity elements in B-cell functional responses. 
 Mapping of the human VLA-alpha 4 gene to chromosome 2q31-q32. The integrin VLA-4 (alpha 4: beta 1; CD49d/CD29) is involved in both cellular adhesion to   extracellular matrix   and cell-cell interactions. We have determined that the human gene coding for VLA-alpha 4 is located on the long arm of chromosome 2 by using fluorescence in situ hybridization. The VLA-alpha 4 gene has been more precisely mapped to the 2q31-q32 region after GTG banding (G-bands by trypsin using Giemsa). These data suggest that the VLA-4 gene belongs to the COL3A1-(ELN-FN)-COL6A3 linkage group and establishes a potential genetic relationship between the alpha 4 and alpha v integrin subunits. 
 Dynamic alterations in integrin α4 expression by hypoxia are involved in trophoblast invasion during early implantation. Implantation of the blastocyst into the maternal endometrium is mediated by a population of well-differentiated primary cells of the placenta known as trophoblasts, which grow in an invasive and destructive fashion similar to tumor cells. Interactions between the endometrium and trophoblasts are regulated by a coordinated interplay of   extracellular matri  x (ECM) proteins secreted by the invading extravillous trophoblasts. Integrins act as adhesion receptors and mediate both cell-ECM and cell-cell interactions. However, the correlation between integrin expression and trophoblast invasion under hypoxia is unclear. Here, we analyzed the expression of integrins in HTR-8/SVneo trophoblast cells exposed to hypoxic conditions in order to demonstrate an association between invasion activity and integrin expression in trophoblasts. Trophoblasts were examined by microarray analysis, RT-PCR, western blotting, and zymography after 1% hypoxic treatment, and cell invasion was estimated. The dynamic expression of integrins and human matrix metalloproteinases (MMPs) was observed under hypoxic conditions. The invasiveness of trophoblasts cultured under 1% hypoxic conditions was significantly greater than that of trophoblasts cultured under normoxic conditions through alterations in MMP-2 and -9 (P < 0.05). Notably, integrin α4 expression during early hypoxia was negatively regulated by hypoxia-inducible factor-1alpha (HIF-1alpha) expression in trophoblasts. The downregulation of integrin α4 expression by siRNA treatment controlled trophoblast invasion activity (P < 0.05). Taken together, we suggest that dynamic changes in integrins, including those in integrin α4 expression by hypoxia, play a regulatory role in trophoblast invasion. These findings expand our understanding of the potential roles of integrin α4 in implantation. 
 Fibulin-5 binds human smooth-muscle cells through alpha5beta1 and alpha4beta1 integrins, but does not support receptor activation. Fibulin-5, an   extracellular matrix   glycoprotein expressed in elastin-rich tissues, regulates vascular cell behaviour and elastic fibre deposition. Recombinant full-length human fibulin-5 supported primary human aortic SMC (smooth-muscle cell) attachment through alpha5beta1 and alpha4beta1 integrins. Cells on fibulin-5 spread poorly and displayed prominent membrane ruffles but no stress fibres or focal adhesions, unlike cells on fibronectin that also binds these integrins. Cell migration and proliferation were significantly lower on fibulin-5 than on fibronectin. Treatment of cells on fibulin-5 with a beta1 integrin-activating antibody induced stress fibres, increased attachment, migration and proliferation, and stimulated signalling of epidermal growth factor receptor and platelet-derived growth factor receptors alpha and beta. Fibulin-5 also modulated fibronectin-mediated cell spreading and morphology. We have thus identified the beta1 integrins on primary SMCs that fibulin-5 interacts with, and have shown that failure of fibulin-5 to activate these receptors limits cell spreading, migration and proliferation. 
 Activated integrin VLA-4 localizes to the lamellipodia and mediates T cell migration on VCAM-1. Lymphocyte migration from blood into lymphoid tissues or to sites of inflammation occurs through interactions between cell surface integrins and their ligands expressed on the vascular endothelium and the   extracellular matrix  . VLA-4 (alpha(4)beta(1)) is a key integrin in the effective trafficking of lymphocytes. Although it has been well established that integrins undergo functionally significant conformational changes to mediate cell adhesion, there is no mechanistic information that explains how these are dynamically and spatially regulated during lymphocyte polarization and migration. Using dynamic fluorescence resonance energy transfer analysis of a novel VLA-4 FRET sensor under total internal reflection fluorescence microscopy, we show that VLA-4 activation localizes to the lamellipodium in living cells. During T cell migration on VCAM-1, VLA-4 activation concurs with spatial redistribution of chemokine receptor and active Rap1 at the leading edge. Selective inhibition of the activated VLA-4 at the leading edge with a small molecule inhibitor is sufficient to block T cell migration. These data suggest that a subpopulation of activated VLA-4 is mainly localized to the leading edge of polarized human T cells and is critical for T cell migration on VCAM-1. 
 Functional and structural analysis of VLA-4 integrin alpha 4 subunit cleavage. The cell surface heterodimer VLA-4 (alpha 4 beta 1), a member of the integrin family of adhesion receptors, is involved in both cell-  extracellular matrix   and cell-cell adhesion. Unlike any other integrin alpha subunit, the intact (150 kDa) alpha 4 subunit of VLA-4 can sometimes be cleaved into two noncovalently associated fragments (80 and 70 kDa). Using biosynthetic and mixing experiments, we found that human alpha 4 cleavage is a regulated, compartmentalized event, occurring soon after maturation of the beta 1-associated alpha 4 subunit. Cleavage of alpha 4, which is increased following T cell activation, has been suggested to correlate with altered VLA-4 functions. To address directly the functional importance of alpha 4 cleavage, we have studied VLA-4-mediated adhesion functions in cells expressing intact alpha 4 in comparison with cells expressing cleaved alpha 4. For this purpose, we first sequenced the N terminus of the endogenously produced 70-kDa alpha 4 fragment and identified the alpha 4 cleavage site between Lys557-Arg558 and Ser559. To abolish cleavage, we converted Arg558 to Leu or Lys557 to Gln by site-directed mutagenesis of the alpha 4 cDNA and then transfected both mutant and wild type alpha 4 cDNAs into VLA-4-negative K562 cells. Whereas transfection with wild type alpha 4 cDNA yielded predominantly cleaved alpha 4 subunit, the Leu558-alpha 4 yielded only intact alpha 4 subunit, and Gln557-alpha 4 yielded mostly intact alpha 4 subunit. Transfectants with the intact or the cleaved alpha 4 were equally capable of engaging in VLA-4-dependent adhesion to vascular cell adhesion molecule-1 and to the Hep II fragment of fibronectin (40 kDa) and aggregated equally well in response to anti-alpha 4 antibodies. Thus, cleavage of the alpha 4 subunit in these transfectants did not alter any of the known VLA-4-mediated adhesion functions. 
 Expression of functional alpha 4 beta 1 integrin by human dermal fibroblasts. Fibroblasts interact with the   extracellular matrix   through cell-surface receptors belonging to the integrin family. In this report, we present evidence that cultured normal human fibroblasts express the integrin alpha 4 beta 1 and that this receptor facilitates fibroblast attachment to fibronectin. Fluorescence-activated cell sorter analysis and immunoprecipitation with monoclonal antibodies demonstrated that normal dermal fibroblasts express the alpha 4-subunit on the cell surface, primarily in association with the beta 1-subunit. Cell-attachment assays demonstrated that normal human fibroblasts can attach to the 40-kDa fibronectin fragment containing the type III connecting segment domain recognized by alpha 4 beta 1. Adhesion to this fragment was inhibited by anti-alpha 4 antibody. Furthermore, our results indicate that alpha 4 beta 1 collaborates with another fibronectin receptor, alpha 5 beta 1, during fibroblast attachment to full-length fibronectin. The region of fibronectin recognized by alpha 5 beta 1 contains the amino acid sequence arg-gly-asp (RGD). A short synthetic RGD peptide, or the 120-kDa fibronectin fragment containing the RGD sequence, only partially inhibited attachment to full-length fibronectin, suggesting that fibroblasts utilize more than the RGD recognition sequence for binding to fibronectin. Accordingly, RGD peptide combined with anti-alpha 4 antibody produced more potent inhibition of cell attachment than either reagent alone. These observations show for the first time that functional alpha 4 beta 1 fibronectin receptor is not restricted to lymphoid cells and transformed cells. 
 Communication between integrin receptors facilitates epicardial cell adhesion and matrix organization. Formation of the epicardium requires interactions between alpha(4)beta(1) integrin, and the   extracellular matrix  . We investigated the role of other integrins expressed by epicardial cells. We detected transcripts for alpha(5), alpha(8), alpha(v), beta(1), beta(3), and beta(5) integrins in the chick proepicardial organ (PE). We demonstrate that alpha(5)beta(1), alpha(8)beta(1), and alpha(v)beta(3) integrins are expressed by chick epicardial mesothelial cells (EMCs). Migration of EMCs in vitro was reduced by RGD-containing peptides. Using adenoviruses expressing an antisense to chick alpha(4) (AdGFPalpha4AS), full-length (Adhalpha4V5), and C-terminal deleted alpha(4) (Adhalpha4DeltaCV5), we found that EMCs were less able to adhere to vitronectin and fibronectin(120) indicating that alpha(4)beta(1) plays a role in regulating EMC adhesion to ligands of alpha(5)beta(1), alpha(8)beta(1), and alpha(v)beta(3). In Adhalpha4DeltaCV5-infected EMCs, alpha(5)beta(1) was diminished in fibrillar adhesions and new FN matrix assembly was abnormal. We propose that cooperation between alpha(4)beta(1) and RGD integrins is important for EMC adhesion and subepicardial matrix formation. 
 Integrins α4 and αM, collagen1A1, and matrix metalloproteinase 7 are upregulated in acute Kawasaki disease vasculopathy. Kawasaki disease (KD) can result in fatal coronary artery (CA) aneurysms, especially if left untreated. Our recent studies of its vascular pathology revealed subacute/chronic vasculitis that begins early in the illness with the proliferation of smooth muscle cell-derived myofibroblasts in a comple  x extracellular matr  ix (ECM). We hypothesized that a dysregulation of specific ECM and adhesion molecules occurs in KD CAs.Gene expression profiling for ECM and adhesion molecules was performed on six acute KD and eight control CAs using a targeted real-time PCR array approach.Integrins α4 and αM (ITGA4, ITGAM), collagen type I, α1 (COL1A1), and matrix metalloproteinase 7 (MMP7) were significantly upregulated in KD CAs as compared with controls. Immunohistochemistry with anti-ITGAM antibodies revealed expression on inflammatory cells within the CA wall in patients with KD but not in controls.Integrins ITGA4 and ITGAM are upregulated in KD vasculopathy, probably promoting inflammatory recruitment that stimulates smooth muscle cell transition to myofibroblasts and their proliferation. MMP7 probably enhances myofibroblast proliferation and luminal lesion expansion, and overexpression of COL1A1 may lead to CA stenosis. Identification of the molecular pathogenesis of KD vasculopathy may lead to the development of circulating biomarkers and to directed therapeutic interventions. 
 A single mRNA encodes the alpha 150 and alpha 80/70 forms of the alpha subunit of VLA4. VLA4 is a cell surface heterodimer (alpha 4 beta 1, CD49d/CD29) which belongs to the integrin family and is involved in cell-  extracellular matrix   interactions, as well as in intercellular adhesion. Unlike other integrin alpha subunits, the alpha subunit of VLA4 (alpha 4) can appear as a 150-kDa polypeptide (alpha 150), or cleaved into two non-covalently associated polypeptides (alpha 70-80). The relative proportion of each form is highly variable among different cells and is dependent on the state of cellular activation. The alpha 4 cleavage site has recently been shown to occur between residues Arg558-Ser559. We report the isolation of genomic clones encoding the alpha 4 subunit, the location of the cleavage site-encoding nucleotides to a specific exon and the determination of the exon-intron organization around the cleavage site. Comparison with the gpIIb (CD41) and the alpha x (p150,95 alpha, CD11c) genes revealed a similar genomic structure in this region, with exons of similar length separated by introns of identical phase. The structure of the alpha 4 mRNA in cells expressing the alternative forms of alpha 4 has been analyzed by means of reverse transcription-polymerase chain reaction. Our results indicate that the exon encoding the cleavage site is present in alpha 4 mRNA molecules from cells expressing either the alpha 150 or the alpha 70-80 form on the cell surface. Moreover, the structure of the alpha 4 mRNA around the cleavage site does not change during the switch towards the alpha 70-80 form that takes place upon lymphocyte activation. Therefore, both forms of alpha 4 arise from a common mRNA, the alpha 150 form contains the cleavage sequence and the alpha 70-80 form must be generated by a post-translational proteolytic event. 
 Evaluating the performance of fibronectin 1 (FN1), integrin α4β1 (ITGA4), syndecan-2 (SDC2), and glycoprotein CD44 as the potential biomarkers of oral squamous cell carcinoma (OSCC). Som  e extracellular matr  ix genes as prognostic biomarkers for oral squamous cell carcinoma (OSCC) were evaluated.We investigated gene expression of fibronectin 1 (FN1), integrin α4β1 (ITGA4), syndecan-2 (SDC2), and glycoprotein CD44 in matched OSCC/margin tissues.Areas under receiver-operating characteristic curves (AUCs) of relative mRNA expression of FN1, ITGA4, SDC2, and CD44 were 0.700, 0.677, 0.513, and 0.549, respectively. For tongue/mouth floor and edentulous ridge, AUC for FN1 and ITGA4 were 0.827 and 0.725 and sensitivities/specificities were 80%/84% and 88%/52%, respectively.FN1 and ITGA4 are potential OSCC biomarkers for tongue/mouth floor and edentulous ridge. 
 Mechanisms of VCAM-1 and fibronectin binding to integrin alpha 4 beta 1: implications for integrin function and rational drug design. Integrin alpha 4 beta 1 can mediate both cell-cell and cell-  extracellular matrix   adhesion by binding to either fibronectin or vascular cell adhesion molecule 1 (VCAM-1). Both interactions are important for extravasation of leukocytes from the blood implying that rationally designed inhibitors of alpha 4 beta 1 function may be useful for treating a various inflammatory conditions. The mechanisms of ligand binding by alpha 4 beta 1 are complicated by the fact that alternative splicing can generate different isoforms of the receptor-binding domains in both fibronectin and VCAM-1. Therefore, in addition to developing alpha 4 beta 1 antagonists, we have also been interested in identifying isoform-specific functions. Recombinant ligand variants have been tested in adhesion and direct receptor-binding assays and each molecule was found to have a different inherent affinity for alpha 4 beta 1 that endows them with different adhesive activities. This suggests that alternative splicing may regulate alpha 4 beta 1-dependent motility in vivo. The initial strategy that we have adopted to develop alpha 4 beta 1 inhibitors has been to identify key amino acid residues and peptide sequences participating in the receptor-ligand binding event and to use this information to generate synthetic mimetics. Three active sites have been identified in fibronectin by testing truncated proteins, expressing recombinant fragments and screening synthetic peptides. Two of these sites employ versions of a novel integrin-binding motif, LDVP/IDAP. A key active site in VCAM-1 has been identified by similar approaches as the related sequence IDSP. Since IDSP-like sequences are probably used by other integrin-binding immunoglobulins, derivatives of these peptides may turn out to be the forerunners of a new generation of therapeutic agents with multiple applications. 
  [Ii]nvasive  PubMed  
 [Study on the relationship between the expression of adhesion molecules and the   invasive  ness of acute myeloid leukemia cells]. To study the relationship between the expression of adhesion molecules CD49d (VLA-4) and CD11a (LFA-1) and the   invasive  ness of acute myeloid leukemia (AML) cells.Peripheral blood and/or bone marrow samples from 50 AML patients were investigated by APAAP and Western blotting method.Extramedullary invasion developed in 32 of 50 patients (64%). The expression of CD49d and CD11a in the   invasive   group was much higher than that in the non-  invasive   group (P<0.005), while the difference between the leukemic cells from bone marrow and peripheral blood for CD49d/CD11a expression was not significant.AML cells might adhere to and get through vascular endothelium by CD49d/VCAM-1 and CD11a/ICAM-1 adhesion mechanism, and the expressions of CD49d and CD11a were not critically responsible for the release of leukemic cells from bone marrow. 
 Dynamic alterations in integrin α4 expression by hypoxia are involved in trophoblast invasion during early implantation. Implantation of the blastocyst into the maternal endometrium is mediated by a population of well-differentiated primary cells of the placenta known as trophoblasts, which grow in an   invasiv  e and destructive fashion similar to tumor cells. Interactions between the endometrium and trophoblasts are regulated by a coordinated interplay of extracellular matrix (ECM) proteins secreted by the invading extravillous trophoblasts. Integrins act as adhesion receptors and mediate both cell-ECM and cell-cell interactions. However, the correlation between integrin expression and trophoblast invasion under hypoxia is unclear. Here, we analyzed the expression of integrins in HTR-8/SVneo trophoblast cells exposed to hypoxic conditions in order to demonstrate an association between invasion activity and integrin expression in trophoblasts. Trophoblasts were examined by microarray analysis, RT-PCR, western blotting, and zymography after 1% hypoxic treatment, and cell invasion was estimated. The dynamic expression of integrins and human matrix metalloproteinases (MMPs) was observed under hypoxic conditions. The   invasiv  eness of trophoblasts cultured under 1% hypoxic conditions was significantly greater than that of trophoblasts cultured under normoxic conditions through alterations in MMP-2 and -9 (P < 0.05). Notably, integrin α4 expression during early hypoxia was negatively regulated by hypoxia-inducible factor-1alpha (HIF-1alpha) expression in trophoblasts. The downregulation of integrin α4 expression by siRNA treatment controlled trophoblast invasion activity (P < 0.05). Taken together, we suggest that dynamic changes in integrins, including those in integrin α4 expression by hypoxia, play a regulatory role in trophoblast invasion. These findings expand our understanding of the potential roles of integrin α4 in implantation. 
 p38MAPK induces cell surface alpha4 integrin downregulation to facilitate erbB-2-mediated invasion. We have previously shown that human breast cancer cells that overexpress erbB-2 are growth factor-independent. In order to test the contribution of erbB-2 to this and other transformed phenotypes without the genetic instability of cancer cells, erbB-2 was overexpressed in human mammary epithelial (HME) cells. ErbB-2-overexpressing HME cells exhibit several transformed phenotypes including cell surface alpha(4) integrin downregulation and   invasive  ness. We formulated a model for   invasive  ness that depends on a cell's ability to downregulate alpha(4) integrin. As small G-proteins play a role in cytoskeleton remodeling and as this is a likely route for alpha(4) integrin trafficking, we investigated the role of small G-proteins and their downstream signals in mediating alpha(4) integrin downregulation and   invasive  ness using Rac 1. Dominant-negative Rac 1 blocked erbB-2-mediated invasion and reversed erbB-2-mediated alpha(4) integrin downregulation. In addition, constitutively active Rac 1 induced alpha(4) integrin downregulation and   invasive  ness. In erbB-2-overexpressing and in constitutively active Rac 1-expressing cells, a p38MAP kinase (p38MAPK) inhibitor blocked   invasive  ness and reversed alpha(4) integrin downregulation. These data suggest a model in which erbB-2 signaling activates Rac 1, which, in turn, activates p38MAPK, leading to the downregulation of alpha(4) integrin. These data strengthen the model where loss of alpha(4) integrin at the cell surface, leading to reduced alpha(4) integrin binding to plasma fibronectin, plays a role in erbB-2-mediated   invasive  ness. 
 Aberrant DNA methylation of integrin α4 in human breast cancer. Integrins are cell surface receptors that mediate cell-cell/extracellular interactions and have shown an association with metastasis or transformation in several cancers. However, the correlation between the clinicopathological status of breast cancer and the altered integrin α4 status is not clear. In this study, we investigated DNA methylation of integrin α4 in breast cancer. We retrieved 351 cases of surgically resected breast cancer (2  90 invas  ive carcinoma and 61 intraductal carcinoma). Methylation-specific polymerase chain reaction was performed to determine integrin α4 methylation status. Integrin α4 methylation was frequently observed in breast cancer specimens (145/351 cases, 41.3 %). In addition, DNA methylation of integrin α4 showed statistical correlation with HER2 positivity and higher histologic grade (p = 0.001, 0.008 in ductal carcinoma in situ and 0.0  03 in in  vasive ductal carcinoma). However, other clinicopathological data such as estrogen receptor, progesterone receptor, metastasis, and TNM status showed no statistical correlation. Moreover, we found that the downregulated expression of integrin α4 in a heavily methylated breast cancer cell line (ZR-75) was restored by treatment with 5-aza-2'deoxycytidine, a DNA methyltransferase inhibitor, implying transcriptional silencing by DNA methylation. We observed that integrin α4 methylation is associated with the histologic grade of tumors and lymph node metastasis. Also, this data supports a previous study that suggested integrin α4 and HER2 are involved in the same signaling pathway. DNA methylation of integrin α4 may be a poor prognostic factor which affects undifferentiated histologic change of breast cancer. 
  [Mm]etastasize  PubMed  
 Acquisition of resistance toward HYD1 correlates with a reduction in cleaved α4 integrin expression and a compromised CAM-DR phenotype. We recently reported that the β1 integrin antagonist, referred to as HYD1, induces necrotic cell death in myeloma cell lines as a single agent using in vitro and in vivo models. In this article, we sought to delineate the determinants of sensitivity and resistance toward HYD1-induced cell death. To this end, we developed an HYD1 isogenic resistant myeloma cell line by chronically exposing H929 myeloma cells to increasing concentrations of HYD1. Our data indicate that the acquisition of resistance toward HYD1 correlates with reduced levels of the cleaved α4 integrin subunit. Consistent with reduced VLA-4 (α4β1) expression, the resistant variant showed ablated functional binding to fibronectin, VCAM-1, and the bone marrow stroma cell line HS-5. The reduction in binding of the resistant cell line to HS-5 cells translated to a compromised cell adhesion-mediated drug resistant phenotype as shown by increased sensitivity to melphalan- and bortezomib-induced cell death in the bone marrow stroma coculture model of drug resistance. Importantly, we show that HYD1 is more potent in relapsed myeloma specimens than newly diagnosed patients, a finding that correlated with α4 integrin expression. Collectively, these data indicate that this novel d-amino acid peptide may represent a good candidate for pursuing clinical trials in relapsed myeloma and in particular patients with high levels of α4 integrin. Moreover, our data provide further rationale for continued preclinical development of HYD1 and analogues of HYD1 for the treatment of multiple myeloma and potentially other tumors that home   and/or meta  stasize to the bone. 
  [Mm]etastasis  PubMed  
 High affinity interaction of integrin alpha4beta1 (VLA-4) and vascular cell adhesion molecule 1 (VCAM-1) enhances migration of human melanoma cells across activated endothelial cell layers. The capacity of tumor cells to form metastatic foci correlates with their ability to interact with and migrate through endothelial cell layers. This process involves multiple adhesive interactions between tumor cells and the endothelium. Only little is known about the molecular nature of these interactions during extravasation of tumor cells. In human melanoma cells, the integrin alphavbeta3 is involved in transendothelial migration and its expression correlates with   metastasis  . However, many human melanoma cells do not express beta3 integrins. Therefore, it remained unclear how these cells undergo transendothelial migration. In this study we show that human melanoma cells with different metastatic potency, which do not express beta2 or beta3 integrins, express the VCAM-1 receptor alpha4beta1. VCAM-1 is up-regulated on activated endothelial cells and is known to promote transendothelial migration of leukocytes. Interestingly, despite comparable cell surface levels of alpha4beta1, only the highly metastatic melanoma cell lines MV3 and BLM, but not the low metastatic cell lines IF6 and 530, bind VCAM-1 with high affinity without further stimulation, and are therefore able to adhere to and migrate on isolated VCAM-1. Moreover, we demonstrate that function-blocking antibodies against the integrin alpha4beta1, as well as siRNA-mediated knock-down of the alpha4 subunit in these highly metastatic human melanoma cells reduce their transendothelial migration. These data imply that only high affinity interactions between the integrin alpha4beta1 on melanoma cells and VCAM-1 on activated endothelial cells may enhance the metastatic capacity of human beta2/beta3-negative melanoma cells. 
 Integrin VLA-4 enhances sialyl-Lewisx/a-negative melanoma adhesion to and extravasation through the endothelium under low flow conditions. During their passage through the circulatory system, tumor cells undergo extensive interactions with various host cells including endothelial cells. The capacity of tumor cells to form   metastasis   is related to their ability to interact with and extravasate through endothelial cell layers, which involves multiple adhesive interactions between tumor cells and endothelium (EC). Thus it is essential to identify the adhesive receptors on the endothelial and melanoma surface that mediate those specific adhesive interactions. P-selectin and E-selectin have been reported as adhesion molecules that mediate the cell-cell interaction of endothelial cells and melanoma cells. However, not all melanoma cells express ligands for selectins. In this study, we elucidated the molecular constituents involved in the endothelial adhesion and extravasation of sialyl-Lewis(x/a)-negative melanoma cell lines under flow in the presence and absence of polymorphonuclear neutrophils (PMNs). Results show the interactions of alpha(4)beta(1) (VLA-4) on sialyl-Lewis(x/a)-negative melanoma cells and vascular adhesion molecule (VCAM-1) on inflamed EC supported melanoma adhesion to and subsequent extravasation through the EC in low shear flow. These findings provide clear evidence for a direct role of the VLA-4/VCAM-1 pathway in melanoma cell adhesion to and extravasation through the vascular endothelium in a shear flow. PMNs facilitated melanoma cell extravasation under both low and high shear conditions via the involvement of distinct molecular mechanisms. In the low shear regime, beta(2)-integrins were sufficient to enhance melanoma cell extravasation, whereas in the high shear regime, selectin ligands and beta(2)-integrins on PMNs were necessary for facilitating the melanoma extravasation process. 
 Adhesion of osteosarcoma cells to the 70-kDa core region of thrombospondin-1 is mediated by the alpha 4 beta 1 integrin. Thrombospondin-1 (TSP-1) is an extracellular glycoprotein that is involved in a variety of physiological processes such as tumor cell adhesion, invasion, and   metastasis  . It has been hypothesized that TSP-1 provides an adhesive matrix for osteosarcoma cells. Here we present data showing that TSP-1 can promote cell substrate adhesion to U2OS and SAOS cells through the alpha 4 beta 1 integrin. The dose-dependent adhesion to TSP-1 was inhibited by anti-integrin antibodies directed against the alpha 4 or beta 1 subunit, but not by control antibodies against other integrins. To localize the potential alpha 4 beta 1-binding site within the TSP-1 molecule, the protein was subjected to limited proteolysis with chymotrypsin in the absence of calcium. The stable 70-kDa core fragment produced under these conditions promoted alpha 4 beta 1-dependent osteosarcoma cell adhesion in a manner similar to that of the intact protein. Moreover adhesion experiments with neutralizing antibodies revealed that the adhesion was totally dependent on the alpha 4 beta 1 interaction. Further blocking experiments with potential inhibitory peptides revealed that the alpha 4 beta 1-mediated adhesion was not influenced by peptides containing the RGD sequence. Attachment to the 70-kDa fragment was strongly inhibited by the CS-1 peptide, which represents the most active recognition domain for alpha 4 beta 1 integrin in fibronectin. The present data provide evidence that TSP-1 contains an alpha 4 beta 1 integrin-binding site within the 70-kDa core region. 
 Integrin alpha4beta1 signaling is required for lymphangiogenesis and tumor   metastasis  . Recent studies have shown that lymphangiogenesis or the growth of lymphatic vessels at the periphery of tumors promotes tumor   metastasis   to lymph nodes. We show here that the fibronectin-binding integrin alpha4beta1 and its ligand fibronectin are novel functional markers of proliferative lymphatic endothelium. Tumors and lymphangiogenic growth factors, such as vascular endothelial growth factor-C (VEGF-C) and VEGF-A, induce lymphatic vessel expression of integrin alpha4beta1. Integrin alpha4beta1 then promotes growth factor and tumor-induced lymphangiogenesis, as genetic loss of integrin alpha4beta1 expression in Tie2Cre+ alpha4(loxp/loxp) mice or genetic loss of alpha4 signaling in alpha4Y991A knock-in mice blocks growth factor and tumor-induced lymphangiogenesis, as well as tumor   metastasis   to lymph nodes. In addition, antagonists of integrin alpha4beta1 suppress lymphangiogenesis and tumor   metastasis  . Our studies show that integrin alpha4beta1 and the signals it transduces regulate the adhesion, migration, invasion, and survival of proliferating lymphatic endothelial cells. As suppression of alpha4beta1 expression, signal transduction, or function in tumor lymphatic endothelium not only inhibits tumor lymphangiogenesis but also prevents metastatic disease, these results show that integrin alpha4beta1-mediated tumor lymphangiogenesis promotes   metastasis   and is a useful target for the suppression of metastatic disease. 
 IL-18 regulates melanoma VLA-4 integrin activation through a Hierarchized sequence of inflammatory factors. Very late antigen-4 (VLA-4) is frequently overexpressed on melanoma cells contributing to inflammation-dependent   metastasis  . Melanoma cell adhesion to endothelium via VLA-4-vascular cell adhesion molecule-1 (VCAM-1) interaction was used to study VLA-4 activation during melanoma cell response to inflammation. Cooperation among major inflammatory mediators was analyzed in melanoma cells exposed to single inflammatory factors in the presence of inhibitors for other assayed mediators. A stepwise cascade of hierarchized molecules heterogeneously made and used during melanoma response to IL-18, induced hydrogen peroxide (H2O2), in turn activating VLA-4 and melanoma cell adhesion to endothelium. The cascade involved prostaglandin E2 (PGE2) production from melanoma induced by IL-18-dependent tumor necrosis factor-α (TNFα); next, PGE2-induced IL-1β via vascular endothelial growth factor (VEGF) secretion, which in turn induced VLA-4 activation via cyclooxygenase 2-dependent H2O2. This sequence operated in IL-18R/VLA-4/VEGF-expressing murine (B16) and human (A375 and 883) melanomas, but not in those without this phenotype. Separation of active VLA-4-expressing B16 melanoma cells through immobilized VCAM-1 verified their higher IL-18R/TNFR1/VEGFR2 expression and metastatic growth than inactive VLA-4-expressing cells. However, cooperation among melanoma cell sub-populations with heterogeneous cytokine receptor levels may occur through VLA-4-stimulating factors, leading to intratumoral amplification of metastatic potential. Therefore, expression of the VLA-4-stimulating factor sequence may help to predict melanoma prometastatic risk, and offers therapeutic targets for metastatic melanoma deactivation through VLA-4 activation blockade. 
 IL-18 regulates melanoma VLA-4 integrin activation through a Hierarchized sequence of inflammatory factors. Very late antigen-4 (VLA-4) is frequently overexpressed on melanoma cells contributing to inflammation-dependent   metastasis  . Melanoma cell adhesion to endothelium via VLA-4-vascular cell adhesion molecule-1 (VCAM-1) interaction was used to study VLA-4 activation during melanoma cell response to inflammation. Cooperation among major inflammatory mediators was analyzed in melanoma cells exposed to single inflammatory factors in the presence of inhibitors for other assayed mediators. A stepwise cascade of hierarchized molecules heterogeneously made and used during melanoma response to IL-18, induced hydrogen peroxide (H2O2), in turn activating VLA-4 and melanoma cell adhesion to endothelium. The cascade involved prostaglandin E2 (PGE2) production from melanoma induced by IL-18-dependent tumor necrosis factor-α (TNFα); next, PGE2-induced IL-1β via vascular endothelial growth factor (VEGF) secretion, which in turn induced VLA-4 activation via cyclooxygenase 2-dependent H2O2. This sequence operated in IL-18R/VLA-4/VEGF-expressing murine (B16) and human (A375 and 883) melanomas, but not in those without this phenotype. Separation of active VLA-4-expressing B16 melanoma cells through immobilized VCAM-1 verified their higher IL-18R/TNFR1/VEGFR2 expression and metastatic growth than inactive VLA-4-expressing cells. However, cooperation among melanoma cell sub-populations with heterogeneous cytokine receptor levels may occur through VLA-4-stimulating factors, leading to intratumoral amplification of metastatic potential. Therefore, expression of the VLA-4-stimulating factor sequence may help to predict melanoma prometastatic risk, and offers therapeutic targets for metastatic melanoma deactivation through VLA-4 activation blockade. 
 Integrin alpha4beta1 signaling is required for lymphangiogenesis and tumor   metastasis  . Recent studies have shown that lymphangiogenesis or the growth of lymphatic vessels at the periphery of tumors promotes tumor   metastasis   to lymph nodes. We show here that the fibronectin-binding integrin alpha4beta1 and its ligand fibronectin are novel functional markers of proliferative lymphatic endothelium. Tumors and lymphangiogenic growth factors, such as vascular endothelial growth factor-C (VEGF-C) and VEGF-A, induce lymphatic vessel expression of integrin alpha4beta1. Integrin alpha4beta1 then promotes growth factor and tumor-induced lymphangiogenesis, as genetic loss of integrin alpha4beta1 expression in Tie2Cre+ alpha4(loxp/loxp) mice or genetic loss of alpha4 signaling in alpha4Y991A knock-in mice blocks growth factor and tumor-induced lymphangiogenesis, as well as tumor   metastasis   to lymph nodes. In addition, antagonists of integrin alpha4beta1 suppress lymphangiogenesis and tumor   metastasis  . Our studies show that integrin alpha4beta1 and the signals it transduces regulate the adhesion, migration, invasion, and survival of proliferating lymphatic endothelial cells. As suppression of alpha4beta1 expression, signal transduction, or function in tumor lymphatic endothelium not only inhibits tumor lymphangiogenesis but also prevents metastatic disease, these results show that integrin alpha4beta1-mediated tumor lymphangiogenesis promotes   metastasis   and is a useful target for the suppression of metastatic disease. 
 Integrin VLA-4 enhances sialyl-Lewisx/a-negative melanoma adhesion to and extravasation through the endothelium under low flow conditions. During their passage through the circulatory system, tumor cells undergo extensive interactions with various host cells including endothelial cells. The capacity of tumor cells to form   metastasis   is related to their ability to interact with and extravasate through endothelial cell layers, which involves multiple adhesive interactions between tumor cells and endothelium (EC). Thus it is essential to identify the adhesive receptors on the endothelial and melanoma surface that mediate those specific adhesive interactions. P-selectin and E-selectin have been reported as adhesion molecules that mediate the cell-cell interaction of endothelial cells and melanoma cells. However, not all melanoma cells express ligands for selectins. In this study, we elucidated the molecular constituents involved in the endothelial adhesion and extravasation of sialyl-Lewis(x/a)-negative melanoma cell lines under flow in the presence and absence of polymorphonuclear neutrophils (PMNs). Results show the interactions of alpha(4)beta(1) (VLA-4) on sialyl-Lewis(x/a)-negative melanoma cells and vascular adhesion molecule (VCAM-1) on inflamed EC supported melanoma adhesion to and subsequent extravasation through the EC in low shear flow. These findings provide clear evidence for a direct role of the VLA-4/VCAM-1 pathway in melanoma cell adhesion to and extravasation through the vascular endothelium in a shear flow. PMNs facilitated melanoma cell extravasation under both low and high shear conditions via the involvement of distinct molecular mechanisms. In the low shear regime, beta(2)-integrins were sufficient to enhance melanoma cell extravasation, whereas in the high shear regime, selectin ligands and beta(2)-integrins on PMNs were necessary for facilitating the melanoma extravasation process. 
 High affinity interaction of integrin alpha4beta1 (VLA-4) and vascular cell adhesion molecule 1 (VCAM-1) enhances migration of human melanoma cells across activated endothelial cell layers. The capacity of tumor cells to form metastatic foci correlates with their ability to interact with and migrate through endothelial cell layers. This process involves multiple adhesive interactions between tumor cells and the endothelium. Only little is known about the molecular nature of these interactions during extravasation of tumor cells. In human melanoma cells, the integrin alphavbeta3 is involved in transendothelial migration and its expression correlates with   metastasis  . However, many human melanoma cells do not express beta3 integrins. Therefore, it remained unclear how these cells undergo transendothelial migration. In this study we show that human melanoma cells with different metastatic potency, which do not express beta2 or beta3 integrins, express the VCAM-1 receptor alpha4beta1. VCAM-1 is up-regulated on activated endothelial cells and is known to promote transendothelial migration of leukocytes. Interestingly, despite comparable cell surface levels of alpha4beta1, only the highly metastatic melanoma cell lines MV3 and BLM, but not the low metastatic cell lines IF6 and 530, bind VCAM-1 with high affinity without further stimulation, and are therefore able to adhere to and migrate on isolated VCAM-1. Moreover, we demonstrate that function-blocking antibodies against the integrin alpha4beta1, as well as siRNA-mediated knock-down of the alpha4 subunit in these highly metastatic human melanoma cells reduce their transendothelial migration. These data imply that only high affinity interactions between the integrin alpha4beta1 on melanoma cells and VCAM-1 on activated endothelial cells may enhance the metastatic capacity of human beta2/beta3-negative melanoma cells. 
 Adhesion of osteosarcoma cells to the 70-kDa core region of thrombospondin-1 is mediated by the alpha 4 beta 1 integrin. Thrombospondin-1 (TSP-1) is an extracellular glycoprotein that is involved in a variety of physiological processes such as tumor cell adhesion, invasion, and   metastasis  . It has been hypothesized that TSP-1 provides an adhesive matrix for osteosarcoma cells. Here we present data showing that TSP-1 can promote cell substrate adhesion to U2OS and SAOS cells through the alpha 4 beta 1 integrin. The dose-dependent adhesion to TSP-1 was inhibited by anti-integrin antibodies directed against the alpha 4 or beta 1 subunit, but not by control antibodies against other integrins. To localize the potential alpha 4 beta 1-binding site within the TSP-1 molecule, the protein was subjected to limited proteolysis with chymotrypsin in the absence of calcium. The stable 70-kDa core fragment produced under these conditions promoted alpha 4 beta 1-dependent osteosarcoma cell adhesion in a manner similar to that of the intact protein. Moreover adhesion experiments with neutralizing antibodies revealed that the adhesion was totally dependent on the alpha 4 beta 1 interaction. Further blocking experiments with potential inhibitory peptides revealed that the alpha 4 beta 1-mediated adhesion was not influenced by peptides containing the RGD sequence. Attachment to the 70-kDa fragment was strongly inhibited by the CS-1 peptide, which represents the most active recognition domain for alpha 4 beta 1 integrin in fibronectin. The present data provide evidence that TSP-1 contains an alpha 4 beta 1 integrin-binding site within the 70-kDa core region. 
 Integrin α4 Enhances   Metastasi  s and May Be Associated with Poor Prognosis in MYCN-low Neuroblastoma. High-risk neuroblastoma is associated with an overall survival rate of 30-50%. Neuroblastoma-expressed cell adhesion receptors of the integrin family impact cell adhesion, migration, proliferation and survival. Integrin α4 is essential for neural crest cell motility during development, is highly expressed on leukocytes, and is critical for transendothelial migration. Thus, cancer cells that express this receptor may exhibit increased metastatic potential. We show that α4 expression in human and murine neuroblastoma cell lines selectively enhances in vitro interaction with the alternatively spliced connecting segment 1 of fibronectin, as well as vascular cell adhesion molecule-1 and increases migration. Integrin α4 expression enhanced experimen  tal metast  asis in a syngeneic tumor model, reconstituting a pattern of organ involvement similar to that seen in patients. Accordingly, antagonism of integrin α4 blo  cked metas  tasis, suggesting adhesive function of the integrin is required. However, adhesive function was not sufficient, as mutants of integrin α4 that conserved the matrix-adhesive and promigratory function in vitro were compromised in their metastatic capacity in vivo. Clinically, integrin α4 is more frequently expressed in non-MYNC amplified tumors, and is selectively associated with poor prognosis in this subset of disease. These results reveal an unexpected role for integrin α4 in neuroblastoma dissemination and identify α4 as a potential prognostic indicator and therapeutic target. 
 Aberrant DNA methylation of integrin alpha4: a potential novel role for   metastasis   of cholangiocarcinoma. Although the altered expression of integrin alpha4 is known to be associated with transformation or   metastasis   in several human cancers, the information on cholangiocarcinoma (CC) is still poor. In this study, we investigated the promoter methylation status of integrin alpha4 gene in CC.A total of 29 CC, 19 adjacent non-tumor-containing tissue and 15 normal liver specimens were used for identification of gene methylation status by methylation-specific polymerase chain reaction.The frequency of DNA methylation was 55.17% (16 of 29) in the CC specimens (P < 0.001). Also, transcripts of the integrin alpha4 gene were decreased in all CC tissues in which there was DNA methylation of the integrin alpha4 gene. In addition, the downregulated expression of integrin alpha4 in CC cells with hypermethylation of the integrin alpha4 gene was restored by treatment with 5-aza-2'-deoxycytidine, a DNA methyltransferase inhibitor. Moreover, we found that DNA methylation of integrin alpha4 was detected in all CC tissues obtained from patients with LN   metastasis   (7/7). Furthermore, phosphorylation of paxillin, cell migration-related molecule, was regulated by silencing of integrin alpha4.Taken together, these results suggest that loss of the integrin alpha4 gene is caused by aberrant DNA methylation of the 5'-CpG island site of the gene, and methylation of the integrin alpha4 gene can be a useful marker of   metastasis   of CC. 
 The Wilms tumor suppressor Wt1 promotes cell adhesion through transcriptional activation of the alpha4integrin gene. Cell-matrix interaction through specific adhesion molecules is a critical step during organ development. In addition, down-regulation of cell adhesion receptors may promote tumor invasion and   metastasis  . We show here that the Wilms tumor suppressor Wt1, which is necessary for normal development of the epicardium, coronary vessels, genitourinary system, and other tissues, activates transcription of the alpha4integrin gene. Binding of the Wt1(-KTS) form, which is transcriptionally active, to the proximal alpha4integrin promoter was demonstrated by electrophoretic mobility shift assay and chromatin immunoprecipitation. A reporter construct harboring approximately 1.9 kb of the human alpha4integrin gene promoter was activated significantly by transient co-transfection of a Wt1(-KTS) expression plasmid. Introducing mutations in two identified Wt1(-KTS) binding motifs in the proximal promoter of the alpha4integrin gene abrogated this stimulatory effect. Endogenous alpha4integrin transcripts were increased more than 3-fold in human embryonic kidney 293 cells with stable expression of the Wt1(-KTS) protein. Wt1-overexpressing cells showed augmented adhesion to the alpha4integrin ligand vascular cell adhesion molecule-1 that was abolished upon incubation with an inhibitory alpha4integrin antibody. Double immunofluorescent staining revealed co-localization of Wt1 and alpha4integrin in the developing epicardium of mouse embryos. Cardiac expression of alpha4integrin was reduced significantly in embryos with a homozygous Wt1 defect (Wt1-/-). These findings demonstrate that Wt1 can support cell adhesion through enhanced expression of alpha4integrin. This transcriptional activation of the alpha4integrin gene by Wt1(-KTS) might contribute to normal formation of the epicardium and other tissues in the developing embryo. 
 Aberrant DNA methylation of integrin α4 in human breast cancer. Integrins are cell surface receptors that mediate cell-cell/extracellular interactions and have shown an association with   metastasi  s or transformation in several cancers. However, the correlation between the clinicopathological status of breast cancer and the altered integrin α4 status is not clear. In this study, we investigated DNA methylation of integrin α4 in breast cancer. We retrieved 351 cases of surgically resected breast cancer (290 invasive carcinoma and 61 intraductal carcinoma). Methylation-specific polymerase chain reaction was performed to determine integrin α4 methylation status. Integrin α4 methylation was frequently observed in breast cancer specimens (145/351 cases, 41.3 %). In addition, DNA methylation of integrin α4 showed statistical correlation with HER2 positivity and higher histologic grade (p = 0.001, 0.008 in ductal carcinoma in situ and 0.003 in invasive ductal carcinoma). However, other clinicopathological data such as estrogen receptor, progesterone rece  ptor, meta  stasis, and TNM status showed no statistical correlation. Moreover, we found that the downregulated expression of integrin α4 in a heavily methylated breast cancer cell line (ZR-75) was restored by treatment with 5-aza-2'deoxycytidine, a DNA methyltransferase inhibitor, implying transcriptional silencing by DNA methylation. We observed that integrin α4 methylation is associated with the histologic grade of tumors and lym  ph node me  tastasis. Also, this data supports a previous study that suggested integrin α4 and HER2 are involved in the same signaling pathway. DNA methylation of integrin α4 may be a poor prognostic factor which affects undifferentiated histologic change of breast cancer. 
 Contribution of very late antigen-4 (VLA-4) integrin to cancer progression and   metastasis  . The integrin "very late antigen-4" (VLA-4) is expressed by numerous cells of hematopoietic origin and possesses a key function in the cellular immune response, e.g., by mediating leukocyte tethering, rolling, binding, and finally transmigration of the vascular wall at inflammatory sites. Thus, VLA-4 is a valuable target in medical sciences to interfere with pathological inflammations. In addition, leukemic cells and different solid tumors, which express VLA-4, make use of these adhesive functions and confer VLA-4 a progressive role in the metastatic spread. With a growing insight into the molecular mechanisms for creating a tumor-friendly microenvironment at metastatic sites and various tumor host interactions, the multiple functions of VLA-4 became evident recently, e.g., in leukocyte recruitment to micrometastases, the protection of tumors from immune surveillance, or contribution to a chemoresistance. Nevertheless, despite accumulating evidence for several functions of VLA-4 in tumorigenicity, a therapeutic interference with VLA-4 in cancer sciences has not been developed yet to the clinical level, undoubtedly by a marked impact on the physiological immune response. This review gives an up to date insight into the multiple functional role of VLA-4 in cancer and introduces this integrin as a promising target worthwhile to attract attention in biomedical cancer research. 
  GeneRIFs  
 ITGA4 expression enhances   metastasis   in MYCN low neuroblastoma. 
 alpha4beta1-mediated tumor lymphangiogenesis promotes   metastasis   
 Results suggest that loss of the integrin alpha4 gene is caused by aberrant DNA methylation of the 5'-CpG island site of the gene, and methylation of the integrin alpha4 gene can be a useful marker of   metastasis   of CC. 
  [Cc]ell [Ii]nvasion  PubMed  
 Dynamic alterations in integrin α4 expression by hypoxia are involved in trophoblast invasion during early implantation. Implantation of the blastocyst into the maternal endometrium is mediated by a population of well-differentiated primary cells of the placenta known as trophoblasts, which grow in an invasive and destructive fashion similar to tumor cells. Interactions between the endometrium and trophoblasts are regulated by a coordinated interplay of extracellular matrix (ECM) proteins secreted by the invading extravillous trophoblasts. Integrins act as adhesion receptors and mediate both cell-ECM and cell-cell interactions. However, the correlation between integrin expression and trophoblast invasion under hypoxia is unclear. Here, we analyzed the expression of integrins in HTR-8/SVneo trophoblast cells exposed to hypoxic conditions in order to demonstrate an association between invasion activity and integrin expression in trophoblasts. Trophoblasts were examined by microarray analysis, RT-PCR, western blotting, and zymography after 1% hypoxic treatment, and   cell invasio  n was estimated. The dynamic expression of integrins and human matrix metalloproteinases (MMPs) was observed under hypoxic conditions. The invasiveness of trophoblasts cultured under 1% hypoxic conditions was significantly greater than that of trophoblasts cultured under normoxic conditions through alterations in MMP-2 and -9 (P < 0.05). Notably, integrin α4 expression during early hypoxia was negatively regulated by hypoxia-inducible factor-1alpha (HIF-1alpha) expression in trophoblasts. The downregulation of integrin α4 expression by siRNA treatment controlled trophoblast invasion activity (P < 0.05). Taken together, we suggest that dynamic changes in integrins, including those in integrin α4 expression by hypoxia, play a regulatory role in trophoblast invasion. These findings expand our understanding of the potential roles of integrin α4 in implantation. 
   
  
 C3orf33 
 
  NCBI Gene  
  GeneRIF  
  Pubmed  
 
 
  Search Term  Rank  Fields  
  [Ii]nvasive  10  PubMed(1)  
 
 
 hide/show details for C3orf33 
  [Ii]nvasive  PubMed  
 Analysis of AC3-33 gene expression in multiple organ cancer and adjacent normal tissue by RNA in situ hybridization. The AC3-33 gene encodes a secretory protein that can inhibit Elk1 transcriptional activity via the ERK1/2 pathway. In the current study, in situ RNA hybridization was used to detect the AC3-33 gene expression in multiple organ cancer and cancer-adjacent normal tissue. The results showed that the expression level of AC3-33 varies across different tissues. AC3-33 exhibited positive expression in squamous cell carcinoma of the esophagus, adenocarcinoma of the rectum, hepatocellular carcinoma, squamous cell carcinoma (SCC) of the lung, cancer-adjacent normal hepatic tissue, clear cell carcinoma of the kidney,   invasive   ductal carcinoma of the breast, SCC of the uterine cervix and cancer-adjacent normal kidney tissue. Negative expression of AC3-33 was observed in adenocarcinoma of the stomach and colon, cancer-adjacent normal esophageal tissue, cancer-adjacent normal gastric tissue, cancer-adjacent normal colon tissue, cancer-adjacent normal rectal tissue, serous adenocarcinoma of the ovary and cancer-adjacent normal ovarian tissue. However, the expression of AC3-33 in cancer adjacent normal breast tissue was partially positive. In conclusion, the AC3-33 gene does exhibit positive expression in certain carcinomas, which may indicate that AC3-33 has a significant involvement in the development and progression of these carcinomas. 
   
  
 SERPINI2 
 
  NCBI Gene  
  GeneRIF  
  Pubmed  
 
 
  Search Term  Rank  Fields  
  [Ii]nvasive  10  PubMed(1)  
  [Mm]etastasis  12  PubMed(2)  
 
 
 hide/show details for SERPINI2 
  [Ii]nvasive  PubMed  
 Suppression of breast cancer growth and metastasis by a serpin myoepithelium-derived serine proteinase inhibitor expressed in the mammary myoepithelial cells. A serpin was identified in normal mammary gland by differential cDNA sequencing. In situ hybridization has detected this serpin exclusively in the myoepithelial cells on the normal and non  invasive   mammary epithelial side of the basement membrane and thus was named myoepithelium-derived serine proteinase inhibitor (MEPI). No MEPI expression was detected in the malignant breast carcinomas. MEPI encodes a 405-aa precursor, including an 18-residue secretion signal with a calculated molecular mass of 46 kDa. The predicted sequence of the new protein shares 33% sequence identity and 58% sequence similarity to plasminogen activator inhibitor (PAI)-1 and PAI-2. To determine whether MEPI can modulate the in vivo growth and progression of human breast cancers, we transfected a full-length MEPI cDNA into human breast cancer cells and studied the orthotopic growth of MEPI-transfected vs. control clones in the mammary fat pad of athymic nude mice. Overexpression of MEPI inhibited the invasion of the cells in the in vitro invasion assay. When injected orthotopically into nude mice, the primary tumor volumes, axillary lymph node metastasis, and lung metastasis were significantly inhibited in MEPI-transfected clones as compared with controls. The expression of MEPI in myoepithelial cells may prevent breast cancer malignant progression leading to metastasis. 
  [Mm]etastasis  PubMed  
 Update of the human and mouse SERPIN gene superfamily. The serpin family comprises a structurally similar, yet functionally diverse, set of proteins. Named originally for their function as serine proteinase inhibitors, many of its members are not inhibitors but rather chaperones, involved in storage, transport, and other roles. Serpins are found in genomes of all kingdoms, with 36 human protein-coding genes and five pseudogenes. The mouse has 60 Serpin functional genes, many of which are orthologous to human SERPIN genes and some of which have expanded into multiple paralogous genes. Serpins are found in tissues throughout the body; whereas most are extracellular, there is a class of intracellular serpins. Serpins appear to have roles in inflammation, immune function, tumorigenesis, blood clotting, dementia, and cancer   metastasis  . Further characterization of these proteins will likely reveal potential biomarkers and therapeutic targets for disease. 
 Suppression of breast cancer growth and   metastasis   by a serpin myoepithelium-derived serine proteinase inhibitor expressed in the mammary myoepithelial cells. A serpin was identified in normal mammary gland by differential cDNA sequencing. In situ hybridization has detected this serpin exclusively in the myoepithelial cells on the normal and noninvasive mammary epithelial side of the basement membrane and thus was named myoepithelium-derived serine proteinase inhibitor (MEPI). No MEPI expression was detected in the malignant breast carcinomas. MEPI encodes a 405-aa precursor, including an 18-residue secretion signal with a calculated molecular mass of 46 kDa. The predicted sequence of the new protein shares 33% sequence identity and 58% sequence similarity to plasminogen activator inhibitor (PAI)-1 and PAI-2. To determine whether MEPI can modulate the in vivo growth and progression of human breast cancers, we transfected a full-length MEPI cDNA into human breast cancer cells and studied the orthotopic growth of MEPI-transfected vs. control clones in the mammary fat pad of athymic nude mice. Overexpression of MEPI inhibited the invasion of the cells in the in vitro invasion assay. When injected orthotopically into nude mice, the primary tumor volumes, axillary lymph node   metastasis  , and lung   metastasis   were significantly inhibited in MEPI-transfected clones as compared with controls. The expression of MEPI in myoepithelial cells may prevent breast cancer malignant progression leading to   metastasis  . 
   
  
 HSPB2 
 
  NCBI Gene  
  GeneRIF  
  Pubmed  
 
 
  Search Term  Rank  Fields  
  [Mm]igration  6  PubMed(6)  
  [Ii]nvasive  10  PubMed(1)  
  [Mm]etastasis  12  PubMed(4); GeneRIFs(1)  
 
 
 hide/show details for HSPB2 
  [Mm]igration  PubMed  
 Vascular endothelial growth factor induces heat shock protein (HSP) 27 serine 82 phosphorylation and endothelial tubulogenesis via protein kinase D and independent of p38 kinase. Proteomic analysis identified HSP27 phosphorylation as a major change in protein phosphorylation stimulated by Vascular Endothelial Growth Factor (VEGF) in Human Umbilical Vein Endothelial Cells (HUVEC). VEGF-induced HSP27 phosphorylation at serines 15, 78 and 82, but whereas HSP27 phosphorylation induced by H2O2 and TNFalpha was completely blocked by the p38 kinase inhibitor, SB203580, VEGF-stimulated serine 82 phosphorylation was resistant to SB203580 and small interfering(si)RNA-mediated knockdown of p38 kinase and MAPKAPK2. The PKC inhibitor, GF109203X, partially reduced VEGF-induced HSP27 serine 82 phosphorylation, and SB203580 plus GF109203X abolished phosphorylation. VEGF activated Protein Kinase D (PKD) via PKC, and siRNAs targeted to PKD1 and PKD2 inhibited VEGF-induced HSP27 serine 82 phosphorylation. Furthermore recombinant PKD selectively phosphorylated HSP27 at serine 82 in vitro, and PKD2 activated by VEGF in HUVECs also phosphorylated HSP27 selectively at this site. Knockdown of HSP27 and PKDs markedly inhibited VEGF-induced HUVEC   migration   and tubulogenesis, whereas inhibition of the p38 kinase pathway using either SB203580 or siRNAs against p38alpha or MAPKAPK2, had no significant effect on the chemotactic response to VEGF. These findings identify a novel pathway for VEGF-induced HSP27 serine 82 phosphorylation via PKC-mediated PKD activation and direct phosphorylation of HSP27 by PKD, and show that PKDs and HSP27 play major roles in the angiogenic response to VEGF. 
 Regulation of HSP27 on NF-kappaB pathway activation may be involved in metastatic hepatocellular carcinoma cells apoptosis. During the process of metastasis, cells are subjected to various apoptotic stimuli. Aberrant expression of apoptotic regulators often contribute to cell metastasis. Heat shock protein 27(HSP27) is confirmed as an apoptosis regulator, but its antiapoptotic mechanism in metastatic hepatocellular carcinoma (HCC) cells remains unclear.Levels of HSP27 protein and its phosphorylation in Hep3B, MHCC97L to MHCC97H cells with different metastatic potentials were determined by western blot analysis. MHCC97H cells were transfected with specific small interference RNA (siRNA) against HSP27. The in vitro   migration   and invasion potentials of cells were evaluated by Transwell assay. The apoptosis ratio of MHCC97H cells was analyzed by TUNEL staining and Flow Cytometry. Alteration of signal transduction pathway after HSP27 knockdown in MHCC97H cells was evaluated through a Human Q Series Signal Transduction in Cancer Gene Array analysis. Nuclear NF-kappaB contentration and endogenous IKK activity were demonstrated by ELISA assay. The association of IKKalpha, IKKbeta, IkappaBalpha with HSP27 and the association between IKKbeta and IKKalpha in MHCC97H cells were determined by co-immunoprecipitation assay followed by western blot analysis.HSP27 protein and its phosphorylation increased in parallel with enhanced metastatic potentials of HCC cells. siRNA-mediated HSP27 knockdown in MHCC97H significantly suppressed cells   migration   and invasion in vitro and induced cell apoptosis; the prominently altered signal transduction pathway was NF-kappaB pathway after HSP27 knockdown in MHCC97H cells. Furthermore, inhibition of HSP27 expression led to a significant decrease of nuclear NF-kappaB contentration and endogenous IKK activity. In addition, HSP27 was associated with IKKalpha, IKKbeta, IkappaBalpha in three HCC cells above. ELISA assay and western blot analysis also showed a decrease of the association between IKKbeta and IKKalpha, the association between phosphor-HSP27 and IKK complex, and an increase of total IkappaBalpha but reducing tendency of phosphor-IkappaBalpha when HSP27 expression was efficiently knocked down in MHCC97H cells.Altogether, these findings revealed a possible effect of HSP27 on apoptosis in metastatic HCC cells, in which HSP27 may regulate NF-kB pathway activation. 
 Regulation of HSP27 on NF-kappaB pathway activation may be involved in metastatic hepatocellular carcinoma cells apoptosis. During the process of metastasis, cells are subjected to various apoptotic stimuli. Aberrant expression of apoptotic regulators often contribute to cell metastasis. Heat shock protein 27(HSP27) is confirmed as an apoptosis regulator, but its antiapoptotic mechanism in metastatic hepatocellular carcinoma (HCC) cells remains unclear.Levels of HSP27 protein and its phosphorylation in Hep3B, MHCC97L to MHCC97H cells with different metastatic potentials were determined by western blot analysis. MHCC97H cells were transfected with specific small interference RNA (siRNA) against HSP27. The in vitro   migration   and invasion potentials of cells were evaluated by Transwell assay. The apoptosis ratio of MHCC97H cells was analyzed by TUNEL staining and Flow Cytometry. Alteration of signal transduction pathway after HSP27 knockdown in MHCC97H cells was evaluated through a Human Q Series Signal Transduction in Cancer Gene Array analysis. Nuclear NF-kappaB contentration and endogenous IKK activity were demonstrated by ELISA assay. The association of IKKalpha, IKKbeta, IkappaBalpha with HSP27 and the association between IKKbeta and IKKalpha in MHCC97H cells were determined by co-immunoprecipitation assay followed by western blot analysis.HSP27 protein and its phosphorylation increased in parallel with enhanced metastatic potentials of HCC cells. siRNA-mediated HSP27 knockdown in MHCC97H significantly suppressed cells   migration   and invasion in vitro and induced cell apoptosis; the prominently altered signal transduction pathway was NF-kappaB pathway after HSP27 knockdown in MHCC97H cells. Furthermore, inhibition of HSP27 expression led to a significant decrease of nuclear NF-kappaB contentration and endogenous IKK activity. In addition, HSP27 was associated with IKKalpha, IKKbeta, IkappaBalpha in three HCC cells above. ELISA assay and western blot analysis also showed a decrease of the association between IKKbeta and IKKalpha, the association between phosphor-HSP27 and IKK complex, and an increase of total IkappaBalpha but reducing tendency of phosphor-IkappaBalpha when HSP27 expression was efficiently knocked down in MHCC97H cells.Altogether, these findings revealed a possible effect of HSP27 on apoptosis in metastatic HCC cells, in which HSP27 may regulate NF-kB pathway activation. 
 Vascular endothelial growth factor induces heat shock protein (HSP) 27 serine 82 phosphorylation and endothelial tubulogenesis via protein kinase D and independent of p38 kinase. Proteomic analysis identified HSP27 phosphorylation as a major change in protein phosphorylation stimulated by Vascular Endothelial Growth Factor (VEGF) in Human Umbilical Vein Endothelial Cells (HUVEC). VEGF-induced HSP27 phosphorylation at serines 15, 78 and 82, but whereas HSP27 phosphorylation induced by H2O2 and TNFalpha was completely blocked by the p38 kinase inhibitor, SB203580, VEGF-stimulated serine 82 phosphorylation was resistant to SB203580 and small interfering(si)RNA-mediated knockdown of p38 kinase and MAPKAPK2. The PKC inhibitor, GF109203X, partially reduced VEGF-induced HSP27 serine 82 phosphorylation, and SB203580 plus GF109203X abolished phosphorylation. VEGF activated Protein Kinase D (PKD) via PKC, and siRNAs targeted to PKD1 and PKD2 inhibited VEGF-induced HSP27 serine 82 phosphorylation. Furthermore recombinant PKD selectively phosphorylated HSP27 at serine 82 in vitro, and PKD2 activated by VEGF in HUVECs also phosphorylated HSP27 selectively at this site. Knockdown of HSP27 and PKDs markedly inhibited VEGF-induced HUVEC   migration   and tubulogenesis, whereas inhibition of the p38 kinase pathway using either SB203580 or siRNAs against p38alpha or MAPKAPK2, had no significant effect on the chemotactic response to VEGF. These findings identify a novel pathway for VEGF-induced HSP27 serine 82 phosphorylation via PKC-mediated PKD activation and direct phosphorylation of HSP27 by PKD, and show that PKDs and HSP27 play major roles in the angiogenic response to VEGF. 
 Potential role of heat-shock proteins in giant cell tumors. We investigated the differential expression protein profile of giant cell tumors (GCTs), which can be used to monitor the tumor's recurrence and metastasis, to provide preliminary results for further study. We also explored heat-shock protein (HSP) inhibitor that prevents tumors from recurring and migrating. A stable isotope-labeling strategy using isobaric tags for relative and absolute quantitation coupled with two-dimensional liquid chromatography tandem mass spectrometry was used to separate and identify differentially expressed proteins. A total of 467 differentially expressed proteins were identified in GCT tissues. Up to 311 proteins were upregulated, whereas 156 proteins were downregulated in GCT tissues. Three of the differentially expressed HSPs, namely HP90A, HSPB1, and HSPB2, were upregulated. The differentially expressed proteins of GCT tissues will provide a scientific foundation for tumor prognosis, and for further studies exploring HSP inhibitor to prevent tumor recurrence and   migration  . 
 Id-1 promotes TGF-beta1-induced cell motility through HSP27 activation and disassembly of adherens junction in prostate epithelial cells. Id-1 (inhibitor of differentiation or DNA binding-1) has been positively associated with cell proliferation, cell cycle progression, and invasiveness during tumorigenesis. In addition, Id-1 has been shown to modulate cellular sensitivity to TGF-beta1 (transforming growth factor beta1). Here we demonstrate a novel role of Id-1 in promoting TGF-beta1-induced cell motility in a non-malignant prostate epithelial cell line, NPTX. We found that Id-1 promoted F-actin stress fiber formation in response to TGF-beta1, which was associated with increased cell-substrate adhesion and cell   migration   in NPTX cells. In addition, this positive effect of Id-1 on TGF-beta1-induced cell motility was mediated through activation of MEK-ERK signaling pathway and subsequent phosphorylation of HSP27 (heat shock protein 27). Furthermore, Id-1 disrupted the adherens junction complex in TGF-beta1-treated cells through down-regulation of E-cadherin, redistribution of beta-catenin, along with up-regulation of N-cadherin. These lines of evidence reveal a novel tumorigenic role of Id-1 through reorganization of actin cytoskeleton and disassembly of cell-cell adhesion in response to TGF-beta1 in human prostate epithelial cells, and suggest that intracellular Id-1 levels might be a determining factor for switching TGF-beta1 from a growth inhibitor to a tumor promoter during prostate carcinogenesis. 
  [Ii]nvasive  PubMed  
 Id-1 promotes TGF-beta1-induced cell motility through HSP27 activation and disassembly of adherens junction in prostate epithelial cells. Id-1 (inhibitor of differentiation or DNA binding-1) has been positively associated with cell proliferation, cell cycle progression, and   invasive  ness during tumorigenesis. In addition, Id-1 has been shown to modulate cellular sensitivity to TGF-beta1 (transforming growth factor beta1). Here we demonstrate a novel role of Id-1 in promoting TGF-beta1-induced cell motility in a non-malignant prostate epithelial cell line, NPTX. We found that Id-1 promoted F-actin stress fiber formation in response to TGF-beta1, which was associated with increased cell-substrate adhesion and cell migration in NPTX cells. In addition, this positive effect of Id-1 on TGF-beta1-induced cell motility was mediated through activation of MEK-ERK signaling pathway and subsequent phosphorylation of HSP27 (heat shock protein 27). Furthermore, Id-1 disrupted the adherens junction complex in TGF-beta1-treated cells through down-regulation of E-cadherin, redistribution of beta-catenin, along with up-regulation of N-cadherin. These lines of evidence reveal a novel tumorigenic role of Id-1 through reorganization of actin cytoskeleton and disassembly of cell-cell adhesion in response to TGF-beta1 in human prostate epithelial cells, and suggest that intracellular Id-1 levels might be a determining factor for switching TGF-beta1 from a growth inhibitor to a tumor promoter during prostate carcinogenesis. 
  [Mm]etastasis  PubMed  
 Regulation of HSP27 on NF-kappaB pathway activation may be involved in metastatic hepatocellular carcinoma cells apoptosis. During the process of   metastasis  , cells are subjected to various apoptotic stimuli. Aberrant expression of apoptotic regulators often contribute to cell   metastasis  . Heat shock protein 27(HSP27) is confirmed as an apoptosis regulator, but its antiapoptotic mechanism in metastatic hepatocellular carcinoma (HCC) cells remains unclear.Levels of HSP27 protein and its phosphorylation in Hep3B, MHCC97L to MHCC97H cells with different metastatic potentials were determined by western blot analysis. MHCC97H cells were transfected with specific small interference RNA (siRNA) against HSP27. The in vitro migration and invasion potentials of cells were evaluated by Transwell assay. The apoptosis ratio of MHCC97H cells was analyzed by TUNEL staining and Flow Cytometry. Alteration of signal transduction pathway after HSP27 knockdown in MHCC97H cells was evaluated through a Human Q Series Signal Transduction in Cancer Gene Array analysis. Nuclear NF-kappaB contentration and endogenous IKK activity were demonstrated by ELISA assay. The association of IKKalpha, IKKbeta, IkappaBalpha with HSP27 and the association between IKKbeta and IKKalpha in MHCC97H cells were determined by co-immunoprecipitation assay followed by western blot analysis.HSP27 protein and its phosphorylation increased in parallel with enhanced metastatic potentials of HCC cells. siRNA-mediated HSP27 knockdown in MHCC97H significantly suppressed cells migration and invasion in vitro and induced cell apoptosis; the prominently altered signal transduction pathway was NF-kappaB pathway after HSP27 knockdown in MHCC97H cells. Furthermore, inhibition of HSP27 expression led to a significant decrease of nuclear NF-kappaB contentration and endogenous IKK activity. In addition, HSP27 was associated with IKKalpha, IKKbeta, IkappaBalpha in three HCC cells above. ELISA assay and western blot analysis also showed a decrease of the association between IKKbeta and IKKalpha, the association between phosphor-HSP27 and IKK complex, and an increase of total IkappaBalpha but reducing tendency of phosphor-IkappaBalpha when HSP27 expression was efficiently knocked down in MHCC97H cells.Altogether, these findings revealed a possible effect of HSP27 on apoptosis in metastatic HCC cells, in which HSP27 may regulate NF-kB pathway activation. 
 Regulation of HSP27 on NF-kappaB pathway activation may be involved in metastatic hepatocellular carcinoma cells apoptosis. During the process of   metastasis  , cells are subjected to various apoptotic stimuli. Aberrant expression of apoptotic regulators often contribute to cell   metastasis  . Heat shock protein 27(HSP27) is confirmed as an apoptosis regulator, but its antiapoptotic mechanism in metastatic hepatocellular carcinoma (HCC) cells remains unclear.Levels of HSP27 protein and its phosphorylation in Hep3B, MHCC97L to MHCC97H cells with different metastatic potentials were determined by western blot analysis. MHCC97H cells were transfected with specific small interference RNA (siRNA) against HSP27. The in vitro migration and invasion potentials of cells were evaluated by Transwell assay. The apoptosis ratio of MHCC97H cells was analyzed by TUNEL staining and Flow Cytometry. Alteration of signal transduction pathway after HSP27 knockdown in MHCC97H cells was evaluated through a Human Q Series Signal Transduction in Cancer Gene Array analysis. Nuclear NF-kappaB contentration and endogenous IKK activity were demonstrated by ELISA assay. The association of IKKalpha, IKKbeta, IkappaBalpha with HSP27 and the association between IKKbeta and IKKalpha in MHCC97H cells were determined by co-immunoprecipitation assay followed by western blot analysis.HSP27 protein and its phosphorylation increased in parallel with enhanced metastatic potentials of HCC cells. siRNA-mediated HSP27 knockdown in MHCC97H significantly suppressed cells migration and invasion in vitro and induced cell apoptosis; the prominently altered signal transduction pathway was NF-kappaB pathway after HSP27 knockdown in MHCC97H cells. Furthermore, inhibition of HSP27 expression led to a significant decrease of nuclear NF-kappaB contentration and endogenous IKK activity. In addition, HSP27 was associated with IKKalpha, IKKbeta, IkappaBalpha in three HCC cells above. ELISA assay and western blot analysis also showed a decrease of the association between IKKbeta and IKKalpha, the association between phosphor-HSP27 and IKK complex, and an increase of total IkappaBalpha but reducing tendency of phosphor-IkappaBalpha when HSP27 expression was efficiently knocked down in MHCC97H cells.Altogether, these findings revealed a possible effect of HSP27 on apoptosis in metastatic HCC cells, in which HSP27 may regulate NF-kB pathway activation. 
 [Expression and clinical significance of 14-3-3 sigma and heat shock protein 27 in colorectal cancer]. To investigate the expression of 14-3-3 sigma and heat shock protein 27 (HSP27) in colorectal carcinoma(CRC) tissue and its clinical significance.The expression of 14-3-3 sigma and HSP27 was detected by immunohistochemical staining in 50 pathologically verified CRC cases. The association of clinical data with 14-3-3 sigma and HSP27 expression was examined.The positive expression rate of 14-3-3 sigma was 10% in normal control mucosa and 58% in CRC tissue (P<0.01). The positive expression rate of HSP27 was 16% in normal control mucosa and 54% in CRC tissue (P<0.01). No correlation between 14-3-3 sigma and HSP27 expression was found in CRC tissue (P>0.05). The expression of 14-3-3 sigma was associated with patient age, tumor diameter and lymph node   metastasis   (LNM) (P<0.05), but not with gender, tumor differentiation or serous membrane invasion (P>0.05). The expression of HSP27 was associated with LNM (P<0.05), but not with gender, age, differentiation, tumor diameter or serous membrane invasion (P>0.05).The abnormal expression of 14-3-3 sigma and HSP27 is significantly associated with LNM in CRC. 
 Potential role of heat-shock proteins in giant cell tumors. We investigated the differential expression protein profile of giant cell tumors (GCTs), which can be used to monitor the tumor's recurrence and   metastasis  , to provide preliminary results for further study. We also explored heat-shock protein (HSP) inhibitor that prevents tumors from recurring and migrating. A stable isotope-labeling strategy using isobaric tags for relative and absolute quantitation coupled with two-dimensional liquid chromatography tandem mass spectrometry was used to separate and identify differentially expressed proteins. A total of 467 differentially expressed proteins were identified in GCT tissues. Up to 311 proteins were upregulated, whereas 156 proteins were downregulated in GCT tissues. Three of the differentially expressed HSPs, namely HP90A, HSPB1, and HSPB2, were upregulated. The differentially expressed proteins of GCT tissues will provide a scientific foundation for tumor prognosis, and for further studies exploring HSP inhibitor to prevent tumor recurrence and migration. 
  GeneRIFs  
 The abnormal expression of 14-3-3 sigma and HSP27 is significantly associated with lymph node   metastasis   in colorectal cancer. 
   
  
 CRYAB 
 
  NCBI Gene  
  GeneRIF  
  Pubmed  
 
 
  Search Term  Rank  Fields  
  [Mm]igration  6  PubMed(6)  
  ECM  7  PubMed(1)  
  [Ee]xtracellular [Mm]atrix  8  PubMed(4)  
  [Ii]nvasive  10  PubMed(6)  
  [Mm]etastasize  11  PubMed(1); GeneRIFs(1)  
  [Mm]etastasis  12  PubMed(9); GeneRIFs(4)  
 
 
 hide/show details for CRYAB 
  [Mm]igration  PubMed  
 Pivotal role of augmented αB-crystallin in tumor development induced by deficient TSC1/2 complex. Tuberous sclerosis complex 1 (TSC1) and TSC2 are suppressors of mechanistic target of rapamycin (mTOR). mTOR is the major component of two protein complexes: mTOR complex 1 (mTORC1) and mTORC2. Inactive mutation of either TSC1 or TSC2 unleashes mTOR signaling and consequently causes TSC, a benign tumor syndrome affecting multiple organs. We report here that expression of αB-crystallin was upregulated in Tsc1-/- or Tsc2-/- mouse embryonic fibroblasts, Eker rat uterine leiomyoma-derived Tsc2-deficient ELT3 cells, mutant Tsc2-associated mouse kidney tumors, and human lung lymphangioleiomyomatosis nodules. αB-crystallin was transcriptionally activated by mTOR complex 2 (mTORC2): nuclear factor-kappa B (NFκB) signaling cascade. The augmented αB-crystallin was critical for   the migr  ation, invasion and apoptotic resistance of Tsc2-defective cells. Disruption of αB-crystallin suppressed Tsc2-null cell proliferation and tumorigenesis. Therefore, enhanced αB-crystallin has an essential role in TSC1/2 complex deficiency-mediated tumorigenesis, and inhibition of αB-crystallin may complement the current therapy for TSC. 
 ΑB-crystallin in clear cell renal cell carcinoma: tumor progression and prognostic significance. AlphaB-crystallin (αB-crystallin), a small heat shock protein, has been reported to be involved in the growth, antiapoptosis  , migrati  on, and chemoresistance of human malignancies.αB-crystallin expression in normal renal and clear cell renal cell carcinoma (ccRCC) tissues was examined with two-dimensional (2D) gel electrophoresis assays. Immunohistochemistry was conducted to determine the presence of αB-crystallin-positive tumor cells and staining intensity in 50 cases of ccRCC tissue samples. The association of αB-crystallin protein expression, clinicopathogic parameters and prognosis of ccRCC patients was also analyzed with Student's t-test and Kaplan-Meier analysis. Moreover, Western blot assays were performed to detect the protein expression of αB-crystallin in normal and tumor tissues and the alteration of cell cycle regulators in αB-crystallin-overexpressing cells. MTT (3-[4,5-dimethythiazol-2-yl]-2,5-diphenyltetrazolium bromide), BrdU, and transwell assays were performed to demonstrate the effects of αB-crystallin overexpression on cell growth, DNA synthesis a  nd cell m  igration of ccRCC cells, respectively.The results showed the up-regulation of αB-crystallin expression in ccRCC tissues. Overall survival of ccRCC patients was significantly correlated with αB-crystallin expression in tumor tissues. We found that αB-crystallin overexpression increased the expression of cyclin A and the incorporation of BrdU, which may be related to the enhancement of cell growth. Transwell analyses demonstrated that presence of αB-crystallin overexpression en  hanced ce  ll migration in ccRCC cells. Furthermore, rapamycin-resistance of tumor cells was induced when αB-crystallin was overexpressed.Our experimental findings highlight the importance of αB-crystallin in the   tumor gro  wth, migration, and target therapy-resistance of ccRCC cells. 
 Role for phospholipid flippase complex of ATP8A1 and CDC50A proteins in cell   migration  . Type IV P-type ATPases (P4-ATPases) and CDC50 family proteins form a putative phospholipid flippase complex that mediates the translocation of aminophospholipids such as phosphatidylserine (PS) and phosphatidylethanolamine (PE) from the outer to inner leaflets of the plasma membrane. In Chinese hamster ovary (CHO) cells, at least eight members of P4-ATPases were identified, but only a single CDC50 family protein, CDC50A, was expressed. We demonstrated that CDC50A associated with and recruited P4-ATPase ATP8A1 to the plasma membrane. Overexpression of CDC50A induced extensive cell spreading and greatly enhanced cell   migration  . Depletion of either CDC50A or ATP8A1 caused a severe defect in the formation of membrane ruffles, thereby inhibiting cell   migration  . Analyses of phospholipid translocation at the plasma membrane revealed that the depletion of CDC50A inhibited the inward translocation of both PS and PE, whereas the depletion of ATP8A1 inhibited the translocation of PE but not that of PS, suggesting that the inward translocation of cell-surface PE is involved in cell   migration  . This hypothesis was further examined by using a PE-binding peptide and a mutant cell line with defective PE synthesis; either cell-surface immobilization of PE by the PE-binding peptide or reduction in the cell-surface content of PE inhibited the formation of membrane ruffles, causing a severe defect in cell   migration  . These results indicate that the phospholipid flippase complex of ATP8A1 and CDC50A plays a major role in cell   migration   and suggest that the flippase-mediated translocation of PE at the plasma membrane is involved in the formation of membrane ruffles to promote cell   migration  . 
 AlphaB-crystallin is a novel oncoprotein that predicts poor clinical outcome in breast cancer. Recent gene profiling studies have identified a new breast cancer subtype, the basal-like group, which expresses genes characteristic of basal epithelial cells and is associated with poor clinical outcomes. However, the genes responsible for the aggressive behavior observed in this group are largely unknown. Here we report that the small heat shock protein alpha-basic-crystallin (alphaB-crystallin) was commonly expressed in basal-like tumors and predicted poor survival in breast cancer patients independently of other prognostic markers. We also demonstrate that overexpression of alphaB-crystallin transformed immortalized human mammary epithelial cells (MECs). In 3D basement membrane culture, alphaB-crystallin overexpression induced luminal filling and other neoplastic-like changes in mammary acini, while silencing alphaB-crystallin by RNA interference inhibited these abnormalities. alphaB-Crystallin overexpression also induced EGF- and anchorage-independent growth, increased cell   migration   and invasion, and constitutively activated the MAPK kinase/ERK (MEK/ERK) pathway. Moreover, the transformed phenotype conferred by alphaB-crystallin was suppressed by MEK inhibitors. In addition, immortalized human MECs overexpressing alphaB-crystallin formed invasive mammary carcinomas in nude mice that recapitulated aspects of human basal-like breast tumors. Collectively, our results indicate that alphaB-crystallin is a novel oncoprotein expressed in basal-like breast carcinomas that independently predicts shorter survival. Our data also implicate the MEK/ERK pathway as a potential therapeutic target for these tumors. 
 αB-crystallin: a novel regulator of breast cancer metastasis to the brain. Basal-like breast tumors are typically (ER/PR/HER2) triple-negative and are associated with a high incidence of brain metastases and poor clinical outcomes. The molecular chaperone αB-crystallin is predominantly expressed in triple-negative breast cancer (TNBC) and contributes to an aggressive tumor phenotype in preclinical models. We investigated the potential role of αB-crystallin in brain metastasis in TNBCs.αB-crystallin expression in primary breast carcinomas and brain metastases was analyzed by immunohistochemistry among patients with breast cancer with brain metastases. αB-crystallin was overexpressed or silenced in two different TNBC cell lines. The effects on cell adhesion to human brain microvascular endothelial cells (HBMEC) or extracellular matrix proteins, transendothe  lial migr  ation, and   transmigr  ation across a HBMEC/astrocyte coculture blood-brain barrier (BBB) model were examined. In addition, the effects of overexpressing or silencing αB-crystallin on brain metastasis in vivo were investigated using orthotopic TNBC models.In a cohort of women with breast cancer brain metastasis, αB-crystallin expression in primary breast carcinomas was associated with poor overall survival and poor survival after brain metastasis, even among patients with TNBC. Stable overexpression of αB-crystallin in TNBC cells enhanced adhesion to HBMECs, transendo  thelial m  igration, and B  BB transm  igration in vitro, whereas silencing αB-crystallin inhibited these events. αB-crystallin promoted adhesion of TNBC cells to HBMECs, at least in part, through an α3β1 integrin-dependent mechanism. αB-crystallin overexpression promoted brain metastasis, whereas silencing αB-crystallin inhibited brain metastasis in orthotopic TNBC models.αB-crystallin is a novel regulator of brain metastasis in TNBC and represents a potential biomarker and drug target for this aggressive disease. 
 αB-Crystallin Regulates Subretinal Fibrosis by Modulation of Epithelial-Mesenchymal Transition. Subretinal fibrosis is an end stage of neovascular age-related macular degeneration, characterized by fibrous membrane formation after choroidal neovascularization. An initial step of the pathogenesis is an epithelial-mesenchymal transition (EMT) of retinal pigment epithelium cells. αB-crystallin plays multiple roles in age-related macular degeneration, including cytoprotection and angiogenesis. However, the role of αB-crystallin in subretinal EMT and fibrosis is unknown. Herein, we showed attenuation of subretinal fibrosis after regression of laser-induced choroidal neovascularization and a decrease in mesenchymal retinal pigment epithelium cells in αB-crystallin knockout mice compared with wild-type mice. αB-crystallin was prominently expressed in subretinal fibrotic lesions in mice. In vitro, overexpression of αB-crystallin induced EMT, whereas suppression of αB-crystallin induced a mesenchymal-epithelial transition. Transforming growth factor-β2-induced EMT was further enhanced by overexpression of αB-crystallin but was inhibited by suppression of αB-crystallin. Silencing of αB-crystallin inhibited multiple fibrotic processes, including cell prol  iferation  , migration, and fibronectin production. Bone morphogenetic protein 4 up-regulated αB-crystallin, and its EMT induction was inhibited by knockdown of αB-crystallin. Furthermore, inhibition of αB-crystallin enhanced monotetraubiquitination of SMAD4, which can impair its nuclear localization. Overexpression of αB-crystallin enhanced nuclear translocation and accumulation of SMAD4 and SMAD5. Thus, αB-crystallin is an important regulator of EMT, acting as a molecular chaperone for SMAD4 and as its potential therapeutic target for preventing subretinal fibrosis development in neovascular age-related macular degeneration. 
  ECM  PubMed  
 The small heat-shock protein αB-crystallin is essential for the nuclear localization of Smad4: impact on pulmonary fibrosis. Idiopathic pulmonary fibrosis (IPF) is a devastating disease characterized by the proliferation of myofibroblasts and the accumulation of extracellular matrix   (EC  M) in the lungs. TGF-β1 is the major profibrotic cytokine involved in IPF and is responsible for myofibroblast proliferation and differentiation an  d E  CM synthesis. αB-crystallin is constitutively expressed in the lungs and is inducible by stress, acts as a chaperone and is known to play a role in cell cytoskeleton architecture homeostasis. The role of αB-crystallin in fibrogenesis remains unknown. The principal signalling pathway involved in this process is the Smad-dependent pathway. We demonstrate here that αB-crystallin is strongly expressed in fibrotic lung tissue from IPF patients and in vivo rodent models of pulmonary fibrosis. We also show that αB-crystallin-deficient mice are protected from bleomycin-induced fibrosis. Similar protection from fibrosis was observed in αB-crystallin KO mice after transient adenoviral-mediated over-expression of IL-1β or TGF-β1. We show in vitro in primary epithelial cells and fibroblasts that αB-crystallin increases the nuclear localization of Smad4, thereby enhancing the TGF-β1-Smad pathway and the consequent activation of TGF-β1 downstream genes. αB-crystallin over-expression disrupts Smad4 mono-ubiquitination by interacting with its E3-ubiquitin ligase, TIF1γ, thus limiting its nuclear export. Conversely, in the absence of αB-crystallin, TIF1γ can freely interact with Smad4. Consequently, Smad4 mono-ubiquitination and nuclear export are favoured and thus TGF-β1-Smad4 pro-fibrotic activity is inhibited. This study demonstrates that αB-crystallin may be a key target for the development of specific drugs in the treatment of IPF or other fibrotic diseases. 
  [Ee]xtracellular [Mm]atrix  PubMed  
 ERK-regulated αB-crystallin induction by matrix detachment inhibits anoikis and promotes lung metastasis in vivo. Evasion of   extracellular matri  x detachment-induced apoptosis ('anoikis') is a defining characteristic of metastatic tumor cells. The ability of metastatic carcinoma cells to survive matrix detachment and escape anoikis enables them to disseminate as viable circulating tumor cells and seed distant organs. Here we report that αB-crystallin, an antiapoptotic molecular chaperone implicated in the pathogenesis of diverse poor-prognosis solid tumors, is induced by matrix detachment and confers anoikis resistance. Specifically, we demonstrate that matrix detachment downregulates extracellular signal-regulated kinase (ERK) activity and increases αB-crystallin protein and messenger RNA (mRNA) levels. Moreover, we show that ERK inhibition in adherent cancer cells mimics matrix detachment by increasing αB-crystallin protein and mRNA levels, whereas constitutive ERK activation suppresses αB-crystallin induction during matrix detachment. These findings indicate that ERK inhibition is both necessary and sufficient for αB-crystallin induction by matrix detachment. To examine the functional consequences of αB-crystallin induction in anoikis, we stably silenced αB-crystallin in two different metastatic carcinoma cell lines. Strikingly, silencing αB-crystallin increased matrix detachment-induced caspase activation and apoptosis but did not affect cell viability of adherent cancer cells. In addition, silencing αB-crystallin in metastatic carcinoma cells reduced the number of viable circulating tumor cells and inhibited lung metastasis in two orthotopic models, but had little or no effect on primary tumor growth. Taken together, our findings point to αB-crystallin as a novel regulator of anoikis resistance that is induced by matrix detachment-mediated suppression of ERK signaling and promotes lung metastasis. Our results also suggest that αB-crystallin represents a promising molecular target for antimetastatic therapies. 
 Accumulation and aberrant modifications of alpha-crystallins in anterior polar cataracts. Crystallins are the major proteins found in the lens, and the localization of specific crystallins is well known. Overexpression and accumulation of alphaB-crystallin has been observed in response to stress conditions or in certain diseases, such as brain tumors and neurodegenerative diseases. The purpose of this study was to examine whether alpha-crystallins are modified during pathological myofibroblastic changes in lens epithelial cells. Lens epithelial cells attached to the anterior capsules of patients with nuclear or anterior polar cataracts were analyzed quantitatively for alpha-crystallin proteins and mRNAs using Western blot and RT-PCR analysis., respectively. The degree of modification of alpha-crystallins was determined by 2-dimensional gel electrophoresis followed by Western blotting. Higher molecular weight protein bands that were immunoreactive to anti-alphaA- and anti-alphaB-crystallin antibodies around 45 kDa accumulated more in the anterior polar cataract samples than in those with the nuclear type of cataracts. Also monomeric alphaB-crystallins accumulated more in lens epithelial cells of patients with anterior polar cataracts. By comparison, no significant changes were found in the levels of the mRNAs encoding alphaA- and alphaB-crystallins in the different types of cataracts. Both alphaA- and alphaB-crystallin proteins seemed to undergo more extensive modification in anterior polar cataracts. Conclusion. In addition to fibrotic changes, which accompany increased levels of   extracellular matrix   molecules, accumulation and abnormal modification of alpha-crystallins might be implicated in the pathogenic mechanism of this type of cataract. 
 The small heat-shock protein αB-crystallin is essential for the nuclear localization of Smad4: impact on pulmonary fibrosis. Idiopathic pulmonary fibrosis (IPF) is a devastating disease characterized by the proliferation of myofibroblasts and the accumulation of   extracellular matri  x (ECM) in the lungs. TGF-β1 is the major profibrotic cytokine involved in IPF and is responsible for myofibroblast proliferation and differentiation and ECM synthesis. αB-crystallin is constitutively expressed in the lungs and is inducible by stress, acts as a chaperone and is known to play a role in cell cytoskeleton architecture homeostasis. The role of αB-crystallin in fibrogenesis remains unknown. The principal signalling pathway involved in this process is the Smad-dependent pathway. We demonstrate here that αB-crystallin is strongly expressed in fibrotic lung tissue from IPF patients and in vivo rodent models of pulmonary fibrosis. We also show that αB-crystallin-deficient mice are protected from bleomycin-induced fibrosis. Similar protection from fibrosis was observed in αB-crystallin KO mice after transient adenoviral-mediated over-expression of IL-1β or TGF-β1. We show in vitro in primary epithelial cells and fibroblasts that αB-crystallin increases the nuclear localization of Smad4, thereby enhancing the TGF-β1-Smad pathway and the consequent activation of TGF-β1 downstream genes. αB-crystallin over-expression disrupts Smad4 mono-ubiquitination by interacting with its E3-ubiquitin ligase, TIF1γ, thus limiting its nuclear export. Conversely, in the absence of αB-crystallin, TIF1γ can freely interact with Smad4. Consequently, Smad4 mono-ubiquitination and nuclear export are favoured and thus TGF-β1-Smad4 pro-fibrotic activity is inhibited. This study demonstrates that αB-crystallin may be a key target for the development of specific drugs in the treatment of IPF or other fibrotic diseases. 
 αB-crystallin: a novel regulator of breast cancer metastasis to the brain. Basal-like breast tumors are typically (ER/PR/HER2) triple-negative and are associated with a high incidence of brain metastases and poor clinical outcomes. The molecular chaperone αB-crystallin is predominantly expressed in triple-negative breast cancer (TNBC) and contributes to an aggressive tumor phenotype in preclinical models. We investigated the potential role of αB-crystallin in brain metastasis in TNBCs.αB-crystallin expression in primary breast carcinomas and brain metastases was analyzed by immunohistochemistry among patients with breast cancer with brain metastases. αB-crystallin was overexpressed or silenced in two different TNBC cell lines. The effects on cell adhesion to human brain microvascular endothelial cells (HBMEC  ) or extracellular m  atrix proteins, transendothelial migration, and transmigration across a HBMEC/astrocyte coculture blood-brain barrier (BBB) model were examined. In addition, the effects of overexpressing or silencing αB-crystallin on brain metastasis in vivo were investigated using orthotopic TNBC models.In a cohort of women with breast cancer brain metastasis, αB-crystallin expression in primary breast carcinomas was associated with poor overall survival and poor survival after brain metastasis, even among patients with TNBC. Stable overexpression of αB-crystallin in TNBC cells enhanced adhesion to HBMECs, transendothelial migration, and BBB transmigration in vitro, whereas silencing αB-crystallin inhibited these events. αB-crystallin promoted adhesion of TNBC cells to HBMECs, at least in part, through an α3β1 integrin-dependent mechanism. αB-crystallin overexpression promoted brain metastasis, whereas silencing αB-crystallin inhibited brain metastasis in orthotopic TNBC models.αB-crystallin is a novel regulator of brain metastasis in TNBC and represents a potential biomarker and drug target for this aggressive disease. 
  [Ii]nvasive  PubMed  
 Expression of the molecular chaperone αB-crystallin in infiltrating ductal breast carcinomas and the significance thereof: an immunohistochemical and proteomics-based strategy. This study aims to evaluate αB-crystallin expression in infiltrating ductal breast carcinomas (IDCAs), as well as, its prognostic significance. Using a two-dimensional electrophoresis matrix-assisted laser desorption/ionisation-time of flight mass spectrometry investigation coupled to an immunohistochemical approach, we have assessed the expression of αB-crystallin in IDCAs, as well as, in other types of breast tumor  s (invas  ive lobular carcinomas, medullary carcinomas, and in situ ductal carcinomas). Correlation between αB-crystallin expression and clinicopathological parameters of breast cancer has also been investigated. Proteomic analyses revealed an increased expression of αB-crystallin in IDCA tumors compared to adjacent nontumor tissues. Overexpression of this molecular chaperone was further confirmed in 51 tumor specimens. Statistical analyses revealed, however, no significant correlations between αB-crystallin expression and clinicopathological parameters of the disease (tumor stage, patient age, hormone receptors, SBR grade, and lymph node metastases). This study demonstrates the upregulation of αB-crystallin in IDCA tissues which may highlight its possible involvement in breast cancer development. Our findings do not, however, support the involvement of this molecular chaperone in the progression of this disease. 
 Tumor suppressor Alpha B-crystallin (CRYAB) associates with the cadherin/catenin adherens junction and impairs NPC progression-associated properties. Alpha B-crystallin (CRYAB) maps within the nasopharyngeal carcinoma (NPC) tumor-suppressive critical region 11q22-23 and its downregulation is significantly associated with the progression of NPC. However, little is known about the functional impact of CRYAB on NPC progression. In this study we evaluated the NPC tumor-suppressive and progression-associated functions of CRYAB. Activation of CRYAB suppressed NPC tumor formation in nude mice. Overexpression of CRYAB affected NPC progression-associated phenotypes such as loss of cell adhesion, invasion, interaction with the tumor microenvironment,   invasive   protrusion formation in three dimensional Matrigel culture, as well as expression of epithelial-mesenchymal transition-associated markers. CRYAB mediates this ability to suppress cancer progression by inhibition of E-cadherin cytoplasmic internalization and maintenance of β-catenin in the membrane that subsequently reduces the levels of expression of critical downstream targets such as cyclin-D1 and c-myc. Both ectopically expressed and recombinant CRYAB proteins were associated with endogenous E-cadherin and β-catenin, and, thus, the cadherin/catenin adherens junction. The CRYAB α-crystallin core domain is responsible for the interaction of CRYAB with both E-cadherin and β-catenin. Taken together, these results indicate that CRYAB functions to suppress NPC progression by associating with the cadherin/catenin adherens junction and modulating the β-catenin function. 
 AlphaB-crystallin: a novel marker of   invasive   basal-like and metaplastic breast carcinomas. Basal-like tumors are a newly recognized estrogen receptor (ER) negative and HER2 negative breast cancer subtype that express basal epithelial genes and are associated with poor survival. Metaplastic carcinomas are thought to belong within the basal-like group. We have recently demonstrated that the small heat shock protein alphaB-crystallin is commonly expressed in basal-like tumors and contributes to their aggressive phenotype. The current study examined the rates and patterns of alphaB-crystallin expression in whole tissue sections of human breast, including normal tissue, proliferative lesions, in situ and   invasive   carcinomas (ER positive, HER2 positive, basal-like, and metaplastic cancers). In normal breast tissue, proliferative lesions and in situ carcinomas, alphaB-crystallin expression was restricted to the myoepithelial cell compartment of ductal and lobular units. Most basal-like and metaplastic carcinomas demonstrated cytoplasmic expression of alphaB-crystallin (81% and 86%, respectively). Conversely, no staining for alphaB-crystallin was observed in nonbasal-like (ie, ER positive or HER2 positive) breast carcinomas. Taken together, our results indicate that alphaB-crystallin is a sensitive (81%) and specific (100%) marker for basal-like breast carcinomas. Moreover, the high rates of expression of alphaB-crystallin in metaplastic breast carcinomas (86%) suggest that these tumors may represent a histologically distinctive subset of basal-like breast tumors with a similar underlying molecular etiology. 
 AlphaB-crystallin is a novel oncoprotein that predicts poor clinical outcome in breast cancer. Recent gene profiling studies have identified a new breast cancer subtype, the basal-like group, which expresses genes characteristic of basal epithelial cells and is associated with poor clinical outcomes. However, the genes responsible for the aggressive behavior observed in this group are largely unknown. Here we report that the small heat shock protein alpha-basic-crystallin (alphaB-crystallin) was commonly expressed in basal-like tumors and predicted poor survival in breast cancer patients independently of other prognostic markers. We also demonstrate that overexpression of alphaB-crystallin transformed immortalized human mammary epithelial cells (MECs). In 3D basement membrane culture, alphaB-crystallin overexpression induced luminal filling and other neoplastic-like changes in mammary acini, while silencing alphaB-crystallin by RNA interference inhibited these abnormalities. alphaB-Crystallin overexpression also induced EGF- and anchorage-independent growth, increased cell migration and invasion, and constitutively activated the MAPK kinase/ERK (MEK/ERK) pathway. Moreover, the transformed phenotype conferred by alphaB-crystallin was suppressed by MEK inhibitors. In addition, immortalized human MECs overexpressing alphaB-crystallin formed   invasive   mammary carcinomas in nude mice that recapitulated aspects of human basal-like breast tumors. Collectively, our results indicate that alphaB-crystallin is a novel oncoprotein expressed in basal-like breast carcinomas that independently predicts shorter survival. Our data also implicate the MEK/ERK pathway as a potential therapeutic target for these tumors. 
 Expression and prognostic significance of the alpha B-crystallin gene in human hepatocellular carcinoma. The aim of this study was to characterize expression of the alpha B-crystallin gene in human hepatocellular carcinomas, to investigate the relationship between expression of this gene and the prognosis of human hepatocellular carcinoma. Real-time polymerase chain reaction, reverse transcriptase-polymerase chain reaction and immunohistochemistry were used to characterize expression of the alpha B-crystallin gene in human hepatocellular carcinoma. Kaplan-Meier survival and Cox regression analyses were performed to evaluate the prognosis of human hepatocellular carcinoma. We characterized alpha B-crystallin gene expression in human hepatocellular carcinoma. Statistical analysis of hepatocellular carcinoma patients showed that patients expressing alpha B-crystallin have different survival rates relative to those not expressing this gene (P = .041). After 18 months, the survival rate of patients expressing alpha B-crystallin declined, but survival in the alpha B-crystallin-negative group remained stable. COX multi-factor analysis showed that alpha B-crystallin (P = .007) and venous invasion (P = .037) were independent prognosis factors for hepatocellular carcinoma. Expression of the alpha B-crystallin gene, which is related with the transferability and   invasive   capacity of hepatocellular carcinoma cells, can be used as a prognostic indicator in human hepatocellular carcinomas. It may also be involved in the malignant transformation of hepatocytes. 
 Carbonic anhydrase 4 and crystallin α-B immunoreactivity may distinguish benign from malignant thyroid nodules in patients with indeterminate thyroid cytology. Thyroid nodules are present in 19%-67% of the population and carry a 5%-10% risk of malignancy. Unfortunately, fine-needle aspiration biopsies are indeterminate in 20%-30% of patients, often necessitating thyroid surgery for diagnosis. Numerous DNA microarray studies including a recently commercialized molecular classifier have helped to better distinguish benign from malignant thyroid nodules. Unfortunately, these assays often require probes for >100 genes, are expensive, and only available at a few laboratories. We sought to validate these DNA microarray assays at the protein level and determine whether simple and widely available immunohistochemical biomarkers alone could distinguish benign from malignant thyroid nodules.A tissue microarray (TMA) composed of 26 follicular thyroid carcinomas (FTCs) and 53 follicular adenomas (FAs) from patients with indeterminate thyroid nodules was stained with 17 immunohistochemical biomarkers selected based on prior DNA microarray studies. Antibodies used included galectin 3, growth and differentiation factor 15, protein convertase 2, cluster of differentiation 44 (CD44), glutamic oxaloacetic transaminase 1 (GOT1), trefoil factor 3 (TFF3), Friedreich Ataxia gene (X123), fibroblast growth factor 13 (FGF13), carbonic anhydrase 4 (CA4), crystallin alpha-B (CRYAB), peptidylprolyl isomerase F (PPIF), asparagine synthase (ASNS), sodium channel, non-voltage gated, 1 alpha subunit (SCNN1A), frizzled homolog 1 (FZD1), tyrosine related protein 1 (TYRP1), E cadherin, type 1 (ECAD), and thyroid hormone receptor associated protein 220 (TRAP220). Of note, two of these biomarkers (GOT1 and CD44) are now used in the Afirma classifier assay. We chose to compare specifically FTC versus FA rather than include all histologic categories to create a more uniform immunohistochemical comparison. In addition, we have found that most papillary thyroid carcinoma could often be reasonably distinguished from benign disease by morphological cytology findings alone.Increased immunoreactivity of CRYAB was associated with thyroid malignancy (c-statistic, 0.644; negative predictive value [NPV], 0.90) and loss of immunoreactivity of CA4 was also associated with malignancy (c-statistic, 0.715; NPV, 0.90) in indeterminate thyroid specimens. The combination of CA4 and CRYAB for discriminating FTC from FA resulted in a better c-statistic of 0.75, sensitivity of 0.76, specificity of 0.59, positive predictive value (PPV) of 0.32, and NPV of 0.91. When comparing widely angi  oinvasiv  e FTC from FA, the resultant c-statistic improved to 0.84, sensitivity of 0.75, specificity of 0.76, PPV of 0.11, and NPV of 0.99.Loss of CA4 and increase in CRYAB immunoreactivity distinguish FTC from FA in indeterminate thyroid nodules on a thyroid TMA with an NPV of 91%. Further studies in preoperative patient fine needle aspiration (FNAs) are needed to validate these results. 
  [Mm]etastasize  PubMed  
 Identification of metastasis-associated proteins through protein analysis of metastatic MDA-MB-435 and metastasis-suppressed BRMS1 transfected-MDA-MB-435 cells. BRMS1 (breast cancer metastasis suppressor 1) was recently identified as a novel breast cancer metastasis suppressor gene. To further characterize BRMS1-mediated metastasis suppression, we applied two-dimensional proteomic and mass spectrometry (LC-tandem MS and MALDI-TOF) analysis to identify proteins differentially expressed between highly metastatic MDA-MB-435 cells and metastasis-suppressed BRMS1-transfected MDA-MB-435 cells. Quadruplicate independent 2D gels were run and analyzed under identical conditions. Following in-gel trypsin digestion of seven differentially expressed proteins, amino acid sequence and mass profiles of the peptides were generated. Proteins were identified from the NCBI non-redundant database using the search program TurboSequest. Differential expression was confirmed for five proteins, including annexin I and alpha B-crystallin, by Northern blot analysis and immunostaining. Furthermore, we showed that both proteins were expressed in vivo in lungs containing   metastasize  d MDA-MB-435 cells but not expressed in normal lung tissue of athymic mice. Our results suggest that annexin I and alpha B-crystallin are important cellular proteins that are down regulated through BRMS1 mediated metastasis suppression. 
  GeneRIFs  
 CRYAB was expressed in vivo in lungs containing   metastasize  d MDA-MB-435 cells but not in normal lung tissue of athymic mice, suggesting that it is an important cellular protein that is down regulated through BRMS1 mediated metastasis suppression. 
  [Mm]etastasis  PubMed  
 ERK-regulated αB-crystallin induction by matrix detachment inhibits anoikis and promotes lung   metastasi  s in vivo. Evasion of extracellular matrix detachment-induced apoptosis ('anoikis') is a defining characteristic of metastatic tumor cells. The ability of metastatic carcinoma cells to survive matrix detachment and escape anoikis enables them to disseminate as viable circulating tumor cells and seed distant organs. Here we report that αB-crystallin, an antiapoptotic molecular chaperone implicated in the pathogenesis of diverse poor-prognosis solid tumors, is induced by matrix detachment and confers anoikis resistance. Specifically, we demonstrate that matrix detachment downregulates extracellular signal-regulated kinase (ERK) activity and increases αB-crystallin protein and messenger RNA (mRNA) levels. Moreover, we show that ERK inhibition in adherent cancer cells mimics matrix detachment by increasing αB-crystallin protein and mRNA levels, whereas constitutive ERK activation suppresses αB-crystallin induction during matrix detachment. These findings indicate that ERK inhibition is both necessary and sufficient for αB-crystallin induction by matrix detachment. To examine the functional consequences of αB-crystallin induction in anoikis, we stably silenced αB-crystallin in two different metastatic carcinoma cell lines. Strikingly, silencing αB-crystallin increased matrix detachment-induced caspase activation and apoptosis but did not affect cell viability of adherent cancer cells. In addition, silencing αB-crystallin in metastatic carcinoma cells reduced the number of viable circulating tumor cells and inhib  ited lung   metastasis in two orthotopic models, but had little or no effect on primary tumor growth. Taken together, our findings point to αB-crystallin as a novel regulator of anoikis resistance that is induced by matrix detachment-mediated suppression of ERK signaling and pro  motes lung   metastasis. Our results also suggest that αB-crystallin represents a promising molecular target for antimetastatic therapies. 
 Association of Alpha B-Crystallin (CRYAB) genotypes with breast cancer susceptibility in Taiwan. Alpha B-Crystallin (CRYAB) is purported to be a   metastasis   suppressor protein, and lack or lower CRYAB expression is a prognostic biomarker for several types of cancer, such as that of the prostate and head and neck. However, the association of genomic variation of CRYAB and breast cancer is not well studied. The aim of this study was to evaluate the association of polymorphic genotypes of CRYAB with breast cancer within a Taiwanese population.In this hospital-based study, 1232 patients with breast cancer and an equal number of healthy controls in central Taiwan were genotyped via polymerase chain reaction and restriction fragment length polymorphism (PCR-RFLP) and the association of CRYAB A-1215G (rs2228387), C-802G (rs14133) and intron 2 (rs2070894) polymorphisms with breast cancer risk in a central Taiwanese population was investigated.Those individuals with CRYAB C-802G CG and GG genotypes had 1.50- and 2.22-fold risk for breast cancer than those with the CC genotype. As for the A-1215G and intron 2 polymorphisms, there was no significant association of the genotype with breast cancer risk. In allelic frequency analysis, the G allele CRYAB C-802G conferred a significantly (p=5.63×10(-10)) increased risk of breast cancer. Our results provide evidence that the G allele of CRYAB C-802G is correlated with breast cancer risk and this polymorphism may be a useful marker for early detection of breast cancer in clinical practice. 
 Alpha B-crystallin is a new prognostic marker for laryngeal squamous cell carcinoma. Alpha B-crystallin (αB-crystallin) has been suggested to play an important role in the development of solid tumors. However, the association between αB-crystallin expression and clinicopathological characteristics of human laryngeal carcinoma is not well defined. This study aimed to examine the expression of αB-crystallin in human laryngeal squamous cell carcinoma (LSCC) and investigate the relationship between its expression and the prognosis of LSCC.Real-time polymerase chain reaction (six LSCC samples, six tumor-adjacent normal samples) and immunohistochemistry by tissue microarrays (109 LSCC samples and 28 tumor-adjacent normal samples) were performed to characterize expression of the αB-crystallin gene in LSCC. Kaplan-Meier survival and Cox regression analyses were carried out to evaluate the prognosis of LSCC.Real-time polymerase chain reaction and immunohistochemistry analysis showed that the expression of αB-crystallin in LSCC was significantly higher than that in tumor-adjacent normal tissues. Moreover, the expression level of αB-crystallin protein in LSCC was significantly related to alcohol consumption (P = 0.022), tumor differentiation (P = 0.007), pTNM stage (P = 0.041) and 5 years' survival (P =0.030). COX multi-factor analysis showed that αB-crystallin (P = .013), as well as pTNM stage (P =0.027) and lym  phatic met  astasis (P = 0.015) were independent prognosis factors for LSCC.The data suggest that αB-crystallin expression is correlated with malignant phenotypes of LSCC and it may serve as a novel prognostic factor for LSCC. 
 High level of αB-crystallin contributes to the progression of osteosarcoma. Accumulating evidences indicate the elevated expression of αB-Crystallin (Cryab) is implicated in tumorigenesis. However, the expression and biologic role of Cryab in osteosarcoma (OS) are still unknown. In this study, we showed that Cryab expression was elevated in OS tissues and cell lines, and down-regulation of Cryab in MG-63 and U-2OS cells led to a decline in the cells' aggressiveness, and reduced secretion of matrix metalloproteinase-9 (MMP-9) in vitro, and lowe  r metastas  is potential in vivo. Further study indicated that the Cryab expression was positively associated with the activity of ERK1/2 which is responsible for the cells' aggressiveness and MMP-9 secretion. Clinically, our data confirmed that the high level of Cryab was associated with shorten survival and tumor recurrence for the postoperative OS patients. Together, our results indicate that high level of Cryab is a new adverse outcomes marker for OS patients and may be used as a new therapeutic target. 
 Alpha B-crystallin correlates with poor survival in colorectal cancer. Alpha B-crystallin (CRYAB) is primarily found as major structural proteins of the ocular lens and is a principal member of the small heat shock protein (HSP) family. So far, CRYAB has been suggested to play critical roles in the development of several kinds of human cancers. However, the association between CRYAB expression and clinicopathological characteristics of colorectal cancer (CRC) has not been elucidated yet. In the present study, one-step quantitative PCR reverse transcription-polymerase chain reaction (qPCR) analysis of 18 samples of CRC and immunohistochemistry (IHC) analysis with 100 cases of CRC sample in tissue microarrays (TMA) were employed to evaluate the expression of CRYAB in CRC. The results suggested that CRYAB expression in the mRNA and protein levels was significantly higher in CRC tissues than in corresponding non-cancerous tissues (P < 0.05 and P = 0.014, respectively). The expression of CRYAB protein in CRC was significantly associated with distant   metastasis   (P = 0.040) and overall survival (P = 0.003). Kaplan-Meier method and multivariate survival analysis indicated that high expression of CRYAB (P = 0.040) and distant   metastasis   (P = 0.005) showed significant correlations with poor prognosis of CRC patients. The data imply that CRYAB expression is correlated with substantial clinical characteristics of CRC, and it may be identified as an unfavorable prognostic factor for CRC. 
 Alpha B-crystallin - a validated prognostic factor for poor prognosis in squamous cell carcinoma of the oral cavity. Alpha B-crystallin was found to be an independent prognostic marker for poor prognosis in oral cavity tumours. For oropharyngeal cancer, alpha B-crystallin had no prognostic value.The aim of this study was to see if earlier findings of alpha B-crystallin as an independent prognostic marker, and SPARC/osteonectin, PAI-1 and uPA as a prognostic combination for poor outcome in squamous cell carcinoma (SCC) of the head and neck could be confirmed in a new set of tumours.In a consecutive series of patients, assessed and primarily treated at a tertiary referral centre, histological sections from 55 patients with oral and SCC (OOPHSSC) with complete clinical data and follow-up were obtained. Oral and oropharyngeal tumours were studied separately. Immunohistochemical detection of alpha B-crystallin, SPARC/osteonectin, PAI-1 and uPA expression was performed.Thirty-five patients had an oral tumour and 20 patients an oropharyngeal tumour. Twenty-five oral tumours stained negatively and 10 positively for alpha B-crystallin. For oropharyngeal tumours the figures were 15 negatively and 5 positively. Median disease-specific survival (DSS) for both sites was 33.8 and 11.9 months, for negative and positive alpha B-crystallin staining, respectively (p = 0.046). For the oral cavity, median DSS was 27.3 months for negative tumours and 7.5 months for positive tumours (p = 0.012). Corresponding figures for oropharyngeal tumours were 33.8 and 34.1 months (p = 0.95). Thus, significance in survival was only found in oral cavity tumours. In multivariate analyses there were no significant differences in DSS in the oropharyngeal group when adjusted for tumour size (T status) and presence of neck node   metastasis   (N status). In the oral cavity group, the significantly better DSS for negative tumours became even stronger when adjusted for T and N status. No statistical difference was found in DSS between positive and negative staining for SPARC/osteonectin, PAI-1 or uPA. 
 Effect of hypoxia on the expression of αB-crystallin in head and neck squamous cell carcinoma. The presence of hypoxia in head and neck squamous cell carcinoma (HNSCC) is associated with therapeutic resistance and increased risk of   metastasi  s formation. αB-crystallin (HspB5) is a small heat shock protein, which is also associated wit  h metastas  is formation in HNSCC. In this study, we investigated whether αB-crystallin protein expression is increased in hypoxic areas of HNSCC biopsies and analyzed whether hypoxia induces αB-crystallin expression in vitro and in this way may confer hypoxic cell survival.In 38 HNSCC biopsies, the overlap between immunohistochemically stained αB-crystallin and pimonidazole-adducts (hypoxiamarker) was determined. Moreover, expression levels of αB-crystallin were analyzed in HNSCC cell lines under hypoxia and reoxygenation conditions and after exposure to reactive oxygen species (ROS) and the ROS scavenger N-acetylcysteine (NAC). siRNA-mediated knockdown was used to determine the influence of αB-crystallin on cell survival under hypoxic conditions.In all biopsies αB-crystallin was more abundantly present in hypoxic areas than in normoxic areas. Remarkably, hypoxia decreased αB-crystallin mRNA expression in the HNSCC cell lines. Only after reoxygenation, a condition that stimulates ROS formation, αB-crystallin expression was increased. αB-crystallin mRNA levels were also increased by extracellular ROS, and NAC abolished the reoxygenation-induced αB-crystallin upregulation. Moreover, it was found that decreased αB-crystallin levels reduced cell survival under hypoxic conditions.We provide the first evidence that hypoxia stimulates upregulation of αB-crystallin in HNSCC. This upregulation was not caused by the low oxygen pressure, but more likely by ROS formation. The higher expression of αB-crystallin may lead to prolonged survival of these cells under hypoxic conditions. 
 αB-crystallin: a novel regulator of breast cancer   metastasi  s to the brain. Basal-like breast tumors are typically (ER/PR/HER2) triple-negative and are associated with a high incidence of brain metastases and poor clinical outcomes. The molecular chaperone αB-crystallin is predominantly expressed in triple-negative breast cancer (TNBC) and contributes to an aggressive tumor phenotype in preclinical models. We investigated the potential role of αB-crystallin in bra  in metasta  sis in TNBCs.αB-crystallin expression in primary breast carcinomas and brain metastases was analyzed by immunohistochemistry among patients with breast cancer with brain metastases. αB-crystallin was overexpressed or silenced in two different TNBC cell lines. The effects on cell adhesion to human brain microvascular endothelial cells (HBMEC) or extracellular matrix proteins, transendothelial migration, and transmigration across a HBMEC/astrocyte coculture blood-brain barrier (BBB) model were examined. In addition, the effects of overexpressing or silencing αB-crystallin on   brain meta  stasis in vivo were investigated using orthotopic TNBC models.In a cohort of women with breast cancer   brain meta  stasis, αB-crystallin expression in primary breast carcinomas was associated with poor overall survival and poor survival after   brain met  astasis, even among patients with TNBC. Stable overexpression of αB-crystallin in TNBC cells enhanced adhesion to HBMECs, transendothelial migration, and BBB transmigration in vitro, whereas silencing αB-crystallin inhibited these events. αB-crystallin promoted adhesion of TNBC cells to HBMECs, at least in part, through an α3β1 integrin-dependent mechanism. αB-crystallin overexpression pr  omoted bra  in metastasis, whereas silencing αB-crystallin in  hibited br  ain metastasis in orthotopic TNBC models.αB-crystallin is a novel regu  lator of b  rain metastasis in TNBC and represents a potential biomarker and drug target for this aggressive disease. 
 Identification of   metastasis  -associated proteins through protein analysis of metastatic MDA-MB-435 and   metastasis  -suppressed BRMS1 transfected-MDA-MB-435 cells. BRMS1 (breast cancer   metastasis   suppressor 1) was recently identified as a novel breast cancer   metastasis   suppressor gene. To further characterize BRMS1-mediated   metastasis   suppression, we applied two-dimensional proteomic and mass spectrometry (LC-tandem MS and MALDI-TOF) analysis to identify proteins differentially expressed between highly metastatic MDA-MB-435 cells and   metastasis  -suppressed BRMS1-transfected MDA-MB-435 cells. Quadruplicate independent 2D gels were run and analyzed under identical conditions. Following in-gel trypsin digestion of seven differentially expressed proteins, amino acid sequence and mass profiles of the peptides were generated. Proteins were identified from the NCBI non-redundant database using the search program TurboSequest. Differential expression was confirmed for five proteins, including annexin I and alpha B-crystallin, by Northern blot analysis and immunostaining. Furthermore, we showed that both proteins were expressed in vivo in lungs containing metastasized MDA-MB-435 cells but not expressed in normal lung tissue of athymic mice. Our results suggest that annexin I and alpha B-crystallin are important cellular proteins that are down regulated through BRMS1 mediated   metastasis   suppression. 
  GeneRIFs  
 Cryab expression was elevated in osteosarcoma tissues and cell lines, and down-regulation of Cryab in MG-63 and U-2OS cells led to a decline in the cells' aggressiveness, and reduced secretion of matrix metalloproteinase-9 in vitro, and lower   metastasis   potential in vivo. 
 findings point to alphaB-crystallin as a novel regulator of anoikis resistance that is induced by matrix detachment-mediated suppression of ERK signaling and promotes lung   metastasis  . 
 AlphaB-crystallin is a novel regulator of brain   metastasis   in triple-negative breast cancer. 
 CRYAB was expressed in vivo in lungs containing metastasized MDA-MB-435 cells but not in normal lung tissue of athymic mice, suggesting that it is an important cellular protein that is down regulated through BRMS1 mediated   metastasis   suppression. 
   
  
 NEGR1 
 
  NCBI Gene  
  GeneRIF  
  Pubmed  
 
 
  Search Term  Rank  Fields  
  [Ee]xtracellular [Mm]atrix  8  PubMed(1)  
 
 
 hide/show details for NEGR1 
  [Ee]xtracellular [Mm]atrix  PubMed  
 A secreted protein microarray platform for extracellular protein interaction discovery. Characterization of the extracellular protein interactome has lagged far behind that of intracellular proteins, where mass spectrometry and yeast two-hybrid technologies have excelled. Improved methods for identifying receptor-ligand and   extracellular matrix   protein interactions will greatly accelerate biological discovery in cell signaling and cellular communication. These technologies must be able to identify low-affinity binding events that are often observed between membrane-bound coreceptor molecules during cell-cell or cell-  extracellular matrix   contact. Here we demonstrate that functional protein microarrays are particularly well-suited for high-throughput screening of extracellular protein interactions. To evaluate the performance of the platform, we screened a set of 89 immunoglobulin (Ig)-type receptors against a highly diverse extracellular protein microarray with 686 genes represented. To enhance detection of low-affinity interactions, we developed a rapid method to assemble bait Fc fusion proteins into multivalent complexes using protein A microbeads. Based on these screens, we developed a statistical methodology for hit calling and identification of nonspecific interactions on protein microarrays. We found that the Ig receptor interactions identified using our methodology are highly specific and display minimal off-target binding, resulting in a 70% true-positive to false-positive hit ratio. We anticipate that these methods will be useful for a wide variety of functional protein microarray users. 
   
  
 SVIL 
 
  NCBI Gene  
  GeneRIF  
  Pubmed  
 
 
  Search Term  Rank  Fields  
  [Ii]nvadopodia  3  PubMed(1); GeneRIFs(1)  
  [Ii]nvadopodium  4  Component(1)  
  [Pp]odosome(s)?  5  PubMed(3); Component(1); GeneRIFs(1)  
  [Mm]igration  6  PubMed(2)  
  ECM  7  PubMed(1)  
  [Ee]xtracellular [Mm]atrix  8  PubMed(1)  
 
 
 hide/show details for SVIL 
  [Ii]nvadopodia  PubMed  
 Supervillin reorganizes the actin cytoskeleton and increases   invadopodia  l efficiency. Tumor cells use actin-rich protrusions called   invadopodia   to degrade extracellular matrix (ECM) and invade tissues; related structures, termed podosomes, are sites of dynamic ECM interaction. We show here that supervillin (SV), a peripheral membrane protein that binds F-actin and myosin II, reorganizes the actin cytoskeleton and potentiates   invadopodia  l function. Overexpressed SV induces redistribution of lamellipodial cortactin and lamellipodin/RAPH1/PREL1 away from the cell periphery to internal sites and concomitantly increases the numbers of F-actin punctae. Most punctae are highly dynamic and colocalize with the podosome/  invadopodia  l proteins, cortactin, Tks5, and cdc42. Cortactin binds SV sequences in vitro and contributes to the formation of enhanced green fluorescent protein (EGFP)-SV induced punctae. SV localizes to the cores of Src-generated podosomes in COS-7 cells and with   invadopodia   in MDA-MB-231 cells. EGFP-SV overexpression increases average numbers of ECM holes per cell; RNA interference-mediated knockdown of SV decreases these numbers. Although SV knockdown alone has no effect, simultaneous down-regulation of SV and the closely related protein gelsolin reduces invasion through ECM. Together, our results show that SV is a component of podosomes and   invadopodia   and that SV plays a role in   invadopodia  l function, perhaps as a mediator of cortactin localization, activation state, and/or dynamics of metalloproteinases at the ventral cell surface. 
  GeneRIFs  
 SV is a component of podosomes and   invadopodia   and SV plays a role in   invadopodia  l function, perhaps as a mediator of cortactin localization, activation state, and/or dynamics of metalloproteinases at the ventral cell surface. 
  [Ii]nvadopodium  Component  
   invadopodium   
  [Pp]odosome(s)?  PubMed  
 Smooth muscle archvillin: a novel regulator of signaling and contractility in vascular smooth muscle. The mechanisms by which protein kinase C (PKC) and extracellular-signal-regulated kinases (ERK1/2) govern smooth-muscle contractility remain unclear. Calponin (CaP), an actin-binding protein and PKC substrate, mediates signaling through ERK1/2. We report here that CaP sequences containing the CaP homology (CH) domain bind to the C-terminal 251 amino acids of smooth-muscle archvillin (SmAV), a new splice variant of supervillin, which is a known actin- and myosin-II-binding protein. The CaP-SmAV interaction is demonstrated by reciprocal yeast two-hybrid and blot-overlay assays and by colocalization in COS-7 cells. In differentiated smooth muscle, endogenous SmAV and CaP co-fractionate and co-translocate to the cell cortex after stimulation by agonist. Antisense knockdown of SmAV in tissue inhibits both the activation of ERK1/2 and contractions stimulated by either agonist or PKC activation. This ERK1/2 signaling and contractile defect is similar to that observed in CaP knockdown experiments. In A7r5 smooth-muscle cells, PKC activation by phorbol esters induces the reorganization of endogenous, membrane-localized SmAV and microfilament-associated CaP into   podosome  -like structures that also contain F-actin, nonmuscle myosin IIB and ERK1/2. These results indicate that SmAV contributes to the regulation of contractility through a CaP-mediated signaling pathway, involving PKC activation and phosphorylation of ERK1/2. 
 The membrane-associated protein, supervillin, accelerates F-actin-dependent rapid integrin recycling and cell motility. In migrating cells, the cytoskeleton coordinates signal transduction and redistribution of transmembrane proteins, including integrins and growth factor receptors. Supervillin is an F-actin- and myosin II-binding protein that tightly associates with signaling proteins in cholesterol-rich, 'lipid raft' membrane microdomains. We show here that supervillin also can localize with markers for early and sorting endosomes (EE/SE) and with overexpressed components of the Arf6 recycling pathway in the cell periphery. Supervillin tagged with the photoswitchable fluorescent protein, tdEos, moves both into and away from dynamic structures resembling   podosomes   at the basal cell surface. Rapid integrin recycling from EE/SE is inhibited in supervillin-knockdown cells, but the rates of integrin endocytosis and recycling from the perinuclear recycling center (PNRC) are unchanged. A lack of synergy between supervillin knockdown and the actin filament barbed-end inhibitor, cytochalasin D, suggests that both treatments affect actin-dependent rapid recycling. Supervillin also enhances signaling from the epidermal growth factor receptor (EGFR) to extracellular signal-regulated kinases (ERKs) 1 and 2 and increases the velocity of cell translocation. These results suggest that supervillin, F-actin and associated proteins coordinate a rapid, basolateral membrane recycling pathway that contributes to ERK signaling and actin-based cell motility. 
 Supervillin reorganizes the actin cytoskeleton and increases invadopodial efficiency. Tumor cells use actin-rich protrusions called invadopodia to degrade extracellular matrix (ECM) and invade tissues; related structures, termed   podosomes  , are sites of dynamic ECM interaction. We show here that supervillin (SV), a peripheral membrane protein that binds F-actin and myosin II, reorganizes the actin cytoskeleton and potentiates invadopodial function. Overexpressed SV induces redistribution of lamellipodial cortactin and lamellipodin/RAPH1/PREL1 away from the cell periphery to internal sites and concomitantly increases the numbers of F-actin punctae. Most punctae are highly dynamic and colocalize with the   podosome  /invadopodial proteins, cortactin, Tks5, and cdc42. Cortactin binds SV sequences in vitro and contributes to the formation of enhanced green fluorescent protein (EGFP)-SV induced punctae. SV localizes to the cores of Src-generated   podosomes   in COS-7 cells and with invadopodia in MDA-MB-231 cells. EGFP-SV overexpression increases average numbers of ECM holes per cell; RNA interference-mediated knockdown of SV decreases these numbers. Although SV knockdown alone has no effect, simultaneous down-regulation of SV and the closely related protein gelsolin reduces invasion through ECM. Together, our results show that SV is a component of   podosomes   and invadopodia and that SV plays a role in invadopodial function, perhaps as a mediator of cortactin localization, activation state, and/or dynamics of metalloproteinases at the ventral cell surface. 
  Component  
   podosome   
  GeneRIFs  
 SV is a component of   podosomes   and invadopodia and SV plays a role in invadopodial function, perhaps as a mediator of cortactin localization, activation state, and/or dynamics of metalloproteinases at the ventral cell surface. 
  [Mm]igration  PubMed  
 Analysis of the myosin-II-responsive focal adhesion proteome reveals a role for β-Pix in negative regulation of focal adhesion maturation. Focal adhesions undergo myosin-II-mediated maturation wherein they grow and change composition to modulate integrin signalling for cell   migratio  n, growth and differentiation. To determine how focal adhesion composition is affected by myosin II activity, we performed proteomic analysis of isolated focal adhesions and compared protein abundance in focal adhesions from cells with and without myosin II inhibition. We identified 905 focal adhesion proteins, 459 of which changed in abundance with myosin II inhibition, defining the myosin-II-responsive focal adhesion proteome. The abundance of 73% of the proteins in the myosin-II-responsive focal adhesion proteome was enhanced by contractility, including proteins involved in Rho-mediated focal adhesion maturation and endocytosis- and calpain-dependent focal adhesion disassembly. During myosin II inhibition, 27% of proteins in the myosin-II-responsive focal adhesion proteome, including proteins involved in Rac-mediated lamellipodial protrusion, were enriched in focal adhesions, establishing that focal adhesion protein recruitment is also negatively regulated by contractility. We focused on the Rac guanine nucleotide exchange factor β-Pix, documenting its role in the negative regulation of focal adhesion maturation and the promotion of lamellipodial protrusion and focal adhesion turnover to drive cel  l migrati  on. 
 Supervillin slows cell spreading by facilitating myosin II activation at the cell periphery. During cell   migration  , myosin II modulates adhesion, cell protrusion and actin organization at the leading edge. We show that an F-actin- and membrane-associated scaffolding protein, called supervillin (SV, p205), binds directly to the subfragment 2 domains of nonmuscle myosin IIA and myosin IIB and to the N-terminus of the long form of myosin light chain kinase (L-MLCK). SV inhibits cell spreading via an MLCK- and myosin II-dependent mechanism. Overexpression of SV reduces the rate of cell spreading, and RNAi-mediated knockdown of endogenous SV increases it. Endogenous and EGFP-tagged SV colocalize with, and enhance the formation of, cortical bundles of F-actin and activated myosin II during early cell spreading. The effects of SV are reversed by inhibition of myosin heavy chain (MHC) ATPase (blebbistatin), MLCK (ML-7) or MEK (U0126), but not by inhibiting Rho-kinase with Y-27632. Flag-tagged L-MLCK co-localizes in cortical bundles with EGFP-SV, and kinase-dead L-MLCK disorganizes these bundles. The L-MLCK- and myosin-binding site in SV, SV1-171, rearranges and co-localizes with mono- and di-phosphorylated myosin light chain and with L-MLCK, but not with the short form of MLCK (S-MLCK) or with myosin phosphatase. Thus, the membrane protein SV apparently contributes to myosin II assembly during cell spreading by modulating myosin II regulation by L-MLCK. 
  ECM  PubMed  
 Supervillin reorganizes the actin cytoskeleton and increases invadopodial efficiency. Tumor cells use actin-rich protrusions called invadopodia to degrade extracellular matrix (  ECM  ) and invade tissues; related structures, termed podosomes, are sites of dynamic   ECM   interaction. We show here that supervillin (SV), a peripheral membrane protein that binds F-actin and myosin II, reorganizes the actin cytoskeleton and potentiates invadopodial function. Overexpressed SV induces redistribution of lamellipodial cortactin and lamellipodin/RAPH1/PREL1 away from the cell periphery to internal sites and concomitantly increases the numbers of F-actin punctae. Most punctae are highly dynamic and colocalize with the podosome/invadopodial proteins, cortactin, Tks5, and cdc42. Cortactin binds SV sequences in vitro and contributes to the formation of enhanced green fluorescent protein (EGFP)-SV induced punctae. SV localizes to the cores of Src-generated podosomes in COS-7 cells and with invadopodia in MDA-MB-231 cells. EGFP-SV overexpression increases average numbers of   ECM   holes per cell; RNA interference-mediated knockdown of SV decreases these numbers. Although SV knockdown alone has no effect, simultaneous down-regulation of SV and the closely related protein gelsolin reduces invasion through   ECM  . Together, our results show that SV is a component of podosomes and invadopodia and that SV plays a role in invadopodial function, perhaps as a mediator of cortactin localization, activation state, and/or dynamics of metalloproteinases at the ventral cell surface. 
  [Ee]xtracellular [Mm]atrix  PubMed  
 Supervillin reorganizes the actin cytoskeleton and increases invadopodial efficiency. Tumor cells use actin-rich protrusions called invadopodia to degrade   extracellular matrix   (ECM) and invade tissues; related structures, termed podosomes, are sites of dynamic ECM interaction. We show here that supervillin (SV), a peripheral membrane protein that binds F-actin and myosin II, reorganizes the actin cytoskeleton and potentiates invadopodial function. Overexpressed SV induces redistribution of lamellipodial cortactin and lamellipodin/RAPH1/PREL1 away from the cell periphery to internal sites and concomitantly increases the numbers of F-actin punctae. Most punctae are highly dynamic and colocalize with the podosome/invadopodial proteins, cortactin, Tks5, and cdc42. Cortactin binds SV sequences in vitro and contributes to the formation of enhanced green fluorescent protein (EGFP)-SV induced punctae. SV localizes to the cores of Src-generated podosomes in COS-7 cells and with invadopodia in MDA-MB-231 cells. EGFP-SV overexpression increases average numbers of ECM holes per cell; RNA interference-mediated knockdown of SV decreases these numbers. Although SV knockdown alone has no effect, simultaneous down-regulation of SV and the closely related protein gelsolin reduces invasion through ECM. Together, our results show that SV is a component of podosomes and invadopodia and that SV plays a role in invadopodial function, perhaps as a mediator of cortactin localization, activation state, and/or dynamics of metalloproteinases at the ventral cell surface. 
   
  
 CYP4Z1 
 
  NCBI Gene  
  GeneRIF  
  Pubmed  
 
 
  Search Term  Rank  Fields  
  [Mm]igration  6  PubMed(1)  
 
 
 hide/show details for CYP4Z1 
  [Mm]igration  PubMed  
 Increased expression of CYP4Z1 promotes tumor angiogenesis and growth in human breast cancer. Cytochrome P450 (CYP) 4Z1, a novel CYP4 family member, is over-expressed in human mammary carcinoma and associated with high-grade tumors and poor prognosis. However, the precise role of CYP4Z1 in tumor progression is unknown. Here, we demonstrate that CYP4Z1 overexpression promotes tumor angiogenesis and growth in breast cancer. Stable expression of CYP4Z1 in T47D and BT-474 human breast cancer cells significantly increased mRNA expression and production of vascular endothelial growth factor (VEGF)-A, and decreased mRNA levels and secretion of tissue inhibitor of metalloproteinase-2 (TIMP-2), without affecting cell proliferation and anchorage-independent cell growth in vitro. Notably, the conditioned medium from CYP4Z1-expressing cells enhanced proliferation,   migration   and tube formation of human umbilical vein endothelial cells, and promoted angiogenesis in the zebrafish embryo and chorioallantoic membrane of the chick embryo. In addition, there were lower levels of myristic acid and lauric acid, and higher contents of 20-hydroxyeicosatetraenoic acid (20-HETE) in CYP4Z1-expressing T47D cells compared with vector control. CYP4Z1 overexpression significantly increased tumor weight and microvessel density by 2.6-fold and 1.9-fold in human tumor xenograft models, respectively. Moreover, CYP4Z1 transfection increased the phosphorylation of ERK1/2 and PI3K/Akt, while PI3K or ERK inhibitors and siRNA silencing reversed CYP4Z1-mediated changes in VEGF-A and TIMP-2 expression. Conversely, HET0016, an inhibitor of the CYP4 family, potently inhibited the tumor-induced angiogenesis with associated changes in the intracellular levels of myristic acid, lauric acid and 20-HETE. Collectively, these data suggest that increased CYP4Z1 expression promotes tumor angiogenesis and growth in breast cancer partly via PI3K/Akt and ERK1/2 activation. 
   
  
 UNC13D 
 
  NCBI Gene  
  GeneRIF  
  Pubmed  
 
 
  Search Term  Rank  Fields  
  [Ii]nvasive  10  PubMed(1)  
 
 
 hide/show details for UNC13D 
  [Ii]nvasive  PubMed  
 A Hemophagocytic Lymphohistiocytosis Case with Newly Defined UNC13D (c.175G>C; p.Ala59Pro) Mutation and a Rare Complication. Hemophagocytic lymphohistiocytosis (HLH) represents a severe hyperinflammatory condition with cardinal symptoms of prolonged fever, cytopenias, hepatosplenomegaly, and hemophagocytosis by activated, morphologically benign macrophages with impaired function of natural killer cells and cytotoxic T lymphocytes. A 2-month-old girl, who was admitted with fever, was diagnosed with HLH and her genetic examination revealed a newly defined mutation in the UNC13D (c.175G>C; p.Ala59Pro) gene. She was treated with dexamethasone, etoposide, and intrathecal methotrexate. During the second week of treatment, after three doses of etoposide, it was noticed that there was a necrotic plaque lesion on the soft palate. Pathologic examination of debrided material in PAS and Grocott staining revealed lots of septated hyphae, which was consistent with aspergillosis infection. Etoposide was stopped and amphotericin B treatment was given for six weeks. HLH 2004 protocol was completed to eight weeks with cyclosporine A orally. There was no patient with   invasive   aspergillosis infection as severe as causing palate and nasal septum perforation during HLH therapy. In immunocompromised patients, fungal infections may cause nasal septum perforation and treatment could be achieved by antifungal therapy and debridement of necrotic tissue. 
   
  
 FHL2 
 
  NCBI Gene  
  GeneRIF  
  Pubmed  
 
 
  Search Term  Rank  Fields  
  [Mm]igration  6  PubMed(14)  
  ECM  7  PubMed(1)  
  [Ee]xtracellular [Mm]atrix  8  PubMed(4)  
  [Ii]nvasive  10  PubMed(6); GeneRIFs(2)  
  [Mm]etastasis  12  PubMed(7); GeneRIFs(5)  
  [Cc]ell [Ii]nvasion  13  PubMed(1); GeneRIFs(1)  
 
 
 hide/show details for FHL2 
  [Mm]igration  PubMed  
 Analysis of the myosin-II-responsive focal adhesion proteome reveals a role for β-Pix in negative regulation of focal adhesion maturation. Focal adhesions undergo myosin-II-mediated maturation wherein they grow and change composition to modulate integrin signalling for cell   migratio  n, growth and differentiation. To determine how focal adhesion composition is affected by myosin II activity, we performed proteomic analysis of isolated focal adhesions and compared protein abundance in focal adhesions from cells with and without myosin II inhibition. We identified 905 focal adhesion proteins, 459 of which changed in abundance with myosin II inhibition, defining the myosin-II-responsive focal adhesion proteome. The abundance of 73% of the proteins in the myosin-II-responsive focal adhesion proteome was enhanced by contractility, including proteins involved in Rho-mediated focal adhesion maturation and endocytosis- and calpain-dependent focal adhesion disassembly. During myosin II inhibition, 27% of proteins in the myosin-II-responsive focal adhesion proteome, including proteins involved in Rac-mediated lamellipodial protrusion, were enriched in focal adhesions, establishing that focal adhesion protein recruitment is also negatively regulated by contractility. We focused on the Rac guanine nucleotide exchange factor β-Pix, documenting its role in the negative regulation of focal adhesion maturation and the promotion of lamellipodial protrusion and focal adhesion turnover to drive cel  l migrati  on. 
 The LIM-only proteins FHL2 and FHL3 interact with alpha- and beta-subunits of the muscle alpha7beta1 integrin receptor. FHL1, FHL2, and FHL3 are members of the four and one-half LIM domain protein subclass that are expressed in striated muscles. Here we show that FHL2 and FHL3 are novel alpha(7)beta(1) integrin-interacting proteins. They bind both the alpha- and the beta-subunit as well as different splice isoforms. The minimal binding sites for FHL2 and FHL3 on beta(1A)-chain overlap, whereas on alpha(7A) and alpha(7B) subunits they are situated adjacent. Determining the binding sites for integrins on FHL2 or FHL3 revealed that the suprastructure of the whole molecule is important for these associations, rather than any single LIM domain. Immunofluorescence studies with cells expressing full-length FHL proteins or their deletion mutants showed that FHL2 and FHL3 but not FHL1 colocalize with integrins at cell adhesion sites. Further, their recruitment to the membrane results from binding to either the alpha- or the beta-chain of the integrin receptor. The association of FHL2 or FHL3 with integrin receptors neither influences attachment of cells to different substrates nor changes their   migration   capacity. However, in cardiac and skeletal muscles, FHL2 and FHL3, respectively, are colocalized with alpha(7)beta(1) integrin receptor at the periphery of Z-discs, suggesting a role in mechanical stabilization of muscle cells. 
 The LIM-only proteins FHL2 and FHL3 interact with alpha- and beta-subunits of the muscle alpha7beta1 integrin receptor. FHL1, FHL2, and FHL3 are members of the four and one-half LIM domain protein subclass that are expressed in striated muscles. Here we show that FHL2 and FHL3 are novel alpha(7)beta(1) integrin-interacting proteins. They bind both the alpha- and the beta-subunit as well as different splice isoforms. The minimal binding sites for FHL2 and FHL3 on beta(1A)-chain overlap, whereas on alpha(7A) and alpha(7B) subunits they are situated adjacent. Determining the binding sites for integrins on FHL2 or FHL3 revealed that the suprastructure of the whole molecule is important for these associations, rather than any single LIM domain. Immunofluorescence studies with cells expressing full-length FHL proteins or their deletion mutants showed that FHL2 and FHL3 but not FHL1 colocalize with integrins at cell adhesion sites. Further, their recruitment to the membrane results from binding to either the alpha- or the beta-chain of the integrin receptor. The association of FHL2 or FHL3 with integrin receptors neither influences attachment of cells to different substrates nor changes their   migration   capacity. However, in cardiac and skeletal muscles, FHL2 and FHL3, respectively, are colocalized with alpha(7)beta(1) integrin receptor at the periphery of Z-discs, suggesting a role in mechanical stabilization of muscle cells. 
 Deletion of FHL2 gene impaired ischemia-induced blood flow recovery by modulating circulating proangiogenic cells. The four and a half Lin11, Isl-1 and Mec-3 (LIM) domain protein 2 (FHL2) is a member of the four and a half LIM domain-only (FHL) gene family, and has been shown to play an important role in inhibiting inflammatory angiogenesis. Here, we tested the hypothesis that impaired ischemia-induced neovascularization in mice lacking FHL2 is related to a defect in proangiogenic cell mobilization and functions in vasculogenesis.Unilateral hindlimb ischemia surgery was conducted in FHL2(-/-) mice and wild-type (FHL2(+/+)) mice. After hindlimb ischemia surgery, expression of FHL2 protein was noted in ischemic tissues of wild-type mice. All FHL2-null mice (100%) suffered from spontaneous foot amputation, but only 20% of wild-type mice had ischemia-induced foot amputation after ischemic surgery. Blood flow recovery was significantly impaired in FHL2(-/-) mice when compared with that in wild-type mice as determined by laser Doppler imaging. Histological analysis revealed that the capillary density in the ischemic limb was increased in wild-type mice, whereas no such increase was noted in FHL2(-/-) mice. Flow cytometry demonstrated that the number of CD34(+) or CD34(+)/Sca-1(+)/Flk-1(+) in peripheral blood after ischemic surgery significantly decreased in FHL2-null mice than those in wild-type mice after hindlimb ischemia surgery. FHL2 deficiency impaired ex vivo angiogenesis in mouse aortic-ring culture assay, which revealed that the mean density of the microvessels was significantly higher in the wild-type aorta than in the FHL2(-/-) aorta. Western blot analysis showed that vascular endothelial growth factor (VEGF), interleukin-6, matrix metalloproteinase-2, matrix metalloproteinase-9, vascular cell adhesion molecule-1, and intercellular adhesion molecule-1 levels were significantly downregulated in ischemic muscles in FHL2-null mice compared with wild-type mice. Deletion of FHL2 protein by FHL2 small interfering RNA impaired VEGF production under hypoxia conditions, and also suppressed endothelial progenitor cell angiogenic functions, but these effects could be recovered by administration of VEGF.Deficiency of FHL2 impairs ischemia-induced neovascularization, and these suppressive effects may occur through a reduction in proangiogenic cell mobilization,   migration  , and vasculogenesis functions. 
 FHL2 exhibits anti-proliferative and anti-apoptotic activities in liver cancer cells. FHL2 displays tumor promoting or tumor suppressing activities depending on the types of tumor cells. In this study, we demonstrated that FHL2 overexpression inhibits the proliferation of human HCC cells Hep3B through cell cycle regulation by decreasing cyclin D1 expression while increasing the expressions of p21 and p27. FHL2 overexpression also inhibits   migration   and invasion of Hep3B cells through the regulation of epithelial-mesenchymal transition. Surprisingly, we also demonstrated an antiapoptotic function for FHL2 overexpression with increased resistance to doxorubicin-induced apoptosis, which indicates the separation of anti-proliferative and anti-apoptotic role of FHL2. Taken together, our results indicate FHL2 could exert anti-apoptotic effect independent of tumor growth suppression. 
 FHL2 expression in peritumoural fibroblasts correlates with lymphatic metastasis in sporadic but not in HNPCC-associated colon cancer. Four and a half LIM domain protein-2 (FHL2) is a component of the focal adhesion structures and has been suggested to have an important role in cancer progression. This study analyses the role of FHL2 in peritumoural fibroblasts of sporadic and hereditary non-polyposis colorectal cancer (HNPCC). Tissue specimens of 48 sporadic and 49 hereditary colon cancers, respectively, were stained immunohistochemically for FHL2, transforming growth factor (TGF)-β1 ligand and α-SMA. Myofibroblasts at the tumour invasion front co-expressed α-SMA and FHL2. Sporadic colon cancer but not HNPCC cases showed a correlation between TGF-β1 expression of the invading tumour cells and FHL2 staining of peritumoural myofibroblasts. Overexpression of FHL2 in peritumoural myofibroblasts correlated to lymphatic metastasis in sporadic colon cancer but not in HNPCC. In cultured mouse fibroblasts, TGF-β1 treatment induced myofibroblast differentiation, stimulated FHL2 protein expression and elevated number of migratory cells in transwell motility assays, suggesting that FHL2 is regulated downstream of TGF-β. Physical contact of colon cancer cells and myofibroblasts via FHL2-positive focal adhesions was detected in human colon carcinoma tissue and in co-culture assays using sporadic as well as HNPCC-derived tumour cell lines. Our data provide strong evidence for an important role of FHL2 in the progression of colon cancers. Tumour-secreted TGF-β1 stimulates FHL2 protein expression in peritumoural fibroblasts, probably facilitating the invasion of tumour glands into the surrounding tissue by enhanced myofibr  oblast mi  gration and tight connection of fibroblasts to tumour cells via focal adhesions. These findings are absent in HNPCC-associated colon cancers in vivo and may contribute to a less invasive and more protruding tumour margin of microsatellite instable carcinomas. 
 Protein-protein interactions of the LIM-only protein FHL2 and functional implication of the interactions relevant in cardiovascular disease. FHL2 belongs to the LIM-domain only proteins and contains four and a half LIM domains, each of which are composed of two zinc finger structures. FHL2 exhibits specific interaction with proteins exhibiting diverse functions, including transmembrane receptors, transcription factors and transcription co-regulators, enzymes, and structural proteins. The function of these proteins is regulated by FHL2, which modulates intracellular signal transduction pathways involved in a plethora of cellular tasks. The present review summarizes the current knowledge on the protein interactome of FHL2 and provides an overview of the functional implication of these interactions in apoptosis,   migration  , and regulation of nuclear receptor function. FHL2 was originally identified in the heart and there is extensive literature available on the role of FHL2 in the cardiovascular system, which is also summarized in this review. 
 FHL2 silencing reduces Wnt signaling and osteosarcoma tumorigenesis in vitro and in vivo. The molecular mechanisms that are involved in the growth and invasiveness of osteosarcoma, an aggressive and invasive primary bone tumor, are not fully understood. The transcriptional co-factor FHL2 (four and a half LIM domains protein 2) acts as an oncoprotein or as a tumor suppressor depending on the tissue context. In this study, we investigated the role of FHL2 in tumorigenesis in osteosarcoma model.Western blot analyses showed that FHL2 is expressed above normal in most human and murine osteosarcoma cells. Tissue microarray analysis revealed that FHL2 protein expression is high in human osteosarcoma and correlates with osteosarcoma aggressiveness. In murine osteosarcoma cells, FHL2 silencing using shRNA decreased canonical Wnt/β-catenin signaling and reduced the expression of Wnt responsive genes as well as of the key Wnt molecules Wnt5a and Wnt10b. This effect resulted in inhibition of osteosarcoma cell proliferation, invasion and   migratio  n in vitro. Using xenograft experiments, we showed that FHL2 silencing markedly reduced tumor growth and lung metastasis occurence in mice. The anti-oncogenic effect of FHL2 silencing in vivo was associated with reduced cell proliferation and decreased Wnt signaling in the tumors.Our findings demonstrate that FHL2 acts as an oncogene in osteosarcoma cells and contributes to tumorigenesis through Wnt signaling. More importantly, FHL2 depletion greatly reduces tumor cell growth and metastasis, which raises the potential therapeutic interest of targeting FHL2 to efficiently impact primary bone tumors. 
 The four-and-a-half-LIM protein 2 (FHL2) is overexpressed in gliomas and associated with oncogenic activities. Four-and-a-half-LIM protein 2 (FHL2) is a member of FHL protein family, which plays a crucial role in regulating gene expression, cell survival, and   migration  . Although its function in oncogenesis appears to be tumor type-specific, its roles in glioma formation and development are yet to be elucidated. In the present study, we demonstrated that the mRNA level of FHL2 was elevated in both low- and high-grade glioma samples. Overexpression of FHL2 stimulated the proliferation, anchorage-independent growth, and   migration   of human glioblastoma cells. Conversely, FHL2 knockdown by short hairpin RNA (shRNA-FHL2) inhibited glioblastoma cell proliferation and   migration  . Overexpression of FHL2 increased the tumorigenicity of glioblastoma cells in nude mice and decreased the mRNA levels of p53 and its downstream proapoptotic genes, including p21, Bcl2-associated protein X (Bax), and p53-upregulated modulator of apoptosis. It also enhanced the promoter activities of activator protein-1 (AP-1), human telomerase reverse transcriptase, and survivin genes. Together, these results provide the first evidence that FHL2 contributes to glioma carcinogenesis. 
 Deficiency of the LIM-only protein FHL2 reduces intestinal tumorigenesis in Apc mutant mice. The four and a half LIM-only protein 2 (FHL2) is capable of shuttling between focal adhesion and nucleus where it signals through direct interaction with a number of proteins including beta-catenin. Although FHL2 activation has been found in various human cancers, evidence of its functional contribution to carcinogenesis has been lacking.Here we have investigated the role of FHL2 in intestinal tumorigenesis in which activation of the Wnt pathway by mutations in the adenomatous polyposis coli gene (Apc) or in beta-catenin constitutes the primary transforming event. In this murine model, introduction of a biallelic deletion of FHL2 into mutant Apc(Delta14/+) mice substantially reduces the number of intestinal adenomas but not tumor growth, suggesting a role of FHL2 in the initial steps of tumorigenesis. In the lesions, Wnt signalling is not affected by FHL2 deficiency, remaining constitutively active. Nevertheless, loss of FHL2 activity is associated with increased epithelial cell   migration   in intestinal epithelium, which might allow to eliminate more efficiently deleterious cells and reduce the risk of tumorigenesis. This finding may provide a mechanistic basis for tumor suppression by FHL2 deficiency. In human colorectal carcinoma but not in low-grade dysplasia, we detected up-regulation and enhanced nuclear localization of FHL2, indicating the activation of FHL2 during the development of malignancy.Our data demonstrate that FHL2 represents a critical factor in intestinal tumorigenesis. 
 Four-and-a-half LIM domain protein 2 is a novel regulator of sphingosine 1-phosphate receptor 1 in CCL19-induced dendritic cell   migration  . We identified the four-and-a-half LIM domain protein 2 (FHL2) as a novel regulator of CCL19-induced dendritic cell (DC)   migration  . Initiation of   migration   is a hallmark of DC function and plays a central role in the induction and regulation of immune responses. In vivo, DCs continuously acquire Ag in the periphery and migrate to draining lymph nodes, under the influence of local environmental chemotactic factors like CCL19/21 or sphingosine 1-phosphate (S1P). We investigated the role of S1P- and RhoA-regulated FHL2 in this process. We found reduced nuclear localization of FHL2 in mature bone marrow-derived DCs (BMDCs), compared with immature BMDCs, following stimulation with CCL19. Furthermore, in vitro-generated murine FHL2(-/-) BMDCs displayed a significantly increased migratory speed, directionality, and migratory persistence toward the chemokine CCL19 compared with wild-type BMDCs. Moreover, in vivo, FHL2(-/-) BMDCs showed increased   migration   toward lymphoid organs. FHL2(-/-) BMDCs increased the expression of S1PR1, which was associated with greater Rac activation. An S1PR1 antagonist and knock-down of S1PR1 abrogated the increased migratory speed of FHL2(-/-) BMDCs. Our results identify FHL2 as an important novel regulator of DC   migration   via regulation of their sensitivity toward environmental migratory cues like S1P and CCL19. 
 Four-and-a-half LIM protein 2 promotes invasive potential and epithelial-mesenchymal transition in colon cancer. Cancer invasion and metastasis may associate with the phenotype transition called epithelial-mesenchymal transition (EMT). We aim to evaluate the impact of four-and-a-half LIM protein 2 (FHL2) on EMT and invasion of colon cancer.The functional role of FHL2 in EMT was determined by overexpression or small interfering RNA-mediated depletion of FHL2. Mechanisms of FHL2 on expression or activity of E-cadherin and beta-catenin were assessed.FHL2 was highly expressed in primary and metastatic colon cancer but not in normal tissues. FHL2 was critical for cancer cell adhesion to extracellular matrix,   migration   and invasion. FHL2 expression was stimulated by transforming growth factor (TGF)-beta1. Moreover, FHL2 acted as a potent EMT inducer by stimulating vimentin and matrix metalloproteinase-9 expressions and causing a loss of E-cadherin, whereas those alterations of EMT markers were not affected by silencing of Smad molecules (typical TGF-beta signal mediators) in FHL2 stable transfectant cells. Therefore, FHL2 induced EMT in a TGF-beta-dependent and Smad-independent manner. FHL2 downregulated E-cadherin expression and inhibited the formation of membrane-associated E-cadherin-beta-catenin complex. FHL2 also stabilized nuclear beta-catenin, resulting in enforcement of beta-catenin transactivation activity.FHL2 is a potent EMT inducer and might be an important mediator for invasion and/or metastasis of colon cancer. 
 FHL-2 suppresses VEGF-induced phosphatidylinositol 3-kinase/Akt activation via interaction with sphingosine kinase-1. In the functional screening of a human heart cDNA library to identify a novel antiangiogenic factor, the prime candidate gene was "four-and-a-half LIM only protein-2" (FHL-2). The goal of this study is to clear the mechanism of antiangiogenic signaling of FHL-2 in endothelial cells (ECs).Overexpressed FHL-2 strongly inhibited vascular endothelial growth factor (VEGF)-induced EC   migration  . In the angiogenic signaling, we focused on sphingosine kinase-1 (SK1), which produces sphingosine-1-phosphate (S1P), a bioactive sphingolipid, as a potent angiogenic mediator in ECs. Immunoprecipitation and immunostaining analysis showed that FHL-2 might bind to SK1. Importantly, overexpression of FHL-2 in ECs inhibited VEGF-induced SK1 activity, phosphatidylinositol 3-kinase activity, and phosphorylation of Akt and eNOS. In contrast, overexpression of FHL-2 had no effect on S1P-induced Akt phosphorylation. Interestingly, VEGF stimulation decreased the binding of FHL-2 and SK1. Depletion of FHL-2 by siRNA increased EC   migration   accompanied with SK1 and Akt activation, and increased the expression of VEGF receptor-2 which further enhanced VEGF signaling. Furthermore, injection of FHL-2 mRNA into Xenopus embryos resulted in inhibition of vascular network development, assessed by in situ hybridization with endothelial markers.FHL-2 may regulate phosphatidylinositol 3-kinase/Akt via direct suppression of the SK1-S1P pathway in ECs. 
 Four and half lim protein 2 (FHL2) stimulates osteoblast differentiation. FHL2, a molecule that interacts with many integrins and transcription factors, was found to play an important role in osteoblast differentiation. Overexpression of FHL2 increases the accumulation of osteoblast differentiation markers and matrix mineralization, whereas FHL2 deficiency results in inhibition of osteoblast differentiation and decreased bone formation.Integrin-matrix interaction plays a critical role in osteoblast function. It has been shown that the cytoplasmic domains of integrin beta subunits mediate signal transduction induced by integrin-matrix interaction. We reasoned that the identification of proteins interacting with beta-cytoplasmic tails followed by analysis of the function of these proteins would enhance our understanding on integrin signaling and the roles of these proteins in osteoblast activities.Yeast two hybrid assay was used to identify proteins interacting with the cytoplasmic domain of integrin beta5 subunit. The association of these proteins with integrin alphavbeta5 was confirmed by confocal analysis and co-immunoprecipitation. A stable MC3T3-E1 cells line overexpressing Four and Half Lim Protein 2 (FHL2) and mouse osteoblasts deficient in FHL2 were used to study the roles of FHL2 in osteoblast differentiation and bone formation. Matrix protein expression was determined by mRNA analysis and Western blotting. Matrix mineralization was detected by Alizarin red staining. Alkaline phosphatase activity was also measured. muCT was used to determine bone histomorphometry.FHL2 and actin-binding proteins, palladin and filamin A, were identified as proteins interacting with beta5 cytoplasmic domain. FHL2 co-localized with alphavbeta5 at the focal adhesion sites in association with palladin and filamin A. FHL2 was also present in nuclei. Osteoblasts overexpressing FHL2 exhibited increased adhesion to and   migration   on matrix proteins. Conversely, FHL2 stimulation of CREB activity was dependent on integrin function because it was inhibited by Gly-Arg-Gly-Asp-Ser (GRGDS) peptide. The expression of osteoblast differentiation markers and Msx2 was upregulated, and bone matrix mineralization was increased in FHL2 overexpressing cells. In contrast, FHL2-deficient bone marrow cells and osteoblasts displayed decreased osteoblast colony formation and differentiation, respectively, compared with wildtype cells. Moreover, FHL2-deficient female mice exhibited greater bone loss than the wildtype littermates after ovariectomy. Thus, FHL2 plays an important role in osteoblast differentiation and bone formation. 
  ECM  PubMed  
 LIM-only protein FHL2 critically determines survival and radioresistance of pancreatic cancer cells. Numerous factors determine the current poor prognosis of pancreatic ductal adenocarcinoma (PDAC). One of the greatest challenges to overcome is treatment resistance. Among a large repertoire of intrinsic resistance mechanisms, integrin-mediated cell adhesion to extracellular matrix (  ECM  ) has been identified to be fundamental. Coalesced in focal adhesion complexes, integrins, receptor tyrosine kinases, protein kinases and adapter proteins mediate prosurvival signaling. Four and a half LIM domains protein 2 (FHL2) is one of these adapter proteins, which operates through protein-protein interactions and shows tumor-specific expression. Based on this, we investigated FHL2 expression in PDAC specimens and three-dimensionally grown cell lines and how FHL2 mechanistically contributes to cell survival, cell cycling and radiation resistance. PDAC exhibited a significantly increased and heterogeneous FHL2 expression. Upon FHL2 depletion, pancreatic cancer cell lines showed significantly decreased cell survival, proliferation and radioresistance as well as enhanced apoptosis and MEK/ERK signaling and cyclin D1, E, A and B1 expression were strongly induced. Targeting of FHL2 and MEK1 was similarly effective than FHL2 depletion alone, suggesting MEK1 as a downstream signaling mediator of FHL2. Taken together, our results provide evidence for the importance of the focal adhesion protein FHL2 in pancreatic cancer cell survival, proliferation and radiosensitivity. 
  [Ee]xtracellular [Mm]atrix  PubMed  
 Deficiency in the LIM-only protein FHL2 impairs assembly of   extracellular matrix   proteins. We have described the scaffolding protein FHL2 as a component of focal adhesion structures, to which it is recruited via binding to both alpha- or beta-integrin subunits. Using mesenchymal stem cells from wild-type and FHL2-knockout mice, we show here that inactivation of FHL2 leads to impaired assembly of   extracellular matrix   proteins on the cell surface and to impaired bundling of focal adhesions. Both altered properties can be restored by reexpression of recombinant FHL2 protein in FHL2-null cells. Molecular analysis of integrin-mediated signaling revealed a higher phosphorylation of FAK at tyrosine 925 in FHL2-knockout cells compared to their wild-type counterpart. Consequently, the activation of the mitogenic kinase ERK was more pronounced in knockout cells on cell adhesion. The growth factor-induced activation of ERK, however, was not altered. The perturbed organization of   extracellular matrix   on FHL2-null cells was improved when the increased activation of MAPK was inhibited. Our findings point to a role of FHL2 in bundling of focal adhesion structures, in integrin-mediated ERK activation, and subsequently in proper allocation of matrix proteins on the cell surface. 
 FHL2 mediates dexamethasone-induced mesenchymal cell differentiation into osteoblasts by activating Wnt/beta-catenin signaling-dependent Runx2 expression. The differentiation of bone marrow mesenchymal stem cells (MSCs) into osteoblasts is a crucial step in bone formation. However, the mechanisms involved in the early stages of osteogenic differentiation are not well understood. In this study, we identified FHL2, a member of the LIM-only subclass of the LIM protein superfamily, that is up-regulated during early osteoblast differentiation induced by dexamethasone in murine and human MSCs. Gain-of-function studies showed that FHL2 promotes the expression of the osteoblast transcription factor Runx2, alkaline phosphatase, type I collagen, as well as in vitro   extracellular matrix   mineralization in murine and human mesenchymal cells. Knocking down FHL2 using sh-RNA reduces basal and dexamethasone-induced osteoblast marker gene expression in MSCs. We demonstrate that FHL2 interacts with beta-catenin, a key player involved in bone formation induced by Wnt signaling. FHL2-beta-catenin interaction potentiates beta-catenin nuclear translocation and TCF/LEF transcription, resulting in increased Runx2 and alkaline phosphatase expression, which was inhibited by the Wnt inhibitor DKK1. Reduction of Runx2 transcriptional activity using a mutant Runx2 results in inhibition of FHL2-induced alkaline phosphatase expression in MSCs. These findings reveal that FHL2 acts as an endogenous activator of mesenchymal cell differentiation into osteoblasts and mediates osteogenic differentiation induced by dexamethasone in MSCs through activation of Wnt/beta-catenin signaling- dependent Runx2 expression. 
 LIM-only protein FHL2 critically determines survival and radioresistance of pancreatic cancer cells. Numerous factors determine the current poor prognosis of pancreatic ductal adenocarcinoma (PDAC). One of the greatest challenges to overcome is treatment resistance. Among a large repertoire of intrinsic resistance mechanisms, integrin-mediated cell adhesion to   extracellular matrix   (ECM) has been identified to be fundamental. Coalesced in focal adhesion complexes, integrins, receptor tyrosine kinases, protein kinases and adapter proteins mediate prosurvival signaling. Four and a half LIM domains protein 2 (FHL2) is one of these adapter proteins, which operates through protein-protein interactions and shows tumor-specific expression. Based on this, we investigated FHL2 expression in PDAC specimens and three-dimensionally grown cell lines and how FHL2 mechanistically contributes to cell survival, cell cycling and radiation resistance. PDAC exhibited a significantly increased and heterogeneous FHL2 expression. Upon FHL2 depletion, pancreatic cancer cell lines showed significantly decreased cell survival, proliferation and radioresistance as well as enhanced apoptosis and MEK/ERK signaling and cyclin D1, E, A and B1 expression were strongly induced. Targeting of FHL2 and MEK1 was similarly effective than FHL2 depletion alone, suggesting MEK1 as a downstream signaling mediator of FHL2. Taken together, our results provide evidence for the importance of the focal adhesion protein FHL2 in pancreatic cancer cell survival, proliferation and radiosensitivity. 
 Four-and-a-half LIM protein 2 promotes invasive potential and epithelial-mesenchymal transition in colon cancer. Cancer invasion and metastasis may associate with the phenotype transition called epithelial-mesenchymal transition (EMT). We aim to evaluate the impact of four-and-a-half LIM protein 2 (FHL2) on EMT and invasion of colon cancer.The functional role of FHL2 in EMT was determined by overexpression or small interfering RNA-mediated depletion of FHL2. Mechanisms of FHL2 on expression or activity of E-cadherin and beta-catenin were assessed.FHL2 was highly expressed in primary and metastatic colon cancer but not in normal tissues. FHL2 was critical for cancer cell adhesion to   extracellular matrix  , migration and invasion. FHL2 expression was stimulated by transforming growth factor (TGF)-beta1. Moreover, FHL2 acted as a potent EMT inducer by stimulating vimentin and matrix metalloproteinase-9 expressions and causing a loss of E-cadherin, whereas those alterations of EMT markers were not affected by silencing of Smad molecules (typical TGF-beta signal mediators) in FHL2 stable transfectant cells. Therefore, FHL2 induced EMT in a TGF-beta-dependent and Smad-independent manner. FHL2 downregulated E-cadherin expression and inhibited the formation of membrane-associated E-cadherin-beta-catenin complex. FHL2 also stabilized nuclear beta-catenin, resulting in enforcement of beta-catenin transactivation activity.FHL2 is a potent EMT inducer and might be an important mediator for invasion and/or metastasis of colon cancer. 
  [Ii]nvasive  PubMed  
 FHL2 expression in peritumoural fibroblasts correlates with lymphatic metastasis in sporadic but not in HNPCC-associated colon cancer. Four and a half LIM domain protein-2 (FHL2) is a component of the focal adhesion structures and has been suggested to have an important role in cancer progression. This study analyses the role of FHL2 in peritumoural fibroblasts of sporadic and hereditary non-polyposis colorectal cancer (HNPCC). Tissue specimens of 48 sporadic and 49 hereditary colon cancers, respectively, were stained immunohistochemically for FHL2, transforming growth factor (TGF)-β1 ligand and α-SMA. Myofibroblasts at the tumour invasion front co-expressed α-SMA and FHL2. Sporadic colon cancer but not HNPCC cases showed a correlation between TGF-β1 expression of the invading tumour cells and FHL2 staining of peritumoural myofibroblasts. Overexpression of FHL2 in peritumoural myofibroblasts correlated to lymphatic metastasis in sporadic colon cancer but not in HNPCC. In cultured mouse fibroblasts, TGF-β1 treatment induced myofibroblast differentiation, stimulated FHL2 protein expression and elevated number of migratory cells in transwell motility assays, suggesting that FHL2 is regulated downstream of TGF-β. Physical contact of colon cancer cells and myofibroblasts via FHL2-positive focal adhesions was detected in human colon carcinoma tissue and in co-culture assays using sporadic as well as HNPCC-derived tumour cell lines. Our data provide strong evidence for an important role of FHL2 in the progression of colon cancers. Tumour-secreted TGF-β1 stimulates FHL2 protein expression in peritumoural fibroblasts, probably facilitating the invasion of tumour glands into the surrounding tissue by enhanced myofibroblast migration and tight connection of fibroblasts to tumour cells via focal adhesions. These findings are absent in HNPCC-associated colon cancers in vivo and may contribute to   a less i  nvasive and more protruding tumour margin of microsatellite instable carcinomas. 
 FHL2 inhibits the Id3-promoted proliferation and   invasive   growth of human MCF-7 breast cancer cells. Id3 plays a key role in the progression of breast cancer. Previously, four and a half LIM protein (FHL2) was identified as a repressor of Id family proteins by interacting with them. This study aimed to investigate the effects of FHL2 on the transcriptional regulation and oncogenic activities of Id3 in human breast cancer cells.Cell transfection was performed with SuperFect reagent. Stable transfectants that overexpressed Id3 were obtained by selection on G418. The level of Id3 protein was determined by Western blotting analysis. Dual luciferase assays were used to measure the effect of Id3 and FHL2 on E47-mediated transcriptional activity in MCF-7 human breast cancer cells. The MTT assay was used to measure cell proliferation. The transwell assay was used to measure the   invasive   capacity of MCF-7 cancer cells.Id3 markedly repressed transcription mediated by the basic helix-loop-helix (bHLH) factor E47 in MCF-7 cells. This Id3-mediated repression was effectively antagonized by FHL2. Overexpression of Id3 markedly promoted the proliferation and   invasive   capacity of MCF-7 cells; however, these effects were significantly suppressed by the overexpression of FHL2.FHL2 can inhibit the proliferation and   invasive   growth of human breast cancer cells by repressing the functional activity of Id3. The functional roles of FHL2-Id3 signaling in the development of human breast cancer need further research. 
 FHL2 silencing reduces Wnt signaling and osteosarcoma tumorigenesis in vitro and in vivo. The molecular mechanisms that are involved in the growth and   invasive  ness of osteosarcoma, an aggressive and   invasive   primary bone tumor, are not fully understood. The transcriptional co-factor FHL2 (four and a half LIM domains protein 2) acts as an oncoprotein or as a tumor suppressor depending on the tissue context. In this study, we investigated the role of FHL2 in tumorigenesis in osteosarcoma model.Western blot analyses showed that FHL2 is expressed above normal in most human and murine osteosarcoma cells. Tissue microarray analysis revealed that FHL2 protein expression is high in human osteosarcoma and correlates with osteosarcoma aggressiveness. In murine osteosarcoma cells, FHL2 silencing using shRNA decreased canonical Wnt/β-catenin signaling and reduced the expression of Wnt responsive genes as well as of the key Wnt molecules Wnt5a and Wnt10b. This effect resulted in inhibition of osteosarcoma cell proliferation, invasion and migration in vitro. Using xenograft experiments, we showed that FHL2 silencing markedly reduced tumor growth and lung metastasis occurence in mice. The anti-oncogenic effect of FHL2 silencing in vivo was associated with reduced cell proliferation and decreased Wnt signaling in the tumors.Our findings demonstrate that FHL2 acts as an oncogene in osteosarcoma cells and contributes to tumorigenesis through Wnt signaling. More importantly, FHL2 depletion greatly reduces tumor cell growth and metastasis, which raises the potential therapeutic interest of targeting FHL2 to efficiently impact primary bone tumors. 
 Four and a half LIM domains 2 contributes to the development of human tongue squamous cell carcinoma. Four and a half LIM domains 2 (FHL2) is a protein of 279 amino acids in length containing four full LIM-domains and a half LIM-domain at the amino terminus. FHL2 is one transcriptional cofactor that can interact with many different proteins, such as AP-1, BRCA1, IGFBP, and integrin, and involved in organ differentiation, development, cell apoptosis, and carcinogenesis. Recent studies showed that FHL2 could play different roles acting as co-activator or corepressor in different cancer types, depending on the cell types involved. However, no report about FHL2 function in tongue squamous cell carcinoma (TSCC) is available to date. This study aims to determine the FHL2 expression and its biological functions in TSCC via in vitro and in vivo studies. Results show that FHL2 expression was associated with the pathological differentiation of TSCC samples through immunohistochemistry. FHL2 overexpression could stimulate cell proliferation,   invasive  ness, and metastases investigated by MTT, flow cytometry, Transwell and cell scratch methods. FHL2 could also elevate tumor-related molecule nuclear transcription factor-B (NF-кB) and β-catenin expression levels both at transcriptional and translational levels through real-time PCR and Western blot analyses. The in vivo nude mice experiment showed that the tumorigenicity of FHL2 overexpression group was significantly increased compared with control groups. These results suggest that FHL2 overexpression could contribute to the growth, proliferation  , invasi  veness, and metastasis of human tongue squamous cell carcinoma; furthermore, its function in TSCC might be related with the upregulation of NF-кB and β-catenin expressions. 
 Four-and-a-half LIM protein 2 promotes   invasive   potential and epithelial-mesenchymal transition in colon cancer. Cancer invasion and metastasis may associate with the phenotype transition called epithelial-mesenchymal transition (EMT). We aim to evaluate the impact of four-and-a-half LIM protein 2 (FHL2) on EMT and invasion of colon cancer.The functional role of FHL2 in EMT was determined by overexpression or small interfering RNA-mediated depletion of FHL2. Mechanisms of FHL2 on expression or activity of E-cadherin and beta-catenin were assessed.FHL2 was highly expressed in primary and metastatic colon cancer but not in normal tissues. FHL2 was critical for cancer cell adhesion to extracellular matrix, migration and invasion. FHL2 expression was stimulated by transforming growth factor (TGF)-beta1. Moreover, FHL2 acted as a potent EMT inducer by stimulating vimentin and matrix metalloproteinase-9 expressions and causing a loss of E-cadherin, whereas those alterations of EMT markers were not affected by silencing of Smad molecules (typical TGF-beta signal mediators) in FHL2 stable transfectant cells. Therefore, FHL2 induced EMT in a TGF-beta-dependent and Smad-independent manner. FHL2 downregulated E-cadherin expression and inhibited the formation of membrane-associated E-cadherin-beta-catenin complex. FHL2 also stabilized nuclear beta-catenin, resulting in enforcement of beta-catenin transactivation activity.FHL2 is a potent EMT inducer and might be an important mediator for invasion and/or metastasis of colon cancer. 
 Enhanced FHL2 and TGF-β1 Expression Is Associated With   Invasiv  e Growth and Poor Survival in Malignant Melanomas. This study examines the expression and the role of four-and-a-half LIM domains protein 2 (FHL2) and transforming growth factor β1 (TGF-β1) in human malignant melanoma. It is determined whether both proteins influence melanoma survival time.We analyzed the immunohistochemical staining intensities of FHL2 and TGF-β1 in normal skin and in 50 malignant melanomas with different mutation status (BRAF-V600E, NRAS codon 61 mutation, and wild type). Survival data were available for 45 cases.In melanocytes of nonneoplastic human skin, FHL2 expression was absent. In contrast, 38 (76%) of 50 melanomas showed strong cytoplasmic and partly nuclear FHL2 expression. At the invasion front, cytoplasmic TGF-β1 staining was observed in 32 (64%) of 50 melanomas, and a correlation of FHL2 and TGF-β1 staining intensities was detectable. In follow-up analyses, enhanced FHL2 and TGF-β1 staining intensities in the tumor invasion front were associated with poor survival.Enhanced FHL2 and TGF-β1 expression is correlated with poor survival in human malignant melanoma. Protumorigenic effects of autocrine TGF-β1 secretion might be exerted by induction of FHL2 expression in melanoma cells. Since melanomas treated with targeted therapies often do not show sufficient response rates, inhibition of FHL2 and/or TGF-β1 might be a promising therapeutic approach. 
  GeneRIFs  
 FHL2 overexpression could contribute to the growth, proliferation,   invasive  ness, and metastasis of human tongue squamous cell carcinoma. 
 Overexpression of Id3 markedly promoted the proliferation and   invasive   capacity of MCF-7 cells; however, these effects were significantly suppressed by the overexpression of FHL2. 
  [Mm]etastasis  PubMed  
 KLF8 promotes tumorigenesis, invasion and   metastasis   of colorectal cancer cells by transcriptional activation of FHL2. The transcription factor Krüppel-like factor (KLF)8 plays an important role in the formation of several human tumors, including colorectal cancer. We recently identified four-and-a-half LIM protein 2 (FHL2) as a critical inducer of the epithelial-to-mesenchymal transition (EMT) and invasion. However, the molecular mechanism by which KLF8 affects FHL2-mediated tumor proliferation, EMT and   metastasi  s remains unknown. Here, we showed that KLF8 overexpression promoted EMT and metastatic phenotypes. KLF8 expression was stimulated by transforming growth factor (TGF)-β1. Moreover, KLF8 acted as a potential EMT inducer by stimulating vimentin expression and inducing a loss of E-cadherin in stable KLF8-transfected cells. KLF8 overexpression induced a strong increase in FHL2 expression, and a positive correlation between the expression patterns of KLF8 and FHL2 was observed in CRC cells. Promoter reporter and chromatin immunoprecipitation (ChIP) assays demonstrated that KLF8 directly bound to and activated the human FHL2 gene promoter. However, siRNA-mediated repression of FHL2 in KLF8-overexpressing cells reversed the EMT and the proliferative and metastatic phenotypes. In vivo, KLF8 promoted FHL2-mediated proliferation an  d metastas  is via orthotopic implantation. Taken together, this work identified KLF8-induced FHL2 activation as a novel and critical signaling mechanism underlying human breast/colorectal cancer invasion an  d metastas  is. 
 FHL2 expression in peritumoural fibroblasts correlates with lymphatic   metastasis   in sporadic but not in HNPCC-associated colon cancer. Four and a half LIM domain protein-2 (FHL2) is a component of the focal adhesion structures and has been suggested to have an important role in cancer progression. This study analyses the role of FHL2 in peritumoural fibroblasts of sporadic and hereditary non-polyposis colorectal cancer (HNPCC). Tissue specimens of 48 sporadic and 49 hereditary colon cancers, respectively, were stained immunohistochemically for FHL2, transforming growth factor (TGF)-β1 ligand and α-SMA. Myofibroblasts at the tumour invasion front co-expressed α-SMA and FHL2. Sporadic colon cancer but not HNPCC cases showed a correlation between TGF-β1 expression of the invading tumour cells and FHL2 staining of peritumoural myofibroblasts. Overexpression of FHL2 in peritumoural myofibroblasts correlated to lympha  tic metast  asis in sporadic colon cancer but not in HNPCC. In cultured mouse fibroblasts, TGF-β1 treatment induced myofibroblast differentiation, stimulated FHL2 protein expression and elevated number of migratory cells in transwell motility assays, suggesting that FHL2 is regulated downstream of TGF-β. Physical contact of colon cancer cells and myofibroblasts via FHL2-positive focal adhesions was detected in human colon carcinoma tissue and in co-culture assays using sporadic as well as HNPCC-derived tumour cell lines. Our data provide strong evidence for an important role of FHL2 in the progression of colon cancers. Tumour-secreted TGF-β1 stimulates FHL2 protein expression in peritumoural fibroblasts, probably facilitating the invasion of tumour glands into the surrounding tissue by enhanced myofibroblast migration and tight connection of fibroblasts to tumour cells via focal adhesions. These findings are absent in HNPCC-associated colon cancers in vivo and may contribute to a less invasive and more protruding tumour margin of microsatellite instable carcinomas. 
 FHL2 silencing reduces Wnt signaling and osteosarcoma tumorigenesis in vitro and in vivo. The molecular mechanisms that are involved in the growth and invasiveness of osteosarcoma, an aggressive and invasive primary bone tumor, are not fully understood. The transcriptional co-factor FHL2 (four and a half LIM domains protein 2) acts as an oncoprotein or as a tumor suppressor depending on the tissue context. In this study, we investigated the role of FHL2 in tumorigenesis in osteosarcoma model.Western blot analyses showed that FHL2 is expressed above normal in most human and murine osteosarcoma cells. Tissue microarray analysis revealed that FHL2 protein expression is high in human osteosarcoma and correlates with osteosarcoma aggressiveness. In murine osteosarcoma cells, FHL2 silencing using shRNA decreased canonical Wnt/β-catenin signaling and reduced the expression of Wnt responsive genes as well as of the key Wnt molecules Wnt5a and Wnt10b. This effect resulted in inhibition of osteosarcoma cell proliferation, invasion and migration in vitro. Using xenograft experiments, we showed that FHL2 silencing markedly reduced tumor growth and lung   metastasi  s occurence in mice. The anti-oncogenic effect of FHL2 silencing in vivo was associated with reduced cell proliferation and decreased Wnt signaling in the tumors.Our findings demonstrate that FHL2 acts as an oncogene in osteosarcoma cells and contributes to tumorigenesis through Wnt signaling. More importantly, FHL2 depletion greatly reduces tumor cell growth and   metastasi  s, which raises the potential therapeutic interest of targeting FHL2 to efficiently impact primary bone tumors. 
 Tumoral expression of nuclear cofactor FHL2 is associated with lymphatic   metastasis   in sporadic but not in HNPCC-associated colorectal cancer. Four and a half LIM domain protein-2 (FHL2) is part of the focal adhesion structures modulating cell motility. FHL2 may translocate into the nucleus serving as a transcriptional cofactor binding several transcription factors. Overexpression of FHL2 has been linked to cancer progression in various neoplasias. The aim of the present study was to determine, whether FHL2's function as nuclear cofactor plays a prognostic role in invading tumor cells of sporadic and HNPCC-associated colorectal cancer (CRC).Immunohistochemical staining intensity of nuclear FHL2 was quantified by Remmele score analysing 47 sporadic and 42 HNPCC-associated colorectal cancers. Analysis was restricted to carcinoma cells of the tumoral invasion front.Confocal microscopy detected nuclear expression of FHL2 in colon cancer cells and absence of nuclear FHL2 signal in normal colon enterocytes. In colon cancer, nuclear FHL2 expression was predominantly observed in low-differentiated, often mucinous tumor areas. 42.55% of sporadic and 54.76% of HNPCC-associated CRC showed enhanced (Remmele score 6-12) nuclear FHL2 expression in the carcinoma cells of the tumoral advancing edge. Enhanced nuclear FHL2 expression was significantly linked to lymphatic   metastasis   in sporadic CRC (p=0.0197) and almost reached significance in HNPCC-associated CRC (p=0.0545). In contrast, nuclear FHL2 expression was neither associated with hematogenic   metastasis   in sporadic (p=0.7087) nor in HNPCC-associated colorectal cancer (p=0.3007).We recently demonstrated that enhanced nuclear FHL2 expression in tumor stroma of sporadic colon cancer is associated with lymphatic   metastasis  . The results of the present study indicate a synergistic effect of nuclear cofactor FHL2 in tumor cells as well as in peritumoral stroma cells promoting lymphatic   metastasis   in sporadic CRC. As HNPCC-associated tumors did not show a significant association between tumoral nuclear FHL2 expression and lymphatic   metastasis   we speculate, that the intensive lymphocytic immune response in HNPCC precludes a direct contact of tumor cells and stromal cells resulting in reduced lymphatic spread. 
 Four and a half LIM domains 2 contributes to the development of human tongue squamous cell carcinoma. Four and a half LIM domains 2 (FHL2) is a protein of 279 amino acids in length containing four full LIM-domains and a half LIM-domain at the amino terminus. FHL2 is one transcriptional cofactor that can interact with many different proteins, such as AP-1, BRCA1, IGFBP, and integrin, and involved in organ differentiation, development, cell apoptosis, and carcinogenesis. Recent studies showed that FHL2 could play different roles acting as co-activator or corepressor in different cancer types, depending on the cell types involved. However, no report about FHL2 function in tongue squamous cell carcinoma (TSCC) is available to date. This study aims to determine the FHL2 expression and its biological functions in TSCC via in vitro and in vivo studies. Results show that FHL2 expression was associated with the pathological differentiation of TSCC samples through immunohistochemistry. FHL2 overexpression could stimulate cell proliferation, invasiveness, and metastases investigated by MTT, flow cytometry, Transwell and cell scratch methods. FHL2 could also elevate tumor-related molecule nuclear transcription factor-B (NF-кB) and β-catenin expression levels both at transcriptional and translational levels through real-time PCR and Western blot analyses. The in vivo nude mice experiment showed that the tumorigenicity of FHL2 overexpression group was significantly increased compared with control groups. These results suggest that FHL2 overexpression could contribute to the growth, proliferation, invasiveness, an  d metastas  is of human tongue squamous cell carcinoma; furthermore, its function in TSCC might be related with the upregulation of NF-кB and β-catenin expressions. 
 Four-and-a-half LIM protein 2 promotes invasive potential and epithelial-mesenchymal transition in colon cancer. Cancer invasion and   metastasis   may associate with the phenotype transition called epithelial-mesenchymal transition (EMT). We aim to evaluate the impact of four-and-a-half LIM protein 2 (FHL2) on EMT and invasion of colon cancer.The functional role of FHL2 in EMT was determined by overexpression or small interfering RNA-mediated depletion of FHL2. Mechanisms of FHL2 on expression or activity of E-cadherin and beta-catenin were assessed.FHL2 was highly expressed in primary and metastatic colon cancer but not in normal tissues. FHL2 was critical for cancer cell adhesion to extracellular matrix, migration and invasion. FHL2 expression was stimulated by transforming growth factor (TGF)-beta1. Moreover, FHL2 acted as a potent EMT inducer by stimulating vimentin and matrix metalloproteinase-9 expressions and causing a loss of E-cadherin, whereas those alterations of EMT markers were not affected by silencing of Smad molecules (typical TGF-beta signal mediators) in FHL2 stable transfectant cells. Therefore, FHL2 induced EMT in a TGF-beta-dependent and Smad-independent manner. FHL2 downregulated E-cadherin expression and inhibited the formation of membrane-associated E-cadherin-beta-catenin complex. FHL2 also stabilized nuclear beta-catenin, resulting in enforcement of beta-catenin transactivation activity.FHL2 is a potent EMT inducer and might be an important mediator for invasion and/or   metastasis   of colon cancer. 
 Epithelial expression of FHL2 is negatively associated with   metastasis  -free and overall survival in colorectal cancer. Four-and-a-half LIM domains protein 2 (FHL2) is a component of the focal adhesion structures and has been suggested to have a role in cancer progression. It has been shown to be overexpressed in the colorectal cancer (CRC).Here, we examined a possible prognostic value of FHL2 in CRC. Immunohistochemistry for FHL2 was performed on 296 CRCs without distant metastases at the time of surgery. Staining in the epithelial compartment was quantitatively evaluated using image analysis, and results were related to clinical variables. Antibody specificity was tested using small-interfering RNA transfection in hTERT-immortalised myofibroblasts.Varying degrees of cytoplasmic FHL2 expression by neoplastic epithelial cells were detectable in all cases. Higher FHL2 expression in the epithelial compartment was an independent adverse prognostic factor. Multivariate Cox analysis shows that expression in the tumour invasion front (P<0.001) as well as in the centre of the tumour (P<0.001) was associated with metachronous metastases independently of the clinicopathological variables; expression in the tumour invasion front was also associated with overall survival independently of the clinicopathological variables (P<0.01).Higher FHL2 expression is involved in CRC progression and correlates with the development of metachronous metastases and overall survival, suggesting that FHL2 is an independent adverse prognostic indicator for CRC. 
  GeneRIFs  
 FHL2 overexpression could contribute to the growth, proliferation, invasiveness, and   metastasis   of human tongue squamous cell carcinoma. 
 KLF8-induced FHL2 activation is a novel and critical signaling mechanism underlying human colorectal cancer invasion and   metastasis  . 
 Tumoral expression of nuclear cofactor FHL2 is associated with lymphatic   metastasis   in sporadic but not in hereditary nonpolyposis colorectal cancer-associated colorectal cancer. 
 Overexpression of FHL2 in peritumoural myofibroblasts correlated to lymphatic   metastasis   in sporadic colon cancer but not in hereditary non-polyposis colorectal cancer. 
 FHL2 is a potent epithelial-mesenchymal transition (EMT) inducer and might be an important mediator for invasion and/or   metastasis   of colon cancer. 
  [Cc]ell [Ii]nvasion  PubMed  
 Mammalian Actin-binding Protein-1/Hip-55 Interacts with FHL2 and Negatively Regulates   Cell Invasion  . Mammalian actin-binding protein-1 (mAbp1) is an adaptor protein that binds actin and modulates scission during endocytosis. Recent studies suggest that mAbp1 impairs   cell invasion  ; however, the mechanism for the inhibitory effects of mAbp1 remain unclear. We performed a yeast two-hybrid screen and identified the adaptor protein, FHL2, as a novel binding partner that interacts with the N-terminal actin depolymerizing factor homology domain (ADFH) domain of mAbp1. Here we report that depletion of mAbp1 or ectopic expression of the ADFH domain of mAbp1 increased Rho GTPase signaling and breast cancer   cell invasion  . Moreover,   cell invasion   induced by the ADFH domain of mAbp1 required the expression of FHL2. Taken together, our findings show that mAbp1 and FHL2 are novel binding partners that differentially regulate Rho GTPase signaling and MTLn3 breast cancer   cell invasion  . 
  GeneRIFs  
 findings show that mAbp1 and FHL2 are novel binding partners that differentially regulate Rho GTPase signaling and MTLn3 breast cancer   cell invasion   
   
  
 IL16 
 
  NCBI Gene  
  GeneRIF  
  Pubmed  
 
 
  Search Term  Rank  Fields  
  [Mm]igration  6  PubMed(10); GeneRIFs(3)  
  [Ee]xtracellular [Mm]atrix  8  PubMed(2); GeneRIFs(1)  
  [Ii]nvasive  10  PubMed(1)  
 
 
 hide/show details for IL16 
  [Mm]igration  PubMed  
 Chemokine receptor CXCR3 desensitization by IL-16/CD4 signaling is dependent on CCR5 and intact membrane cholesterol. Previous work has shown that IL-16/CD4 induces desensitization of both CCR5- and CXCR4-induced   migration  , with no apparent effect on CCR2b or CCR3. To investigate the functional relationship between CD4 and other chemokine receptors, we determined the effects of IL-16 interaction with CD4 on CXCR3-induced   migration  . In this study we demonstrate that IL-16/CD4 induced receptor desensitization of CXCR3 on primary human T cells. IL-16/CD4 stimulation does not result in surface modulation of CXCR3 or changes in CXCL10 binding affinity. This effect does require p56(lck) enzymatic activity and the presence of CCR5, because desensitization is not transmitted in the absence of CCR5. Treatment of human T cells with methyl-beta-cyclodextrin, a cholesterol chelator, prevented the desensitization of CXCR3 via IL-16/CD4, which was restored after reloading of cholesterol, indicating a requirement for intact cholesterol. These studies demonstrate an intimate functional relationship among CD4, CCR5, and CXCR3, in which CCR5 can act as an adaptor molecule for CD4 signaling. This process of regulating Th1 cell chemoattraction may represent a mechanism for orchestrating cell recruitment in Th1-mediated diseases. 
 A role for IL-16 in the cross-talk between dendritic cells and T cells. Dendritic cells (DCs) in the periphery capture and process Ags, migrate to lymphoid organs, and initiate immune responses in T cells. IL-16, the soluble ligand for CD4, is a potent chemoattractant for CD4+ T cells, eosinophils, and monocytes and is mainly derived from activated T cells. Because   migration   is a fundamental property of DCs, we asked whether IL-16 induces chemotaxis in DCs and whether DCs are a source of IL-16. DCs were generated by culture of monocytes in IL-4 and GM-CSF for 6 days and subsequently highly purified employing magnetic beads.   Migration   was assayed by nitrocellulose and polycarbonate filter-based assays, and distinction of chemotaxis and chemokinesis was performed by a checkerboard analysis. Messenger RNA and protein data revealed constitutive expression and release of IL-16 by day-6 DCs. Gradients of rIL-16 induced a chemotactic response of DCs. Furthermore, the chemotactic activity of DC supernatant toward DCs themselves and T cells was mainly due to IL-16, because the addition of neutralizing Abs completely abrogated the migratory response. However, after induction of maturation by the addition of TNF-alpha and PGE2 DCs, neither expressed IL-16 mRNA nor produced IL-16 protein. We conclude that IL-16 may play a role in the trafficking of DCs and may be a major chemotactic signal from DCs toward themselves and toward T cells. 
 A homologous form of human interleukin 16 is implicated in microglia recruitment following nervous system injury in leech Hirudo medicinalis. In contrast to mammals, the medicinal leech Hirudo medicinalis can completely repair its central nervous system (CNS) after injury. This invertebrate model offers unique opportunities to study the molecular and cellular basis of the CNS repair processes. When the leech CNS is injured, microglial cells migrate and accumulate at the site of lesion, a phenomenon known to be essential for the usual sprouting of injured axons. In the present study, we demonstrate that a new molecule, designated HmIL-16, having functional homologies with human interleukin-16 (IL-16), has chemotactic activity on leech microglial cells as observed using a gradient of human IL-16. Preincubation of microglial cells either with an anti-human IL-16 antibody or with anti-HmIL-16 antibody significantly reduced microglia   migration   induced by leech-conditioned medium. Functional homology was demonstrated further by the ability of HmIL-16 to promote human CD4+ T cell   migration   which was inhibited by antibody against human IL-16, an IL-16 antagonist peptide or soluble CD4. Immunohistochemistry of leech CNS indicates that HmIL-16 protein present in the neurons is rapidly transported and stored along the axonal processes to promote the recruitment of microglial cells to the injured axons. To our knowledge, this is the first identification of a functional interleukin-16 homologue in invertebrate CNS. The ability of HmIL-16 to recruit microglial cells to sites of CNS injury suggests a role for HmIL-16 in the crosstalk between neurons and microglia in the leech CNS repair. 
 IL-16 activates plasminogen-plasmin system and promotes human eosinophil   migration   into extracellular matrix via CCR3-chemokine-mediated signaling and by modulating CD4 eosinophil expression. Increased eosinophil counts are a major feature of asthmatic airways. Eosinophil recruitment requires   migration   through epithelium and tissue extracellular matrix by activation of proteases. We assessed the capacity of IL-16, a CD4(+) cell chemotactic factor, to induce   migration   of eosinophils through a reconstituted basement membrane and evaluated the proteases, mediators, and receptors involved in this   migration  . IL-16 added to lower chambers of Invasion Chambers elicited eosinophil   migration   through Matrigel. This effect was decreased by inhibition of the plasminogen-plasmin system (Abs against urokinase plasminogen activator receptor or plasminogen depletion), but not by anti-matrix metalloproteinase-9 Abs. Abs against CD4 also inhibited IL-16-induced eosinophil   migration  . At the baseline level, few eosinophils (4.6% positive cells with a mean fluorescence of 0.9) expressed surface membrane CD4, while most permeabilized eosinophils (68% positive cells with a mean fluorescence of 18) express the CD4 Ag. TNF-pretreatment increased surface membrane CD4(+) expression by 6-fold as previously described, and increased IL-16-induced cell   migration   by 2.2-fold. Incubation of eosinophils with IL-16 also increased surface membrane CD4 expression by 5.4-fold, supporting the role of CD4 as receptor for IL-16. Abs against CCR3, eotaxin, or RANTES blocked IL-16-induced   migration  . In conclusion, IL-16 promotes eosinophil   migration   in vitro, by activating the plasminogen-plasmin system and increasing the membrane expression of its receptor. This effect is initiated via CD4 and mediated via the release of CCR3 ligand chemokines. Interestingly, most eosinophils express intracellular CD4. Hence, IL-16 may play an important role in the recruitment of blood eosinophils to the bronchial mucosa of asthmatics. 
 The CD4-associated tyrosine kinase p56lck is required for lymphocyte chemoattractant factor-induced T lymphocyte   migration  . Lymphocyte chemoattractant factor (LCF) is a polypeptide cytokine which induces both cell motility and activation of T lymphocytes. These LCF-induced events demonstrate an absolute requirement for the cell surface expression of CD4. Because many CD4-mediated T lymphocyte activation events have been demonstrated to require the association of the src-related tyrosine kinase p56lck with the cytoplasmic domain of CD4, we examined the role of p56lck in LCF-induced lymphocyte   migration   in a murine T cell hybridoma line expressing transfected human CD4. LCF induces the catalytic activity of CD4 associated p56lck at chemoattractant concentrations of cytokine. Hybridoma cells that express CD4 with cytoplasmic point mutations which uncouple the CD4-lck association lack both lck enzymatic activity and chemotactic responses to LCF. The enzymatic activity of lck however does not appear to be required for CD4-mediated migratory signal. First, the protein tyrosine kinase inhibitor herbimycin A blocked LCF-induced p56lck activation but had no effect on the LCF-induced motile response. Second, T cell hybridomas expressing a chimeric receptor combining the extracellular domain of human CD4 and murine p56lck which lacked the kinase domain had a normal LCF-induced motile response. We conclude from these observations that CD4-lck coupling is essential for LCF-induced T lymphocyte   migration   but the motile response is independent of the enzymatic activity of CD4-associated p56lck. 
 Depletion of tristetraprolin in breast cancer cells increases interleukin-16 expression and promotes tumor infiltration with monocytes/macrophages. The RNA-binding protein tristetraprolin (TTP) destabilizes target messenger RNAs (mRNAs) containing AU-rich elements within their 3' untranslated region. Thereby, it controls the expression of multiple inflammatory and tumor-associated transcripts. Moreover, a loss of TTP in tumors predicts disease-associated survival. Although tumor intrinsic functions of TTP have previously been studied, the impact of TTP on the interaction of tumors with their microenvironment remains elusive. As immune cell infiltration into tumors is a critical determinant for tumor progression, this study aimed at determining the influence of tumor cell TTP on the interaction between tumor and immune cells, specifically monocytes (MO)/macrophages (MΦ). Knockdown (k/d) of TTP in T47D breast cancer cells enhanced tumor growth both in vitro and in vivo and increased infiltration of MO into 3D tumor spheroids in vitro and of MΦ into tumor xenografts in vivo. Enhance  d migrati  on of MO toward supernatants of TTP-deficient tumor spheroids was determined as the underlying principle. Interestingly, we noticed interleukin-16 (IL-16) mRNA stabilization when TTP was depleted. In line, IL-16 protein levels were elevated in TTP-deficient spheroids and their supernatants as well as in TTP k/d tumor xenografts and critically contributed to the enhanced chemotactic behavior. In summary, we show that the loss of TTP in tumors not only affects tumor cell proliferation and survival but also enhances infiltration of MO/MΦ into the tumors, which is typically associated with poor prognosis. Moreover, we identified IL-16 as a critical TTP-regulated chemotactic factor that contributes to MO/  MΦ migra  tion. 
 CD4-mediated stimulation of human eosinophils: lymphocyte chemoattractant factor and other CD4-binding ligands elicit eosinophil   migration  . Lymphocyte chemoattractant factor (LCF) is a tetrameric glycoprotein of 56,000 relative molecular mass produced by activated T lymphocytes. LCF binds to CD4 and has previously been found to stimulate   migration   of CD4+ lymphocytes and monocytes. Because human eosinophils, like T cells and monocytes, express CD4, we examined functional responses of eosinophils to LCF. Recombinant LCF (rLCF) expressed in COS cells was purified on a CD4 affinity column.   Migration   of eosinophils was elicited by rLCF at low concentrations: the 50% effective dose (ED50) was 10(-12) to 10(-11) M, concentrations 100- to 1,000-fold lower than the ED50s for the recognized eosinophil chemoattractants C5a and platelet-activating factor. Two other ligands which bound to CD4, human immunodeficiency virus-1 envelope glycoprotein gp120 and monoclonal antibody OKT4, also stimulated eosinophil   migration  . Monovalent OKT4 Fab competitively inhibited eosinophil responses to rLCF. rLCF did not influence other functional responses of eosinophils tested, including degranulation, superoxide generation, leukotriene C4 production, in vitro survival, or surface expression of the adherence receptor CR3 (CD11b), human histocompatibility leukocyte antigen DR, or interleukin 2 receptor p55 (CD25). We conclude that CD4 on eosinophils is capable of transducing a migratory stimulus and serves as a receptor for a chemoattractant lymphokine LCF. T cell-derived LCF may contribute to recruitment of eosinophils and CD4+ mononuclear cells concomitantly at inflammatory reactions. 
 Role of interleukin 16 in multiple myeloma. Multiple myeloma is a malignancy characterized by the expansion of a plasma cell clone that localizes to the human bone marrow. Myeloma cells and bone marrow stromal cells produce soluble factors that promote the survival and progression of multiple myeloma. Interleukin 16 (IL-16) is involved in regulating the   migration   and proliferation of normal leukocytes. However, the role of IL-16 in human cancers, including multiple myeloma, is unclear.We investigated IL-16 expression in cell lines (n = 10) and in the bone marrow of myeloma patients (n = 62) and healthy bone marrow donors (n = 12) by quantitative reverse transcription-polymerase chain reaction, immunoblot analysis, enzyme-linked immunosorbent assay, flow cytometry, and immunohistochemistry. Transfection of two human multiple myeloma cell lines with small interfering RNAs was used to examine the effect of IL-16 gene silencing on apoptosis by flow cytometry, on proliferation by bromodeoxyuridine incorporation, and on colony formation. Protein neutralization assays were performed by treating multiple myeloma cells with a monoclonal antibody against the carboxyl-terminal fragment of IL-16. All statistical tests were two-sided.IL-16 was strongly overexpressed in the bone marrow of myeloma patients compared with healthy donors. Myeloma cell lines as well as primary tumor cells from myeloma patients constitutively expressed IL-16 and its receptors CD4 and/or CD9 and spontaneously secreted soluble IL-16. Silencing of IL-16 reduced the proliferative activity of myeloma cells by approximately 80% compared with untreated cells (mean relative proliferative activity IL-16 siRNA vs untransfected cells, EJM cells: 20.1%, 95% confidence interval [CI] = 14.3% to 26.0%, P = .03; KMS-12-BM cells: 22.8%, 95% CI = 5.5% to 40.0%, P = .04), and addition of a recombinant carboxyl-terminal IL-16 peptide reversed that effect. A monoclonal antibody directed against IL-16 or its receptors had a comparably strong growth-inhibiting effect on the tumor cells.IL-16 is an important growth-promoting factor in multiple myeloma and a candidate for novel diagnostic, prognostic, and therapeutic applications for this incurable human malignancy. 
 Molecular and functional analysis of a lymphocyte chemoattractant factor: association of biologic function with CD4 expression. Lymphocyte chemoattractant factor (LCF) is a lymphocyte cell product that stimulates a migratory response in CD4+ lymphocytes, monocytes, and eosinophils. In concert with its chemoattractant activity, LCF induces human T-lymphocyte expression of interleukin 2 receptor. Here we describe the molecular cloning of cDNA encoding human LCF. It is a novel interleukin with no significant homology to any previously described cytokine families. There is an absolute requirement for both autoaggregation of LCF monomers and for membrane-expressed CD4 molecules for LCF-induced   migration   in lymphocytes. 
 Down-regulation of interleukin-16 in human mast cells HMC-1 by Clostridium difficile toxins A and B. Toxin A (TcdA) and toxin B (TcdB) are the major virulence factors of Clostridium difficile and are the causative agents for clinical symptoms, such as secretory diarrhoea and pseudomembranous colitis. Mast cells are essentially involved in the toxin-induced colonic inflammatory processes. To study the direct effects of these toxins on the expression of inflammatory genes, a DNA microarray containing evaluated probes of 90 selected inflammatory genes was applied to the immature mast cell line HMC-1. TcdA and TcdB induced up-regulation of only a limited number of genes within the early phase of cell treatment. Interleukin-8 (IL-8), transcription factor c-jun and heme oxygenase-1 messenger RNA (mRNA) increased more than 2-fold. In contrast, IL-16, known as a CD4(+) T-cell chemoattractant factor and the chemokine receptor cKit were down-regulated. Stimulation of HMC-1 cells with IL-8 had no effect on IL-16 mRNA level, indicating that both cytokines were independently affected by the toxins. Regulation of both cytokines, however, depended on glucosylation of Rho GTPases as tested by application of enzyme-deficient TcdA or TcdB. Down-regulation of total and secreted IL-16 protein was checked by enzyme-linked immunosorbent assay. The data implicate that TcdA and TcdB affect lymphocyte   migration   by modulating release of the chemoattractant factor IL-16 from mast cells. In addition, this is the first report showing that Rho GTPases are involved in the regulation of IL-16 expression. 
  GeneRIFs  
 study identified IL-16 as a critical tristetraprolin-regulated chemotactic factor that contributes to monocytes/macrophages   migration   
 determined the effects of IL-16 interaction with CD4 on CXCR3-induced   migration   
 IL-16 is a potent promoter of eosinophil   migration   through the extracellular matrix components, an effect that is initiated via CD4 and mediated via the release of CCR3 ligand chemokines. 
  [Ee]xtracellular [Mm]atrix  PubMed  
 IL-16 activates plasminogen-plasmin system and promotes human eosinophil migration into   extracellular matrix   via CCR3-chemokine-mediated signaling and by modulating CD4 eosinophil expression. Increased eosinophil counts are a major feature of asthmatic airways. Eosinophil recruitment requires migration through epithelium and tissue   extracellular matrix   by activation of proteases. We assessed the capacity of IL-16, a CD4(+) cell chemotactic factor, to induce migration of eosinophils through a reconstituted basement membrane and evaluated the proteases, mediators, and receptors involved in this migration. IL-16 added to lower chambers of Invasion Chambers elicited eosinophil migration through Matrigel. This effect was decreased by inhibition of the plasminogen-plasmin system (Abs against urokinase plasminogen activator receptor or plasminogen depletion), but not by anti-matrix metalloproteinase-9 Abs. Abs against CD4 also inhibited IL-16-induced eosinophil migration. At the baseline level, few eosinophils (4.6% positive cells with a mean fluorescence of 0.9) expressed surface membrane CD4, while most permeabilized eosinophils (68% positive cells with a mean fluorescence of 18) express the CD4 Ag. TNF-pretreatment increased surface membrane CD4(+) expression by 6-fold as previously described, and increased IL-16-induced cell migration by 2.2-fold. Incubation of eosinophils with IL-16 also increased surface membrane CD4 expression by 5.4-fold, supporting the role of CD4 as receptor for IL-16. Abs against CCR3, eotaxin, or RANTES blocked IL-16-induced migration. In conclusion, IL-16 promotes eosinophil migration in vitro, by activating the plasminogen-plasmin system and increasing the membrane expression of its receptor. This effect is initiated via CD4 and mediated via the release of CCR3 ligand chemokines. Interestingly, most eosinophils express intracellular CD4. Hence, IL-16 may play an important role in the recruitment of blood eosinophils to the bronchial mucosa of asthmatics. 
 Association of polymorphisms in microRNA-binding sites and colorectal cancer in an Iranian population. MicroRNAs (miRNAs) are agents of post-transcriptional gene expression, and they can affect many functions of an individual cell or tissue from   extracellular matrix   production to inflammatory processes and tumor development. We aimed to determine the possible role of miRNA-binding site polymorphisms located in five cancer-related genes: IL-16, CDKN2A (p16), RAF1, PTGER4, and ITGB4 in colorectal cancer (CRC) risk modification in an Iranian population. This study was performed on 643 individuals (249 CRC cases and 394 healthy controls). We selected five cancer-related genes (IL-16, CDKN2A (p16), RAF1, PTGER4, and ITGB4) and investigated the genotypes of the 3' untranslated region miRNA-binding site polymorphisms in these genes in our study population. The restriction fragment length polymorphism results were confirmed by a direct sequencing method. We found a statistically significant difference between the rs1131445 polymorphism of the IL-16 gene and CRC. The frequencies of the genotypes TT, CT, and CC in controls were 51%, 40.4%, and 8.6%, respectively, and in cases were 41.4%, 44.1%, and 14.5%, respectively, which shows a significant association between the CC genotype of the rs1131445 polymorphism and CRC (P = 0.004). The frequency of the C allele in the CRC group was higher than in the controls, and the C allele of the rs1131445 polymorphism was found to be in association with CRC (P = 0.009). These associations remained significant after Bonferroni's correction for multiple testing. We found that the AA genotype of the rs743554 polymorphism in the ITGB4 gene and the T allele of the rs1051208 polymorphism of the RAF1 gene were associated with the risk of CRC in females; however, after Bonferroni's correction we found that they were non-significant. Finally, we can conclude that a significant relationship exists between the miRNA-binding site polymorphism of the IL-16 gene and CRC risk in the Iranian population. 
  GeneRIFs  
 IL-16 is a potent promoter of eosinophil migration through the   extracellular matrix   components, an effect that is initiated via CD4 and mediated via the release of CCR3 ligand chemokines. 
  [Ii]nvasive  PubMed  
 Increased levels of circulating IL-16 and apoptosis markers are related to the activity of Whipple's disease. Whipple's disease (WD) is an infectious disease caused by Tropheryma whipplei, which replicates in macrophages and induces the release of interleukin (IL)-16, a substrate of caspase 3, and macrophage apoptosis. The disease is characterized by intestinal, cardiac or neurological manifestations; its diagnosis is based on   invasive   analysis requiring tissue biopsies or cerebrospinal fluid puncture. The disease progression is slow and often complicated by relapses despite empirical antibiotic treatment.We monitored circulating levels of IL-16 and nucleosomes in 36 French patients with WD; among them, some patients were enrolled in a longitudinal follow-up. As compared to control subjects, the circulating levels of both IL-16 and nucleosomes were increased in untreated patients with WD presenting as intestinal, cardiac or neurological manifestations. This finding was specific to WD since the circulating levels of IL-16 and nucleosomes were not increased in patients with unrelated inflammatory diseases such as inflammatory bowel disease or Q fever endocarditis. We also found that increased levels of IL-16 and nucleosomes were related to the activity of the disease. Indeed, successful antibiotic treatment decreased those levels down to those of control subjects. In contrast, patients who suffered from relapses exhibited circulating levels of IL-16 and nucleosomes as high as those of untreated patients.Circulating levels of both IL-16 and nucleosomes were increased in WD. This finding provides simple and non-  invasive   tools for the diagnosis and the prognosis of WD. 
   
  
 CD63 
 
  NCBI Gene  
  GeneRIF  
  Pubmed  
 
 
  Search Term  Rank  Fields  
  [Ff]ilopodia  1  PubMed(2); GeneRIFs(1)  
  [Mm]igration  6  Process(1); PubMed(10); GeneRIFs(1)  
  ECM  7  PubMed(2)  
  [Ee]xtracellular [Mm]atrix  8  PubMed(4)  
  [Ii]nvasive  10  PubMed(4)  
  [Mm]etastasize  11  PubMed(2)  
  [Mm]etastasis  12  PubMed(12); GeneRIFs(2)  
  [Cc]ell [Ii]nvasion  13  PubMed(2)  
 
 
 hide/show details for CD63 
  [Ff]ilopodia  PubMed  
 Exosomes surf on   filopodia   to enter cells at endocytic hot spots, traffic within endosomes, and are targeted to the ER. Exosomes are nanovesicles released by virtually all cells, which act as intercellular messengers by transfer of protein, lipid, and RNA cargo. Their quantitative efficiency, routes of cell uptake, and subcellular fate within recipient cells remain elusive. We quantitatively characterize exosome cell uptake, which saturates with dose and time and reaches near 100% transduction efficiency at picomolar concentrations. Highly reminiscent of pathogenic bacteria and viruses, exosomes are recruited as single vesicles to the cell body by surfing on   filopodia   as well as   filopodia   grabbing and pulling motions to reach endocytic hot spots at the   filopodia  l base. After internalization, exosomes shuttle within endocytic vesicles to scan the endoplasmic reticulum before being sorted into the lysosome as their final intracellular destination. Our data quantify and explain the efficiency of exosome internalization by recipient cells, establish a new parallel between exosome and virus host cell interaction, and suggest unanticipated routes of subcellular cargo delivery. 
 Concentration of rafts in platelet   filopodia   correlates with recruitment of c-Src and CD63 to these domains. The molecular mechanism that causes non-adhesive, discoid platelets to transform into sticky dendritic bodies that form blood clumps is a complex series of events. Recently it has become clear that lipid microdomains--also known as rafts--play a crucial role in this process. We have used a non-cytolytic derivative of perfringolysin-O, a cholesterol binding cytolysin, that binds selectively to cholesterol-rich membrane domains, combined with confocal- and immunoelectron microscopy to visualize cholesterol-raft dynamics during platelet adhesion. In resting platelets cholesterol was uniformly distributed on the cell surface and confined to distinct intracellular compartments (i.e. multivesicular bodies, dense granules, and the internal membranes of alpha-granules). Upon interaction with fibrinogen, cholesterol accumulated at the tips of   filopodia   and at the leading edge of spreading cells. Stimulation with thrombin receptor activating peptide (TRAP) resulted in a similar redistribution of cholesterol towards   filopodia  . The adhesion-dependent raft aggregation was accompanied by concentration of the tyrosine kinase c-Src and the tetraspanin CD63 in these domains, whereas glycoprotein Ib (GPIb) was not selectively targeted to the raft clusters. c-Src, the tetraspanin CD63, and GPIb were recovered in biochemically isolated low-density membrane fractions. Disruption of rafts by depleting membrane cholesterol had no effect on platelet shape change but inhibited platelet spreading on fibrinogen and TRAP-induced aggregation. Our results demonstrate that cholesterol rafts in platelets are dynamic entities in the membrane that co-cluster with the tyrosine kinase c-Src and the costimulatory molecule CD63 in specialized domains at the cell surface, thereby providing a possible mechanism in functioning as signaling centres. 
  GeneRIFs  
 Upon platelet interaction with fibrinogen, cholesterol accumulated at the tips of   filopodia   and at the leading edge of spreading cells; cholesterol-rich raft aggregation was accompanied by concentration of c-Src and CD63 in these cell domains 
  [Mm]igration  Process  
 cell   migration   
  PubMed  
 Complementary DNA arrays identify CD63 tetraspanin and alpha3 integrin chain as differentially expressed in low and high metastatic human colon carcinoma cells. Malignant tumor cell invasion is determinant for metastasis to occur. E2 and C5 colon carcinoma cells that were derived from the parental Lovo line and that differ experimentally in spontaneous metastatic ability have been monitored for gene expression by cDNA arrays. Among genes found differentially expressed, the CD63 tetraspanin, not previously recognized in colon cancer progression, and the alpha3 integrin chain were both up-regulated in low metastatic E2 cells and were analyzed for their functional role using adhesion,   migration  , and invasion assays. Cell surface expression of CD63 and alpha3 integrin was about 2-fold higher in E2 than in C5 cells and confocal microscopy showed that CD63 and alpha3 integrin colocalized evenly on C5 cells whereas they concentrated at elongated tips of the low-metastatic more substrate-adhesive E2 cells. Antibody-interference experiments identified laminin-5 (LN-5) as a ligand interacting with the alpha3beta1/CD63 complex. Substrate-immobilized anti-CD63 antibodies enhanced tumor cell   migration   and invasion and induced prominent cell surface protrusions that were repressed by the PI3-kinase LY294002 inhibitor. Our results suggest that changes in the expression of surface CD63 and alpha3beta1 integrin interacting with LN-5 could affect migratory signals and the progression of the metastatic disease. 
 TIMP-1 modulates chemotaxis of human neural stem cells through CD63 and integrin signalling. We recently reported that hNSCs (human neural stem cells) have the interesting characteristic of   migration   towards an intracranial glioma. However, the molecules and mechanisms responsible for tumour tropism are unclear. In the present study, we used microarray and proteomics analyses to identify a novel chemoattractant molecule, TIMP-1 (tissue inhibitor of metalloproteinase-1), secreted from human brain tumour tissues. We demonstrate that TIMP-1 significantly enhances hNSC adhesion and   migration   in a cell culture system. These effects were critically dependent on CD63, as shRNA-mediated ablation of CD63 expression attenuated the response. TIMP-1 significantly increased the number of FAs (focal adhesions) and cytoskeletal reorganization for cell   migration   in hNSCs, whereas knockdown of CD63 resulted in decreased hNSC spreading, FAs and   migration  , even after TIMP-1 treatment. In addition, TIMP-1 binding to CD63 activated β1 integrin-mediated signalling through Akt and FAK phosphorylation, leading to pattern changes in distribution of vinculin and F-actin (filamentous actin). Furthermore, inactivation of β1 integrin by use of a blocking antibody or inhibition of PI3K (phosphoinositide 3-kinase) signalling impaired th  e migrati  on of hNSCs towards TIMP-1. Collectively, our results underline TIMP-1 as a novel and effective key regulator of CD63 and β1 integrin-mediated signalling, which regulates hNSC adhesion a  nd migrat  ion. 
 TIMP-1 modulates chemotaxis of human neural stem cells through CD63 and integrin signalling. We recently reported that hNSCs (human neural stem cells) have the interesting characteristic of   migration   towards an intracranial glioma. However, the molecules and mechanisms responsible for tumour tropism are unclear. In the present study, we used microarray and proteomics analyses to identify a novel chemoattractant molecule, TIMP-1 (tissue inhibitor of metalloproteinase-1), secreted from human brain tumour tissues. We demonstrate that TIMP-1 significantly enhances hNSC adhesion and   migration   in a cell culture system. These effects were critically dependent on CD63, as shRNA-mediated ablation of CD63 expression attenuated the response. TIMP-1 significantly increased the number of FAs (focal adhesions) and cytoskeletal reorganization for cell   migration   in hNSCs, whereas knockdown of CD63 resulted in decreased hNSC spreading, FAs and   migration  , even after TIMP-1 treatment. In addition, TIMP-1 binding to CD63 activated β1 integrin-mediated signalling through Akt and FAK phosphorylation, leading to pattern changes in distribution of vinculin and F-actin (filamentous actin). Furthermore, inactivation of β1 integrin by use of a blocking antibody or inhibition of PI3K (phosphoinositide 3-kinase) signalling impaired th  e migrati  on of hNSCs towards TIMP-1. Collectively, our results underline TIMP-1 as a novel and effective key regulator of CD63 and β1 integrin-mediated signalling, which regulates hNSC adhesion a  nd migrat  ion. 
 Expression of tetraspanin adaptor proteins below defined threshold values is associated with in vitro invasiveness of mammary carcinoma cells. Tetraspanins are transmembrane adaptor proteins involved in the regulation of various fundamental cellular processes. For a number of malignant diseases, the level of expression of members of the tetraspanin family was found to correlate with tumor cell invasiveness, ability to form metastases, and poor clinical outcome. We describe the exact quantification of mRNAs coding for the tetraspanins CD9, CD63, CD82 and CD151 expressed by mammary carcinoma-derived cell lines that were classified as invasive or non-invasive according to their ability to penetrate collagen-fibroblast gels in vitro. The mean of beta2-microglobulin-normalized expression of CD9 was about 10-fold higher than the mean calculated for CD63 and about 20-fold higher than expression of CD82 and CD151. Direct comparison of tetraspanin expression of invasive and non-invasive cell lines with the Mann-Whitney test revealed a significant correlation for CD63. Grouping of cell lines in relation to threshold values of expression resulted in significant correlations for CD63 (Fisher's exact test p=0.004) and CD151 (p=0.02) but not for CD82 (p=0.065) and CD9 (p=0.168). Expression of CD9, C63 and CD151 was found to be coupled whereas CD82 was expressed independently. This highly significant association points to common mechanisms of gene regulation for this subgroup of tetraspanins. We showed that on basis of absolute amounts of tetraspanin mRNAs, at least in vitro invasiveness is clearly predictable. Our results support the assumption that downregulation of tetraspanins in breast cancer cells is an important step of tumor progression to more malignant phenotypes and underline their important role as mediators in multimolecular membrane protein complexes regulating cell adhesion and   migration  . 
 Expression of transmembrane 4 superfamily (TM4SF) proteins and their role in hepatic stellate cell motility and wound healing   migration  .   Migration   of activated hepatic stellate cells (HSC) is a key event in the progression of liver fibrosis. Little is known about transmembrane proteins involved in HSC motility. Tetraspanins (TM4SF) have been implicated in cell development, differentiation, motility and tumor cell invasion. We evaluated the expression and function of four TM4SF, namely CD9, CD81, CD63 and CD151, and their involvement in HSC   migration  , adhesion, and proliferation.All TM4SF investigated were highly expressed at the human HSC surface with different patterns of intracellular distribution. Monoclonal antibodies directed against the four TM4SF inhibited HSC   migration   induced by extracellular matrix proteins in both wound healing and haptotaxis assays. This inhibition was independent of the ECM substrates employed (collagen type I or IV, laminin), and was comparable to that obtained by incubating the cells with an anti-beta1 blocking mAb. Importantly, cell adhesion was unaffected by the incubation with the same antibodies. Co-immunoprecipitation studies revealed different patterns of association between the four TM4SF studied and beta1 integrin. Finally, anti-TM4SF antibodies did not affect HSC growth.These findings provide the first characterization of tetraspanins expression and of their role in HSC   migration  , a key event in liver tissue wound healing and fibrogenesis. 
 Complexes of tetraspanins with integrins: more than meets the eye. The transmembrane proteins of the tetraspanin superfamily are implicated in a diverse range of biological phenomena, including cell motility, metastasis, cell proliferation and differentiation. The tetraspanins are associated with adhesion receptors of the integrin family and regulate integrin-dependent cell   migration  . In cells attached to the extracellular matrix, the integrin-tetraspanin adhesion complexes are clustered into a distinct type of adhesion structure at the cell periphery. Various tetraspanins are associated with phosphatidylinositol 4-kinase and protein kinase C isoforms, and they may facilitate assembly of signalling complexes by tethering these enzymes to integrin heterodimers. At the plasma membrane, integrin-tetraspanin signalling complexes are partitioned into specific microdomains proximal to cholesterol-rich lipid rafts. A substantial fraction of tetraspanins colocalise with integrins in various intracellular vesicular compartments. It is proposed that tetraspanins can influence cell   migration   by one of the following mechanisms: (1) modulation of integrin signalling; (2) compartmentalisation of integrins on the cell surface; or (3) direction of intracellular trafficking and recycling of integrins. 
 CD63 modulates spreading and tyrosine phosphorylation of platelets on immobilized fibrinogen. CD63 is a member of the tetraspanin superfamily of integral membrane proteins. Present on a variety of cells, tetraspanins can form lateral associations with integrins and may act as 'organizers' of multimolecular networks that modulate integrinmediated signaling, cell morphology, motility and   migration  . In resting platelets, CD63 is present on the membranes of dense granules and lysosomes but relocates to the plasma membrane following platelet activation and exocytosis where it associates with the platelet integrin alphaIIBbeta3-CD9 complex and with the actin cytoskeleton in an alphaIIBbeta 3-dependent manner. D545, a monoclonal antibody directed at the second extracellular loop of CD63,was used to investigate the role of CD63 in platelet adhesion, spreading and tyrosine phosphorylation. Using immunofluorescence microscopy and confocal imaging, we have demonstrated that D545 does not alter adhesion of platelets to immobilized fibrinogen, but instead platelet spreading. In the presence of buffer or non-specific mouse IgG, activated platelets showed fully spread morphology, F-actin reorganization, redistribution of vinculin and extensive tyrosine phosphorylation, all of which were inhibited by D545. D545 also inhibited the phosphorylation of focal adhesion kinase in thrombin-activated adherent platelets. These results suggest that CD63 may modulate alphaIIBbeta3-dependent cytoskeletal reorganization. To identify signaling enzymes associated with CD63 that could affect this pathway, lipid kinase assays were performed on D545 immunoprecipitates. CD63 co-immunoprecipitated with a lipid kinase which, on the basis of enzymatic properties(stimulated by nonionic detergents, inhibited by adenosine), is consistent with PI 4-kinase type II. The CD63-PI 4-kinase complex was not activation-dependent as the constituents were co-purified from both resting and activated platelets. The linkage of CD63 with PI 4-kinase may result in the recruitment of this signaling enzyme to specific membrane locations in the platelet where it influences phosphoinositide-dependent signaling and platelet spreading. 
 CD63 tetraspanin slows down cell   migration   and translocates to the endosomal-lysosomal-MIICs route after extracellular stimuli in human immature dendritic cells. We analyzed herein whether members of the tetraspanin superfamily are involved in human immature dendritic cell (DC) functions such as foreign antigen internalization, phagocytosis, and cell   migration  . We show that CD63, CD9, CD81, CD82, and CD151 are present in immature DCs. Whereas CD9 and CD81 are mostly expressed at the cell surface, CD63 and CD82 are also located in intracellular organelles. Complexes of monoclonal antibody (Mab) FC-5.01-CD63 or Fab-5.01-CD63 were rapidly translocated "outside-in" and followed the endocytic pathway through early endosomes and lysosomes, reaching major histocompatibility complex (MHC) class II-enriched compartments (MIICs) in less than one hour. Internalization of CD63 was also observed during Saccharomyces cerevisiae phagocytosis. Moreover, an association of CD63 with the beta-glycan receptor dectin-1 was observed. Mabs against CD9, CD63, CD81, and CD82 enhanced by 50% the   migration   induced by the chemokines macrophage inflammatory protein-5 (MIP-5) and MIP-1alpha. Concomitantly, Mabs against CD63 and CD82 diminished the surface expression of CD29, CD11b, CD18, and alpha5 integrins. By immunoprecipitation experiments we found that CD63 associated with integrins CD11b and CD18. These results suggest that CD9, CD63, CD81, and CD82 could play a role in modulating the interactions between immature DCs and their environment, slowing their migratory ability. However, only CD63 would intervene in the internalization of complex antigens. 
 Correlations between transmembrane 4 L6 family member 5 (TM4SF5), CD151, and CD63 in liver fibrotic phenotypes and hepatic   migration   and invasive capacities. Transmembrane 4 L6 family member 5 (TM4SF5) is overexpressed during CCl4-mediated murine liver fibrosis and in human hepatocellular carcinomas. The tetraspanins form tetraspanin-enriched microdomains (TEMs) consisting of large membrane protein complexes on the cell surface. Thus, TM4SF5 may be involved in the signal coordination that controls liver malignancy. We investigated the relationship between TM4SF5-positive TEMs with liver fibrosis and tumorigenesis, using normal Chang hepatocytes that lack TM4SF5 expression and chronically TGFβ1-treated Chang cells that express TM4SF5. TM4SF5 expression is positively correlated with tumorigenic CD151 expression, but is negatively correlated with tumor-suppressive CD63 expression in mouse fibrotic and human hepatic carcinoma tissues, indicating cooperative roles of the tetraspanins in liver malignancies. Although CD151 did not control the expression of TM4SF5, TM4SF5 appeared to control the expression levels of CD151 and CD63. TM4SF5 interacted with CD151, and caused the internalization of CD63 from the cell surface into late lysosomal membranes, presumably leading to terminating the tumor-suppressive functions of CD63. TM4SF5 could overcome the tumorigenic effects of CD151, especially cell   migratio  n and extracellular matrix (ECM)-degradation. Taken together, TM4SF5 appears to play a role in liver malignancy by controlling the levels of tetraspanins on the cell surface, and could provide a promising therapeutic target for the treatment of liver malignancies. 
 Tetraspanin CD63 promotes vascular endothelial growth factor receptor 2-β1 integrin complex formation, thereby regulating activation and downstream signaling in endothelial cells in vitro and in vivo. CD63 is a member of the transmembrane-4 glycoprotein superfamily (tetraspanins) implicated in the regulation of membrane protein trafficking, leukocyte recruitment, and adhesion processes. We have investigated the involvement of CD63 in endothelial cell (EC) signaling downstream of β1 integrin and VEGF. We report that silencing of CD63 in primary ECs arrested capillary sprouting and tube formation in vitro because of impaired adhesion an  d migrati  on of ECs. Mechanistically, CD63 associated with both β1 integrin and the main VEGF receptor on ECs, VEGFR2. Our data suggest that CD63 serves to bridge between β1 integrin and VEGFR2 because CD63 silencing disrupted VEGFR2-β1 integrin complex formation identified using proximity ligation assays. Signaling downstream of β1 integrin and VEGFR2 was attenuated in CD63-silenced cells, although their cell surface expression levels remained unaffected. CD63 was furthermore required for efficient internalization of VEGFR2 in response to VEGF. Importantly, systemic delivery of VEGF failed to potently induce VEGFR2 phosphorylation and downstream signaling in CD63-deficient mouse lungs. Taken together, our findings demonstrate a previously unrecognized role for CD63 in coordinated integrin and receptor tyrosine kinase signaling in vitro and in vivo. 
  GeneRIFs  
 Data suggest that TIMP1 (tissue inhibitor of metalloproteinase-1) acts as chemoattractant for neural stem cells (NSC); TIMP1 enhances NSC adhesion,   migration  , and cytoskeletal reorganization; these effects are dependent on CD63/CD29 (integrin beta1). 
  ECM  PubMed  
 Expression of transmembrane 4 superfamily (TM4SF) proteins and their role in hepatic stellate cell motility and wound healing migration. Migration of activated hepatic stellate cells (HSC) is a key event in the progression of liver fibrosis. Little is known about transmembrane proteins involved in HSC motility. Tetraspanins (TM4SF) have been implicated in cell development, differentiation, motility and tumor cell invasion. We evaluated the expression and function of four TM4SF, namely CD9, CD81, CD63 and CD151, and their involvement in HSC migration, adhesion, and proliferation.All TM4SF investigated were highly expressed at the human HSC surface with different patterns of intracellular distribution. Monoclonal antibodies directed against the four TM4SF inhibited HSC migration induced by extracellular matrix proteins in both wound healing and haptotaxis assays. This inhibition was independent of the   ECM   substrates employed (collagen type I or IV, laminin), and was comparable to that obtained by incubating the cells with an anti-beta1 blocking mAb. Importantly, cell adhesion was unaffected by the incubation with the same antibodies. Co-immunoprecipitation studies revealed different patterns of association between the four TM4SF studied and beta1 integrin. Finally, anti-TM4SF antibodies did not affect HSC growth.These findings provide the first characterization of tetraspanins expression and of their role in HSC migration, a key event in liver tissue wound healing and fibrogenesis. 
 Correlations between transmembrane 4 L6 family member 5 (TM4SF5), CD151, and CD63 in liver fibrotic phenotypes and hepatic migration and invasive capacities. Transmembrane 4 L6 family member 5 (TM4SF5) is overexpressed during CCl4-mediated murine liver fibrosis and in human hepatocellular carcinomas. The tetraspanins form tetraspanin-enriched microdomains (TEMs) consisting of large membrane protein complexes on the cell surface. Thus, TM4SF5 may be involved in the signal coordination that controls liver malignancy. We investigated the relationship between TM4SF5-positive TEMs with liver fibrosis and tumorigenesis, using normal Chang hepatocytes that lack TM4SF5 expression and chronically TGFβ1-treated Chang cells that express TM4SF5. TM4SF5 expression is positively correlated with tumorigenic CD151 expression, but is negatively correlated with tumor-suppressive CD63 expression in mouse fibrotic and human hepatic carcinoma tissues, indicating cooperative roles of the tetraspanins in liver malignancies. Although CD151 did not control the expression of TM4SF5, TM4SF5 appeared to control the expression levels of CD151 and CD63. TM4SF5 interacted with CD151, and caused the internalization of CD63 from the cell surface into late lysosomal membranes, presumably leading to terminating the tumor-suppressive functions of CD63. TM4SF5 could overcome the tumorigenic effects of CD151, especially cell migration and extracellular matrix   (EC  M)-degradation. Taken together, TM4SF5 appears to play a role in liver malignancy by controlling the levels of tetraspanins on the cell surface, and could provide a promising therapeutic target for the treatment of liver malignancies. 
  [Ee]xtracellular [Mm]atrix  PubMed  
 CD63 associates with the alphaIIb beta3 integrin-CD9 complex on the surface of activated platelets. The tetraspanins are integral membrane proteins expressed on cell surface and granular membranes of hematopoietic cells and have been identified in multi-molecular complexes with specific integrins. In resting platelets, CD63, a member of the tetraspanin superfamily, is present in dense granule and lysosomal membranes and, following platelet activation, translocates to the plasma membrane. In the present study, platelet activation by thrombin leads to incorporation of CD63 into the Triton-insoluble actin cytoskeletal fraction. This incorporation was inhibited by preincubation of platelets with RGDS or EGTA and did not occur in platelets from a patient with Glanzmann's thrombasthenia, suggesting that it was dependent upon alphaIIbbeta3. In activated platelets, the anti-CD63 MoAb, D545, co-immunoprecipitated CD63 with other surface-labeled proteins, including alphaIIbbeta3 and another tetraspanin, CD9. The association of CD63 with CD9 and alphaIIbbeta3, was not inhibited by preincubation of platelets with RGDS or EGTA. D545 did not inhibit the adhesion of activated platelets to purified   extracellular matrix   proteins, but significantly decreased adhesion of thrombin-activated platelets to neutrophils in a rosetting assay. D545 also caused disaggregation of platelets stimulated by ADP, but had no effect on aggregation induced by other agonists. These results are consistent with the proposal that CD63 becomes part of an alphaIIbbeta3-CD9-CD63 integrin-tetraspanin complex in activated platelets--an association that may modulate the function of alphaIIbbeta3-dependent interaction with other cells such as neutrophils. 
 Expression of transmembrane 4 superfamily (TM4SF) proteins and their role in hepatic stellate cell motility and wound healing migration. Migration of activated hepatic stellate cells (HSC) is a key event in the progression of liver fibrosis. Little is known about transmembrane proteins involved in HSC motility. Tetraspanins (TM4SF) have been implicated in cell development, differentiation, motility and tumor cell invasion. We evaluated the expression and function of four TM4SF, namely CD9, CD81, CD63 and CD151, and their involvement in HSC migration, adhesion, and proliferation.All TM4SF investigated were highly expressed at the human HSC surface with different patterns of intracellular distribution. Monoclonal antibodies directed against the four TM4SF inhibited HSC migration induced by   extracellular matrix   proteins in both wound healing and haptotaxis assays. This inhibition was independent of the ECM substrates employed (collagen type I or IV, laminin), and was comparable to that obtained by incubating the cells with an anti-beta1 blocking mAb. Importantly, cell adhesion was unaffected by the incubation with the same antibodies. Co-immunoprecipitation studies revealed different patterns of association between the four TM4SF studied and beta1 integrin. Finally, anti-TM4SF antibodies did not affect HSC growth.These findings provide the first characterization of tetraspanins expression and of their role in HSC migration, a key event in liver tissue wound healing and fibrogenesis. 
 Complexes of tetraspanins with integrins: more than meets the eye. The transmembrane proteins of the tetraspanin superfamily are implicated in a diverse range of biological phenomena, including cell motility, metastasis, cell proliferation and differentiation. The tetraspanins are associated with adhesion receptors of the integrin family and regulate integrin-dependent cell migration. In cells attached to the   extracellular matrix  , the integrin-tetraspanin adhesion complexes are clustered into a distinct type of adhesion structure at the cell periphery. Various tetraspanins are associated with phosphatidylinositol 4-kinase and protein kinase C isoforms, and they may facilitate assembly of signalling complexes by tethering these enzymes to integrin heterodimers. At the plasma membrane, integrin-tetraspanin signalling complexes are partitioned into specific microdomains proximal to cholesterol-rich lipid rafts. A substantial fraction of tetraspanins colocalise with integrins in various intracellular vesicular compartments. It is proposed that tetraspanins can influence cell migration by one of the following mechanisms: (1) modulation of integrin signalling; (2) compartmentalisation of integrins on the cell surface; or (3) direction of intracellular trafficking and recycling of integrins. 
 Correlations between transmembrane 4 L6 family member 5 (TM4SF5), CD151, and CD63 in liver fibrotic phenotypes and hepatic migration and invasive capacities. Transmembrane 4 L6 family member 5 (TM4SF5) is overexpressed during CCl4-mediated murine liver fibrosis and in human hepatocellular carcinomas. The tetraspanins form tetraspanin-enriched microdomains (TEMs) consisting of large membrane protein complexes on the cell surface. Thus, TM4SF5 may be involved in the signal coordination that controls liver malignancy. We investigated the relationship between TM4SF5-positive TEMs with liver fibrosis and tumorigenesis, using normal Chang hepatocytes that lack TM4SF5 expression and chronically TGFβ1-treated Chang cells that express TM4SF5. TM4SF5 expression is positively correlated with tumorigenic CD151 expression, but is negatively correlated with tumor-suppressive CD63 expression in mouse fibrotic and human hepatic carcinoma tissues, indicating cooperative roles of the tetraspanins in liver malignancies. Although CD151 did not control the expression of TM4SF5, TM4SF5 appeared to control the expression levels of CD151 and CD63. TM4SF5 interacted with CD151, and caused the internalization of CD63 from the cell surface into late lysosomal membranes, presumably leading to terminating the tumor-suppressive functions of CD63. TM4SF5 could overcome the tumorigenic effects of CD151, especially cell migration and   extracellular matri  x (ECM)-degradation. Taken together, TM4SF5 appears to play a role in liver malignancy by controlling the levels of tetraspanins on the cell surface, and could provide a promising therapeutic target for the treatment of liver malignancies. 
  [Ii]nvasive  PubMed  
 Expression of tetraspanin adaptor proteins below defined threshold values is associated with in vitro   invasive  ness of mammary carcinoma cells. Tetraspanins are transmembrane adaptor proteins involved in the regulation of various fundamental cellular processes. For a number of malignant diseases, the level of expression of members of the tetraspanin family was found to correlate with tumor cell   invasive  ness, ability to form metastases, and poor clinical outcome. We describe the exact quantification of mRNAs coding for the tetraspanins CD9, CD63, CD82 and CD151 expressed by mammary carcinoma-derived cell lines that were classified as   invasive   or non-  invasive   according to their ability to penetrate collagen-fibroblast gels in vitro. The mean of beta2-microglobulin-normalized expression of CD9 was about 10-fold higher than the mean calculated for CD63 and about 20-fold higher than expression of CD82 and CD151. Direct comparison of tetraspanin expression of   invasive   and non-  invasive   cell lines with the Mann-Whitney test revealed a significant correlation for CD63. Grouping of cell lines in relation to threshold values of expression resulted in significant correlations for CD63 (Fisher's exact test p=0.004) and CD151 (p=0.02) but not for CD82 (p=0.065) and CD9 (p=0.168). Expression of CD9, C63 and CD151 was found to be coupled whereas CD82 was expressed independently. This highly significant association points to common mechanisms of gene regulation for this subgroup of tetraspanins. We showed that on basis of absolute amounts of tetraspanin mRNAs, at least in vitro   invasive  ness is clearly predictable. Our results support the assumption that downregulation of tetraspanins in breast cancer cells is an important step of tumor progression to more malignant phenotypes and underline their important role as mediators in multimolecular membrane protein complexes regulating cell adhesion and migration. 
 CD63 tetraspanin is a negative driver of epithelial-to-mesenchymal transition in human melanoma cells. The CD63 tetraspanin is highly expressed in the early stages of melanoma and decreases in advanced lesions, suggesting it as a possible suppressor of tumor progression. We employed loss- and gain-of-gene-function approaches to investigate the role of CD63 in melanoma progression and acquisition of the epithelial-to-mesenchymal transition (EMT) program. We used two human melanoma cell lines derived from primary tumors and one primary human melanoma cell line isolated from a cutaneous metastasis, differing by levels of CD63 expression. CD63-silenced melanoma cells showed enhanced motility and   invasive  ness with downregulation of E-cadherin and upregulation of N-cadherin and Snail. In parallel experiments, transient and stable ectopic expression of CD63 resulted in a robust reduction of cell motility,   invasive  ness, and protease activities, which was proportional to the increase in CD63 protein level. Transfected cells overexpressing the highest level of CD63 when transplanted into immunodeficient mice showed a reduced incidence and rate of tumor growth. Moreover, these cells showed a reduction of N-cadherin, Vimentin, Zeb1, and a-SMA, and a significant resistance to undergo an EMT program both in basal condition and in the following stimulation with TGFβ. Thus, our results establish a previously unreported mechanistic link between the tetraspanin CD63 and EMT abrogation in melanoma. 
 Correlations between transmembrane 4 L6 family member 5 (TM4SF5), CD151, and CD63 in liver fibrotic phenotypes and hepatic migration and   invasive   capacities. Transmembrane 4 L6 family member 5 (TM4SF5) is overexpressed during CCl4-mediated murine liver fibrosis and in human hepatocellular carcinomas. The tetraspanins form tetraspanin-enriched microdomains (TEMs) consisting of large membrane protein complexes on the cell surface. Thus, TM4SF5 may be involved in the signal coordination that controls liver malignancy. We investigated the relationship between TM4SF5-positive TEMs with liver fibrosis and tumorigenesis, using normal Chang hepatocytes that lack TM4SF5 expression and chronically TGFβ1-treated Chang cells that express TM4SF5. TM4SF5 expression is positively correlated with tumorigenic CD151 expression, but is negatively correlated with tumor-suppressive CD63 expression in mouse fibrotic and human hepatic carcinoma tissues, indicating cooperative roles of the tetraspanins in liver malignancies. Although CD151 did not control the expression of TM4SF5, TM4SF5 appeared to control the expression levels of CD151 and CD63. TM4SF5 interacted with CD151, and caused the internalization of CD63 from the cell surface into late lysosomal membranes, presumably leading to terminating the tumor-suppressive functions of CD63. TM4SF5 could overcome the tumorigenic effects of CD151, especially cell migration and extracellular matrix (ECM)-degradation. Taken together, TM4SF5 appears to play a role in liver malignancy by controlling the levels of tetraspanins on the cell surface, and could provide a promising therapeutic target for the treatment of liver malignancies. 
 High levels of exosomes expressing CD63 and caveolin-1 in plasma of melanoma patients. Metastatic melanoma is an untreatable cancer lacking reliable and non-  invasive   markers of disease progression. Exosomes are small vesicles secreted by normal as well as tumor cells. Human tumor-derived exosomes are involved in malignant progression and we evaluated the presence of exosomes in plasma of melanoma patients as a potential tool for cancer screening and follow-up.We designed an in-house sandwich ELISA (Exotest) to capture and quantify exosomes in plasma based on expression of housekeeping proteins (CD63 and Rab-5b) and a tumor-associated marker (caveolin-1). Western blot and flow cytometry analysis of exosomes were used to confirm the Exotest-based findings. The Exotest allowed sensitive detection and quantification of exosomes purified from human tumor cell culture supernatants and plasma from SCID mice engrafted with human melanoma. Plasma levels of exosomes in melanoma-engrafted SCID mice correlated to tumor size. We evaluated the levels of plasma exosomes expressing CD63 and caveolin-1 in melanoma patients (n = 90) and healthy donors (n = 58). Consistently, plasma exosomes expressing CD63 (504+/-315) or caveolin-1 (619+/-310) were significantly increased in melanoma patients as compared to healthy donors (223+/-125 and 228+/-102, respectively). While the Exotest for CD63+ plasma exosomes had limited sensitivity (43%) the Exotest for detection of caveolin-1+ plasma exosomes showed a higher sensitivity (68%). Moreover, caveolin-1+ plasma exosomes were significantly increased with respect to CD63+ exosomes in the patients group.We describe a new non-  invasive   assay allowing detection and quantification of human exosomes in plasma of melanoma patients. Our results suggest that the Exotest for detection of plasma exosomes carrying tumor-associated antigens may represent a novel tool for clinical management of cancer patients. 
  [Mm]etastasize  PubMed  
 Down-regulation of TM4SF is associated with the metastatic potential of gastric carcinoma TM4SF members in gastric carcinoma. The aim of this study was to clarify the clinical significance of TM4SF members CD9, CD63 and CD82 in human gastric carcinoma.By employing RT-PCR and immunohistochemistry, we studied the expression of CD9, CD63 and CD82 in 49 paired tissue specimens of normal gastric mucosa and carcinoma. All tissues were obtained from patients who underwent curative surgery.All normal gastric epithelium and gastric ulcer tissues strongly expressed transcripts and proteins of CD9, CD63 and CD82 as compared with corresponding controls. We found a significant correlation between CD63 mRNA level and different pM statuses (P = 0.036). Carcinomas in M0 stage revealed a stronger expression of CD63 than carcinomas in M1 stage. Expression of CD9 protein was found significantly stronger in pN0, pM0 than in advanced pN stages (P = 0.03), pM1 (P = 0.013), respectively. We found the relationship between CD63 expression, gender (p = 0.09) and nodal status (p = 0.028), respectively. Additionally, advanced and   metastasize  d tumor tissues revealed significantly down-regulated CD82 protein expression (p = 0.033 and p = 0, respectively), which correlated with the tumor pTNM stage (p = 0.001).The reduction of CD9, CD63 and CD82 expression are indicators for the metastatic potential of gastric carcinoma cells. Unlike their expression in other tumor types, the constitutive expression of CD63 may indicate that this factor does play a direct role in human gastric carcinogenesis. 
 CD82, and CD63 in thyroid cancer. CD82 (KAI1) and CD63 (ME491) are highly glycosylated proteins which belong to the transmembrane 4 superfamily (TM4SF). CD82 has been implicated as a possible prostate cancer metastasis suppressor gene, whereas CD63 is involved in the progression of human melanoma cancer. Down-regulation of both CD82 and CD63 expression has been associated with the metastatic potential of several solid tumors. Currently, information is lacking on the role of CD82 and CD63 during thyroid carcinogenesis. The aim of this study was to determine whether the expression of CD82 and CD63 is a useful prognostic indicator in patients with thyroid carcinoma. The expression of CD82 and CD63 was analysed by reverse transcriptase-PCR (RT-PCR) and immunohistochemistry in benign goiter (n=12) and 75 primary thyroid carcinoma tissue specimens (PTC: 33, FTC: 24, UTC: 18) out of which 36 were non-  metastasize  d primary tumors and 39 were   metastasize  d tumors (regional lymph node and/or distant metastases). All of the benign goiter tissues showed CD82 expression. By contrast, a significant decrease in CD82 mRNA and protein levels was detected in carcinoma tissues as compared to benign goiter tissues (p<0.001). A similar down-regulation was observed in   metastasize  d tumor tissues when compared with non-  metastasize  d tumors (all p<0.05). CD82 expression was correlated with pTNM status of differentiated and undifferentiated thyroid tumor and the pathologic stage of differentiated thyroid tumor. In contrast to CD82, CD63 mRNA and protein expression was unchanged in all thyroid carcinomas. Benign goiter tissues showed weak expression of CD63. There were no significant correlation between CD63 mRNA/protein expression and any clinical/pathological parameters. Our results support the hypothesis that down-regulation of CD82 expression may reflect an increased in vivo metastatic potential of thyroid cancer cells. CD82 may serve as a prognostic marker of metastasis in thyroid cancer. Constitutive expression of CD63 may indicate that this factor does not play a direct role in thyroid carcinogenesis. 
  [Mm]etastasis  PubMed  
 Complementary DNA arrays identify CD63 tetraspanin and alpha3 integrin chain as differentially expressed in low and high metastatic human colon carcinoma cells. Malignant tumor cell invasion is determinant for   metastasis   to occur. E2 and C5 colon carcinoma cells that were derived from the parental Lovo line and that differ experimentally in spontaneous metastatic ability have been monitored for gene expression by cDNA arrays. Among genes found differentially expressed, the CD63 tetraspanin, not previously recognized in colon cancer progression, and the alpha3 integrin chain were both up-regulated in low metastatic E2 cells and were analyzed for their functional role using adhesion, migration, and invasion assays. Cell surface expression of CD63 and alpha3 integrin was about 2-fold higher in E2 than in C5 cells and confocal microscopy showed that CD63 and alpha3 integrin colocalized evenly on C5 cells whereas they concentrated at elongated tips of the low-metastatic more substrate-adhesive E2 cells. Antibody-interference experiments identified laminin-5 (LN-5) as a ligand interacting with the alpha3beta1/CD63 complex. Substrate-immobilized anti-CD63 antibodies enhanced tumor cell migration and invasion and induced prominent cell surface protrusions that were repressed by the PI3-kinase LY294002 inhibitor. Our results suggest that changes in the expression of surface CD63 and alpha3beta1 integrin interacting with LN-5 could affect migratory signals and the progression of the metastatic disease. 
 Timp1 interacts with beta-1 integrin and CD63 along melanoma genesis and confers anoikis resistance by activating PI3-K signaling pathway independently of Akt phosphorylation. Anoikis resistance is one of the abilities acquired along tumor progression. This characteristic is associated with   metastasis   development, since tumorigenic cells must survive independently of cell-matrix interactions in this process. In our laboratory, it was developed a murine melanocyte malignant transformation model associated with a sustained stressful condition. After subjecting melan-a melanocytes to 1, 2, 3 and 4 cycles of anchorage impediment, anoikis resistant cells were established and named 1C, 2C, 3C and 4C, respectively. These cells showed altered morphology and PMA independent cell growth, but were not tumorigenic, corresponding to pre-malignant cells. After limiting dilution of 4C pre-malignant cells, melanoma cell lines with different characteristics were obtained. Previous data from our group showed that increased Timp1 expression correlated with anoikis-resistant phenotype. Timp1 was shown to confer anchorage-independent growth capability to melan-a melanocytes and render melanoma cells more aggressive when injected into mice. However, the mechanisms involved in anoikis regulation by Timp1 in tumorigenic cells are not clear yet.The β1-integrin and Timp1 expression were evaluated by Western blotting and CD63 protein expression by flow cytometry using specific antibodies. To analyze the interaction among Timp1, CD63 and β1-integrin, immunoprecipitation assays were performed, anoikis resistance capability was evaluated in the presence or not of the PI3-K inhibitors, Wortmannin and LY294002. Relative expression of TIMP1 and CD63 in human metastatic melanoma cells was analyzed by real time PCR.Differential association among Timp1, CD63 and β1-integrins was observed in melan-a melanocytes, 4C pre-malignant melanocytes and 4C11- and 4C11+ melanoma cells. Timp1 present in conditioned medium of melanoma cells rendered melan-a melanocytes anoikis-resistant through PI3-K signaling pathway independently of Akt activation. In human melanoma cell lines, in which TIMP1 and beta-1 integrin were also found to be interacting, TIMP1 and CD63 levels together was shown to correlate significantly with colony formation capacity.Our results show that Timp1 is assembled in a supramolecular complex containing CD63 and β1-integrins along melanoma genesis and confers anoikis resistance by activating PI3-K signaling pathway, independently of Akt phosphorylation. In addition, our data point TIMP1, mainly together with CD63, as a potential biomarker of melanoma. 
 Timp1 interacts with beta-1 integrin and CD63 along melanoma genesis and confers anoikis resistance by activating PI3-K signaling pathway independently of Akt phosphorylation. Anoikis resistance is one of the abilities acquired along tumor progression. This characteristic is associated with   metastasis   development, since tumorigenic cells must survive independently of cell-matrix interactions in this process. In our laboratory, it was developed a murine melanocyte malignant transformation model associated with a sustained stressful condition. After subjecting melan-a melanocytes to 1, 2, 3 and 4 cycles of anchorage impediment, anoikis resistant cells were established and named 1C, 2C, 3C and 4C, respectively. These cells showed altered morphology and PMA independent cell growth, but were not tumorigenic, corresponding to pre-malignant cells. After limiting dilution of 4C pre-malignant cells, melanoma cell lines with different characteristics were obtained. Previous data from our group showed that increased Timp1 expression correlated with anoikis-resistant phenotype. Timp1 was shown to confer anchorage-independent growth capability to melan-a melanocytes and render melanoma cells more aggressive when injected into mice. However, the mechanisms involved in anoikis regulation by Timp1 in tumorigenic cells are not clear yet.The β1-integrin and Timp1 expression were evaluated by Western blotting and CD63 protein expression by flow cytometry using specific antibodies. To analyze the interaction among Timp1, CD63 and β1-integrin, immunoprecipitation assays were performed, anoikis resistance capability was evaluated in the presence or not of the PI3-K inhibitors, Wortmannin and LY294002. Relative expression of TIMP1 and CD63 in human metastatic melanoma cells was analyzed by real time PCR.Differential association among Timp1, CD63 and β1-integrins was observed in melan-a melanocytes, 4C pre-malignant melanocytes and 4C11- and 4C11+ melanoma cells. Timp1 present in conditioned medium of melanoma cells rendered melan-a melanocytes anoikis-resistant through PI3-K signaling pathway independently of Akt activation. In human melanoma cell lines, in which TIMP1 and beta-1 integrin were also found to be interacting, TIMP1 and CD63 levels together was shown to correlate significantly with colony formation capacity.Our results show that Timp1 is assembled in a supramolecular complex containing CD63 and β1-integrins along melanoma genesis and confers anoikis resistance by activating PI3-K signaling pathway, independently of Akt phosphorylation. In addition, our data point TIMP1, mainly together with CD63, as a potential biomarker of melanoma. 
 Expression and significance of the protein and mRNA of   metastasis   suppressor gene ME491/CD63 and integrin alpha5 in ovarian cancer tissues. To investigate the expression and significance of the proteins and mRNA of   metastasis   suppressor gene Me491/cd63 and integrin alpha5 in ovarian cancer tissues.RT-PCR and in situ hybridization were used to detect the expression of the proteins and mRNA of ME491/CD63 and integrin alpha5 in normal ovarian tissues (Group I), ovarian benign tumor tissues (Group II), ovarian borderline tumor tissues (Group III) and ovarian cancer tissues (Group IV), and the correlation between the expression and the age of patient, degree of differentiation, lymphatic   metastasis  , stage and pathological type was analyzed.There was a significant change in gene expression between the well and moderately differentiated tumors and poorly differentiated tumors. Gene expression in Groups III and IV was significantly weak, and significantly different from that in Group II and the early-stages of Groups III and IV. There was a significant difference in gene expression between each group.There is low expression of the proteins and mRNA of ME491/CD63 and integrin alpha5 in ovarian cancer. The lower the pathological differentiation is, the more significant the loss of expression is and the more likely   metastasis   is. 
 TIMP-1 signaling via CD63 triggers granulopoiesis and neutrophilia in mice. The homeostasis of neutrophil granulocytes can affect the outcome of several inflammation-associated diseases including cancer. The regulation of this homeostasis is still not completely understood. We previously found that elevated systemic levels of tissue inhibitor of metalloproteinases-1 (TIMP-1) induce an increase of neutrophils in the liver, which in turn strongly promotes liver   metastasis  . Here, we report that increasing systemic TIMP-1 levels were sufficient to induce neutrophilia in mice. This was not attributed to prolonged survival or direct mobilization of neutrophils. However, TIMP-1 induced enrichment of myeloid progenitors and concomitant upregulation of granulopoiesis-associated genes in the bone marrow compartment. BrdU pulse-labeling confirmed that proliferating progenitors accounted for TIMP-1-induced neutrophilia. TIMP-1 variants that dissect its protease-inhibitory from its CD63 binding function relevant for cell signaling revealed that the TIMP-1 signaling domain was necessary and sufficient to augment granulopoiesis. Consequently, ablation of the TIMP-1 receptor CD63 abolished both neutrophilia and TIMP-1-enhanced granulopoiesis in the bone marrow. Our findings reveal that elevated levels of TIMP-1 impact on neutrophil homeostasis via signaling through CD63. This may provide a link to clinical observations, where TIMP-1 correlates with high severity and bad prognosis in inflammation-associated diseases. 
 Complexes of tetraspanins with integrins: more than meets the eye. The transmembrane proteins of the tetraspanin superfamily are implicated in a diverse range of biological phenomena, including cell motility,   metastasis  , cell proliferation and differentiation. The tetraspanins are associated with adhesion receptors of the integrin family and regulate integrin-dependent cell migration. In cells attached to the extracellular matrix, the integrin-tetraspanin adhesion complexes are clustered into a distinct type of adhesion structure at the cell periphery. Various tetraspanins are associated with phosphatidylinositol 4-kinase and protein kinase C isoforms, and they may facilitate assembly of signalling complexes by tethering these enzymes to integrin heterodimers. At the plasma membrane, integrin-tetraspanin signalling complexes are partitioned into specific microdomains proximal to cholesterol-rich lipid rafts. A substantial fraction of tetraspanins colocalise with integrins in various intracellular vesicular compartments. It is proposed that tetraspanins can influence cell migration by one of the following mechanisms: (1) modulation of integrin signalling; (2) compartmentalisation of integrins on the cell surface; or (3) direction of intracellular trafficking and recycling of integrins. 
 The major CD9 and CD81 molecular partner. Identification and characterization of the complexes. By associating with specific partner molecules and with each other, the tetraspanins are thought to assemble multimolecular complexes that may be especially relevant with respect to   metastasis  . We have previously identified a 135-kDa molecule (CD9P-1) as a major molecular partner of CD9 in cancer cell lines. This molecule was identified, after immunoaffinity purification and mass spectrometry analysis, as the protein encoded by the KIAA1436 gene and the human ortholog of a rat protein known as FPRP. Cross-linking experiments detected a complex of the size of CD9 plus CD9P-1, showing that these glycoproteins directly associate with each other, probably in the absence of any other molecule. The use of chimeric CD9/CD82 molecules revealed the role of the second half of CD9, comprising the large extracellular loop and the fourth transmembrane domain. CD9P-1 was also shown to form separate complexes with CD81 and with an unidentified 175-kDa molecule. It also associated with other tetraspanins under conditions maintaining tetraspanin/tetraspanin interactions. The identification of a protein strongly linked to the tetraspanin web and the production of a specific monoclonal antibody will help to further characterize the role of this "web" under physiological and pathological conditions. 
 RPN2-mediated glycosylation of tetraspanin CD63 regulates breast cancer cell malignancy. The tetraspanin CD63 is a highly N-glycosylated protein that is known to regulate cancer malignancy. However, the contribution of glycosylation of CD63 to cancer malignancy remains unclear. Previously, we reported that ribophorin II (RPN2), which is part of an N-oligosaccharyle transferase complex, is responsible for drug resistance in breast cancer cells. In this study, we demonstrate that cancer malignancy associated with the glycosylation of CD63 is regulated by RPN2.Inhibition of RPN2 expression led to a reduction in CD63 glycosylation. In addition, the localization of CD63 was deregulated by knockdown of RPN2. Interestingly, multidrug resistance protein 1 (MDR1) localization was displaced from the cell surface in CD63-silenced cells. CD63 silencing reduced the chemoresistance and invasion ability of malignant breast cancer cells. Furthermore, the enrichment of CD63/MDR1-double positive cells was associated with lymph node   metastasis  . Taken together, these results indicated that high glycosylation of CD63 by RPN2 is implicated in clinical outcomes in breast cancer patients.These findings describe a novel and important function of RPN2-mediated CD63 glycosylation, which regulates MDR1 localization and cancer malignancy, including drug resistance and invasion. 
 CD63 tetraspanin is a negative driver of epithelial-to-mesenchymal transition in human melanoma cells. The CD63 tetraspanin is highly expressed in the early stages of melanoma and decreases in advanced lesions, suggesting it as a possible suppressor of tumor progression. We employed loss- and gain-of-gene-function approaches to investigate the role of CD63 in melanoma progression and acquisition of the epithelial-to-mesenchymal transition (EMT) program. We used two human melanoma cell lines derived from primary tumors and one primary human melanoma cell line isolated from a cutaneous   metastasis  , differing by levels of CD63 expression. CD63-silenced melanoma cells showed enhanced motility and invasiveness with downregulation of E-cadherin and upregulation of N-cadherin and Snail. In parallel experiments, transient and stable ectopic expression of CD63 resulted in a robust reduction of cell motility, invasiveness, and protease activities, which was proportional to the increase in CD63 protein level. Transfected cells overexpressing the highest level of CD63 when transplanted into immunodeficient mice showed a reduced incidence and rate of tumor growth. Moreover, these cells showed a reduction of N-cadherin, Vimentin, Zeb1, and a-SMA, and a significant resistance to undergo an EMT program both in basal condition and in the following stimulation with TGFβ. Thus, our results establish a previously unreported mechanistic link between the tetraspanin CD63 and EMT abrogation in melanoma. 
 Tumor-associated macrophages in oral premalignant lesions coexpress CD163 and STAT1 in a Th1-dominated microenvironment. Tumor-associated macrophages (TAMs) are implicated in the growth, invasion and   metastasis   of various solid tumors. However, the phenotype of TAMs in premalignant lesions of solid tumors has not been clarified. In the present study, we identify the phenotype of TAMs in leukoplakia, an oral premalignant lesion, by immunohistochemical analysis and investigate the involvement of infiltrated T cells that participate in the polarization of TAMs.The subjects included 30 patients with oral leukoplakia and 10 individuals with normal mucosa. Hematoxylin and eosin slides were examined for the histological grades, and immunohistochemical analysis was carried out using antibodies against CD68 (pan-MΦ), CD80 (M1 MΦ), CD163 (M2 MΦ), CD4 (helper T cells: Th), CD8 (cytotoxic T cells), CXCR3, CCR5 (Th1), CCR4 (Th2), signal transducer and activator of transcription (STAT1), phosphorylated STAT1 (pSTAT1) and chemokine CXCL9. The differences in the numbers of positively stained cells among the different histological grades were tested for statistical significance using the Kruskal-Wallis test. Correlations between different types of immune cells were determined using Spearman's rank analysis.An increase in the rate of CD163(+) TAM infiltration was observed in mild and moderate epithelial dysplasia, which positively correlated with the rate of intraepithelial CD4(+) Th cell infiltration. Although CCR4(+) cells rarely infiltrated, CXCR3(+) and CCR5(+) cells were observed in these lesions. Cells positive for STAT1 and chemokine CXCL9, interferon- (IFN)-induced gene products, and pSTAT1 were also observed in the same lesions. Double immunofluorescence staining demonstrated that the cells that were positive for CD163 were also positive for STAT1.CD163(+) TAMs in oral premalignant lesions coexpress CD163 and STAT1, suggesting that the TAMs in oral premalignant lesions possess an M1 phenotype in a Th1-dominated micromilieu. 
 CD82, and CD63 in thyroid cancer. CD82 (KAI1) and CD63 (ME491) are highly glycosylated proteins which belong to the transmembrane 4 superfamily (TM4SF). CD82 has been implicated as a possible prostate cancer   metastasis   suppressor gene, whereas CD63 is involved in the progression of human melanoma cancer. Down-regulation of both CD82 and CD63 expression has been associated with the metastatic potential of several solid tumors. Currently, information is lacking on the role of CD82 and CD63 during thyroid carcinogenesis. The aim of this study was to determine whether the expression of CD82 and CD63 is a useful prognostic indicator in patients with thyroid carcinoma. The expression of CD82 and CD63 was analysed by reverse transcriptase-PCR (RT-PCR) and immunohistochemistry in benign goiter (n=12) and 75 primary thyroid carcinoma tissue specimens (PTC: 33, FTC: 24, UTC: 18) out of which 36 were non-metastasized primary tumors and 39 were metastasized tumors (regional lymph node and/or distant metastases). All of the benign goiter tissues showed CD82 expression. By contrast, a significant decrease in CD82 mRNA and protein levels was detected in carcinoma tissues as compared to benign goiter tissues (p<0.001). A similar down-regulation was observed in metastasized tumor tissues when compared with non-metastasized tumors (all p<0.05). CD82 expression was correlated with pTNM status of differentiated and undifferentiated thyroid tumor and the pathologic stage of differentiated thyroid tumor. In contrast to CD82, CD63 mRNA and protein expression was unchanged in all thyroid carcinomas. Benign goiter tissues showed weak expression of CD63. There were no significant correlation between CD63 mRNA/protein expression and any clinical/pathological parameters. Our results support the hypothesis that down-regulation of CD82 expression may reflect an increased in vivo metastatic potential of thyroid cancer cells. CD82 may serve as a prognostic marker of   metastasis   in thyroid cancer. Constitutive expression of CD63 may indicate that this factor does not play a direct role in thyroid carcinogenesis. 
 Tetraspanin CD63 acts as a pro-metastatic factor via β-catenin stabilization. The tetraspanin CD63 is implicated in pro-metastatic signaling pathways but, so far, it is unclear, how CD63 levels affect the tumor cell phenotype. Here, we investigated the effect of CD63 modulation in different metastatic tumor cell lines. In vitro, knock down of CD63 induced a more epithelial-like phenotype concomitant with increased E-cadherin expression, downregulation of its repressors Slug and Zeb1, and decreased N-cadherin. In addition, β-catenin protein was markedly reduced, negatively affecting expression of the target genes MMP-2 and PAI-1. β-catenin inhibitors mimicked the epithelial phenotype induced by CD63 knock down. Inhibition of β-catenin upstream regulators PI3K/AKT or GSK3β could rescue the mesenchymal phenotype underlining the importance of the β-catenin pathway in CD63-regulated cell plasticity. CD63 knock down-induced phenotypical changes correlated with a decrease of experim  ental meta  stasis whereas CD63 overexpression enhanced the tumor cell-intrinsic metastatic potential. Taken together, our data show that CD63 is a crucial player in the regulation of the tumor cell-intrinsic metastatic potential by affecting cell plasticity. 
  GeneRIFs  
 decreased levels of CD63 were associated with distant and lymph node   metastasis   status and does play a direct role in human gastric carcinogenesis. 
 There is low expression of the proteins and mRNA of ME491/CD63 and integrin alpha5 in ovarian cancer. The lower the pathological differentiation is, the more significant the loss of expression is and the more likely   metastasis   is. 
  [Cc]ell [Ii]nvasion  PubMed  
 Complementary DNA arrays identify CD63 tetraspanin and alpha3 integrin chain as differentially expressed in low and high metastatic human colon carcinoma cells. Malignant tumor   cell invasion   is determinant for metastasis to occur. E2 and C5 colon carcinoma cells that were derived from the parental Lovo line and that differ experimentally in spontaneous metastatic ability have been monitored for gene expression by cDNA arrays. Among genes found differentially expressed, the CD63 tetraspanin, not previously recognized in colon cancer progression, and the alpha3 integrin chain were both up-regulated in low metastatic E2 cells and were analyzed for their functional role using adhesion, migration, and invasion assays. Cell surface expression of CD63 and alpha3 integrin was about 2-fold higher in E2 than in C5 cells and confocal microscopy showed that CD63 and alpha3 integrin colocalized evenly on C5 cells whereas they concentrated at elongated tips of the low-metastatic more substrate-adhesive E2 cells. Antibody-interference experiments identified laminin-5 (LN-5) as a ligand interacting with the alpha3beta1/CD63 complex. Substrate-immobilized anti-CD63 antibodies enhanced tumor cell migration and invasion and induced prominent cell surface protrusions that were repressed by the PI3-kinase LY294002 inhibitor. Our results suggest that changes in the expression of surface CD63 and alpha3beta1 integrin interacting with LN-5 could affect migratory signals and the progression of the metastatic disease. 
 Expression of transmembrane 4 superfamily (TM4SF) proteins and their role in hepatic stellate cell motility and wound healing migration. Migration of activated hepatic stellate cells (HSC) is a key event in the progression of liver fibrosis. Little is known about transmembrane proteins involved in HSC motility. Tetraspanins (TM4SF) have been implicated in cell development, differentiation, motility and tumor   cell invasion  . We evaluated the expression and function of four TM4SF, namely CD9, CD81, CD63 and CD151, and their involvement in HSC migration, adhesion, and proliferation.All TM4SF investigated were highly expressed at the human HSC surface with different patterns of intracellular distribution. Monoclonal antibodies directed against the four TM4SF inhibited HSC migration induced by extracellular matrix proteins in both wound healing and haptotaxis assays. This inhibition was independent of the ECM substrates employed (collagen type I or IV, laminin), and was comparable to that obtained by incubating the cells with an anti-beta1 blocking mAb. Importantly, cell adhesion was unaffected by the incubation with the same antibodies. Co-immunoprecipitation studies revealed different patterns of association between the four TM4SF studied and beta1 integrin. Finally, anti-TM4SF antibodies did not affect HSC growth.These findings provide the first characterization of tetraspanins expression and of their role in HSC migration, a key event in liver tissue wound healing and fibrogenesis. 
   
  
 ARHGEF25 
 
  NCBI Gene  
  GeneRIF  
  Pubmed  
 
 
  Search Term  Rank  Fields  
  [Ff]ilopodia  1  PubMed(1)  
  [Mm]igration  6  PubMed(4); GeneRIFs(1)  
  [Mm]etastasis  12  PubMed(1)  
 
 
 hide/show details for ARHGEF25 
  [Ff]ilopodia  PubMed  
 A Rac/Cdc42-specific exchange factor, GEFT, induces cell proliferation, transformation, and migration. The Rho family of small GTPases, including Rho, Rac, and Cdc42, play essential roles in diverse cellular functions. The ability of Rho family GTPases to participate in signaling events is determined by the ratio of inactive (GDP-bound) and active (GTP-bound) forms in the cell. The activation of Rho family proteins requires the exchange of bound GDP for GTP, a process catalyzed by the Dbl family of guanine nucleotide exchange factors (GEFs). The GEFs have high affinity for the guanine nucleotide-free state of the GTPases and are thought to promote GDP release by stabilizing an intermediate transition state. In this study, we have identified and characterized a new Rac/Cdc42-specific Dbl family guanine nucleotide exchange factor, named GEFT. GEFT is highly expressed in the excitable tissues, including brain, heart, and muscle. Low or very little expression was detected in other nonexcitable tissues. GEFT has specific exchange activity for Rac and Cdc42 in our in vitro GTPase exchange assays and glutathione S-transferase-PAK pull-down assays with GTP-bound Rac1 and Cdc42. Overexpression of GEFT leads to changes in cell morphology and actin cytoskeleton re-organization, including the formation of membrane microspikes,   filopodia  , and lamilliopodia. Furthermore, expression of GEFT in NIH3T3 cells promotes foci formation, cell proliferation, and cell migration, possibly through the activation of transcriptional factors involved in cell growth and proliferation. Together, our data suggest that GEFT is a Rac/Cdc42-specific GEF protein that regulates cell morphology, cell proliferation, and transformation. 
  [Mm]igration  PubMed  
 A Rac/Cdc42-specific exchange factor, GEFT, induces cell proliferation, transformation, and   migration  . The Rho family of small GTPases, including Rho, Rac, and Cdc42, play essential roles in diverse cellular functions. The ability of Rho family GTPases to participate in signaling events is determined by the ratio of inactive (GDP-bound) and active (GTP-bound) forms in the cell. The activation of Rho family proteins requires the exchange of bound GDP for GTP, a process catalyzed by the Dbl family of guanine nucleotide exchange factors (GEFs). The GEFs have high affinity for the guanine nucleotide-free state of the GTPases and are thought to promote GDP release by stabilizing an intermediate transition state. In this study, we have identified and characterized a new Rac/Cdc42-specific Dbl family guanine nucleotide exchange factor, named GEFT. GEFT is highly expressed in the excitable tissues, including brain, heart, and muscle. Low or very little expression was detected in other nonexcitable tissues. GEFT has specific exchange activity for Rac and Cdc42 in our in vitro GTPase exchange assays and glutathione S-transferase-PAK pull-down assays with GTP-bound Rac1 and Cdc42. Overexpression of GEFT leads to changes in cell morphology and actin cytoskeleton re-organization, including the formation of membrane microspikes, filopodia, and lamilliopodia. Furthermore, expression of GEFT in NIH3T3 cells promotes foci formation, cell proliferation, and cell   migration  , possibly through the activation of transcriptional factors involved in cell growth and proliferation. Together, our data suggest that GEFT is a Rac/Cdc42-specific GEF protein that regulates cell morphology, cell proliferation, and transformation. 
 p63RhoGEF-mediated formation of a single polarized lamellipodium is required for chemotactic   migration   in breast carcinoma cells. Short hairpin RNAs targeting 66 Rho-GEFs were screened for inhibition of chemotaxis. Six Rho-GEFs (p63RhoGEF, Trio, Duet, Net1, Frabin/Fgd4, and AAH33666) were found to be required for the serum-induced chemotactic   migration   of MDA-MB-231 human breast carcinoma cells. Knockdown of p63RhoGEF suppressed serum-induced RhoA activation and chemotaxis and caused the aberrant formation of multiple lamellipodial protrusions after serum stimulation while control cells formed a single polarized lamellipodium. These results indicate that p63RhoGEF plays a crucial role in serum-induced chemotaxis by limiting lamellipodial protrusion to one direction via RhoA activation. 
 Plasma membrane restricted RhoGEF activity is sufficient for RhoA-mediated actin polymerization. The small GTPase RhoA is involved in cell morphology and   migration  . RhoA activity is tightly regulated in time and space and depends on guanine exchange factors (GEFs). However, the kinetics and subcellular localization of GEF activity towards RhoA are poorly defined. To study the mechanism underlying the spatiotemporal control of RhoA activity by GEFs, we performed single cell imaging with an improved FRET sensor reporting on the nucleotide loading state of RhoA. By employing the FRET sensor we show that a plasma membrane located RhoGEF, p63RhoGEF, can rapidly activate RhoA through endogenous GPCRs and that localized RhoA activity at the cell periphery correlates with actin polymerization. Moreover, synthetic recruitment of the catalytic domain derived from p63RhoGEF to the plasma membrane, but not to the Golgi apparatus, is sufficient to activate RhoA. The synthetic system enables local activation of endogenous RhoA and effectively induces actin polymerization and changes in cellular morphology. Together, our data demonstrate that GEF activity at the plasma membrane is sufficient for actin polymerization via local RhoA signaling. 
 MLK3 limits activated Galphaq signaling to Rho by binding to p63RhoGEF. Mixed lineage kinase 3 (MLK3) is a MAP3K that activates the JNK-dependent MAPK pathways. Here, we show that MLK3 is required for cell   migration   in a manner independent of its role as a MAP3K or MLK3 kinase activity. Rather, MLK3 functions in a regulated way to limit levels of the activated GTPase Rho by binding to the Rho activator, p63RhoGEF/GEFT, which, in turn, prevents its activation by Galphaq. These findings demonstrate a scaffolding role for MLK3 in controlling the extent of Rho activation that modulates cell   migration  . Moreover, they suggest that MLK3 functions as a network hub that links a number of signaling pathways. 
  GeneRIFs  
 p63RhoGEF-mediated formation of a single polarized lamellipodium is required for chemotactic   migration   in breast carcinoma cells. 
  [Mm]etastasis  PubMed  
 Overexpression of GEFT, a Rho family guanine nucleotide exchange factor, predicts poor prognosis in patients with rhabdomyosarcoma. Rhabdomyosarcoma (RMS) is one of the most common soft-tissue sarcomas in children and adolescents with poor prognosis. Yet, there is lack of effective prognostic biomarkers for RMS. The present study, therefore, aimed to explore potential biomarkers for RMS based on our previous findings using array comparative genomic hybridization. We investigated guanine nucleotide exchange factor, GEFT, at expression level in 45 RMS patients and 36 normal striated muscle controls using immunohistochemistry using tissue microarrays. The expression rate of GEFT in RMS samples (42/45, 93.33%) was significantly higher (P<0.05) than that in normal controls (5/36, 13.89%). Moreover, the overexpression rate of GEFT in RMS (31/45, 68.89%) was also significantly higher (P<0.05) than that in normal controls (0/36, 0.00%). Increased expression of GEFT correlated significantly with advanced disease stages (stages III/IV) (P=0.001), lymph node   metastasis   (P=0.019), and distant   metastasis   (P=0.004), respectively, in RMS patients. In addition, RMS patients having overexpressed GEFT experienced worse overall survival (OS) than those having low levels of GEFT (P=0.001). GEFT overexpression was determined to be an independent prognostic factor for poor OS in RMS patients (hazard ratio: 3.491, 95% confidence interval: 1.121-10.871, P=0.004). In conclusion, these observations provide the first evidence of GEFT overexpression in RMS and its correlations with disease aggressiveness and   metastasis  . These findings suggest that GEFT may serve as a promising biomarker predicting poor prognosis in RMS patients, thus implying its potential as a therapeutic target. 
   
  
 HSPB1 
 
  NCBI Gene  
  GeneRIF  
  Pubmed  
 
 
  Search Term  Rank  Fields  
  [Mm]igration  6  Process(1); PubMed(33); GeneRIFs(7)  
  [Ee]xtracellular [Mm]atrix  8  PubMed(7); Component(1)  
  [Ii]nvasive  10  PubMed(11); GeneRIFs(1)  
  [Mm]etastasis  12  PubMed(26); GeneRIFs(6)  
  [Cc]ell [Ii]nvasion  13  PubMed(4); GeneRIFs(2)  
 
 
 hide/show details for HSPB1 
  [Mm]igration  Process  
 positive regulation of blood vessel endothelial cell   migration   
  PubMed  
 Activation of p38 has opposing effects on the proliferation and   migration   of endothelial cells. Pathological conditions such as hypertension and hyperglycemia as well as abrasions following balloon angioplasty all lead to endothelial dysfunction that impacts disease morbidity. These conditions are associated with the elaboration of a variety of cytokines and increases in p38 activity in endothelial cells. However, the relationship between enhanced p38 activity and endothelial cell function remains poorly understood. To investigate the effect of enhanced p38 MAPK activity on endothelial cell function, we expressed an activated mutant of MEK6 (MEK6E), an upstream regulator of p38. Expression of MEK6E activated p38 and resulted in phosphorylation of its downstream substrate, heat shock protein 27 (Hsp27). Activation of p38 was not sufficient to induce apoptosis; however, it did induce p38-dependent cell cycle arrest. MEK6E expression was sufficient to inhibit ERK phosphorylation triggered by growth factors and integrin engagement. MAPK phosphatase-1 (MKP-1) expression was increased upon p38 activation, and expression of a "substrate-trapping" MKP-1 was sufficient to restore ERK activity. Activation of p38 was sufficient to induce cell   migration  , which was accompanied by alterations in actin architecture characterized by enhanced lamellipodia. Co-expression of a mutant form of Hsp27, lacking all three phosphorylation sites, reversed MEK6E-induced cell   migration   and altered the cytoskeletal changes induced by p38 activation. Collectively, these results suggest that cellular decisions regarding   migration   and proliferation are influenced by p38 activity and that prolonged activation of p38 may result in an anti-angiogenic phenotype that contributes to endothelial dysfunction. 
 Vascular endothelial growth factor induces heat shock protein (HSP) 27 serine 82 phosphorylation and endothelial tubulogenesis via protein kinase D and independent of p38 kinase. Proteomic analysis identified HSP27 phosphorylation as a major change in protein phosphorylation stimulated by Vascular Endothelial Growth Factor (VEGF) in Human Umbilical Vein Endothelial Cells (HUVEC). VEGF-induced HSP27 phosphorylation at serines 15, 78 and 82, but whereas HSP27 phosphorylation induced by H2O2 and TNFalpha was completely blocked by the p38 kinase inhibitor, SB203580, VEGF-stimulated serine 82 phosphorylation was resistant to SB203580 and small interfering(si)RNA-mediated knockdown of p38 kinase and MAPKAPK2. The PKC inhibitor, GF109203X, partially reduced VEGF-induced HSP27 serine 82 phosphorylation, and SB203580 plus GF109203X abolished phosphorylation. VEGF activated Protein Kinase D (PKD) via PKC, and siRNAs targeted to PKD1 and PKD2 inhibited VEGF-induced HSP27 serine 82 phosphorylation. Furthermore recombinant PKD selectively phosphorylated HSP27 at serine 82 in vitro, and PKD2 activated by VEGF in HUVECs also phosphorylated HSP27 selectively at this site. Knockdown of HSP27 and PKDs markedly inhibited VEGF-induced HUVEC   migration   and tubulogenesis, whereas inhibition of the p38 kinase pathway using either SB203580 or siRNAs against p38alpha or MAPKAPK2, had no significant effect on the chemotactic response to VEGF. These findings identify a novel pathway for VEGF-induced HSP27 serine 82 phosphorylation via PKC-mediated PKD activation and direct phosphorylation of HSP27 by PKD, and show that PKDs and HSP27 play major roles in the angiogenic response to VEGF. 
 Analysis of the myosin-II-responsive focal adhesion proteome reveals a role for β-Pix in negative regulation of focal adhesion maturation. Focal adhesions undergo myosin-II-mediated maturation wherein they grow and change composition to modulate integrin signalling for cell   migratio  n, growth and differentiation. To determine how focal adhesion composition is affected by myosin II activity, we performed proteomic analysis of isolated focal adhesions and compared protein abundance in focal adhesions from cells with and without myosin II inhibition. We identified 905 focal adhesion proteins, 459 of which changed in abundance with myosin II inhibition, defining the myosin-II-responsive focal adhesion proteome. The abundance of 73% of the proteins in the myosin-II-responsive focal adhesion proteome was enhanced by contractility, including proteins involved in Rho-mediated focal adhesion maturation and endocytosis- and calpain-dependent focal adhesion disassembly. During myosin II inhibition, 27% of proteins in the myosin-II-responsive focal adhesion proteome, including proteins involved in Rac-mediated lamellipodial protrusion, were enriched in focal adhesions, establishing that focal adhesion protein recruitment is also negatively regulated by contractility. We focused on the Rac guanine nucleotide exchange factor β-Pix, documenting its role in the negative regulation of focal adhesion maturation and the promotion of lamellipodial protrusion and focal adhesion turnover to drive cel  l migrati  on. 
 Plasminogen and angiostatin interact with heat shock proteins. Previous studies from this laboratory have demonstrated that plasminogen and angiostatin bind to endothelial cell (EC) surface-associated actin via their kringles in a specific manner. Heat shock proteins (hsps) like hsp 27 are constitutively expressed by vascular ECs and regulate actin polymerization, cell growth, and   migration  . Since many hsps have also been found to be highly abundant on cell surfaces and there is evidence that bacterial surface hsps may interact with human plasminogen, the purpose of this study was to determine whether human plasminogen and angiostatin would interact with human hsps. ELISAs were developed in our laboratory to assess these interactions. It was observed that plasminogen bound to hsps 27, 60, and 70. In all cases, binding was inhibited (85-90%) by excess (50 mM) lysine indicating kringle involvement. Angiostatin predominantly bound to hsp 27 and to hsp 70 in a concentration- and kringle-dependent manner. As observed previously for actin, there was concentration-dependent inhibition of angiostatin's interaction with hsp 27 by plasminogen. In addition, 30-fold molar excess actin inhibited (up to 50%), the interaction of plasminogen with all hsps. However, 30-fold molar excess actin could only inhibit the interaction of angiostatin with hsp 27 by 15-20%. Collectively, these data indicate that (i) while plasminogen interacts specifically with hsp 27, 60, and 70, angiostatin interacts predominantly with hsp 27 and to some extent with hsp 70; (ii) plasminogen only partially displaces angiostatin's binding to hsp 27 and (iii) actin only partially displaces plasminogen/angiostatin binding to hsps. It is conceivable therefore that surface-associated hsps could mediate the binding of these ligands to cells like ECs. 
 Potential role of heat-shock proteins in giant cell tumors. We investigated the differential expression protein profile of giant cell tumors (GCTs), which can be used to monitor the tumor's recurrence and metastasis, to provide preliminary results for further study. We also explored heat-shock protein (HSP) inhibitor that prevents tumors from recurring and migrating. A stable isotope-labeling strategy using isobaric tags for relative and absolute quantitation coupled with two-dimensional liquid chromatography tandem mass spectrometry was used to separate and identify differentially expressed proteins. A total of 467 differentially expressed proteins were identified in GCT tissues. Up to 311 proteins were upregulated, whereas 156 proteins were downregulated in GCT tissues. Three of the differentially expressed HSPs, namely HP90A, HSPB1, and HSPB2, were upregulated. The differentially expressed proteins of GCT tissues will provide a scientific foundation for tumor prognosis, and for further studies exploring HSP inhibitor to prevent tumor recurrence and   migration  . 
 Analysis of protein-protein interactions in cross-talk pathways reveals CRKL protein as a novel prognostic marker in hepatocellular carcinoma. Deciphering the network of signaling pathways in cancer via protein-protein interactions (PPIs) at the cellular level is a promising approach but remains incomplete. We used an in situ proximity ligation assay to identify and quantify 67 endogenous PPIs among 21 interlinked pathways in two hepatocellular carcinoma (HCC) cells, Huh7 (minimally migratory cells) and Mahlavu (highly migratory cells). We then applied a differential network biology analysis and determined that the novel interaction, CRKL-FLT1, has a high centrality ranking, and the expression of this interaction is strongly correlated with the migratory ability of HCC and other cancer cell lines. Knockdown of CRKL and FLT1 in HCC cells leads to a decrease in cell   migration   via ERK signaling and the epithelial-mesenchymal transition process. Our immunohistochemical analysis shows high expression levels of the CRKL and CRKL-FLT1 pair that strongly correlate with reduced disease-free and overall survival in HCC patient samples, and a multivariate analysis further established CRKL and the CRKL-FLT1 as novel prognosis markers. This study demonstrated that functional exploration of a disease network with interlinked pathways via PPIs can be used to discover novel biomarkers. 
 Potential role of heat-shock proteins in giant cell tumors. We investigated the differential expression protein profile of giant cell tumors (GCTs), which can be used to monitor the tumor's recurrence and metastasis, to provide preliminary results for further study. We also explored heat-shock protein (HSP) inhibitor that prevents tumors from recurring and migrating. A stable isotope-labeling strategy using isobaric tags for relative and absolute quantitation coupled with two-dimensional liquid chromatography tandem mass spectrometry was used to separate and identify differentially expressed proteins. A total of 467 differentially expressed proteins were identified in GCT tissues. Up to 311 proteins were upregulated, whereas 156 proteins were downregulated in GCT tissues. Three of the differentially expressed HSPs, namely HP90A, HSPB1, and HSPB2, were upregulated. The differentially expressed proteins of GCT tissues will provide a scientific foundation for tumor prognosis, and for further studies exploring HSP inhibitor to prevent tumor recurrence and   migration  . 
 Plasminogen and angiostatin interact with heat shock proteins. Previous studies from this laboratory have demonstrated that plasminogen and angiostatin bind to endothelial cell (EC) surface-associated actin via their kringles in a specific manner. Heat shock proteins (hsps) like hsp 27 are constitutively expressed by vascular ECs and regulate actin polymerization, cell growth, and   migration  . Since many hsps have also been found to be highly abundant on cell surfaces and there is evidence that bacterial surface hsps may interact with human plasminogen, the purpose of this study was to determine whether human plasminogen and angiostatin would interact with human hsps. ELISAs were developed in our laboratory to assess these interactions. It was observed that plasminogen bound to hsps 27, 60, and 70. In all cases, binding was inhibited (85-90%) by excess (50 mM) lysine indicating kringle involvement. Angiostatin predominantly bound to hsp 27 and to hsp 70 in a concentration- and kringle-dependent manner. As observed previously for actin, there was concentration-dependent inhibition of angiostatin's interaction with hsp 27 by plasminogen. In addition, 30-fold molar excess actin inhibited (up to 50%), the interaction of plasminogen with all hsps. However, 30-fold molar excess actin could only inhibit the interaction of angiostatin with hsp 27 by 15-20%. Collectively, these data indicate that (i) while plasminogen interacts specifically with hsp 27, 60, and 70, angiostatin interacts predominantly with hsp 27 and to some extent with hsp 70; (ii) plasminogen only partially displaces angiostatin's binding to hsp 27 and (iii) actin only partially displaces plasminogen/angiostatin binding to hsps. It is conceivable therefore that surface-associated hsps could mediate the binding of these ligands to cells like ECs. 
 Activation of p38 has opposing effects on the proliferation and   migration   of endothelial cells. Pathological conditions such as hypertension and hyperglycemia as well as abrasions following balloon angioplasty all lead to endothelial dysfunction that impacts disease morbidity. These conditions are associated with the elaboration of a variety of cytokines and increases in p38 activity in endothelial cells. However, the relationship between enhanced p38 activity and endothelial cell function remains poorly understood. To investigate the effect of enhanced p38 MAPK activity on endothelial cell function, we expressed an activated mutant of MEK6 (MEK6E), an upstream regulator of p38. Expression of MEK6E activated p38 and resulted in phosphorylation of its downstream substrate, heat shock protein 27 (Hsp27). Activation of p38 was not sufficient to induce apoptosis; however, it did induce p38-dependent cell cycle arrest. MEK6E expression was sufficient to inhibit ERK phosphorylation triggered by growth factors and integrin engagement. MAPK phosphatase-1 (MKP-1) expression was increased upon p38 activation, and expression of a "substrate-trapping" MKP-1 was sufficient to restore ERK activity. Activation of p38 was sufficient to induce cell   migration  , which was accompanied by alterations in actin architecture characterized by enhanced lamellipodia. Co-expression of a mutant form of Hsp27, lacking all three phosphorylation sites, reversed MEK6E-induced cell   migration   and altered the cytoskeletal changes induced by p38 activation. Collectively, these results suggest that cellular decisions regarding   migration   and proliferation are influenced by p38 activity and that prolonged activation of p38 may result in an anti-angiogenic phenotype that contributes to endothelial dysfunction. 
 Heat shock protein 27 regulates neutrophil chemotaxis and exocytosis through two independent mechanisms. The targets of the p38 MAPK pathway responsible for regulation of neutrophil chemotaxis and exocytosis are unknown. One target of this pathway is the actin-binding protein, heat shock protein 27 (Hsp27). Therefore, we tested the hypothesis that Hsp27 mediates p38 MAPK-dependent chemotaxis and exocytosis in human neutrophils through regulation of actin reorganization. Sequestration of Hsp27 by introduction of anti-Hsp27 Ab, but not an isotype Ab, inhibited fMLP-stimulated chemotaxis, increased cortical F-actin in the absence of fMLP stimulation, and inhibited fMLP-stimulated exocytosis. Pretreatment with latrunculin A prevented actin reorganization and the changes in fMLP-stimulated exocytosis induced by Hsp27 sequestration. To determine the role of Hsp27 phosphorylation, wild-type, phosphorylation-resistant, or phosphorylation-mimicking recombinant Hsp27 was introduced into neutrophils by electroporation. The phosphorylation-resistant mutant significantly reduced   migration   toward fMLP, whereas none of the Hsp27 proteins affected fMLP-stimulated or TNF-alpha-stimulated exocytosis or actin polymerization. Endogenous Hsp27 colocalized with F-actin in unstimulated and fMLP-stimulated neutrophils, whereas phosphorylated Hsp27 showed cytosolic localization in addition to colocalization with F-actin. Our results suggest that Hsp27 regulates neutrophil chemotaxis and exocytosis in an actin-dependent, phosphorylation-independent manner. Phosphorylation of Hsp27 regulates chemotaxis, but not exocytosis, independent of regulation of actin reorganization. 
 R-Ras Inhibits VEGF-Induced p38MAPK Activation and HSP27 Phosphorylation in Endothelial Cells. R-Ras is a Ras family small GTPase that is highly expressed in mature functional blood vessels in normal tissues. It inhibits pathological angiogenesis and promotes vessel maturation and stabilization. Previous studies suggest that R-Ras affects cellular signaling in endothelial cells, pericytes and smooth-muscle cells to regulate vessel formation and remodeling in adult tissues. R-Ras suppresses VEGF-induced endothelial permeability and vessel sprouting while promoting normalization of pathologically developing vessels in mice. It attenuates VEGF receptor-2 (VEGFR2) activation by inhibiting internalization of the receptor upon VEGF ligand binding, leading to significant reduction of VEGFR2 autophosphorylation. Here, we show that R-Ras strongly suppresses the VEGF-dependent activation of stress-activated protein kinase-2/p38 mitogen-activated protein kinase (SAPK2/p38MAPK) and the phosphorylation of downstream heat-shock protein 27 (HSP27), a regulator of actin cytoskeleton organization, in endothelial cells. The suppression of p38MAPK activation and HSP27 phosphorylation by R-Ras concurred with altered actin cytoskeleton architecture, reduced membrane protrusion and inhibition of endothelial cell   migration   toward VEGF. Silencing of endogenous R-Ras by RNA interference increased membrane protrusion and cell   migration   stimulated by VEGF, and these effects were offset by p38MAPK inhibitor SB203580. These results suggest that R-Ras regulates angiogenic activities of endothelial cells in part via inhibition of the p38MAPK-HSP27 axis of VEGF signaling. 
 Involvement of protein kinase C beta-extracellular signal-regulating kinase 1/2/p38 mitogen-activated protein kinase-heat shock protein 27 activation in hepatocellular carcinoma cell motility and invasion. To understand the molecular mechanism that underlies the role of various prominent signal pathways in hepatocellular carcinoma (HCC) metastasis, a human signal transduction oligonucleotide microarray analysis was carried out in cultured HCC cell models with increasing spontaneous metastatic potential (MHCC97L, MHCC97H, and HCCLM6). The results revealed that the mitogen-activated protein kinase (MAPK) pathway is the prominently upregulated pathway in HCC metastasis. Further study showed that basal phosphorylated levels of extracellular signal-regulating kinase (ERK)(1/2) and p38 MAPK consecutively increased from MHCC97L to MHCC97H to HCCLM6 cells, but not c-Jun N-terminal kinase. The phosphorylation of ERK(1/2) and p38 MAPK was regulated by upregulated protein kinase C beta (PKC beta) in HCC cells through the integrated use of PKC beta RNA interference, the PKC beta specific inhibitor enzastaurin and a PKC activator phorbol-12-myristate-13-acetate. Heat shock protein 27 (HSP27) was also verified as a downstream common activated protein of PKC beta-ERK(1/2) and PKC beta-p38 MAPK. In vitro   migration   and invasion assay further showed that the depletion of PKC beta or inhibition of PKC beta activation effectively decreased HCC cell motility and invasion. Moreover, the motility and invasion of phorbol-12-myristate-13-acetate-stimulated PKC beta-mediated HCC cells was significantly negated by an ERK inhibitor, 1.4-diamino-2.3-dicyano-1.4-bis[2-aminophenylthio] butadiene, or a p38 MAPK inhibitor, 4-(4-Fluorophenyl)-2-(4-methylsulfinylphenyl)-5-(4-pyridyl)1H-imidazole. It also showed that HSP27 is critical in PKC beta-mediated HCC cell motility and invasion. Taken together, this study reveals the important role of this PKC beta-ERK(1/2)/p38MAPK-HSP27 pathway, which was verified for the first time, in modulating HCC cell motility and invasion. 
 Silencing the hsp25 gene eliminates   migration   capability of the highly metastatic murine 4T1 breast adenocarcinoma cell. The 25-kDa heat shock protein (Hsp25) is associated with various malignancies and is expressed at high levels in biopsies as well as circulating in the serum of breast cancer patients. In this study, we used RNA interference technology to silence the hsp25 gene in 4T1 breast adenocarcinoma cells, known as a poorly immunogenic, highly metastatic cell line. We demonstrate that transfection of 4T1 cells with short interference RNA-Hsp25 dramatically inhibits proliferation as compared with control transfected cells. In addition, we show that 4T1 cells transfected with short interference RNA-Hsp25 abrogates tumor   migration   potential by a mechanism that is in part due to the repression of matrix metalloproteinase 9 expression and a concomitant upregulation of its antagonist, tissue inhibitor metalloproteinase 1. Taken together, these findings provide a model system for the study of metastatic potential of tumors and are suggestive of an earlier unrecognized role for Hsp25 in tumor   migration  . 
 Heat shock protein 27 is a potential indicator for response to YangZheng XiaoJi and chemotherapy agents in cancer cells. Heat shock protein 27 (HSP27) is a member of the heat shock protein family which has been linked to tumour progression and, most interestingly, to chemotherapy resistance in cancer patients. The present study examined the potential interplay between HSP27 and YangZheng XiaoJi, a traditional Chinese medicine used in cancer treatment. A range of cell lines from different tumour types including pancreatic, lung, gastric, colorectal, breast, prostate and ovarian cancer (both wild-type and resistant) were used. Levels and activation of HSP27 and its potential associated signalling pathways were evaluated by protein array and western blotting. Knockdown of HSP27 in cancer cells was achieved using siRNA. Localisation and co-localisation of HSP27 and other proteins were carried out by immunofluorescence. Cell growth and   migration   were evaluated in their response to a range of chemotherapeutic agents. The present study first identified, by way of protein array, that YangZheng XiaoJi was able to inhibit the phosphorylation of HSP27 protein in cancer cells. We further demonstrated that HSP27, which is co-localised with caspase-9, can be blocked from localising in focal adhesions and co-localising with caspase-9 by YangZheng XiaoJi. The study also demonstrated that YangZheng XiaoJi was able to sensitise cancer cells including those cells that were resistant to chemotherapy, to chemotherapeutic agents. Finally, knocking down HSP27 markedly reduced the   migration   of cancer cells and increased the sensitivity of cancer cells to the inhibitory effect on cellular   migration   by YangZheng XiaoJi. YangZheng XiaoJi can act as an agent in first sensitising cancer cells to chemotherapy and secondly to overcome, to some degree, chemoresistance when used in an appropriate fashion in patients who have active HSP27. 
 HSP27 regulates cell adhesion and invasion via modulation of focal adhesion kinase and MMP-2 expression. Heat-shock protein 27 (HSP27), a member of the small heat-shock protein family, is a molecule involved in cellular protection in response to a variety of stresses such as heat shock, toxicants, and oxidative stress. HSP27 is also known to modulate cell functions via interaction with the actin cytoskeleton. To elucidate the functions of HSP27 in adhesion and invasion in more detail, we examined NIH3T3 cells overexpressing HSP27. HSP27 overexpression affected FAK phosphorylation and focal adhesion formation, depending on integrin-mediated actin cytoskeleton polymerization. In addition, the HSP27-overexpressing cells showed a retarded cell   migration   and invasion in wound-healing assays. Such HSP27-mediated retarded wound healing was correlated with reduced matrix metalloproteinase-2 (MMP-2) expression. The transcription factor for MMP-2 expression, signal transducer and activator or transcription 3 (STAT3), was correspondingly less phosphorylated. When a phosphomimetic form of HSP27 was transiently transfected,   migration   and invasion were similarly decreased via the regulation of the FAK/STAT3/MMP-2 signaling pathway, whereas a non-phosphorylatable form of HSP27 blocked HSP27-mediated phenotypes probably due to a dominant-negative effect on phosphorylation of endogenous HSP27. Altogether, our results suggest that HSP27 can enhance cell adhesion and modulate cell   migration   and invasion via the coordination of FAK-dependent actin organization and STAT3-dependent MMP-2 expression, and that phosphorylation of HSP27 is indispensable to regulate this signal pathway. 
 Heat shock protein 27 regulates human prostate cancer cell motility and metastatic progression. Prostate cancer (PCa) is the most common form of cancer in American men. Mortality from PCa is caused by the movement of cancer cells from the primary organ to form metastatic tumors at distant sites. Heat shock protein 27 (HSP27) is known to increase human PCa cell invasion and its overexpression is associated with metastatic disease. The role of HSP27 in driving PCa cell movement from the prostate to distant metastatic sites is unknown. Increased HSP27 expression increased metastasis as well as primary tumor mass. In vitro studies further examined the mechanism of HSP27-induced metastatic behavior. HSP27 did not affect cell detachment, adhesion, or   migration  , but did increase cell invasion. Cell invasion was dependent upon matrix metalloproteinase 2 (MMP-2), whose expression was increased by HSP27. In vivo, HSP27 induced commensurate changes in MMP-2 expression in tumors. These findings demonstrate that HSP27 drives metastatic spread of cancer cells from the prostate to distant sites, does so across a continuum of expression levels, and identifies HSP27-driven increases in MMP-2 expression as functionally relevant. These findings add to prior studies demonstrating that HSP27 increases PCa cell motility, growth and survival. Together, they demonstrate that HSP27 plays an important role in PCa progression. 
 The Role of heat shock protein 27 in extravillous trophoblast differentiation. Trophoblast cells from placental explants differentiate in culture to extravillous trophoblast cells (EVT cells). During trophoblast differentiation heat-shock-protein-27 (HSP27) mRNA and multidrug-resistance-protein-5 (MRP5, transporter of cyclic nucleotides) expression are increased. HSP27 is a regulator of actin filaments structure and dynamic, has a role in cell differentiation and may affect NF-kB activity. In this study we aimed to assess HSP27 level in trophoblast cells and its correlation with motility and differentiation related processes [MMPs activity, nitric oxide (NO), inducible nitric oxide synthase (iNOS), proliferation and MRP5 levels]. We evaluated HSP27 expression in a first trimester human trophoblast explants model designed to assess EVT cells differentiation/  migration   with/without 6-mercaptopurine (6MP, an EVT inhibitor of   migration  ). We found that HSP27 level is expressed in the nucleous and cytoplasm of non-proliferting villous-trophoblast cells (negative for Ki67) and in the cell periphery and cytoplasm of motile EVT cells. Moreover, 6MP decreased HSP27 nucleous expression that was associated with inhibited MMP2 activity and NO production. Also decreased iNOS expression and increased MRP5 mRNA levels were observed. In conclusion, HSP27 expression is modulated in concordance with   migration   dependent parameters in trophoblast cells. 
 Hsp27 participates in the maintenance of breast cancer stem cells through regulation of epithelial-mesenchymal transition and nuclear factor-κB. Heat shock proteins (HSPs) are normally induced under environmental stress to serve as chaperones for maintenance of correct protein folding but they are often overexpressed in many cancers, including breast cancer. The expression of Hsp27, an ATP-independent small HSP, is associated with cell   migratio  n and drug resistance of breast cancer cells. Breast cancer stem cells (BCSCs) have been identified as a subpopulation of breast cancer cells with markers of CD24-CD44+ or high intracellular aldehyde dehydrogenase activity (ALDH+) and proved to be associated with radiation resistance and metastasis. However, the involvement of Hsp27 in the maintenance of BCSC is largely unknown.Mitogen-activated protein kinase antibody array and Western blot were used to discover the expression of Hsp27 and its phosphorylation in ALDH + BCSCs. To study the involvement of Hsp27 in BCSC biology, siRNA mediated gene silencing and quercetin treatment were used to inhibit Hsp27 expression and the characters of BCSCs, which include ALDH+ population, mammosphere formation and cell   migratio  n, were analyzed simultaneously. The tumorigenicity of breast cancer cells after knockdown of Hsp27 was analyzed by xenograftment assay in NOD/SCID mice. The epithelial-mesenchymal transition (EMT) of breast cancer cells was analyzed by wound-healing assay and Western blot of snail, vimentin and E-cadherin expression. The activation of nuclear factor kappa B (NF-κB) was analyzed by luciferase-based reporter assay and nuclear translocation.Hsp27 and its phosphorylation were increased in ALDH+ BCSCs in comparison with ALDH- non-BCSCs. Knockdown of Hsp27 in breast cancer cells decreased characters of BCSCs, such as ALDH+ population, mammosphere formation and cel  l migrati  on. In addition, the in vivo CSC frequency could be diminished in Hsp27 knockdown breast cancer cells. The inhibitory effects could also be observed in cells treated with quercetin, a plant flavonoid inhibitor of Hsp27, and it could be reversed by overexpression of Hsp27. Knockdown of Hsp27 also suppressed EMT signatures, such as decreasing the expression of snail and vimentin and increasing the expression of E-cadherin. Furthermore, knockdown of Hsp27 decreased the nuclear translocation as well as the activity of NF-κB in ALDH + BCSCs, which resulted from increasing expression of IκBα. Restored activation of NF-κB by knockdown of IκBα could reverse the inhibitory effect of Hsp27 siRNA in suppression of ALDH+ cells.Our data suggest that Hsp27 regulates the EMT process and NF-κB activity to contribute the maintenance of BCSCs. Targeting Hsp27 may be considered as a novel strategy in breast cancer therapy. 
 miR-17-5p Promotes   migration   of human hepatocellular carcinoma cells through the p38 mitogen-activated protein kinase-heat shock protein 27 pathway. miR-17-5p is overexpressed in hepatocellular carcinoma (HCC), but the specific regulatory mechanisms of miR-17-5p in HCC remain unknown. We investigated the molecular basis of miR-17-5p as an oncogene in human HCC cell lines. Our in vivo and in vitro data indicate that miR-17-5p up-regulates the   migration   and proliferation of HCC cells. Interestingly, proteomic and western blotting assays revealed that miR-17-5p significantly activates the p38 mitogen-activated protein kinase MAPK pathway and increases the phosphorylation of heat shock protein 27 (HSP27). Our results also suggest that E2F1-dependent down-regulation of Wip1 regulates miR-17-5p-p38-HSP27 signaling. Furthermore, suppression of HSP27 expression by small interfering RNA or the p38 MAPK pathway-specific inhibitor SB203580 decreases the   migration   of HCC cells overexpressing miR-17-5p but does not reduce their proliferation. Finally, we show that miR-17-5p expression correlates well with HSP27 status in primary human HCC tissues with metastasis.Our findings suggest that the p38 MAPK pathway plays a crucial role in miR-17-5p-induced phosphorylation of HSP27 and, as a consequence, phosphorylated HSP27 enhances the   migration   of HCC cells. Our data highlight an important role of miR-17-5p in the proliferation and   migration   of HCC cells and support the potential application of miR-17-5p in HCC therapy. 
 Hsp27 regulates EGF/β-catenin mediated epithelial to mesenchymal transition in prostate cancer. Increased expression of the molecular chaperone Hsp27 is associated with the progression of prostate cancer (PCa) to castration-resistant disease, which is lethal due to metastatic spread of the prostate tumor. Metastasis requires epithelial to mesenchymal transition (EMT), which endows cancer cells with the ability to disseminate from the primary tumor and colonize new tissue sites. A wide variety of secreted factors promote EMT, and while overexpression and constitutive activation of epidermal growth factor (EGF) signaling is associated with poor prognosis of PCa, a precise role of EGF in PCa progression to metastasis remains unclear. Here, we show that Hsp27 is required for EGF-induced cell   migratio  n, invasion and MMPs activity as well as the expression of EMT markers including Fibronectin, Vimentin and Slug with concomitant decrease of E-cadherin. Mechanistically, we found that Hsp27 is required for EGF-induced AKT and GSK3β phosphorylation and β-catenin nuclear translocation. Moreover, silencing Hsp27 decreases EGF dependent phosphorylation of β-catenin on tyrosine 142 and 654, enhances β-catenin ubiquitination and degradation, prevents β-catenin nuclear translocation and binding to the Slug promoter. These data suggest that Hsp27 is required for EGF-mediated EMT via modulation of the β-catenin/Slug signaling pathway. Together, our findings underscore the importance of Hsp27 in EGF induced EMT in PCa and highlight the use of Hsp27 knockdown as a useful strategy for patients with advanced disease. 
 HSP27 mediates SPARC-induced changes in glioma morphology,   migration  , and invasion. Secreted protein acidic and rich in cysteine (SPARC) regulates cell-extracellular matrix interactions that influence cell adhesion and   migration  . We have demonstrated that SPARC is highly expressed in human gliomas, and it promotes brain tumor invasion in vitro and in vivo. To further our understanding regarding SPARC function in glioma   migration  , we transfected SPARC-green fluorescent protein (GFP) and control GFP vectors into U87MG cells, and assessed the effects of SPARC on cell morphology,   migration  , and invasion after 24 h. The expression of SPARC was associated with elongated cell morphology, and increased   migration   and invasion. The effects of SPARC on downstream signaling were assessed from 0 to 6 h and 24 h. SPARC increased the levels of total and phosphorylated HSP27; the latter was preceded by activation of p38 MAPK and inhibited by the p38 MAPK inhibitor SB203580. Augmented expression of SPARC was correlated with increased levels of HSP27 mRNA. In a panel of glioma cell lines, increasing levels of SPARC correlated with increasing total and phosphorylated HSP27. SPARC and HSP27 were colocalized to invading cells in vivo. Inhibition of HSP27 mRNA reversed the SPARC-induced changes in cell morphology,   migration  , and invasion in vitro. These data indicate that HSP27, a protein that regulates actin polymerization, cell contraction, and   migration  , is a novel downstream effector of SPARC-regulated cell morphology and   migration  . As such, it is a potential therapeutic target to inhibit SPARC-induced glioma invasion. 
 Hsp27 regulates epithelial mesenchymal transition, metastasis, and circulating tumor cells in prostate cancer. Defining the mechanisms underlying metastatic progression of prostate cancer may lead to insights into how to decrease morbidity and mortality in this disease. An important determinant of metastasis is epithelial-to-mesenchymal transition (EMT), and the mechanisms that control the process of EMT in cancer cells are still emerging. Here, we report that the molecular chaperone Hsp27 (HSPB1) drives EMT in prostate cancer, whereas its attenuation reverses EMT and decreases cell   migration  , invasion, and matrix metalloproteinase activity. Mechanistically, silencing Hsp27 decreased IL-6-dependent STAT3 phosphorylation, nuclear translocation, and STAT3 binding to the Twist promoter, suggesting that Hsp27 is required for IL-6-mediated EMT via modulation of STAT3/Twist signaling. We observed a correlation between Hsp27 and Twist in patients with prostate cancer, with Hsp27 and Twist expression each elevated in high-grade prostate cancer tumors. Hsp27 inhibition by OGX-427, an antisense therapy currently in phase II trials, reduced tumor metastasis in a murine model of prostate cancer. More importantly, OGX-427 treatment decreased the number of circulating tumor cells in patients with metastatic castration-resistant prostate cancer in a phase I clinical trial. Overall, this study defines Hsp27 as a critical regulator of IL-6-dependent and IL-6-independent EMT, validating this chaperone as a therapeutic target to treat metastatic prostate cancer. 
 Heat shock protein 27 phosphorylation is involved in epithelial cell apoptosis as well as epithelial   migration   during corneal epithelial wound healing. We reported the expression of phosphorylated HSP27 during epithelial wound healing in murine corneas (Jain et al., 2012) in July of 2012. This in vivo investigation demonstrated that the expression levels of phosphorylated HSP27 were greater in wounded corneal epithelial cells than in unwounded controls and that the localization of phosphorylated HSP27 was in the basal and superficial epithelia three days following corneal epithelial wounding. We suggested that phosphorylated HSP27 had a role in the early phase of corneal epithelial wound healing. The purpose of this study was to investigate the exact role of heat shock protein 27 (HSP27) phosphorylation for the wound healing of cultured human corneal epithelial cells (HCECs). HSP27-specific siRNAs and control-siRNAs, with no known homologous targets in HCECs, were created. The cultured HCECs were divided into two groups: Scrambled control-siRNA-transfected group vs. HSP27-specific siRNA-transfected group. The scratch-induced directional wounding assay, Western blotting, using antibodies against non-phosphorylated and phosphorylated HSP27, non-phosphorylated and phosphorylated Akt, and Bcl-2-associated X protein (Bax), immunofluorescence staining to determine the filament actin, flow cytometry to measure apoptosis, and proliferation assay were performed to determine the role of HSP27. Western blot assay showed that the expression of phosphorylated HSP27 significantly increased at 5, 10, and 30 min after scratch wounding, compared with those in unwounded HCECs (all p < 0.05). Western blot assay also showed HSP27-specific siRNAs effectively blocked the expression of non-phosphorylated HSP27. The HSP27-specific siRNA-transfected group had more Bax expression, less phosphorylated Akt expression, and less non-phosphorylated and phosphorylated HSP27 expression (all p < 0.05). The scratch-induced directional wounding assay showed the HSP27-specific siRNA-transfected group with a less migrating cell number than the control-siRNA-transfected group (p < 0.05). Immunofluorescence staining showed that reorganization of actin cytoskeleton prominently decreased in the HSP27-specific siRNA-transfected group, compared with the control siRNA-tranfected group. Flow cytometry revealed that the HSP27-specific siRNA-transfected group had more HCEC apoptosis. Proliferation assay showed no difference between the two groups. In conclusion, the role of HSP27 in corneal epithelial wound healing can be epithelial cell apoptosis, as well as epi  thelial m  igration. HSP27 is involved   in HCEC m  igration by the reorganization of actin cytoskeleton. 
 Monocyte Proteomics Reveals Involvement of Phosphorylated HSP27 in the Pathogenesis of Osteoporosis. Peripheral monocytes, precursors of osteoclasts, have emerged as important candidates for identifying proteins relevant to osteoporosis, a condition characterized by low Bone Mineral Density (BMD) and increased susceptibility for fractures. We employed 4-plex iTRAQ (isobaric tags for relative and absolute quantification) coupled with LC-MS/MS (liquid chromatography coupled with tandem mass spectrometry) to identify differentially expressed monocyte proteins from premenopausal and postmenopausal women with low versus high BMD. Of 1801 proteins identified, 45 were differentially abundant in low versus high BMD, with heat shock protein 27 (HSP27) distinctly upregulated in low BMD condition in both premenopausal and postmenopausal categories. Validation in individual samples (n = 80) using intracellular ELISA confirmed that total HSP27 (tHSP27) as well as phosphorylated HSP27 (pHSP27) was elevated in low BMD condition in both categories (P < 0.05). Further, using transwell assays, pHSP27, when placed in the upper chamber, could increase monocyte   migration   (P < 0.0001) and this was additive in combination with RANKL (receptor activator of NFkB ligand) placed in the lower chamber (P = 0.05). Effect of pHSP27 in monocyte   migration   towards bone milieu can result in increased osteoclast formation and thus contribute to pathogenesis of osteoporosis. Overall, this study reveals for the first time a novel link between monocyte HSP27 and BMD. 
 Deletion of the SPARC acidic domain or EGF-like module reduces SPARC-induced   migration   and signaling through p38 MAPK/HSP27 in glioma. We previously demonstrated that secreted protein acidic and rich in cysteine (SPARC) increases heat shock protein 27 (HSP27) expression and phosphorylation and promotes glioma cell   migration   through the p38 mitogen-activated protein kinase (MAPK)/HSP27 signaling pathway. As different regions of the SPARC protein mediate different SPARC functions, elucidating which SPARC domains regulate HSP27 expression, signaling and   migration   might provide potential therapeutic strategies to target these functions. To investigate the roles of specific domains, we used an SPARC-green fluorescent protein (GFP) fusion protein and constructs of SPARC-GFP with deletions of either the acidic domain (ΔAcidic) or the epidermal growth factor (EGF)-like module (ΔEGF). GFP, SPARC-GFP and the two deletion mutants were expressed in U87MG glioma cells. Characterization of the derived stable clones by confocal imaging and western blotting suggests proper folding, processing and secretion of the deletion constructs. Uptake of the constructs by naive cells suggests enhanced internalization of ΔAcidic and reduced internalization of ΔEGF. Wound and transw  ell migra  tion assays and western blot analysis confirm our previous results and indicate that ΔAcidic reduces SPARC-ind  uced migr  ation and p38 MAPK/HSP27 signaling and ΔEGF decreases SPARC-in  duced mig  ration and dramatically decreases the expression and phosphorylation of HSP27 but is poorly internalized. Loss of the EGF-like module suppresses the enhanced HSP27 protein stability conferred by SPARC. In conclusion, deletions of the acidic domain and EGF-like module have differential effects on cell surface binding and HSP27 protein stability; however, both regions regulate SPARC-in  duced mig  ration and signaling through HSP27. Our data link the domains of SPARC with different functions and suggest one or both of the constructs as potential therapeutic agents to inhibit SPARC-in  duced mig  ration. 
 Proteomic Characterization of Annexin l (ANX1) and Heat Shock Protein 27 (HSP27) as Biomarkers for Invasive Hepatocellular Carcinoma Cells. To search for reliable biomarkers and drug targets for management of hepatocellular carcinoma (HCC), we performed a global proteomic analysis of a pair of HCC cell lines with distinct differentiation statuses using 2-DE coupled with MALDI-TOF MS. In total, 106 and 55 proteins were successfully identified from the total cell lysate and the cytosolic, nuclear and membrane fractions in well-differentiated (HepG2) and poorly differentiated (SK-Hep-1) HCC clonal variants, respectively. Among these proteins, nine spots corresponding to proteins differentially expressed between HCC cell types were selected and confirmed by immunofluorescence staining and western blotting. Notably, Annexin 1 (ANX1), ANX-2, vimentin and stress-associated proteins, such as GRP78, HSP75, HSC-70, protein disulfide isomerase (PDI), and heat shock protein-27 (HSP27), were exclusively up-regulated in SK-Hep-1 cells. Elevated levels of ANX-4 and antioxidant/metabolic enzymes, such as MnSOD, peroxiredoxin, NADP-dependent isocitrate dehydrogenase, α-enolase and UDP-glucose dehydrogenase, were observed in HepG2 cells. We functionally demonstrated that ANX1 and HSP27 were abundantly overexpressed only in highly invasive types of HCC cells, such as Mahlavu and SK-Hep-1. Knockdown of ANX1 or HSP27 in HCC cells resulted in a severe reduction in cell   migratio  n. The in-vitro observations of ANX1 and HSP27 expressions in HCC sample was demonstrated by immunohistochemical stains performed on HCC tissue microarrays. Poorly differentiated HCC tended to have stronger ANX1 and HSP27 expressions than well-differentiated or moderately differentiated HCC. Collectively, our findings suggest that ANX1 and HSP27 are two novel biomarkers for predicting invasive HCC phenotypes and could serve as potential treatment targets. 
 Silencing heat shock protein 27 decreases metastatic behavior of human head and neck squamous cell cancer cells in vitro. The small heat shock protein 27 (Hsp27) is a molecular chaperone that is involved in a variety of cellular functions in cancer cells. The purpose of this research is to study Hsp27 in vitro metastatic behaviors of head and neck squamous cell carcinoma cells (HNSCC). The expression of Hsp27 in primary and metastatic cell lines derived from the primary HNSCC and a synchronous lymph node metastasis in the same patient was determined using real-time PCR and Western blotting. Proliferation of the primary and metastatic HNSCC cell lines was evaluated using the MTS proliferation assay. Metastatic behavior was assessed using   migration   and invasion assays. SiRNA knockdown of Hsp27 was performed in the highly migratory metastatic HNSCC cell line. MTS assays showed that the primary (UM-SCC-22A) and metastatic (UM-SCC-22B) HNSCC have similar proliferation rates. However, UM-SCC-22B derived from the metastasis showed 2.3- to 3.6-fold higher   migration   ability and 2-fold higher invasion ability than UM-SCC-22A. Real-time PCR demonstrated that Hsp27 mRNA is 22.4-fold higher in metastatic UM-SCC-22B than primary UM-SCC-22A. Similarly, Western blotting showed that Hsp27 is rarely detectable in UM-SCC-22A whereas UM-SCC-22B expresses a 25-fold higher level of Hsp27 protein. SiRNA-mediated knockdown of Hsp27 in UM-SCC-22B reduced Hsp27 mRNA expression by nearly 6-fold and protein expression by 23-fold. Furthermore, siRNA knockdown of Hsp27 decreased metastatic behaviors of UM-SCC-22B by 3- to 4-fold in   migration   and 2-fold in cell invasion reducing cell invasion and   migration   to levels similar to the primary HNSCC UM-SCC-22A. These data indicate that Hsp27 may regulate metastatic potential of HNSCC cancer cells. Targeting Hsp27 may decrease metastasis in head and neck squamous cell cancer cells. 
 LEDGF gene silencing impairs the tumorigenicity of prostate cancer DU145 cells by abating the expression of Hsp27 and activation of the Akt/ERK signaling pathway. Lens epithelium-derived growth factor (LEDGF) maintains survival pathways by augmenting the transcription of stress-response genes such as small heat-shock protein 27. Recently, aberrant expression of LEDGF was found in prostate cancer (PC). Herein, we showed that LEDGF overexpression upregulated Hsp27 in PC cells, DU145, PC-3 and LNCaP and promoted antiapoptotic pathways in PCs. We found that these cells had higher abundance of Hsp27, which was correlated with the levels of LEDGF expression. Transactivation assay in DU145 cells revealed that transactivation of Hsp27 was related to the magnitude of LEDGF expression. Silencing of LEDGF in DU145 cells abrogated Hsp27 expression and inhibited stimulated cell proliferation, invasiveness and   migration  . These cells were arrested in S and G2 phase, and failed to accumulate cyclin B1, and showed increased apoptosis. Furthermore, LEDGF-depleted DU145 cells displayed elevated Bax and cleaved caspase 9 expression and reduced levels of Bcl2, Bcl-(XL). The activated survival pathway(s), ERK1/2 and Akt, were selectively decreased in these cells, which characteristically have lower tumorigenicity. Conversely, the depleted cells, when re-overexpressed with LEDGF or Hsp27, regained tumorigenic properties. Collectively, results reveal the involvement of LEDGF-mediated elevated expression of Hsp27-dependent survival pathway(s) in PC. Our findings suggest new lines of investigation aimed at developing therapies by targeting LEDGF or its aberrant expression-associated stimulated antiapoptotic pathway(s). 
 Small heat shock proteins are induced during multiple sclerosis lesion development in white but not grey matter. The important protective role of small heat-shock proteins (HSPs) in regulating cellular survival and   migration  , counteracting protein aggregation, preventing apoptosis, and regulating inflammation in the central nervous system is now well-recognized. Yet, their role in the neuroinflammatory disorder multiple sclerosis (MS) is largely undocumented. With the exception of alpha B-crystallin (HSPB5), little is known about the roles of small HSPs in disease.Here, we examined the expression of four small HSPs during lesion development in MS, focussing on their cellular distribution, and regional differences between white matter (WM) and grey matter (GM). It is well known that MS lesions in these areas differ markedly in their pathology, with substantially more intense blood-brain barrier damage, leukocyte infiltration and microglial activation typifying WM but not GM lesions. We analysed transcript levels and protein distribution profiles for HSPB1, HSPB6, HSPB8 and HSPB11 in MS lesions at different stages, comparing them with normal-appearing brain tissue from MS patients and non-neurological controls. During active stages of demyelination in WM, and especially the centre of chronic active MS lesions, we found significantly increased expression of HSPB1, HSPB6 and HSPB8, but not HSPB11. When induced, small HSPs were exclusively found in astrocytes but not in oligodendrocytes, microglia or neurons. Surprisingly, while the numbers of astrocytes displaying high expression of small HSPs were markedly increased in actively demyelinating lesions in WM, no such induction was observed in GM lesions. This difference was particularly obvious in leukocortical lesions covering both WM and GM areas.Since induction of small HSPs in astrocytes is apparently a secondary response to damage, their differential expression between WM and GM likely reflects differences in mediators that accompany demyelination in either WM or GM during MS. Our findings also suggest that during MS, cortical structures fail to benefit from the protective actions of small HSPs. 
 Extracellular HSP27 mediates angiogenesis through Toll-like receptor 3. The heat-shock protein 27 (HSP27) is up-regulated in tumor cells and released in their microenvironment. Here, we show that extracellular HSP27 has a proangiogenic effect evidenced on chick chorioallantoic membrane. To explore this effect, we test the recombinant human protein (rhHSP27) at physiopathological doses (0.1-10 μg/ml) onto human microvascular endothelial cells (HMECs) grown as monolayers or spheroids. When added onto HMECs, rhHSP27 dose-dependently accelerates cell   migratio  n (with a peak at 5 μg/ml) and favors spheroid sprouting within 12-24 h. rhHSP27 increases VEGF gene transcription and promotes secretion of VEGF-activating VEGF receptor type 2. Increased VEGF transcription is related to NF-κB activation in 30 min. All of these effects are initiated by rhHSP27 interaction with Toll-like receptor 3 (TLR3). Such an interaction can be detected by immunoprecipitation but does not seem to be direct, as we failed to detect an interaction between rhHSP27 and monomeric TLR3 by SPR analysis. rhHSP27 is rapidly internalized with a pool of TLR3 to the endosomal compartment (within 15-30 min), which is required for NF-κB activation in a cytosolic Ca(2+)-dependent manner. The HSP27/TLR3 interaction induces NF-κB activation, leading to VEGF-mediated   cell migr  ation and angiogenesis. Such a pathway provides alternative targets for antiangiogenic cancer therapy. 
 Hsp-27 expression at diagnosis predicts poor clinical outcome in prostate cancer independent of ETS-gene rearrangement. This study was performed to test the hypothesis that expression of small heat shock protein Hsp-27 is, at diagnosis, a reliable predictive biomarker of clinically aggressive prostate cancer.A panel of tissue microarrays constructed from a well-characterised cohort of 553 men with conservatively managed prostate cancer was stained immunohistochemically to detect Hsp-27 protein. Hsp-27 expression was compared with a series of pathological and clinical parameters, including outcome.Hsp-27 staining was indicative of higher Gleason score (P<0.001). In tissue cores having a Gleason score >7, the presence of Hsp-27 retained its power to independently predict poor clinical outcome (P<0.002). Higher levels of Hsp-27 staining were almost entirely restricted to cancers lacking ERG rearrangements (chi2 trend=31.4, P<0.001), although this distribution did not have prognostic significance.This study has confirmed that, in prostate cancers managed conservatively over a period of more than 15 years, expression of Hsp-27 is an accurate and independent predictive biomarker of aggressive disease with poor clinical outcome (P<0.001). These findings suggest that apoptotic and cell-  migration   pathways modulated by Hsp-27 may contain targets susceptible to the development of biologically appropriate chemotherapeutic agents that are likely to prove effective in treating aggressive prostate cancers. 
 Heat shock protein-27 (HSP27) regulates STAT3 and eIF4G levels in first trimester human placenta. During placental implantation, cytotrophoblast cells differentiate to extravillous trophoblast (EVT) cells that invade from the placenta into the maternal uterine blood vessels. The heat shock protein-27 (HSP27), the signal transducer and activator of transcription-3 (STAT3) and the eukaryotic translation initiation factor 4E (EIF4E) are involved in regulating EVT cell differentiation/  migration  . EIF4E and EIF4G compose the translation initiation complex, which is a major control point in protein translation. The molecular chaperone distinctiveness of HSP27 implies that it directly interferes with many target proteins. STAT3, EIF4E, and EIF4G were found to be HSP27 client proteins in tumor cells. We aimed to analyze if HSP27 regulate STAT3 and EIF4G levels in first trimester human placenta. We found that like STAT3, EIF4G is highly expressed in the EVT cells (immunohistochemistry). Silencing HSP27 in HTR-8/SVneo cells (siRNA, EVT cell line) and in placental explants reduced STAT3 level (47 and 33 %, respectively, p < 0.05). HSP27 silencing reduced the levels of STAT3 phosphorylation (33 % reduction, p < 0.05) and targets (IRF1, MUC1, MMP2/9 and EIF4E, 30-49 % reduction, p < 0.05) in the HTR-8/SVneo cells. Moreover, HSP27 silencing significantly reduced EIF4G level and elevated the level of its fragments in HTR-8/SVneo cells and in the placental explants (p < 0.05). In conclusion, Placental implantation and development are accompanied by trophoblast cell proliferation and differentiation, which necessitates intense protein translation and STAT3 activation. HSP27 was found to be regulator of translation initiation and STAT3 level. Therefore, it suggests that HSP27 is a key protein during placental development and trophoblast cell differentiation. 
 Functional state of the Hsp27 chaperone as a molecular marker of an unfavorable course of larynx cancer. The small heat shock protein 27 kDA (Hsp27) acts as an ATP-independent chaperone in protein folding, but is also implicated in architecture of the cytoskeleton, cell   migration  , metabolism, cell survival, growth/differentiation, mRNA stabilization, and tumor progression.To study the intracellular localization of phosphorylated and non-phosphorylated forms of Hsp27 in squamous cell carcinoma of the larynx (SCCL) and to evaluate their relationship with regional lymphatic metastasis and overall five-year survival.Tumor biopsies of larynx tissue were collected from 50 patients who were between the ages of 30 to 80 years and had a confirmed diagnosis of squamous cell carcinoma of the larynx. Immunohistochemistry was used to determine the intracellular localization of the phosphorylated and non-phosphorylated forms of Hsp27.The study revealed that the Hsp27 chaperone was expressed in both the cytoplasm and the nucleus of tumor cells in SCCL. The biopsies of patients with lymph node metastases showed significantly higher expression of the phosphorylated and unphosphorylated forms of Hsp27 in the nucleus compared to those of patients without lymph node metastases. At the same time, the cytoplasmic expression of Hsp27 in these patients did not differ statistically. Analysis of the overall five-year survival rates showed that negative Hsp27 expression in the nucleus of tumor cells is associated with the survival rate of patients with SCCL.The nuclear expression of phosphorylated and unphosphorylated forms of Hsp27 is a molecular marker of unfavorable squamous cell carcinoma of the larynx associated with lymphogenous metastasis and decreased total five-year survival. 
  GeneRIFs  
 HSP27 is involved in HCEC   migration   by the reorganization of actin cytoskeleton. 
 This study showed that the HSP27/TLR3 interaction induces NF-kappaB activation, leading to VEGF-mediated cell   migration   and angiogenesis. 
 Data show that siRNA knockdown of Hsp27 decreased metastatic behaviors of UM-SCC-22B by 3- to 4-fold in   migration   and 2-fold in cell invasion reducing cell invasion and   migration   to levels similar to the primary HNSCC UM-SCC-22A. 
 findings suggest that the p38 MAPK pathway plays a crucial role in miR-17-5p-induced phosphorylation of HSP27 and, as a consequence, phosphorylated HSP27 enhances the   migration   of hepatocellular carcinoma cells 
 HSP27 is a novel downstream effector of SPARC-regulated cell morphology and cell   migration  . 
 HSP27 expression is modulated in concordance with   migration   dependent parameters in trophoblast cells. 
 findings provide a model system for the study of metastatic potential of tumors and are suggestive of an earlier unrecognized role for Hsp25 in tumor   migration   
  [Ee]xtracellular [Mm]atrix  PubMed  
 Proteomic analysis of human parotid gland exosomes by multidimensional protein identification technology (MudPIT). Human ductal saliva contributes over a thousand unique proteins to whole oral fluids. The mechanism by which most of these proteins are secreted by salivary glands remains to be determined. The present study used a mass spectrometry-based, shotgun proteomics approach to explore the possibility that a subset of the proteins found in saliva are derived from exosomes, membrane-bound vesicles of endosomal origin within multivesicular endosomes. Using MudPIT (multidimensional protein identification technology) mass spectrometry, we catalogued 491 proteins in the exosome fraction of human parotid saliva. Many of these proteins were previously observed in ductal saliva from parotid glands (265 proteins). Furthermore, 72 of the proteins in parotid exosomes overlap with those previously identified as urinary exosome proteins, proteins which are also frequently associated with exosomes from other tissues and cell types. Gene Ontology (GO) and KEGG pathway analyses found that cytosolic proteins comprise the largest category of proteins in parotid exosomes (43%), involved in such processes as phosphatidylinositol signaling system, calcium signaling pathway, inositol metabolism, protein export, and signal transduction, among others; whereas the integral plasma membrane proteins and associated/peripheral plasma membrane proteins (26%) were associated with   extracellular matrix  -receptor interaction, epithelial cell signaling, T-cell and B-cell receptor signaling, cytokine receptor interaction, and antigen processing and presentation, among other biological functions. In addition, these putative saliva exosomal proteins were linked to specific diseases (e.g., neurodegenerative disorders, prion disease, cancers, type I and II diabetes). Consequently, parotid glands secrete exosomes that reflect the metabolic and functional status of the gland and may also carry informative protein markers useful in the diagnosis and treatment of systemic diseases. 
 Arachidonic acid stimulates cell adhesion through a novel p38 MAPK-RhoA signaling pathway that involves heat shock protein 27. Rho GTPases are critical components of cellular signal transduction pathways. Both hyperactivity and overexpression of these proteins have been observed in human cancers and have been implicated as important factors in metastasis. We previously showed that dietary n-6 fatty acids increase cancer cell adhesion to   extracellular matrix   proteins, such as type IV collagen. Here we report that in MDA-MB-435 human melanoma cells, arachidonic acid activates RhoA, and inhibition of RhoA signaling with either C3 exoenzyme or dominant negative Rho blocked arachidonic acid-induced cell adhesion. Inhibition of the Rho kinase (ROCK) with either small molecule inhibitors or ROCK II-specific small interfering RNA (siRNA) blocked the fatty acid-induced adhesion. However, unlike other systems, inhibition of ROCK did not block the activation of p38 mitogen-activated protein kinase (MAPK); instead, Rho activation depended on p38 MAPK activity and the presence of heat shock protein 27 (HSP27), which is phosphorylated downstream of p38 after arachidonic acid treatment. HSP27 associated with p115RhoGEF in fatty acid-treated cells, and this association was blocked when p38 was inhibited. Furthermore, siRNA knockdown of HSP27 blocked the fatty acid-stimulated Rho activity. Expression of dominant negative p115-RhoGEF or p115RhoGEF-specific siRNA inhibited both RhoA activation and adhesion on type IV collagen, whereas a constitutively active p115RhoGEF restored the arachidonic acid stimulation in cells in which the p38 MAPK had been inhibited. These data suggest that n-6 dietary fatty acids stimulate a set of interactions that regulates cell adhesion through RhoA and ROCK II via a p38 MAPK-dependent association of HSP27 and p115RhoGEF. 
 SILAC-based proteomics of human primary endothelial cell morphogenesis unveils tumor angiogenic markers. Proteomics has been successfully used for cell culture on dishes, but more complex cellular systems have proven to be challenging and so far poorly approached with proteomics. Because of the complexity of the angiogenic program, we still do not have a complete understanding of the molecular mechanisms involved in this process, and there have been no in depth quantitative proteomic studies. Plating endothelial cells on matrigel recapitulates aspects of vessel growth, and here we investigate this mechanism by using a spike-in SILAC quantitative proteomic approach. By comparing proteomic changes in primary human endothelial cells morphogenesis on matrigel to general adhesion mechanisms in cells spreading on culture dish, we pinpoint pathways and proteins modulated by endothelial cells. The cell-  extracellular matrix   adhesion proteome depends on the adhesion substrate, and a detailed proteomic profile of the   extracellular matrix   secreted by endothelial cells identified CLEC14A as a matrix component, which binds to MMRN2. We verify deregulated levels of these proteins during tumor angiogenesis in models of multistage carcinogenesis. This is the most in depth quantitative proteomic study of endothelial cell morphogenesis, which shows the potential of applying high accuracy quantitative proteomics to in vitro models of vessel growth to shed new light on mechanisms that accompany pathological angiogenesis. The mass spectrometry proteomics data have been deposited to the ProteomeXchange Consortium with the data set identifier PXD000359. 
 Skin equivalent tensional force alters keloid fibroblast behavior and phenotype. Skin tension may influence keloid scar behavior, development, and spreading, e.g., butterfly-shaped keloid disease in the sternum. Here, we developed a three-dimensional (3D) in vitro model to mimic in vivo tension and evaluate keloid fibroblast (KF) behavior and   extracellular matrix   synthesis under tension. In vivo skin tension measured in volunteers (n = 4) using 3D image photogrammetry enabled prediction of actual force (35 mN). A novel cell force monitor applied tension in a fibroblast-populated 3D collagen lattice replicating the in vivo force. The effect of tension on keloid (n = 10) fibroblast (KF) and normal skin (n = 10) fibroblasts (NF) at set time points (6, 12, and 24 hours) was measured in Hsp27, PAI-2, and α2β1 integrin, tension-related genes demonstrating significant (p < 0.05) time-dependent regulation of these genes in NF vs. KF with and without tension. KF showed higher (p < 0.05) proliferation post-tension. Knockdown of all three genes in 24 and 48 hours with and without tension showed significant down-regulation in NF vs. KF. Additionally, we show significant (p < 0.05) modi  fication of the expr  ession of extracellular matrix-related genes post-tension following down-regulation of Hsp27, PAI-2, or α2β1 integrin. Finally, we demonstrate significant alteration in NF compared with KF morphology following knockdown. In conclusion, this study shows induction of tension-related genes expression following mechano-regulation in KFs, with potential relevance to its development and therapy. 
 Skin equivalent tensional force alters keloid fibroblast behavior and phenotype. Skin tension may influence keloid scar behavior, development, and spreading, e.g., butterfly-shaped keloid disease in the sternum. Here, we developed a three-dimensional (3D) in vitro model to mimic in vivo tension and evaluate keloid fibroblast (KF) behavior and   extracellular matrix   synthesis under tension. In vivo skin tension measured in volunteers (n = 4) using 3D image photogrammetry enabled prediction of actual force (35 mN). A novel cell force monitor applied tension in a fibroblast-populated 3D collagen lattice replicating the in vivo force. The effect of tension on keloid (n = 10) fibroblast (KF) and normal skin (n = 10) fibroblasts (NF) at set time points (6, 12, and 24 hours) was measured in Hsp27, PAI-2, and α2β1 integrin, tension-related genes demonstrating significant (p < 0.05) time-dependent regulation of these genes in NF vs. KF with and without tension. KF showed higher (p < 0.05) proliferation post-tension. Knockdown of all three genes in 24 and 48 hours with and without tension showed significant down-regulation in NF vs. KF. Additionally, we show significant (p < 0.05) modi  fication of the expr  ession of extracellular matrix-related genes post-tension following down-regulation of Hsp27, PAI-2, or α2β1 integrin. Finally, we demonstrate significant alteration in NF compared with KF morphology following knockdown. In conclusion, this study shows induction of tension-related genes expression following mechano-regulation in KFs, with potential relevance to its development and therapy. 
 Arachidonic acid stimulates cell adhesion through a novel p38 MAPK-RhoA signaling pathway that involves heat shock protein 27. Rho GTPases are critical components of cellular signal transduction pathways. Both hyperactivity and overexpression of these proteins have been observed in human cancers and have been implicated as important factors in metastasis. We previously showed that dietary n-6 fatty acids increase cancer cell adhesion to   extracellular matrix   proteins, such as type IV collagen. Here we report that in MDA-MB-435 human melanoma cells, arachidonic acid activates RhoA, and inhibition of RhoA signaling with either C3 exoenzyme or dominant negative Rho blocked arachidonic acid-induced cell adhesion. Inhibition of the Rho kinase (ROCK) with either small molecule inhibitors or ROCK II-specific small interfering RNA (siRNA) blocked the fatty acid-induced adhesion. However, unlike other systems, inhibition of ROCK did not block the activation of p38 mitogen-activated protein kinase (MAPK); instead, Rho activation depended on p38 MAPK activity and the presence of heat shock protein 27 (HSP27), which is phosphorylated downstream of p38 after arachidonic acid treatment. HSP27 associated with p115RhoGEF in fatty acid-treated cells, and this association was blocked when p38 was inhibited. Furthermore, siRNA knockdown of HSP27 blocked the fatty acid-stimulated Rho activity. Expression of dominant negative p115-RhoGEF or p115RhoGEF-specific siRNA inhibited both RhoA activation and adhesion on type IV collagen, whereas a constitutively active p115RhoGEF restored the arachidonic acid stimulation in cells in which the p38 MAPK had been inhibited. These data suggest that n-6 dietary fatty acids stimulate a set of interactions that regulates cell adhesion through RhoA and ROCK II via a p38 MAPK-dependent association of HSP27 and p115RhoGEF. 
 HSP27 mediates SPARC-induced changes in glioma morphology, migration, and invasion. Secreted protein acidic and rich in cysteine (SPARC) regulates cell-  extracellular matrix   interactions that influence cell adhesion and migration. We have demonstrated that SPARC is highly expressed in human gliomas, and it promotes brain tumor invasion in vitro and in vivo. To further our understanding regarding SPARC function in glioma migration, we transfected SPARC-green fluorescent protein (GFP) and control GFP vectors into U87MG cells, and assessed the effects of SPARC on cell morphology, migration, and invasion after 24 h. The expression of SPARC was associated with elongated cell morphology, and increased migration and invasion. The effects of SPARC on downstream signaling were assessed from 0 to 6 h and 24 h. SPARC increased the levels of total and phosphorylated HSP27; the latter was preceded by activation of p38 MAPK and inhibited by the p38 MAPK inhibitor SB203580. Augmented expression of SPARC was correlated with increased levels of HSP27 mRNA. In a panel of glioma cell lines, increasing levels of SPARC correlated with increasing total and phosphorylated HSP27. SPARC and HSP27 were colocalized to invading cells in vivo. Inhibition of HSP27 mRNA reversed the SPARC-induced changes in cell morphology, migration, and invasion in vitro. These data indicate that HSP27, a protein that regulates actin polymerization, cell contraction, and migration, is a novel downstream effector of SPARC-regulated cell morphology and migration. As such, it is a potential therapeutic target to inhibit SPARC-induced glioma invasion. 
  Component  
   extracellular matrix   
  [Ii]nvasive  PubMed  
 Comparison of tear protein levels in breast cancer patients and healthy controls using a de novo proteomic approach. Non  invasive   biomarkers are urgently needed for early detection of breast cancer since the risk of recurrence, morbidity and mortality are closely related to disease stage at the time of primary surgery. In the past decade, many proteomics-based approaches were developed that utilize the protein profiling of human body fluids or identification of putative biomarkers to obtain more knowledge on the effects of cancer emergence and progression. Herein, we report on an analysis of proteins in the tear fluid from breast carcinoma patients and healthy women using a de novo proteomic approach and 25 mixed samples from each group. This study included 25 patients with primary   invasiv  e breast carcinoma and 25 age-matched healthy controls. We performed a MALDI-TOF-TOF-driven semi-quantitative comparison of tear protein levels in cancer (CA) and control (CTRL) using a de novo approach in pooled samples. Over 150 proteins in the tear fluid of CTRL and CA were identified. Using an in-house-developed algorithm we found more than 20 proteins distinctly upregulated or downregulated in the CTRL and CA groups. We identified several proteins that had modified expression in breast cancer patients. These proteins are involved in host immune system pathways (e.g., C1Q1 or S100A8) and different metabolic cascades (ALDH3A or TPI). Further validation of the results in an independent population combined with individual protein profiling of participants is needed to confirm the specificity of our findings and may lead to a better understanding of the pathological mechanism of breast cancer. 
 Differential expression of ANXA6, HSP27, PRDX2, NCF2, and TPM4 during uterine cervix carcinogenesis: diagnostic and prognostic value. Cytology-based diagnostics of squamous cervical cancer (SCC) precursor lesions is subjective and can be improved by objective markers.IHC-based analysis of ANXA6, HSP27, peroxiredoxin 2 (PRDX2), NCF2, and tropomyosin 4 (TPM4) during SCC carcinogenesis.Expression of ANXA6, HSP27, PRDX2, and NCF2 in the cytoplasm of dysplastic cells increased from cervical intraepithelial neoplasia 2/3 (CIN2/3) to micro  invasive   cancer.   Invasive   SCC showed lower expression of TPM4 than CIN and normal epithelium. CIN2/3 with the highest sensitivity and specificity differed from normal epithelium by cytoplasmic expression of HSP27. Patients with cytoplasmic HSP27 expression in SCC deviating from that observed in normal epithelium had worse relapse-free (P=0.019) and overall (P=0.014) survival.   Invasive   SCC with the highest sensitivity and specificity differed from normal epithelium by expression of PRDX2 and TPM4 in the cytoplasm, from CIN2/3 by the expression of ANXA6 and TPM4 in the cytoplasm, and from micro  invasive   SCC by the expression of PRDX2 and ANXA6 in the cytoplasm. The number of sporadic ANXA6+ cells between the atypical cells increased from CIN2/3 to   invasive   SCC.Detection of expression changes of the proteins ANXA6, HSP27, PRDX2, NCF2, and TPM4 in SCC precursor lesions may aid current cytological and pathological diagnostics and evaluation of prognosis. 
 Heat shock protein 27 and gross cystic disease fluid protein 15 play critical roles in molecular apocrine breast cancer. Molecular apocrine breast cancer (MABC) has a distinct hormonal profile, being estrogen receptor (ER) and progesterone receptor (PR) negative but androgen receptor (AR) positive. The clinical significance of MABC and its relative variables have not been absolutely clarified and remain to be determined. Five hundred cases of   invasive   breast carcinoma were randomly selected in this study, including 158 MABC cases and 342 nonMABC cases. Expression of ER, PR, epidermal growth factor receptor 2 (HER2), Ki67, AR, gross cystic disease fluid protein 15 (GCDFP15), and heat shock protein 27 (HSP27) were analyzed by immunohistochemistry. Differences of continuous variables between MABC and nonMABC subgroups were evaluated by the chi-square test. The Kaplan-Meier method was performed to evaluate disease-free survival (DFS) and overall survival (OS). The MABC subgroup had higher histological grade, bigger tumor size, more lymph node metastasis, and higher pTNM stage than the nonMABC subgroup (P < 0.05), and patients with MABC had poorer prognosis than those of the nonMABC subgroup (P < 0.05). Both GCDFP15 and HSP27 were expressed differently in the MABC and nonMABC subgroups (P < 0.05). Furthermore, in the MABC subgroup, positive HSP27 expression indicated higher risk of recurrence (P < 0.05) and positive GCDFP15 expression was also a poor marker for patient outcome (P < 0.05). MABC patients with HSP27 and GCDFP15 co-expression had worse outcome (P < 0.05). Our data suggested that MABC had a high risk of recurrence. Positive expression of both GCDFP15 and HSP27 were correlated with MABC malignancy. Targeting AR and HSP27 at the same time might offer a useful strategy to MABC. 
 Differential expression of heat shock protein 27 in normal oral mucosa, oral epithelial dysplasia and squamous cell carcinoma. The aim of this study was to evaluate HSP27 expression in fetal, normal and inflamed oral mucosal epithelium and in oral premalignant epithelial lesions and in their ensuing   invasive   cancers. In developing human oral epithelia, immunoreactions for HSP27 were moderately observed in suprabasal keratinocytes of palate and tongue. Normal oral epithelium had an intense suprabasal positivity. In inflamed oral mucosa, HSP27 staining was stronger in basal and suprabasal keratinocytes than in normal epithelium. Most oral premalignant lesions showed no (5 cases, 29%) or low (8 cases, 46.4%) staining. In OSCC both low and high HSP27 levels of expression were observed. HSP27 immunolabelling was down-regulated in poorly differentiated areas and up-regulated in highly differentiated ones. These findings indicated that HSP27 expression seems to protect cells from apoptosis during inflammation, while the down-regulation in dysplasia could impair the protective mechanism against mutagenesis induced by environmental factors and thus enhancing the transformation of oral epithelial dysplasia into OSCCs. 
 The overexpression of 14-3-3ζ and Hsp27 promotes non–small cell lung cancer progression. The 14-3-3ζ protein has been identified as a putative oncoprotein in several cancers, including non–small cell lung cancer (NSCLC). However, the mechanisms underlying its functions have not been well defined.Proteins that interact with 14-3-3ζ were identified through coimmunoprecipitation and mass spectrometry in NSCLC cells. The interaction of 14-3-3ζ with these molecular partners and their roles   in the   invasiveness and metastasis of NSCLC cells were assayed through specific disruptions in the 14-3-3ζ signaling network. In addition, the clinical implications of this 14-3-3ζ complex were examined in samples from patients with NSCLC.Among the identified proteins that interacted with 14-3-3ζ, there were 230 proteins in 95-D cells, 181 proteins in 95-C cells, and 203 proteins in A549 cells; and 16 interacting proteins were identified that overlapped between all cell lines. Further studies revealed 14-3-3ζ complexes within the heat shock protein 27 (Hsp27) protein and demonstrated that the interference of Hsp27 or 14-3-3ζ inhibited the invasion and metastasis of NSCL  C cells.   The invasive and metastatic capabilities of cells with both Hsp27 and 14-3-3ζ interference could be completely restored only by Hsp27 and 14-3-3ζ complementary DNA transfection and not by either agent alone. Clinically, the postoperative 5-year overall survival (OS) in patients who had high expression of both 14-3-3ζ and Hsp27 was significantly lower than the 5-year OS in patients who had low expression of both 14-3-3ζ and Hsp27 (26.5% vs 59.7%, respectively). Multivariate analysis revealed that the combined expression of 14-3-3ζ and Hsp27 was an independent prognostic indicator of OS(P = .036).The current data suggest that the combined expression of 14-3-3ζ and Hsp27 may be a biomarker for predicting survival in patients with NSCLC, and this combination may have potential as a therapeutic target for NSCLC. 
 HSP27 is required for invasion and metastasis triggered by hepatocyte growth factor. The hepatocyte growth factor (HGF) also known as scatter factor activates cancer cell invasion and metastasis. We show that in ovarian cancer cells HGF induced the phosphorylation of the small heat shock protein of 27 kDa (HSP27) by activating the p38MAPK. HSP27 is increased in many cancers at advanced stage including ovarian cancer and associated with cancer resistance to therapy and poor patients' survival. The phosphorylation of HSP27 regulates both its chaperone activity and its control of cytoskeletal stability. We show that HSP27 was necessary for the remodeling of actin filaments induced by HGF and that motility in vitro depended on the p38MAPK-MK2 axis. In vivo, HSP27 silencing impaired the ability of the highly metastatic, HGF-secreting ovarian cancer cells to give rise to spontaneous metastases. This was due to defective motility across the vessel wall and reduced growth. Indeed, HSP27 silencing impaired the ability of circulating ovarian cancer cells to home to the lungs and to form experimental hematogenous metastases and the capability of cancer cells to grow as subcutaneous xenografts. Moreover, HSP27 suppression resulted in the sensitization of xenografts to low doses of the chemotherapeutic paclitaxel, likely because HSP27 protected microtubules from bundling caused by the drug. Altogether, these data show that the HSP27 is required for the pro  invasive   and prometastatic activity of HGF and suggest that HSP27 might be not only a marker of progression of ovarian cancer, but also a suitable target for therapy. 
 Proteomic Characterization of Annexin l (ANX1) and Heat Shock Protein 27 (HSP27) as Biomarkers for   Invasive   Hepatocellular Carcinoma Cells. To search for reliable biomarkers and drug targets for management of hepatocellular carcinoma (HCC), we performed a global proteomic analysis of a pair of HCC cell lines with distinct differentiation statuses using 2-DE coupled with MALDI-TOF MS. In total, 106 and 55 proteins were successfully identified from the total cell lysate and the cytosolic, nuclear and membrane fractions in well-differentiated (HepG2) and poorly differentiated (SK-Hep-1) HCC clonal variants, respectively. Among these proteins, nine spots corresponding to proteins differentially expressed between HCC cell types were selected and confirmed by immunofluorescence staining and western blotting. Notably, Annexin 1 (ANX1), ANX-2, vimentin and stress-associated proteins, such as GRP78, HSP75, HSC-70, protein disulfide isomerase (PDI), and heat shock protein-27 (HSP27), were exclusively up-regulated in SK-Hep-1 cells. Elevated levels of ANX-4 and antioxidant/metabolic enzymes, such as MnSOD, peroxiredoxin, NADP-dependent isocitrate dehydrogenase, α-enolase and UDP-glucose dehydrogenase, were observed in HepG2 cells. We functionally demonstrated that ANX1 and HSP27 were abundantly overexpressed only in highly   invasiv  e types of HCC cells, such as Mahlavu and SK-Hep-1. Knockdown of ANX1 or HSP27 in HCC cells resulted in a severe reduction in cell migration. The in-vitro observations of ANX1 and HSP27 expressions in HCC sample was demonstrated by immunohistochemical stains performed on HCC tissue microarrays. Poorly differentiated HCC tended to have stronger ANX1 and HSP27 expressions than well-differentiated or moderately differentiated HCC. Collectively, our findings suggest that ANX1 and HSP27 are two novel biomarkers for predicting   invasiv  e HCC phenotypes and could serve as potential treatment targets. 
 LEDGF gene silencing impairs the tumorigenicity of prostate cancer DU145 cells by abating the expression of Hsp27 and activation of the Akt/ERK signaling pathway. Lens epithelium-derived growth factor (LEDGF) maintains survival pathways by augmenting the transcription of stress-response genes such as small heat-shock protein 27. Recently, aberrant expression of LEDGF was found in prostate cancer (PC). Herein, we showed that LEDGF overexpression upregulated Hsp27 in PC cells, DU145, PC-3 and LNCaP and promoted antiapoptotic pathways in PCs. We found that these cells had higher abundance of Hsp27, which was correlated with the levels of LEDGF expression. Transactivation assay in DU145 cells revealed that transactivation of Hsp27 was related to the magnitude of LEDGF expression. Silencing of LEDGF in DU145 cells abrogated Hsp27 expression and inhibited stimulated cell proliferation,   invasive  ness and migration. These cells were arrested in S and G2 phase, and failed to accumulate cyclin B1, and showed increased apoptosis. Furthermore, LEDGF-depleted DU145 cells displayed elevated Bax and cleaved caspase 9 expression and reduced levels of Bcl2, Bcl-(XL). The activated survival pathway(s), ERK1/2 and Akt, were selectively decreased in these cells, which characteristically have lower tumorigenicity. Conversely, the depleted cells, when re-overexpressed with LEDGF or Hsp27, regained tumorigenic properties. Collectively, results reveal the involvement of LEDGF-mediated elevated expression of Hsp27-dependent survival pathway(s) in PC. Our findings suggest new lines of investigation aimed at developing therapies by targeting LEDGF or its aberrant expression-associated stimulated antiapoptotic pathway(s). 
 Hsp-27 expression in   invasive   ductal breast carcinoma. The aim of this study was to determine the intensity of Hsp27 protein expression in fibrocystic breast changes (FC) and   invasive   ductal breast carcinoma (IDC) and to examine its impact on patients' clinico-pathological characteristics and overall survival. Immunohistochemical reactions were conducted on archival samples of 20 cases of FC and 101 cases of IDC treated in 1999-2002. Nuclear-cytoplasmic Hsp27 expression was observed in 92 (92.1%) of the examined cases of IDC, and all the cases of FC. Significantly higher Hsp27 expression was observed in G2 (p < 0.01) and G3 cases (p < 0.0001) compared to FC. HER-2 positive cases had higher Hsp27 expression (p = 0.0153), than HER-2 negative cases. Our research showed that Hsp27 could have an impact on tumor malignancy. Moreover, a positive correlation between the expression of Hsp27 and HER-2 positive cases was demonstrated. 
 Relationship between the expression of phosphorylated heat shock protein beta-1 with lymph node metastases of breast cancer. Heat shock protein beta-1 (HspB1) is a chaperone of the sHsp (small heat shock protein). The common functions of sHsps are chaperone activity, inhibition of apoptosis, regulation of cell development, and cell differentiation, take part in signal transduction.To study the intracellular localization of phosphorylated features and non-phosphorylated forms of HspB1 in primary breast cancer cells and to evaluate their relationship with regional lymphatic metastasis.Tumor biopsies of breast tissue were collected from 100 patients with a confirmed diagnosis of   invasive   carcinoma, nonspecific type, between the ages of 31-80 years. Immunohistochemistry was used to determine the intracellular localization of phosphorylated and non-phosphorylated forms of HspB1.The result of this study showed that biopsies from patients with lymph node metastasis exhibited significantly higher levels of phosphorylated forms of HspB1 in the nucleus and cytoplasm compared with the group without lymph node metastasis. Analysis showed that the expression of phosphorylated forms of the chaperone HspB1 correlates with the amount and percentage of lymph node metastases affected.The nuclear expression of phosphorylated and non-phosphorylated forms of the chaperone HspB1 is a marker of tumor cells associated with lymphatic metastasis of breast cancer. 
 Prognostic significance of heat shock proteins in urothelial carcinoma of the urinary bladder. Heat shock proteins (HSPs) are a group of molecules induced by a variety of environmental and pathophysiological stresses, including cancer. The expression of HSPs has been implicated in the regulation of apoptosis and immunity in neoplasia. The purpose of this study was to evaluate the expression and clinicopathological relevance of several HSPs in urothelial carcinomas of the urinary bladder.Immunohistochemical staining for HSP27, HSP60, HSP70 and HSP90 was performed on samples collected from 744 clinical cases. The results were correlated with clinicopathological characteristics using univariate and multivariate analyses. High expression of HSP70 predicted recurrence of non-muscle-  invasive   urothelial carcinoma treated by transurethral resection, and low expression of HSP27 correlated with progression and cancer-specific mortality for non-muscle-  invasive   cancers treated by transurethral resection. Low expression of HSP27 also predicted cancer-specific mortality for patients who underwent cystectomy.Both HSP27 and HSP70 impact on the biological behaviour of urothelial carcinomas of the urinary bladder. Immunohistochemical assessment of HSPs can provide useful prognostic information that could ultimately help to develop individualized surveillance programmes. 
  GeneRIFs  
 ANX1 and HSP27 are two novel biomarkers for predicting   invasive   HCC phenotypes. 
  [Mm]etastasis  PubMed  
 Arachidonic acid stimulates cell adhesion through a novel p38 MAPK-RhoA signaling pathway that involves heat shock protein 27. Rho GTPases are critical components of cellular signal transduction pathways. Both hyperactivity and overexpression of these proteins have been observed in human cancers and have been implicated as important factors in   metastasis  . We previously showed that dietary n-6 fatty acids increase cancer cell adhesion to extracellular matrix proteins, such as type IV collagen. Here we report that in MDA-MB-435 human melanoma cells, arachidonic acid activates RhoA, and inhibition of RhoA signaling with either C3 exoenzyme or dominant negative Rho blocked arachidonic acid-induced cell adhesion. Inhibition of the Rho kinase (ROCK) with either small molecule inhibitors or ROCK II-specific small interfering RNA (siRNA) blocked the fatty acid-induced adhesion. However, unlike other systems, inhibition of ROCK did not block the activation of p38 mitogen-activated protein kinase (MAPK); instead, Rho activation depended on p38 MAPK activity and the presence of heat shock protein 27 (HSP27), which is phosphorylated downstream of p38 after arachidonic acid treatment. HSP27 associated with p115RhoGEF in fatty acid-treated cells, and this association was blocked when p38 was inhibited. Furthermore, siRNA knockdown of HSP27 blocked the fatty acid-stimulated Rho activity. Expression of dominant negative p115-RhoGEF or p115RhoGEF-specific siRNA inhibited both RhoA activation and adhesion on type IV collagen, whereas a constitutively active p115RhoGEF restored the arachidonic acid stimulation in cells in which the p38 MAPK had been inhibited. These data suggest that n-6 dietary fatty acids stimulate a set of interactions that regulates cell adhesion through RhoA and ROCK II via a p38 MAPK-dependent association of HSP27 and p115RhoGEF. 
 Potential role of heat-shock proteins in giant cell tumors. We investigated the differential expression protein profile of giant cell tumors (GCTs), which can be used to monitor the tumor's recurrence and   metastasis  , to provide preliminary results for further study. We also explored heat-shock protein (HSP) inhibitor that prevents tumors from recurring and migrating. A stable isotope-labeling strategy using isobaric tags for relative and absolute quantitation coupled with two-dimensional liquid chromatography tandem mass spectrometry was used to separate and identify differentially expressed proteins. A total of 467 differentially expressed proteins were identified in GCT tissues. Up to 311 proteins were upregulated, whereas 156 proteins were downregulated in GCT tissues. Three of the differentially expressed HSPs, namely HP90A, HSPB1, and HSPB2, were upregulated. The differentially expressed proteins of GCT tissues will provide a scientific foundation for tumor prognosis, and for further studies exploring HSP inhibitor to prevent tumor recurrence and migration. 
 Proteomic profiling of hepatocellular carcinoma in Chinese cohort reveals heat-shock proteins (Hsp27, Hsp70, GRP78) up-regulation and their associated prognostic values. To facilitate the identification of candidate molecular biomarkers that are linked to the pathogenesis of hepatocellular carcinoma (HCC), we investigated protein-expression profiles of 146 tissue specimens including 67 pairs of tumors and adjacent non-tumors resected from HCC patients as well as 12 normal livers by 2-DE. Among the 1800 spots displayed in the liver proteome, a total of 90 protein species were found to be significantly different between the three groups (P < 0.05). Three of the top candidate markers up-regulated in HCC, with high receiver operating characteristic (ROC) curves, were identified by MS/MS analysis and belonged to the chaperone members: heat-shock protein (Hsp)27, Hsp70 and glucose-regulated protein (GRP)78. Over-expression of these chaperone proteins in HCC tissues was confirmed by Western blotting and immunohistochemistry. In correlation with clinico-pathological parameters, expression of Hsp27 was linked to alpha-fetoprotein level (P = 0.007) whereas up-regulation of GRP78 was associated with tumor venous infiltration (P = 0.035). No significant association of Hsp70 with any pathologic features was observed. The present HCC proteome analysis revealed that in response to the stressful cancerous microenvironment, tumor cells strived to increase the expression of chaperone proteins for cyto-protective function and to enhance tumor growth and   metastasis  . 
 Potential role of heat-shock proteins in giant cell tumors. We investigated the differential expression protein profile of giant cell tumors (GCTs), which can be used to monitor the tumor's recurrence and   metastasis  , to provide preliminary results for further study. We also explored heat-shock protein (HSP) inhibitor that prevents tumors from recurring and migrating. A stable isotope-labeling strategy using isobaric tags for relative and absolute quantitation coupled with two-dimensional liquid chromatography tandem mass spectrometry was used to separate and identify differentially expressed proteins. A total of 467 differentially expressed proteins were identified in GCT tissues. Up to 311 proteins were upregulated, whereas 156 proteins were downregulated in GCT tissues. Three of the differentially expressed HSPs, namely HP90A, HSPB1, and HSPB2, were upregulated. The differentially expressed proteins of GCT tissues will provide a scientific foundation for tumor prognosis, and for further studies exploring HSP inhibitor to prevent tumor recurrence and migration. 
 Arachidonic acid stimulates cell adhesion through a novel p38 MAPK-RhoA signaling pathway that involves heat shock protein 27. Rho GTPases are critical components of cellular signal transduction pathways. Both hyperactivity and overexpression of these proteins have been observed in human cancers and have been implicated as important factors in   metastasis  . We previously showed that dietary n-6 fatty acids increase cancer cell adhesion to extracellular matrix proteins, such as type IV collagen. Here we report that in MDA-MB-435 human melanoma cells, arachidonic acid activates RhoA, and inhibition of RhoA signaling with either C3 exoenzyme or dominant negative Rho blocked arachidonic acid-induced cell adhesion. Inhibition of the Rho kinase (ROCK) with either small molecule inhibitors or ROCK II-specific small interfering RNA (siRNA) blocked the fatty acid-induced adhesion. However, unlike other systems, inhibition of ROCK did not block the activation of p38 mitogen-activated protein kinase (MAPK); instead, Rho activation depended on p38 MAPK activity and the presence of heat shock protein 27 (HSP27), which is phosphorylated downstream of p38 after arachidonic acid treatment. HSP27 associated with p115RhoGEF in fatty acid-treated cells, and this association was blocked when p38 was inhibited. Furthermore, siRNA knockdown of HSP27 blocked the fatty acid-stimulated Rho activity. Expression of dominant negative p115-RhoGEF or p115RhoGEF-specific siRNA inhibited both RhoA activation and adhesion on type IV collagen, whereas a constitutively active p115RhoGEF restored the arachidonic acid stimulation in cells in which the p38 MAPK had been inhibited. These data suggest that n-6 dietary fatty acids stimulate a set of interactions that regulates cell adhesion through RhoA and ROCK II via a p38 MAPK-dependent association of HSP27 and p115RhoGEF. 
 Proteomic profiling of hepatocellular carcinoma in Chinese cohort reveals heat-shock proteins (Hsp27, Hsp70, GRP78) up-regulation and their associated prognostic values. To facilitate the identification of candidate molecular biomarkers that are linked to the pathogenesis of hepatocellular carcinoma (HCC), we investigated protein-expression profiles of 146 tissue specimens including 67 pairs of tumors and adjacent non-tumors resected from HCC patients as well as 12 normal livers by 2-DE. Among the 1800 spots displayed in the liver proteome, a total of 90 protein species were found to be significantly different between the three groups (P < 0.05). Three of the top candidate markers up-regulated in HCC, with high receiver operating characteristic (ROC) curves, were identified by MS/MS analysis and belonged to the chaperone members: heat-shock protein (Hsp)27, Hsp70 and glucose-regulated protein (GRP)78. Over-expression of these chaperone proteins in HCC tissues was confirmed by Western blotting and immunohistochemistry. In correlation with clinico-pathological parameters, expression of Hsp27 was linked to alpha-fetoprotein level (P = 0.007) whereas up-regulation of GRP78 was associated with tumor venous infiltration (P = 0.035). No significant association of Hsp70 with any pathologic features was observed. The present HCC proteome analysis revealed that in response to the stressful cancerous microenvironment, tumor cells strived to increase the expression of chaperone proteins for cyto-protective function and to enhance tumor growth and   metastasis  . 
 [Expression of HSP70/HSP27 protein in residual lesion after NPC radiotherapy]. To analyze HSP70/HSP27 protein expression in the nasopharyngeal carcinoma (NPC) primary tissues and the residual lesion, and to explore its predictive value.Immunohistochemical experiment was performed to detect the expression of HSP70 and HSP27 in 58 NPC primary tissues: 28 were in the experimental group with the local residual lesion and 30 in the control group without recurring and   metastasis   within 5 years by conventional radiotherapy.The positive expression of HSP70 and HSP27 in the experimental group was 92.9%(26/28) and 53.6%(15/28), while that in the control group was 53.3%(16/30) and 53.3%(16/30),respectively. There was significant difference in the 2 groups. The common positive expression of HSP70 and HSP27 between the 2 groups had significant difference, 50.0% (14/28) in the experimental group and 16.7% (5/30) in the control group, respectively. There was no significant difference in the negative ratio of HSP70 and HSP27 common expression between the 2 groups, 3.6% (1/28) in the experimental group and 10.0% (3/30) in the control group, respectively.HSP70 may have an important role in the radioresistance of NPC, and may predict the residual lesion after radiotherapy. 
 Involvement of protein kinase C beta-extracellular signal-regulating kinase 1/2/p38 mitogen-activated protein kinase-heat shock protein 27 activation in hepatocellular carcinoma cell motility and invasion. To understand the molecular mechanism that underlies the role of various prominent signal pathways in hepatocellular carcinoma (HCC)   metastasis  , a human signal transduction oligonucleotide microarray analysis was carried out in cultured HCC cell models with increasing spontaneous metastatic potential (MHCC97L, MHCC97H, and HCCLM6). The results revealed that the mitogen-activated protein kinase (MAPK) pathway is the prominently upregulated pathway in HCC   metastasis  . Further study showed that basal phosphorylated levels of extracellular signal-regulating kinase (ERK)(1/2) and p38 MAPK consecutively increased from MHCC97L to MHCC97H to HCCLM6 cells, but not c-Jun N-terminal kinase. The phosphorylation of ERK(1/2) and p38 MAPK was regulated by upregulated protein kinase C beta (PKC beta) in HCC cells through the integrated use of PKC beta RNA interference, the PKC beta specific inhibitor enzastaurin and a PKC activator phorbol-12-myristate-13-acetate. Heat shock protein 27 (HSP27) was also verified as a downstream common activated protein of PKC beta-ERK(1/2) and PKC beta-p38 MAPK. In vitro migration and invasion assay further showed that the depletion of PKC beta or inhibition of PKC beta activation effectively decreased HCC cell motility and invasion. Moreover, the motility and invasion of phorbol-12-myristate-13-acetate-stimulated PKC beta-mediated HCC cells was significantly negated by an ERK inhibitor, 1.4-diamino-2.3-dicyano-1.4-bis[2-aminophenylthio] butadiene, or a p38 MAPK inhibitor, 4-(4-Fluorophenyl)-2-(4-methylsulfinylphenyl)-5-(4-pyridyl)1H-imidazole. It also showed that HSP27 is critical in PKC beta-mediated HCC cell motility and invasion. Taken together, this study reveals the important role of this PKC beta-ERK(1/2)/p38MAPK-HSP27 pathway, which was verified for the first time, in modulating HCC cell motility and invasion. 
 MAPKAPK2 and HSP27 are downstream effectors of p38 MAP kinase-mediated matrix metalloproteinase type 2 activation and cell invasion in human prostate cancer. Although cell invasion is a necessary early step in cancer   metastasis  , its regulation is not well understood. We have previously shown, in human prostate cancer, that transforming growth factor beta (TGFbeta)-mediated increases in cell invasion are dependent upon activation of the serine/threonine kinase, p38 MAP kinase. In the current study, downstream effectors of p38 MAP kinase were sought by first screening for proteins phosphorylated after TGFbeta treatment, only in the absence of chemical inhibitors of p38 MAP kinase. This led us to investigate mitogen-activated protein kinase-activated protein kinase 2 (MAPKAPK2), a known substrate of p38 MAP kinase, as well as heat-shock protein 27 (HSP27), a known substrate of MAPKAPK2, in both PC3 and PC3-M human prostate cells. After transient transfection, wild-type MAPKAPK2 and HSP27 both increased TGFbeta-mediated matrix metalloproteinase type 2 (MMP-2) activity, as well as cell invasion, which in turn was inhibited by SB203580, an inhibitor of p38 MAP kinase. Conversely, dominant-negative MAPKAPK2 blocked phosphorylation of HSP27, whereas dominant-negative MAPKAPK2 or mutant, non-phosphorylateable, HSP27 each blocked TGFbeta-mediated increases in MMP-2, as well as cell invasion. Similarly, knock down of MAPKAPK2, HSP27 or both together, by siRNA, also blocked TGFbeta-mediated cell invasion. This study demonstrates that both MAPKAPK2 and HSP27 are necessary for TGFbeta-mediated increases in MMP-2 and cell invasion in human prostate cancer. 
 Heat shock protein 27 regulates human prostate cancer cell motility and metastatic progression. Prostate cancer (PCa) is the most common form of cancer in American men. Mortality from PCa is caused by the movement of cancer cells from the primary organ to form metastatic tumors at distant sites. Heat shock protein 27 (HSP27) is known to increase human PCa cell invasion and its overexpression is associated with metastatic disease. The role of HSP27 in driving PCa cell movement from the prostate to distant metastatic sites is unknown. Increased HSP27 expression increased   metastasis   as well as primary tumor mass. In vitro studies further examined the mechanism of HSP27-induced metastatic behavior. HSP27 did not affect cell detachment, adhesion, or migration, but did increase cell invasion. Cell invasion was dependent upon matrix metalloproteinase 2 (MMP-2), whose expression was increased by HSP27. In vivo, HSP27 induced commensurate changes in MMP-2 expression in tumors. These findings demonstrate that HSP27 drives metastatic spread of cancer cells from the prostate to distant sites, does so across a continuum of expression levels, and identifies HSP27-driven increases in MMP-2 expression as functionally relevant. These findings add to prior studies demonstrating that HSP27 increases PCa cell motility, growth and survival. Together, they demonstrate that HSP27 plays an important role in PCa progression. 
 Hsp27 participates in the maintenance of breast cancer stem cells through regulation of epithelial-mesenchymal transition and nuclear factor-κB. Heat shock proteins (HSPs) are normally induced under environmental stress to serve as chaperones for maintenance of correct protein folding but they are often overexpressed in many cancers, including breast cancer. The expression of Hsp27, an ATP-independent small HSP, is associated with cell migration and drug resistance of breast cancer cells. Breast cancer stem cells (BCSCs) have been identified as a subpopulation of breast cancer cells with markers of CD24-CD44+ or high intracellular aldehyde dehydrogenase activity (ALDH+) and proved to be associated with radiation resistance and   metastasi  s. However, the involvement of Hsp27 in the maintenance of BCSC is largely unknown.Mitogen-activated protein kinase antibody array and Western blot were used to discover the expression of Hsp27 and its phosphorylation in ALDH + BCSCs. To study the involvement of Hsp27 in BCSC biology, siRNA mediated gene silencing and quercetin treatment were used to inhibit Hsp27 expression and the characters of BCSCs, which include ALDH+ population, mammosphere formation and cell migration, were analyzed simultaneously. The tumorigenicity of breast cancer cells after knockdown of Hsp27 was analyzed by xenograftment assay in NOD/SCID mice. The epithelial-mesenchymal transition (EMT) of breast cancer cells was analyzed by wound-healing assay and Western blot of snail, vimentin and E-cadherin expression. The activation of nuclear factor kappa B (NF-κB) was analyzed by luciferase-based reporter assay and nuclear translocation.Hsp27 and its phosphorylation were increased in ALDH+ BCSCs in comparison with ALDH- non-BCSCs. Knockdown of Hsp27 in breast cancer cells decreased characters of BCSCs, such as ALDH+ population, mammosphere formation and cell migration. In addition, the in vivo CSC frequency could be diminished in Hsp27 knockdown breast cancer cells. The inhibitory effects could also be observed in cells treated with quercetin, a plant flavonoid inhibitor of Hsp27, and it could be reversed by overexpression of Hsp27. Knockdown of Hsp27 also suppressed EMT signatures, such as decreasing the expression of snail and vimentin and increasing the expression of E-cadherin. Furthermore, knockdown of Hsp27 decreased the nuclear translocation as well as the activity of NF-κB in ALDH + BCSCs, which resulted from increasing expression of IκBα. Restored activation of NF-κB by knockdown of IκBα could reverse the inhibitory effect of Hsp27 siRNA in suppression of ALDH+ cells.Our data suggest that Hsp27 regulates the EMT process and NF-κB activity to contribute the maintenance of BCSCs. Targeting Hsp27 may be considered as a novel strategy in breast cancer therapy. 
 miR-17-5p Promotes migration of human hepatocellular carcinoma cells through the p38 mitogen-activated protein kinase-heat shock protein 27 pathway. miR-17-5p is overexpressed in hepatocellular carcinoma (HCC), but the specific regulatory mechanisms of miR-17-5p in HCC remain unknown. We investigated the molecular basis of miR-17-5p as an oncogene in human HCC cell lines. Our in vivo and in vitro data indicate that miR-17-5p up-regulates the migration and proliferation of HCC cells. Interestingly, proteomic and western blotting assays revealed that miR-17-5p significantly activates the p38 mitogen-activated protein kinase MAPK pathway and increases the phosphorylation of heat shock protein 27 (HSP27). Our results also suggest that E2F1-dependent down-regulation of Wip1 regulates miR-17-5p-p38-HSP27 signaling. Furthermore, suppression of HSP27 expression by small interfering RNA or the p38 MAPK pathway-specific inhibitor SB203580 decreases the migration of HCC cells overexpressing miR-17-5p but does not reduce their proliferation. Finally, we show that miR-17-5p expression correlates well with HSP27 status in primary human HCC tissues with   metastasis  .Our findings suggest that the p38 MAPK pathway plays a crucial role in miR-17-5p-induced phosphorylation of HSP27 and, as a consequence, phosphorylated HSP27 enhances the migration of HCC cells. Our data highlight an important role of miR-17-5p in the proliferation and migration of HCC cells and support the potential application of miR-17-5p in HCC therapy. 
 Heat shock protein 27 and gross cystic disease fluid protein 15 play critical roles in molecular apocrine breast cancer. Molecular apocrine breast cancer (MABC) has a distinct hormonal profile, being estrogen receptor (ER) and progesterone receptor (PR) negative but androgen receptor (AR) positive. The clinical significance of MABC and its relative variables have not been absolutely clarified and remain to be determined. Five hundred cases of invasive breast carcinoma were randomly selected in this study, including 158 MABC cases and 342 nonMABC cases. Expression of ER, PR, epidermal growth factor receptor 2 (HER2), Ki67, AR, gross cystic disease fluid protein 15 (GCDFP15), and heat shock protein 27 (HSP27) were analyzed by immunohistochemistry. Differences of continuous variables between MABC and nonMABC subgroups were evaluated by the chi-square test. The Kaplan-Meier method was performed to evaluate disease-free survival (DFS) and overall survival (OS). The MABC subgroup had higher histological grade, bigger tumor size, more lymph node   metastasis  , and higher pTNM stage than the nonMABC subgroup (P < 0.05), and patients with MABC had poorer prognosis than those of the nonMABC subgroup (P < 0.05). Both GCDFP15 and HSP27 were expressed differently in the MABC and nonMABC subgroups (P < 0.05). Furthermore, in the MABC subgroup, positive HSP27 expression indicated higher risk of recurrence (P < 0.05) and positive GCDFP15 expression was also a poor marker for patient outcome (P < 0.05). MABC patients with HSP27 and GCDFP15 co-expression had worse outcome (P < 0.05). Our data suggested that MABC had a high risk of recurrence. Positive expression of both GCDFP15 and HSP27 were correlated with MABC malignancy. Targeting AR and HSP27 at the same time might offer a useful strategy to MABC. 
 Hsp27 regulates EGF/β-catenin mediated epithelial to mesenchymal transition in prostate cancer. Increased expression of the molecular chaperone Hsp27 is associated with the progression of prostate cancer (PCa) to castration-resistant disease, which is lethal due to metastatic spread of the prostate tumor.   Metastasi  s requires epithelial to mesenchymal transition (EMT), which endows cancer cells with the ability to disseminate from the primary tumor and colonize new tissue sites. A wide variety of secreted factors promote EMT, and while overexpression and constitutive activation of epidermal growth factor (EGF) signaling is associated with poor prognosis of PCa, a precise role of EGF in PCa progression to   metastasi  s remains unclear. Here, we show that Hsp27 is required for EGF-induced cell migration, invasion and MMPs activity as well as the expression of EMT markers including Fibronectin, Vimentin and Slug with concomitant decrease of E-cadherin. Mechanistically, we found that Hsp27 is required for EGF-induced AKT and GSK3β phosphorylation and β-catenin nuclear translocation. Moreover, silencing Hsp27 decreases EGF dependent phosphorylation of β-catenin on tyrosine 142 and 654, enhances β-catenin ubiquitination and degradation, prevents β-catenin nuclear translocation and binding to the Slug promoter. These data suggest that Hsp27 is required for EGF-mediated EMT via modulation of the β-catenin/Slug signaling pathway. Together, our findings underscore the importance of Hsp27 in EGF induced EMT in PCa and highlight the use of Hsp27 knockdown as a useful strategy for patients with advanced disease. 
 Heat shock protein 27: a potential biomarker of peritoneal   metastasis   in epithelial ovarian cancer? Ovarian cancer is the major gynaecologic malignancy and the leading cause of death in gynaecological cancer. Heat shock proteins (HSPs) are highly expressed in many malignant cancers and involved in   metastasis   including ovarian cancer. The early detection of peritoneal metastases in epithelial ovarian cancer may be more important in clinical care. HSP27, a small heat shock protein, is correlated with peritoneal metastases in epithelial ovarian cancer tissues. In this study, we investigated whether the levels of total HSP27 were detectable in serum and whether it could be a predictive biomarker for peritoneal metastases in epithelial ovarian cancer. Serum samples from 48 patients with epithelial ovarian cancer, 35 patients with benign ovarian tumours and 24 healthy women were included in this study. The serum levels of total HSP27 were measured by enzyme-linked immunosorbent assay (ELISA). There was no difference in the serum levels of total HSP27 between women with benign ovarian tumours and healthy women. However, the serum levels of total HSP27 were significantly increased in patients with epithelial ovarian cancer. The increased serum levels of total HSP27 were only seen in patients with peritoneal metastases. Furthermore, increased serum levels of total HSP27 were significantly reduced after the combination chemotherapies in patients with peritoneal metastases. These data suggest that circulating HSP27 levels were increased in epithelial ovarian cancer and correlated with peritoneal metastases. The measurement of serum HSP27 levels may be used as a potential additional indicator for peritoneal metastases in epithelial ovarian cancer and response to treatment. 
 Increased expression of heat shock protein 27 correlates with peritoneal   metastasis   in epithelial ovarian cancer. Ovarian cancer is the third most common gynecologic malignancy and the leading cause of death in gynecological cancer. Although the 5-year survival rate is increasing, peritoneal   metastasis   of ovarian cancer is still a problem because of no potential predictor. Heat shock proteins (HSPs) are a class of functionally related proteins that are highly expressed in many malignant cancers. Previous studies suggest high levels of HSP27 present in the serum of patients with ovarian cancer. In this study, we investigated whether the expression of HSP27 in epithelial ovarian cancer tissue was associated with peritoneal   metastasis   and whether HSP27 could be used as a potential predictor of peritoneal   metastasis   in epithelial ovarian cancer. Tissues from epithelial ovarian cancer with or without peritoneal   metastasis   were collected and the levels of HSP27 messenger RNA and protein determined by real-time polymerase chain reaction and Western blotting. Immunohistochemistry was used to determine the subcellular localization of HSP27. Immunohistochemistry images showed that HSP27 was highly expressed in the cytoplasm of epithelial cancer cells with peritoneal   metastasis  . Messenger RNA and protein levels of HSP27 were significantly increased in epithelial ovarian cancer with peritoneal   metastasis   compared with epithelial ovarian cancer without peritoneal   metastasis  . Higher expression of HSP27 correlated with poor clinical outcome. These data suggest that higher level of HSP27 was associated with peritoneal   metastasis   in epithelial ovarian cancer. Heat shock protein 27 may be a useful prognostic marker of poor survival and may provide a basis for the development of molecular therapeutics modulating this survival pathway. 
 Hsp27 regulates epithelial mesenchymal transition,   metastasis  , and circulating tumor cells in prostate cancer. Defining the mechanisms underlying metastatic progression of prostate cancer may lead to insights into how to decrease morbidity and mortality in this disease. An important determinant of   metastasis   is epithelial-to-mesenchymal transition (EMT), and the mechanisms that control the process of EMT in cancer cells are still emerging. Here, we report that the molecular chaperone Hsp27 (HSPB1) drives EMT in prostate cancer, whereas its attenuation reverses EMT and decreases cell migration, invasion, and matrix metalloproteinase activity. Mechanistically, silencing Hsp27 decreased IL-6-dependent STAT3 phosphorylation, nuclear translocation, and STAT3 binding to the Twist promoter, suggesting that Hsp27 is required for IL-6-mediated EMT via modulation of STAT3/Twist signaling. We observed a correlation between Hsp27 and Twist in patients with prostate cancer, with Hsp27 and Twist expression each elevated in high-grade prostate cancer tumors. Hsp27 inhibition by OGX-427, an antisense therapy currently in phase II trials, reduced tumor   metastasis   in a murine model of prostate cancer. More importantly, OGX-427 treatment decreased the number of circulating tumor cells in patients with metastatic castration-resistant prostate cancer in a phase I clinical trial. Overall, this study defines Hsp27 as a critical regulator of IL-6-dependent and IL-6-independent EMT, validating this chaperone as a therapeutic target to treat metastatic prostate cancer. 
 The overexpression of 14-3-3ζ and Hsp27 promotes non–small cell lung cancer progression. The 14-3-3ζ protein has been identified as a putative oncoprotein in several cancers, including non–small cell lung cancer (NSCLC). However, the mechanisms underlying its functions have not been well defined.Proteins that interact with 14-3-3ζ were identified through coimmunoprecipitation and mass spectrometry in NSCLC cells. The interaction of 14-3-3ζ with these molecular partners and their roles in the invasiven  ess and me  tastasis of NSCLC cells were assayed through specific disruptions in the 14-3-3ζ signaling network. In addition, the clinical implications of this 14-3-3ζ complex were examined in samples from patients with NSCLC.Among the identified proteins that interacted with 14-3-3ζ, there were 230 proteins in 95-D cells, 181 proteins in 95-C cells, and 203 proteins in A549 cells; and 16 interacting proteins were identified that overlapped between all cell lines. Further studies revealed 14-3-3ζ complexes within the heat shock protein 27 (Hsp27) protein and demonstrated that the interference of Hsp27 or 14-3-3ζ inhibited the   invasion a  nd metastasis of NSCLC cells. The invasive and metastatic capabilities of cells with both Hsp27 and 14-3-3ζ interference could be completely restored only by Hsp27 and 14-3-3ζ complementary DNA transfection and not by either agent alone. Clinically, the postoperative 5-year overall survival (OS) in patients who had high expression of both 14-3-3ζ and Hsp27 was significantly lower than the 5-year OS in patients who had low expression of both 14-3-3ζ and Hsp27 (26.5% vs 59.7%, respectively). Multivariate analysis revealed that the combined expression of 14-3-3ζ and Hsp27 was an independent prognostic indicator of OS(P = .036).The current data suggest that the combined expression of 14-3-3ζ and Hsp27 may be a biomarker for predicting survival in patients with NSCLC, and this combination may have potential as a therapeutic target for NSCLC. 
 A Targetable Molecular Chaperone Hsp27 Confers Aggressiveness in Hepatocellular Carcinoma. Heat shock protein 27 (Hsp27) is an ATP-independent molecular chaperone and confers survival advantages and resistance to cancer cells under stress conditions. The effects and molecular mechanisms of Hsp27 in HCC invasion and   metastasis   are still unclear. In this study, hepatocellular carcinoma (HCC) tissue array (n = 167) was used to investigate the expression and prognostic relevance of Hsp27 in HCC patients. HCC patients with high expression of Hsp27 exhibited poor prognosis. Overexpression of Hsp27 led to the forced invasion of HCC cells, whereas silencing Hsp27 attenuated invasion and   metastasis   of HCC cells in vitro and in vivo. We revealed that Hsp27 activated Akt signaling, which in turn promoted MMP2 and ITGA7 expression and HCC   metastasis  . We further observed that targeting Hsp27 using OGX-427 obviously suppressed HCC   metastasis   in two metastatic models. These findings indicate that Hsp27 is a useful predictive factor for prognosis of HCC and it facilitates HCC   metastasis   through Akt signaling. Targeting Hsp27 with OGX-427 may represent an attractive therapeutic option for suppressing HCC   metastasis  . 
 Drug-induced Modulation of Heat Shock Protein HSPB1 in an Ovarian Cancer Cell Model. Heat-shock protein HSPB1 (alternative name HSP27) plays a pivotal role in cell survival pathways, apoptosis,   metastasis   and has been frequently linked to treatment resistance in ovarian cancer (OC) and other malignancies. Characteristic HSPB1 induction in different solid tumors is often caused by cytotoxic agents.An in vitro OC cell model system was established to characterize resistance mechanisms during chemotherapy. Human OC cell lines OVCAR-3, SK-OV-3 and TOV-21G were treated with paclitaxel or carboplatin. Cellular growth was analyzed by cell counting. Intra- and extracellular HSPB1 concentrations were assessed by western blot and enzyme-linked immunosorbent assays.Incubation with paclitaxel, and with carboplatin significantly reduced cell growth without a definitive increase of intracellular HSPB1 expression. HSPB1 demonstrated drug-inducible secretion into the extracellular compartment.Despite its current lack of analysis in patient samples, serum soluble HSPB1 may function as a specific biomarker for monitoring response to chemotherapy in patients with OC. 
 Increased expression of HSP27 inhibits invasion and   metastasis   in human esophageal squamous cell carcinoma. Previous studies have indicated that heat shock protein 27 (HSP27) had high correlation with the development and progression in several tumors. However, the roles of HSP27 in esophageal squamous cell carcinoma (ESCC) were uncertain. The aim in this study is to investigate the potential roles of HSP27 in the   metastasis   of ESCC. The expression of HSP27 in ESCC tissues and four human esophageal cancer cell lines were examined by immunohistochemistry and Western blotting, respectively. Wound healing assays, transwell assays, and in vivo assays were used to identify the differences of   metastasis   potential between normal and HSP27 overexpressed cells. HSP27 expression was downregulated in cancer tissue compared to the matched normal tissue. And the positive staining was mainly located in the cytoplasm. Statistical analyses showed that the expression of HSP27 in ESCC was significantly correlated with the tumor differentiation (P = 0.023), the patient's TNM stage (P = 0.013), lymph   metastasis   (P = 0.020), and distant   metastasis   (P = 0.017). HSP27 expression was significantly lower in highly metastatic cells than the less ones. The metastatic potentials of EC9706-H and EC109-H cells were higher than EC9706-L and EC109-L cells. In vitro and in vivo assays showed that overexpression of HSP27 in highly metastatic cells dramatically decreased their metastatic capacity. This study indicated that the expression level of HSP27 may be inversely correlated with the   metastasis   behavior of ESCC, and HSP27 may play an important role in this progression. HSP27 may be a potential molecular target for the therapy and prognosis of patients with ESCC. 
 HSP27 is required for invasion and   metastasis   triggered by hepatocyte growth factor. The hepatocyte growth factor (HGF) also known as scatter factor activates cancer cell invasion and   metastasis  . We show that in ovarian cancer cells HGF induced the phosphorylation of the small heat shock protein of 27 kDa (HSP27) by activating the p38MAPK. HSP27 is increased in many cancers at advanced stage including ovarian cancer and associated with cancer resistance to therapy and poor patients' survival. The phosphorylation of HSP27 regulates both its chaperone activity and its control of cytoskeletal stability. We show that HSP27 was necessary for the remodeling of actin filaments induced by HGF and that motility in vitro depended on the p38MAPK-MK2 axis. In vivo, HSP27 silencing impaired the ability of the highly metastatic, HGF-secreting ovarian cancer cells to give rise to spontaneous metastases. This was due to defective motility across the vessel wall and reduced growth. Indeed, HSP27 silencing impaired the ability of circulating ovarian cancer cells to home to the lungs and to form experimental hematogenous metastases and the capability of cancer cells to grow as subcutaneous xenografts. Moreover, HSP27 suppression resulted in the sensitization of xenografts to low doses of the chemotherapeutic paclitaxel, likely because HSP27 protected microtubules from bundling caused by the drug. Altogether, these data show that the HSP27 is required for the proinvasive and prometastatic activity of HGF and suggest that HSP27 might be not only a marker of progression of ovarian cancer, but also a suitable target for therapy. 
 HSP27 expression levels are associated with the sensitivity of hepatocellular carcinoma cells to 17-allylamino-17-demethoxygeldanamycin. AIMS, MATERIALS & METHODS: As heat-shock proteins are associated with tumor proliferation, differentiation, invasion and   metastasis  , we investigated whether targeting Hsp90 with the geldanamycin derivative 17-allylamino-17-demethoxygeldanamycin (17AAG) can inhibit the viability of hepatocellular carcinoma cell lines with various levels of metastatic potential. In addition, we investigated whether the use of Hsp27-siRNA can decrease resistance to 17AAG.Although 17AAG upregulated the expression of heat-shock proteins, it did not affect the expression of Hsp90 client proteins in normal hepatocytes. Hsp90 inhibition by 17AAG degraded its client proteins in both low- and high-metastatic potential cell lines. siRNA inhibited Hsp27 expression in cell lines and improved the sensitivity of 17AAG.17AAG inhibited the viability of hepatocellular carcinoma cells by degrading Hsp90 client proteins. The sensitivity of cells to 17AAG is associated with the level of Hsp27 expression. 
 Silencing heat shock protein 27 decreases metastatic behavior of human head and neck squamous cell cancer cells in vitro. The small heat shock protein 27 (Hsp27) is a molecular chaperone that is involved in a variety of cellular functions in cancer cells. The purpose of this research is to study Hsp27 in vitro metastatic behaviors of head and neck squamous cell carcinoma cells (HNSCC). The expression of Hsp27 in primary and metastatic cell lines derived from the primary HNSCC and a synchronous lymph node   metastasis   in the same patient was determined using real-time PCR and Western blotting. Proliferation of the primary and metastatic HNSCC cell lines was evaluated using the MTS proliferation assay. Metastatic behavior was assessed using migration and invasion assays. SiRNA knockdown of Hsp27 was performed in the highly migratory metastatic HNSCC cell line. MTS assays showed that the primary (UM-SCC-22A) and metastatic (UM-SCC-22B) HNSCC have similar proliferation rates. However, UM-SCC-22B derived from the   metastasis   showed 2.3- to 3.6-fold higher migration ability and 2-fold higher invasion ability than UM-SCC-22A. Real-time PCR demonstrated that Hsp27 mRNA is 22.4-fold higher in metastatic UM-SCC-22B than primary UM-SCC-22A. Similarly, Western blotting showed that Hsp27 is rarely detectable in UM-SCC-22A whereas UM-SCC-22B expresses a 25-fold higher level of Hsp27 protein. SiRNA-mediated knockdown of Hsp27 in UM-SCC-22B reduced Hsp27 mRNA expression by nearly 6-fold and protein expression by 23-fold. Furthermore, siRNA knockdown of Hsp27 decreased metastatic behaviors of UM-SCC-22B by 3- to 4-fold in migration and 2-fold in cell invasion reducing cell invasion and migration to levels similar to the primary HNSCC UM-SCC-22A. These data indicate that Hsp27 may regulate metastatic potential of HNSCC cancer cells. Targeting Hsp27 may decrease   metastasis   in head and neck squamous cell cancer cells. 
 Relationship between the expression of phosphorylated heat shock protein beta-1 with lymph node metastases of breast cancer. Heat shock protein beta-1 (HspB1) is a chaperone of the sHsp (small heat shock protein). The common functions of sHsps are chaperone activity, inhibition of apoptosis, regulation of cell development, and cell differentiation, take part in signal transduction.To study the intracellular localization of phosphorylated features and non-phosphorylated forms of HspB1 in primary breast cancer cells and to evaluate their relationship with regional lymphatic   metastasis  .Tumor biopsies of breast tissue were collected from 100 patients with a confirmed diagnosis of invasive carcinoma, nonspecific type, between the ages of 31-80 years. Immunohistochemistry was used to determine the intracellular localization of phosphorylated and non-phosphorylated forms of HspB1.The result of this study showed that biopsies from patients with lymph node   metastasis   exhibited significantly higher levels of phosphorylated forms of HspB1 in the nucleus and cytoplasm compared with the group without lymph node   metastasis  . Analysis showed that the expression of phosphorylated forms of the chaperone HspB1 correlates with the amount and percentage of lymph node metastases affected.The nuclear expression of phosphorylated and non-phosphorylated forms of the chaperone HspB1 is a marker of tumor cells associated with lymphatic   metastasis   of breast cancer. 
 Functional state of the Hsp27 chaperone as a molecular marker of an unfavorable course of larynx cancer. The small heat shock protein 27 kDA (Hsp27) acts as an ATP-independent chaperone in protein folding, but is also implicated in architecture of the cytoskeleton, cell migration, metabolism, cell survival, growth/differentiation, mRNA stabilization, and tumor progression.To study the intracellular localization of phosphorylated and non-phosphorylated forms of Hsp27 in squamous cell carcinoma of the larynx (SCCL) and to evaluate their relationship with regional lymphatic   metastasis   and overall five-year survival.Tumor biopsies of larynx tissue were collected from 50 patients who were between the ages of 30 to 80 years and had a confirmed diagnosis of squamous cell carcinoma of the larynx. Immunohistochemistry was used to determine the intracellular localization of the phosphorylated and non-phosphorylated forms of Hsp27.The study revealed that the Hsp27 chaperone was expressed in both the cytoplasm and the nucleus of tumor cells in SCCL. The biopsies of patients with lymph node metastases showed significantly higher expression of the phosphorylated and unphosphorylated forms of Hsp27 in the nucleus compared to those of patients without lymph node metastases. At the same time, the cytoplasmic expression of Hsp27 in these patients did not differ statistically. Analysis of the overall five-year survival rates showed that negative Hsp27 expression in the nucleus of tumor cells is associated with the survival rate of patients with SCCL.The nuclear expression of phosphorylated and unphosphorylated forms of Hsp27 is a molecular marker of unfavorable squamous cell carcinoma of the larynx associated with lymphogenous   metastasis   and decreased total five-year survival. 
  GeneRIFs  
 The nuclear expression of phosphorylated and unphosphorylated forms of Hsp27 is a molecular marker of unfavorable squamous cell carcinoma of the larynx associated with lymphogenous   metastasis   and decreased total five-year survival. 
 Overexpression of Hsp27 led to the forced invasion of hepatocellular carcinoma cells, whereas silencing Hsp27 attenuated invasion and   metastasis   of hepatocellular carcinoma cells in vitro and in vivo. 
 The nuclear expression of phosphorylated and non-phosphorylated forms of the chaperone HspB1 is a marker of tumor cells associated with lymphatic   metastasis   of breast cancer. 
 This study indicated that the expression level of HSP27 may be inversely correlated with the   metastasis   behavior of esophageal squamous cell carcinoma , and HSP27 may play an important role in this progression 
 High HSP27 expression is associated with drug resistance, invasion and   metastasis   in ovarian cancer. 
 Data suggest that higher levels of HSP27 in tumor tissue from patients with epithelial ovarian cancer are associated with peritoneal   metastasis  ; thus, tumor HSP27 level may be useful prognostic marker of poor survival. 
  [Cc]ell [Ii]nvasion  PubMed  
 MAPKAPK2 and HSP27 are downstream effectors of p38 MAP kinase-mediated matrix metalloproteinase type 2 activation and   cell invasion   in human prostate cancer. Although   cell invasion   is a necessary early step in cancer metastasis, its regulation is not well understood. We have previously shown, in human prostate cancer, that transforming growth factor beta (TGFbeta)-mediated increases in   cell invasion   are dependent upon activation of the serine/threonine kinase, p38 MAP kinase. In the current study, downstream effectors of p38 MAP kinase were sought by first screening for proteins phosphorylated after TGFbeta treatment, only in the absence of chemical inhibitors of p38 MAP kinase. This led us to investigate mitogen-activated protein kinase-activated protein kinase 2 (MAPKAPK2), a known substrate of p38 MAP kinase, as well as heat-shock protein 27 (HSP27), a known substrate of MAPKAPK2, in both PC3 and PC3-M human prostate cells. After transient transfection, wild-type MAPKAPK2 and HSP27 both increased TGFbeta-mediated matrix metalloproteinase type 2 (MMP-2) activity, as well as   cell invasion  , which in turn was inhibited by SB203580, an inhibitor of p38 MAP kinase. Conversely, dominant-negative MAPKAPK2 blocked phosphorylation of HSP27, whereas dominant-negative MAPKAPK2 or mutant, non-phosphorylateable, HSP27 each blocked TGFbeta-mediated increases in MMP-2, as well as   cell invasion  . Similarly, knock down of MAPKAPK2, HSP27 or both together, by siRNA, also blocked TGFbeta-mediated   cell invasion  . This study demonstrates that both MAPKAPK2 and HSP27 are necessary for TGFbeta-mediated increases in MMP-2 and   cell invasion   in human prostate cancer. 
 Heat shock protein 27 regulates human prostate cancer cell motility and metastatic progression. Prostate cancer (PCa) is the most common form of cancer in American men. Mortality from PCa is caused by the movement of cancer cells from the primary organ to form metastatic tumors at distant sites. Heat shock protein 27 (HSP27) is known to increase human PCa   cell invasion   and its overexpression is associated with metastatic disease. The role of HSP27 in driving PCa cell movement from the prostate to distant metastatic sites is unknown. Increased HSP27 expression increased metastasis as well as primary tumor mass. In vitro studies further examined the mechanism of HSP27-induced metastatic behavior. HSP27 did not affect cell detachment, adhesion, or migration, but did increase   cell invasion  .   Cell invasion   was dependent upon matrix metalloproteinase 2 (MMP-2), whose expression was increased by HSP27. In vivo, HSP27 induced commensurate changes in MMP-2 expression in tumors. These findings demonstrate that HSP27 drives metastatic spread of cancer cells from the prostate to distant sites, does so across a continuum of expression levels, and identifies HSP27-driven increases in MMP-2 expression as functionally relevant. These findings add to prior studies demonstrating that HSP27 increases PCa cell motility, growth and survival. Together, they demonstrate that HSP27 plays an important role in PCa progression. 
 HSP27 is required for invasion and metastasis triggered by hepatocyte growth factor. The hepatocyte growth factor (HGF) also known as scatter factor activates cancer   cell invasion   and metastasis. We show that in ovarian cancer cells HGF induced the phosphorylation of the small heat shock protein of 27 kDa (HSP27) by activating the p38MAPK. HSP27 is increased in many cancers at advanced stage including ovarian cancer and associated with cancer resistance to therapy and poor patients' survival. The phosphorylation of HSP27 regulates both its chaperone activity and its control of cytoskeletal stability. We show that HSP27 was necessary for the remodeling of actin filaments induced by HGF and that motility in vitro depended on the p38MAPK-MK2 axis. In vivo, HSP27 silencing impaired the ability of the highly metastatic, HGF-secreting ovarian cancer cells to give rise to spontaneous metastases. This was due to defective motility across the vessel wall and reduced growth. Indeed, HSP27 silencing impaired the ability of circulating ovarian cancer cells to home to the lungs and to form experimental hematogenous metastases and the capability of cancer cells to grow as subcutaneous xenografts. Moreover, HSP27 suppression resulted in the sensitization of xenografts to low doses of the chemotherapeutic paclitaxel, likely because HSP27 protected microtubules from bundling caused by the drug. Altogether, these data show that the HSP27 is required for the proinvasive and prometastatic activity of HGF and suggest that HSP27 might be not only a marker of progression of ovarian cancer, but also a suitable target for therapy. 
 Silencing heat shock protein 27 decreases metastatic behavior of human head and neck squamous cell cancer cells in vitro. The small heat shock protein 27 (Hsp27) is a molecular chaperone that is involved in a variety of cellular functions in cancer cells. The purpose of this research is to study Hsp27 in vitro metastatic behaviors of head and neck squamous cell carcinoma cells (HNSCC). The expression of Hsp27 in primary and metastatic cell lines derived from the primary HNSCC and a synchronous lymph node metastasis in the same patient was determined using real-time PCR and Western blotting. Proliferation of the primary and metastatic HNSCC cell lines was evaluated using the MTS proliferation assay. Metastatic behavior was assessed using migration and invasion assays. SiRNA knockdown of Hsp27 was performed in the highly migratory metastatic HNSCC cell line. MTS assays showed that the primary (UM-SCC-22A) and metastatic (UM-SCC-22B) HNSCC have similar proliferation rates. However, UM-SCC-22B derived from the metastasis showed 2.3- to 3.6-fold higher migration ability and 2-fold higher invasion ability than UM-SCC-22A. Real-time PCR demonstrated that Hsp27 mRNA is 22.4-fold higher in metastatic UM-SCC-22B than primary UM-SCC-22A. Similarly, Western blotting showed that Hsp27 is rarely detectable in UM-SCC-22A whereas UM-SCC-22B expresses a 25-fold higher level of Hsp27 protein. SiRNA-mediated knockdown of Hsp27 in UM-SCC-22B reduced Hsp27 mRNA expression by nearly 6-fold and protein expression by 23-fold. Furthermore, siRNA knockdown of Hsp27 decreased metastatic behaviors of UM-SCC-22B by 3- to 4-fold in migration and 2-fold in   cell invasion   reducing   cell invasion   and migration to levels similar to the primary HNSCC UM-SCC-22A. These data indicate that Hsp27 may regulate metastatic potential of HNSCC cancer cells. Targeting Hsp27 may decrease metastasis in head and neck squamous cell cancer cells. 
  GeneRIFs  
 Data show that siRNA knockdown of Hsp27 decreased metastatic behaviors of UM-SCC-22B by 3- to 4-fold in migration and 2-fold in   cell invasion   reducing   cell invasion   and migration to levels similar to the primary HNSCC UM-SCC-22A. 
 Both MAPKAPK2 and HSP27 are necessary for TGFbeta-mediated increases in MMP-2 and   cell invasion   in human prostate cancer. 
   
  
 LSAMP 
 
  NCBI Gene  
  GeneRIF  
  Pubmed  
 
 
  Search Term  Rank  Fields  
  [Mm]igration  6  PubMed(1)  
  [Ee]xtracellular [Mm]atrix  8  PubMed(1)  
 
 
 hide/show details for LSAMP 
  [Mm]igration  PubMed  
 Reexpression of LSAMP inhibits tumor growth in a preclinical osteosarcoma model. Osteosarcomas are the most common primary malignant tumors of bone, showing complex chromosomal rearrangements with multiple gains and losses. A frequent deletion within the chromosomal region 3q13.31 has been identified by us and others, and is mainly reported to be present in osteosarcomas. The purpose of the study was to further characterize the frequency and the extent of the deletion in an extended panel of osteosarcoma samples, and the expression level of the affected genes within the region. We have identified LSAMP as the target gene for the deletion, and have studied the functional implications of LSAMP-reexpression.LSAMP copy number, expression level and protein level were investigated by quantitative PCR and western blotting in an osteosarcoma panel. The expression of LSAMP was restored in an osteosarcoma cell line, and differences in proliferation rate, tumor formation, gene expression,   migration   rate, differentiation capabilities, cell cycle distribution and apoptosis were investigated by metabolic dyes, tumor formation in vivo, gene expression profiling, time-lapse photography, differentiation techniques and flow cytometry, respectively.We found reduced copy number of LSAMP in 45/76 osteosarcoma samples, reduced expression level in 25/42 samples and protein expression in 9/42 samples. By restoring the expression of LSAMP in a cell line with a homozygous deletion of the gene, the proliferation rate in vitro was significantly reduced and tumor growth in vivo was significantly delayed. In response to reexpression of LSAMP, mRNA expression profiling revealed consistent upregulation of the genes hairy and enhancer of split 1 (HES1), cancer/testis antigen 2 (CTAG2) and kruppel-like factor 10 (KLF10).The high frequency and the specificity of the deletion indicate that it is important for the development of osteosarcomas. The deletion targets the tumor suppressor LSAMP, and based on the functional evidence, the tumor suppressor function of LSAMP is most likely exerted by reducing the proliferation rate of the tumor cells, possibly by indirectly upregulating one or more of the genes HES1, CTAG2 or KLF10. To our knowledge, this study describes novel functions of LSAMP, a first step to understanding the functional role of this specific deletion in osteosarcomas. 
  [Ee]xtracellular [Mm]atrix  PubMed  
 A secreted protein microarray platform for extracellular protein interaction discovery. Characterization of the extracellular protein interactome has lagged far behind that of intracellular proteins, where mass spectrometry and yeast two-hybrid technologies have excelled. Improved methods for identifying receptor-ligand and   extracellular matrix   protein interactions will greatly accelerate biological discovery in cell signaling and cellular communication. These technologies must be able to identify low-affinity binding events that are often observed between membrane-bound coreceptor molecules during cell-cell or cell-  extracellular matrix   contact. Here we demonstrate that functional protein microarrays are particularly well-suited for high-throughput screening of extracellular protein interactions. To evaluate the performance of the platform, we screened a set of 89 immunoglobulin (Ig)-type receptors against a highly diverse extracellular protein microarray with 686 genes represented. To enhance detection of low-affinity interactions, we developed a rapid method to assemble bait Fc fusion proteins into multivalent complexes using protein A microbeads. Based on these screens, we developed a statistical methodology for hit calling and identification of nonspecific interactions on protein microarrays. We found that the Ig receptor interactions identified using our methodology are highly specific and display minimal off-target binding, resulting in a 70% true-positive to false-positive hit ratio. We anticipate that these methods will be useful for a wide variety of functional protein microarray users. 
   
  
 WIPF1 
 
  NCBI Gene  
  GeneRIF  
  Pubmed  
 
 
  Search Term  Rank  Fields  
  [Ff]ilopodia  1  PubMed(5)  
  [Ff]ilopodium  2  PubMed(2)  
  [Ii]nvadopodia  3  PubMed(1)  
  [Ii]nvadopodium  4  PubMed(1)  
  [Pp]odosome(s)?  5  PubMed(5); GeneRIFs(1)  
  [Mm]igration  6  PubMed(4)  
  [Ii]nvasive  10  PubMed(1); GeneRIFs(1)  
  [Mm]etastasis  12  PubMed(2)  
  [Cc]ell [Ii]nvasion  13  PubMed(1)  
 
 
 hide/show details for WIPF1 
  [Ff]ilopodia  PubMed  
 Cortactin interacts with WIP in regulating Arp2/3 activation and membrane protrusion. Modulation of actin cytoskeleton assembly is an integral step in many cellular events. A key regulator of actin polymerization is Arp2/3 complex. Cortactin, an F-actin binding protein that localizes to membrane ruffles, is an activator of Arp2/3 complex.A yeast two-hybrid screen revealed the interaction of the cortactin Src homology 3 (SH3) domain with a peptide fragment derived from a cDNA encoding a region of WASp-Interacting Protein (WIP). GST-cortactin interacted with WIP in an SH3-dependent manner. The subcellular localization of cortactin and WIP coincided at the cell periphery. WIP increased the efficiency of cortactin-mediated Arp2/3 complex activation of actin polymerization in a concentration-dependent manner. Lastly, coexpression of cortactin and WIP stimulated membrane protrusions.WIP, a protein involved in   filopodia   formation, binds to both actin monomers and cortactin. Thus, recruitment of actin monomers to a cortactin-activated Arp2/3 complex likely leads to the observed increase in cortactin activation of Arp2/3 complex by WIP. These data suggest that a cortactin-WIP complex functions in regulating actin-based structures at the cell periphery. 
 WIP regulates N-WASP-mediated actin polymerization and filopodium formation. Induction of   filopodia   is dependent on activation of the small GTPase Cdc42 and on neural Wiskott-Aldrich-syndrome protein (N-WASP). Here we show that WASP-interacting protein (WIP) interacts directly with N-WASP and actin. WIP retards N-WASP/Cdc42-activated actin polymerization mediated by the Arp2/3 complex, and stabilizes actin filaments. Microinjection of WIP into NIH 3T3 fibroblasts induces   filopodia  ; this is inhibited by microinjection of anti-N-WASP antibody. Microinjection of anti-WIP antibody inhibits induction of   filopodia   by bradykinin, by an active Cdc42 mutant (Cdc42(V12)) and by N-WASP. Our results indicate that WIP and N-WASP may act as a functional unit in filopodium formation, which is consistent with their role in actin-tail formation in cells infected with vaccinia virus or Shigella. 
 The WH1 and EVH1 domains of WASP and Ena/VASP family members bind distinct sequence motifs. A complex of N-WASP and WASP-interacting protein (WIP) plays an important role in actin-based motility of vaccinia virus and the formation of   filopodia  . WIP is also required to maintain the integrity of the actin cytoskeleton in T and B lymphocytes and is essential for T cell activation. However, in contrast to many other N-WASP binding proteins, WIP does not stimulate the ability of N-WASP to activate the Arp2/3 complex. Although the WASP homology 1 (WH1) domain of N-WASP interacts directly with WIP, we still lack the exact nature of its binding site. We have now identified and characterized the N-WASP WH1 binding motif in WIP in vitro and in vivo using Shigella and vaccinia systems. The WH1 domain, which is predicted to have a similar structural fold to the Ena/VASP homology 1 (EVH1) domain, binds to a sequence motif in WIP (ESRFYFHPISD) that is very different from the EVH1 proline-rich DL/FPPPP ligand. Interaction of the WH1 domain of N-WASP with WIP is dependent on the two highly conserved phenylalanine residues in the motif. The WH1 binding motif we have identified is conserved in WIP, CR16, WICH, and yeast verprolin. 
 The rat homologue of Wiskott-Aldrich syndrome protein (WASP)-interacting protein (WIP) associates with actin filaments, recruits N-WASP from the nucleus, and mediates mobilization of actin from stress fibers in favor of   filopodia   formation. We cloned and characterized the rat homologue of the Wiskott-Aldrich syndrome protein (WASP)-interacting protein (WIP). Rat WIP shows 86% amino acid sequence identity to human WIP. Northern analyses revealed two major mRNA species of 5.0 and 3.8 kb, which were ubiquitously expressed, though predominantly in spleen and lung. Minor species of 2.4, 1.8, 1.4, and 1.1 kb were also detected in some tissues and cell lines. Thus, WIP is subject to tissue-specific alternative splicing. WIP bound to N-WASP in vivo, as revealed by co-immunoprecipitation. Expression of WIP in rat fibroblasts revealed a clear co-localization with actin stress fibers. However, expression in tumor cells lacking actin cables did not restore these structures. Interestingly, co-expression of WIP and N-WASP resulted in redistribution of N-WASP, abrogating its dominant nuclear expression and leading to co-localization with WIP in the perinuclear area and with actin in membrane protrusions. Moreover, stress fibers and, concomitantly, the associated WIP were largely dissolved. Very similar effects were seen upon epidermal growth factor stimulation of serum-starved cells. Our results suggest that WIP might be involved in transmitting mitogenic signals to cytoskeletal functions, perhaps by modulating the subcellular localization of N-WASP. Interaction of N-WASP with WIP may in turn lead to mobilization of actin from stress fibers and nucleation of new actin filaments in   filopodia  . 
 WASP-interacting protein (WIP): working in polymerisation and much more. The migration of cells and the movement of some intracellular pathogens, such as Shigella and Vaccinia, are dependent on the actin-based cytoskeleton. Many proteins are involved in regulating the dynamics of the actin-based microfilaments within cells and, among them, WASP and N-WASP have a significant role in the regulation of actin polymerisation. The activity and stability of WASP is regulated by its cellular partner WASP-interacting protein (WIP) during the formation of actin-rich structures, including the immune synapse,   filopodia  , lamellipodia, stress fibres and podosomes. Here, we review the role of WIP in regulating WASP function by stabilising WASP and shuttling WASP to areas of actin assembly in addition to reviewing the WASP-independent functions of WIP. 
  [Ff]ilopodium  PubMed  
 WIP deficiency reveals a differential role for WIP and the actin cytoskeleton in T and B cell activation. WIP stabilizes actin filaments and is important for   filopodium   formation. To define the role of WIP in immunity, we generated WIP-deficient mice. WIP(minus sign/minus sign) mice have normal lymphocyte development, but their T cells fail to proliferate, secrete IL-2, increase their F-actin content, polarize and extend protrusions following T cell receptor ligation, and are deficient in conjugate formation with superantigen-presenting B cells and anti-CD3 bilayers. In contrast, WIP-deficient B lymphocytes have enhanced proliferation and CD69 expression following B cell receptor ligation and mount normal antibody responses to T-independent antigens. Both WIP-deficient T and B cells show a profound defect in their subcortical actin filament networks. These results suggest that WIP is important for immunologic synapse formation and T cell activation. 
 WIP regulates N-WASP-mediated actin polymerization and   filopodium   formation. Induction of filopodia is dependent on activation of the small GTPase Cdc42 and on neural Wiskott-Aldrich-syndrome protein (N-WASP). Here we show that WASP-interacting protein (WIP) interacts directly with N-WASP and actin. WIP retards N-WASP/Cdc42-activated actin polymerization mediated by the Arp2/3 complex, and stabilizes actin filaments. Microinjection of WIP into NIH 3T3 fibroblasts induces filopodia; this is inhibited by microinjection of anti-N-WASP antibody. Microinjection of anti-WIP antibody inhibits induction of filopodia by bradykinin, by an active Cdc42 mutant (Cdc42(V12)) and by N-WASP. Our results indicate that WIP and N-WASP may act as a functional unit in   filopodium   formation, which is consistent with their role in actin-tail formation in cells infected with vaccinia virus or Shigella. 
  [Ii]nvadopodia  PubMed  
 WIP and WICH/WIRE co-ordinately control invadopodium formation and maturation in human breast cancer cell invasion. Cancer cells form actin-rich degradative protrusions (invasive pseudopods and   invadopodia  ), which allows their efficient dispersal during metastasis. Using biochemical and advanced imaging approaches, we demonstrate that the N-WASP-interactors WIP and WICH/WIRE play non-redundant roles in cancer cell invasion. WIP interacts with N-WASP and cortactin and is essential for invadopodium assembly, whereas WICH/WIRE regulates N-WASP activation to control invadopodium maturation and degradative activity. Our data also show that Nck interaction with WIP and WICH/WIRE modulates invadopodium maturation; changes in WIP and WICH/WIRE levels induce differential distribution of Nck. We show that WIP can replace WICH/WIRE functions and that elevated WIP levels correlate with high invasiveness. These findings identify a role for WICH/WIRE in invasiveness and highlight WIP as a hub for signaling molecule recruitment during invadopodium generation and cancer progression, as well as a potential diagnostic biomarker and an optimal target for therapeutic approaches. 
  [Ii]nvadopodium  PubMed  
 WIP and WICH/WIRE co-ordinately control   invadopodium   formation and maturation in human breast cancer cell invasion. Cancer cells form actin-rich degradative protrusions (invasive pseudopods and invadopodia), which allows their efficient dispersal during metastasis. Using biochemical and advanced imaging approaches, we demonstrate that the N-WASP-interactors WIP and WICH/WIRE play non-redundant roles in cancer cell invasion. WIP interacts with N-WASP and cortactin and is essential for   invadopodium   assembly, whereas WICH/WIRE regulates N-WASP activation to control   invadopodium   maturation and degradative activity. Our data also show that Nck interaction with WIP and WICH/WIRE modulates   invadopodium   maturation; changes in WIP and WICH/WIRE levels induce differential distribution of Nck. We show that WIP can replace WICH/WIRE functions and that elevated WIP levels correlate with high invasiveness. These findings identify a role for WICH/WIRE in invasiveness and highlight WIP as a hub for signaling molecule recruitment during   invadopodium   generation and cancer progression, as well as a potential diagnostic biomarker and an optimal target for therapeutic approaches. 
  [Pp]odosome(s)?  PubMed  
 FBP17 Mediates a Common Molecular Step in the Formation of   Podosomes   and Phagocytic Cups in Macrophages. Macrophages act to protect the body against inflammation and infection by engaging in chemotaxis and phagocytosis. In chemotaxis, macrophages use an actin-based membrane structure, the   podosome  , to migrate to inflamed tissues. In phagocytosis, macrophages form another type of actin-based membrane structure, the phagocytic cup, to ingest foreign materials such as bacteria. The formation of these membrane structures is severely affected in macrophages from patients with Wiskott-Aldrich syndrome (WAS), an X chromosome-linked immunodeficiency disorder. WAS patients lack WAS protein (WASP), suggesting that WASP is required for the formation of   podosomes   and phagocytic cups. Here we have demonstrated that formin-binding protein 17 (FBP17) recruits WASP, WASP-interacting protein (WIP), and dynamin-2 to the plasma membrane and that this recruitment is necessary for the formation of   podosomes   and phagocytic cups. The N-terminal EFC (extended FER-CIP4 homology)/F-BAR (FER-CIP4 homology and Bin-amphiphysin-Rvs) domain of FBP17 was previously shown to have membrane binding and deformation activities. Our results suggest that FBP17 facilitates membrane deformation and actin polymerization to occur simultaneously at the same membrane sites, which mediates a common molecular step in the formation of   podosomes   and phagocytic cups. These results provide a potential mechanism underlying the recurrent infections in WAS patients. 
 Tyrosine phosphorylation of WIP releases bound WASP and impairs   podosome   assembly in macrophages.   Podosomes   are integrin-containing adhesion structures commonly found in migrating leukocytes of the monocytic lineage. The actin cytoskeletal organisation of   podosomes   is based on a WASP- and Arp2/3-mediated mechanism. WASP also associates with a second protein, WIP (also known as WIPF1), and they co-localise in   podosome   cores. Here, we report for the first time that WIP can be phosphorylated on tyrosine residues and that tyrosine phosphorylation of WIP is a trigger for release of WASP from the WIP-WASP complex. Using a knockdown approach together with expression of WIP phosphomimics, we show that in the absence of WIP-WASP binding, cellular WASP is rapidly degraded, leading to disruption of   podosomes   and a failure of cells to degrade an underlying matrix. In the absence of tyrosine phosphorylation, the WIP-WASP complex remains intact and   podosome   lifetimes are extended. A screen of candidate kinases and inhibitor-based assays identified Bruton's tyrosine kinase (Btk) as a regulator of WIP tyrosine phosphorylation. We conclude that tyrosine phosphorylation of WIP is a crucial regulator of WASP stability and function as an actin-nucleation-promoting factor. 
 Phosphatidylinositol 4,5-biphosphate (PIP2)-induced vesicle movement depends on N-WASP and involves Nck, WIP, and Grb2. Wiskott-Aldrich syndrome protein (WASP)/Scar family proteins promote actin polymerization by stimulating the actin-nucleating activity of the Arp2/3 complex. While Scar/WAVE proteins are thought to be involved in lamellipodia protrusion, the hematopoietic WASP has been implicated in various actin-based processes such as chemotaxis,   podosome   formation, and phagocytosis. Here we show that the ubiquitously expressed N-WASP is essential for actin assembly at the surface of endomembranes induced as a consequence of increased phosphatidylinositol 4,5-biphosphate (PIP2) levels. This process resulting in the motility of intracellular vesicles at the tips of actin comets involved the recruitment of the Src homology 3 (SH3)-SH2 adaptor proteins Nck and Grb2 as well as of WASP interacting protein (WIP). Reconstitution of vesicle movement in N-WASP-defective cells by expression of various N-WASP mutant proteins revealed three independent domains capable of interaction with the vesicle surface, of which both the WH1 and the polyproline domains contributed significantly to N-WASP recruitment and/or activation. In contrast, the direct interaction of N-WASP with the Rho-GTPase Cdc42 was not required for reconstitution of vesicle motility. Our data reveal a distinct cellular phenotype for N-WASP loss of function, which adds to accumulating evidence that the proposed link between actin and membrane dynamics may, at least partially, be reflected by the actin-based movement of vesicles through the cytoplasm. 
 Hematopoietic lineage cell-specific protein 1 functions in concert with the Wiskott-Aldrich syndrome protein to promote   podosome   array organization and chemotaxis in dendritic cells. Dendritic cells (DCs) are professional APCs that reside in peripheral tissues and survey the body for pathogens. Upon activation by inflammatory signals, DCs undergo a maturation process and migrate to lymphoid organs, where they present pathogen-derived Ags to T cells. DC migration depends on tight regulation of the actin cytoskeleton to permit rapid adaptation to environmental cues. We investigated the role of hematopoietic lineage cell-specific protein 1 (HS1), the hematopoietic homolog of cortactin, in regulating the actin cytoskeleton of murine DCs. HS1 localized to lamellipodial protrusions and   podosomes  , actin-rich structures associated with adhesion and migration. DCs from HS1(-/-) mice showed aberrant lamellipodial dynamics. Moreover, although these cells formed recognizable   podosomes  , their   podosome   arrays were loosely packed and improperly localized within the cell. HS1 interacts with Wiskott-Aldrich syndrome protein (WASp), another key actin-regulatory protein, through mutual binding to WASp-interacting protein. Comparative analysis of DCs deficient for HS1, WASp or both proteins revealed unique roles for these proteins in regulating   podosomes   with WASp being essential for   podosome   formation and with HS1 ensuring efficient array organization. WASp recruitment to   podosome   cores was independent of HS1, whereas HS1 recruitment required Src homology 3 domain-dependent interactions with the WASp/WASp-interacting protein heterodimer. In migration assays, the phenotypes of HS1- and WASp-deficient DCs were related, but distinct. WASp(-/y) DCs migrating in a chemokine gradient showed a large decrease in velocity and diminished directional persistence. In contrast, HS1(-/-) DCs migrated faster than wild-type cells, but directional persistence was significantly reduced. These studies show that HS1 functions in concert with WASp to fine-tune DC cytoarchitecture and direct cell migration. 
 WASP-interacting protein (WIP): working in polymerisation and much more. The migration of cells and the movement of some intracellular pathogens, such as Shigella and Vaccinia, are dependent on the actin-based cytoskeleton. Many proteins are involved in regulating the dynamics of the actin-based microfilaments within cells and, among them, WASP and N-WASP have a significant role in the regulation of actin polymerisation. The activity and stability of WASP is regulated by its cellular partner WASP-interacting protein (WIP) during the formation of actin-rich structures, including the immune synapse, filopodia, lamellipodia, stress fibres and   podosomes  . Here, we review the role of WIP in regulating WASP function by stabilising WASP and shuttling WASP to areas of actin assembly in addition to reviewing the WASP-independent functions of WIP. 
  GeneRIFs  
 formin-binding protein 17 (FBP17) recruits WASP, WASP-interacting protein (WIP), and dynamin-2 to the plasma membrane and that this recruitment is necessary for the formation of   podosomes   and phagocytic cups. 
  [Mm]igration  PubMed  
 Identification of novel SH3 domain ligands for the Src family kinase Hck. Wiskott-Aldrich syndrome protein (WASP), WASP-interacting protein (WIP), and ELMO1. The importance of the SH3 domain of Hck in kinase regulation, substrate phosphorylation, and ligand binding has been established. However, few in vivo ligands are known for the SH3 domain of Hck. In this study, we used mass spectrometry to identify approximately 25 potential binding partners for the SH3 domain of Hck from the monocyte cell line U937. Two major interacting proteins were the actin binding proteins Wiskott-Aldrich syndrome protein (WASP) and WASP-interacting protein (WIP). We also focused on a novel interaction between Hck and ELMO1, an 84-kDa protein that was recently identified as the mammalian ortholog of the Caenorhabditis elegans gene, ced-12. In mammalian cells, ELMO1 interacts with Dock180 as a component of the CrkII/Dock180/Rac pathway responsible for phagocytosis and cell   migration  . Using purified proteins, we confirmed that WASP-interacting protein and ELMO1 interact directly with the SH3 domain of Hck. We also show that Hck and ELMO1 interact in intact cells and that ELMO1 is heavily tyrosine-phosphorylated in cells that co-express Hck, suggesting that it is a substrate of Hck. The binding of ELMO1 to Hck is specifically dependent on the interaction of a polyproline motif with the SH3 domain of Hck. Our results suggest that these proteins may be novel activators/effectors of Hck. 
 Hematopoietic lineage cell-specific protein 1 functions in concert with the Wiskott-Aldrich syndrome protein to promote podosome array organization and chemotaxis in dendritic cells. Dendritic cells (DCs) are professional APCs that reside in peripheral tissues and survey the body for pathogens. Upon activation by inflammatory signals, DCs undergo a maturation process and migrate to lymphoid organs, where they present pathogen-derived Ags to T cells. DC   migration   depends on tight regulation of the actin cytoskeleton to permit rapid adaptation to environmental cues. We investigated the role of hematopoietic lineage cell-specific protein 1 (HS1), the hematopoietic homolog of cortactin, in regulating the actin cytoskeleton of murine DCs. HS1 localized to lamellipodial protrusions and podosomes, actin-rich structures associated with adhesion and   migration  . DCs from HS1(-/-) mice showed aberrant lamellipodial dynamics. Moreover, although these cells formed recognizable podosomes, their podosome arrays were loosely packed and improperly localized within the cell. HS1 interacts with Wiskott-Aldrich syndrome protein (WASp), another key actin-regulatory protein, through mutual binding to WASp-interacting protein. Comparative analysis of DCs deficient for HS1, WASp or both proteins revealed unique roles for these proteins in regulating podosomes with WASp being essential for podosome formation and with HS1 ensuring efficient array organization. WASp recruitment to podosome cores was independent of HS1, whereas HS1 recruitment required Src homology 3 domain-dependent interactions with the WASp/WASp-interacting protein heterodimer. In   migration   assays, the phenotypes of HS1- and WASp-deficient DCs were related, but distinct. WASp(-/y) DCs migrating in a chemokine gradient showed a large decrease in velocity and diminished directional persistence. In contrast, HS1(-/-) DCs migrated faster than wild-type cells, but directional persistence was significantly reduced. These studies show that HS1 functions in concert with WASp to fine-tune DC cytoarchitecture and direct cell   migration  . 
 Requirements for F-BAR proteins TOCA-1 and TOCA-2 in actin dynamics and membrane trafficking during Caenorhabditis elegans oocyte growth and embryonic epidermal morphogenesis. The TOCA family of F-BAR-containing proteins bind to and remodel lipid bilayers via their conserved F-BAR domains, and regulate actin dynamics via their N-Wasp binding SH3 domains. Thus, these proteins are predicted to play a pivotal role in coordinating membrane traffic with actin dynamics during cell   migration   and tissue morphogenesis. By combining genetic analysis in Caenorhabditis elegans with cellular biochemical experiments in mammalian cells, we showed that: i) loss of CeTOCA proteins reduced the efficiency of Clathrin-mediated endocytosis (CME) in oocytes. Genetic interference with CeTOCAs interacting proteins WSP-1 and WVE-1, and other components of the WVE-1 complex, produced a similar effect. Oocyte endocytosis defects correlated well with reduced egg production in these mutants. ii) CeTOCA proteins localize to cell-cell junctions and are required for proper embryonic morphogenesis, to position hypodermal cells and to organize junctional actin and the junction-associated protein AJM-1. iii) Double mutant analysis indicated that the toca genes act in the same pathway as the nematode homologue of N-WASP/WASP, wsp-1. Furthermore, mammalian TOCA-1 and C. elegans CeTOCAs physically associated with N-WASP and WSP-1 directly, or WAVE2 indirectly via ABI-1. Thus, we propose that TOCA proteins control tissues morphogenesis by coordinating Clathrin-dependent membrane trafficking with WAVE and N-WASP-dependent actin-dynamics. 
 WASP-interacting protein (WIP): working in polymerisation and much more. The   migration   of cells and the movement of some intracellular pathogens, such as Shigella and Vaccinia, are dependent on the actin-based cytoskeleton. Many proteins are involved in regulating the dynamics of the actin-based microfilaments within cells and, among them, WASP and N-WASP have a significant role in the regulation of actin polymerisation. The activity and stability of WASP is regulated by its cellular partner WASP-interacting protein (WIP) during the formation of actin-rich structures, including the immune synapse, filopodia, lamellipodia, stress fibres and podosomes. Here, we review the role of WIP in regulating WASP function by stabilising WASP and shuttling WASP to areas of actin assembly in addition to reviewing the WASP-independent functions of WIP. 
  [Ii]nvasive  PubMed  
 WIP and WICH/WIRE co-ordinately control invadopodium formation and maturation in human breast cancer cell invasion. Cancer cells form actin-rich degradative protrusions (  invasive   pseudopods and invadopodia), which allows their efficient dispersal during metastasis. Using biochemical and advanced imaging approaches, we demonstrate that the N-WASP-interactors WIP and WICH/WIRE play non-redundant roles in cancer cell invasion. WIP interacts with N-WASP and cortactin and is essential for invadopodium assembly, whereas WICH/WIRE regulates N-WASP activation to control invadopodium maturation and degradative activity. Our data also show that Nck interaction with WIP and WICH/WIRE modulates invadopodium maturation; changes in WIP and WICH/WIRE levels induce differential distribution of Nck. We show that WIP can replace WICH/WIRE functions and that elevated WIP levels correlate with high   invasive  ness. These findings identify a role for WICH/WIRE in   invasive  ness and highlight WIP as a hub for signaling molecule recruitment during invadopodium generation and cancer progression, as well as a potential diagnostic biomarker and an optimal target for therapeutic approaches. 
  GeneRIFs  
 Study provides evidence that WIP and WIRE contribute to breast cancer cell   invasive  ness through coordinated roles. WIP seems necessary for the assembly of   invasive   protrusions, whereas WIRE regulates their maturation, which leads to matrix degradation. 
  [Mm]etastasis  PubMed  
 WIP and WICH/WIRE co-ordinately control invadopodium formation and maturation in human breast cancer cell invasion. Cancer cells form actin-rich degradative protrusions (invasive pseudopods and invadopodia), which allows their efficient dispersal during   metastasis  . Using biochemical and advanced imaging approaches, we demonstrate that the N-WASP-interactors WIP and WICH/WIRE play non-redundant roles in cancer cell invasion. WIP interacts with N-WASP and cortactin and is essential for invadopodium assembly, whereas WICH/WIRE regulates N-WASP activation to control invadopodium maturation and degradative activity. Our data also show that Nck interaction with WIP and WICH/WIRE modulates invadopodium maturation; changes in WIP and WICH/WIRE levels induce differential distribution of Nck. We show that WIP can replace WICH/WIRE functions and that elevated WIP levels correlate with high invasiveness. These findings identify a role for WICH/WIRE in invasiveness and highlight WIP as a hub for signaling molecule recruitment during invadopodium generation and cancer progression, as well as a potential diagnostic biomarker and an optimal target for therapeutic approaches. 
 WIP remodeling actin behind the scenes: how WIP reshapes immune and other functions. Actin polymerization is a fundamental cellular process regulating immune cell functions and the immune response. The Wiskott-Aldrich syndrome protein (WASp) is an actin nucleation promoting factor, which is exclusively expressed in hematopoietic cells, where it plays a key regulatory role in cytoskeletal dynamics. WASp interacting protein (WIP) was first discovered as the binding partner of WASp, through the use of the yeast two hybrid system. WIP was later identified as a chaperone of WASp, necessary for its stability. Mutations occurring at the WASp homology 1 domain (WH1), which serves as the WIP binding site, were found to cause the Wiskott-Aldrich syndrome (WAS) and X-linked thrombocytopenia (XLT). WAS manifests as an immune deficiency characterized by eczema, thrombocytopenia, recurrent infections, and hematopoietic malignancies, demonstrating the importance of WIP for WASp complex formation and for a proper immune response. WIP deficiency was found to lead to different abnormalities in the activity of various lymphocytes, suggesting differential cell-dependent roles for WIP. Additionally, WIP deficiency causes cellular abnormalities not found in WASp-deficient cells, indicating that WIP fulfills roles beyond stabilizing WASp. Indeed, WIP was shown to interact with various binding partners, including the signaling proteins Nck, CrkL and cortactin. Recent studies have demonstrated that WIP also takes part in non immune cellular processes such as cancer invasion and   metastasis  , in addition to cell subversion by intracellular pathogens. Understanding of numerous functions of WIP can enhance our current understanding of activation and function of immune and other cell types. 
  [Cc]ell [Ii]nvasion  PubMed  
 WIP and WICH/WIRE co-ordinately control invadopodium formation and maturation in human breast cancer   cell invasion  . Cancer cells form actin-rich degradative protrusions (invasive pseudopods and invadopodia), which allows their efficient dispersal during metastasis. Using biochemical and advanced imaging approaches, we demonstrate that the N-WASP-interactors WIP and WICH/WIRE play non-redundant roles in cancer   cell invasion  . WIP interacts with N-WASP and cortactin and is essential for invadopodium assembly, whereas WICH/WIRE regulates N-WASP activation to control invadopodium maturation and degradative activity. Our data also show that Nck interaction with WIP and WICH/WIRE modulates invadopodium maturation; changes in WIP and WICH/WIRE levels induce differential distribution of Nck. We show that WIP can replace WICH/WIRE functions and that elevated WIP levels correlate with high invasiveness. These findings identify a role for WICH/WIRE in invasiveness and highlight WIP as a hub for signaling molecule recruitment during invadopodium generation and cancer progression, as well as a potential diagnostic biomarker and an optimal target for therapeutic approaches. 
   
  
 ACYP2 
 
  NCBI Gene  
  GeneRIF  
  Pubmed  
 
 
  Search Term  Rank  Fields  
  [Mm]igration  6  PubMed(1)  
  [Ee]xtracellular [Mm]atrix  8  PubMed(1)  
 
 
 hide/show details for ACYP2 
  [Mm]igration  PubMed  
 Differential   migration   of acylphosphatase isoenzymes from cytoplasm to nucleus during apoptotic cell death. The subcellular distribution of both muscle and common type acylphosphatases was studied during apoptosis. Both isoenzymes were previously shown to have in vitro nucleolytic activity having a pH optimum at 6.8. In this paper we demonstrate a nuclear   migration   of the muscle type isoenzyme in response to various apoptotic stimuli either in K562 or in Jurkat cells, while the common type acylphosphatase isoform remains cytoplasmatic under the same conditions. Furthermore, we present evidences of a direct in vivo interaction between muscle acylphosphatase and two other DNAses of about 60 and 80 kDa in size. Our results are consistent with an in vivo involvement of the muscle isoform in apoptosis, possibly as a part of a multimeric protein complex that binds and hydrolyses DNA during this process. 
  [Ee]xtracellular [Mm]atrix  PubMed  
 Rapid oligomer formation of human muscle acylphosphatase induced by heparan sulfate. Many human diseases are caused by the conversion of proteins from their native state into amyloid fibrils that deposit in the extracellular space. Heparan sulfate, a component of the   extracellular matrix  , is universally associated with amyloid deposits and promotes fibril formation. The formation of cytotoxic prefibrillar oligomers is challenging to study because of its rapidity, transient appearance and the heterogeneity of species generated. The process is even more complex with agents such as heparan sulfate. Here we have used a stopped-flow device coupled to turbidometry detection to monitor the rapid conversion of human muscle acylphosphatase into oligomers with varying heparan sulfate and protein concentrations. We also analyzed mutants of the 15 basic amino acids of acylphosphatase, identifying the residues primarily involved in heparan sulfate-induced oligomerization of this protein and tracing the process with unprecedented molecular detail. 
   
  
 TLR3 
 
  NCBI Gene  
  GeneRIF  
  Pubmed  
 
 
  Search Term  Rank  Fields  
  [Ff]ilopodia  1  PubMed(1)  
  [Mm]igration  6  PubMed(15); GeneRIFs(3)  
  ECM  7  PubMed(2); GeneRIFs(1)  
  [Ee]xtracellular [Mm]atrix  8  PubMed(7)  
  [Ii]nvasive  10  PubMed(6); GeneRIFs(1)  
  [Mm]etastasis  12  PubMed(6); GeneRIFs(1)  
  [Cc]ell [Ii]nvasion  13  PubMed(1)  
 
 
 hide/show details for TLR3 
  [Ff]ilopodia  PubMed  
 Toll-like receptor 3 expression inhibits cell invasion and migration and predicts a favorable prognosis in neuroblastoma. To evaluate the clinical significance of TLR3 expression on neuroblastomas, we performed immunohistochemical study on archival tissues and in vitro studies on neuroblastoma cell lines. The results showed that positive TLR3 expression was associated with favorable histology and prognosis. Activation of TLR3 by polyinosinic:polycytidylic acid [poly(I:C)] treatment is effective to suppress cell migration and invasion and to decrease organized assembly of F-actin and   filopodia   formation, in TLR3-expressing SK-N-AS cells, which could be reversed by TLR3-targeting siRNA treatment. TLR3 agonist poly(I:C) promotes GAP-43 expression also in SK-N-AS cells only. Taken together, TLR3 could serve to predict favorable behavior in neuroblastomas. 
  [Mm]igration  PubMed  
 Up-regulation of galectin-9 induces cell   migration   in human dendritic cells infected with dengue virus. Galectin-9 (Gal-9) exerts immunosuppressive effects by inducing apoptosis in T cells that produce interferon-γ and interleukin (IL)-17. However, Gal-9 can be pro-inflammatory in lipopolysaccharide-stimulated monocytes. Using microarray analysis, we observed that Gal-9 was up-regulated in human dendritic cells (DCs) after dengue virus (DV) infection. The investigation into the immunomodulatory effects and mechanisms of Gal-9 in DCs exposed to DV revealed that DV infection specifically increased mRNA and protein levels of Gal-9 but not those of Gal-1 or Gal-3. Blocking p38, but not c-Jun N-terminal kinase or extracellular signal-regulated kinase (ERK), inhibited DV-induced expression of Gal-9. Reduction in Gal-9 by small interference RNA treatment suppressed DV-stimulated   migratio  n of DCs towards the chemoattractants CCL19 and CCL21. In addition, DV-induced IL-12p40 production was reduced after knockdown of Gal-9 in DCs. Furthermore, Gal-9 deficiency suppressed DV-induced activation of nuclear factor-κB. Inhibition of DV-induced D  C migrati  on under conditions of Gal-9 deficiency was mediated through suppressing ERK activation but not by regulating the expression of CCR7, the receptor for CCL19 and CCL21. Both the reduction in IL-12 production and the suppression of ERK activity might account for the inhibition of DV-induced D  C migrati  on after knockdown of Gal-9. In summary, this study reveals the roles of Gal-9 in DV-induce  d migrati  on of DCs. The findings indicate that Gal-9 might be a therapeutic target for preventing immunopathogenesis induced by DV infection. 
 Toll-like receptor 3-mediated tumor invasion in head and neck cancer. Chronic inflammation associated with some infectious agents can lead to cancer. The Toll-like receptor (TLR) family is one of the largest and best-studied families of pathogen-associated molecular patterns. TLR3 recognizes double-stranded RNA and is a major effector of the immune response against viral pathogens.We investigated TLR3 protein expression in 153 oral squamous cell carcinoma (OSCC) specimens using tissue microarray. Furthermore, we used polyinosinic-polycytidylic acid (poly I:C) to stimulate head and neck cancer cells and an inhibitor of endosomal acidification bafilomycin A1 to block the TLR 3 signaling pathway to clarify the role of TLR 3 in OSCC.Cytoplasmic TLR3 staining was observed in the vast majority of OSCC tissues (73.2%). Strong TLR3 expression was significantly correlated with patients whose tumors were poorly differentiated (P=0.028) and with perineural invasion (P=0.023). Three of the four head and neck cell lines tested (Fadu, OC2, and SCC4) expressed TLR3 mRNA, although at various levels. The stimulation of TLR3-expressing OC2 cells with poly I:C caused the phosphorylation of IFN regulatory factor 3 and IκB and sequentially induced the secretion of interleukin-6 and chemokine (C-C motif) ligand 5 (CCL5) in a dose- and time-dependent manner. Moreover, poly I:C stimulation promoted CCL5-mediated   migratio  n in OC2 cells.In this report, we provide a novel mechanism for tumor invasion and the TLR3-dependent inflammatory response that could have therapeutic implications for OSCC. 
 dsRNA-activation of TLR3 and RLR signaling: gene induction-dependent and independent effects. Double-stranded (ds) RNA has diverse roles in host defense and disease prevention. dsRNA, produced by viral replication, elicits strong antiviral responses in host; similar protective responses can also be triggered by cellular dsRNA produced by necrotic, apoptotic, or otherwise stressed, uninfected cells. dsRNA is recognized in the cell by a large family of dsRNA-binding proteins, among which are the pattern recognition receptors (PRRs), toll-like receptor 3 (TLR3), and retinoic acid-inducible gene I (RIG-I)-like receptors (RLRs). TLR3 signals from the endosomal membrane where it senses extracellular dsRNA that has been endocytosed, whereas RLRs signal from the cytoplasm using a mitochondrial adaptor protein. In this review, we will summarize the signaling pathways used by these 2 PRRs, which lead to the activation of specific transcription factors and the induction of many proinflammatory and antiviral genes. However, it is becoming increasingly clear that all host responses are not mediated by the products of these induced genes; signal-dependent post-translational modifications of existing proteins can also profoundly change cellular properties. We will discuss how Src activation by TLR3 changes cell   migration  , adhesion, and proliferation rates and how IRF-3 activation by RLR triggers a gene induction-independent pro-apoptotic pathway that provides strong antiviral protection. 
 Expression of IL-12-related molecules in human intestinal microvascular endothelial cells is regulated by TLR3. Members of the interleukin (IL)-12 family constitute subunits of IL-12, -23, and -27. These ILs represent pivotal mediators in the regulation of cell-mediated immune responses and in animal models of human inflammatory bowel disease. Recent work has suggested that intestinal endothelial cells might serve as a second line of defense in bacterial sensing of invading pathogens. The purpose of this study was to examine the production of IL-12 family members in intestinal endothelial cells (HIMEC). HIMEC were stimulated with proinflammatory agents (TNF-alpha, IFN-gamma, IL-1beta) and microbial antigens [LPS, lipoteichoic acid, peptidoglycan, CpG-DNA, flagellin, poly(I:C)]. Expression of IL-12 family members and of Toll-like receptor (TLR)3 in HIMEC was assessed by real-time RT-PCR, immunostaining, flow cytometry, and immunoblot analysis. HIMEC display an induction of Epstein-Barr virus-induced gene 3 (EBI3), IL-12p35, and IL-23p19, whereas no expression of IL-12p40 and IL-27p28 was detectable. The strongest induction was induced by proinflammatory factors known to utilize the NF-kappaB pathway, and expression of EBI3 and IL-23p19 was diminished by an NF-kappaB inhibitor. HIMEC display regulated expression of TLR3. Adhesion and trans  migration   assays showed proinflammatory responses after HIMEC stimulation. HIMEC are capable of producing IL-12 family members as a response to microbial stimuli. The TLR3 agonist, poly(I:C), was shown to enhance leukocyte adhesion in vitro in HIMEC. Our data suggest that the intestinal microvasculature is responsive to ligands of TLR3 expressed on intestinal endothelial cells, thereby adding to the regulation of adaptive immunity and leukocyte recruitment. 
 Activation of Toll-like receptors 2, 3, and 4 on human melanoma cells induces inflammatory factors. Toll-like receptors (TLR) have been shown to be expressed on various types of cancers; however, their functional activity is not known. We examined TLR profiles of human melanoma cells and showed that TLR2, TLR3, and TLR4 were found to be highly expressed. By PCR array analysis, specific stimulation of TLR2, TLR3, and TLR4 on melanoma cells showed significant activation of the adaptor protein MyD88, as well as downstream signal transduction factors nuclear factor-kappaB and inflammatory response-related factors. Specific ligand activation of TLR2, TLR3, and TLR4 was shown to induce cell   migration  . Peripheral blood lymphocytes and melanoma purified RNA was shown to activate TLR3 on melanoma cells. These studies show expression and functional activity of specific TLRs on melanoma cells and as potential therapeutic targets to control tumor progression. 
 The dual role of TLR3 in metastatic cell line. Toll-like receptors (TLRs) are members of transmembrane proteins that recognize conserved molecular motifs of viral and bacterial origin and initiate innate immune response. As the role of TLRs in tumors cells is still not clear, our aim was to investigate the role of TLR3 in primary tumor and metastatic cells (SW480, SW620, FaDu and Detroit 562). We have reported here on the dual role of TLR3 in pharynx metastatic cell line (Detroit 562); on one hand TLR3 activation drove cells to apoptosis while on the other its stimulation contributed to tumor progression by altering the expression of tumor promoting genes (PLAUR, RORB) and enhancing the cell   migration   potential. In addition, we have shown TLR3 signaling pathway is functional in another metastatic cancer cell line (SW620) suggesting TLR3 might be important in the process of tumor metastasis. Since TLR3 agonists have been used in tumor therapy with the aim to activate immune system, scientific contribution of this work is drawing attention to the importance of further work on this topic, especially pro-tumor effect of TLR3, in order to avoid possible side-effects. 
 Autophagy facilitates TLR4- and TLR3-triggered   migration   and invasion of lung cancer cells through the promotion of TRAF6 ubiquitination. Autophagy contributes to the pathogenesis of cancer, whereas toll-like receptors (TLRs) also play an important role in cancer development and immune escape. However, little is known about the potential interaction between TLR signaling and autophagy in cancer cells. Here we show that autophagy induced by TLR4 or TLR3 activation enhances various cytokine productions through promoting TRAF6 (TNF receptor-associated factor 6, E3 ubiquitin protein ligase) ubiquitination and thus facilitates   migration   and invasion of lung cancer cells. Stimulation of TLR4 and TLR3 with lipopolysaccharide (LPS) and polyinosinic-polycytidylic acid [poly(I:C)] respectively triggered autophagy in lung cancer cells. This was mediated by the adaptor protein, toll-like receptor adaptor molecule 1 (TICAM1/TRIF), and was required for TLR4- and TLR3-induced increases in the production of IL6, CCL2/MCP-1 [chemokine (C-C motif) ligand 2], CCL20/MIP-3α [chemokine (C-C motif) ligand 20], VEGFA (vascular endothelial growth factor A), and MMP2 [matrix metallopeptidase 2 (gelatinase A, 72 kDa gelatinase, 72 kDa type IV collagenase)]. These cytokines appeared to be necessary for enhanced   migratio  n and invasion of lung cancer cells upon TLR activation. Remarkably, inhibition of autophagy by chemical or genetic approaches blocked TLR4- or TLR3-induced Lys63 (K63)-linked ubiquitination of TRAF6 that was essential for activation of MAPK and NFKB (nuclear factor of kappa light polypeptide gene enhancer in B-cells) pathways, both of which were involved in the increased production of the cytokines. Collectively, these results identify induction of autophagy by TLR4 and TLR3 as an important mechanism that drives lung cancer progression, and indicate that inhibition of autophagy may be a useful strategy in the treatment of lung cancer. 
 TLR3-dependent immune regulatory functions of human mesangial cells. In glomerulonephritis, the   migration   of inflammatory cells into the glomerulus is an important step in disease initiation and progression. The viral receptor Toll-like receptor 3 (TLR3) is known to play a role in virus-associated glomerulonephritis. Based on this knowledge, this study aimed to define the effects of the TLR3 ligand polyriboinosinic:polyribocytidylic acid (poly(I:C)) on the expression of adhesion molecules and macrophage colony-stimulating factor (M-CSF) on resident glomerular cells. Experiments in MCs demonstrated that the activation of viral receptors by poly(I:C) leads to a time- and dose-dependent induction of intercellular adhesion molecule 1 (ICAM-1), vascular cell adhesion molecule 1 (VCAM-1) and M-CSF at both the mRNA and protein levels; these results were confirmed by incubating MCs with HCV RNA. As shown in knockdown experiments, this effect is specifically mediated by TLR3. The prestimulation of MCs with proinflammatory cytokines increases the effects of poly(I:C), except for its induction of VCAM-1. Tumor-necrosis factor (TNF)-α, likewise, induces ICAM-1, VCAM-1 and M-CSF, and amplifies the mesangial response to poly(I:C). These results were confirmed by incubating MCs with HCV RNA. We thus provide evidence that human MCs represent a potential target of the leukocytes and monocytes that infiltrate the glomerulus in viral disease-associated GN, highlighting the possibility that MCs may act as resident antigen-presenting cells. 
 Baculovirus transduction of mesenchymal stem cells triggers the toll-like receptor 3 pathway. Human mesenchymal stem cells (hMSCs) can be genetically modified with viral vectors and hold promise as a cell source for regenerative medicine, yet how hMSCs respond to viral vector transduction remains poorly understood, leaving the safety concerns unaddressed. Here, we explored the responses of hMSCs against an emerging DNA viral vector, baculovirus (BV), and discovered that BV transduction perturbed the transcription of 816 genes associated with five signaling pathways. Surprisingly, Toll-like receptor-3 (TLR3), a receptor that generally recognizes double-stranded RNA, was apparently upregulated by BV transduction, as confirmed by microarray, PCR array, flow cytometry, and confocal microscopy. Cytokine array data showed that BV transduction triggered robust secretion of interleukin-6 (IL-6) and IL-8 but not of other inflammatory cytokines and beta interferon (IFN-beta). BV transduction activated the signaling molecules (e.g., Toll/interleukin-1 receptor domain-containing adaptor-inducing IFN-beta, NF-kappaB, and IFN regulatory factor 3) downstream of TLR3, while silencing the TLR3 gene with small interfering RNA considerably abolished cytokine expression and promoted cell   migration  . These data demonstrate, for the first time, that a DNA viral vector can activate the TLR3 pathway in hMSCs and lead to a cytokine expression profile distinct from that in immune cells. These findings underscore the importance of evaluating whether the TLR3 signaling cascade plays roles in the immune response provoked by other DNA vectors (e.g., adenovirus). Nonetheless, BV transduction barely disturbed surface marker expression and induced only transient and mild cytokine responses, thereby easing the safety concerns of using BV for hMSCs engineering. 
 A TRIF-independent branch of TLR3 signaling. dsRNA is a common pathogen-associated molecular pattern that is recognized by cellular TLR3 and used by virus-infected cells to activate specific transcription factors and trigger induction of antiviral genes. In this article, we report a new branch of TLR3 signaling that does not lead to gene induction but affects many cellular properties, such as cell   migration  , adhesion, and proliferation. We demonstrated that the   migration   of multiple cell lineages was affected by dsRNA treatment or influenza virus infection in a TLR3-dependent fashion. Surprisingly, for this effect of TLR3 signaling, the adaptor proteins, TRIF and MyD88, were not required. The effects of the new pathway were mediated by the proto-oncoprotein c-Src, which bound to TLR3 after dsRNA stimulation of cells. The response was biphasic: upon dsRNA treatment, we observed an immediate increase in cell motility followed by its strong inhibition. Our results indicate that the first phase was mediated by dsRNA-induced phosphorylation and activation of Src, whereas the second phase resulted from the sequestration of activated Src in lipid rafts, thus decreasing its active cytoplasmic pool. As expected, two other functions of Src, its effect on cell adhesion and cell proliferation, were also inhibited by dsRNA treatment. These results demonstrate that activated TLR3 can engage Src to trigger multiple cellular effects and reveal a possible link between innate immune response and cell growth regulation. This study also provides a rare example of TLR-mediated cellular effects that do not require gene induction and the first example, to our knowledge, of an adaptor-independent effect of any TLR. 
 Toll-like receptor 3 expression inhibits cell invasion and   migration   and predicts a favorable prognosis in neuroblastoma. To evaluate the clinical significance of TLR3 expression on neuroblastomas, we performed immunohistochemical study on archival tissues and in vitro studies on neuroblastoma cell lines. The results showed that positive TLR3 expression was associated with favorable histology and prognosis. Activation of TLR3 by polyinosinic:polycytidylic acid [poly(I:C)] treatment is effective to suppress cell   migration   and invasion and to decrease organized assembly of F-actin and filopodia formation, in TLR3-expressing SK-N-AS cells, which could be reversed by TLR3-targeting siRNA treatment. TLR3 agonist poly(I:C) promotes GAP-43 expression also in SK-N-AS cells only. Taken together, TLR3 could serve to predict favorable behavior in neuroblastomas. 
 Activation of toll-like receptors 2, 3 or 5 induces matrix metalloproteinase-1 and -9 expression with the involvement of MAPKs and NF-kappaB in human epidermal keratinocytes. Toll-like receptors (TLRs) on epidermal keratinocytes are the first line of defense against microbe invasion, and matrix metalloproteases (MMPs) regulate inflammation, cell   migration   and wound healing. In this study, we demonstrate that the mRNA and protein expressions of MMP-1 and MMP-9 in human epidermal keratinocytes are induced by ligands for TLR2, TLR3 and TLR5 [Pam3CSK4, Poly(I:C) and flagellin, respectively] in a dose-dependent manner. We also found that the ligands for TLR2, TLR3 and TLR5 activate the MAP kinases, JNK and p38 MAPK, but not ERK1/2. Furthermore, treatment with the ligands for TLR2, TLR3 and TLR5 also induced the degradation of IkappaB-alpha and activated the nuclear translocation of NF-kappaB. MMP-1 induction by the ligands for TLR2, TLR3 and TLR5 was inhibited by pretreatment with BAY11-7082 (NF-kappaB inhibitor) or SP600125 (JNK inhibitor), whereas MMP-9 expression was inhibited by pretreatment with BAY11-7082, SP600125 or SB203580. These findings demonstrate that the activation of TLR2, TLR3 or TLR5 induces the expression of MMP-1 and MMP-9 in human epidermal keratinocytes. In addition, NF-kappaB or JNK mediated the MMP-1 expression induced by TLR2, TLR3 and TLR5, whereas NF-kappaB, JNK or p38 MAPK mediated the MMP-9 expression induced by TLR2, TLR3 and TLR5. 
 Toll-like receptor 3 on adult human astrocytes triggers production of neuroprotective mediators. Toll-like receptors (TLRs) are innate immunity receptors that are expressed on a wide range of cell types, including CNS glial cells. In general, TLR engagement by specific sets of microbial ligands triggers production of pro-inflammatory factors and enhances antigen-presenting cell functions. The functional roles of TLR in the CNS, however, are still poorly understood. While adult human astrocytes in culture dominantly express TLR4, they display a strikingly strong and selective induction of TLR3 when activated by pro-inflammatory cytokines, TLR3 or TLR4 agonists, or oxidative stress. Gene profiling analysis of the astrocyte response to either TLR3 or TLR4 activation revealed that TLR3, but not TLR4, induces expression of a range of neuroprotective mediators and several other molecules that regulate cellular growth, differentiation, and   migration  . Also, TLR3 triggered enhanced production of anti-inflammatory cytokines including interleukin-9 (IL-9), IL-10, and IL-11 and downregulation of the p40 subunit of IL-12 and IL-23. The collective TLR3-induced products were found in functional assays to inhibit astrocyte growth, promote human endothelial cell growth, and importantly, to enhance neuronal survival in organotypic human brain slice cultures. Together, our data indicate that TLR3 is induced on human astrocytes upon inflammation and when activated, mediates a comprehensive neuroprotective response rather than a polarized pro-inflammatory reaction. 
 High basal levels of functional toll-like receptor 3 (TLR3) and noncanonical Wnt5a are expressed in papillary thyroid cancer and are coordinately decreased by phenylmethimazole together with cell proliferation and   migration  . High basal levels of TLR3 and Wnt5a RNA are present in papillary thyroid carcinoma (PTC) cell lines consistent with their overexpression and colocalization in PTC cells in vivo. This is not the case in thyrocytes from normal tissue and in follicular carcinoma (FC) or anaplastic carcinoma (AC) cells or tissues. The basally expressed TLR3 are functional in PTC cells as evidenced by the ability of double-strand RNA (polyinosine-polycytidylic acid) to significantly increase the activity of transfected NF-kappaB and IFN-beta luciferase reporter genes and the levels of two end products of TLR3 signaling, IFN-beta and CXCL10. Phenylmethimazole (C10), a drug that decreases TLR3 expression and signaling in FRTL-5 thyrocytes, decreases TLR3 levels and signaling in PTC cells in a concentration-dependent manner. C10 also decreased Wnt5a RNA levels coordinate with decreases in TLR3. E-cadherin RNA levels, whose suppression may be associated with high Wnt5a, increased with C10 treatment. C10 simultaneously decreased PTC proliferation and cell   migration   but had no effect on the growth and   migration   of FC, AC, or FRTL-5 cells. C10 decreases high basal phosphorylation of Tyr705 and Ser727 on Stat3 in PTC cells and inhibits IL-6-induced Stat3 phosphorylation. IL-6-induced Stat3 phosphorylation is important both in up-regulating Wnt5a levels and in cell growth. In sum, high Wnt5a levels in PTC cells may be related to high TLR3 levels and signaling; and the ability of phenylmethimazole (C10) to decrease growth and   migration   of PTC cells may be related to its suppressive effect on TLR3 and Wnt5a signaling, particularly Stat3 activation. 
 Arthritis is associated with T-cell-induced upregulation of Toll-like receptor 3 on synovial fibroblasts. Toll-like receptors (TLRs) are likely to play crucial roles in the pathogenesis of rheumatoid arthritis (RA). The aim of this study was to determine the key TLRs in synovium and explore their roles in the activation of fibroblast-like synoviocytes (FLSs) mediated by T cells in arthritis.Pristane-induced arthritis (PIA) was established by subcutaneous injection with pristane at the base of the rat's tail. TLR expression in synovium from PIA rats was detected at different time points by performing real-time PCR. Polyinosinic:polycytidylic acid (poly(I:C)) was intra-articularly administrated to PIA rats, and arthritis was monitored macroscopically and microscopically. Synovial TLR3 was detected by immunohistochemical staining. Rat FLSs were stimulated with pristane-primed T cells or pristane-primed, T-cell conditioned medium. The intervention of TLR3 in FLSs was achieved by specific short-hairpin RNA (shRNA) or an antibody. The   migration   ability of FLSs was measured by using the scratch test, and gene expression was detected by using real-time PCR. FLSs from RA patients were stimulated with various cytokines and TLR ligands, and TLR3 expression was detected by performing real-time PCR. In addition, with different concentrations of poly(I:C) stimulation, TLR3 expression of FLSs from RA patients and patients with osteoarthritis (OA) was compared.Synovium TLR3 displayed early and persistent overexpression in PIA rats. TLR3 was expressed in FLSs, and local treatment with poly(I:C) synergistically aggravated the arthritis. Rat FLSs co-cultured with pristane-primed T cells showed strengthened   migration   ability and significant upregulation of TLR3, IFN-β, IL-6 and matrix metalloproteinase 3 (MMP3) expression, which could also be induced by pristane-primed, T-cell conditioned medium. The upregulation of cytokines and MMPs was blocked by shRNA or TLR3 antibodies. In RA FLSs with cytokine or TLR ligand stimulation, TLR3 expression exhibited remarkable upregulation. Furthermore, RA FLSs showed higher reactivity than OA FLSs to poly(I:C).TLR3 in the synovium of PIA rats was overexpressed, and activation of the TLR3 signaling pathway could aggravate this arthritis. The induction of TLR3 in FLSs resulted from T cell-derived inflammatory stimulation and could further mediate FLS activation in arthritis. We conclude that TLR3 upregulation of FLSs activated by T cells results in articular inflammation. 
  GeneRIFs  
 The Src activation by TLR3 changes cell   migration  , adhesion, and proliferation rates and how IRF-3 activation by RLR triggers a gene induction-independent pro-apoptotic pathway that provides strong antiviral protection. 
 TLR3 has a dual role in pharynx metastatic cell line: on one hand TLR3 activation drove cells to apoptosis while on the other it contributed to tumor progression by altering the expression of tumor promoting genes and enhancing the cell   migration  . 
 Ability of phenylmethimazole (C10) to decrease growth and   migration   of papillary thyroid cancer cells may be related to its suppressive effect on TLR3 and Wnt5a signaling. 
  ECM  PubMed  
 Activation of Toll-like receptor 3 augments myofibroblast differentiation. Airway remodeling is observed in the airways of patients with asthma, and differentiation of fibroblasts to myofibroblasts plays a critical role in the progress of airway remodeling. Viral infection induces not only the disease development and exacerbations but also airway remodeling. The aim of this study was to evaluate whether the activation of Toll-like receptor 3 (TLR3) can affect the differentiation of fibroblasts to myofibroblasts and the extracellular matrix (  ECM  ) protein production. Human fetal lung fibroblasts (HFL-1) and adult lung fibroblasts were treated with a synthetic double-stranded RNA, polyinosine-polycytidylic acid (poly[I:C]) and the expression of alpha-smooth muscle actin (alpha-SMA), a marker of myofibroblast differentiation, was evaluated. The release of transforming growth factor-beta(1) (TGF-beta(1)) and   ECM   protein production were assessed. The effect of anti-TGF-beta antibody on the alpha-SMA and   ECM   production was also assessed. Poly(I:C) significantly augmented the alpha-SMA expression (P < 0.01) and release of TGF-beta(1) (P < 0.01) compared with control. Bafilomycin, an inhibitor of TLR3 signaling, diminished poly(I:C)-augmented TGF-beta(1) release. Anti-TGF-beta(1) antibody inhibited the poly(I:C)-augmented alpha-SMA expression. Poly(I:C) enhanced translocation of nuclear factor-kB (NF-kappaB) and interferon regulatory factor-3 (IRF-3) into the nucleus. Poly(I:C)-augmented TGF-beta(1) release was almost completely blocked by NF-kappaB inhibitors, but not by silencing IRF-3. The production of fibronectin and collagen I expression were significantly increased by poly(I:C) (P < 0.01) and they were inhibited by anti-TGF-beta antibody. These results suggest that activation of TLR3 can affect the differentiation to myofibroblasts and enhance   ECM   production via the NF-kappaB-TGF-beta(1)-dependent pathway. 
 Activation of Toll-like receptor 3 augments myofibroblast differentiation. Airway remodeling is observed in the airways of patients with asthma, and differentiation of fibroblasts to myofibroblasts plays a critical role in the progress of airway remodeling. Viral infection induces not only the disease development and exacerbations but also airway remodeling. The aim of this study was to evaluate whether the activation of Toll-like receptor 3 (TLR3) can affect the differentiation of fibroblasts to myofibroblasts and the extracellular matrix (  ECM  ) protein production. Human fetal lung fibroblasts (HFL-1) and adult lung fibroblasts were treated with a synthetic double-stranded RNA, polyinosine-polycytidylic acid (poly[I:C]) and the expression of alpha-smooth muscle actin (alpha-SMA), a marker of myofibroblast differentiation, was evaluated. The release of transforming growth factor-beta(1) (TGF-beta(1)) and   ECM   protein production were assessed. The effect of anti-TGF-beta antibody on the alpha-SMA and   ECM   production was also assessed. Poly(I:C) significantly augmented the alpha-SMA expression (P < 0.01) and release of TGF-beta(1) (P < 0.01) compared with control. Bafilomycin, an inhibitor of TLR3 signaling, diminished poly(I:C)-augmented TGF-beta(1) release. Anti-TGF-beta(1) antibody inhibited the poly(I:C)-augmented alpha-SMA expression. Poly(I:C) enhanced translocation of nuclear factor-kB (NF-kappaB) and interferon regulatory factor-3 (IRF-3) into the nucleus. Poly(I:C)-augmented TGF-beta(1) release was almost completely blocked by NF-kappaB inhibitors, but not by silencing IRF-3. The production of fibronectin and collagen I expression were significantly increased by poly(I:C) (P < 0.01) and they were inhibited by anti-TGF-beta antibody. These results suggest that activation of TLR3 can affect the differentiation to myofibroblasts and enhance   ECM   production via the NF-kappaB-TGF-beta(1)-dependent pathway. 
  GeneRIFs  
 activation of TLR3 can affect the differentiation to myofibroblasts and enhance   ECM   production via the NF-kappaB-TGF-beta(1)-dependent pathway 
  [Ee]xtracellular [Mm]atrix  PubMed  
 Activation of Toll-like receptor 3 augments myofibroblast differentiation. Airway remodeling is observed in the airways of patients with asthma, and differentiation of fibroblasts to myofibroblasts plays a critical role in the progress of airway remodeling. Viral infection induces not only the disease development and exacerbations but also airway remodeling. The aim of this study was to evaluate whether the activation of Toll-like receptor 3 (TLR3) can affect the differentiation of fibroblasts to myofibroblasts and the   extracellular matrix   (ECM) protein production. Human fetal lung fibroblasts (HFL-1) and adult lung fibroblasts were treated with a synthetic double-stranded RNA, polyinosine-polycytidylic acid (poly[I:C]) and the expression of alpha-smooth muscle actin (alpha-SMA), a marker of myofibroblast differentiation, was evaluated. The release of transforming growth factor-beta(1) (TGF-beta(1)) and ECM protein production were assessed. The effect of anti-TGF-beta antibody on the alpha-SMA and ECM production was also assessed. Poly(I:C) significantly augmented the alpha-SMA expression (P < 0.01) and release of TGF-beta(1) (P < 0.01) compared with control. Bafilomycin, an inhibitor of TLR3 signaling, diminished poly(I:C)-augmented TGF-beta(1) release. Anti-TGF-beta(1) antibody inhibited the poly(I:C)-augmented alpha-SMA expression. Poly(I:C) enhanced translocation of nuclear factor-kB (NF-kappaB) and interferon regulatory factor-3 (IRF-3) into the nucleus. Poly(I:C)-augmented TGF-beta(1) release was almost completely blocked by NF-kappaB inhibitors, but not by silencing IRF-3. The production of fibronectin and collagen I expression were significantly increased by poly(I:C) (P < 0.01) and they were inhibited by anti-TGF-beta antibody. These results suggest that activation of TLR3 can affect the differentiation to myofibroblasts and enhance ECM production via the NF-kappaB-TGF-beta(1)-dependent pathway. 
 A genetic association study of maternal and fetal candidate genes that predispose to preterm prelabor rupture of membranes (PROM). We sought to determine whether maternal/fetal single-nucleotide polymorphisms (SNPs) in candidate genes are associated with preterm prelabor rupture of membranes (pPROM).A case-control study was conducted in patients with pPROM (225 mothers and 155 fetuses) and 599 mothers and 628 fetuses with a normal pregnancy; 190 candidate genes and 775 SNPs were studied. Single locus/haplotype association analyses were performed; false discovery rate was used to correct for multiple testing (q* = 0.15).First, a SNP in tissue inhibitor of metalloproteinase 2 in mothers was significantly associated with pPROM (odds ratio, 2.12; 95% confidence interval, 1.47-3.07; P = .000068), and this association remained significant after correction for multiple comparisons. Second, haplotypes for Alpha 3 type IV collagen isoform precursor in the mother were associated with pPROM (global P = .003). Third, multilocus analysis identified a 3-locus model, which included maternal SNPs in collagen type I alpha 2, defensin alpha 5 gene, and endothelin 1.DNA variants in a maternal gene involved in   extracellular matrix   metabolism doubled the risk of pPROM. 
 Identification of fetal and maternal single nucleotide polymorphisms in candidate genes that predispose to spontaneous preterm labor with intact membranes. The purpose of this study was to determine whether maternal/fetal single nucleotide polymorphisms (SNPs) in candidate genes are associated with spontaneous preterm labor/delivery.A genetic association study was conducted in 223 mothers and 179 fetuses (preterm labor with intact membranes who delivered <37 weeks of gestation [preterm birth (PTB)]), and 599 mothers and 628 fetuses (normal pregnancy); 190 candidate genes and 775 SNPs were studied. Single locus/haplotype association analyses were performed; the false discovery rate was used to correct for multiple testing.The strongest single locus associations with PTB were interleukin-6 receptor 1 (fetus; P=.000148) and tissue inhibitor of metalloproteinase 2 (mother; P=.000197), which remained significant after correction for multiple comparisons. Global haplotype analysis indicated an association between a fetal DNA variant in insulin-like growth factor F2 and maternal alpha 3 type IV collagen isoform 1 (global, P=.004 and .007, respectively).An SNP involved in controlling fetal inflammation (interleukin-6 receptor 1) and DNA variants in maternal genes encoding for proteins involved in   extracellular matrix   metabolism approximately doubled the risk of PTB. 
 Activation of Toll-like receptor 3 augments myofibroblast differentiation. Airway remodeling is observed in the airways of patients with asthma, and differentiation of fibroblasts to myofibroblasts plays a critical role in the progress of airway remodeling. Viral infection induces not only the disease development and exacerbations but also airway remodeling. The aim of this study was to evaluate whether the activation of Toll-like receptor 3 (TLR3) can affect the differentiation of fibroblasts to myofibroblasts and the   extracellular matrix   (ECM) protein production. Human fetal lung fibroblasts (HFL-1) and adult lung fibroblasts were treated with a synthetic double-stranded RNA, polyinosine-polycytidylic acid (poly[I:C]) and the expression of alpha-smooth muscle actin (alpha-SMA), a marker of myofibroblast differentiation, was evaluated. The release of transforming growth factor-beta(1) (TGF-beta(1)) and ECM protein production were assessed. The effect of anti-TGF-beta antibody on the alpha-SMA and ECM production was also assessed. Poly(I:C) significantly augmented the alpha-SMA expression (P < 0.01) and release of TGF-beta(1) (P < 0.01) compared with control. Bafilomycin, an inhibitor of TLR3 signaling, diminished poly(I:C)-augmented TGF-beta(1) release. Anti-TGF-beta(1) antibody inhibited the poly(I:C)-augmented alpha-SMA expression. Poly(I:C) enhanced translocation of nuclear factor-kB (NF-kappaB) and interferon regulatory factor-3 (IRF-3) into the nucleus. Poly(I:C)-augmented TGF-beta(1) release was almost completely blocked by NF-kappaB inhibitors, but not by silencing IRF-3. The production of fibronectin and collagen I expression were significantly increased by poly(I:C) (P < 0.01) and they were inhibited by anti-TGF-beta antibody. These results suggest that activation of TLR3 can affect the differentiation to myofibroblasts and enhance ECM production via the NF-kappaB-TGF-beta(1)-dependent pathway. 
 A genetic association study of maternal and fetal candidate genes that predispose to preterm prelabor rupture of membranes (PROM). We sought to determine whether maternal/fetal single-nucleotide polymorphisms (SNPs) in candidate genes are associated with preterm prelabor rupture of membranes (pPROM).A case-control study was conducted in patients with pPROM (225 mothers and 155 fetuses) and 599 mothers and 628 fetuses with a normal pregnancy; 190 candidate genes and 775 SNPs were studied. Single locus/haplotype association analyses were performed; false discovery rate was used to correct for multiple testing (q* = 0.15).First, a SNP in tissue inhibitor of metalloproteinase 2 in mothers was significantly associated with pPROM (odds ratio, 2.12; 95% confidence interval, 1.47-3.07; P = .000068), and this association remained significant after correction for multiple comparisons. Second, haplotypes for Alpha 3 type IV collagen isoform precursor in the mother were associated with pPROM (global P = .003). Third, multilocus analysis identified a 3-locus model, which included maternal SNPs in collagen type I alpha 2, defensin alpha 5 gene, and endothelin 1.DNA variants in a maternal gene involved in   extracellular matrix   metabolism doubled the risk of pPROM. 
 Identification of fetal and maternal single nucleotide polymorphisms in candidate genes that predispose to spontaneous preterm labor with intact membranes. The purpose of this study was to determine whether maternal/fetal single nucleotide polymorphisms (SNPs) in candidate genes are associated with spontaneous preterm labor/delivery.A genetic association study was conducted in 223 mothers and 179 fetuses (preterm labor with intact membranes who delivered <37 weeks of gestation [preterm birth (PTB)]), and 599 mothers and 628 fetuses (normal pregnancy); 190 candidate genes and 775 SNPs were studied. Single locus/haplotype association analyses were performed; the false discovery rate was used to correct for multiple testing.The strongest single locus associations with PTB were interleukin-6 receptor 1 (fetus; P=.000148) and tissue inhibitor of metalloproteinase 2 (mother; P=.000197), which remained significant after correction for multiple comparisons. Global haplotype analysis indicated an association between a fetal DNA variant in insulin-like growth factor F2 and maternal alpha 3 type IV collagen isoform 1 (global, P=.004 and .007, respectively).An SNP involved in controlling fetal inflammation (interleukin-6 receptor 1) and DNA variants in maternal genes encoding for proteins involved in   extracellular matrix   metabolism approximately doubled the risk of PTB. 
 Deleterious role of TLR3 during hyperoxia-induced acute lung injury. Acute respiratory distress syndrome (ARDS) manifests clinically as a consequence of septic and/or traumatic injury in the lung. Oxygen therapy remains a major therapeutic intervention in ARDS, but this can contribute further to lung damage. Patients with ARDS are highly susceptible to viral infection and it may be due to altered Toll-like receptor (TLR) expression.To evaluate the role of TLR3 in ARDS.TLR3 expression and signaling was determined in airway epithelial cells after in vitro hyperoxia challenge. Using a murine model of hyperoxia-induced lung injury, the role of TLR3 was determined using either TLR3-gene deficient mice or a specific neutralizing antibody directed to TLR3.Increased TLR3 expression was observed in airway epithelial cells from patients with ARDS. Further, hyperoxic conditions alone were a major stimulus for increased TLR3 expression and activation in cultured human epithelial cells. Interestingly, TLR3(-/-) mice exhibited less acute lung injury, activation of apoptotic cascades, and   extracellular matrix   deposition after 5 days of 80% oxygen compared with wild-type (TLR3(+/+)) mice under the same conditions. Administration of a monoclonal anti-TLR3 antibody to TLR3(+/+) mice exposed to hyperoxic conditions likewise protected these mice from lung injury and inflammation.The potential for redundancy in function as well as cross-talk between distinct TLRs may indeed contribute to whether the inflammatory cascade can be effectively disrupted once signaling has been initiated. Together, these data show that TLR3 has a major role in the development of ARDS-like pathology in the absence of a viral pathogen. 
  [Ii]nvasive  PubMed  
 Induction of c-Myc-dependent cell proliferation through toll-like receptor 3 in head and neck cancer. Protein expression of human toll-like receptors (TLR) 1-10 was measured in cell lines and solid tumors of head and neck squamous cell carcinoma (HNSCC). All HNSCC cell lines and 80% of solid tumors were found to express TLR3 as a predominantly intracellular protein, while no other TLR proteins were expressed. TLR3 has previously been shown to contribute to the activation of nuclear factor-kappaB (NF-kappaB), a transcription factor which promotes several types of human cancers. Significantly, NF-kappaB expression was strongest in protein extracts from carcinoma tissue in which TLR3 was overexpressed. Inhibition of TLR3 expression in permanent HNSCC cell lines resulted in decreased expression of the oncoprotein c-Myc resulting in decreased cell proliferation. Correspondingly, overexpression of human TLR3 in mouse fibroblasts resulted in an upregulation of c-Myc and increased sensitivity for PolyI:C-induced cell proliferation. Our data suggest that TLR3 contributes to the malignant phenotype leading to   invasive   carcinoma in HNSCC. 
 Induction of c-Myc-dependent cell proliferation through toll-like receptor 3 in head and neck cancer. Protein expression of human toll-like receptors (TLR) 1-10 was measured in cell lines and solid tumors of head and neck squamous cell carcinoma (HNSCC). All HNSCC cell lines and 80% of solid tumors were found to express TLR3 as a predominantly intracellular protein, while no other TLR proteins were expressed. TLR3 has previously been shown to contribute to the activation of nuclear factor-kappaB (NF-kappaB), a transcription factor which promotes several types of human cancers. Significantly, NF-kappaB expression was strongest in protein extracts from carcinoma tissue in which TLR3 was overexpressed. Inhibition of TLR3 expression in permanent HNSCC cell lines resulted in decreased expression of the oncoprotein c-Myc resulting in decreased cell proliferation. Correspondingly, overexpression of human TLR3 in mouse fibroblasts resulted in an upregulation of c-Myc and increased sensitivity for PolyI:C-induced cell proliferation. Our data suggest that TLR3 contributes to the malignant phenotype leading to   invasive   carcinoma in HNSCC. 
 TLR3 and TLR4 as potential clinical biomarkers for in-stent restenosis in drug-eluting stents patients. In-stent restenosis is still a clinic trouble for percutaneous coronary intervention in drug-stent era. The molecular basis of restenosis is intensively associated with inflammation. TLR3 and TLR4 as innate immune factors have been proven to play a key role in atherosclerosis disease. The aim of this study is to study the TLR3 and TLR4 expressions and their downstream signaling proteins in the inflammatory process of restenosis after drug-stent therapy. mRNA and protein expression of TLR3 and TLR4 were detected in peripheral blood monocytes of primary group (n = 38), N-ISR group (n = 36) and ISR group (n = 33). Some inflammatory factors (including TLR3 and TLR4) were evaluated in serum of three groups. mRNA and protein expression of TLR3 and TLR4 and their downstream signaling proteins have shown a higher level in restenosis patients than non-restenosis patients and even primary patients who accepted first stent therapy. In serum, different from some nonspecific and downstream inflammatory factors, TLR3 and TLR4 also show a significantly higher level in ISR group compared with N-ISR group and primary group. This study provides a potential clinical biomarker for in-stent restenosis in drug-stent patients and some interesting data about the role of TLRs and their downstream signaling factors in the inflammatory process of in-stent restenosis. Compared with first stent therapy and non-restenosis patients, it is hopeful that TLR3 and TLR4 are potential non  invasive   biomarkers in prognosis restenosis. 
 Toll-like receptor 4 polymorphisms and aspergillosis in stem-cell transplantation. Toll-like receptors (TLRs) are essential components of the immune response to fungal pathogens. We examined the role of TLR polymorphisms in conferring a risk of   invasive   aspergillosis among recipients of allogeneic hematopoietic-cell transplants.We analyzed 20 single-nucleotide polymorphisms (SNPs) in the toll-like receptor 2 gene (TLR2), the toll-like receptor 3 gene (TLR3), the toll-like receptor 4 gene (TLR4), and the toll-like receptor 9 gene (TLR9) in a cohort of 336 recipients of hematopoietic-cell transplants and their unrelated donors. The risk of   invasive   aspergillosis was assessed with the use of multivariate Cox regression analysis. The analysis was replicated in a validation study involving 103 case patients and 263 matched controls who received hematopoietic-cell transplants from related and unrelated donors.In the discovery study, two donor TLR4 haplotypes (S3 and S4) increased the risk of   invasive   aspergillosis (adjusted hazard ratio for S3, 2.20; 95% confidence interval [CI], 1.14 to 4.25; P=0.02; adjusted hazard ratio for S4, 6.16; 95% CI, 1.97 to 19.26; P=0.002). The haplotype S4 was present in carriers of two SNPs in strong linkage disequilibrium (1063 A/G [D299G] and 1363 C/T [T399I]) that influence TLR4 function. In the validation study, donor haplotype S4 also increased the risk of   invasive   aspergillosis (adjusted odds ratio, 2.49; 95% CI, 1.15 to 5.41; P=0.02); the association was present in unrelated recipients of hematopoietic-cell transplants (odds ratio, 5.00; 95% CI, 1.04 to 24.01; P=0.04) but not in related recipients (odds ratio, 2.29; 95% CI, 0.93 to 5.68; P=0.07). In the discovery study, seropositivity for cytomegalovirus (CMV) in donors or recipients, donor positivity for S4, or both, as compared with negative results for CMV and S4, were associated with an increase in the 3-year probability of   invasive   aspergillosis (12% vs. 1%, P=0.02) and death that was not related to relapse (35% vs. 22%, P=0.02).This study suggests an association between the donor TLR4 haplotype S4 and the risk of   invasive   aspergillosis among recipients of hematopoietic-cell transplants from unrelated donors. 
 Evidence for possible role of toll-like receptor 3 mediating virus-induced progression of pituitary adenomas. Tumor-related viruses are known to be involved in initiation and progression of certain tumors. However, the relationship between virus and pituitary adenomas (PAs) remains unknown. Here, we investigated infection status of three types of viruses (HPV16, HHV6B and HSV1) and expression level of toll-like receptor 3 (TLR3) in 60 human PA samples. We also determined the role of TLR3 signaling pathway on a PA cell line (GH3). We firstly found that positive rates of HPV16 and HHV6B infection were significantly higher in   invasive   PA samples than in non  invasive   samples (P < 0.01). Similarly, TLR3 mRNA and protein expression also increased i  n invasi  ve PA samples (P < 0.01). In vitro analysis indicated that GH3 cell proliferation and survival were enhanced by TLR3 activation, which was accompanied by NF-κB activation. Our data indicate that HPV16 and HHV6B viruses may be involved in promoting the progression of PA by activating the TLR3 signaling pathway. 
 Toll-like receptor 3: implications for proinflammatory microenvironment in human breast cancer. Under many circumstances, the host constituents that are found in the tumor microenvironment support a malignancy network and provide the cancer cells with advantages in proliferation,   invasive  ness and metastasis establishment at remote organs. It is known that Toll like receptors (TLRs) are expressed not only on immune cells but also on cancer cells and it has suggested a deleterious role for TLR3 in inflammatory disease. Hypothesizing that altered IFNγ signaling may be a key mechanism of immune dysfunction common to cancer as well CXCR4 is overexpressed among breast cancer patients, the mRNA expression of TLR3, CXCR4 and IFNγ in breast cancer tumor tissues was investigated. No statistically significant differences in the expression of CXCR4 mRNA, IFNγ and TLR3 between healthy and tumor tissues was observed, however, it was verified a positive correlation between mRNA relative expression of TLR3 and CXCR4 (p < 0.001), and mRNA relative expression of TLR3 was significantly increased in breast cancer tumor tissue when compared to healthy mammary gland tissue among patients expressing high IFNγ (p = 0.001). Since the tumor microenvironment plays important roles in cancer initiation, growth, progression, invasion and metastasis, it is possible to propose that an overexpression of IFNγ mRNA due to the pro-inflammatory microenvironment can lead to an up-regulation of CXCR4 mRNA and consequently to an increased TLR3 mRNA expression even among nodal negative patients. In the future, a comprehensive study of TLR3, CXCR4 and IFNγ axis in primary breast tumors and corresponding healthy tissues will be crucial to further understanding of the cancer network. 
  GeneRIFs  
 These data suggest that TLR3 contributes to the malignant phenotype leading to   invasive   carcinoma in head and neck squamous cell carcinoma. 
  [Mm]etastasis  PubMed  
 TLR-3 polymorphism is an independent prognostic marker for stage II colorectal cancer. Clinicopathologic stage is still the main parameter to evaluate the prognosis of newly diagnosed colorectal cancer (CRC) patients. Although molecular markers have been suggested for follow up of treated CRC patients, their complete clinical application is still under evaluation.To evaluate the association of immune-related genes with CRC prognosis and survival, a total of 19 single nucleotide polymorphisms (SNPs) were genotyped in 614 German patients within the Kiel cohort (POPGEN).A promoter variant (rs1800872) in the Interleukin-10 (IL-10) gene was associated with an increased lymph node   metastasis   involvement [odds ratio (OR) = 2.1, 95% confidence interval (CI) = 1.03-4.2, for carriers of the TT genotype]. More importantly, among 582 followed up patients the SNP rs3775291 in the toll-like receptor 3 (TLR-3) gene was associated with CRC specific survival (150 events). Patients carrying the TT genotype had a 93% increased risk of death compared with the CC carriers [hazard ratio (HR) = 1.93, 95% CI 1.14-3.28]. The observed effect of the TLR-3 variant was restricted to stage II patients (HR = 4.14, 95% CI 1.24-13.84) and to patients who did not receive adjuvant therapy (HR = 3.2, 95% CI 1.4-7.7).Our results may provide additional candidates for risk assessment in stage II CRC patients for treatment decision. Further validation of the presented findings is warranted. 
 Innate adjuvant receptor Toll-like receptor 3 can promote breast cancer through cell surface. Toll-like receptor 3 has been targeted in different cancers for adjuvant therapy. The ligand-mediated effects of TLR-3 on cancer cells are discordant. In the present work, we have addressed the hypothesis possibility of cell membrane-bound action of TLR-3 in breast cancer to justify its pro-tumor effect. TLR-3 was stimulated by Poly (I:C) on the surface of human breast cancer cells MCF-7 and MDA-MB-231 for up to 72 h. To check the cell survival and growth, thiazol blue tetrazolium bromide (MTT) assay, apoptosis assay, and cell cycle analysis were carried out. For changes in the metastatic properties, in vitro colony formation assay, scratch-wound healing assay and adhesion assay were also done. Using real-time PCR and immunocytochemistry, expression of E-cadherin, was studied. To determine the affect of cytoplasmic stimulation, Poly (I:C) was delivered with lipid transfection reagent. The results of the aforesaid experiments showed that there was a gradual increase of cellular survivability, growth, and   metastasis   after the cell surface stimulation of TLR-3 with Poly (I:C). Interestingly, E-cadherin expression was increased both at transcriptional and translational level. On the other hand, when Poly (I:C) was delivered in the cytoplasm by lipid transfecting medium, the cells survivability was decreased. For the first time, in the present work, we are convincingly reporting the functional evidence that TLR-3 induces cell survivability and   metastasis   through cell surface. The present work may help for the proper understanding of the adjuvant therapy of breast cancer. 
 The dual role of TLR3 in metastatic cell line. Toll-like receptors (TLRs) are members of transmembrane proteins that recognize conserved molecular motifs of viral and bacterial origin and initiate innate immune response. As the role of TLRs in tumors cells is still not clear, our aim was to investigate the role of TLR3 in primary tumor and metastatic cells (SW480, SW620, FaDu and Detroit 562). We have reported here on the dual role of TLR3 in pharynx metastatic cell line (Detroit 562); on one hand TLR3 activation drove cells to apoptosis while on the other its stimulation contributed to tumor progression by altering the expression of tumor promoting genes (PLAUR, RORB) and enhancing the cell migration potential. In addition, we have shown TLR3 signaling pathway is functional in another metastatic cancer cell line (SW620) suggesting TLR3 might be important in the process of tumor   metastasis  . Since TLR3 agonists have been used in tumor therapy with the aim to activate immune system, scientific contribution of this work is drawing attention to the importance of further work on this topic, especially pro-tumor effect of TLR3, in order to avoid possible side-effects. 
 Overexpression of TLR3, TLR4, TLR7 and TLR9 in esophageal squamous cell carcinoma. To investigate the expression of Toll-like receptor (TLR) 3, TLR4, TLR7 and TLR9 in esophageal squamous cell carcinoma (ESCC).Reverse transcription-polymerase chain reaction and immunohistochemistry were used to analyze the expression of TLR3, TLR4, TLR7 and TLR9 mRNA and protein in samples from 87 esophageal cancer patients consisting of both tumor and normal tissue.A significant increase in TLR3, TLR4, TLR7 and TLR9 mRNA levels was detected in ESCC samples. Tumors exhibited high TLR protein expression, (70.1%, 72.4%, 66.7% and 78.2% for TLR3, TLR4, TLR7 and TLR9, respectively, P < 0.05). Nevertheless, a significant percentage of tumors also exhibited TLR4 expression in mononuclear inflammatory cells (48.3%) and TLR9 expression in fibroblast-like cells (60.9%). Tumors with high TLR3 expression in tumor cells or high TLR4 expression in mononuclear inflammatory cells were significantly associated with a higher probability of lymph node   metastasis   and increased depth of invasion. However, tumors with high TLR9 expression in fibroblast-like cells were associated with low probabilities of invasion and   metastasis  . There was no significant variation between the expression of TLR3, TLR4, TLR7 and TLR9 among different ethnic groups.TLR3, TLR4, TLR7 and TLR9 expression appears important to the biological pathogenesis of ESCC. TLRs may represent therapeutic targets for ESCC. 
 Study of TLR3, TLR4 and TLR9 in breast carcinomas and their association with   metastasis  . Toll-like receptors (TLRs) have garnered an extraordinary amount of interest in cancer research due to their role in tumor progression. By activating the production of several biological factors, TLRs induce type I interferons and other cytokines, which drive an inflammatory response and activate the adaptive immune system. The aim of this study was to investigate the expression and clinical relevance of TLR3, 4 and 9 in breast cancer.The expression levels of TLR3, TLR4 and TLR9 were analyzed on tumors from 74 patients with breast cancer. The analysis was performed by immunohistochemistry.Samples of carcinomas with recurrence exhibited a significant increase in the mRNA levels of TLR3, TLR4 and TLR9. Tumors showed high expression of TLRs expression levels by cancer cells, especially TLR4 and 9. Nevertheless, a significant percentage of tumors also showed TLR4 expression by mononuclear inflammatory cells (21.6%) and TLR9 expression by fibroblast-like cells (57.5%). Tumors with high TLR3 expression by tumor cell or with high TLR4 expression by mononuclear inflammatory cells were significantly associated with higher probability of   metastasis  . However, tumours with high TLR9 expression by fibroblast-like cells were associated with low probability of   metastasis  .The expression levels of TLR3, TLR4 and TLR9 have clinical interest as indicators of tumor aggressiveness in breast cancer. TLRs may represent therapeutic targets in breast cancer. 
 Toll-like receptor 3: implications for proinflammatory microenvironment in human breast cancer. Under many circumstances, the host constituents that are found in the tumor microenvironment support a malignancy network and provide the cancer cells with advantages in proliferation, invasiveness and   metastasis   establishment at remote organs. It is known that Toll like receptors (TLRs) are expressed not only on immune cells but also on cancer cells and it has suggested a deleterious role for TLR3 in inflammatory disease. Hypothesizing that altered IFNγ signaling may be a key mechanism of immune dysfunction common to cancer as well CXCR4 is overexpressed among breast cancer patients, the mRNA expression of TLR3, CXCR4 and IFNγ in breast cancer tumor tissues was investigated. No statistically significant differences in the expression of CXCR4 mRNA, IFNγ and TLR3 between healthy and tumor tissues was observed, however, it was verified a positive correlation between mRNA relative expression of TLR3 and CXCR4 (p < 0.001), and mRNA relative expression of TLR3 was significantly increased in breast cancer tumor tissue when compared to healthy mammary gland tissue among patients expressing high IFNγ (p = 0.001). Since the tumor microenvironment plays important roles in cancer initiation, growth, progression, invasion   and metast  asis, it is possible to propose that an overexpression of IFNγ mRNA due to the pro-inflammatory microenvironment can lead to an up-regulation of CXCR4 mRNA and consequently to an increased TLR3 mRNA expression even among nodal negative patients. In the future, a comprehensive study of TLR3, CXCR4 and IFNγ axis in primary breast tumors and corresponding healthy tissues will be crucial to further understanding of the cancer network. 
  GeneRIFs  
 For the first time, in the present work, we are convincingly reporting the functional evidence that TLR-3 induces cell survivability and   metastasis   through cell surface. 
  [Cc]ell [Ii]nvasion  PubMed  
 Toll-like receptor 3 expression inhibits   cell invasion   and migration and predicts a favorable prognosis in neuroblastoma. To evaluate the clinical significance of TLR3 expression on neuroblastomas, we performed immunohistochemical study on archival tissues and in vitro studies on neuroblastoma cell lines. The results showed that positive TLR3 expression was associated with favorable histology and prognosis. Activation of TLR3 by polyinosinic:polycytidylic acid [poly(I:C)] treatment is effective to suppress cell migration and invasion and to decrease organized assembly of F-actin and filopodia formation, in TLR3-expressing SK-N-AS cells, which could be reversed by TLR3-targeting siRNA treatment. TLR3 agonist poly(I:C) promotes GAP-43 expression also in SK-N-AS cells only. Taken together, TLR3 could serve to predict favorable behavior in neuroblastomas. 
   
  
 CASP9 
 
  NCBI Gene  
  GeneRIF  
  Pubmed  
 
 
  Search Term  Rank  Fields  
  [Mm]igration  6  PubMed(1)  
  [Ee]xtracellular [Mm]atrix  8  PubMed(2)  
  [Ii]nvasive  10  PubMed(5)  
  [Mm]etastasis  12  PubMed(8); GeneRIFs(1)  
 
 
 hide/show details for CASP9 
  [Mm]igration  PubMed  
 Potential role of Notch1 signaling pathway in laryngeal squamous cell carcinoma cell line Hep-2 involving proliferation inhibition, cell cycle arrest, cell apoptosis, and cell   migration  . Head and neck cancer is the sixth most common cancer worldwide and laryngeal cancer represents the largest subgroup. However, the molecular mechanism underlying its malignant behavior and progression is not clarified. Accumulating evidence has shown that Notch1 signaling pathway plays a central role in carcinogenesis, but its potential role in regulating the development of laryngeal carcinoma, has not been characterized. Here, we identified that Notch1 signaling pathway was activated in laryngeal carcinoma accompanied with up-regulation of Notch1 and Hes1 expression. Overexpression of Notch1 in laryngeal carcinoma cell line Hep-2 led to suppression of tumor cellular proliferation and arrested cell cycle in the G0/G1 phase and induced cell apoptosis, which were coupled with the down-regulation of cyclin D1, cyclin E, cdk2 and bcl-2 and up-regulation of caspase-3, caspase-9 and p53. Most importantly, up-regulation of Notch1 expression also reduced the   migration   of Hep-2 cells, which was closely associated with down-regulation of MMP-2 and MMP-9. The finding may lay a foundation for further investigations into the Notch1 signaling pathway as a potential target for laryngeal carcinoma. 
  [Ee]xtracellular [Mm]atrix  PubMed  
 Integrin-linked kinase interacts with caspase-9 and -8 in an adhesion-dependent manner for promoting radiation-induced apoptosis in human leukemia cells. Integrin-mediated adhesion of leukemia cells to   extracellular matrix   proteins reduces apoptosis following radiation-induced genotoxic injury. To evaluate the role of integrin-linked kinase (ILK) in this process, HL60 human acute promyelocytic leukemia cells were stably transfected with ILK wild-type or kinase-hyperactive overexpression vectors. Suspension or fibronectin (FN) adhesion cultures were irradiated with X-rays and processed for measurement of apoptosis, mitochondrial transmembrane potential and caspase activation. Adhesion to FN pronouncedly reduced radiation-induced apoptosis of HL60 cells and vector controls. Intriguingly, overexpressed ILK enhanced apoptosis after irradiation by combined activation of caspase-3 through caspase-8 and -9 in irradiated FN cultures. Irradiation of ILK suspension cultures lacked caspase-8 activation, but showed serial cleavage of caspase-9, -3 and poly (ADP-ribose) polymerase. These findings further characterize the cell death-promoting function of ILK in DNA-damaged cells. Moreover, ILK might represent a potential therapeutic target for innovative chemo- and radiooncological approaches in hematological malignancies. 
 Recombinant Multivalent EMMPRIN Extracellular Domain Induces U937 Human Leukemia Cell Apoptosis by Downregulation of Monocarboxylate Transporter 1 and Activation of Procaspase-9. This study was carried out to understand the effect of the recombinant multivalent   extracellular matrix   metalloproteinase inducer (EMMPRIN) extracellular domain, designated as rmEMMPRINex, on the apoptotic cell death of human leukemia U937 cells. Expression of monocarboxylate transporter 1 (MCT1) and caspase-9 in U937 treated with rmEMMPRINex was investigated in this study. Levels of membrane MCT1 and intracellular procaspase-9 were decreased in rmEMMPRINex-treated cells in comparison to controls. However, the expression of activated caspase-9 was undetectable. rmEMMPRINex also induced DNA fragmentation and apoptosis in U937 cells. Taken together, we concluded that interaction of rmEMMPRINex with U937 cells leads to inhibition of MCT1 membrane expression, intracellular activation of procaspase-9, followed by DNA fragmentation and apoptosis. This may contribute to the conceptual development of novel cancer drugs in the future. 
  [Ii]nvasive  PubMed  
 Genetic susceptibility to distinct bladder cancer subphenotypes. Clinical, pathologic, and molecular evidence indicate that bladder cancer is heterogeneous with pathologic/molecular features that define distinct subphenotypes with different prognoses. It is conceivable that specific patterns of genetic susceptibility are associated with particular subphenotypes.To examine evidence for the contribution of germline genetic variation to bladder cancer heterogeneity.The Spanish Bladder Cancer/EPICURO Study is a case-control study based in 18 hospitals located in five areas in Spain. Cases were patients with a newly diagnosed, histologically confirmed, urothelial cell carcinoma of the bladder from 1998 to 2001. Case diagnoses were reviewed and uniformly classified by pathologists following the World Health Organisation/International Society of Urological Pathology 1999 criteria. Controls were hospital-matched patients (n=1149).A total of 1526 candidate variants in 423 candidate genes were analysed. Three distinct subphenotypes were defined according to stage and grade: low-grade nonmuscle   invasive   (n=586), high-grade nonmuscle   invasive   (n=219), and muscle   invasive   (n=246). The association between each variant and subphenotype was assessed by polytomous risk models adjusting for potential confounders. Heterogeneity in genetic susceptibility among subphenotypes was also tested.Two established bladder cancer susceptibility genotypes, NAT2 slow-acetylation and GSTM1-null, exhibited similar associations among the subphenotypes, as did VEGF-rs25648, which was previously identified in our study. Other variants conferred risks for specific tumour subphenotypes such as PMS2-rs6463524 and CD4-rs3213427 (respective heterogeneity p values of 0.006 and 0.004), which were associated with muscle-  invasive   tumours (per-allele odds ratios [95% confidence interval] of 0.56 [0.41-0.77] and 0.71 [0.57-0.88], respectively) but not with non-muscle-  invasive   tumours. Heterogeneity p values were not robust in multiple testing according to their false-discovery rate.These exploratory analyses suggest that genetic susceptibility loci might be related to the molecular/pathologic diversity of bladder cancer. Validation through large-scale replication studies and the study of additional genes and single nucleotide polymorphisms are required. 
 Genetic susceptibility to distinct bladder cancer subphenotypes. Clinical, pathologic, and molecular evidence indicate that bladder cancer is heterogeneous with pathologic/molecular features that define distinct subphenotypes with different prognoses. It is conceivable that specific patterns of genetic susceptibility are associated with particular subphenotypes.To examine evidence for the contribution of germline genetic variation to bladder cancer heterogeneity.The Spanish Bladder Cancer/EPICURO Study is a case-control study based in 18 hospitals located in five areas in Spain. Cases were patients with a newly diagnosed, histologically confirmed, urothelial cell carcinoma of the bladder from 1998 to 2001. Case diagnoses were reviewed and uniformly classified by pathologists following the World Health Organisation/International Society of Urological Pathology 1999 criteria. Controls were hospital-matched patients (n=1149).A total of 1526 candidate variants in 423 candidate genes were analysed. Three distinct subphenotypes were defined according to stage and grade: low-grade nonmuscle   invasive   (n=586), high-grade nonmuscle   invasive   (n=219), and muscle   invasive   (n=246). The association between each variant and subphenotype was assessed by polytomous risk models adjusting for potential confounders. Heterogeneity in genetic susceptibility among subphenotypes was also tested.Two established bladder cancer susceptibility genotypes, NAT2 slow-acetylation and GSTM1-null, exhibited similar associations among the subphenotypes, as did VEGF-rs25648, which was previously identified in our study. Other variants conferred risks for specific tumour subphenotypes such as PMS2-rs6463524 and CD4-rs3213427 (respective heterogeneity p values of 0.006 and 0.004), which were associated with muscle-  invasive   tumours (per-allele odds ratios [95% confidence interval] of 0.56 [0.41-0.77] and 0.71 [0.57-0.88], respectively) but not with non-muscle-  invasive   tumours. Heterogeneity p values were not robust in multiple testing according to their false-discovery rate.These exploratory analyses suggest that genetic susceptibility loci might be related to the molecular/pathologic diversity of bladder cancer. Validation through large-scale replication studies and the study of additional genes and single nucleotide polymorphisms are required. 
 [Expressions and clinical significances of iASPP and caspase-9 in esophageal cancer]. To investigate the expressions of inhibitory member of the apoptosis stimulating protein of p53 family (iASPP) and caspase-9 in the esophageal cancer (EC) tissues and their correlations with clinical pathological parameters.Using streptavidin-peroxidase (SP) immunohistochemistry and reverse transcription PCR (RT-PCR), the protein and mRNA expressions of iASPP and caspase-9 were detected in 85 EC samples and 30 normal esophagus tissues to analyze their relationships with the clinicopathologic parameters of EC.The positive expression rate of iASPP was significantly higher in EC than that in normal esophagus tissues by SP (72.9% vs 16.7%, P<0.01) and by RT-PCR (0.657±0.115 vs 0.321±0.065, P<0.01). However, The positive expression rate of caspase-9 was significantly lower in EC than that in normal esophagus tissue by SP method (18.8% vs 76.7%, P<0.01) and by RT-PCR (0.297±0.058 vs 0.593±0.108, P<0.01). The mRNA expression levels of iASPP and caspase-9 in EC were significantly related to   the inva  sive extent, histological differentiation, TNM stages, and lymphatic metastases (P<0.05), but not related to the age, sex, tumor size and pathological type (P>0.05). The expression of iASPP mRNA was negatively correlated with caspase-9 (r=-0.382, P<0.05).The overexpression of iASPP and the low expression of caspase-9 in EC are closely correlated with tumor invasion and metastasis, and iASPP may promote the progression of EC through inhibiting the expression of caspase-9. 
 Simultaneous downregulation of uPAR and MMP-9 induces overexpression of the FADD-associated protein RIP and activates caspase 9-mediated apoptosis in gliomas. We have previously demonstrated the effectiveness of simultaneous RNA interference (RNAi)-mediated downregulation of urokinase-type plasminogen activator receptor (uPAR) and matrix metalloproteinase-9 (MMP-9) in inhibiting tumor invasion in vitro and in vivo. In particular, we have shown that the downregulation of uPAR and MMP-9 inhibits intracranial tumor growth. The mechanism of the inhibition of tumor growth has not yet been determined. In this study, we have attempted to explain the mechanisms involved in the inhibition of   invasive  ness and tumor growth in vitro. SNB19 glioma cells were transfected with scrambled vector plasmid (pSV) and a siRNA-expressing plasmid targeting either uPAR (pU) or MMP-9 (pM) singly or in combination (pUM). Untransfected cells were also used as a control. Western blotting and RT-PCR analyses showed the downregulation of uPAR in pU-transfected cells and MMP-9 in pM-transfected cells. In cells transfected with pUM, we observed down-regulation of both uPAR and MMP-9, thereby indicating the specificity of the siRNA-expressing plasmids. An increase in caspase 9 expression was observed in cells transfected with pUM whereas no change in the level of caspase 9 was observed in pU or pM-transfected cells. Additionally, no change in the expression level of caspase 8 was observed. However, an increase in the expression level of cleaved PARP was observed in the case of cells transfected with pU, pM and pUM. Cells transfected with pUM showed the highest levels of cleaved PARP expression. Expression levels of APAF-1 were also higher in pUM-transfected cells with no change in expression levels of controls and in pU and pM-transfected cells. Total CAD expression levels did not change under any of the transfection conditions. However, immunohistochemical studies demonstrated that CAD was translocated to the nucleus, thereby indicating DNA damage. As determined by Western blot analysis of subcellular fractions, cytoplasmic levels of cytochrome c were also increased. We determined the extent of DNA damage using the TUNEL assay (poly-A termination of free -OH ends of degraded nuclear DNA). Based on our results we conclude that the simultaneous downregulation of uPAR and MMP-9 induces apoptosome-mediated apoptosis. 
 Caspase 9 and caspase 8 gene polymorphisms and susceptibility to bladder cancer in north Indian population. Dysregulation of apoptosis plays a crucial role in carcinogenesis. Our aim was to investigate the association of Caspase 9 and Caspase 8 gene polymorphism with bladder cancer (BC) susceptibility.We undertook a case-control study of 212 (BC) cases and 250 controls to investigate the association between Caspase 9-1263A > G, Caspase 9-293del, and Caspase 8-6 N ins/del polymorphism and BC susceptibility by polymerase chain reaction (PCR) restriction fragment length polymorphism (RFLP) method, and further to study the influence on recurrence in patients after Bacillus Calmette-Guerin (BCG) immunotherapy.Overall, no statistically significant association was observed in Caspase 9-293del and Caspase 8. Nevertheless, Caspase 9-1263GG genotype was at reduced risk of BC [p = 0.010; odds ratio (OR) = 0.487]. Caspase 9-1263AG genotype was also observed to be significantly associated with reduced risk with high-risk non-muscle-  invasive   bladder cancer (NMIBC) (TaG(2-3), T1G(1-3)) and   invasive   tumors (T2 +) of BC (P = 0.042, OR = 0.39 and P = 0.013, OR = 0.028 respectively). Caspase 9-293del, heterozygous (-/+) genotype, too, demonstrated protective effect in high-risk NMIBC (P = 0.017; OR = 0.205). Haplotype analysis revealed variant genotypes Caspase 9AG + GG/Caspase 8 DI + II to be at reduced risk of BC (= 0P.014, OR = 0.47). The GG genotype of Caspase 9-1263 was associated with reduced risk for recurrence in BCG-treated patients [hazard ratio (HR) = 0.217, P = 0.005], thus showing increased recurrence-free survival (log-rank P = 0.024).Polymorphism in Caspase 9-1263 was observed to play a protective role in susceptibility to BC risk. Caspase 9 gene variants were also associated with reduced risk of NMBIC stages. The variant G allele at Caspase 9-1263 may be responsible for increased recurrence-free survival in BCG-treated patients. 
  [Mm]etastasis  PubMed  
 [Expressions and clinical significances of iASPP and caspase-9 in esophageal cancer]. To investigate the expressions of inhibitory member of the apoptosis stimulating protein of p53 family (iASPP) and caspase-9 in the esophageal cancer (EC) tissues and their correlations with clinical pathological parameters.Using streptavidin-peroxidase (SP) immunohistochemistry and reverse transcription PCR (RT-PCR), the protein and mRNA expressions of iASPP and caspase-9 were detected in 85 EC samples and 30 normal esophagus tissues to analyze their relationships with the clinicopathologic parameters of EC.The positive expression rate of iASPP was significantly higher in EC than that in normal esophagus tissues by SP (72.9% vs 16.7%, P<0.01) and by RT-PCR (0.657±0.115 vs 0.321±0.065, P<0.01). However, The positive expression rate of caspase-9 was significantly lower in EC than that in normal esophagus tissue by SP method (18.8% vs 76.7%, P<0.01) and by RT-PCR (0.297±0.058 vs 0.593±0.108, P<0.01). The mRNA expression levels of iASPP and caspase-9 in EC were significantly related to the invasive extent, histological differentiation, TNM stages, and lymphatic metastases (P<0.05), but not related to the age, sex, tumor size and pathological type (P>0.05). The expression of iASPP mRNA was negatively correlated with caspase-9 (r=-0.382, P<0.05).The overexpression of iASPP and the low expression of caspase-9 in EC are closely correlated with tumor invasion   and metast  asis, and iASPP may promote the progression of EC through inhibiting the expression of caspase-9. 
 Effects of caspase-9 and survivin gene polymorphisms in pancreatic cancer risk and tumor characteristics. This case-control study was performed to evaluate the association between a specific caspase-9 polymorphism as well as the genetic polymorphism -31G/C located in the cycle-dependent elements/cell cycle homology regions repressor element of the human survivin promoter and the risk of pancreatic cancer.Eighty patients with pancreatic cancer and 160 healthy controls were investigated for genotype and allelic frequencies of caspase-9 1263A/G and survivin -31G/C polymorphisms by Polymerase Chain Reaction-Restriction Fragment Length Polymorphism.The G carrier group of patients and the G allele of caspase-9 1263A/G were overrepresented among the pancreatic cancer cases. With regard to tumor characteristics, a statistically significant association was detected between the survivin C carrier group of patients and the advanced T stage as well as the presence of lymph node   metastasis  .The caspase-9 G allele confers increased susceptibility to pancreatic cancer development, and the survivin C carriage status may be related to aggressive features of this malignancy. 
 Influence of caspases 8 and 9 gene promoter polymorphism on prostate cancer susceptibility and early development of hormone refractory prostate cancer. To investigate, by genotyping CASP8 (-652 6N del/ins) and CASP9 (-1263 A > G; -293 19N del/ins), whether inactivation of apoptosis by genetic polymorphism of caspases 8 and 9 play an integral role in the mechanism of cancer development. To investigate the role of these polymorphisms in susceptibility to early development of hormone refractory prostate cancer.The study included 175 histologically confirmed cases of prostate cancer and 198 age and ethnicity matched healthy controls. CASP9-1263 A > G polymorphism was genotyped using the polymerase chain reaction-restriction fragment length polymorphism method. CASP9-293 del/ins and CASP8-652 del/ins polymorphisms were genotyped and the deletion pattern analysed by polyacrylamide gel electrophoresis.Our results demonstrated that presence of CASP9-1263 G allele was associated with reduced risk for prostate cancer (odds ratio 0.6, 95%CI 0.39-0.92, P= 0.02). Other variant CASP9 was not associated with prostate cancer risk. Coincidentally, the presence of CASP9-1263 G allele was associated with increased risk for progression of prostate cancer to bone   metastasis   (odds ratio -2.28, 95%CI 1.14-4.53, P= 0.02). CASP8-652 (+/-) genotype was associated with increased hazard for early development of hormone refractory prostate cancer (hazard ratio 2.44, 95%CI 1.2-5.85, P= 0.045).Our results support the hypothesis that variants of CASP9 may influence the susceptibility to prostate cancer and its progression to bone   metastasis  . CASP8 polymorphism may influence the progression of prostate cancer disease to a hormone refractory state. 
 [A study on the expression of CASP9 gene and its polymorphism distribution in non-small cell lung cancer]. To explore the expression of caspase 9(CASP9) gene in non-small cell lung cancer (NSCLC) and single nucleotide polymorphism (SNP) distribution of CASP9 in NSCLC patients and normal people.Reverse transcription-PCR (RT-PCR) was used to analyze the expression of CASP9 in 81 NSCLC and normal lung tissues. Two SNPs in CASP9 gene were chosen to be investigated. Genotypes of rs1052576 and rs1052571 in 81 NSCLC patients and 100 normal people were analyzed by PCR-restriction fragment length polymorphism (PCR-RFLP). Legally constituted authority statistical analysis was applied to analyze SNP genotype frequency and allele frequency in patients and control group.In comparision with normal lung tissues, CASP9 gene expression was obviously down-regulated in 44.4% (36/81) NSCLC tissues. rs1052571 located in exon 1 of CASP9 gene had no significant difference between two groups, rs1052576 located in exon 5 of CASP9 gene had significant difference between two groups, the G allele frequency in NSCLC patients was higher than those in healthy controls (P< 0.05); the AG genotype frequency in patients with lymph node   metastasis   was higher than those without lymph node   metastasis   (P< 0.05).This study confirms the association between CASP9 gene and NSCLC oncogenesis, rs1052576 which locates in exon 5 of CASP9 gene is associated with NSCLC. AG genotype has association with lymph node   metastasis  . 
 Polymorphisms and haplotypes in caspases 8 and 9 genes and risk for prostate cancer: a case-control study in cohort of North India. Despite the potential importance of apoptosis pathways in prostate tumor etiology, little has been published regarding prostate tumor risk associated with common gene variants in caspases (CASP). Normal variations within the sequence of apoptotic genes may lead to suboptimal apoptotic capacity and therefore increased cancer risk.Using data from a hospital-based case-control study conducted by Sanjay Gandhi Post Graduate Institute of Medical Science, India, from 2007 to 2009, we evaluated risk of prostate cancer (CaP) in 165 patients and age-matched 205 healthy controls. We genotyped the functional IVS12-19G/A, D302H, -678del, and -652 6N ins/del polymorphisms in the promoter of CASP 8 and -293del, -1263A/G in CASP 9 genes.A significant increased risk for CaP was found for the CASP 8 IVS12-19G/A heterozygous genotype (P = 0.02; OR = 1.69) as well as for the variant allele carriers (P = 0.04; OR = 1.56). Also the CASP 9 -1263A/G showed lower risk for both heterozygous and variant allele carrier genotypes (P = 0.002; OR = 0.45 and P = 0.05; OR = 0.66 respectively). CASP 9 -1263A/G was also found to be associated with increased risk with bone   metastasis  . Furthermore, a significant additive interaction between CASP 8 IVS12-19G/A polymorphism and tobacco smoking was observed with CaP risk.These results suggested that the CASP 8 IVS12-19G/A and CASP 9 -1263 polymorphism may be involved in etiology of CaP and thus could be implicated as a marker for genetic susceptibility in North Indian population. 
 Autocrine motility factor signaling induces tumor apoptotic resistance by regulations Apaf-1 and Caspase-9 apoptosome expression. Autocrine motility factor (AMF) is a cytokine that regulates locomotion and   metastasis   of tumor cells. It is well known that expression levels of AMF secretion and its receptor (AMF R) are closely related to tumor malignancy and rheumatoid arthritis. We have established that AMF signaling induced anti-apoptotic activity and that human fibrosarcoma HT-1080 line that secreted high levels of AMF were resistant to drug-induced apoptosis. These cells did not express the apoptotic protease activating factor-1 (Apaf-1) and Caspase-9 genes that encode for the proteins that form the "apoptosome" complex. The disappearance of the Apaf-1 and Caspase-9 gene was recovered by a cellular signaling inhibitor of protein kinase C, phosphatidylinositol 3-phosphate kinase and mitogen-activated protein kinase of the in vitro cultured human fibrosarcoma HT-1080 line. Treatment with these inhibitors favored apoptotic cell death induced by anti-cancer drugs of the murine ascites Ehrlich line. Apoptotic resistance of tumor cells allows them to escape death from cancer chemotherapy, so an understanding of malignant anti-apoptotic activities is important. Antibodies against AMF induced Ehrlich ascites apoptosis in vitro, and effectively aided in vivo apoptosis induced by anti-cancer drugs. The results might indicate a novel route by which tumor cells protect themselves with products, such as AMF, and proliferate despite various stresses and chemical insults; AMF regulates expression of Apaf-1 and caspase-9 genes via a complex signaling pathway and indirectly regulates formation of the apoptosome. 
 BL-038, a Benzofuran Derivative, Induces Cell Apoptosis in Human Chondrosarcoma Cells through Reactive Oxygen Species/Mitochondrial Dysfunction and the Caspases Dependent Pathway. Chondrosarcoma is a highly malignant cartilage-forming bone tumor that has the capacity to invade locally and cause distant   metastasis  . Moreover, chondrosarcoma is intrinsically resistant to conventional chemotherapy or radiotherapy. The novel benzofuran derivative, BL-038 (2-amino-3-(2,6-dichlorophenyl)-6-(4-methoxyphenyl)benzofuran-4-yl acetate), has been evaluated for its anticancer effects in human chondrosarcoma cells. BL-038 caused cell apoptosis in two human chondrosarcoma cell lines, JJ012 and SW1353, but not in primary chondrocytes. Treatment of chondrosarcoma with BL-038 also induced reactive oxygen species (ROS) production. Furthermore, BL-038 decreased mitochondrial membrane potential (MMP) and changed mitochondrial-related apoptosis, by downregulating the anti-apoptotic activity members (Bcl-2, Bcl-xL) and upregulating pro-apoptotic members (Bax, Bak) of the B-cell lymphoma 2 (Bcl-2) family of proteins, key regulators of the apoptotic machinery in cells. These results demonstrate that in human chondrosarcoma cells, the apoptotic and cytotoxic effects of BL-038 are mediated by the intrinsic mitochondria-mediated apoptotic pathway, which in turn causes the release of cytochrome c, the activation of caspase-9 and caspase-3, and the cleavage of poly (ADP-ribose) polymerase (PARP), to elicit apoptosis response. Our results show that the benzofuran derivative BL-038 induces apoptosis in chondrosarcoma cells. 
 Influence of survivin (BIRC5) and caspase-9 (CASP9) functional polymorphisms in renal cell carcinoma development: a study in a southern European population. Renal cell carcinoma (RCC) is the most common cancer of the adult kidney and its incidence and mortality has increase in the last 20 years. The disruption of cellular death is one the mechanism involved in cancer development. This process is precise regulated by apoptotic and anti-apoptotic molecules. Survivin (BIRC5) is a member of the inhibitor of apoptosis protein family and has the ability to inhibit the activation of the pro-apoptotic caspase-9 (CASP9). Thus BIRC5 and CASP9 functional polymorphisms might modulate the apoptosis and consequently RCC development. Our purpose was to investigate the potential role of BIRC5-31G/C and CASP9+83C/T functional polymorphisms in the risk for the development of RCC and metastatic disease. We studied the BIRC5-31G/C and CASP9+83C/T functional polymorphisms by PCR-RFLP and allelic discrimination using the 7300 real-time polymerase chain reaction system, respectively, in 178 RCC patients and in 305 healthy individuals. Regarding the BIRC5-31G/C polymorphism, there is a trend to an overrepresentation of CC genotype in RCC group compared with normal controls (aOR, 1.94; P=0.053). We observed, after gender stratification and age-adjustment, that BIRC5-31CC and CASP9+83CT/TT genotypes were associated with an increased risk for RCC development in the female group of our southern European study population (aOR=3.85; P=0.019; aOR=2.98; P=0.028; respectively). Concerning the waiting time for onset of metastatic disease, we observed that BIRC5-31CC homozygous developed   metastasis   8 years earlier than the G carriers using a Cox proportional hazard model with gender as covariate (HR=4.9, P=0.038, P bootstrap=0.009). The Cox regression proportional hazard model was validated using bootstrap statistic with 1,000 samples of the same number of patients as the original dataset. Our results suggest that individual differences influence the susceptibility to RCC and tumor behavior. This genetic profile may help to define higher risk groups that would benefit from individualized chemoprevention strategies and therapies. 
  GeneRIFs  
 overexpression of iASPP and the low expression of caspase-9 in esophageal cancer are closely correlated with tumor invasion and   metastasis   
   
  
 CASP2 
 
  NCBI Gene  
  GeneRIF  
  Pubmed  
 
 
  Search Term  Rank  Fields  
  [Mm]igration  6  PubMed(1)  
  [Ee]xtracellular [Mm]atrix  8  PubMed(1)  
  [Mm]etastasize  11  PubMed(1)  
  [Mm]etastasis  12  PubMed(1)  
 
 
 hide/show details for CASP2 
  [Mm]igration  PubMed  
 TRIM16 overexpression induces apoptosis through activation of caspase-2 in cancer cells. TRIM16 exhibits tumour suppressor functions by interacting with cytoplasmic vimentin and nuclear E2F1 proteins in neuroblastoma and squamous cell carcinoma cells, reducing cell   migration   and replication. Reduced TRIM16 expression in a range of human primary malignant tissues correlates with increased malignant potential. TRIM16 also induces apoptosis in breast and lung cancer cells, by unknown mechanisms. Here we show that overexpression of TRIM16 induces apoptosis in human breast cancer (MCF7) and neuroblastoma (BE(2)-C) cells, but not in non-malignant HEK293 cells. TRIM16 increased procaspase-2 protein levels in MCF7 and induced caspase-2 activity in both MCF7 and BE(2)-C cells. We show that TRIM16 and caspase-2 proteins directly interact in both MCF7 and BE(2)-C cells and co-localise in MCF7 cells. Most importantly, the induction of caspase-2 activity is required for TRIM16 to initiate apoptosis. Our data suggest a novel mechanism by which TRIM16 can promote apoptosis by directly modulating caspase-2 activity. 
  [Ee]xtracellular [Mm]atrix  PubMed  
 Oncogenic ras-induced down-regulation of pro-apoptotic protease caspase-2 is required for malignant transformation of intestinal epithelial cells. Resistance of carcinoma cells to anoikis, apoptosis that is normally induced by loss of cell-to-  extracellular matrix   adhesion, is thought to be essential for the ability of these cells to form primary tumors, invade adjacent tissues, and metastasize to distant organs. Current knowledge about the mechanisms by which cancer cells evade anoikis is far from complete. In an effort to understand these mechanisms, we found that ras, a major oncogene, down-regulates protease caspase-2 (which initiates certain steps of the cellular apoptotic program) in malignant human and rat intestinal epithelial cells. This down-regulation could be reversed by inhibition of a protein kinase Mek, a mediator of Ras signaling. We also found that enforced down-regulation of caspase-2 in nonmalignant intestinal epithelial cells by RNA interference protected them from anoikis. Furthermore, the reversal of the effect of Ras on caspase-2 achieved by the expression of exogenous caspase-2 in detached ras-transformed intestinal epithelial cells promoted well established apoptotic events, such as the release of the pro-apoptotic mitochondrial factors cytochrome c and HtrA2/Omi into the cytoplasm of these cells, significantly enhanced their anoikis susceptibility, and blocked their long term growth in the absence of adhesion to the   extracellular matrix  . Finally, the blockade of the effect of Ras on caspase-2 substantially suppressed growth of tumors formed by the ras-transformed cells in mice. We conclude that ras-induced down-regulation of caspase-2 represents a novel mechanism by which oncogenic Ras protects malignant intestinal epithelial cells from anoikis, promotes their anchorage-independent growth, and allows them to form tumors in vivo. 
  [Mm]etastasize  PubMed  
 Oncogenic ras-induced down-regulation of pro-apoptotic protease caspase-2 is required for malignant transformation of intestinal epithelial cells. Resistance of carcinoma cells to anoikis, apoptosis that is normally induced by loss of cell-to-extracellular matrix adhesion, is thought to be essential for the ability of these cells to form primary tumors, invade adjacent tissues, and   metastasize   to distant organs. Current knowledge about the mechanisms by which cancer cells evade anoikis is far from complete. In an effort to understand these mechanisms, we found that ras, a major oncogene, down-regulates protease caspase-2 (which initiates certain steps of the cellular apoptotic program) in malignant human and rat intestinal epithelial cells. This down-regulation could be reversed by inhibition of a protein kinase Mek, a mediator of Ras signaling. We also found that enforced down-regulation of caspase-2 in nonmalignant intestinal epithelial cells by RNA interference protected them from anoikis. Furthermore, the reversal of the effect of Ras on caspase-2 achieved by the expression of exogenous caspase-2 in detached ras-transformed intestinal epithelial cells promoted well established apoptotic events, such as the release of the pro-apoptotic mitochondrial factors cytochrome c and HtrA2/Omi into the cytoplasm of these cells, significantly enhanced their anoikis susceptibility, and blocked their long term growth in the absence of adhesion to the extracellular matrix. Finally, the blockade of the effect of Ras on caspase-2 substantially suppressed growth of tumors formed by the ras-transformed cells in mice. We conclude that ras-induced down-regulation of caspase-2 represents a novel mechanism by which oncogenic Ras protects malignant intestinal epithelial cells from anoikis, promotes their anchorage-independent growth, and allows them to form tumors in vivo. 
  [Mm]etastasis  PubMed  
 MiR-125a-5p decreases after long non-coding RNA HOTAIR knockdown to promote cancer cell apoptosis by releasing caspase 2. HOTAIR (homeobox transcript antisense RNA), one of the prototypical long non-coding RNAs, has been verified overexpressed in multiple carcinomas and has emerged as a promising novel anticancer target. Its well-established role is acting as a predictor of poor prognosis and promoting cancer cell   metastasis  . Recently, another important mission of HOTAIR was uncovered that targeting HOTAIR caused cancer cell apoptosis. Nevertheless, so far there is no published data elaborating the mechanism. Here, we report that microRNA miR-125a-5p decreases and releases caspase 2 to promote cancer cell apoptosis after HOTAIR knockdown. We applied siRNAs targeting HOTAIR to various cancer cells, and observed apoptosis in all of these cell lines. RNA sequencing detected that miR-125a-5p was decreased after HOTAIR knockdown and miR-125a-5p mimics could rescue the apoptosis induced by HOTAIR deficiency. Luciferase assays identified caspase 2, an initiator caspase, to be a new target of miR-125a-5p. Elevated expression and subsequent cleavage of caspase 2 was observed after HOTAIR knockdown or inhibition of miR-125a-5p. RNAi of caspase 2 could attenuate the apoptosis induced by HOTAIR knockdown. In 80 clinical colon cancer tissues, HOTAIR and miR-125a-5p levels were higher than adjacent tissues, whereas caspase 2 was lower. MiR-125a-5p expression level was significantly correlated with colon tumor size, lymph node   metastasis   and clinical stage. These findings indicate that miR-125a-5p decreases after HOTAIR knockdown to promote cancer cell apoptosis by releasing caspase 2. Our work reveals a previously unidentified apoptotic mechanism, which might be exploitable in anticancer drug development. 
   
  
 CASP8 
 
  NCBI Gene  
  GeneRIF  
  Pubmed  
 
 
  Search Term  Rank  Fields  
  [Mm]igration  6  PubMed(15); GeneRIFs(7)  
  [Ee]xtracellular [Mm]atrix  8  PubMed(7)  
  [Ii]nvasive  10  PubMed(11); GeneRIFs(2)  
  [Mm]etastasis  12  PubMed(21); GeneRIFs(7)  
  [Cc]ell [Ii]nvasion  13  PubMed(4)  
 
 
 hide/show details for CASP8 
  [Mm]igration  PubMed  
 Caspase-8 is involved in neovascularization-promoting progenitor cell functions. Endothelial progenitor cells (EPCs) comprise a heterogeneous population of cells, which improve therapeutic neovascularization after ischemia. The neovascularization-promoting potential of progenitor cells depends on survival and retention of the infused cells to the tissue. Caspases mediate apoptosis but are also involved in other critical biological processes. Therefore, we aimed to address the role of caspases in proangiogenic cells.The caspase-8 inhibitor zIETD abrogated the ex vivo formation of EPCs, inhibited EPC adhesion and   migration  , and reduced their capacity to improve neovascularization in vivo. Consistently, cells isolated from caspase-8-deficient mice exhibited a reduced capacity for enhancing neovascularization when transplanted into mice after hindlimb ischemia. Because inhibition of Caspase-8 reduced the adhesion and homing functions of EPCs, we further determined the surface expression of integrins and receptors involved in cell recruitment to ischemic tissues. Pharmacological inhibition of caspase-8 and genetic depletion of caspase-8 reduced the expression of the fibronectin receptor subunits alpha5 and beta1 and the SDF-1 receptor CXCR4. Moreover, we identified the E3 ubiquitin ligase Cbl-b, which negatively regulates integrin and receptor-mediated signaling, as a potential Caspase-8 substrate.In summary, our data demonstrate a novel apoptosis-unrelated role of caspase-8 in proangiogenic cells. 
 Src-inducible association of CrkL with procaspase-8 promotes cell   migration  . Procaspase-8, the zymogen form of the apoptosis-initiator caspase-8, undergoes phosphorylation following integrin-mediated cell attachment to an extracellular matrix substrate. Concordant with cell attachment to fibronectin, a population of procaspase-8 becomes associated with a peripheral insoluble compartment that includes focal complexes and lamellar microfilaments. Phosphorylation of procaspase-8 both impairs its maturation to the proapoptotic form and can promote cell   migration  . Here we show that the cytoskeletal adaptor protein CrkL promotes caspase-8 recruitment to the peripheral spreading edge of cells, and that the catalytic domain of caspase-8 directly interacts with the SH2 domain of CrkL. We show that the interaction is abolished by shRNA-mediated silencing of Src, in Src-deficient MEFs, and by pharmacologic inhibitors of the kinase. The results provide insight into how tyrosine kinases may act to coordinate the suppression caspase-8 mediated apoptosis, while promoting cell invasion. 
 EMT phenotype is induced by increased Src kinase activity via Src-mediated caspase-8 phosphorylation. Caspase-8 governs multiple cell responses to the microenvironmental cues. However, its integration of "death-life" signalings remains elusive. In our study, the role of caspase-8-Src is well-established as a promoter for   migration   or metastasis in Casp8(+)Src(+) A549/H226 cells in vivo and in vitro. In particular for nude mice models, mice implanted with Casp8(+)Src(+) A459/H226 cells remarkably increased spontaneous tumor metastatic burden with a significant survival disadvantage. Additionally, we detect that Src-mediated caspase-8 phosphorylation stimulates Src phosphorylation at Tyr-416 via the linkage of Src SH2 domain with phosph-Tyr-380 site of caspase-8. In turn, activated Src can efficiently induce epithelial-mesenchymal transition (EMT) phenotypic features to promote tumor cells metastasis. Surprisingly, RXDLL motif deletion in the DEDa of caspase-8 attenuates tumor cell   migration   or metastasis via impairing the recruitment of caspase-8 into the cellular periphery where activated Src is subject to caspase-8 phosphorylation. Together, a simple model is that the peripherization of caspase-8 is well-poised to facilitate Src-mediated caspase-8 phosphrylation at Tyr-380, then binding of phospho-Tyr380 of caspase-8 to Src SH2 domain may maintain Src in an active conformation to induce EMT phenotype, a key step toward cancer metastasis. 
 Molecular determinants of kinase pathway activation by Apo2 ligand/tumor necrosis factor-related apoptosis-inducing ligand. Apo2 ligand/tumor necrosis factor (TNF)-related apoptosis-inducing ligand (Apo2L/TRAIL) mainly activates programmed cell death through caspases. By contrast, TNF primarily induces gene transcription through the inhibitor of kappaB kinase (IKK), c-Jun N-terminal kinase (JNK), and p38 mitogen-activated protein kinase pathways. Apo2L/TRAIL also can stimulate these kinases, albeit less strongly; however, the underlying mechanisms of this stimulation and its relation to apoptosis are not well understood. Here we show that Apo2L/TRAIL activates kinase pathways by promoting the association of a secondary signaling complex, subsequent to assembly of a primary, death-inducing signaling complex (DISC). The secondary complex retained the DISC components FADD and caspase-8, but recruited several factors involved in kinase activation by TNF, namely, RIP1, TRAF2, and NEMO/IKKgamma. Secondary complex formation required Fas-associated death domain (FADD), as well as caspase-8 activity. Apo2L/TRAIL stimulation of JNK and p38 further depended on RIP1 and TRAF2, whereas IKK activation required NEMO. Apo2L/TRAIL induced secretion of interleukin-8 and monocyte chemoattractant protein-1, augmenting macrophage   migration  . Thus, Apo2L/TRAIL and TNF organize common molecular determinants in distinct signaling complexes to stimulate similar kinase pathways. One function of kinase stimulation by Apo2L/TRAIL may be to promote phagocytic engulfment of apoptotic cells. 
 Caspase-8 as a therapeutic target in cancer. Caspase-8 is an apical caspase which initiates programmed cell death following death receptor ligation. This central role in apoptosis has prompted significant clinical interest in regulating caspase-8 expression and proteolytic activity. However, caspase-8 has also been found to play a number of non-apoptotic roles in cells, such as promoting activation NF-κB signaling, regulating autophagy and altering endosomal trafficking, and enhancing cellular adhesion and   migratio  n. Therefore, depending upon the specific cellular context, caspase-8 may either potentiate or suppress tumor malignancy. Accordingly, a marked heterogeneity exists in the expression patterns of caspase-8 among different tumor types. Therapeutics have been developed which can increase caspase-8 expression, yet it remains unclear whether this approach will be beneficial in all cases. Care is warranted, and the role of caspase-8 should be addressed on a case by case basis. 
 Bcl-2 and caspase-8 related anoikis resistance in human osteosarcoma MG-63 cells. Detachment of adherent cells from extracellular matrix results in apoptosis, a process termed "anoikis". Resistance to anoikis is implicated in the progression of many malignancies by facilitating the   migration   and eventual colonization of distant sites. Human kidney epithelial cells 293T, human osteoblast cells hFOB 1.19 and human osteosarcoma cells Saos-2 significantly underwent anoikis when adherence was prevented. But human osteosarcoma MG-63 cells were distinctly anoikis resistant when detached. They formed large aggregates and showed little apoptosis compared to the other cells. When MG-63 cells were in suspension, caspase-8, physically associated with death receptor was activated by cell-matrix detachment, whereas. Caspase-3 and caspase-9 were not activated. Translational level of Bcl-2 significantly increased in a time-dependent manner, but the level of beta-catenin and PI3K did not. Caspase-8 participates in an anoikis-inducing process in MG-63 cells at an early time, and overexpression of Bcl-2 blocks activation of caspase-8 making MG-63 cells anoikis resistant. 
 Caspase 8 promotes peripheral localization and activation of Rab5. Caspase 8 is a cysteine protease that initiates apoptotic signaling via the extrinsic pathway in a manner dependent upon association with early endosomes. Previously, we identified caspase 8 as an effector of   migration  , promoting motility in a manner dependent upon phosphorylation on Tyr-380 by Src family kinases and its subsequent association with Src homology 2 domain-containing proteins. Here we demonstrate the regulation of the small GTPase Rab5, which mediates early endosome formation, homotypic fusion, and maturation by caspase 8. Regulation requires the Tyr-380 phosphorylation site but not caspase proteolytic activity. Tyr-380 is essential for interaction with the Src homology 2 domains of p85alpha, a multifunctional adaptor for phosphatidylinositol 3-kinase, that possesses Rab-GAP activity. Interaction between caspase 8 and p85alpha promotes Rab5 GTP loading, alters endosomal trafficking, and results in the accumulation of Rab5-positive endosomes at the edge of the cell. Conversely, caspase 8-dependent GTP loading of Rab5 is overcome by increased expression of p85alpha in a Rab-GAP-dependent manner. Thus, we demonstrate a novel function for caspase 8 as a modulator of p85alpha Rab-GAP activity and endosomal trafficking. 
 Caspase-8 mutations in head and neck cancer confer resistance to death receptor-mediated apoptosis and enhance   migration  , invasion, and tumor growth. Little is known regarding molecular markers in head and neck squamous cell carcinoma (HNSCC) that predict responsiveness to different therapeutic regimens or predict HNSCC progression. Mutations in procaspase-8 occur in 9% of HNSCC primary tumors, but the functional consequences of these mutations are poorly understood. In this study, we examined the impact of four, representative, HNSCC-associated procaspase-8 mutations on activation of the extrinsic apoptosis pathway, as well as cellular   migration   and invasion, and in vivo tumor growth. All four mutant proteins acted to potently inhibit activation of apoptosis following treatment with TRAIL or agonistic anti-Fas. In contrast to wild-type procaspase-8, the mutant proteins were not recruited to FADD following treatment with TRAIL or anti-Fas, but may be constitutively bound by FADD. Three of the four procaspase-8 mutants promoted enhanced cellular   migration   and invasion through matrigel, relative to that seen with the wild-type procaspase-8 protein. Procaspase-8 mutation also stimulated the growth of HNSCC xenograft tumors. These findings indicate that HNSCC-associated procaspase-8 mutations inhibit activation of the extrinsic apoptosis pathway and are likely to represent markers for resistance to therapeutic regimens incorporating death receptor activators. Moreover, procaspase-8 mutations may serve as markers of HNSCC tumor progression, as exemplified by enhanced   migration  , invasion, and tumor growth. 
 Caspase-8 interacts with the p85 subunit of phosphatidylinositol 3-kinase to regulate cell adhesion and motility. Cell   migration   plays an important role in tumor cell invasion and metastasis. Previously, we reported that caspase-8 contributes to cell   migration   and adhesion, a novel nonapoptotic function of an established apoptotic factor. Herein, we report that pro-caspase-8 is capable of restoring cell   migration  /adhesion to caspase-8-null cells, establishing the first biological function of a pro-caspase. The catalytic activity of caspase-8 was not required for cell motility. Stimulation of motility with epidermal growth factor induced the phosphorylation of caspase-8 on tyrosine-380 and the interaction of caspase-8 with the p85 alpha subunit of phosphatidylinositol 3-kinase. Tyrosine-380 was required for the restoration of cell motility and cell adhesion in caspase-8-null cells, demonstrating the importance of the caspase-8-p85 interaction for these nonapoptotic functions. These results suggest that caspase-8 phosphorylation converts it from a proapoptotic factor to a cell motility factor that, through tyrosine-380, interacts with p85, an established cell   migration   component. 
 Critical role for caspase-8 in epidermal growth factor signaling. Caspase-8 has a well-defined canonical role as an apical protease of the extrinsic apoptosis pathway. Evidence is growing, however, that the protein has numerous other nonapoptotic functions. We have previously shown that caspase-8 is required for efficient adhesion-induced activation of the extracellular signal-regulated kinase (Erk)-1/2 pathway. We now show that caspase-8 is also necessary for the efficient activation of downstream events associated with epidermal growth factor (EGF) signaling. This promotion of EGF-induced Erk1/2 activation is independent of the proteolytic activity of caspase-8 and can be recapitulated using only the pro-domains of the protein. In addition, we identify specific residues within the caspase-8 "RXDLL motif" that are essential for Erk pathway activation. Furthermore, these residues are also involved in forming a complex with the tyrosine kinase Src. Caspase-8 null cells and cells reconstituted with caspase-8 harboring point mutations of these critical amino acids also show defective EGF-induced   migration   as compared with cells reconstituted with the wild-type protein. In sum, we provide the first evidence for caspase-8 as an essential component of growth factor signaling and suggest that this may be due to its association with Src. As the EGF/Src pathway activity has been shown to promote oncogenic events, our findings that caspase-8 is necessary for these activities may help explain why it is rarely deleted or silenced in tumors. 
 Involvement of caspase 8 and c-FLIPL in the proangiogenic effects of the tumour necrosis factor-related apoptosis-inducing ligand (TRAIL). Tumour necrosis factor-related apoptosis-inducing ligand (TRAIL), a cytokine of the tumour necrosis factor superfamily, is a potent cell-apoptosis inducer, although its effects vary as a function of concentration. In fact, low concentrations of TRAIL are associated with non-apoptotic effects, such as cell proliferation. Here, the effects of TRAIL at different concentrations have been evaluated on mitogenesis and   migration   on human umbilical vein endothelial cells (HUVEC) in vitro, as well as in the chick embryo chorioallantoic membrane (CAM) angiogenesis model in vivo. At low concentrations, TRAIL promoted either mitogenesis or   migration   of HUVEC, evaluated using the wound healing method. Cleavage of caspase 8 was evaluated along with expression of the caspase 8-like molecule, cellular FLICE-inhibitory protein (long form) (c-FLIPL ). Low concentrations of TRAIL failed to induce caspase 8 processing, whereas high concentrations induced apoptosis of HUVEC and activation of caspase 8. Moreover, TRAIL induced a significant angiogenic response in the CAM assay in vivo, comparable with that of vascular endothelial growth factor. These data suggest that the non-apoptotic effects of TRAIL include mitogenesis and increased mobility of endothelial cells, and eventually angiogenesis. In addition, the results demonstrate that the c-FLIPL level is also modulated by differences in TRAIL concentration, suggesting its involvement in the divergent effects of TRAIL. In conclusion, this study envisions a proangiogenic role of TRAIL, suggesting that TRAIL may represent a target for pharmacological manipulation. 
 [Expression of caspase-8 in non-small cell lung cancer and its role in invasion and metastasis of lung cancer A549 cells]. To explore the expression of caspase-8 in non-small cell lung cancer (NSCLC) tissue, and its role in invasion and metastasis of lung cancer A549 cells.The expression of caspase-8 in the lung cancer tissues was detected by immunohistochemistry. RNA interference technology was used to silence the expression of caspase-8 in lung cancer A549 cells to explore the function of caspase-8 protein in invasion and metastasis of cancer cells by means of Western blot, Transwell and scratch   migration   assays.The expression of caspase-8 was found in 38/52 cancer tissues, 22/30 adjacent tissues and 34/52 normal tissues. The tissues number of caspase-8 positive-expression in different groups was similar, but the intensity of caspase-8 expression in different groups was statistically different(P<0.05). In cancer tissues, the expression of caspase-8 indicated more likely to have lymph node-metastasis and worse clinical stage(P<0.05). After RNA interference, we successfully silenced the expression of caspase-8 in the lung cancer A549 cell, and this was confirmed by Western blotting. Transwell and cell scratch test showed that without the expression of caspase-8 A549 cells had a lower invasion and metastasis abilities compared to the control cells (P<0.05).Caspase-8 is not only an apoptosis protein, but has non-apoptosis function, such as promoting invasion and metastasis of lung cancer A549 cell. In NSCLC, it more likely locates in nucleus. And its expression in NSCLC may indicate a later clinical stage and a higher probability of lymph node metastasis. 
 Intestinal genetic inactivation of caspase-8 diminishes   migration   of enterocytes. To verify the hypothesis that caspase-8 (Casp8), which regulates cellular apoptosis and necroptosis, is critically involved in enterocyte   migration  .Casp8-silenced Caco2 cells were used in   migration   assays. In addition, enterocyte-specific Casp8 heterozygous (Casp8(+/∆int)) or homozygous knockout mice (Casp8(∆int)) were generated by crossing genetically modified mice carrying loxP recombination sites in intron 2 and 4 of the murine Casp8 gene with transgenic animals expressing a cre-transgene under control of the villin promoter in a pure C57/BL6 genetic background. The nucleoside analog BrdU was injected i.p. in male Casp8(+/∆int) and Casp8(∆int) animals 4 h, 20 h, or 40 h before performing morphometric studies. Locations of anti-BrdU-immunostained cells (cell(max)) in at least 50 hemi-crypts of 6 histoanatomically distinct intestinal mucosal regions were numbered and extracted for statistical procedures. For the mice cohort (n = 28), the walking distance of enterocytes was evaluated from cell(max) within crypt (n = 57), plateau (n = 19), and villus (n = 172) positions, resulting in a total of 6838 observations. Data analysis was performed by fitting a three-level mixed effects model to the data.In cell culture experiments with Caco2 cells, Casp8 knockdown efficiency mediated by RNA interference on Casp8 transcripts was 80% controlled as determined by Western blotting. In the scratch   assay, m  igration of Casp8-deleted Caco2 cells was significantly diminished when compared with controls (Casp8(∆scramble) and Caco2). In BrdU-labeled Casp8(∆int) mice, cell(max) locations were found along the hemi-crypts in a lower position than it was for Casp8(+/∆int) or control (cre-negative) animals. Statistical data analysis with a three-level mixed effects model revealed that in the six different intestinal locations (distinct segments of the small and large intestine), cell movement between the three mice groups differed widely. Especially in duodenal hemi-crypts, enterocyte movement was different between the groups. At 20 h, duodenal cell(max) location was significantly lower in Casp8(∆int) (25.67 ± 2.49) than in Casp8(+/∆int) (35.67 ± 4.78; P < 0.05) or control littermates (44.33 ± 0.94; P < 0  .01).Casp  8-dependent migration of enterocytes is likely involved in intestinal physiology and inflammation-related pathophysiology. 
 Rab5 mediates caspase-8-promoted cell motility and metastasis. Caspase-8 is a key apical sensory protein that governs cell responses to environmental cues, alternatively promoting apoptosis, proliferation, and cell   migration  . The proteins responsible for integration of these pathways, however, have remained elusive. Here, we reveal that Rab5 regulates caspase-8-dependent signaling from integrins. Integrin ligation leads to Rab5 activation, association with integrins, and activation of Rac, in a caspase-8-dependent manner. Rab5 activation promotes colocalization and coprecipitation of integrins with caspase-8, concomitant with Rab5 recruitment to integrin-rich regions such as focal adhesions and membrane ruffles. Moreover, caspase-8 expression promotes Rab5-mediated internalization and the recycling of beta1 integrins, increasing cell   migration   independently of caspase catalytic activity. Conversely, Rab5 knockdown prevented caspase-8-mediated integrin signaling for Rac activation, cell   migration  , and apoptotic signaling, respectively. Similarly, Rab5 was critical for caspase-8-driven cell   migration   in vivo, because knockdown of Rab5 compromised the ability of caspase-8 to promote metastasis under nonapoptotic conditions. These studies identify Rab5 as a key integrator of caspase-8-mediated signal transduction downstream of integrins, regulating cell survival and   migration   in vivo and in vitro. 
 Identification of a critical tyrosine residue in caspase 8 that promotes cell   migration  . Caspase 8 is a critical upstream initiator of programmed cell death but, paradoxically, has also been shown to promote cell   migration  . Here, we show that tyrosine 380 in the linker loop of human caspase 8 is a critical switch determining caspase 8 function. Our studies show that, in addition to its cytosolic distribution, caspase 8 is recruited to lamella of migrating cells. Although the catalytic domain of caspase 8 is sufficient for recruitment and promotion of cell   migration  , catalytic activity per se is not required. Instead, we find that integrin-mediated adhesion promotes caspase 8 phosphorylation on tyrosine 380. Accordingly, mutation of this site compromises localization to the periphery and the potentiation of cell   migration  . Mechanistically, this linker region of caspase 8 acts as a Src homology 2 binding site. In particular, tyrosine 380 is critical for interaction with Src homology 2 domains. The results identify a novel mechanism by which caspase 8 is recruited to the lamella of a migrating cell, promoting cell   migration   independent of its protease activity. 
  GeneRIFs  
 Casp8-dependent   migration   of enterocytes is likely involved in intestinal physiology and inflammation-related pathophysiology. 
 Three of the four procaspase-8 mutants promoted enhanced cellular   migration   and invasion through matrigel, relative to that seen with the wild-type procaspase-8 protein. 
 TRAIL promoted either mitogenesis or   migration   of HUVEC, evaluated using the wound healing method. Cleavage of caspase 8 was evaluated along with expression of the caspase 8-like molecule, cellular FLICE-inhibitory protein (long form) (c-FLIPL). 
 Data show Src-inducible association of CrkL with procaspase-8 promotes cell   migration  . 
 Src is required for caspase-8-imparted cell   migration   or metastasis dependently on Tyr 380 and RXDLL motif of caspase-8. 
 These studies identify Rab5 as a key integrator of caspase-8-mediated signal transduction downstream of integrins, regulating cell survival and   migration   in vivo and in vitro. 
 identify a novel mechanism by which caspase 8 is recruited to the lamella of a migrating cell, promoting cell   migration   independent of its protease activity 
  [Ee]xtracellular [Mm]atrix  PubMed  
 Integrin-linked kinase interacts with caspase-9 and -8 in an adhesion-dependent manner for promoting radiation-induced apoptosis in human leukemia cells. Integrin-mediated adhesion of leukemia cells to   extracellular matrix   proteins reduces apoptosis following radiation-induced genotoxic injury. To evaluate the role of integrin-linked kinase (ILK) in this process, HL60 human acute promyelocytic leukemia cells were stably transfected with ILK wild-type or kinase-hyperactive overexpression vectors. Suspension or fibronectin (FN) adhesion cultures were irradiated with X-rays and processed for measurement of apoptosis, mitochondrial transmembrane potential and caspase activation. Adhesion to FN pronouncedly reduced radiation-induced apoptosis of HL60 cells and vector controls. Intriguingly, overexpressed ILK enhanced apoptosis after irradiation by combined activation of caspase-3 through caspase-8 and -9 in irradiated FN cultures. Irradiation of ILK suspension cultures lacked caspase-8 activation, but showed serial cleavage of caspase-9, -3 and poly (ADP-ribose) polymerase. These findings further characterize the cell death-promoting function of ILK in DNA-damaged cells. Moreover, ILK might represent a potential therapeutic target for innovative chemo- and radiooncological approaches in hematological malignancies. 
 Aberrant integrin activation induces p38 MAPK phosphorylation resulting in suppressed Fas-mediated apoptosis in T cells: implications for rheumatoid arthritis. Delayed Fas-mediated apoptosis in T cells is associated with inflammatory diseases including rheumatoid arthritis (RA). CD3(+) T cells in RA synovia expressed high amounts of phospho-p38 MAPK. Exposure to RA synovial fluid or soluble collagen, a degradation product of   extracellular matrix   abundant in RA synovium, induced the phosphorylation of p38 MAPK in Jurkat T cells accompanied by resistance against Fas-mediated apoptosis. Blocking beta1 integrin by antibody diminished this effect. In addition, ectopic expression of auto-activated beta1 integrin variant in T cells profoundly induced the phosphorylation of p38 MAPK. Suppression of p38 MAPK sensitized T cells to Fas-mediated apoptosis and increased caspase-8 and caspase-3 cleavage. A physical interaction of p38 MAPK and caspase-8 was demonstrated by using confocal microscopic imaging and co-immunoprecipitation assay. RA synovial fluid markedly increased the formation of phospho-p38 MAPK/caspase-8 complex in Jurkat T cells. In conclusion, abnormal activation of p38 MAPK to prevent Fas-mediated apoptosis may represent a common survival mechanism of RA synovial T cells contributing to the persistent inflammation of affected synovium. 
 Aberrant integrin activation induces p38 MAPK phosphorylation resulting in suppressed Fas-mediated apoptosis in T cells: implications for rheumatoid arthritis. Delayed Fas-mediated apoptosis in T cells is associated with inflammatory diseases including rheumatoid arthritis (RA). CD3(+) T cells in RA synovia expressed high amounts of phospho-p38 MAPK. Exposure to RA synovial fluid or soluble collagen, a degradation product of   extracellular matrix   abundant in RA synovium, induced the phosphorylation of p38 MAPK in Jurkat T cells accompanied by resistance against Fas-mediated apoptosis. Blocking beta1 integrin by antibody diminished this effect. In addition, ectopic expression of auto-activated beta1 integrin variant in T cells profoundly induced the phosphorylation of p38 MAPK. Suppression of p38 MAPK sensitized T cells to Fas-mediated apoptosis and increased caspase-8 and caspase-3 cleavage. A physical interaction of p38 MAPK and caspase-8 was demonstrated by using confocal microscopic imaging and co-immunoprecipitation assay. RA synovial fluid markedly increased the formation of phospho-p38 MAPK/caspase-8 complex in Jurkat T cells. In conclusion, abnormal activation of p38 MAPK to prevent Fas-mediated apoptosis may represent a common survival mechanism of RA synovial T cells contributing to the persistent inflammation of affected synovium. 
 Src-inducible association of CrkL with procaspase-8 promotes cell migration. Procaspase-8, the zymogen form of the apoptosis-initiator caspase-8, undergoes phosphorylation following integrin-mediated cell attachment to an   extracellular matrix   substrate. Concordant with cell attachment to fibronectin, a population of procaspase-8 becomes associated with a peripheral insoluble compartment that includes focal complexes and lamellar microfilaments. Phosphorylation of procaspase-8 both impairs its maturation to the proapoptotic form and can promote cell migration. Here we show that the cytoskeletal adaptor protein CrkL promotes caspase-8 recruitment to the peripheral spreading edge of cells, and that the catalytic domain of caspase-8 directly interacts with the SH2 domain of CrkL. We show that the interaction is abolished by shRNA-mediated silencing of Src, in Src-deficient MEFs, and by pharmacologic inhibitors of the kinase. The results provide insight into how tyrosine kinases may act to coordinate the suppression caspase-8 mediated apoptosis, while promoting cell invasion. 
 IPS-1 is crucial for DAP3-mediated anoikis induction by caspase-8 activation. Detachment of adherent epithelial cells from the   extracellular matrix   induces apoptosis, a process known as anoikis. We have shown that DAP3 is critical for anoikis induction. However, the mechanism for anoikis induction mediated by DAP3 is still unclear. Here, we show that interferon-beta promoter stimulator 1 (IPS-1) binds DAP3 and induces anoikis by caspase activation. Recently, IPS-1 has been shown to be critical for antiviral immune responses, although there has been no report of its function in apoptosis induction. We show that overexpression of IPS-1 induces apoptosis by activation of caspase-3, -8, and -9. In addition, IPS-1 knockout mouse embryonic fibroblasts were shown to be resistant to anoikis. Interestingly, IPS-1 expression, recruitment of caspase-8 to IPS-1, and caspase-8 activation were induced after cell detachment. Furthermore, DAP3-mediated anoikis induction was inhibited by knockdown of IPS-1 expression. Therefore, we elucidated a novel function of IPS-1 for anoikis induction by caspase-8 activation. 
 Bcl-2 and caspase-8 related anoikis resistance in human osteosarcoma MG-63 cells. Detachment of adherent cells from   extracellular matrix   results in apoptosis, a process termed "anoikis". Resistance to anoikis is implicated in the progression of many malignancies by facilitating the migration and eventual colonization of distant sites. Human kidney epithelial cells 293T, human osteoblast cells hFOB 1.19 and human osteosarcoma cells Saos-2 significantly underwent anoikis when adherence was prevented. But human osteosarcoma MG-63 cells were distinctly anoikis resistant when detached. They formed large aggregates and showed little apoptosis compared to the other cells. When MG-63 cells were in suspension, caspase-8, physically associated with death receptor was activated by cell-matrix detachment, whereas. Caspase-3 and caspase-9 were not activated. Translational level of Bcl-2 significantly increased in a time-dependent manner, but the level of beta-catenin and PI3K did not. Caspase-8 participates in an anoikis-inducing process in MG-63 cells at an early time, and overexpression of Bcl-2 blocks activation of caspase-8 making MG-63 cells anoikis resistant. 
 Novel noncatalytic role for caspase-8 in promoting SRC-mediated adhesion and Erk signaling in neuroblastoma cells. Neuroblastomas are extremely aggressive, although heterogeneous, cancers with a poor prognosis upon metastasis. Some evidence has suggested a correlative silencing of caspase-8 with MYCN amplification in neuroblastoma. A prognostic effect of this silencing, however, has been disputed. We report here hitherto undescribed roles for caspase-8 in the modulation of cell adhesion and subsequent activation of the Erk signaling pathway. Re-expression of caspase-8 in neuroblastoma cells lacking endogenous caspase-8 expression was found to promote cell adhesion to   extracellular matrix   and to activate adhesion-dependent signaling pathways, such as the Erk kinase cascade. This function of caspase-8 occurred irrespective of its proteolytic activity. Additionally, a pool of caspase-8 was shown to co-localize with the Src tyrosine kinase at the cellular periphery. Furthermore, our studies showed that caspase-8 forms a physical protein complex with Src via its death effector domains (DED) and maintains the complex in a detergent-soluble fraction. We also show that the DEDs of caspase-8 alone are necessary and sufficient to recreate the adhesive and biochemical phenotypes observed with the full-length protein, suggesting that caspase-8 may exert these effects via its association with Src. This protein complex association of caspase-8 and Src, and concomitant downstream signaling events, may help reconcile why a potential tumor suppressor such as caspase-8 is rarely absent in cancers. 
  [Ii]nvasive  PubMed  
 Genetic susceptibility to distinct bladder cancer subphenotypes. Clinical, pathologic, and molecular evidence indicate that bladder cancer is heterogeneous with pathologic/molecular features that define distinct subphenotypes with different prognoses. It is conceivable that specific patterns of genetic susceptibility are associated with particular subphenotypes.To examine evidence for the contribution of germline genetic variation to bladder cancer heterogeneity.The Spanish Bladder Cancer/EPICURO Study is a case-control study based in 18 hospitals located in five areas in Spain. Cases were patients with a newly diagnosed, histologically confirmed, urothelial cell carcinoma of the bladder from 1998 to 2001. Case diagnoses were reviewed and uniformly classified by pathologists following the World Health Organisation/International Society of Urological Pathology 1999 criteria. Controls were hospital-matched patients (n=1149).A total of 1526 candidate variants in 423 candidate genes were analysed. Three distinct subphenotypes were defined according to stage and grade: low-grade nonmuscle   invasive   (n=586), high-grade nonmuscle   invasive   (n=219), and muscle   invasive   (n=246). The association between each variant and subphenotype was assessed by polytomous risk models adjusting for potential confounders. Heterogeneity in genetic susceptibility among subphenotypes was also tested.Two established bladder cancer susceptibility genotypes, NAT2 slow-acetylation and GSTM1-null, exhibited similar associations among the subphenotypes, as did VEGF-rs25648, which was previously identified in our study. Other variants conferred risks for specific tumour subphenotypes such as PMS2-rs6463524 and CD4-rs3213427 (respective heterogeneity p values of 0.006 and 0.004), which were associated with muscle-  invasive   tumours (per-allele odds ratios [95% confidence interval] of 0.56 [0.41-0.77] and 0.71 [0.57-0.88], respectively) but not with non-muscle-  invasive   tumours. Heterogeneity p values were not robust in multiple testing according to their false-discovery rate.These exploratory analyses suggest that genetic susceptibility loci might be related to the molecular/pathologic diversity of bladder cancer. Validation through large-scale replication studies and the study of additional genes and single nucleotide polymorphisms are required. 
 Caspase 9 and caspase 8 gene polymorphisms and susceptibility to bladder cancer in north Indian population. Dysregulation of apoptosis plays a crucial role in carcinogenesis. Our aim was to investigate the association of Caspase 9 and Caspase 8 gene polymorphism with bladder cancer (BC) susceptibility.We undertook a case-control study of 212 (BC) cases and 250 controls to investigate the association between Caspase 9-1263A > G, Caspase 9-293del, and Caspase 8-6 N ins/del polymorphism and BC susceptibility by polymerase chain reaction (PCR) restriction fragment length polymorphism (RFLP) method, and further to study the influence on recurrence in patients after Bacillus Calmette-Guerin (BCG) immunotherapy.Overall, no statistically significant association was observed in Caspase 9-293del and Caspase 8. Nevertheless, Caspase 9-1263GG genotype was at reduced risk of BC [p = 0.010; odds ratio (OR) = 0.487]. Caspase 9-1263AG genotype was also observed to be significantly associated with reduced risk with high-risk non-muscle-  invasive   bladder cancer (NMIBC) (TaG(2-3), T1G(1-3)) and   invasive   tumors (T2 +) of BC (P = 0.042, OR = 0.39 and P = 0.013, OR = 0.028 respectively). Caspase 9-293del, heterozygous (-/+) genotype, too, demonstrated protective effect in high-risk NMIBC (P = 0.017; OR = 0.205). Haplotype analysis revealed variant genotypes Caspase 9AG + GG/Caspase 8 DI + II to be at reduced risk of BC (= 0P.014, OR = 0.47). The GG genotype of Caspase 9-1263 was associated with reduced risk for recurrence in BCG-treated patients [hazard ratio (HR) = 0.217, P = 0.005], thus showing increased recurrence-free survival (log-rank P = 0.024).Polymorphism in Caspase 9-1263 was observed to play a protective role in susceptibility to BC risk. Caspase 9 gene variants were also associated with reduced risk of NMBIC stages. The variant G allele at Caspase 9-1263 may be responsible for increased recurrence-free survival in BCG-treated patients. 
 Caspase 9 and caspase 8 gene polymorphisms and susceptibility to bladder cancer in north Indian population. Dysregulation of apoptosis plays a crucial role in carcinogenesis. Our aim was to investigate the association of Caspase 9 and Caspase 8 gene polymorphism with bladder cancer (BC) susceptibility.We undertook a case-control study of 212 (BC) cases and 250 controls to investigate the association between Caspase 9-1263A > G, Caspase 9-293del, and Caspase 8-6 N ins/del polymorphism and BC susceptibility by polymerase chain reaction (PCR) restriction fragment length polymorphism (RFLP) method, and further to study the influence on recurrence in patients after Bacillus Calmette-Guerin (BCG) immunotherapy.Overall, no statistically significant association was observed in Caspase 9-293del and Caspase 8. Nevertheless, Caspase 9-1263GG genotype was at reduced risk of BC [p = 0.010; odds ratio (OR) = 0.487]. Caspase 9-1263AG genotype was also observed to be significantly associated with reduced risk with high-risk non-muscle-  invasive   bladder cancer (NMIBC) (TaG(2-3), T1G(1-3)) and   invasive   tumors (T2 +) of BC (P = 0.042, OR = 0.39 and P = 0.013, OR = 0.028 respectively). Caspase 9-293del, heterozygous (-/+) genotype, too, demonstrated protective effect in high-risk NMIBC (P = 0.017; OR = 0.205). Haplotype analysis revealed variant genotypes Caspase 9AG + GG/Caspase 8 DI + II to be at reduced risk of BC (= 0P.014, OR = 0.47). The GG genotype of Caspase 9-1263 was associated with reduced risk for recurrence in BCG-treated patients [hazard ratio (HR) = 0.217, P = 0.005], thus showing increased recurrence-free survival (log-rank P = 0.024).Polymorphism in Caspase 9-1263 was observed to play a protective role in susceptibility to BC risk. Caspase 9 gene variants were also associated with reduced risk of NMBIC stages. The variant G allele at Caspase 9-1263 may be responsible for increased recurrence-free survival in BCG-treated patients. 
 Caspase 9 and caspase 8 gene polymorphisms and susceptibility to bladder cancer in north Indian population. Dysregulation of apoptosis plays a crucial role in carcinogenesis. Our aim was to investigate the association of Caspase 9 and Caspase 8 gene polymorphism with bladder cancer (BC) susceptibility.We undertook a case-control study of 212 (BC) cases and 250 controls to investigate the association between Caspase 9-1263A > G, Caspase 9-293del, and Caspase 8-6 N ins/del polymorphism and BC susceptibility by polymerase chain reaction (PCR) restriction fragment length polymorphism (RFLP) method, and further to study the influence on recurrence in patients after Bacillus Calmette-Guerin (BCG) immunotherapy.Overall, no statistically significant association was observed in Caspase 9-293del and Caspase 8. Nevertheless, Caspase 9-1263GG genotype was at reduced risk of BC [p = 0.010; odds ratio (OR) = 0.487]. Caspase 9-1263AG genotype was also observed to be significantly associated with reduced risk with high-risk non-muscle-  invasive   bladder cancer (NMIBC) (TaG(2-3), T1G(1-3)) and   invasive   tumors (T2 +) of BC (P = 0.042, OR = 0.39 and P = 0.013, OR = 0.028 respectively). Caspase 9-293del, heterozygous (-/+) genotype, too, demonstrated protective effect in high-risk NMIBC (P = 0.017; OR = 0.205). Haplotype analysis revealed variant genotypes Caspase 9AG + GG/Caspase 8 DI + II to be at reduced risk of BC (= 0P.014, OR = 0.47). The GG genotype of Caspase 9-1263 was associated with reduced risk for recurrence in BCG-treated patients [hazard ratio (HR) = 0.217, P = 0.005], thus showing increased recurrence-free survival (log-rank P = 0.024).Polymorphism in Caspase 9-1263 was observed to play a protective role in susceptibility to BC risk. Caspase 9 gene variants were also associated with reduced risk of NMBIC stages. The variant G allele at Caspase 9-1263 may be responsible for increased recurrence-free survival in BCG-treated patients. 
 Genetic susceptibility to distinct bladder cancer subphenotypes. Clinical, pathologic, and molecular evidence indicate that bladder cancer is heterogeneous with pathologic/molecular features that define distinct subphenotypes with different prognoses. It is conceivable that specific patterns of genetic susceptibility are associated with particular subphenotypes.To examine evidence for the contribution of germline genetic variation to bladder cancer heterogeneity.The Spanish Bladder Cancer/EPICURO Study is a case-control study based in 18 hospitals located in five areas in Spain. Cases were patients with a newly diagnosed, histologically confirmed, urothelial cell carcinoma of the bladder from 1998 to 2001. Case diagnoses were reviewed and uniformly classified by pathologists following the World Health Organisation/International Society of Urological Pathology 1999 criteria. Controls were hospital-matched patients (n=1149).A total of 1526 candidate variants in 423 candidate genes were analysed. Three distinct subphenotypes were defined according to stage and grade: low-grade nonmuscle   invasive   (n=586), high-grade nonmuscle   invasive   (n=219), and muscle   invasive   (n=246). The association between each variant and subphenotype was assessed by polytomous risk models adjusting for potential confounders. Heterogeneity in genetic susceptibility among subphenotypes was also tested.Two established bladder cancer susceptibility genotypes, NAT2 slow-acetylation and GSTM1-null, exhibited similar associations among the subphenotypes, as did VEGF-rs25648, which was previously identified in our study. Other variants conferred risks for specific tumour subphenotypes such as PMS2-rs6463524 and CD4-rs3213427 (respective heterogeneity p values of 0.006 and 0.004), which were associated with muscle-  invasive   tumours (per-allele odds ratios [95% confidence interval] of 0.56 [0.41-0.77] and 0.71 [0.57-0.88], respectively) but not with non-muscle-  invasive   tumours. Heterogeneity p values were not robust in multiple testing according to their false-discovery rate.These exploratory analyses suggest that genetic susceptibility loci might be related to the molecular/pathologic diversity of bladder cancer. Validation through large-scale replication studies and the study of additional genes and single nucleotide polymorphisms are required. 
 Consortium analysis of 7 candidate SNPs for ovarian cancer. The Ovarian Cancer Association Consortium selected 7 candidate single nucleotide polymorphisms (SNPs), for which there is evidence from previous studies of an association with variation in ovarian cancer or breast cancer risks. The SNPs selected for analysis were F31I (rs2273535) in AURKA, N372H (rs144848) in BRCA2, rs2854344 in intron 17 of RB1, rs2811712 5' flanking CDKN2A, rs523349 in the 3' UTR of SRD5A2, D302H (rs1045485) in CASP8 and L10P (rs1982073) in TGFB1. Fourteen studies genotyped 4,624   invasive   epithelial ovarian cancer cases and 8,113 controls of white non-Hispanic origin. A marginally significant association was found for RB1 when all studies were included [ordinal odds ratio (OR) 0.88 (95% confidence interval (CI) 0.79-1.00) p = 0.041 and dominant OR 0.87 (95% CI 0.76-0.98) p = 0.025]; when the studies that originally suggested an association were excluded, the result was suggestive although no longer statistically significant (ordinal OR 0.92, 95% CI 0.79-1.06). This SNP has also been shown to have an association with decreased risk in breast cancer. There was a suggestion of an association for AURKA, when one study that caused significant study heterogeneity was excluded [ordinal OR 1.10 (95% CI 1.01-1.20) p = 0.027; dominant OR 1.12 (95% CI 1.01-1.24) p = 0.03]. The other 5 SNPs in BRCA2, CDKN2A, SRD5A2, CASP8 and TGFB1 showed no association with ovarian cancer risk; given the large sample size, these results can also be considered to be informative. These null results for SNPs identified from relatively large initial studies shows the importance of replicating associations by a consortium approach. 
 Unusual roles of caspase-8 in triple-negative breast cancer cell line MDA-MB-231. Triple-negative breast cancer (TNBC) is a clinically aggressive form of breast cancer that is unresponsive to endocrine agents or trastuzumab. TNBC accounts for ~10-20% of all breast cancer cases and represents the form with the poorest prognosis. Patients with TNBC are at higher risk of early recurrence, mainly in the lungs, brain and soft tissue, therefore, there is an urgent need for new therapies. The present study was carried out in MDA-MB-231 cells, where we assessed the role of caspase-8 (casp-8), a critical effector of death receptors, also involved in non‑apoptotic functions. Analysis of casp-8 mRNA and protein levels indicated that they were up-regulated with respect to the normal human mammalian epithelial cells. We demonstrated that silencing of casp-8 by small interfering-RNA, strongly decreased MDA-MB-231 cell growth by delaying G0/G1- to S-phase transition and increasing p21, p27 and hypo-phosphorylated/active form of pRb levels. Surprisingly, casp-8-knockdown, also potently increased both the migratory and metastatic capacity of MDA-MB‑231 cells, as shown by both wound healing and Matrigel assay, and by the expression of a number of related-genes and/or proteins such as VEGFA, C-MYC, CTNNB1, HMGA2, CXCR4, KLF4, VERSICAN V1 and MMP2. Among these, KLF4, a transcriptional factor with a dual role (activator and repressor), seemed to play critical roles. We suggest that in MDA-MB‑231 cells, the endogenous expression of casp-8 might keep the cells perpetually cycling through downregulation of KLF4, the subsequent lowering of p21 and p27, and the inactivation by hyperphosphorylation of pRb. Simultaneously, by lowering the expression of some migrator  y and in  vasive genes, casp-8 might restrain the metastatic ability of the cells. Overall, our findings showed that, in MDA-MB-231 cells, casp-8 might play some unusual roles which should be better explored, in order to understand whether it might be identified as a molecular therapeutic target. 
 Potentiation of neuroblastoma metastasis by loss of caspase-8. Neuroblastoma, the most common paediatric solid tumour, arises from defective neural crest cells. Genetic alterations occur frequently in the most aggressive neuroblastomas. In particular, deletion or suppression of the proapoptotic enzyme caspase-8 is common in malignant, disseminated disease, although the effect of this loss on disease progression is unclear. Here we show that suppression of caspase-8 expression occurs during the establishment of neuroblastoma metastases in vivo, and that reconstitution of caspase-8 expression in deficient neuroblastoma cells suppressed their metastases. Caspase-8 status was not a predictor of primary tumour growth; rather, caspase-8 selectively potentiated apoptosis in neuroblastoma cells invading the collagenous stroma at the tumour margin. Apoptosis was initiated by unligated integrins by means of a process known as integrin-mediated death. Loss of caspase-8 or integrin rendered these cells refractory to integrin-mediated death, allowed cellular survival in the stromal microenvironment, and promoted metastases. These findings define caspase-8 as a metastasis suppressor gene that, together with integrins, regulates the survival and   invasive   capacity of neuroblastoma cells. 
 Mutant PIK3CA licenses TRAIL and CD95L to induce non-apoptotic caspase-8-mediated ROCK activation. Constitutively active PI3K catalytic subunit alpha (PIK3CA) interfered with apoptosis induction downstream of death receptor-signaling complex formation allowing robust caspase-8 activation without triggering the execution steps of apoptosis. In mutant PIK3CA-expressing cells, tumor necrosis factor-related apoptosis-inducing ligand (TRAIL) and CD95L stimulated nuclear factor kappaB (NFkappaB) activation, invasion, and transition to an amoeboid-like morphology. NFkappaB activation and adoption of amoeboid shape were inhibited by caspase-8 knockdown or FLIP-S expression, but only the cell morphology alterations required caspase-8 activity. Furthermore, we identified caspase-8-mediated, caspase-3-independent cleavage of the protein kinase rho-associated, coiled-coil containing protein kinase 1 as a novel mechanism for acquiring amoeboid shape and enhanced   invasive  ness in response to TRAIL and CD95L. Taken together, we provide evidence that mutated PIK3CA converts the 'tumor surveillance' activity of cancer cell-expressed death receptors and caspase-8 toward tumor promotion. 
 CASP8 promoter polymorphism is associated with high-risk HPV types and abnormal cytology but not with cervical cancer. Only a small fraction of women infected with human papillomavirus (HPV) progress to cervical cancer pointing to additional risk factors including host genetics that might play a role in development of cervical cancer. Caspase-8 (encoded by CASP8 gene) is crucial in generating cell death signals to eliminate potentially malignant cells. Genetic variation in CASP8 might influence the susceptibility to cancer. CASP8 -652 6N ins/del polymorphism has been previously reported to influence the progression to several cancers including cervical cancer. This polymorphism was investigated in 445 women of black African and Mixed Ancestry origin with   invasive   cervical cancer and 1,221 controls matched (1:3) by age, ethnicity, and domicile status. Genotyping for CASP8 -652 6N ins/del was done by PCR-RFLP. In the control women cervical disease was detected by cervical cytology. The CASP8 -652 6N del/del genotype did not show any significant association (P=0.948) with cervical cancer. Further analysis within the controls showed a weak association (P=0.048) of this polymorphism with abnormal cytology in both ethnicities and high-risk HPV infection (P=0.030) only in the black Africans. This is the first study of the role of CASP8 -652 6N ins/del polymorphism in cervical cancer in an African population. These results show that CASP8 -652 6N del/del genotype increases the risk of abnormal cytology and high-risk HPV infection but does not show an association with cervical cancer. This result points towards an important role of CASP8 in HPV infection and in the development of pre-cancers. 
 The influence of genetic variation in 30 selected genes on the clinical characteristics of early onset breast cancer. Common variants that alter breast cancer risk are being discovered. Here, we determine how these variants influence breast cancer prognosis, risk and tumour characteristics.We selected 1,001 women with early onset nonfamilial   invasive   breast cancer from the Prospective study of Outcomes in Sporadic versus Hereditary breast cancer (POSH) cohort and genotyped 206 single nucleotide polymorphisms (SNPs) across 30 candidate genes. After quality control, 899 cases and 133 SNPs remained. Survival analyses were used to identify SNPs associated with prognosis and determine their interdependency with recognized prognostic factors. To identify SNPs that alter breast cancer risk, association tests were used to compare cases with controls from the Wellcome Trust Case Control Consortium. To search for SNPs affecting tumour biology, cases were stratified into subgroups according to oestrogen receptor (ER) status and grade and tested for association.We confirmed previous associations between increased breast cancer risk and SNPs in CASP8, TOX3 (previously known as TNRC9) and ESR1. Analysis of prognosis identified eight SNPs in six genes (MAP3K1, DAPK1, LSP1, MMP7, TOX3 and ESR1) and one region without genes on 8q24 that are associated with survival. For MMP7, TOX3 and MAP3K1 the effects on survival are independent of the main recognized clinical prognostic factors. The SNP in 8q24 is more weakly associated with independent effects on survival. Once grade and pathological nodal status (pN stage) were taken into account, SNPs in ESR1 and LSP1 showed no independent survival difference, whereas the effects of the DAPK1 SNP were removed when correcting for ER status. Interestingly, effects on survival for SNPs in ESR1 were most significant when only ER-positive tumours were examined. Stratifying POSH cases by tumour characteristics identified SNPs in FGFR2 and TOX3 associated with ER-positive disease and SNPs in ATM associated with ER-negative disease.We have demonstrated that several SNPs are associated with survival. In some cases this appears to be due to an effect on tumour characteristics known to have a bearing on prognosis; in other cases the effect appears to be independent of these prognostic factors. These findings require validation by further studies in similar patient groups. 
  GeneRIFs  
 Mutant PIK3CA interferes with apoptosis downstream of death receptor-signal complex, allowing caspase-8 activation without apoptosis. Cleavage of ROCK-1 by caspase-8 triggers amoeboid shape and   invasive  ness of HCT116 cells in response to TRAIL and CD95L. 
 findings define caspase-8 as a metastasis suppressor gene that, together with integrins, regulates the survival and   invasive   capacity of neuroblastoma cells 
  [Mm]etastasis  PubMed  
 Influence of caspases 8 and 9 gene promoter polymorphism on prostate cancer susceptibility and early development of hormone refractory prostate cancer. To investigate, by genotyping CASP8 (-652 6N del/ins) and CASP9 (-1263 A > G; -293 19N del/ins), whether inactivation of apoptosis by genetic polymorphism of caspases 8 and 9 play an integral role in the mechanism of cancer development. To investigate the role of these polymorphisms in susceptibility to early development of hormone refractory prostate cancer.The study included 175 histologically confirmed cases of prostate cancer and 198 age and ethnicity matched healthy controls. CASP9-1263 A > G polymorphism was genotyped using the polymerase chain reaction-restriction fragment length polymorphism method. CASP9-293 del/ins and CASP8-652 del/ins polymorphisms were genotyped and the deletion pattern analysed by polyacrylamide gel electrophoresis.Our results demonstrated that presence of CASP9-1263 G allele was associated with reduced risk for prostate cancer (odds ratio 0.6, 95%CI 0.39-0.92, P= 0.02). Other variant CASP9 was not associated with prostate cancer risk. Coincidentally, the presence of CASP9-1263 G allele was associated with increased risk for progression of prostate cancer to bone   metastasis   (odds ratio -2.28, 95%CI 1.14-4.53, P= 0.02). CASP8-652 (+/-) genotype was associated with increased hazard for early development of hormone refractory prostate cancer (hazard ratio 2.44, 95%CI 1.2-5.85, P= 0.045).Our results support the hypothesis that variants of CASP9 may influence the susceptibility to prostate cancer and its progression to bone   metastasis  . CASP8 polymorphism may influence the progression of prostate cancer disease to a hormone refractory state. 
 Polymorphisms and haplotypes in caspases 8 and 9 genes and risk for prostate cancer: a case-control study in cohort of North India. Despite the potential importance of apoptosis pathways in prostate tumor etiology, little has been published regarding prostate tumor risk associated with common gene variants in caspases (CASP). Normal variations within the sequence of apoptotic genes may lead to suboptimal apoptotic capacity and therefore increased cancer risk.Using data from a hospital-based case-control study conducted by Sanjay Gandhi Post Graduate Institute of Medical Science, India, from 2007 to 2009, we evaluated risk of prostate cancer (CaP) in 165 patients and age-matched 205 healthy controls. We genotyped the functional IVS12-19G/A, D302H, -678del, and -652 6N ins/del polymorphisms in the promoter of CASP 8 and -293del, -1263A/G in CASP 9 genes.A significant increased risk for CaP was found for the CASP 8 IVS12-19G/A heterozygous genotype (P = 0.02; OR = 1.69) as well as for the variant allele carriers (P = 0.04; OR = 1.56). Also the CASP 9 -1263A/G showed lower risk for both heterozygous and variant allele carrier genotypes (P = 0.002; OR = 0.45 and P = 0.05; OR = 0.66 respectively). CASP 9 -1263A/G was also found to be associated with increased risk with bone   metastasis  . Furthermore, a significant additive interaction between CASP 8 IVS12-19G/A polymorphism and tobacco smoking was observed with CaP risk.These results suggested that the CASP 8 IVS12-19G/A and CASP 9 -1263 polymorphism may be involved in etiology of CaP and thus could be implicated as a marker for genetic susceptibility in North Indian population. 
 Polymorphisms and haplotypes in caspases 8 and 9 genes and risk for prostate cancer: a case-control study in cohort of North India. Despite the potential importance of apoptosis pathways in prostate tumor etiology, little has been published regarding prostate tumor risk associated with common gene variants in caspases (CASP). Normal variations within the sequence of apoptotic genes may lead to suboptimal apoptotic capacity and therefore increased cancer risk.Using data from a hospital-based case-control study conducted by Sanjay Gandhi Post Graduate Institute of Medical Science, India, from 2007 to 2009, we evaluated risk of prostate cancer (CaP) in 165 patients and age-matched 205 healthy controls. We genotyped the functional IVS12-19G/A, D302H, -678del, and -652 6N ins/del polymorphisms in the promoter of CASP 8 and -293del, -1263A/G in CASP 9 genes.A significant increased risk for CaP was found for the CASP 8 IVS12-19G/A heterozygous genotype (P = 0.02; OR = 1.69) as well as for the variant allele carriers (P = 0.04; OR = 1.56). Also the CASP 9 -1263A/G showed lower risk for both heterozygous and variant allele carrier genotypes (P = 0.002; OR = 0.45 and P = 0.05; OR = 0.66 respectively). CASP 9 -1263A/G was also found to be associated with increased risk with bone   metastasis  . Furthermore, a significant additive interaction between CASP 8 IVS12-19G/A polymorphism and tobacco smoking was observed with CaP risk.These results suggested that the CASP 8 IVS12-19G/A and CASP 9 -1263 polymorphism may be involved in etiology of CaP and thus could be implicated as a marker for genetic susceptibility in North Indian population. 
 Influence of caspases 8 and 9 gene promoter polymorphism on prostate cancer susceptibility and early development of hormone refractory prostate cancer. To investigate, by genotyping CASP8 (-652 6N del/ins) and CASP9 (-1263 A > G; -293 19N del/ins), whether inactivation of apoptosis by genetic polymorphism of caspases 8 and 9 play an integral role in the mechanism of cancer development. To investigate the role of these polymorphisms in susceptibility to early development of hormone refractory prostate cancer.The study included 175 histologically confirmed cases of prostate cancer and 198 age and ethnicity matched healthy controls. CASP9-1263 A > G polymorphism was genotyped using the polymerase chain reaction-restriction fragment length polymorphism method. CASP9-293 del/ins and CASP8-652 del/ins polymorphisms were genotyped and the deletion pattern analysed by polyacrylamide gel electrophoresis.Our results demonstrated that presence of CASP9-1263 G allele was associated with reduced risk for prostate cancer (odds ratio 0.6, 95%CI 0.39-0.92, P= 0.02). Other variant CASP9 was not associated with prostate cancer risk. Coincidentally, the presence of CASP9-1263 G allele was associated with increased risk for progression of prostate cancer to bone   metastasis   (odds ratio -2.28, 95%CI 1.14-4.53, P= 0.02). CASP8-652 (+/-) genotype was associated with increased hazard for early development of hormone refractory prostate cancer (hazard ratio 2.44, 95%CI 1.2-5.85, P= 0.045).Our results support the hypothesis that variants of CASP9 may influence the susceptibility to prostate cancer and its progression to bone   metastasis  . CASP8 polymorphism may influence the progression of prostate cancer disease to a hormone refractory state. 
 Influence of caspases 8 and 9 gene promoter polymorphism on prostate cancer susceptibility and early development of hormone refractory prostate cancer. To investigate, by genotyping CASP8 (-652 6N del/ins) and CASP9 (-1263 A > G; -293 19N del/ins), whether inactivation of apoptosis by genetic polymorphism of caspases 8 and 9 play an integral role in the mechanism of cancer development. To investigate the role of these polymorphisms in susceptibility to early development of hormone refractory prostate cancer.The study included 175 histologically confirmed cases of prostate cancer and 198 age and ethnicity matched healthy controls. CASP9-1263 A > G polymorphism was genotyped using the polymerase chain reaction-restriction fragment length polymorphism method. CASP9-293 del/ins and CASP8-652 del/ins polymorphisms were genotyped and the deletion pattern analysed by polyacrylamide gel electrophoresis.Our results demonstrated that presence of CASP9-1263 G allele was associated with reduced risk for prostate cancer (odds ratio 0.6, 95%CI 0.39-0.92, P= 0.02). Other variant CASP9 was not associated with prostate cancer risk. Coincidentally, the presence of CASP9-1263 G allele was associated with increased risk for progression of prostate cancer to bone   metastasis   (odds ratio -2.28, 95%CI 1.14-4.53, P= 0.02). CASP8-652 (+/-) genotype was associated with increased hazard for early development of hormone refractory prostate cancer (hazard ratio 2.44, 95%CI 1.2-5.85, P= 0.045).Our results support the hypothesis that variants of CASP9 may influence the susceptibility to prostate cancer and its progression to bone   metastasis  . CASP8 polymorphism may influence the progression of prostate cancer disease to a hormone refractory state. 
 A novel inflammatory regulator TIPE2 inhibits TLR4-mediated development of colon cancer via caspase-8. TIPE2 is a novel inflammation regulator, and the role of TIPE2 in colitis-induced colon cancer is not clear. The aim of this study was to test whether TIPE2 inhibits TLR4 pathway in colon cancer patients and to explore potential mechanism of TIPE2 in colon cancer by caspase-8.Expression of TIPE2 and TLR4 in human colon cancer tissues and cell line HT-29 was detected by immunohistochemistry or real-time PCR. TIPE2 mRNA was suppressed by siRNA transfection and the transfection efficiency was proved by fluorescence microscopy and real-time PCR. TLR4 pathway was activated by treating the cells with 1 μg/ml LPS for 4 h. Caspase-8 activities were tested by colorimetric assay in four HT-29 cell groups.TIPE2 was expressed in the cytoplasm of colon cancer tissues and HT-29 cells. TIPE2 expression was more pronounced in colon cancer tissues compared to normal controls and it was related with lymph node   metastasi  s and Dukes stage of colon cancer. TIPE2 expression was positively correlated with that of TLR4 in colon cancer (r=0.7354). TIPE2 expression was knocked down successfully by siRNA transfection. Caspase-8 activity was elevated both in TIPE2 knockdown cells and in TLR4 activated cells compared to wild-type cells (P< 0.05). And the caspase-8 activity was further increased in TIPE2 knockdown cells after TLR4 was activated (P < 0.05).TIPE2 can inhibit caspase-8 activity in colon cancer cells. TIPE2 can regulate TLR4 inflammatory effect and inhibit further amplification of cascade reaction via caspase-8, which provides one new therapeutic target for clinical treatment schedule. 
 The combination of the prodrugs perforin-CEBPD and perforin-granzyme B efficiently enhances the activation of caspase signaling and kills prostate cancer. The survival of prostate cancer (PrCa) patients is associated with the transition to hormone-independent tumor growth and   metastasis  . Clinically, the dysregulation of androgen action has been associated with the formation of PrCa and the outcome of androgen deprivation therapy in PrCa. CCAAT/enhancer binding protein delta (CEBPD) is a transcription factor that has been reported to act as an oncogene or tumor suppressor, depending on the extra- and intracellular environments following tumorigenesis. We found that androgen can activate CEBPD transcription by direct binding of the androgen receptor (AR) to the CEBPD promoter region. Increases of suppressor of zeste 12 (SUZ12) and enhancer of zeste homolog 2 (EZH2) attenuated the androgen-induced transcription of CEBPD. Importantly, the increases in E2F1, SUZ12 and EZH2 as well as the inactivation of CEBPD were associated with the clinicopathological variables and survival of PrCa patients. We revealed that caspase 8 (CASP8), an apoptotic initiator, is responsive to CEBPD induction. Reporter and in vivo DNA-binding assays revealed that CEBPD directly binds to and activates CASP8 reporter activity. A prodrug system was developed for therapeutic application in AR-independent or androgen-insensitive PrCa to avoid the epigenetic effects on the suppression of CEBPD expression. Our results showed that the combination of a perforin (PF)-CEBPD prodrug (which increases the level of procaspase-8) and a PF-granzyme B prodrug (which activates CASP8 and caspase 3 (CASP3)) showed an additive effect in triggering the apoptotic pathway and enhancing apoptosis in PrCa cells. 
 EMT phenotype is induced by increased Src kinase activity via Src-mediated caspase-8 phosphorylation. Caspase-8 governs multiple cell responses to the microenvironmental cues. However, its integration of "death-life" signalings remains elusive. In our study, the role of caspase-8-Src is well-established as a promoter for migration or   metastasis   in Casp8(+)Src(+) A549/H226 cells in vivo and in vitro. In particular for nude mice models, mice implanted with Casp8(+)Src(+) A459/H226 cells remarkably increased spontaneous tumor metastatic burden with a significant survival disadvantage. Additionally, we detect that Src-mediated caspase-8 phosphorylation stimulates Src phosphorylation at Tyr-416 via the linkage of Src SH2 domain with phosph-Tyr-380 site of caspase-8. In turn, activated Src can efficiently induce epithelial-mesenchymal transition (EMT) phenotypic features to promote tumor cells   metastasis  . Surprisingly, RXDLL motif deletion in the DEDa of caspase-8 attenuates tumor cell migration or   metastasis   via impairing the recruitment of caspase-8 into the cellular periphery where activated Src is subject to caspase-8 phosphorylation. Together, a simple model is that the peripherization of caspase-8 is well-poised to facilitate Src-mediated caspase-8 phosphrylation at Tyr-380, then binding of phospho-Tyr380 of caspase-8 to Src SH2 domain may maintain Src in an active conformation to induce EMT phenotype, a key step toward cancer   metastasis  . 
 TRAIL negatively regulates VEGF-induced angiogenesis via caspase-8-mediated enzymatic and non-enzymatic functions. Solid tumors supply oxygen and nutrients required for angiogenesis by producing vascular endothelial growth factor (VEGF). Thus, inhibitors of VEGF signaling abrogate tumor angiogenesis, resulting in the suppression of tumor growth and   metastasis  . We here investigated the effects of tumor necrosis factor-related apoptosis-inducing ligand (TRAIL) on VEGF-induced angiogenesis. TRAIL inhibited VEGF-induced in vitro angiogenesis of human umbilical vein endothelial cells (HUVECs) and in vivo neovascularization in chicken embryos and mice. TRAIL blocked VEGF-induced angiogenic signaling by inhibiting ERK, Src, FAK, paxillin, Akt, and eNOS. Further, TRAIL blocked intracellular Ca(2+) elevation and actin reorganization in HUVECs stimulated with VEGF, without inhibiting VEGF receptor-2 tyrosine phosphorylation. TRAIL increased caspase-8 activity, without inducing caspase-9/-3 activation and apoptosis. Moreover, TRAIL resulted in cleavage of FAK into FAK-related non-kinase-like fragments in VEGF-stimulated HUVECs, which was blocked by a caspase-8 inhibitor and cellular caspase-8-like inhibitory protein. Biochemical and pharmacological inhibition of caspase-8 and FAK blocked the inhibitory effects of TRAIL on VEGF-stimulated anti-angiogenic signaling and events. In addition, caspase-8 knockdown also suppressed VEGF-mediated signaling and angiogenesis, suggesting that procaspase-8 plays a role of a non-apoptotic modulator in VEGF-induced angiogenic signaling. These results suggest that TRAIL inhibits VEGF-induced angiogenesis by increasing caspase-8 activity and subsequently decreasing non-apoptotic signaling functions of procaspase-8, without inducing caspase-3 activation and endothelial cell cytotoxicity. These data indicate that caspase-8 may be used as an anti-angiogenic drug for solid tumors resistant to TRAIL and anti-tumor drugs. 
 Potentiation of neuroblastoma   metastasis   by loss of caspase-8. Neuroblastoma, the most common paediatric solid tumour, arises from defective neural crest cells. Genetic alterations occur frequently in the most aggressive neuroblastomas. In particular, deletion or suppression of the proapoptotic enzyme caspase-8 is common in malignant, disseminated disease, although the effect of this loss on disease progression is unclear. Here we show that suppression of caspase-8 expression occurs during the establishment of neuroblastoma metastases in vivo, and that reconstitution of caspase-8 expression in deficient neuroblastoma cells suppressed their metastases. Caspase-8 status was not a predictor of primary tumour growth; rather, caspase-8 selectively potentiated apoptosis in neuroblastoma cells invading the collagenous stroma at the tumour margin. Apoptosis was initiated by unligated integrins by means of a process known as integrin-mediated death. Loss of caspase-8 or integrin rendered these cells refractory to integrin-mediated death, allowed cellular survival in the stromal microenvironment, and promoted metastases. These findings define caspase-8 as a   metastasis   suppressor gene that, together with integrins, regulates the survival and invasive capacity of neuroblastoma cells. 
 Novel noncatalytic role for caspase-8 in promoting SRC-mediated adhesion and Erk signaling in neuroblastoma cells. Neuroblastomas are extremely aggressive, although heterogeneous, cancers with a poor prognosis upon   metastasis  . Some evidence has suggested a correlative silencing of caspase-8 with MYCN amplification in neuroblastoma. A prognostic effect of this silencing, however, has been disputed. We report here hitherto undescribed roles for caspase-8 in the modulation of cell adhesion and subsequent activation of the Erk signaling pathway. Re-expression of caspase-8 in neuroblastoma cells lacking endogenous caspase-8 expression was found to promote cell adhesion to extracellular matrix and to activate adhesion-dependent signaling pathways, such as the Erk kinase cascade. This function of caspase-8 occurred irrespective of its proteolytic activity. Additionally, a pool of caspase-8 was shown to co-localize with the Src tyrosine kinase at the cellular periphery. Furthermore, our studies showed that caspase-8 forms a physical protein complex with Src via its death effector domains (DED) and maintains the complex in a detergent-soluble fraction. We also show that the DEDs of caspase-8 alone are necessary and sufficient to recreate the adhesive and biochemical phenotypes observed with the full-length protein, suggesting that caspase-8 may exert these effects via its association with Src. This protein complex association of caspase-8 and Src, and concomitant downstream signaling events, may help reconcile why a potential tumor suppressor such as caspase-8 is rarely absent in cancers. 
 Caspase-8 interacts with the p85 subunit of phosphatidylinositol 3-kinase to regulate cell adhesion and motility. Cell migration plays an important role in tumor cell invasion and   metastasis  . Previously, we reported that caspase-8 contributes to cell migration and adhesion, a novel nonapoptotic function of an established apoptotic factor. Herein, we report that pro-caspase-8 is capable of restoring cell migration/adhesion to caspase-8-null cells, establishing the first biological function of a pro-caspase. The catalytic activity of caspase-8 was not required for cell motility. Stimulation of motility with epidermal growth factor induced the phosphorylation of caspase-8 on tyrosine-380 and the interaction of caspase-8 with the p85 alpha subunit of phosphatidylinositol 3-kinase. Tyrosine-380 was required for the restoration of cell motility and cell adhesion in caspase-8-null cells, demonstrating the importance of the caspase-8-p85 interaction for these nonapoptotic functions. These results suggest that caspase-8 phosphorylation converts it from a proapoptotic factor to a cell motility factor that, through tyrosine-380, interacts with p85, an established cell migration component. 
 The CASP8 -652 6N insertion/deletion promoter polymorphism is associated with renal cell carcinoma risk and   metastasis  . Caspase-8 is a key regulator of apoptosis. Its cancer cell antigen induced cell death activity is strongly impacted by the insertion/deletion promoter polymorphism CASP8 -652 6N ins/del (rs3834129). We studied the association of this polymorphism with renal cell carcinoma risk and pathology.In this hospital based case-control study 500 Austrian patients were genotyped, including 250 with renal cell carcinoma, and 250 age and gender matched healthy controls. Polymerase chain reaction amplified genomic DNA was evaluated by restriction fragment length polymorphism analysis and automatic sequencing. We assessed associations with renal cell carcinoma risk and pathological factors, and performed a meta-analysis of the literature.The CASP8 -652 6N ins/del polymorphism was significantly linked to renal cell carcinoma (chi-square for trend = 9.50, p = 0.002). Compared with ins/ins, del/del was associated with a 57% decreased risk of the disease (OR 0.43, 95% CI 0.26-0.73, p = 0.002). Furthermore, del/del was associated with a lower risk of distant metastases (p <0.05) but not with T stage, N stage or grade. On meta-analysis the CASP8 -652 6N ins/del polymorphism was associated with renal cell carcinoma risk (p <0.001).The del/del genotype of the CASP8 -652 6N ins/del promoter polymorphism decreases the overall risk of renal cell carcinoma. It may be associated with a decreased risk of   metastasis  . Larger studies are warranted to validate our findings. 
 Alterations of Fas-pathway genes associated with nodal   metastasis   in non-small cell lung cancer. Many types of cancer cells are resistant to Fas-mediated apoptosis by several mechanisms, including the mutations of the genes involved in Fas-mediated apoptosis. In this study, to explore the possibility that the mutations of the genes involved in the proximal pathway of Fas-mediated apoptosis (Fas, FADD, caspase 8 and caspase 10) are involved in cancer   metastasis  , we have analysed somatic mutation and deletion of these genes in 80 non-small cell lung cancers (NSCLCs) with (n=43) and without (n=37)   metastasis   to the regional lymph nodes. We found 12 mutations (four Fas, four FADD, and four caspase 10 mutations) in 11 of 80 NSCLCs (13.8%). Interestingly, of these mutations, most mutations (10 out of 12) were detected in the NSCLCs with   metastasis  , and the frequency in the   metastasis   lesions (23%) was higher than that in the primary lesions of the NSCLCs without   metastasis   (5.4%). Furthermore, transfection study revealed that the tumor-derived mutants have decreased apoptosis inductions compared to the wild types. These data suggest that the inactivating mutations of the genes in the proximal pathway of Fas-mediated apoptosis may lead to a decreased cancer cell death and play a role in the   metastasis   of NSCLC. 
 Analysis of death receptor 5 and caspase-8 expression in primary and metastatic head and neck squamous cell carcinoma and their prognostic impact. Death receptor 5 (DR5) and caspase-8 are major components in the extrinsic apoptotic pathway. The alterations of the expression of these proteins during the   metastasis   of head and neck squamous cell carcinoma (HNSCC) and their prognostic impact have not been reported. The present study analyzes the expression of DR5 and caspase-8 by immunohistochemistry (IHC) in primary and metastatic HNSCCs and their impact on patient survival. Tumor samples in this study included 100 primary HNSCC with no evidence of   metastasis  , 100 primary HNSCC with lymph node   metastasis   (LNM) and 100 matching LNM. IHC analysis revealed a significant loss or downregulation of DR5 expression in primary tumors with   metastasis   and their matching LNM compared to primary tumors with no evidence of   metastasis  . A similar trend was observed in caspase-8 expression although it was not statistically significant. Downregulation of caspase-8 and DR5 expression was significantly correlated with poorly differentiated tumors compared to moderately and well differentiated tumors. Univariate analysis indicates that, in HNSCC with no   metastasis  , higher expression of caspase-8 significantly correlated with better disease-free survival and overall survival. However, in HNSCC with LNM, higher caspase-8 expression significantly correlated with poorer disease-free survival and overall survival. Similar results were also generated when we combined both DR5 and caspase-8. Taken together, we suggest that both DR5 and caspase-8 are involved in regulation of HNSCC   metastasis  . Our findings warrant further investigation on the dual role of caspase-8 in cancer development. 
 Functional polymorphisms in apoptosis pathway genes and survival in patients with gastric cancer. The FAS, FAS ligand (FASL), and CASP8 are key regulators for apoptosis and their deregulations play an important role in carcinogenesis. However, the effects of promoter polymorphisms of the FAS, and FASL, and CASP8 genes on the survival of gastric cancer are unknown. In this study, we investigated the association of four polymorphisms (FAS -1377G>A, -670A>G, FASL -844C>T, and CASP8 -652 6N ins>del) with the clinical outcome of 940 gastric cancer patients in a Chinese population. The correlation between genotype and survival outcomes was assessed by the Kaplan-Meier method, Cox proportional hazards models and the log-rank test. Our results revealed that individuals with CASP8 -652 6N ins/del+del/del genotypes had a decreased risk of death compared with those with ins/ins genotype (log-rank P=0.005; hazard ratio=0.75, 95% confidence interval=0.62-0.92). The protective effect of the del allele was further confirmed in subgroups of patients with tumor size ≤ 5 cm (0.66, 0.50-0.86) and T2 depth invasion (0.59, 0.37-0.94), but no significant association was observed in the subgroups of lymph nod  e metastas  is (0.67, 0.47-0.97), and distanc  e metastas  is (0.73, 0.60-0.90). Our findings suggest that, if validated in different independent populations, the CASP8 -652 6N ins>del polymorphism may serve as a promising genetic marker for gastric cancer prognosis. 
 Association of CASP8 D302H polymorphism with reduced risk of aggressive prostate carcinoma. Because of the dramatically different clinical course of aggressive and indolent prostate carcinoma (PCa), markers that distinguish between these phenotypes are of critical importance. Apoptosis is an important protective mechanism for unrestrained cellular growth and   metastasis  . Therefore, dysfunction in this pathway is a key step in cancer progression. As such, genetic variants in apoptosis genes are potential markers of aggressive PCa. Recent work in breast carcinoma has implicated the histidine variant of CASP8 D302H (rs1045485) as a protective risk allele.We tested the hypothesis that the H variant was protective for aggressive PCa in a pooled analysis of 796 aggressive cases and 2,060 controls.The H allele was associated with a reduced risk of aggressive PCa (OR(per allele) = 0.67, 95% CI: 0.54-0.83, P(trend) = 0.0003). The results were similar for European-Americans (OR(per allele) = 0.68; 95% CI: 0.54-0.86) and African-Americans (OR(per allele) = 0.61; 95% CI: 0.34-1.10). We further determined from the full series of 1,160 cases and 1,166 controls in the Prostate, Lung, Colorectal, Ovarian (PLCO) population that the protective effect of the H allele tended to be limited to high-grade and advanced PCa (all cases OR(per allele) = 0.94; 95% CI: 0.79-1.11; localized, low-grade disease OR(per allele) = 0.98; 95% CI: 0.79-1.23; and aggressive disease OR(per allele) = 0.73; 95% CI: 0.50-1.07).These results suggest that histidine variant of CASP8 D302H is a protective allele for aggressive PCa with potential utility for identification of patients at differential risk for this clinically significant phenotype. 
 [Expression of caspase-8 in non-small cell lung cancer and its role in invasion and   metastasis   of lung cancer A549 cells]. To explore the expression of caspase-8 in non-small cell lung cancer (NSCLC) tissue, and its role in invasion and   metastasis   of lung cancer A549 cells.The expression of caspase-8 in the lung cancer tissues was detected by immunohistochemistry. RNA interference technology was used to silence the expression of caspase-8 in lung cancer A549 cells to explore the function of caspase-8 protein in invasion and   metastasis   of cancer cells by means of Western blot, Transwell and scratch migration assays.The expression of caspase-8 was found in 38/52 cancer tissues, 22/30 adjacent tissues and 34/52 normal tissues. The tissues number of caspase-8 positive-expression in different groups was similar, but the intensity of caspase-8 expression in different groups was statistically different(P<0.05). In cancer tissues, the expression of caspase-8 indicated more likely to have lymph node-  metastasis   and worse clinical stage(P<0.05). After RNA interference, we successfully silenced the expression of caspase-8 in the lung cancer A549 cell, and this was confirmed by Western blotting. Transwell and cell scratch test showed that without the expression of caspase-8 A549 cells had a lower invasion and   metastasis   abilities compared to the control cells (P<0.05).Caspase-8 is not only an apoptosis protein, but has non-apoptosis function, such as promoting invasion and   metastasis   of lung cancer A549 cell. In NSCLC, it more likely locates in nucleus. And its expression in NSCLC may indicate a later clinical stage and a higher probability of lymph node   metastasis  . 
 Rab5 mediates caspase-8-promoted cell motility and   metastasis  . Caspase-8 is a key apical sensory protein that governs cell responses to environmental cues, alternatively promoting apoptosis, proliferation, and cell migration. The proteins responsible for integration of these pathways, however, have remained elusive. Here, we reveal that Rab5 regulates caspase-8-dependent signaling from integrins. Integrin ligation leads to Rab5 activation, association with integrins, and activation of Rac, in a caspase-8-dependent manner. Rab5 activation promotes colocalization and coprecipitation of integrins with caspase-8, concomitant with Rab5 recruitment to integrin-rich regions such as focal adhesions and membrane ruffles. Moreover, caspase-8 expression promotes Rab5-mediated internalization and the recycling of beta1 integrins, increasing cell migration independently of caspase catalytic activity. Conversely, Rab5 knockdown prevented caspase-8-mediated integrin signaling for Rac activation, cell migration, and apoptotic signaling, respectively. Similarly, Rab5 was critical for caspase-8-driven cell migration in vivo, because knockdown of Rab5 compromised the ability of caspase-8 to promote   metastasis   under nonapoptotic conditions. These studies identify Rab5 as a key integrator of caspase-8-mediated signal transduction downstream of integrins, regulating cell survival and migration in vivo and in vitro. 
 Suppression of death receptor 5 enhances cancer cell invasion and   metastasis   through activation of caspase-8/TRAF2-mediated signaling. The role of death receptor 5 (DR5), a well-known cell surface pro-apoptotic protein, in the negative regulation of invasion and   metastasis   of human cancer cells and the underlying mechanisms are largely unknown and were hence the focus of this study. In this report, we have demonstrated that DR5 functions to suppress invasion and   metastasis   of human cancer cells, as evidenced by enhanced cancer cell invasion and   metastasis   upon genetic suppression of DR5 either by gene knockdown or knockout. When DR5 is suppressed, FADD and caspase-8 may recruit and stabilize TRAF2 to form a   metastasis   and invasion signaling complex, resulting in activation of ERK and JNK/AP-1 signaling that mediate the elevation and activation of matrix metalloproteinase-1 (MMP1) and eventual promotion of cancer invasion and   metastasis  . Our findings thus highlight a novel non-apoptotic function of DR5 as a suppressor of human cancer cell invasion and   metastasis   and suggest a basic working model elucidating the underlying biology. 
 [Reduced expression of CASP8 in nasopharyngeal carcinoma and its clinical significance]. To explore the expression of CASP8 and its clinical significance in nasopharyngeal carcinoma (NPC).The differentially expressed genes between pooled NPC tissues and non-cancerous nasopharyngeal (NP) tissues were screened using 8 microarrays. Real-time PCR and immunohistochemistry were used to validate the detection results of CASP8 expression in NPC, and the correlation of CASP8 expression to the clinical characteristics was analyzed in the NPC cases.Real-time PCR confirmed a reduced expression of CASP8 mRNA level in NPC tissues (P<0.0001), which was consistent with the microarray data. Immunohistochemistry indicated that CASP8 protein expression was also significantly down-regulated in NPC tissue compared to that in the non-cancerous nasopharyngeal tissues (P=0.02). The reduction of CASP8 expression was inversely correlated to lymph node   metastasis   (P=0.002) and the clinical stages (P=0.026) of NPC.Decreased CASP8 expression is an unfavorable factor that promotes the development and progression of NPC. 
  GeneRIFs  
 Data show that when death receptor 5 (DR5) is suppressed, caspase-8 may recruit and stabilize TNF receptor-associated factor 2 (TRAF2) to form a   metastasis   and invasion signaling complex, resulting in activation of ERK signaling. 
 The del/del genotype of the CASP8 -652 6N ins/del promoter polymorphism decreases the overall risk of renal cell carcinoma. It may be associated with a decreased risk of   metastasis  . 
 Caspase-8 is not only an apoptosis protein, but has non-apoptosis function, such as promoting invasion and   metastasis   of lung cancer A549 cell. 
 Src is required for caspase-8-imparted cell migration or   metastasis   dependently on Tyr 380 and RXDLL motif of caspase-8. 
 DR5 and caspase-8 are involved in regulation of HNSCC   metastasis  . 
 findings define caspase-8 as a   metastasis   suppressor gene that, together with integrins, regulates the survival and invasive capacity of neuroblastoma cells 
 alteration associated with nodal   metastasis   in non-small cell lung cancer 
  [Cc]ell [Ii]nvasion  PubMed  
 Src-inducible association of CrkL with procaspase-8 promotes cell migration. Procaspase-8, the zymogen form of the apoptosis-initiator caspase-8, undergoes phosphorylation following integrin-mediated cell attachment to an extracellular matrix substrate. Concordant with cell attachment to fibronectin, a population of procaspase-8 becomes associated with a peripheral insoluble compartment that includes focal complexes and lamellar microfilaments. Phosphorylation of procaspase-8 both impairs its maturation to the proapoptotic form and can promote cell migration. Here we show that the cytoskeletal adaptor protein CrkL promotes caspase-8 recruitment to the peripheral spreading edge of cells, and that the catalytic domain of caspase-8 directly interacts with the SH2 domain of CrkL. We show that the interaction is abolished by shRNA-mediated silencing of Src, in Src-deficient MEFs, and by pharmacologic inhibitors of the kinase. The results provide insight into how tyrosine kinases may act to coordinate the suppression caspase-8 mediated apoptosis, while promoting   cell invasion  . 
 Caspase-8 interacts with the p85 subunit of phosphatidylinositol 3-kinase to regulate cell adhesion and motility. Cell migration plays an important role in tumor   cell invasion   and metastasis. Previously, we reported that caspase-8 contributes to cell migration and adhesion, a novel nonapoptotic function of an established apoptotic factor. Herein, we report that pro-caspase-8 is capable of restoring cell migration/adhesion to caspase-8-null cells, establishing the first biological function of a pro-caspase. The catalytic activity of caspase-8 was not required for cell motility. Stimulation of motility with epidermal growth factor induced the phosphorylation of caspase-8 on tyrosine-380 and the interaction of caspase-8 with the p85 alpha subunit of phosphatidylinositol 3-kinase. Tyrosine-380 was required for the restoration of cell motility and cell adhesion in caspase-8-null cells, demonstrating the importance of the caspase-8-p85 interaction for these nonapoptotic functions. These results suggest that caspase-8 phosphorylation converts it from a proapoptotic factor to a cell motility factor that, through tyrosine-380, interacts with p85, an established cell migration component. 
 Suppression of death receptor 5 enhances cancer   cell invasion   and metastasis through activation of caspase-8/TRAF2-mediated signaling. The role of death receptor 5 (DR5), a well-known cell surface pro-apoptotic protein, in the negative regulation of invasion and metastasis of human cancer cells and the underlying mechanisms are largely unknown and were hence the focus of this study. In this report, we have demonstrated that DR5 functions to suppress invasion and metastasis of human cancer cells, as evidenced by enhanced cancer   cell invasion   and metastasis upon genetic suppression of DR5 either by gene knockdown or knockout. When DR5 is suppressed, FADD and caspase-8 may recruit and stabilize TRAF2 to form a metastasis and invasion signaling complex, resulting in activation of ERK and JNK/AP-1 signaling that mediate the elevation and activation of matrix metalloproteinase-1 (MMP1) and eventual promotion of cancer invasion and metastasis. Our findings thus highlight a novel non-apoptotic function of DR5 as a suppressor of human cancer   cell invasion   and metastasis and suggest a basic working model elucidating the underlying biology. 
 UV-induction of keratinocyte endothelin-1 downregulates E-cadherin in melanocytes and melanoma cells. Endothelin-1 (ET-1), a peptide that is secreted by keratinocytes in the skin in response to ultraviolet irradiation, is a ligand for the endothelin-B (ET(B)) receptor. Blockade of this receptor inhibits melanoma cell growth and induces cell death in vivo and in vitro. Additionally, ET(B) is a melanoma progression marker. These findings suggest that the ET-1/ET(B) receptor pathway contributes to melanoma development or progression. Here, we demonstrate that activation of the ET-1/ET(B) pathway downregulates E-cadherin and associated catenin proteins in human melanocytes and melanoma cells. E-cadherin is an established suppressor of melanoma   cell invasion   in vitro and in vivo. Downregulation of E-cadherin by ET-1/ET(B) involves the downstream activation of caspase-8 but not of distal, executioner caspases, and does not lead to apoptosis. ET-1 also induces a transient association between caspase-8 and E-cadherin:beta-catenin complexes. Hence, activation of the ET-1/ET(B) pathway promotes molecular events known to promote melanoma invasion. 
   
  
 CASP10 
 
  NCBI Gene  
  GeneRIF  
  Pubmed  
 
 
  Search Term  Rank  Fields  
  [Mm]igration  6  PubMed(1)  
  [Ii]nvasive  10  PubMed(4); GeneRIFs(1)  
  [Mm]etastasis  12  PubMed(2); GeneRIFs(1)  
 
 
 hide/show details for CASP10 
  [Mm]igration  PubMed  
 Doxorubicin-mediated apoptosis in glioma cells requires NFAT3. Nuclear factor of activated T cells (NFAT), a family of transcription factors, has been implicated in many cellular processes, including some cancers. Here, we characterize, for the first time, the role of NFAT3 in doxorubicin (DOX)-mediated apoptosis,   migration  , and invasion in SNB19 and U87 glioma cells. This study demonstrates that the specific knockdown of NFAT3 results in a dramatic inhibition of the apoptotic effect induced by DOX and favors cell survival. Inhibition of NFAT3 activation by shNFAT3 (shNF3) significantly downregulated tumor necrosis factor (TNF)-alpha induction, its receptor TNFR1, caspase 10, caspase 3, and poly (ADP-ribose) polymerase, abrogating DOX-mediated apoptosis in glioma cells. DOX treatment resulted in NFAT3 translocation to the nucleus. Similarly, shNF3 treatment in SNB19 and U87 cells reversed DOX-induced inhibition of cell   migration   and invasion, as determined by wound healing and matrigel invasion assays. Taken together, these results indicate that NFAT3 is a prerequisite for the induction of DOX-mediated apoptosis in glioma cells. 
  [Ii]nvasive  PubMed  
 Genetic susceptibility to distinct bladder cancer subphenotypes. Clinical, pathologic, and molecular evidence indicate that bladder cancer is heterogeneous with pathologic/molecular features that define distinct subphenotypes with different prognoses. It is conceivable that specific patterns of genetic susceptibility are associated with particular subphenotypes.To examine evidence for the contribution of germline genetic variation to bladder cancer heterogeneity.The Spanish Bladder Cancer/EPICURO Study is a case-control study based in 18 hospitals located in five areas in Spain. Cases were patients with a newly diagnosed, histologically confirmed, urothelial cell carcinoma of the bladder from 1998 to 2001. Case diagnoses were reviewed and uniformly classified by pathologists following the World Health Organisation/International Society of Urological Pathology 1999 criteria. Controls were hospital-matched patients (n=1149).A total of 1526 candidate variants in 423 candidate genes were analysed. Three distinct subphenotypes were defined according to stage and grade: low-grade nonmuscle   invasive   (n=586), high-grade nonmuscle   invasive   (n=219), and muscle   invasive   (n=246). The association between each variant and subphenotype was assessed by polytomous risk models adjusting for potential confounders. Heterogeneity in genetic susceptibility among subphenotypes was also tested.Two established bladder cancer susceptibility genotypes, NAT2 slow-acetylation and GSTM1-null, exhibited similar associations among the subphenotypes, as did VEGF-rs25648, which was previously identified in our study. Other variants conferred risks for specific tumour subphenotypes such as PMS2-rs6463524 and CD4-rs3213427 (respective heterogeneity p values of 0.006 and 0.004), which were associated with muscle-  invasive   tumours (per-allele odds ratios [95% confidence interval] of 0.56 [0.41-0.77] and 0.71 [0.57-0.88], respectively) but not with non-muscle-  invasive   tumours. Heterogeneity p values were not robust in multiple testing according to their false-discovery rate.These exploratory analyses suggest that genetic susceptibility loci might be related to the molecular/pathologic diversity of bladder cancer. Validation through large-scale replication studies and the study of additional genes and single nucleotide polymorphisms are required. 
 Genetic susceptibility to distinct bladder cancer subphenotypes. Clinical, pathologic, and molecular evidence indicate that bladder cancer is heterogeneous with pathologic/molecular features that define distinct subphenotypes with different prognoses. It is conceivable that specific patterns of genetic susceptibility are associated with particular subphenotypes.To examine evidence for the contribution of germline genetic variation to bladder cancer heterogeneity.The Spanish Bladder Cancer/EPICURO Study is a case-control study based in 18 hospitals located in five areas in Spain. Cases were patients with a newly diagnosed, histologically confirmed, urothelial cell carcinoma of the bladder from 1998 to 2001. Case diagnoses were reviewed and uniformly classified by pathologists following the World Health Organisation/International Society of Urological Pathology 1999 criteria. Controls were hospital-matched patients (n=1149).A total of 1526 candidate variants in 423 candidate genes were analysed. Three distinct subphenotypes were defined according to stage and grade: low-grade nonmuscle   invasive   (n=586), high-grade nonmuscle   invasive   (n=219), and muscle   invasive   (n=246). The association between each variant and subphenotype was assessed by polytomous risk models adjusting for potential confounders. Heterogeneity in genetic susceptibility among subphenotypes was also tested.Two established bladder cancer susceptibility genotypes, NAT2 slow-acetylation and GSTM1-null, exhibited similar associations among the subphenotypes, as did VEGF-rs25648, which was previously identified in our study. Other variants conferred risks for specific tumour subphenotypes such as PMS2-rs6463524 and CD4-rs3213427 (respective heterogeneity p values of 0.006 and 0.004), which were associated with muscle-  invasive   tumours (per-allele odds ratios [95% confidence interval] of 0.56 [0.41-0.77] and 0.71 [0.57-0.88], respectively) but not with non-muscle-  invasive   tumours. Heterogeneity p values were not robust in multiple testing according to their false-discovery rate.These exploratory analyses suggest that genetic susceptibility loci might be related to the molecular/pathologic diversity of bladder cancer. Validation through large-scale replication studies and the study of additional genes and single nucleotide polymorphisms are required. 
 RIOK3 interacts with caspase-10 and negatively regulates the NF-kappaB signaling pathway. RIOK3 was initially characterized as a homolog of Aspergillus nidulans sudD and showed down-regulation at the   invasive   front of malignant melanomas, but the molecular mechanism remains elusive. Here, we report that overexpression of RIOK3 inhibits TNFalpha-induced NF-kappaB activation, but down-regulation of endogenous RIOK3 expression by siRNA potentiates it. A yeast two-hybrid experiment revealed that RIOK3 interacted with caspase-10, and further, a GST pull-down assay and endogenous coimmunoprecipitation validated the interaction. We subsequently showed that the interaction was mediated by the RIO domain of RIOK3 and each death effector domain of caspase-10. Interestingly, our data demonstrated that RIOK3 suppressed caspase-10-mediated NF-kappaB activation by competing RIP1 and NIK to bind to caspase-10. Importantly, the kinase activity of RIOK3 was confirmed to be relevant to NF-kappaB signaling. Taken together, our findings strongly suggest that RIOK3 negatively regulates NF-kappaB signaling pathway activated by TNFalpha dependent on its kinase activity and NF-kappaB signaling pathway activated by caspase-10 independent of its kinase activity. 
 Five polymorphisms and breast cancer risk: results from the Breast Cancer Association Consortium. Previous studies have suggested that minor alleles for ERCC4 rs744154, TNF rs361525, CASP10 rs13010627, PGR rs1042838, and BID rs8190315 may influence breast cancer risk, but the evidence is inconclusive due to their small sample size. These polymorphisms were genotyped in more than 30,000 breast cancer cases and 30,000 controls, primarily of European descent, from 30 studies in the Breast Cancer Association Consortium. We calculated odds ratios (OR) and 95% confidence intervals (95% CI) as a measure of association. We found that the minor alleles for these polymorphisms were not related to   invasive   breast cancer risk overall in women of European descent: ECCR4 per-allele OR (95% CI) = 0.99 (0.97-1.02), minor allele frequency = 27.5%; TNF 1.00 (0.95-1.06), 5.0%; CASP10 1.02 (0.98-1.07), 6.5%; PGR 1.02 (0.99-1.06), 15.3%; and BID 0.98 (0.86-1.12), 1.7%. However, we observed significant between-study heterogeneity for associations with risk for single-nucleotide polymorphisms (SNP) in CASP10, PGR, and BID. Estimates were imprecise for women of Asian and African descent due to small numbers and lower minor allele frequencies (with the exception of BID SNP). The ORs for each copy of the minor allele were not significantly different by estrogen or progesterone receptor status, nor were any significant interactions found between the polymorphisms and age or family history of breast cancer. In conclusion, our data provide persuasive evidence against an overall association between   invasive   breast cancer risk and ERCC4 rs744154, TNF rs361525, CASP10 rs13010627, PGR rs1042838, and BID rs8190315 genotypes among women of European descent. 
  GeneRIFs  
 Our data provide persuasive evidence against an overall association between   invasive   breast cancer risk and ERCC4 rs744154, TNF rs361525, CASP10 rs13010627, PGR rs1042838, and BID rs8190315 genotypes among women of European descent. 
  [Mm]etastasis  PubMed  
 Alterations of Fas-pathway genes associated with nodal   metastasis   in non-small cell lung cancer. Many types of cancer cells are resistant to Fas-mediated apoptosis by several mechanisms, including the mutations of the genes involved in Fas-mediated apoptosis. In this study, to explore the possibility that the mutations of the genes involved in the proximal pathway of Fas-mediated apoptosis (Fas, FADD, caspase 8 and caspase 10) are involved in cancer   metastasis  , we have analysed somatic mutation and deletion of these genes in 80 non-small cell lung cancers (NSCLCs) with (n=43) and without (n=37)   metastasis   to the regional lymph nodes. We found 12 mutations (four Fas, four FADD, and four caspase 10 mutations) in 11 of 80 NSCLCs (13.8%). Interestingly, of these mutations, most mutations (10 out of 12) were detected in the NSCLCs with   metastasis  , and the frequency in the   metastasis   lesions (23%) was higher than that in the primary lesions of the NSCLCs without   metastasis   (5.4%). Furthermore, transfection study revealed that the tumor-derived mutants have decreased apoptosis inductions compared to the wild types. These data suggest that the inactivating mutations of the genes in the proximal pathway of Fas-mediated apoptosis may lead to a decreased cancer cell death and play a role in the   metastasis   of NSCLC. 
 Alterations of Fas-pathway genes associated with nodal   metastasis   in non-small cell lung cancer. Many types of cancer cells are resistant to Fas-mediated apoptosis by several mechanisms, including the mutations of the genes involved in Fas-mediated apoptosis. In this study, to explore the possibility that the mutations of the genes involved in the proximal pathway of Fas-mediated apoptosis (Fas, FADD, caspase 8 and caspase 10) are involved in cancer   metastasis  , we have analysed somatic mutation and deletion of these genes in 80 non-small cell lung cancers (NSCLCs) with (n=43) and without (n=37)   metastasis   to the regional lymph nodes. We found 12 mutations (four Fas, four FADD, and four caspase 10 mutations) in 11 of 80 NSCLCs (13.8%). Interestingly, of these mutations, most mutations (10 out of 12) were detected in the NSCLCs with   metastasis  , and the frequency in the   metastasis   lesions (23%) was higher than that in the primary lesions of the NSCLCs without   metastasis   (5.4%). Furthermore, transfection study revealed that the tumor-derived mutants have decreased apoptosis inductions compared to the wild types. These data suggest that the inactivating mutations of the genes in the proximal pathway of Fas-mediated apoptosis may lead to a decreased cancer cell death and play a role in the   metastasis   of NSCLC. 
  GeneRIFs  
 alteration associated with nodal   metastasis   in non-small cell lung cancer 
   
  
 TP53INP2 
 
  NCBI Gene  
  GeneRIF  
  Pubmed  
 
 
  Search Term  Rank  Fields  
  [Mm]igration  6  PubMed(1)  
  [Ee]xtracellular [Mm]atrix  8  PubMed(1); GeneRIFs(1)  
  [Ii]nvasive  10  PubMed(1)  
 
 
 hide/show details for TP53INP2 
  [Mm]igration  PubMed  
 hnRNP A2 regulates alternative mRNA splicing of TP53INP2 to control invasive cell   migration  . Largely owing to widespread deployment of microarray analysis, many of the transcriptional events associated with invasive cell   migration   are becoming clear. However, the transcriptional drives to invasive   migration   are likely modified by alternative splicing of pre-mRNAs to produce functionally distinct patterns of protein expression. Heterogenous nuclear ribonucleoprotein (hnRNP A2) is a known regulator of alternative splicing that is upregulated in a number of invasive cancer types. Here, we report that although siRNA of hnRNP A2 had little influence on the ability of cells to migrate on plastic surfaces, the splicing regulator was clearly required for cells to move effectively on three-dimensional matrices and to invade into plugs of extracellular matrix proteins. We used exon-tiling microarrays to determine that hnRNP A2 controlled approximately six individual splicing events in a three-dimensional matrix-dependent fashion, one of which influenced invasive   migration  . Here, we show that alternative splicing of an exon in the 5' untranslated region of a gene termed TP53INP2 is a key event downstream of hnRNP A2 that is necessary for cells to invade the extracellular matrix. Furthermore, we report that the consequences of altered TP53INP2 splicing on invasion are likely mediated via alterations in Golgi complex integrity during   migration   on three-dimensional matrices. 
  [Ee]xtracellular [Mm]atrix  PubMed  
 hnRNP A2 regulates alternative mRNA splicing of TP53INP2 to control invasive cell migration. Largely owing to widespread deployment of microarray analysis, many of the transcriptional events associated with invasive cell migration are becoming clear. However, the transcriptional drives to invasive migration are likely modified by alternative splicing of pre-mRNAs to produce functionally distinct patterns of protein expression. Heterogenous nuclear ribonucleoprotein (hnRNP A2) is a known regulator of alternative splicing that is upregulated in a number of invasive cancer types. Here, we report that although siRNA of hnRNP A2 had little influence on the ability of cells to migrate on plastic surfaces, the splicing regulator was clearly required for cells to move effectively on three-dimensional matrices and to invade into plugs of   extracellular matrix   proteins. We used exon-tiling microarrays to determine that hnRNP A2 controlled approximately six individual splicing events in a three-dimensional matrix-dependent fashion, one of which influenced invasive migration. Here, we show that alternative splicing of an exon in the 5' untranslated region of a gene termed TP53INP2 is a key event downstream of hnRNP A2 that is necessary for cells to invade the   extracellular matrix  . Furthermore, we report that the consequences of altered TP53INP2 splicing on invasion are likely mediated via alterations in Golgi complex integrity during migration on three-dimensional matrices. 
  GeneRIFs  
 Alternative splicing of an exon in the 5' untranslated region of a gene termed TP53INP2 is a key event downstream of hnRNP A2 that is necessary for cells to invade the   extracellular matrix  . 
  [Ii]nvasive  PubMed  
 hnRNP A2 regulates alternative mRNA splicing of TP53INP2 to control   invasive   cell migration. Largely owing to widespread deployment of microarray analysis, many of the transcriptional events associated with   invasive   cell migration are becoming clear. However, the transcriptional drives to   invasive   migration are likely modified by alternative splicing of pre-mRNAs to produce functionally distinct patterns of protein expression. Heterogenous nuclear ribonucleoprotein (hnRNP A2) is a known regulator of alternative splicing that is upregulated in a number of   invasive   cancer types. Here, we report that although siRNA of hnRNP A2 had little influence on the ability of cells to migrate on plastic surfaces, the splicing regulator was clearly required for cells to move effectively on three-dimensional matrices and to invade into plugs of extracellular matrix proteins. We used exon-tiling microarrays to determine that hnRNP A2 controlled approximately six individual splicing events in a three-dimensional matrix-dependent fashion, one of which influenced   invasive   migration. Here, we show that alternative splicing of an exon in the 5' untranslated region of a gene termed TP53INP2 is a key event downstream of hnRNP A2 that is necessary for cells to invade the extracellular matrix. Furthermore, we report that the consequences of altered TP53INP2 splicing on invasion are likely mediated via alterations in Golgi complex integrity during migration on three-dimensional matrices. 
   
  
 TMSB4X 
 
  NCBI Gene  
  GeneRIF  
  Pubmed  
 
 
  Search Term  Rank  Fields  
  [Mm]igration  6  Process(2); PubMed(19); GeneRIFs(7); Summary(1)  
  [Ee]xtracellular [Mm]atrix  8  PubMed(2)  
  [Ii]nvasive  10  PubMed(9); GeneRIFs(2)  
  [Mm]etastasis  12  PubMed(15); GeneRIFs(2)  
  [Cc]ell [Ii]nvasion  13  PubMed(2)  
 
 
 hide/show details for TMSB4X 
  [Mm]igration  Process  
 positive regulation of blood vessel endothelial cell   migration   
 positive regulation of blood vessel endothelial cell   migration   
  PubMed  
 Thymosin beta4 activates integrin-linked kinase and promotes cardiac cell   migration  , survival and cardiac repair. Heart disease is a leading cause of death in newborn children and in adults. Efforts to promote cardiac repair through the use of stem cells hold promise but typically involve isolation and introduction of progenitor cells. Here, we show that the G-actin sequestering peptide thymosin beta4 promotes myocardial and endothelial cell   migration   in the embryonic heart and retains this property in postnatal cardiomyocytes. Survival of embryonic and postnatal cardiomyocytes in culture was also enhanced by thymosin beta4. We found that thymosin beta4 formed a functional complex with PINCH and integrin-linked kinase (ILK), resulting in activation of the survival kinase Akt (also known as protein kinase B). After coronary artery ligation in mice, thymosin beta4 treatment resulted in upregulation of ILK and Akt activity in the heart, enhanced early myocyte survival and improved cardiac function. These findings suggest that thymosin beta4 promotes cardiomyocyte   migration  , survival and repair and the pathway it regulates may be a new therapeutic target in the setting of acute myocardial damage. 
 Regulation of glycogen synthase kinase-3 by thymosin beta-4 is associated with gastric cancer cell   migration  . Thymosin beta-4 (Tβ4), actin-sequestering protein, plays important roles in many cellular functions including cancer cell   migratio  ns. Glycogen synthase kinase (GSK) in Wnt signaling pathway is a key molecule to control intercellular interaction. Here, we investigated whether GSK-3 activity is regulated by Tβ4 and it is associated with Tβ4-mediat  ed migrat  ion in gastric cancer cells. Various expression level of Tβ4 was observed in human gastric tumor tissu  es. Migra  tion in gastric cancer cells, SNU638 and SNU668, was dependent on a relative expression level of Tβ4.   Cell migr  ation was higher in SNU668 with a higher expression level of Tβ4 than that in SNU638 with a lower Tβ4. Although the level of phosphorylated(p)-GSK-3α (inactive), β-catenin, E-cadherin and E-cadherin:β-catenin complex was relatively higher, p-GSK-3β (inactive) was lower in SNU638 compared to those in SNU668 cells. LiCl, GSK-3α/β inhibitor, reduced lung metastasis of B16F10 mouse melanoma cells and   SNU668 c  ell migration. Small interference (si)RNA of GSK-3α increase  d SNU638   cell migration in accordance with the reduction of E-cadherin:β-catenin complex formation through a decrease in β-catenin and E-cadherin. Expression level of GSK-3α/β, β-catenin and E-cadherin in SNU668 and SNU638 was reversed by Tβ4-siRNA and by the treatment with acetylated-serine-aspartic acid-lysine-proline (SDKP) tetrapeptide of Tβ4, respectively. E-cadherin expression in SNU638 cells was decreased by β-catenin-siRNA. PD98059, MEK inhibitor, or U0126, ERK inhibitor  , reduced   SNU668 cell migration accompanying an increase in p-GSK-3α, β-catenin and E-cadherin. Taken together, data indicated that the expression of GSK-3α, β-catenin and E-cadherin could be negatively regulated by Tβ4-induced ERK phosphorylation. It suggests that Tβ4 could be a novel regulator to control Wnt signaling pathways. 
 Thymosin beta 4 silencing suppresses proliferation and invasion of non-small cell lung cancer cells by repressing Notch1 activation. Thymosin beta 4 (Tβ4), a pleiotropic actin-sequestering polypeptide that is involved in wound healing and developmental processes, has been reported to be strongly associated with tumorigenesis. A recent tissue microarray analysis showed that Tβ4 was highly expressed in certain tumor cells, including lung cancer. However, the exact expression pattern and the role of Tβ4 in non-small cell lung cancer (NSCLC) have not to our knowledge been investigated. In the present study, we confirmed that Tβ4 expression was increased in NSCLC tissues and cell lines. Tβ4 gene silencing in A549 and H1299 cells inhibited cell proliferat  ion, migr  ation, and invasion in vitro and decreased tumor growth in vivo Mechanistic investigations revealed a significant decrease in Notch1 activation in Tβ4 gene-silenced cells. Moreover, restoring the Notch1 expression attenuated the function of Tβ4 silencing in NSCLC cells. Taken together, these findings suggest that Tβ4 may play an oncogenic role in NSCLC progression and may be a novel molecular target for anti-NSCLC therapy. 
 Thymosin-β4 (Tβ4) blunts PDGF-dependent phosphorylation and binding of AKT to actin in hepatic stellate cells. Hepatic stellate cell transdifferentiation is a key event in the fibrogenic cascade. Therefore, attempts to prevent and/or revert the myofibroblastic phenotype could result in novel therapeutic approaches to treat liver cirrhosis. The expression of platelet-derived growth factor (PDGF)-β receptor and the proliferative response to platelet-derived growth factor-ββ (PDGF-ββ) are hallmarks of the transdifferentiation of hepatic stellate cells (HSC). In this communication, we investigated whether thymosin-β4 (Tβ4), a chemokine expressed by HSC could prevent PDGF-BB-mediated prolifera  tion and   migration of cultured HSC. Using early passages of human HSC, we showed that Tβ4 inhibited cell prolifer  ation and   migration and prevented the expression of PDGF-β receptor (PDGF-βr), α-smooth muscle actin and α1(I) collagen mRNAs. Tβ4 also inhibited the reappearance of PDGF-βr after its PDGF-BB-dependent degradation. These PDGF-dependent events were associated with the inhibition of AKT phosphorylation at both T308 and S473 amino acid residues. The lack of AKT phosphorylation was not due to the inhibition of PDGF-βr phosphorylation, the activation of phosphoinositide 3-kinase (PI3K), pyruvate dehydrogenase kinase isozyme 1 (PDK1), and mammalian target of rapamycin (mTOR). We found that PDGF-BB induced AKT binding to actin, and that Tβ4 prevented this effect. Tβ4 also prevented the activation of freshly isolated HSC cultured in the presence of Dulbecco's modified Eagle's medium or Dulbecco's minimal essential medium containing 10% fetal bovine serum. In conclusion, overall, our findings suggest that Tβ4 by sequestering actin prevents binding of AKT, thus inhibiting its phosphorylation. Therefore, Tβ4 has the potential to be an antifibrogenic agent. 
 Hepatocyte growth factor upregulates thymosin beta4 in human umbilical vein endothelial cells. Hepatocyte growth factor (HGF) is a mesenchymal-derived cytokine. It exerts in vitro a motogenic effect on various target cells, which is displayed either by cell scattering, locomotion, and   migration   during the wound repair process of cultured cells, or invasiveness through the extracellular matrix. Although it is known that HGF influences the motogenic effect of endothelial cells, the precise effects of HGF during angiogenesis are still poorly understood. To identify genes regulated via HGF signaling in HUVECs, we used the differential display polymerase chain reaction. In this study, thymosin beta4 was found to be differentially expressed in HGF-treated HUVECs compared with control. Data from HPLC profile and induction of MMPs indicate that HGF may affect the biological behavior of HUVECs through a combination of the direct effects of HGF itself and indirect effects mediated via induction of thymosin beta4 in vitro. 
 Thymosin beta 4 stimulates directional   migration   of human umbilical vein endothelial cells. Thymosin beta 4 (T beta 4) is a 4.9 kDa polypeptide that interacts with G-actin and is thought to be an important mediator in cell proliferation,   migration  , and differentiation. T beta 4 has been identified as a factor involved in the differentiation of human umbilical vein endothelial cells (HUVECs) cultured on Matrigel. Here we have used various in vitro and in vivo   migration   assays to demonstrate the role of T beta 4 in endothelial cell   migration  . Our results demonstrate that T beta 4 acts as a chemoattractant for endothelial cells, stimulating the   migration   of HUVECs in Boyden chambers four- to sixfold over that observed with media alone. Of the primary cell types tested, only human coronary artery cells responded to T beta 4 treatment, suggesting that the   migration   activity of T beta 4 was endothelial cell-specific. T beta 4 significantly accelerated the rate of   migration   into the scratch wounded area of a HUVEC monolayer. T beta 4 treatment also increased the production of matrix metalloproteinases that may degrade the basement membrane during angiogenesis. Additional experiments using subcutaneously implanted Matrigel showed that T beta 4 stimulated cell   migration   in vivo. These results provide the first direct evidence that T beta 4 has chemoattractive activity and promotes angiogenesis by stimulating the   migration   of endothelial cells. 
 Zyxin is upregulated in the nucleus by thymosin beta4 in SiHa cells. Thymosin beta4 is a 43-amino acid actin-binding protein that promotes cell   migration   and is important in angiogenesis, wound healing, and tumor metastasis. We searched for genes upregulated by thymosin beta4 and identified zyxin as increased in SiHa cells in the presence of exogenously added thymosin beta4 and when thymosin beta4 is overexpressed using adenoviral vectors. Both zyxin and thymosin beta4 show increased localization in the nucleus. We conclude that thymosin beta4 may exert some of its   migration   promoting activity via increased zyxin expression. 
 Thymosin β4 promotes the   migratio  n of endothelial cells without intracellular Ca2+ elevation. Numerous studies have demonstrated the effects of Tβ4 on cel  l migrati  on, proliferation, apoptosis and inflammation after exogenous treatment, but the mechanism by which Tβ4 functions is still unclear. Previously, we demonstrated that incubation of endothelial cells with Tβ4 induced synthesis and secretion of various proteins, including plasminogen activator inhibitor type 1 and matrix metaloproteinases. We also showed that Tβ4 interacts with Ku80, which may operate as a novel receptor for Tβ4 and mediates its intracellular activity. In this paper, we provide evidence that Tβ4 induces cellular processes without changes in the intracellular Ca(2+) concentration. External treatment of HUVECs with Tβ4 and its mutants deprived of the N-terminal tetrapeptide AcSDKP (Tβ4(AcSDKPT/4A)) or the actin-binding sequence KLKKTET (Tβ4(KLKKTET/7A)) resulted in enha  nced cell   migration and formation of tubular structures in Matrigel. Surprisingly, the increased cell motility caused by Tβ4 was not associated with the intracellular Ca(2+) elevation monitored with Fluo-4 NW or Fura-2 AM. Therefore, it is unlikely that externally added Tβ4 in  duces HUV  EC migration via the surface membrane receptors known to generate Ca(2+) influx. Our data confirm the concept that externally added Tβ4 must be internalized to induce intracellular mechanisms supporting endo  thelial c  ell migration. 
 Thymosin β 4 gene silencing decreases stemness and invasiveness in glioblastoma. Thymosin beta 4 is a pleiotropic actin-sequestering polypeptide that is involved in wound healing and developmental processes. Thymosin beta 4 gene silencing promotes differentiation of neural stem cells whereas thymosin beta 4 overexpression initiates cortical folding of developing brain hemispheres. A role of thymosin beta 4 in malignant gliomas has not yet been investigated. We analysed thymosin beta 4 staining on tissue microarrays and performed interrogations of the REMBRANDT and the Cancer Genome Atlas databases. We investigated thymosin beta 4 expression in seven established glioma cell lines and seven glioma-initiating cell lines and induced or silenced thymosin beta 4 expression by lentiviral transduction in LNT-229, U87MG and GS-2 cells to study the effects of altered thymosin beta 4 expression on gene expression, growth, clonogenicity,   migratio  n, invasion, self-renewal and differentiation capacity in vitro, and tumorigenicity in vivo. Thymosin beta 4 expression increased with grade of malignancy in gliomas. Thymosin beta 4 gene silencing in LNT-229 and U87MG glioma cells inhibited   migratio  n and invasion, promoted starvation-induced cell death in vitro and enhanced survival of glioma-bearing mice. Thymosin beta 4 gene silencing in GS-2 cells inhibited self-renewal and promoted differentiation in vitro and decreased tumorigenicity in vivo. Gene expression analysis suggested a thymosin beta 4-dependent regulation of mesenchymal signature genes and modulation of TGFβ and p53 signalling networks. We conclude that thymosin beta 4 should be explored as a novel molecular target for anti-glioma therapy. 
 Identification and quantification of thymosin beta4 in human saliva and tears. Thymosin beta(4) (Tbeta(4)) is a ubiquitous, naturally occurring, 43-amino acid peptide that takes part in several biological activities including angiogenesis, inhibition of inflammation, wound healing, chemotaxis, and endothelial cell   migration  . Recent studies also indicate that Tbeta(4) accelerates corneal wound healing and downregulates several proinflamatory chemokines and cytokines. In this study, we sought to determine whether Tbeta(4) is naturally occurring in human tears and other human bodily fluids, such as saliva. Tear and saliva samples were analyzed by EIA to identify and quantify the amount of Tbeta(4) present. Around 10-20 samples were collected from each of three different age groups: 15-20, 25-35, and >50 years old with n = 30 and n = 60 for tears and saliva, respectively. Exclusion criteria included the use of any topical ophthalmic or topical oral medication and/or history of ocular or oral surgery within the past 6 months. Tears were collected from both eyes using Schirmer's strips. Saliva samples were collected in sterile tubes and were then centrifuged to remove solid particles. Tbeta(4) was found in tear and saliva samples in all age groups. The concentrations ranged from 0.5-7 mug/mL in tears and 0.2-3.6 mug/mL in saliva. In both fluids, Tbeta(4) concentration varied with age and appeared to peak at ages 25-35 years. Studies are in progress to determine if Tbeta(4) levels in saliva and tears demonstrate a circadian rhythm during a 24-h period, as well as to confirm that they vary with age and to explore if they vary with diseased states. This is the first study to report the presence of Tbeta(4) in human tears and saliva. This finding raises the possibility that Tbeta(4) acts as an endogenous agent contributing to the rapid healing of corneal and oral wounds. Considering that Tbeta(4) facilitates reepithelialization and modulates anti-inflammatory mediators, Tbeta(4) could potentially be used therapeutically in the treatment of (a) ocular surface disease and injury of eye and (b) various oral disorders, such as periodontal disease. 
 Pressure ulcers: a role for thymosin beta4. Pressure ulcers occur in up to 14% of acute hospitalizations. They cause pain, decreased quality of life, increased morbidity, and prolonged hospitalizations. Treatment includes pain control, nutritional support, relieving pressure, removing devitalized tissue, and by using dressings and medications, providing an environment in which healing can occur. Even with optimal treatment, pressure ulcers may take months to heal. Thymosin beta4 is being investigated as a treatment for pressure ulcers. Thymosin beta4 has wound healing and anti-inflammatory properties. It is thought to exert its therapeutic effect through promotion of keratinocyte and endothelial cell   migration  , increased collagen deposition, and stimulation of angiogenesis. A study in a rat full-thickness wound model showed that treatment with thymosin beta4 increased collagen deposition and angiogenesis and stimulated keratinocyte   migration   and reepithelialization. If thymosin beta4 decreases healing time, this would represent a significant advance in the treatment of pressure ulcers. 
 The actin-sequestering protein thymosin beta-4 is a novel target of hypoxia-inducible nitric oxide and HIF-1α regulation. The actin-sequestering protein thymosin beta-4 (Tβ4) is involved in various cellular and physiological processes such as proliferation, motility, growth and metastasis. Nitric oxide (NO) promotes tumor invasiveness and metastasis by activating various enzymes. Herein, we investigated whether hypoxia-inducible NO regulates Tβ4 expression and cancer ce  ll migrat  ion using HeLa cervical cancer cells. NO production and Tβ4 expression were increased in a hypoxic condition. The treatment with N-(β-D-Glucopyranosyl)-N2-acetyl-S-nitroso-D, L-penicillaminamide (SNAP-1), to generate NO, enhanced the transcription of Tβ4 and cancer   cell mig  ration. SNAP-1-induced   cell mig  ration was decreased by the inhibition of Tβ4 with small interference (si) RNA. In a hypoxic condition, treatment with N(G)-monomethyl-L-arginine (L-NMMA), nitric oxide synthase (NOS) inhibitor, reduced Tβ4 transcriptional activity, and hypoxia-inducible factor (HIF)-1α. Hypoxia-induced can  cer cell   migration was also decreased by L-NMMA treatment. In a normoxic condition, Tβ4 transcriptional activity was decreased in the cells incubated in the presence of L-NMMA after co-transfection with Tβ4 promoter and GST-conjugated HIF-1α. Collectively, these results suggest that NO could regulate the expression of Tβ4 by direct or indirect effect of HIF-1α on Tβ4 promoter. 
 Regenerative protein thymosin beta-4 is a novel regulator of purinergic signaling. By an unknown mechanism, β-thymosins are extracellular modulators of angiogenesis, inflammation, wound healing, and development. We were interested in identifying β-thymosin interactors and determining their importance in β-thymosins signaling in human vein endothelial cells (HUVECs). We performed pulldown experiments with biotinylated thymosin β-4 (Tβ4) in comparison to neutravidin beads alone and used mass spectrometric analysis to identify differentially interacting proteins. By this method, we identified F1-F0 ATP synthase, a known target of antiangiogenic angiostatin. By surface plasmon resonance, we determined for Tβ4 binding to the β subunit of ATP synthase a K(D) of 12 nM. Blocking antibodies and antagonists (oligomycin, IC(50) ∼1.8 μM; piceatannol, IC(50) ∼1.05 μM; and angiostatin, IC(50) ∼2.9 μg/ml) of ATP synthase inhibited the Tβ4-induced increase in cell surface ATP levels, as measured by luciferase assay, and the Tβ4-induced   increase   in HUVEC migration, as meas  ured by t  ranswell migration assay. Silencing of the ATP-responsive purinergic receptor P2X4 with siRNA also blocked   Tβ4-indu  ced HUVEC migration in a transwell assay. Furthermore, in silico we identified common amphiphilic α-helical structural similarities between β-thymosins and the inhibitory factor 1 (IF1), an inhibitor of ATP synthase hydrolysis. In summary, we have identified an extracellular signaling pathway where Tβ4 increases cell surface ATP levels via ATP synthase and have shown further that ATP-responsive P2X4 receptor is required   for Tβ4-  induced HUVEC migration. 
 Actin-sequestering protein, thymosin beta-4, is a novel hypoxia responsive regulator. Angiogenesis is induced by soluble factors such as vascular endothelial growth factor (VEGF) released from tumor cells in hypoxia. It enhances solid tumor growth and provides an ability to establish metastasis at peripheral sites by tumor cell   migration  . Thymosin beta-4 (TB4) is an actin-sequestering protein to control cytoskeletal reorganization. Here, we investigated whether angiogenesis and tumor metastasis are dependent on hypoxia conditioning-induced TB4 expression in B16F10 melanoma cells. TB4 expression in B16F10 cells was increased by hypoxia conditioning in a time-dependent manner. In addition, we found an increase of angiogenesis and HIF-1α expression in TB4-transgenic (Tg) mice as compared to wildtype mice. When wound healing assay was used to assess in vitro tumor cell   migratio  n, hypoxia conditioning for 1 h enhanced B16F10 cell   migratio  n. When TB4 expression in B16F10 cells was inhibited by the infection with small hairpin (sh) RNA of TB4 cloned in lentiviral vector, tumor cell   migratio  n was retarded. In addition, hypoxia conditioning-induced tumor cell   migratio  n was reduced by the infection of lentiviral shRNA of TB4. HIF-1α stabilization and the expression of VEGF isoform 165 and 121 in hypoxia were also reduced by the infection of lentiviral shRNA of TB4 in B16F10 cells. We also found an increase of tumor growth and lung metastasis count in TB4-Tg mice as compared to wildtype mice. Collectively, hypoxia conditioning induced tumor cel  l migrati  on by TB4 expression-dependent HIF-1α stabilization. It suggests that TB4 could be a hypoxia responsive regulator to control tumor ce  ll migrat  ion in angiogenesis and tumor metastasis. 
 Platelet function and thymosin β4. Thymosin β4 (Tβ4) is a small, low-molecular-weight peptide ubiquitously expressed in all cells and extracellular fluids. It is a major actin sequestering protein present in the cells. In addition to this, Tβ4 has also been shown to be involved in endothelial c  ell migra  tion, angiogenesis, corneal wound healing, and stem cell differentiation. It is also released by platelets after activation. The amount of Tβ4 increases at sites of injury and thus suggests an important role of this biopeptide in wound healing. Herein, we provide an overview of the role of Tβ4 in thrombosis and platelet aggregation. 
 Characterization of thymosin β4 in mammals' saliva. Thymosin β4 (Tβ4) is a low molecular weight peptide found in several mammalian tissues and is known mainly by its ability to bind cytoskeletal actin, influencing ce  ll migrat  ion and differentiation, and promoting tissue repair. Considering the functional role of this peptide, the main goal of this work was to characterize Tβ4 in mammals' saliva by using evolutionary and proteomic tools. For this, mammalian Tβ4 sequences were retrieved from NCBI, SwissProt and Ensembl databases. The alignment of Tβ4 amino acid sequences showed a high degree of conservation between species. The gene seems to be evolving under negative selection as indicated by a dN/dS ratio of 0.05. Whole saliva was collected from dog, human, rabbit, cow, horse and sheep and the salivary peptides were isolated through filtration and analyzed by LC-MS/MS. Spectra was processed against the database constructed with the retrieved Tβ4 sequences. For the first time, the identification of this peptide was achieved in rat, dog, horse and bovine saliva. Detection in these mammal species and its amino acid conservation suggest an important role of Tβ4 in the homeostasis of the mammalian oral cavity. 
 Thymosin β4 induces invasion and   migratio  n of human colorectal cancer cells through the ILK/AKT/β-catenin signaling pathway. Thymosin β4 (Tβ4) is a 43-amino-acid peptide involved in many biological processes. However, the precise molecular signaling mechanism(s) of Tβ4 in cell invasion   and migr  ation remain unclear. In this study, we show that Tβ4 was significantly overexpressed in colorectal cancer tissues compared to adjacent normal tissues and high levels of Tβ4 were correlated with stage of colorectal cancer, and that Tβ4 expression was associated with morphogenesis and EMT. Tβ4-upregulated cancer cells showed increased adhesion, inva  sion and   migration activity, whereas Tβ4-downregulated cells showed decreased activities. We also demonstrated that Tβ4 interacts with ILK, which promoted the phosphorylation and activation of AKT, the phosphorylation and inactivation of GSK3β, the expression and nuclear localization of β-catenin, and integrin receptor activation. These results suggest that Tβ4 is an important regulator of the ILK/AKT/β-catenin/Integrin signaling cascade to induce cel  l invasio  n and migration in colorectal cancer cells, and is a potential target for cancer treatment. 
 Potential role of thymosin Beta 4 in liver fibrosis. Liver fibrosis, the main characteristic of chronic liver diseases, is strongly associated with the activation of hepatic stellate cells (HSCs), which are responsible for extracellular matrix production. As such, investigating the effective regulators controlling HSC activation provides important clues for developing therapeutics to inhibit liver fibrosis. Thymosin beta 4 (Tβ4), a major actin-sequestering protein, is known to be involved in various cellular responses. A growing body of evidence suggests that Tβ4 has a potential role in the pathogenesis of liver fibrosis and that it is especially associated with the activation of HSCs. However, it remains unclear whether Tβ4 promotes or suppresses the activation of HSCs. Herein, we review the potential role of Tβ4 in liver fibrosis by describing the effects of exogenous and endogenous Tβ4, and we discuss the possible signaling pathway regulated by Tβ4. Exogenous Tβ4 reduces liver fibrosis by inhibiting the proliferati  on and mi  gration of HSCs. Tβ4 is expressed endogenously in the activated HSCs, but this endogenous Tβ4 displays opposite effects in HSC activation, either as an activator or an inhibitor. Although the role of Tβ4 has not been established, it is apparent that Tβ4 influences HSC activation, suggesting that Tβ4 is a potential therapeutic target for treating liver diseases. 
 Thymosin beta 4 expression and nuclear transport are regulated by hMLH1. For hMLH1, a key enzyme of DNA mismatch repair and frequently mutated in human cancers, several additional functions have been suggested. We now identified Thymosin beta4 (Tbeta4), an actin-binding and cell motility regulating protein, by bacterial two-hybrid screening. Interaction was confirmed by coimmunoprecipitation. Tbeta4 was weakly expressed in the hMLH1-deficient cell lines 293T and HCT-116. Reconstitution of hMLH1 resulted in strong expression of Tbeta4. Confocal laser microscopy revealed nuclear colocalization of both proteins. Reconstitution with hMLH1 mutants lacking a functional nuclear localization sequence resulted in cytoplasmatic retention of both proteins. After Tbeta4- or hMLH1-siRNA treatment, cell   migration   of hMLH1-proficient cells was markedly decreased. Our results show that hMLH1 interacts with Tbeta4 and regulates its expression and nuclear transport. Moreover, loss of hMLH1 causes Tbeta4 deprivation and results in reduced migratory activity in vitro. These data give insight into novel functions of hMLH1 and probably disease related dysregulated mechanisms. 
  GeneRIFs  
 Tbeta4 gene silencing in A549 and H1299 cells inhibited cell proliferation,   migration  , and invasion in vitro and decreased tumor growth in vivo. Mechanistic investigations revealed a significant decrease in Notch1 activation in Tbeta4 gene-silenced cells. 
 Thymosin beta4 induces invasion and   migration   of human colorectal cancer cells through the ILK/AKT/beta-catenin signaling pathway. 
 Thymosin beta 4 silencing in glioblastoma cells inhibits cell   migration   and invasion. 
 investigated whether GSK-3 activity is regulated by thymosin beta-4 (Tbeta4) and associated with Tbeta4-mediated   migration   in gastric cancer cells; expression of GSK-3alpha, beta-catenin and E-cadherin could be negatively regulated by Tbeta4 induced ERK phosphorylation; suggests that Tbeta4 could be a novel regulator to control Wnt signal pathway 
 The Our data confirm the concept that externally added Tbeta4 must be internalized to induce intracellular mechanisms supporting endothelial cell   migration  . 
 we have identified an extracellular signaling pathway where Tbeta4 increases cell surface ATP levels via ATP synthase and have shown further that ATP-responsive P2X4 receptor is required for Tbeta4-induced cell   migration   
 The TB4 could be a hypoxia responsive regulator to control tumor cell   migration   in angiogenesis and tumor metastasis. 
  Summary  
 This gene encodes an actin sequestering protein which plays a role in regulation of actin polymerization. The protein is also involved in cell proliferation,   migration  , and differentiation. This gene escapes X inactivation and has a homolog on chromosome Y. [provided by RefSeq, Jul 2008] 
  [Ee]xtracellular [Mm]atrix  PubMed  
 Hepatocyte growth factor upregulates thymosin beta4 in human umbilical vein endothelial cells. Hepatocyte growth factor (HGF) is a mesenchymal-derived cytokine. It exerts in vitro a motogenic effect on various target cells, which is displayed either by cell scattering, locomotion, and migration during the wound repair process of cultured cells, or invasiveness through the   extracellular matrix  . Although it is known that HGF influences the motogenic effect of endothelial cells, the precise effects of HGF during angiogenesis are still poorly understood. To identify genes regulated via HGF signaling in HUVECs, we used the differential display polymerase chain reaction. In this study, thymosin beta4 was found to be differentially expressed in HGF-treated HUVECs compared with control. Data from HPLC profile and induction of MMPs indicate that HGF may affect the biological behavior of HUVECs through a combination of the direct effects of HGF itself and indirect effects mediated via induction of thymosin beta4 in vitro. 
 Potential role of thymosin Beta 4 in liver fibrosis. Liver fibrosis, the main characteristic of chronic liver diseases, is strongly associated with the activation of hepatic stellate cells (HSCs), which are responsible for   extracellular matrix   production. As such, investigating the effective regulators controlling HSC activation provides important clues for developing therapeutics to inhibit liver fibrosis. Thymosin beta 4 (Tβ4), a major actin-sequestering protein, is known to be involved in various cellular responses. A growing body of evidence suggests that Tβ4 has a potential role in the pathogenesis of liver fibrosis and that it is especially associated with the activation of HSCs. However, it remains unclear whether Tβ4 promotes or suppresses the activation of HSCs. Herein, we review the potential role of Tβ4 in liver fibrosis by describing the effects of exogenous and endogenous Tβ4, and we discuss the possible signaling pathway regulated by Tβ4. Exogenous Tβ4 reduces liver fibrosis by inhibiting the proliferation and migration of HSCs. Tβ4 is expressed endogenously in the activated HSCs, but this endogenous Tβ4 displays opposite effects in HSC activation, either as an activator or an inhibitor. Although the role of Tβ4 has not been established, it is apparent that Tβ4 influences HSC activation, suggesting that Tβ4 is a potential therapeutic target for treating liver diseases. 
  [Ii]nvasive  PubMed  
 Elevated expression of thymosin β4, vascular endothelial growth factor (VEGF), and hypoxia inducible factor (HIF)-1α in early-stage cervical cancers. Recent studies have shown that thymosin β4 (TB-4) is highly related with tumor metastasis and angiogenesis. In addition, TB-4 induced the expression of VEGF in melanoma cells. We investigated the expression patterns of TB-4 and related angiogenic proteins, VEGF, and HIF-1α, at various stages of cervical cancers and also identified the expression pattern of these proteins in metastatic cervical cancers. Expression patterns of TB-4, VEGF, and HIF-1α were studied with tissue microarray containing 42 samples of cervical cancers. In addition, 15 cervical cancers and metastatic tumors in lymph nodes from patients who have metastatic tumors were also analyzed to confirm the role of TB-4, VEGF, and HIF -1α in cervical cancer metastasis. The expression levels of TB-4, VEGF, and HIF-1α were very weak at early cancer stages (stages 0 to 1A) but significantly increased at stage 1B. The numbers of blood vessels in tumors were also increased at stage 1B. The expression patterns of TB-4, VEGF, and HIF-1α were compared in tumors without lymph node metastasis, primary tumors with lymph node metastasis, and metastatic tumors in lymph nodes. The expression levels of TB-4, VEGF, and HIF-1α in primary tumors with lymph node metastasis and their metastatic tumors in lymph node were less than in tumors without lymph node metastasis. These data suggest that TB-4, VEGF, and HIF-1α triggered angiogensis   and tumo  r invasiveness to surrounding tissues at early stage of cervical carcinoma but have a negative or no effect on the metastatic potential. 
 Immunohistochemical expression of thymosin β4 in ameloblastomas and odontomas. Ameloblastoma is regarded to be a benign odontogenic tumor, but it is destructive, locally   invasiv  e and presents a high rate of recurrence. Thymosin β4 (Tβ4) is closely associated with tooth germ development. Tβ4 also plays a role in malignant progression and invasion. However, little is known about the function of Tβ4 in odontogenic tumors. Thus, we investigated Tβ4 expression in ameloblastomas and compared it with odontomas. We immunohistochemically evaluated the expression of Tβ4, ameloblastin (AMBN), amelogenin (AMEL) and enamelin (ENAM) in 57 samples of ameloblastomas from 40 patients, and also assessed the expression of these molecules in 11 cases of odontomas, two of ameloblastic fibro-odontomas and one of tooth germ-like structures without the formation of enamel and dentin. Tβ4 signals were observed in almost all of the ameloblastomas. The signals were observed in both peripheral columnar cells and central polyhedral/angular cells. Similar findings were observed in tooth germ-like structures, and in the ameloblastomatous nests in the ameloblastic fibro-odontomas. These samples had negative results for AMBN, AMEL and ENAM. Meanwhile, Tβ4 signals were not seen in the odontomas, although immunolabeling for AMBN, AMEL and ENAM was observed in the enamel matrix and in some ameloblasts. Ectomesenhymal regions in the odontomas were negative for staining with the antibodies for AMBN, AMEL and ENAM. These results suggest that Tβ4 could be associated with morphogenesis and tumor invasion in the ameloblastoma, and that Tβ4 may play a role in the behavior of ameloblastoma. 
 Hepatocyte growth factor upregulates thymosin beta4 in human umbilical vein endothelial cells. Hepatocyte growth factor (HGF) is a mesenchymal-derived cytokine. It exerts in vitro a motogenic effect on various target cells, which is displayed either by cell scattering, locomotion, and migration during the wound repair process of cultured cells, or   invasive  ness through the extracellular matrix. Although it is known that HGF influences the motogenic effect of endothelial cells, the precise effects of HGF during angiogenesis are still poorly understood. To identify genes regulated via HGF signaling in HUVECs, we used the differential display polymerase chain reaction. In this study, thymosin beta4 was found to be differentially expressed in HGF-treated HUVECs compared with control. Data from HPLC profile and induction of MMPs indicate that HGF may affect the biological behavior of HUVECs through a combination of the direct effects of HGF itself and indirect effects mediated via induction of thymosin beta4 in vitro. 
 Thymosin beta4 is a determinant of the transformed phenotype and   invasive  ness of S-adenosylmethionine decarboxylase-transfected fibroblasts. S-adenosylmethionine decarboxylase (AdoMetDC) is a key enzyme in the synthesis of polyamines essential for cell growth and proliferation. Its overexpression induces the transformation of murine fibroblasts in both sense and antisense orientations, yielding highly   invasive   tumors in nude mice. These cell lines hence provide a good model to study cell invasion. Here, the gene expression profiles of these cells were compared with their normal counterpart by microarray analyses (Incyte Genomics, Palo Alto, CA, and Affymetrix, Santa Clara, CA). Up-regulation of the actin sequestering molecule thymosin beta4 was the most prominent change in both cell lines. Tetracycline-inducible expression of thymosin beta4 antisense RNA caused a partial reversal of the transformed phenotype. Further, reversal of transformation by dominant-negative mutant of c-Jun (TAM67) caused reduction in thymosin beta4 mRNA. Interestingly, a sponge toxin, latrunculin A, which inhibits the binding of thymosin beta4 to actin, was found to profoundly affect the morphology and proliferation of the AdoMetDC transformants and to block their invasion in three-dimensional Matrigel. Thus, thymosin beta4 is a determinant of AdoMetDC-induced transformed phenotype and   invasive  ness. Up-regulation of thymosin beta4 was also found in ras-transformed fibroblasts and metastatic human melanoma cells. These data encourage testing latrunculin A-like and other agents interfering with thymosin beta4 for treatment of thymosin beta4-overexpressing tumors with high   invasive   and metastatic potential. 
 Thymosin β 4 gene silencing decreases stemness and   invasiv  eness in glioblastoma. Thymosin beta 4 is a pleiotropic actin-sequestering polypeptide that is involved in wound healing and developmental processes. Thymosin beta 4 gene silencing promotes differentiation of neural stem cells whereas thymosin beta 4 overexpression initiates cortical folding of developing brain hemispheres. A role of thymosin beta 4 in malignant gliomas has not yet been investigated. We analysed thymosin beta 4 staining on tissue microarrays and performed interrogations of the REMBRANDT and the Cancer Genome Atlas databases. We investigated thymosin beta 4 expression in seven established glioma cell lines and seven glioma-initiating cell lines and induced or silenced thymosin beta 4 expression by lentiviral transduction in LNT-229, U87MG and GS-2 cells to study the effects of altered thymosin beta 4 expression on gene expression, growth, clonogenicity, migration, invasion, self-renewal and differentiation capacity in vitro, and tumorigenicity in vivo. Thymosin beta 4 expression increased with grade of malignancy in gliomas. Thymosin beta 4 gene silencing in LNT-229 and U87MG glioma cells inhibited migration and invasion, promoted starvation-induced cell death in vitro and enhanced survival of glioma-bearing mice. Thymosin beta 4 gene silencing in GS-2 cells inhibited self-renewal and promoted differentiation in vitro and decreased tumorigenicity in vivo. Gene expression analysis suggested a thymosin beta 4-dependent regulation of mesenchymal signature genes and modulation of TGFβ and p53 signalling networks. We conclude that thymosin beta 4 should be explored as a novel molecular target for anti-glioma therapy. 
 The actin-sequestering protein thymosin beta-4 is a novel target of hypoxia-inducible nitric oxide and HIF-1α regulation. The actin-sequestering protein thymosin beta-4 (Tβ4) is involved in various cellular and physiological processes such as proliferation, motility, growth and metastasis. Nitric oxide (NO) promotes tumo  r invasi  veness and metastasis by activating various enzymes. Herein, we investigated whether hypoxia-inducible NO regulates Tβ4 expression and cancer cell migration using HeLa cervical cancer cells. NO production and Tβ4 expression were increased in a hypoxic condition. The treatment with N-(β-D-Glucopyranosyl)-N2-acetyl-S-nitroso-D, L-penicillaminamide (SNAP-1), to generate NO, enhanced the transcription of Tβ4 and cancer cell migration. SNAP-1-induced cell migration was decreased by the inhibition of Tβ4 with small interference (si) RNA. In a hypoxic condition, treatment with N(G)-monomethyl-L-arginine (L-NMMA), nitric oxide synthase (NOS) inhibitor, reduced Tβ4 transcriptional activity, and hypoxia-inducible factor (HIF)-1α. Hypoxia-induced cancer cell migration was also decreased by L-NMMA treatment. In a normoxic condition, Tβ4 transcriptional activity was decreased in the cells incubated in the presence of L-NMMA after co-transfection with Tβ4 promoter and GST-conjugated HIF-1α. Collectively, these results suggest that NO could regulate the expression of Tβ4 by direct or indirect effect of HIF-1α on Tβ4 promoter. 
 From proteomic multimarker profiling to interesting proteins: thymosin-β(4) and kininogen-1 as new potential biomarkers for inflammatory hepatic lesions. Despite tremendous efforts in disclosing the pathophysiological and epidemiological factors associated with liver fibrogenesis, non  -invasiv  e diagnostic measures to estimate the clinical outcome and progression of liver fibrogenesis are presently limited. Therefore, there is a mandatory need for methodologies allowing the reasonable and reliable assessment of the severity and/or progression of hepatic fibrogenesis. We here performed proteomic serum profiling by matrix-assisted laser desorption ionization time-of-flight mass spectrometry in 179 samples of patients chronically infected with hepatitis C virus and 195 control sera. Multidimensional analysis of spectra allowed the definition of algorithms capable to distinguish class-specific protein expression profiles in serum samples. Overall about 100 peaks could be detected per single spectrum. Different algorithms including protein peaks in the range of 2000 and 10,000 Da were generated after pre-fractionation on a weak cation exchange surface. A specificity of 93% with a sensitivity of 86% as mean of the test set results was found, respectively. The nature of three of these protein peaks that belonged to kininogen-1 and thymosin-β(4) was further analysed by tandem mass spectrometry (MS)/MS. We further found that kininogen-1 mRNA was significantly down-regulated in cirrhotic livers. We have identified kininogen-1 and thymosin-β(4) as potential new biomarkers for human chronic hepatitis C and conclude that serum profiling is a reliable technique to identify hepatitis-associated expression patterns. Based on the high throughput capability, the identified differential protein panel may serve as a diagnostic marker and warrants further validation in larger cohorts. 
 Thymosin β4 induces proliferation, invasion, and epithelial-to-mesenchymal transition of oral squamous cell carcinoma. The epithelial-to-mesenchymal transition (EMT) plays a vital role in carcinogenesis, invasion, and metastasis of many epithelial tumors including oral squamous cell carcinoma (OSCC), a common malignancy of the head and neck. However, the functional role of the actin-sequestering protein thymosin β4 (Tβ4) in the EMT in OSCCs remains unclear. Thus, we investigated whether overexpression of Tβ4 could induce in vitro tumorigenesis such as cell proliferation and anchorage independency and an EMT-like phenotype in OSCCs. Also, we examined whether it affe  cts inva  siveness and cell motility-associated signaling molecules. Tβ4-overexpressing OSCCs, SCC-15_Tβ4 and SCC-25_Tβ4, enhanced cell proliferation and colony formation. In addition, we observed that Tβ4 overexpression induced an EMT-like phenotype, accompanied by a decrease in expression of the epithelial cell marker E-cadherin and an increase in expression of mesenchymal cell markers vimentin and N-cadherin. Also, the expression level of Twist1, an EMT-inducing transcription factor, was significantly enhanced in SCC-15_Tβ4 and SCC-25_Tβ4 cells. Tβ4 overexpression augmented in vitro invasion and MMP-2 activity and enhanced the phosphorylation of paxillin and cortactin and expression of LIMK1. Taken together, these results suggest that Tβ4 overexpression could be one of the causes of tumorigenesis and progression in OSCCs. Further investigation on the Tβ4 molecule would encourage the development of specific targets for cancer treatment. 
 Overexpression of the thymosin beta-4 gene is associated with increased invasion of SW480 colon carcinoma cells and the distant metastasis of human colorectal carcinoma. Cell-matrix and cell-cell adhesive interactions play important roles in the normal organization and stabilization of the cell layer in epithelial tissue. Alterations in the expression and function of these adhesion systems that cause a switch to a migratory phenotype in tumor invasion and metastasis are critical for the malignant conversion of epithelial cells. Thymosin beta-4 (Tbeta-4) is the major actin-sequestering protein that has been shown to be upregulated in a wide variety of human carcinomas and has been implicated to be involved in altering the motility of certain tumors. We have recently demonstrated that the growth rate, colony formation in soft agar, and motility, all good indicators for malignant progression, of SW480 colon carcinoma cells are dramatically increased by enforced Tbeta-4 expression. To test the hypothesis that overexpression of this G-actin sequestering peptide also promotes tumor invasion, we examined not only the invasion capability of Tbeta-4-overexpressing SW480 cells, but also the expression levels of Tbeta-4 as well as several proteins that participate in different stages of tumor progression in matched samples of human primary colorectal adenocarcinoma and liver metastases from several patients. A marked increase on the   invasive  ness in Tbeta-4-overexpressing SW480 cells with increased levels and activity of matrix metalloproteinase-7 (MMP-7) was observed. Furthermore, the levels of Fas as well as the susceptibility to Fas ligand-mediated apoptosis in Tbeta-4-overexpressing cells were significantly decreased. Interestingly, the levels of Tbeta-4 mRNA, beta-catenin, c-Myc, and MMP-7 in metastatic liver lesions were relatively higher, whereas the levels of E-cadherin and Fas were significantly lower than those in the matched primary colorectal tumors. These results suggest that upregulation of Tbeta-4, by promoting the disruption of cell-cell adhesion and a consequential activation of the beta-catenin signaling, could be a key event in the acquisition of growth advantages as well as   invasive   phenotypes in human colorectal carcinomas. 
  GeneRIFs  
 upregulation of Tbeta-4, by promoting the disruption of cell-cell adhesion and consequential activation of the beta-catenin signaling, could be event in the acquisition of growth advantages as well as   invasive   phenotypes in human colorectal carcinomas 
 thymosin beta4 is a determinant of AdoMetDC-induced transformed phenotype and   invasive  ness; up-regulation of thymosin beta4 found in ras-transformed fibroblasts and metastatic human melanoma cells 
  [Mm]etastasis  PubMed  
 Regulation of glycogen synthase kinase-3 by thymosin beta-4 is associated with gastric cancer cell migration. Thymosin beta-4 (Tβ4), actin-sequestering protein, plays important roles in many cellular functions including cancer cell migrations. Glycogen synthase kinase (GSK) in Wnt signaling pathway is a key molecule to control intercellular interaction. Here, we investigated whether GSK-3 activity is regulated by Tβ4 and it is associated with Tβ4-mediated migration in gastric cancer cells. Various expression level of Tβ4 was observed in human gastric tumor tissues. Migration in gastric cancer cells, SNU638 and SNU668, was dependent on a relative expression level of Tβ4. Cell migration was higher in SNU668 with a higher expression level of Tβ4 than that in SNU638 with a lower Tβ4. Although the level of phosphorylated(p)-GSK-3α (inactive), β-catenin, E-cadherin and E-cadherin:β-catenin complex was relatively higher, p-GSK-3β (inactive) was lower in SNU638 compared to those in SNU668 cells. LiCl, GSK-3α/β inhibitor,   reduced lu  ng metastasis of B16F10 mouse melanoma cells and SNU668 cell migration. Small interference (si)RNA of GSK-3α increased SNU638 cell migration in accordance with the reduction of E-cadherin:β-catenin complex formation through a decrease in β-catenin and E-cadherin. Expression level of GSK-3α/β, β-catenin and E-cadherin in SNU668 and SNU638 was reversed by Tβ4-siRNA and by the treatment with acetylated-serine-aspartic acid-lysine-proline (SDKP) tetrapeptide of Tβ4, respectively. E-cadherin expression in SNU638 cells was decreased by β-catenin-siRNA. PD98059, MEK inhibitor, or U0126, ERK inhibitor, reduced SNU668 cell migration accompanying an increase in p-GSK-3α, β-catenin and E-cadherin. Taken together, data indicated that the expression of GSK-3α, β-catenin and E-cadherin could be negatively regulated by Tβ4-induced ERK phosphorylation. It suggests that Tβ4 could be a novel regulator to control Wnt signaling pathways. 
 Elevated expression of thymosin β4, vascular endothelial growth factor (VEGF), and hypoxia inducible factor (HIF)-1α in early-stage cervical cancers. Recent studies have shown that thymosin β4 (TB-4) is highly related with tum  or metasta  sis and angiogenesis. In addition, TB-4 induced the expression of VEGF in melanoma cells. We investigated the expression patterns of TB-4 and related angiogenic proteins, VEGF, and HIF-1α, at various stages of cervical cancers and also identified the expression pattern of these proteins in metastatic cervical cancers. Expression patterns of TB-4, VEGF, and HIF-1α were studied with tissue microarray containing 42 samples of cervical cancers. In addition, 15 cervical cancers and metastatic tumors in lymph nodes from patients who have metastatic tumors were also analyzed to confirm the role of TB-4, VEGF, and HIF -1α in cervical c  ancer meta  stasis. The expression levels of TB-4, VEGF, and HIF-1α were very weak at early cancer stages (stages 0 to 1A) but significantly increased at stage 1B. The numbers of blood vessels in tumors were also increased at stage 1B. The expression patterns of TB-4, VEGF, and HIF-1α were compared in tumors without lym  ph node me  tastasis, primary tumors with lym  ph node me  tastasis, and metastatic tumors in lymph nodes. The expression levels of TB-4, VEGF, and HIF-1α in primary tumors with ly  mph node m  etastasis and their metastatic tumors in lymph node were less than in tumors without ly  mph node m  etastasis. These data suggest that TB-4, VEGF, and HIF-1α triggered angiogensis and tumor invasiveness to surrounding tissues at early stage of cervical carcinoma but have a negative or no effect on the metastatic potential. 
 Expression patterns of Thymosin β4 and cancer stem cell marker CD133 in ovarian cancers. Thymosin β4 (Tβ4), a small acidic actin binding peptide, is overexpressed in a side population of cancer stem cells and CD133-positive colorectal cancer stem cells. In order to understand the relationship between Tβ4 and CD133, we studied the expression patterns of Tβ4 and CD133 in ovarian cancers. The expression patterns of Tβ4 and CD133 were studied in normal ovaries, primary ovarian cancers, metastatic ovarian cancers, primary stomach cancers, and normal stomachs by Western blot and immunohistochemistry. Expression patterns and co-localization of Tβ4 and CD133 were examined by immunofluorescence and confocal laser-scanning microscopy. Tβ4 is overexpressed in primary ovarian cancers, but not in primary stomach cancers, when compared with normal controls. However, Tβ4 levels in metastatic stomach cancers to the ovary are significantly upregulated compared with levels in normal stomachs and primary stomach cancers. These results suggest that Tβ4 levels are related to tumorigenesis in ovarian ca  ncers and   metastasis in stomach cancers. The expression of Tβ4 in normal ovaries and normal stomachs was weak, but was co-localized with CD133 expression. Tβ4 expression was also co-localized with CD133 expression in primary ovarian carcinomas, metastatic ovarian cancers from stomach cancers and primary stomach cancers. These data suggest that Tβ4 expression is strongly related to CD133 expression and is a characteristic of stem cells or cancer stem cells. 
 Overexpression of thymosin beta-4 renders SW480 colon carcinoma cells more resistant to apoptosis triggered by FasL and two topoisomerase II inhibitors via downregulating Fas and upregulating Survivin expression, respectively. The present work was conducted to further examine the effects of thymosin beta-4 (Tbeta4) upregulation on the apoptosis of SW480 colon cancer cells induced by T cells and various chemotherapeutic agents because reduced susceptibility to the cytotoxicity of an anti-Fas IgM (CH-11) in Tbeta4-overexpressing cells has previously been reported by us. As expected, Tbeta4 overexpressers were also more resistant to the killing effect of FasL-bearing Jurkat T cells. On the other hand, pretreating these cells with an MMP inhibitor restored not only their Fas levels but also their sensitivity to CH-11, suggesting a pivotal role of MMP in downregulating Fas in Tbeta4 overexpressers. Interestingly, while the susceptibilities of Tbeta4 overexpressers to 5-FU and irinotecan remained unchanged, they were more resistant to doxorubicin and etoposide which triggered apoptosis via a mitochondrial pathway. Concordantly, activation of both caspases 9 and 3 in Tbeta4 overexpressers by the two aforementioned topoisomerase II inhibitors was dramatically abrogated which could be accounted mainly by an increased expression of Survivin, a critical anti-apoptotic factor. Finally, poor survival was found in stage III colon cancer patients whose tumors were stained positively by the anti-Survivin antibody. Thus, advantages such as immune evasion and resistance to anticancer drug-induced apoptosis acquired by colon cancer cells through Tbeta4 overexpression might facilitate their survival during   metastasis   and chemotherapy. 
 Thymosin β4 expression in human tissues and in tumors using tissue microarrays. Thymosin β4 has been reported to play the key roles in tumor growth  , metastas  is, and angiogenesis. Although the importance of thymosin β4 in angiogenesis a  nd metasta  sis is known, few studies to show the expression patterns of thymosin β4 in human tissues including tumors have been conducted. The comparisons of the expression of thymosin β4 between the normal and tumor tissues are also needed to study the role of thymosin β4 in tumor formation. Using tissue microarray analysis, we compared the expression patterns of thymosin β4 in the normal human tissues and in the tumors to screen certain tumors and upregulated the expression of thymosin β4 by tumorigenesis. Thymosin β4 was highly expressed in the hepatic cells in the normal adult liver, duct, and acinar cells in pancreas, and muscle cells in the heart and also expressed highly in certain tumor cells, including osteosarcoma, colon adenocarcinoma, esophageal squamous cell carcinoma, kidney and urinary bladder transitional carcinoma, lung cancer, and liver cancer. Comparing the thymosin β4 expression between normal and tumors, thymosin β4 was upregulated specifically in osteosarcoma, colorectal carcinoma, and esophageal cancer. To confirm the over-expression of thymosin β4 in these tumors, we analyzed expression of thymosin β4 with each additional microarray of osteosarcoma, colorectal carcinoma, and esophageal cancer. The significant increased expression of thymosin β4 was observed in osteosarcoma and in colorectal cancer. These results suggest that the expression of thymosin β4 is highly related with tumorigenesis of certain tumors including the osteosarcoma and colorectal cancers. 
 Thymosin Beta 4 is overexpressed in human pancreatic cancer cells and stimulates proinflammatory cytokine secretion and JNK activation. Thymosin beta 4 (T beta 4) has been shown to be associated with tumor   metastasis   and angiogenesis; however, its role in pancreatic cancer has not been understood. In the current study, we examined the expression of T beta 4 in pancreatic cancer cells, and determined the effect of exogenous T beta 4 on cytokine secretion, and signal transduction in human pancreatic cancer cells.Pancreatic cancer cell lines expressed higher amount of T beta 4 mRNA than normal human pancreatic ductal epithelium (HPDE) cells. Exogenous T beta 4 increased the secretion of proinflammatory cytokines IL-6, IL-8 and MCP-1 in Panc-1 cells. In addition, T beta 4 activated Jun N-terminal Kinase (JNK) signaling pathways in pancreatic cancer cells.The mRNA levels of T beta 4 were determined by real-time RT PCR. Phosphorylation of JNK in pancreatic cancer cells was determined using Bio-Plex phosphoprotein assay. The expression of cytokines in human pancreatic cancer cell lines was determined with Bio-Plex cytokine assay.T beta 4 might be involved in stimulating human pancreatic cancer progression by promoting proinflammatory cytokine environment and activating JNK signaling pathway. Targeting T beta 4 and related molecules may be a novel therapeutic strategy for pancreatic cancer. 
 Thymosin β4 promotes hepatoblastoma   metastasi  s via the induction of epithelial-mesenchymal transition. Hepatoblastoma (HB) is the most common malignant hepatic tumor in children and complete surgical resection offers the highest possibility for cure in this disease. Tumor   metastasi  s is the principle obstacle to the development of efficient treatments for patients with HB. The present study aimed to measure the expression levels of thymosin β4 (Tβ4) in liver samples from patients with HB and to investigate the involvement of Tβ4 in   HB metast  asis. The expression of Tβ4 was significantly higher in liver samples from patients with metastatic HB and in the HepG2 metastatic HB cell line, compared with that in adjacent healthy liver samples and in the L02 healthy hepatic cell line. By contrast, the expression levels of epithelial-cadherin (E-cadherin) and cytosolic accumulation of β-catenin, the two most prominent markers involved in epithelial-mesenchymal transition (EMT), were reduced in liver specimens from patients with metastatic HB compared with that of healthy adjacent control tissue. HepG2 cells were transfected with small interfering-RNA in order to downregulate Tβ4 gene expression. This resulted in a reduced cell migratory capacity compared with control cells. Tβ4 gene expression knockdown significantly inhibited transforming growth factor β1-mediated-EMT in vitro by upregulating the expression of E-cadherin. The results of the present study suggested that Tβ4 may p  romote HB   metastasis via the induction of EMT, and that Tβ4 may therefore be a target for the development of novel treatments for patients with HB. 
 Zyxin is upregulated in the nucleus by thymosin beta4 in SiHa cells. Thymosin beta4 is a 43-amino acid actin-binding protein that promotes cell migration and is important in angiogenesis, wound healing, and tumor   metastasis  . We searched for genes upregulated by thymosin beta4 and identified zyxin as increased in SiHa cells in the presence of exogenously added thymosin beta4 and when thymosin beta4 is overexpressed using adenoviral vectors. Both zyxin and thymosin beta4 show increased localization in the nucleus. We conclude that thymosin beta4 may exert some of its migration promoting activity via increased zyxin expression. 
 beta-Thymosins, small acidic peptides with multiple functions. The beta-thymosins are a family of highly conserved polar 5 kDa peptides originally thought to be thymic hormones. About 10 years ago, thymosin beta(4) as well as other members of this ubiquitous peptide family were identified as the main intracellular G-actin sequestering peptides, being present in high concentrations in almost every cell. beta-Thymosins bind monomeric actin in a 1:1 complex and act as actin buffers, preventing polymerization into actin filaments but supplying a pool of actin monomers when the cell needs filaments. Changes in the expression of beta-thymosins appear to be related to the differentiation of cells. Increased expression of beta-thymosins or even the synthesis of a beta-thymosin normally not expressed might promote   metastasis   possibly by increasing mobility of the cells. Thymosin beta(4) is detected outside of cells in blood plasma or in wound fluid. Several biological effects are attributed to thymosin beta(4), oxidized thymosin beta(4), or to the fragment, acSDKP, possibly generated from thymosin beta(4). Among the effects are induction of metallo-proteinases, chemotaxis, angiogenesis and inhibition of inflammation as well as the inhibition of bone marrow stem cell proliferation. However, nothing is known about the molecular mechanisms mediating the effects attributed to extracellular beta-thymosins. 
 The actin-sequestering protein thymosin beta-4 is a novel target of hypoxia-inducible nitric oxide and HIF-1α regulation. The actin-sequestering protein thymosin beta-4 (Tβ4) is involved in various cellular and physiological processes such as proliferation, motility, growth an  d metastas  is. Nitric oxide (NO) promotes tumor invasiveness an  d metastas  is by activating various enzymes. Herein, we investigated whether hypoxia-inducible NO regulates Tβ4 expression and cancer cell migration using HeLa cervical cancer cells. NO production and Tβ4 expression were increased in a hypoxic condition. The treatment with N-(β-D-Glucopyranosyl)-N2-acetyl-S-nitroso-D, L-penicillaminamide (SNAP-1), to generate NO, enhanced the transcription of Tβ4 and cancer cell migration. SNAP-1-induced cell migration was decreased by the inhibition of Tβ4 with small interference (si) RNA. In a hypoxic condition, treatment with N(G)-monomethyl-L-arginine (L-NMMA), nitric oxide synthase (NOS) inhibitor, reduced Tβ4 transcriptional activity, and hypoxia-inducible factor (HIF)-1α. Hypoxia-induced cancer cell migration was also decreased by L-NMMA treatment. In a normoxic condition, Tβ4 transcriptional activity was decreased in the cells incubated in the presence of L-NMMA after co-transfection with Tβ4 promoter and GST-conjugated HIF-1α. Collectively, these results suggest that NO could regulate the expression of Tβ4 by direct or indirect effect of HIF-1α on Tβ4 promoter. 
 Thymosin β4 induces proliferation, invasion, and epithelial-to-mesenchymal transition of oral squamous cell carcinoma. The epithelial-to-mesenchymal transition (EMT) plays a vital role in carcinogenesis, invasion, and   metastasi  s of many epithelial tumors including oral squamous cell carcinoma (OSCC), a common malignancy of the head and neck. However, the functional role of the actin-sequestering protein thymosin β4 (Tβ4) in the EMT in OSCCs remains unclear. Thus, we investigated whether overexpression of Tβ4 could induce in vitro tumorigenesis such as cell proliferation and anchorage independency and an EMT-like phenotype in OSCCs. Also, we examined whether it affects invasiveness and cell motility-associated signaling molecules. Tβ4-overexpressing OSCCs, SCC-15_Tβ4 and SCC-25_Tβ4, enhanced cell proliferation and colony formation. In addition, we observed that Tβ4 overexpression induced an EMT-like phenotype, accompanied by a decrease in expression of the epithelial cell marker E-cadherin and an increase in expression of mesenchymal cell markers vimentin and N-cadherin. Also, the expression level of Twist1, an EMT-inducing transcription factor, was significantly enhanced in SCC-15_Tβ4 and SCC-25_Tβ4 cells. Tβ4 overexpression augmented in vitro invasion and MMP-2 activity and enhanced the phosphorylation of paxillin and cortactin and expression of LIMK1. Taken together, these results suggest that Tβ4 overexpression could be one of the causes of tumorigenesis and progression in OSCCs. Further investigation on the Tβ4 molecule would encourage the development of specific targets for cancer treatment. 
 Actin-sequestering protein, thymosin beta-4, is a novel hypoxia responsive regulator. Angiogenesis is induced by soluble factors such as vascular endothelial growth factor (VEGF) released from tumor cells in hypoxia. It enhances solid tumor growth and provides an ability to establish   metastasis   at peripheral sites by tumor cell migration. Thymosin beta-4 (TB4) is an actin-sequestering protein to control cytoskeletal reorganization. Here, we investigated whether angiogenesis and tumor   metastasis   are dependent on hypoxia conditioning-induced TB4 expression in B16F10 melanoma cells. TB4 expression in B16F10 cells was increased by hypoxia conditioning in a time-dependent manner. In addition, we found an increase of angiogenesis and HIF-1α expression in TB4-transgenic (Tg) mice as compared to wildtype mice. When wound healing assay was used to assess in vitro tumor cell migration, hypoxia conditioning for 1 h enhanced B16F10 cell migration. When TB4 expression in B16F10 cells was inhibited by the infection with small hairpin (sh) RNA of TB4 cloned in lentiviral vector, tumor cell migration was retarded. In addition, hypoxia conditioning-induced tumor cell migration was reduced by the infection of lentiviral shRNA of TB4. HIF-1α stabilization and the expression of VEGF isoform 165 and 121 in hypoxia were also reduced by the infection of lentiviral shRNA of TB4 in B16F10 cells. We also found an increase of tumor growth and lun  g metastas  is count in TB4-Tg mice as compared to wildtype mice. Collectively, hypoxia conditioning induced tumor cell migration by TB4 expression-dependent HIF-1α stabilization. It suggests that TB4 could be a hypoxia responsive regulator to control tumor cell migration in angiogenesis and tum  or metasta  sis. 
 Overexpression of the thymosin beta-4 gene is associated with increased invasion of SW480 colon carcinoma cells and the distant   metastasis   of human colorectal carcinoma. Cell-matrix and cell-cell adhesive interactions play important roles in the normal organization and stabilization of the cell layer in epithelial tissue. Alterations in the expression and function of these adhesion systems that cause a switch to a migratory phenotype in tumor invasion and   metastasis   are critical for the malignant conversion of epithelial cells. Thymosin beta-4 (Tbeta-4) is the major actin-sequestering protein that has been shown to be upregulated in a wide variety of human carcinomas and has been implicated to be involved in altering the motility of certain tumors. We have recently demonstrated that the growth rate, colony formation in soft agar, and motility, all good indicators for malignant progression, of SW480 colon carcinoma cells are dramatically increased by enforced Tbeta-4 expression. To test the hypothesis that overexpression of this G-actin sequestering peptide also promotes tumor invasion, we examined not only the invasion capability of Tbeta-4-overexpressing SW480 cells, but also the expression levels of Tbeta-4 as well as several proteins that participate in different stages of tumor progression in matched samples of human primary colorectal adenocarcinoma and liver metastases from several patients. A marked increase on the invasiveness in Tbeta-4-overexpressing SW480 cells with increased levels and activity of matrix metalloproteinase-7 (MMP-7) was observed. Furthermore, the levels of Fas as well as the susceptibility to Fas ligand-mediated apoptosis in Tbeta-4-overexpressing cells were significantly decreased. Interestingly, the levels of Tbeta-4 mRNA, beta-catenin, c-Myc, and MMP-7 in metastatic liver lesions were relatively higher, whereas the levels of E-cadherin and Fas were significantly lower than those in the matched primary colorectal tumors. These results suggest that upregulation of Tbeta-4, by promoting the disruption of cell-cell adhesion and a consequential activation of the beta-catenin signaling, could be a key event in the acquisition of growth advantages as well as invasive phenotypes in human colorectal carcinomas. 
 Thymosin β4 expression correlates with lymph node   metastasi  s through hypoxia inducible factor-α induction in breast cancer. Intratumoral hypoxia has been correlated with distant metastatic potential. Two hypoxia inducible factors (HIFs), HIF-1α and HIF-2α, are induced by hypoxia, and high expression of these proteins has been correlated to angiogenesis and dist  ant metast  asis. Thymosin β4 (Tβ4) is frequently highly expressed in cancer, and this overexpression correlates with malignant progression. The objective of this study was to investigate the clinical correlation of HIF-α with Tβ4 and the intracellular functional roles of Tβ4 on HIF-α activation. We examined HIF-1α, HIF-2α and Tβ4 expressions in clinical human breast carcinoma (n=70) by immunohistochemistry. We show that high expression of HIF-1α and HIF-2α strongly correlates with Tβ4 expression (P≤0.0001) and overexpression of Tβ4 correlates significantly with patien  ts with ly  mph node metastasis (P<0.05) of human breast cancer. Additionally, we demonstrate that hypoxia up-regulates intracellular Tβ4 protein, which then affects HIF-α activity, which is the key in regulating VEGF expression. We confirmed that hypoxia-induced intracellular Tβ4 and HIF-α activities were reduced by interference of Tβ4 expression using Tβ4 shRNA lentivirus. Vascular epidermal growth factor (VEGF)-A, a well-recognized lymphangiogenic cytokine, was also down-regulated, but VEGF-C and VEGF-D expressions were not affected. These findings suggest that the overexpression of Tβ4 is strongly associated with HIF-1α and HIF-2α expression and is also clinicopathologically involved with lymph node metastatic potential of breast cancer through the modulation of HIF-αactivation and induction of VEGF-A. Ultimately, these results highlight Tβ4 as a potentially therapeutic target in malignant cancers. 
 ERK activation by thymosin-beta-4 (TB4) overexpression induces paclitaxel-resistance. The development of paclitaxel-resistance in tumors is one of the most significant obstacles to successful therapy. Thymosin-beta-4 (TB4) has been known as actin-sequestering protein and functions in tumor   metastasis  . Here, we overexpressed TB4 in HeLa cells (TB4-HeLa) and examined the effect of TB4 in paclitaxel-induced cell death. TB4-HeLa cells showed a higher growth rate and a lower percentage of basal apoptosis than HeLa cells. TB4-HeLa cells were more resistant to paclitaxel-induced cell death than HeLa cells. TB4 transcript expression with paclitaxel treatment was dose-dependently increased in HeLa cells but that was not in TB4-HeLa cells. Small interfering RNA (siRNA) of TB4 inhibited HeLa cell growth and enhanced paclitaxel-induced cell death. Basal ERK phosphorylation was elevated and basal p38 kinase phosphorylation was reduced in paclitaxel non-treated TB4-HeLa cells. When treated with paclitaxel, cell death and resistance-induction were independent of ERK and p38 kinase activation. Paclitaxel-resistance of TB4-HeLa cells was overcome by the inhibition of basal ERK activity with PD98059 pre-treatment. The inhibition of basal p38 kinase activity with SB203580 pre-treatment attenuated the paclitaxel-induced HeLa cell death. In conclusion, TB4 induced paclitaxel-resistance through the elevation of basal level of ERK phosphorylation. Therefore, TB4 could be a novel target to regulate paclitaxel-resistance. 
  GeneRIFs  
 The results of the present study suggested that Tb4 may promote HB   metastasis   via the induction of EMT, and that Tb4 may therefore be a target for the development of novel treatments for patients with Hepatoblastoma . 
 The TB4 could be a hypoxia responsive regulator to control tumor cell migration in angiogenesis and tumor   metastasis  . 
  [Cc]ell [Ii]nvasion  PubMed  
 Thymosin beta4 is a determinant of the transformed phenotype and invasiveness of S-adenosylmethionine decarboxylase-transfected fibroblasts. S-adenosylmethionine decarboxylase (AdoMetDC) is a key enzyme in the synthesis of polyamines essential for cell growth and proliferation. Its overexpression induces the transformation of murine fibroblasts in both sense and antisense orientations, yielding highly invasive tumors in nude mice. These cell lines hence provide a good model to study   cell invasion  . Here, the gene expression profiles of these cells were compared with their normal counterpart by microarray analyses (Incyte Genomics, Palo Alto, CA, and Affymetrix, Santa Clara, CA). Up-regulation of the actin sequestering molecule thymosin beta4 was the most prominent change in both cell lines. Tetracycline-inducible expression of thymosin beta4 antisense RNA caused a partial reversal of the transformed phenotype. Further, reversal of transformation by dominant-negative mutant of c-Jun (TAM67) caused reduction in thymosin beta4 mRNA. Interestingly, a sponge toxin, latrunculin A, which inhibits the binding of thymosin beta4 to actin, was found to profoundly affect the morphology and proliferation of the AdoMetDC transformants and to block their invasion in three-dimensional Matrigel. Thus, thymosin beta4 is a determinant of AdoMetDC-induced transformed phenotype and invasiveness. Up-regulation of thymosin beta4 was also found in ras-transformed fibroblasts and metastatic human melanoma cells. These data encourage testing latrunculin A-like and other agents interfering with thymosin beta4 for treatment of thymosin beta4-overexpressing tumors with high invasive and metastatic potential. 
 Thymosin β4 induces invasion and migration of human colorectal cancer cells through the ILK/AKT/β-catenin signaling pathway. Thymosin β4 (Tβ4) is a 43-amino-acid peptide involved in many biological processes. However, the precise molecular signaling mechanism(s) of Tβ  4 in cell inv  asion and migration remain unclear. In this study, we show that Tβ4 was significantly overexpressed in colorectal cancer tissues compared to adjacent normal tissues and high levels of Tβ4 were correlated with stage of colorectal cancer, and that Tβ4 expression was associated with morphogenesis and EMT. Tβ4-upregulated cancer cells showed increased adhesion, invasion and migration activity, whereas Tβ4-downregulated cells showed decreased activities. We also demonstrated that Tβ4 interacts with ILK, which promoted the phosphorylation and activation of AKT, the phosphorylation and inactivation of GSK3β, the expression and nuclear localization of β-catenin, and integrin receptor activation. These results suggest that Tβ4 is an important regulator of the ILK/AKT/β-catenin/Integrin signaling cas  cade to induc  e cell invasion and migration in colorectal cancer cells, and is a potential target for cancer treatment. 
   
  
 ARHGAP20 
 
  NCBI Gene  
  GeneRIF  
  Pubmed  
 
 
  Search Term  Rank  Fields  
  [Ee]xtracellular [Mm]atrix  8  PubMed(1)  
 
 
 hide/show details for ARHGAP20 
  [Ee]xtracellular [Mm]atrix  PubMed  
 Genome-wide association analyses identify multiple loci associated with central corneal thickness and keratoconus. Central corneal thickness (CCT) is associated with eye conditions including keratoconus and glaucoma. We performed a meta-analysis on >20,000 individuals in European and Asian populations that identified 16 new loci associated with CCT at genome-wide significance (P < 5 × 10(-8)). We further showed that 2 CCT-associated loci, FOXO1 and FNDC3B, conferred relatively large risks for keratoconus in 2 cohorts with 874 cases and 6,085 controls (rs2721051 near FOXO1 had odds ratio (OR) = 1.62, 95% confidence interval (CI) = 1.4-1.88, P = 2.7 × 10(-10), and rs4894535 in FNDC3B had OR = 1.47, 95% CI = 1.29-1.68, P = 4.9 × 10(-9)). FNDC3B was also associated with primary open-angle glaucoma (P = 5.6 × 10(-4); tested in 3 cohorts with 2,979 cases and 7,399 controls). Further analyses implicate the collagen   and extracellular ma  trix pathways in the regulation of CCT. 
   
  
 TXNIP 
 
  NCBI Gene  
  GeneRIF  
  Pubmed  
 
 
  Search Term  Rank  Fields  
  [Mm]igration  6  PubMed(6); GeneRIFs(2)  
  [Ii]nvasive  10  PubMed(1)  
  [Mm]etastasis  12  PubMed(10); GeneRIFs(2)  
  [Cc]ell [Ii]nvasion  13  PubMed(1)  
 
 
 hide/show details for TXNIP 
  [Mm]igration  PubMed  
 The cyclin-dependent kinase inhibitor, p21(WAF1), promotes angiogenesis by repressing gene transcription of thioredoxin-binding protein 2 in cancer cells. The cyclin-dependent kinase inhibitor, p21(WAF1), induces cell-cycle arrest and can act as a tumor suppressor. However, increasing evidence indicates that p21(WAF1) can also increase resistance to some anticancer therapies and thus promote tumor growth. The mechanisms explaining this paradox have not been explained. We found that conditioned media from MCF-7 breast cancer cells transfected with a p21(WAF1)-specific small interfering RNA (siRNA) significantly reduced endothelial cell   migration  , invasion and vascular sprouting. Liquid chromatography/mass spectrometry analysis of the conditioned media revealed that p21(WAF1) knockdown significantly reduced secretion of thioredoxin (Trx), a redox protein known to promote tumor angiogenesis. p21(WAF1) knockdown decreased Trx enzymatic activity in cancer cells, by effects on the expression levels of intracellular thioredoxin-binding protein 2 (TBP2), known to bind and inactivate Trx. Consistent with these findings, media from cancer cells transfected with TBP2 siRNA promoted endothelial cell invasion and blocked the anti-angiogenic effect of p21(WAF1) siRNA. Addition of Trx siRNA blocked the pro-angiogenic effects of TBP2 siRNA. Chromatin immunoprecipitation assays showed p21(WAF1) bound TBP2 gene promoter. Taken together, our data suggests that p21(WAF1) can induce Trx secretion and angiogenesis in cancer cells, by direct transcriptional repression of the TBP2 promoter. 
 INFLUENCE OF miR-373 ON THE INVASION AND MIGRATION OF BREAST CANCER AND THE EXPRESSION LEVEL OF TARGET GENES TXNIP. An increasing number of people die from breast cancer every year. Consequently, more research has been concentrated on the study of this type of tumour, and miR-373 resulted as an important gene for treating breast cancer. To explore the influence of miR-373 on the invasion and   migration   of breast cancer and the expression level of target gene TXNIP, a set of therapeutic methods were designed based on miR-373. The transfection was performed using miR-373 inhibitor; the concentration of miR-373 was controlled by inhibitor, and it was transfected into MCF-7 cell by lipofectin. Fluorescent quantitative polymerase chain reaction was used to detect the expression level of miR-373 in cells after transfection as well as that of Caspase-3 and Caspase-8. MTT assay was used to detect the influence of miR-373 inhibitor on MCF-7 cells. The expression quantity of miR-373 in cell and tissue of breast cancer with high-low invasion and   migration   ability was detected by qRT-PCR (quantitative real-time polymerase chain reaction), thus the influence of the expression quantity of miR-373 on the invasion and   migration   of cell was determined. The expression of miR-373, EMT and TXNIP was determined by Western blot. Through the identification of proteomics and bioinformatics, it was finally found that TXNIP was regulated by miR-373. The protein expression level of TXNIP was negatively correlated with the level of miR-373. Thus it was concluded that miR-373 could promote the invasion and   migration   of breast cancer. In addition, in the tissue and cell of breast cancer with different invasion and   migration   abilities, the expression level of TXNIP was negatively correlated with the level of miR-373. 
 E2F1 induces miR-224/452 expression to drive EMT through TXNIP downregulation. Malignant melanoma is highly lethal due to its aggressive invasive properties and metastatic dissemination. The transcription factor E2F1 is crucial for melanoma progression through poorly understood mechanisms. Here, we show that the miR-224/miR-452 cluster is significantly increased in advanced melanoma and invasive/metastatic cell lines that express high levels of E2F1. miR-224/miR-452 expression is directly activated by E2F1 through transactivation of the GABRE gene. Ectopic expression of miR-224/miR-452 in less aggressive cells induces EMT and cytoskeletal rearrangements and enhances   migration  /invasion. Conversely, miR-224/miR-452 depletion in metastatic cells induces the reversal of EMT, inhibition of motility, loss of the invasive phenotype and an absence of lung metastases in mice. We identify the metastasis suppressor TXNIP as new target of miR-224/miR-452 that induces feedback inhibition of E2F1 and show that miR-224/452-mediated downregulation of TXNIP is essential for E2F1-induced EMT and invasion. The E2F1-miR-224/452-TXNIP axis constitutes a molecular signature that predicts patient survival and may help to set novel therapies. 
 MiR-373 drives the epithelial-to-mesenchymal transition and metastasis via the miR-373-TXNIP-HIF1α-TWIST signaling axis in breast cancer. Our previous proteomics study revealed that thioredoxin-interacting protein (TXNIP) was down-regulated by miR-373. However, little is known of the mechanism by which miR-373 decreases TXNIP to stimulate metastasis. In this study, we show that miR-373 promotes the epithelial-to-mesenchymal transition (EMT) in breast cancer. MiR-373 suppresses TXNIP by binding to the 3'UTR of TXNIP, which in turn, induces cancer cell EMT and metastasis. TXNIP co-expression, but not the TXNIP-3'UTR, reverses the enhancement of EMT,   migratio  n, invasion and metastasis induced by miR-373. MiR-373 stimulates EMT,   migratio  n and invasion through TXNIP-dependent reactive oxygen species (ROS) reduction. Mechanistically, miR-373 up-regulates and activates the HIF1α-TWIST signaling axis via the TXNIP pathway. Consequently, TWIST induces miR-373 expression by binding to the promoter of the miR-371-373 cluster. Clinically, miR-373 is negatively associated with TXNIP and positively associated with HIF1α and TWIST, and activation of the miR-373-TXNIP-HIF1α-TWIST signaling axis is correlated with a worse outcome in patients with breast cancer. This signaling axis may be an independent prognostic factor for patients with breast cancer. 
 Nitrosative/oxidative stress conditions regulate thioredoxin-interacting protein (TXNIP) expression and thioredoxin-1 (TRX-1) nuclear localization. Thioredoxin (TRX-1) is a multifunctional protein that controls the redox status of other proteins. TRX-1 can be found in the extracellular milieu, cytoplasm and nucleus, and it has distinct functions in each environment. Previously, we studied the intracellular localization of TRX-1 and its relationship with the activation of the p21Ras-ERK1/2 MAP Kinases signaling pathway. In situations where this pathway was activated by stress conditions evoked by a nitrosothiol, S-nitroso-N-acetylpenicillamine (SNAP), TRX-1 accumulated in the nuclear compartment due to nitrosylation of p21Ras and activation of downstream ERK1/2 MAP kinases. Presently, we demonstrate that ERK1/2 MAP Kinases activation and spatial distribution within cells trigger TRX-1 nuclear translocation through down-regulation of the physiological inhibitor of TRX-1, Thioredoxin Interacting Protein (TXNIP). Once activated by the oxidants, SNAP and H₂O₂, the ERK1/2 MAP kinases migrate to the nucleus. This is correlated with down-regulation of TXNIP. In the presence of the MEK inhibitors (PD98059 or UO126), or in cells transfected with the Protein Enriched in Astrocytes (PEA-15), a cytoplasmic anchor of ERK1/2 MAP kinases, TRX-1 nucl  ear migra  tion and TXNIP down-regulation are no longer observed in cells exposed to oxidants. On the other hand, over-expression of TXNIP abolishes nucl  ear migra  tion of TRX-1 under nitrosative/oxidative stress conditions, whereas gene silencing of TXNIP facilitates nucl  ear migra  tion even in the absence of stress conditions. Studies based on the TXNIP promoter support this regulation. In conclusion, changes in TRX-1 compartmentalization under nitrosative/oxidative stress conditions are dependent on the expression levels of TXNIP, which are regulated by cellular compartmentalization and activation of the ERK1/2 MAP kinases. 
 Thioredoxin-interacting protein mediates TRX1 translocation to the plasma membrane in response to tumor necrosis factor-α: a key mechanism for vascular endothelial growth factor receptor-2 transactivation by reactive oxygen species. Thioredoxin-interacting protein (TXNIP) promotes inflammation in endothelial cells (EC) by binding to thioredoxin-1 (TRX1) in a redox-dependent manner. Formation of the TXNIP-TRX1 complex relieves inhibition of the apoptosis signal-regulating kinase 1-c-Jun N-terminal kinase-vascular cell adhesion molecule-1 pathway. Because TXNIP is an α-arrestin with numerous protein-protein interacting domains, we hypothesized that TXNIP-TRX1 trafficking should alter function of EC exposed to reactive oxygen species (ROS).In response to physiological levels of ROS (10 ng/mL tumor necrosis factor-α and 30 μmol/L H(2)O(2)), TXNIP-TRX1 translocated to the plasma membrane in human umbilical vein EC, with a peak at 30 minutes, as measured by immunofluorescence colocalization with vascular endothelial-cadherin, cell fractionation, and membrane sheet assay. TXNIP-mediated translocation of TRX1 to the membrane required TXNIP and TRX1 binding, as evidenced by inability of the ROS-insensitive TXNIP-Cys247Ser mutant to promote membrane localization. Vascular endothelial growth factor signaling required TXNIP, as shown by significant decreases in plasma membrane tyrosine phosphorylation and   EC migra  tion after TRX1 knockdown. Furthermore, TXNIP knockdown increased human umbilical vein EC apoptosis induced by tumor necrosis factor. Rescue with TXNIP-wild-type but not TXNIP-Cys247Ser prevented cell death.These findings suggest a novel role for the TXNIP-TRX1 complex to enable inflammation by promoting EC survival and vascular endothelial growth factor signaling under conditions of physiological oxidative stress. 
  GeneRIFs  
 The protein expression level of TXNIP was negatively correlated with the level of miR-373 in MCF-7 cells and in breast cancer tissue of various   migration   and invasion abilities. TXNIP was regulated by miR-373. 
 Over-expression of TXNIP abolishes nuclear   migration   of TRX-1 under nitrosative/oxidative stress conditions. 
  [Ii]nvasive  PubMed  
 E2F1 induces miR-224/452 expression to drive EMT through TXNIP downregulation. Malignant melanoma is highly lethal due to its aggressive   invasive   properties and metastatic dissemination. The transcription factor E2F1 is crucial for melanoma progression through poorly understood mechanisms. Here, we show that the miR-224/miR-452 cluster is significantly increased in advanced melanoma and   invasive  /metastatic cell lines that express high levels of E2F1. miR-224/miR-452 expression is directly activated by E2F1 through transactivation of the GABRE gene. Ectopic expression of miR-224/miR-452 in less aggressive cells induces EMT and cytoskeletal rearrangements and enhances migration/invasion. Conversely, miR-224/miR-452 depletion in metastatic cells induces the reversal of EMT, inhibition of motility, loss of the   invasive   phenotype and an absence of lung metastases in mice. We identify the metastasis suppressor TXNIP as new target of miR-224/miR-452 that induces feedback inhibition of E2F1 and show that miR-224/452-mediated downregulation of TXNIP is essential for E2F1-induced EMT and invasion. The E2F1-miR-224/452-TXNIP axis constitutes a molecular signature that predicts patient survival and may help to set novel therapies. 
  [Mm]etastasis  PubMed  
 Metabolic reprogramming in triple-negative breast cancer through Myc suppression of TXNIP. Triple-negative breast cancers (TNBCs) are aggressive and lack targeted therapies. Understanding how nutrients are used in TNBCs may provide new targets for therapeutic intervention. We demonstrate that the transcription factor c-Myc drives glucose metabolism in TNBC cells but does so by a previously unappreciated mechanism that involves direct repression of thioredoxin-interacting protein (TXNIP). TXNIP is a potent negative regulator of glucose uptake, aerobic glycolysis, and glycolytic gene expression; thus its repression by c-Myc provides an alternate route to c-Myc-driven glucose metabolism. c-Myc reduces TXNIP gene expression by binding to an E-box-containing region in the TXNIP promoter, possibly competing with the related transcription factor MondoA. TXNIP suppression increases glucose uptake and drives a dependence on glycolysis. Ectopic TXNIP expression decreases glucose uptake, reduces cell proliferation, and increases apoptosis. Supporting the biological significance of the reciprocal relationship between c-Myc and TXNIP, a Mychigh/TXNIPlow gene signature correlates with decreased overall survival and decreased   metastasis  -free survival in breast cancer. The correlation between the Mychigh/TXNIPlow gene signature and poor clinical outcome is evident only in TNBC, not in other breast cancer subclasses. Mutation of TP53, which is a defining molecular feature of TNBC, enhances the correlation between the Mychigh/TXNIPlow gene signature and death from breast cancer. Because Myc drives nutrient utilization and TXNIP restricts glucose availability, we propose that the Mychigh/TXNIPlow gene signature coordinates nutrient utilization with nutrient availability. Further, our data suggest that loss of the p53 tumor suppressor cooperates with Mychigh/TXNIPlow-driven metabolic dysregulation to drive the aggressive clinical behavior of TNBC. 
 Metabolic reprogramming in triple-negative breast cancer through Myc suppression of TXNIP. Triple-negative breast cancers (TNBCs) are aggressive and lack targeted therapies. Understanding how nutrients are used in TNBCs may provide new targets for therapeutic intervention. We demonstrate that the transcription factor c-Myc drives glucose metabolism in TNBC cells but does so by a previously unappreciated mechanism that involves direct repression of thioredoxin-interacting protein (TXNIP). TXNIP is a potent negative regulator of glucose uptake, aerobic glycolysis, and glycolytic gene expression; thus its repression by c-Myc provides an alternate route to c-Myc-driven glucose metabolism. c-Myc reduces TXNIP gene expression by binding to an E-box-containing region in the TXNIP promoter, possibly competing with the related transcription factor MondoA. TXNIP suppression increases glucose uptake and drives a dependence on glycolysis. Ectopic TXNIP expression decreases glucose uptake, reduces cell proliferation, and increases apoptosis. Supporting the biological significance of the reciprocal relationship between c-Myc and TXNIP, a Mychigh/TXNIPlow gene signature correlates with decreased overall survival and decreased   metastasis  -free survival in breast cancer. The correlation between the Mychigh/TXNIPlow gene signature and poor clinical outcome is evident only in TNBC, not in other breast cancer subclasses. Mutation of TP53, which is a defining molecular feature of TNBC, enhances the correlation between the Mychigh/TXNIPlow gene signature and death from breast cancer. Because Myc drives nutrient utilization and TXNIP restricts glucose availability, we propose that the Mychigh/TXNIPlow gene signature coordinates nutrient utilization with nutrient availability. Further, our data suggest that loss of the p53 tumor suppressor cooperates with Mychigh/TXNIPlow-driven metabolic dysregulation to drive the aggressive clinical behavior of TNBC. 
 E2F1 induces miR-224/452 expression to drive EMT through TXNIP downregulation. Malignant melanoma is highly lethal due to its aggressive invasive properties and metastatic dissemination. The transcription factor E2F1 is crucial for melanoma progression through poorly understood mechanisms. Here, we show that the miR-224/miR-452 cluster is significantly increased in advanced melanoma and invasive/metastatic cell lines that express high levels of E2F1. miR-224/miR-452 expression is directly activated by E2F1 through transactivation of the GABRE gene. Ectopic expression of miR-224/miR-452 in less aggressive cells induces EMT and cytoskeletal rearrangements and enhances migration/invasion. Conversely, miR-224/miR-452 depletion in metastatic cells induces the reversal of EMT, inhibition of motility, loss of the invasive phenotype and an absence of lung metastases in mice. We identify the   metastasis   suppressor TXNIP as new target of miR-224/miR-452 that induces feedback inhibition of E2F1 and show that miR-224/452-mediated downregulation of TXNIP is essential for E2F1-induced EMT and invasion. The E2F1-miR-224/452-TXNIP axis constitutes a molecular signature that predicts patient survival and may help to set novel therapies. 
 MiR-373 drives the epithelial-to-mesenchymal transition and   metastasis   via the miR-373-TXNIP-HIF1α-TWIST signaling axis in breast cancer. Our previous proteomics study revealed that thioredoxin-interacting protein (TXNIP) was down-regulated by miR-373. However, little is known of the mechanism by which miR-373 decreases TXNIP to stimulate   metastasi  s. In this study, we show that miR-373 promotes the epithelial-to-mesenchymal transition (EMT) in breast cancer. MiR-373 suppresses TXNIP by binding to the 3'UTR of TXNIP, which in turn, induces cancer cell EMT and   metastasi  s. TXNIP co-expression, but not the TXNIP-3'UTR, reverses the enhancement of EMT, migration, invasion and   metastasi  s induced by miR-373. MiR-373 stimulates EMT, migration and invasion through TXNIP-dependent reactive oxygen species (ROS) reduction. Mechanistically, miR-373 up-regulates and activates the HIF1α-TWIST signaling axis via the TXNIP pathway. Consequently, TWIST induces miR-373 expression by binding to the promoter of the miR-371-373 cluster. Clinically, miR-373 is negatively associated with TXNIP and positively associated with HIF1α and TWIST, and activation of the miR-373-TXNIP-HIF1α-TWIST signaling axis is correlated with a worse outcome in patients with breast cancer. This signaling axis may be an independent prognostic factor for patients with breast cancer. 
 Deficiency of thioredoxin binding protein-2 (TBP-2) enhances TGF-β signaling and promotes epithelial to mesenchymal transition. Transforming growth factor beta (TGF-β) has critical roles in regulating cell growth, differentiation, apoptosis, invasion and epithelial-mesenchymal transition (EMT) of various cancer cells. TGF-β-induced EMT is an important step during carcinoma progression to invasion state. Thioredoxin binding protein-2 (TBP-2, also called Txnip or VDUP1) is downregulated in various types of human cancer, and its deficiency results in the earlier onset of cancer. However, it remains unclear how TBP-2 suppresses the invasion a  nd metasta  sis of cancer.In this study, we demonstrated that TBP-2 deficiency increases the transcriptional activity in response to TGF-β and also enhances TGF-β-induced Smad2 phosphorylation levels. Knockdown of TBP-2 augmented the TGF-β-responsive expression of Snail and Slug, transcriptional factors related to TGF-β-mediated induction of EMT, and promoted TGF-β-induced spindle-like morphology consistent with the depletion of E-Cadherin in A549 cells.Our results indicate that TBP-2 deficiency enhances TGF-β signaling and promotes TGF-β-induced EMT. The control of TGF-β-induced EMT is critical for the inhibition of the in  vasion and   metastasis. Thus TBP-2, as a novel regulatory molecule of TGF-β signaling, is likely to be a prognostic indicator or a potential therapeutic target for preventing tumor progression. 
 The ubiquitin ligase itch regulates apoptosis by targeting thioredoxin-interacting protein for ubiquitin-dependent degradation. Thioredoxin interacting protein (TXNIP) was originally characterized as an endogenous inhibitor of thioredoxin, a key regulator in cellular redox homeostasis. TXNIP is also known to play important roles in tumor growth and   metastasis  , glucose and lipid metabolism. TXNIP expression is induced by various stress stimuli. However, it has been unclear how TXNIP is down-regulated. Here, we report that TXNIP undergoes proteasomal degradation in cells. We identify Itch as the E3 ubiquitin ligase for TXNIP. We demonstrate that Itch mediates polyubiquitination of TXNIP both in vitro and in vivo. Overexpression of Itch leads to TXNIP proteasomal degradation. Knockdown of Itch by small interfering RNA causes an accumulation of the steady-state level of TXNIP. We also show that the PPXY motifs of TXNIP and the WW domains of Itch mediate their interaction. Furthermore, the Itch-TXNIP interaction regulates intracellular reactive oxygen species levels and apoptosis. These findings establish a new mechanism for the negative regulation of TXNIP by Itch and shed new light on the regulation of cellular redox homeostasis. 
 Global identification of miR-373-regulated genes in breast cancer by quantitative proteomics. Although microRNAs (miRNAs) have been reported to play an important role in carcinogenesis, their molecular mechanism remains largely unknown because of our limited understanding of miRNA target genes. miR-373 was found to be capable of promoting breast cancer invasion and   metastasis  , but only a target gene was experimentally identified on the basis of mRNA expression analysis. In this study, we used SILAC-based quantitative proteomics to globally identify the genes regulated by miR-373. Totally, 3666 proteins were identified, and 335 proteins were found to be regulated by miR-373. Among the 192 proteins that were downregulated by miR-373, 27 (14.1%) were predicted to have at least one potential match site at their 3'-UTR for miR-373 seed sequence. However, miR-373 did not affect the mRNA level of the five selected candidate targets, TXNIP, TRPS1, RABEP1, GRHL2 and HIP1, suggesting that the protein expressions were regulated by miR-373 via translational inhibition instead of mRNA degradation. Luciferase and mutation assays validated that TXNIP and RABEP1 were the direct target genes of miR-373. More than 30 proteins reported to be involved in cancer invasion and   metastasis   were found to be regulated by miR-373 in breast cancer for the first time. 
 Thioredoxin interacting protein (TXNIP) is a novel tumor suppressor in thyroid cancer. Thyroid cancer is the most common endocrine malignancy, and many patients with metastatic differentiated thyroid cancer (DTC), poorly differentiated thyroid cancer (PDTC), and anaplastic thyroid cancer (ATC) fail to respond to conventional therapies, resulting in morbidity and mortality. Additional therapeutic targets and treatment options are needed for these patients. We recently reported that peroxisome proliferator-activated receptor gamma (PPARγ) is highly expressed in ATC and confers an aggressive phenotype when overexpressed in DTC cells.Microarray analysis was used to identify downstream targets of PPARγ in ATC cells. Western blot analysis and immunohistochemistry (IHC) were used to assess thioredoxin interacting protein (TXNIP) expression in thyroid cancer cell lines and primary tumor specimens. Retroviral transduction was used to generate ATC cell lines that overexpress TXNIP, and assays that assess glucose uptake, viable cell proliferation, and invasion were used to characterize the in vitro properties of these cells. An orthotopic thyroid cancer mouse model was used to assess the effect of TXNIP overexpression in ATC cell lines in vivo.Using microarray analysis, we show that TXNIP is highly upregulated when PPARγ is depleted from ATC cells. Using Western blot analysis and IHC, we show that DTC and ATC cells exhibit differential TXNIP expression patterns. DTC cell lines and patient tumors have high TXNIP expression in contrast to low or absent expression in ATC cell lines and tumors. Overexpression of TXNIP decreases the growth of HTh74 cells compared to vector controls and inhibits glucose uptake in the ATC cell lines HTh74 and T238. Importantly, TXNIP overexpression in T238 cells results in attenuated tumor growth and decreas  ed metasta  sis in an orthotopic thyroid cancer mouse model.Our findings indicate that TXNIP functions as a tumor suppressor in thyroid cells, and its downregulation is likely important in the transition from differentiated to advanced thyroid cancer. These studies underscore the potential of TXNIP as a novel therapeutic target and prognostic indicator in advanced thyroid cancer. 
 Importin alpha1 (Rch1) mediates nuclear translocation of thioredoxin-binding protein-2/vitamin D(3)-up-regulated protein 1. Thioredoxin-binding protein-2 (TBP-2)/vitamin D(3) up-regulated protein 1 is an endogenous molecule interacting with thioredoxin (TRX), negatively regulating TRX function, and being implicated in the suppression of tumor development and   metastasis  . We found that TBP-2 ectopically expressed in the breast cancer cell line MCF-7 was localized predominantly in the nucleus exhibiting growth suppressive activity. The nuclear accumulation of endogenous TBP-2 protein was also demonstrated when the cells were treated with an anti-cancer drug, suberoylanilide hydroxamic acid. To investigate the mechanism underlying the nuclear localization, we performed a yeast two-hybrid screening and identified importin alpha(1) (Rch1) as a protein interacting with TBP-2. The physical interaction between TBP-2 and Rch1 was confirmed with a glutathione S-transferase pull-down assay. The interaction of TBP-2 was specific to Rch1 among other importin alpha subfamilies (Qip1 and NPI-1), and amino acids 1-227 of TBP-2 were sufficient for both the interaction with Rch1 and the nuclear localization, although there is no typical nuclear localization signal in this sequence. The expression of short interfering RNA of Rch1 suppressed suberoylanilide hydroxamic acid-induced nuclear accumulation of TBP-2. Collectively, our results strongly suggest that an interaction with importin system is required for TBP-2 nuclear translocation and growth control tightly associated with TRX-dependent redox regulation of transcription factors. 
 Role of thioredoxin reductase 1 and thioredoxin interacting protein in prognosis of breast cancer. The purpose of this work was to study the prognostic influence in breast cancer of thioredoxin reductase 1 (TXNRD1) and thioredoxin interacting protein (TXNIP), key players in oxidative stress control that are currently evaluated as possible therapeutic targets.Analysis of the association of TXNRD1 and TXNIP RNA expression with the   metastasis  -free interval (MFI) was performed in 788 patients with node-negative breast cancer, consisting of three individual cohorts (Mainz, Rotterdam and Transbig). Correlation with metagenes and conventional clinical parameters (age, pT stage, grading, hormone and ERBB2 status) was explored. MCF-7 cells with a doxycycline-inducible expression of an oncogenic ERBB2 were used to investigate the influence of ERBB2 on TXNRD1 and TXNIP transcription.TXNRD1 was associated with worse MFI in the combined cohort (hazard ratio = 1.955; P < 0.001) as well as in all three individual cohorts. In contrast, TXNIP was associated with better prognosis (hazard ratio = 0.642; P < 0.001) and similar results were obtained in all three subcohorts. Interestingly, patients with ERBB2-status-positive tumors expressed higher levels of TXNRD1. Induction of ERBB2 in MCF-7 cells caused not only an immediate increase in TXNRD1 but also a strong decrease in TXNIP. A subsequent upregulation of TXNIP as cells undergo senescence was accompanied by a strong increase in levels of reactive oxygen species.TXNRD1 and TXNIP are associated with prognosis in breast cancer, and ERBB2 seems to be one of the factors shifting balances of both factors of the redox control system in a prognostic unfavorable manner. 
  GeneRIFs  
 Data identify the   metastasis   suppressor TXNIP as new target of miR-224/miR-452 that induces feedback inhibition of E2F1 and show that miR-224/452-mediated downregulation of TXNIP is essential for E2F1-induced EMT and invasion 
 Breast cancer cell line studies showed that microRNA, miR-373, was capable of promoting breast cancer invasion and   metastasis   via translational inhibition of TXNIP and RABEP1 that were the direct target genes of miR-373. 
  [Cc]ell [Ii]nvasion  PubMed  
 The cyclin-dependent kinase inhibitor, p21(WAF1), promotes angiogenesis by repressing gene transcription of thioredoxin-binding protein 2 in cancer cells. The cyclin-dependent kinase inhibitor, p21(WAF1), induces cell-cycle arrest and can act as a tumor suppressor. However, increasing evidence indicates that p21(WAF1) can also increase resistance to some anticancer therapies and thus promote tumor growth. The mechanisms explaining this paradox have not been explained. We found that conditioned media from MCF-7 breast cancer cells transfected with a p21(WAF1)-specific small interfering RNA (siRNA) significantly reduced endothelial cell migration, invasion and vascular sprouting. Liquid chromatography/mass spectrometry analysis of the conditioned media revealed that p21(WAF1) knockdown significantly reduced secretion of thioredoxin (Trx), a redox protein known to promote tumor angiogenesis. p21(WAF1) knockdown decreased Trx enzymatic activity in cancer cells, by effects on the expression levels of intracellular thioredoxin-binding protein 2 (TBP2), known to bind and inactivate Trx. Consistent with these findings, media from cancer cells transfected with TBP2 siRNA promoted endothelial   cell invasion   and blocked the anti-angiogenic effect of p21(WAF1) siRNA. Addition of Trx siRNA blocked the pro-angiogenic effects of TBP2 siRNA. Chromatin immunoprecipitation assays showed p21(WAF1) bound TBP2 gene promoter. Taken together, our data suggests that p21(WAF1) can induce Trx secretion and angiogenesis in cancer cells, by direct transcriptional repression of the TBP2 promoter. 
   
  
 ARRDC3 
 
  NCBI Gene  
  GeneRIF  
  Pubmed  
 
 
  Search Term  Rank  Fields  
  [Mm]igration  6  PubMed(1)  
  [Ii]nvasive  10  PubMed(1); GeneRIFs(1)  
 
 
 hide/show details for ARRDC3 
  [Mm]igration  PubMed  
 ARRDC3 suppresses breast cancer progression by negatively regulating integrin beta4. Large-scale genetic analyses of human tumor samples have been used to identify novel oncogenes, tumor suppressors and prognostic factors, but the functions and molecular interactions of many individual genes have not been determined. In this study we examined the cellular effects and molecular mechanism of the arrestin family member, ARRDC3, a gene preferentially lost in a subset of breast cancers. Oncomine data revealed that the expression of ARRDC3 decreases with tumor grade, metastases and recurrences. ARRDC3 overexpression represses cancer cell proliferation,   migration  , invasion, growth in soft agar and in vivo tumorigenicity, whereas downregulation of ARRCD3 has the opposite effects. Mechanistic studies showed that ARRDC3 functions in a novel regulatory pathway that controls the cell surface adhesion molecule, beta-4 integrin (ITGbeta4), a protein associated with aggressive tumor behavior. Our data indicates ARRDC3 directly binds to a phosphorylated form of ITGbeta4 leading to its internalization, ubiquitination and ultimate degradation. The results identify the ARRCD3-ITGbeta4 pathway as a new therapeutic target in breast cancer and show the importance of connecting genetic arrays with mechanistic studies in the search for new treatments. 
  [Ii]nvasive  PubMed  
 Promoter hypermethylation may be an important mechanism of the transcriptional inactivation of ARRDC3, GATA5, and ELP3 in   invasive   ductal breast carcinoma. Hypermethylation of promoter CpG islands represents an alternative mechanism to inactivate tumor suppressor genes. This study was to detect promoter methylation status and mRNA expression levels of ARRDC3, ELP3, GATA5, and PAX6, and to explore the association between methylation and expression in   invasive   ductal carcinomas (IDCs) and matched normal tissues (MNTs) from breast cancer patients. Aberrant gene methylation was observed as follows: ARRDC3 in 38.5 %, ELP3 in 73.1 %, GATA5 in 48.1 %, and PAX6 in 50.0 % of IDCs. mRNA expression of ARRDC3, ELP3, and GATA5 in IDCs showed a lower level than that in MNTs (P < 0.001, P = 0.001 and P < 0.001, respectively). For ARRDC3, both methylated and unmethylated IDCs showed significantly lower expression values compared to MNTs (P = 0.001 and P = 0.007, respectively). For ELP3 and GATA5, methylated tumors only showed significantly lower expression values compared to MNTs (P = 0.001 and P < 0.001, respectively). For ARRDC3 and GATA5, methylation was associated with their less fold change in IDCs (P = 0.049 and P = 0.020, respectively). Methylation of ARRDC3 was significantly associated with grades and lymph node status of IDCs (P = 0.036 and P = 0.002, respectively). Methylation frequency of ELP3 was higher in lymph node positive versus lymph node negative tumors (P = 0.020); whereas methylation frequency of PAX6 was lower in tumors with the ER negative samples (P = 0.025). Our data suggested that promoter hypermethylation may be an important mechanism of the transcriptional inactivation of ARRDC3, GATA5, and ELP3 in IDCs. 
  GeneRIFs  
 Promoter hypermethylation is an important mechanism of the transcriptional inactivation of ARRDC3 in   invasive   ductal breast carcinoma. 
   
  
 SERPINH1 
 
  NCBI Gene  
  GeneRIF  
  Pubmed  
 
 
  Search Term  Rank  Fields  
  [Mm]igration  6  PubMed(4); GeneRIFs(1)  
  ECM  7  PubMed(2)  
  [Ee]xtracellular [Mm]atrix  8  PubMed(2); GeneRIFs(1)  
  [Ii]nvasive  10  PubMed(3); GeneRIFs(1)  
  [Mm]etastasis  12  PubMed(3)  
  [Cc]ell [Ii]nvasion  13  PubMed(1)  
 
 
 hide/show details for SERPINH1 
  [Mm]igration  PubMed  
 Regulation of LOXL2 and SERPINH1 by antitumor microRNA-29a in lung cancer with idiopathic pulmonary fibrosis. Idiopathic pulmonary fibrosis (IPF) is a chronic, progressive lung disease that is refractory to treatment and carries a high mortality rate. IPF is frequently associated with lung cancer. Identification of molecular targets involved in both diseases may elucidate novel molecular mechanisms contributing to their pathology. Recent studies of microRNA (miRNA) expression signatures showed that microRNA-29a (miR-29a) was downregulated in IPF and lung cancer. The aim of this study was to investigate the functional significance of miR-29a in lung cancer cells (A549 and EBC-1) and lung fibroblasts (MRC-5) and to identify molecular targets modulated by miR-29a in these cells. We confirmed the downregulation of miR-29a in clinical specimens of IPF and lung cancer. Restoration of miR-29a suppressed cancer cell aggressiveness and fibroblast   migration  . A combination of gene expression data and in silico analysis showed that a total of 24 genes were putative targets of miR-29a. Among them, lysyl oxidase-like 2 (LOXL2) and serpin peptidase inhibitor clade H, member 1 (SERPINH1) were direct targets of miR-29a by luciferase reporter assays. The functions of LOXL2 and SERPINH1 contribute significantly to collagen biosynthesis. Overexpression of LOXL2 and SERPINH1 was observed in clinical specimens of lung cancer and fibrotic lesions. Downregulation of miR-29a caused overexpression of LOXL2 and SERPINH1 in lung cancer and IPF, suggesting that these genes are involved in the pathogenesis of these two diseases. 
 Tumor-suppressive microRNA-29a inhibits cancer cell   migration   and invasion via targeting HSP47 in cervical squamous cell carcinoma. Our recent studies of microRNA (miRNA) expression signatures indicated that microRNA-29a (miR-29a) was significantly downregulated in several types of human cancers, suggesting that miR-29a may be a putative tumor-suppressive miRNA in human cancers. The aim of this study was to investigate the functional significance of miR-29a in cervical squamous cell carcinoma (SCC) and to identify novel miR-29a-regulated cancer pathways and target genes involved in cervical SCC oncogenesis and metastasis. Restoration of miR-29a in cervical cancer cell lines (CaSKi, HeLa, ME180 and Yumoto) revealed that this miRNA significantly inhibited cancer cell   migration   and invasion. Gene expression data and in silico analysis demonstrated that heat-shock protein 47 (HSP47), a member of the serpin superfamily of serine proteinase inhibitors and a molecular chaperone involved in the maturation of collagen molecules, was a potential target of miR-29a regulation. Luciferase reporter assays showed that miR-29a directly regulated HSP47. Moreover, silencing of the HSP47 gene significantly inhibited cell   migration   and invasion in cancer cells and the expression of HSP47 was upregulated in cancer tissues and cervical intraepithelial neoplasia (CIN), as demonstrated by immunostaining. Downregulation of miR-29a was a frequent event in cervical SCC and miR-29a acted as a tumor suppressor by directly targeting HSP47. Recognition of tumor-suppressive miRNA-regulated molecular targets provides new insights into the potential mechanisms of cervical SCC oncogenesis and metastasis and suggests novel therapeutic strategies for treatment of this disease. 
 Heat Shock Protein 47 Promotes Glioma Angiogenesis. Heat shock protein 47 (HSP47) is a collagen-binding protein, which has been recently found to express in glioma vessels. However, the expression profile of HSP47 in glioma patients and the underlying mechanisms of HSP47 on glioma angiogenesis are not fully explored. In the current study, we found that expression of HSP47 in glioma vessels was correlated with the grades of gliomas. HSP47 knockdown by siRNAs significantly decreased cell viability in vitro and tumor volume in vivo; moreover, it reduced the microvessel density (MVD) by CD31 immunohistochemistry in vivo. HSP47 knockdown significantly inhibited tube formation, invasion and proliferation of human umbilical vein endothelial cells (HUVECs). Furthermore, conditional medium derived from HSP47 knockdown cells significantly inhibited HUVECs tube formation and   migration  , while it increased chemosensitivity of HUVECs cells to Avastin. Silencing of HSP47 decreased VEGF expression in glioma cells consistently, and reduced glioma vasculature. Furthermore, HSP47 promoted glioma angiogenesis through HIF1α-VEGFR2 signaling. The present study demonstrates that HSP47 promotes glioma angiogenesis and highlights the importance of HSP47 as an attractive therapeutic target of GBM. 
 Cell surface colligin/Hsp47 associates with tetraspanin protein CD9 in epidermoid carcinoma cell lines. Hsps expressed on the cell surface have been associated with tumor invasiveness and used as targets for molecular surveillance. The present study utilized four human oral squamous cell carcinoma cells lines, SCC-4, SCC-9, SCC-15, SCC-25, the murine epidermoid carcinoma cell line LL/2, and primary cultures of human gingival fibroblasts to assess the cell surface expression of colligin/Hsp47, a proposed marker for malignancy. Immunoprecipitation studies following protein crosslinking revealed that Hsp47 was associated with a number of membrane proteins including the tetraspanin CD9. Cytometric analyses were performed to determine the distribution of cell surface colligin/Hsp47 during the phases of the cell cycle. These studies showed that colligin/Hsp47 was not limited to any phase of the cell cycle in epidermoid carcinoma cells. Boyden chamber tumor invasion assays and colloidal gold   migration   assays utilizing a reconstituted basement membrane (Matrigel), collagen type I, and laminin-5 substrates revealed that cell lines expressing constitutive high levels of colligin/Hsp47 manifested the lowest invasion and   migration   indices. The incorporation of antibodies against Hsps into the   migration   and invasion assays, likewise, increased the invasion indices and the phagokinetic   migration   indices. These data indicate that colligin/Hsp47 is anchored to the cell membrane in a complex with CD9 where it moderates tumor cell invasion and motility possibly by acting as a serpin protein inhibitor or as a receptor for collagen. 
  GeneRIFs  
 silencing of the HSP47 gene significantly inhibited cell   migration   and invasion in cancer cells and the expression of HSP47 was upregulated in cancer tissues and cervical intraepithelial neoplasia, as demonstrated by immunostaining. 
  ECM  PubMed  
 MiR-29b inhibits collagen maturation in hepatic stellate cells through down-regulating the expression of HSP47 and lysyl oxidase. Altered expression of miR-29b is implicated in the pathogenesis and progression of liver fibrosis. We and others previously demonstrated that miR-29b down-regulates the expression of several extracellular-matrix (  ECM  ) genes including Col 1A1, Col 3A1 and Elastin via directly targeting their 3'-UTRs. However, whether or not miR-29b plays a role in the post-translational regulation of   ECM   biosynthesis has not been reported. Heat shock protein 47 (HSP47) and lysyl oxidase (LOX) are known to be essential for   ECM   maturation. In this study we have demonstrated that expression of HSP47 and LOX was significantly up-regulated in culture-activated primary rat hepatic stellate cells (HSCs), TGF-β stimulated LX-2 cells and liver tissue of CCl4-treated mice, which was accompanied by a decrease of miR-29b level. In addition, over-expression of miR-29b in LX-2 cells resulted in significant inhibition on HSP47 and LOX expression. Mechanistically, miR-29b inhibited the expression of a reporter gene that contains the respective full-length 3'-UTR from HSP47 and LOX gene, and this inhibitory effect was abolished by the deletion of a putative miR-29b targeting sequence from the 3'-UTRs. Transfection of LX-2 cells with miR-29b led to abnormal collagen structure as shown by electron-microscopy, presumably through down-regulation of the expression of molecules involved in   EC  M maturation including HSP47 and LOX. These results demonstrated that miR-29b is involved in regulating the post-translational processing of   EC  M and fibril formation. 
 Chaperone Hsp47 Drives Malignant Growth and Invasion by Modulating an   ECM   Gene Network. The extracellular matrix (  ECM  ) is a determining factor in the tumor microenvironment that restrains or promotes malignant growth. In this report, we show how the molecular chaperone protein Hsp47 functions as a nodal hub in regulating an   ECM   gene transcription network. A transcription network analysis showed that Hsp47 expression was activated during breast cancer development and progression. Hsp47 silencing reprogrammed human breast cancer cells to form growth-arrested and/or noninvasive structures in 3D cultures, and to limit tumor growth in xenograft assays by reducing deposition of collagen and fibronectin. Coexpression network analysis also showed that levels of microRNA(miR)-29b and -29c were inversely correlated with expression of Hsp47 and   ECM   network genes in human breast cancer tissues. We found that miR-29 repressed expression of Hsp47 along with multiple   ECM   network genes. Ectopic expression of miR-29b suppressed malignant phenotypes of breast cancer cells in 3D culture. Clinically, increased expression of Hsp47 and reduced levels of miR-29b and -29c were associated with poor survival outcomes in breast cancer patients. Our results show that Hsp47 is regulated by miR-29 during breast cancer development and progression, and that increased Hsp47 expression promotes cancer progression in part by enhancing deposition of   ECM   proteins. 
  [Ee]xtracellular [Mm]atrix  PubMed  
 Involvement of collagen-binding heat shock protein 47 in scleroderma-associated fibrosis. Uncontrolled fibrosis of skin and internal organs is the main characteristic of scleroderma, and collagen is a major   extracellular matrix   protein that deposits in the fibrotic organs. As the chaperone of collagen, heat shock protein 47 (HSP47) is closely related with the development of fibrosis. To explore the potential function of HSP47 in the pathogenesis of scleroderma, the clinical, in vivo and in vitro studies were performed. In clinical, the increased mRNA level of HSP47 was observed in the skin fibroblasts and PBMC from scleroderma patients, and the enhanced protein level of HSP47 was also detected in the skin biopsy and plasma of the above patients. Unexpectedly, the enhanced levels of HSP47 were positively correlated with the presence of anti-centromere antibody in scleroderma patients. Moreover, a high expression of HSP47 was found in the skin lesion of BLM-induced scleroderma mouse model. Further in vitro studies demonstrated that HSP47 knockdown could block the intracellular and extracellular collagen over-productions induced by exogenous TGF-β. Therefore, the results in this study provide direct evidence that HSP47 is involved in the pathogenesis of scleroderma. The high expression of HSP47 can be detected in the circulatory system of scleroderma patients, indicating that HSP47 may become a pathological marker to assess the progression of scleroderma, and also explain the systemic fibrosis of scleroderma. Meanwhile, collagen over-expression is blocked by HSP47 knockdown, suggesting the possibility that HSP47 can be a potential therapeutic target for scleroderma. 
 Chaperone Hsp47 Drives Malignant Growth and Invasion by Modulating an ECM Gene Network. The   extracellular matrix   (ECM) is a determining factor in the tumor microenvironment that restrains or promotes malignant growth. In this report, we show how the molecular chaperone protein Hsp47 functions as a nodal hub in regulating an ECM gene transcription network. A transcription network analysis showed that Hsp47 expression was activated during breast cancer development and progression. Hsp47 silencing reprogrammed human breast cancer cells to form growth-arrested and/or noninvasive structures in 3D cultures, and to limit tumor growth in xenograft assays by reducing deposition of collagen and fibronectin. Coexpression network analysis also showed that levels of microRNA(miR)-29b and -29c were inversely correlated with expression of Hsp47 and ECM network genes in human breast cancer tissues. We found that miR-29 repressed expression of Hsp47 along with multiple ECM network genes. Ectopic expression of miR-29b suppressed malignant phenotypes of breast cancer cells in 3D culture. Clinically, increased expression of Hsp47 and reduced levels of miR-29b and -29c were associated with poor survival outcomes in breast cancer patients. Our results show that Hsp47 is regulated by miR-29 during breast cancer development and progression, and that increased Hsp47 expression promotes cancer progression in part by enhancing deposition of ECM proteins. 
  GeneRIFs  
 Hsp47 expression promotes cancer progression in part by enhancing deposition of   extracellular matrix   proteins. 
  [Ii]nvasive  PubMed  
 Comparison of the desmoplastic reaction and invading ability in   invasive   ductal carcinoma of the breast and prostatic adenocarcinoma based on the expression of heat shock protein 47 and fascin. To investigate the diversity within   invasive   ductal carcinoma (IDC) and prostatic adenocarcinoma (PCa) by evaluating immunohistochemical expression of heat shock protein 47 (HSP47) and fascin, the molecules that are related to desmoplasia and invasion, and analyze its correlation with clinicopathologic parameters.HSP47 and fascin immunoreactivity (IR) was evaluated in 49 mastectomies diagnosed as IDC and 57 radical prostatectomies diagnosed as PCa. IR was evaluated as: 0: < 5%, 1+: 5-25%, 2+: 25-50%, 3+: > 50%.HSP47 and fascin were localized to cytoplasm, and HSP47 and fascin IR were higher in IDC and PCa than benign groups (p < 0.05). HSP47 IR in neoplastic cells was 42.1% and 28.6%, in stroma was 81.6% and 15.8% in IDC and PCa, respectively; fascin IR in neoplastic cells was 65.3% in IDC and 15.8% in PCa. Fascin expression correlated with estrogen receptor and progesterone receptor negativity, tumor size and stage in IDC and surgical margin status in PCa. HSP47 expression correlated bilaterality in PCa. HSP47 positively correlated with survival in IDC.HSP47 and fascin expression may play role in the pathogenesis of IDC and PCa because their expression is significantly higher in IDC and PCa than their normal counterpart. Although there is no relationship with recurrence or metastatic status, fascin overexpression correlated with tumor size, which may prompt its use as a prognostic factor in IDC. 
 Cell surface colligin/Hsp47 associates with tetraspanin protein CD9 in epidermoid carcinoma cell lines. Hsps expressed on the cell surface have been associated with tumor   invasive  ness and used as targets for molecular surveillance. The present study utilized four human oral squamous cell carcinoma cells lines, SCC-4, SCC-9, SCC-15, SCC-25, the murine epidermoid carcinoma cell line LL/2, and primary cultures of human gingival fibroblasts to assess the cell surface expression of colligin/Hsp47, a proposed marker for malignancy. Immunoprecipitation studies following protein crosslinking revealed that Hsp47 was associated with a number of membrane proteins including the tetraspanin CD9. Cytometric analyses were performed to determine the distribution of cell surface colligin/Hsp47 during the phases of the cell cycle. These studies showed that colligin/Hsp47 was not limited to any phase of the cell cycle in epidermoid carcinoma cells. Boyden chamber tumor invasion assays and colloidal gold migration assays utilizing a reconstituted basement membrane (Matrigel), collagen type I, and laminin-5 substrates revealed that cell lines expressing constitutive high levels of colligin/Hsp47 manifested the lowest invasion and migration indices. The incorporation of antibodies against Hsps into the migration and invasion assays, likewise, increased the invasion indices and the phagokinetic migration indices. These data indicate that colligin/Hsp47 is anchored to the cell membrane in a complex with CD9 where it moderates tumor cell invasion and motility possibly by acting as a serpin protein inhibitor or as a receptor for collagen. 
 Chaperone Hsp47 Drives Malignant Growth and Invasion by Modulating an ECM Gene Network. The extracellular matrix (ECM) is a determining factor in the tumor microenvironment that restrains or promotes malignant growth. In this report, we show how the molecular chaperone protein Hsp47 functions as a nodal hub in regulating an ECM gene transcription network. A transcription network analysis showed that Hsp47 expression was activated during breast cancer development and progression. Hsp47 silencing reprogrammed human breast cancer cells to form growth-arrested and/or non  invasive   structures in 3D cultures, and to limit tumor growth in xenograft assays by reducing deposition of collagen and fibronectin. Coexpression network analysis also showed that levels of microRNA(miR)-29b and -29c were inversely correlated with expression of Hsp47 and ECM network genes in human breast cancer tissues. We found that miR-29 repressed expression of Hsp47 along with multiple ECM network genes. Ectopic expression of miR-29b suppressed malignant phenotypes of breast cancer cells in 3D culture. Clinically, increased expression of Hsp47 and reduced levels of miR-29b and -29c were associated with poor survival outcomes in breast cancer patients. Our results show that Hsp47 is regulated by miR-29 during breast cancer development and progression, and that increased Hsp47 expression promotes cancer progression in part by enhancing deposition of ECM proteins. 
  GeneRIFs  
 HSP47 and fascin expression may play role in the pathogenesis of   invasive   ductal carcinoma of the breast and prostatic adenocarcinoma because their expression is significantly higher than their normal counterpart. 
  [Mm]etastasis  PubMed  
 Update of the human and mouse SERPIN gene superfamily. The serpin family comprises a structurally similar, yet functionally diverse, set of proteins. Named originally for their function as serine proteinase inhibitors, many of its members are not inhibitors but rather chaperones, involved in storage, transport, and other roles. Serpins are found in genomes of all kingdoms, with 36 human protein-coding genes and five pseudogenes. The mouse has 60 Serpin functional genes, many of which are orthologous to human SERPIN genes and some of which have expanded into multiple paralogous genes. Serpins are found in tissues throughout the body; whereas most are extracellular, there is a class of intracellular serpins. Serpins appear to have roles in inflammation, immune function, tumorigenesis, blood clotting, dementia, and cancer   metastasis  . Further characterization of these proteins will likely reveal potential biomarkers and therapeutic targets for disease. 
 Detailed map of a region commonly amplified at 11q13-->q14 in human breast carcinoma. Amplification of loci present on band q13 of human chromosome 11 is a feature of a subset of estrogen receptor positive breast carcinomas prone to   metastasis  . As many as five distinct amplification units have been described on 11q13. They include particularly a genomic area encompassing the GARP gene at 11q13.5-->q14.1. We have reassessed our current knowledge of this region, located telomeric to CCND1 and EMS1, which is amplified in 7-10% of mammary tumors. The loose definition of the driving forces of these amplification events led us to map accurately the boundaries of the amplifiable region, and thus to contribute a physical and transcriptional map of a 3-Mb region of chromosome 11. Four new genes were placed on the regional map, namely CBP2, CLNS1A, UVRAG, and PAK1. We have narrowed the core of the 11q13-->q14 amplicon to a 350-kb area encompassing D11S533, mostly on its telomeric side. The map reported here represents an indispensable step toward sequencing the entire region, and thus toward uncovering gene(s) which play(s) a critical role in breast cancer progression. 
 Tumor-suppressive microRNA-29a inhibits cancer cell migration and invasion via targeting HSP47 in cervical squamous cell carcinoma. Our recent studies of microRNA (miRNA) expression signatures indicated that microRNA-29a (miR-29a) was significantly downregulated in several types of human cancers, suggesting that miR-29a may be a putative tumor-suppressive miRNA in human cancers. The aim of this study was to investigate the functional significance of miR-29a in cervical squamous cell carcinoma (SCC) and to identify novel miR-29a-regulated cancer pathways and target genes involved in cervical SCC oncogenesis and   metastasis  . Restoration of miR-29a in cervical cancer cell lines (CaSKi, HeLa, ME180 and Yumoto) revealed that this miRNA significantly inhibited cancer cell migration and invasion. Gene expression data and in silico analysis demonstrated that heat-shock protein 47 (HSP47), a member of the serpin superfamily of serine proteinase inhibitors and a molecular chaperone involved in the maturation of collagen molecules, was a potential target of miR-29a regulation. Luciferase reporter assays showed that miR-29a directly regulated HSP47. Moreover, silencing of the HSP47 gene significantly inhibited cell migration and invasion in cancer cells and the expression of HSP47 was upregulated in cancer tissues and cervical intraepithelial neoplasia (CIN), as demonstrated by immunostaining. Downregulation of miR-29a was a frequent event in cervical SCC and miR-29a acted as a tumor suppressor by directly targeting HSP47. Recognition of tumor-suppressive miRNA-regulated molecular targets provides new insights into the potential mechanisms of cervical SCC oncogenesis and   metastasis   and suggests novel therapeutic strategies for treatment of this disease. 
  [Cc]ell [Ii]nvasion  PubMed  
 Cell surface colligin/Hsp47 associates with tetraspanin protein CD9 in epidermoid carcinoma cell lines. Hsps expressed on the cell surface have been associated with tumor invasiveness and used as targets for molecular surveillance. The present study utilized four human oral squamous cell carcinoma cells lines, SCC-4, SCC-9, SCC-15, SCC-25, the murine epidermoid carcinoma cell line LL/2, and primary cultures of human gingival fibroblasts to assess the cell surface expression of colligin/Hsp47, a proposed marker for malignancy. Immunoprecipitation studies following protein crosslinking revealed that Hsp47 was associated with a number of membrane proteins including the tetraspanin CD9. Cytometric analyses were performed to determine the distribution of cell surface colligin/Hsp47 during the phases of the cell cycle. These studies showed that colligin/Hsp47 was not limited to any phase of the cell cycle in epidermoid carcinoma cells. Boyden chamber tumor invasion assays and colloidal gold migration assays utilizing a reconstituted basement membrane (Matrigel), collagen type I, and laminin-5 substrates revealed that cell lines expressing constitutive high levels of colligin/Hsp47 manifested the lowest invasion and migration indices. The incorporation of antibodies against Hsps into the migration and invasion assays, likewise, increased the invasion indices and the phagokinetic migration indices. These data indicate that colligin/Hsp47 is anchored to the cell membrane in a complex with CD9 where it moderates tumor   cell invasion   and motility possibly by acting as a serpin protein inhibitor or as a receptor for collagen. 
   
  
 NLGN2 
 
  NCBI Gene  
  GeneRIF  
  Pubmed  
 
 
  Search Term  Rank  Fields  
  [Mm]igration  6  PubMed(1)  
 
 
 hide/show details for NLGN2 
  [Mm]igration  PubMed  
 MDGAs interact selectively with neuroligin-2 but not other neuroligins to regulate inhibitory synapse development. The MAM domain-containing GPI anchor proteins MDGA1 and MDGA2 are Ig superfamily adhesion molecules composed of six IG domains, a fibronectin III domain, a MAM domain, and a GPI anchor. MDGAs contribute to the radial   migration   and positioning of a subset of cortical neurons during early neural development. However, MDGAs continue to be expressed in postnatal brain, and their functions during postnatal neural development remain unknown. Here, we demonstrate that MDGAs specifically and with a nanomolar affinity bind to neuroligin-2, a cell-adhesion molecule of inhibitory synapses, but do not bind detectably to neuroligin-1 or neuroligin-3. We observed no cell adhesion between cells expressing neuroligin-2 and MDGA1, suggesting a cis interaction. Importantly, RNAi-mediated knockdown of MDGAs increased the abundance of inhibitory but not excitatory synapses in a neuroligin-2-dependent manner. Conversely, overexpression of MDGA1 decreased the numbers of functional inhibitory synapses. Likewise, coexpression of both MDGA1 and neuroligin-2 reduced the synaptogenic capacity of neuroligin-2 in an artificial synapse-formation assay by abolishing the ability of neuroligin-2 to form an adhesion complex with neurexins. Taken together, our data suggest that MDGAs inhibit the activity of neuroligin-2 in controlling the function of inhibitory synapses and that MDGAs do so by binding to neuroligin-2. 
   
  
 NLGN1 
 
  NCBI Gene  
  GeneRIF  
  Pubmed  
 
 
  Search Term  Rank  Fields  
  [Ff]ilopodium  2  Process(1); Component(1)  
 
 
 hide/show details for NLGN1 
  [Ff]ilopodium  Process  
 positive regulation of   filopodium   assembly 
  Component  
   filopodium   tip 
   
  
 PTGS2 
 
  NCBI Gene  
  GeneRIF  
  Pubmed  
 
 
  Search Term  Rank  Fields  
  [Mm]igration  6  Process(1); PubMed(100); GeneRIFs(28)  
  ECM  7  PubMed(5)  
  [Ee]xtracellular [Mm]atrix  8  PubMed(27); GeneRIFs(2)  
  [Ii]nvasive  10  PubMed(141); GeneRIFs(26)  
  [Mm]etastasize  11  PubMed(7)  
  [Mm]etastasis  12  PubMed(205); GeneRIFs(53)  
  [Cc]ell [Ii]nvasion  13  PubMed(31); GeneRIFs(9)  
 
 
 hide/show details for PTGS2 
  [Mm]igration  Process  
 positive regulation of cell   migration   involved in sprouting angiogenesis 
  PubMed  
 Triggering of DC   migration   by dengue virus stimulation of COX-2-dependent signaling cascades in vitro highlights the significance of these cascades beyond inflammation. A term "bone-breaking fever" is used in Chinese medicine to describe the symptoms of patients infected with dengue virus (DV). We examined the significance of the COX-prostaglandin pathway in human DC infected by DV. We show that DV infection induced the expression of COX-2 and the production of prostaglandin E2 (PGE2) in DC, and stimulated the DNA binding of NF-kappaB and the kinase activity of both IkappaBalpha kinase (IKK) alpha and beta. DV infection also activated MAPK and AP-1 signaling. Both IkappaBalpha kinase-NF-kappaB and MAPK-AP-1 were upstream of COX-2 activation. Our investigation into the significance of COX-2-PGE2 pathway also revealed that DV infection enhances DC   migration   by inducing CC chemokine receptor 7 (CCR7) expression, and that blocking COX-2 or MAPK activity suppresses DV-induced DC   migration  . Our data also suggest that PGE2 can induce CCR7 expression on DC and that antagonists of the PGE2 receptors EP2 and EP4 suppress DV-induced DC   migration  . We further show that the increased CCR7 expression was observed in both DV-infected and bystander DC, suggesting the presence of secondary effects in inducing CCR7 expression. Collectively, this study reveals not only the pathways involved in COX-2 synthesis in DV-infected DC but also the autocrine action of PGE2 on the   migration   of DV-infected DC. 
 Prostaglandin E2 stimulates β1-integrin expression in hepatocellular carcinoma through the EP1 receptor/PKC/NF-κB pathway. Prostaglandin E2 (PGE2) has been implicated in cell invasion in hepatocellular carcinoma (HCC), via increased β1-integrin expression and ce  ll migrat  ion; however, the mechanism remains unclear. PGE2 exerts its effects via four subtypes of the E prostanoid receptor (EP receptor 1-4). The present study investigated the effect of EP1 receptor activation on β1-integrin expression and c  ell migra  tion in HCC. C  ell migra  tion increased by 60% in cells treated with 17-PT-PGE2 (EP1 agonist), which was suppressed by pretreatment with a β1-integrin polyclonal antibody. PGE2 increased β1-integrin expression by approximately 2-fold. EP1 receptor transfection or treatment with 17-PT-PGE2 mimicked the effect of PGE2 treatment. EP1 siRNA blocked PGE2-mediated β1-integrin expression. 17-PT-PGE2 treatment induced PKC and NF-κB activation; PKC and NF-κB inhibitors suppressed 17-PT-PGE2-mediated β1-integrin expression. FoxC2, a β1-integrin transcription factor, was also upregulated by 17-PT-PGE2. NF-κB inhibitor suppressed 17-PT-PGE2-mediated FoxC2 upregulation. Immunohistochemistry showed p65, FoxC2, EP1 receptor and β1-integrin were all highly expressed in the HCC cases. This study suggested that PGE2 upregulates β1-integrin expres  sion and   cell migration in HCC cells by activating the PKC/NF-κB signaling pathway. Targeting PGE2/EP1/PKC/NF-κB/FoxC2/β1-integrin pathway may represent a new therapeutic strategy for the prevention and treatment of this cancer. 
 Alpha2beta1 integrin signalling enhances cyclooxygenase-2 expression in intestinal epithelial cells. Inflammatory bowel diseases (IBD) are linked to an increased risk of developing colon cancer, by inflammatory mediators and alterations to the extracellular matrix (ECM). The events induced by inflammatory mediators lead to dysregulated activation and induction of inflammatory genes such as cyclooxygenase-2 (COX-2). COX-2 is involved in the conversion of arachidonic acid to biologically active prostanoids and is highly upregulated in colon cancer. Since inflammation-induced changes to the extracellular matrix could affect integrin activities, we here investigated the effect of integrin signalling on the level of COX-2 expression in the non-transformed intestinal epithelial cell lines, Int 407 and IEC-6. Adhesion of these cells to a collagen I- or IV-coated surface, increased surface expression of alpha2beta1 integrin. Activation of integrins with collagen caused an increased cox-2 promoter activity, with a subsequent increase in COX-2 expression. The signalling cascade leading to this increased expression and promoter activity of cox-2, involves PKCalpha, the small GTPase Ras and NFkappaB but not Erk1/2 or Src activity. The integrin-induced increase in cellular COX-2 activity is responsible for an elevated generation of reactive oxygen species (ROS) and increased cell   migration  . This signalling pathway suggests a mechanism whereby inflammation-induced modulations of the ECM, can promote cancer transformation in the intestinal epithelial cells. 
 COX-2 promotes breast cancer cell radioresistance via p38/MAPK-mediated cellular anti-apoptosis and invasiveness. Radioresistance is one of the major barriers to improve the survival rate of breast cancer patients. Cyclooxygenase 2 (COX-2) is usually overexpressed in highly invasive and metastatic breast cancer, which may indicate an association with breast cancer radioresistance. The function role of COX-2 was investigated by using a radioresistant breast cancer cell line MDA-MB-231/RR10 and its parental cell line MDA-MB-231 cells before or after COX-2 was silenced by a specific small hairpin RNA (shRNA). The cell proliferation,   migration  , invasion, colony formation, and apoptosis were measured by CCK-8, scratch-wound, transwell, clone formation assay, and flow cytometry. Protein and mRNA expression were analyzed by Western blot and quantitative reverse transcriptase-polymerase chain reaction. COX-2 is upregulated in MDA-MB-231/RR10 cells compared with in MDA-MB-231 cells, and silencing of COX-2 expression by shRNA in MDA-MB-231/RR10 cells decreases the expression of Bcl-2 and Bcl-XL, but increases the proapoptotic protein BAK, leading to the increased apoptosis following treatment with γ-irradiation in comparison with those in control cells. Silencing of COX-2 also increases the expression of β-catenin and E-cadherin, two anti-invasion proteins, resulting in reduced cel  l migrati  on and invasion tested by transwell chambers and wound-healing assays. Further study demonstrated that COX-2-induced radioresistance is negatively regulated through the phosphorylation of p38 at Tyr182, and that the phosphorylation of p38 induced by TNF-alpha reduces the expression of Bcl-2, BCL-XL, but increases β-catenin and E-cadherin, leading to the decreased invasiveness of cells. Our data suggest that COX-2, p38, Bcl-2, Bcl-XL, β-catenin, and E-cadherin may be considered as potential therapeutic targets against radioresistant breast cancer. 
 Lysophosphatidic acid upregulates vascular endothelial growth factor-C and tube formation in human endothelial cells through LPA(1/3), COX-2, and NF-kappaB activation- and EGFR transactivation-dependent mechanisms. Lysophosphatidic acid (LPA) is a lipid bioactive mediator which binds to G-protein-coupled receptors and activates a variety of cellular functions. LPA modulates multiple behaviors in endothelial cells, including cell proliferation and   migration  , capillary-like tube formation in vitro, activation of proteases, interactions with leukocytes, and expressions of inflammation-related genes, thereby regulating vessel formation. LPA has been reported to modulate the angiogenesis process. However, the role of LPA in the lymphangiogenesis process has not been studied. In this study, we showed that LPA upregulated vascular endothelial growth factor-C (VEGF-C) mRNA expression in human umbilical vein endothelial cells (HUVECs) and subsequent endothelial cell tube formation in vitro and in vivo. These enhancement effects were LPA(1)- and LPA(3)-dependent and required cyclooxygenase-2 (COX-2), endothelial growth factor receptor (EGFR) transactivation and activation of nuclear factor kappaB (NF-kappaB). Moreover, LPA induced the protein expressions of the lymphatic markers, Prox-1, LYVE-1, and podoplanin, in HUVECs, and these enhancement effects were dependent on LPA(1) and LPA(3) activation and EGFR transactivation. Our results demonstrated that LPA might regulate VEGF-C and lymphatic marker expression in endothelial cells, which contributes to endothelial cell tube formation in vitro and in vivo, thus facilitating endothelial cell participation in the lymphangiogenesis process. This study clarifies the signaling mechanism of LPA-regulated VEGF-C expression and lymphatic marker expressions in endothelial cells, which suggest that LPA may be a suitable target for generating therapeutics against lymphangiogenesis and tumor metastasis. 
 Tanshinone IIA decreases the migratory ability of AGS cells by decreasing the protein expression of matrix metalloproteinases, nuclear factor κB-p65 and cyclooxygenase-2. During progression of gastric cancer, degradation of the extracellular matrix by matrix metalloproteinases (MMPs) has been associated with poor prognosis. Tanshinone IIA (Tan-IIA) exerts antitumor activity in a variety of human cancer cells. It is extracted from Danshen (Salviae miltiorrhizae radix), and induces apoptosis and inhibits the proliferation of gastric cancer cells. However, the molecular mechanisms underlying the inhibition of   migratio  n in gastric cancer by Tan-IIA have not been fully elucidated. In the present study, AGS cell   migratio  n ability was evaluated using a wound-healing assay. The protein expression levels of nuclear factor (NF)-κB-p65, cyclooxygenase (COX)-2, MMP-2, -7, and -9 and β-actin in AGS cells were measured by western blotting. The results demonstrated that AGS cells treated with Tan-IIA exhibit decreased protein expression levels of NF-κB-p65, COX-2, and MMP-2, -7 and -9. The results also indicate that Tan-IIA inhib  its migra  tion ability in a dose- and time-dependent manner. These findings demonstrate that Tan-IIA inhibits   the migra  tion ability of AGS human gastric cancer cells and that decreasing the protein expression of NF-κB-p65, COX-2, and MMP-2, -7 and -9 may be an underlying molecular mechanism. 
 Glucose-regulated protein 78 mediates hormone-independent prostate cancer progression and metastasis through maspin and COX-2 expression. Glucose-regulated protein 78 (GRP78) plays an essential role in embryonic development and in the progression and therapeutic resistance of many cancers. However, little is known about the function of GRP78 in hormone-independent prostate cancer. Here, we found that the expression levels of GRP78 were higher in PC-3 cells than in DU-145 cells. When the expression of GRP78 was silenced using a GRP78-specific small interfering RNA in PC-3 cells, the growth rate and adhesive ability were reduced. Cell   migration   was dramatically decreased in GRP78-depleted cells. Dissection of the involved signal pathways revealed that maspin expression was upregulated after silencing GRP78 expression. The upregulation of maspin and downregulation of COX-2 may cause the decrease in cell proliferation and   migration   observed after silencing GRP78 expression. Silencing GRP78 expression may suppress the proliferation, adhesion, and   migration   of prostate cancer cells via maspin and COX-2 regulation. 
 Cyclooxygenase-2 enhances alpha2beta1 integrin expression and cell   migration   via EP1 dependent signaling pathway in human chondrosarcoma cells. Cyclooxygenase (COX)-2, the inducible isoform of prostaglandin (PG) synthase, has been implicated in tumor metastasis. Interaction of COX-2 with its specific EP receptors on the surface of cancer cells has been reported to induce cancer invasion. However, the effects of COX-2 on   migration   activity in human chondrosarcoma cells are mostly unknown. In this study, we examined whether COX-2 and EP interaction are involved in metastasis of human chondrosarcoma.We found that over-expression of COX-2 or exogenous PGE2 increased the   migration   of human chondrosarcoma cells. We also found that human chondrosarcoma tissues and chondrosarcoma cell lines had significant expression of the COX-2 which was higher than that in normal cartilage. By using pharmacological inhibitors or activators or genetic inhibition by the EP receptors, we discovered that the EP1 receptor but not other PGE receptors is involved in PGE2-mediated cell   migration   and alpha2beta1 integrin expression. Furthermore, we found that human chondrosarcoma tissues expressed a higher level of EP1 receptor than normal cartilage. PGE2-mediated   migration   and integrin up-regulation were attenuated by phospholipase C (PLC), protein kinase C (PKC) and c-Src inhibitor. Activation of the PLCbeta, PKCalpha, c-Src and NF-kappaB signaling pathway after PGE2 treatment was demonstrated, and PGE2-induced expression of integrin and   migration   activity were inhibited by the specific inhibitor, siRNA and mutants of PLC, PKC, c-Src and NF-kappaB cascades.Our results indicated that PGE2 enhances the   migration   of chondrosarcoma cells by increasing alpha2beta1 integrin expression through the EP1/PLC/PKCalpha/c-Src/NF-kappaB signal transduction pathway. 
 Galectin-1 promotes lung cancer progression and chemoresistance by upregulating p38 MAPK, ERK, and cyclooxygenase-2. This study is aimed at investigating the role and novel molecular mechanisms of galectin-1 in lung cancer progression.The role of galectin-1 in lung cancer progression was evaluated both in vitro and in vivo by short hairpin RNA (shRNA)-mediated knockdown of galectin-1 in lung adenocarcinoma cell lines. To explore novel molecular mechanisms underlying galectin-1-mediated tumor progression, we analyzed gene expression profiles and signaling pathways using reverse transcription PCR and Western blotting. A tissue microarray containing samples from patients with lung cancer was used to examine the expression of galectin-1 in lung cancer.We found overexpression of galectin-1 in non-small cell lung cancer (NSCLC) cell lines. Suppression of endogenous galectin-1 in lung adenocarcinoma resulted in reduction of the cell   migration  , invasion, and anchorage-independent growth in vitro and tumor growth in mice. In particular, COX-2 was downregulated in galectin-1-knockdown cells. The decreased tumor invasion and anchorage-independent growth abilities were rescued after reexpression of COX-2 in galectin-1-knockdown cells. Furthermore, we found that TGF-β1 promoted COX-2 expression through galectin-1 interaction with Ras and subsequent activation of p38 mitogen-activated protein kinase (MAPK), extracellular signal-regulated kinase (ERK), and NF-κB pathway. Galectin-1 knockdown sensitized lung cancer cells to platinum-based chemotherapy (cisplatin). In addition, galectin-1 and COX-2 expression was correlated with the progression of lung adenocarcinoma, and high clinical relevance of both proteins was evidenced (n = 47).p38 MAPK, ERK, and COX-2 activation are novel mediators for the galectin-1-promoted tumor progression and chemoresistance in lung cancer. Galectin-1 may be an innovative target for combined modality therapy for lung cancer. 
 Palbociclib inhibits epithelial-mesenchymal transition and metastasis in breast cancer via c-Jun/COX-2 signaling pathway. Palbociclib, a highly selective CDK4/6 inhibitor, has been shown to be a novel anti-tumor agent that suppresses breast cancer cell proliferation. However, its anti-metastasis activity remains controversial. In the present study, we evaluated whether palbociclib prevented breast cancer cell metastasis and revealed its regulatory mechanism. We found that palbociclib inhibited   migration   and invasion in the breast cancer cells MDA-MB-231 and T47D. The epithelial-mesenchymal transition (EMT) markers, vimentin and Snail, were down-regulated with palbociclib treatment. Moreover, we revealed that this inhibition was mediated by the c-Jun/COX-2 pathway. COX-2 was decreased after palbociclib treatment. The production of PGE2 was also reduced along with COX-2. Additionally, our data showed that c-Jun, a crucial transcriptional regulator of COX-2, was down-regulated by palbociclib. We found that palbociclib weakened the COX-2 promoter binding activity of c-Jun and prevented its translocation from the cytoplasm to cell nuclei. Bioluminescence imaging and tail intravenous injection were used to evaluate the anti-metastasis effect of palbociclib in vivo. The data demonstrated that palbociclib reduced breast cancer metastasis to the lung. These results therefore demonstrated that the anti-metastasis activity of palbociclib is mediated via the c-Jun/COX-2 signaling pathway by inhibiting EMT in breast cancer cells. 
 Hepatitis C virus NS3 protein enhances cancer cell invasion by activating matrix metalloproteinase-9 and cyclooxygenase-2 through ERK/p38/NF-κB signal cascade. Hepatitis C virus (HCV) infection causes acute and chronic hepatitis, liver cirrhosis, and hepatocellular carcinoma (HCC). However, the mechanisms by which HCV causes the diseases are largely unknown. Here, we elucidated the effects of HCV on the invasion and   migratio  n of hepatoma cells, with the aim to reveal the mechanism by which HCV infection induces HCC. We initially showed that matrix metalloproteinase-9 (MMP-9) was elevated in the sera of HCV-infected patients, and demonstrated that HCV nonstructural protein 3 (NS3) activated MMP-9 transcription through nuclear factor-κB (NF-κB) by stimulating translocation of NF-κB from cytosol to the nucleus to enhance its binding to MMP-9 promoter. In addition, cyclooxygenase-2 (COX-2) and extracellular signal-regulated kinase (ERK1/2)/mitogen-activated protein kinase (p38) pathway were involved in HCV-activated MMP-9 expression. Moreover, NS3 enhanced hepatoma cell invasion   and migra  tion through MMP-9 and COX-2. Thus, this study provides new insights into the roles of HCV NS3, MMP-9 and COX-2 in regulating cancer cell invasion. 
 p38 Mitogen-activated protein kinase stabilizes mRNAs that contain cyclooxygenase-2 and tumor necrosis factor AU-rich elements by inhibiting deadenylation. AU-rich elements (AREs) in 3'-untranslated regions of mRNAs confer instability. They target mRNAs for rapid deadenylation and degradation and may enhance decapping. The p38 MAPK pathway stabilizes many otherwise unstable ARE-containing mRNAs encoding proteins involved in inflammation; however, the mRNA decay step(s) regulated by the signaling pathway are unknown. To investigate whether it regulates deadenylation or the decay of the mRNA body, we used a tetracycline-regulated beta-globin mRNA reporter system to transcribe pulses of mRNA of uniform length. We measured on Northern gels the   migration   of reporter mRNAs isolated from cells transfected only with reporter plasmid or co-transfected with an active mutant of MAPK kinase-6, and treated either with or without the p38 MAPK inhibitor SB 203580. Differences in   migration   were shown by RNase H mapping with oligo(dT) to be due to poly(A) shortening. Insertion of an ARE into the beta-globin reporter mRNA promoted rapid deadenylation and decay of hypo-adenylated reporter mRNA. p38 MAPK activation inhibited the deadenylation of reporter mRNAs containing either the cyclooxygenase-2 or tumor necrosis factor AREs. The regulation of deadenylation by p38 MAPK was found to be specific because deadenylation of the beta-globin reporter mRNA either lacking an ARE or containing the c-Myc 3'-untranslated region (which is not p38 MAPK-responsive) was unaffected by p38 MAPK. It was concluded that the p38 MAPK pathway predominantly regulates deadenylation, rather than decay of the mRNA body, and this provides an explanation for why p38 MAPK regulates mRNA stability in some situations and translation in others. 
 Tanshinone IIA decreases the migratory ability of AGS cells by decreasing the protein expression of matrix metalloproteinases, nuclear factor κB-p65 and cyclooxygenase-2. During progression of gastric cancer, degradation of the extracellular matrix by matrix metalloproteinases (MMPs) has been associated with poor prognosis. Tanshinone IIA (Tan-IIA) exerts antitumor activity in a variety of human cancer cells. It is extracted from Danshen (Salviae miltiorrhizae radix), and induces apoptosis and inhibits the proliferation of gastric cancer cells. However, the molecular mechanisms underlying the inhibition of   migratio  n in gastric cancer by Tan-IIA have not been fully elucidated. In the present study, AGS cell   migratio  n ability was evaluated using a wound-healing assay. The protein expression levels of nuclear factor (NF)-κB-p65, cyclooxygenase (COX)-2, MMP-2, -7, and -9 and β-actin in AGS cells were measured by western blotting. The results demonstrated that AGS cells treated with Tan-IIA exhibit decreased protein expression levels of NF-κB-p65, COX-2, and MMP-2, -7 and -9. The results also indicate that Tan-IIA inhib  its migra  tion ability in a dose- and time-dependent manner. These findings demonstrate that Tan-IIA inhibits   the migra  tion ability of AGS human gastric cancer cells and that decreasing the protein expression of NF-κB-p65, COX-2, and MMP-2, -7 and -9 may be an underlying molecular mechanism. 
 Palbociclib inhibits epithelial-mesenchymal transition and metastasis in breast cancer via c-Jun/COX-2 signaling pathway. Palbociclib, a highly selective CDK4/6 inhibitor, has been shown to be a novel anti-tumor agent that suppresses breast cancer cell proliferation. However, its anti-metastasis activity remains controversial. In the present study, we evaluated whether palbociclib prevented breast cancer cell metastasis and revealed its regulatory mechanism. We found that palbociclib inhibited   migration   and invasion in the breast cancer cells MDA-MB-231 and T47D. The epithelial-mesenchymal transition (EMT) markers, vimentin and Snail, were down-regulated with palbociclib treatment. Moreover, we revealed that this inhibition was mediated by the c-Jun/COX-2 pathway. COX-2 was decreased after palbociclib treatment. The production of PGE2 was also reduced along with COX-2. Additionally, our data showed that c-Jun, a crucial transcriptional regulator of COX-2, was down-regulated by palbociclib. We found that palbociclib weakened the COX-2 promoter binding activity of c-Jun and prevented its translocation from the cytoplasm to cell nuclei. Bioluminescence imaging and tail intravenous injection were used to evaluate the anti-metastasis effect of palbociclib in vivo. The data demonstrated that palbociclib reduced breast cancer metastasis to the lung. These results therefore demonstrated that the anti-metastasis activity of palbociclib is mediated via the c-Jun/COX-2 signaling pathway by inhibiting EMT in breast cancer cells. 
 Prostaglandin E2 stimulates β1-integrin expression in hepatocellular carcinoma through the EP1 receptor/PKC/NF-κB pathway. Prostaglandin E2 (PGE2) has been implicated in cell invasion in hepatocellular carcinoma (HCC), via increased β1-integrin expression and ce  ll migrat  ion; however, the mechanism remains unclear. PGE2 exerts its effects via four subtypes of the E prostanoid receptor (EP receptor 1-4). The present study investigated the effect of EP1 receptor activation on β1-integrin expression and c  ell migra  tion in HCC. C  ell migra  tion increased by 60% in cells treated with 17-PT-PGE2 (EP1 agonist), which was suppressed by pretreatment with a β1-integrin polyclonal antibody. PGE2 increased β1-integrin expression by approximately 2-fold. EP1 receptor transfection or treatment with 17-PT-PGE2 mimicked the effect of PGE2 treatment. EP1 siRNA blocked PGE2-mediated β1-integrin expression. 17-PT-PGE2 treatment induced PKC and NF-κB activation; PKC and NF-κB inhibitors suppressed 17-PT-PGE2-mediated β1-integrin expression. FoxC2, a β1-integrin transcription factor, was also upregulated by 17-PT-PGE2. NF-κB inhibitor suppressed 17-PT-PGE2-mediated FoxC2 upregulation. Immunohistochemistry showed p65, FoxC2, EP1 receptor and β1-integrin were all highly expressed in the HCC cases. This study suggested that PGE2 upregulates β1-integrin expres  sion and   cell migration in HCC cells by activating the PKC/NF-κB signaling pathway. Targeting PGE2/EP1/PKC/NF-κB/FoxC2/β1-integrin pathway may represent a new therapeutic strategy for the prevention and treatment of this cancer. 
 Hepatitis C virus NS3 protein enhances cancer cell invasion by activating matrix metalloproteinase-9 and cyclooxygenase-2 through ERK/p38/NF-κB signal cascade. Hepatitis C virus (HCV) infection causes acute and chronic hepatitis, liver cirrhosis, and hepatocellular carcinoma (HCC). However, the mechanisms by which HCV causes the diseases are largely unknown. Here, we elucidated the effects of HCV on the invasion and   migratio  n of hepatoma cells, with the aim to reveal the mechanism by which HCV infection induces HCC. We initially showed that matrix metalloproteinase-9 (MMP-9) was elevated in the sera of HCV-infected patients, and demonstrated that HCV nonstructural protein 3 (NS3) activated MMP-9 transcription through nuclear factor-κB (NF-κB) by stimulating translocation of NF-κB from cytosol to the nucleus to enhance its binding to MMP-9 promoter. In addition, cyclooxygenase-2 (COX-2) and extracellular signal-regulated kinase (ERK1/2)/mitogen-activated protein kinase (p38) pathway were involved in HCV-activated MMP-9 expression. Moreover, NS3 enhanced hepatoma cell invasion   and migra  tion through MMP-9 and COX-2. Thus, this study provides new insights into the roles of HCV NS3, MMP-9 and COX-2 in regulating cancer cell invasion. 
 Glucose-regulated protein 78 mediates hormone-independent prostate cancer progression and metastasis through maspin and COX-2 expression. Glucose-regulated protein 78 (GRP78) plays an essential role in embryonic development and in the progression and therapeutic resistance of many cancers. However, little is known about the function of GRP78 in hormone-independent prostate cancer. Here, we found that the expression levels of GRP78 were higher in PC-3 cells than in DU-145 cells. When the expression of GRP78 was silenced using a GRP78-specific small interfering RNA in PC-3 cells, the growth rate and adhesive ability were reduced. Cell   migration   was dramatically decreased in GRP78-depleted cells. Dissection of the involved signal pathways revealed that maspin expression was upregulated after silencing GRP78 expression. The upregulation of maspin and downregulation of COX-2 may cause the decrease in cell proliferation and   migration   observed after silencing GRP78 expression. Silencing GRP78 expression may suppress the proliferation, adhesion, and   migration   of prostate cancer cells via maspin and COX-2 regulation. 
 COX-2 promotes breast cancer cell radioresistance via p38/MAPK-mediated cellular anti-apoptosis and invasiveness. Radioresistance is one of the major barriers to improve the survival rate of breast cancer patients. Cyclooxygenase 2 (COX-2) is usually overexpressed in highly invasive and metastatic breast cancer, which may indicate an association with breast cancer radioresistance. The function role of COX-2 was investigated by using a radioresistant breast cancer cell line MDA-MB-231/RR10 and its parental cell line MDA-MB-231 cells before or after COX-2 was silenced by a specific small hairpin RNA (shRNA). The cell proliferation,   migration  , invasion, colony formation, and apoptosis were measured by CCK-8, scratch-wound, transwell, clone formation assay, and flow cytometry. Protein and mRNA expression were analyzed by Western blot and quantitative reverse transcriptase-polymerase chain reaction. COX-2 is upregulated in MDA-MB-231/RR10 cells compared with in MDA-MB-231 cells, and silencing of COX-2 expression by shRNA in MDA-MB-231/RR10 cells decreases the expression of Bcl-2 and Bcl-XL, but increases the proapoptotic protein BAK, leading to the increased apoptosis following treatment with γ-irradiation in comparison with those in control cells. Silencing of COX-2 also increases the expression of β-catenin and E-cadherin, two anti-invasion proteins, resulting in reduced cel  l migrati  on and invasion tested by transwell chambers and wound-healing assays. Further study demonstrated that COX-2-induced radioresistance is negatively regulated through the phosphorylation of p38 at Tyr182, and that the phosphorylation of p38 induced by TNF-alpha reduces the expression of Bcl-2, BCL-XL, but increases β-catenin and E-cadherin, leading to the decreased invasiveness of cells. Our data suggest that COX-2, p38, Bcl-2, Bcl-XL, β-catenin, and E-cadherin may be considered as potential therapeutic targets against radioresistant breast cancer. 
 Galectin-1 promotes lung cancer progression and chemoresistance by upregulating p38 MAPK, ERK, and cyclooxygenase-2. This study is aimed at investigating the role and novel molecular mechanisms of galectin-1 in lung cancer progression.The role of galectin-1 in lung cancer progression was evaluated both in vitro and in vivo by short hairpin RNA (shRNA)-mediated knockdown of galectin-1 in lung adenocarcinoma cell lines. To explore novel molecular mechanisms underlying galectin-1-mediated tumor progression, we analyzed gene expression profiles and signaling pathways using reverse transcription PCR and Western blotting. A tissue microarray containing samples from patients with lung cancer was used to examine the expression of galectin-1 in lung cancer.We found overexpression of galectin-1 in non-small cell lung cancer (NSCLC) cell lines. Suppression of endogenous galectin-1 in lung adenocarcinoma resulted in reduction of the cell   migration  , invasion, and anchorage-independent growth in vitro and tumor growth in mice. In particular, COX-2 was downregulated in galectin-1-knockdown cells. The decreased tumor invasion and anchorage-independent growth abilities were rescued after reexpression of COX-2 in galectin-1-knockdown cells. Furthermore, we found that TGF-β1 promoted COX-2 expression through galectin-1 interaction with Ras and subsequent activation of p38 mitogen-activated protein kinase (MAPK), extracellular signal-regulated kinase (ERK), and NF-κB pathway. Galectin-1 knockdown sensitized lung cancer cells to platinum-based chemotherapy (cisplatin). In addition, galectin-1 and COX-2 expression was correlated with the progression of lung adenocarcinoma, and high clinical relevance of both proteins was evidenced (n = 47).p38 MAPK, ERK, and COX-2 activation are novel mediators for the galectin-1-promoted tumor progression and chemoresistance in lung cancer. Galectin-1 may be an innovative target for combined modality therapy for lung cancer. 
 Cyclooxygenase-2 enhances alpha2beta1 integrin expression and cell   migration   via EP1 dependent signaling pathway in human chondrosarcoma cells. Cyclooxygenase (COX)-2, the inducible isoform of prostaglandin (PG) synthase, has been implicated in tumor metastasis. Interaction of COX-2 with its specific EP receptors on the surface of cancer cells has been reported to induce cancer invasion. However, the effects of COX-2 on   migration   activity in human chondrosarcoma cells are mostly unknown. In this study, we examined whether COX-2 and EP interaction are involved in metastasis of human chondrosarcoma.We found that over-expression of COX-2 or exogenous PGE2 increased the   migration   of human chondrosarcoma cells. We also found that human chondrosarcoma tissues and chondrosarcoma cell lines had significant expression of the COX-2 which was higher than that in normal cartilage. By using pharmacological inhibitors or activators or genetic inhibition by the EP receptors, we discovered that the EP1 receptor but not other PGE receptors is involved in PGE2-mediated cell   migration   and alpha2beta1 integrin expression. Furthermore, we found that human chondrosarcoma tissues expressed a higher level of EP1 receptor than normal cartilage. PGE2-mediated   migration   and integrin up-regulation were attenuated by phospholipase C (PLC), protein kinase C (PKC) and c-Src inhibitor. Activation of the PLCbeta, PKCalpha, c-Src and NF-kappaB signaling pathway after PGE2 treatment was demonstrated, and PGE2-induced expression of integrin and   migration   activity were inhibited by the specific inhibitor, siRNA and mutants of PLC, PKC, c-Src and NF-kappaB cascades.Our results indicated that PGE2 enhances the   migration   of chondrosarcoma cells by increasing alpha2beta1 integrin expression through the EP1/PLC/PKCalpha/c-Src/NF-kappaB signal transduction pathway. 
 Triggering of DC   migration   by dengue virus stimulation of COX-2-dependent signaling cascades in vitro highlights the significance of these cascades beyond inflammation. A term "bone-breaking fever" is used in Chinese medicine to describe the symptoms of patients infected with dengue virus (DV). We examined the significance of the COX-prostaglandin pathway in human DC infected by DV. We show that DV infection induced the expression of COX-2 and the production of prostaglandin E2 (PGE2) in DC, and stimulated the DNA binding of NF-kappaB and the kinase activity of both IkappaBalpha kinase (IKK) alpha and beta. DV infection also activated MAPK and AP-1 signaling. Both IkappaBalpha kinase-NF-kappaB and MAPK-AP-1 were upstream of COX-2 activation. Our investigation into the significance of COX-2-PGE2 pathway also revealed that DV infection enhances DC   migration   by inducing CC chemokine receptor 7 (CCR7) expression, and that blocking COX-2 or MAPK activity suppresses DV-induced DC   migration  . Our data also suggest that PGE2 can induce CCR7 expression on DC and that antagonists of the PGE2 receptors EP2 and EP4 suppress DV-induced DC   migration  . We further show that the increased CCR7 expression was observed in both DV-infected and bystander DC, suggesting the presence of secondary effects in inducing CCR7 expression. Collectively, this study reveals not only the pathways involved in COX-2 synthesis in DV-infected DC but also the autocrine action of PGE2 on the   migration   of DV-infected DC. 
 Lysophosphatidic acid upregulates vascular endothelial growth factor-C and tube formation in human endothelial cells through LPA(1/3), COX-2, and NF-kappaB activation- and EGFR transactivation-dependent mechanisms. Lysophosphatidic acid (LPA) is a lipid bioactive mediator which binds to G-protein-coupled receptors and activates a variety of cellular functions. LPA modulates multiple behaviors in endothelial cells, including cell proliferation and   migration  , capillary-like tube formation in vitro, activation of proteases, interactions with leukocytes, and expressions of inflammation-related genes, thereby regulating vessel formation. LPA has been reported to modulate the angiogenesis process. However, the role of LPA in the lymphangiogenesis process has not been studied. In this study, we showed that LPA upregulated vascular endothelial growth factor-C (VEGF-C) mRNA expression in human umbilical vein endothelial cells (HUVECs) and subsequent endothelial cell tube formation in vitro and in vivo. These enhancement effects were LPA(1)- and LPA(3)-dependent and required cyclooxygenase-2 (COX-2), endothelial growth factor receptor (EGFR) transactivation and activation of nuclear factor kappaB (NF-kappaB). Moreover, LPA induced the protein expressions of the lymphatic markers, Prox-1, LYVE-1, and podoplanin, in HUVECs, and these enhancement effects were dependent on LPA(1) and LPA(3) activation and EGFR transactivation. Our results demonstrated that LPA might regulate VEGF-C and lymphatic marker expression in endothelial cells, which contributes to endothelial cell tube formation in vitro and in vivo, thus facilitating endothelial cell participation in the lymphangiogenesis process. This study clarifies the signaling mechanism of LPA-regulated VEGF-C expression and lymphatic marker expressions in endothelial cells, which suggest that LPA may be a suitable target for generating therapeutics against lymphangiogenesis and tumor metastasis. 
 p38 Mitogen-activated protein kinase stabilizes mRNAs that contain cyclooxygenase-2 and tumor necrosis factor AU-rich elements by inhibiting deadenylation. AU-rich elements (AREs) in 3'-untranslated regions of mRNAs confer instability. They target mRNAs for rapid deadenylation and degradation and may enhance decapping. The p38 MAPK pathway stabilizes many otherwise unstable ARE-containing mRNAs encoding proteins involved in inflammation; however, the mRNA decay step(s) regulated by the signaling pathway are unknown. To investigate whether it regulates deadenylation or the decay of the mRNA body, we used a tetracycline-regulated beta-globin mRNA reporter system to transcribe pulses of mRNA of uniform length. We measured on Northern gels the   migration   of reporter mRNAs isolated from cells transfected only with reporter plasmid or co-transfected with an active mutant of MAPK kinase-6, and treated either with or without the p38 MAPK inhibitor SB 203580. Differences in   migration   were shown by RNase H mapping with oligo(dT) to be due to poly(A) shortening. Insertion of an ARE into the beta-globin reporter mRNA promoted rapid deadenylation and decay of hypo-adenylated reporter mRNA. p38 MAPK activation inhibited the deadenylation of reporter mRNAs containing either the cyclooxygenase-2 or tumor necrosis factor AREs. The regulation of deadenylation by p38 MAPK was found to be specific because deadenylation of the beta-globin reporter mRNA either lacking an ARE or containing the c-Myc 3'-untranslated region (which is not p38 MAPK-responsive) was unaffected by p38 MAPK. It was concluded that the p38 MAPK pathway predominantly regulates deadenylation, rather than decay of the mRNA body, and this provides an explanation for why p38 MAPK regulates mRNA stability in some situations and translation in others. 
 Alpha2beta1 integrin signalling enhances cyclooxygenase-2 expression in intestinal epithelial cells. Inflammatory bowel diseases (IBD) are linked to an increased risk of developing colon cancer, by inflammatory mediators and alterations to the extracellular matrix (ECM). The events induced by inflammatory mediators lead to dysregulated activation and induction of inflammatory genes such as cyclooxygenase-2 (COX-2). COX-2 is involved in the conversion of arachidonic acid to biologically active prostanoids and is highly upregulated in colon cancer. Since inflammation-induced changes to the extracellular matrix could affect integrin activities, we here investigated the effect of integrin signalling on the level of COX-2 expression in the non-transformed intestinal epithelial cell lines, Int 407 and IEC-6. Adhesion of these cells to a collagen I- or IV-coated surface, increased surface expression of alpha2beta1 integrin. Activation of integrins with collagen caused an increased cox-2 promoter activity, with a subsequent increase in COX-2 expression. The signalling cascade leading to this increased expression and promoter activity of cox-2, involves PKCalpha, the small GTPase Ras and NFkappaB but not Erk1/2 or Src activity. The integrin-induced increase in cellular COX-2 activity is responsible for an elevated generation of reactive oxygen species (ROS) and increased cell   migration  . This signalling pathway suggests a mechanism whereby inflammation-induced modulations of the ECM, can promote cancer transformation in the intestinal epithelial cells. 
 COX-2 expression is correlated with VEGF-C, lymphangiogenesis and lymph node metastasis in human cervical cancer. Lymphangiogenesis has been shown to promote lymph node metastasis in cancers, making it an important target in cancer therapy. Vascular endothelial growth factor (VEGF)-C is upregulated in various tumors/cancers and is one of the most potent growth factors for inducing lymphangiogenesis and promoting lymph node metastasis (LNM). Likewise, cyclooxygenase (COX)-2 plays major roles in carcinogenesis, tumor growth and metastasis via multiple mechanisms including inactivation of host antitumor immunity and promotion of tumor cell   migration  , tumor cell invasiveness and tumor-associated angiogenesis and lymphangiogenesis. We previously demonstrated an association between COX-2 and VEGF-C in an in vitro model of lung cancer. However, little is known about the regulation of VEGF-C by COX-2 in cervical cancer. In this study, we measured the COX-2 and VEGF-C expressions by immunohistochemistry in 23 LNM-positive and 20 LNM-negative cervical cancer specimens. We then examined the correlations among the expressions and the lymphatic microvessel density (LMVD) and ultrastructural changes to the lymphatic vessel walls by enzyme histochemical staining and electron microscopy. In addition, we used the HeLa cervical cancer cell line to explore the in vitro regulation of VEGF-C by COX-2 and its metabolite, PGE(2), using siRNA-mediated gene silencing and EP receptor blockade. The LNM-positive specimens exhibited significantly higher VEGF-C expression, COX-2 expression and LMVD than the LNM-negative specimens. Furthermore, there were strong correlations between the levels of COX-2 expression and the levels of VEGF-C expression and secretion and a significant positive association between the LMVD and LNM. siRNA-mediated knockdown of COX-2 expression inhibited VEGF-C mRNA expression while EP1 and EP4 receptor antagonists reduced the VEGF-C protein level and tyrosine phosphorylation of Src kinase. Moreover, inhibition of Src kinase with the tyrosine kinase inhibitor PP1 attenuated VEGF-C expression. Collectively, our data provide evidence for a clinical association between COX-2 and VEGF-C expressions in cervical cancer. EP1 and EP4 receptors may be involved in the COX-2-mediated regulation of VEGF-C protein and mRNA expressions. Src may be a downstream mediator of EP1 and EP4 receptors. COX-2 inhibition may diminish LNM by suppressing VEGF-C-mediated lymphangiogenesis. 
 MicroRNA-101 inhibits cell proliferation, invasion, and promotes apoptosis by regulating cyclooxygenase-2 in Hela cervical carcinoma cells. Although aberrant miRNA expression has been documented, altered miR-101 expression in cervical cancer and its carcinogenic effects and mechanisms remain unexplored. The aim of our study was to investigate the role of miR-101 alteration in cervical carcinogenesis.Expression of miR-101 was examined by quantitative real-time reverse transcriptase PCR (qRT-PCR) in Hela cells. After modulating miR-101 expression using miR-101 mimics, cell growth, apoptosis and proliferation, and   migration   were tested separately by MTT or flow cytometry and cell wound healing assay and protein expression was detected by qRT-PCR. The expression of COX-2 in Hela cell was also examined by immunohistochemical staining and the correlation with miR-101 expression was analysed.The miR-101 demonstrated significantly low expression in Hela cell. When we transfected miR-101 mimics into Hela cells, the modulation of miR-101 expression remarkably influenced cell proliferation, cycling and apoptosis: 1) The expression of microRNA-101 tended to increase after transfection; 2) Overexpression of miR-101 was able to promote cell apoptosis, the apoptosis rate being markedly higher (97.6%) than that seen pre-transfection (12.2%) (P <0.05); 3) The miR-101 negatively regulates cell   migration   and invasion, scratch results being lower (42.7um±2um) than that observed pre-transfection (181.4 um±2 um); 4) miRNA-101 inhibits the proliferation of Hela cells as well as the level of COX-2 protein, which was negatively correlated with miR-101 expression.Overexpression of miR-101 has obvious inhibitory effects on cell proliferation  , migrati  on and invasion. Thus reduced miR-101 expression could participate in the development of cervical cancer at least partly through loss of inhibition of target gene COX-2, which probably occurs in a relative late phase of carcinogenesis. Our data suggest an important role of miR-101 in the molecular etiology of cancer and indicate potential application of miR-101 in cancer therapy. 
 Over-expression of COX-2 induces human ovarian cancer cells (CAOV-3) viability,   migration   and proliferation in association with PI3-k/Akt activation. COX-2 is involved in several pathological processes. The action of COX-2 on human ovarian cancer cell line (CAOV-3) and relative signal pathway has not been demonstrated. We explored the effects of COX-2 on the CAOV-3 viability and   migration  , moreover the proliferation of CAOV-3, and then ascertained the roles of PI3-k/Akt in these processes. The results showed that COX-2 increased the cells viability,   migration   and augment the number of CAOV-3. LY294002 could reduce COX-2 induce cells viability,   migration   and proliferation. These data indicate that COX-2 induce cells viability and   migration  , moreover promote the proliferation of CAOV-3 via PI3-k/Akt signal pathway. 
 Snail and Cox-2 expressions are associated with WHO tumor grade and survival rate of patients with gliomas. The transcriptional factor Snail and enzyme cyclo-oxygenase-2 (Cox-2) are suggested to be important effectors of invasiveness and tumorigenesis in various tumors. Tumors of higher grade have the propensity for tumor cell   migration   and invasiveness. This study was performed in order to evaluate the association between Snail and Cox-2 expressions and their values as prognostic factors in various grades of glioma, Specimens of 56 patients with glioma were used in the study. Univariate analysis showed that WHO tumor grade, and expressions of Snail and Cox-2 were significant prognostic factors affecting overall and disease progression-free survival rates. In the multivariate analysis by Cox regression model, only WHO tumor grade was shown to be a significant independent prognostic factor of overall and progression-free survival rates. In conclusion, Snail and Cox-2 expressions were associated with WHO grade in gliomas and may be used as prognostic indicators. 
 Potential involvement of the cyclooxygenase-2 pathway in the regulation of tumor-associated angiogenesis and growth in pancreatic cancer. Angiogenesis plays a crucial role in tumor development and growth. The present investigation was undertaken to test the potential involvement of the cyclooxygenase-2 (COX-2) pathway in the regulation of angiogenesis and growth in pancreatic cancer. We compared the angiogenic characteristics of a COX-2-positive human pancreatic tumor cell line, BxPC-3, with those of a COX-2-negative pancreatic tumor cell line, AsPC-1. Cultured BxPC-3 cells promoted a marked increase of endothelial cell   migration   in comparison with   migration   that occurred in the absence of cancer cells. Furthermore, BxPC-3 cell culture supernatants induced endothelial cell capillary morphogenesis in vitro and neovascularization in vivo. In contrast, cultured AsPC-1 cells elicited a modest effect on endothelial cell   migration   and neovascularization in vivo. Pretreatment of BxPC-3 cells with the selective COX-2 inhibitor NS-398 (50 micro M) dramatically decreased angiogenic responses of endothelial cells. NS-398 (25-100 micro M) caused inhibition of BxPC-3 cell proliferation but had no effect on AsPC-1 cell growth. SC-560, a selective COX-1 inhibitor, had no effect on growth of either cell lines. These results suggest an involvement of COX-2 in the control of tumor-dependent angiogenesis and growth in certain pancreatic cancers and provide the rational for inhibition of the COX pathway as an effective therapeutic approach for pancreatic tumors. 
 COX-2 dependent regulation of mechanotransduction in human breast cancer cells. The ability of living cells to exert physical forces upon their surrounding is a necessary prerequisite for diverse biological processes, such as local cellular   migration  s in wound healing to metastatic-invasion of cancer. How forces are coopted in metastasis has remained unclear, however, because the mechanical interplay between cancer cells and the various stromal components has not been experimentally accessible. Current dogma implicates inflammation in these mechanical processes. Using Fourier transform traction microscopy, we measured the force-generating capacity of human breast cancer cells occupying a spectrum of invasiveness as well as basal and inducible COX-2 expression (MCF-7<SUM-149<MDA-MB-231). Compared with non-invasive MCF-7 and moderately-invasive SUM-149, poorly-differentiated MDA-MB-231 cells showed increased cellular dispersion on collagen matrix that was accompanied by emergent distribution of contractile stresses at the interface between the adherent cell and its substrate, defined herein as the traction field. In metastatic MDA-MB-231 cells, the local tractions were precisely tuned to the surrounding matrix rigidity in a physiologic range with the concomitant expression of mechanosensitive integrin β1. These discrete responses at the single-cell resolution were correlated with PGE2 secretion and were ablated by shRNA-mediated knockdown of COX-2. Both COX-2-silenced and COX-2-expressing cells expressed EP2 and EP4 receptors, but not EP1 and EP3. Exogenous addition of PGE2 increased cell tractions and stiffened the underlying cytoskeletal network. To our knowledge this is the first report linking the expression of COX-2 with mechanotransduction of human breast cancer cells, and the regulation of COX-2-PGE2-EP signaling with physical properties of the tumor microenvironment. Drug treatments aimed at reducing this mechanical interplay may have therapeutic potential in the treatment of breast cancer. 
 The activated Notch1 signal pathway is associated with gastric cancer progression through cyclooxygenase-2. Gastric carcinoma is one of the most common cancers and lethal malignancies worldwide. Thus far, the regulatory mechanisms of its aggressiveness are still poorly understood. To understand the pathogenesis and to develop new therapeutic strategies, it is essential to dissect the molecular mechanisms that regulate progression of gastric cancer. Herein, we sought to address whether Notch1 signal pathway is involved in the control of progression in gastric cancer. We found that expression of Notch ligand Jagged1 was correlated with aggressiveness of human gastric cancer. Patients with Jagged1 expression in gastric cancer tissues had a poor survival rate compared with those without Jagged1 expression. The Notch1 receptor intracellular domain (N1IC), the activated form of Notch1 receptor, promoted the colony-forming ability and xenografted tumor growth of human stomach adenocarcinoma SC-M1 cells.   Migration   and invasion abilities of SC-M1 cells were enhanced by N1IC. Furthermore, N1IC and C promoter-binding factor 1 (CBF1) bound to cyclooxygenase-2 (COX-2) promoter and elevated COX-2 expression in SC-M1 cells through a CBF1-dependent manner. The colony-forming,   migration  , and invasion abilities enhanced by N1IC were suppressed in SC-M1 cells after treatment with the COX-2 inhibitor NS-398 or knockdown of COX-2. These cellular processes inhibited by Notch1 knockdown were restored by prostaglandin E(2) or exogenous COX-2. Taken together, these results suggest that activation of Notch1 signal pathway promotes progression of gastric cancer, at least in part through COX-2. 
 Cyclooxygenase-2 is involved in S100A2-mediated tumor suppression in squamous cell carcinoma. S100A2 is considered a putative tumor suppressor due to its loss or down-regulation in several cancer types. However, no mechanism has been described for the tumor suppressor role of S100A2. In this study, ectopic expression of S100A2 in the human malignant squamous cell carcinoma cell line KB resulted in a significant inhibition of proliferation,   migration  , and invasion. Moreover, S100A2 significantly reduced the number of colonies (>or=0.5 mm) formed in semisolid agar and decreased tumor growth and burden in nude mice. cDNA microarray analysis was used to compare mRNA expression profiles of vector- and S100A2-expressing isogenic cells. Among the genes deregulated by S100A2, the expression of cyclooxygenase-2 (COX-2) mRNA was significantly suppressed by S100A2 (2.4-fold). Western blot analysis confirmed that S100A2 reduced the expression of COX-2 protein in stably and transiently transfected KB and RPMI-2650 cells. COX-2 is frequently overexpressed in various types of cancer and plays an important role in tumor progression. Partial restoration of COX-2 expression attenuated the antitumor effect of S100A2 both in vitro and in vivo. Although the interplay between S100A2 and COX-2 remains to be clarified, these findings first showed a potent antitumor role of S100A2 in squamous cell carcinoma partly via reduced expression of COX-2. 
 Human brain endothelial cell-derived COX-2 facilitates extravasation of breast cancer cells across the blood-brain barrier. With improvements in systemic control, metastasis to the brain has been more frequently found in patients with breast cancer. In order to gain access to the brain, breast cancer cells must overcome the blood-brain barrier (BBB), a highly selective filter against cellular and soluble substances. Human brain endothelial cells (HBECs) comprise a major element of the BBB, and breast cancer cells first encounter and pass through them for extravasation. To date, however, the precise role of HBECs in metastasis to the brain is unknown. In this study, we examined how HBECs take part in the extravasation process. In an established in vitro model of the BBB, unexpectedly, the trans  migration   of breast cancer cells was markedly enhanced in the presence of HBECs than in their absence, suggesting that HBECs facilitate the trans  migration   of breast cancer cells rather than acting as a barrier against them. We then showed that cyclooxygenase (COX-2) induced from HBECs rather than that from breast cancer cells plays a key role in the trans  migration  . Moreover, expression of matrix metalloproteinase (MMP-2) mediating the trans  migration   was induced in HBECs by COX-2 after co-culture with breast cancer cells. Taken together, our results suggest that COX-2 and MMP-2 produced from HBECs facilitate the extravasation of breast cancer cells across the BBB. 
 Cyclooxygenase-2-derived prostaglandin E2 stimulates Id-1 transcription. Cyclooxygenase-2 (COX-2) and Id-1 are overexpressed in a variety of human malignancies. Recently, each of these genes was found to play a role in mediating breast cancer metastasis to the lungs, but their potential interdependence was not evaluated. Hence, the main objective of the current study was to determine whether COX-2-derived prostaglandin (PGE(2)) activated Id-1 transcription, leading in turn to increased invasiveness of mammary epithelial cells. In MDA-MB-231 cells, treatment with PGE(2) induced Id-1, an effect that was mimicked by an EP(4) agonist. PGE(2) via EP(4) activated the epidermal growth factor receptor (EGFR) --> ERK1/2 pathway, which led to increased expression of Egr-1. PGE(2) stimulated EGFR signaling by inducing the release of amphiregulin, an EGFR ligand. The ability of PGE(2) to activate Id-1 transcription was mediated by enhanced binding of Egr-1 to the Id-1 promoter. Silencing of COX-2 or pharmacological inhibition of COX-2 led to reduced PGE(2) production, decreased Id-1 expression, and reduced   migration   of cells through extracellular matrix. A similar decrease in cell   migration   was found when Id-1 was silenced. The interrelationship between COX-2, PGE(2), Id-1, and cell invasiveness was also compared in nontumorigenic SCp2 and tumorigenic SCg6 mammary epithelial cells. Consistent with the findings in MDA-MB-231 cells, COX-2-derived PGE(2) induced Id-1, leading in turn to increased cell invasiveness. Taken together, these results suggest that PGE(2) via EP(4) activated the EGFR --> ERK1/2 --> Egr-1 pathway, leading to increased Id-1 transcription and cell invasion. These findings provide new insights into the relationship between COX-2 and Id-1 and their potential role in metastasis. 
 Stable transfection of estrogen receptor-alpha suppresses expression of cyclooxygenase-2 and vascular endothelial growth factor-C in MDA-MB-231 breast cancer cells. Estrogen receptor (ER)-negative breast cancer cells are more aggressive than ER-positive cells. Elevated levels of cyclooxygenase-2 (COX-2) and vascular endothelial growth factor-C (VEGF-C) expression have been detected in cultured human breast cancer cells and are associated with negative hormone receptor status. In this study, we created ERalpha stable transfectants in MDA-MB-231 cells to explore the effect of ERalpha on cell growth and COX-2 and VEGF-C expression.The green fluorescent protein (GFP)-ERalpha plasmids were stably transfected into ER-negative MDA-MB-231 cells. The proliferation and   migration   of untransfected MDA-MB-231 cells, ERalpha-transfected MDA-MB-231 cells and ER-positive MCF-7 cells were determined. The expression of COX-2, and the levels of VEGF-C mRNA and the VEGF-C secretion concentration were assayed in these cell lines.The proliferation and   migration   capacities of ERalpha-tranfected MDA-MB-231 cells were significantly decreased (P < 0.05). The expression of COX-2 was significantly lower in ERalpha-tranfected MDA-MB-231 cells than in untranfected MDA-MB-231 cells. The mRNA and protein levels of VEGF-C were lower in ERalpha-tranfected MDA-MB-231 cells than in untransfected MDA-MB-231 cells (P < 0.05).ERalpha stable transfection inhibits proliferation and   migration   capacities of MDA-MB-231 cells and decreases expression of COX-2 and VEGF-C. The decreases of proliferation and   migration   capacities may be related to suppression of COX-2 and VEGF-C expression. 
 Integrin-mediated adhesion and soluble ligand binding stabilize COX-2 protein levels in endothelial cells by inducing expression and preventing degradation. Cyclooxygenase-2 (COX-2), a key enzyme in prostaglandin synthesis, is highly expressed during inflammation and cellular transformation and promotes tumor progression and angiogenesis. We have previously demonstrated that endothelial cell COX-2 is required for integrin alphaVbeta3-dependent activation of Rac-1 and Cdc-42 and for endothelial cell spreading,   migration  , and angiogenesis (Dormond, O., Foletti, A., Paroz, C., and Ruegg, C. (2001) Nat. Med. 7, 1041-1047; Dormond, O., Bezzi, M., Mariotti, A., and Ruegg, C. (2002) J. Biol. Chem. 277, 45838-45846). In this study, we addressed the question of whether integrin-mediated cell adhesion may regulate COX-2 expression in endothelial cells. We report that cell detachment from the substrate caused rapid degradation of COX-2 protein in human umbilical vein endothelial cells (HUVEC) independent of serum stimulation. This effect was prevented by broad inhibition of cellular proteinases and by neutralizing lysosomal activity but not by inhibiting the proteasome. HUVEC adhesion to laminin, collagen I, fibronectin, or vitronectin induced rapid COX-2 protein expression with peak levels reached within 2 h and increased COX-2-dependent prostaglandin E2 production. In contrast, nonspecific adhesion to poly-L-lysine was ineffective in inducing COX-2 expression. Furthermore, the addition of matrix proteins in solution promoted COX-2 protein expression in suspended or poly-L-lysine-attached HUVEC. Adhesion-induced COX-2 expression was strongly suppressed by pharmacological inhibition of c-Src, phosphatidylinositol 3-kinase, p38, extracellular-regulated kinase 1/2, and, to a lesser extent, protein kinase C and by the inhibition of mRNA or protein synthesis. In conclusion, this work demonstrates that integrin-mediated cell adhesion and soluble integrin ligands contribute to maintaining COX-2 steady-state levels in endothelial cells by the combined prevention of lysosomal-dependent degradation and the stimulation of mRNA synthesis involving multiple signaling pathways. 
 Endothelin-1 enhances cell   migration   through COX-2 up-regulation in human chondrosarcoma. Chondrosarcoma is a type of highly malignant tumor with a potent capacity of local invasion and distant metastasis. The effect of endothelin-1 (ET-1) on   migration   activity in human chondrosarcoma cells is not clearly understood. Here, we found that ET-1 increased the   migration   and expression of cyclooxygenase (COX)-2 in human chondrosarcoma cells.ET-1-mediated COX-2 expression was assessed by qPCR and Western blot analysis. The mechanisms of action of ET-1 in different signaling pathways were studied using Western blotting. Knockdown of proteins was achieved by transfection with siRNA. Chromatin immunoprecipitation assays were used to study in vivo binding of c-Jun to the COX-2 promoter.Human chondrosarcoma tissues had significant expression levels of ET-1 and COX-2, which were higher than that in normal cartilage. Exogenous ET-1 increased cell   migration   and the expression of COX-2. In addition, COX-2 protein levels and cell   migration   ability were abolished by ET receptor antagonists. Activation of the mitogen-activated protein kinase (MAPK) and activator protein-1 (AP-1) pathways after ET-1 treatment was demonstrated, and ET-1-induced COX-2 expression and cell   migration   activity were inhibited by the specific inhibitor and mutant of MAPK and AP-1 cascades. ET-1 increased the binding of c-Jun to the AP-1 element on the COX-2 promoter. Furthermore, knockdown of ET-1 decreased cell metastasis in vitro and in vivo.Our results indicated that ET-1 enhances the cell   migration   of chondrosarcoma by increasing COX-2 expression through the ET receptors, MAPK, and AP-1 signal transduction pathway.We link high ET-1 and COX-2 expression to chondrosarcoma. 
 Regulation of COX-2 expression and epithelial-to-mesenchymal transition by hypoxia-inducible factor-1α is associated with poor prognosis in hepatocellular carcinoma patients post TACE surgery. Currently, it is not entirely clear whether hypoxia-inducible factor-1α (HIF-1α) is involved in the regulation of COX-2 expression and epithelial-to-mesenchymal transition (EMT), and whether these events affect the prognosis of hepatocellular carcinoma (HCC) patients treated with transcatheter arterial chemoembolization (TACE). In this report the relationship between HIF-1α and COX-2 protein expression, EMT in tumor specimens from HCC patients after TACE surgery and the clinical significance of HIF-1α and COX-2 expression were analyzed using statistical approaches. HepG2 cells treated with CoCl2 was employed as a hypoxia cell model in vitro to study hypoxia-induced HIF-1α, COX-2 expression, and EMT alteration. The results showed that HIF-1α and COX-2 protein expression increased in HCC tissues after TACE surgery. Moreover, there was positive correlation between upregulation of HIF-1α and COX-2. Elevated expression of HIF-1α increased both Snail and Vimentin protein expression, while it reduced E-cadherin protein expression. It was further verified that hypoxia enhanced protein expression of HIF-1α and COX-2 in HepG2 cells treated with CoCl2. Upregulation of HIF-1α and COX-2, together with EMT alteration resulted in   increase  d migration and invasion of HepG2 cells under hypoxia. In conclusion, TACE surgery results in aggravated hypoxia status, leading to increased HIF-1α protein expression in HCC tissue. To adapt to hypoxic environment, HIF-1α stimulates COX-2 protein expression and promotes EMT process in hepatocellular cancer cells, which enhances HCC invasion and metastasis, and might contribute to poor prognosis in HCC patients post TACE treatment. 
 Interleukin-17 and prostaglandin E2 are involved in formation of an M2 macrophage-dominant microenvironment in lung cancer. Tumor-associated macrophages (TAMs) are divided into M1 and M2 macrophages. M1 macrophages inhibit tumor growth, whereas M2 macrophages promote tumor growth and metastasis. The aim of this study was to examine the possible causes leading to the formation of an M2-macrophage-dominant tumor microenvironment in non-small-cell lung cancer.Forty-eight archived lung tumor samples were examined for the expression of interleukin-17 (IL-17) receptors, IL-17 receptor A (IL-17RA) and IL-17 receptor C (IL-17RC), and the number of TAMs using immunohistochemical staining. Twenty fresh lung tumors and matched normal lung tissues were examined for expression of IL-17, cyclooxygenase-2, and prostaglandin E2 (PGE2), using enzyme-linked immunosorbent assay and Western blot analysis. Macrophage-  migration   assays were performed using fresh lung tumor tissues and IL-17 as chemoattractants. Induction of M2-macrophage differentiation was analyzed using real-time quantitative polymerase chain reaction.TAMs expressed IL-17RA and IL-17RC. Lung tumors expressed higher levels of IL-17, cyclooxygenase-2, and PGE2, compared with normal lung tissues. Lung tumor tissues attracted   migration   of mouse RAW264.7 macrophages and primary peritoneal macrophages through IL-17, which was mediated by IL-17RA and IL-17RC. IL-17 did not induce either M1- or M2-macrophage differentiation. However, human lung cancer A549 cells strongly induced M2-macrophage differentiation of RAW264.7 macrophages when the two cell lines were cocultured. The inductive factor secreted by A549 cells was identified to be PGE2.IL-17 recruits macrophages, and PGE2 induces M2-macrophage differentiation, hence the increased levels of IL-17 and PGE2 in lung cancer contribute to the formation of an M2-macrophage-dominant tumor microenvironment. 
 Biosynthesis of hemiketal eicosanoids by cross-over of the 5-lipoxygenase and cyclooxygenase-2 pathways. The prostaglandin and leukotriene families of lipid mediators are formed via two distinct biosynthetic pathways that are initiated by the oxygenation of arachidonic acid by either cyclooxygenase-2 (COX-2) or 5-lipoxygenase (5-LOX), respectively. The 5-LOX product 5S-hydroxyeicosatetraenoic acid, however, can also serve as an efficient substrate for COX-2, forming a bicyclic diendoperoxide with structural similarities to the arachidonic acid-derived prostaglandin endoperoxide PGH(2) [Schneider C, et al. (2006) J Am Chem Soc 128:720-721]. Here we identify two cyclic hemiketal (HK) eicosanoids, HKD(2) and HKE(2), as the major nonenzymatic rearrangement products of the diendoperoxide using liquid chromatography-mass spectrometry analyses as well as UV and NMR spectroscopy. HKD(2) and HKE(2) are furoketals formed by spontaneous cyclization of their respective 8,9-dioxo-5S,11R,12S,15S-tetrahydroxy- or 11,12-dioxo-5S,8S,9S,15S-tetrahydroxy-eicosadi-6E,13E-enoic acid precursors, resulting from opening of the 9S,11R- and 8S,12S-peroxide rings of the diendoperoxide. Furthermore, the diendoperoxide is an efficient substrate for the hematopoietic type of prostaglandin D synthase resulting in formation of HKD(2), equivalent to the enzymatic transformation of PGH(2) to PGD(2). HKD(2) and HKE(2) were formed in human blood leukocytes activated with bacterial lipopolysaccharide and calcium ionophore A23187, and biosynthesis was blocked by inhibitors of 5-LOX or COX-2. HKD(2) and HKE(2) stimulated   migration   and tubulogenesis of microvascular endothelial cells, implicating a proangiogenic role of the hemiketals in inflammatory sites that involve expression of 5-LOX and COX-2. Identification of the highly oxygenated hemiketal eicosanoids provides evidence for a previously unrecognized biosynthetic cross-over of the 5-LOX and COX-2 pathways. 
 Cyclooxygenase-2 induced by zymosan in human monocyte-derived dendritic cells shows high stability, and its expression is enhanced by atorvastatin. Cyclooxygenase (COX)-2 is a central enzyme of arachidonic acid metabolism, and its modulation by statins may explain some of the myocardial protective effects of these drugs. Dendritic cells (DCs) play a central role in microbial defense and in atherogenesis, and COX-2 expression in DCs is important for their   migration   to lymph nodes and antibody response, thus explaining why prostaglandin E(2) is a main component of the cocktails used to prepare DCs for clinical applications. On this basis, we addressed the effect of atorvastatin (ATV) on the release of arachidonic acid and on the expression of COX-2 in human monocyte-derived DCs. Although ATV on its own lacked any effect on COX-2 protein induction expression, it enhanced the release of arachidonic acid, the expression of COX-2 protein, and the production of prostaglandin E(2) induced by the fungal wall extract zymosan, and to a lower extent the effect of peptidoglycan. The effect on COX-2 protein was observed mainly 24 h after stimulation by zymosan and was not reverted by mevalonate, thus pointing to an effect unrelated to cholesterol metabolism. It is noteworthy that COX-2 protein showed a great stability, with a t((1/2)) of approximately 12 h, which was enhanced in the presence of ATV. In view of the important role played by COX-2 on DC function, these data indicate that ATV, by enhancing COX-2 stability, may increase DC function after infectious bouts and also counteract some of the risks associated with sustained inhibition of COX-2. 
 NFATc3 regulates the transcription of genes involved in T-cell activation and angiogenesis. The nuclear factor of activated T cells (NFAT) family of transcription factors plays important roles in many biologic processes, including the development and function of the immune and vascular systems. Cells usually express more than one NFAT member, raising the question of whether NFATs play overlapping roles or if each member has selective functions. Using mRNA knock-down, we show that NFATc3 is specifically required for IL2 and cyclooxygenase-2 (COX2) gene expression in transformed and primary T cells and for T-cell proliferation. We also show that NFATc3 regulates COX2 in endothelial cells, where it is required for COX2, dependent   migration   and angiogenesis in vivo. These results indicate that individual NFAT members mediate specific functions through the differential regulation of the transcription of target genes. These effects, observed on short-term suppression by mRNA knock-down, are likely to have been masked by compensatory effects in gene-knockout studies. 
 Cyclooxygenase-2 up-regulates CCR7 via EP2/EP4 receptor signaling pathways to enhance lymphatic invasion of breast cancer cells. Recent studies demonstrate that cyclooxygenase-2 (COX-2) expression is frequently associated with lymph node metastasis. However, the mechanism by which COX-2 increases the invasion of cancer cells to lymph node is unclear. CCR7 is a chemokine receptor that plays important roles in the mediation of   migration   of leukocytes and dendritic cells toward lymphatic endothelial cells (LECs) that express receptor ligand CCL21. We found that treatment of prostaglandin E(2) or ectopic expression of COX-2 in MCF-7 cells up-regulated CCR7 expression. On the contrary, knockdown of COX-2 by small hairpin RNA reduced CCR7 in COX-2-overexpressing MDA-MB-231 cells. Interaction of CCR7 and CCL21 was important for the   migration   of breast cancer cells toward LECs because antibodies against these two molecules inhibited the   migration  . We also found that COX-2 increased CCR7 expression via the EP2 and EP4 receptor in breast cancer cells. EP2 and EP4 agonists stimulated CCR7 in MCF-7 cells, whereas antagonists or small hairpin RNA of EP2 and EP4 attenuated CCR7 in MDA-MB-231 cells. Protein kinase A and AKT kinase were involved in COX-2-induced CCR7. Pathological analysis demonstrated that COX-2 overexpression was associated with CCR7, EP2, and EP4 expressions in breast tumor tissues. In addition, CCR7 expression in COX-2-overexpressing tumors was significantly correlated with lymph node metastasis. Collectively, we suggest that CCR7 is a down-stream target for COX-2 to enhance the   migration   of breast cancer cells toward LECs and to promote lymphatic invasion. 
 Cox2 and β-catenin/T-cell factor signaling intestinalize human esophageal keratinocytes when cultured under organotypic conditions. The incidence of esophageal adenocarcinoma (EAC) is rising in the United States. An important risk factor for EAC is the presence of Barrett esophagus (BE). BE is the replacement of normal squamous esophageal epithelium with a specialized columnar epithelium in response to chronic acid and bile reflux. However, the emergence of BE from squamous keratinocytes has not yet been demonstrated. Our research has focused on this. Wnt and cyclooxygenase 2 (Cox2) are two pathways whose activation has been associated with BE and progression to EAC, but their role has not been tested experimentally. To explore their contribution, we engineered a human esophageal keratinocyte cell line to express either a dominant-active Wnt effector CatCLef or a Cox2 complementary DNA. In a two-dimensional culture environment, Cox2 expression increases cell proliferation and   migratio  n, but neither transgene induces known BE markers. In contrast, when these cells were placed into three-dimensional organotypic culture conditions, we observed more profound effects. CatCLef-expressing cells were more proliferative, developed a thicker epithelium, and upregulated Notch signaling and several BE markers including NHE2. Cox2 expression also increased cell proliferation and induced a thicker epithelium. More importantly, we observed cysts form within the epithelium, filled with intestinal mucins including Muc5B and Muc17. This suggests that Cox2 expression in a three-dimensional culture environment induces a lineage of mucin-secreting cells and supports an important causal role for Cox2 in BE pathogenesis. We conclude that in vitro modeling of BE pathogenesis can be improved by enhancing Wnt signaling and Cox2 activity and using three-dimensional organotypic culture conditions. 
 Epstein-Barr virus protein can upregulate cyclo-oxygenase-2 expression through association with the suppressor of metastasis Nm23-H1. Previous studies have demonstrated the interaction between the Epstein-Barr virus (EBV) nuclear antigen 3C (EBNA3C) and the metastatic suppressor Nm23-H1 both in vitro and in vivo (C. Subramanian, M. A. Cotter II, and E. S. Robertson, Nat. Med. 7:350-355, 2001). EBNA3C can reverse the ability of Nm23-H1 to suppress   migration   of Burkitt's lymphoma and breast carcinoma cell lines in vitro. EBNA3C contributes to EBV-associated human cancers by regulating transcription of a number of cellular and viral promoters and by targeting and altering the transcription activities of the metastasis suppressor Nm23-H1. Cyclo-oxygenase-2 (COX-2), an inducible enzyme important in inflammation, is overexpressed in a variety of cancers and can influence cell   migration  . In this report we show that Nm23-H1 and EBNA3C can modulate expression of COX-2 in the context of EBV infection and transformation. The levels of COX-2 were consistently higher in EBV-positive cells than in EBV-negative cells. Additionally, we show that Nm23-H1 can upregulate the COX-2 promoter element in luciferase reporter assays, whereas EBNA3C alone did not affect the level of response but clearly contributed to an additive increase when coexpressed with Nm23-H1. The downstream effect of COX-2 expression was also evaluated and showed that prostaglandin E(2) levels increased with Nm23-H1 and that there was some level of cooperativity in the presence of EBNA3C. The majority of this response was mediated through the cyclic AMP response element and NF-kappaB sites. These studies suggest a potential role for COX-2 in EBV-associated human cancers. 
 Endothelial cell confluence regulates cyclooxygenase-2 and prostaglandin E2 production that modulate motility. Endothelial cells line the vasculature and, after mechanical denudation during invasive procedures or cellular loss from natural causes, migrate to reestablish a confluent monolayer. We find confluent monolayers of human umbilical vein endothelial cells were quiescent and expressed low levels of cyclooxygenase-2, but expressed cyclooxygenase-2 at levels comparable with cytokine-stimulated cells when present in a subconfluent culture. Mechanically wounding endothelial cell monolayers stimulated rapid cyclooxygenase-2 expression that increased with the level of wounding. Cyclooxygenase-2 re-expression occurred throughout the culture, suggesting signaling from cells proximal to the wound to distal cells. Media from wounded monolayers stimulated cyclooxygenase-2 expression in confluent monolayers, which correlated with the level of wounding of the donor monolayer. Wounded monolayers and cells in subconfluent cultures secreted enhanced levels of prostaglandin (PG) E(2) that depended on cyclooxygenase-2 activity, and PGE(2) stimulated cyclooxygenase-2 expression in confluent endothelial cell monolayers. Cells from subconfluent monolayers migrated through filters more readily than those from confluent monolayers, and the cyclooxygenase-2-selective inhibitor NS-398 suppressed   migration  . Adding PGE(2) to NS-398-treated cells augmented   migration  . Endothelial cells also migrated into mechanically denuded areas of confluent monolayers, and this too was suppressed by NS-398. We conclude that endothelial cells not in contact with neighboring cells express cyclooxygenase-2 that results in enhanced release of PGE(2), and that this autocrine and paracrine loop enhances endothelial cell   migration   to cover denuded areas of the endothelium. 
 Expression of miR-143 reduces growth and   migration   of human bladder carcinoma cells by targeting cyclooxygenase-2. Systemic chemotherapy is the only current modality that provides the potential for long-term survival in bladder carcinoma patients with metastatic disease. Overexpression of cyclooxygenase-2 COX-2 induces expression of immune- and cell proliferation-related genes and is associated with the grade, prognosis and recurrence of transitional cell carcinoma of the bladder. There is abundant evidence that aberrant expression of microRNAs (miRNAs) is implicated in numerous disease states and miRNAs have the potential to be used for cancer therapeutics. Here, we found expression of miR-143 to be low in a series of human bladder carcinomas as compared to background tissue. In addition, restoration of miR-143 by cell transfection in T24 cancer cells led to decreased COX-2 expression, reduced proliferation and mobility. Our findings will help to further understand the functions of miRNAs in cancer cells and point to a specific potential of miR-143 may be employed as a therapeutic agent for bladder carcinoma. The results provide insights into the development of novel tumor markers or new therapeutic strategies. 
 The MIF homologue D-dopachrome tautomerase promotes COX-2 expression through β-catenin-dependent and -independent mechanisms. The cytokine/growth factor, macrophage   migratio  n inhibitory factor (MIF), contributes to pathologies associated with immune, inflammatory, and neoplastic disease processes. Several studies have shown an important contributing role for MIF-dependent COX-2 expression in the progression of these disorders. We now report that the MIF homologue, D-dopachrome tautomerase (D-DT), is both sufficient and necessary for maximal COX-2 expression in colorectal adenocarcinoma cell lines. D-DT-dependent COX-2 transcription is mediated in part by β-catenin protein stabilization and subsequent transcription. Also contributing to D-DTs regulation of COX-2 expression are the activities of both c-jun-N-terminal kinase and the MIF-interacting protein, Jab1/CSN5. Interestingly, D-DT-dependent β-catenin stabilization is regulated by COX-2 expression, suggesting the existence of an amplification loop between COX-2- and β-catenin-mediated transcription in these cells. Because both COX-2- and β-catenin-mediated transcription are important contributors to colorectal cancer (CRC) disease maintenance and progression, these findings suggest a unique and novel regulatory role for MIF family members in CRC pathogenesis. 
 Up-regulation of cyclooxygenase-2 expression and prostaglandin E2 production in human endometriotic cells by macrophage   migration   inhibitory factor: involvement of novel kinase signaling pathways. Cyclooxygenase (COX) is the rate-limiting enzyme in the metabolic conversion of arachidonic acid to prostaglandins (PGs), including prostaglandin E(2) (PGE(2)), a major mediator of inflammation and angiogenesis. Herein, we report that macrophage   migration   inhibitory factor (MIF), a potent proinflammatory and growth-promoting factor found at elevated concentrations in the peritoneal fluid of women with endometriosis and active endometriosis lesions, acts directly on ectopic endometrial cells to stimulate the synthesis of COX-2, the inducible form of COX, and the release of PGE(2). MIF treatment strongly activated p38 and ERK MAPK, and specific inhibitors of both pathways completely blocked basal and MIF-induced PGE(2) synthesis. Whereas p38 inhibitors negatively affected the stimulated synthesis of COX-2 and that of PGE(2), ERK inhibitors only decreased the production of PGE(2). These findings show for the first time a direct role for MIF in the up-regulation of COX-2 synthesis and PGE(2) secretion in ectopic endometrial cells. They further indicate that whereas p38 and ERK MAPK signaling pathways both play a significant role in the regulation of basal and MIF-induced synthesis of PGE(2) by ectopic endometrial cells, only p38 kinase is involved in the regulation of COX-2 expression in these cells. This suggests that MIF acts at more than one level to stimulate the synthesis of PGE(2) and triggers the coordinate activation of multiple enzymes in the biosynthesis pathway. Our data provide evidence for a novel mechanism by which MIF can induce a proinflammatory phenotype in ectopic endometrial cells, and favor the establishment of endometriosis and its related clinical symptoms. 
 Cyclooxygenase-2 expression during immortalization and breast cancer progression. Identification of molecular aberrations in premalignant human mammary epithelial cells (hMEC), the precursors for breast cancers, is a central goal in breast cancer biology. Recent studies implicated expression of cyclooxygenase 2 (COX-2) as a marker to identify precursor cells for breast cancer. In this study, we analyzed COX-2 expression in preselection and postselection hMEC cells and observed similar COX-2 levels in both cells. Interestingly, immortalization of postselection cells using various methods leads to a dramatic decrease in COX-2 expression. Similar to immortal cells, the majority of breast cancer cell lines expressed low levels of COX-2 protein. Finally, analyses of COX-2 expression in a series of specimens from reduction mammoplasty, adenosis, ductal carcinoma in situ, and infiltrating ductal carcinoma showed down-regulation of COX-2 expression during tumor progression. Importantly, down-regulation of COX-2 using small interfering RNA in cells showed no effect on cell proliferation, anchorage-independent growth,   migration  , or invasion. These results show that (a) COX-2 overexpression does not seem to predict a breast cancer precursor cell and does not provide advantage for the cell to be transformed; (b) inhibition of COX-2 does not affect hMEC growth and oncogenic behavior in the conditions analyzed; and (c) COX-2 expression is decreased in breast cancer cell lines and cancer specimens as compared with normal mammary epithelial cells. 
 Glutathione Peroxidase 2 Inhibits Cyclooxygenase-2-Mediated   Migration   and Invasion of HT-29 Adenocarcinoma Cells but Supports Their Growth as Tumors in Nude Mice. The selenoprotein gastrointestinal glutathione peroxidase 2 (GPx2) is up-regulated in a variety of cancer cells with thus far unknown consequences. Therefore, two clones of a human colon cancer cell line (HT-29) in which GPx2 was stably knocked down by small interfering RNA (siRNA; siGPx2) were used to test whether cancer-relevant processes are affected by GPx2. The capacity to grow anchorage independently in soft agar was significantly reduced in siGPx2 cells when compared with controls (i.e., HT-29 cells stably transfected with a scramble siRNA). The weight of tumors derived from siGPx2 cells injected into nude mice was lower in 9 of 10 animals. In contrast, in a wound-healing assay, wound closure was around 50% in controls and 80% in siGPx2 cells, indicating an enhanced capacity of the knockdown cells to migrate. Similarly, invasion of siGPx2 cells in a Transwell assay was significantly increased.   Migration   and invasion of siGPx2 cells were inhibited by celecoxib, a cyclooxygenase-2 (COX-2)-specific inhibitor, but not by alpha-tocopherol. Selenium supplementation of cell culture medium did not influence the results obtained with siGPx2 cells, showing that none of the other selenoproteins could replace GPx2 regarding the described effects. The data show that GPx2 inhibits malignant characteristics of tumor cells, such as   migration   and invasion, obviously by counteracting COX-2 expression but is required for the growth of transformed intestinal cells and may, therefore, facilitate tumor cell growth. The data also shed new light on the use of selenium as a chemopreventive trace element: a beneficial effect may depend on the stage of tumor development. 
 Cyclooxygenase regulates angiogenesis induced by colon cancer cells. To explore the role of cyclooxygenase (COX) in endothelial cell   migration   and angiogenesis, we have used two in vitro model systems involving coculture of endothelial cells with colon carcinoma cells. COX-2-overexpressing cells produce prostaglandins, proangiogenic factors, and stimulate both endothelial   migration   and tube formation, while control cells have little activity. The effect is inhibited by antibodies to combinations of angiogenic factors, by NS-398 (a selective COX-2 inhibitor), and by aspirin. NS-398 does not inhibit production of angiogenic factors or angiogenesis induced by COX-2-negative cells. Treatment of endothelial cells with aspirin or a COX-1 antisense oligonucleotide inhibits COX-1 activity/expression and suppresses tube formation. Cyclooxygenase regulates colon carcinoma-induced angiogenesis by two mechanisms: COX-2 can modulate production of angiogenic factors by colon cancer cells, while COX-1 regulates angiogenesis in endothelial cells. 
 Cyclooxygenase-2 is involved in the up-regulation of matrix metalloproteinase-9 in cholangiocarcinoma induced by tumor necrosis factor-alpha. Matrix metalloproteinase-9 (MMP-9) is an important enzyme in tumor invasion and metastasis in malignant tumors, including cholangiocarcinoma (CC). Tumor necrosis factor-alpha (TNF-alpha), a proinflammatory cytokine, was recently reported to induce the up-regulation of MMP-9 in cultured CC cells. We examined whether cyclooxygenase-2 (COX-2) and prostaglandin-E2 (PGE2), another endogenous tumor promoter, are involved in the up-regulation of MMP-9 in CC using CC tissue specimens and a CC cell line, HuCCT-1. MMP-9 and COX-2 were immunohistochemically expressed in 58% and 89% of 110 CC cases, respectively; the expression of MMP-9 and COX-2 was correlated (r = 0.32, P = 0.00072). Using zymography, latent MMP-9 was detectable in all cases and active MMP-9 was detected in 24% of cases of the CC specimens. The TNF-alpha/TNF-receptor 1 (TNF-R1) interaction induced MMP-9 production and activation, as well as COX-2 overexpression and PGE2 production, and increased the   migration   of CC cells. MMP-9 up-regulation was inhibited by COX inhibitors, antagonists of EP2/4 (receptors of PGE2), and COX-1 and COX-2 siRNAs. Inhibitors of both MMP-9 and MMP-9 siRNA treatment abrogated the increase in the   migration   of CC cells induced by TNF-alpha. In conclusion, we propose a novel signaling pathway of MMP-9 up-regulation in CC cells such that TNF-alpha induces the activation of COX-2 and PGE2 via TNF-R1 followed by the up-regulation of MMP-9 via the PGE2 (EP2/4) receptor. 
 Dual inhibition of COX-2/5-LOX blocks colon cancer proliferation,   migration   and invasion in vitro. Inflammation is emerging as a new hallmark of cancer. Arachidonic acid (AA) metabolism, the family of cyclooxygenases (COXs) and lipoxygenase (LOX) play important roles in AA-related inflammatory cascades. In 94 colorectal cancer samples collected from the Han population, the immunohistochemical results indicated that 68% of the patients with colorectal cancer had a co-expression of both COX-2 and 5-LOX, while both displayed low expression in the matched normal tissues. In cell lines, three colorectal cancer cell lines exhibited high expression of COX-2 and 5-LOX. During stable silencing of the expression of COX-2 or 5-LOX in LoVo cancer cells, we found that downregulation of either COX-2 or 5-LOX significantly diminished the growth,   migration   and invasion of the colon cancer cells and specifically, downregulation of COX-2 could elicit upregulation of 5-LOX protein and vice versa. The above results suggested that the simultaneous blocking of COX-2 and 5-LOX activity may bring more potential benefits in managing the progression of colon cancer. Therefore, we sought to explore the effectiveness of a dual COX-2/5-LOX inhibitor darbufelone on the proliferation,   migration  , invasion and apoptosis of colon cancer cells, as well as the underlying mechanism of action. The results indicated that darbufelone significantly decreased the proliferative and invasive abilities of the colon cancer cells, in a dose-dependent manner. During the study of the related mechanisms, we found an upregulation of p27 and downregulation of cyclin D1 as well as CDK4 after darbufelone treatment, which indicated that darbufelone could arrest the cell cycle of LoVo cells at the G0/G1 phase. Furthermore, the activation of caspase-3 and -9, upregulation of Bax and downregulation of Bcl-2 demonstrated the occurrence of apoptosis by darbufelone. Finally, darbufelone also prevented the   migration   and invasion of LoVo cells, which may be ascribed to the upregulation of E-cadherin and ZO-1. In summary, our data suggest that the inhibition of both COX-2/5-LOX may be an effective therapeutic approach for colon cancer management, particularly for those patients with high expression of COX-2/5-LOX. 
 Notch2-induced COX-2 expression enhancing gastric cancer progression. Gastric carcinoma is one of the most common and mortal types of malignancy worldwide. To date, the mechanisms controlling its aggressiveness are not yet fully understood. Notch signal pathway can function as either an oncogene or a tumor suppressor in tumorigenesis. Four members (Notch1-4) of Notch receptors were found in mammals and each exhibits distinct roles in tumor progression. Previous study showed that the activated Notch1 receptor promoted gastric cancer progression through cyclooxygenase-2 (COX-2). This study addressed whether Notch2 signal pathway is also involved in gastric cancer progression. Constitutive expression of Notch2 intracellular domain (N2IC), the activated form of Notch2 receptor, promoted both cell proliferation and xenografted tumor growth of human stomach adenocarcinoma SC-M1 cells. The colony formation,   migration  , invasion, and wound-healing abilities of SC-M1 cells were enhanced by N2IC expression, whereas these abilities were suppressed by Notch2 knockdown. Similarly, Notch2 knockdown inhibited cancer progressions of AGS and AZ521 gastric cancer cells. Expression of N2IC also caused epithelial-mesenchymal transition in SC-M1 cells. Furthermore, N2IC bound to COX-2 promoter and induced COX-2 expression through a CBF1-dependent manner in SC-M1 cells. The ability of N2IC to enhance tumor progression in SC-M1 cells was suppressed by knockdown of COX-2 or treatment with NS-398, a COX-2 inhibitor. Moreover, the suppression of tumor progression by Notch2 knockdown in SC-M1 cells was reversed by exogenous COX-2 or its major enzymatic product PGE(2) . Taken together, this study is the first to demonstrate that the Notch2-COX-2 signaling axis plays an important role in controlling gastric cancer progression. 
 Frequent co-localization of Cox-2 and laminin-5 gamma2 chain at the invasive front of early-stage lung adenocarcinomas. Laminin-5 is an extracellular matrix protein that plays a key role in cell   migration   and tumor invasion. Cox-2 is an induced isoform of cyclooxygenases that plays an important role in carcinogenesis, suppression of apoptosis, angiogenesis, and metastasis of colon cancer. We report frequent co-expression of cox-2 and laminin-5 at the invasive front of early-stage lung adenocarcinomas. We investigated the expression of cox-2 and laminin-5 immunohistochemically in 102 cases of small-sized lung adenocarcinoma (maximum dimension, 2 cm or less). Cox-2 and laminin-5 were expressed in 97 (95.1%) and 82 (80.4%) cases, respectively. Both were preferentially localized in cancer cells at the cancer-stroma interface, although cox-2 tended to show a diffuse staining pattern in some cases. A comparison of their staining patterns revealed a striking similarity in their distribution in 24 cases, and a partial overlap between their localization in another 20 cases. Moreover, an overall correlation was found between the expression levels of cox-2 and laminin-5 (P = 0.018). To gain insight into the mechanisms that regulate the expression of these proteins, we additionally studied their expression in 58 cases of stage I lung adenocarcinoma, in which p53 status was determined by immunohistochemistry, polymerase chain reaction-single strand conformation polymorphism analysis, and direct sequencing. The results showed that tumors with mutant p53 tended to express more cox-2 than those with wild-type p53 (P = 0.080). Also, tumors that overexpressed p53 had higher levels of cox-2 and laminin-5 than those without p53 overexpression (P = 0.032 and 0.047, respectively). Further immunohistochemical analysis showed that tumors that overexpressed both epidermal growth factor receptor (EGFR) and erbB-2 had higher levels of cox-2 and laminin-5 than those without concomitant overexpression of these proteins (P = 0.014 and P = 0.018, respectively). To see whether EGFR signaling is involved in cox-2 and laminin-5 expression, we further conducted in vitro analyses using six lung adenocarcinoma cell lines (A549, HLC-1, ABC-1, LC-2/ad, VMRC-LCD, and L27). Western blot analyses showed that cox-2 mRNA levels, and to a lesser extent laminin-5 gamma2 mRNA levels, correlated with the expression levels of erbB-2 and the phosphorylated form of MAPK/ERK-1/2 protein. The addition of transforming growth factor-alpha increased both cox-2 and laminin-5 gamma2 mRNA levels in A549, ABC-1, and L27 with different kinetics; the induction of cox-2 occurred earlier than that of laminin-5 gamma2. Finally, the   migration   of ABC-1 cells was inhibited by MAP kinase kinase inhibitor PD98059 and a selective cox-2 inhibitor NS-398. In contrast, the   migration   of A549 cells was inhibited by PD98059, but much less effectively by NS-398. These results suggest that co-stimulatory mechanisms may exist that increase the expression of cox-2 and laminin-5 at the invasive front of lung adenocarcinomas and that EGFR signaling could be one of the mechanisms. Further investigations are warranted concerning the role of cox-2 and laminin-5 in cancer cell invasion and the significance of p53 and EGFR signaling in the regulation of cox-2 and laminin-5 expression. 
 Anti-angiogenic and anti-inflammatory properties of kahweol, a coffee diterpene. Epidemiological studies have shown that unfiltered coffee consumption is associated with a low incidence of cancer. This study aims to identify the effects of kahweol, an antioxidant diterpene contained in unfiltered coffee, on angiogenesis and key inflammatory molecules.The experimental procedures included in vivo angiogenesis assays (both the chicken and quail choriallantoic membrane assay and the angiogenesis assay with fluorescent zebrafish), the ex vivo mouse aortic ring assay and the in vitro analysis of the effects of treatment of human endothelial cells with kahweol in cell growth, cell viability, cell   migration   and zymographic assays, as well as the tube formation assay on Matrigel. Additionally, two inflammation markers were determined, namely, the expression levels of cyclooxygenase 2 and the levels of secreted monocyte chemoattractant protein-1. We show for the first time that kahweol is an anti-angiogenic compound with inhibitory effects in two in vivo and one ex vivo angiogenesis models, with effects on specific steps of the angiogenic process: endothelial cell proliferation,   migration  , invasion and tube formation on Matrigel. We also demonstrate the inhibitory effect of kahweol on the endothelial cell potential to remodel extracellular matrix by targeting two key molecules involved in the process, MMP-2 and uPA. Finally, the anti-inflammatory potential of this compound is demonstrated by its inhibition of both COX-2 expression and MCP-1 secretion in endothelial cells.Taken together, our data indicate that, indeed, kahweol behaves as an anti-inflammatory and anti-angiogenic compound with potential use in antitumoral therapies. These data may contribute to the explanation of the reported antitumoral effects of kahweol, including the recent epidemiological meta-analysis showing that drinking coffee could decrease the risk of certain cancers. 
 Discoidin domain receptor 1 receptor tyrosine kinase induces cyclooxygenase-2 and promotes chemoresistance through nuclear factor-kappaB pathway activation. Discoidin domain receptor 1 (DDR1) is a receptor tyrosine kinase activated by various types of collagens and is known to play a role in cell attachment,   migration  , survival, and proliferation. However, little is known about the molecular mechanism(s) underlying the role of DDR1 in cancer. We report here that DDR1 induces cyclooxygenase-2 (Cox-2) expression resulting in enhanced chemoresistance. Depletion of DDR1-mediated Cox-2 induction using short hairpin RNA (shRNA) results in increased chemosensitivity. We also show that DDR1 activates the nuclear factor-kappaB (NF-kappaB) pathway and blocking this activation by an I kappaB superrepressor mutant results in the ablation of DDR1-induced Cox-2, leading to enhanced chemosensitivity, indicating that DDR1-mediated Cox-2 induction is NF-kappaB dependent. We identify the upstream activating kinases of the NF-kappaB pathway, IKK beta and IKK gamma, as essential for DDR1-mediated NF-kappaB activation, whereas IKK alpha seems to be dispensable. Finally, shRNA-mediated inhibition of DDR1 expression significantly enhanced chemosensitivity to genotoxic drugs in breast cancer cells. Thus, DDR1 signaling provides a novel target for therapeutic intervention with the prosurvival/antiapoptotic machinery of tumor cells. 
 VEGF gene expression in adult human thymus fat: a correlative study with hypoxic induced factor and cyclooxygenase-2. It is well known that the adult human thymus degenerates into fat tissue; however, it has never been considered as a potential source of angiogenic factors. Recently, we have described that this fat (TAT) produces angiogenic factors and induces human endothelial cell proliferation and   migration  , indicating its potential angiogenic properties.Adult thymus fat and subcutaneous adipose tissue specimens were obtained from 28 patients undergoing cardiac surgery, making this tissue readily available as a prime source of adipose tissue. We focused our investigation on determining VEGF gene expression and characterizing the different genes, mediators of inflammation and adipogenesis, and which are known to play a relevant role in angiogenesis regulation.We found that VEGF-A was the isoform most expressed in TAT. This expression was accompanied by an upregulation of HIF-1alpha, COX-2 and HO-1 proteins, and by increased HIF-1 DNA binding activity, compared to SAT. Furthermore, we observed that TAT contains a high percentage of mature adipocytes, 0.25% of macrophage cells, 15% of endothelial cells and a very low percentage of thymocyte cells, suggesting the cellular variability of TAT, which could explain the differences in gene expression observed in TAT. Subsequently, we showed that the expression of genes known as adipogenic mediators, including PPARgamma1/gamma2, FABP-4 and adiponectin was similar in both TAT and SAT. Moreover the expression of these latter genes presented a significantly positive correlation with VEGF, suggesting the potential association between VEGF and the generation of adipose tissue in adult thymus.Here we suggest that this fat has a potential angiogenic function related to ongoing adipogenesis, which substitutes immune functions within the adult thymus. The expression of VEGF seems to be associated with COX-2, HO-1 and adipogenesis related genes, suggesting the importance that this new fat has acquired in research in relation to adipogenesis and angiogenesis. 
 COX2 expression and Erk1/Erk2 activity mediate Cot-induced cell   migration  . The MAPKKK8 Cot/tpl-2, identified as an oncogene (Cot-T), participates in the intracellular signaling activated by members of the TLR and TNFalpha receptor superfamilies. Here we demonstrate that Cot promotes cell   migration   by regulating different steps involved in this process, such as cell adhesion and metalloproteinase activity. Indeed, Cot also regulates the cytoskeleton and Cot-T overexpression provokes the polarization of microtubules and the loss of stress fibers. Moreover, and in accordance with the increased Rac-GTP levels observed, Cot-T overexpressing cells develop more lamellipodia than control cells. Conversely, depletion of endogenous Cot increases the formation of stress fibers which is correlated with the high levels of Rho-GTP observed in these cells. In addition, the increase in COX2 expression and the activation of Erk1/2 regulated by Cot are essential for the induction of cell   migration  . Together, these data provide evidence of a new role for both proto-oncogenic and oncogenic Cot. 
 Alpha-linolenic acid regulates Cox2/VEGF/MAP kinase pathway and decreases the expression of HPV oncoproteins E6/E7 through restoration of p53 and Rb expression in human cervical cancer cell lines. Cervical cancer represents the largest cause of mortality in women worldwide. In our previous report, we have shown how alpha-linolenic acid (ALA), an omega-3 fatty acid, regulated the growth of cervical cancer cells. The present study aimed to explore mechanistic details for the anticancer activity of ALA in cervical cancer cell lines, SiHa and HeLa. ALA significantly modulated the growth kinetics of the cells and reduced cell   migration   with concomitant decrease in the expression of VEGF, MMP-2, and MMP-9 proteins. Besides this, ALA significantly decreased the expression of phosphorylated p38, pERK1/2, c-JUN, NFκB, and COX2, proteins. Most importantly, ALA reduced the expression of HPV onco-proteins E6 and E7, resulting into restoration of expression of tumor suppressor proteins, p53 and Rb. These results suggested that ALA could be explored for its therapeutic potential in cervical cancer. 
 Interleukin-1β induces cyclooxygenase-2 expression and promotes the invasive ability of human mesenchymal stem cells derived from ovarian endometrioma. To elucidate the role of interleukin-1β (IL-1β) on cyclooxygenase-2 (COX-2) expression and invasion of endometrioma-derived ectopic endometrial mesenchymal stem cells (EN-MSCs) and to develop an organoid method to study the invasive ability of endometrial cells.Gene expression and cell functions.Kaohsiung Medical University, Kaohsiung, Taiwan.Human eutopic and endometrioma-derived ectopic EN-MSCs were isolated from different endometrium biopsy samples after surgery for treatment of endometriosis.Chemical treatment of cell culture.Comparative analysis of genomewide messenger RNA (mRNA) expression, ce  ll migrat  ion, and invasion abilities in cell culture and organoid culture.Gene expression profiles revealed that the expression of IL-1β and COX-2 were statistically significantly higher in ectopic EN-MSCs compared with eutopic EN-MSCs. These enhanced expressions coincided with a greater ability for c  ell migra  tion and invasion in ectopic EN-MSCs and were found to be distinctly regulated by IL-1β which up-regulates COX-2 expression. Furthermore, IL-1β treatment of ectopic EN-MSCs in organoids was found to induce tentacle-like structures that mimicked cell invasion.These results indicate that COX-2 and IL-1β regulate the invasion ability of ectopic EN-MSCs. The information may be useful for developing a new therapeutic strategy for endometriosis. The ex vivo invasion model will be useful for characterization of EN-MSCs. 
 Thrombin receptor PAR-1 activation on endothelial progenitor cells enhances chemotaxis-associated genes expression and leukocyte recruitment by a COX-2-dependent mechanism. Endothelial colony forming cells (ECFC) represent a subpopulation of endothelial progenitor cells involved in endothelial repair. The activation of procoagulant mechanisms associated with the vascular wall's inflammatory responses to injury plays a crucial role in the induction and progression of atherosclerosis. However, little is known about ECFC proinflammatory potential.To explore the role of the thrombin receptor PAR-1 proinflammatory effects on ECFC chemotaxis/recruitment capacity.The expression of 30 genes known to be associated with inflammation and chemotaxis was quantified in ECFC by real-time qPCR. PAR-1 activation with the SFLLRN peptide (PAR-1-ap) resulted in a significant increase in nine chemotaxis-associated genes expression, including CCL2 and CCL3 whose receptors are present on ECFC. Furthermore, COX-2 expression was found to be dramatically up-regulated consequently to PAR-1 activation. COX-2 silencing with the specific COX-2-siRNA also triggered down-regulation of the nine target genes. Conditioned media (c.m.) from control-siRNA- and COX-2-siRNA-transfected ECFC, stimulated or not with PAR-1-ap, were produced and tested on ECFC capacity to recruit leukocytes in vitro as well in the muscle of ischemic hindlimb in a preclinical model. The capacity of the c.m. from ECFC stimulated with PAR-1-ap to recruit leukocytes was abrogated when COX-2 gene expression was silenced in vitro (in terms of U937 cells   migration   and adhesion to endothelial cells) as well as in vivo. Finally, the postnatal vasculogenic stem cell derived from infantile hemangioma tumor (HemSC) incubated with PAR-1-ap increased leukocyte recruitment in Matrigel(®) implant.PAR-1 activation in ECFC increases chemotactic gene expression and leukocyte recruitment at ischemic sites through a COX-2-dependent mechanism. 
 Epidermal growth factor-induced cyclooxygenase-2 enhances head and neck squamous cell carcinoma metastasis through fibronectin up-regulation. Epidermal growth factor receptor (EGFR) activation is a major cause of metastasis in many cancers, such as head and neck squamous cell carcinoma (HNSCC). However, whether the induction of cyclooxygenase-2 (COX-2) mediates EGF-enhanced HNSCC metastasis remains unclear. Interestingly, we found that EGF induced COX-2 expression mainly in HNSCC. The tumor cell transformation induced by EGF was repressed by COX-2 knockdown, and this repression was reversed by simultaneously treating the cells with EGF and prostaglandin E₂ (PGE₂). The down-regulation of COX-2 expression or inhibition of COX-2 activity significantly blocked EGF enhancement of c  ell migra  tion and invasion, but the addition of PGE₂ compensated for this inhibitory effect in COX-2-knockdown cells. COX-2 depletion inhibited EGF-induced matrix metalloproteinase (MMP)-1, MMP-2, MMP-3, MMP-9, and fibronectin expression and Rac1/cdc42 activation. The inhibitory effect of COX-2 depletion on MMPs and the fibronectin/Rac1/cdc42 axis were reversed by co-treatment with PGE₂. Furthermore, depletion of fibronectin impeded the COX-2-enhanced binding of HNSCC cells to endothelial cells and tumor cells metastatic seeding of the lungs. These results demonstrate that EGF-induced COX-2 expression enhances HNSCC metastasis via activation of the fibronectin signaling pathway. The inhibition of COX-2 expression and activation may be a potential strategy for the treatment of EGFR-mediated HNSCC metastasis. 
 Involvement of COX-2/PGE2 signalling in hypoxia-induced angiogenic response in endothelial cells. To evaluate the impact of hypoxia on the angiogenic capability of endothelial cells (ECs), and further investigate whether the cyclooxygenase-2 (COX-2)/prostaglandin E(2) (PGE(2)) signalling is involved in the angiogenic response of ECs to hypoxia. We explored the impact of various periods (1, 3, 6, 12, 24 hrs) of hypoxia (2% O(2)) on human umbilical vein endothelial cells (HUVECs) in vitro. We observed cell viability,   migration  , tube formation, analysed COX-2, vascular endothelial growth factor (VEGF), AQP1 mRNA transcription, protein expression and measured PGE(2), VEGF protein concentration in cell supernatants. Then we treated HUVECs with COX-2 selective inhibitor NS398, EP1/2 combined antagonist AH6809 and exogenous PGE(2) to investigate the role of COX-2/PGE(2) signalling in the angiogenic response of ECs to hypoxia. The results demonstrated that short-term hypoxic treatment enhanced HUVECs proliferation,   migration  , tube formation, significantly up-regulated COX-2, VEGF, AQP1 mRNA level, protein expression and promoted PGE(2) , VEGF release. The pharmacological inhibition study revealed that exposure of HUVEC to NS398 and AH6809 under hypoxia impaired the biological responses of ECs to hypoxia. Exogenous PGE(2) augments the effects of hypoxia on HUVECs, and partially reversed the inhibitory effects of NS398 on HUVECs proliferation and angiogenic capability. Short-term hypoxic treatment enhanced angiogenic capability of ECs, and COX-2/PGE(2) signalling may play a critical role in the biological response of ECs to hypoxia. 
 High glucose-induced hyperosmolarity contributes to COX-2 expression and angiogenesis: implications for diabetic retinopathy. We tested the hypothesis that glucose-induced hyperosmolarity, occurring in diabetic hyperglycemia, promotes retinal angiogenesis, and that interference with osmolarity signaling ameliorates excessive angiogenesis and retinopathy in vitro and in vivo.We incubated human aortic (HAECs) and dermal microvascular endothelial cells (HMVECs) with glucose or mannitol for 24 h and tested them for protein levels and in vitro angiogenesis. We used the Ins2 Akita mice as a model of type 1 diabetes to test the in vivo relevance of in vitro observations. Compared to incubations with normal (5 mmol/L) glucose concentrations, cells exposed to both high glucose and high mannitol (at 30.5 or 50.5 mmol/L) increased expression of the water channel aquaporin-1 (AQP1) and cyclooxygenase (COX)-2. This was preceded by increased activity of the osmolarity-sensitive transcription factor Tonicity enhancer binding protein (TonEBP), and enhanced endothelial   migration   and tubulization in Matrigel, reverted by treatment with AQP1 and TonEBP siRNA. Retinas of Ins2 Akita mice showed increased levels of AQP1 and COX-2, as well as angiogenesis, all reverted by AQP1 siRNA intravitreal injections.Glucose-related hyperosmolarity seems to be able to promote angiogenesis and retinopathy through activation of TonEBP and possibly increasing expression of AQP1 and COX-2. Osmolarity signaling may be a target for therapy. 
 Kinin b2 receptor mediates induction of cyclooxygenase-2 and is overexpressed in head and neck squamous cell carcinomas. Bradykinin has been shown to promote growth and   migration   of head and neck squamous cell carcinoma (HNSCC) cells via epidermal growth factor receptor (EGFR) transactivation. It has also been reported that bradykinin can cause the induction of cyclooxygenase-2 (COX-2), a protumorigenic enzyme, via the mitogen-activated protein kinase (MAPK) pathway in human airway cells. To determine whether COX-2 is up-regulated by bradykinin in HNSCC, the current study investigated bradykinin-induced EGFR transactivation, MAPK activation, and COX-2 expression in human HNSCC cells. Bradykinin induced a concentration- and time-dependent induction of COX-2 protein in HNSCC, which was preceded by phosphorylation of EGFR and MAPK. These effects were abolished by the B2 receptor (B2R) antagonist HOE140 but not by the B1 receptor (B1R) antagonist Lys-[Leu(8)]des-Arg(9)-bradykinin. COX-2 induction was accompanied by increased release of prostaglandin E(2). No effect of a B1R agonist (des-Arg(9)-bradykinin) on p-MAPK or COX-2 expression was observed. B2R protein was found to be expressed in all four head and neck cell lines tested. Immunohistochemical analysis and immunoblot analysis revealed that B2R, but not B1R, was significantly overexpressed in HNSCC tumors compared with levels in normal mucosa from the same patient. In HNSCC cells, the bradykinin-induced expression of COX-2 was inhibited by the EGFR kinase inhibitor gefitinib or mitogen-activated protein kinase kinase inhibitors (PD98059 or U0126). These results suggest that EGFR and MAPK are required for COX-2 induction by bradykinin. Up-regulation of the B2R in head and neck cancers suggests that this pathway is involved in HNSCC tumorigenesis. 
 Focal adhesion kinase: important to prostaglandin E2-mediated adhesion,   migration   and invasion in hepatocellular carcinoma cells. Prostaglandin E2 has been implicated in cell growth and metastasis in many types of cancers. However, the effects of PGE2 and its mechanism on cell adhesion,   migration  , and invasion have not been clarified yet. In this study, we found PGE2 treatment significantly increased the cell adhesion,   migration  , and invasion in hepatocellular carcinoma (HCC) cells. In addition, the effects of PGE2 were found to be associated with focal adhesion kinase (FAK). PGE2 treatment increased the phosphorylation and synthesis of FAK in a dose-dependent manner. RNA interference targeting FAK suppressed PGE2-mediated cell adhesion and   migration  . Furthermore, the downstream proteins of FAK, paxillin and Erk2, were also activated by PGE2. PGE2 treatment increased the phosphorylation and synthesis of paxillin in a dose-dependent manner. PGE2 treatment also induced the phosphorylation of Erk2. PD98059, the specific inhibitor of MEK, suppressed PGE2-mediated cell adhesion and   migration  . However, it had no effect on PGE2-induced activation and synthesis of FAK. These results demonstrated that PGE2 greatly induced HCC cell adhesion,   migration  , and invasion by activating FAK/paxillin/Erk pathway. 
 COX2 expression in neuroblastoma increases tumorigenicity but does not affect cell death in response to the COX2 inhibitor celecoxib. COX2 is an inducible cyclooxygenase implicated in the metastasis and   migration   of tumour cells. In neuroblastoma, COX2 expression has been detected in both cell lines and tumours. The treatment of neuroblastoma cells in vitro with celecoxib, a COX2 inhibitor, induces apoptosis. The aim of this study was to investigate the role of COX2 in neuroblastoma tumour biology by creating a cell line in which COX2 could be conditionally expressed. Xenograft studies showed that the conditional expression of COX2 enhanced tumour growth and malignancy. Elevated COX2 expression enhanced the proliferation and   migration   of neuroblastoma cells in vitro. However, elevated COX2 expression or variation between cell lines did not affect sensitivity to the COX2 inhibitor celecoxib, indicating that celecoxib does not promote cell death through COX2 inhibition. These data show that increased COX2 expression alone can enhance the tumorigenic properties of neuroblastoma cells; however, high levels of COX2 may not be a valid biomarker of sensitivity to non-steroidal anti-inflammatory drugs such as celecoxib. 
 Inhibition of PKC-Induced COX-2 and IL-8 Expression in Human Breast Cancer Cells by Glucosamine. Breast cancer is a common cancer leading to many deaths among females. Cyclooxygenase-2 (COX-2) and interleukin-8 (IL-8) are two highly expressed inflammatory mediators to be induced by the protein kinase C (PKC) signaling via various inflammatory stimuli and both contribute significantly to cancer metastasis/progression. Glucosamine has been shown to act as an anti-inflammation molecule. The aim of this study was to clarify the role and acting mechanism of glucosamine during the PKC-regulation of COX-2/IL-8 expression and the associated impact on breast cancer. In MCF-7 breast cancer cells, glucosamine effectively suppresses the PKC induction of COX-2 and IL-8 promoter activity, mRNA and protein levels, as well as the production of prostaglandin E(2) (PGE(2)) and IL-8. Glucosamine is able to promote COX-2 protein degradation in a calpain-dependent manner and IL-8 protein degradation in calpain-dependent and proteasome-dependent manners. The MAPK and NF-κB pathways are involved in PKC-induced COX-2 expression, but only the NF-κB pathway is involved in PKC-induced IL-8 expression. Glucosamine attenuates PKC-mediated IκBα phosphorylation, nuclear NF-κB translocation, and NF-κB reporter activation. Both PGE(2) and IL-8 promote cell proliferation and IL-8 induces   cell mig  ration; thus, glucosamine appears to suppress PKC-induced cell proliferatio  n and mig  ration. Furthermore, glucosamine significantly inhibits the growth of breast cancer xenografts and this is accompanied by a reduction in COX-2 and IL-8 expression. In conclusion, glucosamine seems to attenuate the inflammatory response in vitro and in vivo and this occurs, at least in part by targeting to the NF-κB signaling pathway, resulting in an inhibition of breast cancer cell growth. 
 Epidermal growth factor-activated aryl hydrocarbon receptor nuclear translocator/HIF-1{beta} signal pathway up-regulates cyclooxygenase-2 gene expression associated with squamous cell carcinoma. Hypoxia-inducible factor (HIF) accumulates when tumors grow under hypoxic conditions. The genesis of tumors, however, usually involves normoxic conditions. In this study, we were interested in examining the potential role of aryl hydrocarbon receptor nuclear translocator (ARNT)/HIF-1beta in tumor growth under normoxic conditions, specifically when cells are treated with epidermal growth factor (EGF), which is known to affect the gene expression of tumor growth-related protein COX-2 (cyclooxygenase-2). The results showed that EGF receptor inhibitor, AG1478, abolished EGF-induced nuclear accumulation of ARNT as well as the expression of COX-2. ARNT small interfering RNA inhibited the promoter activity, mRNA level, and protein expression of COX-2 in cells treated with EGF. In contrast, CoCl(2)-induced HIF-1alpha exhibited no effect on COX-2 expression. EGF also stimulated the formation of the ARNT.c-Jun complex as well as the complex binding to the COX-2 promoter. ARNT small interfering RNAs blocked EGF-activated cell   migration  . Moreover, COX-2 and ARNT were cohorts present distinctively in clinical specimens of human cervical squamous cell carcinoma and were almost nondetectable in adjacent normal or noncancerous cervical tissues. Our results revealed that ARNT plays an important role in EGF-regulated COX-2 gene expression and may thus be related to either a cause or a consequence of tumorigenesis in cervical cancer. 
 Interaction between head and neck squamous cell carcinoma cells and fibroblasts in the biosynthesis of PGE2. Prostaglandin (PG)E(2) is relevant in tumor biology, and interactions between tumor and stroma cells dramatically influence tumor progression. We tested the hypothesis that cross-talk between head and neck squamous cell carcinoma (HNSCC) cells and fibroblasts could substantially enhance PGE(2) biosynthesis. We observed an enhanced production of PGE(2) in cocultures of HNSCC cell lines and fibroblasts, which was consistent with an upregulation of COX-2 and microsomal PGE-synthase-1 (mPGES-1) in fibroblasts. In cultured endothelial cells, medium from fibroblasts treated with tumor cell-conditioned medium induced in vitro angiogenesis, and in tumor cell induced   migration   and proliferation, these effects were sensitive to PGs inhibition. Proteomic analysis shows that tumor cells released IL-1, and tumor cell-induced COX-2 and mPGES-1 were suppressed by the IL-1-receptor antagonist. IL-1α levels were higher than those of IL-1β in the tumor cell-conditioning medium and in the secretion from samples obtained from 20 patients with HNSCC. Fractionation of tumor cell-conditioning media indicated that tumor cells secreted mature and unprocessed forms of IL-1. Our results support the concept that tumor-associated fibroblasts are a relevant source of PGE(2) in the tumor mass. Because mPGES-1 seems to be essential for a substantial biosynthesis of PGE(2), these findings also strengthen the concept that mPGES-1 may be \a target for therapeutic intervention in patients with HNSCC. 
 [Small interfering RNA-mediated COX-2 gene silencing inhibits the proliferation and   migration   of human ovarian cancer cell line CAOV-3]. To investigate the changes in the proliferation and   migration   of human ovarian cancer cells (CAOV-3) after knocking down COX-2 gene by RNA interference.The recombinant plasmid of pGenesil-1-siRNA-COX-2 was constructed and transfected into CAOV-3 cells. The transcription of COX-2 gene was detected by semi-quantitative reverse transcription-polymerase chain reaction (RT-PCR), and the protein expression of COX-2 was determined by Western blotting . MTT assay was used to investigate the proliferation of the transfected CAOV-3 cells, and the cell   migration   was evaluated using a transwell   migration   assay.COX-2 mRNA and protein levels were significantly reduced after pGenesil-1-siRNA-COX-2 transfection into CAOV-3 cells, which showed obvious reduction in the cell proliferation and   migration  .RNA interference allows obvious COX-2 gene knocking down in human ovarian cancer cells to result in lowered cell growth rate and   migration   ability. COX-2 gene may become a new therapeutic target for ovarian cancer. 
 PGE(2) is generated by specific COX-2 activity and increases VEGF production in COX-2-expressing human pancreatic cancer cells. In some cancers cyclooxygenase (COX) inhibition appears to be anti-mitogenic and anti-angiogenic, but the actions of COX-derived prostaglandins in pancreatic cancer (PaCa) are unknown. In this study COX-2 was detected in three of six PaCa cell lines while COX-1 was identified in all cell lines. COX-2 expression correlated with basal and arachidonic acid (AA) stimulated PGE(2) production. PGE(2) production was inhibited by the COX-2 inhibitor nimesulide. In COX-2 expressing cells, exogenous AA and PGE(2) increased VEGF synthesis via the EP(2) receptor. Whereas PGE(2) stimulated intracellular cAMP formation in COX-2 positive and negative cells, 8-bromo cAMP stimulated VEGF production only in COX-2 expressing cells. Stimulating COX-2 expressing PaCa cell lines with AA enhanced   migration   of endothelial cells, an effect which was inhibited by a COX-2 inhibitor and EP(2) receptor antagonist. These data identify a subset of human PaCa cell lines that express functional COX-2 enzyme. PGE(2) generated by specific COX-2 activity increases VEGF secretion in human PaCa cells through an autocrine mechanism. 
 TGF-beta1 causes epithelial-mesenchymal transition in HaCaT derivatives, but induces expression of COX-2 and   migration   only in benign, not in malignant keratinocytes. Transforming growth factor beta (TGF-beta) acts as a tumor promoter by inducing epithelial-mesenchymal transition (EMT), which leads to a motile phenotype, enabling invasion and metastasis of cancer cells. Cancer-related inflammation, mediated by prostaglandins, has been proposed as a critical mechanism in conversion of benign cells to malignant.Induction of cyclooxygenase 2 (COX-2), producer of prostaglandins, is thought to be a prerequisite for TGF-beta-induced EMT in benign cells. We used HaCaT derivatives, representative of skin cancer progression, to investigate TGF-beta1 mediated EMT response, and the role of COX-2 in it.Effect of TGF-beta1 was investigated by analyzing cell proliferation, morphology and protein expression. Chemotaxis and scratch-wound assays were used to study   migration  .TGF-beta1 caused proliferation arrest of benign and malignant HaCaT cells, and changed the epithelial morphology of benign and low-grade malignant cells, but not metastatic cells, to mesenchymal spindle-shape. Epithelial junction proteins ZO-1 and E-cadherin were downregulated in all cell lines in response to TGF-beta1, but mesenchymal markers were not induced, suggesting a partial EMT response. COX-2 and   migration   were induced only in benign HaCaT derivatives. Malignant derivatives did not induce COX-2 in response to TGF-beta 1 treatment, thus emphasizing the role of inflammation in EMT response of benign cells.TGF-beta1 operates via distinct mechanisms in inducing EMT and metastasis, and supporting this we show that TGF-beta1 induces COX-2 and promotes the   migration   of benign cells, but does not further augment the   migration   of malignant cells, indicating their resistance to TGF-beta1 in the context of motility. 
 Papillary carcinoma of the thyroid: high expression of COX-2 and low expression of KAI-1/CD82 are associated with increased tumor invasiveness. We have previously demonstrated that expression of COX-2 is upregulated by hepatocyte growth factor in thyroid papillary carcinoma (TPC) cells and is associated with increased invasiveness of tumor cells. COX-2 upregulation was associated with downregulation of KAI-1/CD82, a metastasis suppressor molecule that has been associated with the metastatic potential of several solid tumors. In the present study, we have investigated the possibility that downregulation of KAI-1/CD82 may contribute to the invasiveness of papillary carcinoma cells.Expression of KAI-1/CD82 and its relation to COX-2 levels were investigated in 6 primary cultures of TPC, in 2 tumor cell lines (TPC-1 and K1), and in 55 tumor samples of TPC. The biological role of KAI-1/CD82 in regulating tumor invasiveness was investigated in TPC cell lines and primary cultures transfected with a pCDNA3.1/Hygro.KAI-1; transfected cells were tested in functional studies of cell   migration   and invasiveness. Finally, the role of KAI-1/CD82 in influencing TPC metastasis was investigated in vivo using nu/nu mice injected with K1-transfected cells.We provide evidence that COX-2 and KAI-1/CD82 are inversely regulated in TPC primary cultures and in TPC-1 tumor cells. In fact, inhibition of COX-2 with NS398 is associated with a 2-9-fold upregulation of KAI-1/CD82 RNA. Moreover, a possible relation between COX-2 and KAI-1/CD82 was confirmed by the presence of a statistically significant inverse correlation in the expression of the two genes in 55 tumor samples of TPC (r = -0.513; p = 0.001). In 36 of 55 cases, tumor areas contained lower levels of KAI-1/CD82 RNA as compared with the corresponding normal tissue. Low expression of KAI-1/CD82 RNA in the tumor area was associated with extrathyroid extension of the disease in 16 of 19 cases (p < 0.04) and with lymph node metastasis in 11 of 14 cases (not significant). KAI-1/CD82 re-expression in tumor cells was associated with a significant decrease in their migratory (50-76% reduction) and invasive (46-65% reduction) capacity, even after hepatocyte growth factor stimulation. Finally, nu/nu mice injected with KAI-1/CD82-transfected K1 TPC cells developed fewer and smaller metastasis as compared with mice injected with vector-transfected K1 cells (p=0.016).Our findings raise the possibility that downregulation of KAI-1/CD82 in TPC cells is one of the molecular mechanisms regulating their invasive and metastatic potential. 
 Role of LOXs and COX-2 on FAK activation and cell   migration   induced by linoleic acid in MDA-MB-231 breast cancer cells. Epidemiological studies and animal models suggest a link between high levels of dietary fat intake and an increased risk of developing breast cancer. Particularly, free fatty acids (FFAs) are involved in several processes, including proliferation,   migration   and invasion, in breast cancer cells. Linoleic acid (LA) is a dietary n-6 polyunsaturated fatty acid that is known to induce proliferation and invasion in breast cancer cells. So far, however, the contribution of LA to focal adhesion kinase (FAK) activation and cell   migration   in breast cancer cells has not been studied.Here, we show that LA promotes FAK and Src activation, as well as cell   migration  , in MDA-MB-231 breast cancer cells. FAK activation and cell   migration   require Src, Gi/Go, COX-2 and LOXs activities, whereas both are independent of Δ6 desaturase activity. In addition, we show that cell   migratio  n requires FAK activity, whereas FAK activation requires Src activity, thus suggesting a reciprocal catalytic activation mechanism of FAK and Src.In summary, our findings show that LA induces FAK activation and cell   migratio  n in MDA-MB-231 breast cancer cells. 
 Bisphenol A-induced epithelial to mesenchymal transition is mediated by cyclooxygenase-2 up-regulation in human endometrial carcinoma cells. Many studies have highlighted the correlation between the increase of bisphenol A (BPA) level in the environment and the incidence of tumor in humans. In human carcinogenesis, the overexpression of cyclooxygenase-2 (COX-2) and epithelial-mesenchymal transition (EMT) are closely related with tumor development. In this study, human endometrial carcinoma cells line (RL95-2) was used to investigate whether BPA can induce EMT and COX-2 expression. The results show that BPA increased growth rate and colony-forming efficiency in a dose-dependent manner, induced EMT and COX-2 gene expression and promoted the   migration   and invasion ability of RL95-2 cells. Furthermore, our study showed that the expression of COX-2 was essential for BPA-induced cell   migration   and invasion. The results of this study provide new insights into the mechanism of endometrial cancer cell growth and invasion and potential therapeutic strategy. 
 Lysophosphatidic acid receptor 2 and Gi/Src pathway mediate cell motility through cyclooxygenase 2 expression in CAOV-3 ovarian cancer cells. Lysophosphatidic acid (LPA) is a bioactive phospholipids and involves in various cellular events, including tumor cell   migration  . In the present study, we investigated LPA receptor and its transactivation to EGFR for cyclooxygenase-2 (COX-2) expression and cell   migration   in CAOV-3 ovarian cancer cells. LPA induced COX-2 expression in a dose-dependent manner, and pretreatment of the cells with pharmacological inhibitors of Gi (pertussis toxin), Src (PP2), EGF receptor (EGFR) (AG1478), ERK (PD98059) significantly inhibited LPA- induced COX-2 expression. Consistent to these results, transfection of the cells with selective Src siRNA attenuated COX-2 expression by LPA. LPA stimulated CAOV-3 cell   migration   that was abrogated by pharmacological inhibitors and antibody of EP2. Higher expression of LPA2 mRNA was observed in CAOV-3 cells, and transfection of the cells with a selective LPA2 siRNA significantly inhibited LPA-induced activation of EGFR and ERK, as well as COX-2 expression. Importantly, LPA2 siRNA also blocked LPA-induced ovarian cancer cell   migration  . Collectively, our results clearly show the significance of LPA2 and Gi/Src pathway for LPA-induced COX-2 expression and cell   migration   that could be a promising drug target for ovarian cancer cell metastasis. 
 Androgen receptor promotes the   migration   and invasion of upper urinary tract urothelial carcinoma cells through the upregulation of MMP-9 and COX-2. Dysregulated androgen receptor (AR) signaling is implicated in several types of tumor, including carcinomas of the prostate, breast, liver and bladder. However, the contribution of AR to the progression of upper urinary tract urothelial carcinomas (UUTUC) has not been fully investigated. In the present study, we demonstrated that the AR is involved in the metastasis and invasiveness of UUTUC cells. We investigated the role of the AR in UUTUC by using UUTUC-derived BFTC 909 cells. The overexpression of AR promotes the   migration   and invasion of BFTC 909 cells. Expression of   migration  /invasion-related genes was increased in BFTC 909 cells overexpressing AR determined by qPCR and western blot analyses. The results showed that AR-enhanced   migration   and invasion of UUTUC cells are linked to the upregulation of the matrix-degrading enzyme MMP-9 and cyclooxygenase (COX)-2. Subsequently, the blocking of MMP-9 and COX-2 signaling by inhibitors suppressed AR-enhanced cell   migration   and invasion. The results of the present study provide evidence for the first time of the role of AR in the motility and invasion of UUT cancer cells and support the hypothesis that the AR may play a critical role in the establishment of the invasive phenotype in urothelial neoplasia of UUT. Thus, the AR may also serve as a novel biomarker and potential therapeutic target for UUT cancer. 
 Sodium butyrate inhibits angiogenesis of human intestinal microvascular endothelial cells through COX-2 inhibition. We examined the effect of sodium butyrate on in vitro angiogenesis and cyclooxygenase (COX) expression using primary cultures of human intestinal microvascular endothelial cells (HIMEC). Butyrate inhibited VEGF-induced cellular proliferation, trans  migration   and tube formation of HIMEC. Butyrate also inhibited COX-2 expression as well as prostaglandin (PG)E2 and PGI2 production, and administration of PGI2 analog partially reversed the effect of butyrate on HIMEC angiogenesis. These results indicate that sodium butyrate inhibits HIMEC angiogenesis through down-regulation of COX-2 expression and PG production, and suggest that anti-angiogenic mechanisms may also be involved in the inhibitory effect of sodium butyrate on tumor growth. 
 NFATc1 activation promotes the invasion of U251 human glioblastoma multiforme cells through COX-2. Recent studies have revealed that the nuclear factor of activated T-cells (NFAT) is a transcription factor that is highly expressed in aggressive cancer cells and tissues, and mediates invasion through the transcriptional induction of pro-invasion and pro-  migration   genes. However, the mechanisms through which nuclear factor of activated T-cells, cytoplasmic 1 (NFATc1), in particular, translocates to the nucleus and regulates the invasion of human glioblastoma multiforme (GBM) cells have not yet been fully elucidated. In the present study, to investigate the role of NFATc1 in GBM cells, we established a U251 cell line expressing a constitutively active form of NFATc1 (CA-NFATc1). On the other hand, RNA interference was used to knock down NFATc1 expression in the U251 cell line. Our results demonstrated that the expression of CA-NFATc1 promoted cancer cell invasion, while small interfering RNA (siRNA) against NFATc1 successfully inhibited the invasion ability of the U251 cell line. Moreover, we demonstrated that NFATc1 promoted U251 cell invasion through the induction of cyclooxygenase-2 (COX‑2). NFAT transcriptionally regulates the induction of COX-2 induction in U251 cells and binds to the promoter. We also demonstrated that a large proportion of GBM specimens expressed NFATc1. NFATc1 expression increased according to the histopathological grade of the glioma. However, no NFATc1 staining was observed in the non-neoplastic brain tissues. These findings suggest that the inhibition of the activation of the NFATc1 pathway is an effective therapeutic strategy for the clinical management of GBM. 
 Aromatic-turmerone attenuates invasion and expression of MMP-9 and COX-2 through inhibition of NF-κB activation in TPA-induced breast cancer cells. Recent evidence suggests that breast cancer is one of the most common forms of malignancy in females, and metastasis from the primary cancer site is the main cause of death. Aromatic (ar)-turmerone is present in Curcuma longa and is a common remedy and food. In the present study, we investigated the inhibitory effects of ar-turmerone on expression and enzymatic activity levels of 12-O-tetradecanoylphorbol-13-acetate (TPA)-induced matrix metalloproteinase (MMP)-9 and cyclooxygenaase-2 (COX-2) in breast cancer cells. Our data indicated that ar-turmerone treatment significantly inhibited enzymatic activity and expression of MMP-9 and COX-2 at non-cytotoxic concentrations. However, the expression of tissue inhibitor of metalloproteinase (TIMP)-1, TIMP-2, MMP-2, and COX-1 did not change upon ar-turmerone treatment. We found that ar-turmerone inhibited the activation of NF-κB, whereas it did not affect AP-1 activation. Moreover, The ChIP assay revealed that in vivo binding activities of NF-κB to the MMP-9 and COX-2 promoter were significantly inhibited by ar-turmerone. Our data showed that ar-turmerone reduced the phosphorylation of PI3K/Akt and ERK1/2 signaling, whereas it did not affect phosphorylation of JNK or p38 MAPK. Thus, transfection of breast cancer cells with PI3K/Akt and ERK1/2 siRNAs significantly decreased TPA-induced MMP-9 and COX-2 expression. These results suggest that ar-turmerone suppressed the TPA-induced up-regulation of MMP-9 and COX-2 expression by blocking NF-κB, PI3K/Akt, and ERK1/2 signaling in human breast cancer cells. Furthermore, ar-turmerone significantly inhibited TPA-induced invasi  on, migra  tion, and colony formation in human breast cancer cells. 
 Cyclooxygenase-2, a Potential Therapeutic Target, Is Regulated by miR-101 in Esophageal Squamous Cell Carcinoma. Cyclooxygenase-2 (COX-2) is known to promote the carcinogenesis of esophageal squamous cell carcinoma (ESCC). There are no reports on whether microRNAs (miRNAs) regulate COX-2 expression in ESCC. This study investigated the effect of miR-101 on ESCC through modulating COX-2 expression in ESCC.Real-time quantitative reverse transcription-polymerase chain reaction (RT-PCR) was used to quantify miR-101 expression in ESCC clinical tissues and cell lines. The effects of miR-101 on ESCC progression were evaluated by cell counting kit-8 (CCK8), transwell   migration   and invasion assays, as well as by flow cytometry. The COX-2 and PEG2 levels were determined by western blot and enzyme-linked immunosorbent assays (ELISA). The luciferase reporter assay was used to verify COX-2 as a direct target of miR-101. The anti-tumor activity of miR-101 in vivo was investigated in a xenograft nude mouse model of ESCC.Downregulation of miR-101 was confirmed through comparison of 30 pairs of ESCC tumor and adjacent normal tissues (P < 0.001), as well as in 11 ESCC cell lines and a human immortalized esophageal cell line (P < 0.001). Transfection of miR-101 in ESCC cell lines significantly suppressed cell proliferation,   migration  , and invasion (all P < 0.001). The antitumor effect of miR-101 was verified in a xenograft model. Furthermore, COX-2 was shown to be a target of miR-101.Overexpression of miR-101 in ESCC inhibits proliferation and metastasis. Therefore, the miR-101/COX-2 pathway might be a therapeutic target in ESCC. 
 Nitric oxide synthase-cyclooxygenase interactions are involved in tumor cell angiogenesis and   migration  . Nitric oxide (NO), produced by distinct nitric oxide synthase (NOS) isoforms, and prostaglandins generated by expression of cyclooxygenases are important mediators in tumor progression. Previous studies have shown that NO can influence the formation of prostaglandin E2 (PGE2). We provide evidence that NO, derived from iNOS and eNOS activity in LMM3 murine mammary adenocarcinoma cell line, is involved in tumor angiogenesis and in tumor cell   migration  . LMM3 cells that also stimulate their neovascularization activity and   migration   liberate high basal amounts of PGE2. There is large amount of evidence that postulates positive regulatory interactions between NOS and cyclooxygenase (COX) isoforms. We here show that, in the LMM3 cell line, while PGE2 exerts a positive modulation on NOS activity, NO closes the loop with a negative feed back on COX activity. We also provide evidence of a positive regulatory effect of protein tyrosine kinases on NOS as well as on COX enzymatic functions affecting tumor induced angiogenesis and cell   migration  . 
 MBP-1 suppresses growth and metastasis of gastric cancer cells through COX-2. The c-Myc promoter binding protein 1 (MBP-1) is a transcriptional suppressor of c-myc expression and involved in control of tumorigenesis. Gastric cancer is one of the most frequent neoplasms and lethal malignancies worldwide. So far, the regulatory mechanism of its aggressiveness has not been clearly characterized. Here we studied roles of MBP-1 in gastric cancer progression. We found that cell proliferation was inhibited by MBP-1 overexpression in human stomach adenocarcinoma SC-M1 cells. Colony formation,   migration  , and invasion abilities of SC-M1 cells were suppressed by MBP-1 overexpression but promoted by MBP-1 knockdown. Furthermore, the xenografted tumor growth of SC-M1 cells was suppressed by MBP-1 overexpression. Metastasis in lungs of mice was inhibited by MBP-1 after tail vein injection with SC-M1 cells. MBP-1 also suppressed epithelial-mesenchymal transition in SC-M1 cells. Additionally, MBP-1 bound on cyclooxygenase 2 (COX-2) promoter and downregulated COX-2 expression. The MBP-1-suppressed tumor progression in SC-M1 cells were through inhibition of COX-2 expression. MBP-1 also exerted a suppressive effect on tumor progression of other gastric cancer cells such as AGS and NUGC-3 cells. Taken together, these results suggest that MBP-1-suppressed COX-2 expression plays an important role in the inhibition of growth and progression of gastric cancer. 
 Contribution of reactive oxygen species to   migration  /invasion of human glioblastoma cells U87 via ERK-dependent COX-2/PGE(2) activation. In the presence of 12-O-tetradecanoylphorbol-13-acetate (TPA) stimulation, an increase in the   migration  /invasion of U87 glioblastoma cells was detected by a wound healing assay, transwell analysis, and spheroid formation assay by inducing matrix metalloproteinase-9 (MMP-9) enzyme activity via a gelatin zymographic analysis. A dose- and time-dependent increase in cyclooxygenase-2 (COX-2) gene expression with elevated prostaglandin E(2) (PGE(2)) production was identified in TPA- but not in 4alpha-TPA (a respective inactive compound)-treated U87 cells TPA-induced   migration  /invasion was significantly blocked by adding the COX-2-specific inhibitor, NS398, through a reduction in PGE(2) production. Data from the pharmacological studies using specific chemical inhibitors showed that activation of protein kinase C (PKC) and extracellular signal-regulated kinases (ERKs) was involved in TPA-induced   migration  /invasion, COX-2 protein expression, and MMP-9 activation. Stimulation of intracellular peroxide production by TPA was detected by a DCHF-DA assay, and the addition of superoxide dismutase (SOD) or tempol significantly inhibited TPA-induced   migration  /invasion and COX-2 protein expression accompanied by a decrease in peroxide production. An increase in NADPH oxidase activity by TPA was examined, and TPA-induced   migration  /invasion was blocked by adding DPI, an NADPH oxidase inhibitor. Additionally, the natural flavonoids quercetin (QE), baicalein (BE), and myricetin (ME) effectively blocked TPA-induced   migration  /invasion while simultaneously inhibiting COX-2/PGE(2) production, MMP-9 enzyme activity, and peroxide production in U87 cells. The contribution of ROS production to the   migration  /invasion of U87 glioblastoma cells via ERK-activated COX-2/PGE(2) and MMP-9 induction was first investigated here, and agents such as QE, BE, and ME with the ability to block these events possess the potential to be developed for use against   migration  /invasion by glioblastomas. 
 COX-2 overexpression increases motility and invasion of breast cancer cells. Cyclooxygenase-2 (COX-2), an inducible enzyme involved in prostaglandin (including PGE(2)) biosynthesis, is overexpressed in several epithelial malignancies including breast cancer. We tested the hypothesis that COX-2 overexpression in breast cancer cells results in increased cell motility and invasion. COX-2 overproducing cells were generated by stable transfection of several human breast cancer cells with pSG5-COX2 vector. We confirmed the overexpression of COX-2 protein by western blotting, and by measuring PGE(2) in the medium with an immunoassay. We measured cell motility by counting the number of cells crossing an 8-micron pore size PET membrane, and cell invasion by counting the number of cells invading through a Matrigel-coated membrane that simulates basement membrane. COX-2 transfected MDA-231 cells produced 30-43-fold more PGE2 as compared to parental cells. COX-2 overexpression increased cell   migration   approximately 2.2-fold and cell invasion through Matrigel approximately 5.1-fold. Addition of 50 microM NS-398, a COX-2 inhibitor, inhibited Matrigel invasion of MDA-231 cells by 54% as compared to solvent confirming the role of COX-2 in cell invasion. It is known that an increase in cell   migration   and invasion can be brought about by cytoskeletal alterations and basement membrane degradation due to increased expression of pro-urokinase plasminogen activator (pro-uPA). To investigate the mechanism of our observed increase in cell invasion by COX-2, we found by western blotting that the level of pro-uPA was significantly higher (approximately 5-fold) in COX-2 transfected MDA-231 cells than untransfected MDA-231 cells. Similar to our observations in cell culture, we found evidence that increased COX-2 activity correlates with uPA in a mouse model of breast cancer metastasis to bone. In this study, we conclude that COX-2 overexpression in human breast cancer cells enhances cell motility and invasiveness thus suggesting a mechanism of COX-2 mediated metastasis. 
 Sphingosine 1-phosphate (S1P) induces COX-2 expression and PGE2 formation via S1P receptor 2 in renal mesangial cells. Understanding the mechanisms of sphingosine 1-phosphate (S1P)-induced cyclooxygenase (COX)-2 expression and prostaglandin E2 (PGE2) formation in renal mesangial cells may provide potential therapeutic targets to treat inflammatory glomerular diseases. Thus, we evaluated the S1P-dependent signaling mechanisms which are responsible for enhanced COX-2 expression and PGE2 formation in rat mesangial cells under basal conditions. Furthermore, we investigated whether these mechanisms are operative in the presence of angiotensin II (Ang II) and of the pro-inflammatory cytokine interleukin-1β (IL-1β). Treatment of rat and human mesangial cells with S1P led to concentration-dependent enhanced expression of COX-2. Pharmacological and molecular biology approaches revealed that the S1P-dependent increase of COX-2 mRNA and protein expression was mediated via activation of S1P receptor 2 (S1P2). Further, inhibition of Gi and p42/p44 MAPK signaling, both downstream of S1P2, abolished the S1P-induced COX-2 expression. In addition, S1P/S1P2-dependent upregulation of COX-2 led to significantly elevated PGE2 levels, which were further potentiated in the presence of Ang II and IL-1β. A functional consequence downstream of S1P/S1P2 signaling is mesangial ce  ll migrat  ion that is stimulated by S1P. Interestingly, inhibition of COX-2 by celecoxib and SC-236 completely abolished the migratory response. Overall, our results demonstrate that extracellular S1P induces COX-2 expression via activation of S1P2 and subsequent Gi and p42/p44 MAPK-dependent signaling in renal mesangial cells leading to enhanced PGE2 formation and ce  ll migrat  ion that essentially requires COX-2. Thus, targeting S1P/S1P2 signaling pathways might be a novel strategy to treat renal inflammatory diseases. 
 Docosahexaenoic acid inhibits insulin-induced activation of sterol regulatory-element binding protein 1 and cyclooxygenase-2 expression through upregulation of SIRT1 in human colon epithelial cells. Multiple lines of compelling evidence from clinical and population-based studies support that hyperinsulinemia often accompanying obesity-associated insulin insensitivity promotes colon carcinogenesis. Insulin can acetylate, thereby activating sterol regulator element-binding protein 1 (SREBP-1), a prime transcription factor responsible for expression of genes involved in lipogenesis. Moreover, SREBP-1 upregulates cyclooxygenase-2 (COX-2), a key player in inflammatory signaling. Docosahexaenoic acid (DHA), a representative omega-3 polyunsaturated fatty acid, has been known to negatively regulate SREBP-1, but the underlying molecular mechanism is not fully clarified yet. This prompted us to investigate whether DHA could inhibit insulin-induced activation of SREBP-1 and COX-2 expression in the context of its potential protective effect on obesity-induced inflammation and carcinogenesis. SIRT1, a NAD(+)-dependent histone/non-histone protein deacetylase, has been reported to inhibit intracellular signaling mediated by SREBP-1 through deacetylation of this transcription factor. We found that DHA induced SIRT1 expression in CCD841CoN human colon epithelial cells. DHA abrogated insulin-induced acetylation as well as expression of SREBP-1 and COX-2 upregulation. Insulin-induced stimulation of CCD841CoN cell   migration   was also inhibited by DHA. These effects mediated by DHA were attenuated by pharmacologic inhibition of SIRT1. Hyperinsulinemia or insulin resistance is considered to be associated with obesity-associated inflammation. Genetically obese (ob/ob) mice showed higher colonic expression levels of both SREBP-1 and COX-2 than did normal lean mice. Likewise, expression of SREBP-1 and COX-2 was elevated in human colon tumor specimens compared with surrounding normal tissues. In conclusion, DHA may protect against obesity-associated inflammation and colon carcinogenesis by suppressing insulin-induced activation of SREBP-1 and expression of COX-2 through up-regulation of SIRT1. 
 COX-2 Promotes   Migration   and Invasion by the Side Population of Cancer Stem Cell-Like Hepatocellular Carcinoma Cells. Cancer stem cells (CSCs) are thought to be responsible for tumor relapse and metastasis due to their abilities to self-renew, differentiate, and give rise to new tumors. Cyclooxygenase-2 (COX-2) is highly expressed in several kinds of CSCs, and it helps promote stem cell renewal, proliferation, and radioresistance. Whether and how COX-2 contributes to CSC   migration   and invasion is unclear. In this study, COX-2 was overexpressed in the CSC-like side population (SP) of the human hepatocellular carcinoma (HCC) cell line HCCLM3. COX-2 overexpression significantly enhanced   migration   and invasion of SP cells, while reducing expression of metastasis-related proteins PDCD4 and PTEN. Treating SP cells with the selective COX-2 inhibitor celecoxib down-regulated COX-2 and caused a dose-dependent reduction in cell   migration   and invasion, which was associated with up-regulation of PDCD4 and PTEN. These results suggest that COX-2 exerts pro-metastatic effects on SP cells, and that these effects are mediated at least partly through regulation of PDCD4 and PTEN expression. These results further suggest that celecoxib may be a promising anti-metastatic agent to reduce   migration   and invasion by hepatic CSCs. 
 STIM1 overexpression promotes colorectal cancer progression, cell motility and COX-2 expression. Tumor metastasis is the major cause of death among cancer patients, with >90% of cancer-related death attributable to the spreading of metastatic cells to secondary organs. Store-operated Ca(2+) entry (SOCE) is the predominant Ca(2+) entry mechanism in most cancer cells, and stromal interaction molecule 1 (STIM1) is the endoplasmic reticulum (ER) Ca(2+) sensor for store-operated channels. Here we reported that the STIM1 was overexpressed in colorectal cancer (CRC) patients. STIM1 overexpression in CRC was significantly associated with tumor size, depth of invasion, lymph node metastasis status and serum levels of carcinoembryonic antigen. Furthermore, ectopic expression of STIM1 promoted CRC cell motility, while depletion of STIM1 with short hairpin RNA inhibited CRC cell   migration  . Our data further suggested that STIM1 promoted CRC cell   migration   through increasing the expression of cyclooxygenase-2 (COX-2) and production of prostaglandin E2 (PGE2). Importantly, ectopically expressed COX-2 or exogenous PGE2 were able to rescue   migration   defect in STIM1 knockdown CRC cells, and inhibition of COX-2 with ibuprofen and indomethacin abrogated STIM1-mediated CRC cell motility. In short, our data provided clinicopathological significance for STIM1 and SOCE in CRC progression, and implicated a role for COX-2 in STIM1-mediated CRC metastasis. Our studies also suggested a new approach to inhibit STIM1-mediated metastasis with COX-2 inhibitors. 
 The Notch1/cyclooxygenase-2/Snail/E-cadherin pathway is associated with hypoxia-induced hepatocellular carcinoma cell invasion and   migration  . Hepatocellular carcinoma (HCC) is one of the most common malignancies worldwide; however, the prognosis of HCC patients remains poor. This poor prognosis is mainly attributed to the high rate of intrahepatic and distant metastasis. HCC often occurs in a hypoxic environment and hypoxia can activate metastatic programs, ultimately leading to tumor recurrence or metastasis. Thus, the discovery and subsequent development of novel agents to block HCC invasion and   migration   are the primary objectives of hepatic cancer research. The Notch1 signaling pathway might be involved in hypoxia-induced carcinoma metastasis. However, the mechanisms by which Notch1 mediates cell metastasis, particularly in hepatocellular carcinoma, are not yet entirely clear. The results of the present study show that hypoxia increases the invasion and   migration   capacities of different HCC cells. Activation of the Notch1 signaling pathway contributes to hypoxia-induced invasion and   migration   in HCC cells. The activated Notch1 signaling pathway can regulate Snail/E-cadherin through cyclooxygenase-2 (COX-2) under hypoxic conditions. The above results suggest that the Notch1/COX-2/Snail/E-cadherin pathway is possibly associated with hypoxia-induced invasion and   migration   in HCC cells. Thus, targeting Notch1 may be useful for devising novel preventive and therapeutic strategies for HCC. 
 Cyclooxygenase-2 regulates survival,   migration  , and invasion of human endometriotic cells through multiple mechanisms. Endometriosis is a debilitating disease characterized by the presence of functional endometrial glandular epithelium and stroma outside the uterine cavity that affects up to 20% of women of child-bearing age. Cyclooxygenase-2 (COX-2), a rate-limiting enzyme in the biosynthesis of prostaglandin E(2) (PGE(2)), is highly expressed in endometriotic tissues and results in increased concentrations of peritoneal PGE(2) in women. In this study, we determined the expression of COX-2 protein in ectopic and eutopic endometria in humans and the role of COX-2 in endometriotic cell survival,   migration  , and invasion in humans. Our results indicate that COX-2 protein is abundantly expressed in ectopic endometria compared with eutopic endometria. Comparatively, expression of COX-2 protein is higher in eutopic endometria from women with endometriosis compared with women without endometriosis. Inhibition of COX-2 decreases survival,   migration  , and invasion of endometriotic cells that are associated with decreased production of PGE(2). Cell growth inhibitory effects of COX-2 inhibition/silencing are mediated through nuclear poly (ADP-ribose) polymerase-mediated apoptosis. Cell motility and invasion inhibitory effects of COX-2 inhibition/silencing are mediated through matrix metalloproteinase-2 and -9 activities. Interestingly, effects of COX-2 inhibition is more profound in endometriotic epithelial than in stromal cells. Furthermore, inhibition of COX-2 affects invasion rather than   migration   of endometriotic epithelial and stromal cells. It is the first evidence showing that inhibition of COX-2 decreases endometriotic epithelial and stromal cell survival,   migration  , and invasion in humans. Our results support the emerging concept that COX-2/PGE(2) promotes the pathophysiology and pathogenesis of endometriosis in humans. 
 Cyclooxygenase-2 is a target gene of rho GDP dissociation inhibitor beta in breast cancer cells. Rho GDP dissociation inhibitor beta (Rho-GDI beta), an inhibitor of Rho GTPases, is primarily expressed by hematopoietic cells but is also found in epithelial cancer cells. Recently, we have identified Rho-GDI beta as a target of the transcription factor Ets1. Here, we show that, in breast cancer cells, Ets1 regulates Rho-GDI beta expression and binds to the upstream region of the Rho-GDI beta gene. Furthermore, in primary breast cancer, Rho-GDI beta is coexpressed with Ets1. Studying the function of Rho-GDI beta in breast cancer, we found that a Rho-GD beta-specific small interfering RNA increased cellular   migration   but also decreased the expression of cyclooxygenase-2 (Cox-2) oncogene as shown by microarray, quantitative reverse transcription-PCR, and Western blot analyses. Further studies revealed that Rho-GDI beta regulates Cox-2 gene at least partly on the transcriptional level, most likely by activating nuclear factor of activated T cells 1 (NFAT-1). Vav-1, an interaction partner of Rho-GDI beta, was also found to interfere with Cox-2 expression and NFAT-1 cellular distribution, suggesting a cooperative action of Rho-GDI beta and Vav-1 on Cox-2 expression. To explore the importance of Rho-GDI beta for the survival of breast cancer patients, two cohorts, including 263 and 117 patients, were analyzed for clinical outcome in relation to Rho-GDI beta RNA and protein levels, respectively. Expression of Rho-GDI beta was not associated with either disease-free or overall survival in the two patient population. Our data suggest that the expression of Rho-GDI beta in breast cancer is neither beneficial nor disadvantageous to the patient. This may be the net effect of two opposing activities of Rho-GDI beta, one that suppresses tumor progression by inhibiting   migration   and the other that stimulates it by enhancing Cox-2 expression. 
 Matrix metalloproteinase-19 is a key regulator of lung fibrosis in mice and humans. Idiopathic pulmonary fibrosis (IPF) is a devastating disease characterized by epithelial phenotypic changes and fibroblast activation. Based on the temporal heterogeneity of IPF, we hypothesized that hyperplastic alveolar epithelial cells regulate the fibrotic response.To identify novel mediators of fibrosis comparing the transcriptional signature of hyperplastic epithelial cells and conserved epithelial cells in the same lung.Laser capture microscope and microarrays analysis were used to identify differentially expressed genes in IPF lungs. Bleomycin-induced lung fibrosis was evaluated in Mmp19-deficient and wild-type (WT) mice. The role of matrix metalloproteinase (MMP)-19 was additionally studied by transfecting the human MMP19 in alveolar epithelial cells.Laser capture microscope followed by microarray analysis revealed a novel mediator, MMP-19, in hyperplastic epithelial cells adjacent to fibrotic regions. Mmp19(-/-) mice showed a significantly increased lung fibrotic response to bleomycin compared with WT mice. A549 epithelial cells transfected with human MMP19 stimulated wound healing and cell   migration  , whereas silencing MMP19 had the opposite effect. Gene expression microarray of transfected A549 cells showed that PTGS2 (prostaglandin-endoperoxide synthase 2) was one of the highly induced genes. PTGS2 was overexpressed in IPF lungs and colocalized with MMP-19 in hyperplastic epithelial cells. In WT mice, PTGS2 was significantly increased in bronchoalveolar lavage and lung tissues after bleomycin-induced fibrosis, but not in Mmp19(-/-) mice. Inhibition of Mmp-19 by siRNA resulted in inhibition of Ptgs2 at mRNA and protein levels.Up-regulation of MMP19 induced by lung injury may play a protective role in the development of fibrosis through the induction of PTGS2. 
 Elevated cell   migration  , invasion and tumorigenicity in human KB carcinoma cells transfected with COX-2 cDNA. In order to investigate the involvement of cyclooxygenase (COX)-2 in cell growth and invasion of oral cancer, a human epidermoid carcinoma cell line KB minimally expressing COX-2 protein was transfected with COX-2 cDNA and these activities were compared with mock-transfected KB in vitro and in vivo. KB/COX-2 clones showed a similar growth rate in vitro compared to KB/neo clones, but demonstrated significantly increased PGE2 production, cell   migration   and invasion. These KB/COX-2 clones markedly expressed MMP-9, pro-MMP-2 and activated-MMP-2 as compared to KB/neo clones in gelatin zymography. Western blot analysis showed that expression of MT1-MMP, Rho and Rac 1 in KB/COX-2 clones were stronger than that in KB/neo clones, but expression of TIMP-1 and TIMP-2 were weaker in KB/COX-2 clones than in KB/neo clones. When these cells were inoculated subcutaneously into nude mice, tumorigenicity and tumor growth were significantly elevated in KB/COX-2 tumors than in KB/neo tumors, and the gelatinase activity was much stronger in KB/COX-2 tumor tissues than in KB/neo tumor tissues in film in situ zymography. The orthotopic inoculation of cells to the oral floor showed that local invasion was pronounced in KB/COX-2 tumors. These results indicated that overexpression of COX-2 elevated tumorigenicity, tumor growth and invasion of human KB carcinoma cells via up-regulated MMP and Rho family small GTPases and down-regulated TIMP activities. 
 Induction of prostaglandin E2 production by leukemia inhibitory factor promotes   migration   of first trimester extravillous trophoblast cell line, HTR-8/SVneo. The invasion of first trimester extravillous trophoblast (EVT) to decidua is an important event in placentation. Leukemia inhibitory factor (LIF) is an essential factor for mouse implantation, and it is reported that LIF may be involved in human first trimester EVT invasion. Prostaglandin E2 (PGE2) is also known as a critical factor for first trimester EVT invasion. In this study, we investigated the role of LIF in PGE2 production and EVT invasion using a human first trimester EVT cell line, HTR-8/SVneo.Co-stimulation with LIF and IL-1beta induced higher amounts of PGE2 production and further   migration   of HTR-8/SVneo cells compared with that by stimulation with LIF or IL-1beta alone. Enhanced PGE2 production was most probably due to the enhanced expression of cyclooxygenase-2 (COX-2) and microsomal PGE synthase-1 (mPGES-1). PGE2 produced by HTR-8/SVneo cells promoted the   migration   of HTR-8/SVneo cells. A COX-2 inhibitor suppressed PGE2 production and the   migration   of HTR-8/SVneo cells. Agonists to PGE2 receptors, EP1, EP2 and EP4, promoted the   migration   of HTR-8/SVneo cells. Moreover, stimulation with LIF up-regulated EP1, EP2 and EP4 expression in HTR-8/SVneo cells.It is suggested that LIF participates in placentation through EVT invasion by up-regulating PGE2 production and PGE2 receptor expression in first trimester EVT. 
 High expression of PGE2 enzymatic pathways in cervical (pre)neoplastic lesions and functional consequences for antigen-presenting cells. Although human papillomavirus (HPV) DNA is detected in the majority of squamous intraepithelial lesions (SIL) and carcinoma (SCC) of the uterine cervix, the persistence or progression of cervical lesions suggest that viral antigens are not adequately presented to the immune system. This hypothesis is reinforced by the observation that most SIL show quantitative and functional alterations of Langerhans cells (LC). The aim of this study was to determine whether prostaglandins (PG) may affect LC density in the cervical (pre)neoplastic epithelium. We first demonstrated that the epithelial expression of PGE(2) enzymatic pathways, including cyclooxygenase-2 (COX-2) and microsomal prostaglandin E synthase 1 (mPGES-1), is higher in SIL and SCC compared to the normal exocervical epithelium and inversely correlated to the density of CD1a-positive LC. By using cell   migration   assays, we next showed that the motility of immature dendritic cells (DC) and DC partially differentiated in vitro in the presence of PGE(2) are differentially affected by PGE(2). Immature DC had a lower ability to migrate in the presence of PGE(2) compared to DC generated in vitro in the presence of PGE(2). Finally, we showed that PGE(2) induced a cytokine production profile and phenotypical features of tolerogenic DC, suggesting that the altered expression of PGE(2) enzymatic pathways may promote the cervical carcinogenesis by favouring (pre)cancer immunotolerance. 
 Expression of COX-2 and VEGF-C in cholangiocarcinomas at different clinical and pathological stages. The aim of this study was to investigate the expression and clinical significance of cyclooxygenase 2 (COX-2) and vascular en-dothelial growth factor C (VEGF-C) in cholangiocarcinomas at differ-ent clinical and pathological stages. Eighty cholangiocarcinoma sam-ples of patients treated with surgery between January 2012 and January 2014 were collected. Immunohistochemistry was used to detect COX-2 and VEGF-C expression at different clinical and pathological stages. ELISA, real-time PCR, invasive chambers, and MTT assay were ap-plied in cultured cholangiocarcinoma cells treated with a COX-2 inhib-itor. Expression of COX-2 and VEGF-C correlated positively with the clinical TNM stage but did not correlate with the differentiation status. Inhibition of COX-2 activity reduced VEGF-C mRNA expression and secretion in cholangiocarcinoma cells and decreased their   migration   but not proliferation. Because of its ability to inhibit invasion, COX-2 could be a new target for treatment of cholangiocarcinoma. 
  GeneRIFs  
 Data suggest that transcatheter arterial chemoembolization produced-hypoxic environment induces HIF-1alpha expression upregulating COX-2 to promote cancer angiogenesis, inhibiting cancer cell apoptosis and enhancing cancer invasion and   migration   in hepatocellular carcinoma patients. 
 These findings demonstrate that Tan-IIA inhibits the   migration   ability of AGS human gastric cancer cells and that decreasing the protein expression of NF-kappaB-p65, COX-2, and MMP-2, -7 and -9 may be an underlying molecular mechanism 
 Inhibition of COX-2 activity reduced VEGF-C mRNA expression and secretion in cholangiocarcinoma cells and decreased their   migration   but not proliferation 
 Treating SP cells with the selective COX-2 inhibitor celecoxib down-regulated COX-2 and caused a dose-dependent reduction in cell   migration   and invasion. 
 This study suggested that PGE2 upregulates beta1-integrin expression and cell   migration   in HCC cells by activating the PKC/NF-kappaB signaling pathway 
 High PTGS2 expression enhanced hepatoma cell invasion and   migration  . 
 Silencing GRP78 expression may suppress the proliferation, adhesion, and   migration   of prostate cancer cells via maspin and COX-2 regulation. 
 The blocking of MMP-9 and COX-2 signaling by inhibitors suppressed AR-enhanced cell   migration   and invasion. 
 extracellular S1P induces COX-2 expression via activation of S1P2 and subsequent Gi and p42/p44 MAPK-dependent signaling in renal mesangial cells leading to enhanced PGE2 formation and cell   migration   that essentially requires COX-2. 
 High PTGS2 expression is associated with hypoxia-induced hepatocellular carcinoma cell invasion and   migration  . 
 ET-1 enhances the cell   migration   of chondrosarcoma by increasing COX-2 expression through the ET receptors, MAPK, and AP-1 signal transduction pathway. 
 Data indicate that focal adhesion kinase (FAK) activation and cell   migration   require Src, Gi/Go, COX-2 and LOXs activities. 
 Gene knockdown of COX2 in human ovarian cancer cells results in lowered cell growth rate and   migration   ability. 
 ERalpha stable transfection inhibits proliferation and   migration   capacities of MDA-MB-231 cells and decreases expression of COX-2 and VEGF-C. 
 TGF-beta1 causes epithelial-mesenchymal transition in HaCaT derivatives, but induces expression of COX-2 and   migration   only in benign, not in malignant keratinocytes 
 Prostaglandin E2 enhances the   migration   of chondrosarcoma cells by increasing alpha2beta1 integrin expression through the EP1/PLC/PKCalpha/c-Src/NF-kappaB signal transduction pathway. 
 stimulation of COX-2/PGE2 and MMP-9 by reactive oxygen species-activated ERKs is involved in the invasion/  migration   of U87 glioma cells 
 Data revealed that DV infection enhances DC   migration   by inducing CCR7 expression, and that blocking COX-2 or MAPK activity suppresses DV-induced DC   migration  . 
 findings show a direct role for macrophage   migration   inhibitory factor in the up-regulation of COX-2 synthesis and PGE(2) secretion in ectopic endometrial cells 
 LPA2 and Gi/Src pathways are significant for LPA-induced COX-2 expression and cell   migration   that could be a promising drug target for ovarian cancer cell metastasis. 
 The increase in COX2 expression and the activation of Erk1/2 regulated by Cot are essential for the induction of cell   migration  . 
 PGE2 greatly induced hepatocellular carcinoma cell adhesion,   migration  , and invasion by activating FAK/paxillin/Erk pathway. 
 Data show that GPx2 inhibits malignant characteristics of tumor cells,   migration   and invasion, obviously by counteracting COX-2 expression but is required for the growth of transformed intestinal cells and may, therefore, facilitate tumor cell growth. 
 COX-2 induces cell viability and   migration  , & promotes the proliferation of CAOV-3 cells via the PI3-kinase/Akt signal pathway. 
 CCR7 and the EP2/EP4 receptor signaling pathway are down-stream targets for COX-2 to enhance the   migration   of breast cancer cells toward LECs and to promote lymphatic invasion 
 Inhibition of COX-2 decreases endometriotic epithelial and stromal cell survival,   migration  , and invasion in humans. 
 Rho-GDI beta has two roles: one that suppresses tumor progression by inhibiting   migration   and the other that stimulates it by enhancing Cox-2 expression 
 inducible nitric oxide synthase-cyclooxygenase 2 interactions are involved in tumor cell angiogenesis and   migration   
  ECM  PubMed  
 Alpha2beta1 integrin signalling enhances cyclooxygenase-2 expression in intestinal epithelial cells. Inflammatory bowel diseases (IBD) are linked to an increased risk of developing colon cancer, by inflammatory mediators and alterations to the extracellular matrix (  ECM  ). The events induced by inflammatory mediators lead to dysregulated activation and induction of inflammatory genes such as cyclooxygenase-2 (COX-2). COX-2 is involved in the conversion of arachidonic acid to biologically active prostanoids and is highly upregulated in colon cancer. Since inflammation-induced changes to the extracellular matrix could affect integrin activities, we here investigated the effect of integrin signalling on the level of COX-2 expression in the non-transformed intestinal epithelial cell lines, Int 407 and IEC-6. Adhesion of these cells to a collagen I- or IV-coated surface, increased surface expression of alpha2beta1 integrin. Activation of integrins with collagen caused an increased cox-2 promoter activity, with a subsequent increase in COX-2 expression. The signalling cascade leading to this increased expression and promoter activity of cox-2, involves PKCalpha, the small GTPase Ras and NFkappaB but not Erk1/2 or Src activity. The integrin-induced increase in cellular COX-2 activity is responsible for an elevated generation of reactive oxygen species (ROS) and increased cell migration. This signalling pathway suggests a mechanism whereby inflammation-induced modulations of the   ECM  , can promote cancer transformation in the intestinal epithelial cells. 
 Alpha2beta1 integrin signalling enhances cyclooxygenase-2 expression in intestinal epithelial cells. Inflammatory bowel diseases (IBD) are linked to an increased risk of developing colon cancer, by inflammatory mediators and alterations to the extracellular matrix (  ECM  ). The events induced by inflammatory mediators lead to dysregulated activation and induction of inflammatory genes such as cyclooxygenase-2 (COX-2). COX-2 is involved in the conversion of arachidonic acid to biologically active prostanoids and is highly upregulated in colon cancer. Since inflammation-induced changes to the extracellular matrix could affect integrin activities, we here investigated the effect of integrin signalling on the level of COX-2 expression in the non-transformed intestinal epithelial cell lines, Int 407 and IEC-6. Adhesion of these cells to a collagen I- or IV-coated surface, increased surface expression of alpha2beta1 integrin. Activation of integrins with collagen caused an increased cox-2 promoter activity, with a subsequent increase in COX-2 expression. The signalling cascade leading to this increased expression and promoter activity of cox-2, involves PKCalpha, the small GTPase Ras and NFkappaB but not Erk1/2 or Src activity. The integrin-induced increase in cellular COX-2 activity is responsible for an elevated generation of reactive oxygen species (ROS) and increased cell migration. This signalling pathway suggests a mechanism whereby inflammation-induced modulations of the   ECM  , can promote cancer transformation in the intestinal epithelial cells. 
 Up-regulation of cyclooxygenase-2 expression and prostaglandin synthesis in endometrial stromal cells by malignant endometrial epithelial cells. A paracrine effect mediated by prostaglandin E2 and nuclear factor-kappa B. We investigated the regulation of prostaglandin production in normal endometrial stromal cells (ESC) by malignant endometrial epithelial cells. We found that cyclooxygenase (COX)-2 mRNA and protein levels and prostaglandin (PG)E(2) production in ESC were significantly increased by Ishikawa malignant endometrial epithelial cell conditioned medium (M  ECM  ). By using transient transfection assays, we found that the -360/-218-bp region of the COX-2 promoter gene was critical for M  ECM   induction of promoter activity. This M  ECM  -responsive region contained a variant nuclear factor (NF)-kappa B site at -222 to -213 that, when mutated, completely abolished COX-2 promoter activation by M  ECM  . Employing electrophoretic mobility shift assays, we further demonstrated that binding of NF-kappa B p65 to this NF-kappa B-binding site is, in part, responsible for the COX-2 promoter activation by M  ECM  . To investigate further the potential effects of M  ECM   on COX-2 mRNA stability, ESC were treated with M  ECM   in the absence or presence of actinomycin D, a general transcription inhibitor. We found that M  ECM   significantly increased COX-2 mRNA stability. Intriguingly, we found that PGE(2) was one of the major factors in M  ECM  , which was responsible for up-regulating COX-2 expression in ESC. ECC-1 and HEC-1A malignant endometrial epithelial cell lines also produced significantly increased quantities of PGE(2). In conclusion, malignant endometrial epithelial cells secrete PGE(2) that induces COX-2 expression in normal endometrial stromal cells in a paracrine fashion through activation of transcription and stabilization of COX-2 mRNA. 
 COX-2 induction by heparanase in the progression of breast cancer. Breast cancer confined within the lactiferous duct or lobule, without invading the stroma, is called ductal carcinoma in situ (DCIS), whereas breast cancer that has invaded the stroma through the basal membrane is called invasive cancer. Heparanase, an endo-beta-D-glucuronidase that specifically degrades heparan sulfate proteoglycans (HSPGs) in the extracellular matrix (  ECM  ), plays an important role when breast cancer cells breach the basal membrane. Recently, we have reported that heparanase is involved in angiogenesis through direct induction of cyclo-oxygenase-2 (COX-2). COX-2 induces vascular endothelial growth factor (VEGF) and basic fibroblast growth factor (bFGF) and is thus involved in neovascularization. The present study was undertaken to analyze surgically resected breast cancer specimens for heparanase and COX-2 expression, using specimens from 59 patients with invasive cancer and 85 patients with DCIS (including 41 cases of DCIS adjacent to invasive cancer). This study yielded the following results: a) the distribution of heparanase within tumor tissue was identical to that of COX-2; b) heparanase expression was more frequent in invasive cancer than in non-invasive cancer; c) a close positive correlation was noted between heparanase and COX-2 expression (this correlation was particularly strong in cases of invasive cancer); and d) COX-2 expression was always seen in cases positive for heparanase expression. Our results indicate that heparanase expression increases during the progression of breast cancer into invasive cancer, and that this change is accompanied by increased COX-2 expression. They also suggest that heparanase may play a novel role for COX-2 mediated tumor angiogenesis in breast-cancer progression. 
 Induction of cyclooxygenase-2 in human endometrial stromal cells by malignant endometrial epithelial cells: evidence for the involvement of extracellularly regulated kinases and CCAAT/enhancer binding proteins. We previously reported that human malignant endometrial epithelial cell conditioned medium (M  ECM  ) up-regulated cyclooxygenase (COX)-2 mRNA and protein levels in human normal endometrial stromal cells (ESC). Here we showed that pretreatment with a selective inhibitor of the extracellularly regulated kinase (ERK)1/2 signaling pathway blocked the M  ECM  -induced COX-2 expression in ESC. Transient transfection assays indicated critical roles of a cAMP response element (CRE,-59/-53 bp) and a nuclear factor for interleukin (IL)-6 expression (NF-IL6) site (-132/-124 bp) in the regulation of basal and M  ECM  -induced activity of COX-2 gene promoter in ESC. Employing electrophoretic mobility shift assays, we demonstrated that increased functional binding of CCAAT/enhancer binding protein (C/EBP)alpha, C/EBPbeta and upstream stimulatory factor-2 to the CRE and C/EBPalpha and C/EBPbeta to the NF-IL6 site were, at least in part, responsible for M  ECM  -induced COX-2 expression in ESC. Moreover, overexpression of C/EBPalpha and C/EBPbeta significantly induced COX-2 promoter activity in ESC. Collectively, these results suggest that the basal and M  ECM  -induced transcription of the COX-2 gene in ESC is regulated through a combination of the CRE and the NF-IL6 site by functional interactions of C/EBPalpha and C/EBPbeta. 
  [Ee]xtracellular [Mm]atrix  PubMed  
 Alpha2beta1 integrin signalling enhances cyclooxygenase-2 expression in intestinal epithelial cells. Inflammatory bowel diseases (IBD) are linked to an increased risk of developing colon cancer, by inflammatory mediators and alterations to the   extracellular matrix   (ECM). The events induced by inflammatory mediators lead to dysregulated activation and induction of inflammatory genes such as cyclooxygenase-2 (COX-2). COX-2 is involved in the conversion of arachidonic acid to biologically active prostanoids and is highly upregulated in colon cancer. Since inflammation-induced changes to the   extracellular matrix   could affect integrin activities, we here investigated the effect of integrin signalling on the level of COX-2 expression in the non-transformed intestinal epithelial cell lines, Int 407 and IEC-6. Adhesion of these cells to a collagen I- or IV-coated surface, increased surface expression of alpha2beta1 integrin. Activation of integrins with collagen caused an increased cox-2 promoter activity, with a subsequent increase in COX-2 expression. The signalling cascade leading to this increased expression and promoter activity of cox-2, involves PKCalpha, the small GTPase Ras and NFkappaB but not Erk1/2 or Src activity. The integrin-induced increase in cellular COX-2 activity is responsible for an elevated generation of reactive oxygen species (ROS) and increased cell migration. This signalling pathway suggests a mechanism whereby inflammation-induced modulations of the ECM, can promote cancer transformation in the intestinal epithelial cells. 
 Identification of fetal and maternal single nucleotide polymorphisms in candidate genes that predispose to spontaneous preterm labor with intact membranes. The purpose of this study was to determine whether maternal/fetal single nucleotide polymorphisms (SNPs) in candidate genes are associated with spontaneous preterm labor/delivery.A genetic association study was conducted in 223 mothers and 179 fetuses (preterm labor with intact membranes who delivered <37 weeks of gestation [preterm birth (PTB)]), and 599 mothers and 628 fetuses (normal pregnancy); 190 candidate genes and 775 SNPs were studied. Single locus/haplotype association analyses were performed; the false discovery rate was used to correct for multiple testing.The strongest single locus associations with PTB were interleukin-6 receptor 1 (fetus; P=.000148) and tissue inhibitor of metalloproteinase 2 (mother; P=.000197), which remained significant after correction for multiple comparisons. Global haplotype analysis indicated an association between a fetal DNA variant in insulin-like growth factor F2 and maternal alpha 3 type IV collagen isoform 1 (global, P=.004 and .007, respectively).An SNP involved in controlling fetal inflammation (interleukin-6 receptor 1) and DNA variants in maternal genes encoding for proteins involved in   extracellular matrix   metabolism approximately doubled the risk of PTB. 
 Tanshinone IIA decreases the migratory ability of AGS cells by decreasing the protein expression of matrix metalloproteinases, nuclear factor κB-p65 and cyclooxygenase-2. During progression of gastric cancer, degradation of the   extracellular matri  x by matrix metalloproteinases (MMPs) has been associated with poor prognosis. Tanshinone IIA (Tan-IIA) exerts antitumor activity in a variety of human cancer cells. It is extracted from Danshen (Salviae miltiorrhizae radix), and induces apoptosis and inhibits the proliferation of gastric cancer cells. However, the molecular mechanisms underlying the inhibition of migration in gastric cancer by Tan-IIA have not been fully elucidated. In the present study, AGS cell migration ability was evaluated using a wound-healing assay. The protein expression levels of nuclear factor (NF)-κB-p65, cyclooxygenase (COX)-2, MMP-2, -7, and -9 and β-actin in AGS cells were measured by western blotting. The results demonstrated that AGS cells treated with Tan-IIA exhibit decreased protein expression levels of NF-κB-p65, COX-2, and MMP-2, -7 and -9. The results also indicate that Tan-IIA inhibits migration ability in a dose- and time-dependent manner. These findings demonstrate that Tan-IIA inhibits the migration ability of AGS human gastric cancer cells and that decreasing the protein expression of NF-κB-p65, COX-2, and MMP-2, -7 and -9 may be an underlying molecular mechanism. 
 A genetic association study of maternal and fetal candidate genes that predispose to preterm prelabor rupture of membranes (PROM). We sought to determine whether maternal/fetal single-nucleotide polymorphisms (SNPs) in candidate genes are associated with preterm prelabor rupture of membranes (pPROM).A case-control study was conducted in patients with pPROM (225 mothers and 155 fetuses) and 599 mothers and 628 fetuses with a normal pregnancy; 190 candidate genes and 775 SNPs were studied. Single locus/haplotype association analyses were performed; false discovery rate was used to correct for multiple testing (q* = 0.15).First, a SNP in tissue inhibitor of metalloproteinase 2 in mothers was significantly associated with pPROM (odds ratio, 2.12; 95% confidence interval, 1.47-3.07; P = .000068), and this association remained significant after correction for multiple comparisons. Second, haplotypes for Alpha 3 type IV collagen isoform precursor in the mother were associated with pPROM (global P = .003). Third, multilocus analysis identified a 3-locus model, which included maternal SNPs in collagen type I alpha 2, defensin alpha 5 gene, and endothelin 1.DNA variants in a maternal gene involved in   extracellular matrix   metabolism doubled the risk of pPROM. 
 Tanshinone IIA decreases the migratory ability of AGS cells by decreasing the protein expression of matrix metalloproteinases, nuclear factor κB-p65 and cyclooxygenase-2. During progression of gastric cancer, degradation of the   extracellular matri  x by matrix metalloproteinases (MMPs) has been associated with poor prognosis. Tanshinone IIA (Tan-IIA) exerts antitumor activity in a variety of human cancer cells. It is extracted from Danshen (Salviae miltiorrhizae radix), and induces apoptosis and inhibits the proliferation of gastric cancer cells. However, the molecular mechanisms underlying the inhibition of migration in gastric cancer by Tan-IIA have not been fully elucidated. In the present study, AGS cell migration ability was evaluated using a wound-healing assay. The protein expression levels of nuclear factor (NF)-κB-p65, cyclooxygenase (COX)-2, MMP-2, -7, and -9 and β-actin in AGS cells were measured by western blotting. The results demonstrated that AGS cells treated with Tan-IIA exhibit decreased protein expression levels of NF-κB-p65, COX-2, and MMP-2, -7 and -9. The results also indicate that Tan-IIA inhibits migration ability in a dose- and time-dependent manner. These findings demonstrate that Tan-IIA inhibits the migration ability of AGS human gastric cancer cells and that decreasing the protein expression of NF-κB-p65, COX-2, and MMP-2, -7 and -9 may be an underlying molecular mechanism. 
 Identification of fetal and maternal single nucleotide polymorphisms in candidate genes that predispose to spontaneous preterm labor with intact membranes. The purpose of this study was to determine whether maternal/fetal single nucleotide polymorphisms (SNPs) in candidate genes are associated with spontaneous preterm labor/delivery.A genetic association study was conducted in 223 mothers and 179 fetuses (preterm labor with intact membranes who delivered <37 weeks of gestation [preterm birth (PTB)]), and 599 mothers and 628 fetuses (normal pregnancy); 190 candidate genes and 775 SNPs were studied. Single locus/haplotype association analyses were performed; the false discovery rate was used to correct for multiple testing.The strongest single locus associations with PTB were interleukin-6 receptor 1 (fetus; P=.000148) and tissue inhibitor of metalloproteinase 2 (mother; P=.000197), which remained significant after correction for multiple comparisons. Global haplotype analysis indicated an association between a fetal DNA variant in insulin-like growth factor F2 and maternal alpha 3 type IV collagen isoform 1 (global, P=.004 and .007, respectively).An SNP involved in controlling fetal inflammation (interleukin-6 receptor 1) and DNA variants in maternal genes encoding for proteins involved in   extracellular matrix   metabolism approximately doubled the risk of PTB. 
 A genetic association study of maternal and fetal candidate genes that predispose to preterm prelabor rupture of membranes (PROM). We sought to determine whether maternal/fetal single-nucleotide polymorphisms (SNPs) in candidate genes are associated with preterm prelabor rupture of membranes (pPROM).A case-control study was conducted in patients with pPROM (225 mothers and 155 fetuses) and 599 mothers and 628 fetuses with a normal pregnancy; 190 candidate genes and 775 SNPs were studied. Single locus/haplotype association analyses were performed; false discovery rate was used to correct for multiple testing (q* = 0.15).First, a SNP in tissue inhibitor of metalloproteinase 2 in mothers was significantly associated with pPROM (odds ratio, 2.12; 95% confidence interval, 1.47-3.07; P = .000068), and this association remained significant after correction for multiple comparisons. Second, haplotypes for Alpha 3 type IV collagen isoform precursor in the mother were associated with pPROM (global P = .003). Third, multilocus analysis identified a 3-locus model, which included maternal SNPs in collagen type I alpha 2, defensin alpha 5 gene, and endothelin 1.DNA variants in a maternal gene involved in   extracellular matrix   metabolism doubled the risk of pPROM. 
 Alpha2beta1 integrin signalling enhances cyclooxygenase-2 expression in intestinal epithelial cells. Inflammatory bowel diseases (IBD) are linked to an increased risk of developing colon cancer, by inflammatory mediators and alterations to the   extracellular matrix   (ECM). The events induced by inflammatory mediators lead to dysregulated activation and induction of inflammatory genes such as cyclooxygenase-2 (COX-2). COX-2 is involved in the conversion of arachidonic acid to biologically active prostanoids and is highly upregulated in colon cancer. Since inflammation-induced changes to the   extracellular matrix   could affect integrin activities, we here investigated the effect of integrin signalling on the level of COX-2 expression in the non-transformed intestinal epithelial cell lines, Int 407 and IEC-6. Adhesion of these cells to a collagen I- or IV-coated surface, increased surface expression of alpha2beta1 integrin. Activation of integrins with collagen caused an increased cox-2 promoter activity, with a subsequent increase in COX-2 expression. The signalling cascade leading to this increased expression and promoter activity of cox-2, involves PKCalpha, the small GTPase Ras and NFkappaB but not Erk1/2 or Src activity. The integrin-induced increase in cellular COX-2 activity is responsible for an elevated generation of reactive oxygen species (ROS) and increased cell migration. This signalling pathway suggests a mechanism whereby inflammation-induced modulations of the ECM, can promote cancer transformation in the intestinal epithelial cells. 
 Differential expression of cyclooxygenases in hypertrophic scar and keloid tissues. Hypertrophic scar (HS) and keloid (KL) are two forms of an abnormal cutaneous scarring process, mainly characterized by excessive   extracellular matrix   deposition and fibroblast proliferation. Despite the increased understanding of the molecular and cellular events leading to HS and KL, the pathogenesis of these lesions remains poorly understood. A pivotal role in the formation of abnormal scars has been ascribed to transforming growth factor-beta, whose activity appears to be mediated through a link with pathways acting via cyclooxygenases (COX-1 and COX-2). To date, there is no report on the in vivo expression of COX-1 and COX-2 in human HS and KL tissues. Therefore, using immunohistochemistry and Western blot analysis, we investigated 36 cases of KL, 32 cases of HS, and 25 cases of normal skin in order to define the localization and distribution of COX-1 and COX-2 in the tissues of these scar lesions and the overlying epidermis. The results mainly show the following: (a) a significant overexpression of COX-1 in HS tissues and the overlying epidermis as compared with normal skin and KL tissues and (b) a significant overexpression of COX-2 in KL tissue and the overlying epidermis in contrast to normal skin and HS tissues. Our data support the hypothesis that both COXs are involved in the pathogenesis of scar lesions in different ways and, particularly, COX-1 in the formation of HS and COX-2 in the formation of KL. In addition, the overexpression of COX-1 and COX-2 in the epidermis overlying HS and KL tissues, respectively, underlines the importance of epithelial-mesenchymal interactions in the pathogenesis of scar lesions. 
 Heparanase and COX-2 expression as predictors of lymph node metastasis in large, high-grade breast tumors. Heparanase (HPA) contributes to breast cancer metastasis by facilitating the breakdown of the basement membrane and   extracellular matrix  . High expression of HPA is thought to be associated with increased nodal involvement and poor survival in patients with breast cancer. Overexpression of cyclooxygenase-2 (COX-2) in breast cancer is associated with indicators of poor prognosis such as lymph node metastasis, poor differentiation, and large tumor size. The underlying mechanism by which HPA and COX-2 overexpression increases the metastatic potential of breast cancer is not fully-understood. To enhance our understanding over these mechanisms, we aimed to investigate the relationship between the size of the tumor and HPA expression, tumor grade as well as lymph node status in patients with breast cancer.Immunohistochemical analysis of HPA and COX-2 expression was performed on 246 breast tumor samples. The expression of HPA was correlated with COX-2 expression, tumor grade, lymph node status, oestrogen receptor status.The overexpression of HPA and COX-2 was associated with increased likelihood of lymph node positivity in large, high-grade tumors. High-grade tumors with size greater than 20 mm, that overexpressed HPA, were 4-times more likely to be associated with lymph node involvement (OR 4.71, CI 1.21-18.25). Whereas, tumors greater than 20 mm in size were 5-times more likely to metastasize to the regional lymph nodes, if associated with overexpression of COX-2 (OR 5.5, CI 1.2-24.8).Expression of HPA appears to be a key mechanism by which large, high-grade breast tumors metastasize to regional lymph nodes, while COX-2 overexpression may be an independent predictor of lymph node positivity. 
 Heterogeneous nuclear ribonucleoprotein K (hnRNP-K) promotes tumor metastasis by induction of genes involved in   extracellular matrix  , cell movement, and angiogenesis. Cancer is a leading cause of death and still awaits effective therapies. Rapid industrialization has contributed to increase in incidence of cancer. One of the reasons why most of the cancers fail therapy is due to their metastatic property. Hence identification of factors leading to metastasis is highly important to design effective and novel anti-cancer therapeutics. In our earlier study (Inoue, A., Sawata, S. Y., Taira, K., and Wadhwa, R. (2007) Loss-of-function screening by randomized intracellular antibodies: identification of hnRNP-K as a potential target for metastasis. Proc. Natl. Acad. Sci. U.S.A. 104, 8983-8988), we had reported that the involvement of heterogeneous nuclear ribonucleoprotein K (hnRNP-K) in metastasis. Here, we established hnRNP-K-overexpressing and -underexpressing derivative cell lines and examined their proliferation and metastatic properties in vitro and in vivo. Whereas hnRNP-K compromised cells showed delayed tumor growth, its overexpression resulted in enhanced malignancy and metastasis. Molecular basis of the hnRNP-K induced malignant and metastatic phenotypes was dissected by cDNA microarray and pathway analyses. We found that the hnRNP-K regulates   extracellular matrix  , cell motility, and angiogenesis pathways. Involvement of the selected genes (Cck, Mmp-3, Ptgs2, and Ctgf) and pathways was validated by gene-specific expression analysis. Our results demonstrated that the hnRNP-K is a potential target for metastasis therapy. 
 Cyclooxygenase-2/carbonic anhydrase-IX up-regulation promotes invasive potential and hypoxia survival in colorectal cancer cells. Inflammation promotes colorectal carcinogenesis. Tumour growth often generates a hypoxic environment in the inner tumour mass. We here report that, in colon cancer cells, the expression of the pro-inflammatory enzyme cyclooxygenase-2 (COX-2) associates with that of the hypoxia response gene carbonic anhydrase-IX (CA-IX). The COX-2 knockdown, achieved by the stable infection of a COX-2 specific short harpin RNA interference (shCOX-2), down-regulates CA-IX gene expression. In colorectal cancer (CRC) cells, PGE(2), the main COX-2 gene products, promotes CA-IX gene expression by ERK1/2 activation. In normoxic environment, shCOX-2 infected/CA-IX siRNA transfected CRC cells show a reduced level of active metalloproteinase-2 (MMP-2) that associates with a decreased   extracellular matrix   invasion capacity. In presence of hypoxia, COX-2 gene expression and PGE(2) production increase. The knockdown of COX-2/CA-IX blunts the survival capability of CRC cells in hypoxia. At a high cell density, a culture condition that creates a mild pericellular hypoxic environment, the expression of COX-2/CA-IX genes is increased and triggers the invasive potential of colon cancer cells. In human colon cancer tissues, COX-2/CA-IX protein expression levels, assessed by Western blot and immunohistochemistry, correlate each other and increase with tumour stage. In conclusion, these data indicate that COX-2/CA-IX interplay promotes the aggressive behaviour of CRC cells. 
 Cyclooxygenase-2-derived prostaglandin E2 stimulates Id-1 transcription. Cyclooxygenase-2 (COX-2) and Id-1 are overexpressed in a variety of human malignancies. Recently, each of these genes was found to play a role in mediating breast cancer metastasis to the lungs, but their potential interdependence was not evaluated. Hence, the main objective of the current study was to determine whether COX-2-derived prostaglandin (PGE(2)) activated Id-1 transcription, leading in turn to increased invasiveness of mammary epithelial cells. In MDA-MB-231 cells, treatment with PGE(2) induced Id-1, an effect that was mimicked by an EP(4) agonist. PGE(2) via EP(4) activated the epidermal growth factor receptor (EGFR) --> ERK1/2 pathway, which led to increased expression of Egr-1. PGE(2) stimulated EGFR signaling by inducing the release of amphiregulin, an EGFR ligand. The ability of PGE(2) to activate Id-1 transcription was mediated by enhanced binding of Egr-1 to the Id-1 promoter. Silencing of COX-2 or pharmacological inhibition of COX-2 led to reduced PGE(2) production, decreased Id-1 expression, and reduced migration of cells through   extracellular matrix  . A similar decrease in cell migration was found when Id-1 was silenced. The interrelationship between COX-2, PGE(2), Id-1, and cell invasiveness was also compared in nontumorigenic SCp2 and tumorigenic SCg6 mammary epithelial cells. Consistent with the findings in MDA-MB-231 cells, COX-2-derived PGE(2) induced Id-1, leading in turn to increased cell invasiveness. Taken together, these results suggest that PGE(2) via EP(4) activated the EGFR --> ERK1/2 --> Egr-1 pathway, leading to increased Id-1 transcription and cell invasion. These findings provide new insights into the relationship between COX-2 and Id-1 and their potential role in metastasis. 
 Changes in   extracellular matrix   composition regulate cyclooxygenase-2 expression in human mesangial cells. Glomerular diseases are characterized by a sustained synthesis and accumulation of abnormal   extracellular matrix   proteins, such as collagen type I. The   extracellular matrix   transmits information to cells through interactions with membrane components, which directly activate many intracellular signaling events. Moreover, accumulating evidence suggests that eicosanoids derived from cyclooxygenase (COX)-2 participate in a number of pathological processes in immune-mediated renal diseases, and it is known that protein kinase B (AKT) may act through different transcription factors in the regulation of the COX-2 promoter. The present results show that progressive accumulation of collagen I in the extracellular medium induces a significant increase of COX-2 expression in human mesangial cells, resulting in an enhancement in PGE(2) production. COX-2 overexpression is due to increased COX-2 mRNA levels. The study of the mechanism implicated in COX-2 upregulation by collagen I showed focal adhesion kinase (FAK) activation. Furthermore, we observed that the activation of the phosphatidylinositol 3-kinase (PI3K)/AKT pathway by collagen I and collagen I-induced COX-2 overexpression was abolished by PI3K and AKT inhibitors. Additionally, we showed that the cAMP response element (CRE) transcription factor is implicated. Finally, we studied COX-2 expression in an animal model, N(G)-nitro-l-arginine methyl ester hypertensive rats. In renal tissue and vascular walls, COX-2 and collagen type I content were upregulated. In summary, our results provide evidence that collagen type I increases COX-2 expression via the FAK/PI3K/AKT/cAMP response element binding protein signaling pathway. 
 Noninvasive imaging identifies new roles for cyclooxygenase-2 in choline and lipid metabolism of human breast cancer cells. The expression of cyclooxygenase-2 (COX-2) is observed in approximately 40% of breast cancers. A major product of the COX-2-catalyzed reaction, prostaglandin E(2), is an inflammatory mediator that participates in several biological processes, and influences invasion, vascularization and metastasis. Using noninvasive MRI and MRS, we determined the effect of COX-2 downregulation on the metabolism and invasion of intact poorly differentiated MDA-MB-231 human breast cancer cells stably expressing COX-2 short hairpin RNA. Dynamic tracking of invasion,   extracellular matrix   degradation and metabolism was performed with an MRI- and MRS-compatible cell perfusion assay under controlled conditions of pH, temperature and oxygenation over the course of 48  h. COX-2-silenced cells exhibited a significant decrease in invasion relative to parental cells that was consistent with the reduced expression of invasion-associated matrix metalloproteinase genes and an increased level of the tissue inhibitor of metalloproteinase-1. We identified, for the first time, a role for COX-2 in mediating changes in choline phospholipid metabolism, and established that choline kinase expression is partly dependent on COX-2 function. COX-2 silencing resulted in a significant decrease in phosphocholine and total choline that was detected by MRS. In addition, a significant increase in lipids, as well as lipid droplet formation, was observed. COX-2 silencing transformed parental cell metabolite patterns to those characteristic of less aggressive cancer cells. These new functional roles of COX-2 may identify new biomarkers and new targets for use in combination with COX-2 targeting to prevent invasion and metastasis. 
 The effects of cyclooxygenase2-prostaglandinE2 pathway on Helicobacter pylori-induced urokinase-type plasminogen activator system in the gastric cancer cells. Urokinase-type plasminogen activator (uPA) and its receptor (uPAR) play an important role in the destruction of the   extracellular matrix   and basement membrane. The induction of uPA and uPAR in the gastric cancer cells with H. pylori has been demonstrated previously. The involvement of COX-2-PGE2 pathway in the uPA system (uPA and uPAR) expression is unclear.Gastric cancer cells (MKN45) were co-cultured with H. pylori standard strain (NCTC11637). The specific inductions of uPA and uPAR mRNA were examined by reverse transcription-polymerase chain reaction amplification. The secreted uPA antigen was measured by ELISA. To evaluate the involvement of COX-2 and PGE2 pathway in H. pylori-induced uPA and uPAR expressions, we examined the effects of COX-2 inhibitor and PGE2 receptor antagonist on H. pylori-induced uPA and uPAR expression in the gastric cancer cells.The expressions of both uPA and uPAR mRNAs in the gastric cancer cells increased obviously (12-fold and 3-fold, respectively) with H. pylori stimulation. The amount of uPA antigen into the culture medium increased dramatically with H. pylori stimulation. The COX-2 expression level in the gastric cancer cells increased remarkably with H. pylori stimulation. H. pylori-induced uPA and uPAR expression levels were suppressed with COX2 inhibitor treatment. The amount of PGE2 antigen into the culture medium increased dramatically 24 hours after H. pylori stimulation. The gastric cancer cells expressed EP2 and EP4 subtypes. EP2 receptor antagonist suppressed the H. pylori-induced uPA and uPAR expressions in the gastric cancer cells.Our results indicated that COX2-PGE2 pathway may be involved in H. pylori-associated uPA and uPAR induction, and that COX-2 inhibitor or EP2 receptor antagonist may inhibit angiogenesis and tumor invasion via suppression of the uPA system. 
 ATP modulates load-inducible IL-1beta, COX 2, and MMP-3 gene expression in human tendon cells. Tendon cells receive mechanical signals from the load bearing matrices. The response to mechanical stimulation is crucial for tendon function. However, overloading tendon cells may deteriorate   extracellular matrix   integrity by activating intrinsic factors such as matrix metalloproteinases (MMPs) that trigger matrix destruction. We hypothesized that mechanical loading might induce interleukin-1beta (IL-1beta) in tendon cells, which can induce MMPs, and that extracellular ATP might inhibit the load-inducible gene expression. Human tendon cells isolated from flexor digitorum profundus tendons (FDPs) of four patients were made quiescent and treated with ATP (10 or 100 microM) for 5 min, then stretched equibiaxially (1 Hz, 3.5% elongation) for 2 h followed by an 18-h-rest period. Stretching induced IL-1beta, cyclooxygenase 2 (COX 2), and MMP-3 genes but not MMP-1. ATP reduced the load-inducible gene expression but had no effect alone. A medium change caused tendon cells to secrete ATP into the medium, as did exogenous UTP. The data demonstrate that mechanical loading induces ATP release in tendon cells and stimulates expression of IL-1beta, COX 2, and MMP-3. Load-induced endogenous IL-1beta may trigger matrix remodeling or a more destructive pathway(s) involving IL-1beta, COX 2, and MMP-3. Concomitant autocrine and paracrine release of ATP may serve as a negative feedback mechanism to limit activation of such an injurious pathway. Attenuation or failure of this negative feedback mechanism may result in the progression to tendinosis. 
 Oncogenic RAS mutations in myeloma cells selectively induce cox-2 expression, which participates in enhanced adhesion to fibronectin and chemoresistance. Oncogenic RAS expression occurs in up to 40% of multiple myeloma (MM) cases and correlates with aggressive disease. Since activated RAS induces cyclooxygenase-2 (cox-2) expression in other tumor models, we tested a role for cox-2 in mutant RAS-containing MM cells. We used the ANBL-6 isogenic MM cell lines in which the IL-6-dependent parental line becomes cytokine independent following transfection with mutated N-RAS or K-RAS. Both mutated N-RAS- and K-RAS-expressing ANBL-6 cells demonstrated a selective up-regulation of cox-2 expression and enhanced secretion of PGE2, a product of cox-2. Furthermore, in 3 primary marrow specimens, which contained MM cells expressing mutated RAS, 15% to 40% of tumor cells were positive for cox-2 expression by immunohistochemistry. We used cox-2 inhibitors, NS398 and celecoxib, and neutralizing anti-PGE2 antibody to test whether cox-2/PGE2 was involved in the aggressive phenotype of MM ANBL-6 cells containing mutated RAS. Although these interventions had no effect on IL-6-independent growth or adhesion to marrow stromal cells, they significantly inhibited the enhanced binding of mutant RAS-containing MM cells to fibronectin and the enhanced resistance to melphalan. These results indicate a selective induction of cox-2 in MM cells containing RAS mutations, which results in heightened binding to   extracellular matrix   protein and chemotherapeutic drug resistance. 
 The effect of the expression of angiotensin II on   extracellular matrix   metalloproteinase inducer (EMMPRIN) in macrophages is mediated via the AT1/COX-2/PGE2 pathway. To explore the expression of   extracellular matrix   metalloproteinase inducer (EMMPRIN) in THP-1 macrophages induced by angiotensin II (Ang II) and the mechanism of EMMPRIN expression.THP-1 cells were cultured and induced into macrophages, then stimulated with 10(-6) mol/L Ang II. Levels of EMMPRIN gene and its protein were measured by real-time polymerase chain reaction and western blotting. Prostaglandin E(2) (PGE(2)) expression was assayed by enzyme-linked immunosorbent assay. Antagonists of the angiotensin type-1 receptor (AT(1)R) and angiotensin type-2 receptor (AT(2)R) were used to inhibit the effect of Ang II, and PGE(2) added to detail the mechanism of Ang II-induced EMMPRIN expression.Ang II clearly induced the expression of EMMPRIN mRNA and protein in macrophages; this expression peaked at 12 h and declined after 24 h. The tendency of enhancement of the levels of cyclooxygenase-2 (COX-2) and PGE(2) was coincident with EMMPRIN expression. AT(1)-receptor antagonists and COX-2 inhibitors inhibited the effect of Ang II, but AT(2)-receptor antagonists did not.Ang II can up-regulate EMMPRIN expression in THP-1 macrophages via the AT(1)/COX-2/PGE(2) signal transduction pathway, and the effect can be inhibited by losartan and NS-398. 
 Frequent co-localization of Cox-2 and laminin-5 gamma2 chain at the invasive front of early-stage lung adenocarcinomas. Laminin-5 is an   extracellular matrix   protein that plays a key role in cell migration and tumor invasion. Cox-2 is an induced isoform of cyclooxygenases that plays an important role in carcinogenesis, suppression of apoptosis, angiogenesis, and metastasis of colon cancer. We report frequent co-expression of cox-2 and laminin-5 at the invasive front of early-stage lung adenocarcinomas. We investigated the expression of cox-2 and laminin-5 immunohistochemically in 102 cases of small-sized lung adenocarcinoma (maximum dimension, 2 cm or less). Cox-2 and laminin-5 were expressed in 97 (95.1%) and 82 (80.4%) cases, respectively. Both were preferentially localized in cancer cells at the cancer-stroma interface, although cox-2 tended to show a diffuse staining pattern in some cases. A comparison of their staining patterns revealed a striking similarity in their distribution in 24 cases, and a partial overlap between their localization in another 20 cases. Moreover, an overall correlation was found between the expression levels of cox-2 and laminin-5 (P = 0.018). To gain insight into the mechanisms that regulate the expression of these proteins, we additionally studied their expression in 58 cases of stage I lung adenocarcinoma, in which p53 status was determined by immunohistochemistry, polymerase chain reaction-single strand conformation polymorphism analysis, and direct sequencing. The results showed that tumors with mutant p53 tended to express more cox-2 than those with wild-type p53 (P = 0.080). Also, tumors that overexpressed p53 had higher levels of cox-2 and laminin-5 than those without p53 overexpression (P = 0.032 and 0.047, respectively). Further immunohistochemical analysis showed that tumors that overexpressed both epidermal growth factor receptor (EGFR) and erbB-2 had higher levels of cox-2 and laminin-5 than those without concomitant overexpression of these proteins (P = 0.014 and P = 0.018, respectively). To see whether EGFR signaling is involved in cox-2 and laminin-5 expression, we further conducted in vitro analyses using six lung adenocarcinoma cell lines (A549, HLC-1, ABC-1, LC-2/ad, VMRC-LCD, and L27). Western blot analyses showed that cox-2 mRNA levels, and to a lesser extent laminin-5 gamma2 mRNA levels, correlated with the expression levels of erbB-2 and the phosphorylated form of MAPK/ERK-1/2 protein. The addition of transforming growth factor-alpha increased both cox-2 and laminin-5 gamma2 mRNA levels in A549, ABC-1, and L27 with different kinetics; the induction of cox-2 occurred earlier than that of laminin-5 gamma2. Finally, the migration of ABC-1 cells was inhibited by MAP kinase kinase inhibitor PD98059 and a selective cox-2 inhibitor NS-398. In contrast, the migration of A549 cells was inhibited by PD98059, but much less effectively by NS-398. These results suggest that co-stimulatory mechanisms may exist that increase the expression of cox-2 and laminin-5 at the invasive front of lung adenocarcinomas and that EGFR signaling could be one of the mechanisms. Further investigations are warranted concerning the role of cox-2 and laminin-5 in cancer cell invasion and the significance of p53 and EGFR signaling in the regulation of cox-2 and laminin-5 expression. 
 Anti-angiogenic and anti-inflammatory properties of kahweol, a coffee diterpene. Epidemiological studies have shown that unfiltered coffee consumption is associated with a low incidence of cancer. This study aims to identify the effects of kahweol, an antioxidant diterpene contained in unfiltered coffee, on angiogenesis and key inflammatory molecules.The experimental procedures included in vivo angiogenesis assays (both the chicken and quail choriallantoic membrane assay and the angiogenesis assay with fluorescent zebrafish), the ex vivo mouse aortic ring assay and the in vitro analysis of the effects of treatment of human endothelial cells with kahweol in cell growth, cell viability, cell migration and zymographic assays, as well as the tube formation assay on Matrigel. Additionally, two inflammation markers were determined, namely, the expression levels of cyclooxygenase 2 and the levels of secreted monocyte chemoattractant protein-1. We show for the first time that kahweol is an anti-angiogenic compound with inhibitory effects in two in vivo and one ex vivo angiogenesis models, with effects on specific steps of the angiogenic process: endothelial cell proliferation, migration, invasion and tube formation on Matrigel. We also demonstrate the inhibitory effect of kahweol on the endothelial cell potential to remodel   extracellular matrix   by targeting two key molecules involved in the process, MMP-2 and uPA. Finally, the anti-inflammatory potential of this compound is demonstrated by its inhibition of both COX-2 expression and MCP-1 secretion in endothelial cells.Taken together, our data indicate that, indeed, kahweol behaves as an anti-inflammatory and anti-angiogenic compound with potential use in antitumoral therapies. These data may contribute to the explanation of the reported antitumoral effects of kahweol, including the recent epidemiological meta-analysis showing that drinking coffee could decrease the risk of certain cancers. 
 COX-2 induction by heparanase in the progression of breast cancer. Breast cancer confined within the lactiferous duct or lobule, without invading the stroma, is called ductal carcinoma in situ (DCIS), whereas breast cancer that has invaded the stroma through the basal membrane is called invasive cancer. Heparanase, an endo-beta-D-glucuronidase that specifically degrades heparan sulfate proteoglycans (HSPGs) in the   extracellular matrix   (ECM), plays an important role when breast cancer cells breach the basal membrane. Recently, we have reported that heparanase is involved in angiogenesis through direct induction of cyclo-oxygenase-2 (COX-2). COX-2 induces vascular endothelial growth factor (VEGF) and basic fibroblast growth factor (bFGF) and is thus involved in neovascularization. The present study was undertaken to analyze surgically resected breast cancer specimens for heparanase and COX-2 expression, using specimens from 59 patients with invasive cancer and 85 patients with DCIS (including 41 cases of DCIS adjacent to invasive cancer). This study yielded the following results: a) the distribution of heparanase within tumor tissue was identical to that of COX-2; b) heparanase expression was more frequent in invasive cancer than in non-invasive cancer; c) a close positive correlation was noted between heparanase and COX-2 expression (this correlation was particularly strong in cases of invasive cancer); and d) COX-2 expression was always seen in cases positive for heparanase expression. Our results indicate that heparanase expression increases during the progression of breast cancer into invasive cancer, and that this change is accompanied by increased COX-2 expression. They also suggest that heparanase may play a novel role for COX-2 mediated tumor angiogenesis in breast-cancer progression. 
 EC-SOD induces apoptosis through COX-2 and galectin-7 in the epidermis. Extracellular superoxide dismutase (EC-SOD) is an anti-oxidant enzyme found in the   extracellular matrix   of tissues, and plays an important role in the prevention of many diseases caused by oxidative stress. However, other functions of EC-SOD in epidermis are not well known.We investigated the functions of EC-SOD in epidermis using keratinocyte cell line and EC-SOD transgenic mice.Expression of galectin-7 in pEC-SOD transfected cells or skin of EC-SOD transgenic mice was detected by western blot analysis. The percentage of apoptotic cells was determined by propidium iodide staining and subsequent FACS analysis. COX-2 siRNA or scrambled siRNA was transfected into HaCaT cells and western blot analysis was performed to detect pro-apoptotic protein levels.The epidermis of EC-SOD transgenic mice was thinner than wild type mice. In addition, we showed that the thin epidermis of EC-SOD transgenic mice results from the apoptosis of epidermal cells. To elucidate which molecules are involved in EC-SOD-induced apoptosis, we utilized two-dimensional electrophoresis; the results showed that the epidermis of EC-SOD transgenic mice produces more galectin-7, a pro-apoptotic factor, than the wild type. Furthermore, we showed that the transfection of EC-SOD-expressing plasmids induces the production of galectin-7, and pro-apoptotic proteins in keratinocytes. This suggests that EC-SOD induces apoptosis through increased galectin-7 expression. Finally, we demonstrated that EC-SOD-induced galectin-7 results from the production of COX-2.Our results imply that EC-SOD plays a role not only as a reactive oxygen species scavenger, but also as a pro-apoptotic factor via COX-2/galectin-7 pathways in the epidermis. 
 Silencing of cyclooxygenase-2 inhibits metastasis and delays tumor onset of poorly differentiated metastatic breast cancer cells. Cyclooxygenases (COX) are rate-limiting enzymes involved in the conversion of PLA(2)-mobilized arachidonic acid into prostaglandins and thromboxanes. COX-2 is a key mediator of inflammation during both physiologic and pathologic responses to endogenous stimuli and infectious agents. Its overexpression has been detected in different cancers, including that of the breast. Using RNA interference, we have reduced the expression of COX-2 in the highly malignant breast cancer cell line MDA-MB-231 below detectable levels in response to interleukin-1 beta or 12-O-tetradecanoylphorbol-13-acetate treatment. Microarray analysis showed that COX-2 silencing resulted in the loss of mRNA expression of several oncogenic markers, such as matrix metalloproteinase-1, chemokine (C-X-C motif) receptor 4, and interleukin-11, which have been correlated with poor disease outcome, and in the up-regulation of antimetastatic transcripts, such as thrombospondin-1 and Epstein-Barr-Induced 3. Cells lacking COX-2 were less able to invade reconstituted   extracellular matrix   than parental cells in vitro. Consistent with these changes, loss of COX-2 resulted in the abolition or the significant delay of tumor onset when the cells were injected in the mammary fat pad of severe combined immunodeficient mice. Finally, silencing of COX-2 resulted in the inhibition of metastasis to the lungs of severe combined immunodeficient mice after intravenous injection. These data show that silencing of COX-2 abolishes the metastatic potential of MDA-MB-231 cells in vivo. 
 Expression of gelatinase (MMP-2 and MMP-9) and cyclooxygenase-2 (COX-2) in endometrial carcinoma. Matrix metalloproteinases (MMPs) are key players in the degradation of   extracellular matrix   and basement membranes, and are thus important in tumor invasion. Gelatinases (MMP-2 and MMP-9) in particular are prognostic factors in many solid tumors. In this study the immunohistochemical expression of both COX-2 and matrix metalloproteinases has been shown for the first time in endometrium carcinoma.Forty-two endometrial carcinoma tissues were immunostained for MMP2 antibody (1:100, Rabbit polyclonal), MMP9 antibody (1:100, Rabbit polyclonal) and CoX2 antibody (1:100, Epitope specific rabbit antibody).90.5% of the cases were positive for MMP-2 and MMP-9, and 83.3% of the cases were positive for COX-2. A statistically significant association was found between COX-2 overexpression and FIGO stage (p = 0.001). A positive correlation was also found with histological grade (p = 0.006), myometrial invasion (p = 0.033), vascular invasion (p = 0.017), and lymphatic invasion (p = 0.007). A positive correlation was found between MMP-2 overexpression and vascular and lymphatic invasion (p = 0.030 and p = 0.003, respectively). MMP-9 overexpression was also found to be correlated with vascular and lymphatic invasion (p = 0.001 and p = 0.012, respectively). Furthermore, there was a statistically significant correlation between MMP-2 and MMP-9 overexpression (p = 0.0001).The results showed that COX-2, MMP-2 and MMP-9 were expressed in a high percentage of primary endometrial carcinomas and their expressions may be associated closely with parameters of tumor aggressiveness. 
 Cyclooxygenase-2 and matrix metalloproteinase-9 expressions correlate with tissue inflammation degree in periodontal disease. Cyclooxygenase-2 (Cox-2) and matrix metalloproteinase-9 (MMP-9) have synergistic effects in the degradation of the extra-cellular matrix.The aim of our study was to correlate the intensity of inflammation with MMP-9 and Cox-2 expression in the periodontal tissue of patients with chronic inflammatory disease (gingivitis and chronic periodontitis) in order to determine the role of these two biomarkers in the progression of periodontal disease.To conduct this study we analyzed the gingival biopsies taken from patients clinically divided into three study groups: Group I (control): Patients free of periodontal disease (seven biopsies); Group II: Patients with gingivitis (10 biopsies); Group III: Patients with chronic periodontitis (10 biopsies). In these three groups, we graded the intensity of inflammation in the lamina propria and the immunohistochemical expression of MMP-9 and Cox-2.The presence of a large number of inflammatory cells in the lamina propria in patients with gingivitis or chronic periodontitis (Groups II and III) correlated with the clinically diagnosed inflammation of the gingival tissue. The expression of MMP-9 was higher in patients with chronic periodontitis than in those with gingivitis, showing a trend towards statistical significance (p=0.07, Mann-Whitney U-test). The expression of Cox-2 in periodontitis was also higher compared to gingivitis (p=0.05, Mann-Whitney U-test) and to controls (p=0.001, Mann-Whitney U-test).The inflammation score could be positively correlated to the MMP-9 and Cox-2 expression scores at the overall study group, but not separately on gingivitis and periodontitis patients.The presence of an intensive inflammatory infiltrate is characteristic both for periodontitis and gingivitis. MMP-9 and Cox-2 show higher expression in periodontitis, than in gingivitis and healthy controls, but MMP-9 and Cox-2 expression scores cannot be directly correlated to the grade of inflammatory infiltrate in the two different disease entities. As biomarkers of chronic inflammation activity, angiogenesis, and degradation of the   extracellular matrix  , MMP-9 and Cox-2 can be used in clinical practice for the detection of patients with chronic periodontitis risk, at whom treatment with Cox-2 and MMP-9 inhibitors may be considered. 
 Association of cyclooxygenase-2 and matrix metalloproteinase-2 expression in human breast cancer. Expression of cyclooxygenase-2 (COX-2) and matrix metalloproteinase-2 (MMP-2) associates with reduced survival in human breast cancer. COX-2 may be directly involved with mammary carcinogenesis, since expression of COX-2 is sufficient for formation of breast tumors in transgenic mice, and COX-2 selective inhibitors can suppress tumorigenesis in rodent models of breast cancer. MMP-2 is an   extracellular matrix   degrading proteolytic enzyme that bas been linked to invasion and metastasis. A direct link between COX-2 and MMP-2 may exist, since inhibition of COX-2 activity can result in reduction of MMP-2 expression and activity. In this study we analyzed protein expression of COX-2 and MMP-2 in tissue array specimens of 278 invasive breast cancers by immunohistochemistry. Immunopositivity of these two markers was correlated with each other and with various clinicopathological parameters including survival. We found high COX-2 expression in 30% and high MMP-2 expression in 83% of the breast cancer specimens, and there was a positive association between the expression of these two factors (p = 0.003). It was especially evident that whenever COX-2 expression was high, MMP-2 expression was almost invariably high (95%). Furthermore, high expression of either COX-2 or MMP-2 associated with decreased disease specific survival when compared with the COX-2 or MPP-2 low group (p = 0.026 and p = 0.021, respectively). Taken together, our results indicate that expression of COX-2 protein is associated with expression of MMP-2 protein in human breast cancer and that both COX-2 and MMP-2 are markers of poor prognosis in breast cancer. 
  GeneRIFs  
 hnRNP-K regulates   extracellular matrix  , cell motility, and angiogenesis pathways. Involvement of the selected genes (Cck, Mmp-3, Ptgs2, and Ctgf) and pathways was validated by gene-specific expression analysis 
 a selective induction of cox-2 in multiple myeloma cells containing RAS mutations, results in heightened binding to   extracellular matrix   protein and chemotherapeutic drug resistance 
  [Ii]nvasive  PubMed  
 The hepatitis B virus X protein promotes tumor cell invasion by inducing membrane-type matrix metalloproteinase-1 and cyclooxygenase-2 expression. Hepatocellular carcinoma is strongly associated with chronic infection by the hepatitis B virus (HBV) and has poor prognosis due to intrahepatic metastasis. HBx is often the only HBV protein detected in hepatic tumor cells; however, its contribution to tumor invasion and metastasis has not been established so far. In this work, we show that HBx enhances tumor cell invasion, both in vivo and in vitro. The increased   invasive   capacity induced by HBx is mediated by an upregulation of membrane-type 1 matrix metalloproteinase (MT1-MMP) expression, which in turn activates matrix metalloproteinase-2. Induction of both MT1-MMP expression and cell invasion by HBx is dependent on cyclooxygenase-2 (COX-2) activity. In addition, HBx upregulates the expression of COX-2, which is mediated by the transcriptional activation of the COX-2 gene promoter in a nuclear factor of activated T cell-dependent (NF-AT-dependent) manner. These results demonstrate the ability of HBx to promote tumor cell invasion by a mechanism involving the upregulation of MT1-MMP and COX-2 and provide new insights into the mechanism of action of this viral protein and its involvement in tumor metastasis and recurrence of hepatocellular carcinoma. 
 COX-2 promotes breast cancer cell radioresistance via p38/MAPK-mediated cellular anti-apoptosis and   invasive  ness. Radioresistance is one of the major barriers to improve the survival rate of breast cancer patients. Cyclooxygenase 2 (COX-2) is usually overexpressed in highly   invasive   and metastatic breast cancer, which may indicate an association with breast cancer radioresistance. The function role of COX-2 was investigated by using a radioresistant breast cancer cell line MDA-MB-231/RR10 and its parental cell line MDA-MB-231 cells before or after COX-2 was silenced by a specific small hairpin RNA (shRNA). The cell proliferation, migration, invasion, colony formation, and apoptosis were measured by CCK-8, scratch-wound, transwell, clone formation assay, and flow cytometry. Protein and mRNA expression were analyzed by Western blot and quantitative reverse transcriptase-polymerase chain reaction. COX-2 is upregulated in MDA-MB-231/RR10 cells compared with in MDA-MB-231 cells, and silencing of COX-2 expression by shRNA in MDA-MB-231/RR10 cells decreases the expression of Bcl-2 and Bcl-XL, but increases the proapoptotic protein BAK, leading to the increased apoptosis following treatment with γ-irradiation in comparison with those in control cells. Silencing of COX-2 also increases the expression of β-catenin and E-cadherin, two anti-invasion proteins, resulting in reduced cell migration and invasion tested by transwell chambers and wound-healing assays. Further study demonstrated that COX-2-induced radioresistance is negatively regulated through the phosphorylation of p38 at Tyr182, and that the phosphorylation of p38 induced by TNF-alpha reduces the expression of Bcl-2, BCL-XL, but increases β-catenin and E-cadherin, leading to the decreas  ed invas  iveness of cells. Our data suggest that COX-2, p38, Bcl-2, Bcl-XL, β-catenin, and E-cadherin may be considered as potential therapeutic targets against radioresistant breast cancer. 
 Genetic susceptibility to distinct bladder cancer subphenotypes. Clinical, pathologic, and molecular evidence indicate that bladder cancer is heterogeneous with pathologic/molecular features that define distinct subphenotypes with different prognoses. It is conceivable that specific patterns of genetic susceptibility are associated with particular subphenotypes.To examine evidence for the contribution of germline genetic variation to bladder cancer heterogeneity.The Spanish Bladder Cancer/EPICURO Study is a case-control study based in 18 hospitals located in five areas in Spain. Cases were patients with a newly diagnosed, histologically confirmed, urothelial cell carcinoma of the bladder from 1998 to 2001. Case diagnoses were reviewed and uniformly classified by pathologists following the World Health Organisation/International Society of Urological Pathology 1999 criteria. Controls were hospital-matched patients (n=1149).A total of 1526 candidate variants in 423 candidate genes were analysed. Three distinct subphenotypes were defined according to stage and grade: low-grade nonmuscle   invasive   (n=586), high-grade nonmuscle   invasive   (n=219), and muscle   invasive   (n=246). The association between each variant and subphenotype was assessed by polytomous risk models adjusting for potential confounders. Heterogeneity in genetic susceptibility among subphenotypes was also tested.Two established bladder cancer susceptibility genotypes, NAT2 slow-acetylation and GSTM1-null, exhibited similar associations among the subphenotypes, as did VEGF-rs25648, which was previously identified in our study. Other variants conferred risks for specific tumour subphenotypes such as PMS2-rs6463524 and CD4-rs3213427 (respective heterogeneity p values of 0.006 and 0.004), which were associated with muscle-  invasive   tumours (per-allele odds ratios [95% confidence interval] of 0.56 [0.41-0.77] and 0.71 [0.57-0.88], respectively) but not with non-muscle-  invasive   tumours. Heterogeneity p values were not robust in multiple testing according to their false-discovery rate.These exploratory analyses suggest that genetic susceptibility loci might be related to the molecular/pathologic diversity of bladder cancer. Validation through large-scale replication studies and the study of additional genes and single nucleotide polymorphisms are required. 
 Evaluation of candidate stromal epithelial cross-talk genes identifies association between risk of serous ovarian cancer and TERT, a cancer susceptibility "hot-spot". We hypothesized that variants in genes expressed as a consequence of interactions between ovarian cancer cells and the host micro-environment could contribute to cancer susceptibility. We therefore used a two-stage approach to evaluate common single nucleotide polymorphisms (SNPs) in 173 genes involved in stromal epithelial interactions in the Ovarian Cancer Association Consortium (OCAC). In the discovery stage, cases with epithelial ovarian cancer (n=675) and controls (n=1,162) were genotyped at 1,536 SNPs using an Illumina GoldenGate assay. Based on Positive Predictive Value estimates, three SNPs-PODXL rs1013368, ITGA6 rs13027811, and MMP3 rs522616-were selected for replication using TaqMan genotyping in up to 3,059 serous   invasive   cases and 8,905 controls from 16 OCAC case-control studies. An additional 18 SNPs with Pper-allele<0.05 in the discovery stage were selected for replication in a subset of five OCAC studies (n=1,233 serous   invasive   cases; n=3,364 controls). The discovery stage associations in PODXL, ITGA6, and MMP3 were attenuated in the larger replication set (adj. Pper-allele>or=0.5). However genotypes at TERT rs7726159 were associated with ovarian cancer risk in the smaller, five-study replication study (Pper-allele=0.03). Combined analysis of the discovery and replication sets for this TERT SNP showed an increased risk of serous ovarian cancer among non-Hispanic whites [adj. ORper-allele 1.14 (1.04-1.24) p=0.003]. Our study adds to the growing evidence that, like the 8q24 locus, the telomerase reverse transcriptase locus at 5p15.33, is a general cancer susceptibility locus. 
 Inhibition of cyclooxygenase-2 suppresses the   invasive  ness of oral squamous cell carcinoma cell lines via down-regulation of matrix metalloproteinase-2 production and activation. Increased cyclooxygenase (COX-2) expression in tumors is known to be correlated with tumor invasion, angiogenesis, resistance to apoptosis, and suppression of host immunity. We previously reported that the   invasive  ness of human oral squamous cell carcinoma (OSCC) cell lines NA and HSC-4 was suppressed by treatment with either NS-398, a selective COX-2 inhibitor, or COX-2 antisense oligonucleotide (AS). In the present study, to explore the effects of COX-2 inhibition on the interaction between cancer cells and fibroblasts, we examined the effects of these anti-COX-2 reagents on the expression of matrix metalloproteinases (MMPs) in fibroblast cell lines WI-38 and MRC-5. Western blotting and enzyme-linked immunosorbent assay revealed that NS-398 and COX-2 AS down-regulated the expression and secretion of MMP-2 and the tissue inhibitor of matrix metalloproteinase-2 (TIMP-2) in human fibroblast cell lines. Furthermore, invasion activity of OSCC cells was down-regulated by the addition of culture supernatant from fibroblasts treated with anti-COX-2 reagents in a Matrigel invasion assay. These results suggest that selective COX-2 inhibition suppresses the invasion activity of OSCC cells via down-regulation of an MMP-2-activating mechanism involving TIMP-2 and production of the MMP-2 protein by an interaction between cancer cells and stromal fibroblasts. Genetic or pharmacological inhibition of COX-2 may therefore be a beneficial strategy in the treatment of OSCC. 
 COX-2 promotes breast cancer cell radioresistance via p38/MAPK-mediated cellular anti-apoptosis and   invasive  ness. Radioresistance is one of the major barriers to improve the survival rate of breast cancer patients. Cyclooxygenase 2 (COX-2) is usually overexpressed in highly   invasive   and metastatic breast cancer, which may indicate an association with breast cancer radioresistance. The function role of COX-2 was investigated by using a radioresistant breast cancer cell line MDA-MB-231/RR10 and its parental cell line MDA-MB-231 cells before or after COX-2 was silenced by a specific small hairpin RNA (shRNA). The cell proliferation, migration, invasion, colony formation, and apoptosis were measured by CCK-8, scratch-wound, transwell, clone formation assay, and flow cytometry. Protein and mRNA expression were analyzed by Western blot and quantitative reverse transcriptase-polymerase chain reaction. COX-2 is upregulated in MDA-MB-231/RR10 cells compared with in MDA-MB-231 cells, and silencing of COX-2 expression by shRNA in MDA-MB-231/RR10 cells decreases the expression of Bcl-2 and Bcl-XL, but increases the proapoptotic protein BAK, leading to the increased apoptosis following treatment with γ-irradiation in comparison with those in control cells. Silencing of COX-2 also increases the expression of β-catenin and E-cadherin, two anti-invasion proteins, resulting in reduced cell migration and invasion tested by transwell chambers and wound-healing assays. Further study demonstrated that COX-2-induced radioresistance is negatively regulated through the phosphorylation of p38 at Tyr182, and that the phosphorylation of p38 induced by TNF-alpha reduces the expression of Bcl-2, BCL-XL, but increases β-catenin and E-cadherin, leading to the decreas  ed invas  iveness of cells. Our data suggest that COX-2, p38, Bcl-2, Bcl-XL, β-catenin, and E-cadherin may be considered as potential therapeutic targets against radioresistant breast cancer. 
 Inhibition of cyclooxygenase-2 suppresses the   invasive  ness of oral squamous cell carcinoma cell lines via down-regulation of matrix metalloproteinase-2 production and activation. Increased cyclooxygenase (COX-2) expression in tumors is known to be correlated with tumor invasion, angiogenesis, resistance to apoptosis, and suppression of host immunity. We previously reported that the   invasive  ness of human oral squamous cell carcinoma (OSCC) cell lines NA and HSC-4 was suppressed by treatment with either NS-398, a selective COX-2 inhibitor, or COX-2 antisense oligonucleotide (AS). In the present study, to explore the effects of COX-2 inhibition on the interaction between cancer cells and fibroblasts, we examined the effects of these anti-COX-2 reagents on the expression of matrix metalloproteinases (MMPs) in fibroblast cell lines WI-38 and MRC-5. Western blotting and enzyme-linked immunosorbent assay revealed that NS-398 and COX-2 AS down-regulated the expression and secretion of MMP-2 and the tissue inhibitor of matrix metalloproteinase-2 (TIMP-2) in human fibroblast cell lines. Furthermore, invasion activity of OSCC cells was down-regulated by the addition of culture supernatant from fibroblasts treated with anti-COX-2 reagents in a Matrigel invasion assay. These results suggest that selective COX-2 inhibition suppresses the invasion activity of OSCC cells via down-regulation of an MMP-2-activating mechanism involving TIMP-2 and production of the MMP-2 protein by an interaction between cancer cells and stromal fibroblasts. Genetic or pharmacological inhibition of COX-2 may therefore be a beneficial strategy in the treatment of OSCC. 
 Evaluation of candidate stromal epithelial cross-talk genes identifies association between risk of serous ovarian cancer and TERT, a cancer susceptibility "hot-spot". We hypothesized that variants in genes expressed as a consequence of interactions between ovarian cancer cells and the host micro-environment could contribute to cancer susceptibility. We therefore used a two-stage approach to evaluate common single nucleotide polymorphisms (SNPs) in 173 genes involved in stromal epithelial interactions in the Ovarian Cancer Association Consortium (OCAC). In the discovery stage, cases with epithelial ovarian cancer (n=675) and controls (n=1,162) were genotyped at 1,536 SNPs using an Illumina GoldenGate assay. Based on Positive Predictive Value estimates, three SNPs-PODXL rs1013368, ITGA6 rs13027811, and MMP3 rs522616-were selected for replication using TaqMan genotyping in up to 3,059 serous   invasive   cases and 8,905 controls from 16 OCAC case-control studies. An additional 18 SNPs with Pper-allele<0.05 in the discovery stage were selected for replication in a subset of five OCAC studies (n=1,233 serous   invasive   cases; n=3,364 controls). The discovery stage associations in PODXL, ITGA6, and MMP3 were attenuated in the larger replication set (adj. Pper-allele>or=0.5). However genotypes at TERT rs7726159 were associated with ovarian cancer risk in the smaller, five-study replication study (Pper-allele=0.03). Combined analysis of the discovery and replication sets for this TERT SNP showed an increased risk of serous ovarian cancer among non-Hispanic whites [adj. ORper-allele 1.14 (1.04-1.24) p=0.003]. Our study adds to the growing evidence that, like the 8q24 locus, the telomerase reverse transcriptase locus at 5p15.33, is a general cancer susceptibility locus. 
 Genetic susceptibility to distinct bladder cancer subphenotypes. Clinical, pathologic, and molecular evidence indicate that bladder cancer is heterogeneous with pathologic/molecular features that define distinct subphenotypes with different prognoses. It is conceivable that specific patterns of genetic susceptibility are associated with particular subphenotypes.To examine evidence for the contribution of germline genetic variation to bladder cancer heterogeneity.The Spanish Bladder Cancer/EPICURO Study is a case-control study based in 18 hospitals located in five areas in Spain. Cases were patients with a newly diagnosed, histologically confirmed, urothelial cell carcinoma of the bladder from 1998 to 2001. Case diagnoses were reviewed and uniformly classified by pathologists following the World Health Organisation/International Society of Urological Pathology 1999 criteria. Controls were hospital-matched patients (n=1149).A total of 1526 candidate variants in 423 candidate genes were analysed. Three distinct subphenotypes were defined according to stage and grade: low-grade nonmuscle   invasive   (n=586), high-grade nonmuscle   invasive   (n=219), and muscle   invasive   (n=246). The association between each variant and subphenotype was assessed by polytomous risk models adjusting for potential confounders. Heterogeneity in genetic susceptibility among subphenotypes was also tested.Two established bladder cancer susceptibility genotypes, NAT2 slow-acetylation and GSTM1-null, exhibited similar associations among the subphenotypes, as did VEGF-rs25648, which was previously identified in our study. Other variants conferred risks for specific tumour subphenotypes such as PMS2-rs6463524 and CD4-rs3213427 (respective heterogeneity p values of 0.006 and 0.004), which were associated with muscle-  invasive   tumours (per-allele odds ratios [95% confidence interval] of 0.56 [0.41-0.77] and 0.71 [0.57-0.88], respectively) but not with non-muscle-  invasive   tumours. Heterogeneity p values were not robust in multiple testing according to their false-discovery rate.These exploratory analyses suggest that genetic susceptibility loci might be related to the molecular/pathologic diversity of bladder cancer. Validation through large-scale replication studies and the study of additional genes and single nucleotide polymorphisms are required. 
 The hepatitis B virus X protein promotes tumor cell invasion by inducing membrane-type matrix metalloproteinase-1 and cyclooxygenase-2 expression. Hepatocellular carcinoma is strongly associated with chronic infection by the hepatitis B virus (HBV) and has poor prognosis due to intrahepatic metastasis. HBx is often the only HBV protein detected in hepatic tumor cells; however, its contribution to tumor invasion and metastasis has not been established so far. In this work, we show that HBx enhances tumor cell invasion, both in vivo and in vitro. The increased   invasive   capacity induced by HBx is mediated by an upregulation of membrane-type 1 matrix metalloproteinase (MT1-MMP) expression, which in turn activates matrix metalloproteinase-2. Induction of both MT1-MMP expression and cell invasion by HBx is dependent on cyclooxygenase-2 (COX-2) activity. In addition, HBx upregulates the expression of COX-2, which is mediated by the transcriptional activation of the COX-2 gene promoter in a nuclear factor of activated T cell-dependent (NF-AT-dependent) manner. These results demonstrate the ability of HBx to promote tumor cell invasion by a mechanism involving the upregulation of MT1-MMP and COX-2 and provide new insights into the mechanism of action of this viral protein and its involvement in tumor metastasis and recurrence of hepatocellular carcinoma. 
 Cyclo-oxygenase-2 expression is associated with vascular endothelial growth factor C expression and lymph node metastasis in oral squamous cell carcinoma. Cervical lymph node metastasis in oral squamous cell carcinoma (OSCC) is recognized as a poor prognostic factor, although its mechanism remains unclear. Recently, cyclo-oxygenase-2 (COX-2) level has been found to correlate highly with vascular endothelial growth factor C (VEGF-C) and lymph node metastasis, as in other solid tumors. However, there has been no report of this correlation in OSCC. Therefore, the aim of this study was to investigate whether COX-2 immunohistochemical expression in OSCC was associated with VEGF-C expression, histopathologic parameters, and lymph node metastasis.Lymphatic vessel density, VEGF-C, and COX-2 immunohistochemical expression were examined pathologically in 60 specimens of   invasive   OSCC. Relations of histopathologic parameters to lymph node metastasis were analyzed.Expression levels of VEGF-C and COX-2 and lymphatic vessel density in the lymph node metastatic group were significantly higher than in the nonmetastatic group (P < .01). A significant correlation was found between the expression levels of VEGF-C and COX-2 (r = 0.512; P < .001). COX-2 expression was significantly related to lymph node metastasis (P = .004) and VEGF-C expression (P = .005). Univariate analysis showed that survival time was impaired by higher COX-2 and VEGF-C expression levels. Multivariate survival analysis showed that COX-2 expression was an independent prognostic factor.This study showed that VEGF-C expression was upregulated by COX-2 in OSCC. High VEGF-C expression appears to promote peritumoral lymphangiogenesis. These data indicated that lymph node metastasis is promoted by COX-2 and VEGF-C in OSCC. 
 Autocrine/paracrine prostaglandin E2 production by non-small cell lung cancer cells regulates matrix metalloproteinase-2 and CD44 in cyclooxygenase-2-dependent invasion. Tumor cyclooxygenase-2 (COX-2) expression is known to be associated with enhanced tumor   invasive  ness. In the present study, we evaluated the importance of the COX-2 product prostaglandin E2 (PGE2) and its signaling through the EP4 receptor in mediating non-small cell lung cancer (NSCLC)   invasive  ness. Genetic inhibition of tumor COX-2 led to diminished matrix metalloproteinase (MMP)-2, CD44, and EP4 receptor expression and invasion. Treatment of NSCLC cells with exogenous 16,16-dimethylprostaglandin E2 significantly increased EP4 receptor, CD44, and MMP-2 expression and matrigel invasion. In contrast, anti-PGE2 decreased EP4 receptor, CD44, and MMP-2 expression in NSCLC cells. EP4 receptor signaling was found to be central to this process, because antisense oligonucleotide-mediated inhibition of tumor cell EP4 receptors significantly decreased CD44 expression. In addition, agents that increased intracellular cAMP, as is typical of EP4 receptor signaling, markedly increased CD44 expression. Moreover, MMP-2-AS treatment decreased PGE2-mediated CD44 expression, and CD44-AS treatment decreased MMP-2 expression. Thus, PGE2-mediated effects through EP4 required the parallel induction of both CD44 and MMP-2 expression because genetic inhibition of either MMP-2 or CD44 expression effectively blocked PGE2-mediated invasion in NSCLC. These findings indicate that PGE2 regulates COX-2-dependent, CD44- and MMP-2-mediated invasion in NSCLC in an autocrine/paracrine manner via EP receptor signaling. Thus, blocking PGE2 production or activity by genetic or pharmacological interventions may prove to be beneficial in chemoprevention or treatment of NSCLC. 
 COX-2 expression is correlated with VEGF-C, lymphangiogenesis and lymph node metastasis in human cervical cancer. Lymphangiogenesis has been shown to promote lymph node metastasis in cancers, making it an important target in cancer therapy. Vascular endothelial growth factor (VEGF)-C is upregulated in various tumors/cancers and is one of the most potent growth factors for inducing lymphangiogenesis and promoting lymph node metastasis (LNM). Likewise, cyclooxygenase (COX)-2 plays major roles in carcinogenesis, tumor growth and metastasis via multiple mechanisms including inactivation of host antitumor immunity and promotion of tumor cell migration, tumor cell   invasive  ness and tumor-associated angiogenesis and lymphangiogenesis. We previously demonstrated an association between COX-2 and VEGF-C in an in vitro model of lung cancer. However, little is known about the regulation of VEGF-C by COX-2 in cervical cancer. In this study, we measured the COX-2 and VEGF-C expressions by immunohistochemistry in 23 LNM-positive and 20 LNM-negative cervical cancer specimens. We then examined the correlations among the expressions and the lymphatic microvessel density (LMVD) and ultrastructural changes to the lymphatic vessel walls by enzyme histochemical staining and electron microscopy. In addition, we used the HeLa cervical cancer cell line to explore the in vitro regulation of VEGF-C by COX-2 and its metabolite, PGE(2), using siRNA-mediated gene silencing and EP receptor blockade. The LNM-positive specimens exhibited significantly higher VEGF-C expression, COX-2 expression and LMVD than the LNM-negative specimens. Furthermore, there were strong correlations between the levels of COX-2 expression and the levels of VEGF-C expression and secretion and a significant positive association between the LMVD and LNM. siRNA-mediated knockdown of COX-2 expression inhibited VEGF-C mRNA expression while EP1 and EP4 receptor antagonists reduced the VEGF-C protein level and tyrosine phosphorylation of Src kinase. Moreover, inhibition of Src kinase with the tyrosine kinase inhibitor PP1 attenuated VEGF-C expression. Collectively, our data provide evidence for a clinical association between COX-2 and VEGF-C expressions in cervical cancer. EP1 and EP4 receptors may be involved in the COX-2-mediated regulation of VEGF-C protein and mRNA expressions. Src may be a downstream mediator of EP1 and EP4 receptors. COX-2 inhibition may diminish LNM by suppressing VEGF-C-mediated lymphangiogenesis. 
 Significance of COX-2 expression in human esophageal squamous cell carcinoma. Cyclooxygenase-2 (COX-2) is well established to play an important role in the tumorigenesis of a variety of human cancers; however, the function of COX-2 in the development of esophageal squamous cell carcinoma (ESCC) remains less clear. Here, we determined, first, the pattern of COX-2 expression in normal esophageal mucosa, dysplasia, carcinoma in situ (CIS) and   invasive   SCC. Immunohistochemical analysis showed that, while COX-2 was weakly expressed, if at all, in normal squamous epithelium, strong COX-2 expression was detected as early as the stage of dysplasia and frequently in 20 of 26 (77%) CIS and 86 of 111 (77%)   invasive   SCC. Upregulation of COX-2 in ESCC was found to be significantly associated with tumor progression (R = 0.493, P < 0.01). Further, treatment of human ESCC cell lines (KYSE450 and KYSE510) with NS-398, a COX-2 specific chemical inhibitor, suppressed the production of prostaglandin E2 (PGE2) and induced cell growth inhibition, cell cycle arrest at the G1-S checkpoint, and the expression of cyclin-dependent kinase inhibitors p21waf1/cip1 and p27kip1. Finally, knockdown expression of COX-2 in KYSE450 cells by a specific COX-2 siRNA dramatically inhibited PGE2 production, cell growth and, more importantly, colony formation and tumorigenesis in nude mice. Together, this study suggested that COX-2 may be involved in an early stage of squamous cell carcinogenesis of the esophagus and has a non-redundant role in the regulation of cellular proliferation and tumorigenesis of esophageal epithelial cells. 
 [Expression of COX-2 in different human prostate cancer cell lines and its significance]. To investigate the expression of cyclooxygenase-2 (COX-2) in different prostate cancer (PCa) cell lines and its role in the acquisition of   invasive   and metastatic potentials of PCa.We detected the expressions of COX-2 in prostate cancer cell lines LNCaP, C4-2, IF11, IA8 and PC-3 with different metastatic potentials by Western blotting and RT-PCR, and analyzed their roles in the invasion and metastasis of different PCa cell lines.Western blotting and RT-PCR showed that the expression of the COX-2 protein was high in PC-3, but absent in IF11, IA8, LNCaP and C4-2 (P < 0.05), and it was consistent with the expression of COX-2 mRNA.COX-2 expresses differently in PCa cell lines with different metastatic potentials. The overexpression of COX-2 may be associated with the high invasion and metastasis of PC-3, but not with the metastasis of other cell lines. 
 Snail and Cox-2 expressions are associated with WHO tumor grade and survival rate of patients with gliomas. The transcriptional factor Snail and enzyme cyclo-oxygenase-2 (Cox-2) are suggested to be important effectors of   invasive  ness and tumorigenesis in various tumors. Tumors of higher grade have the propensity for tumor cell migration and   invasive  ness. This study was performed in order to evaluate the association between Snail and Cox-2 expressions and their values as prognostic factors in various grades of glioma, Specimens of 56 patients with glioma were used in the study. Univariate analysis showed that WHO tumor grade, and expressions of Snail and Cox-2 were significant prognostic factors affecting overall and disease progression-free survival rates. In the multivariate analysis by Cox regression model, only WHO tumor grade was shown to be a significant independent prognostic factor of overall and progression-free survival rates. In conclusion, Snail and Cox-2 expressions were associated with WHO grade in gliomas and may be used as prognostic indicators. 
 Significance of cyclooxygenase 2, EZH-2 polycomb group and p53 expression in actinic keratosis and squamous cell carcinomas of the skin. The development and progression of squamous cell carcinoma (SCC) of the skin is characterized by an accumulation of molecular changes. The aim of this study was to investigate the association of the immunohistochemical expression of cyclooxygenase-2 (COX-2), enhancer of zeste homolog 2 (EZH-2), and p53 in actinic keratosis (AK) and SCC and detect any differences between   invasive   and pre  invasive   squamous epidermal lesions. Forty-three cases with AK, 38 with SCC, and 9 with SCC arising on AK (SCC/AK) were studied. For COX-2 immunostaining, weak or no reaction was associated with AK (58.10% of cases), whereas moderate or strong reaction with SCCs (34.2% and 39.5%, respectively). Furthermore, 88.9% of the "mixed" SCC/AK specimens demonstrated moderate reaction (χ2 = 29.924, P < 0.0001). For EZH-2 immunostaining, a weak or no reaction was observed in 62.8% of AK cases, whereas a moderate reaction was observed in 42.1% of SCCs and 77.8% of "mixed" SCC/AK cases (χ2 = 18.91, P = 0.001). Weak immunoreactivity of p53 was associated with AK (58.1%), moderate with SCC (44.7%), and strong with SCC/AK lesions (66.7%) (χ2 = 15.999, P = 0.003). COX-2, p53, but mainly EZH-2 immune expression seems to be strongly associated with the biological potential of squamous epidermal cells and seems to be differentiating SCC by comparison to AK of the skin. The value of the combined expression of these markers is worth being further investigated as an additional tool for diagnostic, prognostic, and possibly, therapeutic use. 
 Hepatocyte growth factor induces anoikis resistance by up-regulation of cyclooxygenase-2 expression in uterine endometrial cancer cells. Cyclooxygenase-2 (COX-2) has been implicated in the promotion of carcinogenesis. Although the role of COX-2 in endometrial cancer remains unclear, recent experiments suggest that COX-2 antagonizes cell apoptosis, increases the   invasive  ness of malignant cells, and promotes angiogenesis. Hepatocyte growth factor (HGF) is a mesenchymal-derived cytokine and the interaction between HGF and its tyrosine kinase receptor, c-Met proto-oncogene, is associated with tumor progression and metastasis. To investigate the molecular mechanism of HGF-induced anoikis resistance, we analyzed the signal transduction and COX-2 expression in endometrial cancer cells. Here, we show i) the expression of COX-2 protein significantly increased in a dose-dependent manner after HGF stimulation in endometrial cancer cell lines (HEC-IB and RL95-2), reaching 200-270% stimulation at the highest doses of HGF tested (40 ng/ml); ii) flow cytometry and TUNEL analyses revealed that HGF significantly inhibited anoikis of RL95-2 cells; iii) phosphatidylinositol 3-kinase (PI3K) inhibitor (LY294002), but not mitogen-activated protein kinase/ERK kinase (MEK) inhibitor (PD98059), specifically blocked HGF-mediated anoikis resistance in RL95-2 cells; and iv) COX-2 inhibitor, Meloxicam, abrogated HGF-mediated anoikis resistance. Our data suggest that HGF induces anoikis resistance in endometrial cancer cells possibly through PI3K/Akt pathway-dependent up-regulation of COX-2 expression. 
 MiR-101 downregulation is involved in cyclooxygenase-2 overexpression in human colon cancer cells. Overexpressed cyclooxygenase-2 (COX-2) strongly contributes to the growth and   invasive  ness of tumoral cells in patients affected by colorectal cancer (CRC). It has been demonstrated that COX-2 overexpression depends on different cellular pathways involving both transcriptional and post-transcriptional regulations. We assumed that COX-2 expression could be regulated also by microRNAs (miRNAs) since these short RNA molecules participate to the fine regulation of several genes implicated in cell growth and differentiation. In this paper, we report the inverse correlation between COX-2 and miR-101 expression in colon cancer cell lines and we demonstrated in vitro the direct inhibition of COX-2 mRNA translation mediated by miR-101. Moreover, this correlation was supported by data collected ex vivo, in which colon cancer tissues and liver metastases derived from CRC patients were analyzed. These findings provide a novel molecular insight in the modulation of COX-2 at post-transcriptional level by miR-101 and strengthen the observation that miRNAs are highly implicated in the control of gene expression. An impairment of miR-101 levels could represent one of the leading causes of COX-2 overexpression in colon cancer cells. 
 Interplay between membrane lipid peroxidation, transglutaminase activity, and cyclooxygenase 2 expression in the tissue adjoining to breast cancer. Breast cancer, a leading cause of cancer related deaths worldwide, is one of the most common neoplasms in women. The increased generation of reactive oxygen species (ROS) in breast lesion is critically involved in the mutagenic processes that drive to breast carcinoma initiation and progression. To date, the molecular events occurring in the tissue adjoin the cancer lesion have not been elucidated. Here, we investigated the role of excess ROS generation during human breast carcinogenesis by evaluating oxidative stress biomarkers, tissue transglutaminase (t-TGase) activity, and expression levels of ubiquitin and cyclooxygenase-2 (COX-2) in the normal tissue adjoin to fibroadenoma (nFA), atypical ductal hyperplasia (nADH), and   invasive   ductal carcinoma (nIDC) from 45 breast cancer patients. We found that lipid peroxidation and nitric oxide production significantly increased in nIDC respect to nFA and nADH (P < 0.005) whereas the 4-hydroxy-2-nonenal (HNE) protein-adducts increased only in nADH (P < 0.005). The increased lipid damage observed in nIDC correlates with estrogen receptor exposure in IDC (R(2) = 0.89). Moreover, nIDC and   invasive   ductal carcinoma (IDC) showed a 10-fold higher t-TGase activity compared to nFA and nADH. Contrary, COX-2 expression levels significantly decreased nIDC and IDC respect to the nFA and nADH (P < 0.001). The analysis of the free ubiquitin expression revealed equal levels in nADH and nIDC samples whereas high molecular weight-ubiquitin conjugate increased about fivefold only in nIDC (P < 0.01 vs. nADH). These novel findings reveal an interplay between membrane lipid peroxidation, t-TGase activity, and COX-2 expression levels in the tissue adjoining to neoplastic lesion during breast cancer progression. 
 COX-2 dependent regulation of mechanotransduction in human breast cancer cells. The ability of living cells to exert physical forces upon their surrounding is a necessary prerequisite for diverse biological processes, such as local cellular migrations in wound healing to metastatic-invasion of cancer. How forces are coopted in metastasis has remained unclear, however, because the mechanical interplay between cancer cells and the various stromal components has not been experimentally accessible. Current dogma implicates inflammation in these mechanical processes. Using Fourier transform traction microscopy, we measured the force-generating capacity of human breast cancer cells occupying a spectrum of   invasive  ness as well as basal and inducible COX-2 expression (MCF-7<SUM-149<MDA-MB-231). Compared with non-  invasive   MCF-7 and moderately-  invasive   SUM-149, poorly-differentiated MDA-MB-231 cells showed increased cellular dispersion on collagen matrix that was accompanied by emergent distribution of contractile stresses at the interface between the adherent cell and its substrate, defined herein as the traction field. In metastatic MDA-MB-231 cells, the local tractions were precisely tuned to the surrounding matrix rigidity in a physiologic range with the concomitant expression of mechanosensitive integrin β1. These discrete responses at the single-cell resolution were correlated with PGE2 secretion and were ablated by shRNA-mediated knockdown of COX-2. Both COX-2-silenced and COX-2-expressing cells expressed EP2 and EP4 receptors, but not EP1 and EP3. Exogenous addition of PGE2 increased cell tractions and stiffened the underlying cytoskeletal network. To our knowledge this is the first report linking the expression of COX-2 with mechanotransduction of human breast cancer cells, and the regulation of COX-2-PGE2-EP signaling with physical properties of the tumor microenvironment. Drug treatments aimed at reducing this mechanical interplay may have therapeutic potential in the treatment of breast cancer. 
 Cyclooxygenase-2/carbonic anhydrase-IX up-regulation promotes   invasive   potential and hypoxia survival in colorectal cancer cells. Inflammation promotes colorectal carcinogenesis. Tumour growth often generates a hypoxic environment in the inner tumour mass. We here report that, in colon cancer cells, the expression of the pro-inflammatory enzyme cyclooxygenase-2 (COX-2) associates with that of the hypoxia response gene carbonic anhydrase-IX (CA-IX). The COX-2 knockdown, achieved by the stable infection of a COX-2 specific short harpin RNA interference (shCOX-2), down-regulates CA-IX gene expression. In colorectal cancer (CRC) cells, PGE(2), the main COX-2 gene products, promotes CA-IX gene expression by ERK1/2 activation. In normoxic environment, shCOX-2 infected/CA-IX siRNA transfected CRC cells show a reduced level of active metalloproteinase-2 (MMP-2) that associates with a decreased extracellular matrix invasion capacity. In presence of hypoxia, COX-2 gene expression and PGE(2) production increase. The knockdown of COX-2/CA-IX blunts the survival capability of CRC cells in hypoxia. At a high cell density, a culture condition that creates a mild pericellular hypoxic environment, the expression of COX-2/CA-IX genes is increased and triggers the   invasive   potential of colon cancer cells. In human colon cancer tissues, COX-2/CA-IX protein expression levels, assessed by Western blot and immunohistochemistry, correlate each other and increase with tumour stage. In conclusion, these data indicate that COX-2/CA-IX interplay promotes the aggressive behaviour of CRC cells. 
 Expression of cyclooxygenase-2 in breast carcinogenesis and its relation to HER-2/neu and p53 protein expression in   invasive   ductal carcinoma. The purpose of this study was to evaluate cyclooxygenase-2 (COX-2) expression in the successive steps of breast carcinogenesis and to determine its correlation with HER-2/neu and p53 expression in   invasive   ductal carcinomas of the breast. Immunohistochemical staining with anti-COX-2 antibody was performed in normal breast tissue, usual hyperplasia, ductal carcinoma in situ, and   invasive   ductal carcinoma. Expression of COX-2 in   invasive   ductal carcinoma was correlated with immunohistochemical expression of HER-2/neu and p53 protein. COX-2 expression was found to be progressively elevated along the continuum from normal breast tissue to   invasive   ductal carcinoma (P<0.001). COX-2 expression significantly correlated with p53 and HER-2/neu protein expression (P<0.05 and P<0.001). On multivariate analysis, only TNM stage and elevated COX-2 expression correlated with survival. Our results suggest that COX-2 may be involved in the carcinogenesis of the breast and may be an independent prognostic indicator in patients with   invasive   ductal carcinoma. HER-2/neu and p53 are likely to be involved in the regulation of COX-2 expression in   invasive   ductal carcinomas of the breast. 
 Overexpression of cyclooxygenase-2 in urothelial carcinoma in conjunction with tumor-associated-macrophage infiltration, hypoxia-inducible factor-1alpha expression, and tumor angiogenesis. This study examines whether the expression of cyclooxgenase-2 (COX-2) in urothelial carcinoma (UC) is associated with macrophage infiltration, hypoxia-inducible factor-1alpha (HIF-1alpha) expression and angiogenesis. We investigated the expression of COX-2 associated with HIF-1alpha and performed double immunohistochemical analysis of 216 UCs for COX-2 expression and the correlation with tumor-associated-macrophage (TAM) density and microvessel density (MVD) in situ. A high expression of COX-2 was positively correlated with tumor   invasive  ness, histologic grade and HIF-1alpha expression in UC (p<0.0001, p=0.003, p<0.0001, respectively). Quantification of double staining of COX-2/CD34 and COX-2/CD68 showed that a higher MVD and TAM density was found in COX-2 high-expression than in COX-2 low-expression tumor fields (p<0.0001). Adjacent to the principal of COX-2 expression areas, MVD value and TAM density were significantly increased in HIF-1alpha high-expression specimens compared with HIF-1alpha low-expression ones (p<0.0001). Interestingly, our data revealed that high COX-2 expression (p=0.002), high HIF-1alpha expression (p<0.0001) and TAM density (p<0.0001) were all associated with high MVD value. Our results suggest that COX-2 may produce a cooperative effect in promoting tumor progression and may be involved in the process of angiogenesis through increasing TAM infiltration or HIF-1alpha regulation by hypoxia. 
 Immunohistochemical COX-2 overexpression correlates with HER-2/neu overexpression in   invasive   breast carcinomas: a pilot study. Cyclooxygenase-2 (COX-2) is a prostaglandin synthase that catalyzes the synthesis of prostaglandin G2 and H2. It has been shown that COX-2 plays an important role in tumorigenesis of different tumor types and it is thought to take part in breast carcinogenesis. In the present study, we aimed to investigate the relationship of immunohistochemical COX-2 expression with clinicopathological parameters, including HER-2/neu overexpression in   invasive   breast carcinoma (IBC). Our study population comprised 10 normal breasts, 25 ductal carcinomas in situ (DCIS), and 51   invasive   breast carcinomas. Immunohistochemical overexpressions of COX-2 and HER-2/neu were investigated in sections of formalin-fixed, paraffin-embedded blocks by 3 observers. In normal breast, DCIS and IBC, the COX-2 overexpression rate was 0%, 84%, and 58.8%, respectively. In IBC, COX-2 overexpression had a significant relationship with HER-2/neu overexpression (p=0.026) and a high histological grade (p=0.026). COX-2 expression in both DCIS (n=25) and IBC (n=51) was significantly higher than in normal breast tissue (p<0.0001). In addition, the COX-2 expression rate was significantly higher in DCIS than in IBC (p=0.042). Our results indicated that COX-2 overexpression correlates with aggressive phenotypic features, such as HER-2/neu overexpression and high histological grade in IBC. Increased expression of COX-2 in both DCIS and IBC in comparison to normal breast could indicate a role in breast carcinogenesis. COX-2 overexpression may provide a clinically useful biomarker for estimating tumor aggressiveness. 
 TNFα-activated stromal COX-2 signalling promotes proliferative and   invasiv  e potential of colon cancer epithelial cells. Up to now it has been unclear whether stromal/epithelial interaction affects progression of colon cancer. This study was designed to examine effects of tumour necrosis factor alpha (TNFα)-activated stromal cyclooxygenase-2 (COX-2) signalling on proliferation an  d invasi  veness of colon cancer epithelial cells.Cyclooxygenase-2 mRNA and protein were determined by real-time PCR and western blotting and prostaglandin E2 (PGE2 ) was assayed by radioimmunoassay. Cell proliferation an  d invasi  veness were determined by transwell chamber assays and protein kinase C (PKC) was assayed by Biotrak(™) PKC Assay System.Our results indicated that TNFα, a powerful inflammatory cytokine, strongly promoted COX-2 expression and PGE2 production in colon cancer-associated fibroblasts. Using in vitro assays for estimating proliferative   and inv  asive potential, we discovered that activation of stromal COX-2 signalling significantly promoted proliferation   and inv  asiveness of colon cancer epithelial cells. In addition, selective COX-2 inhibitor N-[2-(Cyclohexyloxy)-4-nitrophenyl]methanesulfonamide, blocked such proliferative   and inv  asive effects on the cancer epithelial cells. In this process, PKC was involved in activation of COX-2 signalling in the fibroblasts.We conclude that activation of stromal COX-2 signalling by TNFα played a major role in promoting proliferatio  n and in  vasiveness of colon cancer epithelial cells. 
 IL1β-mediated Stromal COX-2 signaling mediates proliferation and   invasiv  eness of colonic epithelial cancer cells. COX-2 is a major inflammatory mediator implicated in colorectal inflammation and cancer. However, the exact origin and role of COX-2 on colorectal inflammation and carcinogenesis are still not well defined. Recently, we reported that COX-2 and iNOS signalings interact in colonic CCD18Co fibroblasts. In this article, we investigated whether activation of COX-2 signaling by IL1β in primary colonic fibroblasts obtained from normal and cancer patients play a critical role in regulation of proliferation an  d invasi  veness of human colonic epithelial cancer cells. Our results demonstrated that COX-2 level was significantly higher in cancer associated fibroblasts than that in normal fibroblasts with or without stimulation of IL-1β, a powerful stimulator of COX-2. Using in vitro assays for estimating proliferative a  nd invas  ive potential, we discovered that the proliferation a  nd invas  iveness of the epithelial cancer cells were much greater when the cells were co-cultured with cancer associated fibroblasts than with normal fibroblasts, with or without stimulation of IL1β. Further analysis indicated that the major COX-2 product, prostaglandin E(2), directly enhanced proliferation   and inva  siveness of the epithelial cancer cells in the absence of fibroblasts. Moreover, a selective COX-2 inhibitor, NS-398, blocked the proliferative   and inva  sive effect of both normal and cancer associate fibroblasts on the epithelial cancer cells, with or without stimulation of IL-1β. Those results indicate that activation of COX-2 signaling in the fibroblasts plays a major role in promoting proliferation   and inv  asiveness of the epithelial cancer cells. In this process, PKC is involved in the activation of COX-2 signaling induced by IL-1β in the fibroblasts. 
 Up-regulation of VEGF, c-fms and COX-2 expression correlates with severity of cervical cancer precursor (CIN) lesions and   invasive   disease. To describe the expression of vascular endothelial growth factor (VEGF), proto-oncogene macrophage colony-stimulating factor receptor (c-fms) and cyclooxygenase-2 (COX-2) in cervical carcinogenesis and to analyze the correlation of VEGF with c-fms and COX-2 expression.In this study, 26 cases of benign cervix, 28 low-grade cervical intraepithelial neoplasia (CIN; CIN 1), 30 high-grade CIN (CIN 2/3) and 28 squamous cervical carcinomas (SCC) were examined by immunohistochemistry (IHC) and analysis was performed separately for epithelium and stroma.Positive epithelial expressions in normal cervix, low-grade CIN, high-grade CIN and SCC were, respectively: VEGF - 11.5%, 39.3%, 53.3% and 75% (P<0.001); c-fms - 0%, 10.7%, 40% and 67.9% (P<0.001); COX-2 - 7.7%, 39.3%, 80% and 100% (P<0.001). Stromal VEGF expression was higher than epithelial expression in all CIN grades and was also associated with the lesion grade, while c-fms and COX-2 stromal expression was weak. VEGF expression was statistically correlated to c-fms and COX-2 expression in high-grade CIN (P=0.020 and P=0.027, respectively) and SCC (P=0.015 and P=0.005, respectively).On the basis of our findings, these factors may participate in the development and progression of CIN lesions, with possible interaction of c-fms and COX-2 on VEGF expression, and may be potential molecular targets for studies of cervical cancer prevention and treatment. 
 Cyclooxygenase-2-derived prostaglandin E2 stimulates Id-1 transcription. Cyclooxygenase-2 (COX-2) and Id-1 are overexpressed in a variety of human malignancies. Recently, each of these genes was found to play a role in mediating breast cancer metastasis to the lungs, but their potential interdependence was not evaluated. Hence, the main objective of the current study was to determine whether COX-2-derived prostaglandin (PGE(2)) activated Id-1 transcription, leading in turn to increased   invasive  ness of mammary epithelial cells. In MDA-MB-231 cells, treatment with PGE(2) induced Id-1, an effect that was mimicked by an EP(4) agonist. PGE(2) via EP(4) activated the epidermal growth factor receptor (EGFR) --> ERK1/2 pathway, which led to increased expression of Egr-1. PGE(2) stimulated EGFR signaling by inducing the release of amphiregulin, an EGFR ligand. The ability of PGE(2) to activate Id-1 transcription was mediated by enhanced binding of Egr-1 to the Id-1 promoter. Silencing of COX-2 or pharmacological inhibition of COX-2 led to reduced PGE(2) production, decreased Id-1 expression, and reduced migration of cells through extracellular matrix. A similar decrease in cell migration was found when Id-1 was silenced. The interrelationship between COX-2, PGE(2), Id-1, and cell   invasive  ness was also compared in nontumorigenic SCp2 and tumorigenic SCg6 mammary epithelial cells. Consistent with the findings in MDA-MB-231 cells, COX-2-derived PGE(2) induced Id-1, leading in turn to increased cell   invasive  ness. Taken together, these results suggest that PGE(2) via EP(4) activated the EGFR --> ERK1/2 --> Egr-1 pathway, leading to increased Id-1 transcription and cell invasion. These findings provide new insights into the relationship between COX-2 and Id-1 and their potential role in metastasis. 
 [COX-2-expression in extragenital endometriosis lesions as a novel therapeutical approach?]. Extragenital endometriosis is a rare form of endometriosis. Due to its   invasive   and metastatic properties it resembles some features of malignant tumours. Cyclooxygenase (COX)-2 is a rate-limiting enzyme in the biosynthesis of prostaglandins and is mainly expressed in inflammatory and malignant processes. In this study we investigated the COX-2 expression in extragenital endometriosis.Tissue was obtained of 13 women with rectal and vaginal endometriosis, scar endometriosis and endometriosis of the omentum majus. The COX-2 expression was investigated by immunohistochemistry.In the glandular epithelium a COX-2 overexpression was found in all cases and in the stroma a weak to moderate COX-2 expression was found in half of the cases. A hormonal therapy at the time of surgery had no influence on the COX-2 expression in extragenital endometriosis.The high COX-2 expression in extragenital endometriosis is believed to be strongly correlated with the pathological abnormalities this of disease. 
 Functional polymorphisms of cyclooxygenase-2 (COX-2) gene and risk for urinary bladder cancer in North India. Cyclooxygenase-2 (COX-2) is an enzyme involved in the synthesis of prostaglandins and thromboxanes, which are regulators of processes that are relevant to cancer development. It is involved in carcinogenesis, immune response suppression, apoptosis inhibition, angiogenesis, and tumor cell invasion and metastasis. The gene for COX-2, designated as prostaglandin-endoperoxide synthase 2 (PTGS-2), carries polymorphisms, such as -765G>C, 1195G>A in the promoter region, and 8473T>C in the 3'-untranslated region (UTR), which have been associated with susceptibility to malignant disease.We undertook a case-control study of 212 urothelial bladder cancer (UBC) cases and 250 controls to investigate the association between COX-2 polymorphism and bladder cancer susceptibility, using the polymerase chain reaction restriction fragment length polymorphism (PCR-RFLP) method and also investigated gene-environment interactions.Cox-2 765G>C, a variant(C) allele carrier, was at an increased risk of UBC (odds ratio [OR] = 1.90; P = .004); however, -1195G>A; -1290A>G; and 3'UTR 8473T>C polymorphisms of COX-2 gene were not significantly associated with UBC. 765G>C also was associated with the   invasive   stage of a bladder tumor (OR = 2.73; P = .033). High risk for UBC also was observed with respect to COX-2 haplotypes C-765T8473A-1195A-1290 (OR = 3.47; P = .014). In case-only analysis, COX-2 765 variant allele carrier genotypes also showed an increased risk among former and current smokers (OR = 3.06; P = .041 and OR = 4.39; P = .032, respectively).COX-2 -765G>C polymorphism confers UBC susceptibility particularly in smokers and in patients with   invasive   tumors. 765C allele carrier genotypes also are influenced with a high risk of recurrence in Bacillus Calmette-Guérin-treated patients. Collectively, these findings confirm that the COX-2 -765G>C polymorphism is a risk factor for the development of bladder cancer and can provide a plausible mechanistic explanation. Further validation in large population-based studies is needed. 
 The transcription factor Snail expressed in cutaneous squamous cell carcinoma induces epithelial-mesenchymal transition and down-regulates COX-2. Cutaneous spindle cell squamous cell carcinoma (SCC) is a rare, but highly malignant variant of SCC. The presence of spindle-shaped cells with a sarcomatous appearance, which are derived from squamous cells, suggests that these cells are produced as a result of epithelial-mesenchymal transition (EMT). EMT is a complex process in which epithelial cells lose their polarity and cell-cell contacts, while also acquiring increased motility and   invasive  ness. Snail regulates EMT by binding to proximal E-boxes in the promoter region of E-cadherin and repressing its transcription. When examining the expression of EMT markers and Snail in spindle cell SCCs, we found that cyclooxygenase-2 (COX-2) expression was down-regulated. Since it has been shown that COX-2 is constitutively overexpressed in a variety of malignancies, including colon, gastric, and lung carcinomas, the down-regulation of COX-2 expression was unexpected. The presence of E-box-like sequences in the promoter region of COX-2 prompted us to perform a more detailed analysis. We introduced a Snail expression vector into keratinocyte-derived cell lines (HaKaT, HSC5, and A431 cells), and isolated stable transfectants. We determined that COX-2 expression was down-regulated in cells expressing Snail. Consistent with these observations, reporter assays revealed that COX-2 promoter activity was repressed upon Snail overexpression. Thus Snail down-regulates COX-2 in these cells. 
 Correlation between expression of cyclooxygenase-2 and angiogenesis in human gastric adenocarcinoma. To evaluate the expression of cyclooxygenase (COX-2) and the relationship with tumor angiogenesis and advancement in gastric adenocarcinoma.Immunohistochemical stain was used for detecting the expression of COX-2 in 45 resected specimens of gastric adenocarcinoma; the monoclonal antibody against CD34 was used for displaying vascular endothelial cells, and microvascular density (MVD) was detected by counting of CD34-positive vascular endothelial cells. Paracancerous tissues were examined as control.Immunohistological staining with COX-2-specific polyclonal antibody showed cytoplasmic staining in the cancer cells, some atypical hyperplasia and intestinal metaplasia,as well as angiogenic vasculature present within the tumors and prexisting vasculature adjacent to cancer lesions. The rate of expression of COX-2 and MVD index in gastric cancers were significantly increased, compared with those in the paracancerous tissues (77.78 vs 33.33 %, 58.13+/-19.99 vs 24.02+/-10.28, P<0.01, P<0.05, respectively). In 36 gastric carcinoma specimens with lymph node metastasis, the rate of COX-2 expression and MVD were higher than those in the specimens without metostasis (86.11 vs 44.44 %, 58.60+/-18.24 vs 43.54+/-15.05, P<0.05, P<0.05, respectively). The rate of COX-2 expression and MVD in the specimens with   invasive   serosa were significantly higher than those in the specimens without invasion to serosa (87.88 vs 50.0 %, 57.01+/-18.79 vs 42.35+/-14.65, P<0.05, P<0.05). Moreover, MVD in COX-2-positive specimens was higher than that in COX-2-negative specimens (61.29+/-14.31 vs 45.38+/-12.42,P<0.05). COX-2 expression was positively correlated with MVD (r=0.63, P<0.05).COX-2 expression might correlate with the occurance and advancement of gastric carcinoma and is involved in tumor angiogenesis in gastric carcinoma. It is likely that COX-2 by inducing angiogenesis can be one of mechanisms which promotes invasion and metastasis of gastric carcinoma. It may become a new therapeutic target for anti-angiogenesis. 
 High-throughput tissue microarray analysis of COX2 expression in urinary bladder cancer. To investigate whether protein expression of cyclooxygenase 2 (COX2) is associated with tumor phenotype, immunohistochemical alterations, and clinical outcome in urinary bladder cancer (BC). Tissue microarrays (n = 776) were used to analyze COX2, P53 and the Ki-67 labeling index immunohistochemically. A monoclonal mouse antibody was used after heat-induced antigen retrieval. COX2 expression was scored semiquantitatively (0-3+). COX2 expression was detected in 60% (368/617) of urothelial BC. Positive COX2 staining was seen in 77.8% (140/182) of muscle   invasive   urothelial BC, compared to 35% (7/20) of muscle   invasive   squamous cell carcinomas (p < 0.001). COX2 protein expression was associated with advanced tumor stage (p < 0.0001), high-grade histology (p < 0.0001), solid growth pattern in   invasive   BC (pT1-4, p = 0.02), high Ki-67 labeling index (p < 0.0001), and positive P53 IHC (p < 0.001). COX2 expression was not associated with survival, recurrence, and progression in clinically relevant subgroups (pTa, pT1, pT2-4). Expression of COX2 is common in advanced BC with poor prognostic characteristics, supporting efforts to initiate clinical trials on the efficacy of COX2 inhibitors in the adjuvant treatment of high-risk urinary BC. 
 Down-regulation of CacyBP is associated with poor prognosis and the effects on COX-2 expression in breast cancer. Calcyclin-binding protein (CacyBP) is a tumor suppressor in gastric and renal cell carcinoma, but an oncogene in pancreatic cancer. However, the function of CacyBP in breast cancer has not been well elucidated. In this study, we explored the clinical relevance of CacyBP and investigated the relationship between CacyBP and COX-2 in breast cancer. Immunohistochemical analysis in 172 cases of breast tissues showed that the positive rate of CacyBP protein expression in normal breast tissues (NBT) (89.3%) was higher than that in   invasive   ductal carcinoma (IDC) (56.1%) (P<0.05). RT-PCR and Western blot analysis showed that CacyBP mRNA and protein expression were significantly lower in tumor tissues as compared to those in the corresponding non-tumorous tissues (P<0.05). The expression trend of COX-2 was opposite with CacyBP in breast carcinogenesis. Moreover, the CacyBP expression was significantly negatively associated with the COX expression in the 132 breast cancer samples (correlation coefficient = 0.505, P<0.001). The clinicopathological data analysis in 132 breast cancer samples showed that CacyBP expression was positively correlated with well differentiated samples (P=0.021), low pathologic TNM stage (P=0.009), and no lymphatic metastasis (P=0.027) of patients with breast cancer. Furthermore, reduced CacyBP expression was associated with poor prognosis. Knockdown of CacyBP gene using siRNA enhanced the proliferation and invasion ability of breast cancer cells, which was dependent on COX-2 expression. In conclusion, CacyBP regulation of COX-2 expression may play an important role in human breast carcinogenesis. Restoration of CacyBP gene is a potential therapeutic target of breast cancer. 
 Cyclooxygenase-2 immunoexpression in breast cancer: progesterone receptor influence. In breast cancer cyclooxygenase-2 overexpression is related with poor outcomes, but the clinical relevance of cyclooxygenase-2 is still unclear. Progesterone receptor is a regulator of cyclooxygenase-2 expression. We postulate that progesterone receptor was associated to cyclooxygenase-2 expression and clinicopathologic factors in breast cancer.Cyclooxygenase-2 and progesterone receptor expression was analyzed, by immunohistochemistry, in 31 cases of   invasive   ductal carcinoma.The expression of cyclooxygenase-2 and progesterone receptor was observed in 64.5% and 45.2% of the tumors, respectively. We found 11 tumors with both cyclooxygenase-2 and progesterone receptor overexpression and they are related with tumor lower size and lower axillary node metastasis.These results suggest that progesterone receptor has a protective role in breast cancer by inhibiting chronic inflammatory pathway. 
 Difference in prognostic values of maximal standardized uptake value on fluorodeoxyglucose-positron emission tomography and cyclooxygenase-2 expression between lung adenocarcinoma and squamous cell carcinoma. The maximal standardized uptake value (SUVmax) on fluorodeoxyglucose-positron emission tomography (FDG-PET) for primary tumors is correlated with clinicopathological and prognostic factors in patients with non-small cell lung cancer. However, previous investigations have discussed the role of SUVmax without distinguishing among the histological subtypes of lung cancer. Herein, we investigated the correlations among the SUVmax on FDG-PET, clinicopathological or prognostic factors, and the expression of tumor angiogenic biomarkers according to histological subtypes.We conducted a retrospective review of data from 52 patients with   invasive   adenocarcinoma (ADC) and 32 patients with squamous cell carcinoma (SQC) measuring less than 3 cm in diameter. Immunohistochemical staining for cyclooxygenase-2 (Cox-2), Ki-67, and vascular endothelial growth factor, which might influence cancer progression, was performed and the correlations between the expressions of these biomarkers and the SUVmax were evaluated.Among ADC patients, a statistically significant correlation was observed between the SUVmax and the major clinicopathological factors; among SQC patients, however, no statistically significant association was observed. The disease-free survival (DFS) period of the ADC patients with a high SUVmax was significantly poorer than that of the patients with a low SUVmax, but the DFS of the SQC patients with a high SUVmax was not significantly poorer. In a multivariate analysis, the pathological stage and the SUVmax were independent prognostic factors of the DFS among the ADC patients. Among the SQC patients, however, only Cox-2 expression was an independent prognostic factor of DFS.Some clear differences in prognostic values of the SUVmax on FDG-PET and Cox-2 expression exist between patients with ADC and those with SQC. Based on these relationships between the SUVmax and clinicopathological or biological factors that influence cancer progression, the importance of the SUVmax appears to be quite different for patients with ADC and those with SQC. 
 Non  invasive   imaging identifies new roles for cyclooxygenase-2 in choline and lipid metabolism of human breast cancer cells. The expression of cyclooxygenase-2 (COX-2) is observed in approximately 40% of breast cancers. A major product of the COX-2-catalyzed reaction, prostaglandin E(2), is an inflammatory mediator that participates in several biological processes, and influences invasion, vascularization and metastasis. Using non  invasive   MRI and MRS, we determined the effect of COX-2 downregulation on the metabolism and invasion of intact poorly differentiated MDA-MB-231 human breast cancer cells stably expressing COX-2 short hairpin RNA. Dynamic tracking of invasion, extracellular matrix degradation and metabolism was performed with an MRI- and MRS-compatible cell perfusion assay under controlled conditions of pH, temperature and oxygenation over the course of 48  h. COX-2-silenced cells exhibited a significant decrease in invasion relative to parental cells that was consistent with the reduced expression of invasion-associated matrix metalloproteinase genes and an increased level of the tissue inhibitor of metalloproteinase-1. We identified, for the first time, a role for COX-2 in mediating changes in choline phospholipid metabolism, and established that choline kinase expression is partly dependent on COX-2 function. COX-2 silencing resulted in a significant decrease in phosphocholine and total choline that was detected by MRS. In addition, a significant increase in lipids, as well as lipid droplet formation, was observed. COX-2 silencing transformed parental cell metabolite patterns to those characteristic of less aggressive cancer cells. These new functional roles of COX-2 may identify new biomarkers and new targets for use in combination with COX-2 targeting to prevent invasion and metastasis. 
 A study of phosphorylated ERK1/2 and COX-2 in early stage (T1-T2) oral squamous cell carcinomas. Histomorphological grading at the   invasive   front of oral squamous cell carcinomas (OSCCs) may provide useful prognostic information. In the present study, we investigated the presence and prognostic value of activated phosphorylated extracellular signal-regulated kinases 1 and 2 (p-ERK1/2) and cyclo-oxygenase-2 (COX-2) both at the   invasive   front and in central/superficial parts of OSCCs.Using immunohistochemistry, we assessed the presence of p-ERK1/2 and COX-2 in 53 early stage OSCCs. Clinical data were recorded prospectively. The end point was disease-free survival.p-ERK1/2 staining was present in almost all tumours. The staining was mostly nuclear in the cells of the   invasive   front and either nuclear or nuclear/cytoplasmic in central/superficial tumour parts. COX-2 was observed in almost all tumours (98%) and the staining was often restricted to focal areas. Most tumours were COX-2 negative at the   invasive   front. The lowest P-value in survival analyses was P = 0.06 for p-ERK1/2 at the   invasive   front. COX-2, the histomorphological grading systems and TNM stage were of no prognostic value.p-ERK1/2 was present in almost all tumours and p-ERK1/2 may be a prognostic marker at the   invasive   front of OSCCs. In early stage OSCCs, most tumours did not express COX-2 at the   invasive   front. 
 An immunohistochemical survey of nine cases of medullary carcinoma of thyroid including reactivity for Cox-1 and Cox-2 enzymes. There has been much recent investigation of the cyclooxygenase (Cox) enzymes in tumor biology, but, to our knowledge, no study has yet been published describing Cox activity in medullary carcinoma of the thyroid (MTC). Nine cases of MTC from the past 10 yr were retrieved from our hospital archives. Slides cut from formalin-fixed paraffin-embedded tumor tissue from these cases were assessed for the activities of Cox-1 and Cox-2 enzymes by immunohistochemistry as well as by a battery of immunohistochemical stains for intermediate filaments, peptide hormone, and proliferation and promoter antigens. The staining reactions were semiquantitatively assessed and scored for comparison with each other as well as with each patient s clinical presentation and course. Staining for Cox-1 and Cox-2 enzymes was present only in tumorous tissue, not in nontumorous thyroid tissue or C-cells. Cox-2 staining was not consistently increased over Cox-1 staining; however, Cox-2 staining bore statistically significant correlations with the expression of low molecular weight keratin, thyroid-transforming factor-1, topoisomerase, and MIB1. Hyperplastic C-cells from patients with diverse physiologic conditions and from three patients with C-cell hyperplasia accompanying medullary carcinoma or multiple endocrine neoplasia type IIa showed no reactivity for the Cox antibodies. It appears that Cox enzyme immunoreactivity is present only in the neoplastic C-cells of medullary carcinoma, but with variable expression. A practical application of the preceding finding might involve the use of Cox staining to distinguish   invasive   medullary carcinoma cells from hyperplastic C-cells. 
 LKB1/STK11 suppresses cyclooxygenase-2 induction and cellular invasion through PEA3 in lung cancer. We showed that the PEA3 transcriptional factor interacted with LKB1, a serine/threonine kinase, which is somatically mutated in lung cancer. This interaction occurred through the ETS domain of PEA3 and the kinase domain of LKB1. Mutation of LKB1 in lung cancer cells stabilized PEA3. Reintroduction of wild-type (WT) LKB1 into cells induced down-regulation of PEA3 and subsequently resulted in reduced cyclooxygenase-2 RNA and protein expression, whereas germ-line and somatic LKB1 mutants were defective in this activity. LKB1 phosphorylated PEA3 and promoted its degradation through a proteasome-mediated mechanism. Cells expressing mutant LKB1 possessed greater   invasive   potential compared with cells expressing WT LKB1. Increased invasion of cells with mutant LKB1 was partly due to PEA3 expression, as RNA interference inhibition of PEA3 resulted in dramatic decrease of Matrigel invasion. However, forced expression of PEA3 resulted in down-regulation of epithelial markers and induction of mesenchymal markers. These results suggest that PEA3 stabilization due to LKB1 inactivation could lead to epithelial/mesenchymal transition and greater lung cancer invasion potential. 
 Inhibitory effect of a selective cyclooxygenase-2 inhibitor on liver metastasis of colon cancer. COX-2 overexpression is recognized in various cancers, but the role of COX-2 in the progression of cancer, including the liver metastasis of colon cancer, is not clearly understood. We examined the role of COX-2 in the mechanism of liver metastasis of colon cancer, using a highly metastasizable colon carcinoma cell line, LM-H3. A COX-2 inhibitor, JTE-522, inhibited cell proliferation and invasion of LM-H3 in vitro and clearly reduced the number of metastatic nodules on the surface of nude mouse livers in vivo. We also examined the effects of JTE-522 on the production of growth factors and MMPs through the use of ELISA and gelatin zymography, respectively. JTE-522 downregulated PDGF production by LM-H3 but had no influence on VEGF production. JTE-522 also inhibited MMP-2 secretion by LM-H3. JTE-522 downregulated PGE(2) production, but the associated changes in PGE(2) did not affect PDGF and VEGF production by LM-H3. We conclude that JTE-522 downregulated the cell proliferation and   invasive   potential of LM-H3 by reducing the production of PDGF and MMP-2 and hypothesize that these inhibitory effects on the production of PDGF and MMP-2 can lead to inhibition of liver metastasis of colon cancer. These data indicate that the COX-2 inhibitor JTE-522 has a high potential for use as a clinical agent for the treatment of liver metastasis of colon cancer. 
 Inhibition of cyclooxygenase-2 suppresses   invasive  ness of oral squamous cell carcinoma cell lines via down-regulation of matrix metalloproteinase-2 and CD44. Expression of cyclooxygenase-2 (COX-2) in tumors is known to be associated with enhanced angiogenesis, suppression of host immunity, and tumor invasion. In the present study, human oral squamous cell carcinoma (OSCC) cell lines NA and HSC-4 were used to evaluate the effects of NS-398, a selective inhibitor of COX-2, and COX-2 antisense oligonucleotide (COX-2 AS) on the invasion activity of OSCC cells. Matrigel invasion assay revealed that the   invasive  ness of NA and HSC-4 was suppressed by treatment with either NS-398 or COX-2 AS. These reagents down-regulated the secretion of matrix metalloproteinase-2 (MMP-2) to culture supernatant as well as the expression of MMP-2 mRNA and protein. Membrane-type 1 matrix metalloproteinase (MT1-MMP), an activator of proMMP-2, was also down-regulated by treatment with these reagents. Furthermore, expression of CD44 on the surface of these cells was reduced by treatment with either NS-398 or COX-2 AS. In addition, MMP-2 antisense oligonucleotides reduced the expression of CD44 on the surface of both OSCC cell lines. These findings suggest that NS-398 and COX-2 AS suppress the   invasive  ness of OSCC cells via down-regulation of MMP-2 and CD44. Genetic or pharmacological inhibition of COX-2 may therefore be a beneficial strategy in the treatment of OSCC patients. 
 MMP1, MMP9, and COX2 expressions in promonocytes are induced by breast cancer cells and correlate with collagen degradation, transformation-like morphological changes in MCF-10A acini, and tumor aggressiveness. Tumor-associated immune cells often lack immune effector activities, and instead they present protumoral functions. To understand how tumors promote this immunological switch,   invasive   and non  invasive   breast cancer cell (BRC) lines were cocultured with a promonocytic cell line in a Matrigel-based 3D system. We hypothesized that if communication exists between tumor and immune cells, coculturing would result in augmented expression of genes associated with tumor malignancy. Upregulation of proteases MMP1 and MMP9 and inflammatory COX2 genes was found likely in response to soluble factors. Interestingly, changes were more apparent in promonocytes and correlated with the aggressiveness of the BRC line. Increased gene expression was confirmed by collagen degradation assays and immunocytochemistry of prostaglandin 2, a product of COX2 activity. Untransformed MCF-10A cells were then used as a sensor of soluble factors with transformation-like capabilities, finding that acini formed in the presence of supernatants of the highly aggressive BRC/promonocyte cocultures often exhibited total loss of the normal architecture. These data support that tumor cells can modify immune cell gene expression and tumor aggressiveness may importantly reside in this capacity. Modeling interactions in the tumor stroma will allow the identification of genes useful as cancer prognostic markers and therapy targets. 
 COX-2 expression is associated with an aggressive phenotype in ductal carcinoma in situ. Cyclooxygenase type-2 (COX-2) is overexpressed in malignant tumours including breast cancers, though the mechanism of upregulation is unclear. This study aimed to determine COX-2 expression in ductal carcinoma in situ (DCIS) in comparison to   invasive   breast cancer (IBC) and normal breast, and also to investigate the relationship of COX-2 expression with HER-2 expression, oestrogen receptor (ER), tumour grade and cellular proliferation (Ki67) in DCIS. Cyclooxygenase type-2, HER-2, ER and Ki67 expression were determined by immunohistochemistry on paraffin tissue sections of DCIS (n=187), IBC (n=65) and normal breast reduction tissue (n=60). Cyclooxygenase type-2 expression in DCIS (67%, P<0.001) and IBC (63%, P<0.001) was significantly greater than in normal breast (23%). There was no difference in COX-2 expression level between DCIS and IBC (P=0.87) or between normal breast from reduction mammoplasty tissue and normal breast ducts around DCIS (22%, P=0.29). In DCIS, COX-2 expression was associated with higher cellular proliferation rates (P<0.0001), nuclear grade (P=0.003), with ER negativity (P=0.003) and with HER-2 positivity (P<0.0001). Cyclooxygenase type-2 expression is upregulated in in situ breast cancer and is associated with surrogate markers of an aggressive DCIS phenotype including nonoestrogen-regulated signalling pathways. Cyclooxygenase type-2 inhibition may potentially prevent the development of ER-positive and ER-negative breast cancers. 
 Prognostic role of host cyclooxygenase and cytokine genotypes in a Caucasian cohort of patients with gastric adenocarcinoma. Genetic factors influencing the prognosis of gastric adenocarcinoma (GAC) are not well known. Given the relevance of cytokines and other pro-inflammatory mediators in cancer progression and   invasive  ness, we aimed to assess the prognostic role of several functional cytokine and cyclooxygenase gene polymorphisms in patients with GAC.Genomic DNA from 380 Spanish Caucasian patients with primary GAC was genotyped for 23 polymorphisms in pro-inflammatory (IL1B, TNFA, LTA, IL6, IL12p40), anti-inflammatory (IL4, IL1RN, IL10, TGFB1) cytokine, and cyclooxygenase (PTGS1 and PTGS2) genes by PCR, RFLP and TaqMan assays. Clinical and histological information was collected prospectively. Survival curves were estimated by the Kaplan-Meier method and compared using the log rank test. Outcome was determined by analysis of Cox proportional hazards, adjusting for confounding factors.The median follow-up period and median overall survival (OS) time were 9.9 months (range 0.4-120.3) and 10.9 months (95% CI: 8.9-14.1), respectively. Multivariate analysis identified tumor stages III (HR, 3.23; 95% CI:2-5.22) and IV (HR, 5.5; 95% CI: 3.51-8.63) as independent factors associated with a significantly reduced OS, whereas surgical treatment (HR: 0.44; 95%CI: 0.3-0.6) was related to a better prognosis of the disease. Concerning genetic factors, none of the 23 polymorphisms evaluated in the current study did influence survival. Moreover, no gene-environment interactions on GAC prognosis were observed.Our results show that, in our population, the panel of selected pro- and anti-inflammatory cytokine, and cyclooxygenase gene polymorphisms are not relevant in determining the prognosis of gastric adenocarcinoma. 
 Methylation status and overexpression of COX-2 in Tunisian patients with ductal   invasive   breast carcinoma. Inflammation and hormonal signalling induce the cyclooxygenase-2 (COX-2) expression in solid tumours including breast cancer, which in turn affects cell proliferation, apoptosis and metastasis. The aim of this study was to investigate the expression of COX-2 and its association with clinical parameters, patient's survival, hormones receptors (oestrogen, progesterone), ERBB2 and TP53 expression in 83 cases of infiltrating ductal breast carcinomas. Moreover, the methylation status at the CpG islands of the COX-2 gene promoter was also explored in 70 specimens. We showed that tumours exhibiting moderate to intense COX-2 immunostaining were significantly more frequent in patients over 45 years old (p = 0.027). Moreover, a high level of COX-2 expression correlated with a shorter survival time (p log-rank = 0.04) and was an independent prognostic factor (p = 0.022; HR 6.4; 95% CI = 1.3-31.4). On the other hand, hypermethylation of the COX-2 gene promoter was observed in 27% of cases and strongly associated with smaller tumours (<5 cm, p = 0.011). Furthermore, patients with methylated COX-2 pattern have a better 4-year disease-free survival (p = 0.022) as well as a prolonged overall survival (p log-rank test = 0.034). In conclusion, we showed that high COX-2 expression was associated with reduced survival and was an independent prognostic factor. However, hypermethylation of the COX-2 promoter correlated with a better overall survival in Tunisian patients with breast carcinoma. 
 Kaposi's sarcoma associated herpes virus (KSHV) induced COX-2: a key factor in latency, inflammation, angiogenesis, cell survival and invasion. Kaposi's sarcoma (KS), an enigmatic endothelial cell vascular neoplasm, is characterized by the proliferation of spindle shaped endothelial cells, inflammatory cytokines (ICs), growth factors (GFs) and angiogenic factors. KSHV is etiologically linked to KS and expresses its latent genes in KS lesion endothelial cells. Primary infection of human micro vascular endothelial cells (HMVEC-d) results in the establishment of latent infection and reprogramming of host genes, and cyclooxygenase-2 (COX-2) is one of the highly up-regulated genes. Our previous study suggested a role for COX-2 in the establishment and maintenance of KSHV latency. Here, we examined the role of COX-2 in the induction of ICs, GFs, angiogenesis and   invasive   events occurring during KSHV de novo infection of endothelial cells. A significant amount of COX-2 was detected in KS tissue sections. Telomerase-immortalized human umbilical vein endothelial cells supporting KSHV stable latency (TIVE-LTC) expressed elevated levels of functional COX-2 and microsomal PGE2 synthase (m-PGES), and secreted the predominant eicosanoid inflammatory metabolite PGE2. Infected HMVEC-d and TIVE-LTC cells secreted a variety of ICs, GFs, angiogenic factors and matrix metalloproteinases (MMPs), which were significantly abrogated by COX-2 inhibition either by chemical inhibitors or by siRNA. The ability of these factors to induce tube formation of uninfected endothelial cells was also inhibited. PGE2, secreted early during KSHV infection, profoundly increased the adhesion of uninfected endothelial cells to fibronectin by activating the small G protein Rac1. COX-2 inhibition considerably reduced KSHV latent ORF73 gene expression and survival of TIVE-LTC cells. Collectively, these studies underscore the pivotal role of KSHV induced COX-2/PGE2 in creating KS lesion like microenvironment during de novo infection. Since COX-2 plays multiple roles in KSHV latent gene expression, which themselves are powerful mediators of cytokine induction, anti-apoptosis, cell survival and viral genome maintainence, effective inhibition of COX-2 via well-characterized clinically approved COX-2 inhibitors could potentially be used in treatment to control latent KSHV infection and ameliorate KS. 
 Co-Expression of p16, Ki67 and COX-2 Is Associated with Basal Phenotype in High-Grade Ductal Carcinoma In Situ of the Breast. We assessed the co-expression of cell cycle-related biomarkers in a series of 121 consecutive cases of high-grade ductal carcinoma in situ (DCIS), pure or associated with   invasive   carcinoma, and their associations with the different immunoprofiles of DCIS. Cases were identified from the histopathology files of the Breast Pathology Laboratory, Federal University of Minas Gerais, Brazil, from 2003 to 2008. The expression of estrogen receptor, progesterone receptor, HER2 overexpression, cytokeratin 5, epidermal growth factor receptor 1, cyclooxygenase-2, p16 and Ki67 were assessed. Tumors were placed into five subgroups according to their immunohistochemical profile: luminal A, luminal B, HER2, basal-like and "not classified". We found that the basal phenotype was associated with a higher frequency of p16-positive cases (83%) and the luminal A phenotype showed a higher frequency of p16-negative cases (93%; p=0.000). The association of biomarkers p16(+)/Ki67(+)/COX2(+) was expressed in 02/06 cases (33.3%) of the basal phenotype but in only 01/70 cases (1.4%) of the luminal A phenotype (p=0.01). The co-expression of p16(+)/Ki67(+)/COX2(-) was associated with a basal phenotype (p=0.004). P16 expression, p16(+)/Ki67(+)/COX2(+) and p16(+)/Ki67(+)/COX2(-) co-expression showed significant associations with the basal phenotype and these profiles could be used to guide more aggressive treatment strategies in patients with high-grade DCIS. 
 Endothelial cell confluence regulates cyclooxygenase-2 and prostaglandin E2 production that modulate motility. Endothelial cells line the vasculature and, after mechanical denudation during   invasive   procedures or cellular loss from natural causes, migrate to reestablish a confluent monolayer. We find confluent monolayers of human umbilical vein endothelial cells were quiescent and expressed low levels of cyclooxygenase-2, but expressed cyclooxygenase-2 at levels comparable with cytokine-stimulated cells when present in a subconfluent culture. Mechanically wounding endothelial cell monolayers stimulated rapid cyclooxygenase-2 expression that increased with the level of wounding. Cyclooxygenase-2 re-expression occurred throughout the culture, suggesting signaling from cells proximal to the wound to distal cells. Media from wounded monolayers stimulated cyclooxygenase-2 expression in confluent monolayers, which correlated with the level of wounding of the donor monolayer. Wounded monolayers and cells in subconfluent cultures secreted enhanced levels of prostaglandin (PG) E(2) that depended on cyclooxygenase-2 activity, and PGE(2) stimulated cyclooxygenase-2 expression in confluent endothelial cell monolayers. Cells from subconfluent monolayers migrated through filters more readily than those from confluent monolayers, and the cyclooxygenase-2-selective inhibitor NS-398 suppressed migration. Adding PGE(2) to NS-398-treated cells augmented migration. Endothelial cells also migrated into mechanically denuded areas of confluent monolayers, and this too was suppressed by NS-398. We conclude that endothelial cells not in contact with neighboring cells express cyclooxygenase-2 that results in enhanced release of PGE(2), and that this autocrine and paracrine loop enhances endothelial cell migration to cover denuded areas of the endothelium. 
 CXCR-4 and COX-2 expression in basal cell carcinomas and well-differentiated squamous cell carcinomas of the skin; their relationship with tumor   invasive  ness and histological subtype. Both CXCR-4 and COX-2 are biological markers that play a significant role in several neoplastic processes. We explored the differences in expression of these markers in certain subtypes of basal cell carcinoma, and squamous cell carcinomas in general.In this study, we investigated the differences between 38 patients with basal cell carcinoma (nodular, infiltrative and micro-nodular subtypes) and 24 patients with well-differentiated squamous cell carcinomas with respect to their depth of invasion, tumor location, age, and CXCR-4 and COX-2 expression.Statistically, we found no significant difference between squamous cell carcinomas and basal cell carcinoma in terms of CXCR-4 and COX-2 expression; however, the degree of marker expression became stronger with increasing depth of invasion in both tumors. The expression of both markers was also higher in infiltrative type basal cell carcinoma compared to all the other subtypes. The results were statistically significant (p<0.05). Additionally, a significantly positive correlation also existed between COX2 and CXCR4 expression (p < 0.05).The degree of expression of CXCR-4 and COX-2 is related to   invasive  ness in both malignancies; thus, infiltrative type of basal cell carcinoma displays the highest degree of CXCR-4 and COX-2 expression among all the subtypes. Furthermore, our results indicate that these two biological markers may both be involved in the process of carcinogenesis and require investigation with further molecular and genetic studies in larger series. 
 Cyclooxygenase-2 expression on urothelial and inflammatory cells of cystoscopic biopsies and urine cytology as a possible predictive marker for bladder carcinoma. Cyclooxygenase-2 (COX-2) is a key inducible enzyme involved in the production of prostaglandins. It contributes to human carcinogenesis by various mechanisms. The aim of the current study was to elucidate the possible involvement of COX-2 in human bladder carcinoma by examining its expression on both urothelial and inflammatory cells in tissue biopsies and urine cytology samples of different urinary bladder lesions. A total of 65 patients were included in the study and were selected from cases admitted to Urology Department, Theodor Bilharz Research Institute (TBRI), Giza, Egypt. They represented seven control cases with almost normal-looking bladder tissue; pure chronic cystitis (n=12); premalignant lesions (18) in the form of squamous metaplasia (n=8) or urothelial dysplasia (n=10) as well as transitional cell carcinoma (TCC) (n=18), and squamous cell carcinoma (SqCC) (n=10). Immunohistochemistry of formalin-fixed, paraffin-embedded tissue sections and urine cytology samples was performed for all cases using COX-2 (H-62): sc-7951, a rabbit polyclonal antibody. The study revealed positive COX-2 expression on the urothelial and inflammatory cells of cystoscopic biopsies from all cases of pure chronic cystitis, squamous metaplasia and SqCC compared with 42.8% and 71.4% of normal controls, respectively. The score of urothelial COX-2 expression was sequentially up-regulated from normal to chronic cystitis (either pure or associated with premalignant changes) (p<0.05) to malignant changes (p<0.05). However, the inflammatory cellular expression was down-regulated with malignant transformation compared with chronic cystitis (p<0.05). In TCC, COX-2 was over-expressed on both urothelial and inflammatory cells in advanced tumors. Urine cytology samples were positive for COX-2 in a comparable manner to that observed in cystoscopic biopsies. Accordingly, the results of the current study have provided new information in two aspects: First, is the possibility of using the differential COX-2 expression on both inflammatory and urothelial cells as markers for premalignant or malignant transformation; second, besides cystoscopy, urine cytology was found to have a high sensitivity for COX-2 expression and hence proved to be valuable in malignancy as a non-  invasive   substitute for cystoscopy. 
 Involvement of Cox-2 in the metastatic potential of chemotherapy-resistant breast cancer cells. A major problem with the use of current chemotherapy regimens for several cancers, including breast cancer, is development of intrinsic or acquired drug resistance, which results in disease recurrence and metastasis. However, the mechanisms underlying this drug resistance are unknown. To study the molecular mechanisms underlying the   invasive   and metastatic activities of drug-resistant cancer cells, we generated a doxorubicin-resistant MCF-7 breast cancer cell line (MCF-7/DOX).We used MTT (3-(4,5-dimethylthiazol-2-yl)-2,5-diphenyltetrazolium bromide) assays, flow cytometry assays, DNA fragmentation assays, Western blot analysis, cell invasion assays, small interfering RNA (siRNA) transfection, reverse transcription-polymerase chain reaction, experimental lung metastasis models, and gelatin and fibrinogen/plasminogen zymography to study the molecular mechanism of metastatic activities in MCF-7/DOX cells.We found that MCF-7/DOX acquired   invasive   activities. In addition, Western blot analysis showed increased expression of epidermal growth factor receptor (EGFR) and Cox-2 in MCF-7/DOX cells. Inhibition of Cox-2, phosphoinositide 3-kinase (PI3K)/Akt, or mitogen-activated protein kinase (MAPK) pathways effectively inhibited the   invasive   activities of MCF-7/DOX cells. Gelatin and fibrinogen/plasminogen zymography analysis showed that the enzymatic activities of matrix metalloproteinase-2 (MMP-2), MMP-9, and urokinase-type plasminogen activator were markedly higher in MCF-7/DOX cells than in the MCF-7 cells. In vitro invasion assays and mouse models of lung metastasis demonstrated that MCF-7/DOX cells acquired   invasive   abilities. Using siRNAs and agonists specific for prostaglandin E (EP) receptors, we found that EP1 and EP3 played important roles in the   invasive  ness of MCF-7/DOX cells.We found that the   invasive   activity of MCF-7/DOX cells is mediated by Cox-2, which is induced by the EGFR-activated PI3K/Akt and MAPK pathways. In addition, EP1 and EP3 are important in the Cox-2-induced invasion of MCF-7/DOX cells. Therefore, not only Cox-2 but also EP1 and EP3 could be important targets for chemosensitization and inhibition of metastasis in breast cancers that are resistant to chemotherapy. 
 Stromal COX-2 signaling activated by deoxycholic acid mediates proliferation and   invasive  ness of colorectal epithelial cancer cells. COX-2 is a major regulator implicated in colonic cancer. However, how COX-2 signaling affects colonic carcinogenesis at cellular level is not clear. In this article, we investigated whether activation of COX-2 signaling by deoxycholic acid (DCA) in primary human normal and cancer associated fibroblasts play a significant role in regulation of proliferation and   invasive  ness of colonic epithelial cancer cells. Our results demonstrated while COX-2 signaling can be activated by DCA in both normal and cancer associated fibroblasts, the level of activation of COX-2 signaling is significantly greater in cancer associated fibroblasts than that in normal fibroblasts. In addition, we discovered that the proliferative and   invasive   potential of colonic epithelial cancer cells were much greater when the cells were co-cultured with cancer associated fibroblasts pre-treated with DCA than with normal fibroblasts pre-treated with DCA. Moreover, COX-2 siRNA attenuated the proliferative and   invasive   effect of both normal and cancer associate fibroblasts pre-treated with DCA on the colonic cancer cells. Further studies indicated that the activation of COX-2 signaling by DCA is through protein kinase C signaling. We speculate that activation of COX-2 signaling especially in cancer associated fibroblasts promotes progression of colonic cancer. 
 Characterization of cyclooxygenase-2 (COX-2) during tumorigenesis in human epithelial cancers: evidence for potential clinical utility of COX-2 inhibitors in epithelial cancers. Increased prostaglandins (PGs) are associated with many inflammatory pathophysiological conditions; and are synthesized from arachidonic acid by either of 2 enzymes, cyclooxygenase-1 (COX-1) or -2 (COX-2). Recent epidemiologic, expression, and pharmacologic studies suggest COX-2 derived metabolites also play a functional role in the maintenance of tumor viability, growth and metastasis. Archival and/or prospectively collected human tissues were prepared for immunohistochemistry, and representative cases assayed via Western blot, RT-PCR, or TAQman analysis. Consistent overexpression of COX-2 was observed in a broad range of premalignant, malignant, and metastatic human epithelial cancers. COX-2 was detected in ca. 85% of the hyperproliferating, dysplastic, and neoplastic epithelial cells, and in the existing and angiogenic vasculature within and adjacent to hyperplastic/neoplastic lesions. These data collectively imply COX-2 may play an important role during premalignant hyperproliferation, as well as the later stages of   invasive   carcinoma and metastasis in various human epithelial cancers. 
 The association between COX-2 polymorphisms and hematologic toxicity in patients with advanced non-small-cell lung cancer treated with platinum-based chemotherapy. Overexpression of COX-2 is proved to contribute to tumor promotion and carcinogenesis through stimulating cell proliferation, inhibiting apoptosis and enhancing the   invasive  ness of cancer cells. Apoptosis-related molecules are potential predictive markers for survival and toxicity in platinum treatment. This study aimed at investigating the association between COX-2 polymorphisms and the occurrence of grade 3 or 4 toxicity in advanced non-small cell lung cancer patients treated with platinum-based chemotherapy.Two hundred and twelve patients with inoperable stage IIIB-IV NSCLC received first-line chemotherapy between 2007 and 2009 were recruited in this study. Four functional COX-2 polymorphisms were genotyped by PCR-based restriction fragment length polymorphism (RFLP) methods.The incidence of grade 3 or 4 hematologic toxicity was significantly higher in G allele carriers of the COX-2 rs689466 (-1195G/A) polymorphism compared with wild-type homozygotes AA (P value = 0.008; odds ratio, 2.47; 95% confidence internal, 1.26-4.84) and the significance still existed after the Bonferroni correction. Statistically significant difference was also found in grade 3 or 4 leukopenia (P value = 0.010; OR = 2.82; 95%CI = 1.28-6.20). No other significant association was observed between genotype and toxicity in the study. The haplotype analysis showed that the haplotype AGG was associated with a reduced risk of grade 3 or 4 hematologic and leukopenia toxicity (P value = 0.009; OR = 0.59; 95%CI = 0.39-0.88 and P value = 0.025; OR = 0.61; 95%CI = 0.39-0.94, respectively) while the haplotype GGG was associated with an increased risk of grade 3 or 4 hematologic and leukopenia toxicity (P value = 0.009; OR = 1.71; 95%CI = 1.14-2.56 and P value = 0.025; OR = 1.65; 95%CI  = 1.06-2.57, respectively).This investigation for the first time suggested that polymorphism in COX-2 rs689466 may be a potent bio-marker in predicting severe hematologic toxicity in NSCLC patients after platinum-based chemotherapy. 
 Cyclooxygenase 2 expression in pancreatic adenocarcinoma and pancreatic intraepithelial neoplasia: an immunohistochemical analysis with automated cellular imaging. We immunohistochemically examined material from 36 pancreata (adenocarcinomas, 30 lesions; pancreatic intraepithelial neoplasia [PanIN], 65; normal pancreatic ducts, 30) for cyclooxygenase 2 (COX-2) with an automated platform. We analyzed 7 to 10 discrete foci and generated an average percentage of positive cells and average staining intensity for each lesion. These 2 values were then multiplied to create an overall "HistoScore" for each lesion. COX-2 demonstrated considerable heterogeneity of expression between and within cases. The overall average percentage of positive cells in adenocarcinomas was 47.3%; in PanINs, 36.3%; and in normal ducts, 19.2%. COX-2 was expressed in more than 20% of cells in 23 adenocarcinomas (77%), 42 PanINs (65%), and 12 normal ducts (40%). The overall average HistoScore for adenocarcinomas was 6.1; for PanINs, 5.4; and for normal ducts, 3.5. Significant differences in COX-2 expression were demonstrable in adenocarcinomas vs normal ducts, PanINs vs normal ducts, and PanIN 2/3 vs PanIN 1a/1b. In general, the pattern of COX-2 expression increased from normal to PanIN to adenocarcinoma. The up-regulation of COX-2 in a subset of non  invasive   precursor lesions makes it a potential target for chemoprevention with selective COX-2 inhibitors. 
 Expression of cyclooxygenase-2 in   invasive   breast carcinomas and its prognostic impact. Representative tumour sections from 468 patients with   invasive   breast cancer were immunostained for cyclooxygenase-2 (COX-2) and evaluated. The relationships between COX-2 expression, clinical outcome and various clinicopathological variables, including tumour vascularity and disseminated tumour cells (DTC) in the bone marrow were examined. COX-2 expression in   invasive   breast carcinoma cells was positively associated with oestrogen receptor and/or progesterone receptor positivity (p<0.001). Triple-negative tumours showed no/low COX-2 expression more frequently than other tumour types (p<0.001). Expression of COX-2 was not associated with breast cancer-specific survival (p=0.49, log-rank) or distant disease-free survival (p=0.67, log-rank) for all patients, including lymph node-negative, untreated patients (p>0.14, log-rank). There was also no significant association between COX-2 expression and histological grade, tumour size, nodal status, DTC in bone marrow, p53, HER2, or tumour vascularity. In conclusion, COX-2 expression in this series was associated with the presence of hormone receptors. Low COX-2 expression was observed in triple-negative breast carcinomas. 
 Significance of COX-2 and VEGF expression in histopathologic grading and   invasive  ness of meningiomas. Meningiomas are slow-growing neoplasms that recur locally. Their morphologic grading does not always correlate with patient outcome. We evaluated the status of several immunohistochemical markers with histopathologic parameters in various grades of meningioma.Eighty-eight meningioma specimens were examined immunohistochemically to determine the status of Ki-67, cyclin D1, epidermal growth factor receptor (EGFR), cyclooxygenase-2 (COX-2), vascular endothelial growth factor (VEGF), and bcl-2. Several clinical and pathological parameters were investigated.Forty-nine Grade I, 33 Grade II, and 6 Grade III meningiomas were observed. VEGF and Ki-67 expression was correlated with higher tumor grade. The association between grade and other immunohistochemical markers expression was not significant. A correlation was observed between COX-2 expression and   invasive  ness to the brain or adjacent soft tissue. Tumor recurrence was correlated with brain or adjacent soft tissue invasion. We also observed a relationship between VEGF level and COX-2 expression, and they were both correlated with necrosis.Immunohistochemical evaluation of VEGF, COX-2, and Ki-67 expression can provide information regarding the behavior of meningiomas, particularly for cases in which histological grading is not straightforward. 
 Increased expression of COX-2 in the development of human lung cancers. It is well accepted that an increase in the expression of cyclooxygenase-2 (COX-2), a key inducible enzyme involved in the production of prostaglandins and other eicosanoids, may play a significant role in carcinogenesis in addition to its well-known role in inflammatory reactions. Whereas previous studies were largely confined to colorectal tumorigenesis, we have shown that a significantly increased expression of COX-2 may also play a role in the development of lung cancer. COX-2 expression was found to be frequently elevated in lung cancer, especially in adenocarcinoma, and the proportion of lung cancer cells with marked COX-2 expression was much higher in lymph node metastases than in the corresponding primary tumors. It was also shown that early stage adenocarcinoma patients with increased COX-2 expression who were surgically treated had a shorter survival. Our studies, which used high- and low-metastatic human lung cancer cell sublines established in our laboratory, revealed an association between metastatic capabilities and COX-2 expression levels: COX-2-specific inhibitors could inhibit in vitro the invasion of the highly metastatic NCI-H460-LNM35 clone through Matrigel-containing basement membrane components as well as the spontaneous in vivo metastasis in SCID mice. Taken together, these findings suggest that an increase in COX-2 expression maybe associated with the development of lung cancer and possibly with the acquisition of an   invasive   and metastatic phenotype. 
 Dual inhibition of COX-2/5-LOX blocks colon cancer proliferation, migration and invasion in vitro. Inflammation is emerging as a new hallmark of cancer. Arachidonic acid (AA) metabolism, the family of cyclooxygenases (COXs) and lipoxygenase (LOX) play important roles in AA-related inflammatory cascades. In 94 colorectal cancer samples collected from the Han population, the immunohistochemical results indicated that 68% of the patients with colorectal cancer had a co-expression of both COX-2 and 5-LOX, while both displayed low expression in the matched normal tissues. In cell lines, three colorectal cancer cell lines exhibited high expression of COX-2 and 5-LOX. During stable silencing of the expression of COX-2 or 5-LOX in LoVo cancer cells, we found that downregulation of either COX-2 or 5-LOX significantly diminished the growth, migration and invasion of the colon cancer cells and specifically, downregulation of COX-2 could elicit upregulation of 5-LOX protein and vice versa. The above results suggested that the simultaneous blocking of COX-2 and 5-LOX activity may bring more potential benefits in managing the progression of colon cancer. Therefore, we sought to explore the effectiveness of a dual COX-2/5-LOX inhibitor darbufelone on the proliferation, migration, invasion and apoptosis of colon cancer cells, as well as the underlying mechanism of action. The results indicated that darbufelone significantly decreased the proliferative and   invasive   abilities of the colon cancer cells, in a dose-dependent manner. During the study of the related mechanisms, we found an upregulation of p27 and downregulation of cyclin D1 as well as CDK4 after darbufelone treatment, which indicated that darbufelone could arrest the cell cycle of LoVo cells at the G0/G1 phase. Furthermore, the activation of caspase-3 and -9, upregulation of Bax and downregulation of Bcl-2 demonstrated the occurrence of apoptosis by darbufelone. Finally, darbufelone also prevented the migration and invasion of LoVo cells, which may be ascribed to the upregulation of E-cadherin and ZO-1. In summary, our data suggest that the inhibition of both COX-2/5-LOX may be an effective therapeutic approach for colon cancer management, particularly for those patients with high expression of COX-2/5-LOX. 
 [Expression of COX-2 and MMP-9 in cervical carcinoma and their clinical significance]. To investigate the expressions of COX-2 and MMP-9 in cervical carcinoma and their clinical significance.Immunohistochemical SP method was used to detect the expressions of COX-2 and MMP-9 in 72 cases of   invasive   carcinoma of cervix (ICC) and 16 cases of normal cervical epithelium remote from tumor (NCE), and the relationship between the expressions of COX-2 and MMP-9 in ICC and some factors relating to clinical pathology of cervical carcinoma such as histopathological grading, lymph node metastasis, stroma involvement and FIGO staging were analyzed statistically.The rates of positive expression of COX-2 and MMP-9 in ICC were significantly higher than those in NCE. COX-2: 88.9% in group ICC and 12.5% in group NCE, P = 0.000. MMP-9: 94.4% in group ICC and 43.8% in group NCE, P = 0.000. The expression of COX-2 was positively correlated with lymph node metastasis and stroma involvement (r = 0.296, P = 0.012 in group ICC and r = 0.257, P = 0.029 in group NCE, respectively). The expression of MMP-9 was positively correlated with FIGO staging (r = 0.329, P = 0.005) and histopathological grading (r = 0.351, P = 0.003). The expression of COX-2 was positively correlated with the expression of MMP-9 in ICC (r = 0.297, P = 0.011).The overexpressions of COX-2 and MMP-9 are closely related to the invasion and growth of cervical carcinoma. The tissue with overexpression of COX-2 has strong invasion ability. COX-2 and MMP-9 have synergistic effect on proliferation, invasion and metastasis of cancer cells. Detecting the expression of both COX-2 and MMP-9 may be of value in further understanding the biological behavior and predicting the prognosis of cervical carcinoma. 
 Histologically normal human mammary epithelia with silenced p16(INK4a) overexpress COX-2, promoting a premalignant program. Breast tissue from healthy women contains variant mammary epithelial cells (vHMEC) exhibiting p16INK4a promoter hypermethylation both in vivo and in vitro. When continuously cultured, vHMEC acquire telomeric dysfunction and produce the types of chromosomal abnormalities seen in premalignant lesions of cancer. We find that late passage vHMEC express elevated prostaglandin cyclo-oxygenase 2 (COX-2), which contributes to increased prostaglandin synthesis, angiogenic activity, and   invasive   ability. These data demonstrate the existence of human mammary epithelial cells with the potential to acquire multiple genomic alterations and phenotypes associated with malignant cells. Moreover, COX-2 overexpression coincides with focal areas of p16INK4a hypermethylation in vivo, creating ideal candidates as precursors to breast cancer. These putative precursors can be selectively eliminated upon exposure to COX-2 inhibitors in vitro. 
 Frequent co-localization of Cox-2 and laminin-5 gamma2 chain at the   invasive   front of early-stage lung adenocarcinomas. Laminin-5 is an extracellular matrix protein that plays a key role in cell migration and tumor invasion. Cox-2 is an induced isoform of cyclooxygenases that plays an important role in carcinogenesis, suppression of apoptosis, angiogenesis, and metastasis of colon cancer. We report frequent co-expression of cox-2 and laminin-5 at the   invasive   front of early-stage lung adenocarcinomas. We investigated the expression of cox-2 and laminin-5 immunohistochemically in 102 cases of small-sized lung adenocarcinoma (maximum dimension, 2 cm or less). Cox-2 and laminin-5 were expressed in 97 (95.1%) and 82 (80.4%) cases, respectively. Both were preferentially localized in cancer cells at the cancer-stroma interface, although cox-2 tended to show a diffuse staining pattern in some cases. A comparison of their staining patterns revealed a striking similarity in their distribution in 24 cases, and a partial overlap between their localization in another 20 cases. Moreover, an overall correlation was found between the expression levels of cox-2 and laminin-5 (P = 0.018). To gain insight into the mechanisms that regulate the expression of these proteins, we additionally studied their expression in 58 cases of stage I lung adenocarcinoma, in which p53 status was determined by immunohistochemistry, polymerase chain reaction-single strand conformation polymorphism analysis, and direct sequencing. The results showed that tumors with mutant p53 tended to express more cox-2 than those with wild-type p53 (P = 0.080). Also, tumors that overexpressed p53 had higher levels of cox-2 and laminin-5 than those without p53 overexpression (P = 0.032 and 0.047, respectively). Further immunohistochemical analysis showed that tumors that overexpressed both epidermal growth factor receptor (EGFR) and erbB-2 had higher levels of cox-2 and laminin-5 than those without concomitant overexpression of these proteins (P = 0.014 and P = 0.018, respectively). To see whether EGFR signaling is involved in cox-2 and laminin-5 expression, we further conducted in vitro analyses using six lung adenocarcinoma cell lines (A549, HLC-1, ABC-1, LC-2/ad, VMRC-LCD, and L27). Western blot analyses showed that cox-2 mRNA levels, and to a lesser extent laminin-5 gamma2 mRNA levels, correlated with the expression levels of erbB-2 and the phosphorylated form of MAPK/ERK-1/2 protein. The addition of transforming growth factor-alpha increased both cox-2 and laminin-5 gamma2 mRNA levels in A549, ABC-1, and L27 with different kinetics; the induction of cox-2 occurred earlier than that of laminin-5 gamma2. Finally, the migration of ABC-1 cells was inhibited by MAP kinase kinase inhibitor PD98059 and a selective cox-2 inhibitor NS-398. In contrast, the migration of A549 cells was inhibited by PD98059, but much less effectively by NS-398. These results suggest that co-stimulatory mechanisms may exist that increase the expression of cox-2 and laminin-5 at the   invasive   front of lung adenocarcinomas and that EGFR signaling could be one of the mechanisms. Further investigations are warranted concerning the role of cox-2 and laminin-5 in cancer cell invasion and the significance of p53 and EGFR signaling in the regulation of cox-2 and laminin-5 expression. 
 Cyclooxygenase-2 and microsomal prostaglandin E synthase-1 are overexpressed in squamous cell carcinoma of the penis. Prostaglandin E2 (PGE2) promotes malignant growth. Cyclooxygenase (COX) catalyzes the synthesis of PGH2, which is converted, in turn, by microsomal prostaglandin E synthase (mPGES-1) to PGE2. One strategy for inhibiting carcinogenesis is to prevent PGE2 production in premalignant and malignant tissues. It is important, therefore, to determine whether enzymes involved in PGE2 biosynthesis are deregulated in neoplasia. The main purpose of this study was to determine whether amounts of COX-2 or mPGES-1 were increased in intraepithelial neoplasia or squamous cell carcinoma (SCC) of the penis. Because human papillomavirus (HPV) has been linked to the development of penile SCC, a secondary objective was to determine whether COX-2 was overexpressed in SCC arising in an HPV16 transgenic mouse.Immunohistochemistry and immunoblotting were used to evaluate the expression of COX-2 and mPGES-1 in benign and malignant lesions including metastases to lymph nodes. Amounts of intratumoral PGE2 were quantified by enzyme immunoassay. Reverse transcription-PCR was used to determine the expression of each of the four known receptors (EP(1-4)) for PGE2.Immunohistochemistry demonstrated increased expression of COX-2 and mPGES-1 in dysplasia, carcinoma in situ,   invasive   SCC, and metastases to lymph nodes. Immunoblot analysis confirmed that COX-2 and mPGES-1 were consistently overexpressed in SCC. PGE2 and all four of the PGE2 receptor subtypes were detected in each of the tumor samples. Elevated levels of COX-2 were also detected in SCC arising in an HPV16 transgenic mouse.Increased amounts of COX-2 and mPGES-1 were detected in penile intraepithelial neoplasia and carcinoma. These findings provide the basis for evaluating whether inhibiting COX-2 will be useful in the prevention or treatment of penile SCC. 
 MMP-7 is upregulated by COX-2 and promotes proliferation and invasion of lung adenocarcinoma cells. Matrix metalloproteinases (MMPs) have been implicated in a variety of pathophysiological conditions, of which MMP-7 is expressed by tumor cells of epithelial and mesenchymal origin. However, the function of MMP-7 in human lung adenocarcinoma (LAC) is unclear. In the present study the expression of MMP-7 in LAC was examined by immunohistochemical assay using a tissue microarray procedure. A loss-of-function experiment was performed to explore the effects and molecular mechanisms of lentiviral vector-mediated MMP-7 siRNA (siMMP-7) on cell proliferation and   invasive   potential in LAC A549 cells, measured by MTT and Transwell assays, respectively. It was found that, the expression of MMP-7 protein in LAC was significantly increased compared with that in adjacent non-cancerous tissues (ANCT) (76.0% vs 44.0%, P&lt;0.001), and positively correlated with lymph node metastases of the tumor (P=0.014). Furthermore, targeted inhibition of cyclooxygenase-2 (COX-2) by siRNA downregulated the expression of MMP-7 and inhibited invasion of LAC cells, and knockdown of MMP-7 suppressed tumor proliferation and invasion in LAC cells. Taken together, our findings indicate that increased expression of MMP-7 is associated with lymph node metastasis and upregulated by COX-2, and promotes the tumorigenesis of LAC, suggesting that MMP-7 may be a potential therapeutic target for the treatment of cancer. 
 COX-2 expression predicts worse breast cancer prognosis and does not modify the association with aspirin. Some previous studies have found worse prognosis among cyclooxygenase-2 (COX-2)-expressing breast cancers. Aspirin and NSAIDs inhibit COX-2. Three studies, including ours, have reported a survival advantage among women with breast cancer who take either aspirin or NSAIDs. Through this study we hypothesized that in the Nurses' Health Study (NHS), COX-2 expression would be associated with worse prognosis, and aspirin use would be associated with better survival particularly among women with COX-2 positive tumors. In this study we investigated 2,001 women presenting with   invasive   breast cancers stained for COX-2 by immunohistochemistry. Tumor prognostic factors were from medical records. Aspirin use was assessed at least 12 months after diagnosis and updated. Cause of death was identified from death certificates. Statistical analyses included logistic regression of prognostic factors with COX-2 status as the outcome, and proportional hazards regression with breast cancer death as the outcome. Tumor COX-2 expression was associated with higher diagnostic stage. Compared with stage I, the RR(95% CI) for stages II-IV were 1.16 (0.93-1.45), 1.68 (1.27-2.22), and 1.76 (0.93-3.32). COX-2 expression was associated with lobular compared with ductal histology (1.40 [1.02-1.92]), and estrogen receptor positive compared with negative (2.22 [1.66-2.95]). The RR(95% CI) of breast cancer death for current aspirin use was similar for women with COX-2-positive and COX-2-negative tumors; 0.64 (0.43-0.96) and 0.57 (0.44-0.74), respectively. In the NHS, COX-2 breast cancer expression was associated with higher stage at diagnosis. The survival benefit associated with aspirin use did not differ by COX-2 status. COX-2 breast cancer expression is associated with worse prognosis. If aspirin truly impacts breast cancer survival, then it is not solely via COX-2. 
 Estrogen-dependent prognostic significance of cyclooxygenase-2 expression in early-stage   invasive   breast cancers treated with breast-conserving surgery and radiation. To evaluate the prognostic significance of cyclooxygenase-2 (COX-2) in breast cancer patients treated with conservative surgery and radiation therapy (CS+RT).Between 1975 and 2003, we retrieved specimens from 504 breast cancer patients treated with CS+RT. The specimens were constructed into tissue microarrays processed and stained for estrogen receptor (ER), progesterone receptor, Her2/neu, and COX-2. Each core was scored as positive or negative. All data including demographics, clinical, pathologic, staging, and outcome variables were entered into a computerized database.Expression of COX-2 was positive in 58% of cases and correlated with younger age (p = 0.01) and larger tumor size (p = 0.001). Expression of COX-2 was predictive of local relapse (relative risk[RR], 3.248; 95% confidence interval [CI], 1.340-7.871; p = 0.0091), distant metastasis (RR, 2.21; 95% CI, 1.259-3.896; p = 0.0058), and decreased survival (RR, 2.321; 95% CI, 1.324-4.071; p = 0.0033). Among ER-positive patients, COX-2 expression was predictive of worse local control (85% vs. 93%, p = 0.04), distant metastasis (75% vs. 95%, p = 0.002) and worse survival (65% vs. 94%, p = 0.002). Among ER-negative tumors COX-2 expression was not significantly correlated with local control (87 vs. 95%, p = 0.12), distant metastasis (73% vs. 78%, p = 0.39), or survival (77% vs. 87%, p = 0.15).In breast cancer patients treated with CS+RT, COX-2 expression is associated with younger age, larger tumor size, worse local control, distant metastasis, and worse overall survival. The significance is limited to hormone receptor-positive tumors, consistent with the known effect of COX-2/PGE2 on aromatase activity. Use of COX-2 inhibitors in estrogen-dependent breast cancers warrants further investigation. 
 Translationally regulated C/EBP beta isoform expression upregulates metastatic genes in hormone-independent prostate cancer cells. C/EBP beta is a transcription factor regulating key biological processes including cellular growth and differentiation and its increased expression correlates with tumor   invasive  ness. Recently, the increased expression of C/EBP beta was reported in proliferative inflammatory atrophy of the prostate, associating with increased COX-2 expression and androgen receptor (AR) downregulation.C/EBP beta expression was determined in DU-145, PC-3 and LNCaP cells by immunoblotting. Transient transfection of C/EBP beta expression vectors was performed to investigate translational regulation of its isoform expression. Reporter gene analysis was performed to investigate transcriptional activity of C/EBP beta on metastatic gene expression.We determined that transcriptionally active, full-length C/EBP beta isoforms were dominantly expressed in hormone-independent DU-145 and PC-3 cells, while transcription-repressing truncated isoform was dominant in hormone-dependent LNCaP cells. Our results further showed lack of full-length isoform expression from the transiently transfected C/EBP beta expression vector in LNCaP cells compared to that in PC-3 cells transfected with the same vector, while the expression of truncated isoform was comparable in both cell lines. Interestingly, however, the most upstream initiation site A null mutation restored translation of full-length isoform in LNCaP cells. These results suggest that full-length C/EBP beta isoform expression in LNCaP cells may be suppressed at the upstream initiation sites, likely at site A. Most importantly, C/EBP beta overexpression significantly upregulated promoter activities of IL-8, COX-2, and anti-apoptotic Bfl-1 genes.Our study demonstrates that C/EBP beta is an important transcription factor upregulating metastatic gene expression and that its isoform expression is differentially regulated at the translational level in prostate cancer cells. 
 Hydroxytyrosol suppresses MMP-9 and COX-2 activity and expression in activated human monocytes via PKCα and PKCβ1 inhibition. Hydroxytyrosol (HT), the major olive oil antioxidant polyphenol in cardioprotective Mediterranean diets, is endowed with anti-inflammatory and anti-atherosclerotic activity. The production of cyclooxygenase (COX)-2-dependent inflammatory eicosanoids and the functionally linked release of matrix metalloproteinase (MMP)-9 by macrophages likely contribute to plaque instability leading to acute coronary events. Objective of the study was to examine the HT effects on inflammatory markers in human activated monocytes, including MMP-9 and COX-2 activity and expression and explore HT underlying mechanisms.Human peripheral blood mononuclear cells (PBMC) and U937 monocytes were treated with 1-10 μmol/L HT before activation with phorbol myristate acetate (PMA). HT blunted monocyte matr  ix invas  ive potential and reduced MMP-9 release and expression at zymography, ELISA and RT-PCR, with an IC50 = 10 μmol/L ( P< 0.05), without affecting tissue inhibitor of metalloproteinase (TIMP)-1. Moreover, HT inhibited prostaglandin (PG)E2 production and COX-2 expression, without affecting COX-1. These effects were mediated by inhibition of transcription factor nuclear factor (NF)-κB and protein kinase C (PKC)α and PKCβ1 activation.HT, at nutritionally relevant concentrations, reduces MMP-9 and COX-2 induction in activated human monocytes via PKCα and PKCβ1 inhibition, thus featuring novel anti-inflammatory properties. Overall, such results contribute to explaining the vascular protective effects by olive oil polyphenols in Mediterranean diets. 
 Coexpression of VEGF-C and COX-2 and its association with lymphangiogenesis in human breast cancer. Lymphangiogenesis has become a new research frontier in tumor metastasis since the discovery of reliable lymphatic markers that have allowed observation and isolation of lymphatic endothelium. Cyclooxygenase-2 (COX-2) has been reported to be involved in the critical steps in carcinogenesis. However, possible role of COX-2 in lymphangiogenesis and lymphatic metastasis is still poorly understood. In present study, we aimed to investigate the relationship between vascular endothelial growth factor-C (VEGF-C) and COX-2 in human breast cancer, and correlations with lymphangiogenesis and prognosis.Tissue samples of primary tumors from 70 patients undergoing intentionally curative surgical resections for breast cancer were immunohistochemically examined for VEGF-C, COX-2, and D2-40 expressions. The association between COX-2 and VEGF-C expressions and clinicopathological parameters as well as prognosis were analysised. To demonstrate the presence of proliferating lymphatic endothelial cells, 10 random cases with high LVD counts were selected for D2-40/Ki-67 double immunostaining.A significant correlation was found between the expression of VEGF-C and COX-2 (r = 0.529, P < 0.001), and both elevated VEGF-C expression and elevated COX-2 expression were associated with higher lymph vessel density (LVD), lymph node metastasis and D2-40 positive lymphatic invasion (LVI) as well as worse disease free survival (DFS) and overall survival (OS) in a univariate analysis. In the double immunostain for the lymph vessel marker D2-40 and the proliferation marker Ki-67, the results confirmed Ki-67-positive nuclei in a proportion of lymph vessel endothelial cells.There is indeed lymphangiogenesis in breast cancer, the most compelling evidence being the presence of proliferating lymphatic endothelial cells. VEGF-C and COX-2 are coexpressed and significantly associated with lymphangiogenesis and prognosis in   invasive   breast cancer. Suggesting COX-2 may up-regulate VEGF-C expression and thus promote lymph node metastasis via lymphangiogenesis pathway in human breast cancer. 
 Mucin 1 Regulates Cox-2 Gene in Pancreatic Cancer. Eighty percent of pancreatic ductal adenocarcinomas (PDAs) overexpress mucin 1 (MUC1), a transmembrane mucin glycoprotein. MUC1(high) PDA patients also express high levels of cyclooxygenase 2 (COX-2) and show poor prognosis. The cytoplasmic tail of MUC1 (MUC1-CT) partakes in oncogenic signaling, resulting in accelerated cancer progression. Our aim was to understand the regulation of Cox-2 expression by MUC1.Levels of COX-2 and MUC1 were determined in MUC1(-/-), MUC1(low), and MUC1(high) PDA cells and tumors using reverse transcriptase-polymerase chain reaction, Western blot, and immunohistochemistry. Proliferative and   invasive   potential was assessed using MTT and Boyden chamber assays. Chromatin immunoprecipitation was performed to evaluate binding of MUC1-CT to the promoter of COX-2 gene.Significantly higher levels of COX-2 mRNA and protein were detected in MUC1(high) versus MUC1(low/null) cells, which were recapitulated in vivo. In addition, deletion of MUC1 gene and transient knockdown of MUC1 led to decreased COX-2 level. Also, MUC1-CT associated with the COX-2 promoter at ∼1000 base pairs upstream of the transcription start site, the same gene locus where nuclear factor κB p65 associates with the COX-2 promoter.Data supports a novel regulation of COX-2 gene by MUC1 in PDA, the intervention of which may lead to a better therapeutic targeting in PDA patients. 
 Cyclooxygenase-2 (Cox-2) expression and angiogenesis in glioblastoma. Cyclooxygenase-2 (Cox-2), the key enzyme that catalyzes the first steps in the biosynthesis of the prostaglandins from arachidonic acid, appears to play a role in the regulation of progression,   invasive  ness and angiogenesis of various neoplasms. We analyzed the immunohistochemical expression of Cox-2 and angiogenic parameters (microvessel density (MVD) and vascular patterns) in 54 glioblastomas. We also examined their relation with prognosis. Cox-2 immunohistochemical expression was observed in 48 tumors (89%). There was no staining in six tumors (11%). On univariate analysis, MVD was correlated with a poor outcome (MVD > 70; hazard ratio, 0.441; 95% confidence interval, 0.200-0.975, P = 0.041). But MVD showed no prognostic impact on multivariate analysis. Neither Cox-2 expression nor vascular pattern showed prognostic value. The difference in Cox-2 expression between the classical and bizarre vascular pattern in glioblastomas was statistically significant (P = 0.047). However, no correlation was found between Cox-2 expression and MVD. These findings suggest that Cox-2 is heterogeneously expressed in glioblastomas without a significant association with MVD. However, Cox-2 expression may be related to vascular pattern in glioblastomas. 
 Cyclooxygenase-2 in oncogenesis. Compelling experimental and clinical evidence supports the notion that cyclooxygenase-2, the inducible isoform of cyclooxygenase, plays a crucial role in oncogenesis. Clinical and epidemiological data indicate that aberrant regulation of cyclooxygenase-2 in certain solid tumors and hematological malignancies is associated with adverse clinical outcome. Moreover, findings extrapolated from experimental studies in cultured tumor cells and animal tumor models indicate that cyclooxygenase-2 critically influences all stages of tumor development from tumor initiation to tumor progression. Cyclooxygenase-2 elicits cell-autonomous effects on tumor cells resulting in stimulation of growth, increased cell survival, enhanced tumor cell   invasive  ness, stimulation of neovascularization, and tumor evasion from the host immune system. Additionally, the oncogenic effects of cyclooxygenase-2 stem from its unique ability to impact tumor cell surroundings and create a proinflammatory environment conducive for tumor development, growth and progression. The initial enthusiasm generated by the availability of cyclooxygenase-2 selective inhibitors for cancer prevention and therapy has been lessened by the severe cardiovascular adverse side effects associated with their long-term use, as well as by the mixed results of recent clinical trials evaluating the efficacy of cyclooxygenase-2 inhibitors in adjuvant chemotherapy. Therefore, our ability to efficiently target the oncogenic effects of cyclooxygenase-2 for therapeutic and preventive purposes strictly depends on a better understanding of the spatial and temporal aspects of its activation in tumor cells along with a clearer elucidation of the signaling networks whereby cyclooxygenase-2 affects tumor cells and their interactions with the tumor microenvironment. This knowledge has the potential of leading to the identification of novel cyclooxygenase-2-dependent molecular and signaling networks that can be exploited to improve cancer prevention and therapy. 
 Interleukin-1β induces cyclooxygenase-2 expression and promotes the   invasiv  e ability of human mesenchymal stem cells derived from ovarian endometrioma. To elucidate the role of interleukin-1β (IL-1β) on cyclooxygenase-2 (COX-2) expression and invasion of endometrioma-derived ectopic endometrial mesenchymal stem cells (EN-MSCs) and to develop an organoid method to study t  he invas  ive ability of endometrial cells.Gene expression and cell functions.Kaohsiung Medical University, Kaohsiung, Taiwan.Human eutopic and endometrioma-derived ectopic EN-MSCs were isolated from different endometrium biopsy samples after surgery for treatment of endometriosis.Chemical treatment of cell culture.Comparative analysis of genomewide messenger RNA (mRNA) expression, cell migration, and invasion abilities in cell culture and organoid culture.Gene expression profiles revealed that the expression of IL-1β and COX-2 were statistically significantly higher in ectopic EN-MSCs compared with eutopic EN-MSCs. These enhanced expressions coincided with a greater ability for cell migration and invasion in ectopic EN-MSCs and were found to be distinctly regulated by IL-1β which up-regulates COX-2 expression. Furthermore, IL-1β treatment of ectopic EN-MSCs in organoids was found to induce tentacle-like structures that mimicked cell invasion.These results indicate that COX-2 and IL-1β regulate the invasion ability of ectopic EN-MSCs. The information may be useful for developing a new therapeutic strategy for endometriosis. The ex vivo invasion model will be useful for characterization of EN-MSCs. 
 Cyclooxygenase-2 expression in proliferative Ki-67-positive breast cancers is associated with poor outcomes. In order to investigate the implications of cyclooxygenase-2 (COX-2) expression in combination with Ki-67 on breast cancer outcomes, the COX-2 and Ki-67 expression levels and other clinicopathologic parameters were investigated in 861 breast cancers. Clinicopathological parameters and survival were investigated in association with the expression levels of both COX-2 and Ki-67 using univariate and multivariate analyses. COX-2 expression was positive in 493 (57.3%) of   invasive   tumors. COX-2 was associated with favorable markers, but was not related to survival outcome by itself. However, COX-2 in proliferative tumors [COX-2(+)/Ki-67(+)] were significantly associated with unfavorable factors and the worst survival, but COX-2 in non-proliferative tumors [COX-2(+)/Ki-67(-)] showed significantly favorable parameters and better outcomes. COX-2(-)/Ki-67(any) showed intermediate prognosis. The statistical significance was maintained in stage-matched and multivariate analyses. The results of present study suggest that COX-2 expression is a common event in breast cancers and may play in a different ways by the proliferation status of the tumor cells. Further studies should be carried out to verify the role of COX-2 by proliferative conditions of breast tumor cells. 
 Differential stromal and epithelial localization of cyclooxygenase-2 (COX-2) during colorectal tumorigenesis. The purpose of the following study is to describe the localization of COX-2 protein and COX-2 mRNA during human colorectal tumorigenesis and to identify potential cellular targets for COX-2 inhibition in chemopreventive strategies. Immunohistochemistry with digital image analysis was used to determine COX-2 protein expression in histologic sections containing synchronous normal colorectal mucosa, adenomas and carcinomas, from 17 previously untreated patients. Epithelial and stromal COX-2 mRNA expression was analyzed by reverse transcription-polymerase chain reaction (RT-PCR), on laser-capture microdissected samples from the same histologies. The stromal compartment in normal colorectal mucosa and adenomas showed higher levels of COX-2 protein expression compared to colorectal carcinomas (p < .0001). Conversely, epithelial COX-2 protein was significantly increased only after development of the   invasive   phenotype (p < .0001). RT-PCR demonstrated higher stromal COX-2 mRNA expression compared to that within the epithelium for colorectal adenomas and carcinomas. In conclusion, stromal COX-2 may be the target for chemopreventive agents in the early stages of colorectal carcinogenesis. 
 Correlation of cyclooxygenase-2 pathway and VEGF expression in head and neck squamous cell carcinoma. The purpose of this study was to determine whether cyclooxygenase-2 (COX-2) was overexpressed in head and neck squamous cell carcinoma (HNSCC) in relation to vascular endothelial growth factor (VEGF) expression and lymph node metastasis. COX-2 has an important role in the promotion of carcinogenesis, tumor   invasive  ness and angiogenesis. VEGF is a proangiogenic factor that is up-regulated in various tumors. VEGF has been shown to interact with COX-derived prostaglandins in angiogenesis. To better understand the roles of these genes in HNSCC, we evaluated the immunohistochemical expression pattern of COX-2 and VEGF in relationship with histologic differentiation, clinical stage, and nodal status in 146 HNSCC. The COX-2/VEGF double immunofluorescein staining was evaluated with confocal scanning laser microscope. A positive expression was seen in 96% (140/146: 98 strong positive, 42 weakly positive) for COX-2 and in 98% (143/146: 63 strong positive, 80 weakly positive) for VEGF in HNSCC. A correlation was present in the positive expression of COX-2 with histologic differentiation, clinical stage, and nodal status. VEGF expression was also correlated with nodal status. A significant relation was observed between COX-2 and VEGF expressions. The simultaneous expression of COX-2 and VEGF was statistically significant (p<0.05). These results indicate that elevated COX-2 or VEGF expression is more common in HNSCC with poor prognostic characteristics. The present findings support the efforts to initiate clinical trials on the efficacy of COX-2 inhibitors in adjuvant treatment of HNSCC. 
 Cyclooxygenase-2 parallels   invasive   depth and increased MVD in transitional cell carcinoma. To investigate the relationship between the expression of cyclooxygenase-2 (COX-2), and   invasive   depth and angiogenesis in human transitional cell carcinoma (TCC), the expression of COX-2 and von Willebrand factor (vWF) was detected in paraffin-embedded transitional cell carcinoma specimens from 50 patients with histologically confirmed bladder transitional cell carcinoma. Microvessels were counted and microvessel density (MVD) was calculated. The correlation between COX-2 and   invasive   depth, MVD and   invasive   depth, COX-2 and MVD were analyzed. COX-2 expression was significantly related to the   invasive   depth of tumors; it solely expressed in   invasive   TCC. The ratio of COX-2 positively stained samples in each stage correlated to the   invasive   stages; so did the proportion of COX-2 (+) cells in whole tumor cells. Also, MVD increased with the   invasive   stages of tumors and positively correlated to the   invasive   depth. The COX-2-positive TCC samples were highly vascular, their microvessel density was significantly higher than that of the COX-2-negative TCC samples. There was a positive correlation between MVD and COX-2 (+) cells proportion. Our findings suggested that COX-2 was positively correlated with the   invasive   depth and involved in the process of angiogenesis in human transitional cell carcinoma. 
 Cyclooxygenase-2 uses the protein kinase C/ interleukin-8/urokinase-type plasminogen activator pathway to increase the   invasive  ness of breast cancer cells. Cyclooxygenase-2 (COX-2) increases breast cancer cell invasion. Expression of various pro-angiogenic and pro-  invasive   factors has been correlated with high expression of COX-2. However, whether these factors are essential to COX-2-mediated breast cancer invasion, and the mechanisms by which COX-2 increases the expression of these factors are unknown. Our microarray results indicate that higher COX-2 expression was associated with increased levels of interleukin-8 (IL-8), a key factor in breast cancer invasion and metastasis. COX-2 overexpressing cells (MCF-7/COX-2), generated by transfecting COX-2-encoding plasmids into the poorly   invasive   MCF-7 breast cancer cells, were more   invasive   and produced higher IL-8 levels than the parental cells. To investigate the role of IL-8 in COX-2-mediated invasion, MCF-7 parental cells were incubated with IL-8. Exogenous IL-8 increased the   invasive  ness of MCF-7 cells. IL-8 is one pathway by which COX-2 mediates breast cancer invasion. Protein kinase A (PKA) and protein kinase C (PKC) are activated by COX-2 and are involved in IL-8 regulation. Inhibition of PKC, not PKA, decreased IL-8 production and invasion in MCF-7/COX-2 cells. Activation of PKC, not PKA, increased IL-8 production and invasion in MCF-7 cells. Thus, the   invasive   effects of COX-2 are mediated by PKC, not PKA. Activity of the urokinase-type plasminogen activator (uPA) was increased in MCF-7 cells by COX-2 overexpression or by the addition of a PKC activator or by IL-8. Inhibition of PKC decreased uPA activity in MCF-7/COX-2 cells. Furthermore, inhibition of uPA activity decreased the   invasive  ness of MCF-7/COX-2 cells, indicating that uPA was essential to COX-2-mediated invasion. Herein we demonstrate for the first time a detailed mechanism by which COX-2 increases breast cancer invasion: the PKC/IL-8/uPA pathway. 
 [Analysis of HIF-1α and COX-2 expression in tumor stroma and correlation with the degree of neoplasm   invasiv  eness in laryngeal cancer--preliminary study]. Despite extensive research in the field of molecular biology, immunology and histopathology, prognostically unambiguous morphological indicators of the   invasiv  eness of tumor which allow the prediction of disease course in laryngeal cancer have not yet been identified. The aim of this study was to analyze gene and protein expression of HIF-1a and COX-2 in the tumor stroma and to find relationships between clinical and morphological features (pT, G, depth of tumor invasion, plasmalymphocytic infiltration) and certain markers in squamous cell laryngeal carcinoma.We analyzed a group of 59 patients with verified squamous cell carcinoma of the larynx. The pathological evaluation was included pTNM classification criteria, depth of invasion according to tumor front grading and degree of morphological differentiation. Quantitative analysis of the amplified product in real time (real-time RT2-PCR) for estimation of mRNA HIF-1a and COX-2 expression in tumor cells were used. The level of HIF-1a and COX-2 protein expression by Western blot analysis was determined.In studied group of laryngeal cancers significant differences in expression of certain molecules analyzed in tumor tissue and noncancerous epithelium of the larynx have been shown. Increased expression of HIF-1a and COX-2 at both mRNA and protein level was indicator of greater aggressiveness of the tumor, evaluated on the basis of clinical and morphological features in laryngeal squamous cell carcinomas. Tumors with the most   invasiv  e growth (pT4 stage, low-differentiated tumors G3, neoplastic infiltration with invasion of cartilage of the larynx) had the highest expression of HIF-1a and COX-2 at the mRNA and protein level.The study pointed out the direction for further research to find unambiguous indicators for estimation of tumor   invasiv  eness and the possibility of practical use of HIF-1a and COX-2 mRNA and protein level assessment as important methods for determining the advancement of clinical and morphological changes in laryngeal cancer, thereby selecting an appropriate model of treatment. 
 [Small interfering RNA in silencing cox-2 expression enhances radiosensitivity of esophageal cancer EC9706 cell]. To explore the effects of small interfering RNA (siRNA) specific to cox-2 gene on the radiosensitivity of esophageal cancer cell EC9706.The siRNA vector was established for cox-2 gene and then induced into esophageal cancer cell EC9706 by lipofectamine. G418 screening yielded stably transfected cells. After the irradiation of 0, 2 and 4 Gy, the cellular expression levels of cox-2, matrix metalloproteinase-2 (MMP2), Bax and Bcl-2 were detected by reverse transcriptase-polymerase chain reaction (RT-PCR) and those of cox-2 protein, AKT protein and phosphorylation AKT protein (pAKT) by Western blot. Cell apoptosis was examined by flow cytometer. Invasion of cells was detected by   invasive   assay in vitro. The   invasive   and metastatic capacities of cancer cells were assessed by invasion assay in vitro. Proliferative potential was quantified by clone-forming assay.The sequencing result confirmed that siRNA vector pRNA-U6 for cox-2 gene was established. The results of 1-sinCox214, RT-PCR and Western blot showed that cox-2 gene expression of transfected EC9706 cell was silenced efficiently. After the irradiation of 0, 2 and 4 Gy, the expressions of MMP2, Bcl-2 mRNA, AKT protein and pAKT in silencing cox-2 gene expression significantly decreased. There was an inverse correlation with irradiation dose. The Bax mRNA expression evidently increased directly with irradiation dose; the apoptotic rate in cox-2 silencing groups was evidently higher than the control groups. And the difference was significant (P < 0.01); invasion cells in vitro in Cox-2 silencing groups evidently decreased with significant difference (P < 0.01). The colony formation rate of cells decreased obviously in cox-2 silencing groups after the irradiation of 0, 1, 2, 4, 6, 8 and 10 Gy (P < 0.01).Small interference RNA in silencing cox-2 gene expression can enhance significantly the radiosensitivity of esophageal cancer EC9706 cells. And the mechanism may be related with MMP2, Bax, Bcl-2, AKT protein and pAKT protein. 
 Inducible cyclooxygenase released prostaglandin E2 modulates the severity of infection caused by Streptococcus pyogenes. Streptococcus pyogenes is a significant human pathogen that can cause life-threatening   invasive   infections. Understanding the mechanism of disease is crucial to the development of more effective therapies. In this report, we explored the role of PGE(2), an arachidonic acid metabolite, and its rate-limiting enzyme cyclooxygenase 2 (COX-2) in the pathogenesis of severe S. pyogenes infections. We found that the COX-2 expression levels in tissue biopsies from S. pyogenes-infected patients, as well as in tissue of experimentally infected mice, strongly correlated with the severity of infection. This harmful effect was attributed to PGE(2)-mediated suppression of the bactericidial activity of macrophages through interaction with the G2-coupled E prostanoid receptor. The suppressive effect of PGE(2) was associated with enhanced intracellular cAMP production and was mimicked by the cAMP-elevating agent, forskolin. Activation of protein kinase A (PKA) was the downstream effector mechanisms of cAMP because treatment with PKI(14-22), a highly specific inhibitor of PKA, prevented the PGE(2)-mediated inhibition of S. pyogenes killing in macrophages. The inhibitory effect exerted by PKA in the generation of antimicrobial oxygen radical species seems to be the ultimate effector mechanism responsible for the PGE(2)-mediated downregulation of the macrophage bactericidal activity. Importantly, either genetic ablation of COX-2, pharmacological inhibition of COX-2 or treatment with the G2-coupled E prostanoid antagonist, AH6809, significantly improved the disease outcome in S. pyogenes infected mice. Therefore, the results of this study open up new perspectives on potential molecular pathways that are prone to pharmacological manipulation during severe streptococcal infections. 
 COX-2 induction by heparanase in the progression of breast cancer. Breast cancer confined within the lactiferous duct or lobule, without invading the stroma, is called ductal carcinoma in situ (DCIS), whereas breast cancer that has invaded the stroma through the basal membrane is called   invasive   cancer. Heparanase, an endo-beta-D-glucuronidase that specifically degrades heparan sulfate proteoglycans (HSPGs) in the extracellular matrix (ECM), plays an important role when breast cancer cells breach the basal membrane. Recently, we have reported that heparanase is involved in angiogenesis through direct induction of cyclo-oxygenase-2 (COX-2). COX-2 induces vascular endothelial growth factor (VEGF) and basic fibroblast growth factor (bFGF) and is thus involved in neovascularization. The present study was undertaken to analyze surgically resected breast cancer specimens for heparanase and COX-2 expression, using specimens from 59 patients with   invasive   cancer and 85 patients with DCIS (including 41 cases of DCIS adjacent to   invasive   cancer). This study yielded the following results: a) the distribution of heparanase within tumor tissue was identical to that of COX-2; b) heparanase expression was more frequent in   invasive   cancer than in non-  invasive   cancer; c) a close positive correlation was noted between heparanase and COX-2 expression (this correlation was particularly strong in cases of   invasive   cancer); and d) COX-2 expression was always seen in cases positive for heparanase expression. Our results indicate that heparanase expression increases during the progression of breast cancer into   invasive   cancer, and that this change is accompanied by increased COX-2 expression. They also suggest that heparanase may play a novel role for COX-2 mediated tumor angiogenesis in breast-cancer progression. 
 Association between cyclooxygenase-2 expression in atypical hyperplasia and risk of breast cancer. The cyclooxygenase-2 (COX-2) enzyme, which is induced by inflammatory and mitogenic stimuli, plays a protumorigenic role in several human cancers. COX-2 is overexpressed in   invasive   and in situ breast cancers. Atypical hyperplasia in breast tissue, although benign, is associated with a high risk of breast cancer. We investigated whether COX-2 overexpression in atypical hyperplasia is associated with the risk of subsequent breast cancer.COX-2 expression was assessed immunohistochemically in archival sections from 235 women with atypia whose biopsy specimens were obtained at the Mayo Clinic from January 1, 1967, through December 31, 1991. COX-2 expression was scored as 0 (negative), 1+ (weak), 2+ (moderate), or 3+ (strong). Risk factor information and follow-up for breast cancer events were obtained via a study questionnaire and the medical records. All statistical tests were two-sided.Forty-one (17%) of the 235 women developed breast cancer during a median follow-up of 15 years. Moderate (category 2+) or strong (category 3+) COX-2 expression was identified in 71 (30%) and 34 (14%) of the 235 samples, respectively. The risk for developing breast cancer, relative to a control population (the Iowa Surveillance, Epidemiology, and End Results registry), increased with increasing COX-2 expression (relative risk [RR] = 2.63, 95% confidence interval [CI] = 1.56 to 4.15, for those with negative or weak COX-2 expression; RR = 3.56, 95% CI = 1.94 to 5.97, for those with moderate expression; and RR = 5.66, 95% CI = 2.59 to 10.75, for those with strong expression; P = .07). Overexpression of COX-2 was statistically significantly associated with the type of atypia (lobular vs ductal, P < .001), number of foci of atypia in the biopsy (P = .02), and older age at time of biopsy (>45 years, P = .01).COX-2 appears to be a biomarker that further stratifies breast cancer risk among women with atypia and may be a relevant target for chemoprevention strategies. 
 Heterogeneous expression of cyclooxygenase-2 and inducible nitric oxide synthase within colorectal tumors: correlation with tumor angiogenesis. Recent studies have shown that the cyclooxygenase (COX) and the inducible nitric oxide synthase (iNOS) pathways are involved in the development of tumor angiogenesis in human cancers.To investigate whether a different pattern of COX-2 and iNOS expression/activity exists within different areas of colorectal tumors and to analyze the relationship between these two enzymes and tumor angiogenesis.Microvessel density (MVD) and COX-2, iNOS, vascular endothelial growth factor (VEGF) and VEGF receptor-2 (VEGFR-2) protein expression were evaluated at both the   invasive   front (IF) and the tumor center (TC) in 46 human colorectal cancer specimens. We also investigated the concentration of PGE2 and NO at the same sites.COX-2 and iNOS protein expression and activity were significantly higher within the IF than the TC of the tumor specimens. Similarly, MVD and VEGF/VEGFR-2 expression significantly increased from the TC to the IF. Only COX-2 expression was significantly correlated with MVD and VEGF/VEGFR-2 expression at both the TC and the IF.Our study shows a heterogeneous expression of COX-2 and iNOS in colorectal cancer. The up-regulation of COX-2 at the IF parallels an increase in vessel density and VEGF/VEGFR-2 expression, thus supporting the hypothesis that the tumor periphery is the most aggressive portion of a colorectal tumor. 
 Cyclooxygenase-2 and inflammation mediators have a crucial role in reflux-related esophageal histological changes and Barrett's esophagus. Gastroesophageal reflux (GER) causes injury of the esophageal squamous epithelium, a condition called reflux esophagitis. The sequence reflux-esophagitis-intestinal metaplasia-dysplasia-  invasive   cancer is widely accepted as the main adenocarcinogenetic pathway in the esophagus; however, the mechanisms of this progression need to be better defined.We evaluated COX-2 expression and activity in biopsies from patients affected with GER, and these parameters have been correlated with the stage of the disease, ceramide expression, apoptotic process, and angiogenesis. The effects of celecoxib on bile acid- and EGF-induced mucosal proliferation, apoptosis and angiogenesis have been also investigated.Four groups of patients were distinguished: non esophagitis, non erosive esophagitis, erosive esophagitis, and Barrett's esophagus. COX-2 expression, basal PGE2 levels, proliferative activity, VEGF expression and apoptosis were evaluated in esophageal biopsies.COX-2 expression, basal PGE2 levels, proliferative activity, VEGF expression and apoptosis progressively increase from non esophagitis patients to patients with non erosive and erosive esophagitis, to those with BE. Incubation of the cells with DCA/EGF increases PGE2 production, proliferative activity and VEGF production, effects prevented by celecoxib pretreatment. Ceramide expression increased from non esophagitis patients to patients with non erosive and erosive esophagitis, and decreased in BE; caspase-3 activity progressively decreased from non esophagitis to BE patients, suggesting an impairment of the apoptotic process with disease progression.These results stand for a close relationship between progression of initial steps of gastroesophageal reflux disease (GERD) and COX-2, proliferative activity and EGF/VEGF expression and could have implications in GERD treatment in order to prevent its neoplastic evolution. 
 Clinicopathologic implications of the epidermal growth factor receptor, cyclooxygenase 2 expression, and human papillomavirus status in squamous cell carcinoma of the uterine cervix in the elderly. To find information on   invasive   squamous cervical carcinoma in the elderly, 110   invasive   squamous cervical carcinomas obtained from 2 groups of patients (aged <60 and >60 years) were analyzed for human papillomavirus (HPV) status by polymerase chain reaction study, for immunohistochemical epidermal growth factor receptor (EGFR), cyclooxygenase 2 (Cox-2) expression, and clinicopathologic features.The HPV status and the expression of Cox-2 and EGFR in the younger and older women were compared and correlated with the grading, staging neoplasm, and lymph nodal status, using Fisher test and Spearman nonparametric correlation test. Overall survival curves were drawn using Kaplan-Meier estimates and were compared using log-rank tests in the whole series of 110 patients. Multinomial logistic regression was also used.The number of neoplasms with higher staging was significantly greater than those in the younger women (P = 0.04). The mortality was higher in the older group than in the younger patients (P = 0.006).In the elderly, the presence of HPV DNA in 65% of cases, and in the absence of sexual activity, could be due to reactivation of latent HPV infection, which might be due to an impairment of host immunologic response.The overexpression of Cox-2 in a number of cases was significantly higher in the older group than in the younger group (P = 0.032, Fisher exact test), but this immunoreactivity is not related to the staging, grading, EGFR expression, or to the presence of HPV.The simultaneous expression of Cox-2 and EGFR had a poor prognostic significance, showing lower survival rates than cases without this immunoreactivity (P = 0.002), on univariate analysis.On multivariate analysis, Cox-2 and EGFR immunopositivity did not reveal any correlation between these markers and prognosis probably because the number of cases considered was not particularly high. 
 Cyclooxygenase-2 expression in   invasive   breast carcinomas of no special type and correlation with pathological profiles suggest a role in tumorigenesis rather than cancer progression. COX-2 has been shown to play an important role in the development of breast cancer and increased expression has been mooted as a poor prognostic factor. The purpose of this study was to investigate the relationship between COX-2 immunohistochemical expression and known predictive and prognostic factors in breast cancer in a routine diagnostic histopathology setting.Formalin-fixed paraffin- embedded tumour tissue of 144 no special type (NST)   invasive   breast carcinomas histologically diagnosed between January 2009 and December 2012 in Hospital Sultanah Bahiyah, Alor Setar, Kedah were immunostained with COX-2 antibody. COX-2 overexpression was analysed against demographic data, hormone receptor status, HER2- neu overexpression, histological grade, tumour size and lymph node status.COX-2 was overexpressed in 108/144 (75%) tumours and was significantly more prevalent (87%) in hormone receptor-positive tumours. There was no correlation between COX-2 overexpression and HER2/neu status. Triple negative cancers had the lowest prevalence (46%) (p<0.05). A rising trend of COX-2 overexpression with increasing age was observed. There was a significant inverse relationship with tumour grade (p<0.05), prevalences being 94%, 83% and 66% in grades 1, 2 and 3 tumours, respectively. A higher prevalence of COX-2 overexpression in smaller size tumours was observed but this did not reach statistical significance. There was no relationship between COX-2 expression and lymph node status.This study did not support the generally held notion that COX-2 overexpression is linked to poor prognosis, rather supporting a role in tumorigenesis. Larger scale studies with outcome data and basic studies on cancer pathogenetic pathways will be required to cast further light on whether COX-2 inhibitors would have clinical utility in cancer prevention or blockage of cancer progression. In either setting, the pathological assessment for COX-2 overexpression in breast cancers would have an important role in the selection of cancer patients for personalized therapy with COX-2 inhibitors. 
 Subepithelial myofibroblasts express cyclooxygenase-2 in colorectal tubular adenomas. Recent data support the hypothesis that the inducible isoform of cyclooxygenase (COX-2) plays a role in the early stages of colonic carcinogenesis and that nonsteroidal anti-inflammatory drugs (NSAIDs) retard the development of colon cancer by modulating COX-2. However, the cell types responsible for producing COX-2 in colorectal adenomas remain a subject of controversy.COX-2 expression in normal colonic mucosa (n = 50), hyperplastic polyps (n = 43), sporadic adenomas (n = 67), and   invasive   colonic adenocarcinoma (n = 39) was studied in formalin-fixed and paraffin-embedded tissue sections from endoscopy biopsy and colonic resection specimens. Immunohistochemistry (avidin-biotin complex technique with double immunolabeling) was used to identify the phenotypes of COX-2-producing cells.In colorectal adenomas, increased expression of COX-2 was detected and localized to alpha smooth muscle actin ( proportional, variant SMA)-positive subepithelial stromal cells (myofibroblasts) in the periluminal region of the lamina propria in 63 (94%) of 67 cases. In contrast, in normal colonic mucosa and in hyperplastic polyps with intact epithelium, COX-2 expression was found only in macrophages and endothelial cells. In areas in which the surface epithelium was ulcerated in normal mucosa as well as hyperplastic or neoplastic polyps, COX-2 expression was increased in granulation tissue (and present in macrophages, endothelium, and myofibroblasts). In   invasive   carcinoma, COX-2 expression in myofibroblasts was limited to the adenomatous portion of the tumor and was detected in 62% of cases (n = 39). In addition, focal expression of COX-2 by malignant epithelial cells was observed in 23% of   invasive   adenocarcinoma.These results show that increased COX-2 expression in sporadic adenoma of the colon is common and is localized specifically to subepithelial intestinal myofibroblasts. These findings further support the hypothesis that myofibroblasts are important target cells for NSAID-mediated chemoprevention of colorectal cancer. 
 Prostaglandins induce early growth response 1 transcription factor mediated microsomal prostaglandin E2 synthase up-regulation for colorectal cancer progression. Cyclooxygenase2 (COX2) has been associated with cell growth,   invasive  ness, tumor progression and metastasis of colorectal carcinomas. However, the downstream prostaglandin (PG)-PG receptor pathway involved in these effects is poorly characterized.We studied the PG-pathway in gene expression databases and we found that PTGS2 (prostaglandin G/H synthase and cyclooxygenase) and PTGES (prostaglandin E synthase) are co-expressed in human colorectal tumors. Moreover, we detected that COX2 and microsomal Prostaglandin E2 synthase 1 (mPGES1) proteins are both up-regulated in colorectal human tumor biopsies.Using colon carcinoma cell cultures we found that COX2 overexpression significantly increased mPGES1 mRNA and protein. This up-regulation was due to an increase in early growth response 1 (EGR1) levels and its transcriptional activity. EGR1 was induced by COX2-generated PGF2α. A PGF2α receptor antagonist, or EGR1 silencing, inhibited the mPGES1 induction by COX2 overexpression. Moreover, using immunodeficient mice, we also demonstrated that both COX2- and mPGES1-overexpressing carcinoma cells were more efficient forming tumors.Our results describe for the first time the molecular pathway correlating PTGS2 and PTGES in colon cancer progression. We demonstrated that in this pathway mPGES1 is induced by COX2 overexpression, via autocrine PGs release, likely PGF2α, through an EGR1-dependent mechanism. This signaling provides a molecular explanation to PTGS2 and PTGES association and contribute to colon cancer advance, pointing out novel potential therapeutic targets in this oncological context. 
 Cyclooxygenase-2 positively regulates Akt signalling and enhances survival of erythroleukemia cells exposed to anticancer agents. Cyclooxygenase-2 (COX-2) has been found to be highly expressed in many types of cancers and to contribute to tumorigenesis via the inhibition of apoptosis, increased angiogenesis and   invasive  ness. In hematological malignancies, COX-2 expression was found to correlate with poor patient prognosis. However, the exact role of COX-2 expression in these malignancies, and particularly in erythroleukemias, remains unclear. The aim of this work was to describe and understand the relationships between COX-2 expression and apoptosis rate in erythroleukemia cells after apoptosis induction by several anticancer agents. We used three different erythroleukemia cell lines in which COX-2 expression was modulated by transfection with either COX-2 siRNA or COX-2 cDNA. These cellular models were then treated with apoptosis inducers and apoptosis onset and intensity was followed. Cell signalling was evaluated in unstimulated transfected cells or after apoptosis induction. We found that COX-2 inhibition rendered erythroleukemia cells more sensitive to apoptosis induction and that in cells overexpressing COX-2 apoptosis induction was reduced. We demonstrated that COX-2 inhibition decreased the pro-survival Akt signalling and activated the negative regulator of Akt signalling, phosphatase and tensin homologue deleted on chromosome 10 (PTEN). Conversely, in COX-2 overexpressing cells, Akt signalling was activated and PTEN was inhibited. In these last cells, inhibition of casein kinase 2 or Akt signalling restored sensitivity to apoptotic agents. Our findings highlighted that COX-2 can positively regulate Akt signalling mostly through PTEN inhibition, partly via casein kinase 2 activation, and enhances survival of erythroleukemia cells exposed to anticancer agents. 
 Analysis of Cox-2 and thromboxane synthase expression in pituitary adenomas and carcinomas. Recent studies have examined the role of cyclooxygenase-2 (Cox-2) expression in normal pituitaries and pituitary adenomas and have suggested a role for Cox-2 in the regulation of angiogenesis in the pituitary. Thromboxane synthase (TBXAS), which catalyzes the synthesis of thromboxane A2, is one of the downstream enzymes in Cox metabolism and appears to play a role in the regulation of   invasive  ness and angiogenesis in some neoplasms. To analyze the role of Cox-2 and TBXAS in pituitary tumor progression, we examined normal pituitaries (n = 8), pituitary adenomas (n = 174), and pituitary carcinomas (n = 7) for expression of Cox-2 and TBXAS by immunohistochemistry. Weak Cox-2 and moderate TBXAS expression was present in normal pituitary cells. Most pituitary adenomas showed increased expression of both Cox-2 and TBXAS. Pituitary tumors as a whole, but particularly carcinomas, showed greater Cox-2 expression than did normal pituitaries. Pituitary adenomas and carcinomas also showed greater staining for TBXAS when compared to normal pituitary. Nonfunctional adenomas had significantly higher levels of TBXAS expression compared to functional adenomas (p = 0.017). Adenomas and carcinomas showed similar degrees of staining for TBXAS. In summary, TBXAS appears to be one of the up-regulated downstream enzymes of Cox metabolism in pituitary tumors. Both Cox-2 and TBXAS may play an important role in pituitary tumor development and progression. 
 Cigarette smoke and increased COX-2 and survivin levels in exhaled breath condensate of lung cancer patients: how hot is the link? One of the most current intriguing hypotheses on lung cancerogenesis envisages a role for inflammation as a possible trigger of both epithelial-mesenchymal transition and cancer development. Cigarette smoke has been suggested to be the main factor underlying the inflammation of the airways described in lung cancer patients. Cycloxygenase and survivin, a COX-2 dependent factor of apoptosis resistance, seem to play a key role in this regard.The aim of this study was to study COX-2 and survivin in the airways of lung cancer patients and in those of a group of smokers in a view to increasing our understanding of the link between smoking, airway inflammation and lung cancer.70 NSCLC patients (28 smokers, 26 ex-smokers and 16 non-smokers) and 30 healthy subjects (20 smokers and 10 non-smokers) were enrolled in the study. Both COX-2 and survivin concentrations were measured in the exhaled breath condensates of all the subjects under study using EIA kits.Higher levels of exhaled survivin and COX-2 were found in NSCLC patients compared to healthy smokers and non-smokers. These levels were observed to be significantly elevated in smokers (patients with lung cancer and healthy) and ex-smokers compared to non-smokers and exhibited a positive correlation with the number of cigarettes smoked expressed as pack/year. A correlation was also found between exhaled COX-2 and survivin and the progression of cancer.We support the hypothesis that cigarette smoke be strongly connected to the inflammation of the airways observed in lung cancer patients. On the basis of the results obtained the use of exhaled breath condensate COX-2 and survivin levels could be suggested as two potential markers within an early non-  invasive   screening of populations of smokers at risk of lung cancer. 
 Helicobacter pylori promote gastric cancer cells invasion through a NF-kappaB and COX-2-mediated pathway. To examine the effects of Helicobacter pylori (H pylori) infection on the   invasive  ness of gastric cancer cells, and to elucidate its mechanism.Gastric carcinoma cells, MKN-45, were incubated with CagA-positive H pylori, and cell invasion was determined by Matrigel analysis. The expression of matrix metalloproteinase-9 (MMP-9), vascular endothelial growth factor (VEGF), and cyclooxygenase-2 (COX-2) were assessed by Western-blot analysis, and transcriptional activation of the COX-2 promoter was examined by measuring luciferase and beta-galactosidase activities. Lastly, the protein-DNA interaction was confirmed by an electrophoretic mobility shift assay.The current studies showed that: (1) incubation of CagA-positive H pylori with MKN-45 cells significantly promotes gastric cancer cells invasion, and this effect is attenuated by pre-treatment with NS-398, a COX-2 inhibitor, or PDTC, a nuclear factor kappaB (NF-kappaB) inhibitor; (2) the induction of MKN-45 cells invasion by H pylori is associated with increases in COX-2, MMP-9, and VEGF protein expression, and co-incubation of NS-398 or PDTC significantly reduces these effects; (3) H pylori infection transactivates COX-2 promoter activity and increases the binding of NF-kappaB to this promoter.Our data demonstrate that H pylori infection promotes gastric epithelial cells invasion by activating MMP-9 and VEGF expression. These effects appear to be mediated through a NF-kappaB and COX-2 mediated pathway, as COX-2 or NF-kappaB inhibitor significantly attenuate the   invasive  ness of gastric cancer cells and the expressions of MMP-9 and VEGF protein. 
 Physiological COX-2 expression in breast epithelium associates with COX-2 levels in ductal carcinoma in situ and   invasive   breast cancer in young women. Cyclooxygenase-2 (COX-2) overexpression is implicated in increased risk and poorer outcomes in breast cancer in young women. We investigated COX-2 regulation in normal premenopausal breast tissue and its relationship to malignancy in young women. Quantitative COX-2 immunohistochemistry was performed on adjacent normal and breast cancer tissues from 96 premenopausal women with known clinical reproductive histories, and on rat mammary glands with distinct ovarian hormone exposures. COX-2 expression in the normal breast epithelium varied more than 40-fold between women and was associated with COX-2 expression levels in ductal carcinoma in situ and   invasive   cancer. Normal breast COX-2 expression was independent of known breast cancer prognostic indicators, including tumor stage and clinical subtype, indicating that factors regulating physiological COX-2 expression may be the primary drivers of COX-2 expression in breast cancer. Ovarian hormones, particularly at pregnancy levels, were identified as modulators of COX-2 in normal mammary epithelium. However, serial breast biopsy analysis in nonpregnant premenopausal women suggested relatively stable baseline levels of COX-2 expression, which persisted independent of menstrual cycling. These data provide impetus to investigate how baseline COX-2 expression is regulated in premenopausal breast tissue because COX-2 levels in normal breast epithelium may prove to be an indicator of breast cancer risk in young women, and predict the chemopreventive and therapeutic efficacy of COX-2 inhibitors in this population. 
 Expression of cyclooxygenase-2 (COX-2) in pituitary tumours. Microvessel density in angiogenesis is regarded as a prognostic factor of tumour   invasive  ness, independent of cell proliferation. In recent studies of pituitary tumours, correlation between the expression of cyclooxygenase-2 (COX-2) and micro-vascularization density and microvessel surface density has been established. We studied the expression of COX-2 in different types of pituitary adenomas to determine the usefulness of COX-2 expression as a prognostic factor of tumour progression or recurrence in patients with hypophyseal tumours.We retrospectively studied a group of 60 patients of mean age 46.7±17.6 (range, 18 to 85) years who underwent pituitary tumour surgery. Expression of COX-2, as determined by immunohistochemistry, was analyzed in relation to histopathology features of tumour, clinical symptoms, MR imaging and post-operative recurrence/progression of disease.COX-2 was expressed in adenomas of 87% of patients, with a median index value of 57.5% [IQR=60.5]. Highest COX-2 expression was observed in hormonally inactive adenomas and gonadotropinomas and lowest in prolactinomas. We found no differences in COX-2 expression with respect to patient age, gender, tumour size, degree of tumour   invasiv  eness, or whether tumours were immunopositive or immunonegative for pituitary hormones, nor have we found any relation between COX-2 expression and recurrence or progression of tumour size.COX-2 does not appear to be a predictive factor for recurrence or progression of tumour size. Nevertheless, due to the observed relatively high expression of COX-2 in pituitary adenomas, further studies with COX-2 inhibitors are justified in these tumours. 
 Expression of Cox-2 and Bcl-2 in Paget's disease of the breast. Paget's disease (PD) is a rare form of intraepithelial adenocarcinoma that involves breast and extramammarian tissues. It is often associated with ductal carcinoma in situ and/or   invasive   ductal cancer. Molecular pathways that play a role in development of Paget's disease are stil unclear. Expression patterns of Cox-2 and bcl-2 were therefore assessed.Patients with a histopathological diagnosis of Paget's disease were included in this study. Patient files were analysed retrospectively.  Invasive   cancer was diagnosed in 35 (76.1%) of the patients, 7 (15.2%) had ductal carcinoma in situ and 4 (8.7%) patients had no associated neoplasm. Twenty four (52.2%) patients showed COX-2 expression in Paget cells whereas no expression was seen in 22 (47.8%) patients. No relation was found between COX-2 expression and the lesion underlying Paget's disease (p=0.518). Bcl-2 expression in Paget cells was found positive in 12 (26.1%) and negative in 27 (58,7%) cases. There was no relation between Bcl-2 expression and the lesion accompanying Paget's disease (p=0.412). No relation was observed between COX-2 expression and Bcl-2 expression (p=0.389).In breast cancer, COX-2 expression is associated with poor prognostic factors. As COX-2 expression increases the tendency to metastasize also increases. In our study we found a significantly high COX-2 expression in Paget's disease of the breast. We suggest that COX-2 expression and inflammatory processes may play a role in pathogenesis of the Paget's disease of the breast. 
 Can cyclo-oxygenase-2 be a useful prognostic and risk stratification marker in breast cancer? Cyclo-oxygenase-2 (COX-2) is a prostaglandin synthease that catalyses the synthesis of prostaglandin G2 (PGG2) and PGH2 from arachidonic acid. COX-2 plays an important role in tumourigenesis of different carcinoma types and it is thought to take part in breast carcinoma. In this study, the aim was to investigate the relationship of COX-2 with clinical parameters such as menopausal status, tumour size, grade, nodal status, Nottingham prognostic index (NPI), oestrogen receptor(ER), progesterone receptor (PR), human epidermal growth factor receptor type 2 (HER-2/ neu). The patients were divided into two groups, first group (group A) comprised 57 primary breast cancer patients and the second group (group B) comprised control group 27 patients consisting of fibro-adenoma and benign breast disease. In control groups COX-2 (0%) is not over expressed and we observed that high frequency of COX-2 (73.68%) over expressed in breast carcinoma. In high grade, large tumour size and positive lymph node metastasis, COX-2 expression rate was 78.6%, 59.5% and 90.5% respectively. COX-2 expression is directly correlated with ER negative (88.1%, p = 0.001) and also associated with higher NPI value (78.6%, p = 0.006). In   invasive   ductal carcinoma (IDC) COX-2 over expression had a significant relationship with HER-2/neu over expression (p < 0.001). The results indicated that COX-2 over expression correlates with aggressive phenotypic features, such as high histological grade, large tumour size, higher NPI value, ER negativity and HER-2/neu positivity. 
 Overexpression of cyclooxygenase-2 in human prostate carcinoma and prostatic intraepithelial neoplasia-association with increased expression of Polo-like kinase-1. Cyclooxygenases (COX) as well as Polo-like kinases (PLK) are involved in proliferation and cell cycle regulation and have been suggested for preventive and therapeutic approaches in prostate carcinoma.In this study, we studied expression and prognostic impact of COX-2 in   invasive   prostate carcinoma, prostatic intraepithelial neoplasia (PIN), atrophic glands, and normal prostatic glands, and investigated the association between COX-2 and PLK-1.We observed a positivity for COX-2 in 72.1% of PIN and in 44.7% of prostate carcinomas with an overexpression of COX-2 in prostate cancer and PIN compared to benign prostatic tissue (P < 0.0005). Furthermore, we observed a strong correlation between expression of PLK-1 and COX-2 (P < 0.0005).To our knowledge, this is the first report of a correlation between COX-2 and PLK-1 in a malignant tumor. COX-2 and PLK-1 may be interesting targets for new molecular therapies in prostate cancer. 
 Papillary carcinoma of the thyroid: high expression of COX-2 and low expression of KAI-1/CD82 are associated with increased tumor   invasive  ness. We have previously demonstrated that expression of COX-2 is upregulated by hepatocyte growth factor in thyroid papillary carcinoma (TPC) cells and is associated with increased   invasive  ness of tumor cells. COX-2 upregulation was associated with downregulation of KAI-1/CD82, a metastasis suppressor molecule that has been associated with the metastatic potential of several solid tumors. In the present study, we have investigated the possibility that downregulation of KAI-1/CD82 may contribute to the   invasive  ness of papillary carcinoma cells.Expression of KAI-1/CD82 and its relation to COX-2 levels were investigated in 6 primary cultures of TPC, in 2 tumor cell lines (TPC-1 and K1), and in 55 tumor samples of TPC. The biological role of KAI-1/CD82 in regulating tumor   invasive  ness was investigated in TPC cell lines and primary cultures transfected with a pCDNA3.1/Hygro.KAI-1; transfected cells were tested in functional studies of cell migration and   invasive  ness. Finally, the role of KAI-1/CD82 in influencing TPC metastasis was investigated in vivo using nu/nu mice injected with K1-transfected cells.We provide evidence that COX-2 and KAI-1/CD82 are inversely regulated in TPC primary cultures and in TPC-1 tumor cells. In fact, inhibition of COX-2 with NS398 is associated with a 2-9-fold upregulation of KAI-1/CD82 RNA. Moreover, a possible relation between COX-2 and KAI-1/CD82 was confirmed by the presence of a statistically significant inverse correlation in the expression of the two genes in 55 tumor samples of TPC (r = -0.513; p = 0.001). In 36 of 55 cases, tumor areas contained lower levels of KAI-1/CD82 RNA as compared with the corresponding normal tissue. Low expression of KAI-1/CD82 RNA in the tumor area was associated with extrathyroid extension of the disease in 16 of 19 cases (p < 0.04) and with lymph node metastasis in 11 of 14 cases (not significant). KAI-1/CD82 re-expression in tumor cells was associated with a significant decrease in their migratory (50-76% reduction) and   invasive   (46-65% reduction) capacity, even after hepatocyte growth factor stimulation. Finally, nu/nu mice injected with KAI-1/CD82-transfected K1 TPC cells developed fewer and smaller metastasis as compared with mice injected with vector-transfected K1 cells (p=0.016).Our findings raise the possibility that downregulation of KAI-1/CD82 in TPC cells is one of the molecular mechanisms regulating their   invasive   and metastatic potential. 
 Relationship between the expression of cyclooxygenase 2 and MDR1/P-glycoprotein in   invasive   breast cancers and their prognostic significance. Recent reports suggest that expression of the cyclooxygenase 2 (COX-2) enzyme may up-regulate expression of MDR1/P-glycoprotein (MDR1/P-gp), an exponent of resistance to cytostatic drugs. The present study aimed at examining the relationship between the expression of COX-2 and of MDR1/P-gp in a group of breast cancer cases.Immunohistochemical reactions were performed using monoclonal antibodies against COX-2 and MDR1/P-gp on samples originating from 104 cases of primary   invasive   breast cancer.COX-2-positive cases were shown to demonstrate higher expression of MDR1/P-gp (P < 0.0001). The studies also demonstrate that COX-2 expression was typical for cases of a higher grade (P = 0.01), a shorter overall survival time (P < 0.0001) and a shorter progression-free time (P < 0.0001). In the case of MDR1/P-gp, its higher expression characterised cases of a higher grade (P < 0001), with lymph node involvement (P < 0001), and shorter overall survival (P < 0.0001) and progression-free time (P < 0.0001).Our studies confirmed the unfavourable prognostic significance of COX-2 and MDR1/P-gp. We also document a relationship between COX-2 and MDR1/P-gp, which suggests that COX-2 inhibitors should be investigated in trials as a treatment supplementary to chemotherapy of breast cancers. 
 Functional polymorphisms in the cyclooxygenase 2 (COX-2) gene and risk of breast cancer in a Chinese population. Cyclooxygenase (COX), the rate-limiting enzyme in prostaglandins (PG) synthesis, exists in at least two isoforms, COX-1 and COX-2. COX-2 plays an important role in carcinogenesis, and overexpression may increase proliferation, inhibit apoptosis, and enhance the   invasive  ness of breast cancer cells. Polymorphisms in the regulatory regions of the COX-2 gene may influence function and/or expression and contribute to interindividual variability in susceptibility to cancer. In this study three variants (-1195G/A and -765G/C in the promoter and 8473C/T in 3'UTR) of COX-2 were examined for correlation with breast cancer risk. A case-control study of 615 histologically confirmed breast cancer patients and 643 cancer-free controls frequency-matched for age were selected. Logistic regression analyses revealed that no overall significant associations were detected in the single-locus analysis between three polymorphisms of COX-2 and the risk of breast cancer. However, a significantly increased risk of breast cancer was associated with the combined genotypes containing "more than 3 variant alleles"' (adjusted OR = 1.37, 95% CI 1.01-1.84) compared with the combined genotypes with "0-3 variant alleles." Haplotype analyses showed that haplotypes A-1195G-765T8473 and A-1195C-765T8473 were significantly associated with breast cancer risk (OR = 1.20, 95% CI 1.01-1.43 for A-1195G-765T8473; OR = 9.16, 95% CI 1.14-73.51 for A-1195C-765T8473) compared with the most common haplotype, G-1195G-765T8473. These findings indicate that these three variants in the regulatory regions of COX-2 may contribute to the etiology of breast cancer. 
 Lower cyclooxygenase-2 expression is associated with recurrence of solitary non-muscle   invasive   bladder carcinoma. A new modality is necessary to prevent recurrence of superficial bladder cancer after complete transurethral resection because of the high recurrence rate even with current prophylaxis protocols.In order to analyze the predictive value of cyclooxygenase-2 (COX-2) expression and tumor infiltrating lymphocytes (TILs) in recurrence of this disease tumor specimens from 127 patients with solitary papillary non-muscle   invasive   bladder cancer (NMIBC), 78 with recurrent disease and 49 without recurrence during follow up of minimum 5 years, were retrieved for tissue microarrays construction and immunohistochemical analysis. COX-2 expression was scored according to Allred's scoring protocol, while presence of TILs was categorized as absent (no) or present (yes) on whole tissue sections.COX-2 immunoreactivity was presented in 70 (71%), weak in 16% and strong in 55% of cases, while 29 (29%) tumors were negative. TILs were present in 64 (58%) NMIBC, while 44 cases (41%) did not reveal mononuclear infiltration in tumoral stroma. Statistical analysis demonstrated a higher proportion of patients with recurrence in the group with the COX-2 score 0, and lower in the group with score 2 (p=0.0001, p=0.0101, respectively). In addition, a higher proportion of recurrent patients in the group with no TILs, and lower proportion in the group with TILs were found (p=0.009, p=0.009, respectively). Univariate and multivariate analysis revealed overexpression of COX-2 and presence of TILs as negative predictors.Patients with lower COX-2 expression and absence of TILs in NMIBC need to be followed up more vigorously and probably selected for adjuvant therapy.The virtual slide(s) for this article can be found here: http://www.diagnosticpathology.diagnomx.eu/vs/1411318819790406. 
 Androgen receptor promotes the migration and invasion of upper urinary tract urothelial carcinoma cells through the upregulation of MMP-9 and COX-2. Dysregulated androgen receptor (AR) signaling is implicated in several types of tumor, including carcinomas of the prostate, breast, liver and bladder. However, the contribution of AR to the progression of upper urinary tract urothelial carcinomas (UUTUC) has not been fully investigated. In the present study, we demonstrated that the AR is involved in the metastasis and   invasive  ness of UUTUC cells. We investigated the role of the AR in UUTUC by using UUTUC-derived BFTC 909 cells. The overexpression of AR promotes the migration and invasion of BFTC 909 cells. Expression of migration/invasion-related genes was increased in BFTC 909 cells overexpressing AR determined by qPCR and western blot analyses. The results showed that AR-enhanced migration and invasion of UUTUC cells are linked to the upregulation of the matrix-degrading enzyme MMP-9 and cyclooxygenase (COX)-2. Subsequently, the blocking of MMP-9 and COX-2 signaling by inhibitors suppressed AR-enhanced cell migration and invasion. The results of the present study provide evidence for the first time of the role of AR in the motility and invasion of UUT cancer cells and support the hypothesis that the AR may play a critical role in the establishment of the   invasive   phenotype in urothelial neoplasia of UUT. Thus, the AR may also serve as a novel biomarker and potential therapeutic target for UUT cancer. 
 Requirement of cyclooxygenase-2 expression and prostaglandins for human prostate cancer cell invasion. The PC-3 Low   Invasive   cells and the PC-3 High   Invasive   cells were used to investigate the correlation of the COX-2 expression and its arachidonic acid metabolites, prostaglandins, with their   invasive  ness through Matrigel using a Boyden chamber assay. The COX-2 expression in PC-3 High   Invasive   cells was approximately 3-fold higher than in PC-3 Low   Invasive   cells while the COX-1 expression was similar in both cell sublines. When incubated with arachidonic acid, PGE2 was the major prostaglandin produced by these cells. PC-3 High   Invasive   cells produced PGE2 approximately 2.5-fold higher than PC-3 Low   Invasive   cells. PGD2 was the second most abundant prostaglandin produced by these cells. Both indomethacin (a nonspecific COX inhibitor) and NS-398 (a specific COX-2 inhibitor) inhibited the production of prostaglandins and the cell invasion. PGE2 alone did not induce the cell invasion of PC-3 Low   Invasive   cells. However, PGE2 reversed the inhibition of cell invasion by NS-398 and enhanced the cell invasion of the PC-3 High   Invasive   cells. In contrast, PGD2 slightly inhibited the cell invasion. These results suggest that in the PC-3 Low   Invasive   cells, COX-2-derived PGE2 may not be sufficient to induce cell invasion while in the PC-3 High   Invasive   cells, PGE2 may be sufficient to act as an enhancer for the cell invasion. Further, PGD2 may represent a weak inhibitor and counteracts the effect of PGE2 in the cell invasion. 
 COX-2 Expression in Renal Cell Carcinoma and Correlations with Tumor Grade, Stage and Patient Prognosis. Cyclooxygenase 2 (COX-2) is important as an enzyme in the pathway leading to the production of prostaglandin E2 (PGE2) and arachidonic acid. This pathway is known to play a role in inflammation, tumor growth,   invasive  ness and metastasis, inhibition of apoptosis and angiogenesis. Inhibition of COX-2 has been shown to be a promising antitumor and antiangiogenic strategy in several tumor types, including renal cell carcinoma (RCC). Therefore, we decided to evaluate the immunohistochemical expression of this marker and its association with several clinicopathological characteristics in a series of cases.COX-2 expression was examined immunohistochemically in tumor tissues obtained from 96 patients who underwent radical (94 cases) or partial (2 cases) nephrectomy. Correlations between COX-2 expression and clinicopathologic findings including pathologic stage, nuclear grade and other indicator of prognosis were examined.Of 96 tumors, 20.9% were positive for COX-2 expression. A correlation was found between COX-2 expression and tumor histological subtype (P=0.03).The papillary subtype showed maximum expression of this marker (43.8%) and the clear subtype minimum (14.7%). There were also possible links between COX-2 expression and pathologic stage, nuclear grade and nodal involvement but the results were not statistically significant (P=0.8, P= 0.14 and P=0.06, respectively). No correlation was found between COX2 expression and patient age, gender, tumor size, metastasis or survival.In our study, COX-2 expression was correlated with the histological subtype of RCC. Additional research is required to determine the link between COX-2 expression and prognosis and also evaluation of probable effectiveness of COX-2 inhibitor drugs in treatment of RCC patients. 
 TNF superfamily gene polymorphism as prognostic factor in early breast cancer. Since apoptosis may play a role in the prognosis of breast cancer, the present study analyzed the polymorphisms of apoptosis-related genes and their impact on the survival of 240 patients with early   invasive   ductal breast cancer.The genomic DNA was extracted from paraffin-embedded tumor-free tissue or blood, and 12 single nucleotide polymorphisms (SNPs) of 11 apoptosis-related genes in the apoptosis pathway determined using a Sequenom MassARRAY system.During the median follow-up of 53.4 (range 2.9-205.9) months, 37 relapses and 22 deaths occurred. Among the target polymorphisms, the tumor necrosis factor superfamily member 10 gene polymorphism (TNFSF10 rs1131532) in a recessive model of the T allele and prostaglandin-endoperoxide synthase 2 gene polymorphism (PTGS2 rs5275) in a dominant model of the C allele were associated with survival in a log-rank test. The TT genotype of TNFSF10 (rs1131532) was also significantly correlated with a lower disease-free, distant disease-free, and overall survival in a multivariate analysis (HR = 3.304, 4.757, and 6.459; P = 0.002, 0.001, and 0.009, respectively), while PTGS2 rs5275 was only associated with a higher distant disease-free survival (HR = 0.302; P = 0.041). No clinicopathologic difference was observed according to the genotypes of these two polymorphisms.The TNFSF10 (rs1131532) polymorphism was identified as a possible prognostic factor of survival in patients with operated   invasive   breast cancer. 
 Reciprocal correlation between the expression of cyclooxygenase-2 and E-cadherin in human bladder transitional cell carcinomas. Carcinoma cells become more motile and   invasive   via downmodulation of E-cadherin. Cyclooxygenase-2 (COX-2) expression is associated with tumor invasion and metastasis. The aim of this study is to investigate the relationship between the expression of COX-2 and E-cadherin in a bladder cancer cell line and human bladder transitional cell carcinoma (TCCs). Phorbol 12-myristate 13-acetate (PMA) treatment for 5637 bladder cancer cells increased COX-2 expression, slightly induced Slug expression, and decreased E-cadherin expression. Ectopic expression of COX-2 or prostaglandin E(2) (PGE(2)) treatment for 5637 cells reduced E-cadherin expression. This finding was confirmed by the result that knockdown of COX-2 expression or indomethacin administration increased the expression of E-cadherin. When compared with cells' motility in serum-free medium, the treatment of PMA and PGE(2) increased cell motility, and indomethacin treatment slightly decreased cell motility. In the tissues of bladder TCCs, COX-2 expression was inversely correlated with membranous E-cadherin expression and positively correlated with nuclear beta-catenin expression. The expression of COX-2 and nuclear beta-catenin expression was significantly higher in TCCs of high grade and   invasive   growth than in TCCs of low grade and non  invasive   growth. In contrast, membranous E-cadherin expression was more decreased in tumors of high grade and   invasive   growth. In addition, nuclear beta-catenin expression was significantly related to tumor recurrence. We suggest that COX-2 pathway reduces membranous E-cadherin expression in bladder TCCs and their expression pattern may provide important information in predicting the clinical behavior of bladder TCCs. 
 Taurocholate Induces Cyclooxygenase-2 Expression via the Sphingosine 1-phosphate Receptor 2 in a Human Cholangiocarcinoma Cell Line. Cholangiocarcinoma (CCA) is a rare, but highly malignant primary hepatobiliary cancer with a very poor prognosis and limited treatment options. Our recent studies reported that conjugated bile acids (CBAs) promote the   invasive   growth of CCA via activation of sphingosine 1-phosphate receptor 2 (S1PR2). Cyclooxygenase-2 (COX-2)-derived prostaglandin E2 (PGE2) is the most abundant prostaglandin in various human malignancies including CCA. Previous studies have indicated that COX-2 was highly expressed in CCA tissues, and the survival rate of CCA patients was negatively associated with high COX-2 expression levels. It has also been reported that CBAs induce COX-2 expression, whereas free bile acids inhibit COX-2 expression in CCA mouse models. However, the underlying cellular mechanisms and connection between S1PR2 and COX-2 expression in CCA cells have still not been fully elucidated. In the current study, we examined the role of S1PR2 in conjugated bile acid (taurocholate, (TCA))-induced COX-2 expression in a human HuCCT1 CCA cell line and further identified the potential underlying cellular mechanisms. The results indicated that TCA-induced   invasive   growth of human CCA cells was correlated with S1PR2-medated up-regulation of COX-2 expression and PGE2 production. Inhibition of S1PR2 activation with chemical antagonist (JTE-013) or down-regulation of S1PR2 expression with gene-specific shRNA not only reduced COX-2 expression, but also inhibited TCA-induced activation of EGFR and the ERK1/2/Akt-NF-κB signaling cascade. In conclusion, S1PR2 plays a critical role in TCA-induced COX-2 expression and CCA growth and may represent a novel therapeutic target for CCA. 
 COX2 overexpression is a prognostic marker for Stage III breast cancer. To determine the significance of (Cyclooxygenase 2) COX2 for clinical outcome in breast cancer, we analyzed the correlation between COX2 overexpression and survival in 687 patients with   invasive   breast cancer. Cytoplasmic immunoreactivity of COX2 was determined as positive in 325 of 687 (47.3%)   invasive   breast cancers. COX2 positivity was significantly correlated with high histologic grade, negative estrogen receptor (ER), high Ki67, luminal B and triple-negative tumors, Bcl2 negativity, and p53 overexpression. In univariate analysis, COX2 overexpression resulted in significantly shorter relapse-free survival (RFS) [hazard ratio (HR) 1.487, 95% CI 1.035-2.110, P = 0.032]. Multivariate analysis revealed no significant association between COX2 overexpression and either overall survival (OS) or RFS. Kaplan-Meier analysis of the whole patient group showed significantly reduced RFS in patients with high COX2 expression, compared to those that did not overexpress COX2 (91 vs. 162 months, P = 0.031). Stratified subgroup analysis by TNM stage disclosed marked differences in OS and RFS rates in Stage III patients. We observed a significant association of COX2 overexpression with shorter RFS in the ER-negative subgroup of Stage III patients. The results show that COX2 overexpression is a significant unfavorable prognostic factor in Stage III breast cancer, and provide selective criteria for COX2 inhibitor combinations for   invasive   breast cancer therapy. 
 Oesophageal squamous cell neoplasia in head and neck cancer patients: upregulation of COX-2 during carcinogenesis. Patients with (previous) head and neck cancer (HNC) are at high risk for developing second squamous cell cancer of the oesophagus. The role of cyclooxygenase-2 (COX-2) in oesophageal squamous carcinogenesis has not yet been investigated in this high-risk group. Therefore, this study examined COX-2 mRNA and protein expression in oesophageal biopsies and resected tissues of 44 HNC patients. The evaluation covered 55 oesophageal tissue samples (18   invasive   oesophageal squamous cell cancers, four high- and eight low-grade dysplasias, 25 normal squamous epithelia) from the 44 patients. mRNA levels of COX-2 were measured by real-time PCR using a LightCycler. COX-2 protein expression was studied immunohistochemically and graded by a staining score. COX-2 mRNA was detected in all samples, and its levels correlated positively with the immunohistochemical staining score (P<0.05). COX-2 expression was upregulated during oesophageal squamous carcinogenesis in HNC patients, that is COX-2 expression increased significantly from normal oesophageal squamous epithelium to low- and high-grade dysplasia and finally to   invasive   squamous cell cancer (P<0.001). Our findings suggest that COX-2 upregulation contributes to oesophageal squamous carcinogenesis in HNC patients. Prospective studies are needed to evaluate the chemopreventive potential of COX-2 inhibitors in this high-risk group. 
 Common polymorphisms in the prostaglandin pathway genes and their association with breast cancer susceptibility and survival. There is evidence that genetic variation in the prostaglandin pathway affects cancer susceptibility and progression. Conflicting data from several studies exist for the association of PTGS2 (cyclooxygenase 2) polymorphisms with breast cancer risk. We investigated associations between common germ-line variations in seven genes in the prostaglandin pathway and breast cancer susceptibility and survival among women with   invasive   breast cancer in the SEARCH study.DNA samples from 9,030 cases and controls were genotyped for 64 single nucleotide polymorphisms tagging known common variants (minor allele frequency > 0.05) in PTGS1, PTGS2, TBXAS1, PTGIS, PTGES, PTGDS, and PGDS with a two-stage case-control study design.Four tagging single nucleotide polymorphisms showed modest association with breast cancer susceptibility. All four fit a recessive genetic model. Minor allele homozygotes for PTGISrs5602 [odds ratio (OR), 1.15; 95% confidence interval (95% CI), 1.04-1.27; P = 0.005], PTGISrs8183919 (OR, 1.22; 95% CI, 1.06-1.41; P = 0.006), and TBXASrs41727 (OR, 1.83; 95% CI, 1.22-2.73; P = 0.003) are associated with an increased risk compared with common allele carriers. For PTGISrs44627 minor allele homozygotes (OR, 0.66; 95% CI, 0.5-0.86; P = 0.002), a protective effect was observed.Specific PTGIS and TBXAS1 variants may affect breast cancer susceptibility, but common variants in PTGS1, PTGS2, PTGES, PTGDS, and PGDS have no major role in breast cancer susceptibility. None of the variants in the seven genes studied appear to affect survival. Further larger studies correlating clinical and genotypic data are required to establish if the clinical utility of prostaglandin-targeted therapies, as chemoprevention agents, is influenced by an individual's profile of genetic variants in key prostaglandin pathway genes. 
 The value of COX-2 expression in the prognostic parameters of   invasive   ductal carcinoma of the breast. Cyclooxygenase is an enzyme that changes the immune response to malign cells and catalyzes prostaglandins that may have an impact on cell proliferation. The purpose of this study was to examine the relation between established clinicopathological parameters in breast carcinomas and COX-2 protein expression. COX-2, estrogen receptor (ER), progesterone receptor (PR) and c-erB-2 primary antibodies were assessed in the slides prepared from the paraffin blocks of 62   invasive   ductal carcinoma cases. The relation between ER, PR, and c-erbB-2 positivity, histological grade, nuclear grade, lymphovascular invasion, tumor diameter, lymph node positivity, metastasis, and age were evaluated. The results were analyzed statistically. Cytoplasmic COX-2 expression was seen in 75.8% of all breast carcinomas. In both univariate and multivariate logistic regression analysis, there was a positive correlation between COX-2 expression and negative ER status, respectively (P=0.0173) (P=0.016). There was any statistically significant relation between PR positivity, c-erbB-2 positivity, histological grade, nuclear grade, lymphovascular invasion, tumor diameter, lymph node positivity, metastasis, and age (P≥0.05). Given that there was found a statistically significant relation between COX-2 expression and negative ER status, which is considered a poor prognostic parameter, suggests that COX-2 expression can have a place among the other prognostic parameters of breast carcinoma. 
 Correlation of COX-2 and Ep-CAM overexpression in human   invasive   breast cancer and its impact on survival. Recent studies have demonstrated cyclooxygenase 2 (COX-2) overexpression in various human malignancies, especially in breast cancer, where COX-2 turned out to be a predictor of poor survival. To evaluate the relation of COX-2 and Ep-CAM overexpression and its prognostic significance, we performed a retrospective study on 212 breast cancer patients with a median follow-up time of 10.5 years. Overexpression of COX-2 in tumour tissue samples was assessed by immunohistochemistry. COX-2 overexpression was found in 48.6% of the tumour samples and was predictive for poor disease-free and overall survival. Univariate analysis revealed a strong correlation between COX-2 and Ep-CAM overexpression (P=0.009). Concurrent COX-2 and Ep-CAM overexpression was present in 21.7% of tumour specimens and had an additive negative impact on disease-free and overall survival. Determination of both tumour markers should help in guiding new therapeutic strategies in patients with   invasive   breast cancer. 
 Expression of C-KIT, CD24, CD44s, and COX2 in benign and non-  invasive   apocrine lesions of the breast. Benign apocrine metaplasia (AM) of the adult breast is a very common, but enigmatic lesion. It has been speculated that AM might be a precursor of malignancy or an indicator of a susceptibility of the breast tissue to develop neoplasia, mainly based on comparing the frequency of AM in breast cancer and non-breast cancer patients [1]. Studies using comparative genomic hybridization have supported this by showing similar molecular alterations in benign and malignant apocrine lesions [2]. Few studies, however, have compared expression of biomarkers involved in tumor progression in AM and progressively more advanced atypical apocrine lesions. The expression of C-KIT, COX2, CD24, and CD44s was evaluated by immunohistochemistry in formalin-fixed, paraffin-embedded material of 9 AM, 20 apocrine ductal intraepithelial neoplasia (DIN1c-3) and 40 atypical apocrine lesions (not qualifying for DIN1c-3) and compared to expression of the same biomarkers in adjacent normal ductal epithelium. Of the 66 apocrine lesions, 62 (94 %) did not express C-KIT compared to 4/63 (6 %) of the normal glands (Fisher's exact, p < 0.001). COX2 was expressed in a significantly higher proportion of apocrine lesions than of normal glands (49 vs. 14 %, p < 0.001), and the number of apocrine lesions positive for CD24 was found to be higher with increasing aggressiveness of the lesions (Spearman, p < 0.001). In conclusion,   benign a  nd non-invasive proliferative apocrine lesions of the breast display immuno-phenotypical characteristics previously ascribed mainly to malignant transformation. This could lend support to the theory that AM is an early step towards malignant transformation, albeit associated with slow progression to carcinoma. 
 Heparanase and cyclooxygenase-2 gene and protein expressions during progression of oral epithelial dysplasia to carcinoma. Heparanase and cyclooxygenase-2 (COX-2) are 2 key enzymes that modulate diverse physiological processes during embryonic development and in adult life. Their deregulations have been implicated in the growth and progression of many cancer types. To date, comparatively little is known about the roles of these molecules during oral carcinogenesis. The aim of this study was to investigate the expression patterns of heparanase and COX-2 during progression of oral epithelial dysplasia (OED) to carcinoma. In situ hybridization and immunohistochemistry were performed on 5 cases of normal mucosa, 15 cases of OED, 5 cases of carcinoma in situ and/or micro  invasive   carcinoma, and 40 cases of oral squamous cell carcinoma (OSCC). Results demonstrated that heparanase and COX-2 messenger RNA and protein were absent in normal oral mucosa but were coexpressed in increasing intensity as OED progressed to OSCC. Concomitant heparanase- and COX-2-positive staining in the stromal cells suggests that OED/OSCC progression may be modulated by stromal-cancer cell interactions. Diffuse intense staining of poorly differentiated OSCC compared with staining localized to tumor nest periphery in well- and moderately differentiated OSCC suggests that heparanase and COX-2 overexpressions correlated with tumor grade. Strong expression of these enzymes in tumor cells at the advancing front suggests a role in local tumor spread. These results, taken together, suggest that heparanase and COX-2 might play complementary roles in the stepwise progression of OED to carcinoma. 
 COX-2 overexpression increases motility and invasion of breast cancer cells. Cyclooxygenase-2 (COX-2), an inducible enzyme involved in prostaglandin (including PGE(2)) biosynthesis, is overexpressed in several epithelial malignancies including breast cancer. We tested the hypothesis that COX-2 overexpression in breast cancer cells results in increased cell motility and invasion. COX-2 overproducing cells were generated by stable transfection of several human breast cancer cells with pSG5-COX2 vector. We confirmed the overexpression of COX-2 protein by western blotting, and by measuring PGE(2) in the medium with an immunoassay. We measured cell motility by counting the number of cells crossing an 8-micron pore size PET membrane, and cell invasion by counting the number of cells invading through a Matrigel-coated membrane that simulates basement membrane. COX-2 transfected MDA-231 cells produced 30-43-fold more PGE2 as compared to parental cells. COX-2 overexpression increased cell migration approximately 2.2-fold and cell invasion through Matrigel approximately 5.1-fold. Addition of 50 microM NS-398, a COX-2 inhibitor, inhibited Matrigel invasion of MDA-231 cells by 54% as compared to solvent confirming the role of COX-2 in cell invasion. It is known that an increase in cell migration and invasion can be brought about by cytoskeletal alterations and basement membrane degradation due to increased expression of pro-urokinase plasminogen activator (pro-uPA). To investigate the mechanism of our observed increase in cell invasion by COX-2, we found by western blotting that the level of pro-uPA was significantly higher (approximately 5-fold) in COX-2 transfected MDA-231 cells than untransfected MDA-231 cells. Similar to our observations in cell culture, we found evidence that increased COX-2 activity correlates with uPA in a mouse model of breast cancer metastasis to bone. In this study, we conclude that COX-2 overexpression in human breast cancer cells enhances cell motility and   invasive  ness thus suggesting a mechanism of COX-2 mediated metastasis. 
 Polymorphisms in the xenobiotic transporter Multidrug Resistance 1 (MDR1) and interaction with meat intake in relation to risk of colorectal cancer in a Danish prospective case-cohort study. The xenobiotic transporters, Multidrug Resistance 1 (MDR1/ABCB1) and Breast Cancer Resistance Protein (BCRP/ABCG2) may restrict intestinal absorption of various carcinogens, including heterocyclic amines (HCA) and polycyclic aromatic hydrocarbons (PAH). Cyclooxygenase-2 (COX-2) derived prostaglandins promote gastrointestinal carcinogenesis, affecting angiogenesis, apoptosis, and   invasive  ness.The aim of this study was to investigate if polymorphisms in these genes were associated with risk of colorectal cancer (CRC), and to investigate possible interactions with lifestyle factors such as smoking, meat consumption, and NSAID use.The following polymorphisms were analyzed; a synonymous MDR1 C3435T (rs1045642) in exon26, G-rs3789243-A in intron3, the functional BCRP C421A (rs2231142), the two COX-2 A-1195G (rs689466) and G-765C (rs20417) in the promoter region, and the COX-2 T8473C (rs5275) polymorphisms in the 3'-untranslated region. The polymorphisms were assessed together with lifestyle factors in a nested case-cohort study of 359 cases and a random cohort sample of 765 participants from the Danish prospective Diet, Cancer and Health study.Carriers of the variant allele of MDR1 intron 3 polymorphism were at 1.52-fold higher risk of CRC than homozygous wild type allele carriers (Incidence rate ratio (IRR) = 1.52, 95% Confidence Interval (CI): 1.12-2.06). Carriers of the variant allele of MDR1 C3435T exon 26 had a lower risk of CRC than homozygous C-allele carriers (IRR = 0.71 (CI:0.50-1.00)). There was interaction between these MDR1 polymorphisms and intake of red and processed meat in relation to CRC risk. Homozygous MDR1 C3435T C-allele carriers were at 8% increased risk pr 25 gram meat per day (CI: 1.00-1.16) whereas variant allele carriers were not at increased risk (p for interaction = 0.02). COX-2 and BCRP polymorphisms were not associated with CRC risk. There was interaction between NSAID use and MDR1 C3435T and COX-2 T8473C (p-values for interaction 0.001 and 0.04, respectively).Two polymorphisms in MDR1 were associated with CRC risk and there was interaction between these polymorphisms and meat intake in relation to CRC risk. Our results suggest that MDR1 polymorphisms affect the relationship between meat and CRC risk. 
 Immunohistochemical expression of the cyclooxygenase-2 (COX-2) in gastric cancer. The correlations with the tumor angiogenesis and patients' survival. The mechanisms by which COX-2 contributes to the carcinogenesis are not known until present. It seems that the COX-2 enzyme stimulates the cell proliferation, inhibits the apoptosis, increases the malignant cells'   invasive  ness and induces the angiogenesis by elaborating some angiogenic factors.In the present study, we intend to evaluate the immunohistochemical expression of COX-2 in gastric carcinomas, keeping track of the correlations between the clinicopathologic factors, the tumor angiogenesis (evaluated by microvascular density--MVD--determination and by VEGF expression) and the patients' survival. In addition, we have tracked the immunoreactions' positivation in the peritumoral mucosa with various lesions, with the purpose to establish the contribution of COX-2 to the gastric carcinogenesis during the pre-  invasive   stages. A prospective study was realized, regarding the evolution and aggressiveness of the gastric cancer, with a duration of five years, 61 patients operated of gastric cancer being included.The COX-2 immunoreactions have been significantly more frequent noticed in the gastric carcinomas included in the study (57.4%) and in the epithelial dysplasia areas adjacent to the carcinomas of intestinal type (35.5% of the cases), than in the normal peritumoral mucosa (4.9%) (p<0.001 ES). The COX-2 immunoreactions have turned positive more frequently in gastric carcinomas of intestinal type (68.4%), in comparison to the carcinomas of diffuse type (29.4%) (p<0.001 ES). The COX-2 expression is significantly correlated with the invasion level, the presence of the metastases in the regional lymph nodes and the pTNM stage, but without influencing the prognosis of the gastric cancer patients. The negative VEGF carcinomas have turned positive for COX-2 only for 19% of the cases. Different from those, the positive VEGF carcinomas have associated COX-2 immunoreactivity in 77.5% of the cases.The results obtained are suggestive for the predominant expression of COX-2 in the carcinomas of intestinal type and its precursory lesions. Our results show a tight correlation between the immunohistochemical expressions of COX-2 and VEGF in gastric carcinomas (r = 0.562, p < 0.001 ES) and also a MVD average value significantly higher in the positive COX-2 carcinomas, suggesting an intense angiogenesis activity in that group of tumors (p < 0.001 ES). 
 Expression of HuR is associated with increased cyclooxygenase-2 expression in uterine cervical carcinoma. The human family embryonic-lethal abnormal vision-like protein Hu antigen R (HuR) serves as a messenger RNA (mRNA)-binding protein and stabilizes a certain group of cellular mRNAs that contain adenylate/uridylate-rich elements in their 3-untranslated region. The HuR is predominantly located in the nucleus, but it shuttles between the nucleus and the cytoplasm. In the cytoplasm, it can stabilize certain transcripts. Reportedly, the mRNA of cyclooxygenase-2 (COX-2) can be stabilized by HuR in breast, ovary, colon, stomach, lung, and brain cancers. We investigated the expression of HuR and COX-2 in 308 primary uterine cervical carcinomas using immunohistochemistry. Nuclear HuR expression was seen in 280 (90.9%) of the cases, and cytoplasmic HuR expression was observed in 61 (19.8%) of the cases. The expression of nuclear HuR was significantly associated with stage (P = 0.031). We found that 135 patients (43.8%) showed positive reaction for the COX-2 protein. In the COX-2 positive cases, nuclear HuR expression was higher, but the difference was not statistically significant. Cytoplasmic HuR expression was significantly associated with tumor size (P = 0.029), stage (P = 0.005), and lymphatic/vascular invasion (P < 0.001). Considering only squamous cell lesions (carcinoma in situ, micro  invasive   squamous cell carcinoma [SCC], and   invasive   SCC), the cytoplasmic expression of HuR significantly increased in   invasive   SCC compared with micro  invasive   SCC or carcinoma in situ (P = 0.005). Cytoplasm HuR expression correlated with COX-2 (P < 0.001). There was no significant correlation between HuR (nuclear or cytoplasmic) expression and patient survival. Our results suggest that the cytoplasmic overexpression of HuR is associated with uterine cervical carcinomas with aggressive clinicopathologic features and that HuR might contribute to the stabilization of COX-2 mRNA in some uterine cervical carcinomas. 
 [Expression and clinical significance of cyclooxygenase-2 in nasal and paranasal sinus carcinoma]. To investigate the relationship between the expression of cyclooxygenase-2 (COX-2) and the carcinogenesis and   invasive   behavior of nasal and paranasal sinus carcinoma (NPSC).The expression of COX-2 were detected in 69 case NPSC tissues, paracancerous atypical hyperplasia tissue and normal tissue by using immunohistochemical techniques of SP method.The positive rate of COX-2 in NPSC tissues and atypical hyperplasia tissue and normal tissue was 65.22% and 42.31% and 3.33%, respectively. The positive rate of COX-2 in NPSC tissues was significantly higher than that in normal tissue (P < 0.01), and increased apparently with the lesion progressing from normal to atypical hyperplasia tissue to NPSC tissues, and also correlated with the increasing of tumor (P < 0.05).COX-2 may play an important role in the carcinogenesis and invasion of NPSC. 
 Lack of association of the cyclooxygenase-2 and inducible nitric oxide synthase gene polymorphism with risk of cervical cancer in Korean population. Currently, many studies have shown that nitric oxide (NO) and prostaglandin (PG) are main inflammatory mediators involved in a variety of pathophysiological processes including inflammation and carcinogenesis. In this article, we explored the possible association of polymorphisms, of two representative inflammatory mediators, with the risk for cervical cancer. This study included 176 cases of histologically confirmed   invasive   cervical cancer, and 172 healthy controls. Allele frequency of 12 single-nucleotide polymorphisms (SNPs) of cyclooxygenase-2 (COX-2) and COX5 of the inducible nitric oxide synthase (iNOS) genes in 43 different normal populations were analyzed. We found that 14 of 17 showed a monomorphic or minimal minor allele frequency; therefore, we did not continue with additional analysis. Three SNPs (2 for COX-2, 1 for iNOS) were chosen for the study. Genotyping of three SNPs (SNP-rs5275 in the untranslated region of exon 10 and rs5277 in the coding region of exon 3 of COX-2, and iNOS Ser(608)Leu allele C/T polymorphism within exon 16 of the iNOS reductase domain) was performed. No significant increase was found in any of the genotypes of the COX-2 or iNOS in the cancer group. We investigated the possible correlation between the genotypes and the clinicopathologic parameters of cervical cancer. No significant association of the genotypes studied was found with respect to clinical stage, lymph node (LN) status, histologic type, or parametrial invasion. Our data did not reveal an association between COX-2 and iNOS polymorphisms with cervical cancer in Korean women. 
 Overexpression of the embryonic-lethal abnormal vision-like protein HuR in ovarian carcinoma is a prognostic factor and is associated with increased cyclooxygenase 2 expression. The human embryonic-lethal abnormal vision-like protein HuR is involved in the regulation of mRNA turnover and serves as a shuttling protein between the nucleus and the cytoplasm that stabilizes mRNAs containing adenine- and uridine-rich elements in their 3' untranslated region. We have shown recently that expression of cyclooxygenase (COX)-2 is related to poor prognosis in ovarian carcinoma. Other studies have shown that the COX-2 mRNA contains an adenine- and uridine-rich element and is stabilized by HuR. In this study, we investigated the expression and cellular distribution of HuR in 83 primary ovarian carcinomas, 16 borderline tumors of the ovary, 3 normal ovaries, and 9 ovarian carcinoma cell lines. Expression of HuR was detected in all cell lines on the mRNA and protein level and showed a predominantly nuclear staining in OVCAR-3 cells by confocal microscopy. In an immunohistochemical evaluation of human ovarian carcinomas, HuR showed a nuclear expression in 81% of tumors. In addition, a cytoplasmic expression of HuR was observed in a subgroup of 45% of ovarian carcinomas. Nuclear as well as cytoplasmic expression of HuR was significantly increased in ovarian carcinomas compared with borderline tumors or normal ovaries. In univariate analysis, a significant association between cytoplasmic HuR expression and increased COX-2 expression (P = 0.025) as well as between histological grade (P = 0.008) and mitotic activity (P = 0.002) was observed, although nuclear expression of HuR was not correlated with COX-2 expression or other clinicopathological parameters. In Kaplan-Meier survival analysis, increased cytoplasmic expression of HuR was a significant prognostic indicator for progression-free survival (P = 0.03) as well as overall survival (P = 0.007). In multivariate analysis using the Cox regression model, cytoplasmic expression of HuR was an independent prognostic parameter for reduced overall survival with a relative risk of 2.62 (95% confidence interval, 1.32-5.19). Our results suggest that there is a dysregulation of cellular distribution of the mRNA stability factor HuR in a subset of   invasive   ovarian carcinomas. This dysregulation appears to result in an increased expression of COX-2, an increased proliferative rate, and may lead to a reduced survival time. Additional studies are required to analyze the downstream effects of increased cytoplasmic expression of HuR. In addition, it would be interesting to investigate the prognostic role of increased cytoplasmic expression of HuR in prospective studies. 
 COX-2 (PTGS2) gene methylation in epithelial, subepithelial lymphocyte and stromal tissue compartments in a spectrum of esophageal squamous neoplasia. Previous studies have shown important effects of stromal elements in carcinogenesis. To explore the tumor-stromal relationship in esophageal neoplasia, we examined methylation of COX-2 (PTGS2), a gene etiologically associated with the development of gastrointestinal cancers, in adjacent foci of epithelium, subepithelial lymphocytes and non-lymphocytic stromal cells found in sections of normal squamous epithelium, squamous dysplasia and   invasive   esophageal squamous cell carcinoma.Adjacent foci of epithelium, subepithelial lymphocytic aggregates and non-lymphocytic stromal tissues were laser microdissected from six fully embedded, ethanol fixed, esophagectomy samples from Shanxi, China, a high-risk region for esophageal cancer. Promoter CpG site-specific hypermethylation status of COX-2 was determined using real-time methylation-specific PCR (qMS-PCR) based on Taqman Chemistry. The methylation status of a subset of samples was confirmed by pyrosequencing.Forty-nine microdissected foci were analyzed. COX-2 gene methylation was significantly more common in subepithelial lymphocytes (12/16 (75% of all foci)) than in epithelial foci (3/16 (19%)) or foci of non-lymphocytic stromal tissues (3/17 (18%)) (Fisher's exact p=0.05). Two of three epithelial samples and all three stromal samples that showed COX-2 methylation were adjacent to foci of methylated subepithelial lymphocytes. Pyrosequencing confirmed the methylation status in a subset of samples.In these esophageal cancer patients, COX-2 gene methylation was more common in subepithelial lymphocytes than in adjacent epithelial or stromal cells in both grades of dysplasia and in foci of   invasive   cancer. These findings raise the possibility that methylation of subepithelial lymphocytes may be important for tumorigenesis. Future studies of gene methylation should consider separate evaluation of epithelial and non-epithelial cell populations. 
 Cyclooxygenase-2 promotes tumor lymphangiogenesis and lymph node metastasis in oral squamous cell carcinoma. Oral squamous cell carcinoma (OSCC) is the sixth most common cancer and frequently metastasizes to the cervical lymph nodes, leading to poor survival of patients with OSCC. However, the mechanism of lymph node metastasis is not fully understood. To clarify the molecular mechanism underlying OSCC metastasis to regional lymph nodes, the highly metastatic fluorescent labeled OSCC cell line SAS-LM3 was successfully established allowing us to monitor the progression of lymph node metastases in a non-  invasive   manner. SAS-LM3 tumors showed increased lymphangiogenesis and elevated expression of VEGF-C, a potent stimulator of lymphangiogenesis, compared to parental SAS tumors. SAS-LM3 showed high expression of cyclooxygenase-2 (COX-2) compared to parental SAS cells and immunohistochemical analysis demonstrated intense COX-2 expression at the primary site. Inactivation of COX-2 by knockdown or the COX-2 inhibitor NS-398 decreased VEGF-C expression. Administration of COX-2 inhibitor NS-398 in SAS-LM3 tumor-bearing mice suppressed tumor lymphangiogenesis and lymphatic metastases. Collectively, our results indicate that COX-2 promotes tumor lymphangiogenesis and lymph node metastasis of OSCC. COX-2 ablation holds promise as a potential therapeutic approach for lymph node metastasis in OSCC. 
 A novel role for cyclooxygenase-2 in regulating vascular channel formation by human breast cancer cells. Cyclo-oxygenase (COX)-2 expression correlates directly with highly aggressive and metastatic breast cancer, but the mechanism underlying this correlation remains obscure. We hypothesized that   invasive   human breast cancer cells that over-express COX-2 have the unique ability to differentiate into extracellular-matrix-rich vascular channels, also known as vasculogenic mimicry. Vascular channels have been associated with angiogenesis without involvement of endothelial cells, and may serve as another mechanism by which tumor cells obtain nutrients to survive, especially in less vascularized regions of the tumor.To determine whether COX-2 regulates vascular channel formation, we assessed whether treatment with celecoxib (a selective COX-2 inhibitor) or silencing COX-2 synthesis by siRNA inhibits vascular channel formation by breast cancer cell lines. Cell lines were selected based on their   invasive   potential and COX-2 expression. Additionally, gene expression analysis was performed to identify candidate genes involved in COX-2-induced vascular channel formation. Finally, vascular channels were analyzed in surgically resected human breast cancer specimens that expressed varying levels of COX-2.We found that   invasive   human breast cancer cells that over-express COX-2 develop vascular channels when plated on three-dimensional matigel cultures, whereas non-  invasive   cell lines that express low levels of COX-2 did not develop such channels. Similarly, we identified vascular channels in high-grade   invasive   ductal carcinoma of the breast over-expressing COX-2, but not in low-grade breast tumors. Vascular channel formation was significantly suppressed when cells were treated with celecoxib or COX-2 siRNA. Inhibition of channel formation was abrogated by addition of exogenous prostaglandin E2. In vitro results were corroborated in vivo in tumor-bearing mice treated with celecoxib. Using gene expression profiling, we identified several genes in the angiogenic and survival pathways that are engaged in vascular channel formation.Antivascular therapies targeting tumor cell vasculogenic mimicry may be an effective approach to the treatment of patients with highly metastatic breast cancer. 
 Expression of cyclo-oxygenase-2 in human tongue carcinoma and its precursor lesions. The expression of Cox-2 protein was studied by immunohistochemistry in normal oral mucosa and in mucosa with various lesions of oral leukoplakia, including hyperplasia and dysplasia of squamous epithelium and frank   invasive   squamous carcinoma. A gradient of Cox-2 staining was found: the expression of Cox-2 was lowest in normal epithelium, somewhat increased in hyperplastic epithelium, further increased in dysplastic epithelium, and highest in   invasive   squamous cell carcinomas. The presence of Cox-2 in squamous cell carcinomas of the oral mucosa and its precursor lesions indicate that Cox-2 could participate in the carcinogenic process of these oral malignancies. 
 Cyclooxygenase-2 and gastric carcinogenesis. Epidemiological studies have shown that the use of nonsteroid anti-inflammatory drugs (NSAIDs) is associated with reduced risk of gastric cancer. The best-known target of NSAIDs is the cyclooxygenase (Cox) enzyme. Two Cox genes have been cloned, of which Cox-2 has been connected with gastric carcinogenesis. Expression of Cox-2 is elevated in gastric adenocarcinomas, which correlates with several clinicopathological parameters, including depth of invasion and lymph node metastasis. This suggests that Cox-2-derived prostanoids promote aggressive behavior of adenocarcinomas of the stomach. Cox-2 expression is especially prominent in intestinal-type gastric carcinoma and it is already present in dysplastic precursor lesions of this disease, which suggests that Cox-2 contributes to gastric carcinogenesis already at the pre  invasive   stage. Our most recent data show that Cox-2 is expressed in gastric adenomas of trefoil factor 1 deficient mice. Treatment of these mice with a Cox-2 selective inhibitor, celecoxib, reduced the size of the adenomas. Taken together these data support efforts to initiate clinical studies to investigate the effect of Cox-2 inhibitors as chemotherapeutic agents and as adjuvant treatment modalities against gastric neoplasias. 
 Role of cyclooxygenase-2 in colorectal cancer. Cyclooxygenase-2 (COX-2) is an inducible enzyme that regulates prostaglandin synthesis and is overexpressed at sites of inflammation and in several epithelial cancers. Recently, a causal link for COX-2 in epithelial tumorigenesis was shown in genetically-manipulated animal models of colon and breast carcinoma. Data indicate that COX-2 is involved in the regulation of apoptosis, angiogenesis, and tumor cell   invasive  ness, which appear to contribute to its effects on tumorigenesis. Multiple studies have shown that nonselective COX and selective COX-2 inhibitors effectively prevent experimental colon cancer. Furthermore, sulindac and the selective COX-2 inhibitor celecoxib were shown to regress colorectal polyps in patients with familial adenomatous polyposis. Although the exact anti-tumor mechanisms of these agents await further study, data indicate that both COX-dependent and COX-independent mechanisms may be important. In this review, the association between COX-2 and colorectal tumorigenesis and potential mechanisms of this effect are discussed. Additionally, evidence supporting the role of NSAIDs and selective COX-2 inhibitors for the prevention and treatment of human colorectal cancer is reviewed. 
 Expression of cyclooxygenase 2 is an independent prognostic factor in human ovarian carcinoma. Cyclooxygenase-2 (COX-2) is the rate-limiting enzyme in prostanoid biosynthesis and is involved in tumor progression. We investigated expression of COX-1 and COX-2 in cell lines and tumors from ovarian carcinomas. Expression of COX-2 mRNA and protein was detectable in three of five ovarian carcinoma cell lines and was inducible by interleukin-1beta or phorbolester in a subset of cell lines. Prostaglandin E(2) (PGE(2)) production could be inhibited by the selective COX-2 inhibitor NS-398. In malignant ascites of ovarian carcinomas significantly increased levels of PGE(2) were found compared to other carcinomas or nonmalignant ascites (P = 0.03). We investigated expression of COX-2 by immunohistochemistry in 117 ovarian surface epithelial tumors. Expression of COX-2 was detected in 42% of 86 ovarian carcinomas and in 37% of 19 low malignant potential tumors, but not in 12 cystadenomas or 2 normal ovaries. Expression of COX-1 was detected by immunohistochemistry in 75% of 75   invasive   ovarian carcinomas and in 75% of 16 low malignant potential tumors, whereas 2 samples from normal ovaries and 8 cystadenomas were positive for COX-1. In univariate survival analysis of   invasive   carcinomas, expression of COX-2 was associated with a significantly reduced median survival time (log rank test, P = 0.04). For patients younger than 60 years of age, this association was even more significant (P < 0.004). In contrast, expression of COX-1 was no prognostic parameter (P = 0.89). There was no significant correlation between COX-2 or COX-1 expression and other clinicopathological markers. In multivariate analysis expression of COX-2 was an independent prognostic factor for poor survival (relative risk, 2.74; 95% CI, 1.38 to 5.47). Our data indicate that COX-2 expression is an independent prognostic factor in ovarian carcinoma. Based on the results of this study, it would be interesting to investigate whether ovarian carcinoma patients with tumors positive for COX-2 would benefit from treatment with selective COX-2 inhibitors. 
 [Expressions and clinical significances of c-met, c-erbB-2, COX-2, and IL-6 in the biliary tract cancers]. c-met, c-erbB-2, interleukin (IL)-6, and cyclooxygenase (COX)-2 expressions are considered to be implicated in the carcinogenesis and progression of cholangiocarcinoma, but the molecular pathogenesis of cholangiocarcinoma is still poorly understood. We aimed to analyze the expressions of each marker and their relationships with clinicopathologic factors.One hundred and fourteen tissue samples were obtained from surgically resected specimens from patients with biliary tract cancer. The expressions of c-met, c-erbB-2, COX-2, and IL-6 were examined by immunohistochemically. The expression of each marker and correlations between these markers and clinicopathologic factors were analyzed.The expression rates of each maker were as follows: c-met 34/112 (30.4%), c-erbB-2 5/112 (4.5%), COX-2 53/113 (46.9%), and IL-6 68/113 (60.2%), respectively. c-met expression was more frequently observed in cases with invasion through the adjacent connective tissues (p=0.0263). IL-6 overexpression was more frequently observed in cases with absent lymph node metastasis (p=0.0325). Either c-erbB-2 expression or COX-2 expression was significantly associated with lymph node metastasis (p=0.0442).The expression of c-met was closely related to the   invasive  ness of cholangiocarcinoma. Co-expression of c-met, COX-2 and, IL-6 showed a significant correlation with   invasive  ness and lymph node metastasis and these could be useful marker to guide clinical outcome in patients with cholangiocarcinoma. 
 Pooled analysis of the association of PTGS2 rs5275 polymorphism and NSAID use with   invasive   ovarian carcinoma risk. Inflammation is postulated to play an important role in ovarian carcinogenesis. Prostaglandin endoperoxide synthase 2 (PTGS2) is responsible for the conversion of arachidonic acid to prostaglandins in response to inflammation. In a pooled analysis of two population-based studies, the Hawaii Ovarian Cancer Case-Control Study and the New England Case-Control Study, including 1,025 women with   invasive   ovarian carcinoma and 1,687 cancer-free controls, the association of ovarian cancer risk with the PTGS2 rs5275 polymorphism and the use of nonsteroidal antiinflammatory drugs (NSAIDs) were examined. Odds ratios (ORs) and 95% confidence intervals (CIs) were estimated using unconditional logistic regression. In the pooled analysis, the CC genotype was associated with a reduced risk of nonserous ovarian carcinoma (OR = 0.66; CI: 0.44-0.98). In addition, the lowest risk was observed among carriers of the CC genotype who were users of only nonaspirin NSAIDs (OR = 0.43; CI:0.20-0.93) in all women combined. The association of PTGS2 rs5275 with nonserous ovarian carcinoma and possible effect modification by NSAID use needs further validation, preferably in prospective studies. 
 Cyclooxygenase-2 expression in bladder cancer and patient prognosis: results from a large clinical cohort and meta-analysis. Aberrant overexpression of cyclooxygenase-2 (COX2) is observed in urothelial carcinoma of the bladder (UCB). Studies evaluating COX2 as a prognostic marker in UCB report contradictory results. We determined the prognostic potential of COX2 expression in UCB and quantitatively summarize the results with those of the literature through a meta-analysis. Newly diagnosed UCB patients recruited between 1998-2001 in 18 Spanish hospitals were prospectively included in the study and followed-up (median, 70.7 months). Diagnostic slides were reviewed and uniformly classified by expert pathologists. Clinical data was retrieved from hospital charts. Tissue microarrays containing non-muscle   invasive   (n=557) and muscle   invasive   (n=216) tumours were analyzed by immunohistochemistry using quantitative image analysis. Expression was evaluated in Cox regression models to assess the risk of recurrence, progression and disease-specific mortality. Meta-hazard ratios were estimated using our results and those from 11 additional evaluable studies. COX2 expression was observed in 38% (211/557) of non-muscle   invasive   and 63% (137/216) of muscle   invasive   tumors. Expression was associated with advanced pathological stage and grade (p<0.0001). In the univariable analyses, COX2 expression - as a categorical variable - was not associated with any of the outcomes analyzed. As a continuous variable, a weak association with recurrence in non-muscle   invasive   tumors was observed (p-value=0.048). In the multivariable analyses, COX2 expression did not independently predict any of the considered outcomes. The meta-analysis confirmed these results. We did not find evidence that COX2 expression is an independent prognostic marker of recurrence, progression or survival in patients with UCB. 
 Clinicopathological correlations of cyclooxygenase-2, MDM2, and p53 expressions in surgically resectable pancreatic   invasive   ductal adenocarcinoma. Cyclooxygenase-2 (COX-2) and p53 represent molecules linked to oncogenesis of pancreatic cancer, and there is also a known regulatory loop between mouse double minute 2 (MDM2) and p53. The complex cross talks between p53 and COX-2 and scenarios explaining patterns of p53 and COX-2 expressions in precursor and cancer lesions have been recently reported.The expressions of COX-2, p53, and MDM2 were examined using immunohistochemistry in 85 resection specimens of pancreatic ductal adenocarcinoma.The positive tumor expression rates of COX-2, p53, and MDM2 were 69.4%, 60.0%, and 41.2%, respectively. Significant correlations between COX-2 and p53 expressions and between p53 and MDM2 expressions were revealed. In the Kaplan-Meier analysis, no statistically significant correlations were found among the levels of COX-2, p53, and MDM2 expressions and survival rates. In the multivariate Cox proportional hazards regression model, grade and nodal status showed to be a valuable predictor of a worse overall survival.The reported findings confirmed the relationship of p53, MDM2, and COX-2 with the biological process of pancreatic cancer. The expression of none of the examined proteins showed to be a valuable independent prognostic factor. On the contrary, grade and nodal status showed to be a valuable predictor of a worse survival. 
 Expression of integrin α3β1 and cyclooxygenase-2 (COX2) are positively correlated in human breast cancer. Expression of integrin α3β1 is associated with tumor progression, metastasis, and poor prognosis in several cancers, including breast cancer. Moreover, preclinical studies have revealed important pro-tumorigenic and pro-metastatic functions for this integrin, including tumor growth, survival, invasion, and paracrine induction of angiogenesis. Our previously published work in a preclinical breast cancer model showed that integrin α3β1 promotes expression of cyclooxygenase-2 (COX2/PTGS2), a known driver of breast cancer progression. However, the clinical significance of this regulation was unknown. The objective of the current study was to assess the clinical relevance of the relationship between integrin α3β1 and COX2 by testing for their correlated expression among various forms of human breast cancer.Immunohistochemistry was performed to assess co-expression of α3 and COX2 in specimens   of human   invasive ductal carcinoma (IDC), either on a commercial tissue microarray (n = 59 samples) or obtained from Albany Medical Center archives (n = 68 samples). Immunostaining intensity for the integrin α3 subunit or COX2 was scored, and Spearman's rank correlation coefficient analysis was performed to assess their co-expression across and within different tumor subtypes or clinicopathologic criteria.Although expression of integrin α3 or COX2 varied among clinical IDC samples, a statistically significant, positive correlation was detected between α3 and COX2 in both tissue microarrays (r(s) = 0.49, p < 0.001, n = 59) and archived samples (r(s) = 0.59, p < 0.0001, n = 68). In both sample sets, this correlation was independent of hormone receptor status, histological grade, or disease stage.COX2 and α3 are correlated in IDC independently of hormone receptor status or other clinicopathologic features, supporting the hypothesis that integrin α3β1 is a determinant of COX2 expression in human breast cancer. These results support the clinical relevance of α3β1-dependent COX2 gene expression that we reported previously in breast cancer cells. The findings also suggest that COX2-positive breast carcinomas of various subtypes might be vulnerable to therapeutic strategies that target α3β1, and that α3 expression might serve as an independent prognostic biomarker. 
 A study on expression pattern of cyclooxygenase-2 in carcinoma of cervix. The purpose of the present study was to determine the differential expression pattern of cyclooxygenase-2 (COX-2) in patients of carcinoma of uterine cervix and its correlation with tumor differentiation and lymphovascular invasion.Seventy (70) cases of cervical carcinoma were included (20 in-situ, 42   invasive   squamous cell, and 8 cases of adenocarcinoma). Formalin-fixed paraffin-embedded tissue sections were stained by Hematoxylin and Eosin. Immunohistochemistry for COX-2 were performed on these blocks.A higher expression of COX-2 was seen in   invasive   squamous cell carcinoma than in in-situ carcinoma (P = 0.002). Five out of eight cases of adenocarcinoma showed strong positivity for COX-2 antibody. Among the histopathological correlates, tumor differentiation did not show a positive correlation (P = 0.717), while lymphovascular invasion was associated with positive staining in majority of the cases (P = 0.001).Expression of COX-2 is more in cases of   invasive   than in in-situ carcinoma. Adenocarcinomas showed a strong expression of COX-2. A positive association of COX-2 expression and the presence of lymphovascular emboli were found in the present study. COX-2 inhibitors need to be studied as a therapeutic adjunct for the treatment of carcinoma cervix. 
 Expression of cyclooxygenase-2 in colorectal adenocarcinoma is associated with p53 accumulation and hdm2 overexpression. Elevated cyclooxygenase-2 (COX-2) expression has been observed in various types of cancer. Induction of COX-2 expression has been reported to increase   invasive  ness and angiogenesis of tumours. While COX-2 overexpression has been repeatedly proven to promote tumor growth, little is known about what initiates its induction. There has been evidence to suggest that COX-2 expression is normally suppressed by wild-type p53 but not mutant p53, suggesting that loss of p53 function may result in the induction of COX-2 expression. Loss of p53 function is not only caused by gene mutation, but also through the overexpression of its negative regulator, so called human double minute 2 (hdm2). The aim of this study was to examine the correlation between COX-2 overexpression, p53 accumulation and HDM2 overexpression, as indications of p53 anomalies, and their relationship to clinicopathologic features of colorectal adenocarcinoma. Tumor tissues and the adjacent normal mucosa were obtained from 73 colorectal cancer patients who underwent curative resection at Maharaj Nakorn Chiang Mai Hospital. Protein levels of COX-2, p53 and HDM2 were determined by Western blot analysis. No normal colorectal tissues possessed detectable levels of COX-2, p53 or HDM2. In contrast, 38.3% (28 cases), 54.8% (40 cases) and 8.2% (6 cases) of tumour tissues were found to express COX-2, p53 and HDM2, respectively. Interestingly, there was a significantly positive relationship between COX-2 overexpression and p53 accumulation and/or HDM2 overexpression (P=0.007). Higher COX-2 overexpression was observed in p53-accumulated or HDM2 overexpressed-tumours (22/43 cases, 51.1%) in comparison to tumours with no evidence of p53 and HDM2 alterations (6/30 cases, 20%). The results obtained from this study indicate that overexpression of COX-2 is frequently associated with p53 protein accumulation and HDM2 overexpression, therefore the COX-2 overexpression observed in colorectal cancer cells may be partly due to the dysfunction of p53. Although mutation of p53 has been previously reported to be associated with COX-2 induction, to our knowledge, this is the first study to show the relationship between HDM2 overexpression and COX-2 overexpression. 
 Expression of COX-2 and VEGF-C in cholangiocarcinomas at different clinical and pathological stages. The aim of this study was to investigate the expression and clinical significance of cyclooxygenase 2 (COX-2) and vascular en-dothelial growth factor C (VEGF-C) in cholangiocarcinomas at differ-ent clinical and pathological stages. Eighty cholangiocarcinoma sam-ples of patients treated with surgery between January 2012 and January 2014 were collected. Immunohistochemistry was used to detect COX-2 and VEGF-C expression at different clinical and pathological stages. ELISA, real-time PCR,   invasive   chambers, and MTT assay were ap-plied in cultured cholangiocarcinoma cells treated with a COX-2 inhib-itor. Expression of COX-2 and VEGF-C correlated positively with the clinical TNM stage but did not correlate with the differentiation status. Inhibition of COX-2 activity reduced VEGF-C mRNA expression and secretion in cholangiocarcinoma cells and decreased their migration but not proliferation. Because of its ability to inhibit invasion, COX-2 could be a new target for treatment of cholangiocarcinoma. 
 Immunohistochemical analysis and in situ hybridization of cyclooxygenase-2 expression in intraductal papillary-mucinous tumors of the pancreas. The objective of this study was to investigate COX-2 expression in intraductal papillary-mucinous tumor of the pancreas (IPMT) using immunohistochemical staining (IH) and in situ hybridization (ISH).Immunohistochemical staining of COX-2 was performed using samples from 42 patients with IPMT (hyperplasia, 10; adenoma, 13; non  invasive   adenocarcinoma, 13;   invasive   adenocarcinoma, 6) and from 10 patients with ductal pancreatic adenocarcinoma, 10 with chronic pancreatitis, and 6 normal pancreatic tissues as controls. Also, COX-2 was determined in five patients with IPMT non  invasive   adenocarcinoma, in whom all histologic types, hyperplasia, adenoma, and adenocarcinoma were observed in the same excised specimens. Furthermore, IH of proliferating cell nuclear antigen (PCNA) was performed, and the labeling index (LI) was calculated to investigate the correlation with COX-2. To confirm COX-2 mRNA, the authors performed ISH in 20 IPMT patients.COX-2 was positive in 0%, 0%, and 10% of pancreatic duct epithelial cells from normal pancreatic tissue, chronic pancreatitis, and IPMT hyperplasia, respectively. Whereas it was positive in 69%, 77%, 67%, and 80% of IPMT adenoma, IPMT non  invasive   adenocarcinoma, IPMT   invasive   adenocarcinoma, and ductal pancreatic adenocarcinoma, respectively, showing significant differences between IPMT hyperplasia and IPMT adenoma or IPMT adenocarcinoma (non  invasive   and   invasive   adenocarcinoma). In the same patient, COX-2 was negative in the hyperplasia region but positive in adenoma and adenocarcinoma regions, showing results reflecting the progression of the disease. In the COX-2 negative group, PCNA-LI was 19.2 +/- 17.9%, and 33.5 +/- 15.7% in the positive group, a significant difference. On ISH, COX-2 mRNA was expressed in three of four and seven of eight COX-2 positive patients with IPMT adenoma and adenocarcinoma, respectively.COX-2 was highly expressed in adenoma and adenocarcinoma in IPMT, showing a relation to the histologic grade of IPMT. 
 Association of cyclooxygenase-2 and matrix metalloproteinase-2 expression in human breast cancer. Expression of cyclooxygenase-2 (COX-2) and matrix metalloproteinase-2 (MMP-2) associates with reduced survival in human breast cancer. COX-2 may be directly involved with mammary carcinogenesis, since expression of COX-2 is sufficient for formation of breast tumors in transgenic mice, and COX-2 selective inhibitors can suppress tumorigenesis in rodent models of breast cancer. MMP-2 is an extracellular matrix degrading proteolytic enzyme that bas been linked to invasion and metastasis. A direct link between COX-2 and MMP-2 may exist, since inhibition of COX-2 activity can result in reduction of MMP-2 expression and activity. In this study we analyzed protein expression of COX-2 and MMP-2 in tissue array specimens of 278   invasive   breast cancers by immunohistochemistry. Immunopositivity of these two markers was correlated with each other and with various clinicopathological parameters including survival. We found high COX-2 expression in 30% and high MMP-2 expression in 83% of the breast cancer specimens, and there was a positive association between the expression of these two factors (p = 0.003). It was especially evident that whenever COX-2 expression was high, MMP-2 expression was almost invariably high (95%). Furthermore, high expression of either COX-2 or MMP-2 associated with decreased disease specific survival when compared with the COX-2 or MPP-2 low group (p = 0.026 and p = 0.021, respectively). Taken together, our results indicate that expression of COX-2 protein is associated with expression of MMP-2 protein in human breast cancer and that both COX-2 and MMP-2 are markers of poor prognosis in breast cancer. 
  GeneRIFs  
 Using Fourier transform traction microscopy, we measured the force-generating capacity of human breast cancer cells occupying a spectrum of   invasive  ness as well as basal and inducible COX-2 expression 
 Cyclooxygenase-2 expression in   invasive   breast carcinomas of no special type and correlation with pathological profiles suggest a role in tumorigenesis rather than cancer progression. 
 The degree of expression of CXCR-4 and COX-2 is related to   invasive  ness in both basal cell carcinomas and well-differentiated squamous cell carcinomas of the skin. 
 High expression of COX-2 and low expression of KAI-1/CD82 are associated with increased tumor   invasive  ness in papillary thyroid carcinoma. 
 COX2 and alpha3 are correlated in   invasive   ductal carcinoma independently of hormone receptor status or other clinicopathologic features, supporting the hypothesis that integrin alpha3beta1 is a determinant of COX2 expression in human breast cancer. 
 The study pointed out the direction for further research to find indicators for tumor   invasive  ness and the possibility of practical use of COX-2 as important methods for determining changes in laryngeal cancer 
 Analysis of 70 cases of cervical carcinoma showed higher expression of COX-2 in   invasive   squamous cell carcinoma than in in-situ carcinoma. 
 Forty-nine Grade I, 33 Grade II, and 6 Grade III meningiomas were observed. VEGF and Ki-67 expression was correlated with higher tumor grade. A correlation was observed between COX-2 expression and   invasive  ness to the brain or adjacent soft tissue. 
 Activation of stromal COX-2 signalling by TNFalpha played a major role in promoting proliferation and   invasive  ness of colon cancer epithelial cells. 
 Lower cyclooxygenase-2 expression is associated with recurrence of solitary non-muscle   invasive   bladder carcinoma. 
 COX-2 expression in   invasive   breast carcinoma cells was positively associated with oestrogen receptor and/or progesterone receptor positivity (p<0.001). Triple-negative tumours showed no/low COX-2 expression more frequently than other tumour types. 
 COX-2 overexpression correlates with HER2 overexpression in   invasive   breast carcinomas and may provide a clinically useful biomarker for estimating tumor aggressiveness. 
 On the basis of the results obtained the use of exhaled breath condensate COX-2 and survivin levels could be suggested as two potential markers within an early non-  invasive   screening of populations of smokers at risk of lung cancer. 
 In early stage oral squamous cell carcinomas, most tumours did not express COX-2 at the   invasive   front. 
 COX-2 gene methylation was more common in subepithelial lymphocytes than in adjacent epithelial or stromal cells in both grades of dysplasia and in foci of   invasive   cancer 
 The COX-2 expression in PC-3 High   Invasive   cells was approximately 3-fold higher than in PC-3 Low   Invasive   cells while the COX-1 expression was similar in both cell sublines. 
 co-stimulatory mechanisms may exist that increase the expression of cox-2 and laminin-5 at the   invasive   front of lung adenocarcinomas and that EGFR signaling could be one of the mechanisms 
 data collectively imply COX-2 may play an important role during premalignant hyperproliferation, as well as the later stages of   invasive   carcinoma and metastasis in various human epithelial cancers 
 Inhibition of cyclooxygenase-2 suppresses   invasive  ness of oral squamous cell carcinoma cell lines via down-regulation of matrix metalloproteinase-2 and CD44 
 The correlation between COX-2 and   invasive   depth, MVD and   invasive   depth, COX-2 and MVD were analyzed. COX-2 expression was significantly related to the   invasive   depth of tumors. 
 COX-2 may be involved in carcinogenesis of the breast and may be an independent prognostic indicator in patients with   invasive   ductal carcinoma. 
 Correlation of COX-2 and Ep-CAM overexpression in human   invasive   breast cancer and its impact on survival. 
 COX-2 and VEGF are expressed in head and neck squamous cell carcinoma and may have a role in tumor   invasive  ness and angiogenesis 
   invasive   human breast cancer cells that over-express COX-2 develop vascular channels, non-  invasive   cell lines that express low levels of COX-2 did not develop such channels.Celecoxib or COX-2 siRNA suppressed vascular channel formation. 
 increased expression associated with development of lung cancer and possibly acquisition of   invasive  /metastatis phenotype 
 COX-2 overexpression in human breast cancer cells enhances cell motility and   invasive  ness thus suggesting a mechanism of COX-2 mediated metastasis 
  [Mm]etastasize  PubMed  
 Upregulation of COX-2 in the lung cancer promotes overexpression of multidrug resistance protein 4 (MRP4) via PGE2-dependent pathway. It is apparent that lung cancer is associated with inflammation, with accompanying hallmark elevations of cyclooxygenase 2 (COX-2) and prostaglandin E2 (PGE2) levels. However, the effects of these changes on MRP efflux transporters have not been thoroughly investigated before. Here, we report that upregulation of COX-2 can induce overexpression of MRP4 in both A549 non-small-cell lung cancer cell lines and mouse lung cancer models. In A549 cells, phorbol 12-myristate 13-acetate (PMA) treatment induced upregulation of COX-2 and MRP4 together, but not other MRP transporters. Transient overexpression of human COX-2 cDNA also specifically increased COX-2 and MRP4. Moreover, COX inhibitor treatment and COX-2-specific siRNA significantly inhibited the upregulation of MRP4. Additionally, PMA-treatment increased extracellular PGE2 levels, likely due to increased MRP4 function. Likewise, COX-2-specific siRNA reduced extracellular PGE2 levels. Furthermore, COX-2 upregulation resulted in an increase in mPGES-1, an enzyme responsible for PGE2 production. Finally,   metastasize  d lung cancer model mice exhibited increased expression levels of COX-2 and MRP4, as well as mPGES-1. In conclusion, the present study suggests that overexpression of MRP4 in lung cancer may be attributable to COX-2 upregulation via a PGE2-dependent pathway. 
 Upregulation of COX-2 in the lung cancer promotes overexpression of multidrug resistance protein 4 (MRP4) via PGE2-dependent pathway. It is apparent that lung cancer is associated with inflammation, with accompanying hallmark elevations of cyclooxygenase 2 (COX-2) and prostaglandin E2 (PGE2) levels. However, the effects of these changes on MRP efflux transporters have not been thoroughly investigated before. Here, we report that upregulation of COX-2 can induce overexpression of MRP4 in both A549 non-small-cell lung cancer cell lines and mouse lung cancer models. In A549 cells, phorbol 12-myristate 13-acetate (PMA) treatment induced upregulation of COX-2 and MRP4 together, but not other MRP transporters. Transient overexpression of human COX-2 cDNA also specifically increased COX-2 and MRP4. Moreover, COX inhibitor treatment and COX-2-specific siRNA significantly inhibited the upregulation of MRP4. Additionally, PMA-treatment increased extracellular PGE2 levels, likely due to increased MRP4 function. Likewise, COX-2-specific siRNA reduced extracellular PGE2 levels. Furthermore, COX-2 upregulation resulted in an increase in mPGES-1, an enzyme responsible for PGE2 production. Finally,   metastasize  d lung cancer model mice exhibited increased expression levels of COX-2 and MRP4, as well as mPGES-1. In conclusion, the present study suggests that overexpression of MRP4 in lung cancer may be attributable to COX-2 upregulation via a PGE2-dependent pathway. 
 Heparanase and COX-2 expression as predictors of lymph node metastasis in large, high-grade breast tumors. Heparanase (HPA) contributes to breast cancer metastasis by facilitating the breakdown of the basement membrane and extracellular matrix. High expression of HPA is thought to be associated with increased nodal involvement and poor survival in patients with breast cancer. Overexpression of cyclooxygenase-2 (COX-2) in breast cancer is associated with indicators of poor prognosis such as lymph node metastasis, poor differentiation, and large tumor size. The underlying mechanism by which HPA and COX-2 overexpression increases the metastatic potential of breast cancer is not fully-understood. To enhance our understanding over these mechanisms, we aimed to investigate the relationship between the size of the tumor and HPA expression, tumor grade as well as lymph node status in patients with breast cancer.Immunohistochemical analysis of HPA and COX-2 expression was performed on 246 breast tumor samples. The expression of HPA was correlated with COX-2 expression, tumor grade, lymph node status, oestrogen receptor status.The overexpression of HPA and COX-2 was associated with increased likelihood of lymph node positivity in large, high-grade tumors. High-grade tumors with size greater than 20 mm, that overexpressed HPA, were 4-times more likely to be associated with lymph node involvement (OR 4.71, CI 1.21-18.25). Whereas, tumors greater than 20 mm in size were 5-times more likely to   metastasize   to the regional lymph nodes, if associated with overexpression of COX-2 (OR 5.5, CI 1.2-24.8).Expression of HPA appears to be a key mechanism by which large, high-grade breast tumors   metastasize   to regional lymph nodes, while COX-2 overexpression may be an independent predictor of lymph node positivity. 
 Cyclooxygenase-2 expression in primary breast cancers predicts dissemination of cancer cells to the bone marrow. Cyclooxygenase-2 (COX2) plays a role in breast cancer progression at various stages starting from pre-malignant phenotype to clinical metastasis. Breast cancer   metastasize  s commonly to the bone and preclinical studies suggest an involvement of COX2 in this process. Detection of disseminated tumor cells in the bone marrow of patients at the time of surgery correlates with the subsequent development of clinical bone metastasis. Therefore, to investigate whether COX2 is important for breast cancer metastasis in humans, we analyzed COX2 protein expression by immunostaining of primary tumors from 112 operable stages I, II, or III patients and determined its correlation with bone marrow micrometastasis (BMM).We detected COX2 protein in primary tumors by immunostaining with a monoclonal antibody, and tumor cells present in the bone marrow by immunostaining for epithelial cytokeratins and by morphological criteria.COX2 expression in primary breast cancer correlated with BMM in a highly statistically significant manner (P = 0.006). Our statistical analyses of correlations of the COX2 positivity in primary tumor with other clinically relevant indicators revealed that COX2 positivity correlates with high nuclear grade (P = 0.0004). Furthermore, we were able to detect COX2 protein in BMM by immunostaining.These studies indicate that COX2 produced in primary breast cancer cells may be vital to the initial development of BMM that may subsequently lead to osteolytic bone metastases in patients with breast cancer, and that COX2 inhibitors may be useful in halting this process. 
 COX-2 drives metastatic breast cells from brain lesions into the cerebrospinal fluid and systemic circulation. Breast cancer is among the most common malignancies that   metastasize   to the brain, with 15% to 20% of patients with metastatic breast cancer eventually developing brain metastases. We previously reported a method to enumerate tumor cells in the cerebrospinal fluid (CSF) of patients with breast cancer with central nervous system (CNS) metastases, a setting that lacks sufficiently informative biomarkers. Here, we show that breast cancer cells can spontaneously disseminate into the CSF from brain lesions in mice in a COX-2-dependent manner and can escape from the CNS to systemic circulation. Enumeration of tumor cells in the peripheral blood (circulating tumor cells, CTC) and CSF (cerebrospinal fluid tumor cells, CSFTC) of nine breast cancer patients with brain metastases revealed dynamic changes in tumor cell burden in both the peripheral blood and CSF compartments that correlated with clinical disease progression. Interestingly, four of the enrolled patients exhibited rapid intercompartmental transitioning of the disease reflected in the CTC and CSFTC counts that preceded corresponding evidence by clinical imaging or neurologic symptoms. Two of these patients had systemic disease recurrence involving the primary malignant site. Intercompartmental cycling of tumor cells may represent an important mechanism for disease persistence and recurrence that may involve tumor self-seeding. Our findings demonstrate the involvement of COX-2 in the genesis of CSFTCs and suggest that COX-2 inhibitors should be investigated in patients with breast cancer with brain metastases for their ability to reduce CSFTC counts and prevent systemic recurrence. 
 Expression of Cox-2 and Bcl-2 in Paget's disease of the breast. Paget's disease (PD) is a rare form of intraepithelial adenocarcinoma that involves breast and extramammarian tissues. It is often associated with ductal carcinoma in situ and/or invasive ductal cancer. Molecular pathways that play a role in development of Paget's disease are stil unclear. Expression patterns of Cox-2 and bcl-2 were therefore assessed.Patients with a histopathological diagnosis of Paget's disease were included in this study. Patient files were analysed retrospectively.Invasive cancer was diagnosed in 35 (76.1%) of the patients, 7 (15.2%) had ductal carcinoma in situ and 4 (8.7%) patients had no associated neoplasm. Twenty four (52.2%) patients showed COX-2 expression in Paget cells whereas no expression was seen in 22 (47.8%) patients. No relation was found between COX-2 expression and the lesion underlying Paget's disease (p=0.518). Bcl-2 expression in Paget cells was found positive in 12 (26.1%) and negative in 27 (58,7%) cases. There was no relation between Bcl-2 expression and the lesion accompanying Paget's disease (p=0.412). No relation was observed between COX-2 expression and Bcl-2 expression (p=0.389).In breast cancer, COX-2 expression is associated with poor prognostic factors. As COX-2 expression increases the tendency to   metastasize   also increases. In our study we found a significantly high COX-2 expression in Paget's disease of the breast. We suggest that COX-2 expression and inflammatory processes may play a role in pathogenesis of the Paget's disease of the breast. 
 Cyclooxygenase-2 promotes tumor lymphangiogenesis and lymph node metastasis in oral squamous cell carcinoma. Oral squamous cell carcinoma (OSCC) is the sixth most common cancer and frequently   metastasize  s to the cervical lymph nodes, leading to poor survival of patients with OSCC. However, the mechanism of lymph node metastasis is not fully understood. To clarify the molecular mechanism underlying OSCC metastasis to regional lymph nodes, the highly metastatic fluorescent labeled OSCC cell line SAS-LM3 was successfully established allowing us to monitor the progression of lymph node metastases in a non-invasive manner. SAS-LM3 tumors showed increased lymphangiogenesis and elevated expression of VEGF-C, a potent stimulator of lymphangiogenesis, compared to parental SAS tumors. SAS-LM3 showed high expression of cyclooxygenase-2 (COX-2) compared to parental SAS cells and immunohistochemical analysis demonstrated intense COX-2 expression at the primary site. Inactivation of COX-2 by knockdown or the COX-2 inhibitor NS-398 decreased VEGF-C expression. Administration of COX-2 inhibitor NS-398 in SAS-LM3 tumor-bearing mice suppressed tumor lymphangiogenesis and lymphatic metastases. Collectively, our results indicate that COX-2 promotes tumor lymphangiogenesis and lymph node metastasis of OSCC. COX-2 ablation holds promise as a potential therapeutic approach for lymph node metastasis in OSCC. 
  [Mm]etastasis  PubMed  
 The hepatitis B virus X protein promotes tumor cell invasion by inducing membrane-type matrix metalloproteinase-1 and cyclooxygenase-2 expression. Hepatocellular carcinoma is strongly associated with chronic infection by the hepatitis B virus (HBV) and has poor prognosis due to intrahepatic   metastasis  . HBx is often the only HBV protein detected in hepatic tumor cells; however, its contribution to tumor invasion and   metastasis   has not been established so far. In this work, we show that HBx enhances tumor cell invasion, both in vivo and in vitro. The increased invasive capacity induced by HBx is mediated by an upregulation of membrane-type 1 matrix metalloproteinase (MT1-MMP) expression, which in turn activates matrix metalloproteinase-2. Induction of both MT1-MMP expression and cell invasion by HBx is dependent on cyclooxygenase-2 (COX-2) activity. In addition, HBx upregulates the expression of COX-2, which is mediated by the transcriptional activation of the COX-2 gene promoter in a nuclear factor of activated T cell-dependent (NF-AT-dependent) manner. These results demonstrate the ability of HBx to promote tumor cell invasion by a mechanism involving the upregulation of MT1-MMP and COX-2 and provide new insights into the mechanism of action of this viral protein and its involvement in tumor   metastasis   and recurrence of hepatocellular carcinoma. 
 EGF-R is expressed and AP-1 and NF-kappaB are activated in stromal myofibroblasts surrounding colon adenocarcinomas paralleling expression of COX-2 and VEGF. COX-2 and VEGF are important triggers of colon cancer growth,   metastasis   and angiogenesis. Cox-2 promoter contains transcriptional regulatory elements for AP-1 and NF-kappaB transcription factors whilst vegf is a known AP-1 downstream target gene. We investigated whether stromal myofibroblasts surrounding colon adenocarcinomas express COX-2 and VEGF and whether activation of AP-1 and NF-kappaB, as well as expression of EGF-R parallel expression of COX-2 and VEGF in these cells.Immunohistochemical methodology was performed on archival sections from 40 patients with colon adenocarcinomas. We evaluated c-FOS, p-c-JUN (phosphorylated c-JUN), p-IkappaB-alpha (phosphorylated IkappaB-alpha), EGF-R, COX-2, NF-kappaB and VEGF expression in stromal myofibroblasts surrounding colon adenocarcinomas. Double immunostaining with a-smooth muscle actin and each antibody was done to verify the expression of these molecules in stromal myofibroblasts.VEGF, p-IkappaB-alpha, NF-kappaB, c-FOS, p-c-JUN, EGF-R and COX-2 were expressed in stromal myofibroblasts surrounding colon adenocarcinomas in the majority of cases. EGF-R, p-IkappaB-alpha, NF-kappaB, c-FOS and p-c-JUN correlated positively with COX-2 and VEGF expression.Stromal myofibroblasts surrounding colon adenocarcinomas are an important source of VEGF and COX-2 production, while AP-1 and NF-kappaB transcription factors are activated and EGF-R is expressed in these cells and associated with COX-2 and VEGF production. 
 Clinical relevance of cyclooxygenase-2 and matrix metalloproteinases (MMP-2 and MT1-MMP) in human breast cancer tissue. Breast cancer (BC) is the most common neoplasm among women in most developed countries, including Egypt. Elevated levels of certain proteins in human BC are associated with unfavorable prognosis and progressive stages of the disease. The aim of our study was to evaluate the protein expression profile and prognostic significance of cyclooxygenase-2 (COX-2), matrix metalloproteinase-2 (MMP-2), MMP-9 and membrane type 1-MMP (MT1-MMP) and their interaction in operable BC patients. The protein expression of COX-2, MMP-2 and MT1-MMP were evaluated by western blot technique, whereas enzymatic activity of MMP-2 and MMP-9 was determined by zymography in 47 breast cancer patients as well as normal adjacent tissues. Also, the correlation between these proteins and age, tumor size, LN stage, TNM stage, estrogen receptor, progesterone receptor, disease-free survival, and overall survival (OS) has been investigated. As compared to adjacent normal tissues, COX-2, MMP-2 and MT1-MMP were over-expressed in 43, 64, and 60 % of tumor tissues, respectively. In the same pattern, the activity of MMP-2 (62 %) and MMP-9 (45 %) was elevated in BC tissues. Multivariate analysis showed a positive correlation between the protein expression of COX-2, MMP-2, and MT1-MMP and the activity of MMP-2 and MMP-9 in BC patients. However, the enzymatic activity showed no correlation with clinicopathological features. This study confirms the preclinical evidence that COX-2 increased the expression of MT1-MMP, which in turn activates MMP-2. The lack of correlation with clinicopathological features, OS or disease-free survival ascertains the complexity of tumor progression and   metastasis   with many pro- and counter regulatory factors. 
 Lysophosphatidic acid upregulates vascular endothelial growth factor-C and tube formation in human endothelial cells through LPA(1/3), COX-2, and NF-kappaB activation- and EGFR transactivation-dependent mechanisms. Lysophosphatidic acid (LPA) is a lipid bioactive mediator which binds to G-protein-coupled receptors and activates a variety of cellular functions. LPA modulates multiple behaviors in endothelial cells, including cell proliferation and migration, capillary-like tube formation in vitro, activation of proteases, interactions with leukocytes, and expressions of inflammation-related genes, thereby regulating vessel formation. LPA has been reported to modulate the angiogenesis process. However, the role of LPA in the lymphangiogenesis process has not been studied. In this study, we showed that LPA upregulated vascular endothelial growth factor-C (VEGF-C) mRNA expression in human umbilical vein endothelial cells (HUVECs) and subsequent endothelial cell tube formation in vitro and in vivo. These enhancement effects were LPA(1)- and LPA(3)-dependent and required cyclooxygenase-2 (COX-2), endothelial growth factor receptor (EGFR) transactivation and activation of nuclear factor kappaB (NF-kappaB). Moreover, LPA induced the protein expressions of the lymphatic markers, Prox-1, LYVE-1, and podoplanin, in HUVECs, and these enhancement effects were dependent on LPA(1) and LPA(3) activation and EGFR transactivation. Our results demonstrated that LPA might regulate VEGF-C and lymphatic marker expression in endothelial cells, which contributes to endothelial cell tube formation in vitro and in vivo, thus facilitating endothelial cell participation in the lymphangiogenesis process. This study clarifies the signaling mechanism of LPA-regulated VEGF-C expression and lymphatic marker expressions in endothelial cells, which suggest that LPA may be a suitable target for generating therapeutics against lymphangiogenesis and tumor   metastasis  . 
 Toll-like receptor 9 agonists up-regulates the expression of cyclooxygenase-2 via activation of NF-kappaB in prostate cancer cells. CpG-oligonucleotides (CpG-ODNs), mimicking bacterial DNA, have recently been shown to stimulate prostate cancer invasion in vitro via Toll-like receptor 9 (TLR9). Since cyclooxygenase 2 (COX-2), frequently overexpressed in multiple tumor types including prostate cancer, is a causal factor for tumor development, invasion and   metastasis  , an interesting question is raised whether TLR9 regulates COX-2 expression in prostate cancer cells. To address this question, herein we examined COX-2 expression in PC-3 cells stimulated with different doses and time courses of CpG-ODNs. The regulatory role of NF-kappaB in TLR9-mediated COX-2 expression was also investigated. CpG-ODN was found to up-regulate the expression of COX-2 in PC-3 cells in a dose- and time-dependent manner, but have little impact on COX-1 expression. Moreover, CpG-ODN also promoted nuclear translocation and activation of NF-kappaB, which appeared to be required for COX-2 induction by CpG-ODN. Overall, TLR9 up-regulates COX-2 expression in prostate cancer cells, at least partially through the activation of NF-kappaB, which may be implicated in tumor invasion and   metastasis  . 
 Glucose-regulated protein 78 mediates hormone-independent prostate cancer progression and   metastasis   through maspin and COX-2 expression. Glucose-regulated protein 78 (GRP78) plays an essential role in embryonic development and in the progression and therapeutic resistance of many cancers. However, little is known about the function of GRP78 in hormone-independent prostate cancer. Here, we found that the expression levels of GRP78 were higher in PC-3 cells than in DU-145 cells. When the expression of GRP78 was silenced using a GRP78-specific small interfering RNA in PC-3 cells, the growth rate and adhesive ability were reduced. Cell migration was dramatically decreased in GRP78-depleted cells. Dissection of the involved signal pathways revealed that maspin expression was upregulated after silencing GRP78 expression. The upregulation of maspin and downregulation of COX-2 may cause the decrease in cell proliferation and migration observed after silencing GRP78 expression. Silencing GRP78 expression may suppress the proliferation, adhesion, and migration of prostate cancer cells via maspin and COX-2 regulation. 
 Cyclooxygenase-2 enhances alpha2beta1 integrin expression and cell migration via EP1 dependent signaling pathway in human chondrosarcoma cells. Cyclooxygenase (COX)-2, the inducible isoform of prostaglandin (PG) synthase, has been implicated in tumor   metastasis  . Interaction of COX-2 with its specific EP receptors on the surface of cancer cells has been reported to induce cancer invasion. However, the effects of COX-2 on migration activity in human chondrosarcoma cells are mostly unknown. In this study, we examined whether COX-2 and EP interaction are involved in   metastasis   of human chondrosarcoma.We found that over-expression of COX-2 or exogenous PGE2 increased the migration of human chondrosarcoma cells. We also found that human chondrosarcoma tissues and chondrosarcoma cell lines had significant expression of the COX-2 which was higher than that in normal cartilage. By using pharmacological inhibitors or activators or genetic inhibition by the EP receptors, we discovered that the EP1 receptor but not other PGE receptors is involved in PGE2-mediated cell migration and alpha2beta1 integrin expression. Furthermore, we found that human chondrosarcoma tissues expressed a higher level of EP1 receptor than normal cartilage. PGE2-mediated migration and integrin up-regulation were attenuated by phospholipase C (PLC), protein kinase C (PKC) and c-Src inhibitor. Activation of the PLCbeta, PKCalpha, c-Src and NF-kappaB signaling pathway after PGE2 treatment was demonstrated, and PGE2-induced expression of integrin and migration activity were inhibited by the specific inhibitor, siRNA and mutants of PLC, PKC, c-Src and NF-kappaB cascades.Our results indicated that PGE2 enhances the migration of chondrosarcoma cells by increasing alpha2beta1 integrin expression through the EP1/PLC/PKCalpha/c-Src/NF-kappaB signal transduction pathway. 
 Peroxisome-proliferator-activated receptor alpha agonists inhibit cyclo-oxygenase 2 and vascular endothelial growth factor transcriptional activation in human colorectal carcinoma cells via inhibition of activator protein-1. Recent evidence indicates that PPAR (peroxisome-proliferator-activated receptor) alpha ligands possess anti-inflammatory and antitumoural properties owing to their inhibitory effects on the expression of genes that are involved in the inflammatory response. However, the precise molecular mechanisms underlying these effects are poorly understood. In the present study, we show that tumour promoter PMA-mediated induction of genes that are significantly associated with inflammation, tumour growth and   metastasis  , such as COX-2 (cyclo-oxygenase 2) and VEGF (vascular endothelial growth factor), is inhibited by PPARalpha ligands in the human colorectal carcinoma cell line SW620. PPARalpha activators LY-171883 and WY-14,643 were able to diminish transcriptional induction of COX-2 and VEGF by inhibiting AP-1 (activator protein-1)-mediated transcriptional activation induced by PMA or by c-Jun overexpression. The actions of these ligands on AP-1 activation and COX-2 and VEGF transcriptional induction were found to be dependent on PPARalpha expression. Our studies demonstrate the existence of a negative cross-talk between the PPARalpha- and AP-1-dependent signalling pathways in these cells. PPARalpha interfered with at least two steps within the pathway leading to AP-1 activation. First, PPARalpha activation impaired AP-1 binding to a consensus DNA sequence. Secondly, PPARalpha ligands inhibited c-Jun transactivating activity. Taken together, these findings provide new insight into the anti-inflammatory and anti-tumoural properties of PPARalpha activation, through the inhibition of the induction of AP-1-dependent genes that are involved in inflammation and tumour progression. 
 [Expression and clinical significance of COX-2, p-Stat3, and p-Stat5 in esophageal carcinoma]. Cyclooxygenase-2 (COX-2) and signal transducers and activators of transcription (STAT) are closely correlated to the genesis of tumors. This study was to investigate the expression and clinical significance of COX-2, p-Stat3 and p-Stat5 (the activated forms of Stat3 and Stat5) in various lesions of esophageal tissues, and to analyze their correlations to clinicopathologic features of esophageal squamous cell carcinoma (ESCC).The expression of COX-2, p-Stat3, and p-Stat5 in 59 specimens of ESCC, 24 specimens of squamous dysplasia, and 18 specimens of normal squamous epithelium was examined by SP immunohistochemistry. Their correlations to clinicopathologic features of ESCC were analyzed.The protein level of COX-2 was significantly higher in ESCC and squamous dysplasia than in normal squamous epithelium (2.10+/-1.77 and 1.85+/-1.24 vs. 0.83+/-0.46, P<0.05). The protein level of p-Stat3 was 0 in normal squamous epithelium, 0.76+/-0.59 in squamous dysplasia, and 2.83+/-1.27 in ESCC. The protein level of p-Stat5 was 1.98+/-0.78 in normal squamous epithelium, 3.92+/-0.41 in squamous dysplasia, and 5.02+/-0.34 in ESCC. There were significant differences among the 3 groups (P<0.05). In ESCC, COX-2 expression was correlated to lymph node   metastasis   and differentiation (P<0.05); p-Stat3 expression was correlated to tumor invasion depth (P<0.05); but p-Stat5 expression had no correlation to clinicopathologic features. COX-2 expression was positively correlated to both p-Stat3 expression and p-Stat5 expression in ESCC.The up-regulation of COX-2, p-Stat3, and p-Stat5 may be correlated to the carcinogenesis of ESCC. The activation of Stat3 is correlated to the aggressive behavior of ESCC. 
 Palbociclib inhibits epithelial-mesenchymal transition and   metastasis   in breast cancer via c-Jun/COX-2 signaling pathway. Palbociclib, a highly selective CDK4/6 inhibitor, has been shown to be a novel anti-tumor agent that suppresses breast cancer cell proliferation. However, its anti-  metastasis   activity remains controversial. In the present study, we evaluated whether palbociclib prevented breast cancer cell   metastasis   and revealed its regulatory mechanism. We found that palbociclib inhibited migration and invasion in the breast cancer cells MDA-MB-231 and T47D. The epithelial-mesenchymal transition (EMT) markers, vimentin and Snail, were down-regulated with palbociclib treatment. Moreover, we revealed that this inhibition was mediated by the c-Jun/COX-2 pathway. COX-2 was decreased after palbociclib treatment. The production of PGE2 was also reduced along with COX-2. Additionally, our data showed that c-Jun, a crucial transcriptional regulator of COX-2, was down-regulated by palbociclib. We found that palbociclib weakened the COX-2 promoter binding activity of c-Jun and prevented its translocation from the cytoplasm to cell nuclei. Bioluminescence imaging and tail intravenous injection were used to evaluate the anti-  metastasis   effect of palbociclib in vivo. The data demonstrated that palbociclib reduced breast cancer   metastasis   to the lung. These results therefore demonstrated that the anti-  metastasis   activity of palbociclib is mediated via the c-Jun/COX-2 signaling pathway by inhibiting EMT in breast cancer cells. 
 Expression of STAT5, COX-2 and PIAS3 in correlation with NSCLC histhopathological features. Signal transducers and activators of transcription (STATs), their inhibitors and cyclooxygenase-2 (COX-2) participate in transformations of many various types of cancers. The aim of the present study was to evaluate the relationship between STAT5A/B, COX-2, and PIAS3 mRNA expression and tumor staging,   metastasis   status, and histopathological subtype in 71 patients with confirmed non-small cell lung cancer (NSCLC) diagnosis. Total RNA was isolated from NSCLC tissue samples and the expression of the studied genes was assessed using TaqMan probes in real-time PCR assay. The expression levels of STAT5A, STAT5B, and COX-2 genes were increased in 69%, 79%, and 71% NSCLC samples respectively, while PIAS3 expression was decreased in the majority (69%) of the studied tissues. Statistically significant differences were observed between STAT5 isoforms (P = 0.0008), with higher expression of STAT5B. We found statistically significant positive correlation between STAT5B and COX-2 (rho = 0.045), and significant negative correlation between STAT5B and PIAS3 (rho = -0.049). The negative correlation between STAT5B and PIAS3 (rho = -0.43) was also observed in T2a+T2b tumor group. Additionally, STAT5B and COX-2 expression levels were significantly different between T1a+T1b and T2a+T2b tumors (P = 0.002 and P = 0.041, respectively), with higher expression of both genes in T2 tumor stage. PIAS3 expression was significantly lower in NSCC subtype as compared with SCC subtype (P = 0.017). Also, STAT5A and STAT5B immunoexpression was assessed, and the results indicated significantly higher protein levels in NSCLC patients as compared with controls (P = 0.048 and P = 0.034, respectively). High STAT5B immunoexpression was positively correlated with STAT5B gene expression in tumors (rho = 0.755). STAT5B protein level was also significantly higher in T2a+T2b tumors, reflecting high STAT5B gene expression in this group. There was no statistically significant association between mRNA and protein expression levels of the studied genes and patients' characteristics: age, gender, smoking. The obtained results highlight the importance of the genes STAT5B and COX-2 in lung cancer progression. 
 Palbociclib inhibits epithelial-mesenchymal transition and   metastasis   in breast cancer via c-Jun/COX-2 signaling pathway. Palbociclib, a highly selective CDK4/6 inhibitor, has been shown to be a novel anti-tumor agent that suppresses breast cancer cell proliferation. However, its anti-  metastasis   activity remains controversial. In the present study, we evaluated whether palbociclib prevented breast cancer cell   metastasis   and revealed its regulatory mechanism. We found that palbociclib inhibited migration and invasion in the breast cancer cells MDA-MB-231 and T47D. The epithelial-mesenchymal transition (EMT) markers, vimentin and Snail, were down-regulated with palbociclib treatment. Moreover, we revealed that this inhibition was mediated by the c-Jun/COX-2 pathway. COX-2 was decreased after palbociclib treatment. The production of PGE2 was also reduced along with COX-2. Additionally, our data showed that c-Jun, a crucial transcriptional regulator of COX-2, was down-regulated by palbociclib. We found that palbociclib weakened the COX-2 promoter binding activity of c-Jun and prevented its translocation from the cytoplasm to cell nuclei. Bioluminescence imaging and tail intravenous injection were used to evaluate the anti-  metastasis   effect of palbociclib in vivo. The data demonstrated that palbociclib reduced breast cancer   metastasis   to the lung. These results therefore demonstrated that the anti-  metastasis   activity of palbociclib is mediated via the c-Jun/COX-2 signaling pathway by inhibiting EMT in breast cancer cells. 
 Expression of STAT5, COX-2 and PIAS3 in correlation with NSCLC histhopathological features. Signal transducers and activators of transcription (STATs), their inhibitors and cyclooxygenase-2 (COX-2) participate in transformations of many various types of cancers. The aim of the present study was to evaluate the relationship between STAT5A/B, COX-2, and PIAS3 mRNA expression and tumor staging,   metastasis   status, and histopathological subtype in 71 patients with confirmed non-small cell lung cancer (NSCLC) diagnosis. Total RNA was isolated from NSCLC tissue samples and the expression of the studied genes was assessed using TaqMan probes in real-time PCR assay. The expression levels of STAT5A, STAT5B, and COX-2 genes were increased in 69%, 79%, and 71% NSCLC samples respectively, while PIAS3 expression was decreased in the majority (69%) of the studied tissues. Statistically significant differences were observed between STAT5 isoforms (P = 0.0008), with higher expression of STAT5B. We found statistically significant positive correlation between STAT5B and COX-2 (rho = 0.045), and significant negative correlation between STAT5B and PIAS3 (rho = -0.049). The negative correlation between STAT5B and PIAS3 (rho = -0.43) was also observed in T2a+T2b tumor group. Additionally, STAT5B and COX-2 expression levels were significantly different between T1a+T1b and T2a+T2b tumors (P = 0.002 and P = 0.041, respectively), with higher expression of both genes in T2 tumor stage. PIAS3 expression was significantly lower in NSCC subtype as compared with SCC subtype (P = 0.017). Also, STAT5A and STAT5B immunoexpression was assessed, and the results indicated significantly higher protein levels in NSCLC patients as compared with controls (P = 0.048 and P = 0.034, respectively). High STAT5B immunoexpression was positively correlated with STAT5B gene expression in tumors (rho = 0.755). STAT5B protein level was also significantly higher in T2a+T2b tumors, reflecting high STAT5B gene expression in this group. There was no statistically significant association between mRNA and protein expression levels of the studied genes and patients' characteristics: age, gender, smoking. The obtained results highlight the importance of the genes STAT5B and COX-2 in lung cancer progression. 
 Glucose-regulated protein 78 mediates hormone-independent prostate cancer progression and   metastasis   through maspin and COX-2 expression. Glucose-regulated protein 78 (GRP78) plays an essential role in embryonic development and in the progression and therapeutic resistance of many cancers. However, little is known about the function of GRP78 in hormone-independent prostate cancer. Here, we found that the expression levels of GRP78 were higher in PC-3 cells than in DU-145 cells. When the expression of GRP78 was silenced using a GRP78-specific small interfering RNA in PC-3 cells, the growth rate and adhesive ability were reduced. Cell migration was dramatically decreased in GRP78-depleted cells. Dissection of the involved signal pathways revealed that maspin expression was upregulated after silencing GRP78 expression. The upregulation of maspin and downregulation of COX-2 may cause the decrease in cell proliferation and migration observed after silencing GRP78 expression. Silencing GRP78 expression may suppress the proliferation, adhesion, and migration of prostate cancer cells via maspin and COX-2 regulation. 
 Clinical relevance of cyclooxygenase-2 and matrix metalloproteinases (MMP-2 and MT1-MMP) in human breast cancer tissue. Breast cancer (BC) is the most common neoplasm among women in most developed countries, including Egypt. Elevated levels of certain proteins in human BC are associated with unfavorable prognosis and progressive stages of the disease. The aim of our study was to evaluate the protein expression profile and prognostic significance of cyclooxygenase-2 (COX-2), matrix metalloproteinase-2 (MMP-2), MMP-9 and membrane type 1-MMP (MT1-MMP) and their interaction in operable BC patients. The protein expression of COX-2, MMP-2 and MT1-MMP were evaluated by western blot technique, whereas enzymatic activity of MMP-2 and MMP-9 was determined by zymography in 47 breast cancer patients as well as normal adjacent tissues. Also, the correlation between these proteins and age, tumor size, LN stage, TNM stage, estrogen receptor, progesterone receptor, disease-free survival, and overall survival (OS) has been investigated. As compared to adjacent normal tissues, COX-2, MMP-2 and MT1-MMP were over-expressed in 43, 64, and 60 % of tumor tissues, respectively. In the same pattern, the activity of MMP-2 (62 %) and MMP-9 (45 %) was elevated in BC tissues. Multivariate analysis showed a positive correlation between the protein expression of COX-2, MMP-2, and MT1-MMP and the activity of MMP-2 and MMP-9 in BC patients. However, the enzymatic activity showed no correlation with clinicopathological features. This study confirms the preclinical evidence that COX-2 increased the expression of MT1-MMP, which in turn activates MMP-2. The lack of correlation with clinicopathological features, OS or disease-free survival ascertains the complexity of tumor progression and   metastasis   with many pro- and counter regulatory factors. 
 Toll-like receptor 9 agonists up-regulates the expression of cyclooxygenase-2 via activation of NF-kappaB in prostate cancer cells. CpG-oligonucleotides (CpG-ODNs), mimicking bacterial DNA, have recently been shown to stimulate prostate cancer invasion in vitro via Toll-like receptor 9 (TLR9). Since cyclooxygenase 2 (COX-2), frequently overexpressed in multiple tumor types including prostate cancer, is a causal factor for tumor development, invasion and   metastasis  , an interesting question is raised whether TLR9 regulates COX-2 expression in prostate cancer cells. To address this question, herein we examined COX-2 expression in PC-3 cells stimulated with different doses and time courses of CpG-ODNs. The regulatory role of NF-kappaB in TLR9-mediated COX-2 expression was also investigated. CpG-ODN was found to up-regulate the expression of COX-2 in PC-3 cells in a dose- and time-dependent manner, but have little impact on COX-1 expression. Moreover, CpG-ODN also promoted nuclear translocation and activation of NF-kappaB, which appeared to be required for COX-2 induction by CpG-ODN. Overall, TLR9 up-regulates COX-2 expression in prostate cancer cells, at least partially through the activation of NF-kappaB, which may be implicated in tumor invasion and   metastasis  . 
 Cyclooxygenase-2 enhances alpha2beta1 integrin expression and cell migration via EP1 dependent signaling pathway in human chondrosarcoma cells. Cyclooxygenase (COX)-2, the inducible isoform of prostaglandin (PG) synthase, has been implicated in tumor   metastasis  . Interaction of COX-2 with its specific EP receptors on the surface of cancer cells has been reported to induce cancer invasion. However, the effects of COX-2 on migration activity in human chondrosarcoma cells are mostly unknown. In this study, we examined whether COX-2 and EP interaction are involved in   metastasis   of human chondrosarcoma.We found that over-expression of COX-2 or exogenous PGE2 increased the migration of human chondrosarcoma cells. We also found that human chondrosarcoma tissues and chondrosarcoma cell lines had significant expression of the COX-2 which was higher than that in normal cartilage. By using pharmacological inhibitors or activators or genetic inhibition by the EP receptors, we discovered that the EP1 receptor but not other PGE receptors is involved in PGE2-mediated cell migration and alpha2beta1 integrin expression. Furthermore, we found that human chondrosarcoma tissues expressed a higher level of EP1 receptor than normal cartilage. PGE2-mediated migration and integrin up-regulation were attenuated by phospholipase C (PLC), protein kinase C (PKC) and c-Src inhibitor. Activation of the PLCbeta, PKCalpha, c-Src and NF-kappaB signaling pathway after PGE2 treatment was demonstrated, and PGE2-induced expression of integrin and migration activity were inhibited by the specific inhibitor, siRNA and mutants of PLC, PKC, c-Src and NF-kappaB cascades.Our results indicated that PGE2 enhances the migration of chondrosarcoma cells by increasing alpha2beta1 integrin expression through the EP1/PLC/PKCalpha/c-Src/NF-kappaB signal transduction pathway. 
 [Expression and clinical significance of COX-2, p-Stat3, and p-Stat5 in esophageal carcinoma]. Cyclooxygenase-2 (COX-2) and signal transducers and activators of transcription (STAT) are closely correlated to the genesis of tumors. This study was to investigate the expression and clinical significance of COX-2, p-Stat3 and p-Stat5 (the activated forms of Stat3 and Stat5) in various lesions of esophageal tissues, and to analyze their correlations to clinicopathologic features of esophageal squamous cell carcinoma (ESCC).The expression of COX-2, p-Stat3, and p-Stat5 in 59 specimens of ESCC, 24 specimens of squamous dysplasia, and 18 specimens of normal squamous epithelium was examined by SP immunohistochemistry. Their correlations to clinicopathologic features of ESCC were analyzed.The protein level of COX-2 was significantly higher in ESCC and squamous dysplasia than in normal squamous epithelium (2.10+/-1.77 and 1.85+/-1.24 vs. 0.83+/-0.46, P<0.05). The protein level of p-Stat3 was 0 in normal squamous epithelium, 0.76+/-0.59 in squamous dysplasia, and 2.83+/-1.27 in ESCC. The protein level of p-Stat5 was 1.98+/-0.78 in normal squamous epithelium, 3.92+/-0.41 in squamous dysplasia, and 5.02+/-0.34 in ESCC. There were significant differences among the 3 groups (P<0.05). In ESCC, COX-2 expression was correlated to lymph node   metastasis   and differentiation (P<0.05); p-Stat3 expression was correlated to tumor invasion depth (P<0.05); but p-Stat5 expression had no correlation to clinicopathologic features. COX-2 expression was positively correlated to both p-Stat3 expression and p-Stat5 expression in ESCC.The up-regulation of COX-2, p-Stat3, and p-Stat5 may be correlated to the carcinogenesis of ESCC. The activation of Stat3 is correlated to the aggressive behavior of ESCC. 
 Lysophosphatidic acid upregulates vascular endothelial growth factor-C and tube formation in human endothelial cells through LPA(1/3), COX-2, and NF-kappaB activation- and EGFR transactivation-dependent mechanisms. Lysophosphatidic acid (LPA) is a lipid bioactive mediator which binds to G-protein-coupled receptors and activates a variety of cellular functions. LPA modulates multiple behaviors in endothelial cells, including cell proliferation and migration, capillary-like tube formation in vitro, activation of proteases, interactions with leukocytes, and expressions of inflammation-related genes, thereby regulating vessel formation. LPA has been reported to modulate the angiogenesis process. However, the role of LPA in the lymphangiogenesis process has not been studied. In this study, we showed that LPA upregulated vascular endothelial growth factor-C (VEGF-C) mRNA expression in human umbilical vein endothelial cells (HUVECs) and subsequent endothelial cell tube formation in vitro and in vivo. These enhancement effects were LPA(1)- and LPA(3)-dependent and required cyclooxygenase-2 (COX-2), endothelial growth factor receptor (EGFR) transactivation and activation of nuclear factor kappaB (NF-kappaB). Moreover, LPA induced the protein expressions of the lymphatic markers, Prox-1, LYVE-1, and podoplanin, in HUVECs, and these enhancement effects were dependent on LPA(1) and LPA(3) activation and EGFR transactivation. Our results demonstrated that LPA might regulate VEGF-C and lymphatic marker expression in endothelial cells, which contributes to endothelial cell tube formation in vitro and in vivo, thus facilitating endothelial cell participation in the lymphangiogenesis process. This study clarifies the signaling mechanism of LPA-regulated VEGF-C expression and lymphatic marker expressions in endothelial cells, which suggest that LPA may be a suitable target for generating therapeutics against lymphangiogenesis and tumor   metastasis  . 
 EGF-R is expressed and AP-1 and NF-kappaB are activated in stromal myofibroblasts surrounding colon adenocarcinomas paralleling expression of COX-2 and VEGF. COX-2 and VEGF are important triggers of colon cancer growth,   metastasis   and angiogenesis. Cox-2 promoter contains transcriptional regulatory elements for AP-1 and NF-kappaB transcription factors whilst vegf is a known AP-1 downstream target gene. We investigated whether stromal myofibroblasts surrounding colon adenocarcinomas express COX-2 and VEGF and whether activation of AP-1 and NF-kappaB, as well as expression of EGF-R parallel expression of COX-2 and VEGF in these cells.Immunohistochemical methodology was performed on archival sections from 40 patients with colon adenocarcinomas. We evaluated c-FOS, p-c-JUN (phosphorylated c-JUN), p-IkappaB-alpha (phosphorylated IkappaB-alpha), EGF-R, COX-2, NF-kappaB and VEGF expression in stromal myofibroblasts surrounding colon adenocarcinomas. Double immunostaining with a-smooth muscle actin and each antibody was done to verify the expression of these molecules in stromal myofibroblasts.VEGF, p-IkappaB-alpha, NF-kappaB, c-FOS, p-c-JUN, EGF-R and COX-2 were expressed in stromal myofibroblasts surrounding colon adenocarcinomas in the majority of cases. EGF-R, p-IkappaB-alpha, NF-kappaB, c-FOS and p-c-JUN correlated positively with COX-2 and VEGF expression.Stromal myofibroblasts surrounding colon adenocarcinomas are an important source of VEGF and COX-2 production, while AP-1 and NF-kappaB transcription factors are activated and EGF-R is expressed in these cells and associated with COX-2 and VEGF production. 
 The hepatitis B virus X protein promotes tumor cell invasion by inducing membrane-type matrix metalloproteinase-1 and cyclooxygenase-2 expression. Hepatocellular carcinoma is strongly associated with chronic infection by the hepatitis B virus (HBV) and has poor prognosis due to intrahepatic   metastasis  . HBx is often the only HBV protein detected in hepatic tumor cells; however, its contribution to tumor invasion and   metastasis   has not been established so far. In this work, we show that HBx enhances tumor cell invasion, both in vivo and in vitro. The increased invasive capacity induced by HBx is mediated by an upregulation of membrane-type 1 matrix metalloproteinase (MT1-MMP) expression, which in turn activates matrix metalloproteinase-2. Induction of both MT1-MMP expression and cell invasion by HBx is dependent on cyclooxygenase-2 (COX-2) activity. In addition, HBx upregulates the expression of COX-2, which is mediated by the transcriptional activation of the COX-2 gene promoter in a nuclear factor of activated T cell-dependent (NF-AT-dependent) manner. These results demonstrate the ability of HBx to promote tumor cell invasion by a mechanism involving the upregulation of MT1-MMP and COX-2 and provide new insights into the mechanism of action of this viral protein and its involvement in tumor   metastasis   and recurrence of hepatocellular carcinoma. 
 Peroxisome-proliferator-activated receptor alpha agonists inhibit cyclo-oxygenase 2 and vascular endothelial growth factor transcriptional activation in human colorectal carcinoma cells via inhibition of activator protein-1. Recent evidence indicates that PPAR (peroxisome-proliferator-activated receptor) alpha ligands possess anti-inflammatory and antitumoural properties owing to their inhibitory effects on the expression of genes that are involved in the inflammatory response. However, the precise molecular mechanisms underlying these effects are poorly understood. In the present study, we show that tumour promoter PMA-mediated induction of genes that are significantly associated with inflammation, tumour growth and   metastasis  , such as COX-2 (cyclo-oxygenase 2) and VEGF (vascular endothelial growth factor), is inhibited by PPARalpha ligands in the human colorectal carcinoma cell line SW620. PPARalpha activators LY-171883 and WY-14,643 were able to diminish transcriptional induction of COX-2 and VEGF by inhibiting AP-1 (activator protein-1)-mediated transcriptional activation induced by PMA or by c-Jun overexpression. The actions of these ligands on AP-1 activation and COX-2 and VEGF transcriptional induction were found to be dependent on PPARalpha expression. Our studies demonstrate the existence of a negative cross-talk between the PPARalpha- and AP-1-dependent signalling pathways in these cells. PPARalpha interfered with at least two steps within the pathway leading to AP-1 activation. First, PPARalpha activation impaired AP-1 binding to a consensus DNA sequence. Secondly, PPARalpha ligands inhibited c-Jun transactivating activity. Taken together, these findings provide new insight into the anti-inflammatory and anti-tumoural properties of PPARalpha activation, through the inhibition of the induction of AP-1-dependent genes that are involved in inflammation and tumour progression. 
 Cyclo-oxygenase-2 expression is associated with vascular endothelial growth factor C expression and lymph node   metastasis   in oral squamous cell carcinoma. Cervical lymph node   metastasis   in oral squamous cell carcinoma (OSCC) is recognized as a poor prognostic factor, although its mechanism remains unclear. Recently, cyclo-oxygenase-2 (COX-2) level has been found to correlate highly with vascular endothelial growth factor C (VEGF-C) and lymph node   metastasis  , as in other solid tumors. However, there has been no report of this correlation in OSCC. Therefore, the aim of this study was to investigate whether COX-2 immunohistochemical expression in OSCC was associated with VEGF-C expression, histopathologic parameters, and lymph node   metastasis  .Lymphatic vessel density, VEGF-C, and COX-2 immunohistochemical expression were examined pathologically in 60 specimens of invasive OSCC. Relations of histopathologic parameters to lymph node   metastasis   were analyzed.Expression levels of VEGF-C and COX-2 and lymphatic vessel density in the lymph node metastatic group were significantly higher than in the nonmetastatic group (P < .01). A significant correlation was found between the expression levels of VEGF-C and COX-2 (r = 0.512; P < .001). COX-2 expression was significantly related to lymph node   metastasis   (P = .004) and VEGF-C expression (P = .005). Univariate analysis showed that survival time was impaired by higher COX-2 and VEGF-C expression levels. Multivariate survival analysis showed that COX-2 expression was an independent prognostic factor.This study showed that VEGF-C expression was upregulated by COX-2 in OSCC. High VEGF-C expression appears to promote peritumoral lymphangiogenesis. These data indicated that lymph node   metastasis   is promoted by COX-2 and VEGF-C in OSCC. 
 COX-2 expression is correlated with VEGF-C, lymphangiogenesis and lymph node   metastasis   in human cervical cancer. Lymphangiogenesis has been shown to promote lymph node   metastasis   in cancers, making it an important target in cancer therapy. Vascular endothelial growth factor (VEGF)-C is upregulated in various tumors/cancers and is one of the most potent growth factors for inducing lymphangiogenesis and promoting lymph node   metastasis   (LNM). Likewise, cyclooxygenase (COX)-2 plays major roles in carcinogenesis, tumor growth and   metastasis   via multiple mechanisms including inactivation of host antitumor immunity and promotion of tumor cell migration, tumor cell invasiveness and tumor-associated angiogenesis and lymphangiogenesis. We previously demonstrated an association between COX-2 and VEGF-C in an in vitro model of lung cancer. However, little is known about the regulation of VEGF-C by COX-2 in cervical cancer. In this study, we measured the COX-2 and VEGF-C expressions by immunohistochemistry in 23 LNM-positive and 20 LNM-negative cervical cancer specimens. We then examined the correlations among the expressions and the lymphatic microvessel density (LMVD) and ultrastructural changes to the lymphatic vessel walls by enzyme histochemical staining and electron microscopy. In addition, we used the HeLa cervical cancer cell line to explore the in vitro regulation of VEGF-C by COX-2 and its metabolite, PGE(2), using siRNA-mediated gene silencing and EP receptor blockade. The LNM-positive specimens exhibited significantly higher VEGF-C expression, COX-2 expression and LMVD than the LNM-negative specimens. Furthermore, there were strong correlations between the levels of COX-2 expression and the levels of VEGF-C expression and secretion and a significant positive association between the LMVD and LNM. siRNA-mediated knockdown of COX-2 expression inhibited VEGF-C mRNA expression while EP1 and EP4 receptor antagonists reduced the VEGF-C protein level and tyrosine phosphorylation of Src kinase. Moreover, inhibition of Src kinase with the tyrosine kinase inhibitor PP1 attenuated VEGF-C expression. Collectively, our data provide evidence for a clinical association between COX-2 and VEGF-C expressions in cervical cancer. EP1 and EP4 receptors may be involved in the COX-2-mediated regulation of VEGF-C protein and mRNA expressions. Src may be a downstream mediator of EP1 and EP4 receptors. COX-2 inhibition may diminish LNM by suppressing VEGF-C-mediated lymphangiogenesis. 
 Circulating cycloxygenase-2 in patients with tobacco-related intraoral squamous cell carcinoma and evaluation of its peptide inhibitors as potential antitumor agent. The aim of this study was to quantitate circulating COX-2 levels in patients with tobacco-related intraoral cancer and to evaluate antitumor activities of COX-2 peptide inhibitors in vitro on KB cell lines.We used a novel biosensor-based surface plasmon resonance (SPR) technique for estimation of circulating COX-2 levels in 76 patients with oral cancer and 43 normal individuals. Antitumor activities of five COX-2 inhibitory peptides were evaluated using propidium iodide labeling and flow cytometry, alamar blue, MTS, and annexin-V binding assays.Patients with oral cancer showed threefold increase in serum COX-2 level when compared to normal controls (P < 0.0001). Further, late-stage tumors and lymph node   metastasis   were associated with significant increase in serum COX-2 levels. Patients with higher circulating COX-2 also showed higher immunoreactivity to anti-COX-2 antibody in the lesions. The peptides significantly reduced viability and inhibited growth/proliferation, induced cytotoxicity and apoptosis in tumor cells. However, no such effect was observed either on normal human leukocytes or on MCF-7 cell line that did not over express COX-2.Our results indicate that SPR may be a useful proteomic technique for quantitative assessment of COX-2 and to identify patients with high-risk oral premalignant or occult cancer, as well as in monitoring response to novel COX-2 targeting strategies. Furthermore, COX-2 peptide inhibitors appear to be a new class of potent anticancer agent for human oral carcinoma. 
 Cyclooxygenase-2 expression in squamous dysplasia and squamous cell carcinoma of the esophagus. Cyclooxygenase-2 (cox-2) overexpression has been observed in several types of human cancers and has been implicated in carcinogenesis. To elucidate the role of cox-2 in esophageal carcinogenesis, we evaluated the expression of cox-2 in normal squamous epithelium squamous epithelial dysplasia (n=47), and squamous cell carcinoma of the esophagus (n=86) by immunohistochemistry, reverse transcription-PCR assay, and western blotting. A significant overexpression of cox-2 was observed in esophageal squamous dysplasia and squamous cell carcinoma compared with normal squamous epithelium. The immunoreactive score of cox-2 expression, an index determined by intensity and positivity of cox-2 staining, was 0.71 +/- 0.46 (mean +/- SD) in normal squamous esophagus, 2.19 +/- 1.79 in squamous epithelial dysplasia, and 2.67 +/- 1.77 in squamous cell carcinoma. The results of immunohistochemistry were confirmed by a reverse transcription-PCR assay and western blotting analysis. Cox-2 expression level was correlated with proliferation activity assessed by proliferating cell nuclear antigen (PCNA) index and MIB-1 index in dysplastic lesion (r=0.55, P<0.01 with PCNA and r=0.72, P<0.01 with MIB-1) and carcinoma (r=0.56, P<0.01 with PCNA and r=0.72, P<0.01 with MIB-1). Elevated cox-2 expression was associated with high p53 expression (p<0.001) but not with clinicopathological features including age, sex, tumor size, histological grade, lymph node   metastasis  , and TNM stage. The results indicated that cox-2 may be involved in an early stage of squamous carcinogenesis of the esophagus, and that cox-2 overexpression was related to cell proliferation in esophageal squamous dysplasia and squamous cell carcinoma. 
 Prognostic importance of COX-2 expression in patients with colorectal cancer. A relationship between cyclooxygenase-2 (COX-2) expression and the pathogenesis of colorectal cancer has been reported in recent studies. Moreover, it has been indicated that COX-2 expression may have a prognostic role in colorectal cancer patients. In this study, we investigated the prognostic significance of COX-2 expression in 83 patients with colorectal cancer. COX-2 expression was assessed using immunohistochemical methods and was evaluated by grading both staining intensity and staining extension. The relationships between COX-2 expression and clinicopathological features of the patients and patient survival were evaluated. There was no relationships between COX-2 expression and tumor size (tm < 3 cm or tm > or = 3 cm), tumor histopathological differentiation (poorly differentiated or moderately + well differentiated), number of metastatic lymph nodes (< 4 or 3 > or = 4), histopathology of the tumor, localization of the tumor (colon or rectum), distant   metastasis  , and vascular invasion of the tumor. In the multivariate analysis, COX-2 expression was not found as an independent prognostic factor. We demonstrated that COX-2 expression was not correlated with clinicopathological characteristics of colon carcinoma and disease outcome. 
 Cyclooxygenase-2 expression is associated with vascular endothelial growth factor-C and lymph node metastases in human prostate cancer. Recent observations suggest an implication of cyclooxygenase-2 (COX-2) in tumor lymphangiogenesis through an upregulation of vascular endothelial growth factor-C (VEGF-C) expression. However, it is unclear whether COX-2 is also associated with VEGF-C expression, tumor lymphangiogenesis and lymph node   metastasis   in human prostate cancer.COX-2 and VEGF-C expression were examined in tumor tissues from 58 prostate cancer patients using immunohistochemical staining. We also analyzed the association of COX-2 and VEGF-C expression with tumor lymphangiogenesis quantified as lymphatic vessel density (LVD), lymph node   metastasis  , and patients' biochemical progression-free survival (b-PFS).High expression of either COX-2 or VEGF-C was correlated with tumor lymphangiogenesis and lymph node   metastasis  , as well as poor b-PFS. Moreover, a strong correlation was found between expression of COX-2 and VEGF-C (r = 0.631, p <0.001).COX-2 is positively associated with VEGF-C expression, tumor lymphangiogenesis and lymphatic   metastasis   in prostate cancer. These findings suggest that COX-2 may play a pivotal role in lymphangiogenesis and lymph node   metastasis   of prostate cancer via the regulation of VEGF-C expression. 
 Total cyclooxygenase-2 mRNA levels correlate with vascular endothelial growth factor mRNA levels, tumor angiogenesis and prognosis in non-small cell lung cancer patients. Interaction between cancer cells and adjacent stromal cells is important to promote tumor development. Our aim was to study total COX-2 mRNA expression in both cancer cells and surrounding stromal cells and its association with angiogenic factor VEGF mRNA expression, tumor angiogenesis and prognosis in patients with NSCLC. COX-2 mRNA expression in both cancer cells and stromal tissue was analyzed using real-time quantitative (RTQ) RT-PCR in 60 NSCLC surgical specimens. Immunohistochemistry (IHC) was used to localize COX-2 protein in tumor specimens. Correlations between tumoral total COX-2 mRNA expression and VEGF mRNA expression (measured by RTQ RT-PCR), intratumoral microvessel counts (evaluated by IHC), other clinicopathologic variables, survival and relapse were tested. COX-2 protein expression was found in cancer as well as the surrounding stromal cells (including infiltrating inflammatory cells and endothelial cells of tumor-associated microvessels). VEGF protein expression was mainly located in cancer cells. There was a significant association between high tumoral total COX-2 mRNA expression and high VEGF mRNA expression (p = 0.01) or high intratumoral MVC (p < 0.001) but not other clinicopathologic variables, including tumor status and lymph node   metastasis  . Patients with higher tumoral total COX-2 mRNA expression had a statistically shorter survival time (median 15.0 +/- 2.61 months) and relapse time (median 5.0 +/- 1.37 months) than those with lower tumoral total COX-2 mRNA expression (median 40.0 +/- 3.12 and 34.0 +/- 3.11 months; p < 0.0001 and p < 0.0001, respectively, log-rank test). A significant difference in survival and relapse time was also seen between patients with high and low tumoral VEGF mRNA expression and between those with high and low intratumoral MVC (p = 0.0046 and p = 0.0038, respectively). After stratification by disease stage or histologic subtype, the prognostic significance of high total COX-2 mRNA expression was still apparent in both stage I and stage II-IV and in both squamous cell carcinoma and adenocarcinoma (p < or = 0.01 for all). Multivariate analysis using the Cox regression model with backward elimination showed that tumoral total COX-2 mRNA expression and lymph node status were the 2 most important independent prognostic predictors for survival and disease relapse. We report that total COX-2 mRNA expression in cancer cells and surrounding stromal cells correlates strongly and positively with VEGF mRNA expression, intratumoral MVC and adverse prognosis in NSCLC patients. This implies that COX-2 expression in both cancer cells and stromal cells within the tumor microenvironment may play an important role in upregulating the expression of the angiogenic factor VEGF and tumor angiogenesis in NSCLC and explains, in part, the adverse prognostic effect of COX-2 overexpression in patients with NSCLC. 
 [Expression of COX-2 in different human prostate cancer cell lines and its significance]. To investigate the expression of cyclooxygenase-2 (COX-2) in different prostate cancer (PCa) cell lines and its role in the acquisition of invasive and metastatic potentials of PCa.We detected the expressions of COX-2 in prostate cancer cell lines LNCaP, C4-2, IF11, IA8 and PC-3 with different metastatic potentials by Western blotting and RT-PCR, and analyzed their roles in the invasion and   metastasis   of different PCa cell lines.Western blotting and RT-PCR showed that the expression of the COX-2 protein was high in PC-3, but absent in IF11, IA8, LNCaP and C4-2 (P < 0.05), and it was consistent with the expression of COX-2 mRNA.COX-2 expresses differently in PCa cell lines with different metastatic potentials. The overexpression of COX-2 may be associated with the high invasion and   metastasis   of PC-3, but not with the   metastasis   of other cell lines. 
 Heparanase and COX-2 expression as predictors of lymph node   metastasis   in large, high-grade breast tumors. Heparanase (HPA) contributes to breast cancer   metastasis   by facilitating the breakdown of the basement membrane and extracellular matrix. High expression of HPA is thought to be associated with increased nodal involvement and poor survival in patients with breast cancer. Overexpression of cyclooxygenase-2 (COX-2) in breast cancer is associated with indicators of poor prognosis such as lymph node   metastasis  , poor differentiation, and large tumor size. The underlying mechanism by which HPA and COX-2 overexpression increases the metastatic potential of breast cancer is not fully-understood. To enhance our understanding over these mechanisms, we aimed to investigate the relationship between the size of the tumor and HPA expression, tumor grade as well as lymph node status in patients with breast cancer.Immunohistochemical analysis of HPA and COX-2 expression was performed on 246 breast tumor samples. The expression of HPA was correlated with COX-2 expression, tumor grade, lymph node status, oestrogen receptor status.The overexpression of HPA and COX-2 was associated with increased likelihood of lymph node positivity in large, high-grade tumors. High-grade tumors with size greater than 20 mm, that overexpressed HPA, were 4-times more likely to be associated with lymph node involvement (OR 4.71, CI 1.21-18.25). Whereas, tumors greater than 20 mm in size were 5-times more likely to metastasize to the regional lymph nodes, if associated with overexpression of COX-2 (OR 5.5, CI 1.2-24.8).Expression of HPA appears to be a key mechanism by which large, high-grade breast tumors metastasize to regional lymph nodes, while COX-2 overexpression may be an independent predictor of lymph node positivity. 
 Inactivation of RASSF1A, RARbeta2 and DAP-kinase by promoter methylation correlates with lymph node   metastasis   in nasopharyngeal carcinoma. Epigenetic modification is one of the mechanisms leading to gene silencing in neoplastic cells. By methylation-specific PCR, we analyzed the promoter methylation of three cancer-related genes: Ras Association domain Family 1A (RASSF1A), Death Associated Protein kinase (DAP-kinase) and Retinoic Acid Receptor beta2 (RARbeta2) in two NPC xenografts (C15 and C17), 68 primary NPC tumors, and nine normal nasopharyngeal epithelia. We showed that C15 and C17 displayed a complete promoter methylation of RASSF1A, RARbeta2 and DAP-kinase genes. In primary NPC tumors, the incidence of promoter methylation was very high for all three tested genes: 91% for RASSF1A, 88% for both RARbeta2 and DAP-kinase whereas all normal nasopharyngeal epithelia were unmethylated. Interestingly, our study revealed that aberrant promoter methylation of the three genes were statistically associated with the lymph node involvement (p < 0.0001). In addition, hypermethylation of RASSF1A was correlated with age at diagnosis (p = 0.047) and T stage (p = 0.037) while the RARbeta2 hypermethylation was associated with histological type (p = 0.011). Taken together, our results demonstrate that silencing of RASSF1A and RARbeta2 expression by promoter hypermethylation is associated with highly differentiated tumors, advanced tumor stage and the presence of lymph node   metastasis  . To assess the functional significance of the epigenetic silencing of RARbeta2 and DAP-kinase in NPC, we analysed the expression of two downstream target genes COX-2 and p53 by reverse PCR (RT-PCR) and immunohistochemistry (IHC). We revealed a significant association between expression of COX-2 and loss of RARbeta2 through aberrant methylation (p = 0.003) in NPC biopsies. We concluded that the inactivation of RASSF1A, RARbeta2 and DAP-Kinase by hypermethylation is a key step in NPC tumorigenesis and progression. 
 Effects of COX-2 inhibition on expression of vascular endothelial growth factor and interleukin-8 in lung cancer cells. Cyclooxygenase (COX)-2 has been implicated in tumour progression, angiogenesis and   metastasis   in non-small cell lung cancer (NSCLC). We speculated that inhibition of COX-2 activity might reduce expression of the pro-angiogenic factors vascular endothelial growth factor (VEGF) and interleukin-8 (IL-8) in lung cancer cells.The levels of IL-8, VEGF and prostaglandin E2 (PGE2) were measured by ELISA. Expression of COX-1 and COX-2 was determined by Western blotting. Inhibition or knockdown of COX-2 was achieved by treating NSCLC cells with specific COX-2 inhibitor NS-398 or COX-2 siRNA, respectively.We found that NSCLC cell lines produced more IL-8 than VEGF (p < 0.001). In contrast, small cell lung cancer (SCLC) cell lines produced more VEGF than IL-8 (p < 0.001). COX-1 was expressed in all cell lines, but COX-2 was expressed only in NSCLC cell lines. Consistent with this, PGE2 was significantly higher in NSCLC cell lines than SCLC cell lines (p < 0.001). We tested these cell lines with a potent specific COX-2 inhibitor NS-398 at concentrations of 0.02, 0.2, 2, 20 microM for 24 or 48 h. The COX-2 activity was reduced in a dose-dependent fashion as shown by reduced PGE2 production. VEGF was significantly reduced following the treatment of NS-398 in A549 (by 31%) and MOR/P (by 47%) cells lines which expressing strong COX-2, but not in H460 cell line which expressing very low COX-2. However, IL-8 was not reduced in these cell lines. To confirm these results, we knocked down COX-2 expression with COX-2 siRNA in these cell lines. VEGF was significantly decreased in A549 (by 24%) and in MOR/P (by 53%), but not in H460 whereas IL-8 was not affected in any cell line.We conclude that NSCLC cells produce much higher levels of IL-8 than SCLC cells whereas both NSCLC and SCLC cells produce similar levels of VEGF. COX-2 is only expressed in NSCLC cells, but not in SCLC cells. VEGF is produced in both NSCLC and SCLC cells regardless of COX-2 expression. However, VEGF production is, at least partly, COX-2 dependent in NSCLC cells expressing COX-2. In contrast, IL-8 production is COX-2 independent in both NSCLC and SCLC cells. We speculate that combined targeting of COX-2 and IL-8 may be useful in the treatment of patients with NSCLC and targeting VEGF may be useful in the treatment of patients with SCLC. 
 [The expression and clinical significance of HIF-1α, COX-2 and E-cadherin in patients with lung adenocarcinoma]. The incidence of lung adenocarcinoma increases rapidly, and hypoxia-inducible factor-1α (HIF-1α), cyclooxygenase-2 (COX-2), E-cadherin play an important role in the proliferation and differentiation of cancer cell. The aim of this study is to investigate the clinical significance of the expression of HIF-1α, COX-2, E-cadherin in patients with lung adenocarcinoma and the internal relationship among them.The expression levels of HIF-1α, COX-2 and E-cadherin were determined in 10 cases of normal lung issue and in 45 cases of issue of lung adenocarcinoma by immunohistochemical method.The positive expression rate of HIF-1α and COX-2 was 60% (27/45) and 40% (18/45) respectively in 45 cases of lung adenocarcinoma, was null in 10 cases of normal lung issue. The positive expression rate of E-cadherin was 48.9% (22/45) in 45 cases of lung adenocarcinoma, was 100% in 10 cases of normal lung issue. The expression of HIF-1α was positively correlated with the size of the cancer (P < 0.05), but not with age, smoking, lym  phatic met  astasis, differentiated degree and surgical-pathologic staging (P > 0.05). The expression of COX-2 was positively correlated with the size of the cancer, lym  phatic met  astasis, surgical-pathologic and expression of HIF-1α (P < 0.05), but not with age, smoking and differentiated degree (P > 0.05). The expression of E-cadherin was positively correlated with differentiated degree and ly  mphatic me  tastasis (P < 0.05), but not with age, smoking, the size of the cancer, surgical-pathologic and expression of HIF-1α (P > 0.05).The overexpression of HIF-1α and COX-2 and the downregulation of E-cadherin is found in lung adenocarcinoma. The overexpression of HIF-1α may induce the overexpression of COX-2, and is non-correlated with expression of E-cadherin. 
 Hepatocyte growth factor induces anoikis resistance by up-regulation of cyclooxygenase-2 expression in uterine endometrial cancer cells. Cyclooxygenase-2 (COX-2) has been implicated in the promotion of carcinogenesis. Although the role of COX-2 in endometrial cancer remains unclear, recent experiments suggest that COX-2 antagonizes cell apoptosis, increases the invasiveness of malignant cells, and promotes angiogenesis. Hepatocyte growth factor (HGF) is a mesenchymal-derived cytokine and the interaction between HGF and its tyrosine kinase receptor, c-Met proto-oncogene, is associated with tumor progression and   metastasis  . To investigate the molecular mechanism of HGF-induced anoikis resistance, we analyzed the signal transduction and COX-2 expression in endometrial cancer cells. Here, we show i) the expression of COX-2 protein significantly increased in a dose-dependent manner after HGF stimulation in endometrial cancer cell lines (HEC-IB and RL95-2), reaching 200-270% stimulation at the highest doses of HGF tested (40 ng/ml); ii) flow cytometry and TUNEL analyses revealed that HGF significantly inhibited anoikis of RL95-2 cells; iii) phosphatidylinositol 3-kinase (PI3K) inhibitor (LY294002), but not mitogen-activated protein kinase/ERK kinase (MEK) inhibitor (PD98059), specifically blocked HGF-mediated anoikis resistance in RL95-2 cells; and iv) COX-2 inhibitor, Meloxicam, abrogated HGF-mediated anoikis resistance. Our data suggest that HGF induces anoikis resistance in endometrial cancer cells possibly through PI3K/Akt pathway-dependent up-regulation of COX-2 expression. 
 Heparanase promotes angiogenesis through Cox-2 and HIF1alpha. Heparanase has been given attention for its role in the invasion and   metastasis   of various cancers for years. We have also investigated and reported the role of heparanase in several human cancers, including gastric, esophageal and colon carcinomas. Other than the critical role of heparanase in tumor invasion and   metastasis  , it is also believed that heparanase is involved in angiogenesis, another feature of tumor progression which is complicatedly mediated by many molecules, including cyclooxygenese-2 (Cox-2). Thus, our recent study elucidated a possible relationship of heparanase with Cox-2 upon tumor angiogenesis. Based upon our study, three major transcription factor binding sites containing NF-kappaB, NF-IL-6 and CRE sites seemed to have a compensative and cooperative role in heparanase-induced Cox-2 upregulation. On the other hand, tumor hypoxia often occurs in most tumors and Cox-2-induced HIF1alpha overexpression has recently been shown in various cancers. Here we believe that heparanase may also be involved in tumor hypoxia through the induction of HIFalpha either directly or indirectly through the Cox-2 pathway. This hypothesis indicates a possible novel function of heparanase and its link to HIF1alpha and Cox-2, and therefore this function would give us a clue about potential new strategies for cancer therapy. 
 Expression of COX-2 and Bcl-2 in primary fallopian tube carcinoma: correlations with clinicopathologic features. The aim of this study was to evaluate the expression of COX-2 and Bcl-2 in primary fallopian tube carcinoma (PFTC), as well as their correlations with clinicopathologic features. We studied a cohort of 33 patients with a pathological diagnosis of PFTC. Thirty normal tubal tissues used for controls were obtained from patients diagnosed with uterine myomas. Expression analysis for COX-2 and Bcl-2 was performed using the immunohistochemical technique. The rate of preoperative diagnosis was 18.2%. With a median survival of 61.0 months (95% CI: 43.2 to 78.8 months), the estimated five-year overall survival rate in the 33 patients was 39.0%. Increased expression of COX-2 and Bcl-2 was observed in tumor specimens compared to normal controls (p = 0.026; p = 0.003). The expression rate of COX-2 in node-positive tumors was significantly higher than that of node-negative tumors (p = 0.024). Moreover, the expression rate of COX-2 was statistically significantly higher in patients with infiltration through the serosa (p = 0.019). Positive significant associations were observed between Bcl-2 staining index and FIGO stage (p = 0.015), and between Bcl-2 staining and lymph node   metastasis   (p = 0.010). There was a significant correlation between COX-2 expression and Bcl-2 staining index (r = 0.517, p = 0.002). We conclude that COX-2 and Bcl-2 may potentially be useful prognostic markers for PFTC. The exact molecular mechanism for correlations between COX-2 and Bcl-2 remains to be elucidated. 
 Prognostic significance of cyclooxygenase-2 in cervical cancer: a meta-analysis. Published data on the prognostic value of cyclooxygenase-2 (COX-2) overexpression in cervical cancer are conflicting and heterogeneous. We performed a meta-analysis to more precisely estimate its prognostic significance. The pooled hazard ratios (HRs) or odds ratios (ORs) with 95% confidence intervals (CIs) were used to estimate the effects. Twenty-three studies with 1,477 cervical cancer patients were selected to evaluate the association between COX-2 and overall survival (OS), disease-free survival (DFS), response to chemoradiation (RC) and clinicopathological parameters. High COX-2 expression predicted poor OS (HR: 2.53, 95% CI: 1.54-4.18), DFS (HR: 2.41, 95% CI: 1.58-3.69) and RC (OR: 3.03, 95% CI: 1.97-4.64). Subgroup analyses showed that COX-2 overexpression was related significantly with poor OS in patients treated by chemoradiation or surgery, and in patients with squamous cell carcinoma, respectively. Besides, COX-2 overexpression was related significantly with poor DFS in chemoradiation subgroup. Furthermore, COX-2 overexpression was associated with poor RC in patients who received "FP" regimen or "P" regimen. Additionally, there were significant associations between COX-2 expression and all clinicopathological parameters except tumor grade. The pooled ORs (95% CI) were as follows: 1.49 (1.09-2.04) for age, 1.77 (1.22-2.56) for lymph node   metastasis  , 1.04 (0.74-1.47) for tumor grade, 1.71 (1.12-2.64) for tumor size, 2.38 (1.28-4.45) for FIGO stage, 3.96 (2.32-6.77) for histological type, 2.45(1.10-5.42) for parametrical involvement. This meta-analysis indicated that COX-2 overexpression might be an unfavorable prognostic and a chemoradiation resistance predictive factor for cervical cancer; it could potentially help to stratify patients further in clinical treatment. 
 [The expression of hepatitis B virus X protein and cyclooxygenase-2 in hepatitis B virus-related hepatocellular carcinoma: correlation with microangiogenesis and   metastasis  , and what is the possible mechanism]. To investigate the expression of HBx and COX-2 in hepatitis B virus-related hepatocellular carcinoma, and Its correlation with microangiogenesis and   metastasis  , and possible mechanism.Immunohistochemistry was used to detect the expression of hepatitis B virus X, cyclooxygenase-2 and CD34 in hepatitis B virus related hepatic carcinoma and 22 non-HBV related hepatic carcinoma tissues. The expression of hepatitis B virus x protein and cyclooxygenase-2 in hepatitis B virus-related hepatocellular carcinoma correlated with microangiogenesis and   metastasis   was tested by Spearman correlation analysis. The expression of COX-2 in HepG2-X was detected by Western blot and RT-PCR. PGE2 was detected by ELISA in clear supernatant liquid of HepG2-X. Trypan blue exclusion was performed to examine the inhibitory effects of COX-2 selective inhibitor (celecoxib).In Hepatitis B carcinoma tissue, HBx and COX-2 were expressed at high level. The positive rate of COX-2 expression was 88.87% (55/62) in HBx positive expression group, which was significantly higher than that of the positive expression 31.82% (7/22, x2=27.188, P<0.01) in HBx negative expression group and 40.91% (9/22, x2=20.453, P<0.01) in non-HBV related hepatic carcinoma tissues, but it had no statistical difference (x2=0.393, P=0.531) between the HBx negative expression group and non-HBV related hepatic carcinoma tissue group. The expressions of HBx and COX-2 in   metastasis   group were higher than that in non-  metastasis   group (P<0.01), MVD in HBx or COX-2 positive expression group was significantly higher than that in negative expression group and non-HBV related hepatic carcinoma tissues (P is less than 0.01). MVD with   metastasis   was higher than that without   metastasis   (P<0.01) and MVD with portal vein invasion was higher than that without invasion (P<0.05). Spearman correlation analysis showed that the expression of COX-2 was significantly correlated with the expression of HBx (Rs=0.568, P<0.01). Celecoxib suppressed the growth of both cells in a dose-dependent manner. HepG2-X was significantly susceptible to celecoxib as compared to the HepG2-PC cells. COX-2 protein and mRNA were upregulated in HepG2-X cells than in HepG2-PC. Moreover, PGE2 was upregulated in clear supernatant liquid of HepG2-X than in HepG2-PC.The expressions of HBx and COX-2 were higher in HBV-related hepatocellular carcinoma. COX-2 was significantly correlated with HBx in HCC and it could be a key factor involved in HBx contributed hepatocellular carcinoma's microangiogenesis and   metastasis  . The possible mechanism of the dual effects might be through HBx, COX-2 and PGE2 pathways in infiltration involved   metastasis   and microangiogenesis involved   metastasis  . 
 Heterogeneous nuclear ribonucleoprotein K (hnRNP-K) promotes tumor   metastasis   by induction of genes involved in extracellular matrix, cell movement, and angiogenesis. Cancer is a leading cause of death and still awaits effective therapies. Rapid industrialization has contributed to increase in incidence of cancer. One of the reasons why most of the cancers fail therapy is due to their metastatic property. Hence identification of factors leading to   metastasis   is highly important to design effective and novel anti-cancer therapeutics. In our earlier study (Inoue, A., Sawata, S. Y., Taira, K., and Wadhwa, R. (2007) Loss-of-function screening by randomized intracellular antibodies: identification of hnRNP-K as a potential target for   metastasis  . Proc. Natl. Acad. Sci. U.S.A. 104, 8983-8988), we had reported that the involvement of heterogeneous nuclear ribonucleoprotein K (hnRNP-K) in   metastasis  . Here, we established hnRNP-K-overexpressing and -underexpressing derivative cell lines and examined their proliferation and metastatic properties in vitro and in vivo. Whereas hnRNP-K compromised cells showed delayed tumor growth, its overexpression resulted in enhanced malignancy and   metastasis  . Molecular basis of the hnRNP-K induced malignant and metastatic phenotypes was dissected by cDNA microarray and pathway analyses. We found that the hnRNP-K regulates extracellular matrix, cell motility, and angiogenesis pathways. Involvement of the selected genes (Cck, Mmp-3, Ptgs2, and Ctgf) and pathways was validated by gene-specific expression analysis. Our results demonstrated that the hnRNP-K is a potential target for   metastasis   therapy. 
 COX-2 dependent regulation of mechanotransduction in human breast cancer cells. The ability of living cells to exert physical forces upon their surrounding is a necessary prerequisite for diverse biological processes, such as local cellular migrations in wound healing to metastatic-invasion of cancer. How forces are coopted in   metastasis   has remained unclear, however, because the mechanical interplay between cancer cells and the various stromal components has not been experimentally accessible. Current dogma implicates inflammation in these mechanical processes. Using Fourier transform traction microscopy, we measured the force-generating capacity of human breast cancer cells occupying a spectrum of invasiveness as well as basal and inducible COX-2 expression (MCF-7<SUM-149<MDA-MB-231). Compared with non-invasive MCF-7 and moderately-invasive SUM-149, poorly-differentiated MDA-MB-231 cells showed increased cellular dispersion on collagen matrix that was accompanied by emergent distribution of contractile stresses at the interface between the adherent cell and its substrate, defined herein as the traction field. In metastatic MDA-MB-231 cells, the local tractions were precisely tuned to the surrounding matrix rigidity in a physiologic range with the concomitant expression of mechanosensitive integrin β1. These discrete responses at the single-cell resolution were correlated with PGE2 secretion and were ablated by shRNA-mediated knockdown of COX-2. Both COX-2-silenced and COX-2-expressing cells expressed EP2 and EP4 receptors, but not EP1 and EP3. Exogenous addition of PGE2 increased cell tractions and stiffened the underlying cytoskeletal network. To our knowledge this is the first report linking the expression of COX-2 with mechanotransduction of human breast cancer cells, and the regulation of COX-2-PGE2-EP signaling with physical properties of the tumor microenvironment. Drug treatments aimed at reducing this mechanical interplay may have therapeutic potential in the treatment of breast cancer. 
 Coexpression of VEGF-C and Cox-2 in human colorectal cancer and its association with lymph node   metastasis  . Several lines of experimental evidence indicated that over-expression of vascular endothelial growth factor-C and cyclooxygenase-2 genes promotes angiogenesis and lymphangiogenesis, both of which are essential for the growth and spreading of tumor cells. This study was designed to evaluate the coexpression of vascular endothelial growth factor-C and cyclooxygenase-2 in human colorectal carcinoma to determine their relationships and correlations with lymph node   metastasis   and prognosis.Tissue samples of primary tumors and metastatic lymph nodes from 150 patients undergoing intentionally curative surgical resections for colorectal adenocarcinoma were immunohistochemically examined for vascular endothelial growth factor-C, cyclooxygenase-2, and CD34 expressions. Then, we analyzed their relationships and correlations with clinicopathologic findings and patients' survival time.The positivity rate of vascular endothelial growth factor-C and cyclooxygenase-2 in the primary tumor was 68 and 72.7 percent, respectively, and in the metastatic lymph nodes was 93.3 and 80 percent, respectively. A significant correlation was found between the expression scores of vascular endothelial growth factor-C and cyclooxygenase-2 (P < 0.0001), and both also were correlated to microvessels density and several clinicopathologic parameters, including primary tumor size, lymph node   metastasis  , lymphatic invasion, and TNM stage. Patients with vascular endothelial growth factor-C-positive and/or cyclooxygenase-2-positive tumors had a significant shorter survival time than those with negative tumors did. However, in a multivariate analysis, only cyclooxygenase-2 expression was recognized as an independent prognostic factor (P = 0.0412; relative risk ratio, 3.067; 95 percent confidence interval, 1.046-8.994).These data show that in human colorectal carcinoma, vascular endothelial growth factor-C and cyclooxygenase-2 are coexpressed and significantly associated with lymph node   metastasis   and prognosis. 
 A prognostic classifier for patients with colorectal cancer liver   metastasis  , based on AURKA, PTGS2 and MMP9. Prognosis of patients with colorectal cancer liver   metastasis   (CRCLM) is estimated based on clinicopathological models. Stratifying patients based on tumor biology may have additional value.Tissue micro-arrays (TMAs), containing resected CRCLM and corresponding primary tumors from a multi-institutional cohort of 507 patients, were immunohistochemically stained for 18 candidate biomarkers. Cross-validated hazard rate ratios (HRRs) for overall survival (OS) and the proportion of HRRs with opposite effect (P(HRR < 1) or P(HRR > 1)) were calculated. A classifier was constructed by classification and regression tree (CART) analysis and its prognostic value determined by permutation analysis. Correlations between protein expression in primary tumor-CRCLM pairs were calculated.Based on their putative prognostic value, EGFR (P(HRR < 1) = .02), AURKA (P(HRR < 1) = .02), VEGFA (P(HRR < 1) = .02), PTGS2 (P(HRR < 1) = .01), SLC2A1 (P(HRR > 1) < 01), HIF1α (P(HRR > 1) = .06), KCNQ1 (P(HRR > 1) = .09), CEA (P (HRR > 1) = .05) and MMP9 (P(HRR < 1) = .07) were included in the CART analysis (n = 201). The resulting classifier was based on AURKA, PTGS2 and MMP9 expression and was associated with OS (HRR 2.79, p < .001), also after multivariate analysis (HRR 3.57, p < .001). The prognostic value of the biomarker-based classifier was superior to the clinicopathological model (p = .001). Prognostic value was highest for colon cancer patients (HRR 5.71, p < .001) and patients not treated with systemic therapy (HRR 3.48, p < .01). Classification based on protein expression in primary tumors could be based on AURKA expression only (HRR 2.59, p = .04).A classifier was generated for patients with CRCLM with improved prognostic value compared to the standard clinicopathological prognostic parameters, which may aid selection of patients who may benefit from adjuvant systemic therapy. 
 Expression of embryonic lethal abnormal vision (ELAV)-like protein HuR and cyclooxygenase-2 (COX-2) in Ewing sarcoma. HuR is a member of the family of ELAV (embryonic lethal abnormal vision)-like proteins that stabilize several cellular mRNAs by binding to AU-rich elements in the 3' untranslated region of the mRNA. Cyclooxygenase-2 (COX-2) is a well known enzyme that promotes tumor growth and   metastasis  . Recent studies have shown that HuR can stabilize the mRNA of COX-2, and cytoplasmic expression of HuR is associated with increased COX-2 expression in some cancers. The aim of this study was to investigate the correlation between COX-2 and HuR in Ewing sarcoma.The expression patterns for HuR and COX-2 were assessed via immunochemical analysis of 70 Ewing sarcoma samples.Nuclear HuR expression was observed in 12 of 70 (17.1%) cases, but cytoplasmic expression was not observed. COX-2 expression was seen in 25 of 70 (35.7%) samples. Nuclear HuR and COX-2 were simultaneously expressed in 8 of 70 (11.4%) samples. The expression of nuclear HuR was significantly associated with COX-2 expression (P = 0.014). Neither HuR nor COX-2 expression showed a correlation with age or sex.COX-2 expression in Ewing sarcoma may not be directly related to mRNA stabilization by HuR. However, a correlation between COX-2 expression and nuclear HuR expression through indirect mRNA stabilization can be suggested. 
 Expression and Significance of COX-2 and Ki-67 in Hepatolithiasis with Bile Duct Carcinoma. As an induced enzyme, COX-2 expression is elevated under stimuli from inflammatory mediator or growth factor product. Ki-67, a cell cycle-related proliferative antigen, reflects the tissue proliferative activity. This study analyzed the expressional profile of cyclooxygenase-2 (COX-2) and Ki-67 in hepatolithiasis and bile duct carcinoma tissues, in an attempt to provide evidence for diagnosis and prognosis prediction of disease.A cohort of tissue samples from hepatolithiasis with bile duct carcinoma (N=47) patients were analyzed using immunohistochemical (IHC) staining method for the expression of COX-2 and Ki-67, in parallel with hepatolithiasis (N=44) and normal bile duct tissues (N=30). The relationship between expression pattern of COX-2 and Ki-67 and pathological conditions was also analyzed, in addition to the correlation with positive expression in hepatolithiasis samples.The positive expression rate of COX-2 and Ki-67 in bile duct carcinoma was 76.6% and 80.9%, respectively, and was significantly higher than those in the hepatolithiasis group, which was also higher than the control group. Expression of both COX-2 and Ki-67 is closely related to TNM staging, lymph node   metastasis  , and differentiation stage. They were also correlated with the mortality rate of patients.Both COX-2 and Ki-67 are abundantly expressed in hepatolithiasis and bile duct carcinoma tissues and may play an important role in the disease occurrence, progression, and   metastasis  . 
 Human brain endothelial cell-derived COX-2 facilitates extravasation of breast cancer cells across the blood-brain barrier. With improvements in systemic control,   metastasis   to the brain has been more frequently found in patients with breast cancer. In order to gain access to the brain, breast cancer cells must overcome the blood-brain barrier (BBB), a highly selective filter against cellular and soluble substances. Human brain endothelial cells (HBECs) comprise a major element of the BBB, and breast cancer cells first encounter and pass through them for extravasation. To date, however, the precise role of HBECs in   metastasis   to the brain is unknown. In this study, we examined how HBECs take part in the extravasation process. In an established in vitro model of the BBB, unexpectedly, the transmigration of breast cancer cells was markedly enhanced in the presence of HBECs than in their absence, suggesting that HBECs facilitate the transmigration of breast cancer cells rather than acting as a barrier against them. We then showed that cyclooxygenase (COX-2) induced from HBECs rather than that from breast cancer cells plays a key role in the transmigration. Moreover, expression of matrix metalloproteinase (MMP-2) mediating the transmigration was induced in HBECs by COX-2 after co-culture with breast cancer cells. Taken together, our results suggest that COX-2 and MMP-2 produced from HBECs facilitate the extravasation of breast cancer cells across the BBB. 
 Cyclooxygenase-2 expression in urinary bladder transitional cell carcinoma and its association with clinicopathological characteristics. Transitional cell carcinoma (TCC) is the most predominant type of urinary bladder tumor. As cyclooxygenase (COX)-2 is recently introduced as an attractive target molecule in bladder TCC, we evaluated the immunohistochemical expression of this marker and its association with several clinicopathological characteristics.This cross-sectional study was performed in the Pathology department of Sina Hospital in Tehran, Iran during 2006-2011. Ninety-two paraffin embedded blocks were selected from patients with urinary bladder TCC who underwent cystectomy or transurethral resection (TUR). Then, we assessed COX-2 expression by immunohistochemical staining using antibody against COX-2. Staining in more than 5% of tumor cells was considered as positive expression.COX-2 was expressed in 50 % of our patients. This marker was markedly expressed in high grade bladder TCC (62.1%) versus other grades and there was statistically a significant difference in COX-2 expression between various grades (p=0.008). In addition, patients' age, lymphatic and perineurial invasion were associated with the expression of COX-2 (p=0.001, 0.015 and 0.039, respectively). However, other parameters such as stage, tumor size, venous invasion and lymph node   metastasis   did not show any significant relationship with this marker (all, p>0.05).COX-2 was expressed in urinary bladder TCC especially in high grade forms, advocating its probable role in the differentiation of this tumor. Accordingly, COX-2 could be a valuable biological target molecule in the evaluation and treatment of patients with bladder TCC. 
 miR-26b is downregulated in human tongue squamous cell carcinoma and regulates cell proliferation and   metastasis   through a COX-2-dependent mechanism. MicroRNAs (miRNAs) are important gene regulators that play a profound role in tumorigenesis. Previous studies have revealed that miR-26b is downregulated in a wide range of malignant tumors and plays an important role in the regulation of carcinogenesis and tumor progression. In the present study, we revealed that miR-26b expression was decreased in human tongue squamous cell carcinoma and was associated with clinical stage, lymph node   metastasis   and survival prognosis. Ectopic expression of miR-26b suppressed the proliferation and   metastasis   of human tongue squamous cell carcinoma cells. Using a luciferase reporter assay, combined with western blot analysis results, we identified PTGS2 (prostaglandin-endoperoxide synthase-2, encoding COX-2) as the functional target of miR-26b. Specific inhibition of COX-2 activity by nimesulide further confirmed that miR-26b was able to regulate the cell proliferation and   metastasis   of the human tongue squamous cell carcinoma cells through a COX-2-dependent mechanism. Taken together, these results suggest that miR-26b serves as a tumor suppressor by targeting COX-2 and calls for the use of miR-26b as a potential therapeutic tool for human tongue squamous cell carcinoma, where COX-2 is often hyperactivated. 
 [The expression of COX-2 mRNA and protein in human laryngeal squamous cell carcinoma]. To investigate the expression of COX-2 in both molecule and protein levels in laryngeal squamous cell carcinoma and the relationship between its intensity and histological grade, clinical stage and lymphoma   metastasis  .By using emi-quantify RT-PCR and Immunohistochemistry, the expression of the COX-2 mRNA and COX-2 protein in laryngeal squamous cell carcinoma and polyp of vocal cord were examined.Compared with the polyp of vocal cord tissue, the expression level of COX-2 mRNA and COX-2 protein in human laryngeal squamous cell carcinoma were significantly increased (P < 0.01). The intensity of COX-2 mRNA and protein expression increased as the laryngeal squamous cell carcinoma progresses developed.COX-2 is high expressed in human laryngeal squamous cell carcinoma, and it may play an significant role in the growth, invasion and   metastasis   of human laryngeal squamous cell carcinoma. A combination of emi-quantify RT-PCR and immunohistochemistry is sensitive and specific for laryngeal neoplasms detection. 
 DNA damage drives an activin a-dependent induction of cyclooxygenase-2 in premalignant cells and lesions. Cyclooxygenase-2 (COX-2) catalyzes the rate-limiting step in the synthesis of prostaglandins. Its overexpression induces numerous tumor-promoting phenotypes and is associated with cancer   metastasis   and poor clinical outcome. Although COX-2 inhibitors are promising chemotherapeutic and chemopreventative agents for cancer, the risk of significant cardiovascular and gastrointestinal complications currently outweighs their potential benefits. Systemic complications of COX-2 inhibition could be avoided by specifically decreasing COX-2 expression in epithelial cells. To that end, we have investigated the signal transduction pathway regulating the COX-2 expression in response to DNA damage in breast epithelial cells. In variant human mammary epithelial cells that have silenced p16 (vHMEC), double-strand DNA damage or telomere malfunction results in a p53- and activin A-dependent induction of COX-2 and continued proliferation. In contrast, telomere malfunction in HMEC with an intact p16/Rb pathway induces cell cycle arrest. Importantly, in ductal carcinoma in situ lesions, high COX-2 expression is associated with high gammaH2AX, TRF2, activin A, and telomere malfunction. These data show that DNA damage and telomere malfunction can have both cell-autonomous and cell-nonautonomous consequences and can provide a novel mechanism for the propagation of tumorigenesis. 
 Cyclooxygenase-2-derived prostaglandin E2 stimulates Id-1 transcription. Cyclooxygenase-2 (COX-2) and Id-1 are overexpressed in a variety of human malignancies. Recently, each of these genes was found to play a role in mediating breast cancer   metastasis   to the lungs, but their potential interdependence was not evaluated. Hence, the main objective of the current study was to determine whether COX-2-derived prostaglandin (PGE(2)) activated Id-1 transcription, leading in turn to increased invasiveness of mammary epithelial cells. In MDA-MB-231 cells, treatment with PGE(2) induced Id-1, an effect that was mimicked by an EP(4) agonist. PGE(2) via EP(4) activated the epidermal growth factor receptor (EGFR) --> ERK1/2 pathway, which led to increased expression of Egr-1. PGE(2) stimulated EGFR signaling by inducing the release of amphiregulin, an EGFR ligand. The ability of PGE(2) to activate Id-1 transcription was mediated by enhanced binding of Egr-1 to the Id-1 promoter. Silencing of COX-2 or pharmacological inhibition of COX-2 led to reduced PGE(2) production, decreased Id-1 expression, and reduced migration of cells through extracellular matrix. A similar decrease in cell migration was found when Id-1 was silenced. The interrelationship between COX-2, PGE(2), Id-1, and cell invasiveness was also compared in nontumorigenic SCp2 and tumorigenic SCg6 mammary epithelial cells. Consistent with the findings in MDA-MB-231 cells, COX-2-derived PGE(2) induced Id-1, leading in turn to increased cell invasiveness. Taken together, these results suggest that PGE(2) via EP(4) activated the EGFR --> ERK1/2 --> Egr-1 pathway, leading to increased Id-1 transcription and cell invasion. These findings provide new insights into the relationship between COX-2 and Id-1 and their potential role in   metastasis  . 
 Correlation between spiral CT preoperative staging of pancreatic cancer and PTEN and COX-2 expression. The aim of the present study was to evaluate the efficacy of TNM staging of pancreatic cancer by multilayer spiral computed tomography (MSCT) and its correlation with tissue PTEN and COX-2 expression.Fifty-two patients with pancreatic cancer had MSCT for TNM staging and PTEN and COX-2 expression were detected in pancreatic cancer tissue by immunohistochemistry.The total accuracy of MSCT was 79.5% in diagnosis of TMN staging of pancreatic cancer, with 73.1% for T staging (70.0% in T1, 65.0% in T2, 82.4% in T3 and 80.0% in T4), 76.9% for N staging (81.8% in N0 and 73.3% in N1) and 88.5% for M staging (89.7% in M0 and 84.6% in M1). The PTEN expression was not related to tumor size (p>0.05) but correlated with T staging, lymph node   metastasis   and clinical staging (p<0.05). COX-2 expression was not related to tumor size and T staging (p>0.05) but correlated with lymph node   metastasis   and clinical staging (p<0.05).TNM staging of pancreatic cancer was accurately conducted by MSCT-thin-layer-imaging. The correlation with PTEN and COX-2 expression implied that combined application of both approaches can improve the accuracy of diagnosis and staging in pancreatic cancer. 
 COX-2 expression correlates with VEGF-C and lymph node metastases in patients with head and neck squamous cell carcinoma. Recent observations suggest an implication of the cyclooxygenase-2 (COX-2) in tumor lymphangiogenesis through an upregulation of vascular endothelial growth factor-C expression. It is unknown whether this mechanism also acts in squamous cell carcinoma of the head and neck region. We performed a retrospective study of 70 patients with head and neck squamous cell carcinoma in order to investigate whether COX-2 immunohistochemical expression correlates with vascular endothelial growth factor-C expression. We also examined the association of the expression of these molecules with clinicopathologic parameters (especially lymph node status) and outcome for these patients. We performed immunostaining on formalin-fixed, paraffin-embedded tissue sections by the routine streptavidin-biotin peroxidase labeled procedure. Increased cyclooxygenase-2 expression was observed in 30 of the 68 tumor samples (44%), while high vascular endothelial growth factor-C expression occurred in 26 of the 68 tumor samples (38%). High expression of the two proteins was correlated with the presence of lymph node   metastasis   at the time of diagnosis, and the observed association was even stronger when there was overexpression for both the antibodies (P<0.001). High expression of vascular endothelial growth factor-C, but not of COX-2 was correlated with increased mortality in patients with oral-larynx squamous cell carcinoma. When multivariate Cox regression model was applied, the presence of lymph node   metastasis   at the time of diagnosis, combined with overexpression of both the antibodies, was the only independent prognostic factor for mortality of these patients. Our results suggest that a lymphangiogenic pathway, in which COX-2 overexpression stimulates vascular endothelial growth factor-C upregulation, probably exists in head and neck squamous cell carcinoma. Also, the predictive ability for mortality of regional lymph node   metastasis   can be improved with the combined evaluation of the immunohistochemical expression of these two proteins. 
 Cyclooxygenase-2 expression in primary breast cancers predicts dissemination of cancer cells to the bone marrow. Cyclooxygenase-2 (COX2) plays a role in breast cancer progression at various stages starting from pre-malignant phenotype to clinical   metastasis  . Breast cancer metastasizes commonly to the bone and preclinical studies suggest an involvement of COX2 in this process. Detection of disseminated tumor cells in the bone marrow of patients at the time of surgery correlates with the subsequent development of clinical bone   metastasis  . Therefore, to investigate whether COX2 is important for breast cancer   metastasis   in humans, we analyzed COX2 protein expression by immunostaining of primary tumors from 112 operable stages I, II, or III patients and determined its correlation with bone marrow micro  metastasis   (BMM).We detected COX2 protein in primary tumors by immunostaining with a monoclonal antibody, and tumor cells present in the bone marrow by immunostaining for epithelial cytokeratins and by morphological criteria.COX2 expression in primary breast cancer correlated with BMM in a highly statistically significant manner (P = 0.006). Our statistical analyses of correlations of the COX2 positivity in primary tumor with other clinically relevant indicators revealed that COX2 positivity correlates with high nuclear grade (P = 0.0004). Furthermore, we were able to detect COX2 protein in BMM by immunostaining.These studies indicate that COX2 produced in primary breast cancer cells may be vital to the initial development of BMM that may subsequently lead to osteolytic bone metastases in patients with breast cancer, and that COX2 inhibitors may be useful in halting this process. 
 Functional polymorphisms of cyclooxygenase-2 (COX-2) gene and risk for urinary bladder cancer in North India. Cyclooxygenase-2 (COX-2) is an enzyme involved in the synthesis of prostaglandins and thromboxanes, which are regulators of processes that are relevant to cancer development. It is involved in carcinogenesis, immune response suppression, apoptosis inhibition, angiogenesis, and tumor cell invasion and   metastasis  . The gene for COX-2, designated as prostaglandin-endoperoxide synthase 2 (PTGS-2), carries polymorphisms, such as -765G>C, 1195G>A in the promoter region, and 8473T>C in the 3'-untranslated region (UTR), which have been associated with susceptibility to malignant disease.We undertook a case-control study of 212 urothelial bladder cancer (UBC) cases and 250 controls to investigate the association between COX-2 polymorphism and bladder cancer susceptibility, using the polymerase chain reaction restriction fragment length polymorphism (PCR-RFLP) method and also investigated gene-environment interactions.Cox-2 765G>C, a variant(C) allele carrier, was at an increased risk of UBC (odds ratio [OR] = 1.90; P = .004); however, -1195G>A; -1290A>G; and 3'UTR 8473T>C polymorphisms of COX-2 gene were not significantly associated with UBC. 765G>C also was associated with the invasive stage of a bladder tumor (OR = 2.73; P = .033). High risk for UBC also was observed with respect to COX-2 haplotypes C-765T8473A-1195A-1290 (OR = 3.47; P = .014). In case-only analysis, COX-2 765 variant allele carrier genotypes also showed an increased risk among former and current smokers (OR = 3.06; P = .041 and OR = 4.39; P = .032, respectively).COX-2 -765G>C polymorphism confers UBC susceptibility particularly in smokers and in patients with invasive tumors. 765C allele carrier genotypes also are influenced with a high risk of recurrence in Bacillus Calmette-Guérin-treated patients. Collectively, these findings confirm that the COX-2 -765G>C polymorphism is a risk factor for the development of bladder cancer and can provide a plausible mechanistic explanation. Further validation in large population-based studies is needed. 
 Endothelin-1 enhances cell migration through COX-2 up-regulation in human chondrosarcoma. Chondrosarcoma is a type of highly malignant tumor with a potent capacity of local invasion and distant   metastasis  . The effect of endothelin-1 (ET-1) on migration activity in human chondrosarcoma cells is not clearly understood. Here, we found that ET-1 increased the migration and expression of cyclooxygenase (COX)-2 in human chondrosarcoma cells.ET-1-mediated COX-2 expression was assessed by qPCR and Western blot analysis. The mechanisms of action of ET-1 in different signaling pathways were studied using Western blotting. Knockdown of proteins was achieved by transfection with siRNA. Chromatin immunoprecipitation assays were used to study in vivo binding of c-Jun to the COX-2 promoter.Human chondrosarcoma tissues had significant expression levels of ET-1 and COX-2, which were higher than that in normal cartilage. Exogenous ET-1 increased cell migration and the expression of COX-2. In addition, COX-2 protein levels and cell migration ability were abolished by ET receptor antagonists. Activation of the mitogen-activated protein kinase (MAPK) and activator protein-1 (AP-1) pathways after ET-1 treatment was demonstrated, and ET-1-induced COX-2 expression and cell migration activity were inhibited by the specific inhibitor and mutant of MAPK and AP-1 cascades. ET-1 increased the binding of c-Jun to the AP-1 element on the COX-2 promoter. Furthermore, knockdown of ET-1 decreased cell   metastasis   in vitro and in vivo.Our results indicated that ET-1 enhances the cell migration of chondrosarcoma by increasing COX-2 expression through the ET receptors, MAPK, and AP-1 signal transduction pathway.We link high ET-1 and COX-2 expression to chondrosarcoma. 
 Regulation of COX-2 expression and epithelial-to-mesenchymal transition by hypoxia-inducible factor-1α is associated with poor prognosis in hepatocellular carcinoma patients post TACE surgery. Currently, it is not entirely clear whether hypoxia-inducible factor-1α (HIF-1α) is involved in the regulation of COX-2 expression and epithelial-to-mesenchymal transition (EMT), and whether these events affect the prognosis of hepatocellular carcinoma (HCC) patients treated with transcatheter arterial chemoembolization (TACE). In this report the relationship between HIF-1α and COX-2 protein expression, EMT in tumor specimens from HCC patients after TACE surgery and the clinical significance of HIF-1α and COX-2 expression were analyzed using statistical approaches. HepG2 cells treated with CoCl2 was employed as a hypoxia cell model in vitro to study hypoxia-induced HIF-1α, COX-2 expression, and EMT alteration. The results showed that HIF-1α and COX-2 protein expression increased in HCC tissues after TACE surgery. Moreover, there was positive correlation between upregulation of HIF-1α and COX-2. Elevated expression of HIF-1α increased both Snail and Vimentin protein expression, while it reduced E-cadherin protein expression. It was further verified that hypoxia enhanced protein expression of HIF-1α and COX-2 in HepG2 cells treated with CoCl2. Upregulation of HIF-1α and COX-2, together with EMT alteration resulted in increased migration and invasion of HepG2 cells under hypoxia. In conclusion, TACE surgery results in aggravated hypoxia status, leading to increased HIF-1α protein expression in HCC tissue. To adapt to hypoxic environment, HIF-1α stimulates COX-2 protein expression and promotes EMT process in hepatocellular cancer cells, which enhances HCC   invasion a  nd metastasis, and might contribute to poor prognosis in HCC patients post TACE treatment. 
 Expression of COX-2 and HER-2 in colorectal cancer and their correlation. To detect the expression of COX-2 and HER-2 in colorectal cancer and to analyze their correlation and clinical significance.A total of 1026 colorectal cancer surgical specimens were collected from patients treated from December 2002 to December 2007 at the First Affiliated Hospital of Anhui Medical University. All specimens were made into 4-μm slices. The expression of COX-2 and HER-2 were detected by immunohistochemistry using the streptavidin-biotin-peroxidase method. The correlations between COX-2 and HER-2 expression and colorectal cancer clinical features were analyzed.The positive rates of COX-2 and HER-2 expression in colorectal cancer were 77.97% (800/1026) and 46.20% (474/1026), respectively. There was a significant correlation between COX-2 and HER-2 expression in colorectal cancer (P < 0.05). In patients with tumor size ≥ 5 cm, the positive rates of COX-2 and HER-2 expression were 81.48% (308/378) and 57.94% (219/378), respectively. In patients with serosal invasion, the positive COX-2 and HER-2 expression rates were 80.53% (612/760) and 49.21% (374/760), respectively. In patients with lymph no  de metasta  sis, the positive expression rates were 85.04% (506/595) and 54.62% (325/595), respectively, and the positive expression rates differed significantly between patients with lymph no  de metasta  sis and those without (P < 0.05). In patients with Duke's C and D colorectal cancer, the positive COX-2 and HER-2 expression rates were 82.80% (443/535) and 57.94% (310/535), respectively. In patients with poorly differentiated colorectal cancer, the positive expression rates were 74.49% (210/282) and 52.84% (149/282), respectively (P < 0.05). In patients with dista  nt metasta  sis, the positive expression rates were 82.27% (116/141) and 53.90% (76/141), respectively (P < 0.05). These findings suggest that COX-2 and HER-2 have synergistic effects in colorectal cancer. COX-2 and HER-2 expression had no significant correlation with sex, age, or tumor location.COX-2 and HER-2 are important markers for invasion a  nd metasta  sis of colorectal cancer, and they act together to regulate the invasion a  nd metasta  sis of colorectal cancer. 
 Correlation between expression of cyclooxygenase-2 and angiogenesis in human gastric adenocarcinoma. To evaluate the expression of cyclooxygenase (COX-2) and the relationship with tumor angiogenesis and advancement in gastric adenocarcinoma.Immunohistochemical stain was used for detecting the expression of COX-2 in 45 resected specimens of gastric adenocarcinoma; the monoclonal antibody against CD34 was used for displaying vascular endothelial cells, and microvascular density (MVD) was detected by counting of CD34-positive vascular endothelial cells. Paracancerous tissues were examined as control.Immunohistological staining with COX-2-specific polyclonal antibody showed cytoplasmic staining in the cancer cells, some atypical hyperplasia and intestinal metaplasia,as well as angiogenic vasculature present within the tumors and prexisting vasculature adjacent to cancer lesions. The rate of expression of COX-2 and MVD index in gastric cancers were significantly increased, compared with those in the paracancerous tissues (77.78 vs 33.33 %, 58.13+/-19.99 vs 24.02+/-10.28, P<0.01, P<0.05, respectively). In 36 gastric carcinoma specimens with lymph node   metastasis  , the rate of COX-2 expression and MVD were higher than those in the specimens without metostasis (86.11 vs 44.44 %, 58.60+/-18.24 vs 43.54+/-15.05, P<0.05, P<0.05, respectively). The rate of COX-2 expression and MVD in the specimens with invasive serosa were significantly higher than those in the specimens without invasion to serosa (87.88 vs 50.0 %, 57.01+/-18.79 vs 42.35+/-14.65, P<0.05, P<0.05). Moreover, MVD in COX-2-positive specimens was higher than that in COX-2-negative specimens (61.29+/-14.31 vs 45.38+/-12.42,P<0.05). COX-2 expression was positively correlated with MVD (r=0.63, P<0.05).COX-2 expression might correlate with the occurance and advancement of gastric carcinoma and is involved in tumor angiogenesis in gastric carcinoma. It is likely that COX-2 by inducing angiogenesis can be one of mechanisms which promotes invasion and   metastasis   of gastric carcinoma. It may become a new therapeutic target for anti-angiogenesis. 
 RhoA mediates cyclooxygenase-2 signaling to disrupt the formation of adherens junctions and increase cell motility. Cyclooxygenase-2 (COX-2) represents an important target for treatment and prevention of colorectal cancer. Although COX-2 signaling is implicated in promoting tumor cell growth and invasion, the molecular mechanisms that mediate these processes are largely unknown. In this study, we show that the RhoA pathway mediates COX-2 signaling to disrupt the formation of adherens junctions and increase cell motility. Disruption of adherens junctions promotes tumor cell invasion and   metastasis   and is often associated with tumor progression. We detected high levels of RhoA activity in HCA-7 colon carcinoma cells that constitutively express COX-2. Inhibition of COX-2 significantly reduced the levels of RhoA activity in HCA-7 cells, suggesting that constitutive expression of COX-2 stimulates RhoA activity. Interestingly, inhibition of COX-2 or silencing of COX-2 expression with small interfering RNA (siRNA) stimulated the formation of adherens junctions, concomitant with increased protein levels of E-cadherin and alpha-catenin. Furthermore, inhibition of RhoA or silencing of RhoA expression with siRNA increased the levels of E-cadherin and alpha-catenin. Inhibition of Rho kinases (ROCK), the RhoA effector proteins, also increased levels of E-cadherin and alpha-catenin and stimulated formation of adherens junctions. The motility of HCA-7 cells was significantly decreased when COX-2 or RhoA was inhibited. Therefore, our data reveal a novel molecular mechanism that links COX-2 signaling to disrupt the formation of adherens junctions; COX-2 stimulates the RhoA/ROCK pathway, which reduces levels of E-cadherin and alpha-catenin leading to disruption of adherens junction formation and increased motility. Understanding of COX-2 downstream signaling pathways that promote tumor progression is crucial for the development of novel therapeutic strategies. 
 Quantitative assessment of the association of COX-2 (Cyclooxygenase-2) immunoexpression with prognosis in human osteosarcoma: a meta-analysis. Numerous studies examining the relationship between Cyclooxygenase-2 (COX-2) immunoexpression and clinical outcome in osteosarcoma patients have yielded inconclusive results.We accordingly conducted a meta-analysis of 9 studies (442 patients) that evaluated the correlation between COX-2 immunoexpression and clinical prognosis (death). Pooled odds ratios (OR) and risk ratios (RR) with 95% confidence intervals (95% CI) were calculated using the random-effects or fixed-effects model.Meta-analysis showed no significant association between COX-2 positivity and age, gender, tumor location, histology, stage,   metastasis   or 90% necrosis. Conversely, COX-2 immunoexpression was associated with overall survival rate (RR=2.12; 95% CI: 1.10-3.74; P=0.009) and disease-free survival rate (RR=1.63; 95% CI: 1.17-2.28; P=0.004) at 2 years. Sensitivity analysis performed by omitting low quality studies showed that the pooled results were stable.COX-2 positivity was associated with a lower 2-year overall survival rate and disease-free survival rate. COX-2 expression change is an independent prognostic factor in patients with osteosarcoma. 
 Expression of cyclooxygenase-2 in gastric cancer and its relation to liver   metastasis   and long-term prognosis. To investigate the expression of cyclooxygenase-2 (COX-2) in gastric cancer and its relation with the liver   metastasis   and prognosis.Expression of COX-2 mRNA and protein was examined in gastric cancer and its paired substantial normal tissue by semi-quantitative reverse transcription-polymerase chain reaction and immunohistochemistry. The relation between COX-2 expression and prognosis was investigated in 195 cases.The expression of COX-2 mRNA in gastric cancer tissue was significantly higher than that in normal tissue in 47 cases (w = 792, P<0.01). The COX-2 mRNA in pT3-4 tissue expressed higher than that in pT1-2 tissue (w = 204, P<0.05). The positive expression rate of COX-2 protein was 57.9% (113/195). The COX-2 expression was significantly related to histological type, lymphnode   metastasis  , venous invasion and liver   metastasis   (P<0.05). No relation was found between COX-2 expression and invasion depth, peritoneal   metastasis   and International Union against Cancer TNM stage. The multiple regression analysis showed that the COX-2 expression and venous invasion were obviously associated with liver   metastasis   (P<0.05). However, there was no significant correlation between COX-2 immunoreactivity and prognosis.COX-2 may play an important role in the development of gastric cancer, and the over-expression of COX-2 protein may be a high risk factor for liver   metastasis  . 
 Cyclooxygenase-2 expression as a predictor of outcome in colorectal carcinoma. To correlate cyclooxygenase-2 (COX-2) expression profile with clinical and pathological variables to assess their prognostic/predictive value in colorectal carcinoma (CRC).Archival tumor samples were analyzed using immunohistochemistry for COX-2 expression in 94 patients with CRC. Patients were diagnosed and treated at the Departments of Surgery and Oncology, King Abdulaziz University Hospital, Saudi Arabia.Fifty-six percent of the tumors showed positive cytoplasmic COX-2 expression, whereas 44% of cases were completely COX-2-negative. There were no significant correlations between COX-2 expression and sex, age, grade or tumor location. However, COX-2 expression revealed a significant correlation with tumor stage (P = 0.01) and distant   metastasis   (P = 0.02), and a borderline association with lymph node involvement (P = 0.07). Tumors with high COX-2 expression showed a higher recurrence rate than tumors with no expression (P < 0.009). In univariate Kaplan-Meier survival analysis, there was a significant (P = 0.026) difference in disease-free survival between COX-2-positive and negative tumors in favor of the latter. COX-2 expression did not significantly predict disease-specific survival, which was much shorter for COX-2-positive tumors. In multivariate (COX) models, COX-2 did not appear among the independent predictors of disease-free survival or disease-specific survival.COX-2 expression seems to provide useful prognostic information in CRC, while predicting the patients at high risk for recurrent disease. 
 [Expression of cyclooxygenase-2 in esophageal squamous cell carcinogenesis]. To investigate the expression of cyclooxygenase-2 (cox-2) protein in normal squamous epithelium, squamous dysplasia and squamous cell carcinoma of the esophagus, and to elucidate the role of cox-2 in esophageal carcinogenesis.Biopsy specimens of atypical esophageal dysplasia (n = 47) and surgical resection of squamous cell carcinoma (n = 86) were compared with normal esophageal specimens (n = 42) and the expression of cox-2 in those specimens was analyzed by immunohistochemistry and western blotting.A significant elevated cox-2 expression was shown in atypical esophageal squamous dysplasia and squamous cell carcinoma, as compared to that in normal esophageal squamous epithelium, with immunohistochemical stain scores of 2.67 +/- 1.77, 2.19 +/- 1.79 and 0.71 +/- 0.46, respectively. Results of western blotting analysis confirmed those obtained by immunohistochemistry. Cox-2 expression significantly correlated with proliferation activity assessed by proliferating cell nuclear antigen index in dysplastic and carcinomous lesions, respectively, and no such correlation could be found in normal esophageal mucosa. Elevated cox-2 expression was not associated with clinical-pathological features of esophageal squamous carcinoma, including age, gender, tumor size, histological grade, lymph node   metastasis   and clinical stage.Elevated expression of cox-2 in atypical squamous dysplasia and squamous cell carcinoma of the esophagus, which correlated with cell proliferation activity, indicated that cox-2 may be involved in the early stage of squamous carcinogenesis of the esophagus, and may be a target of prevention and treatment for esophageal squamous cell carcinoma. 
 Interleukin-17 and prostaglandin E2 are involved in formation of an M2 macrophage-dominant microenvironment in lung cancer. Tumor-associated macrophages (TAMs) are divided into M1 and M2 macrophages. M1 macrophages inhibit tumor growth, whereas M2 macrophages promote tumor growth and   metastasis  . The aim of this study was to examine the possible causes leading to the formation of an M2-macrophage-dominant tumor microenvironment in non-small-cell lung cancer.Forty-eight archived lung tumor samples were examined for the expression of interleukin-17 (IL-17) receptors, IL-17 receptor A (IL-17RA) and IL-17 receptor C (IL-17RC), and the number of TAMs using immunohistochemical staining. Twenty fresh lung tumors and matched normal lung tissues were examined for expression of IL-17, cyclooxygenase-2, and prostaglandin E2 (PGE2), using enzyme-linked immunosorbent assay and Western blot analysis. Macrophage-migration assays were performed using fresh lung tumor tissues and IL-17 as chemoattractants. Induction of M2-macrophage differentiation was analyzed using real-time quantitative polymerase chain reaction.TAMs expressed IL-17RA and IL-17RC. Lung tumors expressed higher levels of IL-17, cyclooxygenase-2, and PGE2, compared with normal lung tissues. Lung tumor tissues attracted migration of mouse RAW264.7 macrophages and primary peritoneal macrophages through IL-17, which was mediated by IL-17RA and IL-17RC. IL-17 did not induce either M1- or M2-macrophage differentiation. However, human lung cancer A549 cells strongly induced M2-macrophage differentiation of RAW264.7 macrophages when the two cell lines were cocultured. The inductive factor secreted by A549 cells was identified to be PGE2.IL-17 recruits macrophages, and PGE2 induces M2-macrophage differentiation, hence the increased levels of IL-17 and PGE2 in lung cancer contribute to the formation of an M2-macrophage-dominant tumor microenvironment. 
 The expression of both peroxisome proliferator-activated receptor delta and cyclooxygenase-2 in tissues is associated with poor prognosis in colorectal cancer patients. The role of peroxisome proliferator-activated receptor delta (PPAR δ) in the development and progression of colorectal cancer (CRC) remains controversial.We investigated the impact of PPAR δ expression in tissues on live  r metastas  is of CRC.We analyzed samples of primary CRC and matched normal adjacent tissues from 52 patients for the expression of PPAR δ, cyclooxygenase (COX)-2, vascular endothelial growth factor (VEGF)-A, and CXC chemokine receptor 4 (CXCR4). Correlations of the molecules expressions with clinical characteristics and prognosis of patients were studied.The number of patients positive for PPAR δ, COX-2, CXCR4, and VEGF-A was 25, 33, 18, and 19, respectively. Among the PPAR δ (+)/COX-2 (+), PPAR δ (-)/COX-2 (+), PPAR δ (+)/COX-2 (-), and PPAR δ (-)/COX-2 (-) patient groups, PPAR δ (+)/COX-2 (+) patients had the highest incidence   of liver m  etastasis (p<0.01). PPAR δ (+)/COX-2 (+) expression was a significant independent prognostic factor (HR=7.108, 95% CI 1.231-41.029, p=0.0283) by Cox proportional analysis. PPAR δ (+)/COX-2 (+) patients had the highest positivity for CXCR4 or VEGF-A in tissues (p<0.01). Among the patients in the CXCR4 (+)/VEGF-A (+), CXCR4 (+)/VEGF-A (-), CXCR4 (-)/VEGF-A (+), and CXCR4 (-)/VEGF-A (-) groups, CXCR4 (+)/VEGF-A (+) patients had the highest incidenc  e of liver   metastasis (p<0.01).The expression of both PPAR δ and COX-2 in tissues may le  ad to live  r metastasis and consequent poor prognosis in CRC patients. 
 [Correlation of cyclooxygenase 2 expression with microlymphatic density and its clinical significance]. To study the expression of cyclooxygenase-2 (COX-2) in esophageal carcinoma and its correlation with microlymphatic density (MLD), and to investigate the clinicopathological and prognostic significance of COX-2 and MLD in patients with esophageal cancer.COX-2 expression and MLD were detected by immunohistochemistry in 100 cases of esophageal squamous cell carcinoma. Follow up was available in 76 patients. Multivariable Cox regression was used to analyze the association between the laboratory indices and overall survival of patients with esophageal carcinoma.COX-2 expression was present in 73% of patients. MLD in patients with high COX-2 expression (99.71±39.62) was significantly higher than that in those with low or no COX-2 expression (80.22±30.36) (P<0.05). No correlations were observed between the over expression of COX-2 and clinicopathologic parameters including tumor size and lymphati  c metastas  is (P<0.05). However, MLD was associated with lymphati  c metastas  is and the depth of invasion (P<0.05, P<0.01). In the 76 patients with followed up, the median survival was 25.5 months. Cox regression showed that the COX-2 expression, histological grade of the tumor and MLD were risk factors of overall survival of esophageal carcinoma.COX-2 may contribute to the lymphangiogenesis in the tumor. COX-2 may be a new target point for the treatment of esophageal carcinoma. COX-2 expression and microlymphatic vessel density are of significant prognostic value for esophageal carcinoma. 
 Down-regulation of CacyBP is associated with poor prognosis and the effects on COX-2 expression in breast cancer. Calcyclin-binding protein (CacyBP) is a tumor suppressor in gastric and renal cell carcinoma, but an oncogene in pancreatic cancer. However, the function of CacyBP in breast cancer has not been well elucidated. In this study, we explored the clinical relevance of CacyBP and investigated the relationship between CacyBP and COX-2 in breast cancer. Immunohistochemical analysis in 172 cases of breast tissues showed that the positive rate of CacyBP protein expression in normal breast tissues (NBT) (89.3%) was higher than that in invasive ductal carcinoma (IDC) (56.1%) (P<0.05). RT-PCR and Western blot analysis showed that CacyBP mRNA and protein expression were significantly lower in tumor tissues as compared to those in the corresponding non-tumorous tissues (P<0.05). The expression trend of COX-2 was opposite with CacyBP in breast carcinogenesis. Moreover, the CacyBP expression was significantly negatively associated with the COX expression in the 132 breast cancer samples (correlation coefficient = 0.505, P<0.001). The clinicopathological data analysis in 132 breast cancer samples showed that CacyBP expression was positively correlated with well differentiated samples (P=0.021), low pathologic TNM stage (P=0.009), and no lymphatic   metastasis   (P=0.027) of patients with breast cancer. Furthermore, reduced CacyBP expression was associated with poor prognosis. Knockdown of CacyBP gene using siRNA enhanced the proliferation and invasion ability of breast cancer cells, which was dependent on COX-2 expression. In conclusion, CacyBP regulation of COX-2 expression may play an important role in human breast carcinogenesis. Restoration of CacyBP gene is a potential therapeutic target of breast cancer. 
 Cyclooxygenase-2 immunoexpression in breast cancer: progesterone receptor influence. In breast cancer cyclooxygenase-2 overexpression is related with poor outcomes, but the clinical relevance of cyclooxygenase-2 is still unclear. Progesterone receptor is a regulator of cyclooxygenase-2 expression. We postulate that progesterone receptor was associated to cyclooxygenase-2 expression and clinicopathologic factors in breast cancer.Cyclooxygenase-2 and progesterone receptor expression was analyzed, by immunohistochemistry, in 31 cases of invasive ductal carcinoma.The expression of cyclooxygenase-2 and progesterone receptor was observed in 64.5% and 45.2% of the tumors, respectively. We found 11 tumors with both cyclooxygenase-2 and progesterone receptor overexpression and they are related with tumor lower size and lower axillary node   metastasis  .These results suggest that progesterone receptor has a protective role in breast cancer by inhibiting chronic inflammatory pathway. 
 Prognostic significance of VEGF-C expression in correlation with COX-2, lymphatic microvessel density, and clinicopathologic characteristics in human non-small cell lung cancer. Lung cancer is one of the most lethal cancers in China because of high incidence and high mortality. Cyclooxygenase-2 (COX-2) and vessel endothelial growth factor C (VEGF-C) were found to play an important role in lymphangiogenesis of malignant tumors. In this study, we investigated whether lymphatic microvessel density (LMVD) is related to the prognosis in non-small cell lung cancer (NSCLC) patients, and the expressions of COX-2 and VEGF-C so as to determine the possible role of COX-2 and VEGF-C in NSCLC lymphangiogenesis. Sixty-five formalin-fixed paraffin embedded tissue samples of NSCLC were evaluated for COX-2 and VEGF-C by immunohistochemical staining. To assess tumor lymphangiogenesis, LMVD was determined by immunohistochemical staining of VEGFR-3 polyclonal antibody. The relationship among COX-2 and VEGF-C expression, LMVD, and clinicopathologic parameters was analyzed. Among the 65 samples, high LMVD was significantly associated with lymph node   metastasis   and poor survival. Multivariate survival analysis showed that LMVD value and lymph node   metastasis   were independent prognostic factors. The expression level of COX-2 and VEGF-C was significantly higher than those of the adjacent tissues. COX-2 and VEGF-C expressions in NSCLC significantly correlated with lymph node   metastasis  , but not with patient gender, age, tumor size, or tumor, nodes,   metastasis   classification stage. The mean LMVD value of COX-2- or VEGF-C-positive tumors was higher than that of COX-2- or VEGF-C-negative tumors. A significant correlation was found between the expressions of COX-2 and VEGF-C. This study suggests that LMVD may be one of the important prognostic factors for NSCLC patients. VEGF-C might play an important role in the COX-2 lymphangiogenic pathway. COX-2 and VEGF-C may play an important role in tumor progression by stimulating lymphangiogenesis. The inhibition of lymphangiogenesis, COX-2, or VEGF-C activity may have an important therapeutic benefit in the control of NSCLC. 
 Prognostic relevance of cyclooxygenase-2 (COX-2) expression in Chinese patients with prostate cancer. Cyclooxygenase-2 (COX-2), an inducible isoform of cyclooxygenase, has been reported to be correlated with tumorigenesis, tumor progression and   metastasis  . The present study was designed to investigate the clinicopathological and prognostic significance of COX-2 in Chinese patients with prostate cancer. Firstly, RT-PCR and Western blot assays were performed to detect the expression of COX-2 mRNA and protein in prostate cancer cell lines and 20 tissue samples (tumor or corresponding non-tumor). Next, immunohistochemistry was performed to detect the expression of COX-2 protein in 88 prostate cancer tissue samples. Finally, the correlation between COX-2 expression and clinicopathological factors and patient survival was evaluated. We found that the expression levels of COX-2 mRNA and protein showed significant difference among four prostate cancer cell lines. Moreover, the levels of COX-2 mRNA and protein were significantly higher in prostate cancer tissues than in corresponding non-tumor tissues. COX-2 staining was positive in the cytoplasm of prostate cancer cells. High-COX-2 expression was correlated with the Gleason score (P=0.009), tumor stage (P=0.012), and lymph-node status (P=0.036). Furthermore, patients with high-COX-2 expression showed lower disease-free (P=0.014) and overall survival (P=0.047) rates than those with low-COX-2 expression. Univariate and multivariate analyses suggested that the status of COX-2 protein expression was an independent prognostic indicator for patients' survival. Taken together, higher COX-2 protein expression might provide an independent prognostic marker for Chinese patients with prostate cancer who have undergone surgery. 
 Expression of cyclooxygenase-2 and clinicopathologic features in human gastric adenocarcinoma. To study the expression of cyclooxygenase-2 (COX-2) gene in gastric cancer and the relationship between COX-2 expression and clinicopathologic features of gastric cancer.With reference to the expression of beta-actin gene, COX-2 mRNA level was examined in cancerous tissues and adjacent noncancerous mucosa from 33 patients by semiquantitative reverse transcription-polymerase chain reaction (RT-PCR). Quantitation of relative band Adj volume counts was performed using molecular Analyst for windows software. The COX-2 index was determined from the band Adj volume counts ratio of COX-2 to constitutively expressed actin.The COX-2 index in gastric carcinoma was significantly higher than that in normal mucosa (0.5966+/-0.2659 vs 0.2979+/-0.171, u=5.4309, P<0.01). Significantly higher expression of COX-2 mRNA was also observed in patients with lymph node involvement than that in those without (0.6775+/-0.2486 vs 0.4105+/-0.2182, t=2.9341, P<0.01). Furthermore, the staging in the UICC TNM classification significantly correlated with COX-2 overexpression (F=3.656, P<0.05), the COX-2 index in stage III and IV was significantly higher than those in stage I and II (q=3.2728 and q=3.4906, P<0.05). The COX-2 index showed no correlation with patient's age, sex, blood group, tumor location, gross typing, depth of invasion, differentiation, and the greatest tumor dimension (P>0.05).Expression of COX-2 mRNA in gastric carcinoma was significantly higher, which may enhance lymphatic   metastasis   in patients with gastric carcinoma. The staging in the UICC TNM classification was significantly correlated with COX-2 over-expression. COX-2 may contribute to progression of tumor in human gastric adenocarcinoma. 
 shRNA-targeted cyclooxygenase (COX)-2 inhibits proliferation, reduces invasion and enhances chemosensitivity in laryngeal carcinoma cells. Cyclooxygenase-2 (COX-2), one isoform of cyclooxygenase proinflammatary enzymes, is a causal factor for tumor development, invasion,   metastasis  , and chemoresistance. It is frequently overexpressed in a variety of human malignancies, including laryngeal carcinoma. To investigate its possibility as a therapeutic target for the treatment of laryngeal carcinoma, we employed RNA interference technology to downregulate endogenous gene COX-2 expression in laryngeal carcinoma cells and analyzed its phenotypical changes. Results showed that shRNA-mediated downregulation of COX-2 expression in human laryngeal carcinoma cells significantly inhibited cell proliferation and colony formation in vitro and reduced the potential of tumorigenicity in vivo. The specific downregulation led to cell arrest in the G(0)/G(1) phase of cell cycle and final apoptosis induction. The increased apoptosis was associated with the ratios of Bcl-2 or Bcl-xL/Bax. In the present study, we also observed that the downregulation of COX-2 could obviously enhanced the cytotoxic effect of Taxanes both in vitro and in vivo. All these results suggest that knockdown of COX-2 expression can lead to potent antitumor activity and chemosensitizing activity to taxanes in human laryngeal carcinomas. 
 Cyclooxygenase-2 (COX-2) overexpression in meningiomas: real time PCR and immunohistochemistry. Cyclooxygenase-2 (COX-2) is the inducible form of the enzyme involved in the first steps of the prostaglandins and thromboxane synthesis. COX-2 up-regulation is demonstrated in tumors where it can modulate tumoral progression,   metastasis  , multidrug resistance, and angiogenesis. Experimental data suggest a possible therapeutic use of the COX-inhibitors nonsteroidal antiinflammatory drugs (NSAIDs). NSAIDs can block tumor growth through many mechanisms, especially through antiangiogenic and proapoptotic effects. Moreover, NSAIDs can also improve the efficacy of radiotherapy, chemotherapy, and hormonal therapy. This study reviews the COX-2 expression as evaluated through immunohistochemistry and real time polymerase chain reaction (RT-PCR) in 23 meningiomas [14 World Health Organization (WHO) grade I; 5 WHO grade II; 3 WHO grade III; 1 oncocytic meningioma]. At immunohistochemistry all the lesions but 4 (83%) were COX-2 positive. At RT-PCR 9 meningiomas, 8 WHO grade I and 1 WHO grade II, showed a COX-2 expression greater than the reference value (average expression of all meningiomas that we studied). The association between tumor grade and immunohistochemical or RT-PCR COX-2 expression was not significant (P=0.427 and P=0.251, respectively). In conclusion, even if further studies on larger series are necessary, the common COX-2 overexpression in meningiomas may suggest considering the COX-2 inhibitors, alone or in combination with radiotherapy, a potential area of therapeutic intervention in some selected meningiomas. 
 Noninvasive imaging identifies new roles for cyclooxygenase-2 in choline and lipid metabolism of human breast cancer cells. The expression of cyclooxygenase-2 (COX-2) is observed in approximately 40% of breast cancers. A major product of the COX-2-catalyzed reaction, prostaglandin E(2), is an inflammatory mediator that participates in several biological processes, and influences invasion, vascularization and   metastasis  . Using noninvasive MRI and MRS, we determined the effect of COX-2 downregulation on the metabolism and invasion of intact poorly differentiated MDA-MB-231 human breast cancer cells stably expressing COX-2 short hairpin RNA. Dynamic tracking of invasion, extracellular matrix degradation and metabolism was performed with an MRI- and MRS-compatible cell perfusion assay under controlled conditions of pH, temperature and oxygenation over the course of 48  h. COX-2-silenced cells exhibited a significant decrease in invasion relative to parental cells that was consistent with the reduced expression of invasion-associated matrix metalloproteinase genes and an increased level of the tissue inhibitor of metalloproteinase-1. We identified, for the first time, a role for COX-2 in mediating changes in choline phospholipid metabolism, and established that choline kinase expression is partly dependent on COX-2 function. COX-2 silencing resulted in a significant decrease in phosphocholine and total choline that was detected by MRS. In addition, a significant increase in lipids, as well as lipid droplet formation, was observed. COX-2 silencing transformed parental cell metabolite patterns to those characteristic of less aggressive cancer cells. These new functional roles of COX-2 may identify new biomarkers and new targets for use in combination with COX-2 targeting to prevent invasion an  d metastas  is. 
 Cyclooxygenase-2 up-regulates CCR7 via EP2/EP4 receptor signaling pathways to enhance lymphatic invasion of breast cancer cells. Recent studies demonstrate that cyclooxygenase-2 (COX-2) expression is frequently associated with lymph node   metastasis  . However, the mechanism by which COX-2 increases the invasion of cancer cells to lymph node is unclear. CCR7 is a chemokine receptor that plays important roles in the mediation of migration of leukocytes and dendritic cells toward lymphatic endothelial cells (LECs) that express receptor ligand CCL21. We found that treatment of prostaglandin E(2) or ectopic expression of COX-2 in MCF-7 cells up-regulated CCR7 expression. On the contrary, knockdown of COX-2 by small hairpin RNA reduced CCR7 in COX-2-overexpressing MDA-MB-231 cells. Interaction of CCR7 and CCL21 was important for the migration of breast cancer cells toward LECs because antibodies against these two molecules inhibited the migration. We also found that COX-2 increased CCR7 expression via the EP2 and EP4 receptor in breast cancer cells. EP2 and EP4 agonists stimulated CCR7 in MCF-7 cells, whereas antagonists or small hairpin RNA of EP2 and EP4 attenuated CCR7 in MDA-MB-231 cells. Protein kinase A and AKT kinase were involved in COX-2-induced CCR7. Pathological analysis demonstrated that COX-2 overexpression was associated with CCR7, EP2, and EP4 expressions in breast tumor tissues. In addition, CCR7 expression in COX-2-overexpressing tumors was significantly correlated with lymph node   metastasis  . Collectively, we suggest that CCR7 is a down-stream target for COX-2 to enhance the migration of breast cancer cells toward LECs and to promote lymphatic invasion. 
 Epidermal growth factor receptor (EGFR) and prostaglandin-endoperoxide synthase 2 (PTGS2) are prognostic biomarkers for patients with resected colorectal cancer liver metastases. Resection of colorectal cancer liver   metastasis   (CRCLM) with curative intent has long-term benefit in ~40% of cases. Prognostic biomarkers are needed to improve clinical management and reduce futile surgeries. Expression of epidermal growth factor receptor (EGFR) and prostaglandin-endoperoxide synthase 2 (PTGS2; also known as cyclooxygenase-2) has been associated with carcinogenesis and survival. We investigated the prognostic value of EGFR and PTGS2 expression in patients with resected CRCLM.Formalin-fixed paraffin-embedded CRCLM tissue and corresponding primary tumour specimens from a multi-institutional cohort of patients who underwent liver resection between 1990 and 2010 were incorporated into tissue microarrays (TMAs). TMAs were stained for EGFR and PTGS2 by immunohistochemistry. The hazard rate ratio (HRR) for the association between expression in CRCLM and overall survival was calculated using a 500-fold cross-validation procedure.EGFR and PTGS2 expression could be evaluated in 323 and 351 patients, respectively. EGFR expression in CRCLM was associated with poor prognosis (HRR 1.54; P<0.01) with a cross-validated HRR of 1.47 (P=0.03). PTGS2 expression was also associated with poor prognosis (HRR 1.60; P<0.01) with a cross-validated HRR of 1.63 (P<0.01). Expression of EGFR and PTGS2 remained prognostic after multivariate analysis with standard clinicopathological variables (cross-validated HRR 1.51; P=0.02 and cross-validated HRR 1.59; P=0.01, respectively). Stratification for the commonly applied systemic therapy regimens demonstrated prognostic value for EGFR and PTGS2 only in the subgroup of patients who were not treated with systemic therapy (HRR 1.78; P<0.01 and HRR 1.64; P=0.04, respectively), with worst prognosis when both EGFR and PTGS2 were highly expressed (HRR 3.08; P<0.01). Expression of PTGS2 in CRCLM was correlated to expression in patient-matched primary tumours (P=0.02, 69.2% concordance).EGFR and PTGS2 expressions are prognostic molecular biomarkers with added value to standard clinicopathological variables for patients with resectable CRCLM. 
 [Expression of COX-2 and VEGF-C in squamous cell carcinoma of the tongue and its correlation to lymph node   metastasis  ]. To evaluate the coexpression of cyclooxygenase-2 (COX-2) and vascular endothelial growth factor-C (VEGF-C) in human squamous cell carcinoma of the tongue (SCCT) and assess their correlations to neoangiogenesis and lymph node   metastasis   of the tumor.Tissue samples of primary SCCT and the metastatic lymph nodes were obtained from 46 patients undergoing surgical resections of SCCT for immunohistochemical detection of COX-2 and VEGF-C expressions.The over-expression rates of COX-2 and VEGF-C was 82.61% and 73.91% in the primary tumor, respectively, significantly higher than those in normal oral mucosa. A significant correlation was found between the expression scores of COX-2 and VEGF-C with a concordance rate of 82.61% (P<0.01) and a kappa value of 0.495 according to Cohen's kappa test (P<0.01). High expression of VEGF-C, but not COX-2, was correlated to the presence of lymph node   metastasis   (P<0.05). The mean microvessel density was significant higher in tumors with strong positivity for COX-2 or VEGF-C than in tumors with only weak or negative expressions (P<0.001).The expressions of COX-2 and VEGF-C are closely correlated in SCCT, and may have clinical value for assessing the prognosis,   metastasis   and invasion of SCCT, but the mechanism of these proteins in carcinogenesis in SCCT needs further investigation. 
 Prognostic significance of cyclooxygenase-2 in osteosarcoma: a meta-analysis. Published studies researching the prognostic significance of cyclooxygenase-2 (COX-2) expression in patients with osteosarcoma are inconclusive and heterogeneous. We conducted a meta-analysis to assess its prognostic value more precisely. The pooled odds ratios (ORs) or hazard ratios (HRs) with corresponding 95 % confidence intervals (CIs) were calculated to evaluate the effects. Fourteen studies with 735 osteosarcoma patients were included to estimate the relationship between COX-2 and   metastasis   of tumor, clinical stage, and 3-year overall survival. High expressions of COX-2 predicted neoplasm   metastasis   (OR = 1.891, 95 % CI 1.276-2.803, P = 0.002), advanced clinical stage (OR = 1.801, 95 % CI 1.257-2.581, P = 0.001). In addition, high COX-2 expression tended to be associated with a poor 3-year survival (HR = 1.741, 95 % CI 0.762-3.979, P = 0.188), but the difference was not significant. Therefore, this meta-analysis demonstrated that high COX-2 expression might be an unfavorable prognostic effect in osteosarcoma. 
 Cyclooxygenase-2--An Imperative Prognostic Biomarker in Oral Squamous Cell Carcinoma--An Immunohistochemical Study. Oral squamous cell carcinoma (OSCC) is the most common head and neck squamous cell carcinoma (HNSCC) with   metastasis   and tumor recurrence resulting in 90 % of cancer associated mortality. COX-2, an inflammatory biomarker, has been shown to play a significant role in tumorigenesis of OSCC. To study the expression of COX-2 in OSCC by immunohistochemistry and investigate its association with the clinicopathological parameters including patient survival. A cross sectional study was carried out in 75 histologically confirmed cases of OSCC. COX-2 expression was evaluated by indirect streptavidin biotin method. The expression was semi-quantitatively assessed using established criteria. The expression profile of COX-2 was correlated with the clinicopathological details like tumor size, regional lymphnode   metastasis  , distant   metastasis  , clinical stage, local recurrence of tumor, histological grade, and survival of patient. Chi square and Kaplan Meier statistical tests were applied for assessing this association. COX-2 expression was absent in normal oral mucosa. Over expression of COX-2 was seen in 58 out of 75 specimens of OSCC. Overexpression of COX-2 was significantly associated with the lymphnode involvement, histological grade, local recurrence of tumor and patient survival. COX-2 expression represents an important biomarker of prognostic significance that may be used to identify a subset of patients at high risk and to predict patient survival. 
 [Expression and relationship of EBV LMP1, COX-2 and VEGF-C in nasopharyngeal carcinoma]. To evaluate the expression of vascular endothelial growth factor C (VEGF-C), Epstein-Barr virus (EBV) latent membrane protein 1 (LMP1), cyclooxygenase-2 (COX-2) in nasopharyngeal carcinoma (NPC).LMP1, COX-2 and VEGF-C were detects by immunohistochemical staining for 57 case NPC tissue.The positives rates of LMP1, COX-2 and VEGF-C detected by immunohistochemical staining were 49.1% (28/57), 75.4% (43/57) and 59.6% (34/57), respectively. The expression of LMP1, COX-2 and VEGF-C were correlated to each other in NPC (P < 0.05).LMP1 and COX-2 may induce expression of VEGF-C directly or LMP1 induce expression of VEGF-C by induce COX-2 expression, may contribute to lymph   metastasis   and develop NPC. 
 Epstein-Barr virus protein can upregulate cyclo-oxygenase-2 expression through association with the suppressor of   metastasis   Nm23-H1. Previous studies have demonstrated the interaction between the Epstein-Barr virus (EBV) nuclear antigen 3C (EBNA3C) and the metastatic suppressor Nm23-H1 both in vitro and in vivo (C. Subramanian, M. A. Cotter II, and E. S. Robertson, Nat. Med. 7:350-355, 2001). EBNA3C can reverse the ability of Nm23-H1 to suppress migration of Burkitt's lymphoma and breast carcinoma cell lines in vitro. EBNA3C contributes to EBV-associated human cancers by regulating transcription of a number of cellular and viral promoters and by targeting and altering the transcription activities of the   metastasis   suppressor Nm23-H1. Cyclo-oxygenase-2 (COX-2), an inducible enzyme important in inflammation, is overexpressed in a variety of cancers and can influence cell migration. In this report we show that Nm23-H1 and EBNA3C can modulate expression of COX-2 in the context of EBV infection and transformation. The levels of COX-2 were consistently higher in EBV-positive cells than in EBV-negative cells. Additionally, we show that Nm23-H1 can upregulate the COX-2 promoter element in luciferase reporter assays, whereas EBNA3C alone did not affect the level of response but clearly contributed to an additive increase when coexpressed with Nm23-H1. The downstream effect of COX-2 expression was also evaluated and showed that prostaglandin E(2) levels increased with Nm23-H1 and that there was some level of cooperativity in the presence of EBNA3C. The majority of this response was mediated through the cyclic AMP response element and NF-kappaB sites. These studies suggest a potential role for COX-2 in EBV-associated human cancers. 
 Inhibitory effect of a selective cyclooxygenase-2 inhibitor on liver   metastasis   of colon cancer. COX-2 overexpression is recognized in various cancers, but the role of COX-2 in the progression of cancer, including the liver   metastasis   of colon cancer, is not clearly understood. We examined the role of COX-2 in the mechanism of liver   metastasis   of colon cancer, using a highly metastasizable colon carcinoma cell line, LM-H3. A COX-2 inhibitor, JTE-522, inhibited cell proliferation and invasion of LM-H3 in vitro and clearly reduced the number of metastatic nodules on the surface of nude mouse livers in vivo. We also examined the effects of JTE-522 on the production of growth factors and MMPs through the use of ELISA and gelatin zymography, respectively. JTE-522 downregulated PDGF production by LM-H3 but had no influence on VEGF production. JTE-522 also inhibited MMP-2 secretion by LM-H3. JTE-522 downregulated PGE(2) production, but the associated changes in PGE(2) did not affect PDGF and VEGF production by LM-H3. We conclude that JTE-522 downregulated the cell proliferation and invasive potential of LM-H3 by reducing the production of PDGF and MMP-2 and hypothesize that these inhibitory effects on the production of PDGF and MMP-2 can lead to inhibition of liver   metastasis   of colon cancer. These data indicate that the COX-2 inhibitor JTE-522 has a high potential for use as a clinical agent for the treatment of liver   metastasis   of colon cancer. 
 Expression of COX-2, iNOS, p53 and Ki-67 in gastric mucosa-associated lymphoid tissue lymphoma. To assess the expression of cyclooxygenase-2 (COX-2), nitric oxide synthase (iNOS), p53 and Ki-67 in gastric mucosa-associated lymphoid tissue (MALT) lymphoma and clarify the relationship between COX-2 expression and iNOS or p53 expression in these patients.The expressions of COX-2, iNOS, p53 and Ki-67 were detected in 32 gastric MALT lymphoma specimens and 10 adjacent mucosal specimens by immunohistochemical Envision method.COX-2 and iNOS expressions were significantly higher in gastric MALT lymphoma tissues than those in adjacent normal tissues. The expression of COX-2 was observed in 22 of 32 cases of MALT lymphoma tissues (68.8%). A positive cytoplasmic immunoreactivity for iNOS was detected in 17 of 31 cases (53.1%). COX-2 expression in gastric MALT lymphoma tissues was positively correlated with iNOS expression (r=0.448, P=0.010) and cell proliferative activity analyzed by Ki-67 labeling index (r=0.410, P=0.020). The expression of COX-2 protein did not correlate with age, sex, stage of disease, lymph node   metastasis   or differentiation. The accumulation of p53 nuclear phosphoprotein was detected in 19(59.4%) of tumors. p53 protein was expressed in 11 of 23 assessed LG tumors and in 8 of 9 assessed HG tumors. The difference of p53 positivity was found statistically significant between LG and HG cases (P=0.0302). The p53 accumulation correlated with advanced clinical stage (stage III+IV vs stage I+II, P=0.017). There was a significant positive correlation between COX-2 expression and p53 accumulation status (r=0.403, P=0.022). The mean PI of Ki-67 in each grade group were 36.0+/-7.73% in HG and 27.4+/-9.21% in LG. High-proliferation rate correlated with HG tumors (r=0.419, P=0.017). The correlation coefficient showed a significant positive correlation between PI and COX-2 expression in MALT lymphoma patients (r=0.410, P=0.020).COX-2 expresses in the majority of gastric MALT lymphoma tissues and correlates with cellular proliferation and iNOS expression. COX-2 overexpression is closely associated with p53 accumulation status. iNOS and COX-2 may play a synergistic role in the pathogenesis of gastric MALT lymphoma. 
 Methylation status and overexpression of COX-2 in Tunisian patients with ductal invasive breast carcinoma. Inflammation and hormonal signalling induce the cyclooxygenase-2 (COX-2) expression in solid tumours including breast cancer, which in turn affects cell proliferation, apoptosis and   metastasis  . The aim of this study was to investigate the expression of COX-2 and its association with clinical parameters, patient's survival, hormones receptors (oestrogen, progesterone), ERBB2 and TP53 expression in 83 cases of infiltrating ductal breast carcinomas. Moreover, the methylation status at the CpG islands of the COX-2 gene promoter was also explored in 70 specimens. We showed that tumours exhibiting moderate to intense COX-2 immunostaining were significantly more frequent in patients over 45 years old (p = 0.027). Moreover, a high level of COX-2 expression correlated with a shorter survival time (p log-rank = 0.04) and was an independent prognostic factor (p = 0.022; HR 6.4; 95% CI = 1.3-31.4). On the other hand, hypermethylation of the COX-2 gene promoter was observed in 27% of cases and strongly associated with smaller tumours (<5 cm, p = 0.011). Furthermore, patients with methylated COX-2 pattern have a better 4-year disease-free survival (p = 0.022) as well as a prolonged overall survival (p log-rank test = 0.034). In conclusion, we showed that high COX-2 expression was associated with reduced survival and was an independent prognostic factor. However, hypermethylation of the COX-2 promoter correlated with a better overall survival in Tunisian patients with breast carcinoma. 
 Cyclooxygenase 2 (rs2745557) Polymorphism and the Susceptibility to Benign Prostate Hyperplasia and Prostate Cancer in Egyptians. Cyclooxygenase-2 (COX-2), an inducible isoform of cyclooxygenase, has been reported to be correlated with tumorigenesis, tumor progression, and   metastasis  . We aimed to evaluate the association between COX-2 (rs2745557) polymorphism and prostate cancer (PCa), benign prostate hyperplasia (BPH) risk. We also assessed the influence of other risk factors such as obesity, smoking, diabetes in modulating the risk of PCa in Egyptian men. COX-2 (rs2745557) was genotyped in 112 PC patients, 111 BPH and 120 subjects as a control group. COX-2 and PSA levels were measured by ELISA. We found that GG genotype was associated with a 17-fold increased risk for PCa and 20-fold increased the risk for BPH more than AA genotype. Also, G allele carriers of COX-2 were associated with metastatic cancer (OR = 1.3, P < 0.05) and disease aggressiveness (OR = 3.5, P < 0.001). The coexistence of obesity, smoking, or diabetes with GG genotype may lead to increasing the risk of developing BPH (OR = 3.3, 4, and 2.7, respectively) and of developing PCa (OR = 2.9, 4.9, and 3.2, respectively). Our results showed evidence suggesting the involvement of the COX-2 (rs2745557) polymorphism and its protein in PCa or BPH initiation and progression. Also, the coexistence of COX-2 (rs2745557) and obesity, smoking, or diabetes may lead to the development of PCa or BPH. 
 The malignant phenotype of breast cancer cells is reduced by COX-2 silencing. The cyclooxygenase (COX) pathway is currently targeted for therapeutic intervention in different cancers. We have previously shown that silencing of COX-2 in the poorly differentiated metastatic breast cell line MDA-MB-231 by RNA interference markedly delayed tumor onset and inhibited   metastasis  . To understand the functional effects of COX-2 silencing underlying the inhibition of tumor growth and   metastasis   previously reported, we investigated changes in these cells for a number of cancer-associated phenotypes. Cyclooxygenase-2-silenced cells were less able to acidify tissue culture medium, a response that could partly be attributed to decreased lactate production or export detected by reduced lactate in the medium. Consistent with the significantly reduced transcript levels of hyaluronan synthase 2, an enzyme responsible for the total level of hyaluronan secreted by these cells, COX-2 silencing resulted in lower hyaluronan levels secreted in culture medium. Inhibition of human umbilical vein endothelial cell network association in a coculture assay was also observed in COX-2-silenced cells. These data highlight the functional role of COX-2 in pathways that mediate increased malignancy. 
 The VEGFR2, COX-2 and MMP-2 polymorphisms are associated with clinical outcome of patients with inoperable non-small cell lung cancer. Certain common inherited variations in genes involved in tumor angiogenesis, progression and   metastasis   may contribute to cancer therapy outcome and prognosis by altering the gene expression and protein activity. In this report, we examined the effect of functional polymorphisms in MMP-1, MMP-2, MMP-3, VEGF, VEGFR2, FGFR4 and COX-2 genes on overall (OS) and progression-free survival (PFS) of 350 Caucasian patients with inoperable non-small cell lung cancer (NSCLC). The results of multivariate analysis indicated that VEGFR2 -906C and COX-2 -1195G alleles were strongly associated with poor OS and PFS (p = 0.002 and 0.015, respectively, for OS; p = 0.009 and 0.015, respectively, for PFS), while MMP-2 -1306 T allele carriers had significantly reduced PFS (p = 0.010). Moreover, an increased risk of death and progression was significantly associated with the number of adverse alleles for VEGFR2/COX-2 (p = 0.0005 for OS and 0.0006 for PFS in >1 adverse allele carriers) and VEGFR2/COX-2/MMP-2 combinations (p = 0.0003 for OS and 0.0001 for PFS in patients with >2 adverse alleles). Finally, VEGFR2 TC/CC, COX-2 AG/GG and MMP-2 CT/TT genotypes as well as "at risk" allele combinations were identified as independent predictors of unfavorable OS and PFS in the group. In conclusion, the data suggest that selected VEGFR2, COX-2 and MMP-2 polymorphisms may be potential prognostic markers in unresectable NSCLC treated with radiotherapy with or without chemotherapy, although further validation studies are warranted to confirm our observations. 
 [The expression of cyclooxygenase-2 in laryngeal squamous cell carcinoma and its relationship with MVD]. To investigate the expression of cyclooxygenase-2 in laryngeal squamous cell carcinoma and the correlation with tumor angiogenesis.The COX-2 and FVIII of 42 cases of laryngeal squamous cell carcinoma were detected by immunohistochemical SABC method,and the correlation between the expression rates, clinical significance and microvessel density(MVD) was analyzed.The positive rate of COX-2 in laryngeal squamous cell carcinoma tissues and adjacent normal tissues were 71.43% and 19.05% respectively. The expression of COX-2 were not associated with age, sex, tumor site and T stage. The tumors of grade III approximately IV showed a higher COX-2 expression than tumors of grade I approximately II did and the expression of COX-2 in the cervical lymph node   metastasis   group was higher than that in the non-  metastasis   group. The expression of COX-2 was closely associated with microvessel density(MVD) of laryngeal squamous cell carcinoma.The expression of COX-2 may play a crucial role in the carcinogenesis and development of laryngeal squamous cell carcinoma and cervical lymph node   metastasis   in patients with laryngeal squamous cell carcinoma. COX-2 may be a important factor in tumor angiogenesis. 
 Involvement of Cox-2 in the metastatic potential of chemotherapy-resistant breast cancer cells. A major problem with the use of current chemotherapy regimens for several cancers, including breast cancer, is development of intrinsic or acquired drug resistance, which results in disease recurrence and   metastasis  . However, the mechanisms underlying this drug resistance are unknown. To study the molecular mechanisms underlying the invasive and metastatic activities of drug-resistant cancer cells, we generated a doxorubicin-resistant MCF-7 breast cancer cell line (MCF-7/DOX).We used MTT (3-(4,5-dimethylthiazol-2-yl)-2,5-diphenyltetrazolium bromide) assays, flow cytometry assays, DNA fragmentation assays, Western blot analysis, cell invasion assays, small interfering RNA (siRNA) transfection, reverse transcription-polymerase chain reaction, experimental lung   metastasis   models, and gelatin and fibrinogen/plasminogen zymography to study the molecular mechanism of metastatic activities in MCF-7/DOX cells.We found that MCF-7/DOX acquired invasive activities. In addition, Western blot analysis showed increased expression of epidermal growth factor receptor (EGFR) and Cox-2 in MCF-7/DOX cells. Inhibition of Cox-2, phosphoinositide 3-kinase (PI3K)/Akt, or mitogen-activated protein kinase (MAPK) pathways effectively inhibited the invasive activities of MCF-7/DOX cells. Gelatin and fibrinogen/plasminogen zymography analysis showed that the enzymatic activities of matrix metalloproteinase-2 (MMP-2), MMP-9, and urokinase-type plasminogen activator were markedly higher in MCF-7/DOX cells than in the MCF-7 cells. In vitro invasion assays and mouse models of lung   metastasis   demonstrated that MCF-7/DOX cells acquired invasive abilities. Using siRNAs and agonists specific for prostaglandin E (EP) receptors, we found that EP1 and EP3 played important roles in the invasiveness of MCF-7/DOX cells.We found that the invasive activity of MCF-7/DOX cells is mediated by Cox-2, which is induced by the EGFR-activated PI3K/Akt and MAPK pathways. In addition, EP1 and EP3 are important in the Cox-2-induced invasion of MCF-7/DOX cells. Therefore, not only Cox-2 but also EP1 and EP3 could be important targets for chemosensitization and inhibition of   metastasis   in breast cancers that are resistant to chemotherapy. 
 [Expression and significance of COX-2 and S-100 positive dendritic cell in laryngeal carcinoma]. To explore the expression of cyclooxygenase-2 (COX-2) and S-100 positive dendritic cell in laryngeal carcinoma tissue and their clinical significance.Sixty-five samples of laryngeal carcinoma and thirty-four biopsies of adjacent noncancerous tissue were obtained. Immunohistochemical technique (SP method) was used to detect the expression of COX-2 and S-100 positive dendritic cell, and the relationship of their expression with clinical pathological parameters and prognosis was analyzed.The rates of COX-2 expression were 63.08% (41/65)and 14.70% (5/34) in laryngeal carcinoma and control group, respectively. The difference was significant (P < 0.05). The positive expression of COX-2 was correlated with T and clinical stage in laryngeal carcinoma(all P < 0.05). The rates of S-100 positive dendritic cell expression were 61.54% (40/65)and 0 in laryngeal carcinoma and control group, respectively. The difference was significant (P < 0.05). S-100 positive dendritic cells showed significant differences between early and late clinical stage and lymph node   metastasis   (all P < 0.05). Kaplan-Meier survival analysis showed that patients with positive expression of COX-2 and S-100 positive dendritic cell had worse disease-free and overall survival (P < 0.05). Cox regression analysis showed that S-100 positive dendritic cell was indicated as an independent prognostic factor for survival(P < 0.05).COX-2 and S-100 positive dendritic cell are highly expressed in laryngeal carcinoma tissue. It suggests that the expression of COX-2 and S-100 positive dendritic cell is related to the process of carcinogenesis and may be the important indicators in laryngeal carcinoma for prognosis. 
 Cyclooxygenase-2 induces EP1- and HER-2/Neu-dependent vascular endothelial growth factor-C up-regulation: a novel mechanism of lymphangiogenesis in lung adenocarcinoma. Cyclooxygenase (COX)-2, the inducible isoform of prostaglandin H synthase, has been implicated in the progression of human lung adenocarcinoma. However, the mechanism underlying COX-2's effect on tumor progression remains largely unknown. Lymphangiogenesis, the formation of new lymphatic vessels, has recently received considerable attention and become a new frontier of tumor   metastasis   research. Here, we study the interaction between COX-2 and the lymphangiogenic factor, vascular endothelial growth factor (VEGF)-C, in human lung cancer cells and their implication in patient outcomes. We developed an isopropyl-beta-D-thiogalactopyranoside-inducible COX-2 gene expression system in human lung adenocarcinoma CL1.0 cells. We found that VEGF-C gene expression but not VEGF-D was significantly elevated in cells overexpressing COX-2. COX-2-mediated VEGF-C up-regulation was commonly observed in a broad array of non-small cell lung cancer cell lines. The use of pharmacological inhibitors or activators and genetic inhibition by EP receptor-antisense oligonucleotides revealed that prostaglandin EP(1) receptor but not other prostaglandin receptors is involved in COX-2-mediated VEGF-C up-regulation. At the mechanistic level, we found that COX-2 expression or prostaglandin E(2) (PGE(2)) treatment could activate the HER-2/Neu tyrosine kinase receptor through the EP(1) receptor-dependent pathway and that this activation was essential for VEGF-C induction. The transactivation of HER-2/Neu by PGE(2) was inhibited by way of blocking the Src kinase signaling using the specific Src family inhibitor, PP1, or transfection with the mutant dominant negative src plasmid. Src kinase was involved in not only the HER-2/Neu transactivation but also the following VEGF-C up-regulation by PGE(2) treatment. In addition, immunohistochemical staining of 59 lung adenocarcinoma specimens showed that COX-2 level was highly correlated with VEGF-C, lymphatic vessels density, and other clinicopathological parameters. Taken together, our results provided evidence that COX-2 up-regulated VEGF-C and promotes lymphangiogenesis in human lung adenocarcinoma via the EP(1)/Src/HER-2/Neu signaling pathway. 
 Characterization of cyclooxygenase-2 (COX-2) during tumorigenesis in human epithelial cancers: evidence for potential clinical utility of COX-2 inhibitors in epithelial cancers. Increased prostaglandins (PGs) are associated with many inflammatory pathophysiological conditions; and are synthesized from arachidonic acid by either of 2 enzymes, cyclooxygenase-1 (COX-1) or -2 (COX-2). Recent epidemiologic, expression, and pharmacologic studies suggest COX-2 derived metabolites also play a functional role in the maintenance of tumor viability, growth and   metastasis  . Archival and/or prospectively collected human tissues were prepared for immunohistochemistry, and representative cases assayed via Western blot, RT-PCR, or TAQman analysis. Consistent overexpression of COX-2 was observed in a broad range of premalignant, malignant, and metastatic human epithelial cancers. COX-2 was detected in ca. 85% of the hyperproliferating, dysplastic, and neoplastic epithelial cells, and in the existing and angiogenic vasculature within and adjacent to hyperplastic/neoplastic lesions. These data collectively imply COX-2 may play an important role during premalignant hyperproliferation, as well as the later stages of invasive carcinoma and   metastasis   in various human epithelial cancers. 
 Cyclooxygenase-2 expression in human thyroid disease. Cyclooxygenase (COX)-2, which is the inducible form of the COX enzyme for prostaglandin synthesis and a key mediator of epithelial cell growth, has been shown to be up-regulated in gastrointestinal cancers. Additionally, regular intake of other non-steroidal anti-inflammatory drugs (NSAID) is known to decrease the incidence of these cancers. Therefore, the goals of the present study were to determine the possible involvement of COX-2 in human thyroid diseases.We used immunohistochemical staining and Western blot analysis to characterize the expression of COX-2 proteins in thyroid tissues from 64 patients with thyroiditis, benign tumors, and malignant tumors with or without   metastasis  . Immunoreactivity scores were calculated by multiplication of the determined grades.COX-2 proteins were not expressed in normal thyroid tissues. However, each type of tumor tissue showed intense bands of COX-2 protein expression in Western blot analyses, and the immunoreactivity scores were 7.67+/-1.17 (SD) for thyroiditis, 7.87+/-0.9 for benign tumors, 7.53+/-1.53 for follicular cancer, 7.63+/-1.11 for papillary cancer without   metastasis  , and 7.17+/-1.55 for papillary cancer with   metastasis  . No significant differences were found in the levels of COX-2 expression between different tumor tissue types.No significant correlations were observed between clinical and/or pathological characteristics of thyroid tumors and the intensity of COX-2 protein expression. In addition, we found no difference in COX-2 protein expression between thyroiditis and thyroid tumors. Thus, up-regulation of COX-2 protein synthesis in human thyroid diseases does not appear to be of clinical significance. 
 Nuclear co-localization and functional interaction of COX-2 and HIF-1α characterize bone   metastasi  s of human breast carcinoma. The aim of this article is to identify nuclear co-localization of COX-2 and HIF-1α in human-bon  e metastas  is of breast cancer, index of transcriptionally activated cells and functional for gene expression. In particular, we verified whether hypoxia exerted a direct role o  n metastas  is-gene expression or through COX-2 signaling, due to the relevance for clinical implications to individuate molecular targets for diagnosis and therapy. The experiments were performed in vitro with two metastatic clones, 1833 and MDA-231BO, and the parental MDA-MB231 cells, in vivo (1833-xenograft model), and in human-bon  e metastas  is specimens. In 1833 cells in vitro, COX-2 signaling pathway was critical for nuclear HIF-1α-protein expression/translocation, mechanisms determining HIF-1 activity and gene expression. The data were corroborated by immunohistochemistry in human-bo  ne metasta  sis specimens. COX-2 and HIF-1α showed wide co-localization in the nucleus, indicative of COX-2-nuclear import in transcriptionally activated metastatic cells and consistent with COX-2-HIF-1α functional interaction. A network of microenvironmental signals controlled COX-2 induction and HIF-1 activation downstream. In fact, hypoxia through HGF and TGF-β1 autoregulatory loops triggered a specific array of transcription factors responsible for COX-2 transactivation. The novelty was that HGF and TGF-β1 biological signals were produced by hypoxic metastatic cells and, therefore, the microenvironment seemed to be modified by metastatic-cell engraftment in the bone. In agreement, HIF-1α expression in bone marrow supportive cells occu  rred in me  tastasis-bearing animals. Altogether, the data supported the pre-metastatic-niche theory. Our observations might be useful to design therapies again  st bone me  tastasis, by affecting the phenotype changes of metastatic cells occurring at the secondary growth site through COX-2-HIF-1 interaction. 
 Epithelial to mesenchymal transition in cutaneous squamous cell carcinoma is correlated with COX-2 expression but not with the presence of stromal macrophages or CD10-expressing cells. Epithelial to mesenchymal transition (EMT) is an intricate process by which epithelial cells loose epithelial characteristics and acquire a mesenchymal-like phenotype. EMT and cyclooxygenase 2 (COX-2) expression are related to tumor invasion and   metastasis  . The tumor microenvironment plays a major role in tumor progression and the induction of EMT. Here, we investigated the relationship between EMT and COX-2 expression as well as tumor-associated macrophages (TAM) and CD10-positive stromal cells during the development of cutaneous squamous neoplastic lesion. We performed immunohistochemical staining for vimentin, E-cadherin, β-catenin, COX-2, CD68, and CD10 in 41 cases of squamous cell cancers (SCC), 20 of Bowen's disease, 30 of actinic keratosis, and 30 samples of normal skin. SCC cells showed significantly increased vimentin expression and reduced expression of membranous E-cadherin and β-catenin compared with cells in precursor lesions and in normal skin. COX-2 expression was also markedly increased in SCC cells. E-cadherin expression was positively correlated with β-catenin expression and inversely correlated with COX-2 expression in SCC cells. The number of TAM and CD10-positive stromal cells increased from the normal skin to precursor lesions and SCC cells. The number of TAM and of CD10-positive stromal cells did not correlate with the expression of E-cadherin, β-catenin, COX-2, and vimentin in SCC cells. We suggest that cutaneous SCC cells show EMT, which appears to be correlated with COX-2 expression but not with stromal CD10 expression and TAM. 
 [Clinical significance and relationship between HIF-1alpha, COX-2, VEGF and microvessel density expression in laryngeal carcinoma]. To evaluate the role of HIF-1alpha, COX-2, VEGF proteins and Microvessel density in angiogenesis and   metastasis   of laryngeal carcinoma.Surgically resected specimens of 64 laryngeal carcinomas were studied by immunohistochemical staining for HIF-1alpha protein, COX-2 protein, VEGF and MVD.Positive expressions of HIF-1alpha, COX-2 protein and VEGF were found in 65.63%, 68.75% and 71.88% of laryngeal carcinoma specimens, respectively. The mean MVD in this series was 35.79 +/- 9.49. The expression of HIF-1alpha, COX-2 and VEGF positively correlated with MVD expression. There was a positive relationship between HIF-1alpha, COX-2 and VEGF expression. The microvessel density (MVD) in HIF-1alpha, COX-2 and VEGF positive tumors was significantly higher in patients with cervical lymph node metastases than that in patients without metastases.HIF-1alpha, COX-2 and VEGF proteins may play an important role in the angiogenesis of laryngeal carcinoma. HIF-1alpha regulate the expression and function of its downstream target genes (COX-2 and VEGF), which play a critical role in the angiogenesis of laryngeal carcinoma, metastases in the process of laryngeal cancer cell spreading to cervical lymph nodes. 
 Clinical and prognosis relevance of COX-2 expression in Tunisian patients with primary gastric adenocarcinoma. Inflammation and hormonal signalling induce the cyclooxygenase-2 (COX-2) expression in various human cancers including Gastric Cancer (GC). GC remains among the human malignancies diagnosticated at advanced tumor stage and thus having a poor prognosis. COX-2 is a key protein in cancer progression which is involved in proliferation, invasion, and   metastasis   of tumor cells.The aim of this study was to investigate the expression of COX-2 and its association with clinico-patholocigal parameters and survival in Tunisian GC patients and to correlate COX-2 expression with others cancer-related proteins.The immunohistochemistry was used to study the expression of COX-2 on 93 patients with gastric adenocarcinoma.Our results show that COX-2 immunostaining is negative to weak in 51.6%, moderate in 33.3%, and intense in 15.1% of tumor tissues. The expression of COX-2 associated significantly with tumor differentiation (p = 0.003), and histological type (p = 0.039). Furthermore, lack of COX-2 expression is significantly associated with 1-year (p= 0.005), 2-years (p= 0.000), and 5-years (p= 0.042) relapse free survival. In addition, Cox regression model, revealed that   metastasis   (p= 0.014), tumor site (p= 0.013), histotype (p = 0.02), and COX-2 expression (p = 0.003) are independent factors for prognosis. Regarding the relationship between COX-2 and cancer related proteins, we found that COX-2 expression is positively associated with APC (p = 0.006), and P53 (p = 0.026), supporting a cross link between these proteins in gastric carcinogenesis.Our findings emphasize the importance of COX-2 as a potential marker of tumor progression and prognosis in GC, and that the inhibition of COX-2 activity may have a therapeutic benefit in GC. 
 Elevated cyclooxygenase-2 expression correlates with distant metastases in breast cancer. Cyclooxygenase-2 (COX-2) overexpression clearly plays an important role in the pathogenesis of breast cancer. In this study, we analysed the relationship between COX-2 expression and various clinicopathological factors in human breast cancer.Using immunohistochemistry, we analysed archival specimens of human breast cancer (n=29) using antibodies to COX-2, ER, PgR and HER2 and, from medical records, obtained clinicopathological data.We observed a significant association between COX-2 overexpression and distant   metastasis  . COX-2 expression was not significantly associated with any other clinical or pathological variable.These findings lend support to the hypothesis that COX-2 overexpression represents an adverse prognostic event in human breast cancer and are encouraging for proposed strategies of COX-2 suppression to treat the disease. 
 Increased expression of COX-2 in the development of human lung cancers. It is well accepted that an increase in the expression of cyclooxygenase-2 (COX-2), a key inducible enzyme involved in the production of prostaglandins and other eicosanoids, may play a significant role in carcinogenesis in addition to its well-known role in inflammatory reactions. Whereas previous studies were largely confined to colorectal tumorigenesis, we have shown that a significantly increased expression of COX-2 may also play a role in the development of lung cancer. COX-2 expression was found to be frequently elevated in lung cancer, especially in adenocarcinoma, and the proportion of lung cancer cells with marked COX-2 expression was much higher in lymph node metastases than in the corresponding primary tumors. It was also shown that early stage adenocarcinoma patients with increased COX-2 expression who were surgically treated had a shorter survival. Our studies, which used high- and low-metastatic human lung cancer cell sublines established in our laboratory, revealed an association between metastatic capabilities and COX-2 expression levels: COX-2-specific inhibitors could inhibit in vitro the invasion of the highly metastatic NCI-H460-LNM35 clone through Matrigel-containing basement membrane components as well as the spontaneous in vivo   metastasis   in SCID mice. Taken together, these findings suggest that an increase in COX-2 expression maybe associated with the development of lung cancer and possibly with the acquisition of an invasive and metastatic phenotype. 
 Cyclooxygenase-2 expression and angiogenesis in colorectal cancer. Cyclooxygenase-2 is involved in a variety of important cellular functions, including cell growth and differentiation, cancer cell motility and invasion, angiogenesis and immune function. However, the role of cyclooxygenase-2 as an angiogenic factor in colorectal cancer tissue is still unclear. We investigated the relationship between cyclooxygenase-2 and angiogenesis by analyzing the expression of cyclooxygenase-2 in colorectal cancer tissue, as well as its association with vascular endothelial growth factor (VEGF) and microvascular density (MVD).The expression of cyclooxygenase-2, VEGF, as well as MVD was detected in 128 cases of colorectal cancer by immunohistochemical staining. The relationship between the cyclooxygenase-2 and VEGF expression and MVD was evaluated. Our objective was to determine the effect of cyclooxygenase-2 on the angiogenesis of colorectal cancer tissue.Among 128 cases of colorectal cancer, 87 were positive for cyclooxygenase-2 (67.9 %), and 49 for VEGF (38.3 %), respectively. The microvessel counts ranged from 23 to 142, with a mean of 51.7 (standard deviation, 19.8). The expression of cyclooxygenase-2 was correlated significantly with the depth of invasion, stage of disease,   metastasis   (lymph node and liver), VEGF expression and MVD. Patients in T3-T4, stage III-IV and with   metastasis   had much higher expression of cyclooxygenase-2 than patients in T1-T2, stage I-II and without   metastasis   (P<0.05). The positive expression rate of VEGF (81.6 %) in the cyclooxygenase-2 positive group was higher than that in the cyclooxygenase-2 negative group (18.4 %, P<0.05). Also, the microvessel count (56+/-16) in cyclooxygenase-2 positive group was significantly higher than that in cyclooxygenase-2 negative group (43+/-12, P<0.05). The microvessel count in tumors with positive cyclooxygenase-2 and VEGF was the highest (60+/-18, 41-142, P<0.05), whereas that in tumors with negative cyclooxygenase-2 and VEGF was the lowest (39+/-16, 23-68, P<0.05).Cyclooxygenase-2 may be associated with tumor progression by madulating the angiogenesis in colorectal cancer tissue and used as a possible biomarker. 
 Cyclooxygenase-2 is involved in the up-regulation of matrix metalloproteinase-9 in cholangiocarcinoma induced by tumor necrosis factor-alpha. Matrix metalloproteinase-9 (MMP-9) is an important enzyme in tumor invasion and   metastasis   in malignant tumors, including cholangiocarcinoma (CC). Tumor necrosis factor-alpha (TNF-alpha), a proinflammatory cytokine, was recently reported to induce the up-regulation of MMP-9 in cultured CC cells. We examined whether cyclooxygenase-2 (COX-2) and prostaglandin-E2 (PGE2), another endogenous tumor promoter, are involved in the up-regulation of MMP-9 in CC using CC tissue specimens and a CC cell line, HuCCT-1. MMP-9 and COX-2 were immunohistochemically expressed in 58% and 89% of 110 CC cases, respectively; the expression of MMP-9 and COX-2 was correlated (r = 0.32, P = 0.00072). Using zymography, latent MMP-9 was detectable in all cases and active MMP-9 was detected in 24% of cases of the CC specimens. The TNF-alpha/TNF-receptor 1 (TNF-R1) interaction induced MMP-9 production and activation, as well as COX-2 overexpression and PGE2 production, and increased the migration of CC cells. MMP-9 up-regulation was inhibited by COX inhibitors, antagonists of EP2/4 (receptors of PGE2), and COX-1 and COX-2 siRNAs. Inhibitors of both MMP-9 and MMP-9 siRNA treatment abrogated the increase in the migration of CC cells induced by TNF-alpha. In conclusion, we propose a novel signaling pathway of MMP-9 up-regulation in CC cells such that TNF-alpha induces the activation of COX-2 and PGE2 via TNF-R1 followed by the up-regulation of MMP-9 via the PGE2 (EP2/4) receptor. 
 [Expression of COX-2 and MMP-9 in cervical carcinoma and their clinical significance]. To investigate the expressions of COX-2 and MMP-9 in cervical carcinoma and their clinical significance.Immunohistochemical SP method was used to detect the expressions of COX-2 and MMP-9 in 72 cases of invasive carcinoma of cervix (ICC) and 16 cases of normal cervical epithelium remote from tumor (NCE), and the relationship between the expressions of COX-2 and MMP-9 in ICC and some factors relating to clinical pathology of cervical carcinoma such as histopathological grading, lymph node   metastasis  , stroma involvement and FIGO staging were analyzed statistically.The rates of positive expression of COX-2 and MMP-9 in ICC were significantly higher than those in NCE. COX-2: 88.9% in group ICC and 12.5% in group NCE, P = 0.000. MMP-9: 94.4% in group ICC and 43.8% in group NCE, P = 0.000. The expression of COX-2 was positively correlated with lymph node   metastasis   and stroma involvement (r = 0.296, P = 0.012 in group ICC and r = 0.257, P = 0.029 in group NCE, respectively). The expression of MMP-9 was positively correlated with FIGO staging (r = 0.329, P = 0.005) and histopathological grading (r = 0.351, P = 0.003). The expression of COX-2 was positively correlated with the expression of MMP-9 in ICC (r = 0.297, P = 0.011).The overexpressions of COX-2 and MMP-9 are closely related to the invasion and growth of cervical carcinoma. The tissue with overexpression of COX-2 has strong invasion ability. COX-2 and MMP-9 have synergistic effect on proliferation, invasion and   metastasis   of cancer cells. Detecting the expression of both COX-2 and MMP-9 may be of value in further understanding the biological behavior and predicting the prognosis of cervical carcinoma. 
 Expression of cyclooxygenase 2 and vascular endothelial growth factor in gastric carcinoma: Relationship with clinicopathological parameters. Gastric cancer is one of the most common cancers and the second most common cause of cancer-related death worldwide. Identification of specific prognostic indicators might allow a better prognostic stratification and more effective therapy.To assess the expression and relationship between COX-2 and VEGF protein in gastric adenocarcinoma and whether these markers are useful in predicting clinicopathological prognostic parameters.The study included 83 formalin-fixed paraffin embedded tissue samples of excised gastric adenocarcinoma and 20 non tumorous tissue controls. The slides were subjected to COX-2 and VEGF immunohistochemical staining using a streptavidin-biotinperoxidase according to the manufacturer's protocol. The results were assessed independently by two pathologists. The relationships among COX-2 and VEGF expression and clinicopathological parameters were statistically analyzed.COX-2 and VEGF expressions were obviously higher in carcinoma tissues compared to normal mucosae (p<0.001). The expression rate of COX-2 was 54.2% and of VEGF was 68.7%. COX-2 positive tumors were significantly correlated with Lauren classification, tumor depth and Helicobacter pylori infection (p<0.001, p=0.008, p=0.035). VEGF was significantly associated with lymph node   metastasis   and tumor depth (p<0.001). There was a positive association between VEGF and COX-2 expression in gastric adenocarcinoma (Kappa value=0.55).In gastric adenocarcinoma, COX-2 expression might serve as a powerful indicator for intestinal type carcinoma, locally advanced disease and H. pylori infection, while VEGF was related to loco-regional progression. COX-2 might be involved in the development of angiogenesis in gastric carcinoma through VEGF upregulation. 
 [Expression of P53, COX2 and CD44V6 in early-stage squamous carcinoma of cervix with lymph vascular space invasion positive and negative and its relationship with prognosis]. To investigate the relationship between lymph vascular space invasion (LVSI) and various clinicopathologic factors in early-stage cervical squamous carcinoma; to explore the expression of P53, COX2 and CD44V6 in early stage squamous carcinoma of cervix and its relationship with prognosis.A case-controlled study was conducted to compare LVSI positive group (n = 44) in early stage squamous carcinoma of cervix with LVSI negative group (n = 44). Expression of P53, COX2 and CD44V6 were evaluated by immunohistochemical method to analyze the relationship between LVSI, P53, COX2 and CD44V6 and various clinicopathologic factors and to investigate their prognostic influences.LVSI positive was correlated with lymph node   metastasis   and deep stromal invasion. The 5-year disease-free survival was 68.9% in LVSI positive group versus 94.7% in LVSI negative group (P < 0.01). And the 5-year overall survival was 70.2% in LVSI positive group versus 100% in LVSI negative group (P < 0.01) with a significant difference. COX2 was correlated with clinical stage and tumor volume while there was no correlation with P53, CD44V6 and clinical factors. The overall survival of P53, COX2 and CD44V6 positive cases were significantly shorter than that of negative ones, especially in constant positive cases of 3 markers (disease-free survival 60.0 months shorter than constant negative cases with an overall survival of 91.3 months, but there was no significant difference).LVSI is correlated with lymph node   metastasis   and deep stromal invasion in the early-stage squamous carcinoma of cervix while it is negatively correlated with disease-free survival and overall survival. COX2 is correlated with clinical stage and tumor volume. Furthermore P53 and CD44V6 are not correlated with clinical factors. The overall survival of P53, COX2 and CD44V6 positive cases are significantly shorter than that of negative ones. 
 [Relationship between carcinoembryonic antigen and cyclooxygenase 2 expression and colorectal cancer]. Some research showed that carcinoembryonic antigen(CEA) related to cell adhesion and cyclooxygenesis 2(COX2) may be related to colorectal carcinogenesis. This study was designed to investigate the expression of CEA and COX2 to evaluate their effects on the tumorigenesis and progression in the colorectal cancer.The immunohistochemistry was used to determine the expression of CEA and COX2 in the 34 tissues of colorectal cancer,border of cancer, normal mucosa,and 19 metastatic lymph nodes.CEA expression level had a correlation with the differentiation of the tumor tissue (P< 0.05), but had no correlation with lymph node   metastasis  ,Dukes' stage,the patients' age,sex,and the location of tumor (P >0.05). The level of COX2 increased in the majority of tumor tissues (31/34) and all of the lymph node metastases, but had no correlation with the tumor differentiation, stage,location,patients' age and sex(P >0.05). There was significant difference of expression of CEA and COX2 between tumor and nontumor tissues (P< 0.01).The abnormal expression of CEA and COX2 plays a role in the carcinogenesis and development of colorectal cancer. 
 Frequent co-localization of Cox-2 and laminin-5 gamma2 chain at the invasive front of early-stage lung adenocarcinomas. Laminin-5 is an extracellular matrix protein that plays a key role in cell migration and tumor invasion. Cox-2 is an induced isoform of cyclooxygenases that plays an important role in carcinogenesis, suppression of apoptosis, angiogenesis, and   metastasis   of colon cancer. We report frequent co-expression of cox-2 and laminin-5 at the invasive front of early-stage lung adenocarcinomas. We investigated the expression of cox-2 and laminin-5 immunohistochemically in 102 cases of small-sized lung adenocarcinoma (maximum dimension, 2 cm or less). Cox-2 and laminin-5 were expressed in 97 (95.1%) and 82 (80.4%) cases, respectively. Both were preferentially localized in cancer cells at the cancer-stroma interface, although cox-2 tended to show a diffuse staining pattern in some cases. A comparison of their staining patterns revealed a striking similarity in their distribution in 24 cases, and a partial overlap between their localization in another 20 cases. Moreover, an overall correlation was found between the expression levels of cox-2 and laminin-5 (P = 0.018). To gain insight into the mechanisms that regulate the expression of these proteins, we additionally studied their expression in 58 cases of stage I lung adenocarcinoma, in which p53 status was determined by immunohistochemistry, polymerase chain reaction-single strand conformation polymorphism analysis, and direct sequencing. The results showed that tumors with mutant p53 tended to express more cox-2 than those with wild-type p53 (P = 0.080). Also, tumors that overexpressed p53 had higher levels of cox-2 and laminin-5 than those without p53 overexpression (P = 0.032 and 0.047, respectively). Further immunohistochemical analysis showed that tumors that overexpressed both epidermal growth factor receptor (EGFR) and erbB-2 had higher levels of cox-2 and laminin-5 than those without concomitant overexpression of these proteins (P = 0.014 and P = 0.018, respectively). To see whether EGFR signaling is involved in cox-2 and laminin-5 expression, we further conducted in vitro analyses using six lung adenocarcinoma cell lines (A549, HLC-1, ABC-1, LC-2/ad, VMRC-LCD, and L27). Western blot analyses showed that cox-2 mRNA levels, and to a lesser extent laminin-5 gamma2 mRNA levels, correlated with the expression levels of erbB-2 and the phosphorylated form of MAPK/ERK-1/2 protein. The addition of transforming growth factor-alpha increased both cox-2 and laminin-5 gamma2 mRNA levels in A549, ABC-1, and L27 with different kinetics; the induction of cox-2 occurred earlier than that of laminin-5 gamma2. Finally, the migration of ABC-1 cells was inhibited by MAP kinase kinase inhibitor PD98059 and a selective cox-2 inhibitor NS-398. In contrast, the migration of A549 cells was inhibited by PD98059, but much less effectively by NS-398. These results suggest that co-stimulatory mechanisms may exist that increase the expression of cox-2 and laminin-5 at the invasive front of lung adenocarcinomas and that EGFR signaling could be one of the mechanisms. Further investigations are warranted concerning the role of cox-2 and laminin-5 in cancer cell invasion and the significance of p53 and EGFR signaling in the regulation of cox-2 and laminin-5 expression. 
 Cyclooxygenase-2 expression correlates with uPAR levels and is responsible for poor prognosis of colorectal cancer. Considering recent findings that cyclooxygensase-2 (COX-2) is involved in the progression of colorectal carcinoma (CRC), the role of COX-2 in promoting invasion and angiogenesis was investigated by evaluating the relationship of COX-2 expression to various clinicopathological variables, including plasminogen activating system (PA system) and vascular endothelial growth factor (VEGF). Tumor tissues from 71 patients with CRC were assayed to determine the antigen levels of urokinase-type plasminogen activator (uPA), uPA receptor (uPAR), and plasminogen activator inhibitor-1 and -2 (PAI-1 and PAI-2), as well as immunohistochemical expression of VEGF. COX-2 was assayed immunohistochemically in 56 patients. COX-2 expression was detected in cancer cells and it was also expressed by stromal cells in some patients. Fourteen patients (25%) were COX-2 positive, whereas 42 were negative. COX-2 expression was significantly related to lymphatic invasion (P = 0.0317), but was not related to microvessel density or VEGF expression. In the PA system, uPAR antigen levels were significantly higher in tumors with COX-2 expression than in tumors without (P = 0.0233). Univariate analysis showed that significant prognostic variables for survival were tumor size, lymph node involvement, lymphatic invasion, vascular invasion, liver   metastasis  , high uPAR level, and COX-2 expression, but only liver   metastasis   was an independent prognostic factor (P = 0.0065) in multivariate analysis. COX-2 expression was a more important prognostic indicator than any other factor except liver   metastasis   (P = 0.0526). The significant relationship between the presence of COX-2 protein and uPAR antigen levels contributed to the enhancement of tumor invasion and the poor outcome in patients with CRC. 
 MMP-7 is upregulated by COX-2 and promotes proliferation and invasion of lung adenocarcinoma cells. Matrix metalloproteinases (MMPs) have been implicated in a variety of pathophysiological conditions, of which MMP-7 is expressed by tumor cells of epithelial and mesenchymal origin. However, the function of MMP-7 in human lung adenocarcinoma (LAC) is unclear. In the present study the expression of MMP-7 in LAC was examined by immunohistochemical assay using a tissue microarray procedure. A loss-of-function experiment was performed to explore the effects and molecular mechanisms of lentiviral vector-mediated MMP-7 siRNA (siMMP-7) on cell proliferation and invasive potential in LAC A549 cells, measured by MTT and Transwell assays, respectively. It was found that, the expression of MMP-7 protein in LAC was significantly increased compared with that in adjacent non-cancerous tissues (ANCT) (76.0% vs 44.0%, P&lt;0.001), and positively correlated with lymph node metastases of the tumor (P=0.014). Furthermore, targeted inhibition of cyclooxygenase-2 (COX-2) by siRNA downregulated the expression of MMP-7 and inhibited invasion of LAC cells, and knockdown of MMP-7 suppressed tumor proliferation and invasion in LAC cells. Taken together, our findings indicate that increased expression of MMP-7 is associated with lymph node   metastasis   and upregulated by COX-2, and promotes the tumorigenesis of LAC, suggesting that MMP-7 may be a potential therapeutic target for the treatment of cancer. 
 The cyclooxygenase-2 (PTGS2) 8473T>C polymorphism is associated with breast cancer risk. Cyclooxygenase-2 (COX-2) is involved in carcinogenesis, immune response suppression, apoptosis inhibition, angiogenesis, and tumor cell invasion and   metastasis  . The gene for COX-2, designated as PTGS2, carries a common polymorphism at position 8473 in the 3'-untranslated region (PTGS2 8473T>C), which has been associated with susceptibility to malignant disease. To investigate the role of this polymorphism for breast cancer, we determined the prevalence of PTGS2 genotypes in 500 women with breast cancer and 500 sex- and age-matched healthy control subjects. Homozygous carriers of the 8473-CC genotype were more frequent among patients (12.4%) than among controls (6.6%; P = 0.002). The odds ratio for carriers of this genotype for breast cancer was 2.1 (95% confidence interval, 1.3-3.3). Among patients, estrogen receptor positivity was less frequent among carriers of a CC genotype (63.9%) than among carriers of a TT or TC genotype (76.9%; P = 0.028). Tumor size, histologic grade, presence of primary lymph node metastases, progesterone receptor positivity, or age at diagnosis were not associated with PTGS2 genotypes. We conclude that the homozygous PTGS2 8473-CC genotype may be associated with breast cancer risk. 
 Coexpression of cyclooxygenase-2 and vascular endothelial growth factor in gastrointestinal stromal tumor: possible relations to pathological parameters and clinical behavior. Currently, the clinicopathological significance of coexpression of cyclooxygenase-2 (COX-2) and vascular endothelial growth factor (VEGF) in gastrointestinal stromal tumors (GISTs) has not been fully described.The present study was designed to investigate the co-expression of the two markers and its significance in 54 patients with GIST by means of immunohistochemistry.We demonstrated that the expressions of COX-2 and VEGF were significantly higher in malignant GIST than those in benign and potentially malignant GIST (p<0.01). A significant correlation was found between COX-2 and VEGF expression. In addition, the expressions of COX-2 and VEGF were significantly correlated with pattern of tumor growth, the size of tumors, and the central necrosis of tumors (p<0.01 or p<0.05). Five-year survival rates were much lower in patterns with high expression of COX-2 and VEGF than those with low expression (p<0.01). Overexpression of COX-2 and VEGF in GIST may enhance the possibility of growth, invasion, and   metastasis  .The levels of COX-2 and VEGF expression can be used as objective parameters in distinguishing benign from malignant, judging the malignant degree, and predicting the prognosis of patients with GIST. 
 Overexpression of COX-2 gene in oral cancer is independent of stage of disease and degree of differentiation. The incidence of oral cancer is high in certain parts of the world including Southeast Asia. Smokeless tobacco and areca nut chewing is proposed as a possible factor. Cyclooxygenase 2 (COX-2) receptors are present on neoplastic cells and are proposed to participate in initiation, transformation, progression and   metastasis   of cancer. In a prospective case-controlled study, 42 cases of squamous cell carcinoma of the oral cavity, 13 cases of oral premalignant lesions, and oral mucosa from 32 normal subjects were evaluated for COX-2 gene expression using reverse transcriptase polymerase chain reaction. The mean age of the patients with oral cancer was 60.2 years. The majority of cancer patients were males while the majority of controls were females. A significantly higher expression of COX-2 was found in cancer patients compared to both normal controls (p=0.0001) and patients with premalignant lesions (0.015). The expression in premalignant lesions was higher compared to healthy subjects (p=0.05). COX-2 expression in oral cancer was found to be independent of grade of tumor and stage of disease. These results show up-regulation of the COX-2 gene in oral cancer and precancer. This suggests a role for COX2 receptors in oral cancer carcinogenesis, and provides the foundation for a large randomized trial to determine the role COX2 inhibitors may play in prevention of oral carcinogenesis. 
 Possible predictive markers related to micro-  metastasis   in breast cancer patients. Despite significant advances in micro-  metastasis   detection methods, little is known about the relationship between micro-  metastasis   and primary tumors. The purpose of this study was to assess the ability of expression of the breast cancer-related markers, HER-2/neu, COX-2, VEGF and PDGF-B, as a predictor for micro-  metastasis  . As destination sites for micro-  metastasis  , we examined the peripheral blood (BD), bone marrow (BM) and sentinel lymph node (SLN) from 53 breast cancer patients. Protein and gene expression of the markers at the primary site were determined by immunohistochemistry (IHC) and quantitative RT-PCR. BD and BM samples were processed using magnetic-activated cell separation and immunocytochemistry. SLNs were examined by hematoxylin and eosin (H&E) staining and IHC. The percentages of patients with micro-  metastasis   were 24.5% in BD, 56.6% in BM, 26.4% in SLN by H&E and 41.5% in SLN by IHC. COX-2 gene amplification was significantly associated with SLN micro-  metastasis   by H&E (P=0.03). Overexpression of HER-2/neu predicts the presence of SLN micro-  metastasis   as detected by H&E (P=0.005) and COX-2 overexpression predicts the presence of micro-  metastasis   in BM (P=0.005) and SLN by H&E (P<0.001) and IHC (P<0.001). Similarly, PDGF-B overexpression predicts micro-  metastasis   in BD (P=0.002), BM (P=0.003) and SLN by H&E (P=0.017), whereas VEGF overexpression predicts only the presence of SLN micro-  metastasis   by IHC (P=0.001). Our results indicate the possible value of using these markers to predict the risk of micro-  metastasis   in breast cancer. 
 Elevation of cyclooxygenase-2 is related to lymph node   metastasis   in adenocarcinoma of uterine cervix. In a previous study, we demonstrated that elevation of COX-2 is significantly associated with lymph node   metastasis   in squamous cell carcinoma (SCC) of cervix. The main objective of this study is to characterize the relationship between elevation of COX-2 and its possible clinical role in adenocarcinoma (AC) of cervix. Using immunohistochemistry, we examined COX-2 expression levels in 84 patients with AC of uterine cervix [71 ACs, 13 adenosquamous carcinomas (ASCs)]. Elevation of COX-2 was correlated with clinicopathological variables and p53 expression, as detected by immunohistochemistry. Elevation of COX-2 was detected in 13.0% (11 of 84) of the tumors. Elevation of COX-2 was significantly correlated with histologic type (AC 8.5% vs. ASC 38.5%, P=0.011). Both tumor stage and lymph node   metastasis   were correlated with elevation of COX-2 with a borderline significance (P=0.062 and 0.068, respectively). Elevation of p53 was not associated with elevation of COX-2. The association between lymph node   metastasis   and elevation of COX-2 was stronger in cases of AC than in cases of ASC (28.4 vs. 4.3%, P=0.023). According to the results of univariate analysis, elevation of COX-2 was significantly associated with decreased overall survival (P=0.003, log-rank test). However, multivariate analyses revealed that only tumor stage was independently associated with overall survival, suggesting that elevation of COX-2 itself may not be an independent prognostic factor. The present study shows that elevation of COX-2 may contribute to lymph node   metastasis   in AC of uterine cervix. This suggests that the potential therapeutic role of COX-2 inhibitors should be validated, not only in SCC, but also in AC of uterine cervix. 
 Estrogen-dependent prognostic significance of cyclooxygenase-2 expression in early-stage invasive breast cancers treated with breast-conserving surgery and radiation. To evaluate the prognostic significance of cyclooxygenase-2 (COX-2) in breast cancer patients treated with conservative surgery and radiation therapy (CS+RT).Between 1975 and 2003, we retrieved specimens from 504 breast cancer patients treated with CS+RT. The specimens were constructed into tissue microarrays processed and stained for estrogen receptor (ER), progesterone receptor, Her2/neu, and COX-2. Each core was scored as positive or negative. All data including demographics, clinical, pathologic, staging, and outcome variables were entered into a computerized database.Expression of COX-2 was positive in 58% of cases and correlated with younger age (p = 0.01) and larger tumor size (p = 0.001). Expression of COX-2 was predictive of local relapse (relative risk[RR], 3.248; 95% confidence interval [CI], 1.340-7.871; p = 0.0091), distant   metastasis   (RR, 2.21; 95% CI, 1.259-3.896; p = 0.0058), and decreased survival (RR, 2.321; 95% CI, 1.324-4.071; p = 0.0033). Among ER-positive patients, COX-2 expression was predictive of worse local control (85% vs. 93%, p = 0.04), distant   metastasis   (75% vs. 95%, p = 0.002) and worse survival (65% vs. 94%, p = 0.002). Among ER-negative tumors COX-2 expression was not significantly correlated with local control (87 vs. 95%, p = 0.12), distant   metastasis   (73% vs. 78%, p = 0.39), or survival (77% vs. 87%, p = 0.15).In breast cancer patients treated with CS+RT, COX-2 expression is associated with younger age, larger tumor size, worse local control, distant   metastasis  , and worse overall survival. The significance is limited to hormone receptor-positive tumors, consistent with the known effect of COX-2/PGE2 on aromatase activity. Use of COX-2 inhibitors in estrogen-dependent breast cancers warrants further investigation. 
 Clinicopathological significance of cyclooxygenase-2 expression in hypopharyngeal squamous cell carcinoma. The purpose of this study was to determine the expression of cyclooxygenase-2 (COX-2) in normal epithelium, dysplasia and squamous cell carcinoma of the hypopharynx and to investigate associations with clinicopathological factors and survival. Seventy-five patients with hypopharyngeal squamous cell carcinomas (HPSCC) who underwent surgical treatment at the Department of Otolaryngology, Osaka Medical Center for Cancer and Cardiovascular Diseases, were investigated. COX-2 expression was determined by immunohistochemistry and 97.3% (73/75) of samples displayed immunostaining in tumor cells. COX-2 staining was localized mainly in the cytoplasm (73/75) and was rare in stromal cells (2/75). Over half of the areas of dysplastic cells adjacent to carcinomas also showed COX-2 staining (41/70, 58.6%). There were no significant correlations between the COX-2 expression and tumor size, location and tumor growth type, T- and N-stage, tumor recurrence, lymph node   metastasis   and survival in this study. COX-2 expression thus does not appear to have a prognostic significance for hypopharyngeal SCC although there was a tendency for higher values in T3/T4 than T1/T2 cases. Furthermore, COX-2 was found to be more strongly expressed in poorly-differentiated than in moderately/well-differentiated carcinomas. In this study group, COX-2 was up-regulated not only in SCCs but also in the dysplastic lesions of the hypopharynx, suggesting that COX-2 inhibition may be a useful chemopreventive strategy. 
 The relationship between cyclooxygenase-2, CD44v6, and nm23H1 in esophageal squamous cell carcinoma. Esophageal cancer is the fourth most prevalent malignancy in China. The relationship between COX-2, CD44v6, and nm23H1 in esophageal squamous cell carcinoma (ESCC) remains unclear.Expression of COX-2, CD44v6, and nm23H1 was examined, using the streptavidin-peroxidase method, in 82 ESCC and 30 normal esophageal mucosa (NEM) samples from the Shaanxi Province in China.The positive rates of COX-2, CD44v6, and nm23H1 were 73.2% (60/82), 64.6% (53/82), and 24.4% (31/82), respectively in ESCC, but 6.7% (2/30), 3.3% (1/30), and 90% (27/30), respectively in NEMs. There was a statistically significant difference between NEMs and ESCCs (p < 0.05). Expression of COX-2 showed a positive statistical correlation with expression of CD44v6 (r = 0.4732, p < 0.0001), and an inverse correlation with nm23H1 (r = -0.3226, p = 0.0035). Expression of COX-2, CD44v6, and nm23H1 had no significant correlation with gender or age (p > 0.05), but increased expression of COX-2 and CD44v6 showed statistical correlation with invasion and lymph node   metastasis  , respectively (p < 0.05). Decreased expression of nm23H1 was statistically correlated with lymph node   metastasis   (p = 0.0007) but had no correlation with invasion (p = 0.8221).This is the first report of a significant correlation between COX-2, CD44v6, and nm23H1 in ESCC. This knowledge might help us to further understand the molecular mechanisms of carcinogenesis and progression of ESCC. 
 Coexpression of VEGF-C and COX-2 and its association with lymphangiogenesis in human breast cancer. Lymphangiogenesis has become a new research frontier in tumor   metastasis   since the discovery of reliable lymphatic markers that have allowed observation and isolation of lymphatic endothelium. Cyclooxygenase-2 (COX-2) has been reported to be involved in the critical steps in carcinogenesis. However, possible role of COX-2 in lymphangiogenesis and lymphatic   metastasis   is still poorly understood. In present study, we aimed to investigate the relationship between vascular endothelial growth factor-C (VEGF-C) and COX-2 in human breast cancer, and correlations with lymphangiogenesis and prognosis.Tissue samples of primary tumors from 70 patients undergoing intentionally curative surgical resections for breast cancer were immunohistochemically examined for VEGF-C, COX-2, and D2-40 expressions. The association between COX-2 and VEGF-C expressions and clinicopathological parameters as well as prognosis were analysised. To demonstrate the presence of proliferating lymphatic endothelial cells, 10 random cases with high LVD counts were selected for D2-40/Ki-67 double immunostaining.A significant correlation was found between the expression of VEGF-C and COX-2 (r = 0.529, P < 0.001), and both elevated VEGF-C expression and elevated COX-2 expression were associated with higher lymph vessel density (LVD), lymph node   metastasis   and D2-40 positive lymphatic invasion (LVI) as well as worse disease free survival (DFS) and overall survival (OS) in a univariate analysis. In the double immunostain for the lymph vessel marker D2-40 and the proliferation marker Ki-67, the results confirmed Ki-67-positive nuclei in a proportion of lymph vessel endothelial cells.There is indeed lymphangiogenesis in breast cancer, the most compelling evidence being the presence of proliferating lymphatic endothelial cells. VEGF-C and COX-2 are coexpressed and significantly associated with lymphangiogenesis and prognosis in invasive breast cancer. Suggesting COX-2 may up-regulate VEGF-C expression and thus promote lymph node   metastasis   via lymphangiogenesis pathway in human breast cancer. 
 Expression of VEGF, VEGFR, EGFR, COX-2 and MVD in cervical carcinoma, in relation with the response to radio-chemotherapy. Despite the improvement in the treatment results due to modern irradiation techniques and to the association of chemo-radiotherapy, cervical cancer remains an unsolved problem of oncology both due to the increased rate of local failures and of the distant   metastasis  . Efforts to implement new therapeutic strategies in order to obtain better results in patients with cervical cancer appear justified. Neovascularization is an important step in the tumor progression and the therapeutic targeting of the tumor blood vessels appears to be a good strategy to follow in the anti-cancer treatment. Thus, even in an incipient phase of the clinical research process, the combination between the anti-angiogenic aimed therapies and the current radio-chemotherapy seems to represent a new, feasible and promising approach. The aim of the present study was to determine the prognostic and/or predictive value of some biological markers of tumor angiogenesis and of their implication in increasing the efficacy of current treatments for this cancer.So far, 54 women were included in a prospective trial: 44 having an advanced cervical carcinoma and 10 healthy women, as controls. A tumor biopsy and a blood sample were obtained from each patient before the start of therapy. The density of microvascularization was assessed using CD34 monoclonal antibody (hot spot technique), the expression of angiogenic factors VEGFR, EGFR and COX-2 were determined in tumor biopsies by specific immunohistochemistry techniques, using primary antibodies anti-EGFR, anti-VEGF and anti-COX-2 respectively. The quantitative polymerase chain reaction (Real Time PCR) was employed for assessing the expression level of the genes involved. Serum VEGF was determined by quantitative ELISA technique.Among the studied clinical and molecular factors, we found to be predictive for the type of response the following factors: tumor size at diagnosis (p=0.01), VEGFR2 expression (p=0.02) and a tendency to significance for patients' age (p=0.06). From the large panel of studied markers it was observed correlation between MVD expression with stromal COX-2 (p=0.01) and a tendency with epithelial COX-2 (p=0.06). Stromal COX-2 has higher correlation with VEGFR2 (p=0.01) and MVD (p=0.01) and also has a lower correlation with tumor size (p=0.08).Univariate analysis demonstrates that the response to radio-chemotherapy in cervical cancer is related to a set of clinical and molecular factors as: the tumor size, the expression of VEGFR2 as mRNA level and the patients' age. Unfortunately, the multivariate analysis by logistic model selects only VEGFR2 expression for prediction of tumor response. The interrelations between the different biomarkers demonstrate the complexity of the tumor progression process and the necessity of further studies to identify new therapeutic targets. 
 [Expressions of COX-2 and MMP-2 in nasopharyngeal carcinoma and the their relationship with lymph node   metastasis  ]. To study the expression of cyclooxygenase-2 (COX-2) and matrix metalloproteinase-2 (MMP-2) in nasopharyngeal carcinoma (NPC) and their relationship with lymph node   metastasis  .The expression of COX-2 and MMP-2 in 86 NPC tissues and 30 normal nasopharyngeal tissues were evaluated by immunohistochemical analysis.The positive rate of COX-2 and MMP-2 in NPC tissue was 75.58% and 66.28% respectively, which was significantly higher than that in normal nasopharyngeal (P < 0.01). The expression of both COX-2 and MMP-2 were positively correlated with lymph node   metastasis   (P < 0.01). There was a positive correlation between the expression of COX-2 and MMP-2 (P < 0.01).COX-2 and MMP-2 expression are increased in NPC, and they may cooperate in the course of lymph   metastasis   of NPC. 
 HGF/Met signalling promotes PGE(2) biogenesis via regulation of COX-2 and 15-PGDH expression in colorectal cancer cells. Evidence points towards a pivotal role for cyclooxygenase (COX)-2 in promoting colorectal tumorigenesis through increasing prostaglandin E(2) (PGE(2)) levels. PGE(2) signalling is closely associated with the survival, proliferation and invasion of colorectal cancer cells. Recently, a reduction in PGE(2) inactivation, a process mediated by the nicotinamide adenine dinucleotide (NAD+)-dependent 15-hydroxyprostaglandin dehydrogenase (15-PGDH), has also been shown to promote tumoral PGE(2) accumulation. The hepatocyte growth factor (HGF) receptor, Met, is frequently over-expressed in colorectal tumours and promotes cancer growth,   metastasis   and resistance to therapy, although the mechanisms for this have not been fully elucidated. Here, we report that HGF/Met signalling can promote PGE(2) biogenesis in colorectal cancer cells via COX-2 up-regulation and 15-PGDH down-regulation at the protein and messenger RNA level. Pharmacological inhibition of MEK and PI3K suggested that both extracellular signal-regulated kinase (ERK) and AKT signalling are required for COX-2 protein up-regulation and 15-PGDH down-regulation downstream of Met. Notably, inhibition of Met with the small molecule inhibitor SU11274 reduced COX-2 expression and increased 15-PGDH expression in high Met-expressing cells. We also show that hypoxia potentiated HGF-driven COX-2 expression and enhanced PGE(2) release. Furthermore, inhibition of COX-2 impeded the growth-promoting effects of HGF, suggesting that the COX-2/PGE(2) pathway is an important mediator of HGF/Met signalling. These data reveal a critical role for HGF/Met signalling in promoting PGE(2) biogenesis in colorectal cancer cells. Targeting the crosstalk between these two important pathways may be useful for therapeutic treatment of colorectal cancer. 
 Cyclooxygenase-2 up-regulates CCR7 expression via AKT-mediated phosphorylation and activation of Sp1 in breast cancer cells. Up-regulation of cyclooxygenase-2 (COX-2) is frequently found in human cancers and is significantly associated with tumor   metastasis  . Our previous results demonstrate that COX-2 and its metabolite prostaglandin E2 (PGE2) stimulate the expression of CCR7 chemokine receptor via EP2/EP4 receptors to promote lymphatic invasion in breast cancer cells. In this study, we address the underlying mechanism of COX-2/PGE2-induced CCR7 expression. We find that COX-2/PGE2 increase CCR7 expression via the AKT signaling pathway in breast cancer cells. Promoter deletion and mutation assays identify the Sp1 site located at the -60/-57 region of CCR7 gene promoter is critical for stimulation. Chromatin immunoprecipitation (ChIP) assay confirms that in vivo binding of Sp1 to human CCR7 promoter is increased by COX-2 and PGE2. Knockdown of Sp1 by shRNA reduces the induction of CCR7 by PGE2. We demonstrate for the first time that AKT may directly phosphorylate Sp1 at S42, T679, and S698. Phosphorylation-mimic Sp1 protein harboring S42D, T679D, and S698D mutation strongly activates CCR7 expression. In contrast, change of these three residues to alanine completely blocks the induction of CCR7 by PGE2. Pathological investigation demonstrates that CCR7 expression is strongly associated with phospho-AKT and Sp1 in 120 breast cancer tissues. Collectively, our results demonstrate that COX-2 up-regulates CCR7 expression via AKT-mediated phosphorylation and activation of Sp1 and this pathway is highly activated in metastatic breast cancer. 
 Correlation of cyclooxygenase-2 pathway and VEGF expression in head and neck squamous cell carcinoma. The purpose of this study was to determine whether cyclooxygenase-2 (COX-2) was overexpressed in head and neck squamous cell carcinoma (HNSCC) in relation to vascular endothelial growth factor (VEGF) expression and lymph node   metastasis  . COX-2 has an important role in the promotion of carcinogenesis, tumor invasiveness and angiogenesis. VEGF is a proangiogenic factor that is up-regulated in various tumors. VEGF has been shown to interact with COX-derived prostaglandins in angiogenesis. To better understand the roles of these genes in HNSCC, we evaluated the immunohistochemical expression pattern of COX-2 and VEGF in relationship with histologic differentiation, clinical stage, and nodal status in 146 HNSCC. The COX-2/VEGF double immunofluorescein staining was evaluated with confocal scanning laser microscope. A positive expression was seen in 96% (140/146: 98 strong positive, 42 weakly positive) for COX-2 and in 98% (143/146: 63 strong positive, 80 weakly positive) for VEGF in HNSCC. A correlation was present in the positive expression of COX-2 with histologic differentiation, clinical stage, and nodal status. VEGF expression was also correlated with nodal status. A significant relation was observed between COX-2 and VEGF expressions. The simultaneous expression of COX-2 and VEGF was statistically significant (p<0.05). These results indicate that elevated COX-2 or VEGF expression is more common in HNSCC with poor prognostic characteristics. The present findings support the efforts to initiate clinical trials on the efficacy of COX-2 inhibitors in adjuvant treatment of HNSCC. 
 Cyclooxygenase-2 uses the protein kinase C/ interleukin-8/urokinase-type plasminogen activator pathway to increase the invasiveness of breast cancer cells. Cyclooxygenase-2 (COX-2) increases breast cancer cell invasion. Expression of various pro-angiogenic and pro-invasive factors has been correlated with high expression of COX-2. However, whether these factors are essential to COX-2-mediated breast cancer invasion, and the mechanisms by which COX-2 increases the expression of these factors are unknown. Our microarray results indicate that higher COX-2 expression was associated with increased levels of interleukin-8 (IL-8), a key factor in breast cancer invasion and   metastasis  . COX-2 overexpressing cells (MCF-7/COX-2), generated by transfecting COX-2-encoding plasmids into the poorly invasive MCF-7 breast cancer cells, were more invasive and produced higher IL-8 levels than the parental cells. To investigate the role of IL-8 in COX-2-mediated invasion, MCF-7 parental cells were incubated with IL-8. Exogenous IL-8 increased the invasiveness of MCF-7 cells. IL-8 is one pathway by which COX-2 mediates breast cancer invasion. Protein kinase A (PKA) and protein kinase C (PKC) are activated by COX-2 and are involved in IL-8 regulation. Inhibition of PKC, not PKA, decreased IL-8 production and invasion in MCF-7/COX-2 cells. Activation of PKC, not PKA, increased IL-8 production and invasion in MCF-7 cells. Thus, the invasive effects of COX-2 are mediated by PKC, not PKA. Activity of the urokinase-type plasminogen activator (uPA) was increased in MCF-7 cells by COX-2 overexpression or by the addition of a PKC activator or by IL-8. Inhibition of PKC decreased uPA activity in MCF-7/COX-2 cells. Furthermore, inhibition of uPA activity decreased the invasiveness of MCF-7/COX-2 cells, indicating that uPA was essential to COX-2-mediated invasion. Herein we demonstrate for the first time a detailed mechanism by which COX-2 increases breast cancer invasion: the PKC/IL-8/uPA pathway. 
 [The expression and relationship of cyclooxygenase-2 and latent membrane protein-1 in nasopharyngeal carcinoma]. To discuss the expression and correlation of COX2 and LMP1 in NPC.Fifty-three nasopharyngeal biopsy specimens of NPC patients who had been diagnosed definitely with pathology in our department from 2000 to 2005, and 8 nasopharyngeal biopsy specimens of chronic nasopharyngitis patients were collected. The expression of COX2, LMP1 were detected with streptavidin peroxidase immunohistochemistry staining. All the datas were analyzed with SPSS 12.0.The positive expression rate of COX2 was 71.70% (38/53), and that of LMP1 was 66.04% (35/53) of Nasopharyngeal carcinoma tissue. The higher expression rate was detected in neck lymph nodes   metastasis   group of nasopharyngeal carcinoma (P<0.05). While the expression rate of COX2, LMP1 was no significant relation with age, gender, clinical stage and pathological classification (P>0.05). The expression of COX2 and LMP1 showed positive correlation (gamma = 0.797, P<0.01).COX2 and LMP1 were highly expressed in nasopharyngeal carcinoma cells. The expressions of COX2 and LMP1 was significantly associated with neck lymph nodes   metastasis  , and was none-significant relation with age, gender, clinical stage and pathological classification. The expression of LMP1 showed a significant positive correlation with that of COX2. LMP1 could enhance NPC neck lymph nodes   metastasis   by up-regulating the expression of COX2. 
 COX-2-mediated regulation of VEGF-C in association with lymphangiogenesis and lymph node   metastasis   in lung cancer. The mechanisms underlying the effects of COX-2 on tumor lymphangiogenesis remain largely undefined. Here, the human lung cancer cell lines A549, 95D, Anip973, and AGZY83-a with different metastatic capacities were investigated by immunostaining, western blotting, and real-time RT-PCR. We observed increased expressions of COX-2 and VEGF-C in the three highly metastatic cell lines compared with the less metastatic AGZY83-a cell line. The COX-2-specific inhibitor Celecoxib suppressed VEGF-C expression whereas the main COX-2 metabolite PGE(2) elevated VEGF-C expression in Anip973 and AGZY83-a cells in positive and negative experiments. To determine the functional link to COX-2 more specifically and elucidate the mechanistic pathway, we used a siRNA to knock down the high COX-2 expression in Anip973 cells and transfected a COX-2 cDNA to enhance the low COX-2 expression in AGZY83-a cells, and then treated the cells with EP1/EP4 agonists or antagonists, respectively. The results revealed that the EP1/EP4 agonists significantly increased VEGF-C production in the COX-2-knockdown Anip973 cells. In contrast, the EP1/EP4 antagonists diminished VEGF-C production in the COX-2-overexpressing AGZY83-a cells. Furthermore, animal models provided evidence that Celecoxib decreased VEGF-C expression, lymphangiogenesis, and lymph node metastases in Anip973 cells, whereas PGE(2) treatment increased the same factors in the parental AGZY83-a cells. A positive correlation between COX-2 and VEGF-C was also confirmed in vivo. The present data suggest that COX-2 regulates VEGF-C expression via the PGE(2) pathway, and that EP1/EP4 receptors are involved in PGE(2)-mediated VEGF-C production. Thus, COX-2 may represent a candidate gene for blocking tumor lymphangiogenesis and lymph node   metastasis  . 
 Epidermal growth factor-induced cyclooxygenase-2 enhances head and neck squamous cell carcinoma   metastasis   through fibronectin up-regulation. Epidermal growth factor receptor (EGFR) activation is a major cause of   metastasis   in many cancers, such as head and neck squamous cell carcinoma (HNSCC). However, whether the induction of cyclooxygenase-2 (COX-2) mediates EGF-enhanced HNSCC   metastasis   remains unclear. Interestingly, we found that EGF induced COX-2 expression mainly in HNSCC. The tumor cell transformation induced by EGF was repressed by COX-2 knockdown, and this repression was reversed by simultaneously treating the cells with EGF and prostaglandin E₂ (PGE₂). The down-regulation of COX-2 expression or inhibition of COX-2 activity significantly blocked EGF enhancement of cell migration and invasion, but the addition of PGE₂ compensated for this inhibitory effect in COX-2-knockdown cells. COX-2 depletion inhibited EGF-induced matrix metalloproteinase (MMP)-1, MMP-2, MMP-3, MMP-9, and fibronectin expression and Rac1/cdc42 activation. The inhibitory effect of COX-2 depletion on MMPs and the fibronectin/Rac1/cdc42 axis were reversed by co-treatment with PGE₂. Furthermore, depletion of fibronectin impeded the COX-2-enhanced binding of HNSCC cells to endothelial cells and tumor cells metastatic seeding of the lungs. These results demonstrate that EGF-induced COX-2 expression enhance  s HNSCC me  tastasis via activation of the fibronectin signaling pathway. The inhibition of COX-2 expression and activation may be a potential strategy for the treatment of EGFR-mediate  d HNSCC me  tastasis. 
 Prognostic evaluation of COX-2 expression in renal cell carcinoma. Prognosis of renal cell carcinoma (RCC) differs within the same stage and grade. Our aim was to investigate the incidence of COX-2 in primary RCC tumors at different stages according to the occurrence of   metastasis  , and the impact of this biomarker on the survival of RCC patients.The cytoplasmic/membranous COX-2 protein expression was examined by immunohistochemistry in RCC tumors from 102 patients. The patients were divided into those with: no   metastasis   during 7.5 years' follow-up (nm), no   metastasis   at the time of nephrectomy but who later developed metastases (lm), and those with   metastasis   at presentation (pm). The immunoreactivity of COX-2 was classified as none (absent/weak intensity in fewer than 10% of the cancer cells), low (weak intensity in over 10% of the cancer cells) or high immunostaining (strong intensity in the majority of the cancer cells). In addition p53 and Ki-67 immunostaining was also assessed in tumors.Percentages of COX-2 reaction were (no/low/high): 78/16/7 in the nm, 53/28/19 in the lm, 92/8/0 in the pm groups (p=0.014). Median   metastasis  -free survival was shorter in lm patients with COX-2-negative tumors when compared to those with COX-2-positive ones (15 vs. 46 months; p=0.020). Median overall survival was shorter in pm/lm patients with COX-2-negative tumors when compared to those with COX-2-positive ones (28 vs. 94 months; p=0.027), and with COX-2-negative/Ki-67-positive tumors when compared to COX-2-positive/Ki-67-negative ones (19 vs. 97 months; p=0.004). Findings for patients with COX-2-negative/p53-positive tumors were similar, with shorter survival compared to those with COX-2-positive/p53-negative ones (19 vs. 97; p=0.006).COX-2 protein expression is associated with slow development of metastases, and favourable prognosis in metastatic RCC. 
 Functional polymorphisms of cyclooxygenase-2 gene and risk for hepatocellular carcinoma. Cyclooxygenase-2 (COX-2) influences carcinogenesis through immune response suppression, apoptosis inhibition, regulation of angiogenesis and tumor cell invasion, and   metastasis  . It is now well established that COX-2 is overexpressed in many premalignant, malignant, and metastatic cancers, including hepatocellular carcinoma (HCC). DNA sequence variations in the COX-2 gene may lead to altered COX-2 production and/or activity, and so they cause inter-individual differences in the susceptibility to HCC. Functional coding region polymorphisms -1195A>G (rs689466), -765G>C (rs20417), and +8473T>C (rs5275) in the COX-2 gene have recently been shown to be associated with several human cancers but their association with HCC has yet to be investigated. We used hospital-based case-control study to assess the hypothesis that the functional COX-2 variation may affect individual susceptibility to the HCC. COX-2 polymorphisms were investigated in 129 confirmed subjects with HCC and 129 cancer-free control subjects matched on age, gender, smoking, and alcohol consumption using a polymerase chain reaction-restriction fragment length polymorphism (PCR-RFLP) assay. The distribution of the COX-2 -1195A>G and +8473T>C genotypes were not significantly different between HCC cases and control. However, proportion of the COX-2 -765CC genotype which leads to a 30% reduction of the COX-2 promoter activity was significantly lower in patients with HCC (3.1%) when compared to control subjects (11.6%) (P < 0.05). Logistic regression analyses revealed that the COX-2 -765G>C variant genotype (-765CC) was associated with a significantly decreased risk of HCC compared with the -765GG wild-type homozygotes [P < 0.05, odds ratio (OR) = 0.25, 95% confidence interval (CI) = 0.08-0.79]. Our results suggest for the first time that the -765CC genotype of COX-2 -765G>C polymorphism, causing lower COX-2 gen expression, is a genetic protective factor for HCC. However, because this is the first report concerning the COX-2 -1195A>G, -765G>C, and +8473T>C polymorphisms and the risk of HCC, independent studies are needed to validate our findings. 
 Vascular endothelial growth factor regulates melanoma cell adhesion and growth in the bone marrow microenvironment via tumor cyclooxygenase-2. Human melanoma frequently colonizes bone marrow (BM) since its earliest stage of systemic dissemination, prior to clinical   metastasis   occurrence. However, how melanoma cell adhesion and proliferation mechanisms are regulated within bone marrow stromal cell (BMSC) microenvironment remain unclear. Consistent with the prometastatic role of inflammatory and angiogenic factors, several studies have reported elevated levels of cyclooxygenase-2 (COX-2) in melanoma although its pathogenic role in bone marrow melanoma   metastasis   is unknown.Herein we analyzed the effect of cyclooxygenase-2 (COX-2) inhibitor celecoxib in a model of generalized BM dissemination of left cardiac ventricle-injected B16 melanoma (B16M) cells into healthy and bacterial endotoxin lipopolysaccharide (LPS)-pretreated mice to induce inflammation. In addition, B16M and human A375 melanoma (A375M) cells were exposed to conditioned media from basal and LPS-treated primary cultured murine and human BMSCs, and the contribution of COX-2 to the adhesion and proliferation of melanoma cells was also studied.Mice given one single intravenous injection of LPS 6 hour prior to cancer cells significantly increased B16M   metastasis   in BM compared to untreated mice; however, administration of oral celecoxib reduced BM   metastasis   incidence and volume in healthy mice, and almost completely abrogated LPS-dependent melanoma metastases. In vitro, untreated and LPS-treated murine and human BMSC-conditioned medium (CM) increased VCAM-1-dependent BMSC adherence and proliferation of B16M and A375M cells, respectively, as compared to basal medium-treated melanoma cells. Addition of celecoxib to both B16M and A375M cells abolished adhesion and proliferation increments induced by BMSC-CM. TNFα and VEGF secretion increased in the supernatant of LPS-treated BMSCs; however, anti-VEGF neutralizing antibodies added to B16M and A375M cells prior to LPS-treated BMSC-CM resulted in a complete abrogation of both adhesion- and proliferation-stimulating effect of BMSC on melanoma cells. Conversely, recombinant VEGF increased adherence to BMSC and proliferation of both B16M and A375M cells, compared to basal medium-treated cells, while addition of celecoxib neutralized VEGF effects on melanoma. Recombinant TNFα induced B16M production of VEGF via COX-2-dependent mechanism. Moreover, exogenous PGE2 also increased B16M cell adhesion to immobilized recombinant VCAM-1.We demonstrate the contribution of VEGF-induced tumor COX-2 to the regulation of adhesion- and proliferation-stimulating effects of TNFα, from endotoxin-activated bone marrow stromal cells, on VLA-4-expressing melanoma cells. These data suggest COX-2 neutralization as a potential anti-metastatic therapy in melanoma patients at high risk of systemic and bone dissemination due to intercurrent infectious and inflammatory diseases. 
 Tumor cells induce COX-2 and mPGES-1 expression in microvascular endothelial cells mainly by means of IL-1 receptor activation. Prostaglandin (PG) E(2) plays a key role in immune response, tumor progression and   metastasis  . We previously showed that macrovessel-derived endothelial cells do not produce PGE(2) enzymatically because they do not express the inducible microsomal PGE-synthase-1 (mPGES-1). Nevertheless, differences between macro- and micro-vessel-derived endothelial cells regarding arachidonic acid (AAc) metabolism profile have been reported. The present work was conducted to evaluate the expression of PGE(2)-pathway-related enzymes in human microvascular endothelial cells (HMVEC) in culture and to test the hypothesis that the tumor cell-HMVEC cross talk could increase mPGES-1 expression in HMVEC. We treated HMVEC in culture with human recombinant IL-1β. IL-1β induced PGE(2) release and COX-2 and mPGES-1 expression in terms of mRNA and protein, determined by real-time PCR and immunoblotting, respectively. HMVEC constitutively expressed mPGES-2 and cytosolic PGES (cPGES) and the IL-1β treatment did not modify their expression. PGE(2) synthesized by HMVEC from exogenous AAc was linked to mPGES-1 expression. Immunohistochemistry analysis confirmed mPGES-1 expression in microvessels in vivo. COX-2 and mPGES-1 were also induced in HMVEC by the conditioned medium from two squamous head and neck carcinoma cell lines. Conditioned medium from tumor cell cultures contained several cytokines including the IL-1β and IL-1α. Tumor cell-induced COX-2 and mPGES-1 in HMVEC was strongly inhibited by the IL-1-receptor antagonist, indicating the important implication of IL-1 in this effect. HMVEC could therefore contribute directly to PGE(2) formed in the tumor. Our findings support the concept that mPGES-1 could be a target for therapeutic intervention in patients with cancer. 
 Messenger RNA expression of COX-2 and angiogenic factors in primary colorectal cancer and corresponding liver   metastasis  . Several new drugs that are targeted towards various angiogenic factors have shown considerable potential for controlling tumor proliferation and metastases. Expression levels of the targeted genes in primary tumors and metastases should be understood to maximize the use of such drugs. The present study aimed to clarify associations between mRNA levels of cyclooxygenase 2 (COX-2) and angiogenic factors [vascular endothelial growth factor (VEGF) and interleukin-8 (IL-8)] in primary colorectal cancer and in corresponding liver   metastasis  . We also compared these gene expressions of primary colorectal cancer between patients with and without liver   metastasis  . In 31 pairs of formalin-fixed and paraffin-embedded primary and metastatic liver tumors as well as 27 specimens of consecutive stage II patients without recurrence, mRNA was quantified by real-time reverse transcription-polymerase chain reaction following the laser capture microdissection. We found a significantly positive correlation in IL-8 between primary tumors and matched liver metastases (p=0.034, rs=0.39) and in VEGF (p=0.0083, rs=0.48), but not in COX-2, which was associated with both VEGF (p=0.044, rs=0.37) and IL-8 (p=0.0004, rs=0.64) in primary colorectal cancers. Multiple regression analysis revealed that COX-2 was independently associated with IL-8 (p<0.0001). There were no differences in mRNA levels between patients with and without liver   metastasis  . The mRNA levels of VEGF and IL-8 in liver   metastasis   can be predicted from those in primary colorectal cancer. COX-2 might exert angiogenic activity more through the IL-8, than the VEGF pathway. These angiogenic factors were sufficiently up-regulated before hematogenous   metastasis  . These preliminary data merit further validation studies. 
 Mediators of vascular remodelling co-opted for sequential steps in lung   metastasis  .   Metastasis   entails numerous biological functions that collectively enable cancerous cells from a primary site to disseminate and overtake distant organs. Using genetic and pharmacological approaches, we show that the epidermal growth factor receptor ligand epiregulin, the cyclooxygenase COX2, and the matrix metalloproteinases 1 and 2, when expressed in human breast cancer cells, collectively facilitate the assembly of new tumour blood vessels, the release of tumour cells into the circulation, and the breaching of lung capillaries by circulating tumour cells to seed pulmonary   metastasis  . These findings reveal how aggressive primary tumorigenic functions can be mechanistically coupled to greater lung metastatic potential, and how such biological activities may be therapeutically targeted with specific drug combinations. 
 Antitumor effects of inhibitors of nitric oxide synthase or cyclooxygenase-2 on human KB carcinoma cells overexpressing COX-2. Inducible nitric oxide synthase (iNOS) and cyclooxygenase (COX)-2 are major inflammatory mediators. Nitric oxide (NO) produced by iNOS has been shown to have an important role in carcinogenesis. Recent studies have suggested that COX-2 expression also contributes to carcinogenesis, as well as tumor growth, invasion, and   metastasis  . COX-2 inhibitors such as celecoxib are widely recognized to have antitumor activity, but can cause adverse effects. We investigated possible relations between COX-2 and NO with the use of a human epidermoid carcinoma cell line, designated KB, in which overexpression of COX-2 protein was induced by gene transfer. We also assessed the possibility of using NOS inhibitor as an antitumor drug. We isolated a COX-2 transfected clone (KB/COX-2) and used a neomycin-transfected clone (KB/neo) as control. NG-nitro-L-arginine-methyl ester (L-NAME) was used as a NOS inhibitor, dihydrochloride (1400W) as an iNOS inhibitor, and celecoxib as a selective COX-2 inhibitor. All agents inhibited the cell growth of both clones to similar extents in a dose-dependent manner. Prostaglandin E2 (PGE2) production and COX-2 expression in KB/COX-2 were inhibited not only by celecoxib, but also by L-NAME and 1400W. The decreases in PGE2 production and COX-2 expression were most prominent with celecoxib and L-NAME. In vivo, L-NAME and celecoxib significantly inhibited the proliferation of KB/COX-2-xenografted tumors. Tumor weight was reduced by L-NAME (60.6% decrease), 1400W (38.0% decrease), and celecoxib (74.5% decrease) as compared with the control after 21 days of treatment. Immunohistochemically, xenografted tumors expressed COX-2, iNOS, and eNOS. Such expression was suppressed by treatment with L-NAME and celecoxib. These results suggest that L-NAME and celecoxib significantly inhibit the proliferation of murine squamous cell carcinoma in vivo. L-NAME as well as celecoxib might thus be useful for the design and development of new antitumor drugs. 
 Cyclooxygenase-2-dependent lymphangiogenesis promotes nodal   metastasis   of postpartum breast cancer. Breast involution following pregnancy has been implicated in the high rates of   metastasis   observed in postpartum breast cancers; however, it is not clear how this remodeling process promotes   metastasis  . Here, we demonstrate that human postpartum breast cancers have increased peritumor lymphatic vessel density that correlates with increased frequency of lymph node metastases. Moreover, lymphatic vessel density was increased in normal postpartum breast tissue compared with tissue from nulliparous women. In rodents, mammary lymphangiogenesis was upregulated during weaning-induced mammary gland involution. Furthermore, breast cancer cells exposed to the involuting mammary microenvironment acquired prolymphangiogenic properties that contributed to peritumor lymphatic expansion, tumor size, invasion, and distant metastases. Finally, in rodent models of postpartum breast cancer, cyclooxygenase-2 (COX-2) inhibition during the involution window decreased normal mammary gland lymphangiogenesis, mammary tumor-associated lymphangiogenesis, tumor cell invasion into lymphatics, and   metastasis  . Our data indicate that physiologic COX-2-dependent lymphangiogenesis occurs in the postpartum mammary gland and suggest that tumors within this mammary microenvironment acquire enhanced prolymphangiogenic activity. Further, our results suggest that the prolymphangiogenic microenvironment of the postpartum mammary gland has potential as a target to inhibit   metastasis   and suggest that further study of the therapeutic efficacy of COX-2 inhibitors in postpartum breast cancer is warranted. 
 [Significance of COX-2, p53, proliferating cell nuclear antigen and nm23 expressions in gastric cancer and its behavior]. Molecular biological studies indicated that abnormal expression of cyclooxygenase-2(COX-2), p53, proliferating cell nuclear antigen(PCNA), and nm23 correlate with the development of gastric cancer, but the relationship between abnormal expression of above gene proteins and the biological behavior of gastric cancer was not yet clear. This study was designed to investigate the relationship between expressions of COX-2, p53, PCNA, and nm23 and the clinicopathological behavior in gastric cancer.The expressions of COX-2, p53, PCNA, and nm23 were detected by using immunohistochemical(SP) method.(1) The overexpression rate of COX-2 in gastric cancer was 70.4% (50/71), and the overexpression of COX-2 was correlated to tumor size, gross morphology, histological type, lymph node metastases, and clinicopathological stage of gastric cancer (P < 0.05, or P < 0.01). (2) The overexpression rates of p53 and PCNA in 71 surgically removed specimens from the patients with gastric cancer were 74.6% and 78.9%, respectively. And the expressions of p53 (86.7%) and PCNA (88.8%) in gastric cancer patients with lymph node   metastasis   were significantly higher than those in patients without lymph node   metastasis   (53.8% and 61.5%) (both, P < 0.05), respectively. Moreover, expressions of p53(52.2%) and PCNA(60.9%) in stage I + II patients were lower than those(85.4% and 87.5%) in stage III + IV (both, P < 0.05). (3) The low-expression of nm23 in gastric cancer was 71.8%, and its low-expression rate of nm23 in patients without lymph node   metastasis  (88.5%) and in stage I + II (91.3%) were much higher than those in the patients with lymph node   metastasis   (62.2%) and in stage III + IV (62.5%) (respectively, P < 0.05). (4) The differences of histological types, depth of invasion, lymph node   metastasis   and clinical stage were significantly different(P < 0.05 or P < 0.001), between the patients with two or more genes expression of COX-2, p53, PCNA, and nm23 and those with less one gene expression.Our results suggest that abnormal expressions of COX-2, p53, PCNA, and nm23 associate with malignant potential, lymph node   metastasis   and clinical stage, and they might therefore play a role in development of gastric cancer. 
 The establishment of supramolecular immunobead real-time PCR and the identification of Cox-2 as a   metastasis  -related marker in colorectal carcinoma. Cyclooxygenase-2 (Cox-2) is an inducible enzyme that converts arachidonic acid to prostaglandins, and it is hypothesized to induce carcinogenesis and   metastasis   in colorectal cancer. Our previous data also indicated that a higher expression level of Cox-2 was correlated with colorectal cancer   metastasis  . The Cox-2 protein was detected in the glandular cavity of colorectal cancer and the surrounding interstitial tissues. The usefulness of the Cox-2 gene as a gene therapy target and diagnostic marker remains unknown. In this study, a method using immuno-PCR and real-time PCR followed by supramolecular immunobead real-time PCR was established and used to detect the expression of Cox-2 in serum samples of nude mice with colorectal carcinoma. In addition, we established a Cox-2 gene stable knockdown colorectal cell line (SW480-EGFP-Cox-2 shRNA) using lentiviral vector-mediated RNA interference (RNAi) technology and established an imageable colorectal cancer   metastasis   mouse model. We found that the proliferation, invasion and tumorigenesis of SW480-EGFP-Cox-2 shRNA cells were attenuated compared with SW480 cells. In vivo experiments also demonstrated that angiogenesis in the Cox-2 knockdown colorectal cancer cells was decreased. The whole body optical imaging revealed that the SW480-EGFP-Cox-2 shRNA cells had an abrogated ability to develop metastases in the lymph nodes, lungs or liver in vivo. The improved immunobead PCR assay detected significantly lower Cox-2 protein levels in the serum samples of the SW480-EGFP-Cox-2 shRNA group compared with those of the SW480-EGFP-Cox-2-Ctrl shRNA group. In conclusion, our results indicated that the knockdown of Cox-2 expression suppressed the proliferation and invasion of colorectal cancer cells both in vitro and in vivo. This study also demonstrated that silencing Cox-2 in vivo reduced the metastastic potential of colorectal cancer. Thus, Cox-2 is a promising marker for the diagnosis of colorec  tal metast  asis and a potential therapeutic target for colorectal cancer. 
 Prostaglandins induce early growth response 1 transcription factor mediated microsomal prostaglandin E2 synthase up-regulation for colorectal cancer progression. Cyclooxygenase2 (COX2) has been associated with cell growth, invasiveness, tumor progression and   metastasis   of colorectal carcinomas. However, the downstream prostaglandin (PG)-PG receptor pathway involved in these effects is poorly characterized.We studied the PG-pathway in gene expression databases and we found that PTGS2 (prostaglandin G/H synthase and cyclooxygenase) and PTGES (prostaglandin E synthase) are co-expressed in human colorectal tumors. Moreover, we detected that COX2 and microsomal Prostaglandin E2 synthase 1 (mPGES1) proteins are both up-regulated in colorectal human tumor biopsies.Using colon carcinoma cell cultures we found that COX2 overexpression significantly increased mPGES1 mRNA and protein. This up-regulation was due to an increase in early growth response 1 (EGR1) levels and its transcriptional activity. EGR1 was induced by COX2-generated PGF2α. A PGF2α receptor antagonist, or EGR1 silencing, inhibited the mPGES1 induction by COX2 overexpression. Moreover, using immunodeficient mice, we also demonstrated that both COX2- and mPGES1-overexpressing carcinoma cells were more efficient forming tumors.Our results describe for the first time the molecular pathway correlating PTGS2 and PTGES in colon cancer progression. We demonstrated that in this pathway mPGES1 is induced by COX2 overexpression, via autocrine PGs release, likely PGF2α, through an EGR1-dependent mechanism. This signaling provides a molecular explanation to PTGS2 and PTGES association and contribute to colon cancer advance, pointing out novel potential therapeutic targets in this oncological context. 
 Focal adhesion kinase: important to prostaglandin E2-mediated adhesion, migration and invasion in hepatocellular carcinoma cells. Prostaglandin E2 has been implicated in cell growth and   metastasis   in many types of cancers. However, the effects of PGE2 and its mechanism on cell adhesion, migration, and invasion have not been clarified yet. In this study, we found PGE2 treatment significantly increased the cell adhesion, migration, and invasion in hepatocellular carcinoma (HCC) cells. In addition, the effects of PGE2 were found to be associated with focal adhesion kinase (FAK). PGE2 treatment increased the phosphorylation and synthesis of FAK in a dose-dependent manner. RNA interference targeting FAK suppressed PGE2-mediated cell adhesion and migration. Furthermore, the downstream proteins of FAK, paxillin and Erk2, were also activated by PGE2. PGE2 treatment increased the phosphorylation and synthesis of paxillin in a dose-dependent manner. PGE2 treatment also induced the phosphorylation of Erk2. PD98059, the specific inhibitor of MEK, suppressed PGE2-mediated cell adhesion and migration. However, it had no effect on PGE2-induced activation and synthesis of FAK. These results demonstrated that PGE2 greatly induced HCC cell adhesion, migration, and invasion by activating FAK/paxillin/Erk pathway. 
 Orthotopic implantation of a colon cancer xenograft induces high expression of cyclooxygenase-2. The aim of the present study is to investigate the relation of cyclooxygenase (COX)-2 with liver   metastasis  . Pieces of human colon cancer xenograft were implanted orthotopically (CI), as well as intraperitoneally, and subcutaneously. Liver   metastasis   developed most frequently, and the COX-2 expression of both mRNA and protein of the tumors was the most dominant in the CI group. In contrast, the expression of COX-1 or vascular endothelial cell growth factor mRNAs had no significant differences among the groups. The intensity of COX-2 mRNA was negatively correlated with the apoptotic index. In conclusion, COX-2 plays an important role in organ-specific   metastasis   of the colon cancer. 
 [Correlation of cyclooxygenase-2 expression to P-glycoprotein expression in B-cell non-Hodgkin's lymphoma]. The overexpression of P-glycoprotein (P-gp) plays an important role in the chemoresistance of relapsed lymphoma. It is up-regulated in a cyclooxygenase-2 (COX-2)-dependent manner. This study was to observe the expression and correlation of COX-2 and P-gp in B-cell non-Hodgkin's lymphoma (B-NHL), and to investigate their correlations to clinicopathologic features of B-NHL.The expression of COX-2 and P-gp in 43 specimens of naive B-NHL and 27 specimens of relapsed B-NHL were detected by SP immunohistochemistry and reverse transcription-polymerase chain reaction (RT-PCR).The positive rates of COX-2 and P-gp were significantly lower in naive B-NHL than in relapsed B-NHL (23.3% vs. 74.1%, 11.6% vs. 70.4%, P<0.01). COX-2 expression was positively correlated to P-gp expression in relapsed B-NHL (r=0.998, P<0.01). The positive rates of COX-2 and P-gp were significantly higher in aggressive B-NHL than in indolent B-NHL (64.3% vs. 10.7%, P=0.002; 52.4% vs. 7.1%, P=0.012).COX-2 and P-gp are highly expressed in relapsed B-NHL, and they may play a role in tumor   metastasis   of B-NHL. 
 The angiogenic switch for vascular endothelial growth factor-A and cyclooxygenase-2 in prostate carcinoma: correlation with microvessel density, androgen receptor content and Gleason grade. Angiogenesis is essential for tumor growth and   metastasis  ; however, angiogenic factors are not uniformly expressed in prostate carcinoma. Our aim was to determine the expression of vascular endothelial growth factor-A (VEGF-A) and cyclooxygenase-2 (COX-2) in prostate carcinomas in relation to intratumoral microvessel density (MVD), tumor grade and androgen receptor (AR) status.The expression of AR, VEGF-A and COX-2 was immunohistochemically evaluated in 24 benign prostatic hyperplasia (BPH) and 139 prostate carcinoma cases. MVD was evaluated by CD34 immunostaining.Nuclear AR expression was inversely related to tumor grade (p < 0.001). MVD was strongly related to tumor grade, VEGF-A and COX-2 (p < 0.001 in all comparisons). VEGF-A expression increased with tumor grade (p < 0.01) and was inversely related to stromal AR expression. COX-2 was present in both BPH and prostate carcinoma, but its expression increased with tumor grade (p < 0.01). High-grade neoplasms presented low-to-moderate VEGF staining intensity compared to strong COX-2 expression.Both VEGF-A and COX-2 expression is positively correlated with tumor grade and MVD. However, in Gleason 8-10 tumors, VEGF expression is moderate while COX-2 immunostaining is intense, suggesting a possible switch in the role of these two angiogenic factors in poorly differentiated neoplasms. 
 Analysis of common germline polymorphisms as prognostic factors in patients with lymph node-positive breast cancer. Women with breast cancer that initially involves local lymph nodes have a higher risk for local recurrence or developing metastases. Recent data suggest that germline polymorphism is a significant, previously unrecognized factor in breast cancer progression and   metastasis  . We assessed the influence of 16 selected common germline polymorphisms in disease-free survival and overall survival among 216 women diagnosed with lymph node-positive breast cancer.The rare allele of FAS 1377G>A was significantly associated with prolonged disease-free survival (P = 0.012, risk ratio of recurrence (RR) = 0.557, 95% confidence interval (CI) = 0.353-0.878) in univariate analysis. After adjusting for known breast cancer prognostic factors the association remained significant (P = 0.050, RR = 0.500, CI = 0.309-0.809). In overall survival analysis we found a significant association of the FAS 1377G>A (P = 0.040, RR = 0.451, CI = 0.496-1.188) and IL10 592C>A polymorphisms (P = 0.020, RR = 1.707, CI = 1.087-2.680) in the univariate Cox regression. The effect remained statistically significant in the multivariate analysis for the IL10 592C>A polymorphism (P = 0.013, RR 1.841, CI 1.140-2.973). No association was found for MTHFR 677C>T, VEGF 936C>T, CCND1 870G>A, TGFB1 29T>C, FASLG 844C>T, FAS 670A>G, GPB3 825C>T, ITGA2 807C>T, ITGA2 1648G>A, ITGB3 176T>C, MMP1 -1607 1G/2G, MMP3 5A/6A, PTGS2 8473T>C, IL10 592C>A and SULT1A1 638G>A polymorphisms and disease-free survival or overall survival.Our data suggest that the FAS 1377G>A and IL10 592C>A polymorphisms could modify disease-free and overall survival in women with lymph node-positive breast cancer. 
 COX2 expression in neuroblastoma increases tumorigenicity but does not affect cell death in response to the COX2 inhibitor celecoxib. COX2 is an inducible cyclooxygenase implicated in the   metastasis   and migration of tumour cells. In neuroblastoma, COX2 expression has been detected in both cell lines and tumours. The treatment of neuroblastoma cells in vitro with celecoxib, a COX2 inhibitor, induces apoptosis. The aim of this study was to investigate the role of COX2 in neuroblastoma tumour biology by creating a cell line in which COX2 could be conditionally expressed. Xenograft studies showed that the conditional expression of COX2 enhanced tumour growth and malignancy. Elevated COX2 expression enhanced the proliferation and migration of neuroblastoma cells in vitro. However, elevated COX2 expression or variation between cell lines did not affect sensitivity to the COX2 inhibitor celecoxib, indicating that celecoxib does not promote cell death through COX2 inhibition. These data show that increased COX2 expression alone can enhance the tumorigenic properties of neuroblastoma cells; however, high levels of COX2 may not be a valid biomarker of sensitivity to non-steroidal anti-inflammatory drugs such as celecoxib. 
 Increasing CD44+/CD24(-) tumor stem cells, and upregulation of COX-2 and HDAC6, as major functions of HER2 in breast tumorigenesis. Cancer cells are believed to arise primarily from stem cells. CD44+/CD24(-) have been identified as markers for human breast cancer stem cells. Although, HER2 is a well known breast cancer oncogene, the mechanisms of action of this gene are not completely understood. Previously, we have derived immortal (M13SV1), weakly tumorigenic (M13SV1R2) and highly tumorigenic (M13SV1R2N1) cell lines from a breast epithelial cell type with stem cell phenotypes after successive SV40 large T-antigen transfection, X-ray irradiation and ectopic expression of HER2/C-erbB2/neu. Recently, we found that M13SV1R2 cells became non-tumorigenic after growing in a growth factor/hormone-deprived medium (R2d cells).In this study, we developed M13SV1R2N1 under the same growth factor/hormone-deprived condition (R2N1d cells). This provides an opportunity to analyze HER2 effect on gene expression associated with tumorigenesis by comparative study of R2d and R2N1d cells with homogeneous genetic background except HER2 expression. The results reveal distinct characters of R2N1d cells that can be ascribed to HER2: 1) development of fast-growing tumors; 2) high frequency of CD44+/CD24(-) cells (~50% for R2N1d vs. ~10% for R2d); 3) enhanced expression of COX-2, HDAC6 mediated, respectively, by MAPK and PI3K/Akt pathways, and many genes associated with inflammation,   metastasis  , and angiogenesis. Furthermore, HER2 expression can be down regulated in non-adhering R2N1d cells. These cells showed longer latent period and lower rate of tumor development compared with adhering cells.HER2 may induce breast cancer by increasing the frequency of tumor stem cells and upregulating the expression of COX-2 and HDAC6 that play pivotal roles in tumor progression. 
 Inhibition of PKC-Induced COX-2 and IL-8 Expression in Human Breast Cancer Cells by Glucosamine. Breast cancer is a common cancer leading to many deaths among females. Cyclooxygenase-2 (COX-2) and interleukin-8 (IL-8) are two highly expressed inflammatory mediators to be induced by the protein kinase C (PKC) signaling via various inflammatory stimuli and both contribute significantly to cancer   metastasis  /progression. Glucosamine has been shown to act as an anti-inflammation molecule. The aim of this study was to clarify the role and acting mechanism of glucosamine during the PKC-regulation of COX-2/IL-8 expression and the associated impact on breast cancer. In MCF-7 breast cancer cells, glucosamine effectively suppresses the PKC induction of COX-2 and IL-8 promoter activity, mRNA and protein levels, as well as the production of prostaglandin E(2) (PGE(2)) and IL-8. Glucosamine is able to promote COX-2 protein degradation in a calpain-dependent manner and IL-8 protein degradation in calpain-dependent and proteasome-dependent manners. The MAPK and NF-κB pathways are involved in PKC-induced COX-2 expression, but only the NF-κB pathway is involved in PKC-induced IL-8 expression. Glucosamine attenuates PKC-mediated IκBα phosphorylation, nuclear NF-κB translocation, and NF-κB reporter activation. Both PGE(2) and IL-8 promote cell proliferation and IL-8 induces cell migration; thus, glucosamine appears to suppress PKC-induced cell proliferation and migration. Furthermore, glucosamine significantly inhibits the growth of breast cancer xenografts and this is accompanied by a reduction in COX-2 and IL-8 expression. In conclusion, glucosamine seems to attenuate the inflammatory response in vitro and in vivo and this occurs, at least in part by targeting to the NF-κB signaling pathway, resulting in an inhibition of breast cancer cell growth. 
 Cyclooxygenase-2 is an independent predictor of poor prognosis in uterine leiomyosarcomas. Cyclooxygenase-2 (COX-2) is a well-known enzyme that promotes tumor growth and   metastasis  . Cyclooxygenase-2 is upregulated in a number of human epithelial tumors, but data about the significance of COX-2 in mesenchymal tumors are lacking. The purpose of this study was to determine COX-2 expression in uterine sarcomas and whether a relationship exists between COX-2 expression and clinicopathologic outcomes.Immunohistochemical staining for COX-2 was performed on paraffin-embedded tissue blocks of 49 uterine sarcomas (30 leiomyosarcomas, 14 endometrial stromal sarcomas, and 5 carcinosarcomas). Positive staining was defined as moderate or strong staining in 5% or more of tumor cells.Four of 30 leiomyosarcomas, 1 of 14 endometrial stromal sarcomas, and 2 of 5 carcinosarcomas were positive for COX-2 expression. In leiomyosarcomas, COX-2 expression correlated with tumor stage with marginal significance (P = 0.058). Patients with leiomyosarcoma positive for COX-2 expression had a lower overall survival rate than those without COX-2 expression (P = 0.025). In the multivariate Cox proportional hazards regression model, COX-2 expression, tumor stage, and mitotic count were independently associated with overall survival in leiomyosarcomas.Our data suggest that immunohistochemically determined COX-2 expression is an independent prognostic factor in uterine leiomyosarcomas. Assessment of COX-2 status might be useful for determining the prognosis in patients with uterine leiomyosarcomas. 
 Can cyclo-oxygenase-2 be a useful prognostic and risk stratification marker in breast cancer? Cyclo-oxygenase-2 (COX-2) is a prostaglandin synthease that catalyses the synthesis of prostaglandin G2 (PGG2) and PGH2 from arachidonic acid. COX-2 plays an important role in tumourigenesis of different carcinoma types and it is thought to take part in breast carcinoma. In this study, the aim was to investigate the relationship of COX-2 with clinical parameters such as menopausal status, tumour size, grade, nodal status, Nottingham prognostic index (NPI), oestrogen receptor(ER), progesterone receptor (PR), human epidermal growth factor receptor type 2 (HER-2/ neu). The patients were divided into two groups, first group (group A) comprised 57 primary breast cancer patients and the second group (group B) comprised control group 27 patients consisting of fibro-adenoma and benign breast disease. In control groups COX-2 (0%) is not over expressed and we observed that high frequency of COX-2 (73.68%) over expressed in breast carcinoma. In high grade, large tumour size and positive lymph node   metastasis  , COX-2 expression rate was 78.6%, 59.5% and 90.5% respectively. COX-2 expression is directly correlated with ER negative (88.1%, p = 0.001) and also associated with higher NPI value (78.6%, p = 0.006). In invasive ductal carcinoma (IDC) COX-2 over expression had a significant relationship with HER-2/neu over expression (p < 0.001). The results indicated that COX-2 over expression correlates with aggressive phenotypic features, such as high histological grade, large tumour size, higher NPI value, ER negativity and HER-2/neu positivity. 
 TGF-beta1 causes epithelial-mesenchymal transition in HaCaT derivatives, but induces expression of COX-2 and migration only in benign, not in malignant keratinocytes. Transforming growth factor beta (TGF-beta) acts as a tumor promoter by inducing epithelial-mesenchymal transition (EMT), which leads to a motile phenotype, enabling invasion and   metastasis   of cancer cells. Cancer-related inflammation, mediated by prostaglandins, has been proposed as a critical mechanism in conversion of benign cells to malignant.Induction of cyclooxygenase 2 (COX-2), producer of prostaglandins, is thought to be a prerequisite for TGF-beta-induced EMT in benign cells. We used HaCaT derivatives, representative of skin cancer progression, to investigate TGF-beta1 mediated EMT response, and the role of COX-2 in it.Effect of TGF-beta1 was investigated by analyzing cell proliferation, morphology and protein expression. Chemotaxis and scratch-wound assays were used to study migration.TGF-beta1 caused proliferation arrest of benign and malignant HaCaT cells, and changed the epithelial morphology of benign and low-grade malignant cells, but not metastatic cells, to mesenchymal spindle-shape. Epithelial junction proteins ZO-1 and E-cadherin were downregulated in all cell lines in response to TGF-beta1, but mesenchymal markers were not induced, suggesting a partial EMT response. COX-2 and migration were induced only in benign HaCaT derivatives. Malignant derivatives did not induce COX-2 in response to TGF-beta 1 treatment, thus emphasizing the role of inflammation in EMT response of benign cells.TGF-beta1 operates via distinct mechanisms in inducing EMT and   metastasis  , and supporting this we show that TGF-beta1 induces COX-2 and promotes the migration of benign cells, but does not further augment the migration of malignant cells, indicating their resistance to TGF-beta1 in the context of motility. 
 Papillary carcinoma of the thyroid: high expression of COX-2 and low expression of KAI-1/CD82 are associated with increased tumor invasiveness. We have previously demonstrated that expression of COX-2 is upregulated by hepatocyte growth factor in thyroid papillary carcinoma (TPC) cells and is associated with increased invasiveness of tumor cells. COX-2 upregulation was associated with downregulation of KAI-1/CD82, a   metastasis   suppressor molecule that has been associated with the metastatic potential of several solid tumors. In the present study, we have investigated the possibility that downregulation of KAI-1/CD82 may contribute to the invasiveness of papillary carcinoma cells.Expression of KAI-1/CD82 and its relation to COX-2 levels were investigated in 6 primary cultures of TPC, in 2 tumor cell lines (TPC-1 and K1), and in 55 tumor samples of TPC. The biological role of KAI-1/CD82 in regulating tumor invasiveness was investigated in TPC cell lines and primary cultures transfected with a pCDNA3.1/Hygro.KAI-1; transfected cells were tested in functional studies of cell migration and invasiveness. Finally, the role of KAI-1/CD82 in influencing TPC   metastasis   was investigated in vivo using nu/nu mice injected with K1-transfected cells.We provide evidence that COX-2 and KAI-1/CD82 are inversely regulated in TPC primary cultures and in TPC-1 tumor cells. In fact, inhibition of COX-2 with NS398 is associated with a 2-9-fold upregulation of KAI-1/CD82 RNA. Moreover, a possible relation between COX-2 and KAI-1/CD82 was confirmed by the presence of a statistically significant inverse correlation in the expression of the two genes in 55 tumor samples of TPC (r = -0.513; p = 0.001). In 36 of 55 cases, tumor areas contained lower levels of KAI-1/CD82 RNA as compared with the corresponding normal tissue. Low expression of KAI-1/CD82 RNA in the tumor area was associated with extrathyroid extension of the disease in 16 of 19 cases (p < 0.04) and with lymph node   metastasis   in 11 of 14 cases (not significant). KAI-1/CD82 re-expression in tumor cells was associated with a significant decrease in their migratory (50-76% reduction) and invasive (46-65% reduction) capacity, even after hepatocyte growth factor stimulation. Finally, nu/nu mice injected with KAI-1/CD82-transfected K1 TPC cells developed fewer and smaller   metastasis   as compared with mice injected with vector-transfected K1 cells (p=0.016).Our findings raise the possibility that downregulation of KAI-1/CD82 in TPC cells is one of the molecular mechanisms regulating their invasive and metastatic potential. 
 PTGS2 (COX-2) -765 G > C functional promoter polymorphism and its association with risk and lymph node   metastasis   in nasopharyngeal carcinoma. Cyclooxygenase-2 (Cox-2) is a key enzyme in the conversion of arachidonic acid to prostaglandins that has been shown to have a particular importance in the progression of several malignancies including nasopharyngeal carcinoma (NPC). In the current report, we designed a case-controlled study to evaluate the susceptibility and prognostic implications of the functional -765 G > C genetic variation in NPC. A PCR and restriction fragment length polymorphism analysis was used to determine the polymorphism in a Tunisian population of patients with NPC (n = 180) and in healthy control subjects (n = 169). A higher risk for NPC was observed for carriers of COX-2 -765 C allele (OR = 1.76; P = 0.01). This association remains significant after adjustments for age and sex (OR = 1.89; P = 0.008). Regarding prognostic indicators, a significant association was found between -765 C allele carriers and the presence of lymph node   metastasis   (OR = 2.28; P = 0.01), as well as, with tumor stage (OR = 2.73; P = 0.03). This is the first report on the studies of COX-2 SNPs in NPC and our data suggest that this genetic variant may play a role in mediating susceptibility to NPC, as well as, in neoplastic progression, a finding which further supports the involvement of COX-2 in NPC etiology. 
 A novel single nucleotide polymorphism of the cyclooxygenase-2 gene associated with breast cancer. Cyclooxygenase-2 (COX-2) is involved in carcinogenesis, immune response suppression, apoptosis inhibition, angiogenesis, and tumour cell invasion and   metastasis  . The gene for COX-2, designated as PTGS2, carries several polymorphisms, such as -765G >C, 1195G/A in the promoter region, and 8473T >C in the 3' UTR, which have been associated with susceptibility to malignant disease. The aim of this study was to search for new mutations and polymorphisms in the COX-2 gene and to assess the relationship with breast cancer.In the present study, we identified a novel single nucleotide polymorphism, 169C >G, in exon 2 using polymerase chain reaction-single-strand conformation polymorphism analysis. This nucleotide change causes the amino acid change from proline to alanine at codon 57. To investigate the role of this polymorphism for breast cancer, we determined the prevalence of PTGS2 genotypes in 310 women with breast cancer and 310 gender- and age-matched healthy control subjects.Homozygous carriers of the 169-GG genotype were more frequent among patients (15.16%) than among controls (9.35%; P = 0.03). The odds ratio for carriers of this genotype for breast cancer was 1.76 (95% confidence interval, 1.20-3.05). Among patients, oestrogen receptor positivity was less frequent among carriers of a GG genotype (61.7%) than among carriers of a CC or GC genotype (72.3%; P = 0.02). Tumour size, histological grade, presence of primary lymph node metastases, progesterone receptor positivity, or age at diagnosis were not associated with PTGS2 genotypes.We conclude that the homozygous PTGS2 169-GG genotype may be associated with breast cancer risk. 
 Cyclooxygenase-2 expression in lymph node   metastasis   of cervical and vulvar cancer. Overexpression of cyclooxygenase-2 (COX-2), characterizes tumors with high potential for local invasion and lymph node involvement. We investigated the expression of COX-2 in primary tumors and metastatic regional lymph nodes (TDL) from untreated and chemotherapy treated cervical cancer, as well as vulvar cancer. Immunostaining of COX-2, expressed as values of COX-2 intensity density (COX-2 IDV) was performed on 57 metastatic TDL and 24 corresponding primary rumors from 14 cervical and 9 vulvar cancer patients admitted to the Department of Obstetrics and Gynecology, Catholic University of Rome. In 6 locally advanced cervical cancer tissue samples, from both primary tumor and TDL, were obtained after chemotherapy treatment. In untreated cervical cancer, COX-2 IDV in tumor cells from positive TDL were significantly lower (median 0.69, range 0.22-0.92) than those from primary tumors (median = 3.84, range 0.19-7.67) (p=0.011). In cervical cancer exposed to chemotherapy, COX-2 IDV in tumor cells from positive TDL were significantly lower (median = 2.06, range 1.48-6.52) than those from primary tumors (median = 6.4, range 4.5-13.7) (p=0.037). In vulvar cancer COX-2 IDV in tumor cells from positive TDL were lower (median = 0.39, range 0.02-6.09) than those from primary tumors (median = 2.49, range 0.71-8.10) (p=0.04). In conclusion, we showed that COX-2 expression is down-regulated in cervical and vulvar tumor cells invading the regional lymph nodes with respect to primary tumors, thus emphasizing the need for deeper insight into the tissue specific relation between tumor cells and node microenvironment. 
 Lysophosphatidic acid receptor 2 and Gi/Src pathway mediate cell motility through cyclooxygenase 2 expression in CAOV-3 ovarian cancer cells. Lysophosphatidic acid (LPA) is a bioactive phospholipids and involves in various cellular events, including tumor cell migration. In the present study, we investigated LPA receptor and its transactivation to EGFR for cyclooxygenase-2 (COX-2) expression and cell migration in CAOV-3 ovarian cancer cells. LPA induced COX-2 expression in a dose-dependent manner, and pretreatment of the cells with pharmacological inhibitors of Gi (pertussis toxin), Src (PP2), EGF receptor (EGFR) (AG1478), ERK (PD98059) significantly inhibited LPA- induced COX-2 expression. Consistent to these results, transfection of the cells with selective Src siRNA attenuated COX-2 expression by LPA. LPA stimulated CAOV-3 cell migration that was abrogated by pharmacological inhibitors and antibody of EP2. Higher expression of LPA2 mRNA was observed in CAOV-3 cells, and transfection of the cells with a selective LPA2 siRNA significantly inhibited LPA-induced activation of EGFR and ERK, as well as COX-2 expression. Importantly, LPA2 siRNA also blocked LPA-induced ovarian cancer cell migration. Collectively, our results clearly show the significance of LPA2 and Gi/Src pathway for LPA-induced COX-2 expression and cell migration that could be a promising drug target for ovarian cancer cell   metastasis  . 
 Androgen receptor promotes the migration and invasion of upper urinary tract urothelial carcinoma cells through the upregulation of MMP-9 and COX-2. Dysregulated androgen receptor (AR) signaling is implicated in several types of tumor, including carcinomas of the prostate, breast, liver and bladder. However, the contribution of AR to the progression of upper urinary tract urothelial carcinomas (UUTUC) has not been fully investigated. In the present study, we demonstrated that the AR is involved in the   metastasis   and invasiveness of UUTUC cells. We investigated the role of the AR in UUTUC by using UUTUC-derived BFTC 909 cells. The overexpression of AR promotes the migration and invasion of BFTC 909 cells. Expression of migration/invasion-related genes was increased in BFTC 909 cells overexpressing AR determined by qPCR and western blot analyses. The results showed that AR-enhanced migration and invasion of UUTUC cells are linked to the upregulation of the matrix-degrading enzyme MMP-9 and cyclooxygenase (COX)-2. Subsequently, the blocking of MMP-9 and COX-2 signaling by inhibitors suppressed AR-enhanced cell migration and invasion. The results of the present study provide evidence for the first time of the role of AR in the motility and invasion of UUT cancer cells and support the hypothesis that the AR may play a critical role in the establishment of the invasive phenotype in urothelial neoplasia of UUT. Thus, the AR may also serve as a novel biomarker and potential therapeutic target for UUT cancer. 
 Expression of COX-2, MMP-2 and VEGF in stage II and III colorectal cancer and the clinical significance. To investigate the expression of COX-2, MMP-2 and VEGF in colorectal cancer and the clinical/pathological significance.Stage II and III colorectal cancer patients (149 cases) that received radical resection between May 2003 and November 2008 and who had complete clinical and pathological data, were recruited in this study. Expression of COX-2, MMP-2 and VEGF were detected by immunohistochemistry.The positive rate of COX-2, MMP-2, and VEGF expression was 60.4%, 50.3% and 69.1%, respectively. COX-2 correlated with stage, lymph node   metastasis  , postoperative recurrence and   metastasis  , and survival rate; MMP-2 correlated with intestinal wall invasion, stage, number of lymph node   metastasis  , postoperative recurrence and   metastasis  , and survival rate; VEGF correlated with preoperative serum levels of CEA and CA199, postoperative recurrence and   metastasis  , and survival rate; the positive rate of COX-2, MMP-2 and VEGF co-expression was 32.9%, which correlated with stage, number of lymph node   metastasis  , preoperative serum level of CEA, postoperative recurrence and   metastasis  , and survival rate.The expression of COX-2, MMP-2 and VEGF in colorectal cancer plays a synergistic promoting effect on the malignant biological behavior of tumors, which could be used as a marker to determine the malignant progression, invasion and   metastasis  , and prognosis of the tumor. 
 Monocyte chemoattractant protein 1 and CD40 ligation have a synergistic effect on vascular endothelial growth factor production through cyclooxygenase 2 upregulation in gastric cancer. Recent studies have reported that expression of monocyte chemoattractant protein 1 (MCP-1) and its receptor (CCR2) and CD40 ligation on mesenchymal cells play important roles in tumor development. Cyclooxygenase 2 (COX-2) has also been shown to contribute to tumor angiogenesis. We examined the interaction between MCP-1 and CD40 ligation in mesenchymal cells in gastric cancer to determine the effect of these factors on vascular endothelial growth factor (VEGF) production via upregulation of COX-2 expression.COX-2, prostaglandin E2 (PGE2), and VEGF production were evaluated in CD40 ligand (CD40L)-stimulated macrophages. CD40L and MCP-1 mRNA levels in gastric cancer tissues were evaluated by real-time polymerase chain reaction (PCR). Localizations of MCP-1, CD40L, CD34, CD40, and CCR2 in 34 gastric cancer tissue specimens were evaluated by single-or double-label immunohistochemistry.COX-2 expression levels were significantly higher in CD40L-stimulated macrophages and correlated with increased PGE2 and VEGF production. Addition of MCP-1 to CD40L-stimulated macrophages had a synergistic effect on COX-2 expression and subsequent PGE2 and VEGF production. CD40L and MCP-1 mRNA levels were significantly higher in poorly differentiated gastric cancers than in H. pylori-infected gastritis patients. High microvessel density was significantly associated with MCP-1 and CCR2 scores and lymph node   metastasis  .MCP-1 and CD40L had a synergistic effect on COX-2 expression and subsequent VEGF production in gastric cancer. 
 Cyclooxygenase-2 promotes angiogenesis by increasing vascular endothelial growth factor and predicts prognosis in gallbladder carcinoma. To investigate the relationships between the expression of cyclooxygenase-2 (COX-2), vascular endothelial growth factor (VEGF) and the degree of vascularization, clinicopathologic feature, survival time of patients with gallbladder carcinomas.Sixty-four gallbladder carcinoma specimens were evaluated for COX-2, VEGF expression by immunohistochemical methods. Microvessel counts (MVC) were determined using CD(34). The relationships between COX-2, VEGF expression, CD(34)-stained MVC, clinicopathologic features and survival time were analyzed. The correlations between COX-2 and VEGF expression, CD(34)-stained MVC were also investigated.COX-2, VEGF immunoreactivity were observed in 71.9% (46/64) and 54.7% (35/64) specimens, respectively. The average MVC in 64 cases of gallbladder carcinoma was 57+/-14 per high power vision field. The status of MVC was closely correlated with Nevin staging, tumor differentiation and lymph node   metastasis   (P<0.01, 0.002, and 0.003, 0.000, respectively). Increased VEGF expression was significantly correlated with tumor differentiation (poorly and moderately>well differentiated, P<0.05, P = 0.016). Clinical stages had no relation with the expression of VEGF (P>0.05, P = 0.612). There was a positive correlation between COX-2 expression and clinical stages. The positive rate of COX-2 was higher in cases of Nevin stages S(4)-S(5) (81.8%) than in those of Nevin stages S(1)-S(3) (50.0%) with a statistical significance (P<0.01, P = 0.009). The expression of COX-2 did not vary with differentiation (P>0.05, P = 0.067). Statistically significant differences were also observed according to lymph node   metastasis  , COX-2 expression and VEGF expression (P<0.01, 0.000, and 0.001, respectively). There was no relation between VEGF, COX-2 expression, MVC and the age and sex of patients. MVC and VEGF positive rate in the COX-2 positive gallbladder carcinoma tissue was higher than that in the COX-2 negative tissue (P<0.05, 0.000, and 0.032, respectively). Patients with VEGF, COX-2 positive tumors had a significantly shorter survival time than those with negative tumors (P<0.05, 0.004, 0.01, respectively).Augmented tumor neovascularization induced by VEGF may be one of the several effects of COX-2 responsible for poor prognosis of human gallbladder carcinoma. COX-2 inhibitor, either in combination therapy with other agents, or for chemoprevention, may be effective via suppression of angiogenesis in this fatal disease. 
 Cycloxygenase-2 is essential for the survival and proliferation of gastric cancer cells. Cycloxygenase-2 catalyzes the synthesis of prostaglandins from arachidonic acid and this enzyme has been implicated in the   metastasis   of gastric cancer. In order to examine the significance of cycloxygenase-2 (Cox-2) in the survival and proliferation of gastric cancer cells, we have stably overexpressed an antisense Cox-2 in two gastric cancer cell lines, SGC7901 and AGS, in order to reduce the expression of this protein. The sense and antisense Cox-2 expression vectors were created by cloning COX-2 cDNA, in pIRES2-EGFP plasmid. Cox-2 gene expression was monitored by RT-PCR and Western blotting and the results indicated that cells with antisense Cox-2 construct had significantly reduced Cox-2 expression in comparison to the cells that received sense-Cox-2 plasmid. Reduction of Cox-2 expression in SGC7901 and AGS gastric cancer cells led to markedly decreased proliferation. The metastatic capability of the two cell lines, as assessed by in vitro colony formation assay, is also significantly compromised by lowered Cox-2 expression. Thus, this study demonstrates that Cox-2 activity is necessary for the proliferation and   metastasis   of gastric cancer cells. 
 Silencing of cyclooxygenase-2 inhibits   metastasis   and delays tumor onset of poorly differentiated metastatic breast cancer cells. Cyclooxygenases (COX) are rate-limiting enzymes involved in the conversion of PLA(2)-mobilized arachidonic acid into prostaglandins and thromboxanes. COX-2 is a key mediator of inflammation during both physiologic and pathologic responses to endogenous stimuli and infectious agents. Its overexpression has been detected in different cancers, including that of the breast. Using RNA interference, we have reduced the expression of COX-2 in the highly malignant breast cancer cell line MDA-MB-231 below detectable levels in response to interleukin-1 beta or 12-O-tetradecanoylphorbol-13-acetate treatment. Microarray analysis showed that COX-2 silencing resulted in the loss of mRNA expression of several oncogenic markers, such as matrix metalloproteinase-1, chemokine (C-X-C motif) receptor 4, and interleukin-11, which have been correlated with poor disease outcome, and in the up-regulation of antimetastatic transcripts, such as thrombospondin-1 and Epstein-Barr-Induced 3. Cells lacking COX-2 were less able to invade reconstituted extracellular matrix than parental cells in vitro. Consistent with these changes, loss of COX-2 resulted in the abolition or the significant delay of tumor onset when the cells were injected in the mammary fat pad of severe combined immunodeficient mice. Finally, silencing of COX-2 resulted in the inhibition of   metastasis   to the lungs of severe combined immunodeficient mice after intravenous injection. These data show that silencing of COX-2 abolishes the metastatic potential of MDA-MB-231 cells in vivo. 
 Relationship between cyclooxygenase-2 and human epidermal growth factor receptor 2 in vascular endothelial growth factor C up-regulation and lymphangiogenesis in human breast cancer. Both cyclooxygenase (COX)-2 and human epidermal growth factor receptor (HER)-2 promote breast cancer progression; however, the relationship between the two molecules remains unclear. We utilized human breast cancer tissues and cell lines to examine whether COX-2 and HER-2 played independent or interdependent roles in vascular endothelial growth factor (VEGF)-C up-regulation and lymphangiogenesis. A paired correlation of immunodetectable levels of COX-2, VEGF-C, and HER-2 proteins and lymphovascular density (LVD; D2-40-immunolabeled) in 55 breast cancer specimens revealed a positive correlation between COX-2 and HER-2 irrespective of clinicopathological status. However COX-2 alone positively correlated with LVD. In 10 independent specimens, mRNA levels showed a positive correlation between HER-2 and COX-2 or VEGF-C but not LYVE-1 (lymphovascular endothelial marker). These findings implicate COX-2, but not HER-2, in breast cancer-associated lymphangiogenesis. Manipulation of the COX-2 or HER-2 genes in breast cancer cell lines varying widely in COX-2 and HER-2 expression revealed a direct role of COX-2 and an indirect COX-2 dependent role of HER-2 in VEGF-C up-regulation: (i) high VEGF-C expression in high COX-2/low HER-2 expressing MDA-MB-231 cells was reduced by siRNA-mediated down-regulation of COX-2, but not HER-2; (ii) integration of HER-2 in these cells simultaneously up-regulated COX-2 protein as well as VEGF-C secretion; and (iii) low VEGF-C secretion by high HER-2/low COX-2 expressing SK-BR-3 cells was stimulated by COX-2 overexpression. These findings of the primary role of COX-2 and the COX-2-dependent role of HER-2, if any, in VEGF-C up-regulation and lymphangiogenesis suggest that COX-2 inhibitors may abrogate lymphatic   metastasis   in breast cancer irrespective of HER-2 status. 
 Promotion of hematogenous metastatic potentials in human KB carcinoma cells with overexpression of cyclooxygenase-2. To understand the role of cyclooxygenase (COX)-2 in metastatic potential of oral cancer, COX-2 overexpressing KB/COX-2 cells were inoculated orthotopically into the masseter muscle or injected into the left cardiac ventricle of nude mice. KB/COX-2 showed about 4-fold increase of COX-2 protein expression as compared to KB/Neo which was a mock transfected control. In orthotopic inoculation,   metastasis   to the regional lymph nodes occurred in 2 out of 15 mice, and   metastasis   to the lung in 3 out of 15 mice. On the other hand, in intra-cardiac injection, hematogenous   metastasis   to the lung and bone occurred in 8 out of 10 mice in KB/COX-2, but no   metastasis   occurred except for only one   metastasis   to the femur bone out of 10 mice in KB/Neo. Treatment of KB/COX-2 with COX-2 small interfering RNA (siRNA) inhibited the colony formation but not cell growth in vitro, and suppressed tumorigenicity and hematogenous   metastasis   in nude mice. When expression of adhesion molecules such as E-cadherin, alpha-catenin, beta-catenin and CD44 was examined, there was no difference in alpha- and beta-catenin between the cells. However, expression of E-cadherin was detected in KB/Neo, but not in KB/COX-2. In contrast, expression of CD44 was markedly increased in KB/COX-2 as compared to KB/Neo. Treatment with COX-2 siRNA resulted in suppression of CD44 expression and detectable expression of E-cadherin in KB/COX-2. These findings suggested that overexpression of COX-2 increased hematogenous   metastasis  , at least in KB cells, via down-regulating E-cadherin and up-regulating CD44 expression. 
 COX-2 Expression in Renal Cell Carcinoma and Correlations with Tumor Grade, Stage and Patient Prognosis. Cyclooxygenase 2 (COX-2) is important as an enzyme in the pathway leading to the production of prostaglandin E2 (PGE2) and arachidonic acid. This pathway is known to play a role in inflammation, tumor growth, invasiveness and   metastasis  , inhibition of apoptosis and angiogenesis. Inhibition of COX-2 has been shown to be a promising antitumor and antiangiogenic strategy in several tumor types, including renal cell carcinoma (RCC). Therefore, we decided to evaluate the immunohistochemical expression of this marker and its association with several clinicopathological characteristics in a series of cases.COX-2 expression was examined immunohistochemically in tumor tissues obtained from 96 patients who underwent radical (94 cases) or partial (2 cases) nephrectomy. Correlations between COX-2 expression and clinicopathologic findings including pathologic stage, nuclear grade and other indicator of prognosis were examined.Of 96 tumors, 20.9% were positive for COX-2 expression. A correlation was found between COX-2 expression and tumor histological subtype (P=0.03).The papillary subtype showed maximum expression of this marker (43.8%) and the clear subtype minimum (14.7%). There were also possible links between COX-2 expression and pathologic stage, nuclear grade and nodal involvement but the results were not statistically significant (P=0.8, P= 0.14 and P=0.06, respectively). No correlation was found between COX2 expression and patient age, gender, tumor size,   metastasis   or survival.In our study, COX-2 expression was correlated with the histological subtype of RCC. Additional research is required to determine the link between COX-2 expression and prognosis and also evaluation of probable effectiveness of COX-2 inhibitor drugs in treatment of RCC patients. 
 P50-associated COX-2 extragenic RNA (PACER) overexpression promotes proliferation and   metastasis   of osteosarcoma cells by activating COX-2 gene. P50-associated cyclooxygenase-2 (COX-2) extragenic RNA (PACER) is a novel long noncoding RNA that has been found to activate the COX-2 gene, which may function as an oncogene in osteosarcoma. However, the role of PACER and the relationship between PACER and COX-2 in osteosarcoma progression have been unknown until now. Here, we examined the expression levels of PACER in clinical tumor samples and human osteosarcoma cell lines, assessed the functions of PACER in osteosarcoma cell proliferation and invasion, and then explored the mechanism of PACER dysregulation in osteosarcoma. The results showed that PACER was overexpressed in osteosarcoma tissues and cell lines compared with normal tissues and osteoblasts, respectively. PACER knockdown inhibited the proliferation and invasion of human osteosarcoma cells. Downregulation of PACER significantly suppressed the expression of COX-2, and the effects of PACER on cell proliferation and invasion were rescued by COX-2 overexpression. Furthermore, COX-2 activation by PACER was NF-κB-dependent. The regulation of PACER by CCCTC-binding factor (CTCF) was associated with DNA methylation status. Taken together, these findings suggest that PACER promotes proliferation and   metastasi  s of osteosarcoma cells by activating the COX-2 gene and its own expression was influenced by DNA methylation. 
 [Expression and function of cyclooxygenase-2 in endometrial carcinoma]. To evaluate the cyclooxygenase-2(COX-2) expression at mRNA and protein levels, as well as its clinical significance, and to study the correlation of COX-2 with angiogenesis, apoptosis and estrogen or progestron receptors.Forty-one samples of normal endometrium tissues and 52 ones of endometrial carcinomas(EC) were collected, together with their corresponding clinical information. RT-PCR was adopted to determine the expression of COX-2 mRNA. Immunohistochemical staining was engaged to evaluate the protein expression of COX-2, VEGF, Bcl-2 and the MVD marked by CD105. And TUNEL was used to count the apoptosis cells.(1)Significantly higher mRNA and protein levels of COX-2 were found in endometrial carcinomas than in normal endometrial tissues (P<0.001). (2)At protein level, COX-2 expression was related to the grade (P=0.020), but had nothing to do with the stage, pathological type, depth of myometrium invasion and lymph node   metastasis  .Neither did the level of COX-2 mRNA with all the factors above. (3)The level of COX-2 mRAN had nothing to do with that of protein(P=0.125, r=0.222). (4)The higher the expression of COX -2 protein was, the higher that of VEG, MVD or Bcl-2 was, and the lower the count of apoptosis cells was P<0.05. (5)The upregulation of COX-2 protein had no correlation with ER-alpha and ER-beta. But it had positive correlation with PRA and PRB, P was 0.031 and 0.007, respectively.(1)The expression of COX-2 is significantly higher in EC than in normal endometrium tissue, not only at the mRNA level, but also at protein level. (2)At protein level, COX-2 expression is related to the grade, but has nothing to do with the stage, pathological type, depth of myometrium invasion and lymph node   metastasis  . Neither does the level of COX-2 mRNA with all the factors above. (3)The upregulation of COX-2 protein in EC correlates with the increase of VEGF protein and MVD. (4)The upregulation of COX-2 protein in EC correlates with the increase of Bcl-2 protein and the decrease of apoptosis cells. (5)The upregulation of COX-2 protein in EC has no significant correlation with ER expression, but has something to do with the increase of PRA and PRB. 
 Reciprocal correlation between the expression of cyclooxygenase-2 and E-cadherin in human bladder transitional cell carcinomas. Carcinoma cells become more motile and invasive via downmodulation of E-cadherin. Cyclooxygenase-2 (COX-2) expression is associated with tumor invasion and   metastasis  . The aim of this study is to investigate the relationship between the expression of COX-2 and E-cadherin in a bladder cancer cell line and human bladder transitional cell carcinoma (TCCs). Phorbol 12-myristate 13-acetate (PMA) treatment for 5637 bladder cancer cells increased COX-2 expression, slightly induced Slug expression, and decreased E-cadherin expression. Ectopic expression of COX-2 or prostaglandin E(2) (PGE(2)) treatment for 5637 cells reduced E-cadherin expression. This finding was confirmed by the result that knockdown of COX-2 expression or indomethacin administration increased the expression of E-cadherin. When compared with cells' motility in serum-free medium, the treatment of PMA and PGE(2) increased cell motility, and indomethacin treatment slightly decreased cell motility. In the tissues of bladder TCCs, COX-2 expression was inversely correlated with membranous E-cadherin expression and positively correlated with nuclear beta-catenin expression. The expression of COX-2 and nuclear beta-catenin expression was significantly higher in TCCs of high grade and invasive growth than in TCCs of low grade and noninvasive growth. In contrast, membranous E-cadherin expression was more decreased in tumors of high grade and invasive growth. In addition, nuclear beta-catenin expression was significantly related to tumor recurrence. We suggest that COX-2 pathway reduces membranous E-cadherin expression in bladder TCCs and their expression pattern may provide important information in predicting the clinical behavior of bladder TCCs. 
 Increased abundance of cyclooxygenase-2 correlates with vascular endothelial growth factor-A abundance and tumor angiogenesis in gastric cancer. To understand the role of cyclooxygenase-2 (COX-2) in gastric cancer, we examined the abundance of COX-2, vascular endothelial growth factor-A (VEGF-A), and CD34 in 45 surgically resected human gastric cancers and paired normal gastric mucosa by immunohistochemical analysis. In addition, the message RNA (mRNA) expression of COX-2 and VEGF-A was evaluated in ten fresh surgically resected human gastric cancers and paired normal gastric mucosas using semi-quantitative reverse transcriptional polymerase chain reaction analysis. Our results confirmed an increased abundance of COX-2 and VEGF-A, and the microvessel density, which was assessed by CD34 abundance, in gastric cancer tissues compared with normal paired mucosa. Abundance of COX-2 and VEGF-A was significantly associated with tumor-node-  metastasis   (TNM) stage (P<0.05) and lymph node   metastasis   (P<0.001). In addition, abundance of VEGF-A associates with distance   metastasis  . A significant correlation was found between COX-2 and VEGF-A abundances (P<0.001). Both abundance of COX-2 and VEGF-A were significantly correlated with microvessel density (P<0.001, respectively). In six of ten cancerous tissues and in one of ten paired normal mucosas, the mRNA expression of COX-2 and VEGF-A was detected in the same specimen. In one other cancerous tissue, only COX-2 mRNA was detected. This study indicates that COX-2 is related to tumor angiogenesis in gastric cancer. VEGF-A might play a main role in the COX-2 angiogenic pathway. 
 Aromatic-turmerone attenuates invasion and expression of MMP-9 and COX-2 through inhibition of NF-κB activation in TPA-induced breast cancer cells. Recent evidence suggests that breast cancer is one of the most common forms of malignancy in females, and   metastasi  s from the primary cancer site is the main cause of death. Aromatic (ar)-turmerone is present in Curcuma longa and is a common remedy and food. In the present study, we investigated the inhibitory effects of ar-turmerone on expression and enzymatic activity levels of 12-O-tetradecanoylphorbol-13-acetate (TPA)-induced matrix metalloproteinase (MMP)-9 and cyclooxygenaase-2 (COX-2) in breast cancer cells. Our data indicated that ar-turmerone treatment significantly inhibited enzymatic activity and expression of MMP-9 and COX-2 at non-cytotoxic concentrations. However, the expression of tissue inhibitor of metalloproteinase (TIMP)-1, TIMP-2, MMP-2, and COX-1 did not change upon ar-turmerone treatment. We found that ar-turmerone inhibited the activation of NF-κB, whereas it did not affect AP-1 activation. Moreover, The ChIP assay revealed that in vivo binding activities of NF-κB to the MMP-9 and COX-2 promoter were significantly inhibited by ar-turmerone. Our data showed that ar-turmerone reduced the phosphorylation of PI3K/Akt and ERK1/2 signaling, whereas it did not affect phosphorylation of JNK or p38 MAPK. Thus, transfection of breast cancer cells with PI3K/Akt and ERK1/2 siRNAs significantly decreased TPA-induced MMP-9 and COX-2 expression. These results suggest that ar-turmerone suppressed the TPA-induced up-regulation of MMP-9 and COX-2 expression by blocking NF-κB, PI3K/Akt, and ERK1/2 signaling in human breast cancer cells. Furthermore, ar-turmerone significantly inhibited TPA-induced invasion, migration, and colony formation in human breast cancer cells. 
 The value of COX-2 expression in the prognostic parameters of invasive ductal carcinoma of the breast. Cyclooxygenase is an enzyme that changes the immune response to malign cells and catalyzes prostaglandins that may have an impact on cell proliferation. The purpose of this study was to examine the relation between established clinicopathological parameters in breast carcinomas and COX-2 protein expression. COX-2, estrogen receptor (ER), progesterone receptor (PR) and c-erB-2 primary antibodies were assessed in the slides prepared from the paraffin blocks of 62 invasive ductal carcinoma cases. The relation between ER, PR, and c-erbB-2 positivity, histological grade, nuclear grade, lymphovascular invasion, tumor diameter, lymph node positivity,   metastasis  , and age were evaluated. The results were analyzed statistically. Cytoplasmic COX-2 expression was seen in 75.8% of all breast carcinomas. In both univariate and multivariate logistic regression analysis, there was a positive correlation between COX-2 expression and negative ER status, respectively (P=0.0173) (P=0.016). There was any statistically significant relation between PR positivity, c-erbB-2 positivity, histological grade, nuclear grade, lymphovascular invasion, tumor diameter, lymph node positivity,   metastasis  , and age (P≥0.05). Given that there was found a statistically significant relation between COX-2 expression and negative ER status, which is considered a poor prognostic parameter, suggests that COX-2 expression can have a place among the other prognostic parameters of breast carcinoma. 
 Cyclooxygenase-2, a Potential Therapeutic Target, Is Regulated by miR-101 in Esophageal Squamous Cell Carcinoma. Cyclooxygenase-2 (COX-2) is known to promote the carcinogenesis of esophageal squamous cell carcinoma (ESCC). There are no reports on whether microRNAs (miRNAs) regulate COX-2 expression in ESCC. This study investigated the effect of miR-101 on ESCC through modulating COX-2 expression in ESCC.Real-time quantitative reverse transcription-polymerase chain reaction (RT-PCR) was used to quantify miR-101 expression in ESCC clinical tissues and cell lines. The effects of miR-101 on ESCC progression were evaluated by cell counting kit-8 (CCK8), transwell migration and invasion assays, as well as by flow cytometry. The COX-2 and PEG2 levels were determined by western blot and enzyme-linked immunosorbent assays (ELISA). The luciferase reporter assay was used to verify COX-2 as a direct target of miR-101. The anti-tumor activity of miR-101 in vivo was investigated in a xenograft nude mouse model of ESCC.Downregulation of miR-101 was confirmed through comparison of 30 pairs of ESCC tumor and adjacent normal tissues (P < 0.001), as well as in 11 ESCC cell lines and a human immortalized esophageal cell line (P < 0.001). Transfection of miR-101 in ESCC cell lines significantly suppressed cell proliferation, migration, and invasion (all P < 0.001). The antitumor effect of miR-101 was verified in a xenograft model. Furthermore, COX-2 was shown to be a target of miR-101.Overexpression of miR-101 in ESCC inhibits proliferation and   metastasis  . Therefore, the miR-101/COX-2 pathway might be a therapeutic target in ESCC. 
 Role of COX-2, VEGF and cyclin D1 in mammary infiltrating duct carcinoma. Cyclooxygenase-2 (COX-2) has an important role in the promotion of carcinogenesis, tumor invasion and angiogenesis. Vascular endothelial growth factor (VEGF) is a proangiogenic factor that is up-regulated in various tumors. VEGF has been shown to interact with COX-derived prostaglandins in angiogenesis. Cyclin D1 gene overexpression and amplification have been shown to play a role as prognostic factors in many human cancers. To better understand the roles of these genes in mammary carcinoma, the immunohistochemical expression patterns of COX-2 and VEGF were evaluated in relationship with cyclin D1 overexpression, tumor stage, clinicopathologic parameters and patient survival in 128 mammary infiltrating duct carcinomas. The expressions of COX-2/VEGF, COX-2/cyclin D1, and VEGF/cyclin D1 were evaluated using double immunofluorescein staining with a confocal scanning laser microscope. A positive expression was seen in 41% for COX-2, 47% for VEGF, and 66% for cyclin D1 in the cases with breast cancer. There was correlation in positive expression of COX-2 or VEGF with histologic grade, lymph node   metastasis  , and tumor size. Conversely, a significant inverse relation was observed between VEGF and patient age. There was a correlation in overexpression of cyclin D1 with lymph node   metastasis  , survival rate and survival length. Significant correlations were observed between COX-2 and VEGF as well as COX-2 and cyclin D1. Co-expression of only COX-2 and VEGF was detected with significance. These results indicate that elevated COX-2 or VEGF expression or cyclin D1 overexpression is more common in breast cancer patients with poor prognostic characteristics and is partly associated with an unfavorable outcome. The present findings support the efforts to initiate clinical trials on the efficacy of COX-2 inhibitors in adjuvant treatment of breast cancer. 
 COX-2 gene promoter haplotypes and prostate cancer risk. Cyclooxygenase-2 (COX-2) is a key rate-limiting enzyme that converts arachidonic acid into pro-inflammatory prostaglandins. COX-2 expression is strongly correlated with increased tumor microvasculature density and plays an important role in inhibiting apoptosis, stimulating angiogenesis and promoting tumor cell   metastasis   and invasion. However, little is known about the role that sequence variation of the COX-2 gene contributes to prostate cancer. Thus, we searched for polymorphisms in the promoter region of the COX-2 gene using denaturing high-performance liquid chromatography. Four single nucleotide polymorphisms (SNPs), -1285A/G, -1265G/A, -899G/C and -297C/G, were detected and confirmed by direct sequencing. Three of the SNPs in the promoter region of COX-2 gene create at least three putative transcription factor binding sites and eliminate CCAAT/enhancer binding protein alpha (C/EBP alpha) and NF-kappa B binding sites. A case-control study of the four SNPs in African American (n = 288), Bini Nigerian (n = 264) and European American (n = 184) prostate cancer cases and age-matched controls revealed that SNP -297G was associated with a decreased risk for prostate cancer [odds ratio (OR) = 0.49; CI = 0.2-0.9; P = 0.01]. The effect on risk was observed in both African Americans (OR = 0.51; CI = 0.2-0.9; P = 0.01) and European Americans (OR = 0.33; CI = 0.1-0.9; P = 0.02). In addition, SNPs -1265A and -899C were associated with increased prostate cancer risk in African Americans (OR = 2.72; CI = 1.3-5.8; P = 0.007 and OR = 3.67; CI = 1.4-9.9; P = 0.007, respectively). Haplotype analyses revealed modest effects on susceptibility to prostate cancer across populations. Haplotype GGCC conferred increased risk in the African American and Nigerian populations. Conversely, haplotype AGGG exhibited a negative association with prostate cancer risk in African Americans (OR = 0.4; CI = 0.1-0.9; P = 0.02) and European Americans (OR = 0.2; CI = 0.1-0.9; P = 0.03). These data suggest that variation of the COX-2 promoter may influence the risk and development of prostate cancer. 
 MBP-1 suppresses growth and   metastasis   of gastric cancer cells through COX-2. The c-Myc promoter binding protein 1 (MBP-1) is a transcriptional suppressor of c-myc expression and involved in control of tumorigenesis. Gastric cancer is one of the most frequent neoplasms and lethal malignancies worldwide. So far, the regulatory mechanism of its aggressiveness has not been clearly characterized. Here we studied roles of MBP-1 in gastric cancer progression. We found that cell proliferation was inhibited by MBP-1 overexpression in human stomach adenocarcinoma SC-M1 cells. Colony formation, migration, and invasion abilities of SC-M1 cells were suppressed by MBP-1 overexpression but promoted by MBP-1 knockdown. Furthermore, the xenografted tumor growth of SC-M1 cells was suppressed by MBP-1 overexpression.   Metastasis   in lungs of mice was inhibited by MBP-1 after tail vein injection with SC-M1 cells. MBP-1 also suppressed epithelial-mesenchymal transition in SC-M1 cells. Additionally, MBP-1 bound on cyclooxygenase 2 (COX-2) promoter and downregulated COX-2 expression. The MBP-1-suppressed tumor progression in SC-M1 cells were through inhibition of COX-2 expression. MBP-1 also exerted a suppressive effect on tumor progression of other gastric cancer cells such as AGS and NUGC-3 cells. Taken together, these results suggest that MBP-1-suppressed COX-2 expression plays an important role in the inhibition of growth and progression of gastric cancer. 
 Prognostic significance of COX-2 immunohistochemical expression in colorectal cancer: a meta-analysis of the literature. Cyclooxygenase-2 (COX-2) is believed to be an important enzyme in the pathogenesis of colorectal cancer (CRC). Correlations between the expression of COX-2 with tumor growth and distant   metastasis   have become an issue; thus, attention has been paid to COX-2 as a prognostic factor. Various studies examined the relationship between COX-2 immunohistochemistry (IHC) overexpression with the clinical outcome in patients with colorectal cancer, but yielded conflicting results. The prognostic significance of COX-2 overexpression in colorectal cancer remains controversial.Electronic databases updated to October 2012 were searched to find relevant studies. A meta-analysis was conducted with eligible studies which quantitatively evaluated the relationship between COX-2 overexpression and survival of patients with colorectal cancer. Survival data were aggregated and quantitatively analyzed.We performed a meta-analysis of 23 studies (n  =  4567 patients) that evaluated the correlation between COX-2 overexpression detected by IHC and survival in patients with colorectal cancer. Combined hazard ratios suggested that COX-2 overexpression had an unfavorable impact on overall survival (OS) (HR [hazard ratio]  =  1.193, 95% CI [confidence interval]: 1.02 ∼ 1.37), but not disease free survival (DFS) (HR  =  1.25, 95% CI: 0.99 ∼ 1.50) in patients with colorectal cancer.Cox-2 overexpression in colorectal cancer detected by IHC appears to have slightly worse overall survival. However, the prognostic value of COX-2 on survival in colorectal cancer still needs further large-scale prospective trials to be clarified. 
 COX-2 overexpression increases motility and invasion of breast cancer cells. Cyclooxygenase-2 (COX-2), an inducible enzyme involved in prostaglandin (including PGE(2)) biosynthesis, is overexpressed in several epithelial malignancies including breast cancer. We tested the hypothesis that COX-2 overexpression in breast cancer cells results in increased cell motility and invasion. COX-2 overproducing cells were generated by stable transfection of several human breast cancer cells with pSG5-COX2 vector. We confirmed the overexpression of COX-2 protein by western blotting, and by measuring PGE(2) in the medium with an immunoassay. We measured cell motility by counting the number of cells crossing an 8-micron pore size PET membrane, and cell invasion by counting the number of cells invading through a Matrigel-coated membrane that simulates basement membrane. COX-2 transfected MDA-231 cells produced 30-43-fold more PGE2 as compared to parental cells. COX-2 overexpression increased cell migration approximately 2.2-fold and cell invasion through Matrigel approximately 5.1-fold. Addition of 50 microM NS-398, a COX-2 inhibitor, inhibited Matrigel invasion of MDA-231 cells by 54% as compared to solvent confirming the role of COX-2 in cell invasion. It is known that an increase in cell migration and invasion can be brought about by cytoskeletal alterations and basement membrane degradation due to increased expression of pro-urokinase plasminogen activator (pro-uPA). To investigate the mechanism of our observed increase in cell invasion by COX-2, we found by western blotting that the level of pro-uPA was significantly higher (approximately 5-fold) in COX-2 transfected MDA-231 cells than untransfected MDA-231 cells. Similar to our observations in cell culture, we found evidence that increased COX-2 activity correlates with uPA in a mouse model of breast cancer   metastasis   to bone. In this study, we conclude that COX-2 overexpression in human breast cancer cells enhances cell motility and invasiveness thus suggesting a mechanism of COX-2 mediated   metastasis  . 
 Cyclooxygenase-2 and epidermal growth factor receptor expressions in different histological subtypes of cervical carcinomas. This study was designed to evaluate the significance of cyclooxygenase (COX)-2 and epidermal growth factor receptor (EGFR) expression in a series of cervical adenocarcinoma (AC), cervical adenosquamous carcinoma (ASC), and cervical squamous cell carcinoma (SCC). One hundred thirty cases of cervical carcinoma (30 ASC, 50 AC, and 50 SCC) were analyzed for COX-2 and EGFR expressions using specific primary antibodies. Samples were scored semiquantitatively as follows: (-), 0% of immunoreactive cells; (+), <5% of immunoreactive cells; (++), 5% to 50% of immunoreactive cells; and (+++), >50% of immunoreactive cells. The COX-2 expression was more frequently positive than EGFR in all cervical cancers studied. The COX-2 expression was also more prominent in AC than in ASC (P = 0.003). Expression of either COX-2 or EGFR was significantly different when comparing SCC with AC (P < 0.001 and P = 0.04, respectively). There was no significant correlation between COX-2 and EGFR expressions and age at diagnosis, recurrence, distant   metastasis  , and/or positive status of regional lymph nodes, neither between COX-2 and EGFR coexpression and the clinical data analyzed. Nevertheless, our data support that there are significant differences between EGFR and COX-2 expressions in the 3 different histogenetic types of cervical cancer. Also, in terms of therapeutic strategies, our data can be valuable in the selection of patients eligible to receive specific EGFR/COX-2-targeted therapy. 
 The expression of MAGE and SSX, and correlation of COX2, VEGF, and survivin in colorectal cancer. We investigated the expression of melanoma-associated antigen gene (MAGE), human synovial sarcoma on X chromosome (SSX) and their clinical implications in sporadic colon cancer.Fresh tissue samples from 37 patients with colorectal adenocarcinomas were analyzed for MAGE and SSX mRNA by reverse transcription-polymerase chain reaction (RT-PCR) and their paraffin-embedded tissues were used for immunohistochemistry for cyclooxygenase 2 (COX2), vascular endothelial growth factor (VEGF), and survivin.Expression of MAGE and SSX was not detected in normal tissues. Colon cancer expressed SSX in 32.4% and MAGE in 51.4% of cases. Co-expression of MAGE and SSX was directly correlated with liver   metastasis   (p=0.024) and also correlated with nuclear expression of survivin (p=0.016), yet did not correlate with expression of COX2 and VEGF. Nuclear survivin expression (83.3%) was found in the cancer tissues exclusively. No significant relationships between the expression of COX2 (73.9%) and VEGF (72.4%) and other clinicopathologic variables were found.Our results suggest that nuclear expression of survivin, lymph node   metastasis  , vascular and perineural invasion, and co-expression of MAGE and SSX may be associated with the   metastasis   of colorectal cancer to the liver. 
 Increased cyclooxygenase-2 expression associated with inflammatory cellular infiltration in elderly patients with vulvar cancer. As the relationship between inflammation and carcinogenesis grows stronger, the role of cyclooxygenase-2 (COX-2) and epidermal growth factor (EGFR) has been highlighted in the pathogenesis and progression of human cancer. In view of the fact that vulvar cancer is characterized by precancerous inflammatory changes in elderly patients, the expressions of COX-2 and EGFR are expected to show different patterns of distribution according to age and other prognostic factors. To verify whether there was a relationship between their expression and clinicopathologic parameters in vulvar cancer, we investigated the inflammatory cellular infiltration and the expression of COX-2 and EGFR by immunohistochemical analysis. Eleven of 19 samples (57.8%) were stained positive for COX-2, and 17 (89.4%) for EGFR. The portion of inflammatory cellular infiltration in adjacent normal tissue was also higher in the older age group, and showed a strong correlation with COX-2 positivity (P = 0.002). Furthermore, COX-2 expression was significantly more frequent in patients over 60 years of age compared to those under 50 years (P = 0.009). COX-2 expression was noted to be high in moderate and well-differentiated cases, whereas, poorly differentiated carcinoma was negative for COX-2 expression (P = 0.023). However, EGFR expression was not differently distributed on the basis of stage, age, tumor grading, or presence of lymph node   metastasis  . Our article suggests that vulvar cancer in elderly patients may be associated with inflammation, and thus with increased COX-2 expression. In light of these findings, a clinical trial designed to assess the addition of COX-2 targeted therapy to conventional treatment in vulvar cancer would be helpful for consideration of additional treatment options and possibly avoiding the serious surgical morbidity in elderly patients. 
 Expression of cyclooxygenase-1 and -2 in extra-hepatic cholangiocarcinoma. To investigate the expression of cyclooxygenase-1 (COX-1) and cyclooxygenase-2 (COX-2) in extra-hepatic cholangiocarcinoma and the relationship between their expression and clinicopathological parameters.COX-1 and COX-2 were detected in 56 extra-hepatic cholangiocarcinomas, including 31 matched tissues originating from non-tumorous bile ductal tissue adjacent to tumours and 6 normal bile ductal tissues, by immunohistochemistry strept avidin-biotin complex using isozyme selective antibodies.There was no difference in expression of COX-1 between carcinomas (96%, 54/56) and noncancerous specimens (94%, 29/31, P>0.05) or normal bile ductal tissues (100%, 6/6, P>0.05). The positive rate of COX-2 expression in extra-hepatic cholangiocarcinomas (86%, 48/56) was significantly higher than their matched tissues (39%, 12/31, P<0.01) and normal bile ductal tissues (0%, 0/6, P<0.01). Overexpression of COX-2 in extra-hepatic cholangiocarcinoma was related to the   metastasis   of lymph nodes, distant organs or tissues (P<0.05) as well as the degree of tumour differentiation (P<0.05).The overexpression of COX-2 plays a crucial role in the carcinogenesis and development of extra-hepatic cholangiocarcinoma, indicating that COX-2 may serve as a target for chemoprevention of extra-hepatic cholangiocarcinoma. 
 Association between expression of embryonic lethal abnormal vision-like protein HuR and cyclooxygenase-2 in oral squamous cell carcinoma. Increased cytoplasmic HuR expression has been noted in several cancer types, where it may contribute to the increased cyclooxygenase-2 (COX-2) expression observed during tumorigenesis and   metastasis  .To assess the correlation between COX-2 and HuR in oral squamous cell carcinoma (OSCC), the expression patterns of HuR and COX-2 were assessed via immunohistochemistry analyses of 103 OSCC samples.Cytoplasmic HuR expression was significantly associated with COX-2 expression (p < .025) and lymph node   metastasis   (p < .050) and distant   metastasis   (p < .025). In multivariate analysis, cytoplasmic HuR expression was identified as an independent prognostic parameter for reduced overall survival. The inhibition of HuR expression by siRNA or leptomycin B (LMB) caused a reduction in the inducibility of COX-2 in oral cancer cells.Our results indicate that the cytoplasmic expression of HuR is associated with COX-2 expression in OSCCs and HuR can regulate COX-2 expression in oral cancer cell lines. 
 Expression of COX-2 proteins in gastric mucosal lesions. To investigate the expression of COX-2 proteins in gastric mucosal lesions and to assess the relationship between COX-2 expression and type, pathologic stage, differentiation, or lymph node   metastasis   in gastric cancer and the relationship between COX-2 expression and H pylori infection in gastric mucosal lesions.Thirty patients with gastric carcinoma underwent surgical resection. Samples were taken from tumor site and paracancerous tissues, and ABC immunohistochemical staining was used to detect the expression of COX-2 proteins. H pylori was determined by rapid urea test combined with pathological stating/14C urea breath test.The positive rate and staining intensity of mutant COX-2 gene expression in gastric cancer were significantly higher than those in paracancerous tissues (66.7% vs 26.7%) (P<0.01, P<0.001). There was a significant correlation between COX-2 and pathologic stage or lymph node   metastasis   type of gastric carcinoma (76.0% vs 20.0%, 79.2% vs 16.7%) (P<0.05). No correlation was found between COX-2 expression and type or grade of differentiation (P>0.05). COX-2 expression of intestinal metaplasia (IM) or dysplasia (DYS) with positive H pylori was significantly higher than that with negative H pylori (50.6% vs 18.1%, 60.0% vs 33.3%) (P<0.05).COX-2 overexpression was found in a large proportion of gastric cancer tissues compared with matched non-cancerous tissues and was significantly associated with advanced tumor stage and lymph node   metastasis  . Overexpression of COX-2 plays an important role in tumor progression of gastric cancer. COX-2 may also play a role in the early development/promotion of gastric carcinoma and is associated with H pylori infection. 
 COX-2 Promotes Migration and Invasion by the Side Population of Cancer Stem Cell-Like Hepatocellular Carcinoma Cells. Cancer stem cells (CSCs) are thought to be responsible for tumor relapse and   metastasis   due to their abilities to self-renew, differentiate, and give rise to new tumors. Cyclooxygenase-2 (COX-2) is highly expressed in several kinds of CSCs, and it helps promote stem cell renewal, proliferation, and radioresistance. Whether and how COX-2 contributes to CSC migration and invasion is unclear. In this study, COX-2 was overexpressed in the CSC-like side population (SP) of the human hepatocellular carcinoma (HCC) cell line HCCLM3. COX-2 overexpression significantly enhanced migration and invasion of SP cells, while reducing expression of   metastasis  -related proteins PDCD4 and PTEN. Treating SP cells with the selective COX-2 inhibitor celecoxib down-regulated COX-2 and caused a dose-dependent reduction in cell migration and invasion, which was associated with up-regulation of PDCD4 and PTEN. These results suggest that COX-2 exerts pro-metastatic effects on SP cells, and that these effects are mediated at least partly through regulation of PDCD4 and PTEN expression. These results further suggest that celecoxib may be a promising anti-metastatic agent to reduce migration and invasion by hepatic CSCs. 
 STIM1 overexpression promotes colorectal cancer progression, cell motility and COX-2 expression. Tumor   metastasis   is the major cause of death among cancer patients, with >90% of cancer-related death attributable to the spreading of metastatic cells to secondary organs. Store-operated Ca(2+) entry (SOCE) is the predominant Ca(2+) entry mechanism in most cancer cells, and stromal interaction molecule 1 (STIM1) is the endoplasmic reticulum (ER) Ca(2+) sensor for store-operated channels. Here we reported that the STIM1 was overexpressed in colorectal cancer (CRC) patients. STIM1 overexpression in CRC was significantly associated with tumor size, depth of invasion, lymph node   metastasis   status and serum levels of carcinoembryonic antigen. Furthermore, ectopic expression of STIM1 promoted CRC cell motility, while depletion of STIM1 with short hairpin RNA inhibited CRC cell migration. Our data further suggested that STIM1 promoted CRC cell migration through increasing the expression of cyclooxygenase-2 (COX-2) and production of prostaglandin E2 (PGE2). Importantly, ectopically expressed COX-2 or exogenous PGE2 were able to rescue migration defect in STIM1 knockdown CRC cells, and inhibition of COX-2 with ibuprofen and indomethacin abrogated STIM1-mediated CRC cell motility. In short, our data provided clinicopathological significance for STIM1 and SOCE in CRC progression, and implicated a role for COX-2 in STIM1-mediated CRC   metastasis  . Our studies also suggested a new approach to inhibit STIM1-mediated   metastasis   with COX-2 inhibitors. 
 Cyclooxygenase-2 promotes tumor lymphangiogenesis and lymph node   metastasis   in oral squamous cell carcinoma. Oral squamous cell carcinoma (OSCC) is the sixth most common cancer and frequently metastasizes to the cervical lymph nodes, leading to poor survival of patients with OSCC. However, the mechanism of lymph node   metastasis   is not fully understood. To clarify the molecular mechanism underlying OSCC   metastasis   to regional lymph nodes, the highly metastatic fluorescent labeled OSCC cell line SAS-LM3 was successfully established allowing us to monitor the progression of lymph node metastases in a non-invasive manner. SAS-LM3 tumors showed increased lymphangiogenesis and elevated expression of VEGF-C, a potent stimulator of lymphangiogenesis, compared to parental SAS tumors. SAS-LM3 showed high expression of cyclooxygenase-2 (COX-2) compared to parental SAS cells and immunohistochemical analysis demonstrated intense COX-2 expression at the primary site. Inactivation of COX-2 by knockdown or the COX-2 inhibitor NS-398 decreased VEGF-C expression. Administration of COX-2 inhibitor NS-398 in SAS-LM3 tumor-bearing mice suppressed tumor lymphangiogenesis and lymphatic metastases. Collectively, our results indicate that COX-2 promotes tumor lymphangiogenesis and lymph node   metastasis   of OSCC. COX-2 ablation holds promise as a potential therapeutic approach for lymph node   metastasis   in OSCC. 
 The Notch1/cyclooxygenase-2/Snail/E-cadherin pathway is associated with hypoxia-induced hepatocellular carcinoma cell invasion and migration. Hepatocellular carcinoma (HCC) is one of the most common malignancies worldwide; however, the prognosis of HCC patients remains poor. This poor prognosis is mainly attributed to the high rate of intrahepatic and distant   metastasis  . HCC often occurs in a hypoxic environment and hypoxia can activate metastatic programs, ultimately leading to tumor recurrence or   metastasis  . Thus, the discovery and subsequent development of novel agents to block HCC invasion and migration are the primary objectives of hepatic cancer research. The Notch1 signaling pathway might be involved in hypoxia-induced carcinoma   metastasis  . However, the mechanisms by which Notch1 mediates cell   metastasis  , particularly in hepatocellular carcinoma, are not yet entirely clear. The results of the present study show that hypoxia increases the invasion and migration capacities of different HCC cells. Activation of the Notch1 signaling pathway contributes to hypoxia-induced invasion and migration in HCC cells. The activated Notch1 signaling pathway can regulate Snail/E-cadherin through cyclooxygenase-2 (COX-2) under hypoxic conditions. The above results suggest that the Notch1/COX-2/Snail/E-cadherin pathway is possibly associated with hypoxia-induced invasion and migration in HCC cells. Thus, targeting Notch1 may be useful for devising novel preventive and therapeutic strategies for HCC. 
 Expression of COX-2 and E-cadherin in Tunisian patients with colorectal adenocarcinoma. Cyclo-oxygenase 2 (COX-2) and E-cadherin are promising biomarkers for cancer diagnosis and therapy. The aim of this study was to examine the expression of these two proteins in primary colorectal adenocarcinomas and to investigate their association with clinicopathological characteristics including survival of patients. Immunostaining of E-cadherin and COX-2 was assessed in 70 primary colorectal adenocarcinomas from Tunisian patients. Membranous E-cadherin immunostaining and cytoplasmic COX-2 expression were observed in 74.3% and 68.6% of cases respectively. A significant association was found between COX-2 expression and age at diagnosis (P=0.02), and vessel invasion (P=0.037). The expression of E-cadherin correlated with age at diagnosis (P=0.01), and tumor size (P=0.02). In addition, by multivariate analysis, we revealed a significant association with 1-year disease free survival and a tendency with distant   metastasis   (P=0.017 and P=0.065 respectively). On the other hand, tumors exhibiting COX-2+/E-cadherin-profile were larger (P=0.006), and in an advanced stage (P=0.001). Survival analysis showed that COX-2 over-expression confers a reduced overall survival rate (Plog rank=0.036) and is an independent factor predictive for prognosis. 
 [Detection of EGFR and COX-2 expression by immunohistochemical method on a tissue microarray section in lung cancer and biological significance]. Epidermal growth factor receptor (EGFR) and cyclooxygenase-2 (COX-2), which can regulate growth, invasion and   metastasis   of tumor through relevant signaling pathway, have been detected in a variety of solid tumors. The aim of this study is to investigate the biological significance of EGFR and COX-2 expression in lung cancer and the relationship between them.The expression of EGFR and COX-2 was detected in 89 primary lung cancer tissues, 12 premalignant lesions, 12 lymph node metastases, and 10 normal lung tissues as the control by immunohistochemical method on a tissue microarray section.EGFR protein was detectable in 59.6%, 41.7%, and 66.7% of primary lung cancer tissues, premalignant lesions and lymph node metastases, respectively; COX-2 protein was detectable in 52.8%, 41.7%, and 66.7% of primary lung cancer tissues, premalignant lesions and lymph node metastases, respectively, which were significantly higher than those of the control (P < 0.05). The positive ratios and the levels of the expression of EGFR and COX-2 proteins were closely related to histological type, clinical stage and lymph node   metastasis   of lung cancer (P < 0.05), but not to histological grade, sex and age (P > 0.05). COX-2 expression was related to gross type (P < 0.05). A highly positive correlation was observed between EGFR and COX-2 expression (P < 0.01).Overexpression of EGFR and COX-2 may play an important role in the tumorgenesis, progression and malignancy of lung cancer. Detection of EGFR and COX-2 expression might be helpful to diagnosis and prognosis of lung cancer. 
 Cyclooxygenase-2 and gastric carcinogenesis. Epidemiological studies have shown that the use of nonsteroid anti-inflammatory drugs (NSAIDs) is associated with reduced risk of gastric cancer. The best-known target of NSAIDs is the cyclooxygenase (Cox) enzyme. Two Cox genes have been cloned, of which Cox-2 has been connected with gastric carcinogenesis. Expression of Cox-2 is elevated in gastric adenocarcinomas, which correlates with several clinicopathological parameters, including depth of invasion and lymph node   metastasis  . This suggests that Cox-2-derived prostanoids promote aggressive behavior of adenocarcinomas of the stomach. Cox-2 expression is especially prominent in intestinal-type gastric carcinoma and it is already present in dysplastic precursor lesions of this disease, which suggests that Cox-2 contributes to gastric carcinogenesis already at the preinvasive stage. Our most recent data show that Cox-2 is expressed in gastric adenomas of trefoil factor 1 deficient mice. Treatment of these mice with a Cox-2 selective inhibitor, celecoxib, reduced the size of the adenomas. Taken together these data support efforts to initiate clinical studies to investigate the effect of Cox-2 inhibitors as chemotherapeutic agents and as adjuvant treatment modalities against gastric neoplasias. 
 Positive correlation of osteopontin, cyclooxygenase-2 and vascular endothelial growth factor in gastric cancer. Osteopontin (OPN), cyclooxygenase-2 (COX-2) and vascular endothelial growth factor (VEGF) are overexpressed in various experimental models of malignancy. However, the correlation and role of the three molecules in gastric cancer is unclear. In the present study, we found that OPN, COX-2 and VEGF were overexpressed in 53 cancerous tissues with gastric cancer compared with 40 normal mucosa tissues by immunohistochemistry method. Moreover, the results indicated co-expression of OPN, COX-2, and VEGF in gastric cancer. Levels of OPN, COX-2, and VEGF were all significantly correlated with TNM stage, lymph node   metastasis   and distant   metastasis   (P < 0.05), while not related to prognosis of patients. In addition, individual levels of OPN, COX-2, and VEGF were all significantly correlated with microvessel density (MVD), valued by CD34 staining directly with r-values of 0.416, 0.400, and 0.566, respectively (P < 0.01). Both OPN and COX-2 levels showed a positive correlation with VEGF (P < 0.05). Meanwhile, expression of COX-2 is in relation to OPN (P < 0.01). Overall, survival for patients with high MVD was significantly lower than for patients with low MVD (P < 0.05). Our findings indicate that OPN, COX-2, and VEGF synergically promote angiogenesis and   metastasis   in gastric cancer. It may be an important and useful strategy to target these molecules for prevention and therapy of tumor. 
 [Expressions and clinical significances of c-met, c-erbB-2, COX-2, and IL-6 in the biliary tract cancers]. c-met, c-erbB-2, interleukin (IL)-6, and cyclooxygenase (COX)-2 expressions are considered to be implicated in the carcinogenesis and progression of cholangiocarcinoma, but the molecular pathogenesis of cholangiocarcinoma is still poorly understood. We aimed to analyze the expressions of each marker and their relationships with clinicopathologic factors.One hundred and fourteen tissue samples were obtained from surgically resected specimens from patients with biliary tract cancer. The expressions of c-met, c-erbB-2, COX-2, and IL-6 were examined by immunohistochemically. The expression of each marker and correlations between these markers and clinicopathologic factors were analyzed.The expression rates of each maker were as follows: c-met 34/112 (30.4%), c-erbB-2 5/112 (4.5%), COX-2 53/113 (46.9%), and IL-6 68/113 (60.2%), respectively. c-met expression was more frequently observed in cases with invasion through the adjacent connective tissues (p=0.0263). IL-6 overexpression was more frequently observed in cases with absent lymph node   metastasis   (p=0.0325). Either c-erbB-2 expression or COX-2 expression was significantly associated with lymph node   metastasis   (p=0.0442).The expression of c-met was closely related to the invasiveness of cholangiocarcinoma. Co-expression of c-met, COX-2 and, IL-6 showed a significant correlation with invasiveness and lymph node   metastasis   and these could be useful marker to guide clinical outcome in patients with cholangiocarcinoma. 
 Overexpression of cyclooxygenase-2 is associated with breast carcinoma and its poor prognostic factors. Cyclooxygenase is the rate-limiting enzyme that catalyzes the conversion of arachidonic acid to prostaglandins. The inducible form, cyclooxygenase-2, is known to be overexpressed in various human cancers including the colon, stomach, and urinary bladder. In this study, we evaluated the overexpression of cyclooxygenase-2 in 64 cases of breast cancer and correlated the results with clinicopathologic parameters. Immunohistochemical staining for cyclooxygenase-2 demonstrated positivity of the tumor cells in 46 of 64 cases (72%). Cyclooxygenase-2 overexpression was significantly correlated with larger tumor size and advanced clinical stage. Cyclooxygenase-2 overexpression tended to be more frequently observed in cases with presence of lymph node   metastasis   and in cases without expression of estrogen and progesterone; however, there was no significant correlation statistically. Nuclear and histologic grade were not well correlated with cyclooxygenase-2 overexpression. When ductal carcinoma in situ was considered separately, 32 of 42 cases (76%) were positive for cyclooxygenase-2. We conclude that cyclooxygenase-2 is up-regulated in a high proportion of breast cancers. The overexpression of cyclooxygenase-2 was associated with larger tumor size and advanced clinical stage, although lymph node status, estrogen and progesterone expression, and nuclear and histologic grade were not significantly correlated. Therefore, cyclooxygenase-2 overexpression may be a feature of the aggressive phenotype and may be useful as a prognostic indicator in breast cancer. 
 Aberrant methylation of the specific CpG island portion regulates cyclooxygenase-2 gene expression in human gastric carcinomas. Although it has been well established that overexpression of cyclooxygenase-2 (Cox-2) favors tumorigenesis and   metastasis  , the molecular mechanism that regulates Cox-2 expression has not been well defined in gastric carcinoma. Aberrant methylation of the CpG island is known to be one of the powerful mechanisms for the suppression of gene expression, and usually, CpG islands are very rich in promoter region and exon-1 region. But, it is controversial whether Cox-2 gene expression is regulated by methylation of promoter region or exon-1 region. In this study, we examined whether the hyper-methylation mediated transcriptional silencing of Cox-2 also occurred in human gastric carcinoma tissues and which portion of CpG island methylation is important in Cox-2 gene expression. Genomic DNAs from human gastric carcinoma tissues were treated with three methylation-sensitive restriction enzymes and then Southern blot analysis was performed. Out of 30 primary gastric tumor samples, 26 cases (86.6%) showed overexpression of Cox-2. Four cases (13.3%) with relatively decreased Cox-2 gene expression were associated with the presence of aberrant methylation of Cox-2 CpG island. We also found that methylation of promoter region and not exon-1 region is related with the transcriptional silencing of Cox-2 in gastric carcinoma cancer by detailed methylation mapping using bisulfite sequencing analysis. Our results suggest that the DNA methylation-mediated transcriptional silencing of Cox-2 is a predominant mechanism for the down-regulation of Cox-2 expression in human gastric carcinoma. Furthermore, the results suggest that methylation of not exon-1 region but promoter region is important to regulation of Cox-2 gene expression. 
 Hepatocyte growth factor inhibits anoikis by induction of activator protein 1-dependent cyclooxygenase-2. Implication in head and neck squamous cell carcinoma progression. Anoikis, also called suspension-induced apoptosis, plays an important role in tumor development, progression, and   metastasis  . Recently we found that hepatocyte growth factor (HGF) inhibited anoikis of human head and neck squamous cell carcinoma (HNSCC) cells by activating the extracellular signal-regulated kinase (ERK)-signaling pathway. However, the anti-apoptotic effectors that were regulated by the ERK-signaling pathway were unknown. Here we report that HGF-mediated inhibition of anoikis was dependent on activator protein-1 activity through the activation of the ERK-signaling pathway. Using a combination of microarray analysis and Northern blot analysis, we found that an anti-apoptotic gene cyclooxygenase-2 (cox-2) was induced by HGF in an activator protein-1-dependent fashion. Inhibition of Cox-2 activity partially abolished HGF-mediated cell survival, and overexpression of Cox-2 in HNSCC cells provided resistance against anoikis. Moreover, HNSCC cells stably expressing Cox-2 had aggressive tumor growth in a nude mouse model compared with control cells. Taken together, our results demonstrate that Cox-2 plays an important role in HGF-mediated anoikis resistance. HGF may stimulate the progression and growth of HNSCC in vivo by induction of Cox-2. 
 Cyclooxygenase-2 in oligodendroglioma: possible prognostic significance. Cyclooxygenase-2 (COX-2) is the inducible form of the enzyme involved in the first two steps of the prostaglandins and thromboxane synthesis. Up-regulation of COX-2 is demonstrated in tumors where it can modulate tumoral progression,   metastasis  , multidrug resistance and angiogenesis. Selective COX-2 inhibitors are seen with growing interest in the tumors treatment. This present study reviews the COX-2 expression in 32 primary oligodendrogliomas (24 WHO II; eight WHO III) and two glioblastomas with prominent oligodendroglial features (WHO IV). Immunohistochemical results were compared with survival in order to verify the COX-2 prognostic significance. COX-2 positivity was found in 44% tumors. Median survival of the patients with a COX-2 positive lesion was 37 months; median survival of the patients with a COX-2 negative lesion was 93 months (P =0.010). Twenty-nine percent WHO grade II tumors, 87% WHO grade III, 50% WHO grade IV resulted COX-2 positive (P =0.016). In patients affected by WHO grade II oligodendroglioma, median survival was 24 and 96 months, respectively, in COX-2 positive and negative lesions (P =0.012). In conclusion, even if further studies on different, homogeneous and larger series in vivo are certainly necessary, it is believed that COX-2 could really have a prognostic value and can be considered as a possible therapeutic opportunity. 
 Clinicopathological significance of vascular endothelial growth factor-C and cyclooxygenase-2 in esophageal squamous cell carcinoma. Vascular endothelial growth factor-C (VEGF-C) is a specific growth factor of lymphatics, which is known to play some role in tumor growth and   metastasis   to lymph nodes and distant organs in various malignancies. The purpose of the present study was to investigate the expression of VEGF-C in human esophageal squamous cell carcinomas (ESCC) to elucidate its role in tumor progression and lymph node   metastasis  . Another aim of the study was to investigate the relation between VEGF-C and cyclooxygenase-2 (COX-2) in ESCC.The expression of VEGF-C and COX-2 in ESCC was evaluated in 13 endoscopic mucosal resection specimens and in 21 surgical specimens by immunohistochemical staining. Clinical data were obtained from medical records.The degree of VEGF-C expression increased as the depth of primary tumor progressed (r = 0.521, P = 0.002), the stage progressed (r = 0.572, P < 0.001), and the degree of COX-2 expression increased (r = 0.387, P = 0.024). The VEGF-C positive rate was different between early cancers in which regional lymph node   metastasis   was thought to be absent and advanced cancers in which regional lymph node metastases were confirmed after surgery (20.0% vs 100.0%; P < 0.001).The VEGF-C expression in ESCC is related to COX-2 expression, and VEGF-C is also associated with the depth of primary tumor, the stage, and probably lymph node   metastasis  . Thus the investigation of VEGF-C expression in ESCC may assist in management planning. 
 [Relation between COX-2 protein expression and biologic behavior of ovarian carcinoma]. To study the relation between cyclooxygenase-2 (COX-2) protein expression and biologic behavior of ovarian carcinoma.The level of COX-2 protein expression was detected by Western Blot assay in 54 biopsy specimens from ovarian serous tumor patients and 10 normal ovarian samples.The level of COX-2 protein expression and relative quantity in ovarian serous carcinoma (81.8%, 20.08 +/- 3.53) were statistically higher than those in the benign ovarian serous tumor (0, 15.04 +/- 0.12) and in the normal ovary (0, 15.33 +/- 0.60) (P < 0.05). The level of COX-2 protein expression and relative quantity in borderline ovarian serous tumor (90.0%, 20.61 +/- 3.03) were statistically higher than those in benign ovarian serous tumor and the normal ovary (P < 0.05). The level of COX-2 protein expression and relative quantity were not significantly different from ovarian serous carcinoma and borderline ovarian serous tumor (P > 0.05); as they were between the benign ovarian serous tumor and the normal ovary (P > 0.05). The level of COX-2 protein expression and relative quantity were not significantly different among different clinical stages (I + II and III + IV), different histological grades, with or without ascites or lymphatic   metastasis   either.COX-2 overexpression may be significantly related to the oncogenesis and development of ovarian serous carcinoma, which may be an early diagnostic parameter and, hence, an attractive target for chemopreventive strategy in the treatment of ovarian serous carcinoma. 
 [The expression and significance of HIF-1alpha and COX-2 in laryngeal squamous cell carcinoma]. To investigate the protein and mRNA expression of HIF-1alpha and COX-2 in laryngeal squamous carcinoma, and their correlation with clinicopathologic parameters.The protein and mRNA expression of HIF-1alpha and COX-2 in fresh laryngeal squamous carcinoma tissues and adjacent normal tissues from 76 patients were examined by immunohistochemistry and reverse transcription polymerase chain reaction.1) The protein positive expression rate of HIF-1alpha and COX-2 in tumor samples were significantly higher than that in normal tissues (P < 0.05); The protein positive expression rate of HIF-1alpha were higher in moderately and poorly differentiated tumors than in well differentiated tumors (P < 0.05); While the protein positive expression rate of COX-2 were not associated with T stage (P > 0.05); 2) The mRNA positive expression rate of HIF-1alpha and COX-2 were 67.11% and 75.00% respectively, their expression were significantly higher in tumors than that in normal tissues (P < 0.05). The mRNA expression of COX-2 were not detected in normal tissues. The mRNA positive expression rate of HIF-1alpha were associated with T stage, but were not associated with differentiated clinicopathologic stage. 3) The protein positive expression rate of HIF-1alpha were associated with that of COX-2.HIF-1alpha and COX-2 could up regulate each other to promote the development and   metastasis   of laryngeal squamous carcinoma. 
 Relationship between expression and distribution of cyclooxygenase-2 and bcl-2 in human gastric adenocarcinoma. To explore expression and distribution features of COX-2 and bcl-2 in human gastric adenocarcinoma tissues and to study its biological significance.Totally 36 human gastric carcinoma samples were enrolled in this study (cardiac adenocarcinoma 16 cases, distal gastric adenocarcinoma 20 cases). The expressions of COX-2 and bcl-2 in cancerous tissues and corresponding para-cancerous tissues were investigated by immunohistochemistry using COX-2 polyclonal antibody and bcl-2 monoclonal antibody. The normal gastric mucosa tissues were used as control.The expressions of COX-2 and bcl-2 in gastric carcinoma were significantly higher than that in the para-cancerous tissues (77.8% vs 47.2%, P<0.01, 80.56% vs 58.33%, P<0.05). The expression of COX-2 in cardiac adenocarcinoma was remarkably higher than that in the distal gastric carcinoma (93.8% vs 65.0%, P<0.01). The expression of COX-2 was mainly localized in the cytoplasm of tumor cells and partly in the nucleus. There is a transition of the COX-2 cytoplasmic positivity to nucleic in tumor cells with the increase of gastric carcinoma pathological grade. Interstitial macrophages, fibroblasts and vascular endothelial cells also expressed COX-2. The tissues with higher expression of COX-2 also expressed high level of bcl-2 protein.Abnormal expression pattern of COX-2 within the tissues of human gastric cancer is correlated with tumor location and lymph node   metastasis  . COX-2 may regulate expression of apoptosis suppressor gene (bcl-2) through interaction of tumor cells and stromal cells and play an important role in the generation and development of tumors, which will be of great help in developing new methods for antitumor therapy. 
 Involvement of store-operated calcium signaling in EGF-mediated COX-2 gene activation in cancer cells. Growing evidence shows that chronic inflammation drives the progression of colorectal cancer (CRC). Cyclooxygenase-2 (COX-2) is one of the most important inflammatory genes involved in solid tumor   metastasis  . Epidermal growth factor receptor (EGFR) also plays a key role in cancer cell development. We compared the expression levels of EGFR and COX-2 between tumor and normal tissues from 20 CRC patients and studied the molecular mechanism of EGFR-mediated COX-2 gene expression in cancer cells. Our results indicated that COX-2 expression was markedly increased after EGF stimulation. COX-2 promoter analysis indicated the involvement of cyclic AMP-responsive element (CRE) and nuclear factor of activated T cells/nuclear factor interleukin-6 (NFAT/NF-IL6)-binding sites in EGF-mediated signaling pathways. Furthermore, EGF-mediated COX-2 activation was prevented by 2-aminoethoxydiphenyl borate (2-APB), a store-operated Ca(2+) channel inhibitor. Transfection of siRNA against ORAI1 or STIM1, the key regulators of store-operated Ca(2+) channels, showed significant inhibitory effects on EGF-mediated COX-2 expression. In conclusion, store-operated Ca(2+) entry is involved in the activation of transcription factors (CREB/NFAT) that are responsible for delivering EGF-mediated signals to evoke inflammatory cascades and is eventually related to CRC tumorigenesis. 
 [Clinicopathologic significance of cyclooxygenase-2 mRNA expression in human breast carcinoma]. To study the expression of cyclooxygenase-2 (COX-2) gene in breast cancer and its clinicopathologic characteristics.With beta-actin gene as reference, the COX-2 mRNA was monitored in 30 specimens of breast cancer tissue and adjacent normal breast tissue by reverse transcription-polymerase chain reaction (RT-PCR).The COX-2 mRNA expression was significantly upregulated in most breast cancer tissues with range of 0.05 - 0.91 (median 0.56), which was rare in normal breast tissue with range of 0 - 0.09 (median 0). The difference of COX-2 mRNA expression between cancer and normal breast tissue was significant (rank sum test, P < 0.05). COX-2 overexpression in breast cancer was related to its lymph node   metastasis   (P < 0.05) but not to age, tumor size, pathologic grade or pathologic type (P > 0.05).The level of COX-2 mRNA expression is obviously higher in the breast cancer tissue than that in normal breast tissue. COX-2 overexpression may play a crucial role in the carcinogenesis, development of cancer and lymph node   metastasis   in breast cancer patients. 
 Cyclooxygenase-2 mediated regulation of E-cadherin occurs in conventional but not early-onset gastric cancer cell lines. COX-2 and E-cadherin, involved in invasion and   metastasis  , are molecules critical for gastric carcinogenesis. A relationship between them is documented in non-small cell lung and prostate cancer. We present novel evidence of a relationship between COX-2 and E-cadherin expression in gastric cancer.Using qPCR and Western blots analysis on celecoxib and PGE2 treated and untreated gastric cancer cell lines derived from tumours of the intestinal type (MKN45, MKN28, AGS3, MKN7) and immunohistochemistry of 178 gastric cancers on tissue microarrays (TMA), we examined the COX-2/E-cadherin relationship.Down-regulation of COX-2 by celecoxib led to up-regulation of E-cadherin mRNA and protein levels in conventional gastric cancer cell lines, whereas expression was down regulated in the early-onset gastric cancer (EOGC) cell line. Immunohistochemistry on TMAs of 178 gastric cancers showed no correlation between COX-2 and E-cadherin expression in the conventional or early gastric cancer groups.The results suggest that COX-2 has an impact on transcriptional regulation of E-cadherin in gastric cancer and our findings further highlight the intriguing nature of EOGCs which appear to have a molecular phenotype distinct from conventional gastric cancer. In addition, our findings also suggest that reduction of COX-2 using nonsteroidal anti-inflammatory drugs in gastric cancer chemoprevention may only be relevant for older patients. 
 Expression of integrin α3β1 and cyclooxygenase-2 (COX2) are positively correlated in human breast cancer. Expression of integrin α3β1 is associated with tumor progressi  on, metast  asis, and poor prognosis in several cancers, including breast cancer. Moreover, preclinical studies have revealed important pro-tumorigenic and pro-metastatic functions for this integrin, including tumor growth, survival, invasion, and paracrine induction of angiogenesis. Our previously published work in a preclinical breast cancer model showed that integrin α3β1 promotes expression of cyclooxygenase-2 (COX2/PTGS2), a known driver of breast cancer progression. However, the clinical significance of this regulation was unknown. The objective of the current study was to assess the clinical relevance of the relationship between integrin α3β1 and COX2 by testing for their correlated expression among various forms of human breast cancer.Immunohistochemistry was performed to assess co-expression of α3 and COX2 in specimens of human invasive ductal carcinoma (IDC), either on a commercial tissue microarray (n = 59 samples) or obtained from Albany Medical Center archives (n = 68 samples). Immunostaining intensity for the integrin α3 subunit or COX2 was scored, and Spearman's rank correlation coefficient analysis was performed to assess their co-expression across and within different tumor subtypes or clinicopathologic criteria.Although expression of integrin α3 or COX2 varied among clinical IDC samples, a statistically significant, positive correlation was detected between α3 and COX2 in both tissue microarrays (r(s) = 0.49, p < 0.001, n = 59) and archived samples (r(s) = 0.59, p < 0.0001, n = 68). In both sample sets, this correlation was independent of hormone receptor status, histological grade, or disease stage.COX2 and α3 are correlated in IDC independently of hormone receptor status or other clinicopathologic features, supporting the hypothesis that integrin α3β1 is a determinant of COX2 expression in human breast cancer. These results support the clinical relevance of α3β1-dependent COX2 gene expression that we reported previously in breast cancer cells. The findings also suggest that COX2-positive breast carcinomas of various subtypes might be vulnerable to therapeutic strategies that target α3β1, and that α3 expression might serve as an independent prognostic biomarker. 
 Cyclooxygenase-2 expression is a prognostic biomarker for non-small cell lung cancer patients treated with adjuvant platinum-based chemotherapy. Adjuvant chemotherapy after the resection of stage IB-IIIA non-small cell lung cancer (NSCLC) is now the standard of care based on large-scale phase III trials and a meta-analysis. However, chemotherapy has plateaued in terms of its efficacy, and the search for treatment prediction biomarkers is imperative for the further identification of treatable subgroups. Therefore, we investigated the significance of cyclooxygenase-2 (Cox-2) expression and the applicability of a Cox-2 inhibitor in patients who had received adjuvant chemotherapy.We conducted a retrospective review of data from 97 patients who had received adjuvant chemotherapy. The adjuvant chemotherapy consisted of an oral tegafur agent (OT) or platinum-based chemotherapy (PB). The criteria for regimen selection were based on a discussion among the cancer board and enrollment in a clinical trial. Immunohistochemical staining (IHC) for Cox-2 was performed, and the correlation between Cox-2 expression and disease-free survival (DFS) was evaluated.IHC showed that 56 cases (57.7%) were positive for Cox-2. The rate of Cox-2 expression was similar for the PB and OT groups. Among the patients who received PB, the DFS of the patients with Cox-2 expression was significantly poorer than that of the patients without Cox-2 expression (P = 0.017), but there was no significant difference among the patients who received OT (P = 0.617). In a multivariate analysis, Cox-2 expression and lym  ph node me  tastasis were independent predictors of DFS among patients who received PB.Cox-2 expression was a powerful predictor of DFS among patients who received PB as an adjuvant chemotherapy. Further study investigating the use of a Cox-2 inhibitor for adjuvant chemotherapy is needed. 
 Cyclooxygenase-2 expression and its association with angiogenesis, Helicobacter pylori, and clinicopathologic characteristics of gastric carcinoma. Cyclooxygenase-2 (COX-2) is upregulated in gastric carcinoma, and its increased levels were found to have a prognostic significance in some studies. Both angiogenesis and Helicobacter pylori infection have been reported to be associated with COX-2 expression of gastric cancer in recent studies. In this study, COX-2 expression and its association with CD31 staining, H.-pylori infection, and well-known clinicopathological factors were investigated in 65 gastric cancer patients. COX-2 and CD31 expression assessment was done by immunohistochemical methods. Whartin Starry stain was performed for H.-pylori infection. Of 65 patients, 32 (49%) revealed intense COX-2 immunostaining. Among various clinicopathologic characteristics, COX-2 expression was inversely correlated with tumor size, TNM stage, and lymph node status. Thirty-two (49%) patients revealed intense CD31 immunostaining. Among various clinicopathologic characteristics, CD31 expression was associated only with lymph node   metastasis  . COX-2 expression was not correlated with CD31 staining and H.-pylori infection. Both COX-2 and CD31 staining had no prognostic significance. In conclusion, we found that COX-2 expression was significantly higher in earlier stages of gastric cancer. It can be suggested that COX-2 expression may be important in the initial development of gastric cancer but not in progression of the disease. Other factors which may be associated with COX-2 in gastric cancer, including angiogenesis and H.-pylori infection, should be investigated in further studies. 
 Association of cyclooxygenase-2 and matrix metalloproteinase-2 expression in human breast cancer. Expression of cyclooxygenase-2 (COX-2) and matrix metalloproteinase-2 (MMP-2) associates with reduced survival in human breast cancer. COX-2 may be directly involved with mammary carcinogenesis, since expression of COX-2 is sufficient for formation of breast tumors in transgenic mice, and COX-2 selective inhibitors can suppress tumorigenesis in rodent models of breast cancer. MMP-2 is an extracellular matrix degrading proteolytic enzyme that bas been linked to invasion and   metastasis  . A direct link between COX-2 and MMP-2 may exist, since inhibition of COX-2 activity can result in reduction of MMP-2 expression and activity. In this study we analyzed protein expression of COX-2 and MMP-2 in tissue array specimens of 278 invasive breast cancers by immunohistochemistry. Immunopositivity of these two markers was correlated with each other and with various clinicopathological parameters including survival. We found high COX-2 expression in 30% and high MMP-2 expression in 83% of the breast cancer specimens, and there was a positive association between the expression of these two factors (p = 0.003). It was especially evident that whenever COX-2 expression was high, MMP-2 expression was almost invariably high (95%). Furthermore, high expression of either COX-2 or MMP-2 associated with decreased disease specific survival when compared with the COX-2 or MPP-2 low group (p = 0.026 and p = 0.021, respectively). Taken together, our results indicate that expression of COX-2 protein is associated with expression of MMP-2 protein in human breast cancer and that both COX-2 and MMP-2 are markers of poor prognosis in breast cancer. 
  GeneRIFs  
 Findings suggest that P50-associated COX-2 extragenic RNA (PACER) promotes proliferation and   metastasis   of osteosarcoma cells by activating the cyclooxygenase-2 (COX-2) gene and its own expression was influenced by DNA methylation. 
 High expression of PTGS2 is associated with colorectal cancer liver   metastasis  . 
 Palbociclib inhibits epithelial-mesenchymal transition and   metastasis   in breast cancer via c-Jun/COX-2 signaling pathway. 
 COX-2 and Ki-67 are abundantly expressed in hepatolithiasis and bile duct carcinoma tissues and may play an important role in the disease occurrence, progression, and   metastasis   
 COX-2 and HER-2 are important markers for invasion and   metastasis   of colorectal cancer, and they act together to regulate the invasion and   metastasis   of colorectal cancer. 
 Results demonstrate that EGF-induced COX-2 expression enhances HNSCC   metastasis   via activation of the fibronectin signaling pathway. 
 Implicated a role for COX-2 in STIM1-mediated CRC   metastasis  . 
 findings indicate that increased expression of MMP-7 is associated with lymph node   metastasis   and upregulated by COX-2, and promotes the tumorigenesis of LAC 
 Overexpression of PTGS2 is associated with lymph node   metastasis   in large, high-grade breast tumors. 
 breast tumors with both cyclooxygenase-2 and progesterone receptor overexpression were related with tumor lower size and lower axillary node   metastasis   
 Data indicate that not only Cox-2 but also EP1 and EP3 could be important targets for chemosensitization and inhibition of   metastasis   in breast cancers that are resistant to chemotherapy. 
 VEGF-C expression was upregulated by COX-2 in OSCC. High VEGF-C expression appears to promote peritumoral lymphangiogenesis. data indicated lymph node   metastasis   is promoted by COX-2 and VEGF-C in OSCC. 
 results indicate that COX-2 promotes tumor lymphangiogenesis and lymph node   metastasis   of oral squamous cell carcinoma 
 Report nuclear co-localization and functional interaction of COX-2 and HIF-1alpha characterize bone   metastasis   of human breast carcinoma. 
 Cox-2 activity is necessary for the proliferation and   metastasis   of gastric cancer cells 
 COX-2 and MMP-2 expression was increased in nasopharyngeal carcinoma, and was positively correlated with lymph node   metastasis  . 
 COX-2 inhibition may diminish lymph node   metastasis   by suppressing VEGF-C-mediated lymphangiogenesis 
 LMP1 induce expression of VEGFC and COX2 which may contribute to lymphatic   metastasis   and development of nasopharyngeal carcinoma. 
 The expression of COX-2 was positively correlated with the size of the cancer and lymphatic   metastasis  . 
 COX2 and LMP1 were highly expressed in nasopharyngeal carcinoma cells. Expression was significantly associated with neck lymph node   metastasis  . 
 The expression of both PPAR delta and COX-2 in tissues may lead to liver   metastasis   and consequent poor prognosis in colorectal cancer patients. 
 HIF-1alpha and COX-2 could upregulate each other to promote the development and   metastasis   of laryngeal squamous carcinoma. 
 COX-2 and VEGF-C were highly expressed in papillary thyroid carcinoma, with possible interaction of their expressions, and may play a critical role in the cervical lymph node   metastasis  . 
 The expression of COX-2 may play a crucial role in the carcinogenesis and development of laryngeal squamous cell carcinoma and cervical lymph node   metastasis  . 
 COX-2 may represent a candidate gene for blocking tumor lymphangiogenesis and lymph node   metastasis  . 
 Data show that late-stage tumors and lymph node   metastasis   were associated with significant increase in serum COX-2 levels. 
 Overexpression of COX-2 increased hematogenous   metastasis  , at least in KB cells, via down-regulating E-cadherin and up-regulating CD44 expression. 
 COX-2 overexpression in breast cancer (in both lobular & ductal carcinomas) correlates with clinico-pathological parameters of the disease (including lymph node   metastasis  ) & appears to be a negative prognostic marker. 
 TLR9 up-regulates COX-2 expression in prostate cancer cells, at least partially through the activation of NF-kappaB, which may be implicated in tumor invasion and   metastasis  . 
 COX-2 and MMP-9 have synergistic effects on proliferation, invasion and   metastasis   of cancer cells. 
 COX2 produced in primary breast cancer cells may be vital to the initial development of bone marrow micro  metastasis   
 COX-2 and P-glycoprotein are highly expressed in relapsed B-cell non-Hodgkin's lymphoma, and they may play a role in tumor   metastasis  . 
 COX-2 may play a pivotal role in lymphangiogenesis and lymph node   metastasis   of prostate cancer via the regulation of VEGF-C expression. 
 COX-2 and angiogenic factors have roles in primary colorectal cancer and liver   metastasis   
 LPA2 and Gi/Src pathways are significant for LPA-induced COX-2 expression and cell migration that could be a promising drug target for ovarian cancer cell   metastasis  . 
 Cyclooxygenase 2 expression was significantly associated with lymph node   metastasis  . 
 In breast cancer patients treated with CS+RT, COX-2 expression is associated with younger age, larger tumor size, worse local control, distant   metastasis  , and worse overall survival. 
 COX-2 may up-regulate VEGF-C expression and thus promote lymph node   metastasis   via lymphangiogenesis pathway in human breast cancer. 
 Osteopontin, cyclooxygenase-2 and vascular endothelial growth factor synergically promote angiogenesis and   metastasis   in gastric cancer. 
 Inhibitory effect of a selective cyclooxygenase-2 inhibitor on liver   metastasis   of colon cancer. 
 Expression of Cox-2 is elevated in gastric adenocarcinomas, which correlates with several clinicopathological parameters, including depth of invasion and lymph node   metastasis  . 
 cyclooxygenase-2 elevation is related to lymph node   metastasis   in adenocarcinoma of uterine cervix 
 Orthotopic implantation of a colon cancer xenograft induces high expression of cyclooxygenase-2.COX-2 plays an important role in organ-specific liver   metastasis   of colon cancer. 
 Significantly higher expression of cyclooxygenase 2 is associated with   metastasis   in non-small cell lung cancer 
 in human colorectal carcinoma, vascular endothelial growth factor-C and cyclooxygenase-2 are coexpressed and significantly associated with lymph node   metastasis   and prognosis 
 COX-2 up-regulation was significantly associated with sentinel lymph node and bone marrow micro-  metastasis   in breast cancer patients 
 Expression in esophageal squamous cell carcinomas related to COX-2 expression. Also associated with depth of primary tumor, stage, and probably lymph node   metastasis  . May assist in management planning. 
 epiregulin, COX2, and MMP1 and 2 collectively facilitate the assembly of new tumour blood vessels, the release of tumour cells into the circulation, and the breaching of lung capillaries by circulating tumour cells to seed pulmonary   metastasis   
 data collectively imply COX-2 may play an important role during premalignant hyperproliferation, as well as the later stages of invasive carcinoma and   metastasis   in various human epithelial cancers 
 COX-2 may play an important role in the development of gastric cancer, and the over-expression of COX-2 protein may be a high risk factor for liver   metastasis  . 
 abnormal expressions of COX-2, p53, PCNA, and nm23 associate with malignant potential, lymph node   metastasis   and clinical stage, and they might therefore play a role in development of gastric cancer 
 COX-2 overexpression may play a crucial role in the carcinogenesis, development of cancer and lymph node   metastasis   in breast cancer patients. 
 COX-2 overexpression in human breast cancer cells enhances cell motility and invasiveness thus suggesting a mechanism of COX-2 mediated   metastasis   
  [Cc]ell [Ii]nvasion  PubMed  
 Prostaglandin E2 stimulates β1-integrin expression in hepatocellular carcinoma through the EP1 receptor/PKC/NF-κB pathway. Prostaglandin E2 (PGE2) has been implicated i  n cell invasi  on in hepatocellular carcinoma (HCC), via increased β1-integrin expression and cell migration; however, the mechanism remains unclear. PGE2 exerts its effects via four subtypes of the E prostanoid receptor (EP receptor 1-4). The present study investigated the effect of EP1 receptor activation on β1-integrin expression and cell migration in HCC. Cell migration increased by 60% in cells treated with 17-PT-PGE2 (EP1 agonist), which was suppressed by pretreatment with a β1-integrin polyclonal antibody. PGE2 increased β1-integrin expression by approximately 2-fold. EP1 receptor transfection or treatment with 17-PT-PGE2 mimicked the effect of PGE2 treatment. EP1 siRNA blocked PGE2-mediated β1-integrin expression. 17-PT-PGE2 treatment induced PKC and NF-κB activation; PKC and NF-κB inhibitors suppressed 17-PT-PGE2-mediated β1-integrin expression. FoxC2, a β1-integrin transcription factor, was also upregulated by 17-PT-PGE2. NF-κB inhibitor suppressed 17-PT-PGE2-mediated FoxC2 upregulation. Immunohistochemistry showed p65, FoxC2, EP1 receptor and β1-integrin were all highly expressed in the HCC cases. This study suggested that PGE2 upregulates β1-integrin expression and cell migration in HCC cells by activating the PKC/NF-κB signaling pathway. Targeting PGE2/EP1/PKC/NF-κB/FoxC2/β1-integrin pathway may represent a new therapeutic strategy for the prevention and treatment of this cancer. 
 The hepatitis B virus X protein promotes tumor   cell invasion   by inducing membrane-type matrix metalloproteinase-1 and cyclooxygenase-2 expression. Hepatocellular carcinoma is strongly associated with chronic infection by the hepatitis B virus (HBV) and has poor prognosis due to intrahepatic metastasis. HBx is often the only HBV protein detected in hepatic tumor cells; however, its contribution to tumor invasion and metastasis has not been established so far. In this work, we show that HBx enhances tumor   cell invasion  , both in vivo and in vitro. The increased invasive capacity induced by HBx is mediated by an upregulation of membrane-type 1 matrix metalloproteinase (MT1-MMP) expression, which in turn activates matrix metalloproteinase-2. Induction of both MT1-MMP expression and   cell invasion   by HBx is dependent on cyclooxygenase-2 (COX-2) activity. In addition, HBx upregulates the expression of COX-2, which is mediated by the transcriptional activation of the COX-2 gene promoter in a nuclear factor of activated T cell-dependent (NF-AT-dependent) manner. These results demonstrate the ability of HBx to promote tumor   cell invasion   by a mechanism involving the upregulation of MT1-MMP and COX-2 and provide new insights into the mechanism of action of this viral protein and its involvement in tumor metastasis and recurrence of hepatocellular carcinoma. 
 Hepatitis C virus NS3 protein enhances cancer   cell invasion   by activating matrix metalloproteinase-9 and cyclooxygenase-2 through ERK/p38/NF-κB signal cascade. Hepatitis C virus (HCV) infection causes acute and chronic hepatitis, liver cirrhosis, and hepatocellular carcinoma (HCC). However, the mechanisms by which HCV causes the diseases are largely unknown. Here, we elucidated the effects of HCV on the invasion and migration of hepatoma cells, with the aim to reveal the mechanism by which HCV infection induces HCC. We initially showed that matrix metalloproteinase-9 (MMP-9) was elevated in the sera of HCV-infected patients, and demonstrated that HCV nonstructural protein 3 (NS3) activated MMP-9 transcription through nuclear factor-κB (NF-κB) by stimulating translocation of NF-κB from cytosol to the nucleus to enhance its binding to MMP-9 promoter. In addition, cyclooxygenase-2 (COX-2) and extracellular signal-regulated kinase (ERK1/2)/mitogen-activated protein kinase (p38) pathway were involved in HCV-activated MMP-9 expression. Moreover, NS3 enhanced hepat  oma cell inva  sion and migration through MMP-9 and COX-2. Thus, this study provides new insights into the roles of HCV NS3, MMP-9 and COX-2 in regulating can  cer cell inva  sion. 
 Prostaglandin E2 stimulates β1-integrin expression in hepatocellular carcinoma through the EP1 receptor/PKC/NF-κB pathway. Prostaglandin E2 (PGE2) has been implicated i  n cell invasi  on in hepatocellular carcinoma (HCC), via increased β1-integrin expression and cell migration; however, the mechanism remains unclear. PGE2 exerts its effects via four subtypes of the E prostanoid receptor (EP receptor 1-4). The present study investigated the effect of EP1 receptor activation on β1-integrin expression and cell migration in HCC. Cell migration increased by 60% in cells treated with 17-PT-PGE2 (EP1 agonist), which was suppressed by pretreatment with a β1-integrin polyclonal antibody. PGE2 increased β1-integrin expression by approximately 2-fold. EP1 receptor transfection or treatment with 17-PT-PGE2 mimicked the effect of PGE2 treatment. EP1 siRNA blocked PGE2-mediated β1-integrin expression. 17-PT-PGE2 treatment induced PKC and NF-κB activation; PKC and NF-κB inhibitors suppressed 17-PT-PGE2-mediated β1-integrin expression. FoxC2, a β1-integrin transcription factor, was also upregulated by 17-PT-PGE2. NF-κB inhibitor suppressed 17-PT-PGE2-mediated FoxC2 upregulation. Immunohistochemistry showed p65, FoxC2, EP1 receptor and β1-integrin were all highly expressed in the HCC cases. This study suggested that PGE2 upregulates β1-integrin expression and cell migration in HCC cells by activating the PKC/NF-κB signaling pathway. Targeting PGE2/EP1/PKC/NF-κB/FoxC2/β1-integrin pathway may represent a new therapeutic strategy for the prevention and treatment of this cancer. 
 Hepatitis C virus NS3 protein enhances cancer   cell invasion   by activating matrix metalloproteinase-9 and cyclooxygenase-2 through ERK/p38/NF-κB signal cascade. Hepatitis C virus (HCV) infection causes acute and chronic hepatitis, liver cirrhosis, and hepatocellular carcinoma (HCC). However, the mechanisms by which HCV causes the diseases are largely unknown. Here, we elucidated the effects of HCV on the invasion and migration of hepatoma cells, with the aim to reveal the mechanism by which HCV infection induces HCC. We initially showed that matrix metalloproteinase-9 (MMP-9) was elevated in the sera of HCV-infected patients, and demonstrated that HCV nonstructural protein 3 (NS3) activated MMP-9 transcription through nuclear factor-κB (NF-κB) by stimulating translocation of NF-κB from cytosol to the nucleus to enhance its binding to MMP-9 promoter. In addition, cyclooxygenase-2 (COX-2) and extracellular signal-regulated kinase (ERK1/2)/mitogen-activated protein kinase (p38) pathway were involved in HCV-activated MMP-9 expression. Moreover, NS3 enhanced hepat  oma cell inva  sion and migration through MMP-9 and COX-2. Thus, this study provides new insights into the roles of HCV NS3, MMP-9 and COX-2 in regulating can  cer cell inva  sion. 
 The hepatitis B virus X protein promotes tumor   cell invasion   by inducing membrane-type matrix metalloproteinase-1 and cyclooxygenase-2 expression. Hepatocellular carcinoma is strongly associated with chronic infection by the hepatitis B virus (HBV) and has poor prognosis due to intrahepatic metastasis. HBx is often the only HBV protein detected in hepatic tumor cells; however, its contribution to tumor invasion and metastasis has not been established so far. In this work, we show that HBx enhances tumor   cell invasion  , both in vivo and in vitro. The increased invasive capacity induced by HBx is mediated by an upregulation of membrane-type 1 matrix metalloproteinase (MT1-MMP) expression, which in turn activates matrix metalloproteinase-2. Induction of both MT1-MMP expression and   cell invasion   by HBx is dependent on cyclooxygenase-2 (COX-2) activity. In addition, HBx upregulates the expression of COX-2, which is mediated by the transcriptional activation of the COX-2 gene promoter in a nuclear factor of activated T cell-dependent (NF-AT-dependent) manner. These results demonstrate the ability of HBx to promote tumor   cell invasion   by a mechanism involving the upregulation of MT1-MMP and COX-2 and provide new insights into the mechanism of action of this viral protein and its involvement in tumor metastasis and recurrence of hepatocellular carcinoma. 
 Lipopolysaccharide initiates a positive feedback of epidermal growth factor receptor signaling by prostaglandin E2 in human biliary carcinoma cells. Bacterial products (e.g., LPS) are viewed as critical stimuli in inflammation-associated cancer. Cyclooxygenase 2 (COX-2), a major effector of LPS, and EGFR, are key to carcinogenesis, notably in the hepatobiliary tract. In this study, we tested the hypothesis that LPS can initiate an interaction between the epidermal growth factor receptor (EGFR) and COX-2 pathways. We examined the effect of LPS in biliary carcinoma cells that displayed constitutive COX-2 expression and PGE(2) production and in normal human biliary epithelial cells in which COX-2/PGE(2) expression was virtually absent. LPS induced early phosphorylation of EGFR and ERK1/2 in both types of cells, which reached maximum levels within 30 min (first phase). However, only the carcinoma cells showed a second significant rise in both EGFR and ERK phosphorylation 6 h after exposure to LPS (second phase). Inhibition of COX-2/PGE(2) production prevented the second, but not the first, phase of EGFR and ERK1/2 phosphorylation, implicating COX-2/PGE(2) in the second phase of phosphorylation. LPS induced COX-2-derived PGE2 production at 4 h, which was before the rise in the second phosphorylation that occurred at 6 h. Exogenous PGE(2) also caused EGFR activation via a signaling pathway involving TACE-dependent TGF-alpha release. Inhibition of the second phase of EGFR phosphorylation with EGFR or COX-2 inhibitor prevented LPS-induced   cell invasion   in vitro, demonstrating the biological importance of this COX-2 feedback signaling in cancer cells. We conclude that LPS triggers a positive feedback loop involving COX-2/PGE(2) in biliary carcinoma cells and that this second phase of EGFR phosphorylation is implicated in   cell invasion   by LPS. 
 Cyclooxygenase-2-derived prostaglandin E2 stimulates Id-1 transcription. Cyclooxygenase-2 (COX-2) and Id-1 are overexpressed in a variety of human malignancies. Recently, each of these genes was found to play a role in mediating breast cancer metastasis to the lungs, but their potential interdependence was not evaluated. Hence, the main objective of the current study was to determine whether COX-2-derived prostaglandin (PGE(2)) activated Id-1 transcription, leading in turn to increased invasiveness of mammary epithelial cells. In MDA-MB-231 cells, treatment with PGE(2) induced Id-1, an effect that was mimicked by an EP(4) agonist. PGE(2) via EP(4) activated the epidermal growth factor receptor (EGFR) --> ERK1/2 pathway, which led to increased expression of Egr-1. PGE(2) stimulated EGFR signaling by inducing the release of amphiregulin, an EGFR ligand. The ability of PGE(2) to activate Id-1 transcription was mediated by enhanced binding of Egr-1 to the Id-1 promoter. Silencing of COX-2 or pharmacological inhibition of COX-2 led to reduced PGE(2) production, decreased Id-1 expression, and reduced migration of cells through extracellular matrix. A similar decrease in cell migration was found when Id-1 was silenced. The interrelationship between COX-2, PGE(2), Id-1, and cell invasiveness was also compared in nontumorigenic SCp2 and tumorigenic SCg6 mammary epithelial cells. Consistent with the findings in MDA-MB-231 cells, COX-2-derived PGE(2) induced Id-1, leading in turn to increased cell invasiveness. Taken together, these results suggest that PGE(2) via EP(4) activated the EGFR --> ERK1/2 --> Egr-1 pathway, leading to increased Id-1 transcription and   cell invasion  . These findings provide new insights into the relationship between COX-2 and Id-1 and their potential role in metastasis. 
 Functional polymorphisms of cyclooxygenase-2 (COX-2) gene and risk for urinary bladder cancer in North India. Cyclooxygenase-2 (COX-2) is an enzyme involved in the synthesis of prostaglandins and thromboxanes, which are regulators of processes that are relevant to cancer development. It is involved in carcinogenesis, immune response suppression, apoptosis inhibition, angiogenesis, and tumor   cell invasion   and metastasis. The gene for COX-2, designated as prostaglandin-endoperoxide synthase 2 (PTGS-2), carries polymorphisms, such as -765G>C, 1195G>A in the promoter region, and 8473T>C in the 3'-untranslated region (UTR), which have been associated with susceptibility to malignant disease.We undertook a case-control study of 212 urothelial bladder cancer (UBC) cases and 250 controls to investigate the association between COX-2 polymorphism and bladder cancer susceptibility, using the polymerase chain reaction restriction fragment length polymorphism (PCR-RFLP) method and also investigated gene-environment interactions.Cox-2 765G>C, a variant(C) allele carrier, was at an increased risk of UBC (odds ratio [OR] = 1.90; P = .004); however, -1195G>A; -1290A>G; and 3'UTR 8473T>C polymorphisms of COX-2 gene were not significantly associated with UBC. 765G>C also was associated with the invasive stage of a bladder tumor (OR = 2.73; P = .033). High risk for UBC also was observed with respect to COX-2 haplotypes C-765T8473A-1195A-1290 (OR = 3.47; P = .014). In case-only analysis, COX-2 765 variant allele carrier genotypes also showed an increased risk among former and current smokers (OR = 3.06; P = .041 and OR = 4.39; P = .032, respectively).COX-2 -765G>C polymorphism confers UBC susceptibility particularly in smokers and in patients with invasive tumors. 765C allele carrier genotypes also are influenced with a high risk of recurrence in Bacillus Calmette-Guérin-treated patients. Collectively, these findings confirm that the COX-2 -765G>C polymorphism is a risk factor for the development of bladder cancer and can provide a plausible mechanistic explanation. Further validation in large population-based studies is needed. 
 RhoA mediates cyclooxygenase-2 signaling to disrupt the formation of adherens junctions and increase cell motility. Cyclooxygenase-2 (COX-2) represents an important target for treatment and prevention of colorectal cancer. Although COX-2 signaling is implicated in promoting tumor cell growth and invasion, the molecular mechanisms that mediate these processes are largely unknown. In this study, we show that the RhoA pathway mediates COX-2 signaling to disrupt the formation of adherens junctions and increase cell motility. Disruption of adherens junctions promotes tumor   cell invasion   and metastasis and is often associated with tumor progression. We detected high levels of RhoA activity in HCA-7 colon carcinoma cells that constitutively express COX-2. Inhibition of COX-2 significantly reduced the levels of RhoA activity in HCA-7 cells, suggesting that constitutive expression of COX-2 stimulates RhoA activity. Interestingly, inhibition of COX-2 or silencing of COX-2 expression with small interfering RNA (siRNA) stimulated the formation of adherens junctions, concomitant with increased protein levels of E-cadherin and alpha-catenin. Furthermore, inhibition of RhoA or silencing of RhoA expression with siRNA increased the levels of E-cadherin and alpha-catenin. Inhibition of Rho kinases (ROCK), the RhoA effector proteins, also increased levels of E-cadherin and alpha-catenin and stimulated formation of adherens junctions. The motility of HCA-7 cells was significantly decreased when COX-2 or RhoA was inhibited. Therefore, our data reveal a novel molecular mechanism that links COX-2 signaling to disrupt the formation of adherens junctions; COX-2 stimulates the RhoA/ROCK pathway, which reduces levels of E-cadherin and alpha-catenin leading to disruption of adherens junction formation and increased motility. Understanding of COX-2 downstream signaling pathways that promote tumor progression is crucial for the development of novel therapeutic strategies. 
 COX2 and PGE2 mediate EGF-induced E-cadherin-independent human ovarian cancer   cell invasion  . Elevated expression of cyclooxygenase 2 (COX2 (PTGS2)) has been reported to occur in human ovarian cancer and to be associated with poor prognosis. We have previously demonstrated that COX2-derived prostaglandin E2 (PGE2) promotes human ovarian cancer   cell invasion  . We had also demonstrated that epidermal growth factor (EGF) induces human ovarian cancer   cell invasion   by downregulating the expression of E-cadherin through various signaling pathways. However, it remains unclear whether COX2 and PGE2 are involved in the EGF-induced downregulation of E-cadherin expression and   cell invasion   in human ovarian cancer cells. In this study, we showed that EGF treatment induces COX2 expression and PGE2 production in SKOV3 and OVCAR5 human ovarian cancer cell lines. Interestingly, COX2 is not required for the EGF-induced downregulation of E-cadherin expression. In addition, EGF treatment activates the phosphatidylinositol-3-kinase (PI3K)/Akt and cAMP response element-binding protein (CREB) signaling pathways, while only the PI3K/Akt pathway is involved in EGF-induced COX2 expression. Moreover, we also showed that EGF-induced   cell invasion   is attenuated by treatment with a selective COX2 inhibitor, NS-398, as well as PGE2 siRNA. This study demonstrates an important role for COX2 and its derivative, PGE2, in the mediation of the effects of EGF on human ovarian cancer   cell invasion  . 
 The association between cyclooxygenase-2 1195 G/A polymorphism and hepatocellular carcinoma: evidence from a meta-analysis. Cyclooxygenase-2 (COX-2) is proven to influence the carcinogenesis through immune response suppression, apoptosis inhibition, angiogenesis regulation, and tumor   cell invasio  n. Previous studies assessing the association between COX-2 1195 G/A polymorphism and susceptibility to hepatocellular carcinoma (HCC) reported conflicting results. The objective of the study was to investigate the association between COX-2 1195 G/A polymorphism and HCC by a meta-analysis. PubMed, Embase, Web of Science, and Wangfang databases were searched for studies investigating the association between COX-2 1195 G/A polymorphism and HCC risk. The pooled odds ratio (OR) and its 95 % confidence interval (CI) were used to assess the strength of the association. Five studies with a total of 1,690 HCC cases and 1,961 controls were identified. Meta-analyses of total included studies showed that there was an obvious association between COX-2 1195 G/A polymorphism and HCC risk under two main genetic models (for AA versus GG, fixed-effects OR=1.45, 95 % CI 1.15-1.81, P=0.001, I (2) =0.0 %; for AA/GA versus GG, fixed-effects OR=1.26, 95 % CI 1.05-1.51, P=0.011, I (2) =0.0 %). Subgroup analysis by ethnicity showed that association was still obvious in Asians under two genetic models (for AA versus GG, fixed-effects OR=1.45, 95 % CI 1.16-1.82, P=0.001, I (2) =21.7 %; for AA/GA versus GG, fixed-effects OR=1.27, 95 % CI 1.05-1.54, P=0.013, I (2) =0.4 %). The evidence from the meta-analysis supports an association between COX-2 1195 G/A polymorphism and HCC risk in Asians. Further studies with large sample and careful design are needed to identify the possible association in Caucasians. 
 Involvement of Cox-2 in the metastatic potential of chemotherapy-resistant breast cancer cells. A major problem with the use of current chemotherapy regimens for several cancers, including breast cancer, is development of intrinsic or acquired drug resistance, which results in disease recurrence and metastasis. However, the mechanisms underlying this drug resistance are unknown. To study the molecular mechanisms underlying the invasive and metastatic activities of drug-resistant cancer cells, we generated a doxorubicin-resistant MCF-7 breast cancer cell line (MCF-7/DOX).We used MTT (3-(4,5-dimethylthiazol-2-yl)-2,5-diphenyltetrazolium bromide) assays, flow cytometry assays, DNA fragmentation assays, Western blot analysis,   cell invasion   assays, small interfering RNA (siRNA) transfection, reverse transcription-polymerase chain reaction, experimental lung metastasis models, and gelatin and fibrinogen/plasminogen zymography to study the molecular mechanism of metastatic activities in MCF-7/DOX cells.We found that MCF-7/DOX acquired invasive activities. In addition, Western blot analysis showed increased expression of epidermal growth factor receptor (EGFR) and Cox-2 in MCF-7/DOX cells. Inhibition of Cox-2, phosphoinositide 3-kinase (PI3K)/Akt, or mitogen-activated protein kinase (MAPK) pathways effectively inhibited the invasive activities of MCF-7/DOX cells. Gelatin and fibrinogen/plasminogen zymography analysis showed that the enzymatic activities of matrix metalloproteinase-2 (MMP-2), MMP-9, and urokinase-type plasminogen activator were markedly higher in MCF-7/DOX cells than in the MCF-7 cells. In vitro invasion assays and mouse models of lung metastasis demonstrated that MCF-7/DOX cells acquired invasive abilities. Using siRNAs and agonists specific for prostaglandin E (EP) receptors, we found that EP1 and EP3 played important roles in the invasiveness of MCF-7/DOX cells.We found that the invasive activity of MCF-7/DOX cells is mediated by Cox-2, which is induced by the EGFR-activated PI3K/Akt and MAPK pathways. In addition, EP1 and EP3 are important in the Cox-2-induced invasion of MCF-7/DOX cells. Therefore, not only Cox-2 but also EP1 and EP3 could be important targets for chemosensitization and inhibition of metastasis in breast cancers that are resistant to chemotherapy. 
 Frequent co-localization of Cox-2 and laminin-5 gamma2 chain at the invasive front of early-stage lung adenocarcinomas. Laminin-5 is an extracellular matrix protein that plays a key role in cell migration and tumor invasion. Cox-2 is an induced isoform of cyclooxygenases that plays an important role in carcinogenesis, suppression of apoptosis, angiogenesis, and metastasis of colon cancer. We report frequent co-expression of cox-2 and laminin-5 at the invasive front of early-stage lung adenocarcinomas. We investigated the expression of cox-2 and laminin-5 immunohistochemically in 102 cases of small-sized lung adenocarcinoma (maximum dimension, 2 cm or less). Cox-2 and laminin-5 were expressed in 97 (95.1%) and 82 (80.4%) cases, respectively. Both were preferentially localized in cancer cells at the cancer-stroma interface, although cox-2 tended to show a diffuse staining pattern in some cases. A comparison of their staining patterns revealed a striking similarity in their distribution in 24 cases, and a partial overlap between their localization in another 20 cases. Moreover, an overall correlation was found between the expression levels of cox-2 and laminin-5 (P = 0.018). To gain insight into the mechanisms that regulate the expression of these proteins, we additionally studied their expression in 58 cases of stage I lung adenocarcinoma, in which p53 status was determined by immunohistochemistry, polymerase chain reaction-single strand conformation polymorphism analysis, and direct sequencing. The results showed that tumors with mutant p53 tended to express more cox-2 than those with wild-type p53 (P = 0.080). Also, tumors that overexpressed p53 had higher levels of cox-2 and laminin-5 than those without p53 overexpression (P = 0.032 and 0.047, respectively). Further immunohistochemical analysis showed that tumors that overexpressed both epidermal growth factor receptor (EGFR) and erbB-2 had higher levels of cox-2 and laminin-5 than those without concomitant overexpression of these proteins (P = 0.014 and P = 0.018, respectively). To see whether EGFR signaling is involved in cox-2 and laminin-5 expression, we further conducted in vitro analyses using six lung adenocarcinoma cell lines (A549, HLC-1, ABC-1, LC-2/ad, VMRC-LCD, and L27). Western blot analyses showed that cox-2 mRNA levels, and to a lesser extent laminin-5 gamma2 mRNA levels, correlated with the expression levels of erbB-2 and the phosphorylated form of MAPK/ERK-1/2 protein. The addition of transforming growth factor-alpha increased both cox-2 and laminin-5 gamma2 mRNA levels in A549, ABC-1, and L27 with different kinetics; the induction of cox-2 occurred earlier than that of laminin-5 gamma2. Finally, the migration of ABC-1 cells was inhibited by MAP kinase kinase inhibitor PD98059 and a selective cox-2 inhibitor NS-398. In contrast, the migration of A549 cells was inhibited by PD98059, but much less effectively by NS-398. These results suggest that co-stimulatory mechanisms may exist that increase the expression of cox-2 and laminin-5 at the invasive front of lung adenocarcinomas and that EGFR signaling could be one of the mechanisms. Further investigations are warranted concerning the role of cox-2 and laminin-5 in cancer   cell invasion   and the significance of p53 and EGFR signaling in the regulation of cox-2 and laminin-5 expression. 
 The cyclooxygenase-2 (PTGS2) 8473T>C polymorphism is associated with breast cancer risk. Cyclooxygenase-2 (COX-2) is involved in carcinogenesis, immune response suppression, apoptosis inhibition, angiogenesis, and tumor   cell invasion   and metastasis. The gene for COX-2, designated as PTGS2, carries a common polymorphism at position 8473 in the 3'-untranslated region (PTGS2 8473T>C), which has been associated with susceptibility to malignant disease. To investigate the role of this polymorphism for breast cancer, we determined the prevalence of PTGS2 genotypes in 500 women with breast cancer and 500 sex- and age-matched healthy control subjects. Homozygous carriers of the 8473-CC genotype were more frequent among patients (12.4%) than among controls (6.6%; P = 0.002). The odds ratio for carriers of this genotype for breast cancer was 2.1 (95% confidence interval, 1.3-3.3). Among patients, estrogen receptor positivity was less frequent among carriers of a CC genotype (63.9%) than among carriers of a TT or TC genotype (76.9%; P = 0.028). Tumor size, histologic grade, presence of primary lymph node metastases, progesterone receptor positivity, or age at diagnosis were not associated with PTGS2 genotypes. We conclude that the homozygous PTGS2 8473-CC genotype may be associated with breast cancer risk. 
 Relationship between COX-2 and cell cycle-regulatory proteins in patients with esophageal squamous cell carcinoma. To investigate the correlation between cyclooxygenase-2 (COX-2) and cell cycle-regulatory proteins in patients with esophageal squamous cell carcinoma (ESCC).One hundred and two surgically obtained specimens of ESCC were randomly collected. All specimens were obtained from patients who had not received chemo- or radiotherapy prior to surgical resection. Twenty-eight specimens of normal squamous epithelium served as controls. The expression of COX-2, Ki-67, cyclin A and p27 was examined by immunohistochemistry. The Pearson test was used to analyze the relationship between groups.The protein level of COX-2, Ki-67 and cyclin A was significantly higher in ESCC than in normal squamous epithelium (74.7 ± 61.2 vs 30.2 ± 43.4, 64.0 ± 51.6 vs 11.6 ± 2.3, 44.2 ± 32.2 vs 11.7 ± 5.0, respectively, all P < 0.01). In contrast, the protein level of p27 was significantly lower in ESCC than in normal squamous epithelium (182.0 ± 69.0 vs 266.4 ± 28.0, P < 0.01). In ESCC, COX-2 expression was correlated with T stage, the score of T1-T2 stage was lower than that of T3-T4 stage (55.0 ± 42.3 vs 83.0 ± 66.5, P < 0.05), and Ki-67, cyclin A and p27 expressions were correlated with the tumor differentiation (43.8 ± 31.7 vs 98.4 ± 84.8, 32.0 ± 19.0 vs 54.1 ± 53.7, 206.2 ± 61.5 vs 123.5 ± 68.3, respectively, all P < 0.01). COX-2 expression was positively correlated to Ki-67, cyclin A and negatively correlated to p27 expression in ESCC (r = 0.270, 0.233 and -0.311, respectively, all P < 0.05).The expression of COX-2 is correl  ated with tum  or cell invasion and is closely related to the cell proliferation in patients with ESCC. 
 Human VRK2 (vaccinia-related kinase 2) modulates tumor   cell invasion   by hyperactivation of NFAT1 and expression of cyclooxygenase-2. Human VRK2 (vaccinia-related kinase 2), a kinase that emerged late in evolution, affects different signaling pathways, and some carcinomas express high levels of VRK2. Invasion by cancer cells has been associated with NFAT1 (nuclear factor of activated T cells) activation and expression of the COX-2 (cyclooxygenase 2) gene. We hypothesized that VRK proteins might play a regulatory role in NFAT1 activation in tumor cells. We demonstrate that VRK2 directly interacts and phosphorylates NFAT1 in Ser-32 within its N-terminal transactivation domain. VRK2 increases NFAT1-dependent transcription by phosphorylation, and this effect is only detected following cell phorbol 12-myristate 13-acetate and ionomycin stimulation and calcineurin activation. This NFAT1 hyperactivation by VRK2 increases COX-2 gene expression through the proximal NFAT1 binding site in the COX-2 gene promoter. Furthermore, VRK2A down-regulation by RNA interference reduces COX-2 expression at transcriptional and protein levels. Therefore, VRK2 down-regulation reduces   cell invasion   by tumor cells, such as MDA-MB-231 and MDA-MB-435, upon stimulation with phorbol 12-myristate 13-acetate plus ionomycin. These findings identify the first reported target and function of human VRK2 as an active kinase playing a role in regulation of cancer   cell invasion   through the NFAT pathway and COX-2 expression. 
 A genetic variant in 3'-untranslated region of cyclooxygenases-2 gene is associated with risk of gastric cancer in a Chinese population. Cyclooxygenase-2 (COX-2) involves in multiple processes in carcinogenesis, including inflammation, apoptosis inhibition, immune response suppression, tumor   cell invasion  , and angiogenesis. COX-2 is overexpressed in various cancers, including gastric cancer. COX-2 is encoded by prostaglandin endoperoxide synthase 2 (PTGS2) gene. We hypothesized that potentially functional polymorphisms in PTGS2 may contribute to gastric cancer risk. To assess this hypothesis, we conducted a case-control study with 1681 gastric cancer cases and 1916 control subjects in a Chinese population to evaluate the association between a polymorphism in 3'-untranslated region of PTGS2, rs5275, and the risk of gastric cancer. Logistic regression analysis revealed that variant allele (C) of rs5275 was significantly associated with an increased risk of gastric cancer (per allele odds ratio [OR] = 1.14, 95% confidence interval [CI] = 1.01-1.29, p = 0.030). This association was more prominent in females (per allele OR = 1.42, 95% CI = 1.11-1.81, p = 0.005) and nonsmokers (per allele OR = 1.35, 95% CI = 1.14-1.59, p = 0.001). Interestingly, we detected a negative interaction between rs5275 and smoking on the gastric cancer risk (p = 0.007). Our findings indicate that PTGS2 rs5275T/C may be a candidate genetic marker for gastric cancer susceptibility. 
 Interleukin-1β induces cyclooxygenase-2 expression and promotes the invasive ability of human mesenchymal stem cells derived from ovarian endometrioma. To elucidate the role of interleukin-1β (IL-1β) on cyclooxygenase-2 (COX-2) expression and invasion of endometrioma-derived ectopic endometrial mesenchymal stem cells (EN-MSCs) and to develop an organoid method to study the invasive ability of endometrial cells.Gene expression and cell functions.Kaohsiung Medical University, Kaohsiung, Taiwan.Human eutopic and endometrioma-derived ectopic EN-MSCs were isolated from different endometrium biopsy samples after surgery for treatment of endometriosis.Chemical treatment of cell culture.Comparative analysis of genomewide messenger RNA (mRNA) expression, cell migration, and invasion abilities in cell culture and organoid culture.Gene expression profiles revealed that the expression of IL-1β and COX-2 were statistically significantly higher in ectopic EN-MSCs compared with eutopic EN-MSCs. These enhanced expressions coincided with a greater ability for cell migration and invasion in ectopic EN-MSCs and were found to be distinctly regulated by IL-1β which up-regulates COX-2 expression. Furthermore, IL-1β treatment of ectopic EN-MSCs in organoids was found to induce tentacle-like structures that mim  icked cell in  vasion.These results indicate that COX-2 and IL-1β regulate the invasion ability of ectopic EN-MSCs. The information may be useful for developing a new therapeutic strategy for endometriosis. The ex vivo invasion model will be useful for characterization of EN-MSCs. 
 Cyclooxygenase-2 uses the protein kinase C/ interleukin-8/urokinase-type plasminogen activator pathway to increase the invasiveness of breast cancer cells. Cyclooxygenase-2 (COX-2) increases breast cancer   cell invasion  . Expression of various pro-angiogenic and pro-invasive factors has been correlated with high expression of COX-2. However, whether these factors are essential to COX-2-mediated breast cancer invasion, and the mechanisms by which COX-2 increases the expression of these factors are unknown. Our microarray results indicate that higher COX-2 expression was associated with increased levels of interleukin-8 (IL-8), a key factor in breast cancer invasion and metastasis. COX-2 overexpressing cells (MCF-7/COX-2), generated by transfecting COX-2-encoding plasmids into the poorly invasive MCF-7 breast cancer cells, were more invasive and produced higher IL-8 levels than the parental cells. To investigate the role of IL-8 in COX-2-mediated invasion, MCF-7 parental cells were incubated with IL-8. Exogenous IL-8 increased the invasiveness of MCF-7 cells. IL-8 is one pathway by which COX-2 mediates breast cancer invasion. Protein kinase A (PKA) and protein kinase C (PKC) are activated by COX-2 and are involved in IL-8 regulation. Inhibition of PKC, not PKA, decreased IL-8 production and invasion in MCF-7/COX-2 cells. Activation of PKC, not PKA, increased IL-8 production and invasion in MCF-7 cells. Thus, the invasive effects of COX-2 are mediated by PKC, not PKA. Activity of the urokinase-type plasminogen activator (uPA) was increased in MCF-7 cells by COX-2 overexpression or by the addition of a PKC activator or by IL-8. Inhibition of PKC decreased uPA activity in MCF-7/COX-2 cells. Furthermore, inhibition of uPA activity decreased the invasiveness of MCF-7/COX-2 cells, indicating that uPA was essential to COX-2-mediated invasion. Herein we demonstrate for the first time a detailed mechanism by which COX-2 increases breast cancer invasion: the PKC/IL-8/uPA pathway. 
 Functional polymorphisms of cyclooxygenase-2 gene and risk for hepatocellular carcinoma. Cyclooxygenase-2 (COX-2) influences carcinogenesis through immune response suppression, apoptosis inhibition, regulation of angiogenesis and tumor   cell invasion  , and metastasis. It is now well established that COX-2 is overexpressed in many premalignant, malignant, and metastatic cancers, including hepatocellular carcinoma (HCC). DNA sequence variations in the COX-2 gene may lead to altered COX-2 production and/or activity, and so they cause inter-individual differences in the susceptibility to HCC. Functional coding region polymorphisms -1195A>G (rs689466), -765G>C (rs20417), and +8473T>C (rs5275) in the COX-2 gene have recently been shown to be associated with several human cancers but their association with HCC has yet to be investigated. We used hospital-based case-control study to assess the hypothesis that the functional COX-2 variation may affect individual susceptibility to the HCC. COX-2 polymorphisms were investigated in 129 confirmed subjects with HCC and 129 cancer-free control subjects matched on age, gender, smoking, and alcohol consumption using a polymerase chain reaction-restriction fragment length polymorphism (PCR-RFLP) assay. The distribution of the COX-2 -1195A>G and +8473T>C genotypes were not significantly different between HCC cases and control. However, proportion of the COX-2 -765CC genotype which leads to a 30% reduction of the COX-2 promoter activity was significantly lower in patients with HCC (3.1%) when compared to control subjects (11.6%) (P < 0.05). Logistic regression analyses revealed that the COX-2 -765G>C variant genotype (-765CC) was associated with a significantly decreased risk of HCC compared with the -765GG wild-type homozygotes [P < 0.05, odds ratio (OR) = 0.25, 95% confidence interval (CI) = 0.08-0.79]. Our results suggest for the first time that the -765CC genotype of COX-2 -765G>C polymorphism, causing lower COX-2 gen expression, is a genetic protective factor for HCC. However, because this is the first report concerning the COX-2 -1195A>G, -765G>C, and +8473T>C polymorphisms and the risk of HCC, independent studies are needed to validate our findings. 
 NFAT induces breast cancer   cell invasion   by promoting the induction of cyclooxygenase-2. The NFAT (nuclear factor of activated T cells) family of transcription factors plays a fundamental role in the transcriptional regulation of the immune response. However, NFATs are ubiquitously expressed, and recent evidence points to their important functions in human epithelial cells and carcinomas. Specifically, NFAT has been shown to be active in human breast and colon carcinoma cells and to promote their invasion through Matrigel. The mechanisms by which NFAT promotes invasion have not been defined. To identify NFAT target genes that induce carcinoma invasion, we have established stable breast cancer cell lines that inducibly express transcriptionally active NFAT. Gene expression profiling by cDNA microarray of cells induced to express NFAT revealed up-regulation of cyclooxygenase-2 (COX-2). Increased NFAT expression and activity induced COX-2 expression as well as prostaglandin E2 synthesis. This induction was more prominent when NFAT was activated by phorbol 12-myristate 13-acetate and calcium ionophore ionomycin and was blocked by the NFAT antagonist cyclosporin A. Breast cancer cells with elevated COX-2 expression showed increased invasion through Matrigel, and this was reduced in cells treated with COX-2 inhibitors. Conversely, loss of NFAT1 protein expression using small interfering RNA led to a reduction in COX-2 transcription and reduced invasion. Similarly, Matrigel invasion was reduced in cells in which COX-2 expression was reduced using specific siRNA. These findings demonstrate that NFAT promotes breast cancer   cell invasion   through the induction of COX-2 and the synthesis of prostaglandins. 
 Cyclooxygenase-2-dependent lymphangiogenesis promotes nodal metastasis of postpartum breast cancer. Breast involution following pregnancy has been implicated in the high rates of metastasis observed in postpartum breast cancers; however, it is not clear how this remodeling process promotes metastasis. Here, we demonstrate that human postpartum breast cancers have increased peritumor lymphatic vessel density that correlates with increased frequency of lymph node metastases. Moreover, lymphatic vessel density was increased in normal postpartum breast tissue compared with tissue from nulliparous women. In rodents, mammary lymphangiogenesis was upregulated during weaning-induced mammary gland involution. Furthermore, breast cancer cells exposed to the involuting mammary microenvironment acquired prolymphangiogenic properties that contributed to peritumor lymphatic expansion, tumor size, invasion, and distant metastases. Finally, in rodent models of postpartum breast cancer, cyclooxygenase-2 (COX-2) inhibition during the involution window decreased normal mammary gland lymphangiogenesis, mammary tumor-associated lymphangiogenesis, tumor   cell invasion   into lymphatics, and metastasis. Our data indicate that physiologic COX-2-dependent lymphangiogenesis occurs in the postpartum mammary gland and suggest that tumors within this mammary microenvironment acquire enhanced prolymphangiogenic activity. Further, our results suggest that the prolymphangiogenic microenvironment of the postpartum mammary gland has potential as a target to inhibit metastasis and suggest that further study of the therapeutic efficacy of COX-2 inhibitors in postpartum breast cancer is warranted. 
 Helicobacter pylori promote gastric cancer cells invasion through a NF-kappaB and COX-2-mediated pathway. To examine the effects of Helicobacter pylori (H pylori) infection on the invasiveness of gastric cancer cells, and to elucidate its mechanism.Gastric carcinoma cells, MKN-45, were incubated with CagA-positive H pylori, and   cell invasion   was determined by Matrigel analysis. The expression of matrix metalloproteinase-9 (MMP-9), vascular endothelial growth factor (VEGF), and cyclooxygenase-2 (COX-2) were assessed by Western-blot analysis, and transcriptional activation of the COX-2 promoter was examined by measuring luciferase and beta-galactosidase activities. Lastly, the protein-DNA interaction was confirmed by an electrophoretic mobility shift assay.The current studies showed that: (1) incubation of CagA-positive H pylori with MKN-45 cells significantly promotes gastric cancer cells invasion, and this effect is attenuated by pre-treatment with NS-398, a COX-2 inhibitor, or PDTC, a nuclear factor kappaB (NF-kappaB) inhibitor; (2) the induction of MKN-45 cells invasion by H pylori is associated with increases in COX-2, MMP-9, and VEGF protein expression, and co-incubation of NS-398 or PDTC significantly reduces these effects; (3) H pylori infection transactivates COX-2 promoter activity and increases the binding of NF-kappaB to this promoter.Our data demonstrate that H pylori infection promotes gastric epithelial cells invasion by activating MMP-9 and VEGF expression. These effects appear to be mediated through a NF-kappaB and COX-2 mediated pathway, as COX-2 or NF-kappaB inhibitor significantly attenuate the invasiveness of gastric cancer cells and the expressions of MMP-9 and VEGF protein. 
 Modulation of the expression of the invasion-suppressor CRMP-1 by cyclooxygenase-2 inhibition via reciprocal regulation of Sp1 and C/EBPalpha. Collapsin response mediator protein-1 (CRMP-1) controls neural development and axonal growth but also acts as a cancer invasion suppressor. In this study, we investigated the transcriptional regulation of CRMP-1 expression. Using a serial deletion strategy, we identified a basal promoter region between nucleotides -100 and -180 in the 5' flanking region of CRMP-1 (nucleotides -1,920 to +50) that contains multiple putative Sp1 and C/EBPalpha sites. Site-directed mutagenesis and deletion analysis revealed that the two C/EBPalpha sites, from nucleotides -122 to -133 and from nucleotides -101 to -113, are the most important regulatory elements. Gel-shift and antibody supershift assays showed that Sp1 protein was also present at this C/EBPalpha site, which overlaps with a Sp1 site. Overexpression of Sp1 decreased CRMP-1 promoter activity and protein expression, whereas overexpression of C/EBPalpha produced the opposite effect. Chromatin immunoprecipitation assays confirmed that Sp1 and C/EBPalpha compete for binding at the overlapping C/EBPalpha and Sp1 sites and reciprocally regulate CRMP-1 expression. Overexpression of cyclooxygenase-2 (COX-2) decreased CRMP-1 mRNA and protein expression. Conversely, the COX-2 inhibitor, celecoxib, induced a dose-dependent increase in CRMP-1 expression. COX-2 inhibition also decreased Sp1-DNA complex formation and inhibited   cell invasion  . We conclude that transcription of the invasion suppressor, CRMP-1, is reciprocally regulated at the promoter region by C/EBPalpha and Sp1. COX-2 inhibitors increase CRMP-1 expression by inhibiting Sp1-DNA complex formation and enhancing DNA binding of C/EBPalpha at the promoter. 
 A novel single nucleotide polymorphism of the cyclooxygenase-2 gene associated with breast cancer. Cyclooxygenase-2 (COX-2) is involved in carcinogenesis, immune response suppression, apoptosis inhibition, angiogenesis, and tumour   cell invasion   and metastasis. The gene for COX-2, designated as PTGS2, carries several polymorphisms, such as -765G >C, 1195G/A in the promoter region, and 8473T >C in the 3' UTR, which have been associated with susceptibility to malignant disease. The aim of this study was to search for new mutations and polymorphisms in the COX-2 gene and to assess the relationship with breast cancer.In the present study, we identified a novel single nucleotide polymorphism, 169C >G, in exon 2 using polymerase chain reaction-single-strand conformation polymorphism analysis. This nucleotide change causes the amino acid change from proline to alanine at codon 57. To investigate the role of this polymorphism for breast cancer, we determined the prevalence of PTGS2 genotypes in 310 women with breast cancer and 310 gender- and age-matched healthy control subjects.Homozygous carriers of the 169-GG genotype were more frequent among patients (15.16%) than among controls (9.35%; P = 0.03). The odds ratio for carriers of this genotype for breast cancer was 1.76 (95% confidence interval, 1.20-3.05). Among patients, oestrogen receptor positivity was less frequent among carriers of a GG genotype (61.7%) than among carriers of a CC or GC genotype (72.3%; P = 0.02). Tumour size, histological grade, presence of primary lymph node metastases, progesterone receptor positivity, or age at diagnosis were not associated with PTGS2 genotypes.We conclude that the homozygous PTGS2 169-GG genotype may be associated with breast cancer risk. 
 Requirement of cyclooxygenase-2 expression and prostaglandins for human prostate cancer   cell invasion  . The PC-3 Low Invasive cells and the PC-3 High Invasive cells were used to investigate the correlation of the COX-2 expression and its arachidonic acid metabolites, prostaglandins, with their invasiveness through Matrigel using a Boyden chamber assay. The COX-2 expression in PC-3 High Invasive cells was approximately 3-fold higher than in PC-3 Low Invasive cells while the COX-1 expression was similar in both cell sublines. When incubated with arachidonic acid, PGE2 was the major prostaglandin produced by these cells. PC-3 High Invasive cells produced PGE2 approximately 2.5-fold higher than PC-3 Low Invasive cells. PGD2 was the second most abundant prostaglandin produced by these cells. Both indomethacin (a nonspecific COX inhibitor) and NS-398 (a specific COX-2 inhibitor) inhibited the production of prostaglandins and the   cell invasion  . PGE2 alone did not induce the   cell invasion   of PC-3 Low Invasive cells. However, PGE2 reversed the inhibition of   cell invasion   by NS-398 and enhanced the   cell invasion   of the PC-3 High Invasive cells. In contrast, PGD2 slightly inhibited the   cell invasion  . These results suggest that in the PC-3 Low Invasive cells, COX-2-derived PGE2 may not be sufficient to induce   cell invasion   while in the PC-3 High Invasive cells, PGE2 may be sufficient to act as an enhancer for the   cell invasion  . Further, PGD2 may represent a weak inhibitor and counteracts the effect of PGE2 in the   cell invasion  . 
 NFATc1 activation promotes the invasion of U251 human glioblastoma multiforme cells through COX-2. Recent studies have revealed that the nuclear factor of activated T-cells (NFAT) is a transcription factor that is highly expressed in aggressive cancer cells and tissues, and mediates invasion through the transcriptional induction of pro-invasion and pro-migration genes. However, the mechanisms through which nuclear factor of activated T-cells, cytoplasmic 1 (NFATc1), in particular, translocates to the nucleus and regulates the invasion of human glioblastoma multiforme (GBM) cells have not yet been fully elucidated. In the present study, to investigate the role of NFATc1 in GBM cells, we established a U251 cell line expressing a constitutively active form of NFATc1 (CA-NFATc1). On the other hand, RNA interference was used to knock down NFATc1 expression in the U251 cell line. Our results demonstrated that the expression of CA-NFATc1 promoted cancer   cell invasion  , while small interfering RNA (siRNA) against NFATc1 successfully inhibited the invasion ability of the U251 cell line. Moreover, we demonstrated that NFATc1 promoted U251   cell invasion   through the induction of cyclooxygenase-2 (COX‑2). NFAT transcriptionally regulates the induction of COX-2 induction in U251 cells and binds to the promoter. We also demonstrated that a large proportion of GBM specimens expressed NFATc1. NFATc1 expression increased according to the histopathological grade of the glioma. However, no NFATc1 staining was observed in the non-neoplastic brain tissues. These findings suggest that the inhibition of the activation of the NFATc1 pathway is an effective therapeutic strategy for the clinical management of GBM. 
 COX-2 overexpression increases motility and invasion of breast cancer cells. Cyclooxygenase-2 (COX-2), an inducible enzyme involved in prostaglandin (including PGE(2)) biosynthesis, is overexpressed in several epithelial malignancies including breast cancer. We tested the hypothesis that COX-2 overexpression in breast cancer cells results in increased cell motility and invasion. COX-2 overproducing cells were generated by stable transfection of several human breast cancer cells with pSG5-COX2 vector. We confirmed the overexpression of COX-2 protein by western blotting, and by measuring PGE(2) in the medium with an immunoassay. We measured cell motility by counting the number of cells crossing an 8-micron pore size PET membrane, and   cell invasion   by counting the number of cells invading through a Matrigel-coated membrane that simulates basement membrane. COX-2 transfected MDA-231 cells produced 30-43-fold more PGE2 as compared to parental cells. COX-2 overexpression increased cell migration approximately 2.2-fold and   cell invasion   through Matrigel approximately 5.1-fold. Addition of 50 microM NS-398, a COX-2 inhibitor, inhibited Matrigel invasion of MDA-231 cells by 54% as compared to solvent confirming the role of COX-2 in   cell invasion  . It is known that an increase in cell migration and invasion can be brought about by cytoskeletal alterations and basement membrane degradation due to increased expression of pro-urokinase plasminogen activator (pro-uPA). To investigate the mechanism of our observed increase in   cell invasion   by COX-2, we found by western blotting that the level of pro-uPA was significantly higher (approximately 5-fold) in COX-2 transfected MDA-231 cells than untransfected MDA-231 cells. Similar to our observations in cell culture, we found evidence that increased COX-2 activity correlates with uPA in a mouse model of breast cancer metastasis to bone. In this study, we conclude that COX-2 overexpression in human breast cancer cells enhances cell motility and invasiveness thus suggesting a mechanism of COX-2 mediated metastasis. 
 The Notch1/cyclooxygenase-2/Snail/E-cadherin pathway is associated with hypoxia-induced hepatocellular carcinoma   cell invasion   and migration. Hepatocellular carcinoma (HCC) is one of the most common malignancies worldwide; however, the prognosis of HCC patients remains poor. This poor prognosis is mainly attributed to the high rate of intrahepatic and distant metastasis. HCC often occurs in a hypoxic environment and hypoxia can activate metastatic programs, ultimately leading to tumor recurrence or metastasis. Thus, the discovery and subsequent development of novel agents to block HCC invasion and migration are the primary objectives of hepatic cancer research. The Notch1 signaling pathway might be involved in hypoxia-induced carcinoma metastasis. However, the mechanisms by which Notch1 mediates cell metastasis, particularly in hepatocellular carcinoma, are not yet entirely clear. The results of the present study show that hypoxia increases the invasion and migration capacities of different HCC cells. Activation of the Notch1 signaling pathway contributes to hypoxia-induced invasion and migration in HCC cells. The activated Notch1 signaling pathway can regulate Snail/E-cadherin through cyclooxygenase-2 (COX-2) under hypoxic conditions. The above results suggest that the Notch1/COX-2/Snail/E-cadherin pathway is possibly associated with hypoxia-induced invasion and migration in HCC cells. Thus, targeting Notch1 may be useful for devising novel preventive and therapeutic strategies for HCC. 
 Fibronectin stimulates human lung carcinoma cell growth by inducing cyclooxygenase-2 (COX-2) expression. Tobacco use is the most important risk factor for the development of lung carcinoma. One characteristic shared by tobacco-related lung diseases is altered lung connective tissue content and composition. In particular, tobacco results in increased expression of fibronectin (FN), a matrix glycoprotein implicated in lung development, injury and repair and in tumor   cell invasion  . We hypothesized that excessive deposition of FN in lung might promote lung carcinoma cell proliferation. Consistent with this hypothesis, we found that FN stimulated human lung carcinoma cell proliferation and diminished apoptosis in vitro, and that this effect was mediated through the integrin alpha5beta1 and associated with upregulation of cyclooxygenase-2 (COX-2) mRNA and protein expression, and increased prostaglandin E2 (PGE2) biosynthesis. The stimulatory effect of FN on COX-2 was blocked by the specific COX-2 inhibitor NS-398 and by inhibitors of protein kinase C (PKC), Calphostin C, and extracellular signal-regulated kinases (Erks), PD98095. Electrophoretic mobility shift assays revealed that FN increased the nuclear binding activity of cyclic AMP response element binding protein (CREB) and CCAAT/enhancer-binding protein (C/EBP), 2 proteins known to play important roles in the regulation of COX-2 promoter activity. Transient transfection assays with wild-type and mutated constructs of the human COX-2 gene promoter revealed that the stimulatory effect of FN was prevented when either the CRE or the NF-IL6 (C/EBP) sites were mutated. Taken together, the results indicate that FN stimulates human lung carcinoma cell proliferation and diminishes apoptosis by inducing COX-2 gene expression and PGE2 biosynthesis. Activation of PKC and Erk and DNA-protein interactions at CRE and NF-IL6 (C/EBP) sites in the COX-2 gene promoter appear to play key roles in this process. This work demonstrates that signaling through specific matrix-binding beta1 integrins (i.e., alpha5beta1) resulting from exaggerated deposition in lung of the matrix glycoprotein fibronectin might promote lung carcinoma cell growth. 
  GeneRIFs  
 High PTGS2 expression enhanced hepatoma   cell invasion   and migration. 
 COX2 and PGE2 mediate EGF-induced E-cadherin-independent human ovarian cancer   cell invasion   
 High PTGS2 expression is associated with hypoxia-induced hepatocellular carcinoma   cell invasion   and migration. 
 Human VRK2 is an active kinase playing a role in regulation of cancer   cell invasion   through the NFAT pathway and COX-2 expression. 
 Data suggest that the expression of COX-2 is correlated with tumor   cell invasion   and is closely related to the cell proliferation in patients with esophageal squamous cell carcinoma. 
 Bacterial products (e.g., LPS) triggers a positive feedback loop involving COX-2/PGE(2) in biliary carcinoma cells and this second phase of EGFR phosphorylation is implicated in   cell invasion   by LPS 
 PGE(2) via EP(4) activates the EGFR --> ERK1/2 --> Egr-1 pathway, leading to increased Id-1 transcription and   cell invasion   
 Results demonstrate the ability of hepatitis B virus X protein to promote tumor   cell invasion   by a mechanism involving the upregulation of membrane-type 1 matrix metalloproteinase (MT1-MMP) and cyclooxygenase-2 (COX-2). 
 NFAT promotes breast cancer   cell invasion   through the induction of COX-2 and the synthesis of prostaglandins 
   
  
 TRPA1 
 
  NCBI Gene  
  GeneRIF  
  Pubmed  
 
 
  Search Term  Rank  Fields  
  [Mm]igration  6  PubMed(1)  
  [Ee]xtracellular [Mm]atrix  8  PubMed(1)  
  [Ii]nvasive  10  PubMed(1); GeneRIFs(1)  
 
 
 hide/show details for TRPA1 
  [Mm]igration  PubMed  
 The combination of TRPM8 and TRPA1 expression causes an invasive phenotype in lung cancer. Our recent studies have shown that hypothermic microenvironment promotes tumor progression and that the molecular sensors for cold are the transient receptor potential (TRP) channels TRPM8 and TRPA1. To evaluate the contribution of TRPM8 and TRPA1 to cancer malignancy, we screened cell subpopulations from Lewis lung cancer (LLC) using limiting dilutions and Western blotting. We identified that LLC-1 cells express 3-fold more TRPM8 than TRPA1, LLC-2 cells express TRPM8 at levels similar to TRPA1, and LLC-3 cells express TRPM8 at one-third the level of TRPA1. LLC-2 cells showed greater adhesion,   migration  , invasiveness and resistance to hypothermia than LLC-1 and LLC-3 cells, although LLC-2 cells had a longer doubling time. TRPM8 or TRPA1 knockdown using siRNA promoted cell proliferation and decreased adhesion and invasiveness in LLC-2 cells. When assessed for UCP2 staining, LLC-1 cells showed increased staining compared to LLC-2 cells, both of which had more UCP2-positive cells than the LLC-3 subpopulation. In an autophagy assay, hypothermia induced substantially less autophagy in LLC-1 cells than in LLC-2 cells, which displayed decreased autophagy compared to LLC-3 cells. Moreover, mice injected with LLC-2 cells had significantly more spontaneous and experimental lung metastases and a shorter overall survival time than mice injected with LLC-1 or LLC-3 cells. Importantly, LLC-2 cells were also more resistant to activated spleen CTL and the chemotherapeutic drug doxorubicin than LLC-1 and LLC-3 cells in vitro. Collectively, our data suggest that TRPM8 induces UCP2 to trigger metabolic transformation, whereas TRPA1 induces autophagy during adverse conditions, and the combination of both genes contributes directly to an invasive phenotype in lung cancer. 
  [Ee]xtracellular [Mm]atrix  PubMed  
 Down-regulated proteins of mesenchymal tumor cells. To identify proteins that are lost during the establishment of the transformed phenotype of a tumor cell, we have prepared a subtracted cDNA library with mRNA from normal human fibroblasts and from their matched SV40 transformed counterparts. More than 40 clones were obtained that showed a dramatic reduction in their relative expression after oncogenic transformation. The proteins encoded by these clones could be grouped into four distinct classes:   extracellular matrix   proteins (fibronectin, beta ig-h3, collagen VI), enzymes (collagenase, urokinase), cytoskeletal proteins (vinculin, SM22) and regulatory proteins (beta-glycan, integrin-associated protein, myosin kinase, IGFBP-5). Six novel gene products were discovered during these experiments, including a novel serine protease, a zyxin-like protein, an ankyrin-like protein and a GTP-binding protein. Only four of all the transformation-sensitive cDNAs were consistently down-regulated when a variety of cell lines derived from spontaneous mesenchymal tumors was investigated: beta ig-h3, collagen VI, the novel ankyrin-like protein, and IGFBP-5. It is likely that these gene products play an important role in the maintenance of the normal phenotype. 
  [Ii]nvasive  PubMed  
 The combination of TRPM8 and TRPA1 expression causes an   invasive   phenotype in lung cancer. Our recent studies have shown that hypothermic microenvironment promotes tumor progression and that the molecular sensors for cold are the transient receptor potential (TRP) channels TRPM8 and TRPA1. To evaluate the contribution of TRPM8 and TRPA1 to cancer malignancy, we screened cell subpopulations from Lewis lung cancer (LLC) using limiting dilutions and Western blotting. We identified that LLC-1 cells express 3-fold more TRPM8 than TRPA1, LLC-2 cells express TRPM8 at levels similar to TRPA1, and LLC-3 cells express TRPM8 at one-third the level of TRPA1. LLC-2 cells showed greater adhesion, migration,   invasive  ness and resistance to hypothermia than LLC-1 and LLC-3 cells, although LLC-2 cells had a longer doubling time. TRPM8 or TRPA1 knockdown using siRNA promoted cell proliferation and decreased adhesion and   invasive  ness in LLC-2 cells. When assessed for UCP2 staining, LLC-1 cells showed increased staining compared to LLC-2 cells, both of which had more UCP2-positive cells than the LLC-3 subpopulation. In an autophagy assay, hypothermia induced substantially less autophagy in LLC-1 cells than in LLC-2 cells, which displayed decreased autophagy compared to LLC-3 cells. Moreover, mice injected with LLC-2 cells had significantly more spontaneous and experimental lung metastases and a shorter overall survival time than mice injected with LLC-1 or LLC-3 cells. Importantly, LLC-2 cells were also more resistant to activated spleen CTL and the chemotherapeutic drug doxorubicin than LLC-1 and LLC-3 cells in vitro. Collectively, our data suggest that TRPM8 induces UCP2 to trigger metabolic transformation, whereas TRPA1 induces autophagy during adverse conditions, and the combination of both genes contributes directly to an   invasive   phenotype in lung cancer. 
  GeneRIFs  
 our data suggest that TRPM8 induces UCP2 to trigger metabolic transformation, whereas TRPA1 induces autophagy during adverse conditions, and the combination of both genes contributes directly to an   invasive   phenotype in lung cancer. 
   
  
 TFPI2 
 
  NCBI Gene  
  GeneRIF  
  Pubmed  
 
 
  Search Term  Rank  Fields  
  [Mm]igration  6  PubMed(14); GeneRIFs(2)  
  ECM  7  PubMed(5)  
  [Ee]xtracellular [Mm]atrix  8  Function(1); PubMed(20); Component(2); GeneRIFs(1)  
  [Ii]nvasive  10  PubMed(20); GeneRIFs(6)  
  [Mm]etastasize  11  PubMed(1)  
  [Mm]etastasis  12  PubMed(27); GeneRIFs(8)  
  [Cc]ell [Ii]nvasion  13  PubMed(7)  
 
 
 hide/show details for TFPI2 
  [Mm]igration  PubMed  
 Methylation of TFPI-2 is an early event of esophageal carcinogenesis. To explore the epigenetic changes and the function of TFPI-2 in esophageal cancer.Nine esophageal cancer cell lines, nine normal esophageal mucosa, 60 esophageal dysplasia and 106 advanced esophageal cancer samples were included in this study. TFPI-2 methylation was examined by methylation-specific PCR. TFPI-2 expression was evaluated by immunohistochemistry in tissue samples. The effect of TFPI-2 on proliferation, apoptosis, invasion and   migration   was analyzed by colony formation assay, western blot assay, transwell assay and flow cytometric analysis.TFPI-2 expression was regulated by promoter region hypermethylation in human esophageal cancer cell lines, and TFPI-2 expression is inversely correlated with methylation in primary cancer. Methylation was found in 28.2, 33.3 and 33.3% of grade 1, 2 and 3 esophageal dysplasia, and 67% of primary esophageal cancer, but no methylation was found in normal mucosa. Methylation is significantly related to tumor differentiation. Inhibition of invasion,   migration  , colony formation and proliferation, and induction of apoptosis occurred with the restoration of TFPI-2 expression in the KYSE70 cell line.TFPI-2 is frequently methylated in esophageal cancer with a progression tendency. TFPI-2 is a potential tumor suppressor in esophageal cancer. 
 Adenovirus-mediated expression of tissue factor pathway inhibitor-2 inhibits endothelial cell   migration   and angiogenesis. Extracellular matrix (ECM) remodeling during angiogenesis is accomplished through plasmin-dependent pericellular proteolysis and through the action of matrix metalloproteinases (MMPs). Because tissue factor pathway inhibitor-2 (TFPI-2), a Kunitz-type protease inhibitor with prominent ECM localization, inhibits plasmin and MMPs activity, we investigated the role of TFPI-2 in endothelial cell (EC)   migration   and angiogenesis.Real-time polymerase chain reaction and immunostaining showed that the expression of TFPI-2 mRNA and protein was upregulated in migrating ECs. The effect of TFPI-2 on angiogenesis was studied in mouse models of Matrigel and polyvinylalcohol sponge implants by overexpressing TFPI-2 through infection with a replication-deficient adenovirus (AdTFPI-2). Using (immuno)fluorescence and confocal microscopy we observed that TFPI-2 reduced neovascularization and promoted ECM deposition. Lateral cell   migration   and capillary tube formation in vitro also were impaired by TFPI-2, a process reversed by anti-TFPI-2 antibodies. Increased apoptosis occurred both in AdTFPI-2-treated ECs and in the mouse implants. Zymography and assays in the absence of plasminogen confirmed plasmin inhibition as a main mechanism through which TFPI-2 inhibits EC   migration  .Our data suggest that TFPI-2 may be an important regulator of aberrant angiogenesis associated with tumor growth/metastasis, cardiovascular diseases, chronic inflammation, or diabetes. 
 TFPI-2 is a putative tumor suppressor gene frequently inactivated by promoter hypermethylation in nasopharyngeal carcinoma. Epigenetic silencing of tumor suppressor genes play important roles in NPC tumorgenesis. Tissue factor pathway inhibitor-2 (TFPI-2), is a protease inhibitor. Recently, TFPI-2 was suggested to be a tumor suppressor gene involved in tumorigenesis and metastasis in some cancers. In this study, we investigated whether TFPI-2 was inactivated epigenetically in nasopharyngeal carcinoma (NPC).Transcriptional expression levels of TFPI-2 was evaluated by RT-PCR. Methylation status were investigated by methylation specific PCR and bisulfate genomic sequencing. The role of TFPI-2 as a tumor suppressor gene in NPC was addressed by re-introducing TFPI-2 expression into the NPC cell line CNE2.TFPI-2 mRNA transcription was inactivated in NPC cell lines. TFPI-2 was aberrantly methylated in 66.7% (4/6) NPC cell lines and 88.6% (62/70) of NPC primary tumors, but not in normal nasopharyngeal epithelia. TFPI-2 expression could be restored in NPC cells after demethylation treatment. Ectopic expression of TFPI-2 in NPC cells induced apoptosis and inhibited cell proliferation, colony formation and cell   migration  .Epigenetic inactivation of TFPI-2 by promoter hypermethylation is a frequent and tumor specific event in NPC. TFPI-2 might be considering as a putative tumor suppressor gene in NPC. 
 Trypsinogen 4 boosts tumor endothelial cells   migration   through proteolysis of tissue factor pathway inhibitor-2. Proteases contribute to cancer in many ways, including tumor vascularization and metastasis, and their pharmacological inhibition is a potential anticancer strategy. We report that human endothelial cells (EC) express the trypsinogen 4 isoform of the serine protease 3 (PRSS3), and lack both PRSS2 and PRSS1. Trypsinogen 4 expression was upregulated by the combined action of VEGF-A, FGF-2 and EGF, angiogenic factors representative of the tumor microenvironment. Suppression of trypsinogen 4 expression by siRNA inhibited the angiogenic milieu-induced   migration   of EC from cancer specimens (tumor-EC), but did not affect EC from normal tissues. We identified tissue factor pathway inhibitor-2 (TFPI-2), a matrix associated inhibitor of cell motility, as the functional target of trypsinogen 4, which cleaved TFPI-2 and removed it from the matrix put down by tumor-EC. Silencing tumor-EC for trypsinogen 4 accumulated TFPI2 in the matrix. Showing that angiogenic factors stimulate trypsinogen 4 expression, which hydrolyses TFPI-2 favoring a pro-migratory situation, our study suggests a new pathway linking tumor microenvironment signals to endothelial cell   migration  , which is essential for angiogenesis and blood vessel remodeling. Abolishing trypsinogen 4 functions might be an exploitable strategy as anticancer, particularly anti-vascular, therapy. 
 Tissue factor pathway inhibitor-2 is upregulated by vascular endothelial growth factor and suppresses growth factor-induced proliferation of endothelial cells. The purpose of this study is to investigate the expression and regulation of type-2 tissue factor pathway inhibitor (TFPI-2) in endothelial cells, as well as the regulation of human endothelial cell (EC) function by TFPI-2.Real-time polymerase chain reaction (PCR) and Western blot analysis revealed that vascular endothelial growth factor (VEGF) induced both time- and dose-dependent increase in TFPI-2 mRNA and protein expression in endothelial cells. TFPI-2 mRNA expression was also significantly upregulated by IL-1beta, and modestly increased by both tumor necrosis factor (TNF)-alpha and fibroblast growth factor (FGF)-2, but not placental growth factor (PlGF). VEGF upregulation of TFPI-2 was dramatically reduced by inhibition of the MEK pathway. Administration of TFPI-2 protein suppressed both VEGF and FGF-2 stimulation of EC proliferation in a dose-dependent manner. A recombinant preparation of the first Kunitz-type domain of TFPI-2 (KD1) did not suppress growth factor stimulation of EC proliferation, suggesting a mechanism distinct from the proteinase inhibitory activity of TFPI-2. Exogenously added TFPI-2 protein suppressed VEGF-induced EC   migration   in 2 different assays. Recombinant wt-KD1 or the R24K mutant of KD1, but not the R24Q mutant, dramatically suppressed VEGF-induced EC   migration  . TFPI-2 protein, but not recombinant KD1, blocked VEGF-induced activation of both Akt and ERK1/2 in ECs. At higher doses, TFPI-2 protein blocked VEGFR2 activation.Our data suggest that VEGF-upregulation of TFPI-2 expression in endothelial cells may represent a mechanism for negative feedback regulation and modulation of its pro-angiogenic action on endothelial cells. TFPI-2, or derivatives of TFPI-2, may be novel therapeutics for treatment of angiogenic disease processes. 
 Epigenetic inactivation of TFPI-2 as a common mechanism associated with growth and invasion of pancreatic ductal adenocarcinoma. Using microarrays, we have screened for genes reactivated by drugs that modify epigenetic mechanisms in pancreatic cancer cells. One of the genes identified was tissue factor pathway inhibitor 2 (TFPI-2), which encodes for a broad-spectrum serine proteinase inhibitor that negatively regulates the extracellular matrix degradation, an essential step in tumor invasion and metastasis. We therefore investigated the expression and methylation patterns of the TFPI-2 gene in pancreatic adenocarcinoma, and determined its role in tumor growth and invasion. In contrast to its abundant expression in normal pancreas, TFPI-2 mRNA was undetectable in a high fraction of pancreatic cancer cell lines and in primary pancreatic ductal neoplasms (IPMNs). Loss of TFPI-2 expression was associated with aberrant hypermethylation of its promoter CpG island. Treatment with the phorbol ester (PMA), known to stimulate the TFPI-2 promoter activity, augmented the TFPI-2 expression in cell lines with unmethylated or partially methylated TFPI-2, but failed to induce the expression in cell lines that harbored fully methylated TFPI-2. Aberrant methylation of TFPI-2 was also detected in 73% (102/140) of pancreatic cancer xenografts and primary pancreatic adenocarcinomas, was more likely in older patients with pancreatic cancer, and significantly correlated with progression of IPMNs (P=0.0002). Restored expression of the TFPI-2 gene in nonexpressing pancreatic cancer cells resulted in marked suppression in their proliferation,   migration  , and invasive potential in vitro. We thus conclude that epigenetic inactivation of TFPI-2 is a common mechanism that contributes to the aggressive phenotype of pancreatic ductal adenocarcinoma. 
 Effects of tissue factor pathway inhibitor-2 expression on biological behavior of BeWo and JEG-3 cell lines. To investigate the effect of tissue factor pathway inhibitor-2 (TFPI-2) expression on biological behavior of BeWo and JEG-3 cell lines.The expression of TFPI-2 in BeWo and JEG-3 cells was upregulated by pEGFP-N3-TFPI-2 and downregulated by small interference RNA transfection, confirmed by Western blotting assay and real-time polymerase chain reaction (RT-PCR). Boyden chamber, Cell Counting Kit-8 (CCK-8), and Hoechst 33258/terminal deoxynucleotidyltransferase-mediated UTP end labeling (TUNEL) assays were used for   migration  , invasion, and proliferation/apoptosis analysis, respectively.In Western blotting and RT-PCR assay, protein and messenger RNA (mRNA) expression of TFPI-2 in transfected BeWo and JEG-3 cells were confirmed. Expression of TFPI-2 inhibited BeWo and downregulated JEG-3 cell   migration  , invasion, proliferation, and induced apoptosis (P < .05) in Boyden chamber, CCK-8, Hoechst 33258, and TUNEL detection, respectively.TFPI-2 expression caused invasion and proliferation impair and induced apoptosis in TFPI-2 regulated BeWo and JEG-3 cells. It provides a clue for potential role of TFPI-2 in trophoblast. 
 Expression of tissue factor pathway inhibitor 2 in human pancreatic carcinoma and its effect on tumor growth, invasion, and   migration   in vitro and in vivo. Tissue factor pathway inhibitor 2 (TFPI-2), also known as placental protein and matrix-associated serine protease inhibitor, plays an important role in angiogenesis, intravascular fibrinolysis, wound healing, tumor invasion, metastasis by plasmin, and trypsin mediated activation of zymogen matrix metalloproteinases. To detect whether TFPI-2 can be expressed in human pancreatic carcinoma samples and to investigate its role in the growth, invasion, and metastasis of pancreatic carcinoma cell in vitro and in vivo, we collected eight normal pancreatic tissue samples and 50 pancreatic carcinoma samples, and stably transfected the human pancreatic carcinoma cell line Panc-1 with a vector capable of expressing TFPI-2 gene. RT-PCR and Western blot analysis revealed that the levels of TFPI-2 expression were markedly lower in pancreatic carcinoma samples compared with normal pancreas samples. The level of TFPI-2 protein was significantly higher in cells transfected with TFPI-2 gene than that in the untransfected cells. The results of MTT assay showed that TFPI-2 inhibited Panc-1 cells growth in vitro. The invasive capacity of the cells transfected with TFPI-2 gene was also markedly less than that of untransfected cells in vitro as determined by the Matrigel invasion  /migratio  n assay. Moreover, TFPI-2 inhibited tumor growth, invasion, and metastasis in vivo in an orthotopic pancreatic carcinoma model. Our findings suggest that TFPI-2 plays a significant role in the growth, invasion, and metastasis of pancreatic carcinoma cell in vitro and in vivo, and has potential in anticancer therapy. 
 Promoter hypermethylation and silencing of tissue factor pathway inhibitor-2 in oral squamous cell carcinoma. The treatment of oral squamous cell carcinoma (OSCC) following early detection is associated with good outcomes. Therefore, the survival and prognosis of OSCC patients could be hugely improved by identifying reliable biomarkers for the early diagnosis of the disease. Our previous methylation microarray analysis results have suggested that the gene encoding tissue factor pathway inhibitor-2 (TFPI-2) is a potential clinical predictor as well as a key regulator involved in OSCC malignancy.Methylation of the TFPI-2 promoter in oral tissue specimens was evaluated by bisulfite sequencing assay, quantitative methylation-specific PCR, and pyrosequencing assay. The differences in methylation levels among the groups were compared using the Mann-Whitney U test. The area under the receiver operating characteristic curve (AUROC) was used to evaluate the discrimination ability for detecting OSCC. Cellular TFPI-2 expression was analyzed by quantitative reverse-transcription PCR before and after treatment with 5'-aza-2'-deoxycytidine and trichostatin A, to confirm whether TFPI-2 was epigenetically silenced in OSCC cells. We investigated whether TFPI-2 plays a role as a tumor suppressor by establishing TFPI-2-overexpressing OSCC cells and subjecting them to in vitro cellular proliferation,   migration  , and invasion assays, as well as an in vivo metastasis assay.TFPI-2 was hypermethylated in OSCC tissues versus normal oral tissues (P < 0.0001), with AUROC = 0.91, when using a pyrosequencing assay to quantify the methylation level. TFPI-2 silencing in OSCC was regulated by both DNA methylation and chromatin histone modification. Restoration of TFPI-2 counteracted the invasiveness of OSCC by inhibiting the enzymatic activity of matrix metalloproteinase-2, and consequently interfered with OSCC metastasis in vivo.Our data suggest strongly that TFPI-2 is a down-regulated tumor suppressor gene in OSCC, probably involving epigenetic silencing mechanisms. The loss of TFPI-2 expression is a key event for oral tumorigenesis, especially in the process of tumor metastasis. 
 [Role of tissue factor pathway inhibitor-2 in ovarian tumor   migration   and invasion]. Human tissue factor pathway inhibitor-2 (TFPI-2) is a newly discovered serine protease inhibitor, which inhibits plasmin, trypsin, matrix metalloproteinase (MMPs), but not urokinase, tissue-type plasminogen activators and thrombin. Earlier studies have shown that the production of TFPI-2 is downregulated during the progression of various cancers. The aim of this study was to elucidate the relationship between TFPI-2 expression and ovarian tumor   migration   and invasion.Human TFPI-2 expression vector pBos-Cite-neo/TFPI-2 was transfected into ovarian tumor cells line A2780. After the transfected cells were screened by G418, transfected and nontransfected cells were examined for TFPI-2 mRNA and protein by reverse transcription-polymerase chain reaction (RT-PCR) and Western blot analysis, respectively. The number of transfected or nontransfected cells passing through membrane of Boyden chamber was counted as the basis assessing tumor cells migratory and invasive behaviors.Expression of mRNA and protein of TFPI-2 were confirmed in transfected cells. In invasion assay, the number of TFPI-2-expressing cells to traverse a Matrigel-coated membrane was obviously decreased compared with that of nonexpressing cells (59.3+/-6.5 versus 109.7+/-5.5, P< 0.01); while in   migration   assay, no significant difference was observed between transfected and nontransfected cells (114.7+/-8.6 versus 127.3+/-7.1, P >0.05).Expression of TFPI-2 may strongly inhibit the invasive ability of ovarian tumor cells in vitro, but has no effect on the migratory ability, which provides an experimental basis for treating human ovarian tumor with gene therapy. 
 Human tissue factor pathway inhibitor-2 suppresses the wound-healing activities of human Tenon's capsule fibroblasts in vitro. Human tissue Factor Pathway Inhibitor-2 (TFPI-2) is a potent inhibitor of plasmin, which activates metalloproteinases involved in extracellular matrix degradation. Its secretion in the extracellular matrix makes TFPI-2 a potential inhibitor of tumor cell invasion. However, no studies have yet evaluated the wound-healing activities of human Tenon's capsule fibroblasts (hTCFs). The aim of the study is to elucidate the effect of TFPI-2 overexpression on hTCF proliferation and   migration  , to determine whether TFPI-2 may act as an antiscarring agent in vivo after glaucoma filtration surgery.Plasmid vector pBos-Cite-neo/TFPI-2 was transfected into hTCFs with Lipofectamine 2000. After selection by G418, transfected, non-transfected, and mock-transfected cells were screened for TFPI-2 mRNA and protein by reverse transcription-PCR and western blot analysis respectively. Cell proliferation and viability were determined by 3-(4,5-Dimethylthiazol-2-yl)-2,5-diphenyltetrazolium bromide (MTT) assay and flow cytometry. Cell   migration   was studied on restrained collagen gels and with a scratch-wound assay.TFPI-2 expression of mRNA and protein was confirmed in transfected cells. The transfected, non-transfected, and mock-transfected cells showed no significant difference in cell proliferation and apoptosis, with TFPI-2 found not to be cytotoxic in hTCFs. Overexpression of TFPI-2 significantly suppressed cell   migration   three- to four-fold on collagen gel for 2 weeks and in the scratch-wound assay for 2 d (39.27+/-2.40% versus 16.43+/-1.10% at 1 d, and 79.0+/-3.04% versus 30.13+/-2.1% at 2 d).TFPI-2 expression may strongly inhibit the   migration   ability of hTCFs in vitro, making it a promising candidate for novel therapies to minimize scar development after glaucoma drainage surgery. 
 Decreased expression of TFPI-2 correlated with increased expression of CD133 in cholangiocarcinoma. Recent findings suggest decreasing TFPI-2 expression plays a significant role in inhibiting cell   migration   and tumor invasion. The clinicopathological significance of the expression of TFPI-2 and its possible correlation with the expression of CD133 in cholangiocarcinoma remains to be solved.We investigated if TFPI-2 was involved in the clinicopathological significance of cholangiocarcinoma. An immunohistochemical method was used to detect 218 cases of cholangiocarcinoma, 30 para-neoplastic and 20 normal bile ducts for their expression status of TFPI-2 and CD133, and then the results were analyzed with the patient's age, sex, tumor site and the histological grade, clinical stage as well as overall mean survival time.Compared with the para-neoplastic and normal cholangiocytes, the expression of TFPI-2 was obviously decreased while the expression of CD133 in carcinoma cells was increased. Carcinomas with low expression of TFPI-2 were significantly corresponding to the tumor site (P = 0.006), size (P = 0.005), histological grade (P = 0.0001) and clinical stage (P = 0.0001), but not to the age (P = 0.066) and sex (P = 0.411), respectively. By Kaplan-Meier survival analysis, the low expression of TFPI-2 was significantly correlative with the overall survival time (P = 0.0001). Further, the expression of TFPI-2 was found inversely correlative with the expression of CD133 (g = -0.3876, P < 0.0001).Our finding suggests that the decreased expression of TFPI-2 may play an important role in the carcinogenesis and progression, and may become a new adjunct marker in the diagnosis and prognosis in cholangiocarcinoma. The expression of TFPI-2 may be inversely correlative with the expression of CD133. 
 Characterization and functional analysis of TFPI-2 gene promoter in a human choriocarcinoma cell line. Tissue factor pathway Inhibitor-2 (TFPI-2) is associated with extracellular matrices and plays a major role in cell   migration   and tumor invasion. In this study, a 4.8-kb human TFPI-2 gene 5'-flanking region was isolated, cloned and sequenced. Promoter region analysis revealed a high GC-rich content without canonical TATA and CAAT boxes but three transcription initiation sites were identified. Moreover, several putative binding sites for transcription factors were identified (MyoD, LYF1, NF-Y, GATA, oct-1, AP-1, Sp1, NF1, NF-kappa B and egr-1). To characterize potential regulatory regions, TFPI-2/luciferase promoter constructs were then transfected in human choriocarcinoma JEG-3 cells. We first showed that the minimal TFPI-2 promoter is located between -166 and -111 from the translation start site. Luciferase activity consistently increased after stimulation of JEG-3 cells by phorbol 12-myristate 13-acetate indicating that NF1, NF-kappa B and egr-1/Sp1 binding sites are crucial in inducible TFPI-2 expression. Moreover, negative regulatory regions included AP-1 binding sites were identified. This study demonstrates that the TFPI-2 gene promoter exhibits typical features of a housekeeping gene. 
 Knockdown of TFPI-2 promotes   migration   and invasion of glioma cells. Glioblastoma is the most malignant primary brain tumor. Due to its highly promigratory and proinvasive properties, standard therapy including surgery, chemotherapy and radiation fails in eradicating this highly aggressive type of cancer. Here, we evaluated the role of TFPI-2, a Kunitz-type serine protease inhibitor, which has been previously described as a tumor suppressor gene in several types of cancer, including glioma. TFPI-2 expression was absent in five of nine investigated high-grade glioma cell lines. Lentiviral knockdown of TFPI-2 in two of the TFPI-2-expressing cell lines (MZ-18 and Hs 638) was associated with pronounced changes in the cellular behavior: glioma cell proliferation,   migration   and invasion were significantly increased in TFPI-2 knockdown cells in comparison to empty vector-transfected control cells. Since TFPI-2 might exert its tumor suppressor function by inhibiting MMPs, we subsequently analyzed the effects of specific MMP inhibitors on cell invasion of TFPI-2 KD cells vs. control cells. The data obtained from these experiments suggest that the anti-invasive properties of TFPI-2 are associated with inhibition of MMP-1 and MMP-2, while inhibition of MMP-9 seems to play a minor role in this context. Our findings underscore the important role of TFPI-2 as a tumor suppressor gene and indicate that TFPI-2 may be a useful diagnostic marker for the aggressive phenotype of glial tumors. 
  GeneRIFs  
 TFPI-2 expression may strongly inhibit the   migration   ability of human Tenon's capsule fibroblasts in vitro. 
 Restored expression of the TFPI-2 gene in nonexpressing pancreatic cancer cells resulted in marked suppression in their proliferation,   migration  , and invasive potential. 
  ECM  PubMed  
 Adenovirus-mediated expression of tissue factor pathway inhibitor-2 inhibits endothelial cell migration and angiogenesis. Extracellular matrix (  ECM  ) remodeling during angiogenesis is accomplished through plasmin-dependent pericellular proteolysis and through the action of matrix metalloproteinases (MMPs). Because tissue factor pathway inhibitor-2 (TFPI-2), a Kunitz-type protease inhibitor with prominent   ECM   localization, inhibits plasmin and MMPs activity, we investigated the role of TFPI-2 in endothelial cell (EC) migration and angiogenesis.Real-time polymerase chain reaction and immunostaining showed that the expression of TFPI-2 mRNA and protein was upregulated in migrating ECs. The effect of TFPI-2 on angiogenesis was studied in mouse models of Matrigel and polyvinylalcohol sponge implants by overexpressing TFPI-2 through infection with a replication-deficient adenovirus (AdTFPI-2). Using (immuno)fluorescence and confocal microscopy we observed that TFPI-2 reduced neovascularization and promoted   ECM   deposition. Lateral cell migration and capillary tube formation in vitro also were impaired by TFPI-2, a process reversed by anti-TFPI-2 antibodies. Increased apoptosis occurred both in AdTFPI-2-treated ECs and in the mouse implants. Zymography and assays in the absence of plasminogen confirmed plasmin inhibition as a main mechanism through which TFPI-2 inhibits EC migration.Our data suggest that TFPI-2 may be an important regulator of aberrant angiogenesis associated with tumor growth/metastasis, cardiovascular diseases, chronic inflammation, or diabetes. 
 Matrix localization of tissue factor pathway inhibitor-2/matrix-associated serine protease inhibitor (TFPI-2/MSPI) involves arginine-mediated ionic interactions with heparin and dermatan sulfate: heparin accelerates the activity of TFPI-2/MSPI toward plasmin. Human tissue factor pathway inhibitor-2 (TFPI-2)/matrix-associated serine protease inhibitor (MSPI), a Kunitz-type serine protease inhibitor, inhibits plasmin, trypsin, chymotrypsin, plasma kallikrein, cathepsin G, and factor VIIa-tissue factor complex. The mature protein has a molecular mass of 32-33 kDa, but exists in vivo as two smaller, underglycosylated species of 31 and 27 kDa. TFPI-2/MSPI triplet is synthesized and secreted by a variety of cell types that include epithelial, endothelial, and mesenchymal cells. Because the majority (75-90%) of TFPI-2/MSPI is associated with the extracellular matrix (  ECM  ), we examined which components of the   ECM   bind TFPI-2/MSPI. We found that TFPI-2/MSPI bound specifically to heparin and dermatan sulfate. Interaction of these two glycosaminoglycans (GAGs) with TFPI-2/MSPI involved one or more common protein domains, as evidenced by cross-competition experiments. However, binding affinity for TFPI-2/MSPI with heparin was 250-300 times greater than that for TFPI-2/MSPI with dermatan sulfate. Binding of TFPI-2/MSPI to GAGs was inhibited by NaCl or arginine but not by glucose, mannose, galactose, 6-aminohexanoic acid, or urea, suggesting that arginine-mediated ionic interactions participate in the GAG binding of TFPI-2/MSPI. This supposition was supported by the observation that only NaCl or arginine could elute the TFPI-2/MSPI protein triplet from an   ECM   derived from human dermal fibroblasts. Reduced TFPI-2/MSPI did not bind to heparin, suggesting that proper disulfide pairings and conformation are essential for matrix binding. To determine whether heparin modulates the activity of TFPI-2/MSPI, we determined the rate of inhibition of plasmin by the inhibitor with and without heparin and found that TFPI-2/MSPI is more active in the presence of heparin. Collectively, our results demonstrate that conformation-dependent arginine-mediated ionic interactions are responsible for the TFPI-2/MSPI triplet binding to fibroblast   ECM  , heparin, and dermatan sulfate and that heparin augmented the rate of inhibition of plasmin by TFPI-2/MSPI. 
 The effect of human tissue factor pathway inhibitor-2 on the growth and metastasis of fibrosarcoma tumors in athymic mice. Human tissue factor pathway inhibitor-2 (TFPI-2) is a matrix-associated Kunitz inhibitor that inhibits the plasmin- and trypsin-mediated activation of zymogen matrix metalloproteinases involved in tumor progression, invasion, and metastasis. To directly assess its role in tumor growth and metastasis in vivo, we stably transfected HT-1080 fibrosarcoma cells expressing either fully active wild-type human TFPI-2 (WT) or inactive R24Q TFPI-2 (QT) and examined their ability to form tumors and metastasize in athymic mice in comparison to mock-transfected cells (MT). MT and QT fibrosarcoma tumors grew 2 to 3 times larger than WT tumors. Tumor metastasis was confined to the lung and was observed in 75% of mice treated with either MT or QT cells, whereas only 42% of mice treated with WT cells developed lung metastases. Real-time quantitative reverse transcriptase-polymerase chain reaction (RT-PCR) analyses of each tumor group revealed 3- to 6-fold lower levels of murine vascular endothelial growth factor gene expression in WT tumors in relation to either MT or QT tumors. Comparative tumor gene expression analysis revealed that several human genes implicated in oncogenesis, invasion, metastasis, apoptosis, and angiogenesis had significantly altered levels of expression in WT tumors. Our collective data demonstrate that secretion of inhibitory TFPI-2 by a highly metastatic tumor cell markedly inhibits its growth and metastasis in vivo by regulating pericellular extracellular matrix (  ECM  ) remodeling and angiogenesis. 
 Extracellular matrix-associated serine protease inhibitors (Mr 33,000, 31,000, and 27,000) are single-gene products with differential glycosylation: cDNA cloning of the 33-kDa inhibitor reveals its identity to tissue factor pathway inhibitor-2. Recently, we reported the identification and partial characterization of three serine protease inhibitors (M(r) 33,000, 31,000, and 27,000) from the extracellular matrix (  ECM  ) of human umbilical vein endothelial cells and skin cells. Here, we report that a full-length cDNA clone for the 33-kDa inhibitor from SV-40 transformed human skin fibroblasts (t12FB) is identical to a recombinant trypsin/tissue factor pathway inhibitor called TFPI-2 from placenta. By immunoblotting, the three inhibitors from   ECM   and cell lysates demonstrated cross-reactivity with an antiTFPI-2 IgG. To further elucidate how these inhibitors are related, pulse-chase labeling of t12FB with [35S]methionine followed by immunoprecipitation with antiTFPI-2 IgG was performed on   ECM   and cytosolic proteins. A precursor-product relationship did not exist between the three inhibitors from   ECM  . In contrast, the various species of inhibitors from cytosolic fractions demonstrated a precursor-product relationship. Within the cytosolic fraction, 26-, 29-, and 30-kDa inhibitors were detected in the early chases (0 and 15 min) but they form precursors to the synthesis of the 33-kDa inhibitor which accumulated in the later chases (30 min to 1 h). When pulse-chase experiments were performed in the presence of tunicamycin, synthesis as well as sequestration of the three inhibitors into   ECM   was completely inhibited. In the presence of tunicamycin, the cells synthesized and sequestered a single 25.5-kDa inhibitor into   ECM  . Peak quantities of the 25.5-kDa inhibitor appeared in the   ECM   after 6 h chase while they were 1 h for the 27- and 31-kDa inhibitors and 3 h for the 33-kDa inhibitor. To further support that the three inhibitors are related but only differ in the extent of glycosylation, the 33-kDa inhibitor from the t12FB   ECM   was deglycosylated with N-glycosidase F and the products were identified by immunoblotting with antiTFPI-2 IgG. The enzyme released the 31-, 27-, and 25.5-kDa inhibitors from the 33-kDa inhibitor. Collectively, these results demonstrate that the   ECM  -associated 33-, 31-, and 27-kDa inhibitors are biosynthetic products of a single gene with differential glycosylation. The 25.5-kDa inhibitor is unglycosylated, whereas 27- and 30- to 31-kDa inhibitors are partially glycosylated and the 33-kDa inhibitor is fully glycosylated. Inhibition of glycosylation significantly retarded the rate of secretion of the inhibitor but did not prevent its association with   ECM  . Quantitation of the inhibitors with cell-conditioned medium and   ECM   fractions reveals that 70-75% were   ECM  -associated and 25-30% cell-associated. None or very little of the inhibitors (0-2%) remained in a conditioned medium. Because they are primarily associated with   ECM  , the inhibitors may play a major role in   ECM   remodeling and turnover. 
 Quantification and characterization of human endothelial cell-derived tissue factor pathway inhibitor-2. Tissue factor pathway inhibitor-2 (TFPI-2), also known as placental protein 5, is a serine protease inhibitor consisting of three tandemly-arranged Kunitz-type protease inhibitor domains. While TFPI-2 is a potent inhibitor of trypsin, plasmin, kallikrein, and factor XIa in the test tube, the function of this inhibitor in vivo remains unclear. In the present study, we investigated the synthesis and secretion of TFPI-2 by cultured endothelial cells derived from human umbilical vein, aorta, saphenous vein, and dermal microvessels to gain insight into its biological function. While all endothelial cells examined synthesized and secreted TFPI-2, dermal microvascular endothelial cells synthesized threefold to sevenfold higher levels of TFPI-2. Approximately 60% to 90% of the TFPI-2 secreted by endothelial cells was directed to the subendothelial extracellular matrix (  ECM  ). When cultured human umbilical vein endothelial cells were stimulated with inflammatory mediators such as phorbol 12-myristate,13-acetate; endotoxin; and tumor necrosis factor-alpha, TFPI-2 synthesis by these cells increased twofold to 14-fold. Recombinant TFPI-2 bound to dermal microvascular endothelial cell monolayers and its   ECM   in a specific, dose-dependent, and saturable manner with Kd values of 21 and 24 nmol/L, respectively. TFPI-2 interacted with 4.5 X 10(10) sites/cm2 (3 X 10[5] sites/cell) and 2.3 X 10(11) sites/cm2 on endothelial cells and   ECM  , respectively. In the presence of rabbit anti-TFPI-2 IgG, but not preimmune IgG, endothelial cells dissociated from the culture flask in a time- and IgG concentration-dependent manner. Our findings provide evidence that endothelial cell-derived TFPI-2 is primarily secreted into the abluminal space and presumably plays an important role in maintaining the integrity of the   ECM   essential for cell attachment. 
  [Ee]xtracellular [Mm]atrix  Function  
   extracellular matrix   structural constituent 
  PubMed  
 SILAC-based proteomics of human primary endothelial cell morphogenesis unveils tumor angiogenic markers. Proteomics has been successfully used for cell culture on dishes, but more complex cellular systems have proven to be challenging and so far poorly approached with proteomics. Because of the complexity of the angiogenic program, we still do not have a complete understanding of the molecular mechanisms involved in this process, and there have been no in depth quantitative proteomic studies. Plating endothelial cells on matrigel recapitulates aspects of vessel growth, and here we investigate this mechanism by using a spike-in SILAC quantitative proteomic approach. By comparing proteomic changes in primary human endothelial cells morphogenesis on matrigel to general adhesion mechanisms in cells spreading on culture dish, we pinpoint pathways and proteins modulated by endothelial cells. The cell-  extracellular matrix   adhesion proteome depends on the adhesion substrate, and a detailed proteomic profile of the   extracellular matrix   secreted by endothelial cells identified CLEC14A as a matrix component, which binds to MMRN2. We verify deregulated levels of these proteins during tumor angiogenesis in models of multistage carcinogenesis. This is the most in depth quantitative proteomic study of endothelial cell morphogenesis, which shows the potential of applying high accuracy quantitative proteomics to in vitro models of vessel growth to shed new light on mechanisms that accompany pathological angiogenesis. The mass spectrometry proteomics data have been deposited to the ProteomeXchange Consortium with the data set identifier PXD000359. 
 TFPI-2 silencing increases tumour progression and promotes metalloproteinase 1 and 3 induction through tumour-stromal cell interactions. Tissue factor pathway inhibitor-2 (TFPI-2) is a potent inhibitor of plasmin which activates matrix metalloproteinases (MMPs) involved in degradation of the   extracellular matrix  . Its secretion in the tumour microenvironment makes TFPI-2 a potential inhibitor of tumour invasion and metastasis. As demonstrated in aggressive cancers, TFPI-2 is frequently down-regulated in cancer cells, but the mechanisms involved in the inhibition of tumour progression remained unclear. We showed in this study that stable TFPI-2 down-regulation in the National Cancer Institute (NCI)-H460 non-small cell lung cancer cell line using specific micro interfering micro-interfering RNA promoted tumour progression in a nude mice orthotopic model that resulted in an increase in cell invasion. Moreover, TFPI-2 down-regulation enhanced cell adhesion to collagen IV and laminin via an increase in α(1) integrin on cell surface, and increased MMP expression (mainly MMP-1 and -3) contributing to cancer cell invasion through basement membrane components. This study also reveals for the first time that pulmonary fibroblasts incubated with conditioned media from TFPI-2 silencing cancer cells exhibited increased expression of MMPs, particularly MMP-1, -3 and -7, that are likely involved in lung cancer cell invasion through the surrounding stromal tissue, thus enhancing formation of metastases. 
 Physiological and chemical inducers of tissue factor pathway inhibitor-2 in human glioma cells. Tissue factor pathway inhibitor-2 (TFPI-2), a serine protease inhibitor abundant in the   extracellular matrix  , is expressed in high amounts in low-grade, non-invasive glioma cells but in low amounts in high-grade, highly invasive glioma cells. Overexpression of TFPI-2 by highly invasive glioma cells reduces their invasiveness and thus may be useful in cancer therapy. The mechanisms underlying the transcriptional regulation of TFPI-2 are not well elucidated. We previously reported that the -312 to +1 region of TFPI-2 was critical for the minimal, inducible regulation of TFPI-2 in gliomas. This region harbors sites for several transcription factors, including SP1 (-192 to -183 and -135 to -128), AP-1 (-310 to -300, -213 to -204, and -163 to -154), NF-kappaB (-229 to -221), an NF-kappaB-like site (-291 to -281), and Lyf-1 (-260 to -252). Here we transiently transfected low-grade Hs683 glioma cells with mutant constructs to clarify the role of these transcription factors in TFPI-2 regulation. Addition of phorbol 12-myristate 13-acetate, 1,2-diacyl-sn-glycerol, IFN-gamma, or IFN-alpha induced the expression of TFPI-2 wild-type promoter construct as well as TFPI-2 protein and mRNA in Hs683 cells. Mutations at either of two AP-1 sites (-310 to -300 and -163 to -154) or either of two SP1 sites (-192 to -183 and -135 to -128) resulted in reduced TFPI-2 activity, regardless of the presence of stimulator compounds, and reduction in DNA-protein binding (by electrophoretic mobility shift assay). 
 Influence of MMP-2 and MMP-9 promoter polymorphisms on gene expression and clinical outcome of non-small cell lung cancer. Matrix metalloproteinases (MMP) including MMP-2 and MMP-9 play a major role in tumour invasion by proteolysing the   extracellular matrix  . Their activation, particularly that of MMP-9, is partly dependent on plasmin that is inhibited by TFPI-2 (tissue factor pathway inhibitor-2), a serine protease inhibitor whose gene expression is decreased in about one-third of non-small cell lung cancers (NSCLC). In addition, MMP-2 and MMP-9 are essential in the development of NSCLC and can be regulated by functional promoter polymorphisms. In this study, the -1306C/T MMP-2, -735C/T MMP-2 and -1562C/T MMP-9 polymorphisms were analysed in 90 NSCLC patients and 90 controls. In addition, the promoter region of the TFPI-2 gene was screened for sequence variations in both groups by DHPLC. A -167G/A polymorphism was identified in 3% of controls whereas none of the 90 patients exhibited this genetic variation in the TFPI-2 promoter region. Moreover, no difference in -1306C/T MMP-2, -735C/T MMP-2 and -1562C/T MMP-9 genotypes was found between cases and controls. However, the homozygous -1562CC MMP-9 genotype was more frequent in patients with squamous cell carcinoma than in controls (p=0.018). When genotype distributions were compared to MMP-2 and MMP-9 gene expression in tumours, no relationship was found with the -1306 MMP-2 and -1562 MMP-9 polymorphisms. In contrast, tumour MMP-2 gene expression was lower in homozygous -735CC patients than in those with CT or TT genotypes. In addition, the survival time was longer in patients with the MMP-2 -735T allele than in those with the CC genotype (p=0.02). The relative risk of death was increased 2.6-fold in -735CC patients (p=0.045; 95% CI=1.0-6.7). The results of this study suggest that the -735C/T MMP-2 polymorphism might be an independent prognostic marker in NSCLC, but this should be confirmed in a larger cohort of patients. 
 Adenovirus-mediated expression of tissue factor pathway inhibitor-2 inhibits endothelial cell migration and angiogenesis.   Extracellular matrix   (ECM) remodeling during angiogenesis is accomplished through plasmin-dependent pericellular proteolysis and through the action of matrix metalloproteinases (MMPs). Because tissue factor pathway inhibitor-2 (TFPI-2), a Kunitz-type protease inhibitor with prominent ECM localization, inhibits plasmin and MMPs activity, we investigated the role of TFPI-2 in endothelial cell (EC) migration and angiogenesis.Real-time polymerase chain reaction and immunostaining showed that the expression of TFPI-2 mRNA and protein was upregulated in migrating ECs. The effect of TFPI-2 on angiogenesis was studied in mouse models of Matrigel and polyvinylalcohol sponge implants by overexpressing TFPI-2 through infection with a replication-deficient adenovirus (AdTFPI-2). Using (immuno)fluorescence and confocal microscopy we observed that TFPI-2 reduced neovascularization and promoted ECM deposition. Lateral cell migration and capillary tube formation in vitro also were impaired by TFPI-2, a process reversed by anti-TFPI-2 antibodies. Increased apoptosis occurred both in AdTFPI-2-treated ECs and in the mouse implants. Zymography and assays in the absence of plasminogen confirmed plasmin inhibition as a main mechanism through which TFPI-2 inhibits EC migration.Our data suggest that TFPI-2 may be an important regulator of aberrant angiogenesis associated with tumor growth/metastasis, cardiovascular diseases, chronic inflammation, or diabetes. 
 Expression and localization of tissue factor pathway inhibitor-2 in normal and atherosclerotic human vessels. Tissue factor pathway inhibitor-2 (TFPI-2) is a Kunitz-type, serine protease inhibitor with inhibitory activity toward activated factor XI, plasma kallikrein, plasmin, certain matrix metalloproteinases, and the tissue factor:activated factor VII complex. In this study, we investigated TFPI-2 expression and localization in normal and atherosclerotic human arteries by using in situ hybridization and immunohistochemical techniques. In healthy human blood vessels, TFPI-2 was detected in the vascular endothelium alone. In human atherosclerotic tissues, TFPI-2 expression was assigned to macrophages, T cells, endothelial cells, and smooth muscle cells. Western blot analysis for TFPI-2 confirmed its production by cultured human aortic smooth muscle cells, U937 cells (monocytes), and Jurkat (T cell) cell lines. Reverse transcription-polymerase chain reaction revealed similar TFPI-2 expression levels in both monocytes and macrophages in culture. Electron microscopic study with immunogold labeling revealed the association of TFPI-2 antigen with both the   extracellular matrix   and plasma membranes. TFPI-2 antigen was detected in some areas of atheroma that also stained positively for both tissue factor and factor VII. Moreover, detection of TFPI-2 in close spatial proximity to plasmin/plasminogen on macrophages, on endothelial cells, and in matrix-rich areas highlighted its possible functional significance in the regulation of plasmin activity and downstream proteolytic mechanisms that occur in the atherosclerotic lesion. 
 Human tissue factor pathway inhibitor-2 does not bind or inhibit activated matrix metalloproteinase-1. Tissue factor pathway inhibitor-2 (TFPI-2) is a Kunitz-type serine proteinase inhibitor associated with the extracellular matrices of vascular cells. A recent report provided in vitro evidence that TFPI-2 may be a novel inhibitor of the matrix metalloproteinases MMP-1, MMP-13, MMP-2 and MMP-9. In studies aimed at identifying the structural elements of TFPI-2 mediating the putative inhibition of the above MMPs, we re-examined the ability of native TFPI-2 to form complexes with MMP-2, MMP-9 and MMP-1, as well as assess its ability to inhibit the proteolytic activity of the interstitial collagenase, activated MMP-1. We report here that TFPI-2 failed to form complexes with MMP-2, MMP-9 and MMP-1 as revealed in immunoprecipitation and ligand blotting studies. In addition, TFPI-2 had no influence on the proteolytic activity of activated MMP-1 towards triple-helical collagen. These data provide presumptive evidence that TFPI-2 does not bind to MMP-2, MMP-9 and MMP-1, or regulate MMP-1, in the   extracellular matrix  . 
 Matrix localization of tissue factor pathway inhibitor-2/matrix-associated serine protease inhibitor (TFPI-2/MSPI) involves arginine-mediated ionic interactions with heparin and dermatan sulfate: heparin accelerates the activity of TFPI-2/MSPI toward plasmin. Human tissue factor pathway inhibitor-2 (TFPI-2)/matrix-associated serine protease inhibitor (MSPI), a Kunitz-type serine protease inhibitor, inhibits plasmin, trypsin, chymotrypsin, plasma kallikrein, cathepsin G, and factor VIIa-tissue factor complex. The mature protein has a molecular mass of 32-33 kDa, but exists in vivo as two smaller, underglycosylated species of 31 and 27 kDa. TFPI-2/MSPI triplet is synthesized and secreted by a variety of cell types that include epithelial, endothelial, and mesenchymal cells. Because the majority (75-90%) of TFPI-2/MSPI is associated with the   extracellular matrix   (ECM), we examined which components of the ECM bind TFPI-2/MSPI. We found that TFPI-2/MSPI bound specifically to heparin and dermatan sulfate. Interaction of these two glycosaminoglycans (GAGs) with TFPI-2/MSPI involved one or more common protein domains, as evidenced by cross-competition experiments. However, binding affinity for TFPI-2/MSPI with heparin was 250-300 times greater than that for TFPI-2/MSPI with dermatan sulfate. Binding of TFPI-2/MSPI to GAGs was inhibited by NaCl or arginine but not by glucose, mannose, galactose, 6-aminohexanoic acid, or urea, suggesting that arginine-mediated ionic interactions participate in the GAG binding of TFPI-2/MSPI. This supposition was supported by the observation that only NaCl or arginine could elute the TFPI-2/MSPI protein triplet from an ECM derived from human dermal fibroblasts. Reduced TFPI-2/MSPI did not bind to heparin, suggesting that proper disulfide pairings and conformation are essential for matrix binding. To determine whether heparin modulates the activity of TFPI-2/MSPI, we determined the rate of inhibition of plasmin by the inhibitor with and without heparin and found that TFPI-2/MSPI is more active in the presence of heparin. Collectively, our results demonstrate that conformation-dependent arginine-mediated ionic interactions are responsible for the TFPI-2/MSPI triplet binding to fibroblast ECM, heparin, and dermatan sulfate and that heparin augmented the rate of inhibition of plasmin by TFPI-2/MSPI. 
 CD24 promotes tumor cell invasion by suppressing tissue factor pathway inhibitor-2 (TFPI-2) in a c-Src-dependent fashion. CD24 is a glycosyl-phosphatidylinositol-anchored protein with mucin-type structure that resides exclusively in membrane microdomains. CD24 is often highly expressed in carcinomas and correlates with poor prognosis. Experimentally, the over-expression or depletion of CD24 alters cell proliferation, adhesion, and invasion in vitro and tumor growth in vivo. However, little is known about the mechanisms by which CD24 mediates these cellular effects. Here we have studied the mechanism of CD24-dependent cell invasion using transient CD24 knock-down or over-expression in human cancer cell lines. We show that CD24 depletion reduced tumor cell invasion and up-regulated expression of Tissue Factor Pathway Inhibitor 2 (TFPI-2), a potent inhibitor of   extracellular matrix   degradation that can block metastases formation and tumor cell invasion. Over-expression of CD24 in A125 cells resulted in reduced TFPI-2 expression and enhanced invasion. We provide evidence that the activity of c-Src is reduced upon CD24 knock-down. The silencing of c-Src, similar to CD24, was able to enhance TFPI-2 expression and reduce tumor cell invasion. An inverse expression of CD24 and TFPI-2 was observed by immunohistochemical analysis of primary breast cancers (N = 1,174). TFPI-2 expression was highest in CD24 negative samples and lowered with increasing CD24 expression. Patients with a CD24 low/TFPI-2 high phenotype showed significantly better survival compared to CD24 high/TFPI-2 low patients. Our results provide evidence that CD24 can regulate cell invasion via TFPI-2 and suggests a role of c-Src in this process. 
 The effect of human tissue factor pathway inhibitor-2 on the growth and metastasis of fibrosarcoma tumors in athymic mice. Human tissue factor pathway inhibitor-2 (TFPI-2) is a matrix-associated Kunitz inhibitor that inhibits the plasmin- and trypsin-mediated activation of zymogen matrix metalloproteinases involved in tumor progression, invasion, and metastasis. To directly assess its role in tumor growth and metastasis in vivo, we stably transfected HT-1080 fibrosarcoma cells expressing either fully active wild-type human TFPI-2 (WT) or inactive R24Q TFPI-2 (QT) and examined their ability to form tumors and metastasize in athymic mice in comparison to mock-transfected cells (MT). MT and QT fibrosarcoma tumors grew 2 to 3 times larger than WT tumors. Tumor metastasis was confined to the lung and was observed in 75% of mice treated with either MT or QT cells, whereas only 42% of mice treated with WT cells developed lung metastases. Real-time quantitative reverse transcriptase-polymerase chain reaction (RT-PCR) analyses of each tumor group revealed 3- to 6-fold lower levels of murine vascular endothelial growth factor gene expression in WT tumors in relation to either MT or QT tumors. Comparative tumor gene expression analysis revealed that several human genes implicated in oncogenesis, invasion, metastasis, apoptosis, and angiogenesis had significantly altered levels of expression in WT tumors. Our collective data demonstrate that secretion of inhibitory TFPI-2 by a highly metastatic tumor cell markedly inhibits its growth and metastasis in vivo by regulating pericellular   extracellular matrix   (ECM) remodeling and angiogenesis. 
 Epigenetic inactivation of TFPI-2 as a common mechanism associated with growth and invasion of pancreatic ductal adenocarcinoma. Using microarrays, we have screened for genes reactivated by drugs that modify epigenetic mechanisms in pancreatic cancer cells. One of the genes identified was tissue factor pathway inhibitor 2 (TFPI-2), which encodes for a broad-spectrum serine proteinase inhibitor that negatively regulates the   extracellular matrix   degradation, an essential step in tumor invasion and metastasis. We therefore investigated the expression and methylation patterns of the TFPI-2 gene in pancreatic adenocarcinoma, and determined its role in tumor growth and invasion. In contrast to its abundant expression in normal pancreas, TFPI-2 mRNA was undetectable in a high fraction of pancreatic cancer cell lines and in primary pancreatic ductal neoplasms (IPMNs). Loss of TFPI-2 expression was associated with aberrant hypermethylation of its promoter CpG island. Treatment with the phorbol ester (PMA), known to stimulate the TFPI-2 promoter activity, augmented the TFPI-2 expression in cell lines with unmethylated or partially methylated TFPI-2, but failed to induce the expression in cell lines that harbored fully methylated TFPI-2. Aberrant methylation of TFPI-2 was also detected in 73% (102/140) of pancreatic cancer xenografts and primary pancreatic adenocarcinomas, was more likely in older patients with pancreatic cancer, and significantly correlated with progression of IPMNs (P=0.0002). Restored expression of the TFPI-2 gene in nonexpressing pancreatic cancer cells resulted in marked suppression in their proliferation, migration, and invasive potential in vitro. We thus conclude that epigenetic inactivation of TFPI-2 is a common mechanism that contributes to the aggressive phenotype of pancreatic ductal adenocarcinoma. 
   Extracellular matrix  -associated serine protease inhibitors (Mr 33,000, 31,000, and 27,000) are single-gene products with differential glycosylation: cDNA cloning of the 33-kDa inhibitor reveals its identity to tissue factor pathway inhibitor-2. Recently, we reported the identification and partial characterization of three serine protease inhibitors (M(r) 33,000, 31,000, and 27,000) from the   extracellular matrix   (ECM) of human umbilical vein endothelial cells and skin cells. Here, we report that a full-length cDNA clone for the 33-kDa inhibitor from SV-40 transformed human skin fibroblasts (t12FB) is identical to a recombinant trypsin/tissue factor pathway inhibitor called TFPI-2 from placenta. By immunoblotting, the three inhibitors from ECM and cell lysates demonstrated cross-reactivity with an antiTFPI-2 IgG. To further elucidate how these inhibitors are related, pulse-chase labeling of t12FB with [35S]methionine followed by immunoprecipitation with antiTFPI-2 IgG was performed on ECM and cytosolic proteins. A precursor-product relationship did not exist between the three inhibitors from ECM. In contrast, the various species of inhibitors from cytosolic fractions demonstrated a precursor-product relationship. Within the cytosolic fraction, 26-, 29-, and 30-kDa inhibitors were detected in the early chases (0 and 15 min) but they form precursors to the synthesis of the 33-kDa inhibitor which accumulated in the later chases (30 min to 1 h). When pulse-chase experiments were performed in the presence of tunicamycin, synthesis as well as sequestration of the three inhibitors into ECM was completely inhibited. In the presence of tunicamycin, the cells synthesized and sequestered a single 25.5-kDa inhibitor into ECM. Peak quantities of the 25.5-kDa inhibitor appeared in the ECM after 6 h chase while they were 1 h for the 27- and 31-kDa inhibitors and 3 h for the 33-kDa inhibitor. To further support that the three inhibitors are related but only differ in the extent of glycosylation, the 33-kDa inhibitor from the t12FB ECM was deglycosylated with N-glycosidase F and the products were identified by immunoblotting with antiTFPI-2 IgG. The enzyme released the 31-, 27-, and 25.5-kDa inhibitors from the 33-kDa inhibitor. Collectively, these results demonstrate that the ECM-associated 33-, 31-, and 27-kDa inhibitors are biosynthetic products of a single gene with differential glycosylation. The 25.5-kDa inhibitor is unglycosylated, whereas 27- and 30- to 31-kDa inhibitors are partially glycosylated and the 33-kDa inhibitor is fully glycosylated. Inhibition of glycosylation significantly retarded the rate of secretion of the inhibitor but did not prevent its association with ECM. Quantitation of the inhibitors with cell-conditioned medium and ECM fractions reveals that 70-75% were ECM-associated and 25-30% cell-associated. None or very little of the inhibitors (0-2%) remained in a conditioned medium. Because they are primarily associated with ECM, the inhibitors may play a major role in ECM remodeling and turnover. 
 Quantification and characterization of human endothelial cell-derived tissue factor pathway inhibitor-2. Tissue factor pathway inhibitor-2 (TFPI-2), also known as placental protein 5, is a serine protease inhibitor consisting of three tandemly-arranged Kunitz-type protease inhibitor domains. While TFPI-2 is a potent inhibitor of trypsin, plasmin, kallikrein, and factor XIa in the test tube, the function of this inhibitor in vivo remains unclear. In the present study, we investigated the synthesis and secretion of TFPI-2 by cultured endothelial cells derived from human umbilical vein, aorta, saphenous vein, and dermal microvessels to gain insight into its biological function. While all endothelial cells examined synthesized and secreted TFPI-2, dermal microvascular endothelial cells synthesized threefold to sevenfold higher levels of TFPI-2. Approximately 60% to 90% of the TFPI-2 secreted by endothelial cells was directed to the subendothelial   extracellular matrix   (ECM). When cultured human umbilical vein endothelial cells were stimulated with inflammatory mediators such as phorbol 12-myristate,13-acetate; endotoxin; and tumor necrosis factor-alpha, TFPI-2 synthesis by these cells increased twofold to 14-fold. Recombinant TFPI-2 bound to dermal microvascular endothelial cell monolayers and its ECM in a specific, dose-dependent, and saturable manner with Kd values of 21 and 24 nmol/L, respectively. TFPI-2 interacted with 4.5 X 10(10) sites/cm2 (3 X 10[5] sites/cell) and 2.3 X 10(11) sites/cm2 on endothelial cells and ECM, respectively. In the presence of rabbit anti-TFPI-2 IgG, but not preimmune IgG, endothelial cells dissociated from the culture flask in a time- and IgG concentration-dependent manner. Our findings provide evidence that endothelial cell-derived TFPI-2 is primarily secreted into the abluminal space and presumably plays an important role in maintaining the integrity of the ECM essential for cell attachment. 
 Tissue factor pathway inhibitor 2 is induced by thrombin in human macrophages. Tissue factor pathway inhibitor 2 (TFPI2) is a serine protease inhibitor critical for the regulation of   extracellular matrix   remodeling and atherosclerotic plaque stability. Previously, we demonstrated that TFPI2 expression is increased in monocytes from patients with familial combined hyperlipidemia (FCH). To gain insight into the molecular mechanisms responsible for this upregulation, we examined TFPI2 expression in THP-1 macrophages exposed to lipoproteins and thrombin. Our results showed that TFPI2 expression was not affected by treatment with very low density lipoproteins (VLDL), but was induced by thrombin (10 U/ml) in THP-1 (1.9-fold increase, p<0.001) and human monocyte-derived macrophages (2.3-fold increase, p<0.005). The specificity of the inductive effect was demonstrated by preincubation with the thrombin inhibitors hirudin and PPACK, which ablated thrombin effects. TFPI2 induction was prevented by pre-incubation with MEK1/2 and JNK inhibitors, but not by the EGF receptor antagonist AG1478. In the presence of parthenolide, an inhibitor of NFκB, but not of SR-11302, a selective AP-1 inhibitor, thrombin-mediated TFPI2 induction was blunted. Our results also show that thrombin treatment increased ERK1/2, JNK and IκBα phosphorylation. Finally, we ruled out the possibility that TFPI2 induction by thrombin was mediated by COX-2, as preincubation with a selective COX-2 inhibitor did not prevent the inductive effect. In conclusion, thrombin induces TFPI2 expression by a mechanism involving ERK1/2 and JNK phosphorylation, leading finally to NFkB activation. In the context of atherosclerosis, thrombin-induced macrophage TFPI2 expression could represent a means of avoiding excessive activation of matrix metalloproteases at sites of inflammation. 
 Human tissue factor pathway inhibitor-2 suppresses the wound-healing activities of human Tenon's capsule fibroblasts in vitro. Human tissue Factor Pathway Inhibitor-2 (TFPI-2) is a potent inhibitor of plasmin, which activates metalloproteinases involved in   extracellular matrix   degradation. Its secretion in the   extracellular matrix   makes TFPI-2 a potential inhibitor of tumor cell invasion. However, no studies have yet evaluated the wound-healing activities of human Tenon's capsule fibroblasts (hTCFs). The aim of the study is to elucidate the effect of TFPI-2 overexpression on hTCF proliferation and migration, to determine whether TFPI-2 may act as an antiscarring agent in vivo after glaucoma filtration surgery.Plasmid vector pBos-Cite-neo/TFPI-2 was transfected into hTCFs with Lipofectamine 2000. After selection by G418, transfected, non-transfected, and mock-transfected cells were screened for TFPI-2 mRNA and protein by reverse transcription-PCR and western blot analysis respectively. Cell proliferation and viability were determined by 3-(4,5-Dimethylthiazol-2-yl)-2,5-diphenyltetrazolium bromide (MTT) assay and flow cytometry. Cell migration was studied on restrained collagen gels and with a scratch-wound assay.TFPI-2 expression of mRNA and protein was confirmed in transfected cells. The transfected, non-transfected, and mock-transfected cells showed no significant difference in cell proliferation and apoptosis, with TFPI-2 found not to be cytotoxic in hTCFs. Overexpression of TFPI-2 significantly suppressed cell migration three- to four-fold on collagen gel for 2 weeks and in the scratch-wound assay for 2 d (39.27+/-2.40% versus 16.43+/-1.10% at 1 d, and 79.0+/-3.04% versus 30.13+/-2.1% at 2 d).TFPI-2 expression may strongly inhibit the migration ability of hTCFs in vitro, making it a promising candidate for novel therapies to minimize scar development after glaucoma drainage surgery. 
 Identification of a human TFPI-2 splice variant that is upregulated in human tumor tissues. Previous studies have shown that the expression of tissue factor pathway inhibitor-2 (TFPI-2), a matrix-associated Kunitz-type serine proteinase inhibitor, is markedly down-regulated in several tumor cells through hypermethylation of the TFPI-2 gene promoter. In the present study, RT-PCR analysis of total RNA from both human normal and tumor cells revealed a novel 289 nucleotide splice variant of the TFPI-2 transcript designated as aberrantly-spliced TFPI-2 (asTFPI-2).Nucleotide sequence analyses indicated that asTFPI-2 consists of complete exons II and V, fused with several nucleotides derived from exons III and IV, as well as six nucleotides derived from intron C. 5'- and 3'-RACE analyses of total RNA amplified exclusively the wild-type TFPI-2 transcript, indicating that asTFPI-2 lacks either a 5'-untranslated region (UTR) or a 3'-poly (A)+ tail. Quantitative real-time RT-PCR analyses revealed that several human tumor cells contain 4 to 50-fold more copies of asTFPI-2 in comparison to normal cells. In spite of the absence of a 5'-UTR or poly (A)+ tail, the asTFPI-2 variant exhibited a half-life of ~16 h in tumor cells.Our studies reveal the existence of a novel, aberrantly-spliced TFPI-2 transcript predominantly expressed in tumor cells and provides suggestive evidence for an additional mechanism for tumor cells to down-regulate TFPI-2 protein expression enhancing their ability to degrade the   extracellular matrix  . 
 Tissue factor pathway inhibitor-2 inhibits the growth and invasion of hepatocellular carcinoma cells and is inactivated in human hepatocellular carcinoma. Human tissue factor pathway inhibitor-2 (TFPI-2) is an   extracellular matrix  -associated Kunitz-type serine proteinase inhibitor that inhibits the plasmin- and trypsin-mediated activation of matrix metalloproteinases and inhibits tumor progression, invasion and metastasis. Previous studies have shown that TFPI-2 is downregulated in the progression of various tumors. The purpose of this study was to investigate the expression and function of TFPI-2 in hepatocellular carcinoma (HCC). In situ hybridization was used to detect human TFPI-2 mRNA and immunohistochemistry was performed to examine the role of TFPI-2 expression in hepatocarcinoma tissues. Cell proliferation was assessed using MTT assay. In situ hybridization and immunohistochemical analyses revealed that the expression of TFPI-2 in hepatocarcinoma tissues was markedly lower than that in tumor-adjacent normal hepatic tissues. Restored expression of TFPI-2 in HepG(2) cells inhibits cell proliferation and invasion. Taken together, the results suggest that TFPI-2 has a tumor-suppression action and its inactivation may contribute to HCC. 
 Human tissue factor pathway inhibitor-2 induces caspase-mediated apoptosis in a human fibrosarcoma cell line. Human TFPI-2 is an   extracellular matrix  -associated Kunitz-type serine proteinase inhibitor. We previously demonstrated that a human fibrosarcoma cell line, HT-1080, does not express TFPI-2, but genetic restoration of TFPI-2 expression in these cells markedly inhibited their growth and metastasis in vivo. In the present study, either full-length recombinant TFPI-2, or its mutated first Kunitz-type domain (R24K KD1), were offered to HT-1080 cells, and the degree of apoptosis assessed by nuclear fragmentation, ethidium bromide and acridine orange staining, fluorescence activated cell sorting, immunoblotting and gene expression profiling. R24K KD1 induced apoptosis in 69% of HT-1080 cells in a 48 h period compared to 39% for TFPI-2, while a KD1 preparation lacking a reactive site arginine/lysine residue (R24Q KD1) produced only an 18% apoptosis rate, suggesting that the observed apoptosis was related to proteinase inhibition. Immunoblotting experiments indicated increased caspase 3 and 9 activation, up-regulation of pro-apoptotic Bax and suppression of anti-apoptotic Bcl-2 protein. Finally, microarray analyses of R24K KD1-treated cells indicated elevated expression of several pro-apoptotic genes and under-expression of anti-apoptotic genes. Collectively, our results demonstrate that treatment of HT-1080 cells exogenously with either TFPI-2 or R24K KD1 activates caspase-mediated, pro-apoptotic signaling pathways resulting in apoptosis. 
 Regulation of tissue factor pathway inhibitor-2 (TFPI-2) expression by lysine-specific demethylase 1 and 2 (LSD1 and LSD2). Tissue factor pathway inhibitor-2 (TFPI-2) is a major inhibitor of   extracellular matrix   degradation. Decreases in TFPI-2 contribute to malignant tumor cell production, and TFPI-2 is a presumed tumor suppressor. TFPI-2 gene transcription is regulated by two epigenetic mechanisms: DNA methylation of the promoter and K4 methylation of histone 3 (H3). Lysine-specific demethylase 1 (LSD1) and LSD2 demethylate H3K4me2/1. LSD1 has been implicated in TFPI-2 regulation through both epigenetic mechanisms, but the involvement of LSD2 remains unknown. We prepared a monoclonal anti-LSD2 antibody that clearly distinguishes LSD2 from LSD1. Knockdown of LSD1 or LSD2 by siRNAs increased TFPI-2 protein and mRNA. Simultaneous knockdown of both LSD1 and LSD2 showed additive effects. Bisulfite sequencing revealed that CpG sites in the TFPI-2 promoter region were unmethylated. These results indicate that LSD2 also contributes to TFPI-2 regulation through histone modification, and that further studies of the involvement of LSD2 in tumor malignancy are warranted. 
 Reduced expression of tissue factor pathway inhibitor-2 contributes to apoptosis and angiogenesis in cervical cancer. Tissue factor pathway inhibitor-2 (TFPI-2) is an   extracellular matrix   associated broad-spectrum Kunitz-type serine proteinase inhibitor. Recently, down regulation of TFPI-2 was suggested to be involved in tumor invasion and metastasis in some cancers.This study involved 12 normal cervical squamous epithelia, 48 cervical intraepithelial neoplasia (CIN), and 68 cervical cancer. The expression of TFPI-2, Ki-67 and vascular endothelial growth factor (VEGF) were investigated by immunohistochemistry staining. The apoptolic index(AI) was determined with an in situ end-labeling assay(TUNEL). And the marker of CD34 staining was used as an indicator of microvessel density (MVD).TFPI-2 expression has a decreasing trend with the progression of cervical cancer and was significantly correlated with FIGO stage, lymph node metastasis and HPV infection. In addition, there were significant positive correlations between the grading of TFPI-2 expression and AI(P=0.004). In contrast, the expression of TFPI-2 and VEGF or MVD was negatively correlated (both p < 0.001). However, we did not establish any signiﬁcant correlation between Ki-67 and TFPI-2 expression in cervical cancer.The results suggested that the expression of TFPI-2 had a decreasing trend with tumor progression of cervical cancer. There was a close association between the expression of TFPI-2 and tumor cell apoptosis and angiogenesis in patients with cervical cancer. TFPI-2 may play an inhibitive role during the development of cervical cancer. 
  Component  
   extracellular matrix   
 proteinaceous   extracellular matrix   
  GeneRIFs  
 These data provide evidence that tissue factor pathway inhibitor-2 does not bind to matrix metalloproteinases-2, -9 or -1, or regulate matrix metalloproteinase-1, in the   extracellular matrix  . 
  [Ii]nvasive  PubMed  
 Tissue factor pathway inhibitor-2 was repressed by CpG hypermethylation through inhibition of KLF6 binding in highly   invasive   breast cancer cells. Tissue factor pathway inhibitor-2 (TFPI-2) is a matrix-associated Kunitz inhibitor that inhibits plasmin and trypsin-mediated activation of zymogen matrix metalloproteinases involved in tumor progression, invasion and metastasis. Here, we have investigated the mechanism of DNA methylation on the repression of TFPI-2 in breast cancer cell lines.We found that both protein and mRNA of TFPI-2 could not be detected in highly   invasive   breast cancer cell line MDA-MB-435. To further investigate the mechanism of TFPI-2 repression in breast cancer cells, 1.5 Kb TFPI-2 promoter was cloned, and several genetic variations were detected, but the promoter luciferase activities were not affected by the point mutation in the promoter region and the phenomena was further supported by deleted mutation. Scan mutation and informatics analysis identified a potential KLF6 binding site in TFPI-2 promoter. It was revealed, by bisulfite modified sequence, that the CpG island in TFPI-2 promoter region was hypermethylated in MDA-MB-435. Finally, using EMSA and ChIP assay, we demonstrated that the CpG methylation in the binding site of KLF-6 diminished the binding of KLF6 to TFPI-2 promoter.In this study, we found that the CpG islands in TFPI-2 promoter was hypermethylated in highly   invasive   breast cancer cell line, and DNA methylation in the entire promoter region caused TFPI-2 repression by inducing inactive chromatin structure and decreasing KLF6 binding to its DNA binding sequence. 
 Tissue factor pathway inhibitor-2 was repressed by CpG hypermethylation through inhibition of KLF6 binding in highly   invasive   breast cancer cells. Tissue factor pathway inhibitor-2 (TFPI-2) is a matrix-associated Kunitz inhibitor that inhibits plasmin and trypsin-mediated activation of zymogen matrix metalloproteinases involved in tumor progression, invasion and metastasis. Here, we have investigated the mechanism of DNA methylation on the repression of TFPI-2 in breast cancer cell lines.We found that both protein and mRNA of TFPI-2 could not be detected in highly   invasive   breast cancer cell line MDA-MB-435. To further investigate the mechanism of TFPI-2 repression in breast cancer cells, 1.5 Kb TFPI-2 promoter was cloned, and several genetic variations were detected, but the promoter luciferase activities were not affected by the point mutation in the promoter region and the phenomena was further supported by deleted mutation. Scan mutation and informatics analysis identified a potential KLF6 binding site in TFPI-2 promoter. It was revealed, by bisulfite modified sequence, that the CpG island in TFPI-2 promoter region was hypermethylated in MDA-MB-435. Finally, using EMSA and ChIP assay, we demonstrated that the CpG methylation in the binding site of KLF-6 diminished the binding of KLF6 to TFPI-2 promoter.In this study, we found that the CpG islands in TFPI-2 promoter was hypermethylated in highly   invasive   breast cancer cell line, and DNA methylation in the entire promoter region caused TFPI-2 repression by inducing inactive chromatin structure and decreasing KLF6 binding to its DNA binding sequence. 
 Minimal and inducible regulation of tissue factor pathway inhibitor-2 in human gliomas. Tissue factor pathway inhibitor-2 (TFPI-2), a serine protease inhibitor abundant in the extra cellular matrix, is highly expressed in non-  invasive   cells but undetectable levels in highly   invasive   human glioma cells. The mechanisms responsible for its transcriptional regulation are not well elucidated. In this study, we made several deletion constructs from a 3.6 kb genomic fragment from Hs683 cells containing the 5'-flanking region of the TFPI-2 gene, transiently transfected with these constructs into non-  invasive   (Hs683) and highly   invasive   (SNB19) human glioma cells, and assessed their expression by using a luciferase reporter gene. Three constructs showed high promoter activity (pTF5, -670 to +1; pTF6, -312 to +1; pTF2, -1511 to +1). Another construct, pTF8 (-81 to +1), showed no activity. PTF9, a variant of pTF5 in which a further 231 bp fragment (-312 to -81) was deleted, from the [-670 to +1] pTF5 region, also showed no promoter activity. Hence, (-312 to -81) this region is essential for the transcription of TFPI-2 in glioma cells. Sequencing of this promoter region revealed that it has a high G+C content, contains potential SP1 and AP1 binding motifs, and lacks canonical TATA and CAAT boxes immediately upstream of the major transcriptional initiation site, although CAAT boxes were found about -3000 bp upstream of the transcription start site. We also found a strong repressor in the region between -927 to -1181, upstream of the major transcriptional initiation site, followed by positive elements or enhancers between -1511 to -1181. These positive elements masked the silencer effect. Finally TFPI-2 was induced in Hs683 cells transfected with the pTF6 construct (-312 to +1) and stimulated with phorbol-12-myristate-13-acetate (PMA). We conclude that the -312 to +1 region is critical for the minimal and inducible regulation of TFPI-2 in non-  invasive   (Hs683) and highly   invasive   (SNB19) human glioma cell lines. 
 Promoter methylation and silencing of the tissue factor pathway inhibitor-2 (TFPI-2), a gene encoding an inhibitor of matrix metalloproteinases in human glioma cells. We have shown previously that the tissue factor pathway inhibitor-2 (TFPI-2), a broad range proteinase inhibitor, is highly expressed in low-grade gliomas, but, minimally expressed or undetectable in glioblastomas, and that enforced expression of this gene reduces the   invasive   properties of brain tumor cells. Here, we examined the role of promoter methylation as a mechanism of TFPI-2 gene silencing. In SNB19 glioblastoma cells, which have no detectable TFPI-2 expression, 5-aza-2'-deoxycytidine (5aC), an inhibitor of DNA methyltransferase, induced TFPI-2 mRNA in a dose-dependent manner. Trichostatin A (TSA), the histone deacetylase (HDAC) inhibitor, by itself, was more efficient than 5aC in inducing TFPI-2 transcripts, and the 5aC+TSA combination resulted in highly synergistic reactivation of the gene, both at the transcript and protein levels. In Hs683 glioma cells, which express the TFPI-2 gene at high levels, transfection of the in vitro methylated TFPI-2 promoter constructs resulted in a drastic decrease of promoter activity compared to the unmethylated promoter. Further, the methylation-specific PCR in SNB19 and Hs683 cells showed that TFPI-2 gene repression was closely linked with methylation of the CpG islands in the promoter. Finally, the chromatin immunoprecipitation assays in SNB19 cells showed that the methylated and repressed TFPI-2 promoter was associated with the methyl-CpG binding protein 2 (MeCP2), and that gene reactivation resulted in the loss of MeCP2 from this site. These studies establish that TFPI-2 is transcriptionally silenced through promoter methylation in SNB19 cells. 
 Physiological and chemical inducers of tissue factor pathway inhibitor-2 in human glioma cells. Tissue factor pathway inhibitor-2 (TFPI-2), a serine protease inhibitor abundant in the extracellular matrix, is expressed in high amounts in low-grade, non-  invasive   glioma cells but in low amounts in high-grade, highly   invasive   glioma cells. Overexpression of TFPI-2 by highly   invasive   glioma cells reduces their   invasive  ness and thus may be useful in cancer therapy. The mechanisms underlying the transcriptional regulation of TFPI-2 are not well elucidated. We previously reported that the -312 to +1 region of TFPI-2 was critical for the minimal, inducible regulation of TFPI-2 in gliomas. This region harbors sites for several transcription factors, including SP1 (-192 to -183 and -135 to -128), AP-1 (-310 to -300, -213 to -204, and -163 to -154), NF-kappaB (-229 to -221), an NF-kappaB-like site (-291 to -281), and Lyf-1 (-260 to -252). Here we transiently transfected low-grade Hs683 glioma cells with mutant constructs to clarify the role of these transcription factors in TFPI-2 regulation. Addition of phorbol 12-myristate 13-acetate, 1,2-diacyl-sn-glycerol, IFN-gamma, or IFN-alpha induced the expression of TFPI-2 wild-type promoter construct as well as TFPI-2 protein and mRNA in Hs683 cells. Mutations at either of two AP-1 sites (-310 to -300 and -163 to -154) or either of two SP1 sites (-192 to -183 and -135 to -128) resulted in reduced TFPI-2 activity, regardless of the presence of stimulator compounds, and reduction in DNA-protein binding (by electrophoretic mobility shift assay). 
 Hypermethylation of TFPI2 correlates with cervical cancer incidence in the Uygur and Han populations of Xinjiang, China. Tissue factor pathway inhibitor 2 (TFPI2) is a Kunitz-type serine proteinase inhibitor, which plays an important role in the etiology of human malignancies. DNA methylation is a common epigenetic modification of the genome that is involved in regulating many cellular processes. In addition to human papilloma virus (HPV) infection, DNA methylation may play a role in the carcinogenesis of cervical cancer. Methylation of 22 CpG sites in the promoter region of the TFPI2 gene was detected by MassARRAY spectrometry and a gene mass spectrogram was drawn using MALDI-TOF MS. HPV16 was detected by PCR. We show that aberrant methylation of TFPI2 is present in a higher proportion of   invasive   cervical carcinoma (ICC) clinical samples as compared to normal cervical samples in Uygur and Han. Across the four pathologic lesions of the progression of cervical cancer, ICC showed the highest level of aberrant methylation, and with a stronger correlation between CpG site and lesion grade in Uygur than in Han. Moreover, a difference in TFPI2 methylation between Uygur patients positive and negative for HPV16 infection was observed at CpG_6 (P = 0.028) and CpG_15 (P = 0.007). Altogether, these results indicate that DNA methylation of TFPI2 may play an important role in the carcinogenesis of cervical cancer and that the differential methylation of TFPI2 may at least partially explain the disparity in cervical cancer incidence between Uygur and Han women. 
 Detection of methylated tissue factor pathway inhibitor 2 and human long DNA in fecal samples of patients with colorectal cancer in China. To investigate the feasibility of detecting methylated tissue factor pathway inhibitor (TFPI2) and quantifying human long DNA with fluorescent quantitative Alu PCR in fecal DNA as a non-  invasive   screening tool for colorectal cancer (CRC).Methylation-specific PCR (MSP) was performed to analyze TFPI2 gene promoter methylation status in a blinded fashion in stool samples taken from 30 endoscopically diagnosed healthy controls, 20 patients with adenomas, and 60 patients with colorectal cancer. Real-time Alu PCR was used to quantify human long DNA.The specificity of fecal TFPI2 MSP assay and long DNA assay was 100% and 83.3%, respectively. The sensitivity of fecal TFPI2 MSP assay and long DNA assay was 68.3% and 53.3%, respectively. The sensitivity of fecal DNA assay (either marker being positive) was 86.7%, which was high for CRC.Our results have demonstrated the feasibility of using TFPI2 methylation and quantify human long DNA with fluorescent quantitative Alu PCR in fecal samples as a new non  invasive   test for CRC. 
 EGCG inhibits growth and induces apoptosis in renal cell carcinoma through TFPI-2 overexpression. EGCG (epigallocatechin gallate), the major catechin found in green tea, has been shown to inhibit proliferation and induce apoptosis in many tumors. EGCG can inhibit DNMT (DNA methyltransferase) activity, and cause CpG demethylation and reactivation of methylation-silenced genes. Tissue factor pathway inhibitor-2 (TFPI-2), a member of the Kunitz-type serine proteinase inhibitor family, is inversely related to an increasing degree of malignancy. Our previous study showed that the expression of TFPI-2 and   invasive  ness of renal cell carcinoma had a negative correlation. Overexpression of TFPI-2 may induce tumor cell apoptosis in renal cell carcinoma. Lower expression of TFPI-2 in renal cell carcinoma was partly due to hypermethylation of the gene promoter. Herein, using MTT and flow cytometry, we demonstrated that EGCG can inhibit growth and induces apoptosis in renal cell carcinoma cell line 786-0. In addition, Western blotting and real-time RT-PCR showed that EGCG can upregulate expression of TFPI-2. Before and after EGCG treatment, real-time methylation specific PCR could not detect methylation status of TFPI-2 gene promoter in cell line 786-0. In vivo   invasive  ness and metastasis test did not indicate any significant differences between control and treatment group. Our results suggest that EGCG inhibits growth and induces apoptosis in renal cell carcinoma through TFPI-2 overexpression. This is the first report showing that EGCG is likely to be an effective agent for renal cell carcinoma. 
 Silencing of tissue factor pathway inhibitor-2 gene in malignant melanomas. To identify tumor-suppressor genes inactivated by aberrant methylation of promoter CpG islands (CGIs) in human malignant melanomas, genes upregulated by treatment of cells with a demethylating agent, 5-aza-2'-deoxycytidine (5-aza-dC), were searched for using oligonucleotide microarrays in melanoma cell lines, HMV-I, MeWo and WM-115. Seventy-nine known genes with CGIs were identified as being upregulated (>or=16-fold), and 18 of them had methylation of their putative promoter CGIs in 1 or more of 8 melanoma cell lines. Among the 18 genes, TFPI-2, which is involved in repression of the   invasive   potential of malignant melanomas, was further analyzed. Its expression was repressed in a melanoma cell line with its complete methylation, and was restored by 5-aza-dC treatment. It was unmethylated in cultured neonatal normal epidermal melanocyte, and was induced by ultraviolet B. In surgical melanoma specimens, TFPI-2 methylation was detected in 5 of 17 metastatic site specimens (29%), while it was not detected in 20 primary site specimens (0%) (p=0.009). By immunohistochemistry, the 5 specimens with promoter methylation lacked immunoreactivity for TFPI-2. The results showed that TFPI-2 is silenced in human malignant melanomas by methylation of its promoter CGI and suggested that its silencing is involved in melanoma metastasis. 
 A novel role for tissue factor pathway inhibitor-2 in the therapy of human esophageal carcinoma. Esophageal cancer is characterized by rapid clinical progression and poor prognosis, due to early-stage invasion of adjacent tissues and metastasis. Tissue factor pathway inhibitor-2 (TFPI-2) has been implicated as a metastasis-associated gene in many types of tumors. Here we describe the potential involvement of TFPI-2 in the development of esophageal carcinoma. Western blotting revealed that TFPI-2 was downregulated in 75% of esophageal carcinomas and in most esophageal carcinoma cell lines. Immunohistochemistry revealed that TFPI-2 was significantly downregulated in tumor tissues and in lymph node metastases. Experimental overexpression of TFPI-2 in KYSE450, KYSE510, YES2, and EC9706 cells significantly inhibited their   invasive   ability. Overexpression of TFPI-2 in EC9706 cells inhibited xenograft tumor growth and invasion into surrounding tissues, as well as reduced lung metastasis. Further studies demonstrated that recombinant TFPI-2 protein significantly inhibited the activity of matrix metalloproteinases and tumor-related angiogenesis. Parenteral treatment with recombinant TFPI-2 protein significantly suppressed xenograft growth and metastasis. Together, these data indicate that TFPI-2 inhibits tumor invasion and angiogenesis both in vitro and in vivo, and suggest a potentially important therapeutic role for recombinant TFPI-2 in the treatment of malignant esophageal carcinomas. 
 Detection of TFPI2 methylation in the serum of hepatocellular carcinoma patients. DNA methylation plays a key role in hepatocellular carcinogenesis and progression. Analysis of aberrant methylation in serum DNA might provide a strategy for non  invasive   detection of hepatocellular carcinoma (HCC).To explore the feasibility of this approach, we compared TFPI2 methylation status in serum samples of HCC, chronic hepatitis B (CHB) patients and normal control groups using methylation-specific polymerase chain reaction.Our results showed that the percentage of serum TFPI2 promoter methylation was significantly higher in the HCC group (46.5 %, 20/43) compared with the CHB group (16.7 %, 4/24; p = 0.015) and the normal control group (19.2 %, 5/26; p = 0.022), respectively, indicating that TFPI2 methylation frequently existed in the serum of HCC patients. In our study, the detection rate of HCC using serum TFPI2 methylation was 46.5 % (20/43), which was quite close to the reported detection rate of α-fetoprotein (54 %). In cases where we combined both markers, the detection rate was 61.0 %, suggesting that serum TFPI2 methylation could be used as a potential marker for no  ninvasiv  e detection of HCC. Then, we evaluated the correlation between the serum TFPI2 methylation status of HCC patients and their clinicopathological parameters. Patients with advanced TNM stage (III-IV) showed a significantly elevated serum methylation percentage of TFPI2 in comparison with those with early TNM stage (I-II) (p = 0.025). Moreover, TFPI2 methylation was observed more frequently according to the progression of TNM stage.Our present study suggested that TFPI2 methylation in serum tended to be detected more easily in patients with advanced HCC and might be used as a predictor of HCC progression. 
 Epigenetic inactivation of TFPI-2 as a common mechanism associated with growth and invasion of pancreatic ductal adenocarcinoma. Using microarrays, we have screened for genes reactivated by drugs that modify epigenetic mechanisms in pancreatic cancer cells. One of the genes identified was tissue factor pathway inhibitor 2 (TFPI-2), which encodes for a broad-spectrum serine proteinase inhibitor that negatively regulates the extracellular matrix degradation, an essential step in tumor invasion and metastasis. We therefore investigated the expression and methylation patterns of the TFPI-2 gene in pancreatic adenocarcinoma, and determined its role in tumor growth and invasion. In contrast to its abundant expression in normal pancreas, TFPI-2 mRNA was undetectable in a high fraction of pancreatic cancer cell lines and in primary pancreatic ductal neoplasms (IPMNs). Loss of TFPI-2 expression was associated with aberrant hypermethylation of its promoter CpG island. Treatment with the phorbol ester (PMA), known to stimulate the TFPI-2 promoter activity, augmented the TFPI-2 expression in cell lines with unmethylated or partially methylated TFPI-2, but failed to induce the expression in cell lines that harbored fully methylated TFPI-2. Aberrant methylation of TFPI-2 was also detected in 73% (102/140) of pancreatic cancer xenografts and primary pancreatic adenocarcinomas, was more likely in older patients with pancreatic cancer, and significantly correlated with progression of IPMNs (P=0.0002). Restored expression of the TFPI-2 gene in nonexpressing pancreatic cancer cells resulted in marked suppression in their proliferation, migration, and   invasive   potential in vitro. We thus conclude that epigenetic inactivation of TFPI-2 is a common mechanism that contributes to the aggressive phenotype of pancreatic ductal adenocarcinoma. 
 Expression of tissue factor pathway inhibitor 2 in human pancreatic carcinoma and its effect on tumor growth, invasion, and migration in vitro and in vivo. Tissue factor pathway inhibitor 2 (TFPI-2), also known as placental protein and matrix-associated serine protease inhibitor, plays an important role in angiogenesis, intravascular fibrinolysis, wound healing, tumor invasion, metastasis by plasmin, and trypsin mediated activation of zymogen matrix metalloproteinases. To detect whether TFPI-2 can be expressed in human pancreatic carcinoma samples and to investigate its role in the growth, invasion, and metastasis of pancreatic carcinoma cell in vitro and in vivo, we collected eight normal pancreatic tissue samples and 50 pancreatic carcinoma samples, and stably transfected the human pancreatic carcinoma cell line Panc-1 with a vector capable of expressing TFPI-2 gene. RT-PCR and Western blot analysis revealed that the levels of TFPI-2 expression were markedly lower in pancreatic carcinoma samples compared with normal pancreas samples. The level of TFPI-2 protein was significantly higher in cells transfected with TFPI-2 gene than that in the untransfected cells. The results of MTT assay showed that TFPI-2 inhibited Panc-1 cells growth in vitro. The   invasive   capacity of the cells transfected with TFPI-2 gene was also markedly less than that of untransfected cells in vitro as determined by the Matrigel invasion/migration assay. Moreover, TFPI-2 inhibited tumor growth, invasion, and metastasis in vivo in an orthotopic pancreatic carcinoma model. Our findings suggest that TFPI-2 plays a significant role in the growth, invasion, and metastasis of pancreatic carcinoma cell in vitro and in vivo, and has potential in anticancer therapy. 
 Methylation of TFPI2 in stool DNA: a potential novel biomarker for the detection of colorectal cancer. We have used a gene expression array-based strategy to identify the methylation of tissue factor pathway inhibitor 2 (TFPI2), a potential tumor suppressor gene, as a frequent event in human colorectal cancers (CRC). TFPI2 belongs to the recently described group of embryonic cell Polycomb group (PcG)-marked genes that may be predisposed to aberrant DNA methylation in early stages of colorectal carcinogenesis. Aberrant methylation of TFPI2 was detected in almost all CRC adenomas (97%, n = 56) and stages I to IV CRCs (99%, n = 115). We further explored the potential of TFPI2 as a biomarker for the early detection of CRC using stool DNA-based assays in patients with nonmetastatic CRC and average-risk noncancer controls who were candidates for screening. TFPI2 methylation was detected in stool DNA from stage I to III CRC patients with a sensitivity of 76% to 89% and a specificity of 79% to 93%. Detection of TFPI2 methylation in stool DNA may act as a useful adjunct to the non  invasive   strategies for screening of CRCs in the future. 
 Promoter hypermethylation and silencing of tissue factor pathway inhibitor-2 in oral squamous cell carcinoma. The treatment of oral squamous cell carcinoma (OSCC) following early detection is associated with good outcomes. Therefore, the survival and prognosis of OSCC patients could be hugely improved by identifying reliable biomarkers for the early diagnosis of the disease. Our previous methylation microarray analysis results have suggested that the gene encoding tissue factor pathway inhibitor-2 (TFPI-2) is a potential clinical predictor as well as a key regulator involved in OSCC malignancy.Methylation of the TFPI-2 promoter in oral tissue specimens was evaluated by bisulfite sequencing assay, quantitative methylation-specific PCR, and pyrosequencing assay. The differences in methylation levels among the groups were compared using the Mann-Whitney U test. The area under the receiver operating characteristic curve (AUROC) was used to evaluate the discrimination ability for detecting OSCC. Cellular TFPI-2 expression was analyzed by quantitative reverse-transcription PCR before and after treatment with 5'-aza-2'-deoxycytidine and trichostatin A, to confirm whether TFPI-2 was epigenetically silenced in OSCC cells. We investigated whether TFPI-2 plays a role as a tumor suppressor by establishing TFPI-2-overexpressing OSCC cells and subjecting them to in vitro cellular proliferation, migration, and invasion assays, as well as an in vivo metastasis assay.TFPI-2 was hypermethylated in OSCC tissues versus normal oral tissues (P < 0.0001), with AUROC = 0.91, when using a pyrosequencing assay to quantify the methylation level. TFPI-2 silencing in OSCC was regulated by both DNA methylation and chromatin histone modification. Restoration of TFPI-2 counterac  ted the   invasiveness of OSCC by inhibiting the enzymatic activity of matrix metalloproteinase-2, and consequently interfered with OSCC metastasis in vivo.Our data suggest strongly that TFPI-2 is a down-regulated tumor suppressor gene in OSCC, probably involving epigenetic silencing mechanisms. The loss of TFPI-2 expression is a key event for oral tumorigenesis, especially in the process of tumor metastasis. 
 Tissue factor pathway inhibitor-2 as a frequently silenced tumor suppressor gene in hepatocellular carcinoma. In HCC, inactivation of tumor suppressor genes plays a significant role in carcinogenesis. Apart from deletions and mutations, growing evidence has indicated that epigenetic alterations including aberrant promoter methylation and histone deacetylation are also implicated in inactivation of tumor suppressor genes. The goal of this study was to identify epigenetically silenced candidate tumor suppressor genes in human HCC by comparing the changes in oligonucleotide microarray gene expression profiles in HCC cell lines upon pharmacological treatment with the demethylating agent 5-Aza-2'-deoxycytidine (5-Aza-dC). By analyzing the gene expression profiles, we selected tissue factor pathway inhibitor-2 (TFPI-2), a Kunitz-type serine protease inhibitor, for validation and further characterization. Our results showed that TFPI-2 was frequently silenced in human HCC and HCC cell lines. TFPI-2 was significantly underexpressed in approximately 90% of primary HCCs when compared with their corresponding nontumorous livers. TFPI-2 promoter methylation was detected in 80% of HCC cell lines and 47% of human HCCs and was accompanied by reduced TFPI-2 messenger RNA expression. In addition, TFPI-2 expression in HCC cell lines can be robustly restored by combined treatment with 5-Aza-dC and histone deacetylase inhibitor trichostatin A. These findings indicate that TFPI-2 is frequently silenced in human HCC via epigenetic alterations, including promoter methylation and histone deacetylation. Moreover, ectopic overexpression of TFPI-2 significantly suppressed the proliferation and   invasive  ness of HCC cells.Our findings suggest that TFPI-2 is a candidate tumor suppressor gene in human HCC. 
 [Role of tissue factor pathway inhibitor-2 in ovarian tumor migration and invasion]. Human tissue factor pathway inhibitor-2 (TFPI-2) is a newly discovered serine protease inhibitor, which inhibits plasmin, trypsin, matrix metalloproteinase (MMPs), but not urokinase, tissue-type plasminogen activators and thrombin. Earlier studies have shown that the production of TFPI-2 is downregulated during the progression of various cancers. The aim of this study was to elucidate the relationship between TFPI-2 expression and ovarian tumor migration and invasion.Human TFPI-2 expression vector pBos-Cite-neo/TFPI-2 was transfected into ovarian tumor cells line A2780. After the transfected cells were screened by G418, transfected and nontransfected cells were examined for TFPI-2 mRNA and protein by reverse transcription-polymerase chain reaction (RT-PCR) and Western blot analysis, respectively. The number of transfected or nontransfected cells passing through membrane of Boyden chamber was counted as the basis assessing tumor cells migratory and   invasive   behaviors.Expression of mRNA and protein of TFPI-2 were confirmed in transfected cells. In invasion assay, the number of TFPI-2-expressing cells to traverse a Matrigel-coated membrane was obviously decreased compared with that of nonexpressing cells (59.3+/-6.5 versus 109.7+/-5.5, P< 0.01); while in migration assay, no significant difference was observed between transfected and nontransfected cells (114.7+/-8.6 versus 127.3+/-7.1, P >0.05).Expression of TFPI-2 may strongly inhibit the   invasive   ability of ovarian tumor cells in vitro, but has no effect on the migratory ability, which provides an experimental basis for treating human ovarian tumor with gene therapy. 
 Expression and methylation status of tissue factor pathway inhibitor-2 gene in non-small-cell lung cancer. Tissue factor pathway inhibitor-2 (TFPI-2) is a Kunitz-type serine proteinase inhibitor that inhibits plasmin-dependent activation of several metalloproteinases. Downregulation of TFPI-2 could thus enhance the   invasive   potential of neoplastic cells in several cancers, including lung cancer. In this study, TFPI-2 mRNA was measured using a real-time PCR method in tumours of 59 patients with non-small-cell lung cancer (NSCLC). Tumour TFPI-2 mRNA levels appeared well correlated with protein expression evaluated by immunohistochemistry and were 4-120 times lower compared to those of nonaffected lung tissue in 22 cases (37%). Hypermethylation of the TFPI-2 gene promoter was demonstrated by restriction enzyme-polymerase chain reaction in 12 of 40 cases of NSCLC (30%), including nine of 17 for whom tumour TFPI-2 gene expression was lower than in noncancerous tissue. In contrast, this epigenetic modification was shown in only three of 23 tumours in which no decrease in TFPI-2 synthesis was found (P=0.016). Decreased TFPI-2 gene expression and hypermethylation were more frequently associated with stages III or IV NSCLC (eight out of 10, P=0.02) and the TFPI-2 gene promoter was more frequently hypermethylated in patients with lymph node metastases (eight out of 16, P=0.02). These results suggest that silencing of the TFPI-2 gene by hypermethylation might contribute to tumour progression in NSCLC. 
 Keratoepithelin reverts the suppression of tissue factor pathway inhibitor 2 by MYCN in human neuroblastoma: a mechanism to inhibit invasion. Neuroblastoma is the most frequent solid malignancy of children. The most reliable prognostic factor in neuroblastoma is the amplification status of the MYCN oncogene, but exceptions from this rule have been observed. Recently we have demonstrated that keratoepithelin (BIGH3, TGFBI) expression significantly reduces proliferation and invasion of neuroblastomas in vitro and in vivo. In these experiments, we also observed that tissue factor pathway inhibitor 2 (TFPI2, PP5, MSPI), a potent inhibitor of matrix-metalloproteinases, is most prominently up-regulated. As MYCN-amplified neuroblastomas are highly   invasive  , we sought to determine the interaction between MYCN, keratoepithelin and TFPI2. In this study we provide initial evidence that i) keratoepithelin expression in neuroblastoma inversely correlates with MYCN expression; ii) TFPI2 expression in neuroblastoma also correlates inversely with MYCN expression but positively with keratoepithelin expression and iii) keratoepithelin induces elevated TFPI2 transcript levels in neuroblastoma cells without alterations of MYCN expression. 
 Knockdown of TFPI-2 promotes migration and invasion of glioma cells. Glioblastoma is the most malignant primary brain tumor. Due to its highly promigratory and pro  invasive   properties, standard therapy including surgery, chemotherapy and radiation fails in eradicating this highly aggressive type of cancer. Here, we evaluated the role of TFPI-2, a Kunitz-type serine protease inhibitor, which has been previously described as a tumor suppressor gene in several types of cancer, including glioma. TFPI-2 expression was absent in five of nine investigated high-grade glioma cell lines. Lentiviral knockdown of TFPI-2 in two of the TFPI-2-expressing cell lines (MZ-18 and Hs 638) was associated with pronounced changes in the cellular behavior: glioma cell proliferation, migration and invasion were significantly increased in TFPI-2 knockdown cells in comparison to empty vector-transfected control cells. Since TFPI-2 might exert its tumor suppressor function by inhibiting MMPs, we subsequently analyzed the effects of specific MMP inhibitors on cell invasion of TFPI-2 KD cells vs. control cells. The data obtained from these experiments suggest that the anti-  invasive   properties of TFPI-2 are associated with inhibition of MMP-1 and MMP-2, while inhibition of MMP-9 seems to play a minor role in this context. Our findings underscore the important role of TFPI-2 as a tumor suppressor gene and indicate that TFPI-2 may be a useful diagnostic marker for the aggressive phenotype of glial tumors. 
  GeneRIFs  
 Results demonstrate the feasibility of using TFPI2 methylation and quantify human long DNA with fluorescent quantitative Alu PCR in fecal samples as a new non  invasive   test for CRC. 
 Expression of TFPI-2 may strongly inhibit the   invasive   ability of ovarian tumor cells in vitro, but has no effect on the migratory ability. 
 The CpG islands in TFPI-2 promoter was hypermethylated in highly   invasive   breast cancer cell line, and DNA methylation in the entire promoter region caused TFPI-2 repression by inducing inactive chromatin structure and decreasing KLF6 binding. 
 TFPI-2 is frequently silenced in hepatocellular carcinoma via epigenetic alterations, including promoter methylation and histone deacetylation; ectopic overexpression of TFPI-2 significantly suppressed the proliferation and   invasive  ness of HCC cells 
 highly expressed in non-  invasive   cells but undetectable in highly   invasive   human glioma cells 
 Restored expression of the TFPI-2 gene in nonexpressing pancreatic cancer cells resulted in marked suppression in their proliferation, migration, and   invasive   potential. 
  [Mm]etastasize  PubMed  
 The effect of human tissue factor pathway inhibitor-2 on the growth and metastasis of fibrosarcoma tumors in athymic mice. Human tissue factor pathway inhibitor-2 (TFPI-2) is a matrix-associated Kunitz inhibitor that inhibits the plasmin- and trypsin-mediated activation of zymogen matrix metalloproteinases involved in tumor progression, invasion, and metastasis. To directly assess its role in tumor growth and metastasis in vivo, we stably transfected HT-1080 fibrosarcoma cells expressing either fully active wild-type human TFPI-2 (WT) or inactive R24Q TFPI-2 (QT) and examined their ability to form tumors and   metastasize   in athymic mice in comparison to mock-transfected cells (MT). MT and QT fibrosarcoma tumors grew 2 to 3 times larger than WT tumors. Tumor metastasis was confined to the lung and was observed in 75% of mice treated with either MT or QT cells, whereas only 42% of mice treated with WT cells developed lung metastases. Real-time quantitative reverse transcriptase-polymerase chain reaction (RT-PCR) analyses of each tumor group revealed 3- to 6-fold lower levels of murine vascular endothelial growth factor gene expression in WT tumors in relation to either MT or QT tumors. Comparative tumor gene expression analysis revealed that several human genes implicated in oncogenesis, invasion, metastasis, apoptosis, and angiogenesis had significantly altered levels of expression in WT tumors. Our collective data demonstrate that secretion of inhibitory TFPI-2 by a highly metastatic tumor cell markedly inhibits its growth and metastasis in vivo by regulating pericellular extracellular matrix (ECM) remodeling and angiogenesis. 
  [Mm]etastasis  PubMed  
 Tissue factor pathway inhibitor-2 was repressed by CpG hypermethylation through inhibition of KLF6 binding in highly invasive breast cancer cells. Tissue factor pathway inhibitor-2 (TFPI-2) is a matrix-associated Kunitz inhibitor that inhibits plasmin and trypsin-mediated activation of zymogen matrix metalloproteinases involved in tumor progression, invasion and   metastasis  . Here, we have investigated the mechanism of DNA methylation on the repression of TFPI-2 in breast cancer cell lines.We found that both protein and mRNA of TFPI-2 could not be detected in highly invasive breast cancer cell line MDA-MB-435. To further investigate the mechanism of TFPI-2 repression in breast cancer cells, 1.5 Kb TFPI-2 promoter was cloned, and several genetic variations were detected, but the promoter luciferase activities were not affected by the point mutation in the promoter region and the phenomena was further supported by deleted mutation. Scan mutation and informatics analysis identified a potential KLF6 binding site in TFPI-2 promoter. It was revealed, by bisulfite modified sequence, that the CpG island in TFPI-2 promoter region was hypermethylated in MDA-MB-435. Finally, using EMSA and ChIP assay, we demonstrated that the CpG methylation in the binding site of KLF-6 diminished the binding of KLF6 to TFPI-2 promoter.In this study, we found that the CpG islands in TFPI-2 promoter was hypermethylated in highly invasive breast cancer cell line, and DNA methylation in the entire promoter region caused TFPI-2 repression by inducing inactive chromatin structure and decreasing KLF6 binding to its DNA binding sequence. 
 Tissue factor pathway inhibitor-2 was repressed by CpG hypermethylation through inhibition of KLF6 binding in highly invasive breast cancer cells. Tissue factor pathway inhibitor-2 (TFPI-2) is a matrix-associated Kunitz inhibitor that inhibits plasmin and trypsin-mediated activation of zymogen matrix metalloproteinases involved in tumor progression, invasion and   metastasis  . Here, we have investigated the mechanism of DNA methylation on the repression of TFPI-2 in breast cancer cell lines.We found that both protein and mRNA of TFPI-2 could not be detected in highly invasive breast cancer cell line MDA-MB-435. To further investigate the mechanism of TFPI-2 repression in breast cancer cells, 1.5 Kb TFPI-2 promoter was cloned, and several genetic variations were detected, but the promoter luciferase activities were not affected by the point mutation in the promoter region and the phenomena was further supported by deleted mutation. Scan mutation and informatics analysis identified a potential KLF6 binding site in TFPI-2 promoter. It was revealed, by bisulfite modified sequence, that the CpG island in TFPI-2 promoter region was hypermethylated in MDA-MB-435. Finally, using EMSA and ChIP assay, we demonstrated that the CpG methylation in the binding site of KLF-6 diminished the binding of KLF6 to TFPI-2 promoter.In this study, we found that the CpG islands in TFPI-2 promoter was hypermethylated in highly invasive breast cancer cell line, and DNA methylation in the entire promoter region caused TFPI-2 repression by inducing inactive chromatin structure and decreasing KLF6 binding to its DNA binding sequence. 
 TFPI-2 silencing increases tumour progression and promotes metalloproteinase 1 and 3 induction through tumour-stromal cell interactions. Tissue factor pathway inhibitor-2 (TFPI-2) is a potent inhibitor of plasmin which activates matrix metalloproteinases (MMPs) involved in degradation of the extracellular matrix. Its secretion in the tumour microenvironment makes TFPI-2 a potential inhibitor of tumour invasion and   metastasis  . As demonstrated in aggressive cancers, TFPI-2 is frequently down-regulated in cancer cells, but the mechanisms involved in the inhibition of tumour progression remained unclear. We showed in this study that stable TFPI-2 down-regulation in the National Cancer Institute (NCI)-H460 non-small cell lung cancer cell line using specific micro interfering micro-interfering RNA promoted tumour progression in a nude mice orthotopic model that resulted in an increase in cell invasion. Moreover, TFPI-2 down-regulation enhanced cell adhesion to collagen IV and laminin via an increase in α(1) integrin on cell surface, and increased MMP expression (mainly MMP-1 and -3) contributing to cancer cell invasion through basement membrane components. This study also reveals for the first time that pulmonary fibroblasts incubated with conditioned media from TFPI-2 silencing cancer cells exhibited increased expression of MMPs, particularly MMP-1, -3 and -7, that are likely involved in lung cancer cell invasion through the surrounding stromal tissue, thus enhancing formation of metastases. 
 Methylation of TFPI-2 gene is not the sole cause of its silencing. TFPI-2 (tissue factor pathway inhibitor-2) is a serine protease inhibitor that may suppress tumor cell invasion and   metastasis  . TFPI-2 expression is often lost in cells derived from tumors of diverse organs. We have examined whether aberrant hypermethylation of the 5' end of the TFPI-2 gene is associated with its loss of expression. After 5-azacytidine treatment of three cell lines lacking TFPI-2 expression (HT1080 fibrosarcoma cells, MCF-7 breast carcinoma cells, and LNCaP prostate carcinoma cells), TFPI-2 transcripts could be detected by RT-PCR. In these three cell lines, methylation of the 5' end of the TFPI-2 gene was detected, while two prostate carcinoma cell lines in which the TFPI-2 gene was expressed, PC-3 and DU-145, showed no methylation. However, all the three cell lines which lacked TFPI-2 expression also contained unmethylated TFPI-2 alleles. Furthermore, a transiently transfected TFPI-2 promoter was non-functional in the three cell lines, but function was attained following treatment with 5-azacytidine. Our results indicate that while methylation of the TFPI-2 gene is associated with its silencing, it is not the sole cause, and we suggest that one or more components of pathways regulating TFPI-2 expression have also undergone methylation-associated silencing in these cell lines. 
 Adenovirus-mediated expression of tissue factor pathway inhibitor-2 inhibits endothelial cell migration and angiogenesis. Extracellular matrix (ECM) remodeling during angiogenesis is accomplished through plasmin-dependent pericellular proteolysis and through the action of matrix metalloproteinases (MMPs). Because tissue factor pathway inhibitor-2 (TFPI-2), a Kunitz-type protease inhibitor with prominent ECM localization, inhibits plasmin and MMPs activity, we investigated the role of TFPI-2 in endothelial cell (EC) migration and angiogenesis.Real-time polymerase chain reaction and immunostaining showed that the expression of TFPI-2 mRNA and protein was upregulated in migrating ECs. The effect of TFPI-2 on angiogenesis was studied in mouse models of Matrigel and polyvinylalcohol sponge implants by overexpressing TFPI-2 through infection with a replication-deficient adenovirus (AdTFPI-2). Using (immuno)fluorescence and confocal microscopy we observed that TFPI-2 reduced neovascularization and promoted ECM deposition. Lateral cell migration and capillary tube formation in vitro also were impaired by TFPI-2, a process reversed by anti-TFPI-2 antibodies. Increased apoptosis occurred both in AdTFPI-2-treated ECs and in the mouse implants. Zymography and assays in the absence of plasminogen confirmed plasmin inhibition as a main mechanism through which TFPI-2 inhibits EC migration.Our data suggest that TFPI-2 may be an important regulator of aberrant angiogenesis associated with tumor growth/  metastasis  , cardiovascular diseases, chronic inflammation, or diabetes. 
 Low expression of TFPI-2 associated with poor survival outcome in patients with breast cancer. The purpose of this study is to evaluate the prognostic value of TFPI-2 expression in breast cancer patients through examining the correlation between TFPI-2 expression and breast cancer clinicopathologic features.Immunohistochemical staining combined with digital image analysis was used to quantify the expression of TFPI-2 protein in breast tumor tissues. For evaluation of the prognostic value of TFPI-2 expression to each clinicopathologic factor, Kaplan-Meier method and COX's Proportional Hazard Model were employed.TFPI-2 expression was significantly correlated with tumor size, lymph node   metastasis  , histologic grade, clinical stage, and vessel invasion. More importantly, TFPI-2 expression was also associated with disease-free survival (DFS) of breast cancer patients. We found that patients with high TFPI-2 expression had longer DFS compared with those with low or negative expression of TFPI-2 (P <0.05, log-rank test). Cox's regression analysis indicated that TFPI-2 expression, histologic grade, and vessel invasion might be significant prognostic factors for DFS, while TFPI-2 expression and histologic grade were the most significant independent predictors for tumor recurrence. Compared with the group with low/high TFPI-2 expression, the TFPI-2 negative group was more likely to have tumor relapse. The hazard ratio of DFS is 0.316 (P <0.01).Low or negative expression of TFPI-2 is associated with breast cancer progression, recurrence and poor survival outcome after breast cancer surgery. TFPI-2 expression in breast tumors is a potential prognostic tool for breast cancer patients. 
 Methylation of TFPI2 gene is frequently detected in advanced well-differentiated colorectal cancer. Recently, it has been reported that TFPI2 (tissue factor pathway inhibitor-2), a Kunitz-type serine proteinase inhibitor, is frequently methylated in human colorectal cancer using a gene expression array-based strategy. The aim of this study therefore was to examine whether the TFPI2 methylation in surgically removed colorectal cancers was correlated to the clinicopathological features.The methylation status of the TFPI2 gene was examined in primary carcinomas and corresponding normal tissues derived from 50 patients with colorectal cancer using quantitative methylation-specific PCR (qMSP), and the correlation between the methylation status and the clinicopathological findings was evaluated. Results. Methylation of the TFPI2 gene was detected in 31 out of the 50 (62%) primary colon carcinomas, suggesting that the methylation of TFPI2 is frequently observed in colorectal cancer. The clinicopathological data were compared with these results. Significant differences were observed between methylation of TFPI2 and histology (p=0.0053) or lymph node   metastasis   (p=0.0396). These results indicated that TFPI2 was more frequently methylated in well-differentiated advanced colorectal carcinomas.TFPI2 may act as a tumour suppressor in colorectal carcinomas and TFPI2 methylation may present a potential risk of malignancy in colorectal cancer. 
 Associations between TFPI-2 methylation and poor prognosis in glioblastomas. The epigenetic silencing of tumor suppressor genes plays an important role in gliomagenesis. Recently, tissue factor pathway inhibitor 2 (TFPI-2) has been suggested as a tumor suppressor gene involved in tumorigenesis and   metastasis   in some cancers. However, to date, little is known about the methylation status of TFPI-2 gene in glioblastoma tissues. In this study, we aimed to investigate the methylation status of TFPI-2 promoter and its associations with patient prognosis in glioblastoma.The methylation status of TFPI-2 was investigated by methylation-specific polymerase chain reaction in 99 glioblastoma patients. The associations between patients' clinical variables and overall survival time were assessed. RESULTS. TFPI-2 was aberrantly methylated in 22.2% (22/99) of glioblastoma tumors, but was not methylated in normal brain samples. The survival of patients with glioblastoma differed significantly between the methylated and unmethylated TFPI-2 groups (P=0.047). The 2-year survival among patients carrying methylated TFPI-2 tumors was significantly lower compared with that of patients with unmethylated TFPI-2 (27% versus 4.7%, P=0.037).The present work demonstrated that the epigenetic inactivation of TFPI-2 by promoter hypermethylation was a frequent and tumor-specific event in glioblastoma, and TFPI-2 promoter methylation might be considered as a prognostic marker in glioblastoma. 
 EGCG inhibits growth and induces apoptosis in renal cell carcinoma through TFPI-2 overexpression. EGCG (epigallocatechin gallate), the major catechin found in green tea, has been shown to inhibit proliferation and induce apoptosis in many tumors. EGCG can inhibit DNMT (DNA methyltransferase) activity, and cause CpG demethylation and reactivation of methylation-silenced genes. Tissue factor pathway inhibitor-2 (TFPI-2), a member of the Kunitz-type serine proteinase inhibitor family, is inversely related to an increasing degree of malignancy. Our previous study showed that the expression of TFPI-2 and invasiveness of renal cell carcinoma had a negative correlation. Overexpression of TFPI-2 may induce tumor cell apoptosis in renal cell carcinoma. Lower expression of TFPI-2 in renal cell carcinoma was partly due to hypermethylation of the gene promoter. Herein, using MTT and flow cytometry, we demonstrated that EGCG can inhibit growth and induces apoptosis in renal cell carcinoma cell line 786-0. In addition, Western blotting and real-time RT-PCR showed that EGCG can upregulate expression of TFPI-2. Before and after EGCG treatment, real-time methylation specific PCR could not detect methylation status of TFPI-2 gene promoter in cell line 786-0. In vivo invasiveness and   metastasis   test did not indicate any significant differences between control and treatment group. Our results suggest that EGCG inhibits growth and induces apoptosis in renal cell carcinoma through TFPI-2 overexpression. This is the first report showing that EGCG is likely to be an effective agent for renal cell carcinoma. 
 Silencing of tissue factor pathway inhibitor-2 gene in malignant melanomas. To identify tumor-suppressor genes inactivated by aberrant methylation of promoter CpG islands (CGIs) in human malignant melanomas, genes upregulated by treatment of cells with a demethylating agent, 5-aza-2'-deoxycytidine (5-aza-dC), were searched for using oligonucleotide microarrays in melanoma cell lines, HMV-I, MeWo and WM-115. Seventy-nine known genes with CGIs were identified as being upregulated (>or=16-fold), and 18 of them had methylation of their putative promoter CGIs in 1 or more of 8 melanoma cell lines. Among the 18 genes, TFPI-2, which is involved in repression of the invasive potential of malignant melanomas, was further analyzed. Its expression was repressed in a melanoma cell line with its complete methylation, and was restored by 5-aza-dC treatment. It was unmethylated in cultured neonatal normal epidermal melanocyte, and was induced by ultraviolet B. In surgical melanoma specimens, TFPI-2 methylation was detected in 5 of 17 metastatic site specimens (29%), while it was not detected in 20 primary site specimens (0%) (p=0.009). By immunohistochemistry, the 5 specimens with promoter methylation lacked immunoreactivity for TFPI-2. The results showed that TFPI-2 is silenced in human malignant melanomas by methylation of its promoter CGI and suggested that its silencing is involved in melanoma   metastasis  . 
 A novel role for tissue factor pathway inhibitor-2 in the therapy of human esophageal carcinoma. Esophageal cancer is characterized by rapid clinical progression and poor prognosis, due to early-stage invasion of adjacent tissues and   metastasis  . Tissue factor pathway inhibitor-2 (TFPI-2) has been implicated as a   metastasis  -associated gene in many types of tumors. Here we describe the potential involvement of TFPI-2 in the development of esophageal carcinoma. Western blotting revealed that TFPI-2 was downregulated in 75% of esophageal carcinomas and in most esophageal carcinoma cell lines. Immunohistochemistry revealed that TFPI-2 was significantly downregulated in tumor tissues and in lymph node metastases. Experimental overexpression of TFPI-2 in KYSE450, KYSE510, YES2, and EC9706 cells significantly inhibited their invasive ability. Overexpression of TFPI-2 in EC9706 cells inhibited xenograft tumor growth and invasion into surrounding tissues, as well as reduced lung   metastasis  . Further studies demonstrated that recombinant TFPI-2 protein significantly inhibited the activity of matrix metalloproteinases and tumor-related angiogenesis. Parenteral treatment with recombinant TFPI-2 protein significantly suppressed xenograft growth and   metastasis  . Together, these data indicate that TFPI-2 inhibits tumor invasion and angiogenesis both in vitro and in vivo, and suggest a potentially important therapeutic role for recombinant TFPI-2 in the treatment of malignant esophageal carcinomas. 
 TFPI-2 is a putative tumor suppressor gene frequently inactivated by promoter hypermethylation in nasopharyngeal carcinoma. Epigenetic silencing of tumor suppressor genes play important roles in NPC tumorgenesis. Tissue factor pathway inhibitor-2 (TFPI-2), is a protease inhibitor. Recently, TFPI-2 was suggested to be a tumor suppressor gene involved in tumorigenesis and   metastasis   in some cancers. In this study, we investigated whether TFPI-2 was inactivated epigenetically in nasopharyngeal carcinoma (NPC).Transcriptional expression levels of TFPI-2 was evaluated by RT-PCR. Methylation status were investigated by methylation specific PCR and bisulfate genomic sequencing. The role of TFPI-2 as a tumor suppressor gene in NPC was addressed by re-introducing TFPI-2 expression into the NPC cell line CNE2.TFPI-2 mRNA transcription was inactivated in NPC cell lines. TFPI-2 was aberrantly methylated in 66.7% (4/6) NPC cell lines and 88.6% (62/70) of NPC primary tumors, but not in normal nasopharyngeal epithelia. TFPI-2 expression could be restored in NPC cells after demethylation treatment. Ectopic expression of TFPI-2 in NPC cells induced apoptosis and inhibited cell proliferation, colony formation and cell migration.Epigenetic inactivation of TFPI-2 by promoter hypermethylation is a frequent and tumor specific event in NPC. TFPI-2 might be considering as a putative tumor suppressor gene in NPC. 
 Prognostic significance of tissue factor pathway inhibitor-2 in pancreatic carcinoma and its effect on tumor invasion and   metastasis  . Tissue factor pathway inhibitor-2 (TFPI-2) is a matrix-associated kunitz-type serine proteinase inhibitor that plays an important role in plasmin and trypsin-mediated activation of zymogen matrix metalloproteinases involved in tumor angiogenesis, invasion and   metastasis  . Earlier studies have shown that the production of TFPI-2 is downregulated during the progression of various tumors. To detect whether TFPI-2 can be expressed in human pancreatic carcinoma samples, to evaluate its prognostic significance on pancreatic carcinoma and to investigate its effect on tumor invasion and   metastasis  , we collected 9 normal pancreatic tissue samples and 41 pancreatic carcinoma samples and stably transfected the human pancreatic carcinoma cell line Panc-1 with a vector capable of expressing TFPI-2 gene. RT-PCR and Western blot analysis revealed that the expression of TFPI-2 in pancreatic carcinoma samples was markedly lower than that in normal pancreas samples, and there was no TFPI-2 expression in Panc-1 cell. Its expression was related with biological characters of pancreatic carcinoma. The results of Boyden chamber assay and orthotopic pancreatic carcinoma model showed that TFPI-2 could inhibit invasion and   metastasis   ability of pancreatic carcinoma in vitro and in vivo. Kaplan-Meier survival curve and Cox proportional hazards model assay identified TFPI-2 as an independent prognostic factor for pancreatic carcinoma. Our data suggest that TFPI-2 plays a significant role in the invasion and   metastasis   of pancreatic carcinoma cell in vitro and in vivo and is determined to be an important prognostic factor for pancreatic carcinoma patients. 
 Promoter of TFPI-2 is hypermethylated in Chinese pediatric acute myeloid leukemia. Human tissue factor pathway inhibitor-2 (TFPI-2) has been implicated as a   metastasis  -associated gene in many types of tumors. In this study, we investigated whether TFPI-2 was inactivated epigenetically in pediatric acute myeloid leukemia (AML). Methylation status was investigated by methylation-specific polymerase chain reaction and bisulfate genomic sequencing. TFPI-2 was aberrantly methylated in 50% (3/6) of AML cell lines. Aberrant methylation of TFPI-2 promoter was detected in 71.6% (48/67) of the Chinese pediatric AML patients. TFPI-2 transcript was significantly lower in AML group compared with controls (3.44 vs. 32.8, P<0.001). Patients with methylated TFPI-2 gene had significantly lower TFPI-2 transcript than those patients without methylated TFPI-2 (P=0.04). Promoter hypermethylation of TFPI-2 is frequent and specific event in pediatric AML. 
 Trypsinogen 4 boosts tumor endothelial cells migration through proteolysis of tissue factor pathway inhibitor-2. Proteases contribute to cancer in many ways, including tumor vascularization and   metastasis  , and their pharmacological inhibition is a potential anticancer strategy. We report that human endothelial cells (EC) express the trypsinogen 4 isoform of the serine protease 3 (PRSS3), and lack both PRSS2 and PRSS1. Trypsinogen 4 expression was upregulated by the combined action of VEGF-A, FGF-2 and EGF, angiogenic factors representative of the tumor microenvironment. Suppression of trypsinogen 4 expression by siRNA inhibited the angiogenic milieu-induced migration of EC from cancer specimens (tumor-EC), but did not affect EC from normal tissues. We identified tissue factor pathway inhibitor-2 (TFPI-2), a matrix associated inhibitor of cell motility, as the functional target of trypsinogen 4, which cleaved TFPI-2 and removed it from the matrix put down by tumor-EC. Silencing tumor-EC for trypsinogen 4 accumulated TFPI2 in the matrix. Showing that angiogenic factors stimulate trypsinogen 4 expression, which hydrolyses TFPI-2 favoring a pro-migratory situation, our study suggests a new pathway linking tumor microenvironment signals to endothelial cell migration, which is essential for angiogenesis and blood vessel remodeling. Abolishing trypsinogen 4 functions might be an exploitable strategy as anticancer, particularly anti-vascular, therapy. 
 The effect of human tissue factor pathway inhibitor-2 on the growth and   metastasis   of fibrosarcoma tumors in athymic mice. Human tissue factor pathway inhibitor-2 (TFPI-2) is a matrix-associated Kunitz inhibitor that inhibits the plasmin- and trypsin-mediated activation of zymogen matrix metalloproteinases involved in tumor progression, invasion, and   metastasis  . To directly assess its role in tumor growth and   metastasis   in vivo, we stably transfected HT-1080 fibrosarcoma cells expressing either fully active wild-type human TFPI-2 (WT) or inactive R24Q TFPI-2 (QT) and examined their ability to form tumors and metastasize in athymic mice in comparison to mock-transfected cells (MT). MT and QT fibrosarcoma tumors grew 2 to 3 times larger than WT tumors. Tumor   metastasis   was confined to the lung and was observed in 75% of mice treated with either MT or QT cells, whereas only 42% of mice treated with WT cells developed lung metastases. Real-time quantitative reverse transcriptase-polymerase chain reaction (RT-PCR) analyses of each tumor group revealed 3- to 6-fold lower levels of murine vascular endothelial growth factor gene expression in WT tumors in relation to either MT or QT tumors. Comparative tumor gene expression analysis revealed that several human genes implicated in oncogenesis, invasion,   metastasis  , apoptosis, and angiogenesis had significantly altered levels of expression in WT tumors. Our collective data demonstrate that secretion of inhibitory TFPI-2 by a highly metastatic tumor cell markedly inhibits its growth and   metastasis   in vivo by regulating pericellular extracellular matrix (ECM) remodeling and angiogenesis. 
 Tissue factor pathway inhibitor-2 silencing promotes hepatocellular carcinoma cell invasion in vitro. Hepatocellular carcinoma (HCC) is a leading cause of cancer-related death in the world and   metastasis   is an essential aspect of HCC progression. Tissue factor pathway inhibitor-2 (TFPI-2) has been implicated as a potential suppressor gene to regulate tumor invasion and   metastasis  . In this study, we silenced TFPI-2 in the HCC cell line MHCC97-L and evaluated the role of TFPI-2 in cell invasion and its impact on gene expression. We showed in this study that stable TFPI-2 downregulation in MHCC97-L cells resulted in increased cell adhesion and invasion. We also showed that mRNA and protein expression levels of MMP-1/3, CD44, and ICAM-1 were increased, while those of MMP-2/9 were not changed by TFPI-2 silencing. Furthermore, silencing of TFPI-2 caused increased Akt phosphorylation level and NF-κB transcription in MHCC97-L cells. In conclusion, this study confirms that TFPI-2 downregulation can contribute to tumor invasion of HCC cells through alteration in the expression of   metastasi  s-related genes. 
 Epigenetic inactivation of TFPI-2 as a common mechanism associated with growth and invasion of pancreatic ductal adenocarcinoma. Using microarrays, we have screened for genes reactivated by drugs that modify epigenetic mechanisms in pancreatic cancer cells. One of the genes identified was tissue factor pathway inhibitor 2 (TFPI-2), which encodes for a broad-spectrum serine proteinase inhibitor that negatively regulates the extracellular matrix degradation, an essential step in tumor invasion and   metastasis  . We therefore investigated the expression and methylation patterns of the TFPI-2 gene in pancreatic adenocarcinoma, and determined its role in tumor growth and invasion. In contrast to its abundant expression in normal pancreas, TFPI-2 mRNA was undetectable in a high fraction of pancreatic cancer cell lines and in primary pancreatic ductal neoplasms (IPMNs). Loss of TFPI-2 expression was associated with aberrant hypermethylation of its promoter CpG island. Treatment with the phorbol ester (PMA), known to stimulate the TFPI-2 promoter activity, augmented the TFPI-2 expression in cell lines with unmethylated or partially methylated TFPI-2, but failed to induce the expression in cell lines that harbored fully methylated TFPI-2. Aberrant methylation of TFPI-2 was also detected in 73% (102/140) of pancreatic cancer xenografts and primary pancreatic adenocarcinomas, was more likely in older patients with pancreatic cancer, and significantly correlated with progression of IPMNs (P=0.0002). Restored expression of the TFPI-2 gene in nonexpressing pancreatic cancer cells resulted in marked suppression in their proliferation, migration, and invasive potential in vitro. We thus conclude that epigenetic inactivation of TFPI-2 is a common mechanism that contributes to the aggressive phenotype of pancreatic ductal adenocarcinoma. 
 Diagnostic utility of aberrant methylation of tissue factor pathway inhibitor 2 in pure pancreatic juice for pancreatic carcinoma. The tissue factor pathway inhibitor 2 (TFPI-2) is a Kunitz-type serine proteinase inhibitor. Recently, the aberrant methylation of TFPI-2 was detected frequently in pancreatic carcinoma (PCa) tissues but not in normal pancreatic tissues. We analyzed the aberrant methylation of TFPI-2 in the pure pancreatic juice (PPJ) aspirated endoscopically from patients with various pancreatic diseases. Using the highly sensitive methylation-specific polymerase chain reaction (MSP) and quantitative MSP (Q-MSP) assay, we investigated the aberrant methylation of TFPI-2 in nine human PCa cell lines and in the PPJ from patients with PCa, intraductal papillary mucinous neoplasms (IPMN) and chronic pancreatitis (CP). The incidence of aberrant TFPI-2 methylation was seven (77.8%) of nine PCa cell lines by Q-MSP. In cell lines, the expression of TFPI-2 mRNA by quantitative reverse transcription-polymerase chain reaction showed an inverse correlation to the aberrant methylation of TFPI-2. The incidence of aberrant TFPI-2 methylation in the PPJ was 21 (58.3%) of 36 PCa patients, three (17.6%) of 17 IPMN and one (4.8%) of 21 CP by MSP assay. Using a suitable cut-off value of 2.5 according to the receiver operating characteristic curve, the incidence of aberrant TFPI-2 methylation in the PPJ by real-time MSP was 18 (62.1%) of 29 PCa patients, one (5.1%) of 17 IPMN and three (14.3%) of 21 CP, respectively. The incidence of quantitative TFPI-2 hypermethylation in the PPJ with PCa was significantly higher than that with IPMN (P < 0.001) or CP (P < 0.001). Moreover, the aberrant methylation rate of TFPI-2 in the PPJ was 100%, as observed (6/6) in the PCa patients with liver   metastasis  , and 86.7% (26/30) in stages IVa + IVb of PCa by Q-MSP assay. These results suggest that promoter methylation of TFPI-2 in the PPJ may be a useful marker in the diagnosis and progression of PCa using an endoscopically feasible approach. 
 Expression of tissue factor pathway inhibitor 2 in human pancreatic carcinoma and its effect on tumor growth, invasion, and migration in vitro and in vivo. Tissue factor pathway inhibitor 2 (TFPI-2), also known as placental protein and matrix-associated serine protease inhibitor, plays an important role in angiogenesis, intravascular fibrinolysis, wound healing, tumor invasion,   metastasis   by plasmin, and trypsin mediated activation of zymogen matrix metalloproteinases. To detect whether TFPI-2 can be expressed in human pancreatic carcinoma samples and to investigate its role in the growth, invasion, and   metastasis   of pancreatic carcinoma cell in vitro and in vivo, we collected eight normal pancreatic tissue samples and 50 pancreatic carcinoma samples, and stably transfected the human pancreatic carcinoma cell line Panc-1 with a vector capable of expressing TFPI-2 gene. RT-PCR and Western blot analysis revealed that the levels of TFPI-2 expression were markedly lower in pancreatic carcinoma samples compared with normal pancreas samples. The level of TFPI-2 protein was significantly higher in cells transfected with TFPI-2 gene than that in the untransfected cells. The results of MTT assay showed that TFPI-2 inhibited Panc-1 cells growth in vitro. The invasive capacity of the cells transfected with TFPI-2 gene was also markedly less than that of untransfected cells in vitro as determined by the Matrigel invasion/migration assay. Moreover, TFPI-2 inhibited tumor growth, invasion, and   metastasi  s in vivo in an orthotopic pancreatic carcinoma model. Our findings suggest that TFPI-2 plays a significant role in the growth, invasion, and   metastasi  s of pancreatic carcinoma cell in vitro and in vivo, and has potential in anticancer therapy. 
 Detection of TFPI2 methylation in the serum of gastric cancer patients. Methylation of tissue factor pathway inhibitor-2 (TFPI2) has been detected in the stool of colorectal cancer patients. Using quantitative methylation-specific polymerase chain reaction (qMSP), 39 out of 215 (18%) patients exhibited TFPI2 methylation in their serum DNA, suggesting that a significant number of methylated TFPI2 existed in colorectal cancer patients' sera.Methylation status of the TFPI2 gene was examined in sera derived from 73 patients with gastric cancer using qMSP and the correlation between the methylation status and the clinicopathological findings was evaluated.Out of 73 serum samples, 7 (10%) exhibited TFPI2 methylation in their serum DNA by qMSP, suggesting that TFPI2 methylation existed in the serum of gastric cancer patients. After completion of qMSP analysis of all specimens, clinicopathological data were correlated with the molecular analysis. TFPI2 methylation was significantly more frequently found in serum of patients with lymph node   metastasis   (p=0.0040) and distant   metastasis   (p=0.0115).In principle, knowledge of the methylation status of a primary tumor is not required in advance in order to be able to detect circulating tumor DNA. Therefore, qMSP could be used as a cancer screening method. 
 Detection of TFPI2 methylation in the serum of colorectal cancer patients. We examined whether TFPI2 methylation can be used as a molecular marker for colorectal cancers by detecting TFPI2 methylation in colorectal cancer patients' sera by using quantitative methylation-specific polymerase chain reaction (qMSP). The qMSP analysis showed that 39 of 215 (18%) patients exhibited TFPI2 methylation in their serum DNA, suggesting that TFPI2 methylation frequently existed in colorectal cancer patients' sera. After completion of qMSP analysis, clinicopathological data were correlated with molecular data. TFPI2 methylation was significant in the sera of patients with large (p = 0.0022), poorly differentiated carcinoma (p = 0.0164), deep invasion (p = 0.0002), lymph node   metastasis   (p = 0.0147), or distant   metastasis   (p < 0.0001). Moreover, TFPI2 methylation was observed more frequently according to the progression of TNM stage, suggesting that serum TFPI2 methylation could be detected more easily in patients with advanced colorectal cancer. We also examined whether serum TFPI2 methylation would be useful in the detection of colorectal cancer, compared to the conventional tumor markers. Detection rates of colorectal cancer using the tumor markers TFPI2 methylation, CEA and CA19-9, in the serum were 18%, 33%, and 17%, respectively. In cases where we combined all three markers, the detection rate was 42%. High sensitivity of qMSP enables detection of smaller amounts of serum tumor DNA. In principle, the methylation status of a primary tumor is not required in advance to detect circulating tumor DNA, suggesting the potential of qMSP as a cancer screening method. 
 Promoter hypermethylation and silencing of tissue factor pathway inhibitor-2 in oral squamous cell carcinoma. The treatment of oral squamous cell carcinoma (OSCC) following early detection is associated with good outcomes. Therefore, the survival and prognosis of OSCC patients could be hugely improved by identifying reliable biomarkers for the early diagnosis of the disease. Our previous methylation microarray analysis results have suggested that the gene encoding tissue factor pathway inhibitor-2 (TFPI-2) is a potential clinical predictor as well as a key regulator involved in OSCC malignancy.Methylation of the TFPI-2 promoter in oral tissue specimens was evaluated by bisulfite sequencing assay, quantitative methylation-specific PCR, and pyrosequencing assay. The differences in methylation levels among the groups were compared using the Mann-Whitney U test. The area under the receiver operating characteristic curve (AUROC) was used to evaluate the discrimination ability for detecting OSCC. Cellular TFPI-2 expression was analyzed by quantitative reverse-transcription PCR before and after treatment with 5'-aza-2'-deoxycytidine and trichostatin A, to confirm whether TFPI-2 was epigenetically silenced in OSCC cells. We investigated whether TFPI-2 plays a role as a tumor suppressor by establishing TFPI-2-overexpressing OSCC cells and subjecting them to in vitro cellular proliferation, migration, and invasion assays, as well as an in vivo   metastasis   assay.TFPI-2 was hypermethylated in OSCC tissues versus normal oral tissues (P < 0.0001), with AUROC = 0.91, when using a pyrosequencing assay to quantify the methylation level. TFPI-2 silencing in OSCC was regulated by both DNA methylation and chromatin histone modification. Restoration of TFPI-2 counteracted the invasiveness of OSCC by inhibiting the enzymatic activity of matrix metalloproteinase-2, and consequently interfered wi  th OSCC me  tastasis in vivo.Our data suggest strongly that TFPI-2 is a down-regulated tumor suppressor gene in OSCC, probably involving epigenetic silencing mechanisms. The loss of TFPI-2 expression is a key event for oral tumorigenesis, especially in the process o  f tumor me  tastasis. 
 Correlation and prognostic significance of MMP-2 and TFPI-2 differential expression in pancreatic carcinoma. Aberrant expression of matrix metalloproteinase (MMP)-2 and tissue factor pathway inhibitor (TFPI)-2 not only correlate with tumorigenesis, but also with tumor invasion and   metastasis  . This study aims to investigate the correlation and prognostic significance of MMP-2 and TFPI-2 differential expression in pancreatic carcinoma. Immunohistochemistry was used to evaluate MMP-2 and TFPI-2 expression in tumor tissues and corresponding non-tumor tissues from 122 patients with pancreatic carcinoma. The results showed that the expression of MMP-2 was significantly (P < 0.05) higher in tumor tissues (78.7%) than in adjacent non-tumor tissues (27.9%), whereas the expression of TFPI-2 was significantly (P < 0.001) lower in tumor tissues (27.9%) than in adjacent non-tumor tissues (79.5%). Spearman's rank correlation test showed a negative correlation between MMP-2 and TFPI-2 expression (r = -0.346, P < 0.001). Kaplan-Meier survival analysis showed that high MMP-2 expression was significantly correlated with decreased disease-free survival (DFS) (P < 0.001) and overall survival (OS) (P < 0.001), while high TFPI-2 expression was significantly associated with increased DFS (P < 0.001) and OS (P < 0.001) of the patients. Multivariate analysis showed that high MMP-2 expression can act as an independent predictive factor for poor DFS (P = 0.01); and low TFPI-2 expression as an independent prognostic factor for poor DFS (P < 0.001) and OS (P < 0.001). In conclusion, our findings suggested that the differential expression of MMP-2 and TFPI-2 have a negative correlation in pancreatic carcinoma tissues; they may be considered as valuable biomarkers for prognosis of pancreatic carcinoma. 
 Tissue factor pathway inhibitor-2 inhibits the growth and invasion of hepatocellular carcinoma cells and is inactivated in human hepatocellular carcinoma. Human tissue factor pathway inhibitor-2 (TFPI-2) is an extracellular matrix-associated Kunitz-type serine proteinase inhibitor that inhibits the plasmin- and trypsin-mediated activation of matrix metalloproteinases and inhibits tumor progression, invasion and   metastasis  . Previous studies have shown that TFPI-2 is downregulated in the progression of various tumors. The purpose of this study was to investigate the expression and function of TFPI-2 in hepatocellular carcinoma (HCC). In situ hybridization was used to detect human TFPI-2 mRNA and immunohistochemistry was performed to examine the role of TFPI-2 expression in hepatocarcinoma tissues. Cell proliferation was assessed using MTT assay. In situ hybridization and immunohistochemical analyses revealed that the expression of TFPI-2 in hepatocarcinoma tissues was markedly lower than that in tumor-adjacent normal hepatic tissues. Restored expression of TFPI-2 in HepG(2) cells inhibits cell proliferation and invasion. Taken together, the results suggest that TFPI-2 has a tumor-suppression action and its inactivation may contribute to HCC. 
 Human tissue factor pathway inhibitor-2 induces caspase-mediated apoptosis in a human fibrosarcoma cell line. Human TFPI-2 is an extracellular matrix-associated Kunitz-type serine proteinase inhibitor. We previously demonstrated that a human fibrosarcoma cell line, HT-1080, does not express TFPI-2, but genetic restoration of TFPI-2 expression in these cells markedly inhibited their growth and   metastasis   in vivo. In the present study, either full-length recombinant TFPI-2, or its mutated first Kunitz-type domain (R24K KD1), were offered to HT-1080 cells, and the degree of apoptosis assessed by nuclear fragmentation, ethidium bromide and acridine orange staining, fluorescence activated cell sorting, immunoblotting and gene expression profiling. R24K KD1 induced apoptosis in 69% of HT-1080 cells in a 48 h period compared to 39% for TFPI-2, while a KD1 preparation lacking a reactive site arginine/lysine residue (R24Q KD1) produced only an 18% apoptosis rate, suggesting that the observed apoptosis was related to proteinase inhibition. Immunoblotting experiments indicated increased caspase 3 and 9 activation, up-regulation of pro-apoptotic Bax and suppression of anti-apoptotic Bcl-2 protein. Finally, microarray analyses of R24K KD1-treated cells indicated elevated expression of several pro-apoptotic genes and under-expression of anti-apoptotic genes. Collectively, our results demonstrate that treatment of HT-1080 cells exogenously with either TFPI-2 or R24K KD1 activates caspase-mediated, pro-apoptotic signaling pathways resulting in apoptosis. 
 Reduced expression of tissue factor pathway inhibitor-2 contributes to apoptosis and angiogenesis in cervical cancer. Tissue factor pathway inhibitor-2 (TFPI-2) is an extracellular matrix associated broad-spectrum Kunitz-type serine proteinase inhibitor. Recently, down regulation of TFPI-2 was suggested to be involved in tumor invasion and   metastasis   in some cancers.This study involved 12 normal cervical squamous epithelia, 48 cervical intraepithelial neoplasia (CIN), and 68 cervical cancer. The expression of TFPI-2, Ki-67 and vascular endothelial growth factor (VEGF) were investigated by immunohistochemistry staining. The apoptolic index(AI) was determined with an in situ end-labeling assay(TUNEL). And the marker of CD34 staining was used as an indicator of microvessel density (MVD).TFPI-2 expression has a decreasing trend with the progression of cervical cancer and was significantly correlated with FIGO stage, lymph node   metastasis   and HPV infection. In addition, there were significant positive correlations between the grading of TFPI-2 expression and AI(P=0.004). In contrast, the expression of TFPI-2 and VEGF or MVD was negatively correlated (both p < 0.001). However, we did not establish any signiﬁcant correlation between Ki-67 and TFPI-2 expression in cervical cancer.The results suggested that the expression of TFPI-2 had a decreasing trend with tumor progression of cervical cancer. There was a close association between the expression of TFPI-2 and tumor cell apoptosis and angiogenesis in patients with cervical cancer. TFPI-2 may play an inhibitive role during the development of cervical cancer. 
  GeneRIFs  
 TFPI-2 is a down-regulated tumor suppressor gene in oral squamous cell carcinoma, probably involving epigenetic silencing mechanisms. The loss of TFPI-2 expression is a key event for oral tumorigenesis, especially in the process of tumor   metastasis  . 
 TFPI-2 downregulation can contribute to tumor invasion of hepatocellular carcinoma cells through alteration in the expression of   metastasis  -related genes. 
 TFPI2 methylation was significant in the sera of patients with large, poorly differentiated carcinoma, deep invasion, lymph node   metastasis  , or distant   metastasis  . TFPI2 methylation was observed more frequently according to the progression of TNM stage. 
 TFPI-2 plays a significant role in the growth, invasion, and   metastasis   of pancreatic carcinoma cell in vitro and in vivo, and has potential in anticancer therapy. 
 TFPI-2 plays a significant role in the invasion and   metastasis   of pancreatic carcinoma cell in vitro and in vivo. TFPI-2 is an independent prognostic factor for pancreatic carcinoma. 
 Results showed that TFPI-2 is silenced in malignant melanomas by methylation of its promoter CGI and suggested that its silencing is involved in melanoma   metastasis  . 
 secretion of inhibitory TFPI-2 by a highly metastatic tumor cell markedly inhibits its growth and   metastasis   
 Our data suggest that TFPI-2 may be an important regulator of aberrant angiogenesis associated with tumor growth/  metastasis  , cardiovascular diseases, chronic inflammation, or diabetes. 
  [Cc]ell [Ii]nvasion  PubMed  
 TFPI-2 silencing increases tumour progression and promotes metalloproteinase 1 and 3 induction through tumour-stromal cell interactions. Tissue factor pathway inhibitor-2 (TFPI-2) is a potent inhibitor of plasmin which activates matrix metalloproteinases (MMPs) involved in degradation of the extracellular matrix. Its secretion in the tumour microenvironment makes TFPI-2 a potential inhibitor of tumour invasion and metastasis. As demonstrated in aggressive cancers, TFPI-2 is frequently down-regulated in cancer cells, but the mechanisms involved in the inhibition of tumour progression remained unclear. We showed in this study that stable TFPI-2 down-regulation in the National Cancer Institute (NCI)-H460 non-small cell lung cancer cell line using specific micro interfering micro-interfering RNA promoted tumour progression in a nude mice orthotopic model that resulted in an increase in   cell invasion  . Moreover, TFPI-2 down-regulation enhanced cell adhesion to collagen IV and laminin via an increase in α(1) integrin on cell surface, and increased MMP expression (mainly MMP-1 and -3) contributing to cancer   cell invasio  n through basement membrane components. This study also reveals for the first time that pulmonary fibroblasts incubated with conditioned media from TFPI-2 silencing cancer cells exhibited increased expression of MMPs, particularly MMP-1, -3 and -7, that are likely involved in lung cancer   cell invasio  n through the surrounding stromal tissue, thus enhancing formation of metastases. 
 Methylation of TFPI-2 gene is not the sole cause of its silencing. TFPI-2 (tissue factor pathway inhibitor-2) is a serine protease inhibitor that may suppress tumor   cell invasion   and metastasis. TFPI-2 expression is often lost in cells derived from tumors of diverse organs. We have examined whether aberrant hypermethylation of the 5' end of the TFPI-2 gene is associated with its loss of expression. After 5-azacytidine treatment of three cell lines lacking TFPI-2 expression (HT1080 fibrosarcoma cells, MCF-7 breast carcinoma cells, and LNCaP prostate carcinoma cells), TFPI-2 transcripts could be detected by RT-PCR. In these three cell lines, methylation of the 5' end of the TFPI-2 gene was detected, while two prostate carcinoma cell lines in which the TFPI-2 gene was expressed, PC-3 and DU-145, showed no methylation. However, all the three cell lines which lacked TFPI-2 expression also contained unmethylated TFPI-2 alleles. Furthermore, a transiently transfected TFPI-2 promoter was non-functional in the three cell lines, but function was attained following treatment with 5-azacytidine. Our results indicate that while methylation of the TFPI-2 gene is associated with its silencing, it is not the sole cause, and we suggest that one or more components of pathways regulating TFPI-2 expression have also undergone methylation-associated silencing in these cell lines. 
 CD24 promotes tumor   cell invasion   by suppressing tissue factor pathway inhibitor-2 (TFPI-2) in a c-Src-dependent fashion. CD24 is a glycosyl-phosphatidylinositol-anchored protein with mucin-type structure that resides exclusively in membrane microdomains. CD24 is often highly expressed in carcinomas and correlates with poor prognosis. Experimentally, the over-expression or depletion of CD24 alters cell proliferation, adhesion, and invasion in vitro and tumor growth in vivo. However, little is known about the mechanisms by which CD24 mediates these cellular effects. Here we have studied the mechanism of CD24-dependent   cell invasion   using transient CD24 knock-down or over-expression in human cancer cell lines. We show that CD24 depletion reduced tumor   cell invasion   and up-regulated expression of Tissue Factor Pathway Inhibitor 2 (TFPI-2), a potent inhibitor of extracellular matrix degradation that can block metastases formation and tumor   cell invasion  . Over-expression of CD24 in A125 cells resulted in reduced TFPI-2 expression and enhanced invasion. We provide evidence that the activity of c-Src is reduced upon CD24 knock-down. The silencing of c-Src, similar to CD24, was able to enhance TFPI-2 expression and reduce tumor   cell invasion  . An inverse expression of CD24 and TFPI-2 was observed by immunohistochemical analysis of primary breast cancers (N = 1,174). TFPI-2 expression was highest in CD24 negative samples and lowered with increasing CD24 expression. Patients with a CD24 low/TFPI-2 high phenotype showed significantly better survival compared to CD24 high/TFPI-2 low patients. Our results provide evidence that CD24 can regulate   cell invasion   via TFPI-2 and suggests a role of c-Src in this process. 
 Tissue factor pathway inhibitor-2 silencing promotes hepatocellular carcinoma   cell invasion   in vitro. Hepatocellular carcinoma (HCC) is a leading cause of cancer-related death in the world and metastasis is an essential aspect of HCC progression. Tissue factor pathway inhibitor-2 (TFPI-2) has been implicated as a potential suppressor gene to regulate tumor invasion and metastasis. In this study, we silenced TFPI-2 in the HCC cell line MHCC97-L and evaluated the role of TFPI-2 in   cell invasion   and its impact on gene expression. We showed in this study that stable TFPI-2 downregulation in MHCC97-L cells resulted in increased cell adhesion and invasion. We also showed that mRNA and protein expression levels of MMP-1/3, CD44, and ICAM-1 were increased, while those of MMP-2/9 were not changed by TFPI-2 silencing. Furthermore, silencing of TFPI-2 caused increased Akt phosphorylation level and NF-κB transcription in MHCC97-L cells. In conclusion, this study confirms that TFPI-2 downregulation can contribute to tumor invasion of HCC cells through alteration in the expression of metastasis-related genes. 
 Human tissue factor pathway inhibitor-2 suppresses the wound-healing activities of human Tenon's capsule fibroblasts in vitro. Human tissue Factor Pathway Inhibitor-2 (TFPI-2) is a potent inhibitor of plasmin, which activates metalloproteinases involved in extracellular matrix degradation. Its secretion in the extracellular matrix makes TFPI-2 a potential inhibitor of tumor   cell invasion  . However, no studies have yet evaluated the wound-healing activities of human Tenon's capsule fibroblasts (hTCFs). The aim of the study is to elucidate the effect of TFPI-2 overexpression on hTCF proliferation and migration, to determine whether TFPI-2 may act as an antiscarring agent in vivo after glaucoma filtration surgery.Plasmid vector pBos-Cite-neo/TFPI-2 was transfected into hTCFs with Lipofectamine 2000. After selection by G418, transfected, non-transfected, and mock-transfected cells were screened for TFPI-2 mRNA and protein by reverse transcription-PCR and western blot analysis respectively. Cell proliferation and viability were determined by 3-(4,5-Dimethylthiazol-2-yl)-2,5-diphenyltetrazolium bromide (MTT) assay and flow cytometry. Cell migration was studied on restrained collagen gels and with a scratch-wound assay.TFPI-2 expression of mRNA and protein was confirmed in transfected cells. The transfected, non-transfected, and mock-transfected cells showed no significant difference in cell proliferation and apoptosis, with TFPI-2 found not to be cytotoxic in hTCFs. Overexpression of TFPI-2 significantly suppressed cell migration three- to four-fold on collagen gel for 2 weeks and in the scratch-wound assay for 2 d (39.27+/-2.40% versus 16.43+/-1.10% at 1 d, and 79.0+/-3.04% versus 30.13+/-2.1% at 2 d).TFPI-2 expression may strongly inhibit the migration ability of hTCFs in vitro, making it a promising candidate for novel therapies to minimize scar development after glaucoma drainage surgery. 
 Tissue factor pathway inhibitor-2 (TFPI-2) recognizes the complement and kininogen binding protein gC1qR/p33 (gC1qR): implications for vascular inflammation. Evidence is accumulating to suggest that TFPI-2 is involved in regulating pericellular proteases implicated in a variety of physiologic and pathologic processes including cancer   cell invasion  , vascular inflammation, and atherosclerosis. Recent immunohistochemical studies of advanced atherosclerotic lesions, demonstrated a similar tissue distribution for TFPI-2, High Molecular Weight Kininogen (HK), and gC1qR/p33 (gC1qR), a ubiquitously expressed, multicompartmental cellular protein involved in modulating complement, coagulation, and kinin cascades. Further studies to evaluate TFPI-2 interactions with gC1qR demonstrated direct interactions between gC1qR and TFPI-2 using immunoprecipitation and solid phase binding studies. Specific and saturable binding between TFPI-2 and gC1qR (estimated Kd: approximately 70 nM) was observed by ELISA and surface plasmon resonance (Biacore) binding assays. Binding was inhibited by antibodies to gC1qR, and was strongly dependent on the Kunitz-2 domain of TFPI-2, as deletion of this domain reduced gC1qR-TFPI-2 interactions by approximately 75%. Deletion of gC1qR amino acids 74-95, involved in C1q binding, had no effect on gC1qR binding to TFPI-2, although antibodies to this region and purified C1q both inhibited binding, most likely via allosteric effects. In contrast, HK did not affect TFPI-2 binding to gC1qR. Binding of TFPI-2 to gC1qR produced statistically significant but modest reductions in TFPI-2 inhibition of plasmin, but had no effect on kallikrein inhibition in fluid phase chromogenic assays. Taken together, these data suggest that gC1qR may participate in tissue remodeling and inflammation by localizing TFPI-2 to the pericellular environment to modulate local protease activity and regulate HK activation. 
 Knockdown of TFPI-2 promotes migration and invasion of glioma cells. Glioblastoma is the most malignant primary brain tumor. Due to its highly promigratory and proinvasive properties, standard therapy including surgery, chemotherapy and radiation fails in eradicating this highly aggressive type of cancer. Here, we evaluated the role of TFPI-2, a Kunitz-type serine protease inhibitor, which has been previously described as a tumor suppressor gene in several types of cancer, including glioma. TFPI-2 expression was absent in five of nine investigated high-grade glioma cell lines. Lentiviral knockdown of TFPI-2 in two of the TFPI-2-expressing cell lines (MZ-18 and Hs 638) was associated with pronounced changes in the cellular behavior: glioma cell proliferation, migration and invasion were significantly increased in TFPI-2 knockdown cells in comparison to empty vector-transfected control cells. Since TFPI-2 might exert its tumor suppressor function by inhibiting MMPs, we subsequently analyzed the effects of specific MMP inhibitors on   cell invasion   of TFPI-2 KD cells vs. control cells. The data obtained from these experiments suggest that the anti-invasive properties of TFPI-2 are associated with inhibition of MMP-1 and MMP-2, while inhibition of MMP-9 seems to play a minor role in this context. Our findings underscore the important role of TFPI-2 as a tumor suppressor gene and indicate that TFPI-2 may be a useful diagnostic marker for the aggressive phenotype of glial tumors. 
   
  
 ENDOG 
 
  NCBI Gene  
  GeneRIF  
  Pubmed  
 
 
  Search Term  Rank  Fields  
  [Ii]nvasive  10  PubMed(1); GeneRIFs(1)  
  [Mm]etastasis  12  PubMed(1)  
 
 
 hide/show details for ENDOG 
  [Ii]nvasive  PubMed  
 Endonuclease G promotes cell death of non-  invasive   human breast cancer cells. The   invasive  ness of breast cancer cells was shown to be associated with the suppressed ability to develop apoptosis. The role of cell death DNases/endonucleases has not been previously examined in relation with the   invasive  ness of breast cancer cells. We have compared the activity of the endonucleases in seven human breast cancer cell lines different in the level of   invasive  ness and differentiation. The   invasive  ness of cell lines was confirmed by an in vitro Matrigel-based assay. The total endonuclease activity in the differentiated non-  invasive   (WDNI) cell lines was higher than that in the poorly differentiated   invasive   (PDI) cells. The expression of EndoG strongly correlated with the degree of estrogen receptor expression and showed an inverse correlation with vimentin and matrix metalloproteinase-13. The EndoG-positive WDNI cells were more sensitive to etoposide- or camptothecin-induced cell death than EndoG-negative PDI cells. Silencing of EndoG caused inhibited of SK-BR-3 WDNI cell death induced by etoposide. Human ductal carcinomas in situ expressed high levels of EndoG, while   invasive   medullar and ductal carcinomas had significantly decreased expression of EndoG. This correlated with decreased apoptosis as measured by TUNEL assay. Our findings suggest that the presence of EndoG in non-  invasive   breast cancer cells determines their sensitivity to apoptosis, which may be taken into consideration for developing the chemotherapeutic strategy for cancer treatment. 
  GeneRIFs  
 Our findings suggest that the presence of EndoG in non-  invasive   breast cancer cells determines their sensitivity to apoptosis, which may be taken into consideration for developing the chemotherapeutic strategy for cancer treatment. 
  [Mm]etastasis  PubMed  
 Increased expression of endonuclease G in gastric and colorectal carcinomas. Endonuclease G (EndoG) is a mitochondrial protein that plays a role in DNA fragmentation during apoptosis. In addition, EndoG plays a role in cell proliferation and survival. It may be important to identify EndoG protein expression to predict its function in human cancers. The aim of this study was to explore whether alteration of EndoG expression might be a characteristic of colorectal or gastric carcinoma.We investigated EndoG protein expression in 103 colorectal and 60 gastric carcinoma tissues by immunohistochemistry using a tissue microarray approach.Expression of EndoG was detected in 72 (70%) of the colorectal carcinomas and 41 (68%) of the gastric carcinomas in cytoplasm. By contrast, normal mucosal cells of both stomach and colon tissues showed no or very weak expression of EndoG. There was no significant association of EndoG expression with clinocopathological characteristics, including invasion,   metastasis   and stage.Our data indicate that EndoG inactivation by loss of expression may not occur in colorectal and gastric cancers. Rather, increased expression of EndoG in colorectal and gastric cancer cells compared to their normal mucosal epithelial counterparts suggests that neo-expression of EndoG may play a role in both colorectal and gastric tumorigenesis. 
   
  
 CTSH 
 
  NCBI Gene  
  GeneRIF  
  Pubmed  
 
 
  Search Term  Rank  Fields  
  [Mm]igration  6  Process(2); PubMed(2)  
  ECM  7  PubMed(2)  
  [Ee]xtracellular [Mm]atrix  8  PubMed(3)  
  [Ii]nvasive  10  PubMed(2)  
  [Mm]etastasis  12  PubMed(3); GeneRIFs(1)  
 
 
 hide/show details for CTSH 
  [Mm]igration  Process  
 positive regulation of cell   migration   
 positive regulation of epithelial cell   migration   
  PubMed  
 Cathepsin H mediates the processing of talin and regulates   migration   of prostate cancer cells. The cytoskeletal protein talin, an actin- and β-integrin tail-binding protein, plays an important role in cell   migratio  n by promoting integrin activation and focal adhesion formation. Here, we show that talin is a substrate for cathepsin H (CtsH), a lysosomal cysteine protease with a strong aminopeptidase activity. Purified active CtsH sequentially cleaved a synthetic peptide representing the N terminus of the talin F0 head domain. The processing of talin by CtsH was determined also in the metastatic PC-3 prostate cancer cell line, which exhibits increased expression of CtsH. The attenuation of CtsH aminopeptidase activity by a specific inhibitor or siRNA-mediated silencing significantly reduced the   migratio  n of PC-3 cells on fibronectin and invasion through Matrigel. We found that in migrating PC-3 cells, CtsH was co-localized with talin in the focal adhesions. Furthermore, specific inhibition of CtsH increased the activation of α(v)β(3)-integrin on PC-3 cells. We propose that CtsH-mediated processing of talin might promote cancer cell progression by affecting integrin activation and adhesion strength. 
 Cathepsin H regulated by the thyroid hormone receptors associate with tumor invasion in human hepatoma cells. Thyroid hormone, 3, 3', 5-triiodo-L-thyronine (T(3)), mediates cell growth, development and differentiation by binding to its nuclear receptors (TRs). The role of TRs in cancer is still undefined. Notably, hyperthyroxinemia has been reported to influence the rate of colon cancer in an experimental model of carcinogenesis in rats. Previous microarray analysis revealed that cathepsin H (CTSH) is upregulated by T(3) in HepG2-TR cells. We verified that mRNA and protein expression of CTSH are induced by T(3) in HepG2-TR cells and in thyroidectomized rats following administration of T(3). The possible thyroid hormone-responsive elements of the CTSH promoter localized to the nucleotides -2038 to -1966 and -1565 to -1501 regions. An in vitro functional assay showed that CTSH can increase metastasis. J7 cells overexpressing CTSH were inoculated into severe combined immune-deficient mice and these J7-CTSH mice displayed a greater metastatic potential than did J7-control mice. The clinicopathologic significance of CTSH expression in hepatocellular carcinoma (HCC) was also investigated. The CTSH overexpressing in HCC was associated with the presence of microvascular invasion (P=0.037). The microvascular invasion characteristic is closely related to our in vitro characterization of CTSH function. Our results show that T(3)-mediated upregulation of CTSH led to matrix metallopeptidase or extracellular signal-regulated kinase activation and increased cell   migration  . This study demonstrated that CTSH overexpression in a subset hepatoma may be TR dependent and suggests that this overexpression has an important role in hepatoma progression. 
  ECM  PubMed  
 Expression patterns of cathepsins B, H, K, L and S in the human endometrium. Cathepsins B, H, K, L and S belong to the family of lysosomal cysteine proteinases and participate in a variety of proteolytic processes, including degradation of the extracellular matrix (  ECM  ). In the present study, we used Northern hybridization to demonstrate the presence of mRNAs for cathepsins B, H, K, L and S in human endometrium during both the proliferative and secretory phases of menstrual cycle. The mRNA levels for cathepsins H and K were significantly lower in secretory phase endometrium in comparison with proliferative phase endometrium. Immunohistochemical localization of the different cathepsins revealed widespread distribution of all cathepsins in both stroma and epithelial cells. The immunoreactivity for cathepsins B, H and K exhibited changes related to endometrial location and/or to the phase of the cycle. The strongest immunoreactivity for cathepsins B, H, L and S was observed in the surface epithelium of the endometrium. The staining for cathepsins was predominantly intracellular, but immunoreactivity was also detected on the surface of small lymphoid cells in the stroma. The findings of the present study suggest that cysteine cathepsins are needed for normal development and function of human endometrium during both the proliferative and secretory phases. 
 The expression and activity of cathepsins D, H and K in asthmatic airways. Tumstatin is an anti-angiogenic collagen IV α3 fragment, levels of which are reduced in the airways of asthmatics. Its reduction may be due to the degradation by extracellular matrix   (EC  M) proteases. Cathepsins play a role in   EC  M remodelling, with cathepsin D, H and K (CTSD, CTSH and CTSK) being associated with lung diseases. CTSD modulates the NC1 domains of collagen molecules including tumstatin, while CTSH and CTSK are involved in   EC  M degradation. The role of these cathepsins in the regulation of tumstatin in the lung has not previously been examined. We demonstrated that CTSB, D, F, H, K, L and S mRNA was expressed in the airways. Quantification of immunohistochemistry showed that there is no difference in the global expression of CTSD, CTSH and CTSK between asthmatics and non-asthmatics. CTSD and CTSK, but not CTSH had the capacity to degrade tumstatin. No difference was observed in the activity of CTSD and H in bronchoalveolar lavage fluid of asthmatic and non-asthmatics, while CTSK was undetectable. This indicates that while CTSD possesses the potential to directly regulate tumstatin, and thus angiogenesis through this mechanism however, it is not likely to be involved in the dysregulation of tumstatin found in asthmatic airways. 
  [Ee]xtracellular [Mm]atrix  PubMed  
 Expression of cathepsins B, H, K, L, and S during human fetal lung development. Cathepsins are involved in lysosomal protein degradation, proenzyme activation, antigen processing, and hormone maturation. They are secreted by tumor cells and macrophages and catalyze the remodeling of   extracellular matrix   proteins. To gain insight into the expression pattern of cathepsins during fetal lung development, the expression of cathepsins B, H, K, L, and S at protein and mRNA levels were evaluated by using immunohistochemistry and in situ hybridization. Early expression of cathepsins B, H, and K was found in epithelial cells of the branching presumptive bronchi (<12th week of gestation). The most intense cathepsin K-specific immunoreactivity was found in developing airways with a lumen. Cathepsin K was found in epithelial cells only, whereas in contrast, cathepsins B and H were detected both in epithelial and interstitial cells. During fetal maturation, interstitial cells displayed cathepsin L immunoreactivity and, in the saccular phase (>26th week of gestation), both cathepsin L and S immunoreactivities. A continuous decline in the proportion of cathepsin H-positive interstitial CD68-positive cells was observed. These discrete temporal and spatial variations in cathepsin expression during organogenesis of the human lung indicate different physiological roles for the individual enzymes in different cell types and developmental stages. 
 Expression patterns of cathepsins B, H, K, L and S in the human endometrium. Cathepsins B, H, K, L and S belong to the family of lysosomal cysteine proteinases and participate in a variety of proteolytic processes, including degradation of the   extracellular matrix   (ECM). In the present study, we used Northern hybridization to demonstrate the presence of mRNAs for cathepsins B, H, K, L and S in human endometrium during both the proliferative and secretory phases of menstrual cycle. The mRNA levels for cathepsins H and K were significantly lower in secretory phase endometrium in comparison with proliferative phase endometrium. Immunohistochemical localization of the different cathepsins revealed widespread distribution of all cathepsins in both stroma and epithelial cells. The immunoreactivity for cathepsins B, H and K exhibited changes related to endometrial location and/or to the phase of the cycle. The strongest immunoreactivity for cathepsins B, H, L and S was observed in the surface epithelium of the endometrium. The staining for cathepsins was predominantly intracellular, but immunoreactivity was also detected on the surface of small lymphoid cells in the stroma. The findings of the present study suggest that cysteine cathepsins are needed for normal development and function of human endometrium during both the proliferative and secretory phases. 
 The expression and activity of cathepsins D, H and K in asthmatic airways. Tumstatin is an anti-angiogenic collagen IV α3 fragment, levels of which are reduced in the airways of asthmatics. Its reduction may be due to the degradation by   extracellular matri  x (ECM) proteases. Cathepsins play a role in ECM remodelling, with cathepsin D, H and K (CTSD, CTSH and CTSK) being associated with lung diseases. CTSD modulates the NC1 domains of collagen molecules including tumstatin, while CTSH and CTSK are involved in ECM degradation. The role of these cathepsins in the regulation of tumstatin in the lung has not previously been examined. We demonstrated that CTSB, D, F, H, K, L and S mRNA was expressed in the airways. Quantification of immunohistochemistry showed that there is no difference in the global expression of CTSD, CTSH and CTSK between asthmatics and non-asthmatics. CTSD and CTSK, but not CTSH had the capacity to degrade tumstatin. No difference was observed in the activity of CTSD and H in bronchoalveolar lavage fluid of asthmatic and non-asthmatics, while CTSK was undetectable. This indicates that while CTSD possesses the potential to directly regulate tumstatin, and thus angiogenesis through this mechanism however, it is not likely to be involved in the dysregulation of tumstatin found in asthmatic airways. 
  [Ii]nvasive  PubMed  
 Genetic susceptibility to distinct bladder cancer subphenotypes. Clinical, pathologic, and molecular evidence indicate that bladder cancer is heterogeneous with pathologic/molecular features that define distinct subphenotypes with different prognoses. It is conceivable that specific patterns of genetic susceptibility are associated with particular subphenotypes.To examine evidence for the contribution of germline genetic variation to bladder cancer heterogeneity.The Spanish Bladder Cancer/EPICURO Study is a case-control study based in 18 hospitals located in five areas in Spain. Cases were patients with a newly diagnosed, histologically confirmed, urothelial cell carcinoma of the bladder from 1998 to 2001. Case diagnoses were reviewed and uniformly classified by pathologists following the World Health Organisation/International Society of Urological Pathology 1999 criteria. Controls were hospital-matched patients (n=1149).A total of 1526 candidate variants in 423 candidate genes were analysed. Three distinct subphenotypes were defined according to stage and grade: low-grade nonmuscle   invasive   (n=586), high-grade nonmuscle   invasive   (n=219), and muscle   invasive   (n=246). The association between each variant and subphenotype was assessed by polytomous risk models adjusting for potential confounders. Heterogeneity in genetic susceptibility among subphenotypes was also tested.Two established bladder cancer susceptibility genotypes, NAT2 slow-acetylation and GSTM1-null, exhibited similar associations among the subphenotypes, as did VEGF-rs25648, which was previously identified in our study. Other variants conferred risks for specific tumour subphenotypes such as PMS2-rs6463524 and CD4-rs3213427 (respective heterogeneity p values of 0.006 and 0.004), which were associated with muscle-  invasive   tumours (per-allele odds ratios [95% confidence interval] of 0.56 [0.41-0.77] and 0.71 [0.57-0.88], respectively) but not with non-muscle-  invasive   tumours. Heterogeneity p values were not robust in multiple testing according to their false-discovery rate.These exploratory analyses suggest that genetic susceptibility loci might be related to the molecular/pathologic diversity of bladder cancer. Validation through large-scale replication studies and the study of additional genes and single nucleotide polymorphisms are required. 
 Genetic susceptibility to distinct bladder cancer subphenotypes. Clinical, pathologic, and molecular evidence indicate that bladder cancer is heterogeneous with pathologic/molecular features that define distinct subphenotypes with different prognoses. It is conceivable that specific patterns of genetic susceptibility are associated with particular subphenotypes.To examine evidence for the contribution of germline genetic variation to bladder cancer heterogeneity.The Spanish Bladder Cancer/EPICURO Study is a case-control study based in 18 hospitals located in five areas in Spain. Cases were patients with a newly diagnosed, histologically confirmed, urothelial cell carcinoma of the bladder from 1998 to 2001. Case diagnoses were reviewed and uniformly classified by pathologists following the World Health Organisation/International Society of Urological Pathology 1999 criteria. Controls were hospital-matched patients (n=1149).A total of 1526 candidate variants in 423 candidate genes were analysed. Three distinct subphenotypes were defined according to stage and grade: low-grade nonmuscle   invasive   (n=586), high-grade nonmuscle   invasive   (n=219), and muscle   invasive   (n=246). The association between each variant and subphenotype was assessed by polytomous risk models adjusting for potential confounders. Heterogeneity in genetic susceptibility among subphenotypes was also tested.Two established bladder cancer susceptibility genotypes, NAT2 slow-acetylation and GSTM1-null, exhibited similar associations among the subphenotypes, as did VEGF-rs25648, which was previously identified in our study. Other variants conferred risks for specific tumour subphenotypes such as PMS2-rs6463524 and CD4-rs3213427 (respective heterogeneity p values of 0.006 and 0.004), which were associated with muscle-  invasive   tumours (per-allele odds ratios [95% confidence interval] of 0.56 [0.41-0.77] and 0.71 [0.57-0.88], respectively) but not with non-muscle-  invasive   tumours. Heterogeneity p values were not robust in multiple testing according to their false-discovery rate.These exploratory analyses suggest that genetic susceptibility loci might be related to the molecular/pathologic diversity of bladder cancer. Validation through large-scale replication studies and the study of additional genes and single nucleotide polymorphisms are required. 
  [Mm]etastasis  PubMed  
 Cathepsin H regulated by the thyroid hormone receptors associate with tumor invasion in human hepatoma cells. Thyroid hormone, 3, 3', 5-triiodo-L-thyronine (T(3)), mediates cell growth, development and differentiation by binding to its nuclear receptors (TRs). The role of TRs in cancer is still undefined. Notably, hyperthyroxinemia has been reported to influence the rate of colon cancer in an experimental model of carcinogenesis in rats. Previous microarray analysis revealed that cathepsin H (CTSH) is upregulated by T(3) in HepG2-TR cells. We verified that mRNA and protein expression of CTSH are induced by T(3) in HepG2-TR cells and in thyroidectomized rats following administration of T(3). The possible thyroid hormone-responsive elements of the CTSH promoter localized to the nucleotides -2038 to -1966 and -1565 to -1501 regions. An in vitro functional assay showed that CTSH can increase   metastasis  . J7 cells overexpressing CTSH were inoculated into severe combined immune-deficient mice and these J7-CTSH mice displayed a greater metastatic potential than did J7-control mice. The clinicopathologic significance of CTSH expression in hepatocellular carcinoma (HCC) was also investigated. The CTSH overexpressing in HCC was associated with the presence of microvascular invasion (P=0.037). The microvascular invasion characteristic is closely related to our in vitro characterization of CTSH function. Our results show that T(3)-mediated upregulation of CTSH led to matrix metallopeptidase or extracellular signal-regulated kinase activation and increased cell migration. This study demonstrated that CTSH overexpression in a subset hepatoma may be TR dependent and suggests that this overexpression has an important role in hepatoma progression. 
 Cathepsin B and H activities and cystatin C concentrations in cerebrospinal fluid from patients with leptomeningeal   metastasis  . Cysteine proteases are involved in the extension of cancer into the subarachnoid space. The presence of cathepsins B and H along with their potent inhibitor cystatin C in the cerebrospinal fluid (CSF) was investigated in patients with leptomeningeal   metastasis   of cancer (LM).CSF samples were obtained in 16 cases of LM (10 solid tumors and 6 leukemia or lymphoma) and compared with 11 cancer cases without involvement of the central nervous system, 12 multiple sclerosis cases and 34 healthy volunteers. The activity of the enzymes was measured, their molecular forms were analyzed by the Western blotting, and the concentration of cystatin C was measured by ELISA. Immunohistochemistry of the leptomeningeal tissues was also performed in six autopsy cases of LM.High activities of cathepsins B and H along with decreased cystatin C concentration were observed in CSF of LM as compared with three control groups. Western blot analysis revealed higher concentration of the enzyme protein as well as its active forms in samples with higher enzyme activity. Cells metastasizing leptomeningeal tissue were clearly positive in immunohistochemical staining of cathepsins, indicating active production by tumor cells.Production of cathepsins B and H by tumor cells and their high activity along with concomitant decrease of their potent inhibitor, cystatin C, in the CSF might contribute in the process of   metastasis   and spread of the cancer cells in the leptomeningeal tissues. A high enzyme activity/cystatin C concentration ratio in the CSF could be useful when diagnosing LM in combination with other parameters. 
 Cathepsin B, cathepsin H, cathepsin X and cystatin C in sera of patients with early-stage and inflammatory breast cancer. Numerous studies have linked cathepsins and their inhibitor cystatin C to tumor invasion and   metastasis  . We examined whether cathepsin B, cathepsin H, cathepsin X and cystatin C could be detected in sera from women with early stage or inflammatory breast cancer and whether they correlated with clinicopathological characteristics. Preoperative serum was obtained from 176 patients with early-stage breast cancer (tumor size <or= 5 cm, negative lymph nodes) and 31 patients with inflammatory breast cancer. Cathepsin and cystatin C levels were measured by ELISA. The patient and tumor characteristics under study were age at diagnosis, menopausal status, tumor size, tumor grade, and steroid hormone receptor status. Serum cathepsin B levels were significantly lower in patients with poorly differentiated tumors. High cystatin C levels were associated with tumor size, postmenopausal status and patient age. Interestingly, significantly lower levels of cathepsin X and H were found in patients with inflammatory breast cancer, a trend also observed for cathepsin B and cystatin C. In conclusion, our results show a limited association of cathepsins B, H, X and cystatin C with established prognostic parameters. These data are promising and encourage future analysis of the clinical outcome of our patients in order to examine the potential prognostic value of these biomarkers. Further, this study indicates a role for cathepsin X and H in inflammatory breast cancer. 
  GeneRIFs  
 production of cathepsins B and H by tumor cells along with concomitant decrease of their inhibitor, cystatin C, in the CSF might contribute in the process of   metastasis   and spread of the cancer cells in the leptomeningeal tissues 
   
  
 CADM4 
 
  NCBI Gene  
  GeneRIF  
  Pubmed  
 
 
  Search Term  Rank  Fields  
  [Ii]nvasive  10  PubMed(2); GeneRIFs(1)  
  [Mm]etastasis  12  PubMed(1)  
 
 
 hide/show details for CADM4 
  [Ii]nvasive  PubMed  
 Clinicopathological significance of CADM4 expression in   invasive   ductal carcinoma of the breast. Cell adhesion molecule 4 (CADM4) is a novel tumour suppressor involved in cell adhesion. Loss or decreased expression of CADM4 has been associated with the development and progression of some cancers. The purpose of this study was to investigate the clinicopathological significance of CADM4 expression in breast cancer.We constructed tissue microarrays to evaluate the immunohistochemical expression of CADM4 in 256 cases of   invasive   ductal carcinoma (IDC) and 45 cases of ductal carcinoma in situ (DCIS).CADM4 was expressed in 37 (82.2%) DCIS cases, and in 173 (67.6%) IDC cases. CADM4 expression was higher in DCIS than in IDC (p=0.049). Loss or decrease of CADM4 expression was significantly correlated with higher histological grade (p=0.020), absence of oestrogen receptors (p<0.001), absence of progesterone receptors (p=0.024), and overexpression of c-erbB-2 (p=0.018). In univariable and multivariable Cox regression analyses of all 256 IDC cases, CADM4 expression was not significantly associated with overall and disease-free survival. However, it showed a significant positive association with longer disease-free survival in 187 stages I and II IDC cases (p=0.039, log-rank test).Loss or decrease of CADM4 expression seems to play an important role in breast cancer   invasive  ness, and it is associated with poorer biological parameters. CADM4 can be used as a novel marker predicting risk of recurrence and disease outcomes in stages I and II IDC. 
 The cell adhesion nectin-like molecules (Necl) 1 and 4 suppress the growth and tumorigenic ability of colon cancer cells. A key step in human colon cancer development includes the hyperactivation of Wnt/beta-catenin signaling and the induction of beta-catenin-TCF target genes that participate in colon cancer progression. Recent studies identified members of the immunoglobulin-like cell adhesion molecules (IgCAM) of the L1CAM family (L1 and Nr-CAM) as targets of beta-catenin-TCF signaling in colon cancer cells. L1 was detected at the   invasive   front of colon cancer tissue and confers metastasis when overexpressed in cells. In contrast to L1, we did not detect in colon cancer cells significant levels of another IgCAM family of molecules, the nectin-like (Necl) receptors Necl1 and Necl4, while Necl4 was previously found in the normal small intestine and colon tissues. We studied the properties of colon cancer cells in which Necl4 and Necl1 were expressed either alone, or in combination, and found that such cells display a wide range of properties associated with tumor suppression. Expression of both Necl1 and Necl4 was the most efficient in suppressing the tumorigenicity of colon cancer cells. This was associated with enhanced rates of apoptosis and change in several apoptosis-related markers. In contrast to its capacity to suppress tumorigenesis, Necl4 was unable to affect the highly malignant and metastatic capacities of colon cancer cells in which L1 was overexpressed. Our results suggest that various IgCAM receptor families play different roles in affecting the tumorigenic function of the same cells, and that Necl1 and Necl4 can fulfill a tumor suppressive role. 
  GeneRIFs  
 Loss or decrease of CADM4 expression seems to play an important role in breast cancer   invasive  ness, and it is associated with poorer biological parameters. 
  [Mm]etastasis  PubMed  
 The cell adhesion nectin-like molecules (Necl) 1 and 4 suppress the growth and tumorigenic ability of colon cancer cells. A key step in human colon cancer development includes the hyperactivation of Wnt/beta-catenin signaling and the induction of beta-catenin-TCF target genes that participate in colon cancer progression. Recent studies identified members of the immunoglobulin-like cell adhesion molecules (IgCAM) of the L1CAM family (L1 and Nr-CAM) as targets of beta-catenin-TCF signaling in colon cancer cells. L1 was detected at the invasive front of colon cancer tissue and confers   metastasis   when overexpressed in cells. In contrast to L1, we did not detect in colon cancer cells significant levels of another IgCAM family of molecules, the nectin-like (Necl) receptors Necl1 and Necl4, while Necl4 was previously found in the normal small intestine and colon tissues. We studied the properties of colon cancer cells in which Necl4 and Necl1 were expressed either alone, or in combination, and found that such cells display a wide range of properties associated with tumor suppression. Expression of both Necl1 and Necl4 was the most efficient in suppressing the tumorigenicity of colon cancer cells. This was associated with enhanced rates of apoptosis and change in several apoptosis-related markers. In contrast to its capacity to suppress tumorigenesis, Necl4 was unable to affect the highly malignant and metastatic capacities of colon cancer cells in which L1 was overexpressed. Our results suggest that various IgCAM receptor families play different roles in affecting the tumorigenic function of the same cells, and that Necl1 and Necl4 can fulfill a tumor suppressive role. 
   
  
 CADM1 
 
  NCBI Gene  
  GeneRIF  
  Pubmed  
 
 
  Search Term  Rank  Fields  
  [Mm]igration  6  PubMed(6); GeneRIFs(1)  
  ECM  7  PubMed(1)  
  [Ee]xtracellular [Mm]atrix  8  PubMed(3)  
  [Ii]nvasive  10  PubMed(15); GeneRIFs(6)  
  [Mm]etastasize  11  PubMed(1)  
  [Mm]etastasis  12  PubMed(16); GeneRIFs(6)  
  [Cc]ell [Ii]nvasion  13  PubMed(2)  
 
 
 hide/show details for CADM1 
  [Mm]igration  PubMed  
 MicroRNA-1246 enhances   migration   and invasion through CADM1 in hepatocellular carcinoma. The aberrant expression of microRNAs has been demonstrated to play a crucial role in the initiation and progression of hepatocarcinoma. miR-1246 expression in High invasive ability cell line than significantly higher than that in low invasive ability cell line.Transwell chambers (8-uM pore size; Costar) were used in the in vitro   migration   and invison anssay. Dual luciferase reporter gene construct and Dual luciferase reporter assay to identify the target of miR-1246. CADM1 expression was evaluated by immunohistochemistric staining. The clinical manifestations, treatments and survival were collected for statistical analysis.Inhibition of miR-1246 effectively reduced   migration   and invasion of hepatocellular carcinoma cell lines. Bioinformatics and luciferase reporter assay revealed that miR-1246 specifically targeted the 3'-UTR of Cell adhesion molecule 1 and regulated its expression. Down-regulation of CADM1 enhanced   migration   and invasion of HCC cell lines. Furthermore, in tumor tissues obtained from liver cancer patients, the expression of miR-1246 was negatively correlated with CADM1 and the high expression of miR-1246 combined with low expression of CADM1 might serve as a risk factor for stage1 liver cancer patients.Our study showed that miR-1246, by down-regulation CADM1, enhances   migration   and invasion in HCC cells. 
 MicroRNA-126 regulates   migration   and invasion of gastric cancer by targeting CADM1. The aberrant expression of microRNAs has been demonstrated to play a crucial role in the initiation and progression of gastric cancer (GC). We here aimed to investigate the mechanism of microRNAs in the regulation of GC pathogenesis.Transwell chambers (8-μM pore size; Costar) were used in the in vitro   migratio  n and in vision assay. Dual luciferase reporter gene construct and dual luciferase reporter assay to identify the target of miR-126. CADM1 expression was evaluated by immunohistochemical staining. The clinical manifestations, treatments and survival were collected for statistical analysis.Inhibition of miR-126 effectively reduced   migratio  n and invasion of gastric cancer cell lines. Bioinformatics and luciferase reporter assay revealed that miR-126 specifically targeted the 3'UTR of cell adhesion molecule 1 (CADM1) and regulated its expression. Down-regulation of CADM1 enhanced   migratio  n and invasion of GC cell lines. Furthermore, in tumor tissues obtained from gastric cancer patients, the expression of miR-126 was negatively correlated with CADM1 and the high expression of miR-126 combined with low expression of CADM1 might serve as a risk factor for stage1 gastric cancer patients.Our study showed that miR-126, by down-regulation CADM1, enhances   migratio  n and invasion in GC cells. 
 MicroRNA-10b promotes   migration   and invasion through CADM1 in human hepatocellular carcinoma cells. MicroRNA-10b (miR-10b) was recently reported to be dysregulated in some types of cancer and to play a role in invasion and metastasis. However, effects and potential mechanisms of action of miR-10b in the metastasis of hepatocellular carcinoma (HCC) have not been explored. In this study, we confirmed that miR-10b is highly expressed in metastatic HCC tissues and in metastatic HCC cell lines by qRT-PCR. Moreover, patients with higher miR-10b expression had significantly poorer overall survival, and high miR-10b expression was an independent predictor of poor prognosis. Inhibition of miR-10b reduced cell   migration   and invasion in MHCC97H cells, whereas over-expression of miR-10b in HepG2 cells increased cell   migration   and invasion. Bioinformatics and luciferase reporter assays revealed that miR-10b binds the 3'-UTR of CADM1 mRNA and represses its translation. Western blot and qRT-PCR showed that CADM1 is inhibited by miR-10b over-expression. Silencing of CADM1 resulted in substantially increased cell motility and invasion similar to that observed with over-expression of miR-10b in HepG2 cells. These results suggest that miR-10b may positively regulate the invasion and metastasis of HCC through targeting CADM1. 
 LncRNA TSLC1-AS1 is a novel tumor suppressor in glioma. Growing evidence demonstrates that long non coding RNAs (lncRNAs) play an important role in cancer origination and progression. A novel lncRNA, TSLC1-AS1, is the antisense transcript of tumor suppressor TSLC1. The expression profile and function of TSLC1-AS1 in glioma were investigated using Real-Time Quantitative PCR and siRNA knockdown. The data showed that TSLC1-AS1 expression was down-regulated in tumor tissues compared with that in adjacent normal tissues, and negatively associated with the WHO criteria of the tumors. Overexpression of TSLC1-AS1 resulted in up-regulation of TSLC1 and significant inhibition of cell proliferation,   migration   and invasion in U87 cells, while knockdown of TSLC1-AS1 in SNB-19 cells showed the opposite effect. The expression of TSLC1-AS1 was also positively correlated with other tumor suppressors NF1, VHL, PIK3R1 and negatively correlated with the oncogene BRAF. The results suggested that TSLC1-AS1 was a tumor suppressor of glioma and a mediator of TSLC1 expression. LncRNA TSLC1-AS1 may serve as a potential biomarker and therapeutic target for glioma. 
 TGF-β upregulates miR-182 expression to promote gallbladder cancer metastasis by targeting CADM1. Transforming growth factor β (TGF-β) plays important roles in tumor metastasis by regulating miRNAs expression. miR-182 is an important molecule in the regulation of cancer progression. The aim of the study is to assess the role of miR-182 in TGF-β-induced cancer metastasis. In the present study, we found that miR-182 levels are significantly upregulated in GBC tissues compared with normal controls, and miR-182 expression is remarkably increased in primary tumors that subsequently metastasized, when compared to those primary tumors that did not metastasize. TGF-β induces miR-182 expression in GBC cells, and overexpression of miR-182 promotes GBC   cell migr  ation and invasion, whereas miR-182 inhibition suppresses TGF-β-induced cancer   cell mig  ration and invasion. The blockage of miR-182 by a specific inhibitor effectively inhibits pulmonary metastases in vivo. We further identified that the cell adhesion molecule1 (CADM1) is a new target gene of miR-182. miR-182 negatively regulates CADM1 expression in vitro and in vivo. Importantly, re-expression of CADM1 in GBC cells partially abrogates miR-182-induced cell invasion.miR-182 is an important mediator of GBC metastasis, thus offering a new target for the development of therapeutic agents against GBC. 
 Cell adhesion molecule 1: a novel risk factor for venous thrombosis. Protein C (PC) deficiency increases the risk of venous thrombosis (VT) among members of Kindred Vermont II but fails to fully account for the inheritance pattern. A genome scan of the pedigree supported the presence of a prothrombotic gene on chromosome 11q23 (nominal P < .0001), with weaker support on chromosomes 10p12 (P < .0003) and 18p11.2-q11 (P < .0007). Resequencing of 109 genes in the linkage regions identified 5030 variants in a sample of 20 kindred members. Of 16 single nucleotide polymorphisms in 6 genes tested in the larger family set, only single nucleotide polymorphisms in cell adhesion molecule 1 (CADM1) associated with VT. Among the 8 CADM1 single nucleotide polymorphisms genotyped in the complete sample, rs6589488 was most strongly supported (P < .000007), but the association was limited to the PC-deficient subset of the sample (P < .000001). Haplotype analysis narrowed the region containing the causative variant to the coding region of the CADM1 gene. CADM1 gene expression analyzed in blood outgrowth endothelial cells cultured from family members was decreased compared with control subjects, lending phenotypic support to this conclusion. Finally, we have for the first time demonstrated CADM1 in endothelial cells, where it appears to be selectively involved in endothelial cell   migration  , suggesting a role in endothelial barrier repair. 
  GeneRIFs  
 Our study showed that miR-126, by down-regulation CADM1, enhances   migration   and invasion in gastric cancer cells. 
  ECM  PubMed  
 CADM1 controls actin cytoskeleton assembly and regulates extracellular matrix adhesion in human mast cells. CADM1 is a major receptor for the adhesion of mast cells (MCs) to fibroblasts, human airway smooth muscle cells (HASMCs) and neurons. It also regulates E-cadherin and alpha6beta4 integrin in other cell types. Here we investigated a role for CADM1 in MC adhesion to both cells and extracellular matrix (  ECM  ). Downregulation of CADM1 in the human MC line HMC-1 resulted not only in reduced adhesion to HASMCs, but also reduced adhesion to their   ECM  . Time-course studies in the presence of EDTA to inhibit integrins demonstrated that CADM1 provided fast initial adhesion to HASMCs and assisted with slower adhesion to   ECM  . CADM1 downregulation, but not antibody-dependent CADM1 inhibition, reduced MC adhesion to   ECM  , suggesting indirect regulation of   ECM   adhesion. To investigate potential mechanisms, phosphotyrosine signalling and polymerisation of actin filaments, essential for integrin-mediated adhesion, were examined. Modulation of CADM1 expression positively correlated with surface KIT levels and polymerisation of cortical F-actin in HMC-1 cells. It also influenced phosphotyrosine signalling and KIT tyrosine autophosphorylation. CADM1 accounted for 46% of surface KIT levels and 31% of F-actin in HMC-1 cells. CADM1 downregulation resulted in elongation of cortical actin filaments in both HMC-1 cells and human lung MCs and increased cell rigidity of HMC-1 cells. Collectively these data suggest that CADM1 is a key adhesion receptor, which regulates MC net adhesion, both directly through CADM1-dependent adhesion, and indirectly through the regulation of other adhesion receptors. The latter is likely to occur via docking of KIT and polymerisation of cortical F-actin. Here we propose a stepwise model of adhesion with CADM1 as a driving force for net MC adhesion. 
  [Ee]xtracellular [Mm]atrix  PubMed  
 CADM1 controls actin cytoskeleton assembly and regulates   extracellular matrix   adhesion in human mast cells. CADM1 is a major receptor for the adhesion of mast cells (MCs) to fibroblasts, human airway smooth muscle cells (HASMCs) and neurons. It also regulates E-cadherin and alpha6beta4 integrin in other cell types. Here we investigated a role for CADM1 in MC adhesion to both cells and   extracellular matrix   (ECM). Downregulation of CADM1 in the human MC line HMC-1 resulted not only in reduced adhesion to HASMCs, but also reduced adhesion to their ECM. Time-course studies in the presence of EDTA to inhibit integrins demonstrated that CADM1 provided fast initial adhesion to HASMCs and assisted with slower adhesion to ECM. CADM1 downregulation, but not antibody-dependent CADM1 inhibition, reduced MC adhesion to ECM, suggesting indirect regulation of ECM adhesion. To investigate potential mechanisms, phosphotyrosine signalling and polymerisation of actin filaments, essential for integrin-mediated adhesion, were examined. Modulation of CADM1 expression positively correlated with surface KIT levels and polymerisation of cortical F-actin in HMC-1 cells. It also influenced phosphotyrosine signalling and KIT tyrosine autophosphorylation. CADM1 accounted for 46% of surface KIT levels and 31% of F-actin in HMC-1 cells. CADM1 downregulation resulted in elongation of cortical actin filaments in both HMC-1 cells and human lung MCs and increased cell rigidity of HMC-1 cells. Collectively these data suggest that CADM1 is a key adhesion receptor, which regulates MC net adhesion, both directly through CADM1-dependent adhesion, and indirectly through the regulation of other adhesion receptors. The latter is likely to occur via docking of KIT and polymerisation of cortical F-actin. Here we propose a stepwise model of adhesion with CADM1 as a driving force for net MC adhesion. 
 Interaction of nectin-like molecule 2 with integrin alpha6beta4 and inhibition of disassembly of integrin alpha6beta4 from hemidesmosomes. In normal epithelial cells, integrin α(6)β(4) is abundantly expressed and forms hemidesmosomes, which is a cellular structure that mediates cel  l-extracellular matr  ix binding. In many types of cancer cells, integrin α(6)β(4) is up-regulated, laminin is cleaved, and hemidesmosomes are disrupted, eventually causing an enhancement of cancer cell movement and facilitation of their invasion. We previously showed that the immunoglobulin-like cell adhesion molecule Necl-2 (Nectin-like molecule 2), known as a tumor suppressor, inhibits cancer cell movement by suppressing the ErbB3/ErbB2 signaling. We show here that Necl-2 interacts in cis with integrin α(6)β(4). The binding of Necl-2 with integrin β(4) was mediated by its extracellular region. In human colorectal adenocarcinoma Caco-2 cells, integrin α(6)β(4) was localized at hemidesmosomes. Small interfering RNA-mediated suppression of Necl-2 expression enhanced the phorbol ester-induced disruption of the integrin α(6)β(4) complex at hemidesmosomes, whereas expression of Necl-2 suppressed the disruption of this structure. These results indicate that tumor-suppressive functions of Necl-2 are mediated by the stabilization of the hemidesmosome structure in addition to the inhibition of the ErbB3/ErbB2 signaling. 
 Recruitment of nectin-3 to cell-cell junctions through trans-heterophilic interaction with CD155, a vitronectin and poliovirus receptor that localizes to alpha(v)beta3 integrin-containing membrane microdomains. Nectins present a novel class of Ig superfamily adhesion molecules that, cooperatively with cadherins, establish and maintain cell-cell adherens junctions. CD155, the cognate receptor for poliovirus, undergoes cell-matrix contacts by binding to the   extracellular matrix   protein vitronectin. The significant homology of nectins with CD155 prompted us to investigate the possibility of their interaction. We determined that nectin-3 binds CD155 and its putative mouse homologue Tage4 in cell-based ligand binding assays. Coculture of nectin-3- and CD155-expressing HeLa cells led to CD155-dependent recruitment of nectin-3 to cell-cell contacts. In a heterologous coculture system with CD155 expressing mouse neuroblastoma cells, HeLa cell-expressed nectin-3 was recruited to contact sites with CD155 bearing neurites. CD155 and nectin-3 colocalized to epithelial cell-cell junctions in renal proximal tubules and in the amniotic membrane. Efficient interaction depended on CD155 dimerization, which appears to be aided by cell type-specific cofactors. We furthermore found CD155 to codistribute with alpha(v) integrin microdomains on the surface of transfected mouse fibroblasts and at amniotic epithelial cell junctions. Our findings demonstrate the possible trans-interaction between the bona fide cell-cell adherens type adhesion system (cadherin/nectin) and the cell-matrix adhesion system (integrin/CD155) by virtue of their nectin-3 and CD155 components, respectively. 
  [Ii]nvasive  PubMed  
 Tumor suppressor CADM1 is involved in epithelial cell structure. The tumor suppressor, CADM1, is involved in cell adhesion and preferentially inactivated in   invasive   cancer. We have previously reported that CADM1 associates with an actin-binding protein, 4.1B/DAL-1, and a scaffold protein, membrane protein palmitoylated 3 (MPP3)/DLG3. However, underlying mechanism of tumor suppression by CADM1 is not clarified yet. Here, we demonstrate that MPP1/p55 and MPP2/DLG2, as well as MPP3, interact with both CADM1 and 4.1B, forming a tripartite complex. We then examined cell biological roles of CADM1 and its complex in epithelia using HEK293 cells. Among MPP1-3, MPP2 is recruited to the CADM1-4.1B complex in the early process of adhesion in HEK293 cells. By suppression of CADM1 expression using siRNA, HEK293 lose epithelia-like structure and show flat morphology with immature cell adhesion. 4.1B and MPP2, as well as E-cadherin and ZO-1, are mislocalized from the membrane by depletion of CADM1 in HEK293. Mislocalization of MPP2 is also observed in several cancer cells lacking CADM1 expression with the transformed morphology. These findings suggest that CADM1 is involved in the formation of epithelia-like cell structure with 4.1B and MPP2, while loss of its function could cause morphological transformation of cancer cells. 
 CADM1 interacts with Tiam1 and promotes   invasive   phenotype of human T-cell leukemia virus type I-transformed cells and adult T-cell leukemia cells. CADM1 encodes a multifunctional immunoglobulin-like cell adhesion molecule whose cytoplasmic domain contains a type II PSD95/Dlg/ZO-1 (PDZ)-binding motif (BM) for associating with other intracellular proteins. Although CADM1 lacks expression in T lymphocytes of healthy individuals, it is overexpressed in adult T-cell leukemia-lymphoma (ATL) cells. It has been suggested that the expression of CADM1 protein promotes infiltration of leukemic cells into various organs and tissues, which is one of the frequent clinical manifestations of ATL. Amino acid sequence alignment revealed that Tiam1 (T-lymphoma invasion and metastasis 1), a Rac-specific guanine nucleotide exchange factor, has a type II PDZ domain similar to those of membrane-associated guanylate kinase homologs (MAGUKs) that are known to bind to the PDZ-BM of CADM1. In this study, we demonstrated that the cytoplasmic domain of CADM1 directly interacted with the PDZ domain of Tiam1 and induced formation of lamellipodia through Rac activation in HTLV-I-transformed cell lines as well as ATL cell lines. Our results indicate that Tiam1 integrates signals from CADM1 to regulate the actin cytoskeleton through Rac activation, which may lead to tissue infiltration of leukemic cells in ATL patients. 
 Expression of a splicing variant of the CADM1 specific to small cell lung cancer. CADM1, a member of the immunoglobulin superfamily cell adhesion molecule, acts as a tumor suppressor in a variety of human cancers. CADM1 is also ectopically expressed in adult T-cell leukemia (ATL), conferring an   invasive   phenotype characteristic to ATL. Therefore, CADM1 plays dual roles in human oncogenesis. Here, we investigate the roles of CADM1 in small cell lung cancer (SCLC). Immunohistochemistry demonstrates that 10 of 35 (29%) primary SCLC tumors express CADM1 protein. Western blotting and RT-PCR analyses reveal that CADM1 is significantly expressed in 11 of 14 SCLC cells growing in suspension cultures but in neither of 2 SCLC cells showing attached growth to plastic dishes, suggesting that CADM1 is involved in anchorage-independent growth in SCLC. In the present study, we demonstrate that SCLC expresses a unique splicing variant of CADM1 (variant 8/9) containing additional extracellular fragments corresponding to exon 9 in addition to variant 8, a common isoform in epithelia. Variant 8/9 of CADM1 is almost exclusively observed in SCLC and testis, although this variant protein localizes along the membrane and shows similar cell aggregation activity to variant 8. Interestingly, both variant 8/9 and variant 8 of CADM1 show enhanced tumorigenicity in nude mice when transfected into SBC5, a SCLC cell lacking CADM1. Inversely, suppression of CADM1 expression by shRNA reduced spheroid-like cell aggregation of NCI-H69, an SCLC cell expressing a high amount of CADM1. These findings suggest that CADM1 enhances the malignant features of SCLC, as is observed in ATL, and could provide a molecular marker specific to SCLC. 
 CADM1/TSLC1 inhibits melanoma cell line A375 invasion through the suppression of matrix metalloproteinases. Increasing evidence has demonstrated that cell adhesion molecule 1/tumor suppressor in lung cancer 1 (CADM1/TSLC1) is crucially implicated in various biological processes, including proliferation, apoptosis, and invasion during tumorigenesis and development. However, the mechanism underlying its suppression of tumor growth and metastasis in melanoma remains elusive. The aim of the present study was to examine if CADM1/TSLC1 was able to induce growth suppression in melanoma. The plasmid pcDNA3.1‑CADM1/TSLC1 was transfected into A375 cells (a human melanoma cell line). The expression of CADM1/TSLC1 in the transfected cells was determined by RT‑PCR and western blotting analysis. Cell growth was measured by an 3‑(4,5‑dimethylthiazol‑2‑yl)‑2,5‑diphenyl‑tetrazolium bromide assay and cell apoptosis was determined by flow cytometry, while a transwell assay was utilized to measure the ability of invasion. The expression of MMP‑2 and MMP‑9 in the transfected cells was determined by western blotting analysis. RT‑PCR and western blotting revealed that in pcDNA3.1‑CADM1/TSLC1 the protein expression of CADM1/TSLC1 protein was higher than in the pcDNA3.1 and A375 cells. The expression of MMP‑2 and MMP‑9 was lower in the pcDNA3.1‑CADM1/TSLC1 than that in the pcDNA3.1 and A375 cells. The growth of CADM1/TSLC1‑transfected cells was significantly suppressed in vitro and the ability of invasion was also reduced, CADM1/TSLC1 was able to induce cell apoptosis.   Furtherm  ore, CADM1/TSLC1 was an anti‑invasive gene, the overexpression of which inhibited the invasion of A375 cells. This inhibition may be due to the suppression of the MMP‑2 and MMP‑9 expression, which is relative to tumor metastasis and progression. 
 Alterations of ATM and CADM1 in chromosomal 11q22.3-23.2 region are associated with the development of   invasive   cervical carcinoma. To understand the importance of chr11q22.3-23.2 region in the development of cervical cancer, we have studied the genetic and epigenetic alterations of the candidate genes ATM, PPP2R1B, SDHD and CADM1 in cervical intraepithelial neoplasia (CIN) and cervical carcinoma (CACX) samples. Our study revealed low expression and high alterations (methylation/deletion) (55-59%) of ATM and CADM1 genes along with poor patient outcome. The alterations of ATM and CADM1 are associated with the progression of tumor from CIN to Stage I/II, thus implying their role in early   invasive  ness. The two genes, PPP2R1B and SDHD, lying in between ATM and CADM1, have low frequency of alterations, and majority of the alterations are in CACX samples, indicating that their alterations might be associated with disease progression. Expressions (mRNA/protein) of the genes showed concordance with their molecular alterations. Significant co-alteration of ATM and CADM1 points to their synergic action for the development of CACX. Mutation is, however, a rare phenomenon for inactivation of ATM. Association between the alteration of ATM and CHEK1 and poor survival of the patients having co-alterations of ATM and CHEK1 points to the DNA damage response pathway disruption in development of CACX. Thus, our data suggest that inactivation of ATM-CHEK1-associated DNA damage response pathway and CADM1-associated signaling network might have an important role in the development of CACX. 
 MicroRNA-1246 enhances migration and invasion through CADM1 in hepatocellular carcinoma. The aberrant expression of microRNAs has been demonstrated to play a crucial role in the initiation and progression of hepatocarcinoma. miR-1246 expression in High   invasive   ability cell line than significantly higher than that in low   invasive   ability cell line.Transwell chambers (8-uM pore size; Costar) were used in the in vitro migration and invison anssay. Dual luciferase reporter gene construct and Dual luciferase reporter assay to identify the target of miR-1246. CADM1 expression was evaluated by immunohistochemistric staining. The clinical manifestations, treatments and survival were collected for statistical analysis.Inhibition of miR-1246 effectively reduced migration and invasion of hepatocellular carcinoma cell lines. Bioinformatics and luciferase reporter assay revealed that miR-1246 specifically targeted the 3'-UTR of Cell adhesion molecule 1 and regulated its expression. Down-regulation of CADM1 enhanced migration and invasion of HCC cell lines. Furthermore, in tumor tissues obtained from liver cancer patients, the expression of miR-1246 was negatively correlated with CADM1 and the high expression of miR-1246 combined with low expression of CADM1 might serve as a risk factor for stage1 liver cancer patients.Our study showed that miR-1246, by down-regulation CADM1, enhances migration and invasion in HCC cells. 
 Loss/Down-regulation of tumor suppressor in lung cancer 1 expression is associated with tumor progression and is a biomarker of poor prognosis in ovarian carcinoma. The tumor suppressor in lung cancer 1 (TSLC1) has been identified as a putative tumor suppressor gene in non-small cell lung cancer. Although loss of TSLC1 has been observed in a number of human malignancies, the expression levels of TSLC1 gene in ovarian cancer and its clinical or prognostic significance have not been investigated.Protein expression levels of TSLC1 was explored by semiquantitative immunohistochemical staining on archival formalin-fixed, paraffin-embedded pathological specimen consisting of 30 normal ovaries, 30 ovarian cystadenomas, 40 borderline ovarian tumors, and 160   invasive   ovarian carcinomas. The TSLC1 immunohistochemical staining results were then correlated with various clinicopathologic parameters and patient prognosis using various statistical models.Significantly decreased, or complete loss of, protein expression of the TSLC1 gene was observed in 59% ovarian carcinomas, 45% borderline tumors, and 7% cystadenomas, but in none of the normal ovaries (0%). In ovarian carcinomas, decreased TSLC1 expression was significantly correlated with lymph node metastasis (pN, P = 0.001), distant metastasis (pM, P = 0.028), and more advanced International Federation of Gynecology and Obstetrics stages (P = 0.008). By univariate survival analysis on the ovarian carcinoma cohorts, decreased TSLC1 protein expression was significantly associated with shortened patient survival (mean: 26.9 months in tumors with complete loss of TSLC1 vs 63.1 months in tumors with significantly decreased TSLC1 vs 94.3 months in tumors with normal levels of TSLC1; P < 0.001). By multivariate analysis, TSLC1 protein expression remained as a significant and independent prognostic factor for the prediction of patient survival (P = 0.003).Decreased protein expression of the TSLC1 gene might be important in conferring a more aggressive behavior in ovarian carcinoma. Thus, TSLC1 may be used as an independent prognostic molecular marker for patients with ovarian carcinoma. 
 Expression of the TSLC1 adhesion molecule in pulmonary epithelium and its down-regulation in pulmonary adenocarcinoma other than bronchioloalveolar carcinoma. TSLC1 (tumor suppressor in lung cancer-1) is an adhesion molecule of the Ig superfamily that binds homophilically and mediates cell-cell interactions. Originally, TSLC1 was cloned as a candidate tumor suppressor from the genomic region that frequently exhibits loss of heterogeneity in human non-small-cell lung cancer (NSCLC). However, there have been no studies on TSLC1 expression in normal lungs or NSCLC. Here we show that pulmonary epithelial cells express TSLC1 and its expression levels are often decreased or lost in primary pulmonary adenocarcinoma, a major histologic type of NSCLC. Immunohistochemistry revealed that TSLC1 was localized at cell-cell boundaries of all columnar epithelial cells in mouse embryonic lungs of 10.5 and 13 days postcoitus. Similar staining patterns were observed in bronchiolar and alveolar epithelial cells of adult human lungs, suggesting a physiologic role for TSLC1 in interactions of these cells. Next we performed Western blot analyses of TSLC1 in 47 primary pulmonary adenocarcinomas and judged each tumor as either decreased or nondecreased by comparing TSLC1 expression levels of the tumor with the levels of normal lungs. The expression profiles had a significant relation to histologic subtypes but not to other clinicopathologic parameters. Sixteen bronchioloalveolar carcinomas (BACs) were all judged nondecreased, while 19 of 31 (63%) adenocarcinomas other than BAC were judged decreased (p < 0.0001). Immunohistochemistry of tumors judged nondecreased revealed that not only BAC cells but also tumor cells in lepidic growth components of adenocarcinomas other than BAC expressed TSLC1 on their lateral plasma membranes. These tumor cells are considered less   invasive   because they proliferate in a lepidic growth pattern along alveolar walls. Thus, the present results not only support the hypothesis that TSLC1 is a tumor suppressor of NSCLC but also suggest that preserved integrity of TSLC1 may contribute to less   invasive   phenotypes of lepidic growth tumor cells. 
 Loss of TSLC1 expression in lung adenocarcinoma: relationships with histological subtypes, sex and prognostic significance. The TSLC1 (tumor suppressor in lung cancer 1) gene is a tumor suppressor recently identified through functional complementation in a lung adenocarcinoma cell line A549. In this study we immunohistochemically examined the loss of TSLC1 expression in 93 cases of surgically resected lung adenocarcinoma, and investigated its correlation with clinicopathological parameters, including histological subtypes of tumors. The prognostic significance of loss of TSLC1 expression was analyzed by univariate and multivariate analyses, in parallel with other prognostic markers such as p53, p27, and Ki-67. In non-cancerous lung tissue, TSLC1 was weakly positive in bronchial and bronchiolar epithelial cells, type II pneumocytes and bronchial glands. Overall, TSLC1 was negative in 60 of 93 lung adenocarcinomas. TSLC1 was mainly localized in the cytoplasm of the cells, but cell membrane staining was also observed, especially at sites of cell-cell adhesion. TSLC1-negative tumors were more frequently observed in male cases (41/54 cases, 70.0%) than in female cases (19/39 cases, 48.7%) (P < 0.01). Notably, TSLC1 expression was preserved in a non-  invasive  , bronchiolo-alveolar histological pattern of tumor cells (P < 0.0001). Survival analyses showed that loss of TSLC1 expression was associated with lower patient survival in univariate and multivariate analyses (P < 0.05 and P = 0.059, respectively). Subset analyses further showed that the prognostic impact of loss of TSLC1 was significant for male patients (P = 0.0089), but not for female patients. We conclude that TSLC1 is expressed in a subset of lung adenocarcinomas, especially in those with bronchiolo-alveolar spread pattern. Loss of TSLC1 is associated with lower patient survival, supporting its role as a tumor suppressor. 
 TSLC1 gene silencing in cervical cancer cell lines and cervical neoplasia. Cervical carcinogenesis is initiated by infection with high-risk (i.e., carcinogenic) human papillomavirus (HPV) types. The subsequent progression from premalignant cervical intraepithelial neoplasia (CIN) to   invasive   cancer is driven by both genetic and epigenetic processes. We assessed the role of the gene encoding the adhesion molecule tumor suppressor in lung cancer 1 (TSLC1) in this progression.We analyzed TSLC1 gene expression by real-time quantitative reverse transcription-polymerase chain reaction, promoter methylation by sodium bisulfite genomic DNA sequencing, and allelic loss by microsatellite analysis in primary keratinocytes, in four non-tumorigenic HPV-immortalized human keratinocyte cell lines, and in 11 human cervical cancer cell lines that were positive for a high-risk HPV DNA type and in normal cervical epithelial cells. We transfected cervical cancer SiHa cells that did not express TSLC1 mRNA with an expression vector containing the TSLC1 complementary DNA (cDNA) or an empty vector and analyzed transfectants for anchorage-independent growth and tumorigenicity in nude mice. We also examined TSLC1 promoter methylation in premalignant cervical lesions and in cervical carcinomas and smears. All statistical tests were two-sided.TSLC1 mRNA was strongly reduced, relative to levels in primary keratinocytes, or absent in 10 (91%) of 11 cervical carcinoma cell lines but in none (0%) of the four HPV-immortalized cell lines (difference = 91%, 95% confidence interval [CI] = 74% to 100%; P =.004). The TSLC1 promoter was hypermethylated, relative to normal foreskin and cervical epithelial cells, in nine (82%) of the 11 cervical carcinoma cell lines but in none (0%) of the four HPV-immortalized cell lines (difference = 82%, 95 CI = 59% to 100%; P =.01). Seven (88%, 95% CI = 47% to 100%) of the eight SiHa/TSLC1 transfectants displayed a marked reduction in anchorage-independent growth (i.e., 0-100 colonies per 5000 cells) compared with none of the four (0%, 95% CI = 0% to 60%) SiHa transfectants bearing the empty vector (i.e., SiHa/hygro transfectants; difference = 88%, 95% CI = 65% to 100%; P =.01) or untransfected SiHa cells. All seven mice (100%, 95% CI = 59% to 100%) injected with untransfected SiHa cells or SiHa/hygro transfectants displayed tumors of at least 50 mm(3) by 2-6 weeks after injection compared with none of eight mice (0%, 95% CI = 0% to 37%) injected with the SiHa/TSLC1 transfectants (difference = 100%, 95% CI = 68% to 100%; P<.001). We detected TSLC1 promoter hypermethylation in seven (35%, 95% CI = 15% to 59%) of 20 high-grade CIN lesions (i.e., CIN II and III) and in 30 (58%, 95% CI = 43% to 71%) of 52 cervical squamous cell carcinomas compared with none (0%, 95% CI = 0% to 34%) of nine normal cervical epithelial biopsy samples and none (0%, 95% CI = 0% to 22%) of 12 CIN I lesions (P<.001 for cervical squamous cell cancer versus normal epithelial biopsy samples plus CIN I lesions).TSLC1 gene silencing via promoter hypermethylation is a frequent event in the progression from high-risk HPV-containing, high-grade CIN lesions to   invasive   cervical cancer. 
 Tumor Suppressor in Lung Cancer-1 Is a Prognostic Predictor for the Recurrence and Progression of Non-Muscle-  Invasive   Bladder Cancer. To investigate the relationship between the expression of tumor suppressor in lung cancer-1 (TSLC1) and clinicopathological characteristics of patients with non-muscle-  invasive   bladder cancer (NMIBC) and evaluate the prognostic significance of TSLC1.TSLC1 expression in 241 specimens of NMIBC was examined by immunohistochemistry. The correlation between TSLC1 expression and clinicopathological characteristics was evaluated using the chi-square test. The prognostic significance of TSLC1 was analyzed by univariate, multivariate analysis and Kaplan-Meier survival curves.The total negative rate of TSLC1 expression was 47.3% in NMIBC. Decreased expression of TSLC1 was correlated with a higher clinical stage, higher pathological grade, the number of tumors, lager tumor size, tumor recurrence and progression. TSLC1 expression was an independent risk factor for predicting tumor recurrence (p = 0.005) and progression (p < 0.001).Decreased expression of TSLC1 is correlated with the malignancy of NMIBC tissues and low TSLC1 expression may serve as a predictor for bladder cancer recurrence and progression. 
 Methylation of Cervical Neoplastic Cells Infected With Human Papillomavirus 16. This study was conducted to evaluate the role of methylation of adenylate cyclase activating peptide 1 (ADCYAP1), paired box gene 1 (PAX1), cell adhesion molecule 1 (CADM1), and T-lymphocyte maturation-associated protein (MAL) during carcinogenesis.We evaluated the methylation of 4 genes by using the cervical carcinoma cell lines (CaSki, SiHa, HeLa, and C33A) and cervical neoplastic cells from 56 subjects with human papillomavirus 16 (HPV16)-infected low-grade squamous intraepithelial lesions (LSILs), 50 subjects with HPV16-infected high-grade squamous intraepithelial lesions (HSILs), and 24 subjects with HPV16-infected   invasive   cervical cancer who attended Seoul St. Mary's Hospital. Methylation of the 4 genes was evaluated using quantitative bisulfate pyrosequencing.The ADCYAP1 promoter was hypermethylated in the 4 cell lines (CaSki, 97.40 ± 1.39; SiHa, 82.04 ± 17.02; HeLa, 96.14 ± 2.08; and C33A, 78 ± 10.18). PAX1 and CADM1 were hypermethylated in the HPV16/18-infected cell lines CaSki (PAX1, 91.18 ± 9.91; CADM1, 93.5 ± 7.33), SiHa (PAX1, 96.14 ± 2.08; CADM1, 93.15 ± 8.81), and HeLa (PAX1, 82.04 ± 17.02; CADM1, 92.43 ± 9.95). MAL was hypermethylated in the CaSki cell line (96.04 ± 4.74). Among human cervical neoplastic cells, the methylation indices of ADCYAP1 were 7.8 (95% confidence interval [95% CI], 7.0-8.6) in subjects with LSILs and 39.8 (95% CI, 29.0-54.7) in those with cervical cancer (P < 0.001); for PAX1, 7.2 (95% CI, 6.1-8.5) and 37.8 (95% CI, 27.1-52.7), respectively; for CADM1, 3.5 (95% CI, 3.0-4.0) and 17.7 (95% CI, 10.8-29.1), respectively; for MAL, 2.7 (95% CI, 2.5-3.0) and 13.0 (95% CI, 7.6-22.0), respectively (P < 0.001 for each). Immunohistochemical staining results were positive in the cytoplasm of subjects with low methylation of the 4 gene promoters; however, they were negative in the cytoplasm of those with hypermethylation of the 4 gene promoters.The results of this study suggest that the methylation of ADCYAP1, PAX1, CADM1, and MAL may be highly associated with the development of cervical cancer, and that gene expression can be suppressed by gene promoter hypermethylation. 
 Aberrant expression of tumor suppressors CADM1 and 4.1B in   invasive   lesions of primary breast cancer. The tumor suppressor genes CADM1/TSLC1 and DAL-1/4.1B are frequently inactivated by promoter methylation in non-small cell lung cancer. The proteins they encode, CADM1 and 4.1B, form a complex in human epithelial cells and are involved in cell-cell adhesion.Expression of CADM1 and 4.1B proteins was examined by immunohistochemistry in 67 primary breast cancer and adjacent noncancerous tissues. CADM1 and 4.1B messenger RNA (mRNA) was detected by reverse-transcription polymerase chain reaction (RT-PCR). The methylation status of the CADM1 and 4.1B promoters was determined quantitatively by bisulfite treatment followed by pyrosequencing.CADM1 and 4.1B protein signals were detected along the cell membrane in normal mammary epithelia. By contrast, 47 (70%) and 49 (73%) of 67 primary breast cancers showed aberrant CADM1 and 4.1B staining, respectively. Aberrant CADM1 staining was more frequently observed in pT2 and pT3 tumors and for stages II and III (P = 0.045 and P = 0.020, respectively), while aberrant 4.1B staining was more often observed in tumors with lymph node metastasis, for pT2 and pT3 tumors, and for stages II and III (P = 0.0058, P = 0.0098, and P = 0.0007, respectively). Furthermore, aberrant CADM1 and 4.1B expression was preferentially   observed   in invasive rel  ative to   noninvasive lesions from the same specimen (P = 0.036 and P = 0.0009, respectively). Finally, hypermethylation of CADM1 and 4.1B genes was detected in 46% and 42% of primary breast cancers, respectively.Our findings suggest that aberrant CADM1 and 4.1B expression is involved in progression of breast cancer, especially in invasion into the stroma and metastasis. 
 Hypermethylation of IGSF4 gene for non  invasive   prenatal diagnosis of thalassemia. For patients with pregnancy-induced thalassemia, fetal cord blood or amniotic fluid is   invasive  ly collected in the traditional diagnosis and prediction of thalassemia. However, there is no specific molecular target in the diagnosis of thalassemia using fetal DNA from the plasma of pregnant women.The promoter of cell surface adhesion molecule (IGSF4) gene was found to be down-regulated in patients with homozygous thalassemia, and the expression of IGSF4 was closely associated with the methylation of its promoter. In the present study, mass spectrometric sequencing of methylation was performed using MassARRAY to detect the 12 CpG sites in the promoter of IGSF4 gene.The methylation degree of these 12 CpG sites was significantly higher than that in healthy subjects (P<0.05). Hierarchical clustering was done in 23 patients with thalassemia and 5 healthy individuals. Results revealed the promoter of IGSF4 gene was highly methylated in thalassemia patients, which was dramatically different from that in healthy subjects (P<0.05). Methylation-specific PCR (MSP) was employed to confirm the methylation of the promoter of IGSF4 gene and results were consistence with those obtained in sequencing with MassARRAY. Real-time PCR showed, when compared with heterozygous subjects, the expression of IGSF4 was significantly down-regulated in thalassemia patients (ratio=0.18).The expression of IGSF4 was closely related to the methylation of its promoter, suggesting the methylation of IGSF4 gene is tissue-specific for thalassemia. These findings provide evidence for the non-  invasive   prenatal diagnosis of thalassemia in terms of epigenetics. 
 SynCAM, a novel putative tumor suppressor, suppresses growth and   invasive  ness of glioblastoma. SynCAM, also named by TSLC1, SgIGSF and IGSF4, was identified as a neural tissue-specific immunoglobulin-like cell-cell adhesion molecule. However, the role of SynCAM in tumorigenesis remains elusive. We aimed to clarify its epigenetic regulation and biological functions in glioblastoma. SynCAM was silenced in 72 % (5/7) glioblastoma cell lines. A significant downregulation was also detected in paired glioblastoma tumors compared with adjacent non-cancerous tissues. In contrast, SynCAM was readily expressed in various normal adult brain tissues. Ectopic expression of SynCAM in the silenced cancer cell line T98G significantly reduced colony formation and cell proliferation, induced cell cycle arrests and repressed cell   invasiv  e ability. Nude mice were subcutaneously injected into the flank with T98G cells and treated with normal saline, pcDNA3.1 (vector) or pcDNA3.1-SynCAM, respectively. Treatment with pcDNA3.1-SynCAM retarded growth in the xenografts, which contributed to a 58 % decrease in tumor volume compared to controls. In conclusion, our results suggest that SynCAM suppressions growth of glioblastoma and may serve as a novel functional tumor-suppressor gene. 
  GeneRIFs  
 Decreased expression of TSLC1 is correlated with the malignancy of non-muscle   invasive   bladder cancer tissues and low TSLC1 expression may serve as a predictor for bladder cancer recurrence and progression. 
 CADM1/TSLC1 is an anti  invasive   gene, the overexpression of which inhibits the invasion of A375 cells. 
 SynCAM, a novel putative tumor suppressor, suppresses growth and   invasive  ness of glioblastoma. 
 The expression of IGSF4 is closely related to the methylation of its promoter and that is useful for non  invasive   prenatal diagnosis of thalassemia. 
 TSLC1 gene silencing via promoter hypermethylation is a frequent event in the progression from high-risk HPV-containing, high-grade CIN lesions to   invasive   cervical cancer 
 The results support the hypothesis that TSLC1 is a tumor suppressor of NSCLC and also suggest that preserved integrity of TSLC1 may contribute to less   invasive   phenotypes of lepidic growth tumor cells. 
  [Mm]etastasize  PubMed  
 TGF-β upregulates miR-182 expression to promote gallbladder cancer metastasis by targeting CADM1. Transforming growth factor β (TGF-β) plays important roles in tumor metastasis by regulating miRNAs expression. miR-182 is an important molecule in the regulation of cancer progression. The aim of the study is to assess the role of miR-182 in TGF-β-induced cancer metastasis. In the present study, we found that miR-182 levels are significantly upregulated in GBC tissues compared with normal controls, and miR-182 expression is remarkably increased in primary tumors that subsequen  tly metasta  sized, when compared to those primary tumors that did   not metasta  size. TGF-β induces miR-182 expression in GBC cells, and overexpression of miR-182 promotes GBC cell migration and invasion, whereas miR-182 inhibition suppresses TGF-β-induced cancer cell migration and invasion. The blockage of miR-182 by a specific inhibitor effectively inhibits pulmonary metastases in vivo. We further identified that the cell adhesion molecule1 (CADM1) is a new target gene of miR-182. miR-182 negatively regulates CADM1 expression in vitro and in vivo. Importantly, re-expression of CADM1 in GBC cells partially abrogates miR-182-induced cell invasion.miR-182 is an important mediator of GBC metastasis, thus offering a new target for the development of therapeutic agents against GBC. 
  [Mm]etastasis  PubMed  
 CADM1 interacts with Tiam1 and promotes invasive phenotype of human T-cell leukemia virus type I-transformed cells and adult T-cell leukemia cells. CADM1 encodes a multifunctional immunoglobulin-like cell adhesion molecule whose cytoplasmic domain contains a type II PSD95/Dlg/ZO-1 (PDZ)-binding motif (BM) for associating with other intracellular proteins. Although CADM1 lacks expression in T lymphocytes of healthy individuals, it is overexpressed in adult T-cell leukemia-lymphoma (ATL) cells. It has been suggested that the expression of CADM1 protein promotes infiltration of leukemic cells into various organs and tissues, which is one of the frequent clinical manifestations of ATL. Amino acid sequence alignment revealed that Tiam1 (T-lymphoma invasion and   metastasis   1), a Rac-specific guanine nucleotide exchange factor, has a type II PDZ domain similar to those of membrane-associated guanylate kinase homologs (MAGUKs) that are known to bind to the PDZ-BM of CADM1. In this study, we demonstrated that the cytoplasmic domain of CADM1 directly interacted with the PDZ domain of Tiam1 and induced formation of lamellipodia through Rac activation in HTLV-I-transformed cell lines as well as ATL cell lines. Our results indicate that Tiam1 integrates signals from CADM1 to regulate the actin cytoskeleton through Rac activation, which may lead to tissue infiltration of leukemic cells in ATL patients. 
 Involvement of TSLC1 in progression of esophageal squamous cell carcinoma. Frequent allelic losses of 11q23 in esophageal squamous cell carcinoma (ESCC) have been reported previously, but no tumor suppressor genes in this region have been identified in ESCC. TSLC1 was identified on chromosome 11q23.2 as a tumor suppressor gene in non-small cell lung cancer by functional complementation of a lung adenocarcinoma cell line. The purpose of this study is to evaluate the role of TSLC1 in ESCC. Loss of TSLC1 expression was observed by reverse transcription-PCR in 75% of the cell lines (27 of 36) and 50% of the primary tumors from ESCC patients (28 of 56). In a clinicopathological analysis, loss of TSLC1 expression correlated significantly with depth of invasion (pT) and status of   metastasis   (pM; P = 0.012 and 0.036, respectively). Patients with tumors lacking TSLC1 expression tended to have a poorer prognosis than those with tumors expressing TSLC1. (P = 0.079). Moreover, TSLC1 expression was an independent prognostic factor in a multivariate analysis (P = 0.049). Methylation analyses revealed that TSLC1 expression or loss correlated with the promoter methylation status, as determined by bisulfite sequencing, and that TSLC1 expression could be restored by a demethylating agent in certain cell lines. The growth of TSLC1-transfected ESCC cells was significantly suppressed both in vitro and in vivo (P < 0.01), possibly by a G(1) cell cycle arrest. TSLC1 expression also suppressed motility and invasion of ESCC cells in vitro significantly (P < 0.01). These findings suggest that loss of TSLC1 expression has an important role in tumor growth, cell motility, and invasion and is associated with aggressive tumor behavior in ESCC. 
 CADM1/TSLC1 inhibits melanoma cell line A375 invasion through the suppression of matrix metalloproteinases. Increasing evidence has demonstrated that cell adhesion molecule 1/tumor suppressor in lung cancer 1 (CADM1/TSLC1) is crucially implicated in various biological processes, including proliferation, apoptosis, and invasion during tumorigenesis and development. However, the mechanism underlying its suppression of tumor growth an  d metastas  is in melanoma remains elusive. The aim of the present study was to examine if CADM1/TSLC1 was able to induce growth suppression in melanoma. The plasmid pcDNA3.1‑CADM1/TSLC1 was transfected into A375 cells (a human melanoma cell line). The expression of CADM1/TSLC1 in the transfected cells was determined by RT‑PCR and western blotting analysis. Cell growth was measured by an 3‑(4,5‑dimethylthiazol‑2‑yl)‑2,5‑diphenyl‑tetrazolium bromide assay and cell apoptosis was determined by flow cytometry, while a transwell assay was utilized to measure the ability of invasion. The expression of MMP‑2 and MMP‑9 in the transfected cells was determined by western blotting analysis. RT‑PCR and western blotting revealed that in pcDNA3.1‑CADM1/TSLC1 the protein expression of CADM1/TSLC1 protein was higher than in the pcDNA3.1 and A375 cells. The expression of MMP‑2 and MMP‑9 was lower in the pcDNA3.1‑CADM1/TSLC1 than that in the pcDNA3.1 and A375 cells. The growth of CADM1/TSLC1‑transfected cells was significantly suppressed in vitro and the ability of invasion was also reduced, CADM1/TSLC1 was able to induce cell apoptosis. Furthermore, CADM1/TSLC1 was an anti‑invasive gene, the overexpression of which inhibited the invasion of A375 cells. This inhibition may be due to the suppression of the MMP‑2 and MMP�  ��9 expres  sion, which is relative to tumor metastasis and progression. 
 Loss/Down-regulation of tumor suppressor in lung cancer 1 expression is associated with tumor progression and is a biomarker of poor prognosis in ovarian carcinoma. The tumor suppressor in lung cancer 1 (TSLC1) has been identified as a putative tumor suppressor gene in non-small cell lung cancer. Although loss of TSLC1 has been observed in a number of human malignancies, the expression levels of TSLC1 gene in ovarian cancer and its clinical or prognostic significance have not been investigated.Protein expression levels of TSLC1 was explored by semiquantitative immunohistochemical staining on archival formalin-fixed, paraffin-embedded pathological specimen consisting of 30 normal ovaries, 30 ovarian cystadenomas, 40 borderline ovarian tumors, and 160 invasive ovarian carcinomas. The TSLC1 immunohistochemical staining results were then correlated with various clinicopathologic parameters and patient prognosis using various statistical models.Significantly decreased, or complete loss of, protein expression of the TSLC1 gene was observed in 59% ovarian carcinomas, 45% borderline tumors, and 7% cystadenomas, but in none of the normal ovaries (0%). In ovarian carcinomas, decreased TSLC1 expression was significantly correlated with lymph node   metastasis   (pN, P = 0.001), distant   metastasis   (pM, P = 0.028), and more advanced International Federation of Gynecology and Obstetrics stages (P = 0.008). By univariate survival analysis on the ovarian carcinoma cohorts, decreased TSLC1 protein expression was significantly associated with shortened patient survival (mean: 26.9 months in tumors with complete loss of TSLC1 vs 63.1 months in tumors with significantly decreased TSLC1 vs 94.3 months in tumors with normal levels of TSLC1; P < 0.001). By multivariate analysis, TSLC1 protein expression remained as a significant and independent prognostic factor for the prediction of patient survival (P = 0.003).Decreased protein expression of the TSLC1 gene might be important in conferring a more aggressive behavior in ovarian carcinoma. Thus, TSLC1 may be used as an independent prognostic molecular marker for patients with ovarian carcinoma. 
 Re-expression of cell adhesion molecule inhibits growth and induces apoptosis of human pancreatic cancer cell line PANC-1. This study examined the expression of cell adhesion molecule 1 (CADM1) in pancreatic cancer and the possible mechanism. The expression of CADM1 was detected by immunohistochemistry in tissues of pancreatic cancer, pancreatitis, and normal pancreas. The plasmid pcDNA3.1-Hygro(+)/CADM1 was transfected into PANC-1 cells (a pancreatic cancer cell line). The expression of CADM1 in the transfected cells was determined by RT-PCR and Western blotting. Cell growth was measured by the MTT method and cell apoptosis by flow cytometry. The results showed that CADM1 was weakly expressed in tissues of pancreatic cancer in contrast to its high expression in normal pancreatic and pancreatitis tissues. The expression level of CADM in pancreatic caner was intensely correlated with the differentiation degree, lymph node   metastasis   and TNM stages. The growth of CADM1-transfected PANC-1 cells was significantly suppressed in vitro by a G1 cell cycle arrest and apoptosis occurrence. It was concluded that re-expression of CADM1 inhibits the growth of pancreatic cancer cells and induces their apoptosis in vitro. As a tumor suppressor gene, CADM1 plays an important role in the occurrence, progression and   metastasis   of pancreatic cancer. 
 Loss of CADM1 expression is associated with poor prognosis and brain   metastasis   in breast cancer patients. Breast cancer brain metastases (BCBM) are detected with increasing incidence. In order to detect potential genes involved in BCBM, we first screened for genes down-regulated by methylation in cell lines with site-specific metastatic ability. The expression of five genes, CADM1, SPARC, RECK, TNFAIP3 and CXCL14, which were also found down-regulated in gene expression profiling analyses of BCBM tissue samples, was verified by qRT-PCR in a larger patient cohort. CADM1 was chosen for further down-stream analyses. A higher incidence of CADM1 methylation, correlating with lower expression levels, was found in BCBM as compared to primary BC. Loss of CADM1 protein expression was detected most commonly among BCBM samples as well as among primary tumors with subsequent brain relapse. The prognostic role of CADM1 expression was finally verified in four large independent breast cancer cohorts (n=2136). Loss of CADM1 protein expression was associated with disease stage, lymph node status, and tumor size in primary BC. Furthermore, all analyses revealed a significant association between loss of CADM1 and shorter survival. In multivariate analyses, survival was significantly shorter among patients with CADM1-negative tumors. Loss of CADM1 expression is an independent prognostic factor especially associated with the development of brain metastases in breast cancer patients. 
 TSLC1 is a tumor suppressor gene associated with   metastasis   in nasopharyngeal carcinoma. In up to 87% of nasopharyngeal carcinoma (NPC) clinical tumor specimens, there was either down-regulation or loss of TSLC1 gene expression. Using a tissue microarray and immunohistochemical staining, the frequency of down-regulated or loss of expression of TSLC1 in metastatic lymph node NPC was 83% and the frequency of loss of expression of TSLC1 was 35%, which was significantly higher than that in primary NPC (12%). To examine the possible growth-suppressive activity of TSLC1 in NPC, three NPC cell lines, HONE1, HNE1, and CNE2, were transfected with the wild-type TSLC1 gene cloned into the pCR3.1 expression vector; a reduction of colony formation ability was observed for all three cell lines. A tetracycline-inducible expression vector, pETE-Bsd, was also used to obtain stable transfectants of TSLC1. There was a dramatic difference between colony formation ability in the presence or absence of doxycycline when the gene is shut off or expressed, respectively, with the tetracycline-inducible system. Tumorigenicity assay results show that the activation of TSLC1 suppresses tumor formation in nude mice and functional inactivation of this gene is observed in all the tumors derived from tumorigenic transfectants. Further studies indicate that expression of TSLC1 inhibits HONE1 cell growth in vitro by arresting cells in G(0)-G(1) phase in normal culture conditions, whereas in the absence of serum, TSLC1 induced apoptosis. These findings suggest that TSLC1 is a tumor suppressor gene in NPC, which is significantly associated with lymph node metastases. 
 MicroRNA-10b promotes migration and invasion through CADM1 in human hepatocellular carcinoma cells. MicroRNA-10b (miR-10b) was recently reported to be dysregulated in some types of cancer and to play a role in invasion and   metastasis  . However, effects and potential mechanisms of action of miR-10b in the   metastasis   of hepatocellular carcinoma (HCC) have not been explored. In this study, we confirmed that miR-10b is highly expressed in metastatic HCC tissues and in metastatic HCC cell lines by qRT-PCR. Moreover, patients with higher miR-10b expression had significantly poorer overall survival, and high miR-10b expression was an independent predictor of poor prognosis. Inhibition of miR-10b reduced cell migration and invasion in MHCC97H cells, whereas over-expression of miR-10b in HepG2 cells increased cell migration and invasion. Bioinformatics and luciferase reporter assays revealed that miR-10b binds the 3'-UTR of CADM1 mRNA and represses its translation. Western blot and qRT-PCR showed that CADM1 is inhibited by miR-10b over-expression. Silencing of CADM1 resulted in substantially increased cell motility and invasion similar to that observed with over-expression of miR-10b in HepG2 cells. These results suggest that miR-10b may positively regulate the invasion and   metastasis   of HCC through targeting CADM1. 
 Cadm1 is a   metastasis   susceptibility gene that suppresses   metastasis   by modifying tumor interaction with the cell-mediated immunity.   Metastasis   is a complex process utilizing both tumor-cell-autonomous properties and host-derived factors, including cellular immunity. We have previously shown that germline polymorphisms can modify tumor cell metastatic capabilities through cell-autonomous mechanisms. However, how   metastasis   susceptibility genes interact with the tumor stroma is incompletely understood. Here, we employ a complex genetic screen to identify Cadm1 as a novel modifier of   metastasis  . We demonstrate that Cadm1 can specifically suppress   metastasis   without affecting primary tumor growth. Unexpectedly, Cadm1 did not alter tumor-cell-autonomous properties such as proliferation or invasion, but required the host's adaptive immune system to affect   metastasis  . The   metastasis  -suppressing effect of Cadm1 was lost in mice lacking T cell-mediated immunity, which was partially phenocopied by depleting CD8(+) T cells in immune-competent mice. Our data show a novel function for Cadm1 in suppressing   metastasis   by sensitizing tumor cells to immune surveillance mechanisms, and this is the first report of a heritable   metastasis   susceptibility gene engaging tumor non-autonomous factors. 
 Nectin-2 and DDX3 are biomarkers for   metastasis   and poor prognosis of squamous cell/adenosquamous carcinomas and adenocarcinoma of gallbladder. The clinicopathological and biological characteristics of squamous cell/adenosquamous carcinoma (SC/ASC) of gallbladder have not been well documented because it is a rare subtype of gallbladder cancer. In this study, the protein expression of Nectin-2 and DDX3 in 46 SC/ASCs and 80 adenocarcinomas was measured using immunohistochemistry. We demonstrated that positive Nectin-2 and DDX3 expression was significantly associated with large tumor size, high TNM stage, and lymph node   metastasis   of SC/ASC and AC. Positive Nectin-2 and DDX3 expression was significantly associated with invasion and surgical curability of AC. Univariate Kaplan-Meier analysis showed that positive Nectin-2 and DDX3 expression, degree of differentiation, tumor size, TNM stage, invasion, lymph node   metastasis  , and surgical curability were significantly associated with post-operative survival in both SC/ASC and AC patients. Multivariate Cox regression analysis showed that positive Nectin-2 and DDX3 expression, degree of differentiation, tumor size, TNM stage, invasion, lymph node   metastasis  , and no surgical curability are independent poor-prognostic factors in both SC/ASC and AC patients. Our study suggested that positive Nectin-2 and DDx3 expression is closely correlated with clinical, pathological, and biological behaviors as well as poor-prognosis of gallbladder cancer. 
 Structural basis of tumor suppressor in lung cancer 1 (TSLC1) binding to differentially expressed in adenocarcinoma of the lung (DAL-1/4.1B). Perturbed cell adhesion mechanisms are crucial for tumor invasion and   metastasis  . A cell adhesion protein, TSLC1 (tumor suppressor in lung cancer 1), is inactivated in a majority of metastatic cancers. DAL-1 (differentially expressed in adenocarcinoma of the lung protein), another tumor suppressor, binds through its FERM domain to the TSLC1 C-terminal, 4.1 glycophorin C-like, cytoplasmic domain. However, the molecular basis for this interaction is unknown. Here, we describe the crystal structure of a complex between the DAL-1 FERM domain and a portion of the TSLC1 cytoplasmic domain. DAL-1 binds to TSLC1 through conserved residues in a well defined hydrophobic pocket in the structural C-lobe of the DAL-1 FERM domain. From the crystal structure, it is apparent that Tyr(406) and Thr(408) in the TSLC1 cytoplasmic domain form the most important interactions with DAL-1, and this was also confirmed by surface plasmon resonance studies. Our results refute earlier exon deletion experiments that indicated that glycophorin C interacts with the α-lobe of 4.1 FERM domains. 
 Direct association of TSLC1 and DAL-1, two distinct tumor suppressor proteins in lung cancer. The tumor suppressor gene TSLC1, which we recently identified in human non-small cell lung cancer, encodes a membrane glycoprotein of the immunoglobulin superfamily. Here, we report that TSLC1 directly associates with DAL-1, a gene product of another lung tumor suppressor belonging to the protein 4.1 family. TSLC1 additionally interacts with the actin filament through DAL-1 at the cell-cell attached site where the complex formation of TSLC1 and DAL-1 is dependent on the integrity of actin cytoskeleton. Redistribution of both TSLC1 and DAL-1 to the newly generated membrane ruffling areas suggests that these proteins are also involved in cell motility accompanying the actin rearrangement. Furthermore, restoration of TSLC1 expression strongly suppressed the   metastasis   of a human non-small cell lung cancer cell line, A549, from the spleen to the liver in nude mice. These findings, together with frequent loss of their expression in lung cancers, suggest that TSLC1 and DAL-1 play a critical role in the same pathway involved in the suppression of lung tumor formation and   metastasis  . 
 Down-regulation of tumor suppressor in lung cancer 1 (TSLC1) expression correlates with poor prognosis in patients with colon cancer. Tumor suppressor in lung cancer 1 (TSLC1), a novel tumor suppressor gene, has been reported to be frequently inactivated in a variety of human malignant tumors. The aim of this study was to detect TSLC1 expression in human colon cancer and to analyze its association with prognosis of patients with colon cancer. Using quantitative real-time PCR and Western blot analysis, we found significantly decreased expression of TSLC1 in primary colon tumor tissues (n = 30) compared with adjacent normal tissues. Immunohistochemistry analysis also found decreased TSLC1 expression in 41.3 % (33/80) colon tumor tissues. In clinicopathological analysis, loss of TSLC1 expression significantly correlated with female gender and lymph node   metastasis   of colon cancer patients (P < 0.05). In addition, decreased expression of TSLC1 in tumors was found to be closely associated with a poor prognosis (P = 0.037, log-rank test), and multivariate analysis showed that lower TSLC1 protein expression was an independent prognostic factor for colon cancer patients. Our study suggests that down-regulated expression of TSLC1 may play an important role in the progression of colon cancer and TSLC1 expression may serve as a useful marker for the prognostic evaluation of patients with colon cancer. 
 TGF-β upregulates miR-182 expression to promote gallbladder cancer   metastasi  s by targeting CADM1. Transforming growth factor β (TGF-β) plays important roles in tum  or metasta  sis by regulating miRNAs expression. miR-182 is an important molecule in the regulation of cancer progression. The aim of the study is to assess the role of miR-182 in TGF-β-induced can  cer metast  asis. In the present study, we found that miR-182 levels are significantly upregulated in GBC tissues compared with normal controls, and miR-182 expression is remarkably increased in primary tumors that subsequently metastasized, when compared to those primary tumors that did not metastasize. TGF-β induces miR-182 expression in GBC cells, and overexpression of miR-182 promotes GBC cell migration and invasion, whereas miR-182 inhibition suppresses TGF-β-induced cancer cell migration and invasion. The blockage of miR-182 by a specific inhibitor effectively inhibits pulmonary metastases in vivo. We further identified that the cell adhesion molecule1 (CADM1) is a new target gene of miR-182. miR-182 negatively regulates CADM1 expression in vitro and in vivo. Importantly, re-expression of CADM1 in GBC cells partially abrogates miR-182-induced cell invasion.miR-182 is an important mediator o  f GBC meta  stasis, thus offering a new target for the development of therapeutic agents against GBC. 
 Silencing of ErbB3/ErbB2 signaling by immunoglobulin-like Necl-2. ErbB2 and ErbB3, members of the EGF receptor/ErbB family, form a heterodimer upon binding of a ligand, inducing the activation of Rac small G protein and Akt protein kinase for cell movement and survival, respectively. The enhanced ErbB3/ErbB2 signaling causes tumorigenesis, invasion, and   metastasis  . We found here that the ErbB3/ErbB2 signaling is regulated by immunoglobulin-like Necl-2, which is down-regulated in various cancer cells and serves as a tumor suppressor. The extracellular region of ErbB3, but not ErbB2, interacted in cis with that of Necl-2. This interaction reduced the ligand-induced, ErbB2-catalyzed tyrosine phosphorylation of ErbB3 and inhibited the consequent ErbB3-mediated activation of Rac and Akt, resulting in the inhibition of cancer cell movement and survival. These inhibitory effects of Necl-2 were mediated by the protein-tyrosine phosphatase PTPN13 which interacted with the cytoplasmic tail of Necl-2. We describe here this novel mechanism for silencing of the ErbB3/ErbB2 signaling by Necl-2. 
 Aberrant expression of tumor suppressors CADM1 and 4.1B in invasive lesions of primary breast cancer. The tumor suppressor genes CADM1/TSLC1 and DAL-1/4.1B are frequently inactivated by promoter methylation in non-small cell lung cancer. The proteins they encode, CADM1 and 4.1B, form a complex in human epithelial cells and are involved in cell-cell adhesion.Expression of CADM1 and 4.1B proteins was examined by immunohistochemistry in 67 primary breast cancer and adjacent noncancerous tissues. CADM1 and 4.1B messenger RNA (mRNA) was detected by reverse-transcription polymerase chain reaction (RT-PCR). The methylation status of the CADM1 and 4.1B promoters was determined quantitatively by bisulfite treatment followed by pyrosequencing.CADM1 and 4.1B protein signals were detected along the cell membrane in normal mammary epithelia. By contrast, 47 (70%) and 49 (73%) of 67 primary breast cancers showed aberrant CADM1 and 4.1B staining, respectively. Aberrant CADM1 staining was more frequently observed in pT2 and pT3 tumors and for stages II and III (P = 0.045 and P = 0.020, respectively), while aberrant 4.1B staining was more often observed in tumors with lymph   node metas  tasis, for pT2 and pT3 tumors, and for stages II and III (P = 0.0058, P = 0.0098, and P = 0.0007, respectively). Furthermore, aberrant CADM1 and 4.1B expression was preferentially observed in invasive relative to noninvasive lesions from the same specimen (P = 0.036 and P = 0.0009, respectively). Finally, hypermethylation of CADM1 and 4.1B genes was detected in 46% and 42% of primary breast cancers, respectively.Our findings suggest that aberrant CADM1 and 4.1B expression is involved in progression of breast cancer, especially in invasion into   the strom  a and metastasis. 
  GeneRIFs  
 TGF-beta upregulates miR-182 expression to promote gallbladder cancer   metastasis   by targeting CADM1. 
 As a tumor suppressor gene, CADM1 plays an important role in the occurrence, progression and   metastasis   of pancreatic cancer. 
 aberrant CADM1 and 4.1B expression is involved in progression of breast cancer, especially in invasion into the stroma and   metastasis   
 Our data show a novel function for Cadm1 in suppressing   metastasis   by sensitizing tumor cells to immune surveillance mechanisms, and this is the first report of a heritable   metastasis   susceptibility gene engaging tumor non-autonomous factors. 
 miR-10b may positively regulate the invasion and   metastasis   of HCC through targeting CADM1. 
 TSLC1 inhibits nasopharyngeal carcinoma cell growth by arresting cells in G(0)-G(1) phase in normal culture conditions.  Without serum, TSLC1 induced apoptosis. TSLC1 is a tumor suppressor gene in NPC; its loss is seen in lymphatic   metastasis  . 
  [Cc]ell [Ii]nvasion  PubMed  
 Tumor suppressor TSLC1 is implicated in cell proliferation, invasion and apoptosis in laryngeal squamous cell carcinoma by regulating Akt signaling pathway. Overwhelming evidence has demonstrated that TSLC1 (tumor suppressor in lung cancer 1), a novel tumor suppressor, is crucially implicated in various biological processes including progression, proliferation and apoptosis during tumorigenesis. However, the exact functions and molecular details of TSLC1 in laryngeal cancer remain ill-defined. Here, the expression of TSLC1 in laryngeal squamous cell carcinoma (LSCC) tissues and cells was detected, and the biological roles of TSLC1 in LSCC cells were investigated. The results showed that expressions of TSLC1 mRNA and protein were significantly reduced in LSCC tissues with low expression in 18 of 85 (21.18 %) and 16 of 85 (18.82 %), respectively. Additionally, statistical analysis revealed a significant correlation of TSLC1 expression with TNM staging and lymph node metastases (P < 0.05), but not related to age, gender and tumor differentiation (P > 0.05). Elevation of TSLC1 level inhibited cell proliferation, reduced   cell invasion   in vitro and induced cell apoptosis in Hep-2 cells, most importantly, TSLC1 upregulation decreased the level of pAkt, but not changed the level of total Akt in Hep-2 cells. Stepwise investigations demonstrated that overexpression of TSLC1 in Hep-2 cells increased caspase-3 activity and expressions of bax and p21 proteins but decreased the levels of bcl-2, MMP-2 and MMP-9 proteins. These data suggest that TSLC1 may exert essential roles in the progression and development of LSCC, and thus TSLC1 may be a potential molecular target for LSCC treatment. 
 TGF-β upregulates miR-182 expression to promote gallbladder cancer metastasis by targeting CADM1. Transforming growth factor β (TGF-β) plays important roles in tumor metastasis by regulating miRNAs expression. miR-182 is an important molecule in the regulation of cancer progression. The aim of the study is to assess the role of miR-182 in TGF-β-induced cancer metastasis. In the present study, we found that miR-182 levels are significantly upregulated in GBC tissues compared with normal controls, and miR-182 expression is remarkably increased in primary tumors that subsequently metastasized, when compared to those primary tumors that did not metastasize. TGF-β induces miR-182 expression in GBC cells, and overexpression of miR-182 promotes GBC cell migration and invasion, whereas miR-182 inhibition suppresses TGF-β-induced cancer cell migration and invasion. The blockage of miR-182 by a specific inhibitor effectively inhibits pulmonary metastases in vivo. We further identified that the cell adhesion molecule1 (CADM1) is a new target gene of miR-182. miR-182 negatively regulates CADM1 expression in vitro and in vivo. Importantly, re-expression of CADM1 in GBC cells partially abrogates miR-182-in  duced cell in  vasion.miR-182 is an important mediator of GBC metastasis, thus offering a new target for the development of therapeutic agents against GBC. 
   
  
 NECTIN3 
 
  NCBI Gene  
  GeneRIF  
  Pubmed  
 
 
  Search Term  Rank  Fields  
  [Mm]igration  6  PubMed(3)  
  [Ee]xtracellular [Mm]atrix  8  PubMed(2)  
  [Ii]nvasive  10  PubMed(1)  
  [Mm]etastasis  12  PubMed(3)  
 
 
 hide/show details for NECTIN3 
  [Mm]igration  PubMed  
 Silencing of thrombospondin-1 is critical for myc-induced metastatic phenotypes in medulloblastoma. Mechanisms by which c-Myc (Myc) amplification confers aggressive medulloblastoma phenotypes are poorly defined. Here, we show using orthotopic models that high Myc expression promotes cell   migration  /invasion and induces metastatic tumors, which recapitulate aggressive histologic features of Myc-amplified primary human medulloblastoma. Using ChIP-chip analysis, we identified cell   migration   and adhesion genes, including Tsp-1/THBS1, ING4, PVRL3, and PPAP2B, as Myc-bound loci in medulloblastoma cells. Expression of Tsp-1 was most consistently and robustly diminished in medulloblastoma cell lines and primary human tumors with high Myc expression (n = 101, P = 0.032). Strikingly, stable Tsp-1 expression significantly attenuated in vitro transformation and invasive/migratory properties of high Myc-expressing medulloblastoma cells without altering cell proliferation, whereas RNA interference-mediated Myc knockdown was consistently accompanied by increased Tsp-1 levels and reduced cell   migration   and invasion in medulloblastoma cells. Chromatin immunoprecipitation (ChIP) assays revealed colocalization of Myc and obligate partner Max and correlated diminished RNA polymerase II occupancy (∼3-fold decrease, P < 0.01) with increased Myc binding at a core Tsp-1 promoter. Reporter gene and/or gel shift assays confirmed direct repression of Tsp-1 transcription by Myc and also identified JPO2, a Myc interactor associated with metastatic medulloblastoma, as a cofactor in Myc-mediated Tsp-1 repression. These findings indicate the Myc-regulatory network targets Tsp-1 via multiple mechanisms in medulloblastoma transformation, and highlight a novel critical role for Tsp-1 in Myc-mediated aggressive medulloblastoma phenotypes. 
 Nectin-3 (CD113) interacts with Nectin-2 (CD112) to promote lymphocyte transendothelial   migration  . Lymphocyte trafficking and   migration   through vascular endothelial cells (ECs) in secondary lymphoid tissues is critical for immune protection. In the present study, we investigate the role of nectin cell adhesion molecules for the   migration   of lymphocytes through ECs. Nectins are key players for the establishment of homotypic and heterotypic cell to cell contacts; they are required for cell to cell adherens junction formation and take part in the transendothelial   migration   of monocytes during the step of diapedesis, when monocytes migrate through EC junctions. We first show that Nectin-3 (CD113) is the only nectin expressed by T lymphocytes and since nectins are expressed on ECs we explored Nectin-3 potential functions in lymphocyte: EC interactions. We demonstrate that Nectin-2, expressed on ECs, is the major counter-receptor of Nectin-3. A soluble form of Nectin-3 binds to Nectin-2 localized at EC junctions and blocking Nectin-2 trans-interactions with monoclonal antibodies abolishes the binding of soluble Nectin-3 to ECs. Nectin-2 is expressed on High Endothelial venules (HEVs), where lymphocyte homing occurs in vivo. Finally, we show that Nectin-3 trans-interaction with Nectin-2 is essential for the process of lymphocyte transendothelial   migration   in vitro as targeting with blocking monoclonal antibodies either Nectin-3, expressed on lymphocytes, or Nectin-2, expressed on ECs, inhibits lymphocyte extravasation. The nectin family of CAMs is important for the regulation of endothelial barrier functions and transendothelial   migration   of immune cells. Our results demonstrate for the first time that Nectin-3 trans-interacts with Nectin-2 to promote lymphocyte and monocyte extravasation. 
 The expression of the Nectin complex in human breast cancer and the role of Nectin-3 in the control of tight junctions during metastasis. Nectins are a family of integral protein molecules involved in the formation of functioning Adherens and Tight Junctions (TJ). Aberrant expression is associated with cancer progression but little is known how this effects changes in cell behaviour. This study aimed to ascertain the distribution of Nectins-1 to -4 in human breast cancer and the effect on junctional integrity of Nectin-3 modulation in human endothelial and breast cancer cells.A human breast tissue cohort was processed for Q-PCR and immunohistochemistry for analysis of Nectin-1/-2/-3/-4. Nectin-3 over-expression was induced in the human breast cancer cell line MDA-MB-231 and the human endothelial cell line HECV. Functional testing was carried out to ascertain changes in cell behaviour.Q-PCR revealed a distinct reduction in node positive tumours and in patients with poor outcome. There was increased expression of Nectin-1/-2 in patients with metastatic disease, Nectin-3/-4 was reduced. IHC revealed that Nectin-3 expression showed clear changes in distribution between normal and cancerous cells. Nectin-3 over-expression in MDA-MB-231 cells showed reduced invasion and   migration   even when treated with HGF. Changes in barrier function resulted in MDAN3 cells showing less change in resistance after 2h treatment with HGF (p<0.001). Nectin-3 transformed endothelial cells were significantly more adhesive, irrespective of treatment with HGF (p<0.05) and had reduced growth. Barrier function revealed that transformed HECV cells had significantly tighter junctions that wildtype cells when treated with HGF (p<0.0001). HGF-induced changes in permeability were also reduced. Overexpression of Nectin-3 produced endothelial cells with significantly reduced ability to form tubules (p<0.0001). Immunoprecipitation studies discovered hitherto novel associations for Nectin-3. Moreover, HGF appeared to exert an effect on Nectin-3 via tyrosine and threonine phosphorylation.Nectin-3 may be a key component in the formation of cell junctions and be a putative suppressor molecule to the invasion of breast cancer cells. 
  [Ee]xtracellular [Mm]atrix  PubMed  
 A secreted protein microarray platform for extracellular protein interaction discovery. Characterization of the extracellular protein interactome has lagged far behind that of intracellular proteins, where mass spectrometry and yeast two-hybrid technologies have excelled. Improved methods for identifying receptor-ligand and   extracellular matrix   protein interactions will greatly accelerate biological discovery in cell signaling and cellular communication. These technologies must be able to identify low-affinity binding events that are often observed between membrane-bound coreceptor molecules during cell-cell or cell-  extracellular matrix   contact. Here we demonstrate that functional protein microarrays are particularly well-suited for high-throughput screening of extracellular protein interactions. To evaluate the performance of the platform, we screened a set of 89 immunoglobulin (Ig)-type receptors against a highly diverse extracellular protein microarray with 686 genes represented. To enhance detection of low-affinity interactions, we developed a rapid method to assemble bait Fc fusion proteins into multivalent complexes using protein A microbeads. Based on these screens, we developed a statistical methodology for hit calling and identification of nonspecific interactions on protein microarrays. We found that the Ig receptor interactions identified using our methodology are highly specific and display minimal off-target binding, resulting in a 70% true-positive to false-positive hit ratio. We anticipate that these methods will be useful for a wide variety of functional protein microarray users. 
 Recruitment of nectin-3 to cell-cell junctions through trans-heterophilic interaction with CD155, a vitronectin and poliovirus receptor that localizes to alpha(v)beta3 integrin-containing membrane microdomains. Nectins present a novel class of Ig superfamily adhesion molecules that, cooperatively with cadherins, establish and maintain cell-cell adherens junctions. CD155, the cognate receptor for poliovirus, undergoes cell-matrix contacts by binding to the   extracellular matrix   protein vitronectin. The significant homology of nectins with CD155 prompted us to investigate the possibility of their interaction. We determined that nectin-3 binds CD155 and its putative mouse homologue Tage4 in cell-based ligand binding assays. Coculture of nectin-3- and CD155-expressing HeLa cells led to CD155-dependent recruitment of nectin-3 to cell-cell contacts. In a heterologous coculture system with CD155 expressing mouse neuroblastoma cells, HeLa cell-expressed nectin-3 was recruited to contact sites with CD155 bearing neurites. CD155 and nectin-3 colocalized to epithelial cell-cell junctions in renal proximal tubules and in the amniotic membrane. Efficient interaction depended on CD155 dimerization, which appears to be aided by cell type-specific cofactors. We furthermore found CD155 to codistribute with alpha(v) integrin microdomains on the surface of transfected mouse fibroblasts and at amniotic epithelial cell junctions. Our findings demonstrate the possible trans-interaction between the bona fide cell-cell adherens type adhesion system (cadherin/nectin) and the cell-matrix adhesion system (integrin/CD155) by virtue of their nectin-3 and CD155 components, respectively. 
  [Ii]nvasive  PubMed  
 Silencing of thrombospondin-1 is critical for myc-induced metastatic phenotypes in medulloblastoma. Mechanisms by which c-Myc (Myc) amplification confers aggressive medulloblastoma phenotypes are poorly defined. Here, we show using orthotopic models that high Myc expression promotes cell migration/invasion and induces metastatic tumors, which recapitulate aggressive histologic features of Myc-amplified primary human medulloblastoma. Using ChIP-chip analysis, we identified cell migration and adhesion genes, including Tsp-1/THBS1, ING4, PVRL3, and PPAP2B, as Myc-bound loci in medulloblastoma cells. Expression of Tsp-1 was most consistently and robustly diminished in medulloblastoma cell lines and primary human tumors with high Myc expression (n = 101, P = 0.032). Strikingly, stable Tsp-1 expression significantly attenuated in vitro transformation and   invasive  /migratory properties of high Myc-expressing medulloblastoma cells without altering cell proliferation, whereas RNA interference-mediated Myc knockdown was consistently accompanied by increased Tsp-1 levels and reduced cell migration and invasion in medulloblastoma cells. Chromatin immunoprecipitation (ChIP) assays revealed colocalization of Myc and obligate partner Max and correlated diminished RNA polymerase II occupancy (∼3-fold decrease, P < 0.01) with increased Myc binding at a core Tsp-1 promoter. Reporter gene and/or gel shift assays confirmed direct repression of Tsp-1 transcription by Myc and also identified JPO2, a Myc interactor associated with metastatic medulloblastoma, as a cofactor in Myc-mediated Tsp-1 repression. These findings indicate the Myc-regulatory network targets Tsp-1 via multiple mechanisms in medulloblastoma transformation, and highlight a novel critical role for Tsp-1 in Myc-mediated aggressive medulloblastoma phenotypes. 
  [Mm]etastasis  PubMed  
 Differential gene expression of primary cultured lymphatic and blood vascular endothelial cells. Blood vascular endothelial cells (BECs) and the developmentally related lymphatic endothelial cells (LECs) create complementary, yet distinct vascular networks. Each endothelial cell type interacts with flowing fluid and circulating cells, yet each vascular system has evolved specialized gene expression programs and thus both cell types display different phenotypes. BECs and LECs express distinct genes that are unique to their specific vascular microenvironment. Tumors also take advantage of the molecules that are expressed in these vascular systems to enhance their metastatic potential. We completed transcriptome analyses on primary cultured LECs and BECs, where each comparative set was isolated from the same individual. Differences were resolved in the expression of several major categories, such as cell adhesion molecules (CAMs), cytokines, and cytokine receptors. We have identified new molecules that are associated with BECs (e.g., claudin-9, CXCL11, neurexin-1, neurexin-2, and the neuronal growth factor regulator-1) and LECs (e.g., claudin-7, CD58, hyaluronan and proteoglycan link protein 1 (HAPLN1), and the poliovirus receptor-related 3 molecule) that may lead to novel therapeutic treatments for diseases of lymphatic or blood vessels, including   metastasis   of cancer to lymph nodes or distant organs. 
 The expression of the Nectin complex in human breast cancer and the role of Nectin-3 in the control of tight junctions during   metastasis  . Nectins are a family of integral protein molecules involved in the formation of functioning Adherens and Tight Junctions (TJ). Aberrant expression is associated with cancer progression but little is known how this effects changes in cell behaviour. This study aimed to ascertain the distribution of Nectins-1 to -4 in human breast cancer and the effect on junctional integrity of Nectin-3 modulation in human endothelial and breast cancer cells.A human breast tissue cohort was processed for Q-PCR and immunohistochemistry for analysis of Nectin-1/-2/-3/-4. Nectin-3 over-expression was induced in the human breast cancer cell line MDA-MB-231 and the human endothelial cell line HECV. Functional testing was carried out to ascertain changes in cell behaviour.Q-PCR revealed a distinct reduction in node positive tumours and in patients with poor outcome. There was increased expression of Nectin-1/-2 in patients with metastatic disease, Nectin-3/-4 was reduced. IHC revealed that Nectin-3 expression showed clear changes in distribution between normal and cancerous cells. Nectin-3 over-expression in MDA-MB-231 cells showed reduced invasion and migration even when treated with HGF. Changes in barrier function resulted in MDAN3 cells showing less change in resistance after 2h treatment with HGF (p<0.001). Nectin-3 transformed endothelial cells were significantly more adhesive, irrespective of treatment with HGF (p<0.05) and had reduced growth. Barrier function revealed that transformed HECV cells had significantly tighter junctions that wildtype cells when treated with HGF (p<0.0001). HGF-induced changes in permeability were also reduced. Overexpression of Nectin-3 produced endothelial cells with significantly reduced ability to form tubules (p<0.0001). Immunoprecipitation studies discovered hitherto novel associations for Nectin-3. Moreover, HGF appeared to exert an effect on Nectin-3 via tyrosine and threonine phosphorylation.Nectin-3 may be a key component in the formation of cell junctions and be a putative suppressor molecule to the invasion of breast cancer cells. 
 Nectin expression in pancreatic adenocarcinoma: nectin-3 is associated with a poor prognosis. Nectins are cell adhesion molecules that regulate the formation of adherens junctions and are linked with E-cadherin-based cell-cell adherens junctions. In pancreatic cancer, the expression of E-cadherin and nectins is considered to be related to   metastasis  , invasion and prognosis.We evaluated the distribution of cells that were positive for nectin subtypes and E-cadherin using immunohistochemistry in specimens of human pancreatic adenocarcinoma, and correlated these results with the clinicopathological features and patient outcomes.The immunohistochemical distribution of nectin-1 and E-cadherin showed a good correlation (r = 0.523, p < 0.01). Tumors over 4 cm in diameter had more intense staining for nectin-4 than smaller tumors (p = 0.035). Nectin-2 expression correlated with a poorer histological grade (p = 0.04). The cases that showed diffuse nectin-3 expression had a better prognosis than those with negative expression (p = 0.018).Our results showed that the expression of nectin-3 in pancreatic cancer can be a prognostic factor. 
   
  
 PVR 
 
  NCBI Gene  
  GeneRIF  
  Pubmed  
 
 
  Search Term  Rank  Fields  
  [Mm]igration  6  PubMed(10)  
  ECM  7  PubMed(1)  
  [Ee]xtracellular [Mm]atrix  8  PubMed(2); Summary(1)  
  [Ii]nvasive  10  PubMed(2); GeneRIFs(1)  
  [Mm]etastasis  12  PubMed(4)  
  [Cc]ell [Ii]nvasion  13  PubMed(3); GeneRIFs(2)  
 
 
 hide/show details for PVR 
  [Mm]igration  PubMed  
 Analysis of the myosin-II-responsive focal adhesion proteome reveals a role for β-Pix in negative regulation of focal adhesion maturation. Focal adhesions undergo myosin-II-mediated maturation wherein they grow and change composition to modulate integrin signalling for cell   migratio  n, growth and differentiation. To determine how focal adhesion composition is affected by myosin II activity, we performed proteomic analysis of isolated focal adhesions and compared protein abundance in focal adhesions from cells with and without myosin II inhibition. We identified 905 focal adhesion proteins, 459 of which changed in abundance with myosin II inhibition, defining the myosin-II-responsive focal adhesion proteome. The abundance of 73% of the proteins in the myosin-II-responsive focal adhesion proteome was enhanced by contractility, including proteins involved in Rho-mediated focal adhesion maturation and endocytosis- and calpain-dependent focal adhesion disassembly. During myosin II inhibition, 27% of proteins in the myosin-II-responsive focal adhesion proteome, including proteins involved in Rac-mediated lamellipodial protrusion, were enriched in focal adhesions, establishing that focal adhesion protein recruitment is also negatively regulated by contractility. We focused on the Rac guanine nucleotide exchange factor β-Pix, documenting its role in the negative regulation of focal adhesion maturation and the promotion of lamellipodial protrusion and focal adhesion turnover to drive cel  l migrati  on. 
 Overexpression of Necl-5 correlates with unfavorable prognosis in patients with lung adenocarcinoma. Nectin-like molecule-5 (Necl-5) is an immunoglobulin (Ig)-like molecule that is up-regulated in many types of cancer cells. It was shown experimentally that Necl-5 enhances cell   migration  , proliferation, and metastasis, but its clinical significance has not been documented. The aim of this study was to observe the expression of Necl-5 in surgically resected primary lung adenocarcinomas and to investigate its clinical significance. A total of 63 surgically resected primary pulmonary adenocarcinoma tissues were investigated by immunohistochemistry for the expression of Necl-5. The relationship between expression of Necl-5 and clinicopathological features was analyzed, and the influence of Necl-5 expression on outcomes in these patients was assessed. A strong expression of Necl-5 by cancer cells was observed in 43 of the 63 tumors. The overexpression of Necl-5 by cancer cells was significantly associated with lymph node metastasis (P = 0.0398), TNM staging (P = 0.0367), and the bronchioloalveolar carcinoma ratio of tumors (P = 0.0423). Furthermore, the disease-free survival rate in patients with positive Necl-5 overexpression was significantly lower than that in patients with negative Necl-5 overexpression (P = 0.0004). Multivariate survival analysis revealed Necl-5 expression to be an independent risk factor for an unfavorable outcome (P = 0.0294). Additionally, an analysis including only the stage I cases revealed that the disease-free survival rate of the Necl-5-positive group was significantly lower than that of the Necl-5-negative group (P = 0.0192). These results indicate that Necl-5 plays a role in mediating tumor cell invasion and that the overexpression of Necl-5 in cancer cells has clinical significance for prognostic evaluation of patients with primary pulmonary adenocarcinoma. 
 The CD155/poliovirus receptor enhances the proliferation of ras-mutated cells. Stimulation of the CD155/poliovirus receptor, which localizes in the cell-matrix and at cell-cell junctions, inhibits cell adhesion and enhances cell   migration  . Necl-5, a mouse homolog of CD155, is implicated in the formation of adherence junctions. Recently, Necl-5 has also been found to enhance cell proliferation via the stimulation of serum and platelet-derived growth factor through the Ras-Raf-MEK-ERK signaling pathway. In our present study, we find that CD155 significantly enhances the serum-induced cell proliferation of NIH3T3 cells which have been transformed by an oncogenic Ras (V12Ras-NIH3T3), but not the parental cells. CD155 expression in V12Ras-NIH3T3 cells is also found to upregulate cyclin D2, downregulate p27(Kip1) and shorten the G0/G1 phase of the cell cycle. An inhibitor of focal adhesion kinase does not reduce this CD155-mediated enhancement of V12Ras-NIH3T3 cell proliferation. The expression of CD155DeltaCP, which lacks the cytoplasmic region including the immunoreceptor tyrosine-based inhibitory motif (ITIM), has a reduced ability to enhance the serum responsiveness of V12Ras-NIH3T3 cells, suggesting that the ITIM might be required for this effect of CD155. In addition, the overexpression of exogenous CD155 enhances the serum responsiveness of HT1080 cells, which harbor a mutant N-ras gene. On the other hand, siRNA-induced knockdown of endogenous CD155 and/or CD155DeltaCP expression significantly repress the serum responsiveness of DLD-1 cells, which express endogenous CD155 and harbor a mutant K-ras gene, suggesting that this mutant may function in a dominant negative manner. Taken together, our present data suggest that CD155, at least in part, enhances the proliferation of ras-mutated cells. 
 DNAM-1 and PVR regulate monocyte   migration   through endothelial junctions. DNAX accessory molecule 1 (DNAM-1; CD226) is a transmembrane glycoprotein involved in T cell and natural killer (NK) cell cytotoxicity. We demonstrated recently that DNAM-1 triggers NK cell-mediated killing of tumor cells upon engagement by its two ligands, poliovirus receptor (PVR; CD155) and Nectin-2 (CD112). In the present paper, we show that PVR and Nectin-2 are expressed at cell junctions on primary vascular endothelial cells. Moreover, the specific binding of a soluble DNAM-1-Fc molecule was detected at endothelial junctions. This binding was almost completely abrogated by anti-PVR monoclonal antibodies (mAbs), but not modified by anti-Nectin-2 mAbs, which demonstrates that PVR is the major DNAM-1 ligand on endothelial cells. Because DNAM-1 is highly expressed on leukocytes, we investigated the role of the DNAM-1-PVR interaction during the monocyte transendothelial   migration   process. In vitro, both anti-DNAM-1 and anti-PVR mAbs strongly blocked the trans  migration   of monocytes through the endothelium. Moreover, after anti-DNAM-1 or anti-PVR mAb treatment, monocytes were arrested at the apical surface of the endothelium over intercellular junctions, which strongly suggests that the DNAM-1-PVR interaction occurs during the diapedesis step. Altogether, our results demonstrate that DNAM-1 regulates monocyte extravasation via its interaction with PVR expressed at endothelial junctions on normal cells. 
 Cutting edge: CD96 (tactile) promotes NK cell-target cell adhesion by interacting with the poliovirus receptor (CD155). The poliovirus receptor (PVR) belongs to a large family of Ig molecules called nectins and nectin-like proteins, which mediate cell-cell adhesion, cell   migration  , and serve as entry receptors for viruses. It has been recently shown that human NK cells recognize PVR through the receptor DNAM-1, which triggers NK cell stimulation in association with beta(2) integrin. In this study, we show that NK cells recognize PVR through an additional receptor, CD96, or T cell-activated increased late expression (Tactile). CD96 promotes NK cell adhesion to target cells expressing PVR, stimulates cytotoxicity of activated NK cells, and mediates acquisition of PVR from target cells. Thus, NK cells have evolved a dual receptor system that recognizes nectins and nectin-like molecules on target cells and mediates NK cell adhesion and triggering of effector functions. As PVR is highly expressed in certain tumors, this receptor system may be critical for NK cell recognition of tumors. 
 CD155/PVR plays a key role in cell motility during tumor cell invasion and   migration  . Invasion is an important early step of cancer metastasis that is not well understood. Developing therapeutics to limit metastasis requires the identification and validation of candidate proteins necessary for invasion and   migration  .We developed a functional proteomic screen to identify mediators of tumor cell invasion. This screen couples Fluorophore Assisted Light Inactivation (FALI) to a scFv antibody library to systematically inactivate surface proteins expressed by human fibrosarcoma cells followed by a high-throughput assessment of transwell invasion.Using this screen, we have identified CD155 (the poliovirus receptor) as a mediator of tumor cell invasion through its role in   migration  . Knockdown of CD155 by FALI or by RNAi resulted in a significant decrease in transwell   migration   of HT1080 fibrosarcoma cells towards a serum chemoattractant. CD155 was found to be highly expressed in multiple cancer cell lines and primary tumors including glioblastoma (GBM). Knockdown of CD155 also decreased   migration   of U87MG GBM cells. CD155 is recruited to the leading edge of migrating cells where it colocalizes with actin and alphav-integrin, known mediators of motility and adhesion. Knockdown of CD155 also altered cellular morphology, resulting in cells that were larger and more elongated than controls when plated on a Matrigel substrate.These results implicate a role for CD155 in mediating tumor cell invasion and   migration   and suggest that CD155 may contribute to tumorigenesis. 
 Poliovirus receptor (CD155) regulates a step in transendothelial   migration   between PECAM and CD99. The movement of leukocytes across endothelium [referred to as diapedesis or transendothelial   migration   (TEM)] is a critical step in the inflammatory process. Recently, it was demonstrated that treatment of endothelial cells and monocytes with antibodies against poliovirus receptor (PVR; CD155) and DNAX-associated molecule-1 (DNAM-1; CD226) arrested monocytes over endothelial junctions and prevented TEM, suggesting that these molecules are involved in diapedesis. However, nothing was known about the mechanism by which PVR and DNAM-1 work in TEM. Herein, we show that, similar to endothelial PECAM interacting with leukocyte PECAM, activation of endothelial PVR with anti-PVR antibodies or interaction with its ligand, DNAM-1, results in recruitment of the tyrosine phosphatase Shp-2, and this process is dependent on Src kinases. Furthermore, differential and sequential treatment with blocking antibodies directed against PVR, DNAM-1, PECAM, and CD99 showed that endothelial PVR and monocyte DNAM-1 interact at and regulate a step between those regulated by PECAM and CD99. Further studies demonstrate that PVR resides in the recently identified lateral border recycling compartment, similar to PECAM and CD99. These findings suggest that the localization of adhesion/signaling molecules to the lateral border recycling compartment and the recruitment of Shp-2 may be common mechanisms for the regulation of TEM by endothelial cells. 
 Ligand stimulation of CD155alpha inhibits cell adhesion and enhances cell   migration   in fibroblasts. CD155 (poliovirus receptor) localizes in cell-matrix adhesions and cell-cell junctions, but its role in the regulation of cell adhesion and cell motility has not been investigated. We identified a conserved immunoreceptor tyrosine-based inhibitory motif (ITIM) in the cytoplasmic domain of human CD155alpha. The ITIM was tyrosine-phosphorylated upon binding of anti-CD155 monoclonal antibody D171, poliovirus, and DNAM-1 (CD226) to human CD155alpha, and recruited SH2-domain-containing tyrosine phosphatase-2 (SHP-2). After CD155alpha stimulation with its ligands, cell adhesion was inhibited and cell motility was enhanced, effects that were associated with the phosphorylation of ITIM by Src kinases and accompanied by dephosphorylation of focal adhesion kinase and paxillin. These effects were abolished by introducing a point-mutation in Y398F into the ITIM of CD155alpha and by coexpression of a dominant negative SHP-2 mutant with CD155alpha. These results suggest that CD155alpha plays a role in the regulation of cell adhesion and cell motility. 
 The human immunodeficiency virus type 1 Nef and Vpu proteins downregulate the natural killer cell-activating ligand PVR. The human immunodeficiency virus type 1 (HIV-1) evades the immune responses of natural killer (NK) cells through mechanisms that have been partially deciphered. Here we show that in HIV-1-infected T lymphocytes, the early viral Nef protein downmodulates PVR (CD155, Necl-5), a ligand for the activating receptor DNAM-1 (CD226) expressed by all NK cells, CD8(+) T cells, and other cell types. This novel Nef activity is conserved by Nef proteins of laboratory HIV-1 strains (NL4-3, SF2) and of a patient-derived virus, but it is not maintained by HIV-2. Nef uses the same motifs to downregulate PVR and HLA-I molecules, likely by the same mechanisms. Indeed, as previously demonstrated for HLA-I, Nef reduces the total amounts of cell-associated PVR. Optimal downregulation of cell surface PVR by Nef also requires the presence of the late viral factor Vpu. In line with PVR reduction, the NK cell-mediated lysis of T cells infected by a wild-type but not Nef-deficient virus is virtually abrogated upon blocking of both DNAM-1 and another activating receptor, NKG2D, previously shown to mediate killing of HIV-infected cells. Together, these data demonstrate that the PVR downmodulation by Nef and Vpu is a strategy evolved by HIV-1 to prevent NK cell-mediated lysis of infected cells. The PVR downregulation reported here has the potential to affect the immune responses of other DNAM-1-positive cells besides NK cells and to alter multiple PVR-mediated cellular processes, such as adhesion and   migration  , and may thus greatly influence HIV-1 pathogenesis. 
 The role of Necl-5 in the invasive activity of lung adenocarcinoma. Nectin-like molecule-5 (Necl-5) is an immunoglobulin-like molecule that was originally identified as a poliovirus receptor and is often upregulated in cancer cells. It has been said that Necl-5 plays a role in not only cell-cell adhesion, but also cell   migration  , proliferation, and metastasis. In this study, we used a bronchioloalveolar carcinoma (BAC) cell line and fibroblasts to assess the expression of Necl-5 in the development of cancer-stroma communication by using an easy-to-prepare double-layered collagen gel hemisphere (DL-CGH) system that enables visualization of cell   migration   during invasion. The expression of Necl-5 was higher in BAC cells than in fibroblasts. This tendency didn't change even when the BAC cells were mixed with fibroblasts. To assess the role of Necl-5 in the invasive activity of the BAC cells, we knocked down its expression using RNA interference (RNAi). The invasion assay with DL-CGH revealed that inhibition of Necl-5 expression in the BAC cells was associated with suppressed invasiveness. In addition, Necl-5 knockdown inhibited the movement and proliferation of the BAC cells. Necl-5 expression in lung cancer cells is crucial for their invasiveness in the cancer-stromal interaction, suggesting that Necl-5 could be a favorable molecular target for the suppression of invasiveness in lung adenocarcinoma. 
  ECM  PubMed  
 Receptors for hyaluronic acid and poliovirus: a combinatorial role in glioma invasion? CD44 has long been associated with glioma invasion while, more recently, CD155 has been implicated in playing a similar role. Notably, these two receptors have been shown closely positioned on monocytes.In this study, an up-regulation of CD44 and CD155 was demonstrated in established and early-passage cultures of glioblastoma. Total internal reflected fluorescence (TIRF) microscopy revealed close proximity of CD44 and CD155. CD44 antibody blocking and gene silencing (via siRNA) resulted in greater inhibition of invasion than that for CD155. Combined interference resulted in 86% inhibition of invasion, although in these investigations no obvious evidence of synergy between CD44 and CD155 in curbing invasion was shown. Both siRNA-CD44 and siRNA-CD155 treated cells lacked processes and were rounder, while live cell imaging showed reduced motility rate compared to wild type cells. Adhesion assay demonstrated that wild type cells adhered most efficiently to laminin, whereas siRNA-treated cells (p<0.0001 for both CD44 and CD155 expression) showed decreased adhesion on several   ECM  s investigated. BrdU assay showed a higher proliferation of siRNA-CD44 and siRNA-CD155 cells, inversely correlated with reduced invasion. Confocal microscopy revealed overlapping of CD155 and integrins (β(1), α(v)β(1) and α(v)β(3)) on glioblastoma cell processes whereas siRNA-transfected cells showed consequent reduction in integrin expression with no specific staining patterns. Reduced expression of Rho GTPases, Cdc42, Rac1/2/3, RhoA and RhoB, was seen in siRNA-CD44 and siRNA-CD155 cells. In contrast to CD44-knockdown and 'double'-knockdown cells, no obvious decrease in RhoC expression was observed in CD155-knockdown cells.This investigation has enhanced our understanding of cell invasion and confirmed CD44 to play a more significant role in this biological process than CD155. Joint CD44/CD155 approaches may, however, merit further study in therapeutic targeting of infiltrating glioma cells. 
  [Ee]xtracellular [Mm]atrix  PubMed  
 Recruitment of nectin-3 to cell-cell junctions through trans-heterophilic interaction with CD155, a vitronectin and poliovirus receptor that localizes to alpha(v)beta3 integrin-containing membrane microdomains. Nectins present a novel class of Ig superfamily adhesion molecules that, cooperatively with cadherins, establish and maintain cell-cell adherens junctions. CD155, the cognate receptor for poliovirus, undergoes cell-matrix contacts by binding to the   extracellular matrix   protein vitronectin. The significant homology of nectins with CD155 prompted us to investigate the possibility of their interaction. We determined that nectin-3 binds CD155 and its putative mouse homologue Tage4 in cell-based ligand binding assays. Coculture of nectin-3- and CD155-expressing HeLa cells led to CD155-dependent recruitment of nectin-3 to cell-cell contacts. In a heterologous coculture system with CD155 expressing mouse neuroblastoma cells, HeLa cell-expressed nectin-3 was recruited to contact sites with CD155 bearing neurites. CD155 and nectin-3 colocalized to epithelial cell-cell junctions in renal proximal tubules and in the amniotic membrane. Efficient interaction depended on CD155 dimerization, which appears to be aided by cell type-specific cofactors. We furthermore found CD155 to codistribute with alpha(v) integrin microdomains on the surface of transfected mouse fibroblasts and at amniotic epithelial cell junctions. Our findings demonstrate the possible trans-interaction between the bona fide cell-cell adherens type adhesion system (cadherin/nectin) and the cell-matrix adhesion system (integrin/CD155) by virtue of their nectin-3 and CD155 components, respectively. 
 Expression of the human poliovirus receptor/CD155 gene is activated by sonic hedgehog. The human poliovirus receptor/CD155 is a transmembrane glycoprotein belonging to the immunoglobulin superfamily. The ectodomain of CD155 mediates cell attachment to the   extracellular matrix   molecule vitronectin, while its intracellular domain interacts with the dynein light chain Tctex-1. CD155 is a primate-restricted gene that is expressed during development in mesenchymal tissues and ventrally derived structures within the CNS. Its function in adults is as yet unknown, but significantly, CD155 is aberrantly expressed in neuroectodermal tumors. We show that the expression of CD155 mRNA is up-regulated when human Ntera2 cells are treated with purified Sonic hedgehog (Shh) protein. Reporter gene expression driven by the CD155 core promoter is activated by Shh in transient co-transfection assays. Analysis of the CD155 core promoter indicates that an intact GLI binding site is required for Shh activation. In addition, overexpression of Gli1 or Gli3 potently activates reporter gene expression driven by the CD155 core promoter. These data identify the CD155 gene as a transcriptional target of Shh, a finding that has significance for the normal function of CD155 during development and the expression of CD155 in neuroectodermal tumors. 
  Summary  
 The protein encoded by this gene is a transmembrane glycoprotein belonging to the immunoglobulin superfamily. The external domain mediates cell attachment to the   extracellular matrix   molecule vitronectin, while its intracellular domain interacts with the dynein light chain Tctex-1/DYNLT1. The gene is specific to the primate lineage, and serves as a cellular receptor for poliovirus in the first step of poliovirus replication. Multiple transcript variants encoding different isoforms have been found for this gene. [provided by RefSeq, Oct 2008] 
  [Ii]nvasive  PubMed  
 Necl-5/poliovirus receptor interacts in cis with integrin alphaVbeta3 and regulates its clustering and focal complex formation. Integrin alphavbeta3, which forms focal complexes at leading edges in moving cells, is up-regulated in cancer cells and so is implicated in their   invasive  ness. Necl-5, originally identified as a poliovirus receptor and also up-regulated in cancer cells, colocalizes with integrin alphavbeta3 at leading edges in moving cells and enhances growth factor-induced cell movement. Here, we show that Necl-5 interacts directly, in cis, with integrin alphavbeta3, and enhances integrin alphavbeta3 clustering and focal complex formation at leading edges in NIH3T3 cells. The extracellular region of Necl-5, but not the cytoplasmic region, is necessary for its interaction with integrin alphavbeta3; however, both regions are necessary for its action. An interaction between integrin alphavbeta3 and vitronectin and PDGF-induced activation of Rac are also necessary for integrin alphavbeta3 clustering. The interaction between Necl-5 and integrin alphavbeta3 enhances PDGF-induced Rac activation, facilitating integrin alphavbeta3 clustering presumably in a feedback amplification manner. Thus, Necl-5 has a critical role in integrin alphavbeta3 clustering and focal complex formation. 
 The role of Necl-5 in the   invasive   activity of lung adenocarcinoma. Nectin-like molecule-5 (Necl-5) is an immunoglobulin-like molecule that was originally identified as a poliovirus receptor and is often upregulated in cancer cells. It has been said that Necl-5 plays a role in not only cell-cell adhesion, but also cell migration, proliferation, and metastasis. In this study, we used a bronchioloalveolar carcinoma (BAC) cell line and fibroblasts to assess the expression of Necl-5 in the development of cancer-stroma communication by using an easy-to-prepare double-layered collagen gel hemisphere (DL-CGH) system that enables visualization of cell migration during invasion. The expression of Necl-5 was higher in BAC cells than in fibroblasts. This tendency didn't change even when the BAC cells were mixed with fibroblasts. To assess the role of Necl-5 in the   invasive   activity of the BAC cells, we knocked down its expression using RNA interference (RNAi). The invasion assay with DL-CGH revealed that inhibition of Necl-5 expression in the BAC cells was associated with suppressed   invasive  ness. In addition, Necl-5 knockdown inhibited the movement and proliferation of the BAC cells. Necl-5 expression in lung cancer cells is crucial for their   invasive  ness in the cancer-stromal interaction, suggesting that Necl-5 could be a favorable molecular target for the suppression of   invasive  ness in lung adenocarcinoma. 
  GeneRIFs  
 findings suggest Necl-5 expression in lung cancer cells is crucial for their   invasive  ness in the cancer-stromal interaction 
  [Mm]etastasis  PubMed  
 Overexpression of Necl-5 correlates with unfavorable prognosis in patients with lung adenocarcinoma. Nectin-like molecule-5 (Necl-5) is an immunoglobulin (Ig)-like molecule that is up-regulated in many types of cancer cells. It was shown experimentally that Necl-5 enhances cell migration, proliferation, and   metastasis  , but its clinical significance has not been documented. The aim of this study was to observe the expression of Necl-5 in surgically resected primary lung adenocarcinomas and to investigate its clinical significance. A total of 63 surgically resected primary pulmonary adenocarcinoma tissues were investigated by immunohistochemistry for the expression of Necl-5. The relationship between expression of Necl-5 and clinicopathological features was analyzed, and the influence of Necl-5 expression on outcomes in these patients was assessed. A strong expression of Necl-5 by cancer cells was observed in 43 of the 63 tumors. The overexpression of Necl-5 by cancer cells was significantly associated with lymph node   metastasis   (P = 0.0398), TNM staging (P = 0.0367), and the bronchioloalveolar carcinoma ratio of tumors (P = 0.0423). Furthermore, the disease-free survival rate in patients with positive Necl-5 overexpression was significantly lower than that in patients with negative Necl-5 overexpression (P = 0.0004). Multivariate survival analysis revealed Necl-5 expression to be an independent risk factor for an unfavorable outcome (P = 0.0294). Additionally, an analysis including only the stage I cases revealed that the disease-free survival rate of the Necl-5-positive group was significantly lower than that of the Necl-5-negative group (P = 0.0192). These results indicate that Necl-5 plays a role in mediating tumor cell invasion and that the overexpression of Necl-5 in cancer cells has clinical significance for prognostic evaluation of patients with primary pulmonary adenocarcinoma. 
 CD155/PVR plays a key role in cell motility during tumor cell invasion and migration. Invasion is an important early step of cancer   metastasis   that is not well understood. Developing therapeutics to limit   metastasis   requires the identification and validation of candidate proteins necessary for invasion and migration.We developed a functional proteomic screen to identify mediators of tumor cell invasion. This screen couples Fluorophore Assisted Light Inactivation (FALI) to a scFv antibody library to systematically inactivate surface proteins expressed by human fibrosarcoma cells followed by a high-throughput assessment of transwell invasion.Using this screen, we have identified CD155 (the poliovirus receptor) as a mediator of tumor cell invasion through its role in migration. Knockdown of CD155 by FALI or by RNAi resulted in a significant decrease in transwell migration of HT1080 fibrosarcoma cells towards a serum chemoattractant. CD155 was found to be highly expressed in multiple cancer cell lines and primary tumors including glioblastoma (GBM). Knockdown of CD155 also decreased migration of U87MG GBM cells. CD155 is recruited to the leading edge of migrating cells where it colocalizes with actin and alphav-integrin, known mediators of motility and adhesion. Knockdown of CD155 also altered cellular morphology, resulting in cells that were larger and more elongated than controls when plated on a Matrigel substrate.These results implicate a role for CD155 in mediating tumor cell invasion and migration and suggest that CD155 may contribute to tumorigenesis. 
 Nectin like-5 overexpression correlates with the malignant phenotype in cutaneous melanoma. NECL-5 is involved in regulating cell-cell junctions, in cooperation with cadherins, integrins and platelet-derived growth factor receptor, that are essential for intercellular communication. Its role in malignant transformation was previously described. It has been reported that transformation of melanocytes is associated with altered expression of adhesion molecules suggesting the potential involment of NECL-5 in melanoma development and prognosis. To shed light on this issue, the expression and the role of NECL-5 in melanoma tissues was investigated by bioinformatic and molecular approaches. NECL-5 was up-regulated both at the mRNA and the protein levels in WM35, M14 and A375 cell lines compared with normal melanocytes. A subsequent analysis in primary and metastatic melanoma specimens confirmed "in vitro" findings. NECL-5 overexpression was observed in 53 of 59 (89.8%) and 12 of 12 (100%), primary melanoma and melanoma   metastasis  , respectively; while, low expression of NECL-5 was detected in 12 of 20 (60%) benign nevi. A significant correlation of NECL-5 overexpression was observed with most of known negative melanoma prognostic factors, including lymph-node involvement (P = 0.009) and thickness (P = 0.004). Intriguingly, by analyzing the large series of melanoma samples in the Xu dataset, we identified the transcription factor YY1 among genes positively correlated with NECL-5 (r = 0.5). The concordant computational and experimental data of the present study indicate that the extent of NECL-5 expression correlates with melanoma progression. 
 The role of Necl-5 in the invasive activity of lung adenocarcinoma. Nectin-like molecule-5 (Necl-5) is an immunoglobulin-like molecule that was originally identified as a poliovirus receptor and is often upregulated in cancer cells. It has been said that Necl-5 plays a role in not only cell-cell adhesion, but also cell migration, proliferation, and   metastasis  . In this study, we used a bronchioloalveolar carcinoma (BAC) cell line and fibroblasts to assess the expression of Necl-5 in the development of cancer-stroma communication by using an easy-to-prepare double-layered collagen gel hemisphere (DL-CGH) system that enables visualization of cell migration during invasion. The expression of Necl-5 was higher in BAC cells than in fibroblasts. This tendency didn't change even when the BAC cells were mixed with fibroblasts. To assess the role of Necl-5 in the invasive activity of the BAC cells, we knocked down its expression using RNA interference (RNAi). The invasion assay with DL-CGH revealed that inhibition of Necl-5 expression in the BAC cells was associated with suppressed invasiveness. In addition, Necl-5 knockdown inhibited the movement and proliferation of the BAC cells. Necl-5 expression in lung cancer cells is crucial for their invasiveness in the cancer-stromal interaction, suggesting that Necl-5 could be a favorable molecular target for the suppression of invasiveness in lung adenocarcinoma. 
  [Cc]ell [Ii]nvasion  PubMed  
 Overexpression of Necl-5 correlates with unfavorable prognosis in patients with lung adenocarcinoma. Nectin-like molecule-5 (Necl-5) is an immunoglobulin (Ig)-like molecule that is up-regulated in many types of cancer cells. It was shown experimentally that Necl-5 enhances cell migration, proliferation, and metastasis, but its clinical significance has not been documented. The aim of this study was to observe the expression of Necl-5 in surgically resected primary lung adenocarcinomas and to investigate its clinical significance. A total of 63 surgically resected primary pulmonary adenocarcinoma tissues were investigated by immunohistochemistry for the expression of Necl-5. The relationship between expression of Necl-5 and clinicopathological features was analyzed, and the influence of Necl-5 expression on outcomes in these patients was assessed. A strong expression of Necl-5 by cancer cells was observed in 43 of the 63 tumors. The overexpression of Necl-5 by cancer cells was significantly associated with lymph node metastasis (P = 0.0398), TNM staging (P = 0.0367), and the bronchioloalveolar carcinoma ratio of tumors (P = 0.0423). Furthermore, the disease-free survival rate in patients with positive Necl-5 overexpression was significantly lower than that in patients with negative Necl-5 overexpression (P = 0.0004). Multivariate survival analysis revealed Necl-5 expression to be an independent risk factor for an unfavorable outcome (P = 0.0294). Additionally, an analysis including only the stage I cases revealed that the disease-free survival rate of the Necl-5-positive group was significantly lower than that of the Necl-5-negative group (P = 0.0192). These results indicate that Necl-5 plays a role in mediating tumor   cell invasion   and that the overexpression of Necl-5 in cancer cells has clinical significance for prognostic evaluation of patients with primary pulmonary adenocarcinoma. 
 Receptors for hyaluronic acid and poliovirus: a combinatorial role in glioma invasion? CD44 has long been associated with glioma invasion while, more recently, CD155 has been implicated in playing a similar role. Notably, these two receptors have been shown closely positioned on monocytes.In this study, an up-regulation of CD44 and CD155 was demonstrated in established and early-passage cultures of glioblastoma. Total internal reflected fluorescence (TIRF) microscopy revealed close proximity of CD44 and CD155. CD44 antibody blocking and gene silencing (via siRNA) resulted in greater inhibition of invasion than that for CD155. Combined interference resulted in 86% inhibition of invasion, although in these investigations no obvious evidence of synergy between CD44 and CD155 in curbing invasion was shown. Both siRNA-CD44 and siRNA-CD155 treated cells lacked processes and were rounder, while live cell imaging showed reduced motility rate compared to wild type cells. Adhesion assay demonstrated that wild type cells adhered most efficiently to laminin, whereas siRNA-treated cells (p<0.0001 for both CD44 and CD155 expression) showed decreased adhesion on several ECMs investigated. BrdU assay showed a higher proliferation of siRNA-CD44 and siRNA-CD155 cells, inversely correlated with reduced invasion. Confocal microscopy revealed overlapping of CD155 and integrins (β(1), α(v)β(1) and α(v)β(3)) on glioblastoma cell processes whereas siRNA-transfected cells showed consequent reduction in integrin expression with no specific staining patterns. Reduced expression of Rho GTPases, Cdc42, Rac1/2/3, RhoA and RhoB, was seen in siRNA-CD44 and siRNA-CD155 cells. In contrast to CD44-knockdown and 'double'-knockdown cells, no obvious decrease in RhoC expression was observed in CD155-knockdown cells.This investigation has enhanced our understandin  g of cell inv  asion and confirmed CD44 to play a more significant role in this biological process than CD155. Joint CD44/CD155 approaches may, however, merit further study in therapeutic targeting of infiltrating glioma cells. 
 CD155/PVR plays a key role in cell motility during tumor   cell invasion   and migration. Invasion is an important early step of cancer metastasis that is not well understood. Developing therapeutics to limit metastasis requires the identification and validation of candidate proteins necessary for invasion and migration.We developed a functional proteomic screen to identify mediators of tumor   cell invasion  . This screen couples Fluorophore Assisted Light Inactivation (FALI) to a scFv antibody library to systematically inactivate surface proteins expressed by human fibrosarcoma cells followed by a high-throughput assessment of transwell invasion.Using this screen, we have identified CD155 (the poliovirus receptor) as a mediator of tumor   cell invasion   through its role in migration. Knockdown of CD155 by FALI or by RNAi resulted in a significant decrease in transwell migration of HT1080 fibrosarcoma cells towards a serum chemoattractant. CD155 was found to be highly expressed in multiple cancer cell lines and primary tumors including glioblastoma (GBM). Knockdown of CD155 also decreased migration of U87MG GBM cells. CD155 is recruited to the leading edge of migrating cells where it colocalizes with actin and alphav-integrin, known mediators of motility and adhesion. Knockdown of CD155 also altered cellular morphology, resulting in cells that were larger and more elongated than controls when plated on a Matrigel substrate.These results implicate a role for CD155 in mediating tumor   cell invasion   and migration and suggest that CD155 may contribute to tumorigenesis. 
  GeneRIFs  
 This investigation has enhanced understanding of   cell invasion   and confirmed CD44 to play a more significant role in this biological process than CD155. 
 Necl-5 plays a role in mediating tumor   cell invasion   and that the overexpression of Necl-5 in cancer cells has clinical significance for prognostic evaluation of patients with primary pulmonary adenocarcinoma. 
   
  
 CHIT1 
 
  NCBI Gene  
  GeneRIF  
  Pubmed  
 
 
  Search Term  Rank  Fields  
  [Mm]igration  6  PubMed(3)  
  [Mm]etastasis  12  PubMed(1)  
 
 
 hide/show details for CHIT1 
  [Mm]igration  PubMed  
 High prevalence of chitotriosidase deficiency in Peruvian Amerindians exposed to chitin-bearing food and enteroparasites. The human genome encodes a gene for an enzymatically active chitinase (CHIT1) located in a single copy on Chromosome 1, which is highly expressed by activated macrophages and in other cells of the innate immune response. Several dysfunctional mutations are known in CHIT1, including a 24-bp duplication in Exon 10 causing catalytic deficiency. This duplication is a common variant conserved in many human populations, except in West and South Africans. Thus it has been proposed that human   migration   out of Africa and the consequent reduction of exposure to chitin from environmental factors may have enabled the conservation of dysfunctional mutations in human chitinases. Our data obtained from 85 indigenous Amerindians from Peru, representative of populations characterized by high prevalence of chitin-bearing enteroparasites and intense entomophagy, reveal a very high frequency of the 24-bp duplication (47.06%), and of other single nucleotide polymorphisms which are known to partially affect enzymatic activity (G102S: 42.7% and A442G/V: 25.5%). Our finding is in line with a founder effect, but appears to confute our previous hypothesis of a protective role against parasite infection and sustains the discussion on the redundancy of chitinolytic function. 
 Chitinase effects on immune cell response in neuromyelitis optica and multiple sclerosis. Recent studies conducted in arthritis, asthma, and inflammatory bowel disease suggest that chitinases are important in inflammatory processes and tissue remodeling.To investigate the role of chitinases in multiple sclerosis (MS) and neuromyelitis optica (NMO).Levels of chitotriosidase, acid mammalian chitinase (AMCase), and chitinase 3-like-1 (CHI3L1) were measured using ELISA, in cerebrospinal fluid (CSF) and in serum from 24 patients with relapsing remitting (RR) MS, 24 patients with secondary progressive (SP) MS, 12 patients with NMO, 24 patients with other inflammatory neurological diseases (OIND), and 24 healthy controls (HCs). The number of anti-MOG cytokine-secreting cells was studied using ELISPOT. Eotaxins, MCP-1, RANTES, and IL-8 were assessed using ELISA. Cell trans  migration   was determined using an in vitro blood-brain barrier (BBB) model, in the presence and absence of chitinases.CSF chitinase levels were significantly increased in patients with RRMS and NMO compared with HCs and patients with SPMS and OIND. In contrast, no significant differences were detected in serum chitinase levels between groups. Chitinase CSF levels showed correlation with anti-MOG IL-13-producing cells, and eotaxin levels. In vitro experiments showed macrophage chitinase secretion was significantly increased by IL-13, but not by IL-5, IL-6, IL-12, or IFN-γ. Moreover, chitinases enhanced IL-8, RANTES, MCP-1, and eotaxin production, increasing migratory capacity in eosinophils, T cells, and macrophages across an in vitro BBB model.Chitinases increased in the CSF from patients with NMO in response to IL-13. These enhanced levels could contribute to central nervous system inflammation by increasing immune cell   migratio  n across the BBB. 
 Serum YKL-40 levels and chitotriosidase activity as potential biomarkers in primary prostate cancer and benign prostatic hyperplasia. YKL-40, also called human cartilage glycoprotein-39 (HC gp-39) and chitotriosidase are homologs of family 18 glycosyl hydrolases secreted by human macrophages. Although high levels of YKL-40 and chitotriosidase are associated with several diseases, the physiological functions of these enzymes are still unclear. YKL-40, a growth factor for connective tissue cells, a   migration   factor for endothelial and vascular smooth muscle cells, is expressed by several types of solid human carcinoma, including prostate carcinoma.The purpose of this study was to compare serum YKL-40 levels and chitotriosidase activity both in benign prostatic hyperplasia and primary prostate cancer.YKL-40 and chitotriosidase were determined in serum samples from 93 patients with primary prostate cancer and 61 patients with benign prostatic hyperplasia. Serum YKL-40 levels were measured by ELISA and chitotriosidase activity was determined by fluorometer. PSA levels were also measured by using an automated system.Serum YKL-40 levels were significantly higher (P < 0.001) in patients with prostate cancer compared with control group whereas there was no significant difference between BPH and control group. Serum chitotriosidase activities were significantly higher in carcinoma patients with high Gleason score than the control group (P < 0.001). No significant difference was observed in BPH patients (P > 0.05). Both YKL-40 and chitotriosidase were found statistically significant higher in primary prostate cancer and BPH.High serum YKL-40 levels in patients with primary prostate cancer indicate that YKL-40 may have a function in the progression of malignant diseases, whereas no significant elevation was observed in benign prostatic hyperplasia. Meanwhile, high serum chitotriosidase activity was observed only in patients with Gleason high grade, indicating possible macrophage involvement in cancer progression. Further studies are needed to elucidate the biologic role of YKL-40 in cancer aggressiveness and in progression of malignant diseases. 
  [Mm]etastasis  PubMed  
 Determination of chitinases family during osteoclastogenesis. Mammalian chitinases consisting of CHIA, CHIT1, CHI3L1, CHI3L2 and CHID1 exert important biological roles in the monocyte lineage and chronic inflammatory diseases. Pathological bone resorption is a cause of significant morbidity in diseases affecting the skeleton such as rheumatoid arthritis, osteoporosis, periodontitis and cancer   metastasis  . The biologic role of chitinases in bone resorption is poorly understood. In this study, we evaluated the expression of the chitinases family during osteoclast differentiation. The expression of CHIA, CHI3L2 and CHID1 resulted unchanged during osteoclast differentiation, whereas CHIT1 and CHI3L1 increased significantly. We also observed that CHIT1 and CHI3L1 are involved in osteoclast function. Indeed, silencing CHIT1 and CHI3L1 with siRNA resulted in a significant decrease in bone resorption activity. In addition, transfection with CHIT1 or CHI3L1 siRNA and co-transfection with both decreased the levels of the pro-differentiative marker MMP9. Overall, these discoveries reveal a novel and crucial role for both CHIT1 and CHI3L1 in promoting bone resorption and identifying new potential candidate markers for therapeutic targeting. 
   
  
 HES1 
 
  NCBI Gene  
  GeneRIF  
  Pubmed  
 
 
  Search Term  Rank  Fields  
  [Mm]igration  6  Process(1); PubMed(10)  
  [Ii]nvasive  10  PubMed(6); GeneRIFs(1)  
  [Mm]etastasis  12  PubMed(6); GeneRIFs(4)  
 
 
 hide/show details for HES1 
  [Mm]igration  Process  
 cell   migration   
  PubMed  
 Potential role of Notch1 signaling pathway in laryngeal squamous cell carcinoma cell line Hep-2 involving proliferation inhibition, cell cycle arrest, cell apoptosis, and cell   migration  . Head and neck cancer is the sixth most common cancer worldwide and laryngeal cancer represents the largest subgroup. However, the molecular mechanism underlying its malignant behavior and progression is not clarified. Accumulating evidence has shown that Notch1 signaling pathway plays a central role in carcinogenesis, but its potential role in regulating the development of laryngeal carcinoma, has not been characterized. Here, we identified that Notch1 signaling pathway was activated in laryngeal carcinoma accompanied with up-regulation of Notch1 and Hes1 expression. Overexpression of Notch1 in laryngeal carcinoma cell line Hep-2 led to suppression of tumor cellular proliferation and arrested cell cycle in the G0/G1 phase and induced cell apoptosis, which were coupled with the down-regulation of cyclin D1, cyclin E, cdk2 and bcl-2 and up-regulation of caspase-3, caspase-9 and p53. Most importantly, up-regulation of Notch1 expression also reduced the   migration   of Hep-2 cells, which was closely associated with down-regulation of MMP-2 and MMP-9. The finding may lay a foundation for further investigations into the Notch1 signaling pathway as a potential target for laryngeal carcinoma. 
 Reexpression of LSAMP inhibits tumor growth in a preclinical osteosarcoma model. Osteosarcomas are the most common primary malignant tumors of bone, showing complex chromosomal rearrangements with multiple gains and losses. A frequent deletion within the chromosomal region 3q13.31 has been identified by us and others, and is mainly reported to be present in osteosarcomas. The purpose of the study was to further characterize the frequency and the extent of the deletion in an extended panel of osteosarcoma samples, and the expression level of the affected genes within the region. We have identified LSAMP as the target gene for the deletion, and have studied the functional implications of LSAMP-reexpression.LSAMP copy number, expression level and protein level were investigated by quantitative PCR and western blotting in an osteosarcoma panel. The expression of LSAMP was restored in an osteosarcoma cell line, and differences in proliferation rate, tumor formation, gene expression,   migration   rate, differentiation capabilities, cell cycle distribution and apoptosis were investigated by metabolic dyes, tumor formation in vivo, gene expression profiling, time-lapse photography, differentiation techniques and flow cytometry, respectively.We found reduced copy number of LSAMP in 45/76 osteosarcoma samples, reduced expression level in 25/42 samples and protein expression in 9/42 samples. By restoring the expression of LSAMP in a cell line with a homozygous deletion of the gene, the proliferation rate in vitro was significantly reduced and tumor growth in vivo was significantly delayed. In response to reexpression of LSAMP, mRNA expression profiling revealed consistent upregulation of the genes hairy and enhancer of split 1 (HES1), cancer/testis antigen 2 (CTAG2) and kruppel-like factor 10 (KLF10).The high frequency and the specificity of the deletion indicate that it is important for the development of osteosarcomas. The deletion targets the tumor suppressor LSAMP, and based on the functional evidence, the tumor suppressor function of LSAMP is most likely exerted by reducing the proliferation rate of the tumor cells, possibly by indirectly upregulating one or more of the genes HES1, CTAG2 or KLF10. To our knowledge, this study describes novel functions of LSAMP, a first step to understanding the functional role of this specific deletion in osteosarcomas. 
 Reexpression of LSAMP inhibits tumor growth in a preclinical osteosarcoma model. Osteosarcomas are the most common primary malignant tumors of bone, showing complex chromosomal rearrangements with multiple gains and losses. A frequent deletion within the chromosomal region 3q13.31 has been identified by us and others, and is mainly reported to be present in osteosarcomas. The purpose of the study was to further characterize the frequency and the extent of the deletion in an extended panel of osteosarcoma samples, and the expression level of the affected genes within the region. We have identified LSAMP as the target gene for the deletion, and have studied the functional implications of LSAMP-reexpression.LSAMP copy number, expression level and protein level were investigated by quantitative PCR and western blotting in an osteosarcoma panel. The expression of LSAMP was restored in an osteosarcoma cell line, and differences in proliferation rate, tumor formation, gene expression,   migration   rate, differentiation capabilities, cell cycle distribution and apoptosis were investigated by metabolic dyes, tumor formation in vivo, gene expression profiling, time-lapse photography, differentiation techniques and flow cytometry, respectively.We found reduced copy number of LSAMP in 45/76 osteosarcoma samples, reduced expression level in 25/42 samples and protein expression in 9/42 samples. By restoring the expression of LSAMP in a cell line with a homozygous deletion of the gene, the proliferation rate in vitro was significantly reduced and tumor growth in vivo was significantly delayed. In response to reexpression of LSAMP, mRNA expression profiling revealed consistent upregulation of the genes hairy and enhancer of split 1 (HES1), cancer/testis antigen 2 (CTAG2) and kruppel-like factor 10 (KLF10).The high frequency and the specificity of the deletion indicate that it is important for the development of osteosarcomas. The deletion targets the tumor suppressor LSAMP, and based on the functional evidence, the tumor suppressor function of LSAMP is most likely exerted by reducing the proliferation rate of the tumor cells, possibly by indirectly upregulating one or more of the genes HES1, CTAG2 or KLF10. To our knowledge, this study describes novel functions of LSAMP, a first step to understanding the functional role of this specific deletion in osteosarcomas. 
 Potential role of Notch1 signaling pathway in laryngeal squamous cell carcinoma cell line Hep-2 involving proliferation inhibition, cell cycle arrest, cell apoptosis, and cell   migration  . Head and neck cancer is the sixth most common cancer worldwide and laryngeal cancer represents the largest subgroup. However, the molecular mechanism underlying its malignant behavior and progression is not clarified. Accumulating evidence has shown that Notch1 signaling pathway plays a central role in carcinogenesis, but its potential role in regulating the development of laryngeal carcinoma, has not been characterized. Here, we identified that Notch1 signaling pathway was activated in laryngeal carcinoma accompanied with up-regulation of Notch1 and Hes1 expression. Overexpression of Notch1 in laryngeal carcinoma cell line Hep-2 led to suppression of tumor cellular proliferation and arrested cell cycle in the G0/G1 phase and induced cell apoptosis, which were coupled with the down-regulation of cyclin D1, cyclin E, cdk2 and bcl-2 and up-regulation of caspase-3, caspase-9 and p53. Most importantly, up-regulation of Notch1 expression also reduced the   migration   of Hep-2 cells, which was closely associated with down-regulation of MMP-2 and MMP-9. The finding may lay a foundation for further investigations into the Notch1 signaling pathway as a potential target for laryngeal carcinoma. 
 Hes1 triggers epithelial-mesenchymal transition (EMT)-like cellular marker alterations and promotes invasion and metastasis of nasopharyngeal carcinoma by activating the PTEN/AKT pathway. Overexpression of the transcriptional factor Hes1 (hairy and enhancer of split-1) has been observed in numerous cancers, but the precise roles of Hes1 in epithelial-mesenchymal transition (EMT), cancer invasion and metastasis remain unknown. Our current study firstly revealed that Hes1 upregulation in a cohort of human nasopharyngeal carcinoma (NPC) biopsies is significantly associated with the EMT, invasive and metastatic phenotypes of cancer. In the present study, we found that Hes1 overexpression triggered EMT-like cellular marker alterations of NPC cells, whereas knockdown of Hes1 through shRNA reversed the EMT-like phenotypes, as strongly supported by Hes1-mediated EMT in NPC clinical specimens described above. Gain-of-function and loss-of-function experiments demonstrated that Hes1 promoted the   migration   and invasion of NPC cells in vitro. In addition, exogenous expression of Hes1 significantly enhanced the metastatic ability of NPC cells in vivo. Chromatin immunoprecipitation (ChIP) assays showed that Hes1 inhibited PTEN expression in NPC cells through binding to PTEN promoter region. Increased Hes1 expression and decreased PTEN expression were also observed in a cohort of NPC biopsies. Additional studies demonstrated that Hes1-induced EMT-like molecular changes and increased motility and invasion of NPC cells were mediated by PTEN. Taken together, our results suggest, for what we believe is the first time, that Hes1 plays an important role in the invasion and metastasis of NPC through inhibiting PTEN expression to trigger EMT-like phenotypes. 
 Hes1 promotes cell proliferation and   migration   by activating Bmi-1 and PTEN/Akt/GSK3β pathway in human colon cancer. Hes1 is a transcription factor that influences cell proliferation and differentiation. However, the effect of Hes1 on invasiveness and the underlying mechanism remain unknown. In the current study, we found that Hes1 suppressed cell apoptosis, promoted cell growth, induced EMT phenotype and cytoskeleton reconstruction, and enhanced the metastatic potential of colon cancer cells in vitro and in vivo. Furthermore, we indicated that Bmi-1 mediated Hes1-induced cell proliferation and   migratio  n, downregulated PTEN and activated the Akt/GSK3β pathway, consequently induced EMT and cytoskeleton reconstruction, ultimately leading to enhanced invasiveness of cancer cells. In addition, we also found that both Hes1 and Bmi-1 could directly regulate PTEN by associating at the PTEN locus, and played important roles in regulating PTEN/Akt/GSK3β pathway. Our results provide functional and mechanistic links between Hes1 and Bmi-1/PTEN/Akt/GSK3β signaling in the development and progression of colon cancer. 
 HES1 promotes metastasis and predicts poor survival in patients with colorectal cancer. Hairy enhancer of split-1 (HES1) is a transcriptional target of the Notch pathway, and a high level of HES1 is regarded as a marker of activated Notch. The aim of the study was to investigate the role of HES1 in colorectal cancer progression. We used tissue microarrays to analyze the expression and clinical significance of HES1 in 320 colorectal cancer samples. Stable overexpression and knockdown of HES1 were established in three colorectal cancer cell (CRC) lines (RKO, HCT8 and LOVO). We investigated the differentially expressed genes and enriched pathways in HES1 overexpressing CRC cells by gene expression profiling. Also, the role of HES1 in invasion and   migration   were examined in vitro and in vivo. We found that high expression of HES1 was significantly correlated with distal metastasis (P = 0.037) at diagnosis, and HES1 could serve as an unfavorable prognostic factor for colorectal cancer patients (P = 0.034). Gene expression profiling and pathway enrichment analysis revealed that HES1 was related to cellular adherens junction loss. In addition, we showed that HES1 overexpression lead to depressed E-cadherin, and elevated N-cadherin, vimentin and Twist-1 levels. Functionally, HES1 enhanced invasiveness and metastasis of CRC cells. HES1 promotes cancer metastasis via inducing epithelial mesenchymal transition and serves as a poor prognosis factor of colorectal cancer patients. 
 Prognostic significance of Hes-1, a downstream target of notch signaling in hepatocellular carcinoma. Hairy and enhancer of split 1 (Hes-1) protein is a downstream target of Notch signaling and is a basic helix-loop-helix transcriptional repressor. However, definitive evidence for a role in hepatocellular carcinoma (HCC) cells has not been reported. Here, Hes-1 was revealed to an important component of the Notch signaling cascade in HCC cell lines possessing different potential for lung metastasis.RNAi mediated by plasmid constructs was used to analyze the role of Hes-1 in MHCC-97L HCC cells by assessing proliferation, apoptosis, cell   migration   and matrigel invasion following transfection. Hes-1 protein expression analysis in HCC tissue was also conducted by immunohistochemistry.Our studies revealed that Hes-1 was decreased in HCC cell lines with higher lung metastasis potential at both the mRNA and protein levels. Down-regulation of the Hes-1 gene in MHCC-97L cells resulted in increased cell proliferation, reduced apoptosis and increased   migration   and invasion.Hes-1 has potential prognostic value in post-surgical HCC patients and may be an independent prognostic indicator for overall survival and tumor recurrence. These findings have important implications for understanding the mechanisms by which Hes-1 participates in tumor proliferation and invasion. 
 Numb/Notch signaling pathway modulation enhances human pancreatic cancer cell radiosensitivity. The present study aims to evaluate whether repression of the Numb/Notch signaling pathway affects the radiosensitivity of human pancreatic cancer cell lines. Different doses of X-rays (0, 2, 3, 4, and 5 Gy) were applied to the PANC-1, SW1990, and MIA PaCa-2 human pancreatic cancer cell lines, and the Numb/Notch pathway inhibitor DAPT was added at different doses (0, 1, 3, and 5 μmol/l). MTT assay, colony formation assay, flow cytometry, scratch assay, and Transwell experiments were performed, and qRT-PCR and Western blot were conducted for the detection of Numb expression. Tumorigenicity assay in nude mice was carried out to verify the influence of blocker of the Numb/Notch signaling pathway on the radiosensitivity of xenograft tumors. The MTT assay, colony formation assay and flow cytometry experiments revealed that proliferation decreased as radiation dose increased. The viability of PANC-1 cells at 5 Gy, SW 1990 cells at 4 Gy and 5 Gy, and MIA PaCa-2 cells at 2-5 Gy was significantly lower than that of non-irradiated cells (all P < 0.  05). The   migration and invasion assays indicated that the PANC-1 cell line was least radiosensitive, while the MIA PaCa-2 cell line was the most radiosensitive. Numb expression significantly increased with increasing radiation dose, whereas the expression of Hes1, Notch1, and Hes5 significantly decreased compared to non-irradiated cells (P < 0.05). Compared to untreated control cells, DAPT dose dependently increased Numb expression and inhibited Notch1, Hes1, and Hes5 expressions at 2 Gy (P < 0.05). Subcutaneous tumorigenicity assay in nude mice demonstrated that DAPT increased the radiosensitivity of PANC-1, SW 1990, and MIA PaCa-2 cells. These findings suggest that Numb/Notch signaling in pancreatic cancer cells is associated with X-ray radiation and that inhibition of the Numb/Notch signaling pathway can enhance radiosensitivity, suggesting that inhibition of the Numb/Notch signaling pathway may serve as a potential target for clinical improvement of the radiosensitivity of pancreatic cancer. 
 Involvement of Numb-mediated HIF-1α inhibition in anti-proliferative effect of PNA-antimiR-182 in trastuzumab-sensitive and -resistant SKBR3 cells. Trastuzumab is a humanized monoclonal antibody against the human epidermal growth factor receptor 2 (HER2) that is overexpressed in about 25 % of breast cancer patients. However, primary and/or acquired resistance to trastuzumab develops in most affected persons. In this study, we explored the functional role of miR-182 inhibition with aiming the sensitization of SKBR3 cells to trastuzumab. Cell viability, apoptosis, colony formation, an  d migrati  on capacities of SKBR3(S) (sensitive) and SKBR3(R) (resistant) cells were assessed to determine the anti-proliferative effects of PNA-antimiR-182. In addition, the expression levels of miR-182, mRNA of FOXO1, and Bim as well as the protein levels of HER2 and Notch1 signaling factors were evaluated by stem-loop RT-qPCR, RT-qPCR, and Western blot, respectively. The results indicated that miR-182 might play a causal role in the mechanism of trastuzumab. In line with that, PNA-antimiR-182 inhibited synergistically the viability of both the sensitive and resistant cell groups. Furthermore, the inhibitory effect of PNA-anitmiR-182 o  n migrati  on in SKBR3 cells was more than the induction of apoptosis. In addition, PNA-antimiR-182 reduced the levels of NICD, Hes1, HIF-1α, and p-Akt in both cell groups, while it augmented the intracellular content of FOXO1 and Numb suppressor proteins. In other words, PNA-antimiR-182-mediated upregulation of Numb was associated with downregulation of HIF-1α and Hes1. Consequently, downregulation of miR-182 might find therapeutical value for overcoming trastuzumab resistance. Graphical Abstract The crosstalk between HER2 and Notch1 signaling pathway is mediated by miR-182. 
  [Ii]nvasive  PubMed  
 Hes1 triggers epithelial-mesenchymal transition (EMT)-like cellular marker alterations and promotes invasion and metastasis of nasopharyngeal carcinoma by activating the PTEN/AKT pathway. Overexpression of the transcriptional factor Hes1 (hairy and enhancer of split-1) has been observed in numerous cancers, but the precise roles of Hes1 in epithelial-mesenchymal transition (EMT), cancer invasion and metastasis remain unknown. Our current study firstly revealed that Hes1 upregulation in a cohort of human nasopharyngeal carcinoma (NPC) biopsies is significantly associated with the EMT,   invasive   and metastatic phenotypes of cancer. In the present study, we found that Hes1 overexpression triggered EMT-like cellular marker alterations of NPC cells, whereas knockdown of Hes1 through shRNA reversed the EMT-like phenotypes, as strongly supported by Hes1-mediated EMT in NPC clinical specimens described above. Gain-of-function and loss-of-function experiments demonstrated that Hes1 promoted the migration and invasion of NPC cells in vitro. In addition, exogenous expression of Hes1 significantly enhanced the metastatic ability of NPC cells in vivo. Chromatin immunoprecipitation (ChIP) assays showed that Hes1 inhibited PTEN expression in NPC cells through binding to PTEN promoter region. Increased Hes1 expression and decreased PTEN expression were also observed in a cohort of NPC biopsies. Additional studies demonstrated that Hes1-induced EMT-like molecular changes and increased motility and invasion of NPC cells were mediated by PTEN. Taken together, our results suggest, for what we believe is the first time, that Hes1 plays an important role in the invasion and metastasis of NPC through inhibiting PTEN expression to trigger EMT-like phenotypes. 
 Hes1 promotes cell proliferation and migration by activating Bmi-1 and PTEN/Akt/GSK3β pathway in human colon cancer. Hes1 is a transcription factor that influences cell proliferation and differentiation. However, the effect of Hes1 on   invasiv  eness and the underlying mechanism remain unknown. In the current study, we found that Hes1 suppressed cell apoptosis, promoted cell growth, induced EMT phenotype and cytoskeleton reconstruction, and enhanced the metastatic potential of colon cancer cells in vitro and in vivo. Furthermore, we indicated that Bmi-1 mediated Hes1-induced cell proliferation and migration, downregulated PTEN and activated the Akt/GSK3β pathway, consequently induced EMT and cytoskeleton reconstruction, ultimately leading to enhance  d invasi  veness of cancer cells. In addition, we also found that both Hes1 and Bmi-1 could directly regulate PTEN by associating at the PTEN locus, and played important roles in regulating PTEN/Akt/GSK3β pathway. Our results provide functional and mechanistic links between Hes1 and Bmi-1/PTEN/Akt/GSK3β signaling in the development and progression of colon cancer. 
 HES1 promotes metastasis and predicts poor survival in patients with colorectal cancer. Hairy enhancer of split-1 (HES1) is a transcriptional target of the Notch pathway, and a high level of HES1 is regarded as a marker of activated Notch. The aim of the study was to investigate the role of HES1 in colorectal cancer progression. We used tissue microarrays to analyze the expression and clinical significance of HES1 in 320 colorectal cancer samples. Stable overexpression and knockdown of HES1 were established in three colorectal cancer cell (CRC) lines (RKO, HCT8 and LOVO). We investigated the differentially expressed genes and enriched pathways in HES1 overexpressing CRC cells by gene expression profiling. Also, the role of HES1 in invasion and migration were examined in vitro and in vivo. We found that high expression of HES1 was significantly correlated with distal metastasis (P = 0.037) at diagnosis, and HES1 could serve as an unfavorable prognostic factor for colorectal cancer patients (P = 0.034). Gene expression profiling and pathway enrichment analysis revealed that HES1 was related to cellular adherens junction loss. In addition, we showed that HES1 overexpression lead to depressed E-cadherin, and elevated N-cadherin, vimentin and Twist-1 levels. Functionally, HES1 enhanced   invasive  ness and metastasis of CRC cells. HES1 promotes cancer metastasis via inducing epithelial mesenchymal transition and serves as a poor prognosis factor of colorectal cancer patients. 
 Significance of Notch1-signaling pathway in human pancreatic development and carcinogenesis. In animal studies, Notch1-signaling pathway plays an important role in the pancreatic embryogenesis by promoting pancreatic progenitor cells self-renewal and exocrine linage development. The persistent activation of Notch pathway could arrest the organ development and keep cells at an undifferentiated stage. Studies have shown that Notch1-signaling pathway is upregulated in   invasive   pancreatic ductal adenocarcinoma (PDAC). Here we examined the expression pattern of Notch1 and Hes1 in human fetal pancreatic tissues to elucidate the role of Notch1 in human pancreatic embryonic development. We also compared Notch1 expression in tissues from PDAC, chronic pancreatitis and pancreatic intraepithelial neoplasm. Our data show that Notch1/Hes1-signaling pathway is activated during early pancreatic embryogenesis and reaches the highest at birth. After pancreas is fully developed, Notch1/Hes1 pathway is inactivated even though Notch1 protein cell-surface expression is upregulated. We also showed that the expression of both Notch1 and Hes1 are present in 50% (33/66) of PDACs, but not in pancreatic intraepithelial neoplasms. These findings indicate that Notch1 activation is only apparent in late stage of pancreatic carcinogenesis, suggesting that treatment with Notch-signaling inhibitors including γ-secretase should be selectively used for PDACs with confirmed Notch1-signaling activation. 
 Prostate tumor OVerexpressed-1 (PTOV1) down-regulates HES1 and HEY1 notch targets genes and promotes prostate cancer progression. PTOV1 is an adaptor protein with functions in diverse processes, including gene transcription and protein translation, whose overexpression is associated with a higher proliferation index and tumor grade in prostate cancer (PC) and other neoplasms. Here we report its interaction with the Notch pathway and its involvement in PC progression.Stable PTOV1 knockdown or overexpression were performed by lentiviral transduction. Protein interactions were analyzed by co-immunoprecipitation, pull-down and/or immunofluorescence. Endogenous gene expression was analyzed by real time RT-PCR and/or Western blotting. Exogenous promoter activities were studied by luciferase assays. Gene promoter interactions were analyzed by chromatin immunoprecipitation assays (ChIP). In vivo studies were performed in the Drosophila melanogaster wing, the SCID-Beige mouse model, and human prostate cancer tissues and metastasis. The Excel package was used for statistical analysis.Knockdown of PTOV1 in prostate epithelial cells and HaCaT skin keratinocytes caused the upregulation, and overexpression of PTOV1 the downregulation, of the Notch target genes HEY1 and HES1, suggesting that PTOV1 counteracts Notch signaling. Under conditions of inactive Notch signaling, endogenous PTOV1 associated with the HEY1 and HES1 promoters, together with components of the Notch repressor complex. Conversely, expression of active Notch1 provoked the dismissal of PTOV1 from these promoters. The antagonist role of PTOV1 on Notch activity was corroborated in the Drosophila melanogaster wing, where human PTOV1 exacerbated Notch deletion mutant phenotypes and suppressed the effects of constitutively active Notch. PTOV1 was required for optimal in vitro   invasive  ness and anchorage-independent growth of PC-3 cells, activities counteracted by Notch, and for their efficient growth and metastatic spread in vivo. In prostate tumors, the overexpression of PTOV1 was associated with decreased expression of HEY1 and HES1, and this correlation was significant in metastatic lesions.High levels of the adaptor protein PTOV1 counteract the transcriptional activity of Notch. Our evidences link the pro-oncogenic and pro-metastatic effects of PTOV1 in prostate cancer to its inhibitory activity on Notch signaling and are supportive of a tumor suppressor role of Notch in prostate cancer progression. 
 HES1 as an independent prognostic marker in esophageal squamous cell carcinoma. Notch signaling is one of the main involved pathways in cell differentiation and organogenesis, and its deregulation may lead to tumorigenesis. In this pathway, targeted to the CSL (CBF1, Suppressor of Hairless or Lag-1) complex, notch intracellular domain (NICD) releases corepressors and recruits MAML1 as coactivator triggering the activation of notch signaling transcription complex. Hairy enhance of split-1 (HES1) is one of the notch signaling target genes which is a basic helix-loop-helix (bHLH) transcription factor acting as a proliferation stimulator through the suppression of cell cycle inhibitors such as p27 and p21.In this study, we aimed to analyze the role of HES1 in the progression of esophageal squamous cell carcinoma (ESCC).Messenger RNA (mRNA) expression of HES1 in fresh tumoral tissues and their margin normal samples were assessed in 50 ESCC patients by real-time polymerase chain reaction (RT-PCR).Thirteen out of 50 cases (26 %) had HES1 underexpression, while HES1 overexpression was observed only in 4 (8 %) samples. HES1 underexpression was significantly correlated with tumor depth of invasion (P = 0.035).Although we have not observed any significant correlation between the HES1 expression and notch activation in ESCC, this study is the first report that elucidated the HES1 underexpression in ESCC and revealed its correlation wit  h the in  vasiveness of ESCC. 
  GeneRIFs  
 This study is the first report that elucidated the HES1 underexpression in ESCC and revealed its correlation with the   invasive  ness of ESCC. 
  [Mm]etastasis  PubMed  
 Hes1 triggers epithelial-mesenchymal transition (EMT)-like cellular marker alterations and promotes invasion and   metastasis   of nasopharyngeal carcinoma by activating the PTEN/AKT pathway. Overexpression of the transcriptional factor Hes1 (hairy and enhancer of split-1) has been observed in numerous cancers, but the precise roles of Hes1 in epithelial-mesenchymal transition (EMT), cancer invasion and   metastasis   remain unknown. Our current study firstly revealed that Hes1 upregulation in a cohort of human nasopharyngeal carcinoma (NPC) biopsies is significantly associated with the EMT, invasive and metastatic phenotypes of cancer. In the present study, we found that Hes1 overexpression triggered EMT-like cellular marker alterations of NPC cells, whereas knockdown of Hes1 through shRNA reversed the EMT-like phenotypes, as strongly supported by Hes1-mediated EMT in NPC clinical specimens described above. Gain-of-function and loss-of-function experiments demonstrated that Hes1 promoted the migration and invasion of NPC cells in vitro. In addition, exogenous expression of Hes1 significantly enhanced the metastatic ability of NPC cells in vivo. Chromatin immunoprecipitation (ChIP) assays showed that Hes1 inhibited PTEN expression in NPC cells through binding to PTEN promoter region. Increased Hes1 expression and decreased PTEN expression were also observed in a cohort of NPC biopsies. Additional studies demonstrated that Hes1-induced EMT-like molecular changes and increased motility and invasion of NPC cells were mediated by PTEN. Taken together, our results suggest, for what we believe is the first time, that Hes1 plays an important role in the invasion and   metastasis   of NPC through inhibiting PTEN expression to trigger EMT-like phenotypes. 
 HES1 promotes   metastasis   and predicts poor survival in patients with colorectal cancer. Hairy enhancer of split-1 (HES1) is a transcriptional target of the Notch pathway, and a high level of HES1 is regarded as a marker of activated Notch. The aim of the study was to investigate the role of HES1 in colorectal cancer progression. We used tissue microarrays to analyze the expression and clinical significance of HES1 in 320 colorectal cancer samples. Stable overexpression and knockdown of HES1 were established in three colorectal cancer cell (CRC) lines (RKO, HCT8 and LOVO). We investigated the differentially expressed genes and enriched pathways in HES1 overexpressing CRC cells by gene expression profiling. Also, the role of HES1 in invasion and migration were examined in vitro and in vivo. We found that high expression of HES1 was significantly correlated with distal   metastasis   (P = 0.037) at diagnosis, and HES1 could serve as an unfavorable prognostic factor for colorectal cancer patients (P = 0.034). Gene expression profiling and pathway enrichment analysis revealed that HES1 was related to cellular adherens junction loss. In addition, we showed that HES1 overexpression lead to depressed E-cadherin, and elevated N-cadherin, vimentin and Twist-1 levels. Functionally, HES1 enhanced invasiveness and   metastasis   of CRC cells. HES1 promotes cancer   metastasis   via inducing epithelial mesenchymal transition and serves as a poor prognosis factor of colorectal cancer patients. 
 Prognostic significance of Hes-1, a downstream target of notch signaling in hepatocellular carcinoma. Hairy and enhancer of split 1 (Hes-1) protein is a downstream target of Notch signaling and is a basic helix-loop-helix transcriptional repressor. However, definitive evidence for a role in hepatocellular carcinoma (HCC) cells has not been reported. Here, Hes-1 was revealed to an important component of the Notch signaling cascade in HCC cell lines possessing different potential for lung   metastasis  .RNAi mediated by plasmid constructs was used to analyze the role of Hes-1 in MHCC-97L HCC cells by assessing proliferation, apoptosis, cell migration and matrigel invasion following transfection. Hes-1 protein expression analysis in HCC tissue was also conducted by immunohistochemistry.Our studies revealed that Hes-1 was decreased in HCC cell lines with higher lung   metastasis   potential at both the mRNA and protein levels. Down-regulation of the Hes-1 gene in MHCC-97L cells resulted in increased cell proliferation, reduced apoptosis and increased migration and invasion.Hes-1 has potential prognostic value in post-surgical HCC patients and may be an independent prognostic indicator for overall survival and tumor recurrence. These findings have important implications for understanding the mechanisms by which Hes-1 participates in tumor proliferation and invasion. 
 Prostate tumor OVerexpressed-1 (PTOV1) down-regulates HES1 and HEY1 notch targets genes and promotes prostate cancer progression. PTOV1 is an adaptor protein with functions in diverse processes, including gene transcription and protein translation, whose overexpression is associated with a higher proliferation index and tumor grade in prostate cancer (PC) and other neoplasms. Here we report its interaction with the Notch pathway and its involvement in PC progression.Stable PTOV1 knockdown or overexpression were performed by lentiviral transduction. Protein interactions were analyzed by co-immunoprecipitation, pull-down and/or immunofluorescence. Endogenous gene expression was analyzed by real time RT-PCR and/or Western blotting. Exogenous promoter activities were studied by luciferase assays. Gene promoter interactions were analyzed by chromatin immunoprecipitation assays (ChIP). In vivo studies were performed in the Drosophila melanogaster wing, the SCID-Beige mouse model, and human prostate cancer tissues and   metastasis  . The Excel package was used for statistical analysis.Knockdown of PTOV1 in prostate epithelial cells and HaCaT skin keratinocytes caused the upregulation, and overexpression of PTOV1 the downregulation, of the Notch target genes HEY1 and HES1, suggesting that PTOV1 counteracts Notch signaling. Under conditions of inactive Notch signaling, endogenous PTOV1 associated with the HEY1 and HES1 promoters, together with components of the Notch repressor complex. Conversely, expression of active Notch1 provoked the dismissal of PTOV1 from these promoters. The antagonist role of PTOV1 on Notch activity was corroborated in the Drosophila melanogaster wing, where human PTOV1 exacerbated Notch deletion mutant phenotypes and suppressed the effects of constitutively active Notch. PTOV1 was required for optimal in vitro invasiveness and anchorage-independent growth of PC-3 cells, activities counteracted by Notch, and for their efficient growth and metastatic spread in vivo. In prostate tumors, the overexpression of PTOV1 was associated with decreased expression of HEY1 and HES1, and this correlation was significant in metastatic lesions.High levels of the adaptor protein PTOV1 counteract the transcriptional activity of Notch. Our evidences link the pro-oncogenic and pro-metastatic effects of PTOV1 in prostate cancer to its inhibitory activity on Notch signaling and are supportive of a tumor suppressor role of Notch in prostate cancer progression. 
 Hes1: a key role in stemness,   metastasis   and multidrug resistance. Hes1 is one mammalian counterpart of the Hairy and Enhancer of split proteins that play a critical role in many physiological processes including cellular differentiation, cell cycle arrest, apoptosis and self-renewal ability. Recent studies have shown that Hes1 functions in the maintenance of cancer stem cells (CSCs),   metastasis   and antagonizing drug-induced apoptosis. Pathways that are involved in the up-regulation of Hes1 level canonically or non-canonically, such as the Hedgehog, Wnt and hypoxia pathways are frequently aberrant in cancer cells. Here, we summarize the recent data supporting the idea that Hes1 may have an important function in the maintenance of cancer stem cells self-renewal, cancer   metastasis  , and epithelial-mesenchymal transition (EMT) process induction, as well as chemotherapy resistance, and conclude with the possible mechanisms by which Hes1 functions have their effect, as well as their crosstalk with other carcinogenic signaling pathways. 
 Notch-induced transcription factors are predictive of survival and 5-fluorouracil response in colorectal cancer patients. The purpose of this study was to evaluate the expression of Notch-induced transcription factors (NTFs) HEY1, HES1 and SOX9 in colorectal cancer (CRC) patients to determine their clinicopathologic and prognostic significance.Levels of HEY1, HES1 and SOX9 protein were measured by immunohistochemistry in a nonmalignant and malignant tissue microarray of 441 CRC patients, and the findings correlated with pathologic, molecular and clinical variables.The NTFs HEY1, HES1 and SOX9 were overexpressed in tumours relative to colonic mucosa (OR=3.44, P<0.0001; OR=7.40, P<0.0001; OR=4.08 P<0.0001, respectively). HEY1 overexpression was a negative prognostic factor for all CRC patients (HR=1.29, P=0.023) and strongly correlated with perineural and vascular invasion and lymph node (LN)   metastasis  . In 5-fluorouracil (5-FU)-treated patients, the tumour overexpression of SOX9 correlated with markedly poorer survival (HR=8.72, P=0.034), but had no predictive effect in untreated patients (HR=0.70, P=0.29). When HEY1, HES1 and SOX9 expression were combined to predict survival with chemotherapy, in treated patients there was an additive increase in the risk of death with each NTF overexpressed (HR=2.09, P=0.01), but no prognostic import in the untreated patient group (HR=0.74, P=0.19).The present study is the first to discover that HEY1 overexpression correlates with poorer outcome in CRC, and NTF expression is predictive of CRC patient survival with 5-FU chemotherapy. If confirmed in future studies, testing of NTF expression has the potential to enter routine pathological practice for the selection of patients to undergo chemotherapy alone or in combination with Notch inhibitors. 
  GeneRIFs  
 Results suggest that Hes1 plays an important role in the invasion and   metastasis   of NPC through inhibiting PTEN expression to trigger EMT-like phenotypes. 
 Decreased HES1 expression is associated with higher lung   metastasis   in hepatocellular carcinoma 
 we summarize the recent data supporting the idea that Hes1 may have an important function in the maintenance of cancer stem cells self-renewal, cancer   metastasis  , and epithelial-mesenchymal transition (EMT) process induction 
 HES1 has a role in   metastasis   and predicts poor survival in patients with colorectal cancer 
   
  
 BHLHE41 
 
  NCBI Gene  
  GeneRIF  
  Pubmed  
 
 
  Search Term  Rank  Fields  
  [Mm]igration  6  PubMed(3); GeneRIFs(1)  
  [Ii]nvasive  10  PubMed(3)  
  [Mm]etastasis  12  PubMed(4)  
  [Cc]ell [Ii]nvasion  13  PubMed(1)  
 
 
 hide/show details for BHLHE41 
  [Mm]igration  PubMed  
 SHARP1 suppresses breast cancer metastasis by promoting degradation of hypoxia-inducible factors. The molecular determinants of malignant cell behaviours in breast cancer remain only partially understood. Here we show that SHARP1 (also known as BHLHE41 or DEC2) is a crucial regulator of the invasive and metastatic phenotype in triple-negative breast cancer (TNBC), one of the most aggressive types of breast cancer. SHARP1 is regulated by the p63 metastasis suppressor and inhibits TNBC aggressiveness through inhibition of hypoxia-inducible factor 1α (HIF-1α) and HIF-2α (HIFs). SHARP1 opposes HIF-dependent TNBC ce  ll migrat  ion in vitro, and invasive or metastatic behaviours in vivo. SHARP1 is required, and sufficient, to limit expression of HIF-target genes. In primary TNBC, endogenous SHARP1 levels are inversely correlated with those of HIF targets. Mechanistically, SHARP1 binds to HIFs and promotes HIF proteasomal degradation by serving as the HIF-presenting factor to the proteasome. This process is independent of pVHL (von Hippel-Lindau tumour suppressor), hypoxia and the ubiquitination machinery. SHARP1 therefore determines the intrinsic instability of HIF proteins to act in parallel to, and cooperate with, oxygen levels. This work sheds light on the mechanisms and pathways by which TNBC acquires invasiveness and metastatic propensity. 
 Roles of SHARP1 in thyroid cancer. SHARP1 is a basic helix‑loop‑helix transcription factor involved in various cellular processes, including proliferation and differentiation. The present study assessed the role of SHARP1 in the progression and invasion of thyroid cancer. PCR and western blot analysis demonstrated that in thyroid cancer tissues, SHARP1 was significantly downregulated at the mRNA and protein level compared with that in normal tissues. Furthermore, SHARP1 was downregulated in the TT and TPC‑1 thyroid cancer cell lines compared with a normal thyroid cell line, while it was upregulated in other thyroid cancer cell lines. Overexpression of SHARP1 in TT and TPC‑1 cells significantly inhibited the cell via  bility, m  igration and invasion in vitro. Furthermore, the protein and mRNA levels of HIF‑1α were found to be decreased in TT and TPC‑1 cells following forced overexpression of SHARP1. In addition, silencing of HIF‑1α reduce  d the via  bility, migration and invasion of TT and TPC-1 cells. In conclusion, the present study indicated that SHARP1 acts as a tumor suppressor in thyroid cancer and that its downregulation may contribute to th  e prolife  ration, migration and invasion of thyroid cancer cells through mechanisms possibly involving HIF‑1α, suggesting that SHARP1 may be an important therapeutic target for the treatment of thyroid cancer. 
 Suppression of the epithelial-mesenchymal transition by SHARP1 is linked to the NOTCH1 signaling pathway in metastasis of endometrial cancer. Mechanisms governing the metastasis of endometrial cancer (EC) are poorly defined. Recent data support a role for Enhancer-of-split and hairy-related protein 1 (SHARP1), a basic helix-loop-helix transcription repressor, in regulating invasiveness and angiogenesis of several human cancers. However, the role of SHARP1 in metastasis of EC remains unclear.Human EC cell lines (Ishikawa and HEC-1B) were used. SHARP1 was upregulated by lentivirus transduction, while intracellular domain of NOTCH1 (ICN) were upregulated by transient transfection with plasmids. Effects of SHARP1 on cell   migration   and invasion were evaluated by wound healing assay and transwell invasion assay. Experimental metastasis assay were performed in nude mice. Effects of SHAPR1 on protein levels of target genes were detected by western blotting. Furthermore, the association between SHARP1 and the NOTCH1/EMT pathway was further verified in EC tissue specimens by immunohistochemical analysis.Overexpression of SHARP1 in EC cells inhibited cell   migration  , invasion, and metastasis. Exogenous SHARP1 overexpression affected the proteins levels of genes involved in EMT process and NOTCH1 signaling pathway. Upregulation of ICN in SHARP1-overexpressing Ishikawa cells induced cell   migration   and an EMT phenotype. Additionally, immunohistochemical analysis demonstrated that SHARP1 protein levels were lower in metastatic EC than in primary tumors, and statistical analysis revealed correlations between levels of SHARP1 and markers of EMT and NOTCH1 signaling pathway in human EC tissue specimen.This work supports a role for SHARP1 in suppressing EMT and metastasis in EC by attenuating NOTCH1 signaling. Therefore, SHARP1 may be a novel marker for lymphatic metastasis in EC patients. 
  GeneRIFs  
 the present study indicated that SHARP1 acts as a tumor suppressor in thyroid cancer and that its downregulation may contribute to the proliferation,   migration   and invasion of thyroid cancer cells through mechanisms possibly involving HIF1alpha 
  [Ii]nvasive  PubMed  
 DEC2 expression is positively correlated with HIF-1 activation and the   invasive  ness of human osteosarcomas. Osteosarcoma is the most common malignancy of bone. HIF-1 (hypoxia-inducible factor 1) activation is critical for the metabolic reprogramming and progression of solid tumors, and DEC2 (differentiated embryonic chondrocyte gene 2) has been recently reported to suppress HIF-1 in human breast and endometrial cancers. However, the roles of HIF-1 and DEC2 in human osteosarcomas remain unclear.We evaluated the correlation of DEC2 and HIF-1 expression to the prognosis, and studied the roles of DEC2 and HIF-1 activation in the   invasive  ness of osteosarcoma. Multiple approaches including immunohistochemical staining of clinical osteosarcoma tissues, siRNA-based knockdown and other molecular biology techniques were used. Particularly, by using a repetitive trans-well culture-based in vitro evolution system, we selected a more   invasive   subpopulation (U2OS-M) of osteosarcoma cells from U2OS and used it as a model to study the roles of DEC2 and HIF-1 in the   invasive  ness of osteosarcoma.We found that the expression of DEC2 was positively correlated with HIF-1α levels, and HIF-1α expression positively correlated with poor prognosis in osteosarcomas. DEC2 knockdown in osteosarcoma cell lines (U2OS, MNNG and 143B) attenuated HIF-1α accumulation and impaired the up-regulation of HIF-1 target genes in response to hypoxia. Compared with the l  ow invas  ive parental U2OS, U2OS-M showed higher levels of DEC2 expression which were confirmed at both mRNA and protein levels. Importantly, we found that the increased DEC2 expression resulted in a more rapid accumulation of HIF-1α in U2OS-M cells in response to hypoxia. Finally, we found that HIF-1 activation is sufficient to upregulate DEC2 expression in osteosarcoma cells.Taken together, whereas DEC2 was found to promote HIF-1α degradation in other types of tumors, our data indicate that DEC2 facilitates HIF-1α stabilization and promotes HIF-1 activation in osteosarcoma. This implies that DEC2 may contribute to the progression and metastasis of human osteosarcoma by sensitizing tumor cells to hypoxia. On the other hand, HIF-1 activation may contribute to the expression of DEC2 in osteosarcoma. This is the first demonstration of a novel DEC2-HIF-1 vicious cycle in osteosarcoma and a tumor-type specific role for DEC2. 
 SHARP1 suppresses breast cancer metastasis by promoting degradation of hypoxia-inducible factors. The molecular determinants of malignant cell behaviours in breast cancer remain only partially understood. Here we show that SHARP1 (also known as BHLHE41 or DEC2) is a crucial regulator of the   invasive   and metastatic phenotype in triple-negative breast cancer (TNBC), one of the most aggressive types of breast cancer. SHARP1 is regulated by the p63 metastasis suppressor and inhibits TNBC aggressiveness through inhibition of hypoxia-inducible factor 1α (HIF-1α) and HIF-2α (HIFs). SHARP1 opposes HIF-dependent TNBC cell migration in vitro, a  nd invas  ive or metastatic behaviours in vivo. SHARP1 is required, and sufficient, to limit expression of HIF-target genes. In primary TNBC, endogenous SHARP1 levels are inversely correlated with those of HIF targets. Mechanistically, SHARP1 binds to HIFs and promotes HIF proteasomal degradation by serving as the HIF-presenting factor to the proteasome. This process is independent of pVHL (von Hippel-Lindau tumour suppressor), hypoxia and the ubiquitination machinery. SHARP1 therefore determines the intrinsic instability of HIF proteins to act in parallel to, and cooperate with, oxygen levels. This work sheds light on the mechanisms and pathways by which TNBC acquir  es invas  iveness and metastatic propensity. 
 Suppression of the epithelial-mesenchymal transition by SHARP1 is linked to the NOTCH1 signaling pathway in metastasis of endometrial cancer. Mechanisms governing the metastasis of endometrial cancer (EC) are poorly defined. Recent data support a role for Enhancer-of-split and hairy-related protein 1 (SHARP1), a basic helix-loop-helix transcription repressor, in regulating   invasive  ness and angiogenesis of several human cancers. However, the role of SHARP1 in metastasis of EC remains unclear.Human EC cell lines (Ishikawa and HEC-1B) were used. SHARP1 was upregulated by lentivirus transduction, while intracellular domain of NOTCH1 (ICN) were upregulated by transient transfection with plasmids. Effects of SHARP1 on cell migration and invasion were evaluated by wound healing assay and transwell invasion assay. Experimental metastasis assay were performed in nude mice. Effects of SHAPR1 on protein levels of target genes were detected by western blotting. Furthermore, the association between SHARP1 and the NOTCH1/EMT pathway was further verified in EC tissue specimens by immunohistochemical analysis.Overexpression of SHARP1 in EC cells inhibited cell migration, invasion, and metastasis. Exogenous SHARP1 overexpression affected the proteins levels of genes involved in EMT process and NOTCH1 signaling pathway. Upregulation of ICN in SHARP1-overexpressing Ishikawa cells induced cell migration and an EMT phenotype. Additionally, immunohistochemical analysis demonstrated that SHARP1 protein levels were lower in metastatic EC than in primary tumors, and statistical analysis revealed correlations between levels of SHARP1 and markers of EMT and NOTCH1 signaling pathway in human EC tissue specimen.This work supports a role for SHARP1 in suppressing EMT and metastasis in EC by attenuating NOTCH1 signaling. Therefore, SHARP1 may be a novel marker for lymphatic metastasis in EC patients. 
  [Mm]etastasis  PubMed  
 DEC2 expression is positively correlated with HIF-1 activation and the invasiveness of human osteosarcomas. Osteosarcoma is the most common malignancy of bone. HIF-1 (hypoxia-inducible factor 1) activation is critical for the metabolic reprogramming and progression of solid tumors, and DEC2 (differentiated embryonic chondrocyte gene 2) has been recently reported to suppress HIF-1 in human breast and endometrial cancers. However, the roles of HIF-1 and DEC2 in human osteosarcomas remain unclear.We evaluated the correlation of DEC2 and HIF-1 expression to the prognosis, and studied the roles of DEC2 and HIF-1 activation in the invasiveness of osteosarcoma. Multiple approaches including immunohistochemical staining of clinical osteosarcoma tissues, siRNA-based knockdown and other molecular biology techniques were used. Particularly, by using a repetitive trans-well culture-based in vitro evolution system, we selected a more invasive subpopulation (U2OS-M) of osteosarcoma cells from U2OS and used it as a model to study the roles of DEC2 and HIF-1 in the invasiveness of osteosarcoma.We found that the expression of DEC2 was positively correlated with HIF-1α levels, and HIF-1α expression positively correlated with poor prognosis in osteosarcomas. DEC2 knockdown in osteosarcoma cell lines (U2OS, MNNG and 143B) attenuated HIF-1α accumulation and impaired the up-regulation of HIF-1 target genes in response to hypoxia. Compared with the low invasive parental U2OS, U2OS-M showed higher levels of DEC2 expression which were confirmed at both mRNA and protein levels. Importantly, we found that the increased DEC2 expression resulted in a more rapid accumulation of HIF-1α in U2OS-M cells in response to hypoxia. Finally, we found that HIF-1 activation is sufficient to upregulate DEC2 expression in osteosarcoma cells.Taken together, whereas DEC2 was found to promote HIF-1α degradation in other types of tumors, our data indicate that DEC2 facilitates HIF-1α stabilization and promotes HIF-1 activation in osteosarcoma. This implies that DEC2 may contribute to the progressio  n and meta  stasis of human osteosarcoma by sensitizing tumor cells to hypoxia. On the other hand, HIF-1 activation may contribute to the expression of DEC2 in osteosarcoma. This is the first demonstration of a novel DEC2-HIF-1 vicious cycle in osteosarcoma and a tumor-type specific role for DEC2. 
 SHARP1 suppresses breast cancer   metastasis   by promoting degradation of hypoxia-inducible factors. The molecular determinants of malignant cell behaviours in breast cancer remain only partially understood. Here we show that SHARP1 (also known as BHLHE41 or DEC2) is a crucial regulator of the invasive and metastatic phenotype in triple-negative breast cancer (TNBC), one of the most aggressive types of breast cancer. SHARP1 is regulated by the p63   metastasis   suppressor and inhibits TNBC aggressiveness through inhibition of hypoxia-inducible factor 1α (HIF-1α) and HIF-2α (HIFs). SHARP1 opposes HIF-dependent TNBC cell migration in vitro, and invasive or metastatic behaviours in vivo. SHARP1 is required, and sufficient, to limit expression of HIF-target genes. In primary TNBC, endogenous SHARP1 levels are inversely correlated with those of HIF targets. Mechanistically, SHARP1 binds to HIFs and promotes HIF proteasomal degradation by serving as the HIF-presenting factor to the proteasome. This process is independent of pVHL (von Hippel-Lindau tumour suppressor), hypoxia and the ubiquitination machinery. SHARP1 therefore determines the intrinsic instability of HIF proteins to act in parallel to, and cooperate with, oxygen levels. This work sheds light on the mechanisms and pathways by which TNBC acquires invasiveness and metastatic propensity. 
 SHARP1 suppresses angiogenesis of endometrial cancer by decreasing hypoxia-inducible factor-1α level. Recent data support a role for SHARP1, a basic helix-loop-helix transcription repressor, in the regulation of malignant cell behavior in several human cancers. However, the expression and role of SHARP1 during the development of endometrial cancer (EC) remain unclear. Here we show that upregulation of SHARP1 suppressed tumor angiogenesis by decreasing hypoxia-inducible factor-1α (HIF-1α), inhibited cell viability and tumor growth in EC. Immunohistochemical staining showed that the expression of SHARP1 was negatively correlated with tumor stage, histological grade, myometrial invasion, lymph no  de metasta  sis, blood vessel permeation in the myometrium and HIF-1α expression. Mechanistic studies showed that SHARP1 interacted with HIF-1α physically, and the protein level of HIF-1α and the mRNA level of its target genes (VEGFA, ANGPTL4 and CA9) were decreased by SHARP1 under hypoxia. Upregulation of SHARP1 in EC impeded hypoxia-induced angiogenesis by reducing VEGF secretion. Immunohistochemical analysis verified a correlation between decreased SHARP1 expression and increased microvessel density in EC tissues. Additionally, SHARP1 inhibited cell viability in EC cell lines. Overexpression of SHARP1 in vivo inhibited tumor growth and angiogenesis, and decreased HIF-1α expression. In this study, we established SHARP1 as a novel tumor suppressor of EC and shed light on the mechanisms by how SHARP1 inhibited EC progression. Therefore, SHARP1 may be a valuable prognostic biomarker for EC progression and shows promise as a new potential target for antiangiogenic therapeutics in human EC. 
 Suppression of the epithelial-mesenchymal transition by SHARP1 is linked to the NOTCH1 signaling pathway in   metastasis   of endometrial cancer. Mechanisms governing the   metastasis   of endometrial cancer (EC) are poorly defined. Recent data support a role for Enhancer-of-split and hairy-related protein 1 (SHARP1), a basic helix-loop-helix transcription repressor, in regulating invasiveness and angiogenesis of several human cancers. However, the role of SHARP1 in   metastasis   of EC remains unclear.Human EC cell lines (Ishikawa and HEC-1B) were used. SHARP1 was upregulated by lentivirus transduction, while intracellular domain of NOTCH1 (ICN) were upregulated by transient transfection with plasmids. Effects of SHARP1 on cell migration and invasion were evaluated by wound healing assay and transwell invasion assay. Experimental   metastasis   assay were performed in nude mice. Effects of SHAPR1 on protein levels of target genes were detected by western blotting. Furthermore, the association between SHARP1 and the NOTCH1/EMT pathway was further verified in EC tissue specimens by immunohistochemical analysis.Overexpression of SHARP1 in EC cells inhibited cell migration, invasion, and   metastasis  . Exogenous SHARP1 overexpression affected the proteins levels of genes involved in EMT process and NOTCH1 signaling pathway. Upregulation of ICN in SHARP1-overexpressing Ishikawa cells induced cell migration and an EMT phenotype. Additionally, immunohistochemical analysis demonstrated that SHARP1 protein levels were lower in metastatic EC than in primary tumors, and statistical analysis revealed correlations between levels of SHARP1 and markers of EMT and NOTCH1 signaling pathway in human EC tissue specimen.This work supports a role for SHARP1 in suppressing EMT and   metastasis   in EC by attenuating NOTCH1 signaling. Therefore, SHARP1 may be a novel marker for lymphatic   metastasis   in EC patients. 
  [Cc]ell [Ii]nvasion  PubMed  
 Regulation of the Mechanism of TWIST1 Transcription by BHLHE40 and BHLHE41 in Cancer Cells. BHLHE40 and BHLHE41 (BHLHE40/41) are basic helix-loop-helix type transcription factors that play key roles in multiple cell behaviors. BHLHE40/41 were recently shown to be involved in an epithelial-to-mesenchymal transition (EMT). However, the precise mechanism of EMT control by BHLHE40/41 remains unclear. In the present study, we demonstrated that BHLHE40/41 expression was controlled in a pathological stage-dependent manner in human endometrial cancer (HEC). Our in vitro assays showed that BHLHE40/41 suppressed tumor   cell invasion  . BHLHE40/41 also suppressed the transcription of the EMT effectors SNAI1, SNAI2, and TWIST1. We identified the critical promoter regions of TWIST1 for its basal transcriptional activity. We elucidated that the transcription factor SP1 was involved in the basal transcriptional activity of TWIST1 and that BHLHE40/41 competed with SP1 for DNA binding to regulate gene transcription. This study is the first to report the detailed functions of BHLHE40 and BHLHE41 in the suppression of EMT effectors in vitro. Our results suggest that BHLHE40/41 suppress tumor   cell invasion   by inhibiting EMT in tumor cells. We propose that BHLHE40/41 are promising markers to predict the aggressiveness of each HEC case and that molecular targeting strategies involving BHLHE40/41 and SP1 may effectively regulate HEC progression. 
   
  
 BHLHE40 
 
  NCBI Gene  
  GeneRIF  
  Pubmed  
 
 
  Search Term  Rank  Fields  
  [Mm]igration  6  PubMed(1)  
  [Ii]nvasive  10  PubMed(2); GeneRIFs(1)  
  [Mm]etastasis  12  PubMed(2)  
  [Cc]ell [Ii]nvasion  13  PubMed(2)  
 
 
 hide/show details for BHLHE40 
  [Mm]igration  PubMed  
 The BHLH transcription factor DEC1 plays an important role in the epithelial-mesenchymal transition of pancreatic cancer. DEC1 (BHLHE40/Stra13/Sharp2) is a basic helix-loop-helix (bHLH) transcription factor that is involved in the regulation of apoptosis and cell proliferation and the response to hypoxia. Epithelial-mesenchymal transition (EMT) is an important step leading to invasion and   migration   of various tumor cells, and TGF-β treatment has been shown to induce cancer cells to undergo EMT. However, the significance of DEC1 in TGF-β-induced EMT remains unknown. We examined the role of DEC1 in EMT of PANC-1 cells, a human pancreatic cancer cell line. As a result, we found that DEC1 was upregulated by TGF-β in PANC-1 cells, and regulated the expression and the levels of nuclear, cytoplasmic or membrane localization of EMT-related factors, including phosphorylated Smad3 (pSmad3), snail, claudin-4 and N-cadherin. In the presence of TGF-β, DEC1 knockdown by siRNA inhibited morphological changes during EMT processes, while TGF-β induced PANC-1 cells to taken on a spindle-shaped morphology. Furthermore, a combination treatment of DEC1 expression with TGF-β was closely linked t  o the mig  ration and invasion of PANC-1 cells. Immunohistochemically, DEC1 and pSmad3 were detected within pancreatic cancer tissues, whereas claudin-4 expression was weaker in the cancer tissues compared with the adjacent non-cancer pancreatic tissues. These findings suggest that DEC1 plays an important role in the regulation of these EMT-related factors in pancreatic cancer. 
  [Ii]nvasive  PubMed  
 DEC1 is positively associated with the malignant phenotype of   invasive   breast cancers and negatively correlated with the expression of claudin-1. Differentiated embryo-chondrocyte expressed gene 1 (DEC1) is a basic helix-loop-helix transcriptional regulator, reportedly involved in cell growth, differentiation, apoptosis and tumorigenesis. In breast cancer, DEC1 expression correlates with increased malignant potential and   invasive  ness. Nevertheless, the detailed mechanisms by which DEC1 modulates breast cancer progression are still unclear. Claudin-1, an important tight junction protein, functions as a tumor invasion suppressor. In the present study, the relationship between DEC1 and claudin-1 in 147 cases of   invasive   breast ductal carcinomas was examined by immunohistochemistry. Based on the data, DEC1 expression was elevated in   invasive   breast ductal carcinomas and DEC1 levels were positively correlated with tumor grade (P=0.023). Moreover, DEC1 expression was negatively correlated with the claudin-1 level (correlation coefficient =-0.245, P=0.003). We further identified that, in MCF-7 and MDA-MB-231 breast cancer cell lines, DEC1 knockdown led to the enhanced expression of claudin-1 at both the mRNA and protein levels, and reduced cell   invasiv  e capacity. Collectively, our data suggest that overexpression of DEC1 may promote the   invasiv  eness of breast cancer through downregulation of claudin-1. 
 Overexpression of the DEC1 protein induces senescence in vitro and is related to better survival in esophageal squamous cell carcinoma. Esophageal squamous cell carcinoma (ESCC) is a leading cause of cancer-related death in China and has limited effective therapeutic options except for early surgery, since the underlying molecular mechanism driving its precursor lesions towards   invasive   ESCC is not fully understood. Cellular senescence is the state of the permanent growth arrest of a cell, and is considered as the initial barrier of tumor development. Human differentiated embryo chondrocyte expressed gene 1 (Dec1) is an important transcription factor that related to senescence. In this study, DEC1 immunohistochemical analysis was performed on tissue microarray blocks constructed from ESCC combined with adjacent precursor tissues of 241 patients. Compared with normal epithelia, DEC1 expression was significantly increased in intraepithelial neoplasia and DEC1 expression was significantly decreased in ESCC in comparison with intraepithelial neoplasia. In vitro, DEC1 overexpression induced cellular senescence, and it inhibited cell growth and colony formation in ESCC cell line EC9706. Fresh esophagectomy tissue sections from five ESCC patients were detected by immunohistochemistry of DEC1 and senescence-associated β-galactosidase (SA-β-Gal) activity, and strongly positive expression of DEC1 was correlated to more senescent cells in these fresh tissue sections. Kaplan-Meier method analysis of the 241 patients revealed that DEC1 expression levels were significantly correlated with the survival of ESCC patients after surgery. The expression levels of DEC1 were also correlated with age, tumor embolus, depth of invasion of ESCC, lymph metastasis status and pTNMs. These results suggest that DEC1 overexpression in precursor lesions of ESCC is a protective mechanism by inducing cellular senescence in ESCC initiation, and DEC1 may be a potential prognostic marker of ESCC. 
  GeneRIFs  
 Studied DEC1 & claudin-1 in   invasive   breast ductal carcinomas;found DEC1 elevated in   invasive   breast ductal carcinoma. DEC1 knockdown led to the enhanced expression of claudin-1 at both the mRNA and protein levels in breast cancer cell lines. 
  [Mm]etastasis  PubMed  
 Overexpression of the DEC1 protein induces senescence in vitro and is related to better survival in esophageal squamous cell carcinoma. Esophageal squamous cell carcinoma (ESCC) is a leading cause of cancer-related death in China and has limited effective therapeutic options except for early surgery, since the underlying molecular mechanism driving its precursor lesions towards invasive ESCC is not fully understood. Cellular senescence is the state of the permanent growth arrest of a cell, and is considered as the initial barrier of tumor development. Human differentiated embryo chondrocyte expressed gene 1 (Dec1) is an important transcription factor that related to senescence. In this study, DEC1 immunohistochemical analysis was performed on tissue microarray blocks constructed from ESCC combined with adjacent precursor tissues of 241 patients. Compared with normal epithelia, DEC1 expression was significantly increased in intraepithelial neoplasia and DEC1 expression was significantly decreased in ESCC in comparison with intraepithelial neoplasia. In vitro, DEC1 overexpression induced cellular senescence, and it inhibited cell growth and colony formation in ESCC cell line EC9706. Fresh esophagectomy tissue sections from five ESCC patients were detected by immunohistochemistry of DEC1 and senescence-associated β-galactosidase (SA-β-Gal) activity, and strongly positive expression of DEC1 was correlated to more senescent cells in these fresh tissue sections. Kaplan-Meier method analysis of the 241 patients revealed that DEC1 expression levels were significantly correlated with the survival of ESCC patients after surgery. The expression levels of DEC1 were also correlated with age, tumor embolus, depth of invasion of ESCC, lymp  h metastas  is status and pTNMs. These results suggest that DEC1 overexpression in precursor lesions of ESCC is a protective mechanism by inducing cellular senescence in ESCC initiation, and DEC1 may be a potential prognostic marker of ESCC. 
 Identification of genes differentially induced by hypoxia in pancreatic cancer cells. A hypoxic microenvironment is characteristic of many solid tumors, including pancreatic cancer, the fifth leading cause of cancer death in the United States. Hypoxia causes the stabilization of the HIF-1 (hypoxia-inducible factor-1) transcription factor and the induction of many genes that promote angiogenesis, tumor growth, and   metastasis  . We performed representational difference analysis (RDA) using mRNA extracted from hypoxic and normoxic Capan-2, a human pancreatic cancer cell line. cDNAs corresponding to hypoxia-inducible genes were cloned and sequenced. We identified GPI/NLK/AMF (glucose phosphate isomerase/neuroleukin/autocrine motility factor) as a hypoxic inducible gene. In addition, hexokinase II and DEC1/Stra13, genes known to be hypoxia inducible in other systems, were found to be hypoxia inducible in our pancreatic cancer system. We thus identified three genes that are induced by hypoxia in a human pancreatic cancer, including GPI/NLK/AMF, which was not previously known to be hypoxia inducible in any other system. These genes may provide new targets for diagnosis and treatment of pancreatic cancer. 
  [Cc]ell [Ii]nvasion  PubMed  
 Regulation of the Mechanism of TWIST1 Transcription by BHLHE40 and BHLHE41 in Cancer Cells. BHLHE40 and BHLHE41 (BHLHE40/41) are basic helix-loop-helix type transcription factors that play key roles in multiple cell behaviors. BHLHE40/41 were recently shown to be involved in an epithelial-to-mesenchymal transition (EMT). However, the precise mechanism of EMT control by BHLHE40/41 remains unclear. In the present study, we demonstrated that BHLHE40/41 expression was controlled in a pathological stage-dependent manner in human endometrial cancer (HEC). Our in vitro assays showed that BHLHE40/41 suppressed tumor   cell invasion  . BHLHE40/41 also suppressed the transcription of the EMT effectors SNAI1, SNAI2, and TWIST1. We identified the critical promoter regions of TWIST1 for its basal transcriptional activity. We elucidated that the transcription factor SP1 was involved in the basal transcriptional activity of TWIST1 and that BHLHE40/41 competed with SP1 for DNA binding to regulate gene transcription. This study is the first to report the detailed functions of BHLHE40 and BHLHE41 in the suppression of EMT effectors in vitro. Our results suggest that BHLHE40/41 suppress tumor   cell invasion   by inhibiting EMT in tumor cells. We propose that BHLHE40/41 are promising markers to predict the aggressiveness of each HEC case and that molecular targeting strategies involving BHLHE40/41 and SP1 may effectively regulate HEC progression. 
 Regulation of the Mechanism of TWIST1 Transcription by BHLHE40 and BHLHE41 in Cancer Cells. BHLHE40 and BHLHE41 (BHLHE40/41) are basic helix-loop-helix type transcription factors that play key roles in multiple cell behaviors. BHLHE40/41 were recently shown to be involved in an epithelial-to-mesenchymal transition (EMT). However, the precise mechanism of EMT control by BHLHE40/41 remains unclear. In the present study, we demonstrated that BHLHE40/41 expression was controlled in a pathological stage-dependent manner in human endometrial cancer (HEC). Our in vitro assays showed that BHLHE40/41 suppressed tumor   cell invasion  . BHLHE40/41 also suppressed the transcription of the EMT effectors SNAI1, SNAI2, and TWIST1. We identified the critical promoter regions of TWIST1 for its basal transcriptional activity. We elucidated that the transcription factor SP1 was involved in the basal transcriptional activity of TWIST1 and that BHLHE40/41 competed with SP1 for DNA binding to regulate gene transcription. This study is the first to report the detailed functions of BHLHE40 and BHLHE41 in the suppression of EMT effectors in vitro. Our results suggest that BHLHE40/41 suppress tumor   cell invasion   by inhibiting EMT in tumor cells. We propose that BHLHE40/41 are promising markers to predict the aggressiveness of each HEC case and that molecular targeting strategies involving BHLHE40/41 and SP1 may effectively regulate HEC progression. 
   
  
 PDZD2 
 
  NCBI Gene  
  GeneRIF  
  Pubmed  
 
 
  Search Term  Rank  Fields  
  [Mm]etastasis  12  PubMed(1)  
 
 
 hide/show details for PDZD2 
  [Mm]etastasis  PubMed  
 Activated in prostate cancer: a PDZ domain-containing protein highly expressed in human primary prostate tumors. Critical events in prostate tumorigenesis and   metastasis   likely include the abnormal activation and expression of specific genes. Using RNA expression profiling techniques, we have identified a transcript originating from the activated in prostate cancer (AIPC) gene, the expression of which is preferentially up-regulated in several cultured prostate tumor cell lines and human primary prostate tumors. Sequence analysis revealed that the AIPC protein encodes six PDZ domains, which are protein-protein binding domains likely involved in protein clustering and scaffolding. Immunohistochemical analysis of a tissue microarray comprising 158 tumor, 18 high-grade prostatic intraepithelial neoplasia, and 91 normal prostate specimens with an anti-AIPC antibody demonstrated abundant AIPC protein expression in 75% of tumors, 83% of prostatic intraepithelial neoplasia lesions, and 3% of normal tissues (P < 0.0001). These data suggest that the accumulation of AIPC protein may be closely associated with the initiation or early promotion of prostate tumorigenesis. 
   
  
 TRAF6 
 
  NCBI Gene  
  GeneRIF  
  Pubmed  
 
 
  Search Term  Rank  Fields  
  [Mm]igration  6  PubMed(14); GeneRIFs(2)  
  [Ii]nvasive  10  PubMed(5)  
  [Mm]etastasis  12  PubMed(12); GeneRIFs(2)  
  [Cc]ell [Ii]nvasion  13  PubMed(2)  
 
 
 hide/show details for TRAF6 
  [Mm]igration  PubMed  
 Sphingosine 1-phosphate as a regulator of osteoclast differentiation and osteoclast-osteoblast coupling. Sphingosine 1-phosphate (S1P), produced by sphingosine kinase (SPHK), acts both by intracellular and extracellular modes. We evaluated the role of SPHK1 and S1P in osteoclastogenesis using bone marrow-derived macrophage (BMM) single and BMM/osteoblast coculture systems. In BMM single cultures, the osteoclastogenic factor receptor activator of NF-kappaB ligand (RANKL) upregulated SPHK1 and increased S1P production and secretion. SPHK1 siRNA enhanced and SPHK1 overexpression attenuated osteoclastogenesis via modulation of p38 and ERK activities, and NFATc1 and c-Fos levels. Extracellular S1P had no effect in these cultures. These data suggest that intracellular S1P produced in response to RANKL forms a negative feedback loop in BMM single cultures. In contrast, S1P addition to BMM/osteoblast cocultures greatly increased osteoclastogenesis by increasing RANKL in osteoblasts via cyclooxygenase-2 and PGE(2) regulation. S1P also stimulated osteoblast   migration   and survival. The RANKL elevation and chemotactic effects were also observed with T cells. These results indicate that secreted S1P attracts and acts on osteoblasts and T cells to augment osteoclastogenesis. Taken together, S1P plays an important role in osteoclastogenesis regulation and in communication between osteoclasts and osteoblasts or T cells. 
 SASH1 is a scaffold molecule in endothelial TLR4 signaling. Recognition of microbial products by TLRs is critical for mediating innate immune responses to invading pathogens. In this study, we identify a novel scaffold protein in TLR4 signaling called SAM and SH3 domain containing protein 1 (SASH1). Sash1 is expressed across all microvascular beds and functions as a scaffold molecule to independently bind TRAF6, TAK1, IκB kinase α, and IκB kinase β. This interaction fosters ubiquitination of TRAF6 and TAK1 and promotes LPS-induced NF-κB, JNK, and p38 activation, culminating in increased production of proinflammatory cytokines and increased LPS-induced endothe  lial migr  ation. Our findings suggest that SASH1 acts to assemble a signaling complex downstream of TLR4 to activate early endothelial responses to receptor activation. 
 Autophagy facilitates TLR4- and TLR3-triggered   migration   and invasion of lung cancer cells through the promotion of TRAF6 ubiquitination. Autophagy contributes to the pathogenesis of cancer, whereas toll-like receptors (TLRs) also play an important role in cancer development and immune escape. However, little is known about the potential interaction between TLR signaling and autophagy in cancer cells. Here we show that autophagy induced by TLR4 or TLR3 activation enhances various cytokine productions through promoting TRAF6 (TNF receptor-associated factor 6, E3 ubiquitin protein ligase) ubiquitination and thus facilitates   migration   and invasion of lung cancer cells. Stimulation of TLR4 and TLR3 with lipopolysaccharide (LPS) and polyinosinic-polycytidylic acid [poly(I:C)] respectively triggered autophagy in lung cancer cells. This was mediated by the adaptor protein, toll-like receptor adaptor molecule 1 (TICAM1/TRIF), and was required for TLR4- and TLR3-induced increases in the production of IL6, CCL2/MCP-1 [chemokine (C-C motif) ligand 2], CCL20/MIP-3α [chemokine (C-C motif) ligand 20], VEGFA (vascular endothelial growth factor A), and MMP2 [matrix metallopeptidase 2 (gelatinase A, 72 kDa gelatinase, 72 kDa type IV collagenase)]. These cytokines appeared to be necessary for enhanced   migratio  n and invasion of lung cancer cells upon TLR activation. Remarkably, inhibition of autophagy by chemical or genetic approaches blocked TLR4- or TLR3-induced Lys63 (K63)-linked ubiquitination of TRAF6 that was essential for activation of MAPK and NFKB (nuclear factor of kappa light polypeptide gene enhancer in B-cells) pathways, both of which were involved in the increased production of the cytokines. Collectively, these results identify induction of autophagy by TLR4 and TLR3 as an important mechanism that drives lung cancer progression, and indicate that inhibition of autophagy may be a useful strategy in the treatment of lung cancer. 
 Autophagy facilitates TLR4- and TLR3-triggered   migration   and invasion of lung cancer cells through the promotion of TRAF6 ubiquitination. Autophagy contributes to the pathogenesis of cancer, whereas toll-like receptors (TLRs) also play an important role in cancer development and immune escape. However, little is known about the potential interaction between TLR signaling and autophagy in cancer cells. Here we show that autophagy induced by TLR4 or TLR3 activation enhances various cytokine productions through promoting TRAF6 (TNF receptor-associated factor 6, E3 ubiquitin protein ligase) ubiquitination and thus facilitates   migration   and invasion of lung cancer cells. Stimulation of TLR4 and TLR3 with lipopolysaccharide (LPS) and polyinosinic-polycytidylic acid [poly(I:C)] respectively triggered autophagy in lung cancer cells. This was mediated by the adaptor protein, toll-like receptor adaptor molecule 1 (TICAM1/TRIF), and was required for TLR4- and TLR3-induced increases in the production of IL6, CCL2/MCP-1 [chemokine (C-C motif) ligand 2], CCL20/MIP-3α [chemokine (C-C motif) ligand 20], VEGFA (vascular endothelial growth factor A), and MMP2 [matrix metallopeptidase 2 (gelatinase A, 72 kDa gelatinase, 72 kDa type IV collagenase)]. These cytokines appeared to be necessary for enhanced   migratio  n and invasion of lung cancer cells upon TLR activation. Remarkably, inhibition of autophagy by chemical or genetic approaches blocked TLR4- or TLR3-induced Lys63 (K63)-linked ubiquitination of TRAF6 that was essential for activation of MAPK and NFKB (nuclear factor of kappa light polypeptide gene enhancer in B-cells) pathways, both of which were involved in the increased production of the cytokines. Collectively, these results identify induction of autophagy by TLR4 and TLR3 as an important mechanism that drives lung cancer progression, and indicate that inhibition of autophagy may be a useful strategy in the treatment of lung cancer. 
 Role of CYB5A in pancreatic cancer prognosis and autophagy modulation. Loss of 18q22.3 is a prognostic marker in pancreatic ductal adenocarcinoma (PDAC). This study investigated genes encoded by this cytoband.We studied mRNA/protein expression in radically resected (n = 130) and metastatic patients (n = 50). The role of CYB5A was tested in 11 PDAC cell lines and five primary cultures through retrovirus-mediated upregulation and small interfering RNA using wound-healing, invasion, annexin-V, electron microscopy, and autophagic assays, as well as autophagy genes and kinases arrays. CYB5A+ orthotopic models (n = 6 mice/group) were monitored by Firefly and Gaussia-luciferase bioluminescence, magnetic resonance imaging, and high-frequency ultrasound. Data were analyzed by t test, Fisher exact-test, log-rank test and Cox proportional hazards models. All statistical tests were two-sided.Both resected and metastatic patients with low mRNA or protein expression of CYB5A had statistically significantly shorter survival (eg, median = 16.7 months, 95% confidence interval [CI] = 13.5 to 19.9; vs median = 24.8 months, 95% CI = 12.8 to 36.9; P = .02, two-sided log-rank test; n = 82 radically resected PDACs), and multivariable analyses confirmed prognostic relevance. Moreover, we characterized a novel function to CYB5A, autophagy induction, concomitant with reduced proliferation and   migration  /invasion of PDAC cells. Network analysis of proautophagic pathways suggested CYB5A interaction with TRAF6, which was confirmed by TRAF6 downregulation after CYB5A reconstitution (-69% in SU.86.86-CYB5A+; P = .005, two-sided t test). CYB5A silencing had opposite effects, restoring TRAF6 expression and wound healing. In vivo studies showed that CYB5A induced autophagy while inhibiting tumor growth/metastasis and increasing survival (median = 57 days, 95% CI = 52 to 61; vs median = 44 days, 95% CI = 21 to 57; P = .03, two-sided log-rank test).These results define CYB5A as a novel prognostic factor for PDAC that exerts its tumor-suppressor function through autophagy induction and TRAF6 modulation. 
 TRAF6 inhibits proangiogenic signals in endothelial cells and regulates the expression of vascular endothelial growth factor. TNF-family molecules induce the expression Vascular Endothelial Growth Factor (VEGF) in endothelial cells (EC) and elicit signaling responses that result in angiogenesis. However, the role of TNF-receptor associated factors (TRAFs) as upstream regulators of VEGF expression or as mediators of angiogenesis is not known. In this study, HUVEC were cotransfected with a full-length VEGF promoter-luciferase construct and siRNAs to TRAF 1, -2, -3, -5, -6, and promoter activity was measured. Paradoxically, rather than inhibiting VEGF expression, we found that knockdown of TRAF6 resulted in a 4-6-fold increase in basal VEGF promoter activity compared to control siRNA-transfected EC (P<0.0001). In addition, knockdown of TRAF 1, -2, -3 or -5 resulted in a slight increase or no change in VEGF promoter activation. Using [(3)H]thymidine incorporation assays as well as the in vitro wound healing assay, we also found that basal rates of EC proliferation and   migration   were increased following TRAF6 knockdown; and this response was inhibited by the addition of a blocking anti-VEGF antibody into cell cultures. Using a limited protein array to gain insight into TRAF6-dependent intermediary signaling responses, we observed that TRAF6 knockdown resulted in an increase in the activity of Src family kinases. In addition, we found that treatment with AZD-0530, a pharmacological Src inhibitor, reduced the regulatory effect of TRAF6 knockdown on VEGF promoter activity. Collectively, these findings define a novel pro-angiogenic signaling response in EC that is regulated by TRAF6. 
 A novel TNF receptor family member binds TWEAK and is implicated in angiogenesis. TWEAK is a member of the TNF ligand family that induces angiogenesis in vivo. We report cloning of a receptor for TWEAK (TweakR) from a human umbilical vein endothelial cell (HUVEC) library. The mature form of TweakR has only one hundred and two amino acids and six cysteine residues in its extracellular region. Five different assays demonstrate TWEAK-TweakR binding, and the interaction affinity constant (Kd) is within a physiologically relevant range of 2.3 +/- 0.1 nM. The TweakR cytoplasmic domain binds TRAFs 1, 2, and 3. Cross-linking of TweakR induces HUVEC growth, and mRNA levels are upregulated in vitro by a variety of agents and in vivo following arterial injury. Soluble TweakR inhibits endothelial cell   migration   in vitro and corneal angiogenesis in vivo. 
 TRAF6 promoted the metastasis of esophageal squamous cell carcinoma. Esophageal cancer is one of the most aggressive malignancies and has been ranked as the sixth leading cause of cancer-related death in the world. It is very urgent to find new therapeutic targets. Although tumor necrosis factor receptor-associated factor 6 (TRAF6) was initially identified as an adaptor for NF-κB signaling, recently it has been found to be involved in cancer by modulating various signaling pathways. In the previous study, we have found that TRAF6 promoted the growth of esophageal squamous cell carcinoma (ESCC) cell in vitro and in vivo. However, the effects of TRAF6 on the   migratio  n and metastasis of ESCC cells are poorly understood. Here, we found that TRAF6 promoted   migratio  n and metastasis of ESCC cells through modulating Ras signaling. Overexpression of TRAF6 promoted the   migratio  n of ESCC cells and immortalized esophageal epithelial cells, while knock down the expression of TRAF6 inhibited the   migratio  n and metastasis of ESCC cells in vitro and in vivo. Mechanically, TRAF6 binds Ras with its N-terminal and activated Ras signaling. Taken together, TRAF6 played an important role in the metastasis of ESCC and might be a promising therapeutic target. 
 [Effect of TRAF6 Downregulation on Malignant Biological Behavior of Lung Cancer Cell Lines]. It has been proven that tumor necrosis factor receptor-associated factor 6 (TRAF6) was a commonly amplified oncogene in lung cancer. However, the precise role of TRAF6 protein in lung cancer has not been extensively investigated. This study analyzed the effects of TRAF6 on the proliferation, apoptosis, cell cycle  , migrati  on, and invasion capability of lung cancer cell lines, as well as the potential molecular mechanisms involved.To address the expression of TRAF6 in lung cancer cells, four lung cancer cell lines (A549, H1650, SPC-A-1 and Calu-3) were assayed to determine the expression of TRAF6 protein by Western blot and TRAF6 mRNA via qRT-PCR. Moreover, siRNA targeting TRAF6 was introduced into SPC-A-1 and Calu-3 cells. Nuclear factor-қB (NF-қB) DNA-binding activity, apoptosis rates, cell proliferation, cell cyc  le, migra  tion, and invasion were determined by electrophoretic mobility shift assay, flow cytometry, MTS assay, flow cytometry, scratch test, and transwell chamber assay, respectively. Western blot analysis was also performed to evaluate the expression of the following proteins through K63-ubiquitination: P65, CD24 and CXCR4. Whole-genome sequencing analysis was conducted using a second-generation sequencer in SPC-A-1 cells.TRAF6 was highly up-expressed in SPC-A-1 and Calu-3 cell lines than the other two cells, which also showed K63-ubiquitinization in TRAF6. However, constitutive activation of NF-қB was observed only in SPC-A-1 lung cancer cells. Downregulation of TRAF6 suppressed the NF-κB activation,   cell mig  ration, and invasion but promoted the cell apoptosis of SPC-A-1 cells. Markedly decreased expression of CD24 and CXCR4 was observed in SPC-A-1 cells transfected by TRAF6 siRNA. Nevertheless, TRAF6 downregulation did not affect the proliferation and cell cycle of SPC-A-1 cells. Additionally, TRAF6 regulation did not affect the proliferation, apoptosis, cell c  ycle, mig  ration, and invasion of Calu-3 cells. No mutations and no changes in gene copy numbers of TRAF6 were found by whole-exome sequencing of SPC-A-1 cells.TRAF6 may be involved in   cell mig  ration, invasion, and apoptosis of SPC-A-1 cells, possibly through regulating the NF-қB-CD24/CXCR4 pathway. . 
 MiR-146a Regulates Inflammatory Infiltration by Macrophages in Polymyositis/Dermatomyositis by Targeting TRAF6 and Affecting IL-17/ICAM-1 Pathway. The primary objective of this study was to investigate the role of miR-146a in inducing the inflammatory infiltration of macrophages in polymyositis/dermatomyositis (PM/DM) through targeting TNF receptor associated factor 6 (TRAF6), which may further down-regulate the Interleukin-17 (IL-17)/Intercellular Adhesion Molecule 1 (ICAM-1) pathway.Biopsies were collected from PM/DM patients and healthy volunteers. PM/DM model establishment and macrophage isolation were performed on Sprague Dawley (SD) rats. Model rats and macrophages were treated with anti-IL-17, anti-ICAM-1, miR-146a mimics, miR-146a inhibitors, and TRAF6 siRNAs. Serum creatine phosphokinase (S-CK) expression was assessed using double antibody sandwich enzyme-linked immunosorbent assay (ELISA) assay, and immunohistochemistry assay was performed to analyze CD163 expression in muscle samples. Furthermore, we used transwell assay to test cell   migration  ; RT-PCR and western blot were carried out to determine the expression of miR-146a, TRAF6, IL-17, and ICAM-1.The S-CK, TRAF6, IL-17 and ICAM-1 levels were higher in PM/DM patients compared with healthy controls and were down-regulated after the conventional treatment. Treatment with miR-146a mimics, anti-IL-17 and anti-ICAM-1 decreased the expression of IL-17 and ICAM-1, whereas miR-146a inhibitors exerted the opposite effects. The effects of miR-146a inhibitors were suppressed by treatment with TRAF6 siRNA. In addition, the luciferase reporter assay validated the targeting relationship between miR-146a and TRAF6.MiR-146a regulates inflammatory macrophage infiltration in PM/DM by targeting TRAF6 and affecting the IL-17/ICAM-1 pathway. 
 TRAF6 is over-expressed in pancreatic cancer and promotes the tumorigenicity of pancreatic cancer cells. Pancreatic cancer is one of the most lethal malignancies, with a poor response to chemotherapy and therefore it is important to identify novel therapeutic targets. TNF receptor-associated factor 6 (TRAF6) , a regulator of NF-κB signaling, has been found recently to be involved in tumorigenesis. However, its function in pancreatic cancer remains poorly understood. Here, we found that the expression of TRAF6 was up-regulated in pancreatic cancer tissues. Moreover, over-expression of TRAF6 in pancreatic cancer cells promoted cell proliferation and   migratio  n, whereas down-regulation of TRAF6 impaired the tumorigenicity of pancreatic cancer cells in vitro and in vivo. Mechanistically, TRAF6 regulated the expression of multiple genes involved in cell growth, apoptosis and   migratio  n. Our results suggested several important roles of TRAF6 in the pathogenesis of pancreatic cancer. TRAF6 might therefore represent a potential therapeutic target. 
 Functional role of asparaginyl endopeptidase ubiquitination by TRAF6 in tumor invasion and metastasis. Asparaginyl endopeptidase (AEP) has been implicated in human cancer development. However, the molecular mechanisms underlying AEP regulation, including the role of pro-AEP activation, remain elusive.We investigated the regulation of AEP by TRAF6 and its effects on tumor progression and metastasis in cancer cell lines, murine models, and specimens from patients using biochemical analyses, confocal microscopy, immunoelectron microscopy, and   migration  -invasion assays. The sera of healthy donors and breast cancer patients were examined by enzyme-linked immunosorbent assay, and a tissue array of 314 breast cancer specimens was assessed for AEP and TRAF6 by immunohistochemistry. Furthermore, the effects of AEP inhibitors or monoclonal antibodies on pulmonary metastasis were evaluated in murine models. The statistical significance between groups was determined using two-tailed Student t tests.We demonstrate that TRAF6 ubiquitinates the proform of AEP through K63-linked polyubiquitin, reversible by USP17, and forms a complex with HSP90α to subsequently promote pro-AEP intracellular stability as well as secretion. Disrupting the interaction between pro-AEP and TRAF6 or inhibiting HSP90α reduced pro-AEP secretion and consequently reduced tumor metastasis. Higher circulating AEP levels were detected in the sera of breast cancer patients, and AEP inhibitors or neutralizing antibodies remarkably decreased tumor metastasis in murine models. Notably, TRAF6 and AEP were overexpressed in human breast neoplasms and correlated with poor prognosis. Patients with low AEP/TRAF6 expression survived for a mean of 111 months (95% confidence interval [CI] = 108 to 115 months), whereas those with high AEP/TRAF6 expression survived for a mean of only 61 months (95% CI = 42 to 79 months; P < .001).Our study elucidates a novel mechanism of AEP regulation and an alternative oncogenic pathway for TRAF6 in breast cancer, which suggests that AEP and TRAF6 protein levels may have prognostic implications in breast cancer patients. Thus, AEP may serve as a biomarker as well as new therapeutic target. 
 Ubiquitin-specific protease 4 (USP4) targets TRAF2 and TRAF6 for deubiquitination and inhibits TNFα-induced cancer cell   migratio  n. TRAF [TNF (tumour necrosis factor)-receptor-associated factor] 2 and 6 are essential adaptor proteins for the NF-κB (nuclear factor κB) signalling pathway, which play important roles in inflammation and immune response. Polyubiquitination of TRAF2 and TRAF6 is critical to their activities and functions in TNFα- and IL (interleukin)-1β-induced NF-κB activation. However, the regulation of TRAF2 and TRAF6 by deubiquitination remains incompletely understood. In the present study, we identified USP (ubiquitin-specific protease) 4 as a novel deubiquitinase targeting TRAF2 and TRAF6 for deubiquitination. We found that USP4 specifically interacts with TRAF2 and TRAF6, but not TRAF3. Moreover, USP4 associates with TRAF6 both in vitro and in vivo, independent of its deubiquitinase activity. The USP domain is responsible for USP4 to interact with TRAF6. Ectopic expression of USP4 inhibits the TRAF2- and TRAF6-stimulated NF-κB reporter gene and negatively regulates the TNFα-induced IκBα (inhibitor of NF-κBα) degradation and NF-κB activation. Knockdown of USP4 significantly increased TNFα-induced cytokine expression. Furthermore, we found that USP4 deubiquitinates both TRAF2 and TRAF6 in vivo and in vitro in a deubiquitinase activity-dependent manner. Importantly, the results of the present study showed that USP4 is a negative regulator of TNFα- and IL-1β-indu  ced cance  r cell migration. Taken together, the present study provides a novel insight into the regulation of the NF-κB signalling pathway and uncovers a previously unknown function of USP4 in cancer. 
 siRNA-induced TRAF6 knockdown promotes the apoptosis and inhibits the invasion of human lung cancer SPC-A1 cells. Tumor necrosis factor receptor-associated factor 6 (TRAF6) has been found to be involved in multiple cancers. However, the effect of small interfering RNA (siRNA)‑induced knockdown of TRAF6 on the biological behaviors of cancer cells remains unknown. Thus, the present study aimed to investigate the effect of siRNA-induced knockdown of TRAF6 on the biological behaviors of human lung cancer SPC-A1 cells. The expression of TRAF6 was determined in human lung adenocarcinoma A549, non-small cell lung cancer H1650, human airway epithelial Calu-3 and human lung cancer SPC-A1 cell lines using quantitative RT-PCR (qRT‑PCR) and western blotting at the transcriptional and translational levels. TRAF6 expression was knocked down in the SPC-A1 cells using an siRNA technique, and the effects of TRAF6 knockdown on NF-κB activity, cell proliferation, apoptosis, cell cycle, invasion   and migr  ation of the SPC-A1 cells were determined using electrophoretic mobility shift assay (EMSA), cell proliferation assay, flow cytometry, Transwell invasion assay and scratch wound assay. In addition, the protein expression of CD24, CXCR4, MMP1, MMP2, MMP9, TWIST, TIMP-2 and Slug was quantified using western blotting assay. Western blotting and qRT-PCR assays showed upregulation of TRAF6 at both the translational and transcriptional levels in the Calu-3 and SPC-A1 cells, and K63-linked ubiquitination of TRAF6 and constitutive NF-κB activation were detected in the SPC-A1 cells. Knockdown of TRAF6 inhibite  d the mig  ration and invasion and promoted the apoptosis of the SPC-A1 cells, but had little effect on cell proliferation and the cell cycle. In addition, siRNA-induced TRAF6 knockdown caused a marked reduction in the protein expression of CD24 and CXCR4, but had little effect on MMP-1, MMP-2, MMP-9, Twist, TIMP-2 or Slug expression. The present study demonstrated that TRAF6 is upregulated in human lung cancer cells, and siRNA-induced TRAF6 knockdown inhibits the invasion of lung cancer cells and promotes apoptosis. It is suggested that TRAF6 may be a promising target for the therapy of lung cancer. 
  GeneRIFs  
 TRAF6 may be involved in cell   migration  , invasion, and apoptosis of SPC-A-1 cells, possibly through regulating the NF-B-CD24/CXCR4 pathway.. 
 Over-expression of TRAF6 in pancreatic cancer cells promoted cell proliferation and   migration  . 
  [Ii]nvasive  PubMed  
 TRAF6 promotes TGFβ-induced invasion and cell-cycle regulation via Lys63-linked polyubiquitination of Lys178 in TGFβ type I receptor. Transforming growth factor β (TGFβ) can act either as a tumor promoter or a tumor suppressor in a context-dependent manner. High levels of TGFβ are found in prostate cancer tissues and correlate with poor patient prognosis. We recently identified a novel TGFβ-regulated signaling cascade in which TGFβ type I receptor (TβRI) is activated by the E3 ligase TNF-receptor-associated factor 6 (TRAF6) via the Lys63-linked polyubiquitination of TβRI. TRAF6 also contributes to activation of TNF-α-converting enzyme and presenilin-1, resulting in the proteolytic cleavage of TβRI and releasing the intracellular domain of TβRI, which is translocated to the nucleus to pr  omote tu  mor invasiveness. In this report, we provide evidence that Lys178 of TβRI is polyubiquitinated by TRAF6. Moreover, our data suggest that TRAF6-mediated Lys63-linked ubiquitination of the TβRI intracellular domain is a prerequisite for TGFβ regulation of mRNA for cyclin D1 (CCND1), expression, as well as for the regulation of other genes controlling the cell cycle, differ  entiatio  n, and invasiveness of prostate cancer cells. 
 TRAF6 ubiquitinates TGFβ type I receptor to promote its cleavage and nuclear translocation in cancer. Transforming growth factor β (TGFβ) is a pluripotent cytokine promoting epithelial cell plasticity during morphogenesis and tumour progression. TGFβ binding to type II and type I serine/threonine kinase receptors (TβRII and TβRI) causes activation of different intracellular signaling pathways. TβRI is associated with the ubiquitin ligase tumor necrosis factor receptor (TNFR)-associated factor 6 (TRAF6). Here we show that TGFβ, via TRAF6, causes Lys63-linked polyubiquitination of TβRI, promoting cleavage of TβRI by TNF-alpha converting enzyme (TACE), in a PKCζ-dependent manner. The liberated intracellular domain (ICD) of TβRI associates with the transcriptional regulator p300 to activate genes involved in   tumour c  ell invasiveness, such as Snail and MMP2. Moreover, TGFβ-induced invasion of cancer cells is TACE- and PKCζ- dependent and the TβRI ICD is localized in the nuclei of different kinds of tumour cells in tissue sections. Thus, our data reveal a specific role for TβRI in TGFβ mediated tumour invasion. 
 TRAF6 stimulates the tumor-promoting effects of TGFβ type I receptor through polyubiquitination and activation of presenilin 1. Transforming growth factor-β (TGFβ) can be both a tumor promoter and suppressor, although the mechanisms behind the protumorigenic switch remain to be fully elucidated. The TGFβ type I receptor (TβRI) is proteolytically cleaved in the ectodomain region. Cleavage requires the combined activities of tumor necrosis factor (TNF) receptor-associated factor 6 (TRAF6) and TNF-α-converting enzyme (TACE). The cleavage event occurs selectively in cancer cells and generates an intracellular domain (ICD) of TβRI, which enters the nucleus to mediate gene transcription. Presenilin 1 (PS1), a γ-secretase catalytic core component, mediates intramembrane proteolysis of transmembrane receptors, such as Notch. We showed that TGFβ increased both the abundance and activity of PS1. TRAF6 recruited PS1 to the TβRI complex and promoted lysine-63-linked polyubiquitination of PS1, which activated PS1. Furthermore, PS1 cleaved TβRI in the transmembrane domain between valine-129 and isoleucine-130, and ICD generation was inhibited when these residues were mutated to alanine. We also showed that, after entering the nucleus, TβRI-ICD bound to the promoter and increased the transcription of the gene encoding TβRI. The TRAF6- and PS1-induced intramembrane proteolysis of TβRI promoted TGFβ-induced invasion of various cancer cells in vitro. Furthermore, when a mouse xenograft model of prostate cancer was treated with the γ-secretase inhibitor DBZ {(2S)-2-[2-(3,5-difluorophenyl)-acetylamino]-N-(5-methyl-6-oxo-6,7-dihydro-5H-dibenzo[b,d]azepin-7-yl)-propionamide}, generation of TβRI-ICD was prevented, transcription of the gene   encodin  g the proinvasive transcription factor Snail1 was reduced, and tumor growth was inhibited. These results suggest that γ-secretase inhibitors may be useful for treating aggressive prostate cancer. 
 TRAF6 regulates proliferation, apoptosis, and invasion of osteosarcoma cell. TRAF6, a unique tumor necrosis factor receptor-associated factor (TRAF) family member, possesses a unique receptor-binding specificity that results in its crucial role as the signaling mediator for TNF receptor superfamily and interleukin-1 receptor/Toll-like receptor superfamily. TRAF6 plays an important role in tumorigenesis, invasion and metastasis. This study aimed to explore the expression of TRAF6 in osteosarcoma tissues and its correlation to the clinical pathology of osteosarcoma and to discuss the relationship between TRAF6 expression and osteosarcoma invasion. These data will provide the experimental base for the biological treatment of osteosarcoma in the future. Using RT-PCR and Western blot, the results showed that the expression rate of TRAF6 mRNA in osteosarcoma tissues was significantly higher than that in normal bone tissue (p < 0.05), that the expression rate of TRAF6 mRNA in the carcinoma tissues from patients with lung metastasis was significantly higher than that from patients without lung metastasis (p < 0.05), and that the expression rate of TRAF6 mRNA also increased with increasing Enneking stage (p < 0.05). However, the mRNA expression of TRAF6 in osteosarcoma was independent of the patient's gender, age, and tumor size (p > 0.05). The TRAF6 protein displayed an up-regulation in osteosarcoma tissues compared to normal bone tissue (p < 0.05), displayed an up-regulation in osteosarcoma tissues from patients with lung metastasis compared to from patients without lung metastasis (p < 0.05), and displayed a gradual increase with increasing Enneking stage (p < 0.05). By the technique of RNA interference, the expression of TRAF6 in the human osteosarcoma MG-63 cell line was down-regulated, and the   invasive   ability of MG-63 cells was examined. The results showed that TRAF6 protein expression was significantly decreased in the MG-63 cells from TRAF6 siRNA-transfected group (p < 0.05), and the proliferation ability of MG-63 cells and the number of MG-63 cells that passed through the Transwell chamber were significantly lower than that in the non-transfected control group as well as the transfected control group (p < 0.05). In addition, the percentage of MG-63 cells undergoing apoptosis was significantly higher in the TRAF6 siRNA-transfected group compared with the non-transfected control group as well as the transfected control group (p < 0.05). The expression of p-p65, cyclin D1, MMP-9 was down-regulated in the MG-63 cells from TRAF6 siRNA-transfected group. The expression of caspase 3 was up-regulated in the MG-63 cells from TRAF6 siRNA-transfected group compared to the non-transfected control group as well as the transfected control group (p < 0.05). To make a long story short, the overexpression of TRAF6 in osteosarcoma might be related to the tumorigenesis, invasion of osteosarcoma. 
 TRAF6 promotes the invasion and metastasis and predicts a poor prognosis in gastric cancer. This study investigated the relationships of TRAF6 expression with clinical pathologic parameters and the prognosis of patients with gastric cancer. This study also explored the roles of TRAF6 in cell apoptosis and   invasive  ness, as well as underlying molecular mechanism of gastric cancer cell line in vitro.A total of 90 cases of tissue microarrays were immunohistochemically analyzed for TRAF6 expression. Cell proliferation was measured by MTT assay. Flow cytometry was used for analyzing cell apoptosis and cell invasion ability was detected by a Transwell invasion assay. Protein expression was assessed by Western blotting.TRAF6 was expressed in 53 of 90 (58.9%) cases of gastric cancer. TRAF6 expression was significantly positively correlated with advanced N stage, pathological stage and a poor prognosis, but not an independent predictor of a poor prognosis in gastric cancer (p=0.083). The knockdown of TRAF6 increased cell apoptosis and reduced   invasive   ability of BGC-823 cell. Moreover, TRAF6 down-regulation decreased protein levels of phosphor-Akt, Bcl-2 and MMP9 and up-regulation of Bax in BGC-823 cell. Inversely, overexpression of TRAF6 in SGC-7901 cells increased protein levels of phosphor-Akt, Bcl-2 and MMP9 and down-regulation of Bax.The expression of TRAF6 was positively correlated with an advanced N stage and acted as a predictor of a poor prognosis in patients with gastric cancer. Moreover, TRAF6 regulating cell apoptosis and   invasive   ability of gastric cancer cells might be associated with Akt activation and alterations of protein expression of Bcl2, Bax and MMP9. 
  [Mm]etastasis  PubMed  
 Interleukin-8 induces nuclear transcription factor-kappaB through a TRAF6-dependent pathway. Considering the potential role of interleukin-8 (IL-8) in inflammation, angiogenesis, tumorigenesis, and   metastasis  , we investigated the molecular mechanism involved in IL-8-mediated signaling. In this report we provide evidence that like TNF, an inducer of NF-kappaB and also a NF-kappaB-dependent gene product, IL-8 induces NF-kappaB in a unique pathway. IL-8 induces NF-kappaB activation in a dose-dependent manner in different cell types as detected by a DNA-protein binding assay. IL-8 induces NF-kappaB-dependent reporter gene expression as well as ICAM-1, VCAM-1, and Cox-2 expression. IL-8 also induces IkappaBalpha phosphorylation followed by degradation and p65 translocation. IL-8 induces c-Jun N-terminal kinase (JNK) and mitogen-activated protein kinase (MAPK) in a dose- and time-dependent manner. IL-8-induced NF-kappaB activation is for the most part unaltered when cells are transfected with dominant-negative TRADD, FADD, or TRAF2, but is inhibited with dominant-negative TRAF6-, NIK-, IKK-, or IkappaBalpha-transfected cells. The data suggest that IL-8-induced NF-kappaB activation proceeds through a TRAF2-independent but TRAF6-dependent pathway, followed by recruitment of IRAK and activation of IKK. IL-8-induced NF-kappaB activation is not observed in a cell-permeable peptide that has TRAF6 binding motif-treated cells or IRAK-deficient cells. IL-8-induced NF-kappaB activation proceeds mostly through interaction with TRAF6 and partially through the Rho-GTPase pathways. This is the first report that IL-8 induces NF-kappaB in a distinct pathway, and activation of NF-kappaB and its dependent genes may be one of the pathways of IL-8-induced inflammation and angiogenesis. 
 Interleukin-8 induces nuclear transcription factor-kappaB through a TRAF6-dependent pathway. Considering the potential role of interleukin-8 (IL-8) in inflammation, angiogenesis, tumorigenesis, and   metastasis  , we investigated the molecular mechanism involved in IL-8-mediated signaling. In this report we provide evidence that like TNF, an inducer of NF-kappaB and also a NF-kappaB-dependent gene product, IL-8 induces NF-kappaB in a unique pathway. IL-8 induces NF-kappaB activation in a dose-dependent manner in different cell types as detected by a DNA-protein binding assay. IL-8 induces NF-kappaB-dependent reporter gene expression as well as ICAM-1, VCAM-1, and Cox-2 expression. IL-8 also induces IkappaBalpha phosphorylation followed by degradation and p65 translocation. IL-8 induces c-Jun N-terminal kinase (JNK) and mitogen-activated protein kinase (MAPK) in a dose- and time-dependent manner. IL-8-induced NF-kappaB activation is for the most part unaltered when cells are transfected with dominant-negative TRADD, FADD, or TRAF2, but is inhibited with dominant-negative TRAF6-, NIK-, IKK-, or IkappaBalpha-transfected cells. The data suggest that IL-8-induced NF-kappaB activation proceeds through a TRAF2-independent but TRAF6-dependent pathway, followed by recruitment of IRAK and activation of IKK. IL-8-induced NF-kappaB activation is not observed in a cell-permeable peptide that has TRAF6 binding motif-treated cells or IRAK-deficient cells. IL-8-induced NF-kappaB activation proceeds mostly through interaction with TRAF6 and partially through the Rho-GTPase pathways. This is the first report that IL-8 induces NF-kappaB in a distinct pathway, and activation of NF-kappaB and its dependent genes may be one of the pathways of IL-8-induced inflammation and angiogenesis. 
 Role of CYB5A in pancreatic cancer prognosis and autophagy modulation. Loss of 18q22.3 is a prognostic marker in pancreatic ductal adenocarcinoma (PDAC). This study investigated genes encoded by this cytoband.We studied mRNA/protein expression in radically resected (n = 130) and metastatic patients (n = 50). The role of CYB5A was tested in 11 PDAC cell lines and five primary cultures through retrovirus-mediated upregulation and small interfering RNA using wound-healing, invasion, annexin-V, electron microscopy, and autophagic assays, as well as autophagy genes and kinases arrays. CYB5A+ orthotopic models (n = 6 mice/group) were monitored by Firefly and Gaussia-luciferase bioluminescence, magnetic resonance imaging, and high-frequency ultrasound. Data were analyzed by t test, Fisher exact-test, log-rank test and Cox proportional hazards models. All statistical tests were two-sided.Both resected and metastatic patients with low mRNA or protein expression of CYB5A had statistically significantly shorter survival (eg, median = 16.7 months, 95% confidence interval [CI] = 13.5 to 19.9; vs median = 24.8 months, 95% CI = 12.8 to 36.9; P = .02, two-sided log-rank test; n = 82 radically resected PDACs), and multivariable analyses confirmed prognostic relevance. Moreover, we characterized a novel function to CYB5A, autophagy induction, concomitant with reduced proliferation and migration/invasion of PDAC cells. Network analysis of proautophagic pathways suggested CYB5A interaction with TRAF6, which was confirmed by TRAF6 downregulation after CYB5A reconstitution (-69% in SU.86.86-CYB5A+; P = .005, two-sided t test). CYB5A silencing had opposite effects, restoring TRAF6 expression and wound healing. In vivo studies showed that CYB5A induced autophagy while inhibiting tumor growth/  metastasis   and increasing survival (median = 57 days, 95% CI = 52 to 61; vs median = 44 days, 95% CI = 21 to 57; P = .03, two-sided log-rank test).These results define CYB5A as a novel prognostic factor for PDAC that exerts its tumor-suppressor function through autophagy induction and TRAF6 modulation. 
 TRAF6 regulates melanoma invasion and   metastasis   through ubiquitination of Basigin. TRAF6 plays a crucial role in the regulation of the innate and adaptive immune responses. Although studies have shown that TRAF6 has oncogenic activity, the role of TRAF6 in melanoma is unclear. Here, we report that TRAF6 is overexpressed in primary as well as metastatic melanoma tumors and melanoma cell lines. Knockdown of TRAF6 with shRNA significantly suppressed malignant phenotypes including cell proliferation, anchorage-independent cell growth and   metastasis   in vitro and in vivo. Notably, we demonstrated that Basigin (BSG)/CD147, a critical molecule for cancer cell invasion and   metastasis  , is a novel interacting partner of TRAF6. Furthermore, depletion of TRAF6 by shRNA reduced the recruitment of BSG to the plasma membrane and K63-linked ubiquitination, in turn, which impaired BSG-dependent MMP9 induction. Taken together, our findings indicate that TRAF6 is involved in regulating melanoma invasion and   metastasis  , suggesting that TRAF6 may be a potential target for therapy or chemo-prevention in melanoma. 
 TRAF6 promoted the   metastasis   of esophageal squamous cell carcinoma. Esophageal cancer is one of the most aggressive malignancies and has been ranked as the sixth leading cause of cancer-related death in the world. It is very urgent to find new therapeutic targets. Although tumor necrosis factor receptor-associated factor 6 (TRAF6) was initially identified as an adaptor for NF-κB signaling, recently it has been found to be involved in cancer by modulating various signaling pathways. In the previous study, we have found that TRAF6 promoted the growth of esophageal squamous cell carcinoma (ESCC) cell in vitro and in vivo. However, the effects of TRAF6 on the migration and   metastasi  s of ESCC cells are poorly understood. Here, we found that TRAF6 promoted migration and   metastasi  s of ESCC cells through modulating Ras signaling. Overexpression of TRAF6 promoted the migration of ESCC cells and immortalized esophageal epithelial cells, while knock down the expression of TRAF6 inhibited the migration and   metastasi  s of ESCC cells in vitro and in vivo. Mechanically, TRAF6 binds Ras with its N-terminal and activated Ras signaling. Taken together, TRAF6 played an important role in the   metastasi  s of ESCC and might be a promising therapeutic target. 
 Functional role of asparaginyl endopeptidase ubiquitination by TRAF6 in tumor invasion and   metastasis  . Asparaginyl endopeptidase (AEP) has been implicated in human cancer development. However, the molecular mechanisms underlying AEP regulation, including the role of pro-AEP activation, remain elusive.We investigated the regulation of AEP by TRAF6 and its effects on tumor progression and   metastasis   in cancer cell lines, murine models, and specimens from patients using biochemical analyses, confocal microscopy, immunoelectron microscopy, and migration-invasion assays. The sera of healthy donors and breast cancer patients were examined by enzyme-linked immunosorbent assay, and a tissue array of 314 breast cancer specimens was assessed for AEP and TRAF6 by immunohistochemistry. Furthermore, the effects of AEP inhibitors or monoclonal antibodies on pulmonary   metastasis   were evaluated in murine models. The statistical significance between groups was determined using two-tailed Student t tests.We demonstrate that TRAF6 ubiquitinates the proform of AEP through K63-linked polyubiquitin, reversible by USP17, and forms a complex with HSP90α to subsequently promote pro-AEP intracellular stability as well as secretion. Disrupting the interaction between pro-AEP and TRAF6 or inhibiting HSP90α reduced pro-AEP secretion and consequently reduced tumo  r metastas  is. Higher circulating AEP levels were detected in the sera of breast cancer patients, and AEP inhibitors or neutralizing antibodies remarkably decreased tumo  r metastas  is in murine models. Notably, TRAF6 and AEP were overexpressed in human breast neoplasms and correlated with poor prognosis. Patients with low AEP/TRAF6 expression survived for a mean of 111 months (95% confidence interval [CI] = 108 to 115 months), whereas those with high AEP/TRAF6 expression survived for a mean of only 61 months (95% CI = 42 to 79 months; P < .001).Our study elucidates a novel mechanism of AEP regulation and an alternative oncogenic pathway for TRAF6 in breast cancer, which suggests that AEP and TRAF6 protein levels may have prognostic implications in breast cancer patients. Thus, AEP may serve as a biomarker as well as new therapeutic target. 
 TRAF6 regulates proliferation, apoptosis, and invasion of osteosarcoma cell. TRAF6, a unique tumor necrosis factor receptor-associated factor (TRAF) family member, possesses a unique receptor-binding specificity that results in its crucial role as the signaling mediator for TNF receptor superfamily and interleukin-1 receptor/Toll-like receptor superfamily. TRAF6 plays an important role in tumorigenesis, invasion and   metastasis  . This study aimed to explore the expression of TRAF6 in osteosarcoma tissues and its correlation to the clinical pathology of osteosarcoma and to discuss the relationship between TRAF6 expression and osteosarcoma invasion. These data will provide the experimental base for the biological treatment of osteosarcoma in the future. Using RT-PCR and Western blot, the results showed that the expression rate of TRAF6 mRNA in osteosarcoma tissues was significantly higher than that in normal bone tissue (p < 0.05), that the expression rate of TRAF6 mRNA in the carcinoma tissues from patients with lung   metastasis   was significantly higher than that from patients without lung   metastasis   (p < 0.05), and that the expression rate of TRAF6 mRNA also increased with increasing Enneking stage (p < 0.05). However, the mRNA expression of TRAF6 in osteosarcoma was independent of the patient's gender, age, and tumor size (p > 0.05). The TRAF6 protein displayed an up-regulation in osteosarcoma tissues compared to normal bone tissue (p < 0.05), displayed an up-regulation in osteosarcoma tissues from patients with lung   metastasis   compared to from patients without lung   metastasis   (p < 0.05), and displayed a gradual increase with increasing Enneking stage (p < 0.05). By the technique of RNA interference, the expression of TRAF6 in the human osteosarcoma MG-63 cell line was down-regulated, and the invasive ability of MG-63 cells was examined. The results showed that TRAF6 protein expression was significantly decreased in the MG-63 cells from TRAF6 siRNA-transfected group (p < 0.05), and the proliferation ability of MG-63 cells and the number of MG-63 cells that passed through the Transwell chamber were significantly lower than that in the non-transfected control group as well as the transfected control group (p < 0.05). In addition, the percentage of MG-63 cells undergoing apoptosis was significantly higher in the TRAF6 siRNA-transfected group compared with the non-transfected control group as well as the transfected control group (p < 0.05). The expression of p-p65, cyclin D1, MMP-9 was down-regulated in the MG-63 cells from TRAF6 siRNA-transfected group. The expression of caspase 3 was up-regulated in the MG-63 cells from TRAF6 siRNA-transfected group compared to the non-transfected control group as well as the transfected control group (p < 0.05). To make a long story short, the overexpression of TRAF6 in osteosarcoma might be related to the tumorigenesis, invasion of osteosarcoma. 
 Carboxyl terminus of Hsp70-interacting protein regulation of osteoclast formation in mice through promotion of tumor necrosis factor receptor-associated factor 6 protein degradation. Carboxyl terminus of Hsp70-interacting protein (CHIP or STUB1) is an E3 ligase that regulates the stability of several proteins involved in tumor growth and   metastasis  . However, the role of CHIP in bone growth and bone remodeling in vivo has not been reported. This study was undertaken to investigate the role and mechanism of CHIP in regulation of bone mass and bone remodeling.The bone phenotype of Chip(-/-) mice was assessed by histologic, histomorphometric, and micro-computed tomographic analyses. The mechanism by which CHIP regulates the degradation of tumor necrosis factor receptor-associated factor 6 (TRAF6) and the inhibition of NF-κB signaling was examined by immunoprecipitation, Western blot, and luciferase reporter assays.Deletion of the Chip gene led to an osteopenic phenotype and increased osteoclast formation. TRAF6, an adaptor protein that is a key regulator of NF-κB signaling and is critical for RANKL-induced osteoclastogenesis, was up-regulated in osteoclasts from Chip(-/-) mice. CHIP interacted with TRAF6 to promote TRAF6 ubiquitination and proteasome degradation. Further, CHIP inhibited p65 nuclear translocation, leading to the repression of TRAF6-mediated NF-κB transcription.CHIP inhibits NF-κB signaling by promoting TRAF6 degradation and plays an important role in osteoclastogenesis and bone remodeling. These findings suggest that CHIP may be a novel therapeutic target in bone loss-associated disorders. 
 Expression of TRAF6 and ubiquitin mRNA in skeletal muscle of gastric cancer patients. To investigate the prognostic significance of tumor necrosis factor receptor (TNFR),-associated factor 6 (TRAF6),-and ubiquitin in gastric cancer patients.Biopsies of the rectus abdominis muscle were obtained intra operatively from 102 gastric cancer patients and 29 subjects undergoing surgery for benign abdominal diseases, and muscle TRAF6 and ubiquitin mRNA expression and proteasome proteolytic activities were assessed.TRAF6 was significantly upregulated in muscle of gastric cancer compared with the control muscles. TRAF6 was upregulated in 67.65% (69/102) muscle of gastric cancer. Over expression of TRAF6 in muscles of gastric cancer were associated with TNM stage, level of serum albumin and percent of weight loss. Ubiquitin was significantly upregulated in muscle of gastric cancer compared with the control muscles. Ubiquitin was upregulated in 58.82% (60/102) muscles of gastric cancer. Over expression of ubiquitin in muscles of gastric cancer were associated with TNM (Tumor-Node-  Metastasis  ) stage and weight loss. There was significant relation between TRAF6 and ubiquitin expression.We found a positive correlation between TRAF6 and ubiquitin expression, suggesting that TRAF6 may up regulates ubiquitin activity in cancer cachexia. While more investigations are required to understand its mechanisms of TRAF6 and ubiquitin in skeletal muscle. Correct the catabolic-anabolic imbalance is essential for the effective treatment of cancer cachexia. 
 Expression of tumor necrosis factor receptor-associated factor 6 in lung cancer tissues. Tumor necrosis factor (TNF) receptor-associated factor 6 (TRAF6) has been reported to be associated with the development of various cancers. However, the role of TRAF6 in lung cancer remains unclear.To explore the expression and clinicopathological significance of TRAF6 protein in lung cancer tissues.Three hundred and sixty-five lung cancer samples and thirty normal lung tissues were constructed into 3 microarrays. The expression of TRAF6 protein was determined using immunohistochemistry (IHC). Furthermore, correlations between the expression of TRAF6 and clinicopathological parameters were investigated.The expression of TRAF6 in total lung cancer tissues (365 cases), as well as in small cell lung cancer (SCLC, 26 cases) and non-small cell lung cancer (NSCLC, 339 cases) was significantly higher compared with that in normal lung tissues. The ROC curve showed that the area under curve of TRAF6 was 0.663 (95%CI 0.570~0.756) for lung cancer. The diagnostic sensitivity and specificity of TRAF6 were 52.6% and 80%, respectively. In addition, the expression of TRAF6 was correlated with clinical TNM stage, tumor size and lymph node   metastasis   in all lung cancers. Consistent correlations were also observed for NSCLCs.TRAF6 might be an oncogene and the expression of TRAF6 protein is related to the progression of lung cancer. Thus, TRAF6 might become a target for diagnosis and gene therapy for lung cancer patients. 
 miR-146a enhances the oncogenicity of oral carcinoma by concomitant targeting of the IRAK1, TRAF6 and NUMB genes. MicroRNAs are short non-coding RNAs that regulate gene expression and are crucial to tumorigenesis. Oral squamous cell carcinoma (OSCC) is a prevalent malignancy worldwide. Up-regulation of miR-146 has been identified in OSCC tissues. However, the roles of miR-146 in carcinogenesis are controversial as it is suppressive in many other malignancies. The present study investigated the pathogenic implications of miR-146a in oral carcinogenesis. Microdissected OSCC exhibits higher levels of miR-146a expression than matched adjacent mucosal cells. The plasma miR-146a levels of patients are significantly higher than those of control subjects; these levels decrease drastically after tumor resection. miR-146a levels in tumors and in patients' plasma can be used to classify OSCC and non-disease status (sensitivity: >0.72). Exogenous miR-146a expression is significantly increased in vitro oncogenic phenotypes as well as during xenograft tumorigenesis and OSCC   metastasis  . The plasma miR-146a levels of these mice parallel the xenograft tumor burdens of the mice. A miR-146a blocker abrogates the growth of xenograft tumors. miR-146a oncogenic activity is associated with down-regulation of IRAK1, TRAF6 and NUMB expression. Furthermore, miR-146a directly targets the 3'UTR of NUMB and a region within the NUMB coding sequence when suppressing NUMB expression. Exogenous NUMB expression attenuates OSCC oncogenicity. Double knockdown of IRAK1 and TRAF6, and of TRAF6 and NUMB, enhance the oncogenic phenotypes of OSCC cells. Oncogenic enhancement modulated by miR-146a expression is attenuated by exogenous IRAK1 or NUMB expression. This study shows that miR-146a expression contributes to oral carcinogenesis by targeting the IRAK1, TRAF6 and NUMB genes. 
 TRAF6 promotes the invasion and   metastasis   and predicts a poor prognosis in gastric cancer. This study investigated the relationships of TRAF6 expression with clinical pathologic parameters and the prognosis of patients with gastric cancer. This study also explored the roles of TRAF6 in cell apoptosis and invasiveness, as well as underlying molecular mechanism of gastric cancer cell line in vitro.A total of 90 cases of tissue microarrays were immunohistochemically analyzed for TRAF6 expression. Cell proliferation was measured by MTT assay. Flow cytometry was used for analyzing cell apoptosis and cell invasion ability was detected by a Transwell invasion assay. Protein expression was assessed by Western blotting.TRAF6 was expressed in 53 of 90 (58.9%) cases of gastric cancer. TRAF6 expression was significantly positively correlated with advanced N stage, pathological stage and a poor prognosis, but not an independent predictor of a poor prognosis in gastric cancer (p=0.083). The knockdown of TRAF6 increased cell apoptosis and reduced invasive ability of BGC-823 cell. Moreover, TRAF6 down-regulation decreased protein levels of phosphor-Akt, Bcl-2 and MMP9 and up-regulation of Bax in BGC-823 cell. Inversely, overexpression of TRAF6 in SGC-7901 cells increased protein levels of phosphor-Akt, Bcl-2 and MMP9 and down-regulation of Bax.The expression of TRAF6 was positively correlated with an advanced N stage and acted as a predictor of a poor prognosis in patients with gastric cancer. Moreover, TRAF6 regulating cell apoptosis and invasive ability of gastric cancer cells might be associated with Akt activation and alterations of protein expression of Bcl2, Bax and MMP9. 
  GeneRIFs  
 High TRAF6 expression is associated with melanoma invasion and   metastasis  . 
 TRAF6 promoted the   metastasis   of esophageal squamous cell carcinoma. 
  [Cc]ell [Ii]nvasion  PubMed  
 TRAF6 regulates melanoma invasion and metastasis through ubiquitination of Basigin. TRAF6 plays a crucial role in the regulation of the innate and adaptive immune responses. Although studies have shown that TRAF6 has oncogenic activity, the role of TRAF6 in melanoma is unclear. Here, we report that TRAF6 is overexpressed in primary as well as metastatic melanoma tumors and melanoma cell lines. Knockdown of TRAF6 with shRNA significantly suppressed malignant phenotypes including cell proliferation, anchorage-independent cell growth and metastasis in vitro and in vivo. Notably, we demonstrated that Basigin (BSG)/CD147, a critical molecule for cancer   cell invasion   and metastasis, is a novel interacting partner of TRAF6. Furthermore, depletion of TRAF6 by shRNA reduced the recruitment of BSG to the plasma membrane and K63-linked ubiquitination, in turn, which impaired BSG-dependent MMP9 induction. Taken together, our findings indicate that TRAF6 is involved in regulating melanoma invasion and metastasis, suggesting that TRAF6 may be a potential target for therapy or chemo-prevention in melanoma. 
 TRAF6 promotes the invasion and metastasis and predicts a poor prognosis in gastric cancer. This study investigated the relationships of TRAF6 expression with clinical pathologic parameters and the prognosis of patients with gastric cancer. This study also explored the roles of TRAF6 in cell apoptosis and invasiveness, as well as underlying molecular mechanism of gastric cancer cell line in vitro.A total of 90 cases of tissue microarrays were immunohistochemically analyzed for TRAF6 expression. Cell proliferation was measured by MTT assay. Flow cytometry was used for analyzing cell apoptosis and   cell invasion   ability was detected by a Transwell invasion assay. Protein expression was assessed by Western blotting.TRAF6 was expressed in 53 of 90 (58.9%) cases of gastric cancer. TRAF6 expression was significantly positively correlated with advanced N stage, pathological stage and a poor prognosis, but not an independent predictor of a poor prognosis in gastric cancer (p=0.083). The knockdown of TRAF6 increased cell apoptosis and reduced invasive ability of BGC-823 cell. Moreover, TRAF6 down-regulation decreased protein levels of phosphor-Akt, Bcl-2 and MMP9 and up-regulation of Bax in BGC-823 cell. Inversely, overexpression of TRAF6 in SGC-7901 cells increased protein levels of phosphor-Akt, Bcl-2 and MMP9 and down-regulation of Bax.The expression of TRAF6 was positively correlated with an advanced N stage and acted as a predictor of a poor prognosis in patients with gastric cancer. Moreover, TRAF6 regulating cell apoptosis and invasive ability of gastric cancer cells might be associated with Akt activation and alterations of protein expression of Bcl2, Bax and MMP9. 
   
  
 RAB31 
 
  NCBI Gene  
  GeneRIF  
  Pubmed  
 
 
  Search Term  Rank  Fields  
  [Ee]xtracellular [Mm]atrix  8  PubMed(1)  
  [Ii]nvasive  10  PubMed(1); GeneRIFs(1)  
  [Mm]etastasis  12  PubMed(3)  
 
 
 hide/show details for RAB31 
  [Ee]xtracellular [Mm]atrix  PubMed  
 Rab31 expression levels modulate tumor-relevant characteristics of breast cancer cells. Rab proteins constitute a large family of monomeric GTP-binding proteins that regulate intracellular vesicle transport. Several Rab proteins, including rab31, have been shown to affect cancer progression and are related with prognosis in various types of cancer including breast cancer. Recently, the gene encoding rab31 was found to be overexpressed in estrogen receptor-positive breast cancer tissue. In a previous study we found a significant association of high rab31 mRNA expression with poor prognosis in node-negative breast cancer patients. In the present study, we aimed to investigate the impact of rab31 (over)-expression on important aspects of tumor progression in vitro and in vivo.Breast cancer cells displaying low (MDA-MB-231) or no (CAMA-1) endogenous rab31 expression were stably transfected with a rab31 expression plasmid. Batch-transfected cells as well as selected cell clones, expressing different levels of rab31 protein, were analyzed with regard to proliferation, cell adhesion, the invasive capacity of tumor cells, and in vivo in a xenograft tumor model. Polyclonal antibodies directed to recombinantly expressed rab31 were generated and protein expression analyzed by immunohistochemistry, Western blot analysis, and a newly developed sensitive ELISA.Elevated rab31 protein levels were associated with enhanced proliferation of breast cancer cells. Interestingly, weak to moderate overexpression of rab31 in cell lines with no detectable endogenous rab31 expression was already sufficient to elicit distinct effects on cell proliferation. By contrast, increased expression of rab31 in breast cancer cells led to reduced adhesion towards several   extracellular matrix   proteins and decreased invasive capacity through Matrigel(TM). Again, the rab31-mediated effects on cell adhesion and invasion were dose-dependent. Finally, in a xenograft mouse model, we observed a significantly impaired metastatic dissemination of rab31 overexpressing MDA-MB-231 breast cancer cells to the lung.Overexpression of rab31 in breast cancer cells leads to a switch from an invasive to a proliferative phenotype as indicated by an increased cell proliferation, reduced adhesion and invasion in vitro, and a reduced capacity to form lung metastases in vivo. 
  [Ii]nvasive  PubMed  
 Rab31 expression levels modulate tumor-relevant characteristics of breast cancer cells. Rab proteins constitute a large family of monomeric GTP-binding proteins that regulate intracellular vesicle transport. Several Rab proteins, including rab31, have been shown to affect cancer progression and are related with prognosis in various types of cancer including breast cancer. Recently, the gene encoding rab31 was found to be overexpressed in estrogen receptor-positive breast cancer tissue. In a previous study we found a significant association of high rab31 mRNA expression with poor prognosis in node-negative breast cancer patients. In the present study, we aimed to investigate the impact of rab31 (over)-expression on important aspects of tumor progression in vitro and in vivo.Breast cancer cells displaying low (MDA-MB-231) or no (CAMA-1) endogenous rab31 expression were stably transfected with a rab31 expression plasmid. Batch-transfected cells as well as selected cell clones, expressing different levels of rab31 protein, were analyzed with regard to proliferation, cell adhesion, the   invasive   capacity of tumor cells, and in vivo in a xenograft tumor model. Polyclonal antibodies directed to recombinantly expressed rab31 were generated and protein expression analyzed by immunohistochemistry, Western blot analysis, and a newly developed sensitive ELISA.Elevated rab31 protein levels were associated with enhanced proliferation of breast cancer cells. Interestingly, weak to moderate overexpression of rab31 in cell lines with no detectable endogenous rab31 expression was already sufficient to elicit distinct effects on cell proliferation. By contrast, increased expression of rab31 in breast cancer cells led to reduced adhesion towards several extracellular matrix proteins and decreased   invasive   capacity through Matrigel(TM). Again, the rab31-mediated effects on cell adhesion and invasion were dose-dependent. Finally, in a xenograft mouse model, we observed a significantly impaired metastatic dissemination of rab31 overexpressing MDA-MB-231 breast cancer cells to the lung.Overexpression of rab31 in breast cancer cells leads to a switch from an   invasive   to a proliferative phenotype as indicated by an increased cell proliferation, reduced adhesion and invasion in vitro, and a reduced capacity to form lung metastases in vivo. 
  GeneRIFs  
 Overexpression of rab31 in breast cancer cells leads to a switch from an   invasive   to a proliferative phenotype as indicated by an increased cell proliferation, reduced adhesion and invasion in vitro, and a reduced capacity to form lung metastases in vivo. 
  [Mm]etastasis  PubMed  
 Urokinase receptor splice variant uPAR-del4/5-associated gene expression in breast cancer: identification of rab31 as an independent prognostic factor. To evaluate the pure prognostic impact of the uPA-receptor splice variant uPAR-del4/5 for lymph node-negative breast cancer patients, and to identify differentially expressed genes associated with high or low uPAR-del4/5 mRNA levels.mRNA transcript levels were measured by real-time PCR in tumor samples from 280 node-negative breast cancer patients who had not received adjuvant systemic therapy. Endpoints were distant   metastasis  -free survival (DMFS) and overall survival (OS). Gene expression analysis was performed with RNA isolated from breast cancer tissue and breast cancer cell lines using Affymetrix U133a GeneChips.In multivariate analysis, uPAR-del4/5 significantly contributed to the base model of traditional prognostic factors for DMFS (HR = 3.29, P < 0.001) and OS (HR = 2.87, P = 0.002). Using microarrays, seven genes were found to be up-regulated in tumor samples and cancer cell lines with high uPAR-del4/5 mRNA expression. The gene encoding rab31, a member of the Ras oncogene family, was selected for quantitative analysis of mRNA expression in the set of 280 patients. High rab31 values were significantly associated with worse outcome of patients for DMFS (HR = 2.27, P < 0.001) and OS (HR = 2.01, P = 0.008) in multivariate analysis, independent from uPAR-del4/5. The patient subgroup with high uPAR-del4/5 and rab31 levels showed the worst DMFS and OS (P < 0.001, both) compared with tumors with low values of both factors.Our results suggest that uPAR-del4/5 and rab31 mRNA represent independent prognostic markers in breast cancer and may be components of different, but possibly associated, tumor-relevant signaling pathways. 
 mRNA expression levels of the biological factors uPAR, uPAR-del4/5, and rab31, displaying prognostic value in breast cancer, are not clinically relevant in advanced ovarian cancer. High tumor tissue mRNA expression of the tumor biological factors uPAR, uPAR-del4/5, or rab31 is associated with shorter distant   metastasis  -free and overall survival in breast cancer patients. To evaluate whether these factors are also clinically relevant in ovarian cancer, we quantified the respective mRNA levels in primary tumor tissue of advanced ovarian cancer patients (n=103) and evaluated their association with clinicopathological parameters and patients' prognosis. mRNA expression levels of all three markers did not show any significant association with overall or progression-free survival, demonstrating that these factors have no prognostic value in advanced ovarian cancer. 
 Rab31 promoted hepatocellular carcinoma (HCC) progression via inhibition of cell apoptosis induced by PI3K/AKT/Bcl-2/BAX pathway. Rab31 belongs to the Ras superfamily of small GTP-binding proteins, which has been found to regulate the vesicle transport from the Golgi apparatus to early and late endosomes. The investigation here detected the expression of Rab31 in 96 patients with hepatocellular carcinoma (HCC) and tried to identify its significance on outcome of HCCs after liver resection. By immunohistochemistry staining, it was found that Rab31 expression in HCC tissues was remarkably higher than that in adjacent liver tissues. Aberrant Rab31 overexpression in HCC tissues was identified to be associated with worse prognosis after liver resection. Univariate analysis showed that advanced tumor-nodes-  metastasis   (TNM) staging of HCC, intrahepatic metastases, portal vein invasion, and higher Rab31 were the predictive factors of poor prognosis. Multivariate analysis demonstrated that intrahepatic metastases and higher Rab31 were the independent prognostic factors. Furthermore, forced expression of Rab31 in Huh7 cells was found to promote cell growth via upregulation of Bcl-2/BAX ratio induced by PI3K/AKT. Correspondingly, silencing Rab31 induced cell apoptosis and in turn suppressed the growth capacity of MHCC97 cells in vitro. Taken together, this study provides the evidence of Rab31 overexpression in HCC, and Rab31 is potentially used as a novel biomarker of poor prognosis in patients with HCC. PI3K/AKT/Bcl-2/BAX axis was involved in Rab31-promoting HCC progression. 
   
  
 RAB5A 
 
  NCBI Gene  
  GeneRIF  
  Pubmed  
 
 
  Search Term  Rank  Fields  
  [Ff]ilopodia  1  PubMed(3)  
  [Ff]ilopodium  2  Process(1)  
  [Pp]odosome(s)?  5  PubMed(2)  
  [Mm]igration  6  PubMed(22); GeneRIFs(9)  
  ECM  7  PubMed(1)  
  [Ee]xtracellular [Mm]atrix  8  PubMed(3)  
  [Ii]nvadosome  9  PubMed(1)  
  [Ii]nvasive  10  PubMed(6); GeneRIFs(1)  
  [Mm]etastasize  11  PubMed(1)  
  [Mm]etastasis  12  PubMed(13); GeneRIFs(4)  
  [Cc]ell [Ii]nvasion  13  PubMed(4); GeneRIFs(1)  
 
 
 hide/show details for RAB5A 
  [Ff]ilopodia  PubMed  
 The TRE17 oncogene encodes a component of a novel effector pathway for Rho GTPases Cdc42 and Rac1 and stimulates actin remodeling. The Rho family GTPases Cdc42 and Rac1 play fundamental roles in transformation and actin remodeling. Here, we demonstrate that the TRE17 oncogene encodes a component of a novel effector pathway for these GTPases. TRE17 coprecipitated specifically with the active forms of Cdc42 and Rac1 in vivo. Furthermore, the subcellular localization of TRE17 was dramatically regulated by these GTPases and mitogens. Under serum-starved conditions, TRE17 localized predominantly to filamentous structures within the cell. Epidermal growth factor (EGF) induced relocalization of TRE17 to the plasma membrane in a Cdc42-/Rac1-dependent manner. Coexpression of activated alleles of Cdc42 or Rac1 also caused complete redistribution of TRE17 to the plasma membrane, where it partially colocalized with the GTPases in   filopodia   and ruffles, respectively. Membrane recruitment of TRE17 by EGF or the GTPases was dependent on actin polymerization. Finally, we found that a C-terminal truncation mutant of TRE17 induced the accumulation of cortical actin, mimicking the effects of activated Cdc42. Together, these results identify TRE17 as part of a novel effector complex for Cdc42 and Rac1, potentially contributing to their effects on actin remodeling. The present study provides insights into the regulation and cellular function of this previously uncharacterized oncogene. 
 Regulation of dendritic branching and   filopodia   formation in hippocampal neurons by specific acylated protein motifs. Although neuronal axons and dendrites with their associated   filopodia   and spines exhibit a profound cell polarity, the mechanism by which they develop is largely unknown. Here, we demonstrate that specific palmitoylated protein motifs, characterized by two adjacent cysteines and nearby basic residues, are sufficient to induce   filopodia  l extensions in heterologous cells and to increase the number of   filopodia   and the branching of dendrites and axons in neurons. Such motifs are present at the N-terminus of GAP-43 and the C-terminus of paralemmin, two neuronal proteins implicated in cytoskeletal organization and   filopodia  l outgrowth.   Filopodia   induction is blocked by mutations of the palmitoylated sites or by treatment with 2-bromopalmitate, an agent that inhibits protein palmitoylation. Moreover, overexpression of a constitutively active form of ARF6, a GTPase that regulates membrane cycling and dendritic branching reversed the effects of the acylated protein motifs.   Filopodia   induction by the specific palmitoylated motifs was also reduced upon overexpression of a dominant negative form of the GTPase cdc42. These results demonstrate that select dually lipidated protein motifs trigger changes in the development and growth of neuronal processes. 
 Knockdown of Rab5a expression decreases cancer cell motility and invasion through integrin-mediated signaling pathway. Rab GTPases function as modulators in intracellular transport. Rab5a, a member of the Rab subfamily of small GTPases, is an important regulator of vesicle traffic from the plasma membrane to early endosomes. Recent findings have reported that Rab5a gene was involved in the progression of cancer. In the present study, we investigated the effect of Rab5a on cervical cancer invasion and metastasis and the molecular mechanism underlying the involvement of Rab5a.Rab5a expression was assessed by immunohistochemical analysis on a cervical cancer tissue microarray. RNA interference (RNAi) was performed to knock down the endogenous expression of Rab5a gene in HeLa and SiHa cells. Cell motility was evaluated using invasion assay and wound migration assay in vitro. The expression levels of integrin-associated molecules were detected by Western blot and immunofluorescence.We found that Rab5a was expressed at a high level in cervical cancer tissues. Silencing of Rab5a expression significantly decreased cancer cell motility and invasiveness. The down-regulation of integrin-associated focal adhesion signaling molecules was further detected in Rab5a knockdown cells. Meanwhile, active GTP-bound Rac1, Cdc42, and RhoA were also down-regulated, accompanied with the reduction in the number and size of   filopodia   and lamellipodia.Taken together, these data suggest that Rab5a functions in regulating the invasion phenotype, and we propose that this regulation may be via integrin-mediated signaling pathway in cervical cancer cells. 
  [Ff]ilopodium  Process  
 regulation of   filopodium   assembly 
  [Pp]odosome(s)?  PubMed  
 A specific subset of RabGTPases controls cell surface exposure of MT1-MMP, extracellular matrix degradation and three-dimensional invasion of macrophages. The matrix metalloproteinase MT1-MMP has a major impact on invasive cell migration in both physiological and pathological settings such as immune cell extravasation or metastasis of cancer cells. Surface-associated MT1-MMP is able to cleave components of the extracellular matrix, which is a prerequisite for proteolytic invasive migration. However, current knowledge on the molecular mechanisms that regulate MT1-MMP trafficking to and from the cell surface is limited. We have identified three members of the RabGTPase family, Rab5a, Rab8a and Rab14, as crucial regulators of MT1-MMP trafficking and function in primary human macrophages. Both overexpressed and endogenous forms show prominent colocalisation with MT1-MMP-positive vesicles, whereas expression of mutant constructs, as well as siRNA-induced knockdown, reveal that these RabGTPases are crucial in the regulation of MT1-MMP surface exposure, contact of MT1-MMP-positive vesicles with   podosomes  , extracellular matrix degradation in two and three dimensions, as well as three-dimensional proteolytic invasion of macrophages. Collectively, our results identify Rab5a, Rab8a and Rab14 as major regulators of MT1-MMP trafficking and invasive migration of primary human macrophages, which could be promising potential targets for manipulation of immune cell invasion. 
 A specific subset of RabGTPases controls cell surface exposure of MT1-MMP, extracellular matrix degradation and three-dimensional invasion of macrophages. The matrix metalloproteinase MT1-MMP has a major impact on invasive cell migration in both physiological and pathological settings such as immune cell extravasation or metastasis of cancer cells. Surface-associated MT1-MMP is able to cleave components of the extracellular matrix, which is a prerequisite for proteolytic invasive migration. However, current knowledge on the molecular mechanisms that regulate MT1-MMP trafficking to and from the cell surface is limited. We have identified three members of the RabGTPase family, Rab5a, Rab8a and Rab14, as crucial regulators of MT1-MMP trafficking and function in primary human macrophages. Both overexpressed and endogenous forms show prominent colocalisation with MT1-MMP-positive vesicles, whereas expression of mutant constructs, as well as siRNA-induced knockdown, reveal that these RabGTPases are crucial in the regulation of MT1-MMP surface exposure, contact of MT1-MMP-positive vesicles with   podosomes  , extracellular matrix degradation in two and three dimensions, as well as three-dimensional proteolytic invasion of macrophages. Collectively, our results identify Rab5a, Rab8a and Rab14 as major regulators of MT1-MMP trafficking and invasive migration of primary human macrophages, which could be promising potential targets for manipulation of immune cell invasion. 
  [Mm]igration  PubMed  
 Rab5 mediates caspase-8-promoted cell motility and metastasis. Caspase-8 is a key apical sensory protein that governs cell responses to environmental cues, alternatively promoting apoptosis, proliferation, and cell   migration  . The proteins responsible for integration of these pathways, however, have remained elusive. Here, we reveal that Rab5 regulates caspase-8-dependent signaling from integrins. Integrin ligation leads to Rab5 activation, association with integrins, and activation of Rac, in a caspase-8-dependent manner. Rab5 activation promotes colocalization and coprecipitation of integrins with caspase-8, concomitant with Rab5 recruitment to integrin-rich regions such as focal adhesions and membrane ruffles. Moreover, caspase-8 expression promotes Rab5-mediated internalization and the recycling of beta1 integrins, increasing cell   migration   independently of caspase catalytic activity. Conversely, Rab5 knockdown prevented caspase-8-mediated integrin signaling for Rac activation, cell   migration  , and apoptotic signaling, respectively. Similarly, Rab5 was critical for caspase-8-driven cell   migration   in vivo, because knockdown of Rab5 compromised the ability of caspase-8 to promote metastasis under nonapoptotic conditions. These studies identify Rab5 as a key integrator of caspase-8-mediated signal transduction downstream of integrins, regulating cell survival and   migration   in vivo and in vitro. 
 Rab5 isoforms orchestrate a "division of labor" in the endocytic network; Rab5C modulates Rac-mediated cell motility. Rab5, the prototypical Rab GTPase and master regulator of the endocytic pathway, is encoded as three differentially expressed isoforms, Rab5A, Rab5B and Rab5C. Here, we examined the differential effects of Rab5 isoform silencing on cell motility and report that Rab5C, but neither Rab5A nor Rab5B, is selectively associated with the growth factor-activation of Rac1 and with enhanced cell motility. Initial observations revealed that silencing of Rab5C expression, but neither Rab5A nor Rab5C, led to spindle-shaped cells that displayed reduced formation of membrane ruffles. When subjected to a scratch wound assay, cells depleted of Rab5C, but not Rab5A or Rab5B, demonstrated reduced cell   migration  . U937 cells depleted of Rab5C also displayed reduced cell motility in a Transwell plate   migration   assay. To examine activation of Rac, HeLa cells stably expressing GFP-Rac1 were independently depleted of Rab5A, Rab5B or Rab5C and seeded onto coverslips imprinted with a crossbow pattern. 3-D GFP-Rac1 images of micro-patterned cells show that GFP-Rac1 was less localized to the cell periphery in the absence of Rab5C. To confirm the connection between Rab5C and Rac activation, HeLa cells depleted of Rab5 isoforms were starved and then stimulated with EGF. Rac1 pull-down assays revealed that EGF-stimulated Rac1 activity was significantly suppressed in Rab5C-suppressed cells. To determine whether events upstream of Rac activation were affected by Rab5C, we observed that EGF-stimulated Akt phosphorylation was suppressed in cells depleted of Rab5C. Finally, since spatio-temporal assembly/disassembly of adhesion complexes are essential components of cell   migration  , we examined the effect of Rab5 isoform depletion on the formation of focal adhesion complexes. Rab5C-depleted HeLa cells have significantly fewer focal adhesion foci, in accordance with the lack of persistent lamellipodial protrusions and reduced directional   migration  . We conclude that Rab5 isoforms selectively oversee the multiple signaling and trafficking events associated with the endocytic network. 
 A specific subset of RabGTPases controls cell surface exposure of MT1-MMP, extracellular matrix degradation and three-dimensional invasion of macrophages. The matrix metalloproteinase MT1-MMP has a major impact on invasive cell   migration   in both physiological and pathological settings such as immune cell extravasation or metastasis of cancer cells. Surface-associated MT1-MMP is able to cleave components of the extracellular matrix, which is a prerequisite for proteolytic invasive   migration  . However, current knowledge on the molecular mechanisms that regulate MT1-MMP trafficking to and from the cell surface is limited. We have identified three members of the RabGTPase family, Rab5a, Rab8a and Rab14, as crucial regulators of MT1-MMP trafficking and function in primary human macrophages. Both overexpressed and endogenous forms show prominent colocalisation with MT1-MMP-positive vesicles, whereas expression of mutant constructs, as well as siRNA-induced knockdown, reveal that these RabGTPases are crucial in the regulation of MT1-MMP surface exposure, contact of MT1-MMP-positive vesicles with podosomes, extracellular matrix degradation in two and three dimensions, as well as three-dimensional proteolytic invasion of macrophages. Collectively, our results identify Rab5a, Rab8a and Rab14 as major regulators of MT1-MMP trafficking and invasive   migration   of primary human macrophages, which could be promising potential targets for manipulation of immune cell invasion. 
 Small GTPase Rab21 regulates cell adhesion and controls endosomal traffic of beta1-integrins. Dynamic turnover of integrin cell adhesion molecules to and from the cell surface is central to cell   migration  . We report for the first time an association between integrins and Rab proteins, which are small GTPases involved in the traffic of endocytotic vesicles. Rab21 (and Rab5) associate with the cytoplasmic domains of alpha-integrin chains, and their expression influences the endo/exocytic traffic of integrins. This function of Rab21 is dependent on its GTP/GDP cycle and proper membrane targeting. Knock down of Rab21 impairs integrin-mediated cell adhesion and motility, whereas its overexpression stimulates cell   migration   and cancer cell adhesion to collagen and human bone. Finally, overexpression of Rab21 fails to induce cell adhesion via an integrin point mutant deficient in Rab21 association. These data provide mechanistic insight into how integrins are targeted to intracellular compartments and how their traffic regulates cell adhesion. 
 Caspase 8 promotes peripheral localization and activation of Rab5. Caspase 8 is a cysteine protease that initiates apoptotic signaling via the extrinsic pathway in a manner dependent upon association with early endosomes. Previously, we identified caspase 8 as an effector of   migration  , promoting motility in a manner dependent upon phosphorylation on Tyr-380 by Src family kinases and its subsequent association with Src homology 2 domain-containing proteins. Here we demonstrate the regulation of the small GTPase Rab5, which mediates early endosome formation, homotypic fusion, and maturation by caspase 8. Regulation requires the Tyr-380 phosphorylation site but not caspase proteolytic activity. Tyr-380 is essential for interaction with the Src homology 2 domains of p85alpha, a multifunctional adaptor for phosphatidylinositol 3-kinase, that possesses Rab-GAP activity. Interaction between caspase 8 and p85alpha promotes Rab5 GTP loading, alters endosomal trafficking, and results in the accumulation of Rab5-positive endosomes at the edge of the cell. Conversely, caspase 8-dependent GTP loading of Rab5 is overcome by increased expression of p85alpha in a Rab-GAP-dependent manner. Thus, we demonstrate a novel function for caspase 8 as a modulator of p85alpha Rab-GAP activity and endosomal trafficking. 
 Rab GTPase regulation of VEGFR2 trafficking and signaling in endothelial cells. Vascular endothelial growth factor receptor 2 (VEGFR2) is a receptor tyrosine kinase that regulates vascular physiology. However, mechanism(s) by which VEGFR2 signaling and trafficking is coordinated are not clear. Here, we have tested endocytic Rab GTPases for regulation of VEGFR2 trafficking and signaling linked to endothelial cell   migration  .Quiescent VEGFR2 displays endosomal localization and colocalization with the Rab5a GTPase, an early endosome fusion regulator. Expression of GTP or GDP-bound Rab5a mutants block activated VEGFR2 trafficking and degradation. Manipulation of Rab7a GTPase activity associated with late endosomes using overexpression of wild-type or mutant proteins blocks activated VEGFR2 trafficking and degradation. Depletion of Rab7a decreased VEGFR2 Y1175 phosphorylation but increased p42/44 (pERK1/2) MAPK phosphorylation. Endothelial cell   migration   was increased by Rab5a depletion but decreased by Rab7a depletion.Rab5a and Rab7a regulate VEGFR2 trafficking toward early and late endosomes. Our data suggest that VEGFR2-mediated regulation of endothelial function is dependent on different but specific Rab-mediated GTP hydrolysis activity required for endosomal trafficking. 
 Rab5 isoforms orchestrate a "division of labor" in the endocytic network; Rab5C modulates Rac-mediated cell motility. Rab5, the prototypical Rab GTPase and master regulator of the endocytic pathway, is encoded as three differentially expressed isoforms, Rab5A, Rab5B and Rab5C. Here, we examined the differential effects of Rab5 isoform silencing on cell motility and report that Rab5C, but neither Rab5A nor Rab5B, is selectively associated with the growth factor-activation of Rac1 and with enhanced cell motility. Initial observations revealed that silencing of Rab5C expression, but neither Rab5A nor Rab5C, led to spindle-shaped cells that displayed reduced formation of membrane ruffles. When subjected to a scratch wound assay, cells depleted of Rab5C, but not Rab5A or Rab5B, demonstrated reduced cell   migration  . U937 cells depleted of Rab5C also displayed reduced cell motility in a Transwell plate   migration   assay. To examine activation of Rac, HeLa cells stably expressing GFP-Rac1 were independently depleted of Rab5A, Rab5B or Rab5C and seeded onto coverslips imprinted with a crossbow pattern. 3-D GFP-Rac1 images of micro-patterned cells show that GFP-Rac1 was less localized to the cell periphery in the absence of Rab5C. To confirm the connection between Rab5C and Rac activation, HeLa cells depleted of Rab5 isoforms were starved and then stimulated with EGF. Rac1 pull-down assays revealed that EGF-stimulated Rac1 activity was significantly suppressed in Rab5C-suppressed cells. To determine whether events upstream of Rac activation were affected by Rab5C, we observed that EGF-stimulated Akt phosphorylation was suppressed in cells depleted of Rab5C. Finally, since spatio-temporal assembly/disassembly of adhesion complexes are essential components of cell   migration  , we examined the effect of Rab5 isoform depletion on the formation of focal adhesion complexes. Rab5C-depleted HeLa cells have significantly fewer focal adhesion foci, in accordance with the lack of persistent lamellipodial protrusions and reduced directional   migration  . We conclude that Rab5 isoforms selectively oversee the multiple signaling and trafficking events associated with the endocytic network. 
 A specific subset of RabGTPases controls cell surface exposure of MT1-MMP, extracellular matrix degradation and three-dimensional invasion of macrophages. The matrix metalloproteinase MT1-MMP has a major impact on invasive cell   migration   in both physiological and pathological settings such as immune cell extravasation or metastasis of cancer cells. Surface-associated MT1-MMP is able to cleave components of the extracellular matrix, which is a prerequisite for proteolytic invasive   migration  . However, current knowledge on the molecular mechanisms that regulate MT1-MMP trafficking to and from the cell surface is limited. We have identified three members of the RabGTPase family, Rab5a, Rab8a and Rab14, as crucial regulators of MT1-MMP trafficking and function in primary human macrophages. Both overexpressed and endogenous forms show prominent colocalisation with MT1-MMP-positive vesicles, whereas expression of mutant constructs, as well as siRNA-induced knockdown, reveal that these RabGTPases are crucial in the regulation of MT1-MMP surface exposure, contact of MT1-MMP-positive vesicles with podosomes, extracellular matrix degradation in two and three dimensions, as well as three-dimensional proteolytic invasion of macrophages. Collectively, our results identify Rab5a, Rab8a and Rab14 as major regulators of MT1-MMP trafficking and invasive   migration   of primary human macrophages, which could be promising potential targets for manipulation of immune cell invasion. 
 Rab5 mediates caspase-8-promoted cell motility and metastasis. Caspase-8 is a key apical sensory protein that governs cell responses to environmental cues, alternatively promoting apoptosis, proliferation, and cell   migration  . The proteins responsible for integration of these pathways, however, have remained elusive. Here, we reveal that Rab5 regulates caspase-8-dependent signaling from integrins. Integrin ligation leads to Rab5 activation, association with integrins, and activation of Rac, in a caspase-8-dependent manner. Rab5 activation promotes colocalization and coprecipitation of integrins with caspase-8, concomitant with Rab5 recruitment to integrin-rich regions such as focal adhesions and membrane ruffles. Moreover, caspase-8 expression promotes Rab5-mediated internalization and the recycling of beta1 integrins, increasing cell   migration   independently of caspase catalytic activity. Conversely, Rab5 knockdown prevented caspase-8-mediated integrin signaling for Rac activation, cell   migration  , and apoptotic signaling, respectively. Similarly, Rab5 was critical for caspase-8-driven cell   migration   in vivo, because knockdown of Rab5 compromised the ability of caspase-8 to promote metastasis under nonapoptotic conditions. These studies identify Rab5 as a key integrator of caspase-8-mediated signal transduction downstream of integrins, regulating cell survival and   migration   in vivo and in vitro. 
 Rab GTPase regulation of VEGFR2 trafficking and signaling in endothelial cells. Vascular endothelial growth factor receptor 2 (VEGFR2) is a receptor tyrosine kinase that regulates vascular physiology. However, mechanism(s) by which VEGFR2 signaling and trafficking is coordinated are not clear. Here, we have tested endocytic Rab GTPases for regulation of VEGFR2 trafficking and signaling linked to endothelial cell   migration  .Quiescent VEGFR2 displays endosomal localization and colocalization with the Rab5a GTPase, an early endosome fusion regulator. Expression of GTP or GDP-bound Rab5a mutants block activated VEGFR2 trafficking and degradation. Manipulation of Rab7a GTPase activity associated with late endosomes using overexpression of wild-type or mutant proteins blocks activated VEGFR2 trafficking and degradation. Depletion of Rab7a decreased VEGFR2 Y1175 phosphorylation but increased p42/44 (pERK1/2) MAPK phosphorylation. Endothelial cell   migration   was increased by Rab5a depletion but decreased by Rab7a depletion.Rab5a and Rab7a regulate VEGFR2 trafficking toward early and late endosomes. Our data suggest that VEGFR2-mediated regulation of endothelial function is dependent on different but specific Rab-mediated GTP hydrolysis activity required for endosomal trafficking. 
 Caspase 8 promotes peripheral localization and activation of Rab5. Caspase 8 is a cysteine protease that initiates apoptotic signaling via the extrinsic pathway in a manner dependent upon association with early endosomes. Previously, we identified caspase 8 as an effector of   migration  , promoting motility in a manner dependent upon phosphorylation on Tyr-380 by Src family kinases and its subsequent association with Src homology 2 domain-containing proteins. Here we demonstrate the regulation of the small GTPase Rab5, which mediates early endosome formation, homotypic fusion, and maturation by caspase 8. Regulation requires the Tyr-380 phosphorylation site but not caspase proteolytic activity. Tyr-380 is essential for interaction with the Src homology 2 domains of p85alpha, a multifunctional adaptor for phosphatidylinositol 3-kinase, that possesses Rab-GAP activity. Interaction between caspase 8 and p85alpha promotes Rab5 GTP loading, alters endosomal trafficking, and results in the accumulation of Rab5-positive endosomes at the edge of the cell. Conversely, caspase 8-dependent GTP loading of Rab5 is overcome by increased expression of p85alpha in a Rab-GAP-dependent manner. Thus, we demonstrate a novel function for caspase 8 as a modulator of p85alpha Rab-GAP activity and endosomal trafficking. 
 Rab5A is associated with axillary lymph node metastasis in breast cancer patients. The expression of Rab proteins has been associated with cancer. However, few data are available on Rab5A expression in human breast cancer or its impact on disease progression. First, we examined the functional role of Rab5A in breast cancer cells. The expression of Rab5A in MDA-MB-231 cells can be stimulated by epidermal growth factor in a dose-dependent manner. The epidermal growth factor-induced increase of Rab5A expression correlated well with enhanced   migration   in wound healing   migration   assays in these cells. Furthermore, we evaluated the expression of Rab5A in breast cancer specimens using immunohistochemical staining, then analyzed the relationship between the expression of Rab5A and clinicopathological parameters. The increased expression of Rab5A protein in 123 breast cancer samples was associated with higher histological grade (P = 0.004), more lymphovascular invasion (P = 0.027), more axillary lymph node (LN) metastasis (P = 0.008), and a higher number of axillary LN metastases (P = 0.043). Among 218 axillary LNs of more than 10 breast cancer patients with node metastases, 167 metastatic LNs were found to have increased Rab5A expression. Rab5A is associated with axillary LN metastasis in breast cancer patients. 
 Rab5 activation promotes focal adhesion disassembly,   migration   and invasiveness in tumor cells.   Migration   and invasion are essential steps associated with tumor cell metastasis and increasing evidence points towards endosome trafficking being essential in this process. Indeed, the small GTPase Rab5, a crucial regulator of early endosome dynamics, promotes cell   migration   in vitro and in vivo. Precisely how Rab5 participates in these events remains to be determined. Considering that focal adhesions represent structures crucial to cell   migration  , we specifically asked whether Rab5 activation promoted focal adhesion disassembly and thereby facilitated   migration   and invasion of metastatic cancer cells. Pulldown and biosensor assays revealed that Rab5-GTP loading increased at the leading edge of migrating tumor cells. Additionally, targeting of Rab5 by different shRNA sequences, but not control shRNA, decreased Rab5-GTP levels, leading to reduced cell spreading,   migration   and invasiveness. Re-expression in knockdown cells of wild-type Rab5, but not the S34N mutant (GDP-bound), restored these properties. Importantly, Rab5 association with the focal adhesion proteins vinculin and paxillin increased during   migration  , and expression of wild-type, but not GDP-bound Rab5, accelerated focal adhesion disassembly, as well as FAK dephosphorylation on tyrosine 397. Finally, Rab5-driven invasiveness required focal adhesion disassembly, as treatment with the FAK inhibitor number 14 prevented Matrigel invasion and matrix metalloproteinase release. Taken together, these observations show that Rab5 activation is required to enhance cancer cell   migration   and invasion by promoting focal adhesion disassembly. 
 Rab5 activation as a tumor cell   migration   switch. Increased cell   migration   is an acquired feature of metastatic cancer cells and relies on derailed signal transduction pathways. Intracellular vesicular trafficking plays a key role in cell   migration   due to its intricate involvement in cargo transport and membrane composition. In the last decade, endocytosis has been implicated in cell   migration   and found to be responsible for the internalization of membrane receptors at the plasma membrane, where integrin trafficking and fine-tuning of receptor tyrosine kinase signaling by internalization are major mechanisms. Accumulating evidence has suggested a link between endosome dynamics, cell   migration  , and invasion, in which small GTPases of the Rab family have central roles. We have recently determined that Rab5 activation is a crucial event in promoting focal adhesion disassembly, which is concomitant with the   migration   and invasion of metastatic cancer cells. The mechanisms underlying this novel role for Rab5 are currently unclear, and their elucidation will provide insight into the role of Rab5 function in cancer cell metastasis. 
 Rab'ing tumor cell   migration   and invasion: focal adhesion disassembly driven by Rab5. The small GTPase Rab5 has been extensively studied in the context of endocytic trafficking because it is critical in the regulation of early endosome dynamics. In addition to this canonical role, evidence obtained in recent years implicates Rab5 in the regulation of cell   migration  . This novel role of Rab5 is based not only on an indirect relationship between cell   migration   and endosomal trafficking as separate processes, but also on the direct regulation of signaling proteins implicated in cell   migration  . However, the precise mechanisms underlying this connection have remained elusive. Recent studies have shown that the activation of Rab5 is a critical event for maintaining the dynamics of focal adhesions, which is fundamental in regulating not only cell   migration   but also tumor cell invasion. 
 Ligand-induced tyrosine phosphorylation of cysteinyl leukotriene receptor 1 triggers internalization and signaling in intestinal epithelial cells. Leukotriene D(4) (LTD(4)) belongs to the bioactive lipid group known as eicosanoids and has implications in pathological processes such as inflammation and cancer. Leukotriene D(4) exerts its effects mainly through two different G-protein-coupled receptors, CysLT(1) and CysLT(2). The high affinity LTD(4) receptor CysLT(1)R exhibits tumor-promoting properties by triggering cell proliferation, survival, and   migration   in intestinal epithelial cells. In addition, increased expression and nuclear localization of CysLT(1)R correlates with a poorer prognosis for patients with colon cancer.Using a proximity ligation assay and immunoprecipitation, this study showed that endogenous CysLT(1)R formed heterodimers with its counter-receptor CysLT(2)R under basal conditions and that LTD(4) triggers reduced dimerization of CysLTRs in intestinal epithelial cells. This effect was dependent upon a parallel LTD(4)-induced increase in CysLT(1)R tyrosine phosphorylation. Leukotriene D(4) also led to elevated internalization of CysLT(1)Rs from the plasma membrane and a simultaneous increase at the nucleus. Using sucrose, a clathrin endocytic inhibitor, dominant-negative constructs, and siRNA against arrestin-3, we suggest that a clathrin-, arrestin-3, and Rab-5-dependent process mediated the internalization of CysLT(1)R. Altering the CysLT(1)R internalization process at either the clathrin or the arrestin-3 stage led to disruption of LTD(4)-induced Erk1/2 activation and up-regulation of COX-2 mRNA levels.Our data suggests that upon ligand activation, CysLT(1)R is tyrosine-phosphorylated and released from heterodimers with CysLT(2)R and, subsequently, internalizes from the plasma membrane to the nuclear membrane in a clathrin-, arrestin-3-, and Rab-5-dependent manner, thus, enabling Erk1/2 signaling and downstream transcription of the COX-2 gene. 
 Downregulation of miR-101-3p by hepatitis B virus promotes proliferation and   migration   of hepatocellular carcinoma cells by targeting Rab5a. RAB GTPase 5A (RAB5A), a member of the Rab subfamily of small GTPases, acts as an oncogene and has been associated with various key cellular functions, including cell growth, differentiation, apoptosis and angiogenesis. Recently, it has been reported that the Rab5a gene is involved in the progression of cancer. Hepatocellular carcinoma (HCC) is one of the most common and aggressive cancers, and it is usually associated with persistent hepatitis B virus (HBV) infections. Emerging evidence suggests that HBV alters microRNA (miRNA) expression profiles, but the mechanisms underlying this process have not yet been fully elucidated. Here, we examine how HBV affects the production of miR-101-1, which has been shown to be downregulated in HCC. We found that HBV could repress miR-101-3p by inhibiting its promoter activity. Downregulation of miR-101-3p promoted cancer cell growth and   migration  , and a specific miR-101-3p inhibitor was able to enhance proliferation and   migration  . Moreover, we identified Rab5a was one of the target genes of miR-101-3p in HBV-related HCC. Forced expression of miR-101-3p in liver cell lines resulted in a marked reduction of the expression of Rab5a at both the mRNA and protein level by directly targeting the 3'untranslated region of Rab5a. Overexpression of Rab5a resulted in a reversal of the suppression of proliferation and   migration   of SMMC-7721 cells mediated by miR-101-3p. Taken together, our data show that HBV can downregulate miR-101-3p expression by inhibiting its promoter activity and that downregulation of miR-101-3p promotes HCC cell proliferation and   migration   by targeting Rab5a. This provides new insights into the mechanisms of HBV-related HCC pathogenesis. 
 Rab5 is required in metastatic cancer cells for Caveolin-1-enhanced Rac1 activation,   migration   and invasion. Rab5 is a small GTPase that regulates early endosome trafficking and other cellular processes, including cell adhesion and   migration  . Specifically, Rab5 promotes Rac1 activation and cancer cell   migration  , but little is known about the upstream regulators of Rab5. We have previously shown that the scaffolding protein Caveolin-1 (CAV1) promotes Rac1 activation and   migration   of cancer cells. Here, we hypothesized that CAV1 stimulates Rab5 activation, leading to increased Rac1 activity and cell   migration  . Expression of CAV1 in B16-F10 mouse melanoma and HT-29(US) human colon adenocarcinoma cells increased the GTP loading of Rab5, whereas shRNA-mediated targeting of endogenous CAV1 in MDA-MB-231 breast cancer cells decreased Rab5-GTP levels. Accordingly, shRNA-mediated downregulation of Rab5 decreased CAV1-mediated Rac1 activation, cell   migration   and invasion in B16-F10 and HT-29(US) cells. Expression of CAV1 was accompanied by increased recruitment of Tiam1, a Rac1 guanine nucleotide exchange factor (GEF), to Rab5-positive early endosomes. Using the inhibitor NSC23766, Tiam1 was shown to be required for Rac1 activation and cell   migration   induced by CAV1 and Rab5. Mechanistically, we provide evidence implicating p85α (also known as PIK3R1), a Rab5 GTPase-activating protein (GAP), in CAV1-dependent effects, by showing that CAV1 recruits p85α, precluding p85α-mediated Rab5 inactivation and increasing ce  ll migrat  ion. In summary, these studies identify a novel CAV1-Rab5-Rac1 signaling axis, whereby CAV1 prevents Rab5 inactivation, leading to increased Rac1 activity and enhanced tumor ce  ll migrat  ion and invasion. 
 Rab5a‑mediated autophagy regulates the phenotype and behavior of vascular smooth muscle cells. Rab5a, a key member of the Rab family of GTPases, was determined to be a regulator of vascular smooth muscle cell (VSMC) proliferation an  d migrati  on. However, the exact regulatory mechanism remains unclear. As Rab5a has been shown to be associated with autophagy, which is essential for the conversion of VSMCs from a contractile to a synthetic phenotype in order to prevent cell death due to oxidative stress. The present study hypothesized that autophagy may be responsible for the proliferation an  d migrati  on of VSMCs via the Rab5a protein. The aim of the present study was to evaluate the effect of Rab5a on autophagy in VSMCs. The human aorta vascular smooth muscle cell line, T/G HA‑VSMCs, was treated with small interfering (si)RNA against Rab5a and/or platelet‑derived growth factor (PDGF). Following treatment, the phenotype transition of the VSMCs was evaluated by detecting the mRNA and protien expression levels of VSMC molecular markers using reverse transcription‑quantitative polymerase chain reaction and western blotting, respectively. In addition, autophagy in VSMCs was evaluated by western blotting for autophagy‑associated proteins, flow cytometry of acidic vesicular organelles, punctate fluorescence of microtubule associated protein light chain 3 and transmission electron microscopy of typical scattered double‑membrane vacuolar structures. Additionally, the pr  oliferati  on, migration, cell cycle and apoptotic response of VSMCs were detected by sulforhodamine B assay, transwell assay and flow cytometry, respectively. The results revealed that transfection with siRNA against Rab5a led to a significant decrease in Rab5a protein expression, while the reduced expression trend of Rab5a was rescued by intervention with PDGF. Furthermore, cells transfected with siRNA against Rab5a inhibited the autophagy of VSMCs. Downregulated Rab5a inhibited the phenotype transition of VSMCs. Additionally, downregulated Rab5a led to slowed cell growth, decreased numbers of migrated cells, decreased numbers of cells at the G0‑G1 phase and a higher apoptosis rate. However, PDGF significantly rescued these phenomena caused by siRNA against Rab5a. These results indicated that Rab5a‑mediated autophagy may regulate the phenotype transition and cell behavior of VSMCs through the activation of the extracellular‑regulated kinase 1/2 signaling pathway. 
 Phosphorylation of Rab5a protein by protein kinase Cϵ is crucial for T-cell   migratio  n. Rab GTPases control membrane traffic and receptor-mediated endocytosis. Within this context, Rab5a plays an important role in the spatial regulation of intracellular transport and signal transduction processes. Here, we report a previously uncharacterized role for Rab5a in the regulation of T-cell motility. We show that Rab5a physically associates with protein kinase Cϵ (PKCϵ) in migrating T-cells. After stimulation of T-cells through the integrin LFA-1 or the chemokine receptor CXCR4, Rab5a is phosphorylated on an N-terminal Thr-7 site by PKCϵ. Both Rab5a and PKCϵ dynamically interact at the centrosomal region of migrating cells, and PKCϵ-mediated phosphorylation on Thr-7 regulates Rab5a trafficking to the cell leading edge. Furthermore, we demonstrate that Rab5a Thr-7 phosphorylation is functionally necessary for Rac1 activation, actin rearrangement, and T-cell motility. We present a novel mechanism by which a PKCϵ-Rab5a-Rac1 axis regulates cytoskeleton remodeling and   T-cell mi  gration, both of which are central for the adaptive immune response. 
 Knockdown of Rab5a expression decreases cancer cell motility and invasion through integrin-mediated signaling pathway. Rab GTPases function as modulators in intracellular transport. Rab5a, a member of the Rab subfamily of small GTPases, is an important regulator of vesicle traffic from the plasma membrane to early endosomes. Recent findings have reported that Rab5a gene was involved in the progression of cancer. In the present study, we investigated the effect of Rab5a on cervical cancer invasion and metastasis and the molecular mechanism underlying the involvement of Rab5a.Rab5a expression was assessed by immunohistochemical analysis on a cervical cancer tissue microarray. RNA interference (RNAi) was performed to knock down the endogenous expression of Rab5a gene in HeLa and SiHa cells. Cell motility was evaluated using invasion assay and wound   migration   assay in vitro. The expression levels of integrin-associated molecules were detected by Western blot and immunofluorescence.We found that Rab5a was expressed at a high level in cervical cancer tissues. Silencing of Rab5a expression significantly decreased cancer cell motility and invasiveness. The down-regulation of integrin-associated focal adhesion signaling molecules was further detected in Rab5a knockdown cells. Meanwhile, active GTP-bound Rac1, Cdc42, and RhoA were also down-regulated, accompanied with the reduction in the number and size of filopodia and lamellipodia.Taken together, these data suggest that Rab5a functions in regulating the invasion phenotype, and we propose that this regulation may be via integrin-mediated signaling pathway in cervical cancer cells. 
 Overexpression of Rab5a promotes hepatocellular carcinoma cell proliferation and invasion via FAK signaling pathway. Rab5a was reported to be overexpressed in human malignancy and associated with the malignant phenotype. To data, its expression pattern and biological function in hepatocellular carcinoma (HCC) have not been studied. We analyzed Rab5a protein expression in 98 cases of HCC tissues and four HCC cell lines. We found that Rab5a expression was upregulated in HCC tissues and cell lines. Rab5a overexpression correlated with TNM stage and nodal metastasis (p < 0.05). To confirm the biological function of Rab5a in HCC cell lines, Rab5a siRNA was employed in SK-Hep-1 cell line and plasmid transfection was performed in Huh7 cell line. CCK-8 assay showed that Rab5a depletion blocked cell growth rate while Rab5a overexpression facilitated proliferation. Transwell   and migra  tion assay showed that Rab5a positively regulated cell invasion   and migra  tion. To explore the molecular mechanism underlying the biological effects of Rab5a, we checked several signaling pathways and found that Rab5a overexpression upregulated cyclin D1, cyclin E expression, FAK (Tyr397), and AKT (Ser473) phosphorylation. Blockage of FAK using inhibitor PF573228 abolished the role of Rab5a on cyclin D1. In conclusion, Rab5a is overexpressed in human HCC and contributes to cancer cell proliferation and invasion through regulation of FAK and AKT signaling. 
  GeneRIFs  
 Rab5 activation as a tumor cell   migration   switch 
 Recent studies have shown that the activation of Rab5 is a critical event for maintaining the dynamics of focal adhesions, which is fundamental in regulating not only cell   migration   but also tumor cell invasion 
 activation, which is required for cell   migration   is promoted by Caveolin-1 
 Hepatitis B virus can downregulate miR-101-3p expression by inhibiting its promoter activity and that downregulation of miR-101-3p promotes hepatocellular carcinoma cell proliferation and   migration   by targeting Rab5a. 
 a novel mechanism by which a PKC-Rab5a-Rac1 axis regulates cytoskeleton remodeling and T-cell   migration  , both of which are central for the adaptive immune response. 
 Rab5 activation is required to enhance cancer cell   migration   and invasion by promoting focal adhesion disassembly. 
 Rab5a, Rab8a and Rab14 are major regulators of MT1-MMP trafficking and invasive   migration   of primary human macrophages. 
 These studies identify Rab5 as a key integrator of caspase-8-mediated signal transduction downstream of integrins, regulating cell survival and   migration   in vivo and in vitro. 
 Rab GTPase regulation of VEGFR2 trafficking and signaling in endothelial cells; Endothelial cell   migration   was increased by Rab5a depletion but decreased by Rab7a depletion 
  ECM  PubMed  
 A RAB5/RAB4 recycling circuitry induces a proteolytic invasive program and promotes tumor dissemination. The mechanisms by which tumor cells metastasize and the role of endocytic proteins in this process are not well understood. We report that overexpression of the GTPase RAB5A, a master regulator of endocytosis, is predictive of aggressive behavior and metastatic ability in human breast cancers. RAB5A is necessary and sufficient to promote local invasion and distant dissemination of various mammary and nonmammary tumor cell lines, and this prometastatic behavior is associated with increased intratumoral cell motility. Specifically, RAB5A is necessary for the formation of invadosomes, membrane protrusions specialized in extracellular matrix (  ECM  ) degradation. RAB5A promotes RAB4- and RABENOSYN-5-dependent endo/exocytic cycles (EECs) of critical cargos (membrane-type 1 matrix metalloprotease [MT1-MMP] and β3 integrin) required for invadosome formation in response to motogenic stimuli. This trafficking circuitry is necessary for spatially localized hepatocyte growth factor (HGF)/MET signaling that drives invasive, proteolysis-dependent chemotaxis in vitro and for conversion of ductal carcinoma in situ to invasive ductal carcinoma in vivo. Thus, RAB5A/RAB4 EECs promote tumor dissemination by controlling a proteolytic, mesenchymal invasive program. 
  [Ee]xtracellular [Mm]atrix  PubMed  
 A specific subset of RabGTPases controls cell surface exposure of MT1-MMP,   extracellular matrix   degradation and three-dimensional invasion of macrophages. The matrix metalloproteinase MT1-MMP has a major impact on invasive cell migration in both physiological and pathological settings such as immune cell extravasation or metastasis of cancer cells. Surface-associated MT1-MMP is able to cleave components of the   extracellular matrix  , which is a prerequisite for proteolytic invasive migration. However, current knowledge on the molecular mechanisms that regulate MT1-MMP trafficking to and from the cell surface is limited. We have identified three members of the RabGTPase family, Rab5a, Rab8a and Rab14, as crucial regulators of MT1-MMP trafficking and function in primary human macrophages. Both overexpressed and endogenous forms show prominent colocalisation with MT1-MMP-positive vesicles, whereas expression of mutant constructs, as well as siRNA-induced knockdown, reveal that these RabGTPases are crucial in the regulation of MT1-MMP surface exposure, contact of MT1-MMP-positive vesicles with podosomes,   extracellular matrix   degradation in two and three dimensions, as well as three-dimensional proteolytic invasion of macrophages. Collectively, our results identify Rab5a, Rab8a and Rab14 as major regulators of MT1-MMP trafficking and invasive migration of primary human macrophages, which could be promising potential targets for manipulation of immune cell invasion. 
 A specific subset of RabGTPases controls cell surface exposure of MT1-MMP,   extracellular matrix   degradation and three-dimensional invasion of macrophages. The matrix metalloproteinase MT1-MMP has a major impact on invasive cell migration in both physiological and pathological settings such as immune cell extravasation or metastasis of cancer cells. Surface-associated MT1-MMP is able to cleave components of the   extracellular matrix  , which is a prerequisite for proteolytic invasive migration. However, current knowledge on the molecular mechanisms that regulate MT1-MMP trafficking to and from the cell surface is limited. We have identified three members of the RabGTPase family, Rab5a, Rab8a and Rab14, as crucial regulators of MT1-MMP trafficking and function in primary human macrophages. Both overexpressed and endogenous forms show prominent colocalisation with MT1-MMP-positive vesicles, whereas expression of mutant constructs, as well as siRNA-induced knockdown, reveal that these RabGTPases are crucial in the regulation of MT1-MMP surface exposure, contact of MT1-MMP-positive vesicles with podosomes,   extracellular matrix   degradation in two and three dimensions, as well as three-dimensional proteolytic invasion of macrophages. Collectively, our results identify Rab5a, Rab8a and Rab14 as major regulators of MT1-MMP trafficking and invasive migration of primary human macrophages, which could be promising potential targets for manipulation of immune cell invasion. 
 A RAB5/RAB4 recycling circuitry induces a proteolytic invasive program and promotes tumor dissemination. The mechanisms by which tumor cells metastasize and the role of endocytic proteins in this process are not well understood. We report that overexpression of the GTPase RAB5A, a master regulator of endocytosis, is predictive of aggressive behavior and metastatic ability in human breast cancers. RAB5A is necessary and sufficient to promote local invasion and distant dissemination of various mammary and nonmammary tumor cell lines, and this prometastatic behavior is associated with increased intratumoral cell motility. Specifically, RAB5A is necessary for the formation of invadosomes, membrane protrusions specialized in   extracellular matrix   (ECM) degradation. RAB5A promotes RAB4- and RABENOSYN-5-dependent endo/exocytic cycles (EECs) of critical cargos (membrane-type 1 matrix metalloprotease [MT1-MMP] and β3 integrin) required for invadosome formation in response to motogenic stimuli. This trafficking circuitry is necessary for spatially localized hepatocyte growth factor (HGF)/MET signaling that drives invasive, proteolysis-dependent chemotaxis in vitro and for conversion of ductal carcinoma in situ to invasive ductal carcinoma in vivo. Thus, RAB5A/RAB4 EECs promote tumor dissemination by controlling a proteolytic, mesenchymal invasive program. 
  [Ii]nvadosome  PubMed  
 A RAB5/RAB4 recycling circuitry induces a proteolytic invasive program and promotes tumor dissemination. The mechanisms by which tumor cells metastasize and the role of endocytic proteins in this process are not well understood. We report that overexpression of the GTPase RAB5A, a master regulator of endocytosis, is predictive of aggressive behavior and metastatic ability in human breast cancers. RAB5A is necessary and sufficient to promote local invasion and distant dissemination of various mammary and nonmammary tumor cell lines, and this prometastatic behavior is associated with increased intratumoral cell motility. Specifically, RAB5A is necessary for the formation of   invadosome  s, membrane protrusions specialized in extracellular matrix (ECM) degradation. RAB5A promotes RAB4- and RABENOSYN-5-dependent endo/exocytic cycles (EECs) of critical cargos (membrane-type 1 matrix metalloprotease [MT1-MMP] and β3 integrin) required for   invadosom  e formation in response to motogenic stimuli. This trafficking circuitry is necessary for spatially localized hepatocyte growth factor (HGF)/MET signaling that drives invasive, proteolysis-dependent chemotaxis in vitro and for conversion of ductal carcinoma in situ to invasive ductal carcinoma in vivo. Thus, RAB5A/RAB4 EECs promote tumor dissemination by controlling a proteolytic, mesenchymal invasive program. 
  [Ii]nvasive  PubMed  
 A specific subset of RabGTPases controls cell surface exposure of MT1-MMP, extracellular matrix degradation and three-dimensional invasion of macrophages. The matrix metalloproteinase MT1-MMP has a major impact on   invasive   cell migration in both physiological and pathological settings such as immune cell extravasation or metastasis of cancer cells. Surface-associated MT1-MMP is able to cleave components of the extracellular matrix, which is a prerequisite for proteolytic   invasive   migration. However, current knowledge on the molecular mechanisms that regulate MT1-MMP trafficking to and from the cell surface is limited. We have identified three members of the RabGTPase family, Rab5a, Rab8a and Rab14, as crucial regulators of MT1-MMP trafficking and function in primary human macrophages. Both overexpressed and endogenous forms show prominent colocalisation with MT1-MMP-positive vesicles, whereas expression of mutant constructs, as well as siRNA-induced knockdown, reveal that these RabGTPases are crucial in the regulation of MT1-MMP surface exposure, contact of MT1-MMP-positive vesicles with podosomes, extracellular matrix degradation in two and three dimensions, as well as three-dimensional proteolytic invasion of macrophages. Collectively, our results identify Rab5a, Rab8a and Rab14 as major regulators of MT1-MMP trafficking and   invasive   migration of primary human macrophages, which could be promising potential targets for manipulation of immune cell invasion. 
 A specific subset of RabGTPases controls cell surface exposure of MT1-MMP, extracellular matrix degradation and three-dimensional invasion of macrophages. The matrix metalloproteinase MT1-MMP has a major impact on   invasive   cell migration in both physiological and pathological settings such as immune cell extravasation or metastasis of cancer cells. Surface-associated MT1-MMP is able to cleave components of the extracellular matrix, which is a prerequisite for proteolytic   invasive   migration. However, current knowledge on the molecular mechanisms that regulate MT1-MMP trafficking to and from the cell surface is limited. We have identified three members of the RabGTPase family, Rab5a, Rab8a and Rab14, as crucial regulators of MT1-MMP trafficking and function in primary human macrophages. Both overexpressed and endogenous forms show prominent colocalisation with MT1-MMP-positive vesicles, whereas expression of mutant constructs, as well as siRNA-induced knockdown, reveal that these RabGTPases are crucial in the regulation of MT1-MMP surface exposure, contact of MT1-MMP-positive vesicles with podosomes, extracellular matrix degradation in two and three dimensions, as well as three-dimensional proteolytic invasion of macrophages. Collectively, our results identify Rab5a, Rab8a and Rab14 as major regulators of MT1-MMP trafficking and   invasive   migration of primary human macrophages, which could be promising potential targets for manipulation of immune cell invasion. 
 Rab5 activation promotes focal adhesion disassembly, migration and   invasive  ness in tumor cells. Migration and invasion are essential steps associated with tumor cell metastasis and increasing evidence points towards endosome trafficking being essential in this process. Indeed, the small GTPase Rab5, a crucial regulator of early endosome dynamics, promotes cell migration in vitro and in vivo. Precisely how Rab5 participates in these events remains to be determined. Considering that focal adhesions represent structures crucial to cell migration, we specifically asked whether Rab5 activation promoted focal adhesion disassembly and thereby facilitated migration and invasion of metastatic cancer cells. Pulldown and biosensor assays revealed that Rab5-GTP loading increased at the leading edge of migrating tumor cells. Additionally, targeting of Rab5 by different shRNA sequences, but not control shRNA, decreased Rab5-GTP levels, leading to reduced cell spreading, migration and   invasive  ness. Re-expression in knockdown cells of wild-type Rab5, but not the S34N mutant (GDP-bound), restored these properties. Importantly, Rab5 association with the focal adhesion proteins vinculin and paxillin increased during migration, and expression of wild-type, but not GDP-bound Rab5, accelerated focal adhesion disassembly, as well as FAK dephosphorylation on tyrosine 397. Finally, Rab5-driven   invasive  ness required focal adhesion disassembly, as treatment with the FAK inhibitor number 14 prevented Matrigel invasion and matrix metalloproteinase release. Taken together, these observations show that Rab5 activation is required to enhance cancer cell migration and invasion by promoting focal adhesion disassembly. 
 The Autophagic Process Occurs in Human Bone Metastasis and Implicates Molecular Mechanisms Differently Affected by Rab5a in the Early and Late Stages. Autophagy favours metastatic growth through fuelling energy and nutrients and resistance to anoikis, typical of disseminated-tumour cells. The autophagic process, mediated by a unique organelle, the autophagosome, which fuses with lysosomes, is divided into three steps. Several stages, especially early omegasome formation and isolation-membrane initiation, remain controversial; molecular mechanisms involve the small-GTPase Rab5a, which regulates vesicle traffic for autophagosome formation. We examined Rab5a involvement in the function of key members of ubiquitin-conjugation systems, Atg7 and LC3-lipidated, interacting with the scaffold-protein p62. Immunohistochemistry of Rab5a was performed in human specimens of bone metastasis and pair-matched breast carcinoma; the autophagic-molecular mechanisms affected by Rab5a were evaluated in human 1833 bone metastatic cells, derived from breast-carcinoma MDA-MB231 cells. To clarify the role of Rab5a, 1833 cells were transfected transiently with Rab5a-dominant negative, and/or stably with the short-hairpin RNA Atg7, were exposed to two inhibitors of autolysosome function, and LC3II and p62 expression was measured. We showed basal autophagy in bone-metastatic cells and the pivotal role of Rab5a together with Beclin 1 between the early stages, elongation of isolation membrane/closed autophagosome mediated by Atg7, and the late-degradative stages. This regulatory network might occur in bone-metastasis and in high-grade dysplastic lesions, preceding   invasive  -breast carcinoma and conferring phenotypic characteristics for dissemination. 
 A RAB5/RAB4 recycling circuitry induces a proteolytic   invasive   program and promotes tumor dissemination. The mechanisms by which tumor cells metastasize and the role of endocytic proteins in this process are not well understood. We report that overexpression of the GTPase RAB5A, a master regulator of endocytosis, is predictive of aggressive behavior and metastatic ability in human breast cancers. RAB5A is necessary and sufficient to promote local invasion and distant dissemination of various mammary and nonmammary tumor cell lines, and this prometastatic behavior is associated with increased intratumoral cell motility. Specifically, RAB5A is necessary for the formation of invadosomes, membrane protrusions specialized in extracellular matrix (ECM) degradation. RAB5A promotes RAB4- and RABENOSYN-5-dependent endo/exocytic cycles (EECs) of critical cargos (membrane-type 1 matrix metalloprotease [MT1-MMP] and β3 integrin) required for invadosome formation in response to motogenic stimuli. This trafficking circuitry is necessary for spatially localized hepatocyte growth factor (HGF)/MET signaling that drives   invasiv  e, proteolysis-dependent chemotaxis in vitro and for conversion of ductal carcinoma in situ to   invasiv  e ductal carcinoma in vivo. Thus, RAB5A/RAB4 EECs promote tumor dissemination by controlling a proteolytic, mesenchymal   invasiv  e program. 
 Knockdown of Rab5a expression decreases cancer cell motility and invasion through integrin-mediated signaling pathway. Rab GTPases function as modulators in intracellular transport. Rab5a, a member of the Rab subfamily of small GTPases, is an important regulator of vesicle traffic from the plasma membrane to early endosomes. Recent findings have reported that Rab5a gene was involved in the progression of cancer. In the present study, we investigated the effect of Rab5a on cervical cancer invasion and metastasis and the molecular mechanism underlying the involvement of Rab5a.Rab5a expression was assessed by immunohistochemical analysis on a cervical cancer tissue microarray. RNA interference (RNAi) was performed to knock down the endogenous expression of Rab5a gene in HeLa and SiHa cells. Cell motility was evaluated using invasion assay and wound migration assay in vitro. The expression levels of integrin-associated molecules were detected by Western blot and immunofluorescence.We found that Rab5a was expressed at a high level in cervical cancer tissues. Silencing of Rab5a expression significantly decreased cancer cell motility and   invasive  ness. The down-regulation of integrin-associated focal adhesion signaling molecules was further detected in Rab5a knockdown cells. Meanwhile, active GTP-bound Rac1, Cdc42, and RhoA were also down-regulated, accompanied with the reduction in the number and size of filopodia and lamellipodia.Taken together, these data suggest that Rab5a functions in regulating the invasion phenotype, and we propose that this regulation may be via integrin-mediated signaling pathway in cervical cancer cells. 
  GeneRIFs  
 Rab5a, Rab8a and Rab14 are major regulators of MT1-MMP trafficking and   invasive   migration of primary human macrophages. 
  [Mm]etastasize  PubMed  
 A RAB5/RAB4 recycling circuitry induces a proteolytic invasive program and promotes tumor dissemination. The mechanisms by which tumor cells   metastasize   and the role of endocytic proteins in this process are not well understood. We report that overexpression of the GTPase RAB5A, a master regulator of endocytosis, is predictive of aggressive behavior and metastatic ability in human breast cancers. RAB5A is necessary and sufficient to promote local invasion and distant dissemination of various mammary and nonmammary tumor cell lines, and this prometastatic behavior is associated with increased intratumoral cell motility. Specifically, RAB5A is necessary for the formation of invadosomes, membrane protrusions specialized in extracellular matrix (ECM) degradation. RAB5A promotes RAB4- and RABENOSYN-5-dependent endo/exocytic cycles (EECs) of critical cargos (membrane-type 1 matrix metalloprotease [MT1-MMP] and β3 integrin) required for invadosome formation in response to motogenic stimuli. This trafficking circuitry is necessary for spatially localized hepatocyte growth factor (HGF)/MET signaling that drives invasive, proteolysis-dependent chemotaxis in vitro and for conversion of ductal carcinoma in situ to invasive ductal carcinoma in vivo. Thus, RAB5A/RAB4 EECs promote tumor dissemination by controlling a proteolytic, mesenchymal invasive program. 
  [Mm]etastasis  PubMed  
 Rab5 mediates caspase-8-promoted cell motility and   metastasis  . Caspase-8 is a key apical sensory protein that governs cell responses to environmental cues, alternatively promoting apoptosis, proliferation, and cell migration. The proteins responsible for integration of these pathways, however, have remained elusive. Here, we reveal that Rab5 regulates caspase-8-dependent signaling from integrins. Integrin ligation leads to Rab5 activation, association with integrins, and activation of Rac, in a caspase-8-dependent manner. Rab5 activation promotes colocalization and coprecipitation of integrins with caspase-8, concomitant with Rab5 recruitment to integrin-rich regions such as focal adhesions and membrane ruffles. Moreover, caspase-8 expression promotes Rab5-mediated internalization and the recycling of beta1 integrins, increasing cell migration independently of caspase catalytic activity. Conversely, Rab5 knockdown prevented caspase-8-mediated integrin signaling for Rac activation, cell migration, and apoptotic signaling, respectively. Similarly, Rab5 was critical for caspase-8-driven cell migration in vivo, because knockdown of Rab5 compromised the ability of caspase-8 to promote   metastasis   under nonapoptotic conditions. These studies identify Rab5 as a key integrator of caspase-8-mediated signal transduction downstream of integrins, regulating cell survival and migration in vivo and in vitro. 
 A specific subset of RabGTPases controls cell surface exposure of MT1-MMP, extracellular matrix degradation and three-dimensional invasion of macrophages. The matrix metalloproteinase MT1-MMP has a major impact on invasive cell migration in both physiological and pathological settings such as immune cell extravasation or   metastasis   of cancer cells. Surface-associated MT1-MMP is able to cleave components of the extracellular matrix, which is a prerequisite for proteolytic invasive migration. However, current knowledge on the molecular mechanisms that regulate MT1-MMP trafficking to and from the cell surface is limited. We have identified three members of the RabGTPase family, Rab5a, Rab8a and Rab14, as crucial regulators of MT1-MMP trafficking and function in primary human macrophages. Both overexpressed and endogenous forms show prominent colocalisation with MT1-MMP-positive vesicles, whereas expression of mutant constructs, as well as siRNA-induced knockdown, reveal that these RabGTPases are crucial in the regulation of MT1-MMP surface exposure, contact of MT1-MMP-positive vesicles with podosomes, extracellular matrix degradation in two and three dimensions, as well as three-dimensional proteolytic invasion of macrophages. Collectively, our results identify Rab5a, Rab8a and Rab14 as major regulators of MT1-MMP trafficking and invasive migration of primary human macrophages, which could be promising potential targets for manipulation of immune cell invasion. 
 A specific subset of RabGTPases controls cell surface exposure of MT1-MMP, extracellular matrix degradation and three-dimensional invasion of macrophages. The matrix metalloproteinase MT1-MMP has a major impact on invasive cell migration in both physiological and pathological settings such as immune cell extravasation or   metastasis   of cancer cells. Surface-associated MT1-MMP is able to cleave components of the extracellular matrix, which is a prerequisite for proteolytic invasive migration. However, current knowledge on the molecular mechanisms that regulate MT1-MMP trafficking to and from the cell surface is limited. We have identified three members of the RabGTPase family, Rab5a, Rab8a and Rab14, as crucial regulators of MT1-MMP trafficking and function in primary human macrophages. Both overexpressed and endogenous forms show prominent colocalisation with MT1-MMP-positive vesicles, whereas expression of mutant constructs, as well as siRNA-induced knockdown, reveal that these RabGTPases are crucial in the regulation of MT1-MMP surface exposure, contact of MT1-MMP-positive vesicles with podosomes, extracellular matrix degradation in two and three dimensions, as well as three-dimensional proteolytic invasion of macrophages. Collectively, our results identify Rab5a, Rab8a and Rab14 as major regulators of MT1-MMP trafficking and invasive migration of primary human macrophages, which could be promising potential targets for manipulation of immune cell invasion. 
 Rab5 mediates caspase-8-promoted cell motility and   metastasis  . Caspase-8 is a key apical sensory protein that governs cell responses to environmental cues, alternatively promoting apoptosis, proliferation, and cell migration. The proteins responsible for integration of these pathways, however, have remained elusive. Here, we reveal that Rab5 regulates caspase-8-dependent signaling from integrins. Integrin ligation leads to Rab5 activation, association with integrins, and activation of Rac, in a caspase-8-dependent manner. Rab5 activation promotes colocalization and coprecipitation of integrins with caspase-8, concomitant with Rab5 recruitment to integrin-rich regions such as focal adhesions and membrane ruffles. Moreover, caspase-8 expression promotes Rab5-mediated internalization and the recycling of beta1 integrins, increasing cell migration independently of caspase catalytic activity. Conversely, Rab5 knockdown prevented caspase-8-mediated integrin signaling for Rac activation, cell migration, and apoptotic signaling, respectively. Similarly, Rab5 was critical for caspase-8-driven cell migration in vivo, because knockdown of Rab5 compromised the ability of caspase-8 to promote   metastasis   under nonapoptotic conditions. These studies identify Rab5 as a key integrator of caspase-8-mediated signal transduction downstream of integrins, regulating cell survival and migration in vivo and in vitro. 
 [The expression and clinical significance of RABEX-5 and RAB5 in breast cancer]. To study the expression and significance of RABEX-5 and RAB5 in human breast cancer.In this study the expression of RABEX-5 and RAB5 mRNA and protein was detected by Immunohistochemical techniques, the reverse transcriptase polymerase chain reaction (RT-PCR) and Western blot assay in 60 cases of breast cancer, 15 cases of breast fibroadenomas and 15 cases of normal breast tissue. The relationship of RABEX-5, RAB5 and the clinic pathological features was also analyzed.Expression of RABEX-5 and RAB5 in breast cancer was obviously higher than that in breast fibroadenomas and normal breast tissue (P < 0.05). RABEX-5 and RAB5 expression level in tumor diameter (T) >5 cm group of human breast cancer was obviously higher than that in T < or =2 cm and 2 < T < or =5 cm group (P < 0.05). RABEX-5 and RAB5 expression level in axillary lymph node positive group was obviously higher than that in negative group (P < 0.05). However, there were no significant differences of RABEX-5 and RAB5 levels between HER-2 (+) group and HER-2 (-) group (P > 0.05), ER (-) group and ER (+) group (P > 0.05), PR (-) group and PR (+) group (P> 0.05), and in histological grade I, II and III, there also were no significant differences of RABEX-5 and RAB5 levels. The expression of RABEX-5 is correlated with RAB5 in both mRNA and protein level.RABEX-5 and RAB5 may be involved in the development of breast cancer   metastasis  . 
 Rab5A is associated with axillary lymph node   metastasis   in breast cancer patients. The expression of Rab proteins has been associated with cancer. However, few data are available on Rab5A expression in human breast cancer or its impact on disease progression. First, we examined the functional role of Rab5A in breast cancer cells. The expression of Rab5A in MDA-MB-231 cells can be stimulated by epidermal growth factor in a dose-dependent manner. The epidermal growth factor-induced increase of Rab5A expression correlated well with enhanced migration in wound healing migration assays in these cells. Furthermore, we evaluated the expression of Rab5A in breast cancer specimens using immunohistochemical staining, then analyzed the relationship between the expression of Rab5A and clinicopathological parameters. The increased expression of Rab5A protein in 123 breast cancer samples was associated with higher histological grade (P = 0.004), more lymphovascular invasion (P = 0.027), more axillary lymph node (LN)   metastasis   (P = 0.008), and a higher number of axillary LN metastases (P = 0.043). Among 218 axillary LNs of more than 10 breast cancer patients with node metastases, 167 metastatic LNs were found to have increased Rab5A expression. Rab5A is associated with axillary LN   metastasis   in breast cancer patients. 
 Rab5 activation promotes focal adhesion disassembly, migration and invasiveness in tumor cells. Migration and invasion are essential steps associated with tumor cell   metastasis   and increasing evidence points towards endosome trafficking being essential in this process. Indeed, the small GTPase Rab5, a crucial regulator of early endosome dynamics, promotes cell migration in vitro and in vivo. Precisely how Rab5 participates in these events remains to be determined. Considering that focal adhesions represent structures crucial to cell migration, we specifically asked whether Rab5 activation promoted focal adhesion disassembly and thereby facilitated migration and invasion of metastatic cancer cells. Pulldown and biosensor assays revealed that Rab5-GTP loading increased at the leading edge of migrating tumor cells. Additionally, targeting of Rab5 by different shRNA sequences, but not control shRNA, decreased Rab5-GTP levels, leading to reduced cell spreading, migration and invasiveness. Re-expression in knockdown cells of wild-type Rab5, but not the S34N mutant (GDP-bound), restored these properties. Importantly, Rab5 association with the focal adhesion proteins vinculin and paxillin increased during migration, and expression of wild-type, but not GDP-bound Rab5, accelerated focal adhesion disassembly, as well as FAK dephosphorylation on tyrosine 397. Finally, Rab5-driven invasiveness required focal adhesion disassembly, as treatment with the FAK inhibitor number 14 prevented Matrigel invasion and matrix metalloproteinase release. Taken together, these observations show that Rab5 activation is required to enhance cancer cell migration and invasion by promoting focal adhesion disassembly. 
 Rab5 activation as a tumor cell migration switch. Increased cell migration is an acquired feature of metastatic cancer cells and relies on derailed signal transduction pathways. Intracellular vesicular trafficking plays a key role in cell migration due to its intricate involvement in cargo transport and membrane composition. In the last decade, endocytosis has been implicated in cell migration and found to be responsible for the internalization of membrane receptors at the plasma membrane, where integrin trafficking and fine-tuning of receptor tyrosine kinase signaling by internalization are major mechanisms. Accumulating evidence has suggested a link between endosome dynamics, cell migration, and invasion, in which small GTPases of the Rab family have central roles. We have recently determined that Rab5 activation is a crucial event in promoting focal adhesion disassembly, which is concomitant with the migration and invasion of metastatic cancer cells. The mechanisms underlying this novel role for Rab5 are currently unclear, and their elucidation will provide insight into the role of Rab5 function in cancer cell   metastasis  . 
 The Autophagic Process Occurs in Human Bone   Metastasis   and Implicates Molecular Mechanisms Differently Affected by Rab5a in the Early and Late Stages. Autophagy favours metastatic growth through fuelling energy and nutrients and resistance to anoikis, typical of disseminated-tumour cells. The autophagic process, mediated by a unique organelle, the autophagosome, which fuses with lysosomes, is divided into three steps. Several stages, especially early omegasome formation and isolation-membrane initiation, remain controversial; molecular mechanisms involve the small-GTPase Rab5a, which regulates vesicle traffic for autophagosome formation. We examined Rab5a involvement in the function of key members of ubiquitin-conjugation systems, Atg7 and LC3-lipidated, interacting with the scaffold-protein p62. Immunohistochemistry of Rab5a was performed in human specimens of bone   metastasis   and pair-matched breast carcinoma; the autophagic-molecular mechanisms affected by Rab5a were evaluated in human 1833 bone metastatic cells, derived from breast-carcinoma MDA-MB231 cells. To clarify the role of Rab5a, 1833 cells were transfected transiently with Rab5a-dominant negative, and/or stably with the short-hairpin RNA Atg7, were exposed to two inhibitors of autolysosome function, and LC3II and p62 expression was measured. We showed basal autophagy in bone-metastatic cells and the pivotal role of Rab5a together with Beclin 1 between the early stages, elongation of isolation membrane/closed autophagosome mediated by Atg7, and the late-degradative stages. This regulatory network might occur in bone-  metastasis   and in high-grade dysplastic lesions, preceding invasive-breast carcinoma and conferring phenotypic characteristics for dissemination. 
 [Expression and significance of Rab5a and APPL1 in breast cancer]. To investigate the expression of Rab5a and APPL1 in breast cancer and fibroma, and analyze their correlation with HER-2 expression,   metastasis   and development of breast cancer.Rab5a and APPL1 in paraffin embedded tissues of 74 breast carcinomas and 40 breast fibromas were detected by immunohistochemistry. Their relationship with   metastasis  , pathological grade, and HER-2 expression in breast cancer was determined by statistical analysis.There was no expression or low expression of Rab5a and APPL1 in the breast fibroma, but the positive expression rate of Rab5a and APPL1 in the breast carcinomas were 91.9% and 83.8%, respectively. No significant difference in expression of Rab5a and APPL1 was found between metastatic and non-metastatic groups, and pathological grade I/II and grade III groups. But Rab5a was overexpressed in HER-2-positive group compared with that in the HER-2-negative group.Rab5a and APPL1 are overexpressed in breast cancer, and are positively correlated with the HER-2 expression. These proteins may influence the growth and proliferation of breast cancer cells by HER-2 signal transduction. 
 Knockdown of Rab5a expression decreases cancer cell motility and invasion through integrin-mediated signaling pathway. Rab GTPases function as modulators in intracellular transport. Rab5a, a member of the Rab subfamily of small GTPases, is an important regulator of vesicle traffic from the plasma membrane to early endosomes. Recent findings have reported that Rab5a gene was involved in the progression of cancer. In the present study, we investigated the effect of Rab5a on cervical cancer invasion and   metastasis   and the molecular mechanism underlying the involvement of Rab5a.Rab5a expression was assessed by immunohistochemical analysis on a cervical cancer tissue microarray. RNA interference (RNAi) was performed to knock down the endogenous expression of Rab5a gene in HeLa and SiHa cells. Cell motility was evaluated using invasion assay and wound migration assay in vitro. The expression levels of integrin-associated molecules were detected by Western blot and immunofluorescence.We found that Rab5a was expressed at a high level in cervical cancer tissues. Silencing of Rab5a expression significantly decreased cancer cell motility and invasiveness. The down-regulation of integrin-associated focal adhesion signaling molecules was further detected in Rab5a knockdown cells. Meanwhile, active GTP-bound Rac1, Cdc42, and RhoA were also down-regulated, accompanied with the reduction in the number and size of filopodia and lamellipodia.Taken together, these data suggest that Rab5a functions in regulating the invasion phenotype, and we propose that this regulation may be via integrin-mediated signaling pathway in cervical cancer cells. 
 Increased expression of Rab5A predicts   metastasis   and poor prognosis in colorectal cancer patients. Rab5A is reported to correlate with cancer development and progression. The purpose of this study is to explore the association between Rab5A expression and the clinical characteristics of colorectal cancer (CRC). Data containing three independent investigations from Oncomine database demonstrated that Rab5A is overexpression in CRC compared with normal tissue, similar result was also found in 32 matched CRC tissue samples by qPCR. The protein expression of Rab5A was examined in 390 CRC specimens and the results showed that high expression of Rab5A was significantly correlated with tumor size (P = 0.008), serum CEA (P = 0.002), liver   metastasis   (P = 0.014) and clinical stage (P = 0.010). Kaplan-Meier method suggested that overexpression of Rab5A protein expression had shorter overall survival times in CRC patients (P < 0.001). Multivariate Cox regression analysis confirmed Rab5A expression, tumor size and clinical stage as independent prognostic factor in CRC. In conclusion, the data indicated that higher expression of Rab5A was observed in CRC tissues and Rab5A may be identified as a useful predictor of   metastasis   and prognosis for CRC. 
 Overexpression of Rab5a promotes hepatocellular carcinoma cell proliferation and invasion via FAK signaling pathway. Rab5a was reported to be overexpressed in human malignancy and associated with the malignant phenotype. To data, its expression pattern and biological function in hepatocellular carcinoma (HCC) have not been studied. We analyzed Rab5a protein expression in 98 cases of HCC tissues and four HCC cell lines. We found that Rab5a expression was upregulated in HCC tissues and cell lines. Rab5a overexpression correlated with TNM stage and nodal   metastasis   (p < 0.05). To confirm the biological function of Rab5a in HCC cell lines, Rab5a siRNA was employed in SK-Hep-1 cell line and plasmid transfection was performed in Huh7 cell line. CCK-8 assay showed that Rab5a depletion blocked cell growth rate while Rab5a overexpression facilitated proliferation. Transwell and migration assay showed that Rab5a positively regulated cell invasion and migration. To explore the molecular mechanism underlying the biological effects of Rab5a, we checked several signaling pathways and found that Rab5a overexpression upregulated cyclin D1, cyclin E expression, FAK (Tyr397), and AKT (Ser473) phosphorylation. Blockage of FAK using inhibitor PF573228 abolished the role of Rab5a on cyclin D1. In conclusion, Rab5a is overexpressed in human HCC and contributes to cancer cell proliferation and invasion through regulation of FAK and AKT signaling. 
  GeneRIFs  
 Low expression of RAB5A is associated with   metastasis   in prostate cancer. 
 Higher expression of Rab5A was observed in colorectal cancer tissues and Rab5A may be identified as a useful predictor of   metastasis   and prognosis for CRC. 
 RABEX-5 and RAB5 may be involved in the development of breast cancer   metastasis  . 
 Rab5A is associated with axillary lymph node   metastasis   in breast cancer patients. 
  [Cc]ell [Ii]nvasion  PubMed  
 A specific subset of RabGTPases controls cell surface exposure of MT1-MMP, extracellular matrix degradation and three-dimensional invasion of macrophages. The matrix metalloproteinase MT1-MMP has a major impact on invasive cell migration in both physiological and pathological settings such as immune cell extravasation or metastasis of cancer cells. Surface-associated MT1-MMP is able to cleave components of the extracellular matrix, which is a prerequisite for proteolytic invasive migration. However, current knowledge on the molecular mechanisms that regulate MT1-MMP trafficking to and from the cell surface is limited. We have identified three members of the RabGTPase family, Rab5a, Rab8a and Rab14, as crucial regulators of MT1-MMP trafficking and function in primary human macrophages. Both overexpressed and endogenous forms show prominent colocalisation with MT1-MMP-positive vesicles, whereas expression of mutant constructs, as well as siRNA-induced knockdown, reveal that these RabGTPases are crucial in the regulation of MT1-MMP surface exposure, contact of MT1-MMP-positive vesicles with podosomes, extracellular matrix degradation in two and three dimensions, as well as three-dimensional proteolytic invasion of macrophages. Collectively, our results identify Rab5a, Rab8a and Rab14 as major regulators of MT1-MMP trafficking and invasive migration of primary human macrophages, which could be promising potential targets for manipulation of immune   cell invasion  . 
 A specific subset of RabGTPases controls cell surface exposure of MT1-MMP, extracellular matrix degradation and three-dimensional invasion of macrophages. The matrix metalloproteinase MT1-MMP has a major impact on invasive cell migration in both physiological and pathological settings such as immune cell extravasation or metastasis of cancer cells. Surface-associated MT1-MMP is able to cleave components of the extracellular matrix, which is a prerequisite for proteolytic invasive migration. However, current knowledge on the molecular mechanisms that regulate MT1-MMP trafficking to and from the cell surface is limited. We have identified three members of the RabGTPase family, Rab5a, Rab8a and Rab14, as crucial regulators of MT1-MMP trafficking and function in primary human macrophages. Both overexpressed and endogenous forms show prominent colocalisation with MT1-MMP-positive vesicles, whereas expression of mutant constructs, as well as siRNA-induced knockdown, reveal that these RabGTPases are crucial in the regulation of MT1-MMP surface exposure, contact of MT1-MMP-positive vesicles with podosomes, extracellular matrix degradation in two and three dimensions, as well as three-dimensional proteolytic invasion of macrophages. Collectively, our results identify Rab5a, Rab8a and Rab14 as major regulators of MT1-MMP trafficking and invasive migration of primary human macrophages, which could be promising potential targets for manipulation of immune   cell invasion  . 
 Rab'ing tumor cell migration and invasion: focal adhesion disassembly driven by Rab5. The small GTPase Rab5 has been extensively studied in the context of endocytic trafficking because it is critical in the regulation of early endosome dynamics. In addition to this canonical role, evidence obtained in recent years implicates Rab5 in the regulation of cell migration. This novel role of Rab5 is based not only on an indirect relationship between cell migration and endosomal trafficking as separate processes, but also on the direct regulation of signaling proteins implicated in cell migration. However, the precise mechanisms underlying this connection have remained elusive. Recent studies have shown that the activation of Rab5 is a critical event for maintaining the dynamics of focal adhesions, which is fundamental in regulating not only cell migration but also tumor   cell invasion  . 
 Overexpression of Rab5a promotes hepatocellular carcinoma cell proliferation and invasion via FAK signaling pathway. Rab5a was reported to be overexpressed in human malignancy and associated with the malignant phenotype. To data, its expression pattern and biological function in hepatocellular carcinoma (HCC) have not been studied. We analyzed Rab5a protein expression in 98 cases of HCC tissues and four HCC cell lines. We found that Rab5a expression was upregulated in HCC tissues and cell lines. Rab5a overexpression correlated with TNM stage and nodal metastasis (p < 0.05). To confirm the biological function of Rab5a in HCC cell lines, Rab5a siRNA was employed in SK-Hep-1 cell line and plasmid transfection was performed in Huh7 cell line. CCK-8 assay showed that Rab5a depletion blocked cell growth rate while Rab5a overexpression facilitated proliferation. Transwell and migration assay showed that Rab5a positively regula  ted cell inva  sion and migration. To explore the molecular mechanism underlying the biological effects of Rab5a, we checked several signaling pathways and found that Rab5a overexpression upregulated cyclin D1, cyclin E expression, FAK (Tyr397), and AKT (Ser473) phosphorylation. Blockage of FAK using inhibitor PF573228 abolished the role of Rab5a on cyclin D1. In conclusion, Rab5a is overexpressed in human HCC and contributes to cancer cell proliferation and invasion through regulation of FAK and AKT signaling. 
  GeneRIFs  
 Recent studies have shown that the activation of Rab5 is a critical event for maintaining the dynamics of focal adhesions, which is fundamental in regulating not only cell migration but also tumor   cell invasion   
   
  
 RAB5C 
 
  NCBI Gene  
  GeneRIF  
  Pubmed  
 
 
  Search Term  Rank  Fields  
  [Mm]igration  6  PubMed(2)  
  [Ee]xtracellular [Mm]atrix  8  PubMed(1)  
  [Ii]nvasive  10  PubMed(2)  
  [Mm]etastasis  12  PubMed(2)  
  [Cc]ell [Ii]nvasion  13  PubMed(2)  
 
 
 hide/show details for RAB5C 
  [Mm]igration  PubMed  
 Rab5 isoforms orchestrate a "division of labor" in the endocytic network; Rab5C modulates Rac-mediated cell motility. Rab5, the prototypical Rab GTPase and master regulator of the endocytic pathway, is encoded as three differentially expressed isoforms, Rab5A, Rab5B and Rab5C. Here, we examined the differential effects of Rab5 isoform silencing on cell motility and report that Rab5C, but neither Rab5A nor Rab5B, is selectively associated with the growth factor-activation of Rac1 and with enhanced cell motility. Initial observations revealed that silencing of Rab5C expression, but neither Rab5A nor Rab5C, led to spindle-shaped cells that displayed reduced formation of membrane ruffles. When subjected to a scratch wound assay, cells depleted of Rab5C, but not Rab5A or Rab5B, demonstrated reduced cell   migration  . U937 cells depleted of Rab5C also displayed reduced cell motility in a Transwell plate   migration   assay. To examine activation of Rac, HeLa cells stably expressing GFP-Rac1 were independently depleted of Rab5A, Rab5B or Rab5C and seeded onto coverslips imprinted with a crossbow pattern. 3-D GFP-Rac1 images of micro-patterned cells show that GFP-Rac1 was less localized to the cell periphery in the absence of Rab5C. To confirm the connection between Rab5C and Rac activation, HeLa cells depleted of Rab5 isoforms were starved and then stimulated with EGF. Rac1 pull-down assays revealed that EGF-stimulated Rac1 activity was significantly suppressed in Rab5C-suppressed cells. To determine whether events upstream of Rac activation were affected by Rab5C, we observed that EGF-stimulated Akt phosphorylation was suppressed in cells depleted of Rab5C. Finally, since spatio-temporal assembly/disassembly of adhesion complexes are essential components of cell   migration  , we examined the effect of Rab5 isoform depletion on the formation of focal adhesion complexes. Rab5C-depleted HeLa cells have significantly fewer focal adhesion foci, in accordance with the lack of persistent lamellipodial protrusions and reduced directional   migration  . We conclude that Rab5 isoforms selectively oversee the multiple signaling and trafficking events associated with the endocytic network. 
 Rab5 isoforms orchestrate a "division of labor" in the endocytic network; Rab5C modulates Rac-mediated cell motility. Rab5, the prototypical Rab GTPase and master regulator of the endocytic pathway, is encoded as three differentially expressed isoforms, Rab5A, Rab5B and Rab5C. Here, we examined the differential effects of Rab5 isoform silencing on cell motility and report that Rab5C, but neither Rab5A nor Rab5B, is selectively associated with the growth factor-activation of Rac1 and with enhanced cell motility. Initial observations revealed that silencing of Rab5C expression, but neither Rab5A nor Rab5C, led to spindle-shaped cells that displayed reduced formation of membrane ruffles. When subjected to a scratch wound assay, cells depleted of Rab5C, but not Rab5A or Rab5B, demonstrated reduced cell   migration  . U937 cells depleted of Rab5C also displayed reduced cell motility in a Transwell plate   migration   assay. To examine activation of Rac, HeLa cells stably expressing GFP-Rac1 were independently depleted of Rab5A, Rab5B or Rab5C and seeded onto coverslips imprinted with a crossbow pattern. 3-D GFP-Rac1 images of micro-patterned cells show that GFP-Rac1 was less localized to the cell periphery in the absence of Rab5C. To confirm the connection between Rab5C and Rac activation, HeLa cells depleted of Rab5 isoforms were starved and then stimulated with EGF. Rac1 pull-down assays revealed that EGF-stimulated Rac1 activity was significantly suppressed in Rab5C-suppressed cells. To determine whether events upstream of Rac activation were affected by Rab5C, we observed that EGF-stimulated Akt phosphorylation was suppressed in cells depleted of Rab5C. Finally, since spatio-temporal assembly/disassembly of adhesion complexes are essential components of cell   migration  , we examined the effect of Rab5 isoform depletion on the formation of focal adhesion complexes. Rab5C-depleted HeLa cells have significantly fewer focal adhesion foci, in accordance with the lack of persistent lamellipodial protrusions and reduced directional   migration  . We conclude that Rab5 isoforms selectively oversee the multiple signaling and trafficking events associated with the endocytic network. 
  [Ee]xtracellular [Mm]atrix  PubMed  
 Proteomic analysis of human parotid gland exosomes by multidimensional protein identification technology (MudPIT). Human ductal saliva contributes over a thousand unique proteins to whole oral fluids. The mechanism by which most of these proteins are secreted by salivary glands remains to be determined. The present study used a mass spectrometry-based, shotgun proteomics approach to explore the possibility that a subset of the proteins found in saliva are derived from exosomes, membrane-bound vesicles of endosomal origin within multivesicular endosomes. Using MudPIT (multidimensional protein identification technology) mass spectrometry, we catalogued 491 proteins in the exosome fraction of human parotid saliva. Many of these proteins were previously observed in ductal saliva from parotid glands (265 proteins). Furthermore, 72 of the proteins in parotid exosomes overlap with those previously identified as urinary exosome proteins, proteins which are also frequently associated with exosomes from other tissues and cell types. Gene Ontology (GO) and KEGG pathway analyses found that cytosolic proteins comprise the largest category of proteins in parotid exosomes (43%), involved in such processes as phosphatidylinositol signaling system, calcium signaling pathway, inositol metabolism, protein export, and signal transduction, among others; whereas the integral plasma membrane proteins and associated/peripheral plasma membrane proteins (26%) were associated with   extracellular matrix  -receptor interaction, epithelial cell signaling, T-cell and B-cell receptor signaling, cytokine receptor interaction, and antigen processing and presentation, among other biological functions. In addition, these putative saliva exosomal proteins were linked to specific diseases (e.g., neurodegenerative disorders, prion disease, cancers, type I and II diabetes). Consequently, parotid glands secrete exosomes that reflect the metabolic and functional status of the gland and may also carry informative protein markers useful in the diagnosis and treatment of systemic diseases. 
  [Ii]nvasive  PubMed  
 Rab5c promotes AMAP1-PRKD2 complex formation to enhance β1 integrin recycling in EGF-induced cancer invasion. Epidermal growth factor receptor (EGFR) signaling is one of the crucial factors in breast cancer malignancy. Breast cancer cells often overexpress Arf6 and its effector, AMAP1/ASAP1/DDEF1; in these cells, EGFR signaling may activate the Arf6 pathway to induce invasion and metastasis. Active recycling of some integrins is crucial for invasion and metastasis. Here, we show that the Arf6-AMAP1 pathway links to the machinery that recycles β1 integrins, such as α3β1, to promote cell invasion upon EGFR stimulation. We found that AMAP1 had the ability to bind directly to PRKD2 and hence to make a complex with the cytoplasmic tail of the β1 subunit. Moreover, GTP-Rab5c also bound to AMAP1, and activation of Rab5c by EGFR signaling was necessary to promote the intracellular association of AMAP1 and PRKD2. Our results suggest a novel mechanism by which EGFR signaling promotes   the inv  asiveness of some breast cancer cells via integrin recycling. 
 Rab5c promotes AMAP1-PRKD2 complex formation to enhance β1 integrin recycling in EGF-induced cancer invasion. Epidermal growth factor receptor (EGFR) signaling is one of the crucial factors in breast cancer malignancy. Breast cancer cells often overexpress Arf6 and its effector, AMAP1/ASAP1/DDEF1; in these cells, EGFR signaling may activate the Arf6 pathway to induce invasion and metastasis. Active recycling of some integrins is crucial for invasion and metastasis. Here, we show that the Arf6-AMAP1 pathway links to the machinery that recycles β1 integrins, such as α3β1, to promote cell invasion upon EGFR stimulation. We found that AMAP1 had the ability to bind directly to PRKD2 and hence to make a complex with the cytoplasmic tail of the β1 subunit. Moreover, GTP-Rab5c also bound to AMAP1, and activation of Rab5c by EGFR signaling was necessary to promote the intracellular association of AMAP1 and PRKD2. Our results suggest a novel mechanism by which EGFR signaling promotes   the inv  asiveness of some breast cancer cells via integrin recycling. 
  [Mm]etastasis  PubMed  
 Rab5c promotes AMAP1-PRKD2 complex formation to enhance β1 integrin recycling in EGF-induced cancer invasion. Epidermal growth factor receptor (EGFR) signaling is one of the crucial factors in breast cancer malignancy. Breast cancer cells often overexpress Arf6 and its effector, AMAP1/ASAP1/DDEF1; in these cells, EGFR signaling may activate the Arf6 pathway to induce invasion and   metastasi  s. Active recycling of some integrins is crucial for invasion and   metastasi  s. Here, we show that the Arf6-AMAP1 pathway links to the machinery that recycles β1 integrins, such as α3β1, to promote cell invasion upon EGFR stimulation. We found that AMAP1 had the ability to bind directly to PRKD2 and hence to make a complex with the cytoplasmic tail of the β1 subunit. Moreover, GTP-Rab5c also bound to AMAP1, and activation of Rab5c by EGFR signaling was necessary to promote the intracellular association of AMAP1 and PRKD2. Our results suggest a novel mechanism by which EGFR signaling promotes the invasiveness of some breast cancer cells via integrin recycling. 
 Rab5c promotes AMAP1-PRKD2 complex formation to enhance β1 integrin recycling in EGF-induced cancer invasion. Epidermal growth factor receptor (EGFR) signaling is one of the crucial factors in breast cancer malignancy. Breast cancer cells often overexpress Arf6 and its effector, AMAP1/ASAP1/DDEF1; in these cells, EGFR signaling may activate the Arf6 pathway to induce invasion and   metastasi  s. Active recycling of some integrins is crucial for invasion and   metastasi  s. Here, we show that the Arf6-AMAP1 pathway links to the machinery that recycles β1 integrins, such as α3β1, to promote cell invasion upon EGFR stimulation. We found that AMAP1 had the ability to bind directly to PRKD2 and hence to make a complex with the cytoplasmic tail of the β1 subunit. Moreover, GTP-Rab5c also bound to AMAP1, and activation of Rab5c by EGFR signaling was necessary to promote the intracellular association of AMAP1 and PRKD2. Our results suggest a novel mechanism by which EGFR signaling promotes the invasiveness of some breast cancer cells via integrin recycling. 
  [Cc]ell [Ii]nvasion  PubMed  
 Rab5c promotes AMAP1-PRKD2 complex formation to enhance β1 integrin recycling in EGF-induced cancer invasion. Epidermal growth factor receptor (EGFR) signaling is one of the crucial factors in breast cancer malignancy. Breast cancer cells often overexpress Arf6 and its effector, AMAP1/ASAP1/DDEF1; in these cells, EGFR signaling may activate the Arf6 pathway to induce invasion and metastasis. Active recycling of some integrins is crucial for invasion and metastasis. Here, we show that the Arf6-AMAP1 pathway links to the machinery that recycles β1 integrins, such as α3β1, to prom  ote cell inva  sion upon EGFR stimulation. We found that AMAP1 had the ability to bind directly to PRKD2 and hence to make a complex with the cytoplasmic tail of the β1 subunit. Moreover, GTP-Rab5c also bound to AMAP1, and activation of Rab5c by EGFR signaling was necessary to promote the intracellular association of AMAP1 and PRKD2. Our results suggest a novel mechanism by which EGFR signaling promotes the invasiveness of some breast cancer cells via integrin recycling. 
 Rab5c promotes AMAP1-PRKD2 complex formation to enhance β1 integrin recycling in EGF-induced cancer invasion. Epidermal growth factor receptor (EGFR) signaling is one of the crucial factors in breast cancer malignancy. Breast cancer cells often overexpress Arf6 and its effector, AMAP1/ASAP1/DDEF1; in these cells, EGFR signaling may activate the Arf6 pathway to induce invasion and metastasis. Active recycling of some integrins is crucial for invasion and metastasis. Here, we show that the Arf6-AMAP1 pathway links to the machinery that recycles β1 integrins, such as α3β1, to prom  ote cell inva  sion upon EGFR stimulation. We found that AMAP1 had the ability to bind directly to PRKD2 and hence to make a complex with the cytoplasmic tail of the β1 subunit. Moreover, GTP-Rab5c also bound to AMAP1, and activation of Rab5c by EGFR signaling was necessary to promote the intracellular association of AMAP1 and PRKD2. Our results suggest a novel mechanism by which EGFR signaling promotes the invasiveness of some breast cancer cells via integrin recycling. 
   
  
 RAB22A 
 
  NCBI Gene  
  GeneRIF  
  Pubmed  
 
 
  Search Term  Rank  Fields  
  [Mm]igration  6  PubMed(5)  
  [Ee]xtracellular [Mm]atrix  8  PubMed(1)  
  [Mm]etastasis  12  PubMed(4); GeneRIFs(1)  
  [Cc]ell [Ii]nvasion  13  PubMed(2)  
 
 
 hide/show details for RAB22A 
  [Mm]igration  PubMed  
 LncRNA HOTAIR controls the expression of Rab22a by sponging miR-373 in ovarian cancer. Increasing evidence suggests that the long non-coding RNA, HOX transcript antisense intergenic RNA (HOTAIR) is widely involved in the progression and metastasis of cancer. However, the specific role of HOTAIR in ovarian carcinogenesis remains to be fully elucidated. In the present study, the levels of HOTAIR were detected in 30 paired cancer and noncancer tissues using reverse transcription-quantitative polymerase chain reaction analysis. The effect of HOTAIR on the ovarian cancer cells was examined by overexpression or small interfering RNA interference experiments. To examine the competitive endogenous RNA (ceRNAs) mechanism, a luciferase reporter assay was used. In patients with ovarian cancer, HOTAIR was significantly upregulated. Furthermore, the upregulation of HOTAIR increased the proliferation,   migratio  n and invasion of ovarian cancer cells. By contrast, the knockdown of HOTAIR repressed cell invasion and viability. HOTAIR functioned as a ceRNA, and acted as a sink for microRNA (miR)‑373, thereby regulating the expression of Rab22a. The upregulation of HOTAIR contributed to the malignant progression of ovarian cancer cells. Therefore, the positive regulation between HOTAIR and Rab22a can be partially attributed to the ceRNA regulatory network through miR-373. 
 Tumor suppressive microRNA-193b promotes breast cancer progression via targeting DNAJC13 and RAB22A. Breast cancer is still a leading cause of cancer deaths in women. Despite improvements in therapeutic approaches in local control, metastatic relapse is almost always incurable, underlining the importance to better understand the biological bases that contribute to disease progression. In this study, we demonstrated that miR-193b was significantly down-regulated in two primary human breast cancer cell lines (MDA-MB-231 and MCF-7). Reconstitution of miR-193b expression resulted in decreasing cell proliferation, clonogenicity,   migration   and invasion. By using in silico prediction algorithms approach for target identification, we identified DNAJC13 (HPS40) and RAB22A to be direct targets of miR-193b. Concordantly, Re-expression of miR-193b decreased DNAJC13 (HPS40) and RAB22A expression. Luciferase reporter assays confirmed the direct interaction of miR-193b with both DNAJC13 (HPS40) and RAB22A. Our findings have demonstrated that miR-193b as a novel tumor suppressor plays an important role in breast cancer progression, understanding the mechanisms could account for the aggressive behaviour of breast cancer. 
 MiR-373 targeting of the Rab22a oncogene suppresses tumor invasion and metastasis in ovarian cancer. Metastasis is major cause of mortality in patients with ovarian cancer. MiR-373 has been shown to play pivotal roles in tumorigenesis and metastasis; however, a role for miR-373 in ovarian cancer has not been investigated. In this study, we show that the miR-373 expression is down-regulated in human epithelial ovarian cancer (EOC) and inversely correlated with clinical stage and histological grade. Ectopic overexpression of miR-373 in human EOC cells suppressed cell invasion in vitro and metastasis in vivo, and the epithelial-mesenchymal transition process. Silencing the expression of miR-373 resulted in an increased   migration   and invasion of EOC cells. Using integrated bioinformatics analysis, gene expression arrays, and luciferase assay, we identified Rab22a as a direct and functional target of miR-373 in EOC cells. Expression levels of miR-373 were inversely correlated with Rab22a protein levels in human EOC tissues. Rab22a knockdown inhibited invasion and   migration   of EOC cells, increased E-cadherin expression, and suppressed the expression of N-cadherin. Moreover, overexpression of Rab22a abrogated miR-373-induced invasion and   migration   of EOC cells. Taken together, these results demonstrate that miR-373 suppresses EOC invasion and metastasis by directly targeting Rab22a gene, a new potential therapeutic target in EOC. 
 miR-204-5p inhibits proliferation and invasion and enhances chemotherapeutic sensitivity of colorectal cancer cells by downregulating RAB22A. miR-204-5p was found to be downregulated in colorectal cancer tissues in our preliminary microarray analyses. However, the function of miR-204-5p in colorectal cancer remains unknown. We therefore investigated the role, mechanism, and clinical significance of miR-204-5p in colorectal cancer development and progression.We measured the expression of miR-204-5p and determined its correlation with patient prognoses. Ectopic expression in colorectal cancer cells, xenografts, and pulmonary metastasis models was used to evaluate the effects of miR-204-5p on proliferation,   migration  , and chemotherapy sensitivity. Luciferase assay and Western blotting were performed to validate the potential targets of miR-204-5p after the preliminary screening by a microarray analysis and computer-aided algorithms.miR-204-5p is frequently downregulated in colorectal cancer tissues, and survival analysis showed that the downregulation of miR-204-5p in colorectal cancer was associated with poor prognoses. Ectopic miR-204-5p expression repressed colorectal cancer cell growth both in vitro and in vivo. Moreover, restoring miR-204-5p expression inhibited colorectal cancer   migration   and invasion and promoted tumor sensitivity to chemotherapy. Mechanistic investigations revealed that RAB22A, a member of the RAS oncogene family, is a direct functional target of miR-204-5p in colorectal cancer. Furthermore, RAB22A protein levels in colorectal cancer tissues were frequently increased and negatively associated with miR-204-5p levels and survival time.Our results demonstrate for the first time that miR-204-5p acts as a tumor suppressor in colorectal cancer through inhibiting RAB22A and reveal RAB22A to be a new oncogene and prognostic factor for colorectal cancer. 
 miR-203 Acts as a Tumor Suppressor Gene in Osteosarcoma by Regulating RAB22A. microRNAs (miRNAs), small noncoding RNAs of 19-25 nt, play an important roles in the pathological processes of tumorigenesis. The object of this study was to study the expression and function of miR-203 and to found its target gene in osteosarcoma. In our study, we found the expression level of miR-203 was significantly downregulated in osteosarcoma cell lines and tissues. In addition, overexpression of miR-203 inhibited the osteosarcoma cell proliferation and   migration   and inhibited Mesenchymal-to-Epithelial reversion Transition (MErT). Moreover, we identified RAB22A as a direct target of miR-203 and RAB22A overexpression blocks the roles of miR-203 in osteosarcoma cell. Furthermore, we demonstrated that RAB22A expression was upregulated in human osteosarcoma cell lines and tissues. Take together, our results demonstrated that miR-203 act as a tumor suppressor miRNA through regulating RAB22A expression and suggested its involvement in osteosarcoma progression and carcinogenesis. 
  [Ee]xtracellular [Mm]atrix  PubMed  
 Proteomic analysis of human parotid gland exosomes by multidimensional protein identification technology (MudPIT). Human ductal saliva contributes over a thousand unique proteins to whole oral fluids. The mechanism by which most of these proteins are secreted by salivary glands remains to be determined. The present study used a mass spectrometry-based, shotgun proteomics approach to explore the possibility that a subset of the proteins found in saliva are derived from exosomes, membrane-bound vesicles of endosomal origin within multivesicular endosomes. Using MudPIT (multidimensional protein identification technology) mass spectrometry, we catalogued 491 proteins in the exosome fraction of human parotid saliva. Many of these proteins were previously observed in ductal saliva from parotid glands (265 proteins). Furthermore, 72 of the proteins in parotid exosomes overlap with those previously identified as urinary exosome proteins, proteins which are also frequently associated with exosomes from other tissues and cell types. Gene Ontology (GO) and KEGG pathway analyses found that cytosolic proteins comprise the largest category of proteins in parotid exosomes (43%), involved in such processes as phosphatidylinositol signaling system, calcium signaling pathway, inositol metabolism, protein export, and signal transduction, among others; whereas the integral plasma membrane proteins and associated/peripheral plasma membrane proteins (26%) were associated with   extracellular matrix  -receptor interaction, epithelial cell signaling, T-cell and B-cell receptor signaling, cytokine receptor interaction, and antigen processing and presentation, among other biological functions. In addition, these putative saliva exosomal proteins were linked to specific diseases (e.g., neurodegenerative disorders, prion disease, cancers, type I and II diabetes). Consequently, parotid glands secrete exosomes that reflect the metabolic and functional status of the gland and may also carry informative protein markers useful in the diagnosis and treatment of systemic diseases. 
  [Mm]etastasis  PubMed  
 LncRNA HOTAIR controls the expression of Rab22a by sponging miR-373 in ovarian cancer. Increasing evidence suggests that the long non-coding RNA, HOX transcript antisense intergenic RNA (HOTAIR) is widely involved in the progression and   metastasis   of cancer. However, the specific role of HOTAIR in ovarian carcinogenesis remains to be fully elucidated. In the present study, the levels of HOTAIR were detected in 30 paired cancer and noncancer tissues using reverse transcription-quantitative polymerase chain reaction analysis. The effect of HOTAIR on the ovarian cancer cells was examined by overexpression or small interfering RNA interference experiments. To examine the competitive endogenous RNA (ceRNAs) mechanism, a luciferase reporter assay was used. In patients with ovarian cancer, HOTAIR was significantly upregulated. Furthermore, the upregulation of HOTAIR increased the proliferation, migration and invasion of ovarian cancer cells. By contrast, the knockdown of HOTAIR repressed cell invasion and viability. HOTAIR functioned as a ceRNA, and acted as a sink for microRNA (miR)‑373, thereby regulating the expression of Rab22a. The upregulation of HOTAIR contributed to the malignant progression of ovarian cancer cells. Therefore, the positive regulation between HOTAIR and Rab22a can be partially attributed to the ceRNA regulatory network through miR-373. 
 Hypoxia-inducible factors and RAB22A mediate formation of microvesicles that stimulate breast cancer invasion and   metastasis  . Extracellular vesicles such as exosomes and microvesicles (MVs) are shed by cancer cells, are detected in the plasma of cancer patients, and promote cancer progression, but the molecular mechanisms regulating their production are not well understood. Intratumoral hypoxia is common in advanced breast cancers and is associated with an increased risk of   metastasis   and patient mortality that is mediated in part by the activation of hypoxia-inducible factors (HIFs). In this paper, we report that exposure of human breast cancer cells to hypoxia augments MV shedding that is mediated by the HIF-dependent expression of the small GTPase RAB22A, which colocalizes with budding MVs at the cell surface. Incubation of naïve breast cancer cells with MVs shed by hypoxic breast cancer cells promotes focal adhesion formation, invasion, and   metastasi  s. In breast cancer patients, RAB22A mRNA overexpression in the primary tumor is associated with decreased overall and   metastasi  s-free survival and, in an orthotopic mouse model, RAB22A knockdown impairs breast cancer   metastasi  s. 
 MiR-373 targeting of the Rab22a oncogene suppresses tumor invasion and   metastasis   in ovarian cancer.   Metastasis   is major cause of mortality in patients with ovarian cancer. MiR-373 has been shown to play pivotal roles in tumorigenesis and   metastasis  ; however, a role for miR-373 in ovarian cancer has not been investigated. In this study, we show that the miR-373 expression is down-regulated in human epithelial ovarian cancer (EOC) and inversely correlated with clinical stage and histological grade. Ectopic overexpression of miR-373 in human EOC cells suppressed cell invasion in vitro and   metastasis   in vivo, and the epithelial-mesenchymal transition process. Silencing the expression of miR-373 resulted in an increased migration and invasion of EOC cells. Using integrated bioinformatics analysis, gene expression arrays, and luciferase assay, we identified Rab22a as a direct and functional target of miR-373 in EOC cells. Expression levels of miR-373 were inversely correlated with Rab22a protein levels in human EOC tissues. Rab22a knockdown inhibited invasion and migration of EOC cells, increased E-cadherin expression, and suppressed the expression of N-cadherin. Moreover, overexpression of Rab22a abrogated miR-373-induced invasion and migration of EOC cells. Taken together, these results demonstrate that miR-373 suppresses EOC invasion and   metastasis   by directly targeting Rab22a gene, a new potential therapeutic target in EOC. 
 miR-204-5p inhibits proliferation and invasion and enhances chemotherapeutic sensitivity of colorectal cancer cells by downregulating RAB22A. miR-204-5p was found to be downregulated in colorectal cancer tissues in our preliminary microarray analyses. However, the function of miR-204-5p in colorectal cancer remains unknown. We therefore investigated the role, mechanism, and clinical significance of miR-204-5p in colorectal cancer development and progression.We measured the expression of miR-204-5p and determined its correlation with patient prognoses. Ectopic expression in colorectal cancer cells, xenografts, and pulmonary   metastasis   models was used to evaluate the effects of miR-204-5p on proliferation, migration, and chemotherapy sensitivity. Luciferase assay and Western blotting were performed to validate the potential targets of miR-204-5p after the preliminary screening by a microarray analysis and computer-aided algorithms.miR-204-5p is frequently downregulated in colorectal cancer tissues, and survival analysis showed that the downregulation of miR-204-5p in colorectal cancer was associated with poor prognoses. Ectopic miR-204-5p expression repressed colorectal cancer cell growth both in vitro and in vivo. Moreover, restoring miR-204-5p expression inhibited colorectal cancer migration and invasion and promoted tumor sensitivity to chemotherapy. Mechanistic investigations revealed that RAB22A, a member of the RAS oncogene family, is a direct functional target of miR-204-5p in colorectal cancer. Furthermore, RAB22A protein levels in colorectal cancer tissues were frequently increased and negatively associated with miR-204-5p levels and survival time.Our results demonstrate for the first time that miR-204-5p acts as a tumor suppressor in colorectal cancer through inhibiting RAB22A and reveal RAB22A to be a new oncogene and prognostic factor for colorectal cancer. 
  GeneRIFs  
 these results demonstrate that miR-373 suppresses epithelial ovarian cancer invasion and   metastasis   by directly targeting Rab22a gene 
  [Cc]ell [Ii]nvasion  PubMed  
 LncRNA HOTAIR controls the expression of Rab22a by sponging miR-373 in ovarian cancer. Increasing evidence suggests that the long non-coding RNA, HOX transcript antisense intergenic RNA (HOTAIR) is widely involved in the progression and metastasis of cancer. However, the specific role of HOTAIR in ovarian carcinogenesis remains to be fully elucidated. In the present study, the levels of HOTAIR were detected in 30 paired cancer and noncancer tissues using reverse transcription-quantitative polymerase chain reaction analysis. The effect of HOTAIR on the ovarian cancer cells was examined by overexpression or small interfering RNA interference experiments. To examine the competitive endogenous RNA (ceRNAs) mechanism, a luciferase reporter assay was used. In patients with ovarian cancer, HOTAIR was significantly upregulated. Furthermore, the upregulation of HOTAIR increased the proliferation, migration and invasion of ovarian cancer cells. By contrast, the knockdown of HOTAIR repressed   cell invasio  n and viability. HOTAIR functioned as a ceRNA, and acted as a sink for microRNA (miR)‑373, thereby regulating the expression of Rab22a. The upregulation of HOTAIR contributed to the malignant progression of ovarian cancer cells. Therefore, the positive regulation between HOTAIR and Rab22a can be partially attributed to the ceRNA regulatory network through miR-373. 
 MiR-373 targeting of the Rab22a oncogene suppresses tumor invasion and metastasis in ovarian cancer. Metastasis is major cause of mortality in patients with ovarian cancer. MiR-373 has been shown to play pivotal roles in tumorigenesis and metastasis; however, a role for miR-373 in ovarian cancer has not been investigated. In this study, we show that the miR-373 expression is down-regulated in human epithelial ovarian cancer (EOC) and inversely correlated with clinical stage and histological grade. Ectopic overexpression of miR-373 in human EOC cells suppressed   cell invasion   in vitro and metastasis in vivo, and the epithelial-mesenchymal transition process. Silencing the expression of miR-373 resulted in an increased migration and invasion of EOC cells. Using integrated bioinformatics analysis, gene expression arrays, and luciferase assay, we identified Rab22a as a direct and functional target of miR-373 in EOC cells. Expression levels of miR-373 were inversely correlated with Rab22a protein levels in human EOC tissues. Rab22a knockdown inhibited invasion and migration of EOC cells, increased E-cadherin expression, and suppressed the expression of N-cadherin. Moreover, overexpression of Rab22a abrogated miR-373-induced invasion and migration of EOC cells. Taken together, these results demonstrate that miR-373 suppresses EOC invasion and metastasis by directly targeting Rab22a gene, a new potential therapeutic target in EOC. 
   
  
 CYP3A4 
 
  NCBI Gene  
  GeneRIF  
  Pubmed  
 
 
  Search Term  Rank  Fields  
  [Mm]igration  6  GeneRIFs(1)  
  [Ii]nvasive  10  PubMed(9)  
  [Mm]etastasis  12  PubMed(1); GeneRIFs(1)  
 
 
 hide/show details for CYP3A4 
  [Mm]igration  GeneRIFs  
 Data suggest that CYP3A4 (cytochrome P-450 3A4) is involved in proliferation,   migration  , invasion, and colony formation abilities of hepatocellular carcinoma cells; in HEPG2 cells, the naturally occurring flavonoid rutin (an antioxidant anticarcinogen) inhibits progression (inhibits proliferation,   migration  , etc.), promotes apoptosis, and down-regulates expression of CYP3A4. 
  [Ii]nvasive  PubMed  
 Genetic susceptibility to distinct bladder cancer subphenotypes. Clinical, pathologic, and molecular evidence indicate that bladder cancer is heterogeneous with pathologic/molecular features that define distinct subphenotypes with different prognoses. It is conceivable that specific patterns of genetic susceptibility are associated with particular subphenotypes.To examine evidence for the contribution of germline genetic variation to bladder cancer heterogeneity.The Spanish Bladder Cancer/EPICURO Study is a case-control study based in 18 hospitals located in five areas in Spain. Cases were patients with a newly diagnosed, histologically confirmed, urothelial cell carcinoma of the bladder from 1998 to 2001. Case diagnoses were reviewed and uniformly classified by pathologists following the World Health Organisation/International Society of Urological Pathology 1999 criteria. Controls were hospital-matched patients (n=1149).A total of 1526 candidate variants in 423 candidate genes were analysed. Three distinct subphenotypes were defined according to stage and grade: low-grade nonmuscle   invasive   (n=586), high-grade nonmuscle   invasive   (n=219), and muscle   invasive   (n=246). The association between each variant and subphenotype was assessed by polytomous risk models adjusting for potential confounders. Heterogeneity in genetic susceptibility among subphenotypes was also tested.Two established bladder cancer susceptibility genotypes, NAT2 slow-acetylation and GSTM1-null, exhibited similar associations among the subphenotypes, as did VEGF-rs25648, which was previously identified in our study. Other variants conferred risks for specific tumour subphenotypes such as PMS2-rs6463524 and CD4-rs3213427 (respective heterogeneity p values of 0.006 and 0.004), which were associated with muscle-  invasive   tumours (per-allele odds ratios [95% confidence interval] of 0.56 [0.41-0.77] and 0.71 [0.57-0.88], respectively) but not with non-muscle-  invasive   tumours. Heterogeneity p values were not robust in multiple testing according to their false-discovery rate.These exploratory analyses suggest that genetic susceptibility loci might be related to the molecular/pathologic diversity of bladder cancer. Validation through large-scale replication studies and the study of additional genes and single nucleotide polymorphisms are required. 
 Genetic susceptibility to distinct bladder cancer subphenotypes. Clinical, pathologic, and molecular evidence indicate that bladder cancer is heterogeneous with pathologic/molecular features that define distinct subphenotypes with different prognoses. It is conceivable that specific patterns of genetic susceptibility are associated with particular subphenotypes.To examine evidence for the contribution of germline genetic variation to bladder cancer heterogeneity.The Spanish Bladder Cancer/EPICURO Study is a case-control study based in 18 hospitals located in five areas in Spain. Cases were patients with a newly diagnosed, histologically confirmed, urothelial cell carcinoma of the bladder from 1998 to 2001. Case diagnoses were reviewed and uniformly classified by pathologists following the World Health Organisation/International Society of Urological Pathology 1999 criteria. Controls were hospital-matched patients (n=1149).A total of 1526 candidate variants in 423 candidate genes were analysed. Three distinct subphenotypes were defined according to stage and grade: low-grade nonmuscle   invasive   (n=586), high-grade nonmuscle   invasive   (n=219), and muscle   invasive   (n=246). The association between each variant and subphenotype was assessed by polytomous risk models adjusting for potential confounders. Heterogeneity in genetic susceptibility among subphenotypes was also tested.Two established bladder cancer susceptibility genotypes, NAT2 slow-acetylation and GSTM1-null, exhibited similar associations among the subphenotypes, as did VEGF-rs25648, which was previously identified in our study. Other variants conferred risks for specific tumour subphenotypes such as PMS2-rs6463524 and CD4-rs3213427 (respective heterogeneity p values of 0.006 and 0.004), which were associated with muscle-  invasive   tumours (per-allele odds ratios [95% confidence interval] of 0.56 [0.41-0.77] and 0.71 [0.57-0.88], respectively) but not with non-muscle-  invasive   tumours. Heterogeneity p values were not robust in multiple testing according to their false-discovery rate.These exploratory analyses suggest that genetic susceptibility loci might be related to the molecular/pathologic diversity of bladder cancer. Validation through large-scale replication studies and the study of additional genes and single nucleotide polymorphisms are required. 
 Expression of CYP3A4 in human breast tumour and non-tumour tissues. Cytochrome P450 3A4 (CYP3A4), a member of multigene superfamily of enzymes, plays a major role in the activation of procarcinogens such as polycyclic hydrocarbon dihydrodiols, aflatoxins and heterocyclic amines as well as of several drugs including tamoxifen which is used in breast cancer therapy. Using immunohistochemistry, the cellular distribution and the level of expression of CYP3A4 was assessed in breast tumour and surrounding tumour free (control) breast tissue in 25 pairs of samples obtained from females with infiltrating ductal carcinoma. Cells staining with the CYP3A4 antibody showed only cytoplasmic positivity with varying intensities in 100% (25/25) of tumours and 68% (17/25) of normal breast tissues. Cytoplasmic staining was present in   invasive   ductal carcinoma cells but not in tumour stroma. CYP3A4 was detected in normal epithelium and myoepithelium. Only focal staining was noted in some of the non-epithelial cells (e.g. endothelial cells) in normal breast tissues. When CYP3A4 staining was assessed semiquantitatively the mean staining score values of CYP3A4 were found to be significantly higher in tumours compared to that of normal breast tissues. These results show that CYP3A4 protein is expressed in both tumour and normal breast tissue with an increased expression in tumours. 
 Pharmacogenetic analysis of paclitaxel transport and metabolism genes in breast cancer. Paclitaxel is commonly used in the treatment of breast cancer. Variability in paclitaxel clearance may contribute to the unpredictability of clinical outcomes. We assessed genomic DNA from the plasma of 93 patients with high-risk primary or stage IV breast cancer, who received dose-intense paclitaxel, doxorubicin and cyclophosphamide. Eight polymorphisms in six genes associated with metabolism and transport of paclitaxel were analyzed using Pyrosequencing. We found no association between ABCB1, ABCG2, CYP1B1, CYP3A4, CYP3A5 and CYP2C8 genotypes and paclitaxel clearance. However, patients homozygous for the CYP1B1*3 allele had a significantly longer progression-free survival than patients with at least one Valine allele (P=0.037). This finding could reflect altered paclitaxel metabolism, however, the finding was independent of paclitaxel clearance. Alternatively, the role of CYP1B1 in estrogen metabolism may influence the risk of   invasive   or paclitaxel resistant breast cancer in patients carrying the CYP1B1*3 allele. 
 Risk of testicular germ cell cancer in relation to variation in maternal and offspring cytochrome p450 genes involved in catechol estrogen metabolism. The incidence of testicular germ cell carcinoma (TGCC) is highest among men ages 20 to 44 years. Exposure to relatively high circulating maternal estrogen levels during pregnancy has long been suspected as being a risk factor for TGCC. Catechol (hydroxylated) estrogens have carcinogenic potential, thought to arise from reactive catechol intermediates with enhanced capability of forming mutation-inducing DNA adducts. Polymorphisms in maternal or offspring genes encoding estrogen-metabolizing enzymes may influence prenatal catechol estrogen levels and could therefore be biomarkers of TGCC risk. We conducted a population-based, case-parent triad study to evaluate TGCC risk in relation to maternal and/or offspring polymorphisms in CYP1A2, CYP1B1, CYP3A4, and CYP3A5. We identified 18- to 44-year-old men diagnosed with   invasive   TGCC from 1999 to 2004 through a population-based cancer registry in Washington State and recruited cases and their parents (110 case-parent triads, 50 case-parent dyads). Maternal or offspring carriage of CYP1A2 -163A was associated with reduced risk of TGCC [maternal heterozygote relative risk (RR), 0.6; 95% confidence interval (95% CI), 0.2-1.7; offspring heterozygote RR, 0.7; 95% CI, 0.3-1.5)]. Maternal CYP1B1 (48)Gly homozygosity was associated with a 2.7-fold increased risk of TGCC (95% CI, 0.9-7.9), with little evidence that Leu(432)Val or Asn(453)Ser genotypes were related to risk. Men were also at increased risk of TGCC if they carried the CYP3A4 -392G (RR, 7.0; 95% CI, 1.6-31) or CYP3A5 6986G (RR, 2.4; 95% CI, 1.1-5.6) alleles. These results support the hypothesis that maternal and/or offspring catechol estrogen activity may influence sons' risk of TGCC. 
 Novel mutations of CYP3A4 in fine needle aspiration cytology samples of breast cancer patients and its clinical correlations. Human Cytochrome P450 3A4 is a major P450 enzyme in the liver and gastrointestinal tract. It plays an important role in the metabolism of a wide variety of drugs, some endogenous steroids, and harmful environmental contaminants. To investigate the interindividual variation in CYP3A4 levels we have carried out a study on the genetic polymorphism of 100 breast cancer subjects using fine needle aspiration cytology (FNAC) sampling procedure. DNA was extracted from all the samples and PCR was carried out for detecting the CYP3A4 gene polymorphisms. We selected exon-7 and 10 which are present on the 5'-flanking coding region of the gene using the respective primers for PCR followed by direct automated sequencing method for detecting the mutations. These mutations were compared to the wild type sequence structures obtained from GenBank database (accession no. AF209389). We found two novel point mutations which are heterozygous mutant alleles. The two variant alleles were Ile222Arg and Phe175Val occurring in coding region of exon-7. Ile222Arg mutation was found in 3 malignant cases whereas Phe175Val mutation was found in 4 malignant cases. This is the first report of these two novel point mutations in CYP3A4 gene. These mutations in the gene in respective patients were found to relate to drug response in   invasive   ductal carcinomas of breast cancer. 
 Evidence for cytochrome P-450NF, the nifedipine oxidase, being the principal enzyme involved in the bioactivation of aflatoxins in human liver. In vitro studies with human liver indicate that the major catalyst involved in the bioactivation of the hepato-carcinogen aflatoxin B1 (AFB1) to its genotoxic 2,3-epoxide derivative is cytochrome P-450NF (P-450NF), a previously characterized protein that also catalyzes the oxidation of nifedipine and other dihydropyridines, quinidine, macrolide antibiotics, various steroids, and other compounds. Evidence was obtained using activation of AFB1 as monitored by umuC gene expression response in Salmonella typhimurium TA1535/pSK1002 and enzyme reconstitution, immunochemical inhibition, correlation of response with levels of P-450NF and nifedipine oxidase activity in different liver samples, stimulation of activity by 7,8-benzoflavone, and inhibition of activity by troleandomycin. Similar results were obtained when levels of 2,3-dihydro-2-(N7-guanyl)-3-hydroxyaflatoxin B1 formed in DNA were measured. P-450NF or a closely related protein also appears to be the major catalyst involved in the activation of aflatoxin G1 and sterigmatocystin, the latter compound being more genotoxic than AFB1 in these systems. Several drugs and conditions are known to influence the levels and activity of P-450NF in human liver, and the activity of the enzyme can be estimated by non  invasive   assays. These findings provide a test system for the hypothesis that a specific human disease state (liver cancer) is linked to the level of oxidative metabolism in populations in which aflatoxin ingestion is high. 
 Relationship between mRNA expression levels of CYP3A4, CYP3A5 and SXR in peripheral mononuclear blood cells and aging in young kidney transplant recipients under tacrolimus treatment. The activity of several key enzymes involved in the metabolism of many drugs is subject to change closely related to the age of patients. This possibility must also be considered in the case of tacrolimus, the most important calcineurins inhibitor, which is widely used in pediatric kidney transplantation. As well as in the liver and intestine, some of the enzymes involved in the metabolism of tacrolimus were also isolated in the peripheral blood mononuclear cells (PBMCs), where also appear to play an important regulatory action. Therefore, the influence of some external factors on the expression of specific mRNA can be determined non  invasive  ly.The correlation between the levels of mRNA specific for key enzymes SXR, CYP3A and ABCB1 involved in the metabolism of tacrolimus was evaluated in PBMCs obtained from a selected population of 29 young kidney transplant recipients. A possible correlation between the expression of these specific mRNAs and tacrolimus pharmacokinetics was also investigated.The patients' age and their blood concentrations of SXR mRNA were directly correlated with the expression of CYP3A4, CYP3A5 mRNAs, but not of ABCB1 mRNA in the PBMCs. tacrolimus-normalized daily dose was strongly correlated with patient's age and multivariable regression indicates the CYP3A4-specific mRNA as the sole independent variable influencing tacrolimus concentration-to-dose ratio.Aging and SXR mRNA significantly affect the expression of CYP3A4- and CYP3A5-specific mRNA as measured by their concentration in PBMC. 
 Human liver microsomal steroid metabolism: identification of the major microsomal steroid hormone 6 beta-hydroxylase cytochrome P-450 enzyme. Cytochrome P-450-dependent steroid hormone metabolism was studied in isolated human liver microsomal fractions. 6 beta hydroxylation was shown to be the major route of NADPH-dependent oxidative metabolism (greater than or equal to 75% of total hydroxylated metabolites) with each of three steroid substrates, testosterone, androstenedione, and progesterone. With testosterone, 2 beta and 15 beta hydroxylation also occurred, proceeding at approximately 10% and 3-4% the rate of microsomal 6 beta hydroxylation, respectively, in each of the liver samples examined. Rates for the three steroid 6 beta-hydroxylase activities were highly correlated with each other (r = 0.95-0.97 for 25 individual microsomal preparations), suggesting that a single human liver P-450 enzyme is the principal microsomal 6 beta-hydroxylase catalyst with all three steroid substrates. Steroid 6 beta-hydroxylase rates correlated well with the specific content of human P-450NF (r = 0.69-0.83) and with its associated nifedipine oxidase activity (r = 0.80), but not with the rates for debrisoquine 4-hydroxylase, phenacetin O-deethylase, or S-mephenytoin 4-hydroxylase activities or the specific contents of their respective associated P-450 forms in these same liver microsomes (r less than 0.2). These correlative observations were supported by the selective inhibition of human liver microsomal 6 beta hydroxylation by antibody raised to either human P-450NF or a rat homolog, P-450 PB-2a. Anti-P-450NF also inhibited human microsomal testosterone 2 beta and 15 beta hydroxylation in parallel to the 6 beta-hydroxylation reaction. This antibody also inhibited rat P-450 2a-dependent steroid hormone 6 beta hydroxylation in uninduced adult male rat liver microsomes but not the steroid 2 alpha, 16 alpha, or 7 alpha hydroxylation reactions catalyzed by other rat P-450 forms. Finally, steroid 6 beta hydroxylation catalyzed by either human or rat liver microsomes was selectively inhibited by NADPH-dependent complexation of the macrolide antibiotic triacetyloleandomycin, a reaction that is characteristic of members of the P-450NF gene subfamily (P-450 IIIA subfamily). These observations establish that P-450NF or a closely related enzyme is the major catalyst of steroid hormone 6 beta hydroxylation in human liver microsomes, and furthermore suggest that steroid 6 beta hydroxylation may provide a useful, non  invasive   monitor for the monooxygenase activity of this hepatic P-450 form. 
  [Mm]etastasis  PubMed  
 CYP3A isoforms in Ewing's sarcoma tumours: an immunohistochemical study with clinical correlation. Ewing's sarcoma is an aggressive malignancy of bone and soft tissue with high incidence of   metastasis   and resistance to chemotherapy. Cytochrome P450 (CYP) monooxygenases are a family of enzymes that are involved in the metabolism of exogenous and endogenous compounds, including anti-cancer drugs, and have been implicated in the aggressive behaviour of various malignancies. Tumour samples and clinical information including age, sex, tumour site, tumour size, clinical stage and survival were collected from 36 adult and paediatric patients with Ewing's sarcoma family tumours. Tissue microarrays slides were processed for immunohistochemical labelling for CYP3A4, CYP3A5 and CYP3A7 using liver sections as positive control. The intensity of staining was scored as negative, low or high expression and was analysed statistically for any association with patients' clinical information. Four cases were later excluded due to inadequate viable tissue. CYP3A4 staining was present in 26 (81%) cases with high expression noted in 13 (40%) of 32 cases. High expression was significantly associated with distant metastases (P < 0.05). CYP3A5 and CYP3A7 were expressed in 5 and 13 cases respectively (15.6%, 40.6%). There was no association between the expression of CYP3A isoforms and age, sex, tumour size, or location (pelvic or extra-pelvic). None of the biomarkers showed any correlation with overall or disease-free survival. In conclusion, expression of CYP3A isoforms is noted in Ewing's sarcoma tumours and high CYP3A4 expression may be associated with   metastasis  . Additional studies are needed to further investigate the role of CYP3A4 in the prognosis of these tumours. 
  GeneRIFs  
 Expression of CYP3A isoforms is noted in Ewing's sarcoma tumours and high CYP3A4 expression may be associated with   metastasis  . 
   
  
 CYP3A5 
 
  NCBI Gene  
  GeneRIF  
  Pubmed  
 
 
  Search Term  Rank  Fields  
  [Mm]igration  6  PubMed(1)  
  [Ii]nvasive  10  PubMed(6)  
  [Mm]etastasis  12  PubMed(1); GeneRIFs(1)  
 
 
 hide/show details for CYP3A5 
  [Mm]igration  PubMed  
 CYP3A5 Functions as a Tumor Suppressor in Hepatocellular Carcinoma by Regulating mTORC2/Akt Signaling. CYP3A5 is a cytochrome P450 protein that functions in the liver metabolism of many carcinogens and cancer drugs. However, it has not been thought to directly affect cancer progression. In this study, we challenge this perspective by demonstrating that CYP3A5 is downregulated in many hepatocellular carcinomas (HCC), where it has an important role as a tumor suppressor that antagonizes the malignant phenotype. CYP3A5 was downregulated in multiple cohorts of human HCC examined. Lower CYP3A5 levels were associated with more aggressive vascular invasion, poor differentiation, shorter time to disease recurrence after treatment, and worse overall patient survival. Mechanistic investigations showed that CYP3A5 overexpression limited MMP2/9 function and suppressed HCC   migration   and invasion in vitro and in vivo by inhibiting AKT signaling. Notably, AKT phosphorylation at Ser473 was inhibited in CYP3A5-overexpressing HCC cells, an event requiring mTORC2 but not Rictor/mTOR complex formation. CYP3A5-induced ROS accumulation was found to be a critical upstream regulator of mTORC2 activity, consistent with evidence of reduced GSH redox activity in most clinical HCC specimens with reduced metastatic capacity. Taken together, our results defined CYP3A5 as a suppressor of HCC pathogenesis and metastasis with potential utility a prognostic biomarker. 
  [Ii]nvasive  PubMed  
 Pharmacogenetic analysis of paclitaxel transport and metabolism genes in breast cancer. Paclitaxel is commonly used in the treatment of breast cancer. Variability in paclitaxel clearance may contribute to the unpredictability of clinical outcomes. We assessed genomic DNA from the plasma of 93 patients with high-risk primary or stage IV breast cancer, who received dose-intense paclitaxel, doxorubicin and cyclophosphamide. Eight polymorphisms in six genes associated with metabolism and transport of paclitaxel were analyzed using Pyrosequencing. We found no association between ABCB1, ABCG2, CYP1B1, CYP3A4, CYP3A5 and CYP2C8 genotypes and paclitaxel clearance. However, patients homozygous for the CYP1B1*3 allele had a significantly longer progression-free survival than patients with at least one Valine allele (P=0.037). This finding could reflect altered paclitaxel metabolism, however, the finding was independent of paclitaxel clearance. Alternatively, the role of CYP1B1 in estrogen metabolism may influence the risk of   invasive   or paclitaxel resistant breast cancer in patients carrying the CYP1B1*3 allele. 
 Risk of testicular germ cell cancer in relation to variation in maternal and offspring cytochrome p450 genes involved in catechol estrogen metabolism. The incidence of testicular germ cell carcinoma (TGCC) is highest among men ages 20 to 44 years. Exposure to relatively high circulating maternal estrogen levels during pregnancy has long been suspected as being a risk factor for TGCC. Catechol (hydroxylated) estrogens have carcinogenic potential, thought to arise from reactive catechol intermediates with enhanced capability of forming mutation-inducing DNA adducts. Polymorphisms in maternal or offspring genes encoding estrogen-metabolizing enzymes may influence prenatal catechol estrogen levels and could therefore be biomarkers of TGCC risk. We conducted a population-based, case-parent triad study to evaluate TGCC risk in relation to maternal and/or offspring polymorphisms in CYP1A2, CYP1B1, CYP3A4, and CYP3A5. We identified 18- to 44-year-old men diagnosed with   invasive   TGCC from 1999 to 2004 through a population-based cancer registry in Washington State and recruited cases and their parents (110 case-parent triads, 50 case-parent dyads). Maternal or offspring carriage of CYP1A2 -163A was associated with reduced risk of TGCC [maternal heterozygote relative risk (RR), 0.6; 95% confidence interval (95% CI), 0.2-1.7; offspring heterozygote RR, 0.7; 95% CI, 0.3-1.5)]. Maternal CYP1B1 (48)Gly homozygosity was associated with a 2.7-fold increased risk of TGCC (95% CI, 0.9-7.9), with little evidence that Leu(432)Val or Asn(453)Ser genotypes were related to risk. Men were also at increased risk of TGCC if they carried the CYP3A4 -392G (RR, 7.0; 95% CI, 1.6-31) or CYP3A5 6986G (RR, 2.4; 95% CI, 1.1-5.6) alleles. These results support the hypothesis that maternal and/or offspring catechol estrogen activity may influence sons' risk of TGCC. 
 Relationship between mRNA expression levels of CYP3A4, CYP3A5 and SXR in peripheral mononuclear blood cells and aging in young kidney transplant recipients under tacrolimus treatment. The activity of several key enzymes involved in the metabolism of many drugs is subject to change closely related to the age of patients. This possibility must also be considered in the case of tacrolimus, the most important calcineurins inhibitor, which is widely used in pediatric kidney transplantation. As well as in the liver and intestine, some of the enzymes involved in the metabolism of tacrolimus were also isolated in the peripheral blood mononuclear cells (PBMCs), where also appear to play an important regulatory action. Therefore, the influence of some external factors on the expression of specific mRNA can be determined non  invasive  ly.The correlation between the levels of mRNA specific for key enzymes SXR, CYP3A and ABCB1 involved in the metabolism of tacrolimus was evaluated in PBMCs obtained from a selected population of 29 young kidney transplant recipients. A possible correlation between the expression of these specific mRNAs and tacrolimus pharmacokinetics was also investigated.The patients' age and their blood concentrations of SXR mRNA were directly correlated with the expression of CYP3A4, CYP3A5 mRNAs, but not of ABCB1 mRNA in the PBMCs. tacrolimus-normalized daily dose was strongly correlated with patient's age and multivariable regression indicates the CYP3A4-specific mRNA as the sole independent variable influencing tacrolimus concentration-to-dose ratio.Aging and SXR mRNA significantly affect the expression of CYP3A4- and CYP3A5-specific mRNA as measured by their concentration in PBMC. 
 Relationship between mRNA expression levels of CYP3A4, CYP3A5 and SXR in peripheral mononuclear blood cells and aging in young kidney transplant recipients under tacrolimus treatment. The activity of several key enzymes involved in the metabolism of many drugs is subject to change closely related to the age of patients. This possibility must also be considered in the case of tacrolimus, the most important calcineurins inhibitor, which is widely used in pediatric kidney transplantation. As well as in the liver and intestine, some of the enzymes involved in the metabolism of tacrolimus were also isolated in the peripheral blood mononuclear cells (PBMCs), where also appear to play an important regulatory action. Therefore, the influence of some external factors on the expression of specific mRNA can be determined non  invasive  ly.The correlation between the levels of mRNA specific for key enzymes SXR, CYP3A and ABCB1 involved in the metabolism of tacrolimus was evaluated in PBMCs obtained from a selected population of 29 young kidney transplant recipients. A possible correlation between the expression of these specific mRNAs and tacrolimus pharmacokinetics was also investigated.The patients' age and their blood concentrations of SXR mRNA were directly correlated with the expression of CYP3A4, CYP3A5 mRNAs, but not of ABCB1 mRNA in the PBMCs. tacrolimus-normalized daily dose was strongly correlated with patient's age and multivariable regression indicates the CYP3A4-specific mRNA as the sole independent variable influencing tacrolimus concentration-to-dose ratio.Aging and SXR mRNA significantly affect the expression of CYP3A4- and CYP3A5-specific mRNA as measured by their concentration in PBMC. 
 Pharmacogenetic analysis of paclitaxel transport and metabolism genes in breast cancer. Paclitaxel is commonly used in the treatment of breast cancer. Variability in paclitaxel clearance may contribute to the unpredictability of clinical outcomes. We assessed genomic DNA from the plasma of 93 patients with high-risk primary or stage IV breast cancer, who received dose-intense paclitaxel, doxorubicin and cyclophosphamide. Eight polymorphisms in six genes associated with metabolism and transport of paclitaxel were analyzed using Pyrosequencing. We found no association between ABCB1, ABCG2, CYP1B1, CYP3A4, CYP3A5 and CYP2C8 genotypes and paclitaxel clearance. However, patients homozygous for the CYP1B1*3 allele had a significantly longer progression-free survival than patients with at least one Valine allele (P=0.037). This finding could reflect altered paclitaxel metabolism, however, the finding was independent of paclitaxel clearance. Alternatively, the role of CYP1B1 in estrogen metabolism may influence the risk of   invasive   or paclitaxel resistant breast cancer in patients carrying the CYP1B1*3 allele. 
 Risk of testicular germ cell cancer in relation to variation in maternal and offspring cytochrome p450 genes involved in catechol estrogen metabolism. The incidence of testicular germ cell carcinoma (TGCC) is highest among men ages 20 to 44 years. Exposure to relatively high circulating maternal estrogen levels during pregnancy has long been suspected as being a risk factor for TGCC. Catechol (hydroxylated) estrogens have carcinogenic potential, thought to arise from reactive catechol intermediates with enhanced capability of forming mutation-inducing DNA adducts. Polymorphisms in maternal or offspring genes encoding estrogen-metabolizing enzymes may influence prenatal catechol estrogen levels and could therefore be biomarkers of TGCC risk. We conducted a population-based, case-parent triad study to evaluate TGCC risk in relation to maternal and/or offspring polymorphisms in CYP1A2, CYP1B1, CYP3A4, and CYP3A5. We identified 18- to 44-year-old men diagnosed with   invasive   TGCC from 1999 to 2004 through a population-based cancer registry in Washington State and recruited cases and their parents (110 case-parent triads, 50 case-parent dyads). Maternal or offspring carriage of CYP1A2 -163A was associated with reduced risk of TGCC [maternal heterozygote relative risk (RR), 0.6; 95% confidence interval (95% CI), 0.2-1.7; offspring heterozygote RR, 0.7; 95% CI, 0.3-1.5)]. Maternal CYP1B1 (48)Gly homozygosity was associated with a 2.7-fold increased risk of TGCC (95% CI, 0.9-7.9), with little evidence that Leu(432)Val or Asn(453)Ser genotypes were related to risk. Men were also at increased risk of TGCC if they carried the CYP3A4 -392G (RR, 7.0; 95% CI, 1.6-31) or CYP3A5 6986G (RR, 2.4; 95% CI, 1.1-5.6) alleles. These results support the hypothesis that maternal and/or offspring catechol estrogen activity may influence sons' risk of TGCC. 
  [Mm]etastasis  PubMed  
 CYP3A5 Functions as a Tumor Suppressor in Hepatocellular Carcinoma by Regulating mTORC2/Akt Signaling. CYP3A5 is a cytochrome P450 protein that functions in the liver metabolism of many carcinogens and cancer drugs. However, it has not been thought to directly affect cancer progression. In this study, we challenge this perspective by demonstrating that CYP3A5 is downregulated in many hepatocellular carcinomas (HCC), where it has an important role as a tumor suppressor that antagonizes the malignant phenotype. CYP3A5 was downregulated in multiple cohorts of human HCC examined. Lower CYP3A5 levels were associated with more aggressive vascular invasion, poor differentiation, shorter time to disease recurrence after treatment, and worse overall patient survival. Mechanistic investigations showed that CYP3A5 overexpression limited MMP2/9 function and suppressed HCC migration and invasion in vitro and in vivo by inhibiting AKT signaling. Notably, AKT phosphorylation at Ser473 was inhibited in CYP3A5-overexpressing HCC cells, an event requiring mTORC2 but not Rictor/mTOR complex formation. CYP3A5-induced ROS accumulation was found to be a critical upstream regulator of mTORC2 activity, consistent with evidence of reduced GSH redox activity in most clinical HCC specimens with reduced metastatic capacity. Taken together, our results defined CYP3A5 as a suppressor of HCC pathogenesis and   metastasis   with potential utility a prognostic biomarker. 
  GeneRIFs  
 CYP3A5 as a suppressor of hepatocellular carcinoma pathogenesis and   metastasis   with potential utility a prognostic biomarker. 
   
  
 TBXAS1 
 
  NCBI Gene  
  GeneRIF  
  Pubmed  
 
 
  Search Term  Rank  Fields  
  [Mm]igration  6  PubMed(1)  
  [Ee]xtracellular [Mm]atrix  8  PubMed(4)  
  [Ii]nvasive  10  PubMed(6); GeneRIFs(1)  
  [Mm]etastasis  12  GeneRIFs(1)  
 
 
 hide/show details for TBXAS1 
  [Mm]igration  PubMed  
 Prognostic and functional significance of thromboxane synthase gene overexpression in invasive bladder cancer. Thromboxane synthase (TXAS) is one of the enzymes downstream from cyclooxygenase-2 and catalyzes the synthesis of thromboxane A(2) (TXA(2)). TXAS was among the genes we identified based on its overexpression in invasive bladder tumors. TXAS is overexpressed in common forms of bladder tumors: 69 of 97 (71.1%) transitional cell carcinoma (TCC), 38 of 53 (71.6%) squamous cell carcinoma, and 5 of 11 (45.5%) adenocarcinoma relative to nontumor tissue. Overall, 112 of 161 (69.5%) invasive tumors exhibited elevated expression. Significantly, patients with tumors having >4-fold levels of TXAS expression showed significant statistical evidence of lower overall survival expressed by the estimated hazard ratio of 2.74 with P = 0.009 in Cox's regression analysis. TXAS mRNA expression was found to be an independent prognostic marker for patients with bladder cancer. Treatment of bladder cancer cell lines (T24 and TCC-SUP) with TXAS inhibitors and TXA(2) (TP) receptor antagonists reduced cell growth,   migration  , and invasion, whereas TP agonists stimulated cell   migration   and invasion. The positive correlation between elevated TXAS expression and shorter patient survival supports a potential role for TXAS-regulated pathways in tumor invasion and metastases and suggests that modulation of the TXAS pathway may offer a novel therapeutic approach. 
  [Ee]xtracellular [Mm]atrix  PubMed  
 Identification of fetal and maternal single nucleotide polymorphisms in candidate genes that predispose to spontaneous preterm labor with intact membranes. The purpose of this study was to determine whether maternal/fetal single nucleotide polymorphisms (SNPs) in candidate genes are associated with spontaneous preterm labor/delivery.A genetic association study was conducted in 223 mothers and 179 fetuses (preterm labor with intact membranes who delivered <37 weeks of gestation [preterm birth (PTB)]), and 599 mothers and 628 fetuses (normal pregnancy); 190 candidate genes and 775 SNPs were studied. Single locus/haplotype association analyses were performed; the false discovery rate was used to correct for multiple testing.The strongest single locus associations with PTB were interleukin-6 receptor 1 (fetus; P=.000148) and tissue inhibitor of metalloproteinase 2 (mother; P=.000197), which remained significant after correction for multiple comparisons. Global haplotype analysis indicated an association between a fetal DNA variant in insulin-like growth factor F2 and maternal alpha 3 type IV collagen isoform 1 (global, P=.004 and .007, respectively).An SNP involved in controlling fetal inflammation (interleukin-6 receptor 1) and DNA variants in maternal genes encoding for proteins involved in   extracellular matrix   metabolism approximately doubled the risk of PTB. 
 A genetic association study of maternal and fetal candidate genes that predispose to preterm prelabor rupture of membranes (PROM). We sought to determine whether maternal/fetal single-nucleotide polymorphisms (SNPs) in candidate genes are associated with preterm prelabor rupture of membranes (pPROM).A case-control study was conducted in patients with pPROM (225 mothers and 155 fetuses) and 599 mothers and 628 fetuses with a normal pregnancy; 190 candidate genes and 775 SNPs were studied. Single locus/haplotype association analyses were performed; false discovery rate was used to correct for multiple testing (q* = 0.15).First, a SNP in tissue inhibitor of metalloproteinase 2 in mothers was significantly associated with pPROM (odds ratio, 2.12; 95% confidence interval, 1.47-3.07; P = .000068), and this association remained significant after correction for multiple comparisons. Second, haplotypes for Alpha 3 type IV collagen isoform precursor in the mother were associated with pPROM (global P = .003). Third, multilocus analysis identified a 3-locus model, which included maternal SNPs in collagen type I alpha 2, defensin alpha 5 gene, and endothelin 1.DNA variants in a maternal gene involved in   extracellular matrix   metabolism doubled the risk of pPROM. 
 Identification of fetal and maternal single nucleotide polymorphisms in candidate genes that predispose to spontaneous preterm labor with intact membranes. The purpose of this study was to determine whether maternal/fetal single nucleotide polymorphisms (SNPs) in candidate genes are associated with spontaneous preterm labor/delivery.A genetic association study was conducted in 223 mothers and 179 fetuses (preterm labor with intact membranes who delivered <37 weeks of gestation [preterm birth (PTB)]), and 599 mothers and 628 fetuses (normal pregnancy); 190 candidate genes and 775 SNPs were studied. Single locus/haplotype association analyses were performed; the false discovery rate was used to correct for multiple testing.The strongest single locus associations with PTB were interleukin-6 receptor 1 (fetus; P=.000148) and tissue inhibitor of metalloproteinase 2 (mother; P=.000197), which remained significant after correction for multiple comparisons. Global haplotype analysis indicated an association between a fetal DNA variant in insulin-like growth factor F2 and maternal alpha 3 type IV collagen isoform 1 (global, P=.004 and .007, respectively).An SNP involved in controlling fetal inflammation (interleukin-6 receptor 1) and DNA variants in maternal genes encoding for proteins involved in   extracellular matrix   metabolism approximately doubled the risk of PTB. 
 A genetic association study of maternal and fetal candidate genes that predispose to preterm prelabor rupture of membranes (PROM). We sought to determine whether maternal/fetal single-nucleotide polymorphisms (SNPs) in candidate genes are associated with preterm prelabor rupture of membranes (pPROM).A case-control study was conducted in patients with pPROM (225 mothers and 155 fetuses) and 599 mothers and 628 fetuses with a normal pregnancy; 190 candidate genes and 775 SNPs were studied. Single locus/haplotype association analyses were performed; false discovery rate was used to correct for multiple testing (q* = 0.15).First, a SNP in tissue inhibitor of metalloproteinase 2 in mothers was significantly associated with pPROM (odds ratio, 2.12; 95% confidence interval, 1.47-3.07; P = .000068), and this association remained significant after correction for multiple comparisons. Second, haplotypes for Alpha 3 type IV collagen isoform precursor in the mother were associated with pPROM (global P = .003). Third, multilocus analysis identified a 3-locus model, which included maternal SNPs in collagen type I alpha 2, defensin alpha 5 gene, and endothelin 1.DNA variants in a maternal gene involved in   extracellular matrix   metabolism doubled the risk of pPROM. 
  [Ii]nvasive  PubMed  
 Analysis of Cox-2 and thromboxane synthase expression in pituitary adenomas and carcinomas. Recent studies have examined the role of cyclooxygenase-2 (Cox-2) expression in normal pituitaries and pituitary adenomas and have suggested a role for Cox-2 in the regulation of angiogenesis in the pituitary. Thromboxane synthase (TBXAS), which catalyzes the synthesis of thromboxane A2, is one of the downstream enzymes in Cox metabolism and appears to play a role in the regulation of   invasive  ness and angiogenesis in some neoplasms. To analyze the role of Cox-2 and TBXAS in pituitary tumor progression, we examined normal pituitaries (n = 8), pituitary adenomas (n = 174), and pituitary carcinomas (n = 7) for expression of Cox-2 and TBXAS by immunohistochemistry. Weak Cox-2 and moderate TBXAS expression was present in normal pituitary cells. Most pituitary adenomas showed increased expression of both Cox-2 and TBXAS. Pituitary tumors as a whole, but particularly carcinomas, showed greater Cox-2 expression than did normal pituitaries. Pituitary adenomas and carcinomas also showed greater staining for TBXAS when compared to normal pituitary. Nonfunctional adenomas had significantly higher levels of TBXAS expression compared to functional adenomas (p = 0.017). Adenomas and carcinomas showed similar degrees of staining for TBXAS. In summary, TBXAS appears to be one of the up-regulated downstream enzymes of Cox metabolism in pituitary tumors. Both Cox-2 and TBXAS may play an important role in pituitary tumor development and progression. 
 Common polymorphisms in the prostaglandin pathway genes and their association with breast cancer susceptibility and survival. There is evidence that genetic variation in the prostaglandin pathway affects cancer susceptibility and progression. Conflicting data from several studies exist for the association of PTGS2 (cyclooxygenase 2) polymorphisms with breast cancer risk. We investigated associations between common germ-line variations in seven genes in the prostaglandin pathway and breast cancer susceptibility and survival among women with   invasive   breast cancer in the SEARCH study.DNA samples from 9,030 cases and controls were genotyped for 64 single nucleotide polymorphisms tagging known common variants (minor allele frequency > 0.05) in PTGS1, PTGS2, TBXAS1, PTGIS, PTGES, PTGDS, and PGDS with a two-stage case-control study design.Four tagging single nucleotide polymorphisms showed modest association with breast cancer susceptibility. All four fit a recessive genetic model. Minor allele homozygotes for PTGISrs5602 [odds ratio (OR), 1.15; 95% confidence interval (95% CI), 1.04-1.27; P = 0.005], PTGISrs8183919 (OR, 1.22; 95% CI, 1.06-1.41; P = 0.006), and TBXASrs41727 (OR, 1.83; 95% CI, 1.22-2.73; P = 0.003) are associated with an increased risk compared with common allele carriers. For PTGISrs44627 minor allele homozygotes (OR, 0.66; 95% CI, 0.5-0.86; P = 0.002), a protective effect was observed.Specific PTGIS and TBXAS1 variants may affect breast cancer susceptibility, but common variants in PTGS1, PTGS2, PTGES, PTGDS, and PGDS have no major role in breast cancer susceptibility. None of the variants in the seven genes studied appear to affect survival. Further larger studies correlating clinical and genotypic data are required to establish if the clinical utility of prostaglandin-targeted therapies, as chemoprevention agents, is influenced by an individual's profile of genetic variants in key prostaglandin pathway genes. 
 Common polymorphisms in the prostaglandin pathway genes and their association with breast cancer susceptibility and survival. There is evidence that genetic variation in the prostaglandin pathway affects cancer susceptibility and progression. Conflicting data from several studies exist for the association of PTGS2 (cyclooxygenase 2) polymorphisms with breast cancer risk. We investigated associations between common germ-line variations in seven genes in the prostaglandin pathway and breast cancer susceptibility and survival among women with   invasive   breast cancer in the SEARCH study.DNA samples from 9,030 cases and controls were genotyped for 64 single nucleotide polymorphisms tagging known common variants (minor allele frequency > 0.05) in PTGS1, PTGS2, TBXAS1, PTGIS, PTGES, PTGDS, and PGDS with a two-stage case-control study design.Four tagging single nucleotide polymorphisms showed modest association with breast cancer susceptibility. All four fit a recessive genetic model. Minor allele homozygotes for PTGISrs5602 [odds ratio (OR), 1.15; 95% confidence interval (95% CI), 1.04-1.27; P = 0.005], PTGISrs8183919 (OR, 1.22; 95% CI, 1.06-1.41; P = 0.006), and TBXASrs41727 (OR, 1.83; 95% CI, 1.22-2.73; P = 0.003) are associated with an increased risk compared with common allele carriers. For PTGISrs44627 minor allele homozygotes (OR, 0.66; 95% CI, 0.5-0.86; P = 0.002), a protective effect was observed.Specific PTGIS and TBXAS1 variants may affect breast cancer susceptibility, but common variants in PTGS1, PTGS2, PTGES, PTGDS, and PGDS have no major role in breast cancer susceptibility. None of the variants in the seven genes studied appear to affect survival. Further larger studies correlating clinical and genotypic data are required to establish if the clinical utility of prostaglandin-targeted therapies, as chemoprevention agents, is influenced by an individual's profile of genetic variants in key prostaglandin pathway genes. 
 Analysis of Cox-2 and thromboxane synthase expression in pituitary adenomas and carcinomas. Recent studies have examined the role of cyclooxygenase-2 (Cox-2) expression in normal pituitaries and pituitary adenomas and have suggested a role for Cox-2 in the regulation of angiogenesis in the pituitary. Thromboxane synthase (TBXAS), which catalyzes the synthesis of thromboxane A2, is one of the downstream enzymes in Cox metabolism and appears to play a role in the regulation of   invasive  ness and angiogenesis in some neoplasms. To analyze the role of Cox-2 and TBXAS in pituitary tumor progression, we examined normal pituitaries (n = 8), pituitary adenomas (n = 174), and pituitary carcinomas (n = 7) for expression of Cox-2 and TBXAS by immunohistochemistry. Weak Cox-2 and moderate TBXAS expression was present in normal pituitary cells. Most pituitary adenomas showed increased expression of both Cox-2 and TBXAS. Pituitary tumors as a whole, but particularly carcinomas, showed greater Cox-2 expression than did normal pituitaries. Pituitary adenomas and carcinomas also showed greater staining for TBXAS when compared to normal pituitary. Nonfunctional adenomas had significantly higher levels of TBXAS expression compared to functional adenomas (p = 0.017). Adenomas and carcinomas showed similar degrees of staining for TBXAS. In summary, TBXAS appears to be one of the up-regulated downstream enzymes of Cox metabolism in pituitary tumors. Both Cox-2 and TBXAS may play an important role in pituitary tumor development and progression. 
 Prognostic and functional significance of thromboxane synthase gene overexpression in   invasive   bladder cancer. Thromboxane synthase (TXAS) is one of the enzymes downstream from cyclooxygenase-2 and catalyzes the synthesis of thromboxane A(2) (TXA(2)). TXAS was among the genes we identified based on its overexpression in   invasive   bladder tumors. TXAS is overexpressed in common forms of bladder tumors: 69 of 97 (71.1%) transitional cell carcinoma (TCC), 38 of 53 (71.6%) squamous cell carcinoma, and 5 of 11 (45.5%) adenocarcinoma relative to nontumor tissue. Overall, 112 of 161 (69.5%)   invasive   tumors exhibited elevated expression. Significantly, patients with tumors having >4-fold levels of TXAS expression showed significant statistical evidence of lower overall survival expressed by the estimated hazard ratio of 2.74 with P = 0.009 in Cox's regression analysis. TXAS mRNA expression was found to be an independent prognostic marker for patients with bladder cancer. Treatment of bladder cancer cell lines (T24 and TCC-SUP) with TXAS inhibitors and TXA(2) (TP) receptor antagonists reduced cell growth, migration, and invasion, whereas TP agonists stimulated cell migration and invasion. The positive correlation between elevated TXAS expression and shorter patient survival supports a potential role for TXAS-regulated pathways in tumor invasion and metastases and suggests that modulation of the TXAS pathway may offer a novel therapeutic approach. 
 Examination of thromboxane synthase as a prognostic factor and therapeutic target in non-small cell lung cancer. Thromboxane synthase (TXS) metabolises prostaglandin H2 into thromboxanes, which are biologically active on cancer cells. TXS over-expression has been reported in a range of cancers, and associated with a poor prognosis. TXS inhibition induces cell death in-vitro, providing a rationale for therapeutic intervention. We aimed to determine the expression profile of TXS in NSCLC and if it is prognostic and/or a survival factor in the disease.TXS expression was examined in human NSCLC and matched controls by western analysis and IHC. TXS metabolite (TXB2) levels were measured by EIA. A 204-patient NSCLC TMA was stained for COX-2 and downstream TXS expression. TXS tissue expression was correlated with clinical parameters, including overall survival. Cell proliferation/survival and invasion was examined in NSCLC cells following both selective TXS inhibition and stable TXS over-expression.TXS was over-expressed in human NSCLC samples, relative to matched normal controls. TXS and TXB2 levels were increased in protein (p < 0.05) and plasma (p < 0.01) NSCLC samples respectively. TXS tissue expression was higher in adenocarcinoma (p < 0.001) and female patients (p < 0.05). No significant correlation with patient survival was observed. Selective TXS inhibition significantly reduced tumour cell growth and increased apoptosis, while TXS over-expression stimulated cell proliferation and   invasive  ness, and was protective against apoptosis.TXS is over-expressed in NSCLC, particularly in the adenocarcinoma subtype. Inhibition of this enzyme inhibits proliferation and induces apoptosis. Targeting thromboxane synthase alone, or in combination with conventional chemotherapy is a potential therapeutic strategy for NSCLC. 
  GeneRIFs  
 Overexpression of thromboxane synthase is associated with   invasive   bladder cancer 
  [Mm]etastasis  GeneRIFs  
 Significantly higher expression of thromboxane synthase is associated with   metastasis   in non-small cell lung cancer 
   
  
 ZFAND5 
 
  NCBI Gene  
  GeneRIF  
  Pubmed  
 
 
  Search Term  Rank  Fields  
  [Mm]igration  6  Process(1)  
 
 
 hide/show details for ZFAND5 
  [Mm]igration  Process  
 fibroblast   migration   
   
  
 TACR3 
 
  NCBI Gene  
  GeneRIF  
  Pubmed  
 
 
  Search Term  Rank  Fields  
  [Ii]nvasive  10  PubMed(1)  
 
 
 hide/show details for TACR3 
  [Ii]nvasive  PubMed  
 Tachykinin Receptor 3 Distribution in Human Oral Squamous Cell Carcinoma. Tachykinin 3 (TAC3) and its preferred tachykinin receptor 3 (TACR3) that are prominently detected in the central nervous system, play significant roles in physiological development and specifically in the human reproductive system. The roles of TAC3/TACR3 in oral squamous cell carcinoma are unknown.We examined the expression pattern of TAC3/TACR3 in clinically-resected oral squamous cell carcinoma samples using immunohistochemistry and immunofluorescence analysis.We found that even though the expression level of TACR3 was negative in the normal epithelium, it was highly elevated in tumor cells. A more intense signal was observed in the   invasive   front of tumor cells that had migrated into the mandible bone matrix. TAC3 was not detected in tumor cells, but was expressed in PGP-9.5-positive sensory nerves in the mandible.Our results suggest that peripheral sensory nerve-derived TAC3 may affect gingival oral squamous cell carcinoma cells through TACR3 in the bone matrix. 
   
  
 REG1A 
 
  NCBI Gene  
  GeneRIF  
  Pubmed  
 
 
  Search Term  Rank  Fields  
  [Mm]igration  6  PubMed(1); GeneRIFs(1)  
  [Ee]xtracellular [Mm]atrix  8  PubMed(1)  
  [Ii]nvasive  10  PubMed(3); GeneRIFs(1)  
  [Mm]etastasis  12  PubMed(1)  
 
 
 hide/show details for REG1A 
  [Mm]igration  PubMed  
 Decreased REG1α expression suppresses growth, invasion and angiogenesis of bladder cancer. Previous study has indicated association between REG1α and bladder cancer. The aim of this study was to investigate the role of Regenerating gene I alpha (REG1α) in bladder cancer.The role of REG1α in bladder cancer cell proliferati  on, migra  tion and VEGF-induced angiogenesis was explored in vitro and in vivo. Immunohistochemistry (IHC) analysis was assessed to determine the expression of REG1α in ten paired bladder cancer and adjacent non-cancerous tissues, and in 296 bladder cancer samples.Down-regulation of REG1α expression significantly reduced the prolife  ration, m  igration, invasion and VEGF-induced angiogenesis in vitro and the growth of xenograft tumors in vivo. VEGF expression in bladder cancer is associated with REG1α expression and recurrence. REG1α was overexpressed in bladder cancer tissues compared with adjacent normal samples. Patients with elevated REG1α exhibited shorter recurrence times and poor survival.Downregulation of REG1α expression can reduce   tumor gro  wth, migration, invasion and angiogenesis. Our study demonstrates that REG1α can be used as a marker of recurrence and prognosis in bladder cancer. Therefore, REG1α targeting in bladder cancer patients represents a promising therapeutic strategy. 
  GeneRIFs  
 REG1alpha was overexpressed in bladder cancer tissues compared with adjacent normal samples. Downregulation of REG1alpha expression in urothelial bladder cancer can reduce tumor growth,   migration  , invasion and angiogenesis. 
  [Ee]xtracellular [Mm]atrix  PubMed  
 PAP I interacts with itself, PAP II, PAP III, and lithostathine/regIalpha. PAP I, PAP II, PAP III, and lithostathine/regIalpha are members of a multigenic family of proteins expressed in several tissues. PAP I was shown to be antiapoptotic, mitogenic, and anti-inflammatory and can promote cell adhesion to the   extracellular matrix  . Lithostathine/regIalpha can be mitogenic. Because polymerization might regulate activity, we examined the ability of rat PAP I to interact with itself (homodimerization), PAP II, PAP III, and lithostathine/regIalpha (heterodimerization) by the yeast two-hybrid system, affinity experiments, and crosslinking. PAP I interacted significantly with all members of the PAP protein family, homodimerization showing the strongest interaction as judged by the beta-galactosidase test. This was confirmed by showing specific affinity between a MBP-rPAP I fusion protein and the native rPAP I. Finally, crosslinking experiments showed that rPAP I formed dimers in solution. These findings should be taken into account in functional studies involving PAP I and PAP-related proteins. 
  [Ii]nvasive  PubMed  
 Identification of a Three-Biomarker Panel in Urine for Early Detection of Pancreatic Adenocarcinoma. Non  invasive   biomarkers for early detection of pancreatic ductal adenocarcinoma (PDAC) are currently not available. Here, we aimed to identify a set of urine proteins able to distinguish patients with early-stage PDAC from healthy individuals.Proteomes of 18 urine samples from healthy controls, chronic pancreatitis, and patients with PDAC (six/group) were assayed using GeLC/MS/MS analysis. The selected biomarkers were subsequently validated with ELISA assays using multiple logistic regression applied to a training dataset in a multicenter cohort comprising 488 urine samples.LYVE-1, REG1A, and TFF1 were selected as candidate biomarkers. When comparing PDAC (n = 192) with healthy (n = 87) urine specimens, the resulting areas under the receiver-operating characteristic curves (AUC) of the panel were 0.89 [95% confidence interval (CI), 0.84-0.94] in the training (70% of the data) and 0.92 (95% CI, 0.86-0.98) in the validation (30% of the data) datasets. When comparing PDAC stage I-II (n = 71) with healthy urine specimens, the panel achieved AUCs of 0.90 (95% CI, 0.84-0.96) and 0.93 (95% CI, 0.84-1.00) in the training and validation datasets, respectively. In PDAC stage I-II and healthy samples with matching plasma CA19.9, the panel achieved a higher AUC of 0.97 (95% CI, 0.94-0.99) than CA19.9 (AUC = 0.88; 95% CI, 0.81-0.95, P = 0.005). Adding plasma CA19.9 to the panel increased the AUC from 0.97 (95% CI, 0.94-0.99) to 0.99 (95% CI, 0.97-1.00, P = 0.04), but did not improve the comparison of stage I-IIA PDAC (n = 17) with healthy urine.We have established a novel, three-protein biomarker panel that is able to detect patients with early-stage pancreatic cancer in urine specimens. 
 REG Iα gene expression is linked with the poor prognosis of lung adenocarcinoma and squamous cell carcinoma patients via discrete mechanisms. The aim of the present study was to evaluate the effects of the REG Iα and REG Iβ genes on lung cancer cell lines, and thereafter, the expression of REG family genes (REG Iα, REG Iβ, REG III, HIP/PAP and REG IV) in lung cancer in relation to patient prognosis was evaluated. Lung adenocarcinoma (AD) and squamous cell carcinoma (SCC) cell lines expressing REG Iα or REG Iβ (HLC-1 REG Iα/Iβ and EBC-1 REG Iα/Iβ) were established, and cell nu  mber, ce  ll invasive activity, and anchorage-independent cell growth were compared with these variables in the control cells. The expression levels of REG family genes were evaluated by real-time RT-PCR in surgically resected lung cancers, and disease-specific survival (DSS) curves were generated. The HLC-1 REG Iα/Iβ cell line showed significant increases in cell number and anchorage-independent cell growth compared with the control cells. EBC-1 REG Iα/Iβ cells showed significant inc  reases i  n cell invasive activity and anchorage-independent cell growth as compared with the control cells. Except for the REG Iβ gene, expression of other REG family genes was observed in the surgically resected samples; however, DSS was significantly worse only in stage I patients who were positive for REG Iα expression than in patients who were negative for REG Iα expression. The effects of REG Iα on AD and SCC cells were different in the in vitro study, and a correlation between REG Iα expression and patient prognosis was noted in the in vivo study. Therefore, overexpression of REG Iα is a risk factor for poor prognosis caused by discrete mechanisms in AD and SCC patients. 
 Pancreatic stone protein predicts postoperative infection in cardiac surgery patients irrespective of cardiopulmonary bypass or surgical technique. We investigated the role of pancreatic stone protein (PSP) in predicting the occurrence of infection in the postoperative course of cardiac surgery patients. Several biomarkers indicating the presence of inflammation and infection are available in the clinical routine; yet, their utility in the postoperative course of patients following cardiac surgery remains uncertain. Moreover, cardiopulmonary bypass, also referred to as "on-pump surgery", increases the susceptibility to an exaggerated inflammatory state. However, the impact of such extracorporeal circulation on circulating PSP levels remains poorly understood.In a prospective cohort of unselected patients undergoing cardiac surgery, we set out to elucidate the diagnostic accuracy of serum PSP levels as opposed to canonical biomarkers (CRP, WBC) of inflammation to discriminate between the presence of infection and surgical trauma,. In addition, we investigated whether the biomarkers were influenced by the surgical technique employed, i.e. on-pump vs. off-pump and minimally   invasive   surgery vs. sternotomy. Levels of circulating PSP and routine inflammatory biomarkers (CRP, WBC) were measured in samples taken from 120 patients at baseline as well as at postoperative day 1-3.Univariate analysis showed that among the biomarkers investigated, only PSP levels had discriminatory power to differentiate infection from surgical trauma in the postoperative course of the entire cohort of patients following cardiac surgery. With regard to cardiac surgical interventions, there was no significant association between the absence or presence of extracorporeal circulation and PSP levels. However, there was a significant difference in the slope of the rise of postoperative PSP between minimally   invasive   surgery as opposed to patients subjected to sternotomy.In an unselected population of cardiac surgery patients, post-operative serum PSP levels were significantly associated with the presence of infection in both the on-pump and off-pump setting. Of note, the surgical technique employed (sternotomy vs. minimally   invasive   approach) had a significant impact on postoperative PSP levels. 
  GeneRIFs  
 The expression of REG gene is closely related to the carcinoma   invasive  ness of gastric neoplasms. 
  [Mm]etastasis  PubMed  
 Effect of REG Iα protein on angiogenesis in gastric cancer tissues. Regenerating gene (REG) Iα is not only overexpressed in a subset of gastric cancers, but also involved in tumor progression. However, the mechanism by which (REG) Iα promotes tumor growth is not fully understood. In the present study, we investigated whether REG Iα plays a role in angiogenesis during the progression of gastric cancers. Expression of REG Iα and its receptor (EXTL3; exostoses like-3) was examined using immunohistochemistry in specimens of human gastric cancer. Microvessel density (MVD) in gastric cancer tissues was evaluated using an image analysis system after CD34 immunostaining. Relationships among clinicopathological features, REG Iα expression and MVD in gastric cancer tissues were analyzed. Effects of REG Iα protein on HUVEC cells in terms of proliferation and anti-apoptosis were assessed by WST-1 assay and FACS, respectively. Furthermore, the intracellular signaling by which REG Iα exerts its biological roles was examined in vitro. REG Iα expression was significantly relate  d to lymph   node metastasis and its receptor EXTL3 was ubiquitously expressed in not only the tumor cells, but also the tumor vessel cells in the gastric cancer tissues. MVD was significantly higher in gastric cancers that were REG Iα-positive than in those that were negative. Treatment with REG Iα protein promoted growth and anti-apoptosis through activation of the ERK and Akt signaling pathways in HUVEC cells, whereas these effects were attenuated by treatment with anti-REG Iα -antibody. REG Iα protein may play a role in angiogenesis during progression of gastric cancer. 
   
  
 BTBD2 
 
  NCBI Gene  
  GeneRIF  
  Pubmed  
 
 
  Search Term  Rank  Fields  
  [Ii]nvasive  10  PubMed(1)  
 
 
 hide/show details for BTBD2 
  [Ii]nvasive  PubMed  
 Search for cellular partners of human papillomavirus type 16 E2 protein. Human papillomaviruses (HPVs) are small, double-stranded DNA viruses that infect cutaneous and mucosal epithelia. Type 16 (HPV16) displays tropism to genital epithelia, giving rise to genital warts and cervical intraepithelial neoplasia (CIN), which is a precursor lesion to   invasive   carcinoma of the cervix. The great majority of human cervical cancers contain integrated HPV DNA where the E2 gene is usually disrupted, suggesting that the loss of the E2 protein is an important step in HPV-induced carcinogenesis. The HPV16 E2 protein is a regulatory protein that seems to be essential for creating favourable conditions for establishment of infection and proper completion of the viral life cycle. Recently, diverse activities of the E2 proteins have been described, but the molecular basis of these processes has not beenfully elucidated. Using a yeast two-hybrid system, we have identified epithelial cellular proteins that bind to the E2 protein of HPV16. 
   
  
 BTBD1 
 
  NCBI Gene  
  GeneRIF  
  Pubmed  
 
 
  Search Term  Rank  Fields  
  [Ii]nvasive  10  PubMed(1)  
 
 
 hide/show details for BTBD1 
  [Ii]nvasive  PubMed  
 Search for cellular partners of human papillomavirus type 16 E2 protein. Human papillomaviruses (HPVs) are small, double-stranded DNA viruses that infect cutaneous and mucosal epithelia. Type 16 (HPV16) displays tropism to genital epithelia, giving rise to genital warts and cervical intraepithelial neoplasia (CIN), which is a precursor lesion to   invasive   carcinoma of the cervix. The great majority of human cervical cancers contain integrated HPV DNA where the E2 gene is usually disrupted, suggesting that the loss of the E2 protein is an important step in HPV-induced carcinogenesis. The HPV16 E2 protein is a regulatory protein that seems to be essential for creating favourable conditions for establishment of infection and proper completion of the viral life cycle. Recently, diverse activities of the E2 proteins have been described, but the molecular basis of these processes has not beenfully elucidated. Using a yeast two-hybrid system, we have identified epithelial cellular proteins that bind to the E2 protein of HPV16. 
   
  
 LRRC38 
 
  NCBI Gene  
  GeneRIF  
  Pubmed  
 
 
  Search Term  Rank  Fields  
  [Ee]xtracellular [Mm]atrix  8  Component(1)  
 
 
 hide/show details for LRRC38 
  [Ee]xtracellular [Mm]atrix  Component  
 proteinaceous   extracellular matrix   
   
  
 LRRC55 
 
  NCBI Gene  
  GeneRIF  
  Pubmed  
 
 
  Search Term  Rank  Fields  
  [Ee]xtracellular [Mm]atrix  8  Component(1)  
 
 
 hide/show details for LRRC55 
  [Ee]xtracellular [Mm]atrix  Component  
 proteinaceous   extracellular matrix   
   
  
 LRRC3B 
 
  NCBI Gene  
  GeneRIF  
  Pubmed  
 
 
  Search Term  Rank  Fields  
  [Mm]etastasis  12  PubMed(1)  
 
 
 hide/show details for LRRC3B 
  [Mm]etastasis  PubMed  
 LRRC3B is downregulated in non-small-cell lung cancer and inhibits cancer cell proliferation and invasion. LRRC3B has emerged as a tumor suppressor in several human cancers. However, its expression pattern and biological roles in human non-small-cell lung cancer (NSCLC) have not been explored. In the present study, we investigated clinical significance of LRRC3B in 101 NSCLC specimens. We found that LRRC3B expression was downregulated in NSCLC tissues compared with normal bronchial epithelium and that its downregulation significantly correlated with tumor-node-  metastasis   (TNM) stage (p < 0.0001), no  dal metast  asis (p < 0.0001), and poor patient prognosis (p = 0.0016, log-rank test). We also checked LRRC3B levels in several lung cancer cell lines and found that its expression was downregulated in four of nine lung cancer cell lines compared with normal human bronchial epithelial (NHBE) cell line. We further explored the biological role of LRRC3B. LRRC3B plasmid transfection in H460 and A549 cell lines inhibited proliferation, colony formation ability, and invading ability. Furthermore, we identified that LRRC3B could inhibit cell cycle progression with downregulation of cyclin D1 and decreased MMP9 expression. In addition, LRRC3B depletion in HBE cells promoted proliferation and invasion. In conclusion, our data suggested that LRRC3B may serve as an important tumor suppressor in NSCLC. 
   
  
 SH3D19 
 
  NCBI Gene  
  GeneRIF  
  Pubmed  
 
 
  Search Term  Rank  Fields  
  [Mm]igration  6  PubMed(1)  
 
 
 hide/show details for SH3D19 
  [Mm]igration  PubMed  
 Identification and characterization of a set of conserved and new regulators of cytoskeletal organization, cell morphology and   migration  . Cell   migration   is essential during development and in human disease progression including cancer. Most cell   migration   studies concentrate on known or predicted components of   migration   pathways.Here we use data from a genome-wide RNAi morphology screen in Drosophila melanogaster cells together with bioinformatics to identify 26 new regulators of morphology and cytoskeletal organization in human cells. These include genes previously implicated in a wide range of functions, from mental retardation, Down syndrome and Huntington's disease to RNA and DNA-binding genes. We classify these genes into seven groups according to phenotype and identify those that affect cell   migration  . We further characterize a subset of seven genes, FAM40A, FAM40B, ARC, FMNL3, FNBP3/FBP11, LIMD1 and ZRANB1, each of which has a different effect on cell shape, actin filament distribution and cell   migration  . Interestingly, in several instances closely related isoforms with a single Drosophila homologue have distinct phenotypes. For example, FAM40B depletion induces cell elongation and tail retraction defects, whereas FAM40A depletion reduces cell spreading.Our results identify multiple regulators of cell   migration   and cytoskeletal signalling that are highly conserved between Drosophila and humans, and show that closely related paralogues can have very different functions in these processes. 
   
  
 SP1 
 
  NCBI Gene  
  GeneRIF  
  Pubmed  
 
 
  Search Term  Rank  Fields  
  [Mm]igration  6  PubMed(42); GeneRIFs(4)  
  ECM  7  PubMed(6)  
  [Ee]xtracellular [Mm]atrix  8  PubMed(17); GeneRIFs(2)  
  [Ii]nvasive  10  PubMed(28); GeneRIFs(5)  
  [Mm]etastasize  11  PubMed(1)  
  [Mm]etastasis  12  PubMed(61); GeneRIFs(12)  
  [Cc]ell [Ii]nvasion  13  PubMed(13); GeneRIFs(3)  
 
 
 hide/show details for SP1 
  [Mm]igration  PubMed  
 Activation of TGF-β1 promoter by hepatitis C virus-induced AP-1 and Sp1: role of TGF-β1 in hepatic stellate cell activation and invasion. Our previous studies have shown the induction and maturation of transforming growth factor-beta 1 (TGF-β1) in HCV-infected human hepatoma cells. In this study, we have investigated the molecular mechanism of TGF-β1 gene expression in response to HCV infection. We demonstrate that HCV-induced transcription factors AP-1, Sp1, NF-κB and STAT-3 are involved in TGF-β1 gene expression. Using chromatin immunoprecipitation (ChIP) assay, we further show that AP-1 and Sp1 interact with TGF-b1 promoter in vivo in HCV-infected cells. In addition, we demonstrate that HCV-induced TGF-β1 gene expression is mediated by the activation of cellular kinases such as p38 MAPK, Src, JNK, and MEK1/2. Next, we determined the role of secreted bioactive TGF-β1 in human hepatic stellate cells (HSCs) activation and invasion. Using siRNA approach, we show that HCV-induced bioactive TGF-β1 is critical for the induction of alpha smooth muscle actin (α-SMA) and type 1 collagen, the markers of HSCs activation and proliferation. We further demonstrate the potential role of HCV-induced bioactive TGF-β1 in HSCs inv  asion/cel  l migration using a transwell Boyden chamber. Our results also suggest the role of HCV-induced TGF-β1 in HCV replication and release. Collectively, these observations provide insight into the mechanism of TGF-β1 promoter activation, as well as HSCs activation and invasion, which likely manifests in liver fibrosis associated with HCV infection. 
 Differential regulation of MMPs by E2F1, Sp1 and NF-kappa B controls the small cell lung cancer invasive phenotype. E2F1 transcription factor plays a vital role in the regulation of diverse cellular processes including cell proliferation, apoptosis, invasion and metastasis. E2F1 overexpression has been demonstrated in small cell lung cancer (SCLC), and extensive metastasis in early phase is the most important feature of SCLC. In this study, we investigated the involvement of E2F1 in the process of invasion and metastasis in SCLC by regulating the expression of matrix metalloproteinases (MMPs).Immunohistochemistry was performed to evaluate the expression of E2F1 and MMPs in SCLC samples in a Chinese Han population. The impact of E2F1 on invasion and metastasis was observed by transwell and wound healing experiments with depletion of E2F1 by specific siRNA. The target genes regulated by E2F1 were identified by chromatin immunoprecipitation (ChIP)-to-sequence, and the expressions of target genes were detected by real time PCR and western blotting. The dual luciferase reporter system was performed to analyze the regulatory relationship between E2F1 and MMPs.E2F1 is an independent and adverse prognosis factor that is highly expressed in SCLC in a Chinese Han population. Knockdown of E2F1 by specific siRNA resulted in the downregulation of   migration   and invasion in SCLC. The expressions of MMP-9 and -16 in SCLC were higher than other MMPs, and their expressions were most significantly reduced after silencing E2F1. ChIP-to-sequence and promoter-based luciferase analysis demonstrated that E2F1 directly controlled MMP-16 expression via an E2F1 binding motif in the promoter. Although one E2F1 binding site was predicted in the MMP-9 promoter, luciferase analysis indicated that this binding site was not functionally required. Further study demonstrated that E2F1 transcriptionally controlled the expression of Sp1 and p65, which in turn enhanced the MMP-9 promoter activity in SCLC cells. The associations between E2F1, Sp1, p65, and MMP-9 were validated by immunohistochemistry staining in SCLC tumors.E2F1 acts as a transcriptional activator for MMPs and directly enhances MMP transcription by binding to E2F1 binding sequences in the promoter, or indirectly activates MMPs through enhanced Sp1 and NF-kappa B as a consequence of E2F1 activation in SCLC. 
 Differential regulation of MMPs by E2F1, Sp1 and NF-kappa B controls the small cell lung cancer invasive phenotype. E2F1 transcription factor plays a vital role in the regulation of diverse cellular processes including cell proliferation, apoptosis, invasion and metastasis. E2F1 overexpression has been demonstrated in small cell lung cancer (SCLC), and extensive metastasis in early phase is the most important feature of SCLC. In this study, we investigated the involvement of E2F1 in the process of invasion and metastasis in SCLC by regulating the expression of matrix metalloproteinases (MMPs).Immunohistochemistry was performed to evaluate the expression of E2F1 and MMPs in SCLC samples in a Chinese Han population. The impact of E2F1 on invasion and metastasis was observed by transwell and wound healing experiments with depletion of E2F1 by specific siRNA. The target genes regulated by E2F1 were identified by chromatin immunoprecipitation (ChIP)-to-sequence, and the expressions of target genes were detected by real time PCR and western blotting. The dual luciferase reporter system was performed to analyze the regulatory relationship between E2F1 and MMPs.E2F1 is an independent and adverse prognosis factor that is highly expressed in SCLC in a Chinese Han population. Knockdown of E2F1 by specific siRNA resulted in the downregulation of   migration   and invasion in SCLC. The expressions of MMP-9 and -16 in SCLC were higher than other MMPs, and their expressions were most significantly reduced after silencing E2F1. ChIP-to-sequence and promoter-based luciferase analysis demonstrated that E2F1 directly controlled MMP-16 expression via an E2F1 binding motif in the promoter. Although one E2F1 binding site was predicted in the MMP-9 promoter, luciferase analysis indicated that this binding site was not functionally required. Further study demonstrated that E2F1 transcriptionally controlled the expression of Sp1 and p65, which in turn enhanced the MMP-9 promoter activity in SCLC cells. The associations between E2F1, Sp1, p65, and MMP-9 were validated by immunohistochemistry staining in SCLC tumors.E2F1 acts as a transcriptional activator for MMPs and directly enhances MMP transcription by binding to E2F1 binding sequences in the promoter, or indirectly activates MMPs through enhanced Sp1 and NF-kappa B as a consequence of E2F1 activation in SCLC. 
 Activation of TGF-β1 promoter by hepatitis C virus-induced AP-1 and Sp1: role of TGF-β1 in hepatic stellate cell activation and invasion. Our previous studies have shown the induction and maturation of transforming growth factor-beta 1 (TGF-β1) in HCV-infected human hepatoma cells. In this study, we have investigated the molecular mechanism of TGF-β1 gene expression in response to HCV infection. We demonstrate that HCV-induced transcription factors AP-1, Sp1, NF-κB and STAT-3 are involved in TGF-β1 gene expression. Using chromatin immunoprecipitation (ChIP) assay, we further show that AP-1 and Sp1 interact with TGF-b1 promoter in vivo in HCV-infected cells. In addition, we demonstrate that HCV-induced TGF-β1 gene expression is mediated by the activation of cellular kinases such as p38 MAPK, Src, JNK, and MEK1/2. Next, we determined the role of secreted bioactive TGF-β1 in human hepatic stellate cells (HSCs) activation and invasion. Using siRNA approach, we show that HCV-induced bioactive TGF-β1 is critical for the induction of alpha smooth muscle actin (α-SMA) and type 1 collagen, the markers of HSCs activation and proliferation. We further demonstrate the potential role of HCV-induced bioactive TGF-β1 in HSCs inv  asion/cel  l migration using a transwell Boyden chamber. Our results also suggest the role of HCV-induced TGF-β1 in HCV replication and release. Collectively, these observations provide insight into the mechanism of TGF-β1 promoter activation, as well as HSCs activation and invasion, which likely manifests in liver fibrosis associated with HCV infection. 
 Sp1 regulates osteopontin expression in SW480 human colon adenocarcinoma cells. Osteopontin (OPN) mediates cancer metastasis. Mechanisms regulating OPN expression in human colorectal cancer are unknown. Using SW480 colon adenocarcinoma cells, we hypothesized that transcription determines OPN expression.SW480 constitutively express OPN. Transient transfection and deletion analysis of human OPN promoter (full-length 2.1 kb)-luciferase constructs identified cis-regulatory regions. Gelshift and chromatin immunoprecipitation (ChIP) assays identified the trans-regulatory nuclear protein. Using in vitro adhesion,   migration  , and invasion studies, siRNA was used to determine the functional effect of decreased nuclear protein expression.A cis-regulatory promoter region, nt-80 to nt-108, upregulated OPN transcription. Gelshift assays demonstrated specific binding of nuclear proteins. Competition with unlabeled mutant oligonucleotides indicated that the region, nt-94 to nt-104 (TGGGCTGGGC), was essential for protein binding in gelshift assays. Confirmatory ChIP assays showed the corresponding nuclear protein to be Sp1. Sp1 expression was ablated with siRNA (si-Sp1), resulting in decreased OPN-dependent adhesion,   migration  , and invasion by 50%, 70%, and 65%, respectively. Exogenous addition of OPN to si-Sp1 cells restored adhesion,   migration  , and invasion indices.In SW480 human colon cancer cells, we conclude that Sp1 mediated expression of the tumor metastasis protein, OPN, regulates in vitro functional correlates of tumor metastasis. 
 The histone deacetylase 4/SP1/microrna-200a regulatory network contributes to aberrant histone acetylation in hepatocellular carcinoma. As an important epigenetic mechanism, histone acetylation modulates the transcription of many genes and plays important roles in hepatocellular carcinoma (HCC). Aberrations in histone acetylation have been observed in HCC, but the factors that contribute to the aberrations have not been fully elucidated. MicroRNAs (miRNAs), which are noncoding RNAs that regulate gene expression, are involved in important epigenetic mechanisms. In this study, we determined that miR-200a and the level of histone H3 acetylation at its promoter were reduced in human HCC tissues in comparison with adjacent noncancerous hepatic tissues. Furthermore, our results suggested that the histone deacetylase 4 (HDAC4) inhibited the expression of miR-200a and its promoter activity and reduced the histone H3 acetylation level at the mir-200a promoter through a Sp1-dependent pathway. Interestingly, we observed that the miR-200a directly targeted the 3'-untranslated region of the HDAC4 messenger RNA and repressed expression of HDAC4. Therefore, miR-200a ultimately induced its own transcription and increased the histone H3 acetylation level at its own promoter. Through targeting HDAC4, miR-200a also induced the up-regulation of total acetyl-histone H3 levels and increased the histone H3 acetylation level at the p21(WAF/Cip1) promoter. Finally, we determined that miR-200a inhibited the proliferation and   migration   of HCC cells in vivo and in vitro.Our findings suggest that the HDAC4/Sp1/miR-200a regulatory network induces the down-regulation of miR-200a and the up-regulation of HDAC4 in HCC. As a result, down-regulation of miR-200a enhances the proliferation and   migration   of HCC cells and induces aberrant histone acetylation in HCC. These findings highlight a potential therapeutic approach in targeting the HDAC4/Sp1/miR-200a regulatory network for the treatment of HCC. 
 MicroRNA-335 acts as a metastasis suppressor in gastric cancer by targeting Bcl-w and specificity protein 1. Aberrant expression of miR-335 has been frequently reported in cancer studies, suggesting that there is a close correlation between miR-335 and cancer during its development, progression, metastasis and prognosis. The expression of miR-335 in gastric cancer and its effects are not known. Relative expression of miR-335 in 4 gastric cancer cell lines and in 70 gastric cancer tissues was confirmed by real-time quantitative reverse transcriptase-PCR compared with controls. Transwell cell   migration   and Matrigel invasion assay in vitro and metastasis formation assay in vivo were used to examine the effects of miR-335 expression on gastric cancer cell invasion and metastasis. The effect of miR-335 expression on gastric cancer cell proliferation was estimated by the 3-(4,5-dimethylthiazol-2-yl)-2, 5-diphenyltetrazolium bromide assay. Luciferase reporter assay and western blot were used to examine the potential target genes and related pathways. Gene silencing with small-interfering RNA was used to examine the effects of target genes on gastric cancer cell invasion. miR-335 was dramatically downregulated in gastric cancer cell lines than in the normal gastric cell line GES-1. Low expression of miR-335 was significantly associated with lymph-node metastasis, poor pT stage, poor pN stage and invasion of lymphatic vessels. Overexpression of miR-335 suppressed gastric cancer cell invasion and metastasis in vitro and in vivo, but has no significant effects on cell proliferation. Furthermore, miR-335 might suppress gastric cancer invasion and metastasis by targeting Bcl-w and specificity protein 1 (SP1). Taken together, our results provide evidence that miR-335 might function as a metastasis suppressor in gastric cancer by targeting SP1 directly and indirectly through the Bcl-w-induced phosphoinositide 3-kinase-Akt-Sp1 pathway. miR-335 showing altered expression at different stages of gastric cancer could be a target for gastric cancer therapies and could be further developed as a potential prognostic factor. 
 The oncoprotein hepatitis B X-interacting protein promotes the   migration   of ovarian cancer cells through the upregulation of S-phase kinase-associated protein 2 by Sp1. Hepatitis B X-interacting protein (HBXIP) is a novel oncoprotein. We have previously reported that HBXIP promotes the proliferation and   migration   of breast cancer cells. S-phase kinase-associated protein 2 (Skp2) is another oncoprotein which is important for   migration  . In this study, we investigated whether Skp2 is involved in the   migration   enhanced by HBXIP in ovarian cancer. The expression of HBXIP and Skp2 in ovarian cancer tissues was examined by immunohistochemistry using tissue microarrays. The role of HBXIP and Skp2 in the   migration   of ovarian cancer cells was investigated by wound-healing assay and Transwell   migration   assay. The effect of HBXIP on Skp2 was assessed by reverse transcription polymerase chain reaction (RT-PCR), western blot analysis, luciferase reporter gene assays and chromatin immunoprecipitation in ovarian cancer cells (SKOV3 and CAOV3). We found that both HBXIP and Skp2 were highly expressed in ovarian cancer tissues. We observed that the overexpression of HBXIP enhanced the   migration   of ovarian cancer cells, while Skp2 siRNAs decreased the cell   migration   enhanced by HBXIP. The HBXIP siRNAs inhibited ovarian cancer cell   migration   and Skp2 rescued the   migration   inhibition induced by HBXIP siRNA. HBXIP could upregulate Skp2 at the levels of mRNA and protein in ovarian cancer cells. Moreover, HBXIP increased the activity of Skp2 promoter via binding to the transcription factor Sp1. HBXIP is highly expressed in ovarian cancer tissues. HBXIP enhances the   migration   of ovarian cancer cells. HBXIP was able to stimulate the activity of Skp2 promoter via transcription factor Sp1 thus promoting the   migration   of ovarian cancer cells. 
 Expression of alpha V integrin is modulated by Epstein-Barr virus nuclear antigen 3C and the metastasis suppressor Nm23-H1 through interaction with the GATA-1 and Sp1 transcription factors. Epstein-Barr virus (EBV) is a lymphotrophic herpesvirus infecting most of the world's population. It is associated with a number of human lymphoid and epithelial tumors and lymphoproliferative diseases in immunocompromised patients. A subset of latent EBV antigens is required for immortalization of primary B-lymphocytes. The metastatic suppressor Nm23-H1 which is downregulated in human invasive breast carcinoma reduces the   migration   and metastatic activity of breast carcinoma cells when expressed from a heterologous promoter. Interestingly, the EBV nuclear antigen 3C (EBNA3C) reverses these activities of Nm23-H1. The alpha V integrins recognize a variety of ligands for signaling and are involved in cell   migration   and proliferation and also serve as major receptors for extracellular-matrix-mediated cell adhesion and   migration  . The goal of this study was to determine if Nm23-H1 and EBNA3C can modulate alpha V integrin expression and downstream activities. The results of our studies indicate that Nm23-H1 downregulates alpha V intregrin expression in a dose responsive manner. In contrast, EBNA3C can upregulate alpha V integrin expression. Furthermore, the study showed that the association of the Sp1 and GATA transcription factors with Nm23-H1 is required for modulation of the alpha V integrin activity. Thus, these results suggest a direct correlation between the alpha V integrin expression and the interaction of Nm23-H1 with EBNA3C. 
 Butyrate suppresses expression of neuropilin I in colorectal cell lines through inhibition of Sp1 transactivation. Neuropilin is a transmembrane receptor for vascular endothelial growth factor (VEGF) and is expressed in normal endothelial cells and upregulated in cancer cells. Neuropilin-1 (NRP-1) has been shown to promote tumour cell   migration   and survival in colon cancer in response to VEGF binding. The expression profiles of neuropilins, associated co-receptors and known ligands have been mapped in three colorectal cell lines: Caco-2, HCT116 & HT29. We have previously shown that butyrate, a naturally occurring histone deacetylase inhibitor (HDACi) produced by fermentation of fibre in the colon, causes apoptosis of colon cancer cell lines.Here we demonstrate that butyrate down-regulates NRP-1 and VEGF at the mRNA and protein level in colorectal cancer cell lines. NRP-1 is a known transcriptional target of Sp1, whose activity is regulated by acetylation. NRP-1 down-regulation by butyrate was associated with decreased binding affinity of Sp1 for canonical Sp-binding sites in the NRP-1 promoter. siRNA-mediated knock-down of Sp1 implied that Sp1 may have strong DNA binding activity but weak transactivation potential.The downregulation of the key apoptotic and angiogenesis regulator NRP-1 by butyrate suggests a novel contributory mechanism to the chemopreventive effect of dietary fibre. 
 MicroRNA-892b influences proliferation,   migration   and invasion of bladder cancer cells by mediating the p19ARF/cyclin D1/CDK6 and Sp-1/MMP-9 pathways. Cancers often utilize microRNAs to suppress tumor suppressor genes, thus facilitating their potential for growth and invasion. In the present study, we report the novel findings that miR-892b inhibits proliferation,   migration   and invasion of bladder cancer cells. The basal expression level of miR‑892b was significantly lower in 3 different bladder cancer cell lines than in normal human urothelial cells. Transfection of miR-892b mimics to bladder cancer cells resulted in dose‑dependent growth arrest. Flow cytometric analysis of the cell cycle showed that miR-892b-transfected bladder cancer cells were subject to arrest in the G1 phase, which was due to the downregulation of cyclin D1 and CDK6 followed by upregulation of p19ARF. In addition, overexpression of miR-892b imped  ed the mi  gration and invasion of EJ cells. Expression of MMP-9 in EJ cells was blocked by transfection of miR-892b; the effect was regulated, at least in part, by activation of the Sp-1 transcription factor. Overall, we verified that miR-892b regulates the p19ARF/cyclin D1/CDK6 and Sp-1/MMP-9 signaling networks in bladder cancer cells and may provide a treatment option for advanced-stage bladder cancers. 
 Acetylated Sp1 inhibits PTEN expression through binding to PTEN core promoter and recruitment of HDAC1 and promotes cancer cell   migration   and invasion. Specificity protein 1 (Sp1) is often overexpressed in cancer cells. Its binding sites are known to exist in the phosphatase and tension homolog deleted on chromosome 10 (PTEN) promoter. In this study, we hypothesized that Sp1 negatively regulates PTEN expression. We used several cell lines to determine the effects of Sp1. The results showed that Sp1 overexpression inhibited the expression and promoter activity of PTEN and correspondingly upregulated AKT phosphorylation, whereas Sp1 knockdown upregulated the expression and promoter ability of PTEN and downregulated AKT phosphorylation. Moreover, a series of deletion and site-directed mutations of the PTEN promoter indicated that Sp1 can inhibit PTEN promoter activity through a specific Sp1-binding site at the PTEN core promoter in vivo. Meanwhile, non-acetylated Sp1, with its loss of DNA binding activity, failed to inhibit the expression and promoter activity of PTEN. Histone deacetylase 1 was necessary for Sp1 to inhibit PTEN expression. The inverse expression of Sp1 and PTEN was found in tongue cancer cells and salivary adenoid cystic cancer (SACC)-LM cells (possessing higher potential for lung metastasis than SACC-83) as compared with that in adjacent normal tissue and SACC-83 cells, respectively. Sp1 knockdown decreased the   migration   and invasion of SACC-LM cells, whereas Sp1 overexpression increased the   migration   and invasion of SACC-83 cells. Overall, these results suggest that Sp1 is involved in the development and invasiveness of cancer through inhibition of PTEN. 
 17β-estradiol enhances α(5) integrin subunit gene expression through ERα-Sp1 interaction and reduces cell motility and invasion of ERα-positive breast cancer cells. In breast tumors the expression of estrogen receptor alpha (ERα) is known to be associated with a more favorable prognosis. ERα expression has been reported to reduce the metastatic potential of breast cancer cells. Recently, we have observed that extracellular matrix proteins activate ERα and that both liganded and unliganded receptor modulate cell invasiveness acting at nuclear level. To explain the mechanisms by which ERα regulates cell adhesion, we have evaluated the expression of α(5)β(1) integrin, prevalently expressed in stationary cells, in response to 17β-estradiol (E2). Here we show that E2/ERα increases the expression of integrin α(5)β(1) through Sp1-mediated binding to a GC-rich region located upstream of an ERE half-site in the 5' flanking region of the α(5) gene forming a ternary ERα-Sp1-DNA complex. Estrogen responsiveness of the α(5) gene promoter, as observed in HeLa cells, underlies a general mechanism of regulation which is not strictly linked to the cell type. Our data reveal novel insight into the molecular mechanisms sustaining the reduced invasiveness of ERα expressing cells demonstrating that α(5)β(1) integrin expression is related to the maintenance of the stationary status of the cells, counteracting E2/ERα capability to enhanc  e breast   cancer cell migration and invasion. 
 The oncoprotein HBXIP upregulates PDGFB via activating transcription factor Sp1 to promote the proliferation of breast cancer cells. We have reported that the oncoprotein hepatitis B virus X-interacting protein (HBXIP) acts as a novel transcriptional coactivator to promote proliferation and   migration   of breast cancer cells. Previously, we showed that HBXIP was able to activate nuclear factor-κB (NF-κB) in breast cancer cells. As an oncogene, the platelet-derived growth factor beta polypeptide (PDGFB) plays crucial roles in carcinogenesis. In the present study, we found that both HBXIP and PDGFB were highly expressed in breast cancer cell lines. Interestingly, HBXIP was able to increase transcriptional activity of NF-κB through PDGFB, suggesting that HBXIP is associated with PDGFB in the cells. Moreover, HBXIP was able to upregulate PDGFB at the levels of mRNA, protein and promoter in the cells. Then, we identified that HBXIP stimulated the promoter of PDGFB through activating transcription factor Sp1. In function, HBXIP enhanced the proliferation of breast cancer cells through PDGFB in vitro. Thus, we conclude that HBXIP upregulates PDGFB via activating transcription factor Sp1 to promote proliferation of breast cancer cells. 
 ERK3 promotes endothelial cell functions by upregulating SRC-3/SP1-mediated VEGFR2 expression. Despite a regain of interest recently in ERK3 kinase signaling, the molecular regulations of both ERK3 gene expression and protein kinase activity are still largely unknown. While it is shown that disruption of ERK3 gene causes neonatal lethality, cell type-specific functions of ERK3 signaling remain to be explored. In this study, we report that ERK3 gene expression is upregulated by cytokines through c-Jun in endothelial cells; c-Jun binds to the ERK3 gene and regulates its transcription. We further reveal a new role for ERK3 in regulating endothelial cell   migration  , proliferation and tube formation by upregulating SRC-3/SP-1-mediated VEGFR2 expression. The underlying molecular mechanism involves ERK3-stimulated formation of a transcriptional complex involving coactivator SRC-3, transcription factor SP-1 and the secondary coactivator CBP. Taken together, our study identified a molecular regulatory mechanism of ERK3 gene expression and revealed a previously unknown role of ERK3 in regulating endothelial cell functions. 
 Low SP1 expression differentially affects intestinal-type compared with diffuse-type gastric adenocarcinoma. Specificity protein 1 (SP1) is an essential transcription factor that regulates multiple cancer-related genes. Because aberrant expression of SP1 is related to cancer development and progression, we focused on SP1 expression in gastric carcinoma and its correlation with disease outcomes. Although patient survival decreased as SP1 expression increased (P<0.05) in diffuse-type gastric cancer, the lack of SP1 expression in intestinal-type gastric cancer was significantly correlated with poor survival (P<0.05). The knockdown of SP1 in a high SP1-expressing intestinal-type gastric cell line, MKN28, increased   migration   and invasion but decreased proliferation. Microarray data in SP1 siRNA-transfected MKN28 revealed that the genes inhibiting   migration   were downregulated, whereas the genes negatively facilitating proliferation were increased. However, both   migration   and invasion were decreased by forced SP1 expression in a low SP1-expressing intestinal-type gastric cell line, AGS. Unlike the intestinal-type, in a high SP1-expressing diffuse-type gastric cell line, SNU484,   migration   and invasion were decreased by SP1 siRNA. In contrast to previous studies that did not identify differences between the 2 histological types, our results reveal that low expression of SP1 is involved in cancer progression and metastasis and differentially affects intestinal-type compared with diffuse-type gastric adenocarcinoma. 
 Transcription factor Sp1 induces ADAM17 and contributes to tumor cell invasiveness under hypoxia. Expression of the Sp1 transcription factor is induced by hypoxia, and the ADAM17 promoter contains predicted Sp1 binding sites. ADAM17 contributes to hypoxic-induce invasiveness of glioma. In this study, we investigated whether Sp1 transcription factor induces ADAM17 and/or contributes to tumor cell invasiveness in hypoxia.Employing RT-PCR and Western blot, we examined the role of Sp1 in ADAM17 transcription/expression under normoxic and hypoxic conditions, and whether it binds to the ADAM17 GC-rich promoter region using a chromatin immunoprecipitation assay. Additionally, we tested the effect of Sp1 suppression in tumor cell invasion and   migration  , using Matrigel basement membrane invasion chambers, a scratch wound-healing assay, and small interfering RNA.Here, we found that Sp1 binds to the ADAM17 promoter, and that Sp1 regulates ADAM17 expression under hypoxia. Furthermore, suppression of Sp1 decreases invasiveness and   migration   in U87 tumor cells.Our findings suggest the Sp1 transcription factor mediates ADAM17 expression under hypoxia, regulates glioma invasiveness, and thus, may be a target for anti-invasion therapies. 
 Association of Sp1 and survivin in epithelial ovarian cancer: Sp1 inhibitor and cisplatin, a novel combination for inhibiting epithelial ovarian cancer cell proliferation. The expression of specificity protein 1 (Sp1) and survivin was evaluated in clinical specimens of epithelial ovarian cancer (EOC) patients. When compared to normal tissue, EOC samples showed high expression of Sp1 and survivin using qPCR (Sp1: ∼2-fold; survivin: ∼5-fold) and Western blot (Sp1: >2.6-fold; survivin: >100-fold). The Sp1 inhibitor, and anti-cancer small molecule, tolfenamic acid (TA), was tested to enhance the response of Cisplatin (Cis) in EOC cell lines. Cell viability (CellTiter-Glo), combination index (CalcuSyn software), apoptosis (Annexin-V staining), cell cycle analyses (flow cytometry), and reactive oxygen species (flow cytometry) were determined. C  ell migra  tion and invasion was assessed using matrigel coated transwell chambers. Agilent Technologies proteomics analysis identified potential signaling pathways involved. The combination of TA (50 μM) and Cis (5 μM) synergistically increased the growth inhibition in ES2 (∼80 %, p < 0.001) and OVCAR-3 (60 %, p < 0.001) cells. TA or TA + Cis treatment in ES2 cells caused cell cycle arrest in G1 Phase (TA) or S-Phase (TA + Cis) and unregulated reactive o  xygen spe  cies. Invasion and migration was decreased in ES2 cells. Global proteomic profiling showed modulation of proteins associated with oxidative phosphorylation, apoptosis, electron transport chain, DNA damage, and cell cycle proteins. These results demonstrate an association of Sp1 and survivin in EOC and confirm targeting these candidates with TA potentially sensitizes EOC cells to cisplatin. 
 The antimetastatic effects of resveratrol on hepatocellular carcinoma through the downregulation of a metastasis-associated protease by SP-1 modulation. The mortality and morbidity rates from cancer metastasis have not declined in Taiwan, especially because of hepatocellular carcinoma (HCC). Resveratrol has been shown to have benefits such as cardioprotection, providing antioxidative, anti-inflammatory, anti-cancer properties in previous studies. Therefore, HCC cells were subjected to treatment with resveratrol and then analyzed to determine the effects of resveratrol on the   migration   and invasion.Modified Boyden chamber assays revealed that resveratrol treatment significantly inhibited cell   migration   and invasion capacities of Huh7 cell lines that have low cytotoxicity in vitro, even at a high concentration of 100 µM. The results of casein zymography and western blotting revealed that the activities and protein levels of the urokinase-type plasminogen activator (u-PA) were inhibited by resveratrol. Western blot analysis also showed that resveratrol inhibits phosphorylation of JNK1/2. Tests of the mRNA level, real-time PCR, and promoter assays evaluated the inhibitory effects of resveratrol on u-PA expression in HCC cells. The chromatin immunoprecipitation (ChIP) assay showed that reactive in transcription protein of nuclear factor SP-1 was inhibited by resveratrol.Resveratrol inhibits u-PA expression and the metastasis of HCC cells and is a powerful chemopreventive agent. The inhibitory effects were associated with the downregulation of the transcription factors of SP-1 signaling pathways. 
 Regulation of constitutive and microbial pathogen-induced human macrophage   migration   inhibitory factor (MIF) gene expression. The cytokine macrophage   migration   inhibitory factor (MIF) is an important regulator of innate immunity, inflammation and oncogenesis. However, four decades after its identification, the molecular mechanism(s) regulating the expression of the MIF gene remain largely unknown. Analyses of human monocytic (THP-1), epithelial (HeLa and A549) and keratinocytic (HaCat) cells transfected with wild-type, truncated and mutated MIF promoter reporter constructs, and electrophoretic mobility shift assay, chromatin immunoprecipitation, and siRNA inhibition indicated that the transcription factors specificity protein (Sp)1 and cAMP response element-binding protein (CREB) are critical positive regulators of constitutive human MIF gene expression. Albeit located in a cytosine guanine dinucleotide island, the MIF gene was found to be hypomethylated, an observation consistent with high baseline transcriptional activity. Moreover, stimulation of THP-1 cells and of peripheral blood mononuclear cells with microbial products up-regulated phosphorylated Sp1 nuclear content, Sp1 DNA-binding activity, MIF promoter activity and MIF mRNA levels in a MEK1/2-, Sp1-dependent manner. Taken together with previous observations of an important role for MIF in pro-inflammatory macrophage responses, these present findings suggest a key role for Sp1 and CREB in transcriptional regulation of MIF gene expression and MIF-dependent host antimicrobial innate immune defense. 
 Suppression of lung cancer cell invasion by LKB1 is due to the downregulation of tissue factor and vascular endothelial growth factor, partly dependent on SP1. LKB1 encodes a serine/threonine kinase generally inactivated in many human cancers, which mediates cancer cell proliferation,   migration   and differentiation. Recent studies indicated that LKB1 exhibits potent anti-metastatic activity. However, the underlying molecular mechanisms of this activity remain unclear. In this study, we re‑introduced LKB1 into A549 lung cancer cells that lack the LKB1 gene to investigate how LKB1 affects tumor invasiveness and metastasis. We demonstrated that overexpression of the LKB1 protein in lung cancer cells resulted in significant inhibition of invasion. Furthermore, transfected lung cancer cells with LKB1 suppressed tissue factor (TF) and vascular endothelial growth factor (VEGF) expression at both the mRNA and protein levels. Here, we provided evidence showing that downregulation of TF and VEGF by LKB1 is correlated well with the inhibition of cell invasion. Overexpression of the LKB1 protein in human lung cancer is significantly associated with a decrease in activity and expression of the transcription factor SP1. Constitutive activation of the transcription factor Sp1 plays a critical role in TF and VEGF overexpression. We conclude that suppression of lung cancer cell invasion by LKB1 through downregulation of TF and VEGF may partly depend on its inhibitory effect on the transcription factor Sp1. Collectively, our data provide a novel molecular mechanism for the antitumor activity of LKB1 and may help further improve its effectiveness in controlling lung cancer growth and invasion. 
 miR-375 inhibits the invasion and metastasis of colorectal cancer via targeting SP1 and regulating EMT-associated genes. Accumulating evidence has shown that aberrantly expressed microRNAs (miRNAs) are associated with tumor development and progression. Our previous study found that microRNA-375 (miR-375) was downregulated in colorectal cancer (CRC), but little is known concerning the role of miR-375 and the related mechanism in CRC development. The proliferation, invasion and   migration   effects were investigated by Cell Counting Kit-8 (CCK-8), colony formation and Transwell assays with or without Matrigel. In addition, candidate target genes were screened and validated by luciferase reporter and western blot assays. In addition, western blot analysis was performed to explore the molecular mechanisms associated with epithelial‑mesenchymal transition (EMT). It was found that miR-375 inhibited proliferation, invasion an  d migrati  on in DLD1 and HCT8 cells. In addition, miR-375 negatively regulated Sp1 transcription factor (SP1) protein by directly binding to the 3'-untranslated region (3'-UTR). Furthermore, it was found that miR-375 regulated matrix metalloproteinase 2 (MMP2) and EMT-associated genes, E-cadherin, vimentin, snail, N-cadherin and β-catenin. In conclusion, miR-375 inhibited the proliferation, invasion   and migra  tion by directly targeting SP1 and regulating MMP2 and EMT-associated genes. 
 CYP1B1 Enhances Cell Proliferation and Metastasis through Induction of EMT and Activation of Wnt/β-Catenin Signaling via Sp1 Upregulation. Cytochrome P450 1B1 (CYP1B1) is a major E2 hydroxylase involved in the metabolism of potential carcinogens. CYP1B1 expression has been reported to be higher in tumors compared to normal tissues, especially in hormone-related cancers including breast, ovary, and prostate tumors. To explore the role of CYP1B1 in cancer progression, we investigated the action of CYP1B1 in cells with increased CYP1B1 via the inducer 7,12-dimethylbenz[α]anthracene (DMBA) or an overexpression vector, in addition to decreased CYP1B1 via the inhibitor tetramethoxystilbene (TMS) or siRNA knockdown. We observed that CYP1B1 promoted cell proliferation  , migrati  on, and invasion in MCF-7 and MCF-10A cells. To understand its molecular mechanism, we measured key oncogenic proteins including β-catenin, c-Myc, ZEB2, and matrix metalloproteinases following CYP1B1 modulation. CYP1B1 induced epithelial-mesenchymal transition (EMT) and activated Wnt/β-catenin signaling via upregulation of CTNNB1, ZEB2, SNAI1, and TWIST1. Sp1, a transcription factor involved in cell growth and metastasis, was positively regulated by CYP1B1, and suppression of Sp1 expression by siRNA or DNA binding activity using mithramycin A blocked oncogenic transformation by CYP1B1. Therefore, we suggest that Sp1 acts as a key mediator for CYP1B1 action. Treatment with 4-hydroxyestradiol (4-OHE2), a major metabolite generated by CYP1B1, showed similar effects as CYP1B1 overexpression, indicating that CYP1B1 activity mediated various oncogenic events in cells. In conclusion, our data suggests that CYP1B1 promotes cell proliferation and metastasis by inducing EMT and Wnt/β-catenin signaling via Sp1 induction. 
 microRNA-330 inhibits cell motility by downregulating Sp1 in prostate cancer cells. microRNAs (miRNAs), small non-coding RNAs, have emerged as key regulators of a large number of genes. The present study aimed to explore novel biological functions of miR-330 in the human prostate cancer cell lines DU145 and PC3. We confirmed that miR-330 was downregulated and inversely correlated with specificity protein 1 (Sp1) expression. Overexpression of miR-330 by transfection of a chemically synthesized miR-330 mimic induced a reduction in expression levels of the Sp1 protein, accompanied by significant suppression of cellular   migration   and invasion capability. In addition, the Sp1-knockdown experiments presented similar phenomena. Finally, the luciferase reporter assay validated Sp1 as the direct target of miR-330. These findings indicate that miR-330 acts as an anti-metastatic miRNA in prostate cancer. 
 Control of intestinal promoter activity of the cellular migratory regulator gene ELMO3 by CDX2 and SP1. An important aspect of the cellular differentiation in the intestine is the   migration   of epithelial cells from the crypt to the villus tip. As homeodomaine transcription factor CDX2 has been suggested to influence cell   migration  , we performed a genome-wide promoter analysis for CDX2 binding in the differentiated human intestinal cancer cell line Caco-2 in order to identify CDX2-regulated genes involved in cellular   migration  . The engulfment and cell motility 3 (ELMO3) gene was identified as a potential CDX2 target gene. ELMO3 is an essential upstream regulator of the GTP-binding protein RAC during cell   migration  . However, no information is available about the transcriptional regulation of the ELMO3 gene. The aim of this study was to investigate the potential role of CDX2 in the regulation of the ELMO3 promoter activity. Electrophoretic mobility shift assays showed that CDX2 bound to conserved CDX2 sequences and mutations of the CDX2-binding sites, significantly reduced the promoter activity. Reporter gene assays demonstrated that the region mediating ELMO3 basal transcriptional activity to be located between -270 and -31 bp. Sequence analysis revealed no typical TATA-box, but four GC-rich sequences. In vitro analyses (electrophoretic mobility shift assays and promoter analyses) demonstrate that the SP1-binding sites are likely to play an important role in regulating the ELMO3 promoter activity. Furthermore, we showed here that CDX2 and SP1 can activate the ELMO3 promoter. Taken together, the present study reports the first characterization of the ELMO3 promoter and suggests a significant role of CDX2 in the basal transcriptional regulation of the intestine-specific expression of ELMO3, possibly through interaction with SP1. 
 Specificity protein 1 expression contributes to Bcl-w-induced aggressiveness in glioblastoma multiforme. We already had reported that Bcl-w promotes invasion or   migration   in gastric cancer cells and glioblastoma multiforme (GBM) by activating matrix metalloproteinase-2 (MMP-2) via specificity protein 1 (Sp1) or β-cateinin, respectively. High expression of Bcl-w also has been reported in GBM which is the most common malignant brain tumor and exhibits aggressive and invasive behavior. These reports propose that Bcl-w-induced signaling is strongly associated with aggressive characteristic of GBM. We demonstrated that Sp1 protein or mRNA expression is induced by Bcl-w using Western blotting or RT-PCR, respectively, and markedly elevated in high-grade glioma specimens compared with low-grade glioma tissues using tissue array. However, relationship between Bcl-w-related signaling and aggressive characteristic of GBM is poorly characterized. This study suggested that Bcl-w-induced Sp1 activation promoted expression of glioma stem-like cell markers, such as Musashi, Nanog, Oct4 and sox-2, as well as neurosphere formation and invasiveness, using western blotting, neurosphere formation assay, or invasion assay, culminating in their aggressive behavior. Therefore, Bcl-w-induced Sp1 activation is proposed as a putative marker for aggressiveness of GBM. 
 Prostaglandin E2 induces stromal cell-derived factor-1 expression in prostate stromal cells by activating protein kinase A and transcription factor Sp1. Recent reports indicate prostaglandin E2 (PGE2) can modulate tumor environment and promote angiogenesis through induction of stromal cell-derived factor 1 (SDF-1) production. We investigated the mechanism of PGE2-induced SDF-1 regulation in human prostate stromal cell and analyzed the effects in a stromal-epithelial interaction model. PGE2 stimulation increased SDF-1 expression in the prostate stromal cell lines WPMY-1 and NAF. We revealed signaling through the PGE2 receptor EP3 and activation of protein kinase A (PKA) are required. The EP3 agonist sulprostone and the cAMP analog forskolin mimicked and the EP3 siRNA, antagonist L798106 and the PKA inhibitor H89 abrogated the effect of PGE2 on SDF-1 expression. SDF-1 promoter truncation experiments demonstrated a 254 bp (from nt -219 to nt +34) SDF-1 proximal promoter fragment containing 5 putative transcription factor Sp1 binding motifs is sufficient for PGE2 induction. CHIP assays confirmed binding and PGE2 induced recruitment of Sp1 to the SDF-1 promoter. Sp1 motif mutation identified Sp1 motifs -140/-133 and -9/+1 as the crucial elements responsible for PGE2 induction. Moreover, SDF-1 was up- or down-regulated by Sp1 over-expression or knock-down. We also demonstrate stimulation of   migration   of prostate cancer cell lines PC3 and DU145 with conditioned media collected from WPMY-1 or NAF cells stimulated with PGE2 and blockade of enhanced   migration   by a SDF-1 neutralizing antibody. In conclusion, we provide evidence for a paracrine prostate stromal-epithelial interaction induced by upregulation of expression of SDF-1 by PGE2. Our research provides new insights into the mechanism promoting metastasis of prostate carcinoma via stromal-epithelial interaction. 
 Glyceraldehyde-3-phosphate dehydrogenase promotes cancer growth and metastasis through upregulation of SNAIL expression. Glyceraldehyde-3-phosphate dehydrogenase (GAPDH) plays an important role in multiple cellular functions including metabolism and gene transcription. Our previous study showed that GAPDH expression was elevated in colon cancer and further upregulated in liver metastatic tissues, suggesting a possilbe role of GAPDH in promoting cancer metastasis. The present study was designed to investigate the underlying mechanism, using multiple experimental approaches including genetic silencing of GAPDH expression by short hairpin RNA (shRNA) and biochemcial/molecular analyses of the key events involved in glycolytic metabolism and epithelial-mesenchymal transition (EMT). We showed that silencing of GAPDH expression resulted in a significant reduction of glycolysis in colon cancer cell lines, accompanied by a decrease in cell proliferation and an apparent change in cell morphology associated with alterations in actin expression and phalloidine staining patterns. Furthermore, GAPDH suppression also caused a downregulation of gene expression involved in cancer stem-like cells and EMT. CHIP assay and co-immunoprecipitation revealed that GAPDH physically interacted with the transcriptional factor Sp1 and enhance the expression of SNAIL, a major regulator of EMT. Suppression of GAPDH expression resulted in a signficant decrease in SNAIL expression, leading to inhibition of EMT and attenuation of colon cancer cell   migration   in vitro and reduced metastasis in vivo. Overall, the present study suggests that GAPDH plays an important role in cancer metastasis by affecting EMT through regulation of Sp1-mediated SNAIL expression. 
 JMJD2A-dependent silencing of Sp1 in advanced breast cancer promotes metastasis by downregulation of DIRAS3. Specificity protein 1(Sp1) is a ubiquitous transcription factor and is highly expressed in breast cancer. However, its expression pattern and role in breast cancer progression remain unclear. The purpose of this study is to examine the expression pattern of Sp1 and determine its role in breast cancer progression. Immunohistochemistry (IHC) was performed on breast cancer tissues to reveal the expression pattern of Sp1. Spearman rank correlation was used for clinical statistics. Gene and protein expressions were monitored by IHC analysis, quantitative polymerase chain reaction, and Western blot. Wound-healing and Transwell assays were conducted to assess the role of Sp1 in breast cancer. Co-immunoprecipitation, deletion mutagenesis, chromatin immunoprecipitation, and dual luciferase reporter gene assays were used for investigation of the regulatory network. Sp1 expression was downregulated in late stage breast cancer and in highly invasive breast cancer cell lines. Expression of Sp1 was negatively correlated with TNM staging (P = 0.002) and metastasis status (P = 0.023). Overexpression of Sp1 inhibited breast cancer cell migratory and invasive abilities, whereas knockdown of GTP-binding RAS-like 3 (DIRAS3, also known as ARHI, NOEY2) attenuated the inhibitory effects. Moreover, re-expression of DIRAS3 abolished Sp1 knockdown-mediated cell   migration   and invasion. Jumonji domain containing 2A (JMJD2A) inhibited Sp1 autoregulation and explains Sp1 expression pattern in breast cancer. Sp1 negatively regulated breast cancer metastasis by transcriptional activation of DIRAS3. Inhibition of Sp1 autoregulation by JMJD2A contributed to Sp1 expression pattern in breast cancer. Our findings provided evidence that targeted therapy against Sp1 might be useful in early stage breast cancer. However, in late stages, development of Sp1 activator may be more promising for breast cancer treatments. 
 Sp1-driven up-regulation of miR-19a decreases RHOB and promotes pancreatic cancer. Cancer treatment alters microRNA (miRNA) expression, revealing potential therapeutic targets (oncotarget). Here we treated pancreatic cancer (ASPC-1) cells with either recombinant human endostatin (rh-endostatin) or gemcitabine. Then high-throughput sequencing assay was performed to screen for altered miRNAs. Both treatments decreased levels of MiR-19a. We found that miR-19a stimulated cell proliferation,   migration  , invasion in vitro and tumor growth in vivo. High levels of miR-19a correlated with poor prognosis in patients. Ras homolog family member B (RHOB) was identified as a direct target of miR-19a. Furthermore, RHOB was down-regulated in human pancreatic cancer samples. Restoration of RHOB induced apoptosis, inhibited proliferation and   migration   of ASPC-1 cells. SP-1 was identified as an upstream transcription factor of miR-19a gene, promoting miR-19a transcription. Rh-endostatin decreased miR-19a expression by down-regulating SP-1. These findings suggest that miR-19a is a potential therapeutic target in pancreatic cancer. 
 miRNA-411 acts as a potential tumor suppressor miRNA via the downregulation of specificity protein 1 in breast cancer. The expression and functions of microRNA (miR)-411 have been investigated in several types of cancer. However, until now, miR-411 in human breast cancer has not been examined. The present study investigated the expression, biological functions and molecular mechanisms of miR‑411 in human breast cancer, discussing whether it offers potential as a therapeutic biomarker for breast cancer in the future. The expression levels of miR‑411 in human breast cancer tissues and cells were measured using reverse transcription‑quantitative polymerase chain reaction analysis. Following transfection with miR‑411 mimics, an MTT assa  y, cell m  igration and invasion assay, western blot analysis and luciferase assay were performed in human breast cancer cell lines. According to the results, it was found that miR‑411 was significantly downregulated in breast cancer, and associated with lymph node metastasis and histological grade. Additionally, it was observed that miR‑411 suppressed c  ell growt  h, migration and invasion in the breast cancer cells. The present study also provided the first evidence, to the best of our knowledge, that miR‑411 was likely to directly target specificity protein 1 in breast cancer. These findings indicated that miR‑411 may be used a therapeutic biomarker for the treatment of breast cancer in the future. 
 miR-29b suppresses proliferation,   migration  , and invasion of tongue squamous cell carcinoma through PTEN-AKT signaling pathway by targeting Sp1. miR-29b has been implicated in various cancers. However, the role of miR-29b in tongue squamous cell carcinoma (TSCC) remains unclear. This study aimed to investigate the role of miR-29b in TSCC progression.The expression of miR-29b was analyzed in TSCC tissues and cells. Functional studies were performed in TSCC cells. Real time-PCR, Western blot, cell proliferation, transwell, and dual luciferase reporter assays were performed according to standard procedures.miR-29b was significantly decreased in TSCC specimens and cell lines compared with corresponding normal counterparts. Overexpression of miR-29b significantly inhibited the proliferation,   migration  , invasion, and cell-cycle progression of TSCC cells, and promoted apoptosis. Moreover, miR-29b targeted the 3' untranslated region of the Sp1 transcript and resulted in the deregulation of Sp1. The inhibition of Sp1 by miR-29b subsequently resulted in the upregulation of PTEN, leading to a decline of phosphorylated AKT. Knockdown of Sp1 in TSCC cell lines mimicked the effects of miR-29b overexpression. In addition, the expression of miR-29b was inversely correlated with Sp1 and positively correlated with the PTEN in TSCC specimens.miR-29b functions as a tumor suppressor in TSCC, and the miR-29b/Sp1/PTEN/AKT axis might represent a potential therapeutic target for TSCC intervention. 
 The oncoprotein HBXIP up-regulates FGF4 through activating transcriptional factor Sp1 to promote the   migration   of breast cancer cells. We have reported that the oncoprotein hepatitis B X-interacting protein (HBXIP) is able to promote   migration   of breast cancer cells. Fibroblast growth factor 4 (FGF4) is a multipotent growth factor and is highly expressed in various human cancers. However, the regulatory mechanism of FGF4 in breast cancer remains poorly understood. In the present study, we report that HBXIP is able to up-regulate FGF4 to enhance the   migration   of breast cancer cells. Immunohistochemistry staining showed that HBXIP and FGF4 were highly expressed in clinical metastatic lymph nodes of breast tumor. The expression levels of HBXIP were positively related to those of FGF4 in clinical breast cancer tissues. Then, we validated that HBXIP up-regulated the expression of FGF4 at the levels of promoter, mRNA and protein by luciferase reporter gene assays, reverse transcription-polymerase chain reaction and Western blot analysis. Moreover, we found that HBXIP was able to activate FGF4 promoter through transcriptional factor Sp1 by luciferase reporter gene assays. Chromatin immunoprecipitation assays confirmed that HBXIP coactivated Sp1 to stimulate FGF4 promoter. In function, we showed that HBXIP promoted breast cancer cell   migration   through FGF4 by wound healing and transwell cell   migration   assays. Thus, we conclude that the oncoprotein HBXIP up-regulates FGF4 through activating transcriptional factor Sp1 to promote the   migration   of breast cancer cells. Therapeutically, HBXIP may serve as a novel target in breast cancer. 
 MiR-324-5p Suppresses Hepatocellular Carcinoma Cell Invasion by Counteracting ECM Degradation through Post-Transcriptionally Downregulating ETS1 and SP1. Hepatocellular carcinoma (HCC) is one of the common malignancies, which is highly metastatic and the third common cause of cancer deaths in the world. The invasion and metastasis of cancer cells is a multistep and complex process which is mainly initiated by extracellular matrix (ECM) degradation. Aberrant expression of microRNA has been investigated in HCC and shown to play essential roles during HCC progression. In the present study, we found that microRNA-324-5p (miR-324-5p) was downregulated in both HCC cell lines and tissues. Ectopic miR-324-5p led to the reduction of HCC cells invasive and metastatic capacity, whereas inhibition of miR-324-5p promoted the invasion of HCC cells. Matrix metalloproteinase 2 (MMP2) and MMP9, the major regulators of ECM degradation, were found to be downregulated by ectopic miR-324-5p, while upregulated by miR-324-5p inhibitor. E26 transformation-specific 1 (ETS1) and Specificity protein 1 (SP1), both of which could modulate MMP2 and MMP9 expression and activity, were presented as the direct targets of and downregulated by miR-324-5p. Downregulation of ETS1 and SP1 mediated the inhibitory function of miR-324-5p on HCC   migration   and invasion. Our study demonstrates that miR-324-5p suppresses hepatocellular carcinoma cell invasion and might provide new clues to invasive HCC therapy. 
 Mithramycin Depletes Specificity Protein 1 and Activates p53 to Mediate Senescence and Apoptosis of Malignant Pleural Mesothelioma Cells. Specificity protein 1 (SP1) is an oncogenic transcription factor overexpressed in various human malignancies. This study sought to examine SP1 expression in malignant pleural mesotheliomas (MPM) and ascertain the potential efficacy of targeting SP1 in these neoplasms.qRT-PCR, immunoblotting, and immunohistochemical techniques were used to evaluate SP1 expression in cultured MPM cells and MPM specimens and normal mesothelial cells/pleura. MTS, chemotaxis, soft agar, β-galactosidase, and Apo-BrdUrd techniques were used to assess proliferation,   migratio  n, clonogenicity, senescence, and apoptosis in MPM cells following SP1 knockdown, p53 overexpression, or mithramycin treatment. Murine subcutaneous and intraperitoneal xenograft models were used to examine effects of mithramycin on MPM growth in vivo. Microarray, qRT-PCR, immunoblotting, and chromatin immunoprecipitation techniques were used to examine gene expression profiles mediated by mithramycin and combined SP1 knockdown/p53 overexpression and correlate these changes with SP1 and p53 levels within target gene promoters.MPM cells and tumors exhibited higher SP1 mRNA and protein levels relative to control cells/tissues. SP1 knockdown significantly inhibited proliferation,   migratio  n, and clonogenicity of MPM cells. Mithramycin depleted SP1 and activated p53, dramatically inhibiting proliferation and clonogenicity of MPM cells. Intraperitoneal mithramycin significantly inhibited growth of subcutaneous MPM xenografts and completely eradicated mesothelioma carcinomatosis in 75% of mice. Mithramycin modulated genes mediating oncogene signaling, cell-cycle regulation, senescence, and apoptosis in vitro and in vivo. The growth-inhibitory effects of mithramycin in MPM cells were recapitulated by combined SP1 knockdown/p53 overexpression.These findings provide preclinical rationale for phase II evaluation of mithramycin in patients with mesothelioma. 
 Endothelial Aquaporin-1 (AQP1) Expression Is Regulated by Transcription Factor Mef2c. Aquaporin 1 (AQP1) is expressed in most microvasculature endothelial cells and forms water channels that play major roles in a variety of physiologic processes. This study aimed to delineate the transcriptional regulation of AQP1 by Mef2c in endothelial cells. Mef2c cooperated with Sp1 to activate human AQP1 transcription by binding to its proximal promoter in human umbilical cord vein endothelial cells (HUVEC). Over-expression of Mef2c, Sp1, or Mef2c/Sp1 increased HUVEC   migration   and tube-forming ability, which can be abolished AQP1 knockdown. These data indicate that AQP1 is a direct target of Mef2c in regulating angiogenesis and vasculogenesis of endothelial cells. 
 miR-22 is down-regulated in gastric cancer, and its overexpression inhibits cell   migration   and invasion via targeting transcription factor Sp1. Accumulating evidence has shown that microRNAs are involved in multiple processes in cancer development and progression. Recently, miR-22 has been identified as a tumor-suppressing microRNA in many human cancers. However, the specific function of miR-22 in gastric cancer is unclear at this point. In this study, we first measured miR-22 expression level in 30 pairs of gastric cancer and matched normal tissues, two normal and six gastric cancer cell lines by real-time quantitative RT-PCR. We found that the expression of miR-22 in gastric cancer tissues and cell lines was much lower than that in normal control, respectively. Transfection of miR-22 expression plasmid could significantly inhibit the cell   migration   and invasion in SGC-7901 and NCL-N87 gastric cancer cell lines. Moreover, we also showed that Sp1 was negatively regulated by miR-22 at the posttranscriptional level, via a specific target site within the 3'UTR by luciferase reporter assay. The expression of Sp1 was inversely correlated with miR-22 expression in gastric cancer tissues, and knockdown of Sp1 by siRNA inhibited cell malignant behaviors. Thus, our findings suggest that miR-22 acts as tumor suppressor by targeting the Sp1 gene and inhibiting gastric cancer cell   migration   and invasion. The findings of this study contribute to current understanding of the functions of miR-22 in gastric cancer. 
 Laminin reduces expression of the human alpha6 integrin subunit gene by altering the level of the transcription factors Sp1 and Sp3. Damage to the corneal epithelium results in the massive secretion of fibronectin (FN) shortly after corneal injury, which is later replaced by the secretion of laminin (LM). Laminin is recognized by receptors (alpha6beta1 and alpha6beta4) that belong to the integrin family. The authors characterized the regulatory influence exerted by laminin on the Sp1/Sp3-mediated alpha6 gene promoter function.Recombinant plasmids bearing the CAT reporter gene, fused to various segments from the alpha6 promoter, were transfected into rabbit corneal epithelial cells (RCECs) grown on untreated or on LM-coated culture plates or into Sp1-deficient Drosophila Schneider cells. Expression and DNA binding of Sp1/Sp3 was monitored with the use of Western blot and electrophoretic mobility shift assays (EMSAs), respectively. DNA target sites for Sp1/Sp3 in the alpha6 gene promoter were identified in vitro by DNAse I footprinting. Binding of Sp1 and of a few other transcription factors was also examined in vivo by chromatin immunoprecipitation (ChIP) assays. Expression of the alpha6 mRNA in RCECs grown on LM-coated culture plates was also assessed by Northern blot and RT-PCR analyses.Transfection experiments provided evidence that both Sp1 and Sp3 positively influence alpha6 promoter activity in Sp1/Sp3-deficient SL2 Schneider cells. Two GC-rich target sites for Sp1/Sp3 (a proximal and a distal site) were identified in the alpha6 promoter by DNAse I footprinting. Binding of Sp1/Sp3 was further validated in vivo by ChIP. Transfections conducted into RCECs grown on LM-coated culture plates resulted in repression of the activity directed by the alpha6 promoter. This LM-mediated negative regulatory influence also translated into a similar reduction in the expression of the endogenous alpha6 mRNA as revealed by both RT-PCR and Northern blot analyses. Most of all, results from EMSA and Western blot analyses suggested that the LM-mediated repression of the alpha6 promoter activity results in part from the proteolytic cleavage of Sp1/Sp3.Unlike FN, which functions as an activator of alpha5 gene transcription, LM repressed transcription, directed by the alpha6 gene promoter, by altering the nuclear levels of Sp1 and Sp3. Reappearance of LM in the basement membrane after repair of the corneal damage is therefore expected to substantially contribute to the final steps of this process by influencing the degree to which genes that encode protein products requested for cell adhesion and   migration  , such as integrin genes, are expressed during corneal wound healing. 
 Transcriptional repression of protein kinase Calpha via Sp1 by wild type p53 is involved in inhibition of multidrug resistance 1 P-glycoprotein phosphorylation. The protein kinase C (PKC) family consists of serine/threonine protein kinases that play important roles in signal transduction, cell proliferation, and tumor formation. Recent studies found that PKCs are commonly overexpressed in human tumors, including soft tissue sarcoma (STS). Overexpression of PKCs contributes to invasion and   migration   of tumor cells and induction of angiogenesis. PKC can also phosphorylate the multidrug resistance (MDR) gene-encoded P-glycoprotein and induce MDR phenotype. Our previous studies showed that mutation of p53 enhanced STS metastasis and mediated the MDR phenotype. Restoring wild type (WT) p53 in STS cells containing mutant p53 sensitized the cells to chemotherapy. In the present study, we found that PKCalpha protein expression is inhibited by WT p53 partly due to reduced PKCalpha mRNA expression in STS cells, but p53 does not affect PKCalpha mRNA stability. Deletion and mutation analysis of the PKCalpha promoter fused to the luciferase reporter gene identified a Sp1 binding site (-244/-234) in the PKCalpha promoter that is required for p53-mediated inhibition of PKCalpha promoter activity. More importantly, PKCalpha phosphorylates and activates MDR1 P-glycoprotein, whereas inhibition of PKCalpha by p53 leads to decreased MDR1 phosphorylation in STS cells, which sensitizes STS cells to chemotherapeutic agents. These data indicate that WT p53 may resensitize STS to chemotherapeutic agents by reducing MDR1 phosphorylation via transcriptional repression of PKCalpha expression. Thus, molecular-based therapies targeting mutant p53 and PKCalpha may be an effective new strategy to improve chemotherapeutic efficacy in STS. 
 Functional interaction of E1AF and Sp1 in glioma invasion. Transcription factor E1AF is widely known to play critical roles in tumor metastasis via directly binding to the promoters of genes involved in tumor   migration   and invasion. Here, we report for the first time E1AF as a novel binding partner for ubiquitously expressed Sp1 transcription factor. E1AF forms a complex with Sp1, contributes to Sp1 phosphorylation and transcriptional activity, and functions as a mediator between epidermal growth factor and Sp1 phosphorylation and activity. Sp1 functions as a carrier bringing E1AF to the promoter region, thus activating transcription of glioma-related gene for beta1,4-galactosyltransferase V (GalT V; EC 2.4.1.38). Biologically, E1AF functions as a positive invasion regulator in glioma in cooperation with Sp1 partly via up-regulation of GalT V. This report describes a new mechanism of glioma invasion involving a cooperative effort between E1AF and Sp1 transcription factors. 
 Transcription factor Sp1 is involved in expressional regulation of coxsackie and adenovirus receptor in cancer cells. Coxsackie and adenovirus receptor (CAR) was first known as a virus receptor. Recently, it is also known to have tumor suppressive activity such as inhibition of cell proliferation,   migration  , and invasion. It is important to understand how CAR expression can be regulated in cancers. Based on an existence of putative Sp1 binding site within CAR promoter, we investigated whether indeed Sp1 is involved in the regulation of CAR expression. We observed that deletion or mutation of Sp1 binding motif (-503/-498) prominently impaired the Sp1 binding affinity and activity of CAR promoter. Histone deacetylase inhibitor (TSA) treatment enhanced recruitment of Sp1 to the CAR promoter in ChIP assay. Meanwhile, Sp1 binding inhibitor suppressed the recruitment. Exogenous expression of wild-type Sp1 increased CAR expression in CAR-negative cells; meanwhile, dominant negative Sp1 decreased the CAR expression in CAR-positive cells. These results indicate that Sp1 is involved in regulation of CAR expression. 
 Maximal Expression of the Evolutionarily Conserved Slit2 Gene Promoter Requires Sp1. Slit2 is a neural axon guidance and chemorepellent protein that stimulates motility in a variety of cell types. The role of Slit2 in neural development and neoplastic growth and   migration   has been well established, while the genetic mechanisms underlying regulation of the Slit2 gene have not. We identified the core and proximal promoter of Slit2 by mapping multiple transcriptional start sites, analyzing transcriptional activity, and confirming sequence homology for the Slit2 proximal promoter among a number of species. Deletion series and transient transfection identified the Slit2 proximal promoter as within 399 base pairs upstream of the start of transcription. A crucial region for full expression of the Slit2 proximal promoter lies between 399 base pairs and 296 base pairs upstream of the start of transcription. Computer modeling identified three transcription factor-binding consensus sites within this region, of which only site-directed mutagenesis of one of the two identified Sp1 consensus sites inhibited transcriptional activity of the Slit2 proximal promoter (-399 to +253). Bioinformatics analysis of the Slit2 proximal promoter -399 base pair to -296 base pair region shows high sequence conservation over twenty-two species, and that this region follows an expected pattern of sequence divergence through evolution. 
  GeneRIFs  
 It was found that miR411 was significantly downregulated in breast cancer, and associated with lymph node metastasis and histological grade. Additionally, it was observed that miR411 suppressed cell growth,   migration   and invasion in the breast cancer cells. miRNA-411 acts as a potential tumor suppressor miRNA via the downregulation of specificity protein 1 in breast cancer. 
 MiR-375 inhibited the proliferation, invasion and   migration   by directly targeting SP1. 
 Thus, we conclude that the oncoprotein HBXIP up-regulates FGF4 through activating transcriptional factor Sp1 to promote the   migration   of breast cancer cells. Therapeutically, HBXIP may serve as a novel target in breast cancer. 
 ERK3 regulates endothelial cell   migration  , proliferation and tube formation by upregulating SRC-3/SP-1-mediated VEGFR2 expression. 
  ECM  PubMed  
 TGF-beta-induced expression of tissue inhibitor of metalloproteinases-3 gene in chondrocytes is mediated by extracellular signal-regulated kinase pathway and Sp1 transcription factor. Transforming growth factor (TGF-beta1) is a potent inducer of chondrogenesis and stimulant of cartilage extracellular matrix (  ECM  ) synthesis. Tissue inhibitor of metalloproteinases-3 (TIMP-3) is located in   ECM   and is the major inhibitor of matrix metalloproteinases (MMPs) and aggrecanase, the principal enzymes implicated in collagen and aggrecan degradation in arthritis. We investigated the role of extracellular-signal-regulated kinase (ERK)-mitogen-activated protein kinases (MAPK) and Sp1 transcription factor in TGF-beta-induced TIMP-3 gene in chondrocytes and chondrosarcoma cells. TGF-beta time-dependently induced a sustained phosphorylation of ERK-MAPKs in primary human or bovine chondrocytes. Inhibitors of this pathway, PD98059 and U0126, downregulated TGF-beta-induced expression of TIMP-3 RNA and protein. Since the ERKs can phosphorylate Sp1, and the promoter of human TIMP-3 gene contains four Sp1-binding sites, we investigated whether Sp1 is a downstream target of this pathway. Mithramycin and WP631, the agents that prevent binding of Sp1 to its consensus site, downregulated TGF-beta-inducible TIMP-3 expression. Indeed, mithramycin blocked TGF-beta-stimulated Sp1 binding activity. Transfection of cytomegalovirus (CMV) promoter-Sp1 plasmid increased TIMP-3 promoter (-940 to +376)-driven luciferase activity. Depletion of Sp1 by transfection of an antisense phosphorothioate oligonucleotide suppressed TGF-beta-induced TIMP-3 protein expression, while its sense homolog had no effect. These results suggest that activation of ERK-MAPK pathway and Sp1 transcription factor play a pivotal role in the induction of TIMP-3 by TGF-beta in chondrocytes. 
 TGF-beta-induced expression of tissue inhibitor of metalloproteinases-3 gene in chondrocytes is mediated by extracellular signal-regulated kinase pathway and Sp1 transcription factor. Transforming growth factor (TGF-beta1) is a potent inducer of chondrogenesis and stimulant of cartilage extracellular matrix (  ECM  ) synthesis. Tissue inhibitor of metalloproteinases-3 (TIMP-3) is located in   ECM   and is the major inhibitor of matrix metalloproteinases (MMPs) and aggrecanase, the principal enzymes implicated in collagen and aggrecan degradation in arthritis. We investigated the role of extracellular-signal-regulated kinase (ERK)-mitogen-activated protein kinases (MAPK) and Sp1 transcription factor in TGF-beta-induced TIMP-3 gene in chondrocytes and chondrosarcoma cells. TGF-beta time-dependently induced a sustained phosphorylation of ERK-MAPKs in primary human or bovine chondrocytes. Inhibitors of this pathway, PD98059 and U0126, downregulated TGF-beta-induced expression of TIMP-3 RNA and protein. Since the ERKs can phosphorylate Sp1, and the promoter of human TIMP-3 gene contains four Sp1-binding sites, we investigated whether Sp1 is a downstream target of this pathway. Mithramycin and WP631, the agents that prevent binding of Sp1 to its consensus site, downregulated TGF-beta-inducible TIMP-3 expression. Indeed, mithramycin blocked TGF-beta-stimulated Sp1 binding activity. Transfection of cytomegalovirus (CMV) promoter-Sp1 plasmid increased TIMP-3 promoter (-940 to +376)-driven luciferase activity. Depletion of Sp1 by transfection of an antisense phosphorothioate oligonucleotide suppressed TGF-beta-induced TIMP-3 protein expression, while its sense homolog had no effect. These results suggest that activation of ERK-MAPK pathway and Sp1 transcription factor play a pivotal role in the induction of TIMP-3 by TGF-beta in chondrocytes. 
 Targeting of Sp1 transcription factor: a novel therapeutic approach for keloids, an in vitro analysis. Keloid scars are fibroproliferative disorders characterized by the accumulation of extracellular matrix (  ECM  ) components resulting in a fibrotic condition. Several   ECM   promoters are regulated by Sp1. Thus, our aim was to investigate the role of Sp1 in keloid pathogenesis and investigate the antiproliferative and antifibrotic effects of Wp631 and mitoxantrone, potent inhibitors of Sp1-activated transcription. An elevated level of Sp1 was observed in tissue extracts obtained from keloid tissue. Serum stimulation elevated Sp1 levels in keloid fibroblasts (KF). Under coculture conditions Sp1 seemed to be downregulated. Wp631 and mitoxanthrone in serum growth factors resulted in a reduced expression of   ECM   components in KF. Both Wp631 and mitoxanthrone were also able to inhibit the proliferation of normal and keloid keratinocytes and fibroblasts significantly. As Wp631 seems to be potent in downregulating the   ECM   components in KF and also inhibiting the proliferation of these cells it could be explored as a possible therapeutic agent in the treatment of keloids. 
 Sp1 downregulates proinflammatory cytokine‑induced catabolic gene expression in nucleus pulposus cells. During the pathogenesis of intervertebral disc degeneration, pro‑inflammatory cytokines, including tumor necrosis factor‑α (TNF‑α), stimulate the degradation of the extracellula  r m  atrix (ECM) of intervertebral discs via the activity of catabolic enzymes including matrix metalloproteinases (MMPs), disintegrins and metalloproteinases with thrombospondin motifs (ADAMTSs), and cyclooxygenase 2 (Cox2). The transcriptional promoters of the human catabolic enzymes MMPs, ADAMTS, Cox2 and Syndecan 4 contain at least one specificity protein‑1 (Sp1) transcription factor‑binding site. The present study investigated the role of Sp1 in the regulation of the mRNA and protein expression of the aforementioned catabolic enzyme genes in nucleus pulposus cells, using reverse transcription‑quantitative polymerase chain reaction, western blot, transfection and RNA interference. The data demonstrated that Sp1 transcription factor protein expression is induced by TNF‑α and interleukin‑1β. Specific inhibitors of Sp1 DNA binding to its GC‑rich consensus site, WP631 and mithramycin A, partially suppressed TNF‑α‑induced catabolic enzyme expression and activity. Genetic inhibition of Sp1 by small interfering RNA‑mediated Sp1 knockdown partially inhibited catabolic enzyme induction by TNF‑α. In addition, Sp1 transcription factor inhibitors decreased the activity of MMP3, ADAMTS4 and ADAMTS5 promoters. Furthermore, chromatin immunoprecipitation revealed functional Sp1 binding sites at ‑577/‑567 bp within the ADAMTS4 promoter and ‑718/‑708 bp within the ADAMTS5 promoter. These results provide pharmacological and genetic evidence of the importance of Sp1 in catabolic enzyme gene regulation during TNF‑α stimulation. Thus, Sp1 may represent an effective t  arg  et in reducing intervertebral disc‑associated ECM loss. 
 Accessible chromatin structure permits factors Sp1 and Sp3 to regulate human TGFBI gene expression. Transforming growth factor beta 1-induced (TGFBI) protein is an extracellular matrix (  ECM  ) protein that is associated with other   ECM   proteins and functions as a ligand for various types of integrins. In this study, we investigated how human TGFBI expression is regulated in lung and breast cancer cells. We observed that the TGFBI promoter in A549 and MBA-MD-231 cells, which constitutively express TGFBI, existed in an open chromatin conformation associated with transcriptionally permissive histone modifications. Moreover, we found that TGFBI expression required Sp1 transcription elements that can bind transcription factors Sp1 and Sp3 in vitro. Occupancy of the TGFBI promoter by Sp1 and Sp3 in vivo was only observed in TGFBI-expressing cells, indicating that open chromatin conformation might facilitate the binding of Sp1 and Sp3 to the TGFBI promoter region. TGFBI promoter activity was impaired when Sp1 elements were mutated, but was increased when Sp1 or Sp3 factors was overexpressed. Furthermore, Sp1 inhibition in vivo by mithramycin A, as well as knockdown of Sp1 and/or Sp3 expression by short interfering RNA, significantly reduced TGFBI mRNA and protein levels. Thus, our data demonstrated that the expression of TGFBI is well correlated with chromatin conformation at the TGFBI promoter, and that factors Sp1 and Sp3 are the primary determinants for the control of constitutive expression of TGFBI gene. 
 MiR-324-5p Suppresses Hepatocellular Carcinoma Cell Invasion by Counteracting   ECM   Degradation through Post-Transcriptionally Downregulating ETS1 and SP1. Hepatocellular carcinoma (HCC) is one of the common malignancies, which is highly metastatic and the third common cause of cancer deaths in the world. The invasion and metastasis of cancer cells is a multistep and complex process which is mainly initiated by extracellular matrix (  ECM  ) degradation. Aberrant expression of microRNA has been investigated in HCC and shown to play essential roles during HCC progression. In the present study, we found that microRNA-324-5p (miR-324-5p) was downregulated in both HCC cell lines and tissues. Ectopic miR-324-5p led to the reduction of HCC cells invasive and metastatic capacity, whereas inhibition of miR-324-5p promoted the invasion of HCC cells. Matrix metalloproteinase 2 (MMP2) and MMP9, the major regulators of   ECM   degradation, were found to be downregulated by ectopic miR-324-5p, while upregulated by miR-324-5p inhibitor. E26 transformation-specific 1 (ETS1) and Specificity protein 1 (SP1), both of which could modulate MMP2 and MMP9 expression and activity, were presented as the direct targets of and downregulated by miR-324-5p. Downregulation of ETS1 and SP1 mediated the inhibitory function of miR-324-5p on HCC migration and invasion. Our study demonstrates that miR-324-5p suppresses hepatocellular carcinoma cell invasion and might provide new clues to invasive HCC therapy. 
  [Ee]xtracellular [Mm]atrix  PubMed  
 NF-kappaB induced by IL-1beta inhibits elastin transcription and myofibroblast phenotype. Interleukin (IL)-1beta released after lung injury regulates the production of   extracellular matrix   components. We found that IL-1beta treatment reduced the rate of elastin gene transcription by 74% in neonatal rat lung fibroblasts. Deletion analysis of the rat elastin promoter detected a cis-acting element located at -118 to -102 bp that strongly bound Sp1 and Sp3 but not nuclear factor (NF)-kappaB. This element mediated IL-1beta-induced inhibition of the elastin promoter. IL-1beta treatment did not affect the level of Sp1 but did induce translocation of the p65 subunit of NF-kappaB. Overexpression of p65 decreased elastin promoter activity and markedly reduced elastin mRNA. Immunoprecipitation studies indicated an interaction between the p65 subunit and Sp1 protein. Microarray analysis of mRNA isolated after overexpression of p65 or treatment with IL-1beta revealed downregulation of alpha-smooth muscle actin and calponin mRNAs. Expression of these genes is associated with the myofibroblast phenotype. These results indicate that IL-1beta activates the nuclear localization of NF-kappaB that subsequently interacts with Sp1 to downregulate elastin transcription and expression of the myofibroblast phenotype. 
 TGF-beta-induced expression of tissue inhibitor of metalloproteinases-3 gene in chondrocytes is mediated by extracellular signal-regulated kinase pathway and Sp1 transcription factor. Transforming growth factor (TGF-beta1) is a potent inducer of chondrogenesis and stimulant of cartilage   extracellular matrix   (ECM) synthesis. Tissue inhibitor of metalloproteinases-3 (TIMP-3) is located in ECM and is the major inhibitor of matrix metalloproteinases (MMPs) and aggrecanase, the principal enzymes implicated in collagen and aggrecan degradation in arthritis. We investigated the role of extracellular-signal-regulated kinase (ERK)-mitogen-activated protein kinases (MAPK) and Sp1 transcription factor in TGF-beta-induced TIMP-3 gene in chondrocytes and chondrosarcoma cells. TGF-beta time-dependently induced a sustained phosphorylation of ERK-MAPKs in primary human or bovine chondrocytes. Inhibitors of this pathway, PD98059 and U0126, downregulated TGF-beta-induced expression of TIMP-3 RNA and protein. Since the ERKs can phosphorylate Sp1, and the promoter of human TIMP-3 gene contains four Sp1-binding sites, we investigated whether Sp1 is a downstream target of this pathway. Mithramycin and WP631, the agents that prevent binding of Sp1 to its consensus site, downregulated TGF-beta-inducible TIMP-3 expression. Indeed, mithramycin blocked TGF-beta-stimulated Sp1 binding activity. Transfection of cytomegalovirus (CMV) promoter-Sp1 plasmid increased TIMP-3 promoter (-940 to +376)-driven luciferase activity. Depletion of Sp1 by transfection of an antisense phosphorothioate oligonucleotide suppressed TGF-beta-induced TIMP-3 protein expression, while its sense homolog had no effect. These results suggest that activation of ERK-MAPK pathway and Sp1 transcription factor play a pivotal role in the induction of TIMP-3 by TGF-beta in chondrocytes. 
 TGF-beta-induced expression of tissue inhibitor of metalloproteinases-3 gene in chondrocytes is mediated by extracellular signal-regulated kinase pathway and Sp1 transcription factor. Transforming growth factor (TGF-beta1) is a potent inducer of chondrogenesis and stimulant of cartilage   extracellular matrix   (ECM) synthesis. Tissue inhibitor of metalloproteinases-3 (TIMP-3) is located in ECM and is the major inhibitor of matrix metalloproteinases (MMPs) and aggrecanase, the principal enzymes implicated in collagen and aggrecan degradation in arthritis. We investigated the role of extracellular-signal-regulated kinase (ERK)-mitogen-activated protein kinases (MAPK) and Sp1 transcription factor in TGF-beta-induced TIMP-3 gene in chondrocytes and chondrosarcoma cells. TGF-beta time-dependently induced a sustained phosphorylation of ERK-MAPKs in primary human or bovine chondrocytes. Inhibitors of this pathway, PD98059 and U0126, downregulated TGF-beta-induced expression of TIMP-3 RNA and protein. Since the ERKs can phosphorylate Sp1, and the promoter of human TIMP-3 gene contains four Sp1-binding sites, we investigated whether Sp1 is a downstream target of this pathway. Mithramycin and WP631, the agents that prevent binding of Sp1 to its consensus site, downregulated TGF-beta-inducible TIMP-3 expression. Indeed, mithramycin blocked TGF-beta-stimulated Sp1 binding activity. Transfection of cytomegalovirus (CMV) promoter-Sp1 plasmid increased TIMP-3 promoter (-940 to +376)-driven luciferase activity. Depletion of Sp1 by transfection of an antisense phosphorothioate oligonucleotide suppressed TGF-beta-induced TIMP-3 protein expression, while its sense homolog had no effect. These results suggest that activation of ERK-MAPK pathway and Sp1 transcription factor play a pivotal role in the induction of TIMP-3 by TGF-beta in chondrocytes. 
 Role of Sp1 transcription factor in Interleukin-1-induced ADAMTS-4 (aggrecanase-1) gene expression in human articular chondrocytes. Proinflammatory cytokines such as interleukin-1 beta (IL-1β) stimulate cartilage   extracellular matri  x aggrecan degradation by aggrecanases or ADAMTS (a disintegrin and metalloproteinase with thrombospondin motif) during the pathogenesis of arthritis. Human aggrecanase-1 (ADAMTS-4) gene promoter contains at least one specificity protein-1 (Sp1)-transcription factor-binding site. We investigated the previously unknown role of Sp1 in the regulation of ADAMTS-4 gene expression in human articular chondrocytes. Mithramycin and WP631, the specific inhibitors of guanine cytosine (GC)-rich Sp1 DNA binding, partially suppressed IL-1-induced ADAMTS-4 expression and activity. Genetic inhibition of Sp1 by antisense oligonucleotide or by small interfering RNA (siRNA)-mediated Sp1 knockdown partially inhibited ADAMTS-4 induction by IL-1. Sense oligonucleotide and negative control siRNA had no effect. In contrast, cytomegalovirus promoter-driven Sp1 overexpression further enhanced IL-1-induced ADAMTS-4 expression and activity. Constitutively expressed glyceraldehyde-3-phosphate dehydrogenase (GAPDH) was not affected by any of the agents. These results provide pharmacological and genetic evidence for the importance of Sp1 in ADAMTS-4 gene regulation by IL-1. Thus, Sp1 could be potentially targeted to reduce arthritis-associated cartilage aggrecan loss. 
 Targeting of Sp1 transcription factor: a novel therapeutic approach for keloids, an in vitro analysis. Keloid scars are fibroproliferative disorders characterized by the accumulation of   extracellular matrix   (ECM) components resulting in a fibrotic condition. Several ECM promoters are regulated by Sp1. Thus, our aim was to investigate the role of Sp1 in keloid pathogenesis and investigate the antiproliferative and antifibrotic effects of Wp631 and mitoxantrone, potent inhibitors of Sp1-activated transcription. An elevated level of Sp1 was observed in tissue extracts obtained from keloid tissue. Serum stimulation elevated Sp1 levels in keloid fibroblasts (KF). Under coculture conditions Sp1 seemed to be downregulated. Wp631 and mitoxanthrone in serum growth factors resulted in a reduced expression of ECM components in KF. Both Wp631 and mitoxanthrone were also able to inhibit the proliferation of normal and keloid keratinocytes and fibroblasts significantly. As Wp631 seems to be potent in downregulating the ECM components in KF and also inhibiting the proliferation of these cells it could be explored as a possible therapeutic agent in the treatment of keloids. 
 Constitutive connective tissue growth factor expression in scleroderma fibroblasts is dependent on Sp1. Fibrotic diseases such as scleroderma (systemic sclerosis, SSc) are characterized by an excessive production of   extracellular matrix   and profibrotic proteins such as connective tissue growth factor (CTGF). In normal dermal fibroblasts, CTGF is not expressed unless induced by proteins such as tumor growth factor-beta (TGFbeta). Conversely, in fibroblasts cultured from fibrotic lesions CTGF mRNA and protein are constitutively expressed, even in the absence of exogenously added TGFbeta. Thus, studying the mechanism underlying CTGF overexpression in SSc fibroblasts is likely to yield valuable insights into the basis of the fibrotic phenotype of SSc and possibly other scarring disease. CTGF overexpression is mediated primarily by sequences in the CTGF promoter. In this report, we identify the minimal promoter element involved with the overexpression of CTGF in SSc fibroblasts. This element is distinct from the element necessary and sufficient for the induction of CTGF expression by TGFbeta in normal fibroblasts. Within this region is a functional Sp1 binding site. Blocking Sp1 activity reduces the elevated, constitutive levels of CTGF promoter activity and protein expression observed in SSc fibroblasts. Relative to those prepared from normal dermal fibroblasts, nuclear extracts prepared from SSc fibroblasts possess increased Sp1 binding activity. Removal of phosphate groups from nuclear extracts enhanced Sp1 binding activity, suggesting that phosphorylation of Sp1 normally reduces Sp1 binding to DNA. Thus, the constitutive overexpression of CTGF in SSc fibroblasts seems to be independent of TGFbeta signaling but dependent at least in part on Sp1. 
 17β-estradiol enhances α(5) integrin subunit gene expression through ERα-Sp1 interaction and reduces cell motility and invasion of ERα-positive breast cancer cells. In breast tumors the expression of estrogen receptor alpha (ERα) is known to be associated with a more favorable prognosis. ERα expression has been reported to reduce the metastatic potential of breast cancer cells. Recently, we have observed   that extracellular   matrix proteins activate ERα and that both liganded and unliganded receptor modulate cell invasiveness acting at nuclear level. To explain the mechanisms by which ERα regulates cell adhesion, we have evaluated the expression of α(5)β(1) integrin, prevalently expressed in stationary cells, in response to 17β-estradiol (E2). Here we show that E2/ERα increases the expression of integrin α(5)β(1) through Sp1-mediated binding to a GC-rich region located upstream of an ERE half-site in the 5' flanking region of the α(5) gene forming a ternary ERα-Sp1-DNA complex. Estrogen responsiveness of the α(5) gene promoter, as observed in HeLa cells, underlies a general mechanism of regulation which is not strictly linked to the cell type. Our data reveal novel insight into the molecular mechanisms sustaining the reduced invasiveness of ERα expressing cells demonstrating that α(5)β(1) integrin expression is related to the maintenance of the stationary status of the cells, counteracting E2/ERα capability to enhance breast cancer cell migration and invasion. 
 Sp1 family of transcription factors regulates the human alpha2 (XI) collagen gene (COL11A2) in Saos-2 osteoblastic cells. Genes encoding type XI collagen, normally associated with chondrogenesis, are also expressed by osteoblasts. By studying Saos-2 cells, we showed that the transcription factors, Sp1, Sp3, and Sp7 (Osterix), regulate COL11A2 expression through its proximal promoter. The findings indicate both ubiquitous and osteoblast-specific mechanisms of collagen gene regulation.Type XI collagen is essential for skeletal morphogenesis. Collagen XI gene regulation has been studied in chondrocytes but not in osteoblasts.We cultured Saos-2 cells, a human osteosarcoma-derived line of osteoblasts, and analyzed them for alpha2(XI) protein and COL11A2 regulatory mechanisms.Although types I and V were the dominant collagens deposited by Saos-2 cells, they expressed COL11A2 mRNA, and alpha2(XI) chains were present in the   extracellular matrix  . The COL11A2 promoter region (from -149 to -40) containing three Sp1 binding sites was required for promoter activity in transient transfection assays. All three Sp1 sites were critical for binding by nuclear proteins in electrophoretic mobility shift assays. Further analysis using consensus oligonucleotides and specific antibodies as well as chromatin immunoprecipitation assay implicated Sp1 and Sp3 in binding to this promoter region. Overexpressing Sp1 or Sp3 significantly increased COL11A2 promoter activity and endogenous COL11A2 gene expression, an effect that was suppressed by the Sp1-binding inhibitor mithramycin A. Further experiments showed that Sp1, Sp3, CREB-binding protein (CBP), p300, and histone deacetylase (HDAC) were physically associated and HDAC inhibitors (trichostatin A or NaB) upregulated COL11A2 promoter activity and endogenous gene expression. Another Sp1 family member, Sp7 (Osterix), was expressed in Saos-2 cells, but not in chondrocytes, and was shown by chromatin immunoprecipitation to occupy the COL11A2 promoter. Overexpressing Sp7 increased COL11A2 promoter activity and endogenous gene expression, an effect also blocked by mithramycin A. Using siRNA to knockdown Sp1, Sp3, or Sp7, it was shown that depression of any of them decreased COL11A2 promoter activity and endogenous gene expression. Finally, primary cultures of osteoblasts expressed COL11A2 and Sp7, upregulated COL11A2 promoter activity and endogenous gene expression when Sp1, Sp3, or Sp7 were overexpressed, and downregulated them when Sp1, Sp3, or Sp7 were selectively depressed. The results establish that Sp1 proteins regulate COL11A2 transcription by binding to its proximal promoter and directly interacting with CBP, p300, and HDAC. 
 Cellular content of UDP-N-acetylhexosamines controls hyaluronan synthase 2 expression and correlates with O-linked N-acetylglucosamine modification of transcription factors YY1 and SP1. Hyaluronan, a high molecular mass polysaccharide on the vertebrate cell surface and   extracellular matrix  , is produced at the plasma membrane by hyaluronan synthases using UDP-GlcNAc and UDP-GlcUA as substrates. The availability of these UDP-sugar substrates can limit the synthesis rate of hyaluronan. In this study, we show that the cellular level of UDP-HexNAc also controls hyaluronan synthesis by modulating the expression of HAS2 (hyaluronan synthase 2). Increasing UDP-HexNAc in HaCaT keratinocytes by adding glucosamine down-regulated HAS2 gene expression, whereas a decrease in UDP-HexNAc, realized by mannose treatment or siRNA for GFAT1 (glutamine:fructose-6-phosphate amidotransferase 1), enhanced expression of the gene. Tracing the UDP-HexNAc-initiated signal to the HAS2 promoter revealed no change in the binding of STAT3, NF-κB, and cAMP response element-binding protein, shown previously to mediate growth factor and cytokine signals on HAS2 expression. Instead, altered binding of SP1 and YY1 to the promoter correlated with cellular UDP-HexNAc content and inhibition of HAS2 expression. siRNA silencing of YY1 and SP1 confirmed their inhibitory effects on HAS2 expression. Reduced and increased levels of O-GlcNAc-modified SP1 and YY1 proteins were associated with stimulation or inhibition of HAS2 expression, respectively. Our data are consistent with the hypothesis that, by regulating the level of protein O-GlcNAc modifications, cellular UDP-HexNAc content controls HAS2 transcription and decreases the effects on hyaluronan synthesis that would result from cellular fluctuations of this substrate. 
 Sp1 downregulates proinflammatory cytokine‑induced catabolic gene expression in nucleus pulposus cells. During the pathogenesis of intervertebral disc degeneration, pro‑inflammatory cytokines, including tumor necrosis factor‑α (TNF‑α), stimulate the degradati  on of the extracellu  lar matrix (ECM) of intervertebral discs via the activity of catabolic enzymes including matrix metalloproteinases (MMPs), disintegrins and metalloproteinases with thrombospondin motifs (ADAMTSs), and cyclooxygenase 2 (Cox2). The transcriptional promoters of the human catabolic enzymes MMPs, ADAMTS, Cox2 and Syndecan 4 contain at least one specificity protein‑1 (Sp1) transcription factor‑binding site. The present study investigated the role of Sp1 in the regulation of the mRNA and protein expression of the aforementioned catabolic enzyme genes in nucleus pulposus cells, using reverse transcription‑quantitative polymerase chain reaction, western blot, transfection and RNA interference. The data demonstrated that Sp1 transcription factor protein expression is induced by TNF‑α and interleukin‑1β. Specific inhibitors of Sp1 DNA binding to its GC‑rich consensus site, WP631 and mithramycin A, partially suppressed TNF‑α‑induced catabolic enzyme expression and activity. Genetic inhibition of Sp1 by small interfering RNA‑mediated Sp1 knockdown partially inhibited catabolic enzyme induction by TNF‑α. In addition, Sp1 transcription factor inhibitors decreased the activity of MMP3, ADAMTS4 and ADAMTS5 promoters. Furthermore, chromatin immunoprecipitation revealed functional Sp1 binding sites at ‑577/‑567 bp within the ADAMTS4 promoter and ‑718/‑708 bp within the ADAMTS5 promoter. These results provide pharmacological and genetic evidence of the importance of Sp1 in catabolic enzyme gene regulation during TNF‑α stimulation. Thus, Sp1 may represent an effective target in reducing intervertebral disc‑associated ECM loss. 
 Synergistic activation of the rat laminin gamma1 chain promoter by the gut-enriched Kruppel-like factor (GKLF/KLF4) and Sp1. Laminin is a multifunctional heterotrimeric protein present in   extracellular matrix   where it regulates processes that compose tissue architecture including cell differentiation. Laminin gamma1 is the most widely expressed laminin chain and its absence causes early lethality in mouse embryos. Laminin gamma1 chain gene (LAMC1) promoter contains several GC/GT-rich motifs including the bcn-1 element. Using the bcn-1 element as a bait in the yeast one-hybrid screen, we cloned the gut-enriched Kruppel-like factor (GKLF or KLF4) from a rat mesangial cell library. We show that GKLF binds bcn-1, but this binding is not required for the GKLF-mediated activation of the LAMC1 promoter. The activity of GKLF is dependent on a synergism with another Kruppel-like factor, Sp1. The LAMC1 promoter appears to have multiple GKLF- and Sp1-responsive elements which may account for the synergistic activation. We provide evidence that the synergistic action of GKLF and Sp1 is dependent on the promoter context and the integrity of GKLF activation and DNA-binding domain. GKLF is thought to participate in the switch from cell proliferation to differentiation. Thus, the Sp1-GKLF synergistic activation of the LAMC1 promoter may be one of the avenues for expression of laminin gamma1 chain when laminin is needed to regulate cell differentiation. 
 Accessible chromatin structure permits factors Sp1 and Sp3 to regulate human TGFBI gene expression. Transforming growth factor beta 1-induced (TGFBI) protein is an   extracellular matrix   (ECM) protein that is associated with other ECM proteins and functions as a ligand for various types of integrins. In this study, we investigated how human TGFBI expression is regulated in lung and breast cancer cells. We observed that the TGFBI promoter in A549 and MBA-MD-231 cells, which constitutively express TGFBI, existed in an open chromatin conformation associated with transcriptionally permissive histone modifications. Moreover, we found that TGFBI expression required Sp1 transcription elements that can bind transcription factors Sp1 and Sp3 in vitro. Occupancy of the TGFBI promoter by Sp1 and Sp3 in vivo was only observed in TGFBI-expressing cells, indicating that open chromatin conformation might facilitate the binding of Sp1 and Sp3 to the TGFBI promoter region. TGFBI promoter activity was impaired when Sp1 elements were mutated, but was increased when Sp1 or Sp3 factors was overexpressed. Furthermore, Sp1 inhibition in vivo by mithramycin A, as well as knockdown of Sp1 and/or Sp3 expression by short interfering RNA, significantly reduced TGFBI mRNA and protein levels. Thus, our data demonstrated that the expression of TGFBI is well correlated with chromatin conformation at the TGFBI promoter, and that factors Sp1 and Sp3 are the primary determinants for the control of constitutive expression of TGFBI gene. 
 Myocardin-related transcription factor-A complexes activate type I collagen expression in lung fibroblasts. Pulmonary fibrosis is characterized by the excessive deposition of a collagen-rich   extracellular matrix  . The accumulation of collagen within the lung interstitium leads to impaired respiratory function. Furthermore, smooth muscle actin-positive myofibroblasts within the fibrotic lung contribute to disease progression. Because collagen and smooth muscle cell α-actin are coordinately expressed in the setting of fibrosis, the hypothesis was tested that specific transcriptional regulators of the myocardin family might also regulate collagen gene expression in myofibroblasts. Myocardin-related transcription factors (MRTFs), through their interaction with the serum-response factor (SRF) on CArG box regulatory elements (CC(A/T)6GG), are important regulators of myofibroblast differentiation. MRTF-A transactivated type I collagen gene reporters as much as 100-fold in lung myofibroblasts. Loss of functional MRTF-A using either a dominant negative MRTF-A isoform, shRNA targeting MRTF-A, or genetic deletion of MRTF-A in lung fibroblasts significantly disrupted type I collagen synthesis relative to controls. Analysis of the COL1A2 proximal promoter revealed a noncanonical CArG box (CCAAACTTGG), flanked by several Sp1 sites important for MRTF-A activation. Chromatin immunoprecipitation experiments confirmed the co-localization of MRTF-A, SRF, and Sp1 bound to the same region of the COL1A2 promoter. Mutagenesis of either the noncanonical CArG box or the Sp1 sites significantly disrupted MRTF-A activation of COL1A2. Together, our findings show that MRTF-A is an important regulator of collagen synthesis in lung fibroblasts and exhibits a dependence on both SRF and Sp1 function to enhance collagen expression. 
 MiR-324-5p Suppresses Hepatocellular Carcinoma Cell Invasion by Counteracting ECM Degradation through Post-Transcriptionally Downregulating ETS1 and SP1. Hepatocellular carcinoma (HCC) is one of the common malignancies, which is highly metastatic and the third common cause of cancer deaths in the world. The invasion and metastasis of cancer cells is a multistep and complex process which is mainly initiated by   extracellular matrix   (ECM) degradation. Aberrant expression of microRNA has been investigated in HCC and shown to play essential roles during HCC progression. In the present study, we found that microRNA-324-5p (miR-324-5p) was downregulated in both HCC cell lines and tissues. Ectopic miR-324-5p led to the reduction of HCC cells invasive and metastatic capacity, whereas inhibition of miR-324-5p promoted the invasion of HCC cells. Matrix metalloproteinase 2 (MMP2) and MMP9, the major regulators of ECM degradation, were found to be downregulated by ectopic miR-324-5p, while upregulated by miR-324-5p inhibitor. E26 transformation-specific 1 (ETS1) and Specificity protein 1 (SP1), both of which could modulate MMP2 and MMP9 expression and activity, were presented as the direct targets of and downregulated by miR-324-5p. Downregulation of ETS1 and SP1 mediated the inhibitory function of miR-324-5p on HCC migration and invasion. Our study demonstrates that miR-324-5p suppresses hepatocellular carcinoma cell invasion and might provide new clues to invasive HCC therapy. 
 Sp1 and Sp3 mediate constitutive transcription of the human hyaluronan synthase 2 gene. The linear glycosaminoglycan hyaluronan (HA) is synthesized at the plasma membrane by the HA synthase (HAS) enzymes HAS1, -2, and -3 and performs multiple functions as part of the vertebrate   extracellular matrix  . Up-regulation of HA synthesis in the renal corticointerstitium, and the resultant   extracellular matrix   expansion, is a common feature of renal fibrosis. However, the regulation of expression of these HAS isoforms at transcriptional and translational levels is poorly understood. We have recently described the genomic structures of the human HAS genes, thereby identifying putative promoter regions for each isoform. Further analysis of the HAS2 gene identified the transcription initiation site and showed that region F3, comprising the proximal 121 bp of promoter sequence, mediated full constitutive transcription. In the present study, we have analyzed this region in the human renal proximal tubular epithelial cell line HK-2. Electrophoretic mobility shift and promoter assay data demonstrated that transcription factors Sp1 and Sp3 bound to three sites immediately upstream of the HAS2 transcription initiation site and that mutation of the consensus recognition sequences within these sites ablated their transcriptional response. Furthermore, subsequent knockdown of Sp1 or Sp3 using small interfering RNAs decreased constitutive HAS2 mRNA synthesis. In contrast, significant binding of HK-2 nuclear proteins by putative upstream NF-Y, CCAAT, and NF-kappaB recognition sites was not observed. The identification of Sp1 and Sp3 as principal mediators of HAS2 constitutive transcription augments recent findings identifying upstream promoter elements and provides further insights into the mechanism of HAS2 transcriptional activation. 
 Modulation of transforming growth factor beta signalling pathway genes by transforming growth factor beta in human osteoarthritic chondrocytes: involvement of Sp1 in both early and late response cells to transforming growth factor beta. Transforming growth factor beta (TGFβ) plays a central role in morphogenesis, growth, and cell differentiation. This cytokine is particularly important in cartilage where it regulates cell proliferation and   extracellular matri  x synthesis. While the action of TGFβ on chondrocyte metabolism has been extensively catalogued, the modulation of specific genes that function as mediators of TGFβ signalling is poorly defined. In the current study, elements of the Smad component of the TGFβ intracellular signalling system and TGFβ receptors were characterised in human chondrocytes upon TGFβ1 treatment.Human articular chondrocytes were incubated with TGFβ1. Then, mRNA and protein levels of TGFβ receptors and Smads were analysed by RT-PCR and western blot analysis. The role of specific protein 1 (Sp1) was investigated by gain and loss of function (inhibitor, siRNA, expression vector).We showed that TGFβ1 regulates mRNA levels of its own receptors, and of Smad3 and Smad7. It modulates TGFβ receptors post-transcriptionally by affecting their mRNA stability, but does not change the Smad-3 and Smad-7 mRNA half-life span, suggesting a potential transcriptional effect on these genes. Moreover, the transcriptional factor Sp1, which is downregulated by TGFβ1, is involved in the repression of both TGFβ receptors but not in the modulation of Smad3 and Smad7. Interestingly, Sp1 ectopic expression permitted also to maintain a similar expression pattern to early response to TGFβ at 24 hours of treatment. It restored the induction of Sox9 and COL2A1 and blocked the late response (repression of aggrecan, induction of COL1A1 and COL10A1).These data help to better understand the negative feedback loop in the TGFβ signalling system, and enlighten an interesting role of Sp1 to regulate TGFβ response. 
 Peroxisome proliferator-activated receptor-gamma ligands suppress fibronectin gene expression in human lung carcinoma cells: involvement of both CRE and Sp1. Lung carcinoma often occurs in patients with chronic lung disease such as tobacco-related emphysema and asbestos-related pulmonary fibrosis. These diseases are characterized by dramatic alterations in the content and composition of the lung   extracellular matrix  , and we believe this "altered" matrix has the ability to promote lung carcinoma cell growth. One   extracellular matrix   molecule shown to be altered in these lung diseases is fibronectin (Fn). We previously reported increased growth and survival of non-small cell lung carcinoma (NSCLC) cells exposed to Fn. Thus Fn may serve as a mitogen/survival factor for NSCLC and therefore represents a novel target for anti-cancer strategies. To this end, we studied the effects of the PPARgamma ligands 15d-PGJ(2), rosiglitazone (BRL49653), and troglitazone on Fn expression in NSCLC cells and found that they were able to inhibit Fn gene transcription. Inhibition of Fn expression by BRL49653 and troglitazone, but not by 15d-PGJ(2), was prevented by the specific PPARgamma antagonist GW-9662 and by PPARgamma small interfering RNA. Working with Fn deletion and mutated promoter constructs, we found that the region between -170 and -50 bp downstream from the transcriptional start site of the promoter was involved in PPARgamma ligand inhibition. PPARgamma ligands also diminished the phosphorylation of CREB, diminished Sp1 nuclear protein expression, and prevented the binding of these transcription factors to CRE and Sp1 sites, respectively, within the Fn promoter. In summary, our results demonstrate that PPARgamma ligands inhibit Fn gene expression in NSCLC cells through PPARgamma-dependent and -independent pathways that affect both CREB and Sp1. 
  GeneRIFs  
 MiR-324-5p suppresses hepatocellular carcinoma cell invasion by counteracting   extracellular matrix   degradation through post-transcriptionally downregulating ETS1 and SP1. 
 elevated level of Sp1 observed in tissue extracts obtained from keloid tissue; inhibitors of Sp1-activated transcription reduced expression of   extracellular matrix   components in keloid fibroblasts 
  [Ii]nvasive  PubMed  
 Doxorubicin influences the expression of glucosylceramide synthase in   invasive   ductal breast cancer. Glucosylceramide synthase (GCS) is one enzyme that provides a major route for ceramide clearance. Recent evidence has indicated an important role for GCS in multidrug resistance (MDR) tumors. Doxorubicin (DOX)can modulate the expression of GCS in leukemia and ovary cell lines. However, few studies have investigated their relationship in breast cancer;We collected 84 excision biopsies from patients with   invasive   ductal breast cancer of whom 33 patients had undergone preoperative chemotherapy. Immunohistochemistry was used to analyze the expression of GCS protein and significantly showed that the expression of GCS was higher in the samples from patients treated with preoperative chemotherapy(p = 0.018). In order to investigate the underlying mechanism, breast cancer cell lines were cultured with different concentrations of DOX, and mRNA and protein levels of GCS were then detected;DOX significantly upregulated the expression of GCS at both the mRNA and protein level in ERα-positive MCF-7 cells.We then block down the Sp1 site of GCS promoter, which inhibited the DOX-mediated increase in GCS expression; and after Erα was inhibited in MCF-7 cells, the up-regulation of GCS by DOX also been inhibited.In conclusion, our data demonstrated the novel finding that DOX could modulate the expression of GCS through the Sp1 site of GCS promoter in ERα-positive breast cancer cells. 
 KLF6/Sp1 initiates transcription of the tmsg-1 gene in human prostate carcinoma cells: an exon involved mechanism. The tumor metastasis suppressor gene-1 (tmsg-1) was first cloned as a new tumor suppressor gene in our laboratory several years ago. Since then, however, despite the substantial progression that has been made in investigation of the biologic roles played by this gene, the manner in which it exerts its regulatory influence is still unknown. With transfection of various deletion or mutation constructs, we identified a potential enhancer and three potential silencers in the 5'-flanking region. However, it was particularly interesting to find that a region (+59 to +123 bp) of exon 1 exhibited a strong role in initiation of tmsg-1 gene transcription. Deletion of this region led to essentially complete loss of driving activity of exon-1 sequence on luciferase. Further analysis showed that transcription factors KLF6 and Sp1 are able to interact with each other and bind to their elements in this region. Co-transfection of pGL3-114/+123 with KLF6- and/or Sp1-expressing plasmids resulted in an elevation of luciferase activity and transcription level of tmsg-1, which was abolished by knockdown of KLF6 or Sp1. Analysis of metastatic capacity showed that cells with high metastatic capability exhibited a lower level of KLF6/TMSG-1 proteins with highe  r invasi  ve capability and vice versa. Thus, we concluded that interaction of KLF6 and Sp1, together with their binding of elements in exon 1 are critical events in initiation of transcription of the tmsg-1 gene. These results reveal a hitherto unreported mechanism for initiation of transcription of the tmsg-1 gene. 
 [Transcriptional activation of TMSG-1 by complex of KLF6 and Sp1]. To investigate the regulatory mechanism of the transcription of tumor metastasis suppressor gene TMSG-1.Luciferase reporter assay and site-directed mutagenesis were used to analyze the regulatory region of TMSG-1. Electrophoretic mobility shift assay (EMSA) and chromatin immunoprecipitation (ChIP) were carried out to verify the interaction of KLF6 and Sp1 with the regulatory region of TMSG-1. Co-immunoprecipitation (CoIP) was performed to analyze the interaction between KLF6 and Sp1. TMSG-1 and wt-KLF6 mRNA expressions in cells with different metastatic capacities were quantitated by real-time PCR. Cell   invasive   capability was determined by Matrigel invasion assay.A 63 bp inducible regulatory region (+59 bp - +123 bp) in exon 1 was identified by luciferase assay using reporter plasmids with a series of TMSG-1 regulatory region deletions. Mutations in KLF6/Sp1 binding sites of this region resulted in a decrease of luciferase activity, while cotransfection with KLF6 or Sp1 expressing plasmids led to a remarkable increase of luciferase activity. EMSA and ChIP demonstrated that KLF6 as well as Sp1 interacted with this region. CoIP also indicated a possible interaction between KLF6 and Sp1 proteins. In the highly metastatic cell sublines, a low level of wild type KLF6 was associated synchronously with a low TMSG-1 level. Prostate carcinoma cells overexpressing KLF6 exhibited a higher TMSG-1 level and a lower   invasive   capability.Transcription factor complex of KLF6 and Sp1 may participate in the inducible transcriptional regulation of TMSG-1, and a decreased wild type KLF6 expression is likely associated with a low TMSG-1 level in the highly metastatic cell sublines. 
 ZEB2 upregulates integrin α5 expression through cooperation with Sp1 to induce invasion during epithelial-mesenchymal transition of human cancer cells. Epithelial-mesenchymal transition (EMT) is a process implicated in tumor invasion, metastasis, embryonic development and wound healing. ZEB2 is a transcription factor involved in EMT that represses E-cadherin transcription. Although E-cadherin downregulation is a major event during EMT and tumor progression, E-cadherin reduction is probably not sufficient for full   invasiv  eness. The mechanisms by which E-cadherin transcriptional repressors induce mesenchymal genes during EMT remain largely unknown. Here, we investigated the role of ZEB2 in the induction of integrin α5 during cancer EMT and its underlying mechanism. In human cancer cells, ZEB2 was found to directly upregulate integrin α5 transcription in a manner that is independent of the regulation of E-cadherin expression. Conversely, depletion of ZEB2 by small interfering RNA suppressed integrin α5 expression, leading to reduced invasion. Suppression of integrin α5 inhibited cancer cell invasion, suggesting an important role for integrin α5 in cancer progression. Furthermore, ZEB2 was found to activate the integrin α5 and vimentin promoters by interacting with and activating the transcription factor Sp1, suggesting that cooperation between ZEB2 and Sp1 represents a novel mechanism of mesenchymal gene activation during EMT. These findings increase our understanding of the pathways beyond E-cadherin reduction that regulate mesenchymal gene expression during EMT and cancer progression. 
 A regulatory loop involving miR-22, Sp1, and c-Myc modulates CD147 expression in breast cancer invasion and metastasis. Breast cancer is the most common cancer in women for which the metastatic process is still poorly understood. CD147 is upregulated in breast cancer and has been associated with tumor progression, but little is known about its regulatory mechanisms. In this study, we demonstrated that CD147 was overexpressed in breast cancer tissues and cell lines, and the high expression correlated with tumor invasion and metastasis. We also found that the transcription factors Sp1 and c-Myc could bind to the CD147 promoter and enhance its expression. The CD147 mRNA has a 748-bp 3'-untranslated region (UTR) with many miRNA target sites, suggesting possible regulation by miRNAs. We discovered that miR-22 repressed CD147 expression by directly targeting the CD147 3'UTR. We also determined that miR-22 could indirectly participate in CD147 modulation by downregulating Sp1 expression. miR-22 could form an autoregulatory loop with Sp1, which repressed miR-22 transcription by binding to the miR-22 promoter. Together with the c-Myc-mediated inhibition of miR-22 expression, our investigation identified a miR-22/Sp1/c-Myc network that regulates CD147 gene transcription. In addition, miR-22 overexpression suppressed breast cancer cell invasion, metastasis, and proliferation by targeting CD147 in vitro and in vivo. Furthermore, we found that miR-22 was significantly downregulated in breast cancer tissues and that its expression was inversely correlated with the tumor-node-metastasis stage and lymphatic metastasis in patients. Our study provides the first evidence that an miR-22/Sp1/c-Myc network regulates CD147 upregulation in breast cancer and that miR-22 represses breast cancer   invasive   and metastatic capacities. 
 Differential regulation of MMPs by E2F1, Sp1 and NF-kappa B controls the small cell lung cancer   invasive   phenotype. E2F1 transcription factor plays a vital role in the regulation of diverse cellular processes including cell proliferation, apoptosis, invasion and metastasis. E2F1 overexpression has been demonstrated in small cell lung cancer (SCLC), and extensive metastasis in early phase is the most important feature of SCLC. In this study, we investigated the involvement of E2F1 in the process of invasion and metastasis in SCLC by regulating the expression of matrix metalloproteinases (MMPs).Immunohistochemistry was performed to evaluate the expression of E2F1 and MMPs in SCLC samples in a Chinese Han population. The impact of E2F1 on invasion and metastasis was observed by transwell and wound healing experiments with depletion of E2F1 by specific siRNA. The target genes regulated by E2F1 were identified by chromatin immunoprecipitation (ChIP)-to-sequence, and the expressions of target genes were detected by real time PCR and western blotting. The dual luciferase reporter system was performed to analyze the regulatory relationship between E2F1 and MMPs.E2F1 is an independent and adverse prognosis factor that is highly expressed in SCLC in a Chinese Han population. Knockdown of E2F1 by specific siRNA resulted in the downregulation of migration and invasion in SCLC. The expressions of MMP-9 and -16 in SCLC were higher than other MMPs, and their expressions were most significantly reduced after silencing E2F1. ChIP-to-sequence and promoter-based luciferase analysis demonstrated that E2F1 directly controlled MMP-16 expression via an E2F1 binding motif in the promoter. Although one E2F1 binding site was predicted in the MMP-9 promoter, luciferase analysis indicated that this binding site was not functionally required. Further study demonstrated that E2F1 transcriptionally controlled the expression of Sp1 and p65, which in turn enhanced the MMP-9 promoter activity in SCLC cells. The associations between E2F1, Sp1, p65, and MMP-9 were validated by immunohistochemistry staining in SCLC tumors.E2F1 acts as a transcriptional activator for MMPs and directly enhances MMP transcription by binding to E2F1 binding sequences in the promoter, or indirectly activates MMPs through enhanced Sp1 and NF-kappa B as a consequence of E2F1 activation in SCLC. 
 Differential regulation of MMPs by E2F1, Sp1 and NF-kappa B controls the small cell lung cancer   invasive   phenotype. E2F1 transcription factor plays a vital role in the regulation of diverse cellular processes including cell proliferation, apoptosis, invasion and metastasis. E2F1 overexpression has been demonstrated in small cell lung cancer (SCLC), and extensive metastasis in early phase is the most important feature of SCLC. In this study, we investigated the involvement of E2F1 in the process of invasion and metastasis in SCLC by regulating the expression of matrix metalloproteinases (MMPs).Immunohistochemistry was performed to evaluate the expression of E2F1 and MMPs in SCLC samples in a Chinese Han population. The impact of E2F1 on invasion and metastasis was observed by transwell and wound healing experiments with depletion of E2F1 by specific siRNA. The target genes regulated by E2F1 were identified by chromatin immunoprecipitation (ChIP)-to-sequence, and the expressions of target genes were detected by real time PCR and western blotting. The dual luciferase reporter system was performed to analyze the regulatory relationship between E2F1 and MMPs.E2F1 is an independent and adverse prognosis factor that is highly expressed in SCLC in a Chinese Han population. Knockdown of E2F1 by specific siRNA resulted in the downregulation of migration and invasion in SCLC. The expressions of MMP-9 and -16 in SCLC were higher than other MMPs, and their expressions were most significantly reduced after silencing E2F1. ChIP-to-sequence and promoter-based luciferase analysis demonstrated that E2F1 directly controlled MMP-16 expression via an E2F1 binding motif in the promoter. Although one E2F1 binding site was predicted in the MMP-9 promoter, luciferase analysis indicated that this binding site was not functionally required. Further study demonstrated that E2F1 transcriptionally controlled the expression of Sp1 and p65, which in turn enhanced the MMP-9 promoter activity in SCLC cells. The associations between E2F1, Sp1, p65, and MMP-9 were validated by immunohistochemistry staining in SCLC tumors.E2F1 acts as a transcriptional activator for MMPs and directly enhances MMP transcription by binding to E2F1 binding sequences in the promoter, or indirectly activates MMPs through enhanced Sp1 and NF-kappa B as a consequence of E2F1 activation in SCLC. 
 A regulatory loop involving miR-22, Sp1, and c-Myc modulates CD147 expression in breast cancer invasion and metastasis. Breast cancer is the most common cancer in women for which the metastatic process is still poorly understood. CD147 is upregulated in breast cancer and has been associated with tumor progression, but little is known about its regulatory mechanisms. In this study, we demonstrated that CD147 was overexpressed in breast cancer tissues and cell lines, and the high expression correlated with tumor invasion and metastasis. We also found that the transcription factors Sp1 and c-Myc could bind to the CD147 promoter and enhance its expression. The CD147 mRNA has a 748-bp 3'-untranslated region (UTR) with many miRNA target sites, suggesting possible regulation by miRNAs. We discovered that miR-22 repressed CD147 expression by directly targeting the CD147 3'UTR. We also determined that miR-22 could indirectly participate in CD147 modulation by downregulating Sp1 expression. miR-22 could form an autoregulatory loop with Sp1, which repressed miR-22 transcription by binding to the miR-22 promoter. Together with the c-Myc-mediated inhibition of miR-22 expression, our investigation identified a miR-22/Sp1/c-Myc network that regulates CD147 gene transcription. In addition, miR-22 overexpression suppressed breast cancer cell invasion, metastasis, and proliferation by targeting CD147 in vitro and in vivo. Furthermore, we found that miR-22 was significantly downregulated in breast cancer tissues and that its expression was inversely correlated with the tumor-node-metastasis stage and lymphatic metastasis in patients. Our study provides the first evidence that an miR-22/Sp1/c-Myc network regulates CD147 upregulation in breast cancer and that miR-22 represses breast cancer   invasive   and metastatic capacities. 
 Doxorubicin influences the expression of glucosylceramide synthase in   invasive   ductal breast cancer. Glucosylceramide synthase (GCS) is one enzyme that provides a major route for ceramide clearance. Recent evidence has indicated an important role for GCS in multidrug resistance (MDR) tumors. Doxorubicin (DOX)can modulate the expression of GCS in leukemia and ovary cell lines. However, few studies have investigated their relationship in breast cancer;We collected 84 excision biopsies from patients with   invasive   ductal breast cancer of whom 33 patients had undergone preoperative chemotherapy. Immunohistochemistry was used to analyze the expression of GCS protein and significantly showed that the expression of GCS was higher in the samples from patients treated with preoperative chemotherapy(p = 0.018). In order to investigate the underlying mechanism, breast cancer cell lines were cultured with different concentrations of DOX, and mRNA and protein levels of GCS were then detected;DOX significantly upregulated the expression of GCS at both the mRNA and protein level in ERα-positive MCF-7 cells.We then block down the Sp1 site of GCS promoter, which inhibited the DOX-mediated increase in GCS expression; and after Erα was inhibited in MCF-7 cells, the up-regulation of GCS by DOX also been inhibited.In conclusion, our data demonstrated the novel finding that DOX could modulate the expression of GCS through the Sp1 site of GCS promoter in ERα-positive breast cancer cells. 
 ZEB2 upregulates integrin α5 expression through cooperation with Sp1 to induce invasion during epithelial-mesenchymal transition of human cancer cells. Epithelial-mesenchymal transition (EMT) is a process implicated in tumor invasion, metastasis, embryonic development and wound healing. ZEB2 is a transcription factor involved in EMT that represses E-cadherin transcription. Although E-cadherin downregulation is a major event during EMT and tumor progression, E-cadherin reduction is probably not sufficient for full   invasiv  eness. The mechanisms by which E-cadherin transcriptional repressors induce mesenchymal genes during EMT remain largely unknown. Here, we investigated the role of ZEB2 in the induction of integrin α5 during cancer EMT and its underlying mechanism. In human cancer cells, ZEB2 was found to directly upregulate integrin α5 transcription in a manner that is independent of the regulation of E-cadherin expression. Conversely, depletion of ZEB2 by small interfering RNA suppressed integrin α5 expression, leading to reduced invasion. Suppression of integrin α5 inhibited cancer cell invasion, suggesting an important role for integrin α5 in cancer progression. Furthermore, ZEB2 was found to activate the integrin α5 and vimentin promoters by interacting with and activating the transcription factor Sp1, suggesting that cooperation between ZEB2 and Sp1 represents a novel mechanism of mesenchymal gene activation during EMT. These findings increase our understanding of the pathways beyond E-cadherin reduction that regulate mesenchymal gene expression during EMT and cancer progression. 
 KLF6/Sp1 initiates transcription of the tmsg-1 gene in human prostate carcinoma cells: an exon involved mechanism. The tumor metastasis suppressor gene-1 (tmsg-1) was first cloned as a new tumor suppressor gene in our laboratory several years ago. Since then, however, despite the substantial progression that has been made in investigation of the biologic roles played by this gene, the manner in which it exerts its regulatory influence is still unknown. With transfection of various deletion or mutation constructs, we identified a potential enhancer and three potential silencers in the 5'-flanking region. However, it was particularly interesting to find that a region (+59 to +123 bp) of exon 1 exhibited a strong role in initiation of tmsg-1 gene transcription. Deletion of this region led to essentially complete loss of driving activity of exon-1 sequence on luciferase. Further analysis showed that transcription factors KLF6 and Sp1 are able to interact with each other and bind to their elements in this region. Co-transfection of pGL3-114/+123 with KLF6- and/or Sp1-expressing plasmids resulted in an elevation of luciferase activity and transcription level of tmsg-1, which was abolished by knockdown of KLF6 or Sp1. Analysis of metastatic capacity showed that cells with high metastatic capability exhibited a lower level of KLF6/TMSG-1 proteins with highe  r invasi  ve capability and vice versa. Thus, we concluded that interaction of KLF6 and Sp1, together with their binding of elements in exon 1 are critical events in initiation of transcription of the tmsg-1 gene. These results reveal a hitherto unreported mechanism for initiation of transcription of the tmsg-1 gene. 
 [Transcriptional activation of TMSG-1 by complex of KLF6 and Sp1]. To investigate the regulatory mechanism of the transcription of tumor metastasis suppressor gene TMSG-1.Luciferase reporter assay and site-directed mutagenesis were used to analyze the regulatory region of TMSG-1. Electrophoretic mobility shift assay (EMSA) and chromatin immunoprecipitation (ChIP) were carried out to verify the interaction of KLF6 and Sp1 with the regulatory region of TMSG-1. Co-immunoprecipitation (CoIP) was performed to analyze the interaction between KLF6 and Sp1. TMSG-1 and wt-KLF6 mRNA expressions in cells with different metastatic capacities were quantitated by real-time PCR. Cell   invasive   capability was determined by Matrigel invasion assay.A 63 bp inducible regulatory region (+59 bp - +123 bp) in exon 1 was identified by luciferase assay using reporter plasmids with a series of TMSG-1 regulatory region deletions. Mutations in KLF6/Sp1 binding sites of this region resulted in a decrease of luciferase activity, while cotransfection with KLF6 or Sp1 expressing plasmids led to a remarkable increase of luciferase activity. EMSA and ChIP demonstrated that KLF6 as well as Sp1 interacted with this region. CoIP also indicated a possible interaction between KLF6 and Sp1 proteins. In the highly metastatic cell sublines, a low level of wild type KLF6 was associated synchronously with a low TMSG-1 level. Prostate carcinoma cells overexpressing KLF6 exhibited a higher TMSG-1 level and a lower   invasive   capability.Transcription factor complex of KLF6 and Sp1 may participate in the inducible transcriptional regulation of TMSG-1, and a decreased wild type KLF6 expression is likely associated with a low TMSG-1 level in the highly metastatic cell sublines. 
 Expression of alpha V integrin is modulated by Epstein-Barr virus nuclear antigen 3C and the metastasis suppressor Nm23-H1 through interaction with the GATA-1 and Sp1 transcription factors. Epstein-Barr virus (EBV) is a lymphotrophic herpesvirus infecting most of the world's population. It is associated with a number of human lymphoid and epithelial tumors and lymphoproliferative diseases in immunocompromised patients. A subset of latent EBV antigens is required for immortalization of primary B-lymphocytes. The metastatic suppressor Nm23-H1 which is downregulated in human   invasive   breast carcinoma reduces the migration and metastatic activity of breast carcinoma cells when expressed from a heterologous promoter. Interestingly, the EBV nuclear antigen 3C (EBNA3C) reverses these activities of Nm23-H1. The alpha V integrins recognize a variety of ligands for signaling and are involved in cell migration and proliferation and also serve as major receptors for extracellular-matrix-mediated cell adhesion and migration. The goal of this study was to determine if Nm23-H1 and EBNA3C can modulate alpha V integrin expression and downstream activities. The results of our studies indicate that Nm23-H1 downregulates alpha V intregrin expression in a dose responsive manner. In contrast, EBNA3C can upregulate alpha V integrin expression. Furthermore, the study showed that the association of the Sp1 and GATA transcription factors with Nm23-H1 is required for modulation of the alpha V integrin activity. Thus, these results suggest a direct correlation between the alpha V integrin expression and the interaction of Nm23-H1 with EBNA3C. 
 Acetylated Sp1 inhibits PTEN expression through binding to PTEN core promoter and recruitment of HDAC1 and promotes cancer cell migration and invasion. Specificity protein 1 (Sp1) is often overexpressed in cancer cells. Its binding sites are known to exist in the phosphatase and tension homolog deleted on chromosome 10 (PTEN) promoter. In this study, we hypothesized that Sp1 negatively regulates PTEN expression. We used several cell lines to determine the effects of Sp1. The results showed that Sp1 overexpression inhibited the expression and promoter activity of PTEN and correspondingly upregulated AKT phosphorylation, whereas Sp1 knockdown upregulated the expression and promoter ability of PTEN and downregulated AKT phosphorylation. Moreover, a series of deletion and site-directed mutations of the PTEN promoter indicated that Sp1 can inhibit PTEN promoter activity through a specific Sp1-binding site at the PTEN core promoter in vivo. Meanwhile, non-acetylated Sp1, with its loss of DNA binding activity, failed to inhibit the expression and promoter activity of PTEN. Histone deacetylase 1 was necessary for Sp1 to inhibit PTEN expression. The inverse expression of Sp1 and PTEN was found in tongue cancer cells and salivary adenoid cystic cancer (SACC)-LM cells (possessing higher potential for lung metastasis than SACC-83) as compared with that in adjacent normal tissue and SACC-83 cells, respectively. Sp1 knockdown decreased the migration and invasion of SACC-LM cells, whereas Sp1 overexpression increased the migration and invasion of SACC-83 cells. Overall, these results suggest that Sp1 is involved in the development and   invasive  ness of cancer through inhibition of PTEN. 
 17β-estradiol enhances α(5) integrin subunit gene expression through ERα-Sp1 interaction and reduces cell motility and invasion of ERα-positive breast cancer cells. In breast tumors the expression of estrogen receptor alpha (ERα) is known to be associated with a more favorable prognosis. ERα expression has been reported to reduce the metastatic potential of breast cancer cells. Recently, we have observed that extracellular matrix proteins activate ERα and that both liganded and unliganded receptor modulat  e cell i  nvasiveness acting at nuclear level. To explain the mechanisms by which ERα regulates cell adhesion, we have evaluated the expression of α(5)β(1) integrin, prevalently expressed in stationary cells, in response to 17β-estradiol (E2). Here we show that E2/ERα increases the expression of integrin α(5)β(1) through Sp1-mediated binding to a GC-rich region located upstream of an ERE half-site in the 5' flanking region of the α(5) gene forming a ternary ERα-Sp1-DNA complex. Estrogen responsiveness of the α(5) gene promoter, as observed in HeLa cells, underlies a general mechanism of regulation which is not strictly linked to the cell type. Our data reveal novel insight into the molecular mechanisms sustai  ning the   reduced invasiveness of ERα expressing cells demonstrating that α(5)β(1) integrin expression is related to the maintenance of the stationary status of the cells, counteracting E2/ERα capability to enhance breast cancer cell migration and invasion. 
 Inhibition of bladder cancer invasion by Sp1-mediated BTG2 expression via inhibition of DNA methyltransferase 1. Significantly lower endogenous expression of B-cell translocation gene 2 (BTG2) was observed in human muscle-  invasive   bladder cancers (MIBC) than matched normal tissues and non-muscle   invasive   bladder cancers (NMIBC). BTG2 expression was inversely correlated with increased expression of the DNA methyltransferases DNMT1 and DNMT3a in MIBC, but not NMIBC, suggesting a potential role for BTG2 expression in muscle invasion of bladder cancer. Over 90% of tumor tissues revealed strong methylation at CpG islands of the BTG2 gene, compared with no methylation in the normal tissues, implying epigenetic regulation of BTG2 expression in bladder carcinogenesis. By using EJ bladder cancer cells and the demethylating agent decitabine, transcription of BTG2 was shown to be up-regulated by inhibiting DNMT1 expression via modification at CpG islands. DNMT1 binding to the BTG2 gene further regulated BTG2 expression by chromatin remodeling, such as H3K9 dimethylation and H3K4 trimethylation, and Sp1 activation. Induced BTG2 expression significantly reduced EJ cell tumorigenesis and   invasive  ness together with induction of G2 /M arrest. These results demonstrate an important role for the BTG2(/TIS21/PC3) gene in the progression of bladder cancers, and suggest that BTG2(/TIS21/PC3) is a promising epigenetic target for prevention of muscle invasion in human bladder cancers. 
 MicroRNA-377 inhibited proliferation and invasion of human glioblastoma cells by directly targeting specificity protein 1. Increasing evidence has indicated that microRNAs (miRNAs) are strongly implicated in the initiation and progression of glioblastoma multiforme (GBM). Here, we identiﬁed a novel tumor suppressive miRNA, miR-377, and investigated its role and therapeutic effect for GBM.MiRNA global screening was performed on GBM patient samples and adjacent nontumor brain tissues. The expression of miR-377 was detected by real-time reverse-transcription PCR. The effects of miR-377 on GBM cell proliferation, cell cycle progression, invasion, and orthotopic tumorigenicity were investigated The therapeutic effect of miR-377 mimic was explored in a subcutaneous GBM model. Western blot and luciferase reporter assay were used to identify the direct and functional target of miR-377.MiR-377 was markedly downregulated in human GBM tissues and cell lines. Overexpression of miR-377 dramatically inhibited cell growth both in culture and in orthotopic xenograft tumor models, blocked G1/S transition, and suppressed cell invasion in GBM cells. Importantly, introduction of miR-377 could strongly inhibit tumor growth in a subcutaneous GBM model. Subsequent investigation revealed that specificity protein 1 (Sp1) was a direct and functional target of miR-377 in GBM cells. Silencing of Sp1 recapitulated the antiproliferative and ant  i-invasi  ve effects of miR-377, whereas restoring the Sp1 expression antagonized the tumor-suppressive function of miR-377. Finally, analysis of miR-377 and Sp1 levels in human GBM tissues revealed that miR-377 is inversely correlated with Sp1 expression.These findings reveal that miR-377/Sp1 signaling that may be required for GBM development and may consequently serve as a therapeutic target for the treatment of GBM. 
 Transcription factor Sp1 induces ADAM17 and contributes to tumor cell   invasive  ness under hypoxia. Expression of the Sp1 transcription factor is induced by hypoxia, and the ADAM17 promoter contains predicted Sp1 binding sites. ADAM17 contributes to hypoxic-induce   invasive  ness of glioma. In this study, we investigated whether Sp1 transcription factor induces ADAM17 and/or contributes to tumor cell   invasive  ness in hypoxia.Employing RT-PCR and Western blot, we examined the role of Sp1 in ADAM17 transcription/expression under normoxic and hypoxic conditions, and whether it binds to the ADAM17 GC-rich promoter region using a chromatin immunoprecipitation assay. Additionally, we tested the effect of Sp1 suppression in tumor cell invasion and migration, using Matrigel basement membrane invasion chambers, a scratch wound-healing assay, and small interfering RNA.Here, we found that Sp1 binds to the ADAM17 promoter, and that Sp1 regulates ADAM17 expression under hypoxia. Furthermore, suppression of Sp1 decreases   invasive  ness and migration in U87 tumor cells.Our findings suggest the Sp1 transcription factor mediates ADAM17 expression under hypoxia, regulates glioma   invasive  ness, and thus, may be a target for anti-invasion therapies. 
 Transcriptional regulation of KiSS-1 gene expression in metastatic melanoma by specificity protein-1 and its coactivator DRIP-130. Loss of the metastasis suppressor gene, KiSS-1 has been strongly correlated to the progression of metastases in numerous types of cancers. The mechanism through which KiSS-1 is lost during metastasis, however, is still not completely known. Previous studies have shown that genetic material on human chromosome 6q16.3-q23 is essential for KiSS-1 expression in normal tissues. Additionally, microcell-mediated transfer of this chromosome in cancerous tissue results in rescued expression of KiSS-1 and reduced metastatic phenotype. Here, we show that loss of Sp1-coactivator protein DRIP-130, which is encoded by human chromosome 6q16.3-q23, results in reduced KiSS-1 promoter activation in highly malignant melanoma cells. Co-expression of Sp1 and DRIP-130 not only rescues KiSS-1 expression, but also induces an inhibition of the   invasive   and migratory behavior in highly metastatic melanoma cells, similar to the overexpression of KiSS-1 metastasis suppressor gene in those cells. Furthermore, we demonstrate that KiSS-1 expression is regulated by Sp1 elements within the first 100-bp region of the KiSS-1 promoter and that targeted deletion of a single GC-rich region spanning -93 to -58 interrupts Sp1- and DRIP-130-modulated transcriptional control of KiSS-1 expression. Our results thus suggest that DRIP-130 is a key regulator in KiSS-1 transactivation in normal tissue, and that the loss of DRIP-130 expression, as a result of the gross loss of human chromosome 6q16.3-q23, provokes increased tumor metastasis. 
 Sp1-mediated microRNA-182 expression regulates lung cancer progression. Our recent study indicated that overexpression of Sp1 enhances the proliferation of lung cancer cells, while represses metastasis. In this study, we found that the transcriptional activity of FOXO3 was increased, but its protein levels decreased following Sp1 expression. Sp1 increased expression of miR-182, which was then recruited to the 3'-untranslated region of FOXO3 mRNA to silence its translational activity. Knockdown of miR-182 inhibited lung cancer cells growth, but enhanced the   invasive   and migratory abilities of these cells through increased N-cadherin expression. Repression of FOXO3 expression in the miR-182 knockdown cells partially reversed this effect, suggesting that miR-182 promotes cancer cell growth and inhibits cancer metastatic activity by regulating the expression of FOXO3. The expression of several cancer metastasis-related genes such as ADAM9, CDH9 and CD44 was increased following miR-182 knockdown. In conclusion, in the early stages of lung cancer progression, Sp1 stimulates miR-182 expression, which in turn decreases FOXO3 expression. This stimulates proliferation and tumor growth. In the late stages, Sp1 and miR-182 decline, thus increasing FOXO3 expression, which leads to lung metastasis. 
 Sp1 is over-expressed in nasopharyngeal cancer and is a poor prognostic indicator for patients receiving radiotherapy. Nasopharyngeal cancer (NPC) is a tumor of epithelial origin with complex etiology. Currently the standard treatment of NPC is radiotherapy, but therapy failure is quite common, making radioresistance an important issue. This study explores the association of specificity protein 1 (Sp1) protein expression with clinicopathological significance and disease prognosis in NPC patients receiving radiotherapy. A total of 82 NPC patients (55 males and 27 females, median age: 48 years old) were enrolled and received radiotherapy between September 2011 and March 2014. Tumor tissue and grossly adjacent normal mucosa were obtained in each patient. Sp1 expression was detected by western blot and immunohistochemical analysis, and the associations with clinicopathological status and radiotherapy response were analyzed. Our Results showed Sp1 protein expression was higher in CNE-1 and CNE-2 nasopharyngeal cancer cells than in normal nasopharyngeal mucosal NP69 cells. All 82 patients' tissue sections were stained positive for the Sp1 protein, and 39 (47.6%) patients showed higher level than adjacent normal mucosa. Sp1-overexpression in the tumor tissue was correlated with a higher tumor stage, nodal status, clinical stage and distant metastasis (P < 0.01). Patients with higher Sp1 expression in pretreatment biopsies had a lower radiotherapy response compared to those with lower expression. In conclusion, Sp1 may play roles in radioresistance of nasopharyngeal cancer which attributes to tumor   invasive  ness, and serve as a novel prognostic marker of NPC radiotherapy. However, further studies are required to validate our findings in larger samples and explore more detailed mechanisms underlying radioresistance of Sp1. 
 Sp1 is upregulated in human glioma, promotes MMP-2-mediated cell invasion and predicts poor clinical outcome. Sp1, the first identified transcription factor, has been reported to be associated with the development and progression of various human cancer types. However, the clinical significance and biological role of Sp1 in glioma are less well understood. In this study, we found that the expression of Sp1 was markedly elevated in glioma cell lines and tissues. Immunohistochemistry analysis revealed that the vast majority of 222 paraffin-embedded archival glioma specimens tested displayed positive Sp1 expression, and 58.6% exhibited high-level Sp1 expression. Statistical analysis suggested that the high Sp1 expression was correlated strongly with the WHO grading (p < 0.001) and survival status (p < 0.001) of glioma patients. Patients with lower Sp1 expression had better overall survival than those with higher Sp1 expression. Multivariate analysis suggested that Sp1 expression might be an independent prognostic indicator of the survival of patients with glioma. Furthermore, overexpression of Sp1 in glioma cells was found to increase their   invasive  ness, and in contrast, silencing Sp1 by siRNA caused an inhibition of cell invasion. Moreover, we demonstrated that the up-regulation of Sp1 could increase activity and expression of MMP-2. Collectively, our data suggest that Sp1 might represent a valuable prognostic marker for glioma and is involved in modulation of tumor invasion. 
 Suppression of lung cancer cell invasion by LKB1 is due to the downregulation of tissue factor and vascular endothelial growth factor, partly dependent on SP1. LKB1 encodes a serine/threonine kinase generally inactivated in many human cancers, which mediates cancer cell proliferation, migration and differentiation. Recent studies indicated that LKB1 exhibits potent anti-metastatic activity. However, the underlying molecular mechanisms of this activity remain unclear. In this study, we re‑introduced LKB1 into A549 lung cancer cells that lack the LKB1 gene to investigate how LKB1 affects tumo  r invasi  veness and metastasis. We demonstrated that overexpression of the LKB1 protein in lung cancer cells resulted in significant inhibition of invasion. Furthermore, transfected lung cancer cells with LKB1 suppressed tissue factor (TF) and vascular endothelial growth factor (VEGF) expression at both the mRNA and protein levels. Here, we provided evidence showing that downregulation of TF and VEGF by LKB1 is correlated well with the inhibition of cell invasion. Overexpression of the LKB1 protein in human lung cancer is significantly associated with a decrease in activity and expression of the transcription factor SP1. Constitutive activation of the transcription factor Sp1 plays a critical role in TF and VEGF overexpression. We conclude that suppression of lung cancer cell invasion by LKB1 through downregulation of TF and VEGF may partly depend on its inhibitory effect on the transcription factor Sp1. Collectively, our data provide a novel molecular mechanism for the antitumor activity of LKB1 and may help further improve its effectiveness in controlling lung cancer growth and invasion. 
 Specificity protein 1 expression contributes to Bcl-w-induced aggressiveness in glioblastoma multiforme. We already had reported that Bcl-w promotes invasion or migration in gastric cancer cells and glioblastoma multiforme (GBM) by activating matrix metalloproteinase-2 (MMP-2) via specificity protein 1 (Sp1) or β-cateinin, respectively. High expression of Bcl-w also has been reported in GBM which is the most common malignant brain tumor and exhibits aggressive and   invasiv  e behavior. These reports propose that Bcl-w-induced signaling is strongly associated with aggressive characteristic of GBM. We demonstrated that Sp1 protein or mRNA expression is induced by Bcl-w using Western blotting or RT-PCR, respectively, and markedly elevated in high-grade glioma specimens compared with low-grade glioma tissues using tissue array. However, relationship between Bcl-w-related signaling and aggressive characteristic of GBM is poorly characterized. This study suggested that Bcl-w-induced Sp1 activation promoted expression of glioma stem-like cell markers, such as Musashi, Nanog, Oct4 and sox-2, as well as neurosphere formation and   invasiv  eness, using western blotting, neurosphere formation assay, or invasion assay, culminating in their aggressive behavior. Therefore, Bcl-w-induced Sp1 activation is proposed as a putative marker for aggressiveness of GBM. 
 JMJD2A-dependent silencing of Sp1 in advanced breast cancer promotes metastasis by downregulation of DIRAS3. Specificity protein 1(Sp1) is a ubiquitous transcription factor and is highly expressed in breast cancer. However, its expression pattern and role in breast cancer progression remain unclear. The purpose of this study is to examine the expression pattern of Sp1 and determine its role in breast cancer progression. Immunohistochemistry (IHC) was performed on breast cancer tissues to reveal the expression pattern of Sp1. Spearman rank correlation was used for clinical statistics. Gene and protein expressions were monitored by IHC analysis, quantitative polymerase chain reaction, and Western blot. Wound-healing and Transwell assays were conducted to assess the role of Sp1 in breast cancer. Co-immunoprecipitation, deletion mutagenesis, chromatin immunoprecipitation, and dual luciferase reporter gene assays were used for investigation of the regulatory network. Sp1 expression was downregulated in late stage breast cancer and in highly   invasive   breast cancer cell lines. Expression of Sp1 was negatively correlated with TNM staging (P = 0.002) and metastasis status (P = 0.023). Overexpression of Sp1 inhibited breast cancer cell migratory and   invasive   abilities, whereas knockdown of GTP-binding RAS-like 3 (DIRAS3, also known as ARHI, NOEY2) attenuated the inhibitory effects. Moreover, re-expression of DIRAS3 abolished Sp1 knockdown-mediated cell migration and invasion. Jumonji domain containing 2A (JMJD2A) inhibited Sp1 autoregulation and explains Sp1 expression pattern in breast cancer. Sp1 negatively regulated breast cancer metastasis by transcriptional activation of DIRAS3. Inhibition of Sp1 autoregulation by JMJD2A contributed to Sp1 expression pattern in breast cancer. Our findings provided evidence that targeted therapy against Sp1 might be useful in early stage breast cancer. However, in late stages, development of Sp1 activator may be more promising for breast cancer treatments. 
 MiR-324-5p Suppresses Hepatocellular Carcinoma Cell Invasion by Counteracting ECM Degradation through Post-Transcriptionally Downregulating ETS1 and SP1. Hepatocellular carcinoma (HCC) is one of the common malignancies, which is highly metastatic and the third common cause of cancer deaths in the world. The invasion and metastasis of cancer cells is a multistep and complex process which is mainly initiated by extracellular matrix (ECM) degradation. Aberrant expression of microRNA has been investigated in HCC and shown to play essential roles during HCC progression. In the present study, we found that microRNA-324-5p (miR-324-5p) was downregulated in both HCC cell lines and tissues. Ectopic miR-324-5p led to the reduction of HCC cells   invasive   and metastatic capacity, whereas inhibition of miR-324-5p promoted the invasion of HCC cells. Matrix metalloproteinase 2 (MMP2) and MMP9, the major regulators of ECM degradation, were found to be downregulated by ectopic miR-324-5p, while upregulated by miR-324-5p inhibitor. E26 transformation-specific 1 (ETS1) and Specificity protein 1 (SP1), both of which could modulate MMP2 and MMP9 expression and activity, were presented as the direct targets of and downregulated by miR-324-5p. Downregulation of ETS1 and SP1 mediated the inhibitory function of miR-324-5p on HCC migration and invasion. Our study demonstrates that miR-324-5p suppresses hepatocellular carcinoma cell invasion and might provide new clues to   invasive   HCC therapy. 
 Constitutive expression of the Id-1 promoter in human metastatic breast cancer cells is linked with the loss of NF-1/Rb/HDAC-1 transcription repressor complex. The helix-loop-helix protein Id-1 is a dominant negative regulator of basic helix-loop-helix transcription factors, and plays a key role in the control of breast epithelial cell growth, invasion and differentiation. Previous investigations in our laboratory have shown that Id-1 mRNA was constitutively expressed in highly aggressive and   invasive   human breast cancer cells in comparison to non-transformed or non-aggressive cancerous cells, and that this loss of regulation is mediated by a 2.2-kb region of the human Id-1 promoter. Here we show that a 31 bp sequence within this 2.2-kb promoter, located 200 bp upstream of the initiation of transcription, is responsible for the constitutive expression of Id-1 in metastatic human breast cancer cells. Using gel shift experiments, we identified a high molecular weight complex present only in non-aggressive breast cancer cells cultured in serum-free medium and which appear to be necessary for proper Id-1 repression. In contrast, nuclear extracts from highly aggressive and metastatic cell lines do not contain this large molecular weight complex. Using DNA affinity precipitation assays (DAPA), we show that this complex contains SP-1, NF-1, Rb and HDAC-1 proteins. On the basis of these findings, we propose a mechanism for the loss of regulation of Id-1 promoter in   invasive   and metastatic human breast cancer cells. 
 Binding of Sp1 to the proximal promoter links constitutive expression of the human uPA gene and   invasive   potential of PC3 cells. Activated transcription of the urokinase-type plasminogen activator (uPA) gene depends on the enhancer, located approximately 2 kb from the start of transcription. The proximal promoter, driving basal transcription, contains a GC-/GA-rich sequence immediately upstream of the TATA box. We have investigated the role played by this element in the transcription of the uPA gene in HeLa and PC3 cells, which do not express or constitutively express the gene, respectively. This region binds either Sp1 or Sp3, as monomers or multimers, but not a combination of the 2 proteins. The more efficient binding of Sp1 to the proximal promoter in PC3 cells is correlated to its phosphorylation state. Polymerase chain reaction (PCR)-coupled, chromatin immunoprecipitation experiments with anti-Sp1 antibodies indeed show an enrichment of proximal promoter sequences in PC3 cells and support the observed difference in transcription levels from proximal promoter constructs in HeLa versus PC3 cells. Furthermore, overexpression of Sp1 increases transcription from the reporter construct in HeLa cells, whereas in PC3 cells, overexpression of Sp3 does not reduce transcription from the same construct, indicating that the Sp1/Sp3 balance cannot be shifted. We conclude that the GC-/GA-rich element of the uPA regulatory region is an independent functional element, regulated by Sp family proteins. Phosphorylation of Sp1 determines the presence in vivo and the functionality of this element in PC3 cells. Thus, the cellular context determines the relevance of the GC-/GA-rich region in uPA gene transcription, which contributes to constitutive gene expression, related, in turn, to the   invasive   phenotype. 
  GeneRIFs  
 Negative regulation of Sp1 by JMJD2A causes downregulation of Sp1 in highly   invasive   breast cancer cells.JMJD2A-dependent silencing of Sp1 in advanced breast cancer promotes metastasis by downregulation of DIRAS3. 
 Our study provides the first evidence that an miR-22/Sp1/c-Myc network regulates CD147 upregulation in breast cancer and that miR-22 represses breast cancer   invasive   and metastatic capacities. 
 these results suggest that Sp1 is involved in the development and   invasive  ness of cancer through inhibition of PTEN 
 Sp1 transcription factor mediates ADAM17 expression under hypoxia, regulates glioma   invasive  ness, and thus, may be a target for anti-invasion therapies. 
 Co-expression of Sp1 and DRIP-130 not only rescues KiSS-1 expression, but also induces an inhibition of the   invasive   and migratory behavior in highly metastatic melanoma cells 
  [Mm]etastasize  PubMed  
 Starvation after Cobalt-60 γ-Ray Radiation Enhances Metastasis in U251 Glioma Cells by Regulating the Transcription Factor SP1. Radiation is of clinical importance during glioma therapy; however, vasculature damage is observed over the treatment course. This type of tissue damage might lead to starvation conditions, affecting tumor metastasis. To test this possibility, we compared starvation conditions in conjunction with radiation treatment to monitor metastatic ability in the U251 glioma cell line. Transcriptome, western blot, and immunofluorescence analyses were used to measure the RNA and protein expression changes of the U251 cells after various treatments. We found that starvation combined with radiation treatment yielded the most significant expression changes in metastasis-related factors compared to that in the control groups. In addition, a metastasis assay was used to directly measure the metastatic ability of the treated cells, which confirmed that the U251 cells treated with starvation combined with radiation possessed the highest metastatic ability. Furthermore, bioinformatics analysis demonstrated that SP1 represented a common transcription factor associated with changes in metastasis-related factors. Blocking SP1 activity by an inhibitor suppressed the starvation-plus-radiation treatment-mediated enhancement of U251 cell metastasis. Our study provides the first evidence that starvation caused by radiation might play a significant role in enhancing the ability of the glioma cell line U251 to   metastasiz  e via regulation of the transcription factor SP1. 
  [Mm]etastasis  PubMed  
 KLF6/Sp1 initiates transcription of the tmsg-1 gene in human prostate carcinoma cells: an exon involved mechanism. The tumor   metastasis   suppressor gene-1 (tmsg-1) was first cloned as a new tumor suppressor gene in our laboratory several years ago. Since then, however, despite the substantial progression that has been made in investigation of the biologic roles played by this gene, the manner in which it exerts its regulatory influence is still unknown. With transfection of various deletion or mutation constructs, we identified a potential enhancer and three potential silencers in the 5'-flanking region. However, it was particularly interesting to find that a region (+59 to +123 bp) of exon 1 exhibited a strong role in initiation of tmsg-1 gene transcription. Deletion of this region led to essentially complete loss of driving activity of exon-1 sequence on luciferase. Further analysis showed that transcription factors KLF6 and Sp1 are able to interact with each other and bind to their elements in this region. Co-transfection of pGL3-114/+123 with KLF6- and/or Sp1-expressing plasmids resulted in an elevation of luciferase activity and transcription level of tmsg-1, which was abolished by knockdown of KLF6 or Sp1. Analysis of metastatic capacity showed that cells with high metastatic capability exhibited a lower level of KLF6/TMSG-1 proteins with higher invasive capability and vice versa. Thus, we concluded that interaction of KLF6 and Sp1, together with their binding of elements in exon 1 are critical events in initiation of transcription of the tmsg-1 gene. These results reveal a hitherto unreported mechanism for initiation of transcription of the tmsg-1 gene. 
 [Transcriptional activation of TMSG-1 by complex of KLF6 and Sp1]. To investigate the regulatory mechanism of the transcription of tumor   metastasis   suppressor gene TMSG-1.Luciferase reporter assay and site-directed mutagenesis were used to analyze the regulatory region of TMSG-1. Electrophoretic mobility shift assay (EMSA) and chromatin immunoprecipitation (ChIP) were carried out to verify the interaction of KLF6 and Sp1 with the regulatory region of TMSG-1. Co-immunoprecipitation (CoIP) was performed to analyze the interaction between KLF6 and Sp1. TMSG-1 and wt-KLF6 mRNA expressions in cells with different metastatic capacities were quantitated by real-time PCR. Cell invasive capability was determined by Matrigel invasion assay.A 63 bp inducible regulatory region (+59 bp - +123 bp) in exon 1 was identified by luciferase assay using reporter plasmids with a series of TMSG-1 regulatory region deletions. Mutations in KLF6/Sp1 binding sites of this region resulted in a decrease of luciferase activity, while cotransfection with KLF6 or Sp1 expressing plasmids led to a remarkable increase of luciferase activity. EMSA and ChIP demonstrated that KLF6 as well as Sp1 interacted with this region. CoIP also indicated a possible interaction between KLF6 and Sp1 proteins. In the highly metastatic cell sublines, a low level of wild type KLF6 was associated synchronously with a low TMSG-1 level. Prostate carcinoma cells overexpressing KLF6 exhibited a higher TMSG-1 level and a lower invasive capability.Transcription factor complex of KLF6 and Sp1 may participate in the inducible transcriptional regulation of TMSG-1, and a decreased wild type KLF6 expression is likely associated with a low TMSG-1 level in the highly metastatic cell sublines. 
 ZEB2 upregulates integrin α5 expression through cooperation with Sp1 to induce invasion during epithelial-mesenchymal transition of human cancer cells. Epithelial-mesenchymal transition (EMT) is a process implicated in tumor invasion,   metastasi  s, embryonic development and wound healing. ZEB2 is a transcription factor involved in EMT that represses E-cadherin transcription. Although E-cadherin downregulation is a major event during EMT and tumor progression, E-cadherin reduction is probably not sufficient for full invasiveness. The mechanisms by which E-cadherin transcriptional repressors induce mesenchymal genes during EMT remain largely unknown. Here, we investigated the role of ZEB2 in the induction of integrin α5 during cancer EMT and its underlying mechanism. In human cancer cells, ZEB2 was found to directly upregulate integrin α5 transcription in a manner that is independent of the regulation of E-cadherin expression. Conversely, depletion of ZEB2 by small interfering RNA suppressed integrin α5 expression, leading to reduced invasion. Suppression of integrin α5 inhibited cancer cell invasion, suggesting an important role for integrin α5 in cancer progression. Furthermore, ZEB2 was found to activate the integrin α5 and vimentin promoters by interacting with and activating the transcription factor Sp1, suggesting that cooperation between ZEB2 and Sp1 represents a novel mechanism of mesenchymal gene activation during EMT. These findings increase our understanding of the pathways beyond E-cadherin reduction that regulate mesenchymal gene expression during EMT and cancer progression. 
 Cyclooxygenase-2 up-regulates CCR7 expression via AKT-mediated phosphorylation and activation of Sp1 in breast cancer cells. Up-regulation of cyclooxygenase-2 (COX-2) is frequently found in human cancers and is significantly associated with tumor   metastasis  . Our previous results demonstrate that COX-2 and its metabolite prostaglandin E2 (PGE2) stimulate the expression of CCR7 chemokine receptor via EP2/EP4 receptors to promote lymphatic invasion in breast cancer cells. In this study, we address the underlying mechanism of COX-2/PGE2-induced CCR7 expression. We find that COX-2/PGE2 increase CCR7 expression via the AKT signaling pathway in breast cancer cells. Promoter deletion and mutation assays identify the Sp1 site located at the -60/-57 region of CCR7 gene promoter is critical for stimulation. Chromatin immunoprecipitation (ChIP) assay confirms that in vivo binding of Sp1 to human CCR7 promoter is increased by COX-2 and PGE2. Knockdown of Sp1 by shRNA reduces the induction of CCR7 by PGE2. We demonstrate for the first time that AKT may directly phosphorylate Sp1 at S42, T679, and S698. Phosphorylation-mimic Sp1 protein harboring S42D, T679D, and S698D mutation strongly activates CCR7 expression. In contrast, change of these three residues to alanine completely blocks the induction of CCR7 by PGE2. Pathological investigation demonstrates that CCR7 expression is strongly associated with phospho-AKT and Sp1 in 120 breast cancer tissues. Collectively, our results demonstrate that COX-2 up-regulates CCR7 expression via AKT-mediated phosphorylation and activation of Sp1 and this pathway is highly activated in metastatic breast cancer. 
 A regulatory loop involving miR-22, Sp1, and c-Myc modulates CD147 expression in breast cancer invasion and   metastasis  . Breast cancer is the most common cancer in women for which the metastatic process is still poorly understood. CD147 is upregulated in breast cancer and has been associated with tumor progression, but little is known about its regulatory mechanisms. In this study, we demonstrated that CD147 was overexpressed in breast cancer tissues and cell lines, and the high expression correlated with tumor invasion and   metastasis  . We also found that the transcription factors Sp1 and c-Myc could bind to the CD147 promoter and enhance its expression. The CD147 mRNA has a 748-bp 3'-untranslated region (UTR) with many miRNA target sites, suggesting possible regulation by miRNAs. We discovered that miR-22 repressed CD147 expression by directly targeting the CD147 3'UTR. We also determined that miR-22 could indirectly participate in CD147 modulation by downregulating Sp1 expression. miR-22 could form an autoregulatory loop with Sp1, which repressed miR-22 transcription by binding to the miR-22 promoter. Together with the c-Myc-mediated inhibition of miR-22 expression, our investigation identified a miR-22/Sp1/c-Myc network that regulates CD147 gene transcription. In addition, miR-22 overexpression suppressed breast cancer cell invasion,   metastasis  , and proliferation by targeting CD147 in vitro and in vivo. Furthermore, we found that miR-22 was significantly downregulated in breast cancer tissues and that its expression was inversely correlated with the tumor-node-  metastasis   stage and lymphatic   metastasis   in patients. Our study provides the first evidence that an miR-22/Sp1/c-Myc network regulates CD147 upregulation in breast cancer and that miR-22 represses breast cancer invasive and metastatic capacities. 
 Differential regulation of MMPs by E2F1, Sp1 and NF-kappa B controls the small cell lung cancer invasive phenotype. E2F1 transcription factor plays a vital role in the regulation of diverse cellular processes including cell proliferation, apoptosis, invasion and   metastasis  . E2F1 overexpression has been demonstrated in small cell lung cancer (SCLC), and extensive   metastasis   in early phase is the most important feature of SCLC. In this study, we investigated the involvement of E2F1 in the process of invasion and   metastasis   in SCLC by regulating the expression of matrix metalloproteinases (MMPs).Immunohistochemistry was performed to evaluate the expression of E2F1 and MMPs in SCLC samples in a Chinese Han population. The impact of E2F1 on invasion and   metastasis   was observed by transwell and wound healing experiments with depletion of E2F1 by specific siRNA. The target genes regulated by E2F1 were identified by chromatin immunoprecipitation (ChIP)-to-sequence, and the expressions of target genes were detected by real time PCR and western blotting. The dual luciferase reporter system was performed to analyze the regulatory relationship between E2F1 and MMPs.E2F1 is an independent and adverse prognosis factor that is highly expressed in SCLC in a Chinese Han population. Knockdown of E2F1 by specific siRNA resulted in the downregulation of migration and invasion in SCLC. The expressions of MMP-9 and -16 in SCLC were higher than other MMPs, and their expressions were most significantly reduced after silencing E2F1. ChIP-to-sequence and promoter-based luciferase analysis demonstrated that E2F1 directly controlled MMP-16 expression via an E2F1 binding motif in the promoter. Although one E2F1 binding site was predicted in the MMP-9 promoter, luciferase analysis indicated that this binding site was not functionally required. Further study demonstrated that E2F1 transcriptionally controlled the expression of Sp1 and p65, which in turn enhanced the MMP-9 promoter activity in SCLC cells. The associations between E2F1, Sp1, p65, and MMP-9 were validated by immunohistochemistry staining in SCLC tumors.E2F1 acts as a transcriptional activator for MMPs and directly enhances MMP transcription by binding to E2F1 binding sequences in the promoter, or indirectly activates MMPs through enhanced Sp1 and NF-kappa B as a consequence of E2F1 activation in SCLC. 
 Combination analysis of activator protein-1 family members, Sp1 and an activator protein-2alpha-related factor binding to different regions of the urokinase receptor gene in resected colorectal cancers. Studies on the transactivation of genes via promoter elements have mostly been done on cell lines rather than resected tissues. This, however, is essential to address an in vivo or clinical relevance. We have previously shown tumor-specific binding of Sp1 and an activator protein (AP)-2-related factor to promoter region -152/-135 of the   metastasis  -related u-PAR gene in 60% of in vivo-resected cancer tissues. Cell lines have implicated an additional role, and potential synergism, of an AP-1 region (-190/-171) in u-PAR regulation. This study was done to (a) analyze AP-1 binding to this region in resected tumor and normal tissues, and define subgroups in which it is tumor-specific, and (b) to analyze transcription factor-binding patterns to both promoter motifs in resected tissues, supporting synergism, and draw first prognostic conclusions.In 103 patients with colorectal cancer, electrophoretic mobility shift assay/supershift analysis for u-PAR promoter region -190/-171 was done in tumors and normal tissues. In 71 patients, region -152/-135 was also analyzed. U-PAR protein was measured by ELISA.Tumor-specific AP-1 binding to region -190/-171 of the u-PAR promoter was found in 40% of patients. Subgroup analysis showed tumor-specific binding for c-Fos in 58%, for c-Jun in 50%, for JunD in 39%, and for Fra-1 in 4% of cases. AP-1 binding correlated significantly with u-PAR protein amounts in both normal and tumor tissues (P<0.001), in contrast to a tumor-specific correlation with u-PAR of the AP-2/Sp1 region. In analyses for both promoter regions, 62% of cancers showed simultaneous binding for AP-1, AP-2, and Sp1, 11% for AP-1 and AP-2, 16% for AP-2 and Sp1, 4% for AP-2 only, 3% for AP-1 only, and 0% for Sp1 only. The binding of AP-1, AP-2, and Sp1 correlated significantly with each other (P<0.001), the combination of AP-1 and AP-2 showing the highest correlation with u-PAR (P=0.008). Preliminary survival analysis indicated a trend for poorer prognosis for binding of all three transcription factors.This is the first study differentiating transcription factor-binding to two important u-PAR promoter regions in a large series of resected tumors and normal tissues. The AP-1 site seems to be a less tumor-specific regulator than the Sp1/AP-2 motif. Nevertheless, data corroborate the hypothesis of synergism between both elements in resected tumors. 
 Differential regulation of MMPs by E2F1, Sp1 and NF-kappa B controls the small cell lung cancer invasive phenotype. E2F1 transcription factor plays a vital role in the regulation of diverse cellular processes including cell proliferation, apoptosis, invasion and   metastasis  . E2F1 overexpression has been demonstrated in small cell lung cancer (SCLC), and extensive   metastasis   in early phase is the most important feature of SCLC. In this study, we investigated the involvement of E2F1 in the process of invasion and   metastasis   in SCLC by regulating the expression of matrix metalloproteinases (MMPs).Immunohistochemistry was performed to evaluate the expression of E2F1 and MMPs in SCLC samples in a Chinese Han population. The impact of E2F1 on invasion and   metastasis   was observed by transwell and wound healing experiments with depletion of E2F1 by specific siRNA. The target genes regulated by E2F1 were identified by chromatin immunoprecipitation (ChIP)-to-sequence, and the expressions of target genes were detected by real time PCR and western blotting. The dual luciferase reporter system was performed to analyze the regulatory relationship between E2F1 and MMPs.E2F1 is an independent and adverse prognosis factor that is highly expressed in SCLC in a Chinese Han population. Knockdown of E2F1 by specific siRNA resulted in the downregulation of migration and invasion in SCLC. The expressions of MMP-9 and -16 in SCLC were higher than other MMPs, and their expressions were most significantly reduced after silencing E2F1. ChIP-to-sequence and promoter-based luciferase analysis demonstrated that E2F1 directly controlled MMP-16 expression via an E2F1 binding motif in the promoter. Although one E2F1 binding site was predicted in the MMP-9 promoter, luciferase analysis indicated that this binding site was not functionally required. Further study demonstrated that E2F1 transcriptionally controlled the expression of Sp1 and p65, which in turn enhanced the MMP-9 promoter activity in SCLC cells. The associations between E2F1, Sp1, p65, and MMP-9 were validated by immunohistochemistry staining in SCLC tumors.E2F1 acts as a transcriptional activator for MMPs and directly enhances MMP transcription by binding to E2F1 binding sequences in the promoter, or indirectly activates MMPs through enhanced Sp1 and NF-kappa B as a consequence of E2F1 activation in SCLC. 
 A regulatory loop involving miR-22, Sp1, and c-Myc modulates CD147 expression in breast cancer invasion and   metastasis  . Breast cancer is the most common cancer in women for which the metastatic process is still poorly understood. CD147 is upregulated in breast cancer and has been associated with tumor progression, but little is known about its regulatory mechanisms. In this study, we demonstrated that CD147 was overexpressed in breast cancer tissues and cell lines, and the high expression correlated with tumor invasion and   metastasis  . We also found that the transcription factors Sp1 and c-Myc could bind to the CD147 promoter and enhance its expression. The CD147 mRNA has a 748-bp 3'-untranslated region (UTR) with many miRNA target sites, suggesting possible regulation by miRNAs. We discovered that miR-22 repressed CD147 expression by directly targeting the CD147 3'UTR. We also determined that miR-22 could indirectly participate in CD147 modulation by downregulating Sp1 expression. miR-22 could form an autoregulatory loop with Sp1, which repressed miR-22 transcription by binding to the miR-22 promoter. Together with the c-Myc-mediated inhibition of miR-22 expression, our investigation identified a miR-22/Sp1/c-Myc network that regulates CD147 gene transcription. In addition, miR-22 overexpression suppressed breast cancer cell invasion,   metastasis  , and proliferation by targeting CD147 in vitro and in vivo. Furthermore, we found that miR-22 was significantly downregulated in breast cancer tissues and that its expression was inversely correlated with the tumor-node-  metastasis   stage and lymphatic   metastasis   in patients. Our study provides the first evidence that an miR-22/Sp1/c-Myc network regulates CD147 upregulation in breast cancer and that miR-22 represses breast cancer invasive and metastatic capacities. 
 Cyclooxygenase-2 up-regulates CCR7 expression via AKT-mediated phosphorylation and activation of Sp1 in breast cancer cells. Up-regulation of cyclooxygenase-2 (COX-2) is frequently found in human cancers and is significantly associated with tumor   metastasis  . Our previous results demonstrate that COX-2 and its metabolite prostaglandin E2 (PGE2) stimulate the expression of CCR7 chemokine receptor via EP2/EP4 receptors to promote lymphatic invasion in breast cancer cells. In this study, we address the underlying mechanism of COX-2/PGE2-induced CCR7 expression. We find that COX-2/PGE2 increase CCR7 expression via the AKT signaling pathway in breast cancer cells. Promoter deletion and mutation assays identify the Sp1 site located at the -60/-57 region of CCR7 gene promoter is critical for stimulation. Chromatin immunoprecipitation (ChIP) assay confirms that in vivo binding of Sp1 to human CCR7 promoter is increased by COX-2 and PGE2. Knockdown of Sp1 by shRNA reduces the induction of CCR7 by PGE2. We demonstrate for the first time that AKT may directly phosphorylate Sp1 at S42, T679, and S698. Phosphorylation-mimic Sp1 protein harboring S42D, T679D, and S698D mutation strongly activates CCR7 expression. In contrast, change of these three residues to alanine completely blocks the induction of CCR7 by PGE2. Pathological investigation demonstrates that CCR7 expression is strongly associated with phospho-AKT and Sp1 in 120 breast cancer tissues. Collectively, our results demonstrate that COX-2 up-regulates CCR7 expression via AKT-mediated phosphorylation and activation of Sp1 and this pathway is highly activated in metastatic breast cancer. 
 ZEB2 upregulates integrin α5 expression through cooperation with Sp1 to induce invasion during epithelial-mesenchymal transition of human cancer cells. Epithelial-mesenchymal transition (EMT) is a process implicated in tumor invasion,   metastasi  s, embryonic development and wound healing. ZEB2 is a transcription factor involved in EMT that represses E-cadherin transcription. Although E-cadherin downregulation is a major event during EMT and tumor progression, E-cadherin reduction is probably not sufficient for full invasiveness. The mechanisms by which E-cadherin transcriptional repressors induce mesenchymal genes during EMT remain largely unknown. Here, we investigated the role of ZEB2 in the induction of integrin α5 during cancer EMT and its underlying mechanism. In human cancer cells, ZEB2 was found to directly upregulate integrin α5 transcription in a manner that is independent of the regulation of E-cadherin expression. Conversely, depletion of ZEB2 by small interfering RNA suppressed integrin α5 expression, leading to reduced invasion. Suppression of integrin α5 inhibited cancer cell invasion, suggesting an important role for integrin α5 in cancer progression. Furthermore, ZEB2 was found to activate the integrin α5 and vimentin promoters by interacting with and activating the transcription factor Sp1, suggesting that cooperation between ZEB2 and Sp1 represents a novel mechanism of mesenchymal gene activation during EMT. These findings increase our understanding of the pathways beyond E-cadherin reduction that regulate mesenchymal gene expression during EMT and cancer progression. 
 KLF6/Sp1 initiates transcription of the tmsg-1 gene in human prostate carcinoma cells: an exon involved mechanism. The tumor   metastasis   suppressor gene-1 (tmsg-1) was first cloned as a new tumor suppressor gene in our laboratory several years ago. Since then, however, despite the substantial progression that has been made in investigation of the biologic roles played by this gene, the manner in which it exerts its regulatory influence is still unknown. With transfection of various deletion or mutation constructs, we identified a potential enhancer and three potential silencers in the 5'-flanking region. However, it was particularly interesting to find that a region (+59 to +123 bp) of exon 1 exhibited a strong role in initiation of tmsg-1 gene transcription. Deletion of this region led to essentially complete loss of driving activity of exon-1 sequence on luciferase. Further analysis showed that transcription factors KLF6 and Sp1 are able to interact with each other and bind to their elements in this region. Co-transfection of pGL3-114/+123 with KLF6- and/or Sp1-expressing plasmids resulted in an elevation of luciferase activity and transcription level of tmsg-1, which was abolished by knockdown of KLF6 or Sp1. Analysis of metastatic capacity showed that cells with high metastatic capability exhibited a lower level of KLF6/TMSG-1 proteins with higher invasive capability and vice versa. Thus, we concluded that interaction of KLF6 and Sp1, together with their binding of elements in exon 1 are critical events in initiation of transcription of the tmsg-1 gene. These results reveal a hitherto unreported mechanism for initiation of transcription of the tmsg-1 gene. 
 [Transcriptional activation of TMSG-1 by complex of KLF6 and Sp1]. To investigate the regulatory mechanism of the transcription of tumor   metastasis   suppressor gene TMSG-1.Luciferase reporter assay and site-directed mutagenesis were used to analyze the regulatory region of TMSG-1. Electrophoretic mobility shift assay (EMSA) and chromatin immunoprecipitation (ChIP) were carried out to verify the interaction of KLF6 and Sp1 with the regulatory region of TMSG-1. Co-immunoprecipitation (CoIP) was performed to analyze the interaction between KLF6 and Sp1. TMSG-1 and wt-KLF6 mRNA expressions in cells with different metastatic capacities were quantitated by real-time PCR. Cell invasive capability was determined by Matrigel invasion assay.A 63 bp inducible regulatory region (+59 bp - +123 bp) in exon 1 was identified by luciferase assay using reporter plasmids with a series of TMSG-1 regulatory region deletions. Mutations in KLF6/Sp1 binding sites of this region resulted in a decrease of luciferase activity, while cotransfection with KLF6 or Sp1 expressing plasmids led to a remarkable increase of luciferase activity. EMSA and ChIP demonstrated that KLF6 as well as Sp1 interacted with this region. CoIP also indicated a possible interaction between KLF6 and Sp1 proteins. In the highly metastatic cell sublines, a low level of wild type KLF6 was associated synchronously with a low TMSG-1 level. Prostate carcinoma cells overexpressing KLF6 exhibited a higher TMSG-1 level and a lower invasive capability.Transcription factor complex of KLF6 and Sp1 may participate in the inducible transcriptional regulation of TMSG-1, and a decreased wild type KLF6 expression is likely associated with a low TMSG-1 level in the highly metastatic cell sublines. 
 Combination analysis of activator protein-1 family members, Sp1 and an activator protein-2alpha-related factor binding to different regions of the urokinase receptor gene in resected colorectal cancers. Studies on the transactivation of genes via promoter elements have mostly been done on cell lines rather than resected tissues. This, however, is essential to address an in vivo or clinical relevance. We have previously shown tumor-specific binding of Sp1 and an activator protein (AP)-2-related factor to promoter region -152/-135 of the   metastasis  -related u-PAR gene in 60% of in vivo-resected cancer tissues. Cell lines have implicated an additional role, and potential synergism, of an AP-1 region (-190/-171) in u-PAR regulation. This study was done to (a) analyze AP-1 binding to this region in resected tumor and normal tissues, and define subgroups in which it is tumor-specific, and (b) to analyze transcription factor-binding patterns to both promoter motifs in resected tissues, supporting synergism, and draw first prognostic conclusions.In 103 patients with colorectal cancer, electrophoretic mobility shift assay/supershift analysis for u-PAR promoter region -190/-171 was done in tumors and normal tissues. In 71 patients, region -152/-135 was also analyzed. U-PAR protein was measured by ELISA.Tumor-specific AP-1 binding to region -190/-171 of the u-PAR promoter was found in 40% of patients. Subgroup analysis showed tumor-specific binding for c-Fos in 58%, for c-Jun in 50%, for JunD in 39%, and for Fra-1 in 4% of cases. AP-1 binding correlated significantly with u-PAR protein amounts in both normal and tumor tissues (P<0.001), in contrast to a tumor-specific correlation with u-PAR of the AP-2/Sp1 region. In analyses for both promoter regions, 62% of cancers showed simultaneous binding for AP-1, AP-2, and Sp1, 11% for AP-1 and AP-2, 16% for AP-2 and Sp1, 4% for AP-2 only, 3% for AP-1 only, and 0% for Sp1 only. The binding of AP-1, AP-2, and Sp1 correlated significantly with each other (P<0.001), the combination of AP-1 and AP-2 showing the highest correlation with u-PAR (P=0.008). Preliminary survival analysis indicated a trend for poorer prognosis for binding of all three transcription factors.This is the first study differentiating transcription factor-binding to two important u-PAR promoter regions in a large series of resected tumors and normal tissues. The AP-1 site seems to be a less tumor-specific regulator than the Sp1/AP-2 motif. Nevertheless, data corroborate the hypothesis of synergism between both elements in resected tumors. 
 The Sp1 transcription factor contributes to the tumor necrosis factor-induced expression of the angiogenic factor thymidine phosphorylase in human colon carcinoma cells. Thymidine phosphorylase (TP; also known as platelet-derived endothelial cell growth factor, PD-ECGF) is an angiogenic factor that is chemotactic for endothelial cells and has been found to induce neovascularization in vivo. TP is frequently overexpressed in human solid tumors, where its expression has been correlated with increased tumor microvessel density, invasion, and   metastasis  , and shorter patient survival. In this report, TP activity in the WiDr colon carcinoma cell line was found to be induced 100-fold by tumor necrosis factor (TNFalpha), a secretory product of activated macrophages that has indirect angiogenic activities. Increased TP activity was accompanied by increased TP mRNA levels and without an increase in mRNA stability. TNFalpha-induced TP mRNA levels were reduced by mithramycin, a DNA-binding transcription inhibitor specific for GC-rich sequences. Transcriptional regulation by TNFalpha was confirmed by transient transfection of WiDr with upstream TP sequences in a luciferase reporter construct. Deletion analysis of the reporter pinpointed two regions of the TP promoter with regulatory elements for both TNFalpha-inducible and basal expression, and they contained, respectively, three and one consensus binding sites for the Sp1-family of transcription factors. One additional region contributed only to basal TP expression, and it contained three Sp1 sites. TNFalpha-induced TP expression decreased when point mutations were made in three of the four Sp1 sites postulated to contribute to both basal and TNFalpha-inducible expression. Electrophoretic mobility shift assays further demonstrated binding of nuclear Sp1 to these three sites. Sp1-binding activity was also increased in cells treated with TNFalpha. These studies establish a role for Sp1 in the regulation of expression of the angiogenic factor TP in colon cancer WiDr cells. 
 Melatonin suppresses TPA-induced   metastasis   by downregulating matrix metalloproteinase-9 expression through JNK/SP-1 signaling in nasopharyngeal carcinoma. Nasopharyngeal carcinoma (NPC), a disease common in the South-East Asian population, has high lymph node metastatic ability. Melatonin, an endogenously produced substance present in animals, plants, fungi, and bacteria, has oncostatic activity via several mechanisms. The molecular mechanisms involved in melatonin-mediated tumor inhibitory potential are not completely defined. Here, we show that melatonin treatment inhibits TPA-induced cell motility by regulating the matrix metalloproteinase-9 (MMP-9) expression in NPC. We also identified the signaling cascade through which melatonin inhibits MMP-9 expression; this involves melatonin regulating the binding activity of the transcription factor specificity protein-1 (SP-1)-DNA. Our mechanistic analysis further reveals that the c-Jun N-terminal kinase/mitogen-activated protein kinase pathway is involved in the melatonin-mediated tumor suppressor activity. Furthermore, the findings indicate a functional link between melatonin-mediated MMP-9 regulation and tumor suppressing ability and provide new insights into the role of melatonin-induced molecular and epigenetic regulation of tumor growth. Thus, we conclude that melatonin suppresses the motility of NPC by regulating TPA-induced MMP-9 gene expression via inhibiting SP-1-DNA binding ability. The results provide a functional link between melatonin-mediated SP-1 regulation and the antimetastatic actions of melatonin on nasopharyngeal carcinoma. 
 Sp1 regulates osteopontin expression in SW480 human colon adenocarcinoma cells. Osteopontin (OPN) mediates cancer   metastasis  . Mechanisms regulating OPN expression in human colorectal cancer are unknown. Using SW480 colon adenocarcinoma cells, we hypothesized that transcription determines OPN expression.SW480 constitutively express OPN. Transient transfection and deletion analysis of human OPN promoter (full-length 2.1 kb)-luciferase constructs identified cis-regulatory regions. Gelshift and chromatin immunoprecipitation (ChIP) assays identified the trans-regulatory nuclear protein. Using in vitro adhesion, migration, and invasion studies, siRNA was used to determine the functional effect of decreased nuclear protein expression.A cis-regulatory promoter region, nt-80 to nt-108, upregulated OPN transcription. Gelshift assays demonstrated specific binding of nuclear proteins. Competition with unlabeled mutant oligonucleotides indicated that the region, nt-94 to nt-104 (TGGGCTGGGC), was essential for protein binding in gelshift assays. Confirmatory ChIP assays showed the corresponding nuclear protein to be Sp1. Sp1 expression was ablated with siRNA (si-Sp1), resulting in decreased OPN-dependent adhesion, migration, and invasion by 50%, 70%, and 65%, respectively. Exogenous addition of OPN to si-Sp1 cells restored adhesion, migration, and invasion indices.In SW480 human colon cancer cells, we conclude that Sp1 mediated expression of the tumor   metastasis   protein, OPN, regulates in vitro functional correlates of tumor   metastasis  . 
 MicroRNA-335 acts as a   metastasis   suppressor in gastric cancer by targeting Bcl-w and specificity protein 1. Aberrant expression of miR-335 has been frequently reported in cancer studies, suggesting that there is a close correlation between miR-335 and cancer during its development, progression,   metastasis   and prognosis. The expression of miR-335 in gastric cancer and its effects are not known. Relative expression of miR-335 in 4 gastric cancer cell lines and in 70 gastric cancer tissues was confirmed by real-time quantitative reverse transcriptase-PCR compared with controls. Transwell cell migration and Matrigel invasion assay in vitro and   metastasis   formation assay in vivo were used to examine the effects of miR-335 expression on gastric cancer cell invasion and   metastasis  . The effect of miR-335 expression on gastric cancer cell proliferation was estimated by the 3-(4,5-dimethylthiazol-2-yl)-2, 5-diphenyltetrazolium bromide assay. Luciferase reporter assay and western blot were used to examine the potential target genes and related pathways. Gene silencing with small-interfering RNA was used to examine the effects of target genes on gastric cancer cell invasion. miR-335 was dramatically downregulated in gastric cancer cell lines than in the normal gastric cell line GES-1. Low expression of miR-335 was significantly associated with lymph-node   metastasis  , poor pT stage, poor pN stage and invasion of lymphatic vessels. Overexpression of miR-335 suppressed gastric cancer cell invasion and   metastasis   in vitro and in vivo, but has no significant effects on cell proliferation. Furthermore, miR-335 might suppress gastric cancer invasion and   metastasis   by targeting Bcl-w and specificity protein 1 (SP1). Taken together, our results provide evidence that miR-335 might function as a   metastasis   suppressor in gastric cancer by targeting SP1 directly and indirectly through the Bcl-w-induced phosphoinositide 3-kinase-Akt-Sp1 pathway. miR-335 showing altered expression at different stages of gastric cancer could be a target for gastric cancer therapies and could be further developed as a potential prognostic factor. 
 Sp1-mediated ectopic expression of T-cell lymphoma invasion and   metastasis   2 in hepatocellular carcinoma. T-cell lymphoma invasion and   metastasis   2 (TIAM2) is a neuron-specific protein that has been found ectopically expressed in hepatocellular carcinoma (HCC). Results from clinical specimens and cellular and animal models have shown that the short form of TIAM2 (TIAM2S) functions as an oncogene in the tumorigenesis of liver cancer. However, the regulation of TIAM2S ectopic expression in HCC cells remains largely unknown. This study aimed to identify the mechanism underlying the ectopic expression of TIAM2S in liver cancer cells. In this report, we provide evidence illustrating that Sp1 binds directly to the GC box located in the TIAM2S core promoter. We further demonstrated that overexpression of Sp1 in HepaRG cells promotes endogenous TIAM2S mRNA and protein expressions, and knockdown of Sp1 in 2 HCC cell lines, HepG2 and PLC/PRF/5, led to a substantial reduction in TIAM2S mRNA and protein in these cells. Of 60 paired HCC samples, 70% showed a significant increase (from 1.1- to 3.6-fold) in Sp1 protein expression in the tumor cells. The elevated Sp1 expression was highly correlated with both TIAM2S mRNA and protein expressions in these samples. Together, these results illustrate that Sp1 positively controls TIAM2S transcription and that Sp1-mediated transcriptional activation is essential for TIAM2S ectopic expression in liver cancer cells. 
 Expression of alpha V integrin is modulated by Epstein-Barr virus nuclear antigen 3C and the   metastasis   suppressor Nm23-H1 through interaction with the GATA-1 and Sp1 transcription factors. Epstein-Barr virus (EBV) is a lymphotrophic herpesvirus infecting most of the world's population. It is associated with a number of human lymphoid and epithelial tumors and lymphoproliferative diseases in immunocompromised patients. A subset of latent EBV antigens is required for immortalization of primary B-lymphocytes. The metastatic suppressor Nm23-H1 which is downregulated in human invasive breast carcinoma reduces the migration and metastatic activity of breast carcinoma cells when expressed from a heterologous promoter. Interestingly, the EBV nuclear antigen 3C (EBNA3C) reverses these activities of Nm23-H1. The alpha V integrins recognize a variety of ligands for signaling and are involved in cell migration and proliferation and also serve as major receptors for extracellular-matrix-mediated cell adhesion and migration. The goal of this study was to determine if Nm23-H1 and EBNA3C can modulate alpha V integrin expression and downstream activities. The results of our studies indicate that Nm23-H1 downregulates alpha V intregrin expression in a dose responsive manner. In contrast, EBNA3C can upregulate alpha V integrin expression. Furthermore, the study showed that the association of the Sp1 and GATA transcription factors with Nm23-H1 is required for modulation of the alpha V integrin activity. Thus, these results suggest a direct correlation between the alpha V integrin expression and the interaction of Nm23-H1 with EBNA3C. 
 [Expression of   metastasis   associated 1 family member 2 (MTA2) in gastric cancer and its relationship with transcription factor Sp1]. To determine the expression patterns of   metastasis   associated 1 family member 2 (MTA2) in gastric cancer and non-cancerous gastric mucosa, and analyze its relationship with nuclear transcription factor Sp1 expression.Tissue samples and clinicopathological information from 83 gastric cancer patients, who underwent surgery, were collected from Shanghai Rui Jin Hospital. All samples included cancer tissue and non-cancerous mucosa which was 5 cm away from the tumor lesion. The expression of MTA2 and Sp1 were detected by immunohistochemistry (IHC) staining. The mRNA of MTA2 was also detected by reverse transcription-polymerase chain reaction (RT-PCR). SPSS software was used for statistical analysis.The expression of MTA2 protein was significantly higher in primary lesions of the gastric cancer than that in non-cancerous mucosa by IHC (31.3% vs 12.0%, P < 0.01). MTA2 expression was closely related with tumor invasion or T staging (χ(2) = 5.677, P < 0.05). Yet, no significant relationship was observed between MTA2 expression and other clinicopathological parameters, including the age, sex, tumor differentiation, Lauren classification, lymph node   metastasi  s, distant   metastasi  s, as well as pathological staging. Furthermore, MTA2 expression was concomitant with Sp1 expression (r = 0.320, P < 0.05). Elevated MTA2 expression was observed in Sp1 positive cancer tissues (χ(2) = 9.565, P < 0.01). RT-PCR results also demonstrated that MTA2 mRNA was also highly expressed in the tissue samples with Sp1 expression.MTA2 is highly expressed in the primary lesions of gastric cancer than that in adjacent non-cancerous tissues, and is closely related with tumor invasion. MTA2 expression is elevated in Sp1 positive gastric cancer. 
 Sp1 is involved in H2O2-induced PUMA gene expression and apoptosis in colorectal cancer cells. Reactive oxygen species (ROS) are intricately involved in tumor progression through effects on proliferation, apoptosis and   metastasis  . But how ROS works is not well understood. In previous study, we found PUMA (p53-upregulated modulator of apoptosis) played an important role in oxaliplatin-induced apoptosis. In the present study, we detect the role of PUMA in H2O2-induced apoptosis in colorectal cancer cells and investigate the potential mechanism.We showed that H2O2 stimulated the activity of a 493 PUMA promoter reporter gene construct. Suppressing the expression of PUMA abrogated H2O2-induced apoptosis. Deletion of the Sp1-binding sites also decreased the transactivation of PUMA promoter by H2O2. Furthermore, induction of PUMA promoter activity by H2O2 was abrogated by PFT-alpha (a p53 inhibitor) and Mithramycin A (a Sp1 inhibitor), as compared with PFT-alpha alone. To determine the effects of Sp1 on PUMA in H2O2-induced apoptosis, procaspase 3, procaspase 9 and procaspase 8 expression was assessed. Mithramycin A and PFT-alpha also reduced H2O2-induced apoptosis synergistically and abrogated the expression of procaspase 3 and procaspase 9.Our findings suggest that PUMA plays a role in H2O2-induced apoptosis, and that Sp1 works together with p53 in the regulation of H2O2-induced PUMA expression and apoptosis in colorectal cancer cells. This study provides important regulatory insights in the mechanisms of ROS in colorectal cancer. 
 Acetylated Sp1 inhibits PTEN expression through binding to PTEN core promoter and recruitment of HDAC1 and promotes cancer cell migration and invasion. Specificity protein 1 (Sp1) is often overexpressed in cancer cells. Its binding sites are known to exist in the phosphatase and tension homolog deleted on chromosome 10 (PTEN) promoter. In this study, we hypothesized that Sp1 negatively regulates PTEN expression. We used several cell lines to determine the effects of Sp1. The results showed that Sp1 overexpression inhibited the expression and promoter activity of PTEN and correspondingly upregulated AKT phosphorylation, whereas Sp1 knockdown upregulated the expression and promoter ability of PTEN and downregulated AKT phosphorylation. Moreover, a series of deletion and site-directed mutations of the PTEN promoter indicated that Sp1 can inhibit PTEN promoter activity through a specific Sp1-binding site at the PTEN core promoter in vivo. Meanwhile, non-acetylated Sp1, with its loss of DNA binding activity, failed to inhibit the expression and promoter activity of PTEN. Histone deacetylase 1 was necessary for Sp1 to inhibit PTEN expression. The inverse expression of Sp1 and PTEN was found in tongue cancer cells and salivary adenoid cystic cancer (SACC)-LM cells (possessing higher potential for lung   metastasis   than SACC-83) as compared with that in adjacent normal tissue and SACC-83 cells, respectively. Sp1 knockdown decreased the migration and invasion of SACC-LM cells, whereas Sp1 overexpression increased the migration and invasion of SACC-83 cells. Overall, these results suggest that Sp1 is involved in the development and invasiveness of cancer through inhibition of PTEN. 
 Sp1 cooperates with Sp3 to upregulate MALAT1 expression in human hepatocellular carcinoma. Long non-coding RNA (lncRNA)   metastasis  -associated lung adenocarcinoma transcript 1 (MALAT1), also known as nuclear-enriched transcript 2 (NEAT2), is highly conserved among mammals and highly expressed in the nucleus. It was first identified in lung cancer as a prognostic marker for   metastasis   but is also associated with several other solid tumors. In hepatocellular carcinoma (HCC), MALAT1 is a novel biomarker for predicting tumor recurrence after liver transplantation. The mechanism of overexpression in tumor progression remains unclear. In the present study, we investigated the role of specificity protein 1/3 (Sp1/3) in regulation of MALAT1 transcription in HCC cells. The results showed a high expression of Sp1, Sp3 and MALAT1 in HCC vs. paired non-tumor liver tissues, which was associated with the AFP level (Sp1, r=7.44, P=0.0064; MALAT1, r=12.37, P=0.0004). Co-silencing of Sp1 and Sp3 synergistically repressed MALAT1 expression. Sp1 binding inhibitor, mithramycin A (MIT), also inhibited MALAT1 expression in HCC cells. In conclusion, the upstream of MALAT1 contains five Sp1/3 binding sites, which may be responsible for MALAT1 transcription. Inhibitors, such as MIT, provide a potential therapeutic strategy for HCC patients with MALAT1 overexpression. 
 Tumor suppressor Pdcd4 inhibits invasion/intravasation and regulates urokinase receptor (u-PAR) gene expression via Sp-transcription factors. Tumor suppressor Pdcd4 has recently been shown to inhibit invasion by activating activator protein-1 (AP-1); however, little is known of the functionally significant Pdcd4-target genes. The urokinase receptor (u-PAR) promotes invasion/  metastasis  , and is associated with poor cancer-patient survival. The present study was conducted (1) to investigate a role for Pdcd4 in intravasation, invasion and u-PAR regulation, and (2) to describe mechanisms by which this is achieved. Fourteen cell lines showed reciprocal expression of u-PAR/Pdcd4. Resected tumor/normal tissues of 29 colorectal cancer patients demonstrated a significant inverse correlation between Pdcd4/u-PAR. siRNA-Pdcd4-transfected GEO cells significantly increased endogenous u-PAR mRNA/protein. A u-PAR-promoter-chloramphenicol acetyl transferase (CAT)-reporter was reduced in activity with increasing Pdcd4 expression in RKO. Deletion of a putative Sp-1-binding site (-402/-350) inhibited u-PAR promoter regulation by Pdcd4, this being paralleled by a reduction of Sp1 binding to this region in pdcd4-transfected cells. Pdcd4-transfected cells showed an increase in Sp3 binding to u-PAR promoter region -152/-135, the deletion of which reduces the ability of Pdcd4 to suppress u-PAR promoter activity. Surprisingly, the u-PAR-AP-1 site was not targeted by Pdcd4. Finally, RKO cells overexpressing Pdcd4 showed an inhibition of invasion/intravasation (chicken embryo   metastasis   assay). These data suggest Pdcd4 as a new negative regulator of intravasation, and qas the invasion-related gene u-PAR. It is the first study to implicate Pdcd4 regulation of gene expression via Sp1/Sp3. 
 Transcriptional regulation of oncogenic protein kinase Cϵ (PKCϵ) by STAT1 and Sp1 proteins. Overexpression of PKCϵ, a kinase associated with tumor aggressiveness and widely implicated in malignant transformation a  nd metasta  sis, is a hallmark of multiple cancers, including mammary, prostate, and lung cancer. To characterize the mechanisms that control PKCϵ expression and its up-regulation in cancer, we cloned an ∼ 1.6-kb promoter segment of the human PKCϵ gene (PRKCE) that displays elevated transcriptional activity in cancer cells. A comprehensive deletional analysis established two regions rich in Sp1 and STAT1 sites located between -777 and -105 bp (region A) and -921 and -796 bp (region B), respectively, as responsible for the high transcriptional activity observed in cancer cells. A more detailed mutagenesis analysis followed by EMSA and ChIP identified Sp1 sites in positions -668/-659 and -269/-247 as well as STAT1 sites in positions -880/-869 and -793/-782 as the elements responsible for elevated promoter activity in breast cancer cells relative to normal mammary epithelial cells. RNAi silencing of Sp1 and STAT1 in breast cancer cells reduced PKCϵ mRNA and protein expression, as well as PRKCE promoter activity. Moreover, a strong correlation was found between PKCϵ and phospho-Ser-727 (active) STAT1 levels in breast cancer cells. Our results may have significant implications for the development of approaches to target PKCϵ and its effectors in cancer therapeutics. 
 HER-2/neu represses the   metastasis   suppressor RECK via ERK and Sp transcription factors to promote cell invasion. Matrix metalloproteinase (MMP) inhibitory proteins may negatively regulate MMP activity to suppress tumor   metastasis  . In this study, we demonstrate that the HER-2/neu oncogene inhibits the expression of the MMP inhibitor RECK to promote cell invasion. RECK was inhibited via transcriptional repression in B104-1-1 cells, which express constitutively active HER-2/neu. Overexpression of HER-2/neu in NIH/3T3 or HaCaT cells also suppressed RECK expression. Deletion and mutation assays showed that HER-2/neu repressed RECK via the Sp1-binding site localized in the -82/-71 region from the translational start site. DNA affinity precipitation and chromatin immunoprecipitation assays indicated that binding of Sp1 and Sp3 to this consensus site was increased in B104-1-1 cells. We also found that HER-2/neu inhibited RECK via the ERK signaling pathway. Sp1 proteins phosphorylated at Thr453 and Thr739 by ERK bound preferentially to the RECK promoter, and this binding was reversed by HER-2/neu and ERK inhibitors. Furthermore, our data indicate that HER-2/neu obviously increased HDAC1 binding to the Sp1-binding site localized in the -82/-71 region of the RECK promoter. The histone deacetylase inhibitor trichostatin A reversed HER-2/neu-induced inhibition of RECK. HER-2/neu activation was associated with increased MMP-9 secretion and activation. Re-expression of RECK in HER-2/neu-overexpressing cells inhibited MMP-9 secretion and cell invasion. Taken together, our results suggest that HER-2/neu induces the binding of Sp proteins and HDAC1 to the RECK promoter to inhibit RECK expression and to promote cell invasion. Restoration of RECK provides a novel strategy for the inhibition of HER-2/neu-induced   metastasis  . 
 Crosstalk of Sp1 and Stat3 signaling in pancreatic cancer pathogenesis. Pancreatic cancer progression is attributed to genetic and epigenetic alterations and a chaotic tumor microenvironment. Those diverse "upstream signal" factors appear to converge on specific sets of central nuclear regulators, namely, transcription factors. Specificity Protein 1 (Sp1) and signal transducer and activator of transcription 3 (Stat3) are central transcription factors that regulate a number of pathways important to tumorigenesis, including tumor cell-cycle progression, apoptosis, angiogenesis,   metastasis  , and evasion of the immune system. Recently, researchers demonstrated many types of crosstalk of Sp1 and Stat3 in tumor signal transduction and that these factors function cooperatively to activate targeted genes and promote tumorigenesis in pancreatic cancer. Therefore, targeting both Sp1 and Stat3 is a potential preventive and therapeutic strategy for pancreatic cancer. 
 Low SP1 expression differentially affects intestinal-type compared with diffuse-type gastric adenocarcinoma. Specificity protein 1 (SP1) is an essential transcription factor that regulates multiple cancer-related genes. Because aberrant expression of SP1 is related to cancer development and progression, we focused on SP1 expression in gastric carcinoma and its correlation with disease outcomes. Although patient survival decreased as SP1 expression increased (P<0.05) in diffuse-type gastric cancer, the lack of SP1 expression in intestinal-type gastric cancer was significantly correlated with poor survival (P<0.05). The knockdown of SP1 in a high SP1-expressing intestinal-type gastric cell line, MKN28, increased migration and invasion but decreased proliferation. Microarray data in SP1 siRNA-transfected MKN28 revealed that the genes inhibiting migration were downregulated, whereas the genes negatively facilitating proliferation were increased. However, both migration and invasion were decreased by forced SP1 expression in a low SP1-expressing intestinal-type gastric cell line, AGS. Unlike the intestinal-type, in a high SP1-expressing diffuse-type gastric cell line, SNU484, migration and invasion were decreased by SP1 siRNA. In contrast to previous studies that did not identify differences between the 2 histological types, our results reveal that low expression of SP1 is involved in cancer progression and   metastasis   and differentially affects intestinal-type compared with diffuse-type gastric adenocarcinoma. 
 Promoter hypomethylation up-regulates CD147 expression through increasing Sp1 binding and associates with poor prognosis in human hepatocellular carcinoma. CD147 is a transmembrane glycoprotein overexpressed in human hepatocellular carcinoma (HCC) which could promote HCC progression and   metastasis  . Promoter methylation is one of the most important processes in gene regulation. In this study, we aim to investigate CD147 promoter methylation status and the correlation with clinicopathological features and prognosis in HCC. CD147 promoter methylation statuses and expression levels in normal and HCC cell lines and 54 paired HCC and adjacent non-tumour (ANT) tissues were, respectively, examined by bisulphite genomic sequencing, methylation-specific PCR, real-time RT-PCR, Western blot and immunohistochemistry. The correlations of promoter methylation statuses with CD147 expression level and the clinicopathological features were statistically analysed in HCC patients. Significantly higher expression of CD147 and significantly lower promoter methylation level were observed in HCC cell lines compared to normal cell lines and tissues control. In vivo and in vitro analysis indicated that demethylation with 5-Aza-2'-deoxycytidine led to increased CD147 expression through enhancing Sp1 binding affinity, and methylation with methyltransferase reduced CD147 transcriptional activity through interfering Sp1 binding. CD147 promoter methylation level in HCC tissues (22.22%) was lower than that in ANT tissues (46.30%; P < 0.05). Within HCC tissues, a significant inverse correlation was observed between CD147 expression and methylation level (r=-0.615). Moreover, HCC patients with unmethylated CD147 promoter had a significantly higher recurrence rate (88.1%versus 58.3%; P < 0.05) and death rate (83.3%versus 50.0%; P < 0.05) than patients with methylated CD147 promoter. In conclusions, promoter hypomethylation up-regulates CD147 expression primarily through increasing Sp1 binding and associates with poor prognosis in HCC patients. 
 Regulation of KiSS-1   metastasis   suppressor gene expression in breast cancer cells by direct interaction of transcription factors activator protein-2alpha and specificity protein-1. KiSS-1 has been shown to function as a tumor   metastasis   suppressor gene and reduce the number of metastases in different cancers. The expression of KiSS-1 or KiSS1, like other tumor suppressor, is commonly reduced or completely ablated in a variety of cancers via an unknown mechanism. Here we show that the loss of KiSS-1 expression in highly metastatic breast cancer cell lines correlates directly with the expression levels of two transcription factors, activator protein-2alpha (AP-2alpha) and specificity protein 1 (Sp1), which synergistically activate the transcriptional regulation of KiSS-1 in breast cancer cells. Although the KiSS-1 promoter contains multiple AP-2alpha binding elements, AP-2alpha-mediated regulation occurs indirectly through Sp1 sites, as determined by deletion and mutation analysis. Overexpression of AP-2alpha into highly metastatic breast cell lines did not alter KiSS-1 promoter-driven luciferase gene activity. However, co-transfection of AP-2alpha wild-type or the dominant negative form of AP-2 lacking its C-terminal DNA-binding domain, AP-2B, together with Sp1, increased KiSS-1 promoter activity dramatically, suggesting that AP-2alpha regulation of KiSS-1 transcription does not require direct binding to the KiSS-1 promoter. Furthermore, we demonstrated that AP-2alpha directly interacted with Sp1 to form transcription complexes at two tandem Sp1-binding sites of the promoter to activate KiSS-1 transcription. Together, our results indicate that AP-2alpha and Sp1 are strong transcriptional regulators of KiSS-1 and that loss or decreased expression of AP-2alpha in breast cancer may account for the loss of tumor   metastasis   suppressor KiSS-1 expression and thus increased cancer   metastasis  . 
 High co-expression of Sp1 and HER-2 is correlated with poor prognosis of gastric cancer patients. The aim of this study was to investigate co-expression of HER-2 and Sp1 in gastric cancer (GC) so as to determine whether these two proteins may be correlated with poor prognosis of GC patients. We examined the HER-2 overexpression and amplification and expression levels of Sp1 in 227 GC patients using immune-histochemical staining and fluorescence in situ hybridization. Data on clinicopathological features and relevant prognostic factors in these patients were analyzed. Of the 227 gastric cancer samples, 11.89% were positive for HER-2 overexpression/amplification under the new scoring system, and the frequency of negative, weak positive and strong positive expression of Sp1 was 14.98%, 48.01% and 37.0% respectively. No statistically positive correlation was observed between the expression levels of HER-2 and Sp1 in GC tissues. HER-2 overexpression was closely correlated to the Lauren type, degree of differentiation, tumor size and lymph node   metastasis  , and Sp1 as well (P < 0.05). Overexpression of HER-2 and Sp1 predicted poor survival in univariate analysis as well as in a Cox proportional hazards model. 
 Transcriptional regulation of KiSS-1 gene expression in metastatic melanoma by specificity protein-1 and its coactivator DRIP-130. Loss of the   metastasis   suppressor gene, KiSS-1 has been strongly correlated to the progression of metastases in numerous types of cancers. The mechanism through which KiSS-1 is lost during   metastasis  , however, is still not completely known. Previous studies have shown that genetic material on human chromosome 6q16.3-q23 is essential for KiSS-1 expression in normal tissues. Additionally, microcell-mediated transfer of this chromosome in cancerous tissue results in rescued expression of KiSS-1 and reduced metastatic phenotype. Here, we show that loss of Sp1-coactivator protein DRIP-130, which is encoded by human chromosome 6q16.3-q23, results in reduced KiSS-1 promoter activation in highly malignant melanoma cells. Co-expression of Sp1 and DRIP-130 not only rescues KiSS-1 expression, but also induces an inhibition of the invasive and migratory behavior in highly metastatic melanoma cells, similar to the overexpression of KiSS-1   metastasis   suppressor gene in those cells. Furthermore, we demonstrate that KiSS-1 expression is regulated by Sp1 elements within the first 100-bp region of the KiSS-1 promoter and that targeted deletion of a single GC-rich region spanning -93 to -58 interrupts Sp1- and DRIP-130-modulated transcriptional control of KiSS-1 expression. Our results thus suggest that DRIP-130 is a key regulator in KiSS-1 transactivation in normal tissue, and that the loss of DRIP-130 expression, as a result of the gross loss of human chromosome 6q16.3-q23, provokes increased tumor   metastasis  . 
 Starvation after Cobalt-60 γ-Ray Radiation Enhances   Metastasi  s in U251 Glioma Cells by Regulating the Transcription Factor SP1. Radiation is of clinical importance during glioma therapy; however, vasculature damage is observed over the treatment course. This type of tissue damage might lead to starvation conditions, affecting tumor   metastasi  s. To test this possibility, we compared starvation conditions in conjunction with radiation treatment to monitor metastatic ability in the U251 glioma cell line. Transcriptome, western blot, and immunofluorescence analyses were used to measure the RNA and protein expression changes of the U251 cells after various treatments. We found that starvation combined with radiation treatment yielded the most significant expression changes in   metastasi  s-related factors compared to that in the control groups. In addition, a   metastasi  s assay was used to directly measure the metastatic ability of the treated cells, which confirmed that the U251 cells treated with starvation combined with radiation possessed the highest metastatic ability. Furthermore, bioinformatics analysis demonstrated that SP1 represented a common transcription factor associated with changes in   metastasi  s-related factors. Blocking SP1 activity by an inhibitor suppressed the starvation-plus-radiation treatment-mediated enhancement of U251 cell   metastasi  s. Our study provides the first evidence that starvation caused by radiation might play a significant role in enhancing the ability of the glioma cell line U251 to metastasize via regulation of the transcription factor SP1. 
 SP1 is required for basal activation and chromatin accessibility of CD151 promoter in liver cancer cells. CD151 plays an important role in liver cancer   metastasis  . The mechanism on how CD151 is expressed remains unclear. Here we have identified SP1 is a protein functioning in constitutive activation of CD151. Applying a PCR-based chromatin accessibility assay, an open chromatin conformation was discovered localized around the transcription start site of the CD151 gene. Deletion constructs of the 5' flanking region were fused to a luciferase reporter gene. After transient transfection in HepG2 and Hep3B cells, a minimal region -171/-53 bearing three SP1-binding sites was identified as the core promoter. Results obtained from electrophoretic mobility shift and chromatin immunoprecipitation assays demonstrated that SP1 is bound to the core promoter. Deletion of SP1 consensus sequence resulted in the total loss of the promoter activity. Moreover, knockdown of SP1 reduced both CD151 promoter activity and chromatin accessibility. Conclusively, SP1 is pivotal to CD151 transcription partly via the construction of a local open chromatin configuration across the promoter. 
 Sp1-mediated microRNA-182 expression regulates lung cancer progression. Our recent study indicated that overexpression of Sp1 enhances the proliferation of lung cancer cells, while represses   metastasis  . In this study, we found that the transcriptional activity of FOXO3 was increased, but its protein levels decreased following Sp1 expression. Sp1 increased expression of miR-182, which was then recruited to the 3'-untranslated region of FOXO3 mRNA to silence its translational activity. Knockdown of miR-182 inhibited lung cancer cells growth, but enhanced the invasive and migratory abilities of these cells through increased N-cadherin expression. Repression of FOXO3 expression in the miR-182 knockdown cells partially reversed this effect, suggesting that miR-182 promotes cancer cell growth and inhibits cancer metastatic activity by regulating the expression of FOXO3. The expression of several cancer   metastasis  -related genes such as ADAM9, CDH9 and CD44 was increased following miR-182 knockdown. In conclusion, in the early stages of lung cancer progression, Sp1 stimulates miR-182 expression, which in turn decreases FOXO3 expression. This stimulates proliferation and tumor growth. In the late stages, Sp1 and miR-182 decline, thus increasing FOXO3 expression, which leads to lung   metastasis  . 
 The role of Sp1 and EZH2 in the regulation of LMX1A in cervical cancer cells. We have reported previously that LIM homeobox transcription factor 1α (LMX1A) is hypermethylated and functions as a   metastasi  s suppressor in cervical cancer cells. However, the regulation of LMX1A in carcinogenesis has not been reported. We aim to clarify whether specificity protein 1 (Sp1) and enhancer of zeste homolog 2 (EZH2) are involved in the regulation of LMX1A in cervical cancer. First we characterized the LMX1A promoter and used overexpression, knockdown, and reporter assays to show that Sp1 increased LMX1A promoter activity. Next, we used site-directed mutagenesis and electrophoresis mobility shift assays (EMSAs) to demonstrate that Sp1-binding sites were important for Sp1-mediated activation of the LMX1A promoter. Chromatin immunoprecipitation data demonstrated that Sp1 could bind directly to the LMX1A promoter and activate endogenous LMX1A expression in cells pretreated with 5-aza-2'-deoxycytidine (5-aza-dC). Knockdown of EZH2 decreased H3K27me3 histone modification but was insufficient to restore LMX1A expression. To explore the effect of EZH2 on the endogenous LMX1A promoter, we treated EZH2-knockdown cells with 5-aza-dC and trichostatin A (TSA) and then depleted the cells of drugs for 3days. H3K14ac was enriched at the LMX1A promoter in EZH2-knockdown cells and LMX1A mRNA was still expressed. Taken together, these data imply that Sp1 may activate LMX1A expression upon oncogenic stress during cervical cancer development. Moreover, suppression of EZH2 may delay resilencing of LMX1A after the removal of 5-aza-dC and TSA. 
 Sp1 is over-expressed in nasopharyngeal cancer and is a poor prognostic indicator for patients receiving radiotherapy. Nasopharyngeal cancer (NPC) is a tumor of epithelial origin with complex etiology. Currently the standard treatment of NPC is radiotherapy, but therapy failure is quite common, making radioresistance an important issue. This study explores the association of specificity protein 1 (Sp1) protein expression with clinicopathological significance and disease prognosis in NPC patients receiving radiotherapy. A total of 82 NPC patients (55 males and 27 females, median age: 48 years old) were enrolled and received radiotherapy between September 2011 and March 2014. Tumor tissue and grossly adjacent normal mucosa were obtained in each patient. Sp1 expression was detected by western blot and immunohistochemical analysis, and the associations with clinicopathological status and radiotherapy response were analyzed. Our Results showed Sp1 protein expression was higher in CNE-1 and CNE-2 nasopharyngeal cancer cells than in normal nasopharyngeal mucosal NP69 cells. All 82 patients' tissue sections were stained positive for the Sp1 protein, and 39 (47.6%) patients showed higher level than adjacent normal mucosa. Sp1-overexpression in the tumor tissue was correlated with a higher tumor stage, nodal status, clinical stage and distant   metastasis   (P < 0.01). Patients with higher Sp1 expression in pretreatment biopsies had a lower radiotherapy response compared to those with lower expression. In conclusion, Sp1 may play roles in radioresistance of nasopharyngeal cancer which attributes to tumor invasiveness, and serve as a novel prognostic marker of NPC radiotherapy. However, further studies are required to validate our findings in larger samples and explore more detailed mechanisms underlying radioresistance of Sp1. 
 The antimetastatic effects of resveratrol on hepatocellular carcinoma through the downregulation of a   metastasis  -associated protease by SP-1 modulation. The mortality and morbidity rates from cancer   metastasis   have not declined in Taiwan, especially because of hepatocellular carcinoma (HCC). Resveratrol has been shown to have benefits such as cardioprotection, providing antioxidative, anti-inflammatory, anti-cancer properties in previous studies. Therefore, HCC cells were subjected to treatment with resveratrol and then analyzed to determine the effects of resveratrol on the migration and invasion.Modified Boyden chamber assays revealed that resveratrol treatment significantly inhibited cell migration and invasion capacities of Huh7 cell lines that have low cytotoxicity in vitro, even at a high concentration of 100 µM. The results of casein zymography and western blotting revealed that the activities and protein levels of the urokinase-type plasminogen activator (u-PA) were inhibited by resveratrol. Western blot analysis also showed that resveratrol inhibits phosphorylation of JNK1/2. Tests of the mRNA level, real-time PCR, and promoter assays evaluated the inhibitory effects of resveratrol on u-PA expression in HCC cells. The chromatin immunoprecipitation (ChIP) assay showed that reactive in transcription protein of nuclear factor SP-1 was inhibited by resveratrol.Resveratrol inhibits u-PA expression and the   metastasi  s of HCC cells and is a powerful chemopreventive agent. The inhibitory effects were associated with the downregulation of the transcription factors of SP-1 signaling pathways. 
 [Expression and prognostic value of transcriptional factor sp1 in breast cancer]. Transcriptional factor Sp1 is involved in many biological processes, such as cell proliferation, apoptosis, differentiation and transformation, and plays an important role in invasion and   metastasis   of tumors. However, the expression patterns of Sp1 in various tumors are different. This study was to investigate the correlations of Sp1 expression to   metastasis  , invasion, and prognosis of breast cancer.The expression of Sp1 in 60 specimens of breast cancer and 12 specimens of adjacent breast tissue was detected by EnVision immunohistochemistry. Its correlation to clinicopathologic features of breast cancer was analyzed by Cox regression analysis.The positive rate of Sp1 was 71.67% in breast cancer tissues, and 33.33% in adjacent tissues. Sp1 staining in cancer tissue was positively correlated to TNM stage (r=0.349, P<0.05), tumor invasion (r=0.407, P<0.01), and lymph node   metastasis   (r=0.314, P<0.05). Univariate analysis indicated that the overall survival rate was significantly lower in Sp1-positive patients than in Sp1-negative patients (41.86% vs. 82.35%, P<0.05). Cox multivariate analysis showed that Sp1 expression, TNM stage, invasion and lymph node   metastasis   were independent prognostic factors of breast cancer.Sp1 maybe participate in the invasion and   metastasis   of breast cancer, and is one of the valuable markers indicating poor prognosis of breast cancer. Sp1 detection, with consideration of tumor invasion and clinical stage, may increase the accuracy of predicting prognosis of patients with breast cancer. 
 Sp1-mediated transcriptional regulation of MALAT1 plays a critical role in tumor. MALAT1 was discovered as a prognostic marker for lung cancer   metastasis   and has been found upregulated in many types of tumor, but its transcriptional regulation mechanism in tumors remains unclear.A deletion analysis of MALAT1 promoter region was performed to find the cis elements that were critical for the transcriptional activation of MALAT1 gene. Reporter gene assays were employed to analyze the effect of Sp1 on the promoter activity of MALAT1 gene. The binding activity of Sp1 with the promoter of MALAT1 gene was examined by EMSA and ChIP assay. Effects of Sp1 on regulation of MALAT1 were analyzed by RNA interference in vitro and in vivo mouse model.By means of luciferase assay, Sp1 was found to activate the promoter of the human MALAT1 gene. The binding of Sp1 to this region was also detected by electrophoretic mobility shift and chromatin immunoprecipitation assays. Sp1 knockdown also decreased the MALAT1 and inhibited A549 lung cancer cells' growth and invasion in vitro. Furthermore, knockdown of Sp1 also mimicked the inhibition of MALAT1 in A549 lung cancer cells' growth and   metastasis   in vivo.Taken together, our data suggest that upregulation of MALAT1 was mediated by the transcription factor Sp1 in A549 lung cancer cells, and Sp1 could be therapeutic target for cancer. 
 Transcription factor Sp1 regulates expression of cancer-associated molecule CD147 in human lung cancer. CD147 is a novel cancer-associated biomarker that plays an important role in the invasion and   metastasis   of human lung cancer. In spite of its many known functions, little is known about CD147 transcriptional regulation. In this study, we explored the regulation of CD147 in human lung cancer tissues. Over 60% of the human lung cancer tissues expressed differential high levels of CD147. We then cloned the 5'-flanking region of the human CD147 gene and identified a critical promoter region at -108 to -42 which contained one binding site for Sp1, which was essential in up-regulating CD147 promoter activity. These results were proven by blocking Sp1 using RNAi or mithramycin A treatment and up-regulating Sp1 using transfection with eukaryotic expression vector. Consistent with the CD147 transcription activation, a high level of Sp1 expression was detected in lung cancer cell lines overexpressing CD147. Chromatin immunoprecipitation assay showed that much more Sp1 could bind to the CD147 promoter in 95-D with CD147 high expression than in SK-MES-1 with CD147 low expression. There was a significant positive correlation between CD147 expression and Sp1 expression level detected by immunohistochemistry (r = 0.831). Collectively, our results suggest that Sp1 is essential for regulating the CD147 gene expression in human lung cancer. 
 Transcriptional activation of p21(waf1/cip1) by alkylphospholipids: role of the mitogen-activated protein kinase pathway in the transactivation of the human p21(waf1/cip1) promoter by Sp1. Alkylphospholipids (ALKs) are a novel class of antitumor agents with an unknown mechanism of action. The first ALK tested in the clinic, miltefosine, has been approved recently in Europe for the local treatment of patients with cutaneous   metastasis  . Perifosine, the only available oral ALK, is being studied currently in human cancer clinical trials. We have shown previously that perifosine induces p21(waf1/cip1) in a p53-independent fashion and that induction of p21(waf1/cip1) is required for the perifosine-induced cell cycle arrest because cell lines lacking p21(waf1/cip1) are refractory to perifosine. In this report, we investigated the mechanism by which perifosine induces p21(waf1/cip1) protein expression. We observed that perifosine induces the accumulation of p21(waf1/cip1) mRNA without affecting p21(waf1/cip1) mRNA stability. Using several p21(waf1/cip1) promoter-driven luciferase reporter plasmids, we observed that perifosine activates the 2.4-kb full-length p21(waf1/cip1) promoter as well as a p21 promoter construct lacking p53-binding sites, suggesting that perifosine activates the p21(waf1/cip1) promoter independent of p53. The minimal p21 promoter region required for perifosine-induced p21 promoter activation contains four consensus Sp1-binding sites. Mutations in each particular Sp1 site block perifosine-induced p21(waf1/cip1) expression. Moreover, we showed that perifosine activates the mitogen-activated protein/extracellular signal-regulated kinase pathway, and this activation promotes the phosphorylation of Sp1 in known mitogen-activated protein kinase residues (threonine 453 and 739), thereby leading to increased Sp1 binding and enhanced p21(waf1/cip1) transcription. These results represent a novel mechanism by which alkylphospholipids modulate transcription, and may contribute to the discovery of new signal transduction pathways crucial for normal and neoplastic cell cycle control. 
 Egr-1 regulates the transcription of NGX6 gene through a Sp1/Egr-1 overlapping site in the promoter. As a novel candidate   metastasis   suppressor gene, Nasopharyngeal carcinoma-associated gene 6 (NGX6) is involved in cellular growth, cell cycle progression and tumor angiogenesis. Previous studies have shown that NGX6 gene is down-regulated in colorectal cancer (CRC). However, little is known about its transcriptional regulation.We defined the minimal promoter of NGX6 gene in a 186-bp region (from-86 to +100) through mutation construct methods and luciferase assays. Results from Electrophoretic mobility shift assays (EMSA) and Chromatin immunoprecipitation (ChIP) revealed that Early growth response gene 1 (Egr-1) binds to the Sp1/Egr-1 overlapping site of NGX6 minimal promoter. Overexpression of Egr-1 increased the promoter activity and mRNA level of NGX6 gene; while knock-down of endogenous Egr-1 decreased mRNA expression of NGX6 gene.These results demonstrate that Egr-1 regulates NGX6 gene transcription through an overlapping Sp1/Egr-1 binding site as a positive regulator of NGX6 gene. 
 Vascular endothelial growth factor induces multidrug resistance-associated protein 1 overexpression through phosphatidylinositol-3-kinase /protein kinase B signaling pathway and transcription factor specificity protein 1 in BGC823 cell line. Multidrug resistance (MDR) is one of the most important causes of chemotherapy failure and carcinoma recurrence. But the roles of the MDR-associated protein MRP1 in MDR remain poorly understood. Vascular endothelial growth factor (VEGF), one of the most active and specific vascular growth factors, plays a significant role in proliferation, differentiation, and   metastasis   of cancers. To explore the effect of VEGF on the expression of MRP1, we used recombinant human VEGF to stimulate K562 and BGC-823 cell lines. Quantitative real-time polymerase chain reaction and western blot analysis showed that the expression of MRP1 at both mRNA and protein levels was increased. 3-(4,5-Dimethylthiazol-2-yl)-2,5-diphenyl tetrazolium bromide results also showed that VEGF significantly enhanced the IC50 of the cells treated with adriamycin. To explore the underlying regulatory mechanisms, we constructed MRP1 promoter and the luciferase reporter gene recombinant vector. The luciferase reporter gene assay showed that the activity of the MRP1 promoter was markedly increased by VEGF stimulation, while LY294002, an inhibitor of the phosphatidylinositol-3-kinase (PI3K)/protein kinase B (Akt) signaling pathway, reduced this effect. Transcription factor specificity protein 1 (SP1) binding site mutation partially blocked the up-regulation of MRP1 promoter activity by VEGF. In summary, our results demonstrated that VEGF enhanced the expression of MRP1, and the PI3K/Akt signaling pathway and SP1 may be involved in this modulation. 
 Suppression of lung cancer cell invasion by LKB1 is due to the downregulation of tissue factor and vascular endothelial growth factor, partly dependent on SP1. LKB1 encodes a serine/threonine kinase generally inactivated in many human cancers, which mediates cancer cell proliferation, migration and differentiation. Recent studies indicated that LKB1 exhibits potent anti-metastatic activity. However, the underlying molecular mechanisms of this activity remain unclear. In this study, we re‑introduced LKB1 into A549 lung cancer cells that lack the LKB1 gene to investigate how LKB1 affects tumor invasiveness an  d metastas  is. We demonstrated that overexpression of the LKB1 protein in lung cancer cells resulted in significant inhibition of invasion. Furthermore, transfected lung cancer cells with LKB1 suppressed tissue factor (TF) and vascular endothelial growth factor (VEGF) expression at both the mRNA and protein levels. Here, we provided evidence showing that downregulation of TF and VEGF by LKB1 is correlated well with the inhibition of cell invasion. Overexpression of the LKB1 protein in human lung cancer is significantly associated with a decrease in activity and expression of the transcription factor SP1. Constitutive activation of the transcription factor Sp1 plays a critical role in TF and VEGF overexpression. We conclude that suppression of lung cancer cell invasion by LKB1 through downregulation of TF and VEGF may partly depend on its inhibitory effect on the transcription factor Sp1. Collectively, our data provide a novel molecular mechanism for the antitumor activity of LKB1 and may help further improve its effectiveness in controlling lung cancer growth and invasion. 
 PI3K/Akt signaling pathway triggers P2X7 receptor expression as a pro-survival factor of neuroblastoma cells under limiting growth conditions. The expression of purinergic P2X7 receptor (P2X7R) in neuroblastoma cells is associated to accelerated growth rate, angiogenesis,   metastasis   and poor prognosis. Noticeably, P2X7R allows the survival of neuroblastoma cells under restrictive conditions, including serum and glucose deprivation. Previously we identified specificity protein 1 (Sp1) as the main factor involved in the transcriptional regulation of P2rx7 gene, reporting that serum withdrawal triggers the expression of P2X7R in Neuro-2a (N2a) neuroblastoma cell line. Here we demonstrate that PI3K/Akt pathway is crucial for the upregulation of P2X7R expression in serum-deprived neuroblastoma cells, circumstance that facilitates cell proliferation in the absence of trophic support. The effect exerted by PI3K/Akt is independent of both mTOR and GSK3, but requires the activation of EGF receptor (EGFR). Nuclear levels of Sp1 are strongly reduced by inhibition of PI3K/Akt pathway, and blockade of Sp1-dependent transcription with mithramycin A prevents upregulation of P2rx7 gene expression following serum withdrawal. Furthermore, atypical PKCζ plays a key role in the regulation of P2X7R expression by preventing phosphorylation and, consequently, activation of Akt. Altogether, these data indicate that activation of EGFR enhanced the expression of P2X7R in neuroblastoma cells lacking trophic support, being PI3K/Akt/PKCζ signaling pathway and Sp1 mediating this pro-survival outcome. 
 miR-375 inhibits the invasion and   metastasis   of colorectal cancer via targeting SP1 and regulating EMT-associated genes. Accumulating evidence has shown that aberrantly expressed microRNAs (miRNAs) are associated with tumor development and progression. Our previous study found that microRNA-375 (miR-375) was downregulated in colorectal cancer (CRC), but little is known concerning the role of miR-375 and the related mechanism in CRC development. The proliferation, invasion and migration effects were investigated by Cell Counting Kit-8 (CCK-8), colony formation and Transwell assays with or without Matrigel. In addition, candidate target genes were screened and validated by luciferase reporter and western blot assays. In addition, western blot analysis was performed to explore the molecular mechanisms associated with epithelial‑mesenchymal transition (EMT). It was found that miR-375 inhibited proliferation, invasion and migration in DLD1 and HCT8 cells. In addition, miR-375 negatively regulated Sp1 transcription factor (SP1) protein by directly binding to the 3'-untranslated region (3'-UTR). Furthermore, it was found that miR-375 regulated matrix metalloproteinase 2 (MMP2) and EMT-associated genes, E-cadherin, vimentin, snail, N-cadherin and β-catenin. In conclusion, miR-375 inhibited the proliferation, invasion and migration by directly targeting SP1 and regulating MMP2 and EMT-associated genes. 
 CYP1B1 Enhances Cell Proliferation and   Metastasis   through Induction of EMT and Activation of Wnt/β-Catenin Signaling via Sp1 Upregulation. Cytochrome P450 1B1 (CYP1B1) is a major E2 hydroxylase involved in the metabolism of potential carcinogens. CYP1B1 expression has been reported to be higher in tumors compared to normal tissues, especially in hormone-related cancers including breast, ovary, and prostate tumors. To explore the role of CYP1B1 in cancer progression, we investigated the action of CYP1B1 in cells with increased CYP1B1 via the inducer 7,12-dimethylbenz[α]anthracene (DMBA) or an overexpression vector, in addition to decreased CYP1B1 via the inhibitor tetramethoxystilbene (TMS) or siRNA knockdown. We observed that CYP1B1 promoted cell proliferation, migration, and invasion in MCF-7 and MCF-10A cells. To understand its molecular mechanism, we measured key oncogenic proteins including β-catenin, c-Myc, ZEB2, and matrix metalloproteinases following CYP1B1 modulation. CYP1B1 induced epithelial-mesenchymal transition (EMT) and activated Wnt/β-catenin signaling via upregulation of CTNNB1, ZEB2, SNAI1, and TWIST1. Sp1, a transcription factor involved in cell growth   and metast  asis, was positively regulated by CYP1B1, and suppression of Sp1 expression by siRNA or DNA binding activity using mithramycin A blocked oncogenic transformation by CYP1B1. Therefore, we suggest that Sp1 acts as a key mediator for CYP1B1 action. Treatment with 4-hydroxyestradiol (4-OHE2), a major metabolite generated by CYP1B1, showed similar effects as CYP1B1 overexpression, indicating that CYP1B1 activity mediated various oncogenic events in cells. In conclusion, our data suggests that CYP1B1 promotes cell proliferation   and metast  asis by inducing EMT and Wnt/β-catenin signaling via Sp1 induction. 
 Prostaglandin E2 induces stromal cell-derived factor-1 expression in prostate stromal cells by activating protein kinase A and transcription factor Sp1. Recent reports indicate prostaglandin E2 (PGE2) can modulate tumor environment and promote angiogenesis through induction of stromal cell-derived factor 1 (SDF-1) production. We investigated the mechanism of PGE2-induced SDF-1 regulation in human prostate stromal cell and analyzed the effects in a stromal-epithelial interaction model. PGE2 stimulation increased SDF-1 expression in the prostate stromal cell lines WPMY-1 and NAF. We revealed signaling through the PGE2 receptor EP3 and activation of protein kinase A (PKA) are required. The EP3 agonist sulprostone and the cAMP analog forskolin mimicked and the EP3 siRNA, antagonist L798106 and the PKA inhibitor H89 abrogated the effect of PGE2 on SDF-1 expression. SDF-1 promoter truncation experiments demonstrated a 254 bp (from nt -219 to nt +34) SDF-1 proximal promoter fragment containing 5 putative transcription factor Sp1 binding motifs is sufficient for PGE2 induction. CHIP assays confirmed binding and PGE2 induced recruitment of Sp1 to the SDF-1 promoter. Sp1 motif mutation identified Sp1 motifs -140/-133 and -9/+1 as the crucial elements responsible for PGE2 induction. Moreover, SDF-1 was up- or down-regulated by Sp1 over-expression or knock-down. We also demonstrate stimulation of migration of prostate cancer cell lines PC3 and DU145 with conditioned media collected from WPMY-1 or NAF cells stimulated with PGE2 and blockade of enhanced migration by a SDF-1 neutralizing antibody. In conclusion, we provide evidence for a paracrine prostate stromal-epithelial interaction induced by upregulation of expression of SDF-1 by PGE2. Our research provides new insights into the mechanism promoting   metastasis   of prostate carcinoma via stromal-epithelial interaction. 
 Glyceraldehyde-3-phosphate dehydrogenase promotes cancer growth and   metastasis   through upregulation of SNAIL expression. Glyceraldehyde-3-phosphate dehydrogenase (GAPDH) plays an important role in multiple cellular functions including metabolism and gene transcription. Our previous study showed that GAPDH expression was elevated in colon cancer and further upregulated in liver metastatic tissues, suggesting a possilbe role of GAPDH in promoting cancer   metastasis  . The present study was designed to investigate the underlying mechanism, using multiple experimental approaches including genetic silencing of GAPDH expression by short hairpin RNA (shRNA) and biochemcial/molecular analyses of the key events involved in glycolytic metabolism and epithelial-mesenchymal transition (EMT). We showed that silencing of GAPDH expression resulted in a significant reduction of glycolysis in colon cancer cell lines, accompanied by a decrease in cell proliferation and an apparent change in cell morphology associated with alterations in actin expression and phalloidine staining patterns. Furthermore, GAPDH suppression also caused a downregulation of gene expression involved in cancer stem-like cells and EMT. CHIP assay and co-immunoprecipitation revealed that GAPDH physically interacted with the transcriptional factor Sp1 and enhance the expression of SNAIL, a major regulator of EMT. Suppression of GAPDH expression resulted in a signficant decrease in SNAIL expression, leading to inhibition of EMT and attenuation of colon cancer cell migration in vitro and reduced   metastasi  s in vivo. Overall, the present study suggests that GAPDH plays an important role in cance  r metastas  is by affecting EMT through regulation of Sp1-mediated SNAIL expression. 
 JMJD2A-dependent silencing of Sp1 in advanced breast cancer promotes   metastasis   by downregulation of DIRAS3. Specificity protein 1(Sp1) is a ubiquitous transcription factor and is highly expressed in breast cancer. However, its expression pattern and role in breast cancer progression remain unclear. The purpose of this study is to examine the expression pattern of Sp1 and determine its role in breast cancer progression. Immunohistochemistry (IHC) was performed on breast cancer tissues to reveal the expression pattern of Sp1. Spearman rank correlation was used for clinical statistics. Gene and protein expressions were monitored by IHC analysis, quantitative polymerase chain reaction, and Western blot. Wound-healing and Transwell assays were conducted to assess the role of Sp1 in breast cancer. Co-immunoprecipitation, deletion mutagenesis, chromatin immunoprecipitation, and dual luciferase reporter gene assays were used for investigation of the regulatory network. Sp1 expression was downregulated in late stage breast cancer and in highly invasive breast cancer cell lines. Expression of Sp1 was negatively correlated with TNM staging (P = 0.002) and   metastasis   status (P = 0.023). Overexpression of Sp1 inhibited breast cancer cell migratory and invasive abilities, whereas knockdown of GTP-binding RAS-like 3 (DIRAS3, also known as ARHI, NOEY2) attenuated the inhibitory effects. Moreover, re-expression of DIRAS3 abolished Sp1 knockdown-mediated cell migration and invasion. Jumonji domain containing 2A (JMJD2A) inhibited Sp1 autoregulation and explains Sp1 expression pattern in breast cancer. Sp1 negatively regulated breast cancer   metastasis   by transcriptional activation of DIRAS3. Inhibition of Sp1 autoregulation by JMJD2A contributed to Sp1 expression pattern in breast cancer. Our findings provided evidence that targeted therapy against Sp1 might be useful in early stage breast cancer. However, in late stages, development of Sp1 activator may be more promising for breast cancer treatments. 
 miRNA-411 acts as a potential tumor suppressor miRNA via the downregulation of specificity protein 1 in breast cancer. The expression and functions of microRNA (miR)-411 have been investigated in several types of cancer. However, until now, miR-411 in human breast cancer has not been examined. The present study investigated the expression, biological functions and molecular mechanisms of miR‑411 in human breast cancer, discussing whether it offers potential as a therapeutic biomarker for breast cancer in the future. The expression levels of miR‑411 in human breast cancer tissues and cells were measured using reverse transcription‑quantitative polymerase chain reaction analysis. Following transfection with miR‑411 mimics, an MTT assay, cell migration and invasion assay, western blot analysis and luciferase assay were performed in human breast cancer cell lines. According to the results, it was found that miR‑411 was significantly downregulated in breast cancer, and associated with l  ymph node   metastasis and histological grade. Additionally, it was observed that miR‑411 suppressed cell growth, migration and invasion in the breast cancer cells. The present study also provided the first evidence, to the best of our knowledge, that miR‑411 was likely to directly target specificity protein 1 in breast cancer. These findings indicated that miR‑411 may be used a therapeutic biomarker for the treatment of breast cancer in the future. 
 [Expression of transcription factor SP1, vascular endothelial growth factor and CD34 in serosa-infiltrating gastric cancer and their relationship with biological behavior and prognosis]. To investigate the expression of transcription factor SP1, vascular endothelial growth factor(VEGF) and CD34 in serosa-infiltrating gastric cancer and their relationship with biological behavior and survival rate.Immunohistochemical technique was used to detect the expression of SP1, VEGF and CD34(described by microvessel density, MVD) in 68 specimens with serosa-infiltrating gastric cancer.The positive expression rates of SP1 and VEGF in serosa-infiltrating gastric cancer were 50.0% and 52.9% respectively. In positive SP1 specimens, the positive rate of VEGF(73.5%) was significantly higher than that of negative SP1 specimens (32.4%, chi(2)=11.57, P=0.01). The mean tumor MVD was correlated with the expression levels of SP1 and VEGF(P<0.01). There was a significant correlation of the SP1 expression with tumor size and growth pattern(P =0.01). The expression levels of VEGF and MVD were correlated with Borrmann types, cell differentiation, metastatic lymph nodes and growth pattern(P<0.01). Univariate analysis revealed that SP1 and VEGF expression, MVD, Borrmann types, lymph node   metastasis  , tumor size and growth pattern were significant prognostic factors related to survival time. Multivariate analysis showed that SP1 expression, MVD and growth pattern were independently prognostic factors of poor survival.The activation of SP1 contributes to angiogenesis and   metastasis   in gastric cancer through the up-regulation of VEGF. SP1, VEGF and MVD may serve as valuable indicators of biological behavior of gastric cancer. SP1 protein expression is not related with the number of metastatic lymph nodes. SP1 expression and MVD may serve as valuable indicators of prognosis in gastric carcinoma. 
 Melanoma upregulates ICAM-1 expression on endothelial cells through engagement of tumor CD44 with endothelial E-selectin and activation of a PKCα-p38-SP-1 pathway. Cancer   metastasi  s involves multistep adhesive interactions between tumor cells (TCs) and endothelial cells (ECs), but the molecular mechanisms of intercellular communication in the tumor microenvironment remain elusive. Using static and flow coculture systems in conjunction with flow cytometry, we discovered that certain receptors on the ECs are upregulated on melanoma cell adhesion. Direct contact but not separate coculture between human umbilical endothelial cells (HUVECs) and a human melanoma cell line (Lu1205) increased intercellular adhesion molecule 1 (ICAM-1) and E-selectin expression on HUVECs by 3- and 1.5-fold, respectively, compared with HUVECs alone. The nonmetastatic cell line WM35 failed to promote ICAM-1 expression changes in HUVECs on contact. Enzyme-linked immunosorbent assay (ELISA) revealed that EC-TC contact has a synergistic effect on the expression of the cytokines interleukin (IL)-8, IL-6, and growth-related oncogene α (Gro-α). By using E-selectin cross-linking and beads coated with CD44 immunopurified from Lu1205 cells, we showed that CD44/selectin ligation was responsible for the ICAM-1 up-regulation on HUVECs. Protein kinase Cα (PKC-α) activation was found to be the downstream target of the CD44/selectin-initiated signaling, as ICAM-1 elevation was inhibited by siRNA targeting PKCα or a dominant negative form of PKCα (PKCα DN). Western blot analysis and electrophoretic mobility shift assays (EMSAs) showed that TC-EC contact mediated p38 phosphorylation and binding of the transcription factor SP-1 to its regulation site. In conclusion, CD44/selectin binding signals ICAM-1 up-regulation on the EC surface through a PKCα-p38-SP-1 pathway, which further enhances melanoma cell adhesion to EC  s during m  etastasis. 
 p16 inhibits matrix metalloproteinase-2 expression via suppression of Sp1-mediated gene transcription. Previous studies demonstrate that p16, a cyclin-dependent kinase inhibitor and a tumor suppressor, may inhibit matrix metalloproteinase-2 (MMP-2) expression in human cancer cells to suppress tumor invasion and   metastasis  . However, the detailed mechanism is still unclear. Our results show that p16 inhibits MMP-2 expression via transcriptional repression. Promoter deletion and mutation analysis indicates that p16 acts through the Sp1 transcription factor-binding site located between -72 and -64 bp region from the transcriptional start site of the human MMP-2 promoter to repress gene expression. DNA affinity precipitation assay (DAPA) and chromatin immuno-precipitation (CHIP) assay demonstrate that Sp1 proteins constitutively bind to this consensus sequence in vitro and in vivo. p16 attenuates Sp1 binding to the MMP-2 promoter to suppress gene transcription and overexpression of Sp1 may counteract p16-induced downregulation of MMP-2. CyclinA/CDK complex may directly phosphorylate Sp1 and enhance its DNA-binding activity. Thus, we investigated the effect of p16 on the interaction between cyclin A and Sp1. Our results indicate that p16 induces downregulation of cyclin A and CDK2, reduces the interaction between cyclin A and Sp1, and attenuates phosphorylation of Sp1. Ectoexpression of cyclin A counteracts p16-mediated inhibition of DNA binding of Sp1 and activates MMP-2 promoter activity and mRNA expression. Collectively, our results suggest that p16 suppresses MMP-2 by blocking Sp1-mediated gene transcription. 
 NFAT-1, Sp-1, Sp-3, and miR-21: New regulators of chemokine C receptor 7 expression in mature human dendritic cells. The chemokine C receptor 7 (CCR7) is a G-protein-coupled heptahelical receptor (GPCR) that is expressed on a wide variety of cells including memory T cells, B cells, mature dendritic cells, and cancer cells. Activated by its ligands CCL19 or CCL21, CCR7 plays a major role in   metastasis   of cancer cells. Recent studies demonstrated the role of NF-κB and AP-1 transcription factors in addition to let-7 microRNA in CCR7 expression. Our ChIP assays further show the binding of Sp-1, Sp-3 and NFAT-1 transcription factors to their potential binding sites in the 1Kb promoter region with the later found to inhibit whilst Sp-1, and Sp-3 were found to stimulate CCR7 expression as demonstrated by transfection assays. On the other hand, in addition to the known let-7 regulation of CCR7, we found miR-21 to have a highly conserved target region in CCR7 3'UTR and to be significantly down-regulated during the course of dendritic cell maturation, allowing for high expression of CCR7. 
 MiR-324-5p Suppresses Hepatocellular Carcinoma Cell Invasion by Counteracting ECM Degradation through Post-Transcriptionally Downregulating ETS1 and SP1. Hepatocellular carcinoma (HCC) is one of the common malignancies, which is highly metastatic and the third common cause of cancer deaths in the world. The invasion and   metastasis   of cancer cells is a multistep and complex process which is mainly initiated by extracellular matrix (ECM) degradation. Aberrant expression of microRNA has been investigated in HCC and shown to play essential roles during HCC progression. In the present study, we found that microRNA-324-5p (miR-324-5p) was downregulated in both HCC cell lines and tissues. Ectopic miR-324-5p led to the reduction of HCC cells invasive and metastatic capacity, whereas inhibition of miR-324-5p promoted the invasion of HCC cells. Matrix metalloproteinase 2 (MMP2) and MMP9, the major regulators of ECM degradation, were found to be downregulated by ectopic miR-324-5p, while upregulated by miR-324-5p inhibitor. E26 transformation-specific 1 (ETS1) and Specificity protein 1 (SP1), both of which could modulate MMP2 and MMP9 expression and activity, were presented as the direct targets of and downregulated by miR-324-5p. Downregulation of ETS1 and SP1 mediated the inhibitory function of miR-324-5p on HCC migration and invasion. Our study demonstrates that miR-324-5p suppresses hepatocellular carcinoma cell invasion and might provide new clues to invasive HCC therapy. 
 The human receptor tyrosine kinase Axl gene--promoter characterization and regulation of constitutive expression by Sp1, Sp3 and CpG methylation. Axl is a receptor tyrosine kinase which promotes anti-apoptosis, mitogenesis, invasion, angiogenesis and   metastasis  , and is highly expressed in cancers. However, the transcriptional regulation of this important gene has never been characterized. The present study was initiated to characterize the promoter, cis-acting elements and promoter methylation driving expression of Axl. The 2.4 kb sequence upstream of the translational start site, and sequential 5'-deletions were cloned and revealed a minimal GC-rich region (-556 to +7) to be sufficient for basal Axl promoter activity in Rko, HCT116 and HeLa cells. Within this minimal region, five Sp (specificity protein)-binding sites were identified. Two sites (Sp a and Sp b) proximal to the translation start site were indispensable for Axl promoter activity, whereas mutation of three additional upstream motifs (Sp c, Sp d and Sp e) was of additional relevance. Gel-shift assays and chromatin immunoprecipitation identified that Sp1 and Sp3 bound to all five motifs, and mutation of all motifs abolished binding. Mithramycin, which inhibits binding of Sp factors to GC-rich sites, dramatically reduced Axl promoter activity and Axl, Sp1 and Sp3 expression. In Drosophila Schneider SL2-cells, exogenous expression of Sp1/Sp3 increased Axl promoter activity. Use of Sp1/Sp3 siRNAs (small interfering RNAs) significantly reduced Axl promoter activity and protein levels in Rko and HeLa cells. Methylation-bisulfite sequencing detected methylated CpG sites within three Sp motifs (Sp a, Sp b and Sp c) and GC-rich flanking sequences, and demethylation by 5-aza-2'-deoxycytidine up-regulated Axl and Sp3 expression in low-Axl-expressing Colo206f/WiDr cells, but not in high-Axl-expressing Rko cells. The results of the present study suggest that Axl gene expression in cancer cells is (1) constitutively driven by Sp1/Sp3 bound to five core promoter motifs, and (2) restricted by methylation within/around Sp-binding sites. This might enhance the understanding and treatment of essential mechanisms associated with cancer and other diseases. 
 Transcriptional repression of protein kinase Calpha via Sp1 by wild type p53 is involved in inhibition of multidrug resistance 1 P-glycoprotein phosphorylation. The protein kinase C (PKC) family consists of serine/threonine protein kinases that play important roles in signal transduction, cell proliferation, and tumor formation. Recent studies found that PKCs are commonly overexpressed in human tumors, including soft tissue sarcoma (STS). Overexpression of PKCs contributes to invasion and migration of tumor cells and induction of angiogenesis. PKC can also phosphorylate the multidrug resistance (MDR) gene-encoded P-glycoprotein and induce MDR phenotype. Our previous studies showed that mutation of p53 enhanced STS   metastasis   and mediated the MDR phenotype. Restoring wild type (WT) p53 in STS cells containing mutant p53 sensitized the cells to chemotherapy. In the present study, we found that PKCalpha protein expression is inhibited by WT p53 partly due to reduced PKCalpha mRNA expression in STS cells, but p53 does not affect PKCalpha mRNA stability. Deletion and mutation analysis of the PKCalpha promoter fused to the luciferase reporter gene identified a Sp1 binding site (-244/-234) in the PKCalpha promoter that is required for p53-mediated inhibition of PKCalpha promoter activity. More importantly, PKCalpha phosphorylates and activates MDR1 P-glycoprotein, whereas inhibition of PKCalpha by p53 leads to decreased MDR1 phosphorylation in STS cells, which sensitizes STS cells to chemotherapeutic agents. These data indicate that WT p53 may resensitize STS to chemotherapeutic agents by reducing MDR1 phosphorylation via transcriptional repression of PKCalpha expression. Thus, molecular-based therapies targeting mutant p53 and PKCalpha may be an effective new strategy to improve chemotherapeutic efficacy in STS. 
 Functional interaction of E1AF and Sp1 in glioma invasion. Transcription factor E1AF is widely known to play critical roles in tumor   metastasis   via directly binding to the promoters of genes involved in tumor migration and invasion. Here, we report for the first time E1AF as a novel binding partner for ubiquitously expressed Sp1 transcription factor. E1AF forms a complex with Sp1, contributes to Sp1 phosphorylation and transcriptional activity, and functions as a mediator between epidermal growth factor and Sp1 phosphorylation and activity. Sp1 functions as a carrier bringing E1AF to the promoter region, thus activating transcription of glioma-related gene for beta1,4-galactosyltransferase V (GalT V; EC 2.4.1.38). Biologically, E1AF functions as a positive invasion regulator in glioma in cooperation with Sp1 partly via up-regulation of GalT V. This report describes a new mechanism of glioma invasion involving a cooperative effort between E1AF and Sp1 transcription factors. 
  GeneRIFs  
 It was found that miR411 was significantly downregulated in breast cancer, and associated with lymph node   metastasis   and histological grade. Additionally, it was observed that miR411 suppressed cell growth, migration and invasion in the breast cancer cells. miRNA-411 acts as a potential tumor suppressor miRNA via the downregulation of specificity protein 1 in breast cancer. 
 the present study suggests that GAPDH plays an important role in cancer   metastasis   by affecting EMT through regulation of Sp1-mediated SNAIL expression. 
 Bioinformatics analysis demonstrated that SP1 represented a common transcription factor associated with changes in   metastasis  -related factors. Blocking SP1 activity by an inhibitor suppressed the starvation-plus-radiation treatment-mediated enhancement of U251 cell   metastasis  . 
 data suggests that CYP1B1 promotes cell proliferation and   metastasis   by inducing EMT and Wnt/beta-catenin signaling via Sp1 induction 
 Negative regulation of Sp1 by JMJD2A causes downregulation of Sp1 in highly invasive breast cancer cells.JMJD2A-dependent silencing of Sp1 in advanced breast cancer promotes   metastasis   by downregulation of DIRAS3. 
 CD44/selectin binding signals increased intercellular adhesion molecule 1 up-regulation on the endothelial cells (ECs)surface through a PKCalpha-p38-SP-1 pathway, which further enhances melanoma cell adhesion to ECs during   metastasis   
 Resveratrol inhibits u-PA expression and the   metastasis   of HCC cells and is a powerful chemopreventive agent. The inhibitory effects were associated with the downregulation of the transcription factors of SP-1 signaling pathways. 
 low expression of SP1 is involved in cancer progression and   metastasis   and differentially affects intestinal-type compared with diffuse-type gastric adenocarcinoma. 
 our results provide evidence that miR-335 might function as a   metastasis   suppressor in gastric cancer by targeting SP1 directly and indirectly through the Bcl-w-induced phosphoinositide 3-kinase-Akt-Sp1 pathway 
 The activation of SP1 contributes to angiogenesis and   metastasis   in gastric cancer through the up-regulation of VEGF. 
 Sp1 expression, TNM stage, invasion and lymph node   metastasis   were independent prognostic factors of breast cancer. 
 In SW480 human colon cancer cells, we conclude that Sp1 mediated expression of the tumor   metastasis   protein, osteopontin, regulates in vitro functional correlates of tumor   metastasis   
  [Cc]ell [Ii]nvasion  PubMed  
 ZEB2 upregulates integrin α5 expression through cooperation with Sp1 to induce invasion during epithelial-mesenchymal transition of human cancer cells. Epithelial-mesenchymal transition (EMT) is a process implicated in tumor invasion, metastasis, embryonic development and wound healing. ZEB2 is a transcription factor involved in EMT that represses E-cadherin transcription. Although E-cadherin downregulation is a major event during EMT and tumor progression, E-cadherin reduction is probably not sufficient for full invasiveness. The mechanisms by which E-cadherin transcriptional repressors induce mesenchymal genes during EMT remain largely unknown. Here, we investigated the role of ZEB2 in the induction of integrin α5 during cancer EMT and its underlying mechanism. In human cancer cells, ZEB2 was found to directly upregulate integrin α5 transcription in a manner that is independent of the regulation of E-cadherin expression. Conversely, depletion of ZEB2 by small interfering RNA suppressed integrin α5 expression, leading to reduced invasion. Suppression of integrin α5 inhibited ca  ncer cell inv  asion, suggesting an important role for integrin α5 in cancer progression. Furthermore, ZEB2 was found to activate the integrin α5 and vimentin promoters by interacting with and activating the transcription factor Sp1, suggesting that cooperation between ZEB2 and Sp1 represents a novel mechanism of mesenchymal gene activation during EMT. These findings increase our understanding of the pathways beyond E-cadherin reduction that regulate mesenchymal gene expression during EMT and cancer progression. 
 A regulatory loop involving miR-22, Sp1, and c-Myc modulates CD147 expression in breast cancer invasion and metastasis. Breast cancer is the most common cancer in women for which the metastatic process is still poorly understood. CD147 is upregulated in breast cancer and has been associated with tumor progression, but little is known about its regulatory mechanisms. In this study, we demonstrated that CD147 was overexpressed in breast cancer tissues and cell lines, and the high expression correlated with tumor invasion and metastasis. We also found that the transcription factors Sp1 and c-Myc could bind to the CD147 promoter and enhance its expression. The CD147 mRNA has a 748-bp 3'-untranslated region (UTR) with many miRNA target sites, suggesting possible regulation by miRNAs. We discovered that miR-22 repressed CD147 expression by directly targeting the CD147 3'UTR. We also determined that miR-22 could indirectly participate in CD147 modulation by downregulating Sp1 expression. miR-22 could form an autoregulatory loop with Sp1, which repressed miR-22 transcription by binding to the miR-22 promoter. Together with the c-Myc-mediated inhibition of miR-22 expression, our investigation identified a miR-22/Sp1/c-Myc network that regulates CD147 gene transcription. In addition, miR-22 overexpression suppressed breast cancer   cell invasion  , metastasis, and proliferation by targeting CD147 in vitro and in vivo. Furthermore, we found that miR-22 was significantly downregulated in breast cancer tissues and that its expression was inversely correlated with the tumor-node-metastasis stage and lymphatic metastasis in patients. Our study provides the first evidence that an miR-22/Sp1/c-Myc network regulates CD147 upregulation in breast cancer and that miR-22 represses breast cancer invasive and metastatic capacities. 
 Modulation of the expression of the invasion-suppressor CRMP-1 by cyclooxygenase-2 inhibition via reciprocal regulation of Sp1 and C/EBPalpha. Collapsin response mediator protein-1 (CRMP-1) controls neural development and axonal growth but also acts as a cancer invasion suppressor. In this study, we investigated the transcriptional regulation of CRMP-1 expression. Using a serial deletion strategy, we identified a basal promoter region between nucleotides -100 and -180 in the 5' flanking region of CRMP-1 (nucleotides -1,920 to +50) that contains multiple putative Sp1 and C/EBPalpha sites. Site-directed mutagenesis and deletion analysis revealed that the two C/EBPalpha sites, from nucleotides -122 to -133 and from nucleotides -101 to -113, are the most important regulatory elements. Gel-shift and antibody supershift assays showed that Sp1 protein was also present at this C/EBPalpha site, which overlaps with a Sp1 site. Overexpression of Sp1 decreased CRMP-1 promoter activity and protein expression, whereas overexpression of C/EBPalpha produced the opposite effect. Chromatin immunoprecipitation assays confirmed that Sp1 and C/EBPalpha compete for binding at the overlapping C/EBPalpha and Sp1 sites and reciprocally regulate CRMP-1 expression. Overexpression of cyclooxygenase-2 (COX-2) decreased CRMP-1 mRNA and protein expression. Conversely, the COX-2 inhibitor, celecoxib, induced a dose-dependent increase in CRMP-1 expression. COX-2 inhibition also decreased Sp1-DNA complex formation and inhibited   cell invasion  . We conclude that transcription of the invasion suppressor, CRMP-1, is reciprocally regulated at the promoter region by C/EBPalpha and Sp1. COX-2 inhibitors increase CRMP-1 expression by inhibiting Sp1-DNA complex formation and enhancing DNA binding of C/EBPalpha at the promoter. 
 A regulatory loop involving miR-22, Sp1, and c-Myc modulates CD147 expression in breast cancer invasion and metastasis. Breast cancer is the most common cancer in women for which the metastatic process is still poorly understood. CD147 is upregulated in breast cancer and has been associated with tumor progression, but little is known about its regulatory mechanisms. In this study, we demonstrated that CD147 was overexpressed in breast cancer tissues and cell lines, and the high expression correlated with tumor invasion and metastasis. We also found that the transcription factors Sp1 and c-Myc could bind to the CD147 promoter and enhance its expression. The CD147 mRNA has a 748-bp 3'-untranslated region (UTR) with many miRNA target sites, suggesting possible regulation by miRNAs. We discovered that miR-22 repressed CD147 expression by directly targeting the CD147 3'UTR. We also determined that miR-22 could indirectly participate in CD147 modulation by downregulating Sp1 expression. miR-22 could form an autoregulatory loop with Sp1, which repressed miR-22 transcription by binding to the miR-22 promoter. Together with the c-Myc-mediated inhibition of miR-22 expression, our investigation identified a miR-22/Sp1/c-Myc network that regulates CD147 gene transcription. In addition, miR-22 overexpression suppressed breast cancer   cell invasion  , metastasis, and proliferation by targeting CD147 in vitro and in vivo. Furthermore, we found that miR-22 was significantly downregulated in breast cancer tissues and that its expression was inversely correlated with the tumor-node-metastasis stage and lymphatic metastasis in patients. Our study provides the first evidence that an miR-22/Sp1/c-Myc network regulates CD147 upregulation in breast cancer and that miR-22 represses breast cancer invasive and metastatic capacities. 
 ZEB2 upregulates integrin α5 expression through cooperation with Sp1 to induce invasion during epithelial-mesenchymal transition of human cancer cells. Epithelial-mesenchymal transition (EMT) is a process implicated in tumor invasion, metastasis, embryonic development and wound healing. ZEB2 is a transcription factor involved in EMT that represses E-cadherin transcription. Although E-cadherin downregulation is a major event during EMT and tumor progression, E-cadherin reduction is probably not sufficient for full invasiveness. The mechanisms by which E-cadherin transcriptional repressors induce mesenchymal genes during EMT remain largely unknown. Here, we investigated the role of ZEB2 in the induction of integrin α5 during cancer EMT and its underlying mechanism. In human cancer cells, ZEB2 was found to directly upregulate integrin α5 transcription in a manner that is independent of the regulation of E-cadherin expression. Conversely, depletion of ZEB2 by small interfering RNA suppressed integrin α5 expression, leading to reduced invasion. Suppression of integrin α5 inhibited ca  ncer cell inv  asion, suggesting an important role for integrin α5 in cancer progression. Furthermore, ZEB2 was found to activate the integrin α5 and vimentin promoters by interacting with and activating the transcription factor Sp1, suggesting that cooperation between ZEB2 and Sp1 represents a novel mechanism of mesenchymal gene activation during EMT. These findings increase our understanding of the pathways beyond E-cadherin reduction that regulate mesenchymal gene expression during EMT and cancer progression. 
 Modulation of the expression of the invasion-suppressor CRMP-1 by cyclooxygenase-2 inhibition via reciprocal regulation of Sp1 and C/EBPalpha. Collapsin response mediator protein-1 (CRMP-1) controls neural development and axonal growth but also acts as a cancer invasion suppressor. In this study, we investigated the transcriptional regulation of CRMP-1 expression. Using a serial deletion strategy, we identified a basal promoter region between nucleotides -100 and -180 in the 5' flanking region of CRMP-1 (nucleotides -1,920 to +50) that contains multiple putative Sp1 and C/EBPalpha sites. Site-directed mutagenesis and deletion analysis revealed that the two C/EBPalpha sites, from nucleotides -122 to -133 and from nucleotides -101 to -113, are the most important regulatory elements. Gel-shift and antibody supershift assays showed that Sp1 protein was also present at this C/EBPalpha site, which overlaps with a Sp1 site. Overexpression of Sp1 decreased CRMP-1 promoter activity and protein expression, whereas overexpression of C/EBPalpha produced the opposite effect. Chromatin immunoprecipitation assays confirmed that Sp1 and C/EBPalpha compete for binding at the overlapping C/EBPalpha and Sp1 sites and reciprocally regulate CRMP-1 expression. Overexpression of cyclooxygenase-2 (COX-2) decreased CRMP-1 mRNA and protein expression. Conversely, the COX-2 inhibitor, celecoxib, induced a dose-dependent increase in CRMP-1 expression. COX-2 inhibition also decreased Sp1-DNA complex formation and inhibited   cell invasion  . We conclude that transcription of the invasion suppressor, CRMP-1, is reciprocally regulated at the promoter region by C/EBPalpha and Sp1. COX-2 inhibitors increase CRMP-1 expression by inhibiting Sp1-DNA complex formation and enhancing DNA binding of C/EBPalpha at the promoter. 
 MicroRNA-335 acts as a metastasis suppressor in gastric cancer by targeting Bcl-w and specificity protein 1. Aberrant expression of miR-335 has been frequently reported in cancer studies, suggesting that there is a close correlation between miR-335 and cancer during its development, progression, metastasis and prognosis. The expression of miR-335 in gastric cancer and its effects are not known. Relative expression of miR-335 in 4 gastric cancer cell lines and in 70 gastric cancer tissues was confirmed by real-time quantitative reverse transcriptase-PCR compared with controls. Transwell cell migration and Matrigel invasion assay in vitro and metastasis formation assay in vivo were used to examine the effects of miR-335 expression on gastric cancer   cell invasion   and metastasis. The effect of miR-335 expression on gastric cancer cell proliferation was estimated by the 3-(4,5-dimethylthiazol-2-yl)-2, 5-diphenyltetrazolium bromide assay. Luciferase reporter assay and western blot were used to examine the potential target genes and related pathways. Gene silencing with small-interfering RNA was used to examine the effects of target genes on gastric cancer   cell invasion  . miR-335 was dramatically downregulated in gastric cancer cell lines than in the normal gastric cell line GES-1. Low expression of miR-335 was significantly associated with lymph-node metastasis, poor pT stage, poor pN stage and invasion of lymphatic vessels. Overexpression of miR-335 suppressed gastric cancer   cell invasion   and metastasis in vitro and in vivo, but has no significant effects on cell proliferation. Furthermore, miR-335 might suppress gastric cancer invasion and metastasis by targeting Bcl-w and specificity protein 1 (SP1). Taken together, our results provide evidence that miR-335 might function as a metastasis suppressor in gastric cancer by targeting SP1 directly and indirectly through the Bcl-w-induced phosphoinositide 3-kinase-Akt-Sp1 pathway. miR-335 showing altered expression at different stages of gastric cancer could be a target for gastric cancer therapies and could be further developed as a potential prognostic factor. 
 HER-2/neu represses the metastasis suppressor RECK via ERK and Sp transcription factors to promote   cell invasion  . Matrix metalloproteinase (MMP) inhibitory proteins may negatively regulate MMP activity to suppress tumor metastasis. In this study, we demonstrate that the HER-2/neu oncogene inhibits the expression of the MMP inhibitor RECK to promote   cell invasion  . RECK was inhibited via transcriptional repression in B104-1-1 cells, which express constitutively active HER-2/neu. Overexpression of HER-2/neu in NIH/3T3 or HaCaT cells also suppressed RECK expression. Deletion and mutation assays showed that HER-2/neu repressed RECK via the Sp1-binding site localized in the -82/-71 region from the translational start site. DNA affinity precipitation and chromatin immunoprecipitation assays indicated that binding of Sp1 and Sp3 to this consensus site was increased in B104-1-1 cells. We also found that HER-2/neu inhibited RECK via the ERK signaling pathway. Sp1 proteins phosphorylated at Thr453 and Thr739 by ERK bound preferentially to the RECK promoter, and this binding was reversed by HER-2/neu and ERK inhibitors. Furthermore, our data indicate that HER-2/neu obviously increased HDAC1 binding to the Sp1-binding site localized in the -82/-71 region of the RECK promoter. The histone deacetylase inhibitor trichostatin A reversed HER-2/neu-induced inhibition of RECK. HER-2/neu activation was associated with increased MMP-9 secretion and activation. Re-expression of RECK in HER-2/neu-overexpressing cells inhibited MMP-9 secretion and   cell invasion  . Taken together, our results suggest that HER-2/neu induces the binding of Sp proteins and HDAC1 to the RECK promoter to inhibit RECK expression and to promote   cell invasion  . Restoration of RECK provides a novel strategy for the inhibition of HER-2/neu-induced metastasis. 
 MicroRNA-377 inhibited proliferation and invasion of human glioblastoma cells by directly targeting specificity protein 1. Increasing evidence has indicated that microRNAs (miRNAs) are strongly implicated in the initiation and progression of glioblastoma multiforme (GBM). Here, we identiﬁed a novel tumor suppressive miRNA, miR-377, and investigated its role and therapeutic effect for GBM.MiRNA global screening was performed on GBM patient samples and adjacent nontumor brain tissues. The expression of miR-377 was detected by real-time reverse-transcription PCR. The effects of miR-377 on GBM cell proliferation, cell cycle progression, invasion, and orthotopic tumorigenicity were investigated The therapeutic effect of miR-377 mimic was explored in a subcutaneous GBM model. Western blot and luciferase reporter assay were used to identify the direct and functional target of miR-377.MiR-377 was markedly downregulated in human GBM tissues and cell lines. Overexpression of miR-377 dramatically inhibited cell growth both in culture and in orthotopic xenograft tumor models, blocked G1/S transition, and suppresse  d cell invasi  on in GBM cells. Importantly, introduction of miR-377 could strongly inhibit tumor growth in a subcutaneous GBM model. Subsequent investigation revealed that specificity protein 1 (Sp1) was a direct and functional target of miR-377 in GBM cells. Silencing of Sp1 recapitulated the antiproliferative and anti-invasive effects of miR-377, whereas restoring the Sp1 expression antagonized the tumor-suppressive function of miR-377. Finally, analysis of miR-377 and Sp1 levels in human GBM tissues revealed that miR-377 is inversely correlated with Sp1 expression.These findings reveal that miR-377/Sp1 signaling that may be required for GBM development and may consequently serve as a therapeutic target for the treatment of GBM. 
 Transcription factor Sp1 induces ADAM17 and contributes to tumor cell invasiveness under hypoxia. Expression of the Sp1 transcription factor is induced by hypoxia, and the ADAM17 promoter contains predicted Sp1 binding sites. ADAM17 contributes to hypoxic-induce invasiveness of glioma. In this study, we investigated whether Sp1 transcription factor induces ADAM17 and/or contributes to tumor cell invasiveness in hypoxia.Employing RT-PCR and Western blot, we examined the role of Sp1 in ADAM17 transcription/expression under normoxic and hypoxic conditions, and whether it binds to the ADAM17 GC-rich promoter region using a chromatin immunoprecipitation assay. Additionally, we tested the effect of Sp1 suppression in tumor   cell invasion   and migration, using Matrigel basement membrane invasion chambers, a scratch wound-healing assay, and small interfering RNA.Here, we found that Sp1 binds to the ADAM17 promoter, and that Sp1 regulates ADAM17 expression under hypoxia. Furthermore, suppression of Sp1 decreases invasiveness and migration in U87 tumor cells.Our findings suggest the Sp1 transcription factor mediates ADAM17 expression under hypoxia, regulates glioma invasiveness, and thus, may be a target for anti-invasion therapies. 
 Sp1 is upregulated in human glioma, promotes MMP-2-mediated   cell invasion   and predicts poor clinical outcome. Sp1, the first identified transcription factor, has been reported to be associated with the development and progression of various human cancer types. However, the clinical significance and biological role of Sp1 in glioma are less well understood. In this study, we found that the expression of Sp1 was markedly elevated in glioma cell lines and tissues. Immunohistochemistry analysis revealed that the vast majority of 222 paraffin-embedded archival glioma specimens tested displayed positive Sp1 expression, and 58.6% exhibited high-level Sp1 expression. Statistical analysis suggested that the high Sp1 expression was correlated strongly with the WHO grading (p < 0.001) and survival status (p < 0.001) of glioma patients. Patients with lower Sp1 expression had better overall survival than those with higher Sp1 expression. Multivariate analysis suggested that Sp1 expression might be an independent prognostic indicator of the survival of patients with glioma. Furthermore, overexpression of Sp1 in glioma cells was found to increase their invasiveness, and in contrast, silencing Sp1 by siRNA caused an inhibition of   cell invasion  . Moreover, we demonstrated that the up-regulation of Sp1 could increase activity and expression of MMP-2. Collectively, our data suggest that Sp1 might represent a valuable prognostic marker for glioma and is involved in modulation of tumor invasion. 
 Suppression of lung cancer   cell invasion   by LKB1 is due to the downregulation of tissue factor and vascular endothelial growth factor, partly dependent on SP1. LKB1 encodes a serine/threonine kinase generally inactivated in many human cancers, which mediates cancer cell proliferation, migration and differentiation. Recent studies indicated that LKB1 exhibits potent anti-metastatic activity. However, the underlying molecular mechanisms of this activity remain unclear. In this study, we re‑introduced LKB1 into A549 lung cancer cells that lack the LKB1 gene to investigate how LKB1 affects tumor invasiveness and metastasis. We demonstrated that overexpression of the LKB1 protein in lung cancer cells resulted in significant inhibition of invasion. Furthermore, transfected lung cancer cells with LKB1 suppressed tissue factor (TF) and vascular endothelial growth factor (VEGF) expression at both the mRNA and protein levels. Here, we provided evidence showing that downregulation of TF and VEGF by LKB1 is correlated well with the inhibition o  f cell invasi  on. Overexpression of the LKB1 protein in human lung cancer is significantly associated with a decrease in activity and expression of the transcription factor SP1. Constitutive activation of the transcription factor Sp1 plays a critical role in TF and VEGF overexpression. We conclude that suppression of lung cance  r cell invasi  on by LKB1 through downregulation of TF and VEGF may partly depend on its inhibitory effect on the transcription factor Sp1. Collectively, our data provide a novel molecular mechanism for the antitumor activity of LKB1 and may help further improve its effectiveness in controlling lung cancer growth and invasion. 
 MiR-324-5p Suppresses Hepatocellular Carcinoma   Cell Invasion   by Counteracting ECM Degradation through Post-Transcriptionally Downregulating ETS1 and SP1. Hepatocellular carcinoma (HCC) is one of the common malignancies, which is highly metastatic and the third common cause of cancer deaths in the world. The invasion and metastasis of cancer cells is a multistep and complex process which is mainly initiated by extracellular matrix (ECM) degradation. Aberrant expression of microRNA has been investigated in HCC and shown to play essential roles during HCC progression. In the present study, we found that microRNA-324-5p (miR-324-5p) was downregulated in both HCC cell lines and tissues. Ectopic miR-324-5p led to the reduction of HCC cells invasive and metastatic capacity, whereas inhibition of miR-324-5p promoted the invasion of HCC cells. Matrix metalloproteinase 2 (MMP2) and MMP9, the major regulators of ECM degradation, were found to be downregulated by ectopic miR-324-5p, while upregulated by miR-324-5p inhibitor. E26 transformation-specific 1 (ETS1) and Specificity protein 1 (SP1), both of which could modulate MMP2 and MMP9 expression and activity, were presented as the direct targets of and downregulated by miR-324-5p. Downregulation of ETS1 and SP1 mediated the inhibitory function of miR-324-5p on HCC migration and invasion. Our study demonstrates that miR-324-5p suppresses hepatocellular carcinoma   cell invasion   and might provide new clues to invasive HCC therapy. 
  GeneRIFs  
 MiR-324-5p suppresses hepatocellular carcinoma   cell invasion   by counteracting extracellular matrix degradation through post-transcriptionally downregulating ETS1 and SP1. 
 Study concludes that suppression of lung cancer   cell invasion   by LKB1 through downregulation of TF and VEGF may partly depend on its inhibitory effect on the transcription factor Sp1. 
 HER-2/neu induces the binding of Sp proteins and HDAC1 to the RECK promoter to inhibit RECK expression and to promote   cell invasion   
   
  
 PLA1A 
 
  NCBI Gene  
  GeneRIF  
  Pubmed  
 
 
  Search Term  Rank  Fields  
  [Mm]etastasis  12  PubMed(1); GeneRIFs(1)  
 
 
 hide/show details for PLA1A 
  [Mm]etastasis  PubMed  
 Phosphatidylserine-specific phospholipase A1 (PS-PLA1) expression in colorectal cancer correlates with tumor invasion and hematogenous   metastasis  . The function of phosphatidylserine-specific phospholipase A1 (PS-PLA1), a phospholipase that acts specifically on phosphatidylserine and produces lysophosphatidylserine, a lysophospholipid mediator, has not been fully elucidated. We evaluated the role of PS-PLA1 in oncogenesis and   metastasis   of colorectal cancer (CRC).Specimens from 85 patients with CRC were immunostained with a monoclonal antibody against PS-PLA1. The correlation between PS-PLA1 expression and the clinicopathological variables was analyzed.Tumor depth and hematogenous   metastasis   independently positively correlated with PS-PLA1 expression. High PS-PLA1 expression was associated with shorter disease-free survival, although it was not an independent predictive factor.PS-PLA1 expression in CRC is associated with tumor invasion and   metastasis  . 
  GeneRIFs  
 PS-PLA1 expression in colorectal cancer is associated with tumor invasion and   metastasis  . 
   
  
 LPL 
 
  NCBI Gene  
  GeneRIF  
  Pubmed  
 
 
  Search Term  Rank  Fields  
  [Mm]igration  6  PubMed(1)  
  [Ee]xtracellular [Mm]atrix  8  PubMed(7); Component(1)  
  [Ii]nvasive  10  PubMed(6)  
 
 
 hide/show details for LPL 
  [Mm]igration  PubMed  
 Lipoprotein lipase and phospholipid transfer protein overexpression in human glioma cells and their effect on cell growth, apoptosis, and   migration  . Glioma is one of the common tumors in brain. The expression level of lipoprotein lipase (LPL) or phospholipid transfer protein (PLTP) may influence glioma progression and its relationship with clinical and pathological parameters. The clinical significance of LPL or PLTP expression in glioma has not been established. In the present study, the LPL and PLTP levels in glioma tumors were investigated and the relationship between the LPL and PLTP level and the grade of malignant glioma was analyzed, with the aim to provide new ideas for the diagnosis and treatment of gliomas in clinical and basic research settings. LPL and PLTP mRNA and protein levels were significantly higher in Grade IV glioma than those in the lower grade tumors (P < 0.01). Double immunofluorescent staining showed that the levels of LPL and PLTP were significantly associated with the pathological grade of glioma (P = 0.005). The levels of LPL and PLTP were increased with the shortened survival of glioma patients (P < 0.001). Knockdown of LPL and PLTP led to decreased cell growth and   migration   but increased apoptosis in vitro Additionally, cell cycle-related cyclins and their partners were found to be down-regulated while cyclin-dependent kinase inhibitors p16, p21, and Rb were up-regulated. Furthermore, knockdown of LPL or PLTP resulted in the up-regulation of pro-apoptotic molecules and the down-regulation of anti-apoptotic molecules. Ablation of LPL or PLTP in U251 cells resulted in the down-regulation of epithelial mesenchymal transition markers and invasion molecules matrix metalloproteinases. LPL and PLTP appear to be novel glioma-associated proteins and play a role in the progression of human glioma. 
  [Ee]xtracellular [Mm]atrix  PubMed  
 Genetic variants near TIMP3 and high-density lipoprotein-associated loci influence susceptibility to age-related macular degeneration. We executed a genome-wide association scan for age-related macular degeneration (AMD) in 2,157 cases and 1,150 controls. Our results validate AMD susceptibility loci near CFH (P < 10(-75)), ARMS2 (P < 10(-59)), C2/CFB (P < 10(-20)), C3 (P < 10(-9)), and CFI (P < 10(-6)). We compared our top findings with the Tufts/Massachusetts General Hospital genome-wide association study of advanced AMD (821 cases, 1,709 controls) and genotyped 30 promising markers in additional individuals (up to 7,749 cases and 4,625 controls). With these data, we identified a susceptibility locus near TIMP3 (overall P = 1.1 x 10(-11)), a metalloproteinase involved in degradation of the   extracellular matrix   and previously implicated in early-onset maculopathy. In addition, our data revealed strong association signals with alleles at two loci (LIPC, P = 1.3 x 10(-7); CETP, P = 7.4 x 10(-7)) that were previously associated with high-density lipoprotein cholesterol (HDL-c) levels in blood. Consistent with the hypothesis that HDL metabolism is associated with AMD pathogenesis, we also observed association with AMD of HDL-c-associated alleles near LPL (P = 3.0 x 10(-3)) and ABCA1 (P = 5.6 x 10(-4)). Multilocus analysis including all susceptibility loci showed that 329 of 331 individuals (99%) with the highest-risk genotypes were cases, and 85% of these had advanced AMD. Our studies extend the catalog of AMD associated loci, help identify individuals at high risk of disease, and provide clues about underlying cellular pathways that should eventually lead to new therapies. 
 A genetic association study of maternal and fetal candidate genes that predispose to preterm prelabor rupture of membranes (PROM). We sought to determine whether maternal/fetal single-nucleotide polymorphisms (SNPs) in candidate genes are associated with preterm prelabor rupture of membranes (pPROM).A case-control study was conducted in patients with pPROM (225 mothers and 155 fetuses) and 599 mothers and 628 fetuses with a normal pregnancy; 190 candidate genes and 775 SNPs were studied. Single locus/haplotype association analyses were performed; false discovery rate was used to correct for multiple testing (q* = 0.15).First, a SNP in tissue inhibitor of metalloproteinase 2 in mothers was significantly associated with pPROM (odds ratio, 2.12; 95% confidence interval, 1.47-3.07; P = .000068), and this association remained significant after correction for multiple comparisons. Second, haplotypes for Alpha 3 type IV collagen isoform precursor in the mother were associated with pPROM (global P = .003). Third, multilocus analysis identified a 3-locus model, which included maternal SNPs in collagen type I alpha 2, defensin alpha 5 gene, and endothelin 1.DNA variants in a maternal gene involved in   extracellular matrix   metabolism doubled the risk of pPROM. 
 Identification of fetal and maternal single nucleotide polymorphisms in candidate genes that predispose to spontaneous preterm labor with intact membranes. The purpose of this study was to determine whether maternal/fetal single nucleotide polymorphisms (SNPs) in candidate genes are associated with spontaneous preterm labor/delivery.A genetic association study was conducted in 223 mothers and 179 fetuses (preterm labor with intact membranes who delivered <37 weeks of gestation [preterm birth (PTB)]), and 599 mothers and 628 fetuses (normal pregnancy); 190 candidate genes and 775 SNPs were studied. Single locus/haplotype association analyses were performed; the false discovery rate was used to correct for multiple testing.The strongest single locus associations with PTB were interleukin-6 receptor 1 (fetus; P=.000148) and tissue inhibitor of metalloproteinase 2 (mother; P=.000197), which remained significant after correction for multiple comparisons. Global haplotype analysis indicated an association between a fetal DNA variant in insulin-like growth factor F2 and maternal alpha 3 type IV collagen isoform 1 (global, P=.004 and .007, respectively).An SNP involved in controlling fetal inflammation (interleukin-6 receptor 1) and DNA variants in maternal genes encoding for proteins involved in   extracellular matrix   metabolism approximately doubled the risk of PTB. 
 Genetic variants near TIMP3 and high-density lipoprotein-associated loci influence susceptibility to age-related macular degeneration. We executed a genome-wide association scan for age-related macular degeneration (AMD) in 2,157 cases and 1,150 controls. Our results validate AMD susceptibility loci near CFH (P < 10(-75)), ARMS2 (P < 10(-59)), C2/CFB (P < 10(-20)), C3 (P < 10(-9)), and CFI (P < 10(-6)). We compared our top findings with the Tufts/Massachusetts General Hospital genome-wide association study of advanced AMD (821 cases, 1,709 controls) and genotyped 30 promising markers in additional individuals (up to 7,749 cases and 4,625 controls). With these data, we identified a susceptibility locus near TIMP3 (overall P = 1.1 x 10(-11)), a metalloproteinase involved in degradation of the   extracellular matrix   and previously implicated in early-onset maculopathy. In addition, our data revealed strong association signals with alleles at two loci (LIPC, P = 1.3 x 10(-7); CETP, P = 7.4 x 10(-7)) that were previously associated with high-density lipoprotein cholesterol (HDL-c) levels in blood. Consistent with the hypothesis that HDL metabolism is associated with AMD pathogenesis, we also observed association with AMD of HDL-c-associated alleles near LPL (P = 3.0 x 10(-3)) and ABCA1 (P = 5.6 x 10(-4)). Multilocus analysis including all susceptibility loci showed that 329 of 331 individuals (99%) with the highest-risk genotypes were cases, and 85% of these had advanced AMD. Our studies extend the catalog of AMD associated loci, help identify individuals at high risk of disease, and provide clues about underlying cellular pathways that should eventually lead to new therapies. 
 Identification of fetal and maternal single nucleotide polymorphisms in candidate genes that predispose to spontaneous preterm labor with intact membranes. The purpose of this study was to determine whether maternal/fetal single nucleotide polymorphisms (SNPs) in candidate genes are associated with spontaneous preterm labor/delivery.A genetic association study was conducted in 223 mothers and 179 fetuses (preterm labor with intact membranes who delivered <37 weeks of gestation [preterm birth (PTB)]), and 599 mothers and 628 fetuses (normal pregnancy); 190 candidate genes and 775 SNPs were studied. Single locus/haplotype association analyses were performed; the false discovery rate was used to correct for multiple testing.The strongest single locus associations with PTB were interleukin-6 receptor 1 (fetus; P=.000148) and tissue inhibitor of metalloproteinase 2 (mother; P=.000197), which remained significant after correction for multiple comparisons. Global haplotype analysis indicated an association between a fetal DNA variant in insulin-like growth factor F2 and maternal alpha 3 type IV collagen isoform 1 (global, P=.004 and .007, respectively).An SNP involved in controlling fetal inflammation (interleukin-6 receptor 1) and DNA variants in maternal genes encoding for proteins involved in   extracellular matrix   metabolism approximately doubled the risk of PTB. 
 A genetic association study of maternal and fetal candidate genes that predispose to preterm prelabor rupture of membranes (PROM). We sought to determine whether maternal/fetal single-nucleotide polymorphisms (SNPs) in candidate genes are associated with preterm prelabor rupture of membranes (pPROM).A case-control study was conducted in patients with pPROM (225 mothers and 155 fetuses) and 599 mothers and 628 fetuses with a normal pregnancy; 190 candidate genes and 775 SNPs were studied. Single locus/haplotype association analyses were performed; false discovery rate was used to correct for multiple testing (q* = 0.15).First, a SNP in tissue inhibitor of metalloproteinase 2 in mothers was significantly associated with pPROM (odds ratio, 2.12; 95% confidence interval, 1.47-3.07; P = .000068), and this association remained significant after correction for multiple comparisons. Second, haplotypes for Alpha 3 type IV collagen isoform precursor in the mother were associated with pPROM (global P = .003). Third, multilocus analysis identified a 3-locus model, which included maternal SNPs in collagen type I alpha 2, defensin alpha 5 gene, and endothelin 1.DNA variants in a maternal gene involved in   extracellular matrix   metabolism doubled the risk of pPROM. 
 Lipoprotein lipase in the arterial wall: linking LDL to the arterial   extracellular matrix   and much more. For low density lipoprotein (LDL) particles to be atherogenic, increasing evidence indicates that their residence time in the arterial intima must be sufficient to allow their modification into forms capable of triggering extracellular and intracellular lipid accumulation. Recent reports have confirmed the longstanding hypothesis that the major determinant(s) of initial LDL retention in the preatherosclerotic arterial intima is the proteoglycans. However, once the initial atherosclerotic lesions have formed, a shift to retention facilitated by macrophage-derived lipoprotein lipase (LPL) appears, leading to the progression of the lesions. Here, we review recent findings on the mechanisms enabling LPL to promote LDL retention and extracellular lipid accumulation in the arterial intima, and we describe the structures in the   extracellular matrix   that are held to be important in this process. Finally, the potentially harmful consequences of LDL linking by LPL and of other LPL actions in the arterial intima are briefly reviewed. 
  Component  
   extracellular matrix   
  [Ii]nvasive  PubMed  
 Genetic susceptibility to distinct bladder cancer subphenotypes. Clinical, pathologic, and molecular evidence indicate that bladder cancer is heterogeneous with pathologic/molecular features that define distinct subphenotypes with different prognoses. It is conceivable that specific patterns of genetic susceptibility are associated with particular subphenotypes.To examine evidence for the contribution of germline genetic variation to bladder cancer heterogeneity.The Spanish Bladder Cancer/EPICURO Study is a case-control study based in 18 hospitals located in five areas in Spain. Cases were patients with a newly diagnosed, histologically confirmed, urothelial cell carcinoma of the bladder from 1998 to 2001. Case diagnoses were reviewed and uniformly classified by pathologists following the World Health Organisation/International Society of Urological Pathology 1999 criteria. Controls were hospital-matched patients (n=1149).A total of 1526 candidate variants in 423 candidate genes were analysed. Three distinct subphenotypes were defined according to stage and grade: low-grade nonmuscle   invasive   (n=586), high-grade nonmuscle   invasive   (n=219), and muscle   invasive   (n=246). The association between each variant and subphenotype was assessed by polytomous risk models adjusting for potential confounders. Heterogeneity in genetic susceptibility among subphenotypes was also tested.Two established bladder cancer susceptibility genotypes, NAT2 slow-acetylation and GSTM1-null, exhibited similar associations among the subphenotypes, as did VEGF-rs25648, which was previously identified in our study. Other variants conferred risks for specific tumour subphenotypes such as PMS2-rs6463524 and CD4-rs3213427 (respective heterogeneity p values of 0.006 and 0.004), which were associated with muscle-  invasive   tumours (per-allele odds ratios [95% confidence interval] of 0.56 [0.41-0.77] and 0.71 [0.57-0.88], respectively) but not with non-muscle-  invasive   tumours. Heterogeneity p values were not robust in multiple testing according to their false-discovery rate.These exploratory analyses suggest that genetic susceptibility loci might be related to the molecular/pathologic diversity of bladder cancer. Validation through large-scale replication studies and the study of additional genes and single nucleotide polymorphisms are required. 
 Genetic susceptibility to distinct bladder cancer subphenotypes. Clinical, pathologic, and molecular evidence indicate that bladder cancer is heterogeneous with pathologic/molecular features that define distinct subphenotypes with different prognoses. It is conceivable that specific patterns of genetic susceptibility are associated with particular subphenotypes.To examine evidence for the contribution of germline genetic variation to bladder cancer heterogeneity.The Spanish Bladder Cancer/EPICURO Study is a case-control study based in 18 hospitals located in five areas in Spain. Cases were patients with a newly diagnosed, histologically confirmed, urothelial cell carcinoma of the bladder from 1998 to 2001. Case diagnoses were reviewed and uniformly classified by pathologists following the World Health Organisation/International Society of Urological Pathology 1999 criteria. Controls were hospital-matched patients (n=1149).A total of 1526 candidate variants in 423 candidate genes were analysed. Three distinct subphenotypes were defined according to stage and grade: low-grade nonmuscle   invasive   (n=586), high-grade nonmuscle   invasive   (n=219), and muscle   invasive   (n=246). The association between each variant and subphenotype was assessed by polytomous risk models adjusting for potential confounders. Heterogeneity in genetic susceptibility among subphenotypes was also tested.Two established bladder cancer susceptibility genotypes, NAT2 slow-acetylation and GSTM1-null, exhibited similar associations among the subphenotypes, as did VEGF-rs25648, which was previously identified in our study. Other variants conferred risks for specific tumour subphenotypes such as PMS2-rs6463524 and CD4-rs3213427 (respective heterogeneity p values of 0.006 and 0.004), which were associated with muscle-  invasive   tumours (per-allele odds ratios [95% confidence interval] of 0.56 [0.41-0.77] and 0.71 [0.57-0.88], respectively) but not with non-muscle-  invasive   tumours. Heterogeneity p values were not robust in multiple testing according to their false-discovery rate.These exploratory analyses suggest that genetic susceptibility loci might be related to the molecular/pathologic diversity of bladder cancer. Validation through large-scale replication studies and the study of additional genes and single nucleotide polymorphisms are required. 
 Body mass, polymorphisms in obesity-related genes, and the risk of developing breast cancer among women with benign breast disease. A cohort study was conducted among post-menopausal women to determine whether genetic polymorphisms in selected obesity-related genes (PPARG, LPL, LEPR, PON1, PON2, TNF-alpha) were associated with the progression of benign breast disease (BBD) to breast cancer and whether the selected polymorphisms modified the association between body mass and breast cancer among women with BBD.Among participants in an ongoing cohort study, 994 Caucasian post-menopausal women had a breast biopsy for BBD. Of these women, 61 subsequently developed breast cancer. A short questionnaire was administered at baseline in 1989. Genotypes were determined using DNA extracted from blood collected in 1989.In this cohort, body mass index (BMI) was positively associated with the risk of developing breast cancer. In contrast, polymorphisms in PON1 (Gln192Arg) and LEPR (IVS2+6920) were associated with a decreased risk of developing   invasive   breast cancer. No statistically significant associations were observed for polymorphisms in PPARG, PON2, LPL, or TNF and breast cancer risk or for interactions between the polymorphisms and BMI and breast cancer risk.The findings suggest that specific polymorphisms in the PON1 and LEPR genes may play a role in progression of BBD to breast cancer among post-menopausal Caucasian women. 
 Carriers of the frequent lipoprotein lipase S447X variant exhibit enhanced postprandial apoprotein B-48 clearance. The frequent lipoprotein lipase S447X variant (LPLS447X) is firmly associated with a lower incidence of cardiovascular disease, the mechanisms for which remain to be established. To further unravel these beneficial effects, we studied the consequences of LPLS447X heterozygosity on LPL mass and activity, as well as on the postprandial lipoprotein profile. Fifteen male heterozygous LPLS447X carriers and 15 matched control subjects received an oral fat load (30 g/m(2)). Lipid parameters were evaluated at baseline and 2, 3, 4, and 6 hours after fat loading. LPL concentration and activity were analyzed, and endothelial function was evaluated non  invasive  ly as flow-mediated dilation of the brachial artery. Although baseline apoprotein B-48 (apoB48) levels were similar, the rise in apoB48 was attenuated in LPLS447X carriers with 25% lower peak values compared with controls (P=.04). In conjunction, LPLS447X carriers were characterized by a 2.4-fold increase in pre-heparin LPL mass (P<.0001). The decrease in postprandial flow-mediated dilation was comparable in both groups. LPLS447X carriers exhibit enhanced apoB48 clearance with concomitant increase in pre-heparin LPL mass, without changes in LPL activity. This combination might suggest a role for increased ligand action of LPL in LPLS447X carriers contributing to the cardiovascular protection in carriers of this mutation. 
 Lipoprotein lipase and endothelial lipase in human testis and in germ cell neoplasms. The aim of this study was to investigate endothelial lipase (EL, LIPG) and lipoprotein lipase (LPL) mRNA and protein expression in normal human testis and testicular germ cell tumours (GCT). Both EL and LPL were expressed in normal seminiferous tubules and in the interstitial compartment. EL mRNA and protein were found in all germ cells as well as in Sertoli and Leydig cells. EL mRNA was abundant in pre-  invasive   carcinoma in situ (CIS) cells and GCTs, and EL protein was present in the cytoplasm of these cells. LPL mRNA was also relatively abundant in germ cells, Sertoli cells, CIS cells and GCTs. The LPL protein, however, was restricted to the cell membranes of pachytene spermatocytes and spermatids in normal tubules, absent from CIS cells and scarcely represented in tumours. The distribution of LPL protein in non-seminomas resembled the distribution of OCT3/4, a marker of embryonal carcinoma. The results suggest that both EL and LPL participate in the supply of nutrients and steroidogenesis in the testes, and that especially EL may be important for the supply of cholesterol for testosterone production in the Leydig cells. The partial cellular separation of the expression of the two lipases in normal testis suggests the existence of distinct biological roles, perhaps developmentally regulated, as indicated by the LPL expression in GCTs with embryonic features. A high expression of EL and abundance of lipid in tubules with CIS may have a diagnostic value. 
 Lipoprotein lipase (LPL) gene variation and progression of carotid artery plaque. Coding single nucleotide polymorphisms (cSNPs) in the lipoprotein lipase (LPL) gene have been associated with lipoprotein phenotypes and vascular disease risk. We studied the association between LPL cSNPs and a novel non  invasive   measure of disease, namely, cross-sectional carotid plaque area (CPA) on B-mode ultrasound.Four hundred fifty-two patients from an atherosclerosis prevention clinic had determinations of baseline and total CPA. Traditional atherosclerosis risk factors were recorded, and the LPL D9N, N291S, and S447X cSNPs were genotyped. Multiple regression analysis was used to identify determinants of CPA.Minor allele frequencies for LPL D9N, N291S, and S447X were 2.8%, 0.9%, and 4.4%, respectively. There were no significant between-genotype differences in treated fasting lipids. The LPL D9N genotype was a significant predictor of both baseline CPA (P=0.008) and plaque progression from baseline to 1 year later (P=0.001). Heterozygotes for the N9 allele had higher mean baseline CPA and plaque progression than did LPL D9/D9 homozygotes.LPL D9N genotype may be a determinant of atherosclerosis as estimated by static baseline CPA and by progression of CPA. 
   
  
 LIPG 
 
  NCBI Gene  
  GeneRIF  
  Pubmed  
 
 
  Search Term  Rank  Fields  
  [Mm]igration  6  PubMed(1)  
  [Ii]nvasive  10  PubMed(2)  
 
 
 hide/show details for LIPG 
  [Mm]igration  PubMed  
 Expression of endothelial lipase correlates with the size of neointima in a murine model of vascular remodeling. Endothelial lipase (EL) regulates plasma high-density lipoprotein-cholesterol (HDL-C) levels by promoting HDL catabolism. However, it remains unknown whether the inhibition of EL has beneficial effects on the genesis of vascular diseases. Here, we investigated the role of EL on vascular remodeling in mice.Vascular remodeling was developed by ligation of the left common carotid artery and neointimal lesions were histologically compared between EL-knockout (ELKO), EL-transgenic (ELTg), and wild-type (WT) mice. HDL was isolated from these mice, and effects of the HDL on cell growth and Erk activation were evaluated in vitro using cultured vascular smooth muscle cells.Plasma HDL-C levels were 62% higher in ELKO and 13% lower in ELTg than in WT mice, after the carotid ligation. The size of neointimal lesion was significantly larger in ELTg and smaller in ELKO than in WT mice. Vascular expression of adhesion molecules was lower in ELKO and higher in ELTg compared with WT mice. Moreover, oxidative stress was attenuated in ELKO mice. HDL isolated from ELKO, ELTg, and WT mice inhibited expression of intercellular adhesion molecule-1, angiotensin II-induced activation of Erk, and growth of cultured vascular smooth muscle cells, whereas EL expression itself did not affect cell   migration   or growth.EL expression modulates vascular remodeling as well as plasma HDL-C levels. EL inactivation may increase HDL particles that can inhibit smooth muscle cell growth and   migration  . 
  [Ii]nvasive  PubMed  
 Lipoprotein lipase and endothelial lipase in human testis and in germ cell neoplasms. The aim of this study was to investigate endothelial lipase (EL, LIPG) and lipoprotein lipase (LPL) mRNA and protein expression in normal human testis and testicular germ cell tumours (GCT). Both EL and LPL were expressed in normal seminiferous tubules and in the interstitial compartment. EL mRNA and protein were found in all germ cells as well as in Sertoli and Leydig cells. EL mRNA was abundant in pre-  invasive   carcinoma in situ (CIS) cells and GCTs, and EL protein was present in the cytoplasm of these cells. LPL mRNA was also relatively abundant in germ cells, Sertoli cells, CIS cells and GCTs. The LPL protein, however, was restricted to the cell membranes of pachytene spermatocytes and spermatids in normal tubules, absent from CIS cells and scarcely represented in tumours. The distribution of LPL protein in non-seminomas resembled the distribution of OCT3/4, a marker of embryonal carcinoma. The results suggest that both EL and LPL participate in the supply of nutrients and steroidogenesis in the testes, and that especially EL may be important for the supply of cholesterol for testosterone production in the Leydig cells. The partial cellular separation of the expression of the two lipases in normal testis suggests the existence of distinct biological roles, perhaps developmentally regulated, as indicated by the LPL expression in GCTs with embryonic features. A high expression of EL and abundance of lipid in tubules with CIS may have a diagnostic value. 
 Lipoprotein lipase and endothelial lipase in human testis and in germ cell neoplasms. The aim of this study was to investigate endothelial lipase (EL, LIPG) and lipoprotein lipase (LPL) mRNA and protein expression in normal human testis and testicular germ cell tumours (GCT). Both EL and LPL were expressed in normal seminiferous tubules and in the interstitial compartment. EL mRNA and protein were found in all germ cells as well as in Sertoli and Leydig cells. EL mRNA was abundant in pre-  invasive   carcinoma in situ (CIS) cells and GCTs, and EL protein was present in the cytoplasm of these cells. LPL mRNA was also relatively abundant in germ cells, Sertoli cells, CIS cells and GCTs. The LPL protein, however, was restricted to the cell membranes of pachytene spermatocytes and spermatids in normal tubules, absent from CIS cells and scarcely represented in tumours. The distribution of LPL protein in non-seminomas resembled the distribution of OCT3/4, a marker of embryonal carcinoma. The results suggest that both EL and LPL participate in the supply of nutrients and steroidogenesis in the testes, and that especially EL may be important for the supply of cholesterol for testosterone production in the Leydig cells. The partial cellular separation of the expression of the two lipases in normal testis suggests the existence of distinct biological roles, perhaps developmentally regulated, as indicated by the LPL expression in GCTs with embryonic features. A high expression of EL and abundance of lipid in tubules with CIS may have a diagnostic value. 
   
  
 LIPC 
 
  NCBI Gene  
  GeneRIF  
  Pubmed  
 
 
  Search Term  Rank  Fields  
  [Ee]xtracellular [Mm]atrix  8  PubMed(7)  
  [Ii]nvasive  10  PubMed(2)  
 
 
 hide/show details for LIPC 
  [Ee]xtracellular [Mm]atrix  PubMed  
 Seven new loci associated with age-related macular degeneration. Age-related macular degeneration (AMD) is a common cause of blindness in older individuals. To accelerate the understanding of AMD biology and help design new therapies, we executed a collaborative genome-wide association study, including >17,100 advanced AMD cases and >60,000 controls of European and Asian ancestry. We identified 19 loci associated at P < 5 × 10(-8). These loci show enrichment for genes involved in the regulation of complement activity, lipid metabolism,   extracellular matri  x remodeling and angiogenesis. Our results include seven loci with associations reaching P < 5 × 10(-8) for the first time, near the genes COL8A1-FILIP1L, IER3-DDR1, SLC16A8, TGFBR1, RAD51B, ADAMTS9 and B3GALTL. A genetic risk score combining SNP genotypes from all loci showed similar ability to distinguish cases and controls in all samples examined. Our findings provide new directions for biological, genetic and therapeutic studies of AMD. 
 Genetic variants near TIMP3 and high-density lipoprotein-associated loci influence susceptibility to age-related macular degeneration. We executed a genome-wide association scan for age-related macular degeneration (AMD) in 2,157 cases and 1,150 controls. Our results validate AMD susceptibility loci near CFH (P < 10(-75)), ARMS2 (P < 10(-59)), C2/CFB (P < 10(-20)), C3 (P < 10(-9)), and CFI (P < 10(-6)). We compared our top findings with the Tufts/Massachusetts General Hospital genome-wide association study of advanced AMD (821 cases, 1,709 controls) and genotyped 30 promising markers in additional individuals (up to 7,749 cases and 4,625 controls). With these data, we identified a susceptibility locus near TIMP3 (overall P = 1.1 x 10(-11)), a metalloproteinase involved in degradation of the   extracellular matrix   and previously implicated in early-onset maculopathy. In addition, our data revealed strong association signals with alleles at two loci (LIPC, P = 1.3 x 10(-7); CETP, P = 7.4 x 10(-7)) that were previously associated with high-density lipoprotein cholesterol (HDL-c) levels in blood. Consistent with the hypothesis that HDL metabolism is associated with AMD pathogenesis, we also observed association with AMD of HDL-c-associated alleles near LPL (P = 3.0 x 10(-3)) and ABCA1 (P = 5.6 x 10(-4)). Multilocus analysis including all susceptibility loci showed that 329 of 331 individuals (99%) with the highest-risk genotypes were cases, and 85% of these had advanced AMD. Our studies extend the catalog of AMD associated loci, help identify individuals at high risk of disease, and provide clues about underlying cellular pathways that should eventually lead to new therapies. 
 Identification of fetal and maternal single nucleotide polymorphisms in candidate genes that predispose to spontaneous preterm labor with intact membranes. The purpose of this study was to determine whether maternal/fetal single nucleotide polymorphisms (SNPs) in candidate genes are associated with spontaneous preterm labor/delivery.A genetic association study was conducted in 223 mothers and 179 fetuses (preterm labor with intact membranes who delivered <37 weeks of gestation [preterm birth (PTB)]), and 599 mothers and 628 fetuses (normal pregnancy); 190 candidate genes and 775 SNPs were studied. Single locus/haplotype association analyses were performed; the false discovery rate was used to correct for multiple testing.The strongest single locus associations with PTB were interleukin-6 receptor 1 (fetus; P=.000148) and tissue inhibitor of metalloproteinase 2 (mother; P=.000197), which remained significant after correction for multiple comparisons. Global haplotype analysis indicated an association between a fetal DNA variant in insulin-like growth factor F2 and maternal alpha 3 type IV collagen isoform 1 (global, P=.004 and .007, respectively).An SNP involved in controlling fetal inflammation (interleukin-6 receptor 1) and DNA variants in maternal genes encoding for proteins involved in   extracellular matrix   metabolism approximately doubled the risk of PTB. 
 A genetic association study of maternal and fetal candidate genes that predispose to preterm prelabor rupture of membranes (PROM). We sought to determine whether maternal/fetal single-nucleotide polymorphisms (SNPs) in candidate genes are associated with preterm prelabor rupture of membranes (pPROM).A case-control study was conducted in patients with pPROM (225 mothers and 155 fetuses) and 599 mothers and 628 fetuses with a normal pregnancy; 190 candidate genes and 775 SNPs were studied. Single locus/haplotype association analyses were performed; false discovery rate was used to correct for multiple testing (q* = 0.15).First, a SNP in tissue inhibitor of metalloproteinase 2 in mothers was significantly associated with pPROM (odds ratio, 2.12; 95% confidence interval, 1.47-3.07; P = .000068), and this association remained significant after correction for multiple comparisons. Second, haplotypes for Alpha 3 type IV collagen isoform precursor in the mother were associated with pPROM (global P = .003). Third, multilocus analysis identified a 3-locus model, which included maternal SNPs in collagen type I alpha 2, defensin alpha 5 gene, and endothelin 1.DNA variants in a maternal gene involved in   extracellular matrix   metabolism doubled the risk of pPROM. 
 Genetic variants near TIMP3 and high-density lipoprotein-associated loci influence susceptibility to age-related macular degeneration. We executed a genome-wide association scan for age-related macular degeneration (AMD) in 2,157 cases and 1,150 controls. Our results validate AMD susceptibility loci near CFH (P < 10(-75)), ARMS2 (P < 10(-59)), C2/CFB (P < 10(-20)), C3 (P < 10(-9)), and CFI (P < 10(-6)). We compared our top findings with the Tufts/Massachusetts General Hospital genome-wide association study of advanced AMD (821 cases, 1,709 controls) and genotyped 30 promising markers in additional individuals (up to 7,749 cases and 4,625 controls). With these data, we identified a susceptibility locus near TIMP3 (overall P = 1.1 x 10(-11)), a metalloproteinase involved in degradation of the   extracellular matrix   and previously implicated in early-onset maculopathy. In addition, our data revealed strong association signals with alleles at two loci (LIPC, P = 1.3 x 10(-7); CETP, P = 7.4 x 10(-7)) that were previously associated with high-density lipoprotein cholesterol (HDL-c) levels in blood. Consistent with the hypothesis that HDL metabolism is associated with AMD pathogenesis, we also observed association with AMD of HDL-c-associated alleles near LPL (P = 3.0 x 10(-3)) and ABCA1 (P = 5.6 x 10(-4)). Multilocus analysis including all susceptibility loci showed that 329 of 331 individuals (99%) with the highest-risk genotypes were cases, and 85% of these had advanced AMD. Our studies extend the catalog of AMD associated loci, help identify individuals at high risk of disease, and provide clues about underlying cellular pathways that should eventually lead to new therapies. 
 Identification of fetal and maternal single nucleotide polymorphisms in candidate genes that predispose to spontaneous preterm labor with intact membranes. The purpose of this study was to determine whether maternal/fetal single nucleotide polymorphisms (SNPs) in candidate genes are associated with spontaneous preterm labor/delivery.A genetic association study was conducted in 223 mothers and 179 fetuses (preterm labor with intact membranes who delivered <37 weeks of gestation [preterm birth (PTB)]), and 599 mothers and 628 fetuses (normal pregnancy); 190 candidate genes and 775 SNPs were studied. Single locus/haplotype association analyses were performed; the false discovery rate was used to correct for multiple testing.The strongest single locus associations with PTB were interleukin-6 receptor 1 (fetus; P=.000148) and tissue inhibitor of metalloproteinase 2 (mother; P=.000197), which remained significant after correction for multiple comparisons. Global haplotype analysis indicated an association between a fetal DNA variant in insulin-like growth factor F2 and maternal alpha 3 type IV collagen isoform 1 (global, P=.004 and .007, respectively).An SNP involved in controlling fetal inflammation (interleukin-6 receptor 1) and DNA variants in maternal genes encoding for proteins involved in   extracellular matrix   metabolism approximately doubled the risk of PTB. 
 A genetic association study of maternal and fetal candidate genes that predispose to preterm prelabor rupture of membranes (PROM). We sought to determine whether maternal/fetal single-nucleotide polymorphisms (SNPs) in candidate genes are associated with preterm prelabor rupture of membranes (pPROM).A case-control study was conducted in patients with pPROM (225 mothers and 155 fetuses) and 599 mothers and 628 fetuses with a normal pregnancy; 190 candidate genes and 775 SNPs were studied. Single locus/haplotype association analyses were performed; false discovery rate was used to correct for multiple testing (q* = 0.15).First, a SNP in tissue inhibitor of metalloproteinase 2 in mothers was significantly associated with pPROM (odds ratio, 2.12; 95% confidence interval, 1.47-3.07; P = .000068), and this association remained significant after correction for multiple comparisons. Second, haplotypes for Alpha 3 type IV collagen isoform precursor in the mother were associated with pPROM (global P = .003). Third, multilocus analysis identified a 3-locus model, which included maternal SNPs in collagen type I alpha 2, defensin alpha 5 gene, and endothelin 1.DNA variants in a maternal gene involved in   extracellular matrix   metabolism doubled the risk of pPROM. 
  [Ii]nvasive  PubMed  
 Genetic susceptibility to distinct bladder cancer subphenotypes. Clinical, pathologic, and molecular evidence indicate that bladder cancer is heterogeneous with pathologic/molecular features that define distinct subphenotypes with different prognoses. It is conceivable that specific patterns of genetic susceptibility are associated with particular subphenotypes.To examine evidence for the contribution of germline genetic variation to bladder cancer heterogeneity.The Spanish Bladder Cancer/EPICURO Study is a case-control study based in 18 hospitals located in five areas in Spain. Cases were patients with a newly diagnosed, histologically confirmed, urothelial cell carcinoma of the bladder from 1998 to 2001. Case diagnoses were reviewed and uniformly classified by pathologists following the World Health Organisation/International Society of Urological Pathology 1999 criteria. Controls were hospital-matched patients (n=1149).A total of 1526 candidate variants in 423 candidate genes were analysed. Three distinct subphenotypes were defined according to stage and grade: low-grade nonmuscle   invasive   (n=586), high-grade nonmuscle   invasive   (n=219), and muscle   invasive   (n=246). The association between each variant and subphenotype was assessed by polytomous risk models adjusting for potential confounders. Heterogeneity in genetic susceptibility among subphenotypes was also tested.Two established bladder cancer susceptibility genotypes, NAT2 slow-acetylation and GSTM1-null, exhibited similar associations among the subphenotypes, as did VEGF-rs25648, which was previously identified in our study. Other variants conferred risks for specific tumour subphenotypes such as PMS2-rs6463524 and CD4-rs3213427 (respective heterogeneity p values of 0.006 and 0.004), which were associated with muscle-  invasive   tumours (per-allele odds ratios [95% confidence interval] of 0.56 [0.41-0.77] and 0.71 [0.57-0.88], respectively) but not with non-muscle-  invasive   tumours. Heterogeneity p values were not robust in multiple testing according to their false-discovery rate.These exploratory analyses suggest that genetic susceptibility loci might be related to the molecular/pathologic diversity of bladder cancer. Validation through large-scale replication studies and the study of additional genes and single nucleotide polymorphisms are required. 
 Genetic susceptibility to distinct bladder cancer subphenotypes. Clinical, pathologic, and molecular evidence indicate that bladder cancer is heterogeneous with pathologic/molecular features that define distinct subphenotypes with different prognoses. It is conceivable that specific patterns of genetic susceptibility are associated with particular subphenotypes.To examine evidence for the contribution of germline genetic variation to bladder cancer heterogeneity.The Spanish Bladder Cancer/EPICURO Study is a case-control study based in 18 hospitals located in five areas in Spain. Cases were patients with a newly diagnosed, histologically confirmed, urothelial cell carcinoma of the bladder from 1998 to 2001. Case diagnoses were reviewed and uniformly classified by pathologists following the World Health Organisation/International Society of Urological Pathology 1999 criteria. Controls were hospital-matched patients (n=1149).A total of 1526 candidate variants in 423 candidate genes were analysed. Three distinct subphenotypes were defined according to stage and grade: low-grade nonmuscle   invasive   (n=586), high-grade nonmuscle   invasive   (n=219), and muscle   invasive   (n=246). The association between each variant and subphenotype was assessed by polytomous risk models adjusting for potential confounders. Heterogeneity in genetic susceptibility among subphenotypes was also tested.Two established bladder cancer susceptibility genotypes, NAT2 slow-acetylation and GSTM1-null, exhibited similar associations among the subphenotypes, as did VEGF-rs25648, which was previously identified in our study. Other variants conferred risks for specific tumour subphenotypes such as PMS2-rs6463524 and CD4-rs3213427 (respective heterogeneity p values of 0.006 and 0.004), which were associated with muscle-  invasive   tumours (per-allele odds ratios [95% confidence interval] of 0.56 [0.41-0.77] and 0.71 [0.57-0.88], respectively) but not with non-muscle-  invasive   tumours. Heterogeneity p values were not robust in multiple testing according to their false-discovery rate.These exploratory analyses suggest that genetic susceptibility loci might be related to the molecular/pathologic diversity of bladder cancer. Validation through large-scale replication studies and the study of additional genes and single nucleotide polymorphisms are required. 
   
  
 CTRB1 
 
  NCBI Gene  
  GeneRIF  
  Pubmed  
 
 
  Search Term  Rank  Fields  
  [Ee]xtracellular [Mm]atrix  8  Process(1)  
 
 
 hide/show details for CTRB1 
  [Ee]xtracellular [Mm]atrix  Process  
   extracellular matrix   disassembly 
   
  
 CELA1 
 
  NCBI Gene  
  GeneRIF  
  Pubmed  
 
 
  Search Term  Rank  Fields  
  ECM  7  PubMed(1)  
  [Ee]xtracellular [Mm]atrix  8  PubMed(2); GeneRIFs(1)  
 
 
 hide/show details for CELA1 
  ECM  PubMed  
 Extract of the mycelium of T. matsutake inhibits elastase activity and TPA-induced MMP-1 expression in human fibroblasts. Skin aging is induced through complex biological processes in human skin caused by proteolysis of collagen and elastin, two structural proteins of the dermal extracellular matrix (  ECM  ). Collagen and elastin degradation can induce the expression of matrix metalloproteinases (MMPs), as well as that of a family of zinc-dependent endopeptidases that play critical roles in skin aging. Moreover, elastase is a metalloproteinase which acts on the degradation of elastin in skin aging, and is also involved in the inhibition or the repair of wrinkle formation. Extract of the mycelium of Tricholoma matsutake (T. matsutake), or pine mushroom, is widely distributed in Asian countries. The extract is from the natural biomaterial of the mushroom which is rich in polysaccharides, including β-glucan. This extract has shown potent bioactive antioxidant, immunomodulatory and antitumoral properties. In the present study, we investigated whether the extract of the mycelium of T. matsutake has effects on elastase activity, as well as on the expression of tissue inhibitor of matrix metalloproteinases-1 (TIMP-1) and MMP-1 under basal conditions. Our results revealed that the extract of the mycelium of T. matsutake significantly decreased elastase activity in a dose-dependent manner and reduced the levels of MMP-1 and MMP-3. On the other hand, the expression of TIMP-1 and tropoelastin was increased in fibroblasts treated with the extract of the mycelium of T. matsutake. However, collagent expression was not affected. In addition, our results demonstrated that the extract of the mycelium of T. matsutake inhibited the 12-O-tetradecanoylphorbol-13-acetate (TPA)-induced MMP-1 expression and suppressed TPA-induced p38 activity. Therefore, the inhibitory effects of the extract of the mycelium of T. matsutake on MMP-1 induction are mediated by the inhibition of p38 in human fibroblasts. Our data suggest that the extract of the mycelium of T. matsutake may prove to be an effective biomaterial for anti-wrinkle treatment, as it can obstruct the degradation of the dermal   EC  M. 
  [Ee]xtracellular [Mm]atrix  PubMed  
 NGS nominated CELA1, HSPG2, and KCNK5 as candidate genes for predisposition to Balkan endemic nephropathy. Balkan endemic nephropathy (BEN) is a familial chronic tubulointerstitial disease with insidious onset and slow progression leading to terminal renal failure. The results of molecular biological investigations propose that BEN is a multifactorial disease with genetic predisposition to environmental risk agents. Exome sequencing of 22 000 genes with Illumina Nextera Exome Enrichment Kit was performed on 22 DNA samples (11 Bulgarian patients and 11 Serbian patients). Software analysis was performed via NextGene, Provean, and PolyPhen. The frequency of all annotated genetic variants with deleterious/damaging effect was compared with those of European populations. Then we focused on nonannotated variants (with no data available about them and not found in healthy Bulgarian controls). There is no statistically significant difference between annotated variants in BEN patients and European populations. From nonannotated variants with more than 40% frequency in both patients' groups, we nominated 3 genes with possible deleterious/damaging variants--CELA1, HSPG2, and KCNK5. Mutant genes (CELA1, HSPG2, and KCNK5) in BEN patients encode proteins involved in basement membrane/  extracellular matrix   and vascular tone, tightly connected to process of angiogenesis. We suggest that an abnormal process of angiogenesis plays a key role in the molecular pathogenesis of BEN. 
 Extract of the mycelium of T. matsutake inhibits elastase activity and TPA-induced MMP-1 expression in human fibroblasts. Skin aging is induced through complex biological processes in human skin caused by proteolysis of collagen and elastin, two structural proteins of the dermal   extracellular matrix   (ECM). Collagen and elastin degradation can induce the expression of matrix metalloproteinases (MMPs), as well as that of a family of zinc-dependent endopeptidases that play critical roles in skin aging. Moreover, elastase is a metalloproteinase which acts on the degradation of elastin in skin aging, and is also involved in the inhibition or the repair of wrinkle formation. Extract of the mycelium of Tricholoma matsutake (T. matsutake), or pine mushroom, is widely distributed in Asian countries. The extract is from the natural biomaterial of the mushroom which is rich in polysaccharides, including β-glucan. This extract has shown potent bioactive antioxidant, immunomodulatory and antitumoral properties. In the present study, we investigated whether the extract of the mycelium of T. matsutake has effects on elastase activity, as well as on the expression of tissue inhibitor of matrix metalloproteinases-1 (TIMP-1) and MMP-1 under basal conditions. Our results revealed that the extract of the mycelium of T. matsutake significantly decreased elastase activity in a dose-dependent manner and reduced the levels of MMP-1 and MMP-3. On the other hand, the expression of TIMP-1 and tropoelastin was increased in fibroblasts treated with the extract of the mycelium of T. matsutake. However, collagent expression was not affected. In addition, our results demonstrated that the extract of the mycelium of T. matsutake inhibited the 12-O-tetradecanoylphorbol-13-acetate (TPA)-induced MMP-1 expression and suppressed TPA-induced p38 activity. Therefore, the inhibitory effects of the extract of the mycelium of T. matsutake on MMP-1 induction are mediated by the inhibition of p38 in human fibroblasts. Our data suggest that the extract of the mycelium of T. matsutake may prove to be an effective biomaterial for anti-wrinkle treatment, as it can obstruct the degradation of the dermal ECM. 
  GeneRIFs  
 Mutant genes (CELA1, HSPG2, and KCNK5) in Balkan endemic nephropathy patients encode proteins involved in basement membrane/  extracellular matrix   and vascular tone, tightly connected to process of angiogenesis. 
   
  
 CTRB2 
 
  NCBI Gene  
  GeneRIF  
  Pubmed  
 
 
  Search Term  Rank  Fields  
  [Ee]xtracellular [Mm]atrix  8  Process(1)  
 
 
 hide/show details for CTRB2 
  [Ee]xtracellular [Mm]atrix  Process  
   extracellular matrix   disassembly 
   
  
 CTRC 
 
  NCBI Gene  
  GeneRIF  
  Pubmed  
 
 
  Search Term  Rank  Fields  
  [Mm]igration  6  PubMed(1); GeneRIFs(1)  
 
 
 hide/show details for CTRC 
  [Mm]igration  PubMed  
 Effect of chymotrypsin C and related proteins on pancreatic cancer cell   migration  . Pancreatic cancer is a malignant cancer with a high mortality rate. The amount of chymotrypsin C in pancreatic cancer cells is only 20% of that found in normal cells. Chymotrypsin C has been reported to be involved in cancer cell apoptosis, but its effect on pancreatic cancer cell   migration   is unclear. We performed cell   migration   scratch assays and Transwell experiments, and found that cell   migration   ability was downregulated in pancreatic cancer Aspc-1 cells that overexpressed chymotrypsin C, whereas the cell   migration   ability was upregulated in Aspc-1 cells in which chymotrypsin C was suppressed. Two-dimensional fluorescence differential in gel electrophoresis/mass spectrometry method was used to identify the proteins that were differentially expressed in Aspc-1 cells that were transfected with plasmids to induce either overexpression or suppressed expression of chymotrypsin C. Among 26 identified differential proteins, cytokeratin 18 was most obviously correlated with chymotrypsin C expression. Cytokeratin 18 is expressed in developmental tissues in early stages of cancer, and is highly expressed in most carcinomas. We speculated that chymotrypsin C might regulate pancreatic cancer cell   migration   in relation to cytokeratin 18 expression. 
  GeneRIFs  
 speculated that chymotrypsin C might regulate pancreatic cancer cell   migration   in relation to cytokeratin 18 expression 
   
  
 HP 
 
  NCBI Gene  
  GeneRIF  
  Pubmed  
 
 
  Search Term  Rank  Fields  
  [Mm]igration  6  PubMed(4); GeneRIFs(2)  
  [Ee]xtracellular [Mm]atrix  8  PubMed(1)  
  [Ii]nvasive  10  PubMed(5)  
  [Mm]etastasis  12  PubMed(4); GeneRIFs(1)  
  [Cc]ell [Ii]nvasion  13  PubMed(1); GeneRIFs(1)  
 
 
 hide/show details for HP 
  [Mm]igration  PubMed  
 Haptoglobin and CCR2 receptor expression in ovarian cancer cells that were exposed to ascitic fluid: exploring a new role of haptoglobin in the tumoral microenvironment. Haptoglobin (Hp) is an acute-phase protein that is produced by the liver to capture the iron that is present in the blood circulation, thus avoiding its accumulation in the blood. Moreover, Hp has been detected in a wide variety of tissues, in which it performs various functions. In addition, this protein is considered a potential biomarker in many diseases, such as cancer, including ovarian carcinoma; however, its participation in the cancerous processes has not yet been determined. The objective of this work was to demonstrate the expression of Hp and its receptor CCR2 in the ovarian cancer cells and its possible involvement in the process of cell   migration   through changes in the rearrangement of the actin cytoskeleton using western blot and wound-healing assays and confirming by confocal microscopy. Ovarian cancer cells express both Hp and its receptor CCR2 but only after exposure to ascitic fluid, inducing moderated cell   migration  . However, when the cells are exposed to exogenous Hp, the expression of CCR2 is induced together with drastic changes in the actin cytoskeleton rearrangement. At the same time, Hp induced cell   migration   in a much more efficient manner than did ascitic fluid. These effects were blocked when the CCR2 synthetic antagonist RS102895 was used to pretreat the cells. These results suggest that Hp-induced changes in the cell morphology, actin cytoskeleton structure, and   migration   ability of tumor cells, is possibly "preparing" these cells for the potential induction of the metastatic phenotype. 
 Proteomic identification of haptoglobin α2 as a glioblastoma serum biomarker: implications in cancer cell   migratio  n and tumor growth. Glioblastoma (GBM; grade IV astrocytoma) is the most malignant and common primary brain tumor in adults. Using combination of 2-DE and MALDI-TOF MS, we analyzed 14 GBM and 6 normal control sera and identified haptoglobin α2 chain as an up-regulated serum protein in GBM patients. GBM-specific up-regulation was confirmed by ELISA based quantitation of haptoglobin (Hp) in the serum of 99 GBM patients as against lower grades (49 grade III/AA; 26 grade II/DA) and 26 normal individuals (p = 0.0001). Further validation using RT-qPCR on an independent set (n = 78) of tumor and normal brain (n = 4) samples and immunohistochemcial staining on a subset (n = 42) of above samples showed increasing levels of transcript and protein with tumor grade and were highest in GBM (p = <0.0001 and <0.0001, respectively). Overexpression of Hp either by stable integration of Hp cDNA or exogenous addition of purified Hp to immortalized astrocytes resulted in increased cel  l migrati  on. RNAi-mediated silencing of Hp in glioma cells decreased cel  l migrati  on. Further, we demonstrate that both human glioma and mouse melanoma cells overexpressing Hp showed increased tumor growth. Thus, we have identified haptoglobin as a GBM-specific serum marker with a role on glioma tumor growth an  d migrati  on. 
 The obesity and inflammatory marker haptoglobin attracts monocytes via interaction with chemokine (C-C motif) receptor 2 (CCR2). Obesity is a chronic low inflammatory state. In the obesity condition the white adipose tissue (WAT) is massively infiltrated with monocytes/macrophages, and the nature of the signals recruiting these inflammatory cells has yet to be fully elucidated. Haptoglobin (Hp) is an inflammatory marker and its expression is induced in the WAT of obese subjects. In an effort to elucidate the biological significance of Hp presence in the WAT and of its upregulation in obesity we formulated the hypothesis that Hp may serve as a macrophage chemoattractant.We demonstrated by chemotaxis assay that Hp is able to attract chemokine (C-C motif) receptor 2 (CCR2)-transfected pre-B lymphocytes and monocytes in a dose-dependent manner. Moreover, Hp-mediated   migration   of monocytes is impaired by CCR2-specific inhibition or previous cell exposure to monocyte chemoattractant protein 1 (MCP1) (also known as CCR2 ligand or chemokine (C-C motif) ligand 2 (CCL2)). Downstream effects of Hp/CCR2 interaction were also investigated: flow cytometry proved that monocytes treated with Hp show reduced CCR2 expression on their surface; Hp interaction induces calcium release that is reduced upon pretreatment with CCR2 antagonist; extracellular signal-regulated kinase (ERK)1/2, a signal transducer activated by CCR2, is phosphorylated following Hp treatment and this phosphorylation is reduced when cells are pretreated with a specific CCR2 inhibitor. Consistently, blocking the ERK1/2 pathway with U0126, the selective inhibitor of the ERK upstream mitogen-activated protein (MAP)-ERK kinase (MEK), results in a dramatic reduction (by almost 100%) of the capability of Hp to induce monocyte   migration  .Our data show that Hp is a novel monocyte chemoattractant and that its chemotactic potential is mediated, at least in part. by its interaction with CCR2. 
 Haptoglobin polymorphism: a key factor in the proatherogenic role of B cells? B cells play a role in atherosclerosis. B lymphocytes may reduce the progression of vascular disease. Antibody production against modified auto-antigens is an element in the atheroprotective involvement of B lymphocytes. Paradoxical evidence is emerging from animal studies that suggest a proatherogenic B-cell behaviour independently of autoantibody production. One aspect that has received limited consideration is the role of genetic susceptibility modulated by extracellular matrix proteins. Haptoglobin is a polymorphic glycoprotein that binds to CD22 on B lymphocytes. Hp phenotypes show an important molecular heterogeneity. Hp 2-2 has been linked to an increased susceptibility for atherosclerosis. Haptoglobin and its polymorphism play a role in B-cell   migration   and function. Hp phenotypes may influence B-T cell dialogue and T cell activation. Haptoglobin is involved in the interplay of lymphocytes, neutrophils, and monocytes. Haptoglobin binds to the CD11b/CD18 receptor and to mast cells. HDL particles can become pro-inflammatory through interactions of Hp-Hb complexes with apolipoprotein A1. Haptoglobin is a chemoattractant to pre-B lymphocytes and monocytes. Beyond the conventional view of haptoglobin as a marker of hemolysis, several findings point towards an immunomodulatory effect of haptoglobin in B-cell mediated progression of atherosclerosis. The balance between proatherogenic and protective immunological properties of the different Hp phenotypes determines if lesions progress or regress. Clinical studies indicate a strong association between the Hp 2-2 phenotype and a more frequent onset of diabetic complications and cardiovascular disease. Findings in animal models (where no haptoglobin polymorphism is present) cannot always be extrapolated to humans. 
  GeneRIFs  
 Ovarian cancer cells express both Hp and its receptor CCR2 but only after exposure to ascitic fluid, inducing moderated cell   migration  ; results suggest that Hp-induced changes in the cell morphology, actin cytoskeleton structure, and   migration   ability of tumor cells, is possibly "preparing" these cells for the potential induction of the metastatic phenotype. 
 role on glioma tumor growth and   migration   
  [Ee]xtracellular [Mm]atrix  PubMed  
 Haptoglobin polymorphism: a key factor in the proatherogenic role of B cells? B cells play a role in atherosclerosis. B lymphocytes may reduce the progression of vascular disease. Antibody production against modified auto-antigens is an element in the atheroprotective involvement of B lymphocytes. Paradoxical evidence is emerging from animal studies that suggest a proatherogenic B-cell behaviour independently of autoantibody production. One aspect that has received limited consideration is the role of genetic susceptibility modulated by   extracellular matrix   proteins. Haptoglobin is a polymorphic glycoprotein that binds to CD22 on B lymphocytes. Hp phenotypes show an important molecular heterogeneity. Hp 2-2 has been linked to an increased susceptibility for atherosclerosis. Haptoglobin and its polymorphism play a role in B-cell migration and function. Hp phenotypes may influence B-T cell dialogue and T cell activation. Haptoglobin is involved in the interplay of lymphocytes, neutrophils, and monocytes. Haptoglobin binds to the CD11b/CD18 receptor and to mast cells. HDL particles can become pro-inflammatory through interactions of Hp-Hb complexes with apolipoprotein A1. Haptoglobin is a chemoattractant to pre-B lymphocytes and monocytes. Beyond the conventional view of haptoglobin as a marker of hemolysis, several findings point towards an immunomodulatory effect of haptoglobin in B-cell mediated progression of atherosclerosis. The balance between proatherogenic and protective immunological properties of the different Hp phenotypes determines if lesions progress or regress. Clinical studies indicate a strong association between the Hp 2-2 phenotype and a more frequent onset of diabetic complications and cardiovascular disease. Findings in animal models (where no haptoglobin polymorphism is present) cannot always be extrapolated to humans. 
  [Ii]nvasive  PubMed  
 Comparison of tear protein levels in breast cancer patients and healthy controls using a de novo proteomic approach. Non  invasive   biomarkers are urgently needed for early detection of breast cancer since the risk of recurrence, morbidity and mortality are closely related to disease stage at the time of primary surgery. In the past decade, many proteomics-based approaches were developed that utilize the protein profiling of human body fluids or identification of putative biomarkers to obtain more knowledge on the effects of cancer emergence and progression. Herein, we report on an analysis of proteins in the tear fluid from breast carcinoma patients and healthy women using a de novo proteomic approach and 25 mixed samples from each group. This study included 25 patients with primary   invasiv  e breast carcinoma and 25 age-matched healthy controls. We performed a MALDI-TOF-TOF-driven semi-quantitative comparison of tear protein levels in cancer (CA) and control (CTRL) using a de novo approach in pooled samples. Over 150 proteins in the tear fluid of CTRL and CA were identified. Using an in-house-developed algorithm we found more than 20 proteins distinctly upregulated or downregulated in the CTRL and CA groups. We identified several proteins that had modified expression in breast cancer patients. These proteins are involved in host immune system pathways (e.g., C1Q1 or S100A8) and different metabolic cascades (ALDH3A or TPI). Further validation of the results in an independent population combined with individual protein profiling of participants is needed to confirm the specificity of our findings and may lead to a better understanding of the pathological mechanism of breast cancer. 
 Discovery and identification of potential biomarkers of papillary thyroid carcinoma. Thyroid carcinoma is the most common endocrine malignancy and a common cancer among the malignancies of head and neck. Non  invasive   and convenient biomarkers for diagnosis of papillary thyroid carcinoma (PTC) as early as possible remain an urgent need. The aim of this study was to discover and identify potential protein biomarkers for PTC specifically.Two hundred and twenty four (224) serum samples with 108 PTC and 116 controls were randomly divided into a training set and a blind testing set. Serum proteomic profiles were analyzed using SELDI-TOF-MS. Candidate biomarkers were purified by HPLC, identified by LC-MS/MS and validated using ProteinChip immunoassays.A total of 3 peaks (m/z with 9190, 6631 and 8697 Da) were screened out by support vector machine (SVM) to construct the classification model with high discriminatory power in the training set. The sensitivity and specificity of the model were 95.15% and 93.97% respectively in the blind testing set. The candidate biomarker with m/z of 9190 Da was found to be up-regulated in PTC patients, and was identified as haptoglobin alpha-1 chain. Another two candidate biomarkers (6631, 8697 Da) were found down-regulated in PTC and identified as apolipoprotein C-I and apolipoprotein C-III, respectively. In addition, the level of haptoglobin alpha-1 chain (9190 Da) progressively increased with the clinical stage I, II, III and IV, and the expression of apolipoprotein C-I and apolipoprotein C-III (6631, 8697 Da) gradually decreased in higher stages.We have identified a set of biomarkers that could discriminate PTC from non-cancer controls. An efficient strategy, including SELDI-TOF-MS analysis, HPLC purification, MALDI-TOF-MS trace and LC-MS/MS identification, has been proved successful. 
 Serum haptoglobin as a novel molecular biomarker predicting colorectal cancer hepatic metastasis. Early detection of liver metastasis is important for improving colorectal cancer (CRC) patient survival. Our previous studies showed haptoglobin was highly expressed in primary CRC tissues, especially in heterochronous metastatic cases. Here, we assessed the potential of serum haptoglobin (sHP) as a biomarker for early detection of CRC liver metastasis by evaluating the sHP in 475 CRC patients and 152 healthy volunteers. In the training set (250 cases), sHP level in CRC-M1 (1773.18 ± 690.25 ng/mL) were significantly increased as compared to in CRC-M0 (1544.37 ± 1497.65 ng/mL) or healthy (917.76 ± 571.59 ng/mL). And the high sHP level was correlated with poor survival. Logistic regression analysis revealed that sHP, serum carcinoembryonic antigen (sCEA) and serum carbohydrate antigen 19.9 (sCA19.9) level were the significant parameters for detecting liver metastasis. In leave-one-out-cross-validation, these three markers resulted in 89.1% sensitivity and 85.8% specificity for hepatic metastasis detection. In an independent test set (225 cases), receiver operating characteristic curve analysis of sHP in CRC liver metastasis showed an area under the curve of 0.735, with a sensitivity of 87.2% and a specificity of 59.9%. Combination of sHP, sCEA and sCA19.9 improved diagnostic accuracy to 0.880, with a sensitivity of 88.5% and a specificity of 87.8%. Silencing of HP by specific shRNA significantly inhibited the LOVO and SW620 cell invasion, and suppressed xenograft tum  or invas  ive growth. In summary, these results demonstrate that sHP is associated with poor prognosis of CRC patients and that HP promotes colorectal cancer cell invasion. sHP combining with sCA19.9 and sCEA may be used as accurate predictors of CRC liver metastasis. 
 Increased plasma zonulin in patients with sepsis. Zonulin is a eukaryotic protein structurally similar to Vibrio cholerae's zonula occludens toxin. It plays an important role in the opening of small intestine tight junctions. The loss of gut wall integrity during sepsis might be pivotal and has been described in various experimental as well as human studies. Increased levels of zonulin could be demonstrated in diseases associated with increased intestinal inflammation, such as celiac disease and type 1 diabetes. We therefore investigated the role of plasma levels of zonulin in patients with sepsis as a non-  invasive   marker of gut wall integrity.Plasma level of zonulin was measured in 25 patients with sepsis, severe sepsis or septic shock according to ACCP/SCCM criteria at the first day of diagnosed sepsis. 18 non-septic post-surgical ICU-patients and 20 healthy volunteers served as control. Plasma levels were determined by using commercially available ELISA kit. Data are given as median and interquartile range (IQR).Significantly higher plasma concentration of zonulin were found in the sepsis group: 6.61 ng/mL (IQR 3.51-9.46), as compared to the to the post-surgical control group: 3.40 ng/mL (IQR 2.14-5.70) (P = 0.025), as well as to the healthy group: 3.55 ng/mL (IQR 3.14-4.14) (P = 0.008).We were able demonstrate elevated levels of plasma zonulin, a potential marker of intestinal permeability in septic patients. Increased zonulin may serve as an additional mechanism for the observed increased intestinal permeability during sepsis and SIRS. 
 Sex steroid hormones influence the risk for cervical cancer: modulation by haptoglobin genetic polymorphism. Sex steroid hormones ingestion (contraceptives and replacement therapy) may influence cervical carcinogenesis. Haptoglobin (Hp), an acute phase protein that has genetic polymorphism, can influence immune response to tumor. Our objective was to study the influence of haptoglobin genetic polymorphism on the risk for development of cervical cancer dependent on sex steroid hormones. A total of 492 Caucasian women, 196 pathologic [high-grade squamous intraepithelial lesions and   invasive   cervical cancer (HSIL + ICC)], ranging in age from 18 to 81 years, were phenotyped for plasma Hp using a polyacrylamide gel electrophoresis method. The effect of the interaction between the Hp genetic phenotype and steroid hormone therapy was analyzed using a multinomial logistic regression. Hp 1/1 genetic phenotype was associated with the risk for cervical cancer of steroid hormone ingestion: general risk odds ratio (OR)=5.388, P<0.001; for the interaction with carriers of Hp 1/1, OR=6.765, P<0.001; with carriers of Hp 2/1, OR=6.499, P<0.001; and with carriers of Hp 2/2, OR=3.903, P=0.001. The linear trend of risks that result from that interaction is also significant (chi2 =31.8, P<0.001). The higher risk for HSIL + ICC observed in carriers of increasing allele 1 of Haptoglobin probably results from the intense immune suppressor effect of this form of Hp, in addition to that of steroid hormones ingestion. 
  [Mm]etastasis  PubMed  
 Expression of haptoglobin predicts recurrence in head and neck squamous cell carcinoma. We determined whether expression of haptoglobin by head and neck squamous cell carcinoma (HNSCC) cells is associated with prognosis.Western blotting was carried out to investigate the expression of haptoglobin in oral cancer cell lines. We study patients with HNSCC without distant   metastasis   at diagnosis. Correlation between cellular haptoglobin and clinical characteristics of HNSCC was analyzed to assess the prognostic value of cellular haptoglobin level. Kaplan-Meier survival curves and log-rank test were used to evaluate differences in recurrence, distant   metastasis  , and overall survival rates between patients grouped according to cellular haptoglobin level in cancer tissues. The relationship of haptoglobin expression with survival was assessed using Cox proportional hazard models.Western blotting analysis showed that haptoglobin protein was expressed in 4 oral cancer cell lines. The recurrence rate was higher in HNSCC patients with over-expression of haptoglobin (>50%) (P=0.045). Over-expression of haptoglobin was also associated with an increased risk for recurrence (hazard ratio [HR] 3.2; 95% confidence interval [CI], 1.127-8.895; P=0.029) after adjusting for age, gender, disease site, stage, and treatment modality.Altogether, the data presented show that cellular expression of haptoglobin is closely related to recurrence rate in HNSCC patients. The elevated risk of relapse was confirmed in a multivariate analysis. The cellular expression of haptoglobin may be a prognostic factor in HNSCC. 
 The prognostic utility of haptoglobin genotypes in squamous cell carcinoma of the head and neck. The aim of this study was to determine whether haptoglobin (Hp) genotypes are associated with prognosis in patients with squamous cell carcinoma of the head and neck (HNSCC).We studied patients with HNSCC without distant   metastasis   at diagnosis. The Hp genotype of each patient was determined and the prognostic significance of the Hp genotype was further analyzed. Pearson's chi(2)-test or Fisher's exact test were used to analyze correlations between Hp genotype and clinical characteristics of HNSCC. Eighty patients with newly diagnosed HNSCC who were treated with curative modality were enrolled in this study. Kaplan-Meier plots and log-rank test were used to compare locoregional recurrence-free survival, distant-  metastasis  -free survival and overall survival of patients according to Hp genotype. Survival analysis was performed using Cox proportional hazard models.Eighty patients with newly diagnosed HNSCC were enrolled in this study. There was no significant difference in the distribution of Hp genotypes in HNSCC patients and healthy individuals (p=0.959). Matched-pair analysis showed that locoregional recurrence-free survival was poor (p=0.02) for HNSCC patients with Hp 2-2 or 2-0. There was no significant difference in distant   metastasis  -free survival and overall survival (p=0.422 and 0.509, respectively). Multivariate analysis showed that Hp 2-2 or 2-0 was associated with an increased risk of locoregional recurrence [Hazard ratio (HR) 5.9; 95% confidence interval (CI), 1.1-6.65; p=0.038]. The risk was still higher in patients with Hp 2-2 or 2-0 after further adjusting for age and treatment modality (HR 7.6; 95% CI, 1.2-46; p=0.028) in locoregional recurrence-free survival.The present data show that the Hp genotype is closely related to recurrence rate in patients with HNSCC. Patients with Hp 2-2 or 2-0 have greater locoregional recurrence and significantly increased HRs in multivariate analysis. The Hp genotype may be a prognostic factor in patients with HNSCC. 
 Serum haptoglobin as a novel molecular biomarker predicting colorectal cancer hepatic   metastasis  . Early detection of liver   metastasis   is important for improving colorectal cancer (CRC) patient survival. Our previous studies showed haptoglobin was highly expressed in primary CRC tissues, especially in heterochronous metastatic cases. Here, we assessed the potential of serum haptoglobin (sHP) as a biomarker for early detection of CRC liver   metastasis   by evaluating the sHP in 475 CRC patients and 152 healthy volunteers. In the training set (250 cases), sHP level in CRC-M1 (1773.18 ± 690.25 ng/mL) were significantly increased as compared to in CRC-M0 (1544.37 ± 1497.65 ng/mL) or healthy (917.76 ± 571.59 ng/mL). And the high sHP level was correlated with poor survival. Logistic regression analysis revealed that sHP, serum carcinoembryonic antigen (sCEA) and serum carbohydrate antigen 19.9 (sCA19.9) level were the significant parameters for detecting liv  er metasta  sis. In leave-one-out-cross-validation, these three markers resulted in 89.1% sensitivity and 85.8% specificity for hepat  ic metasta  sis detection. In an independent test set (225 cases), receiver operating characteristic curve analysis of sHP in CRC liv  er metasta  sis showed an area under the curve of 0.735, with a sensitivity of 87.2% and a specificity of 59.9%. Combination of sHP, sCEA and sCA19.9 improved diagnostic accuracy to 0.880, with a sensitivity of 88.5% and a specificity of 87.8%. Silencing of HP by specific shRNA significantly inhibited the LOVO and SW620 cell invasion, and suppressed xenograft tumor invasive growth. In summary, these results demonstrate that sHP is associated with poor prognosis of CRC patients and that HP promotes colorectal cancer cell invasion. sHP combining with sCA19.9 and sCEA may be used as accurate predictors of CRC liv  er metasta  sis. 
 Combination of haptoglobin and osteopontin could predict colorectal cancer hepatic   metastasis  . This study aimed to identify novel molecular markers for the early detection of colorectal cancer liver   metastasis  .Genes related to hepatic   metastasis   were screened from the Oncomine database. The candidate markers were tested by immunohistochemistry, and their predictive accuracy was assessed by the cross-validation method and an independent test set.We got three datasets containing 2,973 genes that were highly expressed in primary colon cancer tissues compared with non-metastatic colon cancer tissues and identified 7 candidate molecules for immunohistochemical validation. A total of 213 colorectal cancer samples were randomly divided into a training set (113 cases) and a blind testing set (100 cases). In the training set, immunohistochemical analysis showed that HP, OPN, and PTGIS expression were significantly higher in the hepatic   metastasis   group than in the non-  metastasis   group. Logistic regression analysis showed that HP and OPN levels in primary tumors and lymph node   metastasis   status were the only significant (P<0.05) parameters for detecting liver   metastasis  . The predictive accuracy of markers was assessed by the cross-validation method and an independent test set. In leave-one-out cross-validation, the two markers combined with clinicopathologic features resulted in 91.2% sensitivity and 96.4% specificity for hepatic   metastasis   detection. In an independent test set, the combination achieved 94.5% sensitivity and 88.9% specificity for predicting the hepatic   metastasis   of colorectal cancer.Our results suggest that combined HP and OPN expression levels are significantly related to liver   metastasis   and prognosis, and, if this is validated, they could be used as accurate predictors of liver   metastasis   in colorectal cancer. 
  GeneRIFs  
 High haptoglobin expression is associated with colorectal cancer hepatic   metastasis  . 
  [Cc]ell [Ii]nvasion  PubMed  
 Serum haptoglobin as a novel molecular biomarker predicting colorectal cancer hepatic metastasis. Early detection of liver metastasis is important for improving colorectal cancer (CRC) patient survival. Our previous studies showed haptoglobin was highly expressed in primary CRC tissues, especially in heterochronous metastatic cases. Here, we assessed the potential of serum haptoglobin (sHP) as a biomarker for early detection of CRC liver metastasis by evaluating the sHP in 475 CRC patients and 152 healthy volunteers. In the training set (250 cases), sHP level in CRC-M1 (1773.18 ± 690.25 ng/mL) were significantly increased as compared to in CRC-M0 (1544.37 ± 1497.65 ng/mL) or healthy (917.76 ± 571.59 ng/mL). And the high sHP level was correlated with poor survival. Logistic regression analysis revealed that sHP, serum carcinoembryonic antigen (sCEA) and serum carbohydrate antigen 19.9 (sCA19.9) level were the significant parameters for detecting liver metastasis. In leave-one-out-cross-validation, these three markers resulted in 89.1% sensitivity and 85.8% specificity for hepatic metastasis detection. In an independent test set (225 cases), receiver operating characteristic curve analysis of sHP in CRC liver metastasis showed an area under the curve of 0.735, with a sensitivity of 87.2% and a specificity of 59.9%. Combination of sHP, sCEA and sCA19.9 improved diagnostic accuracy to 0.880, with a sensitivity of 88.5% and a specificity of 87.8%. Silencing of HP by specific shRNA significantly inhibited the LOVO and SW6  20 cell invas  ion, and suppressed xenograft tumor invasive growth. In summary, these results demonstrate that sHP is associated with poor prognosis of CRC patients and that HP promotes colorectal canc  er cell invas  ion. sHP combining with sCA19.9 and sCEA may be used as accurate predictors of CRC liver metastasis. 
  GeneRIFs  
 these results demonstrate that sHP is associated with poor prognosis of colorectal cancer patients and that HP promotes colorectal cancer   cell invasion  . 
   
  
 PRSS1 
 
  NCBI Gene  
  GeneRIF  
  Pubmed  
 
 
  Search Term  Rank  Fields  
  [Mm]igration  6  PubMed(1)  
  [Ee]xtracellular [Mm]atrix  8  Process(1)  
  [Ii]nvasive  10  PubMed(2)  
  [Mm]etastasis  12  PubMed(1)  
 
 
 hide/show details for PRSS1 
  [Mm]igration  PubMed  
 Limited tryptic digestion near the amino terminus of bovine liver rhodanese produces active electrophoretic variants with altered refolding. When the enzyme rhodanese was partially digested by immobilized trypsin, it retained greater than 50% of its original activity although less than 10% of the undigested enzyme remained. The predominant daughter species were two 31-kDa polypeptides whose amino termini corresponded to either residue 44 or 45 of the enzyme's sequence. Following digestion, charged species were isolated by ion exchange chromatography. Denaturing electrophoresis revealed that a 4-kDa peptide remained associated with the 31-kDa fragment. This 4-kDa peptide appears to correspond to the amino-terminal 45 residues of rhodanese. Further proteolysis gave a 2.5-kDa peptide that dissociated under non-denaturing conditions without apparent change in   migration   of the 31-kDa fragment on SDS gels. Refolding of undigested, urea-denatured rhodanese restored much of its activity. Similar treatment of rhodanese following limited tryptic digestion resulted in no regain of activity. Refolding of a mixture of intact and digested rhodanese resulted in regain of activity appropriate for the amount of intact rhodanese in the sample, indicating that clipped rhodanese does not inhibit refolding of intact rhodanese. It is concluded that portions of the amino terminus of rhodanese are important in the enzyme's folding, but are not essential for the enzyme's sulfurtransferase activity. 
  [Ee]xtracellular [Mm]atrix  Process  
   extracellular matrix   disassembly 
  [Ii]nvasive  PubMed  
 Differential expression of trypsinogen and tumor-associated trypsin inhibitor (TATI) in bladder cancer. Tumor-associated trypsin inhibitor (TATI) is a marker of mucinous ovarian carcinoma, but it is also widely expressed in other malignant tumors and normal human tissues. Elevated serum concentrations of TATI are of prognostic value in ovarian, kidney, and bladder cancer. Tumor-associated trypsin is co-expressed with TATI in many malignancies and is thought to be involved in tumor invasion. TATI mRNA has been shown to be overexpressed in bladder cancer. We therefore studied whether trypsinogen expression also can be detected in bladder cancer and how this and TATI expression are associated with the clinicopathological characteristics of the tumors. We used RT-PCR, in situ hybridization and immunohistochemistry to detect trypsinogen- and TATI mRNA and protein in tissue samples from 28 bladder cancer patients and ten benign urothelia. TATI expression was detected in all benign tissues and non-  invasive   tumors. However, the expression was lower in the muscle-  invasive   tumors (pT2; n=5), whereas trypsinogen expression was seen in all but one non-  invasive   tumor. We conclude that trypsinogen is expressed in both malignant and benign bladder epithelium, whereas TATI expression decreases with increasing stage and grade of the tumor. This may suggest that a balanced expression of TATI and trypsinogen is required in normal tissue and that this balance is disrupted during tumor progression. 
 Expression of tumor-associated trypsinogens (TAT-1 and TAT-2) in prostate cancer. Trypsinogens are pancreatic serine proteinases and expressed in several cancers as tumor-associated trypsinogens (TAT). Trypsin mediates activation of pro-uPA and pro-MMPs, thus promoting angiogenesis and tumor invasion. Recently, we described expression of TAT in the human male genital tract and now we studied TAT in relation to PSA in PCa.TAT expression was studied by immunohistochemistry, in situ hybridization, RT-PCR, DNA-sequencing and IFMA. LNCaP cells were used to study secretion of TAT and PSA after androgen stimulation.Immunoreactive TAT was localized in all prostatic tumors (n = 109), lymph node (n = 16), and bone metastases (n = 17). Immunostaining intensity increased with higher Gleason's grade, whereas PSA immunostaining decreased significantly. PSA and TAT were not identically distributed in benign and malignant cells. Androgen stimulation of LNCaP cells decreased secretion of TAT and increased that of PSA. TAT mRNA was demonstrated in tissue sections and identified as TAT-1 and -2 by RT-PCR and DNA-sequencing.Expression of TAT is better preserved than PSA in high-grade PCa. Expression of TAT and PSA is regulated by different mechanisms as demonstrated in tissue sections and in vitro. Locally produced TAT may act in a paracrine mode to promote angiogenesis and tumor invasion in PCa by both activating and degrading of other proteinases. Further studies on the role of TAT in   invasive   PCa and on the mechanisms involved in the regulation of TAT expression are warranted. 
  [Mm]etastasis  PubMed  
 Three female familial cases of solid pseudopapillary tumors with a protease serine 1 gene mutation. Solid pseudopapillary tumors (SPTs) are a rare pancreatic neoplastic lesion. Familial aggregation has not been reported in this disease. The objectives of this study were to report the history, clinicopathological features, and gene mutations of 3 familial cases of SPT. Three female cases of SPT presented in 1 family. Eight family relatives, 5 healthy volunteers, and 8 patients with SPT acted as controls. Histological examination and immunohistochemistry were performed on the surgical tumor specimens. Polymerase chain reaction-single-strand conformation polymorphism and gene sequencing were performed on genomic DNA extracted from blood. All 3 patients underwent surgical treatment, 2 patients died (3 months and 5 months after surgery), whereas neither recurrence nor   metastasis   was observed in the other patient during 2-year follow-up. The tumors from the 3 cases had identical immunoreactivity to a series of molecular markers. A Leu104Val mutation of protease serine 1 (PRSS1) was observed in the familial patients and 2 healthy male family members; no β-catenin or adenomatous polyposis coli mutations were detected in the familial cases. This study indicates the possibility of genetic involvement in the pathogenesis of SPT. Family history may be a positive predictive factor for malignancy in SPT. 
   
  
 CORIN 
 
  NCBI Gene  
  GeneRIF  
  Pubmed  
 
 
  Search Term  Rank  Fields  
  [Mm]igration  6  PubMed(1)  
 
 
 hide/show details for CORIN 
  [Mm]igration  PubMed  
 Corin, an enzyme with a putative role in spiral artery remodeling, is up-regulated in late secretory endometrium and first trimester decidua. What is the nature of cellular Corin expression in human gestational tissues?CORIN is expressed in non-pregnant late secretory phase endometrium, first trimester human implantation sites and is up-regulated with decidualization ex vivo.Adequate trophoblast invasion and spiral artery remodeling/transformation is critical for successful implantation. CORIN, best known for its role in activating atrial natruietic peptide (ANP) to regulate blood pressure, has recently been proposed to be centrally involved in trophoblast invasion and spiral artery remodeling. It is postulated that ANP, activated by CORIN, promotes trophoblast invasion and that a deficiency causes pre-eclampsia. Mice deficient in either Corin or ANP displayed poor trophoblast invasion, impaired spiral artery remodeling and phenocopied human pre-eclampsia. However, the precise cellular localization of CORIN within human gestational tissues has not been well characterized.We measured CORIN protein localization in a number of human gestational tissues relevant to early embryo/placental implantation: non-pregnant (NP) endometrial biopsies (n = 5 per phase of the menstrual cycle), first trimester placental bed biopsies (n = 12) and pre-term control (n = 10) and severe early onset preeclamptic placentas (n = 15). Endometrial stromal cells were isolated from human endometrial biopsies (n = 5) and induced to decidualize ex vivo. Finally, CORIN concentrations were measured in serum obtained from pregnant women during the first trimester of whom, 56 subsequently ended up with a healthy term delivery (controls), 18 developed fetal growth restriction (FGR) and 21 had a miscarriage.We performed immunohistochemistry to assess CORIN localization. Changes in Corin mRNA expression in human endometrial stromal cells decidualized ex vivo were measured by quantitative RT-PCR, and levels of CORIN within human sera were measured by ELISA.CORIN was expressed in both NP late secretory phase endometrium and first trimester decidua within placental bed biopsies. Importantly, decidualization of primary human endometrial cells ex vivo significantly increased Corin expression (P < 0.05). CORIN was also detected within the villous cytotrophoblast, but there was no change in mRNA levels in placentas complicated by severe preterm pre-eclampsia when compared with pre-term controls. Although CORIN was detected in first trimester serum, levels did not change across gestation, nor could they predict miscarriage or FGR (other disorders of impaired placental invasion).Owing to the fact that we utilized early pregnancy human specimens, this is mainly a descriptive study with a limited amount of functional experiments.This is the first study to thoroughly characterize Corin mRNA and protein expression in human gestational tissue. Our findings support recent data from murine studies collectively suggesting that CORIN plays a critical role in trophoblast   migration   and spiral artery remodeling during early pregnancy in humans. Therefore, further studies of CORIN biology in early pregnancy may identify new therapeutic targets to improve implantation quality in early pregnancy and potentially reduce the rates of pregnancy complications caused by inadequate implantation (pre-eclampsia, FGR and miscarriage).This study was supported by The National Health and Medical Research Council of Australia (Salary support #490970, #490995). The funders had no role in study design, data collection and analysis, decision to publish or preparation of the manuscript. The authors declare that no competing interests exist. 
   
  
 GZMB 
 
  NCBI Gene  
  GeneRIF  
  Pubmed  
 
 
  Search Term  Rank  Fields  
  [Mm]igration  6  PubMed(7); GeneRIFs(2)  
  ECM  7  PubMed(2); GeneRIFs(1)  
  [Ee]xtracellular [Mm]atrix  8  PubMed(12); GeneRIFs(1); Summary(1)  
  [Ii]nvasive  10  PubMed(7); GeneRIFs(2)  
  [Mm]etastasis  12  PubMed(5); GeneRIFs(3)  
  [Cc]ell [Ii]nvasion  13  PubMed(2); GeneRIFs(1)  
 
 
 hide/show details for GZMB 
  [Mm]igration  PubMed  
 Extracellular matrix remodeling by human granzyme B via cleavage of vitronectin, fibronectin, and laminin. Human granzyme B (GrB) released from cytotoxic lymphocytes plays a key role in the induction of target cell apoptosis when internalized in the presence of perforin. Here we demonstrate that GrB also possesses a potent extracellular matrix remodeling activity. Both native and recombinant GrB caused detachment of immortalized and transformed cell lines, primary endothelial cells, and chondrocytes. Cell detachment by GrB induced endothelial cell death (anoikis). GrB also inhibited tumor cell spreading,   migration  , and invasion in vitro. Investigation into the underlying mechanism revealed that GrB efficiently cleaves three proteins involved in extracellular matrix structure and function: vitronectin, fibronectin, and laminin. In vitronectin, GrB cleaves after an Arg-Lys-Asp (RGD) motif, which is part of the integrin-binding site found in matrix proteins. We propose that targeting of the integrin-extracellular matrix interface by GrB may allow perforin-independent killing of target cells via anoikis, restrict motility of tumor cells, facilitate lymphocyte   migration  , or directly reduce virus infectivity. It may also contribute to tissue destruction in diseases in which extracellular GrB is evident, such as rheumatoid arthritis and atherosclerosis. 
 Target cell apoptosis induced by cytotoxic T cells and natural killer cells involves synergy between the pore-forming protein, perforin, and the serine protease, granzyme B. Cytotoxic lymphocytes (CL) comprise two effector cell populations with the ability to eliminate unwanted or harmful cells. Cytotoxic T cells (CTLs) demonstrate both an exquisite specificity and memory in recognising target cell oligopeptides presented within the groove of major histocompatibility complex class I antigens. By contrast, natural killer (NK) cells mediate "innate' immunity against virus-infected cells and surveillance against neoplastic transformation, and do not require presensitisation. Despite recognising target cells in very different ways, CTLs and NK cells both utilise a pore-forming protein, perforin, and a battery of serine proteases as a principal means of inflicting cell death. The action of both types of CL results in death by apoptosis. Recently, we and others have accumulated evidence that perforin and serine proteases synergistically trigger an endogenous pathway of programmed cell death that results in dissolution of the nuclear membrane, chromatin condensation and DNA fragmentation. These changes are secondary to inappropriate activation of p34, a kinase whose activation and   migration   from the cytoplasm to the nucleus normally controls a cell's entry into mitosis. Therefore, CI, may exert their actions through the derangement of cell cycle control. The downstream molecular targets of perforin/granzyme-mediated apoptosis (especially the physiological ligand/substrate of granzyme B) are still unclear, though candidate molecules with homology to products of cell death genes found in primitive organisms such as the nematode, C. elegans, are currently under investigation. 
 Granzyme B cleavage of fibronectin disrupts endothelial cell adhesion,   migration   and capillary tube formation. Dysregulated angiogenesis contributes to the pathogenesis of chronic inflammatory diseases. Modulation of the extracellular matrix by immune-derived proteases can alter endothelial cell-matrix interactions as well as endothelial cell sprouting,   migration   and capillary formation. Granzyme B is a serine protease that is expressed by a variety of immune cells, and accumulates in the extracellular milieu in many chronic inflammatory disorders that are associated with dysregulated angiogenesis. Although granzyme B is known to cleave fibronectin, an essential glycoprotein in vascular morphogenesis, the role of granzyme B in modulating angiogenesis is unknown. In the present study, granzyme B cleaved both plasma fibronectin and cell-derived fibronectin, resulting in the release of multiple fibronectin fragments. Granzyme B cleavage of fibronectin resulted in a dose-dependent reduction in endothelial cell adhesion to fibronectin as well as reduced endothelial cell   migration   and tubular formation. These events were prevented when granzyme B activity was inhibited by a small molecule inhibitor. In summary, granzyme B-mediated cleavage of fibronectin contributes to attenuated angiogenesis through the disruption of endothelial cell - fibronectin interaction resulting in impaired endothelial cell   migration   and tubular formation. 
 Cytohesin-associated scaffolding protein (CASP) is a substrate for granzyme B and ubiquitination. Natural killer (NK) cells are a sub-population of cytotoxic lymphocytes that can kill tumor or infected cells without prior exposure, by secreting the contents of preformed cytotoxic vesicles, containing perforin and granzymes, at the immune synapse. Cytohesin-associated scaffolding protein (CASP) is an adaptor molecule uniquely expressed in lymphocytes that forms complexes with both vesicle-initiating and sorting proteins, and has roles in NK cell   migration  , cytotoxicity, and cytokine secretion. In this study, we show that CASP contains a conserved granzyme B cleavage site that could modify its intracellular localization and interaction with sorting nexin 27. We also provide evidence that CASP is modified by ubiquitination. Both of these post-translational modifications could rapidly modify CASP function and highlight the dynamic regulatory mechanisms that direct its role in NK cell functions. 
 Polarized granzyme release is required for antigen-driven transendothelial   migration   of human effector memory CD4 T cells. Human effector memory CD4 T cells may transmigrate across endothelial cell (EC) monolayers either in response to inflammatory chemokines or in response to TCR recognition of Ag presented on the surface of the EC. The kinetics, morphologic manifestations, and molecular requirements of chemokine- and TCR-driven transendothelial   migration   (TEM) differ significantly. In this study, we report that, whereas the microtubule organizing center (MTOC) and cytosolic granules follow the nucleus across the endothelium in a uropod during chemokine-driven TEM, MTOC reorientation to the contact region between the T cell and the EC, accompanied by dynein-driven transport of granzyme-containing granules to and exocytosis at the contact region, are early events in TCR-driven, but not chemokine-driven TEM. Inhibitors of either granule function or granzyme proteolytic activity can arrest TCR-driven TEM, implying a requirement for granule discharge in the process. In the final stages of TCR-driven TEM, the MTOC precedes, rather than follows, the nucleus across the endothelium. Thus, TCR-driven TEM of effector memory CD4 T cells appears to be a novel process that more closely resembles immune synapse formation than it does conventional chemotaxis. 
 UVA induces granzyme B in human keratinocytes through MIF: implication in extracellular matrix remodeling. In a previous study, we have described that UVB induces granzyme B (GrB) in human keratinocyte cells, and that confers potent cellular cytotoxicity against various cellular models, including immune cells (Hernandez-Pigeon, H., Jean, C., Charruyer, A., Haure, M. J., Titeux, M., Tonasso, L., Quillet-Mary, A., Baudouin, C., Charveron, M., and Laurent, G. (2006) J. Biol. Chem. 281, 13525-13532). Herein, we have found that, in contrast to UVB, UVA failed to enhance keratinocyte cellular cytotoxicity but was still able to trigger GrB production. We show that GrB is accumulated through a p38 MAPK-dependent transcriptional mechanism stimulated by redox-dependent   migration   inhibitory factor release. Moreover, GrB purified from UVA-treated cellular extracts was found to degrade fibronectin in vitro. Treatment with antisense oligonucleotide directed against GrB resulted in the inhibition of UVA-induced cell detachment and cell death and facilitated cell   migration   through fibronectin and vitronectin matrix upon UVA exposure. Altogether, these results suggest another function for GrB in the context of the UV response. Indeed, combined with our previous study, it appears that, whereas this enzyme mediates keratinocyte cellular cytotoxicity following UVB irradiation, GrB supports the capacity of keratinocyte to degrade extracellular matrix components following UVA irradiation. UV-mediated GrB production may thus have important consequences in photoaging and photocarcinogenesis. 
 TCR activation eliminates glutamate receptor GluR3 from the cell surface of normal human T cells, via an autocrine/paracrine granzyme B-mediated proteolytic cleavage. The majority of resting normal human T cells, like neuronal cells, express functional receptors for glutamate (the major excitatory neurotransmitter in the CNS) of the ionotropic alpha-amino-3-hydroxy-5-methyl-4-isoxazolepropionic acid (AMPA)-receptor subtype 3 (GluR3). Glutamate by itself ( approximately 10 nM) activates key T cell functions, including adhesion to fibronectin and laminin and chemotactic   migration   toward CXCL12/stromal cell-derived factor 1. In this study, we found by GluR3-specific immunostaining, flow cytometry, and Western blots that GluR3 cell surface expression decreases dramatically following TCR activation of human T cells. CXCR4, VLA-4, and VLA-6 also decrease substantially, whereas CD147 increases as expected, after TCR activation. Media of TCR-activated cells "eliminates" intact GluR3 (but not CXCR4 and VLA-6) from the cell surface of resting T cells, suggesting GluR3 cleavage by a soluble factor. We found that this factor is granzyme B (GB), a serine protease released by TCR-activated cells, because the extent of GluR3 elimination correlated with the active GB levels, and because three highly specific GB inhibitors blocked GluR3 down-regulation. Media of TCR-activated cells, presumably containing cleaved GluR3B peptide (GluR3 aa 372-388), inhibited the specific binding of anti-GluR3B mAb to synthetic GluR3B peptide. In parallel to losing intact GluR3, TCR-activated cells lost glutamate-induced adhesion to laminin. Taken together, our study shows that "classical immunological" TCR activation, via autocrine/paracrine GB, down-regulates substantially the expression of specific neurotransmitter receptors. Accordingly, glutamate T cell neuroimmune interactions are influenced by the T cell activation state, and glutamate, via AMPA-GluR3, may activate only resting, but not TCR-activated, T cells. Finally, the cleavage and release to the extracellular milieu of the GluR3B peptide may in principle increase its antigenicity, and thus the production, of anti-self GluR3B autoantibodies, which activate and kill neurons, found in patients with various types of epilepsy. 
  GeneRIFs  
 Polarized granzyme release is required for antigen-driven transendothelial   migration   of human effector memory CD4 T cells. 
 Granzyme B cleavage of fibronectin disrupts endothelial cell adhesion,   migration   and capillary tube formation. 
  ECM  PubMed  
 Granzyme B is expressed in urothelial carcinoma and promotes cancer cell invasion. Granzyme B (GrB) is a serine proteinase known to be expressed by cytotoxic lymphocytes and to induce, in presence of perforin (Pf), apoptosis in target cells. Recently, GrB expression has been shown (often in absence of Pf) in nonlymphoid cells, but its function is not defined. In our study, we investigated GrB and Pf expression in bladder cancer cell lines and in urothelial carcinoma (UC) tissues by reverse transcription-polymerase chain reaction (RT-PCR), Western blot, ELISA, immunofluorescence and immunohistochemistry. We also assessed the function of GrB in UC cells; the in vitro function of GrB was examined by loss-of-function experiments. Our results revealed that GrB is expressed, in absence of Pf, in UC cells. Significant differences were found between GrB expression and both increasing pathological tumor spreading and high-grade vs. low-grade pTa tumors. Notably, GrB in UC tissues was concentrated at the cancer invasion front and was expressed in neoplastic cells undergoing epithelial-mesenchymal transition, a key event in carcinoma invasion. Indeed, GrB-positive cells also expressed Snail, N-cadherin or were negative for E-cadherin. GrB expressed in tumor cell lines was enzymatically active and capable of vitronectin cleavage, implying extracellular matrix (  ECM  ) remodeling by GrB. Inhibition of GrB activity or Stealth RNA interference-mediated GrB gene silencing markedly suppressed bladder cancer cell invasion through matrigel. This data provides the first evidence for a role of GrB in promoting cancer cell invasion. Taken together, our findings suggest that GrB, via   ECM   degradation, contributes to the establishment of the UC invasive phenotype. 
 Granzyme B cleaves decorin, biglycan and soluble betaglycan, releasing active transforming growth factor-β1. Granzyme B (GrB) is a pro-apoptotic serine protease that contributes to immune-mediated target cell apoptosis. However, during inflammation, GrB accumulates in the extracellular space, retains its activity, and is capable of cleaving extracellular matrix   (EC  M) proteins. Recent studies have implicated a pathogenic extracellular role for GrB in cardiovascular disease, yet the pathophysiological consequences of extracellular GrB activity remain largely unknown. The objective of this study was to identify proteoglycan (PG) substrates of GrB and examine the ability of GrB to release PG-sequestered TGF-β1 into the extracellular milieu.Three extracellular GrB PG substrates were identified; decorin, biglycan and betaglycan. As all of these PGs sequester active TGF-β1, cytokine release assays were conducted to establish if GrB-mediated PG cleavage induced TGF-β1 release. Our data confirmed that GrB liberated TGF-β1 from all three substrates as well as from endoge  nou  s ECM and this process was inhibited by the GrB inhibitor 3,4-dichloroisocoumarin. The released TGF-β1 retained its activity as indicated by the induction of SMAD-3 phosphorylation in human coronary artery smooth muscle cells.In addition to contributi  ng   to ECM degradation and the loss of tissue structural integrity in vivo, increased extracellular GrB activity is also capable of inducing the release of active TGF-β1 from PGs. 
  GeneRIFs  
 data provides the first evidence for a role of GrB in promoting cancer cell invasion. Taken together, our findings suggest that Granzyme B (GrB), via   ECM   degradation, contributes to the establishment of the urothelial carcinoma invasive phenotype 
  [Ee]xtracellular [Mm]atrix  PubMed  
 [Rapid biosynthesis and release of 35 kD granzyme B by NK92 cells bypassing secretory lysosomes]. To investigate the possibility of the biosynthesis and release of granzyme B (GZB) by NK92 cells bypassing the way of secretory lysosomes (SLs) and the possible mechanism.As cell models, NK92 cells were activated by the phorbol myristate acetate (PMA) and ionomycin (ION). Within 4 hours following the activation, immuno- fluorescence and electron microscopy were used to detect the content and distribution of 35 000 (Mr) and 32 000 (Mr) GZB in the cytoplasm of NK92 before and after the protein synthesis was inhibited; Western blotting was performed to detect GZB inside and outside the SLs. After blocking the release of 32 000 (Mr) GZB by inhibiting the exocytosis of SLs with EDTA, we tested the content of Mr 35 000 GZB in activated NK92 supernatant. Activated NK92 cells were co-cultured with K562 cells to observe whether the Mr 35 000 GZB could enter the K562 cells. Activated NK92 cell death rate was determined and the enzyme activity of secreted Mr 35 000 GZB was examined.Four hours after stimulated by PMA/ION, NK92 cells generated large amount of Mr 35 000 GZB in the cytoplasm outside SLs where Mr 32 000 GZB was located. Immunoelectron microscope and immunofluorescence further approved that Mr 35 000 GZB outside SLs was located in vesicles. In addition, Mr 35 000 GZB could be secreted outside NK92 cells. Further investigation found that GZB/Serpinb9 composite and Mr 35 000 GZB could simultaneously emerge in the cytoplasm outside SLs. However, activated NK92 cell death rate did not rise. Mr 32 000 GZB inside SLs had enzyme activity in contrast with the Mr 35 000 GZB in zymogen form outside SLs, which suggested that Mr 35 000 GZB was not originated from the SLs.The activated human NK cell lines could secreted rapidly inactive Mr 35 000 GZB outside SLs, and the GZB could enter the   extracellular matrix   or target cells bypassing SLs, which provides a part of the extracellular GZB. 
 Caspases cleave focal adhesion kinase during apoptosis to generate a FRNK-like polypeptide. Focal adhesion kinase (Fak) is a non-receptor protein-tyrosine kinase that stimulates cell spreading and motility by promoting the formation of contact sites between the cell and the   extracellular matrix   (focal adhesions). It suppresses apoptosis by transducing survival signals that emanate from focal adhesions via the clustering of transmembrane integrins by components of the   extracellular matrix  . We demonstrate that Fak is cleaved by caspases at two distinct sites during apoptosis. The sites were mapped to DQTD772, which was preferentially cleaved by caspase-3, and VSWD704, which was preferentially cleaved by caspase-6 and cytotoxic T lymphocyte-derived granzyme B. The cleavage of Fak during apoptosis separates the tyrosine kinase domain from the focal adhesion targeting (FAT) domain. The carboxyl-terminal fragments that are generated suppress phosphorylation of endogenous Fak and thus resemble a natural variant of Fak, FRNK, that inhibits Fak activity by preventing the localization of Fak to focal adhesions. The cleavage of Fak by caspases may thus play an important role in the execution of the suicide program by disabling the anti-apoptotic function of Fak. Interestingly, rodent Fak lacks an optimal caspase-3 consensus cleavage site although it is cleaved in murine cells undergoing apoptosis at an upstream site. This appears to be the first example of a caspase substrate where the cleavage sites are not conserved between species. 
 Granzyme B is expressed in urothelial carcinoma and promotes cancer cell invasion. Granzyme B (GrB) is a serine proteinase known to be expressed by cytotoxic lymphocytes and to induce, in presence of perforin (Pf), apoptosis in target cells. Recently, GrB expression has been shown (often in absence of Pf) in nonlymphoid cells, but its function is not defined. In our study, we investigated GrB and Pf expression in bladder cancer cell lines and in urothelial carcinoma (UC) tissues by reverse transcription-polymerase chain reaction (RT-PCR), Western blot, ELISA, immunofluorescence and immunohistochemistry. We also assessed the function of GrB in UC cells; the in vitro function of GrB was examined by loss-of-function experiments. Our results revealed that GrB is expressed, in absence of Pf, in UC cells. Significant differences were found between GrB expression and both increasing pathological tumor spreading and high-grade vs. low-grade pTa tumors. Notably, GrB in UC tissues was concentrated at the cancer invasion front and was expressed in neoplastic cells undergoing epithelial-mesenchymal transition, a key event in carcinoma invasion. Indeed, GrB-positive cells also expressed Snail, N-cadherin or were negative for E-cadherin. GrB expressed in tumor cell lines was enzymatically active and capable of vitronectin cleavage, implying   extracellular matrix   (ECM) remodeling by GrB. Inhibition of GrB activity or Stealth RNA interference-mediated GrB gene silencing markedly suppressed bladder cancer cell invasion through matrigel. This data provides the first evidence for a role of GrB in promoting cancer cell invasion. Taken together, our findings suggest that GrB, via ECM degradation, contributes to the establishment of the UC invasive phenotype. 
   Extracellular matrix   remodeling by human granzyme B via cleavage of vitronectin, fibronectin, and laminin. Human granzyme B (GrB) released from cytotoxic lymphocytes plays a key role in the induction of target cell apoptosis when internalized in the presence of perforin. Here we demonstrate that GrB also possesses a potent   extracellular matrix   remodeling activity. Both native and recombinant GrB caused detachment of immortalized and transformed cell lines, primary endothelial cells, and chondrocytes. Cell detachment by GrB induced endothelial cell death (anoikis). GrB also inhibited tumor cell spreading, migration, and invasion in vitro. Investigation into the underlying mechanism revealed that GrB efficiently cleaves three proteins involved in   extracellular matrix   structure and function: vitronectin, fibronectin, and laminin. In vitronectin, GrB cleaves after an Arg-Lys-Asp (RGD) motif, which is part of the integrin-binding site found in matrix proteins. We propose that targeting of the integrin-  extracellular matrix   interface by GrB may allow perforin-independent killing of target cells via anoikis, restrict motility of tumor cells, facilitate lymphocyte migration, or directly reduce virus infectivity. It may also contribute to tissue destruction in diseases in which extracellular GrB is evident, such as rheumatoid arthritis and atherosclerosis. 
 Perforin-independent extracellular granzyme B activity contributes to abdominal aortic aneurysm. Granzyme B (GZMB) is a serine protease that is abundantly expressed in advanced human atherosclerotic lesions and may contribute to plaque instability. Perforin is a pore-forming protein that facilitates GZMB internalization and the induction of apoptosis. Recently a perforin-independent, extracellular role for GZMB has been proposed. In the current study, the role of GZMB in abdominal aortic aneurysm (AAA) was assessed. Apolipoprotein E (APOE)(-/-) x GZMB(-/-) and APOE(-/-) x perforin(-/-) double knockout (GDKO, PDKO) mice were generated to test whether GZMB exerted a causative role in aneurysm formation. To induce aneurysm, mice were given angiotensin II (1000 ng/kg/min) for 28 days. GZMB was found to be abundant in both murine and human AAA specimens. GZMB deficiency was associated with a decrease in AAA and increased survival compared with APOE-KO and PDKO mice. Although AAA rupture was observed frequently in APOE-KO (46.7%; n = 15) and PDKO (43.3%; n = 16) mice, rupture was rarely observed in GDKO (7.1%; n = 14) mice. APOE-KO mice exhibited reduced fibrillin-1 staining compared with GDKO mice, whereas in vitro protease assays demonstrated that fibrillin-1 is a substrate of GZMB. As perforin deficiency did not affect the outcome, our results suggest that GZMB contributes to AAA pathogenesis via a perforin-independent mechanism involving   extracellular matrix   degradation and subsequent loss of vessel wall integrity. 
 Granzyme B cleavage of fibronectin disrupts endothelial cell adhesion, migration and capillary tube formation. Dysregulated angiogenesis contributes to the pathogenesis of chronic inflammatory diseases. Modulation of the   extracellular matrix   by immune-derived proteases can alter endothelial cell-matrix interactions as well as endothelial cell sprouting, migration and capillary formation. Granzyme B is a serine protease that is expressed by a variety of immune cells, and accumulates in the extracellular milieu in many chronic inflammatory disorders that are associated with dysregulated angiogenesis. Although granzyme B is known to cleave fibronectin, an essential glycoprotein in vascular morphogenesis, the role of granzyme B in modulating angiogenesis is unknown. In the present study, granzyme B cleaved both plasma fibronectin and cell-derived fibronectin, resulting in the release of multiple fibronectin fragments. Granzyme B cleavage of fibronectin resulted in a dose-dependent reduction in endothelial cell adhesion to fibronectin as well as reduced endothelial cell migration and tubular formation. These events were prevented when granzyme B activity was inhibited by a small molecule inhibitor. In summary, granzyme B-mediated cleavage of fibronectin contributes to attenuated angiogenesis through the disruption of endothelial cell - fibronectin interaction resulting in impaired endothelial cell migration and tubular formation. 
 Granzyme B in injury, inflammation, and repair. Fragile skin and susceptibility to skin tearing are major problems among the elderly and can be complicated further by impaired wound healing. Non-healing wounds fail to progress through the normal stages of healing and enter a state of chronic inflammation featuring increased proteolytic activity. Increased expression of the serine protease granzyme B is observed during prolonged inflammation and is implicated in the pathogenesis of several chronic inflammatory diseases. Although its role in cytotoxic lymphocyte-mediated apoptosis is well established, granzyme B can also degrade   extracellular matrix   proteins and alter inflammation if present in the extracellular milieu. The present review focuses on the emerging evidence for the involvement of granzyme B in chronic inflammation, impaired wound healing, and age-related skin fragility. 
 Elevation of plasma granzyme B levels after acute myocardial infarction. Apoptosis is reported to play an important role in left ventricular (LV) remodeling after acute myocardial infarction (AMI). Granzyme B is a member of the serine esterase family, which has an important role in cellular apoptosis and   extracellular matrix   degradation.Peripheral blood samples were obtained from 33 patients with a first-onset AMI treated by percutaneous coronary intervention (mean age: 61.4+/-8.7 years old) on days 1, 7 and 14 after onset. Plasma levels of tumor necrosis factor (TNF)-alpha, a soluble form of the Fas ligand (sFasL), and granzyme B were measured. TIMI grade 3 recanalization was accomplished in all patients within 12 h after onset. The LV end-diastolic volume index (LVEDVI) was calculated on day 1 and at 6 months after onset. Plasma levels of TNF-alpha, sFasL and granzyme B increased significantly on days 7 and 14 after onset of AMI. Stepwise multivariate regression analysis showed that the plasma granzyme B level on day 14 is a significant explanatory variable for changes in the LVEDVI.Plasma levels of granzyme B increased after AMI, which might be an important factor in the progression of late LV remodeling after AMI. 
 Granzyme B cleaves decorin, biglycan and soluble betaglycan, releasing active transforming growth factor-β1. Granzyme B (GrB) is a pro-apoptotic serine protease that contributes to immune-mediated target cell apoptosis. However, during inflammation, GrB accumulates in the extracellular space, retains its activity, and is capable of cleaving   extracellular matri  x (ECM) proteins. Recent studies have implicated a pathogenic extracellular role for GrB in cardiovascular disease, yet the pathophysiological consequences of extracellular GrB activity remain largely unknown. The objective of this study was to identify proteoglycan (PG) substrates of GrB and examine the ability of GrB to release PG-sequestered TGF-β1 into the extracellular milieu.Three extracellular GrB PG substrates were identified; decorin, biglycan and betaglycan. As all of these PGs sequester active TGF-β1, cytokine release assays were conducted to establish if GrB-mediated PG cleavage induced TGF-β1 release. Our data confirmed that GrB liberated TGF-β1 from all three substrates as well as from endogenous ECM and this process was inhibited by the GrB inhibitor 3,4-dichloroisocoumarin. The released TGF-β1 retained its activity as indicated by the induction of SMAD-3 phosphorylation in human coronary artery smooth muscle cells.In addition to contributing to ECM degradation and the loss of tissue structural integrity in vivo, increased extracellular GrB activity is also capable of inducing the release of active TGF-β1 from PGs. 
 Active and zymogen forms of granzyme B are constitutively released from cytotoxic lymphocytes in the absence of target cell engagement. Granzyme B (GrB) plays a well-established intracellular role in cytotoxic lymphocyte (CL)-mediated killing of abnormal cells; however, emerging evidence suggests that it participates in   extracellular matrix   remodeling and target cell destruction through anoikis. As these processes require the release of GrB from the CL into the extracellular environment, we examined the secretion of GrB from natural killer (NK) cells and cytotoxic T lymphocytes (CTLs). We found that a proportion of GrB is constitutively secreted by both CTLs and NK cells in the absence of target cell engagement. In NK cells, the protease is primarily released in an active form through secretory granules. By contrast, T lymphocytes primarily secrete inactive GrB zymogen, bypassing the granules. The release of GrB through two routes from unconjugated CLs suggests that it functions outside the cell and may contribute to pathology in cases of immune dysregulation, such as familial hemophagocytic lymphohistiocytosis (FHL). Our findings also predict the existence of an extracellular activator of GrB. 
 Cell binding, internalization and cytotoxic activity of human granzyme B expressed in the yeast Pichia pastoris. Granzyme B (GrB) is an apoptosis-inducing protease of cytotoxic lymphocytes. We have investigated intracellular and extracellular effects of human GrB using recombinant protein expressed in the yeast Pichia pastoris. GrB was rapidly taken up by HeLa cells, and accumulated in vesicular structures in the cytoplasm. There it remained inactive and could not be liberated by the endosomolytic reagent chloroquine, indicating that the vesicular structures are distinct from late endosomes and lysosomes. Direct cytosolic delivery of GrB with a cationic lipid-based transduction reagent, however, resulted in the induction of apoptotic cell death. After prolonged incubation at or above 125 nM, GrB on its own induced pronounced morphological changes in human tumour cells, leading to partial loss of contact to the culture support. This extracellular effect was dependent on enzymatic activity and could be reversed by removal of the protein, suggesting GrB-dependent cleavage of   extracellular matrix   components as the underlying mechanism. 
 UVA induces granzyme B in human keratinocytes through MIF: implication in   extracellular matrix   remodeling. In a previous study, we have described that UVB induces granzyme B (GrB) in human keratinocyte cells, and that confers potent cellular cytotoxicity against various cellular models, including immune cells (Hernandez-Pigeon, H., Jean, C., Charruyer, A., Haure, M. J., Titeux, M., Tonasso, L., Quillet-Mary, A., Baudouin, C., Charveron, M., and Laurent, G. (2006) J. Biol. Chem. 281, 13525-13532). Herein, we have found that, in contrast to UVB, UVA failed to enhance keratinocyte cellular cytotoxicity but was still able to trigger GrB production. We show that GrB is accumulated through a p38 MAPK-dependent transcriptional mechanism stimulated by redox-dependent migration inhibitory factor release. Moreover, GrB purified from UVA-treated cellular extracts was found to degrade fibronectin in vitro. Treatment with antisense oligonucleotide directed against GrB resulted in the inhibition of UVA-induced cell detachment and cell death and facilitated cell migration through fibronectin and vitronectin matrix upon UVA exposure. Altogether, these results suggest another function for GrB in the context of the UV response. Indeed, combined with our previous study, it appears that, whereas this enzyme mediates keratinocyte cellular cytotoxicity following UVB irradiation, GrB supports the capacity of keratinocyte to degrade   extracellular matrix   components following UVA irradiation. UV-mediated GrB production may thus have important consequences in photoaging and photocarcinogenesis. 
  GeneRIFs  
 GZMB contributes to abdominal aortic aneurysm pathogenesis via a perforin-independent mechanism involving   extracellular matrix   degradation and subsequent loss of vessel wall integrity. 
  Summary  
 This gene encodes a member of the granzyme subfamily of proteins, part of the peptidase S1 family of serine proteases. The encoded preproprotein is secreted by natural killer (NK) cells and cytotoxic T lymphocytes (CTLs) and proteolytically processed to generate the active protease, which induces target cell apoptosis. This protein also processes cytokines and degrades   extracellular matrix   proteins, and these roles are implicated in chronic inflammation and wound healing. Expression of this gene may be elevated in human patients with cardiac fibrosis. [provided by RefSeq, Sep 2016] 
  [Ii]nvasive  PubMed  
 A Meta-analysis of the Significance of Granzyme B and Perforin in Non  invasive   Diagnosis of Acute Rejection After Kidney Transplantation. Previous studies have reported that granzyme B (GZMB) and perforin (PRF) could serve as non  invasive   biomarkers in the diagnosis of acute rejection (AR) after kidney transplant. Yet, their non  invasive   diagnostic value in clinical practice is still unknown.To assess the non  invasive   diagnostic performance of GZMB and PRF for AR, we performed a systematic search. After reviewing published studies in which both GZMB and PRF were detected, data on the diagnostic accuracy of separate and combined evaluation of GZMB and PRF were pooled.Across 16 studies (680 subjects), summary sensitivity, specificity, positive likelihood ratios, and negative likelihood ratios with 95% confidence intervals were calculated. For overall GZMB analysis, the indices were 0.76 (0.71-0.81), 0.86 (0.82-0.89), 4.58 (3.36-6.25), and 0.32 (0.22-0.47), respectively. For overall PRF analysis, the indices were 0.83 (0.78-0.88), 0.86 (0.82-0.89), 4.82 (3.66-6.35), and 0.26 (0.18-0.37), respectively. Subgroup analyses showed similar results compared to overall study analyses. In analyses of combined evaluation of GZMB and PRF, the above indices were 0.65 (0.53-0.76), 0.96 (0.91-0.98), 12.66 (5.83-27.50), and 0.40 (0.23-0.69), respectively, when both markers were positive. The probability of developing AR in kidney transplant recipients increased from 15% to 73% when both GZMB and PRF tests were positive and was reduced to 2% if that were negative.Currently, neither GZMB nor PRF, if evaluated alone, could be a convincing non  invasive   diagnostic marker for AR in clinical practice. Combined use of PRF and GZMB post-kidney transplant may be a better choice in AR evaluation to direct allograft biopsy execution and earlier therapeutic intervention. 
 Granzyme B is expressed in urothelial carcinoma and promotes cancer cell invasion. Granzyme B (GrB) is a serine proteinase known to be expressed by cytotoxic lymphocytes and to induce, in presence of perforin (Pf), apoptosis in target cells. Recently, GrB expression has been shown (often in absence of Pf) in nonlymphoid cells, but its function is not defined. In our study, we investigated GrB and Pf expression in bladder cancer cell lines and in urothelial carcinoma (UC) tissues by reverse transcription-polymerase chain reaction (RT-PCR), Western blot, ELISA, immunofluorescence and immunohistochemistry. We also assessed the function of GrB in UC cells; the in vitro function of GrB was examined by loss-of-function experiments. Our results revealed that GrB is expressed, in absence of Pf, in UC cells. Significant differences were found between GrB expression and both increasing pathological tumor spreading and high-grade vs. low-grade pTa tumors. Notably, GrB in UC tissues was concentrated at the cancer invasion front and was expressed in neoplastic cells undergoing epithelial-mesenchymal transition, a key event in carcinoma invasion. Indeed, GrB-positive cells also expressed Snail, N-cadherin or were negative for E-cadherin. GrB expressed in tumor cell lines was enzymatically active and capable of vitronectin cleavage, implying extracellular matrix (ECM) remodeling by GrB. Inhibition of GrB activity or Stealth RNA interference-mediated GrB gene silencing markedly suppressed bladder cancer cell invasion through matrigel. This data provides the first evidence for a role of GrB in promoting cancer cell invasion. Taken together, our findings suggest that GrB, via ECM degradation, contributes to the establishment of the UC   invasive   phenotype. 
 Epitelial-to-mesenchimal transition and invasion are upmodulated by tumor-expressed granzyme B and inhibited by docosahexaenoic acid in human colorectal cancer cells. Granzyme B (GrB) is a serine protease, traditionally known as expressed by cytotoxic lymphocytes to induce target cell apoptosis. However, it is emerging that GrB, being also produced by a variety of normal and neoplastic cells and potentially acting on multiple targets, might represent a powerful regulator of a wide range of fundamental biological processes. We have previously shown that GrB is expressed in urothelial carcinoma tissues and its expression is associated to both pathological tumor spreading and EMT. We have also shown that docosahexaenoic acid (DHA), a dietary ω-3 polyunsaturated fatty acid with anti-tumor activity, while inhibiting urothelial and pancreatic carcinoma cell invasion also inhibited their GrB expression in vitro. In this study, we characterized a panel of colorectal carcinoma (CRC) cells, with different   invasiv  e capabilities, for GrB expression and for the contribution of GrB to their EMT and   invasiv  e phenotype. In addition, we investigated the effect of DHA on CRC cell-associated GrB expression, EMT and invasion.The expression levels of GrB and EMT-related markers were evaluated by Western blotting. GrB knockdown was performed by Stealth RNAi small interfering RNA silencing and ectopic GrB expression by transfection of human GrB vector. Cell invasion was determined by the BioCoat Matrigel invasion chamber test.GrB was produced in 57.1% CRC cell lines and 100% CRC-derived Cancer Stem Cells. Although GrB was constitutive expressed in both   invasiv  e and no  ninvasiv  e CRC cells, GrB depletion in   invasiv  e CRC cells downmodulated their invasion in vitro, suggesting a contribution of GrB to CRC   invasiv  eness. GrB loss or gain of function downmodulated or upmodulated EMT, respectively, according to the analysis of cancer cell expression of three EMT biomarkers (Snail1, E-cadherin, N-cadherin). Moreover, TGF-β1-driven EMT was associated to the enhancement of GrB expression in CRC cell lines, and GrB depletion led to downmodulation of TGF-β1-driven EMT. In addition, DHA inhibited GrB expression, EMT and invasion in CRC cells in vitro.These findings present a novel role for GrB as upmodulator of EMT in CRC cells. Moreover, these results support the use of DHA, a dietary compound without toxic effects, as adjuvant in CRC therapy. 
 Serial peripheral blood perforin and granzyme B gene expression measurements for prediction of acute rejection in kidney graft recipients. In the present study we investigated whether peripheral blood gene expression measurements may serve as an early and non-  invasive   tool to predict renal allograft rejection. Peripheral blood was collected twice weekly after transplantation and gene expression was measured using real-time polymerase chain reaction (PCR). Recipients with acute rejection (n = 17) had higher levels of perforin and granzyme B transcript on days 5-7, 8-10, 11-13, 17-19, 20-22, and 26-29, as compared to patients without rejection (n = 50, p < 0.05 in all cases). Rejection diagnosis using gene expression criteria, determined with receiver operating characteristic (ROC) curves, was possible 2-30 days before traditional diagnosis (median 11 days). The best diagnostic result was obtained from samples taken on days 8-10, with a specificity of 90% and a sensitivity of 82% for perforin, and a specificity of 87% and sensitivity of 72% for granzyme B. Decreases in perforin (p < 0.01) and granzyme B expression (p < 0.05) were observed after initiation of anti-rejection therapy. Our data indicate that gene expression measurement is a useful tool for the recognition of graft rejection in its earliest stages. Serial measurements could be implemented as a monitoring system to highlight patients at higher risk of rejection, making them candidates for biopsy or pre-emptive anti-rejection therapy. 
 Elevated mRNA levels of CTLA-4, FoxP3, and granzyme B in BAL, but not in blood, during acute rejection of lung allografts. Regulatory T cells (Tregs) have been related to acute rejection as have the cytotoxic T cells, their immunological counterpart. High expression of cytotoxic markers has been related to acute rejection incidents following both kidney and intestine transplantation, while the correlation between FoxP3 expression and acute rejection is still being debated. Some studies have been performed on blood samples from lung-transplanted patients, while others have investigated the local immune response in the lungs by analysing broncho-alveolar-lavage (BAL) fluids or biopsies. Biopsies are considered the gold standard in diagnosis of acute rejection.The aim was to measure the expression of both Treg (FoxP3, CD25 and CTLA-4) and cytotoxic (granzyme B, granulysin, and perforin) markers in BAL and blood samples from lung-transplanted patients to investigate the possible relation of expression and acute rejection incidents in order to develop a non-  invasive   diagnostic method for acute rejection.24 lung-transplanted patients were included in this 6-month cross-section study. BAL and blood samples were analysed for FoxP3, CD25, CTLA-4, granzyme B, granulysin, perforin, CD4 and CD8 mRNA by QRT-PCR with glyceraldehyde 3-phosphate dehydrogenase (GAPDH) as primary reference.We demonstrate that the mRNA levels in BAL relative to GAPDH of nearly all markers are elevated during acute rejection; CTLA-4, FoxP3, and granzyme B significantly, while a strong tendency is seen among the others. No significant differences were detected in blood.CTLA-4, FoxP3 and Granzyme B mRNA levels are elevated during acute rejection in BAL, but not in blood, following lung transplantation, indicating that evaluation of Treg and cytotoxic marker expression in BAL can be used in the assessment of allograft rejection state. 
 High levels of granzyme B expression in   invasive   cervical carcinoma correlates to poor response to treatment. The present study assessed, in cervical carcinoma, expression levels of seven immune response genes and sought correlation to response to treatment. The expression levels of CD28, CTLA4, ICOS, ICOSL, CD80 and CD86 and granzyme B genes were assessed by real-time RT-PCR in pre-treatment tumor fragments. During the six-month follow-up after treatment, 8 patients presented tumor and 10 survived free of tumor. The only gene whose expression levels were higher in patients with poor outcome (p = 0.03) was granzyme B. Further evaluation, in adequately powered prospective studies is warranted to confirm the data and to translate this observation to the clinical setting. 
 Non  invasive   diagnosis of cellular and antibody-mediated rejection by perforin and granzyme B in renal allografts. A major milestone in transplantation would be the use of biomarkers to monitor rejection. We examined the association between perforin and granzyme-B gene expression detected in the peripheral blood of renal allograft recipients with cellular and antibody-mediated rejection. Furthermore, we judged the appropriateness of assigning negative rejection statuses to persons without a biopsy whose grafts were functioning well clinically. Of the 46 patients who completed the study, recipients with cellular rejection had higher perforin and granzyme-B levels compared with nonrejectors (p = 0.006). Interestingly, recipients with antibody-mediated rejection also had higher perforin and granzyme-B levels compared with nonrejectors (p = 0.04). Patients with high levels of granzyme B had a probability of rejecting that was 26.7 times greater than those patients with low levels of granzyme B. Perforin and granzyme B had sensitivities of 50% and specificities of 95% in predicting rejection (cutoff value = 140). Assigning negative rejection statuses to recipients without a biopsy whose grafts were functioning well did not have a major effect on the direction or significance of covariate values. This study suggests that perforin and granzyme-B gene expressions in peripheral blood are accurate in detecting both cellular and antibody-mediated rejection. 
  GeneRIFs  
 Combined evaluation of granzyme B and perforin may have a role in non  invasive   diagnosis of acute rejection after kidney transplantation. 
 data provides the first evidence for a role of GrB in promoting cancer cell invasion. Taken together, our findings suggest that Granzyme B (GrB), via ECM degradation, contributes to the establishment of the urothelial carcinoma   invasive   phenotype 
  [Mm]etastasis  PubMed  
 Absence of Granzyme B positive tumour-infiltrating lymphocytes in primary melanoma excisional biopsies is strongly associated with the presence of sentinel lymph node   metastasis  . Sentinel Lymph Node (SLN) status is strongly related to clinical outcome in melanoma patients. In this study we investigated the possible association between the presence of activated and/or suppressive Tumour Infiltrating Lymphocytes (TILs) and SLN status in clinically stage I/II melanoma patients.Diagnostic primary melanoma samples from 20 patients with a sentinel lymph node   metastasis   were compared to melanoma samples from 20 patients with a negative sentinel lymph node, who were matched for gender, age and Breslow thickness. Presence of activated Granzyme B positive (GrB+) TILs, presence of suppressive (FoxP3+) TILs and MHC class I antigen expression on tumour cells were analysed by immunohistochemistry.FoxP3 and MHC-I expression had no direct bearing on the presence of melanoma metastases in the SLN. Whereas the presence of activated GrB+ TILs in the primary melanoma had no predictive value for SLN status either, their absence was strongly associated with the presence of   metastasis   in the SLN (p=0.001). While both GrB+ and FoxP3+ TILs could be detected in SLN metastases, a majority did not display MHC-I expression.These data support a role for cytotoxic T cells in the prevention of early   metastasis   of melanoma to the draining lymph nodes. 
 Granzyme B gene polymorphisms, colorectal cancer risk, and   metastasis  . The human granzyme B protein (GrB), which is encoded by granzyme B gene (GZMB), plays a major role in eliminating cancer cells. Polymorphisms of GZMB gene such as Q55R, P94A, and Y247H have been shown to affect GrB activity and the subsequent cancer risk.In this study, we examined possible association between GZMB gene polymorphisms and susceptibility to colorectal cancer (CRC). In addition, the contribution of the examined polymorphisms to colorectal cancer   metastasis   to lymph node and distant organ was investigated.A total of 50 venous blood samples collected from CRC patients were analyzed to identify the Q55R, P94A, and Y247H polymorphisms. As a control group, 20 healthy subjects were enrolled in the study. The Q55R, P94A, and Y247H polymorphisms were genotyped by polymerase chain reaction and sequencing method.Data analysis was carried out using the statistical package SPSS version 17 to compute all descriptive statistics. Chi-square and Fisher exact tests (if the expected value in any cell is less than 5) were used to evaluate the genotype distribution and allele frequencies of the studied polymorphism.The results revealed that the distribution of Q55R, P94A, and Y247H are not significantly different in CRC patients compared to the controls. In addition, no association between Q55R, P94A, and Y247H polymorphisms and its   metastasis   to lymph node and distant organ was detected.These results suggest that GZMB Q55R, P94A, and Y247H polymorphisms are not significantly associated with colon cancer incidence, or   metastasis   to lymph node and distant organ. However, this study was limited by its relatively small sample size; thus, to confirm current findings, a bigger multicenter design study is warranted. 
 Active secretion of CXCL10 and CCL5 from colorectal cancer microenvironments associates with GranzymeB+ CD8+ T-cell infiltration. Transcriptional expression of CXCR3 and CCR5 cognate chemokines correlate with CD8+ T-cell infiltration and prolonged survival in colorectal cancer (CRC). These findings were derived mainly from paraffin embedded tissues; thus little is known about the secretion pattern of CD8+ T-cell targeting chemokines from CRCs. Therefore, we developed and introduced a novel platform that assesses the immune mediators that are secreted from live excised tissues. Transcriptional profiling and unsupervised hierarchical clustering of 43 CRCs based on expression of genes that represent the adaptive immune response were used to predict tumors that are strong secretors of T-cell targeting chemokines. Secretion of these mediators were corroborated using flow cytometric analysis of T-cell lineage markers: CD4, CD8, IFN-γ, and GzmB. We demonstrate that stronger secretion of CXCL10 (CXCR3 ligand) and CCL5 (CCR5 ligand) and infiltration of GzmB+CD8+ cytotoxic T-lymphocytes (CTLs) and IFN-γ+CD4+ helper T-cells can be predicted by transcriptional profiling, and that CRCs with stronger T-cell immunity were proportionally skewed towards early TNM stages and lacked distant orga  n metastas  is. Our study represents the first functional analysis of secreted immune mediators from CRCs beyond immunohistochemistry and real-time PCR, and observed active physiological interactions between the tumor cells and the immune cells in the tumor microenvironment. 
 Low expression of Granzyme B in colorectal cancer is associated with signs of early metastastic invasion. Tumour-infiltrating forkhead box P3 (FoxP3+ ) regulatory T cells (T(regs) ) have stronger prognostic significance than cytotoxic CD8+ T cells in colorectal cancer (CRC). Because there is evidence that some tumour-infiltrating CD8+ T cells may be inactive, the present study aimed to investigate the prognostic significance of Granzyme B, one of the major effector molecules of T cells.A tissue microarray containing 963 CRCs was stained immunohistochemically for Granzyme B and the level of expression quantified by digital image analysis. Granzyme B expression was higher in tumours with microsatellite instability (P < 0.0001), a dense lymphocytic infiltrate (P < 0.0001) and location in the proximal colon (P = 0.009), but lower in tumours with vascular invasion (P = 0.007), perineural invasion (P =0.041) and positive nodal status (P < 0.001). Elevated expression of Granzyme B was associated with improved survival on univariate analysis (hazard ratio = 0.65; 95% confidence interval 0.51-0.84; P = 0.001), but not in a multivariate model that included stage, vascular invasion and FoxP3+ T(reg) cell density.Low expression of Granzyme B was a  ssociated   with early signs of metastasis in CRC. The stronger prognostic significance of FoxP3+ T(regs) is in keeping with animal models that suggest these cells act as gatekeepers for the release of Granzyme B from CD8+ T cells. 
 Targeted apoptosis activation with GrB/scFvMEL modulates melanoma growth, metastatic spread, chemosensitivity, and radiosensitivity. GrB/scFvMEL, a fusion protein composed of human granzyme B (GrB) and the single-chain antibody scFvMEL, targets melanoma gp240 antigen and exerts impressive cytotoxic effects by inducing apoptosis. We evaluated the effects of GrB/scFvMEL on chemotherapy, radiation therapy,   metastasis   in vitro, and the growth of human melanoma A375 xenograft tumors in nude mice. GrB/scFvMEL showed synergistic cytotoxicity when coadministered with doxorubicin, vincristine or cisplatin, and additive effects, in combination with etoposide or cytarabine. Optimal cytotoxic effects were obtained when cells were treated first with GrB/scFvMEL followed by exposure to the agent (rather than the reverse). Pretreatment of A375 cells with GrB/scFvMEL significantly sensitized melanoma cells to ionizing radiation assessed using a clonogenic survival assay. Subtoxic doses of GrB/scFvMEL inhibited the invasion of A375 cells into Matrigel. GrB/scFvMEL (37.5 mg/kg) was administered intravenously to nude mice bearing A375 tumors. Saline-treated tumors increased 24-fold, whereas tumors treated with GrB/scFvMEL showed a significant tumor growth delay increasing four-fold. Tumor tissue displayed an increase in apoptotic nuclei compared to control. Thus, the targeted delivery of GrB to tumors may have a significant potential for cancer treatment. Targeted therapeutic agents specifically designed to impact cellular apoptotic pathways may represent a novel class of therapeutic agents. 
  GeneRIFs  
 These results suggest that GZMB Q55R, P94A, and Y247H polymorphisms are not significantly associated with colon cancer incidence, or   metastasis   to lymph node and distant organ. 
 Low expression of Granzyme B was associated with early signs of   metastasis   in colorectal cancer. 
 absence of GrB+ tumour-infiltrating lymphocytes in primary melanoma excisional biopsies in immunohistochemistry might be used to predict presence of sentinel lymph node   metastasis  . 
  [Cc]ell [Ii]nvasion  PubMed  
 Granzyme B is expressed in urothelial carcinoma and promotes cancer   cell invasion  . Granzyme B (GrB) is a serine proteinase known to be expressed by cytotoxic lymphocytes and to induce, in presence of perforin (Pf), apoptosis in target cells. Recently, GrB expression has been shown (often in absence of Pf) in nonlymphoid cells, but its function is not defined. In our study, we investigated GrB and Pf expression in bladder cancer cell lines and in urothelial carcinoma (UC) tissues by reverse transcription-polymerase chain reaction (RT-PCR), Western blot, ELISA, immunofluorescence and immunohistochemistry. We also assessed the function of GrB in UC cells; the in vitro function of GrB was examined by loss-of-function experiments. Our results revealed that GrB is expressed, in absence of Pf, in UC cells. Significant differences were found between GrB expression and both increasing pathological tumor spreading and high-grade vs. low-grade pTa tumors. Notably, GrB in UC tissues was concentrated at the cancer invasion front and was expressed in neoplastic cells undergoing epithelial-mesenchymal transition, a key event in carcinoma invasion. Indeed, GrB-positive cells also expressed Snail, N-cadherin or were negative for E-cadherin. GrB expressed in tumor cell lines was enzymatically active and capable of vitronectin cleavage, implying extracellular matrix (ECM) remodeling by GrB. Inhibition of GrB activity or Stealth RNA interference-mediated GrB gene silencing markedly suppressed bladder cancer   cell invasion   through matrigel. This data provides the first evidence for a role of GrB in promoting cancer   cell invasion  . Taken together, our findings suggest that GrB, via ECM degradation, contributes to the establishment of the UC invasive phenotype. 
 Epitelial-to-mesenchimal transition and invasion are upmodulated by tumor-expressed granzyme B and inhibited by docosahexaenoic acid in human colorectal cancer cells. Granzyme B (GrB) is a serine protease, traditionally known as expressed by cytotoxic lymphocytes to induce target cell apoptosis. However, it is emerging that GrB, being also produced by a variety of normal and neoplastic cells and potentially acting on multiple targets, might represent a powerful regulator of a wide range of fundamental biological processes. We have previously shown that GrB is expressed in urothelial carcinoma tissues and its expression is associated to both pathological tumor spreading and EMT. We have also shown that docosahexaenoic acid (DHA), a dietary ω-3 polyunsaturated fatty acid with anti-tumor activity, while inhibiting urothelial and pancreatic carcinoma   cell invasio  n also inhibited their GrB expression in vitro. In this study, we characterized a panel of colorectal carcinoma (CRC) cells, with different invasive capabilities, for GrB expression and for the contribution of GrB to their EMT and invasive phenotype. In addition, we investigated the effect of DHA on CRC cell-associated GrB expression, EMT and invasion.The expression levels of GrB and EMT-related markers were evaluated by Western blotting. GrB knockdown was performed by Stealth RNAi small interfering RNA silencing and ectopic GrB expression by transfection of human GrB vector.   Cell invasio  n was determined by the BioCoat Matrigel invasion chamber test.GrB was produced in 57.1% CRC cell lines and 100% CRC-derived Cancer Stem Cells. Although GrB was constitutive expressed in both invasive and noninvasive CRC cells, GrB depletion in invasive CRC cells downmodulated their invasion in vitro, suggesting a contribution of GrB to CRC invasiveness. GrB loss or gain of function downmodulated or upmodulated EMT, respectively, according to the analysis of cancer cell expression of three EMT biomarkers (Snail1, E-cadherin, N-cadherin). Moreover, TGF-β1-driven EMT was associated to the enhancement of GrB expression in CRC cell lines, and GrB depletion led to downmodulation of TGF-β1-driven EMT. In addition, DHA inhibited GrB expression, EMT and invasion in CRC cells in vitro.These findings present a novel role for GrB as upmodulator of EMT in CRC cells. Moreover, these results support the use of DHA, a dietary compound without toxic effects, as adjuvant in CRC therapy. 
  GeneRIFs  
 data provides the first evidence for a role of GrB in promoting cancer   cell invasion  . Taken together, our findings suggest that Granzyme B (GrB), via ECM degradation, contributes to the establishment of the urothelial carcinoma invasive phenotype 
   
  
 GSTT1 
 
  NCBI Gene  
  GeneRIF  
  Pubmed  
 
 
  Search Term  Rank  Fields  
  [Mm]igration  6  PubMed(3)  
  [Ee]xtracellular [Mm]atrix  8  PubMed(1)  
  [Ii]nvasive  10  PubMed(20); GeneRIFs(6)  
  [Mm]etastasis  12  PubMed(6); GeneRIFs(1)  
 
 
 hide/show details for GSTT1 
  [Mm]igration  PubMed  
 Predictive role of glutathione-S-transferase gene polymorphisms in the survival of gastric cancer cases. We conducted a prospective study in an Chinese population to detect the association between GSTM, GSTT and GSTP gene polymorphisms and survival of gastric cancer.A prospective follow-up study with 317 gastric cancer patients was conducted between January 2003 and January 2005. GSTM1, GSTT1 and GSTP1 genotyping was performed using ABI TaqMan Gene Expression assays.Of 317 patients, 5 were lost to follow-up due to   migration  , while the remaining 302 patients completed the study. The median follow-up time was 34.2 months (range: 2 to 60 months), during which a total of 120 (39.1%) died of gastric cancer. The GSTT1-null genotype showed a significant increased risk of death from gastric cancer, with an HR (95% CI) of 1.59 (1.04-3.58). Moreover, we found individuals carrying null-GSTM1 and null-GSTT1 had a moderate higher risk of death from gastric cancer, with an HR of 1.92 (1.05-3.65).This study reported the carriage of null GSTT1 and null GSTM1 might be linked to the higher death risk from gastric cancer in Chinese population. 
 Genetic polymorphisms of CYP2E1, GST, and NAT2 enzymes are not associated with risk of breast cancer in a sample of Lebanese women. Changes in the activity of drug metabolizing enzymes (DMEs) are potentially associated with cancer risk. This relationship is attributed to their involvement in the bioactivation of multiple procarcinogens or the metabolism of multiple substrates including an array of xenobiotics and environmental carcinogens. 326 Lebanese women of whom 99 were cancer free (controls) and 227 were diagnosed with breast cancer (cases) were included. Blood for DNA was collected and medical charts were reviewed. Three genotyping methods were employed including: (1) restriction fragment length polymorphism (RFLP) for CYP2E1*5B, CYP2E1*6, NAT2*5 and NAT2*6; (2) gel electrophoresis for GSTM1 and GSTT1; and (3) real-time PCR for GSTP1 Ile/Val polymorphism. We analyzed the relationship between genetic susceptibilities in selected xenobiotic metabolizing genes and breast cancer risk. Allele frequencies were fairly similar to previously reported values from neighboring populations with relevant   migration   routes. There were no statistically significant differences in the distribution of variant carcinogen metabolizing genes between cases and controls even after adjusting for age at diagnosis, menopausal status, smoking, and alcohol intake. Despite its limitations, this is the first study that assesses the role of genetic polymorphisms in DMEs with breast cancer in a sample of Lebanese women. Further studies are needed to determine the genetic predisposition and gene-environment interactions of breast cancer in this population. 
 Deletion polymorphism of GSTT1 gene as protective marker for allergic rhinitis. Allergic rhinitis (AR) is one of the most common respiratory diseases among human populations. Strong evidence suggests that genetic predisposition and environmental factors could contribute to the development of this complex disease. Glutathione S-transferases (GSTs) are biomarkers of inflammation and oxidative stress. These phase II enzymes play a significant role in detoxifying xenobiotic compounds. To analyze the role of GST gene polymorphisms in AR pathogenesis in a case-control population of 103 patients affected by AR and 200 healthy non-allergic subjects.We screened genomic DNA extracted from buccal cells for GSTM1 positive/null, GSTP1*I105V (rs1695) and GSTT1 positive/null polymorphisms. The X(2) -test, odds ratio (OR) and logistic regression were used as statistical analyses.Significant differences in null genotype distribution between AR patients (13%) and healthy controls (30%) were found for the GSTT1 null genotype (OR = 0.30, 95% confidence interval = 0.14-0.65; P = 0.001). GSTM1 and GSTP1 polymorphisms did not show any significant results.Our data indicated that GSTT1 may be a susceptibility locus for AR. Specifically, the positive/null polymorphism of GSTT1 may be involved in an oxidative stress-related mechanism that may enhance pathogenic pathways related to AR. Moreover, beside GSTT1, this deletion polymorphism affects also another gene potentially related to AR phenotype, LOC391322. This gene belongs to MIF   (macropha  ge migration inhibitory factor) gene family and several studies indicated the role of this gene in several immunology-related phenotypes. Therefore, two different scenarios may explain the observed genetic association. 
  [Ee]xtracellular [Mm]atrix  PubMed  
 Pterygium in patients from Goiânia, Goiás, Brazil. Pterygium is an inflammatory and degenerative ocular surface disease in which the conjunctiva on the cornea grows to form a fibrous tissue in the shape of a triangle. The disorder may be characterized by cell proliferation, inflammatory processes, fibrosis, angiogenesis, and destruction of th  e extracellular matr  ix. The anomaly is considered a degenerative eye disease and is erroneously confused with cataract. It displays similar features to those of tumors, such as local invasion, metaplasia of epithelial cells, presence of oncogenic viruses (human papilloma virus), inactivation of tumor suppressor genes (e.g., p53), and loss of heterozygosity. The treatment of pterygium is based on factors such as the evolution and progression of the disease, risk factors, symptoms, and patient age. Considerations about the best technique for the surgical removal of pterygium remain controversial, and complications and recurrence are very common. The development of new surgical techniques and adjuvant drugs is thus necessary. This study aims to analyze and compare the frequency of the GSTT1 genotypes in relation to pterygium through statistical analyzes in order to build a genotypic profile for the Replicon patients. The genotypic profile of the GSTT1-null polymorphism in Goiânia showed no significant difference when the frequency of the null genotype was compared between the control and experimental groups. The null genotype was more frequent in the population studied. Furthermore, the GSTT1 genotype was not related to the analyzed risk factors for pterygium, namely gender, ethnicity, family history, occupational exposure, smoking, hypertension, and diabetes. 
  [Ii]nvasive  PubMed  
 Genetic susceptibility to distinct bladder cancer subphenotypes. Clinical, pathologic, and molecular evidence indicate that bladder cancer is heterogeneous with pathologic/molecular features that define distinct subphenotypes with different prognoses. It is conceivable that specific patterns of genetic susceptibility are associated with particular subphenotypes.To examine evidence for the contribution of germline genetic variation to bladder cancer heterogeneity.The Spanish Bladder Cancer/EPICURO Study is a case-control study based in 18 hospitals located in five areas in Spain. Cases were patients with a newly diagnosed, histologically confirmed, urothelial cell carcinoma of the bladder from 1998 to 2001. Case diagnoses were reviewed and uniformly classified by pathologists following the World Health Organisation/International Society of Urological Pathology 1999 criteria. Controls were hospital-matched patients (n=1149).A total of 1526 candidate variants in 423 candidate genes were analysed. Three distinct subphenotypes were defined according to stage and grade: low-grade nonmuscle   invasive   (n=586), high-grade nonmuscle   invasive   (n=219), and muscle   invasive   (n=246). The association between each variant and subphenotype was assessed by polytomous risk models adjusting for potential confounders. Heterogeneity in genetic susceptibility among subphenotypes was also tested.Two established bladder cancer susceptibility genotypes, NAT2 slow-acetylation and GSTM1-null, exhibited similar associations among the subphenotypes, as did VEGF-rs25648, which was previously identified in our study. Other variants conferred risks for specific tumour subphenotypes such as PMS2-rs6463524 and CD4-rs3213427 (respective heterogeneity p values of 0.006 and 0.004), which were associated with muscle-  invasive   tumours (per-allele odds ratios [95% confidence interval] of 0.56 [0.41-0.77] and 0.71 [0.57-0.88], respectively) but not with non-muscle-  invasive   tumours. Heterogeneity p values were not robust in multiple testing according to their false-discovery rate.These exploratory analyses suggest that genetic susceptibility loci might be related to the molecular/pathologic diversity of bladder cancer. Validation through large-scale replication studies and the study of additional genes and single nucleotide polymorphisms are required. 
 Genetic susceptibility to distinct bladder cancer subphenotypes. Clinical, pathologic, and molecular evidence indicate that bladder cancer is heterogeneous with pathologic/molecular features that define distinct subphenotypes with different prognoses. It is conceivable that specific patterns of genetic susceptibility are associated with particular subphenotypes.To examine evidence for the contribution of germline genetic variation to bladder cancer heterogeneity.The Spanish Bladder Cancer/EPICURO Study is a case-control study based in 18 hospitals located in five areas in Spain. Cases were patients with a newly diagnosed, histologically confirmed, urothelial cell carcinoma of the bladder from 1998 to 2001. Case diagnoses were reviewed and uniformly classified by pathologists following the World Health Organisation/International Society of Urological Pathology 1999 criteria. Controls were hospital-matched patients (n=1149).A total of 1526 candidate variants in 423 candidate genes were analysed. Three distinct subphenotypes were defined according to stage and grade: low-grade nonmuscle   invasive   (n=586), high-grade nonmuscle   invasive   (n=219), and muscle   invasive   (n=246). The association between each variant and subphenotype was assessed by polytomous risk models adjusting for potential confounders. Heterogeneity in genetic susceptibility among subphenotypes was also tested.Two established bladder cancer susceptibility genotypes, NAT2 slow-acetylation and GSTM1-null, exhibited similar associations among the subphenotypes, as did VEGF-rs25648, which was previously identified in our study. Other variants conferred risks for specific tumour subphenotypes such as PMS2-rs6463524 and CD4-rs3213427 (respective heterogeneity p values of 0.006 and 0.004), which were associated with muscle-  invasive   tumours (per-allele odds ratios [95% confidence interval] of 0.56 [0.41-0.77] and 0.71 [0.57-0.88], respectively) but not with non-muscle-  invasive   tumours. Heterogeneity p values were not robust in multiple testing according to their false-discovery rate.These exploratory analyses suggest that genetic susceptibility loci might be related to the molecular/pathologic diversity of bladder cancer. Validation through large-scale replication studies and the study of additional genes and single nucleotide polymorphisms are required. 
 Mitochondrial DNA copy number and risk of oral cancer: a report from Northeast India. Oral squamous cell carcinoma (OSCC) is the sixth most common cancer globally. Tobacco consumption and HPV infection, both are the major risk factor for the development of oral cancer and causes mitochondrial dysfunction. Genetic polymorphisms in xenobiotic-metabolizing enzymes modify the effect of environmental exposures, thereby playing a significant role in gene-environment interactions and hence contributing to the individual susceptibility to cancer. Here, we have investigated the association of tobacco - betel quid chewing, HPV infection, GSTM1-GSTT1 null genotypes, and tumour stages with mitochondrial DNA (mtDNA) content variation in oral cancer patients.The study comprised of 124 cases of OSCC and 140 control subjects to PCR based detection was done for high-risk HPV using a consensus primer and multiplex PCR was done for detection of GSTM1-GSTT1 polymorphism. A comparative ΔCt method was used for determination of mtDNA content. The risk of OSCC increased with the ceased mtDNA copy number (Ptrend  = 0.003). The association between mtDNA copy number and OSCC risk was evident among tobacco - betel quid chewers rather than tobacco - betel quid non chewers; the interaction between mtDNA copy number and tobacco - betel quid was significant (P = 0.0005). Significant difference was observed between GSTM1 - GSTT1 null genotypes (P = 0.04, P = 0.001 respectively) and HPV infection (P<0.001) with mtDNA content variation in cases and controls. Positive correlation was found with decrease in mtDNA content with the increase in tumour stages (P<0.001). We are reporting for the first time the association of HPV infection and GSTM1-GSTT1 null genotypes with mtDNA content in OSCC.Our results indicate that the mtDNA content in tumour tissues changes with tumour stage and tobacco-betel quid chewing habits while low levels of mtDNA   content   suggests invasive thereby serving as a biomarker in detection of OSCC. 
 SNPs of GSTM1, T1, P1, epoxide hydrolase and DNA repair enzyme XRCC1 and risk of urinary transitional cell carcinoma in southwestern Taiwan. A hospital-based case-control study was conducted near a former black-foot disease (BFD)-endemic area in southwestern Taiwan to examine the possible risk factors and genetic susceptibility for urinary transitional cell carcinoma (TCC). A total of 221 patients with pathologically confirmed TCC and 223 age-sex-matched control subjects from urology outpatient clinics were recruited between 1998 and 2002. The results showed that residency in the BFD area and consumption of well water for more than 10 years was a strong factor on urinary cancer risk (odds ratio [OR],8.16, 95% confidence interval [CI],3.34-19.90, p<0.0001). Dose response relationship between average arsenic concentration in well water and TCC risk was also observed. Cigarette smoking played a relatively minor role in urinary carcinogenesis in this study. The GSTP1 Ile105Val A-->G polymorphism was significantly associated with cancer risk (A/G+G/G: OR=0.60, 95%CI=0.39-0.94, p=0.02), and the effect of Val105 allele was largely confined to the subjects diagnosed earlier than 55 years old (A/G+G/G: OR,0.29; 95% CI, 0.09-0.87, p=0.03). The results suggest that GSTP1 is a candidate for susceptibility locus and Ile105 allele may predispose individuals to early-onset urinary TCC. The GSTM1 null genotype was associated with tumors of high-  invasive  ness (OR,2.21; 95% CI, 1.34-4.73) as well as with early-onset TCC risk (OR,2.53; 95% CI, 0.97-6.59). Our preliminary results showed the XRCC1 Arg194Trp were associated with arsenic-related urinary TCC and the interaction between the genotype and the exposure was statistically significant. The modulating effect of the GSTM1, GSTT1, GSTP1 Ile105Val, EPHX Tyr113His and XRCC1 Arg280His on arsenic-related TCC risk was also suggestive. These observations implied that impaired metabolism of carcinogenic exposure as well as impaired DNA repair function play an important role in arsenic-related urinary transitional cell carcinogenesis. 
 Association of glutathione S-transferase M1 and T1 null polymorphisms with the development of cervical lesions: a meta-analysis. An accumulation of evidence suggests that gene-based self-susceptibility may contribute to the development of cancer. Some studies have found that particular polymorphisms of the glutathione S-transferase M1 and T1 genes are associated with increased risk of cervical lesions, but other studies have had contrary results. The present meta-analysis evaluated the association of glutathione S-transferase M1 and T1 null polymorphisms with the development of cervical lesions. In addition, stratified analyses were performed in an attempt to identify any race-specific effects.Twenty-one related studies were included in the meta-analysis, comprising glutathione S-transferase M1 data from 1423 patients with cervical lesions and 2415 healthy matched controls, and glutathione S-transferase T1 data from 2081 patients with cervical lesions and 2287 healthy matched controls. The fixed-effect model (Mantel-Haenszel method) and the random-effect (DerSimonian and Laird) model were used to examine the difference in frequency of glutathione S-transferase M1 and T1 null polymorphisms between pre- and   invasive   cervical lesions. Subgroup analyses were also conducted to evaluate any race-specific effect on the frequencies of glutathione S-transferase polymorphisms in cervical lesions. Odds ratios (OR) and 95% confidence intervals (CI) were calculated. Heterogeneity was assessed using the heterogeneity metric (I²) and Chi-squared test.The glutathione S-transferase M1 null polymorphism was associated with increased risk of low-grade intra-epithelial lesions (OR 1.37, 95% CI 1.05-1.77), but no increased risk of high-grade intra-epithelial lesions (OR 1.29, 95% CI 0.87-1.8) or   invasiv  e cervical cancer (OR 1.20, 95% CI 0.99-1.46). The association seemed to be confined to Southeast Asians (OR 1.97, 95% CI 1.44-2.71). No significant associations were found for the glutathione S-transferase T1 null polymorphism for any of the populations.The glutathione S-transferase M1 null polymorphism significantly increases susceptibility to early-stage cervical lesions in Southeast Asians. However, the glutathione S-transferase T1 null polymorphism does not appear to be a risk factor for cervical lesions in any population. 
 The predictive value of GSTT1 polymorphisms in predicting the early response to induction BCG therapy in patients with non-muscle   invasive   bladder cancer. We evaluated the predictive value of glutathione S transferase mu (GSTM1) and theta (GSTT1) polymorphisms in early response to bacillus Calmette-Guérin (BCG) induction therapy in patients with primary non-muscle   invasiv  e bladder cancer.GSTM1 and GSTT1 polymorphisms were analyzed by multiplex polymerase chain reaction using blood genomic DNA from 135 patients with primary non-muscle   invasiv  e bladder cancer who were being treated with a single induction course of BCG. BCG nonresponsiveness (early BCG failure) was defined as a tumor recurrence or progression within 12 months after BCG induction therapy. The predictive value of GST polymorphisms was evaluated by Kaplan-Meier analysis and multivariate logistic regression models.Patients carrying a GSTT1-positive genotype demonstrated a higher likelihood of early BCG failure regardless of cigarette smoking. After stratification based on the tumor stage and grade, the high-risk group (T1G3) with a GSTT1-positive genotype showed a 14-fold higher risk of early BCG failure compared with those with a GSTT1-null genotype. In a combined analysis of 2 genes, the GSTT1-positive/GSTM1-null genotype had a higher risk of BCG nonresponsiveness compared with the GSTT1-null/GSTM1-null genotype (odds ratio = 4.17, 95% CI: 1.54-11.26). By multivariate logistic regression analysis, the GSTT1-positive genotype was an independent predictor of early BCG failure (odds ratio = 3.67, 95% CI: 1.61-8.38). Kaplan-Meier estimates revealed a significant difference in disease-free survival depending on the GSTT1 genotype (log rank test, P = 0.038).The results of this study suggest that the GSTT1-positive genotype is an independent predictor of early BCG failure. These results can help determine whether patients would benefit from adjuvant BCG treatment or may require more aggressive alternative therapies. 
 GSTT1 as a prognosticator for recurrence and progression in patients with non-muscle-  invasive   bladder cancer. Although polymorphisms in glutathione S-transferase (GST) have been associated with the risk of bladder cancer (BC), few reports provide information about the development of BC. The aim of the present study was to investigate the effect of homozygous glutathione S-transferase-μ (GSTM1) and glutathione S-transferase-ϕ (GSTT1) deletions as prognostic markers in non-muscl  e-invasi  ve bladder cancer (NMIBC). A total of 241 patients with primary NMIBC were enrolled in this study. GSTM1 and GSTT1 polymorphisms were analyzed by multiplex polymerase chain reaction (PCR) using blood genomic DNA. The results were compared with clinicopathological parameters. The prognostic significance of the GSTs was evaluated by Kaplan-Meier and multivariate Cox regression model. A statistically significant association between genotype and histopathological parameter was not observed. The patients with the GSTT1-positive genotype had significantly reduced recurrence- and progression-free survival than those with the GSTT1-null genotype (log-rank test, p< 0.05, respectively). Recurrence- and progression-free survival were not related to the GSTM1 genotypes. In multivariate regression analysis, the GSTT1-positive genotype was the independent predictor for recurrence [hazard ratio (HR), 1.631; p=0.043] and progression (HR, 3.418; p=0.006). These results suggested that the GSTT1 genotype could be a useful prognostic marker for recurrence and progression in NMIBC. 
 GSTT1: a marker of the aggressiveness of bladder cancer. Glutathione S-transferases have been implicated in the development of bladder cancer (BC). We investigated the genotype and expression of glutathione S-transferase-mu (GSTM1) and glutathione S-transferase-theta (GSTT1) in BC tissue specimens.Tumor samples and matched normal mucosae were obtained from 34 patients. Genomic DNA was used to analyze GSTM1 and GSTT1 genotypes using multiplex polymerase chain reaction. GSTM1 and GSTT1 mRNA levels were measured using real-time reverse transcriptase polymerase chain reaction.GSTM1 mRNA expression was lower in tumor tissues than in matched normal bladder mucosae, whereas GSTT1 mRNA expression was significantly higher. GSTT1 mRNA expression was higher in muscle-  invasive   BC and high-grade cancers than in non-muscle-  invasive   BC and lower-grade tumors.GSTT1 is correlated with characteristics of aggressive BC. GSTT1 may play an important role in tumorigenesis and disease progression in patients with BC. 
 Glutathione S-transferase T1, O1 and O2 polymorphisms are associated with survival in muscle   invasive   bladder cancer patients. To examine the association of six glutathione transferase (GST) gene polymorphisms (GSTT1, GSTP1/rs1695, GSTO1/rs4925, GSTO2/rs156697, GSTM1, GSTA1/rs3957357) with the survival of patients with muscle   invasive   bladder cancer and the genotype modifying effect on chemotherapy.A total of 105 patients with muscle   invasive   bladder cancer were included in the study. The follow-up lasted 5 years. The effect of GSTs polymorphisms on predicting mortality was analyzed by the Cox proportional hazard models, while Kaplan-Meier analysis was performed to assess differences in survival.GSTT1 active, GSTO1 Asp140Asp or GSTO2 Asp142Asp genotypes were independent predictors of a higher risk of death among bladder cancer patients (HR = 2.5, P = 0.028; HR = 2.9, P = 0.022; HR = 3.9, P = 0.001; respectively) and significantly influenced the overall survival. There was no association between GSTP1, GSTM1 and GSTA1 gene variants with overall mortality. Only GSTO2 polymorphism showed a significant effect on the survival in the subgroup of patients who received chemotherapy (P = 0.006).GSTT1 active genotype and GSTO1 Asp140Asp and GSTO2 Asp142Asp genotypes may have a prognostic/pharmacogenomic r  ole in p  atients with muscle invasive bladder cancer. 
 GSTT1, GSTM1, and GSTP1 polymorphisms and chemotherapy response in locally advanced breast cancer. The glutathione S-transferase (GST) family consists of phase II detoxification enzymes that catalyze the conjugation of toxic substances, such as chemotherapeutic agents, to glutathione. We examined whether GSTT1/GSTT1"null", GSTM1/GSTM1"null" and GSTP1Ile105Ile/GSTP1Ile105Val polymorphisms are associated with different response rates to neoadjuvant chemotherapy in the treatment of stage II and III breast cancer. Forty Brazilian women with   invasive   ductal adenocarcinoma of the breast submitted to neoadjuvant chemotherapy, using 5-fluorouracil, epirubicin and cyclophosphamide, were genotyped for the GSTT1, GSTM1 and GSTP1 genes. Clinical response was assessed by RECIST criteria. Comparisons were made for the three genes alone and in pairs, as polymorphic and as wild-type combinations and polymorphic/wild-type combinations. We analyzed all possible combinations and their response rate. Patients with the GSTT1/GSTP1105Ile combination were found to have a significantly better response than GSTT1"null"/GSTP1105Val (P = 0.0209) and GSTT1/GSTM1 (P = 0.0376) combinations. Analysis of all possible combinations showed the GSTM1"null" polymorphic genotype to be present in four, and the wild-type GSTP1105Ile in six of the combinations associated with the largest number of responding patients. We found that patients with the GSTT1/GSTP1105Ile wild-type combination had a significantly higher response rate to chemotherapy than patients with the respective polymorphic GSTT1"null"/GSTP1105Val combination or patients with the wild-type GSTT1/GSTM1. The six gene combinations associated with the largest number of responding patients were found to contain the wild-type GSTP1105Ile and the polymorphic-type GSTM1"null". These specific combinations were virtually absent in the combinations with few responding patients. 
 Polymorphisms at the glutathione S-transferase GSTM1, GSTT1 and GSTP1 loci: risk of ovarian cancer by histological subtype. The phase II glutathione S-transferases (GSTs) GSTT1, GSTM1 and GSTP1 catalyse glutathione-mediated reduction of exogenous and endogenous electrophiles. These GSTs have broad and overlapping substrate specificities and it has been hypothesized that allelic variants associated with less effective detoxification of potential carcinogens may confer an increased susceptibility to cancer. To assess the role of GST gene variants in ovarian cancer development, we screened 285 epithelial ovarian cancer cases and 299 unaffected controls for the GSTT1 deletion (null) variant, the GSTM1 deletion (null) variant and the GSTP1 codon 104 A-->G Ile-->Val amino acid substitution variant. The frequencies of the GSTT1, GSTM1 and GSTP1 polymorphic variants did not vary with tumour behaviour (low malignant potential or   invasive  ) or p53 immunohistochemical status. There was a suggestion that ovarian cancers of the endometrioid or clear cell histological subtype had a higher frequency of the GSTT1 and GSTM1 deletion genotype than other histological subgroups. The GSTT1, GSTM1 and GSTP1 genotype distributions did not differ significantly between unaffected controls and ovarian cancer cases (overall or   invasive   cancers only). However, the GSTM1 null genotype was associated with increased risk of endometrioid/clear cell   invasive   cancer [age-adjusted OR (95% CI) = 2.04 (1.01-4.09), P = 0.05], suggesting that deletion of GSTM1 may increase the risk of ovarian cancer of these histological subtypes specifically. This marginally significant finding will require verification by independent studies. 
 Interaction between glutathione-S-transferase polymorphisms, smoking habit, and HPV infection in cervical cancer risk. Human papillomavirus (HPV) infection is considered the major cause of cervical cancer (CC), but a number of infected women do not develop   invasive   lesions, suggesting the role of genetic susceptibility and environmental co-factors for cancer outbreak. The aim of this study was to investigate whether some GST polymorphisms could influence the risk to develop CC, either by themselves or in combination with smoking habit, in a cohort of high-risk HPV (HR-HPV) infected Italian women.The study population comprises 192 Italian women including 81 HR-HPV infected women bearing cervical lesions and 111 healthy controls. The cases include: 26 low-grade squamous intraepithelial lesions (LSILs), 30 high-grade-SIL, and 25 CCs, while controls were all negative for HPV. DNA was extracted from peripheral blood samples or cytobrush and individuals were genotyped for GSTM1, GSTT1, and GSTP1 polymorphisms using PCR and PCR/RFLP techniques.On studying the association of GSTs gene polymorphisms with cervical cancer lesions, the combination of GSTM1 null, GSTT1 null and GSTP1 AA genotypes, independently on smoking habit, seems to be related to a 5.7-fold increased risk of developing CLs with a considerable statistical significance (P = 0.0091).We suggest that the investigation of multiple gene polymorphisms, versus single genes, could contribute to a better understanding of the effect of susceptibility genes on cancer risk. 
  GSTT1, GSTM1, and GSTP1 polymorphisms as a prognostic factor in women with breast cancer. The glutathione S-transferase (GST) family comprises phase-II cellular detoxification enzymes that catalyze the conjugation of chemotherapy drugs to glutathione and act on the apoptotic pathway. The aim of this study was to determine whether polymorphisms of the GSTT1, GSTM1, and GSTP1 genes are associated with different rates of overall survival (OS) and disease-free survival (DFS) after neoadjuvant chemotherapy in the management of locally advanced breast cancer, using either simple or combined analyses, and in relation to the post-therapy axillary lymph node status. Forty women with   invasiv  e ductal carcinoma of the breast submitted to neoadjuvant chemotherapy with 5-fluorouracil, epirubicin, and cyclophosphamide were genotyped for GSTT1, GSTM1, and GSTP1. Comparisons were performed for the three genes, either isolated or in pairs, in polymorphic or wild-type combinations. Finally, the OS and DFS of patients were analyzed with respect to axillary lymph node status and with respect to wild-type or polymorphic presentations of each gene. No statistically significant difference in OS and DFS was evident between women with wild-type or polymorphic forms of the genes, either isolated or in pairs, after neoadjuvant chemotherapy. By contrast, after treatment, lymph node-negative women had better OS and DFS only in the presence of polymorphisms of GSTP1, and improved DFS only in the presence of the polymorphic types of GSTT1 and GSTM1 compared to women with positive lymph nodes. The presence of polymorphic forms of GSTP1, GSTM1, and GSTT1 was crucial to conferring better OS and DFS among women with negative axillary lymph nodes. 
 Glutathione S-transferase GSTM1 and GSTT1 genotypes in ovarian cancer: association with p53 expression and survival. The objective of this study was to determine whether the association between GSTM1 null/GSTTI null and survival in ovarian cancer is mediated by the influence of these genes on p53 expression. In 81 women with pure   invasive   ovarian cancer, GSTM1 null and GSTT1 null genotypes were identified using polymerase chain reaction and p53 expression was assessed using immunohistochemistry. The association of these factors with survival was examined using Cox's proportional hazards regression models. Performance status (P < 0.001), operative stage (P = 0.004), residual disease (P = 0.001), histologic subtype (P = 0.05), tumor grade (P = 0.007), and the combined GSTMI null/GSTTl null genotype (P = 0.023) were all individually associated with survival. p53 expression was not associated with survival (P = 0.45). In a multivariate analysis, the effects of GSTM1 null/GSTT1 null on survival were lost when residual disease and tumor grade were included. The effects of p53 expression on survival were unchanged when residual disease, tumor grade, operative stage, and performance score were included. GSTM1 null/GSTT1null did not influence the effects of p53 expression on survival and vice versa. The GSTM1 null/GSTT1 null genotype was associated with response to primary chemotherapy (P = 0.007) but p53 expression was not. We conclude that the association of GSTM1 null/GSTTl null with survival appears to be mediated through different mechanisms to p53 expression in ovarian cancer and in addition, may be a better predictor of outcome. 
 Analysis association between mitochondrial genome instability and xenobiotic metabolizing genes in human breast cancer. The aim of this study was to determine the existence of association between the genetic polymorphisms of metabolizing genes GSTM-1, GSTT-1, and NAT-2, and the presence of mitochondrial genome instability (mtGI) in breast cancer cases. Ninety-four pairs of tumoral/nontumoral breast cancer samples were analyzed. Our samples showed 40.42% of mtGI by analysis of two D-loop region markers, a (CA)n mtMS starting at the 514-bp position, and four informative MnlI sites between the 16,108-16,420-bp. GSTM-1 null genotype has shown a significant association with mtGI presence (chi(2) = 7.62; P = 0.006) in breast cancer cases; moreover, these genotypes also are related to an increased risk for mtDNA damage (odds ratio [OR] = 3.71 [1.41-9.88]; 95% Cornfield confidence interval [CI]). These results suggest that the absence of GSTM-1 enzymatic activity favors chemical actions in damaging the mtDNA. Analysis of GSTT-1 and NAT-2 polymorphisms showed no association with mtGI (chi(2) = 0.03; P = 0.87 and chi(2) = 2.76; P = 0.09, respectively). The analysis of   invasive   breast cancer cases showed mtGI in 74.36% of ILC cases (29 of 39 samples), and in only 18.75% (9 out of 48) IDC cases; this result suggests a possible relation between mtDNA mutations and variations in molecular pathways of tumor development. 
 Interaction of passive smoking with GST (GSTM1, GSTT1, and GSTP1) genotypes in the risk of cervical cancer in India. Human papilloma virus (HPV) infection is a major cause of cervix cancer, but a number of infected women do not develop   invasive   lesions, suggesting that HPV infection in itself is not a sufficient factor and that other cofactors, such as smoking, play an important role in development of cervix cancer. Alongside active cigarette smoking, passive smoking is an independent risk factor for cervix cancer. Smoking maintains cervical HPV infection longer and decreases potential of clearing an oncogenic infection. Thus, it is quite possible that polymorphism at detoxifying enzyme coding loci such as GSTM1, GSTT1, and GSTP1 may determine susceptibility to cervix cancer. This study evaluates the combined effects of genetic polymorphisms of GSTM1, GSTT1, and GSTP1 on susceptibility to cervical cancer and interaction of these genes with smoking. On individual analysis of GSTM1, GSTT1, and GSTP1, it was observed that passive smokers having genotypes GSTM1 (null) (OR = 7.0, 95% CI = 2.19-22.36, P = 0.0005), GSTT1 (null) (OR = 10.2, 95% CI = 1.23-84.18, P = 0.02), and GSTP1 (ile/val) (OR = 6.4, 95% CI = 2.25-18.38, P = 0.0005) have an increased risk of developing cervix cancer. It is thus concluded that cervical cancer risk is increased in passive smokers with GSTM1 (null), GSTT1 (null), and GSTP1 (ile/val) genotypes. 
 Association of genetic polymorphism of glutathione S-transferase (GSTM1, GSTT1, GSTP1) with bladder cancer susceptibility. The glutathione-S-transferases (GSTs) comprise a class of enzymes that detoxify carcinogenic compounds by conjugating glutathione to facilitate their removal. Polymorphisms in GSTM1, GSTT1, and GSTP1 genes have been related to risk for bladder cancer. Studies focusing on GSTs gene variants relationship with the risk of bladder cancer have produced conflicting and inconsistent results. We examine the association between genetic polymorphism of glutathione S-transferase P1, GSTM1, GSTT1 genes and development of bladder transitional cell carcinoma (TCC). The study population consisted of 166 histologically confirmed male bladder TCC cases and 332 healthy male controls. Genotyping was done using the polymerase chain reaction restriction fragment length polymorphism (PCR-RFLP) method and also investigated combined gene interactions. The GSTP1 Val/Val genotype was significantly associated with bladder cancer (OR = 4.32, 95% CI: 2.64-6.34), whereas the association observed for GSTM1 null (OR = 1.32, 95% CI: 0.82-2.62; P = 0.67) and GSTT1 null genotype (OR = 1.18, 95% CI: 0.79-1.67; P = 0.74) did not reach statistical significance. There was a significant multiple interaction between GSTM1, GSTT1, and GSTP1 genotypes in risk of bladder cancer (P for interaction = 0.02). The risk associated with the concurrent presence of GSTM1 positive and GSTP1 Ile/Val or Val/Val (OR = 3.71, 95% CI: 2.34-5.54) and GSTT1 positive and GSTP1 Ile/Val or Val/Val (OR = 2.66, 95% CI: 1.54-4.72) was statistically significant. Patients carrying GSTP1 Val/Val genotype were at increased risk for developing high-grade (OR = 7.68, 95% CI: 4.73-19.25) and muscle   invasive   (OR = 10.67, 95% CI: 6.34-21.75) bladder cancer. High risk for bladder TCC also was observed with respect to combined GSTT1 null/GSTP1 Ile/Val or Val/Val (OR = 4.76, 95% CI: 2.68-18.72) and GSTM1 null/GSTT1 null/GSTP1 Ile/Val or Val/Val (OR = 6.42, 95% CI: 4.76-14.72) genotype variant. This study suggests that the GSTP1 polymorphism and its combination with GSTM1, and GSTT1 may be associated with bladder cancer susceptibility in the Iranian population. Further confirmation in large population-based studies is needed. 
 Genetic polymorphisms in glutathione-S-transferase genes (GSTM1, GSTT1, GSTP1) and survival after chemotherapy for   invasive   breast carcinoma. It has been suggested that genetic polymorphisms in certain glutathione-S-transferase (GST) genes reduce the effectiveness of detoxifying cytotoxins generated by chemotherapeutic agents, potentially resulting in enhanced clinical responses to chemotherapy.The authors evaluated common polymorphisms in the GSTM1, GSTT1, and GSTP1 genes for associations with overall survival in 1034 patients with   invasive   breast carcinoma who were recruited into the Shanghai Breast Cancer Study between 1996 and 1998, treated with chemotherapy, and followed for a median of 5.3 years.After adjusting for age, tumor stage, and the use of radiotherapy and tamoxifen, women who were homozygous for the variant GSTP1 105Val allele had a 60% reduction in mortality risk compared with women who were homozygous for the Ile allele (hazard ratio, 0.4; 95% confidence interval, 0.2-0.8). No association was found with respect to any of the GSTM1 or GSTT1 genotypes.The results of the current study indicate a potential role for GSTP1 polymorphism in predicting the clinical outcomes of patients with breast carcinoma who are treated with chemotherapy. 
 Glutathione S-transferase M1 and T1 polymorphisms: susceptibility and outcomes in muscle   invasive   bladder cancer patients. We investigated whether genetic polymorphisms in the glutathione S transferase mu (GSTM1) and theta (GSTT1) genes modulated risk, disease progression and survival in primary muscle   invasive   bladder cancer (MIBC).GSTM1 and GSTT1 polymorphisms were analysed by multiplex polymerase chain reaction (PCR) using blood genomic DNA in 110 MIBC patients and 220 gender- and age-matched healthy controls. The influence of the genetic polymorphisms on patient survival was evaluated by Kaplan-Meier survival curves and Cox Proportional Hazard models. We also evaluated whether cigarette smoking and treatment modality modified the association between genotype and prognosis.GSTM1-null individuals exhibited increased risk for MIBC and an association with cigarette smoking. GSTT1-null subjects showed significant disease progression and cancer-specific death. In the combined analysis, GSTT1-null genotype was an independent risk factor for disease progression and cancer specific death regardless of GSTM1 genotype. Significant differences in progression-free survival (PFS) and cancer-specific survival (CSS) were seen based on GSTT1 genotype. The survival impact of the GSTT1 genotype was only valid for smokers. The GSTT1-null genotype was an independent prognostic factor for shorter PFS in patients who received chemotherapy and those who did not undergo radical cystectomy. By multivariate Cox regression analysis, GSTT1-null genotype was a predictive factor for disease progression and cancer specific survival regardless of treatment modality.The GSTM1-null genotype plays an important role in genetic susceptibility to MIBC and the GSTT1-null genotype is associated with disease progression and shorter survival in MIBC. 
 The role of glutathione-S-transferase polymorphisms in ovarian cancer survival. Resistance to chemotherapy represents one of the most important causes of treatment failure in patients with ovarian cancer. Common polymorphisms in the glutathione-S-transferase (GSTM1, GSTP1 and GSTT1) family have been implicated in chemoresistence and ovarian cancer survival. In this study, we have analysed Australian women diagnosed with primary   invasive   epithelial ovarian cancer between 1985 and 1997, using DNA extracted from peripheral blood and archival uninvolved (normal) tissues. GSTP1 genotypes were determined using ABI Prism 7700 Sequence Detection System methodology (n=448) and GSTT1 and GSTM1 genotypes using PCR-agarose methodology (n=239). We observed a significant survival advantage among carriers of GSTP1 Ile105Val GG/GA genotype (HR 0.77, 95% confidence interval (CI) 0.61-0.99,p=0.04) and a non-significant survival advantage among women who were homozygous for the GSTM1 and GSTT1 deletion variants. There was also evidence of an additive effect, with a stronger survival benefit in women carrying three low function GST genotypes (GSTM1 null, GSTT1 null and GSTP1 GA/GG) (HR 0.47, 95% CI 0.22-1.02). The results of this study, the largest to date, are consistent with a number of previous smaller studies which have also observed that reduced GST function was associated with better survival outcomes in patients with ovarian cancer. 
  GeneRIFs  
 GSTT1 polymorphisms is associated with BCG therapy failure in patients with non-muscle   invasive   bladder cancer. 
 GSTT1 active genotype and GSTO1 Asp140Asp and GSTO2 Asp142Asp genotypes may have a prognostic/pharmacogenomic role in patients with muscle   invasive   bladder cancer. 
 Data indicate that the GSTM1-null genotype plays an important role in genetic susceptibility to muscle   invasive   bladder cancer (MIBC) and the GSTT1-null genotype is associated with disease progression and shorter survival in MIBC. 
 genetic associations: No significant associations were found for GSTT1 null polymorphism with intraepithelial cervical lesions or   invasive   cervical cancer. [Meta-Analysis; REVIEW] 
 In multivariate regression analysis, the GSTT1-positive genotype was the independent predictor for recurrence and progression in non-muscle-  invasive   bladder cancer 
 Variants of the GSTT1 gene may modify the association between genital talc use and risk of total and serous   invasive   ovarian cancer. 
  [Mm]etastasis  PubMed  
 The association between polymorphisms in glutathione S-transferase (GSTM1 and GSTT1) and lung cancer outcome. Polymorphisms in the glutathione S-transferase (GST) family may be associated with increased risk of lung cancer, somatic changes in lung tumour tissue, and survival. We evaluated survival according to GST polymorphism in lung cancer patients.We studied DNA polymorphisms of 81 primary lung cancer patients at 2 glutathione-related loci: GSTM1, and GSTT1 that encode glutathione S-transferase-mu, and glutathione S-transferase- square. The presences of the GSTM1 and GSTT1 genes were assayed by PCR. Kaplan-Meier with log rank tests, and Cox regression models were applied in the analysis.The median age of 75 males and 6 females was 60 years. Median survival of the whole population was 8 months. In the first presentation, none of the patients with GSTT1 null genotype but 30 percent of the patients with GSTT1-positive genotype had liver   metastasis   (p < 0.01) but GSTT1 genotype was not associated with survival. Sputum (p < 0.01) was more common in patients with GSTM1 null genotype. Subjects with the GSTM1-null genotype had shorter survival. Using a Cox proportional hazard model, GSTM1, tumor (T) factor and thoracic irradiation status were identified as independent prognostic factors.Our preliminary results showed that GSTM1-null genotype was associated with shorter survival. 
 Glutathione S-transferase polymorphisms in osteosarcoma patients. Osteosarcoma is the most common malignant bone tumor in children and adolescents. Multidrug resistance and poor clinical outcome are the problems that still affect osteosarcoma patients. The glutathione S-transferase supergene family includes several genes that encode enzymes involved in the detoxification of many xenobiotic agents, including carcinogens and anticancer drugs. The polymorphisms in these genes have already been associated both with cancer susceptibility and anticancer drugs resistance.This study aims to investigate the genotype frequencies of GSTM1, GSTT1 and GSTM3 genes in 80 osteosarcoma patients and 160 normal control participants, and also the influence of these polymorphisms in the clinical outcome of osteosarcoma patients.GSTM1 and GSTT1 deletion polymorphisms were examined through a multiplex-PCR and the GSTM3 polymorphism of three base pair-deletion at intron 6 using PCR-restriction fragments length polymorphism method.We found that GSTM1 null genotype is correlated to poor clinical outcome characterized by the increased lung relapse occurrence [odds ratio (OR)=2.71, P=0.036], while the presence of at least one GSTM1 allele is associated with a good response to treatment and better survival (OR=4.28, P=0.020 and hazards ratio=4.09, P=0.0078, respectively). The GSTT1 null genotype was correlated with a better overall survival (hazards ratio=7.15, P=0.0247), whereas GSTM3*B allele was associated with   metastasis   at diagnosis (OR=2.83, P=0.028).The findings of this study suggest that GST polymorphisms may have a role in treatment response and osteosarcoma progression. 
 Glutathione S-transferase T1 polymorphisms are associated with outcome in colorectal cancer. Colorectal cancer (CRC) remains a significant cause of mortality accounting for approximately 10% of all deaths from malignancy in the western world. Polymorphism in the glutathione S-transferase GSTT1 gene has been associated with CRC risk in some but not all studies. In this study, we examined associations between GSTT1 genotypes and CRC risk, and prognosis in 361 cases and 881 unrelated controls. GSTT1 null was associated with a small but significant increase in risk (P = 0.0006, odds ratio (OR) = 1.65, 95% confidence interval (CI) = 1.22-2.24). GSTT1 null was also associated with a significantly younger age at diagnosis (mean 65.2 years) compared with GSTT1 A (mean 67.6 years, P = 0.031). There were no significant associations between GSTT1 genotypes and clinical factors (e.g. Dukes stage, differentiation and tumour node   metastasis   classification) in the total case group. However, following stratification by age (<70 versus > or =70 years at diagnosis), in the patients diagnosed <70 years of age, GSTT1 null was more common in Dukes grade A/B tumours (P = 0.046), stage T1/T2 tumours (P = 0.053) and those with a pushing margin (P = 0.066). We also identified associations between GSTT1 null and increased prevalence of host lymphocyte response, particularly in the younger patients (P = 0.036). Furthermore, GSTT1 null was associated with improved survival in younger patients (P = 0.017, hazards ratio (HR) = 0.52, 95% CI = 0.31-0.89) but poorer survival in older patients (P = 0.017, HR = 1.89, 95% CI = 1.12-3.20). We proposed a model based on the dual functionality of GSTT1 to explain these contrasting results. We suggest that the null genotype is associated with improved immune response in younger patients, but poorer detoxification in older patients. These findings may also provide an explanation for the contrasting finding of other studies on the role of this gene in CRC. 
 The GSTM1 null genotype confers an increased risk for solar keratosis development in an Australian Caucasian population. Solar keratoses affect approximately 50% of Australian Caucasians aged over 40 y. Solar keratoses can undergo malignant transformation into squamous cell carcinoma followed by possible   metastasis   and are risk factors for basal cell carcinoma, melanoma, and squamous cell carcinoma. The glutathione-S-transferase genes play a part in detoxification of carcinogens and mutagens, including some produced by ultraviolet radiation. This study examined the role of glutathione-S-transferase M1, T1, P1, and Z1 gene polymorphisms in susceptibility to solar keratoses development. Using DNA samples from volunteers involved in the Nambour Skin Cancer Prevention Trial, allele and genotype frequencies were determined using polymerase chain reaction and restriction enzyme digestion. No significant differences were detected in glutathione-S-transferase P1 and glutathione-S-transferase Z1 allele or genotype frequencies; however, a significant association between glutathione-S-transferase M1 genotypes and solar keratoses development was detected (p=0.003) with null individuals having an approximate 2-fold increase in risk for solar keratoses development (odds ratio: 2.1; confidence interval: 1.3-3.5) and a significantly higher increase in risk in conjunction with high outdoor exposure (odds ratio: 3.4; confidence interval: 1.9-6.3). Also, a difference in glutathione-S-transferase T1 genotype frequencies was detected (p=0.039), although considering that multiple testing was undertaken, this was found not to be significant. Fair skin and inability to tan were found to be highly significant risk factors for solar keratoses development with odds ratios of 18.5 (confidence interval: 5.7-59.9) and 7.4 (confidence interval: 2.6-21.0), respectively. Overall, glutathione-S-transferase M1 conferred a significant increase in risk of solar keratoses development, particularly in the presence of high outdoor exposure and synergistically with known phenotypic risk factors of fair skin and inability to tan. 
 Association of GST genotypes with age of onset and lymph node   metastasis   in oral squamous cell carcinoma. Environment-gene interaction in oral carcinogenesis is well demonstrated by phase I and II enzymes that are involved in the metabolism of carcinogens. This study investigated the association of glutathione S-transferase (GST)T1 and GSTM1 genotypes of phase II enzyme genes with risk for, age of onset, and neck lymph node   metastasis   (LNM) in areca-associated oral squamous cell carcinoma (OSCC).A total of 114 OSCC male patients and 100 male controls were recruited. All subjects were areca users and tobacco smokers. DNA was obtained from peripheral blood samples. Genotyping of GSTT1 (non-null/null) and GSTM1 (non-null/null) was determined by polymerase chain reaction (PCR) analysis using specific primers that only amplify non-null alleles.No association was found between GST genotype and the risk of OSCC based on case-controls. Patients with the GSTT1 null genotype were older at onset (P = 0.03). Those with the GSTM1 null genotype had a higher incidence of neck LNM than those with the GSTM1 non-null genotype (P = 0.01). Patients with the GSTM1/GSTT1 null genotype appeared to have later onset and a higher incidence of neck LNM than those carrying the opposite genotype.The GST genotypes may be important markers for the age of onset and risk of   metastasis   in OSCC. The data also suggest that the various GST isoforms may be differentially involved in development or progression of OSCC. 
 Glutathione S-transferase gene polymorphisms and risk and survival of pancreatic cancer. Pancreatic cancer is a multifactorial disease with   metastasis  -prone and therapy-resistant nature. The authors hypothesized that genetic variants of glutathione S-transferase (GST) affect detoxification of carcinogens and anticancer agents in the human pancreas and, thus, the risk and survival of pancreatic cancer.Genotypes of GSTM1, GSTT1, and GSTP1 were determined in 352 patients with pancreatic ductal adenocarcinoma and in a control group of 315 healthy, non-Hispanic whites (frequency-matched by age and sex). Survival analysis was performed in a subset of 290 patients. Epidemiological and clinical information was obtained. A multiple unconditional logistic regression model, a Cox proportional hazards model, and log-rank tests were used for statistical analysis.No significant main effects of any of 3 GST genes on the risk of pancreatic cancer were observed. Subgroup analysis showed that older individuals (aged >or=62 years) who carried the GSTP1*C ((105)Val-(114)Val) containing genotype tended to have a reduced risk compared with younger individuals who carried the non-*C genotype (for sex and pack-years of smoking, the adjusted odd ratio was 0.54; 95% confidence interval [95% CI], 0.29-1.02). In a survival analysis of 138 patients who received 5-flurorouracil, patients who carried the GSTP1*C containing genotype had a significantly longer survival than patients who carried the non-*C genotype (multivariate hazard ratio, 0.45; 95% CI, 0.22-0.94).The GSTP1*C variant conferred a possible protective effect against pancreatic cancer in older individuals and a significant survival advantage in patients who received 5-florouracil. The current findings must be confirmed before further inferences can be made. 
  GeneRIFs  
 genotype may be important marker for the age of onset and risk of   metastasis   in oral squamous cell carcinoma 
   
  
 EEF1G 
 
  NCBI Gene  
  GeneRIF  
  Pubmed  
 
 
  Search Term  Rank  Fields  
  [Mm]etastasis  12  PubMed(1)  
 
 
 hide/show details for EEF1G 
  [Mm]etastasis  PubMed  
 Proteomics analysis of the interactome of N-myc downstream regulated gene 1 and its interactions with the androgen response program in prostate cancer cells. NDRG1 is known to play important roles in both androgen-induced cell differentiation and inhibition of prostate cancer   metastasis  . However, the proteins associated with NDRG1 function are not fully enumerated. Using coimmunoprecipitation and mass spectrometry analysis, we identified 58 proteins that interact with NDRG1 in prostate cancer cells. These proteins include nuclear proteins, adhesion molecules, endoplasmic reticulum (ER) chaperons, proteasome subunits, and signaling proteins. Integration of our data with protein-protein interaction data from the Human Proteome Reference Database allowed us to build a comprehensive interactome map of NDRG1. This interactome map consists of several modules such as a nuclear module and a cell membrane module; these modules explain the reported versatile functions of NDRG1. We also determined that serine 330 and threonine 366 of NDRG1 were phosphorylated and demonstrated that the phosphorylation of NDRG1 was prominently mediated by protein kinase A (PKA). Further, we showed that NDRG1 directly binds to beta-catenin and E-cadherin. However, the phosphorylation of NDRG1 did not interrupt the binding of NDRG1 to E-cadherin and beta-catenin. Finally, we showed that the inhibition of NDRG1 expression by RNA interference decreased the ER inducible chaperon GRP94 expression, directly proving that NDRG1 is involved in the ER stress response. Intriguingly, we observed that many members of the NDRG1 interactome are androgen-regulated and that the NDRG1 interactome links to the androgen response network through common interactions with beta-catenin and heat shock protein 90. Therefore we overlaid the transcriptomic expression changes in the NDRG1 interactome in response to androgen treatment and built a dual dynamic picture of the NDRG1 interactome in response to androgen. This interactome map provides the first road map for understanding the functions of NDRG1 in cells and its roles in human diseases, such as prostate cancer, which can progress from androgen-dependent curable stages to androgen-independent incurable stages. 
   
  
 RHOT1 
 
  NCBI Gene  
  GeneRIF  
  Pubmed  
 
 
  Search Term  Rank  Fields  
  [Ff]ilopodia  1  PubMed(1)  
  [Mm]igration  6  PubMed(3); GeneRIFs(1)  
  [Mm]etastasis  12  PubMed(1)  
  [Cc]ell [Ii]nvasion  13  PubMed(1)  
 
 
 hide/show details for RHOT1 
  [Ff]ilopodia  PubMed  
 Rho GTPases have diverse effects on the organization of the actin filament system. The Rho GTPases are related to the Ras proto-oncogenes and consist of 22 family members. These proteins have important roles in regulating the organization of the actin filament system, and thereby the morphogenesis of vertebrate cells as well as their ability to migrate. In an effort to compare the effects of all members of the Rho GTPase family, active Rho GTPases were transfected into porcine aortic endothelial cells and the effects on the actin filament system were monitored. Cdc42, TCL (TC10-like), Rac1-Rac3 and RhoG induced the formation of lamellipodia, whereas Cdc42, Rac1 and Rac2 also induced the formation of thick bundles of actin filaments. In contrast, transfection with TC10 or Chp resulted in the formation of focal adhesion-like structures, whereas Wrch-1 induced long and thin   filopodia  . Transfection with RhoA, RhoB or RhoC induced the assembly of stress fibres, whereas Rnd1-Rnd3 resulted in the loss of stress fibres, but this effect was associated with the formation of actin- and ezrin-containing dorsal microvilli. Cells expressing RhoD and Rif had extremely long and flexible   filopodia  . None of the RhoBTB or Miro GTPases had any major influence on the organization of the actin filament system; instead, RhoBTB1 and RhoBTB2 were present in vesicular structures, and Miro-1 and Miro-2 were present in mitochondria. Collectively, the data obtained in this study to some extent confirm earlier observations, but also allow the identification of previously undetected roles of the different members of the Rho GTPases. 
  [Mm]igration  PubMed  
 APC binds the Miro/Milton motor complex to stimulate transport of mitochondria to the plasma membrane. Mutations in adenomatous polyposis coli (APC) disrupt regulation of Wnt signaling, mitosis, and the cytoskeleton. We describe a new role for APC in the transport of mitochondria. Silencing of wild-type APC by small interfering RNA caused mitochondria to redistribute from the cell periphery to the perinuclear region. We identified novel APC interactions with the mitochondrial kinesin-motor complex Miro/Milton that were mediated by the APC C-terminus. Truncating mutations in APC abolished its ability to bind Miro/Milton and reduced formation of the Miro/Milton complex, correlating with disrupted mitochondrial distribution in colorectal cancer cells that could be recovered by reconstitution of wild-type APC. Using proximity ligation assays, we identified endogenous APC-Miro/Milton complexes at mitochondria, and live-cell imaging showed that loss of APC slowed the frequency of anterograde mitochondrial transport to the membrane. We propose that APC helps drive mitochondria to the membrane to supply energy for cellular processes such as directed cell   migration  , a process disrupted by cancer mutations. 
 APC binds the Miro/Milton motor complex to stimulate transport of mitochondria to the plasma membrane. Mutations in adenomatous polyposis coli (APC) disrupt regulation of Wnt signaling, mitosis, and the cytoskeleton. We describe a new role for APC in the transport of mitochondria. Silencing of wild-type APC by small interfering RNA caused mitochondria to redistribute from the cell periphery to the perinuclear region. We identified novel APC interactions with the mitochondrial kinesin-motor complex Miro/Milton that were mediated by the APC C-terminus. Truncating mutations in APC abolished its ability to bind Miro/Milton and reduced formation of the Miro/Milton complex, correlating with disrupted mitochondrial distribution in colorectal cancer cells that could be recovered by reconstitution of wild-type APC. Using proximity ligation assays, we identified endogenous APC-Miro/Milton complexes at mitochondria, and live-cell imaging showed that loss of APC slowed the frequency of anterograde mitochondrial transport to the membrane. We propose that APC helps drive mitochondria to the membrane to supply energy for cellular processes such as directed cell   migration  , a process disrupted by cancer mutations. 
 Miro-1 links mitochondria and microtubule Dynein motors to control lymphocyte   migration   and polarity. The recruitment of leukocytes to sites of inflammation is crucial for a functional immune response. In the present work, we explored the role of mitochondria in lymphocyte adhesion, polarity, and   migration  . We show that during adhesion to the activated endothelium under physiological flow conditions, lymphocyte mitochondria redistribute to the adhesion zone together with the microtubule-organizing center (MTOC) in an integrin-dependent manner. Mitochondrial redistribution and efficient lymphocyte adhesion to the endothelium require the function of Miro-1, an adaptor molecule that couples mitochondria to microtubules. Our data demonstrate that Miro-1 associates with the dynein complex. Moreover, mitochondria accumulate around the MTOC in response to the chemokine CXCL12/SDF-1α; this redistribution is regulated by Miro-1. CXCL12-dependent cell polarization and   migratio  n are reduced in Miro-1-silenced cells, due to impaired myosin II activation at the cell uropod and diminished actin polymerization. These data point to a key role of Miro-1 in the control of lymphocyte adhesion and   migratio  n through the regulation of mitochondrial redistribution. 
  GeneRIFs  
 CXCL12-dependent cell polarization and   migration   are reduced in Miro-1-silenced cells 
  [Mm]etastasis  PubMed  
 RhoT1 and Smad4 are correlated with lymph node   metastasis   and overall survival in pancreatic cancer. Cancer cell invasion and   metastasis   are the most important adverse prognostic factors for pancreatic cancer. Identification of biomarkers associated with outcome of pancreatic cancer may provide new approaches and targets for anticancer therapy. The aim of this study is to examine the relationship between the expression of RhoT1, Smad4 and p16 and   metastasis   and survival in patients with pancreatic cancer. The analysis showed that the high cytoplasmic expression levels of RhoT1, Smad4 and p16 in pancreatic cancer tissues had significantly negative correlation with lymph node   metastasis   (LNM) (P = 0.017, P = 0.032, P = 0.042, respectively). However, no significant association was observed between perineural invasion (PNI) and the expression of above three proteins (all P>0.05). Additionally, the survival analysis showed that the low expression levels of RhoT1 and Smad4 were significantly associated with worse survival (P = 0.034, P = 0.047, respectively). In conclusion, these results indicated that the low-expression levels of RhoT1 and Smad4 were significantly associated with LNM and shorter survival. RhoT1 may be considered as a potential novel marker for predicting the outcome in patients with pancreatic cancer. 
  [Cc]ell [Ii]nvasion  PubMed  
 RhoT1 and Smad4 are correlated with lymph node metastasis and overall survival in pancreatic cancer. Cancer   cell invasion   and metastasis are the most important adverse prognostic factors for pancreatic cancer. Identification of biomarkers associated with outcome of pancreatic cancer may provide new approaches and targets for anticancer therapy. The aim of this study is to examine the relationship between the expression of RhoT1, Smad4 and p16 and metastasis and survival in patients with pancreatic cancer. The analysis showed that the high cytoplasmic expression levels of RhoT1, Smad4 and p16 in pancreatic cancer tissues had significantly negative correlation with lymph node metastasis (LNM) (P = 0.017, P = 0.032, P = 0.042, respectively). However, no significant association was observed between perineural invasion (PNI) and the expression of above three proteins (all P>0.05). Additionally, the survival analysis showed that the low expression levels of RhoT1 and Smad4 were significantly associated with worse survival (P = 0.034, P = 0.047, respectively). In conclusion, these results indicated that the low-expression levels of RhoT1 and Smad4 were significantly associated with LNM and shorter survival. RhoT1 may be considered as a potential novel marker for predicting the outcome in patients with pancreatic cancer. 
   
  
 RHOT2 
 
  NCBI Gene  
  GeneRIF  
  Pubmed  
 
 
  Search Term  Rank  Fields  
  [Ff]ilopodia  1  PubMed(1)  
 
 
 hide/show details for RHOT2 
  [Ff]ilopodia  PubMed  
 Rho GTPases have diverse effects on the organization of the actin filament system. The Rho GTPases are related to the Ras proto-oncogenes and consist of 22 family members. These proteins have important roles in regulating the organization of the actin filament system, and thereby the morphogenesis of vertebrate cells as well as their ability to migrate. In an effort to compare the effects of all members of the Rho GTPase family, active Rho GTPases were transfected into porcine aortic endothelial cells and the effects on the actin filament system were monitored. Cdc42, TCL (TC10-like), Rac1-Rac3 and RhoG induced the formation of lamellipodia, whereas Cdc42, Rac1 and Rac2 also induced the formation of thick bundles of actin filaments. In contrast, transfection with TC10 or Chp resulted in the formation of focal adhesion-like structures, whereas Wrch-1 induced long and thin   filopodia  . Transfection with RhoA, RhoB or RhoC induced the assembly of stress fibres, whereas Rnd1-Rnd3 resulted in the loss of stress fibres, but this effect was associated with the formation of actin- and ezrin-containing dorsal microvilli. Cells expressing RhoD and Rif had extremely long and flexible   filopodia  . None of the RhoBTB or Miro GTPases had any major influence on the organization of the actin filament system; instead, RhoBTB1 and RhoBTB2 were present in vesicular structures, and Miro-1 and Miro-2 were present in mitochondria. Collectively, the data obtained in this study to some extent confirm earlier observations, but also allow the identification of previously undetected roles of the different members of the Rho GTPases. 
   
  
 RHOG 
 
  NCBI Gene  
  GeneRIF  
  Pubmed  
 
 
  Search Term  Rank  Fields  
  [Ff]ilopodia  1  PubMed(1)  
  [Mm]igration  6  PubMed(12); GeneRIFs(2); Summary(1)  
  [Ii]nvasive  10  PubMed(3); GeneRIFs(1)  
  [Mm]etastasis  12  PubMed(2)  
  [Cc]ell [Ii]nvasion  13  PubMed(1)  
 
 
 hide/show details for RHOG 
  [Ff]ilopodia  PubMed  
 RhoG GTPase controls a pathway that independently activates Rac1 and Cdc42Hs. RhoG is a member of the Rho family of GTPases that shares 72% and 62% sequence identity with Rac1 and Cdc42Hs, respectively. We have expressed mutant RhoG proteins fused to the green fluorescent protein and analyzed subsequent changes in cell surface morphology and modifications of cytoskeletal structures. In rat and mouse fibroblasts, green fluorescent protein chimera and endogenous RhoG proteins colocalize according to a tubular cytoplasmic pattern, with perinuclear accumulation and local concentration at the plasma membrane. Constitutively active RhoG proteins produce morphological and cytoskeletal changes similar to those elicited by a simultaneous activation of Rac1 and Cdc42Hs, i.e., the formation of ruffles, lamellipodia,   filopodia  , and partial loss of stress fibers. In addition, RhoG and Cdc42Hs promote the formation of microvilli at the cell apical membrane. RhoG-dependent events are not mediated through a direct interaction with Rac1 and Cdc42Hs targets such as PAK-1, POR1, or WASP proteins but require endogenous Rac1 and Cdc42Hs activities: coexpression of a dominant negative Rac1 impairs membrane ruffling and lamellipodia but not   filopodia   or microvilli formation. Conversely, coexpression of a dominant negative Cdc42Hs only blocks microvilli and   filopodia  , but not membrane ruffling and lamellipodia. Microtubule depolymerization upon nocodazole treatment leads to a loss of RhoG protein from the cell periphery associated with a reversal of the RhoG phenotype, whereas PDGF or bradykinin stimulation of nocodazole-treated cells could still promote Rac1- and Cdc42Hs-dependent cytoskeletal reorganization. Therefore, our data demonstrate that RhoG controls a pathway that requires the microtubule network and activates Rac1 and Cdc42Hs independently of their growth factor signaling pathways. 
  [Mm]igration  PubMed  
 Analysis of the myosin-II-responsive focal adhesion proteome reveals a role for β-Pix in negative regulation of focal adhesion maturation. Focal adhesions undergo myosin-II-mediated maturation wherein they grow and change composition to modulate integrin signalling for cell   migratio  n, growth and differentiation. To determine how focal adhesion composition is affected by myosin II activity, we performed proteomic analysis of isolated focal adhesions and compared protein abundance in focal adhesions from cells with and without myosin II inhibition. We identified 905 focal adhesion proteins, 459 of which changed in abundance with myosin II inhibition, defining the myosin-II-responsive focal adhesion proteome. The abundance of 73% of the proteins in the myosin-II-responsive focal adhesion proteome was enhanced by contractility, including proteins involved in Rho-mediated focal adhesion maturation and endocytosis- and calpain-dependent focal adhesion disassembly. During myosin II inhibition, 27% of proteins in the myosin-II-responsive focal adhesion proteome, including proteins involved in Rac-mediated lamellipodial protrusion, were enriched in focal adhesions, establishing that focal adhesion protein recruitment is also negatively regulated by contractility. We focused on the Rac guanine nucleotide exchange factor β-Pix, documenting its role in the negative regulation of focal adhesion maturation and the promotion of lamellipodial protrusion and focal adhesion turnover to drive cel  l migrati  on. 
 RhoG activates Rac1 by direct interaction with the Dock180-binding protein Elmo. The small GTPase Rac has a central role in regulating the actin cytoskeleton during cell   migration   and axon guidance. Elmo has been identified as an upstream regulator of Rac1 that binds to and functionally cooperates with Dock180 (refs 2-4). Dock180 does not contain a conventional catalytic domain for guanine nucleotide exchange on Rac, but possesses a domain that directly binds to and specifically activates Rac1 (refs 5, 6). The small GTPase RhoG mediates several cellular morphological processes, such as neurite outgrowth in neuronal cells, through a signalling cascade that activates Rac1 (refs 7-12); however, the downstream target of RhoG and the mechanism by which RhoG regulates Rac1 activity remain unclear. Here we show that RhoG interacts directly with Elmo in a GTP-dependent manner and forms a ternary complex with Dock180 to induce activation of Rac1. The RhoG-Elmo-Dock180 pathway is required for activation of Rac1 and cell spreading mediated by integrin, as well as for neurite outgrowth induced by nerve growth factor. We conclude that RhoG activates Rac1 through Elmo and Dock180 to control cell morphology. 
 Direct interaction of Rnd1 with Plexin-B1 regulates PDZ-RhoGEF-mediated Rho activation by Plexin-B1 and induces cell contraction in COS-7 cells. Plexins are receptors for the axon guidance molecule semaphorins, and several lines of evidence suggest that Rho family small GTPases are implicated in the downstream signaling of Plexins. Recent studies have demonstrated that Plexin-B1 activates RhoA and induces growth cone collapse through Rho-specific guanine nucleotide exchange factor PDZ-RhoGEF. Here we show that Rnd1, a member of Rho family GTPases, directly interacted with the cytoplasmic domain of Plexin-B1. In COS-7 cells, coexpression of Rnd1 and Plexin-B1 induced cell contraction in response to semaphorin 4D (Sema4D), a ligand for Plexin-B1, whereas expression of Plexin-B1 alone or coexpression of Rnd1 and a Rnd1 interaction-defective mutant of Plexin-B1 did not. The Sema4D-induced contraction in Plexin-B1/Rnd1-expressing COS-7 cells was suppressed by dominant negative RhoA, a Rho-associated kinase inhibitor, a dominant negative form of PDZ-RhoGEF, or deletion of the carboxyl-terminal PDZ-RhoGEF-binding region of Plexin-B1, indicating that the PDZ-RhoGEF/RhoA/Rho-associated kinase pathway is involved in this morphological effect. We also found that Rnd1 promoted the interaction between Plexin-B1 and PDZ-RhoGEF and thereby dramatically potentiated the Plexin-B1-mediated RhoA activation. We propose that Rnd1 plays an important role in the regulation of Plexin-B1 signaling, leading to Rho activation during axon guidance and cell   migration  . 
 Dock4 is regulated by RhoG and promotes Rac-dependent cell   migration  . Cell   migration   is essential for normal development and many pathological processes including tumor metastasis. Rho family GTPases play important roles in this event. In particular, Rac is required for lamellipodia formation at the leading edge during   migration  . Dock4 is a member of the Dock180 family proteins, and Dock4 mutations are present in a subset of human cancer cell lines. However, the function and the regulatory mechanism of Dock4 remain unclear. Here we show that Dock4 is regulated by the small GTPase RhoG and its effector ELMO and promotes cell   migration   by activating Rac1. Dock4 formed a complex with ELMO, and expression of active RhoG induced translocation of the Dock4-ELMO complex from the cytoplasm to the plasma membrane and enhanced the Dock4- and ELMO-dependent Rac1 activation and cell   migration  . On the other hand, RNA interference-mediated knockdown of Dock4 in NIH3T3 cells reduced cell   migration  . Taken together, these results suggest that Dock4 plays an important role in the regulation of cell   migration   through activation of Rac1, and that RhoG is a key upstream regulator for Dock4. 
 Ephexin4 and EphA2 mediate cell   migration   through a RhoG-dependent mechanism. EphA2, a member of the Eph receptor family, is frequently overexpressed in a variety of human cancers, including breast cancers, and promotes cancer cell motility and invasion independently of its ligand ephrin stimulation. In this study, we identify Ephexin4 as a guanine nucleotide exchange factor (GEF) for RhoG that interacts with EphA2 in breast cancer cells, and knockdown and rescue experiments show that Ephexin4 acts downstream of EphA2 to promote ligand-independent breast cancer cell   migration   and invasion toward epidermal growth factor through activation of RhoG. The activation of RhoG recruits its effector ELMO2 and a Rac GEF Dock4 to form a complex with EphA2 at the tips of cortactin-rich protrusions in migrating breast cancer cells. In addition, the Dock4-mediated Rac activation is required for breast cancer cell   migration  . Our findings reveal a novel link between EphA2 and Rac activation that contributes to the cell motility and invasiveness of breast cancer cells. 
 Activation of Rac1 by RhoG regulates cell   migration  . Cell   migration   is essential for normal development and many pathological processes. Rho-family small GTPases play important roles in this event. In particular, Rac regulates lamellipodia formation at the leading edge during   migration  . The small GTPase RhoG activates Rac through its effector ELMO and the ELMO-binding protein Dock180, which functions as a Rac-specific guanine nucleotide exchange factor. Here we investigated the role of RhoG in cell   migration  . RNA interference-mediated knockdown of RhoG in HeLa cells reduced cell   migration   in Transwell and scratch-wound   migration   assays. In RhoG-knockdown cells, activation of Rac1 and formation of lamellipodia at the leading edge in response to wounding were attenuated. By contrast, expression of active RhoG promoted cell   migration   through ELMO and Dock180. However, the interaction of Dock180 with Crk was dispensable for the activation of Rac1 and promotion of cell   migration   by RhoG. Taken together, these results suggest that RhoG contributes to the regulation of Rac activity in migrating cells. 
 p190 Rho-GTPase activating protein associates with plexins and it is required for semaphorin signalling. Plexins are transmembrane receptors for semaphorins, guiding cell   migration   and axon extension. Plexin activation leads to the disassembly of integrin-based focal adhesive structures and to actin cytoskeleton remodelling and inhibition of cell   migration  ; however, the underlying molecular mechanisms are unclear. We consistently observe a transient decrease of cellular RhoA-GTP levels upon plexin activation in adherent cells. One of the main effectors of RhoA downregulation is p190, a ubiquitously expressed GTPase activating protein (GAP). We show that, in p190-deficient fibroblasts, the typical functional activities mediated by plexins (such as cell collapse and inhibition of integrin-based adhesion) are blocked or greatly impaired. Notably, the functional response can be rescued in these cells by re-expressing exogenous p190, but not a mutant form specifically lacking RhoGAP activity. We furthermore demonstrate that semaphorin function is blocked in epithelial cells, primary endothelial cells and neuroblasts upon treatment with small interfering RNAs that knockdown p190 expression. Finally, we show that p190 transiently associates with plexins, and its RhoGAP activity is increased in response to semaphorin stimulation. We conclude that p190-RhoGAP is crucially involved in semaphorin signalling to the actin cytoskeleton, via interaction with plexins. 
 Ephexin4 and EphA2 mediate cell   migration   through a RhoG-dependent mechanism. EphA2, a member of the Eph receptor family, is frequently overexpressed in a variety of human cancers, including breast cancers, and promotes cancer cell motility and invasion independently of its ligand ephrin stimulation. In this study, we identify Ephexin4 as a guanine nucleotide exchange factor (GEF) for RhoG that interacts with EphA2 in breast cancer cells, and knockdown and rescue experiments show that Ephexin4 acts downstream of EphA2 to promote ligand-independent breast cancer cell   migration   and invasion toward epidermal growth factor through activation of RhoG. The activation of RhoG recruits its effector ELMO2 and a Rac GEF Dock4 to form a complex with EphA2 at the tips of cortactin-rich protrusions in migrating breast cancer cells. In addition, the Dock4-mediated Rac activation is required for breast cancer cell   migration  . Our findings reveal a novel link between EphA2 and Rac activation that contributes to the cell motility and invasiveness of breast cancer cells. 
 Dock4 is regulated by RhoG and promotes Rac-dependent cell   migration  . Cell   migration   is essential for normal development and many pathological processes including tumor metastasis. Rho family GTPases play important roles in this event. In particular, Rac is required for lamellipodia formation at the leading edge during   migration  . Dock4 is a member of the Dock180 family proteins, and Dock4 mutations are present in a subset of human cancer cell lines. However, the function and the regulatory mechanism of Dock4 remain unclear. Here we show that Dock4 is regulated by the small GTPase RhoG and its effector ELMO and promotes cell   migration   by activating Rac1. Dock4 formed a complex with ELMO, and expression of active RhoG induced translocation of the Dock4-ELMO complex from the cytoplasm to the plasma membrane and enhanced the Dock4- and ELMO-dependent Rac1 activation and cell   migration  . On the other hand, RNA interference-mediated knockdown of Dock4 in NIH3T3 cells reduced cell   migration  . Taken together, these results suggest that Dock4 plays an important role in the regulation of cell   migration   through activation of Rac1, and that RhoG is a key upstream regulator for Dock4. 
 Activation of Rac1 by RhoG regulates cell   migration  . Cell   migration   is essential for normal development and many pathological processes. Rho-family small GTPases play important roles in this event. In particular, Rac regulates lamellipodia formation at the leading edge during   migration  . The small GTPase RhoG activates Rac through its effector ELMO and the ELMO-binding protein Dock180, which functions as a Rac-specific guanine nucleotide exchange factor. Here we investigated the role of RhoG in cell   migration  . RNA interference-mediated knockdown of RhoG in HeLa cells reduced cell   migration   in Transwell and scratch-wound   migration   assays. In RhoG-knockdown cells, activation of Rac1 and formation of lamellipodia at the leading edge in response to wounding were attenuated. By contrast, expression of active RhoG promoted cell   migration   through ELMO and Dock180. However, the interaction of Dock180 with Crk was dispensable for the activation of Rac1 and promotion of cell   migration   by RhoG. Taken together, these results suggest that RhoG contributes to the regulation of Rac activity in migrating cells. 
 RhoG regulates anoikis through a phosphatidylinositol 3-kinase-dependent mechanism. In normal epithelial cells, cell-matrix interaction is required for cell survival and proliferation, whereas disruption of this interaction causes epithelial cells to undergo apoptosis called anoikis. Here we show that the small GTPase RhoG plays an important role in the regulation of anoikis. HeLa cells are capable of anchorage-independent cell growth and acquire resistance to anoikis. We found that RNA interference-mediated knockdown of RhoG promoted anoikis in HeLa cells. Previous studies have shown that RhoG activates Rac1 and induces several cellular functions including promotion of cell   migration   through its effector ELMO and the ELMO-binding protein Dock180 that function as a Rac-specific guanine nucleotide exchange factor. However, RhoG-induced suppression of anoikis was independent of the ELMO- and Dock180-mediated activation of Rac1. On the other hand, the regulation of anoikis by RhoG required phosphatidylinositol 3-kinase (PI3K) activity, and constitutively active RhoG bound to the PI3K regulatory subunit p85alpha and induced the PI3K-dependent phosphorylation of Akt. Taken together, these results suggest that RhoG protects cells from apoptosis caused by the loss of anchorage through a PI3K-dependent mechanism, independent of its activation of Rac1. 
 RhoG regulates endothelial apical cup assembly downstream from ICAM1 engagement and is involved in leukocyte trans-endothelial   migration  . During trans-endothelial   migration   (TEM), leukocytes use adhesion receptors such as intercellular adhesion molecule-1 (ICAM1) to adhere to the endothelium. In response to this interaction, the endothelium throws up dynamic membrane protrusions, forming a cup that partially surrounds the adherent leukocyte. Little is known about the signaling pathways that regulate cup formation. In this study, we show that RhoG is activated downstream from ICAM1 engagement. This activation requires the intracellular domain of ICAM1. ICAM1 colocalizes with RhoG and binds to the RhoG-specific SH3-containing guanine-nucleotide exchange factor (SGEF). The SH3 domain of SGEF mediates this interaction. Depletion of endothelial RhoG by small interfering RNA does not affect leukocyte adhesion but decreases cup formation and inhibits leukocyte TEM. Silencing SGEF also results in a substantial reduction in RhoG activity, cup formation, and TEM. Together, these results identify a new signaling pathway involving RhoG and its exchange factor SGEF downstream from ICAM1 that is critical for leukocyte TEM. 
  GeneRIFs  
 These results identify a new signaling pathway involving RhoG and its exchange factor SGEF downstream from ICAM1 that is critical for leukocyte trans-endothelial   migration  . 
 Taken together, these results suggest that Dock4 plays an important role in the regulation of cell   migration   through activation of Rac1, and that RhoG is a key upstream regulator for Dock4. 
  Summary  
 This gene encodes a member of the Rho family of small GTPases, which cycle between inactive GDP-bound and active GTP-bound states and function as molecular switches in signal transduction cascades. Rho proteins promote reorganization of the actin cytoskeleton and regulate cell shape, attachment, and motility. The encoded protein facilitates translocation of a functional guanine nucleotide exchange factor (GEF) complex from the cytoplasm to the plasma membrane where ras-related C3 botulinum toxin substrate 1 is activated to promote lamellipodium formation and cell   migration  . Two related pseudogene have been identified on chromosomes 20 and X. [provided by RefSeq, Aug 2011] 
  [Ii]nvasive  PubMed  
 Ephexin4 and EphA2 mediate cell migration through a RhoG-dependent mechanism. EphA2, a member of the Eph receptor family, is frequently overexpressed in a variety of human cancers, including breast cancers, and promotes cancer cell motility and invasion independently of its ligand ephrin stimulation. In this study, we identify Ephexin4 as a guanine nucleotide exchange factor (GEF) for RhoG that interacts with EphA2 in breast cancer cells, and knockdown and rescue experiments show that Ephexin4 acts downstream of EphA2 to promote ligand-independent breast cancer cell migration and invasion toward epidermal growth factor through activation of RhoG. The activation of RhoG recruits its effector ELMO2 and a Rac GEF Dock4 to form a complex with EphA2 at the tips of cortactin-rich protrusions in migrating breast cancer cells. In addition, the Dock4-mediated Rac activation is required for breast cancer cell migration. Our findings reveal a novel link between EphA2 and Rac activation that contributes to the cell motility and   invasive  ness of breast cancer cells. 
 Ephexin4 and EphA2 mediate cell migration through a RhoG-dependent mechanism. EphA2, a member of the Eph receptor family, is frequently overexpressed in a variety of human cancers, including breast cancers, and promotes cancer cell motility and invasion independently of its ligand ephrin stimulation. In this study, we identify Ephexin4 as a guanine nucleotide exchange factor (GEF) for RhoG that interacts with EphA2 in breast cancer cells, and knockdown and rescue experiments show that Ephexin4 acts downstream of EphA2 to promote ligand-independent breast cancer cell migration and invasion toward epidermal growth factor through activation of RhoG. The activation of RhoG recruits its effector ELMO2 and a Rac GEF Dock4 to form a complex with EphA2 at the tips of cortactin-rich protrusions in migrating breast cancer cells. In addition, the Dock4-mediated Rac activation is required for breast cancer cell migration. Our findings reveal a novel link between EphA2 and Rac activation that contributes to the cell motility and   invasive  ness of breast cancer cells. 
 The   invasive   capacity of HPV transformed cells requires the hDlg-dependent enhancement of SGEF/RhoG activity. A major target of the HPV E6 oncoprotein is the human Discs Large (hDlg) tumour suppressor, although how this interaction contributes to HPV-induced malignancy is still unclear. Using a proteomic approach we show that a strong interacting partner of hDlg is the RhoG-specific guanine nucleotide exchange factor SGEF. The interaction between hDlg1 and SGEF involves both PDZ and SH3 domain recognition, and directly contributes to the regulation of SGEF's cellular localization and activity. Consistent with this, hDlg is a strong enhancer of RhoG activity, which occurs in an SGEF-dependent manner. We also show that HPV-18 E6 can interact indirectly with SGEF in a manner that is dependent upon the presence of hDlg and PDZ binding capacity. In HPV transformed cells, E6 maintains a high level of RhoG activity, and this is dependent upon the presence of hDlg and SGEF, which are found in complex with E6. Furthermore, we show that E6, hDlg and SGEF each directly contributes to the   invasive   capacity of HPV-16 and HPV-18 transformed tumour cells. These studies demonstrate that hDlg has a distinct oncogenic function in the context of HPV induced malignancy, one of the outcomes of which is increased RhoG activity and increased   invasive   capacity. 
  GeneRIFs  
 The   invasive   capacity of HPV transformed cells requires the hDlg-dependent enhancement of SGEF/RhoG activity. 
  [Mm]etastasis  PubMed  
 Dock4 is regulated by RhoG and promotes Rac-dependent cell migration. Cell migration is essential for normal development and many pathological processes including tumor   metastasis  . Rho family GTPases play important roles in this event. In particular, Rac is required for lamellipodia formation at the leading edge during migration. Dock4 is a member of the Dock180 family proteins, and Dock4 mutations are present in a subset of human cancer cell lines. However, the function and the regulatory mechanism of Dock4 remain unclear. Here we show that Dock4 is regulated by the small GTPase RhoG and its effector ELMO and promotes cell migration by activating Rac1. Dock4 formed a complex with ELMO, and expression of active RhoG induced translocation of the Dock4-ELMO complex from the cytoplasm to the plasma membrane and enhanced the Dock4- and ELMO-dependent Rac1 activation and cell migration. On the other hand, RNA interference-mediated knockdown of Dock4 in NIH3T3 cells reduced cell migration. Taken together, these results suggest that Dock4 plays an important role in the regulation of cell migration through activation of Rac1, and that RhoG is a key upstream regulator for Dock4. 
 Dock4 is regulated by RhoG and promotes Rac-dependent cell migration. Cell migration is essential for normal development and many pathological processes including tumor   metastasis  . Rho family GTPases play important roles in this event. In particular, Rac is required for lamellipodia formation at the leading edge during migration. Dock4 is a member of the Dock180 family proteins, and Dock4 mutations are present in a subset of human cancer cell lines. However, the function and the regulatory mechanism of Dock4 remain unclear. Here we show that Dock4 is regulated by the small GTPase RhoG and its effector ELMO and promotes cell migration by activating Rac1. Dock4 formed a complex with ELMO, and expression of active RhoG induced translocation of the Dock4-ELMO complex from the cytoplasm to the plasma membrane and enhanced the Dock4- and ELMO-dependent Rac1 activation and cell migration. On the other hand, RNA interference-mediated knockdown of Dock4 in NIH3T3 cells reduced cell migration. Taken together, these results suggest that Dock4 plays an important role in the regulation of cell migration through activation of Rac1, and that RhoG is a key upstream regulator for Dock4. 
  [Cc]ell [Ii]nvasion  PubMed  
 Yersinia enterocolitica differentially modulates RhoG activity in host cells. Pathogenic bacteria of the genus Yersinia (Y. pestis, Y. enterocolitica and Y. pseudotuberculosis) have evolved numerous virulence factors (termed a stratagem) to manipulate the activity of Rho GTPases. Here, we show that Y. enterocolitica modulates RhoG, an upstream regulator of other Rho GTPases. At the contact site of virulent Y. enterocolitica and host cells, we could visualise spatiotemporally organised activation and deactivation of RhoG. On the one hand, the beta1-integrin clustering protein Invasin on the bacterial surface was found to activate RhoG and this promoted   cell invasion  . On the other hand, active RhoG was downregulated by the type III secretion system effector YopE acting as a GTPase-activating protein (GAP). YopE localised to Golgi and endoplasmic reticulum, and this determined its specificity for RhoG and other selected Rho GTPases. RhoG and its downstream effector module Elmo/Dock180 controlled both Rac1 activation by Invasin and Rac1 deactivation by YopE. We propose that RhoG is a central target of the Yersinia stratagem and a major upstream regulator of Rac1 during different phases of the Yersinia infection cycle. 
   
  
 RHOQ 
 
  NCBI Gene  
  GeneRIF  
  Pubmed  
 
 
  Search Term  Rank  Fields  
  [Ff]ilopodia  1  PubMed(2)  
  [Ff]ilopodium  2  Process(1)  
 
 
 hide/show details for RHOQ 
  [Ff]ilopodia  PubMed  
 Small GTPase Tc10 and its homologue RhoT induce N-WASP-mediated long process formation and neurite outgrowth. Rho family small GTPases regulate multiple cellular functions through reorganization of the actin cytoskeleton. Among them, Cdc42 and Tc10 induce   filopodia   or peripheral processes in cultured cells. We have identified a member of the family, designated as RhoT, which is closely related to Tc10. Tc10 was highly expressed in muscular tissues and brain and remarkably induced during differentiation of C2 skeletal muscle cells and neuronal differentiation of PC12 and N1E-115 cells. On the other hand, RhoT was predominantly expressed in heart and uterus and induced during neuronal differentiation of N1E-115 cells. Tc10 exogenously expressed in fibroblasts generated actin-filament-containing peripheral processes longer than the Cdc42-formed   filopodia  , whereas RhoT produced much longer and thicker processes containing actin filaments. Furthermore, both Tc10 and RhoT induced neurite outgrowth in PC12 and N1E-115 cells, but Cdc42 did not do this by itself. Tc10 and RhoT as well as Cdc42 bound to the N-terminal CRIB-motif-containing portion of N-WASP and activated N-WASP to induce Arp2/3-complex-mediated actin polymerization. The formation of peripheral processes and neurites by Tc10 and RhoT was prevented by the coexpression of dominant-negative mutants of N-WASP. Thus, N-WASP is essential for the process formation and neurite outgrowth induced by Tc10 and RhoT. Neuronal differentiation of PC12 and N1E-115 cells induced by dibutyryl cyclic AMP and by serum starvation, respectively, was prevented by dominant-negative Cdc42, Tc10 and RhoT. Taken together, all these Rho family proteins are required for neuronal differentiation, but they exert their functions differentially in process formation and neurite extension. Consequently, N-WASP activated by these small GTPases mediates neuronal differentiation in addition to its recently identified role in glucose uptake. 
 Cellular functions of TC10, a Rho family GTPase: regulation of morphology, signal transduction and cell growth. The small Ras-related GTPase, TC10, has been classified on the basis of sequence homology to be a member of the Rho family. This family, which includes the Rho, Rac and CDC42 subfamilies, has been shown to regulate a variety of apparently diverse cellular processes such as actin cytoskeletal organization, mitogen-activated protein kinase (MAPK) cascades, cell cycle progression and transformation. In order to begin a study of TC10 biological function, we expressed wild type and various mutant forms of this protein in mammalian cells and investigated both the intracellular localization of the expressed proteins and their abilities to stimulate known Rho family-associated processes. Wild type TC10 was located predominantly in the cell membrane (apparently in the same regions as actin filaments), GTPase defective (75L) and GTP-binding defective (31N) mutants were located predominantly in cytoplasmic perinuclear regions, and a deletion mutant lacking the carboxyl terminal residues required for post-translational prenylation was located predominantly in the nucleus. The GTPase defective (constitutively active) TC10 mutant: (1) stimulated the formation of long   filopodia  ; (2) activated c-Jun amino terminal kinase (JNK); (3) activated serum response factor (SRF)-dependent transcription; (4) activated NF-kappaB-dependent transcription; and (5) synergized with an activated Raf-kinase (Raf-CAAX) to transform NIH3T3 cells. In addition, wild type TC10 function is required for full H-Ras transforming potential. We demonstrate that an intact effector domain and carboxyl terminal prenylation signal are required for proper TC10 function and that TC10 signals to at least two separable downstream target pathways. In addition, TC10 interacted with the actin-binding and filament-forming protein, profilin, in both a two-hybrid cDNA library screen, and an in vitro binding assay. Taken together, these data support a classification of TC10 as a member of the Rho family, and in particular, suggest that TC10 functions to regulate cellular signaling to the actin cytoskeleton and processes associated with cell growth. 
  [Ff]ilopodium  Process  
 positive regulation of   filopodium   assembly 
   
  
 RHOA 
 
  NCBI Gene  
  GeneRIF  
  Pubmed  
 
 
  Search Term  Rank  Fields  
  [Ff]ilopodia  1  PubMed(10); GeneRIFs(1)  
  [Ii]nvadopodia  3  PubMed(4); GeneRIFs(1)  
  [Ii]nvadopodium  4  PubMed(1)  
  [Pp]odosome(s)?  5  PubMed(1); GeneRIFs(1)  
  [Mm]igration  6  Process(7); PubMed(227); GeneRIFs(57)  
  ECM  7  PubMed(12)  
  [Ee]xtracellular [Mm]atrix  8  PubMed(34)  
  [Ii]nvasive  10  PubMed(74); GeneRIFs(9)  
  [Mm]etastasize  11  PubMed(1)  
  [Mm]etastasis  12  PubMed(96); GeneRIFs(21); Summary(1)  
  [Cc]ell [Ii]nvasion  13  PubMed(50); GeneRIFs(12)  
 
 
 hide/show details for RHOA 
  [Ff]ilopodia  PubMed  
 DEF6, a novel PH-DH-like domain protein, is an upstream activator of the Rho GTPases Rac1, Cdc42, and RhoA. In this paper, we describe the characterization of DEF6, a novel PH-DH-like protein related to SWAP-70 that functions as an upstream activator of Rho GTPases. In NIH 3T3 cells, stimulation of the PI 3-kinase signaling pathway with either H2O2 or platelet-derived growth factor (PDGF) resulted in the translocation of an overexpressed DEF6-GFP fusion protein to the cell membrane and induced the formation of   filopodia   and lamellipodia. In contrast to full-length DEF6, expression of the DH-like (DHL) domain as a GFP fusion protein potently induced actin polymerization, including stress fiber formation in COS-7 cells, in the absence of PI 3-kinase signaling, indicating that it was constitutively active. The GTP-loading of Cdc42 was strongly enhanced in NIH 3T3 cells expressing the DH domain while   filopodia   formation, membrane ruffling, and stress fiber formation could be inhibited by the co-expression of the DH domain with dominant negative mutants of either N17Rac1, N17Cdc42, or N19RhoA, respectively. This indicated that DEF6 acts upstream of the Rho GTPases resulting in the activation of the Cdc42, Rac1, and RhoA signaling pathways. In vitro, DEF6 specifically interacted with Rac1, Rac2, Cdc42, and RhoA, suggesting a direct role for DEF6 in the activation of Rho GTPases. The ability of DEF6 to both stimulate actin polymerization and bind to filamentous actin suggests a role for DEF6 in regulating cell shape, polarity, and movement. 
 A MAPK-Driven Feedback Loop Suppresses Rac Activity to Promote RhoA-Driven Cancer Cell Invasion. Cell migration in 3D microenvironments is fundamental to development, homeostasis and the pathobiology of diseases such as cancer. Rab-coupling protein (RCP) dependent co-trafficking of α5β1 and EGFR1 promotes cancer cell invasion into fibronectin (FN) containing extracellular matrix (ECM), by potentiating EGFR1 signalling at the front of invasive cells. This promotes a switch in RhoGTPase signalling to inhibit Rac1 and activate a RhoA-ROCK-Formin homology domain-containing 3 (FHOD3) pathway and generat  e filopod  ial actin-spike protrusions which drive invasion. To further understand the signalling network that drives RCP-driven invasive migration, we generated a Boolean logical model based on existing network pathways/models, where each node can be interrogated by computational simulation. The model predicted an unanticipated feedback loop, whereby Raf/MEK/ERK signalling maintains suppression of Rac1 by inhibiting the Rac-activating Sos1-Eps8-Abi1 complex, allowing RhoA activity to predominate in invasive protrusions. MEK inhibition was sufficient to promote lamellipodia formation and oppos  e filopod  ial actin-spike formation, and led to activation of Rac and inactivation of RhoA at the leading edge of cells moving in 3D matrix. Furthermore, MEK inhibition abrogated RCP/α5β1/EGFR1-driven invasive migration. However, upon knockdown of Eps8 (to suppress the Sos1-Abi1-Eps8 complex), MEK inhibition had no effect on RhoGTPase activity and did not oppose invasive migration, suggesting that MEK-ERK signalling suppresses the Rac-activating Sos1-Abi1-Eps8 complex to maintain RhoA activity and prom  ote filop  odial actin-spike formation and invasive migration. Our study highlights the predictive potential of mathematical modelling approaches, and demonstrates that a simple intervention (MEK-inhibition) could be of therapeutic benefit in preventing invasive migration and metastasis. 
 The small GTPase RalA targets filamin to induce   filopodia  . The Ras-related small GTPases Rac, Rho, Cdc42, and RalA bind filamin, an actin filament-crosslinking protein that also links membrane and other intracellular proteins to actin. Of these GTPases only RalA binds filamin in a GTP-specific manner, and GTP-RalA elicits actin-rich filopods on surfaces of Swiss 3T3 cells and recruits filamin into the   filopodia  l cytoskeleton. Either a dominant negative RalA construct or the RalA-binding domain of filamin 1 specifically block Cdc42-induced filopod formation, but a Cdc42 inhibitor does not impair RalA's effects, which, unlike Cdc42, are Rac independent. RalA does not generate   filopodia   in filamin-deficient human melanoma cells, whereas transfection of filamin 1 restores the functional response. RalA therefore is a downstream intermediate in Cdc42-mediated filopod production and uses filamin in this pathway. 
 A MAPK-Driven Feedback Loop Suppresses Rac Activity to Promote RhoA-Driven Cancer Cell Invasion. Cell migration in 3D microenvironments is fundamental to development, homeostasis and the pathobiology of diseases such as cancer. Rab-coupling protein (RCP) dependent co-trafficking of α5β1 and EGFR1 promotes cancer cell invasion into fibronectin (FN) containing extracellular matrix (ECM), by potentiating EGFR1 signalling at the front of invasive cells. This promotes a switch in RhoGTPase signalling to inhibit Rac1 and activate a RhoA-ROCK-Formin homology domain-containing 3 (FHOD3) pathway and generat  e filopod  ial actin-spike protrusions which drive invasion. To further understand the signalling network that drives RCP-driven invasive migration, we generated a Boolean logical model based on existing network pathways/models, where each node can be interrogated by computational simulation. The model predicted an unanticipated feedback loop, whereby Raf/MEK/ERK signalling maintains suppression of Rac1 by inhibiting the Rac-activating Sos1-Eps8-Abi1 complex, allowing RhoA activity to predominate in invasive protrusions. MEK inhibition was sufficient to promote lamellipodia formation and oppos  e filopod  ial actin-spike formation, and led to activation of Rac and inactivation of RhoA at the leading edge of cells moving in 3D matrix. Furthermore, MEK inhibition abrogated RCP/α5β1/EGFR1-driven invasive migration. However, upon knockdown of Eps8 (to suppress the Sos1-Abi1-Eps8 complex), MEK inhibition had no effect on RhoGTPase activity and did not oppose invasive migration, suggesting that MEK-ERK signalling suppresses the Rac-activating Sos1-Abi1-Eps8 complex to maintain RhoA activity and prom  ote filop  odial actin-spike formation and invasive migration. Our study highlights the predictive potential of mathematical modelling approaches, and demonstrates that a simple intervention (MEK-inhibition) could be of therapeutic benefit in preventing invasive migration and metastasis. 
 Wnt5b promotes the cell motility essential for metastasis of oral squamous cell carcinoma through active Cdc42 and RhoA. The activation of Wnt signaling has been reported in many types of squamous cell carcinoma. In this study, using human oral squamous cell carcinoma (OSCC) cells with different metastatic potential, we investigated the involvement of Wnt signaling in metastasis. Further, we aimed to elucidate the characteristic biological features related to high metastatic potential and to identify new target molecules for the suppression of OSCC lymph node metastasis. We compared SAS-Venus (SAS OSCC cells expressing green fluorescent protein) and SAS-LM8, which is a highly metastatic cell line derived from SAS-Venus by in vivo selection. The SAS-LM8 cell line had greater ability of migration and invasion compared to SAS-Venus. Furthermore, a higher number of   filopodia  -like protrusive structures were produced in SAS-LM8 cells compared to SAS-Venus cells, and the levels of active Cdc42 and active RhoA protein were higher in SAS-LM8 cells compared to SAS-Venus cells. We did not observe any differences in the expression of Wnt/β-catenin target genes between the two cell lines; however, the mRNA levels of Wnt5b were higher in SAS-LM8 cells compared to SAS-Venus cells. To confirm the involvement of Wnt5b in migration in OSCC cells, we examined the effects of the siRNA-mediated knockdown of Wnt5b in SAS-Venus cells and SAS-LM8 cells. The siRNA treatment significantly inhibited migration and the formation of   filopodi  a-like protrusive structures. Conversely, when stimulated with Wnt5b, the migration and formation of   filopodi  a-like protrusions were significantly enhanced and the levels of active Cdc42 and active RhoA proteins were also increased. These results indicate that Wnt5b is involved in the migration ability of OSCC cells through active Cdc42 and RhoA. 
 p75 neurotrophin receptor signaling regulates growth cone   filopodia  l dynamics through modulating RhoA activity. The mechanisms by which neurotrophins regulate growth cone motility are unclear. We investigated the role of the p75 neurotrophin receptor (p75NTR) in mediating neurotrophin-induced increases in   filopodia  l length. Our data demonstrate that neurotrophin binding to p75NTR is necessary and sufficient to regulate   filopodia  l dynamics. Furthermore, retinal and dorsal root ganglion growth cones from p75 mutant mice are insensitive to neurotrophins but display enhanced   filopodia  l lengths comparable with neurotrophin-treated wild-type growth cones. This suggests unoccupied p75NTR negatively regulates   filopodia   length. Furthermore, p75NTR regulates RhoA activity to mediate   filopodia  l dynamics. Constitutively active RhoA blocks neurotrophin-induced increases in   filopodia  l length, whereas inhibition of RhoA enhances   filopodia  l lengths, similar to neurotrophin treatment. BDNF treatment of retinal neurons results in reduced RhoA activity. Furthermore, p75 mutant neurons display reduced levels of activated RhoA compared with wild-type counterparts, consistent with the enhanced   filopodia  l lengths observed on mutant growth cones. These observations suggest that neurotrophins regulate   filopodia  l dynamics by depressing the activation of RhoA that occurs through p75NTR signaling. 
 Protease-activated receptor mediated RhoA signaling and cytoskeletal reorganization in LNCaP cells. Thrombin and trypsin induce cell signaling through a subclass of G-protein-coupled receptors called the protease-activated receptors (PARs). In many cells, PAR signaling results in the activation of RhoA and other members of the Rho family of small GTPases which are involved in cytoskeletal reorganization. The expression of PARs and their role in the activation of Rho GTPases in prostate cancer cells are not clearly known. FACS analysis demonstrated that the androgen-dependent LNCaP cells express PAR1, PAR2, and PAR4 but not PAR3. Stimulation with thrombin and trypsin resulted in the rapid activation of RhoA in a dose-dependent manner with an EC(50) of 1.0 and 5 nM, respectively. Activation of RhoA was enhanced by, but not dependent on, the presence of 1 nM dihydrotestosterone. Inhibition of the proteolytic properties of thrombin by hirudin and trypsin by diisopropyl fluorophosphate abolished the observed RhoA activation. Stimulation with 150 microM PAR-activating peptides TFFLRN (PAR1), SLIGKV (PAR2), and AYPGKF (PAR4) demonstrated that PAR1 and PAR2 mediated protease-activated RhoA signaling. Fluorescent microscopy studies showed that LNCaP cells treated with either thrombin (10 nM) or trypsin (10 nM) developed an increased number of   filopodia  , stress fibers, and focal adhesions relative to untreated cells. These observations represent the first report of PAR signaling in prostate cancer cells as well as the ability of PAR2 to mediate RhoA activation. Since the activation of RhoA is important for cytoskeletal reorganization, we postulate that PAR-mediated RhoA activation may be a major signaling pathway in the biology of prostate cancer. 
 Snail regulates the motility of oral cancer cells via RhoA/Cdc42/p-ERM pathway. The transcriptional factor Snail has been reported to possess properties related to cancer progression; however, the mechanism for it is not fully understood. Our data showed that Snail knockdown by small interfering RNA in two OSCC cell lines, WSU-HN6 and CAL27, significantly inhibited cell migration and invasion which also resulted in decreased cell motility, such as impaired cell spreading on type I collagen substrate, reduced   filopodia  , and premature assembly of stress fibers. In addition, Snail-silencing decreased Cdc42 activity but increased RhoA activity, accompanied by the downregulation in both p-ERM expression and cell motility. Meanwhile, endogenous p-ERM was found specifically co-precipitated with activated Cdc42, but not RhoA, and this co-association was decreased by Snail-silencing. The small molecule inhibitors of Rho-associated kinase (Y27632) markedly enhanced Cdc42 activity and the association of p-ERM with activated Cdc42, increasing cell motility remarkably. Using immunohistochemistry, Snail and p-ERM overexpressions were found in OSCC tissues correlated with nodal metastasis and shorter survival. Taken together, these results demonstrate that Snail regulates cell motility through RhoA/Cdc42/p-ERM pathway and may serve as a biomarker to predict prognosis for OSCC patients. Although RhoA and Cdc42 are concurrently regulated downstream of Snail, there is a direct interplay between them, which indicates RhoA has to be inactivated at some point in cell motility cycle. 
 Regulation of cell polarity and protrusion formation by targeting RhoA for degradation. The Rho family of small guanosine triphosphatases regulates actin cytoskeleton dynamics that underlie cellular functions such as cell shape changes, migration, and polarity. We found that Smurf1, a HECT domain E3 ubiquitin ligase, regulated cell polarity and protrusive activity and was required to maintain the transformed morphology and motility of a tumor cell. Atypical protein kinase C zeta (PKCzeta), an effector of the Cdc42/Rac1-PAR6 polarity complex, recruited Smurf1 to cellular protrusions, where it controlled the local level of RhoA. Smurf1 thus links the polarity complex to degradation of RhoA in lamellipodia and   filopodia   to prevent RhoA signaling during dynamic membrane movements. 
 Synaptopodin orchestrates actin organization and cell motility via regulation of RhoA signalling. The Rho family of small GTPases (RhoA, Rac1 and Cdc42) controls signal-transduction pathways that influence many aspects of cell behaviour, including cytoskeletal dynamics. At the leading edge, Rac1 and Cdc42 promote cell motility through the formation of lamellipodia and   filopodia  , respectively. On the contrary, RhoA promotes the formation of contractile actin-myosin-containing stress fibres in the cell body and at the rear. Here, we identify synaptopodin, an actin-associated protein, as a novel regulator of RhoA signalling and cell migration in kidney podocytes. We show that synaptopodin induces stress fibres by competitive blocking of Smurf1-mediated ubiquitination of RhoA, thereby preventing the targeting of RhoA for proteasomal degradation. Gene silencing of synaptopodin in kidney podocytes causes the loss of stress fibres and the formation of aberrant non-polarized   filopodia   and impairment of cell migration. Together, these data show that synaptopodin is essential for the integrity of the podocyte actin cytoskeleton and for the regulation of podocyte cell migration. 
  GeneRIFs  
 Smurf1 links the Cdc42/Rac1-PAR6 polarity complex to degradation of RhoA in lamellipodia and   filopodia   to prevent RhoA signaling during dynamic membrane movements 
  [Ii]nvadopodia  PubMed  
 A novel protein associated with membrane-type 1 matrix metalloproteinase binds p27(kip1) and regulates RhoA activation, actin remodeling, and matrigel invasion. Pericellular proteolysis by membrane-type 1 matrix metalloproteinase (MT1-MMP) plays a pivotal role in tumor cell invasion. Localization of MT1-MMP at the invasion front of cells, e.g. on lamellipodia and   invadopodia  , has to be regulated in coordination with reorganization of the actin cytoskeleton. However, little is known about how such invasion-related actin structures are regulated at the sites where MT1-MMP localizes. During analysis of MT1-MMP-associated proteins, we identified a heretofore uncharacterized protein. This protein, which we call p27RF-Rho, enhances activation of RhoA by releasing it from inhibition by p27(kip1) and thereby regulates actin structures. p27(kip1) is a well known cell cycle regulator in the nucleus. In contrast, cytoplasmic p27(kip1) has been demonstrated to bind GDP-RhoA and inhibit GDP-GTP exchange mediated by guanine nucleotide exchange factors. p27RF-Rho binds p27(kip1) and prevents p27(kip1) from binding to RhoA, thereby freeing the latter for activation. Knockdown of p27RF-Rho expression renders cells resistant to RhoA activation stimuli, whereas overexpression of p27RF-Rho sensitizes cells to such stimulation. p27RF-Rho exhibits a punctate distribution in invasive human tumor cell lines. Stimulation of the cells with lysophosphatidic acid induces activation of RhoA and induces the formation of punctate actin structures within foci of p27RF-Rho localization. Some of the punctate actin structures co-localize with MT1-MMP and cortactin. Down-regulation of p27RF-Rho prevents both redistribution of actin into the punctate structures and tumor cell invasion. Thus, p27RF-Rho is a new potential target for cancer therapy development. 
 A novel protein associated with membrane-type 1 matrix metalloproteinase binds p27(kip1) and regulates RhoA activation, actin remodeling, and matrigel invasion. Pericellular proteolysis by membrane-type 1 matrix metalloproteinase (MT1-MMP) plays a pivotal role in tumor cell invasion. Localization of MT1-MMP at the invasion front of cells, e.g. on lamellipodia and   invadopodia  , has to be regulated in coordination with reorganization of the actin cytoskeleton. However, little is known about how such invasion-related actin structures are regulated at the sites where MT1-MMP localizes. During analysis of MT1-MMP-associated proteins, we identified a heretofore uncharacterized protein. This protein, which we call p27RF-Rho, enhances activation of RhoA by releasing it from inhibition by p27(kip1) and thereby regulates actin structures. p27(kip1) is a well known cell cycle regulator in the nucleus. In contrast, cytoplasmic p27(kip1) has been demonstrated to bind GDP-RhoA and inhibit GDP-GTP exchange mediated by guanine nucleotide exchange factors. p27RF-Rho binds p27(kip1) and prevents p27(kip1) from binding to RhoA, thereby freeing the latter for activation. Knockdown of p27RF-Rho expression renders cells resistant to RhoA activation stimuli, whereas overexpression of p27RF-Rho sensitizes cells to such stimulation. p27RF-Rho exhibits a punctate distribution in invasive human tumor cell lines. Stimulation of the cells with lysophosphatidic acid induces activation of RhoA and induces the formation of punctate actin structures within foci of p27RF-Rho localization. Some of the punctate actin structures co-localize with MT1-MMP and cortactin. Down-regulation of p27RF-Rho prevents both redistribution of actin into the punctate structures and tumor cell invasion. Thus, p27RF-Rho is a new potential target for cancer therapy development. 
 The interaction of IQGAP1 with the exocyst complex is required for tumor cell invasion downstream of Cdc42 and RhoA.   Invadopodia   are actin-based membrane protrusions formed at contact sites between invasive tumor cells and the extracellular matrix with matrix proteolytic activity. Actin regulatory proteins participate in   invadopodia   formation, whereas matrix degradation requires metalloproteinases (MMPs) targeted to   invadopodia  . In this study, we show that the vesicle-tethering exocyst complex is required for matrix proteolysis and invasion of breast carcinoma cells. We demonstrate that the exocyst subunits Sec3 and Sec8 interact with the polarity protein IQGAP1 and that this interaction is triggered by active Cdc42 and RhoA, which are essential for matrix degradation. Interaction between IQGAP1 and the exocyst is necessary for   invadopodia   activity because enhancement of matrix degradation induced by the expression of IQGAP1 is lost upon deletion of the exocyst-binding site. We further show that the exocyst and IQGAP1 are required for the accumulation of cell surface membrane type 1 MMP at   invadopodia  . Based on these results, we propose that   invadopodia   function in tumor cells relies on the coordination of cytoskeletal assembly and exocytosis downstream of Rho guanosine triphosphatases. 
 Macrophage contact induces RhoA GTPase signaling to trigger tumor cell intravasation. Most cancer patients die as a result of metastasis, thus it is important to understand the molecular mechanisms of dissemination, including intra- and extravasation. Although the mechanisms of extravasation have been vastly studied in vitro and in vivo, the process of intravasation is still unclear. Furthermore, how cells in the tumor microenvironment facilitate tumor cell intravasation is still unknown. Using high-resolution imaging, we found that macrophages enhance tumor cell intravasation upon physical contact. Macrophage and tumor cell contact induce RhoA activity in tumor cells, triggering the formation of actin-rich degradative protrusions called   invadopodia  , enabling tumor cells to degrade and break through matrix barriers during tumor cell transendothelial migration. Interestingly, we show that macrophage-induced invadopodium formation and tumor cell intravasation also occur in patient-derived tumor cells and in vivo models, revealing a conserved mechanism of tumor cell intravasation. Our results illustrate a novel heterotypic cell contact-mediated signaling role for RhoA, as well as yield mechanistic insight into the ability of cells within the tumor microenvironment to facilitate steps of the metastatic cascade. 
  GeneRIFs  
 Macrophage and tumor cell contact induce RhoA activity in tumor cells, triggering the formation of actin-rich degradative   invadopodia  . 
  [Ii]nvadopodium  PubMed  
 Macrophage contact induces RhoA GTPase signaling to trigger tumor cell intravasation. Most cancer patients die as a result of metastasis, thus it is important to understand the molecular mechanisms of dissemination, including intra- and extravasation. Although the mechanisms of extravasation have been vastly studied in vitro and in vivo, the process of intravasation is still unclear. Furthermore, how cells in the tumor microenvironment facilitate tumor cell intravasation is still unknown. Using high-resolution imaging, we found that macrophages enhance tumor cell intravasation upon physical contact. Macrophage and tumor cell contact induce RhoA activity in tumor cells, triggering the formation of actin-rich degradative protrusions called invadopodia, enabling tumor cells to degrade and break through matrix barriers during tumor cell transendothelial migration. Interestingly, we show that macrophage-induced   invadopodium   formation and tumor cell intravasation also occur in patient-derived tumor cells and in vivo models, revealing a conserved mechanism of tumor cell intravasation. Our results illustrate a novel heterotypic cell contact-mediated signaling role for RhoA, as well as yield mechanistic insight into the ability of cells within the tumor microenvironment to facilitate steps of the metastatic cascade. 
  [Pp]odosome(s)?  PubMed  
 Actin can reorganize into   podosomes   in aortic endothelial cells, a process controlled by Cdc42 and RhoA. Members of the Rho GTPase family play a central role in the orchestration of cytoskeletal rearrangements, which are of prime importance in endothelial cell physiology. To explore their role in this specialized cell type, we used the bacterial toxin cytotoxic necrotizing factor 1 (CNF1) as a Rho GTPase activator. Punctate filamentous actin structures appeared along the ventral plasma membrane of endothelial cells and were identified as the core of   podosomes   by the distinctive vinculin ring around the F-actin. Rho, Rac, and Cdc42 were all identified as targets of CNF1, but only a constitutively active mutant of Cdc42 could substitute for CNF1 in   podosome   induction. Accordingly, organization of F-actin in these structures was highly dependent on the main Cdc42 cytoskeletal effector N-Wiskott-Aldrich syndrome protein. Other components of the actin machinery such as Arp2/3 and for the first time WIP also colocalized at these sites. Like CNF1 treatment, sustained Cdc42 activity induced a time-dependent F-actin-vinculin reorganization, prevented cytokinesis, and downregulated Rho activity. Finally,   podosomes   were also detected on endothelial cells explanted from patients undergoing cardiac surgery. These data provide the first description of   podosomes   in endothelial cells. The identification of such specialized structures opens up a new field of investigation in terms of endothelium pathophysiology. 
  GeneRIFs  
 The reorganization of actins into   podosomes   is controlled by RHOA. 
  [Mm]igration  Process  
 cell   migration   
 cell   migration   
 endothelial cell   migration   
 negative regulation of cell   migration   involved in sprouting angiogenesis 
 positive regulation of T cell   migration   
 regulation of cell   migration   
 skeletal muscle satellite cell   migration   
  PubMed  
 Oligophrenin-1 encodes a rhoGAP protein involved in X-linked mental retardation. Primary or nonspecific X-linked mental retardation (MRX) is a heterogeneous condition in which affected patients do not have any distinctive clinical or biochemical features in common apart from cognitive impairment. Although it is present in approximately 0.15-0.3% of males, most of the genetic defects associated with MRX, which may involve more than ten different genes, remain unknown. Here we report the characterization of a new gene on the long arm of the X-chromosome (position Xq12) and the identification in unrelated individuals of different mutations that are predicted to cause a loss of function. This gene is highly expressed in fetal brain and encodes a protein of relative molecular mass 91K, named oligophrenin-1, which contains a domain typical of a Rho-GTPase-activating protein (rhoGAP). By enhancing their GTPase activity, GAP proteins inactivate small Rho and Ras proteins, so inactivation of rhoGAP proteins might cause constitutive activation of their GTPase targets. Such activation is known to affect cell   migration   and outgrowth of axons and dendrites in vivo. Our results demonstrate an association between cognitive impairment and a defect in a signalling pathway that depends on a Ras-like GTPase. 
 Maspin controls mammary tumor cell   migration   through inhibiting Rac1 and Cdc42, but not the RhoA GTPase. Rac1 and Cdc42 are members of the Rho family of small GTPases that play essential roles in diverse cellular functions, including cell   migration  . The activities of these Rho family proteins are controlled by growth factor receptor activation and cell-ECM interactions. Here, we show that maspin, a well-documented tumor suppressor gene, also controls cell motility through inhibiting Rac1/Cdc42 activity. Using the GST-PAK and GST-Rho binding protein pull-down assays for GTP-bound Rac1, Cdc42, and RhoA, we showed that treatment of MDA-MB-231 tumor cells with recombinant maspin for a short time period significantly inhibited the activity of Rac1 and Cdc42, but not RhoA. The reactive site loop (RSL) within maspin protein is the functional domain involved in the inhibition. Maspin mutants with the RSL deleted or a point mutation in the RSL region lost their inhibitory activity. We further examined the ability of maspin to inhibit Rac1- and Cdc42-mediated signaling pathways and transcription factors. Treatment of MDA-MB-231 cells with maspin led to the inhibition of JNK kinase activity as assayed by immuno-kinase assays. In addition, the AP-1 transcription activity downstream of JNK kinase pathway was also reduced. Together, we have identified Rac1 and Cdc42 as the downstream targets that mediate the inhibition of mammary tumor cell   migration   by maspin. 
 Asef, a link between the tumor suppressor APC and G-protein signaling. The adenomatous polyposis coli gene (APC) is mutated in familial adenomatous polyposis and in sporadic colorectal tumors. Here the APC gene product is shown to bind through its armadillo repeat domain to a Rac-specific guanine nucleotide exchange factor (GEF), termed Asef. Endogenous APC colocalized with Asef in mouse colon epithelial cells and neuronal cells. Furthermore, APC enhanced the GEF activity of Asef and stimulated Asef-mediated cell flattening, membrane ruffling, and lamellipodia formation in MDCK cells. These results suggest that the APC-Asef complex may regulate the actin cytoskeletal network, cell morphology and   migration  , and neuronal function. 
 Interleukin-8 induces the endothelial cell   migration   through the activation of phosphoinositide 3-kinase-Rac1/RhoA pathway. Endothelial cell   migration   is essential for tumor angiogenesis, and interleukin-8 (IL-8) has been shown to play an important role in tumor growth, angiogenesis, and metastasis. This study aimed to investigate the molecular mechanism of IL-8 induced endothelial cell   migration  . Our results indicated that IL-8 induced a rapid rearrangement of the actin cytoskeleton in EA.Hy926 cells, generating extensions resembling membrane ruffling and stress fibers. These processes required parallel upregulation of the small GTPases Rac1 and RhoA. Moreover, we demonstrated that IL-8 activated PI3K following the same kinetics observed from IL-8 induction of cytoskeletal rearrangement, suggesting the participation of PI3K in these processes. Taken together, our study demonstrates that PI3K-Rac1/RhoA signaling pathway plays a vital role in IL-8 induced endothelial cell   migration  , and provides new insight into the molecular mechanisms by which IL-8 contributes to tumor angiogenesis and metastasis. 
 Interleukin-8 induces the endothelial cell   migration   through the Rac 1/RhoA-p38MAPK pathway. Endothelial cell   migration   is essential for tumor angiogenesis, and interleukin-8 (IL-8) has been shown to play an important role in tumor growth, angiogenesis, and metastasis. The objective of this study was to investigate the molecular mechanism of IL-8 induced endothelial cell   migration   in vitro.Fluorescence microscope was used to study the distribution of cytoskeleton. The expression of Rac1 and RhoA protein was detected by western blotting. After endothelial cells were transfected by lipofectamine 2000 reagent, the Transwell chamber motility assay was applied to observe the   migration   of endothelial cells induced by IL-8. The active p38MAPK (mitogen-activated protein kinase) was evaluated by the p38MAPK activation assay.We demonstrated that IL-8 activated cell   migration   can be impaired by p38MAPK inhibitor, suggesting the participation of p38MAPK in the cell   migration  . Our results indicated that p38MAPK signaling is required for membrane ruffles, lamellipodia extensions, and actin stress fibers formation induced by IL-8. Furthermore, p38MAPK inhibitor led to increased Rac1 and RhoA expression in IL-8 treated EA.hy926 cells. In addition, IL-8 induced p38MAPK activation was suppressed by dominant-negative mutant for Rac1 and RhoA.Our study demonstrates that IL-8-Rac1/RhoA-p38MAPK signaling pathway plays a vital role in the IL-8-induced endothelial cell   migration  , and it provides new insight into the molecular mechanisms by which IL-8 contributes to tumor angiogenesis and metastasis. 
 Abnormal activation of Ras/Raf/MAPK and RhoA/ROCKII signalling pathways in eutopic endometrial stromal cells of patients with endometriosis. BACKGROUND Enhanced proliferation and survival of eutopic endometrial cells from patients with endometriosis compared with healthy women is associated with abnormal activation of extra-cellular signal-regulated kinases 1 and 2 (ERK1/2). Given the role of Ras/Raf/mitogen-activated protein kinase (MAPK) and RhoA/ROCKII signalling pathways in the regulation of cell proliferation and   migration  , we analysed their possible roles in endometriosis. METHODS Primary eutopic endometrial stromal cells of patients with endometriosis (Eu-hESC, n= 16) and endometriosis-free controls (Co-hESC, n= 14) were harvested and subjected to proliferation and   migration   assays as well as kinase activity assays and immunoblot analysis of proteins from the Ras/Raf/MAPK and RhoA/ROCKII signalling pathways. Effects of ROCKII (Y-27632) and MAPK (U0126) inhibitors or siRNA knockdown of ROCKII, Raf-1 and B-Raf were analysed. RESULTS The proliferation rate of Eu-hESC was 54% higher than Co-hESC. Eu-hESC also displayed a 75% higher   migration   rate than Co-hESC. Eu-hESC displayed higher levels of ERK phosphorylation (83%) and p27 expression (61%) and lower levels of Raf-1 protein (47%) compared with controls. In addition to an inhibitory effect on cell proliferation, ROCKII knockdown led to significant down-regulation of cyclinD1 and p27 but did not affect ERK phosphorylation. Down-regulation of Raf-1 by siRNA was dispensable for cell proliferation control but led to an increase in ROCKII activity and a decrease in cell   migration  . B-Raf was shown to act as a regulator of hESC proliferation by modulating cellular ERK1/2 activity and cyclinD1 levels. Eu-hESC displayed 2.4-fold higher B-Raf activity compared with Co-hESC and therefore exhibit abnormally activated Ras/Raf/MAPK signalling. CONCLUSIONS We show that the same molecular mechanisms operate in Co- and Eu-hESC. The differences in cell proliferation and   migration   between both cell types are likely due to increased activation of Ras/Raf/MAPK and RhoA/ROCKII signalling pathways in cells from endometriosis patients. 
 Urokinase-induced   migration   of human vascular smooth muscle cells requires coupling of the small GTPases RhoA and Rac1 to the Tyk2/PI3-K signalling pathway. Urokinase-type plasminogen activator (uPA) facilitates cell   migration   by localizing proteolisys on the cell surface and by inducing intracellular signalling pathways. In human vascular smooth muscle cell (VSMC) uPA stimulates   migration   via the uPA receptor (uPAR) signalling complex containing the Janus kinase Tyk2 and phosphatidylinositol 3-kinase (PI3-K). We report that active GTP-bound forms of small GTPases RhoA and Rac1, but not Cdc42, are directly associated with Tyk2 and PI3-K in an uPA/uPAR-dependent fashion. Endogenous RhoA, but not Rac1 or Cdc42, was significantly activated in response to uPA. RhoA activation was abolished by cell treatment with two unrelated, structurally distinct, specific inhibitors of PI3-K, wortmannin, and LY294002. Downstream of RhoA, phosphorylation of myosin light chain (MLC) was dramatically upregulated by uPA in a Rho kinase- and PI3-K-dependent manner. Thus, selective Rho kinase inhibitor Y27632 and PI3-K inhibitors wortmannin and LY294002 prevented the uPA-induced stimulation of MLC phosphorylation. Rho kinase inhibition also decreased uPA-stimulated VSMC   migration   as observed in a Boyden chamber. VSMC immunocytochemical staining demonstrated redistribution of RhoA and Rac1 active forms to the newly formed leading edge of migrating cell. VSMC microinjection with antibodies to either Rho or Rac1 decreased uPA-stimulated cell   migration  , indicating the involvement of both GTPases in the   migration   process. Our results provide evidence that the small GTPases RhoA and Rac1, together with Rho kinase, are necessary to mediate the uPA/uPAR-directed   migration   via the Tyk2/PI3-K signalling complex in human VSMC. 
 Balanced Tiam1-rac1 and RhoA drives proliferation and invasion of pancreatic cancer cells. Tiam1 is a rac1-specific guanine nucleotide exchange factor, and Tiam1-rac1 is involved in a number of cellular processes. Rac1 and RhoA act as molecular switches that cycle between GTP- and GDP-bound states to balance the activities of rac1 and RhoA. The downregulation of rac1 activity leads to upregulation of RhoA activity, which promotes invasion and   migration   of pancreatic cancers cells. At present, however, the role of Tiam1-rac1 and RhoA in pancreatic cancers is not fully understood. We found that Tiam1 was upregulated in pancreatic cancers and was significantly expressed in tumors without lymph node involvement or distant metastasis compared with cancers where there was involvement. Although Tiam1-rac1 signaling promoted pancreatic cancer cell proliferation and tumor growth via the Wnt signaling pathway in vitro and in vivo, inhibiting Tiam1-rac1 signaling did not prolong the overall survival time in vivo. This provided evidence that there was a balance between rac1 and RhoA activities in pancreatic cancers. Furthermore, only the combined inhibition of Tiam1-rac1 and RhoA had a beneficial effect on the growth of pancreatic cancers in vivo. Taken together, these results suggest that the progression of pancreatic tumors is partially controlled by the balance between Tiam1-rac1 and RhoA. 
 Integrin alpha8beta1 regulates adhesion,   migration   and proliferation of human intestinal crypt cells via a predominant RhoA/ROCK-dependent mechanism. Integrins are transmembrane alphabeta heterodimer receptors that function as structural and functional bridges between the cytoskeleton and ECM (extracellular matrix) molecules. The RGD (arginine-glycine-aspartate tripeptide motif)-dependent integrin alpha8beta1 has been shown to be involved in various cell functions in neuronal and mesenchymal-derived cell types. Its role in epithelial cells remains unknown.Integrin alpha8beta1 was found to be expressed in the crypt cell population of the human intestine but was absent from differentiating and mature epithelial cells of the villus. The function of alpha8beta1 in epithelial crypt cells was investigated at the cellular level using normal HIECs (human intestinal epithelial cells). Specific knockdown of alpha8 subunit expression using an shRNA (small-hairpin RNA) approach showed that alpha8beta1 plays important roles in RGD-dependent cell adhesion,   migration   and proliferation via a RhoA/ROCK (Rho-associated kinase)-dependent mechanism as demonstrated by active RhoA quantification and pharmacological inhibition of ROCK. Moreover, loss of alpha8beta1, through RhoA/ROCK, impairs FA (focal adhesion) complex integrity as demonstrated by faulty vinculin recruitment.Integrin alpha8beta1 is expressed in epithelial cells. In intestinal crypt cells, alpha8beta1 is closely involved in the regulation of adhesion,   migration   and cell proliferation via a predominant RhoA/ROCK-dependent mechanism. These results suggest an important role for this integrin in intestinal crypt cell homoeostasis. 
 Annexin-A1 controls an ERK-RhoA-NFκB activation loop in breast cancer cells. Wound healing is critical for normal development and pathological processes including cancer cell metastasis. MAPK, Rho-GTPases and NFκB are important regulators of wound healing, but mechanisms for their integration are incompletely understood. Annexin-A1 (ANXA1) is upregulated in invasive breast cancer cells resulting in constitutive activation of NFκB. We show here that silencing ANXA1 increases the formation of stress fibers and focal adhesions, which may inhibit wound healing. ANXA1 regulated wound healing is dependent on the activation of ERK1/2. ANXA1 increases the activation of RhoA, which is dependent on ERK activation. Furthermore, active RhoA is important in NF-κB activation, where constitutively active RhoA potentiates NFκB activation, while dominant negative RhoA inhibits NFκB activation in response to CXCL12 stimulation and active MEKK plasmids. These findings establish a central role for ANXA1 in the   cell mig  ration through the activation of NFκB, ERK1/2 and RhoA. 
 Vascular endothelial growth factor C promotes cervical cancer metastasis via up-regulation and activation of RhoA/ROCK-2/moesin cascade. The elevated expression of vascular endothelial growth factor C (VEGF-C) is correlated with clinical cervical cancer metastasis and patient survival, which is interpreted by VEGF-C functions to stimulate angiogenesis and lymphatic genesis. However, the direct impact of VEGF-C on cervical cancer cell motility remains largely unknown.In this study, we investigated the effects of VEGF-C on actin cytoskeleton remodeling and on cervical cancer cell   migration   and invasion and how the actin-regulatory protein, moesin regulated these effects through RhoA/ROCK-2 signaling pathway.On cervical carcinoma cell line SiHa cells, exposure of VEGF-C triggered remodeling of the actin cytoskeleton and the formation of membrane ruffles, which was required for cell movement. VEGF-C significantly enhanced SiHa cells horizontal   migration   and three-dimensional invasion into matrices. These actions were dependent on increased expression and phosphorylation of the actin-regulatory protein moesin and specific moesin siRNA severely impaired VEGF-C stimulated-cell   migration  . The extracellular small GTPase RhoA/ROCK-2 cascade mediated the increased moesin expression and phosphorylation, which was discovered by the use of Y-27632, a specific inhibitor of Rho kinase and by transfected constitutively active, dominant-negative RhoA as well as ROCK-2 SiRNA. Furthermore, in the surgical cervical specimen from the patients with FIGO stage at cervical intra-epithelial neoplasia and I-II cervical squamous cell carcinoma, the expression levels of moesin were found to be significantly correlated with tumor malignancy and metastasis.These results implied that VEGF-C promoted cervical cancer metastasis by upregulation and activation of moesin protein through RhoA/ROCK-2 pathway. Our findings offer new insight into the role of VEGF-C on cervical cancer progression and may provide potential targets for cervical cancer therapy. 
 Mutation of ARHGAP9 in patients with coronary spastic angina. Coronary artery spasm has an important function in the etiology of variant angina and other acute coronary syndromes. Abnormal activation of Rho-family GTPases has been observed in cardiovascular disorders, but the function of genetic variability in Rho-family GTPases remains to be evaluated in cardiovascular disorders. We examined the genetic variability of Rho-family GTPases and their regulators in coronary artery spasm. We performed a comprehensive candidate gene analysis of 67 single nucleotide polymorphisms with amino-acid substitution in Rho-family GTPases and their regulators in 103 unrelated Japanese patients with acetylcholine-induced coronary artery spasm and 102 control Japanese subjects without acetylcholine-induced coronary artery spasm. We noted an association of the single nucleotide polymorphism of ARHGAP9 (rs11544238, Ala370Ser) with coronary artery spasm (odds ratio =2.67). We found that ARHGAP9 inactivated Rac as RacGAP and that the mRNA level of ARHGAP9 was strongly detected in hematopoietic cells. ARHGAP9 negatively regulated cell   migration  . The Ala370Ser polymorphism counteracted ARHGAP9-reduced cell   migration  , spreading and adhesion. The Ala370Ser polymorphism in the ARHGAP9 gene is associated with coronary artery spasm. These data suggest that the polymorphism of ARHGAP9 has a critical function in the infiltration of hematopoietic cells into the endothelium and inflammation leading to endothelial dysfunction. 
 Integrin engagement differentially modulates epithelial cell motility by RhoA/ROCK and PAK1. Integrin-ligand binding regulates tumor cell motility and invasion. Cell   migration   also involves the Rho GTPases that control the interplay between adhesion receptors and the cytoskeleton. We evaluated how specific extracellular matrix ligands modulate Rho GTPases and control motility of human squamous cell carcinoma cells. On laminin-5 substrates, the epithelial cells rapidly spread and migrated, but on type I collagen the cells spread slowly and showed reduced motility. We found that RhoA activity was suppressed in cells attached to laminin-5 through the alpha3 integrin receptor. In contrast, RhoA was strongly activated in cells bound to type I collagen and this was mediated by the alpha2 integrin. Inhibiting the RhoA pathway by expression of a dominant-negative RhoA mutant or by directly inhibiting ROCK, reduced focal adhesion formation and enhanced cell   migration   on type I collagen. Cdc42 and Rac and their downstream target PAK1 were activated following adhesion to laminin-5. PAK1 activation induced by laminin-5 was suppressed by expression of a dominant-negative Cdc42. Moreover, constitutively active PAK1 stimulated   migration   on collagen I substrates. Our results indicate that in squamous epithelial cells, collagen-alpha2beta1 integrin binding activates RhoA, slowing cell locomotion, whereas laminin-5-alpha3beta1 integrin interaction inhibits RhoA and activates PAK1, stimulating cell   migration  . The data demonstrate that specific ligand-integrin pairs regulate cell motility differentially by selectively modulating activities of Rho GTPases and their effectors. 
 [Under hypoxia condition contactin-1 regulates   migration   of MKN45 cells through RhoA pathway]. Recent studies have suggested that contactin-1 has a key role in cancer cell proliferation and   migration  , however the detailed mechanism of this process is still unclear. Here, human gastric cancer cell line MKN45 was employed. It was found that under hypoxia conditions contactin-1 mRNA and protein levels were both up-regulated by HIF-1alpha expression. Furthermore, although hypoxia increased the   migration   rate of MKN45 cells, contactin-1 (CNTN1) shRNA reversed this process. Meanwhile, RhoA V14 and RhoA V14N19 mutation constructs were employed, and it was found that constitutively active form of RhoA reversed the cell   migration   suppression induced by contactin-1 knockdown, while dominant-negative form of RhoA blocked hypoxia induced hyper  migration  . Apart from this, contactin-1 displayed the ability to phosphorylate the RhoA activator p115 RhoGEF. Thus, under hypoxia conditions, elevated HIF-1alpha seems to up-regulate contactin-1 expression and by this activate RhoA and facilitate   migration   of cancer cells. 
 [The Rho protein family and its role in the cellular cytoskeleton]. Proteins of the Rho protein family (RhoA, Rac1, Cdc42) work as molecular switches in the regulation of many cellular processes. They are involved in cell   migration  , cell-cycle control, apoptosis, and the regulation of gene transcription. Rho proteins show their activity mainly in the cell's cytoskeleton by taking part in the reorganization of microfilaments and microtubules. In recent years, significant progress has been made in understanding the biochemical and genetic nature of many processes controlled by Rho proteins. Although there are still seveal unknowns and hypothesizes which require confirmation, newer and more precise experimental results allow us to better understand these processes. It has also been suggested to use Rho family proteins in the therapy of some diseases. The purpose of this study was to characterize the significance of Rho processes in the cellular cytoskeleton. 
 Immunohistochemical expression of Rho GTPases in ameloblastomas. Rho GTPases are proteins that regulate cell cycle, shape, polarization, invasion,   migration  , and apoptosis, which are important characteristics of normal and neoplastic cells. Rho GTPases expression has been reported in normal tooth germ and several pathologies; however, it has not been evaluated in ameloblastomas. The aim of this study was to analyze the expression and distribution of RhoA, RhoB, Rac1, and Cdc42 Rho GTPases in solid and unicystic ameloblastomas. Three-micrometer sections from paraffin-embedded specimens were evaluated by using an avidin-biotin immunohistochemical method with antibodies against the proteins mentioned above. RhoA and RhoB staining was observed in a high number of cells (P < 0.05) and greater intensity in non-polarized ones. Rac1 was not observed, and Cdc42 did not show any statistical differences between the number of non-polarized and basal positive cells (P > 0.05). Upon comparing the studied ameloblastomas, a higher number of positive cells in the unicystic variant was observed than that in the solid one (P < 0,05). The results obtained suggest that these GTPases could play a role in the ameloblastoma neoplastic epithelial cell phenotype determination (polarized or non-polarized), as well as in variant (solid or unicystic) and subtype (follicular or plexiform) determination. Furthermore, they could participate in solid ameloblastoma invasion mechanisms. 
 H2O2 inhibits proliferation and mediates suppression of   migration   via DLC1/RhoA signaling in cancer cells. RhoGTPase-activating proteins (RhoGAPs) regulate RhoGTPases in cells, but whether individual reactive oxygen species (ROS) regulate RhoGAPs is unknown. Our previous published papers have shown that deleted in liver cancer 1 (DLC1) inhibits cancer cell   migration   by its RhoGAP activity. The present study was designed to explore the role of H2O2 in regulation of DLC1.We treated cells with H2O2 for 24h and phenotypic changes were analyzed by MTT, RT-PCR, Western blotting, immunofluorescence staining and wound healing assays.H2O2 downregulated cyclin D1 and cyclin E to inhibit proliferation, and upregulated BAX to induce apoptosis in MCF-7 cells. Compared with non-tumorigenic cells, H2O2 increased expression of DLC1 and reduced activity of RhoA in cancer cells. Stress fiber production and   migration   were also suppressed by H2O2 in MDA-MB-231 cells.Our study suggests that H2O2 inhibits proliferation through modulation of cell cycle and apoptosis-related genes, and inhibits   migration   by decreasing stress fibers via DLC1/RhoA signaling. 
 Fibroblast activation protein (FAP) is essential for the   migration   of bone marrow mesenchymal stem cells through RhoA activation. The ability of human bone marrow mesenchymal stem cells (BM-MSCs) to migrate and localize specifically to injured tissues is central in developing therapeutic strategies for tissue repair and regeneration. Fibroblast activation protein (FAP) is a cell surface serine protease expressed at sites of tissue remodeling during embryonic development. It is also expressed in BM-MSCs, but not in normal tissues or cells. The function of FAP in BM-MSCs is not known.We found that depletion of FAP proteins significantly inhibited the   migration   of BM-MSCs in a transwell chemotaxis assay. Such impaired   migration   ability of BM-MSCs could be rescued by re-expressing FAP in these cells. We then demonstrated that depletion of FAP activated intracellular RhoA GTPase. Consistently, inhibition of RhoA activity using a RhoA inhibitor rescued its   migration   ability. Inhibition of FAP activity with an FAP-specific inhibitor did not affect the activation of RhoA or the   migration   of BM-MSCs. Furthermore, the inflammatory cytokines interleukin-1beta (IL-1β) and transforming growth factor-beta (TGF-β) upregulated FAP expression, which coincided with better BM-MS  C migrati  on.Our results indicate FAP plays an important role in th  e migrati  on of BM-MSCs through modulation of RhoA GTPase activity. The peptidase activity of FAP is not essential for suc  h migrati  on. Cytokines IL-1β and TGF-β upregulate the expression level of FAP and thus enhance BM-  MSC migra  tion. 
 Blockade of the RhoA-JNK-c-Jun-MMP2 cascade by atorvastatin reduces osteosarcoma cell invasion. Osteosarcoma is characterized by a high malignant and metastatic potential, which points to the need for new therapeutic strategies to prevent cell metastasis. In this study, we show that statin-induced HMG-CoA reductase inhibition reduces cell   migration   and invasion in human and murine osteosarcoma cells, independently of the genotype. The statin-induced reduction of cell   migration   and invasion was independent of induction of apoptosis and was geranylgeranylpyrophosphate-dependent. The statin reduced matrix metalloproteinase (MMP) 2, 9, and 14 and TIMP2 expression or activity in invading cells. Forced expression of MMP2 and MMP14 overcame the inhibitory effect of the statin on cell invasion, suggesting a role for these MMPs in invasive potential. We also investigated the mechanisms involved in the reduced MMP2 activity and cell invasion. Inhibition of JNK, but not ERK1/2 signaling, reduced MMP2 activity. Pharmacological or constitutive activation of JNK overcame the reduced MMP2 activity and cell invasion induced by the statin. The statin decreased JNK phosphorylation and c-Jun nuclear translocation, suggesting that HMG-CoA reductase inhibition targets the JNK-c-Jun signaling pathway. We showed that mevalonate or geranylgeranylpyrophosphate treatment prevented the statin-induced reduction in JNK phosphorylation, MMP2 activity, and cell invasion. Forced expression of a constitutively active form of RhoA increased JNK phosphorylation and overcame the inhibitory effect of atorvastatin on MMP2 activity and cell invasion. The data establish a link between RhoA, JNK, c-Jun, and MMP2 activity that is functionally involved in the reduction in osteosarcoma cell invasion by the statin. This suggests a novel strategy targeting RhoA-JNK-c-Jun signaling to reduce osteosarcoma cell tumorigenesis. 
 The Rho-family GEF Asef2 activates Rac to modulate adhesion and actin dynamics and thereby regulate cell   migration  . Asef2 is a recently identified Rho-family guanine nucleotide exchange factor (GEF) that has been implicated in the modulation of actin, but its function in cell   migration   and adhesion dynamics is not well understood. In this study, we show that Asef2 is an important regulator of cell   migration   and adhesion assembly and disassembly (turnover). Asef2 localizes with actin at the leading edge of cells. Knockdown of endogenous Asef2 impairs   migration   and significantly slows the turnover of adhesions. Asef2 enhances both Rac1 and Cdc42 activity in HT1080 cells, but only Rac1 is crucial for the Asef2-promoted increase in   migration   and adhesion turnover. Phosphoinositide 3-kinase (PI3K) and the serine/threonine kinase Akt are also essential for the Asef2-mediated effects on   migration   and adhesion turnover. Consistent with this, Asef2 increases the amount of active Akt at the leading edge of cells. Asef2 signaling leads to an overall decrease in Rho activity, which is crucial for stimulating   migration   and adhesion dynamics. Thus, our results reveal an important new role for Asef2 in promoting cell   migration   and rapid adhesion turnover by coordinately regulating the activities of Rho-family GTPases. 
 S1P(2) receptors mediate inhibition of glioma cell   migration   through Rho signaling pathways independent of PTEN. Sphingosine 1-phosphate (S1P) induced the inhibition of glioma cell   migration  . Here, we characterized the signaling mechanisms involved in the inhibitory action by S1P. In human GNS-3314 glioblastoma cells, the S1P-induced inhibition of cell   migration   was associated with activation of RhoA and suppression of Rac1. The inhibitory action of S1P was recovered by a small interference RNA specific to S1P(2) receptor, a carboxyl-terminal region of Galpha12 or Galpha13, an RGS domain of p115RhoGEF, and a dominant-negative mutant of RhoA. The inhibitory action of S1P through S1P(2) receptors was also observed in both U87MG glioblastoma and 1321N1 astrocytoma cells, which have no protein expression of a phosphatase and tensin homolog deleted on chromosome 10 (PTEN). These results suggest that S1P(2) receptors/G(12/13)-proteins/Rho signaling pathways mediate S1P-induced inhibition of glioma cell   migration  . However, PTEN, recently postulated as an indispensable molecule for the inhibition of cell   migration  , may not be critical for the S1P(2) receptor-mediated action in glioma cells. 
 Regulation of ephexin1, a guanine nucleotide exchange factor of Rho family GTPases, by fibroblast growth factor receptor-mediated tyrosine phosphorylation. Fibroblast growth factor (FGF) signal is implicated in not only cell proliferation, but cell   migration   and morphological changes. Several different Rho family GTPases downstream of the Ras/ERK pathway are postulated to mediate the latter functions. However, none have been recognized to be directly coupled to FGF receptors (FGFRs). We have previously reported that EphA4 and FGFRs hetero-oligomerize through their cytoplasmic domains, trans-activate each other, and transduce a signal for cell proliferation through a docking protein, FRS2alpha (Yokote, H., Fujita, K., Jing, X., Sawada, T., Liang, S., Yao, L., Yan, X., Zhang, Y., Schlessinger, J., and Sakaguchi, K. (2005) Proc. Natl. Acad. Sci. U. S. A. 102, 18866-18871). Here, we have found that ephexin1, a guanine nucleotide exchange factor for Rho family GTPases, constitutes another downstream component of the receptor complex. Ephexin1 directly binds to the kinase domain of FGFR mainly through its DH and PH domains. The binding appears to become weaker and limited to the DH domain when FGFRs become activated. FGFR-mediated phosphorylation of ephexin1 enhances the guanine nucleotide exchange activity toward RhoA without affecting the activity to Rac1 or Cdc42. The FGFR-mediated tyrosine phosphorylation includes, but is not limited to, the residue (Tyr-87) phosphorylated by Src family kinase, which is known to be activated following EphA4 activation. The Tyr-to-Asp mutations that mimic the tyrosine phosphorylation in some of the putative FGFR-mediated phosphorylation sites increase the nucleotide exchange activity for RhoA without changing the activity for Rac1 or Cdc42. From these results, we conclude that ephexin1 is located immediately downstream of the EphA4-FGFR complex and the function is altered by the FGFR-mediated tyrosine phosphorylation at multiple sites. 
 Oncogenic K-Ras down-regulates Rac1 and RhoA activity and enhances   migration   and invasion of pancreatic carcinoma cells through activation of p38. Activating mutations in the K-ras gene are genetic alterations frequently found in human carcinomas, particularly in pancreatic adenocarcinomas. Mutation of the K-ras gene is thought to be an early and important event in pancreatic tumor initiation, but the precise role of the mutant K-Ras proteins in neoplastic progression is still unknown. In the present study, we have characterized the influence of oncogenic K-Ras on the phenotype and on the signal transduction of epitheloid PANC-1 pancreatic carcinoma cells by generating PANC-1 cell clones, which stably express EGFP(enhanced green fluorescent protein)-K-Ras (V12). EGFP-K-Ras (V12)-expressing cells exhibited a more fibroblastoid cellular phenotype with irregular cell shape and disorganized cytokeratin filaments. Moreover, these cells showed a marked enhancement of their migratory and invasive properties. Stable expression of EGFP-K-Ras (V12) down-regulated the activity of Rac1 and RhoA, resulting in reduced subcortical actin filaments and stress fibers, which might contribute to the epithelial dedifferentiation. Characterization of the activity of mitogen-activated protein kinases revealed that EGFP-K-Ras (V12) enhanced the activity of p38, but did not affect the activities of the Raf/MEK/ERK cascade and JNK. While inhibition of either MEK or JNK activity had no effect on EGFP-K-Ras (V12)-induced   migration  , inhibition of p38 activity markedly reduced EGFP-K-Ras (V12)-induced   migration  . Collectively, the results suggest that oncogenic K-Ras enhances the malignant phenotype and identify the mitogen-activated protein kinase p38 as a target to inhibit oncogenic K-Ras-induced pancreatic tumor cell   migration  . 
 RhoG activates Rac1 by direct interaction with the Dock180-binding protein Elmo. The small GTPase Rac has a central role in regulating the actin cytoskeleton during cell   migration   and axon guidance. Elmo has been identified as an upstream regulator of Rac1 that binds to and functionally cooperates with Dock180 (refs 2-4). Dock180 does not contain a conventional catalytic domain for guanine nucleotide exchange on Rac, but possesses a domain that directly binds to and specifically activates Rac1 (refs 5, 6). The small GTPase RhoG mediates several cellular morphological processes, such as neurite outgrowth in neuronal cells, through a signalling cascade that activates Rac1 (refs 7-12); however, the downstream target of RhoG and the mechanism by which RhoG regulates Rac1 activity remain unclear. Here we show that RhoG interacts directly with Elmo in a GTP-dependent manner and forms a ternary complex with Dock180 to induce activation of Rac1. The RhoG-Elmo-Dock180 pathway is required for activation of Rac1 and cell spreading mediated by integrin, as well as for neurite outgrowth induced by nerve growth factor. We conclude that RhoG activates Rac1 through Elmo and Dock180 to control cell morphology. 
 MiR-200b/200c/429 subfamily negatively regulates Rho/ROCK signaling pathway to suppress hepatocellular carcinoma metastasis. MiR-200 family is an important regulator of epithelial-mesenchymal transition and has been implicated in human carcinogenesis. However, their expression and functions in human cancers remain controversial. In the work presented here, we showed that miR-200 family members were frequently down-regulated in hepatocellular carcinoma (HCC). Although all five members of miR-200 family inhibited ZEB1/2 expression in HCC cell lines, we showed that overexpression only of the miR-200b/200c/429 subfamily, but not the miR-200a/141 subfamily, resulted in impeded HCC cell   migration  . Further investigations led to the identification of RhoA and ROCK2 as specific down-stream targets of the miR-200b/200c/429 subfamily. We demonstrated that the miR-200b/200c/429 subfamily inhibited HCC cell   migration   through modulating Rho/ROCK mediated cell cytoskeletal reorganization and cell-substratum adhesion. Re-expression of miR-200b significantly suppressed lung metastasis of HCC cells in an orthotopic liver implantation model in vivo. In conclusion, our findings identified the miR-200b/200c/429 subfamily as metastasis suppressor microRNAs in human HCC and highlighted the functional discrepancy among miR-200 family members. 
 mTOR regulate EMT through RhoA and Rac1 pathway in prostate cancer. Recently, an increasing number of studies have suggested that mTOR plays a critical role in the regulation of tumor cell motility, invasion and cancer metastasis. However, little is known about the signaling mechanisms in regulating epithelial-mesenchymal transition (EMT) of prostate cancer. In this study, we found that the expression levels of Raptor and Rictor in prostate cancer tissues were elevated, which may suggest that Raptor and Rictor signaling pathways are associated with prostate cancer progression and metastasis. Inhibition of mTORC1 or mTORC2 by knock down of Raptor or Rictor, respectively,   migration   and invasion of prostate cancer were attenuated. Furthermore, EMT, a characterized by the changed expression levels of various markers like E-cadherin, β-catenin, N-cadherin, and vimentin emergend following inhibition of Raptor or Rictor. Finally, the small GTPases (RhoA and Rac1) which were crucial regulatory proteins in cell   migratio  n and invasion were inactivited after downregulating Raptor and Rictor. These results suggest that mTOR regulate EMT at least in part by down regulation of RhoA and Rac1 signaling pathways. Our findings provide novel very attractive target strategies that the inhibition of mTOR signaling pathways may retard prostate cancer   migratio  n and invasion at early stages. 
 A MAPK-Driven Feedback Loop Suppresses Rac Activity to Promote RhoA-Driven Cancer Cell Invasion. Cell   migration   in 3D microenvironments is fundamental to development, homeostasis and the pathobiology of diseases such as cancer. Rab-coupling protein (RCP) dependent co-trafficking of α5β1 and EGFR1 promotes cancer cell invasion into fibronectin (FN) containing extracellular matrix (ECM), by potentiating EGFR1 signalling at the front of invasive cells. This promotes a switch in RhoGTPase signalling to inhibit Rac1 and activate a RhoA-ROCK-Formin homology domain-containing 3 (FHOD3) pathway and generate filopodial actin-spike protrusions which drive invasion. To further understand the signalling network that drives RCP-driven invasiv  e migrati  on, we generated a Boolean logical model based on existing network pathways/models, where each node can be interrogated by computational simulation. The model predicted an unanticipated feedback loop, whereby Raf/MEK/ERK signalling maintains suppression of Rac1 by inhibiting the Rac-activating Sos1-Eps8-Abi1 complex, allowing RhoA activity to predominate in invasive protrusions. MEK inhibition was sufficient to promote lamellipodia formation and oppose filopodial actin-spike formation, and led to activation of Rac and inactivation of RhoA at the leading edge of cells moving in 3D matrix. Furthermore, MEK inhibition abrogated RCP/α5β1/EGFR1-driven invas  ive migra  tion. However, upon knockdown of Eps8 (to suppress the Sos1-Abi1-Eps8 complex), MEK inhibition had no effect on RhoGTPase activity and did not oppose invas  ive migra  tion, suggesting that MEK-ERK signalling suppresses the Rac-activating Sos1-Abi1-Eps8 complex to maintain RhoA activity and promote filopodial actin-spike formation and invas  ive migra  tion. Our study highlights the predictive potential of mathematical modelling approaches, and demonstrates that a simple intervention (MEK-inhibition) could be of therapeutic benefit in preventing invas  ive migra  tion and metastasis. 
 RhoA and Rac1 are both required for efficient wound closure of airway epithelial cells. Repair of the airway epithelium after injury is critical for restoring normal lung. The reepithelialization process involves spreading and   migration   followed later by cell proliferation. Rho-GTPases are key components of the wound healing process in many different types of tissues, but the specific roles for RhoA and Rac1 vary and have not been identified in lung epithelial cells. We investigated whether RhoA and Rac1 regulate wound closure of bronchial epithelial cells. RhoA and Rac1 proteins were efficiently expressed in a cell line of human bronchial epithelial cells (16HBE) by adenovirus-based gene transfer. We found that both constitutively active RhoA and dominant negative RhoA inhibited wound healing, suggesting that both activation and inhibition of RhoA interfere with normal wound healing. Overexpression of wild-type Rac1 induced upregulation of RhoA, disrupted intercellular junctions, and inhibited wound closure. Dominant negative Rac1 also inhibited wound closure. Inhibition of the downstream effector of RhoA, Rho-kinase, with Y-27632 suppressed actin stress fibers and focal adhesion formation, increased Rac1 activity, and stimulated wound closure. The activity of both RhoA and Rac1 are influenced by the polymerization state of microtubules, and cell   migration   involves coordinated action of actin and microtubules. Microtubule depolymerization upon nocodazole treatment led to an increase in focal adhesions and decreased wound closure. We conclude that coordination of both RhoA and Rac1 activity contributes to bronchial epithelial wound repair mechanisms in vitro, that inhibition of Rho-kinase accelerates wound closure, and that efficient repair involves intact microtubules. 
 Analysis of the myosin-II-responsive focal adhesion proteome reveals a role for β-Pix in negative regulation of focal adhesion maturation. Focal adhesions undergo myosin-II-mediated maturation wherein they grow and change composition to modulate integrin signalling for cell   migratio  n, growth and differentiation. To determine how focal adhesion composition is affected by myosin II activity, we performed proteomic analysis of isolated focal adhesions and compared protein abundance in focal adhesions from cells with and without myosin II inhibition. We identified 905 focal adhesion proteins, 459 of which changed in abundance with myosin II inhibition, defining the myosin-II-responsive focal adhesion proteome. The abundance of 73% of the proteins in the myosin-II-responsive focal adhesion proteome was enhanced by contractility, including proteins involved in Rho-mediated focal adhesion maturation and endocytosis- and calpain-dependent focal adhesion disassembly. During myosin II inhibition, 27% of proteins in the myosin-II-responsive focal adhesion proteome, including proteins involved in Rac-mediated lamellipodial protrusion, were enriched in focal adhesions, establishing that focal adhesion protein recruitment is also negatively regulated by contractility. We focused on the Rac guanine nucleotide exchange factor β-Pix, documenting its role in the negative regulation of focal adhesion maturation and the promotion of lamellipodial protrusion and focal adhesion turnover to drive cel  l migrati  on. 
 The prion protein inhibits monocytic cell   migration   by stimulating β1 integrin adhesion and uropod formation. The broad tissue distribution and evolutionary conservation of the glycosylphosphatidylinositol (GPI)-anchored prion protein (PrP, also known as PRNP) suggests that it plays a role in cellular homeostasis. Given that integrin adhesion determines cell behavior, the proposed role of PrP in cell adhesion might underlie the various in vitro and in vivo effects associated with PrP loss-of-function, including the immune phenotypes described in PrP(-/-) mice. Here, we investigated the role of PrP in the adhesion and (transendothelial)   migratio  n of human (pro)monocytes. We found that PrP regulates β1-integrin-mediated adhesion of monocytes. Additionally, PrP controls the cell morphology and migratory behavior of monocytes: PrP-silenced cells show deficient uropod formation on immobilized VCAM and display bleb-like protrusions on the endothelium. Our data further show that PrP regulates ligand-induced integrin activation. Finally, we found that PrP controls the activation of several proteins involved in cell adhesion an  d migrati  on, including RhoA and its effector cofilin, as well as proteins of the ERM family. We propose that PrP modulates β1 integrin adhesion a  nd migrat  ion of monocytes through RhoA-induced actin remodeling mediated by cofilin, and through the regulation of ERM-mediated membrane-cytoskeleton linkage. 
 KiSS1 suppresses TNFalpha-induced breast cancer cell invasion via an inhibition of RhoA-mediated NF-kappaB activation. Tumor necrosis factor-alpha (TNFalpha) induces cancer development and metastasis, which is prominently achieved by nuclear factor-kappa B (NF-kappaB) activation. TNFalpha-induced NF-kappaB activation enhances cellular mechanisms including proliferation,   migration  , and invasion. KiSS1, a key regulator of puberty, was initially discovered as a tumor metastasis suppressor. The expression of KiSS1 was lost or down-regulated in different metastatic tumors. However, it is unclear whether KiSS1 regulates TNFalpha-induced NF-kappaB activation and further tumor cell   migration  . In this study, we demonstrate that KiSS1 suppresses the   migration   of breast cancer cells by inhibiting TNFalpha-induced NF-kappaB pathway and RhoA activation. Both KiSS1 overexpression and KP10 (kisspeptin-10) stimulation inhibited TNFalpha-induced NF-kappaB activity, suppressed TNFalpha-induced cell   migration   and cell attachment to fibronectin in breast cancer cells while KP10 has little effect on cancer cell proliferation. Furthermore, KP10 inhibited TNFalpha-induced cell   migration   and RhoA GTPase activation. Therefore, our data demonstrate that KiSS1 inhibits TNFalpha-induced NF-kappaB activation via downregulation of RhoA activation and suppression of breast cancer cell   migration   and invasion. 
 Integrin α6β4 cooperates with LPA signaling to stimulate Rac through AKAP-Lbc-mediated RhoA activation. The α(6)β(4) integrin promotes carcinoma invasion through its ability to promote direc  ted migra  tion and polarization of carcinoma cells. In this study, we explore how the α(6)β(4) integrin cooperates with lysophosphatidic acid (LPA) to activate Rho and Rac small GTPases. Through the use of dominant negative Rho constructs, C3 exotransferase, and Rho kinase inhibitor, we find that Rho is critical for LPA-dependent chemotaxis and lamellae formation. However, utilization of specific Rho isoforms depends on integrin α(6)β(4) expression status. Integrin α(6)β(4)-negative MDA-MB-435 cells utilize only RhoC for motility, whereas integrin α(6)β(4)-expressing cells utilize RhoC but additionally activate and utilize RhoA for LPA-dependent cell motility and lamellae formation. Notably, the activation of RhoA by cooperative LPA and integrin α(6)β(4) signaling requires the Rho guanine nucleotide exchange factor AKAP-Lbc. We also determine that integrin α(6)β(4) cannot activate Rac1 directly but promotes LPA-mediated Rac1 activation that is dependent on RhoA activity and de novo β(1) integrin ligation. Finally, we find that the regulation of Rac1 and RhoA in response to LPA is differentially regulated by phosphodiesterases, PKA, and phosphatidylinositol 3-kinase, thus supporting their spatially distinct compartmentalization. In summary, signaling from integrin α(6)β(4) facilitates LPA-stimulated chemotaxis through preferential activation of RhoA, which, in turn, facilitates activation of Rac1. 
 p190 Rho-GTPase activating protein associates with plexins and it is required for semaphorin signalling. Plexins are transmembrane receptors for semaphorins, guiding cell   migration   and axon extension. Plexin activation leads to the disassembly of integrin-based focal adhesive structures and to actin cytoskeleton remodelling and inhibition of cell   migration  ; however, the underlying molecular mechanisms are unclear. We consistently observe a transient decrease of cellular RhoA-GTP levels upon plexin activation in adherent cells. One of the main effectors of RhoA downregulation is p190, a ubiquitously expressed GTPase activating protein (GAP). We show that, in p190-deficient fibroblasts, the typical functional activities mediated by plexins (such as cell collapse and inhibition of integrin-based adhesion) are blocked or greatly impaired. Notably, the functional response can be rescued in these cells by re-expressing exogenous p190, but not a mutant form specifically lacking RhoGAP activity. We furthermore demonstrate that semaphorin function is blocked in epithelial cells, primary endothelial cells and neuroblasts upon treatment with small interfering RNAs that knockdown p190 expression. Finally, we show that p190 transiently associates with plexins, and its RhoGAP activity is increased in response to semaphorin stimulation. We conclude that p190-RhoGAP is crucially involved in semaphorin signalling to the actin cytoskeleton, via interaction with plexins. 
 Role of isoprenylcysteine carboxylmethyltransferase-catalyzed methylation in Rho function and   migration  . A number of proteins that play key roles in biological regulatory events undergo a process of post-translational modifications termed prenylation. The prenylation pathway consists of three enzymatic steps; the final processed protein is isoprenoid-modified and methylated on the C-terminal cysteine. This protein modification pathway plays a significant role in cancer biology because many oncogenic proteins undergo prenylation. Methylation of the C terminus by isoprenylcysteine carboxylmethyltransferase (Icmt) is the final step in the prenylation pathway. Cysmethynil, a specific Icmt inhibitor discovered in our laboratory, is able to inhibit Ras-mediated signaling, cell growth, and oncogenesis. We sought to examine the role of Icmt-mediated methylation on the behaviors of cancer cells associated with metastatic potential. Our results indicate that inhibition of methylation reduces   migration   of the highly metastatic MDA-MB-231 breast cancer cell line. In addition, cell adhesion and cell spreading are also significantly impacted by cysmethynil. To examine the mechanism of Icmt-dependent   migration   we focused on RhoA and Rac1, prenylated proteins that are important mediators of cell   migration   through their control of the actin cytoskeleton. Inhibition of Icmt significantly decreases the activation of both RhoA and Rac1; an increase in Rho GDP-dissociation inhibitor (RhoGDI) binding in the absence of methylation appears to contribute to this effect. Furthermore, in the absence of Icmt activity the addition of exogenous RhoA or Rac1 is able to partially rescue directed and random   migration  , respectively. These findings establish a role for Icmt-mediated methylation in cell   migration   and advance our understanding of the biological consequences of Rho methylation. 
 Type II cGMP‑dependent protein kinase inhibits RhoA activation in gastric cancer cells. Small GTPase RhoA is a key signaling component regulating cel  l migrati  on and stress fiber formation. Previous studies have shown that RhoA activity is regulated by protein kinases, such as cAMP‑dependent protein kinase (PKA) and type I cGMP‑dependent protein kinase (PKGI), which phosphorylate the protein. This study was designed to investigate the effect of type II cGMP‑dependent protein kinase (PKGII) on RhoA activity. Cells of the human gastric cancer line AGS were infected with adenoviral constructs bearing the PKGII cDNA in order to increase its endogenous expression, and were treated with 8‑pCPT‑cGMP to activate the PKGII enzyme. A transwell assay was performed to measure the migratory activity of the treated cells, and immunofluorescent microscopy was used to observe the formation of stress fibers. The phosphorylation of RhoA was detected by western blotting, and the activity of RhoA was measured by a pull‑down assay. Co‑immunoprecipitation (co‑IP) was performed to detect binding of PKGII to RhoA. Glutathione S‑transferase (GST)‑fused fragments of RhoA and PKGII were expressed in Escherichia coli and used to investigate the domains required for the binding. The results showed that lysophosphatidic acid (LPA) tr  eatment i  ncreased the migration and the formation of stress fibers in AGS cells and that this effect was RhoA‑dependent. An increase in PKGII activity, not onl  y inhibit  ed LPA‑induced migration and stress fiber formation, but also suppressed LPA‑induced activation of RhoA. PKGII caused serine 188 (Ser188) phosphorylation of RhoA, but not the phosphorylation of the mutant RhoA Ser188A, and therefore had no inhibitory effect on the activity of the mutant protein. Co‑IP results showed that there is direct binding of PKGII to RhoA. The GST pull‑down assay showed that the fragment containing RhoA amino acid residues 1‑44 and the N‑terminal fragment of PKGII containing amino acid residues 1‑176 are required for the binding between the two proteins. These results suggested that PKGII inhibits RhoA activity by binding to this small GTPase and causing phosphorylation at its Ser188 site. 
 Extension, retraction and contraction in the formation of a dendritic cell dendrite: distinct roles for Rho GTPases. The morphology of antigen-presenting dendritic cells (DC) is characterized by the possession of numerous long arborizing processes known as dendrites. The formation of these processes by DC, both in the periphery and in lymphoid organs, is believed to contribute to the remarkable efficiency with which they take up, process and present antigen to T cells. However, the process of dendrite formation and the signaling pathways that lead to the formation of these dendrites remain obscure. In this study we describe an in vitro model in which human immature DC form long processes similar to those formed in vivo. The formation of these processes involves initial attachment of a cell protrusion to the extracellular matrix substrate, and subsequent movement of the cell body away from the adhesion site, leaving behind a long slender dendrite. Dendrite formation, but not their maintenance, was found to be dependent on the activity of Rho GTPases. More specifically, Cdc42 and Rac1 were both required for the   migration   step of process formation, promoting cell spreading and extension. In contrast, Rho, and its downstream effector p160ROCK, regulated the release of adhesions to the substratum, and associated cellular contraction. Consequently, inhibition of Rho/p160ROCK leads to the formation of longer dendrites. DC therefore coordinate adhesion and protrusion to perform a specialized process of cellular morphogenesis, which differentiates these cells from all other cells of the immune system and may contribute to their distinctive function. 
 Sonic Hedgehog activates the GTPases Rac1 and RhoA in a Gli-independent manner through coupling of smoothened to Gi proteins. The vertebrate Hedgehog (Hh) pathway has essential functions during development and tissue homeostasis in normal physiology, and its dysregulation is a common theme in cancer. The Hh ligands (Sonic Hh, Indian Hh, and Desert Hh) bind to the receptors Patched1 and Patched2, resulting in inhibition of their repressive effect on Smoothened (Smo). Smo is a seven-transmembrane protein, which was only recently shown to function as a G protein-coupled receptor (GPCR) with specificity toward the heterotrimeric guanine nucleotide-binding protein G(i). In addition to activating G(i), Smo signals through its C-terminal tail to inhibit Suppressor of Fused, resulting in stabilization and activation of the Gli family of transcription factors, which execute a transcriptional response to so-called "canonical Hh signaling." In this Presentation, we illustrate two outcomes of Hh signaling that are independent of Gli transcriptional activity and, thus, are defined as "noncanonical." One outcome is dependent on Smo coupling to G(i) proteins and exerts changes to the actin cytoskeleton through stimulation of the small guanosine triphosphatases (GTPases) RhoA and Rac1. These cytoskeletal changes promote   migration   in fibroblasts and tubulogenesis in endothelial cells. Signaling through the other noncanonical Hh pathway is independent of Smo and inhibits Patched1-induced cell death. 
 MLK3 limits activated Galphaq signaling to Rho by binding to p63RhoGEF. Mixed lineage kinase 3 (MLK3) is a MAP3K that activates the JNK-dependent MAPK pathways. Here, we show that MLK3 is required for cell   migration   in a manner independent of its role as a MAP3K or MLK3 kinase activity. Rather, MLK3 functions in a regulated way to limit levels of the activated GTPase Rho by binding to the Rho activator, p63RhoGEF/GEFT, which, in turn, prevents its activation by Galphaq. These findings demonstrate a scaffolding role for MLK3 in controlling the extent of Rho activation that modulates cell   migration  . Moreover, they suggest that MLK3 functions as a network hub that links a number of signaling pathways. 
 Valvular dystrophy associated filamin A mutations reveal a new role of its first repeats in small-GTPase regulation. Filamin A (FlnA) is a ubiquitous actin binding protein which anchors various transmembrane proteins to the cell cytoskeleton and provides a scaffold to many cytoplasmic signaling proteins involved in actin cytoskeleton remodeling in response to mechanical stress and cytokines stimulation. Although the vast majority of FlnA binding partners interact with the carboxy-terminal immunoglobulin like (Igl) repeats of FlnA, little is known on the role of the amino-N-terminal repeats. Here, using cardiac mitral valvular dystrophy associated FlnA-G288R and P637Q mutations located in the N-terminal Igl repeat 1 and 4 respectively as a model, we identified a new role of FlnA N-terminal repeats in small Rho-GTPases regulation. Using FlnA-deficient melanoma and HT1080 cell lines as expression systems we showed that FlnA mutations reduce cell spreading and   migration   capacities. Furthermore, we defined a signaling network in which FlnA mutations alter the balance between RhoA and Rac1 GTPases activities in favor of RhoA and provided evidences for a role of the Rac1 specific GTPase activating protein FilGAP in this process. Together our work ascribed a new role to the N-terminal repeats of FlnA in Small GTPases regulation and supports a conceptual framework for the role of FlnA mutations in cardiac valve diseases centered around signaling molecules regulating cellular actin cytoskeleton in response to mechanical stress. 
 DLC1 tumor suppressor gene inhibits   migration   and invasion of multiple myeloma cells through RhoA GTPase pathway. DLC1 (deleted in liver cancer 1), a tumor suppressor gene that encodes a RhoGTPase-activating protein, is recurrently downregulated or silenced in various solid tumors and hematological malignancies because of epigenetic modifications or genomic deletion. Here, we identified DLC1 promoter hypermethylation in 43 out of 44 multiple myeloma (MM) cell lines, which resulted in downregulation or silencing of DLC1 in 41 samples. High frequency of tumor-specific methylation and attenuation or silencing of DLC1 expression could serve as an independent diagnostic marker for MM. Combined treatment with demethylating and acetylating agents significantly elevated the expression of DLC1 and suppressed MM cell proliferation. Two cell lines exhibiting complete promoter methylation and the absence of DLC1 expression were transduced by an adenoviral vector containing DLC1 cDNA. In both cell lines, the reexpression of DLC1 inhibited myeloma cell invasion and   migration  , reduced RhoA activity and resulted in the reorganization of actin cytoskeleton. These results provide the first evidence for the antiproliferative effect of DLC1 in a hematological cancer and implicate RhoA pathway in suppression of MM   migration   and invasion. Given the myeloma cells sensitivity to the reactivation of DLC1 function, the potential for molecular targeted therapy of DLC1-mediated pathways as well as epigenetic therapies hold prospects. 
 Rho GTPases RhoA and Rac1 mediate effects of dietary folate on metastatic potential of A549 cancer cells through the control of cofilin phosphorylation. Folate, an important nutrient in the human diet, has been implicated in cancer, but its role in metastasis is not established. We have shown previously that the withdrawal of medium folate leads to the inhibition of   migration   and invasion of A549 lung carcinoma cells. Here we have demonstrated that medium folate regulates the function of Rho GTPases by enabling their carboxyl methylation and translocation to plasma membrane. Conversely, the lack of folate leads to the retention of these proteins in endoplasmic reticulum. Folate also promoted the switch from inactive (GDP-bound) to active (GTP-bound) GTPases, resulting in the activation of downstream kinases p21-activated kinase and LIM kinase and phosphorylation of the actin-depolymerizing factor cofilin. We have further demonstrated that in A549 cells two GTPases, RhoA and Rac1, but not Cdc42, are immediate sensors of folate status: the siRNA silencing of RhoA or Rac1 blocked effects of folate on cofilin phosphorylation and cellular   migration   and invasion. The finding that folate modulates metastatic potential of cancer cells was confirmed in an animal model of lung cancer using tail vein injection of A549 cells in SCID mice. A folate-rich diet enhanced lung colonization and distant metastasis to lymph nodes and decreased overall survival (35 versus 63 days for mice on a folate-restricted diet). High folate also promoted epithelial-mesenchymal transition in cancer cells and experimental mouse tumors. Our study provides experimental evidence for a mechanism of metastasis promotion by dietary folate and highlights the interaction between nutrients and metastasis-related signaling. 
 A MAPK-Driven Feedback Loop Suppresses Rac Activity to Promote RhoA-Driven Cancer Cell Invasion. Cell   migration   in 3D microenvironments is fundamental to development, homeostasis and the pathobiology of diseases such as cancer. Rab-coupling protein (RCP) dependent co-trafficking of α5β1 and EGFR1 promotes cancer cell invasion into fibronectin (FN) containing extracellular matrix (ECM), by potentiating EGFR1 signalling at the front of invasive cells. This promotes a switch in RhoGTPase signalling to inhibit Rac1 and activate a RhoA-ROCK-Formin homology domain-containing 3 (FHOD3) pathway and generate filopodial actin-spike protrusions which drive invasion. To further understand the signalling network that drives RCP-driven invasiv  e migrati  on, we generated a Boolean logical model based on existing network pathways/models, where each node can be interrogated by computational simulation. The model predicted an unanticipated feedback loop, whereby Raf/MEK/ERK signalling maintains suppression of Rac1 by inhibiting the Rac-activating Sos1-Eps8-Abi1 complex, allowing RhoA activity to predominate in invasive protrusions. MEK inhibition was sufficient to promote lamellipodia formation and oppose filopodial actin-spike formation, and led to activation of Rac and inactivation of RhoA at the leading edge of cells moving in 3D matrix. Furthermore, MEK inhibition abrogated RCP/α5β1/EGFR1-driven invas  ive migra  tion. However, upon knockdown of Eps8 (to suppress the Sos1-Abi1-Eps8 complex), MEK inhibition had no effect on RhoGTPase activity and did not oppose invas  ive migra  tion, suggesting that MEK-ERK signalling suppresses the Rac-activating Sos1-Abi1-Eps8 complex to maintain RhoA activity and promote filopodial actin-spike formation and invas  ive migra  tion. Our study highlights the predictive potential of mathematical modelling approaches, and demonstrates that a simple intervention (MEK-inhibition) could be of therapeutic benefit in preventing invas  ive migra  tion and metastasis. 
 The prion protein inhibits monocytic cell   migration   by stimulating β1 integrin adhesion and uropod formation. The broad tissue distribution and evolutionary conservation of the glycosylphosphatidylinositol (GPI)-anchored prion protein (PrP, also known as PRNP) suggests that it plays a role in cellular homeostasis. Given that integrin adhesion determines cell behavior, the proposed role of PrP in cell adhesion might underlie the various in vitro and in vivo effects associated with PrP loss-of-function, including the immune phenotypes described in PrP(-/-) mice. Here, we investigated the role of PrP in the adhesion and (transendothelial)   migratio  n of human (pro)monocytes. We found that PrP regulates β1-integrin-mediated adhesion of monocytes. Additionally, PrP controls the cell morphology and migratory behavior of monocytes: PrP-silenced cells show deficient uropod formation on immobilized VCAM and display bleb-like protrusions on the endothelium. Our data further show that PrP regulates ligand-induced integrin activation. Finally, we found that PrP controls the activation of several proteins involved in cell adhesion an  d migrati  on, including RhoA and its effector cofilin, as well as proteins of the ERM family. We propose that PrP modulates β1 integrin adhesion a  nd migrat  ion of monocytes through RhoA-induced actin remodeling mediated by cofilin, and through the regulation of ERM-mediated membrane-cytoskeleton linkage. 
 MiR-200b/200c/429 subfamily negatively regulates Rho/ROCK signaling pathway to suppress hepatocellular carcinoma metastasis. MiR-200 family is an important regulator of epithelial-mesenchymal transition and has been implicated in human carcinogenesis. However, their expression and functions in human cancers remain controversial. In the work presented here, we showed that miR-200 family members were frequently down-regulated in hepatocellular carcinoma (HCC). Although all five members of miR-200 family inhibited ZEB1/2 expression in HCC cell lines, we showed that overexpression only of the miR-200b/200c/429 subfamily, but not the miR-200a/141 subfamily, resulted in impeded HCC cell   migration  . Further investigations led to the identification of RhoA and ROCK2 as specific down-stream targets of the miR-200b/200c/429 subfamily. We demonstrated that the miR-200b/200c/429 subfamily inhibited HCC cell   migration   through modulating Rho/ROCK mediated cell cytoskeletal reorganization and cell-substratum adhesion. Re-expression of miR-200b significantly suppressed lung metastasis of HCC cells in an orthotopic liver implantation model in vivo. In conclusion, our findings identified the miR-200b/200c/429 subfamily as metastasis suppressor microRNAs in human HCC and highlighted the functional discrepancy among miR-200 family members. 
 H2O2 inhibits proliferation and mediates suppression of   migration   via DLC1/RhoA signaling in cancer cells. RhoGTPase-activating proteins (RhoGAPs) regulate RhoGTPases in cells, but whether individual reactive oxygen species (ROS) regulate RhoGAPs is unknown. Our previous published papers have shown that deleted in liver cancer 1 (DLC1) inhibits cancer cell   migration   by its RhoGAP activity. The present study was designed to explore the role of H2O2 in regulation of DLC1.We treated cells with H2O2 for 24h and phenotypic changes were analyzed by MTT, RT-PCR, Western blotting, immunofluorescence staining and wound healing assays.H2O2 downregulated cyclin D1 and cyclin E to inhibit proliferation, and upregulated BAX to induce apoptosis in MCF-7 cells. Compared with non-tumorigenic cells, H2O2 increased expression of DLC1 and reduced activity of RhoA in cancer cells. Stress fiber production and   migration   were also suppressed by H2O2 in MDA-MB-231 cells.Our study suggests that H2O2 inhibits proliferation through modulation of cell cycle and apoptosis-related genes, and inhibits   migration   by decreasing stress fibers via DLC1/RhoA signaling. 
 mTOR regulate EMT through RhoA and Rac1 pathway in prostate cancer. Recently, an increasing number of studies have suggested that mTOR plays a critical role in the regulation of tumor cell motility, invasion and cancer metastasis. However, little is known about the signaling mechanisms in regulating epithelial-mesenchymal transition (EMT) of prostate cancer. In this study, we found that the expression levels of Raptor and Rictor in prostate cancer tissues were elevated, which may suggest that Raptor and Rictor signaling pathways are associated with prostate cancer progression and metastasis. Inhibition of mTORC1 or mTORC2 by knock down of Raptor or Rictor, respectively,   migration   and invasion of prostate cancer were attenuated. Furthermore, EMT, a characterized by the changed expression levels of various markers like E-cadherin, β-catenin, N-cadherin, and vimentin emergend following inhibition of Raptor or Rictor. Finally, the small GTPases (RhoA and Rac1) which were crucial regulatory proteins in cell   migratio  n and invasion were inactivited after downregulating Raptor and Rictor. These results suggest that mTOR regulate EMT at least in part by down regulation of RhoA and Rac1 signaling pathways. Our findings provide novel very attractive target strategies that the inhibition of mTOR signaling pathways may retard prostate cancer   migratio  n and invasion at early stages. 
 Annexin-A1 controls an ERK-RhoA-NFκB activation loop in breast cancer cells. Wound healing is critical for normal development and pathological processes including cancer cell metastasis. MAPK, Rho-GTPases and NFκB are important regulators of wound healing, but mechanisms for their integration are incompletely understood. Annexin-A1 (ANXA1) is upregulated in invasive breast cancer cells resulting in constitutive activation of NFκB. We show here that silencing ANXA1 increases the formation of stress fibers and focal adhesions, which may inhibit wound healing. ANXA1 regulated wound healing is dependent on the activation of ERK1/2. ANXA1 increases the activation of RhoA, which is dependent on ERK activation. Furthermore, active RhoA is important in NF-κB activation, where constitutively active RhoA potentiates NFκB activation, while dominant negative RhoA inhibits NFκB activation in response to CXCL12 stimulation and active MEKK plasmids. These findings establish a central role for ANXA1 in the   cell mig  ration through the activation of NFκB, ERK1/2 and RhoA. 
 [Under hypoxia condition contactin-1 regulates   migration   of MKN45 cells through RhoA pathway]. Recent studies have suggested that contactin-1 has a key role in cancer cell proliferation and   migration  , however the detailed mechanism of this process is still unclear. Here, human gastric cancer cell line MKN45 was employed. It was found that under hypoxia conditions contactin-1 mRNA and protein levels were both up-regulated by HIF-1alpha expression. Furthermore, although hypoxia increased the   migration   rate of MKN45 cells, contactin-1 (CNTN1) shRNA reversed this process. Meanwhile, RhoA V14 and RhoA V14N19 mutation constructs were employed, and it was found that constitutively active form of RhoA reversed the cell   migration   suppression induced by contactin-1 knockdown, while dominant-negative form of RhoA blocked hypoxia induced hyper  migration  . Apart from this, contactin-1 displayed the ability to phosphorylate the RhoA activator p115 RhoGEF. Thus, under hypoxia conditions, elevated HIF-1alpha seems to up-regulate contactin-1 expression and by this activate RhoA and facilitate   migration   of cancer cells. 
 Rho GTPases RhoA and Rac1 mediate effects of dietary folate on metastatic potential of A549 cancer cells through the control of cofilin phosphorylation. Folate, an important nutrient in the human diet, has been implicated in cancer, but its role in metastasis is not established. We have shown previously that the withdrawal of medium folate leads to the inhibition of   migration   and invasion of A549 lung carcinoma cells. Here we have demonstrated that medium folate regulates the function of Rho GTPases by enabling their carboxyl methylation and translocation to plasma membrane. Conversely, the lack of folate leads to the retention of these proteins in endoplasmic reticulum. Folate also promoted the switch from inactive (GDP-bound) to active (GTP-bound) GTPases, resulting in the activation of downstream kinases p21-activated kinase and LIM kinase and phosphorylation of the actin-depolymerizing factor cofilin. We have further demonstrated that in A549 cells two GTPases, RhoA and Rac1, but not Cdc42, are immediate sensors of folate status: the siRNA silencing of RhoA or Rac1 blocked effects of folate on cofilin phosphorylation and cellular   migration   and invasion. The finding that folate modulates metastatic potential of cancer cells was confirmed in an animal model of lung cancer using tail vein injection of A549 cells in SCID mice. A folate-rich diet enhanced lung colonization and distant metastasis to lymph nodes and decreased overall survival (35 versus 63 days for mice on a folate-restricted diet). High folate also promoted epithelial-mesenchymal transition in cancer cells and experimental mouse tumors. Our study provides experimental evidence for a mechanism of metastasis promotion by dietary folate and highlights the interaction between nutrients and metastasis-related signaling. 
 Type II cGMP‑dependent protein kinase inhibits RhoA activation in gastric cancer cells. Small GTPase RhoA is a key signaling component regulating cel  l migrati  on and stress fiber formation. Previous studies have shown that RhoA activity is regulated by protein kinases, such as cAMP‑dependent protein kinase (PKA) and type I cGMP‑dependent protein kinase (PKGI), which phosphorylate the protein. This study was designed to investigate the effect of type II cGMP‑dependent protein kinase (PKGII) on RhoA activity. Cells of the human gastric cancer line AGS were infected with adenoviral constructs bearing the PKGII cDNA in order to increase its endogenous expression, and were treated with 8‑pCPT‑cGMP to activate the PKGII enzyme. A transwell assay was performed to measure the migratory activity of the treated cells, and immunofluorescent microscopy was used to observe the formation of stress fibers. The phosphorylation of RhoA was detected by western blotting, and the activity of RhoA was measured by a pull‑down assay. Co‑immunoprecipitation (co‑IP) was performed to detect binding of PKGII to RhoA. Glutathione S‑transferase (GST)‑fused fragments of RhoA and PKGII were expressed in Escherichia coli and used to investigate the domains required for the binding. The results showed that lysophosphatidic acid (LPA) tr  eatment i  ncreased the migration and the formation of stress fibers in AGS cells and that this effect was RhoA‑dependent. An increase in PKGII activity, not onl  y inhibit  ed LPA‑induced migration and stress fiber formation, but also suppressed LPA‑induced activation of RhoA. PKGII caused serine 188 (Ser188) phosphorylation of RhoA, but not the phosphorylation of the mutant RhoA Ser188A, and therefore had no inhibitory effect on the activity of the mutant protein. Co‑IP results showed that there is direct binding of PKGII to RhoA. The GST pull‑down assay showed that the fragment containing RhoA amino acid residues 1‑44 and the N‑terminal fragment of PKGII containing amino acid residues 1‑176 are required for the binding between the two proteins. These results suggested that PKGII inhibits RhoA activity by binding to this small GTPase and causing phosphorylation at its Ser188 site. 
 Fibroblast activation protein (FAP) is essential for the   migration   of bone marrow mesenchymal stem cells through RhoA activation. The ability of human bone marrow mesenchymal stem cells (BM-MSCs) to migrate and localize specifically to injured tissues is central in developing therapeutic strategies for tissue repair and regeneration. Fibroblast activation protein (FAP) is a cell surface serine protease expressed at sites of tissue remodeling during embryonic development. It is also expressed in BM-MSCs, but not in normal tissues or cells. The function of FAP in BM-MSCs is not known.We found that depletion of FAP proteins significantly inhibited the   migration   of BM-MSCs in a transwell chemotaxis assay. Such impaired   migration   ability of BM-MSCs could be rescued by re-expressing FAP in these cells. We then demonstrated that depletion of FAP activated intracellular RhoA GTPase. Consistently, inhibition of RhoA activity using a RhoA inhibitor rescued its   migration   ability. Inhibition of FAP activity with an FAP-specific inhibitor did not affect the activation of RhoA or the   migration   of BM-MSCs. Furthermore, the inflammatory cytokines interleukin-1beta (IL-1β) and transforming growth factor-beta (TGF-β) upregulated FAP expression, which coincided with better BM-MS  C migrati  on.Our results indicate FAP plays an important role in th  e migrati  on of BM-MSCs through modulation of RhoA GTPase activity. The peptidase activity of FAP is not essential for suc  h migrati  on. Cytokines IL-1β and TGF-β upregulate the expression level of FAP and thus enhance BM-  MSC migra  tion. 
 Valvular dystrophy associated filamin A mutations reveal a new role of its first repeats in small-GTPase regulation. Filamin A (FlnA) is a ubiquitous actin binding protein which anchors various transmembrane proteins to the cell cytoskeleton and provides a scaffold to many cytoplasmic signaling proteins involved in actin cytoskeleton remodeling in response to mechanical stress and cytokines stimulation. Although the vast majority of FlnA binding partners interact with the carboxy-terminal immunoglobulin like (Igl) repeats of FlnA, little is known on the role of the amino-N-terminal repeats. Here, using cardiac mitral valvular dystrophy associated FlnA-G288R and P637Q mutations located in the N-terminal Igl repeat 1 and 4 respectively as a model, we identified a new role of FlnA N-terminal repeats in small Rho-GTPases regulation. Using FlnA-deficient melanoma and HT1080 cell lines as expression systems we showed that FlnA mutations reduce cell spreading and   migration   capacities. Furthermore, we defined a signaling network in which FlnA mutations alter the balance between RhoA and Rac1 GTPases activities in favor of RhoA and provided evidences for a role of the Rac1 specific GTPase activating protein FilGAP in this process. Together our work ascribed a new role to the N-terminal repeats of FlnA in Small GTPases regulation and supports a conceptual framework for the role of FlnA mutations in cardiac valve diseases centered around signaling molecules regulating cellular actin cytoskeleton in response to mechanical stress. 
 Balanced Tiam1-rac1 and RhoA drives proliferation and invasion of pancreatic cancer cells. Tiam1 is a rac1-specific guanine nucleotide exchange factor, and Tiam1-rac1 is involved in a number of cellular processes. Rac1 and RhoA act as molecular switches that cycle between GTP- and GDP-bound states to balance the activities of rac1 and RhoA. The downregulation of rac1 activity leads to upregulation of RhoA activity, which promotes invasion and   migration   of pancreatic cancers cells. At present, however, the role of Tiam1-rac1 and RhoA in pancreatic cancers is not fully understood. We found that Tiam1 was upregulated in pancreatic cancers and was significantly expressed in tumors without lymph node involvement or distant metastasis compared with cancers where there was involvement. Although Tiam1-rac1 signaling promoted pancreatic cancer cell proliferation and tumor growth via the Wnt signaling pathway in vitro and in vivo, inhibiting Tiam1-rac1 signaling did not prolong the overall survival time in vivo. This provided evidence that there was a balance between rac1 and RhoA activities in pancreatic cancers. Furthermore, only the combined inhibition of Tiam1-rac1 and RhoA had a beneficial effect on the growth of pancreatic cancers in vivo. Taken together, these results suggest that the progression of pancreatic tumors is partially controlled by the balance between Tiam1-rac1 and RhoA. 
 Immunohistochemical expression of Rho GTPases in ameloblastomas. Rho GTPases are proteins that regulate cell cycle, shape, polarization, invasion,   migration  , and apoptosis, which are important characteristics of normal and neoplastic cells. Rho GTPases expression has been reported in normal tooth germ and several pathologies; however, it has not been evaluated in ameloblastomas. The aim of this study was to analyze the expression and distribution of RhoA, RhoB, Rac1, and Cdc42 Rho GTPases in solid and unicystic ameloblastomas. Three-micrometer sections from paraffin-embedded specimens were evaluated by using an avidin-biotin immunohistochemical method with antibodies against the proteins mentioned above. RhoA and RhoB staining was observed in a high number of cells (P < 0.05) and greater intensity in non-polarized ones. Rac1 was not observed, and Cdc42 did not show any statistical differences between the number of non-polarized and basal positive cells (P > 0.05). Upon comparing the studied ameloblastomas, a higher number of positive cells in the unicystic variant was observed than that in the solid one (P < 0,05). The results obtained suggest that these GTPases could play a role in the ameloblastoma neoplastic epithelial cell phenotype determination (polarized or non-polarized), as well as in variant (solid or unicystic) and subtype (follicular or plexiform) determination. Furthermore, they could participate in solid ameloblastoma invasion mechanisms. 
 Interleukin-8 induces the endothelial cell   migration   through the Rac 1/RhoA-p38MAPK pathway. Endothelial cell   migration   is essential for tumor angiogenesis, and interleukin-8 (IL-8) has been shown to play an important role in tumor growth, angiogenesis, and metastasis. The objective of this study was to investigate the molecular mechanism of IL-8 induced endothelial cell   migration   in vitro.Fluorescence microscope was used to study the distribution of cytoskeleton. The expression of Rac1 and RhoA protein was detected by western blotting. After endothelial cells were transfected by lipofectamine 2000 reagent, the Transwell chamber motility assay was applied to observe the   migration   of endothelial cells induced by IL-8. The active p38MAPK (mitogen-activated protein kinase) was evaluated by the p38MAPK activation assay.We demonstrated that IL-8 activated cell   migration   can be impaired by p38MAPK inhibitor, suggesting the participation of p38MAPK in the cell   migration  . Our results indicated that p38MAPK signaling is required for membrane ruffles, lamellipodia extensions, and actin stress fibers formation induced by IL-8. Furthermore, p38MAPK inhibitor led to increased Rac1 and RhoA expression in IL-8 treated EA.hy926 cells. In addition, IL-8 induced p38MAPK activation was suppressed by dominant-negative mutant for Rac1 and RhoA.Our study demonstrates that IL-8-Rac1/RhoA-p38MAPK signaling pathway plays a vital role in the IL-8-induced endothelial cell   migration  , and it provides new insight into the molecular mechanisms by which IL-8 contributes to tumor angiogenesis and metastasis. 
 Integrin α6β4 cooperates with LPA signaling to stimulate Rac through AKAP-Lbc-mediated RhoA activation. The α(6)β(4) integrin promotes carcinoma invasion through its ability to promote direc  ted migra  tion and polarization of carcinoma cells. In this study, we explore how the α(6)β(4) integrin cooperates with lysophosphatidic acid (LPA) to activate Rho and Rac small GTPases. Through the use of dominant negative Rho constructs, C3 exotransferase, and Rho kinase inhibitor, we find that Rho is critical for LPA-dependent chemotaxis and lamellae formation. However, utilization of specific Rho isoforms depends on integrin α(6)β(4) expression status. Integrin α(6)β(4)-negative MDA-MB-435 cells utilize only RhoC for motility, whereas integrin α(6)β(4)-expressing cells utilize RhoC but additionally activate and utilize RhoA for LPA-dependent cell motility and lamellae formation. Notably, the activation of RhoA by cooperative LPA and integrin α(6)β(4) signaling requires the Rho guanine nucleotide exchange factor AKAP-Lbc. We also determine that integrin α(6)β(4) cannot activate Rac1 directly but promotes LPA-mediated Rac1 activation that is dependent on RhoA activity and de novo β(1) integrin ligation. Finally, we find that the regulation of Rac1 and RhoA in response to LPA is differentially regulated by phosphodiesterases, PKA, and phosphatidylinositol 3-kinase, thus supporting their spatially distinct compartmentalization. In summary, signaling from integrin α(6)β(4) facilitates LPA-stimulated chemotaxis through preferential activation of RhoA, which, in turn, facilitates activation of Rac1. 
 Sonic Hedgehog activates the GTPases Rac1 and RhoA in a Gli-independent manner through coupling of smoothened to Gi proteins. The vertebrate Hedgehog (Hh) pathway has essential functions during development and tissue homeostasis in normal physiology, and its dysregulation is a common theme in cancer. The Hh ligands (Sonic Hh, Indian Hh, and Desert Hh) bind to the receptors Patched1 and Patched2, resulting in inhibition of their repressive effect on Smoothened (Smo). Smo is a seven-transmembrane protein, which was only recently shown to function as a G protein-coupled receptor (GPCR) with specificity toward the heterotrimeric guanine nucleotide-binding protein G(i). In addition to activating G(i), Smo signals through its C-terminal tail to inhibit Suppressor of Fused, resulting in stabilization and activation of the Gli family of transcription factors, which execute a transcriptional response to so-called "canonical Hh signaling." In this Presentation, we illustrate two outcomes of Hh signaling that are independent of Gli transcriptional activity and, thus, are defined as "noncanonical." One outcome is dependent on Smo coupling to G(i) proteins and exerts changes to the actin cytoskeleton through stimulation of the small guanosine triphosphatases (GTPases) RhoA and Rac1. These cytoskeletal changes promote   migration   in fibroblasts and tubulogenesis in endothelial cells. Signaling through the other noncanonical Hh pathway is independent of Smo and inhibits Patched1-induced cell death. 
 Interleukin-8 induces the endothelial cell   migration   through the activation of phosphoinositide 3-kinase-Rac1/RhoA pathway. Endothelial cell   migration   is essential for tumor angiogenesis, and interleukin-8 (IL-8) has been shown to play an important role in tumor growth, angiogenesis, and metastasis. This study aimed to investigate the molecular mechanism of IL-8 induced endothelial cell   migration  . Our results indicated that IL-8 induced a rapid rearrangement of the actin cytoskeleton in EA.Hy926 cells, generating extensions resembling membrane ruffling and stress fibers. These processes required parallel upregulation of the small GTPases Rac1 and RhoA. Moreover, we demonstrated that IL-8 activated PI3K following the same kinetics observed from IL-8 induction of cytoskeletal rearrangement, suggesting the participation of PI3K in these processes. Taken together, our study demonstrates that PI3K-Rac1/RhoA signaling pathway plays a vital role in IL-8 induced endothelial cell   migration  , and provides new insight into the molecular mechanisms by which IL-8 contributes to tumor angiogenesis and metastasis. 
 Abnormal activation of Ras/Raf/MAPK and RhoA/ROCKII signalling pathways in eutopic endometrial stromal cells of patients with endometriosis. BACKGROUND Enhanced proliferation and survival of eutopic endometrial cells from patients with endometriosis compared with healthy women is associated with abnormal activation of extra-cellular signal-regulated kinases 1 and 2 (ERK1/2). Given the role of Ras/Raf/mitogen-activated protein kinase (MAPK) and RhoA/ROCKII signalling pathways in the regulation of cell proliferation and   migration  , we analysed their possible roles in endometriosis. METHODS Primary eutopic endometrial stromal cells of patients with endometriosis (Eu-hESC, n= 16) and endometriosis-free controls (Co-hESC, n= 14) were harvested and subjected to proliferation and   migration   assays as well as kinase activity assays and immunoblot analysis of proteins from the Ras/Raf/MAPK and RhoA/ROCKII signalling pathways. Effects of ROCKII (Y-27632) and MAPK (U0126) inhibitors or siRNA knockdown of ROCKII, Raf-1 and B-Raf were analysed. RESULTS The proliferation rate of Eu-hESC was 54% higher than Co-hESC. Eu-hESC also displayed a 75% higher   migration   rate than Co-hESC. Eu-hESC displayed higher levels of ERK phosphorylation (83%) and p27 expression (61%) and lower levels of Raf-1 protein (47%) compared with controls. In addition to an inhibitory effect on cell proliferation, ROCKII knockdown led to significant down-regulation of cyclinD1 and p27 but did not affect ERK phosphorylation. Down-regulation of Raf-1 by siRNA was dispensable for cell proliferation control but led to an increase in ROCKII activity and a decrease in cell   migration  . B-Raf was shown to act as a regulator of hESC proliferation by modulating cellular ERK1/2 activity and cyclinD1 levels. Eu-hESC displayed 2.4-fold higher B-Raf activity compared with Co-hESC and therefore exhibit abnormally activated Ras/Raf/MAPK signalling. CONCLUSIONS We show that the same molecular mechanisms operate in Co- and Eu-hESC. The differences in cell proliferation and   migration   between both cell types are likely due to increased activation of Ras/Raf/MAPK and RhoA/ROCKII signalling pathways in cells from endometriosis patients. 
 Vascular endothelial growth factor C promotes cervical cancer metastasis via up-regulation and activation of RhoA/ROCK-2/moesin cascade. The elevated expression of vascular endothelial growth factor C (VEGF-C) is correlated with clinical cervical cancer metastasis and patient survival, which is interpreted by VEGF-C functions to stimulate angiogenesis and lymphatic genesis. However, the direct impact of VEGF-C on cervical cancer cell motility remains largely unknown.In this study, we investigated the effects of VEGF-C on actin cytoskeleton remodeling and on cervical cancer cell   migration   and invasion and how the actin-regulatory protein, moesin regulated these effects through RhoA/ROCK-2 signaling pathway.On cervical carcinoma cell line SiHa cells, exposure of VEGF-C triggered remodeling of the actin cytoskeleton and the formation of membrane ruffles, which was required for cell movement. VEGF-C significantly enhanced SiHa cells horizontal   migration   and three-dimensional invasion into matrices. These actions were dependent on increased expression and phosphorylation of the actin-regulatory protein moesin and specific moesin siRNA severely impaired VEGF-C stimulated-cell   migration  . The extracellular small GTPase RhoA/ROCK-2 cascade mediated the increased moesin expression and phosphorylation, which was discovered by the use of Y-27632, a specific inhibitor of Rho kinase and by transfected constitutively active, dominant-negative RhoA as well as ROCK-2 SiRNA. Furthermore, in the surgical cervical specimen from the patients with FIGO stage at cervical intra-epithelial neoplasia and I-II cervical squamous cell carcinoma, the expression levels of moesin were found to be significantly correlated with tumor malignancy and metastasis.These results implied that VEGF-C promoted cervical cancer metastasis by upregulation and activation of moesin protein through RhoA/ROCK-2 pathway. Our findings offer new insight into the role of VEGF-C on cervical cancer progression and may provide potential targets for cervical cancer therapy. 
 KiSS1 suppresses TNFalpha-induced breast cancer cell invasion via an inhibition of RhoA-mediated NF-kappaB activation. Tumor necrosis factor-alpha (TNFalpha) induces cancer development and metastasis, which is prominently achieved by nuclear factor-kappa B (NF-kappaB) activation. TNFalpha-induced NF-kappaB activation enhances cellular mechanisms including proliferation,   migration  , and invasion. KiSS1, a key regulator of puberty, was initially discovered as a tumor metastasis suppressor. The expression of KiSS1 was lost or down-regulated in different metastatic tumors. However, it is unclear whether KiSS1 regulates TNFalpha-induced NF-kappaB activation and further tumor cell   migration  . In this study, we demonstrate that KiSS1 suppresses the   migration   of breast cancer cells by inhibiting TNFalpha-induced NF-kappaB pathway and RhoA activation. Both KiSS1 overexpression and KP10 (kisspeptin-10) stimulation inhibited TNFalpha-induced NF-kappaB activity, suppressed TNFalpha-induced cell   migration   and cell attachment to fibronectin in breast cancer cells while KP10 has little effect on cancer cell proliferation. Furthermore, KP10 inhibited TNFalpha-induced cell   migration   and RhoA GTPase activation. Therefore, our data demonstrate that KiSS1 inhibits TNFalpha-induced NF-kappaB activation via downregulation of RhoA activation and suppression of breast cancer cell   migration   and invasion. 
 Mutation of ARHGAP9 in patients with coronary spastic angina. Coronary artery spasm has an important function in the etiology of variant angina and other acute coronary syndromes. Abnormal activation of Rho-family GTPases has been observed in cardiovascular disorders, but the function of genetic variability in Rho-family GTPases remains to be evaluated in cardiovascular disorders. We examined the genetic variability of Rho-family GTPases and their regulators in coronary artery spasm. We performed a comprehensive candidate gene analysis of 67 single nucleotide polymorphisms with amino-acid substitution in Rho-family GTPases and their regulators in 103 unrelated Japanese patients with acetylcholine-induced coronary artery spasm and 102 control Japanese subjects without acetylcholine-induced coronary artery spasm. We noted an association of the single nucleotide polymorphism of ARHGAP9 (rs11544238, Ala370Ser) with coronary artery spasm (odds ratio =2.67). We found that ARHGAP9 inactivated Rac as RacGAP and that the mRNA level of ARHGAP9 was strongly detected in hematopoietic cells. ARHGAP9 negatively regulated cell   migration  . The Ala370Ser polymorphism counteracted ARHGAP9-reduced cell   migration  , spreading and adhesion. The Ala370Ser polymorphism in the ARHGAP9 gene is associated with coronary artery spasm. These data suggest that the polymorphism of ARHGAP9 has a critical function in the infiltration of hematopoietic cells into the endothelium and inflammation leading to endothelial dysfunction. 
 Integrin alpha8beta1 regulates adhesion,   migration   and proliferation of human intestinal crypt cells via a predominant RhoA/ROCK-dependent mechanism. Integrins are transmembrane alphabeta heterodimer receptors that function as structural and functional bridges between the cytoskeleton and ECM (extracellular matrix) molecules. The RGD (arginine-glycine-aspartate tripeptide motif)-dependent integrin alpha8beta1 has been shown to be involved in various cell functions in neuronal and mesenchymal-derived cell types. Its role in epithelial cells remains unknown.Integrin alpha8beta1 was found to be expressed in the crypt cell population of the human intestine but was absent from differentiating and mature epithelial cells of the villus. The function of alpha8beta1 in epithelial crypt cells was investigated at the cellular level using normal HIECs (human intestinal epithelial cells). Specific knockdown of alpha8 subunit expression using an shRNA (small-hairpin RNA) approach showed that alpha8beta1 plays important roles in RGD-dependent cell adhesion,   migration   and proliferation via a RhoA/ROCK (Rho-associated kinase)-dependent mechanism as demonstrated by active RhoA quantification and pharmacological inhibition of ROCK. Moreover, loss of alpha8beta1, through RhoA/ROCK, impairs FA (focal adhesion) complex integrity as demonstrated by faulty vinculin recruitment.Integrin alpha8beta1 is expressed in epithelial cells. In intestinal crypt cells, alpha8beta1 is closely involved in the regulation of adhesion,   migration   and cell proliferation via a predominant RhoA/ROCK-dependent mechanism. These results suggest an important role for this integrin in intestinal crypt cell homoeostasis. 
 Role of isoprenylcysteine carboxylmethyltransferase-catalyzed methylation in Rho function and   migration  . A number of proteins that play key roles in biological regulatory events undergo a process of post-translational modifications termed prenylation. The prenylation pathway consists of three enzymatic steps; the final processed protein is isoprenoid-modified and methylated on the C-terminal cysteine. This protein modification pathway plays a significant role in cancer biology because many oncogenic proteins undergo prenylation. Methylation of the C terminus by isoprenylcysteine carboxylmethyltransferase (Icmt) is the final step in the prenylation pathway. Cysmethynil, a specific Icmt inhibitor discovered in our laboratory, is able to inhibit Ras-mediated signaling, cell growth, and oncogenesis. We sought to examine the role of Icmt-mediated methylation on the behaviors of cancer cells associated with metastatic potential. Our results indicate that inhibition of methylation reduces   migration   of the highly metastatic MDA-MB-231 breast cancer cell line. In addition, cell adhesion and cell spreading are also significantly impacted by cysmethynil. To examine the mechanism of Icmt-dependent   migration   we focused on RhoA and Rac1, prenylated proteins that are important mediators of cell   migration   through their control of the actin cytoskeleton. Inhibition of Icmt significantly decreases the activation of both RhoA and Rac1; an increase in Rho GDP-dissociation inhibitor (RhoGDI) binding in the absence of methylation appears to contribute to this effect. Furthermore, in the absence of Icmt activity the addition of exogenous RhoA or Rac1 is able to partially rescue directed and random   migration  , respectively. These findings establish a role for Icmt-mediated methylation in cell   migration   and advance our understanding of the biological consequences of Rho methylation. 
 DLC1 tumor suppressor gene inhibits   migration   and invasion of multiple myeloma cells through RhoA GTPase pathway. DLC1 (deleted in liver cancer 1), a tumor suppressor gene that encodes a RhoGTPase-activating protein, is recurrently downregulated or silenced in various solid tumors and hematological malignancies because of epigenetic modifications or genomic deletion. Here, we identified DLC1 promoter hypermethylation in 43 out of 44 multiple myeloma (MM) cell lines, which resulted in downregulation or silencing of DLC1 in 41 samples. High frequency of tumor-specific methylation and attenuation or silencing of DLC1 expression could serve as an independent diagnostic marker for MM. Combined treatment with demethylating and acetylating agents significantly elevated the expression of DLC1 and suppressed MM cell proliferation. Two cell lines exhibiting complete promoter methylation and the absence of DLC1 expression were transduced by an adenoviral vector containing DLC1 cDNA. In both cell lines, the reexpression of DLC1 inhibited myeloma cell invasion and   migration  , reduced RhoA activity and resulted in the reorganization of actin cytoskeleton. These results provide the first evidence for the antiproliferative effect of DLC1 in a hematological cancer and implicate RhoA pathway in suppression of MM   migration   and invasion. Given the myeloma cells sensitivity to the reactivation of DLC1 function, the potential for molecular targeted therapy of DLC1-mediated pathways as well as epigenetic therapies hold prospects. 
 Blockade of the RhoA-JNK-c-Jun-MMP2 cascade by atorvastatin reduces osteosarcoma cell invasion. Osteosarcoma is characterized by a high malignant and metastatic potential, which points to the need for new therapeutic strategies to prevent cell metastasis. In this study, we show that statin-induced HMG-CoA reductase inhibition reduces cell   migration   and invasion in human and murine osteosarcoma cells, independently of the genotype. The statin-induced reduction of cell   migration   and invasion was independent of induction of apoptosis and was geranylgeranylpyrophosphate-dependent. The statin reduced matrix metalloproteinase (MMP) 2, 9, and 14 and TIMP2 expression or activity in invading cells. Forced expression of MMP2 and MMP14 overcame the inhibitory effect of the statin on cell invasion, suggesting a role for these MMPs in invasive potential. We also investigated the mechanisms involved in the reduced MMP2 activity and cell invasion. Inhibition of JNK, but not ERK1/2 signaling, reduced MMP2 activity. Pharmacological or constitutive activation of JNK overcame the reduced MMP2 activity and cell invasion induced by the statin. The statin decreased JNK phosphorylation and c-Jun nuclear translocation, suggesting that HMG-CoA reductase inhibition targets the JNK-c-Jun signaling pathway. We showed that mevalonate or geranylgeranylpyrophosphate treatment prevented the statin-induced reduction in JNK phosphorylation, MMP2 activity, and cell invasion. Forced expression of a constitutively active form of RhoA increased JNK phosphorylation and overcame the inhibitory effect of atorvastatin on MMP2 activity and cell invasion. The data establish a link between RhoA, JNK, c-Jun, and MMP2 activity that is functionally involved in the reduction in osteosarcoma cell invasion by the statin. This suggests a novel strategy targeting RhoA-JNK-c-Jun signaling to reduce osteosarcoma cell tumorigenesis. 
 MLK3 limits activated Galphaq signaling to Rho by binding to p63RhoGEF. Mixed lineage kinase 3 (MLK3) is a MAP3K that activates the JNK-dependent MAPK pathways. Here, we show that MLK3 is required for cell   migration   in a manner independent of its role as a MAP3K or MLK3 kinase activity. Rather, MLK3 functions in a regulated way to limit levels of the activated GTPase Rho by binding to the Rho activator, p63RhoGEF/GEFT, which, in turn, prevents its activation by Galphaq. These findings demonstrate a scaffolding role for MLK3 in controlling the extent of Rho activation that modulates cell   migration  . Moreover, they suggest that MLK3 functions as a network hub that links a number of signaling pathways. 
 [The Rho protein family and its role in the cellular cytoskeleton]. Proteins of the Rho protein family (RhoA, Rac1, Cdc42) work as molecular switches in the regulation of many cellular processes. They are involved in cell   migration  , cell-cycle control, apoptosis, and the regulation of gene transcription. Rho proteins show their activity mainly in the cell's cytoskeleton by taking part in the reorganization of microfilaments and microtubules. In recent years, significant progress has been made in understanding the biochemical and genetic nature of many processes controlled by Rho proteins. Although there are still seveal unknowns and hypothesizes which require confirmation, newer and more precise experimental results allow us to better understand these processes. It has also been suggested to use Rho family proteins in the therapy of some diseases. The purpose of this study was to characterize the significance of Rho processes in the cellular cytoskeleton. 
 S1P(2) receptors mediate inhibition of glioma cell   migration   through Rho signaling pathways independent of PTEN. Sphingosine 1-phosphate (S1P) induced the inhibition of glioma cell   migration  . Here, we characterized the signaling mechanisms involved in the inhibitory action by S1P. In human GNS-3314 glioblastoma cells, the S1P-induced inhibition of cell   migration   was associated with activation of RhoA and suppression of Rac1. The inhibitory action of S1P was recovered by a small interference RNA specific to S1P(2) receptor, a carboxyl-terminal region of Galpha12 or Galpha13, an RGS domain of p115RhoGEF, and a dominant-negative mutant of RhoA. The inhibitory action of S1P through S1P(2) receptors was also observed in both U87MG glioblastoma and 1321N1 astrocytoma cells, which have no protein expression of a phosphatase and tensin homolog deleted on chromosome 10 (PTEN). These results suggest that S1P(2) receptors/G(12/13)-proteins/Rho signaling pathways mediate S1P-induced inhibition of glioma cell   migration  . However, PTEN, recently postulated as an indispensable molecule for the inhibition of cell   migration  , may not be critical for the S1P(2) receptor-mediated action in glioma cells. 
 Regulation of ephexin1, a guanine nucleotide exchange factor of Rho family GTPases, by fibroblast growth factor receptor-mediated tyrosine phosphorylation. Fibroblast growth factor (FGF) signal is implicated in not only cell proliferation, but cell   migration   and morphological changes. Several different Rho family GTPases downstream of the Ras/ERK pathway are postulated to mediate the latter functions. However, none have been recognized to be directly coupled to FGF receptors (FGFRs). We have previously reported that EphA4 and FGFRs hetero-oligomerize through their cytoplasmic domains, trans-activate each other, and transduce a signal for cell proliferation through a docking protein, FRS2alpha (Yokote, H., Fujita, K., Jing, X., Sawada, T., Liang, S., Yao, L., Yan, X., Zhang, Y., Schlessinger, J., and Sakaguchi, K. (2005) Proc. Natl. Acad. Sci. U. S. A. 102, 18866-18871). Here, we have found that ephexin1, a guanine nucleotide exchange factor for Rho family GTPases, constitutes another downstream component of the receptor complex. Ephexin1 directly binds to the kinase domain of FGFR mainly through its DH and PH domains. The binding appears to become weaker and limited to the DH domain when FGFRs become activated. FGFR-mediated phosphorylation of ephexin1 enhances the guanine nucleotide exchange activity toward RhoA without affecting the activity to Rac1 or Cdc42. The FGFR-mediated tyrosine phosphorylation includes, but is not limited to, the residue (Tyr-87) phosphorylated by Src family kinase, which is known to be activated following EphA4 activation. The Tyr-to-Asp mutations that mimic the tyrosine phosphorylation in some of the putative FGFR-mediated phosphorylation sites increase the nucleotide exchange activity for RhoA without changing the activity for Rac1 or Cdc42. From these results, we conclude that ephexin1 is located immediately downstream of the EphA4-FGFR complex and the function is altered by the FGFR-mediated tyrosine phosphorylation at multiple sites. 
 Urokinase-induced   migration   of human vascular smooth muscle cells requires coupling of the small GTPases RhoA and Rac1 to the Tyk2/PI3-K signalling pathway. Urokinase-type plasminogen activator (uPA) facilitates cell   migration   by localizing proteolisys on the cell surface and by inducing intracellular signalling pathways. In human vascular smooth muscle cell (VSMC) uPA stimulates   migration   via the uPA receptor (uPAR) signalling complex containing the Janus kinase Tyk2 and phosphatidylinositol 3-kinase (PI3-K). We report that active GTP-bound forms of small GTPases RhoA and Rac1, but not Cdc42, are directly associated with Tyk2 and PI3-K in an uPA/uPAR-dependent fashion. Endogenous RhoA, but not Rac1 or Cdc42, was significantly activated in response to uPA. RhoA activation was abolished by cell treatment with two unrelated, structurally distinct, specific inhibitors of PI3-K, wortmannin, and LY294002. Downstream of RhoA, phosphorylation of myosin light chain (MLC) was dramatically upregulated by uPA in a Rho kinase- and PI3-K-dependent manner. Thus, selective Rho kinase inhibitor Y27632 and PI3-K inhibitors wortmannin and LY294002 prevented the uPA-induced stimulation of MLC phosphorylation. Rho kinase inhibition also decreased uPA-stimulated VSMC   migration   as observed in a Boyden chamber. VSMC immunocytochemical staining demonstrated redistribution of RhoA and Rac1 active forms to the newly formed leading edge of migrating cell. VSMC microinjection with antibodies to either Rho or Rac1 decreased uPA-stimulated cell   migration  , indicating the involvement of both GTPases in the   migration   process. Our results provide evidence that the small GTPases RhoA and Rac1, together with Rho kinase, are necessary to mediate the uPA/uPAR-directed   migration   via the Tyk2/PI3-K signalling complex in human VSMC. 
 Extension, retraction and contraction in the formation of a dendritic cell dendrite: distinct roles for Rho GTPases. The morphology of antigen-presenting dendritic cells (DC) is characterized by the possession of numerous long arborizing processes known as dendrites. The formation of these processes by DC, both in the periphery and in lymphoid organs, is believed to contribute to the remarkable efficiency with which they take up, process and present antigen to T cells. However, the process of dendrite formation and the signaling pathways that lead to the formation of these dendrites remain obscure. In this study we describe an in vitro model in which human immature DC form long processes similar to those formed in vivo. The formation of these processes involves initial attachment of a cell protrusion to the extracellular matrix substrate, and subsequent movement of the cell body away from the adhesion site, leaving behind a long slender dendrite. Dendrite formation, but not their maintenance, was found to be dependent on the activity of Rho GTPases. More specifically, Cdc42 and Rac1 were both required for the   migration   step of process formation, promoting cell spreading and extension. In contrast, Rho, and its downstream effector p160ROCK, regulated the release of adhesions to the substratum, and associated cellular contraction. Consequently, inhibition of Rho/p160ROCK leads to the formation of longer dendrites. DC therefore coordinate adhesion and protrusion to perform a specialized process of cellular morphogenesis, which differentiates these cells from all other cells of the immune system and may contribute to their distinctive function. 
 Maspin controls mammary tumor cell   migration   through inhibiting Rac1 and Cdc42, but not the RhoA GTPase. Rac1 and Cdc42 are members of the Rho family of small GTPases that play essential roles in diverse cellular functions, including cell   migration  . The activities of these Rho family proteins are controlled by growth factor receptor activation and cell-ECM interactions. Here, we show that maspin, a well-documented tumor suppressor gene, also controls cell motility through inhibiting Rac1/Cdc42 activity. Using the GST-PAK and GST-Rho binding protein pull-down assays for GTP-bound Rac1, Cdc42, and RhoA, we showed that treatment of MDA-MB-231 tumor cells with recombinant maspin for a short time period significantly inhibited the activity of Rac1 and Cdc42, but not RhoA. The reactive site loop (RSL) within maspin protein is the functional domain involved in the inhibition. Maspin mutants with the RSL deleted or a point mutation in the RSL region lost their inhibitory activity. We further examined the ability of maspin to inhibit Rac1- and Cdc42-mediated signaling pathways and transcription factors. Treatment of MDA-MB-231 cells with maspin led to the inhibition of JNK kinase activity as assayed by immuno-kinase assays. In addition, the AP-1 transcription activity downstream of JNK kinase pathway was also reduced. Together, we have identified Rac1 and Cdc42 as the downstream targets that mediate the inhibition of mammary tumor cell   migration   by maspin. 
 Oncogenic K-Ras down-regulates Rac1 and RhoA activity and enhances   migration   and invasion of pancreatic carcinoma cells through activation of p38. Activating mutations in the K-ras gene are genetic alterations frequently found in human carcinomas, particularly in pancreatic adenocarcinomas. Mutation of the K-ras gene is thought to be an early and important event in pancreatic tumor initiation, but the precise role of the mutant K-Ras proteins in neoplastic progression is still unknown. In the present study, we have characterized the influence of oncogenic K-Ras on the phenotype and on the signal transduction of epitheloid PANC-1 pancreatic carcinoma cells by generating PANC-1 cell clones, which stably express EGFP(enhanced green fluorescent protein)-K-Ras (V12). EGFP-K-Ras (V12)-expressing cells exhibited a more fibroblastoid cellular phenotype with irregular cell shape and disorganized cytokeratin filaments. Moreover, these cells showed a marked enhancement of their migratory and invasive properties. Stable expression of EGFP-K-Ras (V12) down-regulated the activity of Rac1 and RhoA, resulting in reduced subcortical actin filaments and stress fibers, which might contribute to the epithelial dedifferentiation. Characterization of the activity of mitogen-activated protein kinases revealed that EGFP-K-Ras (V12) enhanced the activity of p38, but did not affect the activities of the Raf/MEK/ERK cascade and JNK. While inhibition of either MEK or JNK activity had no effect on EGFP-K-Ras (V12)-induced   migration  , inhibition of p38 activity markedly reduced EGFP-K-Ras (V12)-induced   migration  . Collectively, the results suggest that oncogenic K-Ras enhances the malignant phenotype and identify the mitogen-activated protein kinase p38 as a target to inhibit oncogenic K-Ras-induced pancreatic tumor cell   migration  . 
 RhoA and Rac1 are both required for efficient wound closure of airway epithelial cells. Repair of the airway epithelium after injury is critical for restoring normal lung. The reepithelialization process involves spreading and   migration   followed later by cell proliferation. Rho-GTPases are key components of the wound healing process in many different types of tissues, but the specific roles for RhoA and Rac1 vary and have not been identified in lung epithelial cells. We investigated whether RhoA and Rac1 regulate wound closure of bronchial epithelial cells. RhoA and Rac1 proteins were efficiently expressed in a cell line of human bronchial epithelial cells (16HBE) by adenovirus-based gene transfer. We found that both constitutively active RhoA and dominant negative RhoA inhibited wound healing, suggesting that both activation and inhibition of RhoA interfere with normal wound healing. Overexpression of wild-type Rac1 induced upregulation of RhoA, disrupted intercellular junctions, and inhibited wound closure. Dominant negative Rac1 also inhibited wound closure. Inhibition of the downstream effector of RhoA, Rho-kinase, with Y-27632 suppressed actin stress fibers and focal adhesion formation, increased Rac1 activity, and stimulated wound closure. The activity of both RhoA and Rac1 are influenced by the polymerization state of microtubules, and cell   migration   involves coordinated action of actin and microtubules. Microtubule depolymerization upon nocodazole treatment led to an increase in focal adhesions and decreased wound closure. We conclude that coordination of both RhoA and Rac1 activity contributes to bronchial epithelial wound repair mechanisms in vitro, that inhibition of Rho-kinase accelerates wound closure, and that efficient repair involves intact microtubules. 
 Integrin engagement differentially modulates epithelial cell motility by RhoA/ROCK and PAK1. Integrin-ligand binding regulates tumor cell motility and invasion. Cell   migration   also involves the Rho GTPases that control the interplay between adhesion receptors and the cytoskeleton. We evaluated how specific extracellular matrix ligands modulate Rho GTPases and control motility of human squamous cell carcinoma cells. On laminin-5 substrates, the epithelial cells rapidly spread and migrated, but on type I collagen the cells spread slowly and showed reduced motility. We found that RhoA activity was suppressed in cells attached to laminin-5 through the alpha3 integrin receptor. In contrast, RhoA was strongly activated in cells bound to type I collagen and this was mediated by the alpha2 integrin. Inhibiting the RhoA pathway by expression of a dominant-negative RhoA mutant or by directly inhibiting ROCK, reduced focal adhesion formation and enhanced cell   migration   on type I collagen. Cdc42 and Rac and their downstream target PAK1 were activated following adhesion to laminin-5. PAK1 activation induced by laminin-5 was suppressed by expression of a dominant-negative Cdc42. Moreover, constitutively active PAK1 stimulated   migration   on collagen I substrates. Our results indicate that in squamous epithelial cells, collagen-alpha2beta1 integrin binding activates RhoA, slowing cell locomotion, whereas laminin-5-alpha3beta1 integrin interaction inhibits RhoA and activates PAK1, stimulating cell   migration  . The data demonstrate that specific ligand-integrin pairs regulate cell motility differentially by selectively modulating activities of Rho GTPases and their effectors. 
 Roles of Rho guanosine 5'-triphosphatase A, Rho kinases, and extracellular signal regulated kinase (1/2) in prostaglandin E2-mediated   migration   of first-trimester human extravillous trophoblast. Prostaglandin (PG) E(2) may regulate invasiveness of human placenta because we previously reported stimulation of   migration   of placental trophoblasts by PGE(2) acting through PGE receptor (EP)-1 and activating calpain. RhoA GTPase and its important effector Rho kinase (ROCK) have also been previously shown to regulate trophoblast   migration  . Using immortalized HTR-8/SVneo trophoblast cells and first-trimester human chorionic villus explant cultures on matrigel, we further examined the role of RhoA/ROCK and MAPK (ERK1/2) pathways on PGE(2)-mediated stimulation of trophoblast   migration  .   Migration   of cytotrophoblasts was shown to be inhibited by treatment of the trophoblast cell line and chorionic villus explants with either cell-permeable C3 transferase or selective RhoA small interfering RNA. These inhibitions were significantly mitigated by the addition of PGE(2), an EP1/EP3 agonist or an EP3/EP4 agonist, suggesting that RhoA plays an important role in trophoblast   migration   but may not be obligatory for PGE(2) action. Treatment of HTR-8/SVneo cells with nonselective ROCK inhibitor Y27632 or ROCK small interfering RNAs inhibited   migration   of these cells, which could not be rescued with PGE(2) or the other two EP agonists, suggesting the obligatory role of ROCK in PGE(2)-induced migratory response. Furthermore, U0126, an inhibitor of MAPK kinases MEK1 and MEK2, abrogated PGE(2)-induced   migration   of trophoblasts, and PGE(2) or the other two EP agonists stimulated ERK1/2 activation in trophoblasts, which was not abrogated by pretreatment with C3 transferase, indicating that ERK signaling pathway is an efficient alternate pathway for RhoA in PGE(2)-mediated   migration   of trophoblasts. These results suggest that ROCK and ERK1/2 play more important roles than RhoA in PGE(2)-mediated   migration   stimulation of first-trimester trophoblasts. 
 Celiac disease IgA modulates vascular permeability in vitro through the activity of transglutaminase 2 and RhoA. Celiac disease is characterized by the presence of specific autoantibodies targeted against transglutaminase 2 (TG2) in untreated patients' serum and at their production site in the small-bowel mucosa below the basement membrane and around the blood vessels. As these autoantibodies have biological activity in vitro, such as inhibition of angiogenesis, we studied if they might also modulate the endothelial barrier function. Our results show that celiac disease patient autoantibodies increase endothelial permeability for macromolecules, and enhance the binding of lymphocytes to the endothelium and their transendothelial   migration   when compared to control antibodies in an endothelial cell-based in vitro model. We also demonstrate that these effects are mediated by increased activities of TG2 and RhoA. Since the small bowel mucosal endothelium serves as a "gatekeeper" in inflammatory processes, the disease-specific autoantibodies targeted against TG2 could thus contribute to the pathogenic cascade of celiac disease by increasing blood vessel permeability. 
 Smurf1 regulates tumor cell plasticity and motility through degradation of RhoA leading to localized inhibition of contractility. Rho GTPases participate in various cellular processes, including normal and tumor cell   migration  . It has been reported that RhoA is targeted for degradation at the leading edge of migrating cells by the E3 ubiquitin ligase Smurf1, and that this is required for the formation of protrusions. We report that Smurf1-dependent RhoA degradation in tumor cells results in the down-regulation of Rho kinase (ROCK) activity and myosin light chain 2 (MLC2) phosphorylation at the cell periphery. The localized inhibition of contractile forces is necessary for the formation of lamellipodia and for tumor cell motility in 2D tissue culture assays. In 3D invasion assays, and in in vivo tumor cell   migration  , the inhibition of Smurf1 induces a mesenchymal-amoeboid-like transition that is associated with a more invasive phenotype. Our results suggest that Smurf1 is a pivotal regulator of tumor cell movement through its regulation of RhoA signaling. 
 CD44 acts through RhoA to regulate YAP signaling. The Hippo pathway plays an important role in both physical and pathogenesis processes. As crucial downstream effectors of Hippo pathway, YAP is inhibited by Lats1/2 through phosphorylation. However, upstream signals that regulate the Hippo pathway have been still poorly understood. Here, we found that knockdown of CD44 reduced YAP expression and nuclear localization, but nearly had no effect on its upstream effectors, Mst1 and Lats1. Downregulated CD44 expression also significantly decreased the expression of YAP downstream effectors CTGF, Cyr61 and EDN1 at mRNA level. Our next study showed that knockdown of CD44 inhibited RhoA expression, which was consistent with RhoA knockdown mediated YAP downregulation. Furthermore, we demonstrated that over expression of the constitutively active RhoA (RhoA-V14) could block the YAP expression decrease mediated by CD44 knockdown. Moreover, downregulation of CD44 significantly promoted cell apoptosis and inhibited cell proliferation, cell cycle progression and   migration  , which were consistent with the effects of RNAi-mediated YAP knockdown in both A549 and HepG2 cells. Overall, data are presented showing that CD44 could act through RhoA signaling to regulate YAP expression and this study also provide new insights into the regulatory mechanisms of the Hippo-YAP pathway. 
 mTORC2 regulates neutrophil chemotaxis in a cAMP- and RhoA-dependent fashion. We studied the role of the target of rapamycin complex 2 (mTORC2) during neutrophil chemotaxis, a process that is mediated through the polarization of actin and myosin filament networks. We show that inhibition of mTORC2 activity, achieved via knock down (KD) of Rictor, severely inhibits neutrophil polarization and directed   migration   induced by chemoattractants, independently of Akt. Rictor KD also abolishes the ability of chemoattractants to induce cAMP production, a process mediated through the activation of the adenylyl cyclase 9 (AC9). Cells with either reduced or higher AC9 levels also exhibit specific and severe tail retraction defects that are mediated through RhoA. We further show that cAMP is excluded from extending pseudopods and remains restricted to the cell body of migrating neutrophils. We propose that the mTORC2-dependent regulation of MyoII occurs through a cAMP/RhoA-signaling axis, independently of actin reorganization during neutrophil chemotaxis. 
 Acute activation of β2-adrenergic receptor regulates focal adhesions through βArrestin2- and p115RhoGEF protein-mediated activation of RhoA. β(2)-Adrenergic receptors (β(2)ARs) regulate cellular functions through G protein-transduced and βArrestin-transduced signals. β(2)ARs have been shown to regulate cancer   cell mig  ration, but the underlying mechanisms are not well understood. Here, we report that β(2)AR regulates formation of focal adhesions, whose dynamic remodeling is critical for directe  d cell mi  gration. β(2)ARs induce activation of RhoA, which is dependent on βArrestin2 but not G(s). βArrestin2 forms a complex with p115RhoGEF, a guanine nucleotide exchange factor for RhoA that is well known to be activated by G(12/13)-coupled receptors. Our results show that βArrestin2 forms a complex with p115RhoGEF in the cytosol in resting cells. Upon β(2)AR activation, both βArrestin2 and p115RhoGEF translocate to the plasma membrane, with concomitant activation of RhoA and formation of focal adhesions and stress fibers. Activation of RhoA and focal adhesion remodeling may explain, at least in part, the role of β(  2)ARs in   cell migration. These results suggest that βArrestin2 may serve as a convergence point for non-G(12/13) and non-G(q) protein-coupled receptors to activate RhoA. 
 Possible Involvement of Sphingosine-1-Phosphate/G(i)/RhoA pathways in adherence of eosinophils to pulmonary endothelium. Sphingosine-1-phosphate (S1P), a lysophospholipid released from inflammatory cells, causes cell   migration   by increasing cytokines and chemokines. This study was designed to determine whether S1P causes adherence of eosinophils to pulmonary endothelial cells via enhancement of adhesion molecule expression.Expression of VCAM-1 and ICAM-1 was assessed by RT-PCR and Western blot analysis in human pulmonary microvasucular endothelial cells (HPMVECs). The number of adherent eosinophils to HPMVECs was calculated according to adhesion assay.Pre-treatment of HPMVECs with S1P increased mRNA and protein expression of VCAM-1, in contrast, did not dramatically increase those expression of ICAM-1. The maximal expression of these adhesion molecules in mRNA and protein was observed 4 and 8h after exposure to S1P, respectively. Pre-treatment with S1P also activated RhoA, a monomeric G protein; the ability of S1P to enhance the expression of VCAM-1 was attenuated by RhoA related inhibitors such as Y-27632, C3 exoenzyme, and GGTI-286. The effects of S1P on VCAM-1 were attenuated by pre-incubation with pertussis toxin, which catalyzes the ADP-ribosylation of G(i), a heterotrimeric G protein. After HPMVECs were treated with S1P, adhesion of human eosinophilic leukemic cell line (EoL-1) cells to HPMVECs was enhanced in a concentration-dependent manner. Augmented adherence of EoL-1 cells by S1P was also attenuated by Y-27632 and pertussis toxin. S1P causes adherence of eosinophils to pulmonary endothelium via RhoA activation.S1P may act as a lipid mediator in asthma. The RhoA/Rho-kinase pathway may be a therapeutic target for preventing eosinophil infiltration to the airway. 
 MiRNA-125a-3p is a negative regulator of the RhoA-actomyosin pathway in A549 cells. MicroRNAs (miRNAs) function as genetic modulators that regulate gene expression, and are, thus, involved in a wide range of biological roles, including tumor cell   migration   and invasion. MiR-125a-3p is a mature form of miR-125a, derived from the 3'-end of pre-miR-125a. Our group has previously reported that miR-125a-3p functions as a tumor suppressor gene that inhibits the   migration   and invasion of lung cancer cells. Here, we report the discovery of a new regulatory layer of the RhoA-actomyosin pathway through which miR-125a-3p controls tumor cell   migration  . Overexpression of miR-125a-3p by transfection of sense‑miR‑125a-3p resulted in decreased RhoA protein levels, while the levels of RhoA mRNA remained constant. The concentrations of both RhoA-GTP protein and actin filaments decreased after miR-125a-3p overexpression in the A549 lung cancer cell line. Conversely, knockdown of miR-125a-3p by transfection of antisense-miR-125a-3p resulted in increased RhoA protein levels while the levels of RhoA mRNA remained unchanged. However, the concentration of both RhoA-GTP protein and actin filaments increased. To further demonstrate that RhoA is a potential target of miR‑125a-3p, luciferase reporter constructs containing the RhoA 3'UTR demonstrated reduced reporter activity after ectopic expression of miR-125a-3p. Moreover, luciferase reporter constructs containing the RhoA 3'UTR mutant did not show significantly changed reporter activity. Furthermore, A549 cells demonstrated reduced migratory capacity after treatment with the Rho inhibitor CT04. Our results indicate that the loss of miR-125a‑3p-controlled regulation of the RhoA-actomyosin pathway can lead to in  creased m  igration of tumor cells because of the upregulation of RhoA expression. In particular, an increased intracellular concentration of RhoA-GTP protein in A549 cells leads to the accumulation of actin filaments. These results provide new insights into the role of the miR-125a family in lung cancer. 
 RhoGDIα-dependent balance between RhoA and RhoC is a key regulator of cancer cell tumorigenesis. RhoGTPases are key signaling molecules regulating main cellular functions such as   migratio  n, proliferation, survival, and gene expression through interactions with various effectors. Within the RhoA-related subclass, RhoA and RhoC contribute to several steps of tumor growth, and the regulation of their expression affects cancer progression. Our aim is to investigate their respective contributions to the acquisition of an invasive phenotype by using models of reduced or forced expression. The silencing of RhoC, but not of RhoA, increased the expression of genes encoding tumor suppressors, such as nonsteroidal anti-inflammatory drug-activated gene 1 (NAG-1), and decreased   migratio  n and the anchorage-independent growth in vitro. In vivo, RhoC small interfering RNA (siRhoC) impaired tumor growth. Of interest, the simultaneous knockdown of RhoC and NAG-1 repressed most of the siRhoC-related effects, demonstrating the central role of NAG-1. In addition of being induced by RhoC silencing, NAG-1 was also largely up-regulated in cells overexpressing RhoA. The silencing of RhoGDP dissociation inhibitor α (RhoGDIα) and the overexpression of a RhoA mutant unable to bind RhoGDIα suggested that the effect of RhoC silencing is indirect and results from the up-regulation of the RhoA level through competition for RhoGDIα. This study demonstrates the dynamic balance inside the RhoGTPase network and illustrates its biological relevance in cancer progression. 
 Vaccinia virus-induced cell motility requires F11L-mediated inhibition of RhoA signaling. RhoA signaling plays a critical role in many cellular processes, including cell   migration  . Here we show that the vaccinia F11L protein interacts directly with RhoA, inhibiting its signaling by blocking the interaction with its downstream effectors Rho-associated kinase (ROCK) and mDia. RNA interference-mediated depletion of F11L during infection resulted in an absence of vaccinia-induced cell motility and inhibition of viral morphogenesis. Disruption of the RhoA binding site in F11L, which resembles that of ROCK, led to an identical phenotype. Thus, inhibition of RhoA signaling is required for both vaccinia morphogenesis and virus-induced cell motility. 
 Rho A participates in the regulation of phosphatidylserine-dependent procoagulant activity at the surface of megakaryocytic cells. Once exposed at the external surface of activated platelets or apoptotic cells, phosphatidylserine, an anionic phospholipid mostly sequestered in the inner leaflet of the plasma membrane, plays essential roles in hemostasis and phagocytosis. The mechanism governing the   migration   of the phosphatidylserine to the exoplasmic leaflet is not yet fully understood. We have proposed that store-operated calcium entry (SOCE) constitutes a key step of this process. ERK pathway is among the elements modulating SOCE and phosphatidylserine externalization in megakaryocytic HEL cells. Here, we investigated the role of small GTPase Rho A, which may interact with the ERK pathway. Specific inhibitors of Rho A (exoenzyme C3 and toxin B) reduced both SOCE and phosphatidylserine-dependent procoagulant activity. Simultaneous inhibition of Rho A and extracellular signal-regulated kinase (ERK) pathways did not elicit further reduction with respect to each individual one. Rho A can regulate SOCE and phosphatidylserine exposure through the reorganization of actin cytoskeleton, but not through ROCK pathway. Hence, Rho A is another regulatory element for the completion of SOCE-induced phosphatidylserine transmembrane redistribution in HEL cells. 
 Deciphering the transcriptional complex critical for RhoA gene expression and cancer metastasis. The RhoA GTPase is crucial in numerous biological functions and is linked to cancer metastasis. However, the understanding of the molecular mechanism responsible for RhoA transcription is still very limited. Here we show that RhoA transcription is orchestrated by the Myc-Skp2-Miz1-p300 transcriptional complex. Skp2 cooperates with Myc to induce RhoA transcription by recruiting Miz1 and p300 to the RhoA promoter independently of Skp1-Cullin-F-box protein containing complex (SCF)-Skp2 E3 ligase activity. Deficiency of this complex results in impairment in RhoA expression, cell   migration  , invasion, and breast cancer metastasis, recapitulating the phenotypes observed in RhoA knockdown, and RhoA restoration rescues the defect in cell invasion. Overexpression of the Myc-Skp2-Miz1 complex is found in metastatic human cancers and is correlated with RhoA expression. Our study provides insight into how oncogenic Skp2 and Myc coordinate to induce RhoA transcription and establishes a novel SCF-Skp2 E3-ligase-independent function for oncogenic Skp2 in transcription and cancer metastasis. 
 Overexpression of E-cadherin on melanoma cells inhibits chemokine-promoted invasion involving p190RhoGAP/p120ctn-dependent inactivation of RhoA. Melanoma cells express the chemokine receptor CXCR4 that confers high invasiveness upon binding to its ligand CXCL12. Melanoma cells at initial stages of the disease show reduction or loss of E-cadherin expression, but recovery of its expression is frequently found at advanced phases. We overexpressed E-cadherin in the highly invasive BRO lung metastatic cell melanoma cell line to investigate whether it could influence CXCL12-promoted cell invasion. Overexpression of E-cadherin led to defective invasion of melanoma cells across Matrigel and type I collagen in response to CXCL12. A decrease in individual cell   migration   directionality toward the chemokine and reduced adhesion accounted for the impaired invasion. A p190RhoGAP-dependent inhibition of RhoA activation was responsible for the impairment in chemokine-stimulated E-cadherin melanoma transfectant invasion. Furthermore, we show that p190RhoGAP and p120ctn associated predominantly on the plasma membrane of cells overexpressing E-cadherin, and that E-cadherin-bound p120ctn contributed to RhoA inactivation by favoring p190RhoGAP-RhoA association. These results suggest that melanoma cells at advanced stages of the disease could have reduced metastatic potency in response to chemotactic stimuli compared with cells lacking E-cadherin, and the results indicate that p190RhoGAP is a central molecule controlling melanoma cell invasion. 
 The role of RhoA in vulvar squamous cell carcinoma: a carcinogenesis, progression, and target therapy marker. Ras homologue gene family member A (RhoA) is involved in tumor mobility, invasion, and metastasis. We detected RhoA expression in vulvar squamous cell carcinoma (VSCC) tissue, measured RhoA expression in the VSCC cell phenotype, and measured the expression of the relevant molecules after RhoA small interfering RNA (siRNA) transfection in SW962 cells. RhoA has a higher expression level in VSCC than normal vulva skin tissue and was positively associated with the International Federation of Gynecology and Obstetrics (FIGO) stage and differentiation; besides, VSCC patients with lymph node metastasis had higher positive RhoA expression. RhoA messenger RNA and protein expression was significantly reduced in the RhoA siRNA transfectants as compared with the negative control (NC) and mock-transfected cells (p < 0.05). The RhoA siRNA transfectants lead to low growth, G1 arrest, high apoptosis,   low migra  tion and invasion (p < 0.05), and suppressed lamellipodia formation as compared to NC and mock-transfected cells. Besides, matrix metalloproteinase-2 (MMP2), MMP9, and cyclinA1 protein expression was downregulated, while that of Bax was upregulated in the RhoA siRNA transfectants (p < 0.05). SW962 cell proliferation rates were significantly lovastatin dose-dependent. Lovastatin caused G1 arrest, high apo  ptosis, l  ow migration and invasion (p < 0.05), and suppression of lamellipodia formation. Similar to the RhoA siRNA transfectants, lovastatin treatment downregulated RhoA, MMP2, MMP9, and cyclinA1 protein expression, while upregulating that of Bax as compared to that of the NC (p < 0.05). Abnormal RhoA expression in vulvar carcinoma is involved in tumor proliferation and invasion and may be a treatment target. The RhoA inhibitor lovastat  in alters   VSCC cell migration and proliferation and may be effective for treating VSCC. 
 [Role of RhoA in hypoxia-induced breast cancer cell VEGF secretion and proliferation,   migration   and tube formation of HUVECs]. To explore the role of RhoA in regulating vascular endothelial growth factor (VEGF) secretion level in breast cancer cells and in the proliferation,   migration   and tube formation of human umbilical vascular endothelial cells (HUVECs) under hypoxia.Enzyme-linked immunosorbent assay was used to examine the effect of V14RhoA plasmid transfection-induced RhoA activation and RhoA knockdown on VEGF secretion level in breast cancer MCF-7 cells under hypoxic condition. A MCF-7/HUVEC co-culture model was established to assess the effect of the changes in RhoA expressions in MCF-7 cells on HUVEC proliferation,   migration  , and tube formation under hypoxia.Under hypoxic condition, RhoA activation promoted VEGF secretion in MCF-7 cells, and RhoA knockdown inhibited VEGF secretion. In the co-culture model, RhoA activation in the MCF-7 cells enhanced HUVEC proliferation,   migration  , and tube formation, and RhoA knockdown inhibited these changes.Under hypoxic condition, RhoA indirectly influences HUVECs to affect tumor angiogenesis by regulating VEGF level in breast cancer cells. 
 ODAM inhibits RhoA-dependent invasion in breast cancer. Odontogenic ameloblast-associated protein (ODAM) contributes to cell adhesion. In human cancer, ODAM is down-regulated, and the overexpression of ODAM results in a favourable prognosis; however, the molecular mechanisms underlying ODAM-mediated inhibition of cancer invasion and metastasis remain unclear. Here, we identify a critical role for ODAM in inducing cancer cell adhesion. ODAM induced RhoA activity and the expression of downstream factors, including Rho-associated kinase (ROCK). ODAM-mediated RhoA signalling resulted in actin filament rearrangement by activating PTEN and inhibiting the phosphorylation of AKT. When ODAM is overexpressed in MCF7 breast cancer cells and AGS gastric cancer cells that activate RhoA at high levels, it decreases motility, increases adhesion and inhibits the metastasis of MCF7 cells. Conversely, depletion of ODAM in cancer cells inhibits Rho GTPase activation, resulting in increased cancer   migration   and invasion. These results suggest that ODAM expression in cells maintains their adhesion, resulting in the prevention of their metastasis via the regulation of RhoA signalling in breast cancer cells. SIGNIFICANCE Breast cancer represents the first most frequent cancer, and the ratio of mortality is high in women. Of utmost importance for reducing risk by breast cancer are their anti-invasion mechanisms, particularly in the non-invasive cancer cells because metastasis is the principal cause of death among cancer patients. ODAM induced RhoA activity. ODAM-mediated RhoA signalling resulted in actin filament rearrangement, increased cell adhesion and inhibited the   migration  /invasion of MCF7 cells. These results suggest that ODAM expression maintains their adhesion, resulting in the prevention of their metastasis via the regulation of RhoA signalling in breast cancer cells. 
 RhoA and RhoC have distinct roles in   migration   and invasion by acting through different targets. Several studies suggest that RhoA and RhoC, despite their sequence similarity, have different roles in cell   migration   and invasion, but the molecular basis for this is not known. Using RNAi, we show that RhoA-depleted cells became elongated and extended multiple Rac1-driven narrow protrusions in 2D and 3D environments, leading to increased invasion. These phenotypes were caused by combined but distinct effects of the Rho-regulated kinases ROCK1 and ROCK2. Depletion of ROCK2 induced multiple delocalized protrusions and reduced migratory polarity, whereas ROCK1 depletion selectively led to cell elongation and defective tail retraction. In contrast, RhoC depletion increased cell spreading and induced Rac1 activation around the periphery in broad lamellipodia, thereby inhibiting directed   migration   and invasion. These effects of RhoC depletion are mediated by the formin FMNL3, which we identify as a new target of RhoC but not RhoA. We propose that RhoA contributes to migratory cell polarity through ROCK2-mediated suppression of Rac1 activity in lamellipodia, whereas RhoC promotes polarized   migration   through FMNL3 by restricting lamellipodial broadening. 
 S100B Protein Regulates Astrocyte Shape and   Migration   via Interaction with Src Kinase: IMPLICATIONS FOR ASTROCYTE DEVELOPMENT, ACTIVATION, AND TUMOR GROWTH. S100B is a Ca(2+)-binding protein of the EF-hand type that is abundantly expressed in astrocytes and has been implicated in the regulation of several intracellular activities, including proliferation and differentiation. We show here that reducing S100B levels in the astrocytoma cell line GL15 and the Müller cell line MIO-M1 by small interference RNA technique results in a rapid disassembly of stress fibers, collapse of F-actin onto the plasma membrane and reduced   migratio  n, and acquisition of a stellate shape. Also, S100B-silenced GL15 and MIO-M1 Müller cells show a higher abundance of glial fibrillary acidic protein filaments, which mark differentiated astrocytes, compared with control cells. These effects are dependent on reduced activation of the phosphatidylinositol 3-kinase (PI3K) downstream effectors, Akt and RhoA, and consequently elevated activity of GSK3beta and Rac1 and decreased activity of the RhoA-associated kinase. Also, rat primary astrocytes transiently down-regulate S100B expression when exposed to the differentiating agent dibutyryl cyclic AMP and re-express S100B at later stages of dibutyryl cyclic AMP-induced differentiation. Moreover, reducing S100B levels results in a remarkably slow resumption of S100B expression, suggesting the S100B might regulate its own expression. Finally, we show that S100B interacts with Src kinase, thereby stimulating the PI3K/Akt and PI3K/RhoA pathways. These results suggest that S100B might contribute to reduce the differentiation potential of cells of the astrocytic lineage and participate in the astrocyte activation process in the case of brain insult and in invasive properties of glioma cells. 
 p27Kip1 promotes   migration   of metastatic hepatocellular carcinoma cells. p27(Kip1) (p27) is a member of the Cip/Kip family of cyclin/cyclin-dependent kinase inhibitors (CKIs), and its CKI-independent function regarding cell motility modulation has been discovered. However, it is controversial whether p27 promotes or inhibits cell   migration  . This study investigates the   migration   regulatory role of p27 in metastatic hepatocellular carcinoma (HCC) cells.RNA interference, RhoA-GTP pull-down assay, Western blots, immunostaining, transwell and wound-healing assays were used.High levels of p27 and phosphorylated p27 (Ser10) were detected in metastatic HCC cells, MHCC97L and MHCC97H, when compared with nonmetastatic HCC cells, PLC and Hep3B. We hypothesized that p27 is responsible for metastasis-related   migration   in HCC cells and tested the hypothesis by using the p27 RNA interference approach. Increased RhoA activity was observed when p27 was knocked down in MHCC97L cells, which further led to stress fiber formation and decreased cell   migration   and wound healing. Moreover, high p27 and low stathmin expression of metastatic HCC cells indicated that   migration   inhibition by p27-stathmin interaction might not be the major regulatory pathway in metastatic HCC cells.p27 promotes cell   migration   in metastatic HCC cells through the regulation of RhoA activity. 
 Interleukin-1β-induced Wnt5a enhances human corneal endothelial cell   migratio  n through regulation of Cdc42 and RhoA. Wnt5a can activate β-catenin-independent pathways for regulation of various cellular functions, such a  s migrati  on, that play critical roles in wound repair. Investigation of Wnt5a signaling may help identify therapeutic targets for enhancing corneal endothelial wound healing that could provide an alternative to corneal transplantation in patients with blindness from endothelial dysfunction. However, Wnt5a signaling in corneal endothelial cells (CECs) has not been well characterized. In this study, we show transient induction of Wnt5a by interleukin-1β (IL-1β) stimulation proceeds through NF-κB in human CECs. This leads to binding of Fzd5 to Ror2, resulting in activation of disheveled protein (Dvl) and subsequently disheveled-associated activator of morphogenesis 1 (DAAM1). This leads to activation of Cdc42 and subsequent inhibition of RhoA. Inhibition of RhoA leads to parallel dephosphorylation and inactivation of LIM domain kinase 2 along with dephosphorylation and activation of slingshot 1, resulting in dephosphorylation and activation of cofilin and leading to enhanced   cell migr  ation. These findings suggest that Wnt5a enhances   cell migr  ation through activation of Cdc42 and inactivation of RhoA in human CECs. 
 Coordinated RhoA signaling at the leading edge and uropod is required for T cell transendothelial   migration  . Transendothelial   migration   (TEM) is a tightly regulated process whereby leukocytes migrate from the vasculature into tissues. Rho guanosine triphosphatases (GTPases) are implicated in TEM, but the contributions of individual Rho family members are not known. In this study, we use an RNA interference screen to identify which Rho GTPases affect T cell TEM and demonstrate that RhoA is critical for this process. RhoA depletion leads to loss of migratory polarity; cells lack both leading edge and uropod structures and, instead, have stable narrow protrusions with delocalized protrusions and contractions. By imaging a RhoA activity biosensor in transmigrating T cells, we find that RhoA is locally and dynamically activated at the leading edge, where its activation precedes both extension and retraction events, and in the uropod, where it is associated with ROCK-mediated contraction. The Rho guanine nucleotide exchange factor (GEF) GEF-H1 contributes to uropod contraction but does not affect the leading edge. Our data indicate that RhoA activity is dynamically regulated at the front and back of T cells to coordinate TEM. 
 Degradation of RhoA by Smurf1 ubiquitin ligase. The Rho family of small GTPases plays a key role in the dynamic regulation of the actin cytoskeleton that underlies various important cellular functions such as shape changes,   migration  , and polarity. We found that Smurf1, a HECT domain E3 ubiquitin ligase, could specifically target RhoA but not Cdc42 or Rac1 for degradation. Smurf1 interacts with the dominant inactive form of RhoA, RhoA N19, which binds constitutively to guanine nucleotide exchange factors (GEFs) in vivo. Smurf1 also interacts directly with either nucleotide-free or GDP-bound RhoA in vitro; however, loading with GTPgammaS inhibits the interaction. RhoA is ubiquitinated by wild-type Smurf1 but not the catalytic mutant of Smurf1 (C699A) in vivo and in vitro, indicating that RhoA is a direct substrate of Smurf1. In this chapter, we summarize the systems and methods used in the analyses of Smurf1-regulated RhoA ubiquitination and degradation. 
 High targeted   migration   of human mesenchymal stem cells grown in hypoxia is associated with enhanced activation of RhoA. A feature which makes stem cells promising candidates for cell therapy is their ability to migrate effectively into damaged or diseased tissues. Recent reports demonstrated the increased motility of human mesenchymal stem cells (hMSC) grown under hypoxic conditions compared to normoxic cells. However, the directional   migration   of hMSC cultured in hypoxia has not been investigated. In this study we examined the in vitro transmembrane   migration   of hMSC permanently cultured in hypoxia in response to various cytokines. We also studied the involvement of RhoA, a molecule believed to play an essential role in the   migration   of MSC via reorganization of the cytoskeleton.We compared the directional   migration   of human hMSCs grown permanently under normal (21%, normoxic) and low O2 (5%, hypoxic) conditions until passage 4 using an in vitro transmembrane   migration   assay. A series of 17 cytokines was used to induce chemotaxis. We also compared the level of GTP-bound RhoA in the cell extracts of calpeptin-activated hypoxic and normoxic hMSC.We found that hMSC cultured in hypoxia demonstrate markedly higher targeted   migration   activity compared to normoxic cells, particularly towards wound healing cytokines, including those found in ischemic and myocardial infarction. We also demonstrated for the first time that hMSC are dramatically more sensitive to activation of RhoA.The results of this study indicate that high directional   migration   of hMSCs permanently grown in hypoxia is associated with the enhanced activation of RhoA. The enhanced migratory capacity of hypoxic hMSC would further suggest their potential advantages for clinical applications. 
 The inositol 5-phosphatase SHIP2 is an effector of RhoA and is involved in cell polarity and   migration  . Cell   migration   is essential for various physiological and pathological processes. Polarization in motile cells requires the coordination of several key signaling molecules, including RhoA small GTPases and phosphoinositides. Although RhoA participates in a front-rear polarization in migrating cells, little is known about the functional interaction between RhoA and lipid turnover. We find here that src-homology 2-containing inositol-5-phosphatase 2 (SHIP2) interacts with RhoA in a GTP-dependent manner. The association between SHIP2 and RhoA is observed in spreading and migrating U251 glioma cells. The depletion of SHIP2 attenuates cell polarization and   migration  , which is rescued by wild-type SHIP2 but not by a mutant defective in RhoA binding. In addition, the depletion of SHIP2 impairs the proper localization of phosphatidylinositol 3,4,5-trisphosphate, which is not restored by a mutant defective in RhoA binding. These results suggest that RhoA associates with SHIP2 to regulate cell polarization and   migration  . 
 Direct activation of RhoA by reactive oxygen species requires a redox-sensitive motif. Rho family GTPases are critical regulators of the cytoskeleton and affect cell   migration  , cell-cell adhesion, and cell-matrix adhesion. As with all GTPases, their activity is determined by their guanine nucleotide-bound state. Understanding how Rho proteins are activated and inactivated has largely focused on regulatory proteins such as guanine nucleotide exchange factors (GEFs) and GTPase activating proteins (GAPs). However, recent in vitro studies have indicated that GTPases may also be directly regulated by redox agents. We hypothesized that this redox-based mechanism occurs in cells and affects cytoskeletal dynamics, and in this report we conclude this is indeed a novel mechanism of regulating the GTPase RhoA.In this report, we show that RhoA can be directly activated by reactive oxygen species (ROS) in cells, and that this requires two critical cysteine residues located in a unique redox-sensitive motif within the phosphoryl binding loop. First, we show that ROS can reversibly activate RhoA and induce stress fiber formation, a well characterized readout of RhoA activity. To determine the role of cysteine residues in this mechanism of regulation, we generated cysteine to alanine RhoA mutants. Mutation of these cysteines abolishes ROS-mediated activation and stress fiber formation, indicating that these residues are critical for redox-regulation of RhoA. Importantly, these mutants maintain the ability to be activated by GEFs.Our findings identify a novel mechanism for the regulation of RhoA in cells by ROS, which is independent of classical regulatory proteins. This mechanism of regulation may be particularly relevant in pathological conditions where ROS are generated and the cellular redox-balance altered, such as in asthma and ischemia-reperfusion injury. 
 Neuropeptide-stimulated cell   migration   in prostate cancer cells is mediated by RhoA kinase signaling and inhibited by neutral endopeptidase. The neuropeptides bombesin and endothelin-1 stimulate prostate cancer (PC) cell   migration   and invasion (J Clin Invest, 2000; 106: 1399-1407). The intracellular signaling pathways that direct this cell movement are not well delineated. The monomeric GTPase RhoA is required for   migration   in several cell types including neutrophils, monocytes and fibroblasts. We demonstrate that bombesin-stimulated PC cell   migration   occurs via the heterotrimeric G-protein-coupled receptors (G-protein) G alpha 13 subunit leading to activation of RhoA, and Rho-associated coiled-coil forming protein kinase (ROCK). Using siRNA to suppress expression of the three known G-protein alpha-subunit-associated RhoA guanine nucleotide exchange factors (GEFs), we also show that two of these RhoA GEFs, PDZ-RhoGEF and leukemia-associated RhoGEF (LARG), link bombesin receptors to RhoA in a non-redundant manner in PC cells. We next show that focal adhesion kinase, which activates PDZ-RhoGEF and LARG, is required for bombesin-stimulated RhoA activation. Neutral endopeptidase (NEP) is expressed on normal prostate epithelium whereas loss of NEP expression contributes to PC progression. We also demonstrate that NEP inhibits neuropeptide activation of RhoA. Together, these results establish a contiguous signaling pathway from the bombesin receptor to ROCK in PC cells, and they implicate NEP as a major regulator of neuropeptide-stimulated RhoA in these cells. This work also identifies members of this signaling pathway as potential targets for rational pharmacologic manipulation of neuropeptide-stimulated   migration   of PC cells. 
 [Effect of beta-elemene on the proliferation,   migration   and RhoA expression of hepatic stellate cells induced by angiotensin II]. To explore the influence of beta-elemene on the proliferation,   migration   and RhoA expression of hepatic stellate cells (HSC) induced by angiotensin II (ANG II).HSC were incubated in vitro. Proliferation and   migration   of the HSC were induced by ANG II. The effect on the proliferation of HSC was determined by MTT colorimetry. The   migration   ability was detected by transwell chamber cultures. Total RNA was extracted by TRizol reagent and gene levels were determined by semi-quantitative RT-PCR. Protein levels were determined by Western blot.Different concentrations (from 1 to 10 micromol/L) of ANG II markedly promoted the growth of the HSC in a concentration dependent way (0 micromol/L ANG II, F = 112.640, P less than 0.01). 10, 8, 4 micromol/L ANGII significantly induced HSC   migration  , F = 117.496, P less than 0.01. Compared with the 4 micromol/L ANG II group, 10 mg/L, 5 mg/L, 2.5 mg/L beta-elemene markedly inhibited HSC proliferation and   migration   induced by 4 micromol/L ANG II (F values were 95.706 and 55.600 and P less than 0.01). 4 micromol/L ANG II markedly promoted the protein and mRNA expressions of RhoA in HSC. 10 mg/L, 5 mg/L and 2.5 mg/L beta-elemene notably inhibited the expressions of RhoA protein and mRNA (F values were 217.119 and 18.010).ANG II can significantly induce the proliferation and   migration   of HSC. Beta-elemene can inhibit the proliferation and   migration   of HSC induced by ANG II. The effects of beta-elemene are mediated through inhibiting the RhoA signal transduction pathway and are associated with RhoA. 
 Wnt5b promotes the cell motility essential for metastasis of oral squamous cell carcinoma through active Cdc42 and RhoA. The activation of Wnt signaling has been reported in many types of squamous cell carcinoma. In this study, using human oral squamous cell carcinoma (OSCC) cells with different metastatic potential, we investigated the involvement of Wnt signaling in metastasis. Further, we aimed to elucidate the characteristic biological features related to high metastatic potential and to identify new target molecules for the suppression of OSCC lymph node metastasis. We compared SAS-Venus (SAS OSCC cells expressing green fluorescent protein) and SAS-LM8, which is a highly metastatic cell line derived from SAS-Venus by in vivo selection. The SAS-LM8 cell line had greater ability of   migration   and invasion compared to SAS-Venus. Furthermore, a higher number of filopodia-like protrusive structures were produced in SAS-LM8 cells compared to SAS-Venus cells, and the levels of active Cdc42 and active RhoA protein were higher in SAS-LM8 cells compared to SAS-Venus cells. We did not observe any differences in the expression of Wnt/β-catenin target genes between the two cell lines; however, the mRNA levels of Wnt5b were higher in SAS-LM8 cells compared to SAS-Venus cells. To confirm the involvement of Wnt5b in   migratio  n in OSCC cells, we examined the effects of the siRNA-mediated knockdown of Wnt5b in SAS-Venus cells and SAS-LM8 cells. The siRNA treatment significantly inhibited   migratio  n and the formation of filopodia-like protrusive structures. Conversely, when stimulated with Wnt5b, the   migratio  n and formation of filopodia-like protrusions were significantly enhanced and the levels of active Cdc42 and active RhoA proteins were also increased. These results indicate that Wnt5b is involved in the   migratio  n ability of OSCC cells through active Cdc42 and RhoA. 
 Proteomic identification of RhoA as a potential biomarker for proliferation and metastasis in hepatocellular carcinoma. Hepatocellular carcinoma (HCC) is one of the most common malignancies in the world, and there is an urgent need to discover novel factors that can act as biomarkers for prognostic assessment and therapeutic targets of HCC. In this study, highly purified plasma membrane proteins from clinical tissue samples were obtained using a strategy combining sucrose density gradient centrifugation and subsequent phase partition. Using a two-dimensional gel electrophoresis and MALDI-Q-TOF MS/MS-based proteomics approach, we identified 13 plasma membrane-associated proteins that were differentially expressed in HCC and normal liver tissues. Of those, RhoA was one of the most significantly upregulated proteins in HCC, and its overexpression was confirmed using Western blotting. Immunohistochemistry suggested a link between RhoA expression and poor differentiation and clinicopathologic stage. Suppression of RhoA expression in HepG2 and Hep3B cells by RNA interference led to significant inhibition of cell growth, induction of apoptosis, and a decrease in   migration  . Our data suggest that RhoA may serve as a potential biomarker and an attractive therapeutic target for HCC. 
 PRL-1 protein promotes ERK1/2 and RhoA protein activation through a non-canonical interaction with the Src homology 3 domain of p115 Rho GTPase-activating protein. Phosphatases of the regenerating liver (PRL) play oncogenic roles in cancer development and metastasis. Although previous studies indicate that PRL-1 promotes cell growth and   migration   by activating both the ERK1/2 and RhoA pathways, the mechanism by which it activates these signaling events remains unclear. We have identified a PRL-1-binding peptide (Peptide 1) that shares high sequence identity with a conserved motif in the Src homology 3 (SH3) domain of p115 Rho GTPase-activating protein (GAP). p115 RhoGAP directly binds PRL-1 in vitro and in cells via its SH3 domain. Structural analyses of the PRL-1·Peptide 1 complex revealed a novel protein-protein interaction whereby a sequence motif within the PxxP ligand-binding site of the p115 RhoGAP SH3 domain occupies a folded groove within PRL-1. This prevents the canonical interaction between the SH3 domain of p115 RhoGAP and MEKK1 and results in activation of ERK1/2. Furthermore, PRL-1 binding activates RhoA signaling by inhibiting the catalytic activity of p115 RhoGAP. The results demonstrate that PRL-1 binding to p115 RhoGAP provides a coordinated mechanism underlying ERK1/2 and RhoA activation. 
 AAMP Regulates Endothelial Cell   Migration   and Angiogenesis Through RhoA/Rho Kinase Signaling. Angiogenesis is a complicated process including endothelial cell proliferation,   migration   and tube formation. AAMP plays a role in regulating cell   migration   of multiple cell types. The purpose of this study was to investigate whether AAMP regulates angiogenesis, and to clarify the role of AAMP in the VEGF-induced angiogenesis. We found that AAMP expressed in multiple cell types and mainly localized in cytoplasm and membrane in vascular endothelial cells. Using tube formation assay in vitro and aortic ring assay, siRNA-mediated knockdown and antibody blockade of AAMP impaired VEGF-induced endothelial cell tube formation and aortic ring angiogenic sprouting. Mechanistic studies showed that AAMP expression was significantly upregulated by VEGF in a concentration and time-dependent manner. Moreover, VEGF recruited AAMP to the cell membrane protrusions. AAMP regulates angiogenesis by mediating the spreading and   migration   of vascular endothelial cells. AAMP knock-down reduced VEGF-induced actin stress fibers and collagen gel contraction. Furthermore, we identified RhoA/Rho kinase signaling as an important factor that contributes to the action of AAMP in regulating endothelial cell   migration   and angiogenesis. Altogether, these data demonstrated the critical role of AAMP in angiogenesis and suggested blocking AAMP could serve as a potential therapeutic strategy for angiogenesis-related diseases. 
 RHPN2 drives mesenchymal transformation in malignant glioma by triggering RhoA activation. Mesenchymal transformation is a hallmark of aggressive glioblastoma (GBM). Here, we report the development of an unbiased method for computational integration of copy number variation, expression, and mutation data from large datasets. Using this method, we identified rhophilin 2 (RHPN2) as a central genetic determinant of the mesenchymal phenotype of human GBM. Notably, amplification of the human RHPN2 gene on chromosome 19 correlates with a dramatic decrease in the survival of patients with glioma. Ectopic expression of RHPN2 in neural stem cells and astrocytes triggered the expression of mesenchymal genes and promoted an invasive phenotype without impacting cell proliferation. Mechanistically, these effects were implemented through RHPN2-mediated activation of RhoA, a master regulator of cell   migration   and invasion. Our results define RHPN2 amplification as a central genetic determinant of a highly aggressive phenotype that directs the worst clinical outcomes in patients with GBM. 
 SMURF1 plays a role in EGF-induced breast cancer cell   migration   and invasion. Epidermal growth factor (EGF) is a well-known growth factor that induces cancer cell   migration   and invasion. Previous studies have shown that SMAD ubiquitination regulatory factor 1 (SMURF1), an E3 ubiquitin ligase, regulates cell motility by inducing RhoA degradation. Therefore, we examined the role of SMURF1 in EGF-induced cell   migration   and invasion using MDA-MB-231 cells, a human breast cancer cell line. EGF increased SMURF1 expression at both the mRNA and protein levels. All ErbB family members were expressed in MDA-MB-231 cells and receptor tyrosine kinase inhibitors specific for the EGF receptor (EGFR) or ErbB2 blocked the EGF-mediated induction of SMURF1 expression. Within the signaling pathways examined, ERK1/2 and protein kinase C activity were required for EGF-induced SMURF1 expression. The overexpression of constitutively active MEK1 increased the SMURF1 to levels similar to those induced by EGF. SMURF1 induction by EGF treatment or by the overexpression of MEK1 or SMURF1 resulted in enhanced cell   migration   and invasion, whereas SMURF1 knockdown suppressed EGF- or MEK1-induced cell   migration   and invasion. EGF treatment or SMURF1 overexpression decreased the endogenous RhoA protein levels. The overexpression of constitutively active RhoA prevented EGF- or SMURF1-induced cell   migration   and invasion. These results suggest that EGFinduced SMURF1 plays a role in breast cancer cell   migration   and invasion through the downregulation of RhoA. 
 Signal transduction in neuronal   migration  : roles of GTPase activating proteins and the small GTPase Cdc42 in the Slit-Robo pathway. The Slit protein guides neuronal and leukocyte   migration   through the transmembrane receptor Roundabout (Robo). We report here that the intracellular domain of Robo interacts with a novel family of Rho GTPase activating proteins (GAPs). Two of the Slit-Robo GAPs (srGAPs) are expressed in regions responsive to Slit. Slit increased srGAP1-Robo1 interaction and inactivated Cdc42. A dominant negative srGAP1 blocked Slit inactivation of Cdc42 and Slit repulsion of migratory cells from the anterior subventricular zone (SVZa) of the forebrain. A constitutively active Cdc42 blocked the repulsive effect of Slit. These results have demonstrated important roles for GAPs and Cdc42 in neuronal   migration  . We propose a signal transduction pathway from the extracellular guidance cue to intracellular actin polymerization. 
 Protective effects of the mTOR inhibitor everolimus on cytoskeletal injury in human podocytes are mediated by RhoA signaling. Podocytes are highly differentiated kidney cells playing an important role in maintaining the glomerular filtration barrier. Particularly, the integrity of the actin cytoskeleton is crucial as cytoskeletal damage associated with foot process effacement and loss of slit diaphragms constitutes a major aspect of proteinuria. Previously, the mammalian target of rapamycin (mTOR) was linked to actin regulation and aberrant activity of the kinase was associated with renal disease. In this study, actin-related effects of mTOR inhibition by the immunosuppressant everolimus (EV) were investigated in human podocytes using an in vitro model of puromycin aminonucleoside (PAN) induced proteinuria. EV substantially recovered aberrant podocyte behavior by re-establishing a stationary phenotype with decreased   migration   efficiency, enhanced cell adhesion and recovery of actin stress fibers. Biochemical studies revealed substantial increase in the activity of RhoA and the effector pathway Rho-associated protein kinase (ROCK) and myosin light chain (MLC) by EV, all known regulators of stress fiber generation. Taken together, we show for the first time cytoskeleton stabilizing effects of the mTOR inhibitor EV and establish RhoA signaling as a key mediator in this process. 
 Snail regulates the motility of oral cancer cells via RhoA/Cdc42/p-ERM pathway. The transcriptional factor Snail has been reported to possess properties related to cancer progression; however, the mechanism for it is not fully understood. Our data showed that Snail knockdown by small interfering RNA in two OSCC cell lines, WSU-HN6 and CAL27, significantly inhibited cell   migration   and invasion which also resulted in decreased cell motility, such as impaired cell spreading on type I collagen substrate, reduced filopodia, and premature assembly of stress fibers. In addition, Snail-silencing decreased Cdc42 activity but increased RhoA activity, accompanied by the downregulation in both p-ERM expression and cell motility. Meanwhile, endogenous p-ERM was found specifically co-precipitated with activated Cdc42, but not RhoA, and this co-association was decreased by Snail-silencing. The small molecule inhibitors of Rho-associated kinase (Y27632) markedly enhanced Cdc42 activity and the association of p-ERM with activated Cdc42, increasing cell motility remarkably. Using immunohistochemistry, Snail and p-ERM overexpressions were found in OSCC tissues correlated with nodal metastasis and shorter survival. Taken together, these results demonstrate that Snail regulates cell motility through RhoA/Cdc42/p-ERM pathway and may serve as a biomarker to predict prognosis for OSCC patients. Although RhoA and Cdc42 are concurrently regulated downstream of Snail, there is a direct interplay between them, which indicates RhoA has to be inactivated at some point in cell motility cycle. 
 Deoxypodophyllotoxin suppresses tumor vasculature in HUVECs by promoting cytoskeleton remodeling through LKB1-AMPK dependent Rho A activatio. Angiogenesis plays a critical role in the growth and metastasis of tumors, which makes it an attractive target for anti-tumor drug development. Deoxypodophyllotoxin (DPT), a natural product isolated from Anthriscus sylvestris, inhibits cell proliferation and   migration   in various cancer cell types. Our previous studies indicate that DPT possesses both anti-angiogenic and vascular-disrupting activities. Although the RhoA/ RhoA kinase (ROCK) signaling pathway is implicated in DPT-stimulated cytoskeleton remodeling and tumor vasculature suppressing, the detailed mechanisms by which DPT mediates these effects are poorly understood. In the current study, we found that DPT promotes cytoskeleton remodeling in human umbilical vein endothelial cells (HUVECs) via stimulation of AMP-activated protein kinase (AMPK) and that this effect is abolished by either treatment with a selective AMPK inhibitor or knockdown. Moreover, the cellular levels of LKB1, a kinase upstream of AMPK, were enhanced following DPT exposure. DPT-induced activation of AMPK in tumor vasculature effect was also verified by transgenic zebrafish (VEGFR2:GFP), Matrigel plug assay, and xenograft model in nude mice. The present findings may lay the groundwork for a novel therapeutic approach in treating cancer. 
 Polarization and   migration   of hematopoietic stem and progenitor cells rely on the RhoA/ROCK I pathway and an active reorganization of the microtubule network. Understanding the physiological   migration   of hematopoietic progenitors is important, not only for basic stem cell research, but also in view of their therapeutic relevance. Here, we investigated the role of the Rho kinase pathway in the morphology and   migration   of hematopoietic progenitors using an ex vivo co-culture consisting of human primary CD34(+) progenitors and mesenchymal stromal cells. The addition of the Rho kinase inhibitor Y-27632 led to the abolishment of the uropod and microvillar-like structures of hematopoietic progenitors, concomitant with a redistribution of proteins found therein (prominin-1 and ezrin). Y-27632-treated cells displayed a deficiency in   migration  . Time-lapse video microscopy revealed impairment of the rear pole retraction. Interestingly, the knockdown of ROCK I, but not ROCK II, using RNA interference (RNAi) was sufficient to cause the referred morphological and   migration  al changes. Unexpectedly, the addition of nocodazole to either Y-27632- or ROCK I RNAi-treated cells could restore their polarized morphology and   migration   suggesting an active role for the microtubule network in tail retraction. Finally, we could demonstrate using RNAi that RhoA, the upstream regulator of ROCK, is involved in these processes. Collectively, our data provide new insights regarding the role of RhoA/ROCK I and the microtubules in the   migration   of stem cells. 
 Geranylgeranylation but not GTP loading determines rho migratory function in T cells. Rho GTPases orchestrate signaling pathways leading to cell   migration  . Their function depends on GTP loading and isoprenylation by geranylgeranyl pyrophosphate (GGpp). In this study, we show that in human T cells, geranylgeranylation-and not GTP loading-is necessary for RhoA-mediated downstream events. As a result of GGpp depletion with the 3-hydroxy-3-methylglutaryl-CoA reductase inhibitor atorvastatin, RhoA was sequestered from the membrane to the cytosol and, notwithstanding increased GTP loading, the constitutive activation of its substrate Rho-associated coiled-coil protein kinase-1 was blocked. In line with this, T cells expressing increased GTP-RhoA failed to form an intact cytoskeleton and to migrate toward a chemokine gradient. In vivo treatment with atorvastatin in the rodent model of multiple sclerosis markedly decreased the capacity of activated T cells to traffic within the brain, as demonstrated by multiphoton analysis. Thus, tethering of RhoA to the membrane by GGpp is determinant for T cell   migration   and provides a mechanism for preventing T cell infiltration into inflamed compartments by 3-hydroxy-3-methylglutaryl-CoA reductase inhibitors. 
 Integrin-specific control of focal adhesion kinase and RhoA regulates membrane protrusion and invasion. Cell invasion through extracellular matrix (ECM) is a hallmark of the metastatic cascade. Cancer cells require adhesion to surrounding tissues for efficient   migration   to occur, which is mediated through the integrin family of receptors. Alterations in expression levels of β1 and β3 integrins have previously been reported in a number of human cancers. However, whether there are specific roles for these ubiquitous receptors in mediating cell invasion remains unclear. Here we demonstrate that loss of β1 but not β3 integrins leads to increased spread cell area and focal adhesion number in cells on 2D immobilized fibronectin. Increased adhesion numbers in β1 knockdown cells correlated with decreased   cell migr  ation on 2D surfaces. Conversely, cells depleted of β1 integrins showed incr  eased mig  ration speed on 3D cell-derived matrix as well as in 3D organotypic cultures and inverted invasion assays. This increased invasive potential was also seen in cells lacking β3 integrin but only in 3D cultures containing fibroblasts. Mechanistically, in situ analysis using FRET biosensors revealed that enhanced invasion in cells lacking β1 integrins was directly coupled with reduced activation of focal adhesion kinase (FAK) and the small GTPase RhoA resulting in formation of enhanced dynamic protrusions and increased invasion. These reductions in FAK-RhoA signal activation were not detected in β3 knockdown cells under the same conditions. This data demonstrates a specific role for β1 integrins in the modulation of a FAK-RhoA-actomyosin signaling axis to regulate cell invasion through complex ECM environments. 
 CD24 associates with EGFR and supports EGF/EGFR signaling via RhoA in gastric cancer cells. CD24, a mucin-like membrane glycoprotein, plays a critical role in carcinogenesis, but its role in human gastric cancer and the underlying mechanism remains undefined.The contents of CD24 and epidermal growth factor receptor (EGFR) in gastric cancer cells (SGC-7901 and BGC-823) and non-malignant gastric epithelial cells (GES-1) were evaluated by Western blotting assay. Cellular EGFR staining was examined by immunofluorescence assay. Cell   migration   rate was measured by wound healing assay. The effects of depletion/overexperssion of CD24 on EGFR expression and activation of EGF/EGFR singaling pathways were evaluated by immunofluorescence, qPCR, Western blotting and flow cytometry techniques. RhoA activity was assessed by pulldown assay. CD24 and EGFR expression patterns in human gastric tumor samples were also investigated by immunohistochemistry staining.CD24 was overexpressed in human gastric cancer cells. Ectopic expression of CD24 in gastric epithelial cells augmented the expression of EGFR, while knockdown of CD24 in gastric cancer cells decreased the level of EGFR and cell   migration   velocity. To further explore the mechanisms, we investigated the effect of CD24 expression on EGF/EGFR signaling. We noticed that this effect of CD24 on EGFR expression was dependent on promoting EGFR internalization and degradation. Lower ERK and Akt phosphorylations in response to EGF stimulation were observed in CD24-depleted cells. In addition, we noticed that the effect of CD24 on EGFR stability was mediated by RhoA activity in SGC-7901 gastric cancer cells. Analysis of gastric cancer specimens revealed a positive correlation between CD24 and EGFR levels and an association between CD24 expression and worse prognosis.Thus, these findings suggest for the first time that CD24 regulates EGFR signaling by inhibiting EGFR internalization and degradation in a RhoA-dependent manner in gastric cancer cells. 
 Epigenetic silencing of TPM2 contributes to colorectal cancer progression upon RhoA activation. Beta-tropomyosin (β-tropomyosin, TPM2) has been found to be downregulated in colorectal cancer (CRC) in previous studies. In this study, we aimed to investigate the mechanisms and potential biological consequences of the downregulation of TPM2 in colorectal cancer. TPM2 expression in colorectal cancer was assessed by qRT-PCR and immunostaining. The biological functions of TPM2 were assessed in cell lines either overexpressing or underexpressingTPM2. Aberrant DNA methylation in the promoter region is associated with suppression of TPM2 expression in primary colorectal cancer tissue samples. Treatment with the demethylation agent 5-AZA can induceTPM2 expression in colorectal cancer cell lines. Reconstitution of TPM2 suppresses cell proliferation and   migratio  n in colorectal cancer cell lines, whereas the loss of TPM2 expression is associated with increased tumor proliferation and   migratio  n in vitro, which was accompanied by RhoA activation. In summary, our findings indicate that TPM2 appears to be commonly silenced by aberrant DNA methylation in colon cancer. TPM2 loss is associated with RhoA activation and tumor proliferation. 
 The regulation of RhoA at focal adhesions by StarD13 is important for astrocytoma cell motility. Malignant astrocytomas are highly invasive into adjacent and distant regions of the normal brain. Rho GTPases are small monomeric G proteins that play important roles in cytoskeleton rearrangement, cell motility, and tumor invasion. In the present study, we show that the knock down of StarD13, a GTPase activating protein (GAP) for RhoA and Cdc42, inhibits astrocytoma cell   migration   through modulating focal adhesion dynamics and cell adhesion. This effect is mediated by the resulting constitutive activation of RhoA and the subsequent indirect inhibition of Rac. Using Total Internal Reflection Fluorescence (TIRF)-based Förster Resonance Energy Transfer (FRET), we show that RhoA activity localizes with focal adhesions at the basal surface of astrocytoma cells. Moreover, the knock down of StarD13 inhibits the cycling of RhoA activation at the rear edge of cells, which makes them defective in retracting their tail. This study highlights the importance of the regulation of RhoA activity in focal adhesions of astrocytoma cells and establishes StarD13 as a GAP playing a major role in this process. 
 Loss of RhoA expression prevents proliferation and metastasis of SPCA1 lung cancer cells in vitro. Lung cancer is the leading cause of cancer-related death worldwide, mainly due to its highly metastatic properties. Previously, we reported an inverse correlation between Rho-kinase inhibitor and the progression of the lung cancer cells. The purpose of this study was to investigate the effects of RhoA on the proliferation, adhesion, invasion, and   migration   of SPCA1 lung carcinoma cells and to explore the underlying molecular mechanisms. RNA interference was used to downregulate RhoA expression in these cells. Through G418 screening, we generated SPCA1 lung cancer cell lines with stable RhoA silencing. We then observed the cell behaviour, and used matrix metalloproteinase (MMP) activity and western blot assays to evaluate the underlying molecular mechanisms. The proliferation, adhesion,   migration  , and invasion of SPCA1 lung cancer cells were decreased after knockdown of RhoA. At the molecular level, the total amounts of active MMP2 and MMP9 were decreased by about 17.21% (P<0.05) and 45.32% (P<0.01), respectively. Myosin phosphatase targeting subunit 1 phosphorylation (P-MYPT1) was reduced by 36.16% (P<0.05). Taken together, our findings show that the knockdown of RhoA prevents proliferation and metastasis in SPCA1 lung cancer cells. Changes in MMP2, MMP9, and P-MYPT1 levels and activity might be some of the molecular mechanisms underlying these effects. 
 The Semaphorin 4D-Plexin-B1-RhoA signaling axis recruits pericytes and regulates vascular permeability through endothelial production of PDGF-B and ANGPTL4. Semaphorin 4D (SEMA4D) is a member of a family of transmembrane and secreted proteins that have been shown to act through its receptor Plexin-B1 to regulate axon growth cone guidance, lymphocyte activation, and bone density. SEMA4D is also overexpressed by some malignancies and plays a role in tumor-induced angiogenesis similar to vascular endothelial growth factor (VEGF), a protein that has been targeted as part of some cancer therapies. In an attempt to examine the different effects on tumor growth and vascularity for these two pro-angiogenic factors, we previously noted that while inhibition of both VEGF and SEMA4D restricted tumor vascularity and size, vessels forming under conditions of VEGF blockade retained their association with pericytes while those arising in a background of SEMA4D/Plexin-B1 deficiency did not, an intriguing finding considering that alteration in pericyte association with endothelial cells is an emerging aspect of anti-angiogenic intervention in the treatment of cancer. Here we show through array analysis, immunoblots,   migration   and co-culture assays and VE-cadherin immunohistochemistry that SEMA4D production by head and neck carcinoma tumor cells induces expression of platelet-derived growth factor-B and angiopoietin-like protein 4 from endothelial cells in a Plexin-B1/Rho-dependent manner, thereby influencing proliferation and differentiation of pericytes and vascular permeability, whereas VEGF lacks these effects. These results partly explain the differences observed between SEMA4D and VEGF in pathological angiogenesis and suggest that targeting SEMA4D function along with VEGF could represent a novel anti-angiogenic therapeutic strategy for the treatment of solid tumors. 
 TGFBR1*6A enhances the   migration   and invasion of MCF-7 breast cancer cells through RhoA activation. TGFBR1*6A is a common hypomorphic variant of the type 1 transforming growth factor beta receptor (TGFBR1), which has been associated with increased cancer risk in some studies. Although TGFBR1*6A is capable of switching TGF-beta growth-inhibitory signals into growth-stimulatory signals when stably transfected into MCF-7 breast cancer cells, the biological effects of TGFBR1*6A are largely unknown. To broadly explore the potential oncogenic properties of TGFBR1*6A, we assessed its effects on NIH-3T3 cells as well as its effect on the   migration   and invasion of MCF-7 cells. We found that TGFBR1*6A has decreased oncogenic properties compared with TGFBR1. However, TGFBR1*6A significantly enhances MCF-7 cell   migration   and invasion in a TGF-beta signaling-independent manner. Gene expression profiling studies identified two down-regulated genes involved in cell   migration   and invasion: ARHGAP5, encoding ARHGAP5, and FN1, encoding fibronectin-1 (FN1). ARHGAP5 and FN1 expression was similarly down-regulated in MCF-7 cells stably transfected with a kinase-inactivated TGFBR1*6A construct. Functional assays show that TGFBR1*6A-mediated decreased ARHGAP5 expression is associated with higher RhoA activation, a crucial mediator of cell   migration  . Extracellular signal-regulated kinase (ERK) activation is also higher in cells that harbor the TGFBR1*6A allele. We conclude that TGFBR1*6A is not an oncogene but enhances MCF-7 cell   migration   and invasion through RhoA and ERK pathway activation and down-regulates two crucial mediators of this phenotype. These results provide the first evidence that TGFBR1*6A may contribute to cancer progression in a TGF-beta signaling-independent manner. 
 Estrogen-related receptor α decreases RHOA stability to induce orientated cell   migratio  n. Several physiopathological processes require orientated cellular   migratio  n. This phenomenon highly depends on members of the RHO family of GTPases. Both excessive and deficient RHO activity impair directional   migratio  n. A tight control is thus exerted on these proteins through the regulation of their activation and of their stability. Here we show that the estrogen-related receptor α (ERRα) directly activates the expression of TNFAIP1, the product of which [BTB/POZ domain-containing adapter for Cullin3-mediated RhoA degradation 2 (BACURD2)] regulates RHOA protein turnover. Inactivation of the receptor leads to enhanced RHOA stability and activation. This results in cell disorientation, increased actin network, and inability to form a lamellipodium at t  he migrat  ion edge. As a consequence, direction  al migrat  ion, but not cell motility per se, is impaired in the absence of the receptor, under pathological as well as physiological conditions. Altogether, our results show that the control exerted by ERRα on RHOA stability is required for directio  nal migra  tion. 
 Cannabinoid receptor type 1 (CB1) activation inhibits small GTPase RhoA activity and regulates motility of prostate carcinoma cells. The cannabinoid receptor type 1 (CB1) is a G protein-coupled receptor that is activated in an autocrine fashion by the endocannabinoids (EC), N-arachidonoylethanolamine (AEA) and 2-arachidonoylglycerol (2-AG). The CB1 and its endogenous and synthetic agonists are emerging as therapeutic targets in several cancers due to their ability to suppress carcinoma cell invasion and   migration  . However, the mechanisms that the CB1 regulates cell motility are not well understood. In this study, we examined the molecular mechanisms that diminish cell   migration   upon the CB1 activation in prostate carcinoma cells. The CB1 activation with the agonist WIN55212 significantly diminishes the small GTPase RhoA activity but modestly increases the Rac1 and Cdc42 activity. The diminished RhoA activity is accompanied by the loss of actin/myosin microfilaments, cell spreading, and cell   migration  . Interestingly, the CB1 inactivation with the selective CB1 antagonist AM251 significantly increases RhoA activity, enhances microfilament formation and cell spreading, and promotes cell   migration  . This finding suggests that endogenously produced EC activate the CB1, resulting in chronic repression of RhoA activity and cell   migration  . Consistent with this possibility, RhoA activity is significantly diminished by the exogenous application of AEA but not by 2-AG in PC-3 cells (cells with very low AEA hydrolysis). Pretreatment of cells with a monoacylglycerol lipase inhibitor, JZL184, which blocks 2-AG hydrolysis, decreases the RhoA activity. These results indicate the unique CB1 signaling and support the model that EC, through their autocrine activation of CB1 and subsequent repression of RhoA activity, suppress   migration   in prostate carcinoma cells. 
 Hypoxic regulation of mesenchymal stem cell   migration  : the role of RhoA and HIF-1α. A variety of pathologies such as skeletal fracture, neoplasia and inflammation compromise tissue perfusion and thereby decrease tissue oxygen tension. We and others have demonstrated that hypoxia is a potent stimulant for MSC (mesenchymal stem cell) recruitment and differentiation, yet to date little research has focused on the effects of oxygen tension on MSC   migratio  n. In the present study, we examined the effects of hypoxia and the potential role of the GTPase RhoA and HIF-1α (hypoxia-inducible factor 1α) on M  SC migrat  ion. Our results demonstrate that hypoxia decreases M  SC migrat  ion through an HIF-1α and RhoA-mediated pathway. The active GTP-bound form of RhoA was reduced in 1% oxygen, whereas activation of RhoA under hypoxic conditions resc  ued migra  tion. Furthermore, stabilization of HIF-1α under normoxic conditions attenuated   cell migr  ation similar to that of hypoxia. These results suggest that hypoxia negatively affects   MSC migr  ation by regulating activation of GTPases. These results highlight the importance of oxygen in regulating the recruitment of progenitor cells to areas of ischaemic tissue damage. 
 Gastrokine 1 inhibits gastric cancer cell   migration   and invasion by downregulating RhoA expression. We investigated whether GKN1, a gastric tumor suppressor, contributes to the progression of gastric cancer by regulating RhoA expression.We analyzed the expression of GKN1, RhoA, miR-185, and miR-34a in 35 gastric cancer tissues, and compared their expression with T category and TNM stage. Cell   migration   and invasion, as well as the expression of epithelial-to-mesenchymal transition (EMT)-related proteins, were assessed in GKN1- and RhoA small interfering RNA (siRhoA)-transfected and recombinant-GKN1-treated AGS and MKN1 gastric cancer cells.Expression of RhoA protein and messenger RNA (mRNA) was increased in 15 (42.9 %) and 17 (48.6 %) of 35 gastric cancer tissues respectively, and was associated with higher T category and TNM stage. GKN1 expression was significantly decreased in 27 gastric cancers (77.1 %) with a higher T category, and was inversely correlated with RhoA mRNA expression. In AGS and MKN1 cells, GKN1 expression increased miR-185 and miR-34a expression and reduced RhoA mRNA and protein expression. A positive relationship between GKN1 and miR-34a and miR-185 expression and an inverse relationship between miR-34a and RhoA expression were observed in gastric cancer tissues. Ce  ll migrat  ion and invasiveness were markedly decreased in GKN1- and siRhoA-transfected cells. GKN1 expression and silencing of RhoA decreased the expression of the proteins Snail, Slug, and vimentin. Furthermore, miR-185 and miR-34a silencing in MKN1 cells transfected with GKN1 stimulated ce  ll migrat  ion and invasion, and increased the expression of EMT-related proteins.Our data suggest that GKN1 may inhibit gastric cancer ce  ll migrat  ion and invasion by downregulating RhoA expression in a miR-185- and miR-34a-dependent manner. 
 SOX2-RNAi attenuates S-phase entry and induces RhoA-dependent switch to protease-independent amoeboid   migration   in human glioma cells. SOX2, a high mobility group (HMG)-box containing transcription factor, is a key regulator during development of the nervous system and a persistent marker of neural stem cells. Recent studies suggested a role of SOX2 in tumor progression. In our previous work we detected SOX2 in glioma cells and glioblastoma specimens. Herein, we aim to explore the role of SOX2 for glioma malignancy in particular its role in cell proliferation and   migration  .Retroviral shRNA-vectors were utilized to stably knockdown SOX2 in U343-MG and U373-MG cells. The resulting phenotype was investigated by Western blot,   migration  /invasion assays, RhoA G-LISA, time lapse video imaging, and orthotopic xenograft experiments.SOX2 depletion results in pleiotropic effects including attenuated cell proliferation caused by decreased levels of cyclinD1. Also an increased TCF/LEF-signaling and concomitant decrease in Oct4 and Nestin expression was noted. Furthermore, down-regulation of focal adhesion kinase (FAK) signaling and of downstream proteins such as HEF1/NEDD9, matrix metalloproteinases pro-MMP-1 and -2 impaired invasive proteolysis-dependent   migration  . Yet, cells with knockdown of SOX2 switched to a RhoA-dependent amoeboid-like   migration   mode which could be blocked by the ROCK inhibitor Y27632 downstream of RhoA-signaling. Orthotopic xenograft experiments revealed a higher tumorigenicity of U343-MG glioma cells transduced with shRNA targeting SOX2 which was characterized by increased dissemination of glioma cells.Our findings suggest that SOX2 plays a role in the maintenance of a less differentiated glioma cell phenotype. In addition, the results indicate a critical role of SOX2 in adhesion and   migration   of malignant gliomas. 
 RhoA GTPase regulates radiation-induced alterations in endothelial cell adhesion and   migration  . Endothelial cells of the microvasculature are major target of ionizing radiation, responsible of the radiation-induced vascular early dysfunctions. Molecular signaling pathways involved in endothelial responses to ionizing radiation, despite being increasingly investigated, still need precise characterization. Small GTPase RhoA and its effector ROCK are crucial signaling molecules involved in many endothelial cellular functions. Recent studies identified implication of RhoA/ROCK in radiation-induced increase in endothelial permeability but other endothelial functions altered by radiation might also require RhoA proteins. Human microvascular endothelial cells HMEC-1, either treated with Y-27632 (inhibitor of ROCK) or invalidated for RhoA by RNA interference were exposed to 15Gy. We showed a rapid radiation-induced activation of RhoA, leading to a deep reorganisation of actin cytoskeleton with rapid formation of stress fibers. Endothelial early apoptosis induced by ionizing radiation was not affected by Y-27632 pre-treatment or RhoA depletion. Endothelial adhesion to fibronectin and formation of focal adhesions increased in response to radiation in a RhoA/ROCK-dependent manner. Consistent with its pro-adhesive role, ionizing radiation also decreased endothelial cells   migration   and RhoA was required for this inhibition. These results highlight the role of RhoA GTPase in ionizing radiation-induced deregulation of essential endothelial functions linked to actin cytoskeleton. 
 Specific induction of   migration   and invasion of pancreatic carcinoma cells by RhoC, which differs from RhoA in its localisation and activity. RhoA and RhoC are highly related Rho GTPases, but differentially control cellular behaviour. We combined molecular, cellular, and biochemical experiments to characterise differences between these highly similar GTPases. Our findings demonstrate that enhanced expression of RhoC results in a striking increase in the   migration   and invasion of pancreatic carcinoma cells, whereas forced expression of RhoA decreases these actions. These isoform-specific functions correlate with differences in the cellular activity of RhoA and RhoC in human cells, with RhoC being more active than RhoA in activity assays and serum-response factor-dependent gene transcription. Subcellular localisation studies revealed that RhoC is predominantly localised in the membrane-containing fraction, whereas RhoA is mainly localised in the cytoplasmic fraction. These differences are not mediated by a different interaction with RhoGDIs. In vitro GTP/GDP binding analyses demonstrate different affinity of RhoC for GTP[S] and faster intrinsic and guanine nucleotide exchange factor (GEF)-stimulated GDP/GTP exchange rates compared to RhoA. Moreover, the catalytic domains of SopE and Dbs are efficacious GEFs for RhoC. mRNA expression of RhoC is markedly enhanced in advanced pancreatic cancer stages, and thus the differences discovered between RhoA and RhoC might provide explanations for their different influences on cell   migration   and tumour invasion. 
 Chemokine receptor 7 promotes tumor   migration   and invasiveness via the RhoA/ROCK pathway in metastatic squamous cell carcinoma of the head and neck. Metastatic squamous cell carcinoma of the head and neck (SCCHN) has been shown to express chemokine receptor 7 (CCR7), which can activate signaling pathways to promote invasion and survival of SCCHN cells. We hypothesized that the RhoA/Rho-associated kinase (ROCK) pathway is involved in the CCR7-induced invasion and   migration   of metastatic SCCHN cells. Thus, using   migration  , matrigel invasion and scrape wound-healing assays, we elucidated the role of RhoA in mediating CCR7-associated cellular mobility. Pull-down assays and western blotting were used to measure RhoA and its downstream expression. Immunohistochemical staining and analysis were useful in identifying the correlation between CCR7 and RhoA expression and clinicopathological factors. The results showed that inhibition of RhoA/ROCK reduced the tumor cell   migration   and invasiveness induced by CCL19. Activated RhoA, proline-rich tyrosine kinase-2 (Pyk2) and cofilin induced by CCL19 were elevated, and increased RhoA, Pyk2 and cofilin activity was eliminated by CCR7mAb, RhoA/ROCK and Pyk2 inhibitors, indicating involvement of the RhoA/ROCK-Pyk2-cofilin cascade. In summary, CCR7 via RhoA/ROCK-Pyk2 cofilin pathway promotes invasion and   migration   of metastatic SCCHN cells. 
 Regulation of cell polarity and protrusion formation by targeting RhoA for degradation. The Rho family of small guanosine triphosphatases regulates actin cytoskeleton dynamics that underlie cellular functions such as cell shape changes,   migration  , and polarity. We found that Smurf1, a HECT domain E3 ubiquitin ligase, regulated cell polarity and protrusive activity and was required to maintain the transformed morphology and motility of a tumor cell. Atypical protein kinase C zeta (PKCzeta), an effector of the Cdc42/Rac1-PAR6 polarity complex, recruited Smurf1 to cellular protrusions, where it controlled the local level of RhoA. Smurf1 thus links the polarity complex to degradation of RhoA in lamellipodia and filopodia to prevent RhoA signaling during dynamic membrane movements. 
 Fam65b is a new transcriptional target of FOXO1 that regulates RhoA signaling for T lymphocyte   migration  . Forkhead box O (FOXO) transcription factors favor both T cell quiescence and trafficking through their control of the expression of genes involved in cell cycle progression, adhesion, and homing. In this article, we report that the product of the fam65b gene is a new transcriptional target of FOXO1 that regulates RhoA activity. We show that family with sequence similarity 65 member b (Fam65b) binds the small GTPase RhoA via a noncanonical domain and represses its activity by decreasing its GTP loading. As a consequence, Fam65b negatively regulates chemokine-induced responses, such as adhesion, morphological polarization, and   migration  . These results show the existence of a new functional link between FOXO1 and RhoA pathways, through which the FOXO1 target Fam65b tonically dampens chemokine-induced   migration   by repressing RhoA activity. 
 Involvement of MicroRNA-133a in the Development of Arteriosclerosis Obliterans of the Lower Extremities via RhoA Targeting. RhoA is a critical factor in regulating the proliferation and   migration   of arterial smooth muscle cells (ASMCs) in patients with arteriosclerosis obliterans (ASO). RhoA is modulated by microRNA-133a (miR-133a) in cardiac myocytes and bronchial smooth muscle cells. However, the relationship between miR-133a and RhoA with respect to the onset of ASO in the lower extremities is uncertain.We employed in situ hybridization (ISH) and immunohistochemistry (IHC) to detect the location of miR-133a and RhoA in ASO clinical samples, respectively. 5-ethynyl-2'-deoxyuridine (EdU), cell counting kit-8 (CCK-8), Transwell and wound closure assays were utilized to determine the features of human ASMC (HASMC) proliferation and   migration  . The expression of miR-133a in the HASMCs was assessed using quantitative real-time PCR (qRT-PCR), while that of RhoA was examined via qRT-PCR and Western blotting.We found miR-133a and RhoA to be primarily located in the ASMCs of ASO. miR-133a was significantly downregulated in the ASO tissues and proliferating HASMCs. In contrast, RhoA was upregulated in the ASO samples. The proliferation and   migration   of HASMCs was markedly promoted by the downregulation of miR-133a and inhibited by the upregulation of miR-133a. The Luciferase assay confirmed that RhoA was a direct target of miR-133a. The upregulation of miR-133a in the HASMCs decreased the RhoA expression at the protein level. Inversely, the downregulation of miR-133a increased the RhoA protein expression. Of note, the overexpression of RhoA in the HASMCs attenuated the anti-proliferative and anti-migratory effects of miR-133a.Our data indicate that miR-133a regulates the functions of HASMCs by targeting RhoA and may be involved in the pathogenesis of ASO. These findings may lead to the development of potential therapeutic targets for ASO of the lower extremities. 
 The UDP-sugar-sensing P2Y(14) receptor promotes Rho-mediated signaling and chemotaxis in human neutrophils. The G(i)-coupled P2Y(14) receptor (P2Y(14)-R) is potently activated by UDP-sugars and UDP. Although P2Y(14)-R mRNA is prominently expressed in circulating neutrophils, the signaling pathways and functional responses associated with this receptor are undefined. In this study, we illustrate that incubation of isolated human neutrophils with UDP-glucose resulted in cytoskeleton rearrangement, change of cell shape, and enhanced cell   migration  . We also demonstrate that UDP-glucose promotes rapid, robust, and concentration-dependent activation of RhoA in these cells. Ecto-nucleotidases expressed on neutrophils rapidly hydrolyzed extracellular ATP, but incubation with UDP-glucose for up to 1 h resulted in negligible metabolism of the nucleotide-sugar. HL60 human promyelocytic leukemia cells do not express the P2Y(14)-R, but neutrophil differentiation of HL60 cells with DMSO resulted in markedly enhanced P2Y(14)-R expression. Accordingly, UDP-glucose, UDP-galactose, and UDP-N-acetylglucosamine promoted Rho activation in differentiated but not in undifferentiated HL60 cells. Stable expression of recombinant human P2Y(14)-R conferred UDP-sugar-promoted responses to undifferentiated HL60 cells. UDP-glucose-promoted RhoA activation also was accompanied by enhanced cell   migration   in differentiated HL60 cells, and these responses were blocked by Rho kinase inhibitors. These results support the notion that UDP-glucose is a stable and potent proinflammatory mediator that promotes P2Y(14)-R-mediated neutrophil motility via Rho/Rho kinase activation. 
 The Sprouty-related protein, Spred, inhibits cell motility, metastasis, and Rho-mediated actin reorganization. Sprouty and the Sprouty-related protein, Spred (Sprouty-related Ena/vasodilator-stimulated phosphoprotein homology-1 (EVH1) domain-containing protein), inhibit Ras-dependent extracellular signal-regulated kinase (ERK) signaling induced by a variety of growth factors. Since Sprouty proteins have been shown to inhibit not only ERK activation but also cell   migration  , we postulated that Spreds also inhibit cellular   migration  . Using stably highly metastatic LM8 cells infected with the Spred1-Sendai virus vector, we demonstrated that Spred1 inhibits the metastasis of LM8 cells in nude mice. Spred1 overexpression also inhibited   migration   of cells in vitro in response to chemokines, CCL19 and CCL21. We also found that Spred1 overexpression dissolved actin-stress fibers. Both EVH1 domain and C-terminal Sprouty-related domain were required for actin reassembly. Spred1 and Spred2 suppressed constitutively activated RhoA (V14RhoA)-induced stress fiber formation and serum response factor activation. Spred1 bound to activated RhoA, but not cdc42 and Rac. Spred1 also inhibited chemokine-induced RhoA activation and active RhoA-induced Rho-kinase activation. These data suggest that Spreds are key regulators of RhoA-mediated cell motility and signal transduction. Furthermore, our study suggests that the induction of Spreds could be a novel strategy for preventing cancer cell metastasis. 
 MicroRNA-146a inhibits cell   migration   and invasion by targeting RhoA in breast cancer. MicroRNAs (miRNAs) function as genetic modulators that regulate gene expression and are involved in a wide range of biological roles, including tumor cell   migration   and invasion. In the present study, we demonstrated that the   migration   and invasion activity in MDA-MB-231 breast cancer cells could be directly influenced by altering miR-146a expression. The expression of RhoA and miR-146a in the breast cancer cells showed an inverse correlation. Upregulation of miR-146a in the MDA-MB‑231 breast cancer cells by transfection of miR-146a mimics resulted in decreased RhoA protein levels. Conversely, downregulation of miR-146a by transfection of miR-146a inhibitor resulted in increased RhoA protein levels. To confirm the fact that RhoA is a potential target of miR-146a, luciferase reporter containing the RhoA 3' untranslated region (3'UTR) was constructed. The results demonstrated that the luciferase reporter activity was reduced after overexpression of miR-146a. Moreover, the luciferase reporter which was constructed with the RhoA 3'UTR mutant did not show significantly altered luciferase reporter activity. Furthermore, after treatment with the RhoA inhibitor exoenzyme C3 transferase protein, the migratory capacity of the MDA-MB-231 cells was not significantly altered even though the amount of miR-146a was changed. Our results indicate that miR-146a functions as a tumor suppressor in breast cancer cells. Downregulation of the expression of miR-146a increased t  he migrat  ion of MDA-MB-231 cells, due to the upregulation of RhoA expression. 
 Fbxw7 regulates tumor apoptosis, growth arrest and the epithelial-to-mesenchymal transition in part through the RhoA signaling pathway in gastric cancer. F-box and WD repeat domain-containing7 (Fbxw7), a member of the F-box family of proteins, which are components of an E3 ubiquitin ligase complex, plays an important role as a general tumor suppressor in regulating the effects of various oncoproteins. Recently, accumulating studies have shown that Fbxw7 plays an important role in tumor cell motility, invasion and cancer metastasis. However, little is known about the signaling mechanisms that regulate tumor apoptosis, growth arrest and the epithelial-to-mesenchymal transition (EMT) in gastric cancer. In our study, we confirmed that Fbxw7 expression was decreased in gastric cancer tissues, and that Fbxw7 inhibited gastric cancer progression by inducing apoptosis and growth arrest. Furthermore, gastric cancer   migration   and invasion were decreased or increased following Fbxw7 overexpression or knockdown, respectively, and the expressions of various EMT markers, such as E-cadherin, N-cadherin and vimentin, were altered after Fbxw7 inhibition or overexpression. Furthermore, we demonstrated that Fbxw7 inhibits the EMT via the down-regulation of Snail 1 and ZEB 1, which are upstream transcription factors that promote this process. Additionally, RhoA showed higher expression in the same gastric cancer tissues than in normal tumor-adjacent samples. We found that Fbxw7 expression was negatively correlated with RhoA protein expression in gastric cancer tissues based on Pearson's correlation coefficient analysis. Moreover, we found that RhoA protein abundance was regulated by Fbxw7 via ubiquitination and proteasomal degradation in gastric cancer. We further demonstrated the effects of RhoA re-expression or inhibition on stable Fbxw7-overexpressing or Fbxw7-silenced cell lines in vitro and in vivo. These results suggest that Fbxw7 induces apoptosis and growth arrest and inhibits the EMT in part by down-regulating the RhoA signaling pathway. 
 Structural insights into the interaction of ROCKI with the switch regions of RhoA. The Rho-ROCK pathway modulates the phosphorylation level of a variety of important signaling proteins and is thereby involved in miscellaneous cellular processes including cell   migration  , neurite outgrowth, and smooth muscle contraction. The observation of the involvement of the Rho-ROCK pathway in tumor invasion and in diseases such as hypertension and bronchial asthma makes it an interesting target for drug development. We herein present the crystal structure of the complex between active RhoA and the Rho-binding domain of ROCKI. The Rho-binding domain structure forms a parallel alpha-helical coiled-coil dimer and, in contrast to the published Rho-protein kinase N structure, binds exclusively to the switch I and II regions of the guanosine 5'-(beta,gamma-imido)triphosphate-bound RhoA. The switch regions of two different RhoA molecules form a predominantly hydrophobic patch, which is complementarily bound by two identical short helices of 13 residues (amino acids 998-1010). The identified ROCK-binding site of RhoA strikingly supports the assumption of a common consensus-binding site for effector recognition. 
 Adenovirus-mediated RhoA shRNA suppresses growth of esophageal squamous cell carcinoma cells in vitro and in vivo. Over-expression of RhoA in esophageal squamous cell carcinoma (ESCC) indicates a poor prognosis and is correlated with the tumor-node-metastasis (TNM) clinical classification. However, until now RhoA function in the ESCC progression remains to be established. We employed adenovirus-mediated small hairpin RNA (shRNA) against human RhoA (Ad-sh-RhoA) to efficiently silence target gene expression in RhoA-expressing Eca-109 ESCC cells at both protein and mRNA levels. Consequently, Ad-sh-RhoA reduced the proliferation and   migration   of Eca-109 cells assayed by MTT assay and cell wound healing, respectively. Moreover, Ad-sh-RhoA increased cell apoptosis and inhibited the cell cycle G1-S-phase progression of Eca-109 cells assessed by flow cytometry. Finally, in a nude mouse model, intratumoral injections of adenovirus-delivered RhoA shRNA every 3 days for 20 days significantly inhibited the growth and angiogenesis of xenografted Eca-109 tumors. In summary, these data indicate that RhoA may be a key molecule in ESCC cells, and thus, specific inhibition of the Rho signaling pathway with adenovirus-delivered shRNA represents a promising approach for the treatment of aggressive ESCC. 
 Small guanosine triphospatase RhoA and Rho-associated kinase as regulators of trophoblast   migration  . The small guanosine triphosphatase Rho controls cell adhesion and motility through reorganization of the actin cyto-skeleton and regulation of actomyosin contractility. Among the putative target molecules of Rho, a Rho-associated coiled coil-forming protein kinase (ROCK) is thought to participate in Rho-mediated cell adhesion and motility. In the present study, we explored the expression and function of RhoA and ROCK in human trophoblast cells. The colocalization of RhoA, cytokeratin 8/18, and cytokeratin 7 in some cells located in the decidual stromal region indicated that extravillous trophoblast cells expressed RhoA. In double staining for RhoA and ROCK in human chorionic villi, RhoA staining was strongly positive in the cytoplasm of cytotrophoblasts, whereas ROCK stained in the cytoplasm of cytotrophoblasts and syncytiotrophoblasts. Both RhoA and ROCK were stained in cytoplasma of cultured human cytotrophoblast. Cultured human trophoblast cells contained actin stress fibers that were lost after treatment with C3, an exoenzyme produced by Clostridium botulinum. Y-27632, a selective ROCK inhibitor, suppressed RhoA-induced formation of actin stress fibers and formation of focal contact in trophoblast cells. The trophoblast reacquired actin stress fibers and focal contact after withdrawal of Y-27632. Cultured human cytotrophoblast cells from 7-9 wk of gestation migrated into a fibronectin-coated membrane. Both C3 exoenzyme and Y-27632 inhibited cytotrophoblast   migration   in a dose-dependent manner. In conclusion, cyto-trophoblasts express RhoA and ROCK in their cytoplasm, and RhoA-ROCK is involved in their assembly of actin stress fibers. Suppression of RhoA-ROCK reduces trophoblast   migration  . These findings suggest that RhoA-ROCK signaling is a key regulator of trophoblast cell   migration  . 
 Structural determinants of RhoA binding and nucleotide exchange in leukemia-associated Rho guanine-nucleotide exchange factor. Rho guanine-nucleotide exchange factors (RhoGEFs) activate Rho GTPases, and thereby regulate cytoskeletal structure, gene transcription, and cell   migration  . Leukemia-associated RhoGEF (LARG) belongs to a small subfamily of RhoGEFs that are RhoA-selective and directly activated by the Galpha12/13 family of heterotrimeric G proteins. Herein we describe the atomic structures of the catalytic Dbl homology (DH) and pleckstrin homology (PH) domains of LARG alone and in complex with RhoA. These structures demonstrate that the DH/PH domains of LARG can undergo a dramatic conformational change upon binding RhoA, wherein both the DH and PH domains directly engage RhoA. Through mutational analysis we show that full nucleotide exchange activity requires a novel N-terminal extension on the DH domain that is predicted to exist in a broader family of RhoGEFs that includes p115-RhoGEF, Lbc, Lfc, Net1, and Xpln, and identify regions within the LARG PH domain that contribute to its ability to facilitate nucleotide exchange in vitro. In crystals of the DH/PH-RhoA complex, the active site of RhoA adopts two distinct GDP-excluding conformations among the four unique complexes in the asymmetric unit. Similar changes were previously observed in structures of nucleotide-free Ras and Ef-Tu. A potential protein-docking site on the LARG PH domain is also evident and appears to be conserved throughout the Lbc subfamily of RhoGEFs. 
 Lysophosphatidic acid regulates trafficking of beta2-adrenergic receptors: the Galpha13/p115RhoGEF/JNK pathway stimulates receptor internalization. Lysophosphatidic acid is an important lipid ligand regulating many aspects of cell function, including proliferation and   migration  . Operating via heterotrimeric G proteins to downstream effectors, lysophosphatidic acid was shown to regulate the function and trafficking of the G protein-coupled beta(2)-adrenergic receptor. C3 exotoxin, expression of dominant negative RhoA, and inhibition of c-Jun N-terminal kinase blocked the ability of lysophosphatidic acid to sequester the beta(2)-adrenergic receptor, whereas expression of constitutively active Galpha(13), p115RhoGEF, or RhoA mimicked lysophosphatidic acid (LPA) action, stimulating the internalization of the Galpha(s)-coupled beta(2)-adrenergic receptor. This study revealed a novel cross-talk exerted from the LPA/Galpha(13)/p115RhoGEF/RhoA pathway to the beta(2)-adrenergic receptor/Galpha(s)/adenylyl cyclase pathway, attenuating the ability of beta-adrenergic agonists to act following stimulation of cells by LPA as may occur during beta-adrenergic therapy of an inflammatory response. 
 Synaptopodin orchestrates actin organization and cell motility via regulation of RhoA signalling. The Rho family of small GTPases (RhoA, Rac1 and Cdc42) controls signal-transduction pathways that influence many aspects of cell behaviour, including cytoskeletal dynamics. At the leading edge, Rac1 and Cdc42 promote cell motility through the formation of lamellipodia and filopodia, respectively. On the contrary, RhoA promotes the formation of contractile actin-myosin-containing stress fibres in the cell body and at the rear. Here, we identify synaptopodin, an actin-associated protein, as a novel regulator of RhoA signalling and cell   migration   in kidney podocytes. We show that synaptopodin induces stress fibres by competitive blocking of Smurf1-mediated ubiquitination of RhoA, thereby preventing the targeting of RhoA for proteasomal degradation. Gene silencing of synaptopodin in kidney podocytes causes the loss of stress fibres and the formation of aberrant non-polarized filopodia and impairment of cell   migration  . Together, these data show that synaptopodin is essential for the integrity of the podocyte actin cytoskeleton and for the regulation of podocyte cell   migration  . 
 GEF-H1 over-expression in hepatocellular carcinoma promotes cell motility via activation of RhoA signalling. The interstitial chromosome (chr.) 1q21-q22 region is frequently amplified in human cancers, where it has been reported to carry prognostic significance for patients. We attempted to delineate chr. 1q21-q22 for affected gene(s) in hepatocellular carcinoma (HCC) by array-CGH and detected copy number gains of ρ-guanine nucleotide exchange factor-H1 (GEF-H1) as most significant event. Gene expression evaluation in the HCC cohort indicated common up-regulations of GEF-H1 in 64% tumours compared to adjacent non-tumoural liver (64/100; paired t-test p < 0.0001). Moreover, GEF-H1 over-expressions correlated with microvascular invasion and advanced-stage tumours (p < 0.05). High GEF-H1 levels also predict shorter disease-free and overall survival of HCC patients (p < 0.03). Functional knock-down of GEF-H1 by RNAi indicated marked reduction in cell invasion through matrigel and an inhibition of cell   migratio  n (p < 0.035), but an effect on cell viability was not apparent. More interestingly, a mesenchymal-epithelial transition (MET) was readily observed in GEF-H1 knock-down cells, where a concomitant re-expression of epithelial markers (E-cadherin and cytokeratin 18) and cell adhesion proteins (α-catenin and γ-catenin) was found but down-regulation of mesenchymal features (N-cadherin, vimentin and fibronectin). This phenotype was accompanied by reduced filamentous actin polymerizations and diminution of the stress fibre formation. In addition, reduced active form of GTP-RhoA, together with its downstream effectors, including cleaved ROCK1 and phosphorylated MLC2, were also detected in GEF-H1-depleted cells. Taken together, our findings underscore a potent oncogenic role for GEF-H1 in promoting the metastatic potentials of HCC, possibly through activation of RhoA signalling and the EMT phenomenon. 
 The trio guanine nucleotide exchange factor is a RhoA target. Binding of RhoA to the trio immunoglobulin-like domain. Trio is a complex protein containing two guanine nucleotide exchange factor domains each with associated pleckstrin homology domains, a serine/threonine kinase domain, two SH3 domains, an immunoglobulin-like domain, and spectrin-like repeats. Trio was originally identified as a LAR tyrosine phosphatase-binding protein and is involved in actin remodeling, cell   migration  , and cell growth. Herein we provide evidence that Trio not only activates RhoA but is also a RhoA target. The RhoA-binding site was mapped to the Trio immunoglobulin-like domain. RhoA isoprenylation is necessary for the RhoA-Trio interaction, because mutation of the RhoA carboxyl-terminal cysteine residue blocked binding. The existence of an intramolecular functional link between RhoA activation and RhoA binding is suggested by the finding that Trio exchange activity enhanced RhoA binding to Trio. Furthermore, immunofluorescence studies of HeLa cells showed that although ectopically expressed Trio was evenly distributed within the cell, co-expression of Trio with RhoA resulted in relocalization of Trio into punctate structures. Relocalization was not observed with Trio constructs lacking the immunoglobulin-like domain, indicating that RhoA acts to regulate Trio localization via binding to the immunoglobulin-like domain. We propose that Trio-mediated RhoA activation and subsequent RhoA-mediated relocalization of Trio functions to modulate and coordinate Trio signaling. 
 Role of km23-1 in RhoA/actin-based cell   migration  . km23-1 was originally identified as a TGFß receptor-interacting protein that plays an important role in TGFß signaling. Moreover, km23-1 is actually part of an ancient superfamily of NTPase-regulatory proteins, widely represented in archaea and bacteria. To further elucidate the function of km23-1, we identified novel protein interacting partners for km23-1 by using tandem affinity purification (TAP) and tandem mass spectrometry (MS). Here we show that km23-1 interacted with a class of proteins involved in actin-based cell motility and modulation of the actin cytoskeleton. We further showed that km23-1 modulates the formation of a highly organized stress fiber network. More significantly, we demonstrated that knockdown (KD) of km23-1 decreased RhoA activation in Mv1Lu epithelial cells. Finally, our results demonstrated for the first time that depletion of km23-1 inhibited cel  l migrati  on of human colon carcinoma cells (HCCCs) in wound-healing assays. Overall, our findings demonstrate that km23-1 regulates RhoA and motility-associated actin modulating proteins, suggesting that km23-1 may represent a novel target for anti-metastatic therapy. 
 The Amot/Patj/Syx signaling complex spatially controls RhoA GTPase activity in migrating endothelial cells. Controlled regulation of Rho GTPase activity is an essential component mediating growth factor-stimulated   migration  . We have previously shown that angiomotin (Amot), a membrane-associated scaffold protein, plays a critical role during vascular patterning and endothelial   migration   during embryogenesis. However, the signaling pathways by which Amot controls directional   migration   are not known. Here we have used peptide pull-down and yeast 2-hybrid (Y2H) screening to identify proteins that interact with the C-terminal PDZ-binding motifs of Amot and its related proteins AmotL1 and 2. We report that Amot and its related proteins bind to the RhoA GTPase exchange factor (RhoGEF) protein Syx. We show that Amot forms a ternary complex together with Patj (or its paralogue Mupp1) and Syx. Using FRET analysis, we provide evidence that Amot controls targeting of RhoA activity to lamellipodia in vitro. We also report that, similar to Amot, morpholino knockdown of Syx in zebrafish results in inhibition of   migration   of intersegmental arteries. Taken together, our results indicate that the directional   migration   of capillaries in the embryo is governed by the Amot:Patj/Mupp1:Syx signaling that controls local GTPase activity. 
 RhoA-mediated signaling in Notch-induced senescence-like growth arrest and endothelial barrier dysfunction. Notch signaling has a critical role in vascular development and morphogenesis. Activation of Notch in endothelial cells led to a senescence-like phenotype with loss of barrier function. Our objective was to understand the molecular pathways mediating this phenotype.Human primary endothelial cells increase expression of Notch receptors and ligands during propagation in vitro toward natural senescence. This senescence was induced at low passage with Notch activation. We characterized the pathways activated downstream of Notch signaling. Notch was activated by Delta-like 4 ligand or constitutively active Notch receptors and measured for cell proliferation,   migration  , and sprouting. Notch signaling triggered early senescence in low-passage cells, characterized by increased p53 and p21 expression. The senescence phenotype was associated with hyperpermeability of the monolayer, with disrupted vascular endothelial cadherin and β-catenin levels and localization. Consistent with changes in cell shape and contact, we demonstrated that Notch activation increases myosin light chain phosphorylation by activating Rho kinase. Inhibition of Rho abrogated Notch-induced myosin light chain phosphorylation and led to enhanced barrier function by reorganizing F-actin to β-catenin-containing cell-cell adherens junctions.Our findings show that RhoA/Rho kinase regulation by Notch signaling in endothelial cells triggers a senescence phenotype associated with endothelial barrier dysfunction. 
 Breast tumor kinase phosphorylates p190RhoGAP to regulate rho and ras and promote breast carcinoma growth,   migration  , and invasion. Breast tumor kinase (Brk), an Src-like nonreceptor tyrosine kinase, is overexpressed in breast cancer and several other cancer types. Our previous study indicates that Brk promotes cell   migration   and tumor invasion by phosphorylating the focal adhesion protein paxillin. Here, we report the identification of p190RhoGAP-A (p190) as a Brk substrate. Brk phosphorylates p190 at the Y(1105) residue both in vitro and in vivo, thereby promoting the association of p190 with p120RasGAP (p120). As a consequence, Brk stimulates p190 and attenuates p120 functions, leading to RhoA inactivation and Ras activation, respectively. In carcinoma cells expressing high levels of Brk, endogenous Brk functions as a key contributor to epidermal growth factor-induced p190 tyrosine phosphorylation. We present evidence showing that p190 phosphorylation plays essential roles in both migratory and proliferative effects of Brk. Furthermore, disruption of p190 phosphorylation-induced p190/p120 complex in breast cancer cells abolishes not only the abilities of Brk to regulate RhoA and Ras but also the stimulatory effects of Brk on proliferation,   migration  , invasion, transformation, and tumorigenicity. Together, our findings reveal a previously unknown function of Brk in regulating both RhoA and Ras by phosphorylating p190 and provide evidence for the crucial roles of this Brk-elicited signaling pathway in promoting breast malignancy. 
 Chemotherapy Resistance in Diffuse-Type Gastric Adenocarcinoma Is Mediated by RhoA Activation in Cancer Stem-Like Cells. The Lauren diffuse type of gastric adenocarcinoma (DGA), as opposed to the intestinal type (IGA), often harbors mutations in RHOA, but little is known about the role of RhoA in DGA.We examined RhoA activity and RhoA pathway inhibition in DGA cell lines and in two mouse xenograft models. RhoA activity was also assessed in patient tumor samples.RhoA activity was higher in DGA compared with IGA cell lines and was further increased when grown as spheroids to enrich for cancer stem-like cells (CSCs) or when sorted using the gastric CSC marker CD44. RhoA shRNA or the RhoA inhibitor Rhosin decreased expression of the stem cell transcription factor, Sox2, and decreased spheroid formation by 78% to 81%. DGA spheroid cells had 3- to 5-fold greater   migration   and invasion than monolayer cells, and this activity was Rho-dependent. Diffuse GA spheroid cells were resistant in a cytotoxicity assay to 5-fluorouracil and cisplatin chemotherapy, and this resistance could be reversed with RhoA pathway inhibition. In two xenograft models, cisplatin inhibited tumor growth by 40% to 50%, RhoA inhibition by 32% to 60%, and the combination by 77% to 83%. In 288 patient tumors, increased RhoA activity correlated with worse overall survival in DGA patients (P = 0.017) but not in IGA patients (P = 0.612).RhoA signaling promotes CSC phenotypes in DGA cells. Increased RhoA activity is correlated with worse overall survival in DGA patients, and RhoA inhibition can reverse chemotherapy resistance in DGA CSC and in tumor xenografts. Thus, the RhoA pathway is a promising new target in DGA patients. 
 RhoA, RhoB and RhoC have different roles in cancer cell   migration  . Rho GTPases are well known to regulate cell motility through activation of a variety of downstream effector proteins, including enzymes, adaptor proteins and actin nucleators. The three closely related Rho GTPases RhoA, RhoB and RhoC all have the potential to interact with the same downstream effectors, yet they have substantially different effects on cell shape and migratory properties. Here I review the different ways in which RhoA, RhoB and RhoC expression is regulated in cancer and how they play distinct roles in cancer progression. I describe their main effectors known to contribute to cell motility. Recent results from our laboratory and others indicate that RhoA, RhoB and RhoC can be activated by specific stimuli and act through different effectors to control distinct aspects of cancer cell   migration   and invasion. This suggests that they each make unique contributions to cancer by participating in different protein complexes. 
 Overexpression of RhoA enhances peritoneal dissemination: RhoA suppression with Lovastatin may be useful for ovarian cancer. Small guanosine triphosphatase RhoA has been known to re-organize cytoskeletons and regulate cell   migration  . The present authors have previously reported that expression of RhoA is significantly increased in advanced ovarian carcinomas and also in the peritoneal disseminated lesions. The present study investigated whether overexpression of RhoA could alter the progressive behavior of ovarian cancer cells. The effect of various Rho inhibitors on the biological behavior of ovarian cancer cells in vitro and in vivo was also examined. A stable RhoA-transfectant of an ovarian cancer cell line SKOV3 was generated and examined in vitro for alterations of proliferative activity and invasiveness, and also in the nude mice model for peritoneal dissemination. In addition, the effect of a specific Rho inhibitor (C3 exoenzyme), Rho kinase inhibitor (Y27632) and hydroxymethylglutaryl coenzyme A (HMG-CoA) reductase inhibitor (Lovastatin and Pravastatin) were studied in vitro and in vivo. Forced overexpression of RhoA did not alter proliferative activity but significantly increased the invasiveness in vitro, which was suppressed by addition of C3 exoenzyme, Y27834, Lovastatin and Pravastatin. In the nude mice model, the frequency of dissemination and the number of disseminated lesions were significantly increased in the RhoA transfectant than in the control. In addition, oral administration of Lovastatin significantly decreased the number of metastatic sites compared with the control. These findings suggest that upregulation and/or activation of RhoA play an important role in the peritoneal dissemination of ovarian carcinoma, and that Lovastatin might be a candidate for the possible, novel treatment for ovarian carcinoma patients with peritoneal dissemination. 
 Rho isoforms have distinct and specific functions in the process of epithelial to mesenchymal transition in renal proximal tubular cells. Epithelial to mesenchymal transition (EMT) is involved in embryological development, cancerous metastatic spread and organ fibrosis, including the kidney. This process is largely driven by transforming growth factor-beta and recent evidence has implicated Rho as a key intracellular signalling molecule. In this study we have used RNA interference to silence the genetically distinct Rho (A, B and C) isoforms to define their individual functions in human kidney epithelial cells undergoing EMT. We demonstrate that the downregulation of the epithelial cell marker E-cadherin is dependent upon the Rho effector, Rho-kinase. However, silencing RhoA or RhoC expression also results in E-cadherin loss, though each by different mechanisms. Loss of RhoA leads to an upregulation of Snail1 and a reduction in the transcription of E-cadherin whereas loss of RhoC upregulates its breakdown via proteasomal degradation. During EMT, the upregulation of alpha-smooth muscle actin can be blocked by inhibiting the expression of RhoA, but not by that of RhoB or RhoC. This effect is independent of Rho-kinase activity. RhoC is the isoform solely responsible for stress fibre formation and inhibiting its expression reduces EMT-induced   migration   by 50%. RhoB appears to play a role in cell survival as inhibiting its expression leads to >300% increase in cell apoptosis and a relocalization of focal adhesion kinase. We conclude that Rho is a key signalling molecule in the process of EMT but that each isoform has a distinct and specific role. 
 Autocrine Semaphorin3A stimulates eukaryotic initiation factor 4E-dependent RhoA translation in breast tumor cells. Translation of the small G protein RhoA in neurons is regulated by the eukaryotic translation initiation factor eIF4E. Here we show that this translation factor also regulates RhoA expression and activity in breast cancer cells. The introduction of eIF4E into breast tumor cells increased RhoA protein levels, while expression of an eIF4E siRNA reduced RhoA expression. Previous studies indicate that the axon repulsion factor Semaphorin3A (Sema3A) stimulates the eIF4E-dependent translation of RhoA in neurons, and breast tumor cells support autocrine Sema3A signaling. Accordingly, we next examined if autocrine Sema3A signaling drives eIF4E-dependent RhoA translation in breast cancer cells. The incubation of breast tumor cells with recombinant Sema3A rapidly increased eIF4E activity, RhoA protein levels, and RhoA activity. This Sema3A activity was blocked in tumor cells expressing an shRNA-specific for the Sema3A receptor, Neuropilin-1 (NP-1), as well as in cells incubated with an eIF4E inhibitor. Importantly, RhoA protein levels were reduced in Sema3A shRNA-expressing compared to control shRNA-expressing breast tumor cells, demonstrating that autocrine Sema3A increases RhoA expression in breast cancer. Considering that Sema3A suppresses axon extension by stimulating RhoA translation, we next examined if the Sema3A/RhoA axis impacts breast tumor cell   migration  . The incubation of control breast tumor cells, but not RhoA shRNA-expressing cells, with rSema3A significantly reduced their   migration  . Collectively, these studies indicate that Sema3A impedes breast tumor cell   migration   in part by stimulating RhoA. These findings identify common signaling pathways that regulate the navigation of neurons and breast cancer cells, thus suggesting novel targets for suppressing breast tumor cell   migration  . 
 Dvl2-dependent activation of Daam1 and RhoA regulates Wnt5a-induced breast cancer cell   migration  . The Dishevelled (Dvl) and Dishevelled-associated activator of morphogenesis 1 (Daam1) pathway triggered by Wnt5a regulates cellular polarity during development and tissue homoeostasis. However, Wnt5a signaling in breast cancer progression remains poorly defined.We showed here that Wnt5a activated Dvl2, Daam1 and RhoA, and promoted   migration   of breast cancer cells, which was, however, abolished by Secreted Frizzled-related protein 2 (sFRP2) pretreatment. Dominant negative Dvl2 mutants or Dvl2 siRNA significantly decreased Wnt5a-induced Daam1/RhoA activation and cell   migration  . Ectopic expression of N-Daam1, a dominant negative mutant, or Daam1 siRNA remarkably inhibited Wnt5a-induced RhoA activation, stress fiber formation and cell   migration  . Ectopic expression of dominant negative RhoA (N19) or C3 exoenzyme transferase, a Rho inhibitor, decreased Wnt5a-induced stress fiber formation and cell   migration  .Taken together, we demonstrated for the first time that Wnt5a promotes breast cancer cell   migration   via Dvl2/Daam1/RhoA. 
 GCF2/LRRFIP1 promotes colorectal cancer metastasis and liver invasion through integrin-dependent RhoA activation. The precise relationship between GCF2 expression and carcinogenesis has not yet been established. To clarify the metastatic potential of GCF2 in colorectal cancer, HT-29 cells stably suppressing GCF2 expression were injected into the spleens of severe combined immunodeficient (SCID) mice. GCF2 suppression reduced the number of metastatic foci in the liver and reduced fibronectin-induced cell adhesion,   migration  , and invasion. Downstream from the integrin signaling pathways, GCF2 regulates RhoA interaction with the RGS domain of Leukemia associated RhoGEF (LARG). Altogether, our results suggest that GCF2 plays an important role in colorectal cancer metastasis by regulating RhoA-induced cell adhesion,   migration  , and invasion. 
 ABL2/ARG tyrosine kinase mediates SEMA3F-induced RhoA inactivation and cytoskeleton collapse in human glioma cells. Class three semaphorins (SEMAs) were originally shown to be mediators of axon guidance that repelled axons and collapsed growth cones, but it is now evident that SEMA3F, for example, has similar effects on tumor cells and endothelial cells (EC). In both human U87MG glioma cells and human umbilical vein EC, SEMA3F induced rapid cytoskeletal collapse, suppressed cell contractility, decreased phosphorylation of cofilin, and inhibited cell   migration   in culture. Analysis of the signaling pathways showed that SEMA3F formed a complex with NRP2 (neuropilin-2) and plexin A1. These interactions eventually led to inactivation of the small GTPase, RhoA, which is necessary for stress fiber formation and cytoskeleton integrity. A novel upstream RhoA mediator was shown to be ABL2, also known as ARG, a membrane-anchored nonreceptor tyrosine kinase. Within minutes after the addition of SEMA3F, ABL2 directly bound plexin A1 but not to a plexin A1 mutant lacking the cytoplasmic domain. In addition, ABL2 phosphorylated and thereby activated p190RhoGAP, which inactivated RhoA (GTP to GDP), resulting in cytoskeleton collapse and inhibition of cell   migration  . On the other hand, cells overexpressing an ABL2 inactive kinase mutant or treated with ABL2 small interfering RNA did not inactivate RhoA. Cells treated with p190RhoGAP small interfering RNA also did not inactivate RhoA. Together, these results suggested that ABL2/ARG is a novel mediator of SEMA3F-induced RhoA inactivation and collapsing activity. 
 Guanine nucleotide exchange factor-H1 regulates cell   migration   via localized activation of RhoA at the leading edge. Cell   migration   involves the cooperative reorganization of the actin and microtubule cytoskeletons, as well as the turnover of cell-substrate adhesions, under the control of Rho family GTPases. RhoA is activated at the leading edge of motile cells by unknown mechanisms to control actin stress fiber assembly, contractility, and focal adhesion dynamics. The microtubule-associated guanine nucleotide exchange factor (GEF)-H1 activates RhoA when released from microtubules to initiate a RhoA/Rho kinase/myosin light chain signaling pathway that regulates cellular contractility. However, the contributions of activated GEF-H1 to coordination of cytoskeletal dynamics during cell   migration   are unknown. We show that small interfering RNA-induced GEF-H1 depletion leads to decreased HeLa cell directional   migration   due to the loss of the Rho exchange activity of GEF-H1. Analysis of RhoA activity by using a live cell biosensor revealed that GEF-H1 controls localized activation of RhoA at the leading edge. The loss of GEF-H1 is associated with altered leading edge actin dynamics, as well as increased focal adhesion lifetimes. Tyrosine phosphorylation of focal adhesion kinase and paxillin at residues critical for the regulation of focal adhesion dynamics was diminished in the absence of GEF-H1/RhoA signaling. This study establishes GEF-H1 as a critical organizer of key structural and signaling components of cell   migration   through the localized regulation of RhoA activity at the cell leading edge. 
 Arylsulfatase B regulates colonic epithelial cell   migration   by effects on MMP9 expression and RhoA activation. Arylsulfatase B (ASB; N-acetylgalactosamine-4-sulfatase; 4-sulfatase; ARSB) is the enzyme that removes 4-sulfate groups from N-acetylgalactosamine 4-sulfate, which combines with glucuronate to form the disaccharide unit of chondroitin-4-sulfate (C4S). In this study, we report how variation in expression of ASB affected the   migration   of human colonic epithelial cells. In the T84 cell line, derived from lung metastasis of malignant colonic epithelial cells, the activity of ASB, as well as steroid sulfatase, arylsulfatase A, and galactose-6-sulfatase, were significantly less than in normal, primary colonic epithelial cells and in the NCM460 cell line which was derived from normal colonocytes. In the T84 cells, matrix metalloproteinase 9 (MMP9), activated RhoA, and cell   migration  , as well as C4S content, were significantly more than in the NCM460 cells. Silencing and overexpression of ASB had inverse effects on MMP9, activated RhoA, and cell   migration  , as well as the C4S content, in the NCM460 and T84 cells. When ASB expression was silenced by siRNA in the NCM460 cells, MMP9 secretion increased to over 3 times the basal level, activated RhoA increased * 85%, and cell   migration   increased * 52%. Following overexpression of ASB, MMP9 declined 51%, activated RhoA declined * 51%, and cell   migration   decreased * 37%. These findings demonstrate marked effects of ASB expression on the migratory activity of colonic epithelial cells, activated RhoA, and MMP9, and suggest a potential vital role of ASB, due to its impact on chondroitin sulfation, on determination of the invasive phenotype of colonic epithelial cells. 
 Senescent stromal cells induce cancer cell   migration   via inhibition of RhoA/ROCK/myosin-based cell contractility. Cells induced into senescence exhibit a marked increase in the secretion of pro-inflammatory cytokines termed senescence-associated secretory phenotype (SASP). Here we report that SASP from senescent stromal fibroblasts promote spontaneous morphological changes accompanied by an aggressive migratory behavior in originally non-motile human breast cancer cells. This phenotypic switch is coordinated, in space and time, by a dramatic reorganization of the actin and microtubule filament networks, a discrete polarization of EB1 comets, and an unconventional front-to-back inversion of nucleus-MTOC polarity. SASP-induced morphological/migratory changes are critically dependent on microtubule integrity and dynamics, and are coordinated by the inhibition of RhoA and cell contractility. RhoA/ROCK inhibition reduces focal adhesions and traction forces, while promoting a novel gliding mode of   migration  . 
 RhoA as a mediator of clinically relevant androgen action in prostate cancer cells. Recently, we have identified serum response factor (SRF) as a mediator of clinically relevant androgen receptor (AR) action in prostate cancer (PCa). Genes that rely on SRF for androgen responsiveness represent a small fraction of androgen-regulated genes, but distinguish benign from malignant prostate, correlate with aggressive disease, and are associated with biochemical recurrence. Thus, understanding the mechanism(s) by which SRF conveys androgen regulation to its target genes may provide novel opportunities to target clinically relevant androgen signaling. Here, we show that the small GTPase ras homolog family member A (RhoA) mediates androgen-responsiveness of more than half of SRF target genes. Interference with expression of RhoA, activity of the RhoA effector Rho-associated coiled-coil containing protein kinase 1 (ROCK), and actin polymerization necessary for nuclear translocation of the SRF cofactor megakaryocytic acute leukemia (MAL) prevented full androgen regulation of SRF target genes. Androgen treatment induced RhoA activation, increased the nuclear content of MAL, and led to MAL recruitment to the promoter of the SRF target gene FHL2. In clinical specimens RhoA expression was higher in PCa cells than benign prostate cells, and elevated RhoA expression levels were associated with aggressive disease features and decreased disease-free survival after radical prostatectomy. Overexpression of RhoA markedly increased the androgen-responsiveness of select SRF target genes, in a manner that depends on its GTPase activity. The use of isogenic cell lines and a xenograft model that mimics the transition from androgen-stimulated to castration-recurrent PCa indicated that RhoA levels are not altered during disease progression, suggesting that RhoA expression levels in the primary tumor determine disease aggressiveness. Androgen-responsiveness of SRF target genes in castration-recurrent PCa cells continued to rely on AR, RhoA, SRF, and MAL and the presence of intact SRF binding sites. Silencing of RhoA, use of Rho-associated coiled-coil containing protein kinase 1 inhibitors, or an inhibitor of SRF-MAL interaction attenuated (androgen-regulated) cell viability and blunted PCa cell   migration  . Taken together, these studies demonstrate that the RhoA signaling axis mediates clinically relevant AR action in PCa. 
 RHO binding to FAM65A regulates Golgi reorientation during cell   migration  . Directional cell   migration   involves reorientation of the secretory machinery. However, the molecular mechanisms that control this reorientation are not well characterised. Here, we identify a new Rho effector protein, named FAM65A, which binds to active RHOA, RHOB and RHOC. FAM65A links RHO proteins to Golgi-localising cerebral cavernous malformation-3 protein (CCM3; also known as PDCD10) and its interacting proteins mammalian STE20-like protein kinases 3 and 4 (MST3 and MST4; also known as STK24 and STK26, respectively). Binding of active RHO proteins to FAM65A does not affect the kinase activity of MSTs but results in their relocation from the Golgi in a CCM3-dependent manner. This relocation is crucial for reorientation of the Golgi towards the leading edge and subsequent directional cell   migration  . Our results reveal a previously unidentified pathway downstream of RHO that regulates the polarity of migrating cells through Golgi reorientation in a FAM65A-, CCM3- and MST3- and MST4-dependent manner. 
 The orphan adhesion G protein-coupled receptor GPR97 regulates   migration   of lymphatic endothelial cells via the small GTPases RhoA and Cdc42. The important role of the lymphatic vascular system in pathological conditions such as inflammation and cancer has been increasingly recognized, but its potential as a pharmacological target is poorly exploited. Our study aimed at the identification and molecular characterization of lymphatic-specific G protein-coupled receptors (GPCRs) to assess new targets for pharmacological manipulation of the lymphatic vascular system. We used a TaqMan quantitative RT-PCR-based low density array to determine the GPCR expression profiles of ex vivo isolated intestinal mouse lymphatic (LECs) and blood vascular endothelial cells (BECs). GPR97, an orphan adhesion GPCR of unknown function, was the most highly and specifically expressed GPCR in mouse lymphatic endothelium. Using siRNA silencing, we found that GPR97-deficient primary human LECs displayed increased adhesion and collective cell   migration  , whereas single cell   migration   was decreased as compared with nontargeting siRNA-transfected control LECs. Loss of GPR97 shifted the ratio of active Cdc42 and RhoA and initiated cytoskeletal rearrangements, including F-actin redistribution, paxillin and PAK4 phosphorylation, and β1-integrin activation. Our data suggest a possible role of GPR97 in lymphatic remodeling and furthermore provide the first insights into the biological functions of GPR97. 
   Migration   of epithelial cells on laminins: RhoA antagonizes directionally persistent   migration  . Spatial and temporal expression of laminin isoforms is assumed to provide specific local information to neighboring cells. Here, we report the remarkably selective presence of LM-111 at the very tip of hair follicles where LM-332 is absent, suggesting that epithelial cells lining the dermal-epidermal junction at this location may receive different signals from the two laminins. This hypothesis was tested in vitro by characterizing with functional and molecular assays the comportment of keratinocytes exposed to LM-111 and LM-332. The two laminins induced morphologically distinct focal adhesions, and LM-332, but not LM-111, elicited persistent   migration   of keratinocytes. The different impact on cellular behavior was associated with distinct activation patterns of Rho GTPases and other signaling intermediates. In particular, while LM-111 triggered a robust activation of Cdc42, LM-332 provoked a strong and sustained activation of FAK. Interestingly, activation of Rac1 was necessary but not sufficient to promote   migration   because there was no directed   migration   on LM-111 despite Rac1 activation. In contrast, RhoA antagonized directional   migration  , since silencing of RhoA by RNA interference boosted unidirectional   migration   on LM-332. Molecular analysis of the role of RhoA strongly suggested that the mechanisms involve disassembly of cell-cell contacts, loss of the cortical actin network, mobilization of α6β4 integrin out of stable adhesions, and displacement of the integrin from its association with the insoluble pool of intermediate filaments. 
 Annexin A2 is critical for embryo adhesiveness to the human endometrium by RhoA activation through F-actin regulation. Annexin A2 (ANXA2) is present in vivo in the mid- and late-secretory endometria and is mainly localized in the luminal epithelium. Our aim was to evaluate its function in regulating the human implantation process. With an in vitro adhesion model, constructed to evaluate how the mouse embryo and JEG-3 spheroids attach to human endometrial epithelial cells, we demonstrated that ANXA2 inhibition significantly diminishes embryo adhesiveness. ANXA2 is also implicated in endometrial epithelial cell   migration   and trophoblast outgrowth. ANXA2 was seen to be linked to the RhoA/ROCK pathway and to regulate cell adhesion. We noted that ANXA2 inhibition significantly reduces active RhoA, although RhoA inactivation does not alter the ANXA2 levels. RhoA inactivation and ROCK inhibition also moderate embryo adhesiveness to endometrial epithelial cells. We corroborated that the induction of constitutively active RhoA partially reverses the effects of ANXA2 inhibition on endometrial adhesiveness. These molecules colocalize on the plasma membrane of endometrial epithelial cells, and a large proportion of ANXA2 and RhoA are colocalized in the F-actin networks. The functional effects of ANXA2 inhibition and RhoA/ROCK inactivation are associated with significant alterations in F-actin organization and its depolymerization. ANXA2 may act upstream of the RhoA/ROCK pathway by regulating F-actin remodeling and is a key factor in human endometrial adhesiveness. 
 Cannabidiolic acid, a major cannabinoid in fiber-type cannabis, is an inhibitor of MDA-MB-231 breast cancer cell   migration  . Cannabidiol (CBD), a major non-psychotropic constituent of fiber-type cannabis plant, has been reported to possess diverse biological activities, including anti-proliferative effect on cancer cells. Although CBD is obtained from non-enzymatic decarboxylation of its parent molecule, cannabidiolic acid (CBDA), few studies have investigated whether CBDA itself is biologically active. Results of the current investigation revealed that CBDA inhibits   migration   of the highly invasive MDA-MB-231 human breast cancer cells, apparently through a mechanism involving inhibition of cAMP-dependent protein kinase A, coupled with an activation of the small GTPase, RhoA. It is established that activation of the RhoA signaling pathway leads to inhibition of the mobility of various cancer cells, including MDA-MB-231 cells. The data presented in this report suggest for the first time that as an active component in the cannabis plant, CBDA offers potential therapeutic modality in the abrogation of cancer cell   migration  , including aggressive breast cancers. 
 Staphylococcus aureus protein A mediates invasion across airway epithelial cells through activation of RhoA GTPase signaling and proteolytic activity. Staphyococcus aureus and especially the epidemic methicillin-resistant S. aureus strains cause severe necrotizing pneumonia. The mechanisms whereby these organisms invade across the mucosal epithelial barrier to initiate invasive infection are not well understood. Protein A (SpA), a highly conserved and abundant surface protein of S. aureus, activates TNF receptor 1 and EGF receptor (EGFR) signaling cascades that can perturb the cytoskeleton. We demonstrate that wild-type S. aureus, but not spa mutants, invade across polarized airway epithelial cell monolayers via the paracellular junctions. SpA stimulated a RhoA/ROCK/MLC cascade, resulting in the contraction of the cytoskeleton. SpA(+) but not SpA(-) mutants stimulated activation of EGFR and along with subsequent calpain activity cleaved the membrane-spanning junctional proteins occludin and E-cadherin, facilitating staphylococcal trans  migration   through the cell-cell junctions. Treatment of polarized human airway epithelial monolayers with inhibitors of ROCK, EGFR, MAPKs, or calpain prevented staphylococcal penetration through the monolayers. In vivo, blocking calpain activity impeded bacterial invasion into the lung parenchyma. Thus, S. aureus exploits multiple receptors available on the airway mucosal surface to facilitate invasion across epithelial barriers. 
 DLC2 modulates angiogenic responses in vascular endothelial cells by regulating cell attachment and   migration  . Deleted in liver cancer 1 (DLC1) is a RhoGTPase activation protein-containing tumor suppressor that associates with various types of cancer. Although DLC2 shares a similar domain structure with that of DLC1, the function of DLC2 is not well characterized. Here, we describe the expression and ablation of DLC2 in mice using a reporter-knockout approach. DLC2 is expressed in several tissues and in endothelial cells (ECs) of blood vessels. Although ECs and blood vessels show no histological abnormalities and mice appear overall healthy, DLC2-mutant mice display enhanced angiogenic responses induced by matrigel and by tumor cells. Silencing of DLC2 in human ECs has reduced cell attachment, increased   migration  , and tube formation. These changes are rescued by silencing of RhoA, suggesting that the process is RhoA pathway dependent. These results indicate that DLC2 is not required for mouse development and normal vessel formation, but may protect mouse from unwanted angiogenesis induced by, for example, tumor cells. 
 Thy-1-induced   migration   inhibition in vascular endothelial cells through reducing the RhoA activity. Our previous study indicated that Thy-1, which is expressed on blood vessel endothelium in settings of pathological and a specific of physiological, but not during embryonic, angiogenesis, may be used as a marker for angiogenesis. However, the function of Thy-1 during angiogenesis is still not clear. Here, we demonstrate that knock-down of the endogenous Thy-1 expression by Thy-1 siRNA transfection promoted the   migration   of human umbilical vein endothelial cells (HUVEC). In contrast, treatment with interleukin-1β (IL-1β) or phorbol-12-myristate-13-acetate (PMA) increased the level of Thy-1 protein and reduced th  e migrati  on of HUVEC. These effects were abolished by pre-transfection of HUVEC with Thy-1 siRNA to knock-down the expression of Thy-1. Moreover, over-expression of Thy-1 by transfection of HUVEC with Thy-1 pcDNA3.1 decreased the activity of RhoA and Rac-1 and inhibited the adhesion  , migrati  on and capillary-like tube formation of these cells. These effects were prevented by co-transfection of the cell with constitutively active RhoA construct (RhoA V14). On the other hand, pre-treatment with a ROCK (a kinase associated with RhoA for transducing RhoA signaling) inhibitor, Y27632, abolished the RhoA V14-induced prevention effect on the Thy-1-induced inhibition of endothelial cel  l migrati  on and tube formation. Taken together, these results indicate that suppression of the RhoA-mediated pathway might participate in the Thy-1-induce  d migrati  on inhibition in HUVEC. In the present study, we uncover a completely novel role of Thy-1 in endothelial cell behaviors. 
 Human uterine epithelial RL95-2 cells reorganize their cytoplasmic architecture with respect to Rho protein and F-actin in response to trophoblast binding. Embryo implantation is initiated by interaction of trophoblast with uterine epithelium via the apical cell poles of both partners. Using spheroids of human trophoblastoid JAR cells and monolayers of human uterine epithelial RL95-2 cells to simulate this initial interaction, we previously demonstrated that formation of stable cell-to-cell bonds depends on actin cytoskeleton (F-actin) and small GTPases of the Rho family, most likely RhoA. In this study, we determined the apical as well as the basal distribution of these proteins by fluorescence confocal microscopy before and after binding of JAR spheroids. We focussed on changes in cytoplasmic organization with respect to apicobasal polarity of RL95-2 cells. Before binding of spheroids, significantly higher fluorescence signals of RhoA [37 +/- 6 grey scale values (gsv)] and of F-actin (41 +/- 3 gsv) were found in the basal region of RL95-2 cells as compared to the apical pole (RhoA: 24 +/- 3 gsv, F-actin: 28 +/- 2 gsv). After binding of JAR spheroids, this apicobasal asymmetry was inverted (RhoA: 55 +/- 10 gsv apical vs. 25 +/- 3 gsv basal; F-actin: 108 +/- 17 gsv apical vs. 57 +/- 7 gsv basal). Inactivation of Rho GTPases in RL95-2 cells by Clostridium difficile toxin A leads to a loss of their apical adhesion competence, as previously published. Here, we observed a uniform distribution of RhoA and F-actin between apical and basal region rather than an asymmetric one in toxin A-treated cells. These data suggest that activation of Rho GTPases and coordinated rearrangement of F-actin within uterine epithelial cells in response to trophoblast binding are part of a generalized structural and functional reorganization of the cytoplasm. This involves not only the immediate contact zone (apical) but also the opposite (basal) cell pole and may be a critical element of uterine epithelial reactions during transition between trophoblast adhesion and trans  migration  . 
 Syndecan-2 regulation of morphology in breast carcinoma cells is dependent on RhoGTPases. While syndecan-2 is usually considered a mesenchymal transmembrane proteoglycan, it can be upregulated in some tumour cells, such as the malignant breast carcinoma cell line, MDA-MB231. Depletion of this syndecan by siRNA, but not other syndecans, has a marked effect on cell morphology, increasing spreading, microfilament bundle and focal adhesion formation, with reduced cell   migration  .A combination of siRNA transfection, immunofluorescence microscopy, phosphoprotein analysis and   migration   assays was used to determine how syndecan-2 may influence the cytoskeleton.The altered adhesion upon syndecan-2 depletion was dependent on the RhoGTPases. p190ARhoGAP relocated to the margins of spreading cells, where it codistributed with syndecan-4 and active β1-integrin. This was accompanied by increased RhoGAP tyrosine phosphorylation, indicative of activity and RhoGTPase suppression. Consistent with this, GTP-RhoA was strongly present at the edges of control cells, but lost after syndecan-2 reduction by siRNA treatments. Further, RhoA, but not RhoC was shown to be essential for the anchored phenotype of these breast carcinoma cells that accompanied siRNA-mediated loss of syndecan-2.Syndecan-2 has a key role in promoting the invasive activity of these cells, in part by regulating the RhoGTPases.Syndecan-2, as a cell surface receptor is accessible for targeting to determine whether breast tumour progression is altered. This article is part of a Special Issue entitled Matrix-mediated cell behaviour and properties. 
 Passenger strand miRNA miR-31* regulates the phenotypes of oral cancer cells by targeting RhoA. MicroRNAs (miRNAs) are endogenous small non-coding RNAs that negatively regular target gene expression by RNA interference. The processing of the pre-miRNA hairpin generates a miRNA duplex, which consists of a miRNA (guide strand) and a miRNA(*) (passenger strand). miR-31 is an oncogenic miRNA and is up-regulated in oral squamous cell carcinoma (OSCC). miR-31(*) shows a high level of conservation across species and, based on this, this study hypothesized that miR-31(*) is a functional miRNA.The expression of miR-31 and miR-31* in OSCC tissues and oral cells were analyzed. Functional studies were performed on OSCC cells.miR-31(*) is up-regulated in OSCC tissues, but its expression is less abundant than miR-31. miR-31(*) decreases the proliferation and   migration   of both SAS and Fadu cells. Furthermore, miR-31(*) targets the 3'UTR of RhoA and is able to down-regulate RhoA expression. Knockdown of RhoA expression is known to decrease the proliferation and   migration   of OSCC cells. However, up-regulation of both miR-31 and miR-31(*) by delivery of pre-mir-31 does still enhance OSCC oncogenicity.miR-31(*) is a functional miRNA involving in regulating RhoA, and the activity of miR-31(*)'s activity seems to counteract the functions of miR-31 during OSCC tumorigenesis. 
 Prolyl hydroxylase domain (PHD) 2 affects cell   migration   and F-actin formation via RhoA/rho-associated kinase-dependent cofilin phosphorylation. Cells are responding to hypoxia via prolyl-4-hydroxylase domain (PHD) enzymes, which are responsible for oxygen-dependent hydroxylation of the hypoxia-inducible factor (HIF)-1α subunit. To gain further insight into PHD function, we generated knockdown cell models for the PHD2 isoform, which is the main isoform regulating HIF-1α hydroxylation and thus stability in normoxia. Induction of a PHD2 knockdown in tetracycline-inducible HeLa PHD2 knockdown cells resulted in increased F-actin formation as detected by phalloidin staining. A similar effect could be observed in the stably transfected PHD2 knockdown cell clones 1B6 and 3B7. F-actin is at least in part responsible for shaping cell morphology as well as regulating cel  l migrati  on. Cel  l migrati  on was impaired significantly as a consequence of PHD2 knockdown in a scratch assay. Mechanistically, PHD2 knockdown resulted in activation of the RhoA (Ras homolog gene family member A)/Rho-associated kinase pathway with subsequent phosphorylation of cofilin. Because cofilin phosphorylation impairs its actin-severing function, this may explain the F-actin phenotype, thereby providing a functional link between PHD2-dependent signaling and cell motility. 
 NADPH oxidase 1 controls the persistence of directed cell   migration   by a Rho-dependent switch of alpha2/alpha3 integrins. NADPH oxidase 1 (Nox1) is expressed mainly in colon epithelial cells and produces superoxide ions as a primary function. We showed that Nox1 knockdown inhibits directional persistence of   migration   on collagen I. This paper dissects the mechanism by which Nox1 affects the direction of colonic epithelial cell   migration   in a two-dimensional model. Transient activation of Nox1 during cell spreading on collagen 1 temporarily inactivated RhoA and led to efficient exportation of alpha2beta1 integrin to the cell surface, which supported persistent directed   migration  . Nox1 knockdown led to a loss of directional   migration   which takes place through a RhoA-dependent alpha2/alpha3 integrin switch. Transient RhoA overactivation upon Nox1 inhibition led to transient cytoskeletal reorganization and increased cell-matrix contact associated with a stable increase in alpha3 integrin cell surface expression. Blocking of alpha3 integrin completely reversed the loss of directional persistence of   migration  . In this model, Nox1 would represent a switch between random and directional   migration   through RhoA-dependent integrin cell surface expression modulation. 
 Apolipoprotein(a) acts as a chemorepellent to human vascular smooth muscle cells via integrin αVβ3 and RhoA/ROCK-mediated mechanisms. Lipoprotein(a) (Lp(a)) is an independent risk factor for the development of cardiovascular disease. Vascular smooth muscle cell (SMC) motility and plasticity, functions that are influenced by environmental cues, are vital to adaptation and remodelling in vascular physiology and pathophysiology. Lp(a) is reportedly damaging to SMC function via unknown molecular mechanisms. Apolipoprotein(a) (apo(a)), a unique glycoprotein moiety of Lp(a), has been demonstrated as its active component. The aims of this study were to determine functional effects of recombinant apo(a) on human vascular SMC motility and explore the underlying mechanism(s). Exposure of SMC to apo(a) i  n migrati  on assays induced a potent, concentration-dependent chemorepulsion that was RhoA and integrin αVβ3-dependent, but transforming growth factor β-independent. SMC manipulation through RhoA gene silencing, Rho kinase inhibition, statin pre-treatment, αVβ3 neutralising antibody and tyrosine kinase inhibition all markedly inhibited apo(a)-mediat  ed SMC mi  gration. Our data reveal unique and potent activities of apo(a) that may negatively influence SMC remodelling in cardiovascular disease. Circulating levels of Lp(a) are resistant to lipid-lowering strategies and hence a greater understanding of the mechanisms underlying its functional effects on SMC may provide alternative therapeutic targets. 
 Myo9b is a key player in SLIT/ROBO-mediated lung tumor suppression. Emerging evidence indicates that the neuronal guidance molecule SLIT plays a role in tumor suppression, as SLIT-encoding genes are inactivated in several types of cancer, including lung cancer; however, it is not clear how SLIT functions in lung cancer. Here, our data show that SLIT inhibits cancer cell   migration   by activating RhoA and that myosin 9b (Myo9b) is a ROBO-interacting protein that suppresses RhoA activity in lung cancer cells. Structural analyses revealed that the RhoGAP domain of Myo9b contains a unique patch that specifically recognizes RhoA. We also determined that the ROBO intracellular domain interacts with the Myo9b RhoGAP domain and inhibits its activity; therefore, SLIT-dependent activation of RhoA is mediated by ROBO inhibition of Myo9b. In a murine model, compared with control lung cancer cells, SLIT-expressing cells had a decreased capacity for tumor formation and lung metastasis. Evaluation of human lung cancer and adjacent nontumor tissues revealed that Myo9b is upregulated in the cancer tissue. Moreover, elevated Myo9b expression was associated with lung cancer progression and poor prognosis. Together, our data identify Myo9b as a key player in lung cancer and as a ROBO-interacting protein in what is, to the best of our knowledge, a newly defined SLIT/ROBO/Myo9b/RhoA signaling pathway that restricts lung cancer progression and metastasis. Additionally, our work suggests that targeting the SLIT/ROBO/Myo9b/RhoA pathway has potential as a diagnostic and therapeutic strategy for lung cancer. 
 C1q/tumor necrosis factor-related protein-6 attenuates post-infarct cardiac fibrosis by targeting RhoA/MRTF-A pathway and inhibiting myofibroblast differentiation. C1q/tumor necrosis factor-related protein-6 (CTRP6) is a newly identified adiponectin paralog with modulation effects on metabolism and inflammation. However, the cardiovascular function of CTRP6 remains unknown. This study aimed to determine its role in cardiac fibrosis and explore the possible mechanism. Myocardial infarction (MI) was induced by left anterior descending coronary artery ligation in rats. CTRP6 was mainly expressed in the cytoplasm of adult rat cardiomyocytes and significantly decreased in the border and infarct zones post-MI. Adenovirus-mediated CTRP6 delivery improved cardiac function, attenuated cardiac hypertrophy, alleviated cardiac fibrosis, and inhibited myofibroblast differentiation as well as the expression of collagen I, collagen III, and connective tissue growth factor post-MI. In cultured adult rat cardiac fibroblasts (CFs), exogenous or cardiomyocyte-secreted CTRP6 inhibited, whereas knockdown of CTRP6 facilitated transforming growth factor-β1 (TGF-β1)-induced expression of α-smooth muscle actin, smooth muscle 22α, and profibrotic molecules. CTRP6 had no effect on CFs proliferation but attenuated   CFs migra  tion induced by TGF-β1. CTRP6 increased the phosphorylation of AMP-activated protein kinase (AMPK) and Akt in CFs and post-MI hearts. Pretreatment with adenine 9-β-D-arabinofuranoside (AraA), an AMPK inhibitor, or LY294002, a phosphatidylinositol-3-kinase (PI3 K) inhibitor, abolished the protective effect of CTRP6 on TGF-β1-induced profibrotic response. Furthermore, CTRP6 had no effect on TGF-β1-induced Smad3 phosphorylation and nuclear translocation, whereas significantly decreased TGF-β1-induced RhoA activation and myocardin-related transcription factor-A (MRTF-A) nuclear translocation, and these effects were blocked by AMPK or Akt inhibition. In conclusion, CTRP6 attenuates cardiac fibrosis via inhibiting myofibroblast differentiation. AMPK and Akt activation are responsible for the CTRP6-mediated anti-fibrotic effect by targeting RhoA/MRTF-A pathway. 
 Apolipoprotein E Inhibits Cerebrovascular Pericyte Mobility through a RhoA Protein-mediated Pathway. Pericytes play a critical role in the cerebrovasculature within the CNS. These small contractile cells produce large quantities of apolipoprotein E (apoE) whose isoforms influence cerebrovascular functions and determine the genetic risk for Alzheimer disease. Despite extensive studies on astrocyte-secreted apoE, which supports synapses by transporting cholesterol to neurons, the biochemical properties and function of apoE secreted by pericytes are not clear. Because pericytes mediate important functions in the CNS, including the initiation of glial scar formation, angiogenesis, and maintenance of the blood-brain barrier, we investigated the potential role of apoE in pericyte mobility. We found that knockdown of apoE expression significantly accelerates pericyte   migration  , an effect that can be rescued by exogenous apoE3, but not apoE4, a risk factor for Alzheimer disease. ApoE-regulated   migration   of pericytes also requires the function of the low-density lipoprotein receptor-related protein 1 (LRP1), a major apoE receptor in the brain that is abundantly expressed in pericytes. Because apoE-knockdown also leads to enhanced cell adhesion, we investigated the role of apoE in the regulation of the actin cytoskeleton. Interestingly, we found that the levels of active RhoA are increased significantly in apoE knockdown pericytes and that RhoA inhibitors blocked pericyte   migration  . Taken together, our results suggest that apoE has an intrinsic role in pericyte mobility, which is vital in maintaining cerebrovascular function. These findings provide novel insights into the role of apoE in the cerebrovascular system. 
 miR-143 inhibits the metastasis of pancreatic cancer and an associated signaling pathway. Pancreatic cancer is characterized by early metastasis and high mortality. In this study, the role of miR-143 in invasion and metastasis was investigated in pancreatic cancer cells. miR-143 expression was established by an adenovirus-carried miR-143 expression cassette. mRNA and protein levels of gene expression were examined by RT-PCR and Western blot assay, respectively. Rho GTPases activity was measured by the pull down assay. The role of miR-143 in   migration   and invasion of Panc-1 cells was tested in vitro. The antimetastatic effect of miR-143 was tested in a liver metastasis model, while its antitumor growth effect was tested in a xenograft Panc-1 tumor model. Results demonstrated that ARHGEF1 (GEF1), ARHGEF2 (GEF2), and K-RAS genes are the targets of miR-143. miR-143 expression significantly decreased mRNA and protein levels of GEF1, GEF2, and K-RAS genes; lowered the constitutive activities of RhoA, Rac1, and Cdc42 GTPases; decreased the protein levels of MMP-2 and MMP-9; but significantly increased the protein level of E-cadherin. miR-143 expression also significantly inhibited the   migration   and invasion of Panc-1 cells in vitro, liver metastasis, and xenograft tumor growth in vivo. Our study suggested that miR-143 plays a central role in the invasion and metastasis of pancreatic cancer and miR-143 is a potential target for pancreatic cancer therapy. 
 Rapamycin inhibits cytoskeleton reorganization and cell motility by suppressing RhoA expression and activity. The mammalian target of rapamycin (mTOR) functions in cells at least as two complexes, mTORC1 and mTORC2. Intensive studies have focused on the roles of mTOR in the regulation of cell proliferation, growth, and survival. Recently we found that rapamycin inhibits type I insulin-like growth factor (IGF-1)-stimulated lamellipodia formation and cell motility, indicating involvement of mTOR in regulating cell motility. This study was set to further elucidate the underlying mechanism. Here we show that rapamycin inhibited protein synthesis and activities of small GTPases (RhoA, Cdc42, and Rac1), crucial regulatory proteins for cell   migration  . Disruption of mTORC1 or mTORC2 by down-regulation of raptor or rictor, respectively, inhibited the activities of these proteins. However, only disruption of mTORC1 mimicked the effect of rapamycin, inhibiting their protein expression. Ectopic expression of rapamycin-resistant and constitutively active S6K1 partially prevented rapamycin inhibition of RhoA, Rac1, and Cdc42 expression, whereas expression of constitutively hypophosphorylated 4E-BP1 (4EBP1-5A) or down-regulation of S6K1 by RNA interference suppressed expression of the GTPases, suggesting that both mTORC1-mediated S6K1 and 4E-BP1 pathways are involved in protein synthesis of the GTPases. Expression of constitutively active RhoA, but not Cdc42 and Rac1, conferred resistance to rapamycin inhibition of IGF-1-stimulated lamellipodia formation and cell   migration  . The results suggest that rapamycin inhibits cell motility at least in part by down-regulation of RhoA protein expression and activity through mTORC1-mediated S6K1 and 4E-BP1-signaling pathways. 
 Protein kinase D regulates RhoA activity via rhotekin phosphorylation. The members of the protein kinase D (PKD) family of serine/threonine kinases are major targets for tumor-promoting phorbol esters, G protein-coupled receptors, and activated protein kinase C isoforms (PKCs). The expanding list of cellular processes in which PKDs exert their function via phosphorylation of various substrates include proliferation, apoptosis,   migration  , angiogenesis, and vesicle trafficking. Therefore, identification of novel PKD substrates is necessary to understand the profound role of this kinase family in signal transduction. Here, we show that rhotekin, an effector of RhoA GTPase, is a novel substrate of PKD. We identified Ser-435 in rhotekin as the potential site targeted by PKD in vivo. Expression of a phosphomimetic S435E rhotekin mutant resulted in an increase of endogenous active RhoA GTPase levels. Phosphorylation of rhotekin by PKD2 modulates the anchoring of the RhoA in the plasma membrane. Consequently, the S435E rhotekin mutant displayed enhanced stress fiber formation when expressed in serum-starved fibroblasts. Our data thus identify a novel role of PKD as a regulator of RhoA activity and actin stress fiber formation through phosphorylation of rhotekin. 
 Regulation of PTEN by Rho small GTPases. PTEN (phosphatase and tensin homologue) is a phosphatase that dephosphorylates both protein and phosphoinositide substrates. It is mutated in a variety of human tumours and has important roles in a diverse range of biological processes, including cell   migration   and chemotaxis. PTEN's intracellular localization and presumably activity are regulated by chemoattractants in Dictyostelium and mouse neutrophils. However, the mechanisms for its regulation remain elusive. Here we show that RhoA and Cdc42, members of the Rho family of small GTPases, regulate the intracellular localization of PTEN in leukocytes and human transfected embryonic kidney cells. In addition, active RhoA is able to stimulate the phospholipid phosphatase activity of PTEN in human embryonic kidney cells and leukocytes, and this regulation seems to require RhoA's downstream effector, RhoA-associated kinase (Rock). Furthermore, we have identified key residues on PTEN that are required for its regulation by the small GTPase, and show that small GTPase-mediated regulation of PTEN has a significant role in the regulation of chemotaxis. 
 Kindlin-1 Is required for RhoGTPase-mediated lamellipodia formation in keratinocytes. Kindlin-1 is an epithelial-specific member of the novel kindlin protein family, which are regulators of integrin functions. Mutations in the gene that encodes Kindlin-1, FERMT1 (KIND1), cause the Kindler syndrome (KS), a human disorder characterized by mucocutaneous fragility, progressive skin atrophy, ulcerative colitis, photosensitivity, and propensity to skin cancer. Our previous studies indicated that loss of kindlin-1 resulted in abnormalities associated with integrin functions, such as adhesion, proliferation, polarization, and motility of epidermal cells. Here, we disclosed novel FERMT1 mutations in KS and used them, in combination with small-interfering RNA, protein, and imaging studies, to uncover new functions for kindlin-1 in keratinocytes and to discern the molecular pathology of KS. We show that kindlin-1 forms molecular complexes with beta1 integrin, alpha-actinin, migfilin, and focal adhesion kinase and regulates cell shape and   migration   by controlling lamellipodia formation. Kindlin-1 governs these processes by signaling via Rho family GTPases, and it is required to maintain the pool of GTP-bound, active Rac1, RhoA and Cdc42, and the phosphorylation of their downstream effectors p21-activated kinase 1, LIM kinase, and cofilin. Loss of these kindlin-1 functions forms the biological basis for the epithelial cell fragility and atrophy in the pathology of KS. 
 A novel amplification target, ARHGAP5, promotes cell spreading and   migration   by negatively regulating RhoA in Huh-7 hepatocellular carcinoma cells. RhoA, a member of the Rho family of small GTPases, directs the organization of the actin cytoskeleton and is involved in regulating cell shape and movement. Its activity is negatively regulated by p190-B RhoGAP (GTPase-activating protein). We investigated DNA copy number aberrations in human hepatocellular carcinoma and esophageal squamous cell carcinoma cell lines using a high-density oligonucleotide microarray and found a novel amplification at chromosomal region 14q12. We identified ARHGAP5 (the gene encoding p190-B RhoGAP) as a probable target for the amplification at 14q12, and our results showed that p190-B RhoGAP promotes cells spreading and   migration   by negatively regulating RhoA activity in Huh-7 hepatocellular carcinoma cells. 
 Down expression of LRP1B promotes cell   migration   via RhoA/Cdc42 pathway and actin cytoskeleton remodeling in renal cell cancer. The low-density lipoprotein receptor-related protein 1B (LRP1B) is known as a putative tumor suppressor. The decreased expression of LRP1B has been involved in multiple primary cancers in several studies. However, its expression and function in the carcinogenesis of renal cell cancer (RCC) remain unclear. In this study, we investigated the expression of LRP1B in RCC by in situ hybridization (ISH) and real-time polymerase chain reaction (qRT-PCR). Our results indicated that LRP1B was frequently downexpressed in human RCC tissue and cell lines, which involved both epigenetic events (DNA methylation and histone deacetylation) and N-terminal deletion of LRP1B. Moreover, we testified that knockdown of LRP1B by shRNA significantly promoted anchorage-independent growth, cell   migration   and invasion in HEK293 cells and renal cancer cells 127 in vitro. We further found that silencing of LRP1B altered the expression of focal adhesion complex-associated proteins, and Cdc42/RhoA activities, which regulate the cytoskeleton dynamics. Taken together, these results strongly support that LRP1B may function as a tumor suppressor against renal cell cancer, and may regulate cell motility via RhoA/Cdc42 pathway and actin cytoskeleton reorganization in RCC. 
 Memo-RhoA-mDia1 signaling controls microtubules, the actin network, and adhesion site formation in migrating cells. Actin assembly at the cell front drives membrane protrusion and initiates the cell   migration   cycle. Microtubules (MTs) extend within forward protrusions to sustain cell polarity and promote adhesion site turnover. Memo is an effector of the ErbB2 receptor tyrosine kinase involved in breast carcinoma cell   migration  . However, its mechanism of action remained unknown. We report in this study that Memo controls ErbB2-regulated MT dynamics by altering the transition frequency between MT growth and shortening phases. Moreover, although Memo-depleted cells can assemble the Rac1-dependent actin meshwork and form lamellipodia, they show defective localization of lamellipodial markers such as alpha-actinin-1 and a reduced number of short-lived adhesion sites underlying the advancing edge of migrating cells. Finally, we demonstrate that Memo is required for the localization of the RhoA guanosine triphosphatase and its effector mDia1 to the plasma membrane and that Memo-RhoA-mDia1 signaling coordinates the organization of the lamellipodial actin network, adhesion site formation, and MT outgrowth within the cell leading edge to sustain cell motility. 
 Effect of shRNA targeted against RhoA on proliferation and   migration   of human colonic cancer cells. To study the effects of RhoA siRNA on the malignant phenotypes of human colorectal cancer cell line LoVo.The siRNA expression vector pGPU6/GFP/Neo-shRNA-RhoA targeting the mRNA of RhoA and vector pGPU6/GFP/Neo-NC (as a control) were constructed, and then transfected into LoVo cells. The expression of Survivin was detected by real-time fluorescent quantitative polymerase chain reaction and Western blot. The malignant phenotypes of transfected LoVo cells, including invasive activities and adhesive capabilities, were analyzed.RhoA mRNA and protein level were decreased after the pshRNA-RhoA transfection. The cell adhesion rates significantly decreased in the cells transfected with pshRNA-RhoA. The migrating number of LoVo cells (26.5 ± 0.9) transfected with pshRNA-RhoA was also significantly decreased as compared with the control group (53.7 ± 1.4).The sequence specific shRNA against RhoA constructed in the study can block the expression of RhoA in LoVo cell effectively and specifically; Blocking the expression of RhoA in LoVo cells transfected with pshRNA-RhoA can reduce their invasive and adhesive capabilities. 
 Concerted regulation of cell dynamics by BNIP-2 and Cdc42GAP homology/Sec14p-like, proline-rich, and GTPase-activating protein domains of a novel Rho GTPase-activating protein, BPGAP1. RhoA, Cdc42, and Rac1 are small GTPases that regulate cytoskeletal reorganization leading to changes in cell morphology and cell motility. Their signaling pathways are activated by guanine nucleotide exchange factors and inactivated by GTPase-activating proteins (GAPs). We have identified a novel RhoGAP, BPGAP1 (for BNIP-2 and Cdc42GAP Homology (BCH) domain-containing, Proline-rich and Cdc42GAP-like protein subtype-1), that is ubiquitously expressed and shares 54% sequence identity to Cdc42GAP/p50RhoGAP. BP-GAP1 selectively enhanced RhoA GTPase activity in vivo although it also interacted strongly with Cdc42 and Rac1. "Pull-down" and co-immunoprecipitation studies indicated that it formed homophilic or heterophilic complexes with other BCH domain-containing proteins. Fluorescence studies of epitope-tagged BPGAP1 revealed that it induced pseudopodia and increased   migration   of MCF7 cells. Formation of pseudopodia required its BCH and GAP domains but not the proline-rich region, and was differentially inhibited by coexpression of the constitutively active mutant of RhoA, or dominant negative mutants of Cdc42 and Rac1. However, the mutant without the proline-rich region failed to confer any increase in cell   migration   despite the induction of pseudopodia. Our findings provide evidence that cell morphology changes and   migration   are coordinated via multiple domains in BPGAP1 and present a novel mode of regulation for cell dynamics by a RhoGAP protein. 
 SmgGDS is a guanine nucleotide exchange factor that specifically activates RhoA and RhoC. SmgGDS is an atypical guanine nucleotide exchange factor (GEF) that promotes both cell proliferation and   migration   and is up-regulated in several types of cancer. SmgGDS has been previously shown to activate a wide variety of small GTPases, including the Ras family members Rap1a, Rap1b, and K-Ras, as well as the Rho family members Cdc42, Rac1, Rac2, RhoA, and RhoB. In contrast, here we show that SmgGDS exclusively activates RhoA and RhoC among a large panel of purified GTPases. Consistent with the well known properties of GEFs, this activation is catalytic, and SmgGDS preferentially binds to nucleotide-depleted RhoA relative to either GDP- or GTPγS-bound forms. However, mutational analyses indicate that SmgGDS utilizes a distinct exchange mechanism compared with canonical GEFs and in contrast to known GEFs requires RhoA to retain a polybasic region for activation. A homology model of SmgGDS highlights an electronegative surface patch and a highly conserved binding groove. Mutation of either area ablates the ability of SmgGDS to activate RhoA. Finally, the in vitro specificity of SmgGDS for RhoA and RhoC is retained in cells. Together, these results indicate that SmgGDS is a bona fide GEF that specifically activates RhoA and RhoC through a unique mechanism not used by other Rho family exchange factors. 
 The RhoA effector mDia is induced during T cell activation and regulates actin polymerization and cell   migration   in T lymphocytes. Regulation of actin polymerization is critical for many different functions of T lymphocytes, including cell   migration  . Here we show that the RhoA effector mDia is induced in vitro in activated PBL and is highly expressed in vivo in diseased tissue-infiltrating activated lymphocytes. mDia localizes at the leading edge of polarized T lymphoblasts in an area immediately posterior to the leading lamella, in which its effector protein profilin is also concentrated. Overexpression of an activated mutant of mDia results in an inhibition of both spontaneous and chemokine-directed T cell motility. mDia does not regulate the shape of the cell, which involves another RhoA effector, p160 Rho-coiled coil kinase, and is not involved in integrin-mediated cell adhesion. However, mDia activation blocked CD3- and PMA-mediated cell spreading. mDia activation increased polymerized actin levels, which resulted in the blockade of chemokine-induced actin polymerization by depletion of monomeric actin. Moreover, mDia was shown to regulate the function of the small GTPase Rac1 through the control of actin availability. Together, our data demonstrate that RhoA is involved in the control of the filamentous actin/monomeric actin balance through mDia, and that this balance is critical for T cell responses. 
 PAR1-mediated RhoA activation facilitates CCL2-induced chemotaxis in PC-3 cells. Patients with advanced prostate cancer often exhibit increased activation of the coagulation system. The key activator of the coagulation cascade is the serine protease thrombin which is capable of eliciting numerous cellular responses. We previously reported that the thrombin receptor PAR1 is overexpressed in prostate cancer. To investigate further the role of PAR1 in prostate cancer metastasis, we examined the effects of thrombin activation on cell adhesion and motility in PC-3 prostate cancer cells. Activation of PAR1-induced dynamic cytoskeletal reorganization and reduced PC-3 binding to collagen I, collagen IV, and laminin (P < 0.01) but not fibronectin. Expression of the cell surface integrin receptors did not change as assessed by flow cytometry. Immunofluorescence microscopy revealed that PAR1 stimulation caused reorganization of the focal adhesions, suggesting that PAR1 activation in PC-3 cells may be modulating cell adhesion through integrin function but not expression. Furthermore, RhoA was activated upon stimulation with thrombin with subsequent cell contraction, decreased cell adhesion, and induced   migration   towards monocyte chemoattractant protein 1 (MCP-1; CCL2). Thus, it appears that thrombin stimulation plays a role in prostate cancer metastasis by decreasing cell adhesion to the extracellular matrix and positioning the cell in a "ready state" for   migration   in response to a chemotactic signal. Further exploration is needed to determine whether PAR1 activation affects other signaling pathways involved in prostate cancer. 
 The E3 ligase Cdh1-anaphase promoting complex operates upstream of the E3 ligase Smurf1 in the control of axon growth. Axon growth is an essential event during brain development and is extremely limited due to extrinsic and intrinsic inhibition in the adult brain. The E3 ubiquitin ligase Cdh1-anaphase promoting complex (APC) has emerged as an important intrinsic suppressor of axon growth. In this study, we identify in rodents the E3 ligase Smurf1 as a novel substrate of Cdh1-APC and that Cdh1 targets Smurf1 for degradation in a destruction box-dependent manner. We find that Smurf1 acts downstream of Cdh1-APC in axon growth and that the turnover of RhoA by Smurf1 is important in this process. In addition, we demonstrate that acute knockdown of Smurf1 in vivo in the developing cerebellar cortex results in impaired axonal growth and   migration  . Finally, we show that a stabilized form of Smurf1 overrides the inhibition of axon growth by myelin. Taken together, we uncovered a Cdh1-APC/Smurf1/RhoA pathway that mediates axonal growth suppression in the developing mammalian brain. 
 Cerebral cavernous malformation 2 protein promotes smad ubiquitin regulatory factor 1-mediated RhoA degradation in endothelial cells. Mutation of CCM2 predisposes individuals to cerebral cavernous malformations, vascular abnormalities that cause seizures and hemorrhagic stroke. CCM2 has been proposed to regulate the activity of RhoA for maintenance of vascular integrity. Herein, we define a novel mechanism where the CCM2 phosphotyrosine binding (PTB) domain binds the ubiquitin ligase (E3) Smurf1, controlling RhoA degradation. Brain endothelial cells with knockdown of CCM2 have increased RhoA protein and display impaired directed cell   migration  . CCM2 binding of Smurf1 increases Smurf1-mediated degradation of RhoA. CCM2 does not significantly alter the catalytic activity of Smurf1, nor is CCM2 a Smurf1 substrate. Rather the CCM2-Smurf1 interaction functions to localize Smurf1 for RhoA degradation. These findings provide a molecular mechanism for the pathogenesis of cerebral cavernous malformations (CCM) resulting from loss of CCM2-mediated localization of Smurf1, which controls RhoA degradation required for maintenance of normal endothelial cell physiology. 
 Gonadotropin-releasing hormone receptor activates GTPase RhoA and inhibits cell invasion in the breast cancer cell line MDA-MB-231. Gonadotropin-releasing hormone (GnRH) and its receptor (GnRHR) are both expressed by a number of malignant tumors, including those of the breast. In the latter, both behave as potent inhibitors of invasion. Nevertheless, the signaling pathways whereby the activated GnRH/GnRHR system exerts this effect have not been clearly established. In this study, we provide experimental evidence that describes components of the mechanism(s) whereby GnRH inhibits breast cancer cell invasion.Actin polymerization and substrate adhesion was measured in the highly invasive cell line, MDA-MB-231 transiently expressing the wild-type or mutant DesK191 GnRHR by fluorometry, flow cytometric analysis, and confocal microscopy, in the absence or presence of GnRH agonist. The effect of RhoA-GTP on stress fiber formation and focal adhesion assembly was measured in MDA-MB-231 cells co-expressing the GnRHRs and the GAP domain of human p190Rho GAP-A or the dominant negative mutant GAP-Y1284D. Cell invasion was determined by the transwell   migration   assay.Agonist-stimulated activation of the wild-type GnRHR and the highly plasma membrane expressed mutant GnRHR-DesK191 transiently transfected to MDA-MB-231 cells, favored F-actin polymerization and substrate adhesion. Confocal imaging allowed detection of an association between F-actin levels and the increase in stress fibers promoted by exposure to GnRH. Pull-down assays showed that the effects observed on actin cytoskeleton resulted from GnRH-stimulated activation of RhoA GTPase. Activation of this small G protein favored the marked increase in both cell adhesion to Collagen-I and number of focal adhesion complexes leading to inhibition of the invasion capacity of MDA-MB-231 cells as disclosed by assays in Transwell Chambers.We here show that GnRH inhibits invasion of highly invasive breast cancer-derived MDA-MB-231 cells. This effect is mediated through an increase in substrate adhesion promoted by activation of RhoA GTPase and formation of stress fibers and focal adhesions. These observations offer new insights into the molecular mechanisms whereby activation of overexpressed GnRHRs affects cell invasion potential of this malignant cell line, and provide opportunities for designing mechanism-based adjuvant therapies for breast cancer. 
 Macrophages stimulate gastric and colorectal cancer invasion through EGFR Y(1086), c-Src, Erk1/2 and Akt phosphorylation and smallGTPase activity. The interactions between cancer cells and their microenvironment are crucial for malignant progression, as they modulate invasion-related activities. Tumor-associated macrophages are generally considered allies in the process of tumor progression in several types of cancer, although their role on gastric and colorectal carcinomas is still poorly understood. In this report, we studied the influence of primary human macrophages on gastric and colorectal cancer cells, considering invasion, motility/  migration  , proteolysis and activated intracellular signaling pathways. We demonstrated that macrophages stimulate cancer cell invasion, motility and   migration  , and that these effects depend on matrix metalloproteinase (MMP) activity and on the activation of epidermal growth factor receptor (EGFR) (at the residue Y(1086)), PLC-γ (phospholipase C-gamma) and Gab1 (GRB2-associated binding protein-1), as evidenced by siRNA (small interference RNA) experiments. Epidermal growth factor (EGF)-immunodepletion impaired macrophage-mediated cancer cell invasion and motility, suggesting that EGF is the pro-invasive and pro-motile factor produced by macrophages. Macrophages also induced gastric and colorectal cancer cell phosphorylation of Akt, c-Src and ERK1/2, and led to an increase of RhoA and Cdc42 activity. Interestingly, whereas macrophage-mediated cancer cell c-Src and ERK1/2 phosphorylation occurred downstream EGFR activation, Akt phosphorylation seems to be a parallel event, taking place in an EGFR-independent manner. The involvement of EGF, EGFR-downstream signaling partners and MMPs in macrophage-mediated invasion provides novel insights into the molecular crosstalk established between cancer cells and macrophages, opening new perspectives for the design of new and more efficient therapeutic strategies to counteract cancer cell invasion. 
 RhoA is associated with invasion and poor prognosis in colorectal cancer. Colorectal cancer is one of the most common cancers and is the fourth leading cause of cancer death in Korea. Mortality of colorectal cancer is strongly associated with the metastatic spread of the disease. As such, it is important to find and characterize signaling pathways involved in colon cancer metastasis. We investigated the functional importance of RhoA using human cell lines as well as 150 colorectal cancer patient-derived samples as it remains unclear whether RhoA functions as either an oncogene or a tumor suppressor in colon cancer. RhoA was highly expressed in metastatic cancer cell lines. Although cancer cell proliferation was only moderately impaired after depletion of RhoA, RhoA-depleted cancer cells exhibited markedly reduced   migration   and invasion ability in vitro. Furthermore, we found that RhoA is associated with the invasion of lymph nodes and blood vessels in the patient colorectal cancer samples. Most notably, patients with higher RhoA expression had a significantly poorer 5-year survival rate after surgery. These results suggest that RhoA is a marker of poor prognosis in colorectal cancer and may be a promising target for cancer treatment. 
 CCR4 mediates CCL17 (TARC)-induced   migration   of human colon cancer cells via RhoA/Rho-kinase signaling. Accumulating data suggest a role of chemokines in tumor cell metastasis. CCR4 has been implicated in hematologic malignancies and recently also in solid tumors. Herein, we hypothesized that CCR4 might be expressed and support   migration   of colon cancer cells.We used quantitative RT-PCR and flow cytometry to determine mRNA and surface expression of CCR4 on colon cancer cell lines (HT-29) and (AZ-97). Total RhoA and active RhoA protein levels in CCL17-stimulated colon cancer cells were quantified using ELISA and G-LISA assays.   Migration   assays were performed to evaluate colon cancer cells chemotaxis. In vitro tumor growth was assessed using proliferation assay.Our results show clear-cut mRNA levels and surface expression of CCR4 on a colon cancer cell line (HT-29) and on tumor cells (AZ-97). CCR4 ligand CCL17 (TARC) was a potent stimulator of colon cancer cell   migration  . This CCL17-induced colon cancer cell   migration   was inhibited by pre-incubation of the colon cancer cells with an antibody directed against CCR4 or an antagonist against CCR4. CCL17-induced signaling in colon cancer cells revealed that CCL17 increased mRNA formation of RhoA-C in colon cancer cells. Our results also found that CCL17 increased total RhoA and active RhoA protein levels in colon cancer cells. The Rho-kinase inhibitor Y-27632 abolished CCL17-induced colon cancer cell chemotaxis. In addition, inhibition of isoprenylation by GGTI-2133 markedly reduced colon cancer cell   migration   triggered by CCL17.Our novel data indicate for the first time that the CCL17-CCR4 axis might be involved in the spread of colon cancer cells. 
 RACK1 promotes breast carcinoma   migration  /metastasis via activation of the RhoA/Rho kinase pathway. We aimed to gain a mechanistic understanding of the role of RACK1 in breast carcinoma   migration  /metastasis.   Migration   assays were conducted in breast carcinoma cell lines. siRNA targeting RACK1 as well as the Rho kinase inhibitor were also applied. Immunoprecipitation and immunofluorescence were used to study the RACK1/RhoA interaction. GTP-Rho pull-down assays were performed to assess the activation of RhoA. We also conducted immunohistochemistry in 160 breast carcinoma samples. Experiments in vitro showed that RACK1 promotes   migration   via interaction with RhoA and activation of the RhoA/Rho kinase pathway. Immunohistochemistry in 160 samples revealed that RACK1 is strongly correlated with accepted tumor spread indicators and RhoA (all P < 0.05). Kaplan-Meier survival analysis indicated a correlation between higher RACK1 expression and shorter survival times (P < 0.001). RACK1 is a prognostic factor that promotes breast carcinoma   migration  /metastasis by interacting with RhoA and activating the RhoA/Rho kinase pathway. 
 Hax1 regulates neutrophil adhesion and motility through RhoA. Kostmann disease is an inherited severe congenital neutropenia syndrome associated with loss-of-function mutations in an adaptor protein HS1-associated protein X-1 (Hax1). How Hax1 regulates neutrophil function remains largely unknown. In this paper, we use ribonucleic acid interference to deplete Hax1 in the neutrophil-like cell line PLB-985 and identify Hax1 as a negative regulator of integrin-mediated adhesion and chemotaxis. Using microfluidics, we show that depletion of Hax1 impairs neutrophil uropod detachment and directed   migration  . Hax1-deficient cells also display increased integrin-mediated adhesion and reduced RhoA activity. Moreover, depletion of RhoA induces increased neutrophil adhesion and impaired   migration  , suggesting that Hax1 regulates neutrophil adhesion and chemotaxis through RhoA. Accordingly, activation of RhoA is sufficient to rescue adhesion of Hax1-deficient neutrophils. Together, our findings identify Hax1 as a novel regulator of neutrophil uropod detachment and chemotaxis through RhoA. 
 Rho isoform-specific interaction with IQGAP1 promotes breast cancer cell proliferation and   migration  . We performed a proteomics screen for Rho isoform-specific binding proteins to clarify the tumor-promoting effects of RhoA and C that contrast with the tumor-suppressive effects of RhoB. We found that the IQ-motif-containing GTPase-activating protein IQGAP1 interacts directly with GTP-bound, prenylated RhoA and RhoC, but not with RhoB. Co-immunoprecipitation of IQGAP1 with endogenous RhoA/C was enhanced when RhoA/C were activated by epidermal growth factor (EGF) or transfection of a constitutively active guanine nucleotide exchange factor (GEF). Overexpression of IQGAP1 increased GTP-loading of RhoA/C, while siRNA-mediated depletion of IQGAP1 prevented endogenous RhoA/C activation by growth factors. IQGAP1 knockdown also reduced the amount of GTP bound to GTPase-deficient RhoA/C mutants, suggesting that IQGAP enhances Rho activation by GEF(s) or stabilizes Rho-GTP. IQGAP1 depletion in MDA-MB-231 breast cancer cells blocked EGF- and RhoA-induced stimulation of DNA synthesis. Infecting cells with adenovirus encoding constitutively active RhoA(L63) and measuring absolute amounts of RhoA-GTP in infected cells demonstrated that the lack of RhoA(L63)-induced DNA synthesis in IQGAP1-depleted cells was not due to reduced GTP-bound RhoA. These data suggested that IQGAP1 functions downstream of RhoA. Overexpression of IQGAP1 in MDA-MB-231 cells increased DNA synthesis irrespective of siRNA-mediated RhoA knockdown. Breast cancer cell motility was increased by expressing a constitutively-active RhoC(V14) mutant or overexpressing IQGAP1. EGF- or RhoC-induced   migration   required IQGAP1, but IQGAP1-stimulated   migration   independently of RhoC, placing IQGAP1 downstream of RhoC. We conclude that IQGAP1 acts both upstream of RhoA/C, regulating their activation state, and downstream of RhoA/C, mediating their effects on breast cancer cell proliferation and   migration  , respectively. 
 A novel strategy for specifically down-regulating individual Rho GTPase activity in tumor cells. The Rho family GTPases RhoA, RhoB, and RhoC regulate the actin cytoskeleton, cell movement, and cell growth. Unlike Ras, up-regulation or overexpression of these GDP/GTP binding molecular switches, but not activating point mutations, has been associated with human cancer. Although they share over 85% sequence identity, RhoA, RhoB, and RhoC appear to play distinct roles in cell transformation and metastasis. In NIH 3T3 cells, RhoA or RhoB overexpression causes transformation whereas RhoC increases the cell   migration   rate. To specifically target RhoA, RhoB, or RhoC function, we have generated a set of chimeric molecules by fusing the RhoGAP domain of p190, a GTPase-activating protein that accelerates the intrinsic GTPase activity of all three Rho GTPases, with the C-terminal hypervariable sequences of RhoA, RhoB, or RhoC. The p190-Rho chimeras were active as GTPase-activating proteins toward RhoA in vitro, co-localized with the respective active Rho proteins, and specifically down-regulated Rho protein activities in cells depending on which Rho GTPase sequences were included in the chimeras. In particular, the p190-RhoA-C chimera specifically inhibited RhoA-induced transformation whereas p190-RhoC-C specifically reversed the   migration   phenotype induced by the active RhoC. In human mammary epithelial-RhoC breast cancer cells, p190-RhoC-C, but not p190-RhoA-C or p190-RhoB-C, reversed the anchorage-independent growth and invasion phenotypes caused by RhoC overexpression. In the highly metastatic A375-M human melanoma cells, p190-RhoC-C specifically reversed   migration  , and invasion phenotypes attributed to RhoC up-regulation. Thus, we have developed a novel strategy utilizing RhoGAP-Rho chimeras to specifically down-regulate individual Rho activity and demonstrate that this approach may be applied to multiple human tumor cells to reverse the growth and/or invasion phenotypes associated with disregulation of a distinct subtype of Rho GTPase. 
 Overexpression of RhoA induces preneoplastic transformation of primary mammary epithelial cells. Rho family small GTPases serve as molecular switches in the regulation of diverse cellular functions, including actin cytoskeleton remodeling, cell   migration  , gene transcription, and cell proliferation. Importantly, Rho overexpression is frequently seen in many carcinomas. However, published studies have almost invariably used immortal or tumorigenic cell lines to study Rho GTPase functions and there are no studies on the potential of Rho small GTPase to overcome senescence checkpoints and induce preneoplastic transformation of human mammary epithelial cells (hMEC). We show here that ectopic expression of wild-type (WT) RhoA as well as a constitutively active RhoA mutant (G14V) in two independent primary hMEC strains led to their immortalization and preneoplastic transformation. These cells have continued to grow over 300 population doublings (PD) with no signs of senescence, whereas cells expressing the vector or dominant-negative RhoA mutant (T19N) senesced after 20 PDs. Significantly, RhoA-T37A mutant, known to be incapable of interacting with many well-known Rho effectors including Rho kinase, PKN, mDia1, and mDia2, was also capable of immortalizing hMECs. Notably, similar to parental normal cells, Rho-immortalized cells have WT p53 and intact G(1) cell cycle arrest on Adriamycin treatment. Rho-immortalized cells were anchorage dependent and were unable to form tumors when implanted in nude mice. Lastly, microarray expression profiling of Rho-immortalized versus parental cells showed altered expression of several genes previously implicated in immortalization and breast cancer progression. Taken together, these results show that RhoA can induce the preneoplastic transformation of hMECs by altering multiple pathways linked to cellular transformation and breast cancer. 
 RhoA induces MMP-9 expression at CD44 lamellipodial focal complexes and promotes HMEC-1 cell invasion. Much progress has been made in recent years in the understanding of angiogenesis, yet signalling pathways involved remain poorly defined. Here we report that small RhoA GTPase is implicated in the invasion of human microvascular endothelial cells (HMEC-1). Ectopic expression of active-RhoA GTPase induced the expression of MMP-9 metalloproteinase, a key proteinase of the basement membrane, and promoted   migration   of endothelial cells through a 3D-matrix protein gel. MMP-9 was either directed as vesicular-like patches to the apical side of cells, or addressed to specific membrane sites at the cell surface. Confocal microscopy analyses indeed revealed clustering of MMP-9 in advancing lamellipodia at the forefront of endothelial cells, where this proteinase colocalized with RhoA and CD44, a transmembrane receptor known to be proteolysed in tumor cell progression. In addition, TIMP-1, a natural MMP inhibitor, significantly reduced the invasion of RhoAV14 expressing cells, suggesting that MMP-9 was a critical metalloproteinase responsible, at least partly, for the RhoAV14-induced endothelial cell invasion. We propose that RhoA triggers signalling pathways that, upregulating expression of a proteinase at specific membrane localizations, may confer an highly invasive phenotype to endothelial cells. 
 RhoA enhances store-operated Ca2+ entry and intestinal epithelial restitution by interacting with TRPC1 after wounding. Early mucosal restitution occurs as a consequence of epithelial cell   migration   to resealing of superficial wounds after injury. Our previous studies show that canonical transient receptor potential-1 (TRPC1) functions as a store-operated Ca(2+) channel (SOC) in intestinal epithelial cells (IECs) and plays an important role in early epithelial restitution by increasing Ca(2+) influx. Here we further reported that RhoA, a small GTP-binding protein, interacts with and regulates TRPC1, thus enhancing SOC-mediated Ca(2+) entry (SOCE) and epithelial restitution after wounding. RhoA physically associated with TRPC1 and formed the RhoA/TRPC1 complexes, and this interaction increased in stable TRPC1-transfected IEC-6 cells (IEC-TRPC1). Inactivation of RhoA by treating IEC-TRPC1 cells with exoenzyme C3 transferase (C3) or ectopic expression of dominant negative RhoA (DNMRhoA) reduced RhoA/TRPC1 complexes and inhibited Ca(2+) influx after store depletion, which was paralleled by an inhibition of cell   migration   over the wounded area. In contrast, ectopic expression of wild-type (WT)-RhoA increased the levels of RhoA/TRPC1 complexes, induced Ca(2+) influx through activation of SOCE, and promoted cell   migration   after wounding. TRPC1 silencing by transfecting stable WT RhoA-transfected cells with siRNA targeting TRPC1 (siTRPC1) reduced SOCE and repressed epithelial restitution. Moreover, ectopic overexpression of WT-RhoA in polyamine-deficient cells rescued the inhibition of Ca(2+) influx and cell   migration   induced by polyamine depletion. These findings indicate that RhoA interacts with and activates TRPC1 and thus stimulates rapid epithelial restitution after injury by inducing Ca(2+) signaling. 
 The tumor-suppressor gene ARHI (DIRAS3) suppresses ovarian cancer cell   migration   through inhibition of the Stat3 and FAK/Rho signaling pathways. Ovarian cancers migrate and metastasize over the surface of the peritoneal cavity. Consequently, dysregulation of mechanisms that limit cell   migration   may be particularly important in the pathogenesis of the disease. ARHI is an imprinted tumor-suppressor gene that is downregulated in >60% of ovarian cancers, and its loss is associated with decreased progression-free survival. ARHI encodes a 26-kDa GTPase with homology to Ras. In contrast to Ras, ARHI inhibits cell growth, but whether it also regulates cell motility has not been studied previously. Here we report that re-expression of ARHI decreases the motility of IL-6- and epidermal growth factor (EGF)-stimulated SKOv3 and Hey ovarian cancer cells, inhibiting both chemotaxis and haptotaxis. ARHI binds to and sequesters Stat3 in the cytoplasm, preventing its translocation to the nucleus and localization in focal adhesion complexes. Stat3 siRNA or the JAK2 inhibitor AG490 produced similar inhibition of motility. However, the combination of ARHI expression with Stat3 knockdown or inhibition produced greatest inhibition in ovarian cancer cell   migration  , consistent with Stat3-dependent and Stat3-independent mechanisms. Consistent with two distinct signaling pathways, knockdown of Stat3 selectively inhibited IL-6-stimulated   migration  , whereas knockdown of focal adhesion kinase (FAK) preferentially inhibited EGF-stimulated   migration  . In EGF-stimulated ovarian cancer cells, re-expression of ARHI inhibited FAK(Y397) and Src(Y416) phosphorylation, disrupted focal adhesions, and blocked FAK-mediated RhoA signaling, resulting in decreased levels of GTP-RhoA. Re-expression of ARHI also disrupted the formation of actin stress fibers in a FAK- and RhoA-dependent manner. Thus, ARHI has a critical and previously uncharacterized role in the regulation of ovarian cancer cell   migration  , exerting inhibitory effects on two distinct signaling pathways. 
 HMG-CoA reductase regulates CCL17-induced colon cancer cell   migration   via geranylgeranylation and RhoA activation. Simvastatin is widely used to lower cholesterol levels in patients with cardiovascular diseases, although accumulating evidence suggests that statins, such as simvastatin, also exert numerous anti-tumoral effects.The aim of this study was to examine the effect of simvastatin on colon cancer cell   migration  .  Migration   assays were performed to evaluate CCL17-induced colon cancer cell (HT-29) chemotaxis. In vitro tumor growth and apoptosis were assessed using a proliferation assay and annexin V assay, respectively. Active RhoA protein levels in CCL17-stimulated colon cancer cells were quantified using a G-LISA assay.We found that simvastatin dose-dependently decreased CCL17-induced colon cancer cell   migration  . Simvastatin had no effect on colon cancer cell proliferation or apoptosis. Inhibition of beta chemokine receptor 4, CCR4, reduced CCL17-evoked activation of RhoA in colon cancer cells. Moreover, administration of mevalonate reversed the inhibitory effect of simvastatin on CCL17-induced colon cancer cell   migration  . Interestingly, co-incubation with geranylgeranyl pyrophosphate (GGPP) antagonized the inhibitory impact of simvastatin on colon cancer cell   migration   triggered by CCL17. Moreover, we observed that simvastatin decreased CCL17-induced activation of RhoA in colon cancer cells. Administration of mevalonate and GGPP reversed the inhibitory effect of simvastatin on CCL17-provoked RhoA activation in colon cancer cells.Taken together, our findings show for the first time that HMG-CoA reductase regulates CCL17-induced colon cancer cell   migration   via inhibition of geranylgeranylation and RhoA activation. Thus, statins, such as simvastatin, might be effective tools to antagonize CCL17-dependent   migration   and metastasis of colon cancer cells. 
 NET1-mediated RhoA activation facilitates lysophosphatidic acid-induced cell   migration   and invasion in gastric cancer. The most lethal aspects of gastric adenocarcinoma (GA) are its invasive and metastatic properties. This aggressive phenotype remains poorly understood. We have recently identified neuroepithelial cell transforming gene 1 (NET1), a guanine exchange factor (GEF), as a novel GA-associated gene. Neuroepithelial cell transforming gene 1 expression is enhanced in GA and it is of functional importance in cell invasion. In this study, we demonstrate the activity of NET1 in driving cytoskeletal rearrangement, a key pathological mechanism in gastric tumour cell   migration   and invasion. Neuroepithelial cell transforming gene 1 expression was increased 10-fold in response to treatment with lysophosphatidic acid (LPA), resulting in an increase in active levels of RhoA and a 2-fold increase in cell invasion. Lysophosphatidic acid-induced cell invasion and   migration   were significantly inhibited using either NET1 siRNA or a RhoA inhibitor (C3 exoenzyme), thus indicating the activity of both NET1 and RhoA in gastric cancer progression. Furthermore, LPA-induced invasion and   migration   were also significantly reduced in the presence of cytochalasin D, an inhibitor of cytoskeletal rearrangements. Neuroepithelial cell transforming gene 1 knockdown resulted in AGS cell rounding and a loss of actin filament organisation, demonstrating the function of NET1 in actin organisation. These data highlight the importance of NET1 as a driver of tumour cell invasion, an activity mediated by RhoA activation and cytoskeletal reorganisation. 
 The Role of RhoA, RhoB and RhoC GTPases in Cell Morphology, Proliferation and   Migration   in Human Cytomegalovirus (HCMV) Infected Glioblastoma Cells. Rho GTPases are crucial regulators of the actin cytoskeleton, membrane trafficking and cell signaling and their importance in cell   migration   and invasion is well- established. The human cytomegalovirus (HCMV) is a widespread pathogen responsible for generally asymptomatic and persistent infections in healthy people. Recent evidence indicates that HCMV gene products are expressed in over 90% of malignant type glioblastomas (GBM). In addition, the HCMV Immediate Early-1 protein (IE1) is expressed in >90% of tumors analyzed.RhoA, RhoB and RhoC were individually depleted in U373MG glioblastoma cells as well as U373MG cells stably expressing the HCMV IE1 protein (named U373MG-IE1 cells) shRNA lentivirus vectors. Cell proliferation assays,   migration   as well as wound-healing assays were performed in uninfected and HCMV-infected cells.The depletion of RhoA, RhoB and RhoC protein resulted in significant alterations in the morphology of the uninfected cells, which were further enhanced by the cytopathic effect caused by HCMV. Furthermore, in the absence or presence of HCMV, the knockdown of RhoB and RhoC proteins decreased the proliferation rate of the parental and the IE1-expressing glioblastoma cells, whereas the knockdown of RhoA protein in the HCMV infected cell lines restored their proliferation rate. In addition, wound healing assays in U373MG cells revealed that depletion of RhoA, RhoB and RhoC differentially reduced their   migration   rate, even in the presence or the absence of HCMV.Collectively, these data show for the first time a differential implication of Rho GTPases in morphology, proliferation rate and motility of human glioblastoma cells during HCMV infection, further supporting an oncomodulatory role of HCMV depending on the Rho isoforms' state. 
 Effects of peripheral cannabinoid receptor ligands on motility and polarization in neutrophil-like HL60 cells and human neutrophils. The possible role of the peripheral cannabinoid receptor (CB2) in neutrophil   migration   was investigated by using human promyelocytic HL60 cells differentiated into neutrophil-like cells and human neutrophils isolated from whole blood. Cell surface expression of CB2 on HL60 cells, on neutrophil-like HL60 cells, and on human neutrophils was confirmed by flow cytometry. Upon stimulation with either of the CB2 ligands JWH015 and 2-arachidonoylglycerol (2-AG), neutrophil-like HL60 cells rapidly extended and retracted one or more pseudopods containing F-actin in different directions instead of developing front/rear polarity typically exhibited by migrating leukocytes. Activity of the Rho-GTPase RhoA decreased in response to CB2 stimulation, whereas Rac1, Rac2, and Cdc42 activity increased. Moreover, treatment of cells with RhoA-dependent protein kinase (p160-ROCK) inhibitor Y27632 yielded cytoskeletal organization similar to that of CB2-stimulated cells. In human neutrophils, neither JWH015 nor 2-AG induced motility or morphologic alterations. However, pretreatment of neutrophils with these ligands disrupted N-formyl-L-methionyl-L-leucyl-L-phenylalanine (fMLP)-induced front/rear polarization and   migration   and also substantially suppressed fMLP-induced RhoA activity. These results suggest that CB2 might play a role in regulating excessive inflammatory response by controlling RhoA activation, thereby suppressing neutrophil   migration  . 
 Diabetes causes bone marrow endothelial barrier dysfunction by activation of the RhoA-Rho-associated kinase signaling pathway. Diabetes mellitus causes bone marrow (BM) microangiopathy. This study aimed to investigate the mechanisms responsible for BM endothelial dysfunction in diabetes mellitus.The analysis of differentially expressed transcripts in BM endothelial cells (BMECs) from type-1 diabetic and nondiabetic mice showed an effect of diabetes mellitus on signaling pathways controlling cell death,   migration  , and cytoskeletal rearrangement. Type-1 diabetic-BMECs displayed high reactive oxygen species levels, increased expression and activity of RhoA and its associated protein kinases Rho-associated kinase 1/Rho-associated kinase 2, and reduced Akt phosphorylation/activity. Likewise, diabetes mellitus impaired Akt-related BMEC functions, such as   migration  , network formation, and angiocrine factor-releasing activity, and increased vascular permeability. Moreover, high glucose disrupted BMEC contacts through Src tyrosine kinase phosphorylation of vascular endothelial cadherin. These alterations were prevented by constitutively active Akt (myristoylated Akt), Rho-associated kinase inhibitor Y-27632, and Src inhibitors. Insulin replacement restored BMEC abundance, as assessed by flow cytometry analysis of the endothelial marker MECA32, and endothelial barrier function in BM of type-1 diabetic mice.Redox-dependent activation of RhoA/Rho-associated kinase and Src/vascular endothelial cadherin signaling pathways, together with Akt inactivation, contribute to endothelial dysfunction in diabetic BM. Metabolic control is crucial for maintenance of endothelial cell homeostasis and endothelial barrier function in BM of diabetic mice. 
 RhoA protein expression correlates positively with degree of malignancy in astrocytomas. Astrocytic tumors are the most common intracranial neoplasms. Their prognoses correlate with a conventional morphological grading system that suffers from diagnostic subjectivity and hence, inter-observer inconsistency. A molecular marker that provides an objective reference for classification and prognostication of astrocytic tumors would be useful in diagnostic pathology. RhoA, a GTPase protein involved in cell   migration   and adhesion, has been shown to be upregulated in a variety of human cancers. Based on direct analysis of clinical materials, our study demonstrates increased expression of RhoA in high-grade astrocytomas. This observation may be relevant to astrocytoma biology and the development of potential therapeutics against high-grade astrocytomas. Of more immediate consequence, utilization of this marker may aid in the routine pathological grading (and hence prognostication) of astrocytomas. 
 GOLPH3 regulates the   migration   and invasion of glioma cells though RhoA. Golgi phosphoprotein 3 (GOLPH3) has been reported to be involved in the development of several human cancers. However, the biological significance of GOLPH3 in glioma progression remains largely unknown. In this study, we report, for the first time, that downregulation of GOLPH3 led to clear reductions in glioma cell   migration   and invasion. In addition, downregulation of GOLPH3 inhibited the expression of the small GTPase RhoA as well as cytoskeletal reorganization, which are both required for glioma cell   migration  . Furthermore, we found that the observed reductions in glioma cell   migration   and RhoA level could be rescued by RhoA overexpression. Taken together, these results show that GOLPH3 contributes to the motility of glioma cells by regulating the expression of RhoA. 
 The small GTPase RhoA localizes to the nucleus and is activated by Net1 and DNA damage signals. Rho GTPases control many cellular processes, including cell survival, gene expression and   migration  . Rho proteins reside mainly in the cytosol and are targeted to the plasma membrane (PM) upon specific activation by guanine nucleotide exchange factors (GEFs). Accordingly, most GEFs are also cytosolic or associated with the PM. However, Net1, a RhoA-specific GEF predominantly localizes to the cell nucleus at steady-state. Nuclear localization for Net1 has been seen as a mechanism for sequestering the GEF away from RhoA, effectively rendering the protein inactive. However, considering the prominence of nuclear Net1 and the fact that a biological stimulus that promotes Net1 translocation out the nucleus to the cytosol has yet to be discovered, we hypothesized that Net1 might have a previously unidentified function in the nucleus of cells.Using an affinity precipitation method to pulldown the active form of Rho GEFs from different cellular fractions, we show here that nuclear Net1 does in fact exist in an active form, contrary to previous expectations. We further demonstrate that a fraction of RhoA resides in the nucleus, and can also be found in a GTP-bound active form and that Net1 plays a role in the activation of nuclear RhoA. In addition, we show that ionizing radiation (IR) specifically promotes the activation of the nuclear pool of RhoA in a Net1-dependent manner, while the cytoplasmic activity remains unchanged. Surprisingly, irradiating isolated nuclei alone also increases nuclear RhoA activity via Net1, suggesting that all the signals required for IR-induced nuclear RhoA signaling are contained within the nucleus.These results demonstrate the existence of a functional Net1/RhoA signaling pathway within the nucleus of the cell and implicate them in the DNA damage response. 
 Inhibition of RhoA/ROCK signaling pathway promotes the apoptosis of gastric cancer cells. Ras homologue A (RhoA) plays a crucial role in the proliferation, apoptosis,adhesion and   migration   of gastric cancer cells. Rho associated kinase (ROCK) is an effector protein of RhoA.In the present study, RhoA activity was inhibited by siRNA targeting RhoA andY-27632, an inhibitor of ROCK, and the role of RhoA/ROCK signaling pathway in the apoptosis of gastric cancer cells was investigated.RNAi of RhoA inhibited the survival and promoted the apoptotic of AGS cells. RhoA RNAi caused an obvious decrease of ROCK1 expression but an increase of caspase-3/cleaved-caspase-8. Inhibition of ROCK by Y-27632 inhibited the activity of RhoA and promoted the apoptosis.We speculate that RhoA/ROCK signaling pathway plays an important role in the regulation of apoptosis of gastric cancer, and to inhibit this pathway may promote the apoptosis of cancer cells. Thus, inhibition of RhoA/ROCK signaling pathway may become a novel target in the treatment of gastric cancer. 
 Cullin mediates degradation of RhoA through evolutionarily conserved BTB adaptors to control actin cytoskeleton structure and cell movement. Cul3, a Cullin family scaffold protein, is thought to mediate the assembly of a large number of SCF (Skp1-Cullin1-F-box protein)-like ubiquitin ligase complexes through BTB domain substrate-recruiting adaptors. Cul3 controls early embryonic development in several genetic models through mechanisms not understood. Very few functional substrate/adaptor pairs for Cul3 ubiquitin ligases have been identified. Here, we show that Cul3 knockdown in human cells results in abnormal actin stress fibers and distorted cell morphology, owing to impaired ubiquitination and degradation of small GTPase RhoA. We identify a family of RhoA-binding BTB domain adaptors conserved from insects to mammals, designated BACURDs. BACURDs form ubiquitin ligase complexes, which selectively ubiquitinate RhoA, with Cul3. Dysfunction of the Cul3/BACURD complex decreases cell   migration   potential and impairs RhoA-mediated convergent extension movements during Xenopus gastrulation. Our studies reveal a previously unknown mechanism for controlling RhoA degradation and regulating RhoA function in various biological contexts, which involves a Cul3/BACURD ubiquitin ligase complex. 
 Oligodendrocyte lineage transcription factor 2 inhibits the motility of a human glial tumor cell line by activating RhoA. The basic helix-loop-helix transcription factor, oligodendrocyte lineage transcription factor 2 (OLIG2), is specifically expressed in the developing and mature central nervous system and plays an important role in oligodendrogenesis from neural progenitors. It is also expressed in various types of glial tumors, but rarely in glioblastoma. Although we previously showed that OLIG2 expression inhibits glioma cell growth, its role in tumorigenesis remains incompletely understood. Here, we investigated the effect of OLIG2 expression on the   migration   of the human glioblastoma cell line U12-1. In these cells, OLIG2 expression is controlled by the Tet-off system. Induction of OLIG2 expression inhibited both the   migration   and invasiveness of U12-1 cells. OLIG2 expression also increased the activity of the GTPase RhoA as well as inducing the cells to form stress fibers and focal adhesions. Experiments using short interfering RNA against p27(Kip1) revealed that up-regulation of the p27(Kip1) protein was not essential for RhoA activation, rather it contributed independently to the decreased motility of OLIG2-expressing U12-1 cells. Alternatively, semiquantitative reverse transcription-PCR analysis revealed that mRNA expression of RhoGAP8, which regulates cell   migration  , was decreased by OLIG2 expression. Furthermore, expression of C3 transferase, which inhibits Rho via ADP ribosylation, attenuated the OLIG2-induced inhibition of cell motility. Imaging by fluorescence resonance energy transfer revealed that in U12-1 cells lacking OLIG2, the active form of RhoA was localized to protrusions of the cell membrane. In contrast, in OLIG2-expressing cells, it lined almost the entire plasma membrane. Thus, OLIG2 suppresses the motile phenotype of glioblastoma cells by activating RhoA. 
 A role for the Rho-p160 Rho coiled-coil kinase axis in the chemokine stromal cell-derived factor-1alpha-induced lymphocyte actomyosin and microtubular organization and chemotaxis. The possible involvement of the Rho-p160ROCK (Rho coiled-coil kinase) pathway in the signaling induced by the chemokine Stromal cell-derived factor (SDF)-1alpha has been studied in human PBL. SDF-1alpha induced activation of RhoA, but not that of Rac. RhoA activation was followed by p160ROCK activation mediated by RhoA, which led to myosin light chain (MLC) phosphorylation, which was dependent on RhoA and p160ROCK activities. The kinetics of MLC activation was similar to that of RhoA and p160ROCK. The role of this cascade in overall cell morphology and functional responses to the chemokine was examined employing different chemical inhibitors. Inhibition of either RhoA or p160ROCK did not block SDF-1alpha-induced short-term actin polymerization, but induced the formation of long spikes arising from the cell body, which were found to be microtubule based. This morphological change was associated with an increase in microtubule instability, which argues for an active microtubule polymerization in the formation of these spikes. Inhibition of the Rho-p160ROCK-MLC kinase signaling cascade at different steps blocked lymphocyte   migration   and the chemotaxis induced by SDF-1alpha. Our results indicate that the Rho-p160ROCK axis plays a pivotal role in the control of the cell shape as a step before lymphocyte   migration   toward a chemotactic gradient. 
 Thromboxane A2 receptors in prostate carcinoma: expression and its role in regulating cell motility via small GTPase Rho. Thromboxane A(2) (TxA(2)) is a prostanoid formed by thromboxane synthase using the cyclooxygenase product prostaglandin H(2) as the substrate. Previously, increased expression of thromboxane synthase was found in prostate tumors, and tumor cell motility was attenuated by inhibitors of thromboxane synthase. This study was undertaken to elucidate how tumor motility is regulated by TxA(2). Here, we report that human prostate cancer cells express functional receptors for TxA(2) (TP). Ligand binding assay found that PC-3 cells binded to SQ29548, a high-affinity TP antagonist, in a saturable manner with K(d) of 3.64 nmol/L and B(max) of 120.4 fmol per million cells. Treatment of PC-3 cells by U46619, a TP agonist, induced PC-3 cell contraction, which was blocked by pretreatment with the TP antagonist SQ29548 or pinane TxA(2). The   migration   of prostate cancer cells was significantly inhibited either by sustained activation of TP or by blockade of TP activation, suggesting that TP activation must be tightly controlled during cell   migration  . Further studies found that small GTPase RhoA was activated by TP activation, and pretreatment of PC-3 cells with Y27632, a Rho kinase (ROCK) inhibitor, blocked U46619-induced cell contraction. A dominant-negative mutant of RhoA also blocked U46619-induced cell contraction. Taken together, the data suggest that TPs are expressed in prostate cancer and activation of TPs regulates prostate cancer cell motility and cytoskeleton reorganization through activation of Rho. 
 RHOA and PRKCZ control different aspects of cell motility in pancreatic cancer metastatic clones. Our understanding of the mechanism regulating pancreatic cancer metastatic phenotype is limited. We analyzed the role of RHOA and PRKCZ in the motility attitude of two subclones of the pancreatic adenocarcinoma cell line SUIT-2 (S2), with different in vivo metastatic potential in nude mice: S2-m with a low metastatic potential and highly metastatic S2-CP9 using RHOA and PRKCZ cell-permeable inhibitory peptides.Adhesion assays, cell permeable peptides, RHOA activity assay, western blottingWhen used in combination cell-permeable inhibitory peptides partially inhibited cell adhesion by about 50% in clone S2-CP9. In clone S2-m, the effect was limited to 15% inhibition. In a wound healing assay, S2-CP9 was sensitive only to treatment with the combination of both RHOA and PRKCZ inhibitory peptides. Conversely, S2-m was unable to migrate toward both ends of the wound in basal conditions.   Migration   of cells through a membrane with 8 mum pores was completely abolished in both clones by individual treatment with RHOA and PRKCZ inhibitory peptides.Herein, we demonstrate a critical role for RHOA and PRKCZ in the regulation of different aspects of cell motility of pancreatic adenocarcinoma and demonstrate the need to inhibit both pathways to obtain a functionally relevant effect in most assays. These results indicate that RHOA and PRKCZ, and their downstream effectors, can represent important pharmacological targets that could potentially control the highly metastatic attitude of PDAC. 
 Macrophage contact induces RhoA GTPase signaling to trigger tumor cell intravasation. Most cancer patients die as a result of metastasis, thus it is important to understand the molecular mechanisms of dissemination, including intra- and extravasation. Although the mechanisms of extravasation have been vastly studied in vitro and in vivo, the process of intravasation is still unclear. Furthermore, how cells in the tumor microenvironment facilitate tumor cell intravasation is still unknown. Using high-resolution imaging, we found that macrophages enhance tumor cell intravasation upon physical contact. Macrophage and tumor cell contact induce RhoA activity in tumor cells, triggering the formation of actin-rich degradative protrusions called invadopodia, enabling tumor cells to degrade and break through matrix barriers during tumor cell transendothelial   migration  . Interestingly, we show that macrophage-induced invadopodium formation and tumor cell intravasation also occur in patient-derived tumor cells and in vivo models, revealing a conserved mechanism of tumor cell intravasation. Our results illustrate a novel heterotypic cell contact-mediated signaling role for RhoA, as well as yield mechanistic insight into the ability of cells within the tumor microenvironment to facilitate steps of the metastatic cascade. 
 RhoA activation and actin reorganization involved in endothelial CAM-mediated endocytosis of anti-PECAM carriers: critical role for tyrosine 686 in the cytoplasmic tail of PECAM-1. Platelet-endothelial cell adhesion molecule-1 (PECAM-1), a transmembrane glycoprotein involved in leukocyte trans  migration  , represents a good target for endothelial drug delivery (eg, using antibody-directed nanocarriers, anti-PECAM/NCs). Although endothelial cells do not internalize PECAM antibodies, PECAM-1 engagement by multivalent anti-PECAM conjugates and nanocarriers causes endocytosis via a nonclassic CAM-mediated pathway. We found that endothelial uptake of multivalent anti-PECAM complexes is associated with PECAM-1 phosphorylation. Using model REN cells expressing a series of PECAM-1 deletion and point mutants, we found that the PECAM-1 cytoplasmic domain and, more precisely, PECAM-1 tyrosine 686, is critical in mediating RhoA activation and recruitment of EGFP-RhoA to anti-PECAM/NC binding sites at the plasmalemma, actin polymerization into phalloidin-positive stress fibers, and finally CAM endocytosis of anti-PECAM/NCs. Endothelial targeting and endocytosis of anti-PECAM/NCs were markedly efficient and did not compromise endothelial barrier function in vitro (determined by immunostaining of VE-cadherin and (125)I-albumin transport across endothelial monolayers) or in vivo (determined by electron microscopy imaging of pulmonary capillaries and (125)I-albumin transport from the blood into the lung tissue after intravenous injection of anti-PECAM/NCs in mice). These results reveal PECAM-1 signaling and interactions with the cytoskeleton, which are required for CAM-endocytosis, and may provide safe intra-endothelial drug delivery by anti-PECAM/NCs. 
 Kank regulates RhoA-dependent formation of actin stress fibers and cell   migration   via 14-3-3 in PI3K-Akt signaling. Phosphoinositide-3 kinase (PI3K)/Akt signaling is activated by growth factors such as insulin and epidermal growth factor (EGF) and regulates several functions such as cell cycling, apoptosis, cell growth, and cell   migration  . Here, we find that Kank is an Akt substrate located downstream of PI3K and a 14-3-3-binding protein. The interaction between Kank and 14-3-3 is regulated by insulin and EGF and is mediated through phosphorylation of Kank by Akt. In NIH3T3 cells expressing Kank, the amount of actin stress fibers is reduced, and the coexpression of 14-3-3 disrupted this effect. Kank also inhibits insulin-induced cell   migration   via 14-3-3 binding. Furthermore, Kank inhibits insulin and active Akt-dependent activation of RhoA through binding to 14-3-3. Based on these findings, we hypothesize that Kank negatively regulates the formation of actin stress fibers and cell   migration   through the inhibition of RhoA activity, which is controlled by binding of Kank to 14-3-3 in PI3K-Akt signaling. 
 Tetraspanin CD151 regulates RhoA activation and the dynamic stability of carcinoma cell-cell contacts. Tetraspanins regulate integrin-dependent tumor cell interactions with the extracellular matrix. Here we show that tetraspanin CD151, which plays critical roles in regulating the adhesion and motility of individual tumor cells, is also an important regulator of collective tumor cell   migration  . Near total silencing of CD151 destabilizes E-cadherin-dependent carcinoma cell-cell junctions and enhances the collective   migration   of intact tumor cell sheets. This effect does not depend on reduced E-cadherin cell-surface expression or intrinsic adhesivity, or on obvious disruptions in the E-cadherin regulatory complex. Instead, the loss of CD151 causes excessive RhoA activation, loss of actin organization at cell-cell junctions, and increased actin stress fibers at the basal cell surface. Cell-cell contacts within CD151-silenced monolayers display a nearly threefold increase in remodeling rate and a significant reduction in lifespan as compared to cell-cell contacts within wild-type monolayers. CD151 re-expression restores junctional stability, as does acute treatment of CD151-silenced cells with a cell-permeable RhoA inhibitor. However, a CD151 mutant with impaired association with alpha3beta1 integrin fails to restore junctional organization. These data reveal that, in addition to its roles in regulating tumor cell-substrate interactions, CD151 is also an important regulator of the stability of tumor cell-cell interactions, potentially through its interaction with alpha3beta1 integrin. This could help to explain the phenotypes in human patients and mice lacking CD151. 
 Matrix-bound PAI-1 supports cell blebbing via RhoA/ROCK1 signaling. The microenvironment of a tumor can influence both the morphology and the behavior of cancer cells which, in turn, can rapidly adapt to environmental changes. Increasing evidence points to the involvement of amoeboid cell   migration   and thus of cell blebbing in the metastatic process; however, the cues that promote amoeboid cell behavior in physiological and pathological conditions have not yet been clearly identified. Plasminogen Activator Inhibitor type-1 (PAI-1) is found in high amount in the microenvironment of aggressive tumors and is considered as an independent marker of bad prognosis. Here we show by immunoblotting, activity assay and immunofluorescence that, in SW620 human colorectal cancer cells, matrix-associated PAI-1 plays a role in the cell behavior needed for amoeboid   migration   by maintaining cell blebbing, localizing PDK1 and ROCK1 at the cell membrane and maintaining the RhoA/ROCK1/MLC-P pathway activation. The results obtained by modeling PAI-1 deposition around tumors indicate that matrix-bound PAI-1 is heterogeneously distributed at the tumor periphery and that, at certain spots, the elevated concentrations of matrix-bound PAI-1 needed for cancer cells to undergo the mesenchymal-amoeboid transition can be observed. Matrix-bound PAI-1, as a matricellular protein, could thus represent one of the physiopathological requirements to support metastatic formation. 
 Functional proteomics identifies protein-tyrosine phosphatase 1B as a target of RhoA signaling. Rho GTPases are signal transduction effectors that control cell motility, cell attachment, and cell shape by the control of actin polymerization and tyrosine phosphorylation. To identify cellular targets regulated by Rho GTPases, we screened global protein responses to Rac1, Cdc42, and RhoA activation by two-dimensional gel electrophoresis and mass spectrometry. A total of 22 targets were identified of which 19 had never been previously linked to Rho GTPase pathways, providing novel insight into pathway function. One novel target of RhoA was protein-tyrosine phosphatase 1B (PTP1B), which catalyzes dephosphorylation of key signaling molecules in response to activation of diverse pathways. Subsequent analysis demonstrated that RhoA enhances post-translational modification of PTP1B, inactivates phosphotyrosine phosphatase activity, and up-regulates tyrosine phosphorylation of p130Cas, a key mediator of focal adhesion turnover and cell   migration  . Thus, protein profiling reveals a novel role for PTP1B as a mediator of RhoA-dependent phosphorylation of p130Cas. 
 Activated G(alpha)13 impairs cell invasiveness through p190RhoGAP-mediated inhibition of RhoA activity. The GTPase RhoA is a downstream target of heterotrimeric G(13) proteins and plays key roles in cell   migration   and invasion. Here, we show that expression in human melanoma cells of a constitutively active, GTPase-deficient Galpha(13) form (G(alpha)(13)QL) or lysophosphatidylcholine (LPC)-promoted signaling through G(alpha)(13)-coupled receptors led to a blockade of chemokine-stimulated RhoA activation and cell invasion that was rescued by active RhoA. Melanoma cells expressing G(alpha)(13)QL or cells stimulated with LPC displayed an increase in p190RhoGAP activation, and defects in RhoA activation and invasion were recovered by knocking down p190RhoGAP expression, thus identifying this GTPase-activating protein (GAP) protein as a downstream G(alpha)(13) target that is responsible for these inhibitory responses. In addition, defective stress fiber assembly and reduced   migration   speed underlay inefficient invasion of G(alpha)(13)QL melanoma cells. Importantly, G(alpha)(13)QL expression in melanoma cells led to impairment in lung metastasis associated with prolonged survival in SCID mice. The data indicate that G(alpha)(13)-dependent downstream effects on RhoA activation and invasion tightly depend on cell type-specific GAP activities and that G(alpha)(13)-p190RhoGAP signaling might represent a potential target for intervention in melanoma metastasis. 
 IκB kinase γ/nuclear factor-κB-essential modulator (IKKγ/NEMO) facilitates RhoA GTPase activation, which, in turn, activates Rho-associated KINASE (ROCK) to phosphorylate IKKβ in response to transforming growth factor (TGF)-β1. Transforming growth factor (TGF)-β1 plays several roles in a variety of cellular functions. TGF-β1 transmits its signal through Smad transcription factor-dependent and -independent pathways. It was reported that TGF-β1 activates NF-κB and RhoA, and RhoA activates NF-κB in several kinds of cells in a Smad-independent pathway. However, the activation molecular mechanism of NF-κB by RhoA upon TGF-β1 has not been clearly elucidated. We observed that RhoA-GTP level was increased by TGF-β1 in RAW264.7 cells. RhoA-GDP and RhoGDI were bound to N- and C-terminal domains of IKKγ, respectively. Purified IKKγ facilitated GTP binding to RhoA complexed with RhoGDI. Furthermore, Dbs, a guanine nucletotide exchange factor of RhoA much more enhanced GTP binding to RhoA complexed with RhoGDI in the presence of IKKγ. Indeed, si-IKKγ abolished RhoA activation in response to TGF-β1 in cells. However, TGF-β1 stimulated the release of RhoA-GTP from IKKγ and Rho-associated kinase (ROCK), an active RhoA effector protein, directly phosphorylated IKKβ in vitro, whereas TGF-β1-activated kinase 1 activated RhoA upon TGF-β1 stimulation. Taken together, our data indicate that IKKγ facilitates RhoA activation via a guanine nucletotide exchange factor, which in turn activates ROCK to phosphorylate IKKβ, leading to NF-κB activation that induced the che  mokine ex  pression and cell migration upon TGF-β1. 
  GeneRIFs  
 AAMP Regulates Endothelial Cell   Migration   and Angiogenesis Through RhoA/Rho Kinase Signaling. 
 RhoA-depleted cancer cells exhibited markedly reduced   migration   and invasion ability in vitro. 
 ODAM induced RhoA activity. ODAM-mediated RhoA signalling resulted in actin filament rearrangement, increased cell adhesion and inhibited the   migration  /invasion of MCF7 cells. These results suggest that ODAM expression maintains their adhesion, resulting in the prevention of their metastasis via the regulation of RhoA signalling in breast cancer cells. 
 Senescent stromal cells induce cancer cell   migration   via inhibition of RhoA/ROCK/myosin-based cell contractility. 
 We propose that PrP modulates beta1 integrin adhesion and   migration   of monocytes through RhoA-induced actin remodeling mediated by cofilin, and through the regulation of ERM-mediated membrane-cytoskeleton linkage. 
 Results demonstrated that the miR-200b/200c/429 subfamily inhibited HCC cell   migration   through modulating Rho/ROCK mediated cell cytoskeletal reorganization and cell-substratum adhesion. 
 CCR7 via RhoA/ROCK-Pyk2 cofilin pathway promotes invasion and   migration   of metastatic SCCHN cells 
 These findings establish a central role for ANXA1 in the cell   migration   through the activation of NFkappaB, ERK1/2 and RhoA. 
 under hypoxia conditions, elevated HIF-1alpha seems to up-regulate contactin-1 expression and by this activate RhoA and facilitate   migration   of cancer cells. 
 control exerted by ERRalpha on RHOA stability is required for directional   migration   
 FAP is essential for the   migration   of bone marrow mesenchymal stem cells through RhoA activation. 
 Findings suggest that Wnt5a enhances cell   migration   through activation of Cdc42 and inactivation of RhoA in human corneal endothelial cells. 
 These results indicate that Wnt5b is involved in the   migration   ability of OSCC cells through active Cdc42 and RhoA. 
 Results suggest that EGF-induced SMURF1 plays a role in breast cancer cell   migration   and invasion through the downregulation of RhoA. 
 Data show that macrophages on gastric and colorectal cancer cells   migration   through epidermal growth factor receptor and phosphorylation of Akt, c-Src and ERK1/2, and led to an increase of RhoA and Cdc42 activity. 
   Migration   assays were performed to evaluate CCL17-induced colon cancer cell (HT-29) chemotaxis and RhoA protein levels were quantified. 
 CCR4 mediates CCL17 induced cell   migration   via increased total RhoA and active RhoA protein levels in colon cancer cells. 
 IKKgamma facilitates RhoA activation via a guanine nucletotide exchange factor, which in turn activates ROCK to phosphorylate IKKbeta, leading to NF-kappaB activation that induced the chemokine expression and cell   migration   upon TGF-beta1. 
 Exposure of smooth muscle cells to apo(a) in   migration   assays induced a potent, concentration-dependent chemorepulsion that was RhoA and integrin alphaVbeta3-dependent, but transforming growth factor beta-independent. 
 These results indicate that suppression of the RhoA-mediated pathway might participate in the Thy-1-induced   migration   inhibition in human umbilical vein endothelial cells. 
 Our results indicate that the loss of miR-125a3p-controlled regulation of the RhoA-actomyosin pathway can lead to increased   migration   of tumor cells because of the upregulation of RhoA expression. 
 GOLPH3 regulates the   migration   and invasion of glioma cells though regulation of RhoA. 
 Cannabidiolic acid is an inhibitor of breast cell   migration   involving its activation of RhoA by inhibition of cAMP-dependent protein kinase. 
 RhoA associates with SHIP2 to regulate cell polarization and   migration  . 
 Wnt5a promotes breast cancer cell   migration   via Dvl2/Daam1/RhoA. 
 Induction of RhoA-dependent switch to protease-independent amoeboid   migration   in human glioma cells by Sox2 RNAi 
 IL-8-Rac1/RhoA-p38MAPK signaling pathway plays a vital role in the IL-8-induced endothelial cell   migration  . 
 Matrix-associated PAI-1 plays a role in the cell behavior needed for amoeboid   migration   by maintaining cell blebbing, localizing PDK1 and ROCK1 at the cell membrane and maintaining the RhoA/ROCK1/MLC-P pathway activation. 
 endocannabinoids, through their autocrine activation of CB1 and subsequent repression of RhoA activity, suppress   migration   in prostate carcinoma cells 
 hypoxia decreases MSC cell   migration   through an HIF-1alpha and RhoA-mediated pathway 
 The PI3K-Rac1/RhoA signaling pathway plays a vital role in IL-8 induced endothelial cell   migration   
 RACK1 promotes breast carcinoma   migration  /metastasis via activation of the RhoA/Rho kinase pathway 
 findings have shown that interactions of LM-111 and LM-332 with adjacent epithelial cells differentially activate intracellular signaling molecules, in particular RhoA, which is critical for   migration   of epithelial cells on laminins 
 PHD2 affects cell   migration   and F-actin formation via RhoA/rho-associated kinase-dependent cofilin phosphorylation 
 Polarization and   migration   of hematopoietic stem and progenitor cells rely on the RhoA/ROCK I pathway and an active reorganization of the microtubule network. 
 findings demonstrate that enhanced expression of RhoC results in a striking increase in the   migration   and invasion of pancreatic carcinoma cells, whereas forced expression of RhoA decreases these actions 
 This study establishes GEF-H1 as a critical organizer of key structural and signaling components of cell   migration   through the localized regulation of RhoA activity at the cell leading edge. 
 Kindlin-1 forms molecular complexes with beta1 integrin, alpha-actinin, migfilin, and focal adhesion kinase and regulates cell shape and   migration   by controlling lamellipodia formation. 
 KiSS1 inhibits TNFalpha-induced NF-kappaB activation via downregulation of RhoA activation and suppression of breast cancer cell   migration   and invasion 
 In intestinal crypt cells, integrin alpha8beta1 is closely involved in the regulation of adhesion,   migration   and cell proliferation via a predominant RhoA/ROCK-dependent mechanism. 
 Inhibition of isoprenylcysteine carboxylmethyltransferase (Icmt) decreases activation of RhoA and Rac1, and in the absence of Icmt activity, addition of exogenous RhoA or Rac1 partially rescues directed and random   migration  , respectively. 
 Arylsulfatase B regulates colonic epithelial cell   migration   by effects on MMP9 expression and RhoA activation. 
 Nox1 knockdown led to a loss of directional   migration   which takes place through a RhoA-dependent alpha2/alpha3 integrin switch. 
 RhoA pathway is implicated in suppression of MM   migration   and invasion 
 the directional   migration   of capillaries in the embryo is governed by the Amot:Patj/Mupp1:Syx signaling that controls local GTPase activity 
 ARHGAP5 (the gene encoding p190-B RhoGAP) is a probable target for the amplification at 14q12, and p190-B RhoGAP promotes cells spreading and   migration   by negatively regulating RhoA activity in Huh-7 hepatocellular carcinoma cells 
 p27 promotes cell   migration   in metastatic HCC cells through the regulation of RhoA activity. 
 Data suggest that Kank negatively regulates the formation of actin stress fibers and cell   migration   through the inhibition of RhoA activity, which is controlled by binding of Kank to 14-3-3 in PI3K-Akt signaling. 
 Rho kinase and ERK1/2 play more important roles than RhoA in PGE(2)-mediated   migration   stimulation of first-trimester trophoblasts. 
 These results suggest that S1P(2) receptors/G(12/13)-proteins/Rho signaling pathways mediate S1P-induced inhibition of glioma cell   migration  . 
 tethering of RhoA to the membrane by GGpp is determinant for T cell   migration   
 evidence that the small GTPases RhoA and Rac1, but not Cdc42, are directly associated with Tyk2 and PI3-K in an uPA/uPAR-dependent fashion and are necessary to mediate the uPA/uPAR-directed   migration   via the Tyk2/PI3-K signalling complex in human VSMC 
 Synaptopodin, an actin-associated protein, as a novel regulator of RhoA signalling and cell   migration   in kidney podocytes. 
 Maspin control mammary tumor   migration   through inhibiting Rac1 and Cdc42, but not RhoA GTPase. 
 The results suggest that oncogenic K-Ras enhances the malignant phenotype and identify the mitogen-activated protein kinase p38 as a target to inhibit oncogenic K-Ras-induced pancreatic tumor cell   migration  . 
 CB2 might play a role in regulating excessive inflammatory response by controlling RhoA activation, thereby suppressing neutrophil   migration   
 Both neuropeptides that activate G-protein-coupled receptors and growth factors that activate receptor tyrosine kinases require RhoA to induce prostate cancer cell   migration  . 
  ECM  PubMed  
 Maspin controls mammary tumor cell migration through inhibiting Rac1 and Cdc42, but not the RhoA GTPase. Rac1 and Cdc42 are members of the Rho family of small GTPases that play essential roles in diverse cellular functions, including cell migration. The activities of these Rho family proteins are controlled by growth factor receptor activation and cell-  ECM   interactions. Here, we show that maspin, a well-documented tumor suppressor gene, also controls cell motility through inhibiting Rac1/Cdc42 activity. Using the GST-PAK and GST-Rho binding protein pull-down assays for GTP-bound Rac1, Cdc42, and RhoA, we showed that treatment of MDA-MB-231 tumor cells with recombinant maspin for a short time period significantly inhibited the activity of Rac1 and Cdc42, but not RhoA. The reactive site loop (RSL) within maspin protein is the functional domain involved in the inhibition. Maspin mutants with the RSL deleted or a point mutation in the RSL region lost their inhibitory activity. We further examined the ability of maspin to inhibit Rac1- and Cdc42-mediated signaling pathways and transcription factors. Treatment of MDA-MB-231 cells with maspin led to the inhibition of JNK kinase activity as assayed by immuno-kinase assays. In addition, the AP-1 transcription activity downstream of JNK kinase pathway was also reduced. Together, we have identified Rac1 and Cdc42 as the downstream targets that mediate the inhibition of mammary tumor cell migration by maspin. 
 Integrin alpha8beta1 regulates adhesion, migration and proliferation of human intestinal crypt cells via a predominant RhoA/ROCK-dependent mechanism. Integrins are transmembrane alphabeta heterodimer receptors that function as structural and functional bridges between the cytoskeleton and   ECM   (extracellular matrix) molecules. The RGD (arginine-glycine-aspartate tripeptide motif)-dependent integrin alpha8beta1 has been shown to be involved in various cell functions in neuronal and mesenchymal-derived cell types. Its role in epithelial cells remains unknown.Integrin alpha8beta1 was found to be expressed in the crypt cell population of the human intestine but was absent from differentiating and mature epithelial cells of the villus. The function of alpha8beta1 in epithelial crypt cells was investigated at the cellular level using normal HIECs (human intestinal epithelial cells). Specific knockdown of alpha8 subunit expression using an shRNA (small-hairpin RNA) approach showed that alpha8beta1 plays important roles in RGD-dependent cell adhesion, migration and proliferation via a RhoA/ROCK (Rho-associated kinase)-dependent mechanism as demonstrated by active RhoA quantification and pharmacological inhibition of ROCK. Moreover, loss of alpha8beta1, through RhoA/ROCK, impairs FA (focal adhesion) complex integrity as demonstrated by faulty vinculin recruitment.Integrin alpha8beta1 is expressed in epithelial cells. In intestinal crypt cells, alpha8beta1 is closely involved in the regulation of adhesion, migration and cell proliferation via a predominant RhoA/ROCK-dependent mechanism. These results suggest an important role for this integrin in intestinal crypt cell homoeostasis. 
 A MAPK-Driven Feedback Loop Suppresses Rac Activity to Promote RhoA-Driven Cancer Cell Invasion. Cell migration in 3D microenvironments is fundamental to development, homeostasis and the pathobiology of diseases such as cancer. Rab-coupling protein (RCP) dependent co-trafficking of α5β1 and EGFR1 promotes cancer cell invasion into fibronectin (FN) containing extracellular matrix   (E  CM), by potentiating EGFR1 signalling at the front of invasive cells. This promotes a switch in RhoGTPase signalling to inhibit Rac1 and activate a RhoA-ROCK-Formin homology domain-containing 3 (FHOD3) pathway and generate filopodial actin-spike protrusions which drive invasion. To further understand the signalling network that drives RCP-driven invasive migration, we generated a Boolean logical model based on existing network pathways/models, where each node can be interrogated by computational simulation. The model predicted an unanticipated feedback loop, whereby Raf/MEK/ERK signalling maintains suppression of Rac1 by inhibiting the Rac-activating Sos1-Eps8-Abi1 complex, allowing RhoA activity to predominate in invasive protrusions. MEK inhibition was sufficient to promote lamellipodia formation and oppose filopodial actin-spike formation, and led to activation of Rac and inactivation of RhoA at the leading edge of cells moving in 3D matrix. Furthermore, MEK inhibition abrogated RCP/α5β1/EGFR1-driven invasive migration. However, upon knockdown of Eps8 (to suppress the Sos1-Abi1-Eps8 complex), MEK inhibition had no effect on RhoGTPase activity and did not oppose invasive migration, suggesting that MEK-ERK signalling suppresses the Rac-activating Sos1-Abi1-Eps8 complex to maintain RhoA activity and promote filopodial actin-spike formation and invasive migration. Our study highlights the predictive potential of mathematical modelling approaches, and demonstrates that a simple intervention (MEK-inhibition) could be of therapeutic benefit in preventing invasive migration and metastasis. 
 A MAPK-Driven Feedback Loop Suppresses Rac Activity to Promote RhoA-Driven Cancer Cell Invasion. Cell migration in 3D microenvironments is fundamental to development, homeostasis and the pathobiology of diseases such as cancer. Rab-coupling protein (RCP) dependent co-trafficking of α5β1 and EGFR1 promotes cancer cell invasion into fibronectin (FN) containing extracellular matrix   (E  CM), by potentiating EGFR1 signalling at the front of invasive cells. This promotes a switch in RhoGTPase signalling to inhibit Rac1 and activate a RhoA-ROCK-Formin homology domain-containing 3 (FHOD3) pathway and generate filopodial actin-spike protrusions which drive invasion. To further understand the signalling network that drives RCP-driven invasive migration, we generated a Boolean logical model based on existing network pathways/models, where each node can be interrogated by computational simulation. The model predicted an unanticipated feedback loop, whereby Raf/MEK/ERK signalling maintains suppression of Rac1 by inhibiting the Rac-activating Sos1-Eps8-Abi1 complex, allowing RhoA activity to predominate in invasive protrusions. MEK inhibition was sufficient to promote lamellipodia formation and oppose filopodial actin-spike formation, and led to activation of Rac and inactivation of RhoA at the leading edge of cells moving in 3D matrix. Furthermore, MEK inhibition abrogated RCP/α5β1/EGFR1-driven invasive migration. However, upon knockdown of Eps8 (to suppress the Sos1-Abi1-Eps8 complex), MEK inhibition had no effect on RhoGTPase activity and did not oppose invasive migration, suggesting that MEK-ERK signalling suppresses the Rac-activating Sos1-Abi1-Eps8 complex to maintain RhoA activity and promote filopodial actin-spike formation and invasive migration. Our study highlights the predictive potential of mathematical modelling approaches, and demonstrates that a simple intervention (MEK-inhibition) could be of therapeutic benefit in preventing invasive migration and metastasis. 
 Integrin alpha8beta1 regulates adhesion, migration and proliferation of human intestinal crypt cells via a predominant RhoA/ROCK-dependent mechanism. Integrins are transmembrane alphabeta heterodimer receptors that function as structural and functional bridges between the cytoskeleton and   ECM   (extracellular matrix) molecules. The RGD (arginine-glycine-aspartate tripeptide motif)-dependent integrin alpha8beta1 has been shown to be involved in various cell functions in neuronal and mesenchymal-derived cell types. Its role in epithelial cells remains unknown.Integrin alpha8beta1 was found to be expressed in the crypt cell population of the human intestine but was absent from differentiating and mature epithelial cells of the villus. The function of alpha8beta1 in epithelial crypt cells was investigated at the cellular level using normal HIECs (human intestinal epithelial cells). Specific knockdown of alpha8 subunit expression using an shRNA (small-hairpin RNA) approach showed that alpha8beta1 plays important roles in RGD-dependent cell adhesion, migration and proliferation via a RhoA/ROCK (Rho-associated kinase)-dependent mechanism as demonstrated by active RhoA quantification and pharmacological inhibition of ROCK. Moreover, loss of alpha8beta1, through RhoA/ROCK, impairs FA (focal adhesion) complex integrity as demonstrated by faulty vinculin recruitment.Integrin alpha8beta1 is expressed in epithelial cells. In intestinal crypt cells, alpha8beta1 is closely involved in the regulation of adhesion, migration and cell proliferation via a predominant RhoA/ROCK-dependent mechanism. These results suggest an important role for this integrin in intestinal crypt cell homoeostasis. 
 Maspin controls mammary tumor cell migration through inhibiting Rac1 and Cdc42, but not the RhoA GTPase. Rac1 and Cdc42 are members of the Rho family of small GTPases that play essential roles in diverse cellular functions, including cell migration. The activities of these Rho family proteins are controlled by growth factor receptor activation and cell-  ECM   interactions. Here, we show that maspin, a well-documented tumor suppressor gene, also controls cell motility through inhibiting Rac1/Cdc42 activity. Using the GST-PAK and GST-Rho binding protein pull-down assays for GTP-bound Rac1, Cdc42, and RhoA, we showed that treatment of MDA-MB-231 tumor cells with recombinant maspin for a short time period significantly inhibited the activity of Rac1 and Cdc42, but not RhoA. The reactive site loop (RSL) within maspin protein is the functional domain involved in the inhibition. Maspin mutants with the RSL deleted or a point mutation in the RSL region lost their inhibitory activity. We further examined the ability of maspin to inhibit Rac1- and Cdc42-mediated signaling pathways and transcription factors. Treatment of MDA-MB-231 cells with maspin led to the inhibition of JNK kinase activity as assayed by immuno-kinase assays. In addition, the AP-1 transcription activity downstream of JNK kinase pathway was also reduced. Together, we have identified Rac1 and Cdc42 as the downstream targets that mediate the inhibition of mammary tumor cell migration by maspin. 
 Extracellular matrix controls insulin signaling in mammary epithelial cells through the RhoA/Rok pathway. Cellular responses are determined by a number of signaling cues in the local microenvironment, such as growth factors and extracellular matrix (  ECM  ). In cultures of mammary epithelial cells (MECs), functional differentiation requires at least two types of signal, lactogenic hormones (i.e., prolactin, insulin, and hydrocortisone) and the specialized   ECM  , basement membrane (BM). Our previous work has shown that   ECM   affects insulin signaling in mammary cells. Cell adhesion to BM promotes insulin-stimulated tyrosine phosphorylation of insulin receptor substrate-1 (IRS-1) and association of PI3K with IRS-1, whereas cells cultured on stromal   ECM   are inefficient in transducing these post-receptor events. Here we examine the mechanisms underlying   ECM   control of IRS phosphorylation. Compared to cells cultured on BM, cells on plastic exhibit higher level of RhoA activity. The amount and the activity of Rho kinase (Rok) associated with IRS-1 are greater in these cells, leading to serine phosphorylation of IRS-1. Expression of dominant negative RhoA and the application of Rok inhibitor Y27632 in cells cultured on plastic augment tyrosine phosphorylation of IRS-1. Conversely, expression of constitutively active RhoA in cells cultured on BM impedes insulin signaling. These data indicate that RhoA/Rok is involved in substratum-mediated regulation of insulin signaling in MECs, and under the conditions where proper adhesion to BM is missing, such as after wounding and during mammary gland involution, insulin-mediated cellular differentiation and survival would be defective. 
 Regulation of plasticity and fibrogenic activity of trabecular meshwork cells by Rho GTPase signaling. Glaucoma, a prevalent blinding disease is commonly associated with increased intraocular pressure due to impaired aqueous humor (AH) drainage through the trabecular meshwork (TM). Although increased TM tissue contraction and stiffness in association with accumulation of extracellular matrix (  ECM  ) are believed to be partly responsible for increased resistance to AH outflow, the extracellular cues and intracellular mechanisms regulating TM cell contraction and   ECM   production are not well defined. This study tested the hypothesis that sustained activation of Rho GTPase signaling induced by lysophosphatidic acid (LPA), TGF-β, and connective tissue growth factor (CTGF) influences TM cell plasticity and fibrogenic activity which may eventually impact resistance to AH outflow. Various experiments performed using human TM cells revealed that constitutively active RhoA (RhoAV14), TGF-β2, LPA, and CTGF significantly increase the levels and expression of Fibroblast Specific Protein-1 (FSP-1), α-smooth muscle actin (αSMA), collagen-1A1 and secretory total collagen, as determined by q-RT-PCR, immunofluorescence, immunoblot, flow cytometry and the Sircol assay. Significantly, these changes appear to be mediated by Serum Response Factor (SRF), myocardin-related transcription factor (MRTF-A), Slug, and Twist-1, which are transcriptional regulators known to control cell plasticity, myofibroblast generation/activation and fibrogenic activity. Additionally, the Rho kinase inhibitor-Y27632 and anti-fibrotic agent-pirfenidone were both found to suppress the TGF-β2-induced expression of αSMA, FSP-1, and collagen-1A1. Taken together, these observations demonstrate the significance of RhoA/Rho kinase signaling in regulation of TM cell plasticity, fibrogenic activity, and myofibroblast activation, events with potential implications for the pathobiology of elevated intraocular pressure in glaucoma patients. 
 Integrin-specific control of focal adhesion kinase and RhoA regulates membrane protrusion and invasion. Cell invasion through extracellular matrix (  ECM  ) is a hallmark of the metastatic cascade. Cancer cells require adhesion to surrounding tissues for efficient migration to occur, which is mediated through the integrin family of receptors. Alterations in expression levels of β1 and β3 integrins have previously been reported in a number of human cancers. However, whether there are specific roles for these ubiquitous receptors in mediating cell invasion remains unclear. Here we demonstrate that loss of β1 but not β3 integrins leads to increased spread cell area and focal adhesion number in cells on 2D immobilized fibronectin. Increased adhesion numbers in β1 knockdown cells correlated with decreased cell migration on 2D surfaces. Conversely, cells depleted of β1 integrins showed increased migration speed on 3D cell-derived matrix as well as in 3D organotypic cultures and inverted invasion assays. This increased invasive potential was also seen in cells lacking β3 integrin but only in 3D cultures containing fibroblasts. Mechanistically, in situ analysis using FRET biosensors revealed that enhanced invasion in cells lacking β1 integrins was directly coupled with reduced activation of focal adhesion kinase (FAK) and the small GTPase RhoA resulting in formation of enhanced dynamic protrusions and increased invasion. These reductions in FAK-RhoA signal activation were not detected in β3 knockdown cells under the same conditions. This data demonstrates a specific role for β1 integrins in the modulation of a FAK-RhoA-actomyosin signaling axis to regulate cell invasion throug  h c  omplex ECM environments. 
 Rho plays a key role in TGF-beta1-induced cytoskeletal rearrangement in human retinal pigment epithelium. Proliferative vitroretinopathy (PVR) is caused by retinal pigment epithelial (RPE) cell proliferation and transformation into fibrotic cells that produce extracellular matrix (  ECM  ) components. Transforming growth factor beta1 (TGF-beta1) is known to play an important role in PVR pathogenesis. To determine how TGF-beta1 mediates the pathogenic changes in RPE cells, we characterized the effects of TGF-beta1 on the morphology,   ECM   accumulation, and stress fiber formation of ARPE-19 cells, a human RPE cell line. We then elucidated the signaling pathways that mediate these effects. Serum-starved ARPE-19 cells were incubated with 10 ng/ml TGF-beta1 and their morphological changes were examined by phase-contrast microscopy. Actin reorganization was examined by immunochemistry and confocal microscopy. Protein phosphorylation was analyzed by Western blot analysis. TGF-beta1 treatment induced cytoskeleton reorganization, alpha-SMA expression, increased the phosphorylation of ERK, Smad2/3, and AKT, and activated RhoA and Rac1. Cytoskeletal rearrangement was prevented by pretreatment with a Rho inhibitor and by expression of a dominant negative form of Rho. TGF-beta1 also increased LIM kinase and cofilin phosphorylation and the Rho inhibitor blocked this effect. We propose that TGF-beta1 induces human RPE cells to undergo cytoskeletal actin rearrangement via Rho GTPase-dependent pathways that modulate LIM kinase and cofilin activity. This inhibits actin depolymerization and induces the cytoskeletal rearrangements in RPE cells that result in the characteristic features of PVR. 
 RhoA/ROCK signaling mediates plasticity of scirrhous gastric carcinoma motility. The small guanosine triphosphatase (GTPase) Rho and its downstream effector Rho-associated kinase (ROCK) is one of a key mediator involved in controlling focal adhesions and the dynamics of actin stress fibers. The molecular mechanisms for the function of Rho/ROCK pathway leading to the progression in scirrhous gastric carcinoma cells have not been defined. The activation of RhoA in several gastric carcinoma cells was examined. The role of RhoA/ROCK pathway in the metastatic processes of gastric carcinoma cells, using a human scirrhous gastric cancer cell line, OCUM-2MD3 was investigated by in vitro adhesion and invasion assay. The effect of ROCK inhibitor, Y-27632 on the mRNA expression of the integrin family and MMP in gastric carcinoma cells was subsequently examined by Reverse transcriptional (RT)-PCR analysis. Finally, Random OCUM-2MD3 cell motility was evaluated using Time-lapse microscopy. ROCK inhibitor significantly increased the adhesion of OCUM-2MD3 cells to the extracellular matrix (  ECM  ) protein matrigel. Further examination using   ECM   components showed enhanced binding ability was obtained only in laminin and Integrin subunits α3-integrin was clearly up-regulated by treatment with Y-27632 in OCUM-2MD3 cells. ROCK inhibitor also enhanced the invasion of OCUM-2MD3 cells through matrigel and the expression of membrane-type 1 matrix metalloproteinase (MT1-MMP). Time-lapse microscopy showed conversion of OCUM-2MD3 cells from round to more elongated morphology in the presence of Y-27632, suggesting that inhibition of RhoA/ROCK pathway undergo a so-called 'amoeboid to mesenchymal' transition. The fact that Rac1 inhibitor decreased the facilitated invasion by ROCK inhibitor suggested the possibility that increased invasion ability of OCUM-2MD3 cells was related to Rac activity. These data may suggest that RhoA/ROCK regulate plasticity of metastatic gastric carcinoma via mesenchymal-amoeboid transition, leading to provide new insights for designing a new and effective treatment for this type of refractory carcinoma. 
 Characterization of tissue biomechanics and mechanical signaling in uterine leiomyoma. Leiomyoma are common tumors arising within the uterus that feature excessive deposition of a stiff, disordered extracellular matrix (  ECM  ). Mechanical stress is a critical determinant of excessive   ECM   deposition and increased mechanical stress has been shown to be involved in tumorigenesis. Here we tested the viscoelastic properties of leiomyoma and characterized dynamic and static mechanical signaling in leiomyoma cells using three approaches, including measurement of active RhoA. We found that the peak strain and pseudo-dynamic modulus of leiomyoma tissue was significantly increased relative to matched myometrium. In addition, leiomyoma cells demonstrated an attenuated response to applied cyclic uniaxial strain and to variation in substrate stiffness, relative to myometrial cells. However, on a flexible pronectin-coated silicone substrate, basal levels and lysophosphatidic acid-stimulated levels of activated RhoA were similar between leiomyoma and myometrial cells. In contrast, leiomyoma cells plated on a rigid polystyrene substrate had elevated levels of active RhoA, compared to myometrial cells. The results indicate that viscoelastic properties of the   ECM   of leiomyoma contribute significantly to the tumor's inherent stiffness and that leiomyoma cells have an attenuated sensitivity to mechanical cues. The findings suggest there may be a fundamental alteration in the communication between the external mechanical environment (extracellular forces) and reorganization of the actin cytoskeleton mediated by RhoA in leiomyoma cells. Additional research will be needed to elucidate the mechanism(s) responsible for the attenuated mechanical signaling in leiomyoma cells. 
  [Ee]xtracellular [Mm]atrix  PubMed  
 Integrin alpha8beta1 regulates adhesion, migration and proliferation of human intestinal crypt cells via a predominant RhoA/ROCK-dependent mechanism. Integrins are transmembrane alphabeta heterodimer receptors that function as structural and functional bridges between the cytoskeleton and ECM (  extracellular matrix  ) molecules. The RGD (arginine-glycine-aspartate tripeptide motif)-dependent integrin alpha8beta1 has been shown to be involved in various cell functions in neuronal and mesenchymal-derived cell types. Its role in epithelial cells remains unknown.Integrin alpha8beta1 was found to be expressed in the crypt cell population of the human intestine but was absent from differentiating and mature epithelial cells of the villus. The function of alpha8beta1 in epithelial crypt cells was investigated at the cellular level using normal HIECs (human intestinal epithelial cells). Specific knockdown of alpha8 subunit expression using an shRNA (small-hairpin RNA) approach showed that alpha8beta1 plays important roles in RGD-dependent cell adhesion, migration and proliferation via a RhoA/ROCK (Rho-associated kinase)-dependent mechanism as demonstrated by active RhoA quantification and pharmacological inhibition of ROCK. Moreover, loss of alpha8beta1, through RhoA/ROCK, impairs FA (focal adhesion) complex integrity as demonstrated by faulty vinculin recruitment.Integrin alpha8beta1 is expressed in epithelial cells. In intestinal crypt cells, alpha8beta1 is closely involved in the regulation of adhesion, migration and cell proliferation via a predominant RhoA/ROCK-dependent mechanism. These results suggest an important role for this integrin in intestinal crypt cell homoeostasis. 
 Integrin engagement differentially modulates epithelial cell motility by RhoA/ROCK and PAK1. Integrin-ligand binding regulates tumor cell motility and invasion. Cell migration also involves the Rho GTPases that control the interplay between adhesion receptors and the cytoskeleton. We evaluated how specific   extracellular matrix   ligands modulate Rho GTPases and control motility of human squamous cell carcinoma cells. On laminin-5 substrates, the epithelial cells rapidly spread and migrated, but on type I collagen the cells spread slowly and showed reduced motility. We found that RhoA activity was suppressed in cells attached to laminin-5 through the alpha3 integrin receptor. In contrast, RhoA was strongly activated in cells bound to type I collagen and this was mediated by the alpha2 integrin. Inhibiting the RhoA pathway by expression of a dominant-negative RhoA mutant or by directly inhibiting ROCK, reduced focal adhesion formation and enhanced cell migration on type I collagen. Cdc42 and Rac and their downstream target PAK1 were activated following adhesion to laminin-5. PAK1 activation induced by laminin-5 was suppressed by expression of a dominant-negative Cdc42. Moreover, constitutively active PAK1 stimulated migration on collagen I substrates. Our results indicate that in squamous epithelial cells, collagen-alpha2beta1 integrin binding activates RhoA, slowing cell locomotion, whereas laminin-5-alpha3beta1 integrin interaction inhibits RhoA and activates PAK1, stimulating cell migration. The data demonstrate that specific ligand-integrin pairs regulate cell motility differentially by selectively modulating activities of Rho GTPases and their effectors. 
 A MAPK-Driven Feedback Loop Suppresses Rac Activity to Promote RhoA-Driven Cancer Cell Invasion. Cell migration in 3D microenvironments is fundamental to development, homeostasis and the pathobiology of diseases such as cancer. Rab-coupling protein (RCP) dependent co-trafficking of α5β1 and EGFR1 promotes cancer cell invasion into fibronectin (FN) containin  g extracellular matr  ix (ECM), by potentiating EGFR1 signalling at the front of invasive cells. This promotes a switch in RhoGTPase signalling to inhibit Rac1 and activate a RhoA-ROCK-Formin homology domain-containing 3 (FHOD3) pathway and generate filopodial actin-spike protrusions which drive invasion. To further understand the signalling network that drives RCP-driven invasive migration, we generated a Boolean logical model based on existing network pathways/models, where each node can be interrogated by computational simulation. The model predicted an unanticipated feedback loop, whereby Raf/MEK/ERK signalling maintains suppression of Rac1 by inhibiting the Rac-activating Sos1-Eps8-Abi1 complex, allowing RhoA activity to predominate in invasive protrusions. MEK inhibition was sufficient to promote lamellipodia formation and oppose filopodial actin-spike formation, and led to activation of Rac and inactivation of RhoA at the leading edge of cells moving in 3D matrix. Furthermore, MEK inhibition abrogated RCP/α5β1/EGFR1-driven invasive migration. However, upon knockdown of Eps8 (to suppress the Sos1-Abi1-Eps8 complex), MEK inhibition had no effect on RhoGTPase activity and did not oppose invasive migration, suggesting that MEK-ERK signalling suppresses the Rac-activating Sos1-Abi1-Eps8 complex to maintain RhoA activity and promote filopodial actin-spike formation and invasive migration. Our study highlights the predictive potential of mathematical modelling approaches, and demonstrates that a simple intervention (MEK-inhibition) could be of therapeutic benefit in preventing invasive migration and metastasis. 
 Temporal and spatial modulation of Rho GTPases during in vitro formation of capillary vascular network. Adherens junctions and myosin light chain as targets of Rac1 and RhoA. Endothelial cells (ECs) self-organize into capillary networks when plated on   extracellular matrix  . In this process, Rho GTPases-mediated cytoskeletal dynamics control cell movement and organization of cell-to-matrix and cell-to-cell contacts. Time course analysis of RhoA and Rac1 activation matches specific morphological aspects of nascent pattern. RhoA-GTP increases early during EC adhesion and accumulates at sites of membrane ruffling. Rac1 is activated later and localizes in lamellipodia and at cell-to-cell contacts of organized cell chains. When ECs stretch and remodel to form capillary structures, RhoA-GTP increases again and associates with stress fibers running along the major cell axis. N17Rac1 and N19RhoA mutants impair pattern formation. Cell-to-cell contacts and myosin light chains (MLC) are targets of Rac1 and RhoA, respectively. N17Rac1 reduces the shift of beta-catenin and vascular endothelial cadherin to Triton X-100-insoluble fraction and impairs beta-catenin distribution at adherens junctions, suggesting that Rac1 controls the dynamics of cadherin-catenin complex with F-actin. During the remodeling phase of network formation, ECs show an intense staining for phosphorylated MLC along the plasma membrane; in contrast, MLC is less phosphorylated and widely diffused in N19RhoA ECs. Both N17Rac1 and N19RhoA have been used to investigate the role of wild type molecules in the main steps characterizing in vitro angiogenesis: (i) cell adhesion to the substrate, (ii) cell movement, and (iii) mechanical remodeling of matrix. N17Rac1 has a striking inhibitory effect on haptotaxis, whereas N19RhoA slightly inhibits EC adhesion and motility but more markedly Matrigel contraction. We conclude that different Rho GTPases control distinct morphogenetic aspects of vascular morphogenesis. 
 Extension, retraction and contraction in the formation of a dendritic cell dendrite: distinct roles for Rho GTPases. The morphology of antigen-presenting dendritic cells (DC) is characterized by the possession of numerous long arborizing processes known as dendrites. The formation of these processes by DC, both in the periphery and in lymphoid organs, is believed to contribute to the remarkable efficiency with which they take up, process and present antigen to T cells. However, the process of dendrite formation and the signaling pathways that lead to the formation of these dendrites remain obscure. In this study we describe an in vitro model in which human immature DC form long processes similar to those formed in vivo. The formation of these processes involves initial attachment of a cell protrusion to the   extracellular matrix   substrate, and subsequent movement of the cell body away from the adhesion site, leaving behind a long slender dendrite. Dendrite formation, but not their maintenance, was found to be dependent on the activity of Rho GTPases. More specifically, Cdc42 and Rac1 were both required for the migration step of process formation, promoting cell spreading and extension. In contrast, Rho, and its downstream effector p160ROCK, regulated the release of adhesions to the substratum, and associated cellular contraction. Consequently, inhibition of Rho/p160ROCK leads to the formation of longer dendrites. DC therefore coordinate adhesion and protrusion to perform a specialized process of cellular morphogenesis, which differentiates these cells from all other cells of the immune system and may contribute to their distinctive function. 
 Arachidonic acid stimulates cell adhesion through a novel p38 MAPK-RhoA signaling pathway that involves heat shock protein 27. Rho GTPases are critical components of cellular signal transduction pathways. Both hyperactivity and overexpression of these proteins have been observed in human cancers and have been implicated as important factors in metastasis. We previously showed that dietary n-6 fatty acids increase cancer cell adhesion to   extracellular matrix   proteins, such as type IV collagen. Here we report that in MDA-MB-435 human melanoma cells, arachidonic acid activates RhoA, and inhibition of RhoA signaling with either C3 exoenzyme or dominant negative Rho blocked arachidonic acid-induced cell adhesion. Inhibition of the Rho kinase (ROCK) with either small molecule inhibitors or ROCK II-specific small interfering RNA (siRNA) blocked the fatty acid-induced adhesion. However, unlike other systems, inhibition of ROCK did not block the activation of p38 mitogen-activated protein kinase (MAPK); instead, Rho activation depended on p38 MAPK activity and the presence of heat shock protein 27 (HSP27), which is phosphorylated downstream of p38 after arachidonic acid treatment. HSP27 associated with p115RhoGEF in fatty acid-treated cells, and this association was blocked when p38 was inhibited. Furthermore, siRNA knockdown of HSP27 blocked the fatty acid-stimulated Rho activity. Expression of dominant negative p115-RhoGEF or p115RhoGEF-specific siRNA inhibited both RhoA activation and adhesion on type IV collagen, whereas a constitutively active p115RhoGEF restored the arachidonic acid stimulation in cells in which the p38 MAPK had been inhibited. These data suggest that n-6 dietary fatty acids stimulate a set of interactions that regulates cell adhesion through RhoA and ROCK II via a p38 MAPK-dependent association of HSP27 and p115RhoGEF. 
 A MAPK-Driven Feedback Loop Suppresses Rac Activity to Promote RhoA-Driven Cancer Cell Invasion. Cell migration in 3D microenvironments is fundamental to development, homeostasis and the pathobiology of diseases such as cancer. Rab-coupling protein (RCP) dependent co-trafficking of α5β1 and EGFR1 promotes cancer cell invasion into fibronectin (FN) containin  g extracellular matr  ix (ECM), by potentiating EGFR1 signalling at the front of invasive cells. This promotes a switch in RhoGTPase signalling to inhibit Rac1 and activate a RhoA-ROCK-Formin homology domain-containing 3 (FHOD3) pathway and generate filopodial actin-spike protrusions which drive invasion. To further understand the signalling network that drives RCP-driven invasive migration, we generated a Boolean logical model based on existing network pathways/models, where each node can be interrogated by computational simulation. The model predicted an unanticipated feedback loop, whereby Raf/MEK/ERK signalling maintains suppression of Rac1 by inhibiting the Rac-activating Sos1-Eps8-Abi1 complex, allowing RhoA activity to predominate in invasive protrusions. MEK inhibition was sufficient to promote lamellipodia formation and oppose filopodial actin-spike formation, and led to activation of Rac and inactivation of RhoA at the leading edge of cells moving in 3D matrix. Furthermore, MEK inhibition abrogated RCP/α5β1/EGFR1-driven invasive migration. However, upon knockdown of Eps8 (to suppress the Sos1-Abi1-Eps8 complex), MEK inhibition had no effect on RhoGTPase activity and did not oppose invasive migration, suggesting that MEK-ERK signalling suppresses the Rac-activating Sos1-Abi1-Eps8 complex to maintain RhoA activity and promote filopodial actin-spike formation and invasive migration. Our study highlights the predictive potential of mathematical modelling approaches, and demonstrates that a simple intervention (MEK-inhibition) could be of therapeutic benefit in preventing invasive migration and metastasis. 
 Integrin alpha8beta1 regulates adhesion, migration and proliferation of human intestinal crypt cells via a predominant RhoA/ROCK-dependent mechanism. Integrins are transmembrane alphabeta heterodimer receptors that function as structural and functional bridges between the cytoskeleton and ECM (  extracellular matrix  ) molecules. The RGD (arginine-glycine-aspartate tripeptide motif)-dependent integrin alpha8beta1 has been shown to be involved in various cell functions in neuronal and mesenchymal-derived cell types. Its role in epithelial cells remains unknown.Integrin alpha8beta1 was found to be expressed in the crypt cell population of the human intestine but was absent from differentiating and mature epithelial cells of the villus. The function of alpha8beta1 in epithelial crypt cells was investigated at the cellular level using normal HIECs (human intestinal epithelial cells). Specific knockdown of alpha8 subunit expression using an shRNA (small-hairpin RNA) approach showed that alpha8beta1 plays important roles in RGD-dependent cell adhesion, migration and proliferation via a RhoA/ROCK (Rho-associated kinase)-dependent mechanism as demonstrated by active RhoA quantification and pharmacological inhibition of ROCK. Moreover, loss of alpha8beta1, through RhoA/ROCK, impairs FA (focal adhesion) complex integrity as demonstrated by faulty vinculin recruitment.Integrin alpha8beta1 is expressed in epithelial cells. In intestinal crypt cells, alpha8beta1 is closely involved in the regulation of adhesion, migration and cell proliferation via a predominant RhoA/ROCK-dependent mechanism. These results suggest an important role for this integrin in intestinal crypt cell homoeostasis. 
 Arachidonic acid stimulates cell adhesion through a novel p38 MAPK-RhoA signaling pathway that involves heat shock protein 27. Rho GTPases are critical components of cellular signal transduction pathways. Both hyperactivity and overexpression of these proteins have been observed in human cancers and have been implicated as important factors in metastasis. We previously showed that dietary n-6 fatty acids increase cancer cell adhesion to   extracellular matrix   proteins, such as type IV collagen. Here we report that in MDA-MB-435 human melanoma cells, arachidonic acid activates RhoA, and inhibition of RhoA signaling with either C3 exoenzyme or dominant negative Rho blocked arachidonic acid-induced cell adhesion. Inhibition of the Rho kinase (ROCK) with either small molecule inhibitors or ROCK II-specific small interfering RNA (siRNA) blocked the fatty acid-induced adhesion. However, unlike other systems, inhibition of ROCK did not block the activation of p38 mitogen-activated protein kinase (MAPK); instead, Rho activation depended on p38 MAPK activity and the presence of heat shock protein 27 (HSP27), which is phosphorylated downstream of p38 after arachidonic acid treatment. HSP27 associated with p115RhoGEF in fatty acid-treated cells, and this association was blocked when p38 was inhibited. Furthermore, siRNA knockdown of HSP27 blocked the fatty acid-stimulated Rho activity. Expression of dominant negative p115-RhoGEF or p115RhoGEF-specific siRNA inhibited both RhoA activation and adhesion on type IV collagen, whereas a constitutively active p115RhoGEF restored the arachidonic acid stimulation in cells in which the p38 MAPK had been inhibited. These data suggest that n-6 dietary fatty acids stimulate a set of interactions that regulates cell adhesion through RhoA and ROCK II via a p38 MAPK-dependent association of HSP27 and p115RhoGEF. 
 Temporal and spatial modulation of Rho GTPases during in vitro formation of capillary vascular network. Adherens junctions and myosin light chain as targets of Rac1 and RhoA. Endothelial cells (ECs) self-organize into capillary networks when plated on   extracellular matrix  . In this process, Rho GTPases-mediated cytoskeletal dynamics control cell movement and organization of cell-to-matrix and cell-to-cell contacts. Time course analysis of RhoA and Rac1 activation matches specific morphological aspects of nascent pattern. RhoA-GTP increases early during EC adhesion and accumulates at sites of membrane ruffling. Rac1 is activated later and localizes in lamellipodia and at cell-to-cell contacts of organized cell chains. When ECs stretch and remodel to form capillary structures, RhoA-GTP increases again and associates with stress fibers running along the major cell axis. N17Rac1 and N19RhoA mutants impair pattern formation. Cell-to-cell contacts and myosin light chains (MLC) are targets of Rac1 and RhoA, respectively. N17Rac1 reduces the shift of beta-catenin and vascular endothelial cadherin to Triton X-100-insoluble fraction and impairs beta-catenin distribution at adherens junctions, suggesting that Rac1 controls the dynamics of cadherin-catenin complex with F-actin. During the remodeling phase of network formation, ECs show an intense staining for phosphorylated MLC along the plasma membrane; in contrast, MLC is less phosphorylated and widely diffused in N19RhoA ECs. Both N17Rac1 and N19RhoA have been used to investigate the role of wild type molecules in the main steps characterizing in vitro angiogenesis: (i) cell adhesion to the substrate, (ii) cell movement, and (iii) mechanical remodeling of matrix. N17Rac1 has a striking inhibitory effect on haptotaxis, whereas N19RhoA slightly inhibits EC adhesion and motility but more markedly Matrigel contraction. We conclude that different Rho GTPases control distinct morphogenetic aspects of vascular morphogenesis. 
 Extension, retraction and contraction in the formation of a dendritic cell dendrite: distinct roles for Rho GTPases. The morphology of antigen-presenting dendritic cells (DC) is characterized by the possession of numerous long arborizing processes known as dendrites. The formation of these processes by DC, both in the periphery and in lymphoid organs, is believed to contribute to the remarkable efficiency with which they take up, process and present antigen to T cells. However, the process of dendrite formation and the signaling pathways that lead to the formation of these dendrites remain obscure. In this study we describe an in vitro model in which human immature DC form long processes similar to those formed in vivo. The formation of these processes involves initial attachment of a cell protrusion to the   extracellular matrix   substrate, and subsequent movement of the cell body away from the adhesion site, leaving behind a long slender dendrite. Dendrite formation, but not their maintenance, was found to be dependent on the activity of Rho GTPases. More specifically, Cdc42 and Rac1 were both required for the migration step of process formation, promoting cell spreading and extension. In contrast, Rho, and its downstream effector p160ROCK, regulated the release of adhesions to the substratum, and associated cellular contraction. Consequently, inhibition of Rho/p160ROCK leads to the formation of longer dendrites. DC therefore coordinate adhesion and protrusion to perform a specialized process of cellular morphogenesis, which differentiates these cells from all other cells of the immune system and may contribute to their distinctive function. 
 Integrin engagement differentially modulates epithelial cell motility by RhoA/ROCK and PAK1. Integrin-ligand binding regulates tumor cell motility and invasion. Cell migration also involves the Rho GTPases that control the interplay between adhesion receptors and the cytoskeleton. We evaluated how specific   extracellular matrix   ligands modulate Rho GTPases and control motility of human squamous cell carcinoma cells. On laminin-5 substrates, the epithelial cells rapidly spread and migrated, but on type I collagen the cells spread slowly and showed reduced motility. We found that RhoA activity was suppressed in cells attached to laminin-5 through the alpha3 integrin receptor. In contrast, RhoA was strongly activated in cells bound to type I collagen and this was mediated by the alpha2 integrin. Inhibiting the RhoA pathway by expression of a dominant-negative RhoA mutant or by directly inhibiting ROCK, reduced focal adhesion formation and enhanced cell migration on type I collagen. Cdc42 and Rac and their downstream target PAK1 were activated following adhesion to laminin-5. PAK1 activation induced by laminin-5 was suppressed by expression of a dominant-negative Cdc42. Moreover, constitutively active PAK1 stimulated migration on collagen I substrates. Our results indicate that in squamous epithelial cells, collagen-alpha2beta1 integrin binding activates RhoA, slowing cell locomotion, whereas laminin-5-alpha3beta1 integrin interaction inhibits RhoA and activates PAK1, stimulating cell migration. The data demonstrate that specific ligand-integrin pairs regulate cell motility differentially by selectively modulating activities of Rho GTPases and their effectors. 
   Extracellular matrix   controls insulin signaling in mammary epithelial cells through the RhoA/Rok pathway. Cellular responses are determined by a number of signaling cues in the local microenvironment, such as growth factors and   extracellular matrix   (ECM). In cultures of mammary epithelial cells (MECs), functional differentiation requires at least two types of signal, lactogenic hormones (i.e., prolactin, insulin, and hydrocortisone) and the specialized ECM, basement membrane (BM). Our previous work has shown that ECM affects insulin signaling in mammary cells. Cell adhesion to BM promotes insulin-stimulated tyrosine phosphorylation of insulin receptor substrate-1 (IRS-1) and association of PI3K with IRS-1, whereas cells cultured on stromal ECM are inefficient in transducing these post-receptor events. Here we examine the mechanisms underlying ECM control of IRS phosphorylation. Compared to cells cultured on BM, cells on plastic exhibit higher level of RhoA activity. The amount and the activity of Rho kinase (Rok) associated with IRS-1 are greater in these cells, leading to serine phosphorylation of IRS-1. Expression of dominant negative RhoA and the application of Rok inhibitor Y27632 in cells cultured on plastic augment tyrosine phosphorylation of IRS-1. Conversely, expression of constitutively active RhoA in cells cultured on BM impedes insulin signaling. These data indicate that RhoA/Rok is involved in substratum-mediated regulation of insulin signaling in MECs, and under the conditions where proper adhesion to BM is missing, such as after wounding and during mammary gland involution, insulin-mediated cellular differentiation and survival would be defective. 
 Activation of ROCK by RhoA is regulated by cell adhesion, shape, and cytoskeletal tension. Adhesion to the   extracellular matrix   regulates numerous changes in the actin cytoskeleton by regulating the activity of the Rho family of small GTPases. Here, we report that adhesion and the associated changes in cell shape and cytoskeletal tension are all required for GTP-bound RhoA to activate its downstream effector, ROCK. Using an in vitro kinase assay for endogenous ROCK, we found that cells in suspension, attached on substrates coated with low density fibronectin, or on spreading-restrictive micropatterned islands all exhibited low ROCK activity and correspondingly low myosin light chain phosphorylation, in the face of high levels of GTP-bound RhoA. In contrast, allowing cells to spread against substrates rescued ROCK and myosin activity. Interestingly, inhibition of tension with cytochalasin D or blebbistatin also inhibited ROCK activity within 20 min. The abrogation of ROCK activity by cell detachment or inhibition of tension could not be rescued by constitutively active RhoA-V14. These results suggest the existence of a feedback loop between cytoskeletal tension, adhesion maturation, and ROCK signaling that likely contributes to numerous mechanochemical processes. 
 Regulation of plasticity and fibrogenic activity of trabecular meshwork cells by Rho GTPase signaling. Glaucoma, a prevalent blinding disease is commonly associated with increased intraocular pressure due to impaired aqueous humor (AH) drainage through the trabecular meshwork (TM). Although increased TM tissue contraction and stiffness in association with accumulation of   extracellular matrix   (ECM) are believed to be partly responsible for increased resistance to AH outflow, the extracellular cues and intracellular mechanisms regulating TM cell contraction and ECM production are not well defined. This study tested the hypothesis that sustained activation of Rho GTPase signaling induced by lysophosphatidic acid (LPA), TGF-β, and connective tissue growth factor (CTGF) influences TM cell plasticity and fibrogenic activity which may eventually impact resistance to AH outflow. Various experiments performed using human TM cells revealed that constitutively active RhoA (RhoAV14), TGF-β2, LPA, and CTGF significantly increase the levels and expression of Fibroblast Specific Protein-1 (FSP-1), α-smooth muscle actin (αSMA), collagen-1A1 and secretory total collagen, as determined by q-RT-PCR, immunofluorescence, immunoblot, flow cytometry and the Sircol assay. Significantly, these changes appear to be mediated by Serum Response Factor (SRF), myocardin-related transcription factor (MRTF-A), Slug, and Twist-1, which are transcriptional regulators known to control cell plasticity, myofibroblast generation/activation and fibrogenic activity. Additionally, the Rho kinase inhibitor-Y27632 and anti-fibrotic agent-pirfenidone were both found to suppress the TGF-β2-induced expression of αSMA, FSP-1, and collagen-1A1. Taken together, these observations demonstrate the significance of RhoA/Rho kinase signaling in regulation of TM cell plasticity, fibrogenic activity, and myofibroblast activation, events with potential implications for the pathobiology of elevated intraocular pressure in glaucoma patients. 
 The interaction of IQGAP1 with the exocyst complex is required for tumor cell invasion downstream of Cdc42 and RhoA. Invadopodia are actin-based membrane protrusions formed at contact sites between invasive tumor cells and the   extracellular matrix   with matrix proteolytic activity. Actin regulatory proteins participate in invadopodia formation, whereas matrix degradation requires metalloproteinases (MMPs) targeted to invadopodia. In this study, we show that the vesicle-tethering exocyst complex is required for matrix proteolysis and invasion of breast carcinoma cells. We demonstrate that the exocyst subunits Sec3 and Sec8 interact with the polarity protein IQGAP1 and that this interaction is triggered by active Cdc42 and RhoA, which are essential for matrix degradation. Interaction between IQGAP1 and the exocyst is necessary for invadopodia activity because enhancement of matrix degradation induced by the expression of IQGAP1 is lost upon deletion of the exocyst-binding site. We further show that the exocyst and IQGAP1 are required for the accumulation of cell surface membrane type 1 MMP at invadopodia. Based on these results, we propose that invadopodia function in tumor cells relies on the coordination of cytoskeletal assembly and exocytosis downstream of Rho guanosine triphosphatases. 
 [Effects of inhibiting RhoA by Stealth RNA on high glucose-induced RhoA/ROCK signaling pathway in human mesangial cells]. To observe the effect of small interference RNA (Stealth RNAiTM siRNA) of RhoA on the inflammatory response and fibrosis in human mesangial cell (HMC) and explore the role of RhoA/ROCK signaling pathway in the process of diabetic nephropathy.Synchronized HMC were divided into several groups. Lipofectamine(TM)2000 was employed to transfect RhoA-siRNA and RhoA-negative siRNA into the above cells. RhoA-siRNA could inhibit the expression of RhoA. The expressions of RhoA, ROCK-I, fibronectin (FN), connective tissue growth factor (CTGF) and tumor necrosis factor-alpha (TNF-α) were detected by real-time reverse transcription-polymerase chain reaction (RT-PCR) and ELISA (enzyme-linked immunosorbent assay).(1) The expressions of RhoA, ROCK-I and CTGF mRNA were inhibited by RhoA siRNA transfection in high glucose-induced HMC. The expression of each mRNA was reduced 26% - 60% as compared with the high glucose-induced group (P < 0.05); (2) After RhoA siRNA transfection and culturing with high glucose for 48 h, FN, the secretions of CTGF and TNF-α significantly declined [FN: (1.99 ± 0.04) mg/L vs. (4.31 ± 0.13) mg/L, CTGF:(4.98 ± 0.17) mg/L vs. (6.06 ± 0.09) mg/L; TNF-α: (61.17 ± 2.59) ng/L vs.(91.76 ± 2.27) ng/L, all P < 0.05]. The levels of FN and CTGF almost decreased to those of normal glucose-induced HMC.The levels of FN, CTGF and TNF-α in high glucose-induced HMC may be lowered by inhibiting RhoA through RNA interference and reducing the accumu  lation of extracellu  lar matrix, glomerular fibrosis and inflammation. Thus it provides a new intervention target for the prevention of diabetic nephropathy. 
 Arecoline induces HA22T/VGH hepatoma cells to undergo anoikis - involvement of STAT3 and RhoA activation. Our previous study showed that, in basal cell carcinoma cells, arecoline reduces levels of the tumor cell survival factor interleukin-6 (IL-6), increases levels of tumor suppressor factor p53, and elicits cell cycle arrest, followed by apoptosis. In preliminarily studies, we observed that arecoline induces detachment of the human-derived hepatoma cell line HA22T/VGH from the   extracellular matrix  . In the present study, we explored the fate of the detached HA22T/VGH cells and investigated the underlying mechanism.HA22T/VGH cells or primary cultured rat hepatocytes were treated with arecoline, then changes in morphology, viability, apoptosis, and the expression of surface beta1-integrin, apoptosis-related proteins, and IL-6 were examined. Furthermore, activation of the signal transducer and activator of transcription 3 (STAT3) pathway and the RhoA/Rock signaling pathway, including p190RhoGAP and Src homology-2 domain-containing phosphatase SHP2, was examined.A low concentration of arecoline (<or= 100 microg/ml) caused cytoskeletal changes in HA22T/VGH cells, but not hepatocytes, and this was accompanied by decreased beta1-integrin expression and followed by apoptosis, indicating that HA22T/VGH cells undergo anoikis after arecoline treatment. IL-6 expression and phosphorylation of STAT3, which provides protection against anoikis, were inhibited and levels of downstream signaling proteins, including Bcl-XL and Bcl-2, were decreased, while Bax expression, mitochondrial cytochrome c release, and caspase-3 activity were increased. In addition, phosphorylation/activation of p190RhoGAP, a RhoA inhibitor, and of its upstream regulator, SHP2, was inhibited by arecoline treatment, while Rho/Rock activation was increased. Addition of the RhoA inhibitor attenuated the effects of arecoline.This study demonstrated that arecoline induces anoikis of HA22T/VGH cells involving inhibition of STAT3 and increased RhoA/Rock activation and that the STAT3 and RhoA/Rock signaling pathways are connected. 
 Integrin-specific control of focal adhesion kinase and RhoA regulates membrane protrusion and invasion. Cell invasion through   extracellular matrix   (ECM) is a hallmark of the metastatic cascade. Cancer cells require adhesion to surrounding tissues for efficient migration to occur, which is mediated through the integrin family of receptors. Alterations in expression levels of β1 and β3 integrins have previously been reported in a number of human cancers. However, whether there are specific roles for these ubiquitous receptors in mediating cell invasion remains unclear. Here we demonstrate that loss of β1 but not β3 integrins leads to increased spread cell area and focal adhesion number in cells on 2D immobilized fibronectin. Increased adhesion numbers in β1 knockdown cells correlated with decreased cell migration on 2D surfaces. Conversely, cells depleted of β1 integrins showed increased migration speed on 3D cell-derived matrix as well as in 3D organotypic cultures and inverted invasion assays. This increased invasive potential was also seen in cells lacking β3 integrin but only in 3D cultures containing fibroblasts. Mechanistically, in situ analysis using FRET biosensors revealed that enhanced invasion in cells lacking β1 integrins was directly coupled with reduced activation of focal adhesion kinase (FAK) and the small GTPase RhoA resulting in formation of enhanced dynamic protrusions and increased invasion. These reductions in FAK-RhoA signal activation were not detected in β3 knockdown cells under the same conditions. This data demonstrates a specific role for β1 integrins in the modulation of a FAK-RhoA-actomyosin signaling axis to regulate cell invasion through complex ECM environments. 
 Rho plays a key role in TGF-beta1-induced cytoskeletal rearrangement in human retinal pigment epithelium. Proliferative vitroretinopathy (PVR) is caused by retinal pigment epithelial (RPE) cell proliferation and transformation into fibrotic cells that produce   extracellular matrix   (ECM) components. Transforming growth factor beta1 (TGF-beta1) is known to play an important role in PVR pathogenesis. To determine how TGF-beta1 mediates the pathogenic changes in RPE cells, we characterized the effects of TGF-beta1 on the morphology, ECM accumulation, and stress fiber formation of ARPE-19 cells, a human RPE cell line. We then elucidated the signaling pathways that mediate these effects. Serum-starved ARPE-19 cells were incubated with 10 ng/ml TGF-beta1 and their morphological changes were examined by phase-contrast microscopy. Actin reorganization was examined by immunochemistry and confocal microscopy. Protein phosphorylation was analyzed by Western blot analysis. TGF-beta1 treatment induced cytoskeleton reorganization, alpha-SMA expression, increased the phosphorylation of ERK, Smad2/3, and AKT, and activated RhoA and Rac1. Cytoskeletal rearrangement was prevented by pretreatment with a Rho inhibitor and by expression of a dominant negative form of Rho. TGF-beta1 also increased LIM kinase and cofilin phosphorylation and the Rho inhibitor blocked this effect. We propose that TGF-beta1 induces human RPE cells to undergo cytoskeletal actin rearrangement via Rho GTPase-dependent pathways that modulate LIM kinase and cofilin activity. This inhibits actin depolymerization and induces the cytoskeletal rearrangements in RPE cells that result in the characteristic features of PVR. 
 Suppression of RhoA activity by focal adhesion kinase-induced activation of p190RhoGAP: role in regulation of endothelial permeability. The interaction of endothelial cells with   extracellular matrix   proteins at focal adhesions sites contributes to the integrity of vascular endothelial barrier. Although focal adhesion kinase (FAK) activation is required for the recovery of the barrier function after increased endothelial junctional permeability, the basis for the recovery remains unclear. We tested the hypothesis that FAK activates p190RhoGAP and, thus, negatively regulates RhoA activity and promotes endothelial barrier restoration in response to the permeability-increasing mediator thrombin. We observed that thrombin caused a transient activation of RhoA but a more prolonged FAK activation temporally coupled to the recovery of barrier function. Thrombin also induced tyrosine phosphorylation of p190RhoGAP, which coincided with decrease in RhoA activity. We further showed that FAK was associated with p190RhoGAP, and importantly, recombinant FAK phosphorylated p190RhoGAP in vitro. Inhibition of FAK by adenoviral expression of FRNK (a dominant negative FAK construct) in monolayers prevented p190RhoGAP phosphorylation, increased RhoA activity, induced actin stress fiber formation, and produced an irreversible increase in endothelial permeability in response to thrombin. We also observed that p190RhoGAP was unable to attenuate RhoA activation in the absence of FAK activation induced by FRNK. The inhibition of RhoA by the C3 toxin (Clostridium botulinum toxin) restored endothelial barrier function in the FRNK-expressing cells. These findings in endothelial cells were recapitulated in the lung microcirculation in which FRNK expression in microvessel endothelia increased vascular permeability. Our studies demonstrate that FAK-induced down-modulation of RhoA activity via p190RhoGAP is a crucial step in signaling endothelial barrier restoration after increased endothelial permeability. 
 Reactive oxygen species-dependent RhoA activation mediates collagen synthesis in hyperoxic lung fibrosis. Lung fibrosis is an ultimate consequence of pulmonary oxygen toxicity in human and animal models. Excessive production and deposition of   extracellular matrix   proteins, e.g., collagen-I, is the most important feature of pulmonary fibrosis in hyperoxia-induced lung injury. In this study, we investigated the roles of RhoA and reactive oxygen species (ROS) in collagen-I synthesis in hyperoxic lung fibroblasts and in a mouse model of oxygen toxicity. Exposure of human lung fibroblasts to hyperoxia resulted in RhoA activation and an increase in collagen-I synthesis and cell proliferation. Inhibition of RhoA by C3 transferase CT-04, dominant-negative RhoA mutant T19N, or RhoA siRNA prevented hyperoxia-induced collagen-I synthesis. The constitutively active RhoA mutant Q63L mimicked the effect of hyperoxia on collagen-I expression. Moreover, the Rho kinase inhibitor Y27632 inhibited collagen-I synthesis in hyperoxic lung fibroblasts and fibrosis in mouse lungs after oxygen toxicity. Furthermore, the ROS scavenger tiron attenuated hyperoxia-induced increases in RhoA activation and collagen-I synthesis in lung fibroblasts and mouse lungs after oxygen toxicity. More importantly, we found that hyperoxia induced separation of guanine nucleotide dissociation inhibitor (GDI) from RhoA in lung fibroblasts and mouse lungs. Further, tiron prevented the separation of GDI from RhoA in hyperoxic lung fibroblasts and mouse lungs with oxygen toxicity. Together, these results indicate that ROS-induced separation of GDI from RhoA leads to RhoA activation with oxygen toxicity. ROS-dependent RhoA activation is responsible for the increase in collagen-I synthesis in hyperoxic lung fibroblasts and mouse lungs. 
 RhoA/ROCK signaling mediates plasticity of scirrhous gastric carcinoma motility. The small guanosine triphosphatase (GTPase) Rho and its downstream effector Rho-associated kinase (ROCK) is one of a key mediator involved in controlling focal adhesions and the dynamics of actin stress fibers. The molecular mechanisms for the function of Rho/ROCK pathway leading to the progression in scirrhous gastric carcinoma cells have not been defined. The activation of RhoA in several gastric carcinoma cells was examined. The role of RhoA/ROCK pathway in the metastatic processes of gastric carcinoma cells, using a human scirrhous gastric cancer cell line, OCUM-2MD3 was investigated by in vitro adhesion and invasion assay. The effect of ROCK inhibitor, Y-27632 on the mRNA expression of the integrin family and MMP in gastric carcinoma cells was subsequently examined by Reverse transcriptional (RT)-PCR analysis. Finally, Random OCUM-2MD3 cell motility was evaluated using Time-lapse microscopy. ROCK inhibitor significantly increased the adhesion of OCUM-2MD3 cells to the   extracellular matrix   (ECM) protein matrigel. Further examination using ECM components showed enhanced binding ability was obtained only in laminin and Integrin subunits α3-integrin was clearly up-regulated by treatment with Y-27632 in OCUM-2MD3 cells. ROCK inhibitor also enhanced the invasion of OCUM-2MD3 cells through matrigel and the expression of membrane-type 1 matrix metalloproteinase (MT1-MMP). Time-lapse microscopy showed conversion of OCUM-2MD3 cells from round to more elongated morphology in the presence of Y-27632, suggesting that inhibition of RhoA/ROCK pathway undergo a so-called 'amoeboid to mesenchymal' transition. The fact that Rac1 inhibitor decreased the facilitated invasion by ROCK inhibitor suggested the possibility that increased invasion ability of OCUM-2MD3 cells was related to Rac activity. These data may suggest that RhoA/ROCK regulate plasticity of metastatic gastric carcinoma via mesenchymal-amoeboid transition, leading to provide new insights for designing a new and effective treatment for this type of refractory carcinoma. 
 Matrix compliance and RhoA direct the differentiation of mammary progenitor cells. The regenerative capacity of the mammary gland following post-lactational involution depends on the presence of multipotent stem or progenitor cells. Mammary progenitor cells exist as a quiescent and self-renewing population capable of differentiating into luminal epithelial and myoepithelial cells and generating ductal and alveolar structures. The fate choices of these cells are regulated by several soluble signals as well as their surrounding   extracellular matrix  . Whereas matrix stiffness has been implicated in organ-specific differentiation of embryonic and mesenchymal stem cells, the effects of substratum compliance on the more limited fate switches typical of tissue-specific progenitor cells are unknown. Here, we examined how the mechanical properties of the microenvironment affect the differentiation of mammary progenitor cells. Immortalized human mammary progenitor cells were cultured on synthetic hydrogels of varying stiffness, and their self-renewal and fate decisions were quantified. We found that cells cultured on soft substrata differentiated preferentially into luminal epithelial cells, whereas those cultured on stiff substrata differentiated preferentially into myoepithelial cells. Furthermore, pharmacological manipulations of cytoskeletal tension in conjunction with analysis of gene expression revealed that mechanical properties of the microenvironment signal through the small GTPase RhoA and cytoskeletal contractility to modulate the differentiation of mammary progenitor cells. These data suggest that subtle variations in the mechanical compliance of a tissue can direct the fate decisions of its resident progenitor cells. 
 Inadequate activation of the GTPase RhoA contributes to the lack of fibronectin matrix assembly in von Hippel-Lindau protein-defective renal cancer cells. The von Hippel-Lindau (VHL) tumor suppressor gene regulates   extracellular matrix   deposition. In VHL negative renal cancer cells, VHL(-), the lack of fibronectin matrix assembly is thought to promote and maintain tumor angiogenesis allowing vessels to infiltrate tumors. Therefore, and considering the importance of this process in tumor growth, we aimed to study why VHL(-) renal cancer cells fail to form a proper   extracellular matrix  . Our results showed that VHL(-) cells were not defective in fibronectin production and that the fibronectin produced by these cells was equally functional in promoting cell adhesion and matrix assembly as that produced by VHL+ cells. We have previously reported that VHL(-) cells fail to form beta1 integrin fibrillar adhesions and have a diminished organization of actin stress fibers; therefore, we aimed to study if the small GTPase family is involved in this process. We found that activation of the RhoA GTPase was defective in VHL(-) cells, and this was possibly mediated by an increased activation of its inhibitor, p190RhoGAP. Additionally, the expression of constitutively active RhoA in VHL(-) cells resulted in formation of a fibronectin matrix. These results strongly suggest an important role for RhoA in some of the defects observed in renal cancer cells. 
 Activation of vinculin induced by cholinergic stimulation regulates contraction of tracheal smooth muscle tissue. Vinculin localizes to membrane adhesion junctions where it links actin filaments to the   extracellular matrix   by binding to the integrin-binding protein talin at its head domain (Vh) and to actin filaments at its tail domain (Vt). Vinculin can assume an inactive (closed) conformation in which Vh and Vt bind to each other, masking the binding sites for actin and talin, and an active (open) conformation in which the binding sites for talin and actin are exposed. We hypothesized that the contractile activation of smooth muscle tissues might regulate the activation of vinculin and thereby contribute to the regulation of contractile tension. Stimulation of tracheal smooth muscle tissues with acetylcholine (ACh) induced the recruitment of vinculin to cell membrane and its interaction with talin and increased the phosphorylation of membrane-localized vinculin at the C-terminal Tyr-1065. Expression of recombinant vinculin head domain peptide (Vh) in smooth muscle tissues, but not the talin-binding deficient mutant head domain, VhA50I, inhibited the ACh-induced recruitment of endogenous vinculin to the membrane and the interaction of vinculin with talin and also inhibited vinculin phosphorylation. Expression of Vh peptide also inhibited ACh-induced smooth muscle contraction and inhibited ACh-induced actin polymerization; however, it did not affect myosin light chain phosphorylation, which is necessary for cross-bridge cycling. Inactivation of RhoA inhibited vinculin activation in response to ACh. We conclude that ACh stimulation regulates vinculin activation in tracheal smooth muscle via RhoA and that vinculin activation contributes to the regulation of active tension by facilitating connections between actin filaments and talin-integrin adhesion complexes and by mediating the initiation of actin polymerization. 
 Role of RhoA, mDia, and ROCK in cell shape-dependent control of the Skp2-p27kip1 pathway and the G1/S transition. Cell shape-dependent control of cell-cycle progression underlies the spatial differentials of growth that drive tissue morphogenesis, yet little is known about how cell distortion impacts the biochemical signaling machinery that is responsible for growth control. Here we show that the Rho family GTPase, RhoA, conveys the "cell shape signal" to the cell-cycle machinery in human capillary endothelial cells. Cells accumulating p27(kip1) and arrested in mid G(1) phase when spreading were inhibited by restricted   extracellular matrix   adhesion, whereas constitutively active RhoA increased expression of the F-box protein Skp2 required for ubiquitination-dependent degradation of p27(kip1) and restored G(1) progression in these cells. Studies with dominant-negative and constitutively active forms of mDia1, a downstream effector of RhoA, and with a pharmacological inhibitor of ROCK, another RhoA target, revealed that RhoA promoted G(1) progression by altering the balance of activities between these two downstream effectors. These data indicate that signaling proteins such as mDia1 and ROCK, which are thought to be involved primarily in cytoskeletal remodeling, also mediate cell growth regulation by coupling cell shape to the cell-cycle machinery at the level of signal transduction. 
 Kidney expression of RhoA, TGF-beta1, and fibronectin in human IgA nephropathy. The Rho/transforming growth factor-beta (TGF-beta) system plays a crucial role in the progression of renal damage due to stimulation of   extracellular matrix   molecule deposition. In fact, the in vitro TGF-beta-mediated production of fibronectin, one of the major TGF-beta-regulated extracellular components, has recently been correlated with Rho protein signalling molecules. Although a close relationship between increased renal tissue levels of TGF-beta1 and fibronectin has been reported in IgA nephropathy, no data are available on renal tissue expression of Rho proteins.This study was designed to assess in IgA nephropathy patients the kidney tissue immunohistochemical expression of RhoA, TGF-beta1, and fibronectin, and the rate of immunoreactivity for each antigen by image analysis.An increase in RhoA, TGF-beta1, and fibronectin expression was detected in tubulointerstitium and in glomeruli of IgA nephropathy compared to normal kidneys; in particular, RhoA was found also in proximal tubules, unlike control kidneys and mainly at the cell boundary level, which is in keeping with its activated form. The image analysis confirmed that the kidney tissue levels of RhoA, TGF-beta1, and fibronectin were significantly enhanced in the patients.This study suggests that RhoA may represent a key molecule in the signalling transduction pathway of profibrotic signals in IgA nephropathy. 
 Characterization of tissue biomechanics and mechanical signaling in uterine leiomyoma. Leiomyoma are common tumors arising within the uterus that feature excessive deposition of a stiff, disordered   extracellular matrix   (ECM). Mechanical stress is a critical determinant of excessive ECM deposition and increased mechanical stress has been shown to be involved in tumorigenesis. Here we tested the viscoelastic properties of leiomyoma and characterized dynamic and static mechanical signaling in leiomyoma cells using three approaches, including measurement of active RhoA. We found that the peak strain and pseudo-dynamic modulus of leiomyoma tissue was significantly increased relative to matched myometrium. In addition, leiomyoma cells demonstrated an attenuated response to applied cyclic uniaxial strain and to variation in substrate stiffness, relative to myometrial cells. However, on a flexible pronectin-coated silicone substrate, basal levels and lysophosphatidic acid-stimulated levels of activated RhoA were similar between leiomyoma and myometrial cells. In contrast, leiomyoma cells plated on a rigid polystyrene substrate had elevated levels of active RhoA, compared to myometrial cells. The results indicate that viscoelastic properties of the ECM of leiomyoma contribute significantly to the tumor's inherent stiffness and that leiomyoma cells have an attenuated sensitivity to mechanical cues. The findings suggest there may be a fundamental alteration in the communication between the external mechanical environment (extracellular forces) and reorganization of the actin cytoskeleton mediated by RhoA in leiomyoma cells. Additional research will be needed to elucidate the mechanism(s) responsible for the attenuated mechanical signaling in leiomyoma cells. 
 PAR1-mediated RhoA activation facilitates CCL2-induced chemotaxis in PC-3 cells. Patients with advanced prostate cancer often exhibit increased activation of the coagulation system. The key activator of the coagulation cascade is the serine protease thrombin which is capable of eliciting numerous cellular responses. We previously reported that the thrombin receptor PAR1 is overexpressed in prostate cancer. To investigate further the role of PAR1 in prostate cancer metastasis, we examined the effects of thrombin activation on cell adhesion and motility in PC-3 prostate cancer cells. Activation of PAR1-induced dynamic cytoskeletal reorganization and reduced PC-3 binding to collagen I, collagen IV, and laminin (P < 0.01) but not fibronectin. Expression of the cell surface integrin receptors did not change as assessed by flow cytometry. Immunofluorescence microscopy revealed that PAR1 stimulation caused reorganization of the focal adhesions, suggesting that PAR1 activation in PC-3 cells may be modulating cell adhesion through integrin function but not expression. Furthermore, RhoA was activated upon stimulation with thrombin with subsequent cell contraction, decreased cell adhesion, and induced migration towards monocyte chemoattractant protein 1 (MCP-1; CCL2). Thus, it appears that thrombin stimulation plays a role in prostate cancer metastasis by decreasing cell adhesion to the   extracellular matrix   and positioning the cell in a "ready state" for migration in response to a chemotactic signal. Further exploration is needed to determine whether PAR1 activation affects other signaling pathways involved in prostate cancer. 
 A novel function of heparan sulfate in the regulation of cell-cell fusion. Despite the important contribution of cell-cell fusion in the development and physiology of eukaryotes, little is known about the mechanisms that regulate this process. Our study shows that glycosaminoglycans and more specifically heparan sulfate (HS) expressed on the cell surface and   extracellular matrix   may act as negative regulator of cell-cell fusion. Using herpes simplex virus type-1 as a tool to enhance cell-cell fusion, we demonstrate that the absence of HS expression on the cell surface results in a significant increase in cell-cell fusion. An identical phenomenon was observed when other viruses or polyethylene glycol was used as fusion enhancer. Cells deficient in HS biosynthesis showed increased activity of two Rho GTPases, RhoA and Cdc42, both of which showed a correlation between increased activity and increased cell-cell fusion. This could serve as a possible explanation as to why HS-deficient cells showed significantly enhanced cell-cell fusion and suggests that HS could regulate fusion via fine tuning of RhoA and Cdc42 activities. 
 Protein kinase C-related kinase and ROCK are required for thrombin-induced endothelial cell permeability downstream from Galpha12/13 and Galpha11/q. Increase in vascular permeability occurs under many physiological conditions such as wound repair, inflammation, and thrombotic reactions and is central in diverse human pathologies, including tumor-induced angiogenesis, ocular diseases, and septic shock. Thrombin is a pro-coagulant serine protease, which causes the local loss of endothelial barrier integrity thereby enabling the rapid extravasation of plasma proteins and the local formation of fibrin-containing clots. Available information suggests that thrombin induces endothelial permeability by promoting actomyosin contractility through the Rho/ROCK signaling pathway. Here we took advantage of pharmacological inhibitors, knockdown approaches, and the emerging knowledge on how permeability factors affect endothelial junctions to investigate in detail the mechanism underlying thrombin-induced endothelial permeability. We show that thrombin signals through PAR-1 and its coupled G proteins Galpha(12/13) and Galpha(11/q) to induce RhoA activation and intracellular calcium elevation, and that these events are interrelated. In turn, this leads to the stimulation of ROCK, which causes actin stress-fiber formation. However, this alone is not sufficient to account for thrombin-induced permeability. Instead, we found that protein kinase C-related kinase, a Rho-dependent serine/threonine kinase, is activated in endothelial cells upon thrombin stimulation and that its expression is required for endothelial permeability and the remodeling of cell-  extracellular matrix   and cell-cell adhesions. Our results demonstrate that the signal initiated by thrombin bifurcates at the level of RhoA to promote changes in the cytoskeletal architecture through ROCK, and the remodeling of focal adhesion components through protein kinase C-related kinase. Ultimately, both pathways converge to cause cell-cell junction disruption and provoke vascular leakage. 
 Tetraspanin CD151 regulates RhoA activation and the dynamic stability of carcinoma cell-cell contacts. Tetraspanins regulate integrin-dependent tumor cell interactions with the   extracellular matrix  . Here we show that tetraspanin CD151, which plays critical roles in regulating the adhesion and motility of individual tumor cells, is also an important regulator of collective tumor cell migration. Near total silencing of CD151 destabilizes E-cadherin-dependent carcinoma cell-cell junctions and enhances the collective migration of intact tumor cell sheets. This effect does not depend on reduced E-cadherin cell-surface expression or intrinsic adhesivity, or on obvious disruptions in the E-cadherin regulatory complex. Instead, the loss of CD151 causes excessive RhoA activation, loss of actin organization at cell-cell junctions, and increased actin stress fibers at the basal cell surface. Cell-cell contacts within CD151-silenced monolayers display a nearly threefold increase in remodeling rate and a significant reduction in lifespan as compared to cell-cell contacts within wild-type monolayers. CD151 re-expression restores junctional stability, as does acute treatment of CD151-silenced cells with a cell-permeable RhoA inhibitor. However, a CD151 mutant with impaired association with alpha3beta1 integrin fails to restore junctional organization. These data reveal that, in addition to its roles in regulating tumor cell-substrate interactions, CD151 is also an important regulator of the stability of tumor cell-cell interactions, potentially through its interaction with alpha3beta1 integrin. This could help to explain the phenotypes in human patients and mice lacking CD151. 
 RhoA signaling modulates cyclin D1 expression in human lung fibroblasts; implications for idiopathic pulmonary fibrosis. Idiopathic Pulmonary Fibrosis (IPF) is a debilitating disease characterized by exaggerated   extracellular matrix   deposition and aggressive lung structural remodeling. Disease pathogenesis is driven by fibroblastic foci formation, consequent on growth factor overexpression and myofibroblast proliferation. We have previously shown that both CTGF overexpression and myofibroblast formation in IPF cell lines are dependent on RhoA signaling. As RhoA-mediated regulation is also involved in cell cycle progression, we hypothesise that this pathway is key to lung fibroblast turnover through modulation of cyclin D1 kinetic expression.Cyclin D1 expression was compared in primary IPF patient-derived fibroblasts and equivalent normal control cells. Quantitative real time PCR was employed to examine relative expression levels of cyclin D1 mRNA; protein expression was confirmed by western blotting. Effects of Rho signaling were investigated using transient transfection of constitutively active and dominant negative RhoA constructs as well as pharmacological inhibitors. Cellular proliferation of lung fibroblasts was determined by BrdU incorporation ELISA. To further explore RhoA regulation of cyclin D1 in lung fibroblasts and associated cell cycle progression, an established Rho inhibitor, Simvastatin, was incorporated in our studies.Cyclin D1 expression was upregulated in IPF compared to normal lung fibroblasts under exponential growth conditions (p < 0.05). Serum deprivation inhibited cyclin D1 expression, which was restored following treatment with fibrogenic growth factors (TGF-beta1 and CTGF). RhoA inhibition, using a dominant negative mutant and a pharmacological inhibitor (C3 exotoxin), suppressed levels of cyclin D1 mRNA and protein in IPF fibroblasts, with significant abrogation of cell turnover (p < 0.05). Furthermore, Simvastatin dose-dependently inhibited fibroblast cyclin D1 gene and protein expression, inducing G1 cell cycle arrest. Similar trends were observed in control experiments using normal lung fibroblasts, though exhibited responses were lower in magnitude.These findings report for the first time that cyclin D1 expression is deregulated in IPF through a RhoA dependent mechanism that influences lung fibroblast proliferation. This potentially unravels new molecular targets for future anti-IPF strategies; accordingly, Simvastatin inhibition of Rho-mediated cyclin D1 expression in IPF fibroblasts merits further exploitation. 
  [Ii]nvasive  PubMed  
 Activation of Vav/Rho GTPase signaling by CXCL12 controls membrane-type matrix metalloproteinase-dependent melanoma cell invasion. Melanoma cells express the chemokine receptor CXCR4, which confers   invasive   signals on binding to its ligand CXCL12. We show here that knocking down membrane-type matrix metalloproteinase (MT1-MMP) expression translates into a blockade of invasion across reconstituted basement membranes and type I collagen gels in response to CXCL12, which is the result of lack of MMP-2 activation. Interference with MMP-2 expression further confirms its important role during this invasion. Vav proteins are guanine-nucleotide exchange factors for Rho GTPases that regulate actin dynamics and gene expression. We show that melanoma cells express Vav1 and Vav2, which are activated by CXCL12 involving Jak activity. Blocking Vav expression by RNA interference results in impaired activation of Rac and Rho by CXCL12 and in a remarkable inhibition of CXCL12-promoted invasion. Importantly, up-regulation of MT1-MMP expression by CXCL12, a mechanism contributing to melanoma cell invasion, is blocked by knocking down Vav expression or by inhibiting Jak. Together, these data indicate that activation of Jak/Vav/Rho GTPase pathway by CXCL12 is a key signaling event for MT1-MMP/MMP-2-dependent melanoma cell invasion. 
 Protein kinase A gating of a pseudopodial-located RhoA/ROCK/p38/NHE1 signal module regulates invasion in breast cancer cell lines. Metastasis results from a sequence of selective events often involving interactions with elements of the tumor-specific physiological microenvironment. The low-serum component of this microenvironment confers increased motility and invasion in breast cancer cells by activating the Na+/H+ exchanger isoform 1 (NHE1). The present study was undertaken to characterize the signal transduction mechanisms underlying this serum deprivation-dependent activation of both the NHE1 and the concomitant   invasive   characteristics such as leading edge pseudopodia development and penetration of matrigel in breast cancer cell lines representing different stages of metastatic progression. Using pharmacological and genetic manipulation together with transport and kinase activity assays, we observe that the activation of the NHE1 and subsequent invasion by serum deprivation in metastatic human breast cells is coordinated by a sequential RhoA/p160ROCK/p38MAPK signaling pathway gated by direct protein kinase A phosphorylation and inhibition of RhoA. Fluorescence resonance energy transfer imaging of RhoA activity and immunofluorescence analysis of phospho-RhoA and NHE1 show that serum deprivation dynamically remodels the cell, forming long, leading edge pseudopodia and that this signal module is preferentially compartmentalized in these leading edge pseudopodia, suggesting a tight topographic relation of the signaling module to an invasion-specific cell structure. 
 Regulation of CXCR4/AKT-signaling-induced cell invasion and tumor metastasis by RhoA, Rac-1, and Cdc42 in human esophageal cancer. CXC chemokines and their cognate receptors have been implicated wildly in cancer pathogenesis. In the present study, we report a critical cause relationship between CXCR4 expression and tumorigenesis in the setting of human esophageal squamous cell carcinoma (ESCC). In ESCC cells, CXCR4 expression was significantly higher than in human esophageal epithelial cells (HEEC). Reduction of CXCR4 in ESCC cells reduced cell proliferation and invasion in vitro and tumor growth in vivo. Among the potential downstream targets of CXCR4-CXCL12 are RhoA, Rac-1, and Cdc42, which are likely to contribute to the   invasive  ness of ESCC cells. Finally, we found that CXCR4-CXCL12/AKT axis regulates RhoA, Rac-1, and Cdc42 to modulate cell invasion and tumor metastasis. Together, these results demonstrate a role for CXCR4 in ESCC metastasis and progression and suggest potential targets for therapeutic intervention. 
 Increased diacylglycerol kinase ζ expression in human metastatic colon cancer cells augments Rho GTPase activity and contributes to enhanced invasion. Unraveling the signaling pathways responsible for the establishment of a metastatic phenotype in carcinoma cells is critically important for understanding the pathology of cancer. The acquisition of cell motility is a key property of metastatic tumor cells and is a prerequisite for invasion. Rho GTPases regulate actin cytoskeleton reorganization and the cellular responses required for cell motility and invasion. Diacylglycerol kinase ζ (DGKζ), an enzyme that phosphorylates diacylglycerol to yield phosphatidic acid, regulates the activity of the Rho GTPases Rac1 and RhoA. DGKζ mRNA is highly expressed in several different colon cancer cell lines, as well as in colon cancer tissue relative to normal colonic epithelium, and thus may contribute to the metastatic process.To investigate potential roles of DGKζ in cancer metastasis, a cellular, isogenic model of human colorectal cancer metastatic transition was used. DGKζ protein levels, Rac1 and RhoA activity, and PAK phosphorylation were measured in the non-metastatic SW480 adenocarcinoma cell line and its highly metastatic variant, the SW620 line. The effect of DGKζ silencing on Rho GTPase activity and invasion through Matrigel-coated Transwell inserts was studied in SW620   cells. I  nvasiveness was also measured in PC-3 prostate cancer and MDA-MB-231 breast cancer cells depleted of DGKζ.DGKζ protein levels were elevated approximately 3-fold in SW620 cells compared to SW480 cells. There was a concomitant increase in active Rac1 in SW620 cells, as well as substantial increases in the expression and phosphorylation of the Rac1 effector PAK1. Similarly, RhoA activity and expression were increased in SW620 cells. Knockdown of DGKζ expression in SW620 cells by shRNA-mediated silencing significantly reduced Rac1 and RhoA activity and atten  uated th  e invasiveness of SW620 cells in vitro. DGKζ silencing in highly metastatic MDA-MB-231 breast cancer cells and PC-3 prostate cancer cells also significantly attenu  ated the  ir invasiveness.Elevated DGKζ expression contributes to increased Rho GTPase activation and the enhanced motility of metastatic cancer cells. These findings warrant further investigation of the clinical relevance of DGKζ upregulation in colon and other cancers. Interfering with DGKζ function could provide a means of inhibiting invasion and metastasis. 
 A novel protein associated with membrane-type 1 matrix metalloproteinase binds p27(kip1) and regulates RhoA activation, actin remodeling, and matrigel invasion. Pericellular proteolysis by membrane-type 1 matrix metalloproteinase (MT1-MMP) plays a pivotal role in tumor cell invasion. Localization of MT1-MMP at the invasion front of cells, e.g. on lamellipodia and invadopodia, has to be regulated in coordination with reorganization of the actin cytoskeleton. However, little is known about how such invasion-related actin structures are regulated at the sites where MT1-MMP localizes. During analysis of MT1-MMP-associated proteins, we identified a heretofore uncharacterized protein. This protein, which we call p27RF-Rho, enhances activation of RhoA by releasing it from inhibition by p27(kip1) and thereby regulates actin structures. p27(kip1) is a well known cell cycle regulator in the nucleus. In contrast, cytoplasmic p27(kip1) has been demonstrated to bind GDP-RhoA and inhibit GDP-GTP exchange mediated by guanine nucleotide exchange factors. p27RF-Rho binds p27(kip1) and prevents p27(kip1) from binding to RhoA, thereby freeing the latter for activation. Knockdown of p27RF-Rho expression renders cells resistant to RhoA activation stimuli, whereas overexpression of p27RF-Rho sensitizes cells to such stimulation. p27RF-Rho exhibits a punctate distribution in   invasive   human tumor cell lines. Stimulation of the cells with lysophosphatidic acid induces activation of RhoA and induces the formation of punctate actin structures within foci of p27RF-Rho localization. Some of the punctate actin structures co-localize with MT1-MMP and cortactin. Down-regulation of p27RF-Rho prevents both redistribution of actin into the punctate structures and tumor cell invasion. Thus, p27RF-Rho is a new potential target for cancer therapy development. 
 Annexin-A1 controls an ERK-RhoA-NFκB activation loop in breast cancer cells. Wound healing is critical for normal development and pathological processes including cancer cell metastasis. MAPK, Rho-GTPases and NFκB are important regulators of wound healing, but mechanisms for their integration are incompletely understood. Annexin-A1 (ANXA1) is upregulated i  n invasi  ve breast cancer cells resulting in constitutive activation of NFκB. We show here that silencing ANXA1 increases the formation of stress fibers and focal adhesions, which may inhibit wound healing. ANXA1 regulated wound healing is dependent on the activation of ERK1/2. ANXA1 increases the activation of RhoA, which is dependent on ERK activation. Furthermore, active RhoA is important in NF-κB activation, where constitutively active RhoA potentiates NFκB activation, while dominant negative RhoA inhibits NFκB activation in response to CXCL12 stimulation and active MEKK plasmids. These findings establish a central role for ANXA1 in the cell migration through the activation of NFκB, ERK1/2 and RhoA. 
 Blockade of the RhoA-JNK-c-Jun-MMP2 cascade by atorvastatin reduces osteosarcoma cell invasion. Osteosarcoma is characterized by a high malignant and metastatic potential, which points to the need for new therapeutic strategies to prevent cell metastasis. In this study, we show that statin-induced HMG-CoA reductase inhibition reduces cell migration and invasion in human and murine osteosarcoma cells, independently of the genotype. The statin-induced reduction of cell migration and invasion was independent of induction of apoptosis and was geranylgeranylpyrophosphate-dependent. The statin reduced matrix metalloproteinase (MMP) 2, 9, and 14 and TIMP2 expression or activity in invading cells. Forced expression of MMP2 and MMP14 overcame the inhibitory effect of the statin on cell invasion, suggesting a role for these MMPs in   invasive   potential. We also investigated the mechanisms involved in the reduced MMP2 activity and cell invasion. Inhibition of JNK, but not ERK1/2 signaling, reduced MMP2 activity. Pharmacological or constitutive activation of JNK overcame the reduced MMP2 activity and cell invasion induced by the statin. The statin decreased JNK phosphorylation and c-Jun nuclear translocation, suggesting that HMG-CoA reductase inhibition targets the JNK-c-Jun signaling pathway. We showed that mevalonate or geranylgeranylpyrophosphate treatment prevented the statin-induced reduction in JNK phosphorylation, MMP2 activity, and cell invasion. Forced expression of a constitutively active form of RhoA increased JNK phosphorylation and overcame the inhibitory effect of atorvastatin on MMP2 activity and cell invasion. The data establish a link between RhoA, JNK, c-Jun, and MMP2 activity that is functionally involved in the reduction in osteosarcoma cell invasion by the statin. This suggests a novel strategy targeting RhoA-JNK-c-Jun signaling to reduce osteosarcoma cell tumorigenesis. 
 Oncogenic K-Ras down-regulates Rac1 and RhoA activity and enhances migration and invasion of pancreatic carcinoma cells through activation of p38. Activating mutations in the K-ras gene are genetic alterations frequently found in human carcinomas, particularly in pancreatic adenocarcinomas. Mutation of the K-ras gene is thought to be an early and important event in pancreatic tumor initiation, but the precise role of the mutant K-Ras proteins in neoplastic progression is still unknown. In the present study, we have characterized the influence of oncogenic K-Ras on the phenotype and on the signal transduction of epitheloid PANC-1 pancreatic carcinoma cells by generating PANC-1 cell clones, which stably express EGFP(enhanced green fluorescent protein)-K-Ras (V12). EGFP-K-Ras (V12)-expressing cells exhibited a more fibroblastoid cellular phenotype with irregular cell shape and disorganized cytokeratin filaments. Moreover, these cells showed a marked enhancement of their migratory and   invasive   properties. Stable expression of EGFP-K-Ras (V12) down-regulated the activity of Rac1 and RhoA, resulting in reduced subcortical actin filaments and stress fibers, which might contribute to the epithelial dedifferentiation. Characterization of the activity of mitogen-activated protein kinases revealed that EGFP-K-Ras (V12) enhanced the activity of p38, but did not affect the activities of the Raf/MEK/ERK cascade and JNK. While inhibition of either MEK or JNK activity had no effect on EGFP-K-Ras (V12)-induced migration, inhibition of p38 activity markedly reduced EGFP-K-Ras (V12)-induced migration. Collectively, the results suggest that oncogenic K-Ras enhances the malignant phenotype and identify the mitogen-activated protein kinase p38 as a target to inhibit oncogenic K-Ras-induced pancreatic tumor cell migration. 
 A MAPK-Driven Feedback Loop Suppresses Rac Activity to Promote RhoA-Driven Cancer Cell Invasion. Cell migration in 3D microenvironments is fundamental to development, homeostasis and the pathobiology of diseases such as cancer. Rab-coupling protein (RCP) dependent co-trafficking of α5β1 and EGFR1 promotes cancer cell invasion into fibronectin (FN) containing extracellular matrix (ECM), by potentiating EGFR1 signalling at the front o  f invasi  ve cells. This promotes a switch in RhoGTPase signalling to inhibit Rac1 and activate a RhoA-ROCK-Formin homology domain-containing 3 (FHOD3) pathway and generate filopodial actin-spike protrusions which drive invasion. To further understand the signalling network that drives RCP-drive  n invasi  ve migration, we generated a Boolean logical model based on existing network pathways/models, where each node can be interrogated by computational simulation. The model predicted an unanticipated feedback loop, whereby Raf/MEK/ERK signalling maintains suppression of Rac1 by inhibiting the Rac-activating Sos1-Eps8-Abi1 complex, allowing RhoA activity to predominate i  n invasi  ve protrusions. MEK inhibition was sufficient to promote lamellipodia formation and oppose filopodial actin-spike formation, and led to activation of Rac and inactivation of RhoA at the leading edge of cells moving in 3D matrix. Furthermore, MEK inhibition abrogated RCP/α5β1/EGFR1-dri  ven inva  sive migration. However, upon knockdown of Eps8 (to suppress the Sos1-Abi1-Eps8 complex), MEK inhibition had no effect on RhoGTPase activity and did not opp  ose inva  sive migration, suggesting that MEK-ERK signalling suppresses the Rac-activating Sos1-Abi1-Eps8 complex to maintain RhoA activity and promote filopodial actin-spike formation   and inva  sive migration. Our study highlights the predictive potential of mathematical modelling approaches, and demonstrates that a simple intervention (MEK-inhibition) could be of therapeutic benefit in prevent  ing inva  sive migration and metastasis. 
 The NHERF1 PDZ2 domain regulates PKA-RhoA-p38-mediated NHE1 activation and invasion in breast tumor cells. Understanding the signal transduction systems governing invasion is fundamental for the design of therapeutic strategies against metastasis. Na(+)/H(+) exchanger regulatory factor (NHERF1) is a postsynaptic density 95/disc-large/zona occludens (PDZ) domain-containing protein that recruits membrane receptors/transporters and cytoplasmic signaling proteins into functional complexes. NHERF1 expression is altered in breast cancer, but its effective role in mammary carcinogenesis remains undefined. We report here that NHERF1 overexpression in human breast tumor biopsies is associated with metastatic progression, poor prognosis, and hypoxia-inducible factor-1alpha expression. In cultured tumor cells, hypoxia and serum deprivation increase NHERF1 expression, promote the formation of leading-edge pseudopodia, and redistribute NHERF1 to these pseudopodia. This pseudopodial localization of NHERF1 was verified in breast biopsies and in three-dimensional Matrigel culture. Furthermore, serum deprivation and hypoxia stimulate the Na(+)/H(+) exchanger, invasion, and activate a protein kinase A (PKA)-gated RhoA/p38 invasion signal module. Significantly, NHERF1 overexpression was sufficient to induce these morphological and functional changes, and it potentiated their induction by serum deprivation. Functional experiments with truncated and binding groove-mutated PDZ domain constructs demonstrated that NHERF1 regulates these processes through its PDZ2 domain. We conclude that NHERF1 overexpression enhances the   invasive   phenotype in breast cancer cells, both alone and in synergy with exposure to the tumor microenvironment, via the coordination of PKA-gated RhoA/p38 signaling. 
 Inhibition of TMEM45A suppresses proliferation, induces cell cycle arrest and reduces cell invasion in human ovarian cancer cells. The association of TMEM45A with various cancers has been recently reported. However, the biological function of TMEM45A in ovarian cancer remains unclear. The present study aimed to elucidate the role of TMEM45A in regulating the biological behavior of ovarian cancer cells. We compared the expression of TMEM45A between ovarian cancer tissues and normal tissues based on RNA-sequencing data of the ovarian cancer cohort from The Cancer Genome Atlas (TCGA) project and our real-time PCR data from 25 pairs of ovarian cancer and their matched non-cancerous tissue samples. The expression of TMEM45A was then suppressed in two ovarian cancer cell lines, HO-8910 and A2780, by RNA interference. Cell proliferation, cell cycle distribution, adhesion and   invasive   ability were then detected using the Cell Counting Kit-8 assay (CCK-8), propidium iodide (PI) staining, and cell adhesion and Transwell assays, respectively. In addition, the mRNA and protein levels of transforming growth factor-β (TGF-β1 and TGF-β2), Ras homolog family member A (RhoA) and Rho-associated kinase 2 (ROCK2) were detected with real-time PCR and western blotting, respectively. TCGA data and our real-time PCR results demonstrated the overexpression of TMEM45A in ovarian cancer. Silencing of TMEM45A significantly inhibited cell proliferation and significantly increased the cell population in the G1 phase. Moreover, knockdown of TMEM45A also inhibited cell adhesion as well as cell invasion. More importantly, suppression of TMEM45A notably downregulated the expression of TGF-β1, TGF-β2, RhoA and ROCK2. In conclusion, TMEM45A may function as an oncogene for ovarian cancer, and inhibition of TMEM45A may be a therapeutic strategy for ovarian cancer. 
 Rac1, RhoA, and Cdc42 participate in HeLa cell invasion by group B streptococcus. The group B streptococcus (GBS) is an important human pathogen with the ability to cause   invasive   disease. To do so, the bacteria must invade host cells. It has been well documented that GBS are able to invade a variety of nonphagocytic host cell types, and this process is thought to involve a number of pathogen-host cell interactions. While some of the molecular aspects of the GBS-host cell invasion process have been characterized, many events still remain unclear. The objective of this investigation was to evaluate the role of the Rho-family GTPases Rac, Rho, and Cdc42 in GBS invasion into epithelial cells. The epithelial cell invasion process was modeled using HeLa 229 cell culture. Treatment of HeLa cells with 10 microM compactin, a pan-GTPase inhibitor, abolished GBS internalization, suggesting that GTPases are involved in the GBS invasion process. The addition of Toxin B or exoenzyme C3 to HeLa cells before GBS infection reduced invasion by 50%, further suggesting that the Rho-family GTPases are involved in GBS entry. Examining invasion of GBS into HeLa cells with altered genetic backgrounds was used to confirm these findings; GBS invasion into HeLa cells transiently transfected with dominant negative Rac1, Cdc42, or RhoA reduced invasion by 75%, 51%, and 42%, respectively. Results of this study suggest that the Rho-family GTPases are required for efficient invasion of HeLa cells by GBS. 
 The Na+-H+ exchanger-1 induces cytoskeletal changes involving reciprocal RhoA and Rac1 signaling, resulting in motility and invasion in MDA-MB-435 cells. An increasing body of evidence shows that the tumour microenvironment is essential in driving neoplastic progression. The low serum component of this microenvironment stimulates motility/invasion in human breast cancer cells via activation of the Na+-H+ exchanger (NHE) isoform 1, but the signal transduction systems that underlie this process are still poorly understood. We undertook the present study to elucidate the role and pattern of regulation by the Rho GTPases of this serum deprivation-dependent activation of both NHE1 and subsequent   invasive   characteristics, such as pseudopodia and invadiopodia protrusion, directed cell motility and penetration of normal tissues.The present study was performed in a well characterized human mammary epithelial cell line representing late stage metastatic progression, MDA-MB-435. The activity of RhoA and Rac1 was modified using their dominant negative and constitutively active mutants and the activity of NHE1, cell motility/invasion, F-actin content and cell shape were measured.We show for the first time that serum deprivation induces NHE1-dependent morphological and cytoskeletal changes in metastatic cells via a reciprocal interaction of RhoA and Rac1, resulting in increased chemotaxis and invasion. Deprivation changed cell shape by reducing the amount of F-actin and inducing the formation of leading edge pseudopodia. Serum deprivation inhibited RhoA activity and stimulated Rac1 activity. Rac1 and RhoA were antagonistic regulators of both basal and stimulated tumour cell NHE1 activity. The regulation of NHE1 activity by RhoA and Rac1 in both conditions was mediated by an alteration in intracellular proton affinity of the exchanger. Interestingly, the role of each of these G-proteins was reversed during serum deprivation; basal NHE1 activity was regulated positively by RhoA and negatively by Rac1, whereas RhoA negatively and Rac1 positively directed the stimulation of NHE1 during serum deprivation. Importantly, the same pattern of RhoA and Rac1 regulation found for NHE1 activity was observed in both basal and serum deprivation dependent increases in motility, invasion and actin cytoskeletal organization.Our findings suggest that the reported antagonistic roles of RhoA and Rac1 in cell motility/invasion and cytoskeletal organization may be due, in part, to their concerted action on NHE1 activity as a convergence point. 
 A MAPK-Driven Feedback Loop Suppresses Rac Activity to Promote RhoA-Driven Cancer Cell Invasion. Cell migration in 3D microenvironments is fundamental to development, homeostasis and the pathobiology of diseases such as cancer. Rab-coupling protein (RCP) dependent co-trafficking of α5β1 and EGFR1 promotes cancer cell invasion into fibronectin (FN) containing extracellular matrix (ECM), by potentiating EGFR1 signalling at the front o  f invasi  ve cells. This promotes a switch in RhoGTPase signalling to inhibit Rac1 and activate a RhoA-ROCK-Formin homology domain-containing 3 (FHOD3) pathway and generate filopodial actin-spike protrusions which drive invasion. To further understand the signalling network that drives RCP-drive  n invasi  ve migration, we generated a Boolean logical model based on existing network pathways/models, where each node can be interrogated by computational simulation. The model predicted an unanticipated feedback loop, whereby Raf/MEK/ERK signalling maintains suppression of Rac1 by inhibiting the Rac-activating Sos1-Eps8-Abi1 complex, allowing RhoA activity to predominate i  n invasi  ve protrusions. MEK inhibition was sufficient to promote lamellipodia formation and oppose filopodial actin-spike formation, and led to activation of Rac and inactivation of RhoA at the leading edge of cells moving in 3D matrix. Furthermore, MEK inhibition abrogated RCP/α5β1/EGFR1-dri  ven inva  sive migration. However, upon knockdown of Eps8 (to suppress the Sos1-Abi1-Eps8 complex), MEK inhibition had no effect on RhoGTPase activity and did not opp  ose inva  sive migration, suggesting that MEK-ERK signalling suppresses the Rac-activating Sos1-Abi1-Eps8 complex to maintain RhoA activity and promote filopodial actin-spike formation   and inva  sive migration. Our study highlights the predictive potential of mathematical modelling approaches, and demonstrates that a simple intervention (MEK-inhibition) could be of therapeutic benefit in prevent  ing inva  sive migration and metastasis. 
 Regulation of CXCR4/AKT-signaling-induced cell invasion and tumor metastasis by RhoA, Rac-1, and Cdc42 in human esophageal cancer. CXC chemokines and their cognate receptors have been implicated wildly in cancer pathogenesis. In the present study, we report a critical cause relationship between CXCR4 expression and tumorigenesis in the setting of human esophageal squamous cell carcinoma (ESCC). In ESCC cells, CXCR4 expression was significantly higher than in human esophageal epithelial cells (HEEC). Reduction of CXCR4 in ESCC cells reduced cell proliferation and invasion in vitro and tumor growth in vivo. Among the potential downstream targets of CXCR4-CXCL12 are RhoA, Rac-1, and Cdc42, which are likely to contribute to the   invasive  ness of ESCC cells. Finally, we found that CXCR4-CXCL12/AKT axis regulates RhoA, Rac-1, and Cdc42 to modulate cell invasion and tumor metastasis. Together, these results demonstrate a role for CXCR4 in ESCC metastasis and progression and suggest potential targets for therapeutic intervention. 
 Inhibition of TMEM45A suppresses proliferation, induces cell cycle arrest and reduces cell invasion in human ovarian cancer cells. The association of TMEM45A with various cancers has been recently reported. However, the biological function of TMEM45A in ovarian cancer remains unclear. The present study aimed to elucidate the role of TMEM45A in regulating the biological behavior of ovarian cancer cells. We compared the expression of TMEM45A between ovarian cancer tissues and normal tissues based on RNA-sequencing data of the ovarian cancer cohort from The Cancer Genome Atlas (TCGA) project and our real-time PCR data from 25 pairs of ovarian cancer and their matched non-cancerous tissue samples. The expression of TMEM45A was then suppressed in two ovarian cancer cell lines, HO-8910 and A2780, by RNA interference. Cell proliferation, cell cycle distribution, adhesion and   invasive   ability were then detected using the Cell Counting Kit-8 assay (CCK-8), propidium iodide (PI) staining, and cell adhesion and Transwell assays, respectively. In addition, the mRNA and protein levels of transforming growth factor-β (TGF-β1 and TGF-β2), Ras homolog family member A (RhoA) and Rho-associated kinase 2 (ROCK2) were detected with real-time PCR and western blotting, respectively. TCGA data and our real-time PCR results demonstrated the overexpression of TMEM45A in ovarian cancer. Silencing of TMEM45A significantly inhibited cell proliferation and significantly increased the cell population in the G1 phase. Moreover, knockdown of TMEM45A also inhibited cell adhesion as well as cell invasion. More importantly, suppression of TMEM45A notably downregulated the expression of TGF-β1, TGF-β2, RhoA and ROCK2. In conclusion, TMEM45A may function as an oncogene for ovarian cancer, and inhibition of TMEM45A may be a therapeutic strategy for ovarian cancer. 
 Annexin-A1 controls an ERK-RhoA-NFκB activation loop in breast cancer cells. Wound healing is critical for normal development and pathological processes including cancer cell metastasis. MAPK, Rho-GTPases and NFκB are important regulators of wound healing, but mechanisms for their integration are incompletely understood. Annexin-A1 (ANXA1) is upregulated i  n invasi  ve breast cancer cells resulting in constitutive activation of NFκB. We show here that silencing ANXA1 increases the formation of stress fibers and focal adhesions, which may inhibit wound healing. ANXA1 regulated wound healing is dependent on the activation of ERK1/2. ANXA1 increases the activation of RhoA, which is dependent on ERK activation. Furthermore, active RhoA is important in NF-κB activation, where constitutively active RhoA potentiates NFκB activation, while dominant negative RhoA inhibits NFκB activation in response to CXCL12 stimulation and active MEKK plasmids. These findings establish a central role for ANXA1 in the cell migration through the activation of NFκB, ERK1/2 and RhoA. 
 Increased diacylglycerol kinase ζ expression in human metastatic colon cancer cells augments Rho GTPase activity and contributes to enhanced invasion. Unraveling the signaling pathways responsible for the establishment of a metastatic phenotype in carcinoma cells is critically important for understanding the pathology of cancer. The acquisition of cell motility is a key property of metastatic tumor cells and is a prerequisite for invasion. Rho GTPases regulate actin cytoskeleton reorganization and the cellular responses required for cell motility and invasion. Diacylglycerol kinase ζ (DGKζ), an enzyme that phosphorylates diacylglycerol to yield phosphatidic acid, regulates the activity of the Rho GTPases Rac1 and RhoA. DGKζ mRNA is highly expressed in several different colon cancer cell lines, as well as in colon cancer tissue relative to normal colonic epithelium, and thus may contribute to the metastatic process.To investigate potential roles of DGKζ in cancer metastasis, a cellular, isogenic model of human colorectal cancer metastatic transition was used. DGKζ protein levels, Rac1 and RhoA activity, and PAK phosphorylation were measured in the non-metastatic SW480 adenocarcinoma cell line and its highly metastatic variant, the SW620 line. The effect of DGKζ silencing on Rho GTPase activity and invasion through Matrigel-coated Transwell inserts was studied in SW620   cells. I  nvasiveness was also measured in PC-3 prostate cancer and MDA-MB-231 breast cancer cells depleted of DGKζ.DGKζ protein levels were elevated approximately 3-fold in SW620 cells compared to SW480 cells. There was a concomitant increase in active Rac1 in SW620 cells, as well as substantial increases in the expression and phosphorylation of the Rac1 effector PAK1. Similarly, RhoA activity and expression were increased in SW620 cells. Knockdown of DGKζ expression in SW620 cells by shRNA-mediated silencing significantly reduced Rac1 and RhoA activity and atten  uated th  e invasiveness of SW620 cells in vitro. DGKζ silencing in highly metastatic MDA-MB-231 breast cancer cells and PC-3 prostate cancer cells also significantly attenu  ated the  ir invasiveness.Elevated DGKζ expression contributes to increased Rho GTPase activation and the enhanced motility of metastatic cancer cells. These findings warrant further investigation of the clinical relevance of DGKζ upregulation in colon and other cancers. Interfering with DGKζ function could provide a means of inhibiting invasion and metastasis. 
 A novel protein associated with membrane-type 1 matrix metalloproteinase binds p27(kip1) and regulates RhoA activation, actin remodeling, and matrigel invasion. Pericellular proteolysis by membrane-type 1 matrix metalloproteinase (MT1-MMP) plays a pivotal role in tumor cell invasion. Localization of MT1-MMP at the invasion front of cells, e.g. on lamellipodia and invadopodia, has to be regulated in coordination with reorganization of the actin cytoskeleton. However, little is known about how such invasion-related actin structures are regulated at the sites where MT1-MMP localizes. During analysis of MT1-MMP-associated proteins, we identified a heretofore uncharacterized protein. This protein, which we call p27RF-Rho, enhances activation of RhoA by releasing it from inhibition by p27(kip1) and thereby regulates actin structures. p27(kip1) is a well known cell cycle regulator in the nucleus. In contrast, cytoplasmic p27(kip1) has been demonstrated to bind GDP-RhoA and inhibit GDP-GTP exchange mediated by guanine nucleotide exchange factors. p27RF-Rho binds p27(kip1) and prevents p27(kip1) from binding to RhoA, thereby freeing the latter for activation. Knockdown of p27RF-Rho expression renders cells resistant to RhoA activation stimuli, whereas overexpression of p27RF-Rho sensitizes cells to such stimulation. p27RF-Rho exhibits a punctate distribution in   invasive   human tumor cell lines. Stimulation of the cells with lysophosphatidic acid induces activation of RhoA and induces the formation of punctate actin structures within foci of p27RF-Rho localization. Some of the punctate actin structures co-localize with MT1-MMP and cortactin. Down-regulation of p27RF-Rho prevents both redistribution of actin into the punctate structures and tumor cell invasion. Thus, p27RF-Rho is a new potential target for cancer therapy development. 
 Blockade of the RhoA-JNK-c-Jun-MMP2 cascade by atorvastatin reduces osteosarcoma cell invasion. Osteosarcoma is characterized by a high malignant and metastatic potential, which points to the need for new therapeutic strategies to prevent cell metastasis. In this study, we show that statin-induced HMG-CoA reductase inhibition reduces cell migration and invasion in human and murine osteosarcoma cells, independently of the genotype. The statin-induced reduction of cell migration and invasion was independent of induction of apoptosis and was geranylgeranylpyrophosphate-dependent. The statin reduced matrix metalloproteinase (MMP) 2, 9, and 14 and TIMP2 expression or activity in invading cells. Forced expression of MMP2 and MMP14 overcame the inhibitory effect of the statin on cell invasion, suggesting a role for these MMPs in   invasive   potential. We also investigated the mechanisms involved in the reduced MMP2 activity and cell invasion. Inhibition of JNK, but not ERK1/2 signaling, reduced MMP2 activity. Pharmacological or constitutive activation of JNK overcame the reduced MMP2 activity and cell invasion induced by the statin. The statin decreased JNK phosphorylation and c-Jun nuclear translocation, suggesting that HMG-CoA reductase inhibition targets the JNK-c-Jun signaling pathway. We showed that mevalonate or geranylgeranylpyrophosphate treatment prevented the statin-induced reduction in JNK phosphorylation, MMP2 activity, and cell invasion. Forced expression of a constitutively active form of RhoA increased JNK phosphorylation and overcame the inhibitory effect of atorvastatin on MMP2 activity and cell invasion. The data establish a link between RhoA, JNK, c-Jun, and MMP2 activity that is functionally involved in the reduction in osteosarcoma cell invasion by the statin. This suggests a novel strategy targeting RhoA-JNK-c-Jun signaling to reduce osteosarcoma cell tumorigenesis. 
 Rac1, RhoA, and Cdc42 participate in HeLa cell invasion by group B streptococcus. The group B streptococcus (GBS) is an important human pathogen with the ability to cause   invasive   disease. To do so, the bacteria must invade host cells. It has been well documented that GBS are able to invade a variety of nonphagocytic host cell types, and this process is thought to involve a number of pathogen-host cell interactions. While some of the molecular aspects of the GBS-host cell invasion process have been characterized, many events still remain unclear. The objective of this investigation was to evaluate the role of the Rho-family GTPases Rac, Rho, and Cdc42 in GBS invasion into epithelial cells. The epithelial cell invasion process was modeled using HeLa 229 cell culture. Treatment of HeLa cells with 10 microM compactin, a pan-GTPase inhibitor, abolished GBS internalization, suggesting that GTPases are involved in the GBS invasion process. The addition of Toxin B or exoenzyme C3 to HeLa cells before GBS infection reduced invasion by 50%, further suggesting that the Rho-family GTPases are involved in GBS entry. Examining invasion of GBS into HeLa cells with altered genetic backgrounds was used to confirm these findings; GBS invasion into HeLa cells transiently transfected with dominant negative Rac1, Cdc42, or RhoA reduced invasion by 75%, 51%, and 42%, respectively. Results of this study suggest that the Rho-family GTPases are required for efficient invasion of HeLa cells by GBS. 
 Oncogenic K-Ras down-regulates Rac1 and RhoA activity and enhances migration and invasion of pancreatic carcinoma cells through activation of p38. Activating mutations in the K-ras gene are genetic alterations frequently found in human carcinomas, particularly in pancreatic adenocarcinomas. Mutation of the K-ras gene is thought to be an early and important event in pancreatic tumor initiation, but the precise role of the mutant K-Ras proteins in neoplastic progression is still unknown. In the present study, we have characterized the influence of oncogenic K-Ras on the phenotype and on the signal transduction of epitheloid PANC-1 pancreatic carcinoma cells by generating PANC-1 cell clones, which stably express EGFP(enhanced green fluorescent protein)-K-Ras (V12). EGFP-K-Ras (V12)-expressing cells exhibited a more fibroblastoid cellular phenotype with irregular cell shape and disorganized cytokeratin filaments. Moreover, these cells showed a marked enhancement of their migratory and   invasive   properties. Stable expression of EGFP-K-Ras (V12) down-regulated the activity of Rac1 and RhoA, resulting in reduced subcortical actin filaments and stress fibers, which might contribute to the epithelial dedifferentiation. Characterization of the activity of mitogen-activated protein kinases revealed that EGFP-K-Ras (V12) enhanced the activity of p38, but did not affect the activities of the Raf/MEK/ERK cascade and JNK. While inhibition of either MEK or JNK activity had no effect on EGFP-K-Ras (V12)-induced migration, inhibition of p38 activity markedly reduced EGFP-K-Ras (V12)-induced migration. Collectively, the results suggest that oncogenic K-Ras enhances the malignant phenotype and identify the mitogen-activated protein kinase p38 as a target to inhibit oncogenic K-Ras-induced pancreatic tumor cell migration. 
 The Na+-H+ exchanger-1 induces cytoskeletal changes involving reciprocal RhoA and Rac1 signaling, resulting in motility and invasion in MDA-MB-435 cells. An increasing body of evidence shows that the tumour microenvironment is essential in driving neoplastic progression. The low serum component of this microenvironment stimulates motility/invasion in human breast cancer cells via activation of the Na+-H+ exchanger (NHE) isoform 1, but the signal transduction systems that underlie this process are still poorly understood. We undertook the present study to elucidate the role and pattern of regulation by the Rho GTPases of this serum deprivation-dependent activation of both NHE1 and subsequent   invasive   characteristics, such as pseudopodia and invadiopodia protrusion, directed cell motility and penetration of normal tissues.The present study was performed in a well characterized human mammary epithelial cell line representing late stage metastatic progression, MDA-MB-435. The activity of RhoA and Rac1 was modified using their dominant negative and constitutively active mutants and the activity of NHE1, cell motility/invasion, F-actin content and cell shape were measured.We show for the first time that serum deprivation induces NHE1-dependent morphological and cytoskeletal changes in metastatic cells via a reciprocal interaction of RhoA and Rac1, resulting in increased chemotaxis and invasion. Deprivation changed cell shape by reducing the amount of F-actin and inducing the formation of leading edge pseudopodia. Serum deprivation inhibited RhoA activity and stimulated Rac1 activity. Rac1 and RhoA were antagonistic regulators of both basal and stimulated tumour cell NHE1 activity. The regulation of NHE1 activity by RhoA and Rac1 in both conditions was mediated by an alteration in intracellular proton affinity of the exchanger. Interestingly, the role of each of these G-proteins was reversed during serum deprivation; basal NHE1 activity was regulated positively by RhoA and negatively by Rac1, whereas RhoA negatively and Rac1 positively directed the stimulation of NHE1 during serum deprivation. Importantly, the same pattern of RhoA and Rac1 regulation found for NHE1 activity was observed in both basal and serum deprivation dependent increases in motility, invasion and actin cytoskeletal organization.Our findings suggest that the reported antagonistic roles of RhoA and Rac1 in cell motility/invasion and cytoskeletal organization may be due, in part, to their concerted action on NHE1 activity as a convergence point. 
 The NHERF1 PDZ2 domain regulates PKA-RhoA-p38-mediated NHE1 activation and invasion in breast tumor cells. Understanding the signal transduction systems governing invasion is fundamental for the design of therapeutic strategies against metastasis. Na(+)/H(+) exchanger regulatory factor (NHERF1) is a postsynaptic density 95/disc-large/zona occludens (PDZ) domain-containing protein that recruits membrane receptors/transporters and cytoplasmic signaling proteins into functional complexes. NHERF1 expression is altered in breast cancer, but its effective role in mammary carcinogenesis remains undefined. We report here that NHERF1 overexpression in human breast tumor biopsies is associated with metastatic progression, poor prognosis, and hypoxia-inducible factor-1alpha expression. In cultured tumor cells, hypoxia and serum deprivation increase NHERF1 expression, promote the formation of leading-edge pseudopodia, and redistribute NHERF1 to these pseudopodia. This pseudopodial localization of NHERF1 was verified in breast biopsies and in three-dimensional Matrigel culture. Furthermore, serum deprivation and hypoxia stimulate the Na(+)/H(+) exchanger, invasion, and activate a protein kinase A (PKA)-gated RhoA/p38 invasion signal module. Significantly, NHERF1 overexpression was sufficient to induce these morphological and functional changes, and it potentiated their induction by serum deprivation. Functional experiments with truncated and binding groove-mutated PDZ domain constructs demonstrated that NHERF1 regulates these processes through its PDZ2 domain. We conclude that NHERF1 overexpression enhances the   invasive   phenotype in breast cancer cells, both alone and in synergy with exposure to the tumor microenvironment, via the coordination of PKA-gated RhoA/p38 signaling. 
 Protein kinase A gating of a pseudopodial-located RhoA/ROCK/p38/NHE1 signal module regulates invasion in breast cancer cell lines. Metastasis results from a sequence of selective events often involving interactions with elements of the tumor-specific physiological microenvironment. The low-serum component of this microenvironment confers increased motility and invasion in breast cancer cells by activating the Na+/H+ exchanger isoform 1 (NHE1). The present study was undertaken to characterize the signal transduction mechanisms underlying this serum deprivation-dependent activation of both the NHE1 and the concomitant   invasive   characteristics such as leading edge pseudopodia development and penetration of matrigel in breast cancer cell lines representing different stages of metastatic progression. Using pharmacological and genetic manipulation together with transport and kinase activity assays, we observe that the activation of the NHE1 and subsequent invasion by serum deprivation in metastatic human breast cells is coordinated by a sequential RhoA/p160ROCK/p38MAPK signaling pathway gated by direct protein kinase A phosphorylation and inhibition of RhoA. Fluorescence resonance energy transfer imaging of RhoA activity and immunofluorescence analysis of phospho-RhoA and NHE1 show that serum deprivation dynamically remodels the cell, forming long, leading edge pseudopodia and that this signal module is preferentially compartmentalized in these leading edge pseudopodia, suggesting a tight topographic relation of the signaling module to an invasion-specific cell structure. 
 Roles of Rho guanosine 5'-triphosphatase A, Rho kinases, and extracellular signal regulated kinase (1/2) in prostaglandin E2-mediated migration of first-trimester human extravillous trophoblast. Prostaglandin (PG) E(2) may regulate   invasive  ness of human placenta because we previously reported stimulation of migration of placental trophoblasts by PGE(2) acting through PGE receptor (EP)-1 and activating calpain. RhoA GTPase and its important effector Rho kinase (ROCK) have also been previously shown to regulate trophoblast migration. Using immortalized HTR-8/SVneo trophoblast cells and first-trimester human chorionic villus explant cultures on matrigel, we further examined the role of RhoA/ROCK and MAPK (ERK1/2) pathways on PGE(2)-mediated stimulation of trophoblast migration. Migration of cytotrophoblasts was shown to be inhibited by treatment of the trophoblast cell line and chorionic villus explants with either cell-permeable C3 transferase or selective RhoA small interfering RNA. These inhibitions were significantly mitigated by the addition of PGE(2), an EP1/EP3 agonist or an EP3/EP4 agonist, suggesting that RhoA plays an important role in trophoblast migration but may not be obligatory for PGE(2) action. Treatment of HTR-8/SVneo cells with nonselective ROCK inhibitor Y27632 or ROCK small interfering RNAs inhibited migration of these cells, which could not be rescued with PGE(2) or the other two EP agonists, suggesting the obligatory role of ROCK in PGE(2)-induced migratory response. Furthermore, U0126, an inhibitor of MAPK kinases MEK1 and MEK2, abrogated PGE(2)-induced migration of trophoblasts, and PGE(2) or the other two EP agonists stimulated ERK1/2 activation in trophoblasts, which was not abrogated by pretreatment with C3 transferase, indicating that ERK signaling pathway is an efficient alternate pathway for RhoA in PGE(2)-mediated migration of trophoblasts. These results suggest that ROCK and ERK1/2 play more important roles than RhoA in PGE(2)-mediated migration stimulation of first-trimester trophoblasts. 
 Cortactin modulates RhoA activation and expression of Cip/Kip cyclin-dependent kinase inhibitors to promote cell cycle progression in 11q13-amplified head and neck squamous cell carcinoma cells. The cortactin oncoprotein is frequently overexpressed in head and neck squamous cell carcinoma (HNSCC), often due to amplification of the encoding gene (CTTN). While cortactin overexpression enhances   invasive   potential, recent research indicates that it also promotes cell proliferation, but how cortactin regulates the cell cycle machinery is unclear. In this article we report that stable short hairpin RNA-mediated cortactin knockdown in the 11q13-amplified cell line FaDu led to increased expression of the Cip/Kip cyclin-dependent kinase inhibitors (CDKIs) p21(WAF1/Cip1), p27(Kip1), and p57(Kip2) and inhibition of S-phase entry. These effects were associated with increased binding of p21(WAF1/Cip1) and p27(Kip1) to cyclin D1- and E1-containing complexes and decreased retinoblastoma protein phosphorylation. Cortactin regulated expression of p21(WAF1/Cip1) and p27(Kip1) at the transcriptional and posttranscriptional levels, respectively. The direct roles of p21(WAF1/Cip1), p27(Kip1), and p57(Kip2) downstream of cortactin were confirmed by the transient knockdown of each CDKI by specific small interfering RNAs, which led to partial rescue of cell cycle progression. Interestingly, FaDu cells with reduced cortactin levels also exhibited a significant diminution in RhoA expression and activity, together with decreased expression of Skp2, a critical component of the SCF ubiquitin ligase that targets p27(Kip1) and p57(Kip2) for degradation. Transient knockdown of RhoA in FaDu cells decreased expression of Skp2, enhanced the level of Cip/Kip CDKIs, and attenuated S-phase entry. These findings identify a novel mechanism for regulation of proliferation in 11q13-amplified HNSCC cells, in which overexpressed cortactin acts via RhoA to decrease expression of Cip/Kip CDKIs, and highlight Skp2 as a downstream effector for RhoA in this process. 
 Smurf1 regulates tumor cell plasticity and motility through degradation of RhoA leading to localized inhibition of contractility. Rho GTPases participate in various cellular processes, including normal and tumor cell migration. It has been reported that RhoA is targeted for degradation at the leading edge of migrating cells by the E3 ubiquitin ligase Smurf1, and that this is required for the formation of protrusions. We report that Smurf1-dependent RhoA degradation in tumor cells results in the down-regulation of Rho kinase (ROCK) activity and myosin light chain 2 (MLC2) phosphorylation at the cell periphery. The localized inhibition of contractile forces is necessary for the formation of lamellipodia and for tumor cell motility in 2D tissue culture assays. In 3D invasion assays, and in in vivo tumor cell migration, the inhibition of Smurf1 induces a mesenchymal-amoeboid-like transition that is associated with a more   invasive   phenotype. Our results suggest that Smurf1 is a pivotal regulator of tumor cell movement through its regulation of RhoA signaling. 
 PRL-3 facilitates angiogenesis and metastasis by increasing ERK phosphorylation and up-regulating the levels and activities of Rho-A/C in lung cancer. The aim of this study was to investigate the mechanism of PRL-3 in inducing angiogenesis and lymphangiogenesis to promote distant and lymph node metastasis in human lung cancer tissues and cells.We investigated the expression of PRL-3, VEGF, and VEGF-C from 94 patients with non-small cell lung cancer (NSCLC) using immunohistochemical staining. The relationship between PRL-3 expression and microvessel density (MVD), lymphatic vessel density (LVD), clinicopathological factors, and surgical treatment outcome was also studied. Following this, we studied the effect on A549 by blocking PRL-3.PRL-3 expression in NSCLC was high, and this over-expression is correlated with advanced clinical stage (p = 0.019), distant metastasis (p = 0.001), lymph node metastasis (p = 0.001), and poor post-operative survival. PRL-3 over-expression was associated with vascular endothelial growth factor (VEGF; p = 0.000) and VEGF-C (p = 0.008) expressions, MVD and LVD (p = 0.000 and p = 0.000). Blocking PRL-3 expression in A549 cell resulted in decreased cellular proliferative, migratory, and   invasive   abilities, and VEGF, VEGF-C, pERK, Rho-A, and Rho-C expression was inhibited. Following inhibition of either Rho or pERK, VEGF expression was down-regulated.PRL-3 induces microvascular and lymphatic vessel formation by facilitating VEGF and VEGF-C expression in lung cancer tissues, thus promoting distant and lymph node metastasis of lung cancer. PRL-3 up-regulates pERK and Rho expression and activity, facilitating VEGF expression, and accelerating angiogenesis and distant metastasis. How to regulate VEGF-C expression needs to be further studied. 
 RhoGDIα-dependent balance between RhoA and RhoC is a key regulator of cancer cell tumorigenesis. RhoGTPases are key signaling molecules regulating main cellular functions such as migration, proliferation, survival, and gene expression through interactions with various effectors. Within the RhoA-related subclass, RhoA and RhoC contribute to several steps of tumor growth, and the regulation of their expression affects cancer progression. Our aim is to investigate their respective contributions to the acquisition of an   invasiv  e phenotype by using models of reduced or forced expression. The silencing of RhoC, but not of RhoA, increased the expression of genes encoding tumor suppressors, such as nonsteroidal anti-inflammatory drug-activated gene 1 (NAG-1), and decreased migration and the anchorage-independent growth in vitro. In vivo, RhoC small interfering RNA (siRhoC) impaired tumor growth. Of interest, the simultaneous knockdown of RhoC and NAG-1 repressed most of the siRhoC-related effects, demonstrating the central role of NAG-1. In addition of being induced by RhoC silencing, NAG-1 was also largely up-regulated in cells overexpressing RhoA. The silencing of RhoGDP dissociation inhibitor α (RhoGDIα) and the overexpression of a RhoA mutant unable to bind RhoGDIα suggested that the effect of RhoC silencing is indirect and results from the up-regulation of the RhoA level through competition for RhoGDIα. This study demonstrates the dynamic balance inside the RhoGTPase network and illustrates its biological relevance in cancer progression. 
 Therapeutic IMC-C225 antibody inhibits breast cancer cell   invasive  ness via Vav2-dependent activation of RhoA GTPase. Abnormalities in the expression and signaling pathways downstream of epidermal growth factor receptor (EGFR) contribute to progression, invasion, and maintenance of the malignant phenotype in human cancers. Accordingly, biological agents, such as the EGFR-blocking antibody IMC-C225 have promising anticancer potential and are currently in various stages of clinical development. Because use of IMC-C225 is limited, at present, only for treatment of cancer with high EGFR expression, the goal of the present study was to determine the effect of IMC-C225 on the   invasive  ness of breast cancer cells with high and low levels of EGFR expression.The effect of IMC-C225 on invasion was studied using breast cancer cell lines with high and low levels of EGFR expression.The addition of EGF led to progressive stress fiber dissolution. In contrast, cells treated with IMC-C225 showed reduced   invasive  ness and increased stress-fiber formation. Interestingly, IMC-C225 pretreatment was accompanied by EGFR phosphorylation, as detected using an anti-phosphorylated tyrosine antibody (PY99), which correlated with phosphorylation of Vav2 guanine nucleotide exchange factor and activation of RhoA GTPase irrespective of EGFR level, and Vav2 interacted with EGFR only in IMC-C225-treated cells. The underlying mechanism involved an enhanced interaction between beta1 integrins and EGFR upon IMC-C225 treatment.Here, we defined a new mechanism for IMC-C225 that cross-links integrins with EGFR, leading to activation of RhoA and inhibition of breast cancer cell invasion irrespective of the level of EGFR in the cells, thus providing a rationale for using IMC-C225 in the metastatic setting independent of the levels of EGFR. 
 Targeted disruption of protein kinase C epsilon reduces cell invasion and motility through inactivation of RhoA and RhoC GTPases in head and neck squamous cell carcinoma. Over 70% of patients with head and neck squamous cell carcinoma (HNSCC) present with locoregionally advanced stage III and IV disease. In spite of aggressive therapy, locoregional disease recurs in 60% and metastatic disease develops in 15% to 25% of patients causing a major decline in quality and length of life. Therefore, there is a need to identify and understand genes that are responsible for inducing an aggressive HNSCC phenotype. Evidence has shown that protein kinase C (PKC) epsilon is a transforming oncogene and may play a role in HNSCC progression. In this study, we determine the downstream signaling pathway mediated by PKC epsilon to promote an aggressive HNSCC phenotype. RNA interference knockdown of PKC epsilon in UMSCC11A and UMSCC36, two highly   invasive   and motile HNSCC cell lines with elevated endogenous PKC epsilon levels, resulted in cells that were significantly less   invasive   and motile than the small interfering RNA-scrambled control transfectants; 51 +/- 5% (P < 0.006) and 49 +/- 3% (P < 0.010) inhibition in invasion and 69 +/- 1% (P < 0.0005) and 66 +/- 3% (P < 0.0001) inhibition in motility, respectively. PKC epsilon-deficient UMSCC11A clones had reduced levels of active and serine-phosphorylated RhoA and RhoC. Moreover, constitutive active RhoA completely rescued the invasion and motility defect, whereas constitutive active RhoC completely rescued the invasion and partially rescued the motility defect of PKC epsilon-deficient UMSCC11A clones. These results indicate that RhoA and RhoC are downstream of PKC epsilon and critical for PKC epsilon-mediated cell invasion and motility. Our study shows, for the first time, that PKC epsilon is involved in a coordinated regulation of RhoA and RhoC activation, possibly through direct post-translational phosphorylation. 
 Overexpression of E-cadherin on melanoma cells inhibits chemokine-promoted invasion involving p190RhoGAP/p120ctn-dependent inactivation of RhoA. Melanoma cells express the chemokine receptor CXCR4 that confers high   invasive  ness upon binding to its ligand CXCL12. Melanoma cells at initial stages of the disease show reduction or loss of E-cadherin expression, but recovery of its expression is frequently found at advanced phases. We overexpressed E-cadherin in the highly   invasive   BRO lung metastatic cell melanoma cell line to investigate whether it could influence CXCL12-promoted cell invasion. Overexpression of E-cadherin led to defective invasion of melanoma cells across Matrigel and type I collagen in response to CXCL12. A decrease in individual cell migration directionality toward the chemokine and reduced adhesion accounted for the impaired invasion. A p190RhoGAP-dependent inhibition of RhoA activation was responsible for the impairment in chemokine-stimulated E-cadherin melanoma transfectant invasion. Furthermore, we show that p190RhoGAP and p120ctn associated predominantly on the plasma membrane of cells overexpressing E-cadherin, and that E-cadherin-bound p120ctn contributed to RhoA inactivation by favoring p190RhoGAP-RhoA association. These results suggest that melanoma cells at advanced stages of the disease could have reduced metastatic potency in response to chemotactic stimuli compared with cells lacking E-cadherin, and the results indicate that p190RhoGAP is a central molecule controlling melanoma cell invasion. 
 ODAM inhibits RhoA-dependent invasion in breast cancer. Odontogenic ameloblast-associated protein (ODAM) contributes to cell adhesion. In human cancer, ODAM is down-regulated, and the overexpression of ODAM results in a favourable prognosis; however, the molecular mechanisms underlying ODAM-mediated inhibition of cancer invasion and metastasis remain unclear. Here, we identify a critical role for ODAM in inducing cancer cell adhesion. ODAM induced RhoA activity and the expression of downstream factors, including Rho-associated kinase (ROCK). ODAM-mediated RhoA signalling resulted in actin filament rearrangement by activating PTEN and inhibiting the phosphorylation of AKT. When ODAM is overexpressed in MCF7 breast cancer cells and AGS gastric cancer cells that activate RhoA at high levels, it decreases motility, increases adhesion and inhibits the metastasis of MCF7 cells. Conversely, depletion of ODAM in cancer cells inhibits Rho GTPase activation, resulting in increased cancer migration and invasion. These results suggest that ODAM expression in cells maintains their adhesion, resulting in the prevention of their metastasis via the regulation of RhoA signalling in breast cancer cells. SIGNIFICANCE Breast cancer represents the first most frequent cancer, and the ratio of mortality is high in women. Of utmost importance for reducing risk by breast cancer are their anti-invasion mechanisms, particularly in the non-  invasive   cancer cells because metastasis is the principal cause of death among cancer patients. ODAM induced RhoA activity. ODAM-mediated RhoA signalling resulted in actin filament rearrangement, increased cell adhesion and inhibited the migration/invasion of MCF7 cells. These results suggest that ODAM expression maintains their adhesion, resulting in the prevention of their metastasis via the regulation of RhoA signalling in breast cancer cells. 
 S100B Protein Regulates Astrocyte Shape and Migration via Interaction with Src Kinase: IMPLICATIONS FOR ASTROCYTE DEVELOPMENT, ACTIVATION, AND TUMOR GROWTH. S100B is a Ca(2+)-binding protein of the EF-hand type that is abundantly expressed in astrocytes and has been implicated in the regulation of several intracellular activities, including proliferation and differentiation. We show here that reducing S100B levels in the astrocytoma cell line GL15 and the Müller cell line MIO-M1 by small interference RNA technique results in a rapid disassembly of stress fibers, collapse of F-actin onto the plasma membrane and reduced migration, and acquisition of a stellate shape. Also, S100B-silenced GL15 and MIO-M1 Müller cells show a higher abundance of glial fibrillary acidic protein filaments, which mark differentiated astrocytes, compared with control cells. These effects are dependent on reduced activation of the phosphatidylinositol 3-kinase (PI3K) downstream effectors, Akt and RhoA, and consequently elevated activity of GSK3beta and Rac1 and decreased activity of the RhoA-associated kinase. Also, rat primary astrocytes transiently down-regulate S100B expression when exposed to the differentiating agent dibutyryl cyclic AMP and re-express S100B at later stages of dibutyryl cyclic AMP-induced differentiation. Moreover, reducing S100B levels results in a remarkably slow resumption of S100B expression, suggesting the S100B might regulate its own expression. Finally, we show that S100B interacts with Src kinase, thereby stimulating the PI3K/Akt and PI3K/RhoA pathways. These results suggest that S100B might contribute to reduce the differentiation potential of cells of the astrocytic lineage and participate in the astrocyte activation process in the case of brain insult and i  n invasi  ve properties of glioma cells. 
 Vincristine enhances amoeboid-like motility via GEF-H1/RhoA/ROCK/Myosin light chain signaling in MKN45 cells. Anti-cancer drugs are widely used in cancer treatment frequently combined with surgical therapy and/or radiation therapy. Although surgery and radiation have been suggested to facilitate invasion and metastasis of tumor cells in some cases, there is so far little information about the effect of anti-cancer drugs on cellular   invasive   ability and metastasis. In this study, using four different anti-cancer drugs (vincristine, paclitaxel, cisplatin and etoposide), we examined whether these drugs influence the   invasive   ability of tumor cells.Human gastric adenocarcinoma MKN45 cells were used to evaluate the effect of anti-cancer drugs. After drug treatment, cellular   invasive   ability was assessed using the Matrigel invasion chamber. Cytoskeletal changes after treatment were examined microscopically with F-actin staining. In addition, we monitored cellular motility in 3D matrigel environment by time-lapse microscopic analysis. The drug-induced activation of RhoA and ROCK was evaluated by pull-down assay and Western blotting using an antibody against phosphorylated myosin light chain (MLC), respectively. Where necessary, a ROCK inhibitor Y27632 and siRNA for guanine nucleotide exchange factor-H1 (GEF-H1) were applied.Among all drugs tested, only vincristine stimulated the   invasive   ability of MKN45 cells. Microscopic analysis revealed that vincristine induced the formation of non-apoptotic membrane blebs and amoeboid-like motility. Vincristine significantly enhanced RhoA activity and MLC phosphorylation, suggesting the involvement of RhoA/ROCK pathway in the vincristine-induced cytoskeletal reorganization and cellular invasion. Furthermore, we found that Y27632 as well as the siRNA for GEF-H1, a RhoA-specific activator, attenuated MLC phosphorylation, the formation of membrane blebs and the   invasive   ability after vincristine treatment.These results indicate that vincristine activates GEF-H1/RhoA/ROCK/MLC signaling, thereby promoting amoeboid-like motility and the   invasive   ability of MKN45 cells. 
 Involvement of RhoA in progression of human hepatocellular carcinoma. The activation of Rho proteins has been shown to lead to loss of polarity in cancer cells, as well as reorganization of the cytoskeleton and facilitation of cell motility, possibly resulting in their malignant potential. The clinicopathological significance of RhoA, however, is not yet well known in the case of hepatocellular carcinoma (HCC). This study evaluated the clinicopathological correlation of RhoA levels with HCC.The intratumor expression level of Rho was determined and compared with that in adjacent non-cancerous hepatic tissue using quantitative real time RT-PCR and Western blotting in 64 patients with HCC. Relationships between the level of RhoA and clinicopathological factors were examined. RhoA protein expression was confirmed by immunohistochemistry.RhoA immunostaining was strong in malignant tissue whereas it was minimal in benign tissue. Tumor tissue of HCC patients demonstrated a copy number of RhoA mRNA that was well correlated with its protein level in each case and was significantly higher than that found in the corresponding non-cancerous liver tissue (P < 0.01). With regard to venous invasion, satellite lesions and advanced pTNM stage, the RhoA level tended to be higher in HCC than that seen in negative tissue (P < 0.05).This is the first demonstration that the expression level of RhoA is correlated with tumor progression and metastasis in HCC. RhoA in tumor tissue might be expected to be not only a good candidate marker for   invasive   and proliferative tumor cells, but also a molecular target of these cells. 
 Relationship of RhoA signaling activity with ezrin expression and its significance in the prognosis for breast cancer patients. We have recently reported that RhoA may regulate the invasion and metastasis of breast cancer cells as an upstream signal of ezrin in vitro. In this study, we examined the relationship of RhoA signaling activity with ezrin expression in breast cancer and its prognostic significance in patients with breast cancer.Paraffin tumor sections of breast cancer were collected retrospectively from 487 patients diagnosed between 2001 and 2004. Immunohistochemical methods were used to detect the expression of RhoA, phosphorylated (activated) RhoA, and ezrin.Ezrin overexpression was detectable in 15.2% of 487   invasive   breast cancers. The majority (85.1%) of ezrin-overexpressing tumors coexpressed phosphorylated RhoA; 78.8% of tumors with phosphorylated RhoA cooverexpressed ezrin. Patients whose cancers showed overexpression of ezrin or expression of phosphorylated RhoA had shorter survival rates.RhoA activation is important in human breast cancer due to its upregulation of ezrin; thus, agents that target phosphorylated RhoA may be useful in the treatment of tumors with ezrin overexpression. 
 A Blk-p190RhoGAP signaling module downstream of activated Gα13 functionally opposes CXCL12-stimulated RhoA activation and cell invasion. Activation of the GTPase RhoA linked to cell invasion can be tightly regulated following Gα13 stimulation. We have used a cellular model displaying Gα13-dependent inhibition of RhoA activation associated with defective cell invasion to the chemokine CXCL12 to characterize the molecular players regulating these processes. Using both RNAi transfection approaches and protein overexpression experiments here we show that the Src kinase Blk is involved in Gα13-activated tyrosine phosphorylation of p190RhoGAP, which causes RhoA inactivation and ultimately leads to deficient cell invasion. Characterization of molecular interplays between Gα13, Blk and p190RhoGAP revealed that Blk binds Gα13, and that Blk-mediated p190RhoGAP phosphorylation upon Gα13 activation correlates with weakening of Gα13-Blk association connected to increased Blk-p190RhoGAP assembly. These results place Blk upstream of the p190RhoGAP-RhoA pathway in Gα13-activated cells, overall representing an opposing signaling module during CXCL12-triggered invasion. In addition, analyses with Blk- or Gα13-knockdown cells indicated that Blk can also mediate CXCL12-triggered phosphorylation of p190RhoGAP independently of Gα13. However, even if CXCL12 induces the Blk-mediated GAP phosphorylation, the simultaneous stimulation of the guanine-nucleotide exchange factor Vav1 by the chemokine, as earlier reported, leads to a net increase in RhoA activation. Therefore, when Gα13 is concurrently stimulated with CXCL12 there appears to be sufficient Blk activity to promote adequate levels of p190RhoGAP tyrosine phosphorylation to inactivate RhoA and to   impair c  ell invasiveness. 
 The interaction of IQGAP1 with the exocyst complex is required for tumor cell invasion downstream of Cdc42 and RhoA. Invadopodia are actin-based membrane protrusions formed at contact sites between   invasive   tumor cells and the extracellular matrix with matrix proteolytic activity. Actin regulatory proteins participate in invadopodia formation, whereas matrix degradation requires metalloproteinases (MMPs) targeted to invadopodia. In this study, we show that the vesicle-tethering exocyst complex is required for matrix proteolysis and invasion of breast carcinoma cells. We demonstrate that the exocyst subunits Sec3 and Sec8 interact with the polarity protein IQGAP1 and that this interaction is triggered by active Cdc42 and RhoA, which are essential for matrix degradation. Interaction between IQGAP1 and the exocyst is necessary for invadopodia activity because enhancement of matrix degradation induced by the expression of IQGAP1 is lost upon deletion of the exocyst-binding site. We further show that the exocyst and IQGAP1 are required for the accumulation of cell surface membrane type 1 MMP at invadopodia. Based on these results, we propose that invadopodia function in tumor cells relies on the coordination of cytoskeletal assembly and exocytosis downstream of Rho guanosine triphosphatases. 
 Myosin-interacting guanine exchange factor (MyoGEF) regulates the invasion activity of MDA-MB-231 breast cancer cells through activation of RhoA and RhoC. The small guanine triphosphatase (GTPase) proteins RhoA and RhoC are essential for tumor invasion and/or metastasis in breast carcinomas. However, it is poorly understood how RhoA and RhoC are activated in breast cancer cells. Here we describe the role of myosin-interacting guanine nucleotide exchange factor (Myo-GEF) in regulating RhoA and RhoC activation as well as cell polarity and invasion in an   invasive   breast cancer cell line MDA-MB-231. RNA-interference (RNAi)-mediated depletion of MyoGEF in MDA-MB-231 cells not only suppresses the activation of RhoA and RhoC, but also decreases cell polarity and invasion activity. The dominant-negative mutants of RhoA and RhoC, but not Rac1 and Cdc42, dramatically decrease actin polymerization induced by MyoGEF. In addition, MyoGEF co-localizes with nonmuscle myosin IIA (NMIIA) to the front of migrating cells, and depletion of NMIIA by RNAi disrupts the polarized localization of MyoGEF at the cell leading edge, suggesting a role for NMIIA in regulating MyoGEF localization and function. Moreover, MyoGEFprotein levels significantly increase in infiltrating ductal carcinomas as well as in   invasive   breast cancer cell lines. Taken together, our results suggest that MyoGEF cooperates with NMIIA to regulate the polarity and invasion activity of breast cancer cells through activation of RhoA and RhoC. 
 Activin B antagonizes RhoA signaling to stimulate mesenchymal morphology and   invasive  ness of clear cell renal cell carcinomas. Activin B belongs to the TGFβ family of growth factors and is upregulated in clear cell renal cell carcinoma cells by hypoxia inducible factors. Expression of Activin B is required for tumor growth in vivo and tumor cell invasion in vitro. Here we show that activation of RhoA signaling counteracts Activin B mediated disassembly of actin stress fibers, mesenchymal cell morphology and   invasiv  eness, whereas inhibition of RhoA rescues these effects in Activin B knockdown cells. Conversely, Activin B inhibits RhoA signaling suggesting that there is an antagonistic connection between both pathways. In addition we found that Rac1 plays an opposite role to RhoA, i.e. activation of Rac1 initiates loss of actin stress fibers, promotes a mesenchymal cell morphology and induces invasion in Activin B knockown cells, whereas inhibition of Rac1 abolishes these Activin B effects. Collectively, our data provide evidence that reduction of RhoA signaling by Activin B together with persistent Rac1 activity is a prerequisite for inducing an   invasiv  e phenotype in clear cell renal cell carcinoma. 
 RHPN2 drives mesenchymal transformation in malignant glioma by triggering RhoA activation. Mesenchymal transformation is a hallmark of aggressive glioblastoma (GBM). Here, we report the development of an unbiased method for computational integration of copy number variation, expression, and mutation data from large datasets. Using this method, we identified rhophilin 2 (RHPN2) as a central genetic determinant of the mesenchymal phenotype of human GBM. Notably, amplification of the human RHPN2 gene on chromosome 19 correlates with a dramatic decrease in the survival of patients with glioma. Ectopic expression of RHPN2 in neural stem cells and astrocytes triggered the expression of mesenchymal genes and promoted an   invasive   phenotype without impacting cell proliferation. Mechanistically, these effects were implemented through RHPN2-mediated activation of RhoA, a master regulator of cell migration and invasion. Our results define RHPN2 amplification as a central genetic determinant of a highly aggressive phenotype that directs the worst clinical outcomes in patients with GBM. 
 A switch in RND3-RHOA signaling is critical for melanoma cell invasion following mutant-BRAF inhibition. The initial use of BRAF targeted therapeutics in clinical trials has demonstrated encouraging responses in melanoma patients, although a rise in drug-resistant cells capable of advancing malignant disease has been described. The current study uses BRAFV600E expressing WM793 melanoma cells to derive data aimed at investigating the molecular determinant of cell invasion following treatment with clinical BRAF inhibitors.Small-molecule inhibitors targeting BRAF reduced MEK1/2-ERK1/2 pathway activation and cell survival; yet, viable cell subpopulations persisted. The residual cells exhibited an elongated cell shape, prominent actin stress fibers and retained the ability to invade 3-D dermal-like microenvironments. BRAF inhibitor treatments were associated with reduced expression of RND3, an antagonist of RHOA activation, and elevated RHOA-dependent signaling. Restoration of RND3 expression or RHOA knockdown attenuated the migratory ability of residual cells without affecting overall cell survival. The   invasive   ability of BRAF inhibitor treated cells embedded in collagen gels was diminished following RND3 re-expression or RHOA depletion. Conversely, melanoma cell movement in the absence of BRAF inhibition was unaffected by RND3 expression or RHOA depletion.These data reveal a novel switch in the requirement for RND3 and RHOA in coordinating the movement of residual WM793 cells that are initially refractive to BRAF inhibitor therapy. These results have important clinical implications because they suggest that combining BRAF inhibitors with therapies that target the invasion of drug-resistant cells could aid in controlling disease relapse. 
 Integrin-specific control of focal adhesion kinase and RhoA regulates membrane protrusion and invasion. Cell invasion through extracellular matrix (ECM) is a hallmark of the metastatic cascade. Cancer cells require adhesion to surrounding tissues for efficient migration to occur, which is mediated through the integrin family of receptors. Alterations in expression levels of β1 and β3 integrins have previously been reported in a number of human cancers. However, whether there are specific roles for these ubiquitous receptors in mediating cell invasion remains unclear. Here we demonstrate that loss of β1 but not β3 integrins leads to increased spread cell area and focal adhesion number in cells on 2D immobilized fibronectin. Increased adhesion numbers in β1 knockdown cells correlated with decreased cell migration on 2D surfaces. Conversely, cells depleted of β1 integrins showed increased migration speed on 3D cell-derived matrix as well as in 3D organotypic cultures and inverted invasion assays. This incr  eased in  vasive potential was also seen in cells lacking β3 integrin but only in 3D cultures containing fibroblasts. Mechanistically, in situ analysis using FRET biosensors revealed that enhanced invasion in cells lacking β1 integrins was directly coupled with reduced activation of focal adhesion kinase (FAK) and the small GTPase RhoA resulting in formation of enhanced dynamic protrusions and increased invasion. These reductions in FAK-RhoA signal activation were not detected in β3 knockdown cells under the same conditions. This data demonstrates a specific role for β1 integrins in the modulation of a FAK-RhoA-actomyosin signaling axis to regulate cell invasion through complex ECM environments. 
 The regulation of RhoA at focal adhesions by StarD13 is important for astrocytoma cell motility. Malignant astrocytomas are highly   invasive   into adjacent and distant regions of the normal brain. Rho GTPases are small monomeric G proteins that play important roles in cytoskeleton rearrangement, cell motility, and tumor invasion. In the present study, we show that the knock down of StarD13, a GTPase activating protein (GAP) for RhoA and Cdc42, inhibits astrocytoma cell migration through modulating focal adhesion dynamics and cell adhesion. This effect is mediated by the resulting constitutive activation of RhoA and the subsequent indirect inhibition of Rac. Using Total Internal Reflection Fluorescence (TIRF)-based Förster Resonance Energy Transfer (FRET), we show that RhoA activity localizes with focal adhesions at the basal surface of astrocytoma cells. Moreover, the knock down of StarD13 inhibits the cycling of RhoA activation at the rear edge of cells, which makes them defective in retracting their tail. This study highlights the importance of the regulation of RhoA activity in focal adhesions of astrocytoma cells and establishes StarD13 as a GAP playing a major role in this process. 
 Gastrokine 1 inhibits gastric cancer cell migration and invasion by downregulating RhoA expression. We investigated whether GKN1, a gastric tumor suppressor, contributes to the progression of gastric cancer by regulating RhoA expression.We analyzed the expression of GKN1, RhoA, miR-185, and miR-34a in 35 gastric cancer tissues, and compared their expression with T category and TNM stage. Cell migration and invasion, as well as the expression of epithelial-to-mesenchymal transition (EMT)-related proteins, were assessed in GKN1- and RhoA small interfering RNA (siRhoA)-transfected and recombinant-GKN1-treated AGS and MKN1 gastric cancer cells.Expression of RhoA protein and messenger RNA (mRNA) was increased in 15 (42.9 %) and 17 (48.6 %) of 35 gastric cancer tissues respectively, and was associated with higher T category and TNM stage. GKN1 expression was significantly decreased in 27 gastric cancers (77.1 %) with a higher T category, and was inversely correlated with RhoA mRNA expression. In AGS and MKN1 cells, GKN1 expression increased miR-185 and miR-34a expression and reduced RhoA mRNA and protein expression. A positive relationship between GKN1 and miR-34a and miR-185 expression and an inverse relationship between miR-34a and RhoA expression were observed in gastric cancer tissues. Cell migration a  nd invas  iveness were markedly decreased in GKN1- and siRhoA-transfected cells. GKN1 expression and silencing of RhoA decreased the expression of the proteins Snail, Slug, and vimentin. Furthermore, miR-185 and miR-34a silencing in MKN1 cells transfected with GKN1 stimulated cell migration and invasion, and increased the expression of EMT-related proteins.Our data suggest that GKN1 may inhibit gastric cancer cell migration and invasion by downregulating RhoA expression in a miR-185- and miR-34a-dependent manner. 
 SOX2-RNAi attenuates S-phase entry and induces RhoA-dependent switch to protease-independent amoeboid migration in human glioma cells. SOX2, a high mobility group (HMG)-box containing transcription factor, is a key regulator during development of the nervous system and a persistent marker of neural stem cells. Recent studies suggested a role of SOX2 in tumor progression. In our previous work we detected SOX2 in glioma cells and glioblastoma specimens. Herein, we aim to explore the role of SOX2 for glioma malignancy in particular its role in cell proliferation and migration.Retroviral shRNA-vectors were utilized to stably knockdown SOX2 in U343-MG and U373-MG cells. The resulting phenotype was investigated by Western blot, migration/invasion assays, RhoA G-LISA, time lapse video imaging, and orthotopic xenograft experiments.SOX2 depletion results in pleiotropic effects including attenuated cell proliferation caused by decreased levels of cyclinD1. Also an increased TCF/LEF-signaling and concomitant decrease in Oct4 and Nestin expression was noted. Furthermore, down-regulation of focal adhesion kinase (FAK) signaling and of downstream proteins such as HEF1/NEDD9, matrix metalloproteinases pro-MMP-1 and -2 impaired   invasive   proteolysis-dependent migration. Yet, cells with knockdown of SOX2 switched to a RhoA-dependent amoeboid-like migration mode which could be blocked by the ROCK inhibitor Y27632 downstream of RhoA-signaling. Orthotopic xenograft experiments revealed a higher tumorigenicity of U343-MG glioma cells transduced with shRNA targeting SOX2 which was characterized by increased dissemination of glioma cells.Our findings suggest that SOX2 plays a role in the maintenance of a less differentiated glioma cell phenotype. In addition, the results indicate a critical role of SOX2 in adhesion and migration of malignant gliomas. 
 Chemokine receptor 7 promotes tumor migration and   invasive  ness via the RhoA/ROCK pathway in metastatic squamous cell carcinoma of the head and neck. Metastatic squamous cell carcinoma of the head and neck (SCCHN) has been shown to express chemokine receptor 7 (CCR7), which can activate signaling pathways to promote invasion and survival of SCCHN cells. We hypothesized that the RhoA/Rho-associated kinase (ROCK) pathway is involved in the CCR7-induced invasion and migration of metastatic SCCHN cells. Thus, using migration, matrigel invasion and scrape wound-healing assays, we elucidated the role of RhoA in mediating CCR7-associated cellular mobility. Pull-down assays and western blotting were used to measure RhoA and its downstream expression. Immunohistochemical staining and analysis were useful in identifying the correlation between CCR7 and RhoA expression and clinicopathological factors. The results showed that inhibition of RhoA/ROCK reduced the tumor cell migration and   invasive  ness induced by CCL19. Activated RhoA, proline-rich tyrosine kinase-2 (Pyk2) and cofilin induced by CCL19 were elevated, and increased RhoA, Pyk2 and cofilin activity was eliminated by CCR7mAb, RhoA/ROCK and Pyk2 inhibitors, indicating involvement of the RhoA/ROCK-Pyk2-cofilin cascade. In summary, CCR7 via RhoA/ROCK-Pyk2 cofilin pathway promotes invasion and migration of metastatic SCCHN cells. 
 The RhoA pathway mediates MMP-2 and MMP-9-independent   invasive   behavior in a triple-negative breast cancer cell line. Breast cancer is a heterogeneous disease that varies in its biology and response to therapy. A foremost threat to patients is tumor invasion and metastasis, with the greatest risk among patients diagnosed with triple-negative and/or basal-like breast cancers. A greater understanding of the molecular mechanisms underlying cancer cell spreading is needed as 90% of cancer-associated deaths result from metastasis. We previously demonstrated that the Tamoxifen-selected, MCF-7 derivative, TMX2-28, lacks expression of estrogen receptor α (ERα) and is highl  y invasi  ve, yet maintains an epithelial morphology. The present study was designed to further characterize TMX2-28 cells and elucidate their invasion mechanism. We found that TMX2-28 cells do not express human epidermal growth factor receptor 2 (HER2) and progesterone receptor (PR), in addition to lacking ERα, making the cells triple-negative. We then determined that TMX2-28 cells lack expression of active matrix metalloproteinases (MMPs)-1, MMP-2, MMP-9, and other genes involved in epithelial-mesenchymal transition (EMT) suggesting that TMX2-28 may not utilize mesenchymal invasion. In contrast, TMX2-28 cells have high expression of Ras Homolog Gene Family Member, A (RhoA), a protein known to play a critical role in amoeboid invasion. Blocking RhoA activity with the RhoA pathway specific inhibitor H-1152, or a RhoA specific siRNA, resulted in inhibition   of invas  ive behavior. Collectively, these results suggest that TMX2-28 breast cancer cells exploit a RhoA-dependent, proteolytic-independent invasion mechanism. Targeting the RhoA pathway in triple-negative, basal-like breast cancers that have a proteolytic-independent invasion mechanism may provide therapeutic strategies for the treatment of patients with increased risk of metastasis. 
 Lysophosphatidic acid stimulates PC-3 prostate cancer cell Matrigel invasion through activation of RhoA and NF-kappaB activity. This study was performed to determine the relationship of lysophosphatidic acid (LPA) stimulation and increased Ras homolog A (RhoA) activity to nuclear factor kappa B (NF-kappaB) activity, and the role of these factors in regulating prostate cancer cell invasion. PC-3 high   invasive   cells demonstrated constitutively increased RhoA, NF-kappaB, and in vitro Matrigel invasion which were further induced by LPA stimulation or transfection with constitutively active RhoA Q63E mutant. LPA treatment rapidly and transiently induced RhoA activity followed by maximally increased DNA binding of NF-kappaB at 1 h and AP-1 at 4 h. The LPA-induced NF-kappaB DNA binding was preceded by transient IkappaBalpha phosphorylation, and decreased total IkappaBalpha levels. Further demonstrating the relationship between RhoA and NF-kappaB activation, PC-3 cells stably transfected with constitutively active RhoA Q63E demonstrated constitutively increased phospho-IkappaBalpha, while PC-3 cells transfected with dominant negative RhoA N19 exhibited decreased phospho-IkappaBalpha levels. The LPA-induced Matrigel invasion and NF-kappaB DNA binding activity were both inhibited by expression of the RhoA inhibitor C3 exoenzyme or dominant negative mutant NF-kappaB inhibitor IkappaBalpha S32/36A. Similarly, transfection with dominant negative IkappaBalpha S32/36A inhibited PC-3 RhoA Q63E cell in vitro invasion. Treatment of PC-3 high   invasive   and RhoA Q63E cells with sodium salicylate or lactacystin inhibited NF-kappaB and invasion, while pyrrolidine dithiocarbamate (PDTC) treatment of PC-3 high   invasive   cells inhibited NF-kappaB only. Each inhibitor blocked LPA-induced invasion while PDTC inhibited LPA-induced NF-kappaB and invasion to the greatest extent. These results point to a model where LPA stimulates RhoA and increased PC-3 prostate cancer cell invasion activity through an NF-kappaB-dependent pathway. 
 Isoprenylation is necessary for the full   invasive   potential of RhoA overexpression in human melanoma cells. Rho GTPases are signaling molecules known to control cell motility. Several recent studies have suggested a role for Rho proteins in mediating tumor metastasis independent of their affects on cell proliferation. As Rho proteins require post-translational modification with a geranlygeranyl moiety for full activity, we tested the affect of blocking geranylation on localization, downstream signaling, and stimulation of invasion. Expression of a constitutively active Rho construct in A375 melanoma cells dramatically stimulated invasion through Matrigel membranes; however, a constitutively active RhoA mutated so that it cannot be geranylated, failed to stimulate invasion. Moreover, expression of epitope or GFP tagged modifications of this nongeranylatable constitutively active Rho demonstrated that geranylation is necessary for correct cellular localization of Rho. Geranylation was also found to be necessary for full downstream activation of serum response factor mediated transcription. Pharmacologic inhibition of Rho geranylation produced similar inhibition of Rho localization, signaling, and invasion. Our results suggest that inhibition of Rho geranylation may be an attractive pharmacologic target for inhibiting melanoma metastasis. 
 Functional analysis of the contribution of RhoA and RhoC GTPases to   invasive   breast carcinoma. Although the RhoA and RhoC proteins comprise an important subset of the Rho GTPase family that have been implicated in   invasive   breast carcinomas, attributing specific functions to these individual members has been difficult. We have used a stable retroviral RNA interference approach to generate   invasive   breast carcinoma cells (SUM-159 cells) that lack either RhoA or RhoC expression. Analysis of these cells enabled us to deduce that RhoA impedes and RhoC stimulates invasion. Unexpectedly, this analysis also revealed a compensatory relationship between RhoA and RhoC at the level of both their expression and activation, and a reciprocal relationship between RhoA and Rac1 activation. 
 Suppression of MIM by microRNA-182 activates RhoA and promotes breast cancer metastasis. Breast cancer is the most common type of cancer among women worldwide, and metastasis represents the most devastating stage of the disease. Recent studies have revealed that microRNAs (miRNA) have critical roles to regulate cancer cell invasion and metastasis. Here we present evidence to show the role of miR-182 in breast cancer metastasis. miR-182 is upregulated in the malignant cell line variants of both human MCF10 and mouse 4T1 series. Ectopic expression of miR-182 enhanced breast cancer cell motility and   invasive  ness, whereas miR-182 inhibition resulted in opposite changes. In nude mice, miR-182 led to increased pulmonary colonization of cancer cells. We further demonstrated that miR-182 directly targets MIM (Missing in Metastasis), which suppresses metastasis by inhibiting ras homolog family member A (RhoA) activity and stress fiber formation in breast cancer cells. Restoring MIM expression completely blocked the pro-metastasis function of miR-182, while RhoA inhibition reversed the phenotypes of both miR-182 overexpression and MIM knockdown. In breast tumor samples, miR-182 induction is linked to downregulation of MIM, RhoA activation and poor prognosis. Hence, our data delineates the molecular pathway by which miR-182 promotes breast cancer invasion and metastasis, and may have important implication for the treatment of metastatic cancers. 
 Overexpression of RhoA enhances peritoneal dissemination: RhoA suppression with Lovastatin may be useful for ovarian cancer. Small guanosine triphosphatase RhoA has been known to re-organize cytoskeletons and regulate cell migration. The present authors have previously reported that expression of RhoA is significantly increased in advanced ovarian carcinomas and also in the peritoneal disseminated lesions. The present study investigated whether overexpression of RhoA could alter the progressive behavior of ovarian cancer cells. The effect of various Rho inhibitors on the biological behavior of ovarian cancer cells in vitro and in vivo was also examined. A stable RhoA-transfectant of an ovarian cancer cell line SKOV3 was generated and examined in vitro for alterations of proliferative activity and   invasive  ness, and also in the nude mice model for peritoneal dissemination. In addition, the effect of a specific Rho inhibitor (C3 exoenzyme), Rho kinase inhibitor (Y27632) and hydroxymethylglutaryl coenzyme A (HMG-CoA) reductase inhibitor (Lovastatin and Pravastatin) were studied in vitro and in vivo. Forced overexpression of RhoA did not alter proliferative activity but significantly increased the   invasive  ness in vitro, which was suppressed by addition of C3 exoenzyme, Y27834, Lovastatin and Pravastatin. In the nude mice model, the frequency of dissemination and the number of disseminated lesions were significantly increased in the RhoA transfectant than in the control. In addition, oral administration of Lovastatin significantly decreased the number of metastatic sites compared with the control. These findings suggest that upregulation and/or activation of RhoA play an important role in the peritoneal dissemination of ovarian carcinoma, and that Lovastatin might be a candidate for the possible, novel treatment for ovarian carcinoma patients with peritoneal dissemination. 
 Anti-RhoA and anti-RhoC siRNAs inhibit the proliferation and   invasive  ness of MDA-MB-231 breast cancer cells in vitro and in vivo. Overexpression of RhoA or RhoC in breast cancer indicates a poor prognosis, due to increased tumor cell proliferation and invasion and tumor-dependent angiogenesis. Until now, the strategy of blockage of the Rho-signaling pathway has used either GGTI or HMG-CoA reductase inhibitors, but they are not specific to RhoA or RhoC inhibition. In this study, a new approach with anti-RhoA and anti-RhoC siRNAs was used to inhibit specifically RhoA or RhoC synthesis. Two transfections of either RhoA or RhoC siRNA (8.5 nM) into MDA-MB-231 human breast cancer cells or HMEC-1 endothelial cells induced extensive degradation of the target mRNA and led to a dramatic decrease in synthesis of the corresponding protein. In vitro, these siRNAs inhibited cell proliferation and invasion more effectively than conventional blockers of Rho cell signaling. Finally, in a nude mouse model, intratumoral injections of anti-RhoA siRNA (100 microl at 85 nM) every 3 days for 20 days almost totally inhibited the growth and angiogenesis of xenografted MDA-MB-231 tumors. One may infer from these observations that specific inhibition of the Rho-signaling pathway with siRNAs represents a promising approach for the treatment of aggressive breast cancers. 
 Arylsulfatase B regulates colonic epithelial cell migration by effects on MMP9 expression and RhoA activation. Arylsulfatase B (ASB; N-acetylgalactosamine-4-sulfatase; 4-sulfatase; ARSB) is the enzyme that removes 4-sulfate groups from N-acetylgalactosamine 4-sulfate, which combines with glucuronate to form the disaccharide unit of chondroitin-4-sulfate (C4S). In this study, we report how variation in expression of ASB affected the migration of human colonic epithelial cells. In the T84 cell line, derived from lung metastasis of malignant colonic epithelial cells, the activity of ASB, as well as steroid sulfatase, arylsulfatase A, and galactose-6-sulfatase, were significantly less than in normal, primary colonic epithelial cells and in the NCM460 cell line which was derived from normal colonocytes. In the T84 cells, matrix metalloproteinase 9 (MMP9), activated RhoA, and cell migration, as well as C4S content, were significantly more than in the NCM460 cells. Silencing and overexpression of ASB had inverse effects on MMP9, activated RhoA, and cell migration, as well as the C4S content, in the NCM460 and T84 cells. When ASB expression was silenced by siRNA in the NCM460 cells, MMP9 secretion increased to over 3 times the basal level, activated RhoA increased * 85%, and cell migration increased * 52%. Following overexpression of ASB, MMP9 declined 51%, activated RhoA declined * 51%, and cell migration decreased * 37%. These findings demonstrate marked effects of ASB expression on the migratory activity of colonic epithelial cells, activated RhoA, and MMP9, and suggest a potential vital role of ASB, due to its impact on chondroitin sulfation, on determination of the   invasive   phenotype of colonic epithelial cells. 
 Cannabidiolic acid, a major cannabinoid in fiber-type cannabis, is an inhibitor of MDA-MB-231 breast cancer cell migration. Cannabidiol (CBD), a major non-psychotropic constituent of fiber-type cannabis plant, has been reported to possess diverse biological activities, including anti-proliferative effect on cancer cells. Although CBD is obtained from non-enzymatic decarboxylation of its parent molecule, cannabidiolic acid (CBDA), few studies have investigated whether CBDA itself is biologically active. Results of the current investigation revealed that CBDA inhibits migration of the highly   invasive   MDA-MB-231 human breast cancer cells, apparently through a mechanism involving inhibition of cAMP-dependent protein kinase A, coupled with an activation of the small GTPase, RhoA. It is established that activation of the RhoA signaling pathway leads to inhibition of the mobility of various cancer cells, including MDA-MB-231 cells. The data presented in this report suggest for the first time that as an active component in the cannabis plant, CBDA offers potential therapeutic modality in the abrogation of cancer cell migration, including aggressive breast cancers. 
 Staphylococcus aureus protein A mediates invasion across airway epithelial cells through activation of RhoA GTPase signaling and proteolytic activity. Staphyococcus aureus and especially the epidemic methicillin-resistant S. aureus strains cause severe necrotizing pneumonia. The mechanisms whereby these organisms invade across the mucosal epithelial barrier to initiate   invasive   infection are not well understood. Protein A (SpA), a highly conserved and abundant surface protein of S. aureus, activates TNF receptor 1 and EGF receptor (EGFR) signaling cascades that can perturb the cytoskeleton. We demonstrate that wild-type S. aureus, but not spa mutants, invade across polarized airway epithelial cell monolayers via the paracellular junctions. SpA stimulated a RhoA/ROCK/MLC cascade, resulting in the contraction of the cytoskeleton. SpA(+) but not SpA(-) mutants stimulated activation of EGFR and along with subsequent calpain activity cleaved the membrane-spanning junctional proteins occludin and E-cadherin, facilitating staphylococcal transmigration through the cell-cell junctions. Treatment of polarized human airway epithelial monolayers with inhibitors of ROCK, EGFR, MAPKs, or calpain prevented staphylococcal penetration through the monolayers. In vivo, blocking calpain activity impeded bacterial invasion into the lung parenchyma. Thus, S. aureus exploits multiple receptors available on the airway mucosal surface to facilitate invasion across epithelial barriers. 
 Syndecan-2 regulation of morphology in breast carcinoma cells is dependent on RhoGTPases. While syndecan-2 is usually considered a mesenchymal transmembrane proteoglycan, it can be upregulated in some tumour cells, such as the malignant breast carcinoma cell line, MDA-MB231. Depletion of this syndecan by siRNA, but not other syndecans, has a marked effect on cell morphology, increasing spreading, microfilament bundle and focal adhesion formation, with reduced cell migration.A combination of siRNA transfection, immunofluorescence microscopy, phosphoprotein analysis and migration assays was used to determine how syndecan-2 may influence the cytoskeleton.The altered adhesion upon syndecan-2 depletion was dependent on the RhoGTPases. p190ARhoGAP relocated to the margins of spreading cells, where it codistributed with syndecan-4 and active β1-integrin. This was accompanied by increased RhoGAP tyrosine phosphorylation, indicative of activity and RhoGTPase suppression. Consistent with this, GTP-RhoA was strongly present at the edges of control cells, but lost after syndecan-2 reduction by siRNA treatments. Further, RhoA, but not RhoC was shown to be essential for the anchored phenotype of these breast carcinoma cells that accompanied siRNA-mediated loss of syndecan-2.Syndecan-2 has a key role in promoting the   invasiv  e activity of these cells, in part by regulating the RhoGTPases.Syndecan-2, as a cell surface receptor is accessible for targeting to determine whether breast tumour progression is altered. This article is part of a Special Issue entitled Matrix-mediated cell behaviour and properties. 
 Netrin-1 promotes glioblastoma cell   invasive  ness and angiogenesis by multiple pathways including activation of RhoA, cathepsin B, and cAMP-response element-binding protein. Glioblastomas are very difficult tumors to treat because they are highly   invasive   and disseminate within the normal brain, resulting in newly growing tumors. We have identified netrin-1 as a molecule that promotes glioblastoma   invasive  ness. As evidence, netrin-1 stimulates glioblastoma cell invasion directly through Matrigel-coated transwells, promotes tumor cell sprouting and enhances metastasis to lymph nodes in vivo. Furthermore, netrin-1 regulates angiogenesis as shown in specific angiogenesis assays such as enhanced capillary endothelial cells (EC) sprouting and by increased EC infiltration into Matrigel plugs in vivo, as does VEGF-A. This netrin-1 signaling pathway in glioblastoma cells includes activation of RhoA and cyclic AMP response element-binding protein (CREB). A novel finding is that netrin-1-induced glioblastoma   invasive  ness and angiogenesis are mediated by activated cathepsin B (CatB), a cysteine protease that translocates to the cell surface as an active enzyme and co-localizes with cell surface annexin A2 (ANXA2). The specific CatB inhibitor CA-074Me inhibits netrin-1-induced cell invasion, sprouting, and Matrigel plug angiogenesis. Silencing of CREB suppresses netrin-1-induced glioblastoma cell invasion, sprouting, and CatB expression. It is concluded that netrin-1 plays an important dual role in glioblastoma progression by promoting both glioblastoma cell   invasive  ness and angiogenesis in a RhoA-, CREB-, and CatB-dependent manner. Targeting netrin-1 pathways may be a promising strategy for brain cancer therapy. 
 Hypoxia-inducible factors mediate coordinated RhoA-ROCK1 expression and signaling in breast cancer cells. Overexpression of Rho kinase 1 (ROCK1) and the G protein RhoA is implicated in breast cancer progression, but oncogenic mutations are rare, and the molecular mechanisms that underlie increased ROCK1 and RhoA expression have not been determined. RhoA-bound ROCK1 phosphorylates myosin light chain (MLC), which is required for actin-myosin contractility. RhoA also activates focal adhesion kinase (FAK) signaling. Together, these pathways are critical determinants of the motile and   invasive   phenotype of cancer cells. We report that hypoxia-inducible factors coordinately activate RhoA and ROCK1 expression and signaling in breast cancer cells, leading to cell and matrix contraction, focal adhesion formation, and motility through phosphorylation of MLC and FAK. Thus, intratumoral hypoxia acts as an oncogenic stimulus by triggering hypoxia-inducible factor → RhoA → ROCK1 → MLC → FAK signaling in breast cancer cells. 
 Effect of shRNA targeted against RhoA on proliferation and migration of human colonic cancer cells. To study the effects of RhoA siRNA on the malignant phenotypes of human colorectal cancer cell line LoVo.The siRNA expression vector pGPU6/GFP/Neo-shRNA-RhoA targeting the mRNA of RhoA and vector pGPU6/GFP/Neo-NC (as a control) were constructed, and then transfected into LoVo cells. The expression of Survivin was detected by real-time fluorescent quantitative polymerase chain reaction and Western blot. The malignant phenotypes of transfected LoVo cells, including   invasive   activities and adhesive capabilities, were analyzed.RhoA mRNA and protein level were decreased after the pshRNA-RhoA transfection. The cell adhesion rates significantly decreased in the cells transfected with pshRNA-RhoA. The migrating number of LoVo cells (26.5 ± 0.9) transfected with pshRNA-RhoA was also significantly decreased as compared with the control group (53.7 ± 1.4).The sequence specific shRNA against RhoA constructed in the study can block the expression of RhoA in LoVo cell effectively and specifically; Blocking the expression of RhoA in LoVo cells transfected with pshRNA-RhoA can reduce thei  r invasi  ve and adhesive capabilities. 
 Gonadotropin-releasing hormone receptor activates GTPase RhoA and inhibits cell invasion in the breast cancer cell line MDA-MB-231. Gonadotropin-releasing hormone (GnRH) and its receptor (GnRHR) are both expressed by a number of malignant tumors, including those of the breast. In the latter, both behave as potent inhibitors of invasion. Nevertheless, the signaling pathways whereby the activated GnRH/GnRHR system exerts this effect have not been clearly established. In this study, we provide experimental evidence that describes components of the mechanism(s) whereby GnRH inhibits breast cancer cell invasion.Actin polymerization and substrate adhesion was measured in the highly   invasive   cell line, MDA-MB-231 transiently expressing the wild-type or mutant DesK191 GnRHR by fluorometry, flow cytometric analysis, and confocal microscopy, in the absence or presence of GnRH agonist. The effect of RhoA-GTP on stress fiber formation and focal adhesion assembly was measured in MDA-MB-231 cells co-expressing the GnRHRs and the GAP domain of human p190Rho GAP-A or the dominant negative mutant GAP-Y1284D. Cell invasion was determined by the transwell migration assay.Agonist-stimulated activation of the wild-type GnRHR and the highly plasma membrane expressed mutant GnRHR-DesK191 transiently transfected to MDA-MB-231 cells, favored F-actin polymerization and substrate adhesion. Confocal imaging allowed detection of an association between F-actin levels and the increase in stress fibers promoted by exposure to GnRH. Pull-down assays showed that the effects observed on actin cytoskeleton resulted from GnRH-stimulated activation of RhoA GTPase. Activation of this small G protein favored the marked increase in both cell adhesion to Collagen-I and number of focal adhesion complexes leading to inhibition of the invasion capacity of MDA-MB-231 cells as disclosed by assays in Transwell Chambers.We here show that GnRH inhibits invasion of highly   invasive   breast cancer-derived MDA-MB-231 cells. This effect is mediated through an increase in substrate adhesion promoted by activation of RhoA GTPase and formation of stress fibers and focal adhesions. These observations offer new insights into the molecular mechanisms whereby activation of overexpressed GnRHRs affects cell invasion potential of this malignant cell line, and provide opportunities for designing mechanism-based adjuvant therapies for breast cancer. 
 Macrophages stimulate gastric and colorectal cancer invasion through EGFR Y(1086), c-Src, Erk1/2 and Akt phosphorylation and smallGTPase activity. The interactions between cancer cells and their microenvironment are crucial for malignant progression, as they modulate invasion-related activities. Tumor-associated macrophages are generally considered allies in the process of tumor progression in several types of cancer, although their role on gastric and colorectal carcinomas is still poorly understood. In this report, we studied the influence of primary human macrophages on gastric and colorectal cancer cells, considering invasion, motility/migration, proteolysis and activated intracellular signaling pathways. We demonstrated that macrophages stimulate cancer cell invasion, motility and migration, and that these effects depend on matrix metalloproteinase (MMP) activity and on the activation of epidermal growth factor receptor (EGFR) (at the residue Y(1086)), PLC-γ (phospholipase C-gamma) and Gab1 (GRB2-associated binding protein-1), as evidenced by siRNA (small interference RNA) experiments. Epidermal growth factor (EGF)-immunodepletion impaired macrophage-mediated cancer cell invasion and motility, suggesting that EGF is the pro  -invasiv  e and pro-motile factor produced by macrophages. Macrophages also induced gastric and colorectal cancer cell phosphorylation of Akt, c-Src and ERK1/2, and led to an increase of RhoA and Cdc42 activity. Interestingly, whereas macrophage-mediated cancer cell c-Src and ERK1/2 phosphorylation occurred downstream EGFR activation, Akt phosphorylation seems to be a parallel event, taking place in an EGFR-independent manner. The involvement of EGF, EGFR-downstream signaling partners and MMPs in macrophage-mediated invasion provides novel insights into the molecular crosstalk established between cancer cells and macrophages, opening new perspectives for the design of new and more efficient therapeutic strategies to counteract cancer cell invasion. 
 RhoA induces MMP-9 expression at CD44 lamellipodial focal complexes and promotes HMEC-1 cell invasion. Much progress has been made in recent years in the understanding of angiogenesis, yet signalling pathways involved remain poorly defined. Here we report that small RhoA GTPase is implicated in the invasion of human microvascular endothelial cells (HMEC-1). Ectopic expression of active-RhoA GTPase induced the expression of MMP-9 metalloproteinase, a key proteinase of the basement membrane, and promoted migration of endothelial cells through a 3D-matrix protein gel. MMP-9 was either directed as vesicular-like patches to the apical side of cells, or addressed to specific membrane sites at the cell surface. Confocal microscopy analyses indeed revealed clustering of MMP-9 in advancing lamellipodia at the forefront of endothelial cells, where this proteinase colocalized with RhoA and CD44, a transmembrane receptor known to be proteolysed in tumor cell progression. In addition, TIMP-1, a natural MMP inhibitor, significantly reduced the invasion of RhoAV14 expressing cells, suggesting that MMP-9 was a critical metalloproteinase responsible, at least partly, for the RhoAV14-induced endothelial cell invasion. We propose that RhoA triggers signalling pathways that, upregulating expression of a proteinase at specific membrane localizations, may confer an highly   invasive   phenotype to endothelial cells. 
 NET1-mediated RhoA activation facilitates lysophosphatidic acid-induced cell migration and invasion in gastric cancer. The most lethal aspects of gastric adenocarcinoma (GA) are its   invasive   and metastatic properties. This aggressive phenotype remains poorly understood. We have recently identified neuroepithelial cell transforming gene 1 (NET1), a guanine exchange factor (GEF), as a novel GA-associated gene. Neuroepithelial cell transforming gene 1 expression is enhanced in GA and it is of functional importance in cell invasion. In this study, we demonstrate the activity of NET1 in driving cytoskeletal rearrangement, a key pathological mechanism in gastric tumour cell migration and invasion. Neuroepithelial cell transforming gene 1 expression was increased 10-fold in response to treatment with lysophosphatidic acid (LPA), resulting in an increase in active levels of RhoA and a 2-fold increase in cell invasion. Lysophosphatidic acid-induced cell invasion and migration were significantly inhibited using either NET1 siRNA or a RhoA inhibitor (C3 exoenzyme), thus indicating the activity of both NET1 and RhoA in gastric cancer progression. Furthermore, LPA-induced invasion and migration were also significantly reduced in the presence of cytochalasin D, an inhibitor of cytoskeletal rearrangements. Neuroepithelial cell transforming gene 1 knockdown resulted in AGS cell rounding and a loss of actin filament organisation, demonstrating the function of NET1 in actin organisation. These data highlight the importance of NET1 as a driver of tumour cell invasion, an activity mediated by RhoA activation and cytoskeletal reorganisation. 
 RHOA mutation in diffuse-type gastric cancer: a comparative clinicopathology analysis of 87 cases. Recent studies have discovered recurrent RHOA mutations in diffuse-type gastric cancers. These reports show mutant RhoA is an important cancer driver and is a potential therapeutic target. This study aims to investigate the clinicopathological features of diffuse-type gastric cancers with RHOA mutation.We performed a thorough review of 87 diffuse-type gastric cancers, including 22 RHOA-mutated and 65 RHOA wild-type gastric cancers.Most advanced tumors with RHOA mutation appeared as Borrmann type 3 lesions (81 %) developing in the middle (50 %) or distal (32 %) third of the stomach. Histologically, although all of the tumors were predominantly or exclusively composed of poorly cohesive carcinoma, limited tubular differentiation was also observed in 73 % of the RHOA-mutated tumors. Notably, RHOA-mutated tumors more frequently showed a permeative growth pattern at the edge of the mucosal area (59 %) compared with RHOA wild-type tumors (29 %, P = 0.0202). Additionally, the size ratios of the deeply   invasive   components to the mucosal components were significantly lower in RHOA-mutated tumors [less than 1.45 (median) in 68 % of cases] than in RHOA wild-type tumors (less than 1.45 in 42 % of cases, P = 0.0482). RHOA mutation did not significantly impact survival in this study.These observations suggest that RHOA mutation may be associated with the growth patterns of diffuse-type gastric cancer but have a limited prognostic impact in isolation. Further studies, including analyses of the other alterations involving the RhoA pathways, such as CLDN18-ARHGAP fusion, as well as functional studies of mutant RhoA, are necessary to clarify the significance of alterations in the RhoA-signaling pathway in diffuse-type gastric cancers. 
 Extracellular signal-regulated kinase regulates RhoA activation and tumor cell plasticity by inhibiting guanine exchange factor H1 activity. In certain Ras mutant cell lines, the inhibition of extracellular signal-regulated kinase (ERK) signaling increases RhoA activity and inhibits cell motility, which was attributed to a decrease in Fra-1 levels. Here we report a Fra-1-independent augmentation of RhoA signaling during short-term inhibition of ERK signaling. Using mass spectrometry-based proteomics, we identified guanine exchange factor H1 (GEF-H1) as mediating this effect. ERK binds to the Rho exchange factor GEF-H1 and phosphorylates it on S959, causing inhibition of GEF-H1 activity and a consequent decrease in RhoA activity. Knockdown experiments and expression of a nonphosphorylatable S959A GEF-H1 mutant showed that this site is crucial in regulating cell motility and   invasive  ness. Thus, we identified GEF-H1 as a critical ERK effector that regulates motility, cell morphology, and   invasive  ness. 
 Oligodendrocyte lineage transcription factor 2 inhibits the motility of a human glial tumor cell line by activating RhoA. The basic helix-loop-helix transcription factor, oligodendrocyte lineage transcription factor 2 (OLIG2), is specifically expressed in the developing and mature central nervous system and plays an important role in oligodendrogenesis from neural progenitors. It is also expressed in various types of glial tumors, but rarely in glioblastoma. Although we previously showed that OLIG2 expression inhibits glioma cell growth, its role in tumorigenesis remains incompletely understood. Here, we investigated the effect of OLIG2 expression on the migration of the human glioblastoma cell line U12-1. In these cells, OLIG2 expression is controlled by the Tet-off system. Induction of OLIG2 expression inhibited both the migration and   invasive  ness of U12-1 cells. OLIG2 expression also increased the activity of the GTPase RhoA as well as inducing the cells to form stress fibers and focal adhesions. Experiments using short interfering RNA against p27(Kip1) revealed that up-regulation of the p27(Kip1) protein was not essential for RhoA activation, rather it contributed independently to the decreased motility of OLIG2-expressing U12-1 cells. Alternatively, semiquantitative reverse transcription-PCR analysis revealed that mRNA expression of RhoGAP8, which regulates cell migration, was decreased by OLIG2 expression. Furthermore, expression of C3 transferase, which inhibits Rho via ADP ribosylation, attenuated the OLIG2-induced inhibition of cell motility. Imaging by fluorescence resonance energy transfer revealed that in U12-1 cells lacking OLIG2, the active form of RhoA was localized to protrusions of the cell membrane. In contrast, in OLIG2-expressing cells, it lined almost the entire plasma membrane. Thus, OLIG2 suppresses the motile phenotype of glioblastoma cells by activating RhoA. 
 Up-regulation of small GTPases, RhoA and RhoC, is associated with tumor progression in ovarian carcinoma. To clarify the role of small GTPases Rho in the biologic behavior of ovarian carcinoma, we first examined the mRNA expression of RhoA, RhoB, and RhoC in benign, borderline, and malignant ovarian tumors using RT-PCR and real-time RT-PCR. The expression and localization of RhoA protein were also analyzed by Western blotting and immunohistochemistry. Finally, we examined whether up-regulation of Rho enhances the   invasive  ness of ovarian cancer cells in vitro. Analysis of mRNA levels of the Rho family genes revealed that levels of both RhoA and RhoC were significantly higher in carcinomas than in benign tumors (RhoA, p = 0.0035; RhoC, p = 0.0006). According to histologic subtype, both RhoA and RhoC mRNA levels in serous carcinomas were significantly higher than those in other histologic types. With regard to the International Federation of Gynecological and Obstetrics stage classification, both of RhoA and RhoC mRNA levels were significantly higher in tumors of Stages III+IV than in those of Stages I+II (RhoA, p = 0.0200; RhoC, p = 0.0057). In addition, analysis of matched pairs of primary and disseminated lesions demonstrated that expression of both RhoA and RhoC mRNA was significantly higher in metastatic than in primary tumors. Examination of the protein level showed that expression of RhoA was also increased in advanced ovarian carcinomas, especially those of serous histology. Accordingly, we hypothesized that up-regulation of Rho GTPases plays an important role in the progression of ovarian carcinoma. Matrigel invasion assay using the ovarian cancer cell line, SKOV3, showed that up-regulation and activation after treatment with lysophosphatidic acid was associated with enhanced invasion of the cancer cells. This increase in   invasive  ness was suppressed by the addition of C3, a specific inhibitor of Rho. These findings suggest that up-regulation of Rho GTPases is important in the tumor progression of ovarian carcinoma and that Rho family proteins could be a molecular target in cancer therapy. 
 RhoA protein expression in primary breast cancers and matched lymphocytes is associated with progression of the disease. RhoA protein is over-expressed in breast cancer and other solid tumors and has been used in tumor biopsies as a quantitative tumor marker for progression, stage and prognosis in molecular detection strategies. Measuring protein markers in plasma or blood cells is preferred to tumor biopsies as it represents a minimally   invasive  , repeatable measurement that can be followed over time. In this study we evaluated the hypothesis that quantitative RhoA protein expression in circulatory lymphocytes is identically associated with the same tumor clinico-pathological features found in biopsies. RhoA protein levels were analyzed by Western blotting in circulating lymphocytes isolated from 52 consecutive patients with breast cancer and in 34 paired breast tumor biopsies from the same case study, and compared with the following clinico-pathological features of the patients: histological grade, tumor size, steroid receptor status, lymphonode status, proliferative activity and prognosis [Nottingham Prognostic Index (NPI)]. We observed that the level of circulatory, peripheral lymphocyte RhoA expression reflected that found in the matched biopsy of the same patient. Furthermore, similarly to previous reports regarding breast cancer tissue biopsies, the level of RhoA protein expression in both biopsies and in circulatory lymphocytes was positively associated with tumor size, grade, proliferative activity of the tumor biopsy and NPI, while there was no significant association of RhoA protein expression with either estrogen- or progesterone-receptor expression. Our study demonstrated that the association of lymphocyte RhoA protein expression with classical clinico-pathological parameters closely corresponded with that observed for RhoA protein expression in the tumor biopsies. We propose that measurement of RhoA expression in the circulatory lymphocytes of breast cancer patients can be used to predict breast cancer occurrence, progression and prognosis and may prove valuable in the management of cancer patients. 
 GTPases Rho distribution in intraepithelial and   invasive   neoplasias of the uterine cervix. To evaluate the distribution of GTPases RhoA, RhoB, and Cdc42 in cervical intraepithelial neoplasias (CIN) and   invasive   neoplasias of the uterine cervix.samples of neoplastic lesions of the uterine cervix of 44 patients were classified in: CIN I (n = 10), CIN II (n = 10), CIN III (n = 09), and   invasive   carcinoma (n = 15). Antibodies anti-RhoA, anti-RhoB, and anti-Cdc42 were used and staining was classified as: negative, mild, moderate, and intense positive.When compared with dysplastic cells, superficial cells showed: higher expression of RhoB in CIN I (p = 0.0018), and lower expression of Cdc42 in CIN I (p = 0.0225). The authors observed higher expression of RhoA (p = 0.0002) and RhoB (p = 0.0046) in CIN dysplastic cells when compared with   invasive   carcinoma cells.GTPases Rho may be involved with the regulation of biological processes, important to the progression of cervical neoplasias. Probably, RhoA is important for maintenance of cell differentiation and RhoB protects cells from malignant cervical neoplasia. 
 Activated G(alpha)13 impairs cell   invasive  ness through p190RhoGAP-mediated inhibition of RhoA activity. The GTPase RhoA is a downstream target of heterotrimeric G(13) proteins and plays key roles in cell migration and invasion. Here, we show that expression in human melanoma cells of a constitutively active, GTPase-deficient Galpha(13) form (G(alpha)(13)QL) or lysophosphatidylcholine (LPC)-promoted signaling through G(alpha)(13)-coupled receptors led to a blockade of chemokine-stimulated RhoA activation and cell invasion that was rescued by active RhoA. Melanoma cells expressing G(alpha)(13)QL or cells stimulated with LPC displayed an increase in p190RhoGAP activation, and defects in RhoA activation and invasion were recovered by knocking down p190RhoGAP expression, thus identifying this GTPase-activating protein (GAP) protein as a downstream G(alpha)(13) target that is responsible for these inhibitory responses. In addition, defective stress fiber assembly and reduced migration speed underlay inefficient invasion of G(alpha)(13)QL melanoma cells. Importantly, G(alpha)(13)QL expression in melanoma cells led to impairment in lung metastasis associated with prolonged survival in SCID mice. The data indicate that G(alpha)(13)-dependent downstream effects on RhoA activation and invasion tightly depend on cell type-specific GAP activities and that G(alpha)(13)-p190RhoGAP signaling might represent a potential target for intervention in melanoma metastasis. 
  GeneRIFs  
 A MAPK-Driven Feedback Loop Suppresses Rac Activity to Promote RhoA-Driven Cancer Cell Invasion. Our model predicted an unanticipated feedback loop, whereby Raf/MEK/ERK signalling maintains suppression of Rac1 by inhibiting the Rac-activating Sos1-Eps8-Abi1 complex, allowing RhoA activity to predominate in   invasive   protrusions. 
 Blocking the expression of RhoA in colonic tumor cells transfected with pshRNA-RhoA can reduce their   invasive   and adhesive capabilities. 
 reduction of RhoA signaling by Activin B together with persistent Rac1 activity is a prerequisite for inducing an   invasive   phenotype in clear cell renal cell carcinoma. 
 Snail regulates the motility and   invasive  ness of oral cancer cells via RhoA/Cdc42/p-ERM pathway. 
 Study demonstrates that increased DGKzeta protein levels in SW620 cells are associated with increased Rho GTPase activity and downstream signaling; silencing of DGKzeta expression in SW620 cells decreased Rac1 and RhoA activity and attenuated cell invasion; DGKzeta silencing also attenuated the   invasive  ness of PC-3 prostate cancer and MDA-MB-231 breast cancer cells. 
 The TMX2-28 breast cancer cell line RhoA dependent,   invasive   phenotype is mmp2 and mmp9 independent. 
 Netrin-1 promotes glioblastoma cell   invasive  ness and angiogenesis by multiple pathways including activation of RhoA, cathepsin B, and cAMP-response element-binding protein 
 propose that RhoA triggers signalling pathways that, upregulating expression of a proteinase at specific membrane localizations, may confer an highly   invasive   phenotype to endothelial cells 
 NHERF1 overexpression enhances the   invasive   phenotype in breast cancer cells, both alone and in synergy with exposure to the tumor microenvironment, via the coordination of PKA-gated RhoA/p38 signaling. 
  [Mm]etastasize  PubMed  
 The tumor-suppressor gene ARHI (DIRAS3) suppresses ovarian cancer cell migration through inhibition of the Stat3 and FAK/Rho signaling pathways. Ovarian cancers migrate and   metastasize   over the surface of the peritoneal cavity. Consequently, dysregulation of mechanisms that limit cell migration may be particularly important in the pathogenesis of the disease. ARHI is an imprinted tumor-suppressor gene that is downregulated in >60% of ovarian cancers, and its loss is associated with decreased progression-free survival. ARHI encodes a 26-kDa GTPase with homology to Ras. In contrast to Ras, ARHI inhibits cell growth, but whether it also regulates cell motility has not been studied previously. Here we report that re-expression of ARHI decreases the motility of IL-6- and epidermal growth factor (EGF)-stimulated SKOv3 and Hey ovarian cancer cells, inhibiting both chemotaxis and haptotaxis. ARHI binds to and sequesters Stat3 in the cytoplasm, preventing its translocation to the nucleus and localization in focal adhesion complexes. Stat3 siRNA or the JAK2 inhibitor AG490 produced similar inhibition of motility. However, the combination of ARHI expression with Stat3 knockdown or inhibition produced greatest inhibition in ovarian cancer cell migration, consistent with Stat3-dependent and Stat3-independent mechanisms. Consistent with two distinct signaling pathways, knockdown of Stat3 selectively inhibited IL-6-stimulated migration, whereas knockdown of focal adhesion kinase (FAK) preferentially inhibited EGF-stimulated migration. In EGF-stimulated ovarian cancer cells, re-expression of ARHI inhibited FAK(Y397) and Src(Y416) phosphorylation, disrupted focal adhesions, and blocked FAK-mediated RhoA signaling, resulting in decreased levels of GTP-RhoA. Re-expression of ARHI also disrupted the formation of actin stress fibers in a FAK- and RhoA-dependent manner. Thus, ARHI has a critical and previously uncharacterized role in the regulation of ovarian cancer cell migration, exerting inhibitory effects on two distinct signaling pathways. 
  [Mm]etastasis  PubMed  
 Protein kinase A gating of a pseudopodial-located RhoA/ROCK/p38/NHE1 signal module regulates invasion in breast cancer cell lines.   Metastasis   results from a sequence of selective events often involving interactions with elements of the tumor-specific physiological microenvironment. The low-serum component of this microenvironment confers increased motility and invasion in breast cancer cells by activating the Na+/H+ exchanger isoform 1 (NHE1). The present study was undertaken to characterize the signal transduction mechanisms underlying this serum deprivation-dependent activation of both the NHE1 and the concomitant invasive characteristics such as leading edge pseudopodia development and penetration of matrigel in breast cancer cell lines representing different stages of metastatic progression. Using pharmacological and genetic manipulation together with transport and kinase activity assays, we observe that the activation of the NHE1 and subsequent invasion by serum deprivation in metastatic human breast cells is coordinated by a sequential RhoA/p160ROCK/p38MAPK signaling pathway gated by direct protein kinase A phosphorylation and inhibition of RhoA. Fluorescence resonance energy transfer imaging of RhoA activity and immunofluorescence analysis of phospho-RhoA and NHE1 show that serum deprivation dynamically remodels the cell, forming long, leading edge pseudopodia and that this signal module is preferentially compartmentalized in these leading edge pseudopodia, suggesting a tight topographic relation of the signaling module to an invasion-specific cell structure. 
 Interleukin-8 induces the endothelial cell migration through the activation of phosphoinositide 3-kinase-Rac1/RhoA pathway. Endothelial cell migration is essential for tumor angiogenesis, and interleukin-8 (IL-8) has been shown to play an important role in tumor growth, angiogenesis, and   metastasis  . This study aimed to investigate the molecular mechanism of IL-8 induced endothelial cell migration. Our results indicated that IL-8 induced a rapid rearrangement of the actin cytoskeleton in EA.Hy926 cells, generating extensions resembling membrane ruffling and stress fibers. These processes required parallel upregulation of the small GTPases Rac1 and RhoA. Moreover, we demonstrated that IL-8 activated PI3K following the same kinetics observed from IL-8 induction of cytoskeletal rearrangement, suggesting the participation of PI3K in these processes. Taken together, our study demonstrates that PI3K-Rac1/RhoA signaling pathway plays a vital role in IL-8 induced endothelial cell migration, and provides new insight into the molecular mechanisms by which IL-8 contributes to tumor angiogenesis and   metastasis  . 
 The activity of RhoA is correlated with lymph node   metastasis   in human colorectal cancer. Rho family GTPases play a pivotal role in the regulation of numerous cellular functions associated with malignant transformation and   metastasis  . To evaluate the role of these GTPases in colorectal cancer, the protein expression levels and activities of these proteins in matched sets of tumor and non-tumor tissues of surgical specimens were analyzed. The relationship between the protein levels and activities in tumor tissues to the clinicopathological features was also assessed. The expression levels of RhoA, active RhoA, Rac1, and active Rac1 in tumor tissues were higher than in normal tissues. The amounts of active RhoA protein in primary tumors correlated with lymph nodes   metastasis  . No relationship was noted between the protein expression levels and other clinicopathological findings. These findings suggest that the Rho family small GTPases are related to malignant transformation and progression of colorectal cancer and the activation of RhoA is associated with the lymph node   metastasis  . 
 Interleukin-8 induces the endothelial cell migration through the Rac 1/RhoA-p38MAPK pathway. Endothelial cell migration is essential for tumor angiogenesis, and interleukin-8 (IL-8) has been shown to play an important role in tumor growth, angiogenesis, and   metastasis  . The objective of this study was to investigate the molecular mechanism of IL-8 induced endothelial cell migration in vitro.Fluorescence microscope was used to study the distribution of cytoskeleton. The expression of Rac1 and RhoA protein was detected by western blotting. After endothelial cells were transfected by lipofectamine 2000 reagent, the Transwell chamber motility assay was applied to observe the migration of endothelial cells induced by IL-8. The active p38MAPK (mitogen-activated protein kinase) was evaluated by the p38MAPK activation assay.We demonstrated that IL-8 activated cell migration can be impaired by p38MAPK inhibitor, suggesting the participation of p38MAPK in the cell migration. Our results indicated that p38MAPK signaling is required for membrane ruffles, lamellipodia extensions, and actin stress fibers formation induced by IL-8. Furthermore, p38MAPK inhibitor led to increased Rac1 and RhoA expression in IL-8 treated EA.hy926 cells. In addition, IL-8 induced p38MAPK activation was suppressed by dominant-negative mutant for Rac1 and RhoA.Our study demonstrates that IL-8-Rac1/RhoA-p38MAPK signaling pathway plays a vital role in the IL-8-induced endothelial cell migration, and it provides new insight into the molecular mechanisms by which IL-8 contributes to tumor angiogenesis and   metastasis  . 
 Regulation of CXCR4/AKT-signaling-induced cell invasion and tumor   metastasis   by RhoA, Rac-1, and Cdc42 in human esophageal cancer. CXC chemokines and their cognate receptors have been implicated wildly in cancer pathogenesis. In the present study, we report a critical cause relationship between CXCR4 expression and tumorigenesis in the setting of human esophageal squamous cell carcinoma (ESCC). In ESCC cells, CXCR4 expression was significantly higher than in human esophageal epithelial cells (HEEC). Reduction of CXCR4 in ESCC cells reduced cell proliferation and invasion in vitro and tumor growth in vivo. Among the potential downstream targets of CXCR4-CXCL12 are RhoA, Rac-1, and Cdc42, which are likely to contribute to the invasiveness of ESCC cells. Finally, we found that CXCR4-CXCL12/AKT axis regulates RhoA, Rac-1, and Cdc42 to modulate cell invasion and tumor   metastasis  . Together, these results demonstrate a role for CXCR4 in ESCC   metastasis   and progression and suggest potential targets for therapeutic intervention. 
 Balanced Tiam1-rac1 and RhoA drives proliferation and invasion of pancreatic cancer cells. Tiam1 is a rac1-specific guanine nucleotide exchange factor, and Tiam1-rac1 is involved in a number of cellular processes. Rac1 and RhoA act as molecular switches that cycle between GTP- and GDP-bound states to balance the activities of rac1 and RhoA. The downregulation of rac1 activity leads to upregulation of RhoA activity, which promotes invasion and migration of pancreatic cancers cells. At present, however, the role of Tiam1-rac1 and RhoA in pancreatic cancers is not fully understood. We found that Tiam1 was upregulated in pancreatic cancers and was significantly expressed in tumors without lymph node involvement or distant   metastasis   compared with cancers where there was involvement. Although Tiam1-rac1 signaling promoted pancreatic cancer cell proliferation and tumor growth via the Wnt signaling pathway in vitro and in vivo, inhibiting Tiam1-rac1 signaling did not prolong the overall survival time in vivo. This provided evidence that there was a balance between rac1 and RhoA activities in pancreatic cancers. Furthermore, only the combined inhibition of Tiam1-rac1 and RhoA had a beneficial effect on the growth of pancreatic cancers in vivo. Taken together, these results suggest that the progression of pancreatic tumors is partially controlled by the balance between Tiam1-rac1 and RhoA. 
 Increased diacylglycerol kinase ζ expression in human metastatic colon cancer cells augments Rho GTPase activity and contributes to enhanced invasion. Unraveling the signaling pathways responsible for the establishment of a metastatic phenotype in carcinoma cells is critically important for understanding the pathology of cancer. The acquisition of cell motility is a key property of metastatic tumor cells and is a prerequisite for invasion. Rho GTPases regulate actin cytoskeleton reorganization and the cellular responses required for cell motility and invasion. Diacylglycerol kinase ζ (DGKζ), an enzyme that phosphorylates diacylglycerol to yield phosphatidic acid, regulates the activity of the Rho GTPases Rac1 and RhoA. DGKζ mRNA is highly expressed in several different colon cancer cell lines, as well as in colon cancer tissue relative to normal colonic epithelium, and thus may contribute to the metastatic process.To investigate potential roles of DGKζ in ca  ncer metas  tasis, a cellular, isogenic model of human colorectal cancer metastatic transition was used. DGKζ protein levels, Rac1 and RhoA activity, and PAK phosphorylation were measured in the non-metastatic SW480 adenocarcinoma cell line and its highly metastatic variant, the SW620 line. The effect of DGKζ silencing on Rho GTPase activity and invasion through Matrigel-coated Transwell inserts was studied in SW620 cells. Invasiveness was also measured in PC-3 prostate cancer and MDA-MB-231 breast cancer cells depleted of DGKζ.DGKζ protein levels were elevated approximately 3-fold in SW620 cells compared to SW480 cells. There was a concomitant increase in active Rac1 in SW620 cells, as well as substantial increases in the expression and phosphorylation of the Rac1 effector PAK1. Similarly, RhoA activity and expression were increased in SW620 cells. Knockdown of DGKζ expression in SW620 cells by shRNA-mediated silencing significantly reduced Rac1 and RhoA activity and attenuated the invasiveness of SW620 cells in vitro. DGKζ silencing in highly metastatic MDA-MB-231 breast cancer cells and PC-3 prostate cancer cells also significantly attenuated their invasiveness.Elevated DGKζ expression contributes to increased Rho GTPase activation and the enhanced motility of metastatic cancer cells. These findings warrant further investigation of the clinical relevance of DGKζ upregulation in colon and other cancers. Interfering with DGKζ function could provide a means of inhibiting   invasion   and metastasis. 
 Annexin-A1 controls an ERK-RhoA-NFκB activation loop in breast cancer cells. Wound healing is critical for normal development and pathological processes including cancer cell   metastasi  s. MAPK, Rho-GTPases and NFκB are important regulators of wound healing, but mechanisms for their integration are incompletely understood. Annexin-A1 (ANXA1) is upregulated in invasive breast cancer cells resulting in constitutive activation of NFκB. We show here that silencing ANXA1 increases the formation of stress fibers and focal adhesions, which may inhibit wound healing. ANXA1 regulated wound healing is dependent on the activation of ERK1/2. ANXA1 increases the activation of RhoA, which is dependent on ERK activation. Furthermore, active RhoA is important in NF-κB activation, where constitutively active RhoA potentiates NFκB activation, while dominant negative RhoA inhibits NFκB activation in response to CXCL12 stimulation and active MEKK plasmids. These findings establish a central role for ANXA1 in the cell migration through the activation of NFκB, ERK1/2 and RhoA. 
 Vascular endothelial growth factor C promotes cervical cancer   metastasis   via up-regulation and activation of RhoA/ROCK-2/moesin cascade. The elevated expression of vascular endothelial growth factor C (VEGF-C) is correlated with clinical cervical cancer   metastasis   and patient survival, which is interpreted by VEGF-C functions to stimulate angiogenesis and lymphatic genesis. However, the direct impact of VEGF-C on cervical cancer cell motility remains largely unknown.In this study, we investigated the effects of VEGF-C on actin cytoskeleton remodeling and on cervical cancer cell migration and invasion and how the actin-regulatory protein, moesin regulated these effects through RhoA/ROCK-2 signaling pathway.On cervical carcinoma cell line SiHa cells, exposure of VEGF-C triggered remodeling of the actin cytoskeleton and the formation of membrane ruffles, which was required for cell movement. VEGF-C significantly enhanced SiHa cells horizontal migration and three-dimensional invasion into matrices. These actions were dependent on increased expression and phosphorylation of the actin-regulatory protein moesin and specific moesin siRNA severely impaired VEGF-C stimulated-cell migration. The extracellular small GTPase RhoA/ROCK-2 cascade mediated the increased moesin expression and phosphorylation, which was discovered by the use of Y-27632, a specific inhibitor of Rho kinase and by transfected constitutively active, dominant-negative RhoA as well as ROCK-2 SiRNA. Furthermore, in the surgical cervical specimen from the patients with FIGO stage at cervical intra-epithelial neoplasia and I-II cervical squamous cell carcinoma, the expression levels of moesin were found to be significantly correlated with tumor malignancy and   metastasis  .These results implied that VEGF-C promoted cervical cancer   metastasis   by upregulation and activation of moesin protein through RhoA/ROCK-2 pathway. Our findings offer new insight into the role of VEGF-C on cervical cancer progression and may provide potential targets for cervical cancer therapy. 
 MiR-126 suppresses colon cancer cell proliferation and invasion via inhibiting RhoA/ROCK signaling pathway. Recent data strongly suggests the profound role of miRNAs in cancer progression. Here, we showed miR-126 expression was much lower in HCT116, SW620 and HT-29 colon cancer cells with highly metastatic potential and miR-126 downregulation was more frequent in colorectal cancers with   metastasis  . Restored miR-126 expression inhibited HT-29 cell growth, cell-cycle progression and invasion. Mechanically, microarray results combined with bioinformatic and experimental analysis demonstrated miR-126 exerted cancer suppressor role via inhibiting RhoA/ROCK signaling pathway. These results suggest miR-126 function as a potential tumor suppressor in colon cancer progression and miR-126/RhoA/ROCK may be a novel candidate for developing rational therapeutic strategies. 
 Blockade of the RhoA-JNK-c-Jun-MMP2 cascade by atorvastatin reduces osteosarcoma cell invasion. Osteosarcoma is characterized by a high malignant and metastatic potential, which points to the need for new therapeutic strategies to prevent cell   metastasis  . In this study, we show that statin-induced HMG-CoA reductase inhibition reduces cell migration and invasion in human and murine osteosarcoma cells, independently of the genotype. The statin-induced reduction of cell migration and invasion was independent of induction of apoptosis and was geranylgeranylpyrophosphate-dependent. The statin reduced matrix metalloproteinase (MMP) 2, 9, and 14 and TIMP2 expression or activity in invading cells. Forced expression of MMP2 and MMP14 overcame the inhibitory effect of the statin on cell invasion, suggesting a role for these MMPs in invasive potential. We also investigated the mechanisms involved in the reduced MMP2 activity and cell invasion. Inhibition of JNK, but not ERK1/2 signaling, reduced MMP2 activity. Pharmacological or constitutive activation of JNK overcame the reduced MMP2 activity and cell invasion induced by the statin. The statin decreased JNK phosphorylation and c-Jun nuclear translocation, suggesting that HMG-CoA reductase inhibition targets the JNK-c-Jun signaling pathway. We showed that mevalonate or geranylgeranylpyrophosphate treatment prevented the statin-induced reduction in JNK phosphorylation, MMP2 activity, and cell invasion. Forced expression of a constitutively active form of RhoA increased JNK phosphorylation and overcame the inhibitory effect of atorvastatin on MMP2 activity and cell invasion. The data establish a link between RhoA, JNK, c-Jun, and MMP2 activity that is functionally involved in the reduction in osteosarcoma cell invasion by the statin. This suggests a novel strategy targeting RhoA-JNK-c-Jun signaling to reduce osteosarcoma cell tumorigenesis. 
 [Expression and significance of RhoA and NF-ΚB in human gastric carcinoma]. To investigate the expression of RhoA and NF-κB in gastric carcinoma and their correlation with clinicopathological fearures. To determine the effective prognostic factors of long-term suivival of gastric carcinoma patients.The role of RhoA and NF-κB in gastric carcinoma was assessed by tissue array technology and the levels of RhoA and NF-κB expression in paraffin-embedded tissues was quantified by immunohistochemistry from 189 cases of gastric carcinoma, 54 cases of their adjacent tissues, and 32 cases of normal gastric mucosa. The prognosis of gastric carcinoma was evaluated by Kaplan-Meier survival analysis and Cox multivariate regression analysis.The positive rates of RhoA expression were 84.7%, 68.5% and 65.6% in gastric carcinoma, adjacent tissues and normal mucosa, respectively. The expression of RhoA in gasric carcinoma was significantly higher than that in adjacent tissues and normal mucosa (P < 0.05). The positive rates of NF-κB expression were 75.1%, 42.6% and 15.6%% in gastric carcinoma, adjacent tissues and normal mucosa, respectively. The expression of NF-κB in gasric carcinoma was significantly higher than that in adjacent tissues and normal mucosa (P < 0.05). RhoA was positively linked with NF-κB (r = 0.203, P = 0.005). In gastric carcinoma, the expression of RhoA was related with depth of invasion (P < 0.05), and the expression of NF-κB was related with depth of invasion and lym  ph node me  tastasis (P < 0.05). The Kaplan-Meier survival analysis showed that the tumor size, lym  ph node me  tastasis, depth of invasion, expression of RhoA and NF-κB can shorten the cumulative survival rate. With these paramaters entering the Cox multivariate regression analysis mode, it was revealed that expression of NF-κB, l  ymph node   metastasis and depth of invasion are independent prognostic factors.The overexpression of RhoA and NF-κB is involved in the occurrence and development of gastric carcinoma. RhoA is positively linked with NF-κB. They are correlated with the i  nvasion an  d metastasis of gastric carcinoma. The expression of NF-κB  , lymph no  de metastasis, depth of invasion are independent prognostic factors playing an important role in prediction of the clinical outcome after radical resection of gastric carcinoma. 
 MiR-200b/200c/429 subfamily negatively regulates Rho/ROCK signaling pathway to suppress hepatocellular carcinoma   metastasis  . MiR-200 family is an important regulator of epithelial-mesenchymal transition and has been implicated in human carcinogenesis. However, their expression and functions in human cancers remain controversial. In the work presented here, we showed that miR-200 family members were frequently down-regulated in hepatocellular carcinoma (HCC). Although all five members of miR-200 family inhibited ZEB1/2 expression in HCC cell lines, we showed that overexpression only of the miR-200b/200c/429 subfamily, but not the miR-200a/141 subfamily, resulted in impeded HCC cell migration. Further investigations led to the identification of RhoA and ROCK2 as specific down-stream targets of the miR-200b/200c/429 subfamily. We demonstrated that the miR-200b/200c/429 subfamily inhibited HCC cell migration through modulating Rho/ROCK mediated cell cytoskeletal reorganization and cell-substratum adhesion. Re-expression of miR-200b significantly suppressed lung   metastasis   of HCC cells in an orthotopic liver implantation model in vivo. In conclusion, our findings identified the miR-200b/200c/429 subfamily as   metastasis   suppressor microRNAs in human HCC and highlighted the functional discrepancy among miR-200 family members. 
 mTOR regulate EMT through RhoA and Rac1 pathway in prostate cancer. Recently, an increasing number of studies have suggested that mTOR plays a critical role in the regulation of tumor cell motility, invasion and cancer   metastasis  . However, little is known about the signaling mechanisms in regulating epithelial-mesenchymal transition (EMT) of prostate cancer. In this study, we found that the expression levels of Raptor and Rictor in prostate cancer tissues were elevated, which may suggest that Raptor and Rictor signaling pathways are associated with prostate cancer progression and   metastasis  . Inhibition of mTORC1 or mTORC2 by knock down of Raptor or Rictor, respectively, migration and invasion of prostate cancer were attenuated. Furthermore, EMT, a characterized by the changed expression levels of various markers like E-cadherin, β-catenin, N-cadherin, and vimentin emergend following inhibition of Raptor or Rictor. Finally, the small GTPases (RhoA and Rac1) which were crucial regulatory proteins in cell migration and invasion were inactivited after downregulating Raptor and Rictor. These results suggest that mTOR regulate EMT at least in part by down regulation of RhoA and Rac1 signaling pathways. Our findings provide novel very attractive target strategies that the inhibition of mTOR signaling pathways may retard prostate cancer migration and invasion at early stages. 
 A MAPK-Driven Feedback Loop Suppresses Rac Activity to Promote RhoA-Driven Cancer Cell Invasion. Cell migration in 3D microenvironments is fundamental to development, homeostasis and the pathobiology of diseases such as cancer. Rab-coupling protein (RCP) dependent co-trafficking of α5β1 and EGFR1 promotes cancer cell invasion into fibronectin (FN) containing extracellular matrix (ECM), by potentiating EGFR1 signalling at the front of invasive cells. This promotes a switch in RhoGTPase signalling to inhibit Rac1 and activate a RhoA-ROCK-Formin homology domain-containing 3 (FHOD3) pathway and generate filopodial actin-spike protrusions which drive invasion. To further understand the signalling network that drives RCP-driven invasive migration, we generated a Boolean logical model based on existing network pathways/models, where each node can be interrogated by computational simulation. The model predicted an unanticipated feedback loop, whereby Raf/MEK/ERK signalling maintains suppression of Rac1 by inhibiting the Rac-activating Sos1-Eps8-Abi1 complex, allowing RhoA activity to predominate in invasive protrusions. MEK inhibition was sufficient to promote lamellipodia formation and oppose filopodial actin-spike formation, and led to activation of Rac and inactivation of RhoA at the leading edge of cells moving in 3D matrix. Furthermore, MEK inhibition abrogated RCP/α5β1/EGFR1-driven invasive migration. However, upon knockdown of Eps8 (to suppress the Sos1-Abi1-Eps8 complex), MEK inhibition had no effect on RhoGTPase activity and did not oppose invasive migration, suggesting that MEK-ERK signalling suppresses the Rac-activating Sos1-Abi1-Eps8 complex to maintain RhoA activity and promote filopodial actin-spike formation and invasive migration. Our study highlights the predictive potential of mathematical modelling approaches, and demonstrates that a simple intervention (MEK-inhibition) could be of therapeutic benefit in preventing invasive migration   and metast  asis. 
 RhoA mediates cyclooxygenase-2 signaling to disrupt the formation of adherens junctions and increase cell motility. Cyclooxygenase-2 (COX-2) represents an important target for treatment and prevention of colorectal cancer. Although COX-2 signaling is implicated in promoting tumor cell growth and invasion, the molecular mechanisms that mediate these processes are largely unknown. In this study, we show that the RhoA pathway mediates COX-2 signaling to disrupt the formation of adherens junctions and increase cell motility. Disruption of adherens junctions promotes tumor cell invasion and   metastasis   and is often associated with tumor progression. We detected high levels of RhoA activity in HCA-7 colon carcinoma cells that constitutively express COX-2. Inhibition of COX-2 significantly reduced the levels of RhoA activity in HCA-7 cells, suggesting that constitutive expression of COX-2 stimulates RhoA activity. Interestingly, inhibition of COX-2 or silencing of COX-2 expression with small interfering RNA (siRNA) stimulated the formation of adherens junctions, concomitant with increased protein levels of E-cadherin and alpha-catenin. Furthermore, inhibition of RhoA or silencing of RhoA expression with siRNA increased the levels of E-cadherin and alpha-catenin. Inhibition of Rho kinases (ROCK), the RhoA effector proteins, also increased levels of E-cadherin and alpha-catenin and stimulated formation of adherens junctions. The motility of HCA-7 cells was significantly decreased when COX-2 or RhoA was inhibited. Therefore, our data reveal a novel molecular mechanism that links COX-2 signaling to disrupt the formation of adherens junctions; COX-2 stimulates the RhoA/ROCK pathway, which reduces levels of E-cadherin and alpha-catenin leading to disruption of adherens junction formation and increased motility. Understanding of COX-2 downstream signaling pathways that promote tumor progression is crucial for the development of novel therapeutic strategies. 
 KiSS1 suppresses TNFalpha-induced breast cancer cell invasion via an inhibition of RhoA-mediated NF-kappaB activation. Tumor necrosis factor-alpha (TNFalpha) induces cancer development and   metastasis  , which is prominently achieved by nuclear factor-kappa B (NF-kappaB) activation. TNFalpha-induced NF-kappaB activation enhances cellular mechanisms including proliferation, migration, and invasion. KiSS1, a key regulator of puberty, was initially discovered as a tumor   metastasis   suppressor. The expression of KiSS1 was lost or down-regulated in different metastatic tumors. However, it is unclear whether KiSS1 regulates TNFalpha-induced NF-kappaB activation and further tumor cell migration. In this study, we demonstrate that KiSS1 suppresses the migration of breast cancer cells by inhibiting TNFalpha-induced NF-kappaB pathway and RhoA activation. Both KiSS1 overexpression and KP10 (kisspeptin-10) stimulation inhibited TNFalpha-induced NF-kappaB activity, suppressed TNFalpha-induced cell migration and cell attachment to fibronectin in breast cancer cells while KP10 has little effect on cancer cell proliferation. Furthermore, KP10 inhibited TNFalpha-induced cell migration and RhoA GTPase activation. Therefore, our data demonstrate that KiSS1 inhibits TNFalpha-induced NF-kappaB activation via downregulation of RhoA activation and suppression of breast cancer cell migration and invasion. 
 HGF/c-Met overexpressions, but not met mutation, correlates with progression of non-small cell lung cancer. Hepatocyte Growth Factor (HGF) and its receptor c-Met are suggested to play an important role in progression of solid organ tumors by mediating cell motility, invasion and   metastasis  . Overexpression of HGF and c-Met have been shown in non-small-cell lung cancer (NSCLC). However, their role in tumor progression is not clearly defined. The aim of this study is to determine the role of HGF/c-Met pathway and its association with invasion related markers and clinicopathologic parameters in NSCLC. Immunohistochemical analysis was performed on 63 paraffin-embedded NSCLC tumor sections. The expressions of invasion related markers such as Matrix Metalloproteinases (MMPs) 2 and 9, Tissue Inhibitor Metalloproteinase (TIMP) 1 and 3 and RhoA were also examined. Co-expression of HGF/c-Met was significantly associated with lymph node invasion and TIMP-3 and RhoA overexpressions. There were positive correlation between TIMP-3 overexpression and advanced stage and negative correlation between RhoA overexpression and survival. DNA sequencing for Met mutations in both nonkinase and tyrosine kinase (TK) domain was established. A single nucleotide polymorphism (SNP) in sema domain and two SNPs in TK domain of c-Met were found. There was no statistically significant correlation between the presence of c-Met alterations and clinicopathologic parameters except shorter survival time in cases with two SNPs in TK domain. These results suggest that HGF/c-Met might exert their effects in tumor progression in association with RhoA and probably with TIMP-3. The blockade of the HGF/c-Met pathway with RhoA and/or TIMP-3 inhibitors may be an effective therapeutic target for NSCLC treatment. 
 The NHERF1 PDZ2 domain regulates PKA-RhoA-p38-mediated NHE1 activation and invasion in breast tumor cells. Understanding the signal transduction systems governing invasion is fundamental for the design of therapeutic strategies against   metastasis  . Na(+)/H(+) exchanger regulatory factor (NHERF1) is a postsynaptic density 95/disc-large/zona occludens (PDZ) domain-containing protein that recruits membrane receptors/transporters and cytoplasmic signaling proteins into functional complexes. NHERF1 expression is altered in breast cancer, but its effective role in mammary carcinogenesis remains undefined. We report here that NHERF1 overexpression in human breast tumor biopsies is associated with metastatic progression, poor prognosis, and hypoxia-inducible factor-1alpha expression. In cultured tumor cells, hypoxia and serum deprivation increase NHERF1 expression, promote the formation of leading-edge pseudopodia, and redistribute NHERF1 to these pseudopodia. This pseudopodial localization of NHERF1 was verified in breast biopsies and in three-dimensional Matrigel culture. Furthermore, serum deprivation and hypoxia stimulate the Na(+)/H(+) exchanger, invasion, and activate a protein kinase A (PKA)-gated RhoA/p38 invasion signal module. Significantly, NHERF1 overexpression was sufficient to induce these morphological and functional changes, and it potentiated their induction by serum deprivation. Functional experiments with truncated and binding groove-mutated PDZ domain constructs demonstrated that NHERF1 regulates these processes through its PDZ2 domain. We conclude that NHERF1 overexpression enhances the invasive phenotype in breast cancer cells, both alone and in synergy with exposure to the tumor microenvironment, via the coordination of PKA-gated RhoA/p38 signaling. 
 Knockdown of BDNF suppressed invasion of HepG2 and HCCLM3 cells, a mechanism associated with inactivation of RhoA or Rac1 and actin skeleton disorganization. Brain-derived neurotrophic factor (BDNF) and its primary receptor tropomysin-related kinase B (TrkB) mediate critical signalings for supporting survival and growth of neurons. Even though we have previously confirmed that more expressions of BDNF and TrkB were closely correlated with multiple and advanced hepatocellular carcinoma (HCC), the exact mechanisms underlying have not been investigated. The expressions of BDNF and TrkB were examined by western blot and BDNF secretion was evaluated by ELISA in human HCC cell lines of HepG2 and HCCLM3 with high metastatic potential. BDNF knockdown was performed by specific BDNF-siRNA transfection in HCC cells, actin cytoskeleton was shown by FITC-phalloidin staining and the activations of RhoA, Rac1 or Cdc42 were determined using western blot. Cell apoptosis and invasion were examined by flow cytometry and transwell assay, respectively. More expressions of BDNF and TrkB were found in HCCLM3 than in HepG2 cells. Inhibited expression of BDNF by specific siRNA showed impaired actin polymerization and decreased activations of RhoA or Rac1 in both HepG2 and HCCLM3 cells. BDNF knockdown also induced apoptosis and suppressed invasion of both HepG2 and HCCLM3 cells. Our results suggested a role of BDNF/TrkB in confering HCCLM3 cells advantage of   metastasis  , and BDNF knockdown inhibited cell invasion probably through the blocked actin polymerization and the correlated inactivation of RhoA or Rac1. Aiming at BDNF/TrkB signaling interruption may be an effective strategy to prevent HCC progression. 
 Specific upregulation of RHOA and RAC1 in cancer-associated fibroblasts found at primary tumor and lymph node metastatic sites in breast cancer. The importance of tumor-stromal cell interactions in breast tumor progression and invasion is well established. Here, an evaluation of differential genomic profiles of carcinoma-associated fibroblasts (CAFs) compared to fibroblasts derived from tissues adjacent to fibroadenomas (NAFs) revealed altered focal adhesion pathways. These data were validated through confocal assays. To verify the possible role of fibroblasts in lymph node invasion, we constructed a tissue microarray consisting of primary breast cancer samples and corresponding lymph node   metastasis   and compared the expression of adhesion markers RhoA and Rac1 in fibroblasts located at these different locations. Two distinct tissue microarrays were constructed from the stromal component of 43 primary tumors and matched lymph node samples, respectively. Fibroblasts were characterized for their expression of α-smooth muscle actin (α-SMA) and vimentin. Moreover, we verified the level of these proteins in the stromal compartment from normal adjacent tissue and in non-compromised lymph nodes. Our immunohistochemistry revealed that 59 % of fibroblasts associated with primary tumors and 41 % of the respective metastatic lymph nodes (p = 0.271) displayed positive staining for RhoA. In line with this, 57.1 % of fibroblasts associated with primary tumors presented Rac1-positive staining, and the frequency of co-positivity within the lymph nodes was 42.9 % (p = 0.16). Expression of RhoA and Rac1 was absent in fibroblasts of adjacent normal tissue and in compromised lymph nodes. Based on our findings that no significant changes were observed between primary and metastatic lymph nodes, we suggest that fibroblasts are active participants in the invasion of cancer cells to lymph nodes and support the hypothesis that metastatic tumor cells continue to depend on their microenvironment. 
 Rho GTPases RhoA and Rac1 mediate effects of dietary folate on metastatic potential of A549 cancer cells through the control of cofilin phosphorylation. Folate, an important nutrient in the human diet, has been implicated in cancer, but its role in   metastasis   is not established. We have shown previously that the withdrawal of medium folate leads to the inhibition of migration and invasion of A549 lung carcinoma cells. Here we have demonstrated that medium folate regulates the function of Rho GTPases by enabling their carboxyl methylation and translocation to plasma membrane. Conversely, the lack of folate leads to the retention of these proteins in endoplasmic reticulum. Folate also promoted the switch from inactive (GDP-bound) to active (GTP-bound) GTPases, resulting in the activation of downstream kinases p21-activated kinase and LIM kinase and phosphorylation of the actin-depolymerizing factor cofilin. We have further demonstrated that in A549 cells two GTPases, RhoA and Rac1, but not Cdc42, are immediate sensors of folate status: the siRNA silencing of RhoA or Rac1 blocked effects of folate on cofilin phosphorylation and cellular migration and invasion. The finding that folate modulates metastatic potential of cancer cells was confirmed in an animal model of lung cancer using tail vein injection of A549 cells in SCID mice. A folate-rich diet enhanced lung colonization and distant   metastasis   to lymph nodes and decreased overall survival (35 versus 63 days for mice on a folate-restricted diet). High folate also promoted epithelial-mesenchymal transition in cancer cells and experimental mouse tumors. Our study provides experimental evidence for a mechanism of   metastasis   promotion by dietary folate and highlights the interaction between nutrients and   metastasis  -related signaling. 
 Arachidonic acid stimulates cell adhesion through a novel p38 MAPK-RhoA signaling pathway that involves heat shock protein 27. Rho GTPases are critical components of cellular signal transduction pathways. Both hyperactivity and overexpression of these proteins have been observed in human cancers and have been implicated as important factors in   metastasis  . We previously showed that dietary n-6 fatty acids increase cancer cell adhesion to extracellular matrix proteins, such as type IV collagen. Here we report that in MDA-MB-435 human melanoma cells, arachidonic acid activates RhoA, and inhibition of RhoA signaling with either C3 exoenzyme or dominant negative Rho blocked arachidonic acid-induced cell adhesion. Inhibition of the Rho kinase (ROCK) with either small molecule inhibitors or ROCK II-specific small interfering RNA (siRNA) blocked the fatty acid-induced adhesion. However, unlike other systems, inhibition of ROCK did not block the activation of p38 mitogen-activated protein kinase (MAPK); instead, Rho activation depended on p38 MAPK activity and the presence of heat shock protein 27 (HSP27), which is phosphorylated downstream of p38 after arachidonic acid treatment. HSP27 associated with p115RhoGEF in fatty acid-treated cells, and this association was blocked when p38 was inhibited. Furthermore, siRNA knockdown of HSP27 blocked the fatty acid-stimulated Rho activity. Expression of dominant negative p115-RhoGEF or p115RhoGEF-specific siRNA inhibited both RhoA activation and adhesion on type IV collagen, whereas a constitutively active p115RhoGEF restored the arachidonic acid stimulation in cells in which the p38 MAPK had been inhibited. These data suggest that n-6 dietary fatty acids stimulate a set of interactions that regulates cell adhesion through RhoA and ROCK II via a p38 MAPK-dependent association of HSP27 and p115RhoGEF. 
 A MAPK-Driven Feedback Loop Suppresses Rac Activity to Promote RhoA-Driven Cancer Cell Invasion. Cell migration in 3D microenvironments is fundamental to development, homeostasis and the pathobiology of diseases such as cancer. Rab-coupling protein (RCP) dependent co-trafficking of α5β1 and EGFR1 promotes cancer cell invasion into fibronectin (FN) containing extracellular matrix (ECM), by potentiating EGFR1 signalling at the front of invasive cells. This promotes a switch in RhoGTPase signalling to inhibit Rac1 and activate a RhoA-ROCK-Formin homology domain-containing 3 (FHOD3) pathway and generate filopodial actin-spike protrusions which drive invasion. To further understand the signalling network that drives RCP-driven invasive migration, we generated a Boolean logical model based on existing network pathways/models, where each node can be interrogated by computational simulation. The model predicted an unanticipated feedback loop, whereby Raf/MEK/ERK signalling maintains suppression of Rac1 by inhibiting the Rac-activating Sos1-Eps8-Abi1 complex, allowing RhoA activity to predominate in invasive protrusions. MEK inhibition was sufficient to promote lamellipodia formation and oppose filopodial actin-spike formation, and led to activation of Rac and inactivation of RhoA at the leading edge of cells moving in 3D matrix. Furthermore, MEK inhibition abrogated RCP/α5β1/EGFR1-driven invasive migration. However, upon knockdown of Eps8 (to suppress the Sos1-Abi1-Eps8 complex), MEK inhibition had no effect on RhoGTPase activity and did not oppose invasive migration, suggesting that MEK-ERK signalling suppresses the Rac-activating Sos1-Abi1-Eps8 complex to maintain RhoA activity and promote filopodial actin-spike formation and invasive migration. Our study highlights the predictive potential of mathematical modelling approaches, and demonstrates that a simple intervention (MEK-inhibition) could be of therapeutic benefit in preventing invasive migration   and metast  asis. 
 Regulation of CXCR4/AKT-signaling-induced cell invasion and tumor   metastasis   by RhoA, Rac-1, and Cdc42 in human esophageal cancer. CXC chemokines and their cognate receptors have been implicated wildly in cancer pathogenesis. In the present study, we report a critical cause relationship between CXCR4 expression and tumorigenesis in the setting of human esophageal squamous cell carcinoma (ESCC). In ESCC cells, CXCR4 expression was significantly higher than in human esophageal epithelial cells (HEEC). Reduction of CXCR4 in ESCC cells reduced cell proliferation and invasion in vitro and tumor growth in vivo. Among the potential downstream targets of CXCR4-CXCL12 are RhoA, Rac-1, and Cdc42, which are likely to contribute to the invasiveness of ESCC cells. Finally, we found that CXCR4-CXCL12/AKT axis regulates RhoA, Rac-1, and Cdc42 to modulate cell invasion and tumor   metastasis  . Together, these results demonstrate a role for CXCR4 in ESCC   metastasis   and progression and suggest potential targets for therapeutic intervention. 
 MiR-200b/200c/429 subfamily negatively regulates Rho/ROCK signaling pathway to suppress hepatocellular carcinoma   metastasis  . MiR-200 family is an important regulator of epithelial-mesenchymal transition and has been implicated in human carcinogenesis. However, their expression and functions in human cancers remain controversial. In the work presented here, we showed that miR-200 family members were frequently down-regulated in hepatocellular carcinoma (HCC). Although all five members of miR-200 family inhibited ZEB1/2 expression in HCC cell lines, we showed that overexpression only of the miR-200b/200c/429 subfamily, but not the miR-200a/141 subfamily, resulted in impeded HCC cell migration. Further investigations led to the identification of RhoA and ROCK2 as specific down-stream targets of the miR-200b/200c/429 subfamily. We demonstrated that the miR-200b/200c/429 subfamily inhibited HCC cell migration through modulating Rho/ROCK mediated cell cytoskeletal reorganization and cell-substratum adhesion. Re-expression of miR-200b significantly suppressed lung   metastasis   of HCC cells in an orthotopic liver implantation model in vivo. In conclusion, our findings identified the miR-200b/200c/429 subfamily as   metastasis   suppressor microRNAs in human HCC and highlighted the functional discrepancy among miR-200 family members. 
 Specific upregulation of RHOA and RAC1 in cancer-associated fibroblasts found at primary tumor and lymph node metastatic sites in breast cancer. The importance of tumor-stromal cell interactions in breast tumor progression and invasion is well established. Here, an evaluation of differential genomic profiles of carcinoma-associated fibroblasts (CAFs) compared to fibroblasts derived from tissues adjacent to fibroadenomas (NAFs) revealed altered focal adhesion pathways. These data were validated through confocal assays. To verify the possible role of fibroblasts in lymph node invasion, we constructed a tissue microarray consisting of primary breast cancer samples and corresponding lymph node   metastasis   and compared the expression of adhesion markers RhoA and Rac1 in fibroblasts located at these different locations. Two distinct tissue microarrays were constructed from the stromal component of 43 primary tumors and matched lymph node samples, respectively. Fibroblasts were characterized for their expression of α-smooth muscle actin (α-SMA) and vimentin. Moreover, we verified the level of these proteins in the stromal compartment from normal adjacent tissue and in non-compromised lymph nodes. Our immunohistochemistry revealed that 59 % of fibroblasts associated with primary tumors and 41 % of the respective metastatic lymph nodes (p = 0.271) displayed positive staining for RhoA. In line with this, 57.1 % of fibroblasts associated with primary tumors presented Rac1-positive staining, and the frequency of co-positivity within the lymph nodes was 42.9 % (p = 0.16). Expression of RhoA and Rac1 was absent in fibroblasts of adjacent normal tissue and in compromised lymph nodes. Based on our findings that no significant changes were observed between primary and metastatic lymph nodes, we suggest that fibroblasts are active participants in the invasion of cancer cells to lymph nodes and support the hypothesis that metastatic tumor cells continue to depend on their microenvironment. 
 mTOR regulate EMT through RhoA and Rac1 pathway in prostate cancer. Recently, an increasing number of studies have suggested that mTOR plays a critical role in the regulation of tumor cell motility, invasion and cancer   metastasis  . However, little is known about the signaling mechanisms in regulating epithelial-mesenchymal transition (EMT) of prostate cancer. In this study, we found that the expression levels of Raptor and Rictor in prostate cancer tissues were elevated, which may suggest that Raptor and Rictor signaling pathways are associated with prostate cancer progression and   metastasis  . Inhibition of mTORC1 or mTORC2 by knock down of Raptor or Rictor, respectively, migration and invasion of prostate cancer were attenuated. Furthermore, EMT, a characterized by the changed expression levels of various markers like E-cadherin, β-catenin, N-cadherin, and vimentin emergend following inhibition of Raptor or Rictor. Finally, the small GTPases (RhoA and Rac1) which were crucial regulatory proteins in cell migration and invasion were inactivited after downregulating Raptor and Rictor. These results suggest that mTOR regulate EMT at least in part by down regulation of RhoA and Rac1 signaling pathways. Our findings provide novel very attractive target strategies that the inhibition of mTOR signaling pathways may retard prostate cancer migration and invasion at early stages. 
 Annexin-A1 controls an ERK-RhoA-NFκB activation loop in breast cancer cells. Wound healing is critical for normal development and pathological processes including cancer cell   metastasi  s. MAPK, Rho-GTPases and NFκB are important regulators of wound healing, but mechanisms for their integration are incompletely understood. Annexin-A1 (ANXA1) is upregulated in invasive breast cancer cells resulting in constitutive activation of NFκB. We show here that silencing ANXA1 increases the formation of stress fibers and focal adhesions, which may inhibit wound healing. ANXA1 regulated wound healing is dependent on the activation of ERK1/2. ANXA1 increases the activation of RhoA, which is dependent on ERK activation. Furthermore, active RhoA is important in NF-κB activation, where constitutively active RhoA potentiates NFκB activation, while dominant negative RhoA inhibits NFκB activation in response to CXCL12 stimulation and active MEKK plasmids. These findings establish a central role for ANXA1 in the cell migration through the activation of NFκB, ERK1/2 and RhoA. 
 Rho GTPases RhoA and Rac1 mediate effects of dietary folate on metastatic potential of A549 cancer cells through the control of cofilin phosphorylation. Folate, an important nutrient in the human diet, has been implicated in cancer, but its role in   metastasis   is not established. We have shown previously that the withdrawal of medium folate leads to the inhibition of migration and invasion of A549 lung carcinoma cells. Here we have demonstrated that medium folate regulates the function of Rho GTPases by enabling their carboxyl methylation and translocation to plasma membrane. Conversely, the lack of folate leads to the retention of these proteins in endoplasmic reticulum. Folate also promoted the switch from inactive (GDP-bound) to active (GTP-bound) GTPases, resulting in the activation of downstream kinases p21-activated kinase and LIM kinase and phosphorylation of the actin-depolymerizing factor cofilin. We have further demonstrated that in A549 cells two GTPases, RhoA and Rac1, but not Cdc42, are immediate sensors of folate status: the siRNA silencing of RhoA or Rac1 blocked effects of folate on cofilin phosphorylation and cellular migration and invasion. The finding that folate modulates metastatic potential of cancer cells was confirmed in an animal model of lung cancer using tail vein injection of A549 cells in SCID mice. A folate-rich diet enhanced lung colonization and distant   metastasis   to lymph nodes and decreased overall survival (35 versus 63 days for mice on a folate-restricted diet). High folate also promoted epithelial-mesenchymal transition in cancer cells and experimental mouse tumors. Our study provides experimental evidence for a mechanism of   metastasis   promotion by dietary folate and highlights the interaction between nutrients and   metastasis  -related signaling. 
 Increased diacylglycerol kinase ζ expression in human metastatic colon cancer cells augments Rho GTPase activity and contributes to enhanced invasion. Unraveling the signaling pathways responsible for the establishment of a metastatic phenotype in carcinoma cells is critically important for understanding the pathology of cancer. The acquisition of cell motility is a key property of metastatic tumor cells and is a prerequisite for invasion. Rho GTPases regulate actin cytoskeleton reorganization and the cellular responses required for cell motility and invasion. Diacylglycerol kinase ζ (DGKζ), an enzyme that phosphorylates diacylglycerol to yield phosphatidic acid, regulates the activity of the Rho GTPases Rac1 and RhoA. DGKζ mRNA is highly expressed in several different colon cancer cell lines, as well as in colon cancer tissue relative to normal colonic epithelium, and thus may contribute to the metastatic process.To investigate potential roles of DGKζ in ca  ncer metas  tasis, a cellular, isogenic model of human colorectal cancer metastatic transition was used. DGKζ protein levels, Rac1 and RhoA activity, and PAK phosphorylation were measured in the non-metastatic SW480 adenocarcinoma cell line and its highly metastatic variant, the SW620 line. The effect of DGKζ silencing on Rho GTPase activity and invasion through Matrigel-coated Transwell inserts was studied in SW620 cells. Invasiveness was also measured in PC-3 prostate cancer and MDA-MB-231 breast cancer cells depleted of DGKζ.DGKζ protein levels were elevated approximately 3-fold in SW620 cells compared to SW480 cells. There was a concomitant increase in active Rac1 in SW620 cells, as well as substantial increases in the expression and phosphorylation of the Rac1 effector PAK1. Similarly, RhoA activity and expression were increased in SW620 cells. Knockdown of DGKζ expression in SW620 cells by shRNA-mediated silencing significantly reduced Rac1 and RhoA activity and attenuated the invasiveness of SW620 cells in vitro. DGKζ silencing in highly metastatic MDA-MB-231 breast cancer cells and PC-3 prostate cancer cells also significantly attenuated their invasiveness.Elevated DGKζ expression contributes to increased Rho GTPase activation and the enhanced motility of metastatic cancer cells. These findings warrant further investigation of the clinical relevance of DGKζ upregulation in colon and other cancers. Interfering with DGKζ function could provide a means of inhibiting   invasion   and metastasis. 
 MiR-126 suppresses colon cancer cell proliferation and invasion via inhibiting RhoA/ROCK signaling pathway. Recent data strongly suggests the profound role of miRNAs in cancer progression. Here, we showed miR-126 expression was much lower in HCT116, SW620 and HT-29 colon cancer cells with highly metastatic potential and miR-126 downregulation was more frequent in colorectal cancers with   metastasis  . Restored miR-126 expression inhibited HT-29 cell growth, cell-cycle progression and invasion. Mechanically, microarray results combined with bioinformatic and experimental analysis demonstrated miR-126 exerted cancer suppressor role via inhibiting RhoA/ROCK signaling pathway. These results suggest miR-126 function as a potential tumor suppressor in colon cancer progression and miR-126/RhoA/ROCK may be a novel candidate for developing rational therapeutic strategies. 
 Balanced Tiam1-rac1 and RhoA drives proliferation and invasion of pancreatic cancer cells. Tiam1 is a rac1-specific guanine nucleotide exchange factor, and Tiam1-rac1 is involved in a number of cellular processes. Rac1 and RhoA act as molecular switches that cycle between GTP- and GDP-bound states to balance the activities of rac1 and RhoA. The downregulation of rac1 activity leads to upregulation of RhoA activity, which promotes invasion and migration of pancreatic cancers cells. At present, however, the role of Tiam1-rac1 and RhoA in pancreatic cancers is not fully understood. We found that Tiam1 was upregulated in pancreatic cancers and was significantly expressed in tumors without lymph node involvement or distant   metastasis   compared with cancers where there was involvement. Although Tiam1-rac1 signaling promoted pancreatic cancer cell proliferation and tumor growth via the Wnt signaling pathway in vitro and in vivo, inhibiting Tiam1-rac1 signaling did not prolong the overall survival time in vivo. This provided evidence that there was a balance between rac1 and RhoA activities in pancreatic cancers. Furthermore, only the combined inhibition of Tiam1-rac1 and RhoA had a beneficial effect on the growth of pancreatic cancers in vivo. Taken together, these results suggest that the progression of pancreatic tumors is partially controlled by the balance between Tiam1-rac1 and RhoA. 
 Knockdown of BDNF suppressed invasion of HepG2 and HCCLM3 cells, a mechanism associated with inactivation of RhoA or Rac1 and actin skeleton disorganization. Brain-derived neurotrophic factor (BDNF) and its primary receptor tropomysin-related kinase B (TrkB) mediate critical signalings for supporting survival and growth of neurons. Even though we have previously confirmed that more expressions of BDNF and TrkB were closely correlated with multiple and advanced hepatocellular carcinoma (HCC), the exact mechanisms underlying have not been investigated. The expressions of BDNF and TrkB were examined by western blot and BDNF secretion was evaluated by ELISA in human HCC cell lines of HepG2 and HCCLM3 with high metastatic potential. BDNF knockdown was performed by specific BDNF-siRNA transfection in HCC cells, actin cytoskeleton was shown by FITC-phalloidin staining and the activations of RhoA, Rac1 or Cdc42 were determined using western blot. Cell apoptosis and invasion were examined by flow cytometry and transwell assay, respectively. More expressions of BDNF and TrkB were found in HCCLM3 than in HepG2 cells. Inhibited expression of BDNF by specific siRNA showed impaired actin polymerization and decreased activations of RhoA or Rac1 in both HepG2 and HCCLM3 cells. BDNF knockdown also induced apoptosis and suppressed invasion of both HepG2 and HCCLM3 cells. Our results suggested a role of BDNF/TrkB in confering HCCLM3 cells advantage of   metastasis  , and BDNF knockdown inhibited cell invasion probably through the blocked actin polymerization and the correlated inactivation of RhoA or Rac1. Aiming at BDNF/TrkB signaling interruption may be an effective strategy to prevent HCC progression. 
 HGF/c-Met overexpressions, but not met mutation, correlates with progression of non-small cell lung cancer. Hepatocyte Growth Factor (HGF) and its receptor c-Met are suggested to play an important role in progression of solid organ tumors by mediating cell motility, invasion and   metastasis  . Overexpression of HGF and c-Met have been shown in non-small-cell lung cancer (NSCLC). However, their role in tumor progression is not clearly defined. The aim of this study is to determine the role of HGF/c-Met pathway and its association with invasion related markers and clinicopathologic parameters in NSCLC. Immunohistochemical analysis was performed on 63 paraffin-embedded NSCLC tumor sections. The expressions of invasion related markers such as Matrix Metalloproteinases (MMPs) 2 and 9, Tissue Inhibitor Metalloproteinase (TIMP) 1 and 3 and RhoA were also examined. Co-expression of HGF/c-Met was significantly associated with lymph node invasion and TIMP-3 and RhoA overexpressions. There were positive correlation between TIMP-3 overexpression and advanced stage and negative correlation between RhoA overexpression and survival. DNA sequencing for Met mutations in both nonkinase and tyrosine kinase (TK) domain was established. A single nucleotide polymorphism (SNP) in sema domain and two SNPs in TK domain of c-Met were found. There was no statistically significant correlation between the presence of c-Met alterations and clinicopathologic parameters except shorter survival time in cases with two SNPs in TK domain. These results suggest that HGF/c-Met might exert their effects in tumor progression in association with RhoA and probably with TIMP-3. The blockade of the HGF/c-Met pathway with RhoA and/or TIMP-3 inhibitors may be an effective therapeutic target for NSCLC treatment. 
 Interleukin-8 induces the endothelial cell migration through the Rac 1/RhoA-p38MAPK pathway. Endothelial cell migration is essential for tumor angiogenesis, and interleukin-8 (IL-8) has been shown to play an important role in tumor growth, angiogenesis, and   metastasis  . The objective of this study was to investigate the molecular mechanism of IL-8 induced endothelial cell migration in vitro.Fluorescence microscope was used to study the distribution of cytoskeleton. The expression of Rac1 and RhoA protein was detected by western blotting. After endothelial cells were transfected by lipofectamine 2000 reagent, the Transwell chamber motility assay was applied to observe the migration of endothelial cells induced by IL-8. The active p38MAPK (mitogen-activated protein kinase) was evaluated by the p38MAPK activation assay.We demonstrated that IL-8 activated cell migration can be impaired by p38MAPK inhibitor, suggesting the participation of p38MAPK in the cell migration. Our results indicated that p38MAPK signaling is required for membrane ruffles, lamellipodia extensions, and actin stress fibers formation induced by IL-8. Furthermore, p38MAPK inhibitor led to increased Rac1 and RhoA expression in IL-8 treated EA.hy926 cells. In addition, IL-8 induced p38MAPK activation was suppressed by dominant-negative mutant for Rac1 and RhoA.Our study demonstrates that IL-8-Rac1/RhoA-p38MAPK signaling pathway plays a vital role in the IL-8-induced endothelial cell migration, and it provides new insight into the molecular mechanisms by which IL-8 contributes to tumor angiogenesis and   metastasis  . 
 [Expression and significance of RhoA and NF-ΚB in human gastric carcinoma]. To investigate the expression of RhoA and NF-κB in gastric carcinoma and their correlation with clinicopathological fearures. To determine the effective prognostic factors of long-term suivival of gastric carcinoma patients.The role of RhoA and NF-κB in gastric carcinoma was assessed by tissue array technology and the levels of RhoA and NF-κB expression in paraffin-embedded tissues was quantified by immunohistochemistry from 189 cases of gastric carcinoma, 54 cases of their adjacent tissues, and 32 cases of normal gastric mucosa. The prognosis of gastric carcinoma was evaluated by Kaplan-Meier survival analysis and Cox multivariate regression analysis.The positive rates of RhoA expression were 84.7%, 68.5% and 65.6% in gastric carcinoma, adjacent tissues and normal mucosa, respectively. The expression of RhoA in gasric carcinoma was significantly higher than that in adjacent tissues and normal mucosa (P < 0.05). The positive rates of NF-κB expression were 75.1%, 42.6% and 15.6%% in gastric carcinoma, adjacent tissues and normal mucosa, respectively. The expression of NF-κB in gasric carcinoma was significantly higher than that in adjacent tissues and normal mucosa (P < 0.05). RhoA was positively linked with NF-κB (r = 0.203, P = 0.005). In gastric carcinoma, the expression of RhoA was related with depth of invasion (P < 0.05), and the expression of NF-κB was related with depth of invasion and lym  ph node me  tastasis (P < 0.05). The Kaplan-Meier survival analysis showed that the tumor size, lym  ph node me  tastasis, depth of invasion, expression of RhoA and NF-κB can shorten the cumulative survival rate. With these paramaters entering the Cox multivariate regression analysis mode, it was revealed that expression of NF-κB, l  ymph node   metastasis and depth of invasion are independent prognostic factors.The overexpression of RhoA and NF-κB is involved in the occurrence and development of gastric carcinoma. RhoA is positively linked with NF-κB. They are correlated with the i  nvasion an  d metastasis of gastric carcinoma. The expression of NF-κB  , lymph no  de metastasis, depth of invasion are independent prognostic factors playing an important role in prediction of the clinical outcome after radical resection of gastric carcinoma. 
 Interleukin-8 induces the endothelial cell migration through the activation of phosphoinositide 3-kinase-Rac1/RhoA pathway. Endothelial cell migration is essential for tumor angiogenesis, and interleukin-8 (IL-8) has been shown to play an important role in tumor growth, angiogenesis, and   metastasis  . This study aimed to investigate the molecular mechanism of IL-8 induced endothelial cell migration. Our results indicated that IL-8 induced a rapid rearrangement of the actin cytoskeleton in EA.Hy926 cells, generating extensions resembling membrane ruffling and stress fibers. These processes required parallel upregulation of the small GTPases Rac1 and RhoA. Moreover, we demonstrated that IL-8 activated PI3K following the same kinetics observed from IL-8 induction of cytoskeletal rearrangement, suggesting the participation of PI3K in these processes. Taken together, our study demonstrates that PI3K-Rac1/RhoA signaling pathway plays a vital role in IL-8 induced endothelial cell migration, and provides new insight into the molecular mechanisms by which IL-8 contributes to tumor angiogenesis and   metastasis  . 
 Vascular endothelial growth factor C promotes cervical cancer   metastasis   via up-regulation and activation of RhoA/ROCK-2/moesin cascade. The elevated expression of vascular endothelial growth factor C (VEGF-C) is correlated with clinical cervical cancer   metastasis   and patient survival, which is interpreted by VEGF-C functions to stimulate angiogenesis and lymphatic genesis. However, the direct impact of VEGF-C on cervical cancer cell motility remains largely unknown.In this study, we investigated the effects of VEGF-C on actin cytoskeleton remodeling and on cervical cancer cell migration and invasion and how the actin-regulatory protein, moesin regulated these effects through RhoA/ROCK-2 signaling pathway.On cervical carcinoma cell line SiHa cells, exposure of VEGF-C triggered remodeling of the actin cytoskeleton and the formation of membrane ruffles, which was required for cell movement. VEGF-C significantly enhanced SiHa cells horizontal migration and three-dimensional invasion into matrices. These actions were dependent on increased expression and phosphorylation of the actin-regulatory protein moesin and specific moesin siRNA severely impaired VEGF-C stimulated-cell migration. The extracellular small GTPase RhoA/ROCK-2 cascade mediated the increased moesin expression and phosphorylation, which was discovered by the use of Y-27632, a specific inhibitor of Rho kinase and by transfected constitutively active, dominant-negative RhoA as well as ROCK-2 SiRNA. Furthermore, in the surgical cervical specimen from the patients with FIGO stage at cervical intra-epithelial neoplasia and I-II cervical squamous cell carcinoma, the expression levels of moesin were found to be significantly correlated with tumor malignancy and   metastasis  .These results implied that VEGF-C promoted cervical cancer   metastasis   by upregulation and activation of moesin protein through RhoA/ROCK-2 pathway. Our findings offer new insight into the role of VEGF-C on cervical cancer progression and may provide potential targets for cervical cancer therapy. 
 KiSS1 suppresses TNFalpha-induced breast cancer cell invasion via an inhibition of RhoA-mediated NF-kappaB activation. Tumor necrosis factor-alpha (TNFalpha) induces cancer development and   metastasis  , which is prominently achieved by nuclear factor-kappa B (NF-kappaB) activation. TNFalpha-induced NF-kappaB activation enhances cellular mechanisms including proliferation, migration, and invasion. KiSS1, a key regulator of puberty, was initially discovered as a tumor   metastasis   suppressor. The expression of KiSS1 was lost or down-regulated in different metastatic tumors. However, it is unclear whether KiSS1 regulates TNFalpha-induced NF-kappaB activation and further tumor cell migration. In this study, we demonstrate that KiSS1 suppresses the migration of breast cancer cells by inhibiting TNFalpha-induced NF-kappaB pathway and RhoA activation. Both KiSS1 overexpression and KP10 (kisspeptin-10) stimulation inhibited TNFalpha-induced NF-kappaB activity, suppressed TNFalpha-induced cell migration and cell attachment to fibronectin in breast cancer cells while KP10 has little effect on cancer cell proliferation. Furthermore, KP10 inhibited TNFalpha-induced cell migration and RhoA GTPase activation. Therefore, our data demonstrate that KiSS1 inhibits TNFalpha-induced NF-kappaB activation via downregulation of RhoA activation and suppression of breast cancer cell migration and invasion. 
 Arachidonic acid stimulates cell adhesion through a novel p38 MAPK-RhoA signaling pathway that involves heat shock protein 27. Rho GTPases are critical components of cellular signal transduction pathways. Both hyperactivity and overexpression of these proteins have been observed in human cancers and have been implicated as important factors in   metastasis  . We previously showed that dietary n-6 fatty acids increase cancer cell adhesion to extracellular matrix proteins, such as type IV collagen. Here we report that in MDA-MB-435 human melanoma cells, arachidonic acid activates RhoA, and inhibition of RhoA signaling with either C3 exoenzyme or dominant negative Rho blocked arachidonic acid-induced cell adhesion. Inhibition of the Rho kinase (ROCK) with either small molecule inhibitors or ROCK II-specific small interfering RNA (siRNA) blocked the fatty acid-induced adhesion. However, unlike other systems, inhibition of ROCK did not block the activation of p38 mitogen-activated protein kinase (MAPK); instead, Rho activation depended on p38 MAPK activity and the presence of heat shock protein 27 (HSP27), which is phosphorylated downstream of p38 after arachidonic acid treatment. HSP27 associated with p115RhoGEF in fatty acid-treated cells, and this association was blocked when p38 was inhibited. Furthermore, siRNA knockdown of HSP27 blocked the fatty acid-stimulated Rho activity. Expression of dominant negative p115-RhoGEF or p115RhoGEF-specific siRNA inhibited both RhoA activation and adhesion on type IV collagen, whereas a constitutively active p115RhoGEF restored the arachidonic acid stimulation in cells in which the p38 MAPK had been inhibited. These data suggest that n-6 dietary fatty acids stimulate a set of interactions that regulates cell adhesion through RhoA and ROCK II via a p38 MAPK-dependent association of HSP27 and p115RhoGEF. 
 Blockade of the RhoA-JNK-c-Jun-MMP2 cascade by atorvastatin reduces osteosarcoma cell invasion. Osteosarcoma is characterized by a high malignant and metastatic potential, which points to the need for new therapeutic strategies to prevent cell   metastasis  . In this study, we show that statin-induced HMG-CoA reductase inhibition reduces cell migration and invasion in human and murine osteosarcoma cells, independently of the genotype. The statin-induced reduction of cell migration and invasion was independent of induction of apoptosis and was geranylgeranylpyrophosphate-dependent. The statin reduced matrix metalloproteinase (MMP) 2, 9, and 14 and TIMP2 expression or activity in invading cells. Forced expression of MMP2 and MMP14 overcame the inhibitory effect of the statin on cell invasion, suggesting a role for these MMPs in invasive potential. We also investigated the mechanisms involved in the reduced MMP2 activity and cell invasion. Inhibition of JNK, but not ERK1/2 signaling, reduced MMP2 activity. Pharmacological or constitutive activation of JNK overcame the reduced MMP2 activity and cell invasion induced by the statin. The statin decreased JNK phosphorylation and c-Jun nuclear translocation, suggesting that HMG-CoA reductase inhibition targets the JNK-c-Jun signaling pathway. We showed that mevalonate or geranylgeranylpyrophosphate treatment prevented the statin-induced reduction in JNK phosphorylation, MMP2 activity, and cell invasion. Forced expression of a constitutively active form of RhoA increased JNK phosphorylation and overcame the inhibitory effect of atorvastatin on MMP2 activity and cell invasion. The data establish a link between RhoA, JNK, c-Jun, and MMP2 activity that is functionally involved in the reduction in osteosarcoma cell invasion by the statin. This suggests a novel strategy targeting RhoA-JNK-c-Jun signaling to reduce osteosarcoma cell tumorigenesis. 
 The activity of RhoA is correlated with lymph node   metastasis   in human colorectal cancer. Rho family GTPases play a pivotal role in the regulation of numerous cellular functions associated with malignant transformation and   metastasis  . To evaluate the role of these GTPases in colorectal cancer, the protein expression levels and activities of these proteins in matched sets of tumor and non-tumor tissues of surgical specimens were analyzed. The relationship between the protein levels and activities in tumor tissues to the clinicopathological features was also assessed. The expression levels of RhoA, active RhoA, Rac1, and active Rac1 in tumor tissues were higher than in normal tissues. The amounts of active RhoA protein in primary tumors correlated with lymph nodes   metastasis  . No relationship was noted between the protein expression levels and other clinicopathological findings. These findings suggest that the Rho family small GTPases are related to malignant transformation and progression of colorectal cancer and the activation of RhoA is associated with the lymph node   metastasis  . 
 RhoA mediates cyclooxygenase-2 signaling to disrupt the formation of adherens junctions and increase cell motility. Cyclooxygenase-2 (COX-2) represents an important target for treatment and prevention of colorectal cancer. Although COX-2 signaling is implicated in promoting tumor cell growth and invasion, the molecular mechanisms that mediate these processes are largely unknown. In this study, we show that the RhoA pathway mediates COX-2 signaling to disrupt the formation of adherens junctions and increase cell motility. Disruption of adherens junctions promotes tumor cell invasion and   metastasis   and is often associated with tumor progression. We detected high levels of RhoA activity in HCA-7 colon carcinoma cells that constitutively express COX-2. Inhibition of COX-2 significantly reduced the levels of RhoA activity in HCA-7 cells, suggesting that constitutive expression of COX-2 stimulates RhoA activity. Interestingly, inhibition of COX-2 or silencing of COX-2 expression with small interfering RNA (siRNA) stimulated the formation of adherens junctions, concomitant with increased protein levels of E-cadherin and alpha-catenin. Furthermore, inhibition of RhoA or silencing of RhoA expression with siRNA increased the levels of E-cadherin and alpha-catenin. Inhibition of Rho kinases (ROCK), the RhoA effector proteins, also increased levels of E-cadherin and alpha-catenin and stimulated formation of adherens junctions. The motility of HCA-7 cells was significantly decreased when COX-2 or RhoA was inhibited. Therefore, our data reveal a novel molecular mechanism that links COX-2 signaling to disrupt the formation of adherens junctions; COX-2 stimulates the RhoA/ROCK pathway, which reduces levels of E-cadherin and alpha-catenin leading to disruption of adherens junction formation and increased motility. Understanding of COX-2 downstream signaling pathways that promote tumor progression is crucial for the development of novel therapeutic strategies. 
 The NHERF1 PDZ2 domain regulates PKA-RhoA-p38-mediated NHE1 activation and invasion in breast tumor cells. Understanding the signal transduction systems governing invasion is fundamental for the design of therapeutic strategies against   metastasis  . Na(+)/H(+) exchanger regulatory factor (NHERF1) is a postsynaptic density 95/disc-large/zona occludens (PDZ) domain-containing protein that recruits membrane receptors/transporters and cytoplasmic signaling proteins into functional complexes. NHERF1 expression is altered in breast cancer, but its effective role in mammary carcinogenesis remains undefined. We report here that NHERF1 overexpression in human breast tumor biopsies is associated with metastatic progression, poor prognosis, and hypoxia-inducible factor-1alpha expression. In cultured tumor cells, hypoxia and serum deprivation increase NHERF1 expression, promote the formation of leading-edge pseudopodia, and redistribute NHERF1 to these pseudopodia. This pseudopodial localization of NHERF1 was verified in breast biopsies and in three-dimensional Matrigel culture. Furthermore, serum deprivation and hypoxia stimulate the Na(+)/H(+) exchanger, invasion, and activate a protein kinase A (PKA)-gated RhoA/p38 invasion signal module. Significantly, NHERF1 overexpression was sufficient to induce these morphological and functional changes, and it potentiated their induction by serum deprivation. Functional experiments with truncated and binding groove-mutated PDZ domain constructs demonstrated that NHERF1 regulates these processes through its PDZ2 domain. We conclude that NHERF1 overexpression enhances the invasive phenotype in breast cancer cells, both alone and in synergy with exposure to the tumor microenvironment, via the coordination of PKA-gated RhoA/p38 signaling. 
 Protein kinase A gating of a pseudopodial-located RhoA/ROCK/p38/NHE1 signal module regulates invasion in breast cancer cell lines.   Metastasis   results from a sequence of selective events often involving interactions with elements of the tumor-specific physiological microenvironment. The low-serum component of this microenvironment confers increased motility and invasion in breast cancer cells by activating the Na+/H+ exchanger isoform 1 (NHE1). The present study was undertaken to characterize the signal transduction mechanisms underlying this serum deprivation-dependent activation of both the NHE1 and the concomitant invasive characteristics such as leading edge pseudopodia development and penetration of matrigel in breast cancer cell lines representing different stages of metastatic progression. Using pharmacological and genetic manipulation together with transport and kinase activity assays, we observe that the activation of the NHE1 and subsequent invasion by serum deprivation in metastatic human breast cells is coordinated by a sequential RhoA/p160ROCK/p38MAPK signaling pathway gated by direct protein kinase A phosphorylation and inhibition of RhoA. Fluorescence resonance energy transfer imaging of RhoA activity and immunofluorescence analysis of phospho-RhoA and NHE1 show that serum deprivation dynamically remodels the cell, forming long, leading edge pseudopodia and that this signal module is preferentially compartmentalized in these leading edge pseudopodia, suggesting a tight topographic relation of the signaling module to an invasion-specific cell structure. 
 PRL-3 facilitates angiogenesis and   metastasis   by increasing ERK phosphorylation and up-regulating the levels and activities of Rho-A/C in lung cancer. The aim of this study was to investigate the mechanism of PRL-3 in inducing angiogenesis and lymphangiogenesis to promote distant and lymph node   metastasis   in human lung cancer tissues and cells.We investigated the expression of PRL-3, VEGF, and VEGF-C from 94 patients with non-small cell lung cancer (NSCLC) using immunohistochemical staining. The relationship between PRL-3 expression and microvessel density (MVD), lymphatic vessel density (LVD), clinicopathological factors, and surgical treatment outcome was also studied. Following this, we studied the effect on A549 by blocking PRL-3.PRL-3 expression in NSCLC was high, and this over-expression is correlated with advanced clinical stage (p = 0.019), distant   metastasis   (p = 0.001), lymph node   metastasis   (p = 0.001), and poor post-operative survival. PRL-3 over-expression was associated with vascular endothelial growth factor (VEGF; p = 0.000) and VEGF-C (p = 0.008) expressions, MVD and LVD (p = 0.000 and p = 0.000). Blocking PRL-3 expression in A549 cell resulted in decreased cellular proliferative, migratory, and invasive abilities, and VEGF, VEGF-C, pERK, Rho-A, and Rho-C expression was inhibited. Following inhibition of either Rho or pERK, VEGF expression was down-regulated.PRL-3 induces microvascular and lymphatic vessel formation by facilitating VEGF and VEGF-C expression in lung cancer tissues, thus promoting distant and lymph node   metastasis   of lung cancer. PRL-3 up-regulates pERK and Rho expression and activity, facilitating VEGF expression, and accelerating angiogenesis and distant   metastasis  . How to regulate VEGF-C expression needs to be further studied. 
 Loss of p57 expression and RhoA overexpression are associated with poor survival of patients with hepatocellular carcinoma. p57 and Ras homology A (RhoA) have been implicated in the growth and   metastasis   of several types of human cancers. This study aimed to detect their expression in hepatocellular carcinoma (HCC) tissue specimens and to determine a possible association with clinicopathological data and patient survival. A total of 80 HCC and corresponding distant normal tissue specimens were processed for immunohistochemical and qPCR analyses of p57 and RhoA expression. The data showed that expression of p57 mRNA and protein was reduced in HCC tissues when compared to that in distant non-cancer tissues (P<0.05), while expression of RhoA mRNA and protein was significantly higher in HCC tissue specimens when compared to that of the distant normal tissues. Loss of p57 expression was associated with HCC with higher α-fetoprotein (AFP) levels (>400 ng/ml; P=0.044), larger tumor size (>5 cm, P=0.004), poor tumor differentiation (P=0.020), advanced TNM stage (P=0.027), capsule invasion (P=0.018) and tumor thrombosis (P=0.008), whereas expression of RhoA protein was signiﬁcantly associated with poor tumor differentiation (P=0.042), capsule invasion (P=0.022), and tumor thrombosis (P=0.002). Furthermore, there was a strong inverse relationship between p57 and RhoA expression in HCC tissues, indicating that loss of p57 expression may contribute to RhoA overexpression in HCC tissues. The median survival time of HCC patients with p57+ and p57- expression was 13.0 and 9.0 months, respectively, whereas the median survival time of HCC patients with RhoA+ and RhoA- was 9.0 and 15.0 months. Univariate analysis revealed that the levels of AFP, tumor size, TNM stage, histological grade, capsule invasion, tumor thrombosis, p57, RhoA and co-expression of p57 and RhoA were all significant prognostic indicators for overall survival of HCC patients. Multivariate analysis showed that tumor size, TNM stage, p57, RhoA and combined loss of p57 with RhoA were risk factors for poor survival of HCC patients. This study indicates that the abnormal expression of p57 and RhoA contributes to progression of HCC and poor survival of patients. 
 Co-expression of delta-catenin and RhoA is significantly associated with a malignant lung cancer phenotype. Delta-catenin, a member of the p120-catenin subfamily, and the Rho GTPase RhoA both have roles in the regulation of the cytoskeleton. In this study, we found that delta-catenin positive expression and RhoA over-expression is consistently found in non-small cell lung cancer, but not in normal lung tissue, and that their co-expression was significantly associated with histological type, differentiation, pTNM stage, lymphatic   metastasis   and a poor prognosis. We also demonstrate that delta-catenin can directly interact with RhoA and regulate its activity, which in turn mediates tumor invasion and   metastasis  . 
 Deciphering the transcriptional complex critical for RhoA gene expression and cancer   metastasis  . The RhoA GTPase is crucial in numerous biological functions and is linked to cancer   metastasis  . However, the understanding of the molecular mechanism responsible for RhoA transcription is still very limited. Here we show that RhoA transcription is orchestrated by the Myc-Skp2-Miz1-p300 transcriptional complex. Skp2 cooperates with Myc to induce RhoA transcription by recruiting Miz1 and p300 to the RhoA promoter independently of Skp1-Cullin-F-box protein containing complex (SCF)-Skp2 E3 ligase activity. Deficiency of this complex results in impairment in RhoA expression, cell migration, invasion, and breast cancer   metastasis  , recapitulating the phenotypes observed in RhoA knockdown, and RhoA restoration rescues the defect in cell invasion. Overexpression of the Myc-Skp2-Miz1 complex is found in metastatic human cancers and is correlated with RhoA expression. Our study provides insight into how oncogenic Skp2 and Myc coordinate to induce RhoA transcription and establishes a novel SCF-Skp2 E3-ligase-independent function for oncogenic Skp2 in transcription and cancer   metastasis  . 
 The role of RhoA in vulvar squamous cell carcinoma: a carcinogenesis, progression, and target therapy marker. Ras homologue gene family member A (RhoA) is involved in tumor mobility, invasion, and   metastasis  . We detected RhoA expression in vulvar squamous cell carcinoma (VSCC) tissue, measured RhoA expression in the VSCC cell phenotype, and measured the expression of the relevant molecules after RhoA small interfering RNA (siRNA) transfection in SW962 cells. RhoA has a higher expression level in VSCC than normal vulva skin tissue and was positively associated with the International Federation of Gynecology and Obstetrics (FIGO) stage and differentiation; besides, VSCC patients with lymph node   metastasis   had higher positive RhoA expression. RhoA messenger RNA and protein expression was significantly reduced in the RhoA siRNA transfectants as compared with the negative control (NC) and mock-transfected cells (p < 0.05). The RhoA siRNA transfectants lead to low growth, G1 arrest, high apoptosis, low migration and invasion (p < 0.05), and suppressed lamellipodia formation as compared to NC and mock-transfected cells. Besides, matrix metalloproteinase-2 (MMP2), MMP9, and cyclinA1 protein expression was downregulated, while that of Bax was upregulated in the RhoA siRNA transfectants (p < 0.05). SW962 cell proliferation rates were significantly lovastatin dose-dependent. Lovastatin caused G1 arrest, high apoptosis, low migration and invasion (p < 0.05), and suppression of lamellipodia formation. Similar to the RhoA siRNA transfectants, lovastatin treatment downregulated RhoA, MMP2, MMP9, and cyclinA1 protein expression, while upregulating that of Bax as compared to that of the NC (p < 0.05). Abnormal RhoA expression in vulvar carcinoma is involved in tumor proliferation and invasion and may be a treatment target. The RhoA inhibitor lovastatin alters VSCC cell migration and proliferation and may be effective for treating VSCC. 
 ODAM inhibits RhoA-dependent invasion in breast cancer. Odontogenic ameloblast-associated protein (ODAM) contributes to cell adhesion. In human cancer, ODAM is down-regulated, and the overexpression of ODAM results in a favourable prognosis; however, the molecular mechanisms underlying ODAM-mediated inhibition of cancer invasion and   metastasis   remain unclear. Here, we identify a critical role for ODAM in inducing cancer cell adhesion. ODAM induced RhoA activity and the expression of downstream factors, including Rho-associated kinase (ROCK). ODAM-mediated RhoA signalling resulted in actin filament rearrangement by activating PTEN and inhibiting the phosphorylation of AKT. When ODAM is overexpressed in MCF7 breast cancer cells and AGS gastric cancer cells that activate RhoA at high levels, it decreases motility, increases adhesion and inhibits the   metastasis   of MCF7 cells. Conversely, depletion of ODAM in cancer cells inhibits Rho GTPase activation, resulting in increased cancer migration and invasion. These results suggest that ODAM expression in cells maintains their adhesion, resulting in the prevention of their   metastasis   via the regulation of RhoA signalling in breast cancer cells. SIGNIFICANCE Breast cancer represents the first most frequent cancer, and the ratio of mortality is high in women. Of utmost importance for reducing risk by breast cancer are their anti-invasion mechanisms, particularly in the non-invasive cancer cells because   metastasis   is the principal cause of death among cancer patients. ODAM induced RhoA activity. ODAM-mediated RhoA signalling resulted in actin filament rearrangement, increased cell adhesion and inhibited the migration/invasion of MCF7 cells. These results suggest that ODAM expression maintains their adhesion, resulting in the prevention of their   metastasis   via the regulation of RhoA signalling in breast cancer cells. 
 Inhibitory role of RhoA on senescence-like growth arrest by a mechanism involving modulation of phosphatase activity. Recently, negative effects of phosphatase in tumorigenesis and   metastasis   have been suggested in various tumor types. In this study, we showed that RhoA activation modulated phosphatase during senescence-like arrest in human prostate cancer cells. Under senescence-inducing condition, decreased Erk phosphorylation was detected in caRhoA-transfected cells and inactivation of Erk, but not p38, prevented doxorubicin-induced cell senescence. Cells were induced to senescence by inhibition of phosphatase activity (VHR, MKP3, or PP2A) without additional cellular stress. Of interest, caRhoA prevented doxorubicin-induced decrease of phosphatase. Thus, we postulate that RhoA signaling may protect cells against cellular senescence by maintaining phosphatase activity and Erk dephosphorylation. 
 p27Kip1 promotes migration of metastatic hepatocellular carcinoma cells. p27(Kip1) (p27) is a member of the Cip/Kip family of cyclin/cyclin-dependent kinase inhibitors (CKIs), and its CKI-independent function regarding cell motility modulation has been discovered. However, it is controversial whether p27 promotes or inhibits cell migration. This study investigates the migration regulatory role of p27 in metastatic hepatocellular carcinoma (HCC) cells.RNA interference, RhoA-GTP pull-down assay, Western blots, immunostaining, transwell and wound-healing assays were used.High levels of p27 and phosphorylated p27 (Ser10) were detected in metastatic HCC cells, MHCC97L and MHCC97H, when compared with nonmetastatic HCC cells, PLC and Hep3B. We hypothesized that p27 is responsible for   metastasis  -related migration in HCC cells and tested the hypothesis by using the p27 RNA interference approach. Increased RhoA activity was observed when p27 was knocked down in MHCC97L cells, which further led to stress fiber formation and decreased cell migration and wound healing. Moreover, high p27 and low stathmin expression of metastatic HCC cells indicated that migration inhibition by p27-stathmin interaction might not be the major regulatory pathway in metastatic HCC cells.p27 promotes cell migration in metastatic HCC cells through the regulation of RhoA activity. 
 Vincristine enhances amoeboid-like motility via GEF-H1/RhoA/ROCK/Myosin light chain signaling in MKN45 cells. Anti-cancer drugs are widely used in cancer treatment frequently combined with surgical therapy and/or radiation therapy. Although surgery and radiation have been suggested to facilitate invasion and   metastasis   of tumor cells in some cases, there is so far little information about the effect of anti-cancer drugs on cellular invasive ability and   metastasis  . In this study, using four different anti-cancer drugs (vincristine, paclitaxel, cisplatin and etoposide), we examined whether these drugs influence the invasive ability of tumor cells.Human gastric adenocarcinoma MKN45 cells were used to evaluate the effect of anti-cancer drugs. After drug treatment, cellular invasive ability was assessed using the Matrigel invasion chamber. Cytoskeletal changes after treatment were examined microscopically with F-actin staining. In addition, we monitored cellular motility in 3D matrigel environment by time-lapse microscopic analysis. The drug-induced activation of RhoA and ROCK was evaluated by pull-down assay and Western blotting using an antibody against phosphorylated myosin light chain (MLC), respectively. Where necessary, a ROCK inhibitor Y27632 and siRNA for guanine nucleotide exchange factor-H1 (GEF-H1) were applied.Among all drugs tested, only vincristine stimulated the invasive ability of MKN45 cells. Microscopic analysis revealed that vincristine induced the formation of non-apoptotic membrane blebs and amoeboid-like motility. Vincristine significantly enhanced RhoA activity and MLC phosphorylation, suggesting the involvement of RhoA/ROCK pathway in the vincristine-induced cytoskeletal reorganization and cellular invasion. Furthermore, we found that Y27632 as well as the siRNA for GEF-H1, a RhoA-specific activator, attenuated MLC phosphorylation, the formation of membrane blebs and the invasive ability after vincristine treatment.These results indicate that vincristine activates GEF-H1/RhoA/ROCK/MLC signaling, thereby promoting amoeboid-like motility and the invasive ability of MKN45 cells. 
 Involvement of RhoA in progression of human hepatocellular carcinoma. The activation of Rho proteins has been shown to lead to loss of polarity in cancer cells, as well as reorganization of the cytoskeleton and facilitation of cell motility, possibly resulting in their malignant potential. The clinicopathological significance of RhoA, however, is not yet well known in the case of hepatocellular carcinoma (HCC). This study evaluated the clinicopathological correlation of RhoA levels with HCC.The intratumor expression level of Rho was determined and compared with that in adjacent non-cancerous hepatic tissue using quantitative real time RT-PCR and Western blotting in 64 patients with HCC. Relationships between the level of RhoA and clinicopathological factors were examined. RhoA protein expression was confirmed by immunohistochemistry.RhoA immunostaining was strong in malignant tissue whereas it was minimal in benign tissue. Tumor tissue of HCC patients demonstrated a copy number of RhoA mRNA that was well correlated with its protein level in each case and was significantly higher than that found in the corresponding non-cancerous liver tissue (P < 0.01). With regard to venous invasion, satellite lesions and advanced pTNM stage, the RhoA level tended to be higher in HCC than that seen in negative tissue (P < 0.05).This is the first demonstration that the expression level of RhoA is correlated with tumor progression and   metastasis   in HCC. RhoA in tumor tissue might be expected to be not only a good candidate marker for invasive and proliferative tumor cells, but also a molecular target of these cells. 
 Relationship of RhoA signaling activity with ezrin expression and its significance in the prognosis for breast cancer patients. We have recently reported that RhoA may regulate the invasion and   metastasis   of breast cancer cells as an upstream signal of ezrin in vitro. In this study, we examined the relationship of RhoA signaling activity with ezrin expression in breast cancer and its prognostic significance in patients with breast cancer.Paraffin tumor sections of breast cancer were collected retrospectively from 487 patients diagnosed between 2001 and 2004. Immunohistochemical methods were used to detect the expression of RhoA, phosphorylated (activated) RhoA, and ezrin.Ezrin overexpression was detectable in 15.2% of 487 invasive breast cancers. The majority (85.1%) of ezrin-overexpressing tumors coexpressed phosphorylated RhoA; 78.8% of tumors with phosphorylated RhoA cooverexpressed ezrin. Patients whose cancers showed overexpression of ezrin or expression of phosphorylated RhoA had shorter survival rates.RhoA activation is important in human breast cancer due to its upregulation of ezrin; thus, agents that target phosphorylated RhoA may be useful in the treatment of tumors with ezrin overexpression. 
 A p27(kip1)-binding protein, p27RF-Rho, promotes cancer   metastasis   via activation of RhoA and RhoC. Rho family proteins regulate multiple cellular functions including motility and invasion through regulation of the actin cytoskeleton and gene expression. Activation of Rho proteins is controlled precisely by multiple regulators in a spatiotemporal manner. RhoA and/or RhoC are key players that regulate the metastatic activity of malignant tumor cells, and it is therefore of particular interest to understand how activation of these Rho proteins is controlled. We recently identified an upstream regulator of RhoA activation, p27RF-Rho (p27(kip1) releasing factor from RhoA) that acts by freeing RhoA from inhibition by p27(kip1). p27(kip1) is a cell cycle regulator when it is localized to the nucleus, but it binds RhoA and inhibits activation of the latter when it is localized to the cytoplasm. Here, we show that a metastatic variant of mouse melanoma B16 cells (F10) exhibits greater expression of p27RF-Rho, RhoA, and RhoC than the nonmetastatic parental cells (F0). Injection of F10 cells into mouse tail vein resulted in the formation of metastatic lung colonies, whereas prior knockdown of expression of either one of the three proteins using specific shRNA sequences decreased   metastasis   markedly. p27RF-Rho regulated the activation of RhoA and RhoC and thereby modulated cellular adhesion and motility, in addition to pericellular proteolysis. The Rho activities enhanced by p27RF-Rho had a marked effect upon efficiency of lodging of F10 cells in the lung, which represents an early step of   metastasis  . p27RF-Rho also regulated   metastasis   of human melanoma and fibrosarcoma cells. Thus, p27RF-Rho is a key upstream regulator of RhoA and RhoC that controls spreading of tumor cells. 
 Myosin-interacting guanine exchange factor (MyoGEF) regulates the invasion activity of MDA-MB-231 breast cancer cells through activation of RhoA and RhoC. The small guanine triphosphatase (GTPase) proteins RhoA and RhoC are essential for tumor invasion and/or   metastasis   in breast carcinomas. However, it is poorly understood how RhoA and RhoC are activated in breast cancer cells. Here we describe the role of myosin-interacting guanine nucleotide exchange factor (Myo-GEF) in regulating RhoA and RhoC activation as well as cell polarity and invasion in an invasive breast cancer cell line MDA-MB-231. RNA-interference (RNAi)-mediated depletion of MyoGEF in MDA-MB-231 cells not only suppresses the activation of RhoA and RhoC, but also decreases cell polarity and invasion activity. The dominant-negative mutants of RhoA and RhoC, but not Rac1 and Cdc42, dramatically decrease actin polymerization induced by MyoGEF. In addition, MyoGEF co-localizes with nonmuscle myosin IIA (NMIIA) to the front of migrating cells, and depletion of NMIIA by RNAi disrupts the polarized localization of MyoGEF at the cell leading edge, suggesting a role for NMIIA in regulating MyoGEF localization and function. Moreover, MyoGEFprotein levels significantly increase in infiltrating ductal carcinomas as well as in invasive breast cancer cell lines. Taken together, our results suggest that MyoGEF cooperates with NMIIA to regulate the polarity and invasion activity of breast cancer cells through activation of RhoA and RhoC. 
 Wnt5b promotes the cell motility essential for   metastasis   of oral squamous cell carcinoma through active Cdc42 and RhoA. The activation of Wnt signaling has been reported in many types of squamous cell carcinoma. In this study, using human oral squamous cell carcinoma (OSCC) cells with different metastatic potential, we investigated the involvement of Wnt signaling in   metastasis  . Further, we aimed to elucidate the characteristic biological features related to high metastatic potential and to identify new target molecules for the suppression of OSCC lymph node   metastasis  . We compared SAS-Venus (SAS OSCC cells expressing green fluorescent protein) and SAS-LM8, which is a highly metastatic cell line derived from SAS-Venus by in vivo selection. The SAS-LM8 cell line had greater ability of migration and invasion compared to SAS-Venus. Furthermore, a higher number of filopodia-like protrusive structures were produced in SAS-LM8 cells compared to SAS-Venus cells, and the levels of active Cdc42 and active RhoA protein were higher in SAS-LM8 cells compared to SAS-Venus cells. We did not observe any differences in the expression of Wnt/β-catenin target genes between the two cell lines; however, the mRNA levels of Wnt5b were higher in SAS-LM8 cells compared to SAS-Venus cells. To confirm the involvement of Wnt5b in migration in OSCC cells, we examined the effects of the siRNA-mediated knockdown of Wnt5b in SAS-Venus cells and SAS-LM8 cells. The siRNA treatment significantly inhibited migration and the formation of filopodia-like protrusive structures. Conversely, when stimulated with Wnt5b, the migration and formation of filopodia-like protrusions were significantly enhanced and the levels of active Cdc42 and active RhoA proteins were also increased. These results indicate that Wnt5b is involved in the migration ability of OSCC cells through active Cdc42 and RhoA. 
 Proteomic identification of RhoA as a potential biomarker for proliferation and   metastasis   in hepatocellular carcinoma. Hepatocellular carcinoma (HCC) is one of the most common malignancies in the world, and there is an urgent need to discover novel factors that can act as biomarkers for prognostic assessment and therapeutic targets of HCC. In this study, highly purified plasma membrane proteins from clinical tissue samples were obtained using a strategy combining sucrose density gradient centrifugation and subsequent phase partition. Using a two-dimensional gel electrophoresis and MALDI-Q-TOF MS/MS-based proteomics approach, we identified 13 plasma membrane-associated proteins that were differentially expressed in HCC and normal liver tissues. Of those, RhoA was one of the most significantly upregulated proteins in HCC, and its overexpression was confirmed using Western blotting. Immunohistochemistry suggested a link between RhoA expression and poor differentiation and clinicopathologic stage. Suppression of RhoA expression in HepG2 and Hep3B cells by RNA interference led to significant inhibition of cell growth, induction of apoptosis, and a decrease in migration. Our data suggest that RhoA may serve as a potential biomarker and an attractive therapeutic target for HCC. 
 PRL-1 protein promotes ERK1/2 and RhoA protein activation through a non-canonical interaction with the Src homology 3 domain of p115 Rho GTPase-activating protein. Phosphatases of the regenerating liver (PRL) play oncogenic roles in cancer development and   metastasis  . Although previous studies indicate that PRL-1 promotes cell growth and migration by activating both the ERK1/2 and RhoA pathways, the mechanism by which it activates these signaling events remains unclear. We have identified a PRL-1-binding peptide (Peptide 1) that shares high sequence identity with a conserved motif in the Src homology 3 (SH3) domain of p115 Rho GTPase-activating protein (GAP). p115 RhoGAP directly binds PRL-1 in vitro and in cells via its SH3 domain. Structural analyses of the PRL-1·Peptide 1 complex revealed a novel protein-protein interaction whereby a sequence motif within the PxxP ligand-binding site of the p115 RhoGAP SH3 domain occupies a folded groove within PRL-1. This prevents the canonical interaction between the SH3 domain of p115 RhoGAP and MEKK1 and results in activation of ERK1/2. Furthermore, PRL-1 binding activates RhoA signaling by inhibiting the catalytic activity of p115 RhoGAP. The results demonstrate that PRL-1 binding to p115 RhoGAP provides a coordinated mechanism underlying ERK1/2 and RhoA activation. 
 Clinical and prognostic significance of RhoA and RhoC gene expression in esophageal squamous cell carcinoma. Rho GTPases are involved in the organization of a microfilament network, cell-to-cell interaction, and malignant transformation. To elucidate the role of Rho GTPases in esophageal squamous cell carcinoma (ESCC), we compared the levels of RhoA and RhoC mRNA from ESCC with the corresponding normal tissue originating from the same patients.Real-time reverse transcriptase-polymerase chain reaction was performed to observe rhoA and rhoC in esophageal cell lines. Next, the mRNA levels of rhoA and rhoC were evaluated from 50 patients.The rhoA and rhoC were higher in ESCC cell lines than in noncancerous esophageal cell. rhoC was overexpressed in TTn, which was obtained directly from a surgical specimen of a metastatic lesion of ESCC in the mandible. rhoA and rhoC were significantly higher in ESCC patient than in the normal counterparts (P = .0022 and P < .0001, respectively). rhoA correlated with tumor differentiation and rhoC correlated with an advanced tumor, node,   metastasis   system classifications. rhoA and rhoC in ESCC showed a positive correlation (P = .008). Patients with rhoA overexpression showed a significantly poorer prognosis than those with rhoA underexpression (P = .044).To our knowledge, this study is the first in which the expression of RhoA and RhoC at the mRNA level in ESCC was examined and compared with its normal counterpart. Our results suggest that rhoA and rhoC are involved in ESCC progression and useful as prognostic markers. Further study will be needed to examine the therapeutic potential of the Rho GTPase inhibitor as a promising anticancer therapy, especially in ESCC. 
 Snail regulates the motility of oral cancer cells via RhoA/Cdc42/p-ERM pathway. The transcriptional factor Snail has been reported to possess properties related to cancer progression; however, the mechanism for it is not fully understood. Our data showed that Snail knockdown by small interfering RNA in two OSCC cell lines, WSU-HN6 and CAL27, significantly inhibited cell migration and invasion which also resulted in decreased cell motility, such as impaired cell spreading on type I collagen substrate, reduced filopodia, and premature assembly of stress fibers. In addition, Snail-silencing decreased Cdc42 activity but increased RhoA activity, accompanied by the downregulation in both p-ERM expression and cell motility. Meanwhile, endogenous p-ERM was found specifically co-precipitated with activated Cdc42, but not RhoA, and this co-association was decreased by Snail-silencing. The small molecule inhibitors of Rho-associated kinase (Y27632) markedly enhanced Cdc42 activity and the association of p-ERM with activated Cdc42, increasing cell motility remarkably. Using immunohistochemistry, Snail and p-ERM overexpressions were found in OSCC tissues correlated with nodal   metastasis   and shorter survival. Taken together, these results demonstrate that Snail regulates cell motility through RhoA/Cdc42/p-ERM pathway and may serve as a biomarker to predict prognosis for OSCC patients. Although RhoA and Cdc42 are concurrently regulated downstream of Snail, there is a direct interplay between them, which indicates RhoA has to be inactivated at some point in cell motility cycle. 
 Deoxypodophyllotoxin suppresses tumor vasculature in HUVECs by promoting cytoskeleton remodeling through LKB1-AMPK dependent Rho A activatio. Angiogenesis plays a critical role in the growth and   metastasis   of tumors, which makes it an attractive target for anti-tumor drug development. Deoxypodophyllotoxin (DPT), a natural product isolated from Anthriscus sylvestris, inhibits cell proliferation and migration in various cancer cell types. Our previous studies indicate that DPT possesses both anti-angiogenic and vascular-disrupting activities. Although the RhoA/ RhoA kinase (ROCK) signaling pathway is implicated in DPT-stimulated cytoskeleton remodeling and tumor vasculature suppressing, the detailed mechanisms by which DPT mediates these effects are poorly understood. In the current study, we found that DPT promotes cytoskeleton remodeling in human umbilical vein endothelial cells (HUVECs) via stimulation of AMP-activated protein kinase (AMPK) and that this effect is abolished by either treatment with a selective AMPK inhibitor or knockdown. Moreover, the cellular levels of LKB1, a kinase upstream of AMPK, were enhanced following DPT exposure. DPT-induced activation of AMPK in tumor vasculature effect was also verified by transgenic zebrafish (VEGFR2:GFP), Matrigel plug assay, and xenograft model in nude mice. The present findings may lay the groundwork for a novel therapeutic approach in treating cancer. 
 Down-regulated expression of RhoA in human conventional renal cell carcinoma. Renal cell carcinoma (RCC) is the most common cancer in adult kidney. Delayed diagnosis may result in progression and   metastasis  . Markers for early detection of RCC are lacking. The small GTPase RhoA has been implicated in the regulation of cell morphology, motility and transformation, but the role of RhoA in tumorigenesis of RCC remains unclear. In this study, the significance of RhoA expression in human RCC was characterized.RhoA mRNA and protein expressions were examined by real-time polymerase chain reaction (PCR) and immunohistochemistry respectively in 46 pairs of tissues.Real-time PCR revealed that RhoA mRNA expression was significantly lower in cancerous as compared to normal tissues (p = 0.036). RhoA protein expression was significantly higher in normal than in cancerous tissues (p = 0.032). However, RhoA expression did not correlate with age, differentiation grade or TNM stage (all p > 0.05).RhoA down-regulation may be involved in human renal tumorigenesis. 
 Loss of RhoA expression prevents proliferation and   metastasis   of SPCA1 lung cancer cells in vitro. Lung cancer is the leading cause of cancer-related death worldwide, mainly due to its highly metastatic properties. Previously, we reported an inverse correlation between Rho-kinase inhibitor and the progression of the lung cancer cells. The purpose of this study was to investigate the effects of RhoA on the proliferation, adhesion, invasion, and migration of SPCA1 lung carcinoma cells and to explore the underlying molecular mechanisms. RNA interference was used to downregulate RhoA expression in these cells. Through G418 screening, we generated SPCA1 lung cancer cell lines with stable RhoA silencing. We then observed the cell behaviour, and used matrix metalloproteinase (MMP) activity and western blot assays to evaluate the underlying molecular mechanisms. The proliferation, adhesion, migration, and invasion of SPCA1 lung cancer cells were decreased after knockdown of RhoA. At the molecular level, the total amounts of active MMP2 and MMP9 were decreased by about 17.21% (P<0.05) and 45.32% (P<0.01), respectively. Myosin phosphatase targeting subunit 1 phosphorylation (P-MYPT1) was reduced by 36.16% (P<0.05). Taken together, our findings show that the knockdown of RhoA prevents proliferation and   metastasis   in SPCA1 lung cancer cells. Changes in MMP2, MMP9, and P-MYPT1 levels and activity might be some of the molecular mechanisms underlying these effects. 
 The RhoA pathway mediates MMP-2 and MMP-9-independent invasive behavior in a triple-negative breast cancer cell line. Breast cancer is a heterogeneous disease that varies in its biology and response to therapy. A foremost threat to patients is tumor invasion and   metastasis  , with the greatest risk among patients diagnosed with triple-negative and/or basal-like breast cancers. A greater understanding of the molecular mechanisms underlying cancer cell spreading is needed as 90% of cancer-associated deaths result from   metastasis  . We previously demonstrated that the Tamoxifen-selected, MCF-7 derivative, TMX2-28, lacks expression of estrogen receptor α (ERα) and is highly invasive, yet maintains an epithelial morphology. The present study was designed to further characterize TMX2-28 cells and elucidate their invasion mechanism. We found that TMX2-28 cells do not express human epidermal growth factor receptor 2 (HER2) and progesterone receptor (PR), in addition to lacking ERα, making the cells triple-negative. We then determined that TMX2-28 cells lack expression of active matrix metalloproteinases (MMPs)-1, MMP-2, MMP-9, and other genes involved in epithelial-mesenchymal transition (EMT) suggesting that TMX2-28 may not utilize mesenchymal invasion. In contrast, TMX2-28 cells have high expression of Ras Homolog Gene Family Member, A (RhoA), a protein known to play a critical role in amoeboid invasion. Blocking RhoA activity with the RhoA pathway specific inhibitor H-1152, or a RhoA specific siRNA, resulted in inhibition of invasive behavior. Collectively, these results suggest that TMX2-28 breast cancer cells exploit a RhoA-dependent, proteolytic-independent invasion mechanism. Targeting the RhoA pathway in triple-negative, basal-like breast cancers that have a proteolytic-independent invasion mechanism may provide therapeutic strategies for the treatment of patients with increased risk   of metasta  sis. 
 Interplay of RhoA and motility in the programmed spreading of daughter cells postmitosis. Upon cortical retraction in mitosis, mammalian cells have a dramatically decreased physical association with their environment. Hence, mechanisms that prevent mitotic detachment and ensure appropriate positioning of the resulting daughter cells are critical for effective tissue morphogenesis and repair, and are the subject of this study. We find that, unlike low-motility cells, highly motile cells spread isotropically upon division and do not typically reoccupy their mother-cell footprint, and often even disseminate their mitotic cells. To elucidate these different motility-based phenotypes, we investigated their partial recapitulation and rescue using defined molecular perturbations. We show that activated RhoA is localized at the mitotic cell cortex, and Rho-associated kinase inhibition increases the degree of reoccupation of the mother-cell outline in highly motile cells. Conversely, we show that induction of motility in low-motility cells by RasV12 overexpression results in increased isotropic daughter-cell spreading. We thus propose that a balance between cortical retraction forces, which depend in part on RhoA activation, and substrate adhesion forces, which diminish with increasing motility rates, governs the integrity of mitotic actin retraction fibers and influences subsequent daughter-cell spreading. This balance of forces during mitosis has implications for cancer   metastasis  . 
 The Sprouty-related protein, Spred, inhibits cell motility,   metastasis  , and Rho-mediated actin reorganization. Sprouty and the Sprouty-related protein, Spred (Sprouty-related Ena/vasodilator-stimulated phosphoprotein homology-1 (EVH1) domain-containing protein), inhibit Ras-dependent extracellular signal-regulated kinase (ERK) signaling induced by a variety of growth factors. Since Sprouty proteins have been shown to inhibit not only ERK activation but also cell migration, we postulated that Spreds also inhibit cellular migration. Using stably highly metastatic LM8 cells infected with the Spred1-Sendai virus vector, we demonstrated that Spred1 inhibits the   metastasis   of LM8 cells in nude mice. Spred1 overexpression also inhibited migration of cells in vitro in response to chemokines, CCL19 and CCL21. We also found that Spred1 overexpression dissolved actin-stress fibers. Both EVH1 domain and C-terminal Sprouty-related domain were required for actin reassembly. Spred1 and Spred2 suppressed constitutively activated RhoA (V14RhoA)-induced stress fiber formation and serum response factor activation. Spred1 bound to activated RhoA, but not cdc42 and Rac. Spred1 also inhibited chemokine-induced RhoA activation and active RhoA-induced Rho-kinase activation. These data suggest that Spreds are key regulators of RhoA-mediated cell motility and signal transduction. Furthermore, our study suggests that the induction of Spreds could be a novel strategy for preventing cancer cell   metastasis  . 
 Isoprenylation is necessary for the full invasive potential of RhoA overexpression in human melanoma cells. Rho GTPases are signaling molecules known to control cell motility. Several recent studies have suggested a role for Rho proteins in mediating tumor   metastasis   independent of their affects on cell proliferation. As Rho proteins require post-translational modification with a geranlygeranyl moiety for full activity, we tested the affect of blocking geranylation on localization, downstream signaling, and stimulation of invasion. Expression of a constitutively active Rho construct in A375 melanoma cells dramatically stimulated invasion through Matrigel membranes; however, a constitutively active RhoA mutated so that it cannot be geranylated, failed to stimulate invasion. Moreover, expression of epitope or GFP tagged modifications of this nongeranylatable constitutively active Rho demonstrated that geranylation is necessary for correct cellular localization of Rho. Geranylation was also found to be necessary for full downstream activation of serum response factor mediated transcription. Pharmacologic inhibition of Rho geranylation produced similar inhibition of Rho localization, signaling, and invasion. Our results suggest that inhibition of Rho geranylation may be an attractive pharmacologic target for inhibiting melanoma   metastasis  . 
 Fbxw7 regulates tumor apoptosis, growth arrest and the epithelial-to-mesenchymal transition in part through the RhoA signaling pathway in gastric cancer. F-box and WD repeat domain-containing7 (Fbxw7), a member of the F-box family of proteins, which are components of an E3 ubiquitin ligase complex, plays an important role as a general tumor suppressor in regulating the effects of various oncoproteins. Recently, accumulating studies have shown that Fbxw7 plays an important role in tumor cell motility, invasion and cancer   metastasis  . However, little is known about the signaling mechanisms that regulate tumor apoptosis, growth arrest and the epithelial-to-mesenchymal transition (EMT) in gastric cancer. In our study, we confirmed that Fbxw7 expression was decreased in gastric cancer tissues, and that Fbxw7 inhibited gastric cancer progression by inducing apoptosis and growth arrest. Furthermore, gastric cancer migration and invasion were decreased or increased following Fbxw7 overexpression or knockdown, respectively, and the expressions of various EMT markers, such as E-cadherin, N-cadherin and vimentin, were altered after Fbxw7 inhibition or overexpression. Furthermore, we demonstrated that Fbxw7 inhibits the EMT via the down-regulation of Snail 1 and ZEB 1, which are upstream transcription factors that promote this process. Additionally, RhoA showed higher expression in the same gastric cancer tissues than in normal tumor-adjacent samples. We found that Fbxw7 expression was negatively correlated with RhoA protein expression in gastric cancer tissues based on Pearson's correlation coefficient analysis. Moreover, we found that RhoA protein abundance was regulated by Fbxw7 via ubiquitination and proteasomal degradation in gastric cancer. We further demonstrated the effects of RhoA re-expression or inhibition on stable Fbxw7-overexpressing or Fbxw7-silenced cell lines in vitro and in vivo. These results suggest that Fbxw7 induces apoptosis and growth arrest and inhibits the EMT in part by down-regulating the RhoA signaling pathway. 
 Adenovirus-mediated RhoA shRNA suppresses growth of esophageal squamous cell carcinoma cells in vitro and in vivo. Over-expression of RhoA in esophageal squamous cell carcinoma (ESCC) indicates a poor prognosis and is correlated with the tumor-node-  metastasis   (TNM) clinical classification. However, until now RhoA function in the ESCC progression remains to be established. We employed adenovirus-mediated small hairpin RNA (shRNA) against human RhoA (Ad-sh-RhoA) to efficiently silence target gene expression in RhoA-expressing Eca-109 ESCC cells at both protein and mRNA levels. Consequently, Ad-sh-RhoA reduced the proliferation and migration of Eca-109 cells assayed by MTT assay and cell wound healing, respectively. Moreover, Ad-sh-RhoA increased cell apoptosis and inhibited the cell cycle G1-S-phase progression of Eca-109 cells assessed by flow cytometry. Finally, in a nude mouse model, intratumoral injections of adenovirus-delivered RhoA shRNA every 3 days for 20 days significantly inhibited the growth and angiogenesis of xenografted Eca-109 tumors. In summary, these data indicate that RhoA may be a key molecule in ESCC cells, and thus, specific inhibition of the Rho signaling pathway with adenovirus-delivered shRNA represents a promising approach for the treatment of aggressive ESCC. 
 Suppression of MIM by microRNA-182 activates RhoA and promotes breast cancer   metastasis  . Breast cancer is the most common type of cancer among women worldwide, and   metastasis   represents the most devastating stage of the disease. Recent studies have revealed that microRNAs (miRNA) have critical roles to regulate cancer cell invasion and   metastasis  . Here we present evidence to show the role of miR-182 in breast cancer   metastasis  . miR-182 is upregulated in the malignant cell line variants of both human MCF10 and mouse 4T1 series. Ectopic expression of miR-182 enhanced breast cancer cell motility and invasiveness, whereas miR-182 inhibition resulted in opposite changes. In nude mice, miR-182 led to increased pulmonary colonization of cancer cells. We further demonstrated that miR-182 directly targets MIM (Missing in   Metastasis  ), which suppresses   metastasis   by inhibiting ras homolog family member A (RhoA) activity and stress fiber formation in breast cancer cells. Restoring MIM expression completely blocked the pro-  metastasis   function of miR-182, while RhoA inhibition reversed the phenotypes of both miR-182 overexpression and MIM knockdown. In breast tumor samples, miR-182 induction is linked to downregulation of MIM, RhoA activation and poor prognosis. Hence, our data delineates the molecular pathway by which miR-182 promotes breast cancer invasion and   metastasis  , and may have important implication for the treatment of metastatic cancers. 
 Regulation of RhoA activation and cytoskeletal organization by acetylation. Rho GTPases regulate cell motility in a large part through control of actin cytoskeletal organization. The activation state of Rho proteins is regulated by a wide variety of guanine nucleotide exchange factors (GEFs) and GTPase activating proteins that are differentially expressed among cell types and disease states. The RhoA specific GEF neuroepithelial transforming gene 1 (Net1) is highly expressed in many cancer cells and stimulates cell motility, invasion and cell spreading in response to a variety of ligands. A key feature of Net1 proteins is that they are sequestered in the nucleus in the absence of a motility stimulus. We have recently found that accumulation of the Net1A isoform outside the nucleus, which is the primary Net1 isoform controlling cell motility, is regulated by its acetylation status. Here, we describe acetylation as a novel mechanism of RhoGEF regulation in cell motility that can be targeted in cancer and   metastasis  . 
 GCF2/LRRFIP1 promotes colorectal cancer   metastasis   and liver invasion through integrin-dependent RhoA activation. The precise relationship between GCF2 expression and carcinogenesis has not yet been established. To clarify the metastatic potential of GCF2 in colorectal cancer, HT-29 cells stably suppressing GCF2 expression were injected into the spleens of severe combined immunodeficient (SCID) mice. GCF2 suppression reduced the number of metastatic foci in the liver and reduced fibronectin-induced cell adhesion, migration, and invasion. Downstream from the integrin signaling pathways, GCF2 regulates RhoA interaction with the RGS domain of Leukemia associated RhoGEF (LARG). Altogether, our results suggest that GCF2 plays an important role in colorectal cancer   metastasis   by regulating RhoA-induced cell adhesion, migration, and invasion. 
 Arylsulfatase B regulates colonic epithelial cell migration by effects on MMP9 expression and RhoA activation. Arylsulfatase B (ASB; N-acetylgalactosamine-4-sulfatase; 4-sulfatase; ARSB) is the enzyme that removes 4-sulfate groups from N-acetylgalactosamine 4-sulfate, which combines with glucuronate to form the disaccharide unit of chondroitin-4-sulfate (C4S). In this study, we report how variation in expression of ASB affected the migration of human colonic epithelial cells. In the T84 cell line, derived from lung   metastasis   of malignant colonic epithelial cells, the activity of ASB, as well as steroid sulfatase, arylsulfatase A, and galactose-6-sulfatase, were significantly less than in normal, primary colonic epithelial cells and in the NCM460 cell line which was derived from normal colonocytes. In the T84 cells, matrix metalloproteinase 9 (MMP9), activated RhoA, and cell migration, as well as C4S content, were significantly more than in the NCM460 cells. Silencing and overexpression of ASB had inverse effects on MMP9, activated RhoA, and cell migration, as well as the C4S content, in the NCM460 and T84 cells. When ASB expression was silenced by siRNA in the NCM460 cells, MMP9 secretion increased to over 3 times the basal level, activated RhoA increased * 85%, and cell migration increased * 52%. Following overexpression of ASB, MMP9 declined 51%, activated RhoA declined * 51%, and cell migration decreased * 37%. These findings demonstrate marked effects of ASB expression on the migratory activity of colonic epithelial cells, activated RhoA, and MMP9, and suggest a potential vital role of ASB, due to its impact on chondroitin sulfation, on determination of the invasive phenotype of colonic epithelial cells. 
 Long noncoding RNA AFAP1-AS1 indicates a poor prognosis of hepatocellular carcinoma and promotes cell proliferation and invasion via upregulation of the RhoA/Rac2 signaling. It has been shown that long noncoding RNAs (lncRNAs) play a critical role in the regulation of cellular processes including cancer progression and   metastasis  . However, the biological functions and clinical significance of lncRNA AFAP1-AS1 in hepatocellular carcinoma (HCC) remain unclear. Expression of AFAP1-AS1 was analyzed in 78 HCC tissues by real-time PCR. The effect of AFAP1-AS1 on cell proliferation was examined by MTT assay, cell apoptosis was detected by flow cytometric analysis and cell invasion was determined by Transwell assay. RhoA/Rac2 signaling and downstream factors were verified by western blotting. HCC cells infected with si-AFAP1-AS1 were injected into nude mice to investigate the effect of AFAP1-AS1 on the tumorigenesis in vivo. We found that increased expression of AFAP1-AS1 was significantly correlated with pathological staging (P=0.024) and lymph-vascular space invasion (LVSI) in HCC patients (P=0.007). Multivariate analyses indicated that AFAP1-AS1 represented an independent predictor for overall survival of HCC (P=0.029). Further experiments showed that knockdown of AFAP1-AS1 by si-AFAP1-AS1 decreased the proliferation and invasion in vitro and in vivo, induced cell apoptosis and blocked cell cycle in S phase via inhibition of the RhoA/Rac2 signaling. Taken together, our findings indicate that AFAP1-AS1 may promote the HCC development through upregulation of RhoA/Rac2 signaling and provide a potential therapeutic target for HCC. 
 RhoA modulates functional and physical interaction between ROCK1 and Erk1/2 in selenite-induced apoptosis of leukaemia cells. RhoA GTPase dysregulation is frequently reported in various tumours and haematologic malignancies. RhoA, regulating Rho-associated coiled-coil-forming kinase 1 (ROCK1), modulates multiple cell functions, including malignant transformation,   metastasis   and cell death. Therefore, RhoA/ROCK1 could be an ideal candidate target in cancer treatment. However, the roles of RhoA/ROCK1 axis in apoptosis of leukaemia cells remain elusive. In this study, we explored the effects of RhoA/ROCK1 cascade on selenite-induced apoptosis of leukaemia cells and the underlying mechanism. We found selenite deactivated RhoA/ROCK1 and decreased the association between RhoA and ROCK1 in leukaemia NB4 and Jurkat cells. The inhibited RhoA/ROCK1 signalling enhanced the phosphorylation of Erk1/2 in a Mek1/2-independent manner. Erk1/2 promoted apoptosis of leukaemia cells after it was activated. Intriguingly, it was shown that both RhoA and ROCK1 were present in the multimolecular complex containing Erk1/2. GST pull-down analysis showed ROCK1 had a direct interaction with GST-Erk2. In addition, selenite-induced apoptosis in an NB4 xenograft model was also found to be associated with the RhoA/ROCK1/Erk1/2 pathway. Our data demonstrate that the RhoA/ROCK1 signalling pathway has important roles in the determination of cell fates and the modulation of Erk1/2 activity at the Mek-Erk interplay level. 
 Netrin-1 promotes glioblastoma cell invasiveness and angiogenesis by multiple pathways including activation of RhoA, cathepsin B, and cAMP-response element-binding protein. Glioblastomas are very difficult tumors to treat because they are highly invasive and disseminate within the normal brain, resulting in newly growing tumors. We have identified netrin-1 as a molecule that promotes glioblastoma invasiveness. As evidence, netrin-1 stimulates glioblastoma cell invasion directly through Matrigel-coated transwells, promotes tumor cell sprouting and enhances   metastasis   to lymph nodes in vivo. Furthermore, netrin-1 regulates angiogenesis as shown in specific angiogenesis assays such as enhanced capillary endothelial cells (EC) sprouting and by increased EC infiltration into Matrigel plugs in vivo, as does VEGF-A. This netrin-1 signaling pathway in glioblastoma cells includes activation of RhoA and cyclic AMP response element-binding protein (CREB). A novel finding is that netrin-1-induced glioblastoma invasiveness and angiogenesis are mediated by activated cathepsin B (CatB), a cysteine protease that translocates to the cell surface as an active enzyme and co-localizes with cell surface annexin A2 (ANXA2). The specific CatB inhibitor CA-074Me inhibits netrin-1-induced cell invasion, sprouting, and Matrigel plug angiogenesis. Silencing of CREB suppresses netrin-1-induced glioblastoma cell invasion, sprouting, and CatB expression. It is concluded that netrin-1 plays an important dual role in glioblastoma progression by promoting both glioblastoma cell invasiveness and angiogenesis in a RhoA-, CREB-, and CatB-dependent manner. Targeting netrin-1 pathways may be a promising strategy for brain cancer therapy. 
 RHOA inactivation enhances Wnt signalling and promotes colorectal cancer. Activation of the small GTPase RHOA has strong oncogenic effects in many tumour types, although its role in colorectal cancer remains unclear. Here we show that RHOA inactivation contributes to colorectal cancer progression/  metastasis  , largely through the activation of Wnt/β-catenin signalling. RhoA inactivation in the murine intestine accelerates the tumorigenic process and in human colon cancer cells leads to the redistribution of β-catenin from the membrane to the nucleus and enhanced Wnt/β-catenin signalling, resulting in increased proliferation, invasion and de-differentiation. In mice, RHOA inactivation contributes to colon canc  er metasta  sis and reduced RHOA levels were observed at metastatic sites compared with primary human colon tumours. Therefore, we have identified a new mechanism of activation of Wnt/β-catenin signalling and characterized the role of RHOA as a novel tumour suppressor in colorectal cancer. These results constitute a shift from the current paradigm and demonstrate that RHO GTPases can suppress tumour progression   and metast  asis. 
 Effect of RhoA signaling transduction on expression of Ezrin in breast cancer cell lines. Both RhoA and Ezrin are confirmed to play an important role in the development and   metastasis   of tumors. However, the mechanism remains unclear. This study rudimentally investigates the regulatory effect of RhoA on the expression of Ezrin.After MDA-MB-231 cells were treated with epidermal growth factor (EGF) or following pretreatment of fasudil (the special inhibitor of RhoA), the expression of RhoA, p-RhoA and Ezrin in MDA-MB-231 cells was detected by western blot.Stimulation of EGF triggered RhoA phosphorylation in MDA-MB-231 cells which reached the maximum at 30 min; RhoA expression did not change; Ezrin expression was enhanced and reached the maximum at 24 h. However, pretreatment of fasudil before EGF stimulation decreased RhoA phosphorylation and Ezrin expression in MDA-MB-231 cells by 72.73% and 51.28%, respectively.RhoA may regulate the invasion and   metastasis   of breast cancer cells as an upstream signaling of Ezrin. 
 Myo9b is a key player in SLIT/ROBO-mediated lung tumor suppression. Emerging evidence indicates that the neuronal guidance molecule SLIT plays a role in tumor suppression, as SLIT-encoding genes are inactivated in several types of cancer, including lung cancer; however, it is not clear how SLIT functions in lung cancer. Here, our data show that SLIT inhibits cancer cell migration by activating RhoA and that myosin 9b (Myo9b) is a ROBO-interacting protein that suppresses RhoA activity in lung cancer cells. Structural analyses revealed that the RhoGAP domain of Myo9b contains a unique patch that specifically recognizes RhoA. We also determined that the ROBO intracellular domain interacts with the Myo9b RhoGAP domain and inhibits its activity; therefore, SLIT-dependent activation of RhoA is mediated by ROBO inhibition of Myo9b. In a murine model, compared with control lung cancer cells, SLIT-expressing cells had a decreased capacity for tumor formation and lung   metastasis  . Evaluation of human lung cancer and adjacent nontumor tissues revealed that Myo9b is upregulated in the cancer tissue. Moreover, elevated Myo9b expression was associated with lung cancer progression and poor prognosis. Together, our data identify Myo9b as a key player in lung cancer and as a ROBO-interacting protein in what is, to the best of our knowledge, a newly defined SLIT/ROBO/Myo9b/RhoA signaling pathway that restricts lung cancer progression and   metastasis  . Additionally, our work suggests that targeting the SLIT/ROBO/Myo9b/RhoA pathway has potential as a diagnostic and therapeutic strategy for lung cancer. 
 Glutaminase 2 is a novel negative regulator of small GTPase Rac1 and mediates p53 function in suppressing   metastasis  . Glutaminase (GLS) isoenzymes GLS1 and GLS2 are key enzymes for glutamine metabolism. Interestingly, GLS1 and GLS2 display contrasting functions in tumorigenesis with elusive mechanism; GLS1 promotes tumorigenesis, whereas GLS2 exhibits a tumor-suppressive function. In this study, we found that GLS2 but not GLS1 binds to small GTPase Rac1 and inhibits its interaction with Rac1 activators guanine-nucleotide exchange factors, which in turn inhibits Rac1 to suppress cancer   metastasis  . This function of GLS2 is independent of GLS2 glutaminase activity. Furthermore, decreased GLS2 expression is associated with enhanced   metastasis   in human cancer. As a p53 target, GLS2 mediates p53's function in   metastasis   suppression through inhibiting Rac1. In summary, our results reveal that GLS2 is a novel negative regulator of Rac1, and uncover a novel function and mechanism whereby GLS2 suppresses   metastasis  . Our results also elucidate a novel mechanism that contributes to the contrasting functions of GLS1 and GLS2 in tumorigenesis. 
 miR-143 inhibits the   metastasis   of pancreatic cancer and an associated signaling pathway. Pancreatic cancer is characterized by early   metastasis   and high mortality. In this study, the role of miR-143 in invasion and   metastasis   was investigated in pancreatic cancer cells. miR-143 expression was established by an adenovirus-carried miR-143 expression cassette. mRNA and protein levels of gene expression were examined by RT-PCR and Western blot assay, respectively. Rho GTPases activity was measured by the pull down assay. The role of miR-143 in migration and invasion of Panc-1 cells was tested in vitro. The antimetastatic effect of miR-143 was tested in a liver   metastasis   model, while its antitumor growth effect was tested in a xenograft Panc-1 tumor model. Results demonstrated that ARHGEF1 (GEF1), ARHGEF2 (GEF2), and K-RAS genes are the targets of miR-143. miR-143 expression significantly decreased mRNA and protein levels of GEF1, GEF2, and K-RAS genes; lowered the constitutive activities of RhoA, Rac1, and Cdc42 GTPases; decreased the protein levels of MMP-2 and MMP-9; but significantly increased the protein level of E-cadherin. miR-143 expression also significantly inhibited the migration and invasion of Panc-1 cells in vitro, liver   metastasis  , and xenograft tumor growth in vivo. Our study suggested that miR-143 plays a central role in the invasion and   metastasis   of pancreatic cancer and miR-143 is a potential target for pancreatic cancer therapy. 
 Loss of the obscurin-RhoGEF downregulates RhoA signaling and increases microtentacle formation and attachment of breast epithelial cells. Obscurins are RhoGEF-containing proteins whose downregulation has been implicated in the development and progression of breast cancer. Herein, we aim to elucidate the mechanism for increased motility of obscurin-deficient cells. We show that shRNA-mediated obscurin downregulation in MCF10A cells leads to >50% reduction in RhoA activity relative to scramble control (shCtrl), as well as decreased phosphorylation of RhoA effectors, including myosin light chain phosphatase, myosin light chain, lim kinase, and cofilin, in both attached and suspended cells. These alterations result in decreased actomyosin contractility, allowing suspended cells to escape detachment-induced apoptosis. Moreover, ~40% of shObsc-expressing cells, but only ~10% of shCtrl-expressing cells, extend microtentacles, tubulin-based projections that mediate the attachment of circulating tumor cells to endothelium. Indeed, we show that MCF10A cells expressing shObsc attach in vitro more readily than shCtrl cells, an advantage that persists following taxane exposure. Overall, our data suggest that loss of obscurins may represent a substantial selective advantage for breast epithelial cells during   metastasis  , and that treatment with paclitaxel may exacerbate this advantage by preferentially allowing obscurin-deficient, stem-like cells to attach to the endothelium of distant sites, a first step towards colonizing metastatic tumors. 
 PAR1-mediated RhoA activation facilitates CCL2-induced chemotaxis in PC-3 cells. Patients with advanced prostate cancer often exhibit increased activation of the coagulation system. The key activator of the coagulation cascade is the serine protease thrombin which is capable of eliciting numerous cellular responses. We previously reported that the thrombin receptor PAR1 is overexpressed in prostate cancer. To investigate further the role of PAR1 in prostate cancer   metastasis  , we examined the effects of thrombin activation on cell adhesion and motility in PC-3 prostate cancer cells. Activation of PAR1-induced dynamic cytoskeletal reorganization and reduced PC-3 binding to collagen I, collagen IV, and laminin (P < 0.01) but not fibronectin. Expression of the cell surface integrin receptors did not change as assessed by flow cytometry. Immunofluorescence microscopy revealed that PAR1 stimulation caused reorganization of the focal adhesions, suggesting that PAR1 activation in PC-3 cells may be modulating cell adhesion through integrin function but not expression. Furthermore, RhoA was activated upon stimulation with thrombin with subsequent cell contraction, decreased cell adhesion, and induced migration towards monocyte chemoattractant protein 1 (MCP-1; CCL2). Thus, it appears that thrombin stimulation plays a role in prostate cancer   metastasis   by decreasing cell adhesion to the extracellular matrix and positioning the cell in a "ready state" for migration in response to a chemotactic signal. Further exploration is needed to determine whether PAR1 activation affects other signaling pathways involved in prostate cancer. 
 RhoA is associated with invasion and poor prognosis in colorectal cancer. Colorectal cancer is one of the most common cancers and is the fourth leading cause of cancer death in Korea. Mortality of colorectal cancer is strongly associated with the metastatic spread of the disease. As such, it is important to find and characterize signaling pathways involved in colon cancer   metastasis  . We investigated the functional importance of RhoA using human cell lines as well as 150 colorectal cancer patient-derived samples as it remains unclear whether RhoA functions as either an oncogene or a tumor suppressor in colon cancer. RhoA was highly expressed in metastatic cancer cell lines. Although cancer cell proliferation was only moderately impaired after depletion of RhoA, RhoA-depleted cancer cells exhibited markedly reduced migration and invasion ability in vitro. Furthermore, we found that RhoA is associated with the invasion of lymph nodes and blood vessels in the patient colorectal cancer samples. Most notably, patients with higher RhoA expression had a significantly poorer 5-year survival rate after surgery. These results suggest that RhoA is a marker of poor prognosis in colorectal cancer and may be a promising target for cancer treatment. 
 CCR4 mediates CCL17 (TARC)-induced migration of human colon cancer cells via RhoA/Rho-kinase signaling. Accumulating data suggest a role of chemokines in tumor cell   metastasis  . CCR4 has been implicated in hematologic malignancies and recently also in solid tumors. Herein, we hypothesized that CCR4 might be expressed and support migration of colon cancer cells.We used quantitative RT-PCR and flow cytometry to determine mRNA and surface expression of CCR4 on colon cancer cell lines (HT-29) and (AZ-97). Total RhoA and active RhoA protein levels in CCL17-stimulated colon cancer cells were quantified using ELISA and G-LISA assays. Migration assays were performed to evaluate colon cancer cells chemotaxis. In vitro tumor growth was assessed using proliferation assay.Our results show clear-cut mRNA levels and surface expression of CCR4 on a colon cancer cell line (HT-29) and on tumor cells (AZ-97). CCR4 ligand CCL17 (TARC) was a potent stimulator of colon cancer cell migration. This CCL17-induced colon cancer cell migration was inhibited by pre-incubation of the colon cancer cells with an antibody directed against CCR4 or an antagonist against CCR4. CCL17-induced signaling in colon cancer cells revealed that CCL17 increased mRNA formation of RhoA-C in colon cancer cells. Our results also found that CCL17 increased total RhoA and active RhoA protein levels in colon cancer cells. The Rho-kinase inhibitor Y-27632 abolished CCL17-induced colon cancer cell chemotaxis. In addition, inhibition of isoprenylation by GGTI-2133 markedly reduced colon cancer cell migration triggered by CCL17.Our novel data indicate for the first time that the CCL17-CCR4 axis might be involved in the spread of colon cancer cells. 
 RACK1 promotes breast carcinoma migration/  metastasis   via activation of the RhoA/Rho kinase pathway. We aimed to gain a mechanistic understanding of the role of RACK1 in breast carcinoma migration/  metastasis  . Migration assays were conducted in breast carcinoma cell lines. siRNA targeting RACK1 as well as the Rho kinase inhibitor were also applied. Immunoprecipitation and immunofluorescence were used to study the RACK1/RhoA interaction. GTP-Rho pull-down assays were performed to assess the activation of RhoA. We also conducted immunohistochemistry in 160 breast carcinoma samples. Experiments in vitro showed that RACK1 promotes migration via interaction with RhoA and activation of the RhoA/Rho kinase pathway. Immunohistochemistry in 160 samples revealed that RACK1 is strongly correlated with accepted tumor spread indicators and RhoA (all P < 0.05). Kaplan-Meier survival analysis indicated a correlation between higher RACK1 expression and shorter survival times (P < 0.001). RACK1 is a prognostic factor that promotes breast carcinoma migration/  metastasis   by interacting with RhoA and activating the RhoA/Rho kinase pathway. 
 A novel strategy for specifically down-regulating individual Rho GTPase activity in tumor cells. The Rho family GTPases RhoA, RhoB, and RhoC regulate the actin cytoskeleton, cell movement, and cell growth. Unlike Ras, up-regulation or overexpression of these GDP/GTP binding molecular switches, but not activating point mutations, has been associated with human cancer. Although they share over 85% sequence identity, RhoA, RhoB, and RhoC appear to play distinct roles in cell transformation and   metastasis  . In NIH 3T3 cells, RhoA or RhoB overexpression causes transformation whereas RhoC increases the cell migration rate. To specifically target RhoA, RhoB, or RhoC function, we have generated a set of chimeric molecules by fusing the RhoGAP domain of p190, a GTPase-activating protein that accelerates the intrinsic GTPase activity of all three Rho GTPases, with the C-terminal hypervariable sequences of RhoA, RhoB, or RhoC. The p190-Rho chimeras were active as GTPase-activating proteins toward RhoA in vitro, co-localized with the respective active Rho proteins, and specifically down-regulated Rho protein activities in cells depending on which Rho GTPase sequences were included in the chimeras. In particular, the p190-RhoA-C chimera specifically inhibited RhoA-induced transformation whereas p190-RhoC-C specifically reversed the migration phenotype induced by the active RhoC. In human mammary epithelial-RhoC breast cancer cells, p190-RhoC-C, but not p190-RhoA-C or p190-RhoB-C, reversed the anchorage-independent growth and invasion phenotypes caused by RhoC overexpression. In the highly metastatic A375-M human melanoma cells, p190-RhoC-C specifically reversed migration, and invasion phenotypes attributed to RhoC up-regulation. Thus, we have developed a novel strategy utilizing RhoGAP-Rho chimeras to specifically down-regulate individual Rho activity and demonstrate that this approach may be applied to multiple human tumor cells to reverse the growth and/or invasion phenotypes associated with disregulation of a distinct subtype of Rho GTPase. 
 HMG-CoA reductase regulates CCL17-induced colon cancer cell migration via geranylgeranylation and RhoA activation. Simvastatin is widely used to lower cholesterol levels in patients with cardiovascular diseases, although accumulating evidence suggests that statins, such as simvastatin, also exert numerous anti-tumoral effects.The aim of this study was to examine the effect of simvastatin on colon cancer cell migration.Migration assays were performed to evaluate CCL17-induced colon cancer cell (HT-29) chemotaxis. In vitro tumor growth and apoptosis were assessed using a proliferation assay and annexin V assay, respectively. Active RhoA protein levels in CCL17-stimulated colon cancer cells were quantified using a G-LISA assay.We found that simvastatin dose-dependently decreased CCL17-induced colon cancer cell migration. Simvastatin had no effect on colon cancer cell proliferation or apoptosis. Inhibition of beta chemokine receptor 4, CCR4, reduced CCL17-evoked activation of RhoA in colon cancer cells. Moreover, administration of mevalonate reversed the inhibitory effect of simvastatin on CCL17-induced colon cancer cell migration. Interestingly, co-incubation with geranylgeranyl pyrophosphate (GGPP) antagonized the inhibitory impact of simvastatin on colon cancer cell migration triggered by CCL17. Moreover, we observed that simvastatin decreased CCL17-induced activation of RhoA in colon cancer cells. Administration of mevalonate and GGPP reversed the inhibitory effect of simvastatin on CCL17-provoked RhoA activation in colon cancer cells.Taken together, our findings show for the first time that HMG-CoA reductase regulates CCL17-induced colon cancer cell migration via inhibition of geranylgeranylation and RhoA activation. Thus, statins, such as simvastatin, might be effective tools to antagonize CCL17-dependent migration and   metastasis   of colon cancer cells. 
 Macrophage contact induces RhoA GTPase signaling to trigger tumor cell intravasation. Most cancer patients die as a result of   metastasis  , thus it is important to understand the molecular mechanisms of dissemination, including intra- and extravasation. Although the mechanisms of extravasation have been vastly studied in vitro and in vivo, the process of intravasation is still unclear. Furthermore, how cells in the tumor microenvironment facilitate tumor cell intravasation is still unknown. Using high-resolution imaging, we found that macrophages enhance tumor cell intravasation upon physical contact. Macrophage and tumor cell contact induce RhoA activity in tumor cells, triggering the formation of actin-rich degradative protrusions called invadopodia, enabling tumor cells to degrade and break through matrix barriers during tumor cell transendothelial migration. Interestingly, we show that macrophage-induced invadopodium formation and tumor cell intravasation also occur in patient-derived tumor cells and in vivo models, revealing a conserved mechanism of tumor cell intravasation. Our results illustrate a novel heterotypic cell contact-mediated signaling role for RhoA, as well as yield mechanistic insight into the ability of cells within the tumor microenvironment to facilitate steps of the metastatic cascade. 
 Expression of RhoA and RhoC in colorectal carcinoma and its relations with clinicopathological parameters. Ras homologous (Rho) family GTPases play a pivotal role in the regulation of numerous cellular functions associated with malignant transformation and   metastasis  . To evaluate the role of these GTPases in colorectal cancer, the mRNA expression levels in matched sets of tumor and non-tumor tissues from surgical specimens were analyzed. The relationship between the mRNA levels in tumor tissues to the clinicopathological features was also assessed.A total of 68 patients with colorectal carcinoma were recruited and the levels of RhoA and RhoC mRNA transcripts in cancer, paratumoral and normal tissues were characterized by quantitative real-time polymerase chain reaction (QRT-PCR). Their correlation to clinical histopathological parameters was analyzed.The levels of RhoA and RhoC mRNA transcripts in carcinoma tissues were significantly higher than those in the matched paratumor and normal tissues from the same patient (p<0.05). The expression levels of both genes were significantly correlated with   metastasis   of cancer cells to lymph nodes and liver (p<0.05). The levels of RhoA expression were significantly correlated with the histopathological degree of cancer, while the expression of RhoC was correlated with the extent of local invasion to intestine.This is the first study with QRT-PCR to examine the expressions of RhoA and RhoC genes in colorectal carcinoma of Chinese patients. The significantly up-regulated RhoA and RhoC expressions suggest that they may contribute to the initiation, development, invasion and   metastasis   of colorectal carcinoma in Chinese patients. 
 Activated G(alpha)13 impairs cell invasiveness through p190RhoGAP-mediated inhibition of RhoA activity. The GTPase RhoA is a downstream target of heterotrimeric G(13) proteins and plays key roles in cell migration and invasion. Here, we show that expression in human melanoma cells of a constitutively active, GTPase-deficient Galpha(13) form (G(alpha)(13)QL) or lysophosphatidylcholine (LPC)-promoted signaling through G(alpha)(13)-coupled receptors led to a blockade of chemokine-stimulated RhoA activation and cell invasion that was rescued by active RhoA. Melanoma cells expressing G(alpha)(13)QL or cells stimulated with LPC displayed an increase in p190RhoGAP activation, and defects in RhoA activation and invasion were recovered by knocking down p190RhoGAP expression, thus identifying this GTPase-activating protein (GAP) protein as a downstream G(alpha)(13) target that is responsible for these inhibitory responses. In addition, defective stress fiber assembly and reduced migration speed underlay inefficient invasion of G(alpha)(13)QL melanoma cells. Importantly, G(alpha)(13)QL expression in melanoma cells led to impairment in lung   metastasis   associated with prolonged survival in SCID mice. The data indicate that G(alpha)(13)-dependent downstream effects on RhoA activation and invasion tightly depend on cell type-specific GAP activities and that G(alpha)(13)-p190RhoGAP signaling might represent a potential target for intervention in melanoma   metastasis  . 
 Ovarian clear cell carcinomas: RHO GTPases may contribute to explain their singular biologic behavior. Ovarian clear cell carcinoma is found more often confined to the ovary (stage I) than high-grade serous carcinoma. The RHO GTPase family of proteins is involved in tumor invasion and   metastasis   through regulation of the cytoskeleton. The expression of several RHO family genes, including RHOA, RHOC, CDC42, and 3 ARHGDIs (Rho GDP dissociation inhibitors), was studied by real-time polymerase chain reaction in 22 clear cell carcinomas and 31 high-grade serous carcinomas. Immunoreaction for p21-activated kinase 1 (a downstream effector of CDC42) was also investigated on 6 tissue microarrays containing 76 carcinomas (13 clear cell carcinomas and 63 high-grade serous carcinomas). Eight clear cell carcinomas (8/21; 38%) were at stage I, whereas only 3 high-grade serous carcinomas (3/31; 10%) were at stage I. Postoperatively, all patients were treated with taxane and cisplatinum or carboplatinum. ARHGDIA messenger RNA expression was higher in clear cell carcinomas than high-grade serous carcinomas (P = .07). In contrast, CDC42 messenger RNA levels were lower in clear cell carcinomas than high-grade serous carcinomas (P = .02). Immunoreaction for p21-activated kinase 1 was concordant with the results obtained by real-time polymerase chain reaction for CDC42. In clear cell carcinomas, RHOA and RHOC messenger RNA expression was lower in stage I than in advanced-stage tumors (P = .03 and P = .005, respectively). Furthermore, in high-grade serous carcinomas, RHOA expression was higher in patients who did not respond to chemotherapy (P = .04). ARHGDIA, CDC42, RHOA, and RHOC expression may contribute to explain the different stage at diagnosis of clear cell and high-grade serous carcinomas. RHOA may cause resistance of high-grade serous carcinoma to chemotherapy. 
  GeneRIFs  
 It describes acetylation as a novel mechanism of Rhoa guanine nucleotide exchange factors regulation in cell motility that can be targeted in cancer and   metastasis  . 
 we found that CXCR4-CXCL12/AKT axis regulates RhoA, Rac-1, and Cdc42 to modulate cell invasion and tumor   metastasis  . Together, these results demonstrate a role for CXCR4 in ESCC   metastasis   and progression and suggest potential targets for therapeutic intervention. 
 As a p53 target, GLS2 mediates p53's function in   metastasis   suppression through inhibiting Rac1. 
 ODAM induced RhoA activity. ODAM-mediated RhoA signalling resulted in actin filament rearrangement, increased cell adhesion and inhibited the migration/invasion of MCF7 cells. These results suggest that ODAM expression maintains their adhesion, resulting in the prevention of their   metastasis   via the regulation of RhoA signalling in breast cancer cells. 
 RHOA inactivation enhances Wnt signalling and contributes to colon cancer   metastasis  . 
 The knockdown of RhoA prevents proliferation and   metastasis   in SPCA1 lung cancer cells. 
 co-expression of Delta-catenin and RhoA was significantly associated with histological type, differentiation, pTNM stage, lymphatic   metastasis   and a poor prognosis in non-small cell lung cancer 
 Dietary folate affects   metastasis   through the regulation of the carboxyl methylation of Rho family GTPases RhoA and Rac1. 
 Low RHOA expression is associated with   metastasis   of pancreatic cancer. 
 GCF2/LRRFIP1 plays an important role in colorectal cancer   metastasis   by regulating RhoA-induced cell adhesion. 
 RACK1 promotes breast carcinoma migration/  metastasis   via activation of the RhoA/Rho kinase pathway 
 A p27(kip1)-binding protein, p27RF-Rho, promotes cancer   metastasis   via activation of RhoA and RhoC. 
 Results suggest that VEGF-C promoted cervical cancer   metastasis   by upregulation and activation of moesin protein through RhoA/ROCK-2 pathway. 
 These immunohistochemistry results suggest that the overexpression of RhoA protein in breast duct carcinoma, is correlated with TNM clinical classification, presence of regional lymph node   metastasis   and histological grading. 
 oncogenic Skp2 and Myc coordinate to induce RhoA transcription and establish a novel SCF-Skp2 E3-ligase-independent function for oncogenic Skp2 in transcription and cancer   metastasis  . 
 The significantly up-regulated RhoA expression suggest that RhoA may contribute to the initiation, development, invasion and   metastasis   of colorectal carcinoma in Chinese patients. 
 Data show that RhoA may regulate the invasion and   metastasis   of breast cancer cells as an upstream signaling of Ezrin. 
 G(alpha)(13)-dependent downstream effects on RhoA activation and invasion tightly depend on cell type-specific GAP activities and that G(alpha)(13)-p190RhoGAP signaling might represent a potential target for intervention in melanoma   metastasis  . 
 findings suggest that the RhoA is related to malignant transformation and progression of colorectal cancer and the activation of RhoA is associated with lymph node   metastasis  . 
 Expression level of RhoA is correlated with tumor progression and   metastasis   in hepatocellular carcinoma. 
 a role for RhoA in mediating tumor   metastasis   independent of their affects on cell proliferation 
  Summary  
 This gene encodes a member of the Rho family of small GTPases, which cycle between inactive GDP-bound and active GTP-bound states and function as molecular switches in signal transduction cascades. Rho proteins promote reorganization of the actin cytoskeleton and regulate cell shape, attachment, and motility. Overexpression of this gene is associated with tumor cell proliferation and   metastasis  . Multiple alternatively spliced variants have been identified. [provided by RefSeq, Sep 2015] 
  [Cc]ell [Ii]nvasion  PubMed  
 Activation of Vav/Rho GTPase signaling by CXCL12 controls membrane-type matrix metalloproteinase-dependent melanoma   cell invasion  . Melanoma cells express the chemokine receptor CXCR4, which confers invasive signals on binding to its ligand CXCL12. We show here that knocking down membrane-type matrix metalloproteinase (MT1-MMP) expression translates into a blockade of invasion across reconstituted basement membranes and type I collagen gels in response to CXCL12, which is the result of lack of MMP-2 activation. Interference with MMP-2 expression further confirms its important role during this invasion. Vav proteins are guanine-nucleotide exchange factors for Rho GTPases that regulate actin dynamics and gene expression. We show that melanoma cells express Vav1 and Vav2, which are activated by CXCL12 involving Jak activity. Blocking Vav expression by RNA interference results in impaired activation of Rac and Rho by CXCL12 and in a remarkable inhibition of CXCL12-promoted invasion. Importantly, up-regulation of MT1-MMP expression by CXCL12, a mechanism contributing to melanoma   cell invasion  , is blocked by knocking down Vav expression or by inhibiting Jak. Together, these data indicate that activation of Jak/Vav/Rho GTPase pathway by CXCL12 is a key signaling event for MT1-MMP/MMP-2-dependent melanoma   cell invasion  . 
 Involvement of Rac and Rho signaling in cancer cell motility in 3D substrates. The motility of cancer cells in 3D matrices is of two types: mesenchymal motility, in which the cells are elongated and amoeboid motility, in which the cells are round. Amoeboid motility is driven by an actomyosin-based contractile force, which is regulated by the Rho/ROCK pathway. However, the molecular mechanisms underlying the motility of elongated cells remain unknown. Here, we show that the motility of elongated cells is regulated by Rac signaling through the WAVE2/Arp2/3-dependent formation of elongated pseudopodia and cell-substrate adhesion in 3D substrates. The involvement of Rac signaling in cell motility was different in cell lines that displayed an elongated morphology in 3D substrates. In U87MG glioblastoma cells, most of which exhibit mesenchymal motility, inhibition of Rac signaling blocked the invasion of these cells in 3D substrates. In HT1080 fibrosarcoma cells, which display mixed cell motility involving both elongated and rounded cells, inhibition of Rac1 signaling not only blocked mesenchymal motility but also caused a mesenchymal-amoeboid transition. Additionally, Rac1 and RhoA signaling regulated the mesenchymal and amoeboid motility in these cells, respectively, and the inhibition of both pathways dramatically decreased   cell invasion  . Hence, we could conclude that Rac1 and RhoA signaling simultaneously regulate   cell invasion   in 3D matrices. 
 Regulation of CXCR4/AKT-signaling-induced   cell invasion   and tumor metastasis by RhoA, Rac-1, and Cdc42 in human esophageal cancer. CXC chemokines and their cognate receptors have been implicated wildly in cancer pathogenesis. In the present study, we report a critical cause relationship between CXCR4 expression and tumorigenesis in the setting of human esophageal squamous cell carcinoma (ESCC). In ESCC cells, CXCR4 expression was significantly higher than in human esophageal epithelial cells (HEEC). Reduction of CXCR4 in ESCC cells reduced cell proliferation and invasion in vitro and tumor growth in vivo. Among the potential downstream targets of CXCR4-CXCL12 are RhoA, Rac-1, and Cdc42, which are likely to contribute to the invasiveness of ESCC cells. Finally, we found that CXCR4-CXCL12/AKT axis regulates RhoA, Rac-1, and Cdc42 to modulate   cell invasion   and tumor metastasis. Together, these results demonstrate a role for CXCR4 in ESCC metastasis and progression and suggest potential targets for therapeutic intervention. 
 A novel protein associated with membrane-type 1 matrix metalloproteinase binds p27(kip1) and regulates RhoA activation, actin remodeling, and matrigel invasion. Pericellular proteolysis by membrane-type 1 matrix metalloproteinase (MT1-MMP) plays a pivotal role in tumor   cell invasion  . Localization of MT1-MMP at the invasion front of cells, e.g. on lamellipodia and invadopodia, has to be regulated in coordination with reorganization of the actin cytoskeleton. However, little is known about how such invasion-related actin structures are regulated at the sites where MT1-MMP localizes. During analysis of MT1-MMP-associated proteins, we identified a heretofore uncharacterized protein. This protein, which we call p27RF-Rho, enhances activation of RhoA by releasing it from inhibition by p27(kip1) and thereby regulates actin structures. p27(kip1) is a well known cell cycle regulator in the nucleus. In contrast, cytoplasmic p27(kip1) has been demonstrated to bind GDP-RhoA and inhibit GDP-GTP exchange mediated by guanine nucleotide exchange factors. p27RF-Rho binds p27(kip1) and prevents p27(kip1) from binding to RhoA, thereby freeing the latter for activation. Knockdown of p27RF-Rho expression renders cells resistant to RhoA activation stimuli, whereas overexpression of p27RF-Rho sensitizes cells to such stimulation. p27RF-Rho exhibits a punctate distribution in invasive human tumor cell lines. Stimulation of the cells with lysophosphatidic acid induces activation of RhoA and induces the formation of punctate actin structures within foci of p27RF-Rho localization. Some of the punctate actin structures co-localize with MT1-MMP and cortactin. Down-regulation of p27RF-Rho prevents both redistribution of actin into the punctate structures and tumor   cell invasion  . Thus, p27RF-Rho is a new potential target for cancer therapy development. 
 Blockade of the RhoA-JNK-c-Jun-MMP2 cascade by atorvastatin reduces osteosarcoma   cell invasion  . Osteosarcoma is characterized by a high malignant and metastatic potential, which points to the need for new therapeutic strategies to prevent cell metastasis. In this study, we show that statin-induced HMG-CoA reductase inhibition reduces cell migration and invasion in human and murine osteosarcoma cells, independently of the genotype. The statin-induced reduction of cell migration and invasion was independent of induction of apoptosis and was geranylgeranylpyrophosphate-dependent. The statin reduced matrix metalloproteinase (MMP) 2, 9, and 14 and TIMP2 expression or activity in invading cells. Forced expression of MMP2 and MMP14 overcame the inhibitory effect of the statin on   cell invasion  , suggesting a role for these MMPs in invasive potential. We also investigated the mechanisms involved in the reduced MMP2 activity and   cell invasion  . Inhibition of JNK, but not ERK1/2 signaling, reduced MMP2 activity. Pharmacological or constitutive activation of JNK overcame the reduced MMP2 activity and   cell invasion   induced by the statin. The statin decreased JNK phosphorylation and c-Jun nuclear translocation, suggesting that HMG-CoA reductase inhibition targets the JNK-c-Jun signaling pathway. We showed that mevalonate or geranylgeranylpyrophosphate treatment prevented the statin-induced reduction in JNK phosphorylation, MMP2 activity, and   cell invasion  . Forced expression of a constitutively active form of RhoA increased JNK phosphorylation and overcame the inhibitory effect of atorvastatin on MMP2 activity and   cell invasion  . The data establish a link between RhoA, JNK, c-Jun, and MMP2 activity that is functionally involved in the reduction in osteosarcoma   cell invasion   by the statin. This suggests a novel strategy targeting RhoA-JNK-c-Jun signaling to reduce osteosarcoma cell tumorigenesis. 
 A MAPK-Driven Feedback Loop Suppresses Rac Activity to Promote RhoA-Driven Cancer   Cell Invasion  . Cell migration in 3D microenvironments is fundamental to development, homeostasis and the pathobiology of diseases such as cancer. Rab-coupling protein (RCP) dependent co-trafficking of α5β1 and EGFR1 promotes cance  r cell invasi  on into fibronectin (FN) containing extracellular matrix (ECM), by potentiating EGFR1 signalling at the front of invasive cells. This promotes a switch in RhoGTPase signalling to inhibit Rac1 and activate a RhoA-ROCK-Formin homology domain-containing 3 (FHOD3) pathway and generate filopodial actin-spike protrusions which drive invasion. To further understand the signalling network that drives RCP-driven invasive migration, we generated a Boolean logical model based on existing network pathways/models, where each node can be interrogated by computational simulation. The model predicted an unanticipated feedback loop, whereby Raf/MEK/ERK signalling maintains suppression of Rac1 by inhibiting the Rac-activating Sos1-Eps8-Abi1 complex, allowing RhoA activity to predominate in invasive protrusions. MEK inhibition was sufficient to promote lamellipodia formation and oppose filopodial actin-spike formation, and led to activation of Rac and inactivation of RhoA at the leading edge of cells moving in 3D matrix. Furthermore, MEK inhibition abrogated RCP/α5β1/EGFR1-driven invasive migration. However, upon knockdown of Eps8 (to suppress the Sos1-Abi1-Eps8 complex), MEK inhibition had no effect on RhoGTPase activity and did not oppose invasive migration, suggesting that MEK-ERK signalling suppresses the Rac-activating Sos1-Abi1-Eps8 complex to maintain RhoA activity and promote filopodial actin-spike formation and invasive migration. Our study highlights the predictive potential of mathematical modelling approaches, and demonstrates that a simple intervention (MEK-inhibition) could be of therapeutic benefit in preventing invasive migration and metastasis. 
 RhoA mediates cyclooxygenase-2 signaling to disrupt the formation of adherens junctions and increase cell motility. Cyclooxygenase-2 (COX-2) represents an important target for treatment and prevention of colorectal cancer. Although COX-2 signaling is implicated in promoting tumor cell growth and invasion, the molecular mechanisms that mediate these processes are largely unknown. In this study, we show that the RhoA pathway mediates COX-2 signaling to disrupt the formation of adherens junctions and increase cell motility. Disruption of adherens junctions promotes tumor   cell invasion   and metastasis and is often associated with tumor progression. We detected high levels of RhoA activity in HCA-7 colon carcinoma cells that constitutively express COX-2. Inhibition of COX-2 significantly reduced the levels of RhoA activity in HCA-7 cells, suggesting that constitutive expression of COX-2 stimulates RhoA activity. Interestingly, inhibition of COX-2 or silencing of COX-2 expression with small interfering RNA (siRNA) stimulated the formation of adherens junctions, concomitant with increased protein levels of E-cadherin and alpha-catenin. Furthermore, inhibition of RhoA or silencing of RhoA expression with siRNA increased the levels of E-cadherin and alpha-catenin. Inhibition of Rho kinases (ROCK), the RhoA effector proteins, also increased levels of E-cadherin and alpha-catenin and stimulated formation of adherens junctions. The motility of HCA-7 cells was significantly decreased when COX-2 or RhoA was inhibited. Therefore, our data reveal a novel molecular mechanism that links COX-2 signaling to disrupt the formation of adherens junctions; COX-2 stimulates the RhoA/ROCK pathway, which reduces levels of E-cadherin and alpha-catenin leading to disruption of adherens junction formation and increased motility. Understanding of COX-2 downstream signaling pathways that promote tumor progression is crucial for the development of novel therapeutic strategies. 
 KiSS1 suppresses TNFalpha-induced breast cancer   cell invasion   via an inhibition of RhoA-mediated NF-kappaB activation. Tumor necrosis factor-alpha (TNFalpha) induces cancer development and metastasis, which is prominently achieved by nuclear factor-kappa B (NF-kappaB) activation. TNFalpha-induced NF-kappaB activation enhances cellular mechanisms including proliferation, migration, and invasion. KiSS1, a key regulator of puberty, was initially discovered as a tumor metastasis suppressor. The expression of KiSS1 was lost or down-regulated in different metastatic tumors. However, it is unclear whether KiSS1 regulates TNFalpha-induced NF-kappaB activation and further tumor cell migration. In this study, we demonstrate that KiSS1 suppresses the migration of breast cancer cells by inhibiting TNFalpha-induced NF-kappaB pathway and RhoA activation. Both KiSS1 overexpression and KP10 (kisspeptin-10) stimulation inhibited TNFalpha-induced NF-kappaB activity, suppressed TNFalpha-induced cell migration and cell attachment to fibronectin in breast cancer cells while KP10 has little effect on cancer cell proliferation. Furthermore, KP10 inhibited TNFalpha-induced cell migration and RhoA GTPase activation. Therefore, our data demonstrate that KiSS1 inhibits TNFalpha-induced NF-kappaB activation via downregulation of RhoA activation and suppression of breast cancer cell migration and invasion. 
 Ovarian cancer: Stat3, RhoA and IGF-IR as therapeutic targets. Seeking to improve ovarian cancer therapy, we compared biological characteristics of the moderately-aggressive OVCAR-3 cell line with two highly aggressive ovarian cancer cell populations: the SK-OV-3 cell line, and HASCJ primary cells isolated from the ascitic fluid of a patient with FIGO stage IV ovarian cancer. Secretion of angiogenic factors was not discriminative, whereas   cell invasion   through Matrigel and vasculogenic mimicry were much greater in the more aggressive cells. Among 10 agents tested for their ability to decrease cancer cell aggressivity using these two models, inhibitors of Stat3, IGF-IR and Rho GTPase were found to be the most promising. 
 Inhibition of TMEM45A suppresses proliferation, induces cell cycle arrest and reduces   cell invasion   in human ovarian cancer cells. The association of TMEM45A with various cancers has been recently reported. However, the biological function of TMEM45A in ovarian cancer remains unclear. The present study aimed to elucidate the role of TMEM45A in regulating the biological behavior of ovarian cancer cells. We compared the expression of TMEM45A between ovarian cancer tissues and normal tissues based on RNA-sequencing data of the ovarian cancer cohort from The Cancer Genome Atlas (TCGA) project and our real-time PCR data from 25 pairs of ovarian cancer and their matched non-cancerous tissue samples. The expression of TMEM45A was then suppressed in two ovarian cancer cell lines, HO-8910 and A2780, by RNA interference. Cell proliferation, cell cycle distribution, adhesion and invasive ability were then detected using the Cell Counting Kit-8 assay (CCK-8), propidium iodide (PI) staining, and cell adhesion and Transwell assays, respectively. In addition, the mRNA and protein levels of transforming growth factor-β (TGF-β1 and TGF-β2), Ras homolog family member A (RhoA) and Rho-associated kinase 2 (ROCK2) were detected with real-time PCR and western blotting, respectively. TCGA data and our real-time PCR results demonstrated the overexpression of TMEM45A in ovarian cancer. Silencing of TMEM45A significantly inhibited cell proliferation and significantly increased the cell population in the G1 phase. Moreover, knockdown of TMEM45A also inhibited cell adhesion as well   as cell invas  ion. More importantly, suppression of TMEM45A notably downregulated the expression of TGF-β1, TGF-β2, RhoA and ROCK2. In conclusion, TMEM45A may function as an oncogene for ovarian cancer, and inhibition of TMEM45A may be a therapeutic strategy for ovarian cancer. 
 Rac1, RhoA, and Cdc42 participate in HeLa   cell invasion   by group B streptococcus. The group B streptococcus (GBS) is an important human pathogen with the ability to cause invasive disease. To do so, the bacteria must invade host cells. It has been well documented that GBS are able to invade a variety of nonphagocytic host cell types, and this process is thought to involve a number of pathogen-host cell interactions. While some of the molecular aspects of the GBS-host   cell invasion   process have been characterized, many events still remain unclear. The objective of this investigation was to evaluate the role of the Rho-family GTPases Rac, Rho, and Cdc42 in GBS invasion into epithelial cells. The epithelial   cell invasion   process was modeled using HeLa 229 cell culture. Treatment of HeLa cells with 10 microM compactin, a pan-GTPase inhibitor, abolished GBS internalization, suggesting that GTPases are involved in the GBS invasion process. The addition of Toxin B or exoenzyme C3 to HeLa cells before GBS infection reduced invasion by 50%, further suggesting that the Rho-family GTPases are involved in GBS entry. Examining invasion of GBS into HeLa cells with altered genetic backgrounds was used to confirm these findings; GBS invasion into HeLa cells transiently transfected with dominant negative Rac1, Cdc42, or RhoA reduced invasion by 75%, 51%, and 42%, respectively. Results of this study suggest that the Rho-family GTPases are required for efficient invasion of HeLa cells by GBS. 
 LOX-1-MT1-MMP axis is crucial for RhoA and Rac1 activation induced by oxidized low-density lipoprotein in endothelial cells. RhoA and Rac1 activation plays a key role in endothelial dysfunction. Lectin-like oxidized low-density lipoprotein receptor-1 (LOX-1) is a major receptor for oxidized low-density lipoprotein (ox-LDL) in endothelial cells (ECs). Membrane type 1 matrix metalloproteinase (MT1-MMP) has been shown to be involved in atherogenesis. This study was conducted to investigate the role of the LOX-1-MT1-MMP axis in RhoA and Rac1 activation in response to ox-LDL in ECs.Ox-LDL induced rapid RhoA and Rac1 activation as well as MT1-MMP activity in cultured human aortic ECs. Inhibition of LOX-1 prevented ox-LDL-dependent RhoA and Rac1 activation. Knockdown of MT1-MMP by small interfering RNA prevented ox-LDL-induced RhoA and Rac1 activation, indicating that MT1-MMP is upstream of RhoA and Rac1. Fluorescent immunostaining revealed the colocalization of LOX-1 and MT1-MMP, and the formation of a complex of LOX-1 with MT1-MMP was detected by immunoprecipitation. Blockade of LOX-1 or MT1-MMP prevented RhoA-dependent endothelial NO synthase protein downregulation and   cell invasion  , Rac1-mediated NADPH oxidase activity, and reactive oxygen species generation.The present study provides evidence that the LOX-1-MT1-MMP axis plays a crucial role in RhoA and Rac1 activation signalling pathways in ox-LDL stimulation, suggesting that this axis may be a promising target for treating endothelial dysfunction. 
 Knockdown of BDNF suppressed invasion of HepG2 and HCCLM3 cells, a mechanism associated with inactivation of RhoA or Rac1 and actin skeleton disorganization. Brain-derived neurotrophic factor (BDNF) and its primary receptor tropomysin-related kinase B (TrkB) mediate critical signalings for supporting survival and growth of neurons. Even though we have previously confirmed that more expressions of BDNF and TrkB were closely correlated with multiple and advanced hepatocellular carcinoma (HCC), the exact mechanisms underlying have not been investigated. The expressions of BDNF and TrkB were examined by western blot and BDNF secretion was evaluated by ELISA in human HCC cell lines of HepG2 and HCCLM3 with high metastatic potential. BDNF knockdown was performed by specific BDNF-siRNA transfection in HCC cells, actin cytoskeleton was shown by FITC-phalloidin staining and the activations of RhoA, Rac1 or Cdc42 were determined using western blot. Cell apoptosis and invasion were examined by flow cytometry and transwell assay, respectively. More expressions of BDNF and TrkB were found in HCCLM3 than in HepG2 cells. Inhibited expression of BDNF by specific siRNA showed impaired actin polymerization and decreased activations of RhoA or Rac1 in both HepG2 and HCCLM3 cells. BDNF knockdown also induced apoptosis and suppressed invasion of both HepG2 and HCCLM3 cells. Our results suggested a role of BDNF/TrkB in confering HCCLM3 cells advantage of metastasis, and BDNF knockdown inhibited   cell invasion   probably through the blocked actin polymerization and the correlated inactivation of RhoA or Rac1. Aiming at BDNF/TrkB signaling interruption may be an effective strategy to prevent HCC progression. 
 DLC1 tumor suppressor gene inhibits migration and invasion of multiple myeloma cells through RhoA GTPase pathway. DLC1 (deleted in liver cancer 1), a tumor suppressor gene that encodes a RhoGTPase-activating protein, is recurrently downregulated or silenced in various solid tumors and hematological malignancies because of epigenetic modifications or genomic deletion. Here, we identified DLC1 promoter hypermethylation in 43 out of 44 multiple myeloma (MM) cell lines, which resulted in downregulation or silencing of DLC1 in 41 samples. High frequency of tumor-specific methylation and attenuation or silencing of DLC1 expression could serve as an independent diagnostic marker for MM. Combined treatment with demethylating and acetylating agents significantly elevated the expression of DLC1 and suppressed MM cell proliferation. Two cell lines exhibiting complete promoter methylation and the absence of DLC1 expression were transduced by an adenoviral vector containing DLC1 cDNA. In both cell lines, the reexpression of DLC1 inhibited myeloma   cell invasion   and migration, reduced RhoA activity and resulted in the reorganization of actin cytoskeleton. These results provide the first evidence for the antiproliferative effect of DLC1 in a hematological cancer and implicate RhoA pathway in suppression of MM migration and invasion. Given the myeloma cells sensitivity to the reactivation of DLC1 function, the potential for molecular targeted therapy of DLC1-mediated pathways as well as epigenetic therapies hold prospects. 
 A MAPK-Driven Feedback Loop Suppresses Rac Activity to Promote RhoA-Driven Cancer   Cell Invasion  . Cell migration in 3D microenvironments is fundamental to development, homeostasis and the pathobiology of diseases such as cancer. Rab-coupling protein (RCP) dependent co-trafficking of α5β1 and EGFR1 promotes cance  r cell invasi  on into fibronectin (FN) containing extracellular matrix (ECM), by potentiating EGFR1 signalling at the front of invasive cells. This promotes a switch in RhoGTPase signalling to inhibit Rac1 and activate a RhoA-ROCK-Formin homology domain-containing 3 (FHOD3) pathway and generate filopodial actin-spike protrusions which drive invasion. To further understand the signalling network that drives RCP-driven invasive migration, we generated a Boolean logical model based on existing network pathways/models, where each node can be interrogated by computational simulation. The model predicted an unanticipated feedback loop, whereby Raf/MEK/ERK signalling maintains suppression of Rac1 by inhibiting the Rac-activating Sos1-Eps8-Abi1 complex, allowing RhoA activity to predominate in invasive protrusions. MEK inhibition was sufficient to promote lamellipodia formation and oppose filopodial actin-spike formation, and led to activation of Rac and inactivation of RhoA at the leading edge of cells moving in 3D matrix. Furthermore, MEK inhibition abrogated RCP/α5β1/EGFR1-driven invasive migration. However, upon knockdown of Eps8 (to suppress the Sos1-Abi1-Eps8 complex), MEK inhibition had no effect on RhoGTPase activity and did not oppose invasive migration, suggesting that MEK-ERK signalling suppresses the Rac-activating Sos1-Abi1-Eps8 complex to maintain RhoA activity and promote filopodial actin-spike formation and invasive migration. Our study highlights the predictive potential of mathematical modelling approaches, and demonstrates that a simple intervention (MEK-inhibition) could be of therapeutic benefit in preventing invasive migration and metastasis. 
 Regulation of CXCR4/AKT-signaling-induced   cell invasion   and tumor metastasis by RhoA, Rac-1, and Cdc42 in human esophageal cancer. CXC chemokines and their cognate receptors have been implicated wildly in cancer pathogenesis. In the present study, we report a critical cause relationship between CXCR4 expression and tumorigenesis in the setting of human esophageal squamous cell carcinoma (ESCC). In ESCC cells, CXCR4 expression was significantly higher than in human esophageal epithelial cells (HEEC). Reduction of CXCR4 in ESCC cells reduced cell proliferation and invasion in vitro and tumor growth in vivo. Among the potential downstream targets of CXCR4-CXCL12 are RhoA, Rac-1, and Cdc42, which are likely to contribute to the invasiveness of ESCC cells. Finally, we found that CXCR4-CXCL12/AKT axis regulates RhoA, Rac-1, and Cdc42 to modulate   cell invasion   and tumor metastasis. Together, these results demonstrate a role for CXCR4 in ESCC metastasis and progression and suggest potential targets for therapeutic intervention. 
 Inhibition of TMEM45A suppresses proliferation, induces cell cycle arrest and reduces   cell invasion   in human ovarian cancer cells. The association of TMEM45A with various cancers has been recently reported. However, the biological function of TMEM45A in ovarian cancer remains unclear. The present study aimed to elucidate the role of TMEM45A in regulating the biological behavior of ovarian cancer cells. We compared the expression of TMEM45A between ovarian cancer tissues and normal tissues based on RNA-sequencing data of the ovarian cancer cohort from The Cancer Genome Atlas (TCGA) project and our real-time PCR data from 25 pairs of ovarian cancer and their matched non-cancerous tissue samples. The expression of TMEM45A was then suppressed in two ovarian cancer cell lines, HO-8910 and A2780, by RNA interference. Cell proliferation, cell cycle distribution, adhesion and invasive ability were then detected using the Cell Counting Kit-8 assay (CCK-8), propidium iodide (PI) staining, and cell adhesion and Transwell assays, respectively. In addition, the mRNA and protein levels of transforming growth factor-β (TGF-β1 and TGF-β2), Ras homolog family member A (RhoA) and Rho-associated kinase 2 (ROCK2) were detected with real-time PCR and western blotting, respectively. TCGA data and our real-time PCR results demonstrated the overexpression of TMEM45A in ovarian cancer. Silencing of TMEM45A significantly inhibited cell proliferation and significantly increased the cell population in the G1 phase. Moreover, knockdown of TMEM45A also inhibited cell adhesion as well   as cell invas  ion. More importantly, suppression of TMEM45A notably downregulated the expression of TGF-β1, TGF-β2, RhoA and ROCK2. In conclusion, TMEM45A may function as an oncogene for ovarian cancer, and inhibition of TMEM45A may be a therapeutic strategy for ovarian cancer. 
 Knockdown of BDNF suppressed invasion of HepG2 and HCCLM3 cells, a mechanism associated with inactivation of RhoA or Rac1 and actin skeleton disorganization. Brain-derived neurotrophic factor (BDNF) and its primary receptor tropomysin-related kinase B (TrkB) mediate critical signalings for supporting survival and growth of neurons. Even though we have previously confirmed that more expressions of BDNF and TrkB were closely correlated with multiple and advanced hepatocellular carcinoma (HCC), the exact mechanisms underlying have not been investigated. The expressions of BDNF and TrkB were examined by western blot and BDNF secretion was evaluated by ELISA in human HCC cell lines of HepG2 and HCCLM3 with high metastatic potential. BDNF knockdown was performed by specific BDNF-siRNA transfection in HCC cells, actin cytoskeleton was shown by FITC-phalloidin staining and the activations of RhoA, Rac1 or Cdc42 were determined using western blot. Cell apoptosis and invasion were examined by flow cytometry and transwell assay, respectively. More expressions of BDNF and TrkB were found in HCCLM3 than in HepG2 cells. Inhibited expression of BDNF by specific siRNA showed impaired actin polymerization and decreased activations of RhoA or Rac1 in both HepG2 and HCCLM3 cells. BDNF knockdown also induced apoptosis and suppressed invasion of both HepG2 and HCCLM3 cells. Our results suggested a role of BDNF/TrkB in confering HCCLM3 cells advantage of metastasis, and BDNF knockdown inhibited   cell invasion   probably through the blocked actin polymerization and the correlated inactivation of RhoA or Rac1. Aiming at BDNF/TrkB signaling interruption may be an effective strategy to prevent HCC progression. 
 Ovarian cancer: Stat3, RhoA and IGF-IR as therapeutic targets. Seeking to improve ovarian cancer therapy, we compared biological characteristics of the moderately-aggressive OVCAR-3 cell line with two highly aggressive ovarian cancer cell populations: the SK-OV-3 cell line, and HASCJ primary cells isolated from the ascitic fluid of a patient with FIGO stage IV ovarian cancer. Secretion of angiogenic factors was not discriminative, whereas   cell invasion   through Matrigel and vasculogenic mimicry were much greater in the more aggressive cells. Among 10 agents tested for their ability to decrease cancer cell aggressivity using these two models, inhibitors of Stat3, IGF-IR and Rho GTPase were found to be the most promising. 
 KiSS1 suppresses TNFalpha-induced breast cancer   cell invasion   via an inhibition of RhoA-mediated NF-kappaB activation. Tumor necrosis factor-alpha (TNFalpha) induces cancer development and metastasis, which is prominently achieved by nuclear factor-kappa B (NF-kappaB) activation. TNFalpha-induced NF-kappaB activation enhances cellular mechanisms including proliferation, migration, and invasion. KiSS1, a key regulator of puberty, was initially discovered as a tumor metastasis suppressor. The expression of KiSS1 was lost or down-regulated in different metastatic tumors. However, it is unclear whether KiSS1 regulates TNFalpha-induced NF-kappaB activation and further tumor cell migration. In this study, we demonstrate that KiSS1 suppresses the migration of breast cancer cells by inhibiting TNFalpha-induced NF-kappaB pathway and RhoA activation. Both KiSS1 overexpression and KP10 (kisspeptin-10) stimulation inhibited TNFalpha-induced NF-kappaB activity, suppressed TNFalpha-induced cell migration and cell attachment to fibronectin in breast cancer cells while KP10 has little effect on cancer cell proliferation. Furthermore, KP10 inhibited TNFalpha-induced cell migration and RhoA GTPase activation. Therefore, our data demonstrate that KiSS1 inhibits TNFalpha-induced NF-kappaB activation via downregulation of RhoA activation and suppression of breast cancer cell migration and invasion. 
 LOX-1-MT1-MMP axis is crucial for RhoA and Rac1 activation induced by oxidized low-density lipoprotein in endothelial cells. RhoA and Rac1 activation plays a key role in endothelial dysfunction. Lectin-like oxidized low-density lipoprotein receptor-1 (LOX-1) is a major receptor for oxidized low-density lipoprotein (ox-LDL) in endothelial cells (ECs). Membrane type 1 matrix metalloproteinase (MT1-MMP) has been shown to be involved in atherogenesis. This study was conducted to investigate the role of the LOX-1-MT1-MMP axis in RhoA and Rac1 activation in response to ox-LDL in ECs.Ox-LDL induced rapid RhoA and Rac1 activation as well as MT1-MMP activity in cultured human aortic ECs. Inhibition of LOX-1 prevented ox-LDL-dependent RhoA and Rac1 activation. Knockdown of MT1-MMP by small interfering RNA prevented ox-LDL-induced RhoA and Rac1 activation, indicating that MT1-MMP is upstream of RhoA and Rac1. Fluorescent immunostaining revealed the colocalization of LOX-1 and MT1-MMP, and the formation of a complex of LOX-1 with MT1-MMP was detected by immunoprecipitation. Blockade of LOX-1 or MT1-MMP prevented RhoA-dependent endothelial NO synthase protein downregulation and   cell invasion  , Rac1-mediated NADPH oxidase activity, and reactive oxygen species generation.The present study provides evidence that the LOX-1-MT1-MMP axis plays a crucial role in RhoA and Rac1 activation signalling pathways in ox-LDL stimulation, suggesting that this axis may be a promising target for treating endothelial dysfunction. 
 A novel protein associated with membrane-type 1 matrix metalloproteinase binds p27(kip1) and regulates RhoA activation, actin remodeling, and matrigel invasion. Pericellular proteolysis by membrane-type 1 matrix metalloproteinase (MT1-MMP) plays a pivotal role in tumor   cell invasion  . Localization of MT1-MMP at the invasion front of cells, e.g. on lamellipodia and invadopodia, has to be regulated in coordination with reorganization of the actin cytoskeleton. However, little is known about how such invasion-related actin structures are regulated at the sites where MT1-MMP localizes. During analysis of MT1-MMP-associated proteins, we identified a heretofore uncharacterized protein. This protein, which we call p27RF-Rho, enhances activation of RhoA by releasing it from inhibition by p27(kip1) and thereby regulates actin structures. p27(kip1) is a well known cell cycle regulator in the nucleus. In contrast, cytoplasmic p27(kip1) has been demonstrated to bind GDP-RhoA and inhibit GDP-GTP exchange mediated by guanine nucleotide exchange factors. p27RF-Rho binds p27(kip1) and prevents p27(kip1) from binding to RhoA, thereby freeing the latter for activation. Knockdown of p27RF-Rho expression renders cells resistant to RhoA activation stimuli, whereas overexpression of p27RF-Rho sensitizes cells to such stimulation. p27RF-Rho exhibits a punctate distribution in invasive human tumor cell lines. Stimulation of the cells with lysophosphatidic acid induces activation of RhoA and induces the formation of punctate actin structures within foci of p27RF-Rho localization. Some of the punctate actin structures co-localize with MT1-MMP and cortactin. Down-regulation of p27RF-Rho prevents both redistribution of actin into the punctate structures and tumor   cell invasion  . Thus, p27RF-Rho is a new potential target for cancer therapy development. 
 Involvement of Rac and Rho signaling in cancer cell motility in 3D substrates. The motility of cancer cells in 3D matrices is of two types: mesenchymal motility, in which the cells are elongated and amoeboid motility, in which the cells are round. Amoeboid motility is driven by an actomyosin-based contractile force, which is regulated by the Rho/ROCK pathway. However, the molecular mechanisms underlying the motility of elongated cells remain unknown. Here, we show that the motility of elongated cells is regulated by Rac signaling through the WAVE2/Arp2/3-dependent formation of elongated pseudopodia and cell-substrate adhesion in 3D substrates. The involvement of Rac signaling in cell motility was different in cell lines that displayed an elongated morphology in 3D substrates. In U87MG glioblastoma cells, most of which exhibit mesenchymal motility, inhibition of Rac signaling blocked the invasion of these cells in 3D substrates. In HT1080 fibrosarcoma cells, which display mixed cell motility involving both elongated and rounded cells, inhibition of Rac1 signaling not only blocked mesenchymal motility but also caused a mesenchymal-amoeboid transition. Additionally, Rac1 and RhoA signaling regulated the mesenchymal and amoeboid motility in these cells, respectively, and the inhibition of both pathways dramatically decreased   cell invasion  . Hence, we could conclude that Rac1 and RhoA signaling simultaneously regulate   cell invasion   in 3D matrices. 
 DLC1 tumor suppressor gene inhibits migration and invasion of multiple myeloma cells through RhoA GTPase pathway. DLC1 (deleted in liver cancer 1), a tumor suppressor gene that encodes a RhoGTPase-activating protein, is recurrently downregulated or silenced in various solid tumors and hematological malignancies because of epigenetic modifications or genomic deletion. Here, we identified DLC1 promoter hypermethylation in 43 out of 44 multiple myeloma (MM) cell lines, which resulted in downregulation or silencing of DLC1 in 41 samples. High frequency of tumor-specific methylation and attenuation or silencing of DLC1 expression could serve as an independent diagnostic marker for MM. Combined treatment with demethylating and acetylating agents significantly elevated the expression of DLC1 and suppressed MM cell proliferation. Two cell lines exhibiting complete promoter methylation and the absence of DLC1 expression were transduced by an adenoviral vector containing DLC1 cDNA. In both cell lines, the reexpression of DLC1 inhibited myeloma   cell invasion   and migration, reduced RhoA activity and resulted in the reorganization of actin cytoskeleton. These results provide the first evidence for the antiproliferative effect of DLC1 in a hematological cancer and implicate RhoA pathway in suppression of MM migration and invasion. Given the myeloma cells sensitivity to the reactivation of DLC1 function, the potential for molecular targeted therapy of DLC1-mediated pathways as well as epigenetic therapies hold prospects. 
 Blockade of the RhoA-JNK-c-Jun-MMP2 cascade by atorvastatin reduces osteosarcoma   cell invasion  . Osteosarcoma is characterized by a high malignant and metastatic potential, which points to the need for new therapeutic strategies to prevent cell metastasis. In this study, we show that statin-induced HMG-CoA reductase inhibition reduces cell migration and invasion in human and murine osteosarcoma cells, independently of the genotype. The statin-induced reduction of cell migration and invasion was independent of induction of apoptosis and was geranylgeranylpyrophosphate-dependent. The statin reduced matrix metalloproteinase (MMP) 2, 9, and 14 and TIMP2 expression or activity in invading cells. Forced expression of MMP2 and MMP14 overcame the inhibitory effect of the statin on   cell invasion  , suggesting a role for these MMPs in invasive potential. We also investigated the mechanisms involved in the reduced MMP2 activity and   cell invasion  . Inhibition of JNK, but not ERK1/2 signaling, reduced MMP2 activity. Pharmacological or constitutive activation of JNK overcame the reduced MMP2 activity and   cell invasion   induced by the statin. The statin decreased JNK phosphorylation and c-Jun nuclear translocation, suggesting that HMG-CoA reductase inhibition targets the JNK-c-Jun signaling pathway. We showed that mevalonate or geranylgeranylpyrophosphate treatment prevented the statin-induced reduction in JNK phosphorylation, MMP2 activity, and   cell invasion  . Forced expression of a constitutively active form of RhoA increased JNK phosphorylation and overcame the inhibitory effect of atorvastatin on MMP2 activity and   cell invasion  . The data establish a link between RhoA, JNK, c-Jun, and MMP2 activity that is functionally involved in the reduction in osteosarcoma   cell invasion   by the statin. This suggests a novel strategy targeting RhoA-JNK-c-Jun signaling to reduce osteosarcoma cell tumorigenesis. 
 Rac1, RhoA, and Cdc42 participate in HeLa   cell invasion   by group B streptococcus. The group B streptococcus (GBS) is an important human pathogen with the ability to cause invasive disease. To do so, the bacteria must invade host cells. It has been well documented that GBS are able to invade a variety of nonphagocytic host cell types, and this process is thought to involve a number of pathogen-host cell interactions. While some of the molecular aspects of the GBS-host   cell invasion   process have been characterized, many events still remain unclear. The objective of this investigation was to evaluate the role of the Rho-family GTPases Rac, Rho, and Cdc42 in GBS invasion into epithelial cells. The epithelial   cell invasion   process was modeled using HeLa 229 cell culture. Treatment of HeLa cells with 10 microM compactin, a pan-GTPase inhibitor, abolished GBS internalization, suggesting that GTPases are involved in the GBS invasion process. The addition of Toxin B or exoenzyme C3 to HeLa cells before GBS infection reduced invasion by 50%, further suggesting that the Rho-family GTPases are involved in GBS entry. Examining invasion of GBS into HeLa cells with altered genetic backgrounds was used to confirm these findings; GBS invasion into HeLa cells transiently transfected with dominant negative Rac1, Cdc42, or RhoA reduced invasion by 75%, 51%, and 42%, respectively. Results of this study suggest that the Rho-family GTPases are required for efficient invasion of HeLa cells by GBS. 
 RhoA mediates cyclooxygenase-2 signaling to disrupt the formation of adherens junctions and increase cell motility. Cyclooxygenase-2 (COX-2) represents an important target for treatment and prevention of colorectal cancer. Although COX-2 signaling is implicated in promoting tumor cell growth and invasion, the molecular mechanisms that mediate these processes are largely unknown. In this study, we show that the RhoA pathway mediates COX-2 signaling to disrupt the formation of adherens junctions and increase cell motility. Disruption of adherens junctions promotes tumor   cell invasion   and metastasis and is often associated with tumor progression. We detected high levels of RhoA activity in HCA-7 colon carcinoma cells that constitutively express COX-2. Inhibition of COX-2 significantly reduced the levels of RhoA activity in HCA-7 cells, suggesting that constitutive expression of COX-2 stimulates RhoA activity. Interestingly, inhibition of COX-2 or silencing of COX-2 expression with small interfering RNA (siRNA) stimulated the formation of adherens junctions, concomitant with increased protein levels of E-cadherin and alpha-catenin. Furthermore, inhibition of RhoA or silencing of RhoA expression with siRNA increased the levels of E-cadherin and alpha-catenin. Inhibition of Rho kinases (ROCK), the RhoA effector proteins, also increased levels of E-cadherin and alpha-catenin and stimulated formation of adherens junctions. The motility of HCA-7 cells was significantly decreased when COX-2 or RhoA was inhibited. Therefore, our data reveal a novel molecular mechanism that links COX-2 signaling to disrupt the formation of adherens junctions; COX-2 stimulates the RhoA/ROCK pathway, which reduces levels of E-cadherin and alpha-catenin leading to disruption of adherens junction formation and increased motility. Understanding of COX-2 downstream signaling pathways that promote tumor progression is crucial for the development of novel therapeutic strategies. 
 Therapeutic IMC-C225 antibody inhibits breast cancer cell invasiveness via Vav2-dependent activation of RhoA GTPase. Abnormalities in the expression and signaling pathways downstream of epidermal growth factor receptor (EGFR) contribute to progression, invasion, and maintenance of the malignant phenotype in human cancers. Accordingly, biological agents, such as the EGFR-blocking antibody IMC-C225 have promising anticancer potential and are currently in various stages of clinical development. Because use of IMC-C225 is limited, at present, only for treatment of cancer with high EGFR expression, the goal of the present study was to determine the effect of IMC-C225 on the invasiveness of breast cancer cells with high and low levels of EGFR expression.The effect of IMC-C225 on invasion was studied using breast cancer cell lines with high and low levels of EGFR expression.The addition of EGF led to progressive stress fiber dissolution. In contrast, cells treated with IMC-C225 showed reduced invasiveness and increased stress-fiber formation. Interestingly, IMC-C225 pretreatment was accompanied by EGFR phosphorylation, as detected using an anti-phosphorylated tyrosine antibody (PY99), which correlated with phosphorylation of Vav2 guanine nucleotide exchange factor and activation of RhoA GTPase irrespective of EGFR level, and Vav2 interacted with EGFR only in IMC-C225-treated cells. The underlying mechanism involved an enhanced interaction between beta1 integrins and EGFR upon IMC-C225 treatment.Here, we defined a new mechanism for IMC-C225 that cross-links integrins with EGFR, leading to activation of RhoA and inhibition of breast cancer   cell invasion   irrespective of the level of EGFR in the cells, thus providing a rationale for using IMC-C225 in the metastatic setting independent of the levels of EGFR. 
 Deciphering the transcriptional complex critical for RhoA gene expression and cancer metastasis. The RhoA GTPase is crucial in numerous biological functions and is linked to cancer metastasis. However, the understanding of the molecular mechanism responsible for RhoA transcription is still very limited. Here we show that RhoA transcription is orchestrated by the Myc-Skp2-Miz1-p300 transcriptional complex. Skp2 cooperates with Myc to induce RhoA transcription by recruiting Miz1 and p300 to the RhoA promoter independently of Skp1-Cullin-F-box protein containing complex (SCF)-Skp2 E3 ligase activity. Deficiency of this complex results in impairment in RhoA expression, cell migration, invasion, and breast cancer metastasis, recapitulating the phenotypes observed in RhoA knockdown, and RhoA restoration rescues the defect in   cell invasion  . Overexpression of the Myc-Skp2-Miz1 complex is found in metastatic human cancers and is correlated with RhoA expression. Our study provides insight into how oncogenic Skp2 and Myc coordinate to induce RhoA transcription and establishes a novel SCF-Skp2 E3-ligase-independent function for oncogenic Skp2 in transcription and cancer metastasis. 
 Targeted disruption of protein kinase C epsilon reduces   cell invasion   and motility through inactivation of RhoA and RhoC GTPases in head and neck squamous cell carcinoma. Over 70% of patients with head and neck squamous cell carcinoma (HNSCC) present with locoregionally advanced stage III and IV disease. In spite of aggressive therapy, locoregional disease recurs in 60% and metastatic disease develops in 15% to 25% of patients causing a major decline in quality and length of life. Therefore, there is a need to identify and understand genes that are responsible for inducing an aggressive HNSCC phenotype. Evidence has shown that protein kinase C (PKC) epsilon is a transforming oncogene and may play a role in HNSCC progression. In this study, we determine the downstream signaling pathway mediated by PKC epsilon to promote an aggressive HNSCC phenotype. RNA interference knockdown of PKC epsilon in UMSCC11A and UMSCC36, two highly invasive and motile HNSCC cell lines with elevated endogenous PKC epsilon levels, resulted in cells that were significantly less invasive and motile than the small interfering RNA-scrambled control transfectants; 51 +/- 5% (P < 0.006) and 49 +/- 3% (P < 0.010) inhibition in invasion and 69 +/- 1% (P < 0.0005) and 66 +/- 3% (P < 0.0001) inhibition in motility, respectively. PKC epsilon-deficient UMSCC11A clones had reduced levels of active and serine-phosphorylated RhoA and RhoC. Moreover, constitutive active RhoA completely rescued the invasion and motility defect, whereas constitutive active RhoC completely rescued the invasion and partially rescued the motility defect of PKC epsilon-deficient UMSCC11A clones. These results indicate that RhoA and RhoC are downstream of PKC epsilon and critical for PKC epsilon-mediated   cell invasion   and motility. Our study shows, for the first time, that PKC epsilon is involved in a coordinated regulation of RhoA and RhoC activation, possibly through direct post-translational phosphorylation. 
 A mediator of Rho-dependent invasion moonlights as a methionine salvage enzyme. RhoA controls changes in cell morphology and invasion associated with cancer phenotypes. Cell lines derived from melanoma tumors at varying stages revealed that RhoA is selectively activated in cells of metastatic origin. We describe a functional proteomics strategy to identify proteins regulated by RhoA and report a previously uncharacterized human protein, named "mediator of RhoA-dependent invasion (MRDI)," that is induced in metastatic cells by constitutive RhoA activation and promotes   cell invasion  . In human melanomas, MRDI localization correlated with stage, showing nuclear localization in nevi and early stage tumors and cytoplasmic localization with plasma membrane accentuation in late stage tumors. Consistent with its role in promoting   cell invasion  , MRDI localized to cell protrusions and leading edge membranes in cultured cells and was required for cell motility, tyrosine phosphorylation of focal adhesion kinase, and modulation of actin stress fibers. Unexpectedly MRDI had enzymatic function as an isomerase that converts the S-adenosylmethionine catabolite 5-methylribose 1-phosphate into 5-methylribulose 1-phosphate. The enzymatic function of MRDI was required for methionine salvage from S-adenosylmethionine but distinct from its function in   cell invasion  . Thus, mechanisms used by signal transduction pathways to control cell movement have evolved from proteins with ancient function in amino acid metabolism. 
 Overexpression of E-cadherin on melanoma cells inhibits chemokine-promoted invasion involving p190RhoGAP/p120ctn-dependent inactivation of RhoA. Melanoma cells express the chemokine receptor CXCR4 that confers high invasiveness upon binding to its ligand CXCL12. Melanoma cells at initial stages of the disease show reduction or loss of E-cadherin expression, but recovery of its expression is frequently found at advanced phases. We overexpressed E-cadherin in the highly invasive BRO lung metastatic cell melanoma cell line to investigate whether it could influence CXCL12-promoted   cell invasion  . Overexpression of E-cadherin led to defective invasion of melanoma cells across Matrigel and type I collagen in response to CXCL12. A decrease in individual cell migration directionality toward the chemokine and reduced adhesion accounted for the impaired invasion. A p190RhoGAP-dependent inhibition of RhoA activation was responsible for the impairment in chemokine-stimulated E-cadherin melanoma transfectant invasion. Furthermore, we show that p190RhoGAP and p120ctn associated predominantly on the plasma membrane of cells overexpressing E-cadherin, and that E-cadherin-bound p120ctn contributed to RhoA inactivation by favoring p190RhoGAP-RhoA association. These results suggest that melanoma cells at advanced stages of the disease could have reduced metastatic potency in response to chemotactic stimuli compared with cells lacking E-cadherin, and the results indicate that p190RhoGAP is a central molecule controlling melanoma   cell invasion  . 
 A Blk-p190RhoGAP signaling module downstream of activated Gα13 functionally opposes CXCL12-stimulated RhoA activation and   cell invasio  n. Activation of the GTPase RhoA linked to   cell invasio  n can be tightly regulated following Gα13 stimulation. We have used a cellular model displaying Gα13-dependent inhibition of RhoA activation associated with defecti  ve cell invas  ion to the chemokine CXCL12 to characterize the molecular players regulating these processes. Using both RNAi transfection approaches and protein overexpression experiments here we show that the Src kinase Blk is involved in Gα13-activated tyrosine phosphorylation of p190RhoGAP, which causes RhoA inactivation and ultimately leads to defici  ent cell inva  sion. Characterization of molecular interplays between Gα13, Blk and p190RhoGAP revealed that Blk binds Gα13, and that Blk-mediated p190RhoGAP phosphorylation upon Gα13 activation correlates with weakening of Gα13-Blk association connected to increased Blk-p190RhoGAP assembly. These results place Blk upstream of the p190RhoGAP-RhoA pathway in Gα13-activated cells, overall representing an opposing signaling module during CXCL12-triggered invasion. In addition, analyses with Blk- or Gα13-knockdown cells indicated that Blk can also mediate CXCL12-triggered phosphorylation of p190RhoGAP independently of Gα13. However, even if CXCL12 induces the Blk-mediated GAP phosphorylation, the simultaneous stimulation of the guanine-nucleotide exchange factor Vav1 by the chemokine, as earlier reported, leads to a net increase in RhoA activation. Therefore, when Gα13 is concurrently stimulated with CXCL12 there appears to be sufficient Blk activity to promote adequate levels of p190RhoGAP tyrosine phosphorylation to inactivate RhoA and to impair cell invasiveness. 
 The interaction of IQGAP1 with the exocyst complex is required for tumor   cell invasion   downstream of Cdc42 and RhoA. Invadopodia are actin-based membrane protrusions formed at contact sites between invasive tumor cells and the extracellular matrix with matrix proteolytic activity. Actin regulatory proteins participate in invadopodia formation, whereas matrix degradation requires metalloproteinases (MMPs) targeted to invadopodia. In this study, we show that the vesicle-tethering exocyst complex is required for matrix proteolysis and invasion of breast carcinoma cells. We demonstrate that the exocyst subunits Sec3 and Sec8 interact with the polarity protein IQGAP1 and that this interaction is triggered by active Cdc42 and RhoA, which are essential for matrix degradation. Interaction between IQGAP1 and the exocyst is necessary for invadopodia activity because enhancement of matrix degradation induced by the expression of IQGAP1 is lost upon deletion of the exocyst-binding site. We further show that the exocyst and IQGAP1 are required for the accumulation of cell surface membrane type 1 MMP at invadopodia. Based on these results, we propose that invadopodia function in tumor cells relies on the coordination of cytoskeletal assembly and exocytosis downstream of Rho guanosine triphosphatases. 
 Activin B antagonizes RhoA signaling to stimulate mesenchymal morphology and invasiveness of clear cell renal cell carcinomas. Activin B belongs to the TGFβ family of growth factors and is upregulated in clear cell renal cell carcinoma cells by hypoxia inducible factors. Expression of Activin B is required for tumor growth in vivo and tumor   cell invasio  n in vitro. Here we show that activation of RhoA signaling counteracts Activin B mediated disassembly of actin stress fibers, mesenchymal cell morphology and invasiveness, whereas inhibition of RhoA rescues these effects in Activin B knockdown cells. Conversely, Activin B inhibits RhoA signaling suggesting that there is an antagonistic connection between both pathways. In addition we found that Rac1 plays an opposite role to RhoA, i.e. activation of Rac1 initiates loss of actin stress fibers, promotes a mesenchymal cell morphology and induces invasion in Activin B knockown cells, whereas inhibition of Rac1 abolishes these Activin B effects. Collectively, our data provide evidence that reduction of RhoA signaling by Activin B together with persistent Rac1 activity is a prerequisite for inducing an invasive phenotype in clear cell renal cell carcinoma. 
 A switch in RND3-RHOA signaling is critical for melanoma   cell invasion   following mutant-BRAF inhibition. The initial use of BRAF targeted therapeutics in clinical trials has demonstrated encouraging responses in melanoma patients, although a rise in drug-resistant cells capable of advancing malignant disease has been described. The current study uses BRAFV600E expressing WM793 melanoma cells to derive data aimed at investigating the molecular determinant of   cell invasion   following treatment with clinical BRAF inhibitors.Small-molecule inhibitors targeting BRAF reduced MEK1/2-ERK1/2 pathway activation and cell survival; yet, viable cell subpopulations persisted. The residual cells exhibited an elongated cell shape, prominent actin stress fibers and retained the ability to invade 3-D dermal-like microenvironments. BRAF inhibitor treatments were associated with reduced expression of RND3, an antagonist of RHOA activation, and elevated RHOA-dependent signaling. Restoration of RND3 expression or RHOA knockdown attenuated the migratory ability of residual cells without affecting overall cell survival. The invasive ability of BRAF inhibitor treated cells embedded in collagen gels was diminished following RND3 re-expression or RHOA depletion. Conversely, melanoma cell movement in the absence of BRAF inhibition was unaffected by RND3 expression or RHOA depletion.These data reveal a novel switch in the requirement for RND3 and RHOA in coordinating the movement of residual WM793 cells that are initially refractive to BRAF inhibitor therapy. These results have important clinical implications because they suggest that combining BRAF inhibitors with therapies that target the invasion of drug-resistant cells could aid in controlling disease relapse. 
 Integrin-specific control of focal adhesion kinase and RhoA regulates membrane protrusion and invasion.   Cell invasion   through extracellular matrix (ECM) is a hallmark of the metastatic cascade. Cancer cells require adhesion to surrounding tissues for efficient migration to occur, which is mediated through the integrin family of receptors. Alterations in expression levels of β1 and β3 integrins have previously been reported in a number of human cancers. However, whether there are specific roles for these ubiquitous receptors in mediatin  g cell invasi  on remains unclear. Here we demonstrate that loss of β1 but not β3 integrins leads to increased spread cell area and focal adhesion number in cells on 2D immobilized fibronectin. Increased adhesion numbers in β1 knockdown cells correlated with decreased cell migration on 2D surfaces. Conversely, cells depleted of β1 integrins showed increased migration speed on 3D cell-derived matrix as well as in 3D organotypic cultures and inverted invasion assays. This increased invasive potential was also seen in cells lacking β3 integrin but only in 3D cultures containing fibroblasts. Mechanistically, in situ analysis using FRET biosensors revealed that enhanced invasion in cells lacking β1 integrins was directly coupled with reduced activation of focal adhesion kinase (FAK) and the small GTPase RhoA resulting in formation of enhanced dynamic protrusions and increased invasion. These reductions in FAK-RhoA signal activation were not detected in β3 knockdown cells under the same conditions. This data demonstrates a specific role for β1 integrins in the modulation of a FAK-RhoA-actomyosin signaling axis to   regulate cel  l invasion through complex ECM environments. 
 Cannabinoid receptor type 1 (CB1) activation inhibits small GTPase RhoA activity and regulates motility of prostate carcinoma cells. The cannabinoid receptor type 1 (CB1) is a G protein-coupled receptor that is activated in an autocrine fashion by the endocannabinoids (EC), N-arachidonoylethanolamine (AEA) and 2-arachidonoylglycerol (2-AG). The CB1 and its endogenous and synthetic agonists are emerging as therapeutic targets in several cancers due to their ability to suppress carcinoma   cell invasion   and migration. However, the mechanisms that the CB1 regulates cell motility are not well understood. In this study, we examined the molecular mechanisms that diminish cell migration upon the CB1 activation in prostate carcinoma cells. The CB1 activation with the agonist WIN55212 significantly diminishes the small GTPase RhoA activity but modestly increases the Rac1 and Cdc42 activity. The diminished RhoA activity is accompanied by the loss of actin/myosin microfilaments, cell spreading, and cell migration. Interestingly, the CB1 inactivation with the selective CB1 antagonist AM251 significantly increases RhoA activity, enhances microfilament formation and cell spreading, and promotes cell migration. This finding suggests that endogenously produced EC activate the CB1, resulting in chronic repression of RhoA activity and cell migration. Consistent with this possibility, RhoA activity is significantly diminished by the exogenous application of AEA but not by 2-AG in PC-3 cells (cells with very low AEA hydrolysis). Pretreatment of cells with a monoacylglycerol lipase inhibitor, JZL184, which blocks 2-AG hydrolysis, decreases the RhoA activity. These results indicate the unique CB1 signaling and support the model that EC, through their autocrine activation of CB1 and subsequent repression of RhoA activity, suppress migration in prostate carcinoma cells. 
 Lysophosphatidic acid stimulates PC-3 prostate cancer cell Matrigel invasion through activation of RhoA and NF-kappaB activity. This study was performed to determine the relationship of lysophosphatidic acid (LPA) stimulation and increased Ras homolog A (RhoA) activity to nuclear factor kappa B (NF-kappaB) activity, and the role of these factors in regulating prostate cancer   cell invasion  . PC-3 high invasive cells demonstrated constitutively increased RhoA, NF-kappaB, and in vitro Matrigel invasion which were further induced by LPA stimulation or transfection with constitutively active RhoA Q63E mutant. LPA treatment rapidly and transiently induced RhoA activity followed by maximally increased DNA binding of NF-kappaB at 1 h and AP-1 at 4 h. The LPA-induced NF-kappaB DNA binding was preceded by transient IkappaBalpha phosphorylation, and decreased total IkappaBalpha levels. Further demonstrating the relationship between RhoA and NF-kappaB activation, PC-3 cells stably transfected with constitutively active RhoA Q63E demonstrated constitutively increased phospho-IkappaBalpha, while PC-3 cells transfected with dominant negative RhoA N19 exhibited decreased phospho-IkappaBalpha levels. The LPA-induced Matrigel invasion and NF-kappaB DNA binding activity were both inhibited by expression of the RhoA inhibitor C3 exoenzyme or dominant negative mutant NF-kappaB inhibitor IkappaBalpha S32/36A. Similarly, transfection with dominant negative IkappaBalpha S32/36A inhibited PC-3 RhoA Q63E cell in vitro invasion. Treatment of PC-3 high invasive and RhoA Q63E cells with sodium salicylate or lactacystin inhibited NF-kappaB and invasion, while pyrrolidine dithiocarbamate (PDTC) treatment of PC-3 high invasive cells inhibited NF-kappaB only. Each inhibitor blocked LPA-induced invasion while PDTC inhibited LPA-induced NF-kappaB and invasion to the greatest extent. These results point to a model where LPA stimulates RhoA and increased PC-3 prostate cancer   cell invasion   activity through an NF-kappaB-dependent pathway. 
 Suppression of MIM by microRNA-182 activates RhoA and promotes breast cancer metastasis. Breast cancer is the most common type of cancer among women worldwide, and metastasis represents the most devastating stage of the disease. Recent studies have revealed that microRNAs (miRNA) have critical roles to regulate cancer   cell invasion   and metastasis. Here we present evidence to show the role of miR-182 in breast cancer metastasis. miR-182 is upregulated in the malignant cell line variants of both human MCF10 and mouse 4T1 series. Ectopic expression of miR-182 enhanced breast cancer cell motility and invasiveness, whereas miR-182 inhibition resulted in opposite changes. In nude mice, miR-182 led to increased pulmonary colonization of cancer cells. We further demonstrated that miR-182 directly targets MIM (Missing in Metastasis), which suppresses metastasis by inhibiting ras homolog family member A (RhoA) activity and stress fiber formation in breast cancer cells. Restoring MIM expression completely blocked the pro-metastasis function of miR-182, while RhoA inhibition reversed the phenotypes of both miR-182 overexpression and MIM knockdown. In breast tumor samples, miR-182 induction is linked to downregulation of MIM, RhoA activation and poor prognosis. Hence, our data delineates the molecular pathway by which miR-182 promotes breast cancer invasion and metastasis, and may have important implication for the treatment of metastatic cancers. 
 GEF-H1 over-expression in hepatocellular carcinoma promotes cell motility via activation of RhoA signalling. The interstitial chromosome (chr.) 1q21-q22 region is frequently amplified in human cancers, where it has been reported to carry prognostic significance for patients. We attempted to delineate chr. 1q21-q22 for affected gene(s) in hepatocellular carcinoma (HCC) by array-CGH and detected copy number gains of ρ-guanine nucleotide exchange factor-H1 (GEF-H1) as most significant event. Gene expression evaluation in the HCC cohort indicated common up-regulations of GEF-H1 in 64% tumours compared to adjacent non-tumoural liver (64/100; paired t-test p < 0.0001). Moreover, GEF-H1 over-expressions correlated with microvascular invasion and advanced-stage tumours (p < 0.05). High GEF-H1 levels also predict shorter disease-free and overall survival of HCC patients (p < 0.03). Functional knock-down of GEF-H1 by RNAi indicated marked reduction in   cell invasio  n through matrigel and an inhibition of cell migration (p < 0.035), but an effect on cell viability was not apparent. More interestingly, a mesenchymal-epithelial transition (MET) was readily observed in GEF-H1 knock-down cells, where a concomitant re-expression of epithelial markers (E-cadherin and cytokeratin 18) and cell adhesion proteins (α-catenin and γ-catenin) was found but down-regulation of mesenchymal features (N-cadherin, vimentin and fibronectin). This phenotype was accompanied by reduced filamentous actin polymerizations and diminution of the stress fibre formation. In addition, reduced active form of GTP-RhoA, together with its downstream effectors, including cleaved ROCK1 and phosphorylated MLC2, were also detected in GEF-H1-depleted cells. Taken together, our findings underscore a potent oncogenic role for GEF-H1 in promoting the metastatic potentials of HCC, possibly through activation of RhoA signalling and the EMT phenomenon. 
 Divergence of Rho residue 43 impacts GEF activity. RhoA, RhoB and RhoC GTPases are over 85% identical at the amino acid level, with RhoA and RhoC differing at only one residue (43) across the initial two-thirds of their sequences. A putative regulatory distinction between the molecules is their capacity to be uniquely activated by guanine nucleotide exchange factors (GEFs). We hypothesize that variation of amino acid residue 43 between RhoA/B (valine) and RhoC (isoleucine) impacts GEF activity. Direct participation of residue 43 in GEF-catalyzed exchange was confirmed by the observation that mutation of this position to a threonine reduced GEF-catalyzed nucleotide exchange activity in vitro (Vav2, XPLN, GEFT, Dbl and Dbs) and greatly depressed RhoA and RhoC GTP-loading profiles in cell lysates. Using a residue swap approach, substitution of RhoA Val 43 with an Ile was found to significantly promote basal nucleotide exchange activity and enhance GTP-loading in cells. Substitution of Val 43 with an Ile in RhoB negatively affected nucleotide exchange in vitro. Substitution of RhoC Ile 43 with a Val increased GEF-catalyzed exchange in vitro. In addition, RhoC-I43V was more efficacious at driving ovarian cancer   cell invasion   through matrigrel than wild-type RhoC, RhoC-I43T, wild-type RhoA, RhoA-V43I or RhoA-V43T GTPases. These findings suggest that a divergence between RhoA/B and RhoC at residue 43 impacts basal and GEF-stimulated nucleotide exchange activity. 
 Long noncoding RNA AFAP1-AS1 indicates a poor prognosis of hepatocellular carcinoma and promotes cell proliferation and invasion via upregulation of the RhoA/Rac2 signaling. It has been shown that long noncoding RNAs (lncRNAs) play a critical role in the regulation of cellular processes including cancer progression and metastasis. However, the biological functions and clinical significance of lncRNA AFAP1-AS1 in hepatocellular carcinoma (HCC) remain unclear. Expression of AFAP1-AS1 was analyzed in 78 HCC tissues by real-time PCR. The effect of AFAP1-AS1 on cell proliferation was examined by MTT assay, cell apoptosis was detected by flow cytometric analysis and   cell invasion   was determined by Transwell assay. RhoA/Rac2 signaling and downstream factors were verified by western blotting. HCC cells infected with si-AFAP1-AS1 were injected into nude mice to investigate the effect of AFAP1-AS1 on the tumorigenesis in vivo. We found that increased expression of AFAP1-AS1 was significantly correlated with pathological staging (P=0.024) and lymph-vascular space invasion (LVSI) in HCC patients (P=0.007). Multivariate analyses indicated that AFAP1-AS1 represented an independent predictor for overall survival of HCC (P=0.029). Further experiments showed that knockdown of AFAP1-AS1 by si-AFAP1-AS1 decreased the proliferation and invasion in vitro and in vivo, induced cell apoptosis and blocked cell cycle in S phase via inhibition of the RhoA/Rac2 signaling. Taken together, our findings indicate that AFAP1-AS1 may promote the HCC development through upregulation of RhoA/Rac2 signaling and provide a potential therapeutic target for HCC. 
 Netrin-1 promotes glioblastoma cell invasiveness and angiogenesis by multiple pathways including activation of RhoA, cathepsin B, and cAMP-response element-binding protein. Glioblastomas are very difficult tumors to treat because they are highly invasive and disseminate within the normal brain, resulting in newly growing tumors. We have identified netrin-1 as a molecule that promotes glioblastoma invasiveness. As evidence, netrin-1 stimulates glioblastoma   cell invasion   directly through Matrigel-coated transwells, promotes tumor cell sprouting and enhances metastasis to lymph nodes in vivo. Furthermore, netrin-1 regulates angiogenesis as shown in specific angiogenesis assays such as enhanced capillary endothelial cells (EC) sprouting and by increased EC infiltration into Matrigel plugs in vivo, as does VEGF-A. This netrin-1 signaling pathway in glioblastoma cells includes activation of RhoA and cyclic AMP response element-binding protein (CREB). A novel finding is that netrin-1-induced glioblastoma invasiveness and angiogenesis are mediated by activated cathepsin B (CatB), a cysteine protease that translocates to the cell surface as an active enzyme and co-localizes with cell surface annexin A2 (ANXA2). The specific CatB inhibitor CA-074Me inhibits netrin-1-induced   cell invasion  , sprouting, and Matrigel plug angiogenesis. Silencing of CREB suppresses netrin-1-induced glioblastoma   cell invasion  , sprouting, and CatB expression. It is concluded that netrin-1 plays an important dual role in glioblastoma progression by promoting both glioblastoma cell invasiveness and angiogenesis in a RhoA-, CREB-, and CatB-dependent manner. Targeting netrin-1 pathways may be a promising strategy for brain cancer therapy. 
 Gonadotropin-releasing hormone receptor activates GTPase RhoA and inhibits   cell invasion   in the breast cancer cell line MDA-MB-231. Gonadotropin-releasing hormone (GnRH) and its receptor (GnRHR) are both expressed by a number of malignant tumors, including those of the breast. In the latter, both behave as potent inhibitors of invasion. Nevertheless, the signaling pathways whereby the activated GnRH/GnRHR system exerts this effect have not been clearly established. In this study, we provide experimental evidence that describes components of the mechanism(s) whereby GnRH inhibits breast cancer   cell invasion  .Actin polymerization and substrate adhesion was measured in the highly invasive cell line, MDA-MB-231 transiently expressing the wild-type or mutant DesK191 GnRHR by fluorometry, flow cytometric analysis, and confocal microscopy, in the absence or presence of GnRH agonist. The effect of RhoA-GTP on stress fiber formation and focal adhesion assembly was measured in MDA-MB-231 cells co-expressing the GnRHRs and the GAP domain of human p190Rho GAP-A or the dominant negative mutant GAP-Y1284D.   Cell invasion   was determined by the transwell migration assay.Agonist-stimulated activation of the wild-type GnRHR and the highly plasma membrane expressed mutant GnRHR-DesK191 transiently transfected to MDA-MB-231 cells, favored F-actin polymerization and substrate adhesion. Confocal imaging allowed detection of an association between F-actin levels and the increase in stress fibers promoted by exposure to GnRH. Pull-down assays showed that the effects observed on actin cytoskeleton resulted from GnRH-stimulated activation of RhoA GTPase. Activation of this small G protein favored the marked increase in both cell adhesion to Collagen-I and number of focal adhesion complexes leading to inhibition of the invasion capacity of MDA-MB-231 cells as disclosed by assays in Transwell Chambers.We here show that GnRH inhibits invasion of highly invasive breast cancer-derived MDA-MB-231 cells. This effect is mediated through an increase in substrate adhesion promoted by activation of RhoA GTPase and formation of stress fibers and focal adhesions. These observations offer new insights into the molecular mechanisms whereby activation of overexpressed GnRHRs affects   cell invasion   potential of this malignant cell line, and provide opportunities for designing mechanism-based adjuvant therapies for breast cancer. 
 Macrophages stimulate gastric and colorectal cancer invasion through EGFR Y(1086), c-Src, Erk1/2 and Akt phosphorylation and smallGTPase activity. The interactions between cancer cells and their microenvironment are crucial for malignant progression, as they modulate invasion-related activities. Tumor-associated macrophages are generally considered allies in the process of tumor progression in several types of cancer, although their role on gastric and colorectal carcinomas is still poorly understood. In this report, we studied the influence of primary human macrophages on gastric and colorectal cancer cells, considering invasion, motility/migration, proteolysis and activated intracellular signaling pathways. We demonstrated that macrophages stimulate cancer   cell invasion  , motility and migration, and that these effects depend on matrix metalloproteinase (MMP) activity and on the activation of epidermal growth factor receptor (EGFR) (at the residue Y(1086)), PLC-γ (phospholipase C-gamma) and Gab1 (GRB2-associated binding protein-1), as evidenced by siRNA (small interference RNA) experiments. Epidermal growth factor (EGF)-immunodepletion impaired macrophage-mediated cancer   cell invasio  n and motility, suggesting that EGF is the pro-invasive and pro-motile factor produced by macrophages. Macrophages also induced gastric and colorectal cancer cell phosphorylation of Akt, c-Src and ERK1/2, and led to an increase of RhoA and Cdc42 activity. Interestingly, whereas macrophage-mediated cancer cell c-Src and ERK1/2 phosphorylation occurred downstream EGFR activation, Akt phosphorylation seems to be a parallel event, taking place in an EGFR-independent manner. The involvement of EGF, EGFR-downstream signaling partners and MMPs in macrophage-mediated invasion provides novel insights into the molecular crosstalk established between cancer cells and macrophages, opening new perspectives for the design of new and more efficient therapeutic strategies to counteract cancer   cell invasio  n. 
 RhoA induces MMP-9 expression at CD44 lamellipodial focal complexes and promotes HMEC-1   cell invasion  . Much progress has been made in recent years in the understanding of angiogenesis, yet signalling pathways involved remain poorly defined. Here we report that small RhoA GTPase is implicated in the invasion of human microvascular endothelial cells (HMEC-1). Ectopic expression of active-RhoA GTPase induced the expression of MMP-9 metalloproteinase, a key proteinase of the basement membrane, and promoted migration of endothelial cells through a 3D-matrix protein gel. MMP-9 was either directed as vesicular-like patches to the apical side of cells, or addressed to specific membrane sites at the cell surface. Confocal microscopy analyses indeed revealed clustering of MMP-9 in advancing lamellipodia at the forefront of endothelial cells, where this proteinase colocalized with RhoA and CD44, a transmembrane receptor known to be proteolysed in tumor cell progression. In addition, TIMP-1, a natural MMP inhibitor, significantly reduced the invasion of RhoAV14 expressing cells, suggesting that MMP-9 was a critical metalloproteinase responsible, at least partly, for the RhoAV14-induced endothelial   cell invasion  . We propose that RhoA triggers signalling pathways that, upregulating expression of a proteinase at specific membrane localizations, may confer an highly invasive phenotype to endothelial cells. 
 NET1-mediated RhoA activation facilitates lysophosphatidic acid-induced cell migration and invasion in gastric cancer. The most lethal aspects of gastric adenocarcinoma (GA) are its invasive and metastatic properties. This aggressive phenotype remains poorly understood. We have recently identified neuroepithelial cell transforming gene 1 (NET1), a guanine exchange factor (GEF), as a novel GA-associated gene. Neuroepithelial cell transforming gene 1 expression is enhanced in GA and it is of functional importance in   cell invasion  . In this study, we demonstrate the activity of NET1 in driving cytoskeletal rearrangement, a key pathological mechanism in gastric tumour cell migration and invasion. Neuroepithelial cell transforming gene 1 expression was increased 10-fold in response to treatment with lysophosphatidic acid (LPA), resulting in an increase in active levels of RhoA and a 2-fold increase in   cell invasion  . Lysophosphatidic acid-induced   cell invasion   and migration were significantly inhibited using either NET1 siRNA or a RhoA inhibitor (C3 exoenzyme), thus indicating the activity of both NET1 and RhoA in gastric cancer progression. Furthermore, LPA-induced invasion and migration were also significantly reduced in the presence of cytochalasin D, an inhibitor of cytoskeletal rearrangements. Neuroepithelial cell transforming gene 1 knockdown resulted in AGS cell rounding and a loss of actin filament organisation, demonstrating the function of NET1 in actin organisation. These data highlight the importance of NET1 as a driver of tumour   cell invasion  , an activity mediated by RhoA activation and cytoskeletal reorganisation. 
 Characterization of the roles of RHOC and RHOA GTPases in invasion, motility, and matrix adhesion in inflammatory and aggressive breast cancers. The 2 closely related small GTPases, RHOC and RHOA, are involved in mammary gland carcinogenesis; however, their specific roles in determining cancer cell adhesion and invasion have not been elucidated.RHOA and RHOC are highly homologous, thereby posing a major challenge to study their individual functions in cancer cells. By selectively knocking down these proteins, we have been able to alternatively inhibit RHOC and RHOA, while preserving expression of the other rho protein. Quantitative analyses of the growth patterns and invasion in the aggressive estrogen receptor negative cell lines MDA-231 and SUM149 were carried out on collagen I and Matrigel substrates.RHOC, and not RHOA, modulates surface expression and colocalization of alpha2 and beta1 integrins in MDA-MB-231 on collagen I. Neither RHOC or RHOA affected integrin expression in the inflammatory breast cancer cell line SUM149, further highlighting the different regulation of adhesion and motility in inflammatory breast cancer.This work shows that RHOC and RHOA play different roles in cell-matrix adhesion, motility, and invasion of MDA-MB-231 and reaffirms the crucial role of RHOC-GTPase in inflammatory breast cancer   cell invasion  . 
 Activated G(alpha)13 impairs cell invasiveness through p190RhoGAP-mediated inhibition of RhoA activity. The GTPase RhoA is a downstream target of heterotrimeric G(13) proteins and plays key roles in cell migration and invasion. Here, we show that expression in human melanoma cells of a constitutively active, GTPase-deficient Galpha(13) form (G(alpha)(13)QL) or lysophosphatidylcholine (LPC)-promoted signaling through G(alpha)(13)-coupled receptors led to a blockade of chemokine-stimulated RhoA activation and   cell invasion   that was rescued by active RhoA. Melanoma cells expressing G(alpha)(13)QL or cells stimulated with LPC displayed an increase in p190RhoGAP activation, and defects in RhoA activation and invasion were recovered by knocking down p190RhoGAP expression, thus identifying this GTPase-activating protein (GAP) protein as a downstream G(alpha)(13) target that is responsible for these inhibitory responses. In addition, defective stress fiber assembly and reduced migration speed underlay inefficient invasion of G(alpha)(13)QL melanoma cells. Importantly, G(alpha)(13)QL expression in melanoma cells led to impairment in lung metastasis associated with prolonged survival in SCID mice. The data indicate that G(alpha)(13)-dependent downstream effects on RhoA activation and invasion tightly depend on cell type-specific GAP activities and that G(alpha)(13)-p190RhoGAP signaling might represent a potential target for intervention in melanoma metastasis. 
  GeneRIFs  
 A MAPK-Driven Feedback Loop Suppresses Rac Activity to Promote RhoA-Driven Cancer   Cell Invasion  . Our model predicted an unanticipated feedback loop, whereby Raf/MEK/ERK signalling maintains suppression of Rac1 by inhibiting the Rac-activating Sos1-Eps8-Abi1 complex, allowing RhoA activity to predominate in invasive protrusions. 
 we found that CXCR4-CXCL12/AKT axis regulates RhoA, Rac-1, and Cdc42 to modulate   cell invasion   and tumor metastasis. Together, these results demonstrate a role for CXCR4 in ESCC metastasis and progression and suggest potential targets for therapeutic intervention. 
 knockdown of TMEM45A also inhibited cell adhesion as well as   cell invasion  . More importantly, suppression of TMEM45A notably downregulated the expression of TGF-b1, TGF-b2, RhoA and ROCK2. 
 Study demonstrates that increased DGKzeta protein levels in SW620 cells are associated with increased Rho GTPase activity and downstream signaling; silencing of DGKzeta expression in SW620 cells decreased Rac1 and RhoA activity and attenuated   cell invasion  ; DGKzeta silencing also attenuated the invasiveness of PC-3 prostate cancer and MDA-MB-231 breast cancer cells. 
 BDNF knockdown inhibited   cell invasion   probably through the blocked actin polymerization and the correlated inactivation of RhoA or Rac1. 
 This work shows that RHOC and RHOA play different roles in cell-matrix adhesion, motility, and invasion of MDA-MB-231 and reaffirms the crucial role of RHOC-GTPase in inflammatory breast cancer   cell invasion  . 
 Data describe a proteomics strategy to identify proteins regulated by RhoA and report a new human protein, named "mediator of RhoA-dependent invasion," that is induced in metastatic cells by constitutive RhoA activation and promotes   cell invasion  . 
 Rac1 and RhoA signaling simultaneously regulate   cell invasion   in 3D matrices. 
 a link between RhoA, JNK, c-Jun, and MMP2 activity that is functionally involved in the reduction in osteosarcoma   cell invasion   by the statin. This suggests a novel strategy targeting RhoA-JNK-c-Jun signaling to reduce osteosarcoma cell tumorigenesis. 
 the importance of NET1 as a driver of tumour   cell invasion  , an activity mediated by RhoA activation and cytoskeletal reorganisation. 
 RhoA and RhoC are downstream of PKC epsilon & critical for PKC-epsilon-mediated   cell invasion   & motility. PKC epsilon coordinately regulates RhoA & RhoC activation, possibly through direct post-translational phosphorylation. 
 Lysoophosphatidic acid stimulates RhoA and increased PC-3 prostate cancer   cell invasion   activity through an NF-kappaB-dependent pathway. 
   
  
 RHOBTB1 
 
  NCBI Gene  
  GeneRIF  
  Pubmed  
 
 
  Search Term  Rank  Fields  
  [Ff]ilopodia  1  PubMed(1)  
 
 
 hide/show details for RHOBTB1 
  [Ff]ilopodia  PubMed  
 Rho GTPases have diverse effects on the organization of the actin filament system. The Rho GTPases are related to the Ras proto-oncogenes and consist of 22 family members. These proteins have important roles in regulating the organization of the actin filament system, and thereby the morphogenesis of vertebrate cells as well as their ability to migrate. In an effort to compare the effects of all members of the Rho GTPase family, active Rho GTPases were transfected into porcine aortic endothelial cells and the effects on the actin filament system were monitored. Cdc42, TCL (TC10-like), Rac1-Rac3 and RhoG induced the formation of lamellipodia, whereas Cdc42, Rac1 and Rac2 also induced the formation of thick bundles of actin filaments. In contrast, transfection with TC10 or Chp resulted in the formation of focal adhesion-like structures, whereas Wrch-1 induced long and thin   filopodia  . Transfection with RhoA, RhoB or RhoC induced the assembly of stress fibres, whereas Rnd1-Rnd3 resulted in the loss of stress fibres, but this effect was associated with the formation of actin- and ezrin-containing dorsal microvilli. Cells expressing RhoD and Rif had extremely long and flexible   filopodia  . None of the RhoBTB or Miro GTPases had any major influence on the organization of the actin filament system; instead, RhoBTB1 and RhoBTB2 were present in vesicular structures, and Miro-1 and Miro-2 were present in mitochondria. Collectively, the data obtained in this study to some extent confirm earlier observations, but also allow the identification of previously undetected roles of the different members of the Rho GTPases. 
   
  
 RHOD 
 
  NCBI Gene  
  GeneRIF  
  Pubmed  
 
 
  Search Term  Rank  Fields  
  [Ff]ilopodia  1  PubMed(1)  
  [Pp]odosome(s)?  5  PubMed(1)  
  [Mm]igration  6  Process(1); PubMed(6); GeneRIFs(1)  
  [Ii]nvasive  10  PubMed(3); GeneRIFs(1)  
  [Mm]etastasis  12  PubMed(4)  
  [Cc]ell [Ii]nvasion  13  PubMed(1)  
 
 
 hide/show details for RHOD 
  [Ff]ilopodia  PubMed  
 RhoD is a Golgi component with a role in anterograde protein transport from the ER to the plasma membrane. RhoD is a member of the Rho GTPase family and it coordinates actin dynamics and membrane trafficking. Activation of RhoD results in formation of   filopodia  , dissolution of stress fibers, and the subsequent formation of short actin bundles. In addition, RhoD localizes to early endosomes and recycling endosomes, and has a regulatory role in endosome trafficking. In this study, we report on a function of RhoD in the regulation of Golgi homeostasis. We show that manipulation of protein and activation levels of RhoD, as well as of its binding partner WHAMM, result in derailed localization of Golgi stacks. Moreover, vesicle trafficking from the endoplasmic reticulum to the plasma membrane via the Golgi apparatus measured by the VSV-G protein is severely hampered by manipulation of RhoD or WHAMM. In summary, our studies demonstrate a novel role for this member of the Rho GTPases in the regulation of Golgi function. 
  [Pp]odosome(s)?  PubMed  
 Rho signaling, ROCK and mDia1, in transformation, metastasis and invasion. The Rho subgroup of the Rho GTPases consisting of RhoA, RhoB and RhoC induces a specific type of actin cytoskeleton and carry out a variety of functions in the cell. mDia and ROCK are downstream effectors of Rho mediating Rho action on the actin cytoskeleton; mDia produces actin filaments by nucleation and polymerization and ROCK activate myosin to cross-link them for induction of actomyosin bundles and contractility. mDia is potentially linked to Rac activation and membrane ruffle formation through c-Src-induced phosphorylation of focal adhesion proteins, and ROCK antagonizes this mDia action. Thus, cell morphogenesis, adhesion, and motility can be determined by the balance between mDia and ROCK activities. Though they are not oncogenes by themselves, overexpression of RhoA and RhoC are often found in clinical cancers, and RhoC has been repeatedly identified as a gene associated with metastasis. The Rho-ROCK pathway is implicated in Ras-mediated transformation, the amoeboid movement of tumor cells in the three-dimensional matrix, and transmigration of tumor cells through the mesothelial monolayer. On the other hand, the Rho-mDia1 pathway is implicated in Src-mediated remodeling of focal adhesions and migration of tumor cells. There is also an indication that the Rho pathway other than ROCK is involved in Src-mediated induction of   podosome   and regulation of matrix metalloproteases. Thus, Rho mediates various phenotypes of malignant transformation by Ras and Src through its effectors, ROCK and mDia. 
  [Mm]igration  Process  
 positive regulation of cell   migration   
  PubMed  
 Breast tumor kinase phosphorylates p190RhoGAP to regulate rho and ras and promote breast carcinoma growth,   migration  , and invasion. Breast tumor kinase (Brk), an Src-like nonreceptor tyrosine kinase, is overexpressed in breast cancer and several other cancer types. Our previous study indicates that Brk promotes cell   migration   and tumor invasion by phosphorylating the focal adhesion protein paxillin. Here, we report the identification of p190RhoGAP-A (p190) as a Brk substrate. Brk phosphorylates p190 at the Y(1105) residue both in vitro and in vivo, thereby promoting the association of p190 with p120RasGAP (p120). As a consequence, Brk stimulates p190 and attenuates p120 functions, leading to RhoA inactivation and Ras activation, respectively. In carcinoma cells expressing high levels of Brk, endogenous Brk functions as a key contributor to epidermal growth factor-induced p190 tyrosine phosphorylation. We present evidence showing that p190 phosphorylation plays essential roles in both migratory and proliferative effects of Brk. Furthermore, disruption of p190 phosphorylation-induced p190/p120 complex in breast cancer cells abolishes not only the abilities of Brk to regulate RhoA and Ras but also the stimulatory effects of Brk on proliferation,   migration  , invasion, transformation, and tumorigenicity. Together, our findings reveal a previously unknown function of Brk in regulating both RhoA and Ras by phosphorylating p190 and provide evidence for the crucial roles of this Brk-elicited signaling pathway in promoting breast malignancy. 
 Breast tumor kinase phosphorylates p190RhoGAP to regulate rho and ras and promote breast carcinoma growth,   migration  , and invasion. Breast tumor kinase (Brk), an Src-like nonreceptor tyrosine kinase, is overexpressed in breast cancer and several other cancer types. Our previous study indicates that Brk promotes cell   migration   and tumor invasion by phosphorylating the focal adhesion protein paxillin. Here, we report the identification of p190RhoGAP-A (p190) as a Brk substrate. Brk phosphorylates p190 at the Y(1105) residue both in vitro and in vivo, thereby promoting the association of p190 with p120RasGAP (p120). As a consequence, Brk stimulates p190 and attenuates p120 functions, leading to RhoA inactivation and Ras activation, respectively. In carcinoma cells expressing high levels of Brk, endogenous Brk functions as a key contributor to epidermal growth factor-induced p190 tyrosine phosphorylation. We present evidence showing that p190 phosphorylation plays essential roles in both migratory and proliferative effects of Brk. Furthermore, disruption of p190 phosphorylation-induced p190/p120 complex in breast cancer cells abolishes not only the abilities of Brk to regulate RhoA and Ras but also the stimulatory effects of Brk on proliferation,   migration  , invasion, transformation, and tumorigenicity. Together, our findings reveal a previously unknown function of Brk in regulating both RhoA and Ras by phosphorylating p190 and provide evidence for the crucial roles of this Brk-elicited signaling pathway in promoting breast malignancy. 
 The Galpha13-Rho signaling axis is required for SDF-1-induced   migration   through CXCR4. The CXC chemokine stromal cell-derived factor-1alpha (SDF-1) binds to CXCR4, a seven-transmembrane G protein-coupled receptor that plays a critical role in many physiological processes that involve cell   migration   and cell fate decisions, ranging from stem cell homing, angiogenesis, and neuronal development to immune cell trafficking. CXCR4 is also implicated in various pathological conditions, including metastatic spread and human immunodeficiency virus infection. Although SDF-1-induced cell   migration   in CXCR4-expressing cells is sensitive to pertussis toxin treatment, hence involving heterotrimeric G proteins of the G(i) family, whether other G proteins participate in the chemotactic response to SDF-1 is still unknown. In this study, we took advantage of the potent chemotactic activity of SDF-1 in Jurkat T-cells to examine the nature of the heterotrimeric G protein subunits contributing to CXCR4-mediated cell   migration  . We observed that whereas G(i) and Gbetagamma subunits are involved in SDF-1-induced Rac activation and cell   migration  , CXCR4 can also stimulate Rho potently leading to the phosphorylation of myosin light chain through the Rho effector, Rho kinase, but independently of G(i). Furthermore, we found that Galpha(13) mediates the activation of Rho by CXCR4 and that the functional activity of both Galpha(13) and Rho is required for directional cell   migration   in response to SDF-1. Collectively, our data indicate that signaling by CXCR4 to Rho through Galpha(13) contributes to cell   migration   when stimulated by SDF-1, thus identifying the Galpha(13)-Rho signaling axis as a potential pharmacological target in many human diseases that involve the aberrant function of CXCR4. 
 RhoD regulates cytoskeletal dynamics via the actin nucleation-promoting factor WASp homologue associated with actin Golgi membranes and microtubules. The Rho GTPases have mainly been studied in association with their roles in the regulation of actin filament organization. These studies have shown that the Rho GTPases are essential for basic cellular processes, such as cell   migration  , contraction, and division. In this paper, we report that RhoD has a role in the organization of actin dynamics that is distinct from the roles of the better-studied Rho members Cdc42, RhoA, and Rac1. We found that RhoD binds the actin nucleation-promoting factor WASp homologue associated with actin Golgi membranes and microtubules (WHAMM), as well as the related filamin A-binding protein FILIP1. Of these two RhoD-binding proteins, WHAMM was found to bind to the Arp2/3 complex, while FILIP1 bound filamin A. WHAMM was found to act downstream of RhoD in regulating cytoskeletal dynamics. In addition, cells treated with small interfering RNAs for RhoD and WHAMM showed increased cell attachment and decreased cell   migration  . These major effects on cytoskeletal dynamics indicate that RhoD and its effectors control vital cytoskeleton-driven cellular processes. In agreement with this notion, our data suggest that RhoD coordinates Arp2/3-dependent and FLNa-dependent mechanisms to control the actin filament system, cell adhesion, and cell   migration  . 
 Rho signaling, ROCK and mDia1, in transformation, metastasis and invasion. The Rho subgroup of the Rho GTPases consisting of RhoA, RhoB and RhoC induces a specific type of actin cytoskeleton and carry out a variety of functions in the cell. mDia and ROCK are downstream effectors of Rho mediating Rho action on the actin cytoskeleton; mDia produces actin filaments by nucleation and polymerization and ROCK activate myosin to cross-link them for induction of actomyosin bundles and contractility. mDia is potentially linked to Rac activation and membrane ruffle formation through c-Src-induced phosphorylation of focal adhesion proteins, and ROCK antagonizes this mDia action. Thus, cell morphogenesis, adhesion, and motility can be determined by the balance between mDia and ROCK activities. Though they are not oncogenes by themselves, overexpression of RhoA and RhoC are often found in clinical cancers, and RhoC has been repeatedly identified as a gene associated with metastasis. The Rho-ROCK pathway is implicated in Ras-mediated transformation, the amoeboid movement of tumor cells in the three-dimensional matrix, and trans  migration   of tumor cells through the mesothelial monolayer. On the other hand, the Rho-mDia1 pathway is implicated in Src-mediated remodeling of focal adhesions and   migration   of tumor cells. There is also an indication that the Rho pathway other than ROCK is involved in Src-mediated induction of podosome and regulation of matrix metalloproteases. Thus, Rho mediates various phenotypes of malignant transformation by Ras and Src through its effectors, ROCK and mDia. 
 A novel agent, methylophiopogonanone B, promotes Rho activation and tubulin depolymerization. Cytoskeletal reorganization, including reconstruction of actin fibers and microtubules, is essential for various biological processes, such as cell   migration  , proliferation and dendrite formation. We show here that methylophiopogonanone B (MOPB) induces cell morphological change via melanocyte dendrite retraction and stress fiber formation. Since members of the Rho family of small GTP-binding proteins act as master regulators of dendrite formation and actin cytoskeletal reorganization, and activated Rho promotes dendrite retraction and stress fiber formation, we studied the effects of MOPB on the small GTPases using normal human epidermal melanocytes and HeLa cells. In in vitro binding assay, MOPB significantly increased GTP-Rho, but not GTP-Rac or GTP-CDC42. Furthermore, a Rho inhibitor, a Rho kinase inhibitor and a small GTPase inhibitor each blocked MOPB-induced stress fiber formation. The effect of MOPB on actin reorganization was blocked in a Rho dominant negative mutant. These results suggest MOPB acts via the Rho signaling pathway, and it may directly or indirectly activate Rho. Quantitative Western blot analysis indicated that MOPB also induced microtubule destabilization and tubulin depolymerization. Thus, MOPB appears to induce Rho activation, resulting in actin cytoskeletal reorganization, including dendrite retraction and stress fiber formation. 
  GeneRIFs  
 Galpha13-Rho signaling axis is required for SDF-1-induced   migration   through CXCR4 
  [Ii]nvasive  PubMed  
 A p120 catenin isoform switch affects Rho activity, induces tumor cell invasion, and predicts metastatic disease. p120 catenin is a cadherin-associated protein that regulates Rho GTPases and promotes the   invasive  ness of E-cadherin-deficient cancer cells. Multiple p120 isoforms are expressed in cells via alternative splicing, and all of them are essential for HGF signaling to Rac1. However, only full-length p120 (isoform 1) promotes   invasive  ness. This selective ability of p120 isoform 1 is mediated by reduced RhoA activity, both under basal conditions and following HGF treatment. All p120 isoforms can bind RhoA in vitro, via a central RhoA binding site. However, only the cooperative binding of RhoA to the central p120 domain and to the alternatively spliced p120 N terminus stabilizes RhoA binding and inhibits RhoA activity. Consistent with this, increased expression of p120 isoform 1, when compared with other p120 isoforms, is predictive of renal tumor micrometastasis and systemic progression, following nephrectomy. Furthermore, ectopic expression of the RhoA-binding, N-terminal domain of p120 is sufficient to block the ability of p120 isoform 1 to inhibit RhoA and to promote   invasive  ness. The data indicate that the increased expression of p120 isoform 1 during tumor progression contributes to the   invasive   phenotype of cadherin-deficient carcinomas and that the N-terminal domain of p120 is a valid therapeutic target. 
 Non  invasive   single-exon fetal RHD determination in a routine screening program in early pregnancy. To develop a simple and robust assay suitable for fetal RHD screening in first-trimester pregnancy and to estimate the sensitivity and specificity of the test after its implementation in an unselected pregnant population.Pregnant women attending their first antenatal visit were included, and fetal RHD determination was performed for all women who typed RhD-negative by routine serology. DNA was extracted by an automated system and quantitative polymerase chain reaction was done by an assay based on exon 4. Reporting criteria were simple and strict.Four thousand one hundred eighteen pregnancies, with a median gestational age of 10 weeks, were included. After 211 (5.1%) reanalyses, fetal RHD was reported positive in 2,401 (58.3%), negative in 1,552 (37.7%), and inconclusive in 165 (4.0%) based on the first sample. After a second sample in 147 of 165, only 14 remained inconclusive, all resulting from a weak or silent maternal RHD gene. Using blood group serology of the newborns as the gold standard, the false-negative rate was 55 of 2,297 (2.4%) and the false-positive rate was 15 of 1,355 (1.1%). After exclusion of samples obtained before gestational week 8, the false-negative rate was 23 of 2,073 (1.1%) and the false-positive rate was 14 of 1,218 (1.1%). Both sensitivity and specificity were close to 99% provided samples were not collected before gestational week 8. From gestational week 22, sensitivity was 100%.Fetal RHD detection in early pregnancy using a single-exon assay in a routine clinical setting is feasible and accurate.I. 
 Repression of the estrogen receptor-alpha transcriptional activity by the Rho/megakaryoblastic leukemia 1 signaling pathway. Although involved in processes leading to the emergence and development of hormone-dependent breast cancers, the estrogen receptor alpha (ERalpha) also prevents transformed cells from progressing toward a more aggressive phenotype. The transcriptional activity of ERalpha is mediated through two transactivation functions, called activation function 1 and 2, whose respective involvement varies in a cell-specific manner. Here, we identify the Rho/megakaryoblastic leukemia 1 (MKL1) signaling pathway as a main actor in controlling the cell-specific activity of both transactivation functions of ERalpha. Notably, we show that, when the coregulator MKL1 is sequestered in an inactive form by unpolymerized actin, the transcriptional activity of ERalpha mainly relies on the activation function 1. The activation of MKL1, which results from its dissociation from unpolymerized actin, promoted by the ability of Rho to support polymeric actin accumulation, silences the activation function 1 of ERalpha and allows the receptor to mainly act through its activation function 2. Importantly, this switch in the respective contribution exerted by both transactivation functions is correlated with an impaired ability of ERalpha to efficiently transactivate estrogen-regulated reporter genes. MKL1 is further shown to be present on estrogen-responsive genes in vivo. Interestingly, the Rho/MKL1 signaling pathway is activated during the epithelial-mesenchymal transition. A reduced transactivation efficiency of ERalpha, resulting from the activation of this pathway, may therefore suppress the protective role exerted by ERalpha toward tumor progression and   invasive  ness. 
  GeneRIFs  
 the the increased expression of p120 isoform 1 during tumor progression contributes to the   invasive   phenotype of cadherin-deficient carcinomas and that the N-terminal domain of p120 is a valid therapeutic target 
  [Mm]etastasis  PubMed  
 Deleted in liver cancer 1 (DLC1) negatively regulates Rho/ROCK/MLC pathway in hepatocellular carcinoma. Deleted in liver cancer 1 (DLC1), a member of RhoGTPase activating protein (GAP) family, is known to have suppressive activities in tumorigenicity and cancer   metastasis  . However, the underlying molecular mechanisms of how DLC1 suppresses cell motility have not been fully elucidated. Rho-kinase (ROCK) is an immediate down-stream effector of RhoA in mediating cellular cytoskeletal events and cell motility. In the present study, we aimed to investigate the effects of DLC1 on Rho/ROCK signaling pathway in hepatocellular carcinoma (HCC).We demonstrated that DLC1 negatively regulated ROCK-dependent actomyosin contractility. From immunofluorescence study, we found that ectopic expression of DLC1 abrogated Rho/ROCK-mediated cytoskeletal reorganization including formation of stress fibers and focal adhesions. It also downregulated cortical phosphorylation of myosin light chain 2 (MLC2). These inhibitory events by DLC1 were RhoGAP-dependent, as RhoGAP-deficient mutant of DLC1 (DLC1 K714E) abolished these inhibitory events. In addition, from western study, DLC1 inhibited ROCK-related myosin light chain phosphatase targeting unit 1 (MYPT1) phosphorylation at Threonine 853. By examining cell morphology under microscope, we found that ectopic expression of dominant-active ROCK released cells from DLC1-induced cytoskeletal collapse and cell shrinkage.Our data suggest that DLC1 negatively regulates Rho/ROCK/MLC2. This implicates a ROCK-mediated pathway of DLC1 in suppressing   metastasis   of HCC cells and enriches our understanding in the molecular mechanisms involved in the progression of hepatocellular carcinoma. 
 Deleted in liver cancer 1 (DLC1) negatively regulates Rho/ROCK/MLC pathway in hepatocellular carcinoma. Deleted in liver cancer 1 (DLC1), a member of RhoGTPase activating protein (GAP) family, is known to have suppressive activities in tumorigenicity and cancer   metastasis  . However, the underlying molecular mechanisms of how DLC1 suppresses cell motility have not been fully elucidated. Rho-kinase (ROCK) is an immediate down-stream effector of RhoA in mediating cellular cytoskeletal events and cell motility. In the present study, we aimed to investigate the effects of DLC1 on Rho/ROCK signaling pathway in hepatocellular carcinoma (HCC).We demonstrated that DLC1 negatively regulated ROCK-dependent actomyosin contractility. From immunofluorescence study, we found that ectopic expression of DLC1 abrogated Rho/ROCK-mediated cytoskeletal reorganization including formation of stress fibers and focal adhesions. It also downregulated cortical phosphorylation of myosin light chain 2 (MLC2). These inhibitory events by DLC1 were RhoGAP-dependent, as RhoGAP-deficient mutant of DLC1 (DLC1 K714E) abolished these inhibitory events. In addition, from western study, DLC1 inhibited ROCK-related myosin light chain phosphatase targeting unit 1 (MYPT1) phosphorylation at Threonine 853. By examining cell morphology under microscope, we found that ectopic expression of dominant-active ROCK released cells from DLC1-induced cytoskeletal collapse and cell shrinkage.Our data suggest that DLC1 negatively regulates Rho/ROCK/MLC2. This implicates a ROCK-mediated pathway of DLC1 in suppressing   metastasis   of HCC cells and enriches our understanding in the molecular mechanisms involved in the progression of hepatocellular carcinoma. 
 A p120 catenin isoform switch affects Rho activity, induces tumor cell invasion, and predicts metastatic disease. p120 catenin is a cadherin-associated protein that regulates Rho GTPases and promotes the invasiveness of E-cadherin-deficient cancer cells. Multiple p120 isoforms are expressed in cells via alternative splicing, and all of them are essential for HGF signaling to Rac1. However, only full-length p120 (isoform 1) promotes invasiveness. This selective ability of p120 isoform 1 is mediated by reduced RhoA activity, both under basal conditions and following HGF treatment. All p120 isoforms can bind RhoA in vitro, via a central RhoA binding site. However, only the cooperative binding of RhoA to the central p120 domain and to the alternatively spliced p120 N terminus stabilizes RhoA binding and inhibits RhoA activity. Consistent with this, increased expression of p120 isoform 1, when compared with other p120 isoforms, is predictive of renal tumor micro  metastasis   and systemic progression, following nephrectomy. Furthermore, ectopic expression of the RhoA-binding, N-terminal domain of p120 is sufficient to block the ability of p120 isoform 1 to inhibit RhoA and to promote invasiveness. The data indicate that the increased expression of p120 isoform 1 during tumor progression contributes to the invasive phenotype of cadherin-deficient carcinomas and that the N-terminal domain of p120 is a valid therapeutic target. 
 Rho signaling, ROCK and mDia1, in transformation,   metastasis   and invasion. The Rho subgroup of the Rho GTPases consisting of RhoA, RhoB and RhoC induces a specific type of actin cytoskeleton and carry out a variety of functions in the cell. mDia and ROCK are downstream effectors of Rho mediating Rho action on the actin cytoskeleton; mDia produces actin filaments by nucleation and polymerization and ROCK activate myosin to cross-link them for induction of actomyosin bundles and contractility. mDia is potentially linked to Rac activation and membrane ruffle formation through c-Src-induced phosphorylation of focal adhesion proteins, and ROCK antagonizes this mDia action. Thus, cell morphogenesis, adhesion, and motility can be determined by the balance between mDia and ROCK activities. Though they are not oncogenes by themselves, overexpression of RhoA and RhoC are often found in clinical cancers, and RhoC has been repeatedly identified as a gene associated with   metastasis  . The Rho-ROCK pathway is implicated in Ras-mediated transformation, the amoeboid movement of tumor cells in the three-dimensional matrix, and transmigration of tumor cells through the mesothelial monolayer. On the other hand, the Rho-mDia1 pathway is implicated in Src-mediated remodeling of focal adhesions and migration of tumor cells. There is also an indication that the Rho pathway other than ROCK is involved in Src-mediated induction of podosome and regulation of matrix metalloproteases. Thus, Rho mediates various phenotypes of malignant transformation by Ras and Src through its effectors, ROCK and mDia. 
  [Cc]ell [Ii]nvasion  PubMed  
 A p120 catenin isoform switch affects Rho activity, induces tumor   cell invasion  , and predicts metastatic disease. p120 catenin is a cadherin-associated protein that regulates Rho GTPases and promotes the invasiveness of E-cadherin-deficient cancer cells. Multiple p120 isoforms are expressed in cells via alternative splicing, and all of them are essential for HGF signaling to Rac1. However, only full-length p120 (isoform 1) promotes invasiveness. This selective ability of p120 isoform 1 is mediated by reduced RhoA activity, both under basal conditions and following HGF treatment. All p120 isoforms can bind RhoA in vitro, via a central RhoA binding site. However, only the cooperative binding of RhoA to the central p120 domain and to the alternatively spliced p120 N terminus stabilizes RhoA binding and inhibits RhoA activity. Consistent with this, increased expression of p120 isoform 1, when compared with other p120 isoforms, is predictive of renal tumor micrometastasis and systemic progression, following nephrectomy. Furthermore, ectopic expression of the RhoA-binding, N-terminal domain of p120 is sufficient to block the ability of p120 isoform 1 to inhibit RhoA and to promote invasiveness. The data indicate that the increased expression of p120 isoform 1 during tumor progression contributes to the invasive phenotype of cadherin-deficient carcinomas and that the N-terminal domain of p120 is a valid therapeutic target. 
   
  
 RHOC 
 
  NCBI Gene  
  GeneRIF  
  Pubmed  
 
 
  Search Term  Rank  Fields  
  [Mm]igration  6  Process(1); PubMed(52); GeneRIFs(14)  
  [Ee]xtracellular [Mm]atrix  8  PubMed(2)  
  [Ii]nvasive  10  PubMed(38); GeneRIFs(6)  
  [Mm]etastasize  11  PubMed(3)  
  [Mm]etastasis  12  PubMed(57); GeneRIFs(16); Summary(1)  
  [Cc]ell [Ii]nvasion  13  PubMed(17); GeneRIFs(7)  
 
 
 hide/show details for RHOC 
  [Mm]igration  Process  
 skeletal muscle satellite cell   migration   
  PubMed  
 SmgGDS is a guanine nucleotide exchange factor that specifically activates RhoA and RhoC. SmgGDS is an atypical guanine nucleotide exchange factor (GEF) that promotes both cell proliferation and   migration   and is up-regulated in several types of cancer. SmgGDS has been previously shown to activate a wide variety of small GTPases, including the Ras family members Rap1a, Rap1b, and K-Ras, as well as the Rho family members Cdc42, Rac1, Rac2, RhoA, and RhoB. In contrast, here we show that SmgGDS exclusively activates RhoA and RhoC among a large panel of purified GTPases. Consistent with the well known properties of GEFs, this activation is catalytic, and SmgGDS preferentially binds to nucleotide-depleted RhoA relative to either GDP- or GTPγS-bound forms. However, mutational analyses indicate that SmgGDS utilizes a distinct exchange mechanism compared with canonical GEFs and in contrast to known GEFs requires RhoA to retain a polybasic region for activation. A homology model of SmgGDS highlights an electronegative surface patch and a highly conserved binding groove. Mutation of either area ablates the ability of SmgGDS to activate RhoA. Finally, the in vitro specificity of SmgGDS for RhoA and RhoC is retained in cells. Together, these results indicate that SmgGDS is a bona fide GEF that specifically activates RhoA and RhoC through a unique mechanism not used by other Rho family exchange factors. 
 RhoA, RhoB and RhoC have different roles in cancer cell   migration  . Rho GTPases are well known to regulate cell motility through activation of a variety of downstream effector proteins, including enzymes, adaptor proteins and actin nucleators. The three closely related Rho GTPases RhoA, RhoB and RhoC all have the potential to interact with the same downstream effectors, yet they have substantially different effects on cell shape and migratory properties. Here I review the different ways in which RhoA, RhoB and RhoC expression is regulated in cancer and how they play distinct roles in cancer progression. I describe their main effectors known to contribute to cell motility. Recent results from our laboratory and others indicate that RhoA, RhoB and RhoC can be activated by specific stimuli and act through different effectors to control distinct aspects of cancer cell   migration   and invasion. This suggests that they each make unique contributions to cancer by participating in different protein complexes. 
 RhoGDIα-dependent balance between RhoA and RhoC is a key regulator of cancer cell tumorigenesis. RhoGTPases are key signaling molecules regulating main cellular functions such as   migratio  n, proliferation, survival, and gene expression through interactions with various effectors. Within the RhoA-related subclass, RhoA and RhoC contribute to several steps of tumor growth, and the regulation of their expression affects cancer progression. Our aim is to investigate their respective contributions to the acquisition of an invasive phenotype by using models of reduced or forced expression. The silencing of RhoC, but not of RhoA, increased the expression of genes encoding tumor suppressors, such as nonsteroidal anti-inflammatory drug-activated gene 1 (NAG-1), and decreased   migratio  n and the anchorage-independent growth in vitro. In vivo, RhoC small interfering RNA (siRhoC) impaired tumor growth. Of interest, the simultaneous knockdown of RhoC and NAG-1 repressed most of the siRhoC-related effects, demonstrating the central role of NAG-1. In addition of being induced by RhoC silencing, NAG-1 was also largely up-regulated in cells overexpressing RhoA. The silencing of RhoGDP dissociation inhibitor α (RhoGDIα) and the overexpression of a RhoA mutant unable to bind RhoGDIα suggested that the effect of RhoC silencing is indirect and results from the up-regulation of the RhoA level through competition for RhoGDIα. This study demonstrates the dynamic balance inside the RhoGTPase network and illustrates its biological relevance in cancer progression. 
 Rho isoform-specific interaction with IQGAP1 promotes breast cancer cell proliferation and   migration  . We performed a proteomics screen for Rho isoform-specific binding proteins to clarify the tumor-promoting effects of RhoA and C that contrast with the tumor-suppressive effects of RhoB. We found that the IQ-motif-containing GTPase-activating protein IQGAP1 interacts directly with GTP-bound, prenylated RhoA and RhoC, but not with RhoB. Co-immunoprecipitation of IQGAP1 with endogenous RhoA/C was enhanced when RhoA/C were activated by epidermal growth factor (EGF) or transfection of a constitutively active guanine nucleotide exchange factor (GEF). Overexpression of IQGAP1 increased GTP-loading of RhoA/C, while siRNA-mediated depletion of IQGAP1 prevented endogenous RhoA/C activation by growth factors. IQGAP1 knockdown also reduced the amount of GTP bound to GTPase-deficient RhoA/C mutants, suggesting that IQGAP enhances Rho activation by GEF(s) or stabilizes Rho-GTP. IQGAP1 depletion in MDA-MB-231 breast cancer cells blocked EGF- and RhoA-induced stimulation of DNA synthesis. Infecting cells with adenovirus encoding constitutively active RhoA(L63) and measuring absolute amounts of RhoA-GTP in infected cells demonstrated that the lack of RhoA(L63)-induced DNA synthesis in IQGAP1-depleted cells was not due to reduced GTP-bound RhoA. These data suggested that IQGAP1 functions downstream of RhoA. Overexpression of IQGAP1 in MDA-MB-231 cells increased DNA synthesis irrespective of siRNA-mediated RhoA knockdown. Breast cancer cell motility was increased by expressing a constitutively-active RhoC(V14) mutant or overexpressing IQGAP1. EGF- or RhoC-induced   migration   required IQGAP1, but IQGAP1-stimulated   migration   independently of RhoC, placing IQGAP1 downstream of RhoC. We conclude that IQGAP1 acts both upstream of RhoA/C, regulating their activation state, and downstream of RhoA/C, mediating their effects on breast cancer cell proliferation and   migration  , respectively. 
 A novel strategy for specifically down-regulating individual Rho GTPase activity in tumor cells. The Rho family GTPases RhoA, RhoB, and RhoC regulate the actin cytoskeleton, cell movement, and cell growth. Unlike Ras, up-regulation or overexpression of these GDP/GTP binding molecular switches, but not activating point mutations, has been associated with human cancer. Although they share over 85% sequence identity, RhoA, RhoB, and RhoC appear to play distinct roles in cell transformation and metastasis. In NIH 3T3 cells, RhoA or RhoB overexpression causes transformation whereas RhoC increases the cell   migration   rate. To specifically target RhoA, RhoB, or RhoC function, we have generated a set of chimeric molecules by fusing the RhoGAP domain of p190, a GTPase-activating protein that accelerates the intrinsic GTPase activity of all three Rho GTPases, with the C-terminal hypervariable sequences of RhoA, RhoB, or RhoC. The p190-Rho chimeras were active as GTPase-activating proteins toward RhoA in vitro, co-localized with the respective active Rho proteins, and specifically down-regulated Rho protein activities in cells depending on which Rho GTPase sequences were included in the chimeras. In particular, the p190-RhoA-C chimera specifically inhibited RhoA-induced transformation whereas p190-RhoC-C specifically reversed the   migration   phenotype induced by the active RhoC. In human mammary epithelial-RhoC breast cancer cells, p190-RhoC-C, but not p190-RhoA-C or p190-RhoB-C, reversed the anchorage-independent growth and invasion phenotypes caused by RhoC overexpression. In the highly metastatic A375-M human melanoma cells, p190-RhoC-C specifically reversed   migration  , and invasion phenotypes attributed to RhoC up-regulation. Thus, we have developed a novel strategy utilizing RhoGAP-Rho chimeras to specifically down-regulate individual Rho activity and demonstrate that this approach may be applied to multiple human tumor cells to reverse the growth and/or invasion phenotypes associated with disregulation of a distinct subtype of Rho GTPase. 
 RHO binding to FAM65A regulates Golgi reorientation during cell   migration  . Directional cell   migration   involves reorientation of the secretory machinery. However, the molecular mechanisms that control this reorientation are not well characterised. Here, we identify a new Rho effector protein, named FAM65A, which binds to active RHOA, RHOB and RHOC. FAM65A links RHO proteins to Golgi-localising cerebral cavernous malformation-3 protein (CCM3; also known as PDCD10) and its interacting proteins mammalian STE20-like protein kinases 3 and 4 (MST3 and MST4; also known as STK24 and STK26, respectively). Binding of active RHO proteins to FAM65A does not affect the kinase activity of MSTs but results in their relocation from the Golgi in a CCM3-dependent manner. This relocation is crucial for reorientation of the Golgi towards the leading edge and subsequent directional cell   migration  . Our results reveal a previously unidentified pathway downstream of RHO that regulates the polarity of migrating cells through Golgi reorientation in a FAM65A-, CCM3- and MST3- and MST4-dependent manner. 
 Specific induction of   migration   and invasion of pancreatic carcinoma cells by RhoC, which differs from RhoA in its localisation and activity. RhoA and RhoC are highly related Rho GTPases, but differentially control cellular behaviour. We combined molecular, cellular, and biochemical experiments to characterise differences between these highly similar GTPases. Our findings demonstrate that enhanced expression of RhoC results in a striking increase in the   migration   and invasion of pancreatic carcinoma cells, whereas forced expression of RhoA decreases these actions. These isoform-specific functions correlate with differences in the cellular activity of RhoA and RhoC in human cells, with RhoC being more active than RhoA in activity assays and serum-response factor-dependent gene transcription. Subcellular localisation studies revealed that RhoC is predominantly localised in the membrane-containing fraction, whereas RhoA is mainly localised in the cytoplasmic fraction. These differences are not mediated by a different interaction with RhoGDIs. In vitro GTP/GDP binding analyses demonstrate different affinity of RhoC for GTP[S] and faster intrinsic and guanine nucleotide exchange factor (GEF)-stimulated GDP/GTP exchange rates compared to RhoA. Moreover, the catalytic domains of SopE and Dbs are efficacious GEFs for RhoC. mRNA expression of RhoC is markedly enhanced in advanced pancreatic cancer stages, and thus the differences discovered between RhoA and RhoC might provide explanations for their different influences on cell   migration   and tumour invasion. 
 RhoA and RhoC have distinct roles in   migration   and invasion by acting through different targets. Several studies suggest that RhoA and RhoC, despite their sequence similarity, have different roles in cell   migration   and invasion, but the molecular basis for this is not known. Using RNAi, we show that RhoA-depleted cells became elongated and extended multiple Rac1-driven narrow protrusions in 2D and 3D environments, leading to increased invasion. These phenotypes were caused by combined but distinct effects of the Rho-regulated kinases ROCK1 and ROCK2. Depletion of ROCK2 induced multiple delocalized protrusions and reduced migratory polarity, whereas ROCK1 depletion selectively led to cell elongation and defective tail retraction. In contrast, RhoC depletion increased cell spreading and induced Rac1 activation around the periphery in broad lamellipodia, thereby inhibiting directed   migration   and invasion. These effects of RhoC depletion are mediated by the formin FMNL3, which we identify as a new target of RhoC but not RhoA. We propose that RhoA contributes to migratory cell polarity through ROCK2-mediated suppression of Rac1 activity in lamellipodia, whereas RhoC promotes polarized   migration   through FMNL3 by restricting lamellipodial broadening. 
 The Role of RhoA, RhoB and RhoC GTPases in Cell Morphology, Proliferation and   Migration   in Human Cytomegalovirus (HCMV) Infected Glioblastoma Cells. Rho GTPases are crucial regulators of the actin cytoskeleton, membrane trafficking and cell signaling and their importance in cell   migration   and invasion is well- established. The human cytomegalovirus (HCMV) is a widespread pathogen responsible for generally asymptomatic and persistent infections in healthy people. Recent evidence indicates that HCMV gene products are expressed in over 90% of malignant type glioblastomas (GBM). In addition, the HCMV Immediate Early-1 protein (IE1) is expressed in >90% of tumors analyzed.RhoA, RhoB and RhoC were individually depleted in U373MG glioblastoma cells as well as U373MG cells stably expressing the HCMV IE1 protein (named U373MG-IE1 cells) shRNA lentivirus vectors. Cell proliferation assays,   migration   as well as wound-healing assays were performed in uninfected and HCMV-infected cells.The depletion of RhoA, RhoB and RhoC protein resulted in significant alterations in the morphology of the uninfected cells, which were further enhanced by the cytopathic effect caused by HCMV. Furthermore, in the absence or presence of HCMV, the knockdown of RhoB and RhoC proteins decreased the proliferation rate of the parental and the IE1-expressing glioblastoma cells, whereas the knockdown of RhoA protein in the HCMV infected cell lines restored their proliferation rate. In addition, wound healing assays in U373MG cells revealed that depletion of RhoA, RhoB and RhoC differentially reduced their   migration   rate, even in the presence or the absence of HCMV.Collectively, these data show for the first time a differential implication of Rho GTPases in morphology, proliferation rate and motility of human glioblastoma cells during HCMV infection, further supporting an oncomodulatory role of HCMV depending on the Rho isoforms' state. 
 RhoC and ROCKs regulate cancer cell interactions with endothelial cells. RhoC is a member of the Rho GTPase family that is implicated in cancer progression by stimulating cancer cell invasiveness. Here we report that RhoC regulates the interaction of cancer cells with vascular endothelial cells (ECs), a crucial step in the metastatic process. RhoC depletion by RNAi reduces PC3 prostate cancer cell adhesion to ECs, intercalation between ECs as well as transendothelial   migration   in vitro. Depletion of the kinases ROCK1 and ROCK2, two known RhoC downstream effectors, similarly decreases cancer interaction with ECs. RhoC also regulates the extension of protrusions made by cancer cells on vascular ECs in vivo. Transient RhoC depletion is sufficient to reduce both early PC3 cell retention in the lungs and experimental metastasis formation in vivo. Our results indicate RhoC plays a central role in cancer cell interaction with vascular ECs, which is a critical event for cancer progression. 
 Rho isoforms have distinct and specific functions in the process of epithelial to mesenchymal transition in renal proximal tubular cells. Epithelial to mesenchymal transition (EMT) is involved in embryological development, cancerous metastatic spread and organ fibrosis, including the kidney. This process is largely driven by transforming growth factor-beta and recent evidence has implicated Rho as a key intracellular signalling molecule. In this study we have used RNA interference to silence the genetically distinct Rho (A, B and C) isoforms to define their individual functions in human kidney epithelial cells undergoing EMT. We demonstrate that the downregulation of the epithelial cell marker E-cadherin is dependent upon the Rho effector, Rho-kinase. However, silencing RhoA or RhoC expression also results in E-cadherin loss, though each by different mechanisms. Loss of RhoA leads to an upregulation of Snail1 and a reduction in the transcription of E-cadherin whereas loss of RhoC upregulates its breakdown via proteasomal degradation. During EMT, the upregulation of alpha-smooth muscle actin can be blocked by inhibiting the expression of RhoA, but not by that of RhoB or RhoC. This effect is independent of Rho-kinase activity. RhoC is the isoform solely responsible for stress fibre formation and inhibiting its expression reduces EMT-induced   migration   by 50%. RhoB appears to play a role in cell survival as inhibiting its expression leads to >300% increase in cell apoptosis and a relocalization of focal adhesion kinase. We conclude that Rho is a key signalling molecule in the process of EMT but that each isoform has a distinct and specific role. 
 Syndecan-2 regulation of morphology in breast carcinoma cells is dependent on RhoGTPases. While syndecan-2 is usually considered a mesenchymal transmembrane proteoglycan, it can be upregulated in some tumour cells, such as the malignant breast carcinoma cell line, MDA-MB231. Depletion of this syndecan by siRNA, but not other syndecans, has a marked effect on cell morphology, increasing spreading, microfilament bundle and focal adhesion formation, with reduced cell   migration  .A combination of siRNA transfection, immunofluorescence microscopy, phosphoprotein analysis and   migration   assays was used to determine how syndecan-2 may influence the cytoskeleton.The altered adhesion upon syndecan-2 depletion was dependent on the RhoGTPases. p190ARhoGAP relocated to the margins of spreading cells, where it codistributed with syndecan-4 and active β1-integrin. This was accompanied by increased RhoGAP tyrosine phosphorylation, indicative of activity and RhoGTPase suppression. Consistent with this, GTP-RhoA was strongly present at the edges of control cells, but lost after syndecan-2 reduction by siRNA treatments. Further, RhoA, but not RhoC was shown to be essential for the anchored phenotype of these breast carcinoma cells that accompanied siRNA-mediated loss of syndecan-2.Syndecan-2 has a key role in promoting the invasive activity of these cells, in part by regulating the RhoGTPases.Syndecan-2, as a cell surface receptor is accessible for targeting to determine whether breast tumour progression is altered. This article is part of a Special Issue entitled Matrix-mediated cell behaviour and properties. 
 Mutation of ARHGAP9 in patients with coronary spastic angina. Coronary artery spasm has an important function in the etiology of variant angina and other acute coronary syndromes. Abnormal activation of Rho-family GTPases has been observed in cardiovascular disorders, but the function of genetic variability in Rho-family GTPases remains to be evaluated in cardiovascular disorders. We examined the genetic variability of Rho-family GTPases and their regulators in coronary artery spasm. We performed a comprehensive candidate gene analysis of 67 single nucleotide polymorphisms with amino-acid substitution in Rho-family GTPases and their regulators in 103 unrelated Japanese patients with acetylcholine-induced coronary artery spasm and 102 control Japanese subjects without acetylcholine-induced coronary artery spasm. We noted an association of the single nucleotide polymorphism of ARHGAP9 (rs11544238, Ala370Ser) with coronary artery spasm (odds ratio =2.67). We found that ARHGAP9 inactivated Rac as RacGAP and that the mRNA level of ARHGAP9 was strongly detected in hematopoietic cells. ARHGAP9 negatively regulated cell   migration  . The Ala370Ser polymorphism counteracted ARHGAP9-reduced cell   migration  , spreading and adhesion. The Ala370Ser polymorphism in the ARHGAP9 gene is associated with coronary artery spasm. These data suggest that the polymorphism of ARHGAP9 has a critical function in the infiltration of hematopoietic cells into the endothelium and inflammation leading to endothelial dysfunction. 
 Downregulation of the Rho GTPase signaling pathway is involved in the microRNA-138-mediated inhibition of cell   migration   and invasion in tongue squamous cell carcinoma. Tumor metastasis is the dominant cause of death in cancer patients, including patients with oral tongue squamous cell carcinoma (TSCC). Previously, we reported that reduced miR-138 level is correlated with enhanced metastatic potential in TSCC cells. Here, we demonstrate that miR-138 suppresses TSCC cell   migration   and invasion by regulating 2 key genes in the Rho GTPase signaling pathway: RhoC and ROCK2. Direct targeting of miR-138 to specific sequences located in the 3'-untranslated regions of both RhoC and ROCK2 mRNAs was confirmed using luciferase reporter gene assays. Ectopic transfection of miR-138 reduced the expression of both RhoC and ROCK2 in TSCC cells. These reduced expressions, in consequence, led to the reorganization of the stress fibers and the subsequent cell morphology change to a round bleb-like shape as well as the suppression of cell   migration   and invasion. In contrast, knockdown of miR-138 in TSCC cells enhanced the expression of RhoC and ROCK2, which resulted in an altered, elongated cell morphology, enhanced cell stress fiber formation and accelerated cell   migration   and invasion. Taken together, our results suggest that miR-138 plays an important role in TSCC cell   migration   and invasion by concurrently targeting RhoC and ROCK2, and miR-138 may serve as a novel therapeutic target for TSCC patients at risk of metastatic disease. 
 The Role of RhoA, RhoB and RhoC GTPases in Cell Morphology, Proliferation and   Migration   in Human Cytomegalovirus (HCMV) Infected Glioblastoma Cells. Rho GTPases are crucial regulators of the actin cytoskeleton, membrane trafficking and cell signaling and their importance in cell   migration   and invasion is well- established. The human cytomegalovirus (HCMV) is a widespread pathogen responsible for generally asymptomatic and persistent infections in healthy people. Recent evidence indicates that HCMV gene products are expressed in over 90% of malignant type glioblastomas (GBM). In addition, the HCMV Immediate Early-1 protein (IE1) is expressed in >90% of tumors analyzed.RhoA, RhoB and RhoC were individually depleted in U373MG glioblastoma cells as well as U373MG cells stably expressing the HCMV IE1 protein (named U373MG-IE1 cells) shRNA lentivirus vectors. Cell proliferation assays,   migration   as well as wound-healing assays were performed in uninfected and HCMV-infected cells.The depletion of RhoA, RhoB and RhoC protein resulted in significant alterations in the morphology of the uninfected cells, which were further enhanced by the cytopathic effect caused by HCMV. Furthermore, in the absence or presence of HCMV, the knockdown of RhoB and RhoC proteins decreased the proliferation rate of the parental and the IE1-expressing glioblastoma cells, whereas the knockdown of RhoA protein in the HCMV infected cell lines restored their proliferation rate. In addition, wound healing assays in U373MG cells revealed that depletion of RhoA, RhoB and RhoC differentially reduced their   migration   rate, even in the presence or the absence of HCMV.Collectively, these data show for the first time a differential implication of Rho GTPases in morphology, proliferation rate and motility of human glioblastoma cells during HCMV infection, further supporting an oncomodulatory role of HCMV depending on the Rho isoforms' state. 
 RhoC and ROCKs regulate cancer cell interactions with endothelial cells. RhoC is a member of the Rho GTPase family that is implicated in cancer progression by stimulating cancer cell invasiveness. Here we report that RhoC regulates the interaction of cancer cells with vascular endothelial cells (ECs), a crucial step in the metastatic process. RhoC depletion by RNAi reduces PC3 prostate cancer cell adhesion to ECs, intercalation between ECs as well as transendothelial   migration   in vitro. Depletion of the kinases ROCK1 and ROCK2, two known RhoC downstream effectors, similarly decreases cancer interaction with ECs. RhoC also regulates the extension of protrusions made by cancer cells on vascular ECs in vivo. Transient RhoC depletion is sufficient to reduce both early PC3 cell retention in the lungs and experimental metastasis formation in vivo. Our results indicate RhoC plays a central role in cancer cell interaction with vascular ECs, which is a critical event for cancer progression. 
 Syndecan-2 regulation of morphology in breast carcinoma cells is dependent on RhoGTPases. While syndecan-2 is usually considered a mesenchymal transmembrane proteoglycan, it can be upregulated in some tumour cells, such as the malignant breast carcinoma cell line, MDA-MB231. Depletion of this syndecan by siRNA, but not other syndecans, has a marked effect on cell morphology, increasing spreading, microfilament bundle and focal adhesion formation, with reduced cell   migration  .A combination of siRNA transfection, immunofluorescence microscopy, phosphoprotein analysis and   migration   assays was used to determine how syndecan-2 may influence the cytoskeleton.The altered adhesion upon syndecan-2 depletion was dependent on the RhoGTPases. p190ARhoGAP relocated to the margins of spreading cells, where it codistributed with syndecan-4 and active β1-integrin. This was accompanied by increased RhoGAP tyrosine phosphorylation, indicative of activity and RhoGTPase suppression. Consistent with this, GTP-RhoA was strongly present at the edges of control cells, but lost after syndecan-2 reduction by siRNA treatments. Further, RhoA, but not RhoC was shown to be essential for the anchored phenotype of these breast carcinoma cells that accompanied siRNA-mediated loss of syndecan-2.Syndecan-2 has a key role in promoting the invasive activity of these cells, in part by regulating the RhoGTPases.Syndecan-2, as a cell surface receptor is accessible for targeting to determine whether breast tumour progression is altered. This article is part of a Special Issue entitled Matrix-mediated cell behaviour and properties. 
 RhoA, RhoB and RhoC have different roles in cancer cell   migration  . Rho GTPases are well known to regulate cell motility through activation of a variety of downstream effector proteins, including enzymes, adaptor proteins and actin nucleators. The three closely related Rho GTPases RhoA, RhoB and RhoC all have the potential to interact with the same downstream effectors, yet they have substantially different effects on cell shape and migratory properties. Here I review the different ways in which RhoA, RhoB and RhoC expression is regulated in cancer and how they play distinct roles in cancer progression. I describe their main effectors known to contribute to cell motility. Recent results from our laboratory and others indicate that RhoA, RhoB and RhoC can be activated by specific stimuli and act through different effectors to control distinct aspects of cancer cell   migration   and invasion. This suggests that they each make unique contributions to cancer by participating in different protein complexes. 
 Rho isoform-specific interaction with IQGAP1 promotes breast cancer cell proliferation and   migration  . We performed a proteomics screen for Rho isoform-specific binding proteins to clarify the tumor-promoting effects of RhoA and C that contrast with the tumor-suppressive effects of RhoB. We found that the IQ-motif-containing GTPase-activating protein IQGAP1 interacts directly with GTP-bound, prenylated RhoA and RhoC, but not with RhoB. Co-immunoprecipitation of IQGAP1 with endogenous RhoA/C was enhanced when RhoA/C were activated by epidermal growth factor (EGF) or transfection of a constitutively active guanine nucleotide exchange factor (GEF). Overexpression of IQGAP1 increased GTP-loading of RhoA/C, while siRNA-mediated depletion of IQGAP1 prevented endogenous RhoA/C activation by growth factors. IQGAP1 knockdown also reduced the amount of GTP bound to GTPase-deficient RhoA/C mutants, suggesting that IQGAP enhances Rho activation by GEF(s) or stabilizes Rho-GTP. IQGAP1 depletion in MDA-MB-231 breast cancer cells blocked EGF- and RhoA-induced stimulation of DNA synthesis. Infecting cells with adenovirus encoding constitutively active RhoA(L63) and measuring absolute amounts of RhoA-GTP in infected cells demonstrated that the lack of RhoA(L63)-induced DNA synthesis in IQGAP1-depleted cells was not due to reduced GTP-bound RhoA. These data suggested that IQGAP1 functions downstream of RhoA. Overexpression of IQGAP1 in MDA-MB-231 cells increased DNA synthesis irrespective of siRNA-mediated RhoA knockdown. Breast cancer cell motility was increased by expressing a constitutively-active RhoC(V14) mutant or overexpressing IQGAP1. EGF- or RhoC-induced   migration   required IQGAP1, but IQGAP1-stimulated   migration   independently of RhoC, placing IQGAP1 downstream of RhoC. We conclude that IQGAP1 acts both upstream of RhoA/C, regulating their activation state, and downstream of RhoA/C, mediating their effects on breast cancer cell proliferation and   migration  , respectively. 
 RhoGDIα-dependent balance between RhoA and RhoC is a key regulator of cancer cell tumorigenesis. RhoGTPases are key signaling molecules regulating main cellular functions such as   migratio  n, proliferation, survival, and gene expression through interactions with various effectors. Within the RhoA-related subclass, RhoA and RhoC contribute to several steps of tumor growth, and the regulation of their expression affects cancer progression. Our aim is to investigate their respective contributions to the acquisition of an invasive phenotype by using models of reduced or forced expression. The silencing of RhoC, but not of RhoA, increased the expression of genes encoding tumor suppressors, such as nonsteroidal anti-inflammatory drug-activated gene 1 (NAG-1), and decreased   migratio  n and the anchorage-independent growth in vitro. In vivo, RhoC small interfering RNA (siRhoC) impaired tumor growth. Of interest, the simultaneous knockdown of RhoC and NAG-1 repressed most of the siRhoC-related effects, demonstrating the central role of NAG-1. In addition of being induced by RhoC silencing, NAG-1 was also largely up-regulated in cells overexpressing RhoA. The silencing of RhoGDP dissociation inhibitor α (RhoGDIα) and the overexpression of a RhoA mutant unable to bind RhoGDIα suggested that the effect of RhoC silencing is indirect and results from the up-regulation of the RhoA level through competition for RhoGDIα. This study demonstrates the dynamic balance inside the RhoGTPase network and illustrates its biological relevance in cancer progression. 
 RhoA and RhoC have distinct roles in   migration   and invasion by acting through different targets. Several studies suggest that RhoA and RhoC, despite their sequence similarity, have different roles in cell   migration   and invasion, but the molecular basis for this is not known. Using RNAi, we show that RhoA-depleted cells became elongated and extended multiple Rac1-driven narrow protrusions in 2D and 3D environments, leading to increased invasion. These phenotypes were caused by combined but distinct effects of the Rho-regulated kinases ROCK1 and ROCK2. Depletion of ROCK2 induced multiple delocalized protrusions and reduced migratory polarity, whereas ROCK1 depletion selectively led to cell elongation and defective tail retraction. In contrast, RhoC depletion increased cell spreading and induced Rac1 activation around the periphery in broad lamellipodia, thereby inhibiting directed   migration   and invasion. These effects of RhoC depletion are mediated by the formin FMNL3, which we identify as a new target of RhoC but not RhoA. We propose that RhoA contributes to migratory cell polarity through ROCK2-mediated suppression of Rac1 activity in lamellipodia, whereas RhoC promotes polarized   migration   through FMNL3 by restricting lamellipodial broadening. 
 SmgGDS is a guanine nucleotide exchange factor that specifically activates RhoA and RhoC. SmgGDS is an atypical guanine nucleotide exchange factor (GEF) that promotes both cell proliferation and   migration   and is up-regulated in several types of cancer. SmgGDS has been previously shown to activate a wide variety of small GTPases, including the Ras family members Rap1a, Rap1b, and K-Ras, as well as the Rho family members Cdc42, Rac1, Rac2, RhoA, and RhoB. In contrast, here we show that SmgGDS exclusively activates RhoA and RhoC among a large panel of purified GTPases. Consistent with the well known properties of GEFs, this activation is catalytic, and SmgGDS preferentially binds to nucleotide-depleted RhoA relative to either GDP- or GTPγS-bound forms. However, mutational analyses indicate that SmgGDS utilizes a distinct exchange mechanism compared with canonical GEFs and in contrast to known GEFs requires RhoA to retain a polybasic region for activation. A homology model of SmgGDS highlights an electronegative surface patch and a highly conserved binding groove. Mutation of either area ablates the ability of SmgGDS to activate RhoA. Finally, the in vitro specificity of SmgGDS for RhoA and RhoC is retained in cells. Together, these results indicate that SmgGDS is a bona fide GEF that specifically activates RhoA and RhoC through a unique mechanism not used by other Rho family exchange factors. 
 Downregulation of the Rho GTPase signaling pathway is involved in the microRNA-138-mediated inhibition of cell   migration   and invasion in tongue squamous cell carcinoma. Tumor metastasis is the dominant cause of death in cancer patients, including patients with oral tongue squamous cell carcinoma (TSCC). Previously, we reported that reduced miR-138 level is correlated with enhanced metastatic potential in TSCC cells. Here, we demonstrate that miR-138 suppresses TSCC cell   migration   and invasion by regulating 2 key genes in the Rho GTPase signaling pathway: RhoC and ROCK2. Direct targeting of miR-138 to specific sequences located in the 3'-untranslated regions of both RhoC and ROCK2 mRNAs was confirmed using luciferase reporter gene assays. Ectopic transfection of miR-138 reduced the expression of both RhoC and ROCK2 in TSCC cells. These reduced expressions, in consequence, led to the reorganization of the stress fibers and the subsequent cell morphology change to a round bleb-like shape as well as the suppression of cell   migration   and invasion. In contrast, knockdown of miR-138 in TSCC cells enhanced the expression of RhoC and ROCK2, which resulted in an altered, elongated cell morphology, enhanced cell stress fiber formation and accelerated cell   migration   and invasion. Taken together, our results suggest that miR-138 plays an important role in TSCC cell   migration   and invasion by concurrently targeting RhoC and ROCK2, and miR-138 may serve as a novel therapeutic target for TSCC patients at risk of metastatic disease. 
 Specific induction of   migration   and invasion of pancreatic carcinoma cells by RhoC, which differs from RhoA in its localisation and activity. RhoA and RhoC are highly related Rho GTPases, but differentially control cellular behaviour. We combined molecular, cellular, and biochemical experiments to characterise differences between these highly similar GTPases. Our findings demonstrate that enhanced expression of RhoC results in a striking increase in the   migration   and invasion of pancreatic carcinoma cells, whereas forced expression of RhoA decreases these actions. These isoform-specific functions correlate with differences in the cellular activity of RhoA and RhoC in human cells, with RhoC being more active than RhoA in activity assays and serum-response factor-dependent gene transcription. Subcellular localisation studies revealed that RhoC is predominantly localised in the membrane-containing fraction, whereas RhoA is mainly localised in the cytoplasmic fraction. These differences are not mediated by a different interaction with RhoGDIs. In vitro GTP/GDP binding analyses demonstrate different affinity of RhoC for GTP[S] and faster intrinsic and guanine nucleotide exchange factor (GEF)-stimulated GDP/GTP exchange rates compared to RhoA. Moreover, the catalytic domains of SopE and Dbs are efficacious GEFs for RhoC. mRNA expression of RhoC is markedly enhanced in advanced pancreatic cancer stages, and thus the differences discovered between RhoA and RhoC might provide explanations for their different influences on cell   migration   and tumour invasion. 
 Rho isoforms have distinct and specific functions in the process of epithelial to mesenchymal transition in renal proximal tubular cells. Epithelial to mesenchymal transition (EMT) is involved in embryological development, cancerous metastatic spread and organ fibrosis, including the kidney. This process is largely driven by transforming growth factor-beta and recent evidence has implicated Rho as a key intracellular signalling molecule. In this study we have used RNA interference to silence the genetically distinct Rho (A, B and C) isoforms to define their individual functions in human kidney epithelial cells undergoing EMT. We demonstrate that the downregulation of the epithelial cell marker E-cadherin is dependent upon the Rho effector, Rho-kinase. However, silencing RhoA or RhoC expression also results in E-cadherin loss, though each by different mechanisms. Loss of RhoA leads to an upregulation of Snail1 and a reduction in the transcription of E-cadherin whereas loss of RhoC upregulates its breakdown via proteasomal degradation. During EMT, the upregulation of alpha-smooth muscle actin can be blocked by inhibiting the expression of RhoA, but not by that of RhoB or RhoC. This effect is independent of Rho-kinase activity. RhoC is the isoform solely responsible for stress fibre formation and inhibiting its expression reduces EMT-induced   migration   by 50%. RhoB appears to play a role in cell survival as inhibiting its expression leads to >300% increase in cell apoptosis and a relocalization of focal adhesion kinase. We conclude that Rho is a key signalling molecule in the process of EMT but that each isoform has a distinct and specific role. 
 Mutation of ARHGAP9 in patients with coronary spastic angina. Coronary artery spasm has an important function in the etiology of variant angina and other acute coronary syndromes. Abnormal activation of Rho-family GTPases has been observed in cardiovascular disorders, but the function of genetic variability in Rho-family GTPases remains to be evaluated in cardiovascular disorders. We examined the genetic variability of Rho-family GTPases and their regulators in coronary artery spasm. We performed a comprehensive candidate gene analysis of 67 single nucleotide polymorphisms with amino-acid substitution in Rho-family GTPases and their regulators in 103 unrelated Japanese patients with acetylcholine-induced coronary artery spasm and 102 control Japanese subjects without acetylcholine-induced coronary artery spasm. We noted an association of the single nucleotide polymorphism of ARHGAP9 (rs11544238, Ala370Ser) with coronary artery spasm (odds ratio =2.67). We found that ARHGAP9 inactivated Rac as RacGAP and that the mRNA level of ARHGAP9 was strongly detected in hematopoietic cells. ARHGAP9 negatively regulated cell   migration  . The Ala370Ser polymorphism counteracted ARHGAP9-reduced cell   migration  , spreading and adhesion. The Ala370Ser polymorphism in the ARHGAP9 gene is associated with coronary artery spasm. These data suggest that the polymorphism of ARHGAP9 has a critical function in the infiltration of hematopoietic cells into the endothelium and inflammation leading to endothelial dysfunction. 
 A novel strategy for specifically down-regulating individual Rho GTPase activity in tumor cells. The Rho family GTPases RhoA, RhoB, and RhoC regulate the actin cytoskeleton, cell movement, and cell growth. Unlike Ras, up-regulation or overexpression of these GDP/GTP binding molecular switches, but not activating point mutations, has been associated with human cancer. Although they share over 85% sequence identity, RhoA, RhoB, and RhoC appear to play distinct roles in cell transformation and metastasis. In NIH 3T3 cells, RhoA or RhoB overexpression causes transformation whereas RhoC increases the cell   migration   rate. To specifically target RhoA, RhoB, or RhoC function, we have generated a set of chimeric molecules by fusing the RhoGAP domain of p190, a GTPase-activating protein that accelerates the intrinsic GTPase activity of all three Rho GTPases, with the C-terminal hypervariable sequences of RhoA, RhoB, or RhoC. The p190-Rho chimeras were active as GTPase-activating proteins toward RhoA in vitro, co-localized with the respective active Rho proteins, and specifically down-regulated Rho protein activities in cells depending on which Rho GTPase sequences were included in the chimeras. In particular, the p190-RhoA-C chimera specifically inhibited RhoA-induced transformation whereas p190-RhoC-C specifically reversed the   migration   phenotype induced by the active RhoC. In human mammary epithelial-RhoC breast cancer cells, p190-RhoC-C, but not p190-RhoA-C or p190-RhoB-C, reversed the anchorage-independent growth and invasion phenotypes caused by RhoC overexpression. In the highly metastatic A375-M human melanoma cells, p190-RhoC-C specifically reversed   migration  , and invasion phenotypes attributed to RhoC up-regulation. Thus, we have developed a novel strategy utilizing RhoGAP-Rho chimeras to specifically down-regulate individual Rho activity and demonstrate that this approach may be applied to multiple human tumor cells to reverse the growth and/or invasion phenotypes associated with disregulation of a distinct subtype of Rho GTPase. 
 RhoC is a major target of microRNA-93-5P in epithelial ovarian carcinoma tumorigenesis and progression. An increasing amount of evidence has revealed that microRNAs regulate various biological processes, including cell differentiation, cell proliferation, apoptosis, drug resistance, and fat metabolism. Studies have shown that miR-93's targetome in cancer has not been fully defined. Moreover, the role of miR-93 in epithelial ovarian carcinoma (EOC) remains largely unknown.MIR-93 mRNA expression in normal ovarian tissue, benign tumors, borderline tumors, primary ovarian carcinomas, and metastatic omentum was quantified. The ovarian carcinoma cell lines OVCAR3, SKOV3/DDP, and HO8910-PM were transfected with miR-93-5P, after which cell phenotype and expression of relevant molecules were assayed. Dual-luciferase reporter assay and a xenograft mouse model were used to examine miR-93 and its target gene RHOC (Ras homolog gene family member C).MIR-93 mRNA expression was significantly lower in ovarian carcinomas and borderline tumors than in normal ovarian tissues (p < 0.05), and was lower in metastatic omentum than in relative primary ovarian carcinomas (p < 0.05). MIR-93 mRNA expression was also negatively associated with differentiation (well vs. poor and moderate) and International Federation of Gynecology and Obstetrics staging (FIGO stage I/II vs. stage III/IV) in ovarian carcinoma (p < 0.05), besides, miR-93 was higher expressed in mucinous adenocarcinoma than the other types (p < 0.05). MiR-93-5P overexpression reduced proliferation (p < 0.05); promoted G1 or S arrest and apoptosis (p�  ��< 0.0  5); suppressed migration and invasion (p < 0.05); and reduced RhoC, P70S6 kinase, Bcl-xL, matrix metalloproteinase 9 (MMP9) mRNA or protein expression; conversely, it induced P53 and cleaved PARP expression (p < 0.05). Dual-luciferase reporter assay indicated that miR-93 directly targeted RhoC by binding its 3' untranslated region. MiR-93-5P transfection also suppressed tumor development and RhoC expression (determined by immunohistochemistry) in vivo in the xenograft mouse model (p < 0.05).This is the first demonstration that miR-93-5P may inhibit EOC tumorigenesis and progression by targeting RhoC. These findings indicate that miR-93-5P is a potential suppressor of ovarian cellular proliferation. The involvement of miR-93-5P-mediated RhoC downregulation in inhibiting EOC aggressiveness may provide extended insight into the molecular mechanisms underlying cancer aggressiveness. 
 RhoC mediates epidermal growth factor-stimulated   migration   and invasion in head and neck squamous cell carcinoma. Epidermal growth factor receptor (EGFR) is overexpressed in head and neck squamous cell carcinoma (HNSCC) where it has been shown to promote tumor cell invasion upon phosphorylation. One mechanism by which EGFR promotes tumor progression is by activating signal cascades that lead to loss of E-cadherin, a transmembrane glycoprotein of the cell-cell adherence junctions; however mediators of these signaling cascades are not fully understood. One such mediator, RhoC, is activated upon a number of external stimuli, such as epidermal growth factor (EGF), but its role as a mediator of EGF-stimulated   migration   and invasion has not been elucidated in HNSCC. In the present study, we investigate the role of RhoC as a mediator of EGF-stimulated   migration   and invasion in HNSCC. We show that upon EGF stimulation, EGFR and RhoC were strongly activated in HNSCC. This resulted in activation of the phosphatidylinositol 3-Kinase Akt pathway (PI3K-Akt), phosphorylation of GSK-3β at the Ser(9) residue, and subsequent down regulation of E-cadherin cell surface expression resulting in increased tumor cell invasion. Knockdown of RhoC restored E-cadherin expression and inhibited EGF-stimulated   migratio  n and invasion. This is the first report in HNSCC demonstrating the role RhoC plays in mediating EGF-stimulated   migratio  n and invasion by down-regulating the PI3K-Akt pathway and E-cadherin expression. RhoC may serve as a treatment target for HNSCC. 
 Regulation of pancreatic cancer cell   migration   and invasion by RhoC GTPase and caveolin-1. In the current study we investigated the role of caveolin-1 (cav-1) in pancreatic adenocarcinoma (PC) cell   migration   and invasion; initial steps in metastasis. Cav-1 is the major structural protein in caveolae; small Omega-shaped invaginations within the plasma membrane. Caveolae are involved in signal transduction, wherein cav-1 acts as a scaffolding protein to organize multiple molecular complexes regulating a variety of cellular events. Recent evidence suggests a role for cav-1 in promoting cancer cell   migration  , invasion and metastasis; however, the molecular mechanisms have not been described. The small monomeric GTPases are among several molecules which associate with cav-1. Classically, the Rho GTPases control actin cytoskeletal reorganization during cell   migration   and invasion. RhoC GTPase is overexpressed in aggressive cancers that metastasize and is the predominant GTPase in PC. Like several GTPases, RhoC contains a putative cav-1 binding motif.Analysis of 10 PC cell lines revealed high levels of cav-1 expression in lines derived from primary tumors and low expression in those derived from metastases. Comparison of the BxPC-3 (derived from a primary tumor) and HPAF-II (derived from a metastasis) demonstrates a reciprocal relationship between cav-1 expression and p42/p44 Erk activation with PC cell   migration  , invasion, RhoC GTPase and p38 MAPK activation. Furthermore, inhibition of RhoC or p38 activity in HPAF-II cells leads to partial restoration of cav-1 expression.Cav-1 expression inhibits RhoC GTPase activation and subsequent activation of the p38 MAPK pathway in primary PC cells thus restricting   migration   and invasion. In contrast, loss of cav-1 expression leads to RhoC-mediated   migration   and invasion in metastatic PC cells. 
 RhoC is essential for the metastasis of gastric cancer. Rho family members are known to regulate malignant transformation and motility of cancer cells, but the clinicopathological significance of RhoC remains unclear yet in the case of gastric cancer. In this study, we evaluated the protein expression level of RhoC in gastric cancer tissues and cell lines. Results showed that only weak staining of RhoC was detected in 3 of 33 non-tumorous cases by immunohistochemistry. The expression of RhoC was significantly higher in gastric cancer tissues (23/42, 54.8%) than in non-tumorous tissues (p < 0.01). Further analysis demonstrated that RhoC had high specificity (80.0%) in detecting gastric carcinomas with metastatic potential. RhoC was positively expressed in 18 out of 20 metastases (90.0%), even higher than that in primary gastric cancer tissues. Western blot showed that RhoC was up-regulated in five different gastric cancer cell lines but not expressed in SV40-transformed immortal gastric epithelial cell GES-1. Overexpression of RhoC GTPase in GES-1 cells could produce the motile and invasive phenotype but did not alter the monolayer growth rate. To further study the functions of RhoC, we took the powerful siRNA technology to knock down the expression of RhoC in SGC7901 cells. It was shown that down-regulation of RhoC did not affect the proliferation of SGC7901 cells. However, interference of RhoC expression could inhibit   migration  , invasion, and anchorage-independent growth of SGC7901 cells. In conclusion, RhoC may play a very important role in the metastasis of gastric carcinoma. Therapeutic strategies targeting RhoC and RhoC-mediated pathways may be a novel approach for treating metastasis of gastric cancer. 
 [Expressions and significances of PRL-3 and RhoC in A549 cell]. the expression of phosphatase of regenerating liver-3 (PRL-3) is correlated with Ras homologue C (RhoC) in non-small cell lung cancer (NSCLC), suggesting that they have interactions. The aim of this study is to investigate the functions of PRL-3 and RhoC in the   migration   of A549 cell and the potential mechanism of PRL-3 and RhoC in carcinogenesis and cancer development..PRL-3Ab and RhoCAb were used to block the functions of PRL-3 and RhoC respectively. Wound healing assay was applied to detect the   migration   of A549 cell and the expression levels of PRL-3 and RhoC were detected by RT-PCR.the   migration   of A549 cell decreased after blockage of PRL-3 and RhoC. The expression of RhoC decreased when PRL-3 was blocked without any changes on the expression of PRL-3.PRL-3, RhoC could increase cell   migration   in A549 cells. 
 The effect of RhoC siRNA on the invasiveness and proliferation of human cervical cancer cell line SiHa cells. This study investigated the effect of RhoC GTPase on the proliferation and metastasis of cervical cancer cells, SiHa cells, in vitro. RhoC siRNA was introduced into SiHa cells to silence the RhoC gene. The mRNA and protein expression of RhoC, before and after RhoC siRNA transfection, was examined by RT-PCR and Western blotting, respectively. The proliferation and apoptosis of SiHa cells were examined by MTT assay and flow cytometry (FACS), respectively. Adhesive rate was evaluated by Matrigel adhesive assay, and the invasive capability and   migration   capability were assessed by transwell invasive assay and   migration   assay, respectively. The results showed that after the RhoC siRNA transfection, the mRNA and protein expression of RhoC was down-regulated in SiHa cells. The down-regulation of RhoC GTPase did not affect the cell proliferation and apoptosis (P>0.05), but it did suppress SiHa cells' adhesion to matrigel (P<0.01), the invasive capability (P<0.01) and the   migration   capability (P<0.01). It was concluded that RhoC obviously promotes the adhesion, invasion and   migration   of SiHa cells in vitro, but not proliferation and apoptosis, suggesting that RhoC plays an important role in the progression in cervical cancer. 
 Knockdown of Hop downregulates RhoC expression, and decreases pseudopodia formation and   migration   in cancer cell lines. The Hsp90/Hsp70 organising protein (Hop) is a co-chaperone that mediates the interaction of Hsp90 and Hsp70 molecular chaperones during assembly of Hsp90 complexes in cells. Formation of Hsp90 complexes is a key intermediate step in the maturation and homeostasis of oncoproteins and several hormone receptors. In this paper, we demonstrate that knockdown of Hop decreased   migration   of Hs578T and MDA-MB-231 breast cancer cells. Hop was identified in isolated pseudopodia fractions; it colocalised with actin in lamellipodia, and co-sedimented with purified actin in vitro. Knockdown of Hop caused a decrease in the level of RhoC GTPase, and significantly inhibited pseudopodia formation in Hs578T cells. Our data suggest that Hop regulates directional cell   migration   by multiple unknown mechanisms. 
 RhoC interacts with integrin α5β1 and enhances its trafficking in migrating pancreatic carcinoma cells. Human pancreatic ductal adenocarcinoma (PDAC) is characterized by early systemic dissemination. Although RhoC has been implicated in cancer cel  l migrati  on, the relevant underlying molecular mechanisms remain unknown. RhoC has been implicated in the enhancement of cancer cel  l migrati  on and invasion, with actions which are distinct from RhoA (84% homology), and are possibly attributed to the divergent C-terminus domain. Here, we confirm that RhoC significantly enhances the migratory and invasive properties of pancreatic carcinoma cells. In addition, we show that RhoC over-expression decreases cancer cell adhesion and, in turn, accelerates cellular body movement and focal adhesion turnover, especially, on fibronectin-coated surfaces. Whilst RhoC over-expression did not alter integrin expression patterns, we show that it enhanced integrin α5β1 internalization and re-cycling (trafficking), an effect that was dependent specifically on the C-terminus (180-193 amino acids) of RhoC protein. We also report that RhoC and integrin α5β1 co-localize within the peri-nuclear region of pancreatic tumor cells, and by masking the CAAX motif at the C-terminal of RhoC protein, we were able to abolish this interaction in vitro and in vivo. Co-localization of integrin α5β1 and RhoC was demonstrable in invading cancer cells in 3D-organotypic cultures, and further mimicked in vivo analyses of, spontaneous human, (two distinct sources: operated patients and rapid autopsy programme) and transgenic murine (LSL-KrasG12D/+;LSL-Trp53R172H/+;Pdx-1-Cre), pancreatic cancers. In both cases, co-localization of integrin α5β1 and RhoC correlated with poor differentiation status and metastatic potential. We propose that RhoC facilitates tumor cell invasion and promotes subsequent metastasis, in part, by enhancing integrin α5β1 trafficking. Thus, RhoC may serve as a biomarker and a therapeutic target. 
 Notch1 regulates the functional contribution of RhoC to cervical carcinoma progression. The role of Notch signalling in human epithelial cancers is of immense interest. In this study, we examine the interplay between Notch signalling and RhoC, a well-established molecular factor in metastasis. By linking the function of Notch and RhoC, we further strengthen the notion that there is a pro-oncogenic role of Notch signalling in human cervical cancers.RhoC protein expression in cervical carcinoma cell lines was assessed by western blotting. Using CaSki and SiHa cells (cervical carcinoma cells lines), we show that RhoC contributes to wound healing, invasion and   migration  , anoikis resistance, colony formation, in vitro tube formation and tumour formation. Immunohistochemical studies were carried out to assess the co-expression of RhoC, pAkt and Notch1 in clinical sections.An assessment of the changes associated with epithelial-to-mesenchymal transition (EMT) shows that both Notch1 and RhoC have similar phenotypic contribution to EMT. Rho activity assessment on Notch1 inhibition with DAPT shows decreased RhoC activity. We further show that constitutively active RhoC rescues the phenotypic effect of Notch1 inactivation, and a comparison of Notch1 with RhoC expression shows an overlap between the two proteins in the same areas of the tissue.This study has provided evidence to suggest that RhoC is an effector of Notch1 in cervical carcinoma. 
 RhoC protein stimulates   migration   of gastric cancer cells through interaction with scaffold protein IQGAP1. The scaffold protein IQGAP1 is closely related to certain Rho GTPases. Research has revealed that IQGAP1 acts as an effector of Cdc42 and Rac1 in the regulation of cell activity such as proliferation and   migratio  n. However, whether IQGAP1 is associated with RhoC, another important Rho GTPase, is unclear. Previous results from our laboratory indicated that IQGAP1 and RhoC are highly expressed in gastric cancer tissues and cells. This study was designed to investigate the possible interaction between IQGAP1 and RhoC in the regulation of th  e migrati  on of cancer cells. The expression of IQGAP1 and RhoC in gastric cancer tissues and cell lines was detected by Western blotting. siRNAs targeting IQGAP1 or RhoC were transfected into gastric cancer cells to knock down the expression of the proteins. Adenoviral constructs encoding full length IQGAP1, the C‑terminal fragment of IQGAP1, and the constitutively active RhoC gene were used to infect gastric cancer cells to increase the expression of the proteins. The migratory activity of a gastric cancer cell line was measured by a transw  ell migra  tion assay. Western blotting revealed that the IQGAP1 and RhoC proteins were highly expressed in gastric cancer tissues and cells. Spearman's rank correlation analysis indicated that the increases in the expression of IQGAP1 and RhoC were closely correlated. The trans  well migr  ation assay revealed that both IQGAP1 and RhoC stimulated   the migr  ation activity of the gastric cancer cell line AGS. The knockdown of IQGAP1 expression by siRNA blocked   the migr  ation‑stimulating activity of RhoC, while the knockdown of RhoC expression had no effect   on the mi  gration-stimulatory activity of IQGAP1. Co-IP results showed that RhoC and IQGAP1 bound to each other. These results reveal a previously unrecognized interaction between IQGAP1 and RhoC, and demonstrate that IQGAP1 is a downstream effector of RhoC in the regulation   of the mi  gration activity of gastric cancer cells. 
 RhoC is essential for angiogenesis induced by hepatocellular carcinoma cells via regulation of endothelial cell organization. The angiogenesis induced by tumor cells is essential for metastasis of hepatocellular carcinoma. Available information suggests that RhoC participates in angiogenesis through regulation of vascular endothelial growth factor expression in tumor cells. For its broad functions in cell   migration   and cytoskeletal organization, we hypothesized that RhoC regulating angiogenesis does not exclusively depend on regulation of vascular endothelial growth factor expression. To address this question, in the present study, we used a retroviral small interfering RNA approach to selectively knockdown the expression of RhoC in a neovascularization model in vivo and in vitro. Our present results indicate that RhoC is the downstream regulator of vascular endothelial growth factor in endothelial cells and is essential for angiogenesis induced by vascular endothelial growth factor, notwithstanding that RhoC regulates the expression of vascular endothelial growth factor in tumor cells. Furthermore, we show that knockdown of RhoC is associated with the inhibition of invasion and   migration   but not apoptosis of endothelial cells. Knockdown of RhoC results in inhibition of endothelial cell organization through restraining the reorganization of F-actin filaments, which represses endothelial cell network and sprout formation. In conclusion, our results demonstrate that knockdown of RhoC inhibits angiogenesis induced by tumor cells not only through effecting the release of vascular endothelial growth factor, but also through inhibiting endothelial cell   migration   and organization, which implies that it blocks tumor metastasis by specifically inhibiting RhoC in endothelial cells. 
 RhoC mediates invasion and   migration   of CaSki cells through the Rho-associated serine-threonine protein kinase 1 signaling pathway. The small GTPase RhoC in human cancers is up-regulated and correlated with tumor metastasis. However, the role of Rho/Rho-associated serine-threonine protein kinase 1 (ROCK1) signaling pathway in human cervical cancer is still unclear. In this study, we examine the effects of RhoC and its major downstream target, ROCK1, on the invasion and   migration   of CaSki cells to investigate the role of RhoC/ROCK1 signaling pathway in the progression of cervical squamous cell carcinoma.RhoC and ROCK1 protein expression in CaSki cells was detected by Western blotting. Scratch and transwell assays were carried out to assess the effects of RhoC on invasion and   migration   of CaSki cells. Cell viability was assayed by MTT test after adding the ROCK1 inhibitor Y-27632 to CaSki cells.Overexpression of RhoC protein in CaSki cells significantly increases ROCK1 expression and promotes cell invasion and   migration   compared with the control group (P < 0.05). However, in the inhibition of ROCK1 with Y-27632 in CaSki cells when RhoC was overexpressed, the rate of invasiveness and   migration   was reduced remarkably (P < 0.05), dropping to comparable levels as the control.This study suggested that the activation of RhoC/ROCK1 signaling pathways is likely involved in the progression of cervical squamous cell carcinoma. 
 RhoC involved in the   migration   of neural stem/progenitor cells. Alzheimer's disease (AD) is characterized by deposition of beta-amyloid peptides (Aβ) and progressive loss of neurons. Neural stem/progenitor cells (NSPCs) can proliferate and produce immature neurons even in the brain of AD patients. However, Aβ42 significantly decreased the expression of RhoC in NSPCs during the co-incubation (P < 0.01). Treating with RhoC siRNA prevented membrane from protrusion and led to a significant reduction in cel  l migrati  on in responses to SDF-1. Compared with wild-type mice, the numbers of RhoC-immunoreactive cells in hippocampus and cortex were significantly down-regulated in APP/PS1 mice aged 9 months. The results suggest that Aβ42 down-regulates the expression of RhoC in NSPCs in vitro and in vivo; down-regulated RhoC expression results in decreas  ed migrat  ion of NSPCs. 
 Knockdown of RHOC by shRNA suppresses invasion and   migration   of cholangiocellular carcinoma cells via inhibition of MMP2, MMP3, MMP9 and epithelial-mesenchymal transition. Ras homolog family member C (RHOC) is important during the progression of several types of cancer, including prostate, breast and hepatocellular carcinoma. However, the function of RHOC in cholangiocellular carcinoma (CCC), a highly recurrent and metastatic carcinoma with poor prognosis, remains unclear. The aim of the present study was to investigate the involvement of RHOC in CCC tumor progression. RHOC expression levels were examined in CCC tissues and cells, and adjacent nontumorous bile duct tissues. The effects and molecular mechanisms of RHOC expression on cell   migratio  n and invasion were also investigated. The current study demonstrated that RHOC protein was frequently overexpressed in human CCC specimens and CCC cell lines. Downregulation of RHOC inhibited CCC cell invasion and   migratio  n partially via inhibition of matrix metalloproteinase 2, 3 and 9 expression. RHOC also modulated the expression of several epithelial-mesenchymal transition (EMT)-associated proteins, including E‑cadherin, vimentin, Slug and Snail, to promote to EMT progression. The present results demonstrated that RHOC is important for the invasio  n and mig  ration of CCC through simultaneous regulation of MMPs and EMT‑associated protein, suggesting that RHOC is a potential molecular target for CCC treatment. 
 MicroRNA-372 inhibits endometrial carcinoma development by targeting the expression of the Ras homolog gene family member C (RhoC). Here we explore the role of microRNA-372 (miR-372) in tumorigenesis and development of endometrial adenocarcinoma (EC) and analyze the underlying mechanism. We found that miR-372 expression is much lower in EC than normal endometrial specimens. Cell function experiments demonstrated that miR-372 overexpression suppressed cell proliferation,   migration  , and invasion, and led to a G1 phase arrest and promoted the apoptosis of endometrial carcinoma cells in vitro. The nude mouse xenograft assay demonstrated that miR-372 overexpression suppressed tumor growth. RT-PCR and Western blot assays detected the expression of known targets of miR-372 in other malignant tumors and found Cyclin A1 and Cyclin-dependent Kinase 2 (CDK2) was downregulated by miR-372. Bioinformatic predictions and dual-luciferase reporter assays found that RhoC was a possible target of miR-372. RT-PCR and Western blot assays demonstrated that miR-372 transfection reduced the expression of RhoC, matrix metalloproteinase 2 (MMP2) and MMP9, while it increased the expression of cleaved poly (ADP ribose) polymerase (PARP) and bcl-2-associated X protein (Bax). The cell function experiments that transfected siRNA with RhoC showed the same trend as those which were transfected with miR-372. Taken together, our results demonstrated for the first time that miR-372 suppresses tumorigenesis and the development of EC; RhoC is a new and potentially important therapeutic target. 
 Inhibition of Ovarian Epithelial Carcinoma Tumorigenesis and Progression by microRNA 106b Mediated through the RhoC Pathway. Epithelial ovarian cancer (EOC) is the most lethal of the gynecological malignancies. Exploring the molecular mechanisms and major factors of invasion and metastasis could have great significance for the treatment and prognosis of EOC. Studies have demonstrated that microRNA 106b (miR-106b) may be a promising therapeutic target for inhibiting breast cancer bone metastasis, but the role of miR-106b in EOC is largely unknown. In this work, miRNA-106b expression was quantified in various ovarian tissues and tumors. Ovarian carcinoma cell lines were transfected with miR-106b, after which, cell phenotype and expression of relevant molecules was assayed. Dual-luciferase reporter assays and xenograft mouse models were also used to investigate miR-106b and its target gene. MiR-106b mRNA expression was found to be significantly higher in normal ovarian tissues and benign tumors than in ovarian carcinomas and borderline tumors (p < 0.01), and was negatively associated with differentiation (Well vs. Por & Mod) and the International Federation of Gynecology and Obstetrics (FIGO) staging (stage I/II vs. stage III/IV) in ovarian carcinoma (p < 0.05). MiR-106b transfection reduced cell proliferation; promoted G1 or S arrest and apoptosis (p < 0.05); suppressed cell   migration   and invasion (p < 0.05); reduced Ras homolog gene family member C (RhoC), P70 ribosomal S6 kinase (P70S6K), Bcl-xL, Matrix metallopeptidase 2 (MMP2), MMP9 mRNA and protein expression; and induced p53 expression (p < 0.05). Dual-luciferase reporter assays indicated that miR-106b directly targets RhoC by binding its 3'UTR. MiR-106b transfection also suppressed tumor development and RhoC expression in vivo in xenograft mouse models. This is the first demonstration that miR-106b may inhibit tumorigenesis and progression of EOC by targeting RhoC. The involvement of miR-106b-mediated RhoC downregulation in EOC aggression may give extended insights into molecular mechanisms underlying cancer aggression. Approaches aimed at overexpressing miR-106b may serve as promising therapeutic strategies for treating EOC patients. 
 Tumor suppressor microRNA-493 decreases cell motility and   migration   ability in human bladder cancer cells by downregulating RhoC and FZD4. The purpose of this study was to identify new tumor suppressor microRNAs (miRNA; miR) in bladder cancer, conduct functional analysis of their suppressive role, and identify their specific target genes. To explore tumor suppressor miRs in bladder cancer, miR microarray was conducted using SV-HUC-1, T24, J82, and TCCSUP cells. Expression of miR-493 in bladder cancer (T24, J82, and TCCSUP) cells was downregulated compared with normal SV-HUC-1 cells. Also, the expression of miR-493 was significantly lower in bladder cancer tissues than in their corresponding noncancerous tissues. Transfection of miR-493 into T24 or J82 cells decreased their cell growth and   migration   abilities. On the basis of this result, to identify potential miR-493 target genes, we used target scan algorithms to identify target oncogenes related to invasion and   migration  . miR-493 decreased 3'-untranslated region luciferase activity and protein expression of FZD4 and RhoC. miR-493 also decreased binding of RhoC and Rock-1. miR-493 is a new tumor suppressor miRNA in bladder cancer and inhibits cell motility through downregulation of RhoC and FZD4. 
 Overexpression of Ras homologous C (RhoC) induces malignant transformation of hepatocytes in vitro and in nude mouse xenografts. Ras homologous C (RhoC) is expressed in various cancers, including hepatocellular carcinoma (HCC). In this study, we first analyzed RhoC expression in 46 HCC tissue specimens and found that RhoC expression was significantly increased in HCC tissues compared to the adjacent normal liver tissues. Next, we investigated the role of RhoC in malignant transformation of normal hepatocytes. The HL7702 cell line was stably transfected with a RhoC expression vector and then subjected to cell proliferation, differentiation, colony formation,   migration   and invasion assays, as well as nude mouse xenograft assays. Gene expressions in these cells were determined using RT-PCR and Western blot. Overexpression of RhoC significantly promoted proliferation and anchorage-independent growth of HL7702 cells, but suppressed cell differentiation, as compared with the parental cells and the empty vector-transfected control cells. Moreover, RhoC overexpression induced   migration   and invasion of HL7702 cells in vitro. Molecularly, RhoC increased the expression of cell cycle-related genes, matrix metalloprotease 2 (MMP2), MMP9 and vascular endothelial growth factor (VEGF). In addition, RhoC-transfected cells formed tumors in nude mice, whereas vector-transfected HL7702 cells did not form any tumors in nude mice. This study demonstrated the role of RhoC overexpression in malignant transformation of normal human hepatocytes, suggesting that RhoC may function as an oncogene in hepatocytes. 
 miR-10b is overexpressed in hepatocellular carcinoma and promotes cell proliferation,   migration   and invasion through RhoC, uPAR and MMPs. Recently, miR-10b is identified as a miRNA highly expressed in many human cancers, promoting cell   migration   and invasion. However, the specific function of miR-10b in hepatocellular carcinoma (HCC) is unclear at this point.The miR-10b expression levels in 60 paired different TNM Stage HCC tumor tissues compared with adjacent non-tumor (ANT) tissues, normal tissue control (8 benign tumor and 7 normal liver tissues), 3 normal liver and 7 HCC cell lines were measured by real-time quantitative RT-PCR and to evaluate their association with HCC clinicopathologic features. Invasion assay, MTT proliferation assay and wound-healing assay were performed to test the invasion and proliferation of HCC cell after transfection. The effect of miR-10b on HCC in vivo was validated by murine xenograft model.We found that miR-10b expression was increased in human HCC tissues and cell lines compared with normal control, respectively. The expression of miR-10b was correlated with HCC metastatic ability. Overexpression of miR-10b in MHCC-97L cells increased cell motility and invasiveness, whereas inhibition of miR-10b in MHCC-97H cells reduced cell motility and invasiveness in vitro and in vivo. We also showed that HOXD10 was negatively regulated by miR-10b at the posttranscriptional level, via a specific target site within the 3'UTR by luciferase reporter assay. Furthermore, we found that miR-10b induced HCC cell invasion and   migration   by modulating the HOXD10 target gene RhoC, uPAR, MMP-2 and MMP-9 expression.Our results suggested that miR-10b was overexpressed in HCC and promoted HCC cell   migration   and invasion through the HOXD10/ RhoC/ uPAR/ MMPs pathway which may provide a novel bio-target for HCC therapy. 
 The role of RhoC in epithelial-to-mesenchymal transition of ovarian carcinoma cells. RhoC is a small G protein/GTPase and involved in tumor mobility, invasion and metastasis. Previously, up-regulated RhoC expression is found to play an important role in ovarian carcinogenesis and subsequent progression by modulating proliferation, apoptosis,   migration   and invasion.We transfected RhoC-expressing plasmid and RhoC siRNA into CAOV3 and OVCAR3 cells respectively. These cells and transfectants were exposed to vascular epithelial growth factor (VEGF), transforming growth factor (TGF)-β1 or their receptor inhibitors with the phenotypes and their related-molecules examined.TGF-β1R or VEGFR inhibitor suppressed the proliferation  , migrati  on, invasion and lamellipodia formation, the expression of N-cadherin, α-SMA, snail and Notch1 mRNA or protein, and enhanced E-cadherin mRNA and protein expression in CAOV3 and its RhoC-overexpressing transfectants, whereas both growth factors had the opposite effects in OVCAR3 cells and their RhoC-hypoexpressing transfectants. Ectopic RhoC expression enhanc  ed migrat  ion, invasion, lamellipodia formation and the alteration in epithelial to mesenchymal transition (EMT) markers of CAOV3 cells regardless of the treatment of VEGFR or TGF-β1R inhibitor, whereas RhoC knockdown resulted in the converse in OVCAR3 cells even with the exposure to VEGF or TGF-β1.RhoC expression might be involved in EMT of ovarian epithelial carcinoma cells, stimulated by TGF-β1 and VEGF. 
 The role of RhoC in ovarian epithelial carcinoma: a marker for carcinogenesis, progression, prognosis, and target therapy. Ras homolog gene family member C (RhoC) is a small G protein/guanosine triphosphatase involved in tumor mobility, invasion, and metastasis.After RhoC siRNA transfection, we measured the changes in phenotypes and some relevant molecules in ovarian carcinoma cell, OVCAR3. The mRNA and protein expression of RhoC was detected in ovarian tumors.RhoC siRNA transfection resulted in low growth, G1 arrest, and apoptotic induction in the OVCAR3 in comparison with the control and mock. Following RhoC knockdown, there was reduced mRNA or protein expression of protein kinase B (Akt), signal transducer and activator of transcription 3 (stat3), bcl-xL, surviving and phosphorylated p70S6 kinase (p-p70s6k), while the converse was true for Bax and caspase-3. Lovastatin induced apoptosis, suppressed proliferation,   migration   and invasion, and disrupted lamellipodia formation in OVCAR3. Lovastatin exposure induced lower RhoC, bcl-2, matrix metalloproteinase-9 (MMP-9), survivin, Akt, bcl-xL, vascular endothelial growth factor (VEGF), and p-p70s6k expression in OVCAR3 compared to the control, but higher caspase-3 and Bax expression. RhoC mRNA and protein expression was significantly higher in ovarian carcinoma than in benign tumors and normal ovary tissue (p<0.05) and was positively associated with dedifferentiation, FIGO staging and p-p70s6k expression of ovarian carcinoma (p<0.05).The up-regulated RhoC expression may affect ovarian carcinogenesis and should be considered a good biomarker for the differentiation and progression of ovarian carcinoma. RhoC plays an important role in apoptosis by modulating the relevant genes and the phosphorylation of downstream p70s6k. 
 Effects and mechanism of RhoC downregulation in suppressing ovarian cancer stem cell proliferation, drug resistance, invasion and metastasis. Cancer stem cells are considered to be the root cause of tumor initiation, metastasis, recurrence and therapeutic resistance. Recent studies have reported that RhoC plays a critical role in regulating cancer stem cells; however, its function in ovarian cancer stem cells (OCSCs) remains unknown. The ovarian cancer cell line A2780, and the paclitaxel-resistant A2780 cell line (A2780-PTX) were obtained. A2780 cells were used to isolate and identify the highly invasive A2780-PM cells, and A2780-PTX cells were used to isolate and identify the highly drug-resistant and highly invasive A2780-PTX-PM cells by Transwell assay. MTT, Transwell and wound healing assays were used to compare the differences in cell proliferation, invasion and   migration   ability among the four cell lines. Immunofluorescence was used to detect the expression of stem cell markers CD117 and CD133. OCSCs were sorted by flow cytometry. Following si-RhoC transfection of the OCSCs, cell proliferation, drug resistance, invasion and   migration   ability and RhoC, CD117 and CD133 expression levels were assayed. RT-PCR was used to assess RhoC, CD117, CD133 and matrix metalloproteinase 9 (MMP9) mRNA expression levels. A2780-PM and A2780‑PTX-PM cells exhibited higher cell proliferation, drug resistance, and invasion a  nd migrat  ion ability than the A2780 and A2780-PTX cell lines. Furthermore, CD133 and CD117 expression levels were higher in the A2780-PM and A2780‑PTX-PM cells than levels in the A2780 and A2780-PTX cells. Transfection of si-RhoC in OCSCs suppressed the proliferation, drug resistance, invas  ion, migr  ation and CD117 and CD133 expression levels. Furthermore, the expression levels of RhoC, CD117, CD133, MDR1, and MMP9 mRNA were downregulated in the transfected population. Taken together, our results demonstrated that RhoC downregulation may inhibit the proliferation, drug resistance, invasion   and migr  ation of OCSCs, and RhoC may play an important role in the formation of OCSCs. 
 "Smart" Nanoparticles Enhance the Cytoplasmic Delivery of Anti-RhoC Silencing RNA and Inhibit the   Migration   and Invasion of Aggressive Breast Cancer Cells. Rho-GTPases are small GTP-binding proteins that contribute to the epithelial-to-mesenchymal transition by regulating several cellular processes including organization of the actin cytoskeleton, cell motility, transcription, and cell proliferation. Overexpression of RhoC-GTPases (RhoC) in breast cancer has been implicated in poor disease prognosis due to increased cancer cells invasion,   migration  , and motility, which warranted its consideration as a therapeutic target for inhibiting breast cancer metastasis. Using silencing RNA (siRNA) molecules to knockdown RhoC expression is a promising approach to inhibit breast cancer metastases. However, transforming anti-RhoC siRNA molecules into a viable therapy remains a challenge due to the lack of a biocompatible carrier that can selectively deliver the RNA cargo into breast cancer cells. We report the use of a degradable, pH-sensitive, β-cyclodextrin (βCD)-based polymeric carrier that condenses anti-RhoC siRNA forming "smart" particles. These smart anti-RhoC particles were efficiently internalized, successfully escaped the endosome, and delivered the RNA cargo into the cytoplasm of SUM149 and MDA-MB-231 breast cancer cells. Our results show that anti-RhoC particles used at a low N/P ratio of 2.5/1 suppressed RhoC protein levels by 100% and 90% in SUM149 and MDA-MB-231 cells, respectively. Further, anti-RhoC particles inhibited the invasion, motility, an  d migrati  on of SUM149 and MDA-MB-231 cells by 40-47%, 57-60%, and 61.5-73%, respectively. Smart particles encapsulating the scrambled siRNA sequence did not affect RhoC protein expression or the invasion, motility, an  d migrati  on of SUM149 and MDA-MB-231 cells, which indicate the biocompatibility of the polymeric carrier and selectivity of the observed RhoC knockdown. These results collectively indicate the therapeutic potential of smart anti-RhoC particles in arresting the metastatic spread of breast cancer cells. 
 Inhibition of invasion and metastasis of hepatocellular carcinoma cells via targeting RhoC in vitro and in vivo. Hepatocellular carcinoma (HCC) is one of the most deadly human cancers because of its high incidence of metastasis. Our previous work identified a strong correlation between increased expression of RhoC and HCC metastasis. Here, we investigate to define the role of RhoC in HCC metastasis. Furthermore, we sought to determine whether inhibition of the expression of RhoC might block the metastasis of HCC in vivo.A stable retroviral small interfering RNA approach was employed to selectively knockdown the expression of RhoC in vitro and in vivo. Invasion and   migration   assay, MTT and fluorescence-activated cell sorting analysis, Rho activity assay, and immunofluorescence staining were carried out to characterize RhoC in vitro. An anti-RhoC retroviral gene delivery BALB/c nude mice model was established to investigate whether knockdown of the expression of RhoC might inhibit the metastasis of HCC in vivo.We confirmed the correlation of RhoC expression and metastatic potentials of HCC cell lines. We also showed that suppression of RhoC expression resulted in inhibition of invasion and   migration   without an apparent effect on cell survival and proliferation in HCCLM3 cells. Furthermore, a similar effect of RhoC on autotaxin-induced invasion of HCCLM3 cells was also observed. Significantly, we successfully adopted an HCC metastatic mouse model that allowed us to show that knockdown of the RhoC expression resulted in inhibition of metastasis of HCC in vivo for the first time.Our results show a critical role of RhoC in metastasis of HCC, implicating RhoC as a potential therapeutic target to block HCC metastasis. 
 Formin-like 2 drives amoeboid invasive cell motility downstream of RhoC. Invasive cell   migration   is a key step for cancer metastasis and involves Rho GTPase-controlled reorganization of the actin cytoskeleton. Altered Rho GTPase expression is found in various malignancies. Particularly, the closely related GTPases RhoA and RhoC are upregulated in many aggressive tumours, but specific effectors that distinguish between these two GTPases to explain mechanistic differences have not been identified. The formins are by far the largest family of Rho GTPase effectors and are characterized by the actin-nucleating formin homology 2 domain. Using siRNA-based screening against all 15 human formins, we systematically analysed their functions in 3D cell motility using three different cancer cell lines. These results reveal distinct requirements for specific formins in amoeboid versus mesenchymal invasive cell   migration  . Importantly, by knocking down all Rho proteins, we identified formin-like 2 (FMNL2) as a specific RhoC effector, showing selective interaction of FMNL2 with active RhoC, but not RhoA or RhoB. Functional analysis shows that RhoC regulates autoinhibition of FMNL2, whereas suppression of FMNL2 inhibits RhoC-, but not RhoA-dependent, rounded invasive cell   migration  . Thus, our data uncover a novel regulatory and functional interaction between RhoC and FMNL2 for modulating cell shape and invasiveness and provide mechanistic insight into RhoC-specific signalling events. 
  GeneRIFs  
 RhoC downregulation may inhibit the proliferation, drug resistance, invasion and   migration   of ovarian cancer stem cells. 
 Downregulation of RHOC inhibited cholangiocellular carcinoma cells invasion and   migration   partially via inhibition of matrix metalloproteinase 2, 3 and 9 expression. RHOC also modulated the expression of several epithelial-mesenchymal transition (EMT)-associated proteins 
 found that miR-10b induced HCC cell invasion and   migration   by modulating the HOXD10 target gene RhoC, uPAR, MMP-2 and MMP-9 expression 
 This is the first report in HNSCC demonstrating the role RhoC plays in mediating EGF-stimulated   migration   and invasion by down-regulating the PI3K-Akt pathway and E-cadherin expression. 
 Down-regulation of RhoC expression resulted in decreased   migration   of neural stem cells. 
 Knockdown of Hop caused a decrease in the level of RhoC GTPase, and significantly inhibited pseudopodia formation in Hs578T cells. Our data suggest that Hop regulates directional cell   migration   by multiple unknown mechanisms. 
 The knockdown of RhoC expression had no effect on the   migration  -stimulatory activity of IQGAP1. 
 RhoC promotes polarized tumor cell   migration   through FMNL3 by restricting lamellipodial broadening. 
 The   migration   of A549 cell decreased after blockage of PRL-3 and RhoC. PRL-3, RhoC could increase cell   migration   in A549 cells. 
 RhoC promotes the adhesion, invasion and   migration   of SiHa cells in vitro, but not proliferation and apoptosis. 
 results suggest that miR-138 plays an important role in TSCC cell   migration   and invasion by concurrently targeting RhoC and ROCK2, and miR-138 may serve as a novel therapeutic target for TSCC patients at risk of metastatic disease 
 findings demonstrate that enhanced expression of RhoC results in a striking increase in the   migration   and invasion of pancreatic carcinoma cells, whereas forced expression of RhoA decreases these actions 
 In cervical carcinoma cell lines, RhoC contributes to wound healing, invasion &   migration  , anoikis resistance, colony formation, in vitro tube formation & tumour formation. RhoC is a downstream effector of Notch1. 
 caveolin 1 expression inhibits RhoC GTPase activation and subsequent activation of the p38 MAPK pathway in primary pancreatic cancer cells thus restricting   migration   and invasion 
  [Ee]xtracellular [Mm]atrix  PubMed  
 Proteomic analysis of human parotid gland exosomes by multidimensional protein identification technology (MudPIT). Human ductal saliva contributes over a thousand unique proteins to whole oral fluids. The mechanism by which most of these proteins are secreted by salivary glands remains to be determined. The present study used a mass spectrometry-based, shotgun proteomics approach to explore the possibility that a subset of the proteins found in saliva are derived from exosomes, membrane-bound vesicles of endosomal origin within multivesicular endosomes. Using MudPIT (multidimensional protein identification technology) mass spectrometry, we catalogued 491 proteins in the exosome fraction of human parotid saliva. Many of these proteins were previously observed in ductal saliva from parotid glands (265 proteins). Furthermore, 72 of the proteins in parotid exosomes overlap with those previously identified as urinary exosome proteins, proteins which are also frequently associated with exosomes from other tissues and cell types. Gene Ontology (GO) and KEGG pathway analyses found that cytosolic proteins comprise the largest category of proteins in parotid exosomes (43%), involved in such processes as phosphatidylinositol signaling system, calcium signaling pathway, inositol metabolism, protein export, and signal transduction, among others; whereas the integral plasma membrane proteins and associated/peripheral plasma membrane proteins (26%) were associated with   extracellular matrix  -receptor interaction, epithelial cell signaling, T-cell and B-cell receptor signaling, cytokine receptor interaction, and antigen processing and presentation, among other biological functions. In addition, these putative saliva exosomal proteins were linked to specific diseases (e.g., neurodegenerative disorders, prion disease, cancers, type I and II diabetes). Consequently, parotid glands secrete exosomes that reflect the metabolic and functional status of the gland and may also carry informative protein markers useful in the diagnosis and treatment of systemic diseases. 
 Genomic analysis of metastasis reveals an essential role for RhoC. The most damaging change during cancer progression is the switch from a locally growing tumour to a metastatic killer. This switch is believed to involve numerous alterations that allow tumour cells to complete the complex series of events needed for metastasis. Relatively few genes have been implicated in these events. Here we use an in vivo selection scheme to select highly metastatic melanoma cells. By analysing these cells on DNA arrays, we define a pattern of gene expression that correlates with progression to a metastatic phenotype. In particular, we show enhanced expression of several genes involved in   extracellular matrix   assembly and of a second set of genes that regulate, either directly or indirectly, the actin-based cytoskeleton. One of these, the small GTPase RhoC, enhances metastasis when overexpressed, whereas a dominant-negative Rho inhibits metastasis. Analysis of the phenotype of cells expressing dominant-negative Rho or RhoC indicates that RhoC is important in tumour cell invasion. The genomic approach allows us to identify families of genes involved in a process, not just single genes, and can indicate which molecular and cellular events might be important in complex biological processes such as metastasis. 
  [Ii]nvasive  PubMed  
 The MLK-related kinase (MRK) is a novel RhoC effector that mediates lysophosphatidic acid (LPA)-stimulated tumor cell invasion. The small GTPase RhoC is overexpressed in many   invasive   tumors and is essential for metastasis. Despite its high structural homology to RhoA, RhoC appears to perform functions that are different from those controlled by RhoA. The identity of the signaling components that are differentially regulated by these two GTPases is only beginning to emerge. Here, we show that the MAP3K protein MRK directly binds to the GTP-bound forms of both RhoA and RhoC in vitro. However, siRNA-mediated depletion of MRK in cells phenocopies depletion of RhoC, rather than that of RhoA. MRK depletion, like that of RhoC, inhibits LPA-stimulated cell invasion, while depletion of RhoA increases invasion. We also show that active MRK enhances LPA-stimulated invasion, further supporting a role for MRK in the regulation of invasion. Depletion of either RhoC or MRK causes sustained myosin light chain phosphorylation after LPA stimulation. In addition, activation of MRK causes a reduction in myosin light chain phosphorylation. In contrast, as expected, depletion of RhoA inhibits myosin light chain phosphorylation. We also present evidence that both RhoC and MRK are required for LPA-induced stimulation of the p38 and ERK MAP kinases. In conclusion, we have identified MRK as a novel RhoC effector that controls LPA-stimulated cell invasion at least in part by regulating myosin dynamics, ERK and p38. 
 Myosin-interacting guanine exchange factor (MyoGEF) regulates the invasion activity of MDA-MB-231 breast cancer cells through activation of RhoA and RhoC. The small guanine triphosphatase (GTPase) proteins RhoA and RhoC are essential for tumor invasion and/or metastasis in breast carcinomas. However, it is poorly understood how RhoA and RhoC are activated in breast cancer cells. Here we describe the role of myosin-interacting guanine nucleotide exchange factor (Myo-GEF) in regulating RhoA and RhoC activation as well as cell polarity and invasion in an   invasive   breast cancer cell line MDA-MB-231. RNA-interference (RNAi)-mediated depletion of MyoGEF in MDA-MB-231 cells not only suppresses the activation of RhoA and RhoC, but also decreases cell polarity and invasion activity. The dominant-negative mutants of RhoA and RhoC, but not Rac1 and Cdc42, dramatically decrease actin polymerization induced by MyoGEF. In addition, MyoGEF co-localizes with nonmuscle myosin IIA (NMIIA) to the front of migrating cells, and depletion of NMIIA by RNAi disrupts the polarized localization of MyoGEF at the cell leading edge, suggesting a role for NMIIA in regulating MyoGEF localization and function. Moreover, MyoGEFprotein levels significantly increase in infiltrating ductal carcinomas as well as in   invasive   breast cancer cell lines. Taken together, our results suggest that MyoGEF cooperates with NMIIA to regulate the polarity and invasion activity of breast cancer cells through activation of RhoA and RhoC. 
 PRL-3 facilitates angiogenesis and metastasis by increasing ERK phosphorylation and up-regulating the levels and activities of Rho-A/C in lung cancer. The aim of this study was to investigate the mechanism of PRL-3 in inducing angiogenesis and lymphangiogenesis to promote distant and lymph node metastasis in human lung cancer tissues and cells.We investigated the expression of PRL-3, VEGF, and VEGF-C from 94 patients with non-small cell lung cancer (NSCLC) using immunohistochemical staining. The relationship between PRL-3 expression and microvessel density (MVD), lymphatic vessel density (LVD), clinicopathological factors, and surgical treatment outcome was also studied. Following this, we studied the effect on A549 by blocking PRL-3.PRL-3 expression in NSCLC was high, and this over-expression is correlated with advanced clinical stage (p = 0.019), distant metastasis (p = 0.001), lymph node metastasis (p = 0.001), and poor post-operative survival. PRL-3 over-expression was associated with vascular endothelial growth factor (VEGF; p = 0.000) and VEGF-C (p = 0.008) expressions, MVD and LVD (p = 0.000 and p = 0.000). Blocking PRL-3 expression in A549 cell resulted in decreased cellular proliferative, migratory, and   invasive   abilities, and VEGF, VEGF-C, pERK, Rho-A, and Rho-C expression was inhibited. Following inhibition of either Rho or pERK, VEGF expression was down-regulated.PRL-3 induces microvascular and lymphatic vessel formation by facilitating VEGF and VEGF-C expression in lung cancer tissues, thus promoting distant and lymph node metastasis of lung cancer. PRL-3 up-regulates pERK and Rho expression and activity, facilitating VEGF expression, and accelerating angiogenesis and distant metastasis. How to regulate VEGF-C expression needs to be further studied. 
 RhoGDIα-dependent balance between RhoA and RhoC is a key regulator of cancer cell tumorigenesis. RhoGTPases are key signaling molecules regulating main cellular functions such as migration, proliferation, survival, and gene expression through interactions with various effectors. Within the RhoA-related subclass, RhoA and RhoC contribute to several steps of tumor growth, and the regulation of their expression affects cancer progression. Our aim is to investigate their respective contributions to the acquisition of an   invasiv  e phenotype by using models of reduced or forced expression. The silencing of RhoC, but not of RhoA, increased the expression of genes encoding tumor suppressors, such as nonsteroidal anti-inflammatory drug-activated gene 1 (NAG-1), and decreased migration and the anchorage-independent growth in vitro. In vivo, RhoC small interfering RNA (siRhoC) impaired tumor growth. Of interest, the simultaneous knockdown of RhoC and NAG-1 repressed most of the siRhoC-related effects, demonstrating the central role of NAG-1. In addition of being induced by RhoC silencing, NAG-1 was also largely up-regulated in cells overexpressing RhoA. The silencing of RhoGDP dissociation inhibitor α (RhoGDIα) and the overexpression of a RhoA mutant unable to bind RhoGDIα suggested that the effect of RhoC silencing is indirect and results from the up-regulation of the RhoA level through competition for RhoGDIα. This study demonstrates the dynamic balance inside the RhoGTPase network and illustrates its biological relevance in cancer progression. 
 Targeted disruption of protein kinase C epsilon reduces cell invasion and motility through inactivation of RhoA and RhoC GTPases in head and neck squamous cell carcinoma. Over 70% of patients with head and neck squamous cell carcinoma (HNSCC) present with locoregionally advanced stage III and IV disease. In spite of aggressive therapy, locoregional disease recurs in 60% and metastatic disease develops in 15% to 25% of patients causing a major decline in quality and length of life. Therefore, there is a need to identify and understand genes that are responsible for inducing an aggressive HNSCC phenotype. Evidence has shown that protein kinase C (PKC) epsilon is a transforming oncogene and may play a role in HNSCC progression. In this study, we determine the downstream signaling pathway mediated by PKC epsilon to promote an aggressive HNSCC phenotype. RNA interference knockdown of PKC epsilon in UMSCC11A and UMSCC36, two highly   invasive   and motile HNSCC cell lines with elevated endogenous PKC epsilon levels, resulted in cells that were significantly less   invasive   and motile than the small interfering RNA-scrambled control transfectants; 51 +/- 5% (P < 0.006) and 49 +/- 3% (P < 0.010) inhibition in invasion and 69 +/- 1% (P < 0.0005) and 66 +/- 3% (P < 0.0001) inhibition in motility, respectively. PKC epsilon-deficient UMSCC11A clones had reduced levels of active and serine-phosphorylated RhoA and RhoC. Moreover, constitutive active RhoA completely rescued the invasion and motility defect, whereas constitutive active RhoC completely rescued the invasion and partially rescued the motility defect of PKC epsilon-deficient UMSCC11A clones. These results indicate that RhoA and RhoC are downstream of PKC epsilon and critical for PKC epsilon-mediated cell invasion and motility. Our study shows, for the first time, that PKC epsilon is involved in a coordinated regulation of RhoA and RhoC activation, possibly through direct post-translational phosphorylation. 
 Anti-RhoA and anti-RhoC siRNAs inhibit the proliferation and   invasive  ness of MDA-MB-231 breast cancer cells in vitro and in vivo. Overexpression of RhoA or RhoC in breast cancer indicates a poor prognosis, due to increased tumor cell proliferation and invasion and tumor-dependent angiogenesis. Until now, the strategy of blockage of the Rho-signaling pathway has used either GGTI or HMG-CoA reductase inhibitors, but they are not specific to RhoA or RhoC inhibition. In this study, a new approach with anti-RhoA and anti-RhoC siRNAs was used to inhibit specifically RhoA or RhoC synthesis. Two transfections of either RhoA or RhoC siRNA (8.5 nM) into MDA-MB-231 human breast cancer cells or HMEC-1 endothelial cells induced extensive degradation of the target mRNA and led to a dramatic decrease in synthesis of the corresponding protein. In vitro, these siRNAs inhibited cell proliferation and invasion more effectively than conventional blockers of Rho cell signaling. Finally, in a nude mouse model, intratumoral injections of anti-RhoA siRNA (100 microl at 85 nM) every 3 days for 20 days almost totally inhibited the growth and angiogenesis of xenografted MDA-MB-231 tumors. One may infer from these observations that specific inhibition of the Rho-signaling pathway with siRNAs represents a promising approach for the treatment of aggressive breast cancers. 
 RhoC and ROCKs regulate cancer cell interactions with endothelial cells. RhoC is a member of the Rho GTPase family that is implicated in cancer progression by stimulating cancer cell   invasive  ness. Here we report that RhoC regulates the interaction of cancer cells with vascular endothelial cells (ECs), a crucial step in the metastatic process. RhoC depletion by RNAi reduces PC3 prostate cancer cell adhesion to ECs, intercalation between ECs as well as transendothelial migration in vitro. Depletion of the kinases ROCK1 and ROCK2, two known RhoC downstream effectors, similarly decreases cancer interaction with ECs. RhoC also regulates the extension of protrusions made by cancer cells on vascular ECs in vivo. Transient RhoC depletion is sufficient to reduce both early PC3 cell retention in the lungs and experimental metastasis formation in vivo. Our results indicate RhoC plays a central role in cancer cell interaction with vascular ECs, which is a critical event for cancer progression. 
 Syndecan-2 regulation of morphology in breast carcinoma cells is dependent on RhoGTPases. While syndecan-2 is usually considered a mesenchymal transmembrane proteoglycan, it can be upregulated in some tumour cells, such as the malignant breast carcinoma cell line, MDA-MB231. Depletion of this syndecan by siRNA, but not other syndecans, has a marked effect on cell morphology, increasing spreading, microfilament bundle and focal adhesion formation, with reduced cell migration.A combination of siRNA transfection, immunofluorescence microscopy, phosphoprotein analysis and migration assays was used to determine how syndecan-2 may influence the cytoskeleton.The altered adhesion upon syndecan-2 depletion was dependent on the RhoGTPases. p190ARhoGAP relocated to the margins of spreading cells, where it codistributed with syndecan-4 and active β1-integrin. This was accompanied by increased RhoGAP tyrosine phosphorylation, indicative of activity and RhoGTPase suppression. Consistent with this, GTP-RhoA was strongly present at the edges of control cells, but lost after syndecan-2 reduction by siRNA treatments. Further, RhoA, but not RhoC was shown to be essential for the anchored phenotype of these breast carcinoma cells that accompanied siRNA-mediated loss of syndecan-2.Syndecan-2 has a key role in promoting the   invasiv  e activity of these cells, in part by regulating the RhoGTPases.Syndecan-2, as a cell surface receptor is accessible for targeting to determine whether breast tumour progression is altered. This article is part of a Special Issue entitled Matrix-mediated cell behaviour and properties. 
 Up-regulation of small GTPases, RhoA and RhoC, is associated with tumor progression in ovarian carcinoma. To clarify the role of small GTPases Rho in the biologic behavior of ovarian carcinoma, we first examined the mRNA expression of RhoA, RhoB, and RhoC in benign, borderline, and malignant ovarian tumors using RT-PCR and real-time RT-PCR. The expression and localization of RhoA protein were also analyzed by Western blotting and immunohistochemistry. Finally, we examined whether up-regulation of Rho enhances the   invasive  ness of ovarian cancer cells in vitro. Analysis of mRNA levels of the Rho family genes revealed that levels of both RhoA and RhoC were significantly higher in carcinomas than in benign tumors (RhoA, p = 0.0035; RhoC, p = 0.0006). According to histologic subtype, both RhoA and RhoC mRNA levels in serous carcinomas were significantly higher than those in other histologic types. With regard to the International Federation of Gynecological and Obstetrics stage classification, both of RhoA and RhoC mRNA levels were significantly higher in tumors of Stages III+IV than in those of Stages I+II (RhoA, p = 0.0200; RhoC, p = 0.0057). In addition, analysis of matched pairs of primary and disseminated lesions demonstrated that expression of both RhoA and RhoC mRNA was significantly higher in metastatic than in primary tumors. Examination of the protein level showed that expression of RhoA was also increased in advanced ovarian carcinomas, especially those of serous histology. Accordingly, we hypothesized that up-regulation of Rho GTPases plays an important role in the progression of ovarian carcinoma. Matrigel invasion assay using the ovarian cancer cell line, SKOV3, showed that up-regulation and activation after treatment with lysophosphatidic acid was associated with enhanced invasion of the cancer cells. This increase in   invasive  ness was suppressed by the addition of C3, a specific inhibitor of Rho. These findings suggest that up-regulation of Rho GTPases is important in the tumor progression of ovarian carcinoma and that Rho family proteins could be a molecular target in cancer therapy. 
 The influence of genetic variation in 30 selected genes on the clinical characteristics of early onset breast cancer. Common variants that alter breast cancer risk are being discovered. Here, we determine how these variants influence breast cancer prognosis, risk and tumour characteristics.We selected 1,001 women with early onset nonfamilial   invasive   breast cancer from the Prospective study of Outcomes in Sporadic versus Hereditary breast cancer (POSH) cohort and genotyped 206 single nucleotide polymorphisms (SNPs) across 30 candidate genes. After quality control, 899 cases and 133 SNPs remained. Survival analyses were used to identify SNPs associated with prognosis and determine their interdependency with recognized prognostic factors. To identify SNPs that alter breast cancer risk, association tests were used to compare cases with controls from the Wellcome Trust Case Control Consortium. To search for SNPs affecting tumour biology, cases were stratified into subgroups according to oestrogen receptor (ER) status and grade and tested for association.We confirmed previous associations between increased breast cancer risk and SNPs in CASP8, TOX3 (previously known as TNRC9) and ESR1. Analysis of prognosis identified eight SNPs in six genes (MAP3K1, DAPK1, LSP1, MMP7, TOX3 and ESR1) and one region without genes on 8q24 that are associated with survival. For MMP7, TOX3 and MAP3K1 the effects on survival are independent of the main recognized clinical prognostic factors. The SNP in 8q24 is more weakly associated with independent effects on survival. Once grade and pathological nodal status (pN stage) were taken into account, SNPs in ESR1 and LSP1 showed no independent survival difference, whereas the effects of the DAPK1 SNP were removed when correcting for ER status. Interestingly, effects on survival for SNPs in ESR1 were most significant when only ER-positive tumours were examined. Stratifying POSH cases by tumour characteristics identified SNPs in FGFR2 and TOX3 associated with ER-positive disease and SNPs in ATM associated with ER-negative disease.We have demonstrated that several SNPs are associated with survival. In some cases this appears to be due to an effect on tumour characteristics known to have a bearing on prognosis; in other cases the effect appears to be independent of these prognostic factors. These findings require validation by further studies in similar patient groups. 
 RhoC and ROCKs regulate cancer cell interactions with endothelial cells. RhoC is a member of the Rho GTPase family that is implicated in cancer progression by stimulating cancer cell   invasive  ness. Here we report that RhoC regulates the interaction of cancer cells with vascular endothelial cells (ECs), a crucial step in the metastatic process. RhoC depletion by RNAi reduces PC3 prostate cancer cell adhesion to ECs, intercalation between ECs as well as transendothelial migration in vitro. Depletion of the kinases ROCK1 and ROCK2, two known RhoC downstream effectors, similarly decreases cancer interaction with ECs. RhoC also regulates the extension of protrusions made by cancer cells on vascular ECs in vivo. Transient RhoC depletion is sufficient to reduce both early PC3 cell retention in the lungs and experimental metastasis formation in vivo. Our results indicate RhoC plays a central role in cancer cell interaction with vascular ECs, which is a critical event for cancer progression. 
 Syndecan-2 regulation of morphology in breast carcinoma cells is dependent on RhoGTPases. While syndecan-2 is usually considered a mesenchymal transmembrane proteoglycan, it can be upregulated in some tumour cells, such as the malignant breast carcinoma cell line, MDA-MB231. Depletion of this syndecan by siRNA, but not other syndecans, has a marked effect on cell morphology, increasing spreading, microfilament bundle and focal adhesion formation, with reduced cell migration.A combination of siRNA transfection, immunofluorescence microscopy, phosphoprotein analysis and migration assays was used to determine how syndecan-2 may influence the cytoskeleton.The altered adhesion upon syndecan-2 depletion was dependent on the RhoGTPases. p190ARhoGAP relocated to the margins of spreading cells, where it codistributed with syndecan-4 and active β1-integrin. This was accompanied by increased RhoGAP tyrosine phosphorylation, indicative of activity and RhoGTPase suppression. Consistent with this, GTP-RhoA was strongly present at the edges of control cells, but lost after syndecan-2 reduction by siRNA treatments. Further, RhoA, but not RhoC was shown to be essential for the anchored phenotype of these breast carcinoma cells that accompanied siRNA-mediated loss of syndecan-2.Syndecan-2 has a key role in promoting the   invasiv  e activity of these cells, in part by regulating the RhoGTPases.Syndecan-2, as a cell surface receptor is accessible for targeting to determine whether breast tumour progression is altered. This article is part of a Special Issue entitled Matrix-mediated cell behaviour and properties. 
 The MLK-related kinase (MRK) is a novel RhoC effector that mediates lysophosphatidic acid (LPA)-stimulated tumor cell invasion. The small GTPase RhoC is overexpressed in many   invasive   tumors and is essential for metastasis. Despite its high structural homology to RhoA, RhoC appears to perform functions that are different from those controlled by RhoA. The identity of the signaling components that are differentially regulated by these two GTPases is only beginning to emerge. Here, we show that the MAP3K protein MRK directly binds to the GTP-bound forms of both RhoA and RhoC in vitro. However, siRNA-mediated depletion of MRK in cells phenocopies depletion of RhoC, rather than that of RhoA. MRK depletion, like that of RhoC, inhibits LPA-stimulated cell invasion, while depletion of RhoA increases invasion. We also show that active MRK enhances LPA-stimulated invasion, further supporting a role for MRK in the regulation of invasion. Depletion of either RhoC or MRK causes sustained myosin light chain phosphorylation after LPA stimulation. In addition, activation of MRK causes a reduction in myosin light chain phosphorylation. In contrast, as expected, depletion of RhoA inhibits myosin light chain phosphorylation. We also present evidence that both RhoC and MRK are required for LPA-induced stimulation of the p38 and ERK MAP kinases. In conclusion, we have identified MRK as a novel RhoC effector that controls LPA-stimulated cell invasion at least in part by regulating myosin dynamics, ERK and p38. 
 RhoGDIα-dependent balance between RhoA and RhoC is a key regulator of cancer cell tumorigenesis. RhoGTPases are key signaling molecules regulating main cellular functions such as migration, proliferation, survival, and gene expression through interactions with various effectors. Within the RhoA-related subclass, RhoA and RhoC contribute to several steps of tumor growth, and the regulation of their expression affects cancer progression. Our aim is to investigate their respective contributions to the acquisition of an   invasiv  e phenotype by using models of reduced or forced expression. The silencing of RhoC, but not of RhoA, increased the expression of genes encoding tumor suppressors, such as nonsteroidal anti-inflammatory drug-activated gene 1 (NAG-1), and decreased migration and the anchorage-independent growth in vitro. In vivo, RhoC small interfering RNA (siRhoC) impaired tumor growth. Of interest, the simultaneous knockdown of RhoC and NAG-1 repressed most of the siRhoC-related effects, demonstrating the central role of NAG-1. In addition of being induced by RhoC silencing, NAG-1 was also largely up-regulated in cells overexpressing RhoA. The silencing of RhoGDP dissociation inhibitor α (RhoGDIα) and the overexpression of a RhoA mutant unable to bind RhoGDIα suggested that the effect of RhoC silencing is indirect and results from the up-regulation of the RhoA level through competition for RhoGDIα. This study demonstrates the dynamic balance inside the RhoGTPase network and illustrates its biological relevance in cancer progression. 
 Myosin-interacting guanine exchange factor (MyoGEF) regulates the invasion activity of MDA-MB-231 breast cancer cells through activation of RhoA and RhoC. The small guanine triphosphatase (GTPase) proteins RhoA and RhoC are essential for tumor invasion and/or metastasis in breast carcinomas. However, it is poorly understood how RhoA and RhoC are activated in breast cancer cells. Here we describe the role of myosin-interacting guanine nucleotide exchange factor (Myo-GEF) in regulating RhoA and RhoC activation as well as cell polarity and invasion in an   invasive   breast cancer cell line MDA-MB-231. RNA-interference (RNAi)-mediated depletion of MyoGEF in MDA-MB-231 cells not only suppresses the activation of RhoA and RhoC, but also decreases cell polarity and invasion activity. The dominant-negative mutants of RhoA and RhoC, but not Rac1 and Cdc42, dramatically decrease actin polymerization induced by MyoGEF. In addition, MyoGEF co-localizes with nonmuscle myosin IIA (NMIIA) to the front of migrating cells, and depletion of NMIIA by RNAi disrupts the polarized localization of MyoGEF at the cell leading edge, suggesting a role for NMIIA in regulating MyoGEF localization and function. Moreover, MyoGEFprotein levels significantly increase in infiltrating ductal carcinomas as well as in   invasive   breast cancer cell lines. Taken together, our results suggest that MyoGEF cooperates with NMIIA to regulate the polarity and invasion activity of breast cancer cells through activation of RhoA and RhoC. 
 PRL-3 facilitates angiogenesis and metastasis by increasing ERK phosphorylation and up-regulating the levels and activities of Rho-A/C in lung cancer. The aim of this study was to investigate the mechanism of PRL-3 in inducing angiogenesis and lymphangiogenesis to promote distant and lymph node metastasis in human lung cancer tissues and cells.We investigated the expression of PRL-3, VEGF, and VEGF-C from 94 patients with non-small cell lung cancer (NSCLC) using immunohistochemical staining. The relationship between PRL-3 expression and microvessel density (MVD), lymphatic vessel density (LVD), clinicopathological factors, and surgical treatment outcome was also studied. Following this, we studied the effect on A549 by blocking PRL-3.PRL-3 expression in NSCLC was high, and this over-expression is correlated with advanced clinical stage (p = 0.019), distant metastasis (p = 0.001), lymph node metastasis (p = 0.001), and poor post-operative survival. PRL-3 over-expression was associated with vascular endothelial growth factor (VEGF; p = 0.000) and VEGF-C (p = 0.008) expressions, MVD and LVD (p = 0.000 and p = 0.000). Blocking PRL-3 expression in A549 cell resulted in decreased cellular proliferative, migratory, and   invasive   abilities, and VEGF, VEGF-C, pERK, Rho-A, and Rho-C expression was inhibited. Following inhibition of either Rho or pERK, VEGF expression was down-regulated.PRL-3 induces microvascular and lymphatic vessel formation by facilitating VEGF and VEGF-C expression in lung cancer tissues, thus promoting distant and lymph node metastasis of lung cancer. PRL-3 up-regulates pERK and Rho expression and activity, facilitating VEGF expression, and accelerating angiogenesis and distant metastasis. How to regulate VEGF-C expression needs to be further studied. 
 The influence of genetic variation in 30 selected genes on the clinical characteristics of early onset breast cancer. Common variants that alter breast cancer risk are being discovered. Here, we determine how these variants influence breast cancer prognosis, risk and tumour characteristics.We selected 1,001 women with early onset nonfamilial   invasive   breast cancer from the Prospective study of Outcomes in Sporadic versus Hereditary breast cancer (POSH) cohort and genotyped 206 single nucleotide polymorphisms (SNPs) across 30 candidate genes. After quality control, 899 cases and 133 SNPs remained. Survival analyses were used to identify SNPs associated with prognosis and determine their interdependency with recognized prognostic factors. To identify SNPs that alter breast cancer risk, association tests were used to compare cases with controls from the Wellcome Trust Case Control Consortium. To search for SNPs affecting tumour biology, cases were stratified into subgroups according to oestrogen receptor (ER) status and grade and tested for association.We confirmed previous associations between increased breast cancer risk and SNPs in CASP8, TOX3 (previously known as TNRC9) and ESR1. Analysis of prognosis identified eight SNPs in six genes (MAP3K1, DAPK1, LSP1, MMP7, TOX3 and ESR1) and one region without genes on 8q24 that are associated with survival. For MMP7, TOX3 and MAP3K1 the effects on survival are independent of the main recognized clinical prognostic factors. The SNP in 8q24 is more weakly associated with independent effects on survival. Once grade and pathological nodal status (pN stage) were taken into account, SNPs in ESR1 and LSP1 showed no independent survival difference, whereas the effects of the DAPK1 SNP were removed when correcting for ER status. Interestingly, effects on survival for SNPs in ESR1 were most significant when only ER-positive tumours were examined. Stratifying POSH cases by tumour characteristics identified SNPs in FGFR2 and TOX3 associated with ER-positive disease and SNPs in ATM associated with ER-negative disease.We have demonstrated that several SNPs are associated with survival. In some cases this appears to be due to an effect on tumour characteristics known to have a bearing on prognosis; in other cases the effect appears to be independent of these prognostic factors. These findings require validation by further studies in similar patient groups. 
 Targeted disruption of protein kinase C epsilon reduces cell invasion and motility through inactivation of RhoA and RhoC GTPases in head and neck squamous cell carcinoma. Over 70% of patients with head and neck squamous cell carcinoma (HNSCC) present with locoregionally advanced stage III and IV disease. In spite of aggressive therapy, locoregional disease recurs in 60% and metastatic disease develops in 15% to 25% of patients causing a major decline in quality and length of life. Therefore, there is a need to identify and understand genes that are responsible for inducing an aggressive HNSCC phenotype. Evidence has shown that protein kinase C (PKC) epsilon is a transforming oncogene and may play a role in HNSCC progression. In this study, we determine the downstream signaling pathway mediated by PKC epsilon to promote an aggressive HNSCC phenotype. RNA interference knockdown of PKC epsilon in UMSCC11A and UMSCC36, two highly   invasive   and motile HNSCC cell lines with elevated endogenous PKC epsilon levels, resulted in cells that were significantly less   invasive   and motile than the small interfering RNA-scrambled control transfectants; 51 +/- 5% (P < 0.006) and 49 +/- 3% (P < 0.010) inhibition in invasion and 69 +/- 1% (P < 0.0005) and 66 +/- 3% (P < 0.0001) inhibition in motility, respectively. PKC epsilon-deficient UMSCC11A clones had reduced levels of active and serine-phosphorylated RhoA and RhoC. Moreover, constitutive active RhoA completely rescued the invasion and motility defect, whereas constitutive active RhoC completely rescued the invasion and partially rescued the motility defect of PKC epsilon-deficient UMSCC11A clones. These results indicate that RhoA and RhoC are downstream of PKC epsilon and critical for PKC epsilon-mediated cell invasion and motility. Our study shows, for the first time, that PKC epsilon is involved in a coordinated regulation of RhoA and RhoC activation, possibly through direct post-translational phosphorylation. 
 Anti-RhoA and anti-RhoC siRNAs inhibit the proliferation and   invasive  ness of MDA-MB-231 breast cancer cells in vitro and in vivo. Overexpression of RhoA or RhoC in breast cancer indicates a poor prognosis, due to increased tumor cell proliferation and invasion and tumor-dependent angiogenesis. Until now, the strategy of blockage of the Rho-signaling pathway has used either GGTI or HMG-CoA reductase inhibitors, but they are not specific to RhoA or RhoC inhibition. In this study, a new approach with anti-RhoA and anti-RhoC siRNAs was used to inhibit specifically RhoA or RhoC synthesis. Two transfections of either RhoA or RhoC siRNA (8.5 nM) into MDA-MB-231 human breast cancer cells or HMEC-1 endothelial cells induced extensive degradation of the target mRNA and led to a dramatic decrease in synthesis of the corresponding protein. In vitro, these siRNAs inhibited cell proliferation and invasion more effectively than conventional blockers of Rho cell signaling. Finally, in a nude mouse model, intratumoral injections of anti-RhoA siRNA (100 microl at 85 nM) every 3 days for 20 days almost totally inhibited the growth and angiogenesis of xenografted MDA-MB-231 tumors. One may infer from these observations that specific inhibition of the Rho-signaling pathway with siRNAs represents a promising approach for the treatment of aggressive breast cancers. 
 Up-regulation of small GTPases, RhoA and RhoC, is associated with tumor progression in ovarian carcinoma. To clarify the role of small GTPases Rho in the biologic behavior of ovarian carcinoma, we first examined the mRNA expression of RhoA, RhoB, and RhoC in benign, borderline, and malignant ovarian tumors using RT-PCR and real-time RT-PCR. The expression and localization of RhoA protein were also analyzed by Western blotting and immunohistochemistry. Finally, we examined whether up-regulation of Rho enhances the   invasive  ness of ovarian cancer cells in vitro. Analysis of mRNA levels of the Rho family genes revealed that levels of both RhoA and RhoC were significantly higher in carcinomas than in benign tumors (RhoA, p = 0.0035; RhoC, p = 0.0006). According to histologic subtype, both RhoA and RhoC mRNA levels in serous carcinomas were significantly higher than those in other histologic types. With regard to the International Federation of Gynecological and Obstetrics stage classification, both of RhoA and RhoC mRNA levels were significantly higher in tumors of Stages III+IV than in those of Stages I+II (RhoA, p = 0.0200; RhoC, p = 0.0057). In addition, analysis of matched pairs of primary and disseminated lesions demonstrated that expression of both RhoA and RhoC mRNA was significantly higher in metastatic than in primary tumors. Examination of the protein level showed that expression of RhoA was also increased in advanced ovarian carcinomas, especially those of serous histology. Accordingly, we hypothesized that up-regulation of Rho GTPases plays an important role in the progression of ovarian carcinoma. Matrigel invasion assay using the ovarian cancer cell line, SKOV3, showed that up-regulation and activation after treatment with lysophosphatidic acid was associated with enhanced invasion of the cancer cells. This increase in   invasive  ness was suppressed by the addition of C3, a specific inhibitor of Rho. These findings suggest that up-regulation of Rho GTPases is important in the tumor progression of ovarian carcinoma and that Rho family proteins could be a molecular target in cancer therapy. 
 Effect of RhoC on the epithelial-mesenchymal transition process induced by TGF-β1 in lung adenocarcinoma cells. According to recent research, Ras homolog gene family member C (RhoC) is confirmed to have a powerful regulatory effect on cell motility mediated by the cytoskeleton, and this process is closely associated with tumor invasion and metastasis. In addition, the epithelial-mesenchymal transition (EMT) process which causes cytoskeleton rearrangement, also plays a pivotal role in tumor invasion and metastasis.Consequently, in the present study, we aimed to ascertain whether RhoC has an effect on the EMT process induced by TGF-β1 in lung adenocarcinoma cells and whether RhoC promotes tumor invasion by mediating the occurrence of EMT. Based on the findings, we demonstrated that RhoC was an essential mediator of the EMT process in lung adenocarcinoma cell line A549 which was evaluated by observing the morphological characteristics of the cells and by assessing the expression levels of two EMT marker proteins: E-cadherin and vimentin. During the process of EMT in the A549 cells induced by TGF-β1 (5 ng/ml), upregulated RhoC protein and RhoC activity were detected, which was associated with the enha  nced inv  asive capability of the cells in vitro. Conversely, downregulation of the expression of RhoC by shRNA markedly impeded EMT progression as well as the invasion of A549 cells. Our results may provide a novel target towards the prevention of metastasis in advanced lung adenocarcinoma. 
 RhoC is essential for the metastasis of gastric cancer. Rho family members are known to regulate malignant transformation and motility of cancer cells, but the clinicopathological significance of RhoC remains unclear yet in the case of gastric cancer. In this study, we evaluated the protein expression level of RhoC in gastric cancer tissues and cell lines. Results showed that only weak staining of RhoC was detected in 3 of 33 non-tumorous cases by immunohistochemistry. The expression of RhoC was significantly higher in gastric cancer tissues (23/42, 54.8%) than in non-tumorous tissues (p < 0.01). Further analysis demonstrated that RhoC had high specificity (80.0%) in detecting gastric carcinomas with metastatic potential. RhoC was positively expressed in 18 out of 20 metastases (90.0%), even higher than that in primary gastric cancer tissues. Western blot showed that RhoC was up-regulated in five different gastric cancer cell lines but not expressed in SV40-transformed immortal gastric epithelial cell GES-1. Overexpression of RhoC GTPase in GES-1 cells could produce the motile and   invasive   phenotype but did not alter the monolayer growth rate. To further study the functions of RhoC, we took the powerful siRNA technology to knock down the expression of RhoC in SGC7901 cells. It was shown that down-regulation of RhoC did not affect the proliferation of SGC7901 cells. However, interference of RhoC expression could inhibit migration, invasion, and anchorage-independent growth of SGC7901 cells. In conclusion, RhoC may play a very important role in the metastasis of gastric carcinoma. Therapeutic strategies targeting RhoC and RhoC-mediated pathways may be a novel approach for treating metastasis of gastric cancer. 
 The effect of RhoC siRNA on the   invasive  ness and proliferation of human cervical cancer cell line SiHa cells. This study investigated the effect of RhoC GTPase on the proliferation and metastasis of cervical cancer cells, SiHa cells, in vitro. RhoC siRNA was introduced into SiHa cells to silence the RhoC gene. The mRNA and protein expression of RhoC, before and after RhoC siRNA transfection, was examined by RT-PCR and Western blotting, respectively. The proliferation and apoptosis of SiHa cells were examined by MTT assay and flow cytometry (FACS), respectively. Adhesive rate was evaluated by Matrigel adhesive assay, and the   invasive   capability and migration capability were assessed by transwell   invasive   assay and migration assay, respectively. The results showed that after the RhoC siRNA transfection, the mRNA and protein expression of RhoC was down-regulated in SiHa cells. The down-regulation of RhoC GTPase did not affect the cell proliferation and apoptosis (P>0.05), but it did suppress SiHa cells' adhesion to matrigel (P<0.01), the   invasive   capability (P<0.01) and the migration capability (P<0.01). It was concluded that RhoC obviously promotes the adhesion, invasion and migration of SiHa cells in vitro, but not proliferation and apoptosis, suggesting that RhoC plays an important role in the progression in cervical cancer. 
 X-ray crystal structures reveal two activated states for RhoC. RhoC is a member of the Rho family of Ras-related (small) GTPases and shares significant sequence similarity with the founding member of the family, RhoA. However, despite their similarity, RhoA and RhoC exhibit different binding preferences for effector proteins and give rise to distinct cellular outcomes, with RhoC being directly implicated in the   invasive  ness of cancer cells and the development of metastasis. While the structural analyses of the signaling-active and -inactive states of RhoA have been performed, thus far, the work on RhoC has been limited to an X-ray structure for its complex with the effector protein, mDia1 (for mammalian Diaphanous 1). Therefore, in order to gain insights into the molecular basis for RhoC activation, as well as clues regarding how it mediates distinct cellular responses relative to those induced by RhoA, we have undertaken a structural comparison of RhoC in its GDP-bound (signaling-inactive) state versus its GTP-bound (signaling-active) state as induced by the nonhydrolyzable GTP analogues, guanosine 5'-(beta,gamma-iminotriphosphate) (GppNHp) and guanosine 5'-(3-O-thiotriphosphate) (GTPgammaS). Interestingly, we find that GppNHp-bound RhoC only shows differences in its switch II domain, relative to GDP-bound RhoC, whereas GTPgammaS-bound RhoC exhibits differences in both its switch I and switch II domains. Given that each of the nonhydrolyzable GTP analogues is able to promote the binding of RhoC to effector proteins, these results suggest that RhoC can undergo at least two conformational transitions during its conversion from a signaling-inactive to a signaling-active state, similar to what has recently been proposed for the H-Ras and M-Ras proteins. In contrast, the available X-ray structures for RhoA suggest that it undergoes only a single conformational transition to a signaling-active state. These and other differences regarding the changes in the switch domains accompanying the activation of RhoA and RhoC provide plausible explanations for the functional specificity exhibited by the two GTPases. 
 RhoC interacts with integrin α5β1 and enhances its trafficking in migrating pancreatic carcinoma cells. Human pancreatic ductal adenocarcinoma (PDAC) is characterized by early systemic dissemination. Although RhoC has been implicated in cancer cell migration, the relevant underlying molecular mechanisms remain unknown. RhoC has been implicated in the enhancement of cancer cell migration and invasion, with actions which are distinct from RhoA (84% homology), and are possibly attributed to the divergent C-terminus domain. Here, we confirm that RhoC significantly enhances the migratory an  d invasi  ve properties of pancreatic carcinoma cells. In addition, we show that RhoC over-expression decreases cancer cell adhesion and, in turn, accelerates cellular body movement and focal adhesion turnover, especially, on fibronectin-coated surfaces. Whilst RhoC over-expression did not alter integrin expression patterns, we show that it enhanced integrin α5β1 internalization and re-cycling (trafficking), an effect that was dependent specifically on the C-terminus (180-193 amino acids) of RhoC protein. We also report that RhoC and integrin α5β1 co-localize within the peri-nuclear region of pancreatic tumor cells, and by masking the CAAX motif at the C-terminal of RhoC protein, we were able to abolish this interaction in vitro and in vivo. Co-localization of integrin α5β1 and RhoC was demonstrable in invading cancer cells in 3D-organotypic cultures, and further mimicked in vivo analyses of, spontaneous human, (two distinct sources: operated patients and rapid autopsy programme) and transgenic murine (LSL-KrasG12D/+;LSL-Trp53R172H/+;Pdx-1-Cre), pancreatic cancers. In both cases, co-localization of integrin α5β1 and RhoC correlated with poor differentiation status and metastatic potential. We propose that RhoC facilitates tumor cell invasion and promotes subsequent metastasis, in part, by enhancing integrin α5β1 trafficking. Thus, RhoC may serve as a biomarker and a therapeutic target. 
 The interaction of PKN3 with RhoC promotes malignant growth. PKN3 is an AGC-family protein kinase implicated in growth of metastatic prostate cancer cells with phosphoinositide 3-kinase pathway deregulation. The molecular mechanism, however, by which PKN3 contributes to malignant growth and tumorigenesis is not well understood. Using orthotopic mouse tumor models, we now show that inducible knockdown of PKN3 protein not only blocks metastasis, but also impairs primary prostate and breast tumor growth. Correspondingly, overexpression of exogenous PKN3 in breast cancer cells further increases their malignant behavior and   invasive  ness in-vitro. Mechanistically, we demonstrate that PKN3 physically interacts with Rho-family GTPases, and preferentially with RhoC, a known mediator of tumor invasion and metastasis in epithelial cancers. Likewise, RhoC predominantly associates with PKN3 compared to its closely related PKN family members. Unlike the majority of Rho GTPases and PKN molecules, which are ubiquitously expressed, both PKN3 and RhoC show limited expression in normal tissues and become upregulated in late-stage malignancies. Since PKN3 catalytic activity is increased in the presence of Rho GTPases, the co-expression and preferential interaction of PKN3 and RhoC in tumor cells are functionally relevant. Our findings provide novel insight into the regulation and function of PKN3 and suggest that the PKN3-RhoC complex represents an attractive therapeutic target in late-stage malignancies. 
 Higher expression of RhoC is related to   invasive  ness in non-small cell lung carcinoma. The activation of Rho proteins has been shown to lead to loss of polarity in cancer cells, as well as reorganization of the cytoskeleton and facilitation of cell motility, possibly resulting in their malignant potential. The clinicopathological significance of RhoC, however, is not yet well known in the case of non-small cell lung cancer (NSCLC).The intratumor expression level of RhoC mRNA was determined and compared with that in adjacent nontumorous lung tissue using quantitative reverse transcription-PCR in 49 patients with NSCLC. The relationship between the level of RhoC transcript and clinicopathological factors was examined. RhoC protein expression was confirmed by immunohistochemistry and Western blot analysis in several cases.Tumor tissue of NSCLC patients demonstrated a copy number of RhoC mRNA that was well correlated with its protein level in each case and was significantly higher than that found in the corresponding nontumorous lung tissue (2.73 x 10(5) versus 1.13 x 10(4) copies/0.08 micro g mRNA; P < 0.05). Histopathologically positive cases of lymphatic permeation showed a significantly higher copy number of RhoC than negative cases (4.31 x 10(5) versus 1.93 x 10(5) copies/0.08 micro g mRNA; P < 0.05). With regard to venous permeation, the RhoC copy number in positive cases tended to be higher than that seen in negative cases (3.72 x 10(5) versus 2.14 x 10(5) copies/0.08 micro g mRNA; P = 0.06).This is the first demonstration that the expression level of RhoC is correlated to vascular permeation in NSCLC. 
 RhoC mediates invasion and migration of CaSki cells through the Rho-associated serine-threonine protein kinase 1 signaling pathway. The small GTPase RhoC in human cancers is up-regulated and correlated with tumor metastasis. However, the role of Rho/Rho-associated serine-threonine protein kinase 1 (ROCK1) signaling pathway in human cervical cancer is still unclear. In this study, we examine the effects of RhoC and its major downstream target, ROCK1, on the invasion and migration of CaSki cells to investigate the role of RhoC/ROCK1 signaling pathway in the progression of cervical squamous cell carcinoma.RhoC and ROCK1 protein expression in CaSki cells was detected by Western blotting. Scratch and transwell assays were carried out to assess the effects of RhoC on invasion and migration of CaSki cells. Cell viability was assayed by MTT test after adding the ROCK1 inhibitor Y-27632 to CaSki cells.Overexpression of RhoC protein in CaSki cells significantly increases ROCK1 expression and promotes cell invasion and migration compared with the control group (P < 0.05). However, in the inhibition of ROCK1 with Y-27632 in CaSki cells when RhoC was overexpressed, the rate of   invasive  ness and migration was reduced remarkably (P < 0.05), dropping to comparable levels as the control.This study suggested that the activation of RhoC/ROCK1 signaling pathways is likely involved in the progression of cervical squamous cell carcinoma. 
 RhoC promotes metastasis via activation of the Pyk2 pathway in prostate cancer. RhoC is a member of the Ras-homologous family of genes which have been implicated in tumorigenesis and tumor progression. However, the exact role of RhoC is controversial and is yet to be clarified. We have examined the effect of RhoC on prostate tumor cells and found that RhoC had no effect on cell proliferation in vitro or on tumor growth in mice. However, RhoC significantly enhanced the metastatic ability of the tumor cells in these animals, suggesting that RhoC affects only the metastasis but not the growth of prostate tumor cells. The results of our immunohistochemical analyses on tumor specimens from 63 patients with prostate cancer indicate that RhoC expression had no significant correlation with Gleason grade. However, the expression of RhoC showed significant positive correlation with both lymph node and distant metastasis, and it was inversely correlated with patient survival. We also found that RhoC significantly augmented the invasion and motility of prostate tumor cells by activating matrix metalloproteinases 2 and 9 (MMP2 and MMP9) in vitro. The results of our antibody array analysis for signal molecules revealed that RhoC significantly activated kinases including mitogen-activated protein kinase (MAPK), focal adhesion kinase (FAK), Akt, and Pyk2. Inhibition of Pyk2 kinase blocked the RhoC-dependent activation of FAK, MAPK, and Akt, followed by the suppression of MMP2 and MMP9. Inhibitors of both MAPK and Akt also significantly blocked the activities of these MMPs. Therefore, our results indicate that RhoC promotes tumor metastasis in prostate cancer by sequential activation of Pyk2, FAK, MAPK, and Akt followed by the up-regulation of MMP2 and MMP9, which results in the stimulation of   invasive  ness of tumor cells. 
 Antisense RhoC gene suppresses proliferation and invasion capacity of human QBC939 cholangiocarcinoma cells. Cholangiocarcinoma is a highly aggressive, fatal malignancy, which is resistant to all current therapeutic approaches. The recent elevation in the incidence of cholangiocarcinoma has highlighted the need for novel approaches targeting the molecular basis of its   invasive  ness. Previously we reconstructed a RhoC antisense eukaryotic expression vector and transfected it into a cholangiocarcinoma cell line (QBC939) by the lipofectamine method. This study was undertaken to determine the effect of the antisense RhoC gene on the proliferation and invasion capacity of QBC939.Antisense RhoC cDNA was transfected into QBC939 with lipofectin 2000. The cell growth curve was constructed to determine the proliferation rate of cells; flow cytometry was used to analyze cell cycle changes of the tumor cells; and a Boyden chamber was used to assess the   invasive   ability of the cells before and after gene transfection.After the antisense RhoC cDNA was transfected, the number of colonies formed was significantly lower than that in the other two groups (54+/-8 vs. 91+/-11 vs. 90+/-9, P<0. 05) so was the number of the cells which crossed to the lower surface of the matrigel-coater filters (36+/-6 vs. 96+/-12 vs. 95+/-7, P<0.05). There was also a higher percentage of transfected cells in G1 phase than in the other two groups (52.5% vs. 43.4% vs. 43.7%).The antisense RhoC gene can suppress the capacities of proliferation and invasion in a cholangiocarcinoma cell line in vitro. 
 miR-10b promotes cell invasion through RhoC-AKT signaling pathway by targeting HOXD10 in gastric cancer. MicroRNAs play critical roles in tumorigenesis as either oncogenes or tumor suppressors. As a microRNA induced by Twist, miR-10b function as a metastasis driver in different types of cancer, in which the downstream target gene HOXD10 is the main mediator. In gastric tumor species, miR-10b levels were dramatically elevated in lymphoma node metastasis-positive tumor tissues compared with lymphoma node metastasis-free tumor tissues, and were correlated to dowregulation of HOXD10 expression. In gastric cell lines with distinct degrees of differentiation, miR-10b was highly expressed in the cell line with strong metastatic ability. In MNK45 cells, inhibition of miR-10b led to abrogation of cell invasion. While in GES-1 cells, miR-10 overexpression resulted in enhancement of   invasive  ness through translational inhibition of HOXD10, and constitutive expression of HOXD10 reversed the effects of miR-10b on cell invasion. Furthermore, either knockdown of RhoC or inhibition of AKT activation interfered miR-10-induced   invasive  ness in GES-1 cells. In summary, these observations suggest that miR-10b can stimulate the upregulation of RhoC and AKT phosphorylation through targeting HOXD10, thus promoting cell invasion in gastric tumors. 
 Characterization of RhoC expression in benign and malignant breast disease: a potential new marker for small breast carcinomas with metastatic ability. The most important factor in predicting outcome in patients with early breast cancer is the stage of the disease. There is no robust marker capable of identifying   invasive   carcinomas that despite their small size have a high metastatic potential, and that would benefit from more aggressive treatment. RhoC-GTPase is a member of the Ras-superfamily and is involved in cell polarity and motility. We hypothesized that RhoC expression would be a good marker to identify breast cancer patients with high risk of developing metastases, and that it would be a prognostic marker useful in the clinic. We developed a specific anti-RhoC antibody and studied archival breast tissues that comprise a broad spectrum of breast disease. One hundred eighty-two specimens from 164 patients were used. Immunohistochemistry was performed on formalin-fixed tissues. Staining intensity was graded 0 to 3+ (0 to 1+ was considered negative and 2 to 3+ was considered positive). RhoC was not expressed in any of the normal, fibrocystic changes, atypical hyperplasia, or ductal carcinoma in situ, but was expressed in 36 of 118   invasive   carcinomas and strongly correlated with tumor stage (P = 0.01). RhoC had high specificity (88%) in detecting   invasive   carcinomas with metastatic potential. Of the   invasive   carcinomas smaller than 1 cm, RhoC was highly specific in detecting tumors that developed metastases. RhoC expression was associated with negative progesterone receptor and HER-2/neu overexpression. We characterized RhoC expression in human breast tissues. RhoC is specifically expressed in   invasive   breast carcinomas capable of metastasizing, and it may be clinically useful in patients with tumors smaller than 1 cm to guide treatment. 
 miR-10b is overexpressed in hepatocellular carcinoma and promotes cell proliferation, migration and invasion through RhoC, uPAR and MMPs. Recently, miR-10b is identified as a miRNA highly expressed in many human cancers, promoting cell migration and invasion. However, the specific function of miR-10b in hepatocellular carcinoma (HCC) is unclear at this point.The miR-10b expression levels in 60 paired different TNM Stage HCC tumor tissues compared with adjacent non-tumor (ANT) tissues, normal tissue control (8 benign tumor and 7 normal liver tissues), 3 normal liver and 7 HCC cell lines were measured by real-time quantitative RT-PCR and to evaluate their association with HCC clinicopathologic features. Invasion assay, MTT proliferation assay and wound-healing assay were performed to test the invasion and proliferation of HCC cell after transfection. The effect of miR-10b on HCC in vivo was validated by murine xenograft model.We found that miR-10b expression was increased in human HCC tissues and cell lines compared with normal control, respectively. The expression of miR-10b was correlated with HCC metastatic ability. Overexpression of miR-10b in MHCC-97L cells increased cell motility and   invasive  ness, whereas inhibition of miR-10b in MHCC-97H cells reduced cell motility and   invasive  ness in vitro and in vivo. We also showed that HOXD10 was negatively regulated by miR-10b at the posttranscriptional level, via a specific target site within the 3'UTR by luciferase reporter assay. Furthermore, we found that miR-10b induced HCC cell invasion and migration by modulating the HOXD10 target gene RhoC, uPAR, MMP-2 and MMP-9 expression.Our results suggested that miR-10b was overexpressed in HCC and promoted HCC cell migration and invasion through the HOXD10/ RhoC/ uPAR/ MMPs pathway which may provide a novel bio-target for HCC therapy. 
 Effects and mechanism of RhoC downregulation in suppressing ovarian cancer stem cell proliferation, drug resistance, invasion and metastasis. Cancer stem cells are considered to be the root cause of tumor initiation, metastasis, recurrence and therapeutic resistance. Recent studies have reported that RhoC plays a critical role in regulating cancer stem cells; however, its function in ovarian cancer stem cells (OCSCs) remains unknown. The ovarian cancer cell line A2780, and the paclitaxel-resistant A2780 cell line (A2780-PTX) were obtained. A2780 cells were used to isolate and identify the highly   invasive   A2780-PM cells, and A2780-PTX cells were used to isolate and identify the highly drug-resistant and highly   invasive   A2780-PTX-PM cells by Transwell assay. MTT, Transwell and wound healing assays were used to compare the differences in cell proliferation, invasion and migration ability among the four cell lines. Immunofluorescence was used to detect the expression of stem cell markers CD117 and CD133. OCSCs were sorted by flow cytometry. Following si-RhoC transfection of the OCSCs, cell proliferation, drug resistance, invasion and migration ability and RhoC, CD117 and CD133 expression levels were assayed. RT-PCR was used to assess RhoC, CD117, CD133 and matrix metalloproteinase 9 (MMP9) mRNA expression levels. A2780-PM and A2780‑PTX-PM cells exhibited higher cell proliferation, drug resistance, and invasion and migration ability than the A2780 and A2780-PTX cell lines. Furthermore, CD133 and CD117 expression levels were higher in the A2780-PM and A2780‑PTX-PM cells than levels in the A2780 and A2780-PTX cells. Transfection of si-RhoC in OCSCs suppressed the proliferation, drug resistance, invasion, migration and CD117 and CD133 expression levels. Furthermore, the expression levels of RhoC, CD117, CD133, MDR1, and MMP9 mRNA were downregulated in the transfected population. Taken together, our results demonstrated that RhoC downregulation may inhibit the proliferation, drug resistance, invasion and migration of OCSCs, and RhoC may play an important role in the formation of OCSCs. 
 Regulation of inflammatory breast cancer cell invasion through Akt1/PKBα phosphorylation of RhoC GTPase. With a 42% and 18% 5- and 10-year respective disease-free survival rate, inflammatory breast cancer (IBC) is arguably the deadliest form of breast cancer. IBC invades the dermal lymphatic vessels of the skin overlying the breast and as a consequence nearly all women have lymph node involvement and ~1/3 have gross distant metastases at the time of diagnosis. One year after diagnosis ~90% of patients have detectable metastases, making IBC a paradigm for lymphovascular invasion. Understanding the underlying mechanisms of the IBC metastatic phenotype is essential for new therapies. Work from our laboratory and others show distinct molecular differences between IBC and non-IBCs (nIBCs). Previously we showed that RhoC GTPase is a metastatic switch responsible for the   invasiv  e phenotype of IBC. In this study we integrate observations made in IBC patients with in vitro analysis. We show that the PI3K/Akt signaling pathway is crucial in IBC invasion. Key molecules involved in cytoskeletal control and cell motility are specifically upregulated in IBC patients compared with stage and cell-type-of-origin matched nIBCs patients. Distinctively, RhoC GTPase is a substrate for Akt1 and its phosphorylation is absolutely essential for IBC cell invasion. Further our data show that Akt3, not Akt1 has a role in IBC cell survival. Together our data show a unique and targetable pathway for IBC invasion and survival. 
 The role of RhoC in growth and metastatic capacity of melanoma. RhoC overexpression in tumor cells promotes   invasive   and metastatic behavior. RhoC expression levels have been correlated with tumor progression and metastasis in multiple human cancers. In melanoma, RhoC is upregulated in highly metastatic tumors. Induced expression in melanoma cell lines resulted in invasion and metastasis, whereas inhibition of RhoC reversed the metastatic phenotype both in vitro and in vivo.RhoC mRNA and protein expression in two human melanoma cell lines (DX3aza and MeWo) and pooled primary melanocytes were investigated by means of real-time quantitative polymerase chain reaction and western blotting. RhoC protein expression was evaluated in 123 primary cutaneous melanoma samples by the use of immunohistochemistry and correlated with known prognostic features.RhoC upregulation was observed in the highly metastatic DX3aza cell line, whereas in MeWo, only low expression levels could be detected. RhoC expression in primary cutaneous melanoma was strongly associated with thicker and ulcerated tumors. RhoC expression was associated with the presence of lymphatic metastases at the time of diagnosis and shorter disease-free and overall survival rates, without being an independent predictor.These results further support a role for RhoC in growth and metastasis of melanoma. 
 [Correlation of expression of RhoC with   invasive  ness of breast cancer cells in vitro]. To investigate the expression of RhoC in breast cancer cells with different metastatic potential and its correlation with   invasive  ness.Expression of RhoC mRNA and protein in human breast cancer cells MCF-7 with low metastatic potential and MDA-MB-231 with high metastatic potential was detected by RT-PCR, Western blot, immunohistochemistry and immunofluorescence staining. Eukaryotic expression plasmids of RhoC were constructed and transfected into MCF-7 cells. The biological effects were observed, including in vitro invasion by Boyden charmber assay, motility by would healing assay, alteration of microfilament network by TRTIC-phalloidin staining and expression of p-Akt by Western blot assay.The expression levels of RhoC mRNA and protein varied in the two different metastatic breast cancer cell lines. RhoC was significantly up-regulated in the highly metastatic cells in comparison to the weakly metastatic counterpart (P < 0.01). As shown by Boyden charmber assay, the   invasive   capacity of transfected cells overexpressing RhoC was significantly promoted as reflected by more penetrating cells (56.88 +/- 4.18) than that of the antisense transcripts (23.12 +/- 3.22), the negative (23.77 +/- 3.64) and blank controls (28.44 +/- 2.48). Further study by would healing assay indicated that cells overexpressing RhoC were more motile in actin-based active movement. The wound healing ratio after 24 h of the sense transcripts, antisense transcripts, negative controls and blank controls was 58.28% +/- 2.14%, 22.36% +/- 2.73%, 28.23% +/- 2.62%, 30.18% +/- 2.86%, respectively. The TRITC-phalloidin staining revealed less actin filament bundles and a reorganized cytoskeleton within the sense transcripts. In addition, p-Akt expression level was upregulated in the sense transcripts.RhoC overexpression may promote the   invasive   capacity of human breast cancer cells in vitro and its expression level is positively correlated with the metastatic capacity of those cells. So RhoC may be a potential target in the development of a novel strategy for treating metastasis of breast cancer. 
 Formin-like 2 drives amoeboid   invasive   cell motility downstream of RhoC.   Invasive   cell migration is a key step for cancer metastasis and involves Rho GTPase-controlled reorganization of the actin cytoskeleton. Altered Rho GTPase expression is found in various malignancies. Particularly, the closely related GTPases RhoA and RhoC are upregulated in many aggressive tumours, but specific effectors that distinguish between these two GTPases to explain mechanistic differences have not been identified. The formins are by far the largest family of Rho GTPase effectors and are characterized by the actin-nucleating formin homology 2 domain. Using siRNA-based screening against all 15 human formins, we systematically analysed their functions in 3D cell motility using three different cancer cell lines. These results reveal distinct requirements for specific formins in amoeboid versus mesenchymal   invasive   cell migration. Importantly, by knocking down all Rho proteins, we identified formin-like 2 (FMNL2) as a specific RhoC effector, showing selective interaction of FMNL2 with active RhoC, but not RhoA or RhoB. Functional analysis shows that RhoC regulates autoinhibition of FMNL2, whereas suppression of FMNL2 inhibits RhoC-, but not RhoA-dependent, rounded   invasive   cell migration. Thus, our data uncover a novel regulatory and functional interaction between RhoC and FMNL2 for modulating cell shape and   invasive  ness and provide mechanistic insight into RhoC-specific signalling events. 
  GeneRIFs  
 During the process of epithelial-mesenchymal transition in A549 cells induced by TGF-beta1, upregulated RhoC protein and RhoC activity were detected, which was associated with the enhanced   invasive   capability of the cells in vitro. 
 a novel regulatory and functional interaction between RhoC and FMNL2 for modulating cell shape and   invasive  ness and provide mechanistic insight into RhoC-specific signalling events. 
 RhoC overexpression may promote the   invasive   capacity of human breast cancer cells in vitro and its expression level is positively correlated with the metastatic capacity of those cells. 
 Results indicate that RhoC promotes tumor metastasis in prostate cancer by sequential activation of Pyk2, FAK, MAPK, and Akt followed by the up-regulation of MMP2 and MMP9, which results in the stimulation of   invasive  ness of tumor cells. 
 Higher expression of RhoC is related to   invasive  ness in non-small cell lung carcinoma 
 specifically expressed in   invasive   breast carcinomas capable of metastasizing 
  [Mm]etastasize  PubMed  
 Regulation of pancreatic cancer cell migration and invasion by RhoC GTPase and caveolin-1. In the current study we investigated the role of caveolin-1 (cav-1) in pancreatic adenocarcinoma (PC) cell migration and invasion; initial steps in metastasis. Cav-1 is the major structural protein in caveolae; small Omega-shaped invaginations within the plasma membrane. Caveolae are involved in signal transduction, wherein cav-1 acts as a scaffolding protein to organize multiple molecular complexes regulating a variety of cellular events. Recent evidence suggests a role for cav-1 in promoting cancer cell migration, invasion and metastasis; however, the molecular mechanisms have not been described. The small monomeric GTPases are among several molecules which associate with cav-1. Classically, the Rho GTPases control actin cytoskeletal reorganization during cell migration and invasion. RhoC GTPase is overexpressed in aggressive cancers that   metastasize   and is the predominant GTPase in PC. Like several GTPases, RhoC contains a putative cav-1 binding motif.Analysis of 10 PC cell lines revealed high levels of cav-1 expression in lines derived from primary tumors and low expression in those derived from metastases. Comparison of the BxPC-3 (derived from a primary tumor) and HPAF-II (derived from a metastasis) demonstrates a reciprocal relationship between cav-1 expression and p42/p44 Erk activation with PC cell migration, invasion, RhoC GTPase and p38 MAPK activation. Furthermore, inhibition of RhoC or p38 activity in HPAF-II cells leads to partial restoration of cav-1 expression.Cav-1 expression inhibits RhoC GTPase activation and subsequent activation of the p38 MAPK pathway in primary PC cells thus restricting migration and invasion. In contrast, loss of cav-1 expression leads to RhoC-mediated migration and invasion in metastatic PC cells. 
 miR-509 suppresses brain metastasis of breast cancer cells by modulating RhoC and TNF-α. The median survival time of breast cancer patients with brain metastasis is less than 6 months, and even a small metastatic lesion often causes severe neurological disabilities. Because of the location of metastatic lesions, a surgical approach is limited and most chemotherapeutic drugs are ineffective owing to the blood brain barrier (BBB). Despite this clinical importance, the molecular basis of the brain metastasis is poorly understood. In this study, we have isolated RNA from samples obtained from primary breast tumors and also from brain metastatic lesions followed by microRNA profiling analysis. Our results revealed that the miR-509 is highly expressed in the primary tumors, whereas the expression of this microRNA is significantly decreased in the brain metastatic lesions. MicroRNA target prediction and the analysis of cytokine array for the cells ectopically expressed with miR-509 demonstrated that this microRNA was capable of modulating the two genes essential for brain invasion, RhoC and TNF-α that affect the invasion of cancer cells and permeability of BBB, respectively. Importantly, high levels of TNF-α and RhoC-induced MMP9 were significantly correlated with brain metastasis-free survival of breast cancer patients. Furthermore, the results of our in vivo experiments indicate that miR-509 significantly suppressed the ability of cancer cells   to metastas  ize to the brain. These findings suggest that miR-509 has a critical role in brain metastasis of breast cancer by modulating the RhoC-TNF-α network and that this miR-509 axis may represent a potential therapeutic target or serve as a prognostic tool for brain metastasis. 
 RhoC GTPase Is a Potent Regulator of Glutamine Metabolism and N-Acetylaspartate Production in Inflammatory Breast Cancer Cells. Inflammatory breast cancer (IBC) is an extremely lethal cancer that rapidly   metastasize  s. Although the molecular attributes of IBC have been described, little is known about the underlying metabolic features of the disease. Using a variety of metabolic assays, including (13)C tracer experiments, we found that SUM149 cells, the primary in vitro model of IBC, exhibit metabolic abnormalities that distinguish them from other breast cancer cells, including elevated levels of N-acetylaspartate, a metabolite primarily associated with neuronal disorders and gliomas. Here we provide the first evidence of N-acetylaspartate in breast cancer. We also report that the oncogene RhoC, a driver of metastatic potential, modulates glutamine and N-acetylaspartate metabolism in IBC cells in vitro, revealing a novel role for RhoC as a regulator of tumor cell metabolism that extends beyond its well known role in cytoskeletal rearrangement. 
  [Mm]etastasis  PubMed  
 Clinical and prognostic significance of RhoA and RhoC gene expression in esophageal squamous cell carcinoma. Rho GTPases are involved in the organization of a microfilament network, cell-to-cell interaction, and malignant transformation. To elucidate the role of Rho GTPases in esophageal squamous cell carcinoma (ESCC), we compared the levels of RhoA and RhoC mRNA from ESCC with the corresponding normal tissue originating from the same patients.Real-time reverse transcriptase-polymerase chain reaction was performed to observe rhoA and rhoC in esophageal cell lines. Next, the mRNA levels of rhoA and rhoC were evaluated from 50 patients.The rhoA and rhoC were higher in ESCC cell lines than in noncancerous esophageal cell. rhoC was overexpressed in TTn, which was obtained directly from a surgical specimen of a metastatic lesion of ESCC in the mandible. rhoA and rhoC were significantly higher in ESCC patient than in the normal counterparts (P = .0022 and P < .0001, respectively). rhoA correlated with tumor differentiation and rhoC correlated with an advanced tumor, node,   metastasis   system classifications. rhoA and rhoC in ESCC showed a positive correlation (P = .008). Patients with rhoA overexpression showed a significantly poorer prognosis than those with rhoA underexpression (P = .044).To our knowledge, this study is the first in which the expression of RhoA and RhoC at the mRNA level in ESCC was examined and compared with its normal counterpart. Our results suggest that rhoA and rhoC are involved in ESCC progression and useful as prognostic markers. Further study will be needed to examine the therapeutic potential of the Rho GTPase inhibitor as a promising anticancer therapy, especially in ESCC. 
 The MLK-related kinase (MRK) is a novel RhoC effector that mediates lysophosphatidic acid (LPA)-stimulated tumor cell invasion. The small GTPase RhoC is overexpressed in many invasive tumors and is essential for   metastasis  . Despite its high structural homology to RhoA, RhoC appears to perform functions that are different from those controlled by RhoA. The identity of the signaling components that are differentially regulated by these two GTPases is only beginning to emerge. Here, we show that the MAP3K protein MRK directly binds to the GTP-bound forms of both RhoA and RhoC in vitro. However, siRNA-mediated depletion of MRK in cells phenocopies depletion of RhoC, rather than that of RhoA. MRK depletion, like that of RhoC, inhibits LPA-stimulated cell invasion, while depletion of RhoA increases invasion. We also show that active MRK enhances LPA-stimulated invasion, further supporting a role for MRK in the regulation of invasion. Depletion of either RhoC or MRK causes sustained myosin light chain phosphorylation after LPA stimulation. In addition, activation of MRK causes a reduction in myosin light chain phosphorylation. In contrast, as expected, depletion of RhoA inhibits myosin light chain phosphorylation. We also present evidence that both RhoC and MRK are required for LPA-induced stimulation of the p38 and ERK MAP kinases. In conclusion, we have identified MRK as a novel RhoC effector that controls LPA-stimulated cell invasion at least in part by regulating myosin dynamics, ERK and p38. 
 Myosin-interacting guanine exchange factor (MyoGEF) regulates the invasion activity of MDA-MB-231 breast cancer cells through activation of RhoA and RhoC. The small guanine triphosphatase (GTPase) proteins RhoA and RhoC are essential for tumor invasion and/or   metastasis   in breast carcinomas. However, it is poorly understood how RhoA and RhoC are activated in breast cancer cells. Here we describe the role of myosin-interacting guanine nucleotide exchange factor (Myo-GEF) in regulating RhoA and RhoC activation as well as cell polarity and invasion in an invasive breast cancer cell line MDA-MB-231. RNA-interference (RNAi)-mediated depletion of MyoGEF in MDA-MB-231 cells not only suppresses the activation of RhoA and RhoC, but also decreases cell polarity and invasion activity. The dominant-negative mutants of RhoA and RhoC, but not Rac1 and Cdc42, dramatically decrease actin polymerization induced by MyoGEF. In addition, MyoGEF co-localizes with nonmuscle myosin IIA (NMIIA) to the front of migrating cells, and depletion of NMIIA by RNAi disrupts the polarized localization of MyoGEF at the cell leading edge, suggesting a role for NMIIA in regulating MyoGEF localization and function. Moreover, MyoGEFprotein levels significantly increase in infiltrating ductal carcinomas as well as in invasive breast cancer cell lines. Taken together, our results suggest that MyoGEF cooperates with NMIIA to regulate the polarity and invasion activity of breast cancer cells through activation of RhoA and RhoC. 
 PRL-3 facilitates angiogenesis and   metastasis   by increasing ERK phosphorylation and up-regulating the levels and activities of Rho-A/C in lung cancer. The aim of this study was to investigate the mechanism of PRL-3 in inducing angiogenesis and lymphangiogenesis to promote distant and lymph node   metastasis   in human lung cancer tissues and cells.We investigated the expression of PRL-3, VEGF, and VEGF-C from 94 patients with non-small cell lung cancer (NSCLC) using immunohistochemical staining. The relationship between PRL-3 expression and microvessel density (MVD), lymphatic vessel density (LVD), clinicopathological factors, and surgical treatment outcome was also studied. Following this, we studied the effect on A549 by blocking PRL-3.PRL-3 expression in NSCLC was high, and this over-expression is correlated with advanced clinical stage (p = 0.019), distant   metastasis   (p = 0.001), lymph node   metastasis   (p = 0.001), and poor post-operative survival. PRL-3 over-expression was associated with vascular endothelial growth factor (VEGF; p = 0.000) and VEGF-C (p = 0.008) expressions, MVD and LVD (p = 0.000 and p = 0.000). Blocking PRL-3 expression in A549 cell resulted in decreased cellular proliferative, migratory, and invasive abilities, and VEGF, VEGF-C, pERK, Rho-A, and Rho-C expression was inhibited. Following inhibition of either Rho or pERK, VEGF expression was down-regulated.PRL-3 induces microvascular and lymphatic vessel formation by facilitating VEGF and VEGF-C expression in lung cancer tissues, thus promoting distant and lymph node   metastasis   of lung cancer. PRL-3 up-regulates pERK and Rho expression and activity, facilitating VEGF expression, and accelerating angiogenesis and distant   metastasis  . How to regulate VEGF-C expression needs to be further studied. 
 A novel strategy for specifically down-regulating individual Rho GTPase activity in tumor cells. The Rho family GTPases RhoA, RhoB, and RhoC regulate the actin cytoskeleton, cell movement, and cell growth. Unlike Ras, up-regulation or overexpression of these GDP/GTP binding molecular switches, but not activating point mutations, has been associated with human cancer. Although they share over 85% sequence identity, RhoA, RhoB, and RhoC appear to play distinct roles in cell transformation and   metastasis  . In NIH 3T3 cells, RhoA or RhoB overexpression causes transformation whereas RhoC increases the cell migration rate. To specifically target RhoA, RhoB, or RhoC function, we have generated a set of chimeric molecules by fusing the RhoGAP domain of p190, a GTPase-activating protein that accelerates the intrinsic GTPase activity of all three Rho GTPases, with the C-terminal hypervariable sequences of RhoA, RhoB, or RhoC. The p190-Rho chimeras were active as GTPase-activating proteins toward RhoA in vitro, co-localized with the respective active Rho proteins, and specifically down-regulated Rho protein activities in cells depending on which Rho GTPase sequences were included in the chimeras. In particular, the p190-RhoA-C chimera specifically inhibited RhoA-induced transformation whereas p190-RhoC-C specifically reversed the migration phenotype induced by the active RhoC. In human mammary epithelial-RhoC breast cancer cells, p190-RhoC-C, but not p190-RhoA-C or p190-RhoB-C, reversed the anchorage-independent growth and invasion phenotypes caused by RhoC overexpression. In the highly metastatic A375-M human melanoma cells, p190-RhoC-C specifically reversed migration, and invasion phenotypes attributed to RhoC up-regulation. Thus, we have developed a novel strategy utilizing RhoGAP-Rho chimeras to specifically down-regulate individual Rho activity and demonstrate that this approach may be applied to multiple human tumor cells to reverse the growth and/or invasion phenotypes associated with disregulation of a distinct subtype of Rho GTPase. 
 RhoC and ROCKs regulate cancer cell interactions with endothelial cells. RhoC is a member of the Rho GTPase family that is implicated in cancer progression by stimulating cancer cell invasiveness. Here we report that RhoC regulates the interaction of cancer cells with vascular endothelial cells (ECs), a crucial step in the metastatic process. RhoC depletion by RNAi reduces PC3 prostate cancer cell adhesion to ECs, intercalation between ECs as well as transendothelial migration in vitro. Depletion of the kinases ROCK1 and ROCK2, two known RhoC downstream effectors, similarly decreases cancer interaction with ECs. RhoC also regulates the extension of protrusions made by cancer cells on vascular ECs in vivo. Transient RhoC depletion is sufficient to reduce both early PC3 cell retention in the lungs and experimental   metastasis   formation in vivo. Our results indicate RhoC plays a central role in cancer cell interaction with vascular ECs, which is a critical event for cancer progression. 
 A p27(kip1)-binding protein, p27RF-Rho, promotes cancer   metastasis   via activation of RhoA and RhoC. Rho family proteins regulate multiple cellular functions including motility and invasion through regulation of the actin cytoskeleton and gene expression. Activation of Rho proteins is controlled precisely by multiple regulators in a spatiotemporal manner. RhoA and/or RhoC are key players that regulate the metastatic activity of malignant tumor cells, and it is therefore of particular interest to understand how activation of these Rho proteins is controlled. We recently identified an upstream regulator of RhoA activation, p27RF-Rho (p27(kip1) releasing factor from RhoA) that acts by freeing RhoA from inhibition by p27(kip1). p27(kip1) is a cell cycle regulator when it is localized to the nucleus, but it binds RhoA and inhibits activation of the latter when it is localized to the cytoplasm. Here, we show that a metastatic variant of mouse melanoma B16 cells (F10) exhibits greater expression of p27RF-Rho, RhoA, and RhoC than the nonmetastatic parental cells (F0). Injection of F10 cells into mouse tail vein resulted in the formation of metastatic lung colonies, whereas prior knockdown of expression of either one of the three proteins using specific shRNA sequences decreased   metastasis   markedly. p27RF-Rho regulated the activation of RhoA and RhoC and thereby modulated cellular adhesion and motility, in addition to pericellular proteolysis. The Rho activities enhanced by p27RF-Rho had a marked effect upon efficiency of lodging of F10 cells in the lung, which represents an early step of   metastasis  . p27RF-Rho also regulated   metastasis   of human melanoma and fibrosarcoma cells. Thus, p27RF-Rho is a key upstream regulator of RhoA and RhoC that controls spreading of tumor cells. 
 Downregulation of the Rho GTPase signaling pathway is involved in the microRNA-138-mediated inhibition of cell migration and invasion in tongue squamous cell carcinoma. Tumor   metastasis   is the dominant cause of death in cancer patients, including patients with oral tongue squamous cell carcinoma (TSCC). Previously, we reported that reduced miR-138 level is correlated with enhanced metastatic potential in TSCC cells. Here, we demonstrate that miR-138 suppresses TSCC cell migration and invasion by regulating 2 key genes in the Rho GTPase signaling pathway: RhoC and ROCK2. Direct targeting of miR-138 to specific sequences located in the 3'-untranslated regions of both RhoC and ROCK2 mRNAs was confirmed using luciferase reporter gene assays. Ectopic transfection of miR-138 reduced the expression of both RhoC and ROCK2 in TSCC cells. These reduced expressions, in consequence, led to the reorganization of the stress fibers and the subsequent cell morphology change to a round bleb-like shape as well as the suppression of cell migration and invasion. In contrast, knockdown of miR-138 in TSCC cells enhanced the expression of RhoC and ROCK2, which resulted in an altered, elongated cell morphology, enhanced cell stress fiber formation and accelerated cell migration and invasion. Taken together, our results suggest that miR-138 plays an important role in TSCC cell migration and invasion by concurrently targeting RhoC and ROCK2, and miR-138 may serve as a novel therapeutic target for TSCC patients at risk of metastatic disease. 
 Expression of RhoA and RhoC in colorectal carcinoma and its relations with clinicopathological parameters. Ras homologous (Rho) family GTPases play a pivotal role in the regulation of numerous cellular functions associated with malignant transformation and   metastasis  . To evaluate the role of these GTPases in colorectal cancer, the mRNA expression levels in matched sets of tumor and non-tumor tissues from surgical specimens were analyzed. The relationship between the mRNA levels in tumor tissues to the clinicopathological features was also assessed.A total of 68 patients with colorectal carcinoma were recruited and the levels of RhoA and RhoC mRNA transcripts in cancer, paratumoral and normal tissues were characterized by quantitative real-time polymerase chain reaction (QRT-PCR). Their correlation to clinical histopathological parameters was analyzed.The levels of RhoA and RhoC mRNA transcripts in carcinoma tissues were significantly higher than those in the matched paratumor and normal tissues from the same patient (p<0.05). The expression levels of both genes were significantly correlated with   metastasis   of cancer cells to lymph nodes and liver (p<0.05). The levels of RhoA expression were significantly correlated with the histopathological degree of cancer, while the expression of RhoC was correlated with the extent of local invasion to intestine.This is the first study with QRT-PCR to examine the expressions of RhoA and RhoC genes in colorectal carcinoma of Chinese patients. The significantly up-regulated RhoA and RhoC expressions suggest that they may contribute to the initiation, development, invasion and   metastasis   of colorectal carcinoma in Chinese patients. 
 Ovarian clear cell carcinomas: RHO GTPases may contribute to explain their singular biologic behavior. Ovarian clear cell carcinoma is found more often confined to the ovary (stage I) than high-grade serous carcinoma. The RHO GTPase family of proteins is involved in tumor invasion and   metastasis   through regulation of the cytoskeleton. The expression of several RHO family genes, including RHOA, RHOC, CDC42, and 3 ARHGDIs (Rho GDP dissociation inhibitors), was studied by real-time polymerase chain reaction in 22 clear cell carcinomas and 31 high-grade serous carcinomas. Immunoreaction for p21-activated kinase 1 (a downstream effector of CDC42) was also investigated on 6 tissue microarrays containing 76 carcinomas (13 clear cell carcinomas and 63 high-grade serous carcinomas). Eight clear cell carcinomas (8/21; 38%) were at stage I, whereas only 3 high-grade serous carcinomas (3/31; 10%) were at stage I. Postoperatively, all patients were treated with taxane and cisplatinum or carboplatinum. ARHGDIA messenger RNA expression was higher in clear cell carcinomas than high-grade serous carcinomas (P = .07). In contrast, CDC42 messenger RNA levels were lower in clear cell carcinomas than high-grade serous carcinomas (P = .02). Immunoreaction for p21-activated kinase 1 was concordant with the results obtained by real-time polymerase chain reaction for CDC42. In clear cell carcinomas, RHOA and RHOC messenger RNA expression was lower in stage I than in advanced-stage tumors (P = .03 and P = .005, respectively). Furthermore, in high-grade serous carcinomas, RHOA expression was higher in patients who did not respond to chemotherapy (P = .04). ARHGDIA, CDC42, RHOA, and RHOC expression may contribute to explain the different stage at diagnosis of clear cell and high-grade serous carcinomas. RHOA may cause resistance of high-grade serous carcinoma to chemotherapy. 
 RhoC and ROCKs regulate cancer cell interactions with endothelial cells. RhoC is a member of the Rho GTPase family that is implicated in cancer progression by stimulating cancer cell invasiveness. Here we report that RhoC regulates the interaction of cancer cells with vascular endothelial cells (ECs), a crucial step in the metastatic process. RhoC depletion by RNAi reduces PC3 prostate cancer cell adhesion to ECs, intercalation between ECs as well as transendothelial migration in vitro. Depletion of the kinases ROCK1 and ROCK2, two known RhoC downstream effectors, similarly decreases cancer interaction with ECs. RhoC also regulates the extension of protrusions made by cancer cells on vascular ECs in vivo. Transient RhoC depletion is sufficient to reduce both early PC3 cell retention in the lungs and experimental   metastasis   formation in vivo. Our results indicate RhoC plays a central role in cancer cell interaction with vascular ECs, which is a critical event for cancer progression. 
 The MLK-related kinase (MRK) is a novel RhoC effector that mediates lysophosphatidic acid (LPA)-stimulated tumor cell invasion. The small GTPase RhoC is overexpressed in many invasive tumors and is essential for   metastasis  . Despite its high structural homology to RhoA, RhoC appears to perform functions that are different from those controlled by RhoA. The identity of the signaling components that are differentially regulated by these two GTPases is only beginning to emerge. Here, we show that the MAP3K protein MRK directly binds to the GTP-bound forms of both RhoA and RhoC in vitro. However, siRNA-mediated depletion of MRK in cells phenocopies depletion of RhoC, rather than that of RhoA. MRK depletion, like that of RhoC, inhibits LPA-stimulated cell invasion, while depletion of RhoA increases invasion. We also show that active MRK enhances LPA-stimulated invasion, further supporting a role for MRK in the regulation of invasion. Depletion of either RhoC or MRK causes sustained myosin light chain phosphorylation after LPA stimulation. In addition, activation of MRK causes a reduction in myosin light chain phosphorylation. In contrast, as expected, depletion of RhoA inhibits myosin light chain phosphorylation. We also present evidence that both RhoC and MRK are required for LPA-induced stimulation of the p38 and ERK MAP kinases. In conclusion, we have identified MRK as a novel RhoC effector that controls LPA-stimulated cell invasion at least in part by regulating myosin dynamics, ERK and p38. 
 Ovarian clear cell carcinomas: RHO GTPases may contribute to explain their singular biologic behavior. Ovarian clear cell carcinoma is found more often confined to the ovary (stage I) than high-grade serous carcinoma. The RHO GTPase family of proteins is involved in tumor invasion and   metastasis   through regulation of the cytoskeleton. The expression of several RHO family genes, including RHOA, RHOC, CDC42, and 3 ARHGDIs (Rho GDP dissociation inhibitors), was studied by real-time polymerase chain reaction in 22 clear cell carcinomas and 31 high-grade serous carcinomas. Immunoreaction for p21-activated kinase 1 (a downstream effector of CDC42) was also investigated on 6 tissue microarrays containing 76 carcinomas (13 clear cell carcinomas and 63 high-grade serous carcinomas). Eight clear cell carcinomas (8/21; 38%) were at stage I, whereas only 3 high-grade serous carcinomas (3/31; 10%) were at stage I. Postoperatively, all patients were treated with taxane and cisplatinum or carboplatinum. ARHGDIA messenger RNA expression was higher in clear cell carcinomas than high-grade serous carcinomas (P = .07). In contrast, CDC42 messenger RNA levels were lower in clear cell carcinomas than high-grade serous carcinomas (P = .02). Immunoreaction for p21-activated kinase 1 was concordant with the results obtained by real-time polymerase chain reaction for CDC42. In clear cell carcinomas, RHOA and RHOC messenger RNA expression was lower in stage I than in advanced-stage tumors (P = .03 and P = .005, respectively). Furthermore, in high-grade serous carcinomas, RHOA expression was higher in patients who did not respond to chemotherapy (P = .04). ARHGDIA, CDC42, RHOA, and RHOC expression may contribute to explain the different stage at diagnosis of clear cell and high-grade serous carcinomas. RHOA may cause resistance of high-grade serous carcinoma to chemotherapy. 
 A p27(kip1)-binding protein, p27RF-Rho, promotes cancer   metastasis   via activation of RhoA and RhoC. Rho family proteins regulate multiple cellular functions including motility and invasion through regulation of the actin cytoskeleton and gene expression. Activation of Rho proteins is controlled precisely by multiple regulators in a spatiotemporal manner. RhoA and/or RhoC are key players that regulate the metastatic activity of malignant tumor cells, and it is therefore of particular interest to understand how activation of these Rho proteins is controlled. We recently identified an upstream regulator of RhoA activation, p27RF-Rho (p27(kip1) releasing factor from RhoA) that acts by freeing RhoA from inhibition by p27(kip1). p27(kip1) is a cell cycle regulator when it is localized to the nucleus, but it binds RhoA and inhibits activation of the latter when it is localized to the cytoplasm. Here, we show that a metastatic variant of mouse melanoma B16 cells (F10) exhibits greater expression of p27RF-Rho, RhoA, and RhoC than the nonmetastatic parental cells (F0). Injection of F10 cells into mouse tail vein resulted in the formation of metastatic lung colonies, whereas prior knockdown of expression of either one of the three proteins using specific shRNA sequences decreased   metastasis   markedly. p27RF-Rho regulated the activation of RhoA and RhoC and thereby modulated cellular adhesion and motility, in addition to pericellular proteolysis. The Rho activities enhanced by p27RF-Rho had a marked effect upon efficiency of lodging of F10 cells in the lung, which represents an early step of   metastasis  . p27RF-Rho also regulated   metastasis   of human melanoma and fibrosarcoma cells. Thus, p27RF-Rho is a key upstream regulator of RhoA and RhoC that controls spreading of tumor cells. 
 Downregulation of the Rho GTPase signaling pathway is involved in the microRNA-138-mediated inhibition of cell migration and invasion in tongue squamous cell carcinoma. Tumor   metastasis   is the dominant cause of death in cancer patients, including patients with oral tongue squamous cell carcinoma (TSCC). Previously, we reported that reduced miR-138 level is correlated with enhanced metastatic potential in TSCC cells. Here, we demonstrate that miR-138 suppresses TSCC cell migration and invasion by regulating 2 key genes in the Rho GTPase signaling pathway: RhoC and ROCK2. Direct targeting of miR-138 to specific sequences located in the 3'-untranslated regions of both RhoC and ROCK2 mRNAs was confirmed using luciferase reporter gene assays. Ectopic transfection of miR-138 reduced the expression of both RhoC and ROCK2 in TSCC cells. These reduced expressions, in consequence, led to the reorganization of the stress fibers and the subsequent cell morphology change to a round bleb-like shape as well as the suppression of cell migration and invasion. In contrast, knockdown of miR-138 in TSCC cells enhanced the expression of RhoC and ROCK2, which resulted in an altered, elongated cell morphology, enhanced cell stress fiber formation and accelerated cell migration and invasion. Taken together, our results suggest that miR-138 plays an important role in TSCC cell migration and invasion by concurrently targeting RhoC and ROCK2, and miR-138 may serve as a novel therapeutic target for TSCC patients at risk of metastatic disease. 
 Expression of RhoA and RhoC in colorectal carcinoma and its relations with clinicopathological parameters. Ras homologous (Rho) family GTPases play a pivotal role in the regulation of numerous cellular functions associated with malignant transformation and   metastasis  . To evaluate the role of these GTPases in colorectal cancer, the mRNA expression levels in matched sets of tumor and non-tumor tissues from surgical specimens were analyzed. The relationship between the mRNA levels in tumor tissues to the clinicopathological features was also assessed.A total of 68 patients with colorectal carcinoma were recruited and the levels of RhoA and RhoC mRNA transcripts in cancer, paratumoral and normal tissues were characterized by quantitative real-time polymerase chain reaction (QRT-PCR). Their correlation to clinical histopathological parameters was analyzed.The levels of RhoA and RhoC mRNA transcripts in carcinoma tissues were significantly higher than those in the matched paratumor and normal tissues from the same patient (p<0.05). The expression levels of both genes were significantly correlated with   metastasis   of cancer cells to lymph nodes and liver (p<0.05). The levels of RhoA expression were significantly correlated with the histopathological degree of cancer, while the expression of RhoC was correlated with the extent of local invasion to intestine.This is the first study with QRT-PCR to examine the expressions of RhoA and RhoC genes in colorectal carcinoma of Chinese patients. The significantly up-regulated RhoA and RhoC expressions suggest that they may contribute to the initiation, development, invasion and   metastasis   of colorectal carcinoma in Chinese patients. 
 Myosin-interacting guanine exchange factor (MyoGEF) regulates the invasion activity of MDA-MB-231 breast cancer cells through activation of RhoA and RhoC. The small guanine triphosphatase (GTPase) proteins RhoA and RhoC are essential for tumor invasion and/or   metastasis   in breast carcinomas. However, it is poorly understood how RhoA and RhoC are activated in breast cancer cells. Here we describe the role of myosin-interacting guanine nucleotide exchange factor (Myo-GEF) in regulating RhoA and RhoC activation as well as cell polarity and invasion in an invasive breast cancer cell line MDA-MB-231. RNA-interference (RNAi)-mediated depletion of MyoGEF in MDA-MB-231 cells not only suppresses the activation of RhoA and RhoC, but also decreases cell polarity and invasion activity. The dominant-negative mutants of RhoA and RhoC, but not Rac1 and Cdc42, dramatically decrease actin polymerization induced by MyoGEF. In addition, MyoGEF co-localizes with nonmuscle myosin IIA (NMIIA) to the front of migrating cells, and depletion of NMIIA by RNAi disrupts the polarized localization of MyoGEF at the cell leading edge, suggesting a role for NMIIA in regulating MyoGEF localization and function. Moreover, MyoGEFprotein levels significantly increase in infiltrating ductal carcinomas as well as in invasive breast cancer cell lines. Taken together, our results suggest that MyoGEF cooperates with NMIIA to regulate the polarity and invasion activity of breast cancer cells through activation of RhoA and RhoC. 
 PRL-3 facilitates angiogenesis and   metastasis   by increasing ERK phosphorylation and up-regulating the levels and activities of Rho-A/C in lung cancer. The aim of this study was to investigate the mechanism of PRL-3 in inducing angiogenesis and lymphangiogenesis to promote distant and lymph node   metastasis   in human lung cancer tissues and cells.We investigated the expression of PRL-3, VEGF, and VEGF-C from 94 patients with non-small cell lung cancer (NSCLC) using immunohistochemical staining. The relationship between PRL-3 expression and microvessel density (MVD), lymphatic vessel density (LVD), clinicopathological factors, and surgical treatment outcome was also studied. Following this, we studied the effect on A549 by blocking PRL-3.PRL-3 expression in NSCLC was high, and this over-expression is correlated with advanced clinical stage (p = 0.019), distant   metastasis   (p = 0.001), lymph node   metastasis   (p = 0.001), and poor post-operative survival. PRL-3 over-expression was associated with vascular endothelial growth factor (VEGF; p = 0.000) and VEGF-C (p = 0.008) expressions, MVD and LVD (p = 0.000 and p = 0.000). Blocking PRL-3 expression in A549 cell resulted in decreased cellular proliferative, migratory, and invasive abilities, and VEGF, VEGF-C, pERK, Rho-A, and Rho-C expression was inhibited. Following inhibition of either Rho or pERK, VEGF expression was down-regulated.PRL-3 induces microvascular and lymphatic vessel formation by facilitating VEGF and VEGF-C expression in lung cancer tissues, thus promoting distant and lymph node   metastasis   of lung cancer. PRL-3 up-regulates pERK and Rho expression and activity, facilitating VEGF expression, and accelerating angiogenesis and distant   metastasis  . How to regulate VEGF-C expression needs to be further studied. 
 Clinical and prognostic significance of RhoA and RhoC gene expression in esophageal squamous cell carcinoma. Rho GTPases are involved in the organization of a microfilament network, cell-to-cell interaction, and malignant transformation. To elucidate the role of Rho GTPases in esophageal squamous cell carcinoma (ESCC), we compared the levels of RhoA and RhoC mRNA from ESCC with the corresponding normal tissue originating from the same patients.Real-time reverse transcriptase-polymerase chain reaction was performed to observe rhoA and rhoC in esophageal cell lines. Next, the mRNA levels of rhoA and rhoC were evaluated from 50 patients.The rhoA and rhoC were higher in ESCC cell lines than in noncancerous esophageal cell. rhoC was overexpressed in TTn, which was obtained directly from a surgical specimen of a metastatic lesion of ESCC in the mandible. rhoA and rhoC were significantly higher in ESCC patient than in the normal counterparts (P = .0022 and P < .0001, respectively). rhoA correlated with tumor differentiation and rhoC correlated with an advanced tumor, node,   metastasis   system classifications. rhoA and rhoC in ESCC showed a positive correlation (P = .008). Patients with rhoA overexpression showed a significantly poorer prognosis than those with rhoA underexpression (P = .044).To our knowledge, this study is the first in which the expression of RhoA and RhoC at the mRNA level in ESCC was examined and compared with its normal counterpart. Our results suggest that rhoA and rhoC are involved in ESCC progression and useful as prognostic markers. Further study will be needed to examine the therapeutic potential of the Rho GTPase inhibitor as a promising anticancer therapy, especially in ESCC. 
 A novel strategy for specifically down-regulating individual Rho GTPase activity in tumor cells. The Rho family GTPases RhoA, RhoB, and RhoC regulate the actin cytoskeleton, cell movement, and cell growth. Unlike Ras, up-regulation or overexpression of these GDP/GTP binding molecular switches, but not activating point mutations, has been associated with human cancer. Although they share over 85% sequence identity, RhoA, RhoB, and RhoC appear to play distinct roles in cell transformation and   metastasis  . In NIH 3T3 cells, RhoA or RhoB overexpression causes transformation whereas RhoC increases the cell migration rate. To specifically target RhoA, RhoB, or RhoC function, we have generated a set of chimeric molecules by fusing the RhoGAP domain of p190, a GTPase-activating protein that accelerates the intrinsic GTPase activity of all three Rho GTPases, with the C-terminal hypervariable sequences of RhoA, RhoB, or RhoC. The p190-Rho chimeras were active as GTPase-activating proteins toward RhoA in vitro, co-localized with the respective active Rho proteins, and specifically down-regulated Rho protein activities in cells depending on which Rho GTPase sequences were included in the chimeras. In particular, the p190-RhoA-C chimera specifically inhibited RhoA-induced transformation whereas p190-RhoC-C specifically reversed the migration phenotype induced by the active RhoC. In human mammary epithelial-RhoC breast cancer cells, p190-RhoC-C, but not p190-RhoA-C or p190-RhoB-C, reversed the anchorage-independent growth and invasion phenotypes caused by RhoC overexpression. In the highly metastatic A375-M human melanoma cells, p190-RhoC-C specifically reversed migration, and invasion phenotypes attributed to RhoC up-regulation. Thus, we have developed a novel strategy utilizing RhoGAP-Rho chimeras to specifically down-regulate individual Rho activity and demonstrate that this approach may be applied to multiple human tumor cells to reverse the growth and/or invasion phenotypes associated with disregulation of a distinct subtype of Rho GTPase. 
 Effect of RhoC on the epithelial-mesenchymal transition process induced by TGF-β1 in lung adenocarcinoma cells. According to recent research, Ras homolog gene family member C (RhoC) is confirmed to have a powerful regulatory effect on cell motility mediated by the cytoskeleton, and this process is closely associated with tumor invasion an  d metastas  is. In addition, the epithelial-mesenchymal transition (EMT) process which causes cytoskeleton rearrangement, also plays a pivotal role in tumor invasion an  d metastas  is.Consequently, in the present study, we aimed to ascertain whether RhoC has an effect on the EMT process induced by TGF-β1 in lung adenocarcinoma cells and whether RhoC promotes tumor invasion by mediating the occurrence of EMT. Based on the findings, we demonstrated that RhoC was an essential mediator of the EMT process in lung adenocarcinoma cell line A549 which was evaluated by observing the morphological characteristics of the cells and by assessing the expression levels of two EMT marker proteins: E-cadherin and vimentin. During the process of EMT in the A549 cells induced by TGF-β1 (5 ng/ml), upregulated RhoC protein and RhoC activity were detected, which was associated with the enhanced invasive capability of the cells in vitro. Conversely, downregulation of the expression of RhoC by shRNA markedly impeded EMT progression as well as the invasion of A549 cells. Our results may provide a novel target towards the preventi  on of meta  stasis in advanced lung adenocarcinoma. 
 HBx and HBs regulate RhoC expression by upregulating transcription factor Ets-1. The available evidence suggests that HBV proteins play an important role in the development of hepatocellular carcinoma (HCC). RhoC, a member of the Rho subfamily of the Ras superfamily of homologous genes, had been implicated in tumorigenesis and tumor progression. In a previous study, we demonstrated that HBx and HBs could up-regulate RhoC expression by enhancing its promoter activity. However, the specific mechanisms remain unclear. Here, we demonstrate that overexpression of Ets-1 results in upregulation of RhoC promoter activity and mRNA and protein levels. Expression of transcription factor Ets-1 was significantly higher in HepG2.2.15 cells than that in HepG2 cells. Meanwhile, infection of HepG2 cells with an HBV-adenovirus recombinant virus led to up-regulation of Ets-1. Of the four HBV proteins, HBx and HBs, could increase expression of Ets-1, which consequently contributed to the upregulation of RhoC. These findings might provide a novel insight into HBV-induced HCC   metastasis  . 
 Genomic analysis of   metastasis   reveals an essential role for RhoC. The most damaging change during cancer progression is the switch from a locally growing tumour to a metastatic killer. This switch is believed to involve numerous alterations that allow tumour cells to complete the complex series of events needed for   metastasis  . Relatively few genes have been implicated in these events. Here we use an in vivo selection scheme to select highly metastatic melanoma cells. By analysing these cells on DNA arrays, we define a pattern of gene expression that correlates with progression to a metastatic phenotype. In particular, we show enhanced expression of several genes involved in extracellular matrix assembly and of a second set of genes that regulate, either directly or indirectly, the actin-based cytoskeleton. One of these, the small GTPase RhoC, enhances   metastasis   when overexpressed, whereas a dominant-negative Rho inhibits   metastasis  . Analysis of the phenotype of cells expressing dominant-negative Rho or RhoC indicates that RhoC is important in tumour cell invasion. The genomic approach allows us to identify families of genes involved in a process, not just single genes, and can indicate which molecular and cellular events might be important in complex biological processes such as   metastasis  . 
 Regulation of pancreatic cancer cell migration and invasion by RhoC GTPase and caveolin-1. In the current study we investigated the role of caveolin-1 (cav-1) in pancreatic adenocarcinoma (PC) cell migration and invasion; initial steps in   metastasis  . Cav-1 is the major structural protein in caveolae; small Omega-shaped invaginations within the plasma membrane. Caveolae are involved in signal transduction, wherein cav-1 acts as a scaffolding protein to organize multiple molecular complexes regulating a variety of cellular events. Recent evidence suggests a role for cav-1 in promoting cancer cell migration, invasion and   metastasis  ; however, the molecular mechanisms have not been described. The small monomeric GTPases are among several molecules which associate with cav-1. Classically, the Rho GTPases control actin cytoskeletal reorganization during cell migration and invasion. RhoC GTPase is overexpressed in aggressive cancers that metastasize and is the predominant GTPase in PC. Like several GTPases, RhoC contains a putative cav-1 binding motif.Analysis of 10 PC cell lines revealed high levels of cav-1 expression in lines derived from primary tumors and low expression in those derived from metastases. Comparison of the BxPC-3 (derived from a primary tumor) and HPAF-II (derived from a   metastasis  ) demonstrates a reciprocal relationship between cav-1 expression and p42/p44 Erk activation with PC cell migration, invasion, RhoC GTPase and p38 MAPK activation. Furthermore, inhibition of RhoC or p38 activity in HPAF-II cells leads to partial restoration of cav-1 expression.Cav-1 expression inhibits RhoC GTPase activation and subsequent activation of the p38 MAPK pathway in primary PC cells thus restricting migration and invasion. In contrast, loss of cav-1 expression leads to RhoC-mediated migration and invasion in metastatic PC cells. 
 RhoC, vascular endothelial growth factor and microvascular density in esophageal squamous cell carcinoma. To investigate the expression of Ras homolog (Rho)C, vascular endothelial growth factor (VEGF) and CD105 in esophageal squamous cell carcinoma.Semi-quantitative reverse transcriptase polymerase chain reaction, in situ hybridization and immunohistochemical streptavidin-biotin- peroxidase methods were used to detect expression of RhoC mRNA and protein, and VEGF protein in 62 cases with esophageal squamous cell carcinoma, 31 cases with adjacent atypical hyperplastic tissues, and 62 cases with normal esophageal mucosa. CD105 antibody labeling was used to measure microvascular density. Expression levels were compared according to clinicopathologic and patient parameters.Expression of RhoC mRNA showed a positive correlation with the protein level in esophageal squamous cell carcinoma, as well as with VEGF protein levels. RhoC mRNA expression was mainly located within the cytoplasm of the tumor cells, appearing as blue to purple particles by in situ hybridization. The differences in RhoC mRNA expression in esophageal squamous cell carcinoma, adjacent atypical hyperplasia and normal esophageal mucosa were significant (P < 0.05). The relative expression of RhoC mRNA in cancer tissues with lymph node   metastasis   was significantly higher than in the tissues without lymph node   metastasis   (P < 0.05). VEGF protein expression was consistent with microvascular density (t = 25.52, P < 0.05). Positive expression of VEGF protein in esophageal squamous cell carcinoma of different histologic gradings did not differ significantly. Positive expression of VEGF protein in carcinoma tissues with deep infiltration was significantly higher than in tissues with only superficial infiltration (P < 0.05). The positive expression of VEGF protein in cancer tissues with lymph node   metastasis   was significantly higher than in the tissues without lymph node   metastasis   (P < 0.05).RhoC protein may upregulate VEGF expression, thereby promoting tumor angiogenesis. RhoC mRNA and protein expression was correlated with   metastasis  . 
 RhoC is essential for the   metastasis   of gastric cancer. Rho family members are known to regulate malignant transformation and motility of cancer cells, but the clinicopathological significance of RhoC remains unclear yet in the case of gastric cancer. In this study, we evaluated the protein expression level of RhoC in gastric cancer tissues and cell lines. Results showed that only weak staining of RhoC was detected in 3 of 33 non-tumorous cases by immunohistochemistry. The expression of RhoC was significantly higher in gastric cancer tissues (23/42, 54.8%) than in non-tumorous tissues (p < 0.01). Further analysis demonstrated that RhoC had high specificity (80.0%) in detecting gastric carcinomas with metastatic potential. RhoC was positively expressed in 18 out of 20 metastases (90.0%), even higher than that in primary gastric cancer tissues. Western blot showed that RhoC was up-regulated in five different gastric cancer cell lines but not expressed in SV40-transformed immortal gastric epithelial cell GES-1. Overexpression of RhoC GTPase in GES-1 cells could produce the motile and invasive phenotype but did not alter the monolayer growth rate. To further study the functions of RhoC, we took the powerful siRNA technology to knock down the expression of RhoC in SGC7901 cells. It was shown that down-regulation of RhoC did not affect the proliferation of SGC7901 cells. However, interference of RhoC expression could inhibit migration, invasion, and anchorage-independent growth of SGC7901 cells. In conclusion, RhoC may play a very important role in the   metastasis   of gastric carcinoma. Therapeutic strategies targeting RhoC and RhoC-mediated pathways may be a novel approach for treating   metastasis   of gastric cancer. 
 The effect of RhoC siRNA on the invasiveness and proliferation of human cervical cancer cell line SiHa cells. This study investigated the effect of RhoC GTPase on the proliferation and   metastasis   of cervical cancer cells, SiHa cells, in vitro. RhoC siRNA was introduced into SiHa cells to silence the RhoC gene. The mRNA and protein expression of RhoC, before and after RhoC siRNA transfection, was examined by RT-PCR and Western blotting, respectively. The proliferation and apoptosis of SiHa cells were examined by MTT assay and flow cytometry (FACS), respectively. Adhesive rate was evaluated by Matrigel adhesive assay, and the invasive capability and migration capability were assessed by transwell invasive assay and migration assay, respectively. The results showed that after the RhoC siRNA transfection, the mRNA and protein expression of RhoC was down-regulated in SiHa cells. The down-regulation of RhoC GTPase did not affect the cell proliferation and apoptosis (P>0.05), but it did suppress SiHa cells' adhesion to matrigel (P<0.01), the invasive capability (P<0.01) and the migration capability (P<0.01). It was concluded that RhoC obviously promotes the adhesion, invasion and migration of SiHa cells in vitro, but not proliferation and apoptosis, suggesting that RhoC plays an important role in the progression in cervical cancer. 
 RhoC Is an Unexpected Target of RhoGDI2 in Prevention of Lung Colonization of Bladder Cancer. RhoGDI2 (ARHGDIB) suppresses   metastasis   in a variety of cancers but the mechanism is unclear, thus hampering development of human therapeutics. RhoGDI2 is a guanine nucleotide dissociation inhibitor (GDI) for the Rho family of GTPases thought to primarily bind to Rac1; however, Rac1 activation was not decreased by RhoGDI2 expression in bladder cancer cells. To better understand the GTPase-binding partners for RhoGDI2, a mass spectrometry-based proteomic approach was used in bladder cancer cells. As expected, endogenous RhoGDI2 coimmunoprecipitates with Rac1 and unexpectedly also with RhoC. Further analysis demonstrated that RhoGDI2 negatively regulates RhoC, as knockdown of RhoGDI2 increased RhoC activation in response to serum stimulation. Conversely, overexpression of RhoGDI2 decreased RhoC activation. RhoC promoted bladder cancer cell growth and invasion, as knockdown increased cell doubling time, decreased invasion through Matrigel, and decreased colony formation in soft agar. Importantly, RhoC knockdown reduced in vivo lung colonization by bladder cancer cells following tail vein injection in immunocompromised mice. Finally, unbiased transcriptome analysis revealed a set of genes regulated by RhoGDI2 overexpression and RhoC knockdown in bladder cancer cells.RhoGDI2 suppresses bladder cancer metastatic colonization via negative regulation of RhoC activity, providing a rationale for the development of therapeutics that target RhoC signaling. 
 X-ray crystal structures reveal two activated states for RhoC. RhoC is a member of the Rho family of Ras-related (small) GTPases and shares significant sequence similarity with the founding member of the family, RhoA. However, despite their similarity, RhoA and RhoC exhibit different binding preferences for effector proteins and give rise to distinct cellular outcomes, with RhoC being directly implicated in the invasiveness of cancer cells and the development of   metastasis  . While the structural analyses of the signaling-active and -inactive states of RhoA have been performed, thus far, the work on RhoC has been limited to an X-ray structure for its complex with the effector protein, mDia1 (for mammalian Diaphanous 1). Therefore, in order to gain insights into the molecular basis for RhoC activation, as well as clues regarding how it mediates distinct cellular responses relative to those induced by RhoA, we have undertaken a structural comparison of RhoC in its GDP-bound (signaling-inactive) state versus its GTP-bound (signaling-active) state as induced by the nonhydrolyzable GTP analogues, guanosine 5'-(beta,gamma-iminotriphosphate) (GppNHp) and guanosine 5'-(3-O-thiotriphosphate) (GTPgammaS). Interestingly, we find that GppNHp-bound RhoC only shows differences in its switch II domain, relative to GDP-bound RhoC, whereas GTPgammaS-bound RhoC exhibits differences in both its switch I and switch II domains. Given that each of the nonhydrolyzable GTP analogues is able to promote the binding of RhoC to effector proteins, these results suggest that RhoC can undergo at least two conformational transitions during its conversion from a signaling-inactive to a signaling-active state, similar to what has recently been proposed for the H-Ras and M-Ras proteins. In contrast, the available X-ray structures for RhoA suggest that it undergoes only a single conformational transition to a signaling-active state. These and other differences regarding the changes in the switch domains accompanying the activation of RhoA and RhoC provide plausible explanations for the functional specificity exhibited by the two GTPases. 
 RhoC interacts with integrin α5β1 and enhances its trafficking in migrating pancreatic carcinoma cells. Human pancreatic ductal adenocarcinoma (PDAC) is characterized by early systemic dissemination. Although RhoC has been implicated in cancer cell migration, the relevant underlying molecular mechanisms remain unknown. RhoC has been implicated in the enhancement of cancer cell migration and invasion, with actions which are distinct from RhoA (84% homology), and are possibly attributed to the divergent C-terminus domain. Here, we confirm that RhoC significantly enhances the migratory and invasive properties of pancreatic carcinoma cells. In addition, we show that RhoC over-expression decreases cancer cell adhesion and, in turn, accelerates cellular body movement and focal adhesion turnover, especially, on fibronectin-coated surfaces. Whilst RhoC over-expression did not alter integrin expression patterns, we show that it enhanced integrin α5β1 internalization and re-cycling (trafficking), an effect that was dependent specifically on the C-terminus (180-193 amino acids) of RhoC protein. We also report that RhoC and integrin α5β1 co-localize within the peri-nuclear region of pancreatic tumor cells, and by masking the CAAX motif at the C-terminal of RhoC protein, we were able to abolish this interaction in vitro and in vivo. Co-localization of integrin α5β1 and RhoC was demonstrable in invading cancer cells in 3D-organotypic cultures, and further mimicked in vivo analyses of, spontaneous human, (two distinct sources: operated patients and rapid autopsy programme) and transgenic murine (LSL-KrasG12D/+;LSL-Trp53R172H/+;Pdx-1-Cre), pancreatic cancers. In both cases, co-localization of integrin α5β1 and RhoC correlated with poor differentiation status and metastatic potential. We propose that RhoC facilitates tumor cell invasion and promotes s  ubsequent   metastasis, in part, by enhancing integrin α5β1 trafficking. Thus, RhoC may serve as a biomarker and a therapeutic target. 
 Notch1 regulates the functional contribution of RhoC to cervical carcinoma progression. The role of Notch signalling in human epithelial cancers is of immense interest. In this study, we examine the interplay between Notch signalling and RhoC, a well-established molecular factor in   metastasis  . By linking the function of Notch and RhoC, we further strengthen the notion that there is a pro-oncogenic role of Notch signalling in human cervical cancers.RhoC protein expression in cervical carcinoma cell lines was assessed by western blotting. Using CaSki and SiHa cells (cervical carcinoma cells lines), we show that RhoC contributes to wound healing, invasion and migration, anoikis resistance, colony formation, in vitro tube formation and tumour formation. Immunohistochemical studies were carried out to assess the co-expression of RhoC, pAkt and Notch1 in clinical sections.An assessment of the changes associated with epithelial-to-mesenchymal transition (EMT) shows that both Notch1 and RhoC have similar phenotypic contribution to EMT. Rho activity assessment on Notch1 inhibition with DAPT shows decreased RhoC activity. We further show that constitutively active RhoC rescues the phenotypic effect of Notch1 inactivation, and a comparison of Notch1 with RhoC expression shows an overlap between the two proteins in the same areas of the tissue.This study has provided evidence to suggest that RhoC is an effector of Notch1 in cervical carcinoma. 
 Suppression subtractive hybridization profiles of radial growth phase and metastatic melanoma cell lines reveal novel potential targets. Melanoma progression occurs through three major stages: radial growth phase (RGP), confined to the epidermis; vertical growth phase (VGP), when the tumor has invaded into the dermis; and   metastasis  . In this work, we used suppression subtractive hybridization (SSH) to investigate the molecular signature of melanoma progression, by comparing a group of metastatic cell lines with an RGP-like cell line showing characteristics of early neoplastic lesions including expression of the   metastasis   suppressor KISS1, lack of alphavbeta3-integrin and low levels of RHOC.Two subtracted cDNA collections were obtained, one (RGP library) by subtracting the RGP cell line (WM1552C) cDNA from a cDNA pool from four metastatic cell lines (WM9, WM852, 1205Lu and WM1617), and the other (Met library) by the reverse subtraction. Clones were sequenced and annotated, and expression validation was done by Northern blot and RT-PCR. Gene Ontology annotation and searches in large-scale melanoma expression studies were done for the genes identified.We identified 367 clones from the RGP library and 386 from the Met library, of which 351 and 368, respectively, match human mRNA sequences, representing 288 and 217 annotated genes. We confirmed the differential expression of all genes selected for validation. In the Met library, we found an enrichment of genes in the growth factors/receptor, adhesion and motility categories whereas in the RGP library, enriched categories were nucleotide biosynthesis, DNA packing/repair, and macromolecular/vesicular trafficking. Interestingly, 19% of the genes from the RGP library map to chromosome 1 against 4% of the ones from Met library.This study identifies two populations of genes differentially expressed between melanoma cell lines from two tumor stages and suggests that these sets of genes represent profiles of less aggressive versus metastatic melanomas. A search for expression profiles of melanoma in available expression study databases allowed us to point to a great potential of involvement in tumor progression for several of the genes identified here. A few sequences obtained here may also contribute to extend annotated mRNAs or to the identification of novel transcripts. 
 miR-509 suppresses brain   metastasis   of breast cancer cells by modulating RhoC and TNF-α. The median survival time of breast cancer patients with brain   metastasi  s is less than 6 months, and even a small metastatic lesion often causes severe neurological disabilities. Because of the location of metastatic lesions, a surgical approach is limited and most chemotherapeutic drugs are ineffective owing to the blood brain barrier (BBB). Despite this clinical importance, the molecular basis of the brain   metastasi  s is poorly understood. In this study, we have isolated RNA from samples obtained from primary breast tumors and also from brain metastatic lesions followed by microRNA profiling analysis. Our results revealed that the miR-509 is highly expressed in the primary tumors, whereas the expression of this microRNA is significantly decreased in the brain metastatic lesions. MicroRNA target prediction and the analysis of cytokine array for the cells ectopically expressed with miR-509 demonstrated that this microRNA was capable of modulating the two genes essential for brain invasion, RhoC and TNF-α that affect the invasion of cancer cells and permeability of BBB, respectively. Importantly, high levels of TNF-α and RhoC-induced MMP9 were significantly correlated with bra  in metasta  sis-free survival of breast cancer patients. Furthermore, the results of our in vivo experiments indicate that miR-509 significantly suppressed the ability of cancer cells to metastasize to the brain. These findings suggest that miR-509 has a critical role in bra  in metasta  sis of breast cancer by modulating the RhoC-TNF-α network and that this miR-509 axis may represent a potential therapeutic target or serve as a prognostic tool for br  ain metast  asis. 
 RhoC is essential for angiogenesis induced by hepatocellular carcinoma cells via regulation of endothelial cell organization. The angiogenesis induced by tumor cells is essential for   metastasis   of hepatocellular carcinoma. Available information suggests that RhoC participates in angiogenesis through regulation of vascular endothelial growth factor expression in tumor cells. For its broad functions in cell migration and cytoskeletal organization, we hypothesized that RhoC regulating angiogenesis does not exclusively depend on regulation of vascular endothelial growth factor expression. To address this question, in the present study, we used a retroviral small interfering RNA approach to selectively knockdown the expression of RhoC in a neovascularization model in vivo and in vitro. Our present results indicate that RhoC is the downstream regulator of vascular endothelial growth factor in endothelial cells and is essential for angiogenesis induced by vascular endothelial growth factor, notwithstanding that RhoC regulates the expression of vascular endothelial growth factor in tumor cells. Furthermore, we show that knockdown of RhoC is associated with the inhibition of invasion and migration but not apoptosis of endothelial cells. Knockdown of RhoC results in inhibition of endothelial cell organization through restraining the reorganization of F-actin filaments, which represses endothelial cell network and sprout formation. In conclusion, our results demonstrate that knockdown of RhoC inhibits angiogenesis induced by tumor cells not only through effecting the release of vascular endothelial growth factor, but also through inhibiting endothelial cell migration and organization, which implies that it blocks tumor   metastasis   by specifically inhibiting RhoC in endothelial cells. 
 The interaction of PKN3 with RhoC promotes malignant growth. PKN3 is an AGC-family protein kinase implicated in growth of metastatic prostate cancer cells with phosphoinositide 3-kinase pathway deregulation. The molecular mechanism, however, by which PKN3 contributes to malignant growth and tumorigenesis is not well understood. Using orthotopic mouse tumor models, we now show that inducible knockdown of PKN3 protein not only blocks   metastasis  , but also impairs primary prostate and breast tumor growth. Correspondingly, overexpression of exogenous PKN3 in breast cancer cells further increases their malignant behavior and invasiveness in-vitro. Mechanistically, we demonstrate that PKN3 physically interacts with Rho-family GTPases, and preferentially with RhoC, a known mediator of tumor invasion and   metastasis   in epithelial cancers. Likewise, RhoC predominantly associates with PKN3 compared to its closely related PKN family members. Unlike the majority of Rho GTPases and PKN molecules, which are ubiquitously expressed, both PKN3 and RhoC show limited expression in normal tissues and become upregulated in late-stage malignancies. Since PKN3 catalytic activity is increased in the presence of Rho GTPases, the co-expression and preferential interaction of PKN3 and RhoC in tumor cells are functionally relevant. Our findings provide novel insight into the regulation and function of PKN3 and suggest that the PKN3-RhoC complex represents an attractive therapeutic target in late-stage malignancies. 
 RhoC mediates invasion and migration of CaSki cells through the Rho-associated serine-threonine protein kinase 1 signaling pathway. The small GTPase RhoC in human cancers is up-regulated and correlated with tumor   metastasis  . However, the role of Rho/Rho-associated serine-threonine protein kinase 1 (ROCK1) signaling pathway in human cervical cancer is still unclear. In this study, we examine the effects of RhoC and its major downstream target, ROCK1, on the invasion and migration of CaSki cells to investigate the role of RhoC/ROCK1 signaling pathway in the progression of cervical squamous cell carcinoma.RhoC and ROCK1 protein expression in CaSki cells was detected by Western blotting. Scratch and transwell assays were carried out to assess the effects of RhoC on invasion and migration of CaSki cells. Cell viability was assayed by MTT test after adding the ROCK1 inhibitor Y-27632 to CaSki cells.Overexpression of RhoC protein in CaSki cells significantly increases ROCK1 expression and promotes cell invasion and migration compared with the control group (P < 0.05). However, in the inhibition of ROCK1 with Y-27632 in CaSki cells when RhoC was overexpressed, the rate of invasiveness and migration was reduced remarkably (P < 0.05), dropping to comparable levels as the control.This study suggested that the activation of RhoC/ROCK1 signaling pathways is likely involved in the progression of cervical squamous cell carcinoma. 
 Expression and significance of RhoC gene in hepatocellular carcinoma. To investigate the expression of RhoC gene in hepatocellular carcinoma (HCC) and to evaluate the relationship between RhoC gene expression and invasion and   metastasis   of HCC.mRNA expression level of RhoC gene was examined by reverse transcription-polymerase chain reaction (RT-PCR) in 25 cases of HCC and para-cancerous normal liver tissues. In addition, mutation of RhoC gene was examined by polymerase chain reaction-single strand conformational polymorphism (PCR-SSCP).The mRNA expression levels of RhoC in tumor tissues were significantly higher than those in para-cancerous normal liver tissues (1.8+/-1.1 vs 1.0+/-0.7, P<0.01). The metastatic lesions outside of liver also showed significantly higher RhoC mRNA levels than corresponding tumor tissues in liver (3.3+/-0.5 vs 2.0+/-0.7, P<0.01). There were significant associations between RhoC gene expression and certain clinical and pathological findings, including cell differentiation, vein invasion, number of tumor nodes and metastatic lesions. Mutation of RhoC gene was not found by PCR-SSCP.The RhoC gene may be related to malignant transformation and development of HCC and may play an important role in the invasion and   metastasis   of HCC by overexpression but not mutation. 
 RhoC promotes   metastasis   via activation of the Pyk2 pathway in prostate cancer. RhoC is a member of the Ras-homologous family of genes which have been implicated in tumorigenesis and tumor progression. However, the exact role of RhoC is controversial and is yet to be clarified. We have examined the effect of RhoC on prostate tumor cells and found that RhoC had no effect on cell proliferation in vitro or on tumor growth in mice. However, RhoC significantly enhanced the metastatic ability of the tumor cells in these animals, suggesting that RhoC affects only the   metastasis   but not the growth of prostate tumor cells. The results of our immunohistochemical analyses on tumor specimens from 63 patients with prostate cancer indicate that RhoC expression had no significant correlation with Gleason grade. However, the expression of RhoC showed significant positive correlation with both lymph node and distant   metastasis  , and it was inversely correlated with patient survival. We also found that RhoC significantly augmented the invasion and motility of prostate tumor cells by activating matrix metalloproteinases 2 and 9 (MMP2 and MMP9) in vitro. The results of our antibody array analysis for signal molecules revealed that RhoC significantly activated kinases including mitogen-activated protein kinase (MAPK), focal adhesion kinase (FAK), Akt, and Pyk2. Inhibition of Pyk2 kinase blocked the RhoC-dependent activation of FAK, MAPK, and Akt, followed by the suppression of MMP2 and MMP9. Inhibitors of both MAPK and Akt also significantly blocked the activities of these MMPs. Therefore, our results indicate that RhoC promotes tumor   metastasis   in prostate cancer by sequential activation of Pyk2, FAK, MAPK, and Akt followed by the up-regulation of MMP2 and MMP9, which results in the stimulation of invasiveness of tumor cells. 
 p38γ promotes breast cancer cell motility and   metastasi  s through regulation of RhoC GTPase, cytoskeletal architecture, and a novel leading edge behavior. Understanding the molecular alterations that confer cancer cells with motile, metastatic properties is needed to improve patient survival. Here, we report that p38γ motogen-activated protein kinase regulates breast cancer cell motility an  d metastas  is, in part, by controlling expression of th  e metastas  is-associated small GTPase RhoC. This p38γ-RhoC regulatory connection was mediated by a novel mechanism of modulating RhoC ubiquitination. This relationship persisted across multiple cell lines and in clinical breast cancer specimens. Using a computational mechanical model based on the finite element method, we showed that p38γ-mediated cytoskeletal changes are sufficient to control cell motility. This model predicted novel dynamics of leading edge actin protrusions, which were experimentally verified and established to be closely related to cell shape and cytoskeletal morphology. Clinical relevance was supported by evidence that elevated expression of p38γ is associated with lower overall survival of patients with breast cancer. Taken together, our results offer a detailed characterization of how p38γ contributes to breast cancer progression. Herein we present a new mechanics-based analysis of cell motility, and report on the discovery of a leading edge behavior in motile cells to accommodate modified cytoskeletal architecture. In summary, these findings not only identify a novel mechanism for regulating RhoC expression but also advance p38γ as a candidate therapeutic target. 
 Elevated expression of autocrine motility factor receptor correlates with overexpression of RhoC and indicates poor prognosis in hepatocellular carcinoma. Our previous study identified RhoC as an invasion and   metastasis   marker in hepatocellular carcinoma patients. Recent document suggested RhoGTPase is required for autocrine motility factor signaling. In this study, we questioned whether there is a correlation between expression of autocrine motility factor receptor and RhoC. Furthermore, we questioned whether elevated expression of autocrine motility factor correlates with   metastasis   and poor prognosis of HCC. The mRNA expression level of AMFR and RhoC were examined by RT-PCR in 25 cases of HCC and pericarcinomatous liver tissues (PCLT). In addition, the correlation between the expression level of AMFR and clinical pathologic parameters was analyzed. Furthermore, follow-up information was collected to evaluate the prognostic value of AMFR for HCC patients. Our results showed that the expression of AMFR and RhoC significantly increased in HCC compared with PCLT; extrahepatic metastatic lesions expressed significantly higher levels of AMFR and RhoC than corresponding intrahepatic HCC tissues. There is a highly significant correlation of AMFR expression levels with tumor vein invasion, number of tumor nodes, and tumor stage. Anticipatively, positive correlation was observed between mRNA expression of AMFR and RhoC gene. Furthermore, the HCC patients with high-expression of AMFR had significant high recurrence/  metastasis   and shorter survival than those with low-expression of AMFR. Together, our findings suggested a strong correlation between the expression of AMFR and RhoC and also a correlation between overexpression of them and invasion and   metastasis   of HCC. Furthermore, our data indicated AMFR as a potential prognosis for HCC. 
 Expression of G3BP and RhoC in esophageal squamous carcinoma and their effect on prognosis. To investigate the expression and significance of G3BP and RhoC proteins in esophageal squamous carcinoma (ESC).The expression of G3BP and Rhoc proteins in 80 cases of ESC was detected by immunohistochemistry. The relationship was studied between the expression of the two proteins and tumor size, differentiation degree, TNM stage, lymph node   metastasis   and prognosis of ESC.The positive expression rate of G3BP in ESC was 71.25%; and the rate in the lymph node   metastasis   group was significantly higher than that in the non-lymph node   metastasis   group (Z = -2.283, P = 0.022), but no relations were found between G3BP expression and tumor size, differentiation degree and TNM stage (P > 0.05). The group with G3BP positive expression had shorter survival time than the group with G3BP negative expression (P = 0.000). The positive expression rate of RhoC in ESC was 66.25%; and the rate in the lymph node   metastasis   group was significantly higher than that in the non-lymph node   metastasis   group (Z = -2.115, P < 0.05), but no relations were found between RhoC expression and tumor size, differentiation degree and TNM stage (P > 0.05). The RhoC positive expression group had a shorter survival time than the RhoC negative expression group (P < 0.001. The expression of G3BP protein correlated positively with the expression of RhoC in ESC tissues (rs = 0.656, P < 0.001).The expression of G3BP and RhoC protein is closely related to the lymph node   metastasis   and survival in ESC patients. G3BP and RhoC proteins can be considered as predictors of prognosis in ESC patients. 
 Inhibition of Ovarian Epithelial Carcinoma Tumorigenesis and Progression by microRNA 106b Mediated through the RhoC Pathway. Epithelial ovarian cancer (EOC) is the most lethal of the gynecological malignancies. Exploring the molecular mechanisms and major factors of invasion and   metastasis   could have great significance for the treatment and prognosis of EOC. Studies have demonstrated that microRNA 106b (miR-106b) may be a promising therapeutic target for inhibiting breast cancer bone   metastasis  , but the role of miR-106b in EOC is largely unknown. In this work, miRNA-106b expression was quantified in various ovarian tissues and tumors. Ovarian carcinoma cell lines were transfected with miR-106b, after which, cell phenotype and expression of relevant molecules was assayed. Dual-luciferase reporter assays and xenograft mouse models were also used to investigate miR-106b and its target gene. MiR-106b mRNA expression was found to be significantly higher in normal ovarian tissues and benign tumors than in ovarian carcinomas and borderline tumors (p < 0.01), and was negatively associated with differentiation (Well vs. Por & Mod) and the International Federation of Gynecology and Obstetrics (FIGO) staging (stage I/II vs. stage III/IV) in ovarian carcinoma (p < 0.05). MiR-106b transfection reduced cell proliferation; promoted G1 or S arrest and apoptosis (p < 0.05); suppressed cell migration and invasion (p < 0.05); reduced Ras homolog gene family member C (RhoC), P70 ribosomal S6 kinase (P70S6K), Bcl-xL, Matrix metallopeptidase 2 (MMP2), MMP9 mRNA and protein expression; and induced p53 expression (p < 0.05). Dual-luciferase reporter assays indicated that miR-106b directly targets RhoC by binding its 3'UTR. MiR-106b transfection also suppressed tumor development and RhoC expression in vivo in xenograft mouse models. This is the first demonstration that miR-106b may inhibit tumorigenesis and progression of EOC by targeting RhoC. The involvement of miR-106b-mediated RhoC downregulation in EOC aggression may give extended insights into molecular mechanisms underlying cancer aggression. Approaches aimed at overexpressing miR-106b may serve as promising therapeutic strategies for treating EOC patients. 
 miR-10b promotes cell invasion through RhoC-AKT signaling pathway by targeting HOXD10 in gastric cancer. MicroRNAs play critical roles in tumorigenesis as either oncogenes or tumor suppressors. As a microRNA induced by Twist, miR-10b function as a   metastasis   driver in different types of cancer, in which the downstream target gene HOXD10 is the main mediator. In gastric tumor species, miR-10b levels were dramatically elevated in lymphoma node   metastasis  -positive tumor tissues compared with lymphoma node   metastasis  -free tumor tissues, and were correlated to dowregulation of HOXD10 expression. In gastric cell lines with distinct degrees of differentiation, miR-10b was highly expressed in the cell line with strong metastatic ability. In MNK45 cells, inhibition of miR-10b led to abrogation of cell invasion. While in GES-1 cells, miR-10 overexpression resulted in enhancement of invasiveness through translational inhibition of HOXD10, and constitutive expression of HOXD10 reversed the effects of miR-10b on cell invasion. Furthermore, either knockdown of RhoC or inhibition of AKT activation interfered miR-10-induced invasiveness in GES-1 cells. In summary, these observations suggest that miR-10b can stimulate the upregulation of RhoC and AKT phosphorylation through targeting HOXD10, thus promoting cell invasion in gastric tumors. 
 The role of RhoC in epithelial-to-mesenchymal transition of ovarian carcinoma cells. RhoC is a small G protein/GTPase and involved in tumor mobility, invasion and   metastasis  . Previously, up-regulated RhoC expression is found to play an important role in ovarian carcinogenesis and subsequent progression by modulating proliferation, apoptosis, migration and invasion.We transfected RhoC-expressing plasmid and RhoC siRNA into CAOV3 and OVCAR3 cells respectively. These cells and transfectants were exposed to vascular epithelial growth factor (VEGF), transforming growth factor (TGF)-β1 or their receptor inhibitors with the phenotypes and their related-molecules examined.TGF-β1R or VEGFR inhibitor suppressed the proliferation, migration, invasion and lamellipodia formation, the expression of N-cadherin, α-SMA, snail and Notch1 mRNA or protein, and enhanced E-cadherin mRNA and protein expression in CAOV3 and its RhoC-overexpressing transfectants, whereas both growth factors had the opposite effects in OVCAR3 cells and their RhoC-hypoexpressing transfectants. Ectopic RhoC expression enhanced migration, invasion, lamellipodia formation and the alteration in epithelial to mesenchymal transition (EMT) markers of CAOV3 cells regardless of the treatment of VEGFR or TGF-β1R inhibitor, whereas RhoC knockdown resulted in the converse in OVCAR3 cells even with the exposure to VEGF or TGF-β1.RhoC expression might be involved in EMT of ovarian epithelial carcinoma cells, stimulated by TGF-β1 and VEGF. 
 The role of RhoC in ovarian epithelial carcinoma: a marker for carcinogenesis, progression, prognosis, and target therapy. Ras homolog gene family member C (RhoC) is a small G protein/guanosine triphosphatase involved in tumor mobility, invasion, and   metastasis  .After RhoC siRNA transfection, we measured the changes in phenotypes and some relevant molecules in ovarian carcinoma cell, OVCAR3. The mRNA and protein expression of RhoC was detected in ovarian tumors.RhoC siRNA transfection resulted in low growth, G1 arrest, and apoptotic induction in the OVCAR3 in comparison with the control and mock. Following RhoC knockdown, there was reduced mRNA or protein expression of protein kinase B (Akt), signal transducer and activator of transcription 3 (stat3), bcl-xL, surviving and phosphorylated p70S6 kinase (p-p70s6k), while the converse was true for Bax and caspase-3. Lovastatin induced apoptosis, suppressed proliferation, migration and invasion, and disrupted lamellipodia formation in OVCAR3. Lovastatin exposure induced lower RhoC, bcl-2, matrix metalloproteinase-9 (MMP-9), survivin, Akt, bcl-xL, vascular endothelial growth factor (VEGF), and p-p70s6k expression in OVCAR3 compared to the control, but higher caspase-3 and Bax expression. RhoC mRNA and protein expression was significantly higher in ovarian carcinoma than in benign tumors and normal ovary tissue (p<0.05) and was positively associated with dedifferentiation, FIGO staging and p-p70s6k expression of ovarian carcinoma (p<0.05).The up-regulated RhoC expression may affect ovarian carcinogenesis and should be considered a good biomarker for the differentiation and progression of ovarian carcinoma. RhoC plays an important role in apoptosis by modulating the relevant genes and the phosphorylation of downstream p70s6k. 
 RhoC GTPase expression as a potential marker of lymph node   metastasis   in squamous cell carcinomas of the head and neck. Survival rates for squamous cell carcinoma of the head and neck (SCCHN) have remained unchanged for several decades due to local tumor recurrences as well as regional and distant metastases. Recent evidence has shown that RhoC GTPase is overexpressed in stages III and IV regionally metastatic SCCHN compared with stages I and II localized disease. This study evaluated the expression of RhoC in head and neck carcinoma and investigated the prognostic use of this marker on a large cohort of previously untreated patients with SCCHN.Standard Western blot techniques were used to evaluate RhoC protein expression in nine established head and neck cancer cell lines and in normal oral epithelium. In vivo expression of RhoC in metastatic and nonmetastatic SCCHN was investigated using immunohistochemical analysis on a tissue microarray composed of 113 independent tumor samples. RhoC expression was analyzed as it related to clinical and pathologic variables of interest.Levels of RhoC protein were increased in the SCCHN cell lines compared with normal oral epithelium. The in vivo expression of RhoC correlated with advanced clinical stage and lymph node metastases for the entire patient cohort as well as in small primary tumors (T(1) and T(2)).This study is the first to examine the expression of RhoC GTPase protein in SCCHN and normal squamous epithelium. It is clear from the results that RhoC is a specific marker of lymph node metastases in patients with this challenging form of carcinoma. RhoC levels seem to identify a subset of patients with early tumor stage primary tumors and high metastatic potential that might benefit from more aggressive therapy. Through continued investigation, blockade of RhoC activity may be a potential target in the development of novel strategies for treating metastases of head and neck cancer. 
 Effects and mechanism of RhoC downregulation in suppressing ovarian cancer stem cell proliferation, drug resistance, invasion and   metastasis  . Cancer stem cells are considered to be the root cause of tumor initiation,   metastasis  , recurrence and therapeutic resistance. Recent studies have reported that RhoC plays a critical role in regulating cancer stem cells; however, its function in ovarian cancer stem cells (OCSCs) remains unknown. The ovarian cancer cell line A2780, and the paclitaxel-resistant A2780 cell line (A2780-PTX) were obtained. A2780 cells were used to isolate and identify the highly invasive A2780-PM cells, and A2780-PTX cells were used to isolate and identify the highly drug-resistant and highly invasive A2780-PTX-PM cells by Transwell assay. MTT, Transwell and wound healing assays were used to compare the differences in cell proliferation, invasion and migration ability among the four cell lines. Immunofluorescence was used to detect the expression of stem cell markers CD117 and CD133. OCSCs were sorted by flow cytometry. Following si-RhoC transfection of the OCSCs, cell proliferation, drug resistance, invasion and migration ability and RhoC, CD117 and CD133 expression levels were assayed. RT-PCR was used to assess RhoC, CD117, CD133 and matrix metalloproteinase 9 (MMP9) mRNA expression levels. A2780-PM and A2780‑PTX-PM cells exhibited higher cell proliferation, drug resistance, and invasion and migration ability than the A2780 and A2780-PTX cell lines. Furthermore, CD133 and CD117 expression levels were higher in the A2780-PM and A2780‑PTX-PM cells than levels in the A2780 and A2780-PTX cells. Transfection of si-RhoC in OCSCs suppressed the proliferation, drug resistance, invasion, migration and CD117 and CD133 expression levels. Furthermore, the expression levels of RhoC, CD117, CD133, MDR1, and MMP9 mRNA were downregulated in the transfected population. Taken together, our results demonstrated that RhoC downregulation may inhibit the proliferation, drug resistance, invasion and migration of OCSCs, and RhoC may play an important role in the formation of OCSCs. 
 [The expression of RhoC and Ki67 in cervical intraepithelial neoplasia and squamous carcinoma of cervix]. To detect the expression of RhoC and Ki67 in squamous carcinoma of cervix and to reveal the correlation of RhoC and Ki67 with clinic pathological parameters of cervix carcinoma.The expressions of RhoC and Ki67 were evaluated in 26 cases of cervical intraepithelial neoplasia (CIN) and 57 cases of squamous carcinoma of cervix(SCC) by immunohistochemistry, while 14 cases of normal cervical epithelium(NCE) were taken as control.Immunohistochemical staining demonstrated RhoC expression in 82.46% (47/57) of SCC, 15.38% (2/13) of CIN II/III, and negative expression in NCE and CIN I. The positive rate of RhoC expression in SCC was significantly higher than that in NCE and CIN (P < 0.05). The expression of RhoC in SCC was significantly correlated with pelvic lymph node   metastasis   and depth of stroma infiltration, but not correlated with age, FIGO staging,histologic grade and vascular space involvement. The positive rates of Ki67 expression in NCE, CIN I, CIN II/III and SCC were 28.57%, 38.46%, 100% and 96.49% respectively. The positive rates of Ki67 expression in SCC and CIN II/III were significantly higher than those in NCE and CIN I (P < 0.05). The expression of Ki67 in SCC was not correlated with the major clinic pathological parameters. There was no obvious relationship between the expression of RhoC gene and Ki67 antigen in SCC (P > 0. 05).The overexpression of RhoC may play an important role in the invasion and   metastasis   of SCC. RhoC may be a potential marker to identify SCC patients with high risk of invasion and   metastasis  . 
 The role of RhoC in growth and metastatic capacity of melanoma. RhoC overexpression in tumor cells promotes invasive and metastatic behavior. RhoC expression levels have been correlated with tumor progression and   metastasis   in multiple human cancers. In melanoma, RhoC is upregulated in highly metastatic tumors. Induced expression in melanoma cell lines resulted in invasion and   metastasis  , whereas inhibition of RhoC reversed the metastatic phenotype both in vitro and in vivo.RhoC mRNA and protein expression in two human melanoma cell lines (DX3aza and MeWo) and pooled primary melanocytes were investigated by means of real-time quantitative polymerase chain reaction and western blotting. RhoC protein expression was evaluated in 123 primary cutaneous melanoma samples by the use of immunohistochemistry and correlated with known prognostic features.RhoC upregulation was observed in the highly metastatic DX3aza cell line, whereas in MeWo, only low expression levels could be detected. RhoC expression in primary cutaneous melanoma was strongly associated with thicker and ulcerated tumors. RhoC expression was associated with the presence of lymphatic metastases at the time of diagnosis and shorter disease-free and overall survival rates, without being an independent predictor.These results further support a role for RhoC in growth and   metastasis   of melanoma. 
 "Smart" Nanoparticles Enhance the Cytoplasmic Delivery of Anti-RhoC Silencing RNA and Inhibit the Migration and Invasion of Aggressive Breast Cancer Cells. Rho-GTPases are small GTP-binding proteins that contribute to the epithelial-to-mesenchymal transition by regulating several cellular processes including organization of the actin cytoskeleton, cell motility, transcription, and cell proliferation. Overexpression of RhoC-GTPases (RhoC) in breast cancer has been implicated in poor disease prognosis due to increased cancer cells invasion, migration, and motility, which warranted its consideration as a therapeutic target for inhibiting breast cancer   metastasis  . Using silencing RNA (siRNA) molecules to knockdown RhoC expression is a promising approach to inhibit breast cancer metastases. However, transforming anti-RhoC siRNA molecules into a viable therapy remains a challenge due to the lack of a biocompatible carrier that can selectively deliver the RNA cargo into breast cancer cells. We report the use of a degradable, pH-sensitive, β-cyclodextrin (βCD)-based polymeric carrier that condenses anti-RhoC siRNA forming "smart" particles. These smart anti-RhoC particles were efficiently internalized, successfully escaped the endosome, and delivered the RNA cargo into the cytoplasm of SUM149 and MDA-MB-231 breast cancer cells. Our results show that anti-RhoC particles used at a low N/P ratio of 2.5/1 suppressed RhoC protein levels by 100% and 90% in SUM149 and MDA-MB-231 cells, respectively. Further, anti-RhoC particles inhibited the invasion, motility, and migration of SUM149 and MDA-MB-231 cells by 40-47%, 57-60%, and 61.5-73%, respectively. Smart particles encapsulating the scrambled siRNA sequence did not affect RhoC protein expression or the invasion, motility, and migration of SUM149 and MDA-MB-231 cells, which indicate the biocompatibility of the polymeric carrier and selectivity of the observed RhoC knockdown. These results collectively indicate the therapeutic potential of smart anti-RhoC particles in arresting the metastatic spread of breast cancer cells. 
 [Expression of RhoC and ROCK-1 and their effects on MAPK and Akt proteins in prostate carcinoma]. To detect the expression of RhoC and Rho kinase 1 (ROCK-1) in prostate carcinoma, and explore the possible mechanism of RhoC/ROCK-1 in the pathogenesis of prostate carcinoma.Tissue specimens from 73 patients with prostate carcinoma and corresponding paracancerous tissues were obtained by prostate cancer biopsy or radical prostatectomy. The expression of RhoC/ROCK-1 mRNA was detected by RT-PCR. Western blot and immunohistochemistry were performed to dertect the expression of RhoC/ROCK-1 protein. Eukaryotic expression plasmids of RhoC were constructed and transfected into PC-3M-2B4 cells. p-MAPK and p-Akt were detected by Western bolt.The expression levels of RhoC and ROCK-1 mRNA in the prostate carcinomas were significantly higher than those in corresponding paracancerous tissues [72.6% (53/73) vs. 34.2% (25/73); 68.5% (50/73) vs. 38.4% (28/73), P < 0.01], respectively. The results indicated that RhoC/ROCK-1 mRNA expression had no significant correlation with Gleason grade. However, the expression of RhoC/ROCK-1 mRNA showed a significant positive correlation with distant   metastasis  . The RhoC/ROCK-1 protein expression in prostate cancer was also higher than corresponding paracancerous tissues, and showed a significant positive correlation with p-MAPK and p-Akt expression levels. In addition, p-MAPK and p-Akt expression levels were up-regulated in the transcripts.Expression levels of RhoC and ROCK-1 in prostate carcinoma are higher than those in corresponding paracancerous tissues, showing a significant positive correlation with distant   metastasis  . RhoC/ROCK-1 may be involved in the development, invasion and   metastasis   of prostate carcinoma. 
 Inhibition of invasion and   metastasis   of hepatocellular carcinoma cells via targeting RhoC in vitro and in vivo. Hepatocellular carcinoma (HCC) is one of the most deadly human cancers because of its high incidence of   metastasis  . Our previous work identified a strong correlation between increased expression of RhoC and HCC   metastasis  . Here, we investigate to define the role of RhoC in HCC   metastasis  . Furthermore, we sought to determine whether inhibition of the expression of RhoC might block the   metastasis   of HCC in vivo.A stable retroviral small interfering RNA approach was employed to selectively knockdown the expression of RhoC in vitro and in vivo. Invasion and migration assay, MTT and fluorescence-activated cell sorting analysis, Rho activity assay, and immunofluorescence staining were carried out to characterize RhoC in vitro. An anti-RhoC retroviral gene delivery BALB/c nude mice model was established to investigate whether knockdown of the expression of RhoC might inhibit the   metastasis   of HCC in vivo.We confirmed the correlation of RhoC expression and metastatic potentials of HCC cell lines. We also showed that suppression of RhoC expression resulted in inhibition of invasion and migration without an apparent effect on cell survival and proliferation in HCCLM3 cells. Furthermore, a similar effect of RhoC on autotaxin-induced invasion of HCCLM3 cells was also observed. Significantly, we successfully adopted an HCC metastatic mouse model that allowed us to show that knockdown of the RhoC expression resulted in inhibition of   metastasis   of HCC in vivo for the first time.Our results show a critical role of RhoC in   metastasis   of HCC, implicating RhoC as a potential therapeutic target to block HCC   metastasis  . 
 [Correlation of expression of RhoC with invasiveness of breast cancer cells in vitro]. To investigate the expression of RhoC in breast cancer cells with different metastatic potential and its correlation with invasiveness.Expression of RhoC mRNA and protein in human breast cancer cells MCF-7 with low metastatic potential and MDA-MB-231 with high metastatic potential was detected by RT-PCR, Western blot, immunohistochemistry and immunofluorescence staining. Eukaryotic expression plasmids of RhoC were constructed and transfected into MCF-7 cells. The biological effects were observed, including in vitro invasion by Boyden charmber assay, motility by would healing assay, alteration of microfilament network by TRTIC-phalloidin staining and expression of p-Akt by Western blot assay.The expression levels of RhoC mRNA and protein varied in the two different metastatic breast cancer cell lines. RhoC was significantly up-regulated in the highly metastatic cells in comparison to the weakly metastatic counterpart (P < 0.01). As shown by Boyden charmber assay, the invasive capacity of transfected cells overexpressing RhoC was significantly promoted as reflected by more penetrating cells (56.88 +/- 4.18) than that of the antisense transcripts (23.12 +/- 3.22), the negative (23.77 +/- 3.64) and blank controls (28.44 +/- 2.48). Further study by would healing assay indicated that cells overexpressing RhoC were more motile in actin-based active movement. The wound healing ratio after 24 h of the sense transcripts, antisense transcripts, negative controls and blank controls was 58.28% +/- 2.14%, 22.36% +/- 2.73%, 28.23% +/- 2.62%, 30.18% +/- 2.86%, respectively. The TRITC-phalloidin staining revealed less actin filament bundles and a reorganized cytoskeleton within the sense transcripts. In addition, p-Akt expression level was upregulated in the sense transcripts.RhoC overexpression may promote the invasive capacity of human breast cancer cells in vitro and its expression level is positively correlated with the metastatic capacity of those cells. So RhoC may be a potential target in the development of a novel strategy for treating   metastasis   of breast cancer. 
 Formin-like 2 drives amoeboid invasive cell motility downstream of RhoC. Invasive cell migration is a key step for cancer   metastasis   and involves Rho GTPase-controlled reorganization of the actin cytoskeleton. Altered Rho GTPase expression is found in various malignancies. Particularly, the closely related GTPases RhoA and RhoC are upregulated in many aggressive tumours, but specific effectors that distinguish between these two GTPases to explain mechanistic differences have not been identified. The formins are by far the largest family of Rho GTPase effectors and are characterized by the actin-nucleating formin homology 2 domain. Using siRNA-based screening against all 15 human formins, we systematically analysed their functions in 3D cell motility using three different cancer cell lines. These results reveal distinct requirements for specific formins in amoeboid versus mesenchymal invasive cell migration. Importantly, by knocking down all Rho proteins, we identified formin-like 2 (FMNL2) as a specific RhoC effector, showing selective interaction of FMNL2 with active RhoC, but not RhoA or RhoB. Functional analysis shows that RhoC regulates autoinhibition of FMNL2, whereas suppression of FMNL2 inhibits RhoC-, but not RhoA-dependent, rounded invasive cell migration. Thus, our data uncover a novel regulatory and functional interaction between RhoC and FMNL2 for modulating cell shape and invasiveness and provide mechanistic insight into RhoC-specific signalling events. 
 RhoC expression and head and neck cancer   metastasis  . RhoC protein, a known marker of metastases in aggressive breast cancers and melanoma, has also been found to be overexpressed in certain head and neck cancers, thus we investigated the correlation between RhoC expression and the metastatic behavior of head and neck squamous cell carcinoma. Selective inhibition of RhoC expression was achieved using lentiviral small hairpin RNA (shRNA) transduced and tracked with green fluorescent protein to achieve 70% to 80% RhoC inhibition. Fluorescence microscopy of the RhoC knockdown stable clones showed strong green fluorescence in the majority of cells, signifying a high efficiency of transduction. Importantly, quantitative real-time PCR showed no significant decrease in the mRNA expression levels of other members of the Ras superfamily. Cell motility and invasion were markedly diminished in RhoC-depleted cell lines as compared with control transduced lines. H&E staining of lung tissue obtained from severe combined immunodeficiency mice, which had been implanted with RhoC knockdown cells, showed a marked decrease in lung   metastasis   and inflammation of the blood vessels. The cultured lung tissue showed a significant decrease in cell growth in mice implanted with RhoC-depleted cell lines as compared with shRNA-scrambled sequence control lines. Microscopic studies of CD31 expression revealed substantial quantitative and qualitative differences in the primary tumor microvessel density as compared with parental and shRNA-scrambled controls. This study is the first of its kind to establish the involvement of RhoC specifically in head and neck   metastasis  . These findings suggest that RhoC warrants further investigation to delineate its robustness as a novel potentially therapeutic target. 
 Expression of RHOC is associated with   metastasis   of gastric carcinomas. RHOC, a member of the RAS-related small GTPase protein family, regulates cytoskeletal structures and has the potential to transform cultured cells. It has recently been reported that RHOC contributes to the metastatic phenotype of melanoma cells. The purpose of this study was to clarify its biological relevance to gastric carcinogenesis and   metastasis  .We examined the expression of RHOC by quantitative RT-PCR in 51 cases of gastric carcinoma tissues from prior surgical cases (intestinal type: 24 cases, diffuse type: 27 cases) and in 8 gastric carcinoma cell lines.RHOC expression levels in primary tumors were significantly higher in cases with   metastasis   than in those without   metastasis   (p = 0.0202; Mann-Whitney U test). RHOC expression levels in primary tumor and their metastatic tumors were significantly higher than their corresponding nonneoplastic mucosa (p = 0.0357, and 0.0173, respectively; Wilcoxon signed rank test). RHOC mRNA expression was confirmed in the gastric carcinoma cell lines.Our findings suggest that elevated expression of the RHOC gene may be involved in the   metastasis   of gastric carcinomas and may be a good genetic marker for the prediction of a metastatic potential. 
 RhoC impacts the metastatic potential and abundance of breast cancer stem cells. Cancer stem cells (CSCs) have been shown to promote tumorigenesis of many tumor types, including breast, although their relevance to cancer   metastasis   remains unclear. While subpopulations of CSCs required for   metastasis   have been identified, to date there are no known molecular regulators of breast CSC (BCSC)   metastasis  . Here we identify RhoC GTPase as an important regulator of BCSC   metastasis  , and present evidence suggesting that RhoC also modulates the frequency of BCSCs within a population. Using an orthotopic xenograft model of spontaneous   metastasis   we discover that RhoC is both necessary and sufficient to promote SUM149 and MCF-10A BCSC   metastasis  --often independent from primary tumor formation--and can even induce   metastasis   of non-BCSCs within these cell lines. The relationship between RhoC and BCSCs persists in breast cancer patients, as expression of RhoC and the BCSC marker ALDH1 are highly correlated in clinical specimens. These results suggest new avenues to combating the deadliest cells driving the most lethal stage of breast cancer progression. 
  GeneRIFs  
 miR-509 has a critical role in brain   metastasis   of breast cancer by modulating the RhoC-TNF-alpha network. 
 RhoC facilitates tumor cell invasion and promotes subsequent   metastasis  , in part, by enhancing integrin alpha5beta1 trafficking 
 Data demonstrate that PKN3 physically interacts with Rho-family GTPases, and preferentially with RhoC, a known mediator of tumor invasion and   metastasis   in epithelial cancers. 
 Expression levels of RhoC and ROCK-1 in prostate carcinoma are higher than those in corresponding paracancerous tissues, showing a significant positive correlation with distant   metastasis  . 
 p38gamma mitogen-activated protein kinase regulates breast cancer cell motility and   metastasis  , in part, by controlling expression of the   metastasis  -associated small GTPase RhoC. 
 A p27(kip1)-binding protein, p27RF-Rho, promotes cancer   metastasis   via activation of RhoA and RhoC. 
 The overexpression of RhoC may play an important role in the invasion and   metastasis   of squamous carcinoma of cervix. 
 The significantly up-regulated RhoC expression suggest that RhoC may contribute to the initiation, development, invasion and   metastasis   of colorectal carcinoma in Chinese patients. 
 These results further support a role for RhoC in growth and   metastasis   of melanoma 
 knockdown of the RhoC expression resulted in inhibition of   metastasis   of HCC in vivo for the first time. 
 Results indicate that RhoC promotes tumor   metastasis   in prostate cancer by sequential activation of Pyk2, FAK, MAPK, and Akt followed by the up-regulation of MMP2 and MMP9, which results in the stimulation of invasiveness of tumor cells. 
 RhoC may play a very important role in the   metastasis   of gastric carcinoma. Therapeutic strategies targeting RhoC and RhoC-mediated pathways may be a novel approach for treating   metastasis   of gastric cancer. 
 The expression of G3BP and RhoC protein is closely related to the lymph node   metastasis   and survival in esophageal squamous carcinoma (ESC) patients. G3BP and RhoC proteins can be considered as predictors of prognosis in ESC patients. 
 Findings suggest a strong correlation between the expression of autocrine motility factor receptor (AMFR) and RhoC and a correlation between overexpression of them and   metastasis   of hepatocellular carcinoma. 
 elevated expression of the RHOC gene may be involved in the   metastasis   of gastric carcinomas and may be a good genetic marker for the prediction of a metastatic potential 
 The RhoC gene may be related to malignant transformation and development of HCC and may play an important role in the invasion and   metastasis   of HCC by overexpression but not mutation. 
  Summary  
 This gene encodes a member of the Rho family of small GTPases, which cycle between inactive GDP-bound and active GTP-bound states and function as molecular switches in signal transduction cascades. Rho proteins promote reorganization of the actin cytoskeleton and regulate cell shape, attachment, and motility. The protein encoded by this gene is prenylated at its C-terminus, and localizes to the cytoplasm and plasma membrane. It is thought to be important in cell locomotion. Overexpression of this gene is associated with tumor cell proliferation and   metastasis  . Multiple alternatively spliced variants, encoding the same protein, have been identified. [provided by RefSeq, Jul 2008] 
  [Cc]ell [Ii]nvasion  PubMed  
 Divergence of Rho residue 43 impacts GEF activity. RhoA, RhoB and RhoC GTPases are over 85% identical at the amino acid level, with RhoA and RhoC differing at only one residue (43) across the initial two-thirds of their sequences. A putative regulatory distinction between the molecules is their capacity to be uniquely activated by guanine nucleotide exchange factors (GEFs). We hypothesize that variation of amino acid residue 43 between RhoA/B (valine) and RhoC (isoleucine) impacts GEF activity. Direct participation of residue 43 in GEF-catalyzed exchange was confirmed by the observation that mutation of this position to a threonine reduced GEF-catalyzed nucleotide exchange activity in vitro (Vav2, XPLN, GEFT, Dbl and Dbs) and greatly depressed RhoA and RhoC GTP-loading profiles in cell lysates. Using a residue swap approach, substitution of RhoA Val 43 with an Ile was found to significantly promote basal nucleotide exchange activity and enhance GTP-loading in cells. Substitution of Val 43 with an Ile in RhoB negatively affected nucleotide exchange in vitro. Substitution of RhoC Ile 43 with a Val increased GEF-catalyzed exchange in vitro. In addition, RhoC-I43V was more efficacious at driving ovarian cancer   cell invasion   through matrigrel than wild-type RhoC, RhoC-I43T, wild-type RhoA, RhoA-V43I or RhoA-V43T GTPases. These findings suggest that a divergence between RhoA/B and RhoC at residue 43 impacts basal and GEF-stimulated nucleotide exchange activity. 
 The MLK-related kinase (MRK) is a novel RhoC effector that mediates lysophosphatidic acid (LPA)-stimulated tumor   cell invasion  . The small GTPase RhoC is overexpressed in many invasive tumors and is essential for metastasis. Despite its high structural homology to RhoA, RhoC appears to perform functions that are different from those controlled by RhoA. The identity of the signaling components that are differentially regulated by these two GTPases is only beginning to emerge. Here, we show that the MAP3K protein MRK directly binds to the GTP-bound forms of both RhoA and RhoC in vitro. However, siRNA-mediated depletion of MRK in cells phenocopies depletion of RhoC, rather than that of RhoA. MRK depletion, like that of RhoC, inhibits LPA-stimulated   cell invasion  , while depletion of RhoA increases invasion. We also show that active MRK enhances LPA-stimulated invasion, further supporting a role for MRK in the regulation of invasion. Depletion of either RhoC or MRK causes sustained myosin light chain phosphorylation after LPA stimulation. In addition, activation of MRK causes a reduction in myosin light chain phosphorylation. In contrast, as expected, depletion of RhoA inhibits myosin light chain phosphorylation. We also present evidence that both RhoC and MRK are required for LPA-induced stimulation of the p38 and ERK MAP kinases. In conclusion, we have identified MRK as a novel RhoC effector that controls LPA-stimulated   cell invasion   at least in part by regulating myosin dynamics, ERK and p38. 
 Targeted disruption of protein kinase C epsilon reduces   cell invasion   and motility through inactivation of RhoA and RhoC GTPases in head and neck squamous cell carcinoma. Over 70% of patients with head and neck squamous cell carcinoma (HNSCC) present with locoregionally advanced stage III and IV disease. In spite of aggressive therapy, locoregional disease recurs in 60% and metastatic disease develops in 15% to 25% of patients causing a major decline in quality and length of life. Therefore, there is a need to identify and understand genes that are responsible for inducing an aggressive HNSCC phenotype. Evidence has shown that protein kinase C (PKC) epsilon is a transforming oncogene and may play a role in HNSCC progression. In this study, we determine the downstream signaling pathway mediated by PKC epsilon to promote an aggressive HNSCC phenotype. RNA interference knockdown of PKC epsilon in UMSCC11A and UMSCC36, two highly invasive and motile HNSCC cell lines with elevated endogenous PKC epsilon levels, resulted in cells that were significantly less invasive and motile than the small interfering RNA-scrambled control transfectants; 51 +/- 5% (P < 0.006) and 49 +/- 3% (P < 0.010) inhibition in invasion and 69 +/- 1% (P < 0.0005) and 66 +/- 3% (P < 0.0001) inhibition in motility, respectively. PKC epsilon-deficient UMSCC11A clones had reduced levels of active and serine-phosphorylated RhoA and RhoC. Moreover, constitutive active RhoA completely rescued the invasion and motility defect, whereas constitutive active RhoC completely rescued the invasion and partially rescued the motility defect of PKC epsilon-deficient UMSCC11A clones. These results indicate that RhoA and RhoC are downstream of PKC epsilon and critical for PKC epsilon-mediated   cell invasion   and motility. Our study shows, for the first time, that PKC epsilon is involved in a coordinated regulation of RhoA and RhoC activation, possibly through direct post-translational phosphorylation. 
 Characterization of the roles of RHOC and RHOA GTPases in invasion, motility, and matrix adhesion in inflammatory and aggressive breast cancers. The 2 closely related small GTPases, RHOC and RHOA, are involved in mammary gland carcinogenesis; however, their specific roles in determining cancer cell adhesion and invasion have not been elucidated.RHOA and RHOC are highly homologous, thereby posing a major challenge to study their individual functions in cancer cells. By selectively knocking down these proteins, we have been able to alternatively inhibit RHOC and RHOA, while preserving expression of the other rho protein. Quantitative analyses of the growth patterns and invasion in the aggressive estrogen receptor negative cell lines MDA-231 and SUM149 were carried out on collagen I and Matrigel substrates.RHOC, and not RHOA, modulates surface expression and colocalization of alpha2 and beta1 integrins in MDA-MB-231 on collagen I. Neither RHOC or RHOA affected integrin expression in the inflammatory breast cancer cell line SUM149, further highlighting the different regulation of adhesion and motility in inflammatory breast cancer.This work shows that RHOC and RHOA play different roles in cell-matrix adhesion, motility, and invasion of MDA-MB-231 and reaffirms the crucial role of RHOC-GTPase in inflammatory breast cancer   cell invasion  . 
 The MLK-related kinase (MRK) is a novel RhoC effector that mediates lysophosphatidic acid (LPA)-stimulated tumor   cell invasion  . The small GTPase RhoC is overexpressed in many invasive tumors and is essential for metastasis. Despite its high structural homology to RhoA, RhoC appears to perform functions that are different from those controlled by RhoA. The identity of the signaling components that are differentially regulated by these two GTPases is only beginning to emerge. Here, we show that the MAP3K protein MRK directly binds to the GTP-bound forms of both RhoA and RhoC in vitro. However, siRNA-mediated depletion of MRK in cells phenocopies depletion of RhoC, rather than that of RhoA. MRK depletion, like that of RhoC, inhibits LPA-stimulated   cell invasion  , while depletion of RhoA increases invasion. We also show that active MRK enhances LPA-stimulated invasion, further supporting a role for MRK in the regulation of invasion. Depletion of either RhoC or MRK causes sustained myosin light chain phosphorylation after LPA stimulation. In addition, activation of MRK causes a reduction in myosin light chain phosphorylation. In contrast, as expected, depletion of RhoA inhibits myosin light chain phosphorylation. We also present evidence that both RhoC and MRK are required for LPA-induced stimulation of the p38 and ERK MAP kinases. In conclusion, we have identified MRK as a novel RhoC effector that controls LPA-stimulated   cell invasion   at least in part by regulating myosin dynamics, ERK and p38. 
 Divergence of Rho residue 43 impacts GEF activity. RhoA, RhoB and RhoC GTPases are over 85% identical at the amino acid level, with RhoA and RhoC differing at only one residue (43) across the initial two-thirds of their sequences. A putative regulatory distinction between the molecules is their capacity to be uniquely activated by guanine nucleotide exchange factors (GEFs). We hypothesize that variation of amino acid residue 43 between RhoA/B (valine) and RhoC (isoleucine) impacts GEF activity. Direct participation of residue 43 in GEF-catalyzed exchange was confirmed by the observation that mutation of this position to a threonine reduced GEF-catalyzed nucleotide exchange activity in vitro (Vav2, XPLN, GEFT, Dbl and Dbs) and greatly depressed RhoA and RhoC GTP-loading profiles in cell lysates. Using a residue swap approach, substitution of RhoA Val 43 with an Ile was found to significantly promote basal nucleotide exchange activity and enhance GTP-loading in cells. Substitution of Val 43 with an Ile in RhoB negatively affected nucleotide exchange in vitro. Substitution of RhoC Ile 43 with a Val increased GEF-catalyzed exchange in vitro. In addition, RhoC-I43V was more efficacious at driving ovarian cancer   cell invasion   through matrigrel than wild-type RhoC, RhoC-I43T, wild-type RhoA, RhoA-V43I or RhoA-V43T GTPases. These findings suggest that a divergence between RhoA/B and RhoC at residue 43 impacts basal and GEF-stimulated nucleotide exchange activity. 
 Characterization of the roles of RHOC and RHOA GTPases in invasion, motility, and matrix adhesion in inflammatory and aggressive breast cancers. The 2 closely related small GTPases, RHOC and RHOA, are involved in mammary gland carcinogenesis; however, their specific roles in determining cancer cell adhesion and invasion have not been elucidated.RHOA and RHOC are highly homologous, thereby posing a major challenge to study their individual functions in cancer cells. By selectively knocking down these proteins, we have been able to alternatively inhibit RHOC and RHOA, while preserving expression of the other rho protein. Quantitative analyses of the growth patterns and invasion in the aggressive estrogen receptor negative cell lines MDA-231 and SUM149 were carried out on collagen I and Matrigel substrates.RHOC, and not RHOA, modulates surface expression and colocalization of alpha2 and beta1 integrins in MDA-MB-231 on collagen I. Neither RHOC or RHOA affected integrin expression in the inflammatory breast cancer cell line SUM149, further highlighting the different regulation of adhesion and motility in inflammatory breast cancer.This work shows that RHOC and RHOA play different roles in cell-matrix adhesion, motility, and invasion of MDA-MB-231 and reaffirms the crucial role of RHOC-GTPase in inflammatory breast cancer   cell invasion  . 
 Targeted disruption of protein kinase C epsilon reduces   cell invasion   and motility through inactivation of RhoA and RhoC GTPases in head and neck squamous cell carcinoma. Over 70% of patients with head and neck squamous cell carcinoma (HNSCC) present with locoregionally advanced stage III and IV disease. In spite of aggressive therapy, locoregional disease recurs in 60% and metastatic disease develops in 15% to 25% of patients causing a major decline in quality and length of life. Therefore, there is a need to identify and understand genes that are responsible for inducing an aggressive HNSCC phenotype. Evidence has shown that protein kinase C (PKC) epsilon is a transforming oncogene and may play a role in HNSCC progression. In this study, we determine the downstream signaling pathway mediated by PKC epsilon to promote an aggressive HNSCC phenotype. RNA interference knockdown of PKC epsilon in UMSCC11A and UMSCC36, two highly invasive and motile HNSCC cell lines with elevated endogenous PKC epsilon levels, resulted in cells that were significantly less invasive and motile than the small interfering RNA-scrambled control transfectants; 51 +/- 5% (P < 0.006) and 49 +/- 3% (P < 0.010) inhibition in invasion and 69 +/- 1% (P < 0.0005) and 66 +/- 3% (P < 0.0001) inhibition in motility, respectively. PKC epsilon-deficient UMSCC11A clones had reduced levels of active and serine-phosphorylated RhoA and RhoC. Moreover, constitutive active RhoA completely rescued the invasion and motility defect, whereas constitutive active RhoC completely rescued the invasion and partially rescued the motility defect of PKC epsilon-deficient UMSCC11A clones. These results indicate that RhoA and RhoC are downstream of PKC epsilon and critical for PKC epsilon-mediated   cell invasion   and motility. Our study shows, for the first time, that PKC epsilon is involved in a coordinated regulation of RhoA and RhoC activation, possibly through direct post-translational phosphorylation. 
 Genomic analysis of metastasis reveals an essential role for RhoC. The most damaging change during cancer progression is the switch from a locally growing tumour to a metastatic killer. This switch is believed to involve numerous alterations that allow tumour cells to complete the complex series of events needed for metastasis. Relatively few genes have been implicated in these events. Here we use an in vivo selection scheme to select highly metastatic melanoma cells. By analysing these cells on DNA arrays, we define a pattern of gene expression that correlates with progression to a metastatic phenotype. In particular, we show enhanced expression of several genes involved in extracellular matrix assembly and of a second set of genes that regulate, either directly or indirectly, the actin-based cytoskeleton. One of these, the small GTPase RhoC, enhances metastasis when overexpressed, whereas a dominant-negative Rho inhibits metastasis. Analysis of the phenotype of cells expressing dominant-negative Rho or RhoC indicates that RhoC is important in tumour   cell invasion  . The genomic approach allows us to identify families of genes involved in a process, not just single genes, and can indicate which molecular and cellular events might be important in complex biological processes such as metastasis. 
 RhoC mediates epidermal growth factor-stimulated migration and invasion in head and neck squamous cell carcinoma. Epidermal growth factor receptor (EGFR) is overexpressed in head and neck squamous cell carcinoma (HNSCC) where it has been shown to promote tumor   cell invasion   upon phosphorylation. One mechanism by which EGFR promotes tumor progression is by activating signal cascades that lead to loss of E-cadherin, a transmembrane glycoprotein of the cell-cell adherence junctions; however mediators of these signaling cascades are not fully understood. One such mediator, RhoC, is activated upon a number of external stimuli, such as epidermal growth factor (EGF), but its role as a mediator of EGF-stimulated migration and invasion has not been elucidated in HNSCC. In the present study, we investigate the role of RhoC as a mediator of EGF-stimulated migration and invasion in HNSCC. We show that upon EGF stimulation, EGFR and RhoC were strongly activated in HNSCC. This resulted in activation of the phosphatidylinositol 3-Kinase Akt pathway (PI3K-Akt), phosphorylation of GSK-3β at the Ser(9) residue, and subsequent down regulation of E-cadherin cell surface expression resulting in increased tumor   cell invasio  n. Knockdown of RhoC restored E-cadherin expression and inhibited EGF-stimulated migration and invasion. This is the first report in HNSCC demonstrating the role RhoC plays in mediating EGF-stimulated migration and invasion by down-regulating the PI3K-Akt pathway and E-cadherin expression. RhoC may serve as a treatment target for HNSCC. 
 RhoC interacts with integrin α5β1 and enhances its trafficking in migrating pancreatic carcinoma cells. Human pancreatic ductal adenocarcinoma (PDAC) is characterized by early systemic dissemination. Although RhoC has been implicated in cancer cell migration, the relevant underlying molecular mechanisms remain unknown. RhoC has been implicated in the enhancement of cancer cell migration and invasion, with actions which are distinct from RhoA (84% homology), and are possibly attributed to the divergent C-terminus domain. Here, we confirm that RhoC significantly enhances the migratory and invasive properties of pancreatic carcinoma cells. In addition, we show that RhoC over-expression decreases cancer cell adhesion and, in turn, accelerates cellular body movement and focal adhesion turnover, especially, on fibronectin-coated surfaces. Whilst RhoC over-expression did not alter integrin expression patterns, we show that it enhanced integrin α5β1 internalization and re-cycling (trafficking), an effect that was dependent specifically on the C-terminus (180-193 amino acids) of RhoC protein. We also report that RhoC and integrin α5β1 co-localize within the peri-nuclear region of pancreatic tumor cells, and by masking the CAAX motif at the C-terminal of RhoC protein, we were able to abolish this interaction in vitro and in vivo. Co-localization of integrin α5β1 and RhoC was demonstrable in invading cancer cells in 3D-organotypic cultures, and further mimicked in vivo analyses of, spontaneous human, (two distinct sources: operated patients and rapid autopsy programme) and transgenic murine (LSL-KrasG12D/+;LSL-Trp53R172H/+;Pdx-1-Cre), pancreatic cancers. In both cases, co-localization of integrin α5β1 and RhoC correlated with poor differentiation status and metastatic potential. We propose that RhoC facilita  tes tumor cel  l invasion and promotes subsequent metastasis, in part, by enhancing integrin α5β1 trafficking. Thus, RhoC may serve as a biomarker and a therapeutic target. 
 RhoC mediates invasion and migration of CaSki cells through the Rho-associated serine-threonine protein kinase 1 signaling pathway. The small GTPase RhoC in human cancers is up-regulated and correlated with tumor metastasis. However, the role of Rho/Rho-associated serine-threonine protein kinase 1 (ROCK1) signaling pathway in human cervical cancer is still unclear. In this study, we examine the effects of RhoC and its major downstream target, ROCK1, on the invasion and migration of CaSki cells to investigate the role of RhoC/ROCK1 signaling pathway in the progression of cervical squamous cell carcinoma.RhoC and ROCK1 protein expression in CaSki cells was detected by Western blotting. Scratch and transwell assays were carried out to assess the effects of RhoC on invasion and migration of CaSki cells. Cell viability was assayed by MTT test after adding the ROCK1 inhibitor Y-27632 to CaSki cells.Overexpression of RhoC protein in CaSki cells significantly increases ROCK1 expression and promotes   cell invasion   and migration compared with the control group (P < 0.05). However, in the inhibition of ROCK1 with Y-27632 in CaSki cells when RhoC was overexpressed, the rate of invasiveness and migration was reduced remarkably (P < 0.05), dropping to comparable levels as the control.This study suggested that the activation of RhoC/ROCK1 signaling pathways is likely involved in the progression of cervical squamous cell carcinoma. 
 Knockdown of RHOC by shRNA suppresses invasion and migration of cholangiocellular carcinoma cells via inhibition of MMP2, MMP3, MMP9 and epithelial-mesenchymal transition. Ras homolog family member C (RHOC) is important during the progression of several types of cancer, including prostate, breast and hepatocellular carcinoma. However, the function of RHOC in cholangiocellular carcinoma (CCC), a highly recurrent and metastatic carcinoma with poor prognosis, remains unclear. The aim of the present study was to investigate the involvement of RHOC in CCC tumor progression. RHOC expression levels were examined in CCC tissues and cells, and adjacent nontumorous bile duct tissues. The effects and molecular mechanisms of RHOC expression on cell migration and invasion were also investigated. The current study demonstrated that RHOC protein was frequently overexpressed in human CCC specimens and CCC cell lines. Downregulation of RHOC inhibited CCC   cell invasio  n and migration partially via inhibition of matrix metalloproteinase 2, 3 and 9 expression. RHOC also modulated the expression of several epithelial-mesenchymal transition (EMT)-associated proteins, including E‑cadherin, vimentin, Slug and Snail, to promote to EMT progression. The present results demonstrated that RHOC is important for the invasion and migration of CCC through simultaneous regulation of MMPs and EMT‑associated protein, suggesting that RHOC is a potential molecular target for CCC treatment. 
 miR-10b promotes   cell invasion   through RhoC-AKT signaling pathway by targeting HOXD10 in gastric cancer. MicroRNAs play critical roles in tumorigenesis as either oncogenes or tumor suppressors. As a microRNA induced by Twist, miR-10b function as a metastasis driver in different types of cancer, in which the downstream target gene HOXD10 is the main mediator. In gastric tumor species, miR-10b levels were dramatically elevated in lymphoma node metastasis-positive tumor tissues compared with lymphoma node metastasis-free tumor tissues, and were correlated to dowregulation of HOXD10 expression. In gastric cell lines with distinct degrees of differentiation, miR-10b was highly expressed in the cell line with strong metastatic ability. In MNK45 cells, inhibition of miR-10b led to abrogation of   cell invasion  . While in GES-1 cells, miR-10 overexpression resulted in enhancement of invasiveness through translational inhibition of HOXD10, and constitutive expression of HOXD10 reversed the effects of miR-10b on   cell invasion  . Furthermore, either knockdown of RhoC or inhibition of AKT activation interfered miR-10-induced invasiveness in GES-1 cells. In summary, these observations suggest that miR-10b can stimulate the upregulation of RhoC and AKT phosphorylation through targeting HOXD10, thus promoting   cell invasion   in gastric tumors. 
 miR-10b is overexpressed in hepatocellular carcinoma and promotes cell proliferation, migration and invasion through RhoC, uPAR and MMPs. Recently, miR-10b is identified as a miRNA highly expressed in many human cancers, promoting cell migration and invasion. However, the specific function of miR-10b in hepatocellular carcinoma (HCC) is unclear at this point.The miR-10b expression levels in 60 paired different TNM Stage HCC tumor tissues compared with adjacent non-tumor (ANT) tissues, normal tissue control (8 benign tumor and 7 normal liver tissues), 3 normal liver and 7 HCC cell lines were measured by real-time quantitative RT-PCR and to evaluate their association with HCC clinicopathologic features. Invasion assay, MTT proliferation assay and wound-healing assay were performed to test the invasion and proliferation of HCC cell after transfection. The effect of miR-10b on HCC in vivo was validated by murine xenograft model.We found that miR-10b expression was increased in human HCC tissues and cell lines compared with normal control, respectively. The expression of miR-10b was correlated with HCC metastatic ability. Overexpression of miR-10b in MHCC-97L cells increased cell motility and invasiveness, whereas inhibition of miR-10b in MHCC-97H cells reduced cell motility and invasiveness in vitro and in vivo. We also showed that HOXD10 was negatively regulated by miR-10b at the posttranscriptional level, via a specific target site within the 3'UTR by luciferase reporter assay. Furthermore, we found that miR-10b induced HCC   cell invasion   and migration by modulating the HOXD10 target gene RhoC, uPAR, MMP-2 and MMP-9 expression.Our results suggested that miR-10b was overexpressed in HCC and promoted HCC cell migration and invasion through the HOXD10/ RhoC/ uPAR/ MMPs pathway which may provide a novel bio-target for HCC therapy. 
 Regulation of inflammatory breast cancer   cell invasion   through Akt1/PKBα phosphorylation of RhoC GTPase. With a 42% and 18% 5- and 10-year respective disease-free survival rate, inflammatory breast cancer (IBC) is arguably the deadliest form of breast cancer. IBC invades the dermal lymphatic vessels of the skin overlying the breast and as a consequence nearly all women have lymph node involvement and ~1/3 have gross distant metastases at the time of diagnosis. One year after diagnosis ~90% of patients have detectable metastases, making IBC a paradigm for lymphovascular invasion. Understanding the underlying mechanisms of the IBC metastatic phenotype is essential for new therapies. Work from our laboratory and others show distinct molecular differences between IBC and non-IBCs (nIBCs). Previously we showed that RhoC GTPase is a metastatic switch responsible for the invasive phenotype of IBC. In this study we integrate observations made in IBC patients with in vitro analysis. We show that the PI3K/Akt signaling pathway is crucial in IBC invasion. Key molecules involved in cytoskeletal control and cell motility are specifically upregulated in IBC patients compared with stage and cell-type-of-origin matched nIBCs patients. Distinctively, RhoC GTPase is a substrate for Akt1 and its phosphorylation is absolutely essential for IBC   cell invasio  n. Further our data show that Akt3, not Akt1 has a role in IBC cell survival. Together our data show a unique and targetable pathway for IBC invasion and survival. 
 HOXD10 acts as a tumor-suppressive factor via inhibition of the RHOC/AKT/MAPK pathway in human cholangiocellular carcinoma. HOXD10, a key regulator of cell-differentiated phenotype maintainence, has been demonstrated to be involved in the tumorigenesis of many human malignacies. However, the status of HOXD10 expression and its biological function in cholangiocellular carcinoma (CCC) remain to be clarified. In the present study, we investigated the clinical significance and biological functions of HOXD10 in CCC and found that the expression of HOXD10 and its downstream effector RHOC was significantly different in well-differentiated CCC tissues compared with poorly-differentiated lesions. We also observed a significant correlation between low HOXD10 and high RHOC expression levels and worse prognosis. The stable overexpression of HOXD10 by lentivirus vector significantly inhibited   cell invasion   partly by downregulating the expression of MMP2 and MMP9, and significantly increased early apoptosis in CCC cell lines and induced G1 phase cell cycle arrest, contributing to the inhibition of cell proliferation in vitro. Additionally, we demonstrated that the inactivation of the RHOC/AKT/MAPK pathway was involved in the tumor-suppressive functions of HOXD10 in CCC. These results suggested that HOXD10 may be a putative suppressor gene and can act as a prognostic marker and potentially a novel therapeutic target for CCC. 
  GeneRIFs  
 found that miR-10b induced HCC   cell invasion   and migration by modulating the HOXD10 target gene RhoC, uPAR, MMP-2 and MMP-9 expression 
 RhoC facilitates tumor   cell invasion   and promotes subsequent metastasis, in part, by enhancing integrin alpha5beta1 trafficking 
 RhoC GTPase is a substrate for Akt1 and its phosphorylation is absolutely essential for inflammatory breast cancer   cell invasion  . 
 MRK is a novel RhoC effector that controls LPA-stimulated   cell invasion   at least in part by regulating myosin dynamics, ERK and p38 
 Suggest that miR-10b can stimulate the upregulation of RhoC and AKT phosphorylation through targeting HOXD10, thus promoting   cell invasion   in gastric tumors. 
 This work shows that RHOC and RHOA play different roles in cell-matrix adhesion, motility, and invasion of MDA-MB-231 and reaffirms the crucial role of RHOC-GTPase in inflammatory breast cancer   cell invasion  . 
 RhoA and RhoC are downstream of PKC epsilon & critical for PKC-epsilon-mediated   cell invasion   & motility. PKC epsilon coordinately regulates RhoA & RhoC activation, possibly through direct post-translational phosphorylation. 
   
  
 RHOF 
 
  NCBI Gene  
  GeneRIF  
  Pubmed  
 
 
  Search Term  Rank  Fields  
  [Ff]ilopodia  1  PubMed(1); GeneRIFs(1); Alternative Name(3); Description(1)  
  [Mm]igration  6  PubMed(1)  
 
 
 hide/show details for RHOF 
  [Ff]ilopodia  PubMed  
 The novel Rho-family GTPase rif regulates coordinated actin-based membrane rearrangements. Small GTPases of the Rho family have a critical role in controlling cell morphology, motility and adhesion through dynamic regulation of the actin cytoskeleton [1,2]. Individual Rho GTPases have been shown to regulate distinct components of the cytoskeletal architecture; RhoA stimulates the bundling of actin filaments into stress fibres [3], Rac reorganises actin to produce membrane sheets or lamellipodia [4] and Cdc42 causes the formation of thin, actin-rich surface projections called   filopodia   [5]. We have isolated a new Rho-family GTPase, Rif (Rho in   filopodia  ), and shown that it represents an alternative signalling route to the generation of   filopodia  l structures. Coordinated regulation of Rho-family GTPases can be used to generate more complicated actin rearrangements, such as those underlying cell migration [6]. In addition to inducing   filopodia  , Rif functions cooperatively with Cdc42 and Rac to generate additional structures, increasing the diversity of actin-based morphology. 
  GeneRIFs  
 The protein encoded by this gene causes the formation of long, actin-rich   filopodia  .  In combination with RAC1 and CDC42 it causes additional actin rearrangments, suggesting that this protein is intimately involved in regulation of the cytoskeleton. 
  Alternative Name  
 ras homolog family member F (in   filopodia  ) 
 ras homolog gene family, member F (in   filopodia  ) 
 rho in   filopodia   
  Description  
 ras homolog family member F,   filopodia   associated 
  [Mm]igration  PubMed  
 The novel Rho-family GTPase rif regulates coordinated actin-based membrane rearrangements. Small GTPases of the Rho family have a critical role in controlling cell morphology, motility and adhesion through dynamic regulation of the actin cytoskeleton [1,2]. Individual Rho GTPases have been shown to regulate distinct components of the cytoskeletal architecture; RhoA stimulates the bundling of actin filaments into stress fibres [3], Rac reorganises actin to produce membrane sheets or lamellipodia [4] and Cdc42 causes the formation of thin, actin-rich surface projections called filopodia [5]. We have isolated a new Rho-family GTPase, Rif (Rho in filopodia), and shown that it represents an alternative signalling route to the generation of filopodial structures. Coordinated regulation of Rho-family GTPases can be used to generate more complicated actin rearrangements, such as those underlying cell   migration   [6]. In addition to inducing filopodia, Rif functions cooperatively with Cdc42 and Rac to generate additional structures, increasing the diversity of actin-based morphology. 
   
  
 RHOH 
 
  NCBI Gene  
  GeneRIF  
  Pubmed  
 
 
  Search Term  Rank  Fields  
  [Mm]igration  6  PubMed(4)  
 
 
 hide/show details for RHOH 
  [Mm]igration  PubMed  
 RhoH plays distinct roles in T-cell   migration  s induced by different doses of SDF1 alpha. Hematopoietic-specific RhoGTPase RhoH displays an important role in regulating T-cell development, a process that is accompanied by ordered and directed   migration   of progenitors through thymus. While RhoGTPases are key regulators of cytokinesis, the precise role of RhoH in T-cell   migration   is unknown. Here, by using Jurkat cell   migration   as a model, we found that RhoH is required for cell   migration   in response to the high concentration of SDF1 alpha but displays inhibitory roles in regulating   migration   in the absence of SDF1 alpha or in the range of low concentrations. We further found that RhoH-mediated   migration   requires PAK1 but not ITK. Indeed, both the low and high concentrations of SDF1 alpha activate PAK1 but only the high concentration-induced activation of PAK1 requires RhoH. Further study of the RhoH-PAK interaction revealed that RhoH is able to bind and activate PAK1, indicating that RhoH plays a positive role in response to the high concentration of SDF1 alpha. In contrast, at rest, RhoH indirectly inhibits Slp76/ITK activity through its modulation on TCR signaling, supporting a negative regulatory role for RhoH when SDF1 alpha is insufficient to activate the RhoH-PAK1 pathway. Together, our study uncovered a dual role for RhoH in SDF1 alpha signaling in T-cell responses. 
 RhoH is critical for cell-microenvironment interactions in chronic lymphocytic leukemia in mice and humans. Trafficking of B-cell chronic lymphocytic leukemia (CLL) cells to the bone marrow and interaction with supporting stromal cells mediates important survival and proliferation signals. Previous studies have demonstrated that deletion of Rhoh led to a delayed disease onset in a murine model of CLL. Here we assessed the impact of RhoH on homing,   migration  , and cell-contact dependent interactions of CLL cells. Rhoh(-/-) CLL cells exhibited reduced marrow homing and subsequent engraftment. In vitro   migration   toward the chemokines CXCL12 and CXCL13 and cell-cell interactions between Rhoh(-/-) CLL cells and the supporting microenvironment was reduced. In the absence of RhoH the distribution of phosphorylated focal adhesion kinase, a protein known to coordinate activation of the Rho GTPases RhoA and Rac, appeared less polarized in chemokine-stimulated Rhoh(-/-) CLL cells, and activation and localization of RhoA and Rac was dysregulated leading to defective integrin function. These findings in the Rhoh(-/-) CLL cells were subsequently demonstrated to closely resemble changes in GTPase activation observed in human CLL samples after in vitro and in vivo treatment with lenalidomide, an agent with known influence on microenvironment protection, and suggest that RhoH plays a critical role in prosurvival CLL cell-cell and cell-microenvironment interactions with this agent. 
 Opposing roles for RhoH GTPase during T-cell   migration   and activation. T cells spend the majority of their time perusing lymphoid organs in search of cognate antigen presented by antigen presenting cells (APCs) and then quickly recirculate through the bloodstream to another lymph node. Therefore, regulation of a T-cell response is dependent upon the ability of cells to arrive in the correct location following chemokine gradients ("go" signal) as well as to receive appropriate T-cell receptor (TCR) activation signals upon cognate antigen recognition ("stop" signal). However, the mechanisms by which T cells regulate these go and stop signals remain unclear. We found that overexpression of the hematopoietic-specific RhoH protein in the presence of chemokine signals resulted in decreased Rap1-GTP and LFA-1 adhesiveness to ICAM-1, thus impairing T-cell chemotaxis; while in the presence of TCR signals, there were enhanced and sustained Rap1-GTP and LFA-1 activation as well as prolonged T:APC conjugates. RT-PCR analyses of activated CD4(+) T cells and live images of T-cell   migration   and immunological synapse (IS) formation revealed that functions of RhoH took place primarily at the levels of transcription and intracellular distribution. Thus, we conclude that RhoH expression provides a key molecular determinant that allows T cells to switch between sensing chemokine-mediated go signals and TCR-dependent stop signals. 
 Underexpression of RhoH in Hairy Cell Leukemia. The cause of hairy cell leukemia (HCL) is unknown. Current treatments seem effective only for a limited period of time. In addition, a significant proportion of patients remain refractive to all treatment options. These considerations indicate the need to develop alternative therapeutic strategies for HCL. Here, we report that HCL is characterized by underexpression of RhoH. In vitro reconstitution of RhoH expression inhibits the aberrant adhesion and transendothelial   migration   that drives disease pathogenesis. In an in vivo model of HCL, RhoH reconstitution limits malignant progression and protects against mortality. These findings provide the proof of principle that RhoH reconstitution represents a potential new approach to the treatment of HCL. 
   
  
 RAC3 
 
  NCBI Gene  
  GeneRIF  
  Pubmed  
 
 
  Search Term  Rank  Fields  
  [Mm]igration  6  PubMed(10); GeneRIFs(1)  
  [Ii]nvasive  10  PubMed(5); GeneRIFs(1)  
  [Mm]etastasis  12  PubMed(5); GeneRIFs(1)  
  [Cc]ell [Ii]nvasion  13  PubMed(4)  
 
 
 hide/show details for RAC3 
  [Mm]igration  PubMed  
 Rac1 and Rac3 isoform activation is involved in the invasive and metastatic phenotype of human breast cancer cells. The metastatic progression of cancer is a direct result of the disregulation of numerous cellular signaling pathways, including those associated with adhesion,   migration  , and invasion. Members of the Rac family of small GTPases are known to act as regulators of actin cytoskeletal structures and strongly influence the cellular processes of integrin-mediated adhesion and   migration  . Even though hyperactivated Rac proteins have been shown to influence metastatic processes, these proteins have never been directly linked to metastatic progression.To investigate a role for Rac and Cdc42 in metastatic breast cancer cell invasion and   migration  , relative endogenous Rac or Cdc42 activity was determined in a panel of metastatic variants of the MDA-MB-435 metastatic human breast cancer cell line using a p21-binding domain-PAK pull down assay. To investigate the migratory and invasive potential of the Rac isoforms in human breast cancer, namely Rac1 and the subsequently cloned Rac3, we stably expressed either dominant active Rac1 or dominant active Rac3 into the least metastatic cell variant. Dominant negative Rac1 or dominant negative Rac3 were stably expressed in the most metastatic cell variant. Cell lines expressing mutant Rac1 or Rac3 were analyzed using in vitro adhesion,   migration   and invasion assays.We show that increased activation of Rac proteins directly correlates with increasing metastatic potential in a panel of cell variants derived from a single metastatic breast cancer cell line (MDA-MB-435). The same correlation could not be found with activated Cdc42. Expression of a dominant active Rac1 or a dominant active Rac3 resulted in a more invasive and motile phenotype. Moreover, expression of either dominant negative Rac1 or dominant negative Rac3 into the most metastatic cell variant resulted in decreased invasive and motile properties.This study correlates endogenous Rac activity with high metastatic potential and implicates Rac in the regulation of cell   migration   and invasion in metastatic breast cancer cells. Taken together, these results suggest a role for both the Rac1 and Rac3 GTPases in human breast cancer progression. 
 RAC3 is a pro-migratory co-activator of ERα. Estrogen receptor alpha (ERα) is a ligand-dependent nuclear receptor that is important in breast cancer genesis, behavior and response to hormone-based therapies. A T7 phage display screen against full-length human ERα, coupled with genome-wide exon arrays, was used to identify RAC3 as a putative ERα co-regulator. RAC3 is a Rho family small GTPase that is associated with cytoskeletal rearrangement. We demonstrate a novel role for nuclear RAC3 as an ERα transcriptional activator, with prognostic implications for metastatic disease. Through in vitro and cell-based studies, RAC3 was shown to exist in a GTP-bound state and act as a ligand specific ERα co-activator of E2-induced transcription. Overexpression of RAC3 induced pro-growth and pro-migratory genes that resulted in incr  eased mig  ration of ERα-positive breast cancer cells. Chemical inhibition and genetic knockdown of RAC3 antagonized E2-induced cell proliferation  , cell mi  gration and ERα mediated gene expression, indicating that RAC3 is necessary for full ERα transcriptional activity. In agreement with the molecular and cellular data, RAC3 overexpression in ERα-positive breast cancers correlated with a significant decrease in recurrence free survival and a significant increase in the odds ratio of metastasis. In conclusion, RAC3 is a novel ERα co-activator that pro  motes cel  l migration and has prognostic value for ERα-positive breast cancer metastasis. RAC3 may also be a useful therapeutic target for ERα-positive breast cancers. 
 Rac1 and Rac3 have opposing functions in cell adhesion and differentiation of neuronal cells. Rac1 and Rac3 are highly homologous members of the Rho small GTPase family. Rac1 is ubiquitously expressed and regulates cell adhesion,   migration   and differentiation in various cell types. Rac3 is primarily expressed in brain and may therefore have a specific function in neuronal cells. We found that depletion of Rac1 by short interference RNA leads to decreased cell-matrix adhesions and cell rounding in neuronal N1E-115 cells. By contrast, depletion of Rac3 induces stronger cell adhesions and dramatically increases the outgrowth of neurite-like protrusions, suggesting opposite functions for Rac1 and Rac3 in neuronal cells. Consistent with this, overexpression of Rac1 induces cell spreading, whereas overexpression of Rac3 results in a contractile round morphology. Rac1 is mainly found at the plasma membrane, whereas Rac3 is predominantly localized in the perinuclear region. Residues 185-187, present in the variable polybasic rich region at the carboxyl terminus are responsible for the difference in phenotype induced by Rac1 and Rac3 as well as for their different intracellular localization. The Rac1-opposing function of Rac3 is not mediated by or dependent on components of the RhoA signaling pathway. It rather seems that Rac3 exerts its function through negatively affecting integrin-mediated cell-matrix adhesions. Together, our data reveal that Rac3 opposes Rac1 in the regulation of cell adhesion and differentiation of neuronal cells. 
 Roles of the Rac1 and Rac3 GTPases in human tumor cell invasion. Members of the Rho family of small GTPases have been shown to be involved in tumorigenesis and metastasis. Currently, most of the available information on the function of Rho proteins in malignant transformation is based on the use of dominant-negative mutants of these GTPases. The specificity of these dominant-negative mutants is limited however. In this study, we used small interfering RNA directed against either Rac1 or Rac3 to reduce their expression specifically. In line with observations using dominant-negative Rac1 in other cell types, we show that RNA interference-mediated depletion of Rac1 strongly inhibits lamellipodia formation, cell   migration   and invasion in SNB19 glioblastoma cells. Surprisingly however, Rac1 depletion has a much smaller inhibitory effect on SNB19 cell proliferation and survival. Interestingly, whereas depletion of Rac3 strongly inhibits SNB19 cell invasion, it does not affect lamellipodia formation and has only minor effects on cell   migration   and proliferation. Similar results were obtained in BT549 breast carcinoma cells. Thus, functional analysis of Rac1 and Rac3 using RNA interference reveals a critical role for these GTPases in the invasive behavior of glioma and breast carcinoma cells. 
 RAC3 is a pro-migratory co-activator of ERα. Estrogen receptor alpha (ERα) is a ligand-dependent nuclear receptor that is important in breast cancer genesis, behavior and response to hormone-based therapies. A T7 phage display screen against full-length human ERα, coupled with genome-wide exon arrays, was used to identify RAC3 as a putative ERα co-regulator. RAC3 is a Rho family small GTPase that is associated with cytoskeletal rearrangement. We demonstrate a novel role for nuclear RAC3 as an ERα transcriptional activator, with prognostic implications for metastatic disease. Through in vitro and cell-based studies, RAC3 was shown to exist in a GTP-bound state and act as a ligand specific ERα co-activator of E2-induced transcription. Overexpression of RAC3 induced pro-growth and pro-migratory genes that resulted in incr  eased mig  ration of ERα-positive breast cancer cells. Chemical inhibition and genetic knockdown of RAC3 antagonized E2-induced cell proliferation  , cell mi  gration and ERα mediated gene expression, indicating that RAC3 is necessary for full ERα transcriptional activity. In agreement with the molecular and cellular data, RAC3 overexpression in ERα-positive breast cancers correlated with a significant decrease in recurrence free survival and a significant increase in the odds ratio of metastasis. In conclusion, RAC3 is a novel ERα co-activator that pro  motes cel  l migration and has prognostic value for ERα-positive breast cancer metastasis. RAC3 may also be a useful therapeutic target for ERα-positive breast cancers. 
 Rac1 and Rac3 isoform activation is involved in the invasive and metastatic phenotype of human breast cancer cells. The metastatic progression of cancer is a direct result of the disregulation of numerous cellular signaling pathways, including those associated with adhesion,   migration  , and invasion. Members of the Rac family of small GTPases are known to act as regulators of actin cytoskeletal structures and strongly influence the cellular processes of integrin-mediated adhesion and   migration  . Even though hyperactivated Rac proteins have been shown to influence metastatic processes, these proteins have never been directly linked to metastatic progression.To investigate a role for Rac and Cdc42 in metastatic breast cancer cell invasion and   migration  , relative endogenous Rac or Cdc42 activity was determined in a panel of metastatic variants of the MDA-MB-435 metastatic human breast cancer cell line using a p21-binding domain-PAK pull down assay. To investigate the migratory and invasive potential of the Rac isoforms in human breast cancer, namely Rac1 and the subsequently cloned Rac3, we stably expressed either dominant active Rac1 or dominant active Rac3 into the least metastatic cell variant. Dominant negative Rac1 or dominant negative Rac3 were stably expressed in the most metastatic cell variant. Cell lines expressing mutant Rac1 or Rac3 were analyzed using in vitro adhesion,   migration   and invasion assays.We show that increased activation of Rac proteins directly correlates with increasing metastatic potential in a panel of cell variants derived from a single metastatic breast cancer cell line (MDA-MB-435). The same correlation could not be found with activated Cdc42. Expression of a dominant active Rac1 or a dominant active Rac3 resulted in a more invasive and motile phenotype. Moreover, expression of either dominant negative Rac1 or dominant negative Rac3 into the most metastatic cell variant resulted in decreased invasive and motile properties.This study correlates endogenous Rac activity with high metastatic potential and implicates Rac in the regulation of cell   migration   and invasion in metastatic breast cancer cells. Taken together, these results suggest a role for both the Rac1 and Rac3 GTPases in human breast cancer progression. 
 Roles of the Rac1 and Rac3 GTPases in human tumor cell invasion. Members of the Rho family of small GTPases have been shown to be involved in tumorigenesis and metastasis. Currently, most of the available information on the function of Rho proteins in malignant transformation is based on the use of dominant-negative mutants of these GTPases. The specificity of these dominant-negative mutants is limited however. In this study, we used small interfering RNA directed against either Rac1 or Rac3 to reduce their expression specifically. In line with observations using dominant-negative Rac1 in other cell types, we show that RNA interference-mediated depletion of Rac1 strongly inhibits lamellipodia formation, cell   migration   and invasion in SNB19 glioblastoma cells. Surprisingly however, Rac1 depletion has a much smaller inhibitory effect on SNB19 cell proliferation and survival. Interestingly, whereas depletion of Rac3 strongly inhibits SNB19 cell invasion, it does not affect lamellipodia formation and has only minor effects on cell   migration   and proliferation. Similar results were obtained in BT549 breast carcinoma cells. Thus, functional analysis of Rac1 and Rac3 using RNA interference reveals a critical role for these GTPases in the invasive behavior of glioma and breast carcinoma cells. 
 Rac1 and Rac3 have opposing functions in cell adhesion and differentiation of neuronal cells. Rac1 and Rac3 are highly homologous members of the Rho small GTPase family. Rac1 is ubiquitously expressed and regulates cell adhesion,   migration   and differentiation in various cell types. Rac3 is primarily expressed in brain and may therefore have a specific function in neuronal cells. We found that depletion of Rac1 by short interference RNA leads to decreased cell-matrix adhesions and cell rounding in neuronal N1E-115 cells. By contrast, depletion of Rac3 induces stronger cell adhesions and dramatically increases the outgrowth of neurite-like protrusions, suggesting opposite functions for Rac1 and Rac3 in neuronal cells. Consistent with this, overexpression of Rac1 induces cell spreading, whereas overexpression of Rac3 results in a contractile round morphology. Rac1 is mainly found at the plasma membrane, whereas Rac3 is predominantly localized in the perinuclear region. Residues 185-187, present in the variable polybasic rich region at the carboxyl terminus are responsible for the difference in phenotype induced by Rac1 and Rac3 as well as for their different intracellular localization. The Rac1-opposing function of Rac3 is not mediated by or dependent on components of the RhoA signaling pathway. It rather seems that Rac3 exerts its function through negatively affecting integrin-mediated cell-matrix adhesions. Together, our data reveal that Rac3 opposes Rac1 in the regulation of cell adhesion and differentiation of neuronal cells. 
 F-box protein complex FBXL19 regulates TGFβ1-induced E-cadherin down-regulation by mediating Rac3 ubiquitination and degradation. Rac3 is a small GTPase multifunctional protein that regulates cell adhesion,   migratio  n, and differentiation. It has been considered as an oncogene in breast cancer; however, its role in esophageal cancer and the regulation of its stability have not been studied. F-box proteins are major subunits within the Skp1-Cullin-1-F-box (SCF) E3 ubiquitin ligases that recognize particular substrates for ubiquitination and proteasomal degradation. Recently, we have shown that SCFFBXL19 targets Rac1 and RhoA, thus regulating Rac1 and RhoA ubiquitination and degradation. Here, we demonstrate the role of FBXL19 in the regulation of Rac3 site-specific ubiquitination and stability. Expression of TGFβ1 is associated with poor prognosis of esophageal cancer. TGFβ1 reduces tumor suppressor, E-cadherin, expression in various epithelial-derived cancers. Here we investigate the role of FBXL19-mediated Rac3 degradation in TGFβ1-induced E-cadherin down-regulation in esophageal cancer cells.FBXL19-regulated endogenous and over-expressed Rac3 stability were determined by immunoblotting and co-immunoprecipitation. Esophageal cancer cells (OE19 and OE33) were used to investigate TGFβ1-induced E-cadherin down-regulation by Immunoblotting and Immunostaining.Overexpression of FBXL19 decreased endogenous and over-expressed Rac3 expression by interacting and polyubiquitinating Rac3, while down-regulation of FBXL19 suppressed Rac3 degradation. Lysine166 within Rac3 was identified as an ubiquitination acceptor site. The FBXL19 variant with truncation at the N-terminus resulted in an increase in Rac3 degradation; however, the FBXL19 variant with truncation at the C-terminus lost its ability to interact with Rac3 and ubiquitinate Rac3 protein. Further, we found that Rac3 plays a critical role in TGFβ1-induced E-cadherin down-regulation in esophageal cancer cells. Over-expression of FBXL19 attenuated TGFβ1-induced E-cadherin down-regulation and esophageal cancer cells elongation phenotype.Collectively these data unveil that FBXL19 functions as an antagonist of Rac3 by regulating its stability and regulates the TGFβ1-induced E-cadherin down-regulation. This study will provide a new potential therapeutic strategy to regulate TGFβ1 signaling, thus suppressing esophageal tumorigenesis. 
 Silencing of Rac3 inhibits proliferation and induces apoptosis of human lung cancer cells. Rac3, a member of the Rac family of small guanosine triphosphatases (GTPases), regulates a variety of cell functions, including the organization of the cytoskeleton, cell   migration  , and invasion. Overexpression of Rac3 has been reported in several human cancers. However, the role of Rac3 in lung cancer (LC) has not been determined in detail. The purpose of this study was to investigate the effect of silencing of Rac3 expression in human LC cells and the consequences for cell survival.Lentivirus small hairpin RNA (shRNA) interference techniques were utilized to knock down the Rac3 gene. Gene and protein expression was quantified by quantitative real-time polymerase chain reaction (qRT-PCR) and Western blotting. LC cell apoptosis was examined by annexin V-APC /propidium iodide staining.Efficient silencing of Rac3 strongly inhibited A549 cell proliferation and colony formation ability, and significantly decreased tumor growth. Moreover, flow cytometry analysis showed that knockdown of Rac3 led to G2/M phase cell cycle arrest as well as an excess accumulation of cells in the G1 and S phase.Thus, functional analysis using shRNAs revealed a critical role for Rac3 in the tumor growth of LC cells. shRNA silencing of Rac3 could provide an effective strategy to treat LC. 
  GeneRIFs  
 RAC3 is a novel ERalpha co-activator that promotes cell   migration   and has prognostic value for ERalpha-positive breast cancer metastasis. 
  [Ii]nvasive  PubMed  
 Rac1 and Rac3 isoform activation is involved in the   invasive   and metastatic phenotype of human breast cancer cells. The metastatic progression of cancer is a direct result of the disregulation of numerous cellular signaling pathways, including those associated with adhesion, migration, and invasion. Members of the Rac family of small GTPases are known to act as regulators of actin cytoskeletal structures and strongly influence the cellular processes of integrin-mediated adhesion and migration. Even though hyperactivated Rac proteins have been shown to influence metastatic processes, these proteins have never been directly linked to metastatic progression.To investigate a role for Rac and Cdc42 in metastatic breast cancer cell invasion and migration, relative endogenous Rac or Cdc42 activity was determined in a panel of metastatic variants of the MDA-MB-435 metastatic human breast cancer cell line using a p21-binding domain-PAK pull down assay. To investigate the migratory and   invasive   potential of the Rac isoforms in human breast cancer, namely Rac1 and the subsequently cloned Rac3, we stably expressed either dominant active Rac1 or dominant active Rac3 into the least metastatic cell variant. Dominant negative Rac1 or dominant negative Rac3 were stably expressed in the most metastatic cell variant. Cell lines expressing mutant Rac1 or Rac3 were analyzed using in vitro adhesion, migration and invasion assays.We show that increased activation of Rac proteins directly correlates with increasing metastatic potential in a panel of cell variants derived from a single metastatic breast cancer cell line (MDA-MB-435). The same correlation could not be found with activated Cdc42. Expression of a dominant active Rac1 or a dominant active Rac3 resulted in a more   invasive   and motile phenotype. Moreover, expression of either dominant negative Rac1 or dominant negative Rac3 into the most metastatic cell variant resulted in decreased   invasive   and motile properties.This study correlates endogenous Rac activity with high metastatic potential and implicates Rac in the regulation of cell migration and invasion in metastatic breast cancer cells. Taken together, these results suggest a role for both the Rac1 and Rac3 GTPases in human breast cancer progression. 
 Roles of the Rac1 and Rac3 GTPases in human tumor cell invasion. Members of the Rho family of small GTPases have been shown to be involved in tumorigenesis and metastasis. Currently, most of the available information on the function of Rho proteins in malignant transformation is based on the use of dominant-negative mutants of these GTPases. The specificity of these dominant-negative mutants is limited however. In this study, we used small interfering RNA directed against either Rac1 or Rac3 to reduce their expression specifically. In line with observations using dominant-negative Rac1 in other cell types, we show that RNA interference-mediated depletion of Rac1 strongly inhibits lamellipodia formation, cell migration and invasion in SNB19 glioblastoma cells. Surprisingly however, Rac1 depletion has a much smaller inhibitory effect on SNB19 cell proliferation and survival. Interestingly, whereas depletion of Rac3 strongly inhibits SNB19 cell invasion, it does not affect lamellipodia formation and has only minor effects on cell migration and proliferation. Similar results were obtained in BT549 breast carcinoma cells. Thus, functional analysis of Rac1 and Rac3 using RNA interference reveals a critical role for these GTPases in the   invasive   behavior of glioma and breast carcinoma cells. 
 Rac1 and Rac3 isoform activation is involved in the   invasive   and metastatic phenotype of human breast cancer cells. The metastatic progression of cancer is a direct result of the disregulation of numerous cellular signaling pathways, including those associated with adhesion, migration, and invasion. Members of the Rac family of small GTPases are known to act as regulators of actin cytoskeletal structures and strongly influence the cellular processes of integrin-mediated adhesion and migration. Even though hyperactivated Rac proteins have been shown to influence metastatic processes, these proteins have never been directly linked to metastatic progression.To investigate a role for Rac and Cdc42 in metastatic breast cancer cell invasion and migration, relative endogenous Rac or Cdc42 activity was determined in a panel of metastatic variants of the MDA-MB-435 metastatic human breast cancer cell line using a p21-binding domain-PAK pull down assay. To investigate the migratory and   invasive   potential of the Rac isoforms in human breast cancer, namely Rac1 and the subsequently cloned Rac3, we stably expressed either dominant active Rac1 or dominant active Rac3 into the least metastatic cell variant. Dominant negative Rac1 or dominant negative Rac3 were stably expressed in the most metastatic cell variant. Cell lines expressing mutant Rac1 or Rac3 were analyzed using in vitro adhesion, migration and invasion assays.We show that increased activation of Rac proteins directly correlates with increasing metastatic potential in a panel of cell variants derived from a single metastatic breast cancer cell line (MDA-MB-435). The same correlation could not be found with activated Cdc42. Expression of a dominant active Rac1 or a dominant active Rac3 resulted in a more   invasive   and motile phenotype. Moreover, expression of either dominant negative Rac1 or dominant negative Rac3 into the most metastatic cell variant resulted in decreased   invasive   and motile properties.This study correlates endogenous Rac activity with high metastatic potential and implicates Rac in the regulation of cell migration and invasion in metastatic breast cancer cells. Taken together, these results suggest a role for both the Rac1 and Rac3 GTPases in human breast cancer progression. 
 Roles of the Rac1 and Rac3 GTPases in human tumor cell invasion. Members of the Rho family of small GTPases have been shown to be involved in tumorigenesis and metastasis. Currently, most of the available information on the function of Rho proteins in malignant transformation is based on the use of dominant-negative mutants of these GTPases. The specificity of these dominant-negative mutants is limited however. In this study, we used small interfering RNA directed against either Rac1 or Rac3 to reduce their expression specifically. In line with observations using dominant-negative Rac1 in other cell types, we show that RNA interference-mediated depletion of Rac1 strongly inhibits lamellipodia formation, cell migration and invasion in SNB19 glioblastoma cells. Surprisingly however, Rac1 depletion has a much smaller inhibitory effect on SNB19 cell proliferation and survival. Interestingly, whereas depletion of Rac3 strongly inhibits SNB19 cell invasion, it does not affect lamellipodia formation and has only minor effects on cell migration and proliferation. Similar results were obtained in BT549 breast carcinoma cells. Thus, functional analysis of Rac1 and Rac3 using RNA interference reveals a critical role for these GTPases in the   invasive   behavior of glioma and breast carcinoma cells. 
 Rac3 induces a molecular pathway triggering breast cancer cell aggressiveness: differences in MDA-MB-231 and MCF-7 breast cancer cell lines. Rho GTPases are involved in cellular functions relevant to cancer. The roles of RhoA and Rac1 have already been established. However, the role of Rac3 in cancer aggressiveness is less well understood.This work was conducted to analyze the implication of Rac3 in the aggressiveness of two breast cancer cell lines, MDA-MB-231 and MCF-7: both express Rac3, but MDA-MB-231 expresses more activated RhoA. The effect of Rac3 in cancer cells was also compared with its effect on the non-tumorigenic mammary epithelial cells MCF-10A. We analyzed the consequences of Rac3 depletion by anti-Rac3 siRNA.Firstly, we analyzed the effects of Rac3 depletion on the breast cancer cells' aggressiveness. In the   invasive   MDA-MB-231 cells, Rac3 inhibition caused a marked reduction of both invasion (40%) and cell adhesion to collagen (84%), accompanied by an increase in TNF-induced apoptosis (72%). This indicates that Rac3 is involved in the cancer cells' aggressiveness. Secondly, we investigated the effects of Rac3 inhibition on the expression and activation of related signaling molecules, including NF-κB and ERK. Cytokine secretion profiles were also analyzed. In the non  -invasiv  e MCF-7 line; Rac3 did not influence any of the parameters of aggressiveness.This discrepancy between the effects of Rac3 knockdown in the two cell lines could be explained as follows: in the MDA-MB-231 line, the Rac3-dependent aggressiveness of the cancer cells is due to the Rac3/ERK-2/NF-κB signaling pathway, which is responsible for MMP-9, interleukin-6, -8 and GRO secretion, as well as the resistance to TNF-induced apoptosis, whereas in the MCF-7 line, this pathway is not functional because of the low expression of NF-κB subunits in these cells. Rac3 may be a potent target for inhibiting aggressive breast cancer. 
  GeneRIFs  
 functional analysis of Rac1 and Rac3 using RNA interference reveals a critical role for these GTPases in the   invasive   behavior of glioma and breast carcinoma cells 
  [Mm]etastasis  PubMed  
 RAC3 is a pro-migratory co-activator of ERα. Estrogen receptor alpha (ERα) is a ligand-dependent nuclear receptor that is important in breast cancer genesis, behavior and response to hormone-based therapies. A T7 phage display screen against full-length human ERα, coupled with genome-wide exon arrays, was used to identify RAC3 as a putative ERα co-regulator. RAC3 is a Rho family small GTPase that is associated with cytoskeletal rearrangement. We demonstrate a novel role for nuclear RAC3 as an ERα transcriptional activator, with prognostic implications for metastatic disease. Through in vitro and cell-based studies, RAC3 was shown to exist in a GTP-bound state and act as a ligand specific ERα co-activator of E2-induced transcription. Overexpression of RAC3 induced pro-growth and pro-migratory genes that resulted in increased migration of ERα-positive breast cancer cells. Chemical inhibition and genetic knockdown of RAC3 antagonized E2-induced cell proliferation, cell migration and ERα mediated gene expression, indicating that RAC3 is necessary for full ERα transcriptional activity. In agreement with the molecular and cellular data, RAC3 overexpression in ERα-positive breast cancers correlated with a significant decrease in recurrence free survival and a significant increase in the odds   ratio of   metastasis. In conclusion, RAC3 is a novel ERα co-activator that promotes cell migration and has prognostic value for ERα-positive br  east cance  r metastasis. RAC3 may also be a useful therapeutic target for ERα-positive breast cancers. 
 Roles of the Rac1 and Rac3 GTPases in human tumor cell invasion. Members of the Rho family of small GTPases have been shown to be involved in tumorigenesis and   metastasis  . Currently, most of the available information on the function of Rho proteins in malignant transformation is based on the use of dominant-negative mutants of these GTPases. The specificity of these dominant-negative mutants is limited however. In this study, we used small interfering RNA directed against either Rac1 or Rac3 to reduce their expression specifically. In line with observations using dominant-negative Rac1 in other cell types, we show that RNA interference-mediated depletion of Rac1 strongly inhibits lamellipodia formation, cell migration and invasion in SNB19 glioblastoma cells. Surprisingly however, Rac1 depletion has a much smaller inhibitory effect on SNB19 cell proliferation and survival. Interestingly, whereas depletion of Rac3 strongly inhibits SNB19 cell invasion, it does not affect lamellipodia formation and has only minor effects on cell migration and proliferation. Similar results were obtained in BT549 breast carcinoma cells. Thus, functional analysis of Rac1 and Rac3 using RNA interference reveals a critical role for these GTPases in the invasive behavior of glioma and breast carcinoma cells. 
 RAC3 is a pro-migratory co-activator of ERα. Estrogen receptor alpha (ERα) is a ligand-dependent nuclear receptor that is important in breast cancer genesis, behavior and response to hormone-based therapies. A T7 phage display screen against full-length human ERα, coupled with genome-wide exon arrays, was used to identify RAC3 as a putative ERα co-regulator. RAC3 is a Rho family small GTPase that is associated with cytoskeletal rearrangement. We demonstrate a novel role for nuclear RAC3 as an ERα transcriptional activator, with prognostic implications for metastatic disease. Through in vitro and cell-based studies, RAC3 was shown to exist in a GTP-bound state and act as a ligand specific ERα co-activator of E2-induced transcription. Overexpression of RAC3 induced pro-growth and pro-migratory genes that resulted in increased migration of ERα-positive breast cancer cells. Chemical inhibition and genetic knockdown of RAC3 antagonized E2-induced cell proliferation, cell migration and ERα mediated gene expression, indicating that RAC3 is necessary for full ERα transcriptional activity. In agreement with the molecular and cellular data, RAC3 overexpression in ERα-positive breast cancers correlated with a significant decrease in recurrence free survival and a significant increase in the odds   ratio of   metastasis. In conclusion, RAC3 is a novel ERα co-activator that promotes cell migration and has prognostic value for ERα-positive br  east cance  r metastasis. RAC3 may also be a useful therapeutic target for ERα-positive breast cancers. 
 Roles of the Rac1 and Rac3 GTPases in human tumor cell invasion. Members of the Rho family of small GTPases have been shown to be involved in tumorigenesis and   metastasis  . Currently, most of the available information on the function of Rho proteins in malignant transformation is based on the use of dominant-negative mutants of these GTPases. The specificity of these dominant-negative mutants is limited however. In this study, we used small interfering RNA directed against either Rac1 or Rac3 to reduce their expression specifically. In line with observations using dominant-negative Rac1 in other cell types, we show that RNA interference-mediated depletion of Rac1 strongly inhibits lamellipodia formation, cell migration and invasion in SNB19 glioblastoma cells. Surprisingly however, Rac1 depletion has a much smaller inhibitory effect on SNB19 cell proliferation and survival. Interestingly, whereas depletion of Rac3 strongly inhibits SNB19 cell invasion, it does not affect lamellipodia formation and has only minor effects on cell migration and proliferation. Similar results were obtained in BT549 breast carcinoma cells. Thus, functional analysis of Rac1 and Rac3 using RNA interference reveals a critical role for these GTPases in the invasive behavior of glioma and breast carcinoma cells. 
 Matrix compliance regulates Rac1b localization, NADPH oxidase assembly, and epithelial-mesenchymal transition. Epithelial-mesenchymal transition (EMT) is a form of epithelial plasticity implicated in fibrosis and tumor   metastasis  . Here we show that the mechanical rigidity of the microenvironment plays a pivotal role in the promotion of EMT by controlling the subcellular localization and downstream signaling of Rac GTPases. Soft substrata, with compliances comparable to that of normal mammary tissue, are protective against EMT, whereas stiffer substrata, with compliances characteristic of breast tumors, promote EMT. Rac1b, a highly activated splice variant of Rac1 found in tumors, localizes to the plasma membrane in cells cultured on stiff substrata or in collagen-rich regions of human breast tumors. At the membrane, Rac1b forms a complex with NADPH oxidase and promotes the production of reactive oxygen species, expression of Snail, and activation of the EMT program. In contrast, soft microenvironments inhibit the membrane localization of Rac1b and subsequent redox changes. These results reveal a novel mechanotransduction pathway in the regulation of epithelial plasticity via EMT. 
  GeneRIFs  
 RAC3 is a novel ERalpha co-activator that promotes cell migration and has prognostic value for ERalpha-positive breast cancer   metastasis  . 
  [Cc]ell [Ii]nvasion  PubMed  
 Rac1 and Rac3 isoform activation is involved in the invasive and metastatic phenotype of human breast cancer cells. The metastatic progression of cancer is a direct result of the disregulation of numerous cellular signaling pathways, including those associated with adhesion, migration, and invasion. Members of the Rac family of small GTPases are known to act as regulators of actin cytoskeletal structures and strongly influence the cellular processes of integrin-mediated adhesion and migration. Even though hyperactivated Rac proteins have been shown to influence metastatic processes, these proteins have never been directly linked to metastatic progression.To investigate a role for Rac and Cdc42 in metastatic breast cancer   cell invasion   and migration, relative endogenous Rac or Cdc42 activity was determined in a panel of metastatic variants of the MDA-MB-435 metastatic human breast cancer cell line using a p21-binding domain-PAK pull down assay. To investigate the migratory and invasive potential of the Rac isoforms in human breast cancer, namely Rac1 and the subsequently cloned Rac3, we stably expressed either dominant active Rac1 or dominant active Rac3 into the least metastatic cell variant. Dominant negative Rac1 or dominant negative Rac3 were stably expressed in the most metastatic cell variant. Cell lines expressing mutant Rac1 or Rac3 were analyzed using in vitro adhesion, migration and invasion assays.We show that increased activation of Rac proteins directly correlates with increasing metastatic potential in a panel of cell variants derived from a single metastatic breast cancer cell line (MDA-MB-435). The same correlation could not be found with activated Cdc42. Expression of a dominant active Rac1 or a dominant active Rac3 resulted in a more invasive and motile phenotype. Moreover, expression of either dominant negative Rac1 or dominant negative Rac3 into the most metastatic cell variant resulted in decreased invasive and motile properties.This study correlates endogenous Rac activity with high metastatic potential and implicates Rac in the regulation of cell migration and invasion in metastatic breast cancer cells. Taken together, these results suggest a role for both the Rac1 and Rac3 GTPases in human breast cancer progression. 
 Roles of the Rac1 and Rac3 GTPases in human tumor   cell invasion  . Members of the Rho family of small GTPases have been shown to be involved in tumorigenesis and metastasis. Currently, most of the available information on the function of Rho proteins in malignant transformation is based on the use of dominant-negative mutants of these GTPases. The specificity of these dominant-negative mutants is limited however. In this study, we used small interfering RNA directed against either Rac1 or Rac3 to reduce their expression specifically. In line with observations using dominant-negative Rac1 in other cell types, we show that RNA interference-mediated depletion of Rac1 strongly inhibits lamellipodia formation, cell migration and invasion in SNB19 glioblastoma cells. Surprisingly however, Rac1 depletion has a much smaller inhibitory effect on SNB19 cell proliferation and survival. Interestingly, whereas depletion of Rac3 strongly inhibits SNB19   cell invasion  , it does not affect lamellipodia formation and has only minor effects on cell migration and proliferation. Similar results were obtained in BT549 breast carcinoma cells. Thus, functional analysis of Rac1 and Rac3 using RNA interference reveals a critical role for these GTPases in the invasive behavior of glioma and breast carcinoma cells. 
 Rac1 and Rac3 isoform activation is involved in the invasive and metastatic phenotype of human breast cancer cells. The metastatic progression of cancer is a direct result of the disregulation of numerous cellular signaling pathways, including those associated with adhesion, migration, and invasion. Members of the Rac family of small GTPases are known to act as regulators of actin cytoskeletal structures and strongly influence the cellular processes of integrin-mediated adhesion and migration. Even though hyperactivated Rac proteins have been shown to influence metastatic processes, these proteins have never been directly linked to metastatic progression.To investigate a role for Rac and Cdc42 in metastatic breast cancer   cell invasion   and migration, relative endogenous Rac or Cdc42 activity was determined in a panel of metastatic variants of the MDA-MB-435 metastatic human breast cancer cell line using a p21-binding domain-PAK pull down assay. To investigate the migratory and invasive potential of the Rac isoforms in human breast cancer, namely Rac1 and the subsequently cloned Rac3, we stably expressed either dominant active Rac1 or dominant active Rac3 into the least metastatic cell variant. Dominant negative Rac1 or dominant negative Rac3 were stably expressed in the most metastatic cell variant. Cell lines expressing mutant Rac1 or Rac3 were analyzed using in vitro adhesion, migration and invasion assays.We show that increased activation of Rac proteins directly correlates with increasing metastatic potential in a panel of cell variants derived from a single metastatic breast cancer cell line (MDA-MB-435). The same correlation could not be found with activated Cdc42. Expression of a dominant active Rac1 or a dominant active Rac3 resulted in a more invasive and motile phenotype. Moreover, expression of either dominant negative Rac1 or dominant negative Rac3 into the most metastatic cell variant resulted in decreased invasive and motile properties.This study correlates endogenous Rac activity with high metastatic potential and implicates Rac in the regulation of cell migration and invasion in metastatic breast cancer cells. Taken together, these results suggest a role for both the Rac1 and Rac3 GTPases in human breast cancer progression. 
 Roles of the Rac1 and Rac3 GTPases in human tumor   cell invasion  . Members of the Rho family of small GTPases have been shown to be involved in tumorigenesis and metastasis. Currently, most of the available information on the function of Rho proteins in malignant transformation is based on the use of dominant-negative mutants of these GTPases. The specificity of these dominant-negative mutants is limited however. In this study, we used small interfering RNA directed against either Rac1 or Rac3 to reduce their expression specifically. In line with observations using dominant-negative Rac1 in other cell types, we show that RNA interference-mediated depletion of Rac1 strongly inhibits lamellipodia formation, cell migration and invasion in SNB19 glioblastoma cells. Surprisingly however, Rac1 depletion has a much smaller inhibitory effect on SNB19 cell proliferation and survival. Interestingly, whereas depletion of Rac3 strongly inhibits SNB19   cell invasion  , it does not affect lamellipodia formation and has only minor effects on cell migration and proliferation. Similar results were obtained in BT549 breast carcinoma cells. Thus, functional analysis of Rac1 and Rac3 using RNA interference reveals a critical role for these GTPases in the invasive behavior of glioma and breast carcinoma cells. 
   
  
 RHOB 
 
  NCBI Gene  
  GeneRIF  
  Pubmed  
 
 
  Search Term  Rank  Fields  
  [Mm]igration  6  Process(3); PubMed(27); GeneRIFs(1)  
  [Ee]xtracellular [Mm]atrix  8  PubMed(4)  
  [Ii]nvasive  10  PubMed(8); GeneRIFs(2)  
  [Mm]etastasis  12  PubMed(6); GeneRIFs(1)  
  [Cc]ell [Ii]nvasion  13  PubMed(5)  
 
 
 hide/show details for RHOB 
  [Mm]igration  Process  
 negative regulation of cell   migration   
 positive regulation of endothelial cell   migration   
 regulation of cell   migration   
  PubMed  
 Aloe emodin inhibits colon cancer cell   migration  /angiogenesis by downregulating MMP-2/9, RhoB and VEGF via reduced DNA binding activity of NF-κB. Aloe emodin (AE), a natural anthraquinone, is reported to have antiproliferative activity in various cancer cell lines. In this study we analyzed molecular mechanisms involved in the antimigratory and antiangiogenic activity of this hydroxy anthraquinone in colon cancer cell, WiDr. Our results show that a relatively non toxic concentration of AE suppressed the phorbol-12-myristyl-13-acetate (PMA) induced   migratio  n and invasion of tumor cells. On analysis for the molecules involved in the   migratio  n/invasion, we found AE downregulated mRNA expression and promoter/gelatinolytic activity of Matrix Metalloproteinase (MMP)-2/9, as well as the RhoB expression at gene and protein level. It was also a strong inhibitor of Vascular Endothelial Growth Factor (VEGF) expression, promoter activity and endothelial cell   migratio  n/invasion and in vitro angiogenesis. AE suppressed the nuclear translocation and DNA binding of NF-κB, which is an important transcription factor for controlling MMP-2/9 and VEGF gene expression. Taken together these data indicate that AE target multiple molecules responsible for cellular invasion  , migrati  on and angiogenesis. Inhibitory effect on angiogenic and metastatic regulatory processes make AE a sensible candidate as a specific blocker of tumor associated events. 
 RhoA, RhoB and RhoC have different roles in cancer cell   migration  . Rho GTPases are well known to regulate cell motility through activation of a variety of downstream effector proteins, including enzymes, adaptor proteins and actin nucleators. The three closely related Rho GTPases RhoA, RhoB and RhoC all have the potential to interact with the same downstream effectors, yet they have substantially different effects on cell shape and migratory properties. Here I review the different ways in which RhoA, RhoB and RhoC expression is regulated in cancer and how they play distinct roles in cancer progression. I describe their main effectors known to contribute to cell motility. Recent results from our laboratory and others indicate that RhoA, RhoB and RhoC can be activated by specific stimuli and act through different effectors to control distinct aspects of cancer cell   migration   and invasion. This suggests that they each make unique contributions to cancer by participating in different protein complexes. 
 Rho isoform-specific interaction with IQGAP1 promotes breast cancer cell proliferation and   migration  . We performed a proteomics screen for Rho isoform-specific binding proteins to clarify the tumor-promoting effects of RhoA and C that contrast with the tumor-suppressive effects of RhoB. We found that the IQ-motif-containing GTPase-activating protein IQGAP1 interacts directly with GTP-bound, prenylated RhoA and RhoC, but not with RhoB. Co-immunoprecipitation of IQGAP1 with endogenous RhoA/C was enhanced when RhoA/C were activated by epidermal growth factor (EGF) or transfection of a constitutively active guanine nucleotide exchange factor (GEF). Overexpression of IQGAP1 increased GTP-loading of RhoA/C, while siRNA-mediated depletion of IQGAP1 prevented endogenous RhoA/C activation by growth factors. IQGAP1 knockdown also reduced the amount of GTP bound to GTPase-deficient RhoA/C mutants, suggesting that IQGAP enhances Rho activation by GEF(s) or stabilizes Rho-GTP. IQGAP1 depletion in MDA-MB-231 breast cancer cells blocked EGF- and RhoA-induced stimulation of DNA synthesis. Infecting cells with adenovirus encoding constitutively active RhoA(L63) and measuring absolute amounts of RhoA-GTP in infected cells demonstrated that the lack of RhoA(L63)-induced DNA synthesis in IQGAP1-depleted cells was not due to reduced GTP-bound RhoA. These data suggested that IQGAP1 functions downstream of RhoA. Overexpression of IQGAP1 in MDA-MB-231 cells increased DNA synthesis irrespective of siRNA-mediated RhoA knockdown. Breast cancer cell motility was increased by expressing a constitutively-active RhoC(V14) mutant or overexpressing IQGAP1. EGF- or RhoC-induced   migration   required IQGAP1, but IQGAP1-stimulated   migration   independently of RhoC, placing IQGAP1 downstream of RhoC. We conclude that IQGAP1 acts both upstream of RhoA/C, regulating their activation state, and downstream of RhoA/C, mediating their effects on breast cancer cell proliferation and   migration  , respectively. 
 Analysis of the myosin-II-responsive focal adhesion proteome reveals a role for β-Pix in negative regulation of focal adhesion maturation. Focal adhesions undergo myosin-II-mediated maturation wherein they grow and change composition to modulate integrin signalling for cell   migratio  n, growth and differentiation. To determine how focal adhesion composition is affected by myosin II activity, we performed proteomic analysis of isolated focal adhesions and compared protein abundance in focal adhesions from cells with and without myosin II inhibition. We identified 905 focal adhesion proteins, 459 of which changed in abundance with myosin II inhibition, defining the myosin-II-responsive focal adhesion proteome. The abundance of 73% of the proteins in the myosin-II-responsive focal adhesion proteome was enhanced by contractility, including proteins involved in Rho-mediated focal adhesion maturation and endocytosis- and calpain-dependent focal adhesion disassembly. During myosin II inhibition, 27% of proteins in the myosin-II-responsive focal adhesion proteome, including proteins involved in Rac-mediated lamellipodial protrusion, were enriched in focal adhesions, establishing that focal adhesion protein recruitment is also negatively regulated by contractility. We focused on the Rac guanine nucleotide exchange factor β-Pix, documenting its role in the negative regulation of focal adhesion maturation and the promotion of lamellipodial protrusion and focal adhesion turnover to drive cel  l migrati  on. 
 A novel strategy for specifically down-regulating individual Rho GTPase activity in tumor cells. The Rho family GTPases RhoA, RhoB, and RhoC regulate the actin cytoskeleton, cell movement, and cell growth. Unlike Ras, up-regulation or overexpression of these GDP/GTP binding molecular switches, but not activating point mutations, has been associated with human cancer. Although they share over 85% sequence identity, RhoA, RhoB, and RhoC appear to play distinct roles in cell transformation and metastasis. In NIH 3T3 cells, RhoA or RhoB overexpression causes transformation whereas RhoC increases the cell   migration   rate. To specifically target RhoA, RhoB, or RhoC function, we have generated a set of chimeric molecules by fusing the RhoGAP domain of p190, a GTPase-activating protein that accelerates the intrinsic GTPase activity of all three Rho GTPases, with the C-terminal hypervariable sequences of RhoA, RhoB, or RhoC. The p190-Rho chimeras were active as GTPase-activating proteins toward RhoA in vitro, co-localized with the respective active Rho proteins, and specifically down-regulated Rho protein activities in cells depending on which Rho GTPase sequences were included in the chimeras. In particular, the p190-RhoA-C chimera specifically inhibited RhoA-induced transformation whereas p190-RhoC-C specifically reversed the   migration   phenotype induced by the active RhoC. In human mammary epithelial-RhoC breast cancer cells, p190-RhoC-C, but not p190-RhoA-C or p190-RhoB-C, reversed the anchorage-independent growth and invasion phenotypes caused by RhoC overexpression. In the highly metastatic A375-M human melanoma cells, p190-RhoC-C specifically reversed   migration  , and invasion phenotypes attributed to RhoC up-regulation. Thus, we have developed a novel strategy utilizing RhoGAP-Rho chimeras to specifically down-regulate individual Rho activity and demonstrate that this approach may be applied to multiple human tumor cells to reverse the growth and/or invasion phenotypes associated with disregulation of a distinct subtype of Rho GTPase. 
 RHO binding to FAM65A regulates Golgi reorientation during cell   migration  . Directional cell   migration   involves reorientation of the secretory machinery. However, the molecular mechanisms that control this reorientation are not well characterised. Here, we identify a new Rho effector protein, named FAM65A, which binds to active RHOA, RHOB and RHOC. FAM65A links RHO proteins to Golgi-localising cerebral cavernous malformation-3 protein (CCM3; also known as PDCD10) and its interacting proteins mammalian STE20-like protein kinases 3 and 4 (MST3 and MST4; also known as STK24 and STK26, respectively). Binding of active RHO proteins to FAM65A does not affect the kinase activity of MSTs but results in their relocation from the Golgi in a CCM3-dependent manner. This relocation is crucial for reorientation of the Golgi towards the leading edge and subsequent directional cell   migration  . Our results reveal a previously unidentified pathway downstream of RHO that regulates the polarity of migrating cells through Golgi reorientation in a FAM65A-, CCM3- and MST3- and MST4-dependent manner. 
 The Role of RhoA, RhoB and RhoC GTPases in Cell Morphology, Proliferation and   Migration   in Human Cytomegalovirus (HCMV) Infected Glioblastoma Cells. Rho GTPases are crucial regulators of the actin cytoskeleton, membrane trafficking and cell signaling and their importance in cell   migration   and invasion is well- established. The human cytomegalovirus (HCMV) is a widespread pathogen responsible for generally asymptomatic and persistent infections in healthy people. Recent evidence indicates that HCMV gene products are expressed in over 90% of malignant type glioblastomas (GBM). In addition, the HCMV Immediate Early-1 protein (IE1) is expressed in >90% of tumors analyzed.RhoA, RhoB and RhoC were individually depleted in U373MG glioblastoma cells as well as U373MG cells stably expressing the HCMV IE1 protein (named U373MG-IE1 cells) shRNA lentivirus vectors. Cell proliferation assays,   migration   as well as wound-healing assays were performed in uninfected and HCMV-infected cells.The depletion of RhoA, RhoB and RhoC protein resulted in significant alterations in the morphology of the uninfected cells, which were further enhanced by the cytopathic effect caused by HCMV. Furthermore, in the absence or presence of HCMV, the knockdown of RhoB and RhoC proteins decreased the proliferation rate of the parental and the IE1-expressing glioblastoma cells, whereas the knockdown of RhoA protein in the HCMV infected cell lines restored their proliferation rate. In addition, wound healing assays in U373MG cells revealed that depletion of RhoA, RhoB and RhoC differentially reduced their   migration   rate, even in the presence or the absence of HCMV.Collectively, these data show for the first time a differential implication of Rho GTPases in morphology, proliferation rate and motility of human glioblastoma cells during HCMV infection, further supporting an oncomodulatory role of HCMV depending on the Rho isoforms' state. 
 Immunohistochemical expression of Rho GTPases in ameloblastomas. Rho GTPases are proteins that regulate cell cycle, shape, polarization, invasion,   migration  , and apoptosis, which are important characteristics of normal and neoplastic cells. Rho GTPases expression has been reported in normal tooth germ and several pathologies; however, it has not been evaluated in ameloblastomas. The aim of this study was to analyze the expression and distribution of RhoA, RhoB, Rac1, and Cdc42 Rho GTPases in solid and unicystic ameloblastomas. Three-micrometer sections from paraffin-embedded specimens were evaluated by using an avidin-biotin immunohistochemical method with antibodies against the proteins mentioned above. RhoA and RhoB staining was observed in a high number of cells (P < 0.05) and greater intensity in non-polarized ones. Rac1 was not observed, and Cdc42 did not show any statistical differences between the number of non-polarized and basal positive cells (P > 0.05). Upon comparing the studied ameloblastomas, a higher number of positive cells in the unicystic variant was observed than that in the solid one (P < 0,05). The results obtained suggest that these GTPases could play a role in the ameloblastoma neoplastic epithelial cell phenotype determination (polarized or non-polarized), as well as in variant (solid or unicystic) and subtype (follicular or plexiform) determination. Furthermore, they could participate in solid ameloblastoma invasion mechanisms. 
 Rho isoforms have distinct and specific functions in the process of epithelial to mesenchymal transition in renal proximal tubular cells. Epithelial to mesenchymal transition (EMT) is involved in embryological development, cancerous metastatic spread and organ fibrosis, including the kidney. This process is largely driven by transforming growth factor-beta and recent evidence has implicated Rho as a key intracellular signalling molecule. In this study we have used RNA interference to silence the genetically distinct Rho (A, B and C) isoforms to define their individual functions in human kidney epithelial cells undergoing EMT. We demonstrate that the downregulation of the epithelial cell marker E-cadherin is dependent upon the Rho effector, Rho-kinase. However, silencing RhoA or RhoC expression also results in E-cadherin loss, though each by different mechanisms. Loss of RhoA leads to an upregulation of Snail1 and a reduction in the transcription of E-cadherin whereas loss of RhoC upregulates its breakdown via proteasomal degradation. During EMT, the upregulation of alpha-smooth muscle actin can be blocked by inhibiting the expression of RhoA, but not by that of RhoB or RhoC. This effect is independent of Rho-kinase activity. RhoC is the isoform solely responsible for stress fibre formation and inhibiting its expression reduces EMT-induced   migration   by 50%. RhoB appears to play a role in cell survival as inhibiting its expression leads to >300% increase in cell apoptosis and a relocalization of focal adhesion kinase. We conclude that Rho is a key signalling molecule in the process of EMT but that each isoform has a distinct and specific role. 
 Mutation of ARHGAP9 in patients with coronary spastic angina. Coronary artery spasm has an important function in the etiology of variant angina and other acute coronary syndromes. Abnormal activation of Rho-family GTPases has been observed in cardiovascular disorders, but the function of genetic variability in Rho-family GTPases remains to be evaluated in cardiovascular disorders. We examined the genetic variability of Rho-family GTPases and their regulators in coronary artery spasm. We performed a comprehensive candidate gene analysis of 67 single nucleotide polymorphisms with amino-acid substitution in Rho-family GTPases and their regulators in 103 unrelated Japanese patients with acetylcholine-induced coronary artery spasm and 102 control Japanese subjects without acetylcholine-induced coronary artery spasm. We noted an association of the single nucleotide polymorphism of ARHGAP9 (rs11544238, Ala370Ser) with coronary artery spasm (odds ratio =2.67). We found that ARHGAP9 inactivated Rac as RacGAP and that the mRNA level of ARHGAP9 was strongly detected in hematopoietic cells. ARHGAP9 negatively regulated cell   migration  . The Ala370Ser polymorphism counteracted ARHGAP9-reduced cell   migration  , spreading and adhesion. The Ala370Ser polymorphism in the ARHGAP9 gene is associated with coronary artery spasm. These data suggest that the polymorphism of ARHGAP9 has a critical function in the infiltration of hematopoietic cells into the endothelium and inflammation leading to endothelial dysfunction. 
 The Role of RhoA, RhoB and RhoC GTPases in Cell Morphology, Proliferation and   Migration   in Human Cytomegalovirus (HCMV) Infected Glioblastoma Cells. Rho GTPases are crucial regulators of the actin cytoskeleton, membrane trafficking and cell signaling and their importance in cell   migration   and invasion is well- established. The human cytomegalovirus (HCMV) is a widespread pathogen responsible for generally asymptomatic and persistent infections in healthy people. Recent evidence indicates that HCMV gene products are expressed in over 90% of malignant type glioblastomas (GBM). In addition, the HCMV Immediate Early-1 protein (IE1) is expressed in >90% of tumors analyzed.RhoA, RhoB and RhoC were individually depleted in U373MG glioblastoma cells as well as U373MG cells stably expressing the HCMV IE1 protein (named U373MG-IE1 cells) shRNA lentivirus vectors. Cell proliferation assays,   migration   as well as wound-healing assays were performed in uninfected and HCMV-infected cells.The depletion of RhoA, RhoB and RhoC protein resulted in significant alterations in the morphology of the uninfected cells, which were further enhanced by the cytopathic effect caused by HCMV. Furthermore, in the absence or presence of HCMV, the knockdown of RhoB and RhoC proteins decreased the proliferation rate of the parental and the IE1-expressing glioblastoma cells, whereas the knockdown of RhoA protein in the HCMV infected cell lines restored their proliferation rate. In addition, wound healing assays in U373MG cells revealed that depletion of RhoA, RhoB and RhoC differentially reduced their   migration   rate, even in the presence or the absence of HCMV.Collectively, these data show for the first time a differential implication of Rho GTPases in morphology, proliferation rate and motility of human glioblastoma cells during HCMV infection, further supporting an oncomodulatory role of HCMV depending on the Rho isoforms' state. 
 RhoA, RhoB and RhoC have different roles in cancer cell   migration  . Rho GTPases are well known to regulate cell motility through activation of a variety of downstream effector proteins, including enzymes, adaptor proteins and actin nucleators. The three closely related Rho GTPases RhoA, RhoB and RhoC all have the potential to interact with the same downstream effectors, yet they have substantially different effects on cell shape and migratory properties. Here I review the different ways in which RhoA, RhoB and RhoC expression is regulated in cancer and how they play distinct roles in cancer progression. I describe their main effectors known to contribute to cell motility. Recent results from our laboratory and others indicate that RhoA, RhoB and RhoC can be activated by specific stimuli and act through different effectors to control distinct aspects of cancer cell   migration   and invasion. This suggests that they each make unique contributions to cancer by participating in different protein complexes. 
 Rho isoform-specific interaction with IQGAP1 promotes breast cancer cell proliferation and   migration  . We performed a proteomics screen for Rho isoform-specific binding proteins to clarify the tumor-promoting effects of RhoA and C that contrast with the tumor-suppressive effects of RhoB. We found that the IQ-motif-containing GTPase-activating protein IQGAP1 interacts directly with GTP-bound, prenylated RhoA and RhoC, but not with RhoB. Co-immunoprecipitation of IQGAP1 with endogenous RhoA/C was enhanced when RhoA/C were activated by epidermal growth factor (EGF) or transfection of a constitutively active guanine nucleotide exchange factor (GEF). Overexpression of IQGAP1 increased GTP-loading of RhoA/C, while siRNA-mediated depletion of IQGAP1 prevented endogenous RhoA/C activation by growth factors. IQGAP1 knockdown also reduced the amount of GTP bound to GTPase-deficient RhoA/C mutants, suggesting that IQGAP enhances Rho activation by GEF(s) or stabilizes Rho-GTP. IQGAP1 depletion in MDA-MB-231 breast cancer cells blocked EGF- and RhoA-induced stimulation of DNA synthesis. Infecting cells with adenovirus encoding constitutively active RhoA(L63) and measuring absolute amounts of RhoA-GTP in infected cells demonstrated that the lack of RhoA(L63)-induced DNA synthesis in IQGAP1-depleted cells was not due to reduced GTP-bound RhoA. These data suggested that IQGAP1 functions downstream of RhoA. Overexpression of IQGAP1 in MDA-MB-231 cells increased DNA synthesis irrespective of siRNA-mediated RhoA knockdown. Breast cancer cell motility was increased by expressing a constitutively-active RhoC(V14) mutant or overexpressing IQGAP1. EGF- or RhoC-induced   migration   required IQGAP1, but IQGAP1-stimulated   migration   independently of RhoC, placing IQGAP1 downstream of RhoC. We conclude that IQGAP1 acts both upstream of RhoA/C, regulating their activation state, and downstream of RhoA/C, mediating their effects on breast cancer cell proliferation and   migration  , respectively. 
 Immunohistochemical expression of Rho GTPases in ameloblastomas. Rho GTPases are proteins that regulate cell cycle, shape, polarization, invasion,   migration  , and apoptosis, which are important characteristics of normal and neoplastic cells. Rho GTPases expression has been reported in normal tooth germ and several pathologies; however, it has not been evaluated in ameloblastomas. The aim of this study was to analyze the expression and distribution of RhoA, RhoB, Rac1, and Cdc42 Rho GTPases in solid and unicystic ameloblastomas. Three-micrometer sections from paraffin-embedded specimens were evaluated by using an avidin-biotin immunohistochemical method with antibodies against the proteins mentioned above. RhoA and RhoB staining was observed in a high number of cells (P < 0.05) and greater intensity in non-polarized ones. Rac1 was not observed, and Cdc42 did not show any statistical differences between the number of non-polarized and basal positive cells (P > 0.05). Upon comparing the studied ameloblastomas, a higher number of positive cells in the unicystic variant was observed than that in the solid one (P < 0,05). The results obtained suggest that these GTPases could play a role in the ameloblastoma neoplastic epithelial cell phenotype determination (polarized or non-polarized), as well as in variant (solid or unicystic) and subtype (follicular or plexiform) determination. Furthermore, they could participate in solid ameloblastoma invasion mechanisms. 
 Aloe emodin inhibits colon cancer cell   migration  /angiogenesis by downregulating MMP-2/9, RhoB and VEGF via reduced DNA binding activity of NF-κB. Aloe emodin (AE), a natural anthraquinone, is reported to have antiproliferative activity in various cancer cell lines. In this study we analyzed molecular mechanisms involved in the antimigratory and antiangiogenic activity of this hydroxy anthraquinone in colon cancer cell, WiDr. Our results show that a relatively non toxic concentration of AE suppressed the phorbol-12-myristyl-13-acetate (PMA) induced   migratio  n and invasion of tumor cells. On analysis for the molecules involved in the   migratio  n/invasion, we found AE downregulated mRNA expression and promoter/gelatinolytic activity of Matrix Metalloproteinase (MMP)-2/9, as well as the RhoB expression at gene and protein level. It was also a strong inhibitor of Vascular Endothelial Growth Factor (VEGF) expression, promoter activity and endothelial cell   migratio  n/invasion and in vitro angiogenesis. AE suppressed the nuclear translocation and DNA binding of NF-κB, which is an important transcription factor for controlling MMP-2/9 and VEGF gene expression. Taken together these data indicate that AE target multiple molecules responsible for cellular invasion  , migrati  on and angiogenesis. Inhibitory effect on angiogenic and metastatic regulatory processes make AE a sensible candidate as a specific blocker of tumor associated events. 
 Rho isoforms have distinct and specific functions in the process of epithelial to mesenchymal transition in renal proximal tubular cells. Epithelial to mesenchymal transition (EMT) is involved in embryological development, cancerous metastatic spread and organ fibrosis, including the kidney. This process is largely driven by transforming growth factor-beta and recent evidence has implicated Rho as a key intracellular signalling molecule. In this study we have used RNA interference to silence the genetically distinct Rho (A, B and C) isoforms to define their individual functions in human kidney epithelial cells undergoing EMT. We demonstrate that the downregulation of the epithelial cell marker E-cadherin is dependent upon the Rho effector, Rho-kinase. However, silencing RhoA or RhoC expression also results in E-cadherin loss, though each by different mechanisms. Loss of RhoA leads to an upregulation of Snail1 and a reduction in the transcription of E-cadherin whereas loss of RhoC upregulates its breakdown via proteasomal degradation. During EMT, the upregulation of alpha-smooth muscle actin can be blocked by inhibiting the expression of RhoA, but not by that of RhoB or RhoC. This effect is independent of Rho-kinase activity. RhoC is the isoform solely responsible for stress fibre formation and inhibiting its expression reduces EMT-induced   migration   by 50%. RhoB appears to play a role in cell survival as inhibiting its expression leads to >300% increase in cell apoptosis and a relocalization of focal adhesion kinase. We conclude that Rho is a key signalling molecule in the process of EMT but that each isoform has a distinct and specific role. 
 Mutation of ARHGAP9 in patients with coronary spastic angina. Coronary artery spasm has an important function in the etiology of variant angina and other acute coronary syndromes. Abnormal activation of Rho-family GTPases has been observed in cardiovascular disorders, but the function of genetic variability in Rho-family GTPases remains to be evaluated in cardiovascular disorders. We examined the genetic variability of Rho-family GTPases and their regulators in coronary artery spasm. We performed a comprehensive candidate gene analysis of 67 single nucleotide polymorphisms with amino-acid substitution in Rho-family GTPases and their regulators in 103 unrelated Japanese patients with acetylcholine-induced coronary artery spasm and 102 control Japanese subjects without acetylcholine-induced coronary artery spasm. We noted an association of the single nucleotide polymorphism of ARHGAP9 (rs11544238, Ala370Ser) with coronary artery spasm (odds ratio =2.67). We found that ARHGAP9 inactivated Rac as RacGAP and that the mRNA level of ARHGAP9 was strongly detected in hematopoietic cells. ARHGAP9 negatively regulated cell   migration  . The Ala370Ser polymorphism counteracted ARHGAP9-reduced cell   migration  , spreading and adhesion. The Ala370Ser polymorphism in the ARHGAP9 gene is associated with coronary artery spasm. These data suggest that the polymorphism of ARHGAP9 has a critical function in the infiltration of hematopoietic cells into the endothelium and inflammation leading to endothelial dysfunction. 
 A novel strategy for specifically down-regulating individual Rho GTPase activity in tumor cells. The Rho family GTPases RhoA, RhoB, and RhoC regulate the actin cytoskeleton, cell movement, and cell growth. Unlike Ras, up-regulation or overexpression of these GDP/GTP binding molecular switches, but not activating point mutations, has been associated with human cancer. Although they share over 85% sequence identity, RhoA, RhoB, and RhoC appear to play distinct roles in cell transformation and metastasis. In NIH 3T3 cells, RhoA or RhoB overexpression causes transformation whereas RhoC increases the cell   migration   rate. To specifically target RhoA, RhoB, or RhoC function, we have generated a set of chimeric molecules by fusing the RhoGAP domain of p190, a GTPase-activating protein that accelerates the intrinsic GTPase activity of all three Rho GTPases, with the C-terminal hypervariable sequences of RhoA, RhoB, or RhoC. The p190-Rho chimeras were active as GTPase-activating proteins toward RhoA in vitro, co-localized with the respective active Rho proteins, and specifically down-regulated Rho protein activities in cells depending on which Rho GTPase sequences were included in the chimeras. In particular, the p190-RhoA-C chimera specifically inhibited RhoA-induced transformation whereas p190-RhoC-C specifically reversed the   migration   phenotype induced by the active RhoC. In human mammary epithelial-RhoC breast cancer cells, p190-RhoC-C, but not p190-RhoA-C or p190-RhoB-C, reversed the anchorage-independent growth and invasion phenotypes caused by RhoC overexpression. In the highly metastatic A375-M human melanoma cells, p190-RhoC-C specifically reversed   migration  , and invasion phenotypes attributed to RhoC up-regulation. Thus, we have developed a novel strategy utilizing RhoGAP-Rho chimeras to specifically down-regulate individual Rho activity and demonstrate that this approach may be applied to multiple human tumor cells to reverse the growth and/or invasion phenotypes associated with disregulation of a distinct subtype of Rho GTPase. 
 A role for rhoB in the delamination of neural crest cells from the dorsal neural tube. The differentiation of neural crest cells from progenitors located in the dorsal neural tube appears to involve three sequential steps: the specification of premigratory neural crest cell fate, the delamination of these cells from the neural epithelium and the   migration   of neural crest cells in the periphery. BMP signaling has been implicated in the specification of neural crest cell fate but the mechanisms that control the emergence of neural crest cells from the neural tube remain poorly understood. To identify molecules that might function at early steps of neural crest differentiation, we performed a PCR-based screen for genes induced by BMPs in chick neural plate cells. We describe the cloning and characterization of one gene obtained from this screen, rhoB, a member of the rho family GTP-binding proteins. rhoB is expressed in the dorsal neural tube and its expression persists transiently in migrating neural crest cells. BMPs induce the neural expression of rhoB but not the more widely expressed rho family member, rhoA. Inhibition of rho activity by C3 exotoxin prevents the delamination of neural crest cells from neural tube explants but has little effect on the initial specification of premigratory neural crest cell fate or on the later   migration   of neural crest cells. These results suggest that rhoB has a role in the delamination of neural crest cells from the dorsal neural tube. 
 Overexpression of miR-21 promotes an in vitro metastatic phenotype by targeting the tumor suppressor RHOB. Metastasis is a multistep process that involves the deregulation of oncogenes and tumor suppressors beyond changes required for primary tumor formation. RHOB is known to have tumor suppressor activity, and its knockdown is associated with more aggressive tumors as well as changes in cell shape,   migration  , and adhesion. This study shows that oncogenic microRNA, miR-21, represses RHOB expression by directly targeting the 3' untranslated region. Loss of miR-21 is associated with an elevation of RHOB in hepatocellular carcinoma cell lines Huh-7 and HepG2 and in the metastatic breast cancer cell line MDA-MB-231. Using in vitro models of distinct stages of metastasis, we showed that loss of miR-21 also causes a reduction in   migration  , invasion, and cell elongation. The reduction in   migration   and cell elongation can be mimicked by overexpression of RHOB. Furthermore, changes in miR-21 expression lead to alterations in matrix metalloproteinase-9 activity. Therefore, we conclude that miR-21 promotes multiple components of the metastatic phenotype in vitro by regulating several important tumor suppressors, including RHOB. 
 The Rho GTPase RhoB regulates cadherin expression and epithelial cell-cell interaction. The Rho GTPase RhoB has been proposed to be a tumor suppressor in cancer and is downregulated in various tumors including prostate. RhoB has different effects on cell   migration   depending on the cell type and conditions, but the molecular basis for this variability is unclear. RhoB regulates trafficking of membrane receptors and integrins. We have previously shown that RhoB depletion alters focal adhesion dynamics and reduces surface levels of β1 integrin in PC3 prostate cancer cells, correlating with increased   migratio  n speed.Here we show that RhoB depletion reduces cell-cell adhesion and downregulates E-cadherin levels as well as increasing internalized E-cadherin in DU145 prostate cancer cells. This is accompanied by increased   migratio  n speed. RhoB localizes to cell-cell junctions together with E-cadherin in DU145 cells. RhoB depletion also reduces N-cadherin levels in PC3 cells, which do not express E-cadherin.These results indicate that RhoB alters   migratio  n of cells with cell-cell adhesions by regulating cadherin levels. We propose that the relative contribution of integrins and cadherins to cell   migratio  n underlies the variable involvement for RhoB in this process and that the downregulation of RhoB in some epithelial cancers could contribute to the weakening of epithelial cell-cell junction during tumor progression. 
 RhoB enhances   migration   and MMP1 expression of prostate cancer DU145. Rho family protein regulates variety of cellular functions as cytoskeletal organization, cell proliferation and apoptosis. In the present study, we demonstrate that RhoB-overexpressed prostate cancer cells showed an enhanced cell motility and the administration of the GSK-3 inhibitors inhibited this increase in   migration  . Among the extracellular matrix and adhesion-related molecules, MMP1 RNA expression was increased in RhoB-overexpressed cells, administration of MMP inhibitor suppressed the collagen gel invasion in these cells. This is the first report evaluating RhoB function and the downstream signaling events in prostate cancer cell. Our results indicate that RhoB promotes cell motility and invasion in a metastatic prostate cancer cell. 
 Role of RhoB in the regulation of pulmonary endothelial and smooth muscle cell responses to hypoxia. RhoA and Rho kinase contribute to pulmonary vasoconstriction and vascular remodeling in pulmonary hypertension. RhoB, a protein homologous to RhoA and activated by hypoxia, regulates neoplastic growth and vasoconstriction but its role in the regulation of pulmonary vascular function is not known.To determine the role of RhoB in pulmonary endothelial and smooth muscle cell responses to hypoxia and in pulmonary vascular remodeling in chronic hypoxia-induced pulmonary hypertension.Hypoxia increased expression and activity of RhoB in human pulmonary artery endothelial and smooth muscle cells, coincidental with activation of RhoA. Hypoxia or adenoviral overexpression of constitutively activated RhoB increased actomyosin contractility, induced endothelial permeability, and promoted cell growth; dominant negative RhoB or manumycin, a farnesyltransferase inhibitor that targets the vascular function of RhoB, inhibited the effects of hypoxia. Coordinated activation of RhoA and RhoB maximized the hypoxia-induced stress fiber formation caused by RhoB/mammalian homolog of Drosophila diaphanous-induced actin polymerization and RhoA/Rho kinase-induced phosphorylation of myosin light chain on Ser19. Notably, RhoB was specifically required for hypoxia-induced factor-1α stabilization and for hypoxia- and platelet-derived growth factor-induced cell proliferation and   migratio  n. RhoB deficiency in mice markedly attenuated development of chronic hypoxia-induced pulmonary hypertension, despite compensatory expression of RhoA in the lung.RhoB mediates adaptational changes to acute hypoxia in the vasculature, but its continual activation by chronic hypoxia can accentuate vascular remodeling to promote development of pulmonary hypertension. RhoB is a potential target for novel approaches (eg, farnesyltransferase inhibitors) aimed at regulating pulmonary vascular tone and structure. 
 A distinct role of RhoB in gastric cancer suppression. Although Rho family GTPases RhoA, RhoB and RhoC share more than 85% amino acid sequence identity, they may play distinct roles in tumor progression. RhoA and RhoC have been suggested to have positive effects on tumor progression, but the role of RhoB in cancer, particularly in gastric cancer, remains unclear. In our study, we have examined the expression levels of these three Rho GTPases in a large panel of specimens from gastric cancer patients by immunohistochemistry. We found that RhoA and RhoC expression were significantly elevated, while RhoB was reduced or absent, in surgically removed gastric cancer tissues when compared to normal gastric tissues. The significant reduction of RhoB expression was confirmed in another group of gastric cancer samples in comparison to the adjacent non-neoplastic tissues. Then we transfected the plasmids containing RhoA, RhoB or RhoC cDNA into two gastric cancer cell lines, SGC7901 and AGS cells, respectively. By overexpression experiments, we found that RhoA promoted the gastric cancer cell proliferation and RhoC stimulated   migration   and invasion of the cancer cell. RhoB expression, however, significantly inhibited the proliferation,   migration   and invasion of the gastric cancer cells and also enhanced the chemosensitivity of these cells to anticancer drugs. It appears that RhoB plays an opposing role from that of RhoA and/or RhoC in gastric cancer cells. Our work suggests that RhoB may play a tumor suppressor role and subsequently may have potential implications in future targeted therapy. 
 RhoB regulates uPAR signalling. Urokinase-type plasminogen activator (uPA) and its receptor, uPAR, play important roles in promoting cancer cell adhesion,   migration   and invasion. Rho GTPases are key coordinators of these processes; the Rho GTPase Rac1 has previously been implicated in uPA- and/or uPAR-induced migratory or morphological cell responses. We used RNAi to deplete 12 different Rho GTPases to screen for effects on uPA-stimulated   migration  , and found that depletion of RhoB significantly reduces uPA-induced   migration   and invasion of prostate carcinoma cells. RhoB depletion did not affect the expression or surface levels of uPAR but reduced the uPAR-induced increase in levels of several integrins and inhibited uPAR signalling to the actin regulator cofilin, the cell-adhesion signal-transduction adaptor molecule paxillin and the serine/threonine kinase Akt. uPAR rapidly activated RhoB and increased RhoB expression. RhoB depletion also reduced cell adhesion to and spreading on vitronectin, which is a uPAR ligand. This correlated with decreased association between integrins and uPAR and reduced integrin β1 activity. Our results indicate that RhoB is a key regulator of uPAR signalling in cell adhesion,   migratio  n and invasion. 
 MicroRNA-21 exhibits antiangiogenic function by targeting RhoB expression in endothelial cells. MicroRNAs (miRNAs) are endogenously expressed small non-coding RNAs that regulate gene expression at post-transcriptional level. The recent discovery of the involvement of these RNAs in the control of angiogenesis renders them very attractive in the development of new approaches for restoring the angiogenic balance. Whereas miRNA-21 has been demonstrated to be highly expressed in endothelial cells, the potential function of this miRNA in angiogenesis has never been investigated.We first observed in endothelial cells a negative regulation of miR-21 expression by serum and bFGF, two pro-angiogenic factors. Then using in vitro angiogenic assays, we observed that miR-21 acts as a negative modulator of angiogenesis. miR-21 overexpression reduced endothelial cell proliferation,   migration   and the ability of these cells to form tubes whereas miR-21 inhibition using a LNA-anti-miR led to opposite effects. Expression of miR-21 in endothelial cells also led to a reduction in the organization of actin into stress fibers, which may explain the decrease in cell   migration  . Further mechanistic studies showed that miR-21 targets RhoB, as revealed by a decrease in RhoB expression and activity in miR-21 overexpressing cells. RhoB silencing impairs endothelial cell   migration   and tubulogenesis, thus providing a possible mechanism for miR-21 to inhibit angiogenesis. Finally, the therapeutic potential of miR-21 as an angiogenesis inhibitor was demonstrated in vivo in a mouse model of choroidal neovascularization.Our results identify miR-21 as a new angiogenesis inhibitor and suggest that inhibition of cell   migration   and tubulogenesis is mediated through repression of RhoB. 
 RhoB regulates cell   migration   through altered focal adhesion dynamics. The Rho GTPase RhoB has been shown to affect cell   migration  , but how it does this is not clear. Here we show that cells depleted of RhoB by RNAi are rounded and have defects in Rac-mediated spreading and lamellipodium extension, although they have active membrane ruffling around the periphery. Depletion of the exchange factor GEF-H1 induces a similar phenotype. RhoB-depleted cells migrate faster, but less persistently in a chemotactic gradient, and frequently round up during   migration  . RhoB-depleted cells have similar numbers of focal adhesions to control cells during spreading and   migration  , but show more diffuse and patchy contact with the substratum. They have lower levels of surface β1 integrin, and β1 integrin activity is reduced in actin-rich protrusions. We propose that RhoB contributes to directional cel  l migrati  on by regulating β1 integrin surface levels and activity, thereby stabilizing lamellipodial protrusions. 
  GeneRIFs  
 RhoB contributes to directional cell   migration   by regulating beta1 integrin surface levels and activity, thereby stabilizing lamellipodial protrusions. 
  [Ee]xtracellular [Mm]atrix  PubMed  
 RhoB loss induces Rac1-dependent mesenchymal cell invasion in lung cells through PP2A inhibition. Non-small-cell lung cancer (NSCLC) is the leading cause of cancer-related death worldwide, which is mainly due to its high risk of metastatic dissemination. One critical point of this process is the ability of cancer cells to detach from the primary tumor and migrate through the   extracellular matrix  ; however, the underlying molecular mechanisms are not yet fully understood. In the present study, we identified the small GTPase RhoB as a key regulator of bronchial cell morphology in a three-dimensional (3D) matrix. RhoB loss, which is frequently observed during lung cancer progression, induced an epithelial-mesenchymal transition (EMT) characterized by an increased proportion of invasive elongated cells in 3D. The process was mediated by Slug induction and E-cadherin repression. In addition, downregulation of RhoB induced Akt1 activation, which in turn activated Rac1 through the guanine-exchange factor Trio to control cell shape rearrangement. Further, we provide evidence that RhoB interacted with and positively regulates phosphatase PP2A through the recruitment of its regulatory subunit B55, which was found to be crucial for Akt dephosphorylation. B55 inhibition completely suppressed RhoB-mediated PP2A regulation. Finally, we show that PP2A inactivation, by targeting either its catalytic or its regulatory B55 subunit, completely reversed RhoB-dependent morphological changes and also fully prevented the ability of RhoB to decrease the invasiveness of bronchial cells. Altogether, these results highlight a novel signaling axis and describe new molecular mechanisms that could explain the tumor suppressor role of RhoB in lung cancer. Therefore, we propose that RhoB could be responsible for early metastatic prevention by inhibiting the EMT-derived invasiveness of lung cells through the control of PP2A activity. 
 RhoB loss induces Rac1-dependent mesenchymal cell invasion in lung cells through PP2A inhibition. Non-small-cell lung cancer (NSCLC) is the leading cause of cancer-related death worldwide, which is mainly due to its high risk of metastatic dissemination. One critical point of this process is the ability of cancer cells to detach from the primary tumor and migrate through the   extracellular matrix  ; however, the underlying molecular mechanisms are not yet fully understood. In the present study, we identified the small GTPase RhoB as a key regulator of bronchial cell morphology in a three-dimensional (3D) matrix. RhoB loss, which is frequently observed during lung cancer progression, induced an epithelial-mesenchymal transition (EMT) characterized by an increased proportion of invasive elongated cells in 3D. The process was mediated by Slug induction and E-cadherin repression. In addition, downregulation of RhoB induced Akt1 activation, which in turn activated Rac1 through the guanine-exchange factor Trio to control cell shape rearrangement. Further, we provide evidence that RhoB interacted with and positively regulates phosphatase PP2A through the recruitment of its regulatory subunit B55, which was found to be crucial for Akt dephosphorylation. B55 inhibition completely suppressed RhoB-mediated PP2A regulation. Finally, we show that PP2A inactivation, by targeting either its catalytic or its regulatory B55 subunit, completely reversed RhoB-dependent morphological changes and also fully prevented the ability of RhoB to decrease the invasiveness of bronchial cells. Altogether, these results highlight a novel signaling axis and describe new molecular mechanisms that could explain the tumor suppressor role of RhoB in lung cancer. Therefore, we propose that RhoB could be responsible for early metastatic prevention by inhibiting the EMT-derived invasiveness of lung cells through the control of PP2A activity. 
 RhoB enhances migration and MMP1 expression of prostate cancer DU145. Rho family protein regulates variety of cellular functions as cytoskeletal organization, cell proliferation and apoptosis. In the present study, we demonstrate that RhoB-overexpressed prostate cancer cells showed an enhanced cell motility and the administration of the GSK-3 inhibitors inhibited this increase in migration. Among the   extracellular matrix   and adhesion-related molecules, MMP1 RNA expression was increased in RhoB-overexpressed cells, administration of MMP inhibitor suppressed the collagen gel invasion in these cells. This is the first report evaluating RhoB function and the downstream signaling events in prostate cancer cell. Our results indicate that RhoB promotes cell motility and invasion in a metastatic prostate cancer cell. 
 Alphavbeta3 and alphavbeta5 integrins control glioma cell response to ionising radiation through ILK and RhoB. Integrins are   extracellular matrix   receptors involved in tumour invasion and angiogenesis. Although there is evidence that inhibiting integrins might enhance the efficiency of radiotherapy, little is known about the exact mechanisms involved in the integrin-dependent modulation of tumor radiosensitivity. The purpose of this study was to investigate the role of alphavbeta3 and alphavbeta5 integrins in glioblastoma cell radioresistance and overall to decipher the downstream biological pathways. We first demonstrated that silencing alphavbeta3 and alphavbeta5 integrins with specific siRNAs significantly reduced the survival after irradiation of 2 glioblastoma cell lines: U87 and SF763. We then showed that integrin activity and integrin signalling pathways controlled the glioma cell radiosensitivity. This regulation of glioma cell response to ionising radiation was mediated through the integrin-linked kinase, ILK, and the small GTPase, RhoB, by two mechanisms. The first one, independent of ILK, consists in the regulation of the intracellular level of RhoB by alphavbeta3 or alphavbeta5 integrin. The second pathway involved in cell radiosensitivity consists in RhoB activation by ionising radiation through ILK. Furthermore, we demonstrated that the alphavbeta3/alphavbeta5 integrins/ILK/RhoB pathway controlled the glioma cells radiosensitivity by regulating radiation-induced mitotic cell death. This work identifies a new biological pathway controlling glioblastoma cells radioresistance, activated from the membrane through alphavbeta3 and/or alphavbeta5 integrins via ILK and RhoB. Our results are clues that downstream effectors of alphavbeta3 and alphavbeta5 integrins as ILK and RhoB might also be promising candidate targets for improving the efficiency of radiotherapy and thus the clinical outcome of patients with glioblastoma. 
  [Ii]nvasive  PubMed  
 RhoB loss induces Rac1-dependent mesenchymal cell invasion in lung cells through PP2A inhibition. Non-small-cell lung cancer (NSCLC) is the leading cause of cancer-related death worldwide, which is mainly due to its high risk of metastatic dissemination. One critical point of this process is the ability of cancer cells to detach from the primary tumor and migrate through the extracellular matrix; however, the underlying molecular mechanisms are not yet fully understood. In the present study, we identified the small GTPase RhoB as a key regulator of bronchial cell morphology in a three-dimensional (3D) matrix. RhoB loss, which is frequently observed during lung cancer progression, induced an epithelial-mesenchymal transition (EMT) characterized by an increased proportion of   invasive   elongated cells in 3D. The process was mediated by Slug induction and E-cadherin repression. In addition, downregulation of RhoB induced Akt1 activation, which in turn activated Rac1 through the guanine-exchange factor Trio to control cell shape rearrangement. Further, we provide evidence that RhoB interacted with and positively regulates phosphatase PP2A through the recruitment of its regulatory subunit B55, which was found to be crucial for Akt dephosphorylation. B55 inhibition completely suppressed RhoB-mediated PP2A regulation. Finally, we show that PP2A inactivation, by targeting either its catalytic or its regulatory B55 subunit, completely reversed RhoB-dependent morphological changes and also fully prevented the ability of RhoB to decrease the   invasive  ness of bronchial cells. Altogether, these results highlight a novel signaling axis and describe new molecular mechanisms that could explain the tumor suppressor role of RhoB in lung cancer. Therefore, we propose that RhoB could be responsible for early metastatic prevention by inhibiting the EMT-derived   invasive  ness of lung cells through the control of PP2A activity. 
 GTPases Rho distribution in intraepithelial and   invasive   neoplasias of the uterine cervix. To evaluate the distribution of GTPases RhoA, RhoB, and Cdc42 in cervical intraepithelial neoplasias (CIN) and   invasive   neoplasias of the uterine cervix.samples of neoplastic lesions of the uterine cervix of 44 patients were classified in: CIN I (n = 10), CIN II (n = 10), CIN III (n = 09), and   invasive   carcinoma (n = 15). Antibodies anti-RhoA, anti-RhoB, and anti-Cdc42 were used and staining was classified as: negative, mild, moderate, and intense positive.When compared with dysplastic cells, superficial cells showed: higher expression of RhoB in CIN I (p = 0.0018), and lower expression of Cdc42 in CIN I (p = 0.0225). The authors observed higher expression of RhoA (p = 0.0002) and RhoB (p = 0.0046) in CIN dysplastic cells when compared with   invasive   carcinoma cells.GTPases Rho may be involved with the regulation of biological processes, important to the progression of cervical neoplasias. Probably, RhoA is important for maintenance of cell differentiation and RhoB protects cells from malignant cervical neoplasia. 
 RhoB loss induces Rac1-dependent mesenchymal cell invasion in lung cells through PP2A inhibition. Non-small-cell lung cancer (NSCLC) is the leading cause of cancer-related death worldwide, which is mainly due to its high risk of metastatic dissemination. One critical point of this process is the ability of cancer cells to detach from the primary tumor and migrate through the extracellular matrix; however, the underlying molecular mechanisms are not yet fully understood. In the present study, we identified the small GTPase RhoB as a key regulator of bronchial cell morphology in a three-dimensional (3D) matrix. RhoB loss, which is frequently observed during lung cancer progression, induced an epithelial-mesenchymal transition (EMT) characterized by an increased proportion of   invasive   elongated cells in 3D. The process was mediated by Slug induction and E-cadherin repression. In addition, downregulation of RhoB induced Akt1 activation, which in turn activated Rac1 through the guanine-exchange factor Trio to control cell shape rearrangement. Further, we provide evidence that RhoB interacted with and positively regulates phosphatase PP2A through the recruitment of its regulatory subunit B55, which was found to be crucial for Akt dephosphorylation. B55 inhibition completely suppressed RhoB-mediated PP2A regulation. Finally, we show that PP2A inactivation, by targeting either its catalytic or its regulatory B55 subunit, completely reversed RhoB-dependent morphological changes and also fully prevented the ability of RhoB to decrease the   invasive  ness of bronchial cells. Altogether, these results highlight a novel signaling axis and describe new molecular mechanisms that could explain the tumor suppressor role of RhoB in lung cancer. Therefore, we propose that RhoB could be responsible for early metastatic prevention by inhibiting the EMT-derived   invasive  ness of lung cells through the control of PP2A activity. 
 GTPases Rho distribution in intraepithelial and   invasive   neoplasias of the uterine cervix. To evaluate the distribution of GTPases RhoA, RhoB, and Cdc42 in cervical intraepithelial neoplasias (CIN) and   invasive   neoplasias of the uterine cervix.samples of neoplastic lesions of the uterine cervix of 44 patients were classified in: CIN I (n = 10), CIN II (n = 10), CIN III (n = 09), and   invasive   carcinoma (n = 15). Antibodies anti-RhoA, anti-RhoB, and anti-Cdc42 were used and staining was classified as: negative, mild, moderate, and intense positive.When compared with dysplastic cells, superficial cells showed: higher expression of RhoB in CIN I (p = 0.0018), and lower expression of Cdc42 in CIN I (p = 0.0225). The authors observed higher expression of RhoA (p = 0.0002) and RhoB (p = 0.0046) in CIN dysplastic cells when compared with   invasive   carcinoma cells.GTPases Rho may be involved with the regulation of biological processes, important to the progression of cervical neoplasias. Probably, RhoA is important for maintenance of cell differentiation and RhoB protects cells from malignant cervical neoplasia. 
 RASSF1A Suppresses the Invasion and Metastatic Potential of Human Non-Small Cell Lung Cancer Cells by Inhibiting YAP Activation through the GEF-H1/RhoB Pathway. Inactivation of the tumor suppressor gene RASSF1A by promoter hypermethylation represents a key event underlying the initiation and progression of lung cancer. RASSF1A inactivation is also associated with poor prognosis and may promote metastatic spread. In this study, we investigated how RASSF1A inactivation conferred   invasive   phenotypes to human bronchial cells. RNAi-mediated silencing of RASSF1A induced epithelial-to-mesenchymal transition (EMT), fomenting a motile and   invasive   cellular phenotype in vitro and increased metastatic prowess in vivo. Mechanistic investigations revealed that RASSF1A blocked tumor growth by stimulating cofilin/PP2A-mediated dephosphorylation of the guanine nucleotide exchange factor GEF-H1, thereby stimulating its ability to activate the antimetastatic small GTPase RhoB. Furthermore, RASSF1A reduced nuclear accumulation of the Hippo pathway transcriptional cofactor Yes-associated protein (YAP), which was reinforced by RhoB activation. Collectively, our results indicated that RASSF1 acts to restrict EMT and invasion by indirectly controlling YAP nuclear shuttling and activation through a RhoB-regulated cytoskeletal remodeling process, with potential implications to delay the progression of RASSF1-hypermethylated lung tumors. 
 Regulation of glioblastoma cell invasion by PKC iota and RhoB. Glioblastoma multiforme is the most aggressive form of primary brain tumor and remains largely incurable, in large part, due to its highly   invasive   nature. The phosphoinositide (PI) 3-kinase pathway is often constitutively active in these tumors due to activating mutations in the epidermal growth factor receptor, or deletion/loss of function of the tumor suppressor PTEN. Protein kinase C type iota (PKC iota), a member of the atypical protein kinase C family, is activated by the PI 3-kinase pathway and is an important downstream mediator. Here, we have assessed the role of PKC iota in glioblastoma cell invasion. Depletion of PKC iota with RNA interference caused an increase in actin stress fibers and a decrease in cell motility and invasion. Gene expression microarray analysis of U87MG cells showed that PKC iota repressed expression of mRNA for RhoB, which has previously been shown to have a role in actin stress fiber formation. Western blot analysis showed that both PKC iota depletion and pharmacological inhibition of PKC iota caused an increase in the protein levels of RhoB, as did inhibition of PI 3-kinase. Expression of RhoB from a constitutive promoter caused changes in actin stress fibers and cell invasion that were similar to those seen with PKC iota depletion. These data show that PKC iota, activated as a consequence of aberrant upstream PI 3-kinase signaling, mediates glioblastoma cell motility and invasion, and that repression of RhoB is key downstream event in PKC iota signaling leading to enhanced cell motility. In addition, constitutive expression of RhoB repressed PKC iota activity, as assessed by its phosphorylation status on Thr555. PKC iota and RhoB are, therefore, mutually antagonistic, potentially creating a sensitive switch between   invasive   and non-  invasive   phenotypes. 
 RhoB determines tumor aggressiveness in a murine EGFRL858R-induced adenocarcinoma model and is a potential prognostic biomarker for Lepidic lung cancer. A crucial event in lung adenocarcinoma progression is the switch from an aerogenous spread toward an infiltrating tumor. Loss of RhoB expression has been suggested to be critical for lung cancer invasion. Here, we tested RhoB expression as a prognostic biomarker in non-small cell lung cancer (NSCLC) with a special focus on lepidic pattern.We analyzed RhoB expression using both IHC and RT-qPCR in two series of operated patients (n = 100 and 48, respectively) and in a series of advanced lepidic adenocarcinoma (n = 31) from different hospitals. Next, we examined the role of RhoB in lung cancer progression in transgenic mice that express inducible EGFR(L858R) crossed with Rhob null mice.We identified that loss of RhoB expression was strongly associated with worse survival (P = 0.0001) and progression-free survival (P < 0.001) in the first series. We then confirmed these results after multivariate analyses of the second series. In the series of adenocarcinoma with lepidic features issued from a clinical trial (IFCT-0401), we showed that loss of RhoB expression was associated with higher aggressiveness of stage IV. Finally, we showed that EGFR(L858R)/Rhob(+/+) mice developed mainly diffuse lung tumors with a lepidic pattern, whereas EGFR(L858R)/Rhob(+/-) and EGFR(L858R)/Rhob(-/-) developed a greater number of tumors, and aggressive adenocarcinomas with   invasive   properties.We showed that RhoB is not only a strong prognostic factor in NSCLC but it is also critical for the acquisition of an aggressive phenotype of adenocarcinoma. 
 Loss of RhoB expression in human lung cancer progression. RhoB is a low molecular weight GTPase belonging to the Ras protein superfamily. Whereas most Rho proteins have been shown to have a positive role in proliferation and malignant transformation, the specific role of RhoB appears more divergent. We reported previously that RhoB inhibits cell proliferation in various human cancer cells. Here, we studied the specific role played by RhoB in human lung cancer.We analyzed the expression of RhoB protein by immunostaining in human lung tissues ranging from normal to   invasive   carcinoma from different histological types in two large independent studies of, respectively, 94 and 45 samples. We then studied the cellular effect of RhoB overexpression in a model of lung cancer (A549, adenocarcinoma) and tumorigenicity in nude mice.We showed in both studies that RhoB protein was expressed in normal lung and decreased dramatically through lung cancer progression (P < 0.01). Interestingly, RhoB expression was lost in 96% of   invasive   tumors and reduced by 86% in poorly differentiated tumors compared with the nonneoplastic epithelium. Moreover, the loss of expression of RhoB correlated significantly with tumor stage and proliferative index, whereas no correlation was found between RhoB and p53 or Bcl-2 expression. We then showed that ectopic expression of RhoB in lung cancer cell line A549 suppressed cell proliferation, anchorage-independent growth, and xenograft tumor growth in nude mice.RhoB loss of expression occurs very frequently in lung carcinogenesis, reinforcing its putative tumor suppressive activity, and raising the value of its potential use in cancer therapy. 
  GeneRIFs  
 we propose that RhoB could be responsible for early metastatic prevention by inhibiting the EMT-derived   invasive  ness of lung cells through the control of PP2A activity. 
 in glioblastoma, PKC iota and RhoB are mutually antagonistic, potentially creating a sensitive switch between   invasive   and non-  invasive   phenotypes 
  [Mm]etastasis  PubMed  
 A novel strategy for specifically down-regulating individual Rho GTPase activity in tumor cells. The Rho family GTPases RhoA, RhoB, and RhoC regulate the actin cytoskeleton, cell movement, and cell growth. Unlike Ras, up-regulation or overexpression of these GDP/GTP binding molecular switches, but not activating point mutations, has been associated with human cancer. Although they share over 85% sequence identity, RhoA, RhoB, and RhoC appear to play distinct roles in cell transformation and   metastasis  . In NIH 3T3 cells, RhoA or RhoB overexpression causes transformation whereas RhoC increases the cell migration rate. To specifically target RhoA, RhoB, or RhoC function, we have generated a set of chimeric molecules by fusing the RhoGAP domain of p190, a GTPase-activating protein that accelerates the intrinsic GTPase activity of all three Rho GTPases, with the C-terminal hypervariable sequences of RhoA, RhoB, or RhoC. The p190-Rho chimeras were active as GTPase-activating proteins toward RhoA in vitro, co-localized with the respective active Rho proteins, and specifically down-regulated Rho protein activities in cells depending on which Rho GTPase sequences were included in the chimeras. In particular, the p190-RhoA-C chimera specifically inhibited RhoA-induced transformation whereas p190-RhoC-C specifically reversed the migration phenotype induced by the active RhoC. In human mammary epithelial-RhoC breast cancer cells, p190-RhoC-C, but not p190-RhoA-C or p190-RhoB-C, reversed the anchorage-independent growth and invasion phenotypes caused by RhoC overexpression. In the highly metastatic A375-M human melanoma cells, p190-RhoC-C specifically reversed migration, and invasion phenotypes attributed to RhoC up-regulation. Thus, we have developed a novel strategy utilizing RhoGAP-Rho chimeras to specifically down-regulate individual Rho activity and demonstrate that this approach may be applied to multiple human tumor cells to reverse the growth and/or invasion phenotypes associated with disregulation of a distinct subtype of Rho GTPase. 
 A novel strategy for specifically down-regulating individual Rho GTPase activity in tumor cells. The Rho family GTPases RhoA, RhoB, and RhoC regulate the actin cytoskeleton, cell movement, and cell growth. Unlike Ras, up-regulation or overexpression of these GDP/GTP binding molecular switches, but not activating point mutations, has been associated with human cancer. Although they share over 85% sequence identity, RhoA, RhoB, and RhoC appear to play distinct roles in cell transformation and   metastasis  . In NIH 3T3 cells, RhoA or RhoB overexpression causes transformation whereas RhoC increases the cell migration rate. To specifically target RhoA, RhoB, or RhoC function, we have generated a set of chimeric molecules by fusing the RhoGAP domain of p190, a GTPase-activating protein that accelerates the intrinsic GTPase activity of all three Rho GTPases, with the C-terminal hypervariable sequences of RhoA, RhoB, or RhoC. The p190-Rho chimeras were active as GTPase-activating proteins toward RhoA in vitro, co-localized with the respective active Rho proteins, and specifically down-regulated Rho protein activities in cells depending on which Rho GTPase sequences were included in the chimeras. In particular, the p190-RhoA-C chimera specifically inhibited RhoA-induced transformation whereas p190-RhoC-C specifically reversed the migration phenotype induced by the active RhoC. In human mammary epithelial-RhoC breast cancer cells, p190-RhoC-C, but not p190-RhoA-C or p190-RhoB-C, reversed the anchorage-independent growth and invasion phenotypes caused by RhoC overexpression. In the highly metastatic A375-M human melanoma cells, p190-RhoC-C specifically reversed migration, and invasion phenotypes attributed to RhoC up-regulation. Thus, we have developed a novel strategy utilizing RhoGAP-Rho chimeras to specifically down-regulate individual Rho activity and demonstrate that this approach may be applied to multiple human tumor cells to reverse the growth and/or invasion phenotypes associated with disregulation of a distinct subtype of Rho GTPase. 
 Overexpression of miR-21 promotes an in vitro metastatic phenotype by targeting the tumor suppressor RHOB.   Metastasis   is a multistep process that involves the deregulation of oncogenes and tumor suppressors beyond changes required for primary tumor formation. RHOB is known to have tumor suppressor activity, and its knockdown is associated with more aggressive tumors as well as changes in cell shape, migration, and adhesion. This study shows that oncogenic microRNA, miR-21, represses RHOB expression by directly targeting the 3' untranslated region. Loss of miR-21 is associated with an elevation of RHOB in hepatocellular carcinoma cell lines Huh-7 and HepG2 and in the metastatic breast cancer cell line MDA-MB-231. Using in vitro models of distinct stages of   metastasis  , we showed that loss of miR-21 also causes a reduction in migration, invasion, and cell elongation. The reduction in migration and cell elongation can be mimicked by overexpression of RHOB. Furthermore, changes in miR-21 expression lead to alterations in matrix metalloproteinase-9 activity. Therefore, we conclude that miR-21 promotes multiple components of the metastatic phenotype in vitro by regulating several important tumor suppressors, including RHOB. 
 RHOB influences lung adenocarcinoma   metastasis   and resistance in a host-sensitive manner. Lung adenocarcinoma (ADC) is the most common lung cancer subtype and presents a high mortality rate. Clinical recurrence is often associated with the emergence of   metastasis   and treatment resistance. The purpose of this study was to identify genes with high prometastatic activity which could potentially account for treatment resistance. Global transcriptomic profiling was performed by robust microarray analysis in highly metastatic subpopulations. Extensive in vitro and in vivo functional studies were achieved by overexpression and by silencing gene expression. We identified the small GTPase RHOB as a gene that promotes early and late stages of   metastasis   in ADC. Gene silencing of RHOB prevented metastatic activity in a systemic murine model of bone   metastasis  . These effects were highly dependent on tumor-host interactions. Clinical analysis revealed a marked association between high RHOB levels and poor survival. Consistently, high RHOB levels promote   metastasis   progression, taxane-chemoresistance, and contribute to the survival advantage to γ-irradiation. We postulate that RHOB belongs to a novel class of "genes of recurrence" that have a dual role in   metastasi  s and treatment resistance. 
 Histone deacetylase 1 represses the small GTPase RhoB expression in human nonsmall lung carcinoma cell line. The dynamic balance between histone acetylation and deacetylation plays a significant role in the regulation of gene transcription. Much of our current understanding of this transcriptional control comes from the use of HDAC inhibitors such as trapoxin A (TPX), which leads to hyperacetylated histone, alters local chromatin architecture and transcription and results in tumor cell death. In this study, we treated tumor cells with TPX and HDAC1 antisense oligonucleotides, and analysed the transcriptional consequences of HDAC inhibition. Among other genes, the small GTPase RhoB was found to be significantly upregulated by TPX and repressed by HDAC1. The induction of RhoB by HDAC inhibition was mediated by an inverted CCAAT box in the RhoB promoter. Interestingly, measurement of RhoB transcription in approximately 130 tumor-derived cell lines revealed low expression in almost all of these samples, in contrast to RhoA and RhoC. Accumulating evidence indicates that the small GTPase Rho proteins are involved in a variety of important processes in cancer, including cell transformation, survival, invasion,   metastasis   and angiogenesis. This study for the first time demonstrates a link between HDAC inhibition and RhoB expression and provides an important insight into the mechanisms of HDAC-mediated transcriptional control and the potential therapeutic benefit of HDAC inhibition. 
 Critical functions of RhoB in support of glioblastoma tumorigenesis. RhoB is a member of the Rho small GTPase family that regulates cytoskeletal dynamics and vesicle trafficking. The RhoB homologs, RhoA and RhoC, have been shown to promote cancer progression and   metastasis  . In contrast, the functions of RhoB in human cancers are context dependent. Although expression of RhoB inversely correlates with disease progression in several epithelial cancers, recent data suggest that RhoB may support malignant phenotypes in certain cancer types.We assessed RhoB protein levels in glioma surgical specimens and patient-derived xenografts. The roles of RhoB in glioblastoma were determined by loss-of-function and gain-of-function assays in vitro and in vivo. The impact on p53 and STAT3 signaling was investigated.RhoB expression was similar in tumor specimens compared with normal neural tissues obtained from epilepsy surgery. RhoB was expressed in the vast majority of xenograft tumors and spheroid cultures. Knockdown of RhoB induced cell-cycle arrest and apoptosis and compromised in vivo tumorigenic potential. However, overexpression of wild-type RhoB or a constitutively active mutant (RhoB-V14) did not significantly affect cell growth, which suggests that RhoB is not a rate-limiting oncogenic factor and is consistent with the scarcity of RhoB mutations in human cancer. Knockdown of RhoB reduced basal STAT3 activity and impaired cytokine-induced STAT3 activation. In glioblastoma tumors retaining wild-type p53, depletion of RhoB also activated p53 and induced expression of p21(CIP1) (/WAF1).Our data suggest that RhoB belongs to an emerging class of "nononcogene addiction" factors that are essential for maintenance of malignant phenotypes in human cancers. 
  GeneRIFs  
 RHOB belongs to a novel class of "genes of recurrence" that have a dual role in   metastasis   and treatment resistance. 
  [Cc]ell [Ii]nvasion  PubMed  
 Divergence of Rho residue 43 impacts GEF activity. RhoA, RhoB and RhoC GTPases are over 85% identical at the amino acid level, with RhoA and RhoC differing at only one residue (43) across the initial two-thirds of their sequences. A putative regulatory distinction between the molecules is their capacity to be uniquely activated by guanine nucleotide exchange factors (GEFs). We hypothesize that variation of amino acid residue 43 between RhoA/B (valine) and RhoC (isoleucine) impacts GEF activity. Direct participation of residue 43 in GEF-catalyzed exchange was confirmed by the observation that mutation of this position to a threonine reduced GEF-catalyzed nucleotide exchange activity in vitro (Vav2, XPLN, GEFT, Dbl and Dbs) and greatly depressed RhoA and RhoC GTP-loading profiles in cell lysates. Using a residue swap approach, substitution of RhoA Val 43 with an Ile was found to significantly promote basal nucleotide exchange activity and enhance GTP-loading in cells. Substitution of Val 43 with an Ile in RhoB negatively affected nucleotide exchange in vitro. Substitution of RhoC Ile 43 with a Val increased GEF-catalyzed exchange in vitro. In addition, RhoC-I43V was more efficacious at driving ovarian cancer   cell invasion   through matrigrel than wild-type RhoC, RhoC-I43T, wild-type RhoA, RhoA-V43I or RhoA-V43T GTPases. These findings suggest that a divergence between RhoA/B and RhoC at residue 43 impacts basal and GEF-stimulated nucleotide exchange activity. 
 RhoB loss induces Rac1-dependent mesenchymal   cell invasion   in lung cells through PP2A inhibition. Non-small-cell lung cancer (NSCLC) is the leading cause of cancer-related death worldwide, which is mainly due to its high risk of metastatic dissemination. One critical point of this process is the ability of cancer cells to detach from the primary tumor and migrate through the extracellular matrix; however, the underlying molecular mechanisms are not yet fully understood. In the present study, we identified the small GTPase RhoB as a key regulator of bronchial cell morphology in a three-dimensional (3D) matrix. RhoB loss, which is frequently observed during lung cancer progression, induced an epithelial-mesenchymal transition (EMT) characterized by an increased proportion of invasive elongated cells in 3D. The process was mediated by Slug induction and E-cadherin repression. In addition, downregulation of RhoB induced Akt1 activation, which in turn activated Rac1 through the guanine-exchange factor Trio to control cell shape rearrangement. Further, we provide evidence that RhoB interacted with and positively regulates phosphatase PP2A through the recruitment of its regulatory subunit B55, which was found to be crucial for Akt dephosphorylation. B55 inhibition completely suppressed RhoB-mediated PP2A regulation. Finally, we show that PP2A inactivation, by targeting either its catalytic or its regulatory B55 subunit, completely reversed RhoB-dependent morphological changes and also fully prevented the ability of RhoB to decrease the invasiveness of bronchial cells. Altogether, these results highlight a novel signaling axis and describe new molecular mechanisms that could explain the tumor suppressor role of RhoB in lung cancer. Therefore, we propose that RhoB could be responsible for early metastatic prevention by inhibiting the EMT-derived invasiveness of lung cells through the control of PP2A activity. 
 RhoB loss induces Rac1-dependent mesenchymal   cell invasion   in lung cells through PP2A inhibition. Non-small-cell lung cancer (NSCLC) is the leading cause of cancer-related death worldwide, which is mainly due to its high risk of metastatic dissemination. One critical point of this process is the ability of cancer cells to detach from the primary tumor and migrate through the extracellular matrix; however, the underlying molecular mechanisms are not yet fully understood. In the present study, we identified the small GTPase RhoB as a key regulator of bronchial cell morphology in a three-dimensional (3D) matrix. RhoB loss, which is frequently observed during lung cancer progression, induced an epithelial-mesenchymal transition (EMT) characterized by an increased proportion of invasive elongated cells in 3D. The process was mediated by Slug induction and E-cadherin repression. In addition, downregulation of RhoB induced Akt1 activation, which in turn activated Rac1 through the guanine-exchange factor Trio to control cell shape rearrangement. Further, we provide evidence that RhoB interacted with and positively regulates phosphatase PP2A through the recruitment of its regulatory subunit B55, which was found to be crucial for Akt dephosphorylation. B55 inhibition completely suppressed RhoB-mediated PP2A regulation. Finally, we show that PP2A inactivation, by targeting either its catalytic or its regulatory B55 subunit, completely reversed RhoB-dependent morphological changes and also fully prevented the ability of RhoB to decrease the invasiveness of bronchial cells. Altogether, these results highlight a novel signaling axis and describe new molecular mechanisms that could explain the tumor suppressor role of RhoB in lung cancer. Therefore, we propose that RhoB could be responsible for early metastatic prevention by inhibiting the EMT-derived invasiveness of lung cells through the control of PP2A activity. 
 Divergence of Rho residue 43 impacts GEF activity. RhoA, RhoB and RhoC GTPases are over 85% identical at the amino acid level, with RhoA and RhoC differing at only one residue (43) across the initial two-thirds of their sequences. A putative regulatory distinction between the molecules is their capacity to be uniquely activated by guanine nucleotide exchange factors (GEFs). We hypothesize that variation of amino acid residue 43 between RhoA/B (valine) and RhoC (isoleucine) impacts GEF activity. Direct participation of residue 43 in GEF-catalyzed exchange was confirmed by the observation that mutation of this position to a threonine reduced GEF-catalyzed nucleotide exchange activity in vitro (Vav2, XPLN, GEFT, Dbl and Dbs) and greatly depressed RhoA and RhoC GTP-loading profiles in cell lysates. Using a residue swap approach, substitution of RhoA Val 43 with an Ile was found to significantly promote basal nucleotide exchange activity and enhance GTP-loading in cells. Substitution of Val 43 with an Ile in RhoB negatively affected nucleotide exchange in vitro. Substitution of RhoC Ile 43 with a Val increased GEF-catalyzed exchange in vitro. In addition, RhoC-I43V was more efficacious at driving ovarian cancer   cell invasion   through matrigrel than wild-type RhoC, RhoC-I43T, wild-type RhoA, RhoA-V43I or RhoA-V43T GTPases. These findings suggest that a divergence between RhoA/B and RhoC at residue 43 impacts basal and GEF-stimulated nucleotide exchange activity. 
 Regulation of glioblastoma   cell invasion   by PKC iota and RhoB. Glioblastoma multiforme is the most aggressive form of primary brain tumor and remains largely incurable, in large part, due to its highly invasive nature. The phosphoinositide (PI) 3-kinase pathway is often constitutively active in these tumors due to activating mutations in the epidermal growth factor receptor, or deletion/loss of function of the tumor suppressor PTEN. Protein kinase C type iota (PKC iota), a member of the atypical protein kinase C family, is activated by the PI 3-kinase pathway and is an important downstream mediator. Here, we have assessed the role of PKC iota in glioblastoma   cell invasion  . Depletion of PKC iota with RNA interference caused an increase in actin stress fibers and a decrease in cell motility and invasion. Gene expression microarray analysis of U87MG cells showed that PKC iota repressed expression of mRNA for RhoB, which has previously been shown to have a role in actin stress fiber formation. Western blot analysis showed that both PKC iota depletion and pharmacological inhibition of PKC iota caused an increase in the protein levels of RhoB, as did inhibition of PI 3-kinase. Expression of RhoB from a constitutive promoter caused changes in actin stress fibers and   cell invasion   that were similar to those seen with PKC iota depletion. These data show that PKC iota, activated as a consequence of aberrant upstream PI 3-kinase signaling, mediates glioblastoma cell motility and invasion, and that repression of RhoB is key downstream event in PKC iota signaling leading to enhanced cell motility. In addition, constitutive expression of RhoB repressed PKC iota activity, as assessed by its phosphorylation status on Thr555. PKC iota and RhoB are, therefore, mutually antagonistic, potentially creating a sensitive switch between invasive and non-invasive phenotypes. 
   
  
 RND1 
 
  NCBI Gene  
  GeneRIF  
  Pubmed  
 
 
  Search Term  Rank  Fields  
  [Ff]ilopodia  1  PubMed(1)  
  [Mm]igration  6  PubMed(4)  
  [Ii]nvasive  10  PubMed(1)  
  [Mm]etastasis  12  PubMed(2)  
 
 
 hide/show details for RND1 
  [Ff]ilopodia  PubMed  
 Rnd1, a novel rho family GTPase, induces the formation of neuritic processes in PC12 cells. Rho family GTPases have been shown to be involved in the regulation of neuronal cell morphology, including neurite extension and retraction. Rho activation leads to neurite retraction and cell rounding, whereas Rac and Cdc42 are implicated in the promotion of   filopodia   and lamellipodia formation in growth cones and, therefore, in neurite extension. In this study, we examined the morphological role of Rnd1, a new member of Rho family GTPases, in PC12 cells, and found that expression of Rnd1 by itself caused the formation of many neuritic processes from the cell body with disruption of the cortical actin filaments, the processes having microtubules but few filamentous actin and neurofilaments. Treatment with cytochalasin D, an inhibitor of actin polymerization, could mimic the effects of expression of Rnd1, in that this inhibitor disrupted the cortical actin filaments and induced the formation of many thin processes containing microtubules. The process formation induced by Rnd1 was inhibited by dominant negative Rac1. These results suggest that Rnd1 induces the Rac-dependent neuritic process formation in part by disruption of the cortical actin filaments. 
  [Mm]igration  PubMed  
 Direct interaction of Rnd1 with Plexin-B1 regulates PDZ-RhoGEF-mediated Rho activation by Plexin-B1 and induces cell contraction in COS-7 cells. Plexins are receptors for the axon guidance molecule semaphorins, and several lines of evidence suggest that Rho family small GTPases are implicated in the downstream signaling of Plexins. Recent studies have demonstrated that Plexin-B1 activates RhoA and induces growth cone collapse through Rho-specific guanine nucleotide exchange factor PDZ-RhoGEF. Here we show that Rnd1, a member of Rho family GTPases, directly interacted with the cytoplasmic domain of Plexin-B1. In COS-7 cells, coexpression of Rnd1 and Plexin-B1 induced cell contraction in response to semaphorin 4D (Sema4D), a ligand for Plexin-B1, whereas expression of Plexin-B1 alone or coexpression of Rnd1 and a Rnd1 interaction-defective mutant of Plexin-B1 did not. The Sema4D-induced contraction in Plexin-B1/Rnd1-expressing COS-7 cells was suppressed by dominant negative RhoA, a Rho-associated kinase inhibitor, a dominant negative form of PDZ-RhoGEF, or deletion of the carboxyl-terminal PDZ-RhoGEF-binding region of Plexin-B1, indicating that the PDZ-RhoGEF/RhoA/Rho-associated kinase pathway is involved in this morphological effect. We also found that Rnd1 promoted the interaction between Plexin-B1 and PDZ-RhoGEF and thereby dramatically potentiated the Plexin-B1-mediated RhoA activation. We propose that Rnd1 plays an important role in the regulation of Plexin-B1 signaling, leading to Rho activation during axon guidance and cell   migration  . 
 p190 Rho-GTPase activating protein associates with plexins and it is required for semaphorin signalling. Plexins are transmembrane receptors for semaphorins, guiding cell   migration   and axon extension. Plexin activation leads to the disassembly of integrin-based focal adhesive structures and to actin cytoskeleton remodelling and inhibition of cell   migration  ; however, the underlying molecular mechanisms are unclear. We consistently observe a transient decrease of cellular RhoA-GTP levels upon plexin activation in adherent cells. One of the main effectors of RhoA downregulation is p190, a ubiquitously expressed GTPase activating protein (GAP). We show that, in p190-deficient fibroblasts, the typical functional activities mediated by plexins (such as cell collapse and inhibition of integrin-based adhesion) are blocked or greatly impaired. Notably, the functional response can be rescued in these cells by re-expressing exogenous p190, but not a mutant form specifically lacking RhoGAP activity. We furthermore demonstrate that semaphorin function is blocked in epithelial cells, primary endothelial cells and neuroblasts upon treatment with small interfering RNAs that knockdown p190 expression. Finally, we show that p190 transiently associates with plexins, and its RhoGAP activity is increased in response to semaphorin stimulation. We conclude that p190-RhoGAP is crucially involved in semaphorin signalling to the actin cytoskeleton, via interaction with plexins. 
 Structural basis of Rnd1 binding to plexin Rho GTPase binding domains (RBDs). Plexin receptors regulate cell adhesion,   migration  , and guidance. The Rho GTPase binding domain (RBD) of plexin-A1 and -B1 can bind GTPases, including Rnd1. By contrast, plexin-C1 and -D1 reportedly bind Rnd2 but associate with Rnd1 only weakly. The structural basis of this differential Rnd1 GTPase binding to plexin RBDs remains unclear. Here, we solved the structure of the plexin-A2 RBD in complex with Rnd1 and the structures of the plexin-C1 and plexin-D1 RBDs alone, also compared with the previously determined plexin-B1 RBD.Rnd1 complex structure. The plexin-A2 RBD·Rnd1 complex is a heterodimer, whereas plexin-B1 and -A2 RBDs homodimerize at high concentration in solution, consistent with a proposed model for plexin activation. Plexin-C1 and -D1 RBDs are monomeric, consistent with major residue changes in the homodimerization loop. In plexin-A2 and -B1, the RBD β3-β4 loop adjusts its conformation to allow Rnd1 binding, whereas minimal structural changes occur in Rnd1. The plexin-C1 and -D1 RBDs lack several key non-polar residues at the corresponding GTPase binding surface and do not significantly interact with Rnd1. Isothermal titration calorimetry measurements on plexin-C1 and -D1 mutants reveal that the introduction of non-polar residues in this loop generates affinity for Rnd1. Structure and sequence comparisons suggest a similar mode of Rnd1 binding to the RBDs, whereas mutagenesis suggests that the interface with the highly homologous Rnd2 GTPase is different in detail. Our results confirm, from a structural perspective, that Rnd1 does not play a role in the activation of plexin-C1 and -D1. Plexin functions appear to be regulated by subfamily-specific mechanisms, some of which involve different Rho family GTPases. 
 RND1 is up-regulated in esophageal squamous cell carcinoma and promotes the growth and   migration   of cancer cells. RND1 is reported to control the dynamics of cytoskeleton, cell growth, and survival, and dysregulation of RND1 is involved in the tumorigenesis. However, its expression and functions in the esophageal squamous cell carcinoma (ESCC) are unknown. In the present study, it was found that the expression of RND1 was up-regulated in ESCC tissues compared with the adjacent normal tissues. Forced expression of RND1 promoted the growth and   migration   of ESCC cells, while knocking down the expression of RND1 inhibited the growth,   migration  , and metastasis of ESCC cells. Molecular mechanism studies showed that RND1 activated ERK signaling. In summary, our study suggested that RND1 plays an important role in the progression of ESCC, and RND1 might be a promising target for the treatment of ESCC. 
  [Ii]nvasive  PubMed  
 The Rho GTPase Rnd1 suppresses mammary tumorigenesis and EMT by restraining Ras-MAPK signalling. We identified the Rho GTPase Rnd1 as a candidate metastasis suppressor in basal-like and triple-negative breast cancer through bioinformatics analysis. Depletion of Rnd1 disrupted epithelial adhesion and polarity, induced epithelial-to-mesenchymal transition, and cooperated with deregulated expression of c-Myc or loss of p53 to cause neoplastic conversion. Mechanistic studies revealed that Rnd1 suppresses Ras signalling by activating the GAP domain of Plexin B1, which inhibits Rap1. Rap1 inhibition in turn led to derepression of p120 Ras-GAP, which was able to inhibit Ras. Inactivation of Rnd1 in mammary epithelial cells induced highly undifferentiated and   invasive   tumours in mice. Conversely, Rnd1 expression inhibited spontaneous and experimental lung colonization in mouse models of metastasis. Genomic studies indicated that gene deletion in combination with epigenetic silencing or, more rarely, point mutation inactivates RND1 in human breast cancer. These results reveal a previously unappreciated mechanism through which Rnd1 restrains activation of Ras-MAPK signalling and breast tumour initiation and progression. 
  [Mm]etastasis  PubMed  
 RND1 is up-regulated in esophageal squamous cell carcinoma and promotes the growth and migration of cancer cells. RND1 is reported to control the dynamics of cytoskeleton, cell growth, and survival, and dysregulation of RND1 is involved in the tumorigenesis. However, its expression and functions in the esophageal squamous cell carcinoma (ESCC) are unknown. In the present study, it was found that the expression of RND1 was up-regulated in ESCC tissues compared with the adjacent normal tissues. Forced expression of RND1 promoted the growth and migration of ESCC cells, while knocking down the expression of RND1 inhibited the growth, migration, and   metastasis   of ESCC cells. Molecular mechanism studies showed that RND1 activated ERK signaling. In summary, our study suggested that RND1 plays an important role in the progression of ESCC, and RND1 might be a promising target for the treatment of ESCC. 
 The Rho GTPase Rnd1 suppresses mammary tumorigenesis and EMT by restraining Ras-MAPK signalling. We identified the Rho GTPase Rnd1 as a candidate   metastasis   suppressor in basal-like and triple-negative breast cancer through bioinformatics analysis. Depletion of Rnd1 disrupted epithelial adhesion and polarity, induced epithelial-to-mesenchymal transition, and cooperated with deregulated expression of c-Myc or loss of p53 to cause neoplastic conversion. Mechanistic studies revealed that Rnd1 suppresses Ras signalling by activating the GAP domain of Plexin B1, which inhibits Rap1. Rap1 inhibition in turn led to derepression of p120 Ras-GAP, which was able to inhibit Ras. Inactivation of Rnd1 in mammary epithelial cells induced highly undifferentiated and invasive tumours in mice. Conversely, Rnd1 expression inhibited spontaneous and experimental lung colonization in mouse models of   metastasis  . Genomic studies indicated that gene deletion in combination with epigenetic silencing or, more rarely, point mutation inactivates RND1 in human breast cancer. These results reveal a previously unappreciated mechanism through which Rnd1 restrains activation of Ras-MAPK signalling and breast tumour initiation and progression. 
   
  
 RHOU 
 
  NCBI Gene  
  GeneRIF  
  Pubmed  
 
 
  Search Term  Rank  Fields  
  [Ff]ilopodia  1  PubMed(4)  
  [Ff]ilopodium  2  PubMed(1); Summary(1)  
  [Pp]odosome(s)?  5  PubMed(1); Component(1)  
  [Mm]igration  6  PubMed(8); GeneRIFs(3)  
 
 
 hide/show details for RHOU 
  [Ff]ilopodia  PubMed  
 Wrch1 is a GTPase-deficient Cdc42-like protein with unusual binding characteristics and cellular effects. The Rho family of small GTPases controls many biological processes, including cytoskeletal regulation, membrane trafficking, cell adhesion, cell polarization, transcriptional activity, apoptosis, and cell proliferation. Wrch1, which belongs to the Cdc42 subfamily, is one of the least characterized family member. Despite its homology to other Cdc42-like proteins, we found that Wrch1 has unique characteristics. Biochemical experiments showed that Wrch1 has no detectable GTPase activity in vitro and that its intrinsic nucleotide exchange rate is very high in comparison to Cdc42. Furthermore, NIH3T3 cells transiently transfected with Wrch1 showed an up-rounded, retracted phenotype. In addition, Wrch1 was shown to be more efficient than Cdc42 in triggering the formation of   filopodia  . Serum stimulation of cells expressing Wrch1 induces vigorous membrane blebbing, a phenomenon dependent on the activity of ROCK. In a search for proteins interacting with Wrch1, PAK1 and NCKbeta were identified as binding partners. Interestingly, the interaction to NCKbeta was shown to be mediated via PxxP motifs present in an N-terminal extension of Wrch1 to the second and third SH3 domains of NCKbeta. 
 The atypical Rho GTPase Wrch1 collaborates with the nonreceptor tyrosine kinases Pyk2 and Src in regulating cytoskeletal dynamics. The Cdc42-like GTPase Wnt responsive Cdc42 homolog 1 (Wrch1) has several atypical features; it has an N-terminal proline-rich extension that confers binding to SH3 domains, and it harbors an extremely high intrinsic nucleotide exchange activity, which overrides the normal GTPase activity. As a result, Wrch1 resides mainly in the active, GTP-loaded conformation under normal cellular conditions. We have previously shown that ectopic expression of Wrch1 in fibroblasts resulted in an altered cell morphology visible as a formation of   filopodia  , a loss of stress fibers, and a reduction in focal adhesions. Here, we show that Wrch1 binds to the nonreceptor tyrosine kinase Pyk2. The interaction required Wrch1 to be in a GTP conformation and also required an intact N-terminal proline-rich extension as well as an intact effector loop. Wrch1 requires Pyk2 in imposing the cytoskeletal effects, seen as the formation of   filopodia  , since treatment of cells with a Pyk2-specific small interfering RNA abrogated this response. Interestingly, we found that the presence and activity of Src were needed for the formation of a Wrch1-Pyk2 complex as well as for the Wrch1-induced formation of   filopodia  . We propose a model in which Pyk2 and Src function to coordinate the Wrch1-dependent effects on cytoskeletal dynamics. 
 Atypical mechanism of regulation of the Wrch-1 Rho family small GTPase. Rho family GTPases are GDP/GTP-regulated molecular switches that regulate signaling pathways controlling diverse cellular processes. Wrch-1 was identified as a Wnt-1 regulated Cdc42 homolog, upregulated by Wnt1 signaling in Wnt1-transformed mouse mammary cells, and was able to promote formation of   filopodia   and activate the PAK serine/threonine kinase. Wrch-1 shares significant sequence and functional similarity with the Cdc42 small GTPase. However, Wrch-1 possesses a unique N-terminal 46 amino acid sequence extension that contains putative Src homology 3 (SH3) domain-interacting motifs. We determined the contribution of the N terminus to Wrch-1 regulation and activity. We observed that Wrch-1 possesses properties that distinguish it from Cdc42 and other Rho family GTPases. Unlike Cdc42, Wrch-1 possesses an extremely rapid, intrinsic guanine nucleotide exchange activity. Although the N terminus did not influence GTPase or GDP/GTP cycling activity in vitro, N-terminal truncation of Wrch-1 enhanced its ability to interact with and activate PAK and to cause growth transformation. The N terminus associated with the Grb2 SH3 domain-containing adaptor protein, and this association increased the levels of active Wrch-1 in cells. We propose that Grb2 overcomes N-terminal negative regulation to promote Wrch-1 effector interaction. Thus, Wrch-1 exhibits an atypical model of regulation not seen in other Rho family GTPases. 
 Rho GTPases have diverse effects on the organization of the actin filament system. The Rho GTPases are related to the Ras proto-oncogenes and consist of 22 family members. These proteins have important roles in regulating the organization of the actin filament system, and thereby the morphogenesis of vertebrate cells as well as their ability to migrate. In an effort to compare the effects of all members of the Rho GTPase family, active Rho GTPases were transfected into porcine aortic endothelial cells and the effects on the actin filament system were monitored. Cdc42, TCL (TC10-like), Rac1-Rac3 and RhoG induced the formation of lamellipodia, whereas Cdc42, Rac1 and Rac2 also induced the formation of thick bundles of actin filaments. In contrast, transfection with TC10 or Chp resulted in the formation of focal adhesion-like structures, whereas Wrch-1 induced long and thin   filopodia  . Transfection with RhoA, RhoB or RhoC induced the assembly of stress fibres, whereas Rnd1-Rnd3 resulted in the loss of stress fibres, but this effect was associated with the formation of actin- and ezrin-containing dorsal microvilli. Cells expressing RhoD and Rif had extremely long and flexible   filopodia  . None of the RhoBTB or Miro GTPases had any major influence on the organization of the actin filament system; instead, RhoBTB1 and RhoBTB2 were present in vesicular structures, and Miro-1 and Miro-2 were present in mitochondria. Collectively, the data obtained in this study to some extent confirm earlier observations, but also allow the identification of previously undetected roles of the different members of the Rho GTPases. 
  [Ff]ilopodium  PubMed  
 Wrch-1, a novel member of the Rho gene family that is regulated by Wnt-1. We report the isolation and cloning of the Wrch-1 (Wnt-1 responsive Cdc42 homolog) cDNA. Wrch-1 is a novel gene whose mRNA level increases in response to Wnt-1 signaling in Wnt-1 transformed cells, Wnt-1 transgene induced mouse mammary tumors, and Wnt-1 retrovirus infected cells. Wrch-1 encodes a homolog of the Rho family of GTPases. It shares 57% amino acid sequence identity with Cdc42, but possesses a unique N-terminal domain that contains several putative PXXP SH3-binding motifs. Like Cdc42, Wrch-1 can activate PAK-1 and JNK-1, and induce   filopodium   formation and stress fiber dissolution. Active Wrch-1 stimulates quiescent cells to reenter the cell cycle. Moreover, overexpression of Wrch-1 phenocopies Wnt-1 in morphological transformation of mouse mammary epithelial cells. Taken together, Wrch-1 could mediate the effects of Wnt-1 signaling in the regulation of cell morphology, cytoskeletal organization, and cell proliferation. 
  Summary  
 This gene encodes a member of the Rho family of GTPases. This protein can activate PAK1 and JNK1, and can induce   filopodium   formation and stress fiber dissolution. It may also mediate the effects of WNT1 signaling in the regulation of cell morphology, cytoskeletal organization, and cell proliferation. A non-coding transcript variant of this gene results from naturally occurring read-through transcription between this locus and the neighboring DUSP5P (dual specificity phosphatase 5 pseudogene) locus.[provided by RefSeq, Mar 2011] 
  [Pp]odosome(s)?  PubMed  
 The Rho GTPase Wrch1 regulates osteoclast precursor adhesion and migration. An excess of osteoclastic bone resorption relative to osteoblastic bone formation results in progressive bone loss, characteristic of osteoporosis. Understanding the mechanisms of osteoclast differentiation is essential to develop novel therapeutic approaches to prevent and treat osteoporosis. We showed previously that Wrch1/RhoU is the only RhoGTPase whose expression is induced by RANKL during osteoclastogenesis. It associates with   podosomes   and the suppression of Wrch1 in osteoclast precursors leads to defective multinucleated cell formation. Here we further explore the functions of this RhoGTPase in osteoclasts, using RAW264.7 cells and bone marrow macrophages as osteoclast precursors. Suppression of Wrch1 did not prevent induction of classical osteoclastic markers such as NFATc1, Src, TRAP (Tartrate-Resistant Acid Phosphatase) or cathepsin K. ATP6v0d2 and DC-STAMP, which are essential for fusion, were also expressed normally. Similar to the effect of RANKL, we observed that Wrch1 expression increased osteoclast precursor aggregation and reduced their adhesion onto vitronectin but not onto fibronectin. We further found that Wrch1 could bind integrin beta3 cytoplasmic domain and interfered with adhesion-induced Pyk2 and paxillin phosphorylation. Wrch1 also acted as an inhibitor of M-CSF-induced prefusion osteoclast migration. In mature osteoclasts, high Wrch1 activity inhibited   podosome   belt formation. Nevertheless, it had no effect on mineralized matrix resorption. Our observations suggest that during osteoclastogenesis, Wrch1 potentially acts through the modulation of alphav beta3 signaling to regulate osteoclast precursor adhesion and migration and allow fusion. As an essential actor of osteoclast differentiation, the atypical RhoGTPase Wrch1/RhoU could be an interesting target for the development of novel antiresorptive drugs. 
  Component  
   podosome   
  [Mm]igration  PubMed  
 Identification and characterization of a set of conserved and new regulators of cytoskeletal organization, cell morphology and   migration  . Cell   migration   is essential during development and in human disease progression including cancer. Most cell   migration   studies concentrate on known or predicted components of   migration   pathways.Here we use data from a genome-wide RNAi morphology screen in Drosophila melanogaster cells together with bioinformatics to identify 26 new regulators of morphology and cytoskeletal organization in human cells. These include genes previously implicated in a wide range of functions, from mental retardation, Down syndrome and Huntington's disease to RNA and DNA-binding genes. We classify these genes into seven groups according to phenotype and identify those that affect cell   migration  . We further characterize a subset of seven genes, FAM40A, FAM40B, ARC, FMNL3, FNBP3/FBP11, LIMD1 and ZRANB1, each of which has a different effect on cell shape, actin filament distribution and cell   migration  . Interestingly, in several instances closely related isoforms with a single Drosophila homologue have distinct phenotypes. For example, FAM40B depletion induces cell elongation and tail retraction defects, whereas FAM40A depletion reduces cell spreading.Our results identify multiple regulators of cell   migration   and cytoskeletal signalling that are highly conserved between Drosophila and humans, and show that closely related paralogues can have very different functions in these processes. 
 Regulation of the Rho family small GTPase Wrch-1/RhoU by C-terminal tyrosine phosphorylation requires Src. Wrch-1 is an atypical Rho family small GTPase with roles in   migration  , epithelial cell morphogenesis, osteoclastogenesis, and oncogenic transformation. Here, we observed rapid relocalization of Wrch-1 from the plasma membrane upon serum stimulation. Studies revealed a requirement for serum-stimulated tyrosine phosphorylation of Wrch-1 at residue Y254 within its C-terminal membrane targeting domain, mediated by the nonreceptor tyrosine kinase Src. Genetic or pharmacological loss of Src kinase activity blocked both phosphorylation and relocalization of Wrch-1. Functionally, Y254 was required for proper Wrch-1 modulation of cystogenesis in three-dimensional culture, and the phospho-deficient mutant, Y254F, was enhanced in Wrch-1-mediated anchorage-independent growth. Mechanistically, C-terminal tyrosine phosphorylation and subsequent relocalization of Wrch-1 downregulated its ability to interact with and activate its effectors by decreasing active Wrch-1-GTP, perhaps by altering proximity to a GEF or GAP. Phospho-deficient Wrch-1(Y254F) remained at the plasma membrane and GTP bound and continued to recruit and activate its effector PAK, even upon serum stimulation. In contrast, a phospho-mimetic mutant, Y254E, was constitutively endosomally localized and GDP bound and failed to recruit PAK unless mutated to be constitutively active/GAP insensitive. C-terminal tyrosine phosphorylation thus represents a new paradigm in posttranslational control of small GTPase localization, activation, and biological function. 
 PAK4 promotes kinase-independent stabilization of RhoU to modulate cell adhesion. P21-activated kinase 4 (PAK4) is a Cdc42 effector protein thought to regulate cell adhesion disassembly in a kinase-dependent manner. We found that PAK4 expression is significantly higher in high-grade human breast cancer patient samples, whereas depletion of PAK4 modifies cell adhesion dynamics of breast cancer cells. Surprisingly, systematic analysis of PAK4 functionality revealed that PAK4-driven adhesion turnover is neither dependent on Cdc42 binding nor kinase activity. Rather, reduced expression of PAK4 leads to a concomitant loss of RhoU expression. We report that RhoU is targeted for ubiquitination by the Rab40A-Cullin 5 complex and demonstrate that PAK4 protects RhoU from ubiquitination in a kinase-independent manner. Overexpression of RhoU rescues the PAK4 depletion phenotype, whereas loss of RhoU expression reduces cell adhesion turnover and   migration  . These data support a new kinase-independent mechanism for PAK4 function, where an important role of PAK4 in cellular adhesions is to stabilize RhoU protein levels. Thus, PAK4 and RhoU cooperate to drive adhesion turnover and promote cell   migration  . 
 GRB2 couples RhoU to epidermal growth factor receptor signaling and cell   migration  . RhoU is an atypical Rho family member with high homology to CDC42 but containing unique N- and C-terminal extensions. The mechanisms regulating RhoU activation, as well as its downstream effectors, are not fully characterized. We show that after epidermal growth factor (EGF) stimulation RhoU colocalizes with EGF receptor (EGFR) on endosomes, which requires both its N- and C-terminal extension sequences. Moreover, RhoU physically associates with activated EGFR in a GRB2-dependent manner through specific proline-rich motifs within its N-terminus. Mutation of these proline-rich sequences or suppression of GRB2 by RNA interference abrogates the interaction of RhoU with activated EGFR, as well as EGF-stimulated RhoU GTP binding. In addition, RhoU is involved in EGFR-mediated signaling, leading to AP1 transcriptional activity and cell   migration   in pancreatic cancer cells, events that require its interaction with the Grb2-EGFR complex. Taken together, the data suggest a unique regulatory mechanism by which RhoU interaction with SH3 adaptor proteins might serve to integrate growth factor receptor signaling with RhoU activation. 
 The atypical Rho family GTPase Wrch-1 regulates focal adhesion formation and cell   migration  . Wrch-1 (Wnt-regulated Cdc42 homolog) is a new member of the Rho family that was identified as a gene transcriptionally upregulated by Wnt-1. Wrch-1 has no detectable GTPase activity and displays very high intrinsic guanine nucleotide exchange, implying that it is constitutively GTP-bound. The biological functions of Wrch-1 largely remain to be characterized. Here, we report that Wrch-1 prominently localizes to focal adhesions. Depletion of Wrch-1 by small interfering RNA increases focal adhesion formation, whereas Wrch-1 overexpression disassembles focal adhesions. Wrch-1 depletion inhibits myosin-light-chain phosphorylation, which in turn leads to an increase in the number of focal adhesions and inhibits cell   migration   in response to wound healing. Depletion of Wrch-1 also inhibits Akt and JNK activation. Although pharmacological inhibitors of Akt and JNK inhibit cell   migration  , they do not affect focal adhesions. Thus, our data suggest that Wrch-1 regulates cell   migration   by multiple mechanisms: on the one hand Wrch-1 controls focal adhesions by regulating myosin light chain and on the other hand Wrch-1 stimulates the activation of Akt and JNK. 
 ARHGAP30 is a Wrch-1-interacting protein involved in actin dynamics and cell adhesion. The atypical Rho GTPase Wrch-1 has been proposed roles in cell   migration  , focal adhesion dissolution, stress fibre break down and tight junction heterogeneity. A screen for Wrch-1 binding-partners identified the novel RhoGAP protein, ARHGAP30, as a Wrch-1 interactor. ARHGAP30 is related to the Cdc42- and Rac1-specific RhoGAP CdGAP, which was likewise found to bind Wrch-1. In contrast to CdGAP, ARHGAP30 serves as a Rac1- and RhoA-specific RhoGAP. Ectopic expression of ARHGAP30 results in membrane blebbing and dissolution of stress-fibres and focal adhesions. Our data suggest roles for ARHGAP30 and CdGAP in regulation of cell adhesion downstream of Wrch-1. 
 The Rho GTPase Wrch1 regulates osteoclast precursor adhesion and   migration  . An excess of osteoclastic bone resorption relative to osteoblastic bone formation results in progressive bone loss, characteristic of osteoporosis. Understanding the mechanisms of osteoclast differentiation is essential to develop novel therapeutic approaches to prevent and treat osteoporosis. We showed previously that Wrch1/RhoU is the only RhoGTPase whose expression is induced by RANKL during osteoclastogenesis. It associates with podosomes and the suppression of Wrch1 in osteoclast precursors leads to defective multinucleated cell formation. Here we further explore the functions of this RhoGTPase in osteoclasts, using RAW264.7 cells and bone marrow macrophages as osteoclast precursors. Suppression of Wrch1 did not prevent induction of classical osteoclastic markers such as NFATc1, Src, TRAP (Tartrate-Resistant Acid Phosphatase) or cathepsin K. ATP6v0d2 and DC-STAMP, which are essential for fusion, were also expressed normally. Similar to the effect of RANKL, we observed that Wrch1 expression increased osteoclast precursor aggregation and reduced their adhesion onto vitronectin but not onto fibronectin. We further found that Wrch1 could bind integrin beta3 cytoplasmic domain and interfered with adhesion-induced Pyk2 and paxillin phosphorylation. Wrch1 also acted as an inhibitor of M-CSF-induced prefusion osteoclast   migration  . In mature osteoclasts, high Wrch1 activity inhibited podosome belt formation. Nevertheless, it had no effect on mineralized matrix resorption. Our observations suggest that during osteoclastogenesis, Wrch1 potentially acts through the modulation of alphav beta3 signaling to regulate osteoclast precursor adhesion and   migration   and allow fusion. As an essential actor of osteoclast differentiation, the atypical RhoGTPase Wrch1/RhoU could be an interesting target for the development of novel antiresorptive drugs. 
 Analysis of Rho GTPase expression in T-ALL identifies RhoU as a target for Notch involved in T-ALL cell   migration  . NOTCH1 is frequently mutated in T-cell acute lymphoblastic leukaemia (T-ALL), and can stimulate T-ALL cell survival and proliferation. Here we explore the hypothesis that Notch1 also alters T-ALL cell   migration  . Rho GTPases are well known to regulate cell adhesion and   migration  . We have analysed the expression levels of Rho GTPases in primary T-ALL samples compared with normal T cells by quantitative PCR. We found that 5 of the 20 human Rho genes are highly and consistently upregulated in T-ALL, and 3 further Rho genes are expressed in T-ALL but not detectable in normal T cells. Of these, RHOU expression is highly correlated with the expression of the Notch1 target DELTEX-1. Inhibition of Notch1 signalling with a γ-secretase inhibitor (GSI) or Notch1 RNA interference reduced RhoU expression in T-ALL cells, whereas constitutively active Notch1 increased RhoU expression. In addition, Notch1 or RhoU depletion, or GSI treatment, inhibits T-ALL cell adhesion,   migratio  n and chemotaxis. These results indicate that NOTCH1 mutation stimulates T-ALL cell   migratio  n through RhoU upregulation that could contribute to the leukaemia cell dissemination. 
  GeneRIFs  
 PAK4 and RhoU cooperate to drive adhesion turnover and promote cell   migration  . 
 results indicate that NOTCH1 mutation stimulates T-cell acute lymphoblastic leukaemia cell   migration   through RhoU upregulation that could contribute to the leukaemia cell dissemination 
 These data suggest that Wrch-1 regulates cell   migration   by multiple mechanisms: on the one hand Wrch-1 controls focal adhesions by regulating myosin light chain and on the other hand Wrch-1 stimulates the activation of Akt and JNK. 
   
  
 RAC2 
 
  NCBI Gene  
  GeneRIF  
  Pubmed  
 
 
  Search Term  Rank  Fields  
  [Ff]ilopodia  1  PubMed(1)  
  [Pp]odosome(s)?  5  PubMed(2)  
  [Mm]igration  6  Process(1); PubMed(17); GeneRIFs(1)  
  [Ii]nvasive  10  PubMed(4)  
  [Mm]etastasis  12  PubMed(4); GeneRIFs(1)  
  [Cc]ell [Ii]nvasion  13  PubMed(2)  
 
 
 hide/show details for RAC2 
  [Ff]ilopodia  PubMed  
 DEF6, a novel PH-DH-like domain protein, is an upstream activator of the Rho GTPases Rac1, Cdc42, and RhoA. In this paper, we describe the characterization of DEF6, a novel PH-DH-like protein related to SWAP-70 that functions as an upstream activator of Rho GTPases. In NIH 3T3 cells, stimulation of the PI 3-kinase signaling pathway with either H2O2 or platelet-derived growth factor (PDGF) resulted in the translocation of an overexpressed DEF6-GFP fusion protein to the cell membrane and induced the formation of   filopodia   and lamellipodia. In contrast to full-length DEF6, expression of the DH-like (DHL) domain as a GFP fusion protein potently induced actin polymerization, including stress fiber formation in COS-7 cells, in the absence of PI 3-kinase signaling, indicating that it was constitutively active. The GTP-loading of Cdc42 was strongly enhanced in NIH 3T3 cells expressing the DH domain while   filopodia   formation, membrane ruffling, and stress fiber formation could be inhibited by the co-expression of the DH domain with dominant negative mutants of either N17Rac1, N17Cdc42, or N19RhoA, respectively. This indicated that DEF6 acts upstream of the Rho GTPases resulting in the activation of the Cdc42, Rac1, and RhoA signaling pathways. In vitro, DEF6 specifically interacted with Rac1, Rac2, Cdc42, and RhoA, suggesting a direct role for DEF6 in the activation of Rho GTPases. The ability of DEF6 to both stimulate actin polymerization and bind to filamentous actin suggests a role for DEF6 in regulating cell shape, polarity, and movement. 
  [Pp]odosome(s)?  PubMed  
 Rac1 and Rac2 regulate macrophage morphology but are not essential for migration. Rac GTPases are believed to contribute to migration in leukocytes by transducing signals from cell surface receptors to the actin and microtubule cytoskeletons. Mammals have three closely related Rac isoforms, Rac1, Rac2 and Rac3, and it is widely assumed that cell migration requires the activity of these Rac GTPases. We have previously shown that Rac1-null mouse macrophages have altered cell shape and reduced membrane ruffling but normal migration speed. Here we investigate the behaviour of macrophages lacking Rac2 (Rac2(-/-)) or Rac1 and Rac2 (Rac1/2(-/-)). Rac2(-/-) macrophages have reduced F-actin levels and lack   podosomes  , which are integrin-based adhesion sites, and their migration speed is similar to or slightly slower than wild-type macrophages, depending on the substrate. Unexpectedly, Rac1/2(-/-) macrophages, which do not express Rac1, Rac2 or Rac3, migrate at a similar speed to wild-type macrophages on a variety of substrates and perform chemotaxis normally, although their morphology and mode of migration is altered. However, Rac1(-/-) and Rac1/2(-/-) but not Rac2(-/-) macrophages are impaired in their ability to invade through Matrigel. Together, these data show that Rac1 and Rac2 have distinct roles in regulating cell morphology, migration and invasion, but are not essential for macrophage migration or chemotaxis. 
 Rac1 and Rac2 regulate macrophage morphology but are not essential for migration. Rac GTPases are believed to contribute to migration in leukocytes by transducing signals from cell surface receptors to the actin and microtubule cytoskeletons. Mammals have three closely related Rac isoforms, Rac1, Rac2 and Rac3, and it is widely assumed that cell migration requires the activity of these Rac GTPases. We have previously shown that Rac1-null mouse macrophages have altered cell shape and reduced membrane ruffling but normal migration speed. Here we investigate the behaviour of macrophages lacking Rac2 (Rac2(-/-)) or Rac1 and Rac2 (Rac1/2(-/-)). Rac2(-/-) macrophages have reduced F-actin levels and lack   podosomes  , which are integrin-based adhesion sites, and their migration speed is similar to or slightly slower than wild-type macrophages, depending on the substrate. Unexpectedly, Rac1/2(-/-) macrophages, which do not express Rac1, Rac2 or Rac3, migrate at a similar speed to wild-type macrophages on a variety of substrates and perform chemotaxis normally, although their morphology and mode of migration is altered. However, Rac1(-/-) and Rac1/2(-/-) but not Rac2(-/-) macrophages are impaired in their ability to invade through Matrigel. Together, these data show that Rac1 and Rac2 have distinct roles in regulating cell morphology, migration and invasion, but are not essential for macrophage migration or chemotaxis. 
  [Mm]igration  Process  
 regulation of neutrophil   migration   
  PubMed  
 Analysis of the myosin-II-responsive focal adhesion proteome reveals a role for β-Pix in negative regulation of focal adhesion maturation. Focal adhesions undergo myosin-II-mediated maturation wherein they grow and change composition to modulate integrin signalling for cell   migratio  n, growth and differentiation. To determine how focal adhesion composition is affected by myosin II activity, we performed proteomic analysis of isolated focal adhesions and compared protein abundance in focal adhesions from cells with and without myosin II inhibition. We identified 905 focal adhesion proteins, 459 of which changed in abundance with myosin II inhibition, defining the myosin-II-responsive focal adhesion proteome. The abundance of 73% of the proteins in the myosin-II-responsive focal adhesion proteome was enhanced by contractility, including proteins involved in Rho-mediated focal adhesion maturation and endocytosis- and calpain-dependent focal adhesion disassembly. During myosin II inhibition, 27% of proteins in the myosin-II-responsive focal adhesion proteome, including proteins involved in Rac-mediated lamellipodial protrusion, were enriched in focal adhesions, establishing that focal adhesion protein recruitment is also negatively regulated by contractility. We focused on the Rac guanine nucleotide exchange factor β-Pix, documenting its role in the negative regulation of focal adhesion maturation and the promotion of lamellipodial protrusion and focal adhesion turnover to drive cel  l migrati  on. 
 Rac1 and Rac2 regulate macrophage morphology but are not essential for   migration  . Rac GTPases are believed to contribute to   migration   in leukocytes by transducing signals from cell surface receptors to the actin and microtubule cytoskeletons. Mammals have three closely related Rac isoforms, Rac1, Rac2 and Rac3, and it is widely assumed that cell   migration   requires the activity of these Rac GTPases. We have previously shown that Rac1-null mouse macrophages have altered cell shape and reduced membrane ruffling but normal   migration   speed. Here we investigate the behaviour of macrophages lacking Rac2 (Rac2(-/-)) or Rac1 and Rac2 (Rac1/2(-/-)). Rac2(-/-) macrophages have reduced F-actin levels and lack podosomes, which are integrin-based adhesion sites, and their   migration   speed is similar to or slightly slower than wild-type macrophages, depending on the substrate. Unexpectedly, Rac1/2(-/-) macrophages, which do not express Rac1, Rac2 or Rac3, migrate at a similar speed to wild-type macrophages on a variety of substrates and perform chemotaxis normally, although their morphology and mode of   migration   is altered. However, Rac1(-/-) and Rac1/2(-/-) but not Rac2(-/-) macrophages are impaired in their ability to invade through Matrigel. Together, these data show that Rac1 and Rac2 have distinct roles in regulating cell morphology,   migration   and invasion, but are not essential for macrophage   migration   or chemotaxis. 
 The role of SDF-1α/Rac pathway in the regulation of endothelial progenitor cell polarity; homing and expression of Rac1, Rac2 during endothelial repair. The Stromal cell-derived factor-1 (SDF-1)/CXC chemokine receptor 4 (CXCR4) system is considered to be of great importance in diseases involving cardiogenesis and angiogenesis. The SDF-1α-RAC signaling pathway plays a pivotal role in a cell's polarity and serves to activate cell morphology variation and to control the direction o  f migrati  on. We aimed to study whether the polarity of endothelial progenitor cells (EPCs) is changed by the induction of the SDF-1α-RAC signaling pathway, to investigate the mechanism of the effect of polarity on the homing action of EPCs, and to explore the gene and protein expression of Rac1/2 during endothelial repair. We measured the EPC characteristics of polarity induced by various final concentrations of SDF-1α; our observations included morphology variati  on, migra  tion direction, and excursion. Of the dynamic variation and cytoskeleton rearrangement of EPCs induced by different final concentrations of SDF-1α, the most obvious variation was exhibited at the SDF-1α concentration of 200 ng/ml. Obvious polarity variations were also found in the EPCs and signal receptors induced by the SDF-1α concentration of 200 ng/ml. The Western blot analysis of Rac1 and Rac2 showed that the addition of AMD 3100 significantly inhibited the expression of Rac. The SDF-1α pathway potentially regulates the expression of Rac1/2. The actual excursion vector and the direction   of the m  igration of EPCs induced by SDF-1α follows polarity, thus indicating the importance of further exploration regarding the homing induction of EPCs. 
 Association between Gαi2 and ELMO1/Dock180 connects chemokine signalling with Rac activation and metastasis. The chemokine CXCL12 and its G-protein-coupled receptor CXCR4 control the   migratio  n, invasiveness and metastasis of breast cancer cells. Binding of CXCL12 to CXCR4 triggers activation of heterotrimeric Gi proteins that regulate actin polymerization and   migratio  n. However, the pathways linking chemokine G-protein-coupled receptor/Gi signalling to actin polymerization and cancer cell   migratio  n are not known. Here we show that CXCL12 stimulation promotes interaction between Gαi2 and ELMO1. Gi signalling and ELMO1 are both required for CXCL12-mediated actin polymerization  , migrati  on and invasion of breast cancer cells. CXCL12 triggers a Gαi2-dependent membrane translocation of ELMO1, which associates with Dock180 to activate small G-proteins Rac1 and Rac2. In vivo, ELMO1 expression is associated with lymph node and distant metastasis, and knocking down ELMO1 impairs metastasis to the lung. Our findings indicate that a chemokine-controlled pathway, consisting of Gαi2, ELMO1/Dock180, Rac1 and Rac2, regulates the actin cytoskeleton during breast cancer metastasis. 
 Human neutrophils coordinate chemotaxis by differential activation of Rac1 and Rac2. Rac1 and Rac2, members of the small Rho GTPase family, play essential roles in coordinating directional   migration   and superoxide production during neutrophil responses to chemoattractants. Although earlier studies in Rac1 and Rac2 knockout mice have demonstrated unique roles for each Rac isoform in chemotaxis and NADPH oxidase activation, it is still unclear how human neutrophils use Rac1 and Rac2 to achieve their immunological responses to foreign agent stimulation. In the current study, we used TAT dominant-negative Rac1-T17N and Rac2-T17N fusion proteins to acutely alter the activity of Rac1 and Rac2 individually in human neutrophils. We demonstrate distinct activation kinetics and different roles for Rac1 and Rac2 in response to low vs high concentrations of fMLP. These observations were verified using neutrophils from mice in which Rac1 or Rac2 was genetically absent. Based on these results, we propose a model to explain how human neutrophils kill invading microbes while limiting oxidative damage to the adjacent surrounding healthy tissue through the differential activation of Rac1 and Rac2 in response to different concentrations of chemoattractant. 
 Specific roles of Rac1 and Rac2 in motile functions of HT1080 fibrosarcoma cells. Rho family proteins are constitutively activated in the highly invasive human fibrosarcoma HT1080 cells. We now investigated the specific roles of Rac1 and Rac2 in regulating morphology, F-actin organization, adhesion,   migration  , and chemotaxis of HT1080 cells. Downregulation of Rac1 using specific siRNA probes resulted in cell rounding, markedly decreased spreading, adhesion, and chemotaxis of HT1080 cells. 2D   migration   on laminin-coated surfaces in contrast was not markedly affected. Selective Rac2 depletion did not affect cell morphology, cell adhesion, and 2D   migration  , but significantly reduced chemotaxis. Downregulation of both Rac1 and Rac2 resulted in an even more marked reduction, but not complete abolishment, of chemotaxis indicating distinct as well as overlapping roles of both proteins in chemotaxis. Rac1 thus is selectively required for HT1080 cell spreading and adhesion whereas Rac1 and Rac2 are both required for efficient chemotaxis. 
 Association between Gαi2 and ELMO1/Dock180 connects chemokine signalling with Rac activation and metastasis. The chemokine CXCL12 and its G-protein-coupled receptor CXCR4 control the   migratio  n, invasiveness and metastasis of breast cancer cells. Binding of CXCL12 to CXCR4 triggers activation of heterotrimeric Gi proteins that regulate actin polymerization and   migratio  n. However, the pathways linking chemokine G-protein-coupled receptor/Gi signalling to actin polymerization and cancer cell   migratio  n are not known. Here we show that CXCL12 stimulation promotes interaction between Gαi2 and ELMO1. Gi signalling and ELMO1 are both required for CXCL12-mediated actin polymerization  , migrati  on and invasion of breast cancer cells. CXCL12 triggers a Gαi2-dependent membrane translocation of ELMO1, which associates with Dock180 to activate small G-proteins Rac1 and Rac2. In vivo, ELMO1 expression is associated with lymph node and distant metastasis, and knocking down ELMO1 impairs metastasis to the lung. Our findings indicate that a chemokine-controlled pathway, consisting of Gαi2, ELMO1/Dock180, Rac1 and Rac2, regulates the actin cytoskeleton during breast cancer metastasis. 
 The role of SDF-1α/Rac pathway in the regulation of endothelial progenitor cell polarity; homing and expression of Rac1, Rac2 during endothelial repair. The Stromal cell-derived factor-1 (SDF-1)/CXC chemokine receptor 4 (CXCR4) system is considered to be of great importance in diseases involving cardiogenesis and angiogenesis. The SDF-1α-RAC signaling pathway plays a pivotal role in a cell's polarity and serves to activate cell morphology variation and to control the direction o  f migrati  on. We aimed to study whether the polarity of endothelial progenitor cells (EPCs) is changed by the induction of the SDF-1α-RAC signaling pathway, to investigate the mechanism of the effect of polarity on the homing action of EPCs, and to explore the gene and protein expression of Rac1/2 during endothelial repair. We measured the EPC characteristics of polarity induced by various final concentrations of SDF-1α; our observations included morphology variati  on, migra  tion direction, and excursion. Of the dynamic variation and cytoskeleton rearrangement of EPCs induced by different final concentrations of SDF-1α, the most obvious variation was exhibited at the SDF-1α concentration of 200 ng/ml. Obvious polarity variations were also found in the EPCs and signal receptors induced by the SDF-1α concentration of 200 ng/ml. The Western blot analysis of Rac1 and Rac2 showed that the addition of AMD 3100 significantly inhibited the expression of Rac. The SDF-1α pathway potentially regulates the expression of Rac1/2. The actual excursion vector and the direction   of the m  igration of EPCs induced by SDF-1α follows polarity, thus indicating the importance of further exploration regarding the homing induction of EPCs. 
 Human neutrophils coordinate chemotaxis by differential activation of Rac1 and Rac2. Rac1 and Rac2, members of the small Rho GTPase family, play essential roles in coordinating directional   migration   and superoxide production during neutrophil responses to chemoattractants. Although earlier studies in Rac1 and Rac2 knockout mice have demonstrated unique roles for each Rac isoform in chemotaxis and NADPH oxidase activation, it is still unclear how human neutrophils use Rac1 and Rac2 to achieve their immunological responses to foreign agent stimulation. In the current study, we used TAT dominant-negative Rac1-T17N and Rac2-T17N fusion proteins to acutely alter the activity of Rac1 and Rac2 individually in human neutrophils. We demonstrate distinct activation kinetics and different roles for Rac1 and Rac2 in response to low vs high concentrations of fMLP. These observations were verified using neutrophils from mice in which Rac1 or Rac2 was genetically absent. Based on these results, we propose a model to explain how human neutrophils kill invading microbes while limiting oxidative damage to the adjacent surrounding healthy tissue through the differential activation of Rac1 and Rac2 in response to different concentrations of chemoattractant. 
 Specific roles of Rac1 and Rac2 in motile functions of HT1080 fibrosarcoma cells. Rho family proteins are constitutively activated in the highly invasive human fibrosarcoma HT1080 cells. We now investigated the specific roles of Rac1 and Rac2 in regulating morphology, F-actin organization, adhesion,   migration  , and chemotaxis of HT1080 cells. Downregulation of Rac1 using specific siRNA probes resulted in cell rounding, markedly decreased spreading, adhesion, and chemotaxis of HT1080 cells. 2D   migration   on laminin-coated surfaces in contrast was not markedly affected. Selective Rac2 depletion did not affect cell morphology, cell adhesion, and 2D   migration  , but significantly reduced chemotaxis. Downregulation of both Rac1 and Rac2 resulted in an even more marked reduction, but not complete abolishment, of chemotaxis indicating distinct as well as overlapping roles of both proteins in chemotaxis. Rac1 thus is selectively required for HT1080 cell spreading and adhesion whereas Rac1 and Rac2 are both required for efficient chemotaxis. 
 Rac1 and Rac2 regulate macrophage morphology but are not essential for   migration  . Rac GTPases are believed to contribute to   migration   in leukocytes by transducing signals from cell surface receptors to the actin and microtubule cytoskeletons. Mammals have three closely related Rac isoforms, Rac1, Rac2 and Rac3, and it is widely assumed that cell   migration   requires the activity of these Rac GTPases. We have previously shown that Rac1-null mouse macrophages have altered cell shape and reduced membrane ruffling but normal   migration   speed. Here we investigate the behaviour of macrophages lacking Rac2 (Rac2(-/-)) or Rac1 and Rac2 (Rac1/2(-/-)). Rac2(-/-) macrophages have reduced F-actin levels and lack podosomes, which are integrin-based adhesion sites, and their   migration   speed is similar to or slightly slower than wild-type macrophages, depending on the substrate. Unexpectedly, Rac1/2(-/-) macrophages, which do not express Rac1, Rac2 or Rac3, migrate at a similar speed to wild-type macrophages on a variety of substrates and perform chemotaxis normally, although their morphology and mode of   migration   is altered. However, Rac1(-/-) and Rac1/2(-/-) but not Rac2(-/-) macrophages are impaired in their ability to invade through Matrigel. Together, these data show that Rac1 and Rac2 have distinct roles in regulating cell morphology,   migration   and invasion, but are not essential for macrophage   migration   or chemotaxis. 
 Rac2 concentrations in umbilical cord neutrophils. Human newborn infants display a variety of immunodeficiencies of immaturity, including diminished neutrophil adhesion, chemotaxis, and   migration  . Rac2, a guanosine triphosphate-binding protein, is an essential regulator of human neutrophil   migration   and chemotaxis. Since human subjects and mice deficient in Rac2 display deficiencies in neutrophil functions similar to newborn infants, we postulated that newborn neutrophils may be deficient in Rac2.The aim of the study was to measure Rac1 and Rac2 concentrations in neutrophils from umbilical cord blood.Neutrophils from cord and adult blood were isolated, total cell lysates extracted, and Rac protein concentrations determined using Western blot analysis.Rac2 concentrations were significantly lower in the neutrophil protein lysates isolated from cord blood compared to adult blood despite similar levels of Rac1.Diminished Rac2 expression in cord blood neutrophils may contribute to the defects observed in cord blood neutrophil function. 
 Critical roles for Rac1 and Rac2 GTPases in B cell development and signaling. The Rac1 guanosine triphosphatase (GTPase) has been implicated in multiple cellular functions, including actin dynamics, proliferation, apoptosis, adhesion, and   migration   resulting from signaling by multiple receptors, including the B cell antigen receptor (BCR). We used conditional gene targeting to generate mice with specific Rac1 deficiency in the B cell lineage. In the absence of both Rac1 and the highly related Rac2, B cell development was almost completely blocked. Both GTPases were required to transduce BCR signals leading to proliferation, survival and up-regulation of BAFF-R, a receptor for BAFF, a key survival molecule required for B cell development and maintenance. 
 Hematopoietic cell regulation by Rac1 and Rac2 guanosine triphosphatases. The Rho guanosine triphosphatases (GTPases) Rac1 and Rac2 are critical signaling regulators in mammalian cells. The deletion of both Rac1 and Rac2 murine alleles leads to a massive egress of hematopoietic stem/progenitor cells (HSC/Ps) into the blood from the marrow, whereas Rac1-/- but not Rac2-/- HSC/Ps fail to engraft in the bone marrow of irradiated recipient mice. In contrast, Rac2, but not Rac1, regulates superoxide production and directed   migration   in neutrophils, and in each cell type, the two GTPases play distinct roles in actin organization, cell survival, and proliferation. Thus, Rac1 and Rac2 regulate unique aspects of hematopoietic development and function. 
 DOCK2 mediates T cell receptor-induced activation of Rac2 and IL-2 transcription. DOCK2, a CDM family protein exclusively found in hematopoietic cells, has been shown to play a role in lymphocyte   migration   by the regulation of actin cytoskeleton. Although DOCK2 has been shown to induce the activation of Rac1, the regulatory mechanism of Rac2, which is a hematopoietic cell-specific small GTPase, is still unknown. In this study, we examined the role of DOCK2 in the activation of Rac2 in hematopoietic cells. DOCK2 was found to associate with the zeta subunit of the CD3 complex of T cell receptors in Jurkat cells and to activate forced expressed Rac2 in 293T cells. In addition, the stable expression of DOCK2 in Jurkat cells exhibited the elevated activity of endogenous Rac2. Furthermore, the transcriptional activity of interleukin-2 (IL-2) was enhanced in DOCK2-expressing Jurkat cells and the dominant negative form of Rac2 suppressed its elevated IL-2 promoter activity. These results suggest that DOCK2 mediates TCR-dependent activation of Rac2, leading to the regulation of IL-2 promoter activity in T cells. 
 Requirement of Rac1 and Rac2 expression by mature dendritic cells for T cell priming. Upon maturation, dendritic cells (DCs) acquire the unique ability to activate naïve T cells. We used time-lapse video microscopy and two-photon imaging of intact lymph nodes to show that after establishing initial contact between their dendrites and naïve T lymphocytes, mature DCs migrate toward the contacted lymphocytes. Subsequently, the DCs tightly entrap the T cells within a complex net of membrane extensions. The Rho family guanosine triphosphatases Rac1 and Rac2 but not Rho itself control the formation of dendrites in mature DCs, their polarized short-rang  e migrati  on toward T cells, and T cell priming. 
 Ephrin stimulation modulates T cell chemotaxis. Eph receptor tyrosine kinases and their ligands, the ephrins, are known to play an important role in regulating cell   migration   and targeting in neuronal and endothelial cells. Recently, it has been shown that lymphoid cells also express Eph receptors, raising the possibility that Eph receptors may similarly regulate lymphocyte   migration  . Chemotaxis in response to soluble chemokine factors is an essential facet of T cell biology. We demonstrate here that T cell chemotaxis in response to both the stromal cell-derived factor (SDF)-1alpha and macrophage inflammatory protein 3beta chemokines is modulated by costimulation with ephrins. Both ephrin-A and ephrin-B ligands were found to modify the chemotactic responses of a T cell line and primary T cells. Ephrin-A1, in particular, strongly inhibited chemotaxis. In accordance with the tyrosine kinase activity of EphA receptors, ephrin-A1 stimulation induced rapid intracellular tyrosine phosphorylation in T cells. Although strongly inhibiting chemotaxis, ephrin-A1 costimulus did not affect many of the signaling events downstream of the SDF-1alpha receptor CXCR4, including calcium flux and activation of MAPK. Rather, ephrin-A1 altered the balance of small G protein activity in T cells. Ephrin-A1 stimulation prevented SDF-1alpha-induced activation of cdc42, while simultaneously inducing rho activation. Ultimately, ephrin-A1 was found to inhibit chemokine-induced actin polymerization, thereby blocking   migration  . Ubiquitous ephrin expression in vivo creates enormous potential for T cells to encounter these ligands, suggesting that Eph receptors and ephrins may be important regulators of T cell   migration  . 
  GeneRIFs  
 Rac1 and Rac2 have distinct roles in regulating cell morphology,   migration   and invasion, but are not essential for macrophage   migration   or chemotaxis. 
  [Ii]nvasive  PubMed  
 Association between Gαi2 and ELMO1/Dock180 connects chemokine signalling with Rac activation and metastasis. The chemokine CXCL12 and its G-protein-coupled receptor CXCR4 control the migration,   invasiv  eness and metastasis of breast cancer cells. Binding of CXCL12 to CXCR4 triggers activation of heterotrimeric Gi proteins that regulate actin polymerization and migration. However, the pathways linking chemokine G-protein-coupled receptor/Gi signalling to actin polymerization and cancer cell migration are not known. Here we show that CXCL12 stimulation promotes interaction between Gαi2 and ELMO1. Gi signalling and ELMO1 are both required for CXCL12-mediated actin polymerization, migration and invasion of breast cancer cells. CXCL12 triggers a Gαi2-dependent membrane translocation of ELMO1, which associates with Dock180 to activate small G-proteins Rac1 and Rac2. In vivo, ELMO1 expression is associated with lymph node and distant metastasis, and knocking down ELMO1 impairs metastasis to the lung. Our findings indicate that a chemokine-controlled pathway, consisting of Gαi2, ELMO1/Dock180, Rac1 and Rac2, regulates the actin cytoskeleton during breast cancer metastasis. 
 Specific roles of Rac1 and Rac2 in motile functions of HT1080 fibrosarcoma cells. Rho family proteins are constitutively activated in the highly   invasive   human fibrosarcoma HT1080 cells. We now investigated the specific roles of Rac1 and Rac2 in regulating morphology, F-actin organization, adhesion, migration, and chemotaxis of HT1080 cells. Downregulation of Rac1 using specific siRNA probes resulted in cell rounding, markedly decreased spreading, adhesion, and chemotaxis of HT1080 cells. 2D migration on laminin-coated surfaces in contrast was not markedly affected. Selective Rac2 depletion did not affect cell morphology, cell adhesion, and 2D migration, but significantly reduced chemotaxis. Downregulation of both Rac1 and Rac2 resulted in an even more marked reduction, but not complete abolishment, of chemotaxis indicating distinct as well as overlapping roles of both proteins in chemotaxis. Rac1 thus is selectively required for HT1080 cell spreading and adhesion whereas Rac1 and Rac2 are both required for efficient chemotaxis. 
 Association between Gαi2 and ELMO1/Dock180 connects chemokine signalling with Rac activation and metastasis. The chemokine CXCL12 and its G-protein-coupled receptor CXCR4 control the migration,   invasiv  eness and metastasis of breast cancer cells. Binding of CXCL12 to CXCR4 triggers activation of heterotrimeric Gi proteins that regulate actin polymerization and migration. However, the pathways linking chemokine G-protein-coupled receptor/Gi signalling to actin polymerization and cancer cell migration are not known. Here we show that CXCL12 stimulation promotes interaction between Gαi2 and ELMO1. Gi signalling and ELMO1 are both required for CXCL12-mediated actin polymerization, migration and invasion of breast cancer cells. CXCL12 triggers a Gαi2-dependent membrane translocation of ELMO1, which associates with Dock180 to activate small G-proteins Rac1 and Rac2. In vivo, ELMO1 expression is associated with lymph node and distant metastasis, and knocking down ELMO1 impairs metastasis to the lung. Our findings indicate that a chemokine-controlled pathway, consisting of Gαi2, ELMO1/Dock180, Rac1 and Rac2, regulates the actin cytoskeleton during breast cancer metastasis. 
 Specific roles of Rac1 and Rac2 in motile functions of HT1080 fibrosarcoma cells. Rho family proteins are constitutively activated in the highly   invasive   human fibrosarcoma HT1080 cells. We now investigated the specific roles of Rac1 and Rac2 in regulating morphology, F-actin organization, adhesion, migration, and chemotaxis of HT1080 cells. Downregulation of Rac1 using specific siRNA probes resulted in cell rounding, markedly decreased spreading, adhesion, and chemotaxis of HT1080 cells. 2D migration on laminin-coated surfaces in contrast was not markedly affected. Selective Rac2 depletion did not affect cell morphology, cell adhesion, and 2D migration, but significantly reduced chemotaxis. Downregulation of both Rac1 and Rac2 resulted in an even more marked reduction, but not complete abolishment, of chemotaxis indicating distinct as well as overlapping roles of both proteins in chemotaxis. Rac1 thus is selectively required for HT1080 cell spreading and adhesion whereas Rac1 and Rac2 are both required for efficient chemotaxis. 
  [Mm]etastasis  PubMed  
 Long noncoding RNA AFAP1-AS1 indicates a poor prognosis of hepatocellular carcinoma and promotes cell proliferation and invasion via upregulation of the RhoA/Rac2 signaling. It has been shown that long noncoding RNAs (lncRNAs) play a critical role in the regulation of cellular processes including cancer progression and   metastasis  . However, the biological functions and clinical significance of lncRNA AFAP1-AS1 in hepatocellular carcinoma (HCC) remain unclear. Expression of AFAP1-AS1 was analyzed in 78 HCC tissues by real-time PCR. The effect of AFAP1-AS1 on cell proliferation was examined by MTT assay, cell apoptosis was detected by flow cytometric analysis and cell invasion was determined by Transwell assay. RhoA/Rac2 signaling and downstream factors were verified by western blotting. HCC cells infected with si-AFAP1-AS1 were injected into nude mice to investigate the effect of AFAP1-AS1 on the tumorigenesis in vivo. We found that increased expression of AFAP1-AS1 was significantly correlated with pathological staging (P=0.024) and lymph-vascular space invasion (LVSI) in HCC patients (P=0.007). Multivariate analyses indicated that AFAP1-AS1 represented an independent predictor for overall survival of HCC (P=0.029). Further experiments showed that knockdown of AFAP1-AS1 by si-AFAP1-AS1 decreased the proliferation and invasion in vitro and in vivo, induced cell apoptosis and blocked cell cycle in S phase via inhibition of the RhoA/Rac2 signaling. Taken together, our findings indicate that AFAP1-AS1 may promote the HCC development through upregulation of RhoA/Rac2 signaling and provide a potential therapeutic target for HCC. 
 Association between Gαi2 and ELMO1/Dock180 connects chemokine signalling with Rac activation and   metastasi  s. The chemokine CXCL12 and its G-protein-coupled receptor CXCR4 control the migration, invasiveness and   metastasi  s of breast cancer cells. Binding of CXCL12 to CXCR4 triggers activation of heterotrimeric Gi proteins that regulate actin polymerization and migration. However, the pathways linking chemokine G-protein-coupled receptor/Gi signalling to actin polymerization and cancer cell migration are not known. Here we show that CXCL12 stimulation promotes interaction between Gαi2 and ELMO1. Gi signalling and ELMO1 are both required for CXCL12-mediated actin polymerization, migration and invasion of breast cancer cells. CXCL12 triggers a Gαi2-dependent membrane translocation of ELMO1, which associates with Dock180 to activate small G-proteins Rac1 and Rac2. In vivo, ELMO1 expression is associated with lymph node and dista  nt metasta  sis, and knocking down ELMO1 impai  rs metasta  sis to the lung. Our findings indicate that a chemokine-controlled pathway, consisting of Gαi2, ELMO1/Dock180, Rac1 and Rac2, regulates the actin cytoskeleton during breast can  cer metast  asis. 
 Long noncoding RNA AFAP1-AS1 indicates a poor prognosis of hepatocellular carcinoma and promotes cell proliferation and invasion via upregulation of the RhoA/Rac2 signaling. It has been shown that long noncoding RNAs (lncRNAs) play a critical role in the regulation of cellular processes including cancer progression and   metastasis  . However, the biological functions and clinical significance of lncRNA AFAP1-AS1 in hepatocellular carcinoma (HCC) remain unclear. Expression of AFAP1-AS1 was analyzed in 78 HCC tissues by real-time PCR. The effect of AFAP1-AS1 on cell proliferation was examined by MTT assay, cell apoptosis was detected by flow cytometric analysis and cell invasion was determined by Transwell assay. RhoA/Rac2 signaling and downstream factors were verified by western blotting. HCC cells infected with si-AFAP1-AS1 were injected into nude mice to investigate the effect of AFAP1-AS1 on the tumorigenesis in vivo. We found that increased expression of AFAP1-AS1 was significantly correlated with pathological staging (P=0.024) and lymph-vascular space invasion (LVSI) in HCC patients (P=0.007). Multivariate analyses indicated that AFAP1-AS1 represented an independent predictor for overall survival of HCC (P=0.029). Further experiments showed that knockdown of AFAP1-AS1 by si-AFAP1-AS1 decreased the proliferation and invasion in vitro and in vivo, induced cell apoptosis and blocked cell cycle in S phase via inhibition of the RhoA/Rac2 signaling. Taken together, our findings indicate that AFAP1-AS1 may promote the HCC development through upregulation of RhoA/Rac2 signaling and provide a potential therapeutic target for HCC. 
 Association between Gαi2 and ELMO1/Dock180 connects chemokine signalling with Rac activation and   metastasi  s. The chemokine CXCL12 and its G-protein-coupled receptor CXCR4 control the migration, invasiveness and   metastasi  s of breast cancer cells. Binding of CXCL12 to CXCR4 triggers activation of heterotrimeric Gi proteins that regulate actin polymerization and migration. However, the pathways linking chemokine G-protein-coupled receptor/Gi signalling to actin polymerization and cancer cell migration are not known. Here we show that CXCL12 stimulation promotes interaction between Gαi2 and ELMO1. Gi signalling and ELMO1 are both required for CXCL12-mediated actin polymerization, migration and invasion of breast cancer cells. CXCL12 triggers a Gαi2-dependent membrane translocation of ELMO1, which associates with Dock180 to activate small G-proteins Rac1 and Rac2. In vivo, ELMO1 expression is associated with lymph node and dista  nt metasta  sis, and knocking down ELMO1 impai  rs metasta  sis to the lung. Our findings indicate that a chemokine-controlled pathway, consisting of Gαi2, ELMO1/Dock180, Rac1 and Rac2, regulates the actin cytoskeleton during breast can  cer metast  asis. 
  GeneRIFs  
 findings indicate that a chemokine-controlled pathway, consisting of Galphai2, ELMO1/Dock180, Rac1 and Rac2, regulates the actin cytoskeleton during breast cancer   metastasis   
  [Cc]ell [Ii]nvasion  PubMed  
 Long noncoding RNA AFAP1-AS1 indicates a poor prognosis of hepatocellular carcinoma and promotes cell proliferation and invasion via upregulation of the RhoA/Rac2 signaling. It has been shown that long noncoding RNAs (lncRNAs) play a critical role in the regulation of cellular processes including cancer progression and metastasis. However, the biological functions and clinical significance of lncRNA AFAP1-AS1 in hepatocellular carcinoma (HCC) remain unclear. Expression of AFAP1-AS1 was analyzed in 78 HCC tissues by real-time PCR. The effect of AFAP1-AS1 on cell proliferation was examined by MTT assay, cell apoptosis was detected by flow cytometric analysis and   cell invasion   was determined by Transwell assay. RhoA/Rac2 signaling and downstream factors were verified by western blotting. HCC cells infected with si-AFAP1-AS1 were injected into nude mice to investigate the effect of AFAP1-AS1 on the tumorigenesis in vivo. We found that increased expression of AFAP1-AS1 was significantly correlated with pathological staging (P=0.024) and lymph-vascular space invasion (LVSI) in HCC patients (P=0.007). Multivariate analyses indicated that AFAP1-AS1 represented an independent predictor for overall survival of HCC (P=0.029). Further experiments showed that knockdown of AFAP1-AS1 by si-AFAP1-AS1 decreased the proliferation and invasion in vitro and in vivo, induced cell apoptosis and blocked cell cycle in S phase via inhibition of the RhoA/Rac2 signaling. Taken together, our findings indicate that AFAP1-AS1 may promote the HCC development through upregulation of RhoA/Rac2 signaling and provide a potential therapeutic target for HCC. 
 Long noncoding RNA AFAP1-AS1 indicates a poor prognosis of hepatocellular carcinoma and promotes cell proliferation and invasion via upregulation of the RhoA/Rac2 signaling. It has been shown that long noncoding RNAs (lncRNAs) play a critical role in the regulation of cellular processes including cancer progression and metastasis. However, the biological functions and clinical significance of lncRNA AFAP1-AS1 in hepatocellular carcinoma (HCC) remain unclear. Expression of AFAP1-AS1 was analyzed in 78 HCC tissues by real-time PCR. The effect of AFAP1-AS1 on cell proliferation was examined by MTT assay, cell apoptosis was detected by flow cytometric analysis and   cell invasion   was determined by Transwell assay. RhoA/Rac2 signaling and downstream factors were verified by western blotting. HCC cells infected with si-AFAP1-AS1 were injected into nude mice to investigate the effect of AFAP1-AS1 on the tumorigenesis in vivo. We found that increased expression of AFAP1-AS1 was significantly correlated with pathological staging (P=0.024) and lymph-vascular space invasion (LVSI) in HCC patients (P=0.007). Multivariate analyses indicated that AFAP1-AS1 represented an independent predictor for overall survival of HCC (P=0.029). Further experiments showed that knockdown of AFAP1-AS1 by si-AFAP1-AS1 decreased the proliferation and invasion in vitro and in vivo, induced cell apoptosis and blocked cell cycle in S phase via inhibition of the RhoA/Rac2 signaling. Taken together, our findings indicate that AFAP1-AS1 may promote the HCC development through upregulation of RhoA/Rac2 signaling and provide a potential therapeutic target for HCC. 
   
  
 CDC42 
 
  NCBI Gene  
  GeneRIF  
  Pubmed  
 
 
  Search Term  Rank  Fields  
  [Ff]ilopodia  1  PubMed(62); GeneRIFs(5)  
  [Ff]ilopodium  2  Process(3); PubMed(7); Component(1)  
  [Ii]nvadopodia  3  PubMed(7); GeneRIFs(2)  
  [Pp]odosome(s)?  5  PubMed(2); GeneRIFs(1)  
  [Mm]igration  6  Process(2); PubMed(123); GeneRIFs(31); Summary(1)  
  ECM  7  PubMed(4)  
  [Ee]xtracellular [Mm]atrix  8  PubMed(20)  
  [Ii]nvadosome  9  PubMed(3); GeneRIFs(2)  
  [Ii]nvasive  10  PubMed(36); GeneRIFs(2)  
  [Mm]etastasis  12  PubMed(37); GeneRIFs(10)  
  [Cc]ell [Ii]nvasion  13  PubMed(26); GeneRIFs(5)  
 
 
 hide/show details for CDC42 
  [Ff]ilopodia  PubMed  
 Cross-talk between Rac1 and Cdc42 GTPases regulates formation of   filopodia   required for dengue virus type-2 entry into HMEC-1 cells. Infection with dengue virus type-2 (DENV-2) begins with virus adherence to cell surface receptors. In endothelial cells (HMEC-1), a cell model for DENV-2 infection, alpha 5 beta 3 integrin has been identified as a putative receptor for the virus. Previous work had suggested that the actin cytoskeleton of HMEC-1 cells plays an important role in virus entry and infection. In the present work, fixed and living HMEC-1 cells expressing enhanced green fluorescent protein-actin were monitored for actin reorganization after virus inoculation, utilizing fluorescence and time lapse microscopy. Cell infection and production of infective viruses were quantified using an anti-E protein antibody and by measuring the p.f.u. ml(-1). Specific drugs that antagonize actin organization and regulate actin-signalling pathways were tested in viral adhesion and infection assays, as were the expression of dominant-negative Rac1 and Cdc42 proteins. Disorganization of actin precluded infection, while microtubule depolymerization had no effect. Activation of Rac1 and Cdc42 signalling, which occurs upon virus binding, induced reorganization of actin to form   filopodia   in the cellular periphery. Formation of   filopodia   was a requirement for virus entry and further cell infection. Expression of the dominant-negative proteins Rac1 and Cdc42 confirmed the role of these GTPases in the actin reorganization that is required to form   filopodia  . In addition, inhibition of the ATPase activity of myosin II greatly decreased infection, suggesting its participation in   filopodia  l stability. We show here, for the first time, that internalization of DENV-2 into endothelial cells requires viral induction of dynamic   filopodia   regulated by Rac1 and Cdc42 cross-talk and myosin II motor activities. 
 Desmoglein 3 acting as an upstream regulator of Rho GTPases, Rac-1/Cdc42 in the regulation of actin organisation and dynamics. Desmoglein 3 (Dsg3), a member of the desmoglein sub-family, serves as an adhesion molecule in desmosomes. Our previous study showed that overexpression of human Dsg3 in several epithelial lines induces formation of membrane protrusions, a phenotype suggestive of Rho GTPase activation. Here we examined the interaction between Dsg3 and actin in detail and showed that endogenous Dsg3 colocalises and interacts with actin, particularly the junctional actin in a Rac1-dependent manner. Ablation of Rac1 activity by dominant negative Rac1 mutant (N17Rac1) or the Rac1 specific inhibitor (NSC23766) directly disrupts the interaction between Dsg3 and actin. Assembly of the junctional actin at the cell borders is accompanied with enhanced levels of Dsg3, while inhibition of Dsg3 by RNAi results in profound changes in the organisation of actin cytoskeleton. In accordance, overexpression of Dsg3 results in a remarkable increase of Rac1 and Cdc42 activities and to a lesser extent, RhoA. The enhancements in Rho GTPases are accompanied by the pronounced actin-based membrane structures such as lamellipodia and   filopodia  , enhanced rate of actin turnover and cell polarisation. Together, our results reveal an important novel function for Dsg3 in promoting actin dynamics through regulating Rac1 and Cdc42 activation in epithelial cells. 
 Serine-71 phosphorylation of Rac1/Cdc42 diminishes the pathogenic effect of Clostridium difficile toxin A. Clostridium difficile toxin A and B (TcdA/TcdB) are glucosyltransferases that glucosylate GTPases of the Rho family. The epidermal growth factor (EGF) positively modulates C. difficile toxin-induced disturbance of the intestinal barrier function by an unknown mechanism. We found that EGF-treated CaCo-2 monolayers were less susceptible to TcdA-catalysed glucosylation of Rac1 but not of RhoA, which correlated with phosphorylation of Rac1 at Ser-71. Phospho-Rac1/phospho-Cdc42 (Ser-71) still bound to the PAK-CRIB domain indicating an active state. A more detailed characterization of phospho-Rac1 was performed using the phosphomimetic mutant Rac1 S71E. Ectopic expression of Rac1 S71E induced a specific phenotype of cells showing an increase in   filopodia  l structures that were also induced by EGF. Rac1 S71E (and Cdc42 S71E) but not Rac1 S71A was at least fivefold weaker substrate for TcdA-catalysed glucosylation compared with wild type Rac1. The protective effect was checked in transfection experiments where Rac1 S71E and, to a lesser extent, Cdc42 S71E reduced the TcdA-induced cytopathic effect. Thus, Ser-71 phosphorylation of Rac1 might be interesting for modulation of microbial pathogenesis where Rho GTPases, especially Rac1 and Cdc42, are involved. In addition, this is the first description of a specific functional outcome of Rac1 phosphorylation at Ser-71. 
 Cdc42/Rac1-dependent activation of the p21-activated kinase (PAK) regulates human platelet lamellipodia spreading: implication of the cortical-actin binding protein cortactin. Platelet activation by thrombin or thrombin receptor-activating peptide (TRAP) results in extensive actin reorganization that leads to   filopodia   emission and lamellae spreading concomitantly with activation of the Rho family small G proteins, Cdc42 and Rac1. Evidence has been provided that direct binding of Cdc42-guanosine triphosphate (GTP) and Rac1-GTP to the N-terminal regulatory domain of the p21-activated kinase (PAK) stimulates PAK activation and actin reorganization. In the present study, we have investigated the relationship between shape change and PAK activation. We show that thrombin, TRAP, or monoclonal antibody (MoAb) anti-Fc(gamma)RIIA IV.3 induces an activation of Cdc42 and Rac1. The GpVI ligand, convulxin (CVX), that forces platelets to lamellae spreading efficiently activates Rac1. Thrombin, TRAP, MoAb IV.3, and CVX stimulate autophosphorylation and kinase activity of PAK. Inhibition of Cdc42 and Rac1 with clostridial toxin B inhibits PAK activation and lamellae spreading. The cortical-actin binding protein, p80/85 cortactin, is constitutively associated with PAK in resting platelets and dissociates from PAK after thrombin stimulation. Inhibition of PAK autophosphorylation by toxin B prevents the dissociation of cortactin. These results suggest that Cdc42/Rac1-dependent activation of PAK may trigger early platelet shape change, at least in part through the regulation of cortactin binding to PAK. 
 Prostaglandin E2-mediated migration of human trophoblast requires RAC1 and CDC42. The invasion of maternal decidua and uterine spiral arteries by a trophoblast subpopulation called extravillous trophoblast (EVT) is essential for the establishment of a normal placenta and an adequate blood flow toward the fetus. Derangements in these processes underlie pregnancy-related diseases like preeclampsia and intrauterine growth restriction. Many growth factors, growth factor binding proteins, and extracellular matrix components can positively or negatively regulate the proliferation, migration, and/or invasiveness of these EVT cells. RHO GTPases, including RHOA, RAC1, and CDC42, are ubiquitous proteins that control cytoskeletal changes by forming stress fibers and projecting lamellipodia and   filopodia   during cellular migration. We had previously shown that prostaglandin (PG) E(2) produced in abundance by the decidua promotes the migration of first-trimester human EVTs by increasing the intracellular concentration of calcium and activating calpain. Using our well-characterized immortalized EVT cell line, HTR-8/SVneo, as well as villus explants from first-trimester placentae, this study examined the role of RHO GTPases RAC1 and CDC42 in PGE(2)-mediated migratory responses of these cells. Though a RAC1 inhibitor, NSC23766 as well as RAC1 knockdown by siRNA decreased the migration of HTR-8/SVneo cells in a Transwell migration assay, this inhibition could not be restored by PGE(2) or 17-phenyl trinor PGE(2) (PGE receptor PTGER1 agonist) or PGE(1) Alcohol (PGE receptor PTGER4 agonist). Similar results were noted for EVT cell spreading in villus explants. Furthermore, CDC42 silencing using siRNA inhibited PGE(2)-induced migration of HTR-8/SVneo cells. Finally, the treatment of EVT cells with PGE(2), PTGER1 agonist, or PTGER4 agonist activated RAC1 and CDC42 at 10 min, suggesting that RAC1 and CDC42 play an essential role in PGE(2)-mediated migration of human EVTs. 
 Estrogen and resveratrol regulate Rac and Cdc42 signaling to the actin cytoskeleton of metastatic breast cancer cells. Estrogen and structurally related molecules play critical roles in breast cancer. We reported that resveratrol (50 microM), an estrogen-like phytosterol from grapes, acts in an antiestrogenic manner in breast cancer cells to reduce cell migration and to induce a global and sustained extension of actin structures called   filopodia  . Herein, we report that resveratrol-induced   filopodia   formation is time-dependent and concentration-dependent. In contrast to resveratrol at 50 microM, resveratrol at 5 microM acts in a manner similar to estrogen by increasing lamellipodia, as well as cell migration and invasion. Because Rho GTPases regulate the extension of actin structures, we investigated a role for Rac and Cdc42 in estrogen and resveratrol signaling. Our results demonstrate that 50 microM resveratrol decreases Rac and Cdc42 activity, whereas estrogen and 5 microM resveratrol increase Rac activity in breast cancer cells. MDA-MB-231 cells expressing dominant-negative Cdc42 or dominant-negative Rac retain   filopodia   response to 50 microM resveratrol. Lamellipodia response to 5 microM resveratrol, estrogen, or epidermal growth factor is inhibited in cells expressing dominant-negative Rac, indicating that Rac regulates estrogen and resveratrol (5 microM) signaling to the actin cytoskeleton. These results indicate that signaling to the actin cytoskeleton by low and high concentrations of resveratrol may be differentially regulated by Rac and Cdc42. 
 Wnt5b promotes the cell motility essential for metastasis of oral squamous cell carcinoma through active Cdc42 and RhoA. The activation of Wnt signaling has been reported in many types of squamous cell carcinoma. In this study, using human oral squamous cell carcinoma (OSCC) cells with different metastatic potential, we investigated the involvement of Wnt signaling in metastasis. Further, we aimed to elucidate the characteristic biological features related to high metastatic potential and to identify new target molecules for the suppression of OSCC lymph node metastasis. We compared SAS-Venus (SAS OSCC cells expressing green fluorescent protein) and SAS-LM8, which is a highly metastatic cell line derived from SAS-Venus by in vivo selection. The SAS-LM8 cell line had greater ability of migration and invasion compared to SAS-Venus. Furthermore, a higher number of   filopodia  -like protrusive structures were produced in SAS-LM8 cells compared to SAS-Venus cells, and the levels of active Cdc42 and active RhoA protein were higher in SAS-LM8 cells compared to SAS-Venus cells. We did not observe any differences in the expression of Wnt/β-catenin target genes between the two cell lines; however, the mRNA levels of Wnt5b were higher in SAS-LM8 cells compared to SAS-Venus cells. To confirm the involvement of Wnt5b in migration in OSCC cells, we examined the effects of the siRNA-mediated knockdown of Wnt5b in SAS-Venus cells and SAS-LM8 cells. The siRNA treatment significantly inhibited migration and the formation of   filopodi  a-like protrusive structures. Conversely, when stimulated with Wnt5b, the migration and formation of   filopodi  a-like protrusions were significantly enhanced and the levels of active Cdc42 and active RhoA proteins were also increased. These results indicate that Wnt5b is involved in the migration ability of OSCC cells through active Cdc42 and RhoA. 
 Snail regulates the motility of oral cancer cells via RhoA/Cdc42/p-ERM pathway. The transcriptional factor Snail has been reported to possess properties related to cancer progression; however, the mechanism for it is not fully understood. Our data showed that Snail knockdown by small interfering RNA in two OSCC cell lines, WSU-HN6 and CAL27, significantly inhibited cell migration and invasion which also resulted in decreased cell motility, such as impaired cell spreading on type I collagen substrate, reduced   filopodia  , and premature assembly of stress fibers. In addition, Snail-silencing decreased Cdc42 activity but increased RhoA activity, accompanied by the downregulation in both p-ERM expression and cell motility. Meanwhile, endogenous p-ERM was found specifically co-precipitated with activated Cdc42, but not RhoA, and this co-association was decreased by Snail-silencing. The small molecule inhibitors of Rho-associated kinase (Y27632) markedly enhanced Cdc42 activity and the association of p-ERM with activated Cdc42, increasing cell motility remarkably. Using immunohistochemistry, Snail and p-ERM overexpressions were found in OSCC tissues correlated with nodal metastasis and shorter survival. Taken together, these results demonstrate that Snail regulates cell motility through RhoA/Cdc42/p-ERM pathway and may serve as a biomarker to predict prognosis for OSCC patients. Although RhoA and Cdc42 are concurrently regulated downstream of Snail, there is a direct interplay between them, which indicates RhoA has to be inactivated at some point in cell motility cycle. 
 DEF6, a novel PH-DH-like domain protein, is an upstream activator of the Rho GTPases Rac1, Cdc42, and RhoA. In this paper, we describe the characterization of DEF6, a novel PH-DH-like protein related to SWAP-70 that functions as an upstream activator of Rho GTPases. In NIH 3T3 cells, stimulation of the PI 3-kinase signaling pathway with either H2O2 or platelet-derived growth factor (PDGF) resulted in the translocation of an overexpressed DEF6-GFP fusion protein to the cell membrane and induced the formation of   filopodia   and lamellipodia. In contrast to full-length DEF6, expression of the DH-like (DHL) domain as a GFP fusion protein potently induced actin polymerization, including stress fiber formation in COS-7 cells, in the absence of PI 3-kinase signaling, indicating that it was constitutively active. The GTP-loading of Cdc42 was strongly enhanced in NIH 3T3 cells expressing the DH domain while   filopodia   formation, membrane ruffling, and stress fiber formation could be inhibited by the co-expression of the DH domain with dominant negative mutants of either N17Rac1, N17Cdc42, or N19RhoA, respectively. This indicated that DEF6 acts upstream of the Rho GTPases resulting in the activation of the Cdc42, Rac1, and RhoA signaling pathways. In vitro, DEF6 specifically interacted with Rac1, Rac2, Cdc42, and RhoA, suggesting a direct role for DEF6 in the activation of Rho GTPases. The ability of DEF6 to both stimulate actin polymerization and bind to filamentous actin suggests a role for DEF6 in regulating cell shape, polarity, and movement. 
 The small GTPase RalA targets filamin to induce   filopodia  . The Ras-related small GTPases Rac, Rho, Cdc42, and RalA bind filamin, an actin filament-crosslinking protein that also links membrane and other intracellular proteins to actin. Of these GTPases only RalA binds filamin in a GTP-specific manner, and GTP-RalA elicits actin-rich filopods on surfaces of Swiss 3T3 cells and recruits filamin into the   filopodia  l cytoskeleton. Either a dominant negative RalA construct or the RalA-binding domain of filamin 1 specifically block Cdc42-induced filopod formation, but a Cdc42 inhibitor does not impair RalA's effects, which, unlike Cdc42, are Rac independent. RalA does not generate   filopodia   in filamin-deficient human melanoma cells, whereas transfection of filamin 1 restores the functional response. RalA therefore is a downstream intermediate in Cdc42-mediated filopod production and uses filamin in this pathway. 
 PAK5, a new brain-specific kinase, promotes neurite outgrowth in N1E-115 cells. We have characterized a new member of the mammalian PAK family of serine/threonine kinases, PAK5, which is a novel target of the Rho GTPases Cdc42 and Rac. The kinase domain and GTPase-binding domain (GBD) of PAK5 are most closely related in sequence to those of mammalian PAK4. Outside of these domains, however, PAK5 is completely different in sequence from any known mammalian proteins. PAK5 does share considerable sequence homology with the Drosophila MBT protein (for "mushroom body tiny"), however, which is thought to play a role in development of cells in Drosophila brain. Interestingly, PAK5 is highly expressed in mammalian brain and is not expressed in most other tissues. We have found that PAK5, like Cdc42, promotes the induction of   filopodia  . In N1E-115 neuroblastoma cells, expression of PAK5 also triggered the induction of neurite-like processes, and a dominant-negative PAK5 mutant inhibited neurite outgrowth. Expression of activated PAK1 caused no noticeable changes in these cells. An activated mutant of PAK5 had an even more dramatic effect than wild-type PAK5, indicating that the morphologic changes induced by PAK5 are directly related to its kinase activity. Although PAK5 activates the JNK pathway, dominant-negative JNK did not inhibit neurite outgrowth. In contrast, the induction of neurites by PAK5 was abolished by expression of activated RhoA. Previous work has shown that Cdc42 and Rac promote neurite outgrowth by a pathway that is antagonistic to Rho. Our results suggest, therefore, that PAK5 operates downstream to Cdc42 and Rac and antagonizes Rho in the pathway, leading to neurite development. 
 Regulation and functional significance of CDC42 alternative splicing in ovarian cancer. Our previous study found that splicing factor polypyrimidine tract-binding protein 1 (PTBP1) had a role in tumorigenesis but the underlying mechanism remained unclear. In this study, we observed that knockdown of PTBP1 inhibited   filopodia   formation. Subsequently, we found that PTBP1 regulated the alternative splicing of CDC42, a major regulator of   filopodia   formation. Two CDC42 variants, CDC42-v1 and CDC42-v2, can be generated through alternative splicing. Knockdown of PTBP1 increased the expression of CDC42-v2. Ectopic expression of individual variants showed that CDC42-v2 suppressed   filopodia   formation, opposite to the effect of CDC42-v1. Quantitative RT-PCR revealed that CDC42-v2 was expressed at lower levels in ovarian cancer cell lines and ovarian tumor tissues than in normal control cells and tissues. Further, CDC42-v2 was observed to have inhibitory effects on ovarian tumor cell growth, colony formation in soft agar and invasiveness. In contrast, these inhibitory effects were not found with CDC42-v1. Taken together, above results suggest that the role of PTBP1 in tumorigenesis may be partly mediated by its regulation of CDC42 alternative splicing and CDC42-v2 might function as a tumor suppressor. 
 A 68-kDa kinase and NADPH oxidase component p67phox are targets for Cdc42Hs and Rac1 in neutrophils. Cdc42Hs and Rac1 are members of the Ras superfamily of small molecular weight (p21) GTP binding proteins. Cdc42Hs induces   filopodia   formation in Swiss 3T3 fibroblasts while Rac1 induces membrane ruffling. Rac1 also activates superoxide production by the components (cytochrome b, p40phox, p67phox, and p47phox) of the neutrophil oxidase. To isolate target proteins involved in these signaling pathways, we have probed proteins from neutrophil cytosol immobilized on nitrocellulose with Cdc42Hs labeled with [gamma-32P]GTP. Cdc42Hs probe detected binding protein(s) of 66-68 kDa in neutrophil cytosol. Rac1 probe also detected the 66-68-kDa proteins, suggesting the possibility that p67phox may be a binding protein for both of these p21 proteins. Indeed, Cdc42Hs and Rac1 were found to bind specifically to purified recombinant p67phox but not the other oxidase components. A 68-kDa Cdc42Hs binding protein was purified from neutrophil cytosol and found to be related to the recently described p65pak kinase from brain. These results suggest that the p68 kinase and p67phox are targets for Cdc42Hs and Rac1 in neutrophils. 
 cIAP1 regulates TNF-mediated cdc42 activation and   filopodia   formation. Tumour necrosis factor-α (TNF) is a cytokine endowed with multiple functions, depending on the cellular and environmental context. TNF receptor engagement induces the formation of a multimolecular complex including the TNFR-associated factor TRAF2, the receptor-interaction protein kinase RIP1 and the cellular inhibitor of apoptosis cIAP1, the latter being essential for NF-κB activation. Here, we show that cIAP1 also regulates TNF-induced actin cytoskeleton reorganization through a cdc42-dependent, NF-κB-independent pathway. Deletion of cIAP1 prevents TNF-induc  ed filopo  dia and cdc42 activation. The expression of cIAP1 or its E3-ubiquitin ligase-defective mutant restores the ability of cIAP1(-/-) MEFs to produ  ce filopo  dia, whereas a cIAP1 mutant unable to bind TRAF2 does not. Accordingly, the silencing of TRAF2 inhibits TNF-mediat  ed filopo  dia formation, whereas silencing of RIP1 does not. cIAP1 directly binds cdc42 and promotes its RhoGDIα-mediated stabilization. TNF decreases cIAP1-cdc42 interaction, suggesting that TNF-induced recruitment of cIAP1/TRAF2 to the receptor releases cdc42, which in turn triggers actin remodeling. cIAP1 also regulates cdc42 activation in response to EGF and HRas-V12 expression. A downregulation of cIAP1 altered the cell polarization, the cell adhesion to endothelial cells and cell intercalation, which are cdc42-dependent processes. Finally, we demonstrated that the deletion of cIAP1 regulated the HRas-V12-mediated transformation process, including anchorage-dependent cell growth, tumour growth in a xenograft model and the development of experimental metastasis in the lung. 
 Kank attenuates actin remodeling by preventing interaction between IRSp53 and Rac1. In this study, insulin receptor substrate (IRS) p53 is identified as a binding partner for Kank, a kidney ankyrin repeat-containing protein that functions to suppress cell proliferation and regulate the actin cytoskeleton. Kank specifically inhibits the binding of IRSp53 with active Rac1 (Rac1(G12V)) but not Cdc42 (cdc42(G12V)) and thus inhibits the IRSp53-dependent development of lamellipodia without affecting the formation of   filopodia  . Knockdown (KD) of Kank by RNA interference results in increased lamellipodial development, whereas KD of both Kank and IRSp53 has little effect. Moreover, insulin-induced membrane ruffling is inhibited by overexpression of Kank. Kank also suppresses integrin-dependent cell spreading and IRSp53-induced neurite outgrowth. Our results demonstrate that Kank negatively regulates the formation of lamellipodia by inhibiting the interaction between Rac1 and IRSp53. 
 Regulation of dendritic branching and   filopodia   formation in hippocampal neurons by specific acylated protein motifs. Although neuronal axons and dendrites with their associated   filopodia   and spines exhibit a profound cell polarity, the mechanism by which they develop is largely unknown. Here, we demonstrate that specific palmitoylated protein motifs, characterized by two adjacent cysteines and nearby basic residues, are sufficient to induce   filopodia  l extensions in heterologous cells and to increase the number of   filopodia   and the branching of dendrites and axons in neurons. Such motifs are present at the N-terminus of GAP-43 and the C-terminus of paralemmin, two neuronal proteins implicated in cytoskeletal organization and   filopodia  l outgrowth.   Filopodia   induction is blocked by mutations of the palmitoylated sites or by treatment with 2-bromopalmitate, an agent that inhibits protein palmitoylation. Moreover, overexpression of a constitutively active form of ARF6, a GTPase that regulates membrane cycling and dendritic branching reversed the effects of the acylated protein motifs.   Filopodia   induction by the specific palmitoylated motifs was also reduced upon overexpression of a dominant negative form of the GTPase cdc42. These results demonstrate that select dually lipidated protein motifs trigger changes in the development and growth of neuronal processes. 
 The integrin alpha6beta1 modulation of PI3K and Cdc42 activities induces dynamic filopodium formation in human platelets. Platelets are an ideal model for studying a rapid morphological change in response to various signal transduction systems. Morphological changes via the activation of integrin alphaIIbbeta3 in platelets have been investigated intensively. In contrast, activation via integrin alpha6beta1 is less well studied. Here, we provide the first biochemical evidence that integrins alpha6beta1 and alphaIIbbeta3 of platelets are associated with different membrane proteins. We also demonstrate that platelets activated by integrin alpha6beta1 show dynamic change by actively forming   filopodia   and never fully spreading over a period of more than an hour. In addition, platelets activated by integrin alpha6beta1 are different from those activated by integrin alphaIIbbeta3 in terms of cell-substrate contact and in their distribution pattern of actin, Arp2/3 and various phosphotyrosine proteins. The morphological appearance of platelets produced through integrin alpha6beta1 activation is highly dependent on PI3 kinase (PI3K) but less dependent on Src kinase. Suppression of PI3K activity in integrin alpha6beta1 activated platelets induces an increase in Cdc42 activity and more filopodium formation. However, both Cdc42 and PI3K activity are higher in platelets activated by integrin alpha6beta1 than in those activated by integrin alphaIIbbeta3. Taken together, this study demonstrates that the signals induced by integrin alpha6beta1 modulate at the level of PI3K and Cdc42 activity to allow platelets to actively form   filopodia  . 
 Myristoylated Alanine-Rich Protein Kinase Substrate (MARCKS) Regulates Small GTPase Rac1 and Cdc42 Activity and Is a Critical Mediator of Vascular Smooth Muscle Cell Migration in Intimal Hyperplasia Formation. Transcription of the myristoylated alanine-rich C kinase substrate (MARCKS) is upregulated in animal models of intimal hyperplasia. MARCKS knockdown inhibits vascular smooth muscle cell (VSMC) migration in vitro; however, the mechanism is as yet unknown. We sought to elucidate the mechanism of MARCKS-mediated motility and determine whether MARCKS knockdown reduces intimal hyperplasia formation in vivo.MARCKS knockdown blocked platelet-derived growth factor (PDGF)-induced translocation of cortactin to the cell cortex, impaired both lamellipodia and   filopodia   formation, and attenuated motility of human coronary artery smooth muscle cells (CASMCs). Activation of the small GTPases, Rac1 and Cdc42, was prevented by MARCKS knockdown. Phosphorylation of MARCKS resulted in a transient shift of MARCKS from the plasma membrane to the cytosol. MARCKS knockdown significantly decreased membrane-associated phosphatidylinositol 4,5-bisphosphate (PIP2) levels. Cotransfection with an intact, unphosphorylated MARCKS, which has a high binding affinity for PIP2, restored membrane-associated PIP2 levels and was indispensable for activation of Rac1 and Cdc42 and, ultimately, VSMC migration. Overexpression of MARCKS in differentiated VSMCs increased membrane PIP2 abundance, Rac1 and Cdc42 activity, and cell motility. MARCKS protein was upregulated early in the development of intimal hyperplasia in the murine carotid ligation model. Decreased MARKCS expression, but not total knockdown, attenuated intimal hyperplasia formation.MARCKS upregulation increases VSMC motility by activation of Rac1 and Cdc42. These effects are mediated by MARCKS sequestering PIP2 at the plasma membrane. This study delineates a novel mechanism for MARCKS-mediated VSMC migration and supports the rational for MARCKS knockdown to prevent intimal hyperplasia. 
 Formyl peptide receptor-1 activation enhances intestinal epithelial cell restitution through phosphatidylinositol 3-kinase-dependent activation of Rac1 and Cdc42. Inflammatory disorders of the gastrointestinal tract result in the breakdown of the intestinal epithelial barrier in the form of erosion and ulceration. To reestablish the epithelial barrier, the epithelium must efficiently migrate to reseal wounds. Numerous signaling cascades are involved in the induction and regulation of this complex process. N-formyl peptide receptors comprise a group of Gi-coupled receptors that regulate innate immune responses. Previously, we identified the expression of functional N-formyl peptide receptors in model SK-CO15 intestinal epithelial cells and observed a role for activation of these receptors in regulating cellular invasive behavior. In these studies, we performed formyl peptide receptor-1 (FPR) localization and evaluated its role in regulating intestinal epithelial cell wound closure. Immunolocalization studies using a recently developed specific monoclonal anti-FPR Ab demonstrated its localization along the lateral membrane of crypt epithelial cells in normal human colonic epithelium. In vitro studies using the classical FPR agonist fMLF showed that FPR activation significantly enhances model intestinal epithelial cell restitution and that FPR localized along actin filaments in lamellipodial and   filopodia  l extrusions. The increase in cell migration was associated with activation of PI3K, Rac1, and Cdc42. Pharmacologic inhibition of PI3K activity abrogated the fMLF-induced increase in wound closure and activation of both Rac1 and Cdc42. Inhibition of Rac1 and Cdc42 using pharmacologic inhibitors and dominant negative mutants also inhibited the fMLF-induced increase in cell migration. Taken together, theses results support a novel role for FPR stimulation in enhancing intestinal epithelial cell restitution through PI3K-dependent activation of Rac1 and Cdc42. 
 A Rac/Cdc42-specific exchange factor, GEFT, induces cell proliferation, transformation, and migration. The Rho family of small GTPases, including Rho, Rac, and Cdc42, play essential roles in diverse cellular functions. The ability of Rho family GTPases to participate in signaling events is determined by the ratio of inactive (GDP-bound) and active (GTP-bound) forms in the cell. The activation of Rho family proteins requires the exchange of bound GDP for GTP, a process catalyzed by the Dbl family of guanine nucleotide exchange factors (GEFs). The GEFs have high affinity for the guanine nucleotide-free state of the GTPases and are thought to promote GDP release by stabilizing an intermediate transition state. In this study, we have identified and characterized a new Rac/Cdc42-specific Dbl family guanine nucleotide exchange factor, named GEFT. GEFT is highly expressed in the excitable tissues, including brain, heart, and muscle. Low or very little expression was detected in other nonexcitable tissues. GEFT has specific exchange activity for Rac and Cdc42 in our in vitro GTPase exchange assays and glutathione S-transferase-PAK pull-down assays with GTP-bound Rac1 and Cdc42. Overexpression of GEFT leads to changes in cell morphology and actin cytoskeleton re-organization, including the formation of membrane microspikes,   filopodia  , and lamilliopodia. Furthermore, expression of GEFT in NIH3T3 cells promotes foci formation, cell proliferation, and cell migration, possibly through the activation of transcriptional factors involved in cell growth and proliferation. Together, our data suggest that GEFT is a Rac/Cdc42-specific GEF protein that regulates cell morphology, cell proliferation, and transformation. 
 The TRE17 oncogene encodes a component of a novel effector pathway for Rho GTPases Cdc42 and Rac1 and stimulates actin remodeling. The Rho family GTPases Cdc42 and Rac1 play fundamental roles in transformation and actin remodeling. Here, we demonstrate that the TRE17 oncogene encodes a component of a novel effector pathway for these GTPases. TRE17 coprecipitated specifically with the active forms of Cdc42 and Rac1 in vivo. Furthermore, the subcellular localization of TRE17 was dramatically regulated by these GTPases and mitogens. Under serum-starved conditions, TRE17 localized predominantly to filamentous structures within the cell. Epidermal growth factor (EGF) induced relocalization of TRE17 to the plasma membrane in a Cdc42-/Rac1-dependent manner. Coexpression of activated alleles of Cdc42 or Rac1 also caused complete redistribution of TRE17 to the plasma membrane, where it partially colocalized with the GTPases in   filopodia   and ruffles, respectively. Membrane recruitment of TRE17 by EGF or the GTPases was dependent on actin polymerization. Finally, we found that a C-terminal truncation mutant of TRE17 induced the accumulation of cortical actin, mimicking the effects of activated Cdc42. Together, these results identify TRE17 as part of a novel effector complex for Cdc42 and Rac1, potentially contributing to their effects on actin remodeling. The present study provides insights into the regulation and cellular function of this previously uncharacterized oncogene. 
 Cdc42 induces   filopodia   by promoting the formation of an IRSp53:Mena complex. The Rho GTPases Rho, Rac, and Cdc42 regulate the organization of the actin cytoskeleton by interacting with multiple, distinct downstream effector proteins. Cdc42 controls the formation of actin bundle-containing   filopodia   at the cellular periphery. The molecular mechanism for this remains as yet unclear.We report here that Cdc42 interacts with IRSp53/BAP2 alpha, an SH3 domain-containing scaffold protein, at a partial CRIB motif and that an N-terminal fragment of IRSp53 binds, via an intramolecular interaction, to the CRIB motif-containing central region. Overexpression of IRSp53 in fibroblasts leads to the formation of   filopodia  , and both this and Cdc42-induced   filopodia   are inhibited by expression of the N-terminal IRSp53 fragment. Using affinity chromatography, we have identified Mena, an Ena/VASP family member, as interacting with the SH3 domain of IRSp53. Mena and IRSp53 act synergistically to promote   filopodia   formation.We conclude that the interaction of Cdc42 with the partial CRIB motif of IRSp53 relieves an intramolecular, autoinhibitory interaction with the N terminus, allowing the recruitment of Mena to the IRSp53 SH3 domain. This IRSp53:Mena complex initiates actin filament assembly into   filopodia  . 
 CdGAP, a novel proline-rich GTPase-activating protein for Cdc42 and Rac. Cdc42 mediates several signaling pathways leading to actin reorganization, transcriptional activation, and cell cycle control. Mutational analysis of Cdc42 has revealed that actin reorganization and transcriptional activation are induced through independent signaling pathways. The Y40C effector mutant of Cdc42 no longer interacts with many of its known target proteins, such as p65(PAK) and WASP, yet this mutant can still induce   filopodia   formation. To identify Cdc42 targets involved in actin rearrangements, we have screened a yeast two-hybrid cDNA library using the Y40C mutant of Cdc42 as a bait. We report here the identification of a novel serine- and proline-rich GTPase-activating protein, CdGAP, which is active in vitro on both Cdc42 and Rac. Microinjection of CdGAP into serum-starved fibroblasts inhibits both platelet-derived growth factor-induced lamellipodia and bradykinin-induced   filopodia   mediated by Rac and Cdc42, respectively. CdGAP does not show in vitro activity toward Rho, and it has no effect on lysophosphatidic acid-induced stress fiber formation when microinjected into fibroblasts. The carboxyl terminus of CdGAP reveals potential protein kinase C phosphorylation sites and five SH3 binding motifs. Thus, CdGAP is a novel GAP that is likely to participate in Cdc42- and Rac-induced signaling pathways leading to actin reorganization. 
 Regulation and functional significance of CDC42 alternative splicing in ovarian cancer. Our previous study found that splicing factor polypyrimidine tract-binding protein 1 (PTBP1) had a role in tumorigenesis but the underlying mechanism remained unclear. In this study, we observed that knockdown of PTBP1 inhibited   filopodia   formation. Subsequently, we found that PTBP1 regulated the alternative splicing of CDC42, a major regulator of   filopodia   formation. Two CDC42 variants, CDC42-v1 and CDC42-v2, can be generated through alternative splicing. Knockdown of PTBP1 increased the expression of CDC42-v2. Ectopic expression of individual variants showed that CDC42-v2 suppressed   filopodia   formation, opposite to the effect of CDC42-v1. Quantitative RT-PCR revealed that CDC42-v2 was expressed at lower levels in ovarian cancer cell lines and ovarian tumor tissues than in normal control cells and tissues. Further, CDC42-v2 was observed to have inhibitory effects on ovarian tumor cell growth, colony formation in soft agar and invasiveness. In contrast, these inhibitory effects were not found with CDC42-v1. Taken together, above results suggest that the role of PTBP1 in tumorigenesis may be partly mediated by its regulation of CDC42 alternative splicing and CDC42-v2 might function as a tumor suppressor. 
 Myristoylated Alanine-Rich Protein Kinase Substrate (MARCKS) Regulates Small GTPase Rac1 and Cdc42 Activity and Is a Critical Mediator of Vascular Smooth Muscle Cell Migration in Intimal Hyperplasia Formation. Transcription of the myristoylated alanine-rich C kinase substrate (MARCKS) is upregulated in animal models of intimal hyperplasia. MARCKS knockdown inhibits vascular smooth muscle cell (VSMC) migration in vitro; however, the mechanism is as yet unknown. We sought to elucidate the mechanism of MARCKS-mediated motility and determine whether MARCKS knockdown reduces intimal hyperplasia formation in vivo.MARCKS knockdown blocked platelet-derived growth factor (PDGF)-induced translocation of cortactin to the cell cortex, impaired both lamellipodia and   filopodia   formation, and attenuated motility of human coronary artery smooth muscle cells (CASMCs). Activation of the small GTPases, Rac1 and Cdc42, was prevented by MARCKS knockdown. Phosphorylation of MARCKS resulted in a transient shift of MARCKS from the plasma membrane to the cytosol. MARCKS knockdown significantly decreased membrane-associated phosphatidylinositol 4,5-bisphosphate (PIP2) levels. Cotransfection with an intact, unphosphorylated MARCKS, which has a high binding affinity for PIP2, restored membrane-associated PIP2 levels and was indispensable for activation of Rac1 and Cdc42 and, ultimately, VSMC migration. Overexpression of MARCKS in differentiated VSMCs increased membrane PIP2 abundance, Rac1 and Cdc42 activity, and cell motility. MARCKS protein was upregulated early in the development of intimal hyperplasia in the murine carotid ligation model. Decreased MARKCS expression, but not total knockdown, attenuated intimal hyperplasia formation.MARCKS upregulation increases VSMC motility by activation of Rac1 and Cdc42. These effects are mediated by MARCKS sequestering PIP2 at the plasma membrane. This study delineates a novel mechanism for MARCKS-mediated VSMC migration and supports the rational for MARCKS knockdown to prevent intimal hyperplasia. 
 Snail regulates the motility of oral cancer cells via RhoA/Cdc42/p-ERM pathway. The transcriptional factor Snail has been reported to possess properties related to cancer progression; however, the mechanism for it is not fully understood. Our data showed that Snail knockdown by small interfering RNA in two OSCC cell lines, WSU-HN6 and CAL27, significantly inhibited cell migration and invasion which also resulted in decreased cell motility, such as impaired cell spreading on type I collagen substrate, reduced   filopodia  , and premature assembly of stress fibers. In addition, Snail-silencing decreased Cdc42 activity but increased RhoA activity, accompanied by the downregulation in both p-ERM expression and cell motility. Meanwhile, endogenous p-ERM was found specifically co-precipitated with activated Cdc42, but not RhoA, and this co-association was decreased by Snail-silencing. The small molecule inhibitors of Rho-associated kinase (Y27632) markedly enhanced Cdc42 activity and the association of p-ERM with activated Cdc42, increasing cell motility remarkably. Using immunohistochemistry, Snail and p-ERM overexpressions were found in OSCC tissues correlated with nodal metastasis and shorter survival. Taken together, these results demonstrate that Snail regulates cell motility through RhoA/Cdc42/p-ERM pathway and may serve as a biomarker to predict prognosis for OSCC patients. Although RhoA and Cdc42 are concurrently regulated downstream of Snail, there is a direct interplay between them, which indicates RhoA has to be inactivated at some point in cell motility cycle. 
 cIAP1 regulates TNF-mediated cdc42 activation and   filopodia   formation. Tumour necrosis factor-α (TNF) is a cytokine endowed with multiple functions, depending on the cellular and environmental context. TNF receptor engagement induces the formation of a multimolecular complex including the TNFR-associated factor TRAF2, the receptor-interaction protein kinase RIP1 and the cellular inhibitor of apoptosis cIAP1, the latter being essential for NF-κB activation. Here, we show that cIAP1 also regulates TNF-induced actin cytoskeleton reorganization through a cdc42-dependent, NF-κB-independent pathway. Deletion of cIAP1 prevents TNF-induc  ed filopo  dia and cdc42 activation. The expression of cIAP1 or its E3-ubiquitin ligase-defective mutant restores the ability of cIAP1(-/-) MEFs to produ  ce filopo  dia, whereas a cIAP1 mutant unable to bind TRAF2 does not. Accordingly, the silencing of TRAF2 inhibits TNF-mediat  ed filopo  dia formation, whereas silencing of RIP1 does not. cIAP1 directly binds cdc42 and promotes its RhoGDIα-mediated stabilization. TNF decreases cIAP1-cdc42 interaction, suggesting that TNF-induced recruitment of cIAP1/TRAF2 to the receptor releases cdc42, which in turn triggers actin remodeling. cIAP1 also regulates cdc42 activation in response to EGF and HRas-V12 expression. A downregulation of cIAP1 altered the cell polarization, the cell adhesion to endothelial cells and cell intercalation, which are cdc42-dependent processes. Finally, we demonstrated that the deletion of cIAP1 regulated the HRas-V12-mediated transformation process, including anchorage-dependent cell growth, tumour growth in a xenograft model and the development of experimental metastasis in the lung. 
 Wnt5b promotes the cell motility essential for metastasis of oral squamous cell carcinoma through active Cdc42 and RhoA. The activation of Wnt signaling has been reported in many types of squamous cell carcinoma. In this study, using human oral squamous cell carcinoma (OSCC) cells with different metastatic potential, we investigated the involvement of Wnt signaling in metastasis. Further, we aimed to elucidate the characteristic biological features related to high metastatic potential and to identify new target molecules for the suppression of OSCC lymph node metastasis. We compared SAS-Venus (SAS OSCC cells expressing green fluorescent protein) and SAS-LM8, which is a highly metastatic cell line derived from SAS-Venus by in vivo selection. The SAS-LM8 cell line had greater ability of migration and invasion compared to SAS-Venus. Furthermore, a higher number of   filopodia  -like protrusive structures were produced in SAS-LM8 cells compared to SAS-Venus cells, and the levels of active Cdc42 and active RhoA protein were higher in SAS-LM8 cells compared to SAS-Venus cells. We did not observe any differences in the expression of Wnt/β-catenin target genes between the two cell lines; however, the mRNA levels of Wnt5b were higher in SAS-LM8 cells compared to SAS-Venus cells. To confirm the involvement of Wnt5b in migration in OSCC cells, we examined the effects of the siRNA-mediated knockdown of Wnt5b in SAS-Venus cells and SAS-LM8 cells. The siRNA treatment significantly inhibited migration and the formation of   filopodi  a-like protrusive structures. Conversely, when stimulated with Wnt5b, the migration and formation of   filopodi  a-like protrusions were significantly enhanced and the levels of active Cdc42 and active RhoA proteins were also increased. These results indicate that Wnt5b is involved in the migration ability of OSCC cells through active Cdc42 and RhoA. 
 Desmoglein 3 acting as an upstream regulator of Rho GTPases, Rac-1/Cdc42 in the regulation of actin organisation and dynamics. Desmoglein 3 (Dsg3), a member of the desmoglein sub-family, serves as an adhesion molecule in desmosomes. Our previous study showed that overexpression of human Dsg3 in several epithelial lines induces formation of membrane protrusions, a phenotype suggestive of Rho GTPase activation. Here we examined the interaction between Dsg3 and actin in detail and showed that endogenous Dsg3 colocalises and interacts with actin, particularly the junctional actin in a Rac1-dependent manner. Ablation of Rac1 activity by dominant negative Rac1 mutant (N17Rac1) or the Rac1 specific inhibitor (NSC23766) directly disrupts the interaction between Dsg3 and actin. Assembly of the junctional actin at the cell borders is accompanied with enhanced levels of Dsg3, while inhibition of Dsg3 by RNAi results in profound changes in the organisation of actin cytoskeleton. In accordance, overexpression of Dsg3 results in a remarkable increase of Rac1 and Cdc42 activities and to a lesser extent, RhoA. The enhancements in Rho GTPases are accompanied by the pronounced actin-based membrane structures such as lamellipodia and   filopodia  , enhanced rate of actin turnover and cell polarisation. Together, our results reveal an important novel function for Dsg3 in promoting actin dynamics through regulating Rac1 and Cdc42 activation in epithelial cells. 
 Serine-71 phosphorylation of Rac1/Cdc42 diminishes the pathogenic effect of Clostridium difficile toxin A. Clostridium difficile toxin A and B (TcdA/TcdB) are glucosyltransferases that glucosylate GTPases of the Rho family. The epidermal growth factor (EGF) positively modulates C. difficile toxin-induced disturbance of the intestinal barrier function by an unknown mechanism. We found that EGF-treated CaCo-2 monolayers were less susceptible to TcdA-catalysed glucosylation of Rac1 but not of RhoA, which correlated with phosphorylation of Rac1 at Ser-71. Phospho-Rac1/phospho-Cdc42 (Ser-71) still bound to the PAK-CRIB domain indicating an active state. A more detailed characterization of phospho-Rac1 was performed using the phosphomimetic mutant Rac1 S71E. Ectopic expression of Rac1 S71E induced a specific phenotype of cells showing an increase in   filopodia  l structures that were also induced by EGF. Rac1 S71E (and Cdc42 S71E) but not Rac1 S71A was at least fivefold weaker substrate for TcdA-catalysed glucosylation compared with wild type Rac1. The protective effect was checked in transfection experiments where Rac1 S71E and, to a lesser extent, Cdc42 S71E reduced the TcdA-induced cytopathic effect. Thus, Ser-71 phosphorylation of Rac1 might be interesting for modulation of microbial pathogenesis where Rho GTPases, especially Rac1 and Cdc42, are involved. In addition, this is the first description of a specific functional outcome of Rac1 phosphorylation at Ser-71. 
 Cross-talk between Rac1 and Cdc42 GTPases regulates formation of   filopodia   required for dengue virus type-2 entry into HMEC-1 cells. Infection with dengue virus type-2 (DENV-2) begins with virus adherence to cell surface receptors. In endothelial cells (HMEC-1), a cell model for DENV-2 infection, alpha 5 beta 3 integrin has been identified as a putative receptor for the virus. Previous work had suggested that the actin cytoskeleton of HMEC-1 cells plays an important role in virus entry and infection. In the present work, fixed and living HMEC-1 cells expressing enhanced green fluorescent protein-actin were monitored for actin reorganization after virus inoculation, utilizing fluorescence and time lapse microscopy. Cell infection and production of infective viruses were quantified using an anti-E protein antibody and by measuring the p.f.u. ml(-1). Specific drugs that antagonize actin organization and regulate actin-signalling pathways were tested in viral adhesion and infection assays, as were the expression of dominant-negative Rac1 and Cdc42 proteins. Disorganization of actin precluded infection, while microtubule depolymerization had no effect. Activation of Rac1 and Cdc42 signalling, which occurs upon virus binding, induced reorganization of actin to form   filopodia   in the cellular periphery. Formation of   filopodia   was a requirement for virus entry and further cell infection. Expression of the dominant-negative proteins Rac1 and Cdc42 confirmed the role of these GTPases in the actin reorganization that is required to form   filopodia  . In addition, inhibition of the ATPase activity of myosin II greatly decreased infection, suggesting its participation in   filopodia  l stability. We show here, for the first time, that internalization of DENV-2 into endothelial cells requires viral induction of dynamic   filopodia   regulated by Rac1 and Cdc42 cross-talk and myosin II motor activities. 
 Prostaglandin E2-mediated migration of human trophoblast requires RAC1 and CDC42. The invasion of maternal decidua and uterine spiral arteries by a trophoblast subpopulation called extravillous trophoblast (EVT) is essential for the establishment of a normal placenta and an adequate blood flow toward the fetus. Derangements in these processes underlie pregnancy-related diseases like preeclampsia and intrauterine growth restriction. Many growth factors, growth factor binding proteins, and extracellular matrix components can positively or negatively regulate the proliferation, migration, and/or invasiveness of these EVT cells. RHO GTPases, including RHOA, RAC1, and CDC42, are ubiquitous proteins that control cytoskeletal changes by forming stress fibers and projecting lamellipodia and   filopodia   during cellular migration. We had previously shown that prostaglandin (PG) E(2) produced in abundance by the decidua promotes the migration of first-trimester human EVTs by increasing the intracellular concentration of calcium and activating calpain. Using our well-characterized immortalized EVT cell line, HTR-8/SVneo, as well as villus explants from first-trimester placentae, this study examined the role of RHO GTPases RAC1 and CDC42 in PGE(2)-mediated migratory responses of these cells. Though a RAC1 inhibitor, NSC23766 as well as RAC1 knockdown by siRNA decreased the migration of HTR-8/SVneo cells in a Transwell migration assay, this inhibition could not be restored by PGE(2) or 17-phenyl trinor PGE(2) (PGE receptor PTGER1 agonist) or PGE(1) Alcohol (PGE receptor PTGER4 agonist). Similar results were noted for EVT cell spreading in villus explants. Furthermore, CDC42 silencing using siRNA inhibited PGE(2)-induced migration of HTR-8/SVneo cells. Finally, the treatment of EVT cells with PGE(2), PTGER1 agonist, or PTGER4 agonist activated RAC1 and CDC42 at 10 min, suggesting that RAC1 and CDC42 play an essential role in PGE(2)-mediated migration of human EVTs. 
 Formyl peptide receptor-1 activation enhances intestinal epithelial cell restitution through phosphatidylinositol 3-kinase-dependent activation of Rac1 and Cdc42. Inflammatory disorders of the gastrointestinal tract result in the breakdown of the intestinal epithelial barrier in the form of erosion and ulceration. To reestablish the epithelial barrier, the epithelium must efficiently migrate to reseal wounds. Numerous signaling cascades are involved in the induction and regulation of this complex process. N-formyl peptide receptors comprise a group of Gi-coupled receptors that regulate innate immune responses. Previously, we identified the expression of functional N-formyl peptide receptors in model SK-CO15 intestinal epithelial cells and observed a role for activation of these receptors in regulating cellular invasive behavior. In these studies, we performed formyl peptide receptor-1 (FPR) localization and evaluated its role in regulating intestinal epithelial cell wound closure. Immunolocalization studies using a recently developed specific monoclonal anti-FPR Ab demonstrated its localization along the lateral membrane of crypt epithelial cells in normal human colonic epithelium. In vitro studies using the classical FPR agonist fMLF showed that FPR activation significantly enhances model intestinal epithelial cell restitution and that FPR localized along actin filaments in lamellipodial and   filopodia  l extrusions. The increase in cell migration was associated with activation of PI3K, Rac1, and Cdc42. Pharmacologic inhibition of PI3K activity abrogated the fMLF-induced increase in wound closure and activation of both Rac1 and Cdc42. Inhibition of Rac1 and Cdc42 using pharmacologic inhibitors and dominant negative mutants also inhibited the fMLF-induced increase in cell migration. Taken together, theses results support a novel role for FPR stimulation in enhancing intestinal epithelial cell restitution through PI3K-dependent activation of Rac1 and Cdc42. 
 Cdc42/Rac1-dependent activation of the p21-activated kinase (PAK) regulates human platelet lamellipodia spreading: implication of the cortical-actin binding protein cortactin. Platelet activation by thrombin or thrombin receptor-activating peptide (TRAP) results in extensive actin reorganization that leads to   filopodia   emission and lamellae spreading concomitantly with activation of the Rho family small G proteins, Cdc42 and Rac1. Evidence has been provided that direct binding of Cdc42-guanosine triphosphate (GTP) and Rac1-GTP to the N-terminal regulatory domain of the p21-activated kinase (PAK) stimulates PAK activation and actin reorganization. In the present study, we have investigated the relationship between shape change and PAK activation. We show that thrombin, TRAP, or monoclonal antibody (MoAb) anti-Fc(gamma)RIIA IV.3 induces an activation of Cdc42 and Rac1. The GpVI ligand, convulxin (CVX), that forces platelets to lamellae spreading efficiently activates Rac1. Thrombin, TRAP, MoAb IV.3, and CVX stimulate autophosphorylation and kinase activity of PAK. Inhibition of Cdc42 and Rac1 with clostridial toxin B inhibits PAK activation and lamellae spreading. The cortical-actin binding protein, p80/85 cortactin, is constitutively associated with PAK in resting platelets and dissociates from PAK after thrombin stimulation. Inhibition of PAK autophosphorylation by toxin B prevents the dissociation of cortactin. These results suggest that Cdc42/Rac1-dependent activation of PAK may trigger early platelet shape change, at least in part through the regulation of cortactin binding to PAK. 
 Estrogen and resveratrol regulate Rac and Cdc42 signaling to the actin cytoskeleton of metastatic breast cancer cells. Estrogen and structurally related molecules play critical roles in breast cancer. We reported that resveratrol (50 microM), an estrogen-like phytosterol from grapes, acts in an antiestrogenic manner in breast cancer cells to reduce cell migration and to induce a global and sustained extension of actin structures called   filopodia  . Herein, we report that resveratrol-induced   filopodia   formation is time-dependent and concentration-dependent. In contrast to resveratrol at 50 microM, resveratrol at 5 microM acts in a manner similar to estrogen by increasing lamellipodia, as well as cell migration and invasion. Because Rho GTPases regulate the extension of actin structures, we investigated a role for Rac and Cdc42 in estrogen and resveratrol signaling. Our results demonstrate that 50 microM resveratrol decreases Rac and Cdc42 activity, whereas estrogen and 5 microM resveratrol increase Rac activity in breast cancer cells. MDA-MB-231 cells expressing dominant-negative Cdc42 or dominant-negative Rac retain   filopodia   response to 50 microM resveratrol. Lamellipodia response to 5 microM resveratrol, estrogen, or epidermal growth factor is inhibited in cells expressing dominant-negative Rac, indicating that Rac regulates estrogen and resveratrol (5 microM) signaling to the actin cytoskeleton. These results indicate that signaling to the actin cytoskeleton by low and high concentrations of resveratrol may be differentially regulated by Rac and Cdc42. 
 The TRE17 oncogene encodes a component of a novel effector pathway for Rho GTPases Cdc42 and Rac1 and stimulates actin remodeling. The Rho family GTPases Cdc42 and Rac1 play fundamental roles in transformation and actin remodeling. Here, we demonstrate that the TRE17 oncogene encodes a component of a novel effector pathway for these GTPases. TRE17 coprecipitated specifically with the active forms of Cdc42 and Rac1 in vivo. Furthermore, the subcellular localization of TRE17 was dramatically regulated by these GTPases and mitogens. Under serum-starved conditions, TRE17 localized predominantly to filamentous structures within the cell. Epidermal growth factor (EGF) induced relocalization of TRE17 to the plasma membrane in a Cdc42-/Rac1-dependent manner. Coexpression of activated alleles of Cdc42 or Rac1 also caused complete redistribution of TRE17 to the plasma membrane, where it partially colocalized with the GTPases in   filopodia   and ruffles, respectively. Membrane recruitment of TRE17 by EGF or the GTPases was dependent on actin polymerization. Finally, we found that a C-terminal truncation mutant of TRE17 induced the accumulation of cortical actin, mimicking the effects of activated Cdc42. Together, these results identify TRE17 as part of a novel effector complex for Cdc42 and Rac1, potentially contributing to their effects on actin remodeling. The present study provides insights into the regulation and cellular function of this previously uncharacterized oncogene. 
 The integrin alpha6beta1 modulation of PI3K and Cdc42 activities induces dynamic filopodium formation in human platelets. Platelets are an ideal model for studying a rapid morphological change in response to various signal transduction systems. Morphological changes via the activation of integrin alphaIIbbeta3 in platelets have been investigated intensively. In contrast, activation via integrin alpha6beta1 is less well studied. Here, we provide the first biochemical evidence that integrins alpha6beta1 and alphaIIbbeta3 of platelets are associated with different membrane proteins. We also demonstrate that platelets activated by integrin alpha6beta1 show dynamic change by actively forming   filopodia   and never fully spreading over a period of more than an hour. In addition, platelets activated by integrin alpha6beta1 are different from those activated by integrin alphaIIbbeta3 in terms of cell-substrate contact and in their distribution pattern of actin, Arp2/3 and various phosphotyrosine proteins. The morphological appearance of platelets produced through integrin alpha6beta1 activation is highly dependent on PI3 kinase (PI3K) but less dependent on Src kinase. Suppression of PI3K activity in integrin alpha6beta1 activated platelets induces an increase in Cdc42 activity and more filopodium formation. However, both Cdc42 and PI3K activity are higher in platelets activated by integrin alpha6beta1 than in those activated by integrin alphaIIbbeta3. Taken together, this study demonstrates that the signals induced by integrin alpha6beta1 modulate at the level of PI3K and Cdc42 activity to allow platelets to actively form   filopodia  . 
 CDC42 switches IRSp53 from inhibition of actin growth to elongation by clustering of VASP.   Filopodia   explore the environment, sensing soluble and mechanical cues during directional motility and tissue morphogenesis. How   filopodia   are initiated and spatially restricted to specific sites on the plasma membrane is still unclear. Here, we show that the membrane deforming and curvature sensing IRSp53 (Insulin Receptor Substrate of 53 kDa) protein slows down actin filament barbed end growth. This inhibition is relieved by CDC42 and counteracted by VASP, which also binds to IRSp53. The VASP:IRSp53 interaction is regulated by activated CDC42 and promotes high-density clustering of VASP, which is required for processive actin filament elongation. The interaction also mediates VASP recruitment to liposomes. In cells, IRSp53 and VASP accumulate at discrete foci at the leading edge, wher  e filopod  ia are initiated. Genetic removal of IRSp53 impairs the formation of VASP foci  , filopod  ia and chemotactic motility, while IRSp53 null mice display defective wound healing. Thus, IRSp53 dampens barbed end growth. CDC42 activation inhibits this activity and promotes IRSp53-dependent recruitment and clustering of VASP to drive actin assembly. These events result in spatial restriction of VASP filament elongation for initiation o  f filopod  ia during cell migration, invasion, and tissue repair. 
 Intersectin-2L regulates caveola endocytosis secondary to Cdc42-mediated actin polymerization. Here we addressed the role of intersectin-2L (ITSN-2L), a guanine nucleotide exchange factor for the Rho GTPase Cdc42, in the mechanism of caveola endocytosis in endothelial cells (ECs). Immunoprecipitation and co-localization studies showed that ITSN-2L associates with members of the Cdc42-WASp-Arp2/3 actin polymerization pathway. Expression of Dbl homology-pleckstrin homology (DH-PH) region of ITSN-2L (DH-PH(ITSN-2L)) induced specific activation of Cdc42, resulting in formation of extensive   filopodia  , enhanced cortical actin, as well as a shift from G-actin to F-actin. The "catalytically dead" DH-PH domain reversed these effects and induced significant stress fiber formation, without a detectable shift in actin pools. A biotin assay for caveola internalization indicated a significant decrease in the uptake of biotinylated proteins in DH-PH(ITSN-2L)-transfected cells compared with control and 1 microM jasplakinolide-treated cells. ECs depleted of ITSN-2L by small interfering RNA, however, showed decreased Cdc42 activation and actin remodeling similar to the defective DH-PH, resulting in 62% increase in caveola-mediated uptake compared with controls. Thus, ITSN-2L, a guanine nucleotide exchange factor for Cdc42, regulates different steps of caveola endocytosis in ECs by controlling the temporal and spatial actin polymerization and remodeling sub-adjacent to the plasma membrane. 
 PAK4, a novel effector for Cdc42Hs, is implicated in the reorganization of the actin cytoskeleton and in the formation of   filopodia  . The GTPases Rac and Cdc42Hs control diverse cellular functions. In addition to being mediators of intracellular signaling cascades, they have important roles in cell morphogenesis and mitogenesis. We have identified a novel PAK-related kinase, PAK4, as a new effector molecule for Cdc42Hs. PAK4 interacts only with the activated form of Cdc42Hs through its GTPase-binding domain (GBD). Co-expression of PAK4 and the constitutively active Cdc42HsV12 causes the redistribution of PAK4 to the brefeldin A-sensitive compartment of the Golgi membrane and the subsequent induction of   filopodia   and actin polymerization. Importantly, the reorganization of the actin cytoskeleton is dependent on PAK4 kinase activity and on its interaction with Cdc42Hs. Thus, unlike other members of the PAK family, PAK4 provides a novel link between Cdc42Hs and the actin cytoskeleton. The cellular locations of PAK4 and Cdc42Hs suggest a role for the Golgi in cell morphogenesis. 
 Mechanism of IRSp53 inhibition and combinatorial activation by Cdc42 and downstream effectors. The Rho family GTPase effector IRSp53 has essential roles in   filopodia   formation and neuronal development, but its regulatory mechanism is poorly understood. IRSp53 contains a membrane-binding BAR domain followed by an unconventional CRIB motif that overlaps with a proline-rich region (CRIB-PR) and an SH3 domain that recruits actin cytoskeleton effectors. Using a fluorescence reporter assay, we show that human IRSp53 adopts a closed inactive conformation that opens synergistically with the binding of human Cdc42 to the CRIB-PR and effector proteins, such as the tumor-promoting factor Eps8, to the SH3 domain. The crystal structure of Cdc42 bound to the CRIB-PR reveals a new mode of effector binding to Rho family GTPases. Structure-inspired mutations disrupt autoinhibition and Cdc42 binding in vitro and decouple Cdc42- and IRSp53-dependent   filopodia   formation in cells. The data support a combinatorial mechanism of IRSp53 activation. 
 Focal adhesion kinase modulates Cdc42 activity downstream of positive and negative axon guidance cues. There is biochemical, imaging and functional evidence that Rho GTPase signaling is a crucial regulator of actin-based structures such as lamellipodia and   filopodia  . However, although Rho GTPases are believed to serve similar functions in growth cones, the spatiotemporal dynamics of Rho GTPase signaling has not been examined in living growth cones in response to known axon guidance cues. Here we provide the first measurements of Cdc42 activity in living growth cones acutely stimulated with both growth-promoting and growth-inhibiting axon-guidance cues. Interestingly, we find that both permissive and repulsive factors can work by modulating Cdc42 activity, but in opposite directions. We find that the growth-promoting factors laminin and BDNF activate Cdc42, whereas the inhibitor Slit2 reduces Cdc42 activity in growth cones. Remarkably, we find that regulation of focal adhesion kinase (FAK) activity is a common upstream modulator of Cdc42 by BDNF, laminin and Slit. These findings suggest that rapid modulation of Cdc42 signaling through FAK by receptor activation underlies changes in growth cone motility in response to permissive and repulsive guidance cues. 
 The acidic regions of WASp and N-WASP can synergize with CDC42Hs and Rac1 to induce   filopodia   and lamellipodia. The acidic (A) region of WASp family proteins is thought to represent a high-affinity binding site for Arp2/3 complex without activating properties. Here we show that GST-fused WASp-A and N-WASP-A, but not a WASP-A/W500S mutant, several truncated WASp-A constructs or WAVE1-A can pull down Arp2/3 complex from cell lysates. Significantly, WASp-A and N-WASP-A synergistically trigger formation of   filopodia   or lamellipodia when coinjected with sub-effective concentrations of V12CDC42Hs or V12Rac1, respectively, into macrophages. The ability of WASp family A region constructs to induce this effect is closely correlated with their ability to bind Arp2/3 complex in vitro. These results imply that (i) Arp2/3 complex is critically involved in   filopodia   and lamellipodia formation in macrophages and (ii) acidic regions of WASp and N-WASP are not simply binding sites for Arp2/3 complex but can prime it for RhoGTPase-triggered signals leading to actin nucleation. 
 CD28 engagement promotes actin polymerization through the activation of the small Rho GTPase Cdc42 in human T cells. Engagement of the costimulatory molecule CD28 is an important step in the optimal activation of T cells. Nevertheless, the specific role of CD28 in the formation of the immunological synapse and cytoskeletal changes that occur upon TCR/CD3 complex engagement is still poorly understood. Using Ab-coated surfaces, we show that CD28 engagement in the absence of any other signal induced the formation of cytoplasmic elongations enriched in filamentous actin (F-actin), in this work called   filopodia   or microspikes. Such structures were specific for engagement of CD28 on mAb-coated surfaces because they could not be observed in surfaces coated with either poly(L-lysine) or anti-CD3 mAb. The signaling pathway coupling CD28 to cytoskeletal rearrangements required Src-related kinase activity and promoted Vav phosphorylation and Cdc42 activation independently of the zeta-chain-associated kinase (ZAP-70). CD28-induced   filopodia   required Cdc42 GTPase activity, but not the related Rho GTPase Rac1. Moreover, Cdc42 colocalized to areas of increased F-actin. Our results support a specific role for the activation of the small Rho GTPase Cdc42 in the actin reorganization mediated by CD28 in human T cells. 
 RhoGDI-binding-defective mutant of Cdc42Hs targets to membranes and activates   filopodia   formation but does not cycle with the cytosol of mammalian cells. We have identified a mutant of the human G-protein Cdc42Hs, R66E, that fails to form a detectable complex with the GDP-dissociation inhibitor RhoGDI in cell-free systems or in intact cells. This point mutant is prenylated, binds guanine nucleotide and interacts with GTPase-activating protein in a manner indistinguishable from wild-type Cdc42Hs. Immunofluorescence localization studies revealed that this RhoGDI-binding-defective mutant is found predominantly in the Golgi apparatus, with a staining pattern similar to that of the wild-type protein. However, unlike wild-type Cdc42Hs, which is distributed in both the microsomal membrane and cytosolic fractions, studies using differential centrifugation show that prenylated R66E Cdc42Hs is found exclusively in association with lipid bilayers. Additionally, whereas the overexpression of RhoGDI results in an apparent translocation of wild-type Cdc42Hs from the Golgi apparatus into the cytosol, identical RhoGDI-overexpression conditions do not alter the Golgi localization of the R66E mutant. Furthermore, overexpression of this RhoGDI-binding-defective mutant of Cdc42Hs seems to activate redistribution of the actin cytoskeleton and   filopodia   formation in fibroblasts in a manner indistinguishable from the wild-type protein. Taken together, these results suggest that the interaction of Cdc42Hs with RhoGDI is not essential for proper membrane targeting of nascent prenylated Cdc42Hs in mammalian cells; neither is this interaction an essential part of the mechanism by which Cdc42Hs activates   filopodia   formation. However, it does seem that redistribution of Cdc42Hs to the cytosolic compartment is absolutely dependent on RhoGDI interaction. 
 Dual lipidation of the brain-specific Cdc42 isoform regulates its functional properties. Cdc42 (cell division cycle 42) is a member of the Rho GTPase family which regulates a variety of cellular activities by controlling actin cytoskeleton and gene expression. Cdc42 is expressed in the form of two splice variants. The canonical Cdc42 isoform is prenylated (Cdc42-prenyl), whereas the brainspecific isoform can be palmitoylated (Cdc42-palm). In the present study we have demonstrated palmitoylation of endogenous Cdc42 in rodent and human brains and identified Cys(188) and Cys(189) as acylation sites of Cdc42-palm. Moreover, we have shown that Cys(188) can also be prenylated. Analysis of acylation-deficient mutants revealed that lipidation of Cys(188) is essential for proper membrane binding of Cdc42-palm as well as for Cdc42-mediated regulation of gene transcription and induction of densely packed   filopodia   in neuroblastoma cells. We also found that Cdc42-prenyl is a dominant splice variant in a wide range of commonly used cell lines as well as in the cerebellum, whereas Cdc42-palm is the main Cdc42 isoform in hippocampus, where it is critically involved in the formation of dendritic   filopodia   and spines. Replacement of endogenous Cdc42 by its acylation-deficient mutants revealed the importance of Cdc42-palm lipidation for its morphogenic and synaptogenic effects in neurons. These findings demonstrate that dual lipidation of Cdc42-palm represents an important regulator of morphogenic signalling in hippocampal neurons. 
 Cdc42: Role in Cancer Management. Contribution of Cdc42, a member of Rho family, has been characterized for the beginning of variety of cellular responses including cellular transformation, cell division, cell invasion, migration, invadopodia formation, enzyme activity,   filopodia   formation, and cell polarity in cells. Deregulation of Cdc42 can alter the normal functioning of the cells, responsible for the initiation of signaling pathways and is correlated with several pathogenic processes such as cancer. Therefore, maintaining the level of Cdc42 and its effectors in cells, tumor progression can be controlled. Therefore, it can be suggested that deeper understanding about the Cdc42 contribution in cancer cell progression at molecular level can approach to the development of Cdc42 inhibitors in cancer management. 
 Activation of Rho GTPase Cdc42 promotes adhesion and invasion in colorectal cancer cells. The purpose of this study was to investigate the role of activated Rho GTPase cell division control protein 42 homolog (Cdc42) in colorectal cancer cell adhesion, migration, and invasion.The constitutively active form of Cdc42 (GFP-Cdc42L61) or control vector was overexpressed in the colorectal cancer cell line SW480. The localization of active Cdc42 was monitored by immunofluorescence staining, and the effects of active Cdc42 on cell migration and invasion were examined using an attachment assay, a wound healing assay, and a Matrigel migration assay in vitro.Immunofluorescence staining revealed that constitutively active Cdc42 predominately localized to the plasma membrane. Compared to SW480 cells transfected with the control vector, overexpression of constitutively active Cdc42 in SW480 cells promoted   filopodia   formation and cell stretch and dramatically enhanced cell adhesion to the coated plates. The wound healing assay revealed a significant increase of migration capability in SW480 cells expressing active Cdc42 compared to the control cells. Additionally, the Matrigel invasion assay demonstrated that active Cdc42 significantly promoted SW480 cell migration through the chamber.Our results suggest that active Rho GTPase Cdc42 can greatly enhance colorectal cancer cell SW480 to spread, migrate, and invade, which may contribute to colorectal cancer metastasis. 
 Epithelial junction formation requires confinement of Cdc42 activity by a novel SH3BP1 complex. Epithelial cell-cell adhesion and morphogenesis require dynamic control of actin-driven membrane remodeling. The Rho guanosine triphosphatase (GTPase) Cdc42 regulates sequential molecular processes during cell-cell junction formation; hence, mechanisms must exist that inactivate Cdc42 in a temporally and spatially controlled manner. In this paper, we identify SH3BP1, a GTPase-activating protein for Cdc42 and Rac, as a regulator of junction assembly and epithelial morphogenesis using a functional small interfering ribonucleic acid screen. Depletion of SH3BP1 resulted in loss of spatial control of Cdc42 activity, stalled membrane remodeling, and enhanced growth of   filopodia  . SH3BP1 formed a complex with JACOP/paracingulin, a junctional adaptor, and CD2AP, a scaffolding protein; both were required for normal Cdc42 signaling and junction formation. The filamentous actin-capping protein CapZ also associated with the SH3BP1 complex and was required for control of actin remodeling. Epithelial junction formation and morphogenesis thus require a dual activity complex, containing SH3BP1 and CapZ, that is recruited to sites of active membrane remodeling to guide Cdc42 signaling and cytoskeletal dynamics. 
 MUC1 initiates Src-CrkL-Rac1/Cdc42-mediated actin cytoskeletal protrusive motility after ligating intercellular adhesion molecule-1. MUC1, a transmembrane glycoprotein of the mucin family, when aberrantly expressed on breast cancer cells is correlated with increased lymph node metastases. We have previously shown that MUC1 binds intercellular adhesion molecule-1 (ICAM-1) on surrounding accessory cells and facilitates transendothelial migration of MUC1-bearing cells. Nevertheless, the underlying molecular mechanism is still obscure. In the present study, we used a novel assay of actin cytoskeletal reorganization to show that by ligating ICAM-1, MUC1 triggers Rac1- and Cdc42-dependent actin cytoskeletal protrusive activity preferentially at the heterotypic cell-cell contact sites. Further, we show that these MUC1/ICAM-1 interaction-initiated lamellipodial and   filopodia  l protrusions require Src family kinase and CT10 regulator of kinase like (CrkL) accompanied by the rapid formation of a Src-CrkL signaling complex at the MUC1 cytoplasmic domain. Through inhibition of Src kinase activity, we further revealed that Src is required for recruiting CrkL to the MUC1 cytoplasmic domain as well as mediating the observed actin cytoskeleton dynamics. These findings suggest a novel MUC1-Src-CrkL-Rac1/Cdc42 signaling cascade following ICAM-1 ligation, through which MUC1 regulates cytoskeletal reorganization and directed cell motility during cell migration. 
 Coactivation of Rac1 and Cdc42 at lamellipodia and membrane ruffles induced by epidermal growth factor. A major function of Rho-family GTPases is to regulate the organization of the actin cytoskeleton;   filopodia  , lamellipodia, and stress fiber are regarded as typical phenotypes of the activated Cdc42, Rac, and Rho, respectively. Using probes based on fluorescent resonance energy transfer, we report on the spatiotemporal regulation of Rac1 and Cdc42 at lamellipodia and membrane ruffles. In epidermal growth factor (EGF)-stimulated Cos1 and A431 cells, both Rac1 and Cdc42 were activated diffusely at the plasma membrane, followed by lamellipodial protrusion and membrane ruffling. Although Rac1 activity subsided rapidly, Cdc42 activity was sustained at lamellipodia. A critical role of Cdc42 in these EGF-induced morphological changes was demonstrated as follows. First, phorbol 12-myristate 13-acetate, which activated Rac1 but not Cdc42, could not induce full-grown lamellipodia in Cos1 cells. Second, a GTPase-activating protein for Cdc42, KIAA1204/CdGAP, inhibited lamellipodial protrusion and membrane ruffling without interfering with Rac1 activation. Third, expression of the Cdc42-binding domain of N-WASP inhibited the EGF-induced morphological changes. Therefore, Rac1 and Cdc42 seem to synergistically induce lamellipodia and membrane ruffles in EGF-stimulated Cos1 cells and A431 cells. 
 FMNL2 drives actin-based protrusion and migration downstream of Cdc42. Cell migration entails protrusion of lamellipodia, densely packed networks of actin filaments at the cell front. Filaments are generated by nucleation, likely mediated by Arp2/3 complex and its activator Scar/WAVE. It is unclear whether formins contribute to lamellipodial actin filament nucleation or serve as elongators of filaments nucleated by Arp2/3 complex. Here we show that the Diaphanous-related formin FMNL2, also known as FRL3 or FHOD2, accumulates at lamellipodia and   filopodia   tips. FMNL2 is cotranslationally modified by myristoylation and regulated by interaction with the Rho-guanosine triphosphatase Cdc42. Abolition of myristoylation or Cdc42 binding interferes with proper FMNL2 activation, constituting an essential prerequisite for subcellular targeting. In vitro, C-terminal FMNL2 drives elongation rather than nucleation of actin filaments in the presence of profilin. In addition, filament ends generated by Arp2/3-mediated branching are captured and efficiently elongated by the formin. Consistent with these biochemical properties, RNAi-mediated silencing of FMNL2 expression decreases the rate of lamellipodia protrusion and, accordingly, the efficiency of cell migration. Our data establish that the FMNL subfamily member FMNL2 is a novel elongation factor of actin filaments that constitutes the first Cdc42 effector promoting cell migration and actin polymerization at the tips of lamellipodia. 
 Induction of filopodium formation by a WASP-related actin-depolymerizing protein N-WASP. Cdc42 is a small GTPase of the Rho family which regulates the formation of actin filaments to generate   filopodia  . Although there are several proteins such as PAK, ACK and WASP (Wiskott-Aldrich syndrome protein) that bind Cdc42 directly, none of these can account for the filopodium formation induced by Cdc42. Here we demonstrate that before it can induce filopodium formation, Cdc42 must bind a WASP-related protein, N-WASP, that is richest in neural tissues but is expressed ubiquitously. N-WASP induces extremely long actin microspikes only when co-expressed with active Cdc42, whereas WASP, which is expressed in haematopoietic cells, does not, despite the structural similarities between WASP and N-WASP. In a cell-free system, addition of active Cdc42 significantly stimulates the actin-depolymerizing activity of N-WASP, creating free barbed ends from which actin polymerization can then take place. This activation seems to be caused by exposure of N-WASP's actin-depolymerizing region induced by Cdc42 binding. 
 IQGAP1 is a component of Cdc42 signaling to the cytoskeleton. The Ras-GAP related protein IQGAP1 binds several proteins, including actin, calmodulin, E-cadherin and the Rho family GTPase Cdc42. To gain insight into its in vivo function, IQGAP1 was overexpressed in mammalian cells. Transfection of IQGAP1 significantly increased the levels of active, GTP-bound Cdc42, resulting in the formation of peripheral actin microspikes. By contrast, transfection of an IQGAP1 mutant lacking part of the GAP-related domain (IQGAP1deltaGRD) substantially decreased the amount of GTP-bound Cdc42 in cell lysates. Consistent with these findings, IQGAP1DeltaGRD blocked Cdc42 function in cells that stably overexpress constitutively active Cdc42 and abrogated the effect of bradykinin on Cdc42. In cells transfected with IQGAP1deltaGRD, bradykinin was unable to activate Cdc42, translocate Cdc42 to the membrane fraction, or induce   filopodia   production. IQGAP1deltaGRD transfection altered cellular morphology, producing small, round cells that closely resemble Cdc42-/- cells. Some insight into the mechanism was provided by in vitro analysis, which revealed that IQGAP1deltaGRD increased the intrinsic GTPase activity of Cdc42, thereby increasing the amount of inactive, GDP-bound Cdc42. These data imply that IQGAP1 has a crucial role in transducing Cdc42 signaling to the cytoskeleton. 
 Cdc42Hs facilitates cytoskeletal reorganization and neurite outgrowth by localizing the 58-kD insulin receptor substrate to filamentous actin. Cdc42Hs is involved in cytoskeletal reorganization and is required for neurite outgrowth in N1E-115 cells. To investigate the molecular mechanism by which Cdc42Hs regulates these processes, a search for novel Cdc42Hs protein partners was undertaken by yeast two-hybrid assay. Here, we identify the 58-kD substrate of the insulin receptor tyrosine kinase (IRS-58) as a Cdc42Hs target. IRS-58 is a brain-enriched protein comprising at least four protein-protein interaction sites: a Cdc42Hs binding site, an Src homology (SH)3-binding site, an SH3 domain, and a tryptophan, tyrptophan (WW)-binding domain. Expression of IRS-58 in Swiss 3T3 cells leads to reorganization of the filamentous (F)-actin cytoskeleton, involving loss of stress fibers and formation of   filopodia   and clusters. In N1E-115 cells IRS-58 induces neurite outgrowth with high complexity. Expression of a deletion mutant of IRS-58, which lacks the SH3- and WW-binding domains, induced neurite extension without complexity in N1E-115 cells. In Swiss 3T3 cells and N1E-115 cells, IRS-58 colocalizes with F-actin in clusters and   filopodia  . An IRS-58(1267N) mutant unable to bind Cdc42Hs failed to localize with F-actin to induce neurite outgrowth or significant cytoskeletal reorganization. These results suggest that Cdc42Hs facilitates cytoskeletal reorganization and neurite outgrowth by localizing protein complexes via adaptor proteins such as IRS-58 to F-actin. 
 Regulation of cell shape by Cdc42 is mediated by the synergic actin-bundling activity of the Eps8-IRSp53 complex. Actin-crosslinking proteins organize actin into highly dynamic and architecturally diverse subcellular scaffolds that orchestrate a variety of mechanical processes, including lamellipodial and   filopodia  l protrusions in motile cells. How signalling pathways control and coordinate the activity of these crosslinkers is poorly defined. IRSp53, a multi-domain protein that can associate with the Rho-GTPases Rac and Cdc42, participates in these processes mainly through its amino-terminal IMD (IRSp53 and MIM domain). The isolated IMD has actin-bundling activity in vitro and is sufficient to induce   filopodia   in vivo. However, the manner of regulation of this activity in the full-length protein remains largely unknown. Eps8 is involved in actin dynamics through its actin barbed-ends capping activity and its ability to modulate Rac activity. Moreover, Eps8 binds to IRSp53. Here, we describe a novel actin crosslinking activity of Eps8. Additionally, Eps8 activates and synergizes with IRSp53 in mediating actin bundling in vitro, enhancing IRSp53-dependent membrane extensions in vivo. Cdc42 binds to and controls the cellular distribution of the IRSp53-Eps8 complex, supporting the existence of a Cdc42-IRSp53-Eps8 signalling pathway. Consistently, Cdc42-induced   filopodia   are inhibited following individual removal of either IRSp53 or Eps8. Collectively, these results support a model whereby the synergic bundling activity of the IRSp53-Eps8 complex, regulated by Cdc42, contributes to the generation of actin bundles, thus promoting   filopodia  l protrusions. 
 DOCK7 is a critical regulator of the RAGE-Cdc42 signaling axis that induces formation of dendritic pseudopodia in human cancer cells. Cellular migration is a fundamental process linked to cancer metastasis. Growing evidence indicates that the receptor for advanced glycation end products (RAGE) plays a pivotal role in this process. With regard to downstream signal transducers of RAGE, diaphanous-1 and activated small guanine nucleotide triphosphatases, Rac1 and Cdc42, have been identified. To obtain precise insight into the direct downstream signaling mechanism of RAGE, we screened for proteins interacting with the cytoplasmic domain of RAGE employing an immunoprecipitation-liquid chromatography coupled with an electrospray tandem mass spectrometry system. In the present study, we found that the cytoplasmic domain of RAGE interacted with an atypical DOCK180-related guanine nucleotide exchange factor, dedicator of cytokinesis protein 7 (DOCK7). DOCK7 bound to the RAGE cytoplasmic domain and transduced a signal to Cdc42, resulting in the formation of abundant highly branched   filopodi  a-like protrusions, dendritic pseudopodia. Blocking of the function of DOCK7 greatly abrogated the formation of dendritic pseudopodia and suppressed cellular migration. These results indicate that DOCK7 functions as an essential and downstream regulator of RAGE-mediated cellular migration through the formation of dendritic pseudopodia. 
 HIV-1 activates Cdc42 and induces membrane extensions in immature dendritic cells to facilitate cell-to-cell virus propagation. HIV-1 cell-to-cell transmission confers a strong advantage as it increases efficiency of transfer up to 100-fold compared with a cell-free route. Mechanisms of HIV-1 cell-to-cell transmission are still unclear and can in part be explained by the presence of actin-containing cellular protrusions. Such protrusions have been shown to facilitate cell-to-cell viral dissemination. Using fluorescence microscopy, electron tomography, and ion abrasion scanning electron microscopy we show that HIV-1 induces membrane extensions in immature dendritic cells through activation of Cdc42. We demonstrate that these extensions are induced after engagement of DC-SIGN by HIV-1(env) via a cascade that involves Src kinases, Cdc42, Pak1, and Wasp. Silencing of Cdc42 or treatment with a specific Cdc42 inhibitor, Secramine A, dramatically reduced the number of membrane protrusions visualized on the cell surface and decreased HIV-1 transfer via infectious synapses. Ion abrasion scanning electron microscopy of cell-cell contact regions showed that cellular extensions from immature dendritic cells that have the appearance of thin   filopodia   in thin section images are indeed extended membranous sheets with a narrow cross section. Our results demonstrate that HIV-1 binding on immature dendritic cells enhances the formation of membrane extensions that facilitate HIV-1 transfer to CD4(+) T lymphocytes. 
 Cdc42 expression in cervical cancer and its effects on cervical tumor invasion and migration. The aim of the present study was to examine Cdc42 expression in cervical cancer and explore its effects on invasion and migration capability of cervical cancer cells. Immunohistochemistry was used to detect Cdc42 expression in normal cervical tissues as well as CIN I or below, CIN II or above, and cervical cancer tissues. Western blot analysis was used to explore Cdc42 expression in normal cervical cell line Crl-2614 and cervical cancer cell line HeLa. Plasmids of constitutively active Cdc42 (Cdc42 CA), wild-type Cdc42 (Cdc42 WT) and dominant negative Cdc42 (Cdc42 DN) were transfected, respectively, into HeLa cells to investigate the impacts of Cdc42 on migration and invasion of cervical cancer cells using Transwell and on cytoskeleton microfilaments using confocal microscopy after immunofluorescence staining. Cdc42 expression was gradually increased in the order of cervical tissues with CIN I or below, CIN II or above and cancer, showing significant difference (P<0.05), and was significantly higher in HeLa cells than in Crl-2614 cells (P<0.05). Migration ability of HeLa cells transfected with Cdc42 CA was significantly higher than that of non-transfected, as well as Cdc42 WT- or Cdc42 DN-transfected HeLa cells (P<0.05). Overexpression of Cdc42 CA can promote   filopodia   formation in HeLa cells. We concluded that Cdc42 overexpression significantly improved the ability of cervical cancer cells to migrate possibly due to improved pseudopodia formation. 
 Regulation of IRSp53-dependent   filopodia  l dynamics by antagonism between 14-3-3 binding and SH3-mediated localization.   Filopodia   are dynamic structures found at the leading edges of most migrating cells. IRSp53 plays a role in filopodium dynamics by coupling actin elongation with membrane protrusion. IRSp53 is a Cdc42 effector protein that contains an N-terminal inverse-BAR (Bin-amphipysin-Rvs) domain (IRSp53/MIM homology domain [IMD]) and an internal SH3 domain that associates with actin regulatory proteins, including Eps8. We demonstrate that the SH3 domain functions to localize IRSp53 to lamellipodia and that IRSp53 mutated in its SH3 domain fails to induce   filopodia  . Through SH3 domain-swapping experiments, we show that the related IRTKS SH3 domain is not functional in lamellipodial localization. IRSp53 binds to 14-3-3 after phosphorylation in a region that lies between the CRIB and SH3 domains. This association inhibits binding of the IRSp53 SH3 domain to proteins such as WAVE2 and Eps8 and also prevents Cdc42-GTP interaction. The antagonism is achieved by phosphorylation of two related 14-3-3 binding sites at T340 and T360. In the absence of phosphorylation at these sites, filopodium lifetimes in cells expressing exogenous IRSp53 are extended. Our work does not conform to current views that the inverse-BAR domain or Cdc42 controls IRSp53 localization but provides an alternative model of how IRSp53 is recruited (and released) to carry out its functions at lamellipodia and   filopodia  . 
   Filopodia   are induced by aquaporin-9 expression. Understanding   filopodia  l formation in motile cells is a pertinent task in cell biology. In the present study we show that expression of the human water channel aquaporin-9 (AQP9) in different cell lines induces the formation of numerous   filopodia  l extensions. Several lines of evidence support the role of aquaporins functioning both as a water channel and signaling participant. The number of   filopodia   is decreased by site-directed serine substitutions in putative PKC-binding or -phosphorylation sites at amino acid position 11 and 222 in AQP9. The   filopodia  l phenotype obtained with wild-type AQP9 is associated with elevated levels of active Cdc42, while serine-deleted mutants have reduced levels of GTP-Cdc42. Co-transfection with inhibitory N-WASP CRIB completely abolishes wild-type AQP9-induced   filopodia   formation. Active PKC(zeta) phosphorylates wild-type AQP9 and myristoylated PKC(zeta) pseudosubstrate inhibits the formation of   filopodia   in AQP9-expressing cells. Expression of wild-type AQP9, but not mock or serine substituted mutants, increases sensitivity to hypo-osmolaric conditions, yielding a rapid morphological rounding of cells and cell death starting as early as 24 h post-transfection. We propose that increased water influx through AQP9 is critically involved in the formation of membrane protrusions, and that AQP9-induced actin polymerization is augmented by activation of Cdc42 and PKC(zeta). 
 Effect of microRNA-206 on cytoskeleton remodelling by downregulating Cdc42 in MDA-MB-231 cells. MicroRNAs are small, noncoding, single-stranded RNAs that regulate gene expression post-transcriptionally. miR-206 is known to play an important role in breast cancer metastasis. When we sought to predict the target of miR-206 by Targetscan, Pictar and miRanda, we found Cdc42 was a potential one. In this study, we transfected miR-206 into MDA-MB-231 cells and examined Cdc42 protein expression as well as MMP-2 and MMP-9, which are also associated with metastasis of breast cancer. Since Cdc42 is involved in   filopodia   and invadopodia formation, we examined the morphological changes of MDA-MB-231 cells.miR-206 mimics were transfected into MDA-MB-231 cells using LipofectamineTM 2000. Protein expression was detected by Western blot. Cells were stained with FITC-phalloidin to visualize F-actin. Invasive ability and migratory ability were examined by invasion assay and migration assay in vitro.Cdc42, MMP-2 and MMP-9 were downregulated on the protein level. The formation of   filopodia  , which requires Cdc42, was inhibited in miR-206 transfected cells, even under the stimulation of EGF. The invasion and migration of MDA-MB-231 cells in vitro was inhibited by miR-206 too.The results suggest that miR-206 may suppress invasion and migration of MDA-MB-231 cells in vitro partly via regulating actin cytoskeleton remodelling such as   filopodia   formation. 
  GeneRIFs  
 Cdc42 were down-regulated on the protein level.The formation of   filopodia  , which requires Cdc42, was inhibited in miR-206 transfected cells, even under EGF stimulation 
 this work does not conform to current views that the inverse-BAR domain or Cdc42 controls IRSp53 localization but provides an alternative model of how IRSp53 is recruited (and released) to carry out its functions at lamellipodia and   filopodia  . 
 Internalization of DENV-2 into endothelial cells requires viral induction of dynamic   filopodia   regulated by Rac1 and Cdc42 cross-talk and myosin II motor activities. 
 These results support a model whereby the synergic bundling activity of the IRSp53-Eps8 complex, regulated by Cdc42, contributes to the generation of actin bundles, thus promoting   filopodia  l protrusions. 
 the signals induced by integrin alpha6beta1 modulate at the level of PI3K and Cdc42 activity to allow platelets to actively form   filopodia   
  [Ff]ilopodium  Process  
   filopodium   assembly 
 positive regulation of   filopodium   assembly 
 regulation of   filopodium   assembly 
  PubMed  
 The integrin alpha6beta1 modulation of PI3K and Cdc42 activities induces dynamic   filopodium   formation in human platelets. Platelets are an ideal model for studying a rapid morphological change in response to various signal transduction systems. Morphological changes via the activation of integrin alphaIIbbeta3 in platelets have been investigated intensively. In contrast, activation via integrin alpha6beta1 is less well studied. Here, we provide the first biochemical evidence that integrins alpha6beta1 and alphaIIbbeta3 of platelets are associated with different membrane proteins. We also demonstrate that platelets activated by integrin alpha6beta1 show dynamic change by actively forming filopodia and never fully spreading over a period of more than an hour. In addition, platelets activated by integrin alpha6beta1 are different from those activated by integrin alphaIIbbeta3 in terms of cell-substrate contact and in their distribution pattern of actin, Arp2/3 and various phosphotyrosine proteins. The morphological appearance of platelets produced through integrin alpha6beta1 activation is highly dependent on PI3 kinase (PI3K) but less dependent on Src kinase. Suppression of PI3K activity in integrin alpha6beta1 activated platelets induces an increase in Cdc42 activity and more   filopodium   formation. However, both Cdc42 and PI3K activity are higher in platelets activated by integrin alpha6beta1 than in those activated by integrin alphaIIbbeta3. Taken together, this study demonstrates that the signals induced by integrin alpha6beta1 modulate at the level of PI3K and Cdc42 activity to allow platelets to actively form filopodia. 
 IRSp53 is an essential intermediate between Rac and WAVE in the regulation of membrane ruffling. Neural Wiskott-Aldrich syndrome protein (N-WASP) functions in several intracellular events including   filopodium   formation, vesicle transport and movement of Shigella frexneri and vaccinia virus, by stimulating rapid actin polymerization through the Arp2/3 complex. N-WASP is regulated by the direct binding of Cdc42 (refs 7, 8), which exposes the domain in N-WASP that activates the Arp2/3 complex. A WASP-related protein, WAVE/Scar, functions in Rac-induced membrane ruffling; however, Rac does not bind directly to WAVE, raising the question of how WAVE is regulated by Rac. Here we demonstrate that IRSp53, a substrate for insulin receptor with unknown function, is the 'missing link' between Rac and WAVE. Activated Rac binds to the amino terminus of IRSp53, and carboxy-terminal Src-homology-3 domain of IRSp53 binds to WAVE to form a trimolecular complex. From studies of ectopic expression, we found that IRSp53 is essential for Rac to induce membrane ruffling, probably because it recruits WAVE, which stimulates actin polymerization mediated by the Arp2/3 complex. 
 The integrin alpha6beta1 modulation of PI3K and Cdc42 activities induces dynamic   filopodium   formation in human platelets. Platelets are an ideal model for studying a rapid morphological change in response to various signal transduction systems. Morphological changes via the activation of integrin alphaIIbbeta3 in platelets have been investigated intensively. In contrast, activation via integrin alpha6beta1 is less well studied. Here, we provide the first biochemical evidence that integrins alpha6beta1 and alphaIIbbeta3 of platelets are associated with different membrane proteins. We also demonstrate that platelets activated by integrin alpha6beta1 show dynamic change by actively forming filopodia and never fully spreading over a period of more than an hour. In addition, platelets activated by integrin alpha6beta1 are different from those activated by integrin alphaIIbbeta3 in terms of cell-substrate contact and in their distribution pattern of actin, Arp2/3 and various phosphotyrosine proteins. The morphological appearance of platelets produced through integrin alpha6beta1 activation is highly dependent on PI3 kinase (PI3K) but less dependent on Src kinase. Suppression of PI3K activity in integrin alpha6beta1 activated platelets induces an increase in Cdc42 activity and more   filopodium   formation. However, both Cdc42 and PI3K activity are higher in platelets activated by integrin alpha6beta1 than in those activated by integrin alphaIIbbeta3. Taken together, this study demonstrates that the signals induced by integrin alpha6beta1 modulate at the level of PI3K and Cdc42 activity to allow platelets to actively form filopodia. 
 The Arp2/3 complex mediates actin polymerization induced by the small GTP-binding protein Cdc42. The small GTP-binding protein Cdc42 is thought to induce   filopodium   formation by regulating actin polymerization at the cell cortex. Although several Cdc42-binding proteins have been identified and some of them have been implicated in   filopodium   formation, the precise role of Cdc42 in modulating actin polymerization has not been defined. To understand the biochemical pathways that link Cdc42 to the actin cytoskeleton, we have reconstituted Cdc42-induced actin polymerization in Xenopus egg extracts. Using this cell-free system, we have developed a rapid and specific assay that has allowed us to fractionate the extract and isolate factors involved in this activity. We report here that at least two biochemically distinct components are required, based on their chromatographic behavior and affinity for Cdc42. One component is purified to homogeneity and is identified as the Arp2/3 complex, a protein complex that has been shown to nucleate actin polymerization. However, the purified complex alone is not sufficient to mediate the activity; a second component that binds Cdc42 directly and mediates the interaction between Cdc42 and the complex also is required. These results establish an important link between a signaling molecule, Cdc42, and a complex that can directly modulate actin networks in vitro. We propose that activation of the Arp2/3 complex by Cdc42 and other signaling molecules plays a central role in stimulating actin polymerization at the cell surface. 
 Induction of   filopodium   formation by a WASP-related actin-depolymerizing protein N-WASP. Cdc42 is a small GTPase of the Rho family which regulates the formation of actin filaments to generate filopodia. Although there are several proteins such as PAK, ACK and WASP (Wiskott-Aldrich syndrome protein) that bind Cdc42 directly, none of these can account for the   filopodium   formation induced by Cdc42. Here we demonstrate that before it can induce   filopodium   formation, Cdc42 must bind a WASP-related protein, N-WASP, that is richest in neural tissues but is expressed ubiquitously. N-WASP induces extremely long actin microspikes only when co-expressed with active Cdc42, whereas WASP, which is expressed in haematopoietic cells, does not, despite the structural similarities between WASP and N-WASP. In a cell-free system, addition of active Cdc42 significantly stimulates the actin-depolymerizing activity of N-WASP, creating free barbed ends from which actin polymerization can then take place. This activation seems to be caused by exposure of N-WASP's actin-depolymerizing region induced by Cdc42 binding. 
 Phosphorylation of tyrosine 291 enhances the ability of WASp to stimulate actin polymerization and   filopodium   formation. Wiskott-Aldrich Syndrome protein. Wiskott-Aldrich Syndrome protein (WASp) is a key regulator of the Arp2/3 complex and the actin cytoskeleton in hematopoietic cells. WASp is capable of forming an auto-inhibited conformation, which can be disrupted by binding of Cdc42 and phosphatidylinositol 4,5-bisphosphate, leading to its activation. Stimulation of the collagen receptor on platelets and crosslinking the B-cell receptor induce tyrosine phosphorylation of WASp. Here we show that the Src family kinase Hck induces phosphorylation of WASp-Tyr(291) independently of Cdc42 and that this causes a shift in the mobility of WASp upon SDS-PAGE. A phospho-mimicking mutant, WASp-Y291E, exhibited an enhanced ability to stimulate actin polymerization in a cell-free system and when microinjected into primary macrophages induced extensive   filopodium   formation with greater efficiency than wild-type WASp or a Y291F mutant. We propose that phosphorylation of Tyr(291) directly regulates WASp function. 
 Regulation of IRSp53-dependent filopodial dynamics by antagonism between 14-3-3 binding and SH3-mediated localization. Filopodia are dynamic structures found at the leading edges of most migrating cells. IRSp53 plays a role in   filopodium   dynamics by coupling actin elongation with membrane protrusion. IRSp53 is a Cdc42 effector protein that contains an N-terminal inverse-BAR (Bin-amphipysin-Rvs) domain (IRSp53/MIM homology domain [IMD]) and an internal SH3 domain that associates with actin regulatory proteins, including Eps8. We demonstrate that the SH3 domain functions to localize IRSp53 to lamellipodia and that IRSp53 mutated in its SH3 domain fails to induce filopodia. Through SH3 domain-swapping experiments, we show that the related IRTKS SH3 domain is not functional in lamellipodial localization. IRSp53 binds to 14-3-3 after phosphorylation in a region that lies between the CRIB and SH3 domains. This association inhibits binding of the IRSp53 SH3 domain to proteins such as WAVE2 and Eps8 and also prevents Cdc42-GTP interaction. The antagonism is achieved by phosphorylation of two related 14-3-3 binding sites at T340 and T360. In the absence of phosphorylation at these sites,   filopodium   lifetimes in cells expressing exogenous IRSp53 are extended. Our work does not conform to current views that the inverse-BAR domain or Cdc42 controls IRSp53 localization but provides an alternative model of how IRSp53 is recruited (and released) to carry out its functions at lamellipodia and filopodia. 
  Component  
   filopodium   
  [Ii]nvadopodia  PubMed  
 The interaction of IQGAP1 with the exocyst complex is required for tumor cell invasion downstream of Cdc42 and RhoA.   Invadopodia   are actin-based membrane protrusions formed at contact sites between invasive tumor cells and the extracellular matrix with matrix proteolytic activity. Actin regulatory proteins participate in   invadopodia   formation, whereas matrix degradation requires metalloproteinases (MMPs) targeted to   invadopodia  . In this study, we show that the vesicle-tethering exocyst complex is required for matrix proteolysis and invasion of breast carcinoma cells. We demonstrate that the exocyst subunits Sec3 and Sec8 interact with the polarity protein IQGAP1 and that this interaction is triggered by active Cdc42 and RhoA, which are essential for matrix degradation. Interaction between IQGAP1 and the exocyst is necessary for   invadopodia   activity because enhancement of matrix degradation induced by the expression of IQGAP1 is lost upon deletion of the exocyst-binding site. We further show that the exocyst and IQGAP1 are required for the accumulation of cell surface membrane type 1 MMP at   invadopodia  . Based on these results, we propose that   invadopodia   function in tumor cells relies on the coordination of cytoskeletal assembly and exocytosis downstream of Rho guanosine triphosphatases. 
 Podocalyxin-like 1 promotes   invadopodia   formation and metastasis through activation of Rac1/Cdc42/cortactin signaling in breast cancer cells. Metastatic disease is the leading cause of cancer mortality. Identifying biomarkers and regulatory mechanisms is important toward developing diagnostic and therapeutic tools against metastatic cancer. In this study, we demonstrated that podocalyxin-like 1 (PODXL) is overexpressed in breast tumor cells and increased in lymph node metastatic cancer. Mechanistically, we found that the expression of PODXL was associated with cell motility and invasiveness. Suppression of PODXL in MDA-MB-231 cells reduced lamellipodia formation and focal adhesion kinase (FAK) and paxillin phosphorylation. PODXL knockdown reduced the formation of   invadopodia  , such as inhibiting the colocalization of F-actin with cortactin and suppressing phosphorylation of cortactin and neural Wiskott-Aldrich syndrome protein. Conversely, overexpression of PODXL in MCF7 cells induced F-actin/cortactin colocalization and enhanced   invadopodia   formation and activation.   Invadopodia   activity and tumor invasion in PODXL-knockdown cells are similar to that in cortactin-knockdown cells. We further found that the DTHL motif in PODXL is crucial for regulating cortactin phosphorylation and Rac1/Cdc42 activation. Inhibition of Rac1/Cdc42 impeded PODXL-mediated cortactin activation and FAK and paxillin phosphorylation. Moreover, inhibition of PODXL in MDA-MB-231 cells significantly suppressed tumor colonization in the lungs and distant metastases, similar to those in cortactin-knockdown cells. These findings show that overexpression of PODXL enhanced   invadopodia   formation and tumor metastasis by inducing Rac1/Cdc42/cortactin signaling network. 
 Podocalyxin-like 1 promotes   invadopodia   formation and metastasis through activation of Rac1/Cdc42/cortactin signaling in breast cancer cells. Metastatic disease is the leading cause of cancer mortality. Identifying biomarkers and regulatory mechanisms is important toward developing diagnostic and therapeutic tools against metastatic cancer. In this study, we demonstrated that podocalyxin-like 1 (PODXL) is overexpressed in breast tumor cells and increased in lymph node metastatic cancer. Mechanistically, we found that the expression of PODXL was associated with cell motility and invasiveness. Suppression of PODXL in MDA-MB-231 cells reduced lamellipodia formation and focal adhesion kinase (FAK) and paxillin phosphorylation. PODXL knockdown reduced the formation of   invadopodia  , such as inhibiting the colocalization of F-actin with cortactin and suppressing phosphorylation of cortactin and neural Wiskott-Aldrich syndrome protein. Conversely, overexpression of PODXL in MCF7 cells induced F-actin/cortactin colocalization and enhanced   invadopodia   formation and activation.   Invadopodia   activity and tumor invasion in PODXL-knockdown cells are similar to that in cortactin-knockdown cells. We further found that the DTHL motif in PODXL is crucial for regulating cortactin phosphorylation and Rac1/Cdc42 activation. Inhibition of Rac1/Cdc42 impeded PODXL-mediated cortactin activation and FAK and paxillin phosphorylation. Moreover, inhibition of PODXL in MDA-MB-231 cells significantly suppressed tumor colonization in the lungs and distant metastases, similar to those in cortactin-knockdown cells. These findings show that overexpression of PODXL enhanced   invadopodia   formation and tumor metastasis by inducing Rac1/Cdc42/cortactin signaling network. 
 The interaction of IQGAP1 with the exocyst complex is required for tumor cell invasion downstream of Cdc42 and RhoA.   Invadopodia   are actin-based membrane protrusions formed at contact sites between invasive tumor cells and the extracellular matrix with matrix proteolytic activity. Actin regulatory proteins participate in   invadopodia   formation, whereas matrix degradation requires metalloproteinases (MMPs) targeted to   invadopodia  . In this study, we show that the vesicle-tethering exocyst complex is required for matrix proteolysis and invasion of breast carcinoma cells. We demonstrate that the exocyst subunits Sec3 and Sec8 interact with the polarity protein IQGAP1 and that this interaction is triggered by active Cdc42 and RhoA, which are essential for matrix degradation. Interaction between IQGAP1 and the exocyst is necessary for   invadopodia   activity because enhancement of matrix degradation induced by the expression of IQGAP1 is lost upon deletion of the exocyst-binding site. We further show that the exocyst and IQGAP1 are required for the accumulation of cell surface membrane type 1 MMP at   invadopodia  . Based on these results, we propose that   invadopodia   function in tumor cells relies on the coordination of cytoskeletal assembly and exocytosis downstream of Rho guanosine triphosphatases. 
 Cdc42: Role in Cancer Management. Contribution of Cdc42, a member of Rho family, has been characterized for the beginning of variety of cellular responses including cellular transformation, cell division, cell invasion, migration,   invadopodia   formation, enzyme activity, filopodia formation, and cell polarity in cells. Deregulation of Cdc42 can alter the normal functioning of the cells, responsible for the initiation of signaling pathways and is correlated with several pathogenic processes such as cancer. Therefore, maintaining the level of Cdc42 and its effectors in cells, tumor progression can be controlled. Therefore, it can be suggested that deeper understanding about the Cdc42 contribution in cancer cell progression at molecular level can approach to the development of Cdc42 inhibitors in cancer management. 
 Stromal fibroblasts facilitate cancer cell invasion by a novel   invadopodia  -independent matrix degradation process. Metastatic invasion of tumors into peripheral tissues is known to rely upon protease-mediated degradation of the surrounding stroma. This remodeling process uses complex, actin-based, specializations of the plasma membrane termed   invadopodia   that act both to sequester and release matrix metalloproteinases. Here we report that cells of mesenchymal origin, including tumor-associated fibroblasts, degrade substantial amounts of surrounding matrix by a mechanism independent of conventional   invadopodia  . These degradative sites lack the punctate shape of conventional   invadopodia   to spread along the cell base and are reticular and/or fibrous in character. In marked contrast to   invadopodia  , this degradation does not require the action of Src kinase, Cdc42 or Dyn2. Rather, inhibition of Dyn2 causes a marked upregulation of stromal matrix degradation. Further, expression and activity of matrix metalloproteinases are differentially regulated between tumor cells and stromal fibroblasts. This matrix remodeling by fibroblasts increases the invasive capacity of tumor cells, thereby illustrating how the tumor microenvironment can contribute to metastasis. These findings provide evidence for a novel matrix remodeling process conducted by stromal fibroblasts that is substantially more effective than conventional   invadopodia  , distinct in structural organization and regulated by disparate molecular mechanisms. 
 Effect of microRNA-206 on cytoskeleton remodelling by downregulating Cdc42 in MDA-MB-231 cells. MicroRNAs are small, noncoding, single-stranded RNAs that regulate gene expression post-transcriptionally. miR-206 is known to play an important role in breast cancer metastasis. When we sought to predict the target of miR-206 by Targetscan, Pictar and miRanda, we found Cdc42 was a potential one. In this study, we transfected miR-206 into MDA-MB-231 cells and examined Cdc42 protein expression as well as MMP-2 and MMP-9, which are also associated with metastasis of breast cancer. Since Cdc42 is involved in filopodia and   invadopodia   formation, we examined the morphological changes of MDA-MB-231 cells.miR-206 mimics were transfected into MDA-MB-231 cells using LipofectamineTM 2000. Protein expression was detected by Western blot. Cells were stained with FITC-phalloidin to visualize F-actin. Invasive ability and migratory ability were examined by invasion assay and migration assay in vitro.Cdc42, MMP-2 and MMP-9 were downregulated on the protein level. The formation of filopodia, which requires Cdc42, was inhibited in miR-206 transfected cells, even under the stimulation of EGF. The invasion and migration of MDA-MB-231 cells in vitro was inhibited by miR-206 too.The results suggest that miR-206 may suppress invasion and migration of MDA-MB-231 cells in vitro partly via regulating actin cytoskeleton remodelling such as filopodia formation. 
  GeneRIFs  
 In marked contrast to   invadopodia  , this degradation does not require the action of Src kinase, Cdc42 or Dyn2. Rather, inhibition of Dyn2 causes a marked upregulation of stromal matrix degradation 
 These findings show that overexpression of PODXL enhanced   invadopodia   formation and tumor metastasis by inducing Rac1/Cdc42/cortactin signaling network. 
  [Pp]odosome(s)?  PubMed  
 Actin can reorganize into   podosomes   in aortic endothelial cells, a process controlled by Cdc42 and RhoA. Members of the Rho GTPase family play a central role in the orchestration of cytoskeletal rearrangements, which are of prime importance in endothelial cell physiology. To explore their role in this specialized cell type, we used the bacterial toxin cytotoxic necrotizing factor 1 (CNF1) as a Rho GTPase activator. Punctate filamentous actin structures appeared along the ventral plasma membrane of endothelial cells and were identified as the core of   podosomes   by the distinctive vinculin ring around the F-actin. Rho, Rac, and Cdc42 were all identified as targets of CNF1, but only a constitutively active mutant of Cdc42 could substitute for CNF1 in   podosome   induction. Accordingly, organization of F-actin in these structures was highly dependent on the main Cdc42 cytoskeletal effector N-Wiskott-Aldrich syndrome protein. Other components of the actin machinery such as Arp2/3 and for the first time WIP also colocalized at these sites. Like CNF1 treatment, sustained Cdc42 activity induced a time-dependent F-actin-vinculin reorganization, prevented cytokinesis, and downregulated Rho activity. Finally,   podosomes   were also detected on endothelial cells explanted from patients undergoing cardiac surgery. These data provide the first description of   podosomes   in endothelial cells. The identification of such specialized structures opens up a new field of investigation in terms of endothelium pathophysiology. 
 Actin can reorganize into   podosomes   in aortic endothelial cells, a process controlled by Cdc42 and RhoA. Members of the Rho GTPase family play a central role in the orchestration of cytoskeletal rearrangements, which are of prime importance in endothelial cell physiology. To explore their role in this specialized cell type, we used the bacterial toxin cytotoxic necrotizing factor 1 (CNF1) as a Rho GTPase activator. Punctate filamentous actin structures appeared along the ventral plasma membrane of endothelial cells and were identified as the core of   podosomes   by the distinctive vinculin ring around the F-actin. Rho, Rac, and Cdc42 were all identified as targets of CNF1, but only a constitutively active mutant of Cdc42 could substitute for CNF1 in   podosome   induction. Accordingly, organization of F-actin in these structures was highly dependent on the main Cdc42 cytoskeletal effector N-Wiskott-Aldrich syndrome protein. Other components of the actin machinery such as Arp2/3 and for the first time WIP also colocalized at these sites. Like CNF1 treatment, sustained Cdc42 activity induced a time-dependent F-actin-vinculin reorganization, prevented cytokinesis, and downregulated Rho activity. Finally,   podosomes   were also detected on endothelial cells explanted from patients undergoing cardiac surgery. These data provide the first description of   podosomes   in endothelial cells. The identification of such specialized structures opens up a new field of investigation in terms of endothelium pathophysiology. 
  GeneRIFs  
 The reorganization of actins into   podosomes   is controlled by CDC42 GTP-binding protein. 
  [Mm]igration  Process  
 dendritic cell   migration   
 nuclear   migration   
  PubMed  
 CBAP functions as a novel component in chemokine-induced ZAP70-mediated T-cell adhesion and   migration  . Activated chemokine receptor initiates inside-out signaling to transiently trigger activation of integrins, a process involving multiple components that have not been fully characterized. Here we report that GM-CSF/IL-3/IL-5 receptor common beta-chain-associated protein (CBAP) is required to optimize this inside-out signaling and activation of integrins. First, knockdown of CBAP expression in human Jurkat T cells caused attenuated CXC chemokine ligand-12 (CXCL12)-induced cell   migration   and integrin α4β1- and αLβ2-mediated cell adhesion in vitro, which could be rescued sufficiently upon expression of murine CBAP proteins. Freshly isolated CBAP-deficient primary T cells also exhibited diminution of chemotaxis toward CC chemokine ligand-21 (CCL21) and CXCL12, and these chemokines-induced T-cell adhesions in vitro. Adoptive transfer of isolated naive T cells demonstrated that CBAP deficiency significantly reduced lymph node homing ability in vivo. Final  ly, migra  tion of T cell-receptor-activated T cells induced by inflammatory chemokines was also attenuated in CBAP-deficient cells. Further analyses revealed that CBAP constitutively associated with both integrin β1 and ZAP70 and that CBAP is required for chemokine-induced initial binding of the talin-Vav1 complex to integrin β1 and to facilitate subsequent ZAP70-mediated dissociation of the talin-Vav1 complex and Vav1 phosphorylation. Within such an integrin signaling complex, CBAP likely functions as an adaptor and ultimately leads to activation of both integrin α4β1 and Rac1. Taken together, our data suggest that CBAP indeed can function as a novel signaling component within the ZAP70/Vav1/talin complex and plays an important role in regulating chemokine-promoted T-cell trafficking. 
 Oligophrenin-1 encodes a rhoGAP protein involved in X-linked mental retardation. Primary or nonspecific X-linked mental retardation (MRX) is a heterogeneous condition in which affected patients do not have any distinctive clinical or biochemical features in common apart from cognitive impairment. Although it is present in approximately 0.15-0.3% of males, most of the genetic defects associated with MRX, which may involve more than ten different genes, remain unknown. Here we report the characterization of a new gene on the long arm of the X-chromosome (position Xq12) and the identification in unrelated individuals of different mutations that are predicted to cause a loss of function. This gene is highly expressed in fetal brain and encodes a protein of relative molecular mass 91K, named oligophrenin-1, which contains a domain typical of a Rho-GTPase-activating protein (rhoGAP). By enhancing their GTPase activity, GAP proteins inactivate small Rho and Ras proteins, so inactivation of rhoGAP proteins might cause constitutive activation of their GTPase targets. Such activation is known to affect cell   migration   and outgrowth of axons and dendrites in vivo. Our results demonstrate an association between cognitive impairment and a defect in a signalling pathway that depends on a Ras-like GTPase. 
 Distinct predictive performance of Rac1 and Cdc42 in cell   migration  . We propose a new computation-based approach for elucidating how signaling molecules are decoded in cell   migration  . In this approach, we performed FRET time-lapse imaging of Rac1 and Cdc42, members of Rho GTPases which are responsible for cell motility, and quantitatively identified the response functions that describe the conversion from the molecular activities to the morphological changes. Based on the identified response functions, we clarified the profiles of how the morphology spatiotemporally changes in response to local and transient activation of Rac1 and Cdc42, and found that Rac1 and Cdc42 activation triggers laterally propagating membrane protrusion. The response functions were also endowed with property of differentiator, which is beneficial for maintaining sensitivity under adaptation to the mean level of input. Using the response function, we could predict the morphological change from molecular activity, and its predictive performance provides a new quantitative measure of how much the Rho GTPases participate in the cell   migration  . Interestingly, we discovered distinct predictive performance of Rac1 and Cdc42 depending on the   migration   modes, indicating that Rac1 and Cdc42 contribute to persistent and random   migration  , respectively. Thus, our proposed predictive approach enabled us to uncover the hidden information processing rules of Rho GTPases in the cell   migration  . 
 Rac1 and Cdc42 differentially modulate cigarette smoke-induced airway cell   migration   through p120-catenin-dependent and -independent pathways. The adherens junction protein p120-catenin (p120ctn) shuttles between E-cadherin-bound and cytoplasmic pools to regulate E-cadherin/catenin complex stability and cell   migration  , respectively. When released from the adherens junction, p120ctn promotes cell   migration   through modulation of the Rho GTPases Rac1, Cdc42, and RhoA. Accordingly, the down-regulation and cytoplasmic mislocalization of p120ctn has been reported in all subtypes of lung cancers and is associated with grave prognosis. Previously, we reported that cigarette smoke induced cytoplasmic translocation of p120ctn and cell   migration  , but the underlying mechanism was unclear. Using primary human bronchial epithelial cells exposed to smoke-concentrated medium (Smk), we observed the translocation of Rac1 and Cdc42, but not RhoA, to the leading edge of polarized and migrating human bronchial epithelial cells. Rac1 and Cdc42 were robustly activated by smoke, whereas RhoA was inhibited. Accordingly, siRNA knockdown of Rac1 or Cdc42 completely abolished Smk-induced cell   migration  , whereas knockdown of RhoA had no effect. p120ctn/Rac1 double knockdown completely abolished Smk-induced cell   migration  , whereas p120ctn/Cdc42 double knockdown did not. These data suggested that Rac1 and Cdc42 coactivation was essential to smoke-promoted cell   migration   in the presence of p120ctn, whereas   migration   proceeded via Rac1 alone in the absence of p120ctn. Thus, Rac1 may provide an omnipotent therapeutic target in reversing cell   migration   during the early (intact p120ctn) and late (loss of p120ctn) stages of lung carcinogenesis. 
 Interleukin-1β-induced Wnt5a enhances human corneal endothelial cell   migratio  n through regulation of Cdc42 and RhoA. Wnt5a can activate β-catenin-independent pathways for regulation of various cellular functions, such a  s migrati  on, that play critical roles in wound repair. Investigation of Wnt5a signaling may help identify therapeutic targets for enhancing corneal endothelial wound healing that could provide an alternative to corneal transplantation in patients with blindness from endothelial dysfunction. However, Wnt5a signaling in corneal endothelial cells (CECs) has not been well characterized. In this study, we show transient induction of Wnt5a by interleukin-1β (IL-1β) stimulation proceeds through NF-κB in human CECs. This leads to binding of Fzd5 to Ror2, resulting in activation of disheveled protein (Dvl) and subsequently disheveled-associated activator of morphogenesis 1 (DAAM1). This leads to activation of Cdc42 and subsequent inhibition of RhoA. Inhibition of RhoA leads to parallel dephosphorylation and inactivation of LIM domain kinase 2 along with dephosphorylation and activation of slingshot 1, resulting in dephosphorylation and activation of cofilin and leading to enhanced   cell migr  ation. These findings suggest that Wnt5a enhances   cell migr  ation through activation of Cdc42 and inactivation of RhoA in human CECs. 
 Prostaglandin E2-mediated   migration   of human trophoblast requires RAC1 and CDC42. The invasion of maternal decidua and uterine spiral arteries by a trophoblast subpopulation called extravillous trophoblast (EVT) is essential for the establishment of a normal placenta and an adequate blood flow toward the fetus. Derangements in these processes underlie pregnancy-related diseases like preeclampsia and intrauterine growth restriction. Many growth factors, growth factor binding proteins, and extracellular matrix components can positively or negatively regulate the proliferation,   migration  , and/or invasiveness of these EVT cells. RHO GTPases, including RHOA, RAC1, and CDC42, are ubiquitous proteins that control cytoskeletal changes by forming stress fibers and projecting lamellipodia and filopodia during cellular   migration  . We had previously shown that prostaglandin (PG) E(2) produced in abundance by the decidua promotes the   migration   of first-trimester human EVTs by increasing the intracellular concentration of calcium and activating calpain. Using our well-characterized immortalized EVT cell line, HTR-8/SVneo, as well as villus explants from first-trimester placentae, this study examined the role of RHO GTPases RAC1 and CDC42 in PGE(2)-mediated migratory responses of these cells. Though a RAC1 inhibitor, NSC23766 as well as RAC1 knockdown by siRNA decreased the   migration   of HTR-8/SVneo cells in a Transwell   migration   assay, this inhibition could not be restored by PGE(2) or 17-phenyl trinor PGE(2) (PGE receptor PTGER1 agonist) or PGE(1) Alcohol (PGE receptor PTGER4 agonist). Similar results were noted for EVT cell spreading in villus explants. Furthermore, CDC42 silencing using siRNA inhibited PGE(2)-induced   migration   of HTR-8/SVneo cells. Finally, the treatment of EVT cells with PGE(2), PTGER1 agonist, or PTGER4 agonist activated RAC1 and CDC42 at 10 min, suggesting that RAC1 and CDC42 play an essential role in PGE(2)-mediated   migration   of human EVTs. 
 Estrogen and resveratrol regulate Rac and Cdc42 signaling to the actin cytoskeleton of metastatic breast cancer cells. Estrogen and structurally related molecules play critical roles in breast cancer. We reported that resveratrol (50 microM), an estrogen-like phytosterol from grapes, acts in an antiestrogenic manner in breast cancer cells to reduce cell   migration   and to induce a global and sustained extension of actin structures called filopodia. Herein, we report that resveratrol-induced filopodia formation is time-dependent and concentration-dependent. In contrast to resveratrol at 50 microM, resveratrol at 5 microM acts in a manner similar to estrogen by increasing lamellipodia, as well as cell   migration   and invasion. Because Rho GTPases regulate the extension of actin structures, we investigated a role for Rac and Cdc42 in estrogen and resveratrol signaling. Our results demonstrate that 50 microM resveratrol decreases Rac and Cdc42 activity, whereas estrogen and 5 microM resveratrol increase Rac activity in breast cancer cells. MDA-MB-231 cells expressing dominant-negative Cdc42 or dominant-negative Rac retain filopodia response to 50 microM resveratrol. Lamellipodia response to 5 microM resveratrol, estrogen, or epidermal growth factor is inhibited in cells expressing dominant-negative Rac, indicating that Rac regulates estrogen and resveratrol (5 microM) signaling to the actin cytoskeleton. These results indicate that signaling to the actin cytoskeleton by low and high concentrations of resveratrol may be differentially regulated by Rac and Cdc42. 
 Wnt5b promotes the cell motility essential for metastasis of oral squamous cell carcinoma through active Cdc42 and RhoA. The activation of Wnt signaling has been reported in many types of squamous cell carcinoma. In this study, using human oral squamous cell carcinoma (OSCC) cells with different metastatic potential, we investigated the involvement of Wnt signaling in metastasis. Further, we aimed to elucidate the characteristic biological features related to high metastatic potential and to identify new target molecules for the suppression of OSCC lymph node metastasis. We compared SAS-Venus (SAS OSCC cells expressing green fluorescent protein) and SAS-LM8, which is a highly metastatic cell line derived from SAS-Venus by in vivo selection. The SAS-LM8 cell line had greater ability of   migration   and invasion compared to SAS-Venus. Furthermore, a higher number of filopodia-like protrusive structures were produced in SAS-LM8 cells compared to SAS-Venus cells, and the levels of active Cdc42 and active RhoA protein were higher in SAS-LM8 cells compared to SAS-Venus cells. We did not observe any differences in the expression of Wnt/β-catenin target genes between the two cell lines; however, the mRNA levels of Wnt5b were higher in SAS-LM8 cells compared to SAS-Venus cells. To confirm the involvement of Wnt5b in   migratio  n in OSCC cells, we examined the effects of the siRNA-mediated knockdown of Wnt5b in SAS-Venus cells and SAS-LM8 cells. The siRNA treatment significantly inhibited   migratio  n and the formation of filopodia-like protrusive structures. Conversely, when stimulated with Wnt5b, the   migratio  n and formation of filopodia-like protrusions were significantly enhanced and the levels of active Cdc42 and active RhoA proteins were also increased. These results indicate that Wnt5b is involved in the   migratio  n ability of OSCC cells through active Cdc42 and RhoA. 
 Close encounters: regulation of vertebrate skeletal myogenesis by cell-cell contact. Cells of the vertebrate skeletal muscle lineage develop in a highly ordered process that includes specification,   migration   and differentiation into multinucleated myofibers. The changes in gene expression and cell morphology that occur during myogenic differentiation must be coordinated with each other in a spatiotemporal fashion; one way that this might occur is through regulation of these processes by cell-cell adhesion and resultant signaling. The past several years have witnessed the identification of molecules that are likely to be mediators of the promyogenic effects of cell-cell contact and some of the mechanisms by which they work. These include: the community factor, embryonic fibroblast growth factor (eFGF); classical cadherins, which mediate both adhesion and signaling; and cadherin-associated immunoglobulin superfamily members such as CDO, BOC and neogenin. Genetic evidence for the promyogenic roles of some of these factors is emerging. In other cases, potential compensatory or redundant functions necessitate future construction of double or triple mutants. Mechanistic studies in vitro indicate that specific cadherins and immunoglobulin superfamily proteins exert some of their effects in an interdependent fashion by signaling from a multiprotein complex found at sites of cell-cell contact. 
 Signal transduction in neuronal   migration  : roles of GTPase activating proteins and the small GTPase Cdc42 in the Slit-Robo pathway. The Slit protein guides neuronal and leukocyte   migration   through the transmembrane receptor Roundabout (Robo). We report here that the intracellular domain of Robo interacts with a novel family of Rho GTPase activating proteins (GAPs). Two of the Slit-Robo GAPs (srGAPs) are expressed in regions responsive to Slit. Slit increased srGAP1-Robo1 interaction and inactivated Cdc42. A dominant negative srGAP1 blocked Slit inactivation of Cdc42 and Slit repulsion of migratory cells from the anterior subventricular zone (SVZa) of the forebrain. A constitutively active Cdc42 blocked the repulsive effect of Slit. These results have demonstrated important roles for GAPs and Cdc42 in neuronal   migration  . We propose a signal transduction pathway from the extracellular guidance cue to intracellular actin polymerization. 
 Tuberous sclerosis complex 2 (TSC2) regulates cell   migration   and polarity through activation of CDC42 and RAC1. The phosphatidylinositol 3-kinase (PI3K)/AKT pathway plays important roles in regulating cell motility. TSC2, a downstream target of AKT, is a central player in negatively controlling cell proliferation and protein translation through suppressing the activity of mTOR (mammalian target of rapamycin). However, the function of TSC2 in regulating cell   migration   remains unclear. Here, we show that TSC2 plays a critical role in the control of cell spreading, polarity, and   migration  . TSC2-deficient fibroblast cells were impaired in their ability to spread and alter actin cytoskeleton upon stimulation with insulin-like growth factor-1. Using scratch-induced polarization assay, we demonstrate that TSC2((-/-)) fibroblast cells polarized poorly toward the wound compared with wild-type cells. Similarly, knockdown of TSC2 expression in colon cancer cells resulted in a marked decrease in cell motility. Functionally, the activation of CDC42- and RAC1-GTPase was largely reduced in TSC2 knock-out fibroblast and TSC2 knockdown cancer cells. Furthermore, overexpression of an activating p110alpha mutant or short term rapamycin treatment rescued the cell polarization defect in TSC2((-/-)) fibroblast cells. Concurrently, the activation of CDC42 and RAC1 increased. The defect in cell   migration   and CDC42 and RAC1 activation was reversed by reintroducing TSC2 back into TSC2((-/-)) fibroblast cells. Taken together, we identified a novel role of TSC2 in controlling cell polarity and   migration   by regulating CDC42 and RAC1 activation. 
 Novel Activities of Select NSAID R-Enantiomers against Rac1 and Cdc42 GTPases. Rho family GTPases (including Rac, Rho and Cdc42) collectively control cell proliferation, adhesion and   migration   and are of interest as functional therapeutic targets in numerous epithelial cancers. Based on high throughput screening of the Prestwick Chemical Library® and cheminformatics we identified the R-enantiomers of two approved drugs (naproxen and ketorolac) as inhibitors of Rac1 and Cdc42. The corresponding S-enantiomers are considered the active component in racemic drug formulations, acting as non-steroidal anti-inflammatory drugs (NSAIDs) with selective activity against cyclooxygenases. Here, we show that the S-enantiomers of naproxen and ketorolac are inactive against the GTPases. Additionally, more than twenty other NSAIDs lacked inhibitory action against the GTPases, establishing the selectivity of the two identified NSAIDs. R-naproxen was first identified as a lead compound and tested in parallel with its S-enantiomer and the non-chiral 6-methoxy-naphthalene acetic acid (active metabolite of nabumetone, another NSAID) as a structural series. Cheminformatics-based substructure analyses-using the rotationally constrained carboxylate in R-naproxen-led to identification of racemic [R/S] ketorolac as a suitable FDA-approved candidate. Cell based measurement of GTPase activity (in animal and human cell lines) demonstrated that the R-enantiomers specifically inhibit epidermal growth factor stimulated Rac1 and Cdc42 activation. The GTPase inhibitory effects of the R-enantiomers in cells largely mimic those of established Rac1 (NSC23766) and Cdc42 (CID2950007/ML141) specific inhibitors. Docking predicts that rotational constraints position the carboxylate moieties of the R-enantiomers to preferentially coordinate the magnesium ion, thereby destabilizing nucleotide binding to Rac1 and Cdc42. The S-enantiomers can be docked but are less favorably positioned in proximity to the magnesium. R-naproxen and R-ketorolac have potential for rapid translation and efficacy in the treatment of several epithelial cancer types on account of established human toxicity profiles and novel activities against Rho-family GTPases. 
 Snail regulates the motility of oral cancer cells via RhoA/Cdc42/p-ERM pathway. The transcriptional factor Snail has been reported to possess properties related to cancer progression; however, the mechanism for it is not fully understood. Our data showed that Snail knockdown by small interfering RNA in two OSCC cell lines, WSU-HN6 and CAL27, significantly inhibited cell   migration   and invasion which also resulted in decreased cell motility, such as impaired cell spreading on type I collagen substrate, reduced filopodia, and premature assembly of stress fibers. In addition, Snail-silencing decreased Cdc42 activity but increased RhoA activity, accompanied by the downregulation in both p-ERM expression and cell motility. Meanwhile, endogenous p-ERM was found specifically co-precipitated with activated Cdc42, but not RhoA, and this co-association was decreased by Snail-silencing. The small molecule inhibitors of Rho-associated kinase (Y27632) markedly enhanced Cdc42 activity and the association of p-ERM with activated Cdc42, increasing cell motility remarkably. Using immunohistochemistry, Snail and p-ERM overexpressions were found in OSCC tissues correlated with nodal metastasis and shorter survival. Taken together, these results demonstrate that Snail regulates cell motility through RhoA/Cdc42/p-ERM pathway and may serve as a biomarker to predict prognosis for OSCC patients. Although RhoA and Cdc42 are concurrently regulated downstream of Snail, there is a direct interplay between them, which indicates RhoA has to be inactivated at some point in cell motility cycle. 
 Directional sensing requires G beta gamma-mediated PAK1 and PIX alpha-dependent activation of Cdc42. Efficient chemotaxis requires directional sensing and cell polarization. We describe a signaling mechanism involving G beta gamma, PAK-associated guanine nucleotide exchange factor (PIX alpha), Cdc42, and p21-activated kinase (PAK) 1. This pathway is utilized by chemoattractants to regulate directional sensing and directional   migration   of myeloid cells. Our results suggest that G beta gamma binds PAK1 and, via PAK-associated PIX alpha, activates Cdc42, which in turn activates PAK1. Thus, in this pathway, PAK1 is not only an effector for Cdc42, but it also functions as a scaffold protein required for Cdc42 activation. This G beta gamma-PAK1/PIX alpha/Cdc42 pathway is essential for the localization of F-actin formation to the leading edge, the exclusion of PTEN from the leading edge, directional sensing, and the persistent directional   migration   of chemotactic leukocytes. Although ligand-induced production of PIP(3) is not required for activation of this pathway, PIP(3) appears to localize the activation of Cdc42 by the pathway. 
 N-α-acetyltransferase 10 protein suppresses cancer cell metastasis by binding PIX proteins and inhibiting Cdc42/Rac1 activity. N-α-acetyltransferase 10 protein, Naa10p, is an N-acetyltransferase known to be involved in cell cycle control. We found that Naa10p was expressed lower in varieties of malignancies with lymph node metastasis compared with non-lymph node metastasis. Higher Naa10p expression correlates the survival of lung cancer patients. Naa10p significantly suppresse  d migrati  on, tumor growth, and metastasis independent of its enzymatic activity. Instead, Naa10p binds to the GIT-binding domain of PIX, thereby preventing the formation of the GIT-PIX-Paxillin complex, resulting in reduced intrinsic Cdc42/Rac1 activity and decreased cel  l migrati  on. Forced expression of PIX in Naa10-transfected tumor cells restored th  e migrati  on and metastasis ability. We suggest that Naa10p functions as a tumor metastasis suppressor by disrupting the migratory complex, PIX-GIT- Paxillin, in cancer cells. 
 Cdc42 promotes transendothelial   migration   of cancer cells through β1 integrin. Cancer cells interact with endothelial cells during the process of metastatic spreading. Here, we use a small interfering RNA screen targeting Rho GTPases in cancer cells to identify Cdc42 as a critical regulator of cancer cell-endothelial cell interactions and transendothelial   migratio  n. We find that Cdc42 regulates β1 integrin expression at the transcriptional level via the transcription factor serum response factor (SRF). β1 integrin is the main target for Cdc42-mediating interaction of cancer cells with endothelial cells and the underlying extracellular matrix, as exogenous β1 integrin expression was sufficient to rescue the Cdc42-silencing phenotype. We show that Cdc42 was required in vivo for cancer cell spreading and protrusion extension along blood vessels and retention in the lungs. Interestingly, transient Cdc42 depletion was sufficient to decrease experimental lung metastases, which suggests that its role in endothelial attachment is important for metastasis. By identifying β1 integrin as a transcriptional target of Cdc42, our results provide new insight into Cdc42 function. 
 [The Rho protein family and its role in the cellular cytoskeleton]. Proteins of the Rho protein family (RhoA, Rac1, Cdc42) work as molecular switches in the regulation of many cellular processes. They are involved in cell   migration  , cell-cycle control, apoptosis, and the regulation of gene transcription. Rho proteins show their activity mainly in the cell's cytoskeleton by taking part in the reorganization of microfilaments and microtubules. In recent years, significant progress has been made in understanding the biochemical and genetic nature of many processes controlled by Rho proteins. Although there are still seveal unknowns and hypothesizes which require confirmation, newer and more precise experimental results allow us to better understand these processes. It has also been suggested to use Rho family proteins in the therapy of some diseases. The purpose of this study was to characterize the significance of Rho processes in the cellular cytoskeleton. 
 Immunohistochemical expression of Rho GTPases in ameloblastomas. Rho GTPases are proteins that regulate cell cycle, shape, polarization, invasion,   migration  , and apoptosis, which are important characteristics of normal and neoplastic cells. Rho GTPases expression has been reported in normal tooth germ and several pathologies; however, it has not been evaluated in ameloblastomas. The aim of this study was to analyze the expression and distribution of RhoA, RhoB, Rac1, and Cdc42 Rho GTPases in solid and unicystic ameloblastomas. Three-micrometer sections from paraffin-embedded specimens were evaluated by using an avidin-biotin immunohistochemical method with antibodies against the proteins mentioned above. RhoA and RhoB staining was observed in a high number of cells (P < 0.05) and greater intensity in non-polarized ones. Rac1 was not observed, and Cdc42 did not show any statistical differences between the number of non-polarized and basal positive cells (P > 0.05). Upon comparing the studied ameloblastomas, a higher number of positive cells in the unicystic variant was observed than that in the solid one (P < 0,05). The results obtained suggest that these GTPases could play a role in the ameloblastoma neoplastic epithelial cell phenotype determination (polarized or non-polarized), as well as in variant (solid or unicystic) and subtype (follicular or plexiform) determination. Furthermore, they could participate in solid ameloblastoma invasion mechanisms. 
 FAT1 mutations cause a glomerulotubular nephropathy. Steroid-resistant nephrotic syndrome (SRNS) causes 15% of chronic kidney disease (CKD). Here we show that recessive mutations in FAT1 cause a distinct renal disease entity in four families with a combination of SRNS, tubular ectasia, haematuria and facultative neurological involvement. Loss of FAT1 results in decreased cell adhesion and   migration   in fibroblasts and podocytes and the decreased   migration   is partially reversed by a RAC1/CDC42 activator. Podocyte-specific deletion of Fat1 in mice induces abnormal glomerular filtration barrier development, leading to podocyte foot process effacement. Knockdown of Fat1 in renal tubular cells reduces   migration  , decreases active RAC1 and CDC42, and induces defects in lumen formation. Knockdown of fat1 in zebrafish causes pronephric cysts, which is partially rescued by RAC1/CDC42 activators, confirming a role of the two small GTPases in the pathogenesis. These findings provide new insights into the pathogenesis of SRNS and tubulopathy, linking FAT1 and RAC1/CDC42 to podocyte and tubular cell function. 
 Regulation of ephexin1, a guanine nucleotide exchange factor of Rho family GTPases, by fibroblast growth factor receptor-mediated tyrosine phosphorylation. Fibroblast growth factor (FGF) signal is implicated in not only cell proliferation, but cell   migration   and morphological changes. Several different Rho family GTPases downstream of the Ras/ERK pathway are postulated to mediate the latter functions. However, none have been recognized to be directly coupled to FGF receptors (FGFRs). We have previously reported that EphA4 and FGFRs hetero-oligomerize through their cytoplasmic domains, trans-activate each other, and transduce a signal for cell proliferation through a docking protein, FRS2alpha (Yokote, H., Fujita, K., Jing, X., Sawada, T., Liang, S., Yao, L., Yan, X., Zhang, Y., Schlessinger, J., and Sakaguchi, K. (2005) Proc. Natl. Acad. Sci. U. S. A. 102, 18866-18871). Here, we have found that ephexin1, a guanine nucleotide exchange factor for Rho family GTPases, constitutes another downstream component of the receptor complex. Ephexin1 directly binds to the kinase domain of FGFR mainly through its DH and PH domains. The binding appears to become weaker and limited to the DH domain when FGFRs become activated. FGFR-mediated phosphorylation of ephexin1 enhances the guanine nucleotide exchange activity toward RhoA without affecting the activity to Rac1 or Cdc42. The FGFR-mediated tyrosine phosphorylation includes, but is not limited to, the residue (Tyr-87) phosphorylated by Src family kinase, which is known to be activated following EphA4 activation. The Tyr-to-Asp mutations that mimic the tyrosine phosphorylation in some of the putative FGFR-mediated phosphorylation sites increase the nucleotide exchange activity for RhoA without changing the activity for Rac1 or Cdc42. From these results, we conclude that ephexin1 is located immediately downstream of the EphA4-FGFR complex and the function is altered by the FGFR-mediated tyrosine phosphorylation at multiple sites. 
 Invasion of breast cancer cells into collagen matrix requires TGF-α and Cdc42 signaling. Invasion of MDA-MB-231 breast cancer cells into three-dimensional (3-D) type I-collagen matrices depends on TGF-α. We characterized the steps of invasion mediated by TGF-α. Ce  ll migrat  ion, as observed by videomicroscopy, was effectively stimulated by collagen, suggesting that TGF-α may specifically participate in the invasion of a 3-D collagen matrix. We assessed the role of small GTPases of the Rho family in the invasion. Cdc42 was found to be necessary for invasion but dispensable for c  ell migra  tion. These results suggest that TGF-α mediates invasion into 3-D collagen matrices by initiating the formation of protrusions into collagen, likely through activation of Cdc42. 
 RhoG activates Rac1 by direct interaction with the Dock180-binding protein Elmo. The small GTPase Rac has a central role in regulating the actin cytoskeleton during cell   migration   and axon guidance. Elmo has been identified as an upstream regulator of Rac1 that binds to and functionally cooperates with Dock180 (refs 2-4). Dock180 does not contain a conventional catalytic domain for guanine nucleotide exchange on Rac, but possesses a domain that directly binds to and specifically activates Rac1 (refs 5, 6). The small GTPase RhoG mediates several cellular morphological processes, such as neurite outgrowth in neuronal cells, through a signalling cascade that activates Rac1 (refs 7-12); however, the downstream target of RhoG and the mechanism by which RhoG regulates Rac1 activity remain unclear. Here we show that RhoG interacts directly with Elmo in a GTP-dependent manner and forms a ternary complex with Dock180 to induce activation of Rac1. The RhoG-Elmo-Dock180 pathway is required for activation of Rac1 and cell spreading mediated by integrin, as well as for neurite outgrowth induced by nerve growth factor. We conclude that RhoG activates Rac1 through Elmo and Dock180 to control cell morphology. 
 Coronin-1C and RCC2 guide mesenchymal   migration   by trafficking Rac1 and controlling GEF exposure. Sustained forward   migration   through a fibrillar extracellular matrix requires localization of protrusive signals. Contact with fibronectin at the tip of a cell protrusion activates Rac1, and for linear   migration   it is necessary to dampen Rac1 activity in off-axial positions and redistribute Rac1 from non-protrusive membrane to the leading edge. Here, we identify interactions between coronin-1C (Coro1C), RCC2 and Rac1 that focus active Rac1 to a single protrusion. Coro1C mediates release of inactive Rac1 from non-protrusive membrane and is necessary for Rac1 redistribution to a protrusive tip and fibronectin-dependent Rac1 activation. The second component, RCC2, attenuates Rac1 activation outside the protrusive tip by binding to the Rac1 switch regions and competitively inhibiting GEF action, thus preventing off-axial protrusion. Depletion of Coro1C or RCC2 by RNA interference causes loss of cell polarity that results in shunting   migration   in 1D or 3D culture systems. Furthermore, morpholinos against Coro1C or RCC2, or mutation of any of the binding sites in the Rac1-RCC2-Coro1C complex delays the arrival of neural crest derivatives at the correct location in developing zebrafish, demonstrating the crucial role in   migration   guidance in vivo. 
 Myristoylated Alanine-Rich Protein Kinase Substrate (MARCKS) Regulates Small GTPase Rac1 and Cdc42 Activity and Is a Critical Mediator of Vascular Smooth Muscle Cell   Migration   in Intimal Hyperplasia Formation. Transcription of the myristoylated alanine-rich C kinase substrate (MARCKS) is upregulated in animal models of intimal hyperplasia. MARCKS knockdown inhibits vascular smooth muscle cell (VSMC)   migration   in vitro; however, the mechanism is as yet unknown. We sought to elucidate the mechanism of MARCKS-mediated motility and determine whether MARCKS knockdown reduces intimal hyperplasia formation in vivo.MARCKS knockdown blocked platelet-derived growth factor (PDGF)-induced translocation of cortactin to the cell cortex, impaired both lamellipodia and filopodia formation, and attenuated motility of human coronary artery smooth muscle cells (CASMCs). Activation of the small GTPases, Rac1 and Cdc42, was prevented by MARCKS knockdown. Phosphorylation of MARCKS resulted in a transient shift of MARCKS from the plasma membrane to the cytosol. MARCKS knockdown significantly decreased membrane-associated phosphatidylinositol 4,5-bisphosphate (PIP2) levels. Cotransfection with an intact, unphosphorylated MARCKS, which has a high binding affinity for PIP2, restored membrane-associated PIP2 levels and was indispensable for activation of Rac1 and Cdc42 and, ultimately, VSMC   migration  . Overexpression of MARCKS in differentiated VSMCs increased membrane PIP2 abundance, Rac1 and Cdc42 activity, and cell motility. MARCKS protein was upregulated early in the development of intimal hyperplasia in the murine carotid ligation model. Decreased MARKCS expression, but not total knockdown, attenuated intimal hyperplasia formation.MARCKS upregulation increases VSMC motility by activation of Rac1 and Cdc42. These effects are mediated by MARCKS sequestering PIP2 at the plasma membrane. This study delineates a novel mechanism for MARCKS-mediated VSMC   migration   and supports the rational for MARCKS knockdown to prevent intimal hyperplasia. 
 Analysis of the myosin-II-responsive focal adhesion proteome reveals a role for β-Pix in negative regulation of focal adhesion maturation. Focal adhesions undergo myosin-II-mediated maturation wherein they grow and change composition to modulate integrin signalling for cell   migratio  n, growth and differentiation. To determine how focal adhesion composition is affected by myosin II activity, we performed proteomic analysis of isolated focal adhesions and compared protein abundance in focal adhesions from cells with and without myosin II inhibition. We identified 905 focal adhesion proteins, 459 of which changed in abundance with myosin II inhibition, defining the myosin-II-responsive focal adhesion proteome. The abundance of 73% of the proteins in the myosin-II-responsive focal adhesion proteome was enhanced by contractility, including proteins involved in Rho-mediated focal adhesion maturation and endocytosis- and calpain-dependent focal adhesion disassembly. During myosin II inhibition, 27% of proteins in the myosin-II-responsive focal adhesion proteome, including proteins involved in Rac-mediated lamellipodial protrusion, were enriched in focal adhesions, establishing that focal adhesion protein recruitment is also negatively regulated by contractility. We focused on the Rac guanine nucleotide exchange factor β-Pix, documenting its role in the negative regulation of focal adhesion maturation and the promotion of lamellipodial protrusion and focal adhesion turnover to drive cel  l migrati  on. 
 Formyl peptide receptor-1 activation enhances intestinal epithelial cell restitution through phosphatidylinositol 3-kinase-dependent activation of Rac1 and Cdc42. Inflammatory disorders of the gastrointestinal tract result in the breakdown of the intestinal epithelial barrier in the form of erosion and ulceration. To reestablish the epithelial barrier, the epithelium must efficiently migrate to reseal wounds. Numerous signaling cascades are involved in the induction and regulation of this complex process. N-formyl peptide receptors comprise a group of Gi-coupled receptors that regulate innate immune responses. Previously, we identified the expression of functional N-formyl peptide receptors in model SK-CO15 intestinal epithelial cells and observed a role for activation of these receptors in regulating cellular invasive behavior. In these studies, we performed formyl peptide receptor-1 (FPR) localization and evaluated its role in regulating intestinal epithelial cell wound closure. Immunolocalization studies using a recently developed specific monoclonal anti-FPR Ab demonstrated its localization along the lateral membrane of crypt epithelial cells in normal human colonic epithelium. In vitro studies using the classical FPR agonist fMLF showed that FPR activation significantly enhances model intestinal epithelial cell restitution and that FPR localized along actin filaments in lamellipodial and filopodial extrusions. The increase in cell   migration   was associated with activation of PI3K, Rac1, and Cdc42. Pharmacologic inhibition of PI3K activity abrogated the fMLF-induced increase in wound closure and activation of both Rac1 and Cdc42. Inhibition of Rac1 and Cdc42 using pharmacologic inhibitors and dominant negative mutants also inhibited the fMLF-induced increase in cell   migration  . Taken together, theses results support a novel role for FPR stimulation in enhancing intestinal epithelial cell restitution through PI3K-dependent activation of Rac1 and Cdc42. 
 Down expression of LRP1B promotes cell   migration   via RhoA/Cdc42 pathway and actin cytoskeleton remodeling in renal cell cancer. The low-density lipoprotein receptor-related protein 1B (LRP1B) is known as a putative tumor suppressor. The decreased expression of LRP1B has been involved in multiple primary cancers in several studies. However, its expression and function in the carcinogenesis of renal cell cancer (RCC) remain unclear. In this study, we investigated the expression of LRP1B in RCC by in situ hybridization (ISH) and real-time polymerase chain reaction (qRT-PCR). Our results indicated that LRP1B was frequently downexpressed in human RCC tissue and cell lines, which involved both epigenetic events (DNA methylation and histone deacetylation) and N-terminal deletion of LRP1B. Moreover, we testified that knockdown of LRP1B by shRNA significantly promoted anchorage-independent growth, cell   migration   and invasion in HEK293 cells and renal cancer cells 127 in vitro. We further found that silencing of LRP1B altered the expression of focal adhesion complex-associated proteins, and Cdc42/RhoA activities, which regulate the cytoskeleton dynamics. Taken together, these results strongly support that LRP1B may function as a tumor suppressor against renal cell cancer, and may regulate cell motility via RhoA/Cdc42 pathway and actin cytoskeleton reorganization in RCC. 
 The orphan adhesion G protein-coupled receptor GPR97 regulates   migration   of lymphatic endothelial cells via the small GTPases RhoA and Cdc42. The important role of the lymphatic vascular system in pathological conditions such as inflammation and cancer has been increasingly recognized, but its potential as a pharmacological target is poorly exploited. Our study aimed at the identification and molecular characterization of lymphatic-specific G protein-coupled receptors (GPCRs) to assess new targets for pharmacological manipulation of the lymphatic vascular system. We used a TaqMan quantitative RT-PCR-based low density array to determine the GPCR expression profiles of ex vivo isolated intestinal mouse lymphatic (LECs) and blood vascular endothelial cells (BECs). GPR97, an orphan adhesion GPCR of unknown function, was the most highly and specifically expressed GPCR in mouse lymphatic endothelium. Using siRNA silencing, we found that GPR97-deficient primary human LECs displayed increased adhesion and collective cell   migration  , whereas single cell   migration   was decreased as compared with nontargeting siRNA-transfected control LECs. Loss of GPR97 shifted the ratio of active Cdc42 and RhoA and initiated cytoskeletal rearrangements, including F-actin redistribution, paxillin and PAK4 phosphorylation, and β1-integrin activation. Our data suggest a possible role of GPR97 in lymphatic remodeling and furthermore provide the first insights into the biological functions of GPR97. 
 A Rac/Cdc42-specific exchange factor, GEFT, induces cell proliferation, transformation, and   migration  . The Rho family of small GTPases, including Rho, Rac, and Cdc42, play essential roles in diverse cellular functions. The ability of Rho family GTPases to participate in signaling events is determined by the ratio of inactive (GDP-bound) and active (GTP-bound) forms in the cell. The activation of Rho family proteins requires the exchange of bound GDP for GTP, a process catalyzed by the Dbl family of guanine nucleotide exchange factors (GEFs). The GEFs have high affinity for the guanine nucleotide-free state of the GTPases and are thought to promote GDP release by stabilizing an intermediate transition state. In this study, we have identified and characterized a new Rac/Cdc42-specific Dbl family guanine nucleotide exchange factor, named GEFT. GEFT is highly expressed in the excitable tissues, including brain, heart, and muscle. Low or very little expression was detected in other nonexcitable tissues. GEFT has specific exchange activity for Rac and Cdc42 in our in vitro GTPase exchange assays and glutathione S-transferase-PAK pull-down assays with GTP-bound Rac1 and Cdc42. Overexpression of GEFT leads to changes in cell morphology and actin cytoskeleton re-organization, including the formation of membrane microspikes, filopodia, and lamilliopodia. Furthermore, expression of GEFT in NIH3T3 cells promotes foci formation, cell proliferation, and cell   migration  , possibly through the activation of transcriptional factors involved in cell growth and proliferation. Together, our data suggest that GEFT is a Rac/Cdc42-specific GEF protein that regulates cell morphology, cell proliferation, and transformation. 
 Human neutrophils utilize a Rac/Cdc42-dependent MAPK pathway to direct intracellular granule mobilization toward ingested microbial pathogens. Elevated levels of mitogen-activated protein kinase/extracellular regulatory kinase (MAPK/ERK) activity are frequently found in some cancer cells. In efforts to reduce tumor growth, attempts have been made to develop cancer therapeutic agents targeting the MAPK. Here, by use of biologic, biochemical, and gene manipulation methods in human polymorphonuclear neutrophils (PMNs), we have identified a key pathway important in normal cell function involving MAPK/ERK in PMNs for growth inhibition of Candida albicans. Contact with C albicans triggered MAPK/ERK activation in PMNs within 5 minutes, and blocking of MAPK/ERK activation, either by the pharmacologic reagent PD098059 or by dominant-negative MAPK kinase (MEK) expression via vaccinia viral delivery, suppressed antimicrobial activity. Rac and Cdc42, but not Ras or Rho, were responsible for this MAPK/ERK activation. Expression of dominant-negative Rac (N17Rac) or Cdc42 (N17Cdc42) eliminated not only C albicans- mediated ERK phosphorylation but also phagocytosis and granule   migration   toward the ingested microbes, whereas dominant-negative Ras (N17Ras) and Rho (N19Rho) did not. PAK1 (p21-activated kinase 1) activation is induced by C albicans, suggesting that PAK1 may also be involved in the Rac1 activation of MAPK/ERK. We conclude from these data that Rac/Cdc42-dependent activation of MAPK/ERK is a critical event in the immediate phagocytic response of PMNs to microbial challenge. Therefore, use of MAPK pharmacologic inhibitors for the treatment of cancer may result in the interruption of normal neutrophil function. A balance between therapeutic outcome and undesirable side effects must be attained to achieve successful and safe anticancer therapy. 
 Regulation of PTEN by Rho small GTPases. PTEN (phosphatase and tensin homologue) is a phosphatase that dephosphorylates both protein and phosphoinositide substrates. It is mutated in a variety of human tumours and has important roles in a diverse range of biological processes, including cell   migration   and chemotaxis. PTEN's intracellular localization and presumably activity are regulated by chemoattractants in Dictyostelium and mouse neutrophils. However, the mechanisms for its regulation remain elusive. Here we show that RhoA and Cdc42, members of the Rho family of small GTPases, regulate the intracellular localization of PTEN in leukocytes and human transfected embryonic kidney cells. In addition, active RhoA is able to stimulate the phospholipid phosphatase activity of PTEN in human embryonic kidney cells and leukocytes, and this regulation seems to require RhoA's downstream effector, RhoA-associated kinase (Rock). Furthermore, we have identified key residues on PTEN that are required for its regulation by the small GTPase, and show that small GTPase-mediated regulation of PTEN has a significant role in the regulation of chemotaxis. 
 Interaction of the RAGE cytoplasmic domain with diaphanous-1 is required for ligand-stimulated cellular   migration   through activation of Rac1 and Cdc42. Cellular   migration   is a fundamental process linked to diverse pathological states such as diabetes and its complications, atherosclerosis, inflammation, and cancer. The receptor for advanced glycation end products (RAGE) is a multiligand cell surface macromolecule which binds distinct ligands that accumulate in these settings. RAGE-ligand interaction evokes central changes in key biological properties of cells, including proliferation, generation of inflammatory mediators, and   migration  . Although RAGE-dependent signal transduction is critically dependent on its short cytoplasmic domain, to date the proximate mechanism by which this RAGE domain engages and stimulates cytoplasmic signaling pathways has yet to be identified. Here we show that the RAGE cytoplasmic domain interacts with Diaphanous-1 (Dia-1) both in vitro and in vivo. We employed the human RAGE cytoplasmic domain as "bait" in the yeast two-hybrid assay and identified the formin homology (FH1) domain of Dia-1 as a potential binding partner of this RAGE domain. Immunoprecipitation studies revealed that the RAGE cytoplasmic domain interacts with the FH1 domain of Dia-1. Down-regulation of Dia-1 expression by RNA interference blocks RAGE-mediated activation of Rac-1 and Cdc42 and, in parallel, RAGE ligand-stimulated cellular   migration  . Taken together, these findings indicate that the interaction of the RAGE cytoplasmic domain with Dia-1 is required to transduce extracellular environmental cues evoked by binding of RAGE ligands to their cell surface receptor, a chief consequence of which is Rac-1 and Cdc42 activation and cellular   migration  . Because RAGE and Dia-1 are implicated in the regulation of inflammatory, vascular, and transformed cell   migration  , these findings highlight this interaction as a novel target for therapeutic intervention in inflammation, atherosclerosis, diabetes, and cancer. 
 Macrophages stimulate gastric and colorectal cancer invasion through EGFR Y(1086), c-Src, Erk1/2 and Akt phosphorylation and smallGTPase activity. The interactions between cancer cells and their microenvironment are crucial for malignant progression, as they modulate invasion-related activities. Tumor-associated macrophages are generally considered allies in the process of tumor progression in several types of cancer, although their role on gastric and colorectal carcinomas is still poorly understood. In this report, we studied the influence of primary human macrophages on gastric and colorectal cancer cells, considering invasion, motility/  migration  , proteolysis and activated intracellular signaling pathways. We demonstrated that macrophages stimulate cancer cell invasion, motility and   migration  , and that these effects depend on matrix metalloproteinase (MMP) activity and on the activation of epidermal growth factor receptor (EGFR) (at the residue Y(1086)), PLC-γ (phospholipase C-gamma) and Gab1 (GRB2-associated binding protein-1), as evidenced by siRNA (small interference RNA) experiments. Epidermal growth factor (EGF)-immunodepletion impaired macrophage-mediated cancer cell invasion and motility, suggesting that EGF is the pro-invasive and pro-motile factor produced by macrophages. Macrophages also induced gastric and colorectal cancer cell phosphorylation of Akt, c-Src and ERK1/2, and led to an increase of RhoA and Cdc42 activity. Interestingly, whereas macrophage-mediated cancer cell c-Src and ERK1/2 phosphorylation occurred downstream EGFR activation, Akt phosphorylation seems to be a parallel event, taking place in an EGFR-independent manner. The involvement of EGF, EGFR-downstream signaling partners and MMPs in macrophage-mediated invasion provides novel insights into the molecular crosstalk established between cancer cells and macrophages, opening new perspectives for the design of new and more efficient therapeutic strategies to counteract cancer cell invasion. 
 Extension, retraction and contraction in the formation of a dendritic cell dendrite: distinct roles for Rho GTPases. The morphology of antigen-presenting dendritic cells (DC) is characterized by the possession of numerous long arborizing processes known as dendrites. The formation of these processes by DC, both in the periphery and in lymphoid organs, is believed to contribute to the remarkable efficiency with which they take up, process and present antigen to T cells. However, the process of dendrite formation and the signaling pathways that lead to the formation of these dendrites remain obscure. In this study we describe an in vitro model in which human immature DC form long processes similar to those formed in vivo. The formation of these processes involves initial attachment of a cell protrusion to the extracellular matrix substrate, and subsequent movement of the cell body away from the adhesion site, leaving behind a long slender dendrite. Dendrite formation, but not their maintenance, was found to be dependent on the activity of Rho GTPases. More specifically, Cdc42 and Rac1 were both required for the   migration   step of process formation, promoting cell spreading and extension. In contrast, Rho, and its downstream effector p160ROCK, regulated the release of adhesions to the substratum, and associated cellular contraction. Consequently, inhibition of Rho/p160ROCK leads to the formation of longer dendrites. DC therefore coordinate adhesion and protrusion to perform a specialized process of cellular morphogenesis, which differentiates these cells from all other cells of the immune system and may contribute to their distinctive function. 
 Adiponectin promotes   migration   activities of endothelial progenitor cells via Cdc42/Rac1. Adiponectin has anti-atherosclerotic effects through its direct actions on vascular cells. The present study investigates the molecular mechanisms of adiponectin in the   migration   of endothelial progenitor cells (EPCs) which play an important role in neovascularization and re-endothelization. The phosphorylation of Akt and the activations of Cdc42 and Rac1 were significantly increased by adiponectin. Adiponectin increased the   migration   activity of EPCs, which was completely inhibited by a PI3-kinase inhibitor. siRNA of Cdc42 or Rac1 completely inhibited the adiponectin-induced   migration  , but siRNA of Akt had no effects, indicating that adiponectin promotes the   migration   activities of EPCs mainly through PI3-kinase/Cdc42/Rac1. 
 Maspin controls mammary tumor cell   migration   through inhibiting Rac1 and Cdc42, but not the RhoA GTPase. Rac1 and Cdc42 are members of the Rho family of small GTPases that play essential roles in diverse cellular functions, including cell   migration  . The activities of these Rho family proteins are controlled by growth factor receptor activation and cell-ECM interactions. Here, we show that maspin, a well-documented tumor suppressor gene, also controls cell motility through inhibiting Rac1/Cdc42 activity. Using the GST-PAK and GST-Rho binding protein pull-down assays for GTP-bound Rac1, Cdc42, and RhoA, we showed that treatment of MDA-MB-231 tumor cells with recombinant maspin for a short time period significantly inhibited the activity of Rac1 and Cdc42, but not RhoA. The reactive site loop (RSL) within maspin protein is the functional domain involved in the inhibition. Maspin mutants with the RSL deleted or a point mutation in the RSL region lost their inhibitory activity. We further examined the ability of maspin to inhibit Rac1- and Cdc42-mediated signaling pathways and transcription factors. Treatment of MDA-MB-231 cells with maspin led to the inhibition of JNK kinase activity as assayed by immuno-kinase assays. In addition, the AP-1 transcription activity downstream of JNK kinase pathway was also reduced. Together, we have identified Rac1 and Cdc42 as the downstream targets that mediate the inhibition of mammary tumor cell   migration   by maspin. 
 Mammalian target of rapamycin and Rictor control neutrophil chemotaxis by regulating Rac/Cdc42 activity and the actin cytoskeleton. Chemotaxis allows neutrophils to seek out sites of infection and inflammation. The asymmetric accumulation of filamentous actin (F-actin) at the leading edge provides the driving force for protrusion and is essential for the development and maintenance of neutrophil polarity. The mechanism that governs actin cytoskeleton dynamics and assembly in neutrophils has been extensively explored and is still not fully understood. By using neutrophil-like HL-60 cells, we describe a pivotal role for Rictor, a component of mammalian target of rapamycin complex 2 (mTORC2), in regulating assembly of the actin cytoskeleton during neutrophil chemotaxis. Depletion of mTOR and Rictor, but not Raptor, impairs actin polymerization, leading-edge establishment, and directional   migration   in neutrophils stimulated with chemoattractants. Of interest, depletion of mSin1, an integral component of mTORC2, causes no detectable defects in neutrophil polarity and chemotaxis. In addition, experiments with chemical inhibition and kinase-dead mutants indicate that mTOR kinase activity and AKT phosphorylation are dispensable for chemotaxis. Instead, our results suggest that the small Rho GTPases Rac and Cdc42 serve as downstream effectors of Rictor to regulate actin assembly and organization in neutrophils. Together our findings reveal an mTORC2- and mTOR kinase-independent function and mechanism of Rictor in the regulation of neutrophil chemotaxis. 
 Requirements for F-BAR proteins TOCA-1 and TOCA-2 in actin dynamics and membrane trafficking during Caenorhabditis elegans oocyte growth and embryonic epidermal morphogenesis. The TOCA family of F-BAR-containing proteins bind to and remodel lipid bilayers via their conserved F-BAR domains, and regulate actin dynamics via their N-Wasp binding SH3 domains. Thus, these proteins are predicted to play a pivotal role in coordinating membrane traffic with actin dynamics during cell   migration   and tissue morphogenesis. By combining genetic analysis in Caenorhabditis elegans with cellular biochemical experiments in mammalian cells, we showed that: i) loss of CeTOCA proteins reduced the efficiency of Clathrin-mediated endocytosis (CME) in oocytes. Genetic interference with CeTOCAs interacting proteins WSP-1 and WVE-1, and other components of the WVE-1 complex, produced a similar effect. Oocyte endocytosis defects correlated well with reduced egg production in these mutants. ii) CeTOCA proteins localize to cell-cell junctions and are required for proper embryonic morphogenesis, to position hypodermal cells and to organize junctional actin and the junction-associated protein AJM-1. iii) Double mutant analysis indicated that the toca genes act in the same pathway as the nematode homologue of N-WASP/WASP, wsp-1. Furthermore, mammalian TOCA-1 and C. elegans CeTOCAs physically associated with N-WASP and WSP-1 directly, or WAVE2 indirectly via ABI-1. Thus, we propose that TOCA proteins control tissues morphogenesis by coordinating Clathrin-dependent membrane trafficking with WAVE and N-WASP-dependent actin-dynamics. 
 Overexpression of small GTPases directly correlates with expression of δ-catenin and their coexpression predicts a poor clinical outcome in nonsmall cell lung cancer. δ-catenin can affect cytoskeletal assembly, and promote cel  l migrati  on by regulating the activity of small GTPases. While many malignancies have been shown to be positive for δ-catenin, it is still unclear whether δ-catenin and small GTPases are coexpressed in tumor cells, and so is the relationship between their coexpression and prognosis in the tumor patients. In this study, immunohistochemistry was performed to examine expressive levels of δ-catenin, cdc42, and Rac1 in 135 cases of nonsmall cell lung cancer (NSCLC), including 60 cases with follow-up records. Thirty samples of paired lung cancer tissues and adjacent normal lung tissues were collected to analyze mRNA and protein expression of δ-catenin and small GTPases. The effects of δ-catenin on small GTPases expression and invasive ability of lung cancer cells were also evaluated. Compared with normal lung tissues, both mRNA and protein levels of δ-catenin and small GTPases were increased in lung cancer tissues (P < 0.05), and the expression of small GTPases directly correlated with that of δ-catenin (P < 0.001). In addition, δ-catenin and small GTPases tended to be coexpressed in lung adenocarcinoma, advanced stages, and primary tumors with lymph node metastasis (all P < 0.05). The patients with coexpression of δ-catenin and small GTPases had a shorter survival time than those without coexpression (P < 0.05). Furthermore, δ-catenin overexpression could enhance invasive ability of lung cancer cells by upregulating protein and transcriptional level of small GTPases. Therefore, δ-catenin likely upregulates the activity of small GTPases at transcriptional level, and their coexpression may predict a poor clinical outcome in NSCLC patients. 
 A Novel Pharmacologic Activity of Ketorolac for Therapeutic Benefit in Ovarian Cancer Patients. We previously identified the R-enantiomer of ketorolac as an inhibitor of the Rho-family GTPases Rac1 and Cdc42. Rac1 and Cdc42 regulate cancer-relevant functions, including cytoskeleton remodeling necessary for tumor cell adhesion and   migration  . This study investigated whether administration of racemic (R,S) ketorolac after ovarian cancer surgery leads to peritoneal distribution of R-ketorolac, target GTPase inhibition in cells retrieved from the peritoneal cavity, and measureable impact on patient outcomes.Eligible patients had suspected advanced-stage ovarian, fallopian tube or primary peritoneal cancer. Secondary eligibility was met when ovarian cancer was confirmed and optimally debulked, an intraperitoneal port was placed, and there were no contraindications for ketorolac administration. R- and S-ketorolac were measured in serum and peritoneal fluid, and GTPase activity was measured in peritoneal cells. A retrospective study correlated perioperative ketorolac and ovarian cancer-specific survival in ovarian cancer cases.Elevated expression and activity of Rac1 and Cdc42 was detected in ovarian cancer patient tissues, confirming target relevance. Ketorolac in peritoneal fluids was enriched in the R-enantiomer and peritoneal cell GTPase activity was inhibited after ketorolac administration when R-ketorolac was at peak levels. After adjusting for age, AJCC stage, completion of chemotherapy, and neoadjuvant therapy, women given perioperative ketorolac had a lower hazard of death (HR, 0.30; 95% confidence interval, 0.11-0.88).Ketorolac has a novel pharmacologic activity conferred by the R-enantiomer and R-ketorolac achieves sufficient levels in the peritoneal cavity to inhibit Rac1 and Cdc42, potentially contributing to the observed survival benefit in women who received ketorolac. 
 Distinct predictive performance of Rac1 and Cdc42 in cell   migration  . We propose a new computation-based approach for elucidating how signaling molecules are decoded in cell   migration  . In this approach, we performed FRET time-lapse imaging of Rac1 and Cdc42, members of Rho GTPases which are responsible for cell motility, and quantitatively identified the response functions that describe the conversion from the molecular activities to the morphological changes. Based on the identified response functions, we clarified the profiles of how the morphology spatiotemporally changes in response to local and transient activation of Rac1 and Cdc42, and found that Rac1 and Cdc42 activation triggers laterally propagating membrane protrusion. The response functions were also endowed with property of differentiator, which is beneficial for maintaining sensitivity under adaptation to the mean level of input. Using the response function, we could predict the morphological change from molecular activity, and its predictive performance provides a new quantitative measure of how much the Rho GTPases participate in the cell   migration  . Interestingly, we discovered distinct predictive performance of Rac1 and Cdc42 depending on the   migration   modes, indicating that Rac1 and Cdc42 contribute to persistent and random   migration  , respectively. Thus, our proposed predictive approach enabled us to uncover the hidden information processing rules of Rho GTPases in the cell   migration  . 
 A Novel Pharmacologic Activity of Ketorolac for Therapeutic Benefit in Ovarian Cancer Patients. We previously identified the R-enantiomer of ketorolac as an inhibitor of the Rho-family GTPases Rac1 and Cdc42. Rac1 and Cdc42 regulate cancer-relevant functions, including cytoskeleton remodeling necessary for tumor cell adhesion and   migration  . This study investigated whether administration of racemic (R,S) ketorolac after ovarian cancer surgery leads to peritoneal distribution of R-ketorolac, target GTPase inhibition in cells retrieved from the peritoneal cavity, and measureable impact on patient outcomes.Eligible patients had suspected advanced-stage ovarian, fallopian tube or primary peritoneal cancer. Secondary eligibility was met when ovarian cancer was confirmed and optimally debulked, an intraperitoneal port was placed, and there were no contraindications for ketorolac administration. R- and S-ketorolac were measured in serum and peritoneal fluid, and GTPase activity was measured in peritoneal cells. A retrospective study correlated perioperative ketorolac and ovarian cancer-specific survival in ovarian cancer cases.Elevated expression and activity of Rac1 and Cdc42 was detected in ovarian cancer patient tissues, confirming target relevance. Ketorolac in peritoneal fluids was enriched in the R-enantiomer and peritoneal cell GTPase activity was inhibited after ketorolac administration when R-ketorolac was at peak levels. After adjusting for age, AJCC stage, completion of chemotherapy, and neoadjuvant therapy, women given perioperative ketorolac had a lower hazard of death (HR, 0.30; 95% confidence interval, 0.11-0.88).Ketorolac has a novel pharmacologic activity conferred by the R-enantiomer and R-ketorolac achieves sufficient levels in the peritoneal cavity to inhibit Rac1 and Cdc42, potentially contributing to the observed survival benefit in women who received ketorolac. 
 Myristoylated Alanine-Rich Protein Kinase Substrate (MARCKS) Regulates Small GTPase Rac1 and Cdc42 Activity and Is a Critical Mediator of Vascular Smooth Muscle Cell   Migration   in Intimal Hyperplasia Formation. Transcription of the myristoylated alanine-rich C kinase substrate (MARCKS) is upregulated in animal models of intimal hyperplasia. MARCKS knockdown inhibits vascular smooth muscle cell (VSMC)   migration   in vitro; however, the mechanism is as yet unknown. We sought to elucidate the mechanism of MARCKS-mediated motility and determine whether MARCKS knockdown reduces intimal hyperplasia formation in vivo.MARCKS knockdown blocked platelet-derived growth factor (PDGF)-induced translocation of cortactin to the cell cortex, impaired both lamellipodia and filopodia formation, and attenuated motility of human coronary artery smooth muscle cells (CASMCs). Activation of the small GTPases, Rac1 and Cdc42, was prevented by MARCKS knockdown. Phosphorylation of MARCKS resulted in a transient shift of MARCKS from the plasma membrane to the cytosol. MARCKS knockdown significantly decreased membrane-associated phosphatidylinositol 4,5-bisphosphate (PIP2) levels. Cotransfection with an intact, unphosphorylated MARCKS, which has a high binding affinity for PIP2, restored membrane-associated PIP2 levels and was indispensable for activation of Rac1 and Cdc42 and, ultimately, VSMC   migration  . Overexpression of MARCKS in differentiated VSMCs increased membrane PIP2 abundance, Rac1 and Cdc42 activity, and cell motility. MARCKS protein was upregulated early in the development of intimal hyperplasia in the murine carotid ligation model. Decreased MARKCS expression, but not total knockdown, attenuated intimal hyperplasia formation.MARCKS upregulation increases VSMC motility by activation of Rac1 and Cdc42. These effects are mediated by MARCKS sequestering PIP2 at the plasma membrane. This study delineates a novel mechanism for MARCKS-mediated VSMC   migration   and supports the rational for MARCKS knockdown to prevent intimal hyperplasia. 
 FAT1 mutations cause a glomerulotubular nephropathy. Steroid-resistant nephrotic syndrome (SRNS) causes 15% of chronic kidney disease (CKD). Here we show that recessive mutations in FAT1 cause a distinct renal disease entity in four families with a combination of SRNS, tubular ectasia, haematuria and facultative neurological involvement. Loss of FAT1 results in decreased cell adhesion and   migration   in fibroblasts and podocytes and the decreased   migration   is partially reversed by a RAC1/CDC42 activator. Podocyte-specific deletion of Fat1 in mice induces abnormal glomerular filtration barrier development, leading to podocyte foot process effacement. Knockdown of Fat1 in renal tubular cells reduces   migration  , decreases active RAC1 and CDC42, and induces defects in lumen formation. Knockdown of fat1 in zebrafish causes pronephric cysts, which is partially rescued by RAC1/CDC42 activators, confirming a role of the two small GTPases in the pathogenesis. These findings provide new insights into the pathogenesis of SRNS and tubulopathy, linking FAT1 and RAC1/CDC42 to podocyte and tubular cell function. 
 Novel Activities of Select NSAID R-Enantiomers against Rac1 and Cdc42 GTPases. Rho family GTPases (including Rac, Rho and Cdc42) collectively control cell proliferation, adhesion and   migration   and are of interest as functional therapeutic targets in numerous epithelial cancers. Based on high throughput screening of the Prestwick Chemical Library® and cheminformatics we identified the R-enantiomers of two approved drugs (naproxen and ketorolac) as inhibitors of Rac1 and Cdc42. The corresponding S-enantiomers are considered the active component in racemic drug formulations, acting as non-steroidal anti-inflammatory drugs (NSAIDs) with selective activity against cyclooxygenases. Here, we show that the S-enantiomers of naproxen and ketorolac are inactive against the GTPases. Additionally, more than twenty other NSAIDs lacked inhibitory action against the GTPases, establishing the selectivity of the two identified NSAIDs. R-naproxen was first identified as a lead compound and tested in parallel with its S-enantiomer and the non-chiral 6-methoxy-naphthalene acetic acid (active metabolite of nabumetone, another NSAID) as a structural series. Cheminformatics-based substructure analyses-using the rotationally constrained carboxylate in R-naproxen-led to identification of racemic [R/S] ketorolac as a suitable FDA-approved candidate. Cell based measurement of GTPase activity (in animal and human cell lines) demonstrated that the R-enantiomers specifically inhibit epidermal growth factor stimulated Rac1 and Cdc42 activation. The GTPase inhibitory effects of the R-enantiomers in cells largely mimic those of established Rac1 (NSC23766) and Cdc42 (CID2950007/ML141) specific inhibitors. Docking predicts that rotational constraints position the carboxylate moieties of the R-enantiomers to preferentially coordinate the magnesium ion, thereby destabilizing nucleotide binding to Rac1 and Cdc42. The S-enantiomers can be docked but are less favorably positioned in proximity to the magnesium. R-naproxen and R-ketorolac have potential for rapid translation and efficacy in the treatment of several epithelial cancer types on account of established human toxicity profiles and novel activities against Rho-family GTPases. 
 Snail regulates the motility of oral cancer cells via RhoA/Cdc42/p-ERM pathway. The transcriptional factor Snail has been reported to possess properties related to cancer progression; however, the mechanism for it is not fully understood. Our data showed that Snail knockdown by small interfering RNA in two OSCC cell lines, WSU-HN6 and CAL27, significantly inhibited cell   migration   and invasion which also resulted in decreased cell motility, such as impaired cell spreading on type I collagen substrate, reduced filopodia, and premature assembly of stress fibers. In addition, Snail-silencing decreased Cdc42 activity but increased RhoA activity, accompanied by the downregulation in both p-ERM expression and cell motility. Meanwhile, endogenous p-ERM was found specifically co-precipitated with activated Cdc42, but not RhoA, and this co-association was decreased by Snail-silencing. The small molecule inhibitors of Rho-associated kinase (Y27632) markedly enhanced Cdc42 activity and the association of p-ERM with activated Cdc42, increasing cell motility remarkably. Using immunohistochemistry, Snail and p-ERM overexpressions were found in OSCC tissues correlated with nodal metastasis and shorter survival. Taken together, these results demonstrate that Snail regulates cell motility through RhoA/Cdc42/p-ERM pathway and may serve as a biomarker to predict prognosis for OSCC patients. Although RhoA and Cdc42 are concurrently regulated downstream of Snail, there is a direct interplay between them, which indicates RhoA has to be inactivated at some point in cell motility cycle. 
 Interleukin-1β-induced Wnt5a enhances human corneal endothelial cell   migratio  n through regulation of Cdc42 and RhoA. Wnt5a can activate β-catenin-independent pathways for regulation of various cellular functions, such a  s migrati  on, that play critical roles in wound repair. Investigation of Wnt5a signaling may help identify therapeutic targets for enhancing corneal endothelial wound healing that could provide an alternative to corneal transplantation in patients with blindness from endothelial dysfunction. However, Wnt5a signaling in corneal endothelial cells (CECs) has not been well characterized. In this study, we show transient induction of Wnt5a by interleukin-1β (IL-1β) stimulation proceeds through NF-κB in human CECs. This leads to binding of Fzd5 to Ror2, resulting in activation of disheveled protein (Dvl) and subsequently disheveled-associated activator of morphogenesis 1 (DAAM1). This leads to activation of Cdc42 and subsequent inhibition of RhoA. Inhibition of RhoA leads to parallel dephosphorylation and inactivation of LIM domain kinase 2 along with dephosphorylation and activation of slingshot 1, resulting in dephosphorylation and activation of cofilin and leading to enhanced   cell migr  ation. These findings suggest that Wnt5a enhances   cell migr  ation through activation of Cdc42 and inactivation of RhoA in human CECs. 
 Wnt5b promotes the cell motility essential for metastasis of oral squamous cell carcinoma through active Cdc42 and RhoA. The activation of Wnt signaling has been reported in many types of squamous cell carcinoma. In this study, using human oral squamous cell carcinoma (OSCC) cells with different metastatic potential, we investigated the involvement of Wnt signaling in metastasis. Further, we aimed to elucidate the characteristic biological features related to high metastatic potential and to identify new target molecules for the suppression of OSCC lymph node metastasis. We compared SAS-Venus (SAS OSCC cells expressing green fluorescent protein) and SAS-LM8, which is a highly metastatic cell line derived from SAS-Venus by in vivo selection. The SAS-LM8 cell line had greater ability of   migration   and invasion compared to SAS-Venus. Furthermore, a higher number of filopodia-like protrusive structures were produced in SAS-LM8 cells compared to SAS-Venus cells, and the levels of active Cdc42 and active RhoA protein were higher in SAS-LM8 cells compared to SAS-Venus cells. We did not observe any differences in the expression of Wnt/β-catenin target genes between the two cell lines; however, the mRNA levels of Wnt5b were higher in SAS-LM8 cells compared to SAS-Venus cells. To confirm the involvement of Wnt5b in   migratio  n in OSCC cells, we examined the effects of the siRNA-mediated knockdown of Wnt5b in SAS-Venus cells and SAS-LM8 cells. The siRNA treatment significantly inhibited   migratio  n and the formation of filopodia-like protrusive structures. Conversely, when stimulated with Wnt5b, the   migratio  n and formation of filopodia-like protrusions were significantly enhanced and the levels of active Cdc42 and active RhoA proteins were also increased. These results indicate that Wnt5b is involved in the   migratio  n ability of OSCC cells through active Cdc42 and RhoA. 
 Mammalian target of rapamycin and Rictor control neutrophil chemotaxis by regulating Rac/Cdc42 activity and the actin cytoskeleton. Chemotaxis allows neutrophils to seek out sites of infection and inflammation. The asymmetric accumulation of filamentous actin (F-actin) at the leading edge provides the driving force for protrusion and is essential for the development and maintenance of neutrophil polarity. The mechanism that governs actin cytoskeleton dynamics and assembly in neutrophils has been extensively explored and is still not fully understood. By using neutrophil-like HL-60 cells, we describe a pivotal role for Rictor, a component of mammalian target of rapamycin complex 2 (mTORC2), in regulating assembly of the actin cytoskeleton during neutrophil chemotaxis. Depletion of mTOR and Rictor, but not Raptor, impairs actin polymerization, leading-edge establishment, and directional   migration   in neutrophils stimulated with chemoattractants. Of interest, depletion of mSin1, an integral component of mTORC2, causes no detectable defects in neutrophil polarity and chemotaxis. In addition, experiments with chemical inhibition and kinase-dead mutants indicate that mTOR kinase activity and AKT phosphorylation are dispensable for chemotaxis. Instead, our results suggest that the small Rho GTPases Rac and Cdc42 serve as downstream effectors of Rictor to regulate actin assembly and organization in neutrophils. Together our findings reveal an mTORC2- and mTOR kinase-independent function and mechanism of Rictor in the regulation of neutrophil chemotaxis. 
 Macrophages stimulate gastric and colorectal cancer invasion through EGFR Y(1086), c-Src, Erk1/2 and Akt phosphorylation and smallGTPase activity. The interactions between cancer cells and their microenvironment are crucial for malignant progression, as they modulate invasion-related activities. Tumor-associated macrophages are generally considered allies in the process of tumor progression in several types of cancer, although their role on gastric and colorectal carcinomas is still poorly understood. In this report, we studied the influence of primary human macrophages on gastric and colorectal cancer cells, considering invasion, motility/  migration  , proteolysis and activated intracellular signaling pathways. We demonstrated that macrophages stimulate cancer cell invasion, motility and   migration  , and that these effects depend on matrix metalloproteinase (MMP) activity and on the activation of epidermal growth factor receptor (EGFR) (at the residue Y(1086)), PLC-γ (phospholipase C-gamma) and Gab1 (GRB2-associated binding protein-1), as evidenced by siRNA (small interference RNA) experiments. Epidermal growth factor (EGF)-immunodepletion impaired macrophage-mediated cancer cell invasion and motility, suggesting that EGF is the pro-invasive and pro-motile factor produced by macrophages. Macrophages also induced gastric and colorectal cancer cell phosphorylation of Akt, c-Src and ERK1/2, and led to an increase of RhoA and Cdc42 activity. Interestingly, whereas macrophage-mediated cancer cell c-Src and ERK1/2 phosphorylation occurred downstream EGFR activation, Akt phosphorylation seems to be a parallel event, taking place in an EGFR-independent manner. The involvement of EGF, EGFR-downstream signaling partners and MMPs in macrophage-mediated invasion provides novel insights into the molecular crosstalk established between cancer cells and macrophages, opening new perspectives for the design of new and more efficient therapeutic strategies to counteract cancer cell invasion. 
 The orphan adhesion G protein-coupled receptor GPR97 regulates   migration   of lymphatic endothelial cells via the small GTPases RhoA and Cdc42. The important role of the lymphatic vascular system in pathological conditions such as inflammation and cancer has been increasingly recognized, but its potential as a pharmacological target is poorly exploited. Our study aimed at the identification and molecular characterization of lymphatic-specific G protein-coupled receptors (GPCRs) to assess new targets for pharmacological manipulation of the lymphatic vascular system. We used a TaqMan quantitative RT-PCR-based low density array to determine the GPCR expression profiles of ex vivo isolated intestinal mouse lymphatic (LECs) and blood vascular endothelial cells (BECs). GPR97, an orphan adhesion GPCR of unknown function, was the most highly and specifically expressed GPCR in mouse lymphatic endothelium. Using siRNA silencing, we found that GPR97-deficient primary human LECs displayed increased adhesion and collective cell   migration  , whereas single cell   migration   was decreased as compared with nontargeting siRNA-transfected control LECs. Loss of GPR97 shifted the ratio of active Cdc42 and RhoA and initiated cytoskeletal rearrangements, including F-actin redistribution, paxillin and PAK4 phosphorylation, and β1-integrin activation. Our data suggest a possible role of GPR97 in lymphatic remodeling and furthermore provide the first insights into the biological functions of GPR97. 
 Down expression of LRP1B promotes cell   migration   via RhoA/Cdc42 pathway and actin cytoskeleton remodeling in renal cell cancer. The low-density lipoprotein receptor-related protein 1B (LRP1B) is known as a putative tumor suppressor. The decreased expression of LRP1B has been involved in multiple primary cancers in several studies. However, its expression and function in the carcinogenesis of renal cell cancer (RCC) remain unclear. In this study, we investigated the expression of LRP1B in RCC by in situ hybridization (ISH) and real-time polymerase chain reaction (qRT-PCR). Our results indicated that LRP1B was frequently downexpressed in human RCC tissue and cell lines, which involved both epigenetic events (DNA methylation and histone deacetylation) and N-terminal deletion of LRP1B. Moreover, we testified that knockdown of LRP1B by shRNA significantly promoted anchorage-independent growth, cell   migration   and invasion in HEK293 cells and renal cancer cells 127 in vitro. We further found that silencing of LRP1B altered the expression of focal adhesion complex-associated proteins, and Cdc42/RhoA activities, which regulate the cytoskeleton dynamics. Taken together, these results strongly support that LRP1B may function as a tumor suppressor against renal cell cancer, and may regulate cell motility via RhoA/Cdc42 pathway and actin cytoskeleton reorganization in RCC. 
 Rac1 and Cdc42 differentially modulate cigarette smoke-induced airway cell   migration   through p120-catenin-dependent and -independent pathways. The adherens junction protein p120-catenin (p120ctn) shuttles between E-cadherin-bound and cytoplasmic pools to regulate E-cadherin/catenin complex stability and cell   migration  , respectively. When released from the adherens junction, p120ctn promotes cell   migration   through modulation of the Rho GTPases Rac1, Cdc42, and RhoA. Accordingly, the down-regulation and cytoplasmic mislocalization of p120ctn has been reported in all subtypes of lung cancers and is associated with grave prognosis. Previously, we reported that cigarette smoke induced cytoplasmic translocation of p120ctn and cell   migration  , but the underlying mechanism was unclear. Using primary human bronchial epithelial cells exposed to smoke-concentrated medium (Smk), we observed the translocation of Rac1 and Cdc42, but not RhoA, to the leading edge of polarized and migrating human bronchial epithelial cells. Rac1 and Cdc42 were robustly activated by smoke, whereas RhoA was inhibited. Accordingly, siRNA knockdown of Rac1 or Cdc42 completely abolished Smk-induced cell   migration  , whereas knockdown of RhoA had no effect. p120ctn/Rac1 double knockdown completely abolished Smk-induced cell   migration  , whereas p120ctn/Cdc42 double knockdown did not. These data suggested that Rac1 and Cdc42 coactivation was essential to smoke-promoted cell   migration   in the presence of p120ctn, whereas   migration   proceeded via Rac1 alone in the absence of p120ctn. Thus, Rac1 may provide an omnipotent therapeutic target in reversing cell   migration   during the early (intact p120ctn) and late (loss of p120ctn) stages of lung carcinogenesis. 
 Overexpression of small GTPases directly correlates with expression of δ-catenin and their coexpression predicts a poor clinical outcome in nonsmall cell lung cancer. δ-catenin can affect cytoskeletal assembly, and promote cel  l migrati  on by regulating the activity of small GTPases. While many malignancies have been shown to be positive for δ-catenin, it is still unclear whether δ-catenin and small GTPases are coexpressed in tumor cells, and so is the relationship between their coexpression and prognosis in the tumor patients. In this study, immunohistochemistry was performed to examine expressive levels of δ-catenin, cdc42, and Rac1 in 135 cases of nonsmall cell lung cancer (NSCLC), including 60 cases with follow-up records. Thirty samples of paired lung cancer tissues and adjacent normal lung tissues were collected to analyze mRNA and protein expression of δ-catenin and small GTPases. The effects of δ-catenin on small GTPases expression and invasive ability of lung cancer cells were also evaluated. Compared with normal lung tissues, both mRNA and protein levels of δ-catenin and small GTPases were increased in lung cancer tissues (P < 0.05), and the expression of small GTPases directly correlated with that of δ-catenin (P < 0.001). In addition, δ-catenin and small GTPases tended to be coexpressed in lung adenocarcinoma, advanced stages, and primary tumors with lymph node metastasis (all P < 0.05). The patients with coexpression of δ-catenin and small GTPases had a shorter survival time than those without coexpression (P < 0.05). Furthermore, δ-catenin overexpression could enhance invasive ability of lung cancer cells by upregulating protein and transcriptional level of small GTPases. Therefore, δ-catenin likely upregulates the activity of small GTPases at transcriptional level, and their coexpression may predict a poor clinical outcome in NSCLC patients. 
 Cdc42 promotes transendothelial   migration   of cancer cells through β1 integrin. Cancer cells interact with endothelial cells during the process of metastatic spreading. Here, we use a small interfering RNA screen targeting Rho GTPases in cancer cells to identify Cdc42 as a critical regulator of cancer cell-endothelial cell interactions and transendothelial   migratio  n. We find that Cdc42 regulates β1 integrin expression at the transcriptional level via the transcription factor serum response factor (SRF). β1 integrin is the main target for Cdc42-mediating interaction of cancer cells with endothelial cells and the underlying extracellular matrix, as exogenous β1 integrin expression was sufficient to rescue the Cdc42-silencing phenotype. We show that Cdc42 was required in vivo for cancer cell spreading and protrusion extension along blood vessels and retention in the lungs. Interestingly, transient Cdc42 depletion was sufficient to decrease experimental lung metastases, which suggests that its role in endothelial attachment is important for metastasis. By identifying β1 integrin as a transcriptional target of Cdc42, our results provide new insight into Cdc42 function. 
 Immunohistochemical expression of Rho GTPases in ameloblastomas. Rho GTPases are proteins that regulate cell cycle, shape, polarization, invasion,   migration  , and apoptosis, which are important characteristics of normal and neoplastic cells. Rho GTPases expression has been reported in normal tooth germ and several pathologies; however, it has not been evaluated in ameloblastomas. The aim of this study was to analyze the expression and distribution of RhoA, RhoB, Rac1, and Cdc42 Rho GTPases in solid and unicystic ameloblastomas. Three-micrometer sections from paraffin-embedded specimens were evaluated by using an avidin-biotin immunohistochemical method with antibodies against the proteins mentioned above. RhoA and RhoB staining was observed in a high number of cells (P < 0.05) and greater intensity in non-polarized ones. Rac1 was not observed, and Cdc42 did not show any statistical differences between the number of non-polarized and basal positive cells (P > 0.05). Upon comparing the studied ameloblastomas, a higher number of positive cells in the unicystic variant was observed than that in the solid one (P < 0,05). The results obtained suggest that these GTPases could play a role in the ameloblastoma neoplastic epithelial cell phenotype determination (polarized or non-polarized), as well as in variant (solid or unicystic) and subtype (follicular or plexiform) determination. Furthermore, they could participate in solid ameloblastoma invasion mechanisms. 
 N-α-acetyltransferase 10 protein suppresses cancer cell metastasis by binding PIX proteins and inhibiting Cdc42/Rac1 activity. N-α-acetyltransferase 10 protein, Naa10p, is an N-acetyltransferase known to be involved in cell cycle control. We found that Naa10p was expressed lower in varieties of malignancies with lymph node metastasis compared with non-lymph node metastasis. Higher Naa10p expression correlates the survival of lung cancer patients. Naa10p significantly suppresse  d migrati  on, tumor growth, and metastasis independent of its enzymatic activity. Instead, Naa10p binds to the GIT-binding domain of PIX, thereby preventing the formation of the GIT-PIX-Paxillin complex, resulting in reduced intrinsic Cdc42/Rac1 activity and decreased cel  l migrati  on. Forced expression of PIX in Naa10-transfected tumor cells restored th  e migrati  on and metastasis ability. We suggest that Naa10p functions as a tumor metastasis suppressor by disrupting the migratory complex, PIX-GIT- Paxillin, in cancer cells. 
 Invasion of breast cancer cells into collagen matrix requires TGF-α and Cdc42 signaling. Invasion of MDA-MB-231 breast cancer cells into three-dimensional (3-D) type I-collagen matrices depends on TGF-α. We characterized the steps of invasion mediated by TGF-α. Ce  ll migrat  ion, as observed by videomicroscopy, was effectively stimulated by collagen, suggesting that TGF-α may specifically participate in the invasion of a 3-D collagen matrix. We assessed the role of small GTPases of the Rho family in the invasion. Cdc42 was found to be necessary for invasion but dispensable for c  ell migra  tion. These results suggest that TGF-α mediates invasion into 3-D collagen matrices by initiating the formation of protrusions into collagen, likely through activation of Cdc42. 
 Tuberous sclerosis complex 2 (TSC2) regulates cell   migration   and polarity through activation of CDC42 and RAC1. The phosphatidylinositol 3-kinase (PI3K)/AKT pathway plays important roles in regulating cell motility. TSC2, a downstream target of AKT, is a central player in negatively controlling cell proliferation and protein translation through suppressing the activity of mTOR (mammalian target of rapamycin). However, the function of TSC2 in regulating cell   migration   remains unclear. Here, we show that TSC2 plays a critical role in the control of cell spreading, polarity, and   migration  . TSC2-deficient fibroblast cells were impaired in their ability to spread and alter actin cytoskeleton upon stimulation with insulin-like growth factor-1. Using scratch-induced polarization assay, we demonstrate that TSC2((-/-)) fibroblast cells polarized poorly toward the wound compared with wild-type cells. Similarly, knockdown of TSC2 expression in colon cancer cells resulted in a marked decrease in cell motility. Functionally, the activation of CDC42- and RAC1-GTPase was largely reduced in TSC2 knock-out fibroblast and TSC2 knockdown cancer cells. Furthermore, overexpression of an activating p110alpha mutant or short term rapamycin treatment rescued the cell polarization defect in TSC2((-/-)) fibroblast cells. Concurrently, the activation of CDC42 and RAC1 increased. The defect in cell   migration   and CDC42 and RAC1 activation was reversed by reintroducing TSC2 back into TSC2((-/-)) fibroblast cells. Taken together, we identified a novel role of TSC2 in controlling cell polarity and   migration   by regulating CDC42 and RAC1 activation. 
 Adiponectin promotes   migration   activities of endothelial progenitor cells via Cdc42/Rac1. Adiponectin has anti-atherosclerotic effects through its direct actions on vascular cells. The present study investigates the molecular mechanisms of adiponectin in the   migration   of endothelial progenitor cells (EPCs) which play an important role in neovascularization and re-endothelization. The phosphorylation of Akt and the activations of Cdc42 and Rac1 were significantly increased by adiponectin. Adiponectin increased the   migration   activity of EPCs, which was completely inhibited by a PI3-kinase inhibitor. siRNA of Cdc42 or Rac1 completely inhibited the adiponectin-induced   migration  , but siRNA of Akt had no effects, indicating that adiponectin promotes the   migration   activities of EPCs mainly through PI3-kinase/Cdc42/Rac1. 
 Interaction of the RAGE cytoplasmic domain with diaphanous-1 is required for ligand-stimulated cellular   migration   through activation of Rac1 and Cdc42. Cellular   migration   is a fundamental process linked to diverse pathological states such as diabetes and its complications, atherosclerosis, inflammation, and cancer. The receptor for advanced glycation end products (RAGE) is a multiligand cell surface macromolecule which binds distinct ligands that accumulate in these settings. RAGE-ligand interaction evokes central changes in key biological properties of cells, including proliferation, generation of inflammatory mediators, and   migration  . Although RAGE-dependent signal transduction is critically dependent on its short cytoplasmic domain, to date the proximate mechanism by which this RAGE domain engages and stimulates cytoplasmic signaling pathways has yet to be identified. Here we show that the RAGE cytoplasmic domain interacts with Diaphanous-1 (Dia-1) both in vitro and in vivo. We employed the human RAGE cytoplasmic domain as "bait" in the yeast two-hybrid assay and identified the formin homology (FH1) domain of Dia-1 as a potential binding partner of this RAGE domain. Immunoprecipitation studies revealed that the RAGE cytoplasmic domain interacts with the FH1 domain of Dia-1. Down-regulation of Dia-1 expression by RNA interference blocks RAGE-mediated activation of Rac-1 and Cdc42 and, in parallel, RAGE ligand-stimulated cellular   migration  . Taken together, these findings indicate that the interaction of the RAGE cytoplasmic domain with Dia-1 is required to transduce extracellular environmental cues evoked by binding of RAGE ligands to their cell surface receptor, a chief consequence of which is Rac-1 and Cdc42 activation and cellular   migration  . Because RAGE and Dia-1 are implicated in the regulation of inflammatory, vascular, and transformed cell   migration  , these findings highlight this interaction as a novel target for therapeutic intervention in inflammation, atherosclerosis, diabetes, and cancer. 
 Prostaglandin E2-mediated   migration   of human trophoblast requires RAC1 and CDC42. The invasion of maternal decidua and uterine spiral arteries by a trophoblast subpopulation called extravillous trophoblast (EVT) is essential for the establishment of a normal placenta and an adequate blood flow toward the fetus. Derangements in these processes underlie pregnancy-related diseases like preeclampsia and intrauterine growth restriction. Many growth factors, growth factor binding proteins, and extracellular matrix components can positively or negatively regulate the proliferation,   migration  , and/or invasiveness of these EVT cells. RHO GTPases, including RHOA, RAC1, and CDC42, are ubiquitous proteins that control cytoskeletal changes by forming stress fibers and projecting lamellipodia and filopodia during cellular   migration  . We had previously shown that prostaglandin (PG) E(2) produced in abundance by the decidua promotes the   migration   of first-trimester human EVTs by increasing the intracellular concentration of calcium and activating calpain. Using our well-characterized immortalized EVT cell line, HTR-8/SVneo, as well as villus explants from first-trimester placentae, this study examined the role of RHO GTPases RAC1 and CDC42 in PGE(2)-mediated migratory responses of these cells. Though a RAC1 inhibitor, NSC23766 as well as RAC1 knockdown by siRNA decreased the   migration   of HTR-8/SVneo cells in a Transwell   migration   assay, this inhibition could not be restored by PGE(2) or 17-phenyl trinor PGE(2) (PGE receptor PTGER1 agonist) or PGE(1) Alcohol (PGE receptor PTGER4 agonist). Similar results were noted for EVT cell spreading in villus explants. Furthermore, CDC42 silencing using siRNA inhibited PGE(2)-induced   migration   of HTR-8/SVneo cells. Finally, the treatment of EVT cells with PGE(2), PTGER1 agonist, or PTGER4 agonist activated RAC1 and CDC42 at 10 min, suggesting that RAC1 and CDC42 play an essential role in PGE(2)-mediated   migration   of human EVTs. 
 [The Rho protein family and its role in the cellular cytoskeleton]. Proteins of the Rho protein family (RhoA, Rac1, Cdc42) work as molecular switches in the regulation of many cellular processes. They are involved in cell   migration  , cell-cycle control, apoptosis, and the regulation of gene transcription. Rho proteins show their activity mainly in the cell's cytoskeleton by taking part in the reorganization of microfilaments and microtubules. In recent years, significant progress has been made in understanding the biochemical and genetic nature of many processes controlled by Rho proteins. Although there are still seveal unknowns and hypothesizes which require confirmation, newer and more precise experimental results allow us to better understand these processes. It has also been suggested to use Rho family proteins in the therapy of some diseases. The purpose of this study was to characterize the significance of Rho processes in the cellular cytoskeleton. 
 Formyl peptide receptor-1 activation enhances intestinal epithelial cell restitution through phosphatidylinositol 3-kinase-dependent activation of Rac1 and Cdc42. Inflammatory disorders of the gastrointestinal tract result in the breakdown of the intestinal epithelial barrier in the form of erosion and ulceration. To reestablish the epithelial barrier, the epithelium must efficiently migrate to reseal wounds. Numerous signaling cascades are involved in the induction and regulation of this complex process. N-formyl peptide receptors comprise a group of Gi-coupled receptors that regulate innate immune responses. Previously, we identified the expression of functional N-formyl peptide receptors in model SK-CO15 intestinal epithelial cells and observed a role for activation of these receptors in regulating cellular invasive behavior. In these studies, we performed formyl peptide receptor-1 (FPR) localization and evaluated its role in regulating intestinal epithelial cell wound closure. Immunolocalization studies using a recently developed specific monoclonal anti-FPR Ab demonstrated its localization along the lateral membrane of crypt epithelial cells in normal human colonic epithelium. In vitro studies using the classical FPR agonist fMLF showed that FPR activation significantly enhances model intestinal epithelial cell restitution and that FPR localized along actin filaments in lamellipodial and filopodial extrusions. The increase in cell   migration   was associated with activation of PI3K, Rac1, and Cdc42. Pharmacologic inhibition of PI3K activity abrogated the fMLF-induced increase in wound closure and activation of both Rac1 and Cdc42. Inhibition of Rac1 and Cdc42 using pharmacologic inhibitors and dominant negative mutants also inhibited the fMLF-induced increase in cell   migration  . Taken together, theses results support a novel role for FPR stimulation in enhancing intestinal epithelial cell restitution through PI3K-dependent activation of Rac1 and Cdc42. 
 Regulation of ephexin1, a guanine nucleotide exchange factor of Rho family GTPases, by fibroblast growth factor receptor-mediated tyrosine phosphorylation. Fibroblast growth factor (FGF) signal is implicated in not only cell proliferation, but cell   migration   and morphological changes. Several different Rho family GTPases downstream of the Ras/ERK pathway are postulated to mediate the latter functions. However, none have been recognized to be directly coupled to FGF receptors (FGFRs). We have previously reported that EphA4 and FGFRs hetero-oligomerize through their cytoplasmic domains, trans-activate each other, and transduce a signal for cell proliferation through a docking protein, FRS2alpha (Yokote, H., Fujita, K., Jing, X., Sawada, T., Liang, S., Yao, L., Yan, X., Zhang, Y., Schlessinger, J., and Sakaguchi, K. (2005) Proc. Natl. Acad. Sci. U. S. A. 102, 18866-18871). Here, we have found that ephexin1, a guanine nucleotide exchange factor for Rho family GTPases, constitutes another downstream component of the receptor complex. Ephexin1 directly binds to the kinase domain of FGFR mainly through its DH and PH domains. The binding appears to become weaker and limited to the DH domain when FGFRs become activated. FGFR-mediated phosphorylation of ephexin1 enhances the guanine nucleotide exchange activity toward RhoA without affecting the activity to Rac1 or Cdc42. The FGFR-mediated tyrosine phosphorylation includes, but is not limited to, the residue (Tyr-87) phosphorylated by Src family kinase, which is known to be activated following EphA4 activation. The Tyr-to-Asp mutations that mimic the tyrosine phosphorylation in some of the putative FGFR-mediated phosphorylation sites increase the nucleotide exchange activity for RhoA without changing the activity for Rac1 or Cdc42. From these results, we conclude that ephexin1 is located immediately downstream of the EphA4-FGFR complex and the function is altered by the FGFR-mediated tyrosine phosphorylation at multiple sites. 
 Extension, retraction and contraction in the formation of a dendritic cell dendrite: distinct roles for Rho GTPases. The morphology of antigen-presenting dendritic cells (DC) is characterized by the possession of numerous long arborizing processes known as dendrites. The formation of these processes by DC, both in the periphery and in lymphoid organs, is believed to contribute to the remarkable efficiency with which they take up, process and present antigen to T cells. However, the process of dendrite formation and the signaling pathways that lead to the formation of these dendrites remain obscure. In this study we describe an in vitro model in which human immature DC form long processes similar to those formed in vivo. The formation of these processes involves initial attachment of a cell protrusion to the extracellular matrix substrate, and subsequent movement of the cell body away from the adhesion site, leaving behind a long slender dendrite. Dendrite formation, but not their maintenance, was found to be dependent on the activity of Rho GTPases. More specifically, Cdc42 and Rac1 were both required for the   migration   step of process formation, promoting cell spreading and extension. In contrast, Rho, and its downstream effector p160ROCK, regulated the release of adhesions to the substratum, and associated cellular contraction. Consequently, inhibition of Rho/p160ROCK leads to the formation of longer dendrites. DC therefore coordinate adhesion and protrusion to perform a specialized process of cellular morphogenesis, which differentiates these cells from all other cells of the immune system and may contribute to their distinctive function. 
 Maspin controls mammary tumor cell   migration   through inhibiting Rac1 and Cdc42, but not the RhoA GTPase. Rac1 and Cdc42 are members of the Rho family of small GTPases that play essential roles in diverse cellular functions, including cell   migration  . The activities of these Rho family proteins are controlled by growth factor receptor activation and cell-ECM interactions. Here, we show that maspin, a well-documented tumor suppressor gene, also controls cell motility through inhibiting Rac1/Cdc42 activity. Using the GST-PAK and GST-Rho binding protein pull-down assays for GTP-bound Rac1, Cdc42, and RhoA, we showed that treatment of MDA-MB-231 tumor cells with recombinant maspin for a short time period significantly inhibited the activity of Rac1 and Cdc42, but not RhoA. The reactive site loop (RSL) within maspin protein is the functional domain involved in the inhibition. Maspin mutants with the RSL deleted or a point mutation in the RSL region lost their inhibitory activity. We further examined the ability of maspin to inhibit Rac1- and Cdc42-mediated signaling pathways and transcription factors. Treatment of MDA-MB-231 cells with maspin led to the inhibition of JNK kinase activity as assayed by immuno-kinase assays. In addition, the AP-1 transcription activity downstream of JNK kinase pathway was also reduced. Together, we have identified Rac1 and Cdc42 as the downstream targets that mediate the inhibition of mammary tumor cell   migration   by maspin. 
 Estrogen and resveratrol regulate Rac and Cdc42 signaling to the actin cytoskeleton of metastatic breast cancer cells. Estrogen and structurally related molecules play critical roles in breast cancer. We reported that resveratrol (50 microM), an estrogen-like phytosterol from grapes, acts in an antiestrogenic manner in breast cancer cells to reduce cell   migration   and to induce a global and sustained extension of actin structures called filopodia. Herein, we report that resveratrol-induced filopodia formation is time-dependent and concentration-dependent. In contrast to resveratrol at 50 microM, resveratrol at 5 microM acts in a manner similar to estrogen by increasing lamellipodia, as well as cell   migration   and invasion. Because Rho GTPases regulate the extension of actin structures, we investigated a role for Rac and Cdc42 in estrogen and resveratrol signaling. Our results demonstrate that 50 microM resveratrol decreases Rac and Cdc42 activity, whereas estrogen and 5 microM resveratrol increase Rac activity in breast cancer cells. MDA-MB-231 cells expressing dominant-negative Cdc42 or dominant-negative Rac retain filopodia response to 50 microM resveratrol. Lamellipodia response to 5 microM resveratrol, estrogen, or epidermal growth factor is inhibited in cells expressing dominant-negative Rac, indicating that Rac regulates estrogen and resveratrol (5 microM) signaling to the actin cytoskeleton. These results indicate that signaling to the actin cytoskeleton by low and high concentrations of resveratrol may be differentially regulated by Rac and Cdc42. 
 Regulation of PTEN by Rho small GTPases. PTEN (phosphatase and tensin homologue) is a phosphatase that dephosphorylates both protein and phosphoinositide substrates. It is mutated in a variety of human tumours and has important roles in a diverse range of biological processes, including cell   migration   and chemotaxis. PTEN's intracellular localization and presumably activity are regulated by chemoattractants in Dictyostelium and mouse neutrophils. However, the mechanisms for its regulation remain elusive. Here we show that RhoA and Cdc42, members of the Rho family of small GTPases, regulate the intracellular localization of PTEN in leukocytes and human transfected embryonic kidney cells. In addition, active RhoA is able to stimulate the phospholipid phosphatase activity of PTEN in human embryonic kidney cells and leukocytes, and this regulation seems to require RhoA's downstream effector, RhoA-associated kinase (Rock). Furthermore, we have identified key residues on PTEN that are required for its regulation by the small GTPase, and show that small GTPase-mediated regulation of PTEN has a significant role in the regulation of chemotaxis. 
 Human neutrophils utilize a Rac/Cdc42-dependent MAPK pathway to direct intracellular granule mobilization toward ingested microbial pathogens. Elevated levels of mitogen-activated protein kinase/extracellular regulatory kinase (MAPK/ERK) activity are frequently found in some cancer cells. In efforts to reduce tumor growth, attempts have been made to develop cancer therapeutic agents targeting the MAPK. Here, by use of biologic, biochemical, and gene manipulation methods in human polymorphonuclear neutrophils (PMNs), we have identified a key pathway important in normal cell function involving MAPK/ERK in PMNs for growth inhibition of Candida albicans. Contact with C albicans triggered MAPK/ERK activation in PMNs within 5 minutes, and blocking of MAPK/ERK activation, either by the pharmacologic reagent PD098059 or by dominant-negative MAPK kinase (MEK) expression via vaccinia viral delivery, suppressed antimicrobial activity. Rac and Cdc42, but not Ras or Rho, were responsible for this MAPK/ERK activation. Expression of dominant-negative Rac (N17Rac) or Cdc42 (N17Cdc42) eliminated not only C albicans- mediated ERK phosphorylation but also phagocytosis and granule   migration   toward the ingested microbes, whereas dominant-negative Ras (N17Ras) and Rho (N19Rho) did not. PAK1 (p21-activated kinase 1) activation is induced by C albicans, suggesting that PAK1 may also be involved in the Rac1 activation of MAPK/ERK. We conclude from these data that Rac/Cdc42-dependent activation of MAPK/ERK is a critical event in the immediate phagocytic response of PMNs to microbial challenge. Therefore, use of MAPK pharmacologic inhibitors for the treatment of cancer may result in the interruption of normal neutrophil function. A balance between therapeutic outcome and undesirable side effects must be attained to achieve successful and safe anticancer therapy. 
 Directional sensing requires G beta gamma-mediated PAK1 and PIX alpha-dependent activation of Cdc42. Efficient chemotaxis requires directional sensing and cell polarization. We describe a signaling mechanism involving G beta gamma, PAK-associated guanine nucleotide exchange factor (PIX alpha), Cdc42, and p21-activated kinase (PAK) 1. This pathway is utilized by chemoattractants to regulate directional sensing and directional   migration   of myeloid cells. Our results suggest that G beta gamma binds PAK1 and, via PAK-associated PIX alpha, activates Cdc42, which in turn activates PAK1. Thus, in this pathway, PAK1 is not only an effector for Cdc42, but it also functions as a scaffold protein required for Cdc42 activation. This G beta gamma-PAK1/PIX alpha/Cdc42 pathway is essential for the localization of F-actin formation to the leading edge, the exclusion of PTEN from the leading edge, directional sensing, and the persistent directional   migration   of chemotactic leukocytes. Although ligand-induced production of PIP(3) is not required for activation of this pathway, PIP(3) appears to localize the activation of Cdc42 by the pathway. 
 CDC42 switches IRSp53 from inhibition of actin growth to elongation by clustering of VASP. Filopodia explore the environment, sensing soluble and mechanical cues during directional motility and tissue morphogenesis. How filopodia are initiated and spatially restricted to specific sites on the plasma membrane is still unclear. Here, we show that the membrane deforming and curvature sensing IRSp53 (Insulin Receptor Substrate of 53 kDa) protein slows down actin filament barbed end growth. This inhibition is relieved by CDC42 and counteracted by VASP, which also binds to IRSp53. The VASP:IRSp53 interaction is regulated by activated CDC42 and promotes high-density clustering of VASP, which is required for processive actin filament elongation. The interaction also mediates VASP recruitment to liposomes. In cells, IRSp53 and VASP accumulate at discrete foci at the leading edge, where filopodia are initiated. Genetic removal of IRSp53 impairs the formation of VASP foci, filopodia and chemotactic motility, while IRSp53 null mice display defective wound healing. Thus, IRSp53 dampens barbed end growth. CDC42 activation inhibits this activity and promotes IRSp53-dependent recruitment and clustering of VASP to drive actin assembly. These events result in spatial restriction of VASP filament elongation for initiation of filopodia during cel  l migrati  on, invasion, and tissue repair. 
 Prominin-2 expression increases protrusions, decreases caveolae and inhibits Cdc42 dependent fluid phase endocytosis. Membrane protrusions play important roles in biological processes such as cell adhesion, wound healing,   migration  , and sensing of the external environment. Cell protrusions are a subtype of membrane microdomains composed of cholesterol and sphingolipids, and can be disrupted by cholesterol depletion. Prominins are pentaspan membrane proteins that bind cholesterol and localize to plasma membrane (PM) protrusions. Prominin-1 is of great interest as a marker for stem and cancer cells, while Prominin-2 (Prom2) is reportedly restricted to epithelial cells.To characterize the effects of Prom-2 expression on PM microdomain organization.Prom2-fluorescent protein was transfected in human skin fibroblasts (HSF) and Chinese hamster ovary (CHO) cells for PM raft and endocytic studies. Caveolae at PM were visualized using transmission electron microscopy. Cdc42 activation was measured and caveolin-1 knockdown was performed using siRNAs.Prom2 expression in HSF and CHO cells caused extensive Prom2-positive protrusions that co-localized with lipid raft markers. Prom2 expression significantly decreased caveolae at the PM, reduced caveolar endocytosis and increased caveolin-1 phosphorylation. Prom2 expression also inhibited Cdc42-dependent fluid phase endocytosis via decreased Cdc42 activation. Effects on endocytosis were reversed by addition of cholesterol. Knockdown of caveolin-1 by siRNA restored Cdc42 dependent fluid phase endocytosis in Prom2-expressing cells.Prom2 protrusions primarily localize to lipid rafts and recruit cholesterol into protrusions and away from caveolae, leading to increased phosphorylation of caveolin-1, which inhibits Cdc42-dependent endocytosis. This study provides a new insight for the role for prominins in the regulation of PM lipid organization. 
 Polarized   migration   of lymphatic endothelial cells is critically dependent on podoplanin regulation of Cdc42. We have shown previously that T1α/podoplanin is required for capillary tube formation by human lung microvascular lymphatic endothelial cells (HMVEC-LLy) and that cells with decreased podoplanin expression fail to properly activate the small GTPase RhoA shortly after the beginning of the lymphangiogenic process. The objective of this study was to determine whether podoplanin regulates HMVEC-LLy   migratio  n and whether this regulation is via modulation of small GTPase activation. In analysis of scratch wound assays, we found that small interfering RNA (siRNA) depletion of podoplanin expression in HMVEC-LLy inhibits VEGF-induced microtubule-organizing center (MTOC) and Golgi polarization and causes a dramatic reduction in directional   migratio  n compared with control siRNA-transfected cells. In addition, a striking redistribution of cortical actin to fiber networks across the cell body is observed in these cells, and, remarkably, it returns to control levels if the cells are cotransfected with a dominant-negative mutant of Cdc42. Moreover, cotransfection of a dominant-negative construct of Cdc42 into podoplanin knockdown HMVEC-LLy completely abrogated the effect of podoplanin deficiency, rescuing MTOC and Golgi polarization and cell   migratio  n to control level. Importantly, expression of constitutively active Cdc42 construct, like podoplanin knockdown, decreased RhoA-GTP level in HMVEC-LLy, demonstrating cross talk between both GTPases. Taken together, the results indicate that polarized   migratio  n of lymphatic endothelial cells in response to VEGF is mediated via a pathway of podoplanin regulation of small GTPase activities, in particular Cdc42. 
 IQGAP1 promotes cell motility and invasion. The dynamic processes of cell   migration   and invasion are largely coordinated by Rho family GTPases. The scaffolding protein IQGAP1 binds to Cdc42, increasing the amount of active Cdc42 both in vitro and in cells. Here we show that overexpression of IQGAP1 in mammalian cells enhances cell   migration   in a Cdc42- and Rac1-dependent manner. Importantly, cell motility was significantly decreased both by knock down of endogenous IQGAP1 using small interfering RNA and by transfection of a dominant negative IQGAP1 construct, IQGAP1DeltaGRD. Cell invasion was similarly altered by manipulating intracellular IQGAP1 concentrations. Moreover, invasion mediated by constitutively active Cdc42 was attenuated by IQGAP1DeltaGRD. Thus, IQGAP1 has a fundamental role in cell motility and invasion. 
 Multiple factors confer specific Cdc42 and Rac protein activation by dedicator of cytokinesis (DOCK) nucleotide exchange factors. DOCK (dedicator of cytokinesis) guanine nucleotide exchange factors (GEFs) activate the Rho-family GTPases Rac and Cdc42 to control cell   migration  , morphogenesis, and phagocytosis. The DOCK A and B subfamilies activate Rac, whereas the DOCK D subfamily activates Cdc42. Nucleotide exchange is catalyzed by a conserved DHR2 domain (DOCK(DHR2)). Although the molecular basis for DOCK(DHR2)-mediated GTPase activation has been elucidated through structures of a DOCK9(DHR2)-Cdc42 complex, the factors determining recognition of specific GTPases are unknown. To understand the molecular basis for DOCK-GTPase specificity, we have determined the crystal structure of DOCK2(DHR2) in complex with Rac1. DOCK2(DHR2) and DOCK9(DHR2) exhibit similar tertiary structures and homodimer interfaces and share a conserved GTPase-activating mechanism. Multiple structural differences between DOCK2(DHR2) and DOCK9(DHR2) account for their selectivity toward Rac1 and Cdc42. Key determinants of selectivity of Cdc42 and Rac for their cognate DOCK(DHR2) are a Phe or Trp residue within β3 (residue 56) and the ability of DOCK proteins to exploit differences in the GEF-induced conformational changes of switch 1 dependent on a divergent residue at position 27. DOCK proteins, therefore, differ from DH-PH GEFs that select their cognate GTPases through recognition of structural differences within the β2/β3 strands. 
 Activated ezrin promotes cell   migration   through recruitment of the GEF Dbl to lipid rafts and preferential downstream activation of Cdc42. Establishment of polarized cell morphology is a critical factor for   migration   and requires precise spatial and temporal activation of the Rho GTPases. Here, we describe a novel role of the actin-binding ezrin/radixin/moesin (ERM)-protein ezrin to be involved in recruiting Cdc42, but not Rac1, to lipid raft microdomains, as well as the subsequent activation of this Rho GTPase and the downstream effector p21-activated kinase (PAK)1, as shown by fluorescence lifetime imaging microscopy. The establishment of a leading plasma membrane and the polarized morphology necessary for random   migration   are also dependent on ERM function and Cdc42 in motile breast carcinoma cells. Mechanistically, we show that the recruitment of the ERM-interacting Rho/Cdc42-specific guanine nucleotide exchange factor Dbl to the plasma membrane and to lipid raft microdomains requires the phosphorylated, active conformer of ezrin, which serves to tether the plasma membrane or its subdomains to the cytoskeleton. Together these data suggest a mechanism whereby precise spatial guanine nucleotide exchange of Cdc42 by Dbl is dependent on functional ERM proteins and is important for directional cell   migration  . 
 IQGAP1 regulates cell proliferation through a novel CDC42-mTOR pathway. Cell proliferation requires close coordination of cell growth and division to ensure constant cell size through the division cycles. IQGAP1, an effector of CDC42 GTPase has been implicated in the modulation of cell architecture, regulation of exocytosis and in human cancers. The precise mechanism underlying these activities is unclear. Here, we show that IQGAP1 regulates cell proliferation, which requires phosphorylation of IQGAP1 and binding to CDC42. Expression of the C-terminal region of IQGAP1 enhanced cellular transformation and   migration  , but reduced the cell size, whereas expression of the N-terminus increased the cell size, but inhibited cell transformation and   migration  . The N-terminus of IQGAP1 interacts with mTOR, which is required for IQGAP1-mediated cell proliferation. These findings are consistent with a model where IQGAP1 serves as a phosphorylation-sensitive conformation switch to regulate the coupling of cell growth and division through a novel CDC42-mTOR pathway, dysregulation of which generates cellular transformation. 
 The adaptor protein Nck1 mediates endothelin A receptor-regulated cell   migration   through the Cdc42-dependent c-Jun N-terminal kinase pathway. Cell   migration   plays key roles in physiological and pathological phenomena, such as development and oncogenesis. The adaptor proteins Grb2, CrkII, and Nck1 are composed of only a single Src homology 2 domain and some Src homology 3 domains, giving specificity to each signal transduction pathway. However, little is known about the relationships between their adaptor proteins and cell   migration  , which are regulated by the G protein-coupled receptor. Here we showed that Nck1, but not Grb2 or CrkII, mediated the inhibition of cell   migration   induced by the endothelin-1 and endothelin A receptor. The small interference RNA and dominant negative mutants of Nck1 diminished the endothelin-1-induced inhibition of cell   migration  . Although overexpression of wild-type Nck1 was detected in the cytosol and did not affect cell   migration  , expression of the myristoylation signal sequence-conjugated Nck1 was detected in the membrane and induced activation of Cdc42 and c-Jun N-terminal kinase, inhibiting cell   migration  . Taken together, these results suggest that the endothelin A receptor transduces the signal of inhibition of cell   migration   through Cdc42-dependent c-Jun N-terminal kinase activation by using Nck1. 
 p16 Stimulates CDC42-dependent   migration   of hepatocellular carcinoma cells. Hepatocellular carcinoma (HCC) is a leading cause of cancer-related deaths worldwide. Tumor dissemination to the extra-hepatic region of the portal vein, lymph nodes, lungs or bones contributes to the high mortality seen in HCC; yet, the molecular mechanisms responsible for HCC metastasis remain unclear. Prior studies have suggested a potential link between accumulated cytoplasm-localized p16 and tumor progression. Here we report that p16 enhances metastasis-associated phenotypes in HCC cells - ectopic p16 expression increased cell   migration   in vitro, and lung colonization after intravenous injection, whereas knockdown of endogenous p16 reduced cell   migration  . Interestingly, analysis of p16 mutants indicated that the Cdk4 interaction domain is required for stimulation of HCC cell   migration  ; however, knockdown of Cdk4 and Cdk6 showed that these proteins are dispensable for this phenomenon. Intriguingly, we found that in p16-positive HCC samples, p16 protein is predominantly localized in the cytoplasm. In addition, we identified a potential role for nuclear-cytoplasmic shuttling in p16-stimulated   migration  , consistent with the predominantly cytoplasmic localization of p16 in IHC-positive HCC samples. Finally, we determined that p16-stimulated cell   migration   requires the Cdc42 GTPase. Our results demonstrate for the first time a pro-migratory role for p16, and suggest a potential mechanism for the observed association between cytoplasmic p16 and tumor progression in diverse tumor types. 
 Expression analysis of Cdc42 in lung cancer and modulation of its expression by curcumin in lung cancer cell lines. Cdc42, a Rho GTPase family member, is involved in cell transformation, proliferation, survival, invasion and metastasis of human cancer cells. Overexpression of Cdc42 has been reported in several types of human cancer. However, the underlying mechanisms are not well understood. The present study showed that Cdc42 was overexpressed in 80 of 110 primary lung cancer patients, and overexpression of Cdc42 was significantly associated with high TNM stage and lymph node metastasis. Moreover, RNAi-mediated suppression of Cdc42 expression reduced actin ﬁlopodia formation  , migrati  on and invasion potential of a highly metastatic lung cancer cell line, 801D. In parallel, 801D cells were treated with curcumin and the effect on the expression of the Cdc42 gene at the transcriptional and translational levels was analyzed by RT-PCR and Western blotting. Curcumin inhibited cel  l migrati  on, invasion and downregulated Cdc42 gene and Cdc42-related target gene expression in 801D cells. It also induced rearrangements of the actin cytoskeleton. These effects mimicked those of Cdc42 knockdown. Furthermore, xenograft experiments confirmed the suppression of tumor growth and invasion in vivo, which was due to the effect of curcumin and the inhibition of Cdc42 by curcumin. Our results showing the downregulation of Cdc42 expression by curcumin in lung cancer cells taken together with the clinical data suggest a potential therapeutic role for curcumin in inducing Cdc42-mediated inhibition of invasion of lung cancer cells. 
 Cdc42-dependent formation of the ZO-1/MRCKβ complex at the leading edge controls cell   migratio  n. Zonula occludens (ZO)-1 is a multi-domain scaffold protein known to have critical roles in the establishment of cell-cell adhesions and the maintenance of stable tissue structures through the targeting, anchoring, and clustering of transmembrane adhesion molecules and cytoskeletal proteins. Here, we report that ZO-1 directly binds to MRCKβ, a Cdc42 effector kinase that modulates cell protrusion an  d migrati  on, at the leading edge of migrating cells. Structural studies reveal that the binding of a β hairpin from GRINL1A converts ZO-1 ZU5 into a complete ZU5-fold. A similar interaction mode is likely to occur between ZO-1 ZU5 and MRCKβ. The interaction between ZO-1 and MRCKβ requires the kinase to be primed by Cdc42 due to the closed conformation of the kinase. Formation of the ZO-1/MRCKβ complex enriches the kinase at the lamellae of migrating cells. Disruption of the ZO-1/MRCKβ complex inhibits MRCKβ-mediat  ed cell m  igration. These results demonstrate that ZO-1, a classical scaffold protein with accepted roles in maintaining cell-cell adhesions in stable tissues, also has an active role   in cell m  igration during processes such as tissue development and remodelling. 
 Effects of CDC42 on the proliferation and invasion of gastric cancer cells. Cell division cycle 42 (CDC42), which is a member of the Rho GTPase family, has been reported to regulate the metastasis of various human cancer cells; however, the role of CDC42 in gastric cancer (GC) remains unclear. The present study aimed to investigate the effects of CDC42 on the proliferation,   migration   and invasion of GC. Furthermore, the molecular mechanisms underlying the effects of CDC42 on GC were explored. The expression levels of CDC42 in the AGS and SGC7901 human GC cell lines were reduced by RNA interference. Knockdown of CDC42 significantly inhibited the proliferation of AGS and SGC7901 cells, and it was suggested that this inhibitory process may be due to cell cycle arrest at G1/S phase and downregulation of cyclin A, cyclin D1, cyclin E and proliferating cell nuclear antigen. Furthermore, knockdown of CDC42 markedly inhibited the   migration   and invasion of GC cells, and suppressed the expression of matrix metalloproteinase 9. These results indicated that CDC42 is a key regulator involved in regulating the proliferation,   migration   and invasion of GC, and it may be considered a potential therapeutic target in GC. 
 Cdc42: Role in Cancer Management. Contribution of Cdc42, a member of Rho family, has been characterized for the beginning of variety of cellular responses including cellular transformation, cell division, cell invasion,   migration  , invadopodia formation, enzyme activity, filopodia formation, and cell polarity in cells. Deregulation of Cdc42 can alter the normal functioning of the cells, responsible for the initiation of signaling pathways and is correlated with several pathogenic processes such as cancer. Therefore, maintaining the level of Cdc42 and its effectors in cells, tumor progression can be controlled. Therefore, it can be suggested that deeper understanding about the Cdc42 contribution in cancer cell progression at molecular level can approach to the development of Cdc42 inhibitors in cancer management. 
 Blood type B antigen modulates cell   migration   through regulating cdc42 expression and activity in HaCaT cells. ABO blood group is determined by carbohydrate antigens, called ABH antigens. It has been known that the change of carbohydrate antigen expression, including ABH antigens, has correlation with the tumor metastasis and survival; however, the exact mechanism remains to be elucidated. ABH antigens are expressed not only in blood cells but also in several tissues. In epidermis, ABH antigen is expressed in the uppermost spinous and granular layer. We investigated the role of ABH antigens on the cell   migration   of HaCaT keratinocytes, which express B antigen. Knock-down of B antigen expression by small interference RNA of FUT1 inhibited HaCaT cell   migration  . At that time, we found that lamellipodia and actin fiber were also reduced by knock-down of B antigen expression. The transcription of cdc42, a kind of Rho GTPase which plays a key role in actin polymerization, was reduced by down-regulated B antigen expression. Furthermore, the reduced B antigen expression also inhibited the interaction of cdc42 and N-WASP. Collectively, our data provide a clue how ABH antigens regulate the cell   migration   mechanism. 
 Essential role of Cdc42 in Ras-induced transformation revealed by gene targeting. The ras proto-oncogene is one of the most frequently mutated genes in human cancer. However, given the prevalence of activating mutations in Ras and its association with aggressive forms of cancer, attempts to therapeutically target aberrant Ras signaling have been largely disappointing. This lack of progress highlights the deficiency in our understanding of cellular pathways required for Ras-mediated tumorigenesis and suggests the importance of identifying new molecular pathways associated with Ras-driven malignancies. Cdc42 is a Ras-related small GTPase that is known to play roles in oncogenic processes such as cell growth, survival, invasion, and   migration  . A pan-dominant negative mutant overexpression approach to suppress Cdc42 and related pathways has previously shown a requirement for Cdc42 in Ras-induced anchorage-independent cell growth, however the lack of specificity of such approaches make it difficult to determine if effects are directly related to changes in Cdc42 activity or other Rho family members. Therefore, in order to directly and unambiguously address the role of Cdc42 in Ras-mediated transformation, tumor formation and maintenance, we have developed a model of conditional cdc42 gene in Ras-transformed cells. Loss of Cdc42 drastically alters the cell morphology and inhibits proliferation, cell cycle progression and tumorigenicity of Ras-transformed cells, while non-transformed cells or c-Myc transformed cells are largely unaffected. The loss of Cdc42 in Ras-transformed cells results in reduced Akt signaling, restoration of which could partially rescues the proliferation defects associated with Cdc42 loss. Moreover, disruption of Cdc42 function in established tumors inhibited continued tumor growth. These studies implicate Cdc42 in Ras-driven tumor growth and suggest that targeting Cdc42 is beneficial in Ras-mediated malignancies. 
 Impaired NK-cell   migration   in WAS/XLT patients: role of Cdc42/WASp pathway in the control of chemokine-induced beta2 integrin high-affinity state. We analyzed the involvement of Wiskott-Aldrich syndrome protein (WASp), a critical regulator of actin cytoskeleton remodeling, in the control of natural killer (NK)-cell   migration  . NK cells derived from patients with Wiskott-Aldrich syndrome/X-linked thrombocytopenia (WAS/XLT), carrying different mutations in the WASP coding gene, displayed reduced   migration   through intercellular adhesion molecule-1 (ICAM-1), vascular cell adhesion molecule-1 (VCAM-1), or endothelial cells in response to CXCL12/stromal cell-derived factor-1 and CX3CL1/fractalkine. Inhibition of WAS/XLT NK-cell   migration   was associated with reduced ability of these cells to up-regulate the expression of CD18 activation neoepitope and to adhere to ICAM-1 or VCAM-1 following chemokine stimulation. Moreover, chemokine receptor or beta1 or beta2 integrin engagement on NK cells rapidly resulted in Cdc42 activation and WASp tyrosine phosphorylation as well as in WASp association with Fyn and Pyk-2 tyrosine kinases. NK-cell pretreatment with wiskostatin, to prevent Cdc42/WASp association, impaired chemokine-induced NK-cell   migration   through ICAM-1 and beta2 integrin activation-dependent neoepitope expression. These results show that the Cdc42/WASp pathway plays a crucial role in the regulation of NK-cell   migration   by acting as a critical component of the chemokine-induced inside-out signaling that regulates lymphocyte function-associated antigen-1 function and suggest that after integrin or chemokine receptor engagement WASp function is regulated by the coordinate action of both Cdc42 and tyrosine kinases. 
 Epstein-Barr virus-encoded LMP1 interacts with FGD4 to activate Cdc42 and thereby promote   migration   of nasopharyngeal carcinoma cells. Epstein-Barr virus (EBV) is closely associated with nasopharyngeal carcinoma (NPC), a human malignancy notorious for its highly metastatic nature. Among EBV-encoded genes, latent membrane protein 1 (LMP1) is expressed in most NPC tissues and exerts oncogenicity by engaging multiple signaling pathways in a ligand-independent manner. LMP1 expression also results in actin cytoskeleton reorganization, which modulates cell morphology and cell motility- cellular process regulated by RhoGTPases, such as Cdc42. Despite the prominent association of Cdc42 activation with tumorigenesis, the molecular basis of Cdc42 activation by LMP1 in NPC cells remains to be elucidated. Here using GST-CBD (active Cdc42-binding domain) as bait in GST pull-down assays to precipitate active Cdc42 from cell lysates, we demonstrated that LMP1 acts through its transmembrane domains to preferentially induce Cdc42 activation in various types of epithelial cells, including NPC cells. Using RNA interference combined with re-introduction experiments, we identified FGD4 (FYVE, RhoGEF and PH domain containing 4) as the GEF (guanine nucleotide exchange factor) responsible for the activation of Cdc42 by LMP1. Serial deletion experiments and co-immunoprecipitation assays further revealed that ectopically expressed FGD4 modulated LMP1-mediated Cdc42 activation by interacting with LMP1. Moreover, LMP1, through its transmembrane domains, directly bound FGD4 and enhanced FGD4 activity toward Cdc42, leading to actin cytoskeleton rearrangement and increased motility of NPC cells. Depletion of FGD4 or Cdc42 significantly reduced (∼50%) the LMP1-stimulated cell motility, an effect that was partially reversed by expression of a constitutively active mutant of Cdc42. Finally, quantitative RT-PCR and immunohistochemistry analyses showed that FGD4 and LMP1 were expressed in NPC tissues, supporting the potential physiologically relevance of this mechanism in NPC. Collectively, our results not only uncover a novel mechanism underlying LMP1-mediated Cdc42 activation, namely LMP1 interaction with FGD4, but also functionally link FGD4 to NPC tumorigenesis. 
 miR-18a inhibits CDC42 and plays a tumour suppressor role in colorectal cancer cells. The miR-17-92 cluster of microRNAs is elevated in colorectal cancer, and has a causative role in cancer development. Of the six miR-17-92 cluster members, miR-19a and b in particular are key promoters of cancer development and cell proliferation, while preliminary evidence suggests that miR-18a may act in opposition to other cluster members to decrease cell proliferation. It was hypothesised that miR-18a may have a homeostatic function in helping to contain the oncogenic effect of the entire miR-17-92 cluster, and that elevated miR-17-92 cluster activity without a corresponding increase in miR-18a may promote colorectal tumour progression. In colorectal cancer samples and corresponding normal colorectal mucosa, miR-18a displayed lower overall expression than other miR-17-92 cluster members. miR-18a was shown to have an opposing role to other miR-17-92 cluster members, in particular the key oncogenic miRNAs, miR-19a and b. Transfection of HCT116 and LIM1215 colorectal cancer cell lines with miR-18a mimics decreased proliferation, while a miR-18a inhibitor increased proliferation. miR-18a was also responsible for decreasing cell   migration  , altering cell morphology, inducing G1/S phase cell cycle arrest, increasing apoptosis, and enhancing the action of a pro-apoptotic agent. CDC42, a mediator of the PI3K pathway, was identified as a novel miR-18a target. Overexpression of miR-18a reduced CDC42 expression, and a luciferase assay confirmed that miR-18a directly targets the 3'UTR of CDC42. miR-18a mimics had a similar effect on proliferation as a small molecule inhibitor of CDC42. Inhibition of CDC42 expression is likely to be a key mechanism by which miR-18a impairs cancer cell growth, with a target protector experiment revealing miR-18a influences proliferation via direct inhibition of CDC42. Inhibition of CCND1 by miR-18a may also assist in this growth-suppression effect. The homeostatic function of miR-18a within the miR-17-92 cluster in colorectal cancer cells may be achieved through suppression of CDC42 and the PI3K pathway. 
 Cdc42 negatively regulates intrinsic   migration   of highly aggressive breast cancer cells. The small GTPase Cdc42 has been implicated as an important regulator of cell   migration  . However, whether Cdc42 plays similar role in all cancer cells irrespective of metastatic potential remains poorly defined. Here, we show by using three different breast cancer cell lines with different metastatic potential, the role of Cdc42 in cell   migration  /invasion and its relationship with a number of downstream signaling pathways controlling cell   migration  . Small interfering RNA (siRNA)-mediated knockdown of Cdc42 in two highly metastatic breast cancer cell lines (MDA-MB-231 and C3L5) resulted in enhancement, whereas the same in moderately metastatic (Hs578T) cell line resulted in inhibition of intrinsic cellular   migration  /invasion. Furthermore, Cdc42 silencing in MDA-MB-231 and C3L5 but not Hs578T cells was shown to be accompanied by increased RhoA activity and phosphorylation of protein kinase C (PKC)-δ, extracellular signal regulated kinase1/2 (Erk1/2), and protein kinase A (PKA). Pharmacological inhibition of PKCδ, MEK-Erk1/2, or PKA was shown to inhibi  t migrati  on of both control and Cdc42-silenced MDA-MB-231 cells. Furthermore, introduction of constitutively active Cdc42 was shown to decreas  e migrati  on/invasion of MDA-MB-231 and C3L5 but increas  e migrati  on/invasion of Hs578T cells. This decrease  d migrati  on/invasion of MDA-MB-231 and C3L5 cells was also shown to be accompanied by the decrease in the phosphorylations of PKCδ, Erk1/2, and PKA. These results suggested that endogenous Cdc42 could exert a negative regulatory influence on intrins  ic migrat  ion/invasion and some potentially relevant changes in phosphorylation of PKCδ, Erk1/2, and PKA of some aggressive breast cancer cells. 
 Activation of Rho GTPase Cdc42 promotes adhesion and invasion in colorectal cancer cells. The purpose of this study was to investigate the role of activated Rho GTPase cell division control protein 42 homolog (Cdc42) in colorectal cancer cell adhesion,   migration  , and invasion.The constitutively active form of Cdc42 (GFP-Cdc42L61) or control vector was overexpressed in the colorectal cancer cell line SW480. The localization of active Cdc42 was monitored by immunofluorescence staining, and the effects of active Cdc42 on cell   migration   and invasion were examined using an attachment assay, a wound healing assay, and a Matrigel   migration   assay in vitro.Immunofluorescence staining revealed that constitutively active Cdc42 predominately localized to the plasma membrane. Compared to SW480 cells transfected with the control vector, overexpression of constitutively active Cdc42 in SW480 cells promoted filopodia formation and cell stretch and dramatically enhanced cell adhesion to the coated plates. The wound healing assay revealed a significant increase of   migration   capability in SW480 cells expressing active Cdc42 compared to the control cells. Additionally, the Matrigel invasion assay demonstrated that active Cdc42 significantly promoted SW480 cell   migration   through the chamber.Our results suggest that active Rho GTPase Cdc42 can greatly enhance colorectal cancer cell SW480 to spread, migrate, and invade, which may contribute to colorectal cancer metastasis. 
 Locally excitable Cdc42 signals steer cells during chemotaxis. Neutrophils and other amoeboid cells chemotax by steering their front ends towards chemoattractant. Although Ras, Rac, Cdc42 and RhoA small GTPases all regulate chemotaxis, it has been unclear how they spatiotemporally control polarization and steering. Using fluorescence biosensors in neutrophil-like PLB-985 cells and photorelease of chemoattractant, we show that local Cdc42 signals, but not those of Rac, RhoA or Ras, precede cell turning during chemotaxis. Furthermore, pre-existing local Cdc42 signals in morphologically unpolarized cells predict the future direction of movement on uniform stimulation. Moreover, inhibition of actin polymerization uncovers recurring local Cdc42 activity pulses, suggesting that Cdc42 has the excitable characteristic of the compass activity proposed in models of chemotaxis. Globally, Cdc42 antagonizes RhoA, and maintains a steep spatial activity gradient during   migration  , whereas Ras and Rac form shallow gradients. Thus, chemotactic steering and de novo polarization are both directed by locally excitable Cdc42 signals. 
 Cdc42 overexpression induces hyperbranching in the developing mammary gland by enhancing cell   migration  . The Rho GTPase Cdc42 is overexpressed and hyperactivated in breast tumors compared to normal breast tissue. Cdc42 regulates key processes that are critical for mammary gland morphogenesis and become disrupted during the development, progression, and metastasis of breast cancer. However, the contribution of Cdc42 to normal and neoplastic mammary gland development in vivo remains poorly understood. We were therefore interested in investigating the effects of Cdc42 overexpression on mammary gland morphogenesis as a first step toward understanding how its overexpression may contribute to mammary tumorigenesis.We developed a tetracycline-regulatable Cdc42 overexpression mouse model in which Cdc42 can be inducibly overexpressed in the developing mammary gland. The effects of Cdc42 overexpression during postnatal mammary gland development were investigated using in vivo and in vitro approaches, including morphometric analysis of wholemounted mammary glands, quantification of histological markers, and primary mammary epithelial cell (MEC) functional and biochemical assays.Analysis of Cdc42-overexpressing mammary glands revealed abnormal terminal end bud (TEB) morphologies, characterized by hyperbudding and trifurcation, and increased side branching within the ductal tree. Quantification of markers of proliferation and apoptosis suggested that these phenotypes were not due to increased cell proliferation or survival. Rather, Cdc42 overexpressing MECs were more migratory and contractile and formed dysmorphic, invasive acini in three-dimensional cultures. Cdc42 and RhoA activities, phosphorylated myosin light chain, and MAPK signaling, which contribute to   migration   and invasion, were markedly elevated in Cdc42 overexpressing MECs. Interestingly, Cdc42 overexpressing mammary glands displayed several features associated with altered epithelial-stromal interactions, which are known to regulate branching morphogenesis. These included increased stromal thickness and collagen deposition, and stromal cells isolated from Cdc42 overexpressing mammary glands exhibited elevated mRNA expression of extracellular matrix proteins and remodeling enzymes.These data suggest that Cdc42 overexpression disrupts mammary gland branching morphogenesis by altering Rho GTPase and MAPK signaling, leading to increased MEC contractility and   migration   in association with stromal alterations. Our studies provide insight into how aberrant Cdc42 expression may contribute to mammary tumorigenesis. 
 MUC1 initiates Src-CrkL-Rac1/Cdc42-mediated actin cytoskeletal protrusive motility after ligating intercellular adhesion molecule-1. MUC1, a transmembrane glycoprotein of the mucin family, when aberrantly expressed on breast cancer cells is correlated with increased lymph node metastases. We have previously shown that MUC1 binds intercellular adhesion molecule-1 (ICAM-1) on surrounding accessory cells and facilitates transendothelial   migration   of MUC1-bearing cells. Nevertheless, the underlying molecular mechanism is still obscure. In the present study, we used a novel assay of actin cytoskeletal reorganization to show that by ligating ICAM-1, MUC1 triggers Rac1- and Cdc42-dependent actin cytoskeletal protrusive activity preferentially at the heterotypic cell-cell contact sites. Further, we show that these MUC1/ICAM-1 interaction-initiated lamellipodial and filopodial protrusions require Src family kinase and CT10 regulator of kinase like (CrkL) accompanied by the rapid formation of a Src-CrkL signaling complex at the MUC1 cytoplasmic domain. Through inhibition of Src kinase activity, we further revealed that Src is required for recruiting CrkL to the MUC1 cytoplasmic domain as well as mediating the observed actin cytoskeleton dynamics. These findings suggest a novel MUC1-Src-CrkL-Rac1/Cdc42 signaling cascade following ICAM-1 ligation, through which MUC1 regulates cytoskeletal reorganization and directed cell motility during cell   migration  . 
 MALAT1 induced   migration   and invasion of human breast cancer cells by competitively binding miR-1 with cdc42. Competitive endogenous messenger RNAs (ceRNAs) affect other RNAs transcription through competitively binding common microRNAs (miRNAs). In this study we identified long non-coding RNA (lncRNA) MALAT1 can function as a ceRNA of cell division cycle 42 (cdc42) 3'UTR in inducing   migration   and invasion of breast cancer cells via miR-1. We found that miR-1 bound both MALAT1 and cdc42 3'UTR directly. Further study showed that MALAT1 induced   migration   and invasion of breast cancer cells while reduced the level of cdc42. Our results suggest that MALAT1 regulated   migration   and invasion of breast cancer cells via affecting cdc42 through binding miR-1 competitively. 
 The type III TGF-beta receptor regulates epithelial and cancer cell   migration   through beta-arrestin2-mediated activation of Cdc42. Loss of expression of the TGF-beta superfamily coreceptor, the type III TGF-beta receptor (TbetaRIII or betaglycan), occurs in a broad spectrum of human cancers including breast, lung, ovarian, pancreatic, prostate, and renal cell cancer. TbetaRIII suppresses cancer progression in vivo, at least in part, by reducing cancer cell motility. However, the mechanism by which TbetaRIII regulates   migration   is unknown. Here, we demonstrate an unexpected TGF-beta signaling independent role for TbetaRIII in activating Cdc42, altering the actin cytoskeleton and reducing directional persistence to inhibit random   migration   of both cancer and normal epithelial cells. Functionally, TbetaRIII through its interaction with the scaffolding protein beta-arrestin2, activates Cdc42 and inhibits   migration  . These studies identify a TGF-beta independent homeostatic function for TbetaRIII in regulating cell   migration  . 
 Negative pressure accelerated monolayer keratinocyte healing involves Cdc42 mediated cell podia formation. Negative-pressure wound therapy (NPWT) is developed to facilitate wound healing at controlled subatmospheric pressures in modern medicine. Molecular mechanism for this therapy is still undefined.This study highlights the localization and time-course of the cell division control protein 42 (Cdc42) in the cell membrane at ambient pressure (AP) and negative pressures of 75mmHg (NP75), 125mmHg (NP125) and 175mmHg (NP175).The prepared cells were cultured in a negative pressure incubator with the same O2 and CO2 tensions at the four different pressures. The effective time, complete wound closure time, cell volume, cell viability, and the fluorescence of proliferating cell nuclear antigens (PCNA) and actins were evaluated in cells at different pressures. Wound-healing process and Cdc42 fluorescence were examined in cells with the knockdown of Cdc42. Cdc42 pathway proteins in cell membranes were analyzed after incubation at different pressures for 6 and 12h.The cells at NP125 had less wound closure time and obvious cell podia. Similar PCNA fluorescent intensity was observed in cells at different pressures. The Cdc42, neural Wiskott-Aldrich syndrome protein, and actin expression increased significantly (p<0.05) in plasma membranes of cells at NP125 for 12h. The knockdown of active Cdc42 resulted in the absence of Cdc42 expression at the cell leading edge.The activation and localization of Cdc42 pathway proteins in the cell membrane are involved in the cell podia formation in keratinocytes at NP125. NPWT may facilitate cell   migration   to accelerate wound healing. 
 IQGAPs as Key Regulators of Actin-cytoskeleton Dynamics. The actin-cytoskeleton plays a critical role in various biological processes, including cell   migration  , development, tissue remodeling, and memory formation. Both extracellular and intracellular signals regulate reorganization of the actin-cytoskeleton to modulate tissue architecture and cellular morphology in a spatiotemporal manner. Since the discovery that activation of Rho family GTPases induces actin-cytoskeleton reorganization, the mode of action of Rho family GTPases has been extensively studied and individual effectors have been characterized. The actin-binding protein IQGAP1 was identified as an effector of Rac and Cdc42 and is the founding member of the IQGAP family with two additional isoforms. The IQGAP family shows conserved domain organization, and each member displays a specific expression pattern in mammalian tissues. IQGAPs regulate the actin-cytoskeleton alone and with their binding partners, thereby controlling diverse cellular processes, such as cell   migration   and adhesion. Here, we introduce IQGAPs as an actin-cytoskeleton regulator. 
 FMNL2 drives actin-based protrusion and   migration   downstream of Cdc42. Cell   migration   entails protrusion of lamellipodia, densely packed networks of actin filaments at the cell front. Filaments are generated by nucleation, likely mediated by Arp2/3 complex and its activator Scar/WAVE. It is unclear whether formins contribute to lamellipodial actin filament nucleation or serve as elongators of filaments nucleated by Arp2/3 complex. Here we show that the Diaphanous-related formin FMNL2, also known as FRL3 or FHOD2, accumulates at lamellipodia and filopodia tips. FMNL2 is cotranslationally modified by myristoylation and regulated by interaction with the Rho-guanosine triphosphatase Cdc42. Abolition of myristoylation or Cdc42 binding interferes with proper FMNL2 activation, constituting an essential prerequisite for subcellular targeting. In vitro, C-terminal FMNL2 drives elongation rather than nucleation of actin filaments in the presence of profilin. In addition, filament ends generated by Arp2/3-mediated branching are captured and efficiently elongated by the formin. Consistent with these biochemical properties, RNAi-mediated silencing of FMNL2 expression decreases the rate of lamellipodia protrusion and, accordingly, the efficiency of cell   migration  . Our data establish that the FMNL subfamily member FMNL2 is a novel elongation factor of actin filaments that constitutes the first Cdc42 effector promoting cell   migration   and actin polymerization at the tips of lamellipodia. 
 Ack1 mediates Cdc42-dependent cell   migration   and signaling to p130Cas. We previously showed that activation of the small GTPase Cdc42 promotes breast cell   migration   on a collagen matrix. Here we further define the signaling pathways that drive this response and show that Cdc42-mediated   migration   relies on the adaptor molecule p130(Cas). Activated Cdc42 enhanced p130(Cas) phosphorylation and its binding to Crk. Cdc42-driven   migration   and p130(Cas) phosphorylation were dependent on the Cdc42 effector Ack1 (activated Cdc42-associated kinase). Ack1 formed a signaling complex that also included Cdc42, p130(Cas), and Crk, formation of which was regulated by collagen stimulation. The interaction between Ack1 and p130(Cas) occurred through their respective SH3 domains, while the substrate domain of p130(Cas) was the major site of Ack1-dependent phosphorylation. Signaling through this complex is functionally relevant, because treatment with either p130(Cas) or Ack1 siRNA blocked Cdc42-induced   migration  . These results suggest that Cdc42 exerts its effects on cell   migration   in part through its effector Ack1, which regulates p130(Cas) signaling. 
 p210(Bcr-Abl) desensitizes Cdc42 GTPase signaling for SDF-1alpha-directed   migration   in chronic myeloid leukemia cells. Chronic myeloid leukemia (CML) is a lethal hematological disorder caused by the p210(Bcr-Abl) oncogene. Previous studies have suggested that p210(Bcr-Abl) transformation contributes to homing and retention defects, typical of immature myeloid cells in CML, by attenuating chemotactic response to stromal-derived factor-1alpha (SDF-1alpha). As Rho family GTPases are key regulators of the cytoskeleton and have been previously found to interact with p210(Bcr-Abl), this study aimed to determine whether p210(Bcr-Abl) signaling affects SDF-1alpha chemotaxis through Rho GTPase signaling. We found that SDF-1alpha stimulated Cdc42 GTPase activation in myeloid progenitor 32D, but not in p210(Bcr-Abl)-transformed (32Dp210) cells. In fact, the basal level of active Cdc42 was elevated in 32Dp210 cells and mononuclear cells isolated from bone marrow of CML patients. Inhibition of p210(Bcr-Abl) kinase activity decreased basal Cdc42 activity and restored SDF-1alpha-induced Cdc42 and   migration   responses. Transduction of active Tat-Cdc42V12 abolished this reconstituted chemotactic response. As Cdc42 is particularly important in cytoskeletal remodeling and directional sensing, these results suggest that sustained activation of Cdc42 GTPase through p210(Bcr-Abl) tyrosine kinase signaling in CML cells contributes to defects in SDF-1alpha-chemotactic response due to desensitization of the actin polarization signal required for directional   migration  . 
 The suppressor of metastasis Nm23-H1 interacts with the Cdc42 Rho family member and the pleckstrin homology domain of oncoprotein Dbl-1 to suppress cell   migration  . Tumor invasion and metastasis is regulated by a number of cellular molecules known to be involved in signaling and cytoskeletal rearrangement. One of these molecules is the suppressor of tumor metastasis Nm23-H1 which linked to invasiveness and metastatic potential of human cancers. Nm23-H1 expression is down-regulated in human melanoma and invasive breast carcinoma. Recent studies have shown an association between the Nm23-H1 and oncoprotein Dbl-1 which is associated with guanine exchange and belongs to a family of Guanine Exchange Factors (GEF). In this report we show a direct interaction in vitro and in human B cells and specifically identified the pleckstrin homology domain of Dbl-1 as the domain which binds to Nm23-H1. Furthermore, Nm23-H1 and Dbl-1 colocalized in the cytoplasm of COS-7 cells when expressed exogenously and showed predominant signals at the periphery of the cells particularly at the plasma membrane. Interestingly, Dbl-1 and Cdc42 expression rescued the suppressive activities of Nm23-H1 in cell   migration   assays. We show that Cdc42 a regulatory protein involved in cytoskeletal reorganization, cell growth and development can bind to Nm23-H1 and the kinase deficient mutant H118F but only weakly to the mutant P96S which lacks the ability to suppress cell   migration   and metastasis. Cdc42 also colocalized with Nm23-H1 and the Dbl-1 proteins as specific punctate signals in the cytoplasm and at the cell membrane. Nm23-H1 also lead to the reduction in membrane ruffles and protuberances when expressed with Dbl-1 and Cdc42. Surprisingly, Nm23-H1 interacted with Cdc42 as well as Rac1 but somewhat weaker with RhoA. These studies suggests that Nm23-H1 can negatively regulate cell   migration   and tumor metastasis by modulating the activity of Cdc42 and possibly other Rho family members through interaction with Dbl-1. 
 BNIP-2 induces cell elongation and membrane protrusions by interacting with Cdc42 via a unique Cdc42-binding motif within its BNIP-2 and Cdc42GAP homology domain. The Cdc42 small GTPase regulates cytoskeletal reorganization and cell morphological changes that result in cellular extensions,   migration  , or cytokinesis. We previously showed that BNIP-2 interacted with Cdc42 and its cognate inactivator, p50RhoGAP/Cdc42GAP via its BNIP-2 and Cdc42GAP homology (BCH) domain, but its cellular and physiological roles still remain unclear. We report here that following transient expression of BNIP-2 in various cells, the expressed protein was located in irregular spots throughout the cytoplasm and concentrated at the leading edge of cellular extensions. The induced cell elongation and membrane protrusions required an intact BCH domain and were variously inhibited by coexpression of dominant negative mutants of Cdc42 (completely inhibited), Rac1 (partially inhibited), and RhoA (least inhibited). Presence of the Cdc42/Rac1 interactive binding (CRIB) motif alone as the dominant negative mutant of p21-activated kinase also inhibited the BNIP-2 effect. Bioinformatic analyses together with progressive deletional mutagenesis and binding studies revealed that a distal part of the BNIP-2 BCH domain contained a sequence with low homology to CRIB motif. However, in contrary to most effectors, BNIP-2 binding to Cdc42 was mediated exclusively via the unique sequence motif 285VPMEYVGI292. Cells expressing the BNIP-2 mutants devoid of this motif or/and the 34-amino acids immediately upstream to this sequence failed to elicit cell elongation and membrane protrusions despite that the protein still remained in the cytoplasm and interacted with Cdc42GAP. Evidence is presented where BNIP-2 in vivo induces cell dynamics by recruiting Cdc42 via its BCH domain, thus providing a novel mechanism for regulating Cdc42 signaling pathway. 
 The Cdc42 Effector Kinase PAK4 Localizes to Cell-Cell Junctions and Contributes to Establishing Cell Polarity. The serine/threonine kinase PAK4 is a Cdc42 effector whose role is not well understood; overexpression of PAK4 has been associated with some cancers, and there are reports that correlate kinase level with increased cell   migration   in vitro. Here we report that PAK4 is primarily associated with cell-cell junctions in all the cell lines we tested, and fails to accumulate at focal adhesions or at the leading edge of migrating cells. In U2OS osteosarcoma and MCF-7 breast cancer cell lines, PAK4 depletion did not affect collective cell   migration  , but affected cell polarization. By contrast, Cdc42 depletion (as reported by many studies) caused a strong defect in junctional assembly in multiple cells lines. We also report that the depletion of PAK4 protein or treatment of cells with the PAK4 inhibitor PF-3758309 can lead to defects in centrosome reorientation (polarization) after cell monolayer wounding. These experiments are consistent with PAK4 forming part of a conserved cell-cell junctional polarity Cdc42 complex. We also confirm β-catenin as a target for PAK4 in these cells. Treatment of cells with PF-3758309 caused inhibition of β-catenin Ser-675 phosphorylation, which is located predominantly at cell-cell junctions. 
 Modulation of ERK5 is a novel mechanism by which Cdc42 regulates   migration   of breast cancer cells. Members of Rho family GTPases including Cdc42 are known to play pivotal roles in cell   migration  . Cell   migration   is also known to be regulated by many protein kinases. Kinetworks KPSS 11.0 phospho-site screening of Cdc42-silenced Hs578T breast cancer cells revealed most dramatic change in ERK5 MAP kinase. In the present study, we set out to determine the relationship between Cdc42 and ERK5 and its significance in breast cancer cell   migration   and invasion. Specific siRNAs were used for knocking down Cdc42 or ERK5 in breast cancer cells. Increased ERK5 phosphorylation in breast cancer cells was achieved by infection of constitutively active MEK5 adenovirus. The cells were then subjected to cell   migration   or invasion assay without the presence of serum or any growth factor. We found that Cdc42 negatively regulated phosphorylation of ERK5, which in turn exhibited an inverse relationship with   migration   and invasiveness of breast cancer cells. To find out some in vivo relevance of the results of our in vitro experiments we also examined the expression of ERK5 in the breast cancer tissues and their adjacent normal control tissues by real-time RT-PCR and immunocytochemistry. ERK5 expression was found to be reduced in breast cancer tissues as compared with their adjacent uninvolved mammary tissues. Therefore, Cdc42 may promote breast cancer cell   migration   and invasion by inhibiting ERK5 phosphorylation and ERK5 expression may be inversely correlated with the progression of some breast tumors. 
 Cdc42 and RhoA are differentially regulated during arachidonate-mediated HeLa cell adhesion. Cell adhesion to extracellular matrix requires stimulation of an eicosanoid signaling pathway through the metabolism of arachidonate by 5-lipoxygenase to leukotrienes and cyclooxygenase-1/2 to prostaglandins, as well as activation of the small GTPase signaling pathway involving Cdc42 and Rho. These signaling pathways direct remodeling of the actin cytoskeleton during the adhesion process, specifically the polymerization of actin during cell spreading and the bundling of actin filaments when cells migrate. However, few studies linking these signaling pathways have been described in the literature. We have previously shown that HeLa cell adhesion to collagen requires oxidation of arachidonic acid (AA) by lipoxygenase for actin polymerization and cell spreading, and cyclooxygenase for bundling actin filaments during cell   migration  . We demonstrate that small GTPase activity is required for HeLa cell spreading upon gelatin, and that Cdc42 is activated while Rho is downregulated during the spreading process. Using constitutively active and dominant negative expression studies, we show that Cdc42 is required for HeLa cell spreading and   migration  , while activated RhoA is antagonistic towards spreading. Constitutively active RhoA promotes cell   migration   and increases the degree of actin bundling in HeLa cells. Further, we demonstrate that activation of either the AA oxidation pathway or the small GTPase pathway cannot rescue inhibition of spreading when the alternate pathway is blocked. Our results suggest (1) both the eicosanoid signaling pathway and small GTPase activation are required during HeLa cell adhesion, and (2) these signaling pathways converge to properly direct remodeling of the actin cytoskeleton during HeLa cell spreading and   migration   upon collagen. 
 DOCK10-mediated Cdc42 activation is necessary for amoeboid invasion of melanoma cells. Tumor cells can move in a three-dimensional (3D) environment in either mesenchymal-type or amoeboid modes. In mesenchymal-type movement, cells have an elongated morphology with Rac-induced protrusions at the leading edge. Amoeboid cells have high levels of actomyosin contractility, and movement is associated with deformation of the cell body through the matrix without proteolysis. Because signaling pathways that control the activation of GTPases for amoeboid movement are poorly understood, we sought to identify regulators of amoeboid movement by screening an siRNA library targeting guanine nucleotide exchange factors (GEFs) for Rho-family GTPases.We identified DOCK10, a Cdc42 GEF, as a key player in amoeboid   migration  ; accordingly, we find that expression of activated Cdc42 induces a mesenchymal-amoeboid transition and increases cell invasion. Silencing DOCK10 expression promotes conversion to mesenchymal   migration   and is associated with decreased MLC2 phosphorylation and increased Rac1 activation. Consequently, abrogating DOCK10 and Rac1 expression suppresses both amoeboid and mesenchymal   migration   and results in decreased invasion. We show that the Cdc42 effectors N-WASP and Pak2 are required for the maintenance of the rounded-amoeboid phenotype. Blocking Cdc42 results in loss of mesenchymal morphology, arguing that Cdc42 is also involved in mesenchymal morphology through different activation and effector pathways.Previous work has identified roles of Rho and Rac signaling in tumor cell movement, and we now elucidate novel roles of Cdc42 signaling in amoeboid and mesenchymal movement and tumor cell invasion. 
 Cdc42 inhibits ERK-mediated collagenase-1 (MMP-1) expression in collagen-activated human keratinocytes. Following injury, keratinocytes switch gene expression programs from the one that promotes differentiation to the one that supports   migration  . A common feature of human wounds and ulcerations of any form is the expression of matrix metalloproteinase 1 (MMP-1; collagenase-1) by leading-edge basal keratinocytes migrating across the dermal or provisional matrix. Induction of MMP-1 occurs by signaling from the α2β1 integrin in contact with dermal fibrillar type I collagen, and the activity of MMP-1 is required for human keratinocytes to migrate on collagen. Thus, MMP-1 serves a critical role in the repair of damaged human skin. Here, we evaluated the mechanisms controlling MMP-1 expression in primary human keratinocytes from neonatal foreskin and adult female skin. Our results demonstrate that shortly following contact with type I collagen extracellular signal-regulated kinase (ERK) and p38 mitogen-activated protein kinase were markedly activated, whereas c-Jun N-terminal kinase (JNK) phosphorylation remained at basal levels. ERK inhibition markedly blocked collagen-stimulated MMP-1 expression in keratinocytes. In contrast, inhibiting p38 or JNK pathways had no effect on MMP-1 production. Moreover, investigating the role of Rho GTPases revealed that Cdc42 attenuates MMP-1 expression by suppressing ERK activity. Thus, our data indicate that injured keratinocytes induce MMP-1 expression through ERK activation, and this process is negatively regulated by Cdc42 activity. 
 The anaplastic lymphoma kinase controls cell shape and growth of anaplastic large cell lymphoma through Cdc42 activation. Anaplastic large cell lymphoma (ALCL) is a non-Hodgkin's lymphoma that originates from T cells and frequently expresses oncogenic fusion proteins derived from chromosomal translocations or inversions of the anaplastic lymphoma kinase (ALK) gene. The proliferation and survival of ALCL cells are determined by the ALK activity. Here we show that the kinase activity of the nucleophosmin (NPM)-ALK fusion regulated the shape of ALCL cells and F-actin filament assembly in a pattern similar to T-cell receptor-stimulated cells. NPM-ALK formed a complex with the guanine exchange factor VAV1, enhancing its activation through phosphorylation. VAV1 increased Cdc42 activity, and in turn, Cdc42 regulated the shape and   migration   of ALCL cells. In vitro knockdown of VAV1 or Cdc42 by short hairpin RNA, as well as pharmacologic inhibition of Cdc42 activity by secramine, resulted in a cell cycle arrest and apoptosis of ALCL cells. Importantly, the concomitant inhibition of Cdc42 and NPM-ALK kinase acted synergistically to induce apoptosis of ALCL cells. Finally, Cdc42 was necessary for the growth as well as for the maintenance of already established lymphomas in vivo. Thus, our data open perspectives for new therapeutic strategies by revealing a mechanism of regulation of ALCL cell growth through Cdc42. 
 Deregulation of SLIT2-mediated Cdc42 activity is associated with esophageal cancer metastasis and poor prognosis. SLIT2, a secreted protein, has been found to inactivate Cdc42 GTPase to modulate neural cell   migration  . However, alteration of SLIT2-mediated Cdc42 in terms of   migration   regulation remains undefined in esophageal squamous cell carcinoma (ESCC).We report here in ESCC cell, animal, and clinical models that SLIT2 acts as a   migration   suppressor and serves as a prognostic biomarker.The immunohistochemistry data indicated that 31.8% (49 of 154) of tumors from ESCC patients showed low expression of SLIT2 protein which correlated with poor overall survival and disease-free survival. DNA methylation analysis suggested that promoter hypermethylation is responsible for low expression of SLIT2 in ESCC. Knockdown of SLIT2 increased ESCC cell   migration  , while SLIT2 stable overexpression reduced cell   migration  . ESCC cells treated with conditioned media from cells overexpressing SLIT2 also suppressed cell   migration  . Importantly, silencing of SLIT2 decreased the complex formation, and thus induced Cdc42 activity and promoted membrane localization of focal adhesion kinase and Paxillin. Anti-metastatic effect of SLIT2 was confirmed in an experimental metastasis model of SLIT2 knockdown ESCC cells.Our results provide novel evidence that low expression of SLIT2 correlates with poor prognosis and promotes metastasis in ESCC, which may be regulated by the Cdc42-mediated pathways. 
 The small Rho GTPase Cdc42 regulates neutrophil polarity via CD11b integrin signaling. Neutrophil   migration   to sites of infection is the first line of cellular defense. A key event of   migration   is the maintenance of a polarized morphology, which is characterized by a single leading edge of filamentous actin and a contractile uropod devoid of filamentous actin protrusions. Using a mouse model of high Cdc42 activity, we previously demonstrated the importance of Cdc42 activity in neutrophil   migration  . However, the specific functions of Cdc42 in this process remain to be understood. Using neutrophils genetically deficient in Cdc42, we show that Cdc42 regulates directed   migration   by maintaining neutrophil polarity. Although it is known to be activated at the front, Cdc42 suppresses protrusions at the uropod. Interestingly, Cdc42 makes use of the integrin CD11b during this process. Cdc42 determines the redistribution of CD11b at the uropod. In turn, using CD11b-null cells and CD11b crosslinking experiments, we show that CD11b modulates myosin light chain phosphorylation to suppress lateral protrusions. Our results uncover a new mechanism in which Cdc42 regulates the uropod through CD11b signaling to maintain polarity in migrating neutrophils. It also reveals new functions for CD11b in neutrophil polarity. 
 Bif-1 promotes tumor cell   migration   and metastasis via Cdc42 expression and activity. Tumor metastasis is the process by which tumor cells disseminate from tumors and enter nearby and distant microenvironments for new colonization. Bif-1 (BAX-interacting factor 1), which has a BAR domain and an SH3 domain, has been reported to be involved in cell growth, apoptosis and autophagy. However, the influence of Bif-1 on metastasis has been less studied. To understand the role of Bif-1 in metastasis, we studied the expression levels of Bif-1 in human HCC specimens using immunohistochemistry, a tissue microarray and quantitative PCR. The function of Bif-1 was assessed in   migration   and translocation assays and the pulmonary metastatic animal model. The relationship between Bif-1 and the Rho family was determined using immunoblot analyses and chromatin immunoprecipitation. The results showed that the expression of Bif-1 was higher in hepatocellular carcinoma (HCC) than matched adjacent non-tumor liver tissues. Increased Bif-1 expression was associated with tumor size and the intercellular spread and metastasis of HCC. Analysis of the relationship between Bif-1 expression and patients' clinical characteristics revealed that patients with higher levels of Bif-1 had shorter disease-free and overall survival rates. Knockdown of Bif-1 with RNAi suppressed the   migration   of HCC cells and pulmonary metastasis and decreased the expression of Cdc42, a member of the Rho family. Bif-1 localized to the cytosol and nucleus and interacted with the promoter transcription region of Cdc42, which may regulate Cdc42 expression. Our results demonstrate a novel role of Bif-1 in HCC, in which Bif-1 promotes cell metastasis by regulating Cdc42 expression and activity. 
 DOCK7 is a critical regulator of the RAGE-Cdc42 signaling axis that induces formation of dendritic pseudopodia in human cancer cells. Cellular   migration   is a fundamental process linked to cancer metastasis. Growing evidence indicates that the receptor for advanced glycation end products (RAGE) plays a pivotal role in this process. With regard to downstream signal transducers of RAGE, diaphanous-1 and activated small guanine nucleotide triphosphatases, Rac1 and Cdc42, have been identified. To obtain precise insight into the direct downstream signaling mechanism of RAGE, we screened for proteins interacting with the cytoplasmic domain of RAGE employing an immunoprecipitation-liquid chromatography coupled with an electrospray tandem mass spectrometry system. In the present study, we found that the cytoplasmic domain of RAGE interacted with an atypical DOCK180-related guanine nucleotide exchange factor, dedicator of cytokinesis protein 7 (DOCK7). DOCK7 bound to the RAGE cytoplasmic domain and transduced a signal to Cdc42, resulting in the formation of abundant highly branched filopodia-like protrusions, dendritic pseudopodia. Blocking of the function of DOCK7 greatly abrogated the formation of dendritic pseudopodia and suppressed cellular   migratio  n. These results indicate that DOCK7 functions as an essential and downstream regulator of RAGE-mediated cellular   migratio  n through the formation of dendritic pseudopodia. 
 Elevated IQGAP1 and CDC42 levels correlate with tumor malignancy of human glioma. IQGAP1 is a multifunctional scaffold protein involved in cell adhesion and cell   migration  . The abnormal expression of IQGAP1 widely exists in many cancers, but the combined biological roles of IQGAP1 and CDC42 in human glioma remain to be clarified. In this study, we investigated the associated expression level of IQGAP1, CDC42 and clinical significances in human glioma, as well as its biological functions in glioma progression. Our results revealed that IQGAP1 and CDC42 are frequently elevated in glioma tissues compared with their noncancerous counterparts, and a high expression of IQGAP1 and CDC42 correlates with tumor grades and poor overall survival of glioma patients. Moreover, the overexpression of IQGAP1 improves cell proliferation and   migration   ability of human glioma cells, whereas the knockdown of IQGAP1 by siRNA reduces cell growth and cell   migration   in vitro. These results suggest that IQGAP1, CDC42 and their interactions play important roles in human glioma carcinogenesis and progression. 
 CCL19-induced chemokine receptor 7 activates the phosphoinositide-3 kinase-mediated invasive pathway through Cdc42 in metastatic squamous cell carcinoma of the head and neck. Metastatic squamous cell carcinoma of the head and neck (SCCHN) has been shown to express chemokine receptor 7 (CCR7), which activates phosphoinositide-3 kinase (PI3K) to promote invasion and survival of SCCHN cells. We hypothesized that Cdc42 might be involved in the CCR7-PI3K pathway. Adhesion assays,   migration   assays, immunofluorescence staining, Western blotting and immunohistochemical analysis were used to find whether Cdc42 can be activated by CCL19 (the CCR7 ligand) and its role in SCCHN. Results showed that CCL19 induced polarized localization of Cdc42 and actin polymerization in the leading edge of migrating cells. The level of activated membrane-bound Cdc42 was elevated, as measured by the GTPase activity pull-down assay. The increased membrane localization and membrane-bound activity of Cdc42 were abolished by CCR7 and PI3K inhibition, indicating the involvement of Cdc42 in the CCR7-PI3K cascade. Knockdown of Cdc42 by small interfering RNA (siRNA) led to significant reduction in the activation of Rac, filamentous actin (F-actin) accumulation as well as in the   migration   and invasion induced by CCL19. Taken together, our data indicate the important role played by Cdc42 in CCL19-induced   migration   and invasion of SCCHN cells. 
 Differential expression of miR-195 in esophageal squamous cell carcinoma and miR-195 expression inhibits tumor cell proliferation and invasion by targeting of Cdc42. MicroRNAs (miRNA) have played an important role in carcinogenesis. In this study, Agilent miRNA microarray was used to identify differentially expressed miRNAs in esophageal squamous cell carcinoma (ESCC) tissues and miR-195 was downregulated in ESCC compared with normal esophageal tissues. Moreover, Cdc42 was confirmed as target gene of miR-195. Ectopic expression of miR-195 in ESCC cells significantly downregulated Cdc42 by directly binding its 3' untranslated regions, and induced G1 cell cycle arrest, leading to a significant decrease in cell growth,   migration  , and invasion in vitro. Therefore, our findings demonstrated that miR-195 may act as a tumor suppressor in ESCC by targeting Cdc42. 
 Endothelin-1 stimulates human trophoblast cell   migration   through Cdc42 activation. This study investigated the role and mechanism of Cdc42 in Endothelin-1 (ET-1)-induced trophoblast cell   migration  .We examined ET-1-mediated stimulation of trophoblast   migration   with HTR-8/SVneo cells. Cdc42 activation was measured after ET-1 treatment of HTR-8/SVneo cells. To determine the ET receptor subtype involved in ET-1-mediated Cdc42 activation, experiments were performed in the presence of ET(A) and ET(B) receptor antagonists. Finally, using siRNA we knocked down the expression of Cdc42 to examine the involvement of Cdc42 in the regulation of ET-1-stimulated trophoblast cell   migration  .ET-1 was shown to have a dose-dependent effect on trophoblast   migration  . At low concentrations of ET-1 (0.1 nmol/L) ET-1 had a stimulatory effect on cell   migration  . ET-1 (10 nmol/L) increased HTR-8/svneo cell   migration   index by 2.5 fold. ET-1 (10 nmol/L) elevated protein level and activity of Cdc42. ET-1 induced activation of Cdc42 GTPase was mediated by both ET(A) and ET(B). ET-1-induced cell   migration   was shown to be inhibited by Cdc42 siRNA.The inhibition was not mitigated by the addition of ET-1, suggesting that Cdc42 plays an important role in trophoblast   migration   and is obligatory for ET-1 action.ET-1 stimulates EVT   migration   through Cdc42 activation. 
 The mechanism of CSF-1-induced Wiskott-Aldrich syndrome protein activation in vivo: a role for phosphatidylinositol 3-kinase and Cdc42. A role for Wiskott-Aldrich syndrome protein (WASP) in chemotaxis to various agents has been demonstrated in monocyte-derived cell types. Although WASP has been shown to be activated by multiple mechanisms in vitro, it is unclear how WASP is regulated in vivo. A WASP biosensor (WASPbs), which uses intramolecular fluorescence resonance energy transfer to report WASP activation in vivo, was constructed, and following transfection of macrophages, activation of WASPbs upon treatment with colony-stimulating factor-1 (CSF-1) was detected globally as early as 30 s and remained localized to protrusive regions at later time points. Similar results were obtained when endogenous WASP activation was determined using conformation-sensitive antibodies. In vivo CSF-1-induced WASP activation was fully Cdc42-dependent. Activation of WASP in response to treatment with CSF-1 was also shown to be phosphatidylinositol 3-kinase-dependent. However, treatment with the Src family kinase inhibitors PP2 or SU6656 or disruption of the major tyrosine phosphorylation site of WASPbs (Y291F mutation) did not reduce the level of CSF-1-induced WASP activation. Our results indicate that WASP activation downstream of CSF-1R is phosphatidylinositol 3-kinase- and Cdc42-dependent consistent with an involvement of these molecules in macrophage   migration  . However, although tyrosine phosphorylation of WASP has been proposed to stimulate WASP activity, we found no evidence to indicate that this occurs in vivo. 
 MicroRNA-224 suppresses colorectal cancer cell   migration   by targeting Cdc42. The metastatic spread of tumor cells is the major risk factor affecting the clinical prognosis of colorectal cancer (CRC) patients. The metastatic phenotype can be modulated by dysregulating the synthesis of different structural and functional proteins of tumor cells. Micro(mi)RNAs are noncoding RNAs that recognize their cognate messenger (m)RNA targets by sequence-specific interactions with the 3' untranslated region and are involved in the multistep process of CRC development. The objective of this study was to investigate the expression and biological roles of miR-224 in CRC. The miR-224 expression level was assessed by a quantitative real-time PCR in 79 CRC and 18 nontumor tissues. Expression levels of miR-224 in CRC tissues were significantly lower than those in nontumor tissues. Its expression level was associated with the mutation status of the APC gene. Ectopic expression of miR-224 suppressed the migratory ability of CRC cell line, but cell proliferation was less affected. Increased miR-224 diminished Cdc42 and SMAD4 expressions at both the protein and mRNA levels and inhibited the formation of actin filaments. Overall, this study indicated a role of miR-224 in negatively regulating CRC cell   migration  . The expression level of miR-224 may be a useful predictive biomarker for CRC progression. 
 Cdc42 expression in cervical cancer and its effects on cervical tumor invasion and   migration  . The aim of the present study was to examine Cdc42 expression in cervical cancer and explore its effects on invasion and   migration   capability of cervical cancer cells. Immunohistochemistry was used to detect Cdc42 expression in normal cervical tissues as well as CIN I or below, CIN II or above, and cervical cancer tissues. Western blot analysis was used to explore Cdc42 expression in normal cervical cell line Crl-2614 and cervical cancer cell line HeLa. Plasmids of constitutively active Cdc42 (Cdc42 CA), wild-type Cdc42 (Cdc42 WT) and dominant negative Cdc42 (Cdc42 DN) were transfected, respectively, into HeLa cells to investigate the impacts of Cdc42 on   migration   and invasion of cervical cancer cells using Transwell and on cytoskeleton microfilaments using confocal microscopy after immunofluorescence staining. Cdc42 expression was gradually increased in the order of cervical tissues with CIN I or below, CIN II or above and cancer, showing significant difference (P<0.05), and was significantly higher in HeLa cells than in Crl-2614 cells (P<0.05).   Migration   ability of HeLa cells transfected with Cdc42 CA was significantly higher than that of non-transfected, as well as Cdc42 WT- or Cdc42 DN-transfected HeLa cells (P<0.05). Overexpression of Cdc42 CA can promote filopodia formation in HeLa cells. We concluded that Cdc42 overexpression significantly improved the ability of cervical cancer cells to migrate possibly due to improved pseudopodia formation. 
 Zizimin1, a novel Cdc42 activator, reveals a new GEF domain for Rho proteins. The Rho family GTPases Rac, Rho and Cdc42 are critical in regulating the actin-based cytoskeleton, cell   migration  , growth, survival and gene expression. These GTPases are activated by guanine nucleotide-exchange factors (GEFs). A biochemical search for Cdc42 activators led to the cloning of zizimin1, a new protein whose overexpression induces Cdc42 activation. Sequence comparison combined with mutational analysis identified a new domain, which we named CZH2, that mediates direct interaction with Cdc42. CZH2-containing proteins constitute a new superfamily that includes the so-called 'CDM' proteins that bind to and activate Rac. Together, the results suggest that CZH2 is a new GEF domain for the Rho family of proteins. 
 CDC42 Gtpase Activation Affects Hela Cell DNA Repair and Proliferation Following UV Radiation-Induced Genotoxic Stress. Cell division control protein 42 (CDC42) homolog is a small Rho GTPase enzyme that participates in such processes as cell cycle progression,   migration  , polarity, adhesion, and transcription. Recent studies suggest that CDC42 is a potent tumor suppressor in different tissues and is related to aging processes. Although DNA damage is crucial in aging, a potential role for CDC42 in genotoxic stress remains to be explored.   Migration  , survival/proliferation and DNA damage/repair experiments were performed to demonstrate CDC42 involvement in the recovery of HeLa cells exposed to ultraviolet radiation-induced stress. Sub-lines of HeLa cells ectopically expressing the constitutively active CDC42-V12 mutant were generated to examine whether different CDC42-GTP backgrounds might reflect different sensitivities to UV radiation. Our results show that CDC42 constitutive activation does not interfere with HeLa cell   migration   after UV radiation. However, the minor DNA damage exhibited by the CDC42-V12 mutant exposed to UV radiation most likely results in cell cycle arrest at the G2/M checkpoint and reduced proliferation and survival. HeLa cells and Mock clones, which express endogenous wild-type CDC42 and show normal activity, are more resistant to UV radiation. None of these effects are altered by pharmacological CDC42 inhibition. Finally, the phosphorylation status of the DNA damage response proteins γ-H2AX and p-Chk1 was found to be delayed and attenuated, respectively, in CDC42-V12 clones. In conclusion, the sensitivity of HeLa cells to ultraviolet radiation increases with CDC42 over-activation due to inadequate DNA repair signaling, culminating in G2/M cell accumulation, which is translated into reduced cellular proliferation and survival. 
 Effect of microRNA-206 on cytoskeleton remodelling by downregulating Cdc42 in MDA-MB-231 cells. MicroRNAs are small, noncoding, single-stranded RNAs that regulate gene expression post-transcriptionally. miR-206 is known to play an important role in breast cancer metastasis. When we sought to predict the target of miR-206 by Targetscan, Pictar and miRanda, we found Cdc42 was a potential one. In this study, we transfected miR-206 into MDA-MB-231 cells and examined Cdc42 protein expression as well as MMP-2 and MMP-9, which are also associated with metastasis of breast cancer. Since Cdc42 is involved in filopodia and invadopodia formation, we examined the morphological changes of MDA-MB-231 cells.miR-206 mimics were transfected into MDA-MB-231 cells using LipofectamineTM 2000. Protein expression was detected by Western blot. Cells were stained with FITC-phalloidin to visualize F-actin. Invasive ability and migratory ability were examined by invasion assay and   migration   assay in vitro.Cdc42, MMP-2 and MMP-9 were downregulated on the protein level. The formation of filopodia, which requires Cdc42, was inhibited in miR-206 transfected cells, even under the stimulation of EGF. The invasion and   migration   of MDA-MB-231 cells in vitro was inhibited by miR-206 too.The results suggest that miR-206 may suppress invasion and   migration   of MDA-MB-231 cells in vitro partly via regulating actin cytoskeleton remodelling such as filopodia formation. 
  GeneRIFs  
 we discovered distinct predictive performance of Rac1 and Cdc42 depending on the   migration   modes, indicating that Rac1 and Cdc42 contribute to persistent and random   migration  , respectively 
 CDC42 is a key regulator involved in regulating the proliferation,   migration   and invasion of gastric cancer. 
 miR-1 bound both MALAT1 and cdc42 3'UTR directly. Further study showed that MALAT1 induced   migration   and invasion of breast cancer cells while reduced the level of cdc42. 
 Loss of FAT1 results in decreased cell adhesion and   migration   in fibroblasts and podocytes and the decreased   migration   is partially reversed by a RAC1/CDC42 activator. 
 IQGAP1 and its two isoforms regulate the actin-cytoskeleton alone and with their binding partners, Rac and Cdc42, thereby controlling diverse cellular processes, such as cell   migration   and adhesion. (Review) 
 Cdc42 may promote breast cancer cell   migration   and invasion by inhibiting ERK5 phosphorylation and ERK5 expression may be inversely correlated with the progression of some breast tumors. 
 Findings suggest that Wnt5a enhances cell   migration   through activation of Cdc42 and inactivation of RhoA in human corneal endothelial cells. 
 Cdc42 overexpression induces hyperbranching in the developing mammary gland by enhancing cell   migration  . 
 These results indicate that Wnt5b is involved in the   migration   ability of OSCC cells through active Cdc42 and RhoA. 
 Data show that macrophages on gastric and colorectal cancer cells   migration   through epidermal growth factor receptor and phosphorylation of Akt, c-Src and ERK1/2, and led to an increase of RhoA and Cdc42 activity. 
 Study determined that p16-stimulated cell   migration   requires the Cdc42 GTPase. 
 Negative pressure wound therapy activates Cdc42 pathway facilitating   migration   to accelerate wound healing. 
 Cdc42 differentially modulate cigarette smoke-induced airway cell   migration   through p120-catenin-dependent and -independent pathways 
 endothelin 1 stimulates trophoblast cell   migration   through GTP binding protein CDC42 activation 
 FMNL2 is a novel elongation factor of actin filaments that constitutes the first Cdc42 effector promoting cell   migration   and actin polymerization at the tips of lamellipodia. 
 endogenous Cdc42 could exert a negative regulatory influence on intrinsic   migration  /invasion and some potentially relevant changes in phosphorylation of PKCdelta, Erk1/2, and PKA of some aggressive breast cancer cells 
 Important role played by Cdc42 in CCL19-induced   migration   and invasion of squamous cell carcinoma of the head and neck cells. 
 Cdc42-dependent formation of the ZO-1/MRCKbeta complex at the leading edge controls cell   migration  . 
 Naa10p binds to the GIT-binding domain of PIX, thereby preventing the formation of the GIT-PIX-Paxillin complex, resulting in reduced intrinsic Cdc42/Rac1 activity and decreased cell   migration   
 Cdc42 was found to be necessary for breast cancer cell invasion but dispensable for cell   migration   
 TSC2 has a role in controlling cell polarity and   migration   by regulating CDC42 and RAC1 activation 
 Impaired NK-cell   migration   in WAS/XLT patients: role of Cdc42/WASp pathway in the control of chemokine-induced beta2 integrin high-affinity state 
 Activation of Cdc42 GTPase through p210(Bcr-Abl) tyrosine kinase signaling in CML cells contributes to defects in SDF-1alpha-chemotactic response due to desensitization of the actin polarization signal required for directional   migration  . 
 Results suggest that adiponectin promotes the   migration   activities of endothelial progenitor cells mainly through PI3-kinase/Cdc42/Rac1. 
 an unexpected TGF-beta signaling independent role for TbetaRIII in activating Cdc42, altering the actin cytoskeleton and reducing directional persistence to inhibit random   migration   of both cancer and normal epithelial cells 
 the interaction of the RAGE cytoplasmic domain with Dia-1 is required to transduce extracellular environmental cues evoked by binding of RAGE ligands to their cell surface receptor, resulting in Rac-1 and Cdc42 activation and cellular   migration   
 Nm23-H1 can negatively regulate cell   migration   and tumor metastasis by modulating the activity of Cdc42 and possibly other Rho family members through interaction with Dbl-1 
 These findings suggest a novel MUC1-Src-CrkL-Rac1/Cdc42 signaling cascade following ICAM-1 ligation, through which MUC1 regulates cytoskeletal reorganization and directed cell motility during cell   migration  . 
 These data suggest a mechanism whereby precise spatial guanine nucleotide exchange of Cdc42 by Dbl is dependent on functional ERM proteins and is important for directional cell   migration  . 
 Maspin controls mammary tumor cell   migration   through inhibiting Rac1 and Cdc42, but not RhoA GTPase. 
 Cdc42 exerts its effects on cell   migration   in part through its effector Ack1, which regulates p130(Cas) signaling. 
  Summary  
 The protein encoded by this gene is a small GTPase of the Rho-subfamily, which regulates signaling pathways that control diverse cellular functions including cell morphology,   migration  , endocytosis and cell cycle progression. This protein is highly similar to Saccharomyces cerevisiae Cdc 42, and is able to complement the yeast cdc42-1 mutant. The product of oncogene Dbl was reported to specifically catalyze the dissociation of GDP from this protein. This protein could regulate actin polymerization through its direct binding to Neural Wiskott-Aldrich syndrome protein (N-WASP), which subsequently activates Arp2/3 complex. Alternative splicing of this gene results in multiple transcript variants. Pseudogenes of this gene have been identified on chromosomes 3, 4, 5, 7, 8 and 20. [provided by RefSeq, Apr 2013] 
  ECM  PubMed  
 Maspin controls mammary tumor cell migration through inhibiting Rac1 and Cdc42, but not the RhoA GTPase. Rac1 and Cdc42 are members of the Rho family of small GTPases that play essential roles in diverse cellular functions, including cell migration. The activities of these Rho family proteins are controlled by growth factor receptor activation and cell-  ECM   interactions. Here, we show that maspin, a well-documented tumor suppressor gene, also controls cell motility through inhibiting Rac1/Cdc42 activity. Using the GST-PAK and GST-Rho binding protein pull-down assays for GTP-bound Rac1, Cdc42, and RhoA, we showed that treatment of MDA-MB-231 tumor cells with recombinant maspin for a short time period significantly inhibited the activity of Rac1 and Cdc42, but not RhoA. The reactive site loop (RSL) within maspin protein is the functional domain involved in the inhibition. Maspin mutants with the RSL deleted or a point mutation in the RSL region lost their inhibitory activity. We further examined the ability of maspin to inhibit Rac1- and Cdc42-mediated signaling pathways and transcription factors. Treatment of MDA-MB-231 cells with maspin led to the inhibition of JNK kinase activity as assayed by immuno-kinase assays. In addition, the AP-1 transcription activity downstream of JNK kinase pathway was also reduced. Together, we have identified Rac1 and Cdc42 as the downstream targets that mediate the inhibition of mammary tumor cell migration by maspin. 
 Maspin controls mammary tumor cell migration through inhibiting Rac1 and Cdc42, but not the RhoA GTPase. Rac1 and Cdc42 are members of the Rho family of small GTPases that play essential roles in diverse cellular functions, including cell migration. The activities of these Rho family proteins are controlled by growth factor receptor activation and cell-  ECM   interactions. Here, we show that maspin, a well-documented tumor suppressor gene, also controls cell motility through inhibiting Rac1/Cdc42 activity. Using the GST-PAK and GST-Rho binding protein pull-down assays for GTP-bound Rac1, Cdc42, and RhoA, we showed that treatment of MDA-MB-231 tumor cells with recombinant maspin for a short time period significantly inhibited the activity of Rac1 and Cdc42, but not RhoA. The reactive site loop (RSL) within maspin protein is the functional domain involved in the inhibition. Maspin mutants with the RSL deleted or a point mutation in the RSL region lost their inhibitory activity. We further examined the ability of maspin to inhibit Rac1- and Cdc42-mediated signaling pathways and transcription factors. Treatment of MDA-MB-231 cells with maspin led to the inhibition of JNK kinase activity as assayed by immuno-kinase assays. In addition, the AP-1 transcription activity downstream of JNK kinase pathway was also reduced. Together, we have identified Rac1 and Cdc42 as the downstream targets that mediate the inhibition of mammary tumor cell migration by maspin. 
 Cdc42 downregulates MMP-1 expression by inhibiting the ERK1/2 pathway. The small GTPases of the Rho family are key intermediates in cellular signalling triggered by activated cell-adhesion receptors. In this study, we took advantage of RNA interference (RNAi) using small interfering RNAs (siRNAs) to define the roles of the best-characterized members of the RhoGTPase family, RhoA, Rac1 and Cdc42, in the control of MMP-1, MMP-2 and type-I-collagen expression in normal human skin fibroblasts (HSFs). A specific and long-lasting repression, up to 7 days after transfection, of the three GTPases was achieved by transient transfection of specific siRNA. The silencing of Cdc42, but not that of RhoA or Rac1, induced a 15-fold increase in MMP-1 secretion. This upregulation was confirmed at the mRNA level and observed with two different siRNAs targeting Cdc42. Such a regulation was also observed in various human cell lines and was rescued by re-expressing wild-type Cdc42 encoded by a construct bearing silent mutations impeding its recognition by the siRNA. By contrast, MMP-2 and type-I-collagen expression was not affected by the individual silencing of each Rho GTPase. Cytokine protein array, enzyme-linked immunosorbent assays and reverse-transcription PCR measurements revealed that ablation of Cdc42 induced an overexpression of interleukin 8 and MCP-1. Although these cytokines are known to induce the expression of MMP-1, we showed that they were not involved in the Cdc42-mediated upregulation of MMP-1. Silencing of Cdc42 also induced an increased phosphorylation of ERK1/2 and p38 MAP kinase. The use of chemical inhibitors on Cdc42-ablated cells revealed that the upregulation of MMP-1 is dependent on the ERK1/2 pathways, whereas the p38 MAP kinase pathway displayed an inhibitory role. Simultaneous knock-down of two or three Rho GTPases allowed us to demonstrate that the RhoA-ROCK pathway was not involved in this regulation but that the silencing of Rac1 reduced the effect of Cdc42 suppression. These data suggest that, in vivo, when cell/extracellular-matrix interactions via integrins induce cytoskeleton organization, MMP-1 expression is maintained at a low level by Cdc42 via a repression of the Rac1 and ERK1/2 pathways. Therefore, Cdc42 contributes to   ECM   homeostasis and connective tissue integrity. 
 Matrix rigidity regulates spatiotemporal dynamics of Cdc42 activity and vacuole formation kinetics of endothelial colony forming cells. Recent evidence has shown that endothelial colony forming cells (ECFCs) may serve as a cell therapy for improving blood vessel formation in subjects with vascular injury, largely due to their robust vasculogenic potential. The Rho family GTPase Cdc42 is known to play a primary role in this vasculogenesis process, but little is known about how extracellular matrix (  ECM  ) rigidity affects Cdc42 activity during the process. In this study, we addressed two questions: Does matrix rigidity affect Cdc42 activity in ECFC undergoing early vacuole formation? How is the spatiotemporal activation of Cdc42 related to ECFC vacuole formation? A fluorescence resonance energy transfer (FRET)-based Cdc42 biosensor was used to examine the effects of the rigidity of three-dimensional (3D) collagen matrices on spatiotemporal activity of Cdc42 in ECFCs. Collagen matrix stiffness was modulated by varying the collagen concentration and therefore fibril density. The results showed that soft (150 Pa) matrices induced an increased level of Cdc42 activity compared to stiff (1 kPa) matrices. Time-course imaging and colocalization analysis of Cdc42 activity and vacuole formation revealed that Cdc42 activity was colocalized to the periphery of cytoplasmic vacuoles. Moreover, soft matrices generated faster and larger vacuoles than stiff matrices. The matrix-driven vacuole formation was enhanced by a constitutively active Cdc42 mutant, but significantly inhibited by a dominant-negative Cdc42 mutant. Collectively, the results suggest that matrix rigidity is a strong regulator of Cdc42 activity and vacuole formation kinetics, and that enhanced activity of Cdc42 is an important step in early vacuole formation in ECFCs. 
  [Ee]xtracellular [Mm]atrix  PubMed  
 Prostaglandin E2-mediated migration of human trophoblast requires RAC1 and CDC42. The invasion of maternal decidua and uterine spiral arteries by a trophoblast subpopulation called extravillous trophoblast (EVT) is essential for the establishment of a normal placenta and an adequate blood flow toward the fetus. Derangements in these processes underlie pregnancy-related diseases like preeclampsia and intrauterine growth restriction. Many growth factors, growth factor binding proteins, and   extracellular matrix   components can positively or negatively regulate the proliferation, migration, and/or invasiveness of these EVT cells. RHO GTPases, including RHOA, RAC1, and CDC42, are ubiquitous proteins that control cytoskeletal changes by forming stress fibers and projecting lamellipodia and filopodia during cellular migration. We had previously shown that prostaglandin (PG) E(2) produced in abundance by the decidua promotes the migration of first-trimester human EVTs by increasing the intracellular concentration of calcium and activating calpain. Using our well-characterized immortalized EVT cell line, HTR-8/SVneo, as well as villus explants from first-trimester placentae, this study examined the role of RHO GTPases RAC1 and CDC42 in PGE(2)-mediated migratory responses of these cells. Though a RAC1 inhibitor, NSC23766 as well as RAC1 knockdown by siRNA decreased the migration of HTR-8/SVneo cells in a Transwell migration assay, this inhibition could not be restored by PGE(2) or 17-phenyl trinor PGE(2) (PGE receptor PTGER1 agonist) or PGE(1) Alcohol (PGE receptor PTGER4 agonist). Similar results were noted for EVT cell spreading in villus explants. Furthermore, CDC42 silencing using siRNA inhibited PGE(2)-induced migration of HTR-8/SVneo cells. Finally, the treatment of EVT cells with PGE(2), PTGER1 agonist, or PTGER4 agonist activated RAC1 and CDC42 at 10 min, suggesting that RAC1 and CDC42 play an essential role in PGE(2)-mediated migration of human EVTs. 
 The interaction of IQGAP1 with the exocyst complex is required for tumor cell invasion downstream of Cdc42 and RhoA. Invadopodia are actin-based membrane protrusions formed at contact sites between invasive tumor cells and the   extracellular matrix   with matrix proteolytic activity. Actin regulatory proteins participate in invadopodia formation, whereas matrix degradation requires metalloproteinases (MMPs) targeted to invadopodia. In this study, we show that the vesicle-tethering exocyst complex is required for matrix proteolysis and invasion of breast carcinoma cells. We demonstrate that the exocyst subunits Sec3 and Sec8 interact with the polarity protein IQGAP1 and that this interaction is triggered by active Cdc42 and RhoA, which are essential for matrix degradation. Interaction between IQGAP1 and the exocyst is necessary for invadopodia activity because enhancement of matrix degradation induced by the expression of IQGAP1 is lost upon deletion of the exocyst-binding site. We further show that the exocyst and IQGAP1 are required for the accumulation of cell surface membrane type 1 MMP at invadopodia. Based on these results, we propose that invadopodia function in tumor cells relies on the coordination of cytoskeletal assembly and exocytosis downstream of Rho guanosine triphosphatases. 
 The integrin cytoplasmic domain-associated protein ICAP-1 binds and regulates Rho family GTPases during cell spreading. Using two-hybrid screening, we isolated the integrin cytoplasmic domain-associated protein (ICAP-1), an interactor for the COOH terminal region of the beta1A integrin cytoplasmic domain. To investigate the role of ICAP-1 in integrin-mediated adhesive function, we expressed the full-length molecule in NIH3T3 cells. ICAP-1 expression strongly prevents NIH3T3 cell spreading on   extracellular matrix  . This inhibition is transient and can be counteracted by coexpression of a constitutively activated mutant of Cdc42, suggesting that ICAP-1 acts upstream of this GTPase. In addition, we found that ICAP-1 binds both to Cdc42 and Rac1 in vitro, and its expression markedly inhibits activation of these GTPases during integrin-mediated cell adhesion to fibronectin as detected by PAK binding assay. In the attempt to define the molecular mechanism of this inhibition, we show that ICAP-1 reduces both the intrinsic and the exchange factor-induced dissociation of GDP from Cdc42; moreover, purified ICAP-1 displaces this GTPase from cellular membranes. Together, these data show for the first time that ICAP-1 regulates Rho family GTPases during integrin-mediated cell matrix adhesion, acting as guanine dissociation inhibitor. 
 Cdc42 promotes transendothelial migration of cancer cells through β1 integrin. Cancer cells interact with endothelial cells during the process of metastatic spreading. Here, we use a small interfering RNA screen targeting Rho GTPases in cancer cells to identify Cdc42 as a critical regulator of cancer cell-endothelial cell interactions and transendothelial migration. We find that Cdc42 regulates β1 integrin expression at the transcriptional level via the transcription factor serum response factor (SRF). β1 integrin is the main target for Cdc42-mediating interaction of cancer cells with endothelial cells and the underlyi  ng extracellular mat  rix, as exogenous β1 integrin expression was sufficient to rescue the Cdc42-silencing phenotype. We show that Cdc42 was required in vivo for cancer cell spreading and protrusion extension along blood vessels and retention in the lungs. Interestingly, transient Cdc42 depletion was sufficient to decrease experimental lung metastases, which suggests that its role in endothelial attachment is important for metastasis. By identifying β1 integrin as a transcriptional target of Cdc42, our results provide new insight into Cdc42 function. 
 Coronin-1C and RCC2 guide mesenchymal migration by trafficking Rac1 and controlling GEF exposure. Sustained forward migration through a fibrillar   extracellular matrix   requires localization of protrusive signals. Contact with fibronectin at the tip of a cell protrusion activates Rac1, and for linear migration it is necessary to dampen Rac1 activity in off-axial positions and redistribute Rac1 from non-protrusive membrane to the leading edge. Here, we identify interactions between coronin-1C (Coro1C), RCC2 and Rac1 that focus active Rac1 to a single protrusion. Coro1C mediates release of inactive Rac1 from non-protrusive membrane and is necessary for Rac1 redistribution to a protrusive tip and fibronectin-dependent Rac1 activation. The second component, RCC2, attenuates Rac1 activation outside the protrusive tip by binding to the Rac1 switch regions and competitively inhibiting GEF action, thus preventing off-axial protrusion. Depletion of Coro1C or RCC2 by RNA interference causes loss of cell polarity that results in shunting migration in 1D or 3D culture systems. Furthermore, morpholinos against Coro1C or RCC2, or mutation of any of the binding sites in the Rac1-RCC2-Coro1C complex delays the arrival of neural crest derivatives at the correct location in developing zebrafish, demonstrating the crucial role in migration guidance in vivo. 
 Rapid Remodeling of Invadosomes by Gi-coupled Receptors: DISSECTING THE ROLE OF Rho GTPases. Invadosomes are actin-rich membrane protrusions that degrade the   extracellular matrix   to drive tumor cell invasion. Key players in invadosome formation are c-Src and Rho family GTPases. Invadosomes can reassemble into circular rosette-like superstructures, but the underlying signaling mechanisms remain obscure. Here we show that Src-induced invadosomes in human melanoma cells (A375M and MDA-MB-435) undergo rapid remodeling into dynamic   extracellular matrix  -degrading rosettes by distinct G protein-coupled receptor agonists, notably lysophosphatidic acid (LPA; acting through the LPA1 receptor) and endothelin. Agonist-induced rosette formation is blocked by pertussis toxin, dependent on PI3K activity and accompanied by localized production of phosphatidylinositol 3,4,5-trisphosphate, whereas MAPK and Ca(2+) signaling are dispensable. Using FRET-based biosensors, we show that LPA and endothelin transiently activate Cdc42 through Gi, concurrent with a biphasic decrease in Rac activity and differential effects on RhoA. Cdc42 activity is essential for rosette formation, whereas G12/13-mediated RhoA-ROCK signaling suppresses the remodeling process. Our results reveal a Gi-mediated Cdc42 signaling axis by which G protein-coupled receptors trigger invadosome remodeling, the degree of which is dictated by the Cdc42-RhoA activity balance. 
 Proteomic analysis of human parotid gland exosomes by multidimensional protein identification technology (MudPIT). Human ductal saliva contributes over a thousand unique proteins to whole oral fluids. The mechanism by which most of these proteins are secreted by salivary glands remains to be determined. The present study used a mass spectrometry-based, shotgun proteomics approach to explore the possibility that a subset of the proteins found in saliva are derived from exosomes, membrane-bound vesicles of endosomal origin within multivesicular endosomes. Using MudPIT (multidimensional protein identification technology) mass spectrometry, we catalogued 491 proteins in the exosome fraction of human parotid saliva. Many of these proteins were previously observed in ductal saliva from parotid glands (265 proteins). Furthermore, 72 of the proteins in parotid exosomes overlap with those previously identified as urinary exosome proteins, proteins which are also frequently associated with exosomes from other tissues and cell types. Gene Ontology (GO) and KEGG pathway analyses found that cytosolic proteins comprise the largest category of proteins in parotid exosomes (43%), involved in such processes as phosphatidylinositol signaling system, calcium signaling pathway, inositol metabolism, protein export, and signal transduction, among others; whereas the integral plasma membrane proteins and associated/peripheral plasma membrane proteins (26%) were associated with   extracellular matrix  -receptor interaction, epithelial cell signaling, T-cell and B-cell receptor signaling, cytokine receptor interaction, and antigen processing and presentation, among other biological functions. In addition, these putative saliva exosomal proteins were linked to specific diseases (e.g., neurodegenerative disorders, prion disease, cancers, type I and II diabetes). Consequently, parotid glands secrete exosomes that reflect the metabolic and functional status of the gland and may also carry informative protein markers useful in the diagnosis and treatment of systemic diseases. 
 A novel function of heparan sulfate in the regulation of cell-cell fusion. Despite the important contribution of cell-cell fusion in the development and physiology of eukaryotes, little is known about the mechanisms that regulate this process. Our study shows that glycosaminoglycans and more specifically heparan sulfate (HS) expressed on the cell surface and   extracellular matrix   may act as negative regulator of cell-cell fusion. Using herpes simplex virus type-1 as a tool to enhance cell-cell fusion, we demonstrate that the absence of HS expression on the cell surface results in a significant increase in cell-cell fusion. An identical phenomenon was observed when other viruses or polyethylene glycol was used as fusion enhancer. Cells deficient in HS biosynthesis showed increased activity of two Rho GTPases, RhoA and Cdc42, both of which showed a correlation between increased activity and increased cell-cell fusion. This could serve as a possible explanation as to why HS-deficient cells showed significantly enhanced cell-cell fusion and suggests that HS could regulate fusion via fine tuning of RhoA and Cdc42 activities. 
 Extension, retraction and contraction in the formation of a dendritic cell dendrite: distinct roles for Rho GTPases. The morphology of antigen-presenting dendritic cells (DC) is characterized by the possession of numerous long arborizing processes known as dendrites. The formation of these processes by DC, both in the periphery and in lymphoid organs, is believed to contribute to the remarkable efficiency with which they take up, process and present antigen to T cells. However, the process of dendrite formation and the signaling pathways that lead to the formation of these dendrites remain obscure. In this study we describe an in vitro model in which human immature DC form long processes similar to those formed in vivo. The formation of these processes involves initial attachment of a cell protrusion to the   extracellular matrix   substrate, and subsequent movement of the cell body away from the adhesion site, leaving behind a long slender dendrite. Dendrite formation, but not their maintenance, was found to be dependent on the activity of Rho GTPases. More specifically, Cdc42 and Rac1 were both required for the migration step of process formation, promoting cell spreading and extension. In contrast, Rho, and its downstream effector p160ROCK, regulated the release of adhesions to the substratum, and associated cellular contraction. Consequently, inhibition of Rho/p160ROCK leads to the formation of longer dendrites. DC therefore coordinate adhesion and protrusion to perform a specialized process of cellular morphogenesis, which differentiates these cells from all other cells of the immune system and may contribute to their distinctive function. 
 Rapid Remodeling of Invadosomes by Gi-coupled Receptors: DISSECTING THE ROLE OF Rho GTPases. Invadosomes are actin-rich membrane protrusions that degrade the   extracellular matrix   to drive tumor cell invasion. Key players in invadosome formation are c-Src and Rho family GTPases. Invadosomes can reassemble into circular rosette-like superstructures, but the underlying signaling mechanisms remain obscure. Here we show that Src-induced invadosomes in human melanoma cells (A375M and MDA-MB-435) undergo rapid remodeling into dynamic   extracellular matrix  -degrading rosettes by distinct G protein-coupled receptor agonists, notably lysophosphatidic acid (LPA; acting through the LPA1 receptor) and endothelin. Agonist-induced rosette formation is blocked by pertussis toxin, dependent on PI3K activity and accompanied by localized production of phosphatidylinositol 3,4,5-trisphosphate, whereas MAPK and Ca(2+) signaling are dispensable. Using FRET-based biosensors, we show that LPA and endothelin transiently activate Cdc42 through Gi, concurrent with a biphasic decrease in Rac activity and differential effects on RhoA. Cdc42 activity is essential for rosette formation, whereas G12/13-mediated RhoA-ROCK signaling suppresses the remodeling process. Our results reveal a Gi-mediated Cdc42 signaling axis by which G protein-coupled receptors trigger invadosome remodeling, the degree of which is dictated by the Cdc42-RhoA activity balance. 
 Cdc42 promotes transendothelial migration of cancer cells through β1 integrin. Cancer cells interact with endothelial cells during the process of metastatic spreading. Here, we use a small interfering RNA screen targeting Rho GTPases in cancer cells to identify Cdc42 as a critical regulator of cancer cell-endothelial cell interactions and transendothelial migration. We find that Cdc42 regulates β1 integrin expression at the transcriptional level via the transcription factor serum response factor (SRF). β1 integrin is the main target for Cdc42-mediating interaction of cancer cells with endothelial cells and the underlyi  ng extracellular mat  rix, as exogenous β1 integrin expression was sufficient to rescue the Cdc42-silencing phenotype. We show that Cdc42 was required in vivo for cancer cell spreading and protrusion extension along blood vessels and retention in the lungs. Interestingly, transient Cdc42 depletion was sufficient to decrease experimental lung metastases, which suggests that its role in endothelial attachment is important for metastasis. By identifying β1 integrin as a transcriptional target of Cdc42, our results provide new insight into Cdc42 function. 
 A novel function of heparan sulfate in the regulation of cell-cell fusion. Despite the important contribution of cell-cell fusion in the development and physiology of eukaryotes, little is known about the mechanisms that regulate this process. Our study shows that glycosaminoglycans and more specifically heparan sulfate (HS) expressed on the cell surface and   extracellular matrix   may act as negative regulator of cell-cell fusion. Using herpes simplex virus type-1 as a tool to enhance cell-cell fusion, we demonstrate that the absence of HS expression on the cell surface results in a significant increase in cell-cell fusion. An identical phenomenon was observed when other viruses or polyethylene glycol was used as fusion enhancer. Cells deficient in HS biosynthesis showed increased activity of two Rho GTPases, RhoA and Cdc42, both of which showed a correlation between increased activity and increased cell-cell fusion. This could serve as a possible explanation as to why HS-deficient cells showed significantly enhanced cell-cell fusion and suggests that HS could regulate fusion via fine tuning of RhoA and Cdc42 activities. 
 Prostaglandin E2-mediated migration of human trophoblast requires RAC1 and CDC42. The invasion of maternal decidua and uterine spiral arteries by a trophoblast subpopulation called extravillous trophoblast (EVT) is essential for the establishment of a normal placenta and an adequate blood flow toward the fetus. Derangements in these processes underlie pregnancy-related diseases like preeclampsia and intrauterine growth restriction. Many growth factors, growth factor binding proteins, and   extracellular matrix   components can positively or negatively regulate the proliferation, migration, and/or invasiveness of these EVT cells. RHO GTPases, including RHOA, RAC1, and CDC42, are ubiquitous proteins that control cytoskeletal changes by forming stress fibers and projecting lamellipodia and filopodia during cellular migration. We had previously shown that prostaglandin (PG) E(2) produced in abundance by the decidua promotes the migration of first-trimester human EVTs by increasing the intracellular concentration of calcium and activating calpain. Using our well-characterized immortalized EVT cell line, HTR-8/SVneo, as well as villus explants from first-trimester placentae, this study examined the role of RHO GTPases RAC1 and CDC42 in PGE(2)-mediated migratory responses of these cells. Though a RAC1 inhibitor, NSC23766 as well as RAC1 knockdown by siRNA decreased the migration of HTR-8/SVneo cells in a Transwell migration assay, this inhibition could not be restored by PGE(2) or 17-phenyl trinor PGE(2) (PGE receptor PTGER1 agonist) or PGE(1) Alcohol (PGE receptor PTGER4 agonist). Similar results were noted for EVT cell spreading in villus explants. Furthermore, CDC42 silencing using siRNA inhibited PGE(2)-induced migration of HTR-8/SVneo cells. Finally, the treatment of EVT cells with PGE(2), PTGER1 agonist, or PTGER4 agonist activated RAC1 and CDC42 at 10 min, suggesting that RAC1 and CDC42 play an essential role in PGE(2)-mediated migration of human EVTs. 
 The interaction of IQGAP1 with the exocyst complex is required for tumor cell invasion downstream of Cdc42 and RhoA. Invadopodia are actin-based membrane protrusions formed at contact sites between invasive tumor cells and the   extracellular matrix   with matrix proteolytic activity. Actin regulatory proteins participate in invadopodia formation, whereas matrix degradation requires metalloproteinases (MMPs) targeted to invadopodia. In this study, we show that the vesicle-tethering exocyst complex is required for matrix proteolysis and invasion of breast carcinoma cells. We demonstrate that the exocyst subunits Sec3 and Sec8 interact with the polarity protein IQGAP1 and that this interaction is triggered by active Cdc42 and RhoA, which are essential for matrix degradation. Interaction between IQGAP1 and the exocyst is necessary for invadopodia activity because enhancement of matrix degradation induced by the expression of IQGAP1 is lost upon deletion of the exocyst-binding site. We further show that the exocyst and IQGAP1 are required for the accumulation of cell surface membrane type 1 MMP at invadopodia. Based on these results, we propose that invadopodia function in tumor cells relies on the coordination of cytoskeletal assembly and exocytosis downstream of Rho guanosine triphosphatases. 
 Extension, retraction and contraction in the formation of a dendritic cell dendrite: distinct roles for Rho GTPases. The morphology of antigen-presenting dendritic cells (DC) is characterized by the possession of numerous long arborizing processes known as dendrites. The formation of these processes by DC, both in the periphery and in lymphoid organs, is believed to contribute to the remarkable efficiency with which they take up, process and present antigen to T cells. However, the process of dendrite formation and the signaling pathways that lead to the formation of these dendrites remain obscure. In this study we describe an in vitro model in which human immature DC form long processes similar to those formed in vivo. The formation of these processes involves initial attachment of a cell protrusion to the   extracellular matrix   substrate, and subsequent movement of the cell body away from the adhesion site, leaving behind a long slender dendrite. Dendrite formation, but not their maintenance, was found to be dependent on the activity of Rho GTPases. More specifically, Cdc42 and Rac1 were both required for the migration step of process formation, promoting cell spreading and extension. In contrast, Rho, and its downstream effector p160ROCK, regulated the release of adhesions to the substratum, and associated cellular contraction. Consequently, inhibition of Rho/p160ROCK leads to the formation of longer dendrites. DC therefore coordinate adhesion and protrusion to perform a specialized process of cellular morphogenesis, which differentiates these cells from all other cells of the immune system and may contribute to their distinctive function. 
 Discoidin domain receptor 1 controls linear invadosome formation via a Cdc42-Tuba pathway. Accumulation of type I collagen fibrils in tumors is associated with an increased risk of metastasis. Invadosomes are F-actin structures able to degrade the   extracellular matrix  . We previously found that collagen I fibrils induced the formation of peculiar linear invadosomes in an unexpected integrin-independent manner. Here, we show that Discoidin Domain Receptor 1 (DDR1), a collagen receptor overexpressed in cancer, colocalizes with linear invadosomes in tumor cells and is required for their formation and matrix degradation ability. Unexpectedly, DDR1 kinase activity is not required for invadosome formation or activity, nor is Src tyrosine kinase. We show that the RhoGTPase Cdc42 is activated on collagen in a DDR1-dependent manner. Cdc42 and its specific guanine nucleotide-exchange factor (GEF), Tuba, localize to linear invadosomes, and both are required for linear invadosome formation. Finally, DDR1 depletion blocked cell invasion in a collagen gel. Altogether, our data uncover an important role for DDR1, acting through Tuba and Cdc42, in proteolysis-based cell invasion in a collagen-rich environment. 
 Cdc42 and formin activity control non-muscle myosin dynamics during Drosophila heart morphogenesis. During heart formation, a network of transcription factors and signaling pathways guide cardiac cell fate and differentiation, but the genetic mechanisms orchestrating heart assembly and lumen formation remain unclear. Here, we show that the small GTPase Cdc42 is essential for Drosophila melanogaster heart morphogenesis and lumen formation. Cdc42 genetically interacts with the cardiogenic transcription factor tinman; with dDAAM which belongs to the family of actin organizing formins; and with zipper, which encodes nonmuscle myosin II. Zipper is required for heart lumen formation, and its spatiotemporal activity at the prospective luminal surface is controlled by Cdc42. Heart-specific expression of activated Cdc42, or the regulatory formins dDAAM and Diaphanous caused mislocalization of Zipper and induced ectopic heart lumina, as characterized by luminal markers such as the   extracellular matrix   protein Slit. Placement of Slit at the lumen surface depends on Cdc42 and formin function. Thus, Cdc42 and formins play pivotal roles in heart lumen formation through the spatiotemporal regulation of the actomyosin network. 
 Cdc42 overexpression induces hyperbranching in the developing mammary gland by enhancing cell migration. The Rho GTPase Cdc42 is overexpressed and hyperactivated in breast tumors compared to normal breast tissue. Cdc42 regulates key processes that are critical for mammary gland morphogenesis and become disrupted during the development, progression, and metastasis of breast cancer. However, the contribution of Cdc42 to normal and neoplastic mammary gland development in vivo remains poorly understood. We were therefore interested in investigating the effects of Cdc42 overexpression on mammary gland morphogenesis as a first step toward understanding how its overexpression may contribute to mammary tumorigenesis.We developed a tetracycline-regulatable Cdc42 overexpression mouse model in which Cdc42 can be inducibly overexpressed in the developing mammary gland. The effects of Cdc42 overexpression during postnatal mammary gland development were investigated using in vivo and in vitro approaches, including morphometric analysis of wholemounted mammary glands, quantification of histological markers, and primary mammary epithelial cell (MEC) functional and biochemical assays.Analysis of Cdc42-overexpressing mammary glands revealed abnormal terminal end bud (TEB) morphologies, characterized by hyperbudding and trifurcation, and increased side branching within the ductal tree. Quantification of markers of proliferation and apoptosis suggested that these phenotypes were not due to increased cell proliferation or survival. Rather, Cdc42 overexpressing MECs were more migratory and contractile and formed dysmorphic, invasive acini in three-dimensional cultures. Cdc42 and RhoA activities, phosphorylated myosin light chain, and MAPK signaling, which contribute to migration and invasion, were markedly elevated in Cdc42 overexpressing MECs. Interestingly, Cdc42 overexpressing mammary glands displayed several features associated with altered epithelial-stromal interactions, which are known to regulate branching morphogenesis. These included increased stromal thickness and collagen deposition, and stromal cells isolated from Cdc42 overexpressing mammary glands exhibited elevated mRNA expression of   extracellular matrix   proteins and remodeling enzymes.These data suggest that Cdc42 overexpression disrupts mammary gland branching morphogenesis by altering Rho GTPase and MAPK signaling, leading to increased MEC contractility and migration in association with stromal alterations. Our studies provide insight into how aberrant Cdc42 expression may contribute to mammary tumorigenesis. 
 Matrix rigidity regulates spatiotemporal dynamics of Cdc42 activity and vacuole formation kinetics of endothelial colony forming cells. Recent evidence has shown that endothelial colony forming cells (ECFCs) may serve as a cell therapy for improving blood vessel formation in subjects with vascular injury, largely due to their robust vasculogenic potential. The Rho family GTPase Cdc42 is known to play a primary role in this vasculogenesis process, but little is known about how   extracellular matrix   (ECM) rigidity affects Cdc42 activity during the process. In this study, we addressed two questions: Does matrix rigidity affect Cdc42 activity in ECFC undergoing early vacuole formation? How is the spatiotemporal activation of Cdc42 related to ECFC vacuole formation? A fluorescence resonance energy transfer (FRET)-based Cdc42 biosensor was used to examine the effects of the rigidity of three-dimensional (3D) collagen matrices on spatiotemporal activity of Cdc42 in ECFCs. Collagen matrix stiffness was modulated by varying the collagen concentration and therefore fibril density. The results showed that soft (150 Pa) matrices induced an increased level of Cdc42 activity compared to stiff (1 kPa) matrices. Time-course imaging and colocalization analysis of Cdc42 activity and vacuole formation revealed that Cdc42 activity was colocalized to the periphery of cytoplasmic vacuoles. Moreover, soft matrices generated faster and larger vacuoles than stiff matrices. The matrix-driven vacuole formation was enhanced by a constitutively active Cdc42 mutant, but significantly inhibited by a dominant-negative Cdc42 mutant. Collectively, the results suggest that matrix rigidity is a strong regulator of Cdc42 activity and vacuole formation kinetics, and that enhanced activity of Cdc42 is an important step in early vacuole formation in ECFCs. 
 Cdc42 and RhoA are differentially regulated during arachidonate-mediated HeLa cell adhesion. Cell adhesion to   extracellular matrix   requires stimulation of an eicosanoid signaling pathway through the metabolism of arachidonate by 5-lipoxygenase to leukotrienes and cyclooxygenase-1/2 to prostaglandins, as well as activation of the small GTPase signaling pathway involving Cdc42 and Rho. These signaling pathways direct remodeling of the actin cytoskeleton during the adhesion process, specifically the polymerization of actin during cell spreading and the bundling of actin filaments when cells migrate. However, few studies linking these signaling pathways have been described in the literature. We have previously shown that HeLa cell adhesion to collagen requires oxidation of arachidonic acid (AA) by lipoxygenase for actin polymerization and cell spreading, and cyclooxygenase for bundling actin filaments during cell migration. We demonstrate that small GTPase activity is required for HeLa cell spreading upon gelatin, and that Cdc42 is activated while Rho is downregulated during the spreading process. Using constitutively active and dominant negative expression studies, we show that Cdc42 is required for HeLa cell spreading and migration, while activated RhoA is antagonistic towards spreading. Constitutively active RhoA promotes cell migration and increases the degree of actin bundling in HeLa cells. Further, we demonstrate that activation of either the AA oxidation pathway or the small GTPase pathway cannot rescue inhibition of spreading when the alternate pathway is blocked. Our results suggest (1) both the eicosanoid signaling pathway and small GTPase activation are required during HeLa cell adhesion, and (2) these signaling pathways converge to properly direct remodeling of the actin cytoskeleton during HeLa cell spreading and migration upon collagen. 
  [Ii]nvadosome  PubMed  
 Rapid Remodeling of   Invadosome  s by Gi-coupled Receptors: DISSECTING THE ROLE OF Rho GTPases.   Invadosome  s are actin-rich membrane protrusions that degrade the extracellular matrix to drive tumor cell invasion. Key players in   invadosome   formation are c-Src and Rho family GTPases.   Invadosome  s can reassemble into circular rosette-like superstructures, but the underlying signaling mechanisms remain obscure. Here we show that Src-induced   invadosome  s in human melanoma cells (A375M and MDA-MB-435) undergo rapid remodeling into dynamic extracellular matrix-degrading rosettes by distinct G protein-coupled receptor agonists, notably lysophosphatidic acid (LPA; acting through the LPA1 receptor) and endothelin. Agonist-induced rosette formation is blocked by pertussis toxin, dependent on PI3K activity and accompanied by localized production of phosphatidylinositol 3,4,5-trisphosphate, whereas MAPK and Ca(2+) signaling are dispensable. Using FRET-based biosensors, we show that LPA and endothelin transiently activate Cdc42 through Gi, concurrent with a biphasic decrease in Rac activity and differential effects on RhoA. Cdc42 activity is essential for rosette formation, whereas G12/13-mediated RhoA-ROCK signaling suppresses the remodeling process. Our results reveal a Gi-mediated Cdc42 signaling axis by which G protein-coupled receptors trigger   invadosome   remodeling, the degree of which is dictated by the Cdc42-RhoA activity balance. 
 Rapid Remodeling of   Invadosome  s by Gi-coupled Receptors: DISSECTING THE ROLE OF Rho GTPases.   Invadosome  s are actin-rich membrane protrusions that degrade the extracellular matrix to drive tumor cell invasion. Key players in   invadosome   formation are c-Src and Rho family GTPases.   Invadosome  s can reassemble into circular rosette-like superstructures, but the underlying signaling mechanisms remain obscure. Here we show that Src-induced   invadosome  s in human melanoma cells (A375M and MDA-MB-435) undergo rapid remodeling into dynamic extracellular matrix-degrading rosettes by distinct G protein-coupled receptor agonists, notably lysophosphatidic acid (LPA; acting through the LPA1 receptor) and endothelin. Agonist-induced rosette formation is blocked by pertussis toxin, dependent on PI3K activity and accompanied by localized production of phosphatidylinositol 3,4,5-trisphosphate, whereas MAPK and Ca(2+) signaling are dispensable. Using FRET-based biosensors, we show that LPA and endothelin transiently activate Cdc42 through Gi, concurrent with a biphasic decrease in Rac activity and differential effects on RhoA. Cdc42 activity is essential for rosette formation, whereas G12/13-mediated RhoA-ROCK signaling suppresses the remodeling process. Our results reveal a Gi-mediated Cdc42 signaling axis by which G protein-coupled receptors trigger   invadosome   remodeling, the degree of which is dictated by the Cdc42-RhoA activity balance. 
 Discoidin domain receptor 1 controls linear   invadosome   formation via a Cdc42-Tuba pathway. Accumulation of type I collagen fibrils in tumors is associated with an increased risk of metastasis.   Invadosome  s are F-actin structures able to degrade the extracellular matrix. We previously found that collagen I fibrils induced the formation of peculiar linear   invadosome  s in an unexpected integrin-independent manner. Here, we show that Discoidin Domain Receptor 1 (DDR1), a collagen receptor overexpressed in cancer, colocalizes with linear   invadosome  s in tumor cells and is required for their formation and matrix degradation ability. Unexpectedly, DDR1 kinase activity is not required for   invadosome   formation or activity, nor is Src tyrosine kinase. We show that the RhoGTPase Cdc42 is activated on collagen in a DDR1-dependent manner. Cdc42 and its specific guanine nucleotide-exchange factor (GEF), Tuba, localize to linear   invadosome  s, and both are required for linear   invadosome   formation. Finally, DDR1 depletion blocked cell invasion in a collagen gel. Altogether, our data uncover an important role for DDR1, acting through Tuba and Cdc42, in proteolysis-based cell invasion in a collagen-rich environment. 
  GeneRIFs  
 Data suggest LPA (lysophosphatidic acid; acting via LPAR1) and endothelin activate Cdc42, concurrent with biphasic decrease in Rac1 activity, differential effects on RhoA; LPA/endothelin stim. leads to remodeling of   invadosome  s in melanoma cells. 
 Cdc42 and its specific guanine nucleotide-exchange factor (GEF), Tuba, localize to linear   invadosome  s, and both are required for linear   invadosome   formation 
  [Ii]nvasive  PubMed  
 Prostaglandin E2-mediated migration of human trophoblast requires RAC1 and CDC42. The invasion of maternal decidua and uterine spiral arteries by a trophoblast subpopulation called extravillous trophoblast (EVT) is essential for the establishment of a normal placenta and an adequate blood flow toward the fetus. Derangements in these processes underlie pregnancy-related diseases like preeclampsia and intrauterine growth restriction. Many growth factors, growth factor binding proteins, and extracellular matrix components can positively or negatively regulate the proliferation, migration, and/or   invasive  ness of these EVT cells. RHO GTPases, including RHOA, RAC1, and CDC42, are ubiquitous proteins that control cytoskeletal changes by forming stress fibers and projecting lamellipodia and filopodia during cellular migration. We had previously shown that prostaglandin (PG) E(2) produced in abundance by the decidua promotes the migration of first-trimester human EVTs by increasing the intracellular concentration of calcium and activating calpain. Using our well-characterized immortalized EVT cell line, HTR-8/SVneo, as well as villus explants from first-trimester placentae, this study examined the role of RHO GTPases RAC1 and CDC42 in PGE(2)-mediated migratory responses of these cells. Though a RAC1 inhibitor, NSC23766 as well as RAC1 knockdown by siRNA decreased the migration of HTR-8/SVneo cells in a Transwell migration assay, this inhibition could not be restored by PGE(2) or 17-phenyl trinor PGE(2) (PGE receptor PTGER1 agonist) or PGE(1) Alcohol (PGE receptor PTGER4 agonist). Similar results were noted for EVT cell spreading in villus explants. Furthermore, CDC42 silencing using siRNA inhibited PGE(2)-induced migration of HTR-8/SVneo cells. Finally, the treatment of EVT cells with PGE(2), PTGER1 agonist, or PTGER4 agonist activated RAC1 and CDC42 at 10 min, suggesting that RAC1 and CDC42 play an essential role in PGE(2)-mediated migration of human EVTs. 
 The interaction of IQGAP1 with the exocyst complex is required for tumor cell invasion downstream of Cdc42 and RhoA. Invadopodia are actin-based membrane protrusions formed at contact sites between   invasive   tumor cells and the extracellular matrix with matrix proteolytic activity. Actin regulatory proteins participate in invadopodia formation, whereas matrix degradation requires metalloproteinases (MMPs) targeted to invadopodia. In this study, we show that the vesicle-tethering exocyst complex is required for matrix proteolysis and invasion of breast carcinoma cells. We demonstrate that the exocyst subunits Sec3 and Sec8 interact with the polarity protein IQGAP1 and that this interaction is triggered by active Cdc42 and RhoA, which are essential for matrix degradation. Interaction between IQGAP1 and the exocyst is necessary for invadopodia activity because enhancement of matrix degradation induced by the expression of IQGAP1 is lost upon deletion of the exocyst-binding site. We further show that the exocyst and IQGAP1 are required for the accumulation of cell surface membrane type 1 MMP at invadopodia. Based on these results, we propose that invadopodia function in tumor cells relies on the coordination of cytoskeletal assembly and exocytosis downstream of Rho guanosine triphosphatases. 
 Regulation of CXCR4/AKT-signaling-induced cell invasion and tumor metastasis by RhoA, Rac-1, and Cdc42 in human esophageal cancer. CXC chemokines and their cognate receptors have been implicated wildly in cancer pathogenesis. In the present study, we report a critical cause relationship between CXCR4 expression and tumorigenesis in the setting of human esophageal squamous cell carcinoma (ESCC). In ESCC cells, CXCR4 expression was significantly higher than in human esophageal epithelial cells (HEEC). Reduction of CXCR4 in ESCC cells reduced cell proliferation and invasion in vitro and tumor growth in vivo. Among the potential downstream targets of CXCR4-CXCL12 are RhoA, Rac-1, and Cdc42, which are likely to contribute to the   invasive  ness of ESCC cells. Finally, we found that CXCR4-CXCL12/AKT axis regulates RhoA, Rac-1, and Cdc42 to modulate cell invasion and tumor metastasis. Together, these results demonstrate a role for CXCR4 in ESCC metastasis and progression and suggest potential targets for therapeutic intervention. 
 Regulation and functional significance of CDC42 alternative splicing in ovarian cancer. Our previous study found that splicing factor polypyrimidine tract-binding protein 1 (PTBP1) had a role in tumorigenesis but the underlying mechanism remained unclear. In this study, we observed that knockdown of PTBP1 inhibited filopodia formation. Subsequently, we found that PTBP1 regulated the alternative splicing of CDC42, a major regulator of filopodia formation. Two CDC42 variants, CDC42-v1 and CDC42-v2, can be generated through alternative splicing. Knockdown of PTBP1 increased the expression of CDC42-v2. Ectopic expression of individual variants showed that CDC42-v2 suppressed filopodia formation, opposite to the effect of CDC42-v1. Quantitative RT-PCR revealed that CDC42-v2 was expressed at lower levels in ovarian cancer cell lines and ovarian tumor tissues than in normal control cells and tissues. Further, CDC42-v2 was observed to have inhibitory effects on ovarian tumor cell growth, colony formation in soft agar and   invasive  ness. In contrast, these inhibitory effects were not found with CDC42-v1. Taken together, above results suggest that the role of PTBP1 in tumorigenesis may be partly mediated by its regulation of CDC42 alternative splicing and CDC42-v2 might function as a tumor suppressor. 
 Podocalyxin-like 1 promotes invadopodia formation and metastasis through activation of Rac1/Cdc42/cortactin signaling in breast cancer cells. Metastatic disease is the leading cause of cancer mortality. Identifying biomarkers and regulatory mechanisms is important toward developing diagnostic and therapeutic tools against metastatic cancer. In this study, we demonstrated that podocalyxin-like 1 (PODXL) is overexpressed in breast tumor cells and increased in lymph node metastatic cancer. Mechanistically, we found that the expression of PODXL was associated with cell motility and   invasive  ness. Suppression of PODXL in MDA-MB-231 cells reduced lamellipodia formation and focal adhesion kinase (FAK) and paxillin phosphorylation. PODXL knockdown reduced the formation of invadopodia, such as inhibiting the colocalization of F-actin with cortactin and suppressing phosphorylation of cortactin and neural Wiskott-Aldrich syndrome protein. Conversely, overexpression of PODXL in MCF7 cells induced F-actin/cortactin colocalization and enhanced invadopodia formation and activation. Invadopodia activity and tumor invasion in PODXL-knockdown cells are similar to that in cortactin-knockdown cells. We further found that the DTHL motif in PODXL is crucial for regulating cortactin phosphorylation and Rac1/Cdc42 activation. Inhibition of Rac1/Cdc42 impeded PODXL-mediated cortactin activation and FAK and paxillin phosphorylation. Moreover, inhibition of PODXL in MDA-MB-231 cells significantly suppressed tumor colonization in the lungs and distant metastases, similar to those in cortactin-knockdown cells. These findings show that overexpression of PODXL enhanced invadopodia formation and tumor metastasis by inducing Rac1/Cdc42/cortactin signaling network. 
 Differential effects of invasion by and phagocytosis of Salmonella typhimurium on apoptosis in human macrophages: potential role of Rho-GTPases and Akt. In addition to direct activation of caspase-1 and induction of apoptosis by SipB,   invasive   Salmonella stimulates multiple signaling pathways that are key regulators of host cell survival. Nevertheless, little is known about the relative contributions of these pathways to Salmonella-mediated death of macrophages. We studied human monocytic U937 cells and found that apoptosis was induced by invading wild-type Salmonella typhimurium but not by phagocytosed, serum-opsonized, non  invasive   Salmonella mutants. Pretreating U937 cells with inhibitors of tyrosine kinases or phosphatidylinositol-3 kinase (PI-3K) completely blocked phagocytosis of opsonized Salmonella mutants but did not affect invasion by wild-type Salmonella or the apoptosis caused by invasion. However, pretreatment with GGTI-298, a geranylgeranyltransferase-1 inhibitor that prevents prenylation of Cdc42 and Rac1, suppressed Salmonella-induced apoptosis by approximately 70%. Transduction of Tat fusion constructs containing dominant-negative Cdc42 or Rac1 significantly inhibited Salmonella-induced cell death, indicating that the cytotoxicity of Salmonella requires activation of Cdc42 and Rac. In contrast to phagocytosis of opsonized bacteria, invasion by S. typhimurium stimulated Cdc42 and Rac1, regardless of the activities of tyrosine- or PI-3K. Moreover, Salmonella infection activated Akt protein in a tyrosine-kinase or PI-3K-dependent manner, and a reduced expression of Akt by antisense transfection rendered the cells more sensitive to apoptosis induced by opsonized Salmonella. These results indicate that direct activation of Cdc42 and Rac1 by   invasive   Salmonella is a prerequisite of Salmonella-mediated death of U937 cells, whereas the simultaneous activation of Akt by tyrosine kinase and PI-3K during receptor-mediated phagocytosis protects cells from apoptosis. 
 MT1-MMP- and Cdc42-dependent signaling co-regulate cell invasion and tunnel formation in 3D collagen matrices. Complex signaling events control tumor invasion in three-dimensional (3D) extracellular matrices. Recent evidence suggests that cells utilize both matrix metalloproteinase (MMP)-dependent and MMP-independent means to traverse 3D matrices. Herein, we demonstrate that lysophosphatidic-acid-induced HT1080 cell invasion requires membrane-type-1 (MT1)-MMP-mediated collagenolysis to generate matrix conduits the width of a cellular nucleus. We define these spaces as single-cell invasion tunnels (SCITs). Once established, cells can migrate within SCITs in an MMP-independent manner. Endothelial cells, smooth muscle cells and fibroblasts also generate SCITs during   invasive   events, suggesting that SCIT formation represents a fundamental mechanism of cellular motility within 3D matrices. Coordinated cellular signaling events are required during SCIT formation. MT1-MMP, Cdc42 and its associated downstream effectors such as MRCK (myotonic dystrophy kinase-related Cdc42-binding kinase) and Pak4 (p21 protein-activated kinase 4), protein kinase Calpha and the Rho-associated coiled-coil-containing protein kinases (ROCK-1 and ROCK-2) coordinate signaling necessary for SCIT formation. Finally, we show that MT1-MMP and Cdc42 are fundamental components of a co-associated invasion-signaling complex that controls directed single-cell invasion of 3D collagen matrices. 
 Activated Cdc42 sequesters c-Cbl and prevents EGF receptor degradation. Cdc42 is a Ras-related protein that has been implicated in the control of normal cell growth, and when improperly regulated, in cellular transformation and   invasive  ness. A variety of extracellular stimuli, including epidermal growth factor (EGF), activate Cdc42. Here, we show that activation of Cdc42 protects the EGF receptor from the negative regulatory activity of the c-Cbl ubiquitin ligase. Activated Cdc42 binds to p85Cool-1 (for cloned-out-of-library)/beta-Pix (for Pak-interactive exchange factor), a protein that directly associates with c-Cbl. This inhibits the binding of Cbl by the EGF receptor and thus prevents Cbl from catalyzing receptor ubiquitination. The role played by Cdc42 in regulating the timing of EGF receptor-Cbl interactions is underscored by the fact that constitutively active Cdc42(F28L), by persistently blocking the binding of Cbl to these receptors, leads to their aberrant accumulation and sustained EGF-stimulated ERK activation, thus resulting in cellular transformation. 
 Formyl peptide receptor-1 activation enhances intestinal epithelial cell restitution through phosphatidylinositol 3-kinase-dependent activation of Rac1 and Cdc42. Inflammatory disorders of the gastrointestinal tract result in the breakdown of the intestinal epithelial barrier in the form of erosion and ulceration. To reestablish the epithelial barrier, the epithelium must efficiently migrate to reseal wounds. Numerous signaling cascades are involved in the induction and regulation of this complex process. N-formyl peptide receptors comprise a group of Gi-coupled receptors that regulate innate immune responses. Previously, we identified the expression of functional N-formyl peptide receptors in model SK-CO15 intestinal epithelial cells and observed a role for activation of these receptors in regulating cellular   invasive   behavior. In these studies, we performed formyl peptide receptor-1 (FPR) localization and evaluated its role in regulating intestinal epithelial cell wound closure. Immunolocalization studies using a recently developed specific monoclonal anti-FPR Ab demonstrated its localization along the lateral membrane of crypt epithelial cells in normal human colonic epithelium. In vitro studies using the classical FPR agonist fMLF showed that FPR activation significantly enhances model intestinal epithelial cell restitution and that FPR localized along actin filaments in lamellipodial and filopodial extrusions. The increase in cell migration was associated with activation of PI3K, Rac1, and Cdc42. Pharmacologic inhibition of PI3K activity abrogated the fMLF-induced increase in wound closure and activation of both Rac1 and Cdc42. Inhibition of Rac1 and Cdc42 using pharmacologic inhibitors and dominant negative mutants also inhibited the fMLF-induced increase in cell migration. Taken together, theses results support a novel role for FPR stimulation in enhancing intestinal epithelial cell restitution through PI3K-dependent activation of Rac1 and Cdc42. 
 Macrophages stimulate gastric and colorectal cancer invasion through EGFR Y(1086), c-Src, Erk1/2 and Akt phosphorylation and smallGTPase activity. The interactions between cancer cells and their microenvironment are crucial for malignant progression, as they modulate invasion-related activities. Tumor-associated macrophages are generally considered allies in the process of tumor progression in several types of cancer, although their role on gastric and colorectal carcinomas is still poorly understood. In this report, we studied the influence of primary human macrophages on gastric and colorectal cancer cells, considering invasion, motility/migration, proteolysis and activated intracellular signaling pathways. We demonstrated that macrophages stimulate cancer cell invasion, motility and migration, and that these effects depend on matrix metalloproteinase (MMP) activity and on the activation of epidermal growth factor receptor (EGFR) (at the residue Y(1086)), PLC-γ (phospholipase C-gamma) and Gab1 (GRB2-associated binding protein-1), as evidenced by siRNA (small interference RNA) experiments. Epidermal growth factor (EGF)-immunodepletion impaired macrophage-mediated cancer cell invasion and motility, suggesting that EGF is the pro  -invasiv  e and pro-motile factor produced by macrophages. Macrophages also induced gastric and colorectal cancer cell phosphorylation of Akt, c-Src and ERK1/2, and led to an increase of RhoA and Cdc42 activity. Interestingly, whereas macrophage-mediated cancer cell c-Src and ERK1/2 phosphorylation occurred downstream EGFR activation, Akt phosphorylation seems to be a parallel event, taking place in an EGFR-independent manner. The involvement of EGF, EGFR-downstream signaling partners and MMPs in macrophage-mediated invasion provides novel insights into the molecular crosstalk established between cancer cells and macrophages, opening new perspectives for the design of new and more efficient therapeutic strategies to counteract cancer cell invasion. 
 Role of activated Rac1/Cdc42 in mediating endothelial cell proliferation and tumor angiogenesis in breast cancer. Angiogenesis is a well-established target in anti-cancer therapy. Although vascular endothelial growth factor (VEGF)-mediated angiogenesis apparently requires the Rho GTPases Rac1 and Cdc42, the relevant mechanisms are unclear. Here, we determined that activated Rac1/Cdc42 in MCF-7 breast cancer cells could decrease p53 protein levels and increase VEGF secretion to promote proliferation and tube formation of human umbilical vein endothelial cells (HUVECs). However, these effects are reversed after ubiquitin-proteasome breakage. In exploring potential mechanisms for this relationship, we confirmed that activated Rac1/Cdc42 could enhance p53 protein ubiquitination and weaken p53 protein stability to increase VEGF expression. Furthermore, in a xenograft model using nude mice that stably express active Rac1/Cdc42 protein, active Rac1/Cdc42 decreased p53 levels and increased VEGF expression. Additionally, tumor angiogenesis was inhibited, and p53 protein levels were augmented, by intratumoral injection of the ubiquitin-proteasome inhibitor MG132. Finally in 339 human breast cancer tissues, our analyses indicated that Rac1/Cdc42 expression was related to advanced TNM staging, high proliferation index, ER status, and positive   invasive   features. In particular, our data suggests that high Rac1/Cdc42 expression is correlated with low wt-p53 and high VEGF expression. We conclude that activated Rac1/Cdc42 is a vascular regulator of tumor angiogenesis and that it may reduce stability of the p53 protein to promote VEGF expression by enhancing p53 protein ubiquitin. 
 Rac1, RhoA, and Cdc42 participate in HeLa cell invasion by group B streptococcus. The group B streptococcus (GBS) is an important human pathogen with the ability to cause   invasive   disease. To do so, the bacteria must invade host cells. It has been well documented that GBS are able to invade a variety of nonphagocytic host cell types, and this process is thought to involve a number of pathogen-host cell interactions. While some of the molecular aspects of the GBS-host cell invasion process have been characterized, many events still remain unclear. The objective of this investigation was to evaluate the role of the Rho-family GTPases Rac, Rho, and Cdc42 in GBS invasion into epithelial cells. The epithelial cell invasion process was modeled using HeLa 229 cell culture. Treatment of HeLa cells with 10 microM compactin, a pan-GTPase inhibitor, abolished GBS internalization, suggesting that GTPases are involved in the GBS invasion process. The addition of Toxin B or exoenzyme C3 to HeLa cells before GBS infection reduced invasion by 50%, further suggesting that the Rho-family GTPases are involved in GBS entry. Examining invasion of GBS into HeLa cells with altered genetic backgrounds was used to confirm these findings; GBS invasion into HeLa cells transiently transfected with dominant negative Rac1, Cdc42, or RhoA reduced invasion by 75%, 51%, and 42%, respectively. Results of this study suggest that the Rho-family GTPases are required for efficient invasion of HeLa cells by GBS. 
 Phosphatidylinositol 5-phosphate regulates invasion through binding and activation of Tiam1. PtdIns5P is a lipid messenger acting as a stress-response mediator in the nucleus, and known to maintain cell activation through traffic alterations upon bacterial infection. Here, we show that PtdIns5P regulates actin dynamics and invasion via recruitment and activation of the exchange factor Tiam1 and Rac1. Restricted Rac1 activation results from the binding of Tiam1 DH-PH domains to PtdIns5P. Using an assay that mimics Rac1 membrane anchoring by using Rac1-His and liposomes containing Ni(2+)-NTA modified lipids, we demonstrate that intrinsic Tiam1 DH-PH activity increases when Rac1 is anchored in a PtdIns5P-enriched environment. This pathway appears to be general since it is valid in different pathophysiological models: receptor tyrosine kinase activation, bacterial phosphatase IpgD expression and the   invasive   NPM-ALK(+) lymphomas. The discovery that PtdIns5P could be a keystone of GTPases and cytoskeleton spatiotemporal regulation opens important research avenues towards unravelling new strategies counteracting cell invasion. 
 Overexpression of small GTPases directly correlates with expression of δ-catenin and their coexpression predicts a poor clinical outcome in nonsmall cell lung cancer. δ-catenin can affect cytoskeletal assembly, and promote cell migration by regulating the activity of small GTPases. While many malignancies have been shown to be positive for δ-catenin, it is still unclear whether δ-catenin and small GTPases are coexpressed in tumor cells, and so is the relationship between their coexpression and prognosis in the tumor patients. In this study, immunohistochemistry was performed to examine expressive levels of δ-catenin, cdc42, and Rac1 in 135 cases of nonsmall cell lung cancer (NSCLC), including 60 cases with follow-up records. Thirty samples of paired lung cancer tissues and adjacent normal lung tissues were collected to analyze mRNA and protein expression of δ-catenin and small GTPases. The effects of δ-catenin on small GTPases expressi  on and i  nvasive ability of lung cancer cells were also evaluated. Compared with normal lung tissues, both mRNA and protein levels of δ-catenin and small GTPases were increased in lung cancer tissues (P < 0.05), and the expression of small GTPases directly correlated with that of δ-catenin (P < 0.001). In addition, δ-catenin and small GTPases tended to be coexpressed in lung adenocarcinoma, advanced stages, and primary tumors with lymph node metastasis (all P < 0.05). The patients with coexpression of δ-catenin and small GTPases had a shorter survival time than those without coexpression (P < 0.05). Furthermore, δ-catenin overexpression co  uld enha  nce invasive ability of lung cancer cells by upregulating protein and transcriptional level of small GTPases. Therefore, δ-catenin likely upregulates the activity of small GTPases at transcriptional level, and their coexpression may predict a poor clinical outcome in NSCLC patients. 
 Regulation of CXCR4/AKT-signaling-induced cell invasion and tumor metastasis by RhoA, Rac-1, and Cdc42 in human esophageal cancer. CXC chemokines and their cognate receptors have been implicated wildly in cancer pathogenesis. In the present study, we report a critical cause relationship between CXCR4 expression and tumorigenesis in the setting of human esophageal squamous cell carcinoma (ESCC). In ESCC cells, CXCR4 expression was significantly higher than in human esophageal epithelial cells (HEEC). Reduction of CXCR4 in ESCC cells reduced cell proliferation and invasion in vitro and tumor growth in vivo. Among the potential downstream targets of CXCR4-CXCL12 are RhoA, Rac-1, and Cdc42, which are likely to contribute to the   invasive  ness of ESCC cells. Finally, we found that CXCR4-CXCL12/AKT axis regulates RhoA, Rac-1, and Cdc42 to modulate cell invasion and tumor metastasis. Together, these results demonstrate a role for CXCR4 in ESCC metastasis and progression and suggest potential targets for therapeutic intervention. 
 Regulation and functional significance of CDC42 alternative splicing in ovarian cancer. Our previous study found that splicing factor polypyrimidine tract-binding protein 1 (PTBP1) had a role in tumorigenesis but the underlying mechanism remained unclear. In this study, we observed that knockdown of PTBP1 inhibited filopodia formation. Subsequently, we found that PTBP1 regulated the alternative splicing of CDC42, a major regulator of filopodia formation. Two CDC42 variants, CDC42-v1 and CDC42-v2, can be generated through alternative splicing. Knockdown of PTBP1 increased the expression of CDC42-v2. Ectopic expression of individual variants showed that CDC42-v2 suppressed filopodia formation, opposite to the effect of CDC42-v1. Quantitative RT-PCR revealed that CDC42-v2 was expressed at lower levels in ovarian cancer cell lines and ovarian tumor tissues than in normal control cells and tissues. Further, CDC42-v2 was observed to have inhibitory effects on ovarian tumor cell growth, colony formation in soft agar and   invasive  ness. In contrast, these inhibitory effects were not found with CDC42-v1. Taken together, above results suggest that the role of PTBP1 in tumorigenesis may be partly mediated by its regulation of CDC42 alternative splicing and CDC42-v2 might function as a tumor suppressor. 
 Phosphatidylinositol 5-phosphate regulates invasion through binding and activation of Tiam1. PtdIns5P is a lipid messenger acting as a stress-response mediator in the nucleus, and known to maintain cell activation through traffic alterations upon bacterial infection. Here, we show that PtdIns5P regulates actin dynamics and invasion via recruitment and activation of the exchange factor Tiam1 and Rac1. Restricted Rac1 activation results from the binding of Tiam1 DH-PH domains to PtdIns5P. Using an assay that mimics Rac1 membrane anchoring by using Rac1-His and liposomes containing Ni(2+)-NTA modified lipids, we demonstrate that intrinsic Tiam1 DH-PH activity increases when Rac1 is anchored in a PtdIns5P-enriched environment. This pathway appears to be general since it is valid in different pathophysiological models: receptor tyrosine kinase activation, bacterial phosphatase IpgD expression and the   invasive   NPM-ALK(+) lymphomas. The discovery that PtdIns5P could be a keystone of GTPases and cytoskeleton spatiotemporal regulation opens important research avenues towards unravelling new strategies counteracting cell invasion. 
 Podocalyxin-like 1 promotes invadopodia formation and metastasis through activation of Rac1/Cdc42/cortactin signaling in breast cancer cells. Metastatic disease is the leading cause of cancer mortality. Identifying biomarkers and regulatory mechanisms is important toward developing diagnostic and therapeutic tools against metastatic cancer. In this study, we demonstrated that podocalyxin-like 1 (PODXL) is overexpressed in breast tumor cells and increased in lymph node metastatic cancer. Mechanistically, we found that the expression of PODXL was associated with cell motility and   invasive  ness. Suppression of PODXL in MDA-MB-231 cells reduced lamellipodia formation and focal adhesion kinase (FAK) and paxillin phosphorylation. PODXL knockdown reduced the formation of invadopodia, such as inhibiting the colocalization of F-actin with cortactin and suppressing phosphorylation of cortactin and neural Wiskott-Aldrich syndrome protein. Conversely, overexpression of PODXL in MCF7 cells induced F-actin/cortactin colocalization and enhanced invadopodia formation and activation. Invadopodia activity and tumor invasion in PODXL-knockdown cells are similar to that in cortactin-knockdown cells. We further found that the DTHL motif in PODXL is crucial for regulating cortactin phosphorylation and Rac1/Cdc42 activation. Inhibition of Rac1/Cdc42 impeded PODXL-mediated cortactin activation and FAK and paxillin phosphorylation. Moreover, inhibition of PODXL in MDA-MB-231 cells significantly suppressed tumor colonization in the lungs and distant metastases, similar to those in cortactin-knockdown cells. These findings show that overexpression of PODXL enhanced invadopodia formation and tumor metastasis by inducing Rac1/Cdc42/cortactin signaling network. 
 Role of activated Rac1/Cdc42 in mediating endothelial cell proliferation and tumor angiogenesis in breast cancer. Angiogenesis is a well-established target in anti-cancer therapy. Although vascular endothelial growth factor (VEGF)-mediated angiogenesis apparently requires the Rho GTPases Rac1 and Cdc42, the relevant mechanisms are unclear. Here, we determined that activated Rac1/Cdc42 in MCF-7 breast cancer cells could decrease p53 protein levels and increase VEGF secretion to promote proliferation and tube formation of human umbilical vein endothelial cells (HUVECs). However, these effects are reversed after ubiquitin-proteasome breakage. In exploring potential mechanisms for this relationship, we confirmed that activated Rac1/Cdc42 could enhance p53 protein ubiquitination and weaken p53 protein stability to increase VEGF expression. Furthermore, in a xenograft model using nude mice that stably express active Rac1/Cdc42 protein, active Rac1/Cdc42 decreased p53 levels and increased VEGF expression. Additionally, tumor angiogenesis was inhibited, and p53 protein levels were augmented, by intratumoral injection of the ubiquitin-proteasome inhibitor MG132. Finally in 339 human breast cancer tissues, our analyses indicated that Rac1/Cdc42 expression was related to advanced TNM staging, high proliferation index, ER status, and positive   invasive   features. In particular, our data suggests that high Rac1/Cdc42 expression is correlated with low wt-p53 and high VEGF expression. We conclude that activated Rac1/Cdc42 is a vascular regulator of tumor angiogenesis and that it may reduce stability of the p53 protein to promote VEGF expression by enhancing p53 protein ubiquitin. 
 Macrophages stimulate gastric and colorectal cancer invasion through EGFR Y(1086), c-Src, Erk1/2 and Akt phosphorylation and smallGTPase activity. The interactions between cancer cells and their microenvironment are crucial for malignant progression, as they modulate invasion-related activities. Tumor-associated macrophages are generally considered allies in the process of tumor progression in several types of cancer, although their role on gastric and colorectal carcinomas is still poorly understood. In this report, we studied the influence of primary human macrophages on gastric and colorectal cancer cells, considering invasion, motility/migration, proteolysis and activated intracellular signaling pathways. We demonstrated that macrophages stimulate cancer cell invasion, motility and migration, and that these effects depend on matrix metalloproteinase (MMP) activity and on the activation of epidermal growth factor receptor (EGFR) (at the residue Y(1086)), PLC-γ (phospholipase C-gamma) and Gab1 (GRB2-associated binding protein-1), as evidenced by siRNA (small interference RNA) experiments. Epidermal growth factor (EGF)-immunodepletion impaired macrophage-mediated cancer cell invasion and motility, suggesting that EGF is the pro  -invasiv  e and pro-motile factor produced by macrophages. Macrophages also induced gastric and colorectal cancer cell phosphorylation of Akt, c-Src and ERK1/2, and led to an increase of RhoA and Cdc42 activity. Interestingly, whereas macrophage-mediated cancer cell c-Src and ERK1/2 phosphorylation occurred downstream EGFR activation, Akt phosphorylation seems to be a parallel event, taking place in an EGFR-independent manner. The involvement of EGF, EGFR-downstream signaling partners and MMPs in macrophage-mediated invasion provides novel insights into the molecular crosstalk established between cancer cells and macrophages, opening new perspectives for the design of new and more efficient therapeutic strategies to counteract cancer cell invasion. 
 Overexpression of small GTPases directly correlates with expression of δ-catenin and their coexpression predicts a poor clinical outcome in nonsmall cell lung cancer. δ-catenin can affect cytoskeletal assembly, and promote cell migration by regulating the activity of small GTPases. While many malignancies have been shown to be positive for δ-catenin, it is still unclear whether δ-catenin and small GTPases are coexpressed in tumor cells, and so is the relationship between their coexpression and prognosis in the tumor patients. In this study, immunohistochemistry was performed to examine expressive levels of δ-catenin, cdc42, and Rac1 in 135 cases of nonsmall cell lung cancer (NSCLC), including 60 cases with follow-up records. Thirty samples of paired lung cancer tissues and adjacent normal lung tissues were collected to analyze mRNA and protein expression of δ-catenin and small GTPases. The effects of δ-catenin on small GTPases expressi  on and i  nvasive ability of lung cancer cells were also evaluated. Compared with normal lung tissues, both mRNA and protein levels of δ-catenin and small GTPases were increased in lung cancer tissues (P < 0.05), and the expression of small GTPases directly correlated with that of δ-catenin (P < 0.001). In addition, δ-catenin and small GTPases tended to be coexpressed in lung adenocarcinoma, advanced stages, and primary tumors with lymph node metastasis (all P < 0.05). The patients with coexpression of δ-catenin and small GTPases had a shorter survival time than those without coexpression (P < 0.05). Furthermore, δ-catenin overexpression co  uld enha  nce invasive ability of lung cancer cells by upregulating protein and transcriptional level of small GTPases. Therefore, δ-catenin likely upregulates the activity of small GTPases at transcriptional level, and their coexpression may predict a poor clinical outcome in NSCLC patients. 
 MT1-MMP- and Cdc42-dependent signaling co-regulate cell invasion and tunnel formation in 3D collagen matrices. Complex signaling events control tumor invasion in three-dimensional (3D) extracellular matrices. Recent evidence suggests that cells utilize both matrix metalloproteinase (MMP)-dependent and MMP-independent means to traverse 3D matrices. Herein, we demonstrate that lysophosphatidic-acid-induced HT1080 cell invasion requires membrane-type-1 (MT1)-MMP-mediated collagenolysis to generate matrix conduits the width of a cellular nucleus. We define these spaces as single-cell invasion tunnels (SCITs). Once established, cells can migrate within SCITs in an MMP-independent manner. Endothelial cells, smooth muscle cells and fibroblasts also generate SCITs during   invasive   events, suggesting that SCIT formation represents a fundamental mechanism of cellular motility within 3D matrices. Coordinated cellular signaling events are required during SCIT formation. MT1-MMP, Cdc42 and its associated downstream effectors such as MRCK (myotonic dystrophy kinase-related Cdc42-binding kinase) and Pak4 (p21 protein-activated kinase 4), protein kinase Calpha and the Rho-associated coiled-coil-containing protein kinases (ROCK-1 and ROCK-2) coordinate signaling necessary for SCIT formation. Finally, we show that MT1-MMP and Cdc42 are fundamental components of a co-associated invasion-signaling complex that controls directed single-cell invasion of 3D collagen matrices. 
 Prostaglandin E2-mediated migration of human trophoblast requires RAC1 and CDC42. The invasion of maternal decidua and uterine spiral arteries by a trophoblast subpopulation called extravillous trophoblast (EVT) is essential for the establishment of a normal placenta and an adequate blood flow toward the fetus. Derangements in these processes underlie pregnancy-related diseases like preeclampsia and intrauterine growth restriction. Many growth factors, growth factor binding proteins, and extracellular matrix components can positively or negatively regulate the proliferation, migration, and/or   invasive  ness of these EVT cells. RHO GTPases, including RHOA, RAC1, and CDC42, are ubiquitous proteins that control cytoskeletal changes by forming stress fibers and projecting lamellipodia and filopodia during cellular migration. We had previously shown that prostaglandin (PG) E(2) produced in abundance by the decidua promotes the migration of first-trimester human EVTs by increasing the intracellular concentration of calcium and activating calpain. Using our well-characterized immortalized EVT cell line, HTR-8/SVneo, as well as villus explants from first-trimester placentae, this study examined the role of RHO GTPases RAC1 and CDC42 in PGE(2)-mediated migratory responses of these cells. Though a RAC1 inhibitor, NSC23766 as well as RAC1 knockdown by siRNA decreased the migration of HTR-8/SVneo cells in a Transwell migration assay, this inhibition could not be restored by PGE(2) or 17-phenyl trinor PGE(2) (PGE receptor PTGER1 agonist) or PGE(1) Alcohol (PGE receptor PTGER4 agonist). Similar results were noted for EVT cell spreading in villus explants. Furthermore, CDC42 silencing using siRNA inhibited PGE(2)-induced migration of HTR-8/SVneo cells. Finally, the treatment of EVT cells with PGE(2), PTGER1 agonist, or PTGER4 agonist activated RAC1 and CDC42 at 10 min, suggesting that RAC1 and CDC42 play an essential role in PGE(2)-mediated migration of human EVTs. 
 The interaction of IQGAP1 with the exocyst complex is required for tumor cell invasion downstream of Cdc42 and RhoA. Invadopodia are actin-based membrane protrusions formed at contact sites between   invasive   tumor cells and the extracellular matrix with matrix proteolytic activity. Actin regulatory proteins participate in invadopodia formation, whereas matrix degradation requires metalloproteinases (MMPs) targeted to invadopodia. In this study, we show that the vesicle-tethering exocyst complex is required for matrix proteolysis and invasion of breast carcinoma cells. We demonstrate that the exocyst subunits Sec3 and Sec8 interact with the polarity protein IQGAP1 and that this interaction is triggered by active Cdc42 and RhoA, which are essential for matrix degradation. Interaction between IQGAP1 and the exocyst is necessary for invadopodia activity because enhancement of matrix degradation induced by the expression of IQGAP1 is lost upon deletion of the exocyst-binding site. We further show that the exocyst and IQGAP1 are required for the accumulation of cell surface membrane type 1 MMP at invadopodia. Based on these results, we propose that invadopodia function in tumor cells relies on the coordination of cytoskeletal assembly and exocytosis downstream of Rho guanosine triphosphatases. 
 Formyl peptide receptor-1 activation enhances intestinal epithelial cell restitution through phosphatidylinositol 3-kinase-dependent activation of Rac1 and Cdc42. Inflammatory disorders of the gastrointestinal tract result in the breakdown of the intestinal epithelial barrier in the form of erosion and ulceration. To reestablish the epithelial barrier, the epithelium must efficiently migrate to reseal wounds. Numerous signaling cascades are involved in the induction and regulation of this complex process. N-formyl peptide receptors comprise a group of Gi-coupled receptors that regulate innate immune responses. Previously, we identified the expression of functional N-formyl peptide receptors in model SK-CO15 intestinal epithelial cells and observed a role for activation of these receptors in regulating cellular   invasive   behavior. In these studies, we performed formyl peptide receptor-1 (FPR) localization and evaluated its role in regulating intestinal epithelial cell wound closure. Immunolocalization studies using a recently developed specific monoclonal anti-FPR Ab demonstrated its localization along the lateral membrane of crypt epithelial cells in normal human colonic epithelium. In vitro studies using the classical FPR agonist fMLF showed that FPR activation significantly enhances model intestinal epithelial cell restitution and that FPR localized along actin filaments in lamellipodial and filopodial extrusions. The increase in cell migration was associated with activation of PI3K, Rac1, and Cdc42. Pharmacologic inhibition of PI3K activity abrogated the fMLF-induced increase in wound closure and activation of both Rac1 and Cdc42. Inhibition of Rac1 and Cdc42 using pharmacologic inhibitors and dominant negative mutants also inhibited the fMLF-induced increase in cell migration. Taken together, theses results support a novel role for FPR stimulation in enhancing intestinal epithelial cell restitution through PI3K-dependent activation of Rac1 and Cdc42. 
 Rac1, RhoA, and Cdc42 participate in HeLa cell invasion by group B streptococcus. The group B streptococcus (GBS) is an important human pathogen with the ability to cause   invasive   disease. To do so, the bacteria must invade host cells. It has been well documented that GBS are able to invade a variety of nonphagocytic host cell types, and this process is thought to involve a number of pathogen-host cell interactions. While some of the molecular aspects of the GBS-host cell invasion process have been characterized, many events still remain unclear. The objective of this investigation was to evaluate the role of the Rho-family GTPases Rac, Rho, and Cdc42 in GBS invasion into epithelial cells. The epithelial cell invasion process was modeled using HeLa 229 cell culture. Treatment of HeLa cells with 10 microM compactin, a pan-GTPase inhibitor, abolished GBS internalization, suggesting that GTPases are involved in the GBS invasion process. The addition of Toxin B or exoenzyme C3 to HeLa cells before GBS infection reduced invasion by 50%, further suggesting that the Rho-family GTPases are involved in GBS entry. Examining invasion of GBS into HeLa cells with altered genetic backgrounds was used to confirm these findings; GBS invasion into HeLa cells transiently transfected with dominant negative Rac1, Cdc42, or RhoA reduced invasion by 75%, 51%, and 42%, respectively. Results of this study suggest that the Rho-family GTPases are required for efficient invasion of HeLa cells by GBS. 
 Differential effects of invasion by and phagocytosis of Salmonella typhimurium on apoptosis in human macrophages: potential role of Rho-GTPases and Akt. In addition to direct activation of caspase-1 and induction of apoptosis by SipB,   invasive   Salmonella stimulates multiple signaling pathways that are key regulators of host cell survival. Nevertheless, little is known about the relative contributions of these pathways to Salmonella-mediated death of macrophages. We studied human monocytic U937 cells and found that apoptosis was induced by invading wild-type Salmonella typhimurium but not by phagocytosed, serum-opsonized, non  invasive   Salmonella mutants. Pretreating U937 cells with inhibitors of tyrosine kinases or phosphatidylinositol-3 kinase (PI-3K) completely blocked phagocytosis of opsonized Salmonella mutants but did not affect invasion by wild-type Salmonella or the apoptosis caused by invasion. However, pretreatment with GGTI-298, a geranylgeranyltransferase-1 inhibitor that prevents prenylation of Cdc42 and Rac1, suppressed Salmonella-induced apoptosis by approximately 70%. Transduction of Tat fusion constructs containing dominant-negative Cdc42 or Rac1 significantly inhibited Salmonella-induced cell death, indicating that the cytotoxicity of Salmonella requires activation of Cdc42 and Rac. In contrast to phagocytosis of opsonized bacteria, invasion by S. typhimurium stimulated Cdc42 and Rac1, regardless of the activities of tyrosine- or PI-3K. Moreover, Salmonella infection activated Akt protein in a tyrosine-kinase or PI-3K-dependent manner, and a reduced expression of Akt by antisense transfection rendered the cells more sensitive to apoptosis induced by opsonized Salmonella. These results indicate that direct activation of Cdc42 and Rac1 by   invasive   Salmonella is a prerequisite of Salmonella-mediated death of U937 cells, whereas the simultaneous activation of Akt by tyrosine kinase and PI-3K during receptor-mediated phagocytosis protects cells from apoptosis. 
 Cdc42 and the phosphatidylinositol 3-kinase-Akt pathway are essential for PspC-mediated internalization of pneumococci by respiratory epithelial cells. The pneumococcal surface protein C (PspC) is a major adhesin of Streptococcus pneumoniae, the cause of lobar pneumonia and   invasive   diseases. PspC interacts in a human-specific manner with the ectodomain of the human polymeric immunoglobulin receptor (pIgR) produced by respiratory epithelial cells. By adopting the retrograde machinery of human pIgR, this protein-protein interaction promotes colonization and transcytosis across the epithelial layer. Here, we explored the role of Rho family guanosine triphosphatases (GTPases), phosphatidylinositol 3-kinase (PI3K) and protein kinase B (Akt) for ingestion of pneumococci via the human pIgR. Inhibition experiments suggested that the host-cell actin microfilaments and microtubules are essential for this pneumococcal uptake mechanism. By using specific GTPase-modifying toxins, inhibitors, and GTPase expression constructs we demonstrate that Cdc42, but not Rac1 and RhoA are involved in PspC-mediated invasion of pneumococci into host cells. Accordingly, Cdc42 is time-dependently activated during ingestion of pneumococci. In addition, PI3K and Akt are essential for ingestion of pneumococci by respiratory epithelial cells via the PspC-pIgR interaction. The subunit p85alpha of PI3K and Akt was activated during the infection process. Moreover, Akt activation upon pneumococcal invasion depends on PI3K. In conclusion, our results illustrate for the first time key signaling molecules of host cells that are required for PspC-pIgR-mediated invasion of pneumococci into epithelial cells. This unique and specific bacterial entry process is dependent on the cooperation and activation of Rho family GTPase Cdc42, PI3K, and Akt. 
 Cdc42 overexpression induces hyperbranching in the developing mammary gland by enhancing cell migration. The Rho GTPase Cdc42 is overexpressed and hyperactivated in breast tumors compared to normal breast tissue. Cdc42 regulates key processes that are critical for mammary gland morphogenesis and become disrupted during the development, progression, and metastasis of breast cancer. However, the contribution of Cdc42 to normal and neoplastic mammary gland development in vivo remains poorly understood. We were therefore interested in investigating the effects of Cdc42 overexpression on mammary gland morphogenesis as a first step toward understanding how its overexpression may contribute to mammary tumorigenesis.We developed a tetracycline-regulatable Cdc42 overexpression mouse model in which Cdc42 can be inducibly overexpressed in the developing mammary gland. The effects of Cdc42 overexpression during postnatal mammary gland development were investigated using in vivo and in vitro approaches, including morphometric analysis of wholemounted mammary glands, quantification of histological markers, and primary mammary epithelial cell (MEC) functional and biochemical assays.Analysis of Cdc42-overexpressing mammary glands revealed abnormal terminal end bud (TEB) morphologies, characterized by hyperbudding and trifurcation, and increased side branching within the ductal tree. Quantification of markers of proliferation and apoptosis suggested that these phenotypes were not due to increased cell proliferation or survival. Rather, Cdc42 overexpressing MECs were more migratory and contractile and formed dysmorphic,   invasive   acini in three-dimensional cultures. Cdc42 and RhoA activities, phosphorylated myosin light chain, and MAPK signaling, which contribute to migration and invasion, were markedly elevated in Cdc42 overexpressing MECs. Interestingly, Cdc42 overexpressing mammary glands displayed several features associated with altered epithelial-stromal interactions, which are known to regulate branching morphogenesis. These included increased stromal thickness and collagen deposition, and stromal cells isolated from Cdc42 overexpressing mammary glands exhibited elevated mRNA expression of extracellular matrix proteins and remodeling enzymes.These data suggest that Cdc42 overexpression disrupts mammary gland branching morphogenesis by altering Rho GTPase and MAPK signaling, leading to increased MEC contractility and migration in association with stromal alterations. Our studies provide insight into how aberrant Cdc42 expression may contribute to mammary tumorigenesis. 
 An immune escape screen reveals Cdc42 as regulator of cancer susceptibility to lymphocyte-mediated tumor suppression. Adoptive cellular immunotherapy inducing a graft-versus-tumor (GVT) effect is the therapeutic mainstay of allogeneic hematopoietic stem cell transplantation (ASCT) for high-risk leukemias. Autologous immunotherapies using vaccines or adoptive transfer of ex vivo-manipulated lymphocytes are clinically explored in patients with various cancer entities. Main reason for failure of ASCT and cancer immunotherapy is progression of the underlying malignancy, which is more prevalent in patients with advanced disease. Elucidating the molecular mechanisms contributing to immune escape will help to develop strategies for the improvement of immunologic cancer treatment. To this end, we have undertaken functional screening and expression cloning of factors mediating resistance to antigen-specific cytotoxic T lymphocytes (CTLs). We have identified Cdc42, a GTPase regulating actin dynamics and growth factor signaling that is highly expressed in   invasive   cancers, as determinator of cancer cell susceptibility to antigen-specific CTLs in vitro and adoptively transferred immune effectors in vivo. Cdc42 prevents CTL-induced apoptosis via mitogen-activated protein kinase (MAPK) signaling and posttranscriptional stabilization of Bcl-2. Pharmacologic inhibition of MAPK/extracellular signal-regulated kinase (ERK) kinase (MEK) overcomes Cdc42-mediated immunoresistance and activation of Bcl-2 in vivo. In conclusion, Cdc42 signaling contributes to immune escape of cancer. Targeting Cdc42 may improve the efficacy of cancer immunotherapies. 
 The suppressor of metastasis Nm23-H1 interacts with the Cdc42 Rho family member and the pleckstrin homology domain of oncoprotein Dbl-1 to suppress cell migration. Tumor invasion and metastasis is regulated by a number of cellular molecules known to be involved in signaling and cytoskeletal rearrangement. One of these molecules is the suppressor of tumor metastasis Nm23-H1 which linked to   invasive  ness and metastatic potential of human cancers. Nm23-H1 expression is down-regulated in human melanoma and   invasive   breast carcinoma. Recent studies have shown an association between the Nm23-H1 and oncoprotein Dbl-1 which is associated with guanine exchange and belongs to a family of Guanine Exchange Factors (GEF). In this report we show a direct interaction in vitro and in human B cells and specifically identified the pleckstrin homology domain of Dbl-1 as the domain which binds to Nm23-H1. Furthermore, Nm23-H1 and Dbl-1 colocalized in the cytoplasm of COS-7 cells when expressed exogenously and showed predominant signals at the periphery of the cells particularly at the plasma membrane. Interestingly, Dbl-1 and Cdc42 expression rescued the suppressive activities of Nm23-H1 in cell migration assays. We show that Cdc42 a regulatory protein involved in cytoskeletal reorganization, cell growth and development can bind to Nm23-H1 and the kinase deficient mutant H118F but only weakly to the mutant P96S which lacks the ability to suppress cell migration and metastasis. Cdc42 also colocalized with Nm23-H1 and the Dbl-1 proteins as specific punctate signals in the cytoplasm and at the cell membrane. Nm23-H1 also lead to the reduction in membrane ruffles and protuberances when expressed with Dbl-1 and Cdc42. Surprisingly, Nm23-H1 interacted with Cdc42 as well as Rac1 but somewhat weaker with RhoA. These studies suggests that Nm23-H1 can negatively regulate cell migration and tumor metastasis by modulating the activity of Cdc42 and possibly other Rho family members through interaction with Dbl-1. 
 Modulation of ERK5 is a novel mechanism by which Cdc42 regulates migration of breast cancer cells. Members of Rho family GTPases including Cdc42 are known to play pivotal roles in cell migration. Cell migration is also known to be regulated by many protein kinases. Kinetworks KPSS 11.0 phospho-site screening of Cdc42-silenced Hs578T breast cancer cells revealed most dramatic change in ERK5 MAP kinase. In the present study, we set out to determine the relationship between Cdc42 and ERK5 and its significance in breast cancer cell migration and invasion. Specific siRNAs were used for knocking down Cdc42 or ERK5 in breast cancer cells. Increased ERK5 phosphorylation in breast cancer cells was achieved by infection of constitutively active MEK5 adenovirus. The cells were then subjected to cell migration or invasion assay without the presence of serum or any growth factor. We found that Cdc42 negatively regulated phosphorylation of ERK5, which in turn exhibited an inverse relationship with migration and   invasive  ness of breast cancer cells. To find out some in vivo relevance of the results of our in vitro experiments we also examined the expression of ERK5 in the breast cancer tissues and their adjacent normal control tissues by real-time RT-PCR and immunocytochemistry. ERK5 expression was found to be reduced in breast cancer tissues as compared with their adjacent uninvolved mammary tissues. Therefore, Cdc42 may promote breast cancer cell migration and invasion by inhibiting ERK5 phosphorylation and ERK5 expression may be inversely correlated with the progression of some breast tumors. 
 Felic (CIP4b), a novel binding partner with the Src kinase Lyn and Cdc42, localizes to the phagocytic cup. Through its Src homology 3 (SH3) and SH2 domains, the Src kinase Lyn interacts with a small number of phosphoproteins, such as Shc, Cbl, and Vav, which regulate cell cycle and the cytoskeleton. Using Lyn's Unique, SH3, and SH2 domains as bait in a yeast 2-hybrid screen, we isolated a novel gene product with features of a scaffolding protein. We named it Felic because it contains a domain homologous to the tyrosine kinase Fes and the cytoskeletal protein ezrin and forms a Lyn interaction with the GTPase Cdc42 (Felic). Felic was expressed in both hematopoietic and nonhematopoietic tissues. Because it represents an alternative splice product related to the Cdc42-interacting protein 4, CIP4, we also refer to Felic as CIP4b. Felic contains an SH3 recognition site RXPXXP and multiple tyrosine residues. In insulin or serum-stimulated HEK293 cells, Felic became tyrosine phosphorylated. Like CIP4, Felic associated with Cdc42 in its activated form only. Unlike CIP4, Felic does not possess a C-terminal SH3 domain. Coprecipitation studies show that Felic bound to Lyn or activated forms of Cdc42. Overexpression of Felic or CIP4 inhibited NIH 3T3 cell   invasive  ness in a Matrigel assay. Because Lyn and Cdc42 are involved in phagocytosis, we examined the distribution of Felic in RAW macrophages during particle ingestion. Felic was recruited more efficiently than CIP4 to the phagocytic cups. Altogether, these data suggest that CIP4/Felic constitute a novel family of cytoskeletal scaffolding proteins, integrating Src and Cdc42 pathways. The absence of an SH3 domain in Felic provides a structural basis for functional differences. 
 Stromal fibroblasts facilitate cancer cell invasion by a novel invadopodia-independent matrix degradation process. Metastatic invasion of tumors into peripheral tissues is known to rely upon protease-mediated degradation of the surrounding stroma. This remodeling process uses complex, actin-based, specializations of the plasma membrane termed invadopodia that act both to sequester and release matrix metalloproteinases. Here we report that cells of mesenchymal origin, including tumor-associated fibroblasts, degrade substantial amounts of surrounding matrix by a mechanism independent of conventional invadopodia. These degradative sites lack the punctate shape of conventional invadopodia to spread along the cell base and are reticular and/or fibrous in character. In marked contrast to invadopodia, this degradation does not require the action of Src kinase, Cdc42 or Dyn2. Rather, inhibition of Dyn2 causes a marked upregulation of stromal matrix degradation. Further, expression and activity of matrix metalloproteinases are differentially regulated between tumor cells and stromal fibroblasts. This matrix remodeling by fibroblasts increases the   invasive   capacity of tumor cells, thereby illustrating how the tumor microenvironment can contribute to metastasis. These findings provide evidence for a novel matrix remodeling process conducted by stromal fibroblasts that is substantially more effective than conventional invadopodia, distinct in structural organization and regulated by disparate molecular mechanisms. 
 CCL19-induced chemokine receptor 7 activates the phosphoinositide-3 kinase-mediated   invasive   pathway through Cdc42 in metastatic squamous cell carcinoma of the head and neck. Metastatic squamous cell carcinoma of the head and neck (SCCHN) has been shown to express chemokine receptor 7 (CCR7), which activates phosphoinositide-3 kinase (PI3K) to promote invasion and survival of SCCHN cells. We hypothesized that Cdc42 might be involved in the CCR7-PI3K pathway. Adhesion assays, migration assays, immunofluorescence staining, Western blotting and immunohistochemical analysis were used to find whether Cdc42 can be activated by CCL19 (the CCR7 ligand) and its role in SCCHN. Results showed that CCL19 induced polarized localization of Cdc42 and actin polymerization in the leading edge of migrating cells. The level of activated membrane-bound Cdc42 was elevated, as measured by the GTPase activity pull-down assay. The increased membrane localization and membrane-bound activity of Cdc42 were abolished by CCR7 and PI3K inhibition, indicating the involvement of Cdc42 in the CCR7-PI3K cascade. Knockdown of Cdc42 by small interfering RNA (siRNA) led to significant reduction in the activation of Rac, filamentous actin (F-actin) accumulation as well as in the migration and invasion induced by CCL19. Taken together, our data indicate the important role played by Cdc42 in CCL19-induced migration and invasion of SCCHN cells. 
 Effect of microRNA-206 on cytoskeleton remodelling by downregulating Cdc42 in MDA-MB-231 cells. MicroRNAs are small, noncoding, single-stranded RNAs that regulate gene expression post-transcriptionally. miR-206 is known to play an important role in breast cancer metastasis. When we sought to predict the target of miR-206 by Targetscan, Pictar and miRanda, we found Cdc42 was a potential one. In this study, we transfected miR-206 into MDA-MB-231 cells and examined Cdc42 protein expression as well as MMP-2 and MMP-9, which are also associated with metastasis of breast cancer. Since Cdc42 is involved in filopodia and invadopodia formation, we examined the morphological changes of MDA-MB-231 cells.miR-206 mimics were transfected into MDA-MB-231 cells using LipofectamineTM 2000. Protein expression was detected by Western blot. Cells were stained with FITC-phalloidin to visualize F-actin.   Invasive   ability and migratory ability were examined by invasion assay and migration assay in vitro.Cdc42, MMP-2 and MMP-9 were downregulated on the protein level. The formation of filopodia, which requires Cdc42, was inhibited in miR-206 transfected cells, even under the stimulation of EGF. The invasion and migration of MDA-MB-231 cells in vitro was inhibited by miR-206 too.The results suggest that miR-206 may suppress invasion and migration of MDA-MB-231 cells in vitro partly via regulating actin cytoskeleton remodelling such as filopodia formation. 
  GeneRIFs  
 Snail regulates the motility and   invasive  ness of oral cancer cells via RhoA/Cdc42/p-ERM pathway. 
 direct activation of Cdc42 and Rac1 by   invasive   Salmonella is a prerequisite of Salmonella-mediated death of U937 cells. 
  [Mm]etastasis  PubMed  
 Expression of P21-activated kinase 1 and cell division control protein 42 homolog correlates with clinicopathological features and prognosis in cervical carcinoma. This study investigated P21-activated kinase 1 (PAK1) and cell division control protein 42 homolog (CDC42) expression and their correlation with clinicopathological features and prognosis in cervical carcinoma.Cervical carcinoma (n = 55), cervical intraepithelial neoplasias (CIN, n = 93), and normal cervix (n = 26) tissues were sampled. PAK1 and CDC42 expressions were determined using real-time quantitative polymerase chain reaction, Western blot and immunohistochemistry. Correlation analysis of PAK1 and CDC42 expression was performed using a Pearson correlation test. Survival rate was calculated using the Kaplan-Meier method. Independent prognostic factors were analyzed by Cox proportional hazard model.PAK1 and CDC42 expression were positively correlated in all tissues. PAK1 and CDC42 expression in cervical carcinoma tissues were higher than those in normal tissues. PAK1 and CDC42 protein expression in normal cervix, CINI, CINII, CINIII and cervical carcinoma tissues showed a gradually increasing trend. PAK1 and CDC42 expression were correlated with lymph node   metastasis  , the depth of tumor invasion, the degree of tumor differentiation, histological type and lymphovascular space invasion (LVSI; all P < 0.05). Cox regression analysis showed that the degree of tumor differentiation, PAK1 protein expression, histological type and LVSI are independent risk factors for prognosis of cervical carcinoma.High PAK1 and CDC42 expressions are closely related to the clinicopathological features and poor prognosis of cervical carcinoma, serving as unfavorable prognostic factors. 
 Regulation of CXCR4/AKT-signaling-induced cell invasion and tumor   metastasis   by RhoA, Rac-1, and Cdc42 in human esophageal cancer. CXC chemokines and their cognate receptors have been implicated wildly in cancer pathogenesis. In the present study, we report a critical cause relationship between CXCR4 expression and tumorigenesis in the setting of human esophageal squamous cell carcinoma (ESCC). In ESCC cells, CXCR4 expression was significantly higher than in human esophageal epithelial cells (HEEC). Reduction of CXCR4 in ESCC cells reduced cell proliferation and invasion in vitro and tumor growth in vivo. Among the potential downstream targets of CXCR4-CXCL12 are RhoA, Rac-1, and Cdc42, which are likely to contribute to the invasiveness of ESCC cells. Finally, we found that CXCR4-CXCL12/AKT axis regulates RhoA, Rac-1, and Cdc42 to modulate cell invasion and tumor   metastasis  . Together, these results demonstrate a role for CXCR4 in ESCC   metastasis   and progression and suggest potential targets for therapeutic intervention. 
 Wnt5b promotes the cell motility essential for   metastasis   of oral squamous cell carcinoma through active Cdc42 and RhoA. The activation of Wnt signaling has been reported in many types of squamous cell carcinoma. In this study, using human oral squamous cell carcinoma (OSCC) cells with different metastatic potential, we investigated the involvement of Wnt signaling in   metastasis  . Further, we aimed to elucidate the characteristic biological features related to high metastatic potential and to identify new target molecules for the suppression of OSCC lymph node   metastasis  . We compared SAS-Venus (SAS OSCC cells expressing green fluorescent protein) and SAS-LM8, which is a highly metastatic cell line derived from SAS-Venus by in vivo selection. The SAS-LM8 cell line had greater ability of migration and invasion compared to SAS-Venus. Furthermore, a higher number of filopodia-like protrusive structures were produced in SAS-LM8 cells compared to SAS-Venus cells, and the levels of active Cdc42 and active RhoA protein were higher in SAS-LM8 cells compared to SAS-Venus cells. We did not observe any differences in the expression of Wnt/β-catenin target genes between the two cell lines; however, the mRNA levels of Wnt5b were higher in SAS-LM8 cells compared to SAS-Venus cells. To confirm the involvement of Wnt5b in migration in OSCC cells, we examined the effects of the siRNA-mediated knockdown of Wnt5b in SAS-Venus cells and SAS-LM8 cells. The siRNA treatment significantly inhibited migration and the formation of filopodia-like protrusive structures. Conversely, when stimulated with Wnt5b, the migration and formation of filopodia-like protrusions were significantly enhanced and the levels of active Cdc42 and active RhoA proteins were also increased. These results indicate that Wnt5b is involved in the migration ability of OSCC cells through active Cdc42 and RhoA. 
 Snail regulates the motility of oral cancer cells via RhoA/Cdc42/p-ERM pathway. The transcriptional factor Snail has been reported to possess properties related to cancer progression; however, the mechanism for it is not fully understood. Our data showed that Snail knockdown by small interfering RNA in two OSCC cell lines, WSU-HN6 and CAL27, significantly inhibited cell migration and invasion which also resulted in decreased cell motility, such as impaired cell spreading on type I collagen substrate, reduced filopodia, and premature assembly of stress fibers. In addition, Snail-silencing decreased Cdc42 activity but increased RhoA activity, accompanied by the downregulation in both p-ERM expression and cell motility. Meanwhile, endogenous p-ERM was found specifically co-precipitated with activated Cdc42, but not RhoA, and this co-association was decreased by Snail-silencing. The small molecule inhibitors of Rho-associated kinase (Y27632) markedly enhanced Cdc42 activity and the association of p-ERM with activated Cdc42, increasing cell motility remarkably. Using immunohistochemistry, Snail and p-ERM overexpressions were found in OSCC tissues correlated with nodal   metastasis   and shorter survival. Taken together, these results demonstrate that Snail regulates cell motility through RhoA/Cdc42/p-ERM pathway and may serve as a biomarker to predict prognosis for OSCC patients. Although RhoA and Cdc42 are concurrently regulated downstream of Snail, there is a direct interplay between them, which indicates RhoA has to be inactivated at some point in cell motility cycle. 
 N-α-acetyltransferase 10 protein suppresses cancer cell   metastasi  s by binding PIX proteins and inhibiting Cdc42/Rac1 activity. N-α-acetyltransferase 10 protein, Naa10p, is an N-acetyltransferase known to be involved in cell cycle control. We found that Naa10p was expressed lower in varieties of malignancies with lymph nod  e metastas  is compared with non-lymph nod  e metastas  is. Higher Naa10p expression correlates the survival of lung cancer patients. Naa10p significantly suppressed migration, tumor growth, an  d metastas  is independent of its enzymatic activity. Instead, Naa10p binds to the GIT-binding domain of PIX, thereby preventing the formation of the GIT-PIX-Paxillin complex, resulting in reduced intrinsic Cdc42/Rac1 activity and decreased cell migration. Forced expression of PIX in Naa10-transfected tumor cells restored the migration an  d metastas  is ability. We suggest that Naa10p functions as a tumo  r metastas  is suppressor by disrupting the migratory complex, PIX-GIT- Paxillin, in cancer cells. 
 Cdc42 promotes transendothelial migration of cancer cells through β1 integrin. Cancer cells interact with endothelial cells during the process of metastatic spreading. Here, we use a small interfering RNA screen targeting Rho GTPases in cancer cells to identify Cdc42 as a critical regulator of cancer cell-endothelial cell interactions and transendothelial migration. We find that Cdc42 regulates β1 integrin expression at the transcriptional level via the transcription factor serum response factor (SRF). β1 integrin is the main target for Cdc42-mediating interaction of cancer cells with endothelial cells and the underlying extracellular matrix, as exogenous β1 integrin expression was sufficient to rescue the Cdc42-silencing phenotype. We show that Cdc42 was required in vivo for cancer cell spreading and protrusion extension along blood vessels and retention in the lungs. Interestingly, transient Cdc42 depletion was sufficient to decrease experimental lung metastases, which suggests that its role in endothelial attachment is important   for metast  asis. By identifying β1 integrin as a transcriptional target of Cdc42, our results provide new insight into Cdc42 function. 
 Podocalyxin-like 1 promotes invadopodia formation and   metastasis   through activation of Rac1/Cdc42/cortactin signaling in breast cancer cells. Metastatic disease is the leading cause of cancer mortality. Identifying biomarkers and regulatory mechanisms is important toward developing diagnostic and therapeutic tools against metastatic cancer. In this study, we demonstrated that podocalyxin-like 1 (PODXL) is overexpressed in breast tumor cells and increased in lymph node metastatic cancer. Mechanistically, we found that the expression of PODXL was associated with cell motility and invasiveness. Suppression of PODXL in MDA-MB-231 cells reduced lamellipodia formation and focal adhesion kinase (FAK) and paxillin phosphorylation. PODXL knockdown reduced the formation of invadopodia, such as inhibiting the colocalization of F-actin with cortactin and suppressing phosphorylation of cortactin and neural Wiskott-Aldrich syndrome protein. Conversely, overexpression of PODXL in MCF7 cells induced F-actin/cortactin colocalization and enhanced invadopodia formation and activation. Invadopodia activity and tumor invasion in PODXL-knockdown cells are similar to that in cortactin-knockdown cells. We further found that the DTHL motif in PODXL is crucial for regulating cortactin phosphorylation and Rac1/Cdc42 activation. Inhibition of Rac1/Cdc42 impeded PODXL-mediated cortactin activation and FAK and paxillin phosphorylation. Moreover, inhibition of PODXL in MDA-MB-231 cells significantly suppressed tumor colonization in the lungs and distant metastases, similar to those in cortactin-knockdown cells. These findings show that overexpression of PODXL enhanced invadopodia formation and tumor   metastasis   by inducing Rac1/Cdc42/cortactin signaling network. 
 cIAP1 regulates TNF-mediated cdc42 activation and filopodia formation. Tumour necrosis factor-α (TNF) is a cytokine endowed with multiple functions, depending on the cellular and environmental context. TNF receptor engagement induces the formation of a multimolecular complex including the TNFR-associated factor TRAF2, the receptor-interaction protein kinase RIP1 and the cellular inhibitor of apoptosis cIAP1, the latter being essential for NF-κB activation. Here, we show that cIAP1 also regulates TNF-induced actin cytoskeleton reorganization through a cdc42-dependent, NF-κB-independent pathway. Deletion of cIAP1 prevents TNF-induced filopodia and cdc42 activation. The expression of cIAP1 or its E3-ubiquitin ligase-defective mutant restores the ability of cIAP1(-/-) MEFs to produce filopodia, whereas a cIAP1 mutant unable to bind TRAF2 does not. Accordingly, the silencing of TRAF2 inhibits TNF-mediated filopodia formation, whereas silencing of RIP1 does not. cIAP1 directly binds cdc42 and promotes its RhoGDIα-mediated stabilization. TNF decreases cIAP1-cdc42 interaction, suggesting that TNF-induced recruitment of cIAP1/TRAF2 to the receptor releases cdc42, which in turn triggers actin remodeling. cIAP1 also regulates cdc42 activation in response to EGF and HRas-V12 expression. A downregulation of cIAP1 altered the cell polarization, the cell adhesion to endothelial cells and cell intercalation, which are cdc42-dependent processes. Finally, we demonstrated that the deletion of cIAP1 regulated the HRas-V12-mediated transformation process, including anchorage-dependent cell growth, tumour growth in a xenograft model and the development of experimen  tal metast  asis in the lung. 
 Overexpression of small GTPases directly correlates with expression of δ-catenin and their coexpression predicts a poor clinical outcome in nonsmall cell lung cancer. δ-catenin can affect cytoskeletal assembly, and promote cell migration by regulating the activity of small GTPases. While many malignancies have been shown to be positive for δ-catenin, it is still unclear whether δ-catenin and small GTPases are coexpressed in tumor cells, and so is the relationship between their coexpression and prognosis in the tumor patients. In this study, immunohistochemistry was performed to examine expressive levels of δ-catenin, cdc42, and Rac1 in 135 cases of nonsmall cell lung cancer (NSCLC), including 60 cases with follow-up records. Thirty samples of paired lung cancer tissues and adjacent normal lung tissues were collected to analyze mRNA and protein expression of δ-catenin and small GTPases. The effects of δ-catenin on small GTPases expression and invasive ability of lung cancer cells were also evaluated. Compared with normal lung tissues, both mRNA and protein levels of δ-catenin and small GTPases were increased in lung cancer tissues (P < 0.05), and the expression of small GTPases directly correlated with that of δ-catenin (P < 0.001). In addition, δ-catenin and small GTPases tended to be coexpressed in lung adenocarcinoma, advanced stages, and primary tumors with l  ymph node   metastasis (all P < 0.05). The patients with coexpression of δ-catenin and small GTPases had a shorter survival time than those without coexpression (P < 0.05). Furthermore, δ-catenin overexpression could enhance invasive ability of lung cancer cells by upregulating protein and transcriptional level of small GTPases. Therefore, δ-catenin likely upregulates the activity of small GTPases at transcriptional level, and their coexpression may predict a poor clinical outcome in NSCLC patients. 
 Expression of P21-activated kinase 1 and cell division control protein 42 homolog correlates with clinicopathological features and prognosis in cervical carcinoma. This study investigated P21-activated kinase 1 (PAK1) and cell division control protein 42 homolog (CDC42) expression and their correlation with clinicopathological features and prognosis in cervical carcinoma.Cervical carcinoma (n = 55), cervical intraepithelial neoplasias (CIN, n = 93), and normal cervix (n = 26) tissues were sampled. PAK1 and CDC42 expressions were determined using real-time quantitative polymerase chain reaction, Western blot and immunohistochemistry. Correlation analysis of PAK1 and CDC42 expression was performed using a Pearson correlation test. Survival rate was calculated using the Kaplan-Meier method. Independent prognostic factors were analyzed by Cox proportional hazard model.PAK1 and CDC42 expression were positively correlated in all tissues. PAK1 and CDC42 expression in cervical carcinoma tissues were higher than those in normal tissues. PAK1 and CDC42 protein expression in normal cervix, CINI, CINII, CINIII and cervical carcinoma tissues showed a gradually increasing trend. PAK1 and CDC42 expression were correlated with lymph node   metastasis  , the depth of tumor invasion, the degree of tumor differentiation, histological type and lymphovascular space invasion (LVSI; all P < 0.05). Cox regression analysis showed that the degree of tumor differentiation, PAK1 protein expression, histological type and LVSI are independent risk factors for prognosis of cervical carcinoma.High PAK1 and CDC42 expressions are closely related to the clinicopathological features and poor prognosis of cervical carcinoma, serving as unfavorable prognostic factors. 
 Regulation of CXCR4/AKT-signaling-induced cell invasion and tumor   metastasis   by RhoA, Rac-1, and Cdc42 in human esophageal cancer. CXC chemokines and their cognate receptors have been implicated wildly in cancer pathogenesis. In the present study, we report a critical cause relationship between CXCR4 expression and tumorigenesis in the setting of human esophageal squamous cell carcinoma (ESCC). In ESCC cells, CXCR4 expression was significantly higher than in human esophageal epithelial cells (HEEC). Reduction of CXCR4 in ESCC cells reduced cell proliferation and invasion in vitro and tumor growth in vivo. Among the potential downstream targets of CXCR4-CXCL12 are RhoA, Rac-1, and Cdc42, which are likely to contribute to the invasiveness of ESCC cells. Finally, we found that CXCR4-CXCL12/AKT axis regulates RhoA, Rac-1, and Cdc42 to modulate cell invasion and tumor   metastasis  . Together, these results demonstrate a role for CXCR4 in ESCC   metastasis   and progression and suggest potential targets for therapeutic intervention. 
 Podocalyxin-like 1 promotes invadopodia formation and   metastasis   through activation of Rac1/Cdc42/cortactin signaling in breast cancer cells. Metastatic disease is the leading cause of cancer mortality. Identifying biomarkers and regulatory mechanisms is important toward developing diagnostic and therapeutic tools against metastatic cancer. In this study, we demonstrated that podocalyxin-like 1 (PODXL) is overexpressed in breast tumor cells and increased in lymph node metastatic cancer. Mechanistically, we found that the expression of PODXL was associated with cell motility and invasiveness. Suppression of PODXL in MDA-MB-231 cells reduced lamellipodia formation and focal adhesion kinase (FAK) and paxillin phosphorylation. PODXL knockdown reduced the formation of invadopodia, such as inhibiting the colocalization of F-actin with cortactin and suppressing phosphorylation of cortactin and neural Wiskott-Aldrich syndrome protein. Conversely, overexpression of PODXL in MCF7 cells induced F-actin/cortactin colocalization and enhanced invadopodia formation and activation. Invadopodia activity and tumor invasion in PODXL-knockdown cells are similar to that in cortactin-knockdown cells. We further found that the DTHL motif in PODXL is crucial for regulating cortactin phosphorylation and Rac1/Cdc42 activation. Inhibition of Rac1/Cdc42 impeded PODXL-mediated cortactin activation and FAK and paxillin phosphorylation. Moreover, inhibition of PODXL in MDA-MB-231 cells significantly suppressed tumor colonization in the lungs and distant metastases, similar to those in cortactin-knockdown cells. These findings show that overexpression of PODXL enhanced invadopodia formation and tumor   metastasis   by inducing Rac1/Cdc42/cortactin signaling network. 
 Snail regulates the motility of oral cancer cells via RhoA/Cdc42/p-ERM pathway. The transcriptional factor Snail has been reported to possess properties related to cancer progression; however, the mechanism for it is not fully understood. Our data showed that Snail knockdown by small interfering RNA in two OSCC cell lines, WSU-HN6 and CAL27, significantly inhibited cell migration and invasion which also resulted in decreased cell motility, such as impaired cell spreading on type I collagen substrate, reduced filopodia, and premature assembly of stress fibers. In addition, Snail-silencing decreased Cdc42 activity but increased RhoA activity, accompanied by the downregulation in both p-ERM expression and cell motility. Meanwhile, endogenous p-ERM was found specifically co-precipitated with activated Cdc42, but not RhoA, and this co-association was decreased by Snail-silencing. The small molecule inhibitors of Rho-associated kinase (Y27632) markedly enhanced Cdc42 activity and the association of p-ERM with activated Cdc42, increasing cell motility remarkably. Using immunohistochemistry, Snail and p-ERM overexpressions were found in OSCC tissues correlated with nodal   metastasis   and shorter survival. Taken together, these results demonstrate that Snail regulates cell motility through RhoA/Cdc42/p-ERM pathway and may serve as a biomarker to predict prognosis for OSCC patients. Although RhoA and Cdc42 are concurrently regulated downstream of Snail, there is a direct interplay between them, which indicates RhoA has to be inactivated at some point in cell motility cycle. 
 cIAP1 regulates TNF-mediated cdc42 activation and filopodia formation. Tumour necrosis factor-α (TNF) is a cytokine endowed with multiple functions, depending on the cellular and environmental context. TNF receptor engagement induces the formation of a multimolecular complex including the TNFR-associated factor TRAF2, the receptor-interaction protein kinase RIP1 and the cellular inhibitor of apoptosis cIAP1, the latter being essential for NF-κB activation. Here, we show that cIAP1 also regulates TNF-induced actin cytoskeleton reorganization through a cdc42-dependent, NF-κB-independent pathway. Deletion of cIAP1 prevents TNF-induced filopodia and cdc42 activation. The expression of cIAP1 or its E3-ubiquitin ligase-defective mutant restores the ability of cIAP1(-/-) MEFs to produce filopodia, whereas a cIAP1 mutant unable to bind TRAF2 does not. Accordingly, the silencing of TRAF2 inhibits TNF-mediated filopodia formation, whereas silencing of RIP1 does not. cIAP1 directly binds cdc42 and promotes its RhoGDIα-mediated stabilization. TNF decreases cIAP1-cdc42 interaction, suggesting that TNF-induced recruitment of cIAP1/TRAF2 to the receptor releases cdc42, which in turn triggers actin remodeling. cIAP1 also regulates cdc42 activation in response to EGF and HRas-V12 expression. A downregulation of cIAP1 altered the cell polarization, the cell adhesion to endothelial cells and cell intercalation, which are cdc42-dependent processes. Finally, we demonstrated that the deletion of cIAP1 regulated the HRas-V12-mediated transformation process, including anchorage-dependent cell growth, tumour growth in a xenograft model and the development of experimen  tal metast  asis in the lung. 
 Wnt5b promotes the cell motility essential for   metastasis   of oral squamous cell carcinoma through active Cdc42 and RhoA. The activation of Wnt signaling has been reported in many types of squamous cell carcinoma. In this study, using human oral squamous cell carcinoma (OSCC) cells with different metastatic potential, we investigated the involvement of Wnt signaling in   metastasis  . Further, we aimed to elucidate the characteristic biological features related to high metastatic potential and to identify new target molecules for the suppression of OSCC lymph node   metastasis  . We compared SAS-Venus (SAS OSCC cells expressing green fluorescent protein) and SAS-LM8, which is a highly metastatic cell line derived from SAS-Venus by in vivo selection. The SAS-LM8 cell line had greater ability of migration and invasion compared to SAS-Venus. Furthermore, a higher number of filopodia-like protrusive structures were produced in SAS-LM8 cells compared to SAS-Venus cells, and the levels of active Cdc42 and active RhoA protein were higher in SAS-LM8 cells compared to SAS-Venus cells. We did not observe any differences in the expression of Wnt/β-catenin target genes between the two cell lines; however, the mRNA levels of Wnt5b were higher in SAS-LM8 cells compared to SAS-Venus cells. To confirm the involvement of Wnt5b in migration in OSCC cells, we examined the effects of the siRNA-mediated knockdown of Wnt5b in SAS-Venus cells and SAS-LM8 cells. The siRNA treatment significantly inhibited migration and the formation of filopodia-like protrusive structures. Conversely, when stimulated with Wnt5b, the migration and formation of filopodia-like protrusions were significantly enhanced and the levels of active Cdc42 and active RhoA proteins were also increased. These results indicate that Wnt5b is involved in the migration ability of OSCC cells through active Cdc42 and RhoA. 
 Overexpression of small GTPases directly correlates with expression of δ-catenin and their coexpression predicts a poor clinical outcome in nonsmall cell lung cancer. δ-catenin can affect cytoskeletal assembly, and promote cell migration by regulating the activity of small GTPases. While many malignancies have been shown to be positive for δ-catenin, it is still unclear whether δ-catenin and small GTPases are coexpressed in tumor cells, and so is the relationship between their coexpression and prognosis in the tumor patients. In this study, immunohistochemistry was performed to examine expressive levels of δ-catenin, cdc42, and Rac1 in 135 cases of nonsmall cell lung cancer (NSCLC), including 60 cases with follow-up records. Thirty samples of paired lung cancer tissues and adjacent normal lung tissues were collected to analyze mRNA and protein expression of δ-catenin and small GTPases. The effects of δ-catenin on small GTPases expression and invasive ability of lung cancer cells were also evaluated. Compared with normal lung tissues, both mRNA and protein levels of δ-catenin and small GTPases were increased in lung cancer tissues (P < 0.05), and the expression of small GTPases directly correlated with that of δ-catenin (P < 0.001). In addition, δ-catenin and small GTPases tended to be coexpressed in lung adenocarcinoma, advanced stages, and primary tumors with l  ymph node   metastasis (all P < 0.05). The patients with coexpression of δ-catenin and small GTPases had a shorter survival time than those without coexpression (P < 0.05). Furthermore, δ-catenin overexpression could enhance invasive ability of lung cancer cells by upregulating protein and transcriptional level of small GTPases. Therefore, δ-catenin likely upregulates the activity of small GTPases at transcriptional level, and their coexpression may predict a poor clinical outcome in NSCLC patients. 
 Cdc42 promotes transendothelial migration of cancer cells through β1 integrin. Cancer cells interact with endothelial cells during the process of metastatic spreading. Here, we use a small interfering RNA screen targeting Rho GTPases in cancer cells to identify Cdc42 as a critical regulator of cancer cell-endothelial cell interactions and transendothelial migration. We find that Cdc42 regulates β1 integrin expression at the transcriptional level via the transcription factor serum response factor (SRF). β1 integrin is the main target for Cdc42-mediating interaction of cancer cells with endothelial cells and the underlying extracellular matrix, as exogenous β1 integrin expression was sufficient to rescue the Cdc42-silencing phenotype. We show that Cdc42 was required in vivo for cancer cell spreading and protrusion extension along blood vessels and retention in the lungs. Interestingly, transient Cdc42 depletion was sufficient to decrease experimental lung metastases, which suggests that its role in endothelial attachment is important   for metast  asis. By identifying β1 integrin as a transcriptional target of Cdc42, our results provide new insight into Cdc42 function. 
 N-α-acetyltransferase 10 protein suppresses cancer cell   metastasi  s by binding PIX proteins and inhibiting Cdc42/Rac1 activity. N-α-acetyltransferase 10 protein, Naa10p, is an N-acetyltransferase known to be involved in cell cycle control. We found that Naa10p was expressed lower in varieties of malignancies with lymph nod  e metastas  is compared with non-lymph nod  e metastas  is. Higher Naa10p expression correlates the survival of lung cancer patients. Naa10p significantly suppressed migration, tumor growth, an  d metastas  is independent of its enzymatic activity. Instead, Naa10p binds to the GIT-binding domain of PIX, thereby preventing the formation of the GIT-PIX-Paxillin complex, resulting in reduced intrinsic Cdc42/Rac1 activity and decreased cell migration. Forced expression of PIX in Naa10-transfected tumor cells restored the migration an  d metastas  is ability. We suggest that Naa10p functions as a tumo  r metastas  is suppressor by disrupting the migratory complex, PIX-GIT- Paxillin, in cancer cells. 
 p16 Stimulates CDC42-dependent migration of hepatocellular carcinoma cells. Hepatocellular carcinoma (HCC) is a leading cause of cancer-related deaths worldwide. Tumor dissemination to the extra-hepatic region of the portal vein, lymph nodes, lungs or bones contributes to the high mortality seen in HCC; yet, the molecular mechanisms responsible for HCC   metastasis   remain unclear. Prior studies have suggested a potential link between accumulated cytoplasm-localized p16 and tumor progression. Here we report that p16 enhances   metastasis  -associated phenotypes in HCC cells - ectopic p16 expression increased cell migration in vitro, and lung colonization after intravenous injection, whereas knockdown of endogenous p16 reduced cell migration. Interestingly, analysis of p16 mutants indicated that the Cdk4 interaction domain is required for stimulation of HCC cell migration; however, knockdown of Cdk4 and Cdk6 showed that these proteins are dispensable for this phenomenon. Intriguingly, we found that in p16-positive HCC samples, p16 protein is predominantly localized in the cytoplasm. In addition, we identified a potential role for nuclear-cytoplasmic shuttling in p16-stimulated migration, consistent with the predominantly cytoplasmic localization of p16 in IHC-positive HCC samples. Finally, we determined that p16-stimulated cell migration requires the Cdc42 GTPase. Our results demonstrate for the first time a pro-migratory role for p16, and suggest a potential mechanism for the observed association between cytoplasmic p16 and tumor progression in diverse tumor types. 
 Expression analysis of Cdc42 in lung cancer and modulation of its expression by curcumin in lung cancer cell lines. Cdc42, a Rho GTPase family member, is involved in cell transformation, proliferation, survival, invasion and   metastasis   of human cancer cells. Overexpression of Cdc42 has been reported in several types of human cancer. However, the underlying mechanisms are not well understood. The present study showed that Cdc42 was overexpressed in 80 of 110 primary lung cancer patients, and overexpression of Cdc42 was significantly associated with high TNM stage and lymph node   metastasis  . Moreover, RNAi-mediated suppression of Cdc42 expression reduced actin ﬁlopodia formation, migration and invasion potential of a highly metastatic lung cancer cell line, 801D. In parallel, 801D cells were treated with curcumin and the effect on the expression of the Cdc42 gene at the transcriptional and translational levels was analyzed by RT-PCR and Western blotting. Curcumin inhibited cell migration, invasion and downregulated Cdc42 gene and Cdc42-related target gene expression in 801D cells. It also induced rearrangements of the actin cytoskeleton. These effects mimicked those of Cdc42 knockdown. Furthermore, xenograft experiments confirmed the suppression of tumor growth and invasion in vivo, which was due to the effect of curcumin and the inhibition of Cdc42 by curcumin. Our results showing the downregulation of Cdc42 expression by curcumin in lung cancer cells taken together with the clinical data suggest a potential therapeutic role for curcumin in inducing Cdc42-mediated inhibition of invasion of lung cancer cells. 
 Clinicopathological pattern and Annexin A2 and Cdc42 status in patients presenting with differentiation and lymphnode   metastasis   of esophageal squamous cell carcinomas. Annexin A2 and Cdc42 were identified by 2-dimensional electrophoresis (2-DE) and MALDI-TOF-MS between esophageal squamous cell carcinomas (ESCC) and corresponding normal esophagus mucosa in our previous study. To assess clinico-pathological pattern and Annexin A2 and Cdc42 status with respect to cell differentiation and lymphnode   metastasis   in patients with ESCC. The expression of Annexin A2 and Cdc42 in 22 pairs of fresh ESCC and matched tissues were detected by qRT-PCR and western blot, respectively. And it was further confirmed by immunohistochemistry with 175 pairs of formalin-fixed, paraffin-embedded ESCC. Results showed that Annexin A2 expression was significantly down-regulated, and Cdc42 was up-regulated in ESCC compared to matched control on both mRNA and protein level (P < 0.05), which was in accordance with our previous results on proteomics data. Additionally, Annexin A2 and Cdc42 expression was significantly correlated with lymphoid node   metastasis   (P < 0.05) and pathological differentiation (P < 0.05). Taken together, we proposed that the aberrant expression of Annexin A2 and Cdc42 played a role in carcinogenesis, differentiation and   metastasis   of ESCC, which implied its potential target for clinical biomarkers in differentiation and lymph node   metastasis  . 
 Discoidin domain receptor 1 controls linear invadosome formation via a Cdc42-Tuba pathway. Accumulation of type I collagen fibrils in tumors is associated with an increased risk of   metastasis  . Invadosomes are F-actin structures able to degrade the extracellular matrix. We previously found that collagen I fibrils induced the formation of peculiar linear invadosomes in an unexpected integrin-independent manner. Here, we show that Discoidin Domain Receptor 1 (DDR1), a collagen receptor overexpressed in cancer, colocalizes with linear invadosomes in tumor cells and is required for their formation and matrix degradation ability. Unexpectedly, DDR1 kinase activity is not required for invadosome formation or activity, nor is Src tyrosine kinase. We show that the RhoGTPase Cdc42 is activated on collagen in a DDR1-dependent manner. Cdc42 and its specific guanine nucleotide-exchange factor (GEF), Tuba, localize to linear invadosomes, and both are required for linear invadosome formation. Finally, DDR1 depletion blocked cell invasion in a collagen gel. Altogether, our data uncover an important role for DDR1, acting through Tuba and Cdc42, in proteolysis-based cell invasion in a collagen-rich environment. 
 Effects of CDC42 on the proliferation and invasion of gastric cancer cells. Cell division cycle 42 (CDC42), which is a member of the Rho GTPase family, has been reported to regulate the   metastasis   of various human cancer cells; however, the role of CDC42 in gastric cancer (GC) remains unclear. The present study aimed to investigate the effects of CDC42 on the proliferation, migration and invasion of GC. Furthermore, the molecular mechanisms underlying the effects of CDC42 on GC were explored. The expression levels of CDC42 in the AGS and SGC7901 human GC cell lines were reduced by RNA interference. Knockdown of CDC42 significantly inhibited the proliferation of AGS and SGC7901 cells, and it was suggested that this inhibitory process may be due to cell cycle arrest at G1/S phase and downregulation of cyclin A, cyclin D1, cyclin E and proliferating cell nuclear antigen. Furthermore, knockdown of CDC42 markedly inhibited the migration and invasion of GC cells, and suppressed the expression of matrix metalloproteinase 9. These results indicated that CDC42 is a key regulator involved in regulating the proliferation, migration and invasion of GC, and it may be considered a potential therapeutic target in GC. 
 Blood type B antigen modulates cell migration through regulating cdc42 expression and activity in HaCaT cells. ABO blood group is determined by carbohydrate antigens, called ABH antigens. It has been known that the change of carbohydrate antigen expression, including ABH antigens, has correlation with the tumor   metastasis   and survival; however, the exact mechanism remains to be elucidated. ABH antigens are expressed not only in blood cells but also in several tissues. In epidermis, ABH antigen is expressed in the uppermost spinous and granular layer. We investigated the role of ABH antigens on the cell migration of HaCaT keratinocytes, which express B antigen. Knock-down of B antigen expression by small interference RNA of FUT1 inhibited HaCaT cell migration. At that time, we found that lamellipodia and actin fiber were also reduced by knock-down of B antigen expression. The transcription of cdc42, a kind of Rho GTPase which plays a key role in actin polymerization, was reduced by down-regulated B antigen expression. Furthermore, the reduced B antigen expression also inhibited the interaction of cdc42 and N-WASP. Collectively, our data provide a clue how ABH antigens regulate the cell migration mechanism. 
 Activation of Rho GTPase Cdc42 promotes adhesion and invasion in colorectal cancer cells. The purpose of this study was to investigate the role of activated Rho GTPase cell division control protein 42 homolog (Cdc42) in colorectal cancer cell adhesion, migration, and invasion.The constitutively active form of Cdc42 (GFP-Cdc42L61) or control vector was overexpressed in the colorectal cancer cell line SW480. The localization of active Cdc42 was monitored by immunofluorescence staining, and the effects of active Cdc42 on cell migration and invasion were examined using an attachment assay, a wound healing assay, and a Matrigel migration assay in vitro.Immunofluorescence staining revealed that constitutively active Cdc42 predominately localized to the plasma membrane. Compared to SW480 cells transfected with the control vector, overexpression of constitutively active Cdc42 in SW480 cells promoted filopodia formation and cell stretch and dramatically enhanced cell adhesion to the coated plates. The wound healing assay revealed a significant increase of migration capability in SW480 cells expressing active Cdc42 compared to the control cells. Additionally, the Matrigel invasion assay demonstrated that active Cdc42 significantly promoted SW480 cell migration through the chamber.Our results suggest that active Rho GTPase Cdc42 can greatly enhance colorectal cancer cell SW480 to spread, migrate, and invade, which may contribute to colorectal cancer   metastasis  . 
 Cdc42 overexpression induces hyperbranching in the developing mammary gland by enhancing cell migration. The Rho GTPase Cdc42 is overexpressed and hyperactivated in breast tumors compared to normal breast tissue. Cdc42 regulates key processes that are critical for mammary gland morphogenesis and become disrupted during the development, progression, and   metastasis   of breast cancer. However, the contribution of Cdc42 to normal and neoplastic mammary gland development in vivo remains poorly understood. We were therefore interested in investigating the effects of Cdc42 overexpression on mammary gland morphogenesis as a first step toward understanding how its overexpression may contribute to mammary tumorigenesis.We developed a tetracycline-regulatable Cdc42 overexpression mouse model in which Cdc42 can be inducibly overexpressed in the developing mammary gland. The effects of Cdc42 overexpression during postnatal mammary gland development were investigated using in vivo and in vitro approaches, including morphometric analysis of wholemounted mammary glands, quantification of histological markers, and primary mammary epithelial cell (MEC) functional and biochemical assays.Analysis of Cdc42-overexpressing mammary glands revealed abnormal terminal end bud (TEB) morphologies, characterized by hyperbudding and trifurcation, and increased side branching within the ductal tree. Quantification of markers of proliferation and apoptosis suggested that these phenotypes were not due to increased cell proliferation or survival. Rather, Cdc42 overexpressing MECs were more migratory and contractile and formed dysmorphic, invasive acini in three-dimensional cultures. Cdc42 and RhoA activities, phosphorylated myosin light chain, and MAPK signaling, which contribute to migration and invasion, were markedly elevated in Cdc42 overexpressing MECs. Interestingly, Cdc42 overexpressing mammary glands displayed several features associated with altered epithelial-stromal interactions, which are known to regulate branching morphogenesis. These included increased stromal thickness and collagen deposition, and stromal cells isolated from Cdc42 overexpressing mammary glands exhibited elevated mRNA expression of extracellular matrix proteins and remodeling enzymes.These data suggest that Cdc42 overexpression disrupts mammary gland branching morphogenesis by altering Rho GTPase and MAPK signaling, leading to increased MEC contractility and migration in association with stromal alterations. Our studies provide insight into how aberrant Cdc42 expression may contribute to mammary tumorigenesis. 
 Wnt-5a/Ca2+-induced NFAT activity is counteracted by Wnt-5a/Yes-Cdc42-casein kinase 1alpha signaling in human mammary epithelial cells. Wnt-5a has been shown to influence the metastatic behavior of human breast cancer cells, and the loss of Wnt-5a expression is associated with metastatic disease. We show here that NFAT1, a transcription factor connected with breast cancer   metastasis  , is activated by Wnt-5a through a Ca2+ signaling pathway in human breast epithelial cells. This activation was simultaneously counteracted by a Wnt-5a-induced Yes/Cdc42 signaling pathway. The observation that inhibition of the Wnt-5a/Yes/Cdc42 signal prolonged the duration of ionomycin-induced NFAT1 activation revealed the general importance of this pathway. The Wnt-5a-induced inhibition of NFAT1 did not require glycogen synthase kinase 3beta, JNK, or Pak1 activity or modulation of the cytoskeleton. Instead, we observed that Wnt-5a induced a complex formation of NFAT1/casein kinase 1alpha, even upon treatment with ionomycin, which was blocked upon inhibition of the Wnt-5a/Yes/Cdc42 signaling pathway. Our results explain why Wnt-5a/Ca2+-induced NFAT activity is hard to detect and suggest a novel mechanism by which Wnt-5a can suppress tumor-specific, agonist-induced NFAT activity and thus the metastatic behavior of breast cancer cells. 
 The suppressor of   metastasis   Nm23-H1 interacts with the Cdc42 Rho family member and the pleckstrin homology domain of oncoprotein Dbl-1 to suppress cell migration. Tumor invasion and   metastasis   is regulated by a number of cellular molecules known to be involved in signaling and cytoskeletal rearrangement. One of these molecules is the suppressor of tumor   metastasis   Nm23-H1 which linked to invasiveness and metastatic potential of human cancers. Nm23-H1 expression is down-regulated in human melanoma and invasive breast carcinoma. Recent studies have shown an association between the Nm23-H1 and oncoprotein Dbl-1 which is associated with guanine exchange and belongs to a family of Guanine Exchange Factors (GEF). In this report we show a direct interaction in vitro and in human B cells and specifically identified the pleckstrin homology domain of Dbl-1 as the domain which binds to Nm23-H1. Furthermore, Nm23-H1 and Dbl-1 colocalized in the cytoplasm of COS-7 cells when expressed exogenously and showed predominant signals at the periphery of the cells particularly at the plasma membrane. Interestingly, Dbl-1 and Cdc42 expression rescued the suppressive activities of Nm23-H1 in cell migration assays. We show that Cdc42 a regulatory protein involved in cytoskeletal reorganization, cell growth and development can bind to Nm23-H1 and the kinase deficient mutant H118F but only weakly to the mutant P96S which lacks the ability to suppress cell migration and   metastasis  . Cdc42 also colocalized with Nm23-H1 and the Dbl-1 proteins as specific punctate signals in the cytoplasm and at the cell membrane. Nm23-H1 also lead to the reduction in membrane ruffles and protuberances when expressed with Dbl-1 and Cdc42. Surprisingly, Nm23-H1 interacted with Cdc42 as well as Rac1 but somewhat weaker with RhoA. These studies suggests that Nm23-H1 can negatively regulate cell migration and tumor   metastasis   by modulating the activity of Cdc42 and possibly other Rho family members through interaction with Dbl-1. 
 Down-regulation of miRNA-204 by LMP-1 enhances CDC42 activity and facilitates invasion of EBV-associated nasopharyngeal carcinoma cells. Nasopharayngeal carcinoma (NPC) is an Epstein-Barr virus (EBV)-associated malignancy. It is known that microRNAs are implicated in the progression of NPC. However, the role of miR-204 in NPC is poorly understood. In this study, we found that miR-204 was down-regulated in NPC cells and tissues. Low-level expression of miR-204 was significantly associated with a more aggressive and poor prognostic phenotype of NPC. We further found that EBV-encoded latent membrane protein 1 (LMP-1) suppressed miR-204 expression by activating Stat-3. Cdc42 was identified as a direct target of miR-204. Mir-204 inhibited EBV positive C666-1 cell invasion and   metastasis   partly through targeting cdc42. 
 microRNA-195-Cdc42 axis acts as a prognostic factor of esophageal squamous cell carcinoma. Previous studies observed the downregulation of microRNA (miR)-195 in esophageal squamous cell carcinoma (ESCC) tissues, confirmed cell division cycle 42 (Cdc42) as one target gene of miR-195, and demonstrated that miR-195 may act as a tumor suppressor in ESCC by regulating Cdc42 expression. This study aimed to explore the association of miR-195 and Cdc42 combined expression with clinicopathologic factors and prognosis.Expression of miR-195 and Cdc42 mRNA in 98 pairs of ESCC and paracancerous tissues were detected using real-time quantitative RT-PCR.miR-195 downregulation and Cdc42 upregulation were both prevalent in ESCC tissues, and negatively correlated with each other. In addition, miR-195 expression negatively correlated with TNM stage (P=0.008) and lymphatic   metastasis   (P=0.022), while Cdc42 expression positively correlated with TNM stage (P=0.011) and tumor differentiation (P=0.024). Moreover, combined expression of miR-195 and Cdc42 (miR-195/Cdc42) was found to be prognostic indicators for progression-free survival and overall survival of ESCC patients both in univariate and multivariate analyses.The main findings of this study indicate the involvement of miR-195-Cdc42 axis in the progression of ESCC and suggest that the combined aberrant expression of miR-195 and Cdc42 mRNA can serve as a promising unfavorable prognostic biomarker in ESCC. 
 Deregulation of SLIT2-mediated Cdc42 activity is associated with esophageal cancer   metastasis   and poor prognosis. SLIT2, a secreted protein, has been found to inactivate Cdc42 GTPase to modulate neural cell migration. However, alteration of SLIT2-mediated Cdc42 in terms of migration regulation remains undefined in esophageal squamous cell carcinoma (ESCC).We report here in ESCC cell, animal, and clinical models that SLIT2 acts as a migration suppressor and serves as a prognostic biomarker.The immunohistochemistry data indicated that 31.8% (49 of 154) of tumors from ESCC patients showed low expression of SLIT2 protein which correlated with poor overall survival and disease-free survival. DNA methylation analysis suggested that promoter hypermethylation is responsible for low expression of SLIT2 in ESCC. Knockdown of SLIT2 increased ESCC cell migration, while SLIT2 stable overexpression reduced cell migration. ESCC cells treated with conditioned media from cells overexpressing SLIT2 also suppressed cell migration. Importantly, silencing of SLIT2 decreased the complex formation, and thus induced Cdc42 activity and promoted membrane localization of focal adhesion kinase and Paxillin. Anti-metastatic effect of SLIT2 was confirmed in an experimental   metastasis   model of SLIT2 knockdown ESCC cells.Our results provide novel evidence that low expression of SLIT2 correlates with poor prognosis and promotes   metastasis   in ESCC, which may be regulated by the Cdc42-mediated pathways. 
 Stromal fibroblasts facilitate cancer cell invasion by a novel invadopodia-independent matrix degradation process. Metastatic invasion of tumors into peripheral tissues is known to rely upon protease-mediated degradation of the surrounding stroma. This remodeling process uses complex, actin-based, specializations of the plasma membrane termed invadopodia that act both to sequester and release matrix metalloproteinases. Here we report that cells of mesenchymal origin, including tumor-associated fibroblasts, degrade substantial amounts of surrounding matrix by a mechanism independent of conventional invadopodia. These degradative sites lack the punctate shape of conventional invadopodia to spread along the cell base and are reticular and/or fibrous in character. In marked contrast to invadopodia, this degradation does not require the action of Src kinase, Cdc42 or Dyn2. Rather, inhibition of Dyn2 causes a marked upregulation of stromal matrix degradation. Further, expression and activity of matrix metalloproteinases are differentially regulated between tumor cells and stromal fibroblasts. This matrix remodeling by fibroblasts increases the invasive capacity of tumor cells, thereby illustrating how the tumor microenvironment can contribute to   metastasis  . These findings provide evidence for a novel matrix remodeling process conducted by stromal fibroblasts that is substantially more effective than conventional invadopodia, distinct in structural organization and regulated by disparate molecular mechanisms. 
 Bif-1 promotes tumor cell migration and   metastasis   via Cdc42 expression and activity. Tumor   metastasis   is the process by which tumor cells disseminate from tumors and enter nearby and distant microenvironments for new colonization. Bif-1 (BAX-interacting factor 1), which has a BAR domain and an SH3 domain, has been reported to be involved in cell growth, apoptosis and autophagy. However, the influence of Bif-1 on   metastasis   has been less studied. To understand the role of Bif-1 in   metastasis  , we studied the expression levels of Bif-1 in human HCC specimens using immunohistochemistry, a tissue microarray and quantitative PCR. The function of Bif-1 was assessed in migration and translocation assays and the pulmonary metastatic animal model. The relationship between Bif-1 and the Rho family was determined using immunoblot analyses and chromatin immunoprecipitation. The results showed that the expression of Bif-1 was higher in hepatocellular carcinoma (HCC) than matched adjacent non-tumor liver tissues. Increased Bif-1 expression was associated with tumor size and the intercellular spread and   metastasis   of HCC. Analysis of the relationship between Bif-1 expression and patients' clinical characteristics revealed that patients with higher levels of Bif-1 had shorter disease-free and overall survival rates. Knockdown of Bif-1 with RNAi suppressed the migration of HCC cells and pulmonary   metastasis   and decreased the expression of Cdc42, a member of the Rho family. Bif-1 localized to the cytosol and nucleus and interacted with the promoter transcription region of Cdc42, which may regulate Cdc42 expression. Our results demonstrate a novel role of Bif-1 in HCC, in which Bif-1 promotes cell   metastasis   by regulating Cdc42 expression and activity. 
 DOCK7 is a critical regulator of the RAGE-Cdc42 signaling axis that induces formation of dendritic pseudopodia in human cancer cells. Cellular migration is a fundamental process linked to cancer   metastasis  . Growing evidence indicates that the receptor for advanced glycation end products (RAGE) plays a pivotal role in this process. With regard to downstream signal transducers of RAGE, diaphanous-1 and activated small guanine nucleotide triphosphatases, Rac1 and Cdc42, have been identified. To obtain precise insight into the direct downstream signaling mechanism of RAGE, we screened for proteins interacting with the cytoplasmic domain of RAGE employing an immunoprecipitation-liquid chromatography coupled with an electrospray tandem mass spectrometry system. In the present study, we found that the cytoplasmic domain of RAGE interacted with an atypical DOCK180-related guanine nucleotide exchange factor, dedicator of cytokinesis protein 7 (DOCK7). DOCK7 bound to the RAGE cytoplasmic domain and transduced a signal to Cdc42, resulting in the formation of abundant highly branched filopodia-like protrusions, dendritic pseudopodia. Blocking of the function of DOCK7 greatly abrogated the formation of dendritic pseudopodia and suppressed cellular migration. These results indicate that DOCK7 functions as an essential and downstream regulator of RAGE-mediated cellular migration through the formation of dendritic pseudopodia. 
 A polysaccharide from Pinellia ternata inhibits cell proliferation and   metastasis   in human cholangiocarcinoma cells by targeting of Cdc42 and 67kDa Laminin Receptor (LR). In this study, we isolated and purified a polysaccharide (PTPA) from the tubers of Pinellia ternate. We aimed to evaluate the cytotoxic effects of PTPA on human cholangiocarcinoma (CCA) cell lines and to identify the underlying molecular mechanism. PTPA at the dose from 25 to 200μg/mL showed significant inhibitory effect on the proliferation of four cancer cell lines (SNU-245, CL-6, Sk-ChA-1 and MZ-ChA-1), among which Sk-ChA-1 was a most sensitive cell line to PTPA treatment via induction of apoptosis. Interestingly, RNA interference of Sk-ChA-1 cells with 67LR or Cdc42-targeted shRNAs resulted a similar potency in decreasing cell viability and causing apoptotic death. Moreover, PTPA (100μg/mL) or 67LR or Cdc42 special shRNAs increased the ratio of pro-apoptotic Bax to anti-apoptotic Bcl-2, induced the activation of caspase-9 and caspase-3, but not caspsase-8, and inhibited the expression of 67LR or Cdc42 protein in Sk-ChA-1 cells. Taken together, the inhibitory effect of PTPA on the cell growth of Sk-ChA-1 cells was at least in part mediated via the activation of the intrinsic mitochondrial apoptotic pathway and the downregulation of 67LR or Cdc42 protein expression. Thus, PTPA may be developed as a promising candidate for chemopreventive agent in the prevention and treatment of human CCA. 
 Inhibition of cell proliferation and   metastasis   of human hepatocellular carcinoma by miR-137 is regulated by CDC42. In the present study, we evaluated the mechanisms of CDC42 in association with the microRNA-137 (miR-137)-induced inhibition of human hepatocellular carcinoma (HCC). The gene expression levels of miR-137 were evaluated in HCC cell lines. Direct association of miR-137 with its downstream target, cell division control protein 42 (CDC42), was evaluated by dual-luciferase assay, western blot analysis and correlation analysis using clinical tumor samples. In the HCC HuH7 and MHCC97L cell lines, miR-137 was upregulated to inhibit cell proliferation and   metastasi  s in vitro and tumor growth in vivo. CDC42 was overexpressed in the HuH7 and MHCC97L cells to evaluate its effect on the miR-137-mediated antitumor effects. Furthermore, possible crosstalk between CDC42 and another miR-137 target gene, AKT2, was evaluated by co-overexpressing CDC42 and AKT2 in the HuH7 and MHCC97L cells and examining their effects on miR-137-mediated HCC regulation. miR-137 was confirmed to be downregulated in the HCC cell lines. Dual‑luciferase assay showed that CDC42 was directly targeted by miR-137, and western blotting showed that CDC42 was downregulated by miR-137 upregulation in the HuH7 and MHCC97L cells. In human tumors, the expression levels of CDC42 and miR-137 were inversely correlated. The inhibitory effects of miR-137 on HCC prolifera  tion, meta  stasis and in vivo tumor growth were all ameliorated by CDC42 overexpression. Furthermore, co-overexpression of AKT2 in addition to CDC42 additively reduced the inhibition of miR-137 on HCC proliferati  on and met  astasis, suggesting two independent pathways of CDC42 and AKT2 in miR-137-mediated HCC regulation. Our study demonstrated that CDC42 independently regulated the antitumor effects of miR-137 in human HCC. 
 Effect of microRNA-206 on cytoskeleton remodelling by downregulating Cdc42 in MDA-MB-231 cells. MicroRNAs are small, noncoding, single-stranded RNAs that regulate gene expression post-transcriptionally. miR-206 is known to play an important role in breast cancer   metastasis  . When we sought to predict the target of miR-206 by Targetscan, Pictar and miRanda, we found Cdc42 was a potential one. In this study, we transfected miR-206 into MDA-MB-231 cells and examined Cdc42 protein expression as well as MMP-2 and MMP-9, which are also associated with   metastasis   of breast cancer. Since Cdc42 is involved in filopodia and invadopodia formation, we examined the morphological changes of MDA-MB-231 cells.miR-206 mimics were transfected into MDA-MB-231 cells using LipofectamineTM 2000. Protein expression was detected by Western blot. Cells were stained with FITC-phalloidin to visualize F-actin. Invasive ability and migratory ability were examined by invasion assay and migration assay in vitro.Cdc42, MMP-2 and MMP-9 were downregulated on the protein level. The formation of filopodia, which requires Cdc42, was inhibited in miR-206 transfected cells, even under the stimulation of EGF. The invasion and migration of MDA-MB-231 cells in vitro was inhibited by miR-206 too.The results suggest that miR-206 may suppress invasion and migration of MDA-MB-231 cells in vitro partly via regulating actin cytoskeleton remodelling such as filopodia formation. 
  GeneRIFs  
 Our results demonstrate a novel role of Bif-1 in hepatocellular carcinoma (HCC), in which Bif-1 promotes cell   metastasis   by regulating Cdc42 expression and activity. 
 A polysaccharide from Pinellia ternata inhibits cell proliferation and   metastasis   in human cholangiocarcinoma cells by targeting of Cdc42 and 67kDa Laminin Receptor 
 we found that CXCR4-CXCL12/AKT axis regulates RhoA, Rac-1, and Cdc42 to modulate cell invasion and tumor   metastasis  . Together, these results demonstrate a role for CXCR4 in ESCC   metastasis   and progression and suggest potential targets for therapeutic intervention. 
 Results provide novel evidence that low expression of SLIT2 correlates with poor prognosis and promotes   metastasis   in ESCC, which may be regulated by the Cdc42-mediated pathways. 
 These findings show that overexpression of PODXL enhanced invadopodia formation and tumor   metastasis   by inducing Rac1/Cdc42/cortactin signaling network. 
 Active Rho GTPase Cdc42 can greatly enhance colorectal cancer cell SW480 to spread, migrate, and invade, which may contribute to colorectal cancer   metastasis  . 
 The transient Cdc42 depletion was sufficient to decrease experimental lung metastases, which suggests that its role in endothelial attachment is important for   metastasis  . 
 Aberrant expression of Annexin A2 and Cdc42 play a role in carcinogenesis, differentiation and   metastasis   of esophageal squamous cell carcinomas. 
 Nm23-H1 can negatively regulate cell migration and tumor   metastasis   by modulating the activity of Cdc42 and possibly other Rho family members through interaction with Dbl-1 
 NFAT1, a transcription factor connected with breast cancer   metastasis  , is activated by Wnt-5a through a Ca2+ signaling pathway in human breast epithelial cells which was simultaneously counteracted by a Wnt-5a-induced Yes/Cdc42 signaling pathway. 
  [Cc]ell [Ii]nvasion  PubMed  
 The interaction of IQGAP1 with the exocyst complex is required for tumor   cell invasion   downstream of Cdc42 and RhoA. Invadopodia are actin-based membrane protrusions formed at contact sites between invasive tumor cells and the extracellular matrix with matrix proteolytic activity. Actin regulatory proteins participate in invadopodia formation, whereas matrix degradation requires metalloproteinases (MMPs) targeted to invadopodia. In this study, we show that the vesicle-tethering exocyst complex is required for matrix proteolysis and invasion of breast carcinoma cells. We demonstrate that the exocyst subunits Sec3 and Sec8 interact with the polarity protein IQGAP1 and that this interaction is triggered by active Cdc42 and RhoA, which are essential for matrix degradation. Interaction between IQGAP1 and the exocyst is necessary for invadopodia activity because enhancement of matrix degradation induced by the expression of IQGAP1 is lost upon deletion of the exocyst-binding site. We further show that the exocyst and IQGAP1 are required for the accumulation of cell surface membrane type 1 MMP at invadopodia. Based on these results, we propose that invadopodia function in tumor cells relies on the coordination of cytoskeletal assembly and exocytosis downstream of Rho guanosine triphosphatases. 
 Regulation of CXCR4/AKT-signaling-induced   cell invasion   and tumor metastasis by RhoA, Rac-1, and Cdc42 in human esophageal cancer. CXC chemokines and their cognate receptors have been implicated wildly in cancer pathogenesis. In the present study, we report a critical cause relationship between CXCR4 expression and tumorigenesis in the setting of human esophageal squamous cell carcinoma (ESCC). In ESCC cells, CXCR4 expression was significantly higher than in human esophageal epithelial cells (HEEC). Reduction of CXCR4 in ESCC cells reduced cell proliferation and invasion in vitro and tumor growth in vivo. Among the potential downstream targets of CXCR4-CXCL12 are RhoA, Rac-1, and Cdc42, which are likely to contribute to the invasiveness of ESCC cells. Finally, we found that CXCR4-CXCL12/AKT axis regulates RhoA, Rac-1, and Cdc42 to modulate   cell invasion   and tumor metastasis. Together, these results demonstrate a role for CXCR4 in ESCC metastasis and progression and suggest potential targets for therapeutic intervention. 
 MT1-MMP- and Cdc42-dependent signaling co-regulate   cell invasion   and tunnel formation in 3D collagen matrices. Complex signaling events control tumor invasion in three-dimensional (3D) extracellular matrices. Recent evidence suggests that cells utilize both matrix metalloproteinase (MMP)-dependent and MMP-independent means to traverse 3D matrices. Herein, we demonstrate that lysophosphatidic-acid-induced HT1080   cell invasion   requires membrane-type-1 (MT1)-MMP-mediated collagenolysis to generate matrix conduits the width of a cellular nucleus. We define these spaces as single-  cell invasion   tunnels (SCITs). Once established, cells can migrate within SCITs in an MMP-independent manner. Endothelial cells, smooth muscle cells and fibroblasts also generate SCITs during invasive events, suggesting that SCIT formation represents a fundamental mechanism of cellular motility within 3D matrices. Coordinated cellular signaling events are required during SCIT formation. MT1-MMP, Cdc42 and its associated downstream effectors such as MRCK (myotonic dystrophy kinase-related Cdc42-binding kinase) and Pak4 (p21 protein-activated kinase 4), protein kinase Calpha and the Rho-associated coiled-coil-containing protein kinases (ROCK-1 and ROCK-2) coordinate signaling necessary for SCIT formation. Finally, we show that MT1-MMP and Cdc42 are fundamental components of a co-associated invasion-signaling complex that controls directed single-  cell invasion   of 3D collagen matrices. 
 The Vibrio parahaemolyticus effector VopC mediates Cdc42-dependent invasion of cultured cells but is not required for pathogenicity in an animal model of infection. Vibrio parahaemolyticus is a Gram-negative marine bacterium that causes acute gastroenteritis in humans. The virulence of V. parahaemolyticus is dependent upon a type III secretion system (T3SS2). One effector for T3SS2, VopC, is a homologue of the catalytic domain of cytotoxic necrotizing factor (CNF), and was recently reported to be a Rho family GTPase activator and to be linked to internalization of V. parahaemolyticus by non-phagocytic cultured cells. Here, we provide direct evidence that VopC deamidates Rac1 and CDC42, but not RhoA, in vivo. Our results alsosuggest that VopC, through its activation of Rac1, contributes to formation of actin stress fibres in infected cells. Invasion of host cells, which occurs at a low frequency, does not seem linked to Rac1 activation, but instead appears to require CDC42. Finally, using an infant rabbit model of V. parahaemolyticus infection, we show that the virulence of V. parahaemolyticus is not dependent upon VopC-mediated invasion. Genetic inactivation of VopC did not impair intestinal colonization nor reduce signs of disease, including fluid accumulation, diarrhoea and tissue destruction. Thus, although VopC can promo  te host cell   invasion, such internalization is not a critical step of the disease process, consistent with the traditional view of V. parahaemolyticus as an extracellular pathogen. 
 Rapid Remodeling of Invadosomes by Gi-coupled Receptors: DISSECTING THE ROLE OF Rho GTPases. Invadosomes are actin-rich membrane protrusions that degrade the extracellular matrix to drive tumor   cell invasion  . Key players in invadosome formation are c-Src and Rho family GTPases. Invadosomes can reassemble into circular rosette-like superstructures, but the underlying signaling mechanisms remain obscure. Here we show that Src-induced invadosomes in human melanoma cells (A375M and MDA-MB-435) undergo rapid remodeling into dynamic extracellular matrix-degrading rosettes by distinct G protein-coupled receptor agonists, notably lysophosphatidic acid (LPA; acting through the LPA1 receptor) and endothelin. Agonist-induced rosette formation is blocked by pertussis toxin, dependent on PI3K activity and accompanied by localized production of phosphatidylinositol 3,4,5-trisphosphate, whereas MAPK and Ca(2+) signaling are dispensable. Using FRET-based biosensors, we show that LPA and endothelin transiently activate Cdc42 through Gi, concurrent with a biphasic decrease in Rac activity and differential effects on RhoA. Cdc42 activity is essential for rosette formation, whereas G12/13-mediated RhoA-ROCK signaling suppresses the remodeling process. Our results reveal a Gi-mediated Cdc42 signaling axis by which G protein-coupled receptors trigger invadosome remodeling, the degree of which is dictated by the Cdc42-RhoA activity balance. 
 Macrophages stimulate gastric and colorectal cancer invasion through EGFR Y(1086), c-Src, Erk1/2 and Akt phosphorylation and smallGTPase activity. The interactions between cancer cells and their microenvironment are crucial for malignant progression, as they modulate invasion-related activities. Tumor-associated macrophages are generally considered allies in the process of tumor progression in several types of cancer, although their role on gastric and colorectal carcinomas is still poorly understood. In this report, we studied the influence of primary human macrophages on gastric and colorectal cancer cells, considering invasion, motility/migration, proteolysis and activated intracellular signaling pathways. We demonstrated that macrophages stimulate cancer   cell invasion  , motility and migration, and that these effects depend on matrix metalloproteinase (MMP) activity and on the activation of epidermal growth factor receptor (EGFR) (at the residue Y(1086)), PLC-γ (phospholipase C-gamma) and Gab1 (GRB2-associated binding protein-1), as evidenced by siRNA (small interference RNA) experiments. Epidermal growth factor (EGF)-immunodepletion impaired macrophage-mediated cancer   cell invasio  n and motility, suggesting that EGF is the pro-invasive and pro-motile factor produced by macrophages. Macrophages also induced gastric and colorectal cancer cell phosphorylation of Akt, c-Src and ERK1/2, and led to an increase of RhoA and Cdc42 activity. Interestingly, whereas macrophage-mediated cancer cell c-Src and ERK1/2 phosphorylation occurred downstream EGFR activation, Akt phosphorylation seems to be a parallel event, taking place in an EGFR-independent manner. The involvement of EGF, EGFR-downstream signaling partners and MMPs in macrophage-mediated invasion provides novel insights into the molecular crosstalk established between cancer cells and macrophages, opening new perspectives for the design of new and more efficient therapeutic strategies to counteract cancer   cell invasio  n. 
 Rac1, RhoA, and Cdc42 participate in HeLa   cell invasion   by group B streptococcus. The group B streptococcus (GBS) is an important human pathogen with the ability to cause invasive disease. To do so, the bacteria must invade host cells. It has been well documented that GBS are able to invade a variety of nonphagocytic host cell types, and this process is thought to involve a number of pathogen-host cell interactions. While some of the molecular aspects of the GBS-host   cell invasion   process have been characterized, many events still remain unclear. The objective of this investigation was to evaluate the role of the Rho-family GTPases Rac, Rho, and Cdc42 in GBS invasion into epithelial cells. The epithelial   cell invasion   process was modeled using HeLa 229 cell culture. Treatment of HeLa cells with 10 microM compactin, a pan-GTPase inhibitor, abolished GBS internalization, suggesting that GTPases are involved in the GBS invasion process. The addition of Toxin B or exoenzyme C3 to HeLa cells before GBS infection reduced invasion by 50%, further suggesting that the Rho-family GTPases are involved in GBS entry. Examining invasion of GBS into HeLa cells with altered genetic backgrounds was used to confirm these findings; GBS invasion into HeLa cells transiently transfected with dominant negative Rac1, Cdc42, or RhoA reduced invasion by 75%, 51%, and 42%, respectively. Results of this study suggest that the Rho-family GTPases are required for efficient invasion of HeLa cells by GBS. 
 Role of the small Rho GTPases Rac1 and Cdc42 in host   cell invasion   of Campylobacter jejuni. Host   cell invasion   of the food-borne pathogen Campylobacter jejuni is one of the primary reasons of tissue damage in humans but molecular mechanisms are widely unclear. Here, we show that C. jejuni triggers membrane ruffling in the eukaryotic cell followed by invasion in a very specific manner first with its tip followed by the flagellar end. To pinpoint important signalling events involved in the C. jejuni invasion process, we examined the role of small Rho family GTPases. Using specific GTPase-modifying toxins, inhibitors and GTPase expression constructs we show that Rac1 and Cdc42, but not RhoA, are involved in C. jejuni invasion. In agreement with these observations, we found that internalization of C. jejuni is accompanied by a time-dependent activation of both Rac1 and Cdc42. Finally, we show that the activation of these GTPases involves different host cell kinases and the bacterial fibronectin-binding protein CadF. Thus, CadF is a bifunctional protein which triggers bacterial binding to host cells as well as signalling leading to GTPase activation. Collectively, our results suggest that C. jejuni invade host target cells by a unique mechanism and the activation of the Rho GTPase members Rac1 and Cdc42 plays a crucial role in this entry process. 
 Phosphatidylinositol 5-phosphate regulates invasion through binding and activation of Tiam1. PtdIns5P is a lipid messenger acting as a stress-response mediator in the nucleus, and known to maintain cell activation through traffic alterations upon bacterial infection. Here, we show that PtdIns5P regulates actin dynamics and invasion via recruitment and activation of the exchange factor Tiam1 and Rac1. Restricted Rac1 activation results from the binding of Tiam1 DH-PH domains to PtdIns5P. Using an assay that mimics Rac1 membrane anchoring by using Rac1-His and liposomes containing Ni(2+)-NTA modified lipids, we demonstrate that intrinsic Tiam1 DH-PH activity increases when Rac1 is anchored in a PtdIns5P-enriched environment. This pathway appears to be general since it is valid in different pathophysiological models: receptor tyrosine kinase activation, bacterial phosphatase IpgD expression and the invasive NPM-ALK(+) lymphomas. The discovery that PtdIns5P could be a keystone of GTPases and cytoskeleton spatiotemporal regulation opens important research avenues towards unravelling new strategies counteracting   cell invasion  . 
 Regulation of CXCR4/AKT-signaling-induced   cell invasion   and tumor metastasis by RhoA, Rac-1, and Cdc42 in human esophageal cancer. CXC chemokines and their cognate receptors have been implicated wildly in cancer pathogenesis. In the present study, we report a critical cause relationship between CXCR4 expression and tumorigenesis in the setting of human esophageal squamous cell carcinoma (ESCC). In ESCC cells, CXCR4 expression was significantly higher than in human esophageal epithelial cells (HEEC). Reduction of CXCR4 in ESCC cells reduced cell proliferation and invasion in vitro and tumor growth in vivo. Among the potential downstream targets of CXCR4-CXCL12 are RhoA, Rac-1, and Cdc42, which are likely to contribute to the invasiveness of ESCC cells. Finally, we found that CXCR4-CXCL12/AKT axis regulates RhoA, Rac-1, and Cdc42 to modulate   cell invasion   and tumor metastasis. Together, these results demonstrate a role for CXCR4 in ESCC metastasis and progression and suggest potential targets for therapeutic intervention. 
 Rapid Remodeling of Invadosomes by Gi-coupled Receptors: DISSECTING THE ROLE OF Rho GTPases. Invadosomes are actin-rich membrane protrusions that degrade the extracellular matrix to drive tumor   cell invasion  . Key players in invadosome formation are c-Src and Rho family GTPases. Invadosomes can reassemble into circular rosette-like superstructures, but the underlying signaling mechanisms remain obscure. Here we show that Src-induced invadosomes in human melanoma cells (A375M and MDA-MB-435) undergo rapid remodeling into dynamic extracellular matrix-degrading rosettes by distinct G protein-coupled receptor agonists, notably lysophosphatidic acid (LPA; acting through the LPA1 receptor) and endothelin. Agonist-induced rosette formation is blocked by pertussis toxin, dependent on PI3K activity and accompanied by localized production of phosphatidylinositol 3,4,5-trisphosphate, whereas MAPK and Ca(2+) signaling are dispensable. Using FRET-based biosensors, we show that LPA and endothelin transiently activate Cdc42 through Gi, concurrent with a biphasic decrease in Rac activity and differential effects on RhoA. Cdc42 activity is essential for rosette formation, whereas G12/13-mediated RhoA-ROCK signaling suppresses the remodeling process. Our results reveal a Gi-mediated Cdc42 signaling axis by which G protein-coupled receptors trigger invadosome remodeling, the degree of which is dictated by the Cdc42-RhoA activity balance. 
 Phosphatidylinositol 5-phosphate regulates invasion through binding and activation of Tiam1. PtdIns5P is a lipid messenger acting as a stress-response mediator in the nucleus, and known to maintain cell activation through traffic alterations upon bacterial infection. Here, we show that PtdIns5P regulates actin dynamics and invasion via recruitment and activation of the exchange factor Tiam1 and Rac1. Restricted Rac1 activation results from the binding of Tiam1 DH-PH domains to PtdIns5P. Using an assay that mimics Rac1 membrane anchoring by using Rac1-His and liposomes containing Ni(2+)-NTA modified lipids, we demonstrate that intrinsic Tiam1 DH-PH activity increases when Rac1 is anchored in a PtdIns5P-enriched environment. This pathway appears to be general since it is valid in different pathophysiological models: receptor tyrosine kinase activation, bacterial phosphatase IpgD expression and the invasive NPM-ALK(+) lymphomas. The discovery that PtdIns5P could be a keystone of GTPases and cytoskeleton spatiotemporal regulation opens important research avenues towards unravelling new strategies counteracting   cell invasion  . 
 The Vibrio parahaemolyticus effector VopC mediates Cdc42-dependent invasion of cultured cells but is not required for pathogenicity in an animal model of infection. Vibrio parahaemolyticus is a Gram-negative marine bacterium that causes acute gastroenteritis in humans. The virulence of V. parahaemolyticus is dependent upon a type III secretion system (T3SS2). One effector for T3SS2, VopC, is a homologue of the catalytic domain of cytotoxic necrotizing factor (CNF), and was recently reported to be a Rho family GTPase activator and to be linked to internalization of V. parahaemolyticus by non-phagocytic cultured cells. Here, we provide direct evidence that VopC deamidates Rac1 and CDC42, but not RhoA, in vivo. Our results alsosuggest that VopC, through its activation of Rac1, contributes to formation of actin stress fibres in infected cells. Invasion of host cells, which occurs at a low frequency, does not seem linked to Rac1 activation, but instead appears to require CDC42. Finally, using an infant rabbit model of V. parahaemolyticus infection, we show that the virulence of V. parahaemolyticus is not dependent upon VopC-mediated invasion. Genetic inactivation of VopC did not impair intestinal colonization nor reduce signs of disease, including fluid accumulation, diarrhoea and tissue destruction. Thus, although VopC can promo  te host cell   invasion, such internalization is not a critical step of the disease process, consistent with the traditional view of V. parahaemolyticus as an extracellular pathogen. 
 Macrophages stimulate gastric and colorectal cancer invasion through EGFR Y(1086), c-Src, Erk1/2 and Akt phosphorylation and smallGTPase activity. The interactions between cancer cells and their microenvironment are crucial for malignant progression, as they modulate invasion-related activities. Tumor-associated macrophages are generally considered allies in the process of tumor progression in several types of cancer, although their role on gastric and colorectal carcinomas is still poorly understood. In this report, we studied the influence of primary human macrophages on gastric and colorectal cancer cells, considering invasion, motility/migration, proteolysis and activated intracellular signaling pathways. We demonstrated that macrophages stimulate cancer   cell invasion  , motility and migration, and that these effects depend on matrix metalloproteinase (MMP) activity and on the activation of epidermal growth factor receptor (EGFR) (at the residue Y(1086)), PLC-γ (phospholipase C-gamma) and Gab1 (GRB2-associated binding protein-1), as evidenced by siRNA (small interference RNA) experiments. Epidermal growth factor (EGF)-immunodepletion impaired macrophage-mediated cancer   cell invasio  n and motility, suggesting that EGF is the pro-invasive and pro-motile factor produced by macrophages. Macrophages also induced gastric and colorectal cancer cell phosphorylation of Akt, c-Src and ERK1/2, and led to an increase of RhoA and Cdc42 activity. Interestingly, whereas macrophage-mediated cancer cell c-Src and ERK1/2 phosphorylation occurred downstream EGFR activation, Akt phosphorylation seems to be a parallel event, taking place in an EGFR-independent manner. The involvement of EGF, EGFR-downstream signaling partners and MMPs in macrophage-mediated invasion provides novel insights into the molecular crosstalk established between cancer cells and macrophages, opening new perspectives for the design of new and more efficient therapeutic strategies to counteract cancer   cell invasio  n. 
 MT1-MMP- and Cdc42-dependent signaling co-regulate   cell invasion   and tunnel formation in 3D collagen matrices. Complex signaling events control tumor invasion in three-dimensional (3D) extracellular matrices. Recent evidence suggests that cells utilize both matrix metalloproteinase (MMP)-dependent and MMP-independent means to traverse 3D matrices. Herein, we demonstrate that lysophosphatidic-acid-induced HT1080   cell invasion   requires membrane-type-1 (MT1)-MMP-mediated collagenolysis to generate matrix conduits the width of a cellular nucleus. We define these spaces as single-  cell invasion   tunnels (SCITs). Once established, cells can migrate within SCITs in an MMP-independent manner. Endothelial cells, smooth muscle cells and fibroblasts also generate SCITs during invasive events, suggesting that SCIT formation represents a fundamental mechanism of cellular motility within 3D matrices. Coordinated cellular signaling events are required during SCIT formation. MT1-MMP, Cdc42 and its associated downstream effectors such as MRCK (myotonic dystrophy kinase-related Cdc42-binding kinase) and Pak4 (p21 protein-activated kinase 4), protein kinase Calpha and the Rho-associated coiled-coil-containing protein kinases (ROCK-1 and ROCK-2) coordinate signaling necessary for SCIT formation. Finally, we show that MT1-MMP and Cdc42 are fundamental components of a co-associated invasion-signaling complex that controls directed single-  cell invasion   of 3D collagen matrices. 
 The interaction of IQGAP1 with the exocyst complex is required for tumor   cell invasion   downstream of Cdc42 and RhoA. Invadopodia are actin-based membrane protrusions formed at contact sites between invasive tumor cells and the extracellular matrix with matrix proteolytic activity. Actin regulatory proteins participate in invadopodia formation, whereas matrix degradation requires metalloproteinases (MMPs) targeted to invadopodia. In this study, we show that the vesicle-tethering exocyst complex is required for matrix proteolysis and invasion of breast carcinoma cells. We demonstrate that the exocyst subunits Sec3 and Sec8 interact with the polarity protein IQGAP1 and that this interaction is triggered by active Cdc42 and RhoA, which are essential for matrix degradation. Interaction between IQGAP1 and the exocyst is necessary for invadopodia activity because enhancement of matrix degradation induced by the expression of IQGAP1 is lost upon deletion of the exocyst-binding site. We further show that the exocyst and IQGAP1 are required for the accumulation of cell surface membrane type 1 MMP at invadopodia. Based on these results, we propose that invadopodia function in tumor cells relies on the coordination of cytoskeletal assembly and exocytosis downstream of Rho guanosine triphosphatases. 
 Role of the small Rho GTPases Rac1 and Cdc42 in host   cell invasion   of Campylobacter jejuni. Host   cell invasion   of the food-borne pathogen Campylobacter jejuni is one of the primary reasons of tissue damage in humans but molecular mechanisms are widely unclear. Here, we show that C. jejuni triggers membrane ruffling in the eukaryotic cell followed by invasion in a very specific manner first with its tip followed by the flagellar end. To pinpoint important signalling events involved in the C. jejuni invasion process, we examined the role of small Rho family GTPases. Using specific GTPase-modifying toxins, inhibitors and GTPase expression constructs we show that Rac1 and Cdc42, but not RhoA, are involved in C. jejuni invasion. In agreement with these observations, we found that internalization of C. jejuni is accompanied by a time-dependent activation of both Rac1 and Cdc42. Finally, we show that the activation of these GTPases involves different host cell kinases and the bacterial fibronectin-binding protein CadF. Thus, CadF is a bifunctional protein which triggers bacterial binding to host cells as well as signalling leading to GTPase activation. Collectively, our results suggest that C. jejuni invade host target cells by a unique mechanism and the activation of the Rho GTPase members Rac1 and Cdc42 plays a crucial role in this entry process. 
 Rac1, RhoA, and Cdc42 participate in HeLa   cell invasion   by group B streptococcus. The group B streptococcus (GBS) is an important human pathogen with the ability to cause invasive disease. To do so, the bacteria must invade host cells. It has been well documented that GBS are able to invade a variety of nonphagocytic host cell types, and this process is thought to involve a number of pathogen-host cell interactions. While some of the molecular aspects of the GBS-host   cell invasion   process have been characterized, many events still remain unclear. The objective of this investigation was to evaluate the role of the Rho-family GTPases Rac, Rho, and Cdc42 in GBS invasion into epithelial cells. The epithelial   cell invasion   process was modeled using HeLa 229 cell culture. Treatment of HeLa cells with 10 microM compactin, a pan-GTPase inhibitor, abolished GBS internalization, suggesting that GTPases are involved in the GBS invasion process. The addition of Toxin B or exoenzyme C3 to HeLa cells before GBS infection reduced invasion by 50%, further suggesting that the Rho-family GTPases are involved in GBS entry. Examining invasion of GBS into HeLa cells with altered genetic backgrounds was used to confirm these findings; GBS invasion into HeLa cells transiently transfected with dominant negative Rac1, Cdc42, or RhoA reduced invasion by 75%, 51%, and 42%, respectively. Results of this study suggest that the Rho-family GTPases are required for efficient invasion of HeLa cells by GBS. 
 IQGAP1 promotes cell motility and invasion. The dynamic processes of cell migration and invasion are largely coordinated by Rho family GTPases. The scaffolding protein IQGAP1 binds to Cdc42, increasing the amount of active Cdc42 both in vitro and in cells. Here we show that overexpression of IQGAP1 in mammalian cells enhances cell migration in a Cdc42- and Rac1-dependent manner. Importantly, cell motility was significantly decreased both by knock down of endogenous IQGAP1 using small interfering RNA and by transfection of a dominant negative IQGAP1 construct, IQGAP1DeltaGRD.   Cell invasion   was similarly altered by manipulating intracellular IQGAP1 concentrations. Moreover, invasion mediated by constitutively active Cdc42 was attenuated by IQGAP1DeltaGRD. Thus, IQGAP1 has a fundamental role in cell motility and invasion. 
 Discoidin domain receptor 1 controls linear invadosome formation via a Cdc42-Tuba pathway. Accumulation of type I collagen fibrils in tumors is associated with an increased risk of metastasis. Invadosomes are F-actin structures able to degrade the extracellular matrix. We previously found that collagen I fibrils induced the formation of peculiar linear invadosomes in an unexpected integrin-independent manner. Here, we show that Discoidin Domain Receptor 1 (DDR1), a collagen receptor overexpressed in cancer, colocalizes with linear invadosomes in tumor cells and is required for their formation and matrix degradation ability. Unexpectedly, DDR1 kinase activity is not required for invadosome formation or activity, nor is Src tyrosine kinase. We show that the RhoGTPase Cdc42 is activated on collagen in a DDR1-dependent manner. Cdc42 and its specific guanine nucleotide-exchange factor (GEF), Tuba, localize to linear invadosomes, and both are required for linear invadosome formation. Finally, DDR1 depletion blocked   cell invasion   in a collagen gel. Altogether, our data uncover an important role for DDR1, acting through Tuba and Cdc42, in proteolysis-based   cell invasion   in a collagen-rich environment. 
 The C-terminus of IpaC is required for effector activities related to Shigella invasion of host cells. Invasion plasmid antigen C (IpaC) is secreted by the Shigella flexneri type III secretion system (TTSS) as an essential trigger of epithelial   cell invasion  . At the molecular level, IpaC possesses a distinct functional organization. The IpaC C-terminal region between amino acids 319 and 345 is predicted to form a coiled-coil structure. Such alpha-helical motifs appear to be a recurring structural theme among TTSS components. Together with IpaB, this IpaC region is also required for the formation of translocon pores in target cell membranes. In contrast, mutations within the C-terminal tail of IpaC (defined by residues 345-363) have no effect on contact hemolysis (a putative measure of translocon pore formation), but they can contribute significantly to IpaC's ability to trigger S. flexneri entry into cultured cells. Here we describe the molecular dissection of the IpaC C-terminus and how changes in this region affect selected virulence-related activities. IpaC invasion function requires its immediate C-terminus and this general region may be involved in its ability to trigger actin nucleation. In contrast, IpaC could not be shown to interact directly with Cdc42, a host GTPase closely tied to Shigella invasion. 
 Cdc42: Role in Cancer Management. Contribution of Cdc42, a member of Rho family, has been characterized for the beginning of variety of cellular responses including cellular transformation, cell division,   cell invasion  , migration, invadopodia formation, enzyme activity, filopodia formation, and cell polarity in cells. Deregulation of Cdc42 can alter the normal functioning of the cells, responsible for the initiation of signaling pathways and is correlated with several pathogenic processes such as cancer. Therefore, maintaining the level of Cdc42 and its effectors in cells, tumor progression can be controlled. Therefore, it can be suggested that deeper understanding about the Cdc42 contribution in cancer cell progression at molecular level can approach to the development of Cdc42 inhibitors in cancer management. 
 Down-regulation of miRNA-204 by LMP-1 enhances CDC42 activity and facilitates invasion of EBV-associated nasopharyngeal carcinoma cells. Nasopharayngeal carcinoma (NPC) is an Epstein-Barr virus (EBV)-associated malignancy. It is known that microRNAs are implicated in the progression of NPC. However, the role of miR-204 in NPC is poorly understood. In this study, we found that miR-204 was down-regulated in NPC cells and tissues. Low-level expression of miR-204 was significantly associated with a more aggressive and poor prognostic phenotype of NPC. We further found that EBV-encoded latent membrane protein 1 (LMP-1) suppressed miR-204 expression by activating Stat-3. Cdc42 was identified as a direct target of miR-204. Mir-204 inhibited EBV positive C666-1   cell invasion   and metastasis partly through targeting cdc42. 
 DOCK10-mediated Cdc42 activation is necessary for amoeboid invasion of melanoma cells. Tumor cells can move in a three-dimensional (3D) environment in either mesenchymal-type or amoeboid modes. In mesenchymal-type movement, cells have an elongated morphology with Rac-induced protrusions at the leading edge. Amoeboid cells have high levels of actomyosin contractility, and movement is associated with deformation of the cell body through the matrix without proteolysis. Because signaling pathways that control the activation of GTPases for amoeboid movement are poorly understood, we sought to identify regulators of amoeboid movement by screening an siRNA library targeting guanine nucleotide exchange factors (GEFs) for Rho-family GTPases.We identified DOCK10, a Cdc42 GEF, as a key player in amoeboid migration; accordingly, we find that expression of activated Cdc42 induces a mesenchymal-amoeboid transition and increases   cell invasion  . Silencing DOCK10 expression promotes conversion to mesenchymal migration and is associated with decreased MLC2 phosphorylation and increased Rac1 activation. Consequently, abrogating DOCK10 and Rac1 expression suppresses both amoeboid and mesenchymal migration and results in decreased invasion. We show that the Cdc42 effectors N-WASP and Pak2 are required for the maintenance of the rounded-amoeboid phenotype. Blocking Cdc42 results in loss of mesenchymal morphology, arguing that Cdc42 is also involved in mesenchymal morphology through different activation and effector pathways.Previous work has identified roles of Rho and Rac signaling in tumor cell movement, and we now elucidate novel roles of Cdc42 signaling in amoeboid and mesenchymal movement and tumor   cell invasion  . 
 Stromal fibroblasts facilitate cancer   cell invasion   by a novel invadopodia-independent matrix degradation process. Metastatic invasion of tumors into peripheral tissues is known to rely upon protease-mediated degradation of the surrounding stroma. This remodeling process uses complex, actin-based, specializations of the plasma membrane termed invadopodia that act both to sequester and release matrix metalloproteinases. Here we report that cells of mesenchymal origin, including tumor-associated fibroblasts, degrade substantial amounts of surrounding matrix by a mechanism independent of conventional invadopodia. These degradative sites lack the punctate shape of conventional invadopodia to spread along the cell base and are reticular and/or fibrous in character. In marked contrast to invadopodia, this degradation does not require the action of Src kinase, Cdc42 or Dyn2. Rather, inhibition of Dyn2 causes a marked upregulation of stromal matrix degradation. Further, expression and activity of matrix metalloproteinases are differentially regulated between tumor cells and stromal fibroblasts. This matrix remodeling by fibroblasts increases the invasive capacity of tumor cells, thereby illustrating how the tumor microenvironment can contribute to metastasis. These findings provide evidence for a novel matrix remodeling process conducted by stromal fibroblasts that is substantially more effective than conventional invadopodia, distinct in structural organization and regulated by disparate molecular mechanisms. 
 Cdc42-MRCK and Rho-ROCK signalling cooperate in myosin phosphorylation and   cell invasion  . Actomyosin contractility is a mechanism by which cells exert locomotory force against their environment. Signalling downstream of the small GTPase Rho increases contractility through Rho-kinase (ROCK)-mediated regulation of myosin-II light chain (MLC2) phosphorylation. Cdc42 signalling has been shown to control cell polarity. Tumour cells can move through a three-dimensional matrix with either a rounded morphology characterized by Rho-ROCK dependence or with an elongated morphology characterized by Rho-ROCK independence. Here we show that contractility necessary for elongated morphology and invasion can be generated by Cdc42-MRCK signalling. MRCK (myotonic dystrophy kinase-related Cdc42-binding kinase) cooperates with ROCK in the maintenance of elongated morphology and invasion and either MRCK or ROCK is sufficient for MLC2 phosphorylation, through the inhibitory phosphorylation of myosin phosphatase. By contrast, in rounded ROCK-dependent movement, where MLC2 phosphorylation is higher, MRCK has a smaller role. Our data show that a Cdc42-MRCK signal mediates myosin-dependent cell motility and highlight convergence between Rho and Cdc42 signalling. 
  GeneRIFs  
 we found that CXCR4-CXCL12/AKT axis regulates RhoA, Rac-1, and Cdc42 to modulate   cell invasion   and tumor metastasis. Together, these results demonstrate a role for CXCR4 in ESCC metastasis and progression and suggest potential targets for therapeutic intervention. 
 CDC42 is activated by phosphatidylinositol 5-phosphate, regulating actin dynamics and   cell invasion  . 
 Cdc42 was found to be necessary for breast cancer   cell invasion   but dispensable for cell migration 
 MT1-MMP and Cdc42 co-regulate tumor   cell invasion   in 3D collagen matrices. 
 In melanoma, expression of activated Cdc42 induces a mesenchymal-amoeboid transition and increases   cell invasion  . 
   
  
 RND3 
 
  NCBI Gene  
  GeneRIF  
  Pubmed  
 
 
  Search Term  Rank  Fields  
  [Mm]igration  6  PubMed(8); GeneRIFs(1)  
  [Ee]xtracellular [Mm]atrix  8  PubMed(1)  
  [Ii]nvasive  10  PubMed(11); GeneRIFs(3)  
  [Mm]etastasis  12  PubMed(8)  
  [Cc]ell [Ii]nvasion  13  PubMed(8)  
 
 
 hide/show details for RND3 
  [Mm]igration  PubMed  
 Analysis of the myosin-II-responsive focal adhesion proteome reveals a role for β-Pix in negative regulation of focal adhesion maturation. Focal adhesions undergo myosin-II-mediated maturation wherein they grow and change composition to modulate integrin signalling for cell   migratio  n, growth and differentiation. To determine how focal adhesion composition is affected by myosin II activity, we performed proteomic analysis of isolated focal adhesions and compared protein abundance in focal adhesions from cells with and without myosin II inhibition. We identified 905 focal adhesion proteins, 459 of which changed in abundance with myosin II inhibition, defining the myosin-II-responsive focal adhesion proteome. The abundance of 73% of the proteins in the myosin-II-responsive focal adhesion proteome was enhanced by contractility, including proteins involved in Rho-mediated focal adhesion maturation and endocytosis- and calpain-dependent focal adhesion disassembly. During myosin II inhibition, 27% of proteins in the myosin-II-responsive focal adhesion proteome, including proteins involved in Rac-mediated lamellipodial protrusion, were enriched in focal adhesions, establishing that focal adhesion protein recruitment is also negatively regulated by contractility. We focused on the Rac guanine nucleotide exchange factor β-Pix, documenting its role in the negative regulation of focal adhesion maturation and the promotion of lamellipodial protrusion and focal adhesion turnover to drive cel  l migrati  on. 
 Inference of Low and High-Grade Glioma Gene Regulatory Networks Delineates the Role of Rnd3 in Establishing Multiple Hallmarks of Cancer. Gliomas are a highly heterogeneous group of brain tumours that are refractory to treatment, highly invasive and pro-angiogenic. Glioblastoma patients have an average survival time of less than 15 months. Understanding the molecular basis of different grades of glioma, from well differentiated, low-grade tumours to high-grade tumours, is a key step in defining new therapeutic targets. Here we use a data-driven approach to learn the structure of gene regulatory networks from observational data and use the resulting models to formulate hypothesis on the molecular determinants of glioma stage. Remarkably, integration of available knowledge with functional genomics datasets representing clinical and pre-clinical studies reveals important properties within the regulatory circuits controlling low and high-grade glioma. Our analyses first show that low and high-grade gliomas are characterised by a switch in activity of two subsets of Rho GTPases. The first one is involved in maintaining normal glial cell function, while the second is linked to the establishment of multiple hallmarks of cancer. Next, the development and application of a novel data integration methodology reveals novel functions of RND3 in controlling glioma cell   migration  , invasion, proliferation, angiogenesis and clinical outcome. 
 RhoE promotes metastasis in gastric cancer through a mechanism dependent on enhanced expression of CXCR4. RhoE, a novel member of the Rho protein family, is a key regulator of the cytoskeleton and cell   migration  . Our group has previously shown that RhoE as a direct target for HIF-1α and mediates hypoxia-induced epithelial to mesenchymal transition in gastric cancer cells. Therefore, we assumed that RhoE might play an important role in gastric cancer metastasis. In the present study, we have explored the role of RhoE expression in gastric cancer, cell invasion and metastasis, and the influence of RhoE on regulating the potential expression of down-stream genes. RhoE expression was elevated in gastric cancer tissues as compared with normal gastric tissues. We also found a close correlation between the histological grade and the diagnosis of the patient. Up-regulation of RhoE significantly enhanced the migratory and invasive abilities of gastric cancer cells both in vitro and in vivo. Moreover, down-regulation of RhoE diminished the metastatic potential of cancer cells. PCR array and subsequent transwell assay showed that the regulation of gastric cancer metastasis by RhoE was partially mediated by CXCR4. This observation suggested that CXCR4 might be a downstream effector for RhoE. In summary, our study identified RhoE as a novel prognostic biomarker and metastatic-promoting gene of gastric cancer. 
 RhoE Is required for keratinocyte differentiation and stratification. The molecular mechanism via which keratinocyte differentiation assembles multiple layers of cells (stratification) is poorly understood. We describe here a novel function of the Rho family member RhoE as a regulator of epidermal morphogenesis. RhoE protein levels are specifically and transiently up-regulated upon keratinocyte differentiation. RhoE up-regulation requires the activity of Rho kinase (ROCK) I, suggesting that both RhoE and ROCKI are important during keratinocyte differentiation. RhoE overexpression results in a striking enlargement of cell size and the number of stratified cells. In contrast, RhoE depletion induces hyperproliferation and delays initiation of keratinocyte differentiation. Interestingly, up-regulation of RhoE protein is seen primarily in basal, undifferentiated cells, in which commitment to differentiation and stratification takes place. RhoE activation in basal cells negatively modulates integrin adhesion, thereby facilitating detachment from the substratum and   migration   to form suprabasal layers. Thus, RhoE integrates two processes essential for keratinocyte differentiation and stratification: regulation of proliferative status and integrin adhesion. 
 FOXD3 regulates   migration   properties and Rnd3 expression in melanoma cells. Forkhead transcription factor, Foxd3, plays a critical role during development by controlling the lineage specification of neural crest cells. Notably, Foxd3 is highly expressed during the wave of neural crest cell   migration   that forms peripheral neurons and glial cells but is downregulated prior to   migration   of cells that give rise to the melanocytic lineage. Melanoma is the deadliest form of skin cancer and is derived from melanocytes. Recently, we showed that FOXD3 expression is elevated following the targeted inhibition of the B-RAF-MEK (MAP/ERK kinase)-ERK (extracellular signal-regulated kinase)1/2 pathway in mutant B-RAF melanoma cells. Because melanoma cells are highly migratory and invasive in a B-RAF-dependent manner, we explored the role of FOXD3 in these processes. In this study, we show that ectopic FOXD3 expression inhibits the   migration  , invasion, and spheroid outgrowth of mutant B-RAF melanoma cells. Upregulation of FOXD3 expression following inhibition of B-RAF and MEK correlates with the downregulation of Rnd3, a Rho GTPase and inhibitor of RhoA-ROCK signaling. Indeed, expression of FOXD3 alone was sufficient to downregulate Rnd3 expression at the mRNA and protein levels. Mechanistically, FOXD3 was found to be recruited to the Rnd3 promoter. Inhibition of ROCK partially restored   migration   in FOXD3-expressing cells. These data show that FOXD3 expression downregulates   migration   and invasion in melanoma cells and Rnd3, a target known to be involved in these properties. 
 The Rho GTPase RhoE is a p53-regulated candidate tumor suppressor in cancer cells. Previous studies have shown that RhoE, an atypical member of the Rho GTPase family, may play an opposite role to RhoA in regulating cell proliferation and invasion. To explore the relationship between RhoE and the malignant phenotypes of human cancer, we have determined the expression patterns of RhoE in varying grade of human cancer tissues and tested the effects of RhoE expression in several RhoE underexpressing cancer cell lines. Systemic immunocytochemistry analyses of gastric, colorectal, lung and breast carcinomas, respectively, showed that RhoE protein expression was significantly decreased in most cancer cases compared with that of adjacent normal tissues. Enhanced RhoE expression could markedly inhibit proliferation,   migration   and invasion and induce apoptosis of the cancer cells which have relatively low levels of endogenous RhoE expression. Wild-type p53 (wt-p53) could strongly increase RhoE expression in p53-transfected cells. Furthermore, the luciferase assays indicated that wt-p53 significantly enhanced the activities of RhoE promoter compared with mutant p53 (mt-p53) in PC3 cells (p53 null). Collectively, data are presented showing that RhoE may participate in human cancer progression and act as a candidate target of p53, and these findings also strongly suggest that RhoE may be a new candidate tumor suppressor and could serve as a potential target in the gene therapy of cancer. 
 Rnd3 regulation of the actin cytoskeleton promotes melanoma   migration   and invasive outgrowth in three dimensions. The depth of cell invasion into the dermis is a clinical determinant for poor prognosis in cutaneous melanoma. The signaling events that promote the switch from a noninvasive to invasive tumor phenotype remain obscure. Activating mutations in the serine/threonine kinase B-RAF are prevalent in melanoma. Mutant B-RAF is required for melanoma cell invasion. The expression of Rnd3, a Rho family GTPase, is regulated by mutant B-RAF, although its role in melanoma progression is unknown. In this study, we determined the functional contribution of Rnd3 to invasive melanoma. Endogenous Rnd3 was targeted for knockdown using a doxycycline-inducible short hairpin RNA system in invasive human melanoma cells. Depletion of Rnd3 promoted prominent actin stress fibers and enlarged focal adhesions. Mechanistically, stress fiber formation induced by Rnd3 knockdown required the specific involvement of RhoA and ROCK1/2 activity but not RhoB or RhoC. Rnd3 expression in human melanoma cell lines was strongly associated with elevated extracellular signal-regulated kinase phosphorylation and invasive behavior in a three-dimensional dermal-like environment. A functional role for Rnd3 was shown in the invasive outgrowth of melanoma tumor spheroids. Knockdown of Rnd3 reduced the invasive outgrowth of spheroids embedded in collagen gels. Additionally, Rnd3 depletion inhibited collective and border cell movement out from spheroids in a ROCK1/2-dependent manner. Collectively, these findings implicate Rnd3 as a major suppressor of RhoA-mediated actin cytoskeletal organization and in the acquisition of an invasive melanoma phenotype. 
 Genetic deletion of Rnd3 results in aqueductal stenosis leading to hydrocephalus through up-regulation of Notch signaling. Rho family guanosine triphosphatase (GTPase) 3 (Rnd3), a member of the small Rho GTPase family, is involved in the regulation of cell actin cytoskeleton dynamics, cell   migration  , and proliferation through the Rho kinase-dependent signaling pathway. We report a role of Rnd3 in the pathogenesis of hydrocephalus disorder. Mice with Rnd3 genetic deletion developed severe obstructive hydrocephalus with enlargement of the lateral and third ventricles, but not of the fourth ventricles. The cerebral aqueducts in Rnd3-null mice were partially or completely blocked by the overgrowth of ependymal epithelia. We examined the molecular mechanism contributing to this Rnd3-deficiency-mediated hydrocephalus and found that Rnd3 is a regulator of Notch signaling. Rnd3 deficiency, through either gene deletion or siRNA knockdown, resulted in a decrease in Notch intracellular domain (NICD) protein degradation. However, there was no correlated change in mRNA change, which in turn led to an increase in NICD protein levels. Immunoprecipitation analysis demonstrated that Rnd3 and NICD physically interacted, and that down-regulation of Rnd3 attenuated NICD protein ubiquitination. This eventually enhanced Notch signaling activity and promoted aberrant growth of aqueduct ependymal cells, resulting in aqueduct stenosis and the development of congenital hydrocephalus. Inhibition of Notch activity rescued the hydrocephalus disorder in the mutant animals. 
  GeneRIFs  
 the development and application of a novel data integration methodology reveals novel functions of RND3 in controlling glioma cell   migration  , invasion, proliferation, angiogenesis and clinical outcome. 
  [Ee]xtracellular [Mm]atrix  PubMed  
 Reduced expression of the ROCK inhibitor Rnd3 is associated with increased invasiveness and metastatic potential in mesenchymal tumor cells. Mesenchymal and amoeboid movements are two important mechanisms adopted by cancer cells to invade the surrounding environment. Mesenchymal movement depends on   extracellular matrix   protease activity, amoeboid movement on the RhoA-dependent kinase ROCK. Cancer cells can switch from one mechanism to the other in response to different stimuli, limiting the efficacy of antimetastatic therapies.We investigated the acquisition and molecular regulation of the invasion capacity of neoplastically transformed human fibroblasts, which were able to induce sarcomas and metastases when injected into immunocompromised mice. We found that neoplastic transformation was associated with a change in cell morphology (from fibroblastic to polygonal), a reorganization of the actin cytoskeleton, a decrease in the expression of several matrix metalloproteases and increases in cell motility and invasiveness. In a three-dimensional environment, sarcomagenic cells showed a spherical morphology with cortical actin rings, suggesting a switch from mesenchymal to amoeboid movement. Accordingly, cell invasion decreased after treatment with the ROCK inhibitor Y27632, but not with the matrix protease inhibitor Ro 28-2653. The increased invasiveness of tumorigenic cells was associated with reduced expression of Rnd3 (also known as RhoE), a cellular inhibitor of ROCK. Indeed, ectopic Rnd3 expression reduced their invasive ability in vitro and their metastatic potential in vivo.These results indicate that, during neoplastic transformation, cells of mesenchymal origin can switch from a mesenchymal mode of movement to an amoeboid one. In addition, they point to Rnd3 as a possible regulator of mesenchymal tumor cell invasion and to ROCK as a potential therapeutic target for sarcomas. 
  [Ii]nvasive  PubMed  
 A switch in RND3-RHOA signaling is critical for melanoma cell invasion following mutant-BRAF inhibition. The initial use of BRAF targeted therapeutics in clinical trials has demonstrated encouraging responses in melanoma patients, although a rise in drug-resistant cells capable of advancing malignant disease has been described. The current study uses BRAFV600E expressing WM793 melanoma cells to derive data aimed at investigating the molecular determinant of cell invasion following treatment with clinical BRAF inhibitors.Small-molecule inhibitors targeting BRAF reduced MEK1/2-ERK1/2 pathway activation and cell survival; yet, viable cell subpopulations persisted. The residual cells exhibited an elongated cell shape, prominent actin stress fibers and retained the ability to invade 3-D dermal-like microenvironments. BRAF inhibitor treatments were associated with reduced expression of RND3, an antagonist of RHOA activation, and elevated RHOA-dependent signaling. Restoration of RND3 expression or RHOA knockdown attenuated the migratory ability of residual cells without affecting overall cell survival. The   invasive   ability of BRAF inhibitor treated cells embedded in collagen gels was diminished following RND3 re-expression or RHOA depletion. Conversely, melanoma cell movement in the absence of BRAF inhibition was unaffected by RND3 expression or RHOA depletion.These data reveal a novel switch in the requirement for RND3 and RHOA in coordinating the movement of residual WM793 cells that are initially refractive to BRAF inhibitor therapy. These results have important clinical implications because they suggest that combining BRAF inhibitors with therapies that target the invasion of drug-resistant cells could aid in controlling disease relapse. 
 A switch in RND3-RHOA signaling is critical for melanoma cell invasion following mutant-BRAF inhibition. The initial use of BRAF targeted therapeutics in clinical trials has demonstrated encouraging responses in melanoma patients, although a rise in drug-resistant cells capable of advancing malignant disease has been described. The current study uses BRAFV600E expressing WM793 melanoma cells to derive data aimed at investigating the molecular determinant of cell invasion following treatment with clinical BRAF inhibitors.Small-molecule inhibitors targeting BRAF reduced MEK1/2-ERK1/2 pathway activation and cell survival; yet, viable cell subpopulations persisted. The residual cells exhibited an elongated cell shape, prominent actin stress fibers and retained the ability to invade 3-D dermal-like microenvironments. BRAF inhibitor treatments were associated with reduced expression of RND3, an antagonist of RHOA activation, and elevated RHOA-dependent signaling. Restoration of RND3 expression or RHOA knockdown attenuated the migratory ability of residual cells without affecting overall cell survival. The   invasive   ability of BRAF inhibitor treated cells embedded in collagen gels was diminished following RND3 re-expression or RHOA depletion. Conversely, melanoma cell movement in the absence of BRAF inhibition was unaffected by RND3 expression or RHOA depletion.These data reveal a novel switch in the requirement for RND3 and RHOA in coordinating the movement of residual WM793 cells that are initially refractive to BRAF inhibitor therapy. These results have important clinical implications because they suggest that combining BRAF inhibitors with therapies that target the invasion of drug-resistant cells could aid in controlling disease relapse. 
 Reduced expression of the ROCK inhibitor Rnd3 is associated with increased   invasive  ness and metastatic potential in mesenchymal tumor cells. Mesenchymal and amoeboid movements are two important mechanisms adopted by cancer cells to invade the surrounding environment. Mesenchymal movement depends on extracellular matrix protease activity, amoeboid movement on the RhoA-dependent kinase ROCK. Cancer cells can switch from one mechanism to the other in response to different stimuli, limiting the efficacy of antimetastatic therapies.We investigated the acquisition and molecular regulation of the invasion capacity of neoplastically transformed human fibroblasts, which were able to induce sarcomas and metastases when injected into immunocompromised mice. We found that neoplastic transformation was associated with a change in cell morphology (from fibroblastic to polygonal), a reorganization of the actin cytoskeleton, a decrease in the expression of several matrix metalloproteases and increases in cell motility and   invasive  ness. In a three-dimensional environment, sarcomagenic cells showed a spherical morphology with cortical actin rings, suggesting a switch from mesenchymal to amoeboid movement. Accordingly, cell invasion decreased after treatment with the ROCK inhibitor Y27632, but not with the matrix protease inhibitor Ro 28-2653. The increased   invasive  ness of tumorigenic cells was associated with reduced expression of Rnd3 (also known as RhoE), a cellular inhibitor of ROCK. Indeed, ectopic Rnd3 expression reduced their   invasive   ability in vitro and their metastatic potential in vivo.These results indicate that, during neoplastic transformation, cells of mesenchymal origin can switch from a mesenchymal mode of movement to an amoeboid one. In addition, they point to Rnd3 as a possible regulator of mesenchymal tumor cell invasion and to ROCK as a potential therapeutic target for sarcomas. 
 Inference of Low and High-Grade Glioma Gene Regulatory Networks Delineates the Role of Rnd3 in Establishing Multiple Hallmarks of Cancer. Gliomas are a highly heterogeneous group of brain tumours that are refractory to treatment, highly   invasive   and pro-angiogenic. Glioblastoma patients have an average survival time of less than 15 months. Understanding the molecular basis of different grades of glioma, from well differentiated, low-grade tumours to high-grade tumours, is a key step in defining new therapeutic targets. Here we use a data-driven approach to learn the structure of gene regulatory networks from observational data and use the resulting models to formulate hypothesis on the molecular determinants of glioma stage. Remarkably, integration of available knowledge with functional genomics datasets representing clinical and pre-clinical studies reveals important properties within the regulatory circuits controlling low and high-grade glioma. Our analyses first show that low and high-grade gliomas are characterised by a switch in activity of two subsets of Rho GTPases. The first one is involved in maintaining normal glial cell function, while the second is linked to the establishment of multiple hallmarks of cancer. Next, the development and application of a novel data integration methodology reveals novel functions of RND3 in controlling glioma cell migration, invasion, proliferation, angiogenesis and clinical outcome. 
 Genome-wide association study link novel loci to endometriosis. Endometriosis is a common gynecological condition with complex etiology defined by the presence of endometrial glands and stroma outside the womb. Endometriosis is a common cause of both cyclic and chronic pelvic pain, reduced fertility, and reduced quality-of-life. Diagnosis and treatment of endometriosis is, on average, delayed by 7-10 years from the onset of symptoms. Absence of a timely and non-  invasive   diagnostic tool is presently the greatest barrier to the identification and treatment of endometriosis. Twin and family studies have documented an increased relative risk in families. To identify genetic factors that contribute to endometriosis we conducted a two-stage genome-wide association study (GWAS) of a European cohort including 2,019 surgically confirmed endometriosis cases and 14,471 controls. Three of the SNPs we identify associated at P<5×10(-8) in our combined analysis belong to two loci: LINC00339-WNT4 on 1p36.12 (rs2235529; P = 8.65×10(-9), OR = 1.29, CI = 1.18-1.40) and RND3-RBM43 on 2q23.3 (rs1519761; P = 4.70×10(-8), OR = 1.20, Cl = 1.13-1.29, and rs6757804; P = 4.05×10(-8), OR = 1.20, Cl = 1.13-1.29). Using an adjusted Bonferoni significance threshold of 4.51×10(-7) we identify two additional loci in our meta-analysis that associate with endometriosis:, RNF144B-ID4 on 6p22.3 (rs6907340; P = 2.19×10(-7), OR = 1.20, Cl = 1.12-1.28), and HNRNPA3P1-LOC100130539 on 10q11.21 (rs10508881; P = 4.08×10(-7), OR = 1.19, Cl = 1.11-1.27). Consistent with previously suggested associations to WNT4 our study implicate a 150 kb region around WNT4 that also include LINC00339 and CDC42. A univariate analysis of documented infertility, age at menarche, and family history did not show allelic association with these SNP markers. Clinical data from patients in our study reveal an average delay in diagnosis of 8.4 years and confirm a strong correlation between endometriosis severity and infertility (n = 1182, P<0.001, OR = 2.18). This GWAS of endometriosis was conducted with high diagnostic certainty in cases, and with stringent handling of population substructure. Our findings broaden the understanding of the genetic factors that play a role in endometriosis. 
 Rnd3/RhoE Is down-regulated in hepatocellular carcinoma and controls cellular invasion. We performed a review of public microarray data that revealed a significant down-regulation of Rnd3 expression in hepatocellular carcinoma (HCC), as compared to nontumor liver. Rnd3/RhoE is an atypical RhoGTPase family member because it is always under its active GTP-bound conformation and not sensitive to classical regulators. Rnd3 down-regulation was validated by quantitative real-time polymerase chain reaction in 120 independent tumors. Moreover, Rnd3 down-expression was confirmed using immunohistochemistry on tumor sections and western blotting on human tumor and cell-line extracts. Rnd3 expression was significantly lower in   invasive   tumors with satellite nodules. Overexpression and silencing of Rnd3 in Hep3B cells led to decreased and increased three-dimensional cell motility, respectively. The short interfering RNA-mediated down-regulation of Rnd3 expression induced a loss of E-cadherin at cell-cell junctions that was linked to epithelial-mesenchymal transition through the up-regulation of the zinc finger E-box binding homeobox protein, ZEB2, and the down-regulation of miR-200b and miR-200c. Rnd3 knockdown mediated tumor hepatocyte invasion in a matrix-metalloproteinase-independent, and Rac1-dependent manner.Rnd3 down-regulation provides an   invasive   advantage to tumor hepatocytes, suggesting that RND3 might represent a metastasis suppressor gene in HCC. 
 RhoE is frequently down-regulated in hepatocellular carcinoma (HCC) and suppresses HCC invasion through antagonizing the Rho/Rho-kinase/myosin phosphatase target pathway. Deregulation of Rho guanosine triphosphatase (GTPase) pathways plays an important role in tumorigenesis and metastasis of hepatocellular carcinoma (HCC). RhoE/Rnd3 belongs to an atypical subfamily of the RhoGTPase, the Rnd family, as it lacks the intrinsic GTPase activity and remains always in its active GTP-bound form. In this study we investigated the role of RhoE in HCC. We examined the expression of RhoE in primary HCC samples from patients predominantly infected with the hepatitis B virus (HBV) and found that the RhoE messenger RNA (mRNA) level was frequently down-regulated (83.1%, 59/71) in HCCs. Low expression of RhoE in the tumors was significantly associated with shorter disease-free survival (P = 0.020) of the patients. Knockdown of RhoE by short-hairpin RNA using a lentiviral approach led to increased cell motility and   invasive  ness in SMMC7721 and BEL7402 HCC cells. Moreover, in vivo an orthotopic liver injection model in nude mice further demonstrated that knockdown of RhoE enhanced local invasion of HCC cells in the livers, with more   invasive   tumor front and increased incidence of venous invasion. Mechanistically, stable knockdown of RhoE in HCC cells significantly enhanced the phosphorylation of myosin phosphatase, promoted assembly of stress fibers, and increased the formation of plasma membrane blebbings, all these changes and activities being associated with activation of the Rho/Rho-kinase (ROCK) pathway.RhoE was frequently down-regulated in predominantly HBV-associated HCCs and this down-regulation was associated with a more aggressive HCC phenotype. RhoE regulated the cytoskeleton remodeling and suppressed HCC motility and   invasive  ness by way of inhibiting the Rho/ROCK axis. 
 RhoE promotes metastasis in gastric cancer through a mechanism dependent on enhanced expression of CXCR4. RhoE, a novel member of the Rho protein family, is a key regulator of the cytoskeleton and cell migration. Our group has previously shown that RhoE as a direct target for HIF-1α and mediates hypoxia-induced epithelial to mesenchymal transition in gastric cancer cells. Therefore, we assumed that RhoE might play an important role in gastric cancer metastasis. In the present study, we have explored the role of RhoE expression in gastric cancer, cell invasion and metastasis, and the influence of RhoE on regulating the potential expression of down-stream genes. RhoE expression was elevated in gastric cancer tissues as compared with normal gastric tissues. We also found a close correlation between the histological grade and the diagnosis of the patient. Up-regulation of RhoE significantly enhanced the migratory and   invasiv  e abilities of gastric cancer cells both in vitro and in vivo. Moreover, down-regulation of RhoE diminished the metastatic potential of cancer cells. PCR array and subsequent transwell assay showed that the regulation of gastric cancer metastasis by RhoE was partially mediated by CXCR4. This observation suggested that CXCR4 might be a downstream effector for RhoE. In summary, our study identified RhoE as a novel prognostic biomarker and metastatic-promoting gene of gastric cancer. 
 Transcriptional up-regulation of RhoE by hypoxia-inducible factor (HIF)-1 promotes epithelial to mesenchymal transition of gastric cancer cells during hypoxia. Epithelial-mesenchymal transition (EMT) is a key process that drives cancer invasion. Recently, hypoxia has been reported to induce EMT, accompanied by cytoskeleton remodeling. As RhoE is a key regulator in cytoskeleton formation, we hypothesized that RhoE may play a role in hypoxia-induced EMT. For the first time, we report that RhoE protein levels increase in gastric cancer cells under hypoxic conditions. Rigorous analysis revealed that RhoE up-regulation is at the transcriptional levels and requires hypoxia-inducible factor (HIF)-1α induction, and that HIF-1α binds a hypoxia-responsive element (HRE) on the RhoE promoter. Additionally, we discovered that hypoxia or overexpression of RhoE in normoxia up-regulates the mesenchymal marker Vimentin, down-regulates the epithelial marker E-cadherin, and significantly increases cell invasion in vitro. Silencing of HIF-1α or RhoE by specific siRNAs rescued these hypoxia-induced effects. Ectopic expression of RhoE also induced up-regulation of MMP2/MMP-9 in gastric cancer cells. This study identifies RhoE as a direct target for HIF-1 in gastric cancer cells. In addition, RhoE up-regulation represents a pivotal cellular adaptive response to hypoxia with implications in gastric cancer cell EMT and invasion. We propose that RhoE-targeted therapy might inhibit the hi  gh invas  ive potential of gastric cancer cells in hypoxic regions. 
 FOXD3 regulates migration properties and Rnd3 expression in melanoma cells. Forkhead transcription factor, Foxd3, plays a critical role during development by controlling the lineage specification of neural crest cells. Notably, Foxd3 is highly expressed during the wave of neural crest cell migration that forms peripheral neurons and glial cells but is downregulated prior to migration of cells that give rise to the melanocytic lineage. Melanoma is the deadliest form of skin cancer and is derived from melanocytes. Recently, we showed that FOXD3 expression is elevated following the targeted inhibition of the B-RAF-MEK (MAP/ERK kinase)-ERK (extracellular signal-regulated kinase)1/2 pathway in mutant B-RAF melanoma cells. Because melanoma cells are highly migratory and   invasive   in a B-RAF-dependent manner, we explored the role of FOXD3 in these processes. In this study, we show that ectopic FOXD3 expression inhibits the migration, invasion, and spheroid outgrowth of mutant B-RAF melanoma cells. Upregulation of FOXD3 expression following inhibition of B-RAF and MEK correlates with the downregulation of Rnd3, a Rho GTPase and inhibitor of RhoA-ROCK signaling. Indeed, expression of FOXD3 alone was sufficient to downregulate Rnd3 expression at the mRNA and protein levels. Mechanistically, FOXD3 was found to be recruited to the Rnd3 promoter. Inhibition of ROCK partially restored migration in FOXD3-expressing cells. These data show that FOXD3 expression downregulates migration and invasion in melanoma cells and Rnd3, a target known to be involved in these properties. 
 Rnd3 regulation of the actin cytoskeleton promotes melanoma migration and   invasive   outgrowth in three dimensions. The depth of cell invasion into the dermis is a clinical determinant for poor prognosis in cutaneous melanoma. The signaling events that promote the switch from a non  invasive   to   invasive   tumor phenotype remain obscure. Activating mutations in the serine/threonine kinase B-RAF are prevalent in melanoma. Mutant B-RAF is required for melanoma cell invasion. The expression of Rnd3, a Rho family GTPase, is regulated by mutant B-RAF, although its role in melanoma progression is unknown. In this study, we determined the functional contribution of Rnd3 to   invasive   melanoma. Endogenous Rnd3 was targeted for knockdown using a doxycycline-inducible short hairpin RNA system in   invasive   human melanoma cells. Depletion of Rnd3 promoted prominent actin stress fibers and enlarged focal adhesions. Mechanistically, stress fiber formation induced by Rnd3 knockdown required the specific involvement of RhoA and ROCK1/2 activity but not RhoB or RhoC. Rnd3 expression in human melanoma cell lines was strongly associated with elevated extracellular signal-regulated kinase phosphorylation and   invasive   behavior in a three-dimensional dermal-like environment. A functional role for Rnd3 was shown in the   invasive   outgrowth of melanoma tumor spheroids. Knockdown of Rnd3 reduced the   invasive   outgrowth of spheroids embedded in collagen gels. Additionally, Rnd3 depletion inhibited collective and border cell movement out from spheroids in a ROCK1/2-dependent manner. Collectively, these findings implicate Rnd3 as a major suppressor of RhoA-mediated actin cytoskeletal organization and in the acquisition of an   invasive   melanoma phenotype. 
  GeneRIFs  
 Rnd3 expression was significantly lower in   invasive   tumors with satellite nodules 
 Data show that the increased   invasive  ness of tumorigenic cells was associated with reduced expression of Rnd3. 
 Findings implicate Rnd3 as a major suppressor of RhoA-mediated actin cytoskeletal organization and in the acquisition of an   invasive   melanoma phenotype. 
  [Mm]etastasis  PubMed  
 RhoE functions as a tumor suppressor in esophageal squamous cell carcinoma and modulates the PTEN/PI3K/Akt signaling pathway. Emerging evidence indicates that RhoE as novel member of the Rho GTPases family plays an essential role in carcinogenesis and tumor progression of human various tumors, but the functional significance of RhoE in human esophageal squamous cell carcinoma (ESCC) is still unclear. In the current study, RhoE expression in ESCC tissues and cells was examined, and the biological functions of RhoE in ESCC cells were determined. The results revealed that RhoE expression at mRNA and protein levels was significantly downregulated in ESCC tissues and cell lines (P < 0.05). RhoE expression was tightly associated with differentiation degree, clinical staging, and lymph node   metastasis   of the patients with ESCC (P < 0.05), but no significant correlations were found between RhoE expression and gender or age of the patients with ESCC (P > 0.05). Additionally, we found that downregulation of RhoE expression in ESCC cells promoted cell proliferation, cell cycle progression, as well as cell invasion in vitro, and inhibited cell apoptosis. Conversely, upregulation of RhoE expression in ESCC cells inhibited cell proliferation, arrested cell cycle at G0/G1 phase, reduced cell invasion, and promoted cell apoptosis. Furthermore, the downregulation of RhoE expression significantly reduced PTEN level and enhanced pAkt level; however, elevation of RhoE expression markedly increased PTEN level and decreased pAkt level. Stepwise investigations demonstrated that overexpression of RhoE in ESCC cells increased the expressions of p27 and bax proteins but decreased the expressions of cyclin D1 and bcl-2 proteins. These data demonstrate that RhoE may play a driving role in the development and progression of ESCC, and targeting the RhoE may be an effective and feasible approach for treatment of ESCC. 
 Rnd3/RhoE Is down-regulated in hepatocellular carcinoma and controls cellular invasion. We performed a review of public microarray data that revealed a significant down-regulation of Rnd3 expression in hepatocellular carcinoma (HCC), as compared to nontumor liver. Rnd3/RhoE is an atypical RhoGTPase family member because it is always under its active GTP-bound conformation and not sensitive to classical regulators. Rnd3 down-regulation was validated by quantitative real-time polymerase chain reaction in 120 independent tumors. Moreover, Rnd3 down-expression was confirmed using immunohistochemistry on tumor sections and western blotting on human tumor and cell-line extracts. Rnd3 expression was significantly lower in invasive tumors with satellite nodules. Overexpression and silencing of Rnd3 in Hep3B cells led to decreased and increased three-dimensional cell motility, respectively. The short interfering RNA-mediated down-regulation of Rnd3 expression induced a loss of E-cadherin at cell-cell junctions that was linked to epithelial-mesenchymal transition through the up-regulation of the zinc finger E-box binding homeobox protein, ZEB2, and the down-regulation of miR-200b and miR-200c. Rnd3 knockdown mediated tumor hepatocyte invasion in a matrix-metalloproteinase-independent, and Rac1-dependent manner.Rnd3 down-regulation provides an invasive advantage to tumor hepatocytes, suggesting that RND3 might represent a   metastasis   suppressor gene in HCC. 
 RhoE is frequently down-regulated in hepatocellular carcinoma (HCC) and suppresses HCC invasion through antagonizing the Rho/Rho-kinase/myosin phosphatase target pathway. Deregulation of Rho guanosine triphosphatase (GTPase) pathways plays an important role in tumorigenesis and   metastasis   of hepatocellular carcinoma (HCC). RhoE/Rnd3 belongs to an atypical subfamily of the RhoGTPase, the Rnd family, as it lacks the intrinsic GTPase activity and remains always in its active GTP-bound form. In this study we investigated the role of RhoE in HCC. We examined the expression of RhoE in primary HCC samples from patients predominantly infected with the hepatitis B virus (HBV) and found that the RhoE messenger RNA (mRNA) level was frequently down-regulated (83.1%, 59/71) in HCCs. Low expression of RhoE in the tumors was significantly associated with shorter disease-free survival (P = 0.020) of the patients. Knockdown of RhoE by short-hairpin RNA using a lentiviral approach led to increased cell motility and invasiveness in SMMC7721 and BEL7402 HCC cells. Moreover, in vivo an orthotopic liver injection model in nude mice further demonstrated that knockdown of RhoE enhanced local invasion of HCC cells in the livers, with more invasive tumor front and increased incidence of venous invasion. Mechanistically, stable knockdown of RhoE in HCC cells significantly enhanced the phosphorylation of myosin phosphatase, promoted assembly of stress fibers, and increased the formation of plasma membrane blebbings, all these changes and activities being associated with activation of the Rho/Rho-kinase (ROCK) pathway.RhoE was frequently down-regulated in predominantly HBV-associated HCCs and this down-regulation was associated with a more aggressive HCC phenotype. RhoE regulated the cytoskeleton remodeling and suppressed HCC motility and invasiveness by way of inhibiting the Rho/ROCK axis. 
 MicroRNA-200b regulates cyclin D1 expression and promotes S-phase entry by targeting RND3 in HeLa cells. MicroRNAs (miRNAs) are endogenous non-coding small RNAs that inhibit gene expression post-transcriptionally. By regulating their target genes, miRNAs play important roles in tumor generation and development. Recently, the mir-200 family was revealed to inhibit the epithelial-mesenchymal transition, which is viewed as an essential step in early tumor   metastasis  . Here, we used luciferase assays to demonstrate that mir-200b interacts with predicted target sites in the 3' untranslated region of RND3. In HeLa cells, mir-200b directly reduced the expression of RND3 at the mRNA and protein levels, which thereby promoted expression of the downstream protein cyclin D1 and increased S-phase entry. In conclusion, our study demonstrates a novel role for mir-200b in cell cycle progression and identifies RND3 as a novel mir-200b target. 
 RhoE promotes   metastasis   in gastric cancer through a mechanism dependent on enhanced expression of CXCR4. RhoE, a novel member of the Rho protein family, is a key regulator of the cytoskeleton and cell migration. Our group has previously shown that RhoE as a direct target for HIF-1α and mediates hypoxia-induced epithelial to mesenchymal transition in gastric cancer cells. Therefore, we assumed that RhoE might play an important role in gastric cancer   metastasi  s. In the present study, we have explored the role of RhoE expression in gastric cancer, cell invasion and   metastasi  s, and the influence of RhoE on regulating the potential expression of down-stream genes. RhoE expression was elevated in gastric cancer tissues as compared with normal gastric tissues. We also found a close correlation between the histological grade and the diagnosis of the patient. Up-regulation of RhoE significantly enhanced the migratory and invasive abilities of gastric cancer cells both in vitro and in vivo. Moreover, down-regulation of RhoE diminished the metastatic potential of cancer cells. PCR array and subsequent transwell assay showed that the regulation of gastric cancer   metastasi  s by RhoE was partially mediated by CXCR4. This observation suggested that CXCR4 might be a downstream effector for RhoE. In summary, our study identified RhoE as a novel prognostic biomarker and metastatic-promoting gene of gastric cancer. 
 Suppression of RND3 activity by AES downregulation promotes cancer cell proliferation and invasion. Amino-terminal enhancer of split (AES) is a member of the Groucho/TLE family. Although it has no DNA-binding site, AES can regulate transcriptional activity by interacting with transcriptional factors. Emerging evidence indicates that AES may play an important role in tumor   metastasis  , but the molecular mechanism is still poorly understood. In this study, we found that knockdown of AES by RNA interference (RNAi) downregulated RND3 expression at the mRNA and protein levels in MDA-MB-231 and HepG2, two cancer cell lines. Furthermore, luciferase assays showed that overexpression of AES significantly enhanced RND3 promoter activity. Moreover, inhibition of AES both in MDA-MB-231 and HepG2 cells by RNAi significantly promoted cell proliferation, cell cycle progression and invasion, consistent with the effects of RNAi-mediated RND3 knockdown in these cells. For the first time, data are presented showing that alteration of the malignant behavior of cancer cells by AES is related to RND3 regulation, and these findings also provide new insights into the mechanism of AES action in regulating tumor malignancy. 
 The Rho family member RhoE interacts with Skp2 and is degraded at the proteasome during cell cycle progression. RhoE/Rnd3 is an atypical member of the Rho family of small GTPases. In addition to regulating actin cytoskeleton dynamics, RhoE is involved in the regulation of cell proliferation, survival, and   metastasis  . We examined RhoE expression levels during cell cycle and investigated mechanisms controlling them. We show that RhoE accumulates during G1, in contact-inhibited cells, and when the Akt pathway is inhibited. Conversely, RhoE levels rapidly decrease at the G1/S transition and remain low for most of the cell cycle. We also show that the half-life of RhoE is shorter than that of other Rho proteins and that its expression levels are regulated by proteasomal degradation. The expression patterns of RhoE overlap with that of the cell cycle inhibitor p27. Consistently with an involvement of RhoE in cell cycle regulation, RhoE and p27 levels decrease after overexpression of the F-box protein Skp2. We have identified a region between amino acids 231 and 240 of RhoE as the Skp2-interacting domain and Lys(235) as the substrate for ubiquitylation. Based on our results, we propose a mechanism according to which proteasomal degradation of RhoE by Skp2 regulates its protein levels to control cellular proliferation. 
 Rnd3 regulates lung cancer cell proliferation through notch signaling. Rnd3/RhoE is a small Rho GTPase involved in the regulation of different cell behaviors. Dysregulation of Rnd3 has been linked to tumorigenesis and   metastasis  . Lung cancers are the leading cause of cancer-related death in the West and around the world. The expression of Rnd3 and its ectopic role in non-small cell lung cancer (NSCLC) remain to be explored. Here, we reported that Rnd3 was down-regulated in three NSCLC cell lines: H358, H520 and A549. The down-regulation of Rnd3 led to hyper-activation of Rho Kinase and Notch signaling. The reintroduction of Rnd3 or selective inhibition of Notch signaling, but not Rho Kinase signaling, blocked the proliferation of H358 and H520 cells. Mechanistically, Notch intracellular domain (NICD) protein abundance in H358 cells was regulated by Rnd3-mediated NICD proteasome degradation. Rnd3 regulated H358 and H520 cell proliferation through a Notch1/NICD/Hes1 signaling axis independent of Rho Kinase. 
  [Cc]ell [Ii]nvasion  PubMed  
 A switch in RND3-RHOA signaling is critical for melanoma   cell invasion   following mutant-BRAF inhibition. The initial use of BRAF targeted therapeutics in clinical trials has demonstrated encouraging responses in melanoma patients, although a rise in drug-resistant cells capable of advancing malignant disease has been described. The current study uses BRAFV600E expressing WM793 melanoma cells to derive data aimed at investigating the molecular determinant of   cell invasion   following treatment with clinical BRAF inhibitors.Small-molecule inhibitors targeting BRAF reduced MEK1/2-ERK1/2 pathway activation and cell survival; yet, viable cell subpopulations persisted. The residual cells exhibited an elongated cell shape, prominent actin stress fibers and retained the ability to invade 3-D dermal-like microenvironments. BRAF inhibitor treatments were associated with reduced expression of RND3, an antagonist of RHOA activation, and elevated RHOA-dependent signaling. Restoration of RND3 expression or RHOA knockdown attenuated the migratory ability of residual cells without affecting overall cell survival. The invasive ability of BRAF inhibitor treated cells embedded in collagen gels was diminished following RND3 re-expression or RHOA depletion. Conversely, melanoma cell movement in the absence of BRAF inhibition was unaffected by RND3 expression or RHOA depletion.These data reveal a novel switch in the requirement for RND3 and RHOA in coordinating the movement of residual WM793 cells that are initially refractive to BRAF inhibitor therapy. These results have important clinical implications because they suggest that combining BRAF inhibitors with therapies that target the invasion of drug-resistant cells could aid in controlling disease relapse. 
 A switch in RND3-RHOA signaling is critical for melanoma   cell invasion   following mutant-BRAF inhibition. The initial use of BRAF targeted therapeutics in clinical trials has demonstrated encouraging responses in melanoma patients, although a rise in drug-resistant cells capable of advancing malignant disease has been described. The current study uses BRAFV600E expressing WM793 melanoma cells to derive data aimed at investigating the molecular determinant of   cell invasion   following treatment with clinical BRAF inhibitors.Small-molecule inhibitors targeting BRAF reduced MEK1/2-ERK1/2 pathway activation and cell survival; yet, viable cell subpopulations persisted. The residual cells exhibited an elongated cell shape, prominent actin stress fibers and retained the ability to invade 3-D dermal-like microenvironments. BRAF inhibitor treatments were associated with reduced expression of RND3, an antagonist of RHOA activation, and elevated RHOA-dependent signaling. Restoration of RND3 expression or RHOA knockdown attenuated the migratory ability of residual cells without affecting overall cell survival. The invasive ability of BRAF inhibitor treated cells embedded in collagen gels was diminished following RND3 re-expression or RHOA depletion. Conversely, melanoma cell movement in the absence of BRAF inhibition was unaffected by RND3 expression or RHOA depletion.These data reveal a novel switch in the requirement for RND3 and RHOA in coordinating the movement of residual WM793 cells that are initially refractive to BRAF inhibitor therapy. These results have important clinical implications because they suggest that combining BRAF inhibitors with therapies that target the invasion of drug-resistant cells could aid in controlling disease relapse. 
 Reduced expression of the ROCK inhibitor Rnd3 is associated with increased invasiveness and metastatic potential in mesenchymal tumor cells. Mesenchymal and amoeboid movements are two important mechanisms adopted by cancer cells to invade the surrounding environment. Mesenchymal movement depends on extracellular matrix protease activity, amoeboid movement on the RhoA-dependent kinase ROCK. Cancer cells can switch from one mechanism to the other in response to different stimuli, limiting the efficacy of antimetastatic therapies.We investigated the acquisition and molecular regulation of the invasion capacity of neoplastically transformed human fibroblasts, which were able to induce sarcomas and metastases when injected into immunocompromised mice. We found that neoplastic transformation was associated with a change in cell morphology (from fibroblastic to polygonal), a reorganization of the actin cytoskeleton, a decrease in the expression of several matrix metalloproteases and increases in cell motility and invasiveness. In a three-dimensional environment, sarcomagenic cells showed a spherical morphology with cortical actin rings, suggesting a switch from mesenchymal to amoeboid movement. Accordingly,   cell invasion   decreased after treatment with the ROCK inhibitor Y27632, but not with the matrix protease inhibitor Ro 28-2653. The increased invasiveness of tumorigenic cells was associated with reduced expression of Rnd3 (also known as RhoE), a cellular inhibitor of ROCK. Indeed, ectopic Rnd3 expression reduced their invasive ability in vitro and their metastatic potential in vivo.These results indicate that, during neoplastic transformation, cells of mesenchymal origin can switch from a mesenchymal mode of movement to an amoeboid one. In addition, they point to Rnd3 as a possible regulator of mesenchymal tumor   cell invasion   and to ROCK as a potential therapeutic target for sarcomas. 
 RhoE functions as a tumor suppressor in esophageal squamous cell carcinoma and modulates the PTEN/PI3K/Akt signaling pathway. Emerging evidence indicates that RhoE as novel member of the Rho GTPases family plays an essential role in carcinogenesis and tumor progression of human various tumors, but the functional significance of RhoE in human esophageal squamous cell carcinoma (ESCC) is still unclear. In the current study, RhoE expression in ESCC tissues and cells was examined, and the biological functions of RhoE in ESCC cells were determined. The results revealed that RhoE expression at mRNA and protein levels was significantly downregulated in ESCC tissues and cell lines (P < 0.05). RhoE expression was tightly associated with differentiation degree, clinical staging, and lymph node metastasis of the patients with ESCC (P < 0.05), but no significant correlations were found between RhoE expression and gender or age of the patients with ESCC (P > 0.05). Additionally, we found that downregulation of RhoE expression in ESCC cells promoted cell proliferation, cell cycle progression, as well as   cell invasion   in vitro, and inhibited cell apoptosis. Conversely, upregulation of RhoE expression in ESCC cells inhibited cell proliferation, arrested cell cycle at G0/G1 phase, reduced   cell invasion  , and promoted cell apoptosis. Furthermore, the downregulation of RhoE expression significantly reduced PTEN level and enhanced pAkt level; however, elevation of RhoE expression markedly increased PTEN level and decreased pAkt level. Stepwise investigations demonstrated that overexpression of RhoE in ESCC cells increased the expressions of p27 and bax proteins but decreased the expressions of cyclin D1 and bcl-2 proteins. These data demonstrate that RhoE may play a driving role in the development and progression of ESCC, and targeting the RhoE may be an effective and feasible approach for treatment of ESCC. 
 RhoE promotes metastasis in gastric cancer through a mechanism dependent on enhanced expression of CXCR4. RhoE, a novel member of the Rho protein family, is a key regulator of the cytoskeleton and cell migration. Our group has previously shown that RhoE as a direct target for HIF-1α and mediates hypoxia-induced epithelial to mesenchymal transition in gastric cancer cells. Therefore, we assumed that RhoE might play an important role in gastric cancer metastasis. In the present study, we have explored the role of RhoE expression in gastric cancer,   cell invasio  n and metastasis, and the influence of RhoE on regulating the potential expression of down-stream genes. RhoE expression was elevated in gastric cancer tissues as compared with normal gastric tissues. We also found a close correlation between the histological grade and the diagnosis of the patient. Up-regulation of RhoE significantly enhanced the migratory and invasive abilities of gastric cancer cells both in vitro and in vivo. Moreover, down-regulation of RhoE diminished the metastatic potential of cancer cells. PCR array and subsequent transwell assay showed that the regulation of gastric cancer metastasis by RhoE was partially mediated by CXCR4. This observation suggested that CXCR4 might be a downstream effector for RhoE. In summary, our study identified RhoE as a novel prognostic biomarker and metastatic-promoting gene of gastric cancer. 
 B-RAF regulation of Rnd3 participates in actin cytoskeletal and focal adhesion organization. The actin cytoskeleton controls multiple cellular functions, including cell morphology, movement, and growth. Accumulating evidence indicates that oncogenic activation of the mitogen-activated protein kinase kinase/extracellular signal-regulated kinase 1/2 (MEK/ERK1/2) pathway is accompanied by actin cytoskeletal reorganization. However, the signaling events contributing to actin cytoskeleton remodeling mediated by aberrant ERK1/2 activation are largely unknown. Mutant B-RAF is found in a variety of cancers, including melanoma, and it enhances activation of the MEK/ERK1/2 pathway. We show that targeted knockdown of B-RAF with small interfering RNA or pharmacological inhibition of MEK increased actin stress fiber formation and stabilized focal adhesion dynamics in human melanoma cells. These effects were due to stimulation of the Rho/Rho kinase (ROCK)/LIM kinase-2 signaling pathway, cumulating in the inactivation of the actin depolymerizing/severing protein cofilin. The expression of Rnd3, a Rho antagonist, was attenuated after B-RAF knockdown or MEK inhibition, but it was enhanced in melanocytes expressing active B-RAF. Constitutive expression of Rnd3 suppressed the actin cytoskeletal and focal adhesion effects mediated by B-RAF knockdown. Depletion of Rnd3 elevated cofilin phosphorylation and stress fiber formation and reduced   cell invasion  . Together, our results identify Rnd3 as a regulator of cross talk between the RAF/MEK/ERK and Rho/ROCK signaling pathways, and a key contributor to oncogene-mediated reorganization of the actin cytoskeleton and focal adhesions. 
 Transcriptional up-regulation of RhoE by hypoxia-inducible factor (HIF)-1 promotes epithelial to mesenchymal transition of gastric cancer cells during hypoxia. Epithelial-mesenchymal transition (EMT) is a key process that drives cancer invasion. Recently, hypoxia has been reported to induce EMT, accompanied by cytoskeleton remodeling. As RhoE is a key regulator in cytoskeleton formation, we hypothesized that RhoE may play a role in hypoxia-induced EMT. For the first time, we report that RhoE protein levels increase in gastric cancer cells under hypoxic conditions. Rigorous analysis revealed that RhoE up-regulation is at the transcriptional levels and requires hypoxia-inducible factor (HIF)-1α induction, and that HIF-1α binds a hypoxia-responsive element (HRE) on the RhoE promoter. Additionally, we discovered that hypoxia or overexpression of RhoE in normoxia up-regulates the mesenchymal marker Vimentin, down-regulates the epithelial marker E-cadherin, and significantly increase  s cell invasi  on in vitro. Silencing of HIF-1α or RhoE by specific siRNAs rescued these hypoxia-induced effects. Ectopic expression of RhoE also induced up-regulation of MMP2/MMP-9 in gastric cancer cells. This study identifies RhoE as a direct target for HIF-1 in gastric cancer cells. In addition, RhoE up-regulation represents a pivotal cellular adaptive response to hypoxia with implications in gastric cancer cell EMT and invasion. We propose that RhoE-targeted therapy might inhibit the high invasive potential of gastric cancer cells in hypoxic regions. 
 Rnd3 regulation of the actin cytoskeleton promotes melanoma migration and invasive outgrowth in three dimensions. The depth of   cell invasion   into the dermis is a clinical determinant for poor prognosis in cutaneous melanoma. The signaling events that promote the switch from a noninvasive to invasive tumor phenotype remain obscure. Activating mutations in the serine/threonine kinase B-RAF are prevalent in melanoma. Mutant B-RAF is required for melanoma   cell invasion  . The expression of Rnd3, a Rho family GTPase, is regulated by mutant B-RAF, although its role in melanoma progression is unknown. In this study, we determined the functional contribution of Rnd3 to invasive melanoma. Endogenous Rnd3 was targeted for knockdown using a doxycycline-inducible short hairpin RNA system in invasive human melanoma cells. Depletion of Rnd3 promoted prominent actin stress fibers and enlarged focal adhesions. Mechanistically, stress fiber formation induced by Rnd3 knockdown required the specific involvement of RhoA and ROCK1/2 activity but not RhoB or RhoC. Rnd3 expression in human melanoma cell lines was strongly associated with elevated extracellular signal-regulated kinase phosphorylation and invasive behavior in a three-dimensional dermal-like environment. A functional role for Rnd3 was shown in the invasive outgrowth of melanoma tumor spheroids. Knockdown of Rnd3 reduced the invasive outgrowth of spheroids embedded in collagen gels. Additionally, Rnd3 depletion inhibited collective and border cell movement out from spheroids in a ROCK1/2-dependent manner. Collectively, these findings implicate Rnd3 as a major suppressor of RhoA-mediated actin cytoskeletal organization and in the acquisition of an invasive melanoma phenotype. 
   
  
 RHOV 
 
  NCBI Gene  
  GeneRIF  
  Pubmed  
 
 
  Search Term  Rank  Fields  
  [Ff]ilopodia  1  PubMed(1)  
  [Ff]ilopodium  2  PubMed(1)  
  [Mm]igration  6  PubMed(2)  
 
 
 hide/show details for RHOV 
  [Ff]ilopodia  PubMed  
 Critical and distinct roles of amino- and carboxyl-terminal sequences in regulation of the biological activity of the Chp atypical Rho GTPase. Chp (Cdc42 homologous protein) shares significant sequence and functional identity with the human Cdc42 small GTPase, and like Cdc42, promotes formation of   filopodia   and activates the p21-activated kinase serine/threonine kinase. However, unlike Cdc42, Chp contains unique amino- and carboxyl-terminal extensions. Here we determined whether Chp, like Cdc42, can promote growth transformation and evaluated the role of the amino- and carboxyl-terminal sequences in Chp function. Surprisingly, we found that a GTPase-deficient mutant of Chp exhibited low transforming activity but that deletion of the amino terminus of Chp greatly enhanced its transforming activity. Thus, the amino terminus may serve as a negative regulator of Chp function. The carboxyl terminus of Cdc42 contains a CAAX (where C is cysteine, A is aliphatic amino acid, X is terminal amino acid) tetrapeptide sequence that signals for the posttranslational modification critical for Cdc42 membrane association and biological function. Although Chp lacks aCAAXmotif, we found that Chp showed carboxyl terminus-dependent localization to the plasma membrane and to endosomes. Furthermore, an intact carboxyl terminus was required for Chp transforming activity. However, treatment with inhibitors of protein palmitoylation, but not prenylation, caused Chp to mislocalize to the cytoplasm. Thus, Chp depends on palmitoylation, rather than isoprenylation, for membrane association and function. In summary, Chp is implicated in cell transformation, and the unique amino and carboxyl termini of Chp represent atypical mechanisms of regulation of Rho GTPase function. 
  [Ff]ilopodium  PubMed  
 Molecular cloning and characterization of WRCH2 on human chromosome 15q15. WNT signals are transduced to the JNK pathway, the Ca2+-releasing pathway, or the beta-catenin - TCF pathway through seven-transmembrane-type WNT receptors encoded by Frizzled genes (FZD1-FZD10). WRCH1/ARHV and CDC42 are potentially implicated in the WNT-JNK pathway. Here, WRCH2/ARHV cDNAs were isolated by using bioinformatics and cDNA-PCR. WRCH2 gene, consisting of at least 3 exons, encoded a 236-amino-acid protein with proline-rich domain and GTPase domain. WRCH2 was homologous to WRCH1 (55.4% total-amino-acid identity) and CDC42 (43.5% total-amino-acid identity). WRCH2 gene was located on human chromosome 15q15, which is one of fragile sites in the human genome. A single nucleotide substitution (632 Gright curved arrow A) was identified between WRCH2 cDNA and human genome draft sequences, which resulted in Arg177Lys amino-acid substitution. WRCH2 mRNA was relatively highly expressed in pancreas, placenta, and fetal brain. WRCH2 mRNA was over-expressed in TMK1 (gastric cancer), Hs700T (pancreatic cancer), HeLa S3 (cervical cancer), and A549 (lung cancer). WRCH2 mRNA was moderately expressed in MKN74, MKN45, MKN28, KATO-III (gastric cancer), HL-60 (pro-myelocytic leukemia), Raji (Burkitt's lymphoma), and SW480 (colorectal cancer). WRCH2 mRNA was up-regulated in 3 out of 8 cases of primary gastric cancer. Because Wrch1 can activate PAK1 and JNK1, and induce   filopodium   formation and stress fiber dissolution, over-expression of WRCH2 mRNA in human cancer cells might also lead to more malignant phenotype. 
  [Mm]igration  PubMed  
 Autophosphorylation-dependent degradation of Pak1, triggered by the Rho-family GTPase, Chp. The Paks (p21-activated kinases) Pak1, Pak2 and Pak3 are among the most studied effectors of the Rho-family GTPases, Rac, Cdc42 (cell division cycle 42) and Chp (Cdc42 homologous protein). Pak kinases influence a variety of cellular functions, but the process of Pak down-regulation, following activation, is poorly understood. In the present study, we describe for the first time a negative-inhibitory loop generated by the small Rho-GTPases Cdc42 and Chp, resulting in Pak1 inhibition. Upon overexpression of Chp, we unexpectedly observed a T-cell   migration   phenotype consistent with Paks inhibition. In line with this observation, overexpression of either Chp or Cdc42 caused a marked reduction in the level of Pak1 protein in a number of different cell lines. Chp-induced degradation was accompanied by ubiquitination of Pak1, and was dependent on the proteasome. The susceptibility of Pak1 to Chp-induced degradation depended on its p21-binding domain, kinase activity and a number of Pak1 autophosphorylation sites, whereas the PIX- (Pak-interacting exchange factor) and Nck-binding sites were not required. Together, these results implicate Chp-induced kinase autophosphorylation in the degradation of Pak1. The N-terminal domain of Chp was found to be required for Chp-induced degradation, although not for Pak1 activation, suggesting that Chp provides a second function, distinct from kinase activation, to trigger Pak degradation. Collectively, our results demonstrate a novel mechanism of signal termination mediated by the Rho-family GTPases Chp and Cdc42, which results in ubiquitin-mediated degradation of one of their direct effectors, Pak1. 
 Autophosphorylation-dependent degradation of Pak1, triggered by the Rho-family GTPase, Chp. The Paks (p21-activated kinases) Pak1, Pak2 and Pak3 are among the most studied effectors of the Rho-family GTPases, Rac, Cdc42 (cell division cycle 42) and Chp (Cdc42 homologous protein). Pak kinases influence a variety of cellular functions, but the process of Pak down-regulation, following activation, is poorly understood. In the present study, we describe for the first time a negative-inhibitory loop generated by the small Rho-GTPases Cdc42 and Chp, resulting in Pak1 inhibition. Upon overexpression of Chp, we unexpectedly observed a T-cell   migration   phenotype consistent with Paks inhibition. In line with this observation, overexpression of either Chp or Cdc42 caused a marked reduction in the level of Pak1 protein in a number of different cell lines. Chp-induced degradation was accompanied by ubiquitination of Pak1, and was dependent on the proteasome. The susceptibility of Pak1 to Chp-induced degradation depended on its p21-binding domain, kinase activity and a number of Pak1 autophosphorylation sites, whereas the PIX- (Pak-interacting exchange factor) and Nck-binding sites were not required. Together, these results implicate Chp-induced kinase autophosphorylation in the degradation of Pak1. The N-terminal domain of Chp was found to be required for Chp-induced degradation, although not for Pak1 activation, suggesting that Chp provides a second function, distinct from kinase activation, to trigger Pak degradation. Collectively, our results demonstrate a novel mechanism of signal termination mediated by the Rho-family GTPases Chp and Cdc42, which results in ubiquitin-mediated degradation of one of their direct effectors, Pak1. 
   
  
 RHOJ 
 
  NCBI Gene  
  GeneRIF  
  Pubmed  
 
 
  Search Term  Rank  Fields  
  [Mm]igration  6  PubMed(2); GeneRIFs(1)  
  [Mm]etastasis  12  PubMed(1)  
 
 
 hide/show details for RHOJ 
  [Mm]igration  PubMed  
 RhoJ/TCL regulates endothelial motility and tube formation and modulates actomyosin contractility and focal adhesion numbers. RhoJ/TCL was identified by our group as an endothelial-expressed Rho GTPase. The aim of this study was to determine its tissue distribution, subcellular localization, and function in endothelial   migration   and tube formation.Using in situ hybridization, RhoJ was localized to endothelial cells in a set of normal and cancerous tissues and in the vasculature of mouse embryos; endogenous RhoJ was localized to focal adhesions by immunofluorescence. The proangiogenic factor vascular endothelial growth factor activated RhoJ in endothelial cells. Using either small interfering (si)RNA-mediated knockdown of RhoJ expression or overexpression of constitutively active RhoJ (daRhoJ), RhoJ was found to positively regulate endothelial motility and tubule formation. Downregulating RhoJ expression increased focal adhesions and stress fibers in migrating cells, whereas daRhoJ overexpression resulted in the converse. RhoJ downregulation resulted in increased contraction of a collagen gel and increased phospho-myosin light chain, indicative of increased actomyosin contractility. Pharmacological inhibition of Rho-kinase (which phosphorylates myosin light chain) or nonmuscle myosin II reversed the defective tube formation and   migration   of RhoJ knockdown cells.RhoJ is endothelial-expressed in vivo, activated by vascular endothelial growth factor, localizes to focal adhesions, regulates endothelial cell   migration   and tube formation, and modulates actomyosin contractility and focal adhesion numbers. 
 RhoJ modulates melanoma invasion by altering actin cytoskeletal dynamics. Rho family GTPases regulate diverse processes in human melanoma ranging from tumor formation to metastasis and chemoresistance. In this study, a combination of in vitro and in vivo approaches was utilized to determine whether RHOJ, a CDC42 homologue that regulates melanoma chemoresistance, also controls melanoma   migration  . Depletion or overexpression of RHOJ altered cellular morphology, implicating a role for RHOJ in modulating actin cytoskeletal dynamics. RHOJ depletion inhibited melanoma cell   migration   and invasion in vitro and melanoma tumor growth and lymphatic spread in mice. Molecular studies revealed that RHOJ alters actin cytoskeletal dynamics by inducing the phosphorylation of LIMK, cofilin, and p41-ARC (ARP2/3 complex subunit) in a PAK1-dependent manner in vitro and in tumor xenografts. Taken together, these observations identify RHOJ as a melanoma linchpin determinant that regulates both actin cytoskeletal dynamics and chemoresistance by activating PAK1. 
  GeneRIFs  
 RhoJ is endothelial-expressed in vivo, activated by vascular endothelial growth factor, localizes to focal adhesions, regulates endothelial cell   migration   and tube formation, and modulates actomyosin contractility and focal adhesion numbers. 
  [Mm]etastasis  PubMed  
 RhoJ modulates melanoma invasion by altering actin cytoskeletal dynamics. Rho family GTPases regulate diverse processes in human melanoma ranging from tumor formation to   metastasis   and chemoresistance. In this study, a combination of in vitro and in vivo approaches was utilized to determine whether RHOJ, a CDC42 homologue that regulates melanoma chemoresistance, also controls melanoma migration. Depletion or overexpression of RHOJ altered cellular morphology, implicating a role for RHOJ in modulating actin cytoskeletal dynamics. RHOJ depletion inhibited melanoma cell migration and invasion in vitro and melanoma tumor growth and lymphatic spread in mice. Molecular studies revealed that RHOJ alters actin cytoskeletal dynamics by inducing the phosphorylation of LIMK, cofilin, and p41-ARC (ARP2/3 complex subunit) in a PAK1-dependent manner in vitro and in tumor xenografts. Taken together, these observations identify RHOJ as a melanoma linchpin determinant that regulates both actin cytoskeletal dynamics and chemoresistance by activating PAK1. 
   
  
 RHOBTB2 
 
  NCBI Gene  
  GeneRIF  
  Pubmed  
 
 
  Search Term  Rank  Fields  
  [Mm]igration  6  PubMed(3)  
  [Ii]nvasive  10  PubMed(2)  
  [Mm]etastasis  12  PubMed(4)  
  [Cc]ell [Ii]nvasion  13  PubMed(2)  
 
 
 hide/show details for RHOBTB2 
  [Mm]igration  PubMed  
 The atypical Rho GTPase RhoBTB2 is required for expression of the chemokine CXCL14 in normal and cancerous epithelial cells. The Rho family of small GTPases control cell   migration  , cell invasion and cell cycle. Many of these processes are perturbed in cancer and several family members show altered expression in a number of tumor types. RhoBTB2/DBC2 is an atypical member of this family of signaling proteins, containing two BTB domains in addition to its conserved Rho GTPase domain. RhoBTB2 is mutated, deleted or silenced in a large percentage of breast and lung cancers; however, the functional consequences of this loss are unclear. Here we use RNA interference in primary human epithelial cells to mimic the loss of RhoBTB2 seen in cancer cells. Through microarray analysis of global gene expression, we show that loss of RhoBTB2 results in downregulation of CXCL14-a chemokine that controls leukocyte   migration   and angiogenesis, and whose expression is lost through unknown mechanisms in a wide range of epithelial cancers. Loss of RhoBTB2 expression correlates with loss of CXCL14 secretion by head and neck squamous cell carcinoma cell lines, whereas reintroduction of RhoBTB2 restores CXCL14 secretion. Our studies identify CXCL14 as a gene target of RhoBTB2 and support downregulation of CXCL14 as a functional outcome of RhoBTB2 loss in cancer. 
 RhoBTB2 (DBC2) functions as tumor suppressor via inhibiting proliferation, preventing colony formation and inducing apoptosis in breast cancer cells. RhoBTB2 was isolated recently as a tumor suppressor gene from sporadic breast cancer. Although RhoBTB2 was found to be frequently lost in breast cancer and a variety of cancers, its antitumor effect, however, remains unclear. In this study, we constructed a recombinant expression vector pEGFP-N1-RhoBTB2 and transfected it into RhoBTB2-negative breast tumor cell line T-47D. Stable transformanted cells were identified by fluorescence microscope, RT-PCR and Western blot. Cell viability was measured by MTT assay. Colony forming efficiency of breast tumor cells was detected by colony formation assay. Morphological change of apoptotic cells was observed by hematoxylin-eosin staining. Apoptotic ratio was determined by flow cytometry. Cell invasion and   migration   ability assay were performed using transwell system. Overexpression of RhoBTB2 in breast tumor cells significantly inhibited the proliferation and colony formation of tumor cells. In addition, RhoBTB2 also elevated the apoptotic ratio and caused typical changes of apoptotic morphology in breast tumor cells of RhoBTB2 overexpression. But RhoBTB2 did not influence the invasion and   migration   ability of breast tumor cells. Therefore, RhoBTB2 is an important tumor suppressor gene related with breast cancer and may play antitumor roles by inhibiting proliferation, preventing colony formation and promoting the apoptosis of tumor cells. However, the precise mechanism behind the antitumor effects of RhoBTB2 needs to be investigated further. 
 Ectopic expression of RhoBTB2 inhibits   migration   and invasion of human breast cancer cells. RhoBTB2, or Deleted in Breast Cancer 2 (DBC2), identified as a candidate tumor suppressor gene for breast cancer and other human malignancies, is an atypical member of a novel gene family encoding small GTPases. In this study, we found that ectopic expression of RhoBTB2 inhibits the   migration   and invasion of the human metastatic breast cancer cell lines MDA-MB-231 and MDA-MB-435 in a dose-dependent manner. Western blotting analysis revealed that ectopic expression of RhoBTB2 induces a significant increase in the breast cancer metastasis suppressor, BRMS1. siRNA suppression of BRMS1 expression markedly reversed the inhibitory effects of RhoBTB2 on the   migration   and invasion abilities of both cell lines. Ezrin is a member of the ezrin-radixin-moesin cytoskeleton-associated protein family and is a key signaling molecule that regulates cancer   migration   and invasion. Western blotting analysis demonstrated that ectopic expression of RhoBTB2 results in decreased phosphorylation of ezrin and Akt2 in both MDA-MB-231 and MDA-MB-435 cells. Therefore, we conclude that up-regulation of the breast cancer metastasis suppressor BRMS1 and down-regulation of the phosphorylation of the cancer metastasis-related gene, ezrin, contributed to RhoBTB2-induced inhibition of metastatic breast carcinoma cell   migration   and invasion. Our findings suggest that understanding RhoBTB2-mediated   migration   and suppression of invasion is critical to the development of new therapies designed to prevent and treat patients with breast cancer metastasis. 
  [Ii]nvasive  PubMed  
 [Expressions of Fas, CTLA-4 and RhoBTB2 genes in breast carcinoma and their relationship with clinicopathological factors]. The purpose of this study was to explore the clinical significance of expression of Fas, CTLA-4 and RhoBTB2 genes in breast carcinoma.Semi-quantitative reverse transcriptive polymerase chain reaction (RT-PCR) was used to analyze the mRNA expression levels of the three genes in tumor tissues from 60 patients with primary breast cancer and normal breast tissues of 30 cases. The relationship between gene expression and clinicopathological factors were analyzed and determined.The relative expression levels (gray scale ratio between target gene and internal reference gene) of Fas, CTLA-4 and RhoBTB2 genes in breast carcinoma tissues were 0.699 +/- 0.285, 1.045 +/- 0.302 and 0.625 +/- 0.160, respectively. In the normal breast tissues, they were 0.502 +/- 0.178, 0.418 +/- 0.140 and 0.843 +/- 0.218, respectively. There were statistically significant differences of the expression of those three genes between carcinoma tissues and normal breast tissues (P < 0.01). The expression level of Fas in carcinoma tissues was significantly higher in lymph node matastasis positive patients (0.782 +/- 0.313) than that in node-negative patients (0.557 +/- 0.146, P < 0.01). The expression level of CTLA-4 gene in carcinoma tissues was lower in II stage patients (0.978 +/- 0.330) than that in III stage patients (1.134 +/- 0.240, P < 0.05). The expression level of RhoBTB2 gene was lower in   invasive   ductal carcinoma (0.597 +/- 0.157) than that in   invasive   lobular carcinoma (0.717 +/- 0.145, P < 0.05). There were no correlations of expression of the three genes at mRNA level and age, ER, PR, HER2 status and survival time. Furthermore, no correlation was seen among the three genes expression (P > 0.05).The expression of all the three genes at mRNA level is involved in genesis and progression of breast cancer. There exist correlations between Fas expression and axillary lymph node matastasis, CTLA-4 expression and disease stage, and RhoBTB2 expression and pathological type of breast cancer. 
 Loss of DBC2 expression is an early and progressive event in the development of lung adenocarcinoma. DBC2 (Deleted in Breast Cancer 2) has been indicated to be a tumor suppressor gene in many cancers including lung adenocarcinoma recently. In this study, we aimed to explore the expression status of DBC2 in different subtypes of lung adenocarcinoma (from pre-  invasive   to   invasive   lesions), and to determine if downregulation becomes more marked with pathological progression.We collected 172 tissue samples from different subtypes of lung adenocarcinoma and investigated the frequency of DBC2 loss by immunohistochemistry.Our results indicated that DBC2 downregulation is a relatively frequent event in lung adenocarcinoma. Moreover, as the adenocarcinoma subtype turns to be more   invasive  , more downregulation occurred.We conclude that loss of DBC2 expression is an early and progressive event in the pathogenesis of lung adenocarcinoma. Positive DBC2 immunohistochemistry may become an indicator for early stage disease and better prognosis of lung adenocarcinomas. 
  [Mm]etastasis  PubMed  
 The mutation of DBC2 in breast cancer patients from the Han ethnic group in Eastern China. To investigate DBC2 mutations in breast cancer patients and evaluate the relationship between gene mutations and breast cancer susceptibility in an Eastern China population.Mutation analyses of 285bp promoter sequence, coding exon 7 and its exon/intron boundaries of DBC2 were performed in 32 breast cancer specimens by polymerase chain reaction and direct sequencing. Eighteen benign breast tumor specimens were also analyzed as control.No mutation in the promoter or exon 7 was found in either group. An intronic alteration (IVS7+53C>G) was detected in 13 specimens. There was no significant difference in the rate of IVS7+53C>G alteration between the study and control groups (8/32 vs 5/18, respectively, P>0.05). The G allele of IVS7+53 was correlated with HER2 and p53 expression (P<0.05), but not with age, tumor size, lymph node   metastasis  , ER or PR expression (P>0.05).Mutation in the promoter and exon 7 of DBC2 gene is not common in the Chinese population and may not contribute to the susceptibility for breast cancer in China. The intronic alteration IVS7+53C>G is a common polymorphism in the Chinese Han ethnic group. Further research is warranted to evaluate the relationship between IVS7+53C>G and the susceptibility for breast cancer. 
 Decreased expression of the DBC2 gene and its clinicopathological significance in breast cancer: correlation with aberrant DNA methylation. Loss of DBC2 (deleted in breast cancer 2) gene expression is frequent in breast cancer tissues. This can be explained by homozygous deletions or other mutations in a minority of cases but alternative mechanisms need to be investigated. Here, DBC2 expression was significantly suppressed compared with normal breast tissues in breast cancer tissues when analyzed by RT-PCR. Furthermore, DNA methylation on DBC2 was more prevalent in breast tumors than in normal tissues. DBC2 mRNA levels correlated with the degree of DBC2 methylation in breast cancer tissues and in a breast cancer cell line (T47D). Clinico-pathological correlation analysis showed that DBC2 promoter methylation was associated with tumor-node-  metastasis   stages II and III/IV, lymph node   metastasis  , p53 mutation, and HER2-positive status. Thus loss of DBC2 expression is caused by abnormal methylation of DBC2 and might have a role in breast cancer development. 
 Ectopic expression of RhoBTB2 inhibits migration and invasion of human breast cancer cells. RhoBTB2, or Deleted in Breast Cancer 2 (DBC2), identified as a candidate tumor suppressor gene for breast cancer and other human malignancies, is an atypical member of a novel gene family encoding small GTPases. In this study, we found that ectopic expression of RhoBTB2 inhibits the migration and invasion of the human metastatic breast cancer cell lines MDA-MB-231 and MDA-MB-435 in a dose-dependent manner. Western blotting analysis revealed that ectopic expression of RhoBTB2 induces a significant increase in the breast cancer   metastasis   suppressor, BRMS1. siRNA suppression of BRMS1 expression markedly reversed the inhibitory effects of RhoBTB2 on the migration and invasion abilities of both cell lines. Ezrin is a member of the ezrin-radixin-moesin cytoskeleton-associated protein family and is a key signaling molecule that regulates cancer migration and invasion. Western blotting analysis demonstrated that ectopic expression of RhoBTB2 results in decreased phosphorylation of ezrin and Akt2 in both MDA-MB-231 and MDA-MB-435 cells. Therefore, we conclude that up-regulation of the breast cancer   metastasis   suppressor BRMS1 and down-regulation of the phosphorylation of the cancer   metastasis  -related gene, ezrin, contributed to RhoBTB2-induced inhibition of metastatic breast carcinoma cell migration and invasion. Our findings suggest that understanding RhoBTB2-mediated migration and suppression of invasion is critical to the development of new therapies designed to prevent and treat patients with breast cancer   metastasis  . 
 Genetic analysis of the DBC2 gene in gastric cancer. The DBC2 (Deleted in breast cancer, RhoBTB2) has been identified as a tumor suppressor gene that has growth inhibitory function. To investigate whether genetic alterations of the DBC2 gene are involved in the development of gastric cancer, we analyzed mutations and allelic loss in the DBC2 gene in 95 primary gastric cancers by PCR-SSCP, sequencing and LOH analysis. In the mutational analysis, we found one missense somatic mutation (CGG-->TGG, R275W) in the BTB/POZ domain of the gene in a patient with advanced gastric cancer and lymph node   metastasis  . In addition, we found one known polymorphism and three novel polymorphisms in the coding region of DBC2, which showed an amino acid change, and was detected in both the cancer cells and corresponding normal cells. On LOH analysis, 62 cases were heterozygous for at least one marker and 18 cases (29.0%) showed allelic loss at these markers. In conclusion, the mutations and allelic loss in the DBC2 gene are uncommon in gastric cancers in Korean patients. Further studies to identify the target gene at 8q21 responsible for the development of gastric cancer should be explored. 
  [Cc]ell [Ii]nvasion  PubMed  
 The atypical Rho GTPase RhoBTB2 is required for expression of the chemokine CXCL14 in normal and cancerous epithelial cells. The Rho family of small GTPases control cell migration,   cell invasion   and cell cycle. Many of these processes are perturbed in cancer and several family members show altered expression in a number of tumor types. RhoBTB2/DBC2 is an atypical member of this family of signaling proteins, containing two BTB domains in addition to its conserved Rho GTPase domain. RhoBTB2 is mutated, deleted or silenced in a large percentage of breast and lung cancers; however, the functional consequences of this loss are unclear. Here we use RNA interference in primary human epithelial cells to mimic the loss of RhoBTB2 seen in cancer cells. Through microarray analysis of global gene expression, we show that loss of RhoBTB2 results in downregulation of CXCL14-a chemokine that controls leukocyte migration and angiogenesis, and whose expression is lost through unknown mechanisms in a wide range of epithelial cancers. Loss of RhoBTB2 expression correlates with loss of CXCL14 secretion by head and neck squamous cell carcinoma cell lines, whereas reintroduction of RhoBTB2 restores CXCL14 secretion. Our studies identify CXCL14 as a gene target of RhoBTB2 and support downregulation of CXCL14 as a functional outcome of RhoBTB2 loss in cancer. 
 RhoBTB2 (DBC2) functions as tumor suppressor via inhibiting proliferation, preventing colony formation and inducing apoptosis in breast cancer cells. RhoBTB2 was isolated recently as a tumor suppressor gene from sporadic breast cancer. Although RhoBTB2 was found to be frequently lost in breast cancer and a variety of cancers, its antitumor effect, however, remains unclear. In this study, we constructed a recombinant expression vector pEGFP-N1-RhoBTB2 and transfected it into RhoBTB2-negative breast tumor cell line T-47D. Stable transformanted cells were identified by fluorescence microscope, RT-PCR and Western blot. Cell viability was measured by MTT assay. Colony forming efficiency of breast tumor cells was detected by colony formation assay. Morphological change of apoptotic cells was observed by hematoxylin-eosin staining. Apoptotic ratio was determined by flow cytometry.   Cell invasion   and migration ability assay were performed using transwell system. Overexpression of RhoBTB2 in breast tumor cells significantly inhibited the proliferation and colony formation of tumor cells. In addition, RhoBTB2 also elevated the apoptotic ratio and caused typical changes of apoptotic morphology in breast tumor cells of RhoBTB2 overexpression. But RhoBTB2 did not influence the invasion and migration ability of breast tumor cells. Therefore, RhoBTB2 is an important tumor suppressor gene related with breast cancer and may play antitumor roles by inhibiting proliferation, preventing colony formation and promoting the apoptosis of tumor cells. However, the precise mechanism behind the antitumor effects of RhoBTB2 needs to be investigated further. 
   
  
 PLP2 
 
  NCBI Gene  
  GeneRIF  
  Pubmed  
 
 
  Search Term  Rank  Fields  
  [Mm]igration  6  PubMed(2)  
 
 
 hide/show details for PLP2 
  [Mm]igration  PubMed  
 miR-664 negatively regulates PLP2 and promotes cell proliferation and invasion in T-cell acute lymphoblastic leukaemia. MicroRNAs (miRNAs) play important roles in the pathogenesis of many types of cancers by negatively regulating gene expression at posttranscriptional level. However, the role of microRNAs in leukaemia, particularly T-cell acute lymphoblastic leukaemia (T-ALL), has remained elusive. Here, we identified miR-664 and its predicted target gene PLP2 were differentially expressed in T-ALL using bioinformatics methods. In T-ALL cell lines, CCK-8 proliferation assay indicated that the cell proliferation was promoted by miR-664, while miR-664 inhibitor could significantly inhibited the proliferation. Moreover,   migration   and invasion assay showed that overexpression of miR-664 could significantly promoted the   migration   and invasion of T-ALL cells, whereas miR-664 inhibitor could reduce cell   migration   and invasion. luciferase assays confirmed that miR-664 directly bound to the 3'untranslated region of PLP2, and western blotting showed that miR-664 suppressed the expression of PLP2 at the protein levels. This study indicated that miR-664 negatively regulates PLP2 and promotes proliferation and invasion of T-ALL cell lines. Thus, miR-664 may represent a potential therapeutic target for T-ALL intervention. 
 PLP2/A4 interacts with CCR1 and stimulates   migration   of CCR1-expressing HOS cells. Multiple CC chemokines bind to CCR1, which plays important roles in immune and inflammatory responses. To search for proteins involved in the CCR1 signaling pathway, we screened a yeast two-hybrid library using the cytoplasmic tail of CCR1 as the bait. One of the positive clones contained an open reading frame of 456bp, of which the nucleotide sequence was identical to that of proteolipid protein 2 (PLP2), also known as protein A4. Mammalian two-hybrid and coimmunoprecipitation analyses demonstrated the association of PLP2/A4 with CCR1. Indirect immunofluorescence analysis revealed that PLP2/A4 was predominantly located in plasma membrane and colocalized with CCR1 in transfected human HEK293 cells. In addition, focal staining of CCR1 appeared on the periphery of the membrane upon short exposure to Leukotactin-1(Lkn-1)/CCL15, a CCR1 agonist, and was costained with PLP2/A4 on the focal regions. PLP2/A4 mRNAs were detected in various cells such as U-937, HL-60, HEK293, and HOS cells. Overexpression of PLP2/A4 stimulated a twofold increase in the agonist-induced   migration   of HOS/CCR1 cells, implicating a functional role for PLP2/A4 in the chemotactic processes via CCR1. 
   
  
 CMTM1 
 
  NCBI Gene  
  GeneRIF  
  Pubmed  
 
 
  Search Term  Rank  Fields  
  [Cc]ell [Ii]nvasion  13  PubMed(1)  
 
 
 hide/show details for CMTM1 
  [Cc]ell [Ii]nvasion  PubMed  
 Systematic investigation of CMTM family genes suggests relevance to glioblastoma pathogenesis and CMTM1 and CMTM3 as priority targets. The novel CKLF-like Marvel Transmembrane Domain-containing gene family (CMTM) consists of 8 members (CMTM1-8). As little is known about the oncogenic impact of these genes, we aimed to systematically investigate the relevance of CMTMs to glioblastoma pathogenesis. We performed mRNA expression analyses and survival correlations in glioblastoma patients. Moreover, we analyzed the impact of RNAi-based silencing and overexpression of CMTM family genes on tumor cell proliferation and invasion in vitro. CMTMs appeared to be widely regulated in the group of glioblastomas relative to non-neoplastic brain (NB) tissue (significant upregulation for CMTM2, 3, and 6 and significant downregulation for CMTM 4 and 8). For CMTM1, 5 and 7, we found aberrant expression levels in individual tumors. Functionally, CMTM1, 3, and 7 promoted tumor   cell invasion  , while CMTM1 additionally enhanced cell proliferation. In a large clinically annotated dataset, higher CMTM1 and 3 expression was significantly correlated with shorter overall survival. Our data thus suggest CMTM1 and 3 as priority targets in glioblastomas. Using a human phosphokinase protein expression profiling assay, we can provide first insights into signalling of these two genes that might be conveyed by growth factor receptor, Src family kinase and WNT activation. 
   
  
 CMTM3 
 
  NCBI Gene  
  GeneRIF  
  Pubmed  
 
 
  Search Term  Rank  Fields  
  [Mm]igration  6  PubMed(4)  
  [Mm]etastasis  12  PubMed(2); GeneRIFs(1)  
  [Cc]ell [Ii]nvasion  13  PubMed(2)  
 
 
 hide/show details for CMTM3 
  [Mm]igration  PubMed  
 CMTM3 is frequently reduced in clear cell renal cell carcinoma and exhibits tumor suppressor activities. CKLF-like MARVEL transmembrane domain containing member 3 (CMTM3) is silenced in many kinds of cancers and inhibits tumor cells growth. We investigated the expression and role of CMTM3 in clear cell renal cell carcinoma (ccRCC).The expression of CMTM3 was detected in ccRCC tissue microarray, specimens, and cell lines by immunohistochemistry, quantitative real-time polymerase chain reaction (qRT-PCR) and western blot, respectively. After transfected with CMTM3 plasmid or vector, the proliferation and   migration   of ccRCC 786-0 cells were determined by MTT assay and transwell assay, respectively. Furthermore, the anchorage-independent growth of transfected cells was assessed using soft agar colony formation assay.CMTM3 was down-regulated in 84 % (63/75) of ccRCC tissues and its expression had no correlation with the gender, age, clinical staging and histologic grade. CMTM3 protein was undetectable by western blot in most detected ccRCC specimens and two RCC cell lines (786-0 and ACHN). qRT-PCR analysis showed that CMTM3 mRNA was dramatically down-regulated in 40 ccRCC cancer tissues as compared with the paired adjacent normal ones. Restoration of CMTM3 significantly suppressed the anchorage-independent growth, proliferation and   migration   of 786-0 cells.These results indicate that CMTM3 is significantly down-regulated in ccRCC and exerts remarkable tumor-suppressive functions in 786-0 cells. Reduction of CMTM3 expression may contribute to the pathogenesis of ccRCC and CMTM3 may be a potentially target for therapeutic strategy. 
 CMTM3 inhibits cell   migration   and invasion and correlates with favorable prognosis in gastric cancer. The CKLF-like MARVEL transmembrane domain containing 3 (CMTM3) gene is a novel tumor suppressor with frequent epigenetic inactivation. In this study, we showed the role played by CMTM3 in gastric cancer cells as a tumor suppressor gene, and examined the correlation between CMTM3 expression and clinicopathological parameters using immunohistochemistry in gastric cancer patients with different pathological stages (n = 350). We found that CMTM3 expression was reduced or silenced by epigenetic regulation in gastric cell lines, and dramatically downregulated in primary gastric cancer tissues. Restoration of CMTM3 significantly affected   migration   and invasion of AGS and SGC-7901 cells (P < 0.001). In vivo experiments showed that peritoneal disseminated metastases were significantly suppressed by CMTM3 (P < 0.001). We further showed that the expression of MMP2 and the phosphorylation of Erk1/2 were decreased when CMTM3 was restored. In addition, by immunohistochemical staining, we found that the expression of CMTM3 was remarkably weaker in gastric cancer tissues than in normal mucosae (P = 0.008), and was significantly correlated with gender (P = 0.033), tumor depth (P = 0.049), stage (P = 0.021), and histological grade (P = 0.022). More importantly, CMTM3 expression was associated with prognosis in gastric cancer patients (P = 0.041), and was a significant independent prognostic indicator (hazard ratio = 0.704, 95% confidence interval, 0.498-0.994; P = 0.046). Our findings indicate that CMTM3 regulates   migration   and invasion of gastric cancer cells. Moreover, CMTM3 is a candidate marker for prognosis of gastric cancer in the clinic. 
 CMTM3 inhibits cell growth and   migration   and predicts favorable survival in oral squamous cell carcinoma. Downregulation of CKLF-like MARVEL transmembrane domain-containing member 3 (CMTM3) has been reported in a number of human tumors. However, the role of CMTM3 in oral squamous cell carcinoma (OSCC) remains largely unknown. In this study, we showed that the expression of CMTM3 was significantly reduced in OSCC cell lines and primary tumor specimens (P < 0.001). Methylation-specific PCR showed hypermethylation in CMTM3 promoter in a significant proportion of tumor tissues (61 %). The expression of CMTM3 was associated with T stage, lymph node metastasis, tumor node metastasis (TNM) stage, and recurrence of OSCC patients (P < 0.05, n = 201). More importantly, CMTM3 expression was associated with the prognosis of OSCC patients (P < 0.001) and was an independent prognostic factor (hazard ratio = 0.593, 95 % confidence interval, 0.272-1.292; P = 0.039). Overexpression of CMTM3 i  nhibited   the growth and migration of OSCC cells. In vivo experiments also showed that the growth of OSCC xenografts in nude mice was significantly inhibited by CMTM3 overexpression. These findings indicate that downregulation of CMTM3 due to promoter hypermethylation contributed t  o the pro  liferation and migration of OSCC cells and suggest that CMTM3 is an independent prognostic factor for the evaluation of the survival of OSCC patients. 
 CMTM3 is reduced in prostate cancer and inhibits   migration  , invasion and growth of LNCaP cells. A novel tumor suppressor gene CKLF-like MARVEL transmembrane domain-containing member 3 (CMTM3) is reduced or undetectable in many kinds of cancers and relates tumor malignant features. We detected its role in prostate cancer for possibility of target therapy as accumulating evidence has shown that CMTM3 is a promising tumor suppressor gene (TSG) for gene therapy.The expression of CMTM3 detected in prostate tissue microarray, specimens and cell lines were evaluated by immunohistochemistry and semi-quantitative PCR and Western blot, respectively. After being transfected with CMTM3 adenovirus or vector (mock), the proliferation and   migration   and invasion of LNCaP cells were detected by transwell assay and matrigel assay, respectively. Furthermore, the effects of CMTM3 on tumor growth were performed in nude mice xenograft in vivo.We found CMTM3 was reduced in PCa tissues and cells compared with BPH tissues, and its expression in PCa tissues was related to the Gleason score. Moreover, after being transfected with adenovirus, ectopic expression of CMTM3 in LNCaP cells led to significant inhibition of cell proliferation and   migration   and invasion compared with the control (P < 0.05), which may be attributed to decreased Erk1/2 activity as p-Erk1/2 was remarkably reduced when CMTM3 was overexpressed. Finally, restoration of CMTM3 significantly suppressed xenograft tumor growth in vivo (P < 0.01). 
  [Mm]etastasis  PubMed  
 CMTM3 inhibits cell growth and migration and predicts favorable survival in oral squamous cell carcinoma. Downregulation of CKLF-like MARVEL transmembrane domain-containing member 3 (CMTM3) has been reported in a number of human tumors. However, the role of CMTM3 in oral squamous cell carcinoma (OSCC) remains largely unknown. In this study, we showed that the expression of CMTM3 was significantly reduced in OSCC cell lines and primary tumor specimens (P < 0.001). Methylation-specific PCR showed hypermethylation in CMTM3 promoter in a significant proportion of tumor tissues (61 %). The expression of CMTM3 was associated with T stage, lymph n  ode metast  asis, tumor n  ode metast  asis (TNM) stage, and recurrence of OSCC patients (P < 0.05, n = 201). More importantly, CMTM3 expression was associated with the prognosis of OSCC patients (P < 0.001) and was an independent prognostic factor (hazard ratio = 0.593, 95 % confidence interval, 0.272-1.292; P = 0.039). Overexpression of CMTM3 inhibited the growth and migration of OSCC cells. In vivo experiments also showed that the growth of OSCC xenografts in nude mice was significantly inhibited by CMTM3 overexpression. These findings indicate that downregulation of CMTM3 due to promoter hypermethylation contributed to the proliferation and migration of OSCC cells and suggest that CMTM3 is an independent prognostic factor for the evaluation of the survival of OSCC patients. 
 CKLF-Like MARVEL Transmembrane Domain-Containing Member 3 (CMTM3) Inhibits the Proliferation and Tumorigenisis in Hepatocellular Carcinoma Cells. The CKLF-like MARVEL transmembrane domain-containing 3 (CMTM3), a member of the CMTM family, was found in several human tumors and plays an important role in the development and progression of tumors. However, the role of CMTM3 in hepatocellular carcinoma (HCC) remains largely unknown. Thus, in the present study, we explored its expression pattern in human HCC cell lines, as well as its functions in HCC cells. Our results demonstrated that the expression of CMTM3 is lowly expressed in HCC cell lines. In vitro, we found that overexpression of CMTM3 obviously inhibited the proliferation, invasion, and EMT process in HCC cells. Furthermore, overexpression of CMTM3 significantly downregulated the expression levels of phosphorylation of JAK2 and STAT3 in HepG2 cells. In vivo, overexpression of CMTM3 attenuated the tumor growth in Balb/c nude mice. In conclusion, we demonstrated that CMTM3 could play an important role in HCC   metastasis   by EMT induction via, at least partially, suppressing the JAK2/STAT3 signaling pathway. Therefore, CMTM3 may serve as a potential molecular target in the prevention and/or treatment of HCC invasion and   metastasis  . 
  GeneRIFs  
 Low expression of CMTM3 was associated with   metastasis   and recurrence of oral squamous cell carcinoma. 
  [Cc]ell [Ii]nvasion  PubMed  
 Systematic investigation of CMTM family genes suggests relevance to glioblastoma pathogenesis and CMTM1 and CMTM3 as priority targets. The novel CKLF-like Marvel Transmembrane Domain-containing gene family (CMTM) consists of 8 members (CMTM1-8). As little is known about the oncogenic impact of these genes, we aimed to systematically investigate the relevance of CMTMs to glioblastoma pathogenesis. We performed mRNA expression analyses and survival correlations in glioblastoma patients. Moreover, we analyzed the impact of RNAi-based silencing and overexpression of CMTM family genes on tumor cell proliferation and invasion in vitro. CMTMs appeared to be widely regulated in the group of glioblastomas relative to non-neoplastic brain (NB) tissue (significant upregulation for CMTM2, 3, and 6 and significant downregulation for CMTM 4 and 8). For CMTM1, 5 and 7, we found aberrant expression levels in individual tumors. Functionally, CMTM1, 3, and 7 promoted tumor   cell invasion  , while CMTM1 additionally enhanced cell proliferation. In a large clinically annotated dataset, higher CMTM1 and 3 expression was significantly correlated with shorter overall survival. Our data thus suggest CMTM1 and 3 as priority targets in glioblastomas. Using a human phosphokinase protein expression profiling assay, we can provide first insights into signalling of these two genes that might be conveyed by growth factor receptor, Src family kinase and WNT activation. 
 Systematic investigation of CMTM family genes suggests relevance to glioblastoma pathogenesis and CMTM1 and CMTM3 as priority targets. The novel CKLF-like Marvel Transmembrane Domain-containing gene family (CMTM) consists of 8 members (CMTM1-8). As little is known about the oncogenic impact of these genes, we aimed to systematically investigate the relevance of CMTMs to glioblastoma pathogenesis. We performed mRNA expression analyses and survival correlations in glioblastoma patients. Moreover, we analyzed the impact of RNAi-based silencing and overexpression of CMTM family genes on tumor cell proliferation and invasion in vitro. CMTMs appeared to be widely regulated in the group of glioblastomas relative to non-neoplastic brain (NB) tissue (significant upregulation for CMTM2, 3, and 6 and significant downregulation for CMTM 4 and 8). For CMTM1, 5 and 7, we found aberrant expression levels in individual tumors. Functionally, CMTM1, 3, and 7 promoted tumor   cell invasion  , while CMTM1 additionally enhanced cell proliferation. In a large clinically annotated dataset, higher CMTM1 and 3 expression was significantly correlated with shorter overall survival. Our data thus suggest CMTM1 and 3 as priority targets in glioblastomas. Using a human phosphokinase protein expression profiling assay, we can provide first insights into signalling of these two genes that might be conveyed by growth factor receptor, Src family kinase and WNT activation. 
   
  
 CMTM4 
 
  NCBI Gene  
  GeneRIF  
  Pubmed  
 
 
  Search Term  Rank  Fields  
  [Mm]igration  6  PubMed(1)  
 
 
 hide/show details for CMTM4 
  [Mm]igration  PubMed  
 CMTM4 is frequently downregulated and functions as a tumour suppressor in clear cell renal cell carcinoma. Chemokine-like factor (CKLF)-like MARVEL transmembrane domain-containing family (CMTM) is a gene family involved in multiple malignancies. CMTM4 is a member of this family and is located at chromosome 16q22.1, a locus that harbours a number of tumour suppressor genes. It has been defined as a regulator of cell cycle and division in HeLa cells; however, its roles in tumourigenesis remain poorly studied.An integrated bioinformatics analysis based on the array data from the GEO database was conducted to view the differential expression of CMTM4 across multiple cancers and their corresponding control tissues. Primary clear cell renal cell carcinoma (ccRCC) and the paired adjacent non-tumour tissues were then collected to examine the expression of CMTM4 by western blotting, immunohistochemistry, and quantitative RT-PCR. The ccRCC cell lines A498 and 786-O and the normal renal tubular epithelial cell line HK-2 were also tested for CMTM4 expression by western blotting. Cell Counting Kit-8 (CCK-8) and viable cell counting assays were used to delineate the growth curves of 786-O cells after CMTM4 overexpression or knockdown. Wound healing and transwell assays were performed to assess the cells' ability to migrate. The effects of CMTM4 on cellular apoptosis and cell cycle progression were analysed by flow cytometry, and cell cycle hallmarks were detected by western blotting and RT-PCR. The xenograft model in nude mice was used to elucidate the function of CMTM4 in tumourigenesis ex vivo.By omic data analysis, we found a substantial downregulation of CMTM4 in ccRCC. Western blotting then confirmed that CMTM4 was dramatically reduced in 86.9 % (53/61) of ccRCC tissues compared with the paired adjacent non-tumour tissues, as well as in the 786-O and A498 ccRCC cell lines. Restoration of CMTM4 significantly suppressed 786-O cell growth by inducing G2/M cell cycle arrest and p21 upregulation, and cell   migration   was also inhibited. However, knockdown of CMTM4 led to a completely opposite effect on these cell behaviours. Overexpression of CMTM4 also markedly inhibited the tumour xenograft growth in nude mice.CMTM4 is downregulated and exhibits tumour-suppressor activities in ccRCC, and could be exploited as a target for ccRCC treatment. 
   
  
 CMTM5 
 
  NCBI Gene  
  GeneRIF  
  Pubmed  
 
 
  Search Term  Rank  Fields  
  [Mm]igration  6  PubMed(3); GeneRIFs(1)  
  [Mm]etastasis  12  PubMed(1)  
 
 
 hide/show details for CMTM5 
  [Mm]igration  PubMed  
 CMTM5 is reduced in prostate cancer and inhibits cancer cell growth in vitro and in vivo. A novel tumor suppressor CKLF-like MARVEL transmembrane domain-containing member 5 (CMTM5) is reduced or undetectable in many kinds of cancers and inhibits tumor cells' malignant features. To explore its role in prostate cancer (PCa), we detected its expression patterns in prostate tissues and PCa cells, and determined its anti-proliferation functions in PCa cells in vitro and in vivo.The expression of CMTM5 in prostate tissue microarray, specimens and cell lines was evaluated by immunohistochemistry and Western blot, respectively. After being transfected with CMTM5 adenovirus or vector, the proliferation and   migration   of DU145 cells were detected by MTT assay and transwell assay, respectively. Furthermore, the effects of CMTM5 on tumor growth were performed in nude mice xenograft in vivo.We found CMTM5 was reduced in PCa tissues and cells compared with BPH tissues, and its expression in PCa tissues was related to the Gleason score. Moreover, after being transfected with adenovirus, ectopic expression of CMTM5-v1 in DU145 cells led to significant inhibition of cell proliferation and   migration   compared with the control, which may be attributed to decreased Akt activity. Finally, restoration of CMTM5 significantly suppressed tumor growth in vivo.These results indicate that CMTM5 is down-regulated in PCa and exhibit tumor suppressor activities in androgen-independent PCa cells. Loss of CMTM5 protein may be contributed to the development of PCa and it is a potential therapeutic target for castration-resistant prostate cancer. 
 CMTM5 exhibits tumor suppressor activity through promoter methylation in oral squamous cell carcinoma. Oral squamous cell carcinoma (OSCC) is one of the most common types of malignancies in the head and neck region. CKLF-like MARVEL transmembrane domain-containing member 5 (CMTM5) has been recently implicated as a tumor suppressor gene in several cancer types. Herein, we examined the expression and function of CMTM5 in oral squamous cell carcinoma. CMTM5 was down-regulated in oral squamous cell lines and tumor samples from patients with promoter methylation. Treatment with the demethylating agent 5-aza-2'-deoxycytidine restored CMTM5 expression. In the OSCC cell lines CAL27 and GNM, the ectopic expression of CMTM5-v1 strongly inhibited cell proliferation and   migration   and induced apoptosis. In addition, CMTM5-v1 inhibited tumor formation in vivo. Therefore, CMTM5 might act as a putative tumor suppressor gene through promoter methylation in oral squamous cell carcinoma. 
 CMTM5 exhibits tumor suppressor activities and is frequently silenced by methylation in carcinoma cell lines. CMTM5 (CKLF-like MARVEL transmembrane domain containing member 5) is located at 14q11.2, a locus associated with multiple cancers. It has six RNA splicing variants with CMTM5-v1 as the major one. We explored its expression pattern in normal tissues and tumor cell lines, as well as its functions in carcinoma cells.We evaluated CMTM5 expression by semiquantitative reverse transcription-PCR (RT-PCR) in normal tissues and carcinoma cell lines of cervical, breast, nasopharyngeal, lung, hepatocellular, esophageal, gastric, colon, and prostate. We further examined CMTM5 promoter methylation in these cell lines. We also analyzed CMTM5 expression after 5-aza-2'-deoxycytidine treatment and genetic demethylation and the functional consequences of restoring CMTM5 in HeLa and PC-3 cells.CMTM5-v1 is broadly expressed in human normal adult and fetal tissues, but undetectable or down-regulated in most carcinoma cell lines. Its promoter methylation was detected in virtually all the silenced or down-regulated cell lines. The silencing of CMTM5 could be reversed by pharmacologic demethylation or genetic double-knockout of DNMT1 and DNMT3B, indicating methylation-mediated mechanism. Restoration of CMTM5-v1 suppressed carcinoma cell proliferation,   migration  , and invasion.These results indicate that CMTM5 exhibits tumor suppressor activities, but with frequent epigenetic inactivation in carcinoma cell lines. 
  GeneRIFs  
 In the OSCC cell lines CAL27 and GNM, the ectopic expression of CMTM5-v1 strongly inhibited cell proliferation and   migration   and induced apoptosis. 
  [Mm]etastasis  PubMed  
 Reduced CMTM5 expression correlates with carcinogenesis in human epithelial ovarian cancer. Although human chemokinelike factor (CKLF)-like MAL and related proteins for vesicle trafficking transmembrane, domain-containing member 5 (CMTM5) has been proved to play an important role in carcinogenesis and apoptosis in several types of human tumors, the expression of CMTM5 in ovarian cancer remains unclear. We aimed to investigate the association between CMTM5 expression and the survival of patients with epithelial ovarian cancer.Normal surface ovarian epithelium tissues, ovarian cystadenoma tissues, ovarian cancer tissues, and 5 ovarian cancer cell lines were collected. The CMTM5 expressions were determined by reverse transcription polymerase chain reaction, Western blotting, and immunohistochemical staining. The survival information was analyzed by the Kaplan-Meier method.The CMTM5 expression was down-regulated in ovarian cancers. The expression of CMTM5 was absent in 30% (24 of 80) of ovarian cancers compared with 4.55% (1 of 22) of normal surface ovarian epithelium tissues and ovarian cystadenomas by immunohistochemistry. The results from the reverse transcription polymerase chain reaction were consistent with those from Western blotting. Furthermore, we found that although CMTM5 expression has no significant correlation with the age of the patients (P = 0.342), clinical stages (P = 0.155), pathologic types (P = 0.0605), or status of   metastasis   (P = 0.554), it was associated with the 3 groups of different differentiation levels (P = 0.0026) and an increase of CMTM5 loss of expression ratio in patients with preoperative CA125 level more than 500 mIU/mL compared to those with less than 500 mIU/mL (48.57% vs 16.67%, P = 0.0130). Statistical analysis by the Kaplan-Meier method showed that CMTM5 expression had no significant impact on the prognosis of patients with ovarian cancer (P = 0.24).The reduced expression of CMTM5 correlates significantly with poorly differentiated ovarian cancer and high preoperative CA125 level. CMTM5 may contribute to the pathogenesis of human epithelial ovarian cancer. 
   
  
 VAT1 
 
  NCBI Gene  
  GeneRIF  
  Pubmed  
 
 
  Search Term  Rank  Fields  
  [Mm]igration  6  PubMed(1); GeneRIFs(1)  
  [Ee]xtracellular [Mm]atrix  8  PubMed(1)  
  [Ii]nvasive  10  PubMed(1)  
 
 
 hide/show details for VAT1 
  [Mm]igration  PubMed  
 Vesicle amine transport protein-1 (VAT-1) is upregulated in glioblastomas and promotes   migration  . Diffuse invasion of single-glioma cells is the main obstacle to successful therapy of these tumours. After identifying vesicle amine transport protein-1 (VAT-1) as being upregulated in invasive human gliomas, we study its possible function in glioblastoma cell   migration  .Based on data obtained from previous oligonucleotide arrays, we investigated expression of VAT-1 in glioblastoma tissue and cell lines on mRNA levels using reverse transcriptase PCR. Furthermore, we examined the amount and localization of VAT-1 protein using immunoblotting and immunohistochemistry. Using small interfering RNA technology we repressed VAT-1 expression in human glioma cell lines and analysed their   migration   using wound healing and transwell   migration   assays.Increased VAT-1 mRNA and protein levels were found in glioblastoma tissues and cell lines compared with normal human brain. Small interfering RNA-mediated VAT-1 knockdown led to significantly reduced   migration   of human glioma cells.VAT-1 is overexpressed in glioblastomas and functionally involved in glioma cell   migration  , representing a new component involved in glioma invasion 
  GeneRIFs  
 VAT-1 is overexpressed in glioblastomas and functionally involved in glioma cell   migration  , representing a new component involved in glioma invasion 
  [Ee]xtracellular [Mm]atrix  PubMed  
 Proteomic analysis of human parotid gland exosomes by multidimensional protein identification technology (MudPIT). Human ductal saliva contributes over a thousand unique proteins to whole oral fluids. The mechanism by which most of these proteins are secreted by salivary glands remains to be determined. The present study used a mass spectrometry-based, shotgun proteomics approach to explore the possibility that a subset of the proteins found in saliva are derived from exosomes, membrane-bound vesicles of endosomal origin within multivesicular endosomes. Using MudPIT (multidimensional protein identification technology) mass spectrometry, we catalogued 491 proteins in the exosome fraction of human parotid saliva. Many of these proteins were previously observed in ductal saliva from parotid glands (265 proteins). Furthermore, 72 of the proteins in parotid exosomes overlap with those previously identified as urinary exosome proteins, proteins which are also frequently associated with exosomes from other tissues and cell types. Gene Ontology (GO) and KEGG pathway analyses found that cytosolic proteins comprise the largest category of proteins in parotid exosomes (43%), involved in such processes as phosphatidylinositol signaling system, calcium signaling pathway, inositol metabolism, protein export, and signal transduction, among others; whereas the integral plasma membrane proteins and associated/peripheral plasma membrane proteins (26%) were associated with   extracellular matrix  -receptor interaction, epithelial cell signaling, T-cell and B-cell receptor signaling, cytokine receptor interaction, and antigen processing and presentation, among other biological functions. In addition, these putative saliva exosomal proteins were linked to specific diseases (e.g., neurodegenerative disorders, prion disease, cancers, type I and II diabetes). Consequently, parotid glands secrete exosomes that reflect the metabolic and functional status of the gland and may also carry informative protein markers useful in the diagnosis and treatment of systemic diseases. 
  [Ii]nvasive  PubMed  
 Vesicle amine transport protein-1 (VAT-1) is upregulated in glioblastomas and promotes migration. Diffuse invasion of single-glioma cells is the main obstacle to successful therapy of these tumours. After identifying vesicle amine transport protein-1 (VAT-1) as being upregulated in   invasive   human gliomas, we study its possible function in glioblastoma cell migration.Based on data obtained from previous oligonucleotide arrays, we investigated expression of VAT-1 in glioblastoma tissue and cell lines on mRNA levels using reverse transcriptase PCR. Furthermore, we examined the amount and localization of VAT-1 protein using immunoblotting and immunohistochemistry. Using small interfering RNA technology we repressed VAT-1 expression in human glioma cell lines and analysed their migration using wound healing and transwell migration assays.Increased VAT-1 mRNA and protein levels were found in glioblastoma tissues and cell lines compared with normal human brain. Small interfering RNA-mediated VAT-1 knockdown led to significantly reduced migration of human glioma cells.VAT-1 is overexpressed in glioblastomas and functionally involved in glioma cell migration, representing a new component involved in glioma invasion 
   
  
 RTN4IP1 
 
  NCBI Gene  
  GeneRIF  
  Pubmed  
 
 
  Search Term  Rank  Fields  
  [Mm]igration  6  PubMed(1)  
 
 
 hide/show details for RTN4IP1 
  [Mm]igration  PubMed  
 RTN4IP1 is down-regulated in thyroid cancer and has tumor-suppressive function. Previously we identified RTN4IP1 to be differentially expressed in thyroid cancer by sex and the gene is located on chromosome 6q21, a chromosomal region frequently deleted or with loss of heterozygosity in a variety of human malignancies including thyroid cancer.Because the expression and function of this gene is unknown, we sought to characterize its expression in normal, hyperplastic, and benign and malignant thyroid tissue samples and to evaluate its function in cancer cells.RTN4IP1 expression was analyzed in normal and hyperplastic thyroid tissue and benign and malignant thyroid tissue samples. In 3 thyroid cancer cell lines (TPC1 from a papillary thyroid cancer, FTC133 from a follicular thyroid cancer, XTC1 from a Hürthle cell carcinoma), small interfering RNA knockdown of RTN4IP1 was used to determine its role in regulating the hallmarks of malignant cell phenotype (cellular proliferation,   migratio  n, apoptosis, invasion, tumor spheroid formation, anchorage independent growth).We found RTN4IP1 mRNA expression was significantly down-regulated in follicular and papillary thyroid cancer as compared with normal, hyperplastic, and benign thyroid neoplasms (P < .05). Moreover, RTN4IP1 mRNA expression was significantly lower in larger papillary thyroid cancers (P < .05). Small interfering RNA knockdown of RTN4IP1 expression increased cellular proliferation (2- to 4-fold) in all 3 of the cell lines tested and increased cellular invasion (1.5- to 3-fold) and   migratio  n (2- to 7.5-fold), colony formation (3- to 6-fold), and tumor spheroid formation (P < .05) in 2 of the 3 cell lines tested (FTC-133 and XTC1).This is the first study to characterize the expression and function of RTN4IP1 in cancer. Our results demonstrate RTN4IP1 is down-regulated in thyroid cancer and is associated with larger papillary thyroid cancer and that it regulates malignant cell phenotype. These findings, taken together, suggest that RTN4IP1 has a tumor-suppressive function and may regulate thyroid cancer progression. 
   
  
 TP53I3 
 
  NCBI Gene  
  GeneRIF  
  Pubmed  
 
 
  Search Term  Rank  Fields  
  [Mm]igration  6  PubMed(1)  
  [Ee]xtracellular [Mm]atrix  8  PubMed(1)  
  [Ii]nvasive  10  PubMed(4)  
  [Mm]etastasis  12  PubMed(1)  
 
 
 hide/show details for TP53I3 
  [Mm]igration  PubMed  
 Reactivation of p53 by a Cytoskeletal Sensor to Control the Balance Between DNA Damage and Tumor Dissemination. Abnormal cell   migration   and invasion underlie metastasis, and actomyosin contractility is a key regulator of tumor invasion. The links between cancer migratory behavior and DNA damage are poorly understood.Using 3D collagen systems to recapitulate melanoma extracellular matrix, we analyzed the relationship between the actomyosin cytoskeleton of migrating cells and DNA damage. We used multiple melanoma cell lines and microarray analysis to study changes in gene expression and in vivo intravital imaging (n = 7 mice per condition) to understand how DNA damage impacts invasive behavior. We used Protein Tissue Microarrays (n = 164 melanomas) and patient databases (n = 354 melanoma samples) to investigate the associations between markers of DNA damage and actomyosin cytoskeletal features. Data were analyzed with Student's and multiple t tests, Mann-Whitney's test, one-way analysis of variance, and Pearson correlation. All statistical tests were two-sided.Melanoma cells with low levels of Rho-ROCK-driven actomyosin are subjected to oxidative stress-dependent DNA damage and ATM-mediated p53 protein stabilization. This results in a specific transcriptional signature enriched in DNA damage/oxidative stress responsive genes, including Tumor Protein p53 Inducible Protein 3 (TP53I3 or PIG3). PIG3, which functions in DNA damage repair, uses an unexpected catalytic mechanism to suppress Rho-ROCK activity and impair tumor invasion in vivo. This regulation was suppressed by antioxidants. Furthermore, PIG3 levels decreased while ROCK1/2 levels increased in human metastatic melanomas (ROCK1 vs PIG3; r = -0.2261, P < .0001; ROCK2 vs PIG3: r = -0.1381, P = .0093).The results suggest using Rho-kinase inhibitors to reactivate the p53-PIG3 axis as a novel therapeutic strategy; we suggest that the use of antioxidants in melanoma should be very carefully evaluated. 
  [Ee]xtracellular [Mm]atrix  PubMed  
 Reactivation of p53 by a Cytoskeletal Sensor to Control the Balance Between DNA Damage and Tumor Dissemination. Abnormal cell migration and invasion underlie metastasis, and actomyosin contractility is a key regulator of tumor invasion. The links between cancer migratory behavior and DNA damage are poorly understood.Using 3D collagen systems to recapitulate melanoma   extracellular matrix  , we analyzed the relationship between the actomyosin cytoskeleton of migrating cells and DNA damage. We used multiple melanoma cell lines and microarray analysis to study changes in gene expression and in vivo intravital imaging (n = 7 mice per condition) to understand how DNA damage impacts invasive behavior. We used Protein Tissue Microarrays (n = 164 melanomas) and patient databases (n = 354 melanoma samples) to investigate the associations between markers of DNA damage and actomyosin cytoskeletal features. Data were analyzed with Student's and multiple t tests, Mann-Whitney's test, one-way analysis of variance, and Pearson correlation. All statistical tests were two-sided.Melanoma cells with low levels of Rho-ROCK-driven actomyosin are subjected to oxidative stress-dependent DNA damage and ATM-mediated p53 protein stabilization. This results in a specific transcriptional signature enriched in DNA damage/oxidative stress responsive genes, including Tumor Protein p53 Inducible Protein 3 (TP53I3 or PIG3). PIG3, which functions in DNA damage repair, uses an unexpected catalytic mechanism to suppress Rho-ROCK activity and impair tumor invasion in vivo. This regulation was suppressed by antioxidants. Furthermore, PIG3 levels decreased while ROCK1/2 levels increased in human metastatic melanomas (ROCK1 vs PIG3; r = -0.2261, P < .0001; ROCK2 vs PIG3: r = -0.1381, P = .0093).The results suggest using Rho-kinase inhibitors to reactivate the p53-PIG3 axis as a novel therapeutic strategy; we suggest that the use of antioxidants in melanoma should be very carefully evaluated. 
  [Ii]nvasive  PubMed  
 Genetic susceptibility to distinct bladder cancer subphenotypes. Clinical, pathologic, and molecular evidence indicate that bladder cancer is heterogeneous with pathologic/molecular features that define distinct subphenotypes with different prognoses. It is conceivable that specific patterns of genetic susceptibility are associated with particular subphenotypes.To examine evidence for the contribution of germline genetic variation to bladder cancer heterogeneity.The Spanish Bladder Cancer/EPICURO Study is a case-control study based in 18 hospitals located in five areas in Spain. Cases were patients with a newly diagnosed, histologically confirmed, urothelial cell carcinoma of the bladder from 1998 to 2001. Case diagnoses were reviewed and uniformly classified by pathologists following the World Health Organisation/International Society of Urological Pathology 1999 criteria. Controls were hospital-matched patients (n=1149).A total of 1526 candidate variants in 423 candidate genes were analysed. Three distinct subphenotypes were defined according to stage and grade: low-grade nonmuscle   invasive   (n=586), high-grade nonmuscle   invasive   (n=219), and muscle   invasive   (n=246). The association between each variant and subphenotype was assessed by polytomous risk models adjusting for potential confounders. Heterogeneity in genetic susceptibility among subphenotypes was also tested.Two established bladder cancer susceptibility genotypes, NAT2 slow-acetylation and GSTM1-null, exhibited similar associations among the subphenotypes, as did VEGF-rs25648, which was previously identified in our study. Other variants conferred risks for specific tumour subphenotypes such as PMS2-rs6463524 and CD4-rs3213427 (respective heterogeneity p values of 0.006 and 0.004), which were associated with muscle-  invasive   tumours (per-allele odds ratios [95% confidence interval] of 0.56 [0.41-0.77] and 0.71 [0.57-0.88], respectively) but not with non-muscle-  invasive   tumours. Heterogeneity p values were not robust in multiple testing according to their false-discovery rate.These exploratory analyses suggest that genetic susceptibility loci might be related to the molecular/pathologic diversity of bladder cancer. Validation through large-scale replication studies and the study of additional genes and single nucleotide polymorphisms are required. 
 Genetic susceptibility to distinct bladder cancer subphenotypes. Clinical, pathologic, and molecular evidence indicate that bladder cancer is heterogeneous with pathologic/molecular features that define distinct subphenotypes with different prognoses. It is conceivable that specific patterns of genetic susceptibility are associated with particular subphenotypes.To examine evidence for the contribution of germline genetic variation to bladder cancer heterogeneity.The Spanish Bladder Cancer/EPICURO Study is a case-control study based in 18 hospitals located in five areas in Spain. Cases were patients with a newly diagnosed, histologically confirmed, urothelial cell carcinoma of the bladder from 1998 to 2001. Case diagnoses were reviewed and uniformly classified by pathologists following the World Health Organisation/International Society of Urological Pathology 1999 criteria. Controls were hospital-matched patients (n=1149).A total of 1526 candidate variants in 423 candidate genes were analysed. Three distinct subphenotypes were defined according to stage and grade: low-grade nonmuscle   invasive   (n=586), high-grade nonmuscle   invasive   (n=219), and muscle   invasive   (n=246). The association between each variant and subphenotype was assessed by polytomous risk models adjusting for potential confounders. Heterogeneity in genetic susceptibility among subphenotypes was also tested.Two established bladder cancer susceptibility genotypes, NAT2 slow-acetylation and GSTM1-null, exhibited similar associations among the subphenotypes, as did VEGF-rs25648, which was previously identified in our study. Other variants conferred risks for specific tumour subphenotypes such as PMS2-rs6463524 and CD4-rs3213427 (respective heterogeneity p values of 0.006 and 0.004), which were associated with muscle-  invasive   tumours (per-allele odds ratios [95% confidence interval] of 0.56 [0.41-0.77] and 0.71 [0.57-0.88], respectively) but not with non-muscle-  invasive   tumours. Heterogeneity p values were not robust in multiple testing according to their false-discovery rate.These exploratory analyses suggest that genetic susceptibility loci might be related to the molecular/pathologic diversity of bladder cancer. Validation through large-scale replication studies and the study of additional genes and single nucleotide polymorphisms are required. 
 Reactivation of p53 by a Cytoskeletal Sensor to Control the Balance Between DNA Damage and Tumor Dissemination. Abnormal cell migration and invasion underlie metastasis, and actomyosin contractility is a key regulator of tumor invasion. The links between cancer migratory behavior and DNA damage are poorly understood.Using 3D collagen systems to recapitulate melanoma extracellular matrix, we analyzed the relationship between the actomyosin cytoskeleton of migrating cells and DNA damage. We used multiple melanoma cell lines and microarray analysis to study changes in gene expression and in vivo intravital imaging (n = 7 mice per condition) to understand how DNA damage impacts   invasive   behavior. We used Protein Tissue Microarrays (n = 164 melanomas) and patient databases (n = 354 melanoma samples) to investigate the associations between markers of DNA damage and actomyosin cytoskeletal features. Data were analyzed with Student's and multiple t tests, Mann-Whitney's test, one-way analysis of variance, and Pearson correlation. All statistical tests were two-sided.Melanoma cells with low levels of Rho-ROCK-driven actomyosin are subjected to oxidative stress-dependent DNA damage and ATM-mediated p53 protein stabilization. This results in a specific transcriptional signature enriched in DNA damage/oxidative stress responsive genes, including Tumor Protein p53 Inducible Protein 3 (TP53I3 or PIG3). PIG3, which functions in DNA damage repair, uses an unexpected catalytic mechanism to suppress Rho-ROCK activity and impair tumor invasion in vivo. This regulation was suppressed by antioxidants. Furthermore, PIG3 levels decreased while ROCK1/2 levels increased in human metastatic melanomas (ROCK1 vs PIG3; r = -0.2261, P < .0001; ROCK2 vs PIG3: r = -0.1381, P = .0093).The results suggest using Rho-kinase inhibitors to reactivate the p53-PIG3 axis as a novel therapeutic strategy; we suggest that the use of antioxidants in melanoma should be very carefully evaluated. 
 Suppression of p53-inducible gene 3 is significant for glioblastoma progression and predicts poor patient prognosis. Glioblastoma is the most malignant and   invasive   brain tumor with extremely poor prognosis. p53-inducible gene 3, a downstream molecule of the tumor suppressor p53, has been found involved in apoptosis and oxidative stress response. However, the functions of p53-inducible gene 3(PIG3) in cancer are far from clear including glioblastoma. In this study, we found that p53-inducible gene 3 expression was suppressed in glioblastoma tissues compared with normal tissues. And the expression of p53-inducible gene 3 was significantly associated with the World Health Organization grade. Patients with high p53-inducible gene 3 expression have a significantly longer median survival time (15 months) than those with low p53-inducible gene 3 expression (8 months). According to Cox regression analysis, p53-inducible gene 3 was an independent prognostic factor with multivariate hazard ratio of 0.578 (95% confidence interval, 0.352-0.947; p = 0.030) for overall survival. Additionally, gain and loss of function experiments showed that knockdown of p53-inducible gene 3 significantly increased the proliferation and invasion ability of glioblastoma cells while overexpression of p53-inducible gene 3 inhibited the proliferation and invasion ability. The results of in vivo glioblastoma models further confirmed that p53-inducible gene 3 suppression promoted glioblastoma progression. Altogether, our data suggest that high expression of p53-inducible gene 3 is significant for glioblastoma inhibition and p53-inducible gene 3 independently indicates good prognosis in patients, which might be a novel prognostic biomarker or potential therapeutic target in glioblastoma. 
  [Mm]etastasis  PubMed  
 Reactivation of p53 by a Cytoskeletal Sensor to Control the Balance Between DNA Damage and Tumor Dissemination. Abnormal cell migration and invasion underlie   metastasis  , and actomyosin contractility is a key regulator of tumor invasion. The links between cancer migratory behavior and DNA damage are poorly understood.Using 3D collagen systems to recapitulate melanoma extracellular matrix, we analyzed the relationship between the actomyosin cytoskeleton of migrating cells and DNA damage. We used multiple melanoma cell lines and microarray analysis to study changes in gene expression and in vivo intravital imaging (n = 7 mice per condition) to understand how DNA damage impacts invasive behavior. We used Protein Tissue Microarrays (n = 164 melanomas) and patient databases (n = 354 melanoma samples) to investigate the associations between markers of DNA damage and actomyosin cytoskeletal features. Data were analyzed with Student's and multiple t tests, Mann-Whitney's test, one-way analysis of variance, and Pearson correlation. All statistical tests were two-sided.Melanoma cells with low levels of Rho-ROCK-driven actomyosin are subjected to oxidative stress-dependent DNA damage and ATM-mediated p53 protein stabilization. This results in a specific transcriptional signature enriched in DNA damage/oxidative stress responsive genes, including Tumor Protein p53 Inducible Protein 3 (TP53I3 or PIG3). PIG3, which functions in DNA damage repair, uses an unexpected catalytic mechanism to suppress Rho-ROCK activity and impair tumor invasion in vivo. This regulation was suppressed by antioxidants. Furthermore, PIG3 levels decreased while ROCK1/2 levels increased in human metastatic melanomas (ROCK1 vs PIG3; r = -0.2261, P < .0001; ROCK2 vs PIG3: r = -0.1381, P = .0093).The results suggest using Rho-kinase inhibitors to reactivate the p53-PIG3 axis as a novel therapeutic strategy; we suggest that the use of antioxidants in melanoma should be very carefully evaluated. 
   
  
 SERPINF1 
 
  NCBI Gene  
  GeneRIF  
  Pubmed  
 
 
  Search Term  Rank  Fields  
  [Mm]igration  6  Process(1); PubMed(28); GeneRIFs(5)  
  ECM  7  PubMed(2)  
  [Ee]xtracellular [Mm]atrix  8  PubMed(9); Component(1); GeneRIFs(1)  
  [Ii]nvasive  10  PubMed(7); GeneRIFs(1)  
  [Mm]etastasis  12  PubMed(17); GeneRIFs(3)  
 
 
 hide/show details for SERPINF1 
  [Mm]igration  Process  
 negative regulation of endothelial cell   migration   
  PubMed  
 Recombinant pigment epithelium-derived factor PEDF binds vascular endothelial growth factor receptors 1 and 2. Angiogenesis, or the formation of new blood vessels, is stimulated by angiogenic factors such as vascular endothelial growth factor (VEGF). Pigment epithelium-derived factor (PEDF) is a potent inhibitor of angiogenesis. To explore the mechanism by which PEDF acts, recombinant PEDF was expressed with a 6x-His tag (for purification) and a green fluorescent protein (GFP) tag. The PEDF fusion protein was confirmed to be active in inhibition of endothelial cell proliferation and   migration  . Direct binding of PEDF to both vascular endothelial growth factor receptor-1 (VEGFR-1) and VEGFR-2 was demonstrated in an in vitro assay similar to an enzyme-linked immunosorbent assay (ELISA). PEDF was shown by immune-confocal microscopy to be localized within treated endothelial cells. When VEGF-stimulated endothelial cells were incubated with PEDF the VEGF receptors showed intracellular localization. These data suggest that the interaction between PEDF and VEGFR-1 or VEGFR-2 may be a possible mechanism for inhibiting angiogenesis. PEDF may be binding to the VEGF receptors to promote their internalization and/or degradation to limit VEGF responses in treated cells. 
 Recombinant pigment epithelium-derived factor PEDF binds vascular endothelial growth factor receptors 1 and 2. Angiogenesis, or the formation of new blood vessels, is stimulated by angiogenic factors such as vascular endothelial growth factor (VEGF). Pigment epithelium-derived factor (PEDF) is a potent inhibitor of angiogenesis. To explore the mechanism by which PEDF acts, recombinant PEDF was expressed with a 6x-His tag (for purification) and a green fluorescent protein (GFP) tag. The PEDF fusion protein was confirmed to be active in inhibition of endothelial cell proliferation and   migration  . Direct binding of PEDF to both vascular endothelial growth factor receptor-1 (VEGFR-1) and VEGFR-2 was demonstrated in an in vitro assay similar to an enzyme-linked immunosorbent assay (ELISA). PEDF was shown by immune-confocal microscopy to be localized within treated endothelial cells. When VEGF-stimulated endothelial cells were incubated with PEDF the VEGF receptors showed intracellular localization. These data suggest that the interaction between PEDF and VEGFR-1 or VEGFR-2 may be a possible mechanism for inhibiting angiogenesis. PEDF may be binding to the VEGF receptors to promote their internalization and/or degradation to limit VEGF responses in treated cells. 
 Microphthalmia-associated transcription factor acts through PEDF to regulate RPE cell   migration  . Cells of the retinal pigment epithelium (RPE) play major roles in metabolic functions, maintenance of photoreceptor function, and photoreceptor survival in the retina. They normally form a stable monolayer, but migrate during disease states. Although growth factors produced by the RPE cells primarily control these cellular events, how these factors are regulated in RPE cells remain largely unknown. Here we show that the basic-helix-loop-helix-leucine zipper microphthalmia-associated transcription factor (MITF), which plays central roles in the development and function of a variety of cell types including RPE cells, upregulates the expression of a multifunctional factor PEDF in RPE cells. Consequently, the upregulation of PEDF impairs microtubule assembly and thus inhibits RPE cell   migration  . Conversely, specific knockdown of PEDF partially rescues the impairment of microtubule assembly and cell   migration   proceeds in MITF overexpressing stable cells. We conclude that MITF acts through PEDF to inhibit RPE cell   migration   and to play a significant role in regulating RPE cellular function. We suggest that MITF has a novel and important role in maintaining RPE cells as a stable monolayer and the down-regulation of PEDF that may contribute to retinal degenerative diseases. 
 A novel role for microphthalmia-associated transcription factor-regulated pigment epithelium-derived factor during melanoma progression. Microphthalmia-associated transcription factor (MITF) acts via pigment epithelium-derived factor (PEDF), an antiangiogenic protein, to regulate retinal pigment epithelium   migration  . PEDF expression and/or regulation during melanoma development have not been investigated previously. Using immunohistochemistry, we determined expression of PEDF in common and dysplastic melanocytic nevi, melanoma in situ, invasive melanoma, and metastatic melanoma (n = 102). PEDF expression was consistently decreased in invasive and metastatic melanoma, compared with nevi and melanoma in situ (P < 0.0001). PEDF was lost in thicker melanomas (P = 0.003), and correlated with depth of invasion (P = 0.003) and distant metastasis (P = 0.0331), but only marginally with mitotic index, AJCC stage, nodal metastasis, or blood vascular density (0.05 < P < 0.10). Quantitative real-time PCR and microarray analyses confirmed PEDF down-regulation at the mRNA level in several melanoma lines, compared with melanocytes. MITF positively correlated with PEDF expression in invasive melanomas (P = 0.0003). Searching for PEDF regulatory mechanisms revealed two occupied conserved E-boxes (DNA recognition elements) in the first intron of the human and mouse PEDF promoter regions, confirmed by binding assays. Dominant-negative and siRNA approaches in vivo demonstrated direct transcriptional influence of MITF on PEDF, establishing the PEDF gene (SERPINF1) as a MITF target in melanocytes and melanoma cells. These findings suggest that loss of PEDF expression promotes early invasive melanoma growth. 
 Hypoxia negatively regulates antimetastatic PEDF in melanoma cells by a hypoxia inducible factor-independent, autophagy dependent mechanism. Pigment epithelium-derived factor (PEDF), a member of the serine protease inhibitor (SERPIN) superfamily, displays a potent antiangiogenic and antimetastatic activity in a broad range of tumor types. Melanocytes and low aggressive melanoma cells secrete high levels of PEDF, while its expression is lost in highly aggressive melanomas. PEDF efficiently abrogates a number of functional properties critical for the acquisition of metastatic ability by melanoma cells, such as neovascularization, proliferation,   migration  , invasiveness and extravasation. In this study, we identify hypoxia as a relevant negative regulator of PEDF in melanocytes and low aggressive melanoma cells. PEDF was regulated at the protein level. Importantly, although downregulation of PEDF was induced by inhibition of 2-oxoglutarate-dependent dioxygenases, it was independent of the hypoxia inducible factor (HIF), a key mediator of the adaptation to hypoxia. Decreased PEDF protein was not mediated by inhibition of translation through untranslated regions (UTRs) in melanoma cells. Degradation by metalloproteinases, implicated on PEDF degradation in retinal pigment epithelial cells, or by the proteasome, was also excluded as regulatory mechanism in melanoma cells. Instead, we found that degradation by autophagy was critical for PEDF downregulation under hypoxia in human melanoma cells. Our findings show that hypoxic conditions encountered during primary melanoma growth downregulate antiangiogenic and antimetastasic PEDF by a posttranslational mechanism involving degradation by autophagy and could therefore contribute to the acquisition of highly metastatic potential characteristic of aggressive melanoma cells. 
 Late outgrowth endothelial cells derived from Wharton jelly in human umbilical cord reduce neointimal formation after vascular injury: involvement of pigment epithelium-derived factor. The number of endothelial progenitor cells (EPCs) that can be obtained from adult bone marrow and peripheral blood to treat cardiovascular diseases is limited. The goal was to examine the endothelial potential of Wharton jelly in human umbilical cord (WJC)-derived stem cells and evaluate their potential to affect neointimal formation after vascular injury.Mesenchymal cells (MCs) were isolated from WJC and cultured in endothelial growth medium. Differentiation into late outgrowth endothelial cells (WJC-OECs) was demonstrated by incorporation of acetylated low-density lipoprotein and expression of the endothelial-specific markers. Transplantation of these cells into wire-injured femoral arteries in mice led to rapid reendothelialization. At 4 weeks after injury, the neointima/media area ratio was reduced and strong expression of pigment epithelium-derived factor (PEDF) compared to saline-or MC- or cord blood-OEC-treated mice. WJC-OECs-conditioned medium has an extremely strong capacity to inhibit the   migration   and proliferation of smooth muscle cells. The effects were inhibited by neutralizing antibody for PEDF and by siRNA silencing of PEDF.We firstly demonstrated the presence of a cell population within WJC that has the potential to differentiate into OECs. Transplantation of WJC-OECs may play a crucial role in reestablishing endothelial integrity in injured vessels, thereby inhibiting neointimal hyperplasia. These findings have implications for a novel and practical cell-based therapy for vascular diseases. 
 Constitutive overexpression of pigment epithelium-derived factor inhibition of ocular melanoma growth and metastasis. Pigment epithelium-derived factor (PEDF) is known to be an angiogenesis suppressor and to have antitumor effects. This study investigates whether constitutive overexpression of PEDF inhibits the growth and hepatic micrometastasis of ocular melanoma.Real-time RT-PCR was used to detect endogenous PEDF expression in human uveal melanoma cell lines and mouse melanoma cells. A lentiviral vector containing a mouse PEDF expression sequence was constructed and transduced into mouse melanoma cells in vitro. Transgene expression was assessed by Western blot analysis. Angiogenesis and transendothelial   migration   assays were performed in constitutively stable PEDF-overexpressing cells and transduced lentiviral vector control cells. The size and microvessel density of the ocular tumor and the number of hepatic micrometastasis were compared between the mice inoculated with PEDF-overexpressing tumor cells and those mice with the control cell line.Four human uveal melanoma and three mouse melanoma cell lines were found to express PEDF mRNA. Endogenous overexpressing PEDF melanoma cells lost the ability to migrate and form tubes in vitro. In the animal experiment, the size of the ocular melanoma and the number of hepatic micrometastasis were decreased and microvessel density was also reduced in mice inoculated with constitutively overexpressing PEDF melanoma cells.Lentivirus-mediated gene transfer of PEDF decreased the growth of ocular melanoma and its hepatic micrometastasis in a mouse ocular melanoma model. Dual antitumor/antiangiogenic activities of PEDF suggest that PEDF gene therapy may be considered an approach for the treatment of ocular melanoma. 
 Growth factors/chemokines in diabetic vitreous and aqueous alter the function of bone marrow-derived progenitor (CD34⁺) cells in humans. Ocular ischemic microenvironment plays a critical role in the progression of diabetic retinopathy (DR). In this study, we investigated the effect of vitreous and aqueous obtained from proliferative DR patients on the function of CD34⁺ cells derived from healthy humans. Human CD34⁺ cells were incubated with vitreous or aqueous of subjects with PDR. After incubation,   cell mig  ration of CD34⁺ was evaluated with CXCL12. Intracellular levels of nitric oxide (NO) were measured with DAF-FM. Tube formation assay was used to evaluate the effect of treated CD34⁺ cells on in vitro angiogenesis. Angiogenic protein array and mass spectrometry (MS) were performed to ascertain the factors secreted by healthy nondiabetic CD34⁺ cells exposed to diabetic vitreous or aqueous. PDR vitreous/aque  ous reduc  ed migration of CD34⁺ cells (672.45 ± 42.1/736.75 ± 101.7 AFU; P < 0.01) and attenuated intracellular NO levels (182 ± 1.4/184.5 ± 6.3 AFU, P = 0.002). Pretreatment with PDR vitreous suppressed tube formation of human retinal endothelial cells (64 ± 1.6 vs. 80 ± 2.5). CD34⁺ exposed to PDR vitreous resulted in the increased expression of CXCL4 and serpin F1, whereas CD34⁺ exposed to PDR aqueous showed increased expression of CXCL4, serpin F1, and endothelin-1 (ET-1). MS analysis of CD34⁺ (exposed to PDR vitreous) expressed J56 gene segment, isoform 2 of SPARC-related modular calcium-binding protein 2, isoform 1 of uncharacterized protein c1 orf167, integrin α-M, and 40s ribosomal protein s21. Exposure of healthy nondiabetic CD34⁺ cells to PDR vitreous and a  queous re  sulted in decreased migration, reduced generation of NO, and altered paracrine secretory function. Our results suggest that the contribution of CD34⁺ cells to the aberrant neovascularization observed in PDR is driven more by the proangiogenic effects of the retinal cells rather than the influence of the vitreous. 
 Hormonal regulation of pigment epithelium-derived factor (PEDF) in granulosa cells. Angiogenesis is critical for the development of ovarian follicles. Blood vessels are abrogated from the follicle until ovulation, when they invade it to support the developing corpus luteum. Granulosa cells are known to secrete anti-angiogenic factors that shield against premature vascularization; however, their molecular identity is yet to be defined. In this study we address the physiological role of pigment epithelium-derived factor (PEDF), a well-known angiogenic inhibitor, in granulosa cells. We have shown that human and mouse primary granulosa cells express and secrete PEDF, and characterized its hormonal regulation. Stimulation of granulosa cells with increasing doses of estrogen caused a gradual decrease in the PEDF secretion, while stimulation with progesterone caused an abrupt decrease in its secretion. Moreover, We have shown, by time- and dose-response experiments, that the secreted PEDF and vascular endothelial growth factor (VEGF) were inversely regulated by hCG; namely, PEDF level was nearly undetectable under high doses of hCG, while VEGF level was significantly elevated. The anti-angiogenic nature of the PEDF secreted from granulosa cells was examined by   migration  , proliferation and tube formation assays in cultures of human umbilical vein endothelial cells. Depleting PEDF from primary granulosa cells conditioned media accelerated endothelial cells proliferation,   migration   and tube formation. Collectively, the dynamic expression of PEDF that inversely portrays VEGF expression may imply its putative role as a physiological negative regulator of follicular angiogenesis. 
 Proliferative diabetic retinopathy is associated with a low level of the natural ocular anti-angiogenic agent pigment epithelium-derived factor (PEDF) in aqueous humor. a pilot study. Retinopathy is the most common microvascular diabetes complication and represents a major threat to the eyesight. The aim of this study was to address the role of pro- and anti-angiogenic molecules in diabetic retinopathy in the aqueous humor of the eye. Aqueous humor was collected at cataract surgery from 19 diabetic patients and from 13 age- and sex-matched normoglycemic controls. Levels of pro-angiogenic vascular endothelial growth factor (VEGF) and angiogenic inhibitor pigment epithelium-derived factor (PEDF) were determined. Angiogenic activity of the aqueous humor was quantified by measuring its effect on the   migration   of capillary endothelial cells. In the aqueous fluid, VEGF levels were increased in diabetics (mean values: 501 vs. 367 pg/ml; p = 0.05), compared to controls. PEDF was found to be decreased in diabetics (mean values: 2080 vs. 5780 ng/ml; p = 0.04) compared to controls. In seven diabetic patients with proliferative retinopathy, the most profound finding was a significant decrease of the PEDF level (mean value: 237 ng/ml), whereas VEGF levels were comparable to diabetic patients without proliferation (mean value: 3153; p = 0.003). Angiogenic activity in samples of patients from the control group was generally inhibitory due to PEDF, and inhibition was blocked by neutralizing antibodies to PEDF. Likewise, in diabetics without proliferation, angiogenic activity was also blocked by antibodies to PEDF. We will demonstrate here that the level of the natural ocular anti-angiogenic agent PEDF is inversely associated with proliferative retinopathy. PEDF is an important negative regulator of angiogenic activity of aqueous humor. Our data may have implications for the development of novel regimens for diabetic retinopathy. 
 Pigment epithelium-derived factor inhibits neointimal hyperplasia after vascular injury by blocking NADPH oxidase-mediated reactive oxygen species generation. Pigment epithelium-derived factor (PEDF) inhibits cytokine-induced endothelial cell activation through its antioxidative properties. However, the effect of PEDF on restenosis remains to be elucidated. Because the pathophysiological feature of restenosis is characterized by increased superoxide formation and accumulation of smooth muscle cells (SMCs), PEDF may inhibit this process via suppression of reactive oxygen species generation. We investigated here whether PEDF could prevent neointimal formation after balloon injury. PEDF levels were decreased in balloon-injured arteries. Adenoviral vector encoding human PEDF (Ad-PEDF) prevented neointimal formation. Expression and superoxide generation of the membrane components of NADPH oxidase, p22(phox) and gp91(phox), in the neointima were also suppressed by Ad-PEDF. Ad-PEDF reduced G(1) cyclin (cyclin D1 and E) expression and increased p27, a cyclin-dependent kinase inhibitor. In vitro, PEDF inhibited platelet-derived growth factor-BB-induced SMC proliferation and   migration   by blocking reactive oxygen species generation through suppression of NADPH oxidase activity via down-regulation of p22(PHOX) and gp91(PHOX). PEDF down-regulated G(1) cyclins and up-regulated p27 levels in platelet-derived growth factor-BB-exposed SMCs as well. These results demonstrate that PEDF could inhibit neointimal formation via suppression of NADPH oxidase-mediated reactive oxygen species generation. Our present study suggests that substitution of PEDF may be a novel therapeutic strategy for restenosis after balloon angioplasty. 
 Positive correlation between PEDF expression levels and macrophage density in the human prostate. In this study, we investigated the capacity of pigment epithelium-derived factor (PEDF) to modulate the recruitment and the differentiation of monocytes/macrophages both in vitro and in human prostate.Using Boyden chambers, we assessed PEDF effect on the   migration   of monocytes and chemically activated RAW 264.7 macrophages. Normal, prostatitis, and prostate cancer specimens were retrospectively selected and examined by immunohistochemistry for PEDF expression and infiltration of immune CD68 + macrophagic cells. PEDF expression and macrophage density were then correlated with each other and clinicopathological parameters. M1 and M2 differentiation markers were quantified by qRT-PCR, Western blotting, and ELISA.In chemotaxis, PEDF induced   the migra  tion of monocytes/macrophages. In immunohistochemistry, macrophages were markedly increased in prostatitis and malignant compared to normal tissues. PEDF was expressed at variable levels in the stroma and epithelium. PEDF mRNA was down-regulated in both prostate cancer and prostatitis compared to normal tissues. In correlation studies, macrophage density and PEDF expression were respectively positively and negatively associated with prostate size. Most importantly, PEDF expression positively correlated with macrophage density. Finally, PEDF stimulated the expression of iNOS, IL12, and TNFα; and inhibited IL10 and arginase 1 in mouse and human macrophages confirming a M1-type differentiation.Our data demonstrate that PEDF acts directly on monocytes/macrophages by inducing t  heir migr  ation and differentiation into M1-type cells. These findings suggest a possible role of macrophages in PEDF anti-tumor properties and may support further development of PEDF-based anti-cancer therapy. 
 Production and function of pigment epithelium-derived factor in isolated skin keratinocytes. Pigment epithelium-derived factor (PEDF) is a multifunctional factor with potent anti-angiogenic activity that may play a role in skin homoeostasis and wound healing. Analysis of PEDF levels demonstrated that PEDF levels are high in normal skin but quite low in early wounds. As previous studies have suggested that keratinocytes can produce PEDF, we investigated how conditions that mimic those found at sites of injury influence PEDF production by keratinocytes in vitro. Both injury by mechanical disruption (scratch assay) and treatment of human keratinocytes with inflammatory cytokines (IL-1β, IL-6 and TNF-α) inhibited PEDF expression. We next examined how PEDF affects keratinocyte functions that are important in tissue repair. Treatment of keratinocytes with exogenous PEDF enhanced keratinocyte adhesion, therefore impairin  g migrati  on, while having no effect on cell proliferation. The results suggest that modulation of PEDF levels may play a pivotal role in skin homoeostasis and the response of keratinocytes to injury or inflammatory insults. 
 Laminin receptor involvement in the anti-angiogenic activity of pigment epithelium-derived factor. Pigment epithelium-derived factor (PEDF) is a multifunctional protein with neurotrophic, anti-oxidative, and anti-inflammatory properties. It is also one of the most potent endogenous inhibitors of angiogenesis, playing an important role in restricting tumor growth, invasion, and metastasis. Studies show that PEDF binds to cell surface proteins, but little is known about how it exerts its effects. Recently, research identified phospholipase A(2)/nutrin/patatin-like phospholipase domain-containing 2 as one PEDF receptor. To identify other receptors, we performed yeast two-hybrid screening using PEDF as bait and discovered that the non-integrin 37/67-kDa laminin receptor (LR) is another PEDF receptor. Co-immunoprecipitation, His tag pulldown, and surface plasmon resonance assays confirmed the interaction between PEDF and LR. Using the yeast two-hybrid method, we further restricted the LR-interacting domain on PEDF to a 34-amino acid (aa) peptide (aa 44-77) and the PEDF-interacting domain on LR to a 91-aa fragment (aa 120-210). A 25-mer peptide named P46 (aa 46-70), derived from 34-mer, interacts with LR in surface plasmon resonance assays and binds to endothelial cell (EC) membranes. This peptide induces EC apoptosis and inhibits EC   migration  , tube-like network formation in vitro, and retinal angiogenesis ex vivo, like PEDF. Our results suggest that LR is a real PEDF receptor that mediates PEDF angiogenesis inhibition. 
 Changes in the gene expression profile of A375 human melanoma cells induced by overexpression of multifunctional pigment epithelium-derived factor. Pigment epithelium-derived factor (PEDF) is a broad-spectrum angiogenesis inhibitor that displays potent antimetastatic activity in multiple tumor types. We have previously shown that PEDF prevents primary tumor growth and metastatic spread of human melanoma in mouse experimental models. Consistent with these observations, PEDF expression is lost at the late stages of melanoma progression, allowing melanoma cells to become angiogenic, migratory, and invasive. PEDF's ability to modify the interplay between the host and tumor tissues strongly supports its use as a therapeutic agent for the treatment of metastatic melanoma. However, transition to the clinic requires a more detailed knowledge of the molecular mechanisms underpinning PEDF's activity. In this study, we describe changes in the gene expression profile of A375 human melanoma cells induced by PEDF overexpression. PEDF modulated diverse categories of genes known to be involved in angiogenesis and   migration  . It downregulated cytokines such as interleukin-8 and extracellular matrix proteins such as collagen IV, while it upregulated fibronectin. Multiple transcripts previously described as contributing to the acquisition of malignant phenotype by melanoma were also diminished by PEDF overexpression, among which we validated galectin 3 and jagged 1. In addition, PEDF downregulated S100β and melanoma inhibitory activity, which are widely used in the pathological diagnosis of melanoma. Interestingly, PEDF increased the expression of melanophilin and decreased rab27A, which are relevant targets for melanosome transport; suggesting that PEDF could directly impinge on melanocytic lineage-specific processes. Our study identifies new molecular targets and signaling pathways that may potentially contribute to determine PEDF's ability to restrict the aggressiveness of A375 human melanoma cells. 
 Human testicular peritubular cells secrete pigment epithelium-derived factor (PEDF), which may be responsible for the avascularity of the seminiferous tubules. Male fertility depends on spermatogenesis, which takes place in the seminiferous tubules of the testis. This compartment is devoid of blood vessels, which are however found in the wall of the seminiferous tubules. Our proteomic study using cultured human testicular peritubular cells (HTPCs) i.e. the cells, which form this wall, revealed that they constitutively secrete pigment epithelium-derived factor, PEDF, which is known to exert anti-angiogenic actions. Immunohistochemistry supports its presence in vivo, in the human tubular wall. Co-culture studies and analysis of cell   migration   patterns showed that human endothelial cells (HUVECs) are repulsed by HTPCs. The factor involved is likely PEDF, as a PEDF-antiserum blocked the repulsing action. Thus testicular peritubular cells, via PEDF, may prevent vascularization of human seminiferous tubules. Dihydrotestosterone (DHT) increased PEDF (qPCR) in HTPCs, however PEDF expression in the testis of a non-human primate occurs before puberty. Thus PEDF could be involved in the establishment of the avascular nature of seminiferous tubules and after puberty androgens may further reinforce this feature. Testicular microvessels and blood flow are known to contribute to the spermatogonial stem cell niche. Hence HTPCs via control of testicular microvessels may contribute to the regulation of spermatogonial stem cells, as well. 
 Low content of the natural ocular anti-angiogenic agent pigment epithelium-derived factor (PEDF) in aqueous humor predicts progression of diabetic retinopathy. Retinopathy is the most common microvascular complication of diabetes. Our aim was to address the predictive value of pro-angiogenic and anti-angiogenic markers for progression of retinopathy.Aqueous humor was collected at cataract surgery from 32 diabetic patients who had no or very mild retinopathy (ETDRS stage </=20) and 33 normoglycaemic control subjects. Content of pro-angiogenic vascular endothelial growth factor and angiogenic inhibitor pigment epithelium-derived factor were determined. ANGIOGENIC activity was quantified by measuring its effect on the   migration   of capillary endothelial cells. The predictive value of the initial level of these markers for progression of retinopathy was studied by following the probands for a maximum of 75 months.In the aqueous fluid content of vascular endothelial growth factor was increased in diabetic patients (mean values 492 versus 292 pg/ml; p=0.0052), and pigment epithelium-derived factor values were decreased (mean values 1740 versus 3680 ng/ml; p=0.0058) compared to control subjects. Of the diabetic patients ten progressed during follow-up (ETDRS stage >47B). This subgroup showed lower pigment epithelium-derived factor content when compared to non-progressors and control subjects. Migratory activity in samples of patients from the control group and in diabetic patients without progression was generally inhibitory due to pigment epithelium-derived factor. Inhibition was blocked by neutralizing antibodies to pigment epithelium-derived factor. In diabetic patients initial angiogenic activity was higher in those who later developed retinopathy (vs. controls p=0.00005; vs. no progressors p=0.0003). Both pigment epithelium-derived factor and migratory response predicted progression.Pigment epithelium-derived factor is an important negative regulator of angiogenic activity of aqueous humor. Its content in the aqueous humor of diabetic patients strongly predicts who among them will develop progression of retinopathy. 
 Pigment epithelium-derived factor (PEDF) inhibits breast cancer metastasis by down-regulating fibronectin. Pigment epithelium-derived factor (PEDF) plays an important role in the tumor growth and metastasis inhibition. It has been reported that PEDF expression is significantly reduced in breast cancer, and associated with disease progression and poor patient outcome. However, the exact mechanism of PEDF on breast cancer metastasis including liver and lung metastasis remains unclear. The present study aims to reveal the impact of PEDF on breast cancer. The orthotopic tumor mice model inoculated by MDA-MB-231 cells stably expressing PEDF or control cells was used to assess liver and lung metastasis of breast cancer. In vitro,   migration   and invasion experiments were used to detect the metastatic abilities of MDA-MB-231 and SKBR3 breast cancer cells with or without overexpression of PEDF. The metastatic-related molecules including EMT makers, fibronectin, and p-AKT and p-ERK were detected by qRT-PCR, Western blot, and Fluorescent immunocytochemistry. PEDF significantly inhibited breast cancer growth and metastasis in vivo and in vitro. Mechanically, PEDF inhibited breast cancer cell   migration   and invasion by down-regulating fibronectin and subsequent MMP2/MMP9 reduction via p-ERK and p-AKT signaling pathways. However, PEDF had no effect on EMT conversion in the breast cancer cells which was usually involved in cancer metastasis. Furthermore, the study showed that laminin receptor mediated the down-regulation of fibronectin by PEDF. These results reported for the first time that PEDF inhibited breast cancer metastasis by down-regulating fibronectin via laminin receptor/AKT/ERK pathway. Our findings demonstrated PEDF as a dual effector in limiting breast cancer growth and metastasis and highlighted a new avenue to block breast cancer progression. 
 Pigment epithelium-derived factor expression prolongs survival and enhances the cytotoxicity of low-dose chemotherapy in castration-refractory prostate cancer. There is currently no cure for advanced castration-refractory prostate cancer (CRPC) despite the recent approval of several new therapeutic agents. We report here the anti-tumor effect of the angio-inhibitory pigment epithelium-derived factor (PEDF) in the metastatic LNCaP-derivative CRPC CL1 model and explore PEDF anti-neoplasic efficacy in combination with low-dose chemotherapy. Androgen-sensitive LNCaP and CRPC PC3 cell lines were examined as comparison. Using a retroviral expression system, we showed that PEDF limited the proliferation of all prostatic cell lines tested; an effect attributed to interleukin 8 (IL8)-CXCR1/IL8RA inhibition. PEDF also reduced the number and size of 3D tumor spheroids in vitro, but only induced cell differentiation in CRPC spheroids. Similarly, PEDF inhibited the   migration   of CRPC cells suggesting both anti-proliferative and anti-migratory functions. In vivo, PEDF decreased by 85% and 65% the growth of subcutaneous (s.c.) PC3 and CL1 tumors, respectively. In the CL1 orthotopic model, tumor intake with lethal metastases was found in all animals; nevertheless, PEDF prolonged the median survival of tumor-bearing mice (95% confidence interval: 53 ± 0.001 to 57 ± 1 days). Accordingly, PEDF delayed the emergence of skeletal-related event in intra-tibial xenografts. Next, we evaluated low-dose docetaxel (DTX; 5, 1, 0.5 mg/kg) or cyclophosphamide (CTX; 10-20 mg/kg) on established s.c. PC3 tumors that conditionally express PEDF anti-tumoral epitope/NT3. Although NT3-DTX-5 mg/kg combination was inefficient, NT3-DTX-1 mg/kg and -0.5 mg/kg inhibited by 95% and 87.8%, respectively, tumor growth compared with control and induced tumor stasis. Both NT3-CTX combinations were advantageous. Inversely, PEDF-DTX-5 mg/kg and PEDF-CTX-10 mg/kg delayed the most CL1 tumor growth (15, 11 and 5 days for PEDF-DTX-5 mg/kg, PEDF-CTX-10 mg/kg and single treatments, respectively) with elevated apoptosis and serum thrombospondin-1 as possible mechanism and marker, respectively. As well, both PEDF-CTX-10 mg/kg and PEDF-DTX-5 mg/kg prolonged significantly the survival of tumor-bearing mice compared with single treatments. Metastases were reduced in PEDF-DTX-5 mg/kg compared with other treatments, suggesting that PEDF-DTX delayed metastases formation. Our results advocate that PEDF/low-dose chemotherapy may represent a new therapeutic alternative for CRPC. 
 Antiangiogenic property of pigment epithelium-derived factor in hepatocellular carcinoma. Pigment epithelium-derived factor (PEDF) is one of the most powerful endogenous antiangiogenic reagents discovered to date. Its antiangiogenic potential in neoplastic disease remains unclear. In this study, we investigated antiangiogenic property of PEDF in hepatocellular carcinoma (HCC), a typical hypervascular tumor. In HCC cell lines, constitutive messenger RNA and protein expression of PEDF varied. Genomic DNA encoding the PEDF gene was the same in the cell lines examined by Southern blotting. In chemically induced hypoxic conditions, secreted PEDF protein was suppressed in contrast to elevation of vascular endothelial growth factor protein. When PEDF was overexpressed by gene transfer, proliferation and   migration   of endothelial cells were inhibited in conditioned media derived from all HCC cell lines. However, the serum concentration of PEDF, as measured by enzyme-linked immunosorbent assay, was decreased in patients with cirrhosis or HCC complicated by cirrhosis compared to healthy volunteers and patients with chronic hepatitis. According to the endothelial cell proliferation assay, the serum PEDF of patients with HCC had antiangiogenic activity. Moreover, intratumoral injection of a PEDF-expressing plasmid in athymic mouse models caused significant inhibition of preestablished tumor growth. In conclusion, PEDF plays a role in the angiogenic properties of HCC. Reduction of serum PEDF concentration associated with the development of chronic liver diseases may contribute to the progression of HCC. In addition, gene therapy using PEDF may provide an efficient treatment for HCC. 
 Regulatory and functional connection of microphthalmia-associated transcription factor and anti-metastatic pigment epithelium derived factor in melanoma. Pigment epithelium-derived factor (PEDF), a member of the serine protease inhibitor superfamily, has potent anti-metastatic effects in cutaneous melanoma through its direct actions on endothelial and melanoma cells. Here we show that PEDF expression positively correlates with microphthalmia-associated transcription factor (MITF) in melanoma cell lines and human samples. High PEDF and MITF expression is characteristic of low aggressive melanomas classified according to molecular and pathological criteria, whereas both factors are decreased in senescent melanocytes and naevi. Importantly, MITF silencing down-regulates PEDF expression in melanoma cell lines and primary melanocytes, suggesting that the correlation in the expression reflects a causal relationship. In agreement, analysis of Chromatin immunoprecipitation coupled to high throughput sequencing (ChIP-seq) data sets revealed three MITF binding regions within the first intron of SERPINF1, and reporter assays demonstrated that the binding of MITF to these regions is sufficient to drive transcription. Finally, we demonstrate that exogenous PEDF expression efficiently halts in vitro   migration   and invasion, as well as in vivo dissemination of melanoma cells induced by MITF silencing. In summary, these results identify PEDF as a novel transcriptional target of MITF and support a relevant functional role for the MITF-PEDF axis in the biology of melanoma. 
 Pigment Epithelial-Derived Factor Peptide Facilitates the Regeneration of a Functional Limbus in Rabbit Partial Limbal Deficiency. To investigate the potential of a pigment epithelial-derived factor (PEDF) peptide 44-mer to promote limbal regeneration in a rabbit partial limbal deficiency model.Limbal excision (180°) was created surgically, and topical application of 44-mer-containing ointment once a day for 2 weeks was started immediately after injury. Limbal barrier function was inspected at 2 and 6 months after treatment. Corneal neovascularization was observed under slit-lamp microscope. The presence of goblet cells on the corneal surface was examined using impression cytology. The resulting repair tissue was assessed by immunohistochemical staining with antibodies for putative limbal stem cell (LSC) markers ΔNp63α and ABCG2. Cells harvested from the regenerated tissue were analyzed for colony-forming capacity and expression of LSC markers by immunostaining assay and quantitative real-time PCR (qPCR).Eyes treated with the 44-mer blocked vascularization and goblet ce  ll migrat  ion onto the corneal surface. By means of immunohistochemical staining and cell isolation in the repair tissue, we showed that LSCs were widely distributed at the regenerated tissue after 44-mer treatment. The repaired limbus contributed robustly to corneal wound healing as effectively as undamaged limbus.We demonstrated that 44-mer regenerates a functional limbus-like structure on limbal excision wounds. Our finding suggests that the PEDF peptide derivative may be an innovative strategy for tissue engineering and repair therapy in partial LSC deficiency diseases. 
 Pigment epithelium-derived factor binds to cell-surface F(1)-ATP synthase. Pigment epithelium-derived factor (PEDF), a potent blocker of angiogenesis in vivo, and of endothelial cell   migration   and tubule formation, binds with high affinity to an as yet unknown protein on the surfaces of endothelial cells. Given that protein fingerprinting suggested a match of a approximately 60 kDa PEDF-binding protein in bovine retina with Bos taurus F(1)-ATP synthase beta-subunit, and that F(1)F(o)-ATP synthase components have been identified recently as cell-surface receptors, we examined the direct binding of PEDF to F(1). Size-exclusion ultrafiltration assays showed that recombinant human PEDF formed a complex with recombinant yeast F(1). Real-time binding as determined by surface plasmon resonance demonstrated that yeast F(1) interacted specifically and reversibly with human PEDF. Kinetic evaluations revealed high binding affinity for PEDF, in agreement with PEDF affinities for endothelial cell surfaces. PEDF blocked interactions between F(1) and angiostatin, another antiangiogenic factor, suggesting overlapping PEDF-binding and angiostatin-binding sites on F(1). Surfaces of endothelial cells exhibited affinity for PEDF-binding proteins of approximately 60 kDa. Antibodies to F(1)beta-subunit specifically captured PEDF-binding components in endothelial plasma membranes. The extracellular ATP synthesis activity of endothelial cells was examined in the presence of PEDF. PEDF significantly reduced the amount of extracellular ATP produced by endothelial cells, in agreement with direct interactions between cell-surface ATP synthase and PEDF. In addition to demonstrating that PEDF binds to cell-surface F(1), these results show that PEDF is a ligand for endothelial cell-surface F(1)F(o)-ATP synthase. They suggest that PEDF-mediated inhibition of ATP synthase may form part of the biochemical mechanisms by which PEDF exerts its antiangiogenic activity. 
 Pigment epithelium-derived factor and its phosphomimetic mutant induce JNK-dependent apoptosis and p38-mediated   migration   arrest. Pigment epithelium-derived factor (PEDF) is a potent endogenous inhibitor of angiogenesis and a promising anticancer agent. We have previously shown that PEDF can be phosphorylated and that distinct phosphorylations differentially regulate its physiological functions. We also demonstrated that triple phosphomimetic mutant (EEE-PEDF), has significantly increased antiangiogenic activity and is much more efficient than WT-PEDF in inhibiting neovascularization and tumor growth. The enhanced antiangiogenic effect was associated with a direct ability to facilitate apoptosis of tumor-residing endothelial cells (ECs), and subsequently, disruption of intratumoral vascularization. In the present report, we elucidated the molecular mechanism by which EEE-PEDF exerts more profound effects at the cellular level. We found that EEE-PEDF suppresses EC proliferation due to caspase-3-dependent apoptosis and also inhibits   migration   of the EC much better than WT-PEDF. Although WT-PEDF and EEE-PEDF did not affect proliferation and did not induce apoptosis of cancer cells, these agents efficiently inhibited cancer cell motility, with EEE-PEDF showing a stronger effect. The stronger activity of EEE-PEDF was correlated with a better binding to laminin receptors. Furthermore, the proapoptotic and antimigratory activities of WT-PEDF and EEE-PEDF were found regulated by differential activation of two distinct MAPK pathways, namely JNK and p38, respectively. We show that JNK and p38 phosphorylation is much higher in cells treated with EEE-PEDF. JNK leads to apoptosis of ECs, whereas p38 leads to anti-migratory effect in both EC and cancer cells. These results reveal the molecular signaling mechanism by which the phosphorylated PEDF exerts its stronger antiangiogenic, antitumor activities. 
 Pigment epithelium-derived factor plays an inhibitory role in proliferation and   migration   of HaCaT cells. The normal vasculature is maintained by a balance between angiogenic factors and anti-angiogenic factors. Recent studies have shown that pigment epithelium-derived factor (PEDF) can induce differentiation and inhibit angiogenesis of tumors. This study was designed to investigate the expression of PEDF and its roles in proliferation, adhesion and   migration   of HaCaT cells, a human keratinocyte cell line. Our results have shown that PEDF is expressed in HaCaT cells at both mRNA and protein levels determined by RT-PCR and Western blot, separately. PEDF signal mainly localizes in the cytoplasm of HaCaT cell, as determined by immunofluorescence. Furthermore, expression of PEDF is decreased by 50 ng/ml of VEGF(165). Proliferation and   migration   of HaCaT cells are decreased by PEDF, while adhesion of HaCaT cells is upregulated approximately by 29%. PEDF also induce the S phase accumulation of HaCaT cells. In addition, phosphorylation of ERK1/2, not JNK and p38, is decreased by PEDF. These results indicate that PEDF may play an inhibitory role on growth and   migration   of HaCaT cells through dephosphorylation of ERK1/2. 
 Upregulation of PEDF expression by PARP inhibition contributes to the decrease in hyperglycemia-induced apoptosis in HUVECs. Poly(ADP-ribose)polymerase (PARP) inhibitors decrease angiogenesis through reducing vascular endothelium growth factor (VEGF) induced proliferation,   migration  , and tube formation of human umbilical vein endothelial cells (HUVECs). In contrast to VEGF, pigment epithelium-derived factor (PEDF) has been demonstrated to act as a strong endogenous inhibitor of angiogenesis. Here, we show that PARP inhibition with a specific inhibitor PJ-34 or specific PARP antisense oligonucleotide upregulates hyperglycemia-induced PEDF expression in HUVECs in a dose-dependent manner. This results in the retard of activation of p38 MAP kinase and the concomitant decrease in cell apoptosis. These results give the first direct demonstration that PEDF might represent a target for PARP inhibition treatment and the effects of PEDF on endothelial cells growth are context dependent. 
 Antiangiogenic and Neurogenic Activities of Sleeping Beauty-Mediated PEDF-Transfected RPE Cells In Vitro and In Vivo. Pigment epithelium-derived factor (PEDF) is a potent multifunctional protein that inhibits angiogenesis and has neurogenic and neuroprotective properties. Since the wet form of age-related macular degeneration is characterized by choroidal neovascularization (CNV), PEDF would be an ideal candidate to inhibit CNV and support retinal pigment epithelial (RPE) cells. However, its short half-life has precluded its clinical use. To deliver PEDF to the subretinal space, we transfected RPE cells with the PEDF gene using the Sleeping Beauty transposon system. Transfected cells expressed and secreted biologically active recombinant PEDF (rPEDF). In cultures of human umbilical vein endothelial cells, rPEDF reduced VEGF-induced cumulative sprouting by ≥47%, decrease  d migrati  on by 77%, and increased rate of apoptosis at least 3.4 times. rPEDF induced neurite outgrowth in neuroblastoma cells and protected ganglion and photoreceptor cells in organotypic retinal cultures. In a rat model of CNV, subretinal transplantation of PEDF-transfected cells led to a reduction of the CNV area by 48% 14 days after transplantation and decreased clinical significant lesions by 55% and 40% after 7 and 14 days, respectively. We showed that transplantation of pigment epithelial cells overexpressing PEDF can restore a permissive subretinal environment for RPE and photoreceptor maintenance, while inhibiting choroidal blood vessel growth. 
 Mesenchymal stem cells overexpressing PEDF decrease the angiogenesis of gliomas. The present study is an exploration of a novel strategy to target a therapeutic gene to brain tumour tissues. In the present study, we evaluated the feasibility of using hMSCs (human mesenchymal stem cells) to deliver PEDF (pigment epithelium-derived factor), a potent inhibitor of tumour angiogenesis, in a model of intracranial gliomas. To assess its potential of tracking gliomas, MSCs (mesenchymal stem cells) were injected into the cerebral hemisphere and it showed that MSCs infiltrated into the vessel beds and scattered throughout the tumour. In vitro   migratio  n assay showed that the VEGF (vascular endothelial growth factor) enhanced MSC   migratio  n. In contrast, the migratory activity of MSCs was significantly inhibited with the presence of PEDF. Systematic delivery of AAV (adeno-associated virus)-PEDF to established glioma xenografts resulted in increased apoptosis of gliomas. In addition, MSC-PEDF treatment prolonged the survival of mice bearing U87 gliomas. Taken together, these data validate that MSCs-PEDF can migrate and deliver PEDF to target glioma cells, which may be a novel and promising therapeutic approach for refractory brain tumour. 
  GeneRIFs  
 PEDF inhibited breast cancer cell   migration   and invasion by down-regulating fibronectin 
 PEDF acts directly on monocytes/macrophages by inducing their   migration   and differentiation into M1-type cells in prostate. 
 MITF acts through PEDF to inhibit RPE cell   migration   and to play a significant role in regulating RPE cellular function. 
 PEDF modulated diverse categories of genes known to be involved in angiogenesis and   migration   in human melanoma cells. 
 These results indicate that PEDF may play an inhibitory role on growth and   migration   of HaCaT cells through dephosphorylation of ERK1/2. 
  ECM  PubMed  
 Proteomics characterization of extracellular space components in the human aorta. The vascular extracellular matrix (  ECM  ) is essential for the structural integrity of the vessel wall and also serves as a substrate for the binding and retention of secreted products of vascular cells as well as molecules coming from the circulation. Although proteomics has been previously applied to vascular tissues, few studies have specifically targeted the vascular   ECM   and its associated proteins. Thus, its detailed composition remains to be characterized. In this study, we describe a methodology for the extraction of extracellular proteins from human aortas and their identification by proteomics. The approach is based on (a) effective decellularization to enrich for scarce extracellular proteins, (b) successful solubilization and deglycosylation of   ECM   proteins, and (c) relative estimation of protein abundance using spectral counting. Our three-step extraction approach resulted in the identification of 103 extracellular proteins of which one-third have never been reported in the proteomics literature of vascular tissues. In particular, three glycoproteins (podocan, sclerostin, and agrin) were identified for the first time in human aortas at the protein level. We also identified extracellular adipocyte enhancer-binding protein 1, the cartilage glycoprotein asporin, and a previously hypothetical protein, retinal pigment epithelium (RPE) spondin. Moreover, our methodology allowed us to screen for proteolysis in the aortic samples based on the identification of proteolytic enzymes and their corresponding degradation products. For instance, we were able to detect matrix metalloproteinase-9 by mass spectrometry and relate its presence to degradation of fibronectin in a clinical specimen. We expect this proteomics methodology to further our understanding of the composition of the vascular extracellular environment, shed light on   ECM   remodeling and degradation, and provide insights into important pathological processes, such as plaque rupture, aneurysm formation, and restenosis. 
 Involvement of the collagen I-binding motif in the anti-angiogenic activity of pigment epithelium-derived factor. Pigment epithelium-derived factor (PEDF) is the most potent endogenous inhibitor of angiogenesis in age-related macular degeneration and tumors. However, the molecular mechanism of the anti-angiogenic activity of PEDF is poorly understood. PEDF interacts with the extracellular matrix (  ECM  ) in vitro. Here, we investigated the possible involvement of the motif for   ECM   interaction in the anti-angiogenic activity of PEDF. The growth rates of HeLa cells in culture were not affected by transfection of PEDF, indicating that PEDF did not suppress tumor cell growth directly. In tumor xenografts, the overexpression of wild-type PEDF significantly suppressed tumor growth, whereas a mutant of the collagen I-binding site of PEDF (Col-mut PEDF) did not inhibit tumor growth. A mutant of the heparin-binding site of PEDF (Hep-mut PEDF) suppressed tumor growth. Histological analysis showed that the density and area of microvasculatures in either PEDF or Hep-mut PEDF were suppressed when compared with those in either vector or Col-mut PEDF. Our data indicate that PEDF inhibits tumor growth via its anti-angiogenic activity, and the collagen I-binding motif of PEDF is involved in the biological activity. 
  [Ee]xtracellular [Mm]atrix  PubMed  
 Proteomics characterization of extracellular space components in the human aorta. The vascular   extracellular matrix   (ECM) is essential for the structural integrity of the vessel wall and also serves as a substrate for the binding and retention of secreted products of vascular cells as well as molecules coming from the circulation. Although proteomics has been previously applied to vascular tissues, few studies have specifically targeted the vascular ECM and its associated proteins. Thus, its detailed composition remains to be characterized. In this study, we describe a methodology for the extraction of extracellular proteins from human aortas and their identification by proteomics. The approach is based on (a) effective decellularization to enrich for scarce extracellular proteins, (b) successful solubilization and deglycosylation of ECM proteins, and (c) relative estimation of protein abundance using spectral counting. Our three-step extraction approach resulted in the identification of 103 extracellular proteins of which one-third have never been reported in the proteomics literature of vascular tissues. In particular, three glycoproteins (podocan, sclerostin, and agrin) were identified for the first time in human aortas at the protein level. We also identified extracellular adipocyte enhancer-binding protein 1, the cartilage glycoprotein asporin, and a previously hypothetical protein, retinal pigment epithelium (RPE) spondin. Moreover, our methodology allowed us to screen for proteolysis in the aortic samples based on the identification of proteolytic enzymes and their corresponding degradation products. For instance, we were able to detect matrix metalloproteinase-9 by mass spectrometry and relate its presence to degradation of fibronectin in a clinical specimen. We expect this proteomics methodology to further our understanding of the composition of the vascular extracellular environment, shed light on ECM remodeling and degradation, and provide insights into important pathological processes, such as plaque rupture, aneurysm formation, and restenosis. 
 The identification of myocilin-associated proteins in the human trabecular meshwork. Myocilin forms high molecular weight complexes in vivo presumably due to interaction with itself and other myocilin binding proteins. To identify myocilin interacting proteins, yeast 2-hybrid analysis was performed on >1x10(6) human trabecular meshwork cDNA clones. Coimmunoprecipitation and Far Western analysis were also performed on cell lysates obtained from fresh human trabecular meshworks or cultured human monolayer trabecular cell lines. Among the different methods, 46 candidate myocilin-associated proteins were identified, including molecules associated with the   extracellular matrix  , cytoskeleton, signaling, and metabolism. The most consistent interaction was myocilin-myocilin binding. Yeast-2 hybrid and Far Western analysis also found an association between myocilin and glyceraldehyde-3-phosphate dehydrogenase (GAPDH). None of the other candidate myocilin interacting proteins were identified in more than one method. Characterization of these potential interacting proteins may help to better understand the function of myocilin in the trabecular meshwork and aqueous outflow pathway. 
 Pigment epithelium-derived factor (PEDF) binds to glycosaminoglycans: analysis of the binding site. Pigment epithelium-derived factor (PEDF), a neurotrophic protein, is a secreted serpin identified in   extracellular matrix  es. We show that PEDF extractions from the interphotoreceptor matrix are more efficient with increasing NaCl concentrations, indicating that ionic interactions mediate its association with this polyanionic matrix. We have used affinity chromatography and ultrafiltration to probe for direct binding of PEDF to glycosaminoglycans/polyanions. Correctly folded PEDF bound to immobilized heparin, chondroitin sulfate-A, -B, -C, and dextran sulfate columns and eluted from each with an increase in NaCl concentration. However, in the presence of urea, the protein lost its affinity for heparin. Binding of PEDF to heparan sulfate proteoglycan in solution was in a concentration-dependent fashion (half-maximal specific binding EC50 = 40 micrograms/mL) and was sensitive to increasing NaCl concentrations. The glycosaminoglycan-binding region was analyzed using chemical modification and limited proteolysis. PEDF chemically modified on lysine residues by biotinylation lost its capacity for interacting with heparin, implicating the involvement of PEDF lysine residues in heparin binding. Cleavage of the serpin-exposed loop with chymotrypsin did not affect the heparin-binding property. A limited proteolysis product containing residues 21-approximately 260 bound to heparin with similar affinity as the intact PEDF. Homology modeling of PEDF based on the X-ray crystal structures of antithrombin III and ovalbumin shows a region at the center of beta-sheet A-strands 2 and 3- and helix F that has a basic electrostatic surface potential and is densely populated with lysines exposed to the surface (K134, K137, K189, K191, H212, and K214) that are available to interact with various glycosaminoglycans/polyanions. This region represents a novel site for glycosaminoglycan binding in a serpin, which in PEDF, is distinct and nonoverlapping from the PEDF neurotrophic active region. 
 The molecular impact of pigment epithelium-derived factor, PEDF, on lung cancer cells and the clinical significance. Pigment epithelium-derived factor (PEDF) is an endogenous protein factor that has been shown to act as anti-angiognesis factor. The present study aimed to determine the direct biological effects of PEDF on lung cancer cells and deduce a clinical relevance in patients with lung cancer, major cause of death worldwide in which the knowledge of PEDF remains poor. We constructed a mammalian expression system for human PEDF produced recombinant PEDF (rhPEDF) protein from 3T3 cells. The expression of PEDF was examined using SDS-PAGE and Western blot analysis. Using the rhPEDF protein, we investigated the biological function of PEDF in the lung cancer cells as well as endothelial cells. PEDF expression levels were assessed in a cohort of human lung cancer specimen (77 pairs of matched normal and tumour tissues), in association with patient clinical variables and survival, using quantitative analysis of PEDF. In vitro, we found that administration of rhPEDF on two lung cancer cell lines (A549 and SK-MES1) significantly reduced tumour cell growth (P<0.05) with no significant effect on the growth of vascular cell line (HECV). We also found that rhPEDF significantly decreased lung cancer motility and adhesion to   extracellular matrix   (Matrigel) when compared with the control cells (P<0.05). We showed that reduced PEDF levels in lung cancer tissues significantly correlated with lymph node metastasis and an overall poor prognosis in the lung cancer patients. PEDF suppresses the growth and motility of lung cancer cells and has a significant correlation with the clinical outcome of the patients. These results contribute to our understanding of the molecular mechanisms of PEDF and indicates a potential prognostic and therapeutic impact of PEDF in lung cancer. 
 Changes in the gene expression profile of A375 human melanoma cells induced by overexpression of multifunctional pigment epithelium-derived factor. Pigment epithelium-derived factor (PEDF) is a broad-spectrum angiogenesis inhibitor that displays potent antimetastatic activity in multiple tumor types. We have previously shown that PEDF prevents primary tumor growth and metastatic spread of human melanoma in mouse experimental models. Consistent with these observations, PEDF expression is lost at the late stages of melanoma progression, allowing melanoma cells to become angiogenic, migratory, and invasive. PEDF's ability to modify the interplay between the host and tumor tissues strongly supports its use as a therapeutic agent for the treatment of metastatic melanoma. However, transition to the clinic requires a more detailed knowledge of the molecular mechanisms underpinning PEDF's activity. In this study, we describe changes in the gene expression profile of A375 human melanoma cells induced by PEDF overexpression. PEDF modulated diverse categories of genes known to be involved in angiogenesis and migration. It downregulated cytokines such as interleukin-8 and   extracellular matrix   proteins such as collagen IV, while it upregulated fibronectin. Multiple transcripts previously described as contributing to the acquisition of malignant phenotype by melanoma were also diminished by PEDF overexpression, among which we validated galectin 3 and jagged 1. In addition, PEDF downregulated S100β and melanoma inhibitory activity, which are widely used in the pathological diagnosis of melanoma. Interestingly, PEDF increased the expression of melanophilin and decreased rab27A, which are relevant targets for melanosome transport; suggesting that PEDF could directly impinge on melanocytic lineage-specific processes. Our study identifies new molecular targets and signaling pathways that may potentially contribute to determine PEDF's ability to restrict the aggressiveness of A375 human melanoma cells. 
 Cytoplasmic expression and extracellular deposition of an antiangiogenic factor, pigment epithelium-derived factor, in human atherosclerotic plaques. To assess the expression and distribution of a neurotrophic/antiangiogenic factor, pigment epithelium-derived factor (PEDF), related to angiogenesis that is a possibly key event during atherogenesis in human atherosclerotic plaques.Twenty fresh aortic samples were used for reverse-transcription polymerase chain reaction (RT-PCR), Western blot, and immunohistochemistry (IHC). In addition, 80 stocked paraffin blocks of coronary arteries from 40 autopsy cases were also used. IHC revealed divergent staining patterns for PEDF in both the aortas and the coronary arteries tested, ie, "cytoplasmic staining" or "extracellular deposition," were observed, respectively. In the areas showing cytoplasmic staining, double PEDF was expressed in a majority of the foamy macrophages and in some smooth muscle cells, and the PEDF-positive cell frequency was positively correlated with that of microvessels in a cell-rich area in the coronary arteries (P<0.0001). Inversely, extracellular deposition of PEDF was seen in acellular areas and was negatively correlated with the number of microvessels (P=0.0003).These results suggest that PEDF may function as an antiangiogenic factor when it is deposited onto the   extracellular matrix  . Thus, PEDF may play a significant role in determining the balance of angiogenesis/ antiangiogenesis during atherogenesis. 
 Pigment epithelium-derived factor binds to hyaluronan. Mapping of a hyaluronan binding site. Pigment epithelium-derived factor (PEDF) is a multifunctional serpin with antitumorigenic, antimetastatic, and differentiating activities. PEDF is found within tissues rich in the glycosaminoglycan hyaluronan (HA), and its amino acid sequence contains putative HA-binding motifs. We show that PEDF coprecipitation with glycosaminoglycans in media conditioned by human retinoblastoma Y-79 cells decreased after pretreatments with hyaluronidase, implying an association between HA and PEDF. Direct binding of human recombinant PEDF to highly purified HA was demonstrated by coprecipitation in the presence of cetylpyridinium chloride. Binding of PEDF to HA was concentration-dependent and saturable. The PEDF-HA interactions were sensitive to increasing NaCl concentrations, indicating an ionic nature of these interactions and having affinity higher than PEDF-heparin. Competition assays showed that PEDF can bind heparin and HA simultaneously. PEDF chemically modified with fluorescein retained the capacity for interacting with HA but lacked heparin affinity, suggesting one or more distinct HA-binding regions on PEDF. The HA-binding region was examined by site-directed mutagenesis. Single-point and cumulative alterations at basic residues within the putative HA-binding motif K189A/K191A/R194A/K197A drastically reduced the HA-binding activity without affecting heparin- or collagen I binding of PEDF. Cumulative alterations at sites critical for heparin binding (K146A/K147A/R149A) decreased HA affinity but not collagen I binding. Thus these clusters of basic residues (BXBXXBXXB and BX3AB2XB motifs) in PEDF are functional regions for binding HA. In the spatial PEDF structure they are located in distinct areas away from the collagen-binding site. The HA-binding activity of PEDF may contribute to deposition in the   extracellular matrix   and to its reported antitumor/antimetastatic effects. 
 Pigment epithelium-derived factor (PEDF) shares binding sites in collagen with heparin/heparan sulfate proteoglycans. Pigment epithelium-derived factor (PEDF) is a collagen-binding protein that is abundantly distributed in various tissues, including the eye. It exhibits various biological functions, such as anti-angiogenic, neurotrophic, and neuroprotective activities. PEDF also interacts with   extracellular matrix   components such as collagen, heparan sulfate proteoglycans (HSPGs), and hyaluronan. The collagen-binding property has been elucidated to be important for the anti-angiogenic activity in vivo (Hosomichi, J., Yasui, N., Koide, T., Soma, K., and Morita, I. (2005) Biochem. Biophys. Res. Commun. 335, 756-761). Here, we investigated the collagen recognition mechanism by PEDF. We first narrowed down candidate PEDF-binding sequences by taking advantage of previously reported structural requirements in collagen. Subsequent searches for PEDF-binding sequences employing synthetic collagen-like peptides resulted in the identification of one of the critical binding sites for PEDF, human α1(I)(929-938) (IKGHRGFSGL). Further analysis revealed that the collagen recognition by PEDF is sequence- and conformation-specific, and the high affinity binding motif is KGXRGFXGL in the triple helix. The PEDF-binding motif significantly overlapped with the heparin/HSPG-binding motif, KGHRG(F/Y). The interaction of PEDF with collagen I was specifically competed with by heparin but not by chondroitin sulfate-C or hyaluronan. The binding sequences for PEDF and heparin/HSPG also overlapped with the covalent cross-linking sites between collagen molecules. These findings imply a functional relationship between PEDF and HSPGs during angiogenesis, and the interaction of these molecules is regulated by collagen modifications. 
 Involvement of the collagen I-binding motif in the anti-angiogenic activity of pigment epithelium-derived factor. Pigment epithelium-derived factor (PEDF) is the most potent endogenous inhibitor of angiogenesis in age-related macular degeneration and tumors. However, the molecular mechanism of the anti-angiogenic activity of PEDF is poorly understood. PEDF interacts with the   extracellular matrix   (ECM) in vitro. Here, we investigated the possible involvement of the motif for ECM interaction in the anti-angiogenic activity of PEDF. The growth rates of HeLa cells in culture were not affected by transfection of PEDF, indicating that PEDF did not suppress tumor cell growth directly. In tumor xenografts, the overexpression of wild-type PEDF significantly suppressed tumor growth, whereas a mutant of the collagen I-binding site of PEDF (Col-mut PEDF) did not inhibit tumor growth. A mutant of the heparin-binding site of PEDF (Hep-mut PEDF) suppressed tumor growth. Histological analysis showed that the density and area of microvasculatures in either PEDF or Hep-mut PEDF were suppressed when compared with those in either vector or Col-mut PEDF. Our data indicate that PEDF inhibits tumor growth via its anti-angiogenic activity, and the collagen I-binding motif of PEDF is involved in the biological activity. 
  Component  
   extracellular matrix   
  GeneRIFs  
 The HA-binding activity of PEDF may contribute to deposition in the   extracellular matrix   and to its reported antitumor/antimetastatic effects. 
  [Ii]nvasive  PubMed  
 A novel role for microphthalmia-associated transcription factor-regulated pigment epithelium-derived factor during melanoma progression. Microphthalmia-associated transcription factor (MITF) acts via pigment epithelium-derived factor (PEDF), an antiangiogenic protein, to regulate retinal pigment epithelium migration. PEDF expression and/or regulation during melanoma development have not been investigated previously. Using immunohistochemistry, we determined expression of PEDF in common and dysplastic melanocytic nevi, melanoma in situ,   invasive   melanoma, and metastatic melanoma (n = 102). PEDF expression was consistently decreased in   invasive   and metastatic melanoma, compared with nevi and melanoma in situ (P < 0.0001). PEDF was lost in thicker melanomas (P = 0.003), and correlated with depth of invasion (P = 0.003) and distant metastasis (P = 0.0331), but only marginally with mitotic index, AJCC stage, nodal metastasis, or blood vascular density (0.05 < P < 0.10). Quantitative real-time PCR and microarray analyses confirmed PEDF down-regulation at the mRNA level in several melanoma lines, compared with melanocytes. MITF positively correlated with PEDF ex  pression   in invasive melanomas (P = 0.0003). Searching for PEDF regulatory mechanisms revealed two occupied conserved E-boxes (DNA recognition elements) in the first intron of the human and mouse PEDF promoter regions, confirmed by binding assays. Dominant-negative and siRNA approaches in vivo demonstrated direct transcriptional influence of MITF on PEDF, establishing the PEDF gene (SERPINF1) as a MITF target in melanocytes and melanoma cells. These findings suggest that loss of PEDF expression   promotes   early invasive melanoma growth. 
 Hypoxia negatively regulates antimetastatic PEDF in melanoma cells by a hypoxia inducible factor-independent, autophagy dependent mechanism. Pigment epithelium-derived factor (PEDF), a member of the serine protease inhibitor (SERPIN) superfamily, displays a potent antiangiogenic and antimetastatic activity in a broad range of tumor types. Melanocytes and low aggressive melanoma cells secrete high levels of PEDF, while its expression is lost in highly aggressive melanomas. PEDF efficiently abrogates a number of functional properties critical for the acquisition of metastatic ability by melanoma cells, such as neovascularization, proliferation, migration,   invasive  ness and extravasation. In this study, we identify hypoxia as a relevant negative regulator of PEDF in melanocytes and low aggressive melanoma cells. PEDF was regulated at the protein level. Importantly, although downregulation of PEDF was induced by inhibition of 2-oxoglutarate-dependent dioxygenases, it was independent of the hypoxia inducible factor (HIF), a key mediator of the adaptation to hypoxia. Decreased PEDF protein was not mediated by inhibition of translation through untranslated regions (UTRs) in melanoma cells. Degradation by metalloproteinases, implicated on PEDF degradation in retinal pigment epithelial cells, or by the proteasome, was also excluded as regulatory mechanism in melanoma cells. Instead, we found that degradation by autophagy was critical for PEDF downregulation under hypoxia in human melanoma cells. Our findings show that hypoxic conditions encountered during primary melanoma growth downregulate antiangiogenic and antimetastasic PEDF by a posttranslational mechanism involving degradation by autophagy and could therefore contribute to the acquisition of highly metastatic potential characteristic of aggressive melanoma cells. 
 Changes in the gene expression profile of A375 human melanoma cells induced by overexpression of multifunctional pigment epithelium-derived factor. Pigment epithelium-derived factor (PEDF) is a broad-spectrum angiogenesis inhibitor that displays potent antimetastatic activity in multiple tumor types. We have previously shown that PEDF prevents primary tumor growth and metastatic spread of human melanoma in mouse experimental models. Consistent with these observations, PEDF expression is lost at the late stages of melanoma progression, allowing melanoma cells to become angiogenic, migratory, and   invasive  . PEDF's ability to modify the interplay between the host and tumor tissues strongly supports its use as a therapeutic agent for the treatment of metastatic melanoma. However, transition to the clinic requires a more detailed knowledge of the molecular mechanisms underpinning PEDF's activity. In this study, we describe changes in the gene expression profile of A375 human melanoma cells induced by PEDF overexpression. PEDF modulated diverse categories of genes known to be involved in angiogenesis and migration. It downregulated cytokines such as interleukin-8 and extracellular matrix proteins such as collagen IV, while it upregulated fibronectin. Multiple transcripts previously described as contributing to the acquisition of malignant phenotype by melanoma were also diminished by PEDF overexpression, among which we validated galectin 3 and jagged 1. In addition, PEDF downregulated S100β and melanoma inhibitory activity, which are widely used in the pathological diagnosis of melanoma. Interestingly, PEDF increased the expression of melanophilin and decreased rab27A, which are relevant targets for melanosome transport; suggesting that PEDF could directly impinge on melanocytic lineage-specific processes. Our study identifies new molecular targets and signaling pathways that may potentially contribute to determine PEDF's ability to restrict the aggressiveness of A375 human melanoma cells. 
 Pigment epithelium-derived factor is differentially expressed in peripheral neuropathies. Peripheral neuropathies are characterized by asymmetrical slowly progressive weakness with no upper motor neuron signs, and can occur either with or without pain. Due to poor knowledge of the disease mechanisms, available pain treatment is very limited. Because of the difficulties and   invasive  ness involved when performing direct analysis on peripheral and CNS, pathological markers can be searched for in the cerebrospinal fluid (CSF) as an alternative. To investigate pain mechanisms in peripheral neuropathy and find diagnostic markers, CSF samples were analyzed by a differential expression proteomic approach. We studied CSF from: neuropathic patients with pain (PN), without pain (NPN) and healthy controls (CN). 2-DE analysis showed ten protein spots differentially expressed, and six of these were identified by MS. In NPN patients we found an expression level decrease of three pigment epithelium-derived factor (PEDF) protein isoforms. Immunoblot with a specific antibody revealed the presence of additional PEDF isoforms not highlighted by differential expression analysis. Fucose residues on the oligosaccharide chain were found only in the isoforms down regulated in NPN patients. Considered as PEDF has important neurobiological effects, it might be considered an interesting pathology marker. 
 Evaluation of protein pigment epithelium-derived factor (PEDF) and microvessel density (MVD) as prognostic indicators in breast cancer. Angiogenesis, which plays an important role in tumor growth and metastasis, is regulated by a balance between angiogenic stimulators and inhibitors. Pigment epithelium-derived factor (PEDF), a secreted glycoprotein is an important inhibitor of angiogenesis. Although the precise mechanisms by which PEDF exerts its actions remain poorly understood, there is growing evidence supporting the role of PEDF as a candidate antitumor agent. In this study, we investigated the role of PEDF in breast cancer.We investigated the correlation of PEDF protein levels with cancer progression and prognosis in patients with   invasive   ductal breast cancer (IDC). We used immunohistochemistry in a cohort of 119 breast cancer patients to examine the expression of PEDF protein with an anti-PEDF antibody and to measure the microvessel density (MVD) with an anti-CD34 antibody.PEDF was an endogenous inhibitor of angiogenesis in endothelial cells. Decreased intratumoral expression of PEDF was associated with a higher microvessel density (MVD), a more metastatic phenotype, and poorer clinical outcome. PEDF was positive in 43.7% patients. Patients with low PEDF expression had a significantly higher MVD count when compared with patients with high PEDF expression. In univariate and multivariate analysis, PEDF was an independent prognostic factor.The inverse correlation between PEDF expression and MVD in human breast cancer suggests that low PEDF expression is associated with angiogenesis in breast cancer. PEDF expression is therefore a potentially useful prognostic marker for breast cancer. 
 Concurrent PEDF deficiency and Kras mutation induce   invasive   pancreatic cancer and adipose-rich stroma in mice. Pigment epithelium-derived factor (PEDF), a non-inhibitory SERPIN with potent antiangiogenic activity, has been recently implicated in metabolism and adipogenesis, both of which are known to influence pancreatic cancer progression. Increased pancreatic fat in human pancreatic tumour correlates with greater tumour dissemination while PEDF deficiency in mice promotes pancreatic hyperplasia and visceral obesity. Oncogenic Ras, the most common mutation in pancreatic ductal adenocarcinoma (PDAC), has similarly been shown to promote adipogenesis and premalignant lesions.In order to determine whether concurrent loss of PEDF is sufficient to promote adipogenesis and tumorigenesis in the pancreas, the authors ablated PEDF in an EL-Kras(G12D) mouse model of non-  invasive   cystic papillary neoplasms.EL-Kras(G12D)/PEDF deficient mice developed   invasive   PDAC associated with enhanced matrix metalloproteinase (MMP)-2 and MMP-9 expression and increased peripancreatic fat with adipocyte hypertrophy and intrapancreatic adipocyte infiltration (pancreatic steatosis). In support of increased adipogenesis, the stroma of the pancreas of EL-Kras(G12D)/PEDF deficient mice demonstrated higher tissue levels of two lipid droplet associated proteins, tail-interacting protein 47 (TIP47, perilipin 3) and adipose differentiation-related protein (ADRP, Pperilipin 2), while adipose triglyceride lipase, a key factor in lipolysis, was decreased. In patients with PDAC, both tissue and serum levels of PEDF were decreased, stromal TIP47 expression was higher and the tissue VEGF to PEDF ratio was increased (p<0.05).These data highlight the importance of lipid metabolism in the tumour microenvironment and identify PEDF as a critical negative regulator of both adiposity and tumour invasion in the pancreas. 
 PEDF inhibits growth and   invasive  ness of endometrial cancer cells in vitro. Pigment epithelium-derived factor (PEDF) is pluripotent in both antiangiogenesis and direct tumor inhibition. We thus investigated role of PEDF in endometrial cancer cells in vitro.Exogenous PEDF was delivered into Ishikawa and KLE cells by adenovirus system. Realtime RT-PCR was used to detect expressional change of several angiogenesis-related factors. Proliferation and viability tests were performed to establish suitable transduction titre. Cell cycle and apoptosis assays were conducted to evaluate inhibitive effects of PEDF on tumor cell growth. Transwell tests were carried out to investigate the retardation of tumor cell   invasive  ness by PEDF.PEDF induced decrease in viability, proliferation and   invasive  ness of both endometrial tumor cell lines. Incremented cell apoptosis and aggregated population in G1 phase of cell cycle was noted in PEDF groups. PEDF also induced down-regulation of vascular endothelial growth factor (VEGF) and matrix metalloproteinase-9 (MMP-9) and up-regulation of thrombospondin-1 (TSP-1) in both cell lines.Adenovirus mediated PEDF is potent in retarding the growth and   invasive  ness of endometrial cancer cells. PEDF can become promising as novel therapeutic strategy in endometrial carcinoma. 
  GeneRIFs  
 Adenovirus mediated PEDF is potent in retarding the growth and   invasive  ness of endometrial cancer cells. 
  [Mm]etastasis  PubMed  
 Update of the human and mouse SERPIN gene superfamily. The serpin family comprises a structurally similar, yet functionally diverse, set of proteins. Named originally for their function as serine proteinase inhibitors, many of its members are not inhibitors but rather chaperones, involved in storage, transport, and other roles. Serpins are found in genomes of all kingdoms, with 36 human protein-coding genes and five pseudogenes. The mouse has 60 Serpin functional genes, many of which are orthologous to human SERPIN genes and some of which have expanded into multiple paralogous genes. Serpins are found in tissues throughout the body; whereas most are extracellular, there is a class of intracellular serpins. Serpins appear to have roles in inflammation, immune function, tumorigenesis, blood clotting, dementia, and cancer   metastasis  . Further characterization of these proteins will likely reveal potential biomarkers and therapeutic targets for disease. 
 Pigment epithelium-derived factor regulates the vasculature and mass of the prostate and pancreas. Angiogenesis sustains tumor growth and   metastasis  , and recent studies indicate that the vascular endothelium regulates tissue mass. In the prostate, androgens drive angiogenic inducers to stimulate growth, whereas androgen withdrawal leads to decreased vascular endothelial growth factor, vascular regression and epithelial cell apoptosis. Here, we identify the angiogenesis inhibitor pigment epithelium-derived factor (PEDF) as a key inhibitor of stromal vasculature and epithelial tissue growth in mouse prostate and pancreas. In PEDF-deficient mice, stromal vessels were increased and associated with epithelial cell hyperplasia. Androgens inhibited prostatic PEDF expression in cultured cells. In vivo, androgen ablation increased PEDF in normal rat prostates and in human cancer biopsies. Exogenous PEDF induced tumor epithelial apoptosis in vitro and limited in vivo tumor xenograft growth, triggering endothelial apoptosis. Thus, PEDF regulates normal pancreas and prostate mass. Its androgen sensitivity makes PEDF a likely contributor to the anticancer effects of androgen ablation. 
 A novel role for microphthalmia-associated transcription factor-regulated pigment epithelium-derived factor during melanoma progression. Microphthalmia-associated transcription factor (MITF) acts via pigment epithelium-derived factor (PEDF), an antiangiogenic protein, to regulate retinal pigment epithelium migration. PEDF expression and/or regulation during melanoma development have not been investigated previously. Using immunohistochemistry, we determined expression of PEDF in common and dysplastic melanocytic nevi, melanoma in situ, invasive melanoma, and metastatic melanoma (n = 102). PEDF expression was consistently decreased in invasive and metastatic melanoma, compared with nevi and melanoma in situ (P < 0.0001). PEDF was lost in thicker melanomas (P = 0.003), and correlated with depth of invasion (P = 0.003) and di  stant meta  stasis (P = 0.0331), but only marginally with mitotic index, AJCC stage  , nodal me  tastasis, or blood vascular density (0.05 < P < 0.10). Quantitative real-time PCR and microarray analyses confirmed PEDF down-regulation at the mRNA level in several melanoma lines, compared with melanocytes. MITF positively correlated with PEDF expression in invasive melanomas (P = 0.0003). Searching for PEDF regulatory mechanisms revealed two occupied conserved E-boxes (DNA recognition elements) in the first intron of the human and mouse PEDF promoter regions, confirmed by binding assays. Dominant-negative and siRNA approaches in vivo demonstrated direct transcriptional influence of MITF on PEDF, establishing the PEDF gene (SERPINF1) as a MITF target in melanocytes and melanoma cells. These findings suggest that loss of PEDF expression promotes early invasive melanoma growth. 
 Pigment epithelium-derived factor overexpression inhibits orthotopic osteosarcoma growth, angiogenesis and   metastasis  . Despite significant improvements, the current management of primary osteosarcoma is still limited by the development of metastatic disease, which occurs in approximately 30% of patients despite aggressive multiagent chemotherapy and tumor-ablative surgery. Therefore, there is a need for the development of novel agents to improve the outcome of these patients. Pigment epithelium-derived factor (PEDF) has been shown to be one of the most potent inhibitors of angiogenesis, and more recently has demonstrated a functional role in tumor growth, invasion and   metastasis  . In this study we report, for the first time, the multitargeted role of PEDF in the inhibition of growth, angiogenesis and   metastasis   of two orthotopic models of osteosarcoma (rat UMR 106-01 and human SaOS-2). Through stable plasmid-mediated gene transfer of full-length human PEDF, we show that PEDF overexpression significantly reduced tumor cell proliferation (P<0.05) and Matrigel invasion (UMR(PEDF), P<0.001; SaOS(PEDF), P<0.05) and increased adhesion to collagen type-1 (P<0.01), in vitro. In vivo, PEDF overexpression dramatically suppressed orthotopic osteosarcoma growth (P<0.05) and the development of spontaneous pulmonary metastases (UMR(PEDF), P<0.05; SaOS(PEDF), P<0.001). Furthermore, PEDF-overexpressing tumors exhibited reduced intratumoral angiogenesis, evidenced by a significant decrease in microvessel density (P<0.05). Therefore, together these results suggest that PEDF may be a new and promising approach for the treatment of osteosarcoma. 
 The molecular impact of pigment epithelium-derived factor, PEDF, on lung cancer cells and the clinical significance. Pigment epithelium-derived factor (PEDF) is an endogenous protein factor that has been shown to act as anti-angiognesis factor. The present study aimed to determine the direct biological effects of PEDF on lung cancer cells and deduce a clinical relevance in patients with lung cancer, major cause of death worldwide in which the knowledge of PEDF remains poor. We constructed a mammalian expression system for human PEDF produced recombinant PEDF (rhPEDF) protein from 3T3 cells. The expression of PEDF was examined using SDS-PAGE and Western blot analysis. Using the rhPEDF protein, we investigated the biological function of PEDF in the lung cancer cells as well as endothelial cells. PEDF expression levels were assessed in a cohort of human lung cancer specimen (77 pairs of matched normal and tumour tissues), in association with patient clinical variables and survival, using quantitative analysis of PEDF. In vitro, we found that administration of rhPEDF on two lung cancer cell lines (A549 and SK-MES1) significantly reduced tumour cell growth (P<0.05) with no significant effect on the growth of vascular cell line (HECV). We also found that rhPEDF significantly decreased lung cancer motility and adhesion to extracellular matrix (Matrigel) when compared with the control cells (P<0.05). We showed that reduced PEDF levels in lung cancer tissues significantly correlated with lymph node   metastasis   and an overall poor prognosis in the lung cancer patients. PEDF suppresses the growth and motility of lung cancer cells and has a significant correlation with the clinical outcome of the patients. These results contribute to our understanding of the molecular mechanisms of PEDF and indicates a potential prognostic and therapeutic impact of PEDF in lung cancer. 
 Constitutive overexpression of pigment epithelium-derived factor inhibition of ocular melanoma growth and   metastasis  . Pigment epithelium-derived factor (PEDF) is known to be an angiogenesis suppressor and to have antitumor effects. This study investigates whether constitutive overexpression of PEDF inhibits the growth and hepatic micro  metastasis   of ocular melanoma.Real-time RT-PCR was used to detect endogenous PEDF expression in human uveal melanoma cell lines and mouse melanoma cells. A lentiviral vector containing a mouse PEDF expression sequence was constructed and transduced into mouse melanoma cells in vitro. Transgene expression was assessed by Western blot analysis. Angiogenesis and transendothelial migration assays were performed in constitutively stable PEDF-overexpressing cells and transduced lentiviral vector control cells. The size and microvessel density of the ocular tumor and the number of hepatic micro  metastasis   were compared between the mice inoculated with PEDF-overexpressing tumor cells and those mice with the control cell line.Four human uveal melanoma and three mouse melanoma cell lines were found to express PEDF mRNA. Endogenous overexpressing PEDF melanoma cells lost the ability to migrate and form tubes in vitro. In the animal experiment, the size of the ocular melanoma and the number of hepatic micro  metastasis   were decreased and microvessel density was also reduced in mice inoculated with constitutively overexpressing PEDF melanoma cells.Lentivirus-mediated gene transfer of PEDF decreased the growth of ocular melanoma and its hepatic micro  metastasis   in a mouse ocular melanoma model. Dual antitumor/antiangiogenic activities of PEDF suggest that PEDF gene therapy may be considered an approach for the treatment of ocular melanoma. 
 [Inhibition and significance of pigment epithelium-derived factor in the development and   metastasis   of prostate cancer]. To study the inhibition and significance of pigment epithelium-derived factor (PEDF) in the development and   metastasis   of prostate cancer.The expression of PEDF was examined in the normal prostate tissue, benign prostatic hyperplasia, prostate cancer tissue and prostate cancer cell lines, PC-3 and Lncap by immunohistochemical SP method and Western blot. In combination with clinical data, statistical analysis was performed to evaluate the relation of the expression level of PEDF in prostate cancer and the relationship between different histological grades of prostate cancer.In normal prostate tissue and benign prostate tissue, the expression of PEDF were elevated and it was far higher than the prostate cancer and prostate cancer cell line. The expression of PEDF and the pathological grade of prostate cancer were related to the differentiated carcinoma of prostate tissue, and the expression level of PEDF in poorly differentiated carcinoma below the highly differentiated carcinoma of prostate cancer. In metastatic prostate cancer, the expression of PEDF was lower than that of prostate cancer without   metastasis   (12% vs 43.1%).The expression of PEDF and the incidence of prostate cancer have a negative correlation. The lower grade of prostate cancer cells, the less the tissue expression of PEDF and the higher tendency of invasion and   metastasis  . 
 Laminin receptor involvement in the anti-angiogenic activity of pigment epithelium-derived factor. Pigment epithelium-derived factor (PEDF) is a multifunctional protein with neurotrophic, anti-oxidative, and anti-inflammatory properties. It is also one of the most potent endogenous inhibitors of angiogenesis, playing an important role in restricting tumor growth, invasion, and   metastasis  . Studies show that PEDF binds to cell surface proteins, but little is known about how it exerts its effects. Recently, research identified phospholipase A(2)/nutrin/patatin-like phospholipase domain-containing 2 as one PEDF receptor. To identify other receptors, we performed yeast two-hybrid screening using PEDF as bait and discovered that the non-integrin 37/67-kDa laminin receptor (LR) is another PEDF receptor. Co-immunoprecipitation, His tag pulldown, and surface plasmon resonance assays confirmed the interaction between PEDF and LR. Using the yeast two-hybrid method, we further restricted the LR-interacting domain on PEDF to a 34-amino acid (aa) peptide (aa 44-77) and the PEDF-interacting domain on LR to a 91-aa fragment (aa 120-210). A 25-mer peptide named P46 (aa 46-70), derived from 34-mer, interacts with LR in surface plasmon resonance assays and binds to endothelial cell (EC) membranes. This peptide induces EC apoptosis and inhibits EC migration, tube-like network formation in vitro, and retinal angiogenesis ex vivo, like PEDF. Our results suggest that LR is a real PEDF receptor that mediates PEDF angiogenesis inhibition. 
 The reduction in pigment epithelium-derived factor is a sign of malignancy in ovarian cancer expressing low-level of vascular endothelial growth factor. Angiogenesis is a critical factor in the progression of solid tumors and   metastasis  . The aim of this study was to characterise the roles of angiogenic and anti-angiogenic factors on ovarian cancer.The expression levels of vascular endothelial growth factor (VEGF, angiogenic factor) and pigment epithelial growth factor (PEDF, anti-angiogenic factor) were measured by real-time polymerase chain reaction and Western blotting in ovarian tumors. Microvessel density (MVD) was evaluated by the total microvessel length in high-power field of tumor tissue preparations.MVD correlated with tumor malignancy. The tissues with the highest expression levels of VEGF (VEGF-H) were malignant tumors. The VEGF expression levels in some malignant tumors (VEGF-L) were as low as that in benign tumors. Therefore, the expression of PEDF was examined. The PEDF expression levels in VEGF-L malignant tumors were significantly lower than those in benign tumors. On the other hand, the PEDF expression levels in VEGF-H malignant tumor tissues were not significantly different from those in benign tumors.The reduction in PEDF expression levels may be, in part, responsible for tumor malignancy in VEGF-L ovarian tumors. Furthermore, PEDF may be a useful marker of malignancy in VEGF-L ovarian tumors. 
 Melanoma Cells Block PEDF Production in Fibroblasts to Induce the Tumor-Promoting Phenotype of Cancer-Associated Fibroblasts. Loss of pigment epithelium-derived factor (PEDF, SERPINF1) in cancer cells is associated with poor prognosis and   metastasis  , but the contribution of stromal PEDF to cancer evolution is poorly understood. Therefore, we investigated the role of fibroblast-derived PEDF in melanoma progression. We demonstrate that normal dermal fibroblasts expressing high PEDF levels attenuated melanoma growth and angiogenesis in vivo, whereas PEDF-depleted fibroblasts exerted tumor-promoting effects. Accordingly, mice with global PEDF knockout were more susceptible to melanoma   metastasis  . We also demonstrate that normal fibroblasts in close contact with PEDF-null melanoma cells lost PEDF expression and tumor-suppressive properties. Further mechanistic investigations underlying the crosstalk between tumor and stromal cells revealed that melanoma cells produced PDGF-BB and TGFβ, which blocked PEDF production in fibroblasts. Notably, cancer-associated fibroblasts (CAF) isolated from patient-derived tumors expressed markedly low levels of PEDF. Treatment of patient CAF and TGFβ-treated normal fibroblasts with exogenous PEDF decreased the expression of CAF markers and restored PEDF expression. Finally, expression profiling of PEDF-depleted fibroblasts revealed induction of IL8, SERPINB2, hyaluronan synthase-2, and other genes associated with tumor promotion an  d metastas  is. Collectively, our results demonstrate that PEDF maintains tumor-suppressive functions in fibroblasts to prevent CAF conversion and illustrate the mechanisms by which melanoma cells silence stromal PEDF to promote malignancy. Cancer Res; 76(8); 2265-76. ©2016 AACR. 
 Efficacy of continuously administered PEDF-derived synthetic peptides against osteosarcoma growth and   metastasis  . The potent antiangiogenic pigment epithelium-derived factor (PEDF) has shown promise against osteosarcoma, a tumour that originates in the bone and   metastasis  es to the lungs. Neurotrophic, antiangiogenic, antiproliferative, and antimetastatic properties of PEDF have been attributed to a number of functional epitopes on the PEDF glycoprotein. StVOrth-2 (residues 78-102) and StVOrth-3 (residues 90-114) are two PEDF-derived peptides based on these functional epitopes. StVOrth-2 has previously been shown to inhibit osteosarcoma cell proliferation, while StVOrth-3 increased osteosarcoma cell adhesion to collagen I in vitro. In this paper, we have evaluated systemically and continuously delivered StVOrth-2 and StVOrth-3 using a clinically relevant murine model of osteosarcoma with spontaneous   metastasis  . Treatment with StVOrth-2 or StVOrth-3 with microosmotic pumps was initiated after primary osteosarcoma was established in the tibia. While treatment with StVOrth-2 and StVOrth-3 did not appear to affect local tumour invasion, tumour necrosis or apoptosis, StVOrth-2 predominantly restricted the growth of primary tumours, while StVOrth-3 restricted the burden of pulmonary metastatic disease. No peptide caused gross toxicity in mouse tissues as assessed by measuring weight of animals, serum biochemistry, and gross tissue observation. The differential effects exhibited by StVOrth-2 and StVOrth-3 in this orthotopic model of osteosarcoma may be related to the functional epitopes on the PEDF glycoprotein that they represent. 
 Opposing effects of pigment epithelium-derived factor on breast cancer cell versus neuronal survival: implication for brain   metastasis   and   metastasis  -induced brain damage. Brain metastases are a significant cause of morbidity and mortality for patients with cancer, yet preventative and therapeutic options remain an unmet need. The cytokine pigment epithelium-derived factor (PEDF) is downregulated in resected human brain metastases of breast cancer compared with primary breast tumors, suggesting that restoring its expression might limit metastatic spread. Here, we show that outgrowth of large experimental brain metastases from human 231-BR or murine 4T1-BR breast cancer cells was suppressed by PEDF expression, as supported by in vitro analyses as well as direct intracranial implantation. Notably, the suppressive effects of PEDF were not only rapid but independent of the effects of this factor on angiogenesis. Paralleling its cytotoxic effects on breast cancer cells, PEDF also exerted a prosurvival effect on neurons that shielded the brain from tumor-induced damage, as indicated by a relative 3.5-fold reduction in the number of dying neurons adjacent to tumors expressing PEDF. Our findings establish PEDF as both a metastatic suppressor and a neuroprotectant in the brain, highlighting its role as a double agent in limiting brain   metastasis   and its local consequences. 
 Evaluation of protein pigment epithelium-derived factor (PEDF) and microvessel density (MVD) as prognostic indicators in breast cancer. Angiogenesis, which plays an important role in tumor growth and   metastasis  , is regulated by a balance between angiogenic stimulators and inhibitors. Pigment epithelium-derived factor (PEDF), a secreted glycoprotein is an important inhibitor of angiogenesis. Although the precise mechanisms by which PEDF exerts its actions remain poorly understood, there is growing evidence supporting the role of PEDF as a candidate antitumor agent. In this study, we investigated the role of PEDF in breast cancer.We investigated the correlation of PEDF protein levels with cancer progression and prognosis in patients with invasive ductal breast cancer (IDC). We used immunohistochemistry in a cohort of 119 breast cancer patients to examine the expression of PEDF protein with an anti-PEDF antibody and to measure the microvessel density (MVD) with an anti-CD34 antibody.PEDF was an endogenous inhibitor of angiogenesis in endothelial cells. Decreased intratumoral expression of PEDF was associated with a higher microvessel density (MVD), a more metastatic phenotype, and poorer clinical outcome. PEDF was positive in 43.7% patients. Patients with low PEDF expression had a significantly higher MVD count when compared with patients with high PEDF expression. In univariate and multivariate analysis, PEDF was an independent prognostic factor.The inverse correlation between PEDF expression and MVD in human breast cancer suggests that low PEDF expression is associated with angiogenesis in breast cancer. PEDF expression is therefore a potentially useful prognostic marker for breast cancer. 
 Pigment epithelium-derived factor (PEDF) inhibits breast cancer   metastasis   by down-regulating fibronectin. Pigment epithelium-derived factor (PEDF) plays an important role in the tumor growth and   metastasis   inhibition. It has been reported that PEDF expression is significantly reduced in breast cancer, and associated with disease progression and poor patient outcome. However, the exact mechanism of PEDF on breast cancer   metastasis   including liver and lung   metastasis   remains unclear. The present study aims to reveal the impact of PEDF on breast cancer. The orthotopic tumor mice model inoculated by MDA-MB-231 cells stably expressing PEDF or control cells was used to assess liver and lung   metastasis   of breast cancer. In vitro, migration and invasion experiments were used to detect the metastatic abilities of MDA-MB-231 and SKBR3 breast cancer cells with or without overexpression of PEDF. The metastatic-related molecules including EMT makers, fibronectin, and p-AKT and p-ERK were detected by qRT-PCR, Western blot, and Fluorescent immunocytochemistry. PEDF significantly inhibited breast cancer growth and   metastasis   in vivo and in vitro. Mechanically, PEDF inhibited breast cancer cell migration and invasion by down-regulating fibronectin and subsequent MMP2/MMP9 reduction via p-ERK and p-AKT signaling pathways. However, PEDF had no effect on EMT conversion in the breast cancer cells which was usually involved in cancer   metastasis  . Furthermore, the study showed that laminin receptor mediated the down-regulation of fibronectin by PEDF. These results reported for the first time that PEDF inhibited breast cancer   metastasis   by down-regulating fibronectin via laminin receptor/AKT/ERK pathway. Our findings demonstrated PEDF as a dual effector in limiting breast cancer growth and   metastasis   and highlighted a new avenue to block breast cancer progression. 
 In vivo gene transfer of pigment epithelium-derived factor inhibits tumor growth in syngeneic murine models of thoracic malignancies. Pigment epithelium-derived factor is known to be an inhibitor of angiogenesis. We hypothesized that in vivo gene transfer of pigment epithelium-derived factor may inhibit tumor angiogenesis and growth in syngeneic models of thoracic malignancies.An adenovirus vector encoding the human pigment epithelium-derived factor cDNA (AdPEDF) was used to transduce human lung cancer cells in vitro. Transgene expression was assessed using Western analysis. Three different murine flank tumors (2 lung, 1 colon) were then established in syngeneic mice and treated intratumorally with phosphate-buffered saline, AdPEDF, or an empty vector (AdNull). Endpoints measured included transgene expression, tumor size, and animal survival, as well as microvessel density within the tumor. Additionally, a murine pulmonary   metastasis   model was established by intravenous injection of a syngeneic colon adenocarcinoma cell line expressing a marker gene (beta-galactosidase). One day later, treatment (phosphate-buffered saline, AdNull, or AdPEDF) was administered intrapleurally. Tumor burden (gross and histologic inspection, lung weight, and beta-galactosidase expression) was then evaluated 13 days after vector dosing, and survival was recorded.AdPEDF-derived expression of pigment epithelium-derived factor was demonstrated in vitro and in vivo. In syngeneic murine lung cancer flank tumors, intratumoral administration of AdPEDF significantly inhibited tumor growth (P <.01), prolonged mouse survival (P <.01), and decreased microvessel density (P <.01) compared with control groups. In the pulmonary   metastasis   model, AdPEDF-treated mice exhibited significantly reduced lung lesions, lung weight (P <.0005), beta-galactosidase expression (P <.05), and animal survival was prolonged (P <.05).Gene transfer of pigment epithelium-derived factor suppresses tumor vascularization and growth, while prolonging survival in syngeneic murine models of thoracic malignancies. 
 Expression of pigment epithelium-derived factor decreases liver   metastasis   and correlates with favorable prognosis for patients with ductal pancreatic adenocarcinoma. Pigment epithelium-derived factor (PEDF) is expressed in several normal organs and identified as an inhibitor of neovascularization. In the present study, we screened the expression of PEDF immunohistochemically and investigated its correlation with clinicopathological features in patients who underwent surgery for ductal pancreatic adenocarcinoma. Of the 80 patients, 22 cases (27.5%) were positive for PEDF. A significant association was found between the PEDF expression and low microvessel density (P = 0.0003). No correlation was found between PEDF expression and age, gender, depth of invasion, tumor diameter, lymphatic invasion, venous, invasion or histopathological grading. The patients in pathological stage II had a significantly higher incidence of PEDF-positive expression than those in pathological stage III or IVA (P = 0.0418). PEDF immunoreactivity was inversely associated with liver   metastasis   (P = 0.0422). The survival of patients that were PEDF positive was significantly longer than that of those with negative expression (P = 0.0026). Multivariate analysis using the Cox regression model indicated that PEDF-positive expression was an independent favorable prognostic factor (risk ratio, 0.394; P = 0.0016). We conclude that PEDF expression suggests a more favorable prognosis than in patients whose carcinomas lack PEDF expression. 
 Regulation of the expression of pigment epithelium-derived factor, an anti-angiogenic factor in human oral squamous cell carcinoma cell lines. Angiogenesis plays an important role in tumor growth and   metastasis   and is regulated by a balance between angiogenic stimulators and inhibitors. We investigated the gene expression profile of vascular endothelial growth factor (VEGF) and pigment epithelium-derived factor (PEDF), a potent endogenous anti-angiogenic factor, in human oral squamous cell carcinoma (SCC) cell lines. The treatment of SCC cells with hypoxia increased the expression of PEDF as well as VEGF. Moreover, the treatment of SCC cells with VEGF enhanced the expression of PEDF mRNA and secretion of PEDF. In LMF-4, a SCC clone producing abundant VEGF and PEDF, the addition of neutralizing VEGF antibody substantially blocked PEDF expression. These data suggest that human oral squamous cell carcinoma cells produce VEGF, which in turn regulates PEDF production, and this balance may be contributing to neovascularization in tumors. 
  GeneRIFs  
 findings establish PEDF as both a metastatic suppressor and a neuroprotectant in the brain, highlighting its role as a double agent in limiting brain   metastasis   and its local consequences 
 Lentivirus-mediated gene transfer of PEDF decreased the growth of ocular melanoma and its hepatic micro  metastasis   in a mouse ocular melanoma model. 
 Showed that reduced PEDF levels in lung cancer tissues significantly correlated with lymph node   metastasis   and an overall poor prognosis in the lung cancer patients. 
   
  
 SERPINF2 
 
  NCBI Gene  
  GeneRIF  
  Pubmed  
 
 
  Search Term  Rank  Fields  
  [Ee]xtracellular [Mm]atrix  8  PubMed(1)  
  [Mm]etastasis  12  PubMed(3)  
  [Cc]ell [Ii]nvasion  13  PubMed(1)  
 
 
 hide/show details for SERPINF2 
  [Ee]xtracellular [Mm]atrix  PubMed  
 alpha2-antiplasmin is associated with the progression of fibrosis. Systemic sclerosis results in tissue fibrosis due to the activation of fibroblasts and the ensuing overproduction of the   extracellular matrix  . We previously reported that the absence of alpha2-antiplasmin (alpha2AP) attenuated the process of dermal fibrosis; however, the detailed mechanism of how alpha2AP affects the progression of fibrosis remained unclear. The goal of the present study was to examine the role of alpha2AP in fibrotic change. We observed significantly higher levels of alpha2AP expression in the skin of bleomycin-injected systemic sclerosis model mice in comparison with the levels seen in control mice. We also demonstrated that alpha2AP induced myofibroblast differentiation, and the absence of alpha2AP attenuated the induction of myofibroblast differentiation. Moreover, we found that connective tissue growth factor induced the expression of alpha2AP through both the extracellular signal-regulated kinase 1/2 (ERK1/2) and c-Jun N-terminal kinase (JNK) pathways in fibroblasts. Interestingly, alpha2AP also induced transforming growth factor-beta expression through the same pathways, and the inhibition of ERK1/2 and JNK slowed the progression of bleomycin-induced fibrosis. Our findings suggest that alpha2AP is associated with the progression of fibrosis, and regulation of alpha2AP expression by the ERK1/2 and JNK pathways may be an effective antifibrotic therapy for the treatment of systemic sclerosis. 
  [Mm]etastasis  PubMed  
 Update of the human and mouse SERPIN gene superfamily. The serpin family comprises a structurally similar, yet functionally diverse, set of proteins. Named originally for their function as serine proteinase inhibitors, many of its members are not inhibitors but rather chaperones, involved in storage, transport, and other roles. Serpins are found in genomes of all kingdoms, with 36 human protein-coding genes and five pseudogenes. The mouse has 60 Serpin functional genes, many of which are orthologous to human SERPIN genes and some of which have expanded into multiple paralogous genes. Serpins are found in tissues throughout the body; whereas most are extracellular, there is a class of intracellular serpins. Serpins appear to have roles in inflammation, immune function, tumorigenesis, blood clotting, dementia, and cancer   metastasis  . Further characterization of these proteins will likely reveal potential biomarkers and therapeutic targets for disease. 
 Induction of alpha2-antiplasmin inhibits E-cadherin processing mediated by the plasminogen activator/plasmin system, leading to suppression of progression of oral squamous cell carcinoma via upregulation of cell-cell adhesion. The plasminogen activator/plasmin system is one of the main protease systems involved in tumor cell invasion and   metastasis  . Our previous study has shown that plasmin degrades E-cadherin and promotes cell dissemination by downregulation of E-cadherin-mediated cell-cell adhesion in oral squamous cell carcinoma (SCC) cells. To examine the effect of downregulation of the plasminogen activator/plasmin system by alpha2-antiplasmin (alpha2-AP) on cell-cell adhesion mediated by E-cadherin in oral SCC cells, the oral SCC cell line SCCKN was stably transfected with alpha2-AP cDNA. Induction of alpha2-AP expression led to the inhibition of the proteolysis of E-cadherin by plasminogen activator/plasmin in SCC cells, resulting in the enhancement of the cell aggregation and the suppression of the cell motility. Moreover, alpha2-AP also reduced the ability of SCC cells to invade type I collagen gel, and suppressed tumorigenicity in vivo. These results suggested that downregulation of the plasminogen activator/ plasmin system by alpha2-AP might be a potent therapeutic approach to prevent the progression of oral SCC. 
 Characterization of the enzymatic activity of human kallikrein 6: Autoactivation, substrate specificity, and regulation by inhibitors. Human kallikrein 6 (hK6) is a trypsin-like serine protease, member of the human kallikrein gene family. Studies suggested a potential involvement of hK6 in the development and progression of Alzheimer's disease. The serum levels of hK6 might be used as a biomarker for ovarian cancer. To gain insights into the physiological role of this enzyme, we sought to determine its substrate specificity and its interactions with various inhibitors. We produced the proform of hK6 and showed that this enzyme was able to autoactivate, as well as proteolyse itself, leading to inactivation. Kinetic studies indicated that hK6 cleaved with much higher efficiency after Arg than Lys and with a preference for Ser or Pro in the P2 position. The efficient degradation of fibrinogen and collagen types I and IV by hK6 indicated that this kallikrein might play a role in tissue remodeling and/or tumor invasion and   metastasis  . We also demonstrated proteolysis of amyloid precursor protein by hK6 and determined the cleavage sites at the N-terminal end of the protein. Inhibition of hK6 was achieved via binding to different serpins, among which antithrombin III was the most efficient. 
  [Cc]ell [Ii]nvasion  PubMed  
 Induction of alpha2-antiplasmin inhibits E-cadherin processing mediated by the plasminogen activator/plasmin system, leading to suppression of progression of oral squamous cell carcinoma via upregulation of cell-cell adhesion. The plasminogen activator/plasmin system is one of the main protease systems involved in tumor   cell invasion   and metastasis. Our previous study has shown that plasmin degrades E-cadherin and promotes cell dissemination by downregulation of E-cadherin-mediated cell-cell adhesion in oral squamous cell carcinoma (SCC) cells. To examine the effect of downregulation of the plasminogen activator/plasmin system by alpha2-antiplasmin (alpha2-AP) on cell-cell adhesion mediated by E-cadherin in oral SCC cells, the oral SCC cell line SCCKN was stably transfected with alpha2-AP cDNA. Induction of alpha2-AP expression led to the inhibition of the proteolysis of E-cadherin by plasminogen activator/plasmin in SCC cells, resulting in the enhancement of the cell aggregation and the suppression of the cell motility. Moreover, alpha2-AP also reduced the ability of SCC cells to invade type I collagen gel, and suppressed tumorigenicity in vivo. These results suggested that downregulation of the plasminogen activator/ plasmin system by alpha2-AP might be a potent therapeutic approach to prevent the progression of oral SCC. 
   
  
 AGT 
 
  NCBI Gene  
  GeneRIF  
  Pubmed  
 
 
  Search Term  Rank  Fields  
  [Mm]igration  6  Process(2); PubMed(17); GeneRIFs(6)  
  [Ee]xtracellular [Mm]atrix  8  Process(2); PubMed(12); GeneRIFs(1)  
  [Ii]nvasive  10  PubMed(7); GeneRIFs(1)  
  [Mm]etastasis  12  PubMed(4); GeneRIFs(2)  
 
 
 hide/show details for AGT 
  [Mm]igration  Process  
 positive regulation of endothelial cell   migration   
 positive regulation of vascular associated smooth muscle cell   migration   
  PubMed  
 Angiotensin-II and MARCKS: a hydrogen peroxide- and RAC1-dependent signaling pathway in vascular endothelium. MARCKS is an actin-binding protein that modulates vascular endothelial cell   migration   and cytoskeleton signaling (Kalwa, H., and Michel, T. (2011) J. Biol. Chem. 286, 2320-2330). Angiotensin-II is a vasoactive peptide implicated in vascular physiology as well as pathophysiology; the pathways connecting angiotensin-II and cytoskeletal remodeling are incompletely understood. Here we show that MARCKS is expressed in intact arterial preparations, with prominent staining of the endothelium. In endothelial cells, angiotensin-II-promoted MARCKS phosphorylation is abrogated by PEG-catalase, implicating endogenous H(2)O(2) in the angiotensin-II response. Studies using the H(2)O(2) biosensor HyPer2 reveal that angiotensin-II promotes increases in intracellular H(2)O(2). We used a Rac1 FRET biosensor to show that angiotensin-II promotes Rac1 activation that is attenuated by PEG-catalase. siRNA-mediated Rac1 knockdown blocks angiotensin-II-stimulated MARCKS phosphorylation. Cell imaging studies using a phosphoinositide 4,5-bisphosphate (PIP(2)) biosensor revealed that angiotensin-II PIP(2) regulation depends on MARCKS and H(2)O(2). siRNA-mediated knockdown of MARCKS or Rac1 attenuates receptor-mediated activation of the tyrosine kinase c-Abl and disrupts actin fiber formation. These studies establish a critical role for H(2)O(2) in angiotensin-II signaling to the endothelial cytoskeleton in a novel pathway that is critically dependent on MARCKS, Rac1, and c-Abl. 
 Angiotensin II-induced process of angiogenesis is mediated by spleen tyrosine kinase via VEGF receptor-1 phosphorylation. Spleen tyrosine kinase (Syk), expressed in endothelial cells, has been implicated in   migration   and proliferation and in vasculogenesis. This study was conducted to determine the contribution of Syk and the underlying mechanism to the angiogenic effect of ANG II and VEGF. Angiogenesis was determined by tube formation from the endothelial cell line EA.hy926 (EA) and human umbilical vein endothelial cells (HUVECs) and microvessel sprouting in rat aortic rings. ANG II (10 nM), EGF (30 ng/ml), and VEGF (50 ng/ml) stimulated EA cells and HUVECs to form tubular networks and increased aortic sprouting; these effects were blocked by VEGF receptor-1 and Flt-1 antibody (Flt-1/Fc) but not by the VEGF receptor-2 (Flk-1) antagonist SU-1498. ANG II increased the phosphorylation of Flt-1 but not Flk-1, whereas VEGF increased the phosphorylation of both receptors in EA cells and HUVECs. VEGF expression elicited by ANG II was not altered by Flt-1/Fc or SU-1498. EGF stimulated tube formation from EA cells and HUVECs and Flt-1 phosphorylation and aortic sprouting, which were blocked by the EGF receptor antagonist AG-1478 and Flt-1/Fc but not by SU-1498. ANG II-, EGF-, and VEGF-induced tube formation and aortic sprouting were attenuated by the Syk inhibitor piceatannol and by Syk short hairpin interfering (sh)RNA and small interfering RNA, respectively. ANG II, EGF, and VEGF increased Syk phosphorylation, which was inhibited by piceatannol and Syk shRNA in EA cells and HUVECs. Neither piceatannol nor Syk shRNA altered ANG II-, EGF-, or VEGF-induced phosphorylation of Flt-1. These data suggest that ANG II stimulates angiogenesis via transactivation of the EGF receptor, which promotes the phosphorylation of Flt-1 and activation of Syk independent of VEGF expression. 
 Angiotensin-II and MARCKS: a hydrogen peroxide- and RAC1-dependent signaling pathway in vascular endothelium. MARCKS is an actin-binding protein that modulates vascular endothelial cell   migration   and cytoskeleton signaling (Kalwa, H., and Michel, T. (2011) J. Biol. Chem. 286, 2320-2330). Angiotensin-II is a vasoactive peptide implicated in vascular physiology as well as pathophysiology; the pathways connecting angiotensin-II and cytoskeletal remodeling are incompletely understood. Here we show that MARCKS is expressed in intact arterial preparations, with prominent staining of the endothelium. In endothelial cells, angiotensin-II-promoted MARCKS phosphorylation is abrogated by PEG-catalase, implicating endogenous H(2)O(2) in the angiotensin-II response. Studies using the H(2)O(2) biosensor HyPer2 reveal that angiotensin-II promotes increases in intracellular H(2)O(2). We used a Rac1 FRET biosensor to show that angiotensin-II promotes Rac1 activation that is attenuated by PEG-catalase. siRNA-mediated Rac1 knockdown blocks angiotensin-II-stimulated MARCKS phosphorylation. Cell imaging studies using a phosphoinositide 4,5-bisphosphate (PIP(2)) biosensor revealed that angiotensin-II PIP(2) regulation depends on MARCKS and H(2)O(2). siRNA-mediated knockdown of MARCKS or Rac1 attenuates receptor-mediated activation of the tyrosine kinase c-Abl and disrupts actin fiber formation. These studies establish a critical role for H(2)O(2) in angiotensin-II signaling to the endothelial cytoskeleton in a novel pathway that is critically dependent on MARCKS, Rac1, and c-Abl. 
 Angiotensin II-induced process of angiogenesis is mediated by spleen tyrosine kinase via VEGF receptor-1 phosphorylation. Spleen tyrosine kinase (Syk), expressed in endothelial cells, has been implicated in   migration   and proliferation and in vasculogenesis. This study was conducted to determine the contribution of Syk and the underlying mechanism to the angiogenic effect of ANG II and VEGF. Angiogenesis was determined by tube formation from the endothelial cell line EA.hy926 (EA) and human umbilical vein endothelial cells (HUVECs) and microvessel sprouting in rat aortic rings. ANG II (10 nM), EGF (30 ng/ml), and VEGF (50 ng/ml) stimulated EA cells and HUVECs to form tubular networks and increased aortic sprouting; these effects were blocked by VEGF receptor-1 and Flt-1 antibody (Flt-1/Fc) but not by the VEGF receptor-2 (Flk-1) antagonist SU-1498. ANG II increased the phosphorylation of Flt-1 but not Flk-1, whereas VEGF increased the phosphorylation of both receptors in EA cells and HUVECs. VEGF expression elicited by ANG II was not altered by Flt-1/Fc or SU-1498. EGF stimulated tube formation from EA cells and HUVECs and Flt-1 phosphorylation and aortic sprouting, which were blocked by the EGF receptor antagonist AG-1478 and Flt-1/Fc but not by SU-1498. ANG II-, EGF-, and VEGF-induced tube formation and aortic sprouting were attenuated by the Syk inhibitor piceatannol and by Syk short hairpin interfering (sh)RNA and small interfering RNA, respectively. ANG II, EGF, and VEGF increased Syk phosphorylation, which was inhibited by piceatannol and Syk shRNA in EA cells and HUVECs. Neither piceatannol nor Syk shRNA altered ANG II-, EGF-, or VEGF-induced phosphorylation of Flt-1. These data suggest that ANG II stimulates angiogenesis via transactivation of the EGF receptor, which promotes the phosphorylation of Flt-1 and activation of Syk independent of VEGF expression. 
 Hypertension-susceptibility gene prevalence in the Pacific Islands and associations with hypertension in Melanesia. Human essential hypertension is partly caused by genetic factors. Angiotensinogen (AGT), G-protein β3-subunit (GNB3) and cytochrome P450 3A5 (CYP3A5) are candidate hypertension susceptibility genes and risk alleles at these loci have been thought to arise owing to human adaptation to climatic changes following the   migratio  n out-of-Africa. This study aimed to reveal the frequencies of hypertension-susceptibility genotypes in Pacific Island populations and associations of these single-nucleotide polymorphisms (SNPs) to hypertension. Genotyping was conducted for 804 individuals from Melanesian, Micronesian and Polynesian populations at SNPs in the genes encoding AGT (rs699, rs5049 and rs5051), GNB3 (rs5443) and CYP3A5*1/*3 (rs776746). Associations between these SNPs and hypertension were tested for 383 Melanesian Solomon Islanders. We found that the A/A genotype at rs5049 was a risk factor for hypertension (P=0.025) in the Melanesian Solomon Islanders; three SNPs for AGT were in linkage disequilibrium. The ancestral alleles of rs699, rs5051 and rs776746, and the derived allele of rs5443 were as frequent in the populations surveyed here as in other equatorial populations. Although other polymorphisms associated with hypertension and additional populations remain to be studied, these findings suggest that the Pacific Islanders' susceptibility to hypertension arose because of human   migratio  n and adaptation. 
 Angiotensin II facilitates breast cancer cell   migration   and metastasis. Breast cancer metastasis is a leading cause of death by malignancy in women worldwide. Efforts are being made to further characterize the rate-limiting steps of cancer metastasis, i.e. extravasation of circulating tumor cells and colonization of secondary organs. In this study, we investigated whether angiotensin II, a major vasoactive peptide both produced locally and released in the bloodstream, may trigger activating signals that contribute to cancer cell extravasation and metastasis. We used an experimental in vivo model of cancer metastasis in which bioluminescent breast tumor cells (D3H2LN) were injected intra-cardiacally into nude mice in order to recapitulate the late and essential steps of metastatic dissemination. Real-time intravital imaging studies revealed that angiotensin II accelerates the formation of metastatic foci at secondary sites. Pre-treatment of cancer cells with the peptide increases the number of mice with metastases, as well as the number and size of metastases per mouse. In vitro, angiotensin II contributes to each sequential step of cancer metastasis by promoting cancer cell adhesion to endothelial cells, trans-endothelial   migration   and tumor cell   migration   across extracellular matrix. At the molecular level, a total of 102 genes differentially expressed following angiotensin II pre-treatment were identified by comparative DNA microarray. Angiotensin II regulates two groups of connected genes related to its precursor angiotensinogen. Among those, up-regulated MMP2/MMP9 and ICAM1 stand at the crossroad of a network of genes involved in cell adhesion,   migration   and invasion. Our data suggest that targeting angiotensin II production or action may represent a valuable therapeutic option to prevent metastatic progression of invasive breast tumors. 
 Angiotensin II enhances epithelial-to-mesenchymal transition through the interaction between activated hepatic stellate cells and the stromal cell-derived factor-1/CXCR4 axis in intrahepatic cholangiocarcinoma. We previously reported that hepatic stellate cells (HSCs) activated by angiotensin II (AngII) facilitate stromal fibrosis and tumor progression in intrahepatic cholangiocarcinoma (ICC). AngII has been known as a growth factor which can promote epithelial-to-mesenchymal transition (EMT) in renal epithelial cells, alveolar epithelial cells and peritoneal mesothelial cells. However, in the past, the relationship between AngII and stromal cell-derived factor-1 (SDF-1) in the microenvironment around cancer and the role of AngII on EMT of cancer cells has not been reported in detail. SDF-1 and its specific receptor, CXCR4, are now receiving attention as a mechanism of cell progression and metastasis. In this study, we examined whether activated HSCs promote tumor fibrogenesis, tumor progression and distant metastasis by mediating EMT via the AngII/AngII type 1 receptor (AT-1) and the SDF-1/CXCR4 axis. Two human ICC cell lines and a human HSC line, LI-90, express CXCR4. Significantly higher concentration of SDF-1α was released into the supernatant of LI-90 cells to which AngII had been added. SDF-1α increased the proliferative activity of HSCs and enhanced the activation of HSCs as a growth factor. Furthermore, addition of SDF-1α and AngII enhanced the increase of the migratory capability and vimentin expression, reduced E-cadherin expression, and translocated the expression of β-catenin into the nucleus and cytoplasm in ICC cells. Co-culture with HSCs also enhanced the migratory capability of ICC cells. These findings suggest that SDF-1α, released from activated HSCs and AngII, play important roles in cancer progression, tumor fibrogenesis,   and migr  ation in autocrine and paracrine fashion by mediating EMT. Our mechanistic findings may provide pivotal insights into the molecular mechanism of the AngII and SDF-1α-initiated signaling pathway that regulates fibrogenesis in cancerous stroma, tumor progression and meta-stasis of tumor cells expressing AT-1 and CXCR4. 
 From skeleton to cytoskeleton: osteocalcin transforms vascular fibroblasts to myofibroblasts via angiotensin II and Toll-like receptor 4. The expression of osteocalcin is augmented in human atherosclerotic lesions. How osteocalcin triggers vascular pathogenesis and remodeling is unclear.To investigate whether osteocalcin promotes transformation of adventitial fibroblast to myofibroblasts and the molecular mechanism involved.Immunohistochemistry indicated that osteocalcin was expressed in the neointima of renal arteries from diabetic patients. Western blotting and wound-healing assay showed that osteocalcin induced fibroblast transformation and   migration  , which were attenuated by blockers of the renin-angiotensin system and protein kinase Cδ (PKCδ), toll-like receptor 4 (TLR4) neutralizing antibody, and antagonist and inhibitors of free radical production and cyclooxygenase-2. Small interfering RNA silencing of TLR4 and PKCδ abolished fibroblast transformation. Angiotensin II level in the conditioned medium from the osteocalcin-treated fibroblasts was found elevated using enzyme immunoassay. Culturing of fibroblasts in conditioned medium collected from differentiated osteoblasts promoted fibroblast transformation. The expression of fibronectin, TLR4, and cyclooxygenase-2 is augmented in human mesenteric arteries after 5-day in vitro exposure to osteocalcin.Osteocalcin transforms adventitial fibroblasts to myofibroblasts through stimulating angiotensin II release and subsequent activation of PKCδ/TLR4/reactive oxygen species/cyclooxygenase-2 signaling cascade. This study reveals that the skeletal hormone osteocalcin cross-talks with vascular system and contributes to vascular remodeling. 
 Angiotensin II induces focal adhesion kinase/paxillin phosphorylation and cell   migration   in human umbilical vein endothelial cells. In the present study, we demonstrated that Ang II provokes a transitory enhancement of focal adhesion kinase (FAK) and paxillin phosphorylation in human umbilical endothelial cells (HUVEC). Moreover, Ang II induces a time- and dose-dependent augmentation in cell   migration  , but does not affect HUVEC proliferation. The effect of Ang II on FAK and paxillin phosphorylation was markedly attenuated in cells pretreated with wortmannin and LY294002, indicating that phosphoinositide 3-kinase (PI3K) plays an important role in regulating FAK activation. Similar results were observed when HUVEC were pretreated with genistein, a non-selective tyrosine kinases inhibitor, or with the specific inhibitor PP2 for Src family kinases, demonstrating the involvement of protein tyrosine kinases, and particularly Src family of tyrosine kinases, in the downstream signalling pathway of Ang II receptors. Furthermore, FAK and paxillin phosphorylation was markedly blocked after treatment of HUVEC with AG1478, a selective inhibitor of epidermal growth factor receptor (EGFR) phosphorylation. Pretreatment of cells with inhibitors of PI3K, Src family tyrosine kinases, and EGFR also decreased HUVEC   migration  . In conclusion, these results suggest that Ang II mediates an increase in FAK and paxillin phosphorylation and induces HUVEC   migration   through signal transduction pathways dependent on PI3K and Src tyrosine kinase activation and EGFR transactivation. 
 Skeletal muscle-endothelial cell cross talk through angiotensin II. The role of angiotensin II (ANG II) in postnatal vasculogenesis and angiogenesis during skeletal muscle (SKM) regeneration is unknown. We examined the capacity of ANG II to stimulate capillary formation and growth during cardiotoxin-induced muscle regeneration in ACE inhibitor-treated ANG II type 1a receptor knockout (AT1a(-/-)) and C57Bl/6 control mice. Analysis of tibialis anterior (TA) cross-sections revealed 17% and 23% reductions in capillarization in AT1a(-/-) and captopril treated mice, respectively, when compared with controls, 21 days postinjury. Conversely, no differences in capillarization were detected at early time points (7 and 10 days). These results identify ANG II as a regulator of angiogenesis but not vasculogenesis in vivo. In vitro angiogenesis assays of human umbilical vein endothelial cells (HUVECs) further confirmed ANG II as proangiogeneic as 71% and 124% increases in tube length and branch point number were observed following ANG II treatment. Importantly, treatment of HUVECs with conditioned media from differentiated muscle cells resulted in an 84% and 203% increase in tube length and branch point number compared with controls, which was abolished following pretreatment of the cells with an angiotensin-converting enzyme inhibitor. The pro-angiogenic effect of ANG II can be attributed to an enhanced endothelial cell   migration   because both transwell and under agarose   migration   assays revealed a 37% and 101% increase in cell motility, respectively. Collectively, these data highlight ANG II as a proangiogenic regulator during SKM regeneration in vivo and more importantly demonstrates that ANG II released from SKM can signal endothelial cells and regulate angiogenesis through the induction of endothelial cell   migration  . 
 ACE2 and Ang-(1-7) protect endothelial cell function and prevent early atherosclerosis by inhibiting inflammatory response. Angiotensin-converting enzyme 2 (ACE2) is a counter-regulator against ACE by converting angiotensin II (Ang-II) to Ang-(1-7), but the effect of ACE2 and Ang-(1-7) on endothelial cell function and atherosclerotic evolution is unknown. We hypothesized that ACE2 overexpression and Ang-(1-7) may protect endothelial cell function by counterregulation of angiotensin II signaling and inhibition of inflammatory response.We used a recombinant adenovirus vector to locally overexpress ACE2 gene (Ad-ACE2) in human endothelial cells in vitro and in apoE-deficient mice in vivo. The Ang II-induced MCP-1, VCAM-1 and E-selectin expression, endothelial cell   migration   and adhesion of human monocytic cells (U-937) to HUVECs by ACE2 gene transfer were evaluated in vitro. Accelerated atherosclerosis was studied in vivo, and atherosclerosis was induced in apoE-deficient mice which were divided randomly into four groups that received respectively a ACE2 gene transfer, Ad-ACE2, Ad-EGFP, Ad-ACE2 + A779, an Ang-(1-7) receptor antagonist, control group. After a gene transfer for 4 weeks, atherosclerotic pathology was evaluated.ACE2 gene transfer not only promoted HUVECs   migration  , inhibited adhesion of monocyte to HUVECs and decreased Ang II-induced MCP-1, VCAM-1 and E-selectin protein production in vitro, but also decreased the level of MCP-1, VCAM-1 and interleukin 6 and inhibit atherosclerotic plaque evolution in vivo. Further, administration of A779 increased the level of MCP-1, VCAM-1 and interleukin 6 in vivo and led to further advancements in atherosclerotic extent.ACE2 and Ang-(1-7) significantly inhibit early atherosclerotic lesion formation via protection of endothelial function and inhibition of inflammatory response. 
 Enhanced angiotensin converting enzyme 2 regulates the insulin/Akt signalling pathway by blockade of macrophage   migration   inhibitory factor expression. Macrophage   migration   inhibitory factor (MIF) is now known to be a pro-inflammatory cytokine associated with insulin resistance. Our aim was to investigate whether angiotensin converting enzyme 2 (ACE2) could modulate the expression of MIF and the insulin/Akt-endothelial nitric oxide (NO) synthase (eNOS) signalling in a human endothelial cell line (EAhy926).A recombinant plasmid encompassing human ACE2 gene was constructed and transfected into the EAhy926 cells. The mRNA, phosphorylation and protein levels of p22phox, MIF, Akt and eNOS in endothelial cells were determined by real-time PCR and Western blot analysis, respectively.Gene transfer of ACE2 suppressed the expression of p22phox and MIF induced by angiotensin (Ang) II and Ang IV, accompanied by a decreased level of malondialdehyde in cells. In addition, Ang II diminished insulin-stimulated phosphorylation of Akt (at Ser(473)) and eNOS (at Ser(1177)) and NO generation, effects which were reversed by ACE2 gene transfer and anti-MIF treatment in endothelial cells.The results reveal that gene transfer of ACE2 regulated Ang II-mediated impairment of insulin signalling and involved the Akt-eNOS phosphorylation pathway. These beneficial effects of ACE2 overexpression appear to result mainly from blocking MIF expression in endothelial cells, suggesting that the ACE2 gene may be a novel therapeutic target for diseases related to inflammation and insulin resistance. 
 Differential mechanisms of angiotensin II and PDGF-BB on   migration   and proliferation of coronary artery smooth muscle cells. Angiotensin II (Ang II) and platelet-derived growth factor-BB (PDGF-BB) are associated with excessive cell   migration  , proliferation and many growth-related diseases. However, whether these agents utilise similar mechanisms to trigger vascular pathologies remains to be explored. The effects of Ang II and PDGF-BB on coronary artery smooth muscle cell (CASMC)   migration   and proliferation were investigated via Dunn chemotaxis assay and the measurement of [(3)H]thymidine incorporation rates, respectively. Both atherogens produced similar degrees of cell   migration   which were dramatically inhibited by mevastatin (10 nM). However, the inhibitory effects of losartan (10 nM) and MnTBAP (a free radical scavenger; 50 microM) were found to be unique to Ang II-mediated chemotaxis. In contrast, MnTBAP, apocynin (an antioxidant and phagocytic NADPH oxidase inhibitor; 500 microM), mevastatin and pravastatin (100 nM) equally suppressed both Ang II and PDGF-BB-induced cellular growth. Although atherogens produced similar changes in NADPH oxidase, NOS and superoxide dismutase activities, they differentially regulated antioxidant glutathione peroxidase activity which was diminished by Ang II and unaffected by PDGF-BB. Studies with signal transduction pathway inhibitors revealed the involvement of multiple pathways i.e. protein kinase C, tyrosine kinase and MAPK in Ang II- and/or PDGF-BB-induced aforementioned enzyme activity changes. In conclusion, Ang II and PDGF-BB may induce coronary atherosclerotic disease formation by stimulating CASMC   migration   and proliferation through agent-specific regulation of oxidative status and utilisation of different signal transduction pathways. 
 The renin-angiotensin-aldosterone system: a specific target for hypertension management. Angiotensin II plays a central role in the regulation of systemic arterial pressure through its systemic synthesis via the renin-angiotensin-aldosterone cascade. It acts directly on vascular smooth muscle as a potent vasoconstrictor. In addition, it affects cardiac contractility and heart rate through its action on the sympathetic nervous system. Angiotensin II also alters renal sodium and water absorption through its ability to stimulate the zona glomerulosa cells of the adrenal cortex to synthesize and secrete aldosterone. Furthermore, it enhances thirst and stimulates the secretion of the antidiuretic hormone. Consequently, angiotensin II plays a critical role in both the acute and chronic regulation of blood pressure through its systemic endocrine regulation. A potent neurohormone that regulates systemic arterial pressure, angiotensin II also affects vascular structure and function via paracrine and autocrine effects of local tissue-based synthesis. This alternate pathway of angiotensin II production is catalyzed in tissues via enzymes such as cathepsin G, chymostatin-sensitive angiotensin II-generating enzyme, and chymase. Intratissue formation of angiotensin II plays a critical role in cardiovascular remodeling. Upregulation of these alternate pathways may occur through stretch, stress, and turbulence within the blood vessel. Similar processes within the myocardium and glomeruli of the kidney may also lead to restructuring in these target organs, with consequent organ dysfunction. Additionally, angiotensin II may increase receptor density and sensitivity for other factors that modulate growth of vascular smooth muscle, such as fibroblast growth factor, transforming growth factor beta-1, platelet-derived growth factor, and insulin-like growth factors. Atherosclerosis may also be related, in part, to excessive angiotensin II effect on the vessel wall, which causes smooth muscle cell growth and   migration  . It also activates macrophages and increases platelet aggregation. Angiotensin II stimulates plasminogen activator inhibitor 1 and directly causes endothelial dysfunction. Other postulated effects of angiotensin II on vascular structure that could promote atherogenesis include inhibition of apoptosis, increase in oxidative stress, promotion of leukocyte adhesion and   migration  , and stimulation of thrombosis. Inhibition of angiotensin II synthesis with an angiotensin-converting enzyme inhibitor has been demonstrated to be beneficial in modifying human disease progression. This is clearly apparent in clinical trials involving patients with diabetic nephropathy, postmyocardial infarction, or advanced degrees of systolic heart failure. Thus, angiotensin II is an excellent target for pharmacologic blockade. Not only does it play a pivotal role in both the acute and chronic regulation of systemic arterial pressure, but it also is an important modulator of cardiovascular structure and function and may be specifically involved in disease progression. Modification of angiotensin II effect may therefore serve a dual purpose. Not only will blood pressure reduction occur with less stretch, stress, and turbulence of the vascular wall, but there will also be less stimulation, either directly or indirectly, for restructuring and remodeling of the cardiovascular tree. 
 A common effect of angiotensin II and relaxin 2 on the PNT1A normal prostate epithelial cell line. The prostate gland is a part of the male reproductive tract which produces both angiotensin II (Ang II) and relaxin 2 (RLN2). The present study analyzes the effect of both these peptide hormones at concentration 10(-8)M on viability, proliferation, adhesion,   migration  , and invasion of normal prostate epithelial cells (PNT1A). Improved survival in two- and three-dimensional cell cultures was noted as well as visual changes in colony size and structure in Geltrex™. Stimulatory influence on cell viability of each peptide applied single was lower than in combination. Enhanced survival of PNT1A cells appears to be associated with increased BCL2/BAX messenger RNA (mRNA) expression ratio. Modulation of cell spreading and cell-extracellular matrix adhesion dynamics were also altered as an influence of tested hormone application. However, long-term Ang II and RLN2 effects may lead to an increase of normal prostate cel  l migrati  on and invasion abilities. Moreover, gelatin zymography revealed that both gelatinases A and B were augmented by Ang II treatment, whereas RLN2 significantly stimulated only MMP-9 secretion. These results support the hypothesis that deregulation of locally secreted peptide hormones such as Ang II and RLN2 may take part in the development of certain cancers, including prostate cancer. Moreover, the observed ability of relaxin 2 to act as a regulator of mRNA expression levels not only LGR7 but also classic angiotensin receptors suggested that renin-angiotensin system and relaxin family peptide system are functionally linked. 
 Angiotensin II stimulates canonical TGF-β signaling pathway through angiotensin type 1 receptor to induce granulation tissue contraction. Hypertrophic scar contraction (HSc) is caused by granulation tissue contraction propagated by myofibroblast and fibroblast   migratio  n and contractility. Identifying the stimulants that promote   migratio  n and contractility is key to mitigating HSc. Angiotensin II (AngII) promotes   migratio  n and contractility of heart, liver, and lung fibroblasts; thus, we investigated the mechanisms of AngII in HSc. Human scar and unwounded dermis were immunostained for AngII receptors angiotensin type 1 receptor (AT1 receptor) and angiotensin type 2 receptor (AT2 receptor) and analyzed for AT1 receptor expression using Western blot. In vitro assays of fibroblast contraction and   migratio  n under AngII stimulation were conducted with AT1 receptor, AT2 receptor, p38, Jun N-terminal kinase (JNK), MEK, and activin receptor-like kinase 5 (ALK5) antagonism. Excisional wounds were created on AT1 receptor KO and wild-type (WT) mice treated with AngII ± losartan and ALK5 and JNK inhibitors SB-431542 and SP-600125, respectively. Granulation tissue contraction was quantified, and wounds were analyzed by immunohistochemistry. AT1 receptor expression was increased in scar, but not unwounded tissue. AngII induced fibroblast contractio  n and mig  ration through AT1 receptor.   Cell mig  ration was inhibited by ALK5 and JNK, but not p38 or MEK blockade. In vivo experiments determined that absence of AT1 receptor and chemical AT1 receptor antagonism diminished granulation tissue contraction while AngII stimulated wound contraction. AngII granulation tissue contraction was diminished by ALK5 inhibition, but not JNK. AngII promotes granulation tissue contraction through AT1 receptor and downstream canonical transforming growth factor (TGF)-β signaling pathway, ALK5. Further understanding the pathogenesis of HSc as an integrated signaling mechanism could improve our approach to establishing effective therapeutic interventions.AT1 receptor expression is increased in scar tissue compared to unwounded tissue. AngII stimulates expression of proteins that confe  r cell mi  gration and contraction. AngII stimulates fibr  oblast mi  gration and contraction through AT1 receptor, ALK5, and JNK. AngII-stimulated in vivo granulation tissue contraction is AT1 receptor and ALK5 dependent. 
 The Nox1/4 Dual Inhibitor GKT137831 or Nox4 Knockdown Inhibits Angiotensin-II-Induced Adult Mouse Cardiac Fibroblast Proliferation and   Migration  . AT1 Physically Associates With Nox4. Both oxidative stress and inflammation contribute to chronic hypertension-induced myocardial fibrosis and adverse cardiac remodeling. Here we investigated whether angiotensin (Ang)-II-induced fibroblast proliferation and   migration   are NADPH oxidase (Nox) 4/ROS and IL-18 dependent. Our results show that the potent induction of mouse cardiac fibroblast (CF) proliferation and   migration   by Ang-II is markedly attenuated by Nox4 knockdown and the Nox inhibitor DPI. Further, Nox4 knockdown and DPI pre-treatment attenuated Ang-II-induced IL-18, IL-18Rα and collagen expression, and MMP9 and LOX activation. While neutralization of IL-18 blunted Ang-II-induced CF proliferation and   migratio  n, knockdown of MMP9 attenuated CF   migratio  n. The antioxidant NAC and the cell-permeable SOD mimetics Tempol, MnTBAP, and MnTMPyP attenuated oxidative stress and inhibited CF proliferation and   migratio  n. The Nox1/Nox4 dual inhibitor GKT137831 also blunted Ang-II-induced H2 O2 production and CF proliferation and   migratio  n. Further, AT1 bound Nox4, and Ang-II enhanced their physical association. Notably, GKT137831 attenuated the AT1/Nox4 interaction. These results indicate that Ang-II induces CF proliferation and   migratio  n in part via Nox4/ROS-dependent IL-18 induction and MMP9 activation, and may involve AT1/Nox4 physical association. Thus, either (i) neutralizing IL-18, (ii) blocking AT1/Nox4 interaction or (iii) use of the Nox1/Nox4 inhibitor GKT137831 may have therapeutic potential in chronic hypertension-induced adverse cardiac remodeling. 
  GeneRIFs  
 These results indicate that Ang-II induces cardiac fibroblast proliferation and   migration   in part via Nox4/ROS-dependent IL-18 induction and MMP9 activation, and may involve AT1/Nox4 physical association. 
 AngII stimulates fibroblast   migration   and contraction through AT1 receptor, ALK5, and JNK. 
 SDF-1alpha, released from activated hepatic stellate cells and AngII, play important roles in cancer progression, tumor fibrogenesis, and   migration   in autocrine and paracrine fashion 
 Angiotensin II contributes to each sequential step of cancer metastasis by promoting cancer cell adhesion to endothelial cells, trans-endothelial   migration   and tumor cell   migration   across extracellular matrix. 
 ANG II is a proangiogenic regulator during SKM regeneration in vivo; ANG II released from SKM can signal endothelial cells and regulate angiogenesis through the induction of endothelial cell   migration  . 
 May induce coronary atherosclerotic disease formation by stimulating coronary artery smooth muscle cell   migration   and proliferation. 
  [Ee]xtracellular [Mm]atrix  Process  
 positive regulation of   extracellular matrix   constituent secretion 
 regulation of   extracellular matrix   assembly 
  PubMed  
 A genetic association study of maternal and fetal candidate genes that predispose to preterm prelabor rupture of membranes (PROM). We sought to determine whether maternal/fetal single-nucleotide polymorphisms (SNPs) in candidate genes are associated with preterm prelabor rupture of membranes (pPROM).A case-control study was conducted in patients with pPROM (225 mothers and 155 fetuses) and 599 mothers and 628 fetuses with a normal pregnancy; 190 candidate genes and 775 SNPs were studied. Single locus/haplotype association analyses were performed; false discovery rate was used to correct for multiple testing (q* = 0.15).First, a SNP in tissue inhibitor of metalloproteinase 2 in mothers was significantly associated with pPROM (odds ratio, 2.12; 95% confidence interval, 1.47-3.07; P = .000068), and this association remained significant after correction for multiple comparisons. Second, haplotypes for Alpha 3 type IV collagen isoform precursor in the mother were associated with pPROM (global P = .003). Third, multilocus analysis identified a 3-locus model, which included maternal SNPs in collagen type I alpha 2, defensin alpha 5 gene, and endothelin 1.DNA variants in a maternal gene involved in   extracellular matrix   metabolism doubled the risk of pPROM. 
 Identification of fetal and maternal single nucleotide polymorphisms in candidate genes that predispose to spontaneous preterm labor with intact membranes. The purpose of this study was to determine whether maternal/fetal single nucleotide polymorphisms (SNPs) in candidate genes are associated with spontaneous preterm labor/delivery.A genetic association study was conducted in 223 mothers and 179 fetuses (preterm labor with intact membranes who delivered <37 weeks of gestation [preterm birth (PTB)]), and 599 mothers and 628 fetuses (normal pregnancy); 190 candidate genes and 775 SNPs were studied. Single locus/haplotype association analyses were performed; the false discovery rate was used to correct for multiple testing.The strongest single locus associations with PTB were interleukin-6 receptor 1 (fetus; P=.000148) and tissue inhibitor of metalloproteinase 2 (mother; P=.000197), which remained significant after correction for multiple comparisons. Global haplotype analysis indicated an association between a fetal DNA variant in insulin-like growth factor F2 and maternal alpha 3 type IV collagen isoform 1 (global, P=.004 and .007, respectively).An SNP involved in controlling fetal inflammation (interleukin-6 receptor 1) and DNA variants in maternal genes encoding for proteins involved in   extracellular matrix   metabolism approximately doubled the risk of PTB. 
 A genetic association study of maternal and fetal candidate genes that predispose to preterm prelabor rupture of membranes (PROM). We sought to determine whether maternal/fetal single-nucleotide polymorphisms (SNPs) in candidate genes are associated with preterm prelabor rupture of membranes (pPROM).A case-control study was conducted in patients with pPROM (225 mothers and 155 fetuses) and 599 mothers and 628 fetuses with a normal pregnancy; 190 candidate genes and 775 SNPs were studied. Single locus/haplotype association analyses were performed; false discovery rate was used to correct for multiple testing (q* = 0.15).First, a SNP in tissue inhibitor of metalloproteinase 2 in mothers was significantly associated with pPROM (odds ratio, 2.12; 95% confidence interval, 1.47-3.07; P = .000068), and this association remained significant after correction for multiple comparisons. Second, haplotypes for Alpha 3 type IV collagen isoform precursor in the mother were associated with pPROM (global P = .003). Third, multilocus analysis identified a 3-locus model, which included maternal SNPs in collagen type I alpha 2, defensin alpha 5 gene, and endothelin 1.DNA variants in a maternal gene involved in   extracellular matrix   metabolism doubled the risk of pPROM. 
 Identification of fetal and maternal single nucleotide polymorphisms in candidate genes that predispose to spontaneous preterm labor with intact membranes. The purpose of this study was to determine whether maternal/fetal single nucleotide polymorphisms (SNPs) in candidate genes are associated with spontaneous preterm labor/delivery.A genetic association study was conducted in 223 mothers and 179 fetuses (preterm labor with intact membranes who delivered <37 weeks of gestation [preterm birth (PTB)]), and 599 mothers and 628 fetuses (normal pregnancy); 190 candidate genes and 775 SNPs were studied. Single locus/haplotype association analyses were performed; the false discovery rate was used to correct for multiple testing.The strongest single locus associations with PTB were interleukin-6 receptor 1 (fetus; P=.000148) and tissue inhibitor of metalloproteinase 2 (mother; P=.000197), which remained significant after correction for multiple comparisons. Global haplotype analysis indicated an association between a fetal DNA variant in insulin-like growth factor F2 and maternal alpha 3 type IV collagen isoform 1 (global, P=.004 and .007, respectively).An SNP involved in controlling fetal inflammation (interleukin-6 receptor 1) and DNA variants in maternal genes encoding for proteins involved in   extracellular matrix   metabolism approximately doubled the risk of PTB. 
 Important role of the angiotensin II pathway in producing matrix metalloproteinase-9 in human thoracic aortic aneurysms. The precise pathologic mechanisms underlying human thoracic aortic aneurysms (TAAs) remain uncertain, except that matrix metalloproteinase-9 (MMP-9) is considered a key enzyme for the degradation of   extracellular matrix   in aneurysm walls. The aim of this study was to elucidate the significance of the angiotensin II (AngII) pathway to MMP-9 production in human TAA walls.We examined the activation of Smad2, a common downstream molecule of AngII and transforming growth factor β (TGF-β) pathways, and the expression of MMP-9 in human nonsyndromic TAA walls. We observed significant increases in Smad2 activation and MMP-9 expression, associated with disruption of elastic lamellae. Using human TAA walls in ex vivo culture, we investigated whether AngII and/or TGF-β pathways are essential for MMP-9 production. Unexpectedly, TGF-β receptor inhibitor had no effect on MMP-9 production. We used PD98059, an inhibitor of extracellular signal-regulated kinase (ERK) activation, and demonstrated that PD98059 dramatically reduced MMP-9 production with attenuation of Smad2 activation. Moreover, exogenous AngII resulted in increases in Smad2 activation and MMP-9 production, in an ERK-dependent manner.Our findings indicate that the AngII/ERK pathway has an important role in the production of MMP-9 in human nonsyndromic TAA walls. 
 Angiotensin II activates connective tissue growth factor and induces   extracellular matrix   changes involving Smad/activation and p38 mitogen-activated protein kinase signalling pathways in human dermal fibroblasts. Angiotensin II (Ang II) stimulation has been shown to regulate proliferation of skin fibroblasts and the production of   extracellular matrix  , which are very important processes in skin wound healing and fibrosis; however, there is little knowledge about the mechanisms involved in this process. We investigated the molecular aspects of this system with regards to Ang II in human dermal fibroblasts (HDF) and its potential role in fibrosis. Fibroblasts derived from human skin were subjected to examine differential relative gene and protein expression after transfection with specific reporter expression vectors and Ang II in vitro. In growth-arrested HDFs, Ang II treatment for 20 min caused acute activation of Smad2 phosphorylation, Smad overexpression and Smad-dependent gene transcription. The angiotensin type 1 (AT1) antagonist losartan diminished Ang II-induced Smad activation. The blockade of endogenous transforming growth factor-beta1 did modify the activation of Smad caused by Ang II. The p38 mitogen-activated protein kinase (MAPK) inhibitor SB203580 diminished Ang II-induced Smad2 phosphorylation. Transient transfection with Smad7, which interferes with receptor-mediated activation of Smad2, diminished Ang II-induced connective tissue growth factor promoter activation, gene and protein expression and fibronectin, type I procollagen and type III procollagen overexpression, showing that Smad activation is involved in Ang II-induced dermal fibrosis. Our results show that Ang II activation of Smad2 occurs via the AT1 receptor, but not the AT2 receptor. Activation of Smad2 required p38 MAPK but not p42/p44 MAPK or the epidermal growth factor receptor. 
 Angiotensin II facilitates breast cancer cell migration and metastasis. Breast cancer metastasis is a leading cause of death by malignancy in women worldwide. Efforts are being made to further characterize the rate-limiting steps of cancer metastasis, i.e. extravasation of circulating tumor cells and colonization of secondary organs. In this study, we investigated whether angiotensin II, a major vasoactive peptide both produced locally and released in the bloodstream, may trigger activating signals that contribute to cancer cell extravasation and metastasis. We used an experimental in vivo model of cancer metastasis in which bioluminescent breast tumor cells (D3H2LN) were injected intra-cardiacally into nude mice in order to recapitulate the late and essential steps of metastatic dissemination. Real-time intravital imaging studies revealed that angiotensin II accelerates the formation of metastatic foci at secondary sites. Pre-treatment of cancer cells with the peptide increases the number of mice with metastases, as well as the number and size of metastases per mouse. In vitro, angiotensin II contributes to each sequential step of cancer metastasis by promoting cancer cell adhesion to endothelial cells, trans-endothelial migration and tumor cell migration across   extracellular matrix  . At the molecular level, a total of 102 genes differentially expressed following angiotensin II pre-treatment were identified by comparative DNA microarray. Angiotensin II regulates two groups of connected genes related to its precursor angiotensinogen. Among those, up-regulated MMP2/MMP9 and ICAM1 stand at the crossroad of a network of genes involved in cell adhesion, migration and invasion. Our data suggest that targeting angiotensin II production or action may represent a valuable therapeutic option to prevent metastatic progression of invasive breast tumors. 
 Evaluation of angiotensinogen c.1-44G>A and p.M268T variants as risk factors for fibrosis progression in chronic hepatitis C and liver diseases of various etiologies. Hepatic stellate cells express all components of the renin-angiotensinogen (AGT) system and secrete active angiotensin II. Animal studies provided evidence that angiotensin II stimulates the accumulation of   extracellular matrix   by enhancing transforming growth factor beta1 production. A functional genetic alteration in the human AGT promoter (c.1-44G>A) has been linked to accelerated progression of fibrosis in hepatitis C virus infection.We enrolled 2154 patients with chronic liver disease of various etiologies, including 1286 individuals with chronic hepatitis C virus infection as well as 207 healthy volunteers. We performed genotyping for two AGT variants, c.1-44G>A and c.803T>C (p.M268T), by melting curve analysis using fluorescence resonance energy transfer probes.Allele frequencies and genotype distributions of both variants did not differ between patients and controls. Genotype frequencies of the c.1-44G>A variant were GG 31.0%, GA 45.6%, and AA 23.4% in patients and GG 30.0%, GA 47.8%, and AA 22.2% in controls. The genotype frequencies of p.M268T, which is in strong linkage disequilibrium with c.1-44G>A, were MM 30.8%, MT 45.5%, and TT 23.4% in patients and MM 29.0%, MT 48.8%, and TT 22.2% in controls. Both variants were associated with neither higher stages of fibrosis nor requirement for liver transplantation in any of the diagnosis subgroups. Particularly, these genetic alterations were not associated with progressive fibrosis in chronic HCV infection.In contrast to previous reports, both AGT variants do not predispose to the progression of fibrosis in chronic liver disease. 
 Angiotensin II induces connective tissue growth factor gene expression via calcineurin-dependent pathways. Connective tissue growth factor (CTGF) is a polypeptide implicated in the   extracellular matrix   synthesis. Previous studies have provided evidence that angiotensin II (Ang II) promotes collagen synthesis and regulates collagen degradation. We investigated whether or not CTGF mediates the profibrotic effects of Ang II in the heart and kidneys and the role of calcineurin-dependent pathways in CTGF gene regulation. In transgenic rats harboring human renin and angiotensinogen genes, Ang II induced an age-dependent increase in myocardial CTGF expression, which was 3.5-fold greater compared to normotensive Sprague Dawley (SD) rats. CTGF overexpression correlated closely with the Ang II-induced rise in blood pressure. CTGF mRNA and protein were located predominantly in areas with leukocyte infiltration, myocardial, and vascular lesions and co-localized with TGFbeta(1), collagen I, and collagen III mRNA expressions. Ang II induced CTGF mRNA and protein to a lesser extent in the kidneys, predominantly in glomeruli, arterioles, and in the interstitium with ample inflammation. However, no expression was found in the right ventricle or pulmonary arteries. Blockade of calcineurin activity by cyclosporine A completely normalized Ang II-induced CTGF overexpression in heart and kidney, suppressed the inflammatory response, and mitigated Ang II-induced cell proliferation and apoptosis. In contrast, blockade of mTOR (target of rapamycin) pathway by everolimus, further increased the expression of CTGF even though everolimus ameliorated cell proliferation and T-cell-mediated inflammation. Our findings provide evidence that CTGF mediates Ang II-induced fibrosis in the heart and kidneys via blood pressure and calcineurin-dependent pathways. 
 Angiotensin II increases expression and secretion of cathepsin F in cultured human monocyte-derived macrophages: an angiotensin II type 2 receptor-mediated effect. Increasing evidence suggests that cathepsins and angiotensin II (AngII) participate in atherosclerosis, particularly in remodeling of the   extracellular matrix   of the inflamed arterial intima. Here, we show that AngII induces mRNA expression of cathepsin F, a member of the cysteine protease family, in human monocyte-derived macrophages. AngII did not affect the amount of intracellular cathepsin F protein, but significantly enhanced its secretion by the treated cells. The stimulatory effect of AngII was mediated by the AngII type 2 (AT(2)) receptor, as demonstrated by the ability of the AT(2)-receptor antagonist PD123319 to block the AngII-induced increase in cathepsin F secretion. Our present data demonstrate a novel proatherogenic role for AngII, namely its ability to enhance secretion of lysosomal cathepsin F by monocyte-derived macrophages. 
 Role of protein kinase C-angiotensin II pathway for   extracellular matrix   production in cultured human mesangial cells exposed to high glucose levels. The intrarenal renin-angiotensin system has been implicated in the pathogenesis of diabetic nephropathy. This study investigates the mechanisms for glucose-induced increase in angiotensin II (AII) production by human mesangial cells (MCs) in relation to protein kinase C (PKC). We also examine whether locally produced AII mediates   extracellular matrix   protein production in high-glucose conditions. Human MCs were cultured in 5 or 33 mmol/l glucose for 8 days, and were incubated with or without 5 mmol/l GFX, a PKC inhibitor, 0.1 micromol/l candesartan cilexetil (CC), a specific type 1 AII receptor antagonist, for another 24 h. In addition, MCs grown in 5 mmol/l glucose were incubated with 0.1 micromol/l phorbol-12,13-dibutyrate (PDBu) for 24 h. AII, TGF-beta1, fibronectin and type IV collagen in the culture media were measured by ELISA. The amount of AII secreted from MCs exposed to high-glucose levels was significantly greater (P<0.01) than that in normal glucose levels. The increase in AII production was completely prevented by GFX. The addition of PDBu mimicked the effect of glucose on AII production. The glucose-induced increases in the production of TGF-beta1, fibronectin and type IV collagen were partially, but significantly restored (P<0.01) by CC, while GFX totally abolished these effects of glucose. These results suggest that elevated glucose levels stimulate AII production via mechanisms dependent on glucose-induced PKC activation in human MCs, and that locally produced AII partly mediates the increase in mesangial matrix synthesis in high-glucose conditions. 
 A common effect of angiotensin II and relaxin 2 on the PNT1A normal prostate epithelial cell line. The prostate gland is a part of the male reproductive tract which produces both angiotensin II (Ang II) and relaxin 2 (RLN2). The present study analyzes the effect of both these peptide hormones at concentration 10(-8)M on viability, proliferation, adhesion, migration, and invasion of normal prostate epithelial cells (PNT1A). Improved survival in two- and three-dimensional cell cultures was noted as well as visual changes in colony size and structure in Geltrex™. Stimulatory influence on cell viability of each peptide applied single was lower than in combination. Enhanced survival of PNT1A cells appears to be associated with increased BCL2/BAX messenger RNA (mRNA) expression ratio. Modulation of cell spreading and cel  l-extracellular matr  ix adhesion dynamics were also altered as an influence of tested hormone application. However, long-term Ang II and RLN2 effects may lead to an increase of normal prostate cell migration and invasion abilities. Moreover, gelatin zymography revealed that both gelatinases A and B were augmented by Ang II treatment, whereas RLN2 significantly stimulated only MMP-9 secretion. These results support the hypothesis that deregulation of locally secreted peptide hormones such as Ang II and RLN2 may take part in the development of certain cancers, including prostate cancer. Moreover, the observed ability of relaxin 2 to act as a regulator of mRNA expression levels not only LGR7 but also classic angiotensin receptors suggested that renin-angiotensin system and relaxin family peptide system are functionally linked. 
  GeneRIFs  
 Angiotensin II contributes to each sequential step of cancer metastasis by promoting cancer cell adhesion to endothelial cells, trans-endothelial migration and tumor cell migration across   extracellular matrix  . 
  [Ii]nvasive  PubMed  
 Angiotensin II-induced sudden arrhythmic death and electrical remodeling. Rats harboring the human renin and angiotensinogen genes (dTGR) feature angiotensin (ANG) II/hypertension-induced cardiac damage and die suddenly between wk 7 and 8. We observed by electrocardiogram (ECG) telemetry that ventricular tachycardia (VT) is a common terminal event in these animals. Our aim was to investigate electrical remodeling. We used ECG telemetry, non  invasive   cardiac magnetic field mapping (CMFM) at wk 5 and 7, and performed in vivo programmed electrical stimulation at wk 7. We also investigated whether or not losartan (Los; 30 mg x kg(-1) x day(-1)) would prevent electrical remodeling. Cardiac hypertrophy and systolic blood pressure progressively increased in dTGR compared with Sprague-Dawley (SD) controls. Already by wk 5, untreated dTGR showed increased perivascular and interstitial fibrosis, connective tissue growth factor expression, and monocyte infiltration compared with SD rats, differences that progressed through time. Left-ventricular mRNA expression of potassium channel subunit Kv4.3 and gap-junction protein connexin 43 were significantly reduced in dTGR compared with Los-treated dTGR and SD. CMFM showed that depolarization and repolarization were prolonged and inhomogeneous. Los ameliorated all disturbances. VT could be induced in 88% of dTGR but only in 33% of Los-treated dTGR and could not be induced in SD. Untreated dTGR show electrical remodeling and probably die from VT. Los treatment reduces myocardial remodeling and predisposition to arrhythmias. ANG II target organ damage induces VT. 
 Increased urinary angiotensinogen is an effective marker of chronic renal impairment in very low birth weight children. Recent advancements in perinatal and neonatal care have increased the survival of preterm infants with lower birth weight and very low birth weight (VLBW; < 1,500 g) infants. Such infants are exposed to a higher risk of renal insufficiency in later life due to congenitally fewer nephrons; however, urinalysis in order to detect renal insufficiency in those infants at school age has not yet been established. The aim of the study was to assess chronic renal impairment in VLBW infants during their childhood after discharge from the neonatal intensive care unit (NICU) until adolescence using urinary angiotensinogen (uAGT).We compared serum levels of angiotensinogen (sAGT), creatinine, β2-microglobulin (sβ2MG) and cystatin C (sCysC), and urinary levels of uAGT, creatinine (uCre),β2-microglobulin (uβ2MG) and albumin between two infant groups-the VLBW group (50 children who were admitted to our NICU as infants), and a control group of 25 children who were born as full-term infants with birth weight ≥2,500 g. The median age of the VLBW group and control group infants was 60 months (range 7-135) and 57 months (range 5-144), respectively, at the time of evaluation.In the VLBW group, sCysC levels were high (p < 0.05) and estimated glomerular filtration rate (eGFR) was low (p < 0.05). There were no significant differences in the ratios of uβ2MG to creatinine and urinary albumin to creatinine between the two groups. Although there were no differences in concentration of sAGT between the two groups (p = 0.062), the ratio of uAGT to creatinine was significantly higher in the VLBW group (p < 0.01). The examination of 19 VLBW infants (19/50) with eGFR ≤90 ml/min/1.73 m(2) showed a positive correlation between uAGT/creatinine and urinary albumin/creatinine (r = 0.531, p < 0.05). Furthermore, the analysis of correlation between the ratio of uAGT to creatinine and eGFR showed a reverse correlation in 19 VLBW infants (19/50) with eGFR ≤90 ml/min/1.73 m(2), 18 of whom had stage II chronic kidney disease and one who had stage III disease (r = -0.512, p ≤ 0.05).uAGT is an effective marker for predicting the progression of chronic renal impairment in preterm VLBW infants after their growth. uAGT measurement is easier   to condu  ct, less invasive and more sensitive than conventional uβ2MG or urinary albumin measurement. 
 Urinary angiostatin--a novel putative marker of renal pathology chronicity in lupus nephritis. There is a critical need to identify biomarkers for Systemic Lupus Erythematosus (SLE) which has a high prevalence of renal failure. When urine from patients with lupus nephritis was recently screened for the levels of ∼280 molecules using an exploratory array-based proteomic platform, elevated angiostatin levels were noted. Angiostatin is a bioactive fragment of plasminogen, and has been known to have modulatory function in angiogenesis and inflammation. The significant elevation in urinary angiostatin was next validated in an independent cohort of SLE patients (n = 100) using ELISA. Among patients with SLE, urine angiostatin was significantly increased in active SLE compared with inactive SLE, correlating well with the SLEDAI disease activity index and SLICC renal activity score (r = 0.66, p < 0.0001). ROC curve analysis further confirmed that urinary angiostatin had the capacity to discriminate patients with active SLE from those with inactive disease. Patients with Class IV lupus nephritis exhibited the highest levels of urinary angiostatin. Immunohistochemistry staining localized angiostatin expression to the renal tubular cells in these patients. Finally, when paired urine-kidney samples procured concurrently from patients with LN were next examined, urine angiostatin levels correlated strongly with the renal pathology chronicity index, but not with the activity index. Given that Class IV lupus nephritis and renal pathology chronicity changes forebode poor renal and patient survival, urinary angiostatin emerges as a novel n  oninvasi  ve marker of renal disease in SLE. Longitudinal studies are in progress to further assess the disease-predictive potential of urinary angiostatin. 
 Angiotensin II facilitates breast cancer cell migration and metastasis. Breast cancer metastasis is a leading cause of death by malignancy in women worldwide. Efforts are being made to further characterize the rate-limiting steps of cancer metastasis, i.e. extravasation of circulating tumor cells and colonization of secondary organs. In this study, we investigated whether angiotensin II, a major vasoactive peptide both produced locally and released in the bloodstream, may trigger activating signals that contribute to cancer cell extravasation and metastasis. We used an experimental in vivo model of cancer metastasis in which bioluminescent breast tumor cells (D3H2LN) were injected intra-cardiacally into nude mice in order to recapitulate the late and essential steps of metastatic dissemination. Real-time intravital imaging studies revealed that angiotensin II accelerates the formation of metastatic foci at secondary sites. Pre-treatment of cancer cells with the peptide increases the number of mice with metastases, as well as the number and size of metastases per mouse. In vitro, angiotensin II contributes to each sequential step of cancer metastasis by promoting cancer cell adhesion to endothelial cells, trans-endothelial migration and tumor cell migration across extracellular matrix. At the molecular level, a total of 102 genes differentially expressed following angiotensin II pre-treatment were identified by comparative DNA microarray. Angiotensin II regulates two groups of connected genes related to its precursor angiotensinogen. Among those, up-regulated MMP2/MMP9 and ICAM1 stand at the crossroad of a network of genes involved in cell adhesion, migration and invasion. Our data suggest that targeting angiotensin II production or action may represent a valuable therapeutic option to prevent metastatic progression of   invasive   breast tumors. 
 Protein microarrays discover angiotensinogen and PRKRIP1 as novel targets for autoantibodies in chronic renal disease. Biomarkers for early detection of chronic kidney disease are needed, as millions of patients suffer from chronic diseases predisposing them to kidney failure. Protein microarrays may also hold utility in the discovery of auto-antibodies in other conditions not commonly considered auto-immune diseases. We hypothesized that proteins are released as a consequence of damage at a cellular level during end-organ damage from renal injury, not otherwise recognized as self-antigens, and an adaptive humoral immune response to these proteins might be detected in the blood, as a non  invasive   tracker of this injury. The resultant antibodies (Ab) detected in the blood would serve as effective biomarkers for occult renal injury, enabling earlier clinical detection of chronic kidney disease than currently possible, because of the redundancy of the serum creatinine as a biomarker for early kidney injury. To screen for novel autoantibodies in chronic kidney disease, 24 protein microarrays were used to compare serum Ab from patients with chronic kidney disease against matched controls. From a panel of 38 antigens with increased Ab binding, four were validated in 71 individuals, with (n=50) and without (n=21) renal insufficiency. Significant elevations in the titer of novel auto-Ab were noted against angiotensinogen and PRKRIP1 in renal insufficiency. Current validation is underway to evaluate if these auto-Ab can provide means to follow the evolution of chronic kidney disease in patients with early stages of renal insufficiency, and if these rising titers of these auto-Ab correlate with the rate of progression of chronic kidney disease. 
 Influence of polymorphisms in candidate genes on early vascular alterations in obese children. Many studies performed in adults have reported the involvement of genetic determinants in vascular alterations that predispose to cardiovascular diseases later in life. To date, no study has assessed the co-involvement of gene polymorphisms as cardiovascular risk factors in children.To search for variants involved in early vascular alterations in obese children.In 232 obese children, we performed an association study between variables related to endothelial function or arterial mechanical properties and functional variants reported to predispose towards vascular alterations in adults. Candidate polymorphisms were selected in genes involved in the renin-angiotensin system, vascular endothelial cell remodelling and communication, arterial inflammation, adiponectin production and lipoprotein metabolism. Non-  invasive   arterial measurements were performed to evaluate the mechanical characteristics of the common carotid artery and the endothelial function of the brachial artery.We found no association between the polymorphisms studied, taken alone or in combination with the arterial variables measured.Our hypothesis predicting that the tested genetic variants, which are involved in adult cardiovascular diseases, may influence the susceptibility to early vascular alterations in obese children was not validated. Thus, obesity-associated metabolic complications appear to remain the main predictors of arterial alterations in obese children. 
 Single nucleotide polymorphisms in genes of circulatory homeostasis in surviving pediatric intensive care patients with meningococcal infection. In the course of a meningococcal infection,   invasive   and severe disease occurs in a restricted number of individuals. The predominant mechanism of death in case of meningococcal septic shock is circulatory failure. Inotropic requirements between patients vary widely. We investigated whether polymorphisms in genes regulating the hemodynamic response influence the amount of inotropics required or the susceptibility to severe meningococcal disease.Retrospective case control study.Single-center pediatric intensive care unit (PICU).Fifty-six cases (all consecutive patients admitted to the PICU between 1993 and 2001 with a proven meningococcal infection) and 136 controls. Patients were divided into two groups according to their inotropic requirements.DNA analysis was performed to determine the polymorphisms of the beta-adrenergic receptor gene-1, beta-adrenergic receptor gene-2, alpha-adducin, angiotensin converting enzyme, and angiotensin II type-1 receptor-1 genes.For the alpha-adducin gene a significant difference of the genotype distribution was found between the cases and controls. The odds ratio for admission to the PICU with meningococcal sepsis with or without meningitis, for carriers of the variant allele (Gly460Trp or Trp460Trp) was 2.1 (95% confidence interval 1.11-4.04; p < 0.02). Cases, homozygote for the wild-type allele of the beta-1 adrenergic receptor at locus 389, were more likely to have a low pediatric risk of mortality score on admission (odds ratio 3.6, 95% confidence interval 1.11-11.76). No difference was found in the distribution of the beta-adrenergic receptor gene-1, beta-adrenergic receptor gene-2, angiotensin converting enzyme, and angiotensin II type-1 receptor-1 polymorphisms between the two groups of patients or between cases and controls.Among patients admitted to the PICU with a meningococcal infection, the variant allele of the alpha-adducin gene was more prevalent compared with controls. Patients with the variant allele of the beta-adrenergic receptor gene-1 at locus 389 were more likely to have a high pediatric risk of mortality score on admission. The mechanism and clinical relevance of these findings is unclear. 
  GeneRIFs  
 Urinary angiostatin is a novel non  invasive   marker of severity of renal disease in systemic lupus erythematosus. 
  [Mm]etastasis  PubMed  
 Update of the human and mouse SERPIN gene superfamily. The serpin family comprises a structurally similar, yet functionally diverse, set of proteins. Named originally for their function as serine proteinase inhibitors, many of its members are not inhibitors but rather chaperones, involved in storage, transport, and other roles. Serpins are found in genomes of all kingdoms, with 36 human protein-coding genes and five pseudogenes. The mouse has 60 Serpin functional genes, many of which are orthologous to human SERPIN genes and some of which have expanded into multiple paralogous genes. Serpins are found in tissues throughout the body; whereas most are extracellular, there is a class of intracellular serpins. Serpins appear to have roles in inflammation, immune function, tumorigenesis, blood clotting, dementia, and cancer   metastasis  . Further characterization of these proteins will likely reveal potential biomarkers and therapeutic targets for disease. 
 Angiotensin II facilitates breast cancer cell migration and   metastasis  . Breast cancer   metastasis   is a leading cause of death by malignancy in women worldwide. Efforts are being made to further characterize the rate-limiting steps of cancer   metastasis  , i.e. extravasation of circulating tumor cells and colonization of secondary organs. In this study, we investigated whether angiotensin II, a major vasoactive peptide both produced locally and released in the bloodstream, may trigger activating signals that contribute to cancer cell extravasation and   metastasis  . We used an experimental in vivo model of cancer   metastasis   in which bioluminescent breast tumor cells (D3H2LN) were injected intra-cardiacally into nude mice in order to recapitulate the late and essential steps of metastatic dissemination. Real-time intravital imaging studies revealed that angiotensin II accelerates the formation of metastatic foci at secondary sites. Pre-treatment of cancer cells with the peptide increases the number of mice with metastases, as well as the number and size of metastases per mouse. In vitro, angiotensin II contributes to each sequential step of cancer   metastasis   by promoting cancer cell adhesion to endothelial cells, trans-endothelial migration and tumor cell migration across extracellular matrix. At the molecular level, a total of 102 genes differentially expressed following angiotensin II pre-treatment were identified by comparative DNA microarray. Angiotensin II regulates two groups of connected genes related to its precursor angiotensinogen. Among those, up-regulated MMP2/MMP9 and ICAM1 stand at the crossroad of a network of genes involved in cell adhesion, migration and invasion. Our data suggest that targeting angiotensin II production or action may represent a valuable therapeutic option to prevent metastatic progression of invasive breast tumors. 
 Angiotensin II enhances epithelial-to-mesenchymal transition through the interaction between activated hepatic stellate cells and the stromal cell-derived factor-1/CXCR4 axis in intrahepatic cholangiocarcinoma. We previously reported that hepatic stellate cells (HSCs) activated by angiotensin II (AngII) facilitate stromal fibrosis and tumor progression in intrahepatic cholangiocarcinoma (ICC). AngII has been known as a growth factor which can promote epithelial-to-mesenchymal transition (EMT) in renal epithelial cells, alveolar epithelial cells and peritoneal mesothelial cells. However, in the past, the relationship between AngII and stromal cell-derived factor-1 (SDF-1) in the microenvironment around cancer and the role of AngII on EMT of cancer cells has not been reported in detail. SDF-1 and its specific receptor, CXCR4, are now receiving attention as a mechanism of cell progression and   metastasis  . In this study, we examined whether activated HSCs promote tumor fibrogenesis, tumor progression and distant   metastasis   by mediating EMT via the AngII/AngII type 1 receptor (AT-1) and the SDF-1/CXCR4 axis. Two human ICC cell lines and a human HSC line, LI-90, express CXCR4. Significantly higher concentration of SDF-1α was released into the supernatant of LI-90 cells to which AngII had been added. SDF-1α increased the proliferative activity of HSCs and enhanced the activation of HSCs as a growth factor. Furthermore, addition of SDF-1α and AngII enhanced the increase of the migratory capability and vimentin expression, reduced E-cadherin expression, and translocated the expression of β-catenin into the nucleus and cytoplasm in ICC cells. Co-culture with HSCs also enhanced the migratory capability of ICC cells. These findings suggest that SDF-1α, released from activated HSCs and AngII, play important roles in cancer progression, tumor fibrogenesis, and migration in autocrine and paracrine fashion by mediating EMT. Our mechanistic findings may provide pivotal insights into the molecular mechanism of the AngII and SDF-1α-initiated signaling pathway that regulates fibrogenesis in cancerous stroma, tumor progression and meta-stasis of tumor cells expressing AT-1 and CXCR4. 
 Diabetes-associated angiotensin activation enhances liver   metastasis   of colon cancer. We examined the effects of hyperglycemic conditions on liver   metastasis   of colorectal cancer (CRC). Angiotensin (A)-II increased growth, invasion, and anti-apoptotic survival in HT29 and CT26 cells. In contrast, angiotensinogen (ATG) increased these features in HT29 cells but not in CT26 cells. HT29 cells expressed A-II type 1 receptor, chymase, and rennin, whereas CT26 cells did not express renin. Renin expression and ATG-induced cell growth, invasion, and survival induced and increased as glucose concentration increased in HT29 cells and also CT26 cells. An inhibitor of renin or chymase abrogated A-II production in HT29 cells. Reduction of hepatic ATG production by cholesterol-conjugated antisense S-oligodeoxynucleotide suppressed liver   metastasis   of HT29 cells. An examination of 121 CRC patients showed that diabetes in CRC cases was associated with higher blood HbA1c, higher renin and A-II concentrations in the primary tumors, and higher incidence of liver   metastasis   than in nondiabetic cases. These results suggest that diabetes-associated angiotensin activation enhances liver   metastasis   of CRC and may therefore provide a possible target for antimetastatic therapy in CRC. 
  GeneRIFs  
 results suggest that diabetes-associated angiotensin activation enhances liver   metastasis   of colorectal cancer and may therefore provide a possible target for antimetastatic therapy in colorectal cancer 
 Angiotensin II contributes to each sequential step of cancer   metastasis   by promoting cancer cell adhesion to endothelial cells, trans-endothelial migration and tumor cell migration across extracellular matrix. 
   
  
 SERPIND1 
 
  NCBI Gene  
  GeneRIF  
  Pubmed  
 
 
  Search Term  Rank  Fields  
  [Ff]ilopodium  2  PubMed(1)  
  [Mm]igration  6  PubMed(3)  
  [Ee]xtracellular [Mm]atrix  8  PubMed(1)  
  [Ii]nvasive  10  PubMed(1)  
  [Mm]etastasis  12  PubMed(2); GeneRIFs(1); Summary(1)  
 
 
 hide/show details for SERPIND1 
  [Ff]ilopodium  PubMed  
 Heparin co-factor II enhances cell motility and promotes metastasis in non-small cell lung cancer. Using the Serial Analysis of Gene Expression (SAGE) database from the Cancer Genome Anatomy Project, we identified heparin co-factor II (HCII), which is over-expressed in non-small cell lung cancer (NSCLC). Here, we investigated the clinical significance of HCII and provided molecular evidence to support the suggestion that HCII could enhance cancer metastasis in NSCLC. We found that high HCII expression in tumour tissue was associated with increased cancer recurrence and shorter overall survival times in 75 clinically operable NSCLC patients. High pretreatment plasma concentration of HCII was associated with reduced overall survival in 57 consecutive NSCLC patients. We over-expressed and knocked down HCII expression in lung cancer cell lines and confirmed that HCII could promote cell motility, invasion ability and   filopodium   dynamics in NSCLC cells in vitro and increased metastatic colonization in an in vivo mouse model. Exogenous treatment of HCII promoted cancer cell migration, and this promigratory effect of HCII was independent of thrombin. We further showed that HCII could up-regulate cancer cell migration through the activation of PI3K, which acts upstream of Rac1 and Cdc42, and this effect could be blocked by heparin. We suggest that HCII is a novel metastasis enhancer and may be used as a prognostic predictor for heparin treatment in NSCLC. 
  [Mm]igration  PubMed  
 Heparin cofactor II, a serine protease inhibitor, promotes angiogenesis via activation of the AMP-activated protein kinase-endothelial nitric-oxide synthase signaling pathway. We previously clarified that heparin cofactor II (HCII), a serine proteinase inhibitor, exerts various protective actions on cardiovascular diseases in both experimental and clinical studies. In the present study, we aimed to clarify whether HCII participates in the regulation of angiogenesis. Male heterozygous HCII-deficient (HCII(+/-)) mice and male littermate wild-type (HCII(+/+)) mice at the age of 12-16 weeks were subjected to unilateral hindlimb ligation surgery. Laser speckle blood flow analysis showed that blood flow recovery in response to hindlimb ischemia was delayed in HCII(+/-) mice compared with that in HCII(+/+) mice. Capillary number, arteriole number, and endothelial nitric-oxide synthase (eNOS), AMP-activated protein kinase (AMPK), and liver kinase B1 (LKB1) phosphorylation in ischemic muscles were decreased in HCII(+/-) mice. Human purified HCII (h-HCII) administration almost restored blood flow recovery, capillary density, and arteriole number as well as phosphorylation levels of eNOS, AMPK, and LKB1 in ischemic muscles of HCII(+/-) mice. Although treatment with h-HCII increased phosphorylation levels of eNOS, AMPK, and LKB1 in human aortic endothelial cells (HAECs), the h-HCII-induced eNOS phosphorylation was abolished by compound C, an AMPK inhibitor, and by AMPK siRNA. In a similar fashion, tube formation, proliferation, and   migration   of HAECs were also promoted by h-HCII treatment and were abrogated by pretreatment with compound C. HCII potentiates the activation of vascular endothelial cells and the promotion of angiogenesis in response to hindlimb ischemia via an AMPK-eNOS signaling pathway. These findings suggest that HCII is a novel therapeutic target for treatment of patients with peripheral circulation insufficiency. 
 Heparin co-factor II enhances cell motility and promotes metastasis in non-small cell lung cancer. Using the Serial Analysis of Gene Expression (SAGE) database from the Cancer Genome Anatomy Project, we identified heparin co-factor II (HCII), which is over-expressed in non-small cell lung cancer (NSCLC). Here, we investigated the clinical significance of HCII and provided molecular evidence to support the suggestion that HCII could enhance cancer metastasis in NSCLC. We found that high HCII expression in tumour tissue was associated with increased cancer recurrence and shorter overall survival times in 75 clinically operable NSCLC patients. High pretreatment plasma concentration of HCII was associated with reduced overall survival in 57 consecutive NSCLC patients. We over-expressed and knocked down HCII expression in lung cancer cell lines and confirmed that HCII could promote cell motility, invasion ability and filopodium dynamics in NSCLC cells in vitro and increased metastatic colonization in an in vivo mouse model. Exogenous treatment of HCII promoted cancer cell   migration  , and this promigratory effect of HCII was independent of thrombin. We further showed that HCII could up-regulate cancer cell   migration   through the activation of PI3K, which acts upstream of Rac1 and Cdc42, and this effect could be blocked by heparin. We suggest that HCII is a novel metastasis enhancer and may be used as a prognostic predictor for heparin treatment in NSCLC. 
 Leukocyte chemoattractant peptides from the serpin heparin cofactor II. Heparin cofactor II (HC) is a plasma serine proteinase inhibitor (serpin) that inhibits the coagulant proteinase alpha-thrombin. We have recently demonstrated that proteolysis of HC by catalytic amounts of polymorphonuclear leukocyte proteinases (elastase or cathepsin G) generates leukocyte chemotaxins (Hoffman, M., Pratt, C. W., Brown, R. L., and Church, F. C. (1989) Blood 73, 1682-1685). One of four peptides produced when HC is degraded by neutrophil elastase has chemotactic activity for both monocytes and neutrophils with maximal   migration   comparable to formyl-Met-Leu-Phe, the "gold standard" bacterially derived chemotaxin. The amino-terminal sequence of this HC peptide is Asp-Phe-His-Lys-Glu-Asn-Thr-Val-... and the peptide corresponds to Asp-39 to Ile-66 of HC. A variety of synthetic peptides derived from this sequence were evaluated for leukocyte   migration   activity, and a dodecapeptide from Asp-49 to Tyr-60 (Asp-Trp-Ile-Pro-Glu-Gly-Glu-Glu-Asp-Asp-Asp-Tyr) was identified as the active site for leukocyte chemotactic action. The 12-mer synthetic peptide possesses significant neutrophil chemotactic action at 1 nM (60% of the maximal activity of formyl-Met-Leu-Phe), while a peptide with the reverse sequence has essentially no chemotactic activity. Cross-desensitization experiments also show that pretreatment of neutrophils with a 19-mer peptide (Asn-48 to Ile-66) greatly reduces subsequent chemotaxis to HC-neutrophil elastase proteolysis reaction products. When injected intraperitoneally in mice, the HC-neutrophil elastase digest elicits neutrophil   migration  . Our results demonstrate that not only does HC function as a thrombin inhibitor, but that limited proteolysis of HC near the amino terminus yields biologically active peptide(s) which might participate in inflammation and in wound healing and tissue repair processes. 
  [Ee]xtracellular [Mm]atrix  PubMed  
 Effects of fibroblasts and endothelial cells on inactivation of target proteases by protease nexin-1, heparin cofactor II, and C1-inhibitor. Previous studies have shown that glycosaminoglycans in the   extracellular matrix   accelerate the inactivation of target proteases by certain protease inhibitors. It has been suggested that the ability of the matrix of certain cells to accelerate some inhibitors but not others might reflect the site of action of the inhibitors. Previous studies showed that fibroblasts accelerate the inactivation of thrombin by protease nexin-1, an inhibitor that appears to function at the surface of cells in extravascular tissues. The present experiments showed that endothelial cells also accelerate this reaction. The accelerative activity was accounted for by the   extracellular matrix   and was mostly due to heparan sulfate. Fibroblasts but not endothelial cells accelerated the inactivation of thrombin by heparin cofactor II, an abundant inhibitor in plasma. This is consistent with previous suggestions that heparin cofactor II inactivates thrombin when plasma is exposed to fibroblasts and smooth muscle cells. Neither fibroblasts nor endothelial cells accelerated the inactivation of C1s by plasma C1-inhibitor. 
  [Ii]nvasive  PubMed  
 Proteolytic activation transforms heparin cofactor II into a host defense molecule. The abundant serine proteinase inhibitor heparin cofactor II (HCII) has been proposed to inhibit extravascular thrombin. However, the exact physiological role of this plasma protein remains enigmatic. In this study, we demonstrate a previously unknown role for HCII in host defense. Proteolytic cleavage of the molecule induced a conformational change, thereby inducing endotoxin-binding and antimicrobial properties. Analyses employing representative peptide epitopes mapped these effects to helices A and D. Mice deficient in HCII showed increased susceptibility to   invasive   infection by Pseudomonas aeruginosa, along with a significantly increased cytokine response. Correspondingly, decreased levels of HCII were observed in wild-type animals challenged with bacteria or endotoxin. In humans, proteolytically cleaved HCII forms were detected during wounding and in association with bacteria. Thus, the protease-induced uncovering of cryptic epitopes in HCII, which transforms the molecule into a host defense factor, represents a previously unknown regulatory mechanism in HCII biology and innate immunity. 
  [Mm]etastasis  PubMed  
 Update of the human and mouse SERPIN gene superfamily. The serpin family comprises a structurally similar, yet functionally diverse, set of proteins. Named originally for their function as serine proteinase inhibitors, many of its members are not inhibitors but rather chaperones, involved in storage, transport, and other roles. Serpins are found in genomes of all kingdoms, with 36 human protein-coding genes and five pseudogenes. The mouse has 60 Serpin functional genes, many of which are orthologous to human SERPIN genes and some of which have expanded into multiple paralogous genes. Serpins are found in tissues throughout the body; whereas most are extracellular, there is a class of intracellular serpins. Serpins appear to have roles in inflammation, immune function, tumorigenesis, blood clotting, dementia, and cancer   metastasis  . Further characterization of these proteins will likely reveal potential biomarkers and therapeutic targets for disease. 
 Heparin co-factor II enhances cell motility and promotes   metastasis   in non-small cell lung cancer. Using the Serial Analysis of Gene Expression (SAGE) database from the Cancer Genome Anatomy Project, we identified heparin co-factor II (HCII), which is over-expressed in non-small cell lung cancer (NSCLC). Here, we investigated the clinical significance of HCII and provided molecular evidence to support the suggestion that HCII could enhance cancer   metastasis   in NSCLC. We found that high HCII expression in tumour tissue was associated with increased cancer recurrence and shorter overall survival times in 75 clinically operable NSCLC patients. High pretreatment plasma concentration of HCII was associated with reduced overall survival in 57 consecutive NSCLC patients. We over-expressed and knocked down HCII expression in lung cancer cell lines and confirmed that HCII could promote cell motility, invasion ability and filopodium dynamics in NSCLC cells in vitro and increased metastatic colonization in an in vivo mouse model. Exogenous treatment of HCII promoted cancer cell migration, and this promigratory effect of HCII was independent of thrombin. We further showed that HCII could up-regulate cancer cell migration through the activation of PI3K, which acts upstream of Rac1 and Cdc42, and this effect could be blocked by heparin. We suggest that HCII is a novel   metastasis   enhancer and may be used as a prognostic predictor for heparin treatment in NSCLC. 
  GeneRIFs  
 heparin co-factor II is a novel   metastasis   enhancer and may be used as a prognostic predictor for heparin treatment in non-small cell lung cancer 
  Summary  
 This gene belongs to the serpin gene superfamily. Serpins play roles in many processes including inflammation, blood clotting, and cancer   metastasis  . Members of this family have highly conserved secondary structures with a reactive center loop that interacts with the protease active site to inhibit protease activity. This gene encodes a plasma serine protease that functions as a thrombin and chymotrypsin inhibitor. The protein is activated by heparin, dermatan sulfate, and glycosaminoglycans. Allelic variations in this gene are associated with heparin cofactor II deficiency. [provided by RefSeq, Jul 2015] 
   
  
 SERPINB7 
 
  NCBI Gene  
  GeneRIF  
  Pubmed  
 
 
  Search Term  Rank  Fields  
  [Mm]igration  6  PubMed(1)  
  ECM  7  PubMed(1)  
  [Ee]xtracellular [Mm]atrix  8  PubMed(1)  
  [Ii]nvasive  10  PubMed(3)  
  [Mm]etastasis  12  PubMed(2)  
 
 
 hide/show details for SERPINB7 
  [Mm]igration  PubMed  
 Suppression of the invasion and   migration   of cancer cells by SERPINB family genes and their derived peptides. Apart from SERPINB2 and SERPINB5, the roles of the remaining 13 members of the human SERPINB family in cancer metastasis are still unknown. In the present study, we demonstrated that most of these genes are differentially expressed in tumor tissues compared to matched normal tissues from lung or breast cancer patients. Overexpression of each SERPINB gene effectively suppressed the invasiveness and motility of malignant cancer cells. Among all of the genes, the SERPINB1, SERPINB5 and SERPINB7 genes were more potent, and the inhibitory effect was further enhanced by co-expression of any two of them. In addition, single treatment of the synthetic peptides corresponding to the P5-P5' sequences of the reactive center loop (RCL) of SERPINB1, SERPINB5 or SERPINB7 markedly suppressed the invasive and migratory properties of the cancer cells in a dose-dependent manner. More significantly, combination treatment of these peptides in cancer cells further improved the suppressive effect by 20-40%. Here, we determined the expression of all SERPINB family members in lung and breast cancer patients, and identified those members with potent inhibitory ability toward invasion and   migration  , and designed RCL-derived peptides to suppress the malignancy of cancer cells. Forced re-expression of these anti-invasive SERPINB genes or application of the SERPINB RCL-peptides may provide a reasonable strategy against lethal cancer metastasis. 
  ECM  PubMed  
 Overexpression of the serpin megsin induces progressive mesangial cell proliferation and expansion. Mesangial cells maintain normal glomerular function by mediating   ECM   remodeling and immune complex disposal. We have recently identified megsin, a novel member of the serine protease inhibitor (serpin) superfamily predominantly expressed in the mesangium. While our previous studies suggested a role for megsin in the pathogenesis of human glomerular diseases, its exact biological significance remained unknown. Here we produced two lines of megsin transgenic mice. Overexpression of megsin led to progressive mesangial matrix expansion and an increase in the number of mesangial cells. These glomerular lesions were accompanied by an augmented immune complex deposition, together with Ig's and complement. Binding and functional assays in vitro identified plasmin as one biological substrate of megsin and confirmed its activity as a proteinase inhibitor. Transgenic animals exhibiting nephritis as a result of treatment with anti--glomerular basement membrane antiserum showed significantly more persistent expansion of the mesangial   ECM   than was seen in parental mice. Megsin therefore exerts a biologically relevant influence on mesangial function, and on the mesangial microenvironment, such that simple overexpression of this endogenous serpin engenders elementary mesangial lesions. 
  [Ee]xtracellular [Mm]atrix  PubMed  
 Megsin 2093T-2180C haplotype at the 3' untranslated region is associated with poor renal survival in Korean IgA nephropathy patients. Megsin is a mesangial cell-predominant gene which belongs to the serpin superfamily. The expression of megsin was upregulated and coincided with mesangial proliferation and   extracellular matrix   expansion in IgA nephropathy (IgAN). In the present study, we evaluated the influence of the C2093T and C2180T polymorphism within the 3' untranslated region (3'UTR) of megsin gene and its haplotypes on the development and progression of Korean IgAN patients.Korean IgAN patients (n = 260) with a minimal follow-up of 4 years were recruited. Healthy subjects with normal renal function, normal urinalysis and normotension (n = 315) were included as controls. The polymorphisms were determined by the 5' nuclease allelic discrimination assay, and the haplotypes were constructed using the Phase program.The C2093T and C2180T genotype and allele frequencies were not different significantly between IgAN patients and controls. In C2093T polymorphism, patients with CC genotype showed a better renal survival than those with CT or TT genotypes by Kaplan-Meier analysis (p = 0.027). The megsin C2093T polymorphism remained an independent risk factor for progression (Cox regression model, HR for TT genotype: 3.52, 95% CI 1.69 - 7.34; HR for CT genotype: 2.15, 95% CI 1.30 - 3.57). In C2180T polymorphism, patients with TT genotype showed a better outcome than those with CC or CT genotypes (p = 0.025). The C2180T polymorphism was also an independent risk factor for progression (HR for CC genotype: 4.05, 95% CI 1.93 - 8.51; HR for CT genotype: 2.35, 95% CI 1.40 - 3.94). The two alleles showed linkage disequilibrium in phased haplotype. The patients with 2093T-2180C haplotype showed a poor renal survival compared to those with 2093C-2180T haplotype (p = 0.028). The haplotype remained an independent risk factor for progression (HR for 2093T-2180C haplotype: 2.01, 95% CI 1.44 - 2.81).Our results suggest that the 2093T-2180C haplotype at the 3'UTR of megsin gene is associated with rapid disease progression in Korean IgAN patients. This is the reverse of the results from the Chinese IgAN patients. Further studies are strongly needed to elucidate the reasons of disparity. 
  [Ii]nvasive  PubMed  
 Evaluation of candidate stromal epithelial cross-talk genes identifies association between risk of serous ovarian cancer and TERT, a cancer susceptibility "hot-spot". We hypothesized that variants in genes expressed as a consequence of interactions between ovarian cancer cells and the host micro-environment could contribute to cancer susceptibility. We therefore used a two-stage approach to evaluate common single nucleotide polymorphisms (SNPs) in 173 genes involved in stromal epithelial interactions in the Ovarian Cancer Association Consortium (OCAC). In the discovery stage, cases with epithelial ovarian cancer (n=675) and controls (n=1,162) were genotyped at 1,536 SNPs using an Illumina GoldenGate assay. Based on Positive Predictive Value estimates, three SNPs-PODXL rs1013368, ITGA6 rs13027811, and MMP3 rs522616-were selected for replication using TaqMan genotyping in up to 3,059 serous   invasive   cases and 8,905 controls from 16 OCAC case-control studies. An additional 18 SNPs with Pper-allele<0.05 in the discovery stage were selected for replication in a subset of five OCAC studies (n=1,233 serous   invasive   cases; n=3,364 controls). The discovery stage associations in PODXL, ITGA6, and MMP3 were attenuated in the larger replication set (adj. Pper-allele>or=0.5). However genotypes at TERT rs7726159 were associated with ovarian cancer risk in the smaller, five-study replication study (Pper-allele=0.03). Combined analysis of the discovery and replication sets for this TERT SNP showed an increased risk of serous ovarian cancer among non-Hispanic whites [adj. ORper-allele 1.14 (1.04-1.24) p=0.003]. Our study adds to the growing evidence that, like the 8q24 locus, the telomerase reverse transcriptase locus at 5p15.33, is a general cancer susceptibility locus. 
 Evaluation of candidate stromal epithelial cross-talk genes identifies association between risk of serous ovarian cancer and TERT, a cancer susceptibility "hot-spot". We hypothesized that variants in genes expressed as a consequence of interactions between ovarian cancer cells and the host micro-environment could contribute to cancer susceptibility. We therefore used a two-stage approach to evaluate common single nucleotide polymorphisms (SNPs) in 173 genes involved in stromal epithelial interactions in the Ovarian Cancer Association Consortium (OCAC). In the discovery stage, cases with epithelial ovarian cancer (n=675) and controls (n=1,162) were genotyped at 1,536 SNPs using an Illumina GoldenGate assay. Based on Positive Predictive Value estimates, three SNPs-PODXL rs1013368, ITGA6 rs13027811, and MMP3 rs522616-were selected for replication using TaqMan genotyping in up to 3,059 serous   invasive   cases and 8,905 controls from 16 OCAC case-control studies. An additional 18 SNPs with Pper-allele<0.05 in the discovery stage were selected for replication in a subset of five OCAC studies (n=1,233 serous   invasive   cases; n=3,364 controls). The discovery stage associations in PODXL, ITGA6, and MMP3 were attenuated in the larger replication set (adj. Pper-allele>or=0.5). However genotypes at TERT rs7726159 were associated with ovarian cancer risk in the smaller, five-study replication study (Pper-allele=0.03). Combined analysis of the discovery and replication sets for this TERT SNP showed an increased risk of serous ovarian cancer among non-Hispanic whites [adj. ORper-allele 1.14 (1.04-1.24) p=0.003]. Our study adds to the growing evidence that, like the 8q24 locus, the telomerase reverse transcriptase locus at 5p15.33, is a general cancer susceptibility locus. 
 Suppression of the invasion and migration of cancer cells by SERPINB family genes and their derived peptides. Apart from SERPINB2 and SERPINB5, the roles of the remaining 13 members of the human SERPINB family in cancer metastasis are still unknown. In the present study, we demonstrated that most of these genes are differentially expressed in tumor tissues compared to matched normal tissues from lung or breast cancer patients. Overexpression of each SERPINB gene effectively suppressed the   invasive  ness and motility of malignant cancer cells. Among all of the genes, the SERPINB1, SERPINB5 and SERPINB7 genes were more potent, and the inhibitory effect was further enhanced by co-expression of any two of them. In addition, single treatment of the synthetic peptides corresponding to the P5-P5' sequences of the reactive center loop (RCL) of SERPINB1, SERPINB5 or SERPINB7 markedly suppressed the   invasive   and migratory properties of the cancer cells in a dose-dependent manner. More significantly, combination treatment of these peptides in cancer cells further improved the suppressive effect by 20-40%. Here, we determined the expression of all SERPINB family members in lung and breast cancer patients, and identified those members with potent inhibitory ability toward invasion and migration, and designed RCL-derived peptides to suppress the malignancy of cancer cells. Forced re-expression of these anti-  invasive   SERPINB genes or application of the SERPINB RCL-peptides may provide a reasonable strategy against lethal cancer metastasis. 
  [Mm]etastasis  PubMed  
 Update of the human and mouse SERPIN gene superfamily. The serpin family comprises a structurally similar, yet functionally diverse, set of proteins. Named originally for their function as serine proteinase inhibitors, many of its members are not inhibitors but rather chaperones, involved in storage, transport, and other roles. Serpins are found in genomes of all kingdoms, with 36 human protein-coding genes and five pseudogenes. The mouse has 60 Serpin functional genes, many of which are orthologous to human SERPIN genes and some of which have expanded into multiple paralogous genes. Serpins are found in tissues throughout the body; whereas most are extracellular, there is a class of intracellular serpins. Serpins appear to have roles in inflammation, immune function, tumorigenesis, blood clotting, dementia, and cancer   metastasis  . Further characterization of these proteins will likely reveal potential biomarkers and therapeutic targets for disease. 
 Suppression of the invasion and migration of cancer cells by SERPINB family genes and their derived peptides. Apart from SERPINB2 and SERPINB5, the roles of the remaining 13 members of the human SERPINB family in cancer   metastasis   are still unknown. In the present study, we demonstrated that most of these genes are differentially expressed in tumor tissues compared to matched normal tissues from lung or breast cancer patients. Overexpression of each SERPINB gene effectively suppressed the invasiveness and motility of malignant cancer cells. Among all of the genes, the SERPINB1, SERPINB5 and SERPINB7 genes were more potent, and the inhibitory effect was further enhanced by co-expression of any two of them. In addition, single treatment of the synthetic peptides corresponding to the P5-P5' sequences of the reactive center loop (RCL) of SERPINB1, SERPINB5 or SERPINB7 markedly suppressed the invasive and migratory properties of the cancer cells in a dose-dependent manner. More significantly, combination treatment of these peptides in cancer cells further improved the suppressive effect by 20-40%. Here, we determined the expression of all SERPINB family members in lung and breast cancer patients, and identified those members with potent inhibitory ability toward invasion and migration, and designed RCL-derived peptides to suppress the malignancy of cancer cells. Forced re-expression of these anti-invasive SERPINB genes or application of the SERPINB RCL-peptides may provide a reasonable strategy against lethal cancer   metastasis  . 
   
  
 SERPINA3 
 
  NCBI Gene  
  GeneRIF  
  Pubmed  
 
 
  Search Term  Rank  Fields  
  [Ee]xtracellular [Mm]atrix  8  PubMed(3)  
  [Ii]nvasive  10  PubMed(4)  
  [Mm]etastasis  12  PubMed(3)  
 
 
 hide/show details for SERPINA3 
  [Ee]xtracellular [Mm]atrix  PubMed  
 Genetic and epigenetic mechanisms collaborate to control SERPINA3 expression and its association with placental diseases. SERPINA3 (Serpin peptidase inhibitor clade A member 3), also known as a1-antichymotrypsin, is a serine protease inhibitor involved in a wide range of biological processes. Recently, it has been shown to be up-regulated in human placental diseases in association with a hypomethylation of the 5' region of the gene. In the present study, we show that the promoter of SERPINA3 is transcriptionally activated by three transcription factors (TFs) (SP1, MZF1 and ZBTB7B), the level of induction being dependent on the rs1884082 single nucleotide polymorphism (SNP) located inside the promoter, the T allele being consistently induced to a higher level than the G, with or without added TFs. When the promoter was methylated, the response to ZBTB7B was allele specific (the G allele was strongly induced, while the T allele was strongly down-regulated). We propose an adaptive model to explain the interest of such a regulation for placental function and homeostasis. Overexpression of SERPINA3 in JEG-3 cells, a trophoblast cell model, decreased cell adhesion to the   extracellular matrix   and to neighboring cells, but protects them from apoptosis, suggesting a way by which this factor could be deleterious at high doses. In addition, we show in different human populations that the T allele appears to predispose to Intra Uterine Growth Restriction (IUGR), while a G allele at a second SNP located in the second exon (rs4634) increases the risk of preeclampsia. Our results provide mechanistic views inside the involvement of SERPINA3 in placental diseases, through its regulation by a combination of epigenetic, genetic and TF-mediated regulations. 
 alpha 1-Proteinase inhibitor, alpha 1-antichymotrypsin, and alpha 2-macroglobulin are the antiapoptotic factors of vascular smooth muscle cells. Serum depletion induces cell death. Whereas serum contains growth factors and adhesion molecules that are important for survival, serum is also likely to have antiapoptotic factor(s). We show here that the plasma proteinase inhibitors alpha1-proteinase inhibitor, alpha1-antichymotrypsin, and alpha2-macroglobulin function as critical antiapoptotic factors for human vascular smooth muscle cells. Cell survival was assured when serum-free medium was supplemented with any one or all of the above serine proteinase inhibitors. In contrast, the cells were sensitive to apoptosis when cultured in medium containing serum from which the proteinase inhibitors were removed. The antiapoptotic effect conferred by the proteinase inhibitors was proportional to proteinase inhibitory activity. Without proteinase inhibitors, the   extracellular matrix   was degraded, and cells could not attach to the matrix. Cell survival was dependent on the intact   extracellular matrix  . In the presence of the caspase inhibitor z-VAD, the cells detached but did not die. The activity of caspases was elevated without proteinase inhibitors; in contrast, caspases were not activated when medium was supplemented with one of the proteinase inhibitors. In conclusion, the plasma proteinase inhibitors prevent degradation of   extracellular matrix   by proteinases derived from cells. Presumably an intact cell-matrix interaction inhibits caspase activation and supports cell survival. 
 Inflammation-related neutrophil proteases, cathepsin G and elastase, function as insulin-like growth factor binding protein proteases. Over the past few years, several proteolytic enzymes have been identified as insulin-like growth factor binding protein (IGFBP) proteases. It has been suggested that proteolytic cleavage of IGFBPs is associated with regulation of the proliferative effects of IGFs on their target cells. In this study, we have demonstrated that two neutrophil proteases, cathepsin G and elastase, effectively cleave IGFBPs in vitro and in vivo at concentrations lower than previous described IGFBP proteases. Purified leukocyte cathepsin G and elastase cleaved all six well-characterized IGFBPs into distinct fragments in a concentration-dependent manner. Under similar experimental conditions, cathepsin G preferentially cleaved IGFBP-5, followed by BP-2, BP-3, BP-4, BP-1, and BP-6. In comparison, elastase equally preferred IGFBP-3 and IGFBP-4, followed by BP-1, BP-5, BP-6, and BP-2. Proteolysis of rh(125)I-IGFBP-3 by cathepsin G was blocked by alpha(1)-antichymotrypsin, while elastase proteolytic activity was blocked by alpha(1)-proteinase inhibitor as expected. Elastase, but not cathepsin G, cleaved free IGF-I into a smaller molecular weight fragment in vitro, possibly designating unique functions for each protease within the IGF axis. Sequence analysis of IGFBP-3 fragments produced by cathepsin G and elastase demonstrated that each protease cleaved IGFBP-3 at unique sites within its midregion. More importantly, extracts from purified neutrophils have demonstrated significant proteolytic cleavage of IGFBP-3 that resembles elastase proteolysis of IGFBP-3. Recent studies using a monocyte-like cell model have also shown significant cleavage of IGFBP-3. These in vitro and in vivo data suggest that the neutrophil proteases, cathepsin G and elastase, in addition to their previously described functions as   extracellular matrix  -degrading enzymes, may potentially act as IGFBP proteases involved in regulation of IGFs and IGFBPs during inflammation and wound healing. 
  [Ii]nvasive  PubMed  
 c-erbB-2 is of independent prognostic relevance in gastric cancer and is associated with the expression of tumor-associated protease systems. The c-erbB-2 gene (encoding the protein p185) is overexpressed in diverse human cancers and has been implicated to be of prognostic value in gastric cancer. Recent studies suggest a role of p185 in tumor progression by specifically promoting the   invasive   capacity of tumor cells. Therefore, the present study was conducted with the following three objectives: (1) to support the prognostic value of c-erbB-2 in gastric cancer in a large prospective series using a monoclonal antibody and a highly sensitive immunohistochemical method; (2) to determine the association of c-erbB-2 expression with the expression of invasion-related genes; and (3) to perform the first overall multivariate analysis including c-erbB-2 and the invasion-related tumor-associated protease systems.In a consecutive prospective series of 203 gastric cancer patients (median follow-up, 42 months), expression of c-erbB-2 and a panel of tumor-associated proteases and inhibitors by tumor cells were evaluated semiquantitatively (score 0 to 3) and analyzed for correlation (chi(2) test, Bonferroni-corrected). Kaplan-Meier survival analysis and multivariate Cox analysis were performed to determine the relative prognostic impact of c-erbB-2 and the invasion-related parameters.Kaplan-Meier analysis (log-rank statistics) revealed a significant association of increasing expression of c-erbB-2 with shorter disease-free (P =. 0023) and overall survival (P =.0160). High amounts of p185 were significantly associated with a high expression of urokinase-type plasminogen activator (uPA) (P <.010), uPA-receptor (P =.030), type-1 plasminogen activator inhibitor (PAI) (P <.010), type-2 PAI (P =.021), cathepsin D (P =.036), matrix metalloproteinase-2 (P =. 024), alpha-1-antichymotrypsin (P =.025), and alpha-2-macroglobulin (P =.017). Multivariate analysis considering these proteases/protease inhibitors, in addition to alpha-1-antitrypsin, tissue plasminogen activator, plasminogen, alpha-2-antiplasmin, and antithrombin III, and established prognostic parameters revealed that, in addition to surgical curability, pT stage, pN stage, and PAI-1, c-erbB-2 is an independent prognostic factor for overall survival of curatively resected patients (n = 139; P =.049; relative risk, 1.54; 95% confidence interval, 1.08 to 1.67) and all patients (P =.028; relative risk 1.33; 95% CI, 1.28 to 1.38).c-erbB-2 is confirmed as a new independent, functional prognostic parameter for overall survival in gastric cancer, even when a panel of invasion-related factors, including the strong prognostic parameter PAI-1, are considered. The significant correlation of p185 with several tumor-associated proteases supports the hypothesis that c-erbB-2 is a promoter of invasion and metastasis. This strongly suggests that c-erbB-2 may be a promising target for anti-  invasive   therapy in gastric cancer. 
 Immunohistochemical localization of serine-protease inhibitors in the human placenta. Proteases and their inhibitors play a pivotal role in developmental and differentiative processes. In the present report we investigated the immunohistochemical localization of alpha 1-antitrypsin, alpha 1-antichymotrypsin and inter-alpha-trypsin inhibitor in first trimester as well as in term human placentas. For this purpose polyclonal antibodies against these serine-protease inhibitors were used. All inhibitors were expressed in the villous syncytiotrophoblast of first and last trimester placentas. Placental fibrinoid was positively stained for alpha 1-antitrypsin and inter-alpha-trypsin inhibitor throughout gestation. alpha 1-Antitrypsin and alpha 1-antichymotrypsin showed a strong immunostaining in the Hofbauer cells (first trimester and full term placentas). Extravillous cytotrophoblast was negative for the three protease inhibitors throughout gestation. The presence of the three inhibitors in the syncytiotrophoblast suggests a role in coagulative,   invasive   and immunomodulatory processes. Fibrinoid, staining for alpha 1-antitrypsin and inter-alpha-trypsin inhibitor, could also have an important immunoprotective function. The presence of protease inhibitors in the Hofbauer cells suggests an involvement of these cells in villous remodelling and differentiative processes. 
 mRNA expression level of estrogen-inducible gene, alpha 1-antichymotrypsin, is a predictor of early tumor recurrence in patients with   invasive   breast cancers. We previously identified 18 genes that correlated with ER positivity by adapter-tagged competitive-PCR analysis of 2412 genes in human breast cancer tissues. The aim of the present study was to determine the prognostic significance of these genes. mRNA expression levels of 12 of the above 18 genes were quantified in breast cancer tissues by real-time PCR assay, and their association with patients' prognosis (n = 110) was studied according to hormone receptor (HR) status. In addition, the genes found to influence prognosis were further investigated to examine whether their mRNA expression could be induced by estrogen in MCF-7 cells in vitro. Of the 12 genes, mRNA expression levels of two [alpha 1-antichymotrypsin (ACT) and stanniocalcin 2 (STC2)] were significantly (P = 0.002 and P = 0.007, respectively) associated with good prognosis in HR-positive (ER and/or PR positive) breast cancer patients treated with adjuvant hormone therapy. Multivariate analysis showed that ACT mRNA level, but not STC2 mRNA level, in HR-positive patients, was a significant prognostic factor (P = 0.042), which was independent of tumor size and lymph node metastases. On the other hand, mRNA expressions of ACT and STC2 were not significantly associated with prognosis in HR-negative patients. Estradiol treatment resulted in a significant increase in the mRNA levels of both ACT and STC2 in MCF-7 cells. The mRNA level of ACT, which is an estrogen-inducible gene, is a significant predictor of good prognosis in HR-positive, but not HR-negative, patients with breast cancers. Since HR-positive patients were treated with adjuvant hormone therapy, we suggest that ACT mRNA level could potentially be used as a predictor of response to hormone therapy, rather than a prognostic factor (predictor of metastatic potential). 
 Apolipoprotein A1 and C-terminal fragment of α-1 antichymotrypsin are candidate plasma biomarkers associated with acute renal allograft rejection. Current diagnostic methods of renal allograft rejection are neither sensitive nor specific. Needle biopsies are   invasiv  e and associated with patient morbidity. Thus, it is desirable to develop no  ninvasiv  e tests to predict and diagnose rejection.Using a case-control approach, surface-enhanced laser desorption/ionization time-of-flight mass spectrometry was used to identify plasma proteins associated with renal allograft rejection. From each rejection patient (n=16), two plasma samples (one near the biopsy date and the other at a time postbiopsy) were compared. Biopsy-confirmed nonrejection patients (n=48) were further analyzed as controls. Antibody-based quantitative enzyme-linked immunosorbent assay was performed to validate candidate biomarker apolipoprotein A1 (Apo A1) in a subset of the original and a second cohort of biopsy-confirmed rejection (n=40) and nonrejection (n=70) patients.Twenty-two proteins/peptides showed significant differences between rejection and postrejection samples. Peptides 5191 Da and 4467 Da detected rejection with 100% sensitivity and 94% specificity. The 4467 Da peptide was identified as the C-terminal fragment of α-1 antichymotrypsin and a 28 kDa protein was determined as Apo A1. Both protein levels were significantly lower at rejection compared with postrejection. Protein levels of nonrejection patients were similar to the postrejection samples. Apo A1 enzyme-linked immunosorbent assay results showed significantly lower Apo A1 levels (P=0.001 for the original and P=4.14E-11 for the second cohort) at the time of rejection compared with nonrejection which coincides with the SELDI findings.Together α-1 antichymotrypsin, Apo A1, and the unidentified 5191 Da peptide provide a plasma molecular profile, and this is associated with acute cellular renal allograft rejection. 
  [Mm]etastasis  PubMed  
 Update of the human and mouse SERPIN gene superfamily. The serpin family comprises a structurally similar, yet functionally diverse, set of proteins. Named originally for their function as serine proteinase inhibitors, many of its members are not inhibitors but rather chaperones, involved in storage, transport, and other roles. Serpins are found in genomes of all kingdoms, with 36 human protein-coding genes and five pseudogenes. The mouse has 60 Serpin functional genes, many of which are orthologous to human SERPIN genes and some of which have expanded into multiple paralogous genes. Serpins are found in tissues throughout the body; whereas most are extracellular, there is a class of intracellular serpins. Serpins appear to have roles in inflammation, immune function, tumorigenesis, blood clotting, dementia, and cancer   metastasis  . Further characterization of these proteins will likely reveal potential biomarkers and therapeutic targets for disease. 
 c-erbB-2 is of independent prognostic relevance in gastric cancer and is associated with the expression of tumor-associated protease systems. The c-erbB-2 gene (encoding the protein p185) is overexpressed in diverse human cancers and has been implicated to be of prognostic value in gastric cancer. Recent studies suggest a role of p185 in tumor progression by specifically promoting the invasive capacity of tumor cells. Therefore, the present study was conducted with the following three objectives: (1) to support the prognostic value of c-erbB-2 in gastric cancer in a large prospective series using a monoclonal antibody and a highly sensitive immunohistochemical method; (2) to determine the association of c-erbB-2 expression with the expression of invasion-related genes; and (3) to perform the first overall multivariate analysis including c-erbB-2 and the invasion-related tumor-associated protease systems.In a consecutive prospective series of 203 gastric cancer patients (median follow-up, 42 months), expression of c-erbB-2 and a panel of tumor-associated proteases and inhibitors by tumor cells were evaluated semiquantitatively (score 0 to 3) and analyzed for correlation (chi(2) test, Bonferroni-corrected). Kaplan-Meier survival analysis and multivariate Cox analysis were performed to determine the relative prognostic impact of c-erbB-2 and the invasion-related parameters.Kaplan-Meier analysis (log-rank statistics) revealed a significant association of increasing expression of c-erbB-2 with shorter disease-free (P =. 0023) and overall survival (P =.0160). High amounts of p185 were significantly associated with a high expression of urokinase-type plasminogen activator (uPA) (P <.010), uPA-receptor (P =.030), type-1 plasminogen activator inhibitor (PAI) (P <.010), type-2 PAI (P =.021), cathepsin D (P =.036), matrix metalloproteinase-2 (P =. 024), alpha-1-antichymotrypsin (P =.025), and alpha-2-macroglobulin (P =.017). Multivariate analysis considering these proteases/protease inhibitors, in addition to alpha-1-antitrypsin, tissue plasminogen activator, plasminogen, alpha-2-antiplasmin, and antithrombin III, and established prognostic parameters revealed that, in addition to surgical curability, pT stage, pN stage, and PAI-1, c-erbB-2 is an independent prognostic factor for overall survival of curatively resected patients (n = 139; P =.049; relative risk, 1.54; 95% confidence interval, 1.08 to 1.67) and all patients (P =.028; relative risk 1.33; 95% CI, 1.28 to 1.38).c-erbB-2 is confirmed as a new independent, functional prognostic parameter for overall survival in gastric cancer, even when a panel of invasion-related factors, including the strong prognostic parameter PAI-1, are considered. The significant correlation of p185 with several tumor-associated proteases supports the hypothesis that c-erbB-2 is a promoter of invasion and   metastasis  . This strongly suggests that c-erbB-2 may be a promising target for anti-invasive therapy in gastric cancer. 
 Prostatic human kallikrein 2 inactivates and complexes with plasminogen activator inhibitor-1. Human kallikrein 2 (hK2) is a serine protease expressed predominantly in the prostate which has 80% homology to prostate-specific antigen (PSA). hK2 is an active trypsin-like protease which has been shown by immuno-histochemical staining to be more highly expressed in prostate carcinoma than in benign prostate tissue. Unlike PSA, hK2 activates pro-PSA , pro-hK2 and the zymogen form of urokinase-type plasminogen activator (uPA), an extracellular protease correlated with prostate cancer and   metastasis  . We show here that hK2 rapidly forms a complex with plasminogen activator inhibitor-1 (PAI-1), the primary inhibitor of uPA in tissues. In addition, hK2 inactivated 6 to 7 mol of PAI-1 by cleavage at Arg346-Met347 for every mole of hK2-PAI-1 complex formed. In contrast with hK2, PSA neither complexed with nor inactivated PAI-1. PAI-1 inhibited hK2 comparably with protein C inhibitor (PCI) and at least 20 times more rapidly than alpha1-anti-chymotrypsin (ACT). N-Terminal sequencing shows that hK2 forms a covalent complex with PAI-1, PCI and ACT after cleavage at Arg346-Met347, Arg354-Ser355 and Leu358-Ser359, respectively. During complex formation, hK2 inactivated PAI-1 but did not inactivate ACT or PCI. Our current results suggest that the increased hK2 expression in prostate cancer tissues could influence cancer biology not only by activation of uPA but also by inactivation of its primary inhibitor, PAI-1. 
   
  
 FAM89B 
 
  NCBI Gene  
  GeneRIF  
  Pubmed  
 
 
  Search Term  Rank  Fields  
  [Mm]igration  6  PubMed(1)  
 
 
 hide/show details for FAM89B 
  [Mm]igration  PubMed  
 Adaptor protein LRAP25 mediates myotonic dystrophy kinase-related Cdc42-binding kinase (MRCK) regulation of LIMK1 protein in lamellipodial F-actin dynamics. Myotonic dystrophy kinase-related Cdc42-binding kinase (MRCK) has been shown to localize to the lamella of mammalian cells through its interaction with an adaptor protein, leucine repeat adaptor protein 35a (LRAP35a), which links it with myosin 18A (MYO18A) for activation of the lamellar actomyosin network essential for cell   migration  . Here, we report the identification of another adaptor protein LRAP25 that mediates MRCK association with LIM kinase 1 (LIMK1). The lamellipodium-localized LRAP25-MRCK complex is essential for the regulation of local LIMK1 and its downstream F-actin regulatory factor cofilin. Functionally, inhibition of either MRCK or LRAP25 resulted in a marked suppression of LIMK1 activity and down-regulation of cofilin phosphorylation in response to aluminum fluoride induction in B16-F1 cells, which eventually resulted in deregulation of lamellipodial F-actin and reorganization of cytoskeletal structures causing defects in cell polarization and motility. These biochemical and functional characterizations thus underline the functional relevance of the LRAP25-MRCK complex in LIMK1-cofilin signaling and the importance of LRAP adaptors as key determinants of MRCK cellular localization and downstream specificities. 
   
  
 PAPLN 
 
  NCBI Gene  
  GeneRIF  
  Pubmed  
 
 
  Search Term  Rank  Fields  
  [Ee]xtracellular [Mm]atrix  8  PubMed(2); Component(1)  
 
 
 hide/show details for PAPLN 
  [Ee]xtracellular [Mm]atrix  PubMed  
 A disintegrin-like and metalloprotease (reprolysin-type) with thrombospondin type 1 motif (ADAMTS) superfamily: functions and mechanisms. Together with seven ADAMTS-like proteins, the 19 mammalian ADAMTS proteases constitute a superfamily. ADAMTS proteases are secreted zinc metalloproteases whose hallmark is an ancillary domain containing one or more thrombospondin type 1 repeats. ADAMTS-like proteins resemble ADAMTS ancillary domains and lack proteolytic activity. Vertebrate expansion of the superfamily reflects emergence of new substrates, duplication of proteolytic activities in new contexts, and cooperative functions of the duplicated genes. ADAMTS proteases are involved in maturation of procollagen and von Willebrand factor, as well as in   extracellular matrix   proteolysis relating to morphogenesis, angiogenesis, ovulation, cancer, and arthritis. New insights into ADAMTS mechanisms indicate significant regulatory roles for ADAMTS ancillary domains, propeptide processing, and glycosylation. ADAMTS-like proteins appear to have regulatory roles in the   extracellular matrix  . 
 Papilin in development; a pericellular protein with a homology to the ADAMTS metalloproteinases. Papilin is an   extracellular matrix   glycoprotein that we have found to be involved in, (1) thin matrix layers during gastrulation, (2) matrix associated with wandering, phagocytic hemocytes, (3) basement membranes and (4) space-filling matrix during Drosophila development. Determination of its cDNA sequence led to the identification of Caenorhabditis and mammalian papilins. A distinctly conserved 'papilin cassette' of domains at the amino-end of papilins is also the carboxyl-end of the ADAMTS subgroup of secreted, matrix-associated metalloproteinases; this cassette contains one thrombospondin type 1 (TSR) domain, a specific cysteine-rich domain and several partial TSR domains. In vitro, papilin non-competitively inhibits procollagen N-proteinase, an ADAMTS metalloproteinase. Inhibiting papilin synthesis in Drosophila or Caenorhabditis causes defective cell arrangements and embryonic death. Ectopic expression of papilin in Drosophila causes lethal abnormalities in muscle, Malpighian tubule and trachea formation. We suggest that papilin influences cell rearrangements and may modulate metalloproteinases during organogenesis. 
  Component  
 proteinaceous   extracellular matrix   
   
  
 USH2A 
 
  NCBI Gene  
  GeneRIF  
  Pubmed  
 
 
  Search Term  Rank  Fields  
  [Mm]igration  6  PubMed(1); Conserved Domains(1)  
  [Ee]xtracellular [Mm]atrix  8  PubMed(6)  
 
 
 hide/show details for USH2A 
  [Mm]igration  PubMed  
 A common ancestral origin of the frequent and widespread 2299delG USH2A mutation. Usher syndrome type IIa is an autosomal recessive disorder characterized by mild-to-severe hearing loss and progressive visual loss due to retinitis pigmentosa. The mutation that most commonly causes Usher syndrome type IIa is a 1-bp deletion, described as "2299delG," in the USH2A gene. The mutation has been identified in several patients from northern and southern Europe and from North America, and it has been found in single patients from South America, South Africa, and China. Various studies have reported a range of frequencies (.16-.44) among patients with Usher syndrome, depending on the geographic origin of the patients. The 2299delG mutation may be the one that most frequently causes retinitis pigmentosa in humans. Given the high frequencies and the wide geographic distribution of the mutation, it was of interest to determine whether the mutation resulted from an ancestral mutational event or represented a mutational hotspot in the USH2A gene. Haplotype analysis was performed on DNA samples from 116 unrelated patients with Usher syndrome type IIa; the patients were from 14 countries and represented 148 2299delG alleles. On the basis of six single-nucleotide polymorphisms within the USH2A gene, 12 core haplotypes were observed in a panel of normal chromosomes. However, in our analysis, only one core haplotype was found to be associated with the 2299delG mutation. The data indicate that the widespread geographic distribution of the 2299delG mutation is the result of an ancestral mutation that has spread throughout Europe and into the New World as a result of   migration  . 
  Conserved Domains  
 cd00055: EGF_Lam; Laminin-type epidermal growth factor-like domain; laminins are the major noncollagenous components of basement membranes that mediate cell adhesion, growth   migration  , and differentiation; the laminin-type epidermal growth factor-like module occurs in ... 
  [Ee]xtracellular [Mm]atrix  PubMed  
 Four USH2A founder mutations underlie the majority of Usher syndrome type 2 cases among non-Ashkenazi Jews. Type 2 Usher syndrome (USH2) is a recessively inherited disorder, characterized by the combination of early onset, moderate-to-severe, sensorineural hearing loss, and vision impairment due to retinitis pigmentosa. From 74% to 90% of USH2 cases are caused by mutations of the USH2A gene. USH2A is composed of 72 exons, encoding for usherin, an   extracellular matrix   protein, which plays an important role in the development and maintenance of neurosensory cells in both retina and cochlea. To date, over 70 pathogenic mutations of USH2A have been reported in individuals of various ethnicities. Many of these mutations are rare private mutations segregating in single families. The aim of the current work was to investigate the genetic basis for USH2 among Jews of various origins. We found that four USH2A mutations (c.239-240insGTAC, c.1000C>T, c.2209C>T, and c.12067-2A>G) account for 64% of mutant alleles underlying USH2 in Jewish families of non-Ashkenazi descent. Considering the very large size of the USH2A gene and the high number of mutations detected in USH2 patients worldwide, our findings have significant implications for genetic counseling and carrier screening in various Jewish populations. 
 Genomic structure and identification of novel mutations in usherin, the gene responsible for Usher syndrome type IIa. Usher syndrome type IIa (USHIIa) is an autosomal recessive disorder characterized by moderate to severe sensorineural hearing loss and progressive retinitis pigmentosa. This disorder maps to human chromosome 1q41. Recently, mutations in USHIIa patients were identified in a novel gene isolated from this chromosomal region. The USH2A gene encodes a protein with a predicted molecular weight of 171.5 kD and possesses laminin epidermal growth factor as well as fibronectin type III domains. These domains are observed in other protein components of the basal lamina and   extracellular matrix  es; they may also be observed in cell-adhesion molecules. The intron/exon organization of the gene whose protein we name "Usherin" was determined by direct sequencing of PCR products and cloned genomic DNA with cDNA-specific primers. The gene is encoded by 21 exons and spans a minimum of 105 kb. A mutation search of 57 independent USHIIa probands was performed with a combination of direct sequencing and heteroduplex analysis of PCR-amplified exons. Fifteen new mutations were found. Of 114 independent USH2A alleles, 58 harbored probable pathologic mutations. Ten cases of USHIIa were true homozygotes and 10 were compound heterozygotes; 18 heterozygotes with only one identifiable mutation were observed. Sixty-five percent (38/58) of cases had at least one mutation, and 51% (58/114) of the total number of possible mutations were identified. The allele 2299delG (previously reported as 2314delG) was the most frequent mutant allele observed (16%; 31/192). Three new missense mutations (C319Y, N346H, and C419F) were discovered; all were restricted to the previously unreported laminin domain VI region of Usherin. The possible significance of this domain, known to be necessary for laminin network assembly, is discussed in the context of domain VI mutations from other proteins. 
 Mutation of a gene encoding a protein with   extracellular matrix   motifs in Usher syndrome type IIa. Usher syndrome type IIa (OMIM 276901), an autosomal recessive disorder characterized by moderate to severe sensorineural hearing loss and progressive retinitis pigmentosa, maps to the long arm of human chromosome 1q41 between markers AFM268ZD1 and AFM144XF2. Three biologically important mutations in Usher syndrome type IIa patients were identified in a gene (USH2A) isolated from this critical region. The USH2A gene encodes a protein with a predicted size of 171.5 kilodaltons that has laminin epidermal growth factor and fibronectin type III motifs; these motifs are most commonly observed in proteins comprising components of the basal lamina and   extracellular matrix  es and in cell adhesion molecules. 
 Localization and expression of usherin: a novel basement membrane protein defective in people with Usher's syndrome type IIa. People with Usher's syndrome type IIa have mutations in a novel gene encoding a protein with domains commonly found in many types of   extracellular matrix   and cell surface receptor proteins. Here we report that this protein, which we refer to as usherin, is a new basement membrane protein. In the mouse, usherin has a broad, but not ubiquitous, tissue distribution. Usherin is found in all of the capillary and structural basement membranes of the human and mouse retina and in the murine inner ear at both post-natal day 0 and in the adult. High levels of usherin are also observed in tissues not affected in Usher's syndrome type IIa, including spleen, testis, oviduct, epididymis, submaxillary gland, and large and small intestines. Many organs are completely devoid of usherin, including the brain, skin, kidney, lung, liver, and skeletal muscle. Expression was observed in the smooth muscle of the small intestine, colon, and oviduct, however, usherin is not present in cardiac smooth muscle. Usherin is critical for normal development and tissue homeostasis in the inner ear and retina, illustrating yet another example of the importance of basement membranes in the development and function of tissues. 
 Identification of the mouse and rat orthologs of the gene mutated in Usher syndrome type IIA and the cellular source of USH2A mRNA in retina, a target tissue of the disease. Usher syndrome type IIA (MIM: 27601) is an autosomal recessive disorder characterized by moderate to severe congenital deafness and progressive retinitis pigmentosa. We recently identified the human Usher syndrome type IIA gene (USH2A) on chromosome 1q41, which encodes a protein possessing 10 laminin epidermal growth factor and four fibronectin type 3 domains, both commonly observed in   extracellular matrix   proteins. To gain insight into the pathogenesis of Usher syndrome type IIA, we isolated and characterized the murine (Ush2a) and rat (rat Ush2a) orthologs of human USH2A. We mapped mouse Ush2a by fluorescence in situ hybridization to mouse chromosome 1 in the region syntenic to human chromosome 1q41. Rat Ush2a has been localized by radiation hybrid mapping to rat chromosome 13 between d13rat49 and d13rat76. The mouse and rat genes, similar to human USH2A, are expressed primarily in retina and cochlea. Mouse Ush2a encodes a 161-kDa protein that shows 68% identity and 9% similarity to the human USH2A protein. Rat Ush2a encodes a 167-kDa protein with 64% identity and 10% similarity to the human protein and 81% identity and 5% similarity to the mouse USH2A protein. The predicted amino acid sequence of the mouse and rat proteins, like their human counterpart, contains a leader sequence, an amino-terminal globular domain, 10 laminin epidermal growth factor domains, and four carboxy-terminal fibronectin type III motifs. With in situ hybridization, we compared the cellular expression of the USH2A gene in rat, mouse, and human retinas. USH2A mRNA in the adult rat, mouse, and human is expressed in the cells of the outer nuclear layer of the retina, one of the target tissues of the disease. In the developing rat retina, Ush2a mRNA expression appears in the neuroepithelium at embryonic day 17. 
 Three novel mutations and twelve polymorphisms identified in the USH2A gene in Israeli USH2 families. The Usher syndromes are autosomal recessive hereditary disorders characterized by hearing impairment and progressive visual loss due to Retinitis Pigmentosa (RP). Moderate to severe sensorineural hearing loss and progressive RP characterizes Usher syndrome type IIa (USH2A), which maps to the long arm of chromosome 1q41. Recently, three deletions carried by USH2 patients, which were found in a novel gene isolated from the critical 1q41 region, defined this gene as responsible for USH2A. The USH2A gene is predicted to encode a 1546 amino acid protein which possesses domains that are observed in basal lamina and   extracellular matrix   proteins and in cell adhesion molecules. Affected individuals and additional members from eleven USH2 Israeli families of diverse ethnic origin were screened for the presence of changes in all 20 coding exons of the USH2A gene. Three novel mutations (239-242insCGTA, R334W, T1515M) were identified in three families of Jewish Moroccan and Jewish Iranian origins. Twelve polymorphisms were found in the families, four of which are novel. None of the known USH2 mutations were identified in the families studied in this work. Hum Mutat 15:388, 2000. 
   
  
 KIRREL3 
 
  NCBI Gene  
  GeneRIF  
  Pubmed  
 
 
  Search Term  Rank  Fields  
  [Mm]igration  6  Process(1)  
 
 
 hide/show details for KIRREL3 
  [Mm]igration  Process  
 neuron   migration   
   
  
 KIRREL 
 
  NCBI Gene  
  GeneRIF  
  Pubmed  
 
 
  Search Term  Rank  Fields  
  [Mm]igration  6  PubMed(1)  
 
 
 hide/show details for KIRREL 
  [Mm]igration  PubMed  
 Motor protein Myo1c is a podocyte protein that facilitates the transport of slit diaphragm protein Neph1 to the podocyte membrane. The podocyte proteins Neph1 and nephrin organize a signaling complex at the podocyte cell membrane that forms the structural framework for a functional glomerular filtration barrier. Mechanisms regulating the movement of these proteins to and from the membrane are currently unknown. This study identifies a novel interaction between Neph1 and the motor protein Myo1c, where Myo1c plays an active role in targeting Neph1 to the podocyte cell membrane. Using in vivo and in vitro experiments, we provide data supporting a direct interaction between Neph1 and Myo1c which is dynamic and actin dependent. Unlike wild-type Myo1c, the membrane localization of Neph1 was significantly reduced in podocytes expressing dominant negative Myo1c. In addition, Neph1 failed to localize at the podocyte cell membrane and cell junctions in Myo1c-depleted podocytes. We further demonstrate that similarly to Neph1, Myo1c also binds nephrin and reduces its localization at the podocyte cell membrane. A functional analysis of Myo1c knockdown cells showed defects in cell   migration  , as determined by a wound assay. In addition, the ability to form tight junctions was impaired in Myo1c knockdown cells, as determined by transepithelial electric resistance (TER) and bovine serum albumin (BSA) permeability assays. These results identify a novel Myo1c-dependent molecular mechanism that mediates the dynamic organization of Neph1 and nephrin at the slit diaphragm and is critical for podocyte function. 
   
  
 SYDE1 
 
  NCBI Gene  
  GeneRIF  
  Pubmed  
 
 
  Search Term  Rank  Fields  
  [Mm]igration  6  Process(1); PubMed(1); Summary(1)  
 
 
 hide/show details for SYDE1 
  [Mm]igration  Process  
 positive regulation of trophoblast cell   migration   
  PubMed  
 Association of dysfunctional synapse defective 1 (SYDE1) with restricted fetal growth - SYDE1 regulates placental cell   migration   and invasion. The transcription factor glial cells missing 1 (GCM1) regulates trophoblast differentiation and function during placentation. Decreased GCM1 expression is associated with pre-eclampsia, suggesting that abnormal expression of GCM1 target genes may contribute to the pathogenesis of pregnancy complications. Here we identified a novel GCM1 target gene, synapse defective 1 (SYDE1), which encodes a RhoGAP that is highly expressed in human placenta, and demonstrated that SYDE1 promotes cytoskeletal remodelling and cell   migration   and invasion. Importantly, genetic ablation of murine Syde1 results in small fetuses and placentas with aberrant phenotypes in the placental-yolk sac barrier, maternal-trophoblast interface, and placental vascularization. Microarray analysis revealed altered expression of renin-1, angiotensin I converting enzyme 2, angiotensin II type 1a receptor, and membrane metalloendopeptidase of the renin-angiotensin system in Syde1-knockout placenta, which may compensate for the vascular defects to maintain normal blood pressure. As pregnancy proceeds, growth restriction of the Syde1(-/-) fetuses and placentas continues, with elevated expression of the Syde1 homologue Syde2 in placenta. Syde2 may compensate for the loss of Syde1 function because SYDE2, but not the GAP-dead SYDE2 mutant, reverses   migration   and invasion activities of SYDE1-knockdown JAR trophoblast cells. Clinically, we further detected decreased SYDE1 expression in preterm and term IUGR placentas compared with gestational age-matched controls. Our study suggests a novel mechanism for GCM1 and SYDE1 in regulation of trophoblast cell   migration   and invasion during placental development and that decreased SYDE1 expression is associated with IUGR. Copyright © 2016 Pathological Society of Great Britain and Ireland. Published by John Wiley & Sons, Ltd. 
  Summary  
 The protein encoded by this gene is a Rho GTPase-activating protein highly expressed in placenta. The encoded protein is involved in cytoskeletal remodeling and trophoblast cell   migration  . Decreased expression of this gene has been associated with intrauterine growth restriction (IUGR). [provided by RefSeq, Feb 2017] 
   
  
 ARHGAP45 
 
  NCBI Gene  
  GeneRIF  
  Pubmed  
 
 
  Search Term  Rank  Fields  
  [Mm]igration  6  PubMed(2)  
 
 
 hide/show details for ARHGAP45 
  [Mm]igration  PubMed  
 The human minor histocompatibility antigen 1 is a RhoGAP. The human minor Histocompatibility Antigen HMHA-1 is a major target of immune responses after allogeneic stem cell transplantation applied for the treatment of leukemia and solid tumors. The restriction of its expression to hematopoietic cells and many solid tumors raised questions regarding its cellular functions. Sequence analysis of the HMHA-1 encoding HMHA1 protein revealed the presence of a possible C-terminal RhoGTPase Activating Protein (GAP) domain and an N-terminal BAR domain. Rho-family GTPases, including Rac1, Cdc42, and RhoA are key regulators of the actin cytoskeleton and control cell spreading and   migration  . RhoGTPase activity is under tight control as aberrant signaling can lead to pathology, including inflammation and cancer. Whereas Guanine nucleotide Exchange Factors (GEFs) mediate the exchange of GDP for GTP resulting in RhoGTPase activation, GAPs catalyze the low intrinsic GTPase activity of active RhoGTPases, resulting in inactivation. Here we identify the HMHA1 protein as a novel RhoGAP. We show that HMHA1 constructs, lacking the N-terminal region, negatively regulate the actin cytoskeleton as well as cell spreading. Furthermore, we show that HMHA1 regulates RhoGTPase activity in vitro and in vivo. Finally, we demonstrate that the HMHA1 N-terminal BAR domain is auto-inhibitory as HMHA1 mutants lacking this region, but not full-length HMHA1, showed GAP activity towards RhoGTPases. In conclusion, this study shows that HMHA1 acts as a RhoGAP to regulate GTPase activity, cytoskeletal remodeling and cell spreading, which are crucial functions in normal hematopoietic and cancer cells. 
 The human minor histocompatibility antigen 1 is a RhoGAP. The human minor Histocompatibility Antigen HMHA-1 is a major target of immune responses after allogeneic stem cell transplantation applied for the treatment of leukemia and solid tumors. The restriction of its expression to hematopoietic cells and many solid tumors raised questions regarding its cellular functions. Sequence analysis of the HMHA-1 encoding HMHA1 protein revealed the presence of a possible C-terminal RhoGTPase Activating Protein (GAP) domain and an N-terminal BAR domain. Rho-family GTPases, including Rac1, Cdc42, and RhoA are key regulators of the actin cytoskeleton and control cell spreading and   migration  . RhoGTPase activity is under tight control as aberrant signaling can lead to pathology, including inflammation and cancer. Whereas Guanine nucleotide Exchange Factors (GEFs) mediate the exchange of GDP for GTP resulting in RhoGTPase activation, GAPs catalyze the low intrinsic GTPase activity of active RhoGTPases, resulting in inactivation. Here we identify the HMHA1 protein as a novel RhoGAP. We show that HMHA1 constructs, lacking the N-terminal region, negatively regulate the actin cytoskeleton as well as cell spreading. Furthermore, we show that HMHA1 regulates RhoGTPase activity in vitro and in vivo. Finally, we demonstrate that the HMHA1 N-terminal BAR domain is auto-inhibitory as HMHA1 mutants lacking this region, but not full-length HMHA1, showed GAP activity towards RhoGTPases. In conclusion, this study shows that HMHA1 acts as a RhoGAP to regulate GTPase activity, cytoskeletal remodeling and cell spreading, which are crucial functions in normal hematopoietic and cancer cells. 
   
  
 ARHGAP29 
 
  NCBI Gene  
  GeneRIF  
  Pubmed  
 
 
  Search Term  Rank  Fields  
  [Mm]igration  6  PubMed(3)  
  [Mm]etastasis  12  PubMed(1)  
 
 
 hide/show details for ARHGAP29 
  [Mm]igration  PubMed  
 Mutation of ARHGAP9 in patients with coronary spastic angina. Coronary artery spasm has an important function in the etiology of variant angina and other acute coronary syndromes. Abnormal activation of Rho-family GTPases has been observed in cardiovascular disorders, but the function of genetic variability in Rho-family GTPases remains to be evaluated in cardiovascular disorders. We examined the genetic variability of Rho-family GTPases and their regulators in coronary artery spasm. We performed a comprehensive candidate gene analysis of 67 single nucleotide polymorphisms with amino-acid substitution in Rho-family GTPases and their regulators in 103 unrelated Japanese patients with acetylcholine-induced coronary artery spasm and 102 control Japanese subjects without acetylcholine-induced coronary artery spasm. We noted an association of the single nucleotide polymorphism of ARHGAP9 (rs11544238, Ala370Ser) with coronary artery spasm (odds ratio =2.67). We found that ARHGAP9 inactivated Rac as RacGAP and that the mRNA level of ARHGAP9 was strongly detected in hematopoietic cells. ARHGAP9 negatively regulated cell   migration  . The Ala370Ser polymorphism counteracted ARHGAP9-reduced cell   migration  , spreading and adhesion. The Ala370Ser polymorphism in the ARHGAP9 gene is associated with coronary artery spasm. These data suggest that the polymorphism of ARHGAP9 has a critical function in the infiltration of hematopoietic cells into the endothelium and inflammation leading to endothelial dysfunction. 
 Mutation of ARHGAP9 in patients with coronary spastic angina. Coronary artery spasm has an important function in the etiology of variant angina and other acute coronary syndromes. Abnormal activation of Rho-family GTPases has been observed in cardiovascular disorders, but the function of genetic variability in Rho-family GTPases remains to be evaluated in cardiovascular disorders. We examined the genetic variability of Rho-family GTPases and their regulators in coronary artery spasm. We performed a comprehensive candidate gene analysis of 67 single nucleotide polymorphisms with amino-acid substitution in Rho-family GTPases and their regulators in 103 unrelated Japanese patients with acetylcholine-induced coronary artery spasm and 102 control Japanese subjects without acetylcholine-induced coronary artery spasm. We noted an association of the single nucleotide polymorphism of ARHGAP9 (rs11544238, Ala370Ser) with coronary artery spasm (odds ratio =2.67). We found that ARHGAP9 inactivated Rac as RacGAP and that the mRNA level of ARHGAP9 was strongly detected in hematopoietic cells. ARHGAP9 negatively regulated cell   migration  . The Ala370Ser polymorphism counteracted ARHGAP9-reduced cell   migration  , spreading and adhesion. The Ala370Ser polymorphism in the ARHGAP9 gene is associated with coronary artery spasm. These data suggest that the polymorphism of ARHGAP9 has a critical function in the infiltration of hematopoietic cells into the endothelium and inflammation leading to endothelial dysfunction. 
 KIF14 negatively regulates Rap1a-Radil signaling during breast cancer progression. The small GTPase Rap1 regulates inside-out integrin activation and thereby influences cell adhesion,   migration  , and polarity. Several Rap1 effectors have been described to mediate the cellular effects of Rap1 in a context-dependent manner. Radil is emerging as an important Rap effector implicated in cell spreading and   migration  , but the molecular mechanisms underlying its functions are unclear. We report here that the kinesin KIF14 associates with the PDZ domain of Radil and negatively regulates Rap1-mediated inside-out integrin activation by tethering Radil on microtubules. The depletion of KIF14 led to increased cell spreading, altered focal adhesion dynamics, and inhibition of cell   migration   and invasion. We also show that Radil is important for breast cancer cell proliferation and for metastasis in mice. Our findings provide evidence that the concurrent up-regulation of Rap1 activity and increased KIF14 levels in several cancers is needed to reach optimal levels of Rap1-Radil signaling, integrin activation, and cell-matrix adhesiveness required for tumor progression. 
  [Mm]etastasis  PubMed  
 KIF14 negatively regulates Rap1a-Radil signaling during breast cancer progression. The small GTPase Rap1 regulates inside-out integrin activation and thereby influences cell adhesion, migration, and polarity. Several Rap1 effectors have been described to mediate the cellular effects of Rap1 in a context-dependent manner. Radil is emerging as an important Rap effector implicated in cell spreading and migration, but the molecular mechanisms underlying its functions are unclear. We report here that the kinesin KIF14 associates with the PDZ domain of Radil and negatively regulates Rap1-mediated inside-out integrin activation by tethering Radil on microtubules. The depletion of KIF14 led to increased cell spreading, altered focal adhesion dynamics, and inhibition of cell migration and invasion. We also show that Radil is important for breast cancer cell proliferation and for   metastasis   in mice. Our findings provide evidence that the concurrent up-regulation of Rap1 activity and increased KIF14 levels in several cancers is needed to reach optimal levels of Rap1-Radil signaling, integrin activation, and cell-matrix adhesiveness required for tumor progression. 
   
  
 ARHGAP9 
 
  NCBI Gene  
  GeneRIF  
  Pubmed  
 
 
  Search Term  Rank  Fields  
  [Mm]igration  6  PubMed(4)  
  [Ee]xtracellular [Mm]atrix  8  PubMed(1); Summary(1)  
 
 
 hide/show details for ARHGAP9 
  [Mm]igration  PubMed  
 Mutation of ARHGAP9 in patients with coronary spastic angina. Coronary artery spasm has an important function in the etiology of variant angina and other acute coronary syndromes. Abnormal activation of Rho-family GTPases has been observed in cardiovascular disorders, but the function of genetic variability in Rho-family GTPases remains to be evaluated in cardiovascular disorders. We examined the genetic variability of Rho-family GTPases and their regulators in coronary artery spasm. We performed a comprehensive candidate gene analysis of 67 single nucleotide polymorphisms with amino-acid substitution in Rho-family GTPases and their regulators in 103 unrelated Japanese patients with acetylcholine-induced coronary artery spasm and 102 control Japanese subjects without acetylcholine-induced coronary artery spasm. We noted an association of the single nucleotide polymorphism of ARHGAP9 (rs11544238, Ala370Ser) with coronary artery spasm (odds ratio =2.67). We found that ARHGAP9 inactivated Rac as RacGAP and that the mRNA level of ARHGAP9 was strongly detected in hematopoietic cells. ARHGAP9 negatively regulated cell   migration  . The Ala370Ser polymorphism counteracted ARHGAP9-reduced cell   migration  , spreading and adhesion. The Ala370Ser polymorphism in the ARHGAP9 gene is associated with coronary artery spasm. These data suggest that the polymorphism of ARHGAP9 has a critical function in the infiltration of hematopoietic cells into the endothelium and inflammation leading to endothelial dysfunction. 
 Mutation of ARHGAP9 in patients with coronary spastic angina. Coronary artery spasm has an important function in the etiology of variant angina and other acute coronary syndromes. Abnormal activation of Rho-family GTPases has been observed in cardiovascular disorders, but the function of genetic variability in Rho-family GTPases remains to be evaluated in cardiovascular disorders. We examined the genetic variability of Rho-family GTPases and their regulators in coronary artery spasm. We performed a comprehensive candidate gene analysis of 67 single nucleotide polymorphisms with amino-acid substitution in Rho-family GTPases and their regulators in 103 unrelated Japanese patients with acetylcholine-induced coronary artery spasm and 102 control Japanese subjects without acetylcholine-induced coronary artery spasm. We noted an association of the single nucleotide polymorphism of ARHGAP9 (rs11544238, Ala370Ser) with coronary artery spasm (odds ratio =2.67). We found that ARHGAP9 inactivated Rac as RacGAP and that the mRNA level of ARHGAP9 was strongly detected in hematopoietic cells. ARHGAP9 negatively regulated cell   migration  . The Ala370Ser polymorphism counteracted ARHGAP9-reduced cell   migration  , spreading and adhesion. The Ala370Ser polymorphism in the ARHGAP9 gene is associated with coronary artery spasm. These data suggest that the polymorphism of ARHGAP9 has a critical function in the infiltration of hematopoietic cells into the endothelium and inflammation leading to endothelial dysfunction. 
 Mutation of ARHGAP9 in patients with coronary spastic angina. Coronary artery spasm has an important function in the etiology of variant angina and other acute coronary syndromes. Abnormal activation of Rho-family GTPases has been observed in cardiovascular disorders, but the function of genetic variability in Rho-family GTPases remains to be evaluated in cardiovascular disorders. We examined the genetic variability of Rho-family GTPases and their regulators in coronary artery spasm. We performed a comprehensive candidate gene analysis of 67 single nucleotide polymorphisms with amino-acid substitution in Rho-family GTPases and their regulators in 103 unrelated Japanese patients with acetylcholine-induced coronary artery spasm and 102 control Japanese subjects without acetylcholine-induced coronary artery spasm. We noted an association of the single nucleotide polymorphism of ARHGAP9 (rs11544238, Ala370Ser) with coronary artery spasm (odds ratio =2.67). We found that ARHGAP9 inactivated Rac as RacGAP and that the mRNA level of ARHGAP9 was strongly detected in hematopoietic cells. ARHGAP9 negatively regulated cell   migration  . The Ala370Ser polymorphism counteracted ARHGAP9-reduced cell   migration  , spreading and adhesion. The Ala370Ser polymorphism in the ARHGAP9 gene is associated with coronary artery spasm. These data suggest that the polymorphism of ARHGAP9 has a critical function in the infiltration of hematopoietic cells into the endothelium and inflammation leading to endothelial dysfunction. 
 Isolation of a novel human gene, ARHGAP9, encoding a rho-GTPase activating protein. Members of the Rho family of small guanosine triphosphatases (Rho-GTPases) have emerged as key coordinators of signaling pathways leading to remodeling of the actin cytoskeleton, a process that plays a critical role in cell adhesion and   migration  . However, the precise regulatory mechanisms remain to be elucidated. Here we report isolation of a novel human gene, ARHGAP9, which encodes a protein containing a Rho-GTPase activating protein (Rho-GAP) domain, a src-homology 3 (SH3) domain, a pleckstrin homology (PH) region, and a WW domain. In vitro, the recombinant protein revealed substantial GAP activity toward Cdc42Hs and Rac1, and less toward RhoA. The transcript was predominantly expressed in peripheral blood leukocytes, spleen, and thymus. Exogenous expression of the entire coding region of ARHGAP9 into human leukemia KG-1 cells repressed adhesion of the cells to fibronectin and collagen IV. Our results indicate that ARHGAP9 is involved in regulating adhesion of hematopoietic cells to extracellular matrix. 
  [Ee]xtracellular [Mm]atrix  PubMed  
 Isolation of a novel human gene, ARHGAP9, encoding a rho-GTPase activating protein. Members of the Rho family of small guanosine triphosphatases (Rho-GTPases) have emerged as key coordinators of signaling pathways leading to remodeling of the actin cytoskeleton, a process that plays a critical role in cell adhesion and migration. However, the precise regulatory mechanisms remain to be elucidated. Here we report isolation of a novel human gene, ARHGAP9, which encodes a protein containing a Rho-GTPase activating protein (Rho-GAP) domain, a src-homology 3 (SH3) domain, a pleckstrin homology (PH) region, and a WW domain. In vitro, the recombinant protein revealed substantial GAP activity toward Cdc42Hs and Rac1, and less toward RhoA. The transcript was predominantly expressed in peripheral blood leukocytes, spleen, and thymus. Exogenous expression of the entire coding region of ARHGAP9 into human leukemia KG-1 cells repressed adhesion of the cells to fibronectin and collagen IV. Our results indicate that ARHGAP9 is involved in regulating adhesion of hematopoietic cells to   extracellular matrix  . 
  Summary  
 This gene encodes a member of the Rho-GAP family of GTPase activating proteins. The protein has substantial GAP activity towards several Rho-family GTPases in vitro, converting them to an inactive GDP-bound state. It is implicated in regulating adhesion of hematopoietic cells to the   extracellular matrix  . Multiple transcript variants encoding different isoforms have been found for this gene. [provided by RefSeq, Jul 2008] 
   
  
 ARHGAP9 
 
  NCBI Gene  
  GeneRIF  
  Pubmed  
 
 
  Search Term  Rank  Fields  
  [Mm]igration  6  PubMed(1)  
  [Ee]xtracellular [Mm]atrix  8  PubMed(1)  
 
 
 hide/show details for ARHGAP9 
  [Mm]igration  PubMed  
 Isolation of a novel human gene, ARHGAP9, encoding a rho-GTPase activating protein. Members of the Rho family of small guanosine triphosphatases (Rho-GTPases) have emerged as key coordinators of signaling pathways leading to remodeling of the actin cytoskeleton, a process that plays a critical role in cell adhesion and   migration  . However, the precise regulatory mechanisms remain to be elucidated. Here we report isolation of a novel human gene, ARHGAP9, which encodes a protein containing a Rho-GTPase activating protein (Rho-GAP) domain, a src-homology 3 (SH3) domain, a pleckstrin homology (PH) region, and a WW domain. In vitro, the recombinant protein revealed substantial GAP activity toward Cdc42Hs and Rac1, and less toward RhoA. The transcript was predominantly expressed in peripheral blood leukocytes, spleen, and thymus. Exogenous expression of the entire coding region of ARHGAP9 into human leukemia KG-1 cells repressed adhesion of the cells to fibronectin and collagen IV. Our results indicate that ARHGAP9 is involved in regulating adhesion of hematopoietic cells to extracellular matrix. 
  [Ee]xtracellular [Mm]atrix  PubMed  
 Isolation of a novel human gene, ARHGAP9, encoding a rho-GTPase activating protein. Members of the Rho family of small guanosine triphosphatases (Rho-GTPases) have emerged as key coordinators of signaling pathways leading to remodeling of the actin cytoskeleton, a process that plays a critical role in cell adhesion and migration. However, the precise regulatory mechanisms remain to be elucidated. Here we report isolation of a novel human gene, ARHGAP9, which encodes a protein containing a Rho-GTPase activating protein (Rho-GAP) domain, a src-homology 3 (SH3) domain, a pleckstrin homology (PH) region, and a WW domain. In vitro, the recombinant protein revealed substantial GAP activity toward Cdc42Hs and Rac1, and less toward RhoA. The transcript was predominantly expressed in peripheral blood leukocytes, spleen, and thymus. Exogenous expression of the entire coding region of ARHGAP9 into human leukemia KG-1 cells repressed adhesion of the cells to fibronectin and collagen IV. Our results indicate that ARHGAP9 is involved in regulating adhesion of hematopoietic cells to   extracellular matrix  . 
   
  
 ARHGAP12 
 
  NCBI Gene  
  GeneRIF  
  Pubmed  
 
 
  Search Term  Rank  Fields  
  [Ee]xtracellular [Mm]atrix  8  GeneRIFs(1)  
  [Ii]nvasive  10  PubMed(1); GeneRIFs(1)  
  [Cc]ell [Ii]nvasion  13  Summary(1)  
 
 
 hide/show details for ARHGAP12 
  [Ee]xtracellular [Mm]atrix  GeneRIFs  
 ARHGAP12 inactivates RAC1, thereby impairing cell motility, invasion and adhesion to the   extracellular matrix  . The gene is transcriptionally suppressed by Hepatocyte Growth Factor in vitro. 
  [Ii]nvasive  PubMed  
 Met-driven   invasive   growth involves transcriptional regulation of Arhgap12.   Invasive   growth is a complex biological program triggered by hepatocyte growth factor (HGF) through its tyrosine kinase receptor encoded by the Met proto-oncogene. The program involves-besides proliferation-cell dissociation, motility and   invasive  ness, controlled by intracellular signals impinging on PI3K and on the small G-proteins of the Rac/Rho family. The mechanism(s) unbalancing Rac/Rho activation are still not completely clarified. Here, we describe a functional link between HGF and Arhgap12, a gene encoding a previously uncharacterized protein of the RhoGAP family. We identified Arhgap12 as a transcriptional target of HGF, through a novel gene trapping strategy. We found that Arhgap12 mRNA and protein are robustly suppressed by HGF treatment, but not by serum. Arhgap12 displayed GTPase activating protein (GAP) activity towards Rac1 and, upon overexpression, impaired cell scattering, invasion and adhesion to fibronectin in response to HGF. Consistently, Arhgap12 silencing by RNA interference selectively increased the scatter and adhesion responses. These data show that HGF-driven   invasive   growth involves transcriptional regulation of a Rac1-specific GAP. 
  GeneRIFs  
 Met-driven   invasive   growth involves transcriptional regulation of Arhgap12. 
  [Cc]ell [Ii]nvasion  Summary  
 This gene encodes a member of a large family of proteins that activate Rho-type guanosine triphosphate (GTP) metabolizing enzymes. The encoded protein may be involved in suppressing tumor formation by regulating   cell invasion   and adhesion. Alternatively spliced transcript variants encoding multiple isoforms have been observed for this gene. [provided by RefSeq, Jul 2012] 
   
  
 ARHGAP15 
 
  NCBI Gene  
  GeneRIF  
  Pubmed  
 
 
  Search Term  Rank  Fields  
  [Mm]igration  6  PubMed(2); GeneRIFs(1)  
 
 
 hide/show details for ARHGAP15 
  [Mm]igration  PubMed  
 Forkhead box P3 regulates ARHGAP15 expression and affects   migration   of glioma cells through the Rac1 signaling pathway. Forkhead box P3 (FOXP3) plays a crucial role in the development and function of regulatory T cells and was recently identified as a tumor suppressor in different cancer types. Forkhead box P3 is expressed in normal brain tissues, but is strongly downregulated or absent in glioblastomas. In order to understand the FOXP3 adjustment mechanisms in glioma cells, we performed a DNA microarray in U87 cells overexpressing FOXP3 and validated the differences using quantitative real-time PCR, Western blot analysis, and immunohistochemistry in vitro and in vivo. We found that FOXP3 can regulate the expression of ARHGAP15. Expression of FOXP3 was also correlated with ARHGAP15 in glioma samples. Overexpression of FOXP3 inhibited glioma cell   migration   through ARHGAP15 upregulation and Rac1 inactivation. Silencing of FOXP3 promoted   migration   through ARHGAP15 downregulation and Rac1 activation. ARHGAP15, a GTPase-activating protein for Rac1, inhibits small GTPase signaling in a dual negative manner. We found that there is a correlation between expression of ARHGAP15 and glioma level. The small GTPase Rac1 plays an important role in cell   migration  . In addition, we found that FOXP3 regulates expression of epithelial-mesenchymal transition markers E-cadherin and N-cadherin, which is important given that epithelial-mesenchymal transition is critically involved in tumor spreading and dissemination. Thus, FOXP3 or ARHGAP15 may serve as a new molecular target for antimetastatic therapies in treating glioma. 
 Forkhead box P3 regulates ARHGAP15 expression and affects   migration   of glioma cells through the Rac1 signaling pathway. Forkhead box P3 (FOXP3) plays a crucial role in the development and function of regulatory T cells and was recently identified as a tumor suppressor in different cancer types. Forkhead box P3 is expressed in normal brain tissues, but is strongly downregulated or absent in glioblastomas. In order to understand the FOXP3 adjustment mechanisms in glioma cells, we performed a DNA microarray in U87 cells overexpressing FOXP3 and validated the differences using quantitative real-time PCR, Western blot analysis, and immunohistochemistry in vitro and in vivo. We found that FOXP3 can regulate the expression of ARHGAP15. Expression of FOXP3 was also correlated with ARHGAP15 in glioma samples. Overexpression of FOXP3 inhibited glioma cell   migration   through ARHGAP15 upregulation and Rac1 inactivation. Silencing of FOXP3 promoted   migration   through ARHGAP15 downregulation and Rac1 activation. ARHGAP15, a GTPase-activating protein for Rac1, inhibits small GTPase signaling in a dual negative manner. We found that there is a correlation between expression of ARHGAP15 and glioma level. The small GTPase Rac1 plays an important role in cell   migration  . In addition, we found that FOXP3 regulates expression of epithelial-mesenchymal transition markers E-cadherin and N-cadherin, which is important given that epithelial-mesenchymal transition is critically involved in tumor spreading and dissemination. Thus, FOXP3 or ARHGAP15 may serve as a new molecular target for antimetastatic therapies in treating glioma. 
  GeneRIFs  
 FOXP3 can regulate the expression of ARHGAP15 and affects   migration   of glioma cells through the Rac1 signaling pathway 
   
  
 RACGAP1 
 
  NCBI Gene  
  GeneRIF  
  Pubmed  
 
 
  Search Term  Rank  Fields  
  [Ff]ilopodia  1  PubMed(1)  
  [Mm]igration  6  PubMed(8); GeneRIFs(2)  
  [Ee]xtracellular [Mm]atrix  8  PubMed(2)  
  [Ii]nvasive  10  PubMed(4); GeneRIFs(1)  
  [Mm]etastasis  12  PubMed(5); GeneRIFs(1)  
  [Cc]ell [Ii]nvasion  13  PubMed(2)  
 
 
 hide/show details for RACGAP1 
  [Ff]ilopodia  PubMed  
 PAK4, a novel effector for Cdc42Hs, is implicated in the reorganization of the actin cytoskeleton and in the formation of   filopodia  . The GTPases Rac and Cdc42Hs control diverse cellular functions. In addition to being mediators of intracellular signaling cascades, they have important roles in cell morphogenesis and mitogenesis. We have identified a novel PAK-related kinase, PAK4, as a new effector molecule for Cdc42Hs. PAK4 interacts only with the activated form of Cdc42Hs through its GTPase-binding domain (GBD). Co-expression of PAK4 and the constitutively active Cdc42HsV12 causes the redistribution of PAK4 to the brefeldin A-sensitive compartment of the Golgi membrane and the subsequent induction of   filopodia   and actin polymerization. Importantly, the reorganization of the actin cytoskeleton is dependent on PAK4 kinase activity and on its interaction with Cdc42Hs. Thus, unlike other members of the PAK family, PAK4 provides a novel link between Cdc42Hs and the actin cytoskeleton. The cellular locations of PAK4 and Cdc42Hs suggest a role for the Golgi in cell morphogenesis. 
  [Mm]igration  PubMed  
 Vav1 and Rac control chemokine-promoted T lymphocyte adhesion mediated by the integrin alpha4beta1. The chemokine CXCL12 promotes T lymphocyte adhesion mediated by the integrin alpha4beta1. CXCL12 activates the GTPase Rac, as well as Vav1, a guanine-nucleotide exchange factor for Rac, concomitant with up-regulation of alpha4beta1-dependent adhesion. Inhibition of CXCL12-promoted Rac and Vav1 activation by transfection of dominant negative Rac or Vav1 forms, or by transfection of their siRNA, remarkably impaired the increase in T lymphocyte attachment to alpha4beta1 ligands in response to this chemokine. Importantly, inhibition of Vav1 expression by RNA interference resulted in a blockade of Rac activation in response to CXCL12. Adhesions in flow chambers and soluble binding assays using these transfectants indicated that initial ligand binding and adhesion strengthening mediated by alpha4beta1 were dependent on Vav1 and Rac activation by CXCL12. Finally, CXCL12-promoted T-cell transendothelial   migration   involving alpha4beta1-mediated adhesion was notably inhibited by expression of dominant negative Vav1 and Rac. These results indicate that activation of Vav1-Rac signaling pathway by CXCL12 represents an important inside-out event controlling efficient up-regulation of alpha4beta1-dependent T lymphocyte adhesion. 
 H-Ras-specific activation of Rac-MKK3/6-p38 pathway: its critical role in invasion and   migration   of breast epithelial cells. Human tumors frequently exhibit constitutively activated Ras signaling, which contributes to the malignant phenotype. Mounting evidence suggests unique roles of the Ras family members, H-Ras, N-Ras and K-Ras, in normal and pathological conditions. In an effort to dissect distinct Ras isoform-specific functions in malignant phenotypic changes, we previously established H-Ras- and N-Ras-activated MCF10A human breast epithelial cell lines. Using these, we showed that p38 kinase is a key signaling molecule differentially regulated between H-Ras and N-Ras, leading to H-Ras-specific induction of invasive and migrative phenotypes. The present study is to further investigate H-Ras- and N-Ras-mediated signaling pathways and to unveil how these pathways are integrated for regulation of invasive/migrative phenotypic conversion of human breast epithelial cells. Here we report that the Rac-MAPK kinase (MKK)3/6-p38 pathway is a unique signaling pathway activated by H-Ras, leading to the invasive/migrative phenotype. In contrast, Raf-MEK-ERK and phosphatidylinositol 3-kinase-Akt pathways, which are fundamental to proliferation and differentiation, are activated by both H-Ras and N-Ras. A significant role for p38 in cell invasion is further supported by the observation that p38 activation by MKK6 transfection is sufficient to induce invasive and migrative phenotypes in MCF10A cells. Activation of the MKK6-p38 pathway results in a marked induction of matrix metalloproteinase (MMP)-2, whereas it had little effect on MMP-9, suggesting MMP-2 up-regulation by MKK6-p38 pathway as a key step for H-Ras-induced invasion and   migration  . We also provide evidence for cross-talk among the Rac, Raf, and phosphatidylinositol 3-kinase pathways critical for regulation of MMP-2 and MMP-9 expression and invasive phenotype. Taken together, the present study elucidated the role of the Rac-MKK3/6-p38 pathway leading to H-Ras-specific induction of malignant progression in breast epithelial cells, providing implications for developing therapeutic strategies for mammary carcinoma to target Ras downstream signaling molecules required for malignant cancer cell behavior but less critical for normal cell functions. 
 Coronin-1C and RCC2 guide mesenchymal   migration   by trafficking Rac1 and controlling GEF exposure. Sustained forward   migration   through a fibrillar extracellular matrix requires localization of protrusive signals. Contact with fibronectin at the tip of a cell protrusion activates Rac1, and for linear   migration   it is necessary to dampen Rac1 activity in off-axial positions and redistribute Rac1 from non-protrusive membrane to the leading edge. Here, we identify interactions between coronin-1C (Coro1C), RCC2 and Rac1 that focus active Rac1 to a single protrusion. Coro1C mediates release of inactive Rac1 from non-protrusive membrane and is necessary for Rac1 redistribution to a protrusive tip and fibronectin-dependent Rac1 activation. The second component, RCC2, attenuates Rac1 activation outside the protrusive tip by binding to the Rac1 switch regions and competitively inhibiting GEF action, thus preventing off-axial protrusion. Depletion of Coro1C or RCC2 by RNA interference causes loss of cell polarity that results in shunting   migration   in 1D or 3D culture systems. Furthermore, morpholinos against Coro1C or RCC2, or mutation of any of the binding sites in the Rac1-RCC2-Coro1C complex delays the arrival of neural crest derivatives at the correct location in developing zebrafish, demonstrating the crucial role in   migration   guidance in vivo. 
 Vav1 and Rac control chemokine-promoted T lymphocyte adhesion mediated by the integrin alpha4beta1. The chemokine CXCL12 promotes T lymphocyte adhesion mediated by the integrin alpha4beta1. CXCL12 activates the GTPase Rac, as well as Vav1, a guanine-nucleotide exchange factor for Rac, concomitant with up-regulation of alpha4beta1-dependent adhesion. Inhibition of CXCL12-promoted Rac and Vav1 activation by transfection of dominant negative Rac or Vav1 forms, or by transfection of their siRNA, remarkably impaired the increase in T lymphocyte attachment to alpha4beta1 ligands in response to this chemokine. Importantly, inhibition of Vav1 expression by RNA interference resulted in a blockade of Rac activation in response to CXCL12. Adhesions in flow chambers and soluble binding assays using these transfectants indicated that initial ligand binding and adhesion strengthening mediated by alpha4beta1 were dependent on Vav1 and Rac activation by CXCL12. Finally, CXCL12-promoted T-cell transendothelial   migration   involving alpha4beta1-mediated adhesion was notably inhibited by expression of dominant negative Vav1 and Rac. These results indicate that activation of Vav1-Rac signaling pathway by CXCL12 represents an important inside-out event controlling efficient up-regulation of alpha4beta1-dependent T lymphocyte adhesion. 
 H-Ras-specific activation of Rac-MKK3/6-p38 pathway: its critical role in invasion and   migration   of breast epithelial cells. Human tumors frequently exhibit constitutively activated Ras signaling, which contributes to the malignant phenotype. Mounting evidence suggests unique roles of the Ras family members, H-Ras, N-Ras and K-Ras, in normal and pathological conditions. In an effort to dissect distinct Ras isoform-specific functions in malignant phenotypic changes, we previously established H-Ras- and N-Ras-activated MCF10A human breast epithelial cell lines. Using these, we showed that p38 kinase is a key signaling molecule differentially regulated between H-Ras and N-Ras, leading to H-Ras-specific induction of invasive and migrative phenotypes. The present study is to further investigate H-Ras- and N-Ras-mediated signaling pathways and to unveil how these pathways are integrated for regulation of invasive/migrative phenotypic conversion of human breast epithelial cells. Here we report that the Rac-MAPK kinase (MKK)3/6-p38 pathway is a unique signaling pathway activated by H-Ras, leading to the invasive/migrative phenotype. In contrast, Raf-MEK-ERK and phosphatidylinositol 3-kinase-Akt pathways, which are fundamental to proliferation and differentiation, are activated by both H-Ras and N-Ras. A significant role for p38 in cell invasion is further supported by the observation that p38 activation by MKK6 transfection is sufficient to induce invasive and migrative phenotypes in MCF10A cells. Activation of the MKK6-p38 pathway results in a marked induction of matrix metalloproteinase (MMP)-2, whereas it had little effect on MMP-9, suggesting MMP-2 up-regulation by MKK6-p38 pathway as a key step for H-Ras-induced invasion and   migration  . We also provide evidence for cross-talk among the Rac, Raf, and phosphatidylinositol 3-kinase pathways critical for regulation of MMP-2 and MMP-9 expression and invasive phenotype. Taken together, the present study elucidated the role of the Rac-MKK3/6-p38 pathway leading to H-Ras-specific induction of malignant progression in breast epithelial cells, providing implications for developing therapeutic strategies for mammary carcinoma to target Ras downstream signaling molecules required for malignant cancer cell behavior but less critical for normal cell functions. 
 Upregulation of Rac GTPase-activating protein 1 is significantly associated with the early recurrence of human hepatocellular carcinoma. To assess the significance of Rac GTPase-activating protein 1 (RACGAP1) expression in identifying HBV-positive human hepatocellular carcinoma (HCC) patients who are at high risk for recurrent disease.The prognostic significance of RACGAP1 was compared with clinicopathologic parameters available at diagnosis using multivariate and log-rank test. RACGAP1 expression and outcome in recurrence was compared between 35 patients with recurrence and 41 patients without recurrence using Kaplan-Meier analysis. RACGAP1-targeted molecules and pathways were identified and characterized by inhibition with siRNA duplexes.Kaplan-Meier analysis showed that the level of RACGAP1 expression is sufficient to predict the early recurrence of HCC: high RACGAP1 expression correlates with high risk of postresection recurrent HCC (P < 0.0005). Silencing of RACGAP1 in Hep3B and MHCC97-H HCC cells with high endogenous RACGAP1 expression inhibited cell   migration   and invasion. Using Ingenuity Pathway Analysis, the target molecules silenced in the RACGAP1 interactome were mostly genes related to the mitotic roles of the polo-like kinases. These included PRC1, AURKB, CDC2, ECT2, KIF23, PAK1, and PPP2R5E. In providing clinical corroboration of these results, when expression of these transcripts was analyzed in an expression database that we have established previously for HBV-positive HCC patients, these genes was mostly upregulated in patients who exhibited early recurrent disease and hence provided important corroboration of these results.siRNA-silencing RACGAP1 mainly targeted genes in an interactome clinically relevant to early HCC recurrence. Besides being an independent informative prognostic biomarker, RACGAP1 could also be a potential molecular target for designing therapeutic strategies for HCC. 
 RacGAP1-driven focal adhesion formation promotes melanoma transendothelial   migration   through mediating adherens junction disassembly. Melanoma cell   migration   across vascular endothelial cells is an essential step of tumor metastasis. Here, we provide evidence that RacGAP1, a cytokinesis-related Rho GTPase-activating protein, contributed to this process. Depletion of RacGAP1 with RacGAP1-targeting siRNA or overexpression of RacGAP1 mutant (T249A) attenuated melanoma cell transendothelial   migration   and concomitant changes of adherens junctions. In addition, RacGAP1 promoted the activations of RhoA, FAK, paxillin and triggered focal adhesion formation and cytoskeletal rearrangement. By overexpressing FAK-related non-kinase (FRNK) in endothelium, we showed that RacGAP1 mediated endothelial barrier function loss and melanoma trans  migration   in a focal adhesion-dependent manner. These results suggest that endothelial RacGAP1 may play critical roles in pathogenic processes of cancer by regulating endothelial permeability. 
 RacGAP1 expression, increasing tumor malignant potential, as a predictive biomarker for lymph node metastasis and poor prognosis in colorectal cancer. Rac GTPase-activating protein (RacGAP) 1 plays a key role in controlling various cellular phenomena including cytokinesis, transformation, invasive   migration   and metastasis. This study investigated the function and clinical significance of RacGAP1 expression in colorectal cancer (CRC). The intrinsic functions of RacGAP1 in CRC cells were analyzed using small interfering RNA (siRNA). We analyzed RacGAP1 mRNA expression in surgical specimens from 193 CRC patients (Cohort 1) by real-time PCR. Finally, we validated RacGAP1 protein expression using formalin-fixed paraffin-embedded samples from 298 CRC patients (Cohort 2) by immunohistochemistry. Reduced RacGAP1 expression by siRNA in CRC cell lines showed significantly decreased cellular proliferation,   migration   and invasion. In Cohort 1, RacGAP1 expression in CRC was significantly higher than in adjacent normal mucosa and increased according to tumor node metastasis stage progression. High RacGAP1 expression in tumors was significantly associated with progression and prognosis. In Cohort 2, RacGAP1 protein was overexpressed mainly in the nuclei of CRC cells; however, its expression was scarcely observed in normal colorectal mucosa. RacGAP1 protein expression was significantly higher in CRC patients with higher T stage, vessel invasion and lymph node and distant metastasis. Increased expression of RacGAP1 protein was significantly associated with poor disease-free and overall survival. Multivariate analyses revealed that high RacGAP1 expression was an independent predictive marker for lymph node metastasis, recurrence and poor prognosis in CRC. Our data provide novel evidence for the biological and clinical significance of RacGAP1 as a potential biomarker for identifying patients with lymph node metastasis and poor prognosis in CRC. 
  GeneRIFs  
 RacGAP1 mediated endothelial barrier function loss and melanoma trans  migration   in a focal adhesion-dependent manner.RacGAP1 activation mediates focal adhesion assembly and actin stress fiber formation. 
 H-Ras-specific activation of Rac-MKK3/6-p38 pathway has a role in invasion and   migration   of breast epithelial cells 
  [Ee]xtracellular [Mm]atrix  PubMed  
 Proteomic analysis of human parotid gland exosomes by multidimensional protein identification technology (MudPIT). Human ductal saliva contributes over a thousand unique proteins to whole oral fluids. The mechanism by which most of these proteins are secreted by salivary glands remains to be determined. The present study used a mass spectrometry-based, shotgun proteomics approach to explore the possibility that a subset of the proteins found in saliva are derived from exosomes, membrane-bound vesicles of endosomal origin within multivesicular endosomes. Using MudPIT (multidimensional protein identification technology) mass spectrometry, we catalogued 491 proteins in the exosome fraction of human parotid saliva. Many of these proteins were previously observed in ductal saliva from parotid glands (265 proteins). Furthermore, 72 of the proteins in parotid exosomes overlap with those previously identified as urinary exosome proteins, proteins which are also frequently associated with exosomes from other tissues and cell types. Gene Ontology (GO) and KEGG pathway analyses found that cytosolic proteins comprise the largest category of proteins in parotid exosomes (43%), involved in such processes as phosphatidylinositol signaling system, calcium signaling pathway, inositol metabolism, protein export, and signal transduction, among others; whereas the integral plasma membrane proteins and associated/peripheral plasma membrane proteins (26%) were associated with   extracellular matrix  -receptor interaction, epithelial cell signaling, T-cell and B-cell receptor signaling, cytokine receptor interaction, and antigen processing and presentation, among other biological functions. In addition, these putative saliva exosomal proteins were linked to specific diseases (e.g., neurodegenerative disorders, prion disease, cancers, type I and II diabetes). Consequently, parotid glands secrete exosomes that reflect the metabolic and functional status of the gland and may also carry informative protein markers useful in the diagnosis and treatment of systemic diseases. 
 Coronin-1C and RCC2 guide mesenchymal migration by trafficking Rac1 and controlling GEF exposure. Sustained forward migration through a fibrillar   extracellular matrix   requires localization of protrusive signals. Contact with fibronectin at the tip of a cell protrusion activates Rac1, and for linear migration it is necessary to dampen Rac1 activity in off-axial positions and redistribute Rac1 from non-protrusive membrane to the leading edge. Here, we identify interactions between coronin-1C (Coro1C), RCC2 and Rac1 that focus active Rac1 to a single protrusion. Coro1C mediates release of inactive Rac1 from non-protrusive membrane and is necessary for Rac1 redistribution to a protrusive tip and fibronectin-dependent Rac1 activation. The second component, RCC2, attenuates Rac1 activation outside the protrusive tip by binding to the Rac1 switch regions and competitively inhibiting GEF action, thus preventing off-axial protrusion. Depletion of Coro1C or RCC2 by RNA interference causes loss of cell polarity that results in shunting migration in 1D or 3D culture systems. Furthermore, morpholinos against Coro1C or RCC2, or mutation of any of the binding sites in the Rac1-RCC2-Coro1C complex delays the arrival of neural crest derivatives at the correct location in developing zebrafish, demonstrating the crucial role in migration guidance in vivo. 
  [Ii]nvasive  PubMed  
 H-Ras-specific activation of Rac-MKK3/6-p38 pathway: its critical role in invasion and migration of breast epithelial cells. Human tumors frequently exhibit constitutively activated Ras signaling, which contributes to the malignant phenotype. Mounting evidence suggests unique roles of the Ras family members, H-Ras, N-Ras and K-Ras, in normal and pathological conditions. In an effort to dissect distinct Ras isoform-specific functions in malignant phenotypic changes, we previously established H-Ras- and N-Ras-activated MCF10A human breast epithelial cell lines. Using these, we showed that p38 kinase is a key signaling molecule differentially regulated between H-Ras and N-Ras, leading to H-Ras-specific induction of   invasive   and migrative phenotypes. The present study is to further investigate H-Ras- and N-Ras-mediated signaling pathways and to unveil how these pathways are integrated for regulation of   invasive  /migrative phenotypic conversion of human breast epithelial cells. Here we report that the Rac-MAPK kinase (MKK)3/6-p38 pathway is a unique signaling pathway activated by H-Ras, leading to the   invasive  /migrative phenotype. In contrast, Raf-MEK-ERK and phosphatidylinositol 3-kinase-Akt pathways, which are fundamental to proliferation and differentiation, are activated by both H-Ras and N-Ras. A significant role for p38 in cell invasion is further supported by the observation that p38 activation by MKK6 transfection is sufficient to induce   invasive   and migrative phenotypes in MCF10A cells. Activation of the MKK6-p38 pathway results in a marked induction of matrix metalloproteinase (MMP)-2, whereas it had little effect on MMP-9, suggesting MMP-2 up-regulation by MKK6-p38 pathway as a key step for H-Ras-induced invasion and migration. We also provide evidence for cross-talk among the Rac, Raf, and phosphatidylinositol 3-kinase pathways critical for regulation of MMP-2 and MMP-9 expression and   invasive   phenotype. Taken together, the present study elucidated the role of the Rac-MKK3/6-p38 pathway leading to H-Ras-specific induction of malignant progression in breast epithelial cells, providing implications for developing therapeutic strategies for mammary carcinoma to target Ras downstream signaling molecules required for malignant cancer cell behavior but less critical for normal cell functions. 
 H-Ras-specific activation of Rac-MKK3/6-p38 pathway: its critical role in invasion and migration of breast epithelial cells. Human tumors frequently exhibit constitutively activated Ras signaling, which contributes to the malignant phenotype. Mounting evidence suggests unique roles of the Ras family members, H-Ras, N-Ras and K-Ras, in normal and pathological conditions. In an effort to dissect distinct Ras isoform-specific functions in malignant phenotypic changes, we previously established H-Ras- and N-Ras-activated MCF10A human breast epithelial cell lines. Using these, we showed that p38 kinase is a key signaling molecule differentially regulated between H-Ras and N-Ras, leading to H-Ras-specific induction of   invasive   and migrative phenotypes. The present study is to further investigate H-Ras- and N-Ras-mediated signaling pathways and to unveil how these pathways are integrated for regulation of   invasive  /migrative phenotypic conversion of human breast epithelial cells. Here we report that the Rac-MAPK kinase (MKK)3/6-p38 pathway is a unique signaling pathway activated by H-Ras, leading to the   invasive  /migrative phenotype. In contrast, Raf-MEK-ERK and phosphatidylinositol 3-kinase-Akt pathways, which are fundamental to proliferation and differentiation, are activated by both H-Ras and N-Ras. A significant role for p38 in cell invasion is further supported by the observation that p38 activation by MKK6 transfection is sufficient to induce   invasive   and migrative phenotypes in MCF10A cells. Activation of the MKK6-p38 pathway results in a marked induction of matrix metalloproteinase (MMP)-2, whereas it had little effect on MMP-9, suggesting MMP-2 up-regulation by MKK6-p38 pathway as a key step for H-Ras-induced invasion and migration. We also provide evidence for cross-talk among the Rac, Raf, and phosphatidylinositol 3-kinase pathways critical for regulation of MMP-2 and MMP-9 expression and   invasive   phenotype. Taken together, the present study elucidated the role of the Rac-MKK3/6-p38 pathway leading to H-Ras-specific induction of malignant progression in breast epithelial cells, providing implications for developing therapeutic strategies for mammary carcinoma to target Ras downstream signaling molecules required for malignant cancer cell behavior but less critical for normal cell functions. 
 Clinical significance of RacGAP1 expression at the   invasive   front of gastric cancer. Rac GTPase activating protein 1 (RacGAP1) plays a regulatory role in cell growth, transformation and metastasis. The aim of this study was to clarify the association between RacGAP1 expression and clinical outcome in patients with gastric cancer.A total of 232 gastric cancer patients in our institute who underwent surgery without preoperative treatments were enrolled in this study. We investigated RacGAP1 expression using immunohistochemistry (IHC) and evaluated IHC scores calculated by the percentage of positive cells and intensity and its expression at the   invasive   front. RACGAP1 expression was also assessed.RacGAP1 expression was observed in the nuclei of gastric cancer cells. Evaluation by IHC score showed no significant correlations with clinicopathological variables except for histological differentiation. In transcriptional analyses, RACGAP1 expression was elevated in diffuse type gastric cancer than intestinal type without a significant difference. We observed significant correlations of RacGAP1 protein expression at the   invasive   front with older age, tumor size, lymph node metastasis, lymphatic invasion, vascular invasion and advanced stage. Patients with RacGAP1 protein expression at the   invasive   front had significantly poorer prognosis than those without it (P < 0.0001). In multivariate analysis, lymph node metastasis, distant metastasis and positive RacGAP1 expression at the   invasive   front were independent prognostic factors (lymph node metastasis: P = 0.0106; distant metastasis: P = 0.0012; RacGAP1: P = 0.0011).RacGAP1 expression at the   invasive   front in gastric cancer was significantly correlated with factors reflecting tumor progression and poor prognosis. Our data suggest that RacGAP1 might play important roles in the progression of gastric cancer. 
 RacGAP1 expression, increasing tumor malignant potential, as a predictive biomarker for lymph node metastasis and poor prognosis in colorectal cancer. Rac GTPase-activating protein (RacGAP) 1 plays a key role in controlling various cellular phenomena including cytokinesis, transformation,   invasive   migration and metastasis. This study investigated the function and clinical significance of RacGAP1 expression in colorectal cancer (CRC). The intrinsic functions of RacGAP1 in CRC cells were analyzed using small interfering RNA (siRNA). We analyzed RacGAP1 mRNA expression in surgical specimens from 193 CRC patients (Cohort 1) by real-time PCR. Finally, we validated RacGAP1 protein expression using formalin-fixed paraffin-embedded samples from 298 CRC patients (Cohort 2) by immunohistochemistry. Reduced RacGAP1 expression by siRNA in CRC cell lines showed significantly decreased cellular proliferation, migration and invasion. In Cohort 1, RacGAP1 expression in CRC was significantly higher than in adjacent normal mucosa and increased according to tumor node metastasis stage progression. High RacGAP1 expression in tumors was significantly associated with progression and prognosis. In Cohort 2, RacGAP1 protein was overexpressed mainly in the nuclei of CRC cells; however, its expression was scarcely observed in normal colorectal mucosa. RacGAP1 protein expression was significantly higher in CRC patients with higher T stage, vessel invasion and lymph node and distant metastasis. Increased expression of RacGAP1 protein was significantly associated with poor disease-free and overall survival. Multivariate analyses revealed that high RacGAP1 expression was an independent predictive marker for lymph node metastasis, recurrence and poor prognosis in CRC. Our data provide novel evidence for the biological and clinical significance of RacGAP1 as a potential biomarker for identifying patients with lymph node metastasis and poor prognosis in CRC. 
  GeneRIFs  
 RacGAP1 expression at the   invasive   front in gastric cancer was significantly correlated with factors reflecting tumor progression and poor prognosis.RACGAP1 gene expression in diffuse type gastric cancer was elevated. 
  [Mm]etastasis  PubMed  
 Clinical significance of RacGAP1 expression at the invasive front of gastric cancer. Rac GTPase activating protein 1 (RacGAP1) plays a regulatory role in cell growth, transformation and   metastasis  . The aim of this study was to clarify the association between RacGAP1 expression and clinical outcome in patients with gastric cancer.A total of 232 gastric cancer patients in our institute who underwent surgery without preoperative treatments were enrolled in this study. We investigated RacGAP1 expression using immunohistochemistry (IHC) and evaluated IHC scores calculated by the percentage of positive cells and intensity and its expression at the invasive front. RACGAP1 expression was also assessed.RacGAP1 expression was observed in the nuclei of gastric cancer cells. Evaluation by IHC score showed no significant correlations with clinicopathological variables except for histological differentiation. In transcriptional analyses, RACGAP1 expression was elevated in diffuse type gastric cancer than intestinal type without a significant difference. We observed significant correlations of RacGAP1 protein expression at the invasive front with older age, tumor size, lymph node   metastasis  , lymphatic invasion, vascular invasion and advanced stage. Patients with RacGAP1 protein expression at the invasive front had significantly poorer prognosis than those without it (P < 0.0001). In multivariate analysis, lymph node   metastasis  , distant   metastasis   and positive RacGAP1 expression at the invasive front were independent prognostic factors (lymph node   metastasis  : P = 0.0106; distant   metastasis  : P = 0.0012; RacGAP1: P = 0.0011).RacGAP1 expression at the invasive front in gastric cancer was significantly correlated with factors reflecting tumor progression and poor prognosis. Our data suggest that RacGAP1 might play important roles in the progression of gastric cancer. 
 RacGAP1-driven focal adhesion formation promotes melanoma transendothelial migration through mediating adherens junction disassembly. Melanoma cell migration across vascular endothelial cells is an essential step of tumor   metastasis  . Here, we provide evidence that RacGAP1, a cytokinesis-related Rho GTPase-activating protein, contributed to this process. Depletion of RacGAP1 with RacGAP1-targeting siRNA or overexpression of RacGAP1 mutant (T249A) attenuated melanoma cell transendothelial migration and concomitant changes of adherens junctions. In addition, RacGAP1 promoted the activations of RhoA, FAK, paxillin and triggered focal adhesion formation and cytoskeletal rearrangement. By overexpressing FAK-related non-kinase (FRNK) in endothelium, we showed that RacGAP1 mediated endothelial barrier function loss and melanoma transmigration in a focal adhesion-dependent manner. These results suggest that endothelial RacGAP1 may play critical roles in pathogenic processes of cancer by regulating endothelial permeability. 
 RacGAP1 expression, increasing tumor malignant potential, as a predictive biomarker for lymph node   metastasis   and poor prognosis in colorectal cancer. Rac GTPase-activating protein (RacGAP) 1 plays a key role in controlling various cellular phenomena including cytokinesis, transformation, invasive migration and   metastasis  . This study investigated the function and clinical significance of RacGAP1 expression in colorectal cancer (CRC). The intrinsic functions of RacGAP1 in CRC cells were analyzed using small interfering RNA (siRNA). We analyzed RacGAP1 mRNA expression in surgical specimens from 193 CRC patients (Cohort 1) by real-time PCR. Finally, we validated RacGAP1 protein expression using formalin-fixed paraffin-embedded samples from 298 CRC patients (Cohort 2) by immunohistochemistry. Reduced RacGAP1 expression by siRNA in CRC cell lines showed significantly decreased cellular proliferation, migration and invasion. In Cohort 1, RacGAP1 expression in CRC was significantly higher than in adjacent normal mucosa and increased according to tumor node   metastasis   stage progression. High RacGAP1 expression in tumors was significantly associated with progression and prognosis. In Cohort 2, RacGAP1 protein was overexpressed mainly in the nuclei of CRC cells; however, its expression was scarcely observed in normal colorectal mucosa. RacGAP1 protein expression was significantly higher in CRC patients with higher T stage, vessel invasion and lymph node and distant   metastasis  . Increased expression of RacGAP1 protein was significantly associated with poor disease-free and overall survival. Multivariate analyses revealed that high RacGAP1 expression was an independent predictive marker for lymph node   metastasis  , recurrence and poor prognosis in CRC. Our data provide novel evidence for the biological and clinical significance of RacGAP1 as a potential biomarker for identifying patients with lymph node   metastasis   and poor prognosis in CRC. 
 A genome-wide survey of RAS transformation targets. An important aspect of multi-step tumorigenesis is the mutational activation of genes of the RAS family, particularly in sporadic cancers of the pancreas, colon, lung and myeloid system. RAS genes encode small GTP-binding proteins that affect gene expression in a global way by acting as major switches in signal transduction processes, coupling extracellular signals with transcription factors. Oncogenic forms of RAS are locked in their active state and transduce signals essential for transformation, angiogenesis, invasion and   metastasis   via downstream pathways involving the RAF/MEK/ERK cascade of cytoplasmic kinases, the small GTP-binding proteins RAC and RHO, phosphatidylinositol 3-kinase and others. We have used subtractive suppression hybridization (SSH), a PCR-based cDNA subtraction technique, to contrast differential gene expression profiles in immortalized, non-tumorigenic rat embryo fibroblasts and in HRAS- transformed cells. Sequence and expression analysis of more than 1,200 subtracted cDNA fragments revealed transcriptional stimulation or repression of 104 ESTs, 45 novel sequences and 244 known genes in HRAS- transformed cells compared with normal cells. Furthermore, we identified common and distinct targets in cells transformed by mutant HRAS, KRAS and NRAS, as well as 61 putative target genes controlled by the RAF/MEK/ERK pathway in reverted cells treated with the MEK-specific inhibitor PD 98059. 
 Prognostic significance of RACGAP1 mRNA expression in high-risk early breast cancer: a study in primary tumors of breast cancer patients participating in a randomized Hellenic Cooperative Oncology Group trial. RACGAP1 is a Rac GTPase-activating protein involved in cell growth regulation, cell transformation and   metastasis  . The aim of the present study was to explore the prognostic and/or predictive significance of RACGAP1 mRNA expression on disease-free survival (DFS) and overall survival (OS) in high-risk early breast cancer patients and compare it to that of Ki67 protein expression and to the Nottingham prognostic index (NPI).A total of 595 high-risk breast cancer patients were treated in a two-arm trial evaluating postoperative dose-dense sequential chemotherapy with epirubicin followed by CMF with or without paclitaxel. RNA was extracted from 314 formalin-fixed paraffin-embedded primary tumor tissue samples followed by one-step quantitative RT-PCR for assessing RACGAP1 mRNA expression.High RACGAP1 mRNA expression (above the median) was associated with poor DFS (log-rank, p = 0.002) and OS (p < 0.001). High histological grade, as well as high Ki67 protein expression, was more frequent in the high-expression group of RACGAP1. Results of the Cox multivariate regression analysis revealed that high RACGAP1 mRNA expression independently predicted poor overall survival (Wald's p = 0.008). High Ki67 protein expression was also an adverse prognostic factor for death (p = 0.016), while high NPI score values were not.High RACGAP1 mRNA expression, as assessed by qRT-PCR, was found to be of adverse prognostic significance in high-risk early breast cancer patients treated with dose-dense sequential chemotherapy. The utility of RACGAP1 mRNA expression in patient selection for treatment with aggressive chemotherapy regimens should be further explored and validated in larger cohorts. 
  GeneRIFs  
 High RacGAP1 expression was an independent predictive marker for lymph node   metastasis  , recurrence and poor prognosis in colorectal cancer patients. 
  [Cc]ell [Ii]nvasion  PubMed  
 H-Ras-specific activation of Rac-MKK3/6-p38 pathway: its critical role in invasion and migration of breast epithelial cells. Human tumors frequently exhibit constitutively activated Ras signaling, which contributes to the malignant phenotype. Mounting evidence suggests unique roles of the Ras family members, H-Ras, N-Ras and K-Ras, in normal and pathological conditions. In an effort to dissect distinct Ras isoform-specific functions in malignant phenotypic changes, we previously established H-Ras- and N-Ras-activated MCF10A human breast epithelial cell lines. Using these, we showed that p38 kinase is a key signaling molecule differentially regulated between H-Ras and N-Ras, leading to H-Ras-specific induction of invasive and migrative phenotypes. The present study is to further investigate H-Ras- and N-Ras-mediated signaling pathways and to unveil how these pathways are integrated for regulation of invasive/migrative phenotypic conversion of human breast epithelial cells. Here we report that the Rac-MAPK kinase (MKK)3/6-p38 pathway is a unique signaling pathway activated by H-Ras, leading to the invasive/migrative phenotype. In contrast, Raf-MEK-ERK and phosphatidylinositol 3-kinase-Akt pathways, which are fundamental to proliferation and differentiation, are activated by both H-Ras and N-Ras. A significant role for p38 in   cell invasion   is further supported by the observation that p38 activation by MKK6 transfection is sufficient to induce invasive and migrative phenotypes in MCF10A cells. Activation of the MKK6-p38 pathway results in a marked induction of matrix metalloproteinase (MMP)-2, whereas it had little effect on MMP-9, suggesting MMP-2 up-regulation by MKK6-p38 pathway as a key step for H-Ras-induced invasion and migration. We also provide evidence for cross-talk among the Rac, Raf, and phosphatidylinositol 3-kinase pathways critical for regulation of MMP-2 and MMP-9 expression and invasive phenotype. Taken together, the present study elucidated the role of the Rac-MKK3/6-p38 pathway leading to H-Ras-specific induction of malignant progression in breast epithelial cells, providing implications for developing therapeutic strategies for mammary carcinoma to target Ras downstream signaling molecules required for malignant cancer cell behavior but less critical for normal cell functions. 
 H-Ras-specific activation of Rac-MKK3/6-p38 pathway: its critical role in invasion and migration of breast epithelial cells. Human tumors frequently exhibit constitutively activated Ras signaling, which contributes to the malignant phenotype. Mounting evidence suggests unique roles of the Ras family members, H-Ras, N-Ras and K-Ras, in normal and pathological conditions. In an effort to dissect distinct Ras isoform-specific functions in malignant phenotypic changes, we previously established H-Ras- and N-Ras-activated MCF10A human breast epithelial cell lines. Using these, we showed that p38 kinase is a key signaling molecule differentially regulated between H-Ras and N-Ras, leading to H-Ras-specific induction of invasive and migrative phenotypes. The present study is to further investigate H-Ras- and N-Ras-mediated signaling pathways and to unveil how these pathways are integrated for regulation of invasive/migrative phenotypic conversion of human breast epithelial cells. Here we report that the Rac-MAPK kinase (MKK)3/6-p38 pathway is a unique signaling pathway activated by H-Ras, leading to the invasive/migrative phenotype. In contrast, Raf-MEK-ERK and phosphatidylinositol 3-kinase-Akt pathways, which are fundamental to proliferation and differentiation, are activated by both H-Ras and N-Ras. A significant role for p38 in   cell invasion   is further supported by the observation that p38 activation by MKK6 transfection is sufficient to induce invasive and migrative phenotypes in MCF10A cells. Activation of the MKK6-p38 pathway results in a marked induction of matrix metalloproteinase (MMP)-2, whereas it had little effect on MMP-9, suggesting MMP-2 up-regulation by MKK6-p38 pathway as a key step for H-Ras-induced invasion and migration. We also provide evidence for cross-talk among the Rac, Raf, and phosphatidylinositol 3-kinase pathways critical for regulation of MMP-2 and MMP-9 expression and invasive phenotype. Taken together, the present study elucidated the role of the Rac-MKK3/6-p38 pathway leading to H-Ras-specific induction of malignant progression in breast epithelial cells, providing implications for developing therapeutic strategies for mammary carcinoma to target Ras downstream signaling molecules required for malignant cancer cell behavior but less critical for normal cell functions. 
   
  
 BMX 
 
  NCBI Gene  
  GeneRIF  
  Pubmed  
 
 
  Search Term  Rank  Fields  
  [Mm]igration  6  PubMed(10)  
  [Ee]xtracellular [Mm]atrix  8  PubMed(1)  
  [Ii]nvasive  10  PubMed(1)  
  [Mm]etastasis  12  PubMed(3)  
 
 
 hide/show details for BMX 
  [Mm]igration  PubMed  
 Bmx is a downstream Rap1 effector in VEGF-induced endothelial cell activation. We had previously shown that Rap1 mediates certain of the signaling pathways involved in VEGF-induced endothelial cell   migration  , although the downstream Rap1 effectors are not known. Towards the goal of identifying those effectors, we utilized a commercially available antibody array filter to identify proteins that either directly interact with Rap1 or interact indirectly through a multi-protein complex. The protocol identified 10 possible Rap1-interacting proteins, including the Bmx non-receptor tyrosine kinase. The conclusion that VEGF treatment leads to a Rap1/Bmx complex was confirmed by an experiment in which cell lysates from VEGF and control cells were immunoprecipitated with Bmx antibodies and Western blotting was done using anti-Rap1 antibodies. VEGF treatment led to the recruitment of Bmx to the CAS scaffolding protein, and inhibition of the Bmx kinase blocked VEGF-induced cell   migration  . Formation of a Rap1/Bmx complex was not observed in cells transfected with an expression vector for a dominant-negative Rap1, indicating that Bmx is a downstream Rap1 effector in VEGF-induced endothelial cell activation. 
 Bmx is a downstream Rap1 effector in VEGF-induced endothelial cell activation. We had previously shown that Rap1 mediates certain of the signaling pathways involved in VEGF-induced endothelial cell   migration  , although the downstream Rap1 effectors are not known. Towards the goal of identifying those effectors, we utilized a commercially available antibody array filter to identify proteins that either directly interact with Rap1 or interact indirectly through a multi-protein complex. The protocol identified 10 possible Rap1-interacting proteins, including the Bmx non-receptor tyrosine kinase. The conclusion that VEGF treatment leads to a Rap1/Bmx complex was confirmed by an experiment in which cell lysates from VEGF and control cells were immunoprecipitated with Bmx antibodies and Western blotting was done using anti-Rap1 antibodies. VEGF treatment led to the recruitment of Bmx to the CAS scaffolding protein, and inhibition of the Bmx kinase blocked VEGF-induced cell   migration  . Formation of a Rap1/Bmx complex was not observed in cells transfected with an expression vector for a dominant-negative Rap1, indicating that Bmx is a downstream Rap1 effector in VEGF-induced endothelial cell activation. 
 Critical function of Bmx/Etk in ischemia-mediated arteriogenesis and angiogenesis. Bmx/Etk non-receptor tyrosine protein kinase has been implicated in endothelial cell   migration   and tube formation in vitro. However, the role of Bmx in vivo is not known. Bmx is highly induced in the vasculature of ischemic hind limbs. We used both mice with a genetic deletion of Bmx (Bmx-KO mice) and transgenic mice expressing a constitutively active form of Bmx under the endothelial Tie-2 enhancer/promoter (Bmx-SK-Tg mice) to study the role of Bmx in ischemia-mediated arteriogenesis/angiogenesis. In response to ischemia, Bmx-KO mice had markedly reduced, whereas Bmx-SK-Tg mice had enhanced, clinical recovery, limb perfusion, and ischemic reserve capacity when compared with nontransgenic control mice. The functional outcomes in these mice were correlated with ischemia-initiated arteriogenesis, capillary formation, and vessel maturation as well as Bmx-dependent expression/activation of TNF receptor 2- and VEGFR2-mediated (TNFR2/VEGFR2-mediated) angiogenic signaling in both hind limb and bone marrow. More importantly, results of bone marrow transplantation studies showed that Bmx in bone marrow-derived cells plays a critical role in the early phase of ischemic tissue remodeling. Our study provides the first demonstration to our knowledge that Bmx in endothelium and bone marrow plays a critical role in arteriogenesis/angiogenesis in vivo and suggests that Bmx may be a novel target for the treatment of vascular diseases such as coronary artery disease and peripheral arterial disease. 
 Regulation of the PH-domain-containing tyrosine kinase Etk by focal adhesion kinase through the FERM domain. Etk/BMX, a member of the Btk family of tyrosine kinases, is highly expressed in cells with great migratory potential, including endothelial cells and metastatic carcinoma cell lines. Here, we present evidence that Etk is involved in integrin signalling and promotes cell   migration  . The activation of Etk by extracellular matrix proteins is regulated by FAK through an interaction between the PH domain of Etk and the FERM domain of FAK. The lack of Etk activation by extracellular matrix in FAK-null cells could be restored by co-transfection with wild-type FAK. Disrupting the interaction between Etk and FAK diminished the cell   migration   promoted by either kinase. Furthermore, inhibiting Etk expression in metastatic carcinoma cell lines with an antisense oligonucleotide blocks integrin-mediated   migration   of these cells. Taken together, our data indicate the essential role of the interaction of the PH domain of Etk and the FERM domain of FAK in integrin signalling. 
 Etk/Bmx regulates proteinase-activated-receptor1 (PAR1) in breast cancer invasion: signaling partners, hierarchy and physiological significance. While protease-activated-receptor 1 (PAR(1)) plays a central role in tumor progression, little is known about the cell signaling involved.We show here the impact of PAR(1) cellular activities using both an orthotopic mouse mammary xenograft and a colorectal-liver metastasis model in vivo, with biochemical analyses in vitro. Large and highly vascularized tumors were generated by cells over-expressing wt hPar1, Y397Z hPar1, with persistent signaling, or Y381A hPar1 mutant constructs. In contrast, cells over-expressing the truncated form of hPar1, which lacks the cytoplasmic tail, developed small or no tumors, similar to cells expressing empty vector or control untreated cells. Antibody array membranes revealed essential hPar1 partners including Etk/Bmx and Shc. PAR(1) activation induces Etk/Bmx and Shc binding to the receptor C-tail to form a complex. Y/A mutations in the PAR(1) C-tail did not prevent Shc-PAR(1) association, but enhanced the number of liver metastases compared with the already increased metastases obtained with wt hPar1. We found that Etk/Bmx first binds via the PH domain to a region of seven residues, located between C378-S384 in PAR(1) C-tail, enabling subsequent Shc association. Importantly, expression of the hPar1-7A mutant form (substituted A, residues 378-384), which is incapable of binding Etk/Bmx, resulted in inhibition of invasion through Matrigel-coated membranes. Similarly, knocking down Etk/Bmx inhibited PAR(1)-induced MDA-MB-435 cell   migration  . In addition, intact spheroid morphogenesis of MCF10A cells is markedly disrupted by the ectopic expression of wt hPar1. In contrast, the forced expression of the hPar1-7A mutant results in normal ball-shaped spheroids. Thus, by preventing binding of Etk/Bmx to PAR(1) -C-tail, hPar1 oncogenic properties are abrogated.This is the first demonstration that a cytoplasmic portion of the PAR(1) C-tail functions as a scaffold site. We identify here essential signaling partners, determine the hierarchy of binding and provide a platform for therapeutic vehicles via definition of the critical PAR(1)-associating region in the breast cancer signaling niche. 
 Etk/Bmx transactivates vascular endothelial growth factor 2 and recruits phosphatidylinositol 3-kinase to mediate the tumor necrosis factor-induced angiogenic pathway. Tumor necrosis factor (TNF), via its receptor 2 (TNFR2), induces Etk (or Bmx) activation and Etk-dependent endothelial cell (EC)   migration   and tube formation. Because TNF receptor 2 lacks an intrinsic kinase activity, we examined the kinase(s) mediating TNF-induced Etk activation. TNF induces a coordinated phosphorylation of vascular endothelial growth factor (VEGF) receptor 2 (VEGFR2) and Etk, which is blocked by VEGFR2-specific inhibitors. In response to TNF, Etk and VEGFR2 form a complex resulting in a reciprocal activation between the two kinases. Subsequently, the downstream phosphatidylinositol 3-kinase (PI3K)-Akt signaling (but not signaling through phospholipase C-gamma) was initiated and directly led to TNF-induced EC   migration  , which was significantly inhibited by VEGFR2-, PI3K-, or Akt-specific inhibitors. Phosphorylation of VEGFR2 at Tyr-801 and Tyr-1175, the critical sites for VEGF-induced PI3K-Akt signaling, was not involved in TNF-mediated Akt activation. However, TNF induces phosphorylation of Etk at Tyr-566, directly mediating the recruitment of the p85 subunit of PI3K. Furthermore, TNF- but not VEGF-induced activation of VEGFR2, Akt, and EC   migration   are blunted in EC genetically deficient with Etk. Taken together, our data demonstrated that TNF induces transactivation between Etk and VEGFR2, and Etk directly activates PI3K-Akt angiogenic signaling independent of VEGF-induced VEGFR2-PI3K-Akt signaling pathway. 
 Etk/Bmx as a tumor necrosis factor receptor type 2-specific kinase: role in endothelial cell   migration   and angiogenesis. Tumor necrosis factor (TNF) is a cytokine that mediates many pathophysiologial processes, including angiogenesis. However, the molecular signaling involved in TNF-induced angiogenesis has not been determined. In this study, we examined the role of Etk/Bmx, an endothelial/epithelial tyrosine kinase involved in cell adhesion,   migration  , and survival in TNF-induced angiogenesis. We show that TNF activates Etk specifically through TNF receptor type 2 (TNFR2) as demonstrated by studies using a specific agonist to TNFR2 and TNFR2-deficient cells. Etk forms a preexisting complex with TNFR2 in a ligand-independent manner, and the association is through multiple domains (pleckstrin homology domain, TEC homology domain, and SH2 domain) of Etk and the C-terminal domain of TNFR2. The C-terminal 16-amino-acid residues of TNFR2 are critical for Etk association and activation, and this Etk-binding and activating motif in TNFR2 is not overlapped with the TNFR-associated factor type 2 (TRAF2)-binding sequence. Thus, TRAF2 is not involved in TNF-induced Etk activation, suggesting a novel mechanism for Etk activation by cytokine receptors. Moreover, a constitutively active form of Etk enhanced, whereas a dominant-negative Etk blocked, TNF-induced endothelial cell   migration   and tube formation. While most TNF actions have been attributed to TNFR1, our studies demonstrate that Etk is a TNFR2-specific kinase involved in TNF-induced angiogenic events. 
 Bone marrow X kinase-mediated signal transduction in irradiated vascular endothelium. Radiation-induced activation of the phosphatidyl inositol-3 kinase/Akt signal transduction pathway requires Akt binding to phosphatidyl-inositol phosphates (PIP) on the cell membrane. The tyrosine kinase bone marrow X kinase (Bmx) binds to membrane-associated PIPs in a manner similar to Akt. Because Bmx is involved in cell growth and survival pathways, it could contribute to the radiation response within the vascular endothelium. We therefore studied Bmx signaling within the vascular endothelium. Bmx was activated rapidly in response to clinically relevant doses of ionizing radiation. Bmx inhibition enhanced the efficacy of radiotherapy in endothelial cells as well as tumor vascular endothelium in lung cancer tumors in mice. Retroviral shRNA knockdown of Bmx protein enhanced human umbilical vascular endothelial cell (HUVEC) radiosensitization. Furthermore, pretreatment of HUVEC with a pharmacologic inhibitor of Bmx, LFM-A13, produced significant radiosensitization of endothelial cells as measured by clonogenic survival analysis and apoptosis as well as functional assays including cell   migration   and tubule formation. In vivo, LFM-A13, when combined with radiation, resulted in significant tumor microvascular destruction as well as enhanced tumor growth delay. Bmx therefore represents a molecular target for the development of novel radiosensitizing agents. 
 Tyrosine kinase ETK/BMX is up-regulated in bladder cancer and predicts poor prognosis in patients with cystectomy. Deregulation of the non-receptor tyrosine kinase ETK/BMX has been reported in several solid tumors. In this report, we demonstrated that ETK expression is progressively increased during bladder cancer progression. We found that down-regulation of ETK in bladder cancer cells attenuated STAT3 and AKT activity whereas exogenous overexpression of ETK had opposite effects, suggesting that deregulation of ETK may attribute to the elevated activity of STAT3 and AKT frequently detected in bladder cancer. The survival,   migration   and invasion of bladder cancer cells were significantly compromised when ETK expression was knocked down by a specific shRNA. In addition, we showed that ETK localizes to mitochondria in bladder cancer cells through interacting with Bcl-XL and regulating ROS production and drug sensitivity. Therefore, ETK may play an important role in regulating survival,   migration   and invasion by modulating multiple signaling pathways in bladder cancer cells. Immunohistochemistry analysis on tissue microarrays containing 619 human bladder tissue samples shows that ETK is significantly upregulated during bladder cancer development and progression and ETK expression level predicts the survival rate of patients with cystectomy. Taken together, our results suggest that ETK may potentially serve as a new drug target for bladder cancer treatment as well as a biomarker which could be used to identify patients with higher mortality risk, who may be benefited from therapeutics targeting ETK activity. 
 The expression and role of tyrosine kinase ETK/BMX in renal cell carcinoma. Expression of the non-receptor tyrosine kinase ETK/BMX has been reported in several solid tumors, but the underlying molecular mechanisms and its clinical significance in renal cell carcinoma (RCC) remain to be elucidated.ETK expression in 90 human RCC and 30 human normal renal tissue samples was examined by immunohistochemistry and compared with several clinicopathologic parameters. To further demonstrate the biological function of ETK in RCC, Western blot was used to test the expression level of ETK protein in RCC cell lines. Subsequent to the downregulation of ETK by small interfering RNA, the effects of ETK on RCC cell growth, apoptosis,   migration   and invasion were assessed by methyl thiazol tetrazolium assay, flow cytometry and transwell assay. And the varying expression of VEGF, STAT3 and phosphorylated STAT3 (p-STAT3) in RCC were evaluated by Western blot.Immunohistochemistry analysis showed that ETK expression was highly increased in RCC and was positively correlated with clinical stage, grade and metastasis. Simultaneously, the overall survival time in patients with higher ETK expression was obviously shorter than that in patients with lower ETK expression. ETK was also detected in RCC cell lines. Moreover, the down-regulating ETK significantly inhibited RCC cell growth,   migration  , invasion and promoted apoptosis. The expression of VEGF and p-STAT3 were also decreased.Our study suggests that the overexpression of ETK is associated with the malignancy and disease progression of RCC. Since ETK is also involved in RCC cell biological function and VEGF-ETK-STAT3 loop, ETK may be used as a potential therapeutic target for RCC. 
  [Ee]xtracellular [Mm]atrix  PubMed  
 Regulation of the PH-domain-containing tyrosine kinase Etk by focal adhesion kinase through the FERM domain. Etk/BMX, a member of the Btk family of tyrosine kinases, is highly expressed in cells with great migratory potential, including endothelial cells and metastatic carcinoma cell lines. Here, we present evidence that Etk is involved in integrin signalling and promotes cell migration. The activation of Etk by   extracellular matrix   proteins is regulated by FAK through an interaction between the PH domain of Etk and the FERM domain of FAK. The lack of Etk activation by   extracellular matrix   in FAK-null cells could be restored by co-transfection with wild-type FAK. Disrupting the interaction between Etk and FAK diminished the cell migration promoted by either kinase. Furthermore, inhibiting Etk expression in metastatic carcinoma cell lines with an antisense oligonucleotide blocks integrin-mediated migration of these cells. Taken together, our data indicate the essential role of the interaction of the PH domain of Etk and the FERM domain of FAK in integrin signalling. 
  [Ii]nvasive  PubMed  
 Etk/Bmx tyrosine kinase activates Pak1 and regulates tumorigenicity of breast cancer cells. Etk/Bmx, a member of the Tec family of nonreceptor protein-tyrosine kinases, is characterized by an N-terminal pleckstrin homology domain and has been shown to be a downstream effector of phosphatidylinositol 3-kinase. P21-activated kinase 1 (Pak1), another well characterized effector of phosphatidylinositol 3-kinase, has been implicated in the progression of breast cancer cells. In this study, we characterized the role of Etk in mammary development and tumorigenesis and explored the functional interactions between Etk and Pak1. We report that Etk expression is developmentally regulated in the mammary gland. Using transient transfection, coimmunoprecipitation and glutathione S-transferase-pull down assays, we showed that Etk directly associates with Pak1 via its N-terminal pleckstrin homology domain and also phosphorylates Pak1 on tyrosine residues. The expression of wild-type Etk in a non-  invasive   human breast cancer MCF-7 cells significantly increased proliferation and anchorage-independent growth of epithelial cancer cells. Conversely, expression of kinase-inactive mutant Etk-KQ suppressed the proliferation, anchorage-independent growth, and tumorigenicity of human breast cancer MDA-MB435 cells. These results indicate that Pak1 is a target of Etk and that Etk controls the proliferation as well as the anchorage-independent and tumorigenic growth of mammary epithelial cancer cells. 
  [Mm]etastasis  PubMed  
 Etk/Bmx regulates proteinase-activated-receptor1 (PAR1) in breast cancer invasion: signaling partners, hierarchy and physiological significance. While protease-activated-receptor 1 (PAR(1)) plays a central role in tumor progression, little is known about the cell signaling involved.We show here the impact of PAR(1) cellular activities using both an orthotopic mouse mammary xenograft and a colorectal-liver   metastasis   model in vivo, with biochemical analyses in vitro. Large and highly vascularized tumors were generated by cells over-expressing wt hPar1, Y397Z hPar1, with persistent signaling, or Y381A hPar1 mutant constructs. In contrast, cells over-expressing the truncated form of hPar1, which lacks the cytoplasmic tail, developed small or no tumors, similar to cells expressing empty vector or control untreated cells. Antibody array membranes revealed essential hPar1 partners including Etk/Bmx and Shc. PAR(1) activation induces Etk/Bmx and Shc binding to the receptor C-tail to form a complex. Y/A mutations in the PAR(1) C-tail did not prevent Shc-PAR(1) association, but enhanced the number of liver metastases compared with the already increased metastases obtained with wt hPar1. We found that Etk/Bmx first binds via the PH domain to a region of seven residues, located between C378-S384 in PAR(1) C-tail, enabling subsequent Shc association. Importantly, expression of the hPar1-7A mutant form (substituted A, residues 378-384), which is incapable of binding Etk/Bmx, resulted in inhibition of invasion through Matrigel-coated membranes. Similarly, knocking down Etk/Bmx inhibited PAR(1)-induced MDA-MB-435 cell migration. In addition, intact spheroid morphogenesis of MCF10A cells is markedly disrupted by the ectopic expression of wt hPar1. In contrast, the forced expression of the hPar1-7A mutant results in normal ball-shaped spheroids. Thus, by preventing binding of Etk/Bmx to PAR(1) -C-tail, hPar1 oncogenic properties are abrogated.This is the first demonstration that a cytoplasmic portion of the PAR(1) C-tail functions as a scaffold site. We identify here essential signaling partners, determine the hierarchy of binding and provide a platform for therapeutic vehicles via definition of the critical PAR(1)-associating region in the breast cancer signaling niche. 
 Expression of tyrosine kinase Etk/Bmx and its relationship with AP-1- and NF-kappaB-associated proteins in hepatocellular carcinoma. Etk/Bmx is a cytoplasmic tyrosine kinase, which was first identified in human bone marrow cells. It has been found to play an important role in the regulation of differentiation and tumorigenicity in some cancers. The aim of this study was to investigate the significance of Etk/Bmx expression in hepatocellular carcinoma (HCC) and the relationship between Etk/Bmx and activated protein-1 (AP-1)- and nuclear factor-kappaB (NF-kappaB)-associated proteins. We used immunohistochemisty to examine 40 cases of human HCC along with corresponding nontumor tissues to assess Etk/Bmx, Jun family (c-Jun, JunB, JunD), Fos family (c-Fos, FosB, Fra-1) and NF-kappaB p65 expression in these samples. Etk/Bmx expression was present in 12 of 40 (30%) HCC specimens, 4 of which among the 25 well-differentiated tumors and 8 among the 15 poorly differentiated tumors, respectively. In contrast, 6 of 40 (15%) cases expressed Etk/Bmx in adjacent nontumor tissues. Expression level and cellular localization of Etk/Bmx were different in cancer cells and nontumor cells. Etk/Bmx expression was correlated with histological differentiation, but not with clinicopathological features including tumor size, HBV infection, cirrhosis, and   metastasis  . There was a close relationship between Etk/Bmx and c-Fos expression in HCC. Etk/Bmx immunopositivity was independent of c-Jun, JunD, FosB, Fra-1 and NF-kappaB p65. Our results indicated that Etk/Bmx may have different biological roles in tumor and nontumor cells, and may be involved in regulating hepatocyte differentiation by c-Fos activation in HCC. 
 The expression and role of tyrosine kinase ETK/BMX in renal cell carcinoma. Expression of the non-receptor tyrosine kinase ETK/BMX has been reported in several solid tumors, but the underlying molecular mechanisms and its clinical significance in renal cell carcinoma (RCC) remain to be elucidated.ETK expression in 90 human RCC and 30 human normal renal tissue samples was examined by immunohistochemistry and compared with several clinicopathologic parameters. To further demonstrate the biological function of ETK in RCC, Western blot was used to test the expression level of ETK protein in RCC cell lines. Subsequent to the downregulation of ETK by small interfering RNA, the effects of ETK on RCC cell growth, apoptosis, migration and invasion were assessed by methyl thiazol tetrazolium assay, flow cytometry and transwell assay. And the varying expression of VEGF, STAT3 and phosphorylated STAT3 (p-STAT3) in RCC were evaluated by Western blot.Immunohistochemistry analysis showed that ETK expression was highly increased in RCC and was positively correlated with clinical stage, grade and   metastasis  . Simultaneously, the overall survival time in patients with higher ETK expression was obviously shorter than that in patients with lower ETK expression. ETK was also detected in RCC cell lines. Moreover, the down-regulating ETK significantly inhibited RCC cell growth, migration, invasion and promoted apoptosis. The expression of VEGF and p-STAT3 were also decreased.Our study suggests that the overexpression of ETK is associated with the malignancy and disease progression of RCC. Since ETK is also involved in RCC cell biological function and VEGF-ETK-STAT3 loop, ETK may be used as a potential therapeutic target for RCC. 
   
  
 TEC 
 
  NCBI Gene  
  GeneRIF  
  Pubmed  
 
 
  Search Term  Rank  Fields  
  [Mm]igration  6  PubMed(1)  
 
 
 hide/show details for TEC 
  [Mm]igration  PubMed  
 Stem cell factor induces phosphatidylinositol 3'-kinase-dependent Lyn/Tec/Dok-1 complex formation in hematopoietic cells. Stem cell factor (SCF) has an important role in the proliferation, differentiation, survival, and   migration   of hematopoietic cells. SCF exerts its effects by binding to cKit, a receptor with intrinsic tyrosine kinase activity. Activation of phosphatidylinositol 3'-kinase (PI3-K) by cKit was previously shown to contribute to many SCF-induced cellular responses. Therefore, PI3-K-dependent signaling pathways activated by SCF were investigated. The PI3-K-dependent activation and phosphorylation of the tyrosine kinase Tec and the adapter molecule p62Dok-1 are reported. The study shows that Tec and Dok-1 form a stable complex with Lyn and 2 unidentified phosphoproteins of 56 and 140 kd. Both the Tec homology and the SH2 domain of Tec were identified as being required for the interaction with Dok-1, whereas 2 domains in Dok-1 appeared to mediate the association with Tec. In addition, Tec and Lyn were shown to phosphorylate Dok-1, whereas phosphorylated Dok-1 was demonstrated to bind to the SH2 domains of several signaling molecules activated by SCF, including Abl, CrkL, SHIP, and PLCgamma-1, but not those of Vav and Shc. These findings suggest that p62Dok-1 may function as an important scaffold molecule in cKit-mediated signaling. 
   
  
 ITK 
 
  NCBI Gene  
  GeneRIF  
  Pubmed  
 
 
  Search Term  Rank  Fields  
  [Mm]igration  6  PubMed(3); GeneRIFs(1)  
 
 
 hide/show details for ITK 
  [Mm]igration  PubMed  
 IL2 Inducible T-cell Kinase, a Novel Therapeutic Target in Melanoma. IL2 inducible T-cell kinase (ITK) promoter CpG sites are hypomethylated in melanomas compared with nevi. The expression of ITK in melanomas, however, has not been established and requires elucidation.An ITK-specific monoclonal antibody was used to probe sections from deidentified, formalin-fixed paraffin-embedded tumor blocks or cell line arrays and ITK was visualized by IHC. Levels of ITK protein differed among melanoma cell lines and representative lines were transduced with four different lentiviral constructs that each contained an shRNA designed to knockdown ITK mRNA levels. The effects of the selective ITK inhibitor BI 10N on cell lines and mouse models were also determined.ITK protein expression increased with nevus to metastatic melanoma progression. In melanoma cell lines, genetic or pharmacologic inhibition of ITK decreased proliferation and   migration   and increased the percentage of cells in the G0-G1 phase. Treatment of melanoma-bearing mice with BI 10N reduced growth of ITK-expressing xenografts or established autochthonous (Tyr-Cre/Pten(null)/Braf(V600E)) melanomas.We conclude that ITK, formerly considered an immune cell-specific protein, is aberrantly expressed in melanoma and promotes tumor development and progression. Our finding that ITK is aberrantly expressed in most metastatic melanomas suggests that inhibitors of ITK may be efficacious for melanoma treatment. The efficacy of a small-molecule ITK inhibitor in the Tyr-Cre/Pten(null)/Braf(V600E) mouse melanoma model supports this possibility. 
 T cell specific adapter protein (TSAd) interacts with Tec kinase ITK to promote CXCL12 induced   migration   of human and murine T cells. The chemokine CXCL12/SDF-1alpha interacts with its G-protein coupled receptor CXCR4 to induce   migration   of lymphoid and endothelial cells. T cell specific adapter protein (TSAd) has been found to promote   migration   of Jurkat T cells through interaction with the G protein beta subunit. However, the molecular mechanisms for how TSAd influences cellular   migration   have not been characterized in detail.We show that TSAd is required for tyrosine phosphorylation of the Lck substrate IL2-inducible T cell kinase (Itk). Presence of Itk Y511 was necessary to boost TSAd's effect on CXCL12 induced   migration   of Jurkat T cells. In addition, TSAd's ability to promote CXCL12-induced actin polymerization and   migration   of Jurkat T lymphocytes was dependent on the Itk-interaction site in the proline-rich region of TSAd. Furthermore, TSAd-deficient murine thymocytes failed to respond to CXCL12 with increased Itk phosphorylation, and displayed reduced actin polymerization and cell   migration   responses.We propose that TSAd, through its interaction with both Itk and Lck, primes Itk for Lck mediated phosphorylation and thereby regulates CXCL12 induced T cell   migration   and actin cytoskeleton rearrangements. 
 Differential sensitivity to Itk kinase signals for T helper 2 cytokine production and chemokine-mediated   migration  . Allergic asthma is dependent on chemokine-mediated Th2 cell   migration   and Th2 cytokine secretion into the lungs. The inducible T cell tyrosine kinase Itk regulates the production of Th2 cytokines as well as   migration   in response to chemokine gradients. Mice lacking Itk are resistant to developing allergic asthma. However, the role of kinase activity of Itk in the development of this disease is unclear. In addition, whether distinct Itk-derived signals lead to T cell   migration   and secretion of Th2 cytokines is also unknown. Using transgenic mice specifically lacking Itk kinase activity, we show that active kinase signaling is required for control of Th2 responses and development of allergic asthma. Moreover, dominant suppression of kinase Itk activity led to normal Th2 responses, but significantly reduced chemokine-mediated   migration  , resulting in prevention of allergic asthma. These observations indicate that signals required for Th2 responses and   migration   are differentially sensitive to Itk activity. Manipulation of Itk's activity can thus provide a new strategy to treat allergic asthma by differentially affecting   migration   of T cells into the lungs, leaving Th2 responses intact. 
  GeneRIFs  
 TSAd, through its interaction with both Itk and Lck, primes Itk for Lck mediated phosphorylation and thereby regulates CXCL12 induced T cell   migration   and actin cytoskeleton rearrangements 
   
  
 TEK 
 
  NCBI Gene  
  GeneRIF  
  Pubmed  
 
 
  Search Term  Rank  Fields  
  [Ff]ilopodia  1  PubMed(1)  
  [Mm]igration  6  Process(3); PubMed(29); GeneRIFs(4); Conserved Domains(1)  
  ECM  7  PubMed(2)  
  [Ee]xtracellular [Mm]atrix  8  PubMed(6); GeneRIFs(1)  
  [Ii]nvasive  10  PubMed(3); GeneRIFs(1)  
  [Mm]etastasis  12  PubMed(12); GeneRIFs(1)  
  [Cc]ell [Ii]nvasion  13  PubMed(4)  
 
 
 hide/show details for TEK 
  [Ff]ilopodia  PubMed  
 Angiopoietin-1 promotes lymphatic sprouting and hyperplasia. Angiopoietin 1 (Ang1), a ligand for the receptor tyrosine kinase Tie2, regulates the formation and stabilization of the blood vessel network during embryogenesis. In adults, Ang1 is associated with blood vessel stabilization and recruitment of perivascular cells, whereas Ang2 acts to counter these actions. Recent results from gene-targeted mice have shown that Ang2 is also essential for the proper patterning of lymphatic vessels and that Ang1 can be substituted for this function. In order to characterize the effects of the angiopoietins on lymphatic vessels, we employed viral vectors for overexpression of Ang1 in adult mouse tissues. We found that Ang1 activated lymphatic vessel endothelial proliferation, vessel enlargement, and generation of long endothelial cell   filopodia   that eventually fused, leading to new sprouts and vessel development. Cutaneous lymphatic hyperplasia was also detected in transgenic mice expressing Ang1 in the basal epidermal cells. Tie2 was expressed in the lymphatic endothelial cells and Ang1 stimulation of these cells resulted in up-regulation of vascular endothelial growth factor receptor 3 (VEGFR-3). Furthermore, a soluble form of VEGFR-3 inhibited the observed lymphatic sprouting. Our results reinforce the concept that Ang1 therapy may be useful in settings of tissue edema. 
  [Mm]igration  Process  
 leukocyte   migration   
 positive regulation of endothelial cell   migration   
 positive regulation of endothelial cell   migration   
  PubMed  
 Adaptor ShcA protein binds tyrosine kinase Tie2 receptor and regulates   migration   and sprouting but not survival of endothelial cells. Angiopoietin-1 can promote   migration  , sprouting, and survival of endothelial cells through activation of different signaling pathways triggered by the Tie2 tyrosine kinase receptor. ShcA adapter proteins are targets of activated tyrosine kinases and are implicated in the transmission of activation signals to the Ras/mitogen-activated protein kinase pathway. Here we report the identification of an interaction between the adapter protein ShcA and the cytoplasmic domain of Tie2 through in vitro co-immunoprecipitation analysis. Stimulation of endogenous Tie2 in endothelial cells with its ligand angiopoietin-1 increased its association with ShcA and phosphorylation of the adapter protein. The interaction requires the SH2 domain of ShcA and the tyrosine phosphorylation of Tie2 as shown by pull-down experiments. Furthermore, Tyr-1101 of Tie2 was identified as the primary binding site for the SH2 domain of ShcA. Overexpression of a dominant-negative form of ShcA affects angiopoietin-1-induced chemotaxis and sprouting, although it has no effect on survival of endothelial cells. Furthermore, this mutant partially reduces the tyrosine phosphorylation of the regulatory p85 subunit of phosphatidylinositol 3-kinase. Together, our results identified a novel interaction between Tie2 with the adapter molecule ShcA and suggested that this interaction may play a role in the regulation of   migration   and three-dimensional organization of endothelial cells induced by angiopoietin-1. 
 Adaptor ShcA protein binds tyrosine kinase Tie2 receptor and regulates   migration   and sprouting but not survival of endothelial cells. Angiopoietin-1 can promote   migration  , sprouting, and survival of endothelial cells through activation of different signaling pathways triggered by the Tie2 tyrosine kinase receptor. ShcA adapter proteins are targets of activated tyrosine kinases and are implicated in the transmission of activation signals to the Ras/mitogen-activated protein kinase pathway. Here we report the identification of an interaction between the adapter protein ShcA and the cytoplasmic domain of Tie2 through in vitro co-immunoprecipitation analysis. Stimulation of endogenous Tie2 in endothelial cells with its ligand angiopoietin-1 increased its association with ShcA and phosphorylation of the adapter protein. The interaction requires the SH2 domain of ShcA and the tyrosine phosphorylation of Tie2 as shown by pull-down experiments. Furthermore, Tyr-1101 of Tie2 was identified as the primary binding site for the SH2 domain of ShcA. Overexpression of a dominant-negative form of ShcA affects angiopoietin-1-induced chemotaxis and sprouting, although it has no effect on survival of endothelial cells. Furthermore, this mutant partially reduces the tyrosine phosphorylation of the regulatory p85 subunit of phosphatidylinositol 3-kinase. Together, our results identified a novel interaction between Tie2 with the adapter molecule ShcA and suggested that this interaction may play a role in the regulation of   migration   and three-dimensional organization of endothelial cells induced by angiopoietin-1. 
 Expression and function of the angiopoietin receptor Tie-2 in human eosinophils. Increased vascularity of bronchial mucosa is closely related to the expression of angiogenic factors, which contribute to the pathogenesis of diseases such as asthma bronchiale.Here we examine the effects of the angiogenic growth factors angiopoietin 1 and angiopoietin 2 on eosinophil function in vitro and possible involvement of the angiopoietin receptor Tie-2.Eosinophil   migration   was studied by micropore filter assays. Signaling mechanisms required for angiopoietin-dependent   migration   were tested by using signaling enzyme blockers. Tie-2 mRNA and receptor expression on the cell surface of eosinophils was demonstrated in RT-PCR and by fluorescence-activated cell sorting analysis.Angiopoietin 1 significantly stimulated eosinophil chemotaxis via activation of phosphodiesterase, phosphatidylinositol 3'-kinase, and tyrosine kinases. The effect on eosinophil   migration   of angiopoietin 1 was reversed by an antibody against the Tie-2 receptor and by angiopoietin 2. Incubation of eosinophils with angiopoietin 1 abolished the chemotactic effects of vascular endothelial growth factor on human eosinophils via the Tie-2 receptor. Finally, Tie-2 expression by human eosinophils was demonstrated on the transcriptional and protein level.Data suggest that angiopoietin 1 stimulates directed   migration   and possibly inhibits vascular endothelial growth factor-induced eosinophil chemotaxis via its Tie-2 receptor, which is expressed by eosinophils. Thus, angiopoietin 1 may play an important role in the modulation of eosinophilic inflammation. 
 IL-35 Inhibits Angiogenesis through VEGF/Ang2/Tie2 Pathway in Rheumatoid Arthritis. The pro-angiogenic factors vascular endothelial growth factor (VEGF) and angiopoietins (Angs) play a prominent role in synovial angiogenesis, an early and critical event in the pathogenesis of rheumatoid arthritis (RA). Interleukin (IL)-35 is an anti-inflammatory cytokine that attenuates collagen-induced arthritis, however, the mechanisms involved are not fully understood.The effects of IL-35 on endothelial cell   migration  , adhesion, and tube formation were examined using human umbilical vein endothelial cells (HUVEC) in vitro. The effects of IL-35 on vessel formation in vivo were examined using a murine Matrigel plugs model. MMP2/MMP9 and IL-6/IL-8 secretion were assessed by zymography and ELISA, respectively. The crosstalk between IL-35, VEGF, and Ang2 in HUVECs and RA synovial tissue explants was investigated.IL-35 inhibited basal and VEGF-induced HUVEC   migration   and adhesion in vitro as well as tube formation in vitro and in vivo. VEGF increased Ang2 secretion by HUVECs and RA synovial tissue explants, and exogenous Ang2 promoted HUVEC   migration  , adhesion, and tube formation with similar potency to VEGF. Blocking the Ang/Tie2 pathway with a Tie2 kinase antibody inhibited the proangiogenic effects of exogenous Ang2 and VEGF in HUVECs. IL-35 inhibited basal and VEGF-induced Ang2 secretion by HUVECs and RA synovial tissue explants; it also antagonized the proangiogenic effects of exogenous Ang2 in HUVECs. Moreover, IL-35 reduced basal and VEGF/Ang2-induced MMP2/MMP9 and IL-6/IL-8 secretion.These results suggested that IL-35 restrains RA angiogenesis and inflammation by downregulating basal and VEGF-induced Ang2 secretion as well as disrupting Ang2/Tie2 signal transduction. Our findings extend current understanding of mechanisms regulating RA angiogenesis and may support development of novel angiogenesis-targeting therapeutics for RA treatment. 
 A designed angiopoietin-2 variant, pentameric COMP-Ang2, strongly activates Tie2 receptor and stimulates angiogenesis. Despite that angiopoietin-2 (Ang2) produces more versatile and dynamic functions than angiopoietin-1 (Ang1) in angiogenesis and inflammation, the molecular mechanism that underlies this difference is still unknown. To define the role of oligomerization of Ang2 in activation of its receptor, Tie2, we designed and generated different oligomeric forms of Ang2 by replacement of the amino-terminal domains of Ang2 with dimeric, tetrameric, and pentameric short coiled-coil domains derived from GCN4, matrillin-1, and COMP. COMP-Ang2 strongly binds and activates Tie2, whereas GCN4-Ang2 and MAT-Ang2 weakly to moderately bind and activate Tie2. Although native Ang2 strongly binds to Tie2, it does not activate Tie2. Accordingly, COMP-Ang2 strongly promotes endothelial cell survival,   migration  , and tube formation in a Tie2-dependent manner, and the potency of COMP-Ang2 is almost identical to that of COMP-Ang1. Furthermore, the potency of COMP-Ang2-induced enhanced angiogenesis in the wound healing region is almost identical to the potency of COMP-Ang1-induced enhanced angiogenesis. Overall, there is no obvious difference between COMP-Ang2 and COMP-Ang1 in in vitro and in vivo angiogenesis. Our results provide compelling evidence that proper oligomerization of Ang2 is a critical determinant of its binding and activation of Tie2. 
 Tie2-R849W mutant in venous malformations chronically activates a functional STAT1 to modulate gene expression. Tie2 is an endothelial receptor tyrosine kinase. An amino-acid substitution of tryptophan for arginine at residue 849 (Tie2-R849W) leads to a ligand-independent activation of its kinase activity. This mutation has been associated with familial venous malformations (VMs), manifested by variable thickness or lack of smooth-muscle cells in the veins of patient lesions. The underlying mechanism for Tie2-R849W action in endothelial cells remains elusive. In this study, we used adenoviral infection to differentiate the effects of ectopic Tie2 (wild type, kinase-dead K855A, or constitutively active R849W) expression on endothelial cellular behaviors and Tie2-mediated downstream targets. Ectopic Tie2 reduced endothelial cell proliferation and serum withdrawal-induced apoptosis, while stimulating   migration  . When comparing R849W with K855A and its wild-type counterpart, a functional tyrosine kinase activity was required only for   migration  , and constitutively active Tie2-R849W conferred highest resistance to serum-induced apoptosis, but lowest ability to maintain tube-like structures formed on Matrigel. We further demonstrated that Tie2-R849W chronically induced STAT1 tyrosine phosphorylation and the promoter activity of STAT1-responsive IFN-regulatory factor 1 (IRF1). Although STAT1 phosphorylation required JNK and p38MAPK activation, only JNK activation was essential for IRF1 promoter activation by Tie2-R849W. Additional studies are needed to study the role of STAT1 activation in VMs. 
 A unique autophosphorylation site on Tie2/Tek mediates Dok-R phosphotyrosine binding domain binding and function. Tie2/Tek is an endothelial cell receptor tyrosine kinase that induces signal transduction pathways involved in cell   migration   upon angiopoietin-1 (Ang1) stimulation. To address the importance of the various tyrosine residues of Tie2 in signal transduction, we generated a series of Tie2 mutants and examined their signaling properties. Using this approach in conjunction with a phosphorylation state-specific antibody, we identified tyrosine residue 1106 on Tie2 as an Ang1-dependent autophosphorylation site that mediates binding and phosphorylation of the downstream-of-kinase-related (Dok-R) docking protein. This tyrosine residue is contained within a unique interaction motif for the phosphotyrosine binding domain of Dok-R, and the pleckstrin homology domain of Dok-R further contributes to Tie2 binding in a phosphatidylinositol 3'-kinase-dependent manner. Introduction of a Tie2 mutant lacking tyrosine residue 1106 into endothelial cells interferes with Dok-R phosphorylation in response to Ang1. Furthermore, this mutant is unable to restore the   migration   potential of endothelial cells derived from mice lacking Tie2. Together, these findings demonstrate that tyrosine residue 1106 on Tie2 is critical for coupling downstream cell   migration   signal transduction pathways with Ang1 stimulation in endothelial cells. 
 Tie1-Tie2 interactions mediate functional differences between angiopoietin ligands. The Tie family of endothelial-specific receptor tyrosine kinases is essential for cell proliferation,   migration  , and survival during angiogenesis. Despite considerable similarity, experiments with Tie1- or Tie2-deficient mice highlight distinct functions for these receptors in vivo. The Tie2 receptor is further unique with respect to its structurally homologous ligands. Angiopoietin-2 and -3 can function as agonists or antagonists; angiopoietin-1 and -4 are constitutive agonists. To address the role of Tie1 in angiopoietin-mediated Tie2 signaling and determine the basis for the behavior of the individual angiopoietins, we used an in vivo FRET-based proximity assay to monitor Tie1 and -2 localization and association. We provide evidence for Tie1-Tie2 complex formation on the cell surface and identify molecular surface areas essential for receptor-receptor recognition. We further demonstrate that the Tie1-Tie2 interactions are dynamic, inhibitory, and differentially modulated by angiopoietin-1 and -2. Based on the available data, we propose a unified model for angiopoietin-induced Tie2 signaling. 
 STAT1 activation by venous malformations mutant Tie2-R849W antagonizes VEGF-A-mediated angiogenic response partly via reduced bFGF production. A missense mutation from arginine to tryptophan at residue 849 in the kinase domain of Tie2 (Tie2-R849W) is commonly identified in familial venous malformations. The mechanistic action of Tie2-R849W variant expression on angiogenic cascades including smooth muscle cell recruitment, however, remains elusive. To avoid confounding factors from endogenous Tie2 expression, Tie2-depleted endothelial cells (ECs) were used to study the effects of ectopic shRNA-resistant Tie2 variant expression, Tie2-WT* and Tie2-R849W*, on vascular cell proliferation,   migration  , tube formation, and smooth muscle cell (SMC) recruitment. Tie2-R849W* induced STAT1 phosphorylation at Tyr701. Tie2-R849W*-expressing cells had reduced ability to migrate and form tubes on Matrigel than their wildtype counterparts. STAT1 phosphorylation attenuated VEGF-A-induced STAT3 tyrosine phosphorylation in Tie2-R849W*-expressing HUVECs. The induced STAT1 activation also decreased VEGF-A-induced bFGF mRNA expression by competing with activated STAT3 for a direct binding to the consensus STAT-binding site at positions -997 to -989 bp from transcription start site in the bFGF promoter. Depleting STAT1 expression rescued the inability of Tie2-R849W expression to mediate angiogenesis. Moreover, bFGF neutralization or constitutive STAT1 activation, reminiscence of Tie2-R849W* expression, suppressed the smooth muscle cell recruiting ability of endothelial conditioned medium. This work reveals an anti-angiogenic role of STAT1 activation that acts in Tie2-R849W-expressing ECs to impair VEGF-A-mediated STAT3 signaling, bFGF production, and smooth muscle cell recruitment. A balancing activity of STAT1 and STAT3 may be important for Tie2-mediated vascular homeostasis. 
 Regulation of angiopoietin-1/Tie-2 receptor signaling in endothelial cells by dual-specificity phosphatases 1, 4, and 5. Angiopoietin-1 (Ang-1) promotes survival and   migration   of endothelial cells, in part through the activation of mitogen-activated protein kinase (MAPK) pathways downstream of Tie-2 receptors. Dual-specificity phosphatases (DUSPs) dephosphorylate phosphotyrosine and phosphoserine/phosphothreonine residues on target MAPKs. The mechanisms by which DUSPs modulate MAPK activation in Ang-1/Tie-2 receptor signaling are unknown in endothelial cells.Expression of various DUSPs in human umbilical vein endothelial cells exposed to Ang-1 was measured. The functional roles of DUSPs in Ang-1-induced regulation of MAPK activation, endothelial cell survival,   migration  , differentiation, and permeability were measured using selective siRNA oligos. Ang-1 differentially induces DUSP1, DUSP4, and DUSP5 in human umbilical vein endothelial cells through activation of the PI-3 kinase, ERK1/2, p38, and SAPK/JNK pathways. Lack-of-function siRNA screening revealed that DUSP1 preferentially dephosphorylates p38 protein and is involved in Ang-1-induced cell   migration   and differentiation. DUSP4 preferentially dephosphorylates ERK1/2, p38, and SAPK/JNK proteins and, under conditions of serum deprivation, is involved in Ang-1-induced cell   migration  , several antiapoptotic effects, and differentiation. DUSP5 preferentially dephosphorylates ERK1/2 proteins and is involved in cell survival and inhibition of permeability.DUSP1, DUSP4, and DUSP5 differentially modulate MAPK signaling pathways downstream of Tie-2 receptors, thus highlighting the importance of these phosphatases to Ang-1-induced angiogenesis. 
 Roles of reactive oxygen species in angiopoietin-1/tie-2 receptor signaling. In this study we identified the involvement of reactive oxygen species (ROS) in signaling and biological effects of the angiopoietin-1 (Ang-1)/tie-2 receptor pathway. Exposure of human umbilical vein endothelial cells to Ang-1 (50 ng/ml) induced rapid and transient production of ROS, particularly superoxide anions. ROS production was attenuated by preincubation with a peptide (gp91ds-tat) that inhibits the association of the gp91(phox) subunit with the p47(phox) subunit of NADPH oxidase and by the expression of a dominant-negative form of Rac-1 (Rac1N17). These results suggest that ROS production in response to Ang-1 exposure originates mainly from a Rac-1-dependent NADPH oxidase. Overexpression of antioxidants (superoxide dismutase and catalase) and Rac1N17, as well as preincubation with selective inhibitors of NADPH oxidase augmented basal p38 phosphorylation, inhibited Ang-1-induced PAK-1 phosphorylation and potentiated Ang-1-induced Erk1/2 phosphorylation but had no influence on AKT and SAPK/JNK phosphorylation by Ang-1. Exposure to Ang-1 (100 ng/ml) for 5 h induced a threefold increase in endothelial cell   migration  , a response that was strongly inhibited by overexpression of antioxidants, Rac1N17, and selective NADPH oxidase inhibitors. We conclude that activation of tie-2 receptors by Ang-1 triggers the production of ROS through activation of NADPH oxidase and that ROS generation by Ang-1 promotes endothelial cell   migration   while negatively regulating Erk1/2 phosphorylation. 
 Interfering with VE-PTP stabilizes endothelial junctions in vivo via Tie-2 in the absence of VE-cadherin. Vascular endothelial (VE)-protein tyrosine phosphatase (PTP) associates with VE-cadherin, thereby supporting its adhesive activity and endothelial junction integrity. VE-PTP also associates with Tie-2, dampening the tyrosine kinase activity of this receptor that can support stabilization of endothelial junctions. Here, we have analyzed how interference with VE-PTP affects the stability of endothelial junctions in vivo. Blocking VE-PTP by antibodies, a specific pharmacological inhibitor (AKB-9778), and gene ablation counteracted vascular leak induction by inflammatory mediators. In addition, leukocyte trans  migration   through the endothelial barrier was attenuated. Interference with Tie-2 expression in vivo reversed junction-stabilizing effects of AKB-9778 into junction-destabilizing effects. Furthermore, lack of Tie-2 was sufficient to weaken the vessel barrier. Mechanistically, inhibition of VE-PTP stabilized endothelial junctions via Tie-2, which triggered activation of Rap1, which then caused the dissolution of radial stress fibers via Rac1 and suppression of nonmuscle myosin II. Remarkably, VE-cadherin gene ablation did not abolish the junction-stabilizing effect of the VE-PTP inhibitor. Collectively, we conclude that inhibition of VE-PTP stabilizes challenged endothelial junctions in vivo via Tie-2 by a VE-cadherin-independent mechanism. In the absence of Tie-2, however, VE-PTP inhibition destabilizes endothelial barrier integrity in agreement with the VE-cadherin-supportive effect of VE-PTP. 
 NERF2, a member of the Ets family of transcription factors, is increased in response to hypoxia and angiopoietin-1: a potential mechanism for Tie2 regulation during hypoxia. Vascular endothelial growth factor (VEGF) and angiopoietins regulate endothelial cell survival and   migration   and are essential for angiogenesis. Considerable progress has been made towards understanding hypoxia-mediated regulation of VEGF and its receptors. In contrast, little is known about the regulation of angiopoietins and their receptors in hypoxic cells. Using RT-PCR, RNAase protection assay, and Western blotting, we found that Tie1 and Tie2 mRNA and protein levels increased in response to hypoxia in human umbilical vein endothelial cells. Previously, we have shown that NERF2, a member of Ets family of transcription factors that is specifically expressed in endothelial cells, binds to the promoter region of Tie2 and transactivates Tie2 expression. In this study, we show that expression of NERF2 was increased under hypoxia and that this increase temporally correlated with the increase in Tie2 expression. Hypoxia-induced expression of NERF2 and Tie2 was blocked by angiopoietin-2, a competitive inhibitor of angiopoietin-1, and by recombinant soluble extracellular domain of Tie2 but not by VEGF-neutralizing antibodies. In addition, angiopoietin-1 directly induced expression of NERF2 in quiescent cells. These novel findings suggest that angiopoietin-1 regulates expression of NERF2 and its own receptor in hypoxic cells. 
 Wnt5a signaling induces proliferation and survival of endothelial cells in vitro and expression of MMP-1 and Tie-2. Wnts are lipid-modified secreted glycoproteins that regulate diverse biological processes. We report that Wnt5a, which functions in noncanonical Wnt signaling, has activity on endothelial cells. Wnt5a is endogenously expressed in human primary endothelial cells and is expressed in murine vasculature at several sites in mouse embryos and tissues. Expression of exogenous Wnt5a in human endothelial cells promoted angiogenesis. Wnt5a induced noncanonical Wnt signaling in endothelial cells, as measured by Dishevelled and ERK1/2 phosphorylation, and inhibition of canonical Wnt signaling, a known property of Wnt5a. Wnt5a induced endothelial cell proliferation and enhanced cell survival under serum-deprived conditions. The Wnt5a-mediated proliferation was blocked by Frizzled-4 extracellular domain. Wnt5a expression enhanced capillary-like network formation, whereas reduction of Wnt5a expression decreased network formation. Reduced Wnt5a expression inhibited endothelial cell   migration  . Screening for Wnt5a-regulated genes in cultured endothelial cells identified several encoding angiogenic regulators, including matrix metalloproteinase-1, an interstitial collagenase, and Tie-2, a receptor for angiopoietins. Thus, Wnt5a acts through noncanonical Wnt signaling to promote angiogenesis. 
 COMP-Ang1 enhances DNA synthesis and cell cycle progression in human periodontal ligament cells via Tie2-mediated phosphorylation of PI3K/Akt and MAPKs. Recombinant COMP-Ang1, a chimera of angiopoietin-1 (Ang1), and a short coiled-coil domain of cartilage oligomeric matrix protein (COMP) can stimulate multiple cellular processes. Proliferative capacity of periodontal ligament (PDL) fibroblasts (PLFs) is important for maintaining PDL integrity and homeostasis. In this study, we explored whether exogenous COMP-Ang1 addition enhances proliferation of human PLFs and the cellular mechanisms therein. We initially isolated and characterized PLFs, where the cells showed highly positive staining for surface markers, CD90, CD105, and CD146. COMP-Ang1 treatment increased proliferation of PLFs by stimulating   migration   of cells into S and G2/M phases. This increase was coupled with decreased p21(Cip) and p27(Kip) levels and enhanced cyclin D1, cyclin-dependent kinase (CDK) 2, and CDK4 induction. Transfection with si-Tie2 near completely blocked COMP-Ang1-stimulated cell cycle progression in PLFs. Tie2 knockdown also inhibited COMP-Ang1-induced phosphorylation of mitogen-activated protein kinases (MAPKs). In addition, COMP-Ang1-mediated activation of Akt and c-Jun was suppressed by treating each of pharmacological inhibitors specific to phosphoinositide 3-kinase (PI3K) (LY294002 and Wortmannin) or MAPKs (PD98059, SB203580, and SP600125). Similarly, COMP-Ang1-mediated increases in DNA synthesis and cyclin D1 induction were prevented by treating inhibitor of MAPKs and PI3K or by c-Jun knockdown. These results suggest that COMP-Ang1 enhances survival and proliferation of human PLFs through the activation of Tie2-mediated signaling, where PI3K/Akt and MAPK-c-Jun signaling pathways act as downstream effectors. Collectively, COMP-Ang1 may be a useful as a stimulator of human PLFs and therefore improves PDL integrity and homeostasis. 
 Identification of Tek/Tie2 binding partners. Binding to a multifunctional docking site mediates cell survival and   migration  . The Tek/Tie2 receptor tyrosine kinase plays a pivotal role in vascular and hematopoietic development. To study the signal transduction pathways that are mediated by this receptor, we have used the yeast two-hybrid system to identify signaling molecules that associate with the phosphorylated Tek receptor. Using this approach, we demonstrate that five molecules, Grb2, Grb7, Grb14, Shp2, and the p85 subunit of phosphatidylinositol 3-kinase can interact with Tek in a phosphotyrosine-dependent manner through their SH2 domains. Mapping of the binding sites of these molecules on Tek reveals the presence of a multisubstrate docking site in the carboxyl tail of Tek (Tyr(1100)). Mutation of this site abrogates binding of Grb2 and Grb7 to Tek in vivo, and this site is required for tyrosine phosphorylation of Grb7 and p85 in vivo. Furthermore, stimulation of Tek-expressing cells with Angiopoietin-1 results in phosphorylation of both Tek and p85 and in activation of endothelial cell   migration   and survival pathways that are dependent in part on phosphatidylinositol 3-kinase. Taken together, these results demonstrate that Angiopoietin-1-induced signaling from the Tek receptor is mediated by a multifunctional docking site that is responsible for activation of both cell   migration   and cell survival pathways. 
 Biological characterization of angiopoietin-3 and angiopoietin-4. The angiopoietin (Ang) family of growth factors includes Ang1, Ang2, Ang3, and Ang4, all of which bind to the endothelial receptor tyrosine kinase Tie2. Ang3 (mouse) and Ang4 (human) are interspecies orthologs. In experiments with human endothelial cell lines, Ang3 was identified as an antagonist of Tie2 and Ang4 was identified as an agonist of Tie2. However, the biological roles of Ang3 and Ang4 are unknown. We examined the biological effect of recombinant Ang3 and Ang4 proteins in primary cultured endothelial cells and in vivo in mice. Recombinant Ang3 and Ang4 formed disulfide-linked dimers. Ang4 (400 ng/mL) markedly increased Tie2 and Akt phosphorylation in primary cultured HUVECs whereas Ang3 (400 ng/mL) did not produce significant changes. Accordingly, Ang4, but not Ang3, induced survival and   migration   in primary cultured HUVECs. Unexpectedly, intravenously administered Ang3 (30 microg) was more potent than Ang4 (30 microg) in phosphorylating the Tie2 receptor in lung tissue from mice in vivo. Accordingly, Ang3 was more potent than Ang4 in phosphorylating Akt in primary cultured mouse lung microvascular endothelial cells. Ang3 and Ang4 both produced potent corneal angiogenesis extending from the limbus across the mouse cornea in vivo. Thus, Ang3 and Ang4 are agonists of Tie2, but mouse Ang3 has strong activity only on endothelial cells of its own species. 
 The association of the angiopoietin/Tie-2 system with the development of metastasis and leukocyte   migration   in neuroendocrine tumors. The aim of this study was to explore the possible involvement of the angiopoietin (Ang)-1, -2/Tie-2 system in the development, growth, and metastases evolution of gastroenteropancreatic-neuroendocrine tumors (GEP-NETs). We prospectively examined the serum levels of Tie-2, Ang-1, and Ang-2 by ELISA in 42 patients with proven GEP-NETs and 27 controls. We also determined the expression of the Ang/Tie-2 system in freshly isolated peripheral blood monocytes and in tumor cells from malignant primary tumors and/or liver metastases samples from GEP-NET patients by flow cytometry and/or RT-PCR. Furthermore, the function of the Ang/Tie-2 system in monocytes from controls and patients was assessed by a chemotaxis assay. GEP-NET patients showed enhanced serum levels of soluble form of Tie-2 (sTie-2), Ang-1, and Ang-2 (P<0.05 in all cases), compared to controls. sTie-2 and Ang-2 levels were significantly higher in GEP-NETs with metastases compared to those with no metastases. In addition, a significant correlation was detected between Ang-2 levels and chromogranin A or sTie-2 concentrations or 5-hydroxy-indole acetic acid excretion (r=0.71, r=0.60, and r=0.81 respectively, P<0.01 in all cases). Furthermore, we observed an enhanced expression of Ang-1, Ang-2, and Tie-2 in freshly isolated tumor cells from GEP-NET both by immunohistochemistry and by RT-PCR. Interestingly, an enhanced expression and function of Tie-2 was detected in monocytes from GEP-NET patients. Our data suggest that the Ang/Tie-2 system is involved in the growth and development of metastases of GEP-NETs, and that favors the recruitment of Tie-2(+) monocytes to the tumor site, where they can promote inflammation and angiogenesis. 
 Angiopoietin affects neutrophil   migration  . After an ischemic event vascular growth factors are involved in regulating leukocyte infiltration in inflammatory processes. This study focused on effects of 2 other angiogenic growth factors, angiopoietin-1 and angiopoietin-2, on human neutrophils and on the involvement of the angiopoietin receptor Tie-2.Neutrophils were from venous blood of healthy donors and cell   migration   was studied by micropore filter assays. Receptor expression was investigated by reverse transcriptase-polymerase chain reaction (PCR) for mRNA and fluorescence-activated cell-sorter scanner (FACS) analysis. Signaling mechanisms required for angiopoietin-dependent effects were tested functionally by using signaling enzyme blockers.The angiopoietins were chemotactic for neutrophils. They showed antagonistic effects on each other and both inhibited VEGF-directed   migration   of neutrophils. The effects of both angiopoietins were Tie-2 dependent. Tie-2 receptor immunoreactivity was confirmed on neutrophils by FACS. De novo synthesis is suggested by Tie-2 receptor mRNA expression as demonstrated by reverse transcriptase PCR.Data suggest that a Tie-2 receptor is expressed by human neutrophils whose active site ligation with either angiopoietin-1 or angiopoietin-2 exerts migratory effects on the one hand and arrests VEGF-mediated chemotaxis on the other. These effects suggest a role of angiopoietins in modulating neutrophilic inflammation. 
 Critical role for GATA3 in mediating Tie2 expression and function in large vessel endothelial cells. Endothelial phenotypes are highly regulated in space and time by both transcriptional and post-transcriptional mechanisms. There is increasing evidence that the GATA family of transcription factors function as signal transducers, coupling changes in the extracellular environment to changes in downstream target gene expression. Here we show that human primary endothelial cells derived from large blood vessels express GATA2, -3, and -6. Of these factors, GATA3 was expressed at the highest levels. In DNA microarrays of human umbilical vein endothelial cells (HUVEC), small interfering RNA-mediated knockdown of GATA3 resulted in reduced expression of genes associated with angiogenesis, including Tie2. At a functional level, GATA3 knockdown inhibited angiopoietin (Ang)-1-mediated but not vascular endothelial cell growth factor (VEGF)-mediated AKT signaling, cell   migration  , survival, and tube formation. In electrophoretic gel mobility shift assays and chromatin immunoprecipitation, GATA3 was shown to bind to regulatory regions within the 5'-untranslated region of the Tie2 gene. In co-immunoprecipitation and co-transfection assays, GATA3 and the Ets transcription factor, ELF1, physically interacted and synergized to transactivate the Tie2 promoter. GATA3 knockdown blocked the ability of Ang-1 to attenuate vascular endothelial cell growth factor stimulation of vascular cell adhesion molecule-1 expression and monocytic cell adhesion. Moreover, exposure of human umbilical vein endothelial cells to tumor necrosis factor-alpha resulted in marked down-regulation of GATA3 expression and reduction in Tie2 expression. Together, these findings suggest that GATA3 is indispensable for Ang-1-Tie2-mediated signaling in large vessel endothelial cells. 
 Epithin/PRSS14 proteolytically regulates angiopoietin receptor Tie2 during transendothelial   migration  . Epithin/PRSS14, a type II transmembrane serine protease, is involved in normal epithelial development and tumor progression. Here we report, as an interacting substrate of epithin, a receptor tyrosine kinase Tie2 that is well known for important roles in the vessel stability. Epithin interacts with and degrades the Tie2 extracellular portion that contains the ligand-binding domain. Epithin is located in the neighbor of Tie2-expressing vessels in normal tissue. Furthermore, epithin can cleave and degrade Tie2 not only in the same cell but also from neighboring cells nearby, resulting in the degradation of the Tie2 ectodomain. The remaining Tie2 fragment was highly phosphorylated and was able to recruit a downstream effector, phosphatidylinositol 3-kinase. Knocking down epithin expression using short hairpin RNA in thymoma cell severely impaired the   migration   through endothelial cells that show the actin rearrangement during the process. The diminution of epithin protein expression in 4T1 breast cancer cells caused the significant decrease in the number of transendothelial migrating cells in vitro as well as in those of metastasizing tumor nodules in vivo, Therefore, we propose that epithin, which regulates endothelial Tie2 functions, plays a critical role in the fine tuning of transendothelial   migration   for normal and cancer cells. 
 Angiopoietin-2 differentially regulates angiogenesis through TIE2 and integrin signaling. Angiopoietin-2 (ANG-2) is a key regulator of angiogenesis that exerts context-dependent effects on ECs. ANG-2 binds the endothelial-specific receptor tyrosine kinase 2 (TIE2) and acts as a negative regulator of ANG-1/TIE2 signaling during angiogenesis, thereby controlling the responsiveness of ECs to exogenous cytokines. Recent data from tumors indicate that under certain conditions ANG-2 can also promote angiogenesis. However, the molecular mechanisms of dual ANG-2 functions are poorly understood. Here, we identify a model for the opposing roles of ANG-2 in angiogenesis. We found that angiogenesis-activated endothelium harbored a subpopulation of TIE2-negative ECs (TIE2lo). TIE2 expression was downregulated in angiogenic ECs, which abundantly expressed several integrins. ANG-2 bound to these integrins in TIE2lo ECs, subsequently inducing, in a TIE2-independent manner, phosphorylation of the integrin adaptor protein FAK, resulting in RAC1 activation,   migration  , and sprouting angiogenesis. Correspondingly, in vivo ANG-2 blockade interfered with integrin signaling and inhibited FAK phosphorylation and sprouting angiogenesis of TIE2lo ECs. These data establish a contextual model whereby differential TIE2 and integrin expression, binding, and activation control the role of ANG-2 in angiogenesis. The results of this study have immediate translational implications for the therapeutic exploitation of angiopoietin signaling. 
 Isolation and characterization of endothelial cells from intramuscular hemangioma. Intramuscular hemangiomas (IMHs) are benign vascular tumors of deep soft tissue characterized by endothelial cell (EC) proliferation. The purpose of this study was to isolate ECs from IMH, characterize their angiogenic phenotype and functional characteristics, and search for a possible signaling pathway related to IMH development.EC Isolation from IMH was performed by digestion, filtration, washing, incubation, and purification in sequence. Tie2 expression was compared between ECs from IMH and controls using reverse transcriptase polymerase chain reaction (RT-PCR). Cell invasion and proliferation assays were used to analyze functional responses of ECs to angiopoietin 1 (Ang1) and vascular endothelial growth factor (VEGF). Expression of downstream targets was analyzed using Western blot analysis.Isolated ECs showed typical cobblestone appearance under light microscopy and formed capillary-like tubular structures using Matrigel tube-forming assay. RT-PCR of isolated ECs from six patients showed increased expression of Tie2 and VEGF receptor 1 (VEGFR1) compared with control ECs. Tie2 activation by Ang1 compared with VEGFR1 by VEGF resulted in increased EC   migration   and proliferation. Western blot analysis showed increased Tie2 expression in hemangioma samples compared with normal ECs. Phosphorylated Akt and phosphorylated forkhead box O1 (FOXO1) expression was observed in hemangioma samples only.EC isolation from IMH could be a useful tool for further research. These results suggest that increased Tie2 expression, via Akt-FOXO1 pathway activation, may play an important role in IMH pathogenesis. 
 Chemotactic properties of angiopoietin-1 and -2, ligands for the endothelial-specific receptor tyrosine kinase Tie2. Angiopoietin-1 and its putative natural antagonist, angiopoietin-2, were recently isolated, and the critical role of angiopoietin-1 in embryogenic angiogenesis was demonstrated by targeted gene disruption. Specific biological effects of angiopoietin-1, however, have yet to be defined. In this study we demonstrate that angiopoietin-1, but not angiopoietin-2, is chemotactic for endothelial cells. In contrast, angiopoietin-1 as well as angiopoietin-2 exhibit no proliferative effect on endothelial cells. Excess soluble Tie2, but not Tie1 receptor, abolish the chemotactic response of endothelial cells toward angiopoietin-1. Angiopoietin-2 dose-dependently blocks directed   migration   toward angiopoietin-1, consistent with the role of angiopoietin-2 as a naturally occurring inhibitor of angiopoietin-1. Fibroblasts stably transfected with Tie2 receptor exhibit chemotactic responses for both angiopoietin-1 and angiopoietin-2. Fibroblasts stably expressing a transfected chimeric receptor consisting of the ectodomain of TrkC fused to the cytoplasmic domain of Tie2 also exhibit a chemotactic response to neurotrophin 3 (NT-3), a specific ligand for TrkC. Endothelial cells are shown to express angiopoietin-2 mRNA and protein, indicating the potential for autocrine activation of angiopoietin/Tie2. Finally, the demonstration that Tie2 as well as angiopoietin-1 are expressed in normal human arteries and veins suggests that the role of angiopoietin/Tie2 may extend beyond embryonic angiogenesis to maintaining integrity of the adult vasculature. 
 Angiopoietin 2 is a partial agonist/antagonist of Tie2 signaling in the endothelium. Angiopoietin 2 (Ang2) was originally shown to be a competitive antagonist for Ang1 of the receptor tyrosine kinase Tie2 in endothelial cells (ECs). Since then, reports have conflicted on whether Ang2 is an agonist or antagonist of Tie2. Here we show that Ang2 functions as an agonist when Ang1 is absent but as a dose-dependent antagonist when Ang1 is present. Exogenous Ang2 activates Tie2 and the promigratory, prosurvival PI3K/Akt pathway in ECs but with less potency and lower affinity than exogenous Ang1. ECs produce Ang2 but not Ang1. This endogenous Ang2 maintains Tie2, phosphatidylinositol 3-kinase, and Akt activities, and it promotes EC survival,   migration  , and tube formation. However, when ECs are stimulated with Ang1 and Ang2, Ang2 dose-dependently inhibits Ang1-induced Tie2 phosphorylation, Akt activation, and EC survival. We conclude that Ang2 is both an agonist and an antagonist of Tie2. Although Ang2 is a weaker agonist than Ang1, endogenous Ang2 maintains a level of Tie2 activation that is critical to a spectrum of EC functions. These findings may reconcile disparate reports of Ang2's effect on Tie2, impact our understanding of endogenous receptor tyrosine kinase signal transduction mechanisms, and affect how Ang2 and Tie2 are targeted under conditions such as sepsis and cancer. 
 Toll-like receptor 2 induced angiogenesis and invasion is mediated through the Tie2 signalling pathway in rheumatoid arthritis. Angiogenesis is a critical early event in inflammatory arthritis, facilitating leukocyte   migration   into the synovium resulting in invasion and destruction of articular cartilage and bone. This study investigates the effect of TLR2 on angiogenesis, EC adhesion and invasion using microvascular endothelial cells and RA whole tissue synovial explants ex-vivo.Microvascular endothelial cells (HMVEC) and RA synovial explants ex vivo were cultured with the TLR2 ligand, Pam3CSK4 (1 µg/ml). Angiopoietin 2 (Ang2), Tie2 and TLR2 expression in RA synovial tissue was assessed by immunohistology. HMVEC tube formation was assessed using Matrigel matrix assays. Ang2 was measured by ELISA. ICAM-1 cell surface expression was assessed by flow cytometry. Cell   migratio  n was assessed by wound repair scratch assays. ECM invasion, MMP-2 and -9 expression were assessed using transwell invasion chambers and zymography. To examine if the angiopoietin/Tie2 signalling pathway mediates TLR2 induced EC tube formation, invasion and   migratio  n assays were performed in the presence of a specific neutralising anti-Tie2mAb (10 ug/ml) and matched IgG isotype control Ab (10 ug/ml).Ang2 and Tie2 were localised to RA synovial blood vessels, and TLR2 was localised to RA synovial blood vessels, sub-lining infiltrates and the lining layer. Pam3CSK4 significantly increased angiogenic tube formation (p<0.05), and upregulated Ang2 production in HMVEC (p<0.05) and RA synovial explants (p<0.05). Pam3CSK4 induced cell surface expression of ICAM-1, from basal level of 149±54 (MFI) to 617±103 (p<0.01). TLR-2 activation induced an 8.8±2.8 fold increase in cell invasion compared to control (p<0.05). Pam3CSK4 also induced HMVEC c  ell migra  tion and induced MMP-2 and -9 from RA synovial explants. Neutralisation of the Ang2 receptor, Tie2 significantly inhibited Pam3CSK4-induced EC tube formation and invasion (p<0.05).TLR2 activation promotes angiogenesis, cell adhesion and invasion, effects that are in part mediated through the Tie2 signalling pathway, key mechanisms involved in the pathogenesis of RA. 
 Autocrine/paracrine role of the angiopoietin-1 and -2/Tie2 system in cell proliferation and chemotaxis of cultured fibroblastic synoviocytes in rheumatoid arthritis. Hypervascularity is a characteristic synovial feature of rheumatoid arthritis (RA). We previously reported that Tie1 and Tie2, endothelium-specific tyrosine kinase receptors essential for angiogenesis, are expressed not only by vascular cells, but also by a subpopulation of synovial lining and stromal cells in the inflamed RA synovium. The present study used immunohistochemistry and in situ hybridization to examine whether angiopoietin-1 and -2 (Ang1 and Ang2), ligands for Tie2, are also expressed in the RA synovium. Ang1 and Ang2 were expressed in all of 15 RA synovial samples, and their distribution pattern was similar to that of Tie2. Intense staining was noted in synovial lining and stromal cells, as well as in small vessels in areas of papillary projection and high cell density. Double immunohistochemistry revealed coexpression of Ang1, Ang2, and Tie2 in synovial components exhibiting proliferating cell nuclear antigen immunoreactivity. In addition, Ang1 and Ang2 were preferentially expressed in vimentin-positive fibroblastic cells. To address the functional role of Ang/Tie signaling in RA pathophysiology, we carried out [(3)H]thymidine incorporation and transwell chemotaxis assays using cultured fibroblastic synoviocytes obtained from 2 RA patients. Incubation with various concentrations of recombinant Ang1 or Ang2 did not alter DNA synthesis, but the ligands enhanced chemotactic   migration   of RA fibroblastic synoviocytes. Our results suggest that the autocrine/paracrine signaling of the Ang/Tie2 system is important for the up-regulated angiogenesis in the RA synovium, as well as for synoviocyte behavior, by regulating chemotactic cell movement. 
 Venous malformation-causative TIE2 mutations mediate an AKT-dependent decrease in PDGFB. Mutations in the endothelial cell (EC) tyrosine kinase receptor TIE2 cause inherited and sporadic forms of venous malformation. The recurrent somatic mutation L914F and common germline mutation R849W differ in terms of phosphorylation level, as well as sub-cellular localization and trafficking of the receptor. Previous studies have shed light on certain pathogenic properties of R849W, but the mechanisms of action of L914F are unknown. We used global gene expression profiling to study the effects of L914F on ECs. We found that L914F strongly dysregulates genes involved in vascular development, cell   migration   and extracellular matrix processing, while R849W has weak effects. We also demonstrate, for the first time, that TIE2-mutant ECs are deficient in the production of PDGFB, both in vitro and ex vivo in patient tissues. This defect is mediated by the chronic, ligand-independent activation of AKT by the mutant receptors. Inadequate secretion of the major mural cell attractant likely plays an important role in the development of abnormal vascular channels, contributing to the characteristic paucity of surrounding vascular smooth muscle cells. 
 Involvement of the ANGPTs/Tie-2 system in ovarian hyperstimulation syndrome (OHSS). Ovarian hyperstimulation syndrome (OHSS) is a disorder associated with ovarian stimulation. OHSS features are ovarian enlargement with fluid shifting to the third space. Disturbances in the vasculature are considered the main changes that lead to OHSS. Our aim was to analyze the levels of angiopoietins 1 and 2 (ANGPT1 and 2) and their soluble and membrane receptors (s/mTie-2) in follicular fluid (FF) and in granulosa-lutein cells culture (GLCs) from women at risk of developing OHSS. We also evaluated the effect of ANGPT1 on endothelial cell   migration  . In ovaries from an OHSS rat model, we analyzed the protein concentration of ANGPTs, their mTie-2 receptor, and platelet-derived growth factor PDGF-B, -D and PDGFR-β. ANGPT1 levels were increased in both FF and GLCs from women at risk of OHSS. Incubation of these FF with an ANGPT1 neutralizing antibody decreased endothelial cell   migratio  n. In the ovaries of OHSS rat model, mTie-2 protein levels increased and PDGF-B and -D decreased. In summary, these results suggest that ANGPT1 could be another mediator in the development of OHSS. 
  GeneRIFs  
 Epithin, which regulates endothelial Tie2 functions, plays a critical role in the fine tuning of transendothelial   migration   for normal and cancer cells. 
 there is a novel interaction between Tie2 with the adapter molecule ShcA that may play a role in the regulation of   migration   and three-dimensional organization of endothelial cells induced by angiopoietin-1 
 angiopoietin 1 stimulates directed   migration   and possibly inhibits vascular endothelial growth factor-induced eosinophil chemotaxis via its Tie-2 receptor. 
 tyrosine residue 1106 on Tie2 is critical for coupling downstream cell   migration   signal transduction pathways with Angiopoietin 1 stimulation in endothelial cells 
  Conserved Domains  
 cd00055: EGF_Lam; Laminin-type epidermal growth factor-like domain; laminins are the major noncollagenous components of basement membranes that mediate cell adhesion, growth   migration  , and differentiation; the laminin-type epidermal growth factor-like module occurs in ... 
  ECM  PubMed  
 Common and specific effects of TIE2 mutations causing venous malformations. Venous malformations (VMs) are localized defects in vascular morphogenesis frequently caused by mutations in the gene for the endothelial tyrosine kinase receptor TIE2. Here, we report the analysis of a comprehensive collection of 22 TIE2 mutations identified in patients with VM, either as single amino acid substitutions or as double-mutations on the same allele. Using endothelial cell (EC) cultures, mouse models and ultrastructural analysis of tissue biopsies from patients, we demonstrate common as well as mutation-specific cellular and molecular features, on the basis of which mutations cluster into categories that correlate with data from genetic studies. Comparisons of double-mutants with their constituent single-mutant forms identified the pathogenic contributions of individual changes, and their compound effects. We find that defective receptor trafficking and subcellular localization of different TIE2 mutant forms occur via a variety of mechanisms, resulting in attenuated response to ligand. We also demonstrate, for the first time, that TIE2 mutations cause chronic activation of the MAPK pathway resulting in loss of normal EC monolayer due to extracellular matrix (  ECM  ) fibronectin deficiency and leading to upregulation of plasminogen/plasmin proteolytic pathway. Corresponding EC and   ECM   irregularities are observed in affected tissues from mouse models and patients. Importantly, an imbalance between plasminogen activators versus inhibitors would also account for high d-dimer levels, a major feature of unknown cause that distinguishes VMs from other vascular anomalies. 
 Toll-like receptor 2 induced angiogenesis and invasion is mediated through the Tie2 signalling pathway in rheumatoid arthritis. Angiogenesis is a critical early event in inflammatory arthritis, facilitating leukocyte migration into the synovium resulting in invasion and destruction of articular cartilage and bone. This study investigates the effect of TLR2 on angiogenesis, EC adhesion and invasion using microvascular endothelial cells and RA whole tissue synovial explants ex-vivo.Microvascular endothelial cells (HMVEC) and RA synovial explants ex vivo were cultured with the TLR2 ligand, Pam3CSK4 (1 µg/ml). Angiopoietin 2 (Ang2), Tie2 and TLR2 expression in RA synovial tissue was assessed by immunohistology. HMVEC tube formation was assessed using Matrigel matrix assays. Ang2 was measured by ELISA. ICAM-1 cell surface expression was assessed by flow cytometry. Cell migration was assessed by wound repair scratch assays.   EC  M invasion, MMP-2 and -9 expression were assessed using transwell invasion chambers and zymography. To examine if the angiopoietin/Tie2 signalling pathway mediates TLR2 induced EC tube formation, invasion and migration assays were performed in the presence of a specific neutralising anti-Tie2mAb (10 ug/ml) and matched IgG isotype control Ab (10 ug/ml).Ang2 and Tie2 were localised to RA synovial blood vessels, and TLR2 was localised to RA synovial blood vessels, sub-lining infiltrates and the lining layer. Pam3CSK4 significantly increased angiogenic tube formation (p<0.05), and upregulated Ang2 production in HMVEC (p<0.05) and RA synovial explants (p<0.05). Pam3CSK4 induced cell surface expression of ICAM-1, from basal level of 149±54 (MFI) to 617±103 (p<0.01). TLR-2 activation induced an 8.8±2.8 fold increase in cell invasion compared to control (p<0.05). Pam3CSK4 also induced HMVEC cell migration and induced MMP-2 and -9 from RA synovial explants. Neutralisation of the Ang2 receptor, Tie2 significantly inhibited Pam3CSK4-induced EC tube formation and invasion (p<0.05).TLR2 activation promotes angiogenesis, cell adhesion and invasion, effects that are in part mediated through the Tie2 signalling pathway, key mechanisms involved in the pathogenesis of RA. 
  [Ee]xtracellular [Mm]atrix  PubMed  
 Isolation of novel and known genes from a human fetal cochlear cDNA library using subtractive hybridization and differential screening. We used a combination of subtractive hybridization and differential screening strategies to identify genes that may function normally in hearing and, when mutated, result in deafness. A human fetal cochlear (membranous labyrinth) cDNA library was subtracted against total human fetal brain RNAs by an avidin-biotin-based procedure to enrich for cochlear transcripts. Subtracted cochlear clones were differentially screened with 32P-labeled total cochlear and total brain cDNA probes. Sequence analysis of clones that hybridized more intensely with cochlear than with brain cDNA probes revealed some previously characterized genes, including mitochondrial sequences, collagen type I alpha-2 (COL1A2), collagen type II alpha-1 (COL2A1), collagen type III alpha-1 (COL3A1), spermidine/spermine N1-acetyltransferase (SAT), osteonectin (SPARC), and peripheral myelin protein 22 (PMP22). Also identified were clones that are potential novel cochlear genes. Northern blots of cochlear and brain RNAs probed with COL1A2, COL2A1, COL3A1, SAT, SPARC, PMP22, and a novel sequence, designated Coch-5B2, confirm results of the subtractive procedure by showing preferential cochlear expression. A number of these genes serve structural or regulatory functions in   extracellular matrix   or neural conduction; defects in some of these genes are associated with disorders involving hearing loss. Partial sequence analysis of Coch-5B2 reveals a von Willebrand factor type A-like domain in this cDNA. To assess the cochlear specificity of Coch-5B2, a Northern blot panel of 14 human fetal tissue RNAs was probed with Coch-5B2, showing differential expression of this novel gene in the cochlea. 
 Stable interaction between alpha5beta1 integrin and Tie2 tyrosine kinase receptor regulates endothelial cell response to Ang-1. During angiogenic remodeling, Ang-1, the ligand of Tie2 tyrosine kinase, is involved in vessel sprouting and stabilization through unclear effects on nascent capillaries and mural cells. In our study, we hypothesized that the Ang-1/Tie2 system could cross-talk with integrins, and be influenced by the dynamic interactions between   extracellular matrix   and endothelial cells (ECs). Here, we show that alpha5beta1 specifically sensitizes and modulates Tie2 receptor activation and signaling, allowing EC survival at low concentrations of Ang-1 and inducing persistent EC motility. Tie2 and alpha5beta1 interact constitutively; alpha5beta1 binding to fibronectin increases this association, whereas Ang-1 stimulation recruits p85 and FAK to this complex. Furthermore, we demonstrate that Ang-1 is able to mediate selectively alpha5beta1 outside-in FAK phosphorylation. Thus, Ang-1 triggers signaling pathways through Tie2 and alpha5beta1 receptors that could cross-talk when Tie2/alpha5beta1 interaction occurs in ECs plated on fibronectin. By using blocking antibodies, we consistently found that alpha5beta1, but not alphavbeta3 activation, is essential to Ang-1-dependent angiogenesis in vivo. 
 Differential function of Tie2 at cell-cell contacts and cell-substratum contacts regulated by angiopoietin-1. Tie2 belongs to the receptor tyrosine kinase family and functions as a receptor for Angiopoietin-1 (Ang1). Gene-targeting analyses of either Ang1 or Tie2 in mice reveal a critical role of Ang1-Tie2 signalling in developmental vascular formation. It remains elusive how the Tie2 signalling pathway plays distinct roles in both vascular quiescence and angiogenesis. We demonstrate here that Ang1 bridges Tie2 at cell-cell contacts, resulting in trans-association of Tie2 in the presence of cell-cell contacts. In clear contrast, in isolated cells,   extracellular matrix  -bound Ang1 locates Tie2 at cell-substratum contacts. Furthermore, Tie2 activated at cell-cell or cell-substratum contacts leads to preferential activation of Akt and Erk, respectively. Microarray analyses and real-time PCR validation clearly show the differential gene expression profile in vascular endothelial cells upon Ang1 stimulation in the presence or absence of cell-cell contacts, implying downstream signalling is dependent upon the spatial localization of Tie2. 
 Common and specific effects of TIE2 mutations causing venous malformations. Venous malformations (VMs) are localized defects in vascular morphogenesis frequently caused by mutations in the gene for the endothelial tyrosine kinase receptor TIE2. Here, we report the analysis of a comprehensive collection of 22 TIE2 mutations identified in patients with VM, either as single amino acid substitutions or as double-mutations on the same allele. Using endothelial cell (EC) cultures, mouse models and ultrastructural analysis of tissue biopsies from patients, we demonstrate common as well as mutation-specific cellular and molecular features, on the basis of which mutations cluster into categories that correlate with data from genetic studies. Comparisons of double-mutants with their constituent single-mutant forms identified the pathogenic contributions of individual changes, and their compound effects. We find that defective receptor trafficking and subcellular localization of different TIE2 mutant forms occur via a variety of mechanisms, resulting in attenuated response to ligand. We also demonstrate, for the first time, that TIE2 mutations cause chronic activation of the MAPK pathway resulting in loss of normal EC monolayer due to   extracellular matrix   (ECM) fibronectin deficiency and leading to upregulation of plasminogen/plasmin proteolytic pathway. Corresponding EC and ECM irregularities are observed in affected tissues from mouse models and patients. Importantly, an imbalance between plasminogen activators versus inhibitors would also account for high d-dimer levels, a major feature of unknown cause that distinguishes VMs from other vascular anomalies. 
 Angiopoietin-1/Tie2 signal augments basal Notch signal controlling vascular quiescence by inducing delta-like 4 expression through AKT-mediated activation of beta-catenin. Angiopoietin-1 (Ang1) regulates both vascular quiescence and angiogenesis through the receptor tyrosine kinase Tie2. We and another group previously showed that Ang1 and Tie2 form distinct signaling complexes at cell-cell and cell-matrix contacts. We further demonstrated that the former up-regulates Notch ligand delta-like 4 (Dll4) only in the presence of cell-cell contacts. Because Dll4/Notch signal restricts sprouting angiogenesis and promotes vascular stabilization, we investigated the mechanism of how the Ang1/Tie2 signal induces Dll4 expression to clarify the role of the Dll4/Notch signal in Ang1/Tie2 signal-mediated vascular quiescence. Under confluent endothelial cells, the basal Notch signal was observed. Ang1, moreover, induced Dll4 expression and production of the Notch intracellular domain (NICD). Ang1 stimulated transcriptional activity of β-catenin through phosphoinositide 3-kinase (PI3K)/AKT-mediated phosphorylation of glycogen synthase kinase 3β (GSK3β). Correspondingly, the GSK3β inhibitor up-regulated Dll4, whereas depletion of β-catenin by siRNA blocked Ang1-induced Dll4 expression, indicating the indispensability of β-catenin in Ang1-mediated up-regulation of Dll4. In addition, Dll4 expression by the GSK3β inhibitor was only observed in confluent cells, and was impeded by DAPT, a γ-secretase inhibitor, implying requirement of the Notch signal in β-catenin-dependent Dll4 expression. Consistently, we found that either Ang1 or NICD up-regulates Dll4 through the RBP-J binding site within intron 3 of the DLL4 gene and that β-catenin forms a complex with NICD/RBP-J to enhance Dll4 expression. Ang1 induced the depo  sition of extracellu  lar matrix that is preferable for basement membrane formation through Dll4/Notch signaling. Collectively, the Ang1/Tie2 signal potentiates basal Notch signal controlling vascular quiescence by up-regulating Dll4 through AKT-mediated activation of β-catenin. 
 Venous malformation-causative TIE2 mutations mediate an AKT-dependent decrease in PDGFB. Mutations in the endothelial cell (EC) tyrosine kinase receptor TIE2 cause inherited and sporadic forms of venous malformation. The recurrent somatic mutation L914F and common germline mutation R849W differ in terms of phosphorylation level, as well as sub-cellular localization and trafficking of the receptor. Previous studies have shed light on certain pathogenic properties of R849W, but the mechanisms of action of L914F are unknown. We used global gene expression profiling to study the effects of L914F on ECs. We found that L914F strongly dysregulates genes involved in vascular development, cell migration and   extracellular matrix   processing, while R849W has weak effects. We also demonstrate, for the first time, that TIE2-mutant ECs are deficient in the production of PDGFB, both in vitro and ex vivo in patient tissues. This defect is mediated by the chronic, ligand-independent activation of AKT by the mutant receptors. Inadequate secretion of the major mural cell attractant likely plays an important role in the development of abnormal vascular channels, contributing to the characteristic paucity of surrounding vascular smooth muscle cells. 
  GeneRIFs  
 Data show that Angiopoietin 1 bridges Tie2 at cell-cell contacts, resulting in trans-association of Tie2 in the presence of cell-cell contacts, while in isolated cells   extracellular matrix  -bound Ang1 locates Tie2 at cell-substratum contacts. 
  [Ii]nvasive  PubMed  
 Prognostic significance of TIE2-expressing monocytes in hilar cholangiocarcinoma. Angiopoietins (Angs) play a pivotal role in angiogenesis and inflammation, and are associated with prognosis in malignancies. Monocyte express Ang-receptor TIE2 and correlate with prognosis in cancer. We aimed to investigate the prognostic value of Angs and TIE2-expressing monocytes (TEMs) in cholangiocarcinoma.We analyzed surgically resected tumor specimens of hilar cholangiocarcinoma (n = 47) for distribution of Angs (Ang 1/Ang 2) and TEMs, as defined by co-expression of CD14 and Ang receptor TIE2. Ang expression and abundance of TEMs were correlated with clinicopathologic characteristics, tumor recurrence and patients' survival.High Ang 1 expression correlated with reduced metastasis (P < 0.05). Patients characterized by invading Ang-receptor bearing TEMs in tumor showed lower tumor recurrence (P < 0.05). Furthermore, TEMs in tumo  r and tu  mor invasive front correlated with increased survival (P < 0.05)  . TEMs i  n tumor invasive front were confirmed as independent prognosticator in multivariate survival analysis (P < 0.05).High Ang 1 expression in hilar cholangiocarcinoma and infiltration of TEMs defines a subgroup of patients with beneficial tumor characteristics and prolonged survival. Besides suggested functional links between Ang expression and recruitment of TEMs, our data have possible clinical implications as novel diagnostic tools. J. Surg. Oncol. 2016;114:91-98. © 2016 Wiley Periodicals, Inc. 
 Tie2/TEK modulates the interaction of glioma and brain tumor stem cells with endothelial cells and promotes an   invasive   phenotype. Malignant gliomas are the prototype of highly infiltrative tumors and this characteristic is the main factor for the inevitable tumor recurrence and short survival after most aggressive therapies. The aberrant communication between glioma cells and tumor microenvironment represents one of the major factors regulating brain tumor dispersal. Our group has previously reported that the tyrosine kinase receptor Tie2/TEK is expressed in glioma cells and brain tumor stem cells and is associated with the malignant progression of these tumors. In this study, we sought to determine whether the angiopoietin 1 (Ang1)/Tie2 axis regulates crosstalk between glioma cells and endothelial cells. We found that Ang1 enhanced the adhesion of Tie2-expressing glioma and brain tumor stem cells to endothelial cells. Conversely, specific small interfering RNA (siRNA) knockdown of Tie2 expression inhibited the adhesion capability of glioma cells. Tie2 activation induced integrin β1 and N-cadherin upregulation, and neutralizing antibodies against these molecules inhibited the adhesion of Tie2-positive glioma cells to endothelial cells. In 2D and 3D cultures, we observed that Ang1/Tie2 axis activation was related to increased glioma cell invasion, which was inhibited by using Tie2 siRNA. Importantly, intracranial co-implantation of Tie2-positive glioma cells and endothelial cells in a mouse model resulted in diffusely   invasiv  e tumors with cell clusters surrounding glomeruloid vessels mimicking a tumoral niche distribution. Collectively, our results provide new information about the Tie2 signaling in glioma cells that regulates the cross-talk between glioma cells and tumor microenvironment, envisioning Tie2 as a multi-compartmental target for glioma therapy. 
 Angiopoietins 1 and 2 and Tie-2 receptor expression in human ductal breast disease.  This study aimed to identify the involvement of the angiopoietin/Tie-2 receptor system in breast cancer development, progression, metastasis and angiogenesis.We quantified and correlated angiopoietin-1 (Ang-1), Ang-2 and Tie-2 expression in sections of normal human breast, benign and premalignant hyperplastic tissue, pr  e-invasi  ve an  d invasi  ve cancer, and compared these findings with our previously published data on vascular endothelial growth factor (VEGF) and microvessel density (MVD) in the same samples. A breast cancer tissue microarray was used to evaluate the prognostic value of these factors. Histological analysis revealed a significant decrease in Ang-1 expression (P = 0.001) and an inverse correlation with MVD (r = -0.442, P = 0.008) and VEGF (r = -0.510, P =�  ��0.002)   in the non-invasive lesions. In contrast Ang-2 expression increased significantly (P = 0.0004) with increasing severity of lesion and correlated with MVD (r = 0.570; P = 0.0002), while Tie-2 expression remained relatively unchanged. Expression of a  ll three   factors was reduced in invasive breast cancer and did not correlate with oestrogen receptor (ER), progesterone receptor (PR), human epidermal growth factor receptor 2 (HER2), lymph node status or tumour grade.These data suggest that a change in the angiopoietin balance in favour of Ang-2 is associated with the angiogenic switch at the onset of hyperplasia in the breast. However, angiopoietins and the Tie-2 receptor are not related to   known p  rognostic indicators in invasive breast cancer. 
  GeneRIFs  
 Angiopoietins and the Tie-2 receptor are not related to known prognostic indicators in   invasive   breast cancer. 
  [Mm]etastasis  PubMed  
 Expression of Ang-2/Tie-2 and PI3K/AKT in colorectal cancer. To study the expression of angiogenin-2 (Ang-2) and its receptor Tie-2 in colorectal cancer and discuss the possible mechanisms behind this process.Using the streptavidin-peroxidase (SP) immunohistochemical method, paraffin sections from 100 colorectal cancer samples and 10 samples from tumor-adjacent normal tissue (>2 cm from the edge of the gross tumor) were tested for protein expression of Ang-2, Tie-2, PI3K, and AKT. Reverse transcription-polymerase chain reaction and Western blots were further used to measure expression of the 4 genes and proteins in 20 freshly-resected colorectal cancer samples and tumor-adjacent normal tissues.In colorectal cancer tissues, the expression of the Ang-2, Tie-2, PI3K, and AKT genes and their proteins was significantly higher than in tumor-adjacent normal tissues. Protein expression in poorly-differentiated adenocarcinoma was higher than that in well and moderately differentiated adenocarcinoma. According to Duke's classification, the protein expression in Stages C and D was significantly higher than that in Stages A and B. In the group with lymphatic   metastasis  , the protein expression was higher than that without lymphatic   metastasis  .In colorectal cancer, the expression of the Ang-2, Tie-2, PI3K, and AKT genes and their proteins is markedly higher than those in tumor-adjacent normal tissues. No correlation was observed between protein expression and gender, location, or histologic type. Correlations did exist between protein expression and differentiation level, stage of Duke's classification, and lymphatic   metastasis  ; in colorectal cancer tissues with lower differentiation levels, higher stages of Duke's classification, and lymphatic   metastasis  , the expression of all 4 proteins was higher. The study of their expression patterns and relationships with aggression and   metastasis   will provide a valuable experimental foundation for assessing prognosis and targeted therapy of colorectal cancer. 
 Prognostic significance of TIE2-expressing monocytes in hilar cholangiocarcinoma. Angiopoietins (Angs) play a pivotal role in angiogenesis and inflammation, and are associated with prognosis in malignancies. Monocyte express Ang-receptor TIE2 and correlate with prognosis in cancer. We aimed to investigate the prognostic value of Angs and TIE2-expressing monocytes (TEMs) in cholangiocarcinoma.We analyzed surgically resected tumor specimens of hilar cholangiocarcinoma (n = 47) for distribution of Angs (Ang 1/Ang 2) and TEMs, as defined by co-expression of CD14 and Ang receptor TIE2. Ang expression and abundance of TEMs were correlated with clinicopathologic characteristics, tumor recurrence and patients' survival.High Ang 1 expression correlated with redu  ced metast  asis (P < 0.05). Patients characterized by invading Ang-receptor bearing TEMs in tumor showed lower tumor recurrence (P < 0.05). Furthermore, TEMs in tumor and tumor invasive front correlated with increased survival (P < 0.05). TEMs in tumor invasive front were confirmed as independent prognosticator in multivariate survival analysis (P < 0.05).High Ang 1 expression in hilar cholangiocarcinoma and infiltration of TEMs defines a subgroup of patients with beneficial tumor characteristics and prolonged survival. Besides suggested functional links between Ang expression and recruitment of TEMs, our data have possible clinical implications as novel diagnostic tools. J. Surg. Oncol. 2016;114:91-98. © 2016 Wiley Periodicals, Inc. 
 Double antiangiogenic protein, DAAP, targeting VEGF-A and angiopoietins in tumor angiogenesis,   metastasis  , and vascular leakage. Two vascular growth factor families, VEGF and the angiopoietins, play critical and coordinate roles in tumor progression and   metastasis  . A single inhibitor targeting both VEGF and angiopoietins is not available. Here, we developed a chimeric decoy receptor, namely double anti-angiogenic protein (DAAP), which can simultaneously bind VEGF-A and angiopoietins, blocking their actions. Compared to VEGF-Trap or Tie2-Fc, which block either VEGF-A or angiopoietins alone, we believe DAAP is a highly effective molecule for regressing tumor angiogenesis and   metastasis   in implanted and spontaneous solid tumors; it can also effectively reduce ascites formation and vascular leakage in an ovarian carcinoma model. Thus, simultaneous blockade of VEGF-A and angiopoietins with DAAP is an effective therapeutic strategy for blocking tumor angiogenesis,   metastasis  , and vascular leakage. 
 Real-Time Imaging Reveals Local, Transient Vascular Permeability, and Tumor Cell Intravasation Stimulated by TIE2hi Macrophage-Derived VEGFA. Dissemination of tumor cells is an essential step in   metastasis  . Direct contact between a macrophage, mammalian-enabled (MENA)-overexpressing tumor cell, and endothelial cell [Tumor MicroEnvironment of   Metastasis   (TMEM)] correlates with   metastasis   in breast cancer patients. Here we show, using intravital high-resolution two-photon microscopy, that transient vascular permeability and tumor cell intravasation occur simultaneously and exclusively at TMEM. The hyperpermeable nature of tumor vasculature is described as spatially and temporally heterogeneous. Using real-time imaging, we observed that vascular permeability is transient, restricted to the TMEM, and required for tumor cell dissemination. VEGFA signaling from TIE2(hi) TMEM macrophages causes local loss of vascular junctions, transient vascular permeability, and tumor cell intravasation, demonstrating a role for the TMEM within the primary mammary tumor. These data provide insight into the mechanism of tumor cell intravasation and vascular permeability in breast cancer, explaining the value of TMEM density as a predictor of distant metastatic recurrence in patients.Tumor vasculature is abnormal with increased permeability. Here, we show that VEGFA signaling from TIE2(hi) TMEM macrophages results in local, transient vascular permeability and tumor cell intravasation. These data provide evidence for the mechanism underlying the association of TMEM with distant metastatic recurrence, offering a rationale for therapies targeting TMEM. 
 The association of the angiopoietin/Tie-2 system with the development of   metastasis   and leukocyte migration in neuroendocrine tumors. The aim of this study was to explore the possible involvement of the angiopoietin (Ang)-1, -2/Tie-2 system in the development, growth, and metastases evolution of gastroenteropancreatic-neuroendocrine tumors (GEP-NETs). We prospectively examined the serum levels of Tie-2, Ang-1, and Ang-2 by ELISA in 42 patients with proven GEP-NETs and 27 controls. We also determined the expression of the Ang/Tie-2 system in freshly isolated peripheral blood monocytes and in tumor cells from malignant primary tumors and/or liver metastases samples from GEP-NET patients by flow cytometry and/or RT-PCR. Furthermore, the function of the Ang/Tie-2 system in monocytes from controls and patients was assessed by a chemotaxis assay. GEP-NET patients showed enhanced serum levels of soluble form of Tie-2 (sTie-2), Ang-1, and Ang-2 (P<0.05 in all cases), compared to controls. sTie-2 and Ang-2 levels were significantly higher in GEP-NETs with metastases compared to those with no metastases. In addition, a significant correlation was detected between Ang-2 levels and chromogranin A or sTie-2 concentrations or 5-hydroxy-indole acetic acid excretion (r=0.71, r=0.60, and r=0.81 respectively, P<0.01 in all cases). Furthermore, we observed an enhanced expression of Ang-1, Ang-2, and Tie-2 in freshly isolated tumor cells from GEP-NET both by immunohistochemistry and by RT-PCR. Interestingly, an enhanced expression and function of Tie-2 was detected in monocytes from GEP-NET patients. Our data suggest that the Ang/Tie-2 system is involved in the growth and development of metastases of GEP-NETs, and that favors the recruitment of Tie-2(+) monocytes to the tumor site, where they can promote inflammation and angiogenesis. 
 Tie-2 regulates the stemness and metastatic properties of prostate cancer cells. Ample evidence supports that prostate tumor   metastasis   originates from a rare population of cancer cells, known as cancer stem cells (CSCs). Unfortunately, little is known about the identity of these cells, making it difficult to target the metastatic prostate tumor. Here, for the first time, we report the identification of a rare population of prostate cancer cells that express the Tie-2 protein. We found that this Tie-2High population exists mainly in prostate cancer cell lines that are capable of metastasizing to the bone. These cells not only express a higher level of CSC markers but also demonstrate enhanced resistance to the chemotherapeutic drug Cabazitaxel. In addition, knockdown of the expression of the Tie-2 ligand angiopoietin (Ang-1) led to suppression of CSC markers, suggesting that the Ang-1/Tie-2 signaling pathway functions as an autocrine loop for the maintenance of prostate CSCs. More importantly, we found that Tie-2High prostate cancer cells are more adhesive than the Tie-2Low population to both osteoblasts and endothelial cells. Moreover, only the Tie-2High, but not the Tie-2Low cells developed tumor   metastasis   in vivo when injected at a low number. Taken together, our data suggest that Tie-2 may play an important role during the development of prostate tumor   metastasis  . 
 [Role of Ang-2, Tie-2 and VEGFR-2 in angiogenesis in colorectal carcinoma and their prognostic value]. To study the expression of angiotensin-2 (Ang-2), Tie-2 and vascular endothelial growth factor receptor-2 (VEGFR-2) in colorectal cancer and analyze their relationship with the occurrence, recurrence,   metastasis  , angiogenesis and prognosis of colorectal cancer.Immunohistochemistry with SP method was used to detect the expressions of Ang-2, Tie-2 and VEGFR-2 in 118 colorectal cancer, 40 adjacent normal tissue and 40 benign colorectal lesion specimens.The positivity rates of Ang-2, Tie-2 and VEGFR-2 in colorectal cancer tissue were 74.58%, 69.49%, and 61.02%, respectively, significantly higher than those in the adjacent normal tissues (25.00%, 17.50%, and 17.50%, P<0.05) and benign colorectal lesion tissues (35.00%, 32.50%, and 32.50%, P<0.05). The rates of two or three coexpression were significantly higher than that of a single expression in the cancer tissues (61.02% vs 15.25%). The microvascular density (MVD) of colorectal cancer tissues was 31.43∓10.50, significantly higher than that of the adjacent normal tissues (10.61∓3.76) and benign colorectal lesions (16.89∓3.83) (P<0.05). The expressions of Ang-2, Tie-2, and VEGFR-2 were positively correlated with carcinoembryonic antigen (CEA) and MVD (P<0.05). The expression of Ang-2, but not Tie-2 and VEGFR-2, was positively correlated with CA199. Ang-2, Tie-2, and VEGFR-2 expressions showed significant differences between cases with tumor recur  rence/meta  stasis and those without 5 years after radical mastectomy, and were all positively correlated with the 5-year survival rates (P<0.05).Ang-2, Tie-2 and VEGFR-2 are involved in the development, inva  sion, meta  stasis, and prognosis of colorectal cancer, and play important roles in the angiogenesis of the tumors. 
 Serum levels of angiogenic factors and their prognostic relevance in bladder cancer. Angiogenesis plays a critical role in tumor growth. VEGF, angiopoietins (Ang-1, Ang-2) and their tyrosine kinase receptor Tie2 are major regulators of angiogenesis. The aim of this study was to evaluate the prognostic value of the serum levels of these factors in bladder cancer. We analyzed the serum samples of 117 bladder cancer patients and 64 healthy volunteers by enzyme linked immunosorbent assay (ELISA) for Ang-1, Ang-2, VEGF and the extracellular domain of Tie2. The statistical evaluation of the obtained data was performed via Kaplan-Meier log-rank test, univariate Cox analyses as well as Cox proportional hazards regression model. Serum Ang-1 levels of bladder cancer patients were significantly higher (p < 0.001), while soluble Ang-2 and Tie2 levels were significantly lower (p = 0.016 and p = 0.001 respectively) in patients than those in controls. Cox univariate analysis revealed high sTie2 serum level as a risk factor for   metastasis   and as a borderline significant risk factor for disease related death (p = 0.022 and p = 0.081 respectively). These correlations were independent from tumor stage and grade in a Cox multivariate model (p = 0.016 and p = 0.069). These data indicate that the serum levels of analyzed angiogenic factors do change characteristically in bladder cancer. The soluble extracellular serum level of Tie2 may provide a stage and grade independent diagnostic tool to select a high risk group of bladder cancer patients. 
 Angiopoietin-1 overexpression modulates vascular endothelium to facilitate tumor cell dissemination and   metastasis   establishment. The angiopoietin-1 (Ang1)/Tie2 signaling pathway is known to play an important role in the regulation of vascular maturation and maintenance of vessel integrity. In this study, we have investigated the effect of systemic Tie2 activation or inhibition on tumor growth and   metastasis  . We found that treatment with Ang1 delivered via an adenoviral vector promoted s.c. implanted tumor   metastasis   to the lungs. Ang1 treatment did not significantly increase vascular density in the tumors but induced enlargement of blood vessels in both the tumor and normal tissues, which increased tumor cell dissemination into the blood circulation. Ang1 also enhanced the formation of metastatic foci in the lungs when tumor cells were injected into the circulation via the tail vein. The effect of Ang1 on   metastasis   was validated by a simultaneous treatment with a soluble form of Tie2 (sTie2), which led to the suppression of Ang1-induced increase of tumor   metastasis  . Furthermore, using a highly metastatic tumor model, we confirmed that systemic treatment with sTie2 suppressed tumor   metastasis   to the lungs and lymph nodes, whereas tumor-associated angiogenesis and lymphangiogenesis were not significantly affected. This suggests that the Ang1/Tie2 signals contribute to tumor progression by increasing vascular entry and exit of tumor cells to facilitate tumor dissemination and establishment of metastases. 
 Functional significance of Tie2 signaling in the adult vasculature. Abundant data now demonstrate that the growth of new blood vessels, termed angiogenesis, plays both pathological and beneficial roles in human disease. Based on these data, a tremendous effort has been undertaken to understand the molecular mechanisms that drive blood vessel growth in adult tissues. Tie2 recently was identified as a receptor tyrosine kinase expressed principally on vascular endothelium. Disrupting Tie2 function in mice resulted in embryonic lethality with defects in embryonic vasculature, suggesting a role in blood vessel maturation and maintenance. Based on these studies, we undertook a series of studies to probe the function of Tie2 in adult vasculature that will form the focus of this chapter. Consistent with a role in blood vessel growth in adult vasculature, Tie2 was upregulated and activated in the endothelium of rat ovary and in healing rat skin wounds, both areas of active angiogenesis. Moreover, Tie2 was upregulated in the endothelium of vascular "hot spots" in human breast cancer specimens. Surprisingly, Tie2 also was expressed and activated in the endothelium of all normal rat tissues examined, suggesting a role in maintenance of adult vasculature. To determine the functional role of Tie2 in tumor vasculature, a soluble Tie2 extracellular domain (ExTek) was designed that blocked the activation of Tie2 by its activating ligand, angiopoietin 1 (Ang1). Administration of recombinant ExTek protein or an ExTek adenovirus inhibited tumor growth and   metastasis   in rodent tumor models, demonstrating a functional role for Tie2 in pathological angiogenesis in adult tissues. To begin to understand the endothelial signaling pathways and cellular responses that mediate Tie2 function, we identified signaling molecules that are recruited to the activated, autophosphorylated Tie2 kinase domain. Two of these molecules, SHP2 and GRB2, are part of the pathway upstream of mitogen-activated protein kinase (MAPK) activation, a pathway that may be responsible for morphogenetic effects of Tie2 on endothelial cells. Another signaling molecule, p85, is responsible for recruitment of phosphatidylinositol 3 kinase (PI3-K) and activation of the Akt/PI3-K pathway. Akt/PI3-K has emerged as a critical pathway downstream of Tie2 that is necessary for cell survival effects as well as for chemotaxis, activation of endothelial nitric oxide synthase, and perhaps for anti-inflammatory effects of Tie2 activation. Taken together, these studies and many others demonstrate that the Tie2 pathway has important functions in adult tissues, in both quiescent vasculature and during angiogenesis, and help to validate the Tie2 pathway as a therapeutic target. 
 Expression of angiopoietin 1, 2 and their common receptor Tie2 in human gastric carcinoma: implication for angiogenesis. Angiogenesis, formation of new microvessels providing oxygen and nutrient supply, is essential for tumor growth. It is dependent on the production of angiogenic growth factors by tumor cells. Angiopoietin 1 (Ang-1) and 2 (Ang-2) and their common receptor, Tie2, are thought to be critical regulators of tumor angiogenesis. We examined expression of Ang-1, Ang-2, and their common receptor Tie2 mRNAs and proteins in gastric cancers using in situ hybridization and immunohistochemistry. We also investigated the relationship between their expression and differentiation of cancer cells, lymph node   metastasis  , tumor size, depth of cancer cell invasion, TNM staging and microvessel density (MVD). The expression of Ang-1, Ang-2, and Tie2 mRNA in cancer cells significantly correlated with the MVD (p<0.001, <0.001 and =0.019, respectively). Ang-1 and Tie2 positivity correlated with advanced gastric cancers (p<0.05) and larger cancers had higher positive rates of Ang-1, Ang-2, and Tie2 mRNA expression (p<0.001, =0.010 and =0.039, respectively). Significant positive correlations were also found between mRNA expression of Tie2 and those of Ang-1 and Ang-2 (p<0.01 and <0.001, respectively). These findings indicate that the expression of Ang-1 and Ang-2 is important for tumor angiogenesis, and suggest a possible role of autocrine/paracrine function of angiopoietin/Tie2 system in gastric cancer progression. 
 Angiopoietins 1 and 2 and Tie-2 receptor expression in human ductal breast disease.  This study aimed to identify the involvement of the angiopoietin/Tie-2 receptor system in breast cancer development, progression  , metastas  is and angiogenesis.We quantified and correlated angiopoietin-1 (Ang-1), Ang-2 and Tie-2 expression in sections of normal human breast, benign and premalignant hyperplastic tissue, pre-invasive and invasive cancer, and compared these findings with our previously published data on vascular endothelial growth factor (VEGF) and microvessel density (MVD) in the same samples. A breast cancer tissue microarray was used to evaluate the prognostic value of these factors. Histological analysis revealed a significant decrease in Ang-1 expression (P = 0.001) and an inverse correlation with MVD (r = -0.442, P = 0.008) and VEGF (r = -0.510, P = 0.002) in the non-invasive lesions. In contrast Ang-2 expression increased significantly (P = 0.0004) with increasing severity of lesion and correlated with MVD (r = 0.570; P = 0.0002), while Tie-2 expression remained relatively unchanged. Expression of all three factors was reduced in invasive breast cancer and did not correlate with oestrogen receptor (ER), progesterone receptor (PR), human epidermal growth factor receptor 2 (HER2), lymph node status or tumour grade.These data suggest that a change in the angiopoietin balance in favour of Ang-2 is associated with the angiogenic switch at the onset of hyperplasia in the breast. However, angiopoietins and the Tie-2 receptor are not related to known prognostic indicators in invasive breast cancer. 
  GeneRIFs  
 Ang-2, Tie-2 and VEGFR-2 are involved in the development, invasion,   metastasis  , and prognosis of colorectal cancer, and play important roles in angiogenesis. 
  [Cc]ell [Ii]nvasion  PubMed  
 Tie2/TEK modulates the interaction of glioma and brain tumor stem cells with endothelial cells and promotes an invasive phenotype. Malignant gliomas are the prototype of highly infiltrative tumors and this characteristic is the main factor for the inevitable tumor recurrence and short survival after most aggressive therapies. The aberrant communication between glioma cells and tumor microenvironment represents one of the major factors regulating brain tumor dispersal. Our group has previously reported that the tyrosine kinase receptor Tie2/TEK is expressed in glioma cells and brain tumor stem cells and is associated with the malignant progression of these tumors. In this study, we sought to determine whether the angiopoietin 1 (Ang1)/Tie2 axis regulates crosstalk between glioma cells and endothelial cells. We found that Ang1 enhanced the adhesion of Tie2-expressing glioma and brain tumor stem cells to endothelial cells. Conversely, specific small interfering RNA (siRNA) knockdown of Tie2 expression inhibited the adhesion capability of glioma cells. Tie2 activation induced integrin β1 and N-cadherin upregulation, and neutralizing antibodies against these molecules inhibited the adhesion of Tie2-positive glioma cells to endothelial cells. In 2D and 3D cultures, we observed that Ang1/Tie2 axis activation was related to increased glioma   cell invasio  n, which was inhibited by using Tie2 siRNA. Importantly, intracranial co-implantation of Tie2-positive glioma cells and endothelial cells in a mouse model resulted in diffusely invasive tumors with cell clusters surrounding glomeruloid vessels mimicking a tumoral niche distribution. Collectively, our results provide new information about the Tie2 signaling in glioma cells that regulates the cross-talk between glioma cells and tumor microenvironment, envisioning Tie2 as a multi-compartmental target for glioma therapy. 
 Isolation and characterization of endothelial cells from intramuscular hemangioma. Intramuscular hemangiomas (IMHs) are benign vascular tumors of deep soft tissue characterized by endothelial cell (EC) proliferation. The purpose of this study was to isolate ECs from IMH, characterize their angiogenic phenotype and functional characteristics, and search for a possible signaling pathway related to IMH development.EC Isolation from IMH was performed by digestion, filtration, washing, incubation, and purification in sequence. Tie2 expression was compared between ECs from IMH and controls using reverse transcriptase polymerase chain reaction (RT-PCR).   Cell invasion   and proliferation assays were used to analyze functional responses of ECs to angiopoietin 1 (Ang1) and vascular endothelial growth factor (VEGF). Expression of downstream targets was analyzed using Western blot analysis.Isolated ECs showed typical cobblestone appearance under light microscopy and formed capillary-like tubular structures using Matrigel tube-forming assay. RT-PCR of isolated ECs from six patients showed increased expression of Tie2 and VEGF receptor 1 (VEGFR1) compared with control ECs. Tie2 activation by Ang1 compared with VEGFR1 by VEGF resulted in increased EC migration and proliferation. Western blot analysis showed increased Tie2 expression in hemangioma samples compared with normal ECs. Phosphorylated Akt and phosphorylated forkhead box O1 (FOXO1) expression was observed in hemangioma samples only.EC isolation from IMH could be a useful tool for further research. These results suggest that increased Tie2 expression, via Akt-FOXO1 pathway activation, may play an important role in IMH pathogenesis. 
 Toll-like receptor 2 induced angiogenesis and invasion is mediated through the Tie2 signalling pathway in rheumatoid arthritis. Angiogenesis is a critical early event in inflammatory arthritis, facilitating leukocyte migration into the synovium resulting in invasion and destruction of articular cartilage and bone. This study investigates the effect of TLR2 on angiogenesis, EC adhesion and invasion using microvascular endothelial cells and RA whole tissue synovial explants ex-vivo.Microvascular endothelial cells (HMVEC) and RA synovial explants ex vivo were cultured with the TLR2 ligand, Pam3CSK4 (1 µg/ml). Angiopoietin 2 (Ang2), Tie2 and TLR2 expression in RA synovial tissue was assessed by immunohistology. HMVEC tube formation was assessed using Matrigel matrix assays. Ang2 was measured by ELISA. ICAM-1 cell surface expression was assessed by flow cytometry. Cell migration was assessed by wound repair scratch assays. ECM invasion, MMP-2 and -9 expression were assessed using transwell invasion chambers and zymography. To examine if the angiopoietin/Tie2 signalling pathway mediates TLR2 induced EC tube formation, invasion and migration assays were performed in the presence of a specific neutralising anti-Tie2mAb (10 ug/ml) and matched IgG isotype control Ab (10 ug/ml).Ang2 and Tie2 were localised to RA synovial blood vessels, and TLR2 was localised to RA synovial blood vessels, sub-lining infiltrates and the lining layer. Pam3CSK4 significantly increased angiogenic tube formation (p<0.05), and upregulated Ang2 production in HMVEC (p<0.05) and RA synovial explants (p<0.05). Pam3CSK4 induced cell surface expression of ICAM-1, from basal level of 149±54 (MFI) to 617±103 (p<0.01). TLR-2 activation induced an 8.8±2.8 fold increase   in cell inva  sion compared to control (p<0.05). Pam3CSK4 also induced HMVEC cell migration and induced MMP-2 and -9 from RA synovial explants. Neutralisation of the Ang2 receptor, Tie2 significantly inhibited Pam3CSK4-induced EC tube formation and invasion (p<0.05).TLR2 activation promotes angiogenesis, cell adhesion and invasion, effects that are in part mediated through the Tie2 signalling pathway, key mechanisms involved in the pathogenesis of RA. 
 Expression of angiopoietin 1, 2 and their common receptor Tie2 in human gastric carcinoma: implication for angiogenesis. Angiogenesis, formation of new microvessels providing oxygen and nutrient supply, is essential for tumor growth. It is dependent on the production of angiogenic growth factors by tumor cells. Angiopoietin 1 (Ang-1) and 2 (Ang-2) and their common receptor, Tie2, are thought to be critical regulators of tumor angiogenesis. We examined expression of Ang-1, Ang-2, and their common receptor Tie2 mRNAs and proteins in gastric cancers using in situ hybridization and immunohistochemistry. We also investigated the relationship between their expression and differentiation of cancer cells, lymph node metastasis, tumor size, depth of cancer   cell invasion  , TNM staging and microvessel density (MVD). The expression of Ang-1, Ang-2, and Tie2 mRNA in cancer cells significantly correlated with the MVD (p<0.001, <0.001 and =0.019, respectively). Ang-1 and Tie2 positivity correlated with advanced gastric cancers (p<0.05) and larger cancers had higher positive rates of Ang-1, Ang-2, and Tie2 mRNA expression (p<0.001, =0.010 and =0.039, respectively). Significant positive correlations were also found between mRNA expression of Tie2 and those of Ang-1 and Ang-2 (p<0.01 and <0.001, respectively). These findings indicate that the expression of Ang-1 and Ang-2 is important for tumor angiogenesis, and suggest a possible role of autocrine/paracrine function of angiopoietin/Tie2 system in gastric cancer progression. 
   
  
 FGFR4 
 
  NCBI Gene  
  GeneRIF  
  Pubmed  
 
 
  Search Term  Rank  Fields  
  [Mm]igration  6  Process(1); PubMed(7); GeneRIFs(1)  
  [Ee]xtracellular [Mm]atrix  8  Process(1); PubMed(2); GeneRIFs(1)  
  [Ii]nvasive  10  PubMed(11); GeneRIFs(3)  
  [Mm]etastasis  12  PubMed(15); GeneRIFs(3)  
  [Cc]ell [Ii]nvasion  13  PubMed(1)  
 
 
 hide/show details for FGFR4 
  [Mm]igration  Process  
 cell   migration   
  PubMed  
 FGFR4 GLY388 isotype suppresses motility of MDA-MB-231 breast cancer cells by EDG-2 gene repression. Clinical investigations of an FGFR4 germline polymorphism, resulting in substitution of glycine by arginine at codon 388 (G388 to R388), have shown a correlation between FGFR4 R388 and aggressive disease progression in cancer patients. Here, we studied the differential effects of the two FGFR4 isotypes on cellular signalling and motility in the MDA-MB-231 human breast cancer cell model. cDNA array analysis showed the ability of FGFR4 G388 to suppress expression of specific genes involved in invasiveness and motility. Further investigations concentrating on cell signalling and motility revealed an abrogation of phosphatidylinositol-3-kinase-dependent LPA-induced Akt activation and cell   migration   due to downregulation of the LPA receptor Edg-2 in FGFR4 G388-expressing MDA-MB-231 cells. Moreover, FGFR4 G388 expression attenuated the invasivity of the breast cancer cell line and decreased small Rho GTPase activity. We conclude that FGFR4 G388 suppresses cell motility of invasive breast cancer cells by altering signalling pathways and the expression of genes that are required for metastasis. Therefore, the positive effect of FGFR4 R388 on disease progression appears to result from a loss of the tumour suppressor activity displayed by FGFR4 G388 rather than the acquisition or enhancement of oncogenic potential. 
 Functional FGFR4 Gly388Arg polymorphism predicts prognosis in lung adenocarcinoma patients. Fibroblast growth factor receptor 4 (FGFR4) is a member of a family of transmembrane receptors with ligand-induced tyrosine kinase activity. The Gly388Arg polymorphism in the FGFR4 gene was reported to modulate cancer cell   migration   in vitro and to be associated with breast, colon, and prostate cancer prognostic parameters. The purpose of this study was to investigate the involvement of the FGFR4 polymorphism in lung tumorigenesis.A case-control study was performed including 274 patients with histologically confirmed lung adenocarcinoma and 401 healthy control subjects from general population. mRNA expression analysis was carried out in healthy lung of cancer patients.Patients with the Arg/Arg or Gly/Arg genotype compared to those with a Gly/Gly genotype had an earlier age at cancer onset (median age, 60.2 v 63.4 years), higher proportion of poor clinical stage disease (hazard ratio [HR], 2.3; 95% CI, 1.4 to 3.9; P = .002), of nodal involvement (HR, 1.9; 95% CI, 1.1 to 3.2; P = .027), or of short-term survivors (HR, 1.6; 95% CI, 1.1 to 2.3; P = .008). In healthy lungs, FGFR4 did not show allele-specific expression and mRNA levels were not associated with genotype.This study suggests that FGFR4 Gly388Arg polymorphism may predict prognosis in lung adenocarcinoma. 
 Overexpression of fibroblast growth factor receptor 4 in high-grade pancreatic intraepithelial neoplasia and pancreatic ductal adenocarcinoma. The overexpression of fibroblast growth factor receptor (FGFR) 4 has been reported in various human cancers, but it has not been studied in pancreatic ductal adenocarcinoma (PDAC) or its precursor lesion, pancreatic intraepithelial neoplasia (PanIN). Moreover, there is controversy as to whether FGFR4 has a mitogenic role in carcinogenesis or other functions. Therefore, the expression and roles of FGFR4 in pancreatic cancer were investigated. Immunohistochemical staining was performed using an anti-FGFR4 antibody in PDAC and PanIN cases. The expression levels of FGFR4 mRNA and protein were investigated in PDAC cell lines by qRT-PCR and Western blot, respectively. Changes were analyzed in cell morphology, proliferation,   migration  , invasion and attachment in PDAC cell lines with or without the stimulation of FGFR4 by FGF19, as a known specific ligand. The changes in mRNA levels associated with transformation and tumorigenesis as a result of FGF19 administration were also evaluated. FGFR4 was expressed in 39 of 53 PDAC cases (73.6%) and its expression tended to be related to longer overall survival (P=0.068). Moreover, it was frequently expressed in high-grade PanIN lesions [10 of 11 lesions (90.9%)], whereas it was hardly expressed in low-grade PanIN lesions [1 of 10 lesions (10.0%)] (P=0.0003). FGFR4 stimulation of PDAC cells resulted in significantly increased cell adhesion to laminin and fibronectin (P<0.05) and decreased cell   migration   (P<0.05). The results of PCR array analysis indicated that this was a result of up-regulation of the integrin α4 family. In contrast, cell morphology or proliferation in PDAC cells was not affected. We showed that FGFR4 expression is markedly increased in high-grade PanIN and PDAC compared with that in normal and low-grade PanIN, and that FGFR4 stimulation by FGF19 of PDAC cells contributes to tumor suppression by increasing cell adhesion to extracellular matrix. 
 The fibroblast growth factor receptor-4 Arg388 allele is associated with prostate cancer initiation and progression. Increased expression of fibroblast growth factors that can activate the fibroblast growth factor receptor-4 (FGFR-4) occurs in a substantial fraction of human prostate cancers in vivo. A germline polymorphism of the FGFR-4 gene resulting in expression of arginine at codon 388 (Arg388) is associated with aggressive disease in patients with breast and colon cancer. We therefore sought to determine whether the FGFR-4 Arg388 allele was associated with prostate cancer incidence and/or the occurrence of aggressive disease.The FGFR-4 genotype of men undergoing radical prostatectomy and controls of the same race was determined and the genotype correlated with clinical and pathologic markers of disease aggressiveness. PNT1A cell lines expressing predominantly the FGFR-4 Arg388 or Gly388 allele were established, and cell   migration   and invasiveness of these cells were assessed by a wounding assay and by quantitative determination of invasion through Matrigel. Expression of urokinase-type plasminogen activator receptor was determined by quantitative RT-PCR and enzyme-linked immunoabsorption assay.Homozygosity for the FGFR-4 Arg388 allele is strongly associated with the occurrence of prostate cancer in white men. The presence of the FGFR-4 Arg388 allele is also correlated with the occurrence of pelvic lymph node metastasis and biochemical (prostate-specific antigen) recurrence. Expression of FGFR-4 Arg388 in immortalized prostatic epithelial cells results in increased cell motility and invasion through Matrigel and was associated with increased expression of urokinase-type plasminogen activator receptor.The FGFR-4 Arg388 allele is associated with both an increased incidence and clinical aggressiveness of prostate cancer and results in changes in cellular motility and invasiveness in immortalized prostate epithelial cells consistent with the promotion of metastasis. 
 Fibroblast growth factor receptor 4 as a potential prognostic and therapeutic marker in colorectal cancer. The purpose of the study was to explore the significance of FGFR4 protein expression in colorectal cancer. Immunohistochemistry showed 46.8% (148/316) tumors positive for FGFR4 and 7.3% (23/316) for adjacent normal specimens. FGFR4 positivity was correlated with shortened disease free survival (DFS) and overall survival (OS). Multivariate analysis revealed that FGFR4 was an independent prognostic factor. FGFR4 silencing markedly reduced the   migration   and invasion capacity of colorectal cancer cell lines. These results suggest FGFR4 is a potential prognostic and therapeutic marker for colorectal cancer. 
 Differential effects of polymorphic alleles of FGF receptor 4 on colon cancer growth and metastasis. A gly(388)arg polymorphism (rs351855) in the transmembrane domain of the fibroblast growth factor receptor (FGFR4) is associated with increased risk, staging, and metastasis in several different types of cancer. To specifically assess the impact of the polymorphic FGFR4 in colorectal cancer (CRC), we engineered CRC cell lines with distinct endogenous expression patterns to overexpress either the FGFR4(gly) or FGFR4(arg) alleles. The biologic analyses revealed an oncogenic importance for both polymorphic alleles, but FGFR4(gly) was the stronger inducer of tumor growth, whereas FGFR4(arg) was the stronger inducer of   migration  . An evaluation of clinical specimens revealed that FGFR4 was upregulated in 20/71 patients independent of gly(388)arg status. There was no correlation between the presence of an FGFR4(arg) allele and CRC or polyp risk in 3,471 participants of the CORSA study. However, among 182 patients with CRC, FGFR4(arg)-carriers had a fivefold higher risk of tumors that were stage II or greater. Together, our results established that both allelic forms of FGFR4 exert an oncogenic impact and may serve equally well as therapeutic targets in CRC. One important implication of our findings is that FGFR4(arg)-carriers are at a higher risk for more aggressive tumors and therefore may profit from early detection measures. 
 Is fibroblast growth factor receptor 4 a suitable target of cancer therapy? Fibroblast growth factors (FGF) and their tyrosine kinase receptors (FGFR) support cell proliferation, survival and   migration   during embryonic development, organogenesis and tissue maintenance and their deregulation is frequently observed in cancer development and progression. Consequently, increasing efforts are focusing on the development of strategies to target FGF/FGFR signaling for cancer therapy. Among the FGFRs the family member FGFR4 is least well understood and differs from FGFRs1-3 in several aspects. Importantly, FGFR4 deletion does not lead to an embryonic lethal phenotype suggesting the possibility that its inhibition in cancer therapy might not cause grave adverse effects. In addition, the FGFR4 kinase domain differs sufficiently from those of FGFRs1-3 to permit development of highly specific inhibitors. The oncogenic impact of FGFR4, however, is not undisputed, as the FGFR4-mediated hormonal effects of several FGF ligands may also constitute a tissue-protective tumor suppressor activity especially in the liver. Therefore it is the purpose of this review to summarize all relevant aspects of FGFR4 physiology and pathophysiology and discuss the options of targeting this receptor for cancer therapy. 
  GeneRIFs  
 FGFR4 silencing markedly reduces the   migration   and invasion capacity of colorectal cancer cell lines. 
  [Ee]xtracellular [Mm]atrix  Process  
 regulation of   extracellular matrix   disassembly 
  PubMed  
 Fibroblast growth factor receptor 4 regulates tumor invasion by coupling fibroblast growth factor signaling to   extracellular matrix   degradation. Aberrant expression and polymorphism of fibroblast growth factor receptor 4 (FGFR4) has been linked to tumor progression and anticancer drug resistance. We describe here a novel mechanism of tumor progression by matrix degradation involving epithelial-to-mesenchymal transition in response to membrane-type 1 matrix metalloproteinase (MT1-MMP, MMP-14) induction at the edge of tumors expressing the FGFR4-R388 risk variant. Both FGFR4 and MT1-MMP were upregulated in tissue biopsies from several human cancer types including breast adenocarcinomas, where they were partially coexpressed at the tumor/stroma border and tumor invasion front. The strongest overall coexpression was found in prostate carcinoma. Studies with cultured prostate carcinoma cell lines showed that the FGFR4-R388 variant, which has previously been associated with poor cancer prognosis, increased MT1-MMP-dependent collagen invasion. In this experimental model, knockdown of FGFR4-R388 or MT1-MMP by RNA interference blocked tumor cell invasion and growth in collagen. This was coupled with impaired phosphorylation of FGFR substrate 2 and Src, upregulation of E-cadherin, and suppression of cadherin-11 and N-cadherin. These in vitro results were substantiated by reduced MT1-MMP content and in vivo growth of prostate carcinoma cells after the FGFR4-R388 gene silencing. In contrast, knockdown of the alternative FGFR4-G388 allele enhanced MT1-MMP and invasive tumor cell growth in vivo and within three-dimensional collagen. These results will help to explain the reported association of the FGFR4-R388 variant with the progression and poor prognosis of certain types of tumors. 
 Overexpression of fibroblast growth factor receptor 4 in high-grade pancreatic intraepithelial neoplasia and pancreatic ductal adenocarcinoma. The overexpression of fibroblast growth factor receptor (FGFR) 4 has been reported in various human cancers, but it has not been studied in pancreatic ductal adenocarcinoma (PDAC) or its precursor lesion, pancreatic intraepithelial neoplasia (PanIN). Moreover, there is controversy as to whether FGFR4 has a mitogenic role in carcinogenesis or other functions. Therefore, the expression and roles of FGFR4 in pancreatic cancer were investigated. Immunohistochemical staining was performed using an anti-FGFR4 antibody in PDAC and PanIN cases. The expression levels of FGFR4 mRNA and protein were investigated in PDAC cell lines by qRT-PCR and Western blot, respectively. Changes were analyzed in cell morphology, proliferation, migration, invasion and attachment in PDAC cell lines with or without the stimulation of FGFR4 by FGF19, as a known specific ligand. The changes in mRNA levels associated with transformation and tumorigenesis as a result of FGF19 administration were also evaluated. FGFR4 was expressed in 39 of 53 PDAC cases (73.6%) and its expression tended to be related to longer overall survival (P=0.068). Moreover, it was frequently expressed in high-grade PanIN lesions [10 of 11 lesions (90.9%)], whereas it was hardly expressed in low-grade PanIN lesions [1 of 10 lesions (10.0%)] (P=0.0003). FGFR4 stimulation of PDAC cells resulted in significantly increased cell adhesion to laminin and fibronectin (P<0.05) and decreased cell migration (P<0.05). The results of PCR array analysis indicated that this was a result of up-regulation of the integrin α4 family. In contrast, cell morphology or proliferation in PDAC cells was not affected. We showed that FGFR4 expression is markedly increased in high-grade PanIN and PDAC compared with that in normal and low-grade PanIN, and that FGFR4 stimulation by FGF19 of PDAC cells contributes to tumor suppression by increasing cell adhesion to   extracellular matri  x. 
  GeneRIFs  
 FGFR4 expression is markedly increased in high-grade PanIN and PDAC compared with that in normal and low-grade PanIN, and that FGFR4 stimulation by FGF19 of PDAC cells contributes to tumor suppression by increasing cell adhesion to   extracellular matrix  . 
  [Ii]nvasive  PubMed  
 FGFR4 GLY388 isotype suppresses motility of MDA-MB-231 breast cancer cells by EDG-2 gene repression. Clinical investigations of an FGFR4 germline polymorphism, resulting in substitution of glycine by arginine at codon 388 (G388 to R388), have shown a correlation between FGFR4 R388 and aggressive disease progression in cancer patients. Here, we studied the differential effects of the two FGFR4 isotypes on cellular signalling and motility in the MDA-MB-231 human breast cancer cell model. cDNA array analysis showed the ability of FGFR4 G388 to suppress expression of specific genes involved in   invasive  ness and motility. Further investigations concentrating on cell signalling and motility revealed an abrogation of phosphatidylinositol-3-kinase-dependent LPA-induced Akt activation and cell migration due to downregulation of the LPA receptor Edg-2 in FGFR4 G388-expressing MDA-MB-231 cells. Moreover, FGFR4 G388 expression attenuated the invasivity of the breast cancer cell line and decreased small Rho GTPase activity. We conclude that FGFR4 G388 suppresses cell motility of   invasive   breast cancer cells by altering signalling pathways and the expression of genes that are required for metastasis. Therefore, the positive effect of FGFR4 R388 on disease progression appears to result from a loss of the tumour suppressor activity displayed by FGFR4 G388 rather than the acquisition or enhancement of oncogenic potential. 
 Expression of pituitary tumour-derived, N-terminally truncated isoform of fibroblast growth factor receptor 4 (ptd-FGFR4) correlates with tumour   invasive  ness but not with G-protein alpha subunit (gsp) mutation in human GH-secreting pituitary adenomas. Apart from the constitutively activating mutation of the G-protein alpha subunit (Gsalpha) (gsp mutation), factors involved in tumorigenesis or those in tumour behaviour remain elusive in sporadic GH-secreting pituitary adenomas. Recently, the N-terminally truncated form of fibroblast growth factor receptor-4 (ptd-FGFR4) was identified in pituitary adenomas. This aberrant receptor has transforming activity, and causes pituitary adenomas in transgenic mice. The clinical relevance of this receptor warrants investigation. Our objective was twofold: first, to examine how the expression of ptd-FGFR4 relates to gsp mutations; and second, to see whether patients with this receptor have unique clinical characteristics.mRNA was extracted from excised adenomas of 45 Japanese acromegalic patients. ptd-FGFR4 expression and gsp mutations were determined by reverse transcription polymerase chain reaction (RT-PCR) and direct sequencing. Preoperative clinical data were collected by reviewing medical charts and pituitary magnetic resonance imaging (MRI) scans.ptd-FGFR4 mRNA expression was detected in 19 out of 45 tumours (42.2%) while gsp mutations were detected in 25 out of 45 tumours (55.6%). The prevalence of ptd-FGFR4 expression did not differ between gsp-positive (44.0%) and gsp-negative (40.0%) tumours (P = 1.00). ptd-FGFR4-positive tumours invaded the cavernous sinus more frequently (P = 0.0098) than did the ptd-FGFR4-negative tumours. Tumour size was not statistically different between ptd-FGFR4-positive and -negative tumours (P = 0.198). The presence of ptd-FGFR4 did not correlate with age at operation, sex, preoperative serum GH or IGF-1 levels.We found that ptd-FGFR4 expression and gsp mutations occur independently of each other, and that ptd-FGFR4 expression is associated with more   invasive   tumours in patients with GH-secreting pituitary adenomas. 
 Fibroblast growth factor receptor 4 regulates tumor invasion by coupling fibroblast growth factor signaling to extracellular matrix degradation. Aberrant expression and polymorphism of fibroblast growth factor receptor 4 (FGFR4) has been linked to tumor progression and anticancer drug resistance. We describe here a novel mechanism of tumor progression by matrix degradation involving epithelial-to-mesenchymal transition in response to membrane-type 1 matrix metalloproteinase (MT1-MMP, MMP-14) induction at the edge of tumors expressing the FGFR4-R388 risk variant. Both FGFR4 and MT1-MMP were upregulated in tissue biopsies from several human cancer types including breast adenocarcinomas, where they were partially coexpressed at the tumor/stroma border and tumor invasion front. The strongest overall coexpression was found in prostate carcinoma. Studies with cultured prostate carcinoma cell lines showed that the FGFR4-R388 variant, which has previously been associated with poor cancer prognosis, increased MT1-MMP-dependent collagen invasion. In this experimental model, knockdown of FGFR4-R388 or MT1-MMP by RNA interference blocked tumor cell invasion and growth in collagen. This was coupled with impaired phosphorylation of FGFR substrate 2 and Src, upregulation of E-cadherin, and suppression of cadherin-11 and N-cadherin. These in vitro results were substantiated by reduced MT1-MMP content and in vivo growth of prostate carcinoma cells after the FGFR4-R388 gene silencing. In contrast, knockdown of the alternative FGFR4-G388 allele enhanced MT1-MMP and   invasive   tumor cell growth in vivo and within three-dimensional collagen. These results will help to explain the reported association of the FGFR4-R388 variant with the progression and poor prognosis of certain types of tumors. 
 The fibroblast growth factor receptor-4 Arg388 allele is associated with prostate cancer initiation and progression. Increased expression of fibroblast growth factors that can activate the fibroblast growth factor receptor-4 (FGFR-4) occurs in a substantial fraction of human prostate cancers in vivo. A germline polymorphism of the FGFR-4 gene resulting in expression of arginine at codon 388 (Arg388) is associated with aggressive disease in patients with breast and colon cancer. We therefore sought to determine whether the FGFR-4 Arg388 allele was associated with prostate cancer incidence and/or the occurrence of aggressive disease.The FGFR-4 genotype of men undergoing radical prostatectomy and controls of the same race was determined and the genotype correlated with clinical and pathologic markers of disease aggressiveness. PNT1A cell lines expressing predominantly the FGFR-4 Arg388 or Gly388 allele were established, and cell migration and   invasive  ness of these cells were assessed by a wounding assay and by quantitative determination of invasion through Matrigel. Expression of urokinase-type plasminogen activator receptor was determined by quantitative RT-PCR and enzyme-linked immunoabsorption assay.Homozygosity for the FGFR-4 Arg388 allele is strongly associated with the occurrence of prostate cancer in white men. The presence of the FGFR-4 Arg388 allele is also correlated with the occurrence of pelvic lymph node metastasis and biochemical (prostate-specific antigen) recurrence. Expression of FGFR-4 Arg388 in immortalized prostatic epithelial cells results in increased cell motility and invasion through Matrigel and was associated with increased expression of urokinase-type plasminogen activator receptor.The FGFR-4 Arg388 allele is associated with both an increased incidence and clinical aggressiveness of prostate cancer and results in changes in cellular motility and   invasive  ness in immortalized prostate epithelial cells consistent with the promotion of metastasis. 
 Pituitary tumor-derived fibroblast growth factor receptor 4 isoform disrupts neural cell-adhesion molecule/N-cadherin signaling to diminish cell adhesiveness: a mechanism underlying pituitary neoplasia. We previously identified pituitary tumor-derived fibroblast growth factor receptor 4 (ptd-FGFR4), an alternatively transcribed N-terminally truncated cytoplasmic receptor isoform. Unlike wild-type FGFR4, ptd-FGFR4 facilitates cell transformation and results in pituitary tumor formation in transgenic mice. To investigate differences in the tumorigenic properties of FGFR4 and ptd-FGFR4, we examined their abilities to modulate cell adhesiveness. Introduction of ptd-FGFR4 into GH4 pituitary cells or NIH 3T3 fibroblasts resulted in significant reduction in cell adhesion to a collagen IV matrix compared with FGFR4- or empty vector-transfected cells. This adhesive difference was evident in the absence or presence of FGF stimulation. Furthermore, treatment with beta1-integrin neutralizing antibody markedly reduced adhesiveness in FGFR4-transfected cells but had little effect on the depressed adhesiveness of ptd-FGFR4-transfected cells. Unlike wild-type FGFR4, ptd-FGFR4 does not associate with neural cell-adhesion molecule (NCAM). Cells expressing FGFR4 demonstrate membranous N-cadherin with a non  invasive   growth pattern identical to control GH4 cells when injected into immunodeficient mice. In contrast, ptd-FGFR4-expressing cells develop   invasive   tumors in vivo with marked loss of N-cadherin that localizes to the cytoplasm. Consistent with these changes, beta-catenin expression was diminished and its interaction with N-cadherin was disrupted in the presence of ptd-FGFR4, but both were intact in the presence of wild-type FGFR4. These data highlight the importance of membrane-anchored FGFR4 in assembling a multiprotein FGFR4 complex with NCAM and N-cadherin playing pivotal functions in maintaining normal cell adhesion. Disruption of distinct NCAM/N-cadherin proadhesive complexes by a tumor-derived FGFR4 isoform provides a novel mechanism beyond ligand independence that explains the pathobiology of proliferative and infiltrative but nonmetastatic neoplasms. 
 Frequent methylation of the KLOTHO gene and overexpression of the FGFR4 receptor in   invasive   ductal carcinoma of the breast.   Invasive   ductal carcinoma of the breast is the most common cancer affecting women worldwide. The marked heterogeneity of breast cancer is matched only with the heterogeneity in its associated or causative factors. Breast cancer in Saudi Arabia is apparently an early onset with many of the affected females diagnosed before they reach the age of 50 years. One possible rationale underlying this observation is that consanguinity, which is widely spread in the Saudi community, is causing the accumulation of yet undetermined cancer susceptibility mutations. Another factor could be the accumulation of epigenetic aberrations caused by the shift toward a Western-like lifestyle in the past two decades. In order to shed some light into the molecular mechanisms underlying breast cancer in the Saudi community, we identified KLOTHO (KL) as a tumor-specific methylated gene using genome-wide methylation analysis of primary breast tumors utilizing the MBD-seq approach. KL methylation was frequent as it was detected in 55.3 % of breast cancer cases from Saudi Arabia (n = 179) using MethyLight assay. Furthermore, KL is downregulated in breast tumors with its expression induced following treatment with 5-azacytidine. The involvement of KL in breast cancer led us to investigate its relationship in the context of breast cancer, with one of the protagonists of its function, fibroblast growth factor receptor 4 (FGFR4). Overexpression of FGFR4 in breast cancer is frequent in our cohort and this overexpression is associated with poor overall survival. Interestingly, FGFR4 expression is higher in the absence of KL methylation and lower when KL is methylated and presumably silenced, which is suggestive of an intricate relationship between the two factors. In conclusion, our findings further implicate "metabolic" genes or pathways in breast cancer that are disrupted by epigenetic mechanisms and could provide new avenues for understanding this disease in a new context. 
 HFE, MTHFR, and FGFR4 genes polymorphisms and breast cancer in Brazilian women. Genetic factors related to cancer have been extensively studied and several polymorphisms have been associated to breast cancer. The FGFR4, MTHFR, and HFE genes have been associated with neoplastic diseases development. The current report outlines the analysis of the polymorphisms G388A (FGFR4), C677T (MTHFR), C282Y, and H63D (HFE) in Brazilian breast cancer patients. We studied 68 patients with   invasive   ductal and operable breast carcinoma and 85 women as a control group. The polymorphism frequencies in the breast cancer and control groups were analyzed, but no significant difference was observed by comparing the two groups. The presence of each polymorphism was analyzed according to the clinical features and markers already established as prognostic in the breast cancer group. The C677T, H63D, and G388A polymorphisms were not associated to histological grade, age of diagnosis, expression of HER2 receptor, or estrogen and progesterone receptor. The H63D polymorphism showed a significant association (P = 0.02) with the presence of p53 mutations, and C667T showed association to lymph node involvement (P = 0.05). Lymph node involvement, G388A polymorphism, and histological grade were independently associated to metastasis/death. Our data suggests that the polymorphisms G388A, C677T, and H63D are not useful in breast cancer diagnosis, but they may be significant additional prognostic markers related to breast cancer survival. 
 Identification of FGFR4 as a potential therapeutic target for advanced-stage, high-grade serous ovarian cancer. To evaluate the prognostic value of fibroblast growth factor receptor 4 (FGFR4) protein expression in patients with advanced-stage, high-grade serous ovarian cancer, delineate the functional role of FGFR4 in ovarian cancer progression, and evaluate the feasibility of targeting FGFR4 in serous ovarian cancer treatment.Immunolocalization of FGFR4 was conducted on 183 ovarian tumor samples. The collected FGFR4 expression data were correlated with overall survival using Kaplan-Meier and Cox regression analyses. The effects of FGFR4 silencing on ovarian cancer cell growth, survival,   invasive  ness, apoptosis, and FGF1-mediated signaling pathway activation were evaluated by transfecting cells with FGFR4-specific siRNAs. An orthotopic mouse model was used to evaluate the effect of injection of FGFR4-specific siRNAs and FGFR4 trap protein encapsulated in nanoliposomes on ovarian tumor growth in vivo.Overexpression of FGFR4 protein was significantly associated with decreased overall survival durations. FGFR4 silencing significantly decreased the proliferation, survival, and   invasive  ness and increased apoptosis of ovarian cancer cells. Also, downregulation of FGFR4 significantly abrogated the mitogen-activated protein kinase (MAPK), nuclear factor-κB (NF-κB), and WNT signaling pathways, which are activated by FGF1. Targeting FGFR4 with the FGFR4-specific siRNAs and FGFR4 trap protein significantly decreased ovarian tumor growth in vivo.FGFR4 is a prognostic marker for advanced-stage, high-grade serous ovarian carcinoma. Silencing FGFR4 and inhibiting ligand-receptor binding significantly decrease ovarian tumor growth both in vitro and in vivo, suggesting that targeting ovarian cancer cells with high levels of FGFR4 protein expression is a new therapeutic modality for this disease and will improve survival of it. 
 Silencing of FGFR4 could influence the biological features of gastric cancer cells and its therapeutic value in gastric cancer. To clarify the role of fibroblast growth factor receptor 4 (FGFR4) in gastric cancer (GC) and explore the therapeutic value of BGJ398 targeted to FGFR4. We constructed lentivirus vectors to stably knockdown FGFR4 expression in GC cells. Function assays in vitro and in vivo, treated with 5-fluorouracil (5-Fu) and BGJ398, were performed to study the change of biological behaviors of GC cells and related mechanism. The proliferation and   invasive   ability of HGC27 and MKN45 significantly decreased while the apoptosis rate of GC cells obviously increased in shRNA group (P < 0.05). The expressions of Bcl-xl, FLIP, PCNA, vimentin, p-erk, and p-STAT3 significantly reduced while the expressions of caspase-3 and E-cadherin markly enhanced in shRNA group. The proliferation abilities of GC cells were more significantly inhibited by the combination of BGJ398 and 5-Fu in shRNA group (P < 0.05). Compared to negative control (NC), the single and combination of 5-Fu and BGJ398 all significantly increased the apoptosis rate of GC cells, especially in the combination group (P < 0.01). The single and combination of 5-Fu and BGJ398 decreased the expressions of PCNA, Bcl-xl, and FLIP while increased the expression of caspase-3 in GC cells, especially in shRNA groups. Furthermore, knockdown of FGFR4 expression might prevent the growth of GC in vivo. Silencing of FGFR4 expression could   weaken   the invasive ability, increase the apoptosis rate, and decrease the proliferation ability of GC cells in vitro and in vivo. Furthermore, the combination of 5-Fu and BGJ398 had synergy in inhibiting the proliferation ability and increasing apoptosis rate of GC cells, directing a new target drug in GC. 
 Influence of the fibroblast growth factor receptor 4 expression and the G388R functional polymorphism on Cushing's disease outcome. Abnormal FGFR4 expression has been detected in pituitary tumors, especially in larger and   invasive   adenomas. In addition, the FGFR4 functional polymorphism G388R has been associated with poor outcome in several human malignancies. Then, we hypothesized that FGFR4 expression and genotype could be markers of adverse outcome of Cushing's disease after transsphenoidal surgery.The objective was to investigate whether there is an association between the postoperative outcome of Cushing's disease (remission/recurrence) and the FGFR4 G388R genotype or the FGFR4 expression in corticotrophinomas.Clinical, hormonal, and pathological data of 76 patients who underwent the first transsphenoidal surgery were retrospectively reviewed. All patients were genotyped for G388R polymorphism. FGFR4 expression was assessed by real-time PCR in 18 corticotrophinomas.The outcome measures included the FGFR4 G388R genotype and FGFR4 expression in postoperative remission and recurrence of Cushing's disease.Homozygosis for FGFR4 glycine (Gly(388)) allele was associated with reduced disease-free survival, in the univariate analysis (hazard ratio of 6.91; 95% confidence interval of 1.14-11.26; P = 0.028). Male gender (P = 0.036), lack of pathology confirmation (P = 0.009), and cortisol levels more than 2 μg/dl in the early postoperative period (P < 0.001) were also significant predictors of Cushing's disease recurrence in the univariate analysis. FGFR4 overexpression was found in 44% of the corticotrophinomas, and it was associated with lower postoperative remission rate (P = 0.009).Our data suggest that homozygosis for FGFR4 Gly(388) allele and FGFR4 overexpression are associated with higher frequency of postoperative recurrence and persistence of Cushing's disease, respectively. 
 Cytoplasmic expression of fibroblast growth factor receptor-4 in human pituitary adenomas: relation to tumor type, size, proliferation, and   invasive  ness. The pathogenesis of pituitary adenomas remains unknown. A pituitary tumor-derived (ptd) isoform of fibroblast growth factor receptor-4 (ptd-FGFR4) has been implicated in the neoplastic process. To further understand the expression of FGFR4 in sporadic human pituitary adenomas, we studied 137 pituitary adenomas of various types (102 adenomas from Japanese patients and 35 adenomas from Canadian patients) and 10 nontumorous pituitaries using a polyclonal antiserum that recognizes the C terminus of FGFR4 and analyzed possible relationships among expression of FGFR4, patient nationality, tumor type, size, invasion, and the labeling index of the proliferation marker Ki-67 using the MIB-1 antibody. Cytoplasmic expression of FGFR4 protein was observed in 57.8% of Japanese cases and 62.8% of Canadian cases. FGFR4 reactivity was absent in all 10 normal adenohypophysial tissues examined. FGFR4 expression in pituitary adenomas was restricted mainly to the cytoplasm, a pattern similar to that seen in rat pituitary cells transfected with human ptd-FGFR4 but different from that of cells transfected with wild-type FGFR4, which displayed membrane localization of staining. Protein from primary human adenomas migrated as a 65-kDa species consistent with the predicted size of ptd-FGFR4. FGFR4 protein expression was frequently found in adenomas containing GH, ACTH, or FSH/LH and was also found in null cell adenomas, but reactivity was relatively rare in prolactin-containing adenomas in both Japanese and Canadian groups. The expression of FGFR4 protein was stronger in macroadenomas than in microadenomas (P = 0.02) and high levels of FGFR4 expression (moderate or greater density staining) were more frequently observed in macroadenomas than in microadenomas (P < 0.05). High levels of FGFR4 expression also correlated significantly with the proliferation marker Ki-67 (P = 0.002) and tended (but not significantly) to be found in   invasive   tumors. These data are consistent with a role for ptd-FGFR4 in pituitary tumorigenesis in a majority of human pituitary adenomas. Moreover, detection of FGFR4 cytoplasmic staining may provide an ancillary diagnostic tool in the diagnosis of pituitary adenoma, particularly in equivocal cases. 
  GeneRIFs  
 Association of an FGFR4 variant with progression and poor prognosis of tumor   invasive  ness in breast and prostate cancers. 
 FGFR4 expression is associated with   invasive   tumours in patients with GH-secreting pituitary adenomas. 
 We conclude that FGFR4 germline polymorphism G388 suppresses cell motility of   invasive   breast cancer cells by altering signalling pathways and the expression of genes that are required for metastasis. 
  [Mm]etastasis  PubMed  
 FGFR4 GLY388 isotype suppresses motility of MDA-MB-231 breast cancer cells by EDG-2 gene repression. Clinical investigations of an FGFR4 germline polymorphism, resulting in substitution of glycine by arginine at codon 388 (G388 to R388), have shown a correlation between FGFR4 R388 and aggressive disease progression in cancer patients. Here, we studied the differential effects of the two FGFR4 isotypes on cellular signalling and motility in the MDA-MB-231 human breast cancer cell model. cDNA array analysis showed the ability of FGFR4 G388 to suppress expression of specific genes involved in invasiveness and motility. Further investigations concentrating on cell signalling and motility revealed an abrogation of phosphatidylinositol-3-kinase-dependent LPA-induced Akt activation and cell migration due to downregulation of the LPA receptor Edg-2 in FGFR4 G388-expressing MDA-MB-231 cells. Moreover, FGFR4 G388 expression attenuated the invasivity of the breast cancer cell line and decreased small Rho GTPase activity. We conclude that FGFR4 G388 suppresses cell motility of invasive breast cancer cells by altering signalling pathways and the expression of genes that are required for   metastasis  . Therefore, the positive effect of FGFR4 R388 on disease progression appears to result from a loss of the tumour suppressor activity displayed by FGFR4 G388 rather than the acquisition or enhancement of oncogenic potential. 
 Fibroblast growth factor receptor 4 protein expression and clinicopathological features in gastric cancer. To investigate fibroblast growth factor receptor 4 (FGFR4) protein expression in Chinese patients with resectable gastric cancer (GC) and the association with clinicopathological characteristics and survival.One hundred and seventy-five GC patients who underwent curative surgical procedures were enrolled in this study. The protein expression of FGFR4 in formalin-fixed, paraffin-embedded (FFPE) GC tissues was determined by immunohistochemical (IHC) analysis. Patient clinicopathological data and survival information were also collected and χ(2) statistical analysis was performed to analyze FGFR4 protein expression in the subgroups with differing clinicopathological characteristics including; gender, age, tumor location, differentiation, tumor-node  -metastasi  s stage, macroscopic type, depth of invasion, lymph node metastases, distant   metastasi  s, neural invasion and vascular invasion. Furthermore, some common molecular markers of GC in our cancer center, including p53, p27, topoisomerase IIα (Topo IIα) were also determined by IHC and their association with FGFR4 protein expression evaluated. The probability of survival for different subgroups with different clinicopathological characteristics was calculated using the Kaplan-Meier method and survival curves plotted using the log rank test.Seventy seven cases (44%) were found to have high expression of FGFR4 protein. Significantly different FGFR4 expression was observed between gastric cancers with differing expression of Topo IIα (log rank χ(2) = 9.4760, P = 0.0236). No significant differences were observed between subgroups defined by any of the other clinicopathological characteristics. The median survival time of the FGFR4 high expression (77 cases) and low expression groups (98 cases) was 27 mo and 39 mo, respectively. The five-year survival rates and median survival times of gastric cancers with high FGFR4 expression were worse than those with low expression (30.8% vs 39.2%, 27 mo vs 39 mo), respectively, however, no significant difference was observed in survival time (log rank χ(2) = 1.0477, P = 0.3060). Survival analysis revealed that high expression of FGFR4 was a predictor of poor outcome in GC patients if the tumor was small (less than or equal to 3 cm in size) (log rank χ(2) = 5.5033, P = 0.0190), well differentiated (log rank χ(2) = 7.9757, P = 0.0047), and of T1 or T2 stage invasion depth (log rank χ(2) = 4.8827, P = 0.0271).Our results suggest that high tumor expression of FGFR4 protein is not an independent risk factor for GC cancer initiation, but is a useful prognostic marker for GC patients when the tumor is relatively small, well differentiated, or in the early stages of invasion. 
 FGFR-4 Arg³⁸⁸ enhances prostate cancer progression via extracellular signal-related kinase and serum response factor signaling. Increased expression of FGFR-4 and its ligands have been linked to lethal prostate cancer (PCa). Furthermore, a germ line polymorphism in the FGFR-4 gene, resulting in arginine at codon 388 (Arg³⁸⁸) instead of glycine (Gly³⁸⁸), is associated with aggressive disease. The FGFR-4 Arg³⁸⁸ variant results in increased receptor stability, sustained receptor activation, and increased motility and invasion compared with Gly³⁸⁸. However, the impact of sustained signaling on cellular signal transduction pathways is unknown.Expression microarray analysis of immortalized prostatic epithelial cells lines expressing FGFR-4 Arg³⁸⁸ or Gly³⁸⁸ was used to establish a gene signature associated with FGFR-4 Arg³⁸⁸ expression. Transient transfection of reporters and inhibitors was used to establish the pathways activated by FGFR-4 Arg³⁸⁸ expression. The impact of pathway knockdown in vitro and in an orthotopic model was assessed using inhibitors and/or short hairpin RNA (shRNA).Expression of the FGFR-4 Arg³⁸⁸ protein leads to increased activity of the extracellular signal-related kinase (ERK) pathway, increased activity of serum response factor (SRF) and AP1, and transcription of multiple genes that are correlated with aggressive clinical behavior in PCa. Increased expression of SRF is associated with biochemical recurrence in men undergoing radical prostatectomy. Consistent with these observations, knockdown of FGFR-4 Arg³⁸⁸ in PCa cells decreases proliferat  ion and in  vasion in vitro and primary tumor growth and metastasis in vivo.These studies define a signal transduction pathway downstream of FGFR-4 Arg³⁸⁸ that acts via ERK and SRF to promote PCa progression. 
 Cancer progression and tumor cell motility are associated with the FGFR4 Arg(388) allele. Expression analysis of genes encoding components of the phosphotyrosine signaling system by cDNA array hybridization revealed elevated levels of FGFR4 transcripts in several mammary carcinoma cell lines. In the FGFR4 gene transcript from MDA-MB-453 mammary carcinoma cells, a G to A conversion was discovered that results in the substitution of glycine by arginine at position 388 in the transmembrane domain of the receptor. The Arg(388) allele was also found in cell lines derived from a variety of other tumor types as well as in the germ-line of cancer patients and healthy individuals. Analysis of three geographically separated groups indicated that it occurs in approximately 50% of the human population. Investigation of the clinical data of 84 breast cancer patients revealed that homo- or heterozygous carriers of the Arg(388) allele had a significantly reduced disease-free survival time (P = 0.01) within a median follow-up of 62 months. Moreover, the FGFR4 Arg(388) allele was associated with early lymph node   metastasis   and advanced tumor-node-  metastasis   (TNM) stage in 82 colon cancer patients. Consistent with this finding, MDA-MB-231 mammary tumor cells expressing FGFR4 Arg(388) exhibited increased motility relative to cells expressing the FGFR4 Gly(388) isotype. Our results support the conclusion that the FGFR4 Arg(388) allele represents a determinant that is innocuous in healthy individuals but predisposes cancer patients for significantly accelerated disease progression. 
 Identification of FGFR4-activating mutations in human rhabdomyosarcomas that promote   metastasis   in xenotransplanted models. Rhabdomyosarcoma (RMS) is a childhood cancer originating from skeletal muscle, and patient survival is poor in the presence of metastatic disease. Few determinants that regulate   metastasis   development have been identified. The receptor tyrosine kinase FGFR4 is highly expressed in RMS tissue, suggesting a role in tumorigenesis, although its functional importance has not been defined. Here, we report the identification of mutations in FGFR4 in human RMS tumors that lead to its activation and present evidence that it functions as an oncogene in RMS. Higher FGFR4 expression in RMS tumors was associated with advanced-stage cancer and poor survival, while FGFR4 knockdown in a human RMS cell line reduced tumor growth and experimental lung metastases when the cells were transplanted into mice. Moreover, 6 FGFR4 tyrosine kinase domain mutations were found among 7 of 94 (7.5%) primary human RMS tumors. The mutants K535 and E550 increased autophosphorylation, Stat3 signaling, tumor proliferation, and metastatic potential when expressed in a murine RMS cell line. These mutants also transformed NIH 3T3 cells and led to an enhanced metastatic phenotype. Finally, murine RMS cell lines expressing the K535 and E550 FGFR4 mutants were substantially more susceptible to apoptosis in the presence of a pharmacologic FGFR inhibitor than the control cell lines expressing the empty vector or wild-type FGFR4. Together, our results demonstrate that mutationally activated FGFR4 acts as an oncogene, and these are what we believe to be the first known mutations in a receptor tyrosine kinase in RMS. These findings support the potential therapeutic targeting of FGFR4 in RMS. 
 The fibroblast growth factor receptor-4 Arg388 allele is associated with prostate cancer initiation and progression. Increased expression of fibroblast growth factors that can activate the fibroblast growth factor receptor-4 (FGFR-4) occurs in a substantial fraction of human prostate cancers in vivo. A germline polymorphism of the FGFR-4 gene resulting in expression of arginine at codon 388 (Arg388) is associated with aggressive disease in patients with breast and colon cancer. We therefore sought to determine whether the FGFR-4 Arg388 allele was associated with prostate cancer incidence and/or the occurrence of aggressive disease.The FGFR-4 genotype of men undergoing radical prostatectomy and controls of the same race was determined and the genotype correlated with clinical and pathologic markers of disease aggressiveness. PNT1A cell lines expressing predominantly the FGFR-4 Arg388 or Gly388 allele were established, and cell migration and invasiveness of these cells were assessed by a wounding assay and by quantitative determination of invasion through Matrigel. Expression of urokinase-type plasminogen activator receptor was determined by quantitative RT-PCR and enzyme-linked immunoabsorption assay.Homozygosity for the FGFR-4 Arg388 allele is strongly associated with the occurrence of prostate cancer in white men. The presence of the FGFR-4 Arg388 allele is also correlated with the occurrence of pelvic lymph node   metastasis   and biochemical (prostate-specific antigen) recurrence. Expression of FGFR-4 Arg388 in immortalized prostatic epithelial cells results in increased cell motility and invasion through Matrigel and was associated with increased expression of urokinase-type plasminogen activator receptor.The FGFR-4 Arg388 allele is associated with both an increased incidence and clinical aggressiveness of prostate cancer and results in changes in cellular motility and invasiveness in immortalized prostate epithelial cells consistent with the promotion of   metastasis  . 
 Amplification of fgfr4 gene in human breast and gynecological cancers. We have investigated gene amplification of fibroblast growth factor receptor-4 (FGFR4) gene in 30 primary breast tumor samples and 15 gynecological tumor samples. Ten percent of the breast tumors showed 2- to 4-fold amplification. Amplification was found more frequently in estrogen- and progesterone-receptor-positive tumors and in tumors with high lymph-node involvement. Breast tumor samples were also analyzed for the amplification of fgfr3 and erbB2 genes and the chromosome 11q13 located genes hst1/int2/bcl1/sea. erbB2 gene was amplified 2- to 13-fold in 13% of the cases, but no amplification of int2/hst1/bcl1/sea amplicon was found. Gynecological tumors were also analyzed for the amplification of fgfr4 and fgfr3 genes and for int2 and hst1 oncogenes. Eleven of the 15 gynecological tumors were ovarian neoplasms including 2 benign tumors; the remainder comprised 1 ovarian   metastasis   of breast cancer; 1 endometrial cancer; 1 uterine leiomyosarcoma and 1 carcinosarcoma of the fallopian tube. In gynecological tumors, fgfr4 gene was found to be amplified in 2 ovarian tumors. Amplification of hst1 was found in 1 benign ovarian tumor. Thus, the fgfr4 gene may be involved in breast and ovarian tumorigenesis. 
 The FGFR4-G388R single-nucleotide polymorphism alters pancreatic neuroendocrine tumor progression and response to mTOR inhibition therapy. Pancreatic neuroendocrine tumors (pNET), also known as islet cell tumors, exhibit a wide range of biologic behaviors ranging from long dormancy to rapid progression. Currently, there are few molecular biomarkers that can be used to predict recurrence/  metastasis   or response to therapy. This study examined the predictive and prognostic value of a single nucleotide polymorphism substituting an arginine (R) for glycine (G) in codon 388 of the FGFR4 transmembrane domain. We established the FGFR4 genotype of 71 patients with pNETs and correlated genotype with biologic behavior. We created an in vivo model of pNET with BON1 cells and transfected them with either FGFR4-G388 or FGFR4-R388 to determine the mechanism of action and to examine response to the mTOR inhibitor everolimus. We then validated the predictive results of experimental studies in a group of patients treated with everolimus. FGFR4-R388 is associated with more aggressive clinical behavior in patients with pNETs with a statistically significant higher risk of advanced tumor stage and liver   metastasis  . Using an orthotopic mouse xenograft model, we show that FGFR4-R388 promotes tumor progression by increasing intraperitoneal spread and metastatic growth within the liver. Unlike FGFR4-G388, FGFR4-R388 BON1 tumors exhibited diminished responsiveness to everolimus. Concordantly, there was a statistically significant reduction in response to everolimus in patients with FGFR4-R388. Our findings highlight the importance of the FGFR4 allele in pNET progression and identify a predictive marker of potential therapeutic importance in this disease. 
 FGFR4 promotes stroma-induced epithelial-to-mesenchymal transition in colorectal cancer. Tumor cells evolve by interacting with the local microenvironment; however, the tumor-stroma interactions that govern tumor   metastasis   are poorly understood. In this study, proteomic analyses reveal that coculture with tumor-associated fibroblasts (TAF) induces significant overexpression of FGFR4, but not other FGFRs, in colorectal cancer cell lines. Mechanistic study shows that FGFR4 plays crucial roles in TAF-induced epithelial-to-mesenchymal transition (EMT) in colorectal cancer cell lines. Accumulated FGFR4 in cell membrane phosphorylates β-catenin, leading to translocation of β-catenin into the nucleus. Further, TAF-derived CCL2 and its downstream transcription factor, Ets-1, are prerequisites for TAF-induced FGFR4 upregulation. Furthermore, FGFR4-associated pathways are shown to be preferentially activated in colorectal tumor samples, and direct tumo  r metastas  is in a mous  e metastas  is model. Our study shows a pivotal role of FGFR4 in tumor-stroma interactions during colorectal cance  r metastas  is, and suggests novel therapeutic opportunities for the treatment of colorectal cancer. 
 HFE, MTHFR, and FGFR4 genes polymorphisms and breast cancer in Brazilian women. Genetic factors related to cancer have been extensively studied and several polymorphisms have been associated to breast cancer. The FGFR4, MTHFR, and HFE genes have been associated with neoplastic diseases development. The current report outlines the analysis of the polymorphisms G388A (FGFR4), C677T (MTHFR), C282Y, and H63D (HFE) in Brazilian breast cancer patients. We studied 68 patients with invasive ductal and operable breast carcinoma and 85 women as a control group. The polymorphism frequencies in the breast cancer and control groups were analyzed, but no significant difference was observed by comparing the two groups. The presence of each polymorphism was analyzed according to the clinical features and markers already established as prognostic in the breast cancer group. The C677T, H63D, and G388A polymorphisms were not associated to histological grade, age of diagnosis, expression of HER2 receptor, or estrogen and progesterone receptor. The H63D polymorphism showed a significant association (P = 0.02) with the presence of p53 mutations, and C667T showed association to lymph node involvement (P = 0.05). Lymph node involvement, G388A polymorphism, and histological grade were independently associated to   metastasis  /death. Our data suggests that the polymorphisms G388A, C677T, and H63D are not useful in breast cancer diagnosis, but they may be significant additional prognostic markers related to breast cancer survival. 
 FGF receptor-4 (FGFR4) polymorphism acts as an activity switch of a membrane type 1 matrix metalloproteinase-FGFR4 complex. Tumor cells use membrane type 1 matrix metalloproteinase (MT1-MMP) for invasion and   metastasis  . However, the signaling mechanisms that underlie MT1-MMP regulation in cancer have remained unclear. Using a systematic gain-of-function kinome screen for MT1-MMP activity, we have here identified kinases that significantly enhance MT1-MMP activity in tumor cells. In particular, we discovered an MT1-MMP/FGF receptor-4 (FGFR4) membrane complex that either stimulates or suppresses MT1-MMP and FGFR4 activities, depending on a tumor progression-associated polymorphism in FGFR4. The FGFR4-R388 allele, linked to poor cancer prognosis, increased collagen invasion by decreasing lysosomal MT1-MMP degradation. FGFR4-R388 induced MT1-MMP phosphorylation and endosomal stabilization, and surprisingly, the increased MT1-MMP in return enhanced FGFR4-R388 autophosphorylation. A phosphorylation-defective MT1-MMP was stabilized on the cell surface, where it induced simultaneous FGFR4-R388 internalization and dissociation of cell-cell junctions. In contrast, the alternative FGFR4-G388 variant down-regulated MT1-MMP, and the overexpression of MT1-MMP and particularly its phosphorylation-defective mutant vice versa induced FGFR4-G388 degradation. These results provide a mechanistic basis for FGFR4-R388 function in cancer invasion. 
 Differential effects of polymorphic alleles of FGF receptor 4 on colon cancer growth and   metastasis  . A gly(388)arg polymorphism (rs351855) in the transmembrane domain of the fibroblast growth factor receptor (FGFR4) is associated with increased risk, staging, and   metastasis   in several different types of cancer. To specifically assess the impact of the polymorphic FGFR4 in colorectal cancer (CRC), we engineered CRC cell lines with distinct endogenous expression patterns to overexpress either the FGFR4(gly) or FGFR4(arg) alleles. The biologic analyses revealed an oncogenic importance for both polymorphic alleles, but FGFR4(gly) was the stronger inducer of tumor growth, whereas FGFR4(arg) was the stronger inducer of migration. An evaluation of clinical specimens revealed that FGFR4 was upregulated in 20/71 patients independent of gly(388)arg status. There was no correlation between the presence of an FGFR4(arg) allele and CRC or polyp risk in 3,471 participants of the CORSA study. However, among 182 patients with CRC, FGFR4(arg)-carriers had a fivefold higher risk of tumors that were stage II or greater. Together, our results established that both allelic forms of FGFR4 exert an oncogenic impact and may serve equally well as therapeutic targets in CRC. One important implication of our findings is that FGFR4(arg)-carriers are at a higher risk for more aggressive tumors and therefore may profit from early detection measures. 
 ECRG1 and FGFR4 single nucleotide polymorphism as predictive factors for nodal   metastasis   in oral squamous cell carcinoma. The aim of this study was to examine the pattern of SNPs in ECRG1 and FGFR4 gene of oral squamous cell carcinoma, and to evaluate the association between SNPs and prognostic parameters.Total 24 cases of oral squamous cell carcinoma patients were enrolled in this study. We analyzed the pattern of SNPs in ECRG1 and FGFR4 gene using PCR and direct sequence. Also we evaluated the association between SNPs pattern and clinicopathologic parameters of oral squamous cell carcinoma.The allele type Arg/Arg in ECRG1 gene was found in 13 (54.2%) patients, Arg/Gln in 11 (45.8%) patients and Gln/Gln in no patient. No clinical or pathological factor was associated with the SNP pattern in ECRG1. The allele types of FGFR4 amino acid 388 in 24 OSCC patients were Arg/Arg (8.3%), Arg/Gly (54.2%) and Gly/Gly (37.5%). No clinical or pathological factor was significantly associated with the SNP pattern except the nodal stage. The patients carrying FGFR4 allele Arg/Arg or Arg/Gly at amino acid 388 were associated with advanced N stage (pathologic N2+N3), compared to Gly/Gly allele carrying group (p=0.009).This study is the first to describe a SNP pattern of both FGFR4 and ECRG1 gene with OSCC in Asian patients. In this study, FGFR4 Arg allele carrier was associated with higher N stage compared with Gly allele. If the important biological role of FGFR4 and ECRG1 in OSCC can be confirmed in further studies, this might be a rational to consider the evaluation of these genes as a therapeutic target in OSCC. 
 FGFR4 and TGF-β1 expression in hepatocellular carcinoma: correlation with clinicopathological features and prognosis. To investigate the expression and correlation of transforming growth factor-β1 (TGF-β1) and fibroblast growth factor receptor 4 (FGFR4) in human hepatocellular carcinoma (HCC) and the relationship with clinicopathological features and prognosis.The expression of TGF-β1 and FGFR4 in 126 HCC samples was detected immunohistochemically. Combined with clinical postoperative follow-up data, the expression of TGF-β1 and FGFR4 in HCC and the relationship with the prognosis of patients were analyzed by statistically.The positive expression rate of TGF-β1 was 84.1% (106/126) in tumors, and that in peritumoral liver tissues was 64.3% (81/126); the positive expression rate of FGFR4 in tumors was 74.6% (94/126) and that in peritumoral liver tissues was 57.1% (72/126). The expression of TGF-β1 and FGFR4 in the carcinoma tissues was significantly higher than that in peritumoral liver tissues (p < 0.05). Intratumoral TGF-β1 and FGFR4 expression was associated with TNM stage (p < 0.05). TGF-β1 and FGFR4 expression levels didn't significantly correlate with other clinicopathological parameters, including age, sex, tumor size, serum AFP level, tumor differentiation, ly  mph node m  etastasis, etc. (p > 0.05). TGF-β1 expression was positively correlated with FGFR4 expression (r = 0.595, p < 0.05). Patients with positive FGFR4 or TGF-β1 expression had shorter overall survival compared with negative expression (p < 0.05).The expression of TGF-β1 and FGFR4 could make synergy on the occurrence and progression of HCC, and may be used as prognosis indicators for HCC patients. 
 Single nucleotide polymorphism in fibroblast growth factor receptor 4 at codon 388 is associated with prognosis in high-grade soft tissue sarcoma. A recent study revealed that single nucleotide polymorphism (SNP) at codon 388 (Gly or Arg) of fibroblast growth factor receptor 4 (FGFR4) was associated with prognosis in patients with carcinoma of the breast and colorectal carcinoma. The purpose of the current study was to investigate the correlation between codon 388 SNP and clinical prognosis in patients with sarcoma of the bone and soft tissues.Tumor samples were obtained from 143 patients with high-grade bone and soft tissue sarcomas at Okayama University Hospital between 1986-2002, and from 102 healthy volunteers. SNP of codon 388 was detected by sequencing and fragment length of polymerase chain reaction products digested by restriction enzyme. The chi-square test was used to compare genotype distribution and the Kaplan-Meier method was used for survival analysis.With regard to FGFR4 genotypes in the 143 patients studied, 54 (37.8%) were Gly/Gly, 72 (50.3%) were Gly/Arg, and 17 (11.9%) were Arg/Arg, findings that were not significantly different from those of controls (P = 0.97). With regard to cumulative overall and   metastasis  -free survival, patients with the Gly/Gly genotype were found to have a better prognosis (P = 0.085 and P = 0.27, respectively). FGFR4 SNP was found to be correlated significantly with overall and   metastasis  -free survival in patients with soft tissue sarcomas (P = 0.029 and P = 0.045, respectively), but not in those patients with bone sarcomas (P = 0.88 and P = 0.75, respectively).In the current study, the authors found a significant correlation between FGFR4 SNP and prognosis in patients with soft tissue sarcoma, although the samples were comprised of various histologic types. This SNP might be used to improve the prediction of clinical prognosis and lead to new treatment strategies in patients with soft tissue sarcomas. 
  GeneRIFs  
 High FGFR4 expression is associated with stroma-induced epithelial-to-mesenchymal transition resulting in colorectal cancer   metastasis  . 
 knockdown of FGFR-4 Arg388 in PCa cells decreases proliferation and invasion in vitro and primary tumor growth and   metastasis   in vivo. 
 We conclude that FGFR4 germline polymorphism G388 suppresses cell motility of invasive breast cancer cells by altering signalling pathways and the expression of genes that are required for   metastasis  . 
  [Cc]ell [Ii]nvasion  PubMed  
 Fibroblast growth factor receptor 4 regulates tumor invasion by coupling fibroblast growth factor signaling to extracellular matrix degradation. Aberrant expression and polymorphism of fibroblast growth factor receptor 4 (FGFR4) has been linked to tumor progression and anticancer drug resistance. We describe here a novel mechanism of tumor progression by matrix degradation involving epithelial-to-mesenchymal transition in response to membrane-type 1 matrix metalloproteinase (MT1-MMP, MMP-14) induction at the edge of tumors expressing the FGFR4-R388 risk variant. Both FGFR4 and MT1-MMP were upregulated in tissue biopsies from several human cancer types including breast adenocarcinomas, where they were partially coexpressed at the tumor/stroma border and tumor invasion front. The strongest overall coexpression was found in prostate carcinoma. Studies with cultured prostate carcinoma cell lines showed that the FGFR4-R388 variant, which has previously been associated with poor cancer prognosis, increased MT1-MMP-dependent collagen invasion. In this experimental model, knockdown of FGFR4-R388 or MT1-MMP by RNA interference blocked tumor   cell invasion   and growth in collagen. This was coupled with impaired phosphorylation of FGFR substrate 2 and Src, upregulation of E-cadherin, and suppression of cadherin-11 and N-cadherin. These in vitro results were substantiated by reduced MT1-MMP content and in vivo growth of prostate carcinoma cells after the FGFR4-R388 gene silencing. In contrast, knockdown of the alternative FGFR4-G388 allele enhanced MT1-MMP and invasive tumor cell growth in vivo and within three-dimensional collagen. These results will help to explain the reported association of the FGFR4-R388 variant with the progression and poor prognosis of certain types of tumors. 
   
  
 KDR 
 
  NCBI Gene  
  GeneRIF  
  Pubmed  
 
 
  Search Term  Rank  Fields  
  [Ff]ilopodia  1  PubMed(1)  
  [Mm]igration  6  Process(4); PubMed(167); GeneRIFs(14); Summary(1)  
  ECM  7  PubMed(3)  
  [Ee]xtracellular [Mm]atrix  8  Process(1); PubMed(17); GeneRIFs(1)  
  [Ii]nvasive  10  PubMed(31); GeneRIFs(6)  
  [Mm]etastasize  11  PubMed(1)  
  [Mm]etastasis  12  PubMed(71); GeneRIFs(7)  
  [Cc]ell [Ii]nvasion  13  PubMed(2); GeneRIFs(1)  
 
 
 hide/show details for KDR 
  [Ff]ilopodia  PubMed  
 VEGF receptor 2/-3 heterodimers detected in situ by proximity ligation on angiogenic sprouts. The vascular endothelial growth factors VEGFA and VEGFC are crucial regulators of vascular development. They exert their effects by dimerization and activation of the cognate receptors VEGFR2 and VEGFR3. Here, we have used in situ proximity ligation to detect receptor complexes in intact endothelial cells. We show that both VEGFA and VEGFC potently induce formation of VEGFR2/-3 heterodimers. Receptor heterodimers were found in both developing blood vessels and immature lymphatic structures in embryoid bodies. We present evidence that heterodimers frequently localize to tip cell   filopodia  . Interestingly, in the presence of VEGFC, heterodimers were enriched in the leading tip cells as compared with trailing stalk cells of growing sprouts. Neutralization of VEGFR3 to prevent heterodimer formation in response to VEGFA decreased the extent of angiogenic sprouting. We conclude that VEGFR2/-3 heterodimers on angiogenic sprouts induced by VEGFA or VEGFC may serve to positively regulate angiogenic sprouting. 
  [Mm]igration  Process  
 cell   migration   involved in sprouting angiogenesis 
 positive regulation of cell   migration   
 positive regulation of cell   migration   
 positive regulation of endothelial cell   migration   
  PubMed  
 Vascular endothelial-cadherin stimulates syndecan-1-coupled insulin-like growth factor-1 receptor and cross-talk between αVβ3 integrin and vascular endothelial growth factor receptor 2 at the onset of endothelial cell dissemination during angiogenesis. Vascular endothelial growth factor (VEGF)-stimulated angiogenesis depends on a cross-talk mechanism involving VEGF receptor 2 (VEGFR2), vascular endothelial (VE)-cadherin and the αVβ3 integrin. Because we have shown that αVβ3 integrin activation is dependent on its incorporation, along with the insulin-like growth factor-1 receptor (IGF1R) kinase, into a ternary receptor complex organized by the matrix receptor syndecan-1 (Sdc1), we questioned the role of this core complex in VEGF-stimulated angiogenesis. We find that the Sdc1-coupled ternary receptor complex is required for VEGF signalling and for stimulation of vascular endothelial   cell mig  ration by vascular endothelial cadherin (VE-cadherin) engagement. VE-cadherin binding to Fc/VE-cadherin extracellular domain chimera activates Sdc1-coupled IGF1R and αvβ3 integrin; this depends on VEGFR2 and c-Src activated by the cadherin. Blocking homotypic VE-cadherin engagement disrupts VEGF-stimulat  ed cell m  igration, which is restored by clustering the cadherin in the absence of cell-cell adhesion. This cadherin-dependent stimulation requires VEGFR2 and IGF1R and is blocked by synstatin (SSTN)(92-119), a peptide that competitively disrupts the Sdc1-coupled ternary complex and prevents the αVβ3 integrin activation required for VEGFR2 activation. VEGFR2-stimulated angiogenesis in the mouse aortic ring explant assay is disrupted by SSTN, although only early in the process, suggesting that IGF1R coupling to Sdc1 and αVβ3 integrin comprises a core activation mechanism activated by VE-cadherin that is necessary for VEGFR2 and integrin activation in the initial stages of endothelial cell dissemination during angiogenesis. 
 Increased expression of vascular endothelial growth factor and its receptors, VEGFR-1 and VEGFR-2, in medullary thyroid carcinoma. Vascular endothelial growth factor (VEGF-A) expression is upregulated in the majority of human tumors, where it stimulates proliferation,   migration  , and survival of endothelial cells. Studies have suggested that VEGF inhibitors can be used as an alternative therapy in medullary thyroid carcinoma (MTC), but data about expression of VEGF-A and its receptor in this tumor are scarce. The aims of this study were to evaluate VEGF-A, VEGF receptor (VEGFR)-1, VEGFR-2, and microvessel density (MVD) expression in MTC samples and correlate it with clinical parameters.Paraffin-embedded samples from 38 MTC patients were evaluated for VEGF-A, VEGFR-1, VEGFR-2, and MVD expression by immunohistochemistry. Clinical data were retrospectively reviewed in medical records.Thirty-eight patients aged 31.8 +/- 17.1 years were enrolled. Twenty-seven patients had hereditary disease (71.1%). Twenty-five of them were found to have multiple endocrine neoplasia (MEN) 2A and two were found to have MEN 2B. VEGF-A immunohistochemical staining was detected in 95% (36/38), VEGFR-1 in 96% (36/37), and VEGFR-2 in 91% (31/34) of MTC samples. Age at surgery was positively correlated with VEGFR-2 (p = 0.003). There was no correlation between VEGF-A, VEGFR-2, and tumor stage (tumor node metastasis). Nevertheless, VEGFR-1 was found to be inversely correlated with tumor node metastasis (p = 0.034). We also observed a trend toward an association between VEGFR-1 signal intensity and cure of disease, although this did not reach statistical significance (p = 0.054). Neither VEGF-A nor VEGFR-2 was associated with disease outcome after a median follow-up period of 5 years (p = 0.882 and p = 0.236, respectively). As expected, MVD was correlated with age at surgery (p = 0.005) and tumor size (p = 0.03). Patients with the hereditary form of the disease had a stronger intensity for VEGFR-1 (p = 0.039), whereas patients with sporadic disease displayed higher MVD counts (44 [27-63] vs. 21 [9-49], p = 0.018).The VEGF-A, VEGFR-1, and VEGFR-2 immunoreactive proteins are overexpressed in MTC lesions and might be implicated in tumor progression. It is not clear, however, if expression of these molecules provides prognostic information regarding the spread or outcome of MTC. 
 Glioma cells enhance endothelial progenitor cell angiogenesis via VEGFR-2, not VEGFR-1. Although potential contribution of endothelial progenitor cells (EPCs) to angiogenesis in glioma has been proposed, the molecular mechanisms of EPCs recruitment to vasculature have not been fully elucidated. Here, we show that the supernatant from glioma cells promotes EPCs angiogenesis via VEGFR-2, not VEGFR-1. Moreover, VEGFR-2 siRNA inhibits VEGFR-2 expression in EPCs, tube formation on matrigel and cell   migration  . MMP-9 activity and expression and the Akt and ERK phosphorylations are decreased by VEGFR-2 siRNA. Thus, these results indicate that glioma cells enhance EPC angiogenesis via VEGFR-2, not VEGFR-1, mediated by the MMP-9, Akt and ERK signal pathways. 
 Immunolocalization and expression of vascular endothelial growth factor receptors (VEGFRs) and neuropilins (NRPs) on keratinocytes in human epidermis. Vascular endothelial growth factor (VEGF) plays an important role in normal and pathological angiogenesis. VEGF receptors (VEGFRs, including VEGFR-1, VEGFR-2, and VEGFR-3) and neuropilins (NRPs, including NRP-1 and NRP-2) are high-affinity receptors for VEGF and are typically considered to be specific for endothelial cells. Here we showed expression of VEGFRs and NRPs on cultured epidermal keratinocytes at both mRNA and protein levels. We further localized these receptors by immunofluorescence (IF) staining in the epidermis of surgical skin specimens. We found positive staining for VEGFRs and NRPs in all layers of the epidermis except for the stratum corneum. VEGFR-1 and VEGFR-2 are primarily expressed on the cytoplasmic membrane of basal cells and the adjacent spinosum keratinocytes. All layers of the epidermis except for the horny cell layer demonstrated a uniform pattern of VEGFR-3, NRP-1, and NRP-2. Sections staining for NRP-1 and NRP-2 also showed diffuse intense fluorescence and were localized to the cell membrane and cytoplasm of keratinocytes. In another panel of experiments, keratinocytes were treated with different concentrations of VEGF, with or without VEGFR-2 neutralizing antibody in culture. VEGF enhanced the proliferation and   migration   of keratinocytes, and these effects were partially inhibited by pretreatment with VEGFR-2 neutralizing antibody. Adhesion of keratinocytes to type IV collagen-coated culture plates was decreased by VEGF treatment, but this reduction could be completely reversed by pretreatment with VEGFR-2 neutralizing antibody. Taken together, our results suggest that the expression of VEGFRs and NRPs on keratinocytes may constitute important regulators for its activity and may possibly be responsible for the autocrine signaling in the epidermis. 
 The role of heterodimerization between VEGFR-1 and VEGFR-2 in the regulation of endothelial cell homeostasis. VEGF-A activity is tightly regulated by ligand and receptor availability. Here we investigate the physiological function of heterodimers between VEGF receptor-1 (VEGFR-1; Flt-1) and VEGFR-2 (KDR; Flk-1) (VEGFR(1-2)) in endothelial cells with a synthetic ligand that binds specifically to VEGFR(1-2). The dimeric ligand comprises one VEGFR-2-specific monomer (VEGF-E) and a VEGFR-1-specific monomer (PlGF-1). Here we show that VEGFR(1-2) activation mediates VEGFR phosphorylation, endothelial cell   migration  , sustained in vitro tube formation and vasorelaxation via the nitric oxide pathway. VEGFR(1-2) activation does not mediate proliferation or elicit endothelial tissue factor production, confirming that these functions are controlled by VEGFR-2 homodimers. We further demonstrate that activation of VEGFR(1-2) inhibits VEGF-A-induced prostacyclin release, phosphorylation of ERK1/2 MAP kinase and mobilization of intracellular calcium from primary endothelial cells. These findings indicate that VEGFR-1 subunits modulate VEGF activity predominantly by forming heterodimer receptors with VEGFR-2 subunits and such heterodimers regulate endothelial cell homeostasis. 
 Urokinase-type Plasminogen Activator (uPA) Promotes Angiogenesis by Attenuating Proline-rich Homeodomain Protein (PRH) Transcription Factor Activity and De-repressing Vascular Endothelial Growth Factor (VEGF) Receptor Expression. Urokinase-type plasminogen activator (uPA) regulates angiogenesis and vascular permeability through proteolytic degradation of extracellular matrix and intracellular signaling initiated upon its binding to uPAR/CD87 and other cell surface receptors. Here, we describe an additional mechanism by which uPA regulates angiogenesis. Ex vivo VEGF-induced vascular sprouting from Matrigel-embedded aortic rings isolated from uPA knock-out (uPA(-/-)) mice was impaired compared with vessels emanating from wild-type mice. Endothelial cells isolated from uPA(-/-) mice show less proliferation and   migration   in response to VEGF than their wild type counterparts or uPA(-/-) endothelial cells in which expression of wild type uPA had been restored. We reported previously that uPA is transported from cell surface receptors to nuclei through a mechanism that requires its kringle domain. Intranuclear uPA modulates gene transcription by binding to a subset of transcription factors. Here we report that wild type single-chain uPA, but not uPA variants incapable of nuclear transport, increases the expression of cell surface VEGF receptor 1 (VEGFR1) and VEGF receptor 2 (VEGFR2) by translocating to the nuclei of ECs. Intranuclear single-chain uPA binds directly to and interferes with the function of the transcription factor hematopoietically expressed homeodomain protein or proline-rich homeodomain protein (HHEX/PRH), which thereby lose their physiologic capacity to repress the activity of vehgr1 and vegfr2 gene promoters. These studies identify uPA-dependent de-repression of vegfr1 and vegfr2 gene transcription through binding to HHEX/PRH as a novel mechanism by which uPA mediates the pro-angiogenic effects of VEGF and identifies a potential new target for control of pathologic angiogenesis. 
 Hypoxia differentially regulates VEGFR1 and VEGFR2 levels and alters intracellular signaling and cell   migration   in endothelial cells. The role of hypoxia on endothelial cell function and response to growth factors is unknown. Here, we tested the hypothesis that hypoxia re-programs endothelial function by modulating vascular endothelial growth factor receptor levels which in turn alter intracellular signaling and cell function. Hypoxia stimulated VEGF-A and VEGFR1 expression but decreased VEGFR2 levels in endothelial cells. During hypoxia, plasma membrane VEGFR1 levels were elevated whereas VEGFR2 levels were depleted. One functional consequence of hypoxia is a reduction in VEGF-A-stimulated and VEGFR2-regulated intracellular signaling including lowered endothelial nitric oxide synthase activation. Venous, arterial and capillary endothelial cells subjected to hypoxia all exhibited reduced cell   migration   in response to VEGF-A. A mechanistic explanation is that VEGFR1:VEGFR2 ratio is substantially increased during hypoxia to block VEGF-A-stimulated and VEGFR2-regulated endothelial responses to maximize cell viability and recovery. 
 Human immunodeficiency virus tat modulates the Flk-1/KDR receptor, mitogen-activated protein kinases, and components of focal adhesion in Kaposi's sarcoma cells. Kaposi's sarcoma (KS) spindle cell growth and spread have been reported to be modulated by various cytokines as well as the human immunodeficiency virus (HIV) gene product Tat. Recently, HIV-1 Tat has been shown to act like a cytokine and bind to the Flk-1/KDR receptor for the vascular endothelial growth factor A (VEGF-A), which is expressed by KS cells. We have characterized signal transduction pathways stimulated by HIV-1 Tat upon its binding to surface receptors on KS cells. We observed that stimulation in KS 38 spindle cells resulted in tyrosine phosphorylation and activation of the Flk-1/KDR receptor. We also report that HIV-1 Tat treatment enhanced the phosphorylation and association of proteins found in focal adhesions, such as the related adhesion focal tyrosine kinase RAFTK, paxillin, and p130(cas). Further characterization revealed the activation of mitogen-activated protein kinase, c-Jun amino-terminal kinase (JNK), and Src kinase. HIV-1 Tat contains a basic domain which can interact with growth factor tyrosine kinase receptors and a classical RGD sequence which may bind to and activate the surface integrin receptors for fibronectin and vitronectin. We observed that stimulation of KS cells with basic as well as RGD sequence-containing Tat peptides resulted in enhanced phosphorylation of RAFTK and activation of MAP kinase. These studies reveal that Tat stimulation activates a number of signal transduction pathways that are associated with cell growth and   migration  . 
 Integrin activation is required for VEGF and FGF receptor protein presence on human microvascular endothelial cells. Endothelial cell proliferation and   migration   is initiated by growth factors including FGF and VEGF that bind to specific transmembrane receptor tyrosine kinases. Mechanisms that regulate in vivo expression of fibroblast growth factor receptors (FGFR) and vascular endothelial growth factor receptors (VEGFR) are not well understood. Since it is well known that different matrices influence the proliferation and   migration   of endothelial cells in culture, we hypothesized that changes in the extracellular matrix environment can regulate growth factor receptors on endothelial cells. We cultured human microvascular endothelial cells on different matrices (vitronectin, laminin, fibronectin, fibrin, and collagen IV) and examined for the presence of growth factor receptors (FGFR-1, FGFR-2, VEGFR-1, and VEGFR-2). We show that vitronectin increased the presence of all four growth factor receptors and most notably, VEGFR-1. In contrast, fibrin decreased all four receptors, especially FGFR-1 and FGFR-2. Inhibiting phosphotyrosine signaling abolished immunostaining for all four receptors, regardless of the matrix, but was not dependent on activating the Fyn-Shc pathway. Cells plated on vitronectin in the presence of blocking antibodies to integrins alphavbeta3 and alphavbeta5 similarly decreased presence of these growth factor receptors. Our data suggests a possible mechanism of how matrix-integrin interactions regulate endothelial cell responsiveness to growth factors and anchorage-dependent cell growth. 
 ERK3 promotes endothelial cell functions by upregulating SRC-3/SP1-mediated VEGFR2 expression. Despite a regain of interest recently in ERK3 kinase signaling, the molecular regulations of both ERK3 gene expression and protein kinase activity are still largely unknown. While it is shown that disruption of ERK3 gene causes neonatal lethality, cell type-specific functions of ERK3 signaling remain to be explored. In this study, we report that ERK3 gene expression is upregulated by cytokines through c-Jun in endothelial cells; c-Jun binds to the ERK3 gene and regulates its transcription. We further reveal a new role for ERK3 in regulating endothelial cell   migration  , proliferation and tube formation by upregulating SRC-3/SP-1-mediated VEGFR2 expression. The underlying molecular mechanism involves ERK3-stimulated formation of a transcriptional complex involving coactivator SRC-3, transcription factor SP-1 and the secondary coactivator CBP. Taken together, our study identified a molecular regulatory mechanism of ERK3 gene expression and revealed a previously unknown role of ERK3 in regulating endothelial cell functions. 
 Rab GTPase regulation of VEGFR2 trafficking and signaling in endothelial cells. Vascular endothelial growth factor receptor 2 (VEGFR2) is a receptor tyrosine kinase that regulates vascular physiology. However, mechanism(s) by which VEGFR2 signaling and trafficking is coordinated are not clear. Here, we have tested endocytic Rab GTPases for regulation of VEGFR2 trafficking and signaling linked to endothelial cell   migration  .Quiescent VEGFR2 displays endosomal localization and colocalization with the Rab5a GTPase, an early endosome fusion regulator. Expression of GTP or GDP-bound Rab5a mutants block activated VEGFR2 trafficking and degradation. Manipulation of Rab7a GTPase activity associated with late endosomes using overexpression of wild-type or mutant proteins blocks activated VEGFR2 trafficking and degradation. Depletion of Rab7a decreased VEGFR2 Y1175 phosphorylation but increased p42/44 (pERK1/2) MAPK phosphorylation. Endothelial cell   migration   was increased by Rab5a depletion but decreased by Rab7a depletion.Rab5a and Rab7a regulate VEGFR2 trafficking toward early and late endosomes. Our data suggest that VEGFR2-mediated regulation of endothelial function is dependent on different but specific Rab-mediated GTP hydrolysis activity required for endosomal trafficking. 
 Klotho endows hepatoma cells with resistance to anoikis via VEGFR2/PAK1 activation in hepatocellular carcinoma. Klotho was originally characterized as an aging suppressor gene that predisposed Klotho-deficient mice to premature aging-like syndrome. Although Klotho was recently reported to exhibit tumor suppressive properties during various malignant transformations, the functional role and molecular mechanism of Klotho in hepatocarcinogenesis remains poorly understood. In our present study, immunohistochemical Klotho staining levels in a clinical follow-up of 52 hepatoma patients were significantly associated with liver cirrhosis, tumor multiplicity and venous invasion. The overall survival rate of hepatoma patients with high Klotho expression was significantly lower than those patients with low Klotho expression. Moreover, Klotho overexpression increased cellular   migration  , anchorage-independent growth, and anoikis resistance in hepatoma cells. Klotho overexpression elevated p21-activated kinase 1 (PAK1) expression and shRNA-mediated PAK1 knockdown and kinase activity inhibition with kinase dead mutant PAK1 K299R coexpression or allosteric inhibitor IPA3 treatment reversed anoikis resistance in Klotho-overexpressed hepatoma cells. More importantly, the pivotal significance of upregulated VEGFR2 protein levels mediated by Klotho expression was confirmed by VEGFR2 inhibitor Axitinib and blocking antibody treatment in hepatoma cells. Axitinib treatment sensitized anoikis was reversed by constitutive active mutant PAK1 T423E coexpression in Klotho-overexpressed hepatoma cells. Conversely, knockdown of Klotho reduced VEGFR2/PAK1 dependent anoikis resistance, which could be reversed by PAK1 T423E. These results revealed a novel oncogenic function of Klotho in promoting anoikis resistance via activating VEGFR2/PAK1 signaling, thus facilitating tumor   migration   and invasion during hepatoma progression, which could provide a putative molecular mechanism for tumor metastasis. 
 Formation of the collateral circulation is regulated by vascular endothelial growth factor-A and a disintegrin and metalloprotease family members 10 and 17. The density of native (preexisting) collaterals varies widely and is a significant determinant of variation in severity of stroke, myocardial infarction, and peripheral artery disease. However, little is known about mechanisms responsible for formation of the collateral circulation in healthy tissues.We previously found that variation in vascular endothelial growth factor (VEGF) expression causes differences in collateral density of newborn and adult mice. Herein, we sought to determine mechanisms of collaterogenesis in the embryo and the role of VEGF in this process.Pial collaterals begin forming between embryonic day 13.5 and 14.5 as sprout-like extensions from arterioles of existing cerebral artery trees. Global VEGF-A overexpressing mice (Vegf(hi/+)) formed more, and Vegf(lo/+) formed fewer, collaterals during embryogenesis, in association with differences in vascular patterning. Conditional global reduction of Vegf or Flk1 only during collaterogenesis significantly reduced collateral formation, but now without affecting vascular patterning, and the effects remained in adulthood. Endothelial-specific Vegf reduction had no effect on collaterogenesis. Endothelial-specific reduction of a disintegrin-and-metalloprotease-domain-10 (Adam10) and inhibition of γ-secretase increased collateral formation, consistent with their roles in VEGF-induced Notch1 activation and suppression of prosprouting signals. Endothelial-specific knockdown of Adam17 reduced collateral formation, consistent with its roles in endothelial cell   migratio  n and embryonic vascular stabilization, but not in activation of ligand-bound Notch1. These effects also remained in adulthood.Formation of pial collaterals occurs during a narrow developmental window via a sprouting angiogenesis-like mechanism, requires paracrine VEGF stimulation of fetal liver kinase 1-Notch signaling, and adult collateral number is dependent on embryonic collaterogenesis. 
 Analysis of protein-protein interactions in cross-talk pathways reveals CRKL protein as a novel prognostic marker in hepatocellular carcinoma. Deciphering the network of signaling pathways in cancer via protein-protein interactions (PPIs) at the cellular level is a promising approach but remains incomplete. We used an in situ proximity ligation assay to identify and quantify 67 endogenous PPIs among 21 interlinked pathways in two hepatocellular carcinoma (HCC) cells, Huh7 (minimally migratory cells) and Mahlavu (highly migratory cells). We then applied a differential network biology analysis and determined that the novel interaction, CRKL-FLT1, has a high centrality ranking, and the expression of this interaction is strongly correlated with the migratory ability of HCC and other cancer cell lines. Knockdown of CRKL and FLT1 in HCC cells leads to a decrease in cell   migration   via ERK signaling and the epithelial-mesenchymal transition process. Our immunohistochemical analysis shows high expression levels of the CRKL and CRKL-FLT1 pair that strongly correlate with reduced disease-free and overall survival in HCC patient samples, and a multivariate analysis further established CRKL and the CRKL-FLT1 as novel prognosis markers. This study demonstrated that functional exploration of a disease network with interlinked pathways via PPIs can be used to discover novel biomarkers. 
 Recombinant pigment epithelium-derived factor PEDF binds vascular endothelial growth factor receptors 1 and 2. Angiogenesis, or the formation of new blood vessels, is stimulated by angiogenic factors such as vascular endothelial growth factor (VEGF). Pigment epithelium-derived factor (PEDF) is a potent inhibitor of angiogenesis. To explore the mechanism by which PEDF acts, recombinant PEDF was expressed with a 6x-His tag (for purification) and a green fluorescent protein (GFP) tag. The PEDF fusion protein was confirmed to be active in inhibition of endothelial cell proliferation and   migration  . Direct binding of PEDF to both vascular endothelial growth factor receptor-1 (VEGFR-1) and VEGFR-2 was demonstrated in an in vitro assay similar to an enzyme-linked immunosorbent assay (ELISA). PEDF was shown by immune-confocal microscopy to be localized within treated endothelial cells. When VEGF-stimulated endothelial cells were incubated with PEDF the VEGF receptors showed intracellular localization. These data suggest that the interaction between PEDF and VEGFR-1 or VEGFR-2 may be a possible mechanism for inhibiting angiogenesis. PEDF may be binding to the VEGF receptors to promote their internalization and/or degradation to limit VEGF responses in treated cells. 
 Heparanase released from mesenchymal stem cells activates integrin beta1/HIF-2alpha/Flk-1 signaling and promotes endothelial cell   migration   and angiogenesis. Heparanase plays important roles in tumor angiogenesis. Our previous study demonstrated that hypoxic preconditioning (HPC) enhanced the angiogenic and therapeutic effects of mesenchymal stem cells (MSCs), effects that were paralleled by enhanced heparanase expression. This study was designed to elucidate the role of heparanase in the improved therapeutic properties of HPC-MSCs and to explore underlying mechanisms using an ischemic rat hind limb model. MSCs transfected with heparanase (MSC(hpa) ) or empty vector (MSC(null) ) were delivered by intramuscular injections to ischemic hind limbs. Hind limbs that received MSC(hpa) recovered blood flow more rapidly at 7 days and acquired higher capillary density at 14 days compared with MSC(null) . Conditioned medium from MSC(hpa) increased endothelial cell   migration   and promoted greater tube formation relative to that from the MSC(null) groups. Vascular endothelial growth factor receptor 2 (VEGFR2, Flk-1) and its downstream signaling pathway (p38MAPK/HSP27) were significantly increased in human umbilical vein endothelial cells (HUVECs) after treatment with MSC(hpa) conditioned medium. Each of these responses was decreased by cocultured with MSC(hpa-KD) conditioned medium. MSC(hpa) conditioned medium activated hypoxia-inducible factor-2α (HIF-2α) and increased in parallel the transcript level of Flk-1 as determined by chromatin immunoprecipitation-PCR and luciferase assays. Analyses of integrin expression revealed an important role for integrin β1 in the regulation of HIF-2α. All angiogenic effects of MSC(hpa) conditioned medium were abolished by knockdown of integrin β1, HIF-2α, and Flk-1 in HUVECs with selective shRNAs. These findings identify heparanse as a key regulator of angiogenesis by MSCs. We propose a novel pathway wherein heparanse sequentially activates integrin β1, HIF-2α, Flk-1, and p38MAPK/HSP27 with corresponding enhancement of angiogenesis. 
 Thioredoxin-interacting protein mediates TRX1 translocation to the plasma membrane in response to tumor necrosis factor-α: a key mechanism for vascular endothelial growth factor receptor-2 transactivation by reactive oxygen species. Thioredoxin-interacting protein (TXNIP) promotes inflammation in endothelial cells (EC) by binding to thioredoxin-1 (TRX1) in a redox-dependent manner. Formation of the TXNIP-TRX1 complex relieves inhibition of the apoptosis signal-regulating kinase 1-c-Jun N-terminal kinase-vascular cell adhesion molecule-1 pathway. Because TXNIP is an α-arrestin with numerous protein-protein interacting domains, we hypothesized that TXNIP-TRX1 trafficking should alter function of EC exposed to reactive oxygen species (ROS).In response to physiological levels of ROS (10 ng/mL tumor necrosis factor-α and 30 μmol/L H(2)O(2)), TXNIP-TRX1 translocated to the plasma membrane in human umbilical vein EC, with a peak at 30 minutes, as measured by immunofluorescence colocalization with vascular endothelial-cadherin, cell fractionation, and membrane sheet assay. TXNIP-mediated translocation of TRX1 to the membrane required TXNIP and TRX1 binding, as evidenced by inability of the ROS-insensitive TXNIP-Cys247Ser mutant to promote membrane localization. Vascular endothelial growth factor signaling required TXNIP, as shown by significant decreases in plasma membrane tyrosine phosphorylation and   EC migra  tion after TRX1 knockdown. Furthermore, TXNIP knockdown increased human umbilical vein EC apoptosis induced by tumor necrosis factor. Rescue with TXNIP-wild-type but not TXNIP-Cys247Ser prevented cell death.These findings suggest a novel role for the TXNIP-TRX1 complex to enable inflammation by promoting EC survival and vascular endothelial growth factor signaling under conditions of physiological oxidative stress. 
 Angiogenesis in systemic sclerosis: impaired expression of vascular endothelial growth factor receptor 1 in endothelial progenitor-derived cells under hypoxic conditions. To assess angiogenesis and explore the expression and regulation of vascular endothelial growth factor (VEGF), VEGF receptor 1 (VEGFR-1), and VEGFR-2, the leading mediators of angiogenesis, in SSc patients and controls.Late-outgrowth endothelial progenitor cells (EPCs), isolated from the peripheral blood of systemic sclerosis (SSc) patients and controls, and human umbilical vein endothelial cells (HUVECs) were assessed under normal and hypoxic conditions. Genomic background was evaluated in a large case-control study (including 659 patients with SSc and 511 controls) using tag single-nucleotide polymorphisms on VEGFR1 and VEGFR2 genes.EPCs from SSc patients had the phenotype of genuine endothelial cells and displayed in vitro angiogenic properties similar to those of HUVECs and control EPCs under basal conditions, as determined by flow cytometry, tube formation, and   migration   assay. However, after 6 hours of hypoxic exposure, EPCs from SSc patients exhibited lower induced expression of VEGFR-1 at the messenger RNA and protein levels, but similar VEGF and VEGFR-2 expression, compared with HUVECs or EPCs from healthy controls. There was no evidence of defective expression of hypoxia-inducible factor 1alpha. These results were supported by the lower serum levels of soluble VEGFR-1 found in SSc patients (n = 187) compared with healthy controls (n = 48) (mean +/- SD 163.7 +/- 98.5 versus 210.4 +/- 109.5 pg/ml; P = 0.0042). These abnormalities did not seem to be related to genomic background.Our findings shed new light on the possible role of VEGFR-1 in the main vascular disturbances that occur in SSc and lead to more severe disease. 
 Herbal compound farnesiferol C exerts antiangiogenic and antitumor activity and targets multiple aspects of VEGFR1 (Flt1) or VEGFR2 (Flk1) signaling cascades. Farnesiferol C (FC) is one of the major compounds isolated from Ferula assafoetida, an Asian herbal spice used for cancer treatment as a folk remedy. Here, we examined the hypothesis that novel antiangiogenic activities of FC contribute to anticancer efficacy. In human umbilical vein endothelial cells (HUVEC), exposure to the 10 to 40 mumol/L concentration range of FC inhibited vascular endothelial growth factor (VEGF)-induced cell proliferation,   migration  , invasion, tube formation, and the expression of matrix metalloproteinase-2. In addition, FC inhibited the angiogenic sprouting of VEGF-treated rat aorta in an ex vivo model. Furthermore, FC inhibited the in vivo growth of mouse Lewis lung cancer allograft model by 60% (P < 0.001) at a daily i.p. dosage of 1 mg/kg body weight without any negative effect on the weight of the host mice. Immunohistochemistry staining showed decreased microvessel density (CD34) and proliferative index (Ki-67) without affecting the apoptotic (terminal deoxynucleotidyl transferase-mediated dUTP nick end labeling) index. Mechanistically, FC decreased the binding of VEGF to VEGFR1/Flt-1, but not to VEGFR2/KDR/Flk-1. In terms of early signaling, FC exerted a rapid inhibitory action (examined within 10 minutes) on VEGF-induced autophosphorylation of VEGFR1 without affecting that of VEGFR2. Nevertheless, FC decreased the phosphorylation of most of the kinases downstream of VEGFR2: focal adhesion kinase, Src, extracellular signal-regulated kinase 1/2, p38 mitogen-activated protein kinase, and c-jun-NH(2)-kinase without affecting AKT. Computer simulation suggests that FC may inhibit Src or focal adhesion kinase protein activities directly through its docking to their ATP-binding sites. Taken together, the multitargeting actions of FC, particularly VEGFR1 inhibition, may make it a novel drug candidate to complement current VEGF/VEGFR2-targeting antiangiogenic modalities for cancer. 
 MT1-MMP regulates VEGF-A expression through a complex with VEGFR-2 and Src. Membrane-type-1 matrix metalloproteinase (MT1-MMP) is a zinc-dependent type-I transmembrane metalloproteinase involved in pericellular proteolysis,   migration   and invasion, with elevated levels correlating with a poor prognosis in cancer. MT1-MMP-mediated transcriptional regulation of genes in cancer cells can contribute to tumour growth, although this is poorly understood at a mechanistic level. In this study, we investigated the mechanism by which MT1-MMP regulates the expression of VEGF-A in breast cancer cells. We discovered that MT1-MMP regulates VEGFR-2 cell surface localisation and forms a complex with VEGFR-2 and Src that is dependent on the MT1-MMP hemopexin domain and independent of its catalytic activity. Although the localisation of VEGFR-2 was independent of the catalytic and intracellular domain of MT1-MMP, intracellular signalling dependent on VEGFR-2 activity leading to VEGF-A transcription still required the MT1-MMP catalytic and intracellular domain, including residues Y573, C574 and DKV582. However, there was redundancy in the function of the catalytic activity of MT1-MMP, as this could be substituted with MMP-2 or MMP-7 in cells expressing inactive MT1-MMP. The signalling cascade dependent on the MT1-MMP-VEGFR-2-Src complex activated Akt and mTOR, ultimately leading to increased VEGF-A transcription. 
 Gab1 regulates proliferation and   migration   through the PI3K/Akt signaling pathway in intrahepatic cholangiocarcinoma. Intrahepatic cholangiocarcinoma is the second most common primary malignant tumor of the liver, and it originates from the intrahepatic biliary duct epithelium. Prognosis is poor due to lack of effective comprehensive treatments. In this study, we assessed the expression of Gab1, VEGFR-2, and MMP-9 in intrahepatic cholangiocarcinoma solid tumors by immunohistochemistry and determined whether their expression was associated with clinical and pathological features. We found that expression of Gab1, VEGFR-2, and MMP-9 was highly and positively correlated with each other and with lymph node metastasis and TNM stage in intrahepatic cholangiocarcinoma tissues. Interference of Gab1 and VEGFR-2 expression via siRNA in the intrahepatic cholangiocarcinoma cell line RBE resulted in decreased PI3K/Akt pathway activity. Inhibition of Gab1 and VEGFR-2 expression also caused decreased cell proliferation, cell cycle arrested in G1 phase, increased apoptosis, and decreased invasion in RBE cells. These results suggest that Gab1, VEGFR-2, and MMP-9 contribute significantly to the highly malignant behavior of intrahepatic cholangiocarcinoma. The regulation of growth, apoptosis, and invasion by Gab1 through the VEGFR-2/Gab1/PI3K/Akt signaling pathway may represent potential targets for improving the treatment of intrahepatic cholangiocarcinoma. 
 Vascular endothelial growth factor and its receptor system: physiological functions in angiogenesis and pathological roles in various diseases. Vascular endothelial growth factors (VEGFs) belong to the platelet-derived growth factor supergene family, and they play central roles in the regulation of angiogenesis and lymphangiogenesis. VEGF-A, the major factor for angiogenesis, binds to two tyrosine kinase (TK) receptors, VEGFR-1 (Flt-1) and VEGFR-2 (KDR/Flk-1), and regulates endothelial cell proliferation,   migration  , vascular permeability, secretion and other endothelial functions. VEGFR-2 exhibits a strong TK activity towards pro-angiogenic signals, whereas the soluble VEGFR-1 (sFlt-1) functions as an endogenous VEGF inhibitor. sFlt-1 is abnormally overexpressed in the placenta of preeclampsia patients, resulting in the major symptoms of the disease due to abnormal trapping of VEGFs. The VEGF-VEGFR system is crucial for tumour angiogenesis, and anti-VEGF-VEGFR molecules are now widely used in the clinical field to treat cancer patients. The efficacy of these molecules in prolonging the overall survival of patients has been established; however, some cancers do not respond well and reduced tumour sensitivity to anti-VEGF signals may occur after long-term treatment. The molecular basis of tumour refractoriness should be determined to improve anti-angiogenic therapy. 
 Etk/Bmx transactivates vascular endothelial growth factor 2 and recruits phosphatidylinositol 3-kinase to mediate the tumor necrosis factor-induced angiogenic pathway. Tumor necrosis factor (TNF), via its receptor 2 (TNFR2), induces Etk (or Bmx) activation and Etk-dependent endothelial cell (EC)   migration   and tube formation. Because TNF receptor 2 lacks an intrinsic kinase activity, we examined the kinase(s) mediating TNF-induced Etk activation. TNF induces a coordinated phosphorylation of vascular endothelial growth factor (VEGF) receptor 2 (VEGFR2) and Etk, which is blocked by VEGFR2-specific inhibitors. In response to TNF, Etk and VEGFR2 form a complex resulting in a reciprocal activation between the two kinases. Subsequently, the downstream phosphatidylinositol 3-kinase (PI3K)-Akt signaling (but not signaling through phospholipase C-gamma) was initiated and directly led to TNF-induced EC   migration  , which was significantly inhibited by VEGFR2-, PI3K-, or Akt-specific inhibitors. Phosphorylation of VEGFR2 at Tyr-801 and Tyr-1175, the critical sites for VEGF-induced PI3K-Akt signaling, was not involved in TNF-mediated Akt activation. However, TNF induces phosphorylation of Etk at Tyr-566, directly mediating the recruitment of the p85 subunit of PI3K. Furthermore, TNF- but not VEGF-induced activation of VEGFR2, Akt, and EC   migration   are blunted in EC genetically deficient with Etk. Taken together, our data demonstrated that TNF induces transactivation between Etk and VEGFR2, and Etk directly activates PI3K-Akt angiogenic signaling independent of VEGF-induced VEGFR2-PI3K-Akt signaling pathway. 
 Metastatic properties of prostate cancer cells are controlled by VEGF. Mechanisms of metastasis, the major complication of prostate cancer, are poorly understood. In this study, we define molecular mechanisms that may contribute to the highly invasive potential of prostate cancer cells. Vascular endothelial growth factor (VEGF), its receptors (VEGFRs), and alpha5beta1 integrin were expressed by prostate cancer cells in vitro and by prostate tumors in vivo, and their expression was elevated at sites of bone metastasis compared to original prostate tumor. VEGF, through interaction with its receptors, regulated adhesive and migratory properties of the cancer cells. Specifically, the highly metastatic prostate cancer cell subline LNCaP-C4-2 showed a decreased adhesive but an enhanced migratory response to fibronectin, a ligand for alpha5beta1 integrin, compared to its nonmetastatic counterpart. A similar pattern was also observed when bone sialoprotein was used as a ligand in   migration   assays. Increased   migration   of metastatic prostate cancer cells to fibronectin and bone sialoprotein was regulated by VEGF via VEGFR-2. Tumor suppressor PTEN was involved in control of VEGF/VEGFR-2 stimulated prostate cancer cell adhesion as well as proliferation. 
 The inhibition of tyrosine kinase receptor signalling in leiomyosarcoma cells using the small molecule kinase inhibitor PTK787/ZK222584 (Vatalanib®). Leiomyosarcomas remain challenging tumors to manage and novel therapy strategies besides radiation and conventional chemotherapy are needed. Targeting angiogenesis by inhibition of vascular endothelial growth factor (VEGF) receptor tyrosine kinases (RTKs) of the tumor vasculature with small molecules is a promising new therapy. It has been shown recently that these receptors are not only expressed on tumor endothelium but also on tumor cells themselves. Thus, we investigated the expression of members of the VEGF receptor (VEGFR) family and corresponding growth factors in leiomyosarcoma tissue specimens and in the leiomyosarcoma cell lines SK-LMS-1 and SK-UT-1. We evaluated the influence of the VEGFR inhibitor PTK787/ZK222584 (PTK787) on cell growth  , migrati  on, apoptosis and phosphorylation of intracellular signalling molecules. In human leiomyosarcoma tissue specimens VEGFR‑1/-2 and platelet-derived growth factor receptor (PDGFR-β) were strongly expressed. Both leiomyosarcoma cell lines expressed VEGFR‑1/-3 and PDGFR-β but VEGFR-2 protein expression was positive only in SK-UT-1. SK-LMS-1 and SK-UT-1 cells secreted high and low amounts of VEGF-A, respectively, whereas PDGF-BB secretion was similar in both cell lines. Application of PTK787 led to partial inhibition of PDGF-BB-activated AKT/p90RSK and ERK1/2 signalling pathways. In contrast, protein phosphorylation was not affected by PTK787 in VEGF-A-treated cells. PTK787 turned out to inhib  it cell m  igration even though no effects were observed upon stimulation with VEGF-A or PDGF-BB. In line, cell growth in leiomyosarcoma cell lines remained unchanged upon PTK787 treatment alone and with subsequent VEGF-A- or PDGF-BB-stimulation. However, VEGF-A, but not PDGF-BB-treated cells showed increased cell death upon PTK787 treatment. VEGFR family members are expressed in leiomyosarcomas in vivo and in vitro. Upon receptor stimulation, PTK787 is able to inhibit subsequent phosphorylation events and influences cell survival but not metabolic activ  ity and m  igration. Thus, the inhibitor is possibly an additional option in the treatment of leiomyosarcomas. 
 Soluble forms of VEGF receptor-1 and -2 promote vascular maturation via mural cell recruitment. Two soluble forms of vascular endothelial growth factor (VEGF) receptors, sVEGFR-1 and sVEGFR-2, are physiologically released and overproduced in some pathologies. They are known to act as anti-VEGF agents. Here we report that these soluble receptors contribute to vessel maturation by mediating a dialogue between endothelial cells (ECs) and mural cells that leads to blood vessel stabilization. Through a multidisciplinary approach, we provide evidence that these soluble VEGF receptors promote mural cell   migration   through a paracrine mechanism involving interplay in ECs between VEGF/VEGFR-2 and sphingosine-1-phosphate type-1 (S1P)/S1P1 pathways that leads to endothelial nitric oxyde synthase (eNOS) activation. This new paradigm is supported by the finding that sVEGFR-1 and -2 perform the following actions: 1) induce an eNOS-dependent outgrowth of a mural cell network in an ex vivo model of angiogenesis, 2) increase the mural cell coverage of neovessels in vitro and in vivo, 3) promote mural cell   migration   toward ECs, and 4) stimulate endothelial S1P1 overproduction and eNOS activation that promote the   migration   and the recruitment of neighboring mural cells. These findings provide new insights into mechanisms regulating physiological and pathological angiogenesis and vessel stabilization. 
 GEP100-Arf6-AMAP1-cortactin pathway frequently used in cancer invasion is activated by VEGFR2 to promote angiogenesis. Angiogenesis and cancer invasiveness greatly contribute to cancer malignancy.Arf6 and its effector, AMAP1, are frequently overexpressed in breast cancer, and constitute a central pathway to induce the invasion and metastasis. In this pathway, Arf6 is activated by EGFR via GEP100. Arf6 is highly expressed also in human umbilical vein endothelial cells (HUVECs) and is implicated in angiogenesis. Here, we found that HUVECs also highly express AMAP1, and that vascular endothelial growth factor receptor-2 (VEGFR2) recruits GEP100 to activate Arf6. AMAP1 functions by binding to cortactin in cancer invasion and metastasis. We demonstrate that the same GEP100-Arf6-AMAP1-cortactin pathway is essential for angiogenesis activities, including cell   migration   and tubular formation, as well as for the enhancement of cell permeability and VE-cadherin endocytosis of VEGF-stimulated HUVECs. Components of this pathway are highly expressed in pathologic angiogenesis, and blocking of this pathway effectively inhibits VEGF- or tumor-induced angiogenesis and choroidal neovascularization. The GEP100-Arf6-AMAP1-cortactin pathway, activated by receptor tyrosine kinases, appears to be common in angiogenesis and cancer invasion and metastasis, and provides their new therapeutic targets. 
 Early endocardial morphogenesis requires Scl/Tal1. The primitive heart tube is composed of an outer myocardial and an inner endocardial layer that will give rise to the cardiac valves and septa. Specification and differentiation of these two cell layers are among the earliest events in heart development, but the embryonic origins and genetic regulation of early endocardial development remain largely undefined. We have analyzed early endocardial development in the zebrafish using time-lapse confocal microscopy and show that the endocardium seems to originate from a region in the lateral plate mesoderm that will give rise to hematopoietic cells of the primitive myeloid lineage. Endocardial precursors appear to rapidly migrate to the site of heart tube formation, where they arrive prior to the bilateral myocardial primordia. Analysis of a newly discovered zebrafish Scl/Tal1 mutant showed an additional and previously undescribed role of this transcription factor during the development of the endocardium. In Scl/Tal1 mutant embryos, endocardial precursors are specified, but   migration   is severely defective and endocardial cells aggregate at the ventricular pole of the heart. We further show that the initial fusion of the bilateral myocardial precursor populations occurs independently of the endocardium and tal1 function. Our results suggest early separation of the two components of the primitive heart tube and imply Scl/Tal1 as an indispensable component of the molecular hierarchy that controls endocardium morphogenesis. 
 BET Bromodomain Suppression Inhibits VEGF-induced Angiogenesis and Vascular Permeability by Blocking VEGFR2-mediated Activation of PAK1 and eNOS. The tyrosine kinase receptor vascular endothelial growth factor receptor 2 (VEGFR2) is a critical modulator of angiogenesis. Increasing evidence indicate the important role of bromodomain and extra-terminal domain (BET) of chromatin adaptors in regulating tumor growth and inflammatory response. However, whether BET proteins have a role in angiogenesis and endothelial permeability is unclear. In this study, we observed that treatment with JQ1, a specific BET inhibitor, suppressed in vitro tube formation of human umbilical vein endothelial cells (HUVECs) and in vivo angiogenesis in a Matrigel plug and oxygen-induced retinopathy neovascularization. JQ1 attenuated the VEGF-induced decrease in TEER in HUVECs and prevented Evans blue dye leakage in the VEGF-induced Miles assay in athymic Balb/c nude mice. BET inhibition with JQ1 or shRNA for Brd2 or Brd4 suppressed VEGF-induced   migration  , proliferation, and stress fiber formation of HUVECs. Furthermore, BET inhibition suppressed phosphorylation of VEGFR2 and PAK1, as well as eNOS activation in VEGF-stimulated HUVECs. Inhibition with VEGFR2 and PAK1 also reduced   migration   and proliferation, and attenuated the VEGF-induced decrease in TEER. Thus, our observations suggest the important role of BET bromodomain in regulating VEGF-induced angiogenesis. Strategies that target the BET bromodomain may provide a new therapeutic approach for angiogenesis-related diseases. 
 Properties of two VEGF receptors, Flt-1 and KDR, in signal transduction. The properties of two VEGF receptors, Flt-1 and KDR, in the signal transduction of VEGF in human umbilical vein endothelial cells (HUVECs) were investigated by using two newly developed blocking monoclonal antibodies (mAbs) against Flt-1 and KDR. VEGF stimulated the expression of transcription factor Ets-1 as well as matrix metalloproteinase-1 (MMP-1) and Flt-1 in HUVECs. The KDR/Flt-1 heterodimer and the KDR homodimer mediate the expression of Ets-1, MMP-1, and Flt-1. VEGF also stimulated DNA synthesis and   migration   of HUVECs. DNA synthesis is mediated by the same signaling system as the expression of Ets-1. In contrast, cell   migration   is regulated by two distinct signaling systems. The Flt-1 homodimer is required for actin reorganization. The KDR/Flt-1 heterodimer and the KDR homodimer are required for the assembly of vinculin in focal adhesion plaque by regulating the phosphorylation of focal adhesion kinase (FAK) and paxillin. 
 The adaptor protein Gab1 couples the stimulation of vascular endothelial growth factor receptor-2 to the activation of phosphoinositide 3-kinase. Phosphoinositide 3-kinase (PI3K) mediates essential functions of vascular endothelial growth factor (VEGF), including the stimulation of endothelial cell proliferation and   migration  . Nevertheless, the mechanisms coupling the receptor VEGFR-2 to PI3K remain obscure. We observed that the Grb2-bound adapter Gab1 is tyrosine-phosphorylated and relocated to membrane fractions upon VEGF stimulation of endothelial cells. We could detect the PI3K regulatory subunit p85 in immunoprecipitates of endogenous Gab1, and vice versa, and measure a Gab1-associated lipid kinase activity upon VEGF stimulation. Furthermore, transfection of the Gab1-YF3 mutant lacking all p85-binding sites strongly repressed PI3K activation measured in vitro. Moreover, Gab1-YF3 severely decreased the cellular amount of phosphatidylinositol 3,4,5-trisphosphate (PtdIns(3,4,5)P3) generated in response to VEGF. Furthermore, adenoviral expression of Gab1-YF3 suppressed both Akt phosphorylation and recovery of wounded human umbilical vein endothelial cell monolayers, a VEGF-dependent process involving cell   migration   and proliferation under PI3K control. Transfection of other Gab1 mutants, lacking Grb2-binding sites or the pleckstrin homology (PH) domain, also prevented Akt activation, further demonstrating Gab1 involvement in PI3K activation. These mutants were also used to show that interactions with both Grb2 and PtdIns(3,4,5)P3 mediate Gab1 recruitment by VEGFR-2. Importantly, Gab1 mobilization was impaired by (i) PI3K inhibitors, (ii) deletion of Gab1 PH domain, (iii) PTEN (phosphatase and tensin homolog deleted on chromosome 10) overexpression to repress PtdIns(3,4,5)P3 production, and (iv) overexpression of a competitor PH domain for PtdIns(3,4,5)P3 binding, which altogether demonstrated that PI3K is also an upstream regulator of Gab1. Gab1 thus appears as a primary actor in coupling VEGFR-2 to PI3K/Akt, recruited through an amplification loop involving PtdIns(3,4,5)P3 and its PH domain. 
 AIP1 functions as an endogenous inhibitor of VEGFR2-mediated signaling and inflammatory angiogenesis in mice. ASK1-interacting protein-1 (AIP1), a recently identified member of the Ras GTPase-activating protein family, is highly expressed in vascular ECs and regulates EC apoptosis in vitro. However, its function in vivo has not been established. To study this, we generated AIP1-deficient mice (KO mice). Although these mice showed no obvious defects in vascular development, they exhibited dramatically enhanced angiogenesis in 2 models of inflammatory angiogenesis. In one of these models, the enhanced angiogenesis observed in the KO mice was associated with increased VEGF-VEGFR2 signaling. Consistent with this, VEGF-induced ear, cornea, and retina neovascularization were greatly augmented in KO mice and the enhanced retinal angiogenesis was markedly diminished by overexpression of AIP1. In vitro, VEGF-induced EC   migration   was inhibited by AIP1 overexpression, whereas it was augmented by both AIP1 knockout and knockdown, with the enhanced EC   migration   caused by AIP1 knockdown being associated with increased VEGFR2 signaling. We present mechanistic data that suggest AIP1 is recruited to the VEGFR2-PI3K complex, binding to both VEGFR2 and PI3K p85, at a late phase of the VEGF response, and that this leads to inhibition of VEGFR2 signaling. Taken together, our data demonstrate that AIP1 functions as an endogenous inhibitor in VEGFR2-mediated adaptive angiogenesis in mice. 
 Urokinase-type Plasminogen Activator (uPA) Promotes Angiogenesis by Attenuating Proline-rich Homeodomain Protein (PRH) Transcription Factor Activity and De-repressing Vascular Endothelial Growth Factor (VEGF) Receptor Expression. Urokinase-type plasminogen activator (uPA) regulates angiogenesis and vascular permeability through proteolytic degradation of extracellular matrix and intracellular signaling initiated upon its binding to uPAR/CD87 and other cell surface receptors. Here, we describe an additional mechanism by which uPA regulates angiogenesis. Ex vivo VEGF-induced vascular sprouting from Matrigel-embedded aortic rings isolated from uPA knock-out (uPA(-/-)) mice was impaired compared with vessels emanating from wild-type mice. Endothelial cells isolated from uPA(-/-) mice show less proliferation and   migration   in response to VEGF than their wild type counterparts or uPA(-/-) endothelial cells in which expression of wild type uPA had been restored. We reported previously that uPA is transported from cell surface receptors to nuclei through a mechanism that requires its kringle domain. Intranuclear uPA modulates gene transcription by binding to a subset of transcription factors. Here we report that wild type single-chain uPA, but not uPA variants incapable of nuclear transport, increases the expression of cell surface VEGF receptor 1 (VEGFR1) and VEGF receptor 2 (VEGFR2) by translocating to the nuclei of ECs. Intranuclear single-chain uPA binds directly to and interferes with the function of the transcription factor hematopoietically expressed homeodomain protein or proline-rich homeodomain protein (HHEX/PRH), which thereby lose their physiologic capacity to repress the activity of vehgr1 and vegfr2 gene promoters. These studies identify uPA-dependent de-repression of vegfr1 and vegfr2 gene transcription through binding to HHEX/PRH as a novel mechanism by which uPA mediates the pro-angiogenic effects of VEGF and identifies a potential new target for control of pathologic angiogenesis. 
 BET Bromodomain Suppression Inhibits VEGF-induced Angiogenesis and Vascular Permeability by Blocking VEGFR2-mediated Activation of PAK1 and eNOS. The tyrosine kinase receptor vascular endothelial growth factor receptor 2 (VEGFR2) is a critical modulator of angiogenesis. Increasing evidence indicate the important role of bromodomain and extra-terminal domain (BET) of chromatin adaptors in regulating tumor growth and inflammatory response. However, whether BET proteins have a role in angiogenesis and endothelial permeability is unclear. In this study, we observed that treatment with JQ1, a specific BET inhibitor, suppressed in vitro tube formation of human umbilical vein endothelial cells (HUVECs) and in vivo angiogenesis in a Matrigel plug and oxygen-induced retinopathy neovascularization. JQ1 attenuated the VEGF-induced decrease in TEER in HUVECs and prevented Evans blue dye leakage in the VEGF-induced Miles assay in athymic Balb/c nude mice. BET inhibition with JQ1 or shRNA for Brd2 or Brd4 suppressed VEGF-induced   migration  , proliferation, and stress fiber formation of HUVECs. Furthermore, BET inhibition suppressed phosphorylation of VEGFR2 and PAK1, as well as eNOS activation in VEGF-stimulated HUVECs. Inhibition with VEGFR2 and PAK1 also reduced   migration   and proliferation, and attenuated the VEGF-induced decrease in TEER. Thus, our observations suggest the important role of BET bromodomain in regulating VEGF-induced angiogenesis. Strategies that target the BET bromodomain may provide a new therapeutic approach for angiogenesis-related diseases. 
 Recombinant pigment epithelium-derived factor PEDF binds vascular endothelial growth factor receptors 1 and 2. Angiogenesis, or the formation of new blood vessels, is stimulated by angiogenic factors such as vascular endothelial growth factor (VEGF). Pigment epithelium-derived factor (PEDF) is a potent inhibitor of angiogenesis. To explore the mechanism by which PEDF acts, recombinant PEDF was expressed with a 6x-His tag (for purification) and a green fluorescent protein (GFP) tag. The PEDF fusion protein was confirmed to be active in inhibition of endothelial cell proliferation and   migration  . Direct binding of PEDF to both vascular endothelial growth factor receptor-1 (VEGFR-1) and VEGFR-2 was demonstrated in an in vitro assay similar to an enzyme-linked immunosorbent assay (ELISA). PEDF was shown by immune-confocal microscopy to be localized within treated endothelial cells. When VEGF-stimulated endothelial cells were incubated with PEDF the VEGF receptors showed intracellular localization. These data suggest that the interaction between PEDF and VEGFR-1 or VEGFR-2 may be a possible mechanism for inhibiting angiogenesis. PEDF may be binding to the VEGF receptors to promote their internalization and/or degradation to limit VEGF responses in treated cells. 
 Gab1 regulates proliferation and   migration   through the PI3K/Akt signaling pathway in intrahepatic cholangiocarcinoma. Intrahepatic cholangiocarcinoma is the second most common primary malignant tumor of the liver, and it originates from the intrahepatic biliary duct epithelium. Prognosis is poor due to lack of effective comprehensive treatments. In this study, we assessed the expression of Gab1, VEGFR-2, and MMP-9 in intrahepatic cholangiocarcinoma solid tumors by immunohistochemistry and determined whether their expression was associated with clinical and pathological features. We found that expression of Gab1, VEGFR-2, and MMP-9 was highly and positively correlated with each other and with lymph node metastasis and TNM stage in intrahepatic cholangiocarcinoma tissues. Interference of Gab1 and VEGFR-2 expression via siRNA in the intrahepatic cholangiocarcinoma cell line RBE resulted in decreased PI3K/Akt pathway activity. Inhibition of Gab1 and VEGFR-2 expression also caused decreased cell proliferation, cell cycle arrested in G1 phase, increased apoptosis, and decreased invasion in RBE cells. These results suggest that Gab1, VEGFR-2, and MMP-9 contribute significantly to the highly malignant behavior of intrahepatic cholangiocarcinoma. The regulation of growth, apoptosis, and invasion by Gab1 through the VEGFR-2/Gab1/PI3K/Akt signaling pathway may represent potential targets for improving the treatment of intrahepatic cholangiocarcinoma. 
 Heparanase released from mesenchymal stem cells activates integrin beta1/HIF-2alpha/Flk-1 signaling and promotes endothelial cell   migration   and angiogenesis. Heparanase plays important roles in tumor angiogenesis. Our previous study demonstrated that hypoxic preconditioning (HPC) enhanced the angiogenic and therapeutic effects of mesenchymal stem cells (MSCs), effects that were paralleled by enhanced heparanase expression. This study was designed to elucidate the role of heparanase in the improved therapeutic properties of HPC-MSCs and to explore underlying mechanisms using an ischemic rat hind limb model. MSCs transfected with heparanase (MSC(hpa) ) or empty vector (MSC(null) ) were delivered by intramuscular injections to ischemic hind limbs. Hind limbs that received MSC(hpa) recovered blood flow more rapidly at 7 days and acquired higher capillary density at 14 days compared with MSC(null) . Conditioned medium from MSC(hpa) increased endothelial cell   migration   and promoted greater tube formation relative to that from the MSC(null) groups. Vascular endothelial growth factor receptor 2 (VEGFR2, Flk-1) and its downstream signaling pathway (p38MAPK/HSP27) were significantly increased in human umbilical vein endothelial cells (HUVECs) after treatment with MSC(hpa) conditioned medium. Each of these responses was decreased by cocultured with MSC(hpa-KD) conditioned medium. MSC(hpa) conditioned medium activated hypoxia-inducible factor-2α (HIF-2α) and increased in parallel the transcript level of Flk-1 as determined by chromatin immunoprecipitation-PCR and luciferase assays. Analyses of integrin expression revealed an important role for integrin β1 in the regulation of HIF-2α. All angiogenic effects of MSC(hpa) conditioned medium were abolished by knockdown of integrin β1, HIF-2α, and Flk-1 in HUVECs with selective shRNAs. These findings identify heparanse as a key regulator of angiogenesis by MSCs. We propose a novel pathway wherein heparanse sequentially activates integrin β1, HIF-2α, Flk-1, and p38MAPK/HSP27 with corresponding enhancement of angiogenesis. 
 The inhibition of tyrosine kinase receptor signalling in leiomyosarcoma cells using the small molecule kinase inhibitor PTK787/ZK222584 (Vatalanib®). Leiomyosarcomas remain challenging tumors to manage and novel therapy strategies besides radiation and conventional chemotherapy are needed. Targeting angiogenesis by inhibition of vascular endothelial growth factor (VEGF) receptor tyrosine kinases (RTKs) of the tumor vasculature with small molecules is a promising new therapy. It has been shown recently that these receptors are not only expressed on tumor endothelium but also on tumor cells themselves. Thus, we investigated the expression of members of the VEGF receptor (VEGFR) family and corresponding growth factors in leiomyosarcoma tissue specimens and in the leiomyosarcoma cell lines SK-LMS-1 and SK-UT-1. We evaluated the influence of the VEGFR inhibitor PTK787/ZK222584 (PTK787) on cell growth  , migrati  on, apoptosis and phosphorylation of intracellular signalling molecules. In human leiomyosarcoma tissue specimens VEGFR‑1/-2 and platelet-derived growth factor receptor (PDGFR-β) were strongly expressed. Both leiomyosarcoma cell lines expressed VEGFR‑1/-3 and PDGFR-β but VEGFR-2 protein expression was positive only in SK-UT-1. SK-LMS-1 and SK-UT-1 cells secreted high and low amounts of VEGF-A, respectively, whereas PDGF-BB secretion was similar in both cell lines. Application of PTK787 led to partial inhibition of PDGF-BB-activated AKT/p90RSK and ERK1/2 signalling pathways. In contrast, protein phosphorylation was not affected by PTK787 in VEGF-A-treated cells. PTK787 turned out to inhib  it cell m  igration even though no effects were observed upon stimulation with VEGF-A or PDGF-BB. In line, cell growth in leiomyosarcoma cell lines remained unchanged upon PTK787 treatment alone and with subsequent VEGF-A- or PDGF-BB-stimulation. However, VEGF-A, but not PDGF-BB-treated cells showed increased cell death upon PTK787 treatment. VEGFR family members are expressed in leiomyosarcomas in vivo and in vitro. Upon receptor stimulation, PTK787 is able to inhibit subsequent phosphorylation events and influences cell survival but not metabolic activ  ity and m  igration. Thus, the inhibitor is possibly an additional option in the treatment of leiomyosarcomas. 
 ERK3 promotes endothelial cell functions by upregulating SRC-3/SP1-mediated VEGFR2 expression. Despite a regain of interest recently in ERK3 kinase signaling, the molecular regulations of both ERK3 gene expression and protein kinase activity are still largely unknown. While it is shown that disruption of ERK3 gene causes neonatal lethality, cell type-specific functions of ERK3 signaling remain to be explored. In this study, we report that ERK3 gene expression is upregulated by cytokines through c-Jun in endothelial cells; c-Jun binds to the ERK3 gene and regulates its transcription. We further reveal a new role for ERK3 in regulating endothelial cell   migration  , proliferation and tube formation by upregulating SRC-3/SP-1-mediated VEGFR2 expression. The underlying molecular mechanism involves ERK3-stimulated formation of a transcriptional complex involving coactivator SRC-3, transcription factor SP-1 and the secondary coactivator CBP. Taken together, our study identified a molecular regulatory mechanism of ERK3 gene expression and revealed a previously unknown role of ERK3 in regulating endothelial cell functions. 
 Klotho endows hepatoma cells with resistance to anoikis via VEGFR2/PAK1 activation in hepatocellular carcinoma. Klotho was originally characterized as an aging suppressor gene that predisposed Klotho-deficient mice to premature aging-like syndrome. Although Klotho was recently reported to exhibit tumor suppressive properties during various malignant transformations, the functional role and molecular mechanism of Klotho in hepatocarcinogenesis remains poorly understood. In our present study, immunohistochemical Klotho staining levels in a clinical follow-up of 52 hepatoma patients were significantly associated with liver cirrhosis, tumor multiplicity and venous invasion. The overall survival rate of hepatoma patients with high Klotho expression was significantly lower than those patients with low Klotho expression. Moreover, Klotho overexpression increased cellular   migration  , anchorage-independent growth, and anoikis resistance in hepatoma cells. Klotho overexpression elevated p21-activated kinase 1 (PAK1) expression and shRNA-mediated PAK1 knockdown and kinase activity inhibition with kinase dead mutant PAK1 K299R coexpression or allosteric inhibitor IPA3 treatment reversed anoikis resistance in Klotho-overexpressed hepatoma cells. More importantly, the pivotal significance of upregulated VEGFR2 protein levels mediated by Klotho expression was confirmed by VEGFR2 inhibitor Axitinib and blocking antibody treatment in hepatoma cells. Axitinib treatment sensitized anoikis was reversed by constitutive active mutant PAK1 T423E coexpression in Klotho-overexpressed hepatoma cells. Conversely, knockdown of Klotho reduced VEGFR2/PAK1 dependent anoikis resistance, which could be reversed by PAK1 T423E. These results revealed a novel oncogenic function of Klotho in promoting anoikis resistance via activating VEGFR2/PAK1 signaling, thus facilitating tumor   migration   and invasion during hepatoma progression, which could provide a putative molecular mechanism for tumor metastasis. 
 Vascular endothelial-cadherin stimulates syndecan-1-coupled insulin-like growth factor-1 receptor and cross-talk between αVβ3 integrin and vascular endothelial growth factor receptor 2 at the onset of endothelial cell dissemination during angiogenesis. Vascular endothelial growth factor (VEGF)-stimulated angiogenesis depends on a cross-talk mechanism involving VEGF receptor 2 (VEGFR2), vascular endothelial (VE)-cadherin and the αVβ3 integrin. Because we have shown that αVβ3 integrin activation is dependent on its incorporation, along with the insulin-like growth factor-1 receptor (IGF1R) kinase, into a ternary receptor complex organized by the matrix receptor syndecan-1 (Sdc1), we questioned the role of this core complex in VEGF-stimulated angiogenesis. We find that the Sdc1-coupled ternary receptor complex is required for VEGF signalling and for stimulation of vascular endothelial   cell mig  ration by vascular endothelial cadherin (VE-cadherin) engagement. VE-cadherin binding to Fc/VE-cadherin extracellular domain chimera activates Sdc1-coupled IGF1R and αvβ3 integrin; this depends on VEGFR2 and c-Src activated by the cadherin. Blocking homotypic VE-cadherin engagement disrupts VEGF-stimulat  ed cell m  igration, which is restored by clustering the cadherin in the absence of cell-cell adhesion. This cadherin-dependent stimulation requires VEGFR2 and IGF1R and is blocked by synstatin (SSTN)(92-119), a peptide that competitively disrupts the Sdc1-coupled ternary complex and prevents the αVβ3 integrin activation required for VEGFR2 activation. VEGFR2-stimulated angiogenesis in the mouse aortic ring explant assay is disrupted by SSTN, although only early in the process, suggesting that IGF1R coupling to Sdc1 and αVβ3 integrin comprises a core activation mechanism activated by VE-cadherin that is necessary for VEGFR2 and integrin activation in the initial stages of endothelial cell dissemination during angiogenesis. 
 Vascular endothelial growth factor and its receptor system: physiological functions in angiogenesis and pathological roles in various diseases. Vascular endothelial growth factors (VEGFs) belong to the platelet-derived growth factor supergene family, and they play central roles in the regulation of angiogenesis and lymphangiogenesis. VEGF-A, the major factor for angiogenesis, binds to two tyrosine kinase (TK) receptors, VEGFR-1 (Flt-1) and VEGFR-2 (KDR/Flk-1), and regulates endothelial cell proliferation,   migration  , vascular permeability, secretion and other endothelial functions. VEGFR-2 exhibits a strong TK activity towards pro-angiogenic signals, whereas the soluble VEGFR-1 (sFlt-1) functions as an endogenous VEGF inhibitor. sFlt-1 is abnormally overexpressed in the placenta of preeclampsia patients, resulting in the major symptoms of the disease due to abnormal trapping of VEGFs. The VEGF-VEGFR system is crucial for tumour angiogenesis, and anti-VEGF-VEGFR molecules are now widely used in the clinical field to treat cancer patients. The efficacy of these molecules in prolonging the overall survival of patients has been established; however, some cancers do not respond well and reduced tumour sensitivity to anti-VEGF signals may occur after long-term treatment. The molecular basis of tumour refractoriness should be determined to improve anti-angiogenic therapy. 
 Formation of the collateral circulation is regulated by vascular endothelial growth factor-A and a disintegrin and metalloprotease family members 10 and 17. The density of native (preexisting) collaterals varies widely and is a significant determinant of variation in severity of stroke, myocardial infarction, and peripheral artery disease. However, little is known about mechanisms responsible for formation of the collateral circulation in healthy tissues.We previously found that variation in vascular endothelial growth factor (VEGF) expression causes differences in collateral density of newborn and adult mice. Herein, we sought to determine mechanisms of collaterogenesis in the embryo and the role of VEGF in this process.Pial collaterals begin forming between embryonic day 13.5 and 14.5 as sprout-like extensions from arterioles of existing cerebral artery trees. Global VEGF-A overexpressing mice (Vegf(hi/+)) formed more, and Vegf(lo/+) formed fewer, collaterals during embryogenesis, in association with differences in vascular patterning. Conditional global reduction of Vegf or Flk1 only during collaterogenesis significantly reduced collateral formation, but now without affecting vascular patterning, and the effects remained in adulthood. Endothelial-specific Vegf reduction had no effect on collaterogenesis. Endothelial-specific reduction of a disintegrin-and-metalloprotease-domain-10 (Adam10) and inhibition of γ-secretase increased collateral formation, consistent with their roles in VEGF-induced Notch1 activation and suppression of prosprouting signals. Endothelial-specific knockdown of Adam17 reduced collateral formation, consistent with its roles in endothelial cell   migratio  n and embryonic vascular stabilization, but not in activation of ligand-bound Notch1. These effects also remained in adulthood.Formation of pial collaterals occurs during a narrow developmental window via a sprouting angiogenesis-like mechanism, requires paracrine VEGF stimulation of fetal liver kinase 1-Notch signaling, and adult collateral number is dependent on embryonic collaterogenesis. 
 The role of heterodimerization between VEGFR-1 and VEGFR-2 in the regulation of endothelial cell homeostasis. VEGF-A activity is tightly regulated by ligand and receptor availability. Here we investigate the physiological function of heterodimers between VEGF receptor-1 (VEGFR-1; Flt-1) and VEGFR-2 (KDR; Flk-1) (VEGFR(1-2)) in endothelial cells with a synthetic ligand that binds specifically to VEGFR(1-2). The dimeric ligand comprises one VEGFR-2-specific monomer (VEGF-E) and a VEGFR-1-specific monomer (PlGF-1). Here we show that VEGFR(1-2) activation mediates VEGFR phosphorylation, endothelial cell   migration  , sustained in vitro tube formation and vasorelaxation via the nitric oxide pathway. VEGFR(1-2) activation does not mediate proliferation or elicit endothelial tissue factor production, confirming that these functions are controlled by VEGFR-2 homodimers. We further demonstrate that activation of VEGFR(1-2) inhibits VEGF-A-induced prostacyclin release, phosphorylation of ERK1/2 MAP kinase and mobilization of intracellular calcium from primary endothelial cells. These findings indicate that VEGFR-1 subunits modulate VEGF activity predominantly by forming heterodimer receptors with VEGFR-2 subunits and such heterodimers regulate endothelial cell homeostasis. 
 GEP100-Arf6-AMAP1-cortactin pathway frequently used in cancer invasion is activated by VEGFR2 to promote angiogenesis. Angiogenesis and cancer invasiveness greatly contribute to cancer malignancy.Arf6 and its effector, AMAP1, are frequently overexpressed in breast cancer, and constitute a central pathway to induce the invasion and metastasis. In this pathway, Arf6 is activated by EGFR via GEP100. Arf6 is highly expressed also in human umbilical vein endothelial cells (HUVECs) and is implicated in angiogenesis. Here, we found that HUVECs also highly express AMAP1, and that vascular endothelial growth factor receptor-2 (VEGFR2) recruits GEP100 to activate Arf6. AMAP1 functions by binding to cortactin in cancer invasion and metastasis. We demonstrate that the same GEP100-Arf6-AMAP1-cortactin pathway is essential for angiogenesis activities, including cell   migration   and tubular formation, as well as for the enhancement of cell permeability and VE-cadherin endocytosis of VEGF-stimulated HUVECs. Components of this pathway are highly expressed in pathologic angiogenesis, and blocking of this pathway effectively inhibits VEGF- or tumor-induced angiogenesis and choroidal neovascularization. The GEP100-Arf6-AMAP1-cortactin pathway, activated by receptor tyrosine kinases, appears to be common in angiogenesis and cancer invasion and metastasis, and provides their new therapeutic targets. 
 Thioredoxin-interacting protein mediates TRX1 translocation to the plasma membrane in response to tumor necrosis factor-α: a key mechanism for vascular endothelial growth factor receptor-2 transactivation by reactive oxygen species. Thioredoxin-interacting protein (TXNIP) promotes inflammation in endothelial cells (EC) by binding to thioredoxin-1 (TRX1) in a redox-dependent manner. Formation of the TXNIP-TRX1 complex relieves inhibition of the apoptosis signal-regulating kinase 1-c-Jun N-terminal kinase-vascular cell adhesion molecule-1 pathway. Because TXNIP is an α-arrestin with numerous protein-protein interacting domains, we hypothesized that TXNIP-TRX1 trafficking should alter function of EC exposed to reactive oxygen species (ROS).In response to physiological levels of ROS (10 ng/mL tumor necrosis factor-α and 30 μmol/L H(2)O(2)), TXNIP-TRX1 translocated to the plasma membrane in human umbilical vein EC, with a peak at 30 minutes, as measured by immunofluorescence colocalization with vascular endothelial-cadherin, cell fractionation, and membrane sheet assay. TXNIP-mediated translocation of TRX1 to the membrane required TXNIP and TRX1 binding, as evidenced by inability of the ROS-insensitive TXNIP-Cys247Ser mutant to promote membrane localization. Vascular endothelial growth factor signaling required TXNIP, as shown by significant decreases in plasma membrane tyrosine phosphorylation and   EC migra  tion after TRX1 knockdown. Furthermore, TXNIP knockdown increased human umbilical vein EC apoptosis induced by tumor necrosis factor. Rescue with TXNIP-wild-type but not TXNIP-Cys247Ser prevented cell death.These findings suggest a novel role for the TXNIP-TRX1 complex to enable inflammation by promoting EC survival and vascular endothelial growth factor signaling under conditions of physiological oxidative stress. 
 MT1-MMP regulates VEGF-A expression through a complex with VEGFR-2 and Src. Membrane-type-1 matrix metalloproteinase (MT1-MMP) is a zinc-dependent type-I transmembrane metalloproteinase involved in pericellular proteolysis,   migration   and invasion, with elevated levels correlating with a poor prognosis in cancer. MT1-MMP-mediated transcriptional regulation of genes in cancer cells can contribute to tumour growth, although this is poorly understood at a mechanistic level. In this study, we investigated the mechanism by which MT1-MMP regulates the expression of VEGF-A in breast cancer cells. We discovered that MT1-MMP regulates VEGFR-2 cell surface localisation and forms a complex with VEGFR-2 and Src that is dependent on the MT1-MMP hemopexin domain and independent of its catalytic activity. Although the localisation of VEGFR-2 was independent of the catalytic and intracellular domain of MT1-MMP, intracellular signalling dependent on VEGFR-2 activity leading to VEGF-A transcription still required the MT1-MMP catalytic and intracellular domain, including residues Y573, C574 and DKV582. However, there was redundancy in the function of the catalytic activity of MT1-MMP, as this could be substituted with MMP-2 or MMP-7 in cells expressing inactive MT1-MMP. The signalling cascade dependent on the MT1-MMP-VEGFR-2-Src complex activated Akt and mTOR, ultimately leading to increased VEGF-A transcription. 
 Hypoxia differentially regulates VEGFR1 and VEGFR2 levels and alters intracellular signaling and cell   migration   in endothelial cells. The role of hypoxia on endothelial cell function and response to growth factors is unknown. Here, we tested the hypothesis that hypoxia re-programs endothelial function by modulating vascular endothelial growth factor receptor levels which in turn alter intracellular signaling and cell function. Hypoxia stimulated VEGF-A and VEGFR1 expression but decreased VEGFR2 levels in endothelial cells. During hypoxia, plasma membrane VEGFR1 levels were elevated whereas VEGFR2 levels were depleted. One functional consequence of hypoxia is a reduction in VEGF-A-stimulated and VEGFR2-regulated intracellular signaling including lowered endothelial nitric oxide synthase activation. Venous, arterial and capillary endothelial cells subjected to hypoxia all exhibited reduced cell   migration   in response to VEGF-A. A mechanistic explanation is that VEGFR1:VEGFR2 ratio is substantially increased during hypoxia to block VEGF-A-stimulated and VEGFR2-regulated endothelial responses to maximize cell viability and recovery. 
 Increased expression of vascular endothelial growth factor and its receptors, VEGFR-1 and VEGFR-2, in medullary thyroid carcinoma. Vascular endothelial growth factor (VEGF-A) expression is upregulated in the majority of human tumors, where it stimulates proliferation,   migration  , and survival of endothelial cells. Studies have suggested that VEGF inhibitors can be used as an alternative therapy in medullary thyroid carcinoma (MTC), but data about expression of VEGF-A and its receptor in this tumor are scarce. The aims of this study were to evaluate VEGF-A, VEGF receptor (VEGFR)-1, VEGFR-2, and microvessel density (MVD) expression in MTC samples and correlate it with clinical parameters.Paraffin-embedded samples from 38 MTC patients were evaluated for VEGF-A, VEGFR-1, VEGFR-2, and MVD expression by immunohistochemistry. Clinical data were retrospectively reviewed in medical records.Thirty-eight patients aged 31.8 +/- 17.1 years were enrolled. Twenty-seven patients had hereditary disease (71.1%). Twenty-five of them were found to have multiple endocrine neoplasia (MEN) 2A and two were found to have MEN 2B. VEGF-A immunohistochemical staining was detected in 95% (36/38), VEGFR-1 in 96% (36/37), and VEGFR-2 in 91% (31/34) of MTC samples. Age at surgery was positively correlated with VEGFR-2 (p = 0.003). There was no correlation between VEGF-A, VEGFR-2, and tumor stage (tumor node metastasis). Nevertheless, VEGFR-1 was found to be inversely correlated with tumor node metastasis (p = 0.034). We also observed a trend toward an association between VEGFR-1 signal intensity and cure of disease, although this did not reach statistical significance (p = 0.054). Neither VEGF-A nor VEGFR-2 was associated with disease outcome after a median follow-up period of 5 years (p = 0.882 and p = 0.236, respectively). As expected, MVD was correlated with age at surgery (p = 0.005) and tumor size (p = 0.03). Patients with the hereditary form of the disease had a stronger intensity for VEGFR-1 (p = 0.039), whereas patients with sporadic disease displayed higher MVD counts (44 [27-63] vs. 21 [9-49], p = 0.018).The VEGF-A, VEGFR-1, and VEGFR-2 immunoreactive proteins are overexpressed in MTC lesions and might be implicated in tumor progression. It is not clear, however, if expression of these molecules provides prognostic information regarding the spread or outcome of MTC. 
 Herbal compound farnesiferol C exerts antiangiogenic and antitumor activity and targets multiple aspects of VEGFR1 (Flt1) or VEGFR2 (Flk1) signaling cascades. Farnesiferol C (FC) is one of the major compounds isolated from Ferula assafoetida, an Asian herbal spice used for cancer treatment as a folk remedy. Here, we examined the hypothesis that novel antiangiogenic activities of FC contribute to anticancer efficacy. In human umbilical vein endothelial cells (HUVEC), exposure to the 10 to 40 mumol/L concentration range of FC inhibited vascular endothelial growth factor (VEGF)-induced cell proliferation,   migration  , invasion, tube formation, and the expression of matrix metalloproteinase-2. In addition, FC inhibited the angiogenic sprouting of VEGF-treated rat aorta in an ex vivo model. Furthermore, FC inhibited the in vivo growth of mouse Lewis lung cancer allograft model by 60% (P < 0.001) at a daily i.p. dosage of 1 mg/kg body weight without any negative effect on the weight of the host mice. Immunohistochemistry staining showed decreased microvessel density (CD34) and proliferative index (Ki-67) without affecting the apoptotic (terminal deoxynucleotidyl transferase-mediated dUTP nick end labeling) index. Mechanistically, FC decreased the binding of VEGF to VEGFR1/Flt-1, but not to VEGFR2/KDR/Flk-1. In terms of early signaling, FC exerted a rapid inhibitory action (examined within 10 minutes) on VEGF-induced autophosphorylation of VEGFR1 without affecting that of VEGFR2. Nevertheless, FC decreased the phosphorylation of most of the kinases downstream of VEGFR2: focal adhesion kinase, Src, extracellular signal-regulated kinase 1/2, p38 mitogen-activated protein kinase, and c-jun-NH(2)-kinase without affecting AKT. Computer simulation suggests that FC may inhibit Src or focal adhesion kinase protein activities directly through its docking to their ATP-binding sites. Taken together, the multitargeting actions of FC, particularly VEGFR1 inhibition, may make it a novel drug candidate to complement current VEGF/VEGFR2-targeting antiangiogenic modalities for cancer. 
 Soluble forms of VEGF receptor-1 and -2 promote vascular maturation via mural cell recruitment. Two soluble forms of vascular endothelial growth factor (VEGF) receptors, sVEGFR-1 and sVEGFR-2, are physiologically released and overproduced in some pathologies. They are known to act as anti-VEGF agents. Here we report that these soluble receptors contribute to vessel maturation by mediating a dialogue between endothelial cells (ECs) and mural cells that leads to blood vessel stabilization. Through a multidisciplinary approach, we provide evidence that these soluble VEGF receptors promote mural cell   migration   through a paracrine mechanism involving interplay in ECs between VEGF/VEGFR-2 and sphingosine-1-phosphate type-1 (S1P)/S1P1 pathways that leads to endothelial nitric oxyde synthase (eNOS) activation. This new paradigm is supported by the finding that sVEGFR-1 and -2 perform the following actions: 1) induce an eNOS-dependent outgrowth of a mural cell network in an ex vivo model of angiogenesis, 2) increase the mural cell coverage of neovessels in vitro and in vivo, 3) promote mural cell   migration   toward ECs, and 4) stimulate endothelial S1P1 overproduction and eNOS activation that promote the   migration   and the recruitment of neighboring mural cells. These findings provide new insights into mechanisms regulating physiological and pathological angiogenesis and vessel stabilization. 
 Rab GTPase regulation of VEGFR2 trafficking and signaling in endothelial cells. Vascular endothelial growth factor receptor 2 (VEGFR2) is a receptor tyrosine kinase that regulates vascular physiology. However, mechanism(s) by which VEGFR2 signaling and trafficking is coordinated are not clear. Here, we have tested endocytic Rab GTPases for regulation of VEGFR2 trafficking and signaling linked to endothelial cell   migration  .Quiescent VEGFR2 displays endosomal localization and colocalization with the Rab5a GTPase, an early endosome fusion regulator. Expression of GTP or GDP-bound Rab5a mutants block activated VEGFR2 trafficking and degradation. Manipulation of Rab7a GTPase activity associated with late endosomes using overexpression of wild-type or mutant proteins blocks activated VEGFR2 trafficking and degradation. Depletion of Rab7a decreased VEGFR2 Y1175 phosphorylation but increased p42/44 (pERK1/2) MAPK phosphorylation. Endothelial cell   migration   was increased by Rab5a depletion but decreased by Rab7a depletion.Rab5a and Rab7a regulate VEGFR2 trafficking toward early and late endosomes. Our data suggest that VEGFR2-mediated regulation of endothelial function is dependent on different but specific Rab-mediated GTP hydrolysis activity required for endosomal trafficking. 
 Glioma cells enhance endothelial progenitor cell angiogenesis via VEGFR-2, not VEGFR-1. Although potential contribution of endothelial progenitor cells (EPCs) to angiogenesis in glioma has been proposed, the molecular mechanisms of EPCs recruitment to vasculature have not been fully elucidated. Here, we show that the supernatant from glioma cells promotes EPCs angiogenesis via VEGFR-2, not VEGFR-1. Moreover, VEGFR-2 siRNA inhibits VEGFR-2 expression in EPCs, tube formation on matrigel and cell   migration  . MMP-9 activity and expression and the Akt and ERK phosphorylations are decreased by VEGFR-2 siRNA. Thus, these results indicate that glioma cells enhance EPC angiogenesis via VEGFR-2, not VEGFR-1, mediated by the MMP-9, Akt and ERK signal pathways. 
 Angiogenesis in systemic sclerosis: impaired expression of vascular endothelial growth factor receptor 1 in endothelial progenitor-derived cells under hypoxic conditions. To assess angiogenesis and explore the expression and regulation of vascular endothelial growth factor (VEGF), VEGF receptor 1 (VEGFR-1), and VEGFR-2, the leading mediators of angiogenesis, in SSc patients and controls.Late-outgrowth endothelial progenitor cells (EPCs), isolated from the peripheral blood of systemic sclerosis (SSc) patients and controls, and human umbilical vein endothelial cells (HUVECs) were assessed under normal and hypoxic conditions. Genomic background was evaluated in a large case-control study (including 659 patients with SSc and 511 controls) using tag single-nucleotide polymorphisms on VEGFR1 and VEGFR2 genes.EPCs from SSc patients had the phenotype of genuine endothelial cells and displayed in vitro angiogenic properties similar to those of HUVECs and control EPCs under basal conditions, as determined by flow cytometry, tube formation, and   migration   assay. However, after 6 hours of hypoxic exposure, EPCs from SSc patients exhibited lower induced expression of VEGFR-1 at the messenger RNA and protein levels, but similar VEGF and VEGFR-2 expression, compared with HUVECs or EPCs from healthy controls. There was no evidence of defective expression of hypoxia-inducible factor 1alpha. These results were supported by the lower serum levels of soluble VEGFR-1 found in SSc patients (n = 187) compared with healthy controls (n = 48) (mean +/- SD 163.7 +/- 98.5 versus 210.4 +/- 109.5 pg/ml; P = 0.0042). These abnormalities did not seem to be related to genomic background.Our findings shed new light on the possible role of VEGFR-1 in the main vascular disturbances that occur in SSc and lead to more severe disease. 
 Integrin activation is required for VEGF and FGF receptor protein presence on human microvascular endothelial cells. Endothelial cell proliferation and   migration   is initiated by growth factors including FGF and VEGF that bind to specific transmembrane receptor tyrosine kinases. Mechanisms that regulate in vivo expression of fibroblast growth factor receptors (FGFR) and vascular endothelial growth factor receptors (VEGFR) are not well understood. Since it is well known that different matrices influence the proliferation and   migration   of endothelial cells in culture, we hypothesized that changes in the extracellular matrix environment can regulate growth factor receptors on endothelial cells. We cultured human microvascular endothelial cells on different matrices (vitronectin, laminin, fibronectin, fibrin, and collagen IV) and examined for the presence of growth factor receptors (FGFR-1, FGFR-2, VEGFR-1, and VEGFR-2). We show that vitronectin increased the presence of all four growth factor receptors and most notably, VEGFR-1. In contrast, fibrin decreased all four receptors, especially FGFR-1 and FGFR-2. Inhibiting phosphotyrosine signaling abolished immunostaining for all four receptors, regardless of the matrix, but was not dependent on activating the Fyn-Shc pathway. Cells plated on vitronectin in the presence of blocking antibodies to integrins alphavbeta3 and alphavbeta5 similarly decreased presence of these growth factor receptors. Our data suggests a possible mechanism of how matrix-integrin interactions regulate endothelial cell responsiveness to growth factors and anchorage-dependent cell growth. 
 Immunolocalization and expression of vascular endothelial growth factor receptors (VEGFRs) and neuropilins (NRPs) on keratinocytes in human epidermis. Vascular endothelial growth factor (VEGF) plays an important role in normal and pathological angiogenesis. VEGF receptors (VEGFRs, including VEGFR-1, VEGFR-2, and VEGFR-3) and neuropilins (NRPs, including NRP-1 and NRP-2) are high-affinity receptors for VEGF and are typically considered to be specific for endothelial cells. Here we showed expression of VEGFRs and NRPs on cultured epidermal keratinocytes at both mRNA and protein levels. We further localized these receptors by immunofluorescence (IF) staining in the epidermis of surgical skin specimens. We found positive staining for VEGFRs and NRPs in all layers of the epidermis except for the stratum corneum. VEGFR-1 and VEGFR-2 are primarily expressed on the cytoplasmic membrane of basal cells and the adjacent spinosum keratinocytes. All layers of the epidermis except for the horny cell layer demonstrated a uniform pattern of VEGFR-3, NRP-1, and NRP-2. Sections staining for NRP-1 and NRP-2 also showed diffuse intense fluorescence and were localized to the cell membrane and cytoplasm of keratinocytes. In another panel of experiments, keratinocytes were treated with different concentrations of VEGF, with or without VEGFR-2 neutralizing antibody in culture. VEGF enhanced the proliferation and   migration   of keratinocytes, and these effects were partially inhibited by pretreatment with VEGFR-2 neutralizing antibody. Adhesion of keratinocytes to type IV collagen-coated culture plates was decreased by VEGF treatment, but this reduction could be completely reversed by pretreatment with VEGFR-2 neutralizing antibody. Taken together, our results suggest that the expression of VEGFRs and NRPs on keratinocytes may constitute important regulators for its activity and may possibly be responsible for the autocrine signaling in the epidermis. 
 Metastatic properties of prostate cancer cells are controlled by VEGF. Mechanisms of metastasis, the major complication of prostate cancer, are poorly understood. In this study, we define molecular mechanisms that may contribute to the highly invasive potential of prostate cancer cells. Vascular endothelial growth factor (VEGF), its receptors (VEGFRs), and alpha5beta1 integrin were expressed by prostate cancer cells in vitro and by prostate tumors in vivo, and their expression was elevated at sites of bone metastasis compared to original prostate tumor. VEGF, through interaction with its receptors, regulated adhesive and migratory properties of the cancer cells. Specifically, the highly metastatic prostate cancer cell subline LNCaP-C4-2 showed a decreased adhesive but an enhanced migratory response to fibronectin, a ligand for alpha5beta1 integrin, compared to its nonmetastatic counterpart. A similar pattern was also observed when bone sialoprotein was used as a ligand in   migration   assays. Increased   migration   of metastatic prostate cancer cells to fibronectin and bone sialoprotein was regulated by VEGF via VEGFR-2. Tumor suppressor PTEN was involved in control of VEGF/VEGFR-2 stimulated prostate cancer cell adhesion as well as proliferation. 
 The adaptor protein Gab1 couples the stimulation of vascular endothelial growth factor receptor-2 to the activation of phosphoinositide 3-kinase. Phosphoinositide 3-kinase (PI3K) mediates essential functions of vascular endothelial growth factor (VEGF), including the stimulation of endothelial cell proliferation and   migration  . Nevertheless, the mechanisms coupling the receptor VEGFR-2 to PI3K remain obscure. We observed that the Grb2-bound adapter Gab1 is tyrosine-phosphorylated and relocated to membrane fractions upon VEGF stimulation of endothelial cells. We could detect the PI3K regulatory subunit p85 in immunoprecipitates of endogenous Gab1, and vice versa, and measure a Gab1-associated lipid kinase activity upon VEGF stimulation. Furthermore, transfection of the Gab1-YF3 mutant lacking all p85-binding sites strongly repressed PI3K activation measured in vitro. Moreover, Gab1-YF3 severely decreased the cellular amount of phosphatidylinositol 3,4,5-trisphosphate (PtdIns(3,4,5)P3) generated in response to VEGF. Furthermore, adenoviral expression of Gab1-YF3 suppressed both Akt phosphorylation and recovery of wounded human umbilical vein endothelial cell monolayers, a VEGF-dependent process involving cell   migration   and proliferation under PI3K control. Transfection of other Gab1 mutants, lacking Grb2-binding sites or the pleckstrin homology (PH) domain, also prevented Akt activation, further demonstrating Gab1 involvement in PI3K activation. These mutants were also used to show that interactions with both Grb2 and PtdIns(3,4,5)P3 mediate Gab1 recruitment by VEGFR-2. Importantly, Gab1 mobilization was impaired by (i) PI3K inhibitors, (ii) deletion of Gab1 PH domain, (iii) PTEN (phosphatase and tensin homolog deleted on chromosome 10) overexpression to repress PtdIns(3,4,5)P3 production, and (iv) overexpression of a competitor PH domain for PtdIns(3,4,5)P3 binding, which altogether demonstrated that PI3K is also an upstream regulator of Gab1. Gab1 thus appears as a primary actor in coupling VEGFR-2 to PI3K/Akt, recruited through an amplification loop involving PtdIns(3,4,5)P3 and its PH domain. 
 Etk/Bmx transactivates vascular endothelial growth factor 2 and recruits phosphatidylinositol 3-kinase to mediate the tumor necrosis factor-induced angiogenic pathway. Tumor necrosis factor (TNF), via its receptor 2 (TNFR2), induces Etk (or Bmx) activation and Etk-dependent endothelial cell (EC)   migration   and tube formation. Because TNF receptor 2 lacks an intrinsic kinase activity, we examined the kinase(s) mediating TNF-induced Etk activation. TNF induces a coordinated phosphorylation of vascular endothelial growth factor (VEGF) receptor 2 (VEGFR2) and Etk, which is blocked by VEGFR2-specific inhibitors. In response to TNF, Etk and VEGFR2 form a complex resulting in a reciprocal activation between the two kinases. Subsequently, the downstream phosphatidylinositol 3-kinase (PI3K)-Akt signaling (but not signaling through phospholipase C-gamma) was initiated and directly led to TNF-induced EC   migration  , which was significantly inhibited by VEGFR2-, PI3K-, or Akt-specific inhibitors. Phosphorylation of VEGFR2 at Tyr-801 and Tyr-1175, the critical sites for VEGF-induced PI3K-Akt signaling, was not involved in TNF-mediated Akt activation. However, TNF induces phosphorylation of Etk at Tyr-566, directly mediating the recruitment of the p85 subunit of PI3K. Furthermore, TNF- but not VEGF-induced activation of VEGFR2, Akt, and EC   migration   are blunted in EC genetically deficient with Etk. Taken together, our data demonstrated that TNF induces transactivation between Etk and VEGFR2, and Etk directly activates PI3K-Akt angiogenic signaling independent of VEGF-induced VEGFR2-PI3K-Akt signaling pathway. 
 Src homology 2 (SH2) domain containing protein tyrosine phosphatase-1 (SHP-1) dephosphorylates VEGF Receptor-2 and attenuates endothelial DNA synthesis, but not   migration  *. Vascular endothelial growth factor receptor-2 (VEGFR-2, KDR), a receptor tyrosine kinase, regulates mitogenic, chemotactic, hyperpermeability, and survival signals in vascular endothelial cells in response to its ligand vascular permeability factor/ vascular endothelial growth factor (VPF/VEGF). SHP-1 is a protein tyrosine phosphatase known to negatively regulate signaling from receptors such as EGF receptor, IL3 receptor, erythropoietin receptor and also KDR. However, the mechanism by which SHP-1 executes KDR dephosphorylation, the targeted tyrosine residue(s) of KDR and also overall downstream signaling or phenotypic change(s) caused, is not defined.Here, we have demonstrated that KDR and SHP-1 are constitutively associated and upon VEGF treatment, the phosphatase activity of SHP-1 is stimulated in a c-Src kinase dependent manner. Knockdown of SHP-1 by siRNA or inhibition of c-Src by an inhibitor, results in augmented DNA synthesis perhaps due to increased phosphorylation of at least three tyrosine residues of KDR 996, 1059 and 1175. On the other hand, neither tyrosine residue 951 of KDR nor VEGF-mediated   migration   is affected by modulation of SHP-1 function.Taken together our results define the tyrosine residues of KDR that are regulated by SHP-1 and also elucidates a novel feed back loop where SHP-1 is activated upon VEGF treatment through c-Src and controls KDR induced DNA synthesis, eventually leading to controlled angiogenesis. 
 Phosphorylation of Akt and ERK1/2 is required for VEGF-A/VEGFR2-induced proliferation and   migration   of lymphatic endothelium. There is growing evidence that vascular endothelial growth factor-A (VEGF-A), a ligand of the receptor tyrosine kinases VEGFR1 and VEGFR2, promotes lymphangiogenesis. However, the underlying mechanisms by which VEGF-A induces the growth of lymphatic vessels remain poorly defined. Here we report that VEGFR2, not VEGFR1, is the primary receptor regulating VEGF-A-induced lymphangiogenesis. We show that specific inhibition of VEGF-A/VEGFR2 signaling with the fully human monoclonal antibody r84 significantly inhibits lymphangiogenesis in MDA-MB-231 tumors. In vitro experiments with primary human dermal lymphatic endothelial cells (LECs) demonstrate that blocking VEGF-A activation of VEGFR2, not VEGFR1, significantly inhibits VEGF-A-induced proliferation and   migration   of LECs. We show that VEGF-A stimulation of LECs leads to the phosphorylation of VEGFR2 (Tyr 951, 1054, 1059, 1175, and 1214) which subsequently triggers PKC dependent phosphorylation of ERK1/2 and PI3-K dependent phosphorylation of Akt. Additionally, we demonstrate that inhibitors that suppress the phosphorylation of ERK1/2 and Akt significantly block VEGF-A- induced proliferation and   migration   of LECs. Together, these results shed light on the mechanisms regulating VEGF-A-induced proliferation and   migration   of LECs, reveal that VEGFR2 is the primary signaling VEGF-A receptor on lymphatic endothelium, and suggest that therapeutic agents targeting the VEGF-A/VEGFR2 axis could be useful in blocking the pathological formation of lymphatic vessels. 
 Cooperation between integrin alphavbeta3 and VEGFR2 in angiogenesis. The cross-talk between receptor tyrosine kinases and integrin receptors are known to be crucial for a number of cellular functions. On endothelial cells, an interaction between integrin alphavbeta3 and VEGFR2 seems to be particularly important process during vascularization. Importantly, the functional association between VEGFR2 and integrin alphavbeta3 is of reciprocal nature since each receptor is able to promote activation of its counterpart. This mutually beneficial relationship regulates a number of cellular activities involved in angiogenesis, including endothelial cell   migration  , survival and tube formation, and hematopoietic cell functions within vasculature. This article discusses several possible mechanisms reported by different labs which mediate formation of the complex between VEGFR-2 and alphavbeta3 on endothelial cells. The pathological consequences and regulatory events involved in this receptor cross-talk are also presented. 
 The vascular endothelial growth factor VEGF165 induces perlecan synthesis via VEGF receptor-2 in cultured human brain microvascular endothelial cells. A member of the vascular endothelial growth factor (VEGF) family, VEGF165, regulates vascular endothelial cell functions in autocrine and paracrine fashions in microvessels. Proteoglycans are highly glycosylated poly-anionic macromolecules that influence cellular behaviors such as proliferation and   migration   by interacting with cytokines/growth factors. In the present study, we investigated the regulation of proteoglycan synthesis by VEGF165 in cultured human brain microvascular endothelial cells. The cells were exposed to recombinant human VEGF165, and the proteoglycans were then characterized using biochemical techniques. VEGF165 treatment increased the accumulation of proteoglycans 1.4- and 1.6-fold in the cell layer and conditioned medium, respectively. This effect resulted from the activation of VEGFR-2, and was mimicked by vammin, a VEGFR-2 ligand from snake venom but not placenta growth factor, which binds specifically to VEGFR-1. VEGF165 stimulated the production and secretion of perlecan, substituted with shorter heparan sulfate side chains, but with unaltered sulfated disaccharide composition. The perlecan secreted by VEGF165-stimulated endothelial cells may be involved in the regulation of cellular behavior during angiogenesis, in diseases of the brain microvessels, and in the maintenance of the endothelial cell monolayer. 
 Dysregulation of the β3 integrin-VEGFR2 complex in Hantaan virus-directed hyperpermeability upon treatment with VEGF. Hantaviruses infect human endothelial cells (ECs) and are known to cause vascular-permeability-based diseases, including hemorrhagic fever with renal syndrome (HFRS) and hantavirus pulmonary syndrome (HPS). The αvβ3 integrins, which are highly expressed on the surface of ECs, serve as hantavirus receptors. Specifically, the β3 integrin and vascular endothelial growth factor (VEGF) receptor 2 (VEGFR2) form a functional complex and interact with each other. Signaling through this complex causes cytoskeletal reorganization, which is one of the most important mechanisms underlying hyperpermeability. In this study, we show that VEGF dramatically enhances Hantaan virus (HTNV)-directed permeability and increases the reorganization of the cytoskeleton and the disruption of junctional organizations in an EC monolayer at 3 days postinfection. HTNV infection reduced the effect of VEGF on adhesi  on, migra  tion, and the upregulation of β3 expression, but the infection alone upregulated the expression of β3 and VEGFR2. These results indicate that in addition to its role in blocking β3 integrin activation as reported previously, HTNV blocks the function of the complex of VEGFR2 and β3 integrin, and the dysfunction of the complex may contribute to cytoskeletal reorganization in an HTNV-directed hyperpermeability response to VEGF. 
 Biphasic effect of PTK7 on KDR activity in endothelial cells and angiogenesis. Protein tyrosine kinase 7 (PTK7) is a member of the defective receptor protein tyrosine kinase family which lacks catalytic activity. Expression of PTK7 is increased in various cancers but its role in carcinogenesis is not well understood. We previously showed that disruption of PTK7 function suppresses VEGF-induced angiogenic phenotypes in HUVECs and mice. Here, we investigated molecular mechanisms for modulating VEGF-induced physiological effects by PTK7. Treatment with a high concentration of extracellular domain of PTK7 (soluble PTK7; sPTK7) or knockdown of PTK7 inhibited VEGF-induced phosphorylation of kinase insert domain receptor (KDR) but did not inhibit phosphorylation of fms-related tyrosine kinase 1 (FLT-1) in HUVECs. PTK7, more specifically sPTK7, interacted with KDR but not with FLT-1 in HUVECs and HEK293 cells. In vitro binding assay showed that sPTK7 formed oligomers with the extracellular domain of KDR (sKDR) up to an approximately 1:3 molar ratio, and vice versa. sPTK7 at lower molar ratios than sKDR enhanced the binding of VEGF to sKDR. At the same or higher molar ratios, it reduced the binding of VEGF to sKDR. Increasing concentrations of sPTK7 or increasing levels of PTK7 expression first increased and then decreased VEGF-induced KDR phosphorylation,   migration  , and capillary-like tube formation of HUVECs, as well as in vivo angiogenesis. Taken together, our data demonstrates that PTK7 regulates the activity of KDR biphasically by inducing oligomerization of KDR molecules at lower concentrations and by surrounding KDR molecules at higher concentrations. 
 Recruitment and activation of phospholipase Cgamma1 by vascular endothelial growth factor receptor-2 are required for tubulogenesis and differentiation of endothelial cells. Vascular endothelial growth factor-mediated angiogenic signal transduction relay is achieved by coordinated induction of endothelial cell proliferation,   migration  , and differentiation. These complex cellular processes are most likely controlled by activation of both cooperative and antagonistic signals by vascular endothelial growth factor receptors (VEGFRs). Here, we investigated the contribution of tyrosine-phosphorylated residues of VEGFR-2/fetal liver kinase-1 to endothelial cell proliferation and differentiation and activation of signaling proteins. Mutation of tyrosine 1006 of VEGFR-2 to phenylalanine severely impaired the ability of this receptor to stimulate endothelial cell differentiation and tubulogenesis. Paradoxically, the mutant receptor stimulated endothelial cell proliferation far better than the wild-type receptor. Further analysis showed that tyrosine 1006 is responsible for phospholipase Cgamma1 (PLCgamma1) activation and intracellular calcium release in endothelial cells. Activation of PLCgamma1 was selectively mediated by tyrosine 1006. Mutation of tyrosines 799, 820, 949, 994, 1080, 1173, and 1221 had no measurable effect on the ability of VEGFR-2 to stimulate PLCgamma1 activation. Association of VEGFR-2 with PLCgamma1 was mainly established between tyrosine 1006 and the C-terminal SH2 domain of PLCgamma1 in vitro and in vivo. Taken together, the results indicate that phosphorylation of tyrosine 1006 is essential for VEGFR-2-mediated PLCgamma1 activation, calcium flux, and cell differentiation. More importantly, VEGFR-2-mediated endothelial cell proliferation is inversely correlated with the ability of VEGFR-2 to associate with and activate PLCgamma1. 
 The antihypertension drug doxazosin inhibits tumor growth and angiogenesis by decreasing VEGFR-2/Akt/mTOR signaling and VEGF and HIF-1α expression. Doxazosin is an α1 adrenergic receptor blocker that also exerts antitumor effects. However, the underlying mechanisms by which it modulates PI3K/Akt intracellular signaling are poorly understood. In this study, we reveal that doxazosin functions as a novel antiangiogenic agent by inhibiting vascular endothelial growth factor (VEGF)-induced cel  l migrati  on and proliferation. It also inhibited VEGF-induced capillary-like structure tube formation in vitro. Doxazosin inhibited the phosphorylation of VEGF receptor-2 (VEGFR-2) and downstream signaling, including PI3K, Akt, 3-phosphoinositide-dependent protein kinase 1 (PDK1), mammalian target of rapamycin (mTOR), and hypoxia-inducible factor 1 (HIF-1α). However, it had no effect on VEGF-induced extracellular signal-regulated kinase 1/2 (ERK1/2) phosphorylation. Furthermore, doxazosin reduced tumor growth and suppressed tumor vascularization in a xenograft human ovarian cancer model. These results provide evidence that doxazosin functions in the endothelial cell system to modulate angiogenesis by inhibiting Akt and mTOR phosphorylation and interacting with VEGFR-2. 
 Integrin alphavbeta3, requirement for VEGFR2-mediated activation of SAPK2/p38 and for Hsp90-dependent phosphorylation of focal adhesion kinase in endothelial cells activated by VEGF. Endothelial cell   migration  , a key process in angiogenesis, requires the coordinated integration of motogenic signals elicited by the adhesion of endothelial cells to extracellular matrices and by angiogenic cytokines such as the vascular endothelial growth factor (VEGF). In this study, we found that addition of VEGF to human umbilical vein endothelial cells cultivated on vitronectin triggers a synergistic interaction between the VEGF receptor VEGFR2 and the clustered integrin receptor alphavbeta3. The interaction between VEGFR2 and alphavbeta3 is required for full phosphorylation of VEGFR2 and to drive the activation of motogenic pathways involving focal adhesion kinase (FAK) and stress-activated protein kinase-2/p38 (SAPK2/p38). The signal emanating from the VEGFR2 and alphavbeta3 interaction and leading to SAPK2/p38 activation proceeds directly from VEGFR2. The chaperone Hsp90 is found in a complex that coprecipitates with inactivated VEGFR2, and the association is increased by VEGF and decreased by geldanamycin, a specific inhibitor of Hsp90-mediated events. Geldanamycin also impairs the phosphorylation of FAK that results from the interaction between VEGFR2 and alphavbeta3, and this is accompanied by an inhibition of the recruitment of vinculin to VEGFR2. We conclude that a necessary cross talk should occur between VEGFR2 and the integrin alphavbeta3, to transduce the VEGF signals to SAPK2/p38 and FAK and that Hsp90 is instrumental in the building up of focal adhesions by allowing the phosphorylation of FAK and the recruitment of vinculin to VEGFR2. 
 VEGF inhibits tumor cell invasion and mesenchymal transition through a MET/VEGFR2 complex. Inhibition of VEGF signaling leads to a proinvasive phenotype in mouse models of glioblastoma multiforme (GBM) and in a subset of GBM patients treated with bevacizumab. Here, we demonstrate that vascular endothelial growth factor (VEGF) directly and negatively regulates tumor cell invasion through enhanced recruitment of the protein tyrosine phosphatase 1B (PTP1B) to a MET/VEGFR2 heterocomplex, thereby suppressing HGF-dependent MET phosphorylation and tumor cell   migration  . Consequently, VEGF blockade restores and increases MET activity in GBM cells in a hypoxia-independent manner, while inducing a program reminiscent of epithelial-to-mesenchymal transition highlighted by a T-cadherin to N-cadherin switch and enhanced mesenchymal features. Inhibition of MET in GBM mouse models blocks mesenchymal transition and invasion provoked by VEGF ablation, resulting in substantial survival benefit. 
 MicroRNA-16 and microRNA-424 regulate cell-autonomous angiogenic functions in endothelial cells via targeting vascular endothelial growth factor receptor-2 and fibroblast growth factor receptor-1. MicroRNAs play key roles in modulating a variety of cellular processes by posttranscriptional regulation of their target genes. Vascular endothelial growth factor (VEGF), VEGF receptor-2 (VEGFR2), and fibroblast growth factor receptor-1 (FGFR1) were identified by bioinformatic approaches and subsequently validated as targets of microRNA (miR)-16 and miR-424 in endothelial cells (ECs).Mimetics of these microRNAs reduced VEGF, VEGFR2, and FGFR1 expression, whereas specific antagonists enhanced their expression. Expression of mature miR-16 and miR-424 was upregulated on VEGF or basic fibroblast growth factor (bFGF) treatment. This upregulation was accompanied by a parallel increase in primary transcript (pri-miR)-16-1 and pri-miR-16-2 but not in pri-miR-424 levels, indicating a VEGF/bFGF-dependent transcriptional and posttranscriptional regulation of miR-16 and miR-424, respectively. Reduced expression of VEGFR2 and FGFR1 by miR-16 or miR-424 overexpression regulated VEGF and bFGF signaling through these receptors, thereby affecting the activity of downstream components of the pathways. Functionally, miR-16 or miR-424 overexpression reduced proliferation,   migration  , and cord formation of ECs in vitro, and lentiviral overexpression of miR-16 reduced the ability of ECs to form blood vessels in vivo.We conclude that these miRNAs fine-tune the expression of selected endothelial angiogenic mediators in response to these growth factors. Altogether, these findings suggest that miR-16 and miR-424 play important roles in regulating cell-intrinsic angiogenic activity of ECs. 
 Functional and structural characterization of the kinase insert and the carboxy terminal domain in VEGF receptor 2 activation. Vascular endothelial growth factors (VEGFs) regulate blood and lymphatic vessel development and homeostasis. VEGF receptor 2 (VEGFR-2) is the major receptor involved in vasculogenesis and angiogenesis and regulates endothelial cell survival,   migration  , and mitogenesis. Ligand-mediated receptor dimerization instigates transmembrane signaling, thereby promoting activation of the intracellular kinase domain. The intracellular part of the receptor comprises the juxtamembrane domain, the catalytic kinase domain, the kinase insert domain (KID), and the carboxy terminal domain (CD). Here we show that the CD inhibits VEGFR-2 activity in the absence of ligand, whereas the KID, particularly a tyrosine residue in this domain (Y951), is indispensable for downstream signaling by the activated kinase. Because of the lack of crystallographic data for the complete kinase domain, we applied size-exclusion chromatography, multiangle laser scattering, analytical ultracentrifugation, and small-angle X-ray scattering to build and functionally validate structural models. Our data show substantial conformational changes of the kinase when it is switched from the inactive, unphosphorylated state to the active, phosphorylated state. Finally, we structurally characterized recombinantly produced protein complexes between VEGFR-2 and T cell-specific adapter protein, a molecule involved in downstream signaling by VEGFR-2. 
 High blood sugar levels significantly impact the prognosis of colorectal cancer patients through down-regulation of microRNA-16 by targeting Myb and VEGFR2. The high prevalence of type 2 diabetes mellitus in colorectal cancer patients is a crucial public health issue worldwide. The deregulation of microRNAs has been shown to be associated with the progression of CRC; however, the effects of high blood sugar levels on miR deregulation and, in turn, CRC remain unexplored. In this study, 520 CRC patients were classified into two groups according to their blood sugar levels (≧110 or <110 mg/dL). Clinicopathologic features, clinical outcomes, and serum miR-16 levels of the two groups were then analyzed, while cell cycles, cell proliferation  , migrati  on, and cellular miR-16 expression were investigated via D-(+)-glucose administration. Additionally, the target genes of miR-16 were identified. Through multivariate analysis, both the disease-free survival and overall survival of the CRC patients were found to be associated with the UICC stage, perineural invasion, and blood glucose levels (P < 0.05). Serum miR-16 levels were significantly lower in the high blood glucose patients than in the normal blood glucose patients (P = 0.0329). With D-(+)-glucose administration, the proliferation an  d migrati  on of CRC cells in vitro increased remarkably (P < 0.05), while their accumulation in the G1 phase decreased significantly. Cellular miR-16 expression was suppressed by D-(+)-glucose administration. The expression levels of two target genes, Myb and VEGFR2, were affected significantly by miR-16, while glucose administration inhibited miR-16 expression and enhanced tumor cell proliferation an  d migrati  on. Hyperglycemia can impact the clinical outcomes of CRC patients, likely by inhibiting miR-16 expression and the expression of its downstream genes Myb and VEGFR2. 
 Dopamine regulates phosphorylation of VEGF receptor 2 by engaging Src-homology-2-domain-containing protein tyrosine phosphatase 2. Vascular endothelial growth factor (VEGF)-induced receptor phosphorylation is the crucial step for initiating downstream signaling pathways that lead to angiogenesis or related pathophysiological outcomes. Our previous studies have shown that the neurotransmitter dopamine could inhibit VEGF-induced phosphorylation of VEGF receptor 2 (VEGFR-2), endothelial cell proliferation,   migration  , microvascular permeability, and thus, angiogenesis. In this study, we address the mechanism by which VEGFR-2 phosphorylation is regulated by dopamine. Here, we demonstrate that D2 dopamine receptor (D2DR) colocalizes with VEGFR-2 at the cell surface. Dopamine pretreatment increases the translocation and colocalization of Src-homology-2-domain-containing protein tyrosine phosphatase (SHP-2) with D2DR at the cell surface. Dopamine administration leads to increased VEGF-induced phosphorylation of SHP-2 and this increased phosphorylation parallels the increased phosphatase activity of SHP-2. Active SHP-2 then dephosphorylates VEGFR-2 at Y951, Y996 and Y1059, but not Y1175. We also observe that SHP-2 knockdown impairs the dopamine-regulated inhibition of VEGF-induced phosphorylation of VEGFR-2 and, subsequently, Src phosphorylation and   migration  . Our data establish a novel role for SHP-2 phosphatase in the dopamine-mediated regulation of VEGFR-2 phosphorylation. 
 KIF13B regulates angiogenesis through Golgi to plasma membrane trafficking of VEGFR2. Although the trafficking of newly synthesized VEGFR2 to the plasma membrane is a key determinant of angiogenesis, the molecular mechanisms of Golgi to plasma membrane trafficking are unknown. Here, we have identified a key role of the kinesin family plus-end molecular motor KIF13B in delivering VEGFR2 cargo from the Golgi to the endothelial cell surface. KIF13B is shown to interact directly with VEGFR2 on microtubules. We also observed that overexpression of truncated versions of KIF13B containing the binding domains that interact with VEGFR2 inhibited VEGF-induced capillary tube formation. KIF13B depletion prevented VEGF-mediated endothelial   migration  , capillary tube formation and neo-vascularization in mice. Impairment in trafficking induced by knockdown of KIF13B shunted VEGFR2 towards the lysosomal degradation pathway. Thus, KIF13B is an essential molecular motor required for the trafficking of VEGFR2 from the Golgi, and its delivery to the endothelial cell surface mediates angiogenesis. 
 Signal transduction via vascular endothelial growth factor (VEGF) receptors and their roles in atherogenesis. Vascular endothelial growth factor (VEGF)-A plays a critical role in vascular development and angiogenesis through its binding and activation of VEGF receptor-2 (VEGFR-2). The binding of VEGF-A to VEGFR-2 causes receptor dimerization, kinase activation and autophosphorylation of specific tyrosine residues within the dimeric complex. Tyrosine(Y)951 in the kinase-insert domain, Y1054 and Y1059 in the kinase domain and Y1175 and Y1214 in the C-terminal tail have been shown to serve as autophosphorylation sites. Phosphorylated Y1175 creates a binding site for phospholipase Cgamma1 (PLC-gamma1) and Shb. Activation of PLC-gamma1 and Shb regulates VEGF-A-dependent cell proliferation and cell   migration  , respectively. Phosphorylated Y951 binds and mediates tyrosine phosphorylation of the T-cell-specific adaptor protein (TSAd), which is expressed in endothelial cells. Y951-mediated coupling of VEGFR-2 and TSAd is critical for VEGF-A-induced cell   migration   and actin reorganization, and for pathological angiogenesis. These phosphorylation sites may be useful targets for the development of anti-angiogenic therapies to treat atherosclerosis and cancer. 
 Decreased expression of CHIP leads to increased angiogenesis via VEGF-VEGFR2 pathway and poor prognosis in human renal cell carcinoma. CHIP (c-terminal Hsp70-interacting protein) is an E3 ligase which may play different roles in different cancers. The elucidation of the VHL-HIF-1α (hypoxia inducible factor-1α)-VEGF (vascular endothelial growth factor) pathway has led to the development of targeted therapy in renal cell carcinoma (RCC). However, little is known about the role of CHIP and the relationship between CHIP and VEGF-VEGFR2 (VEGF receptor 2) pathway in RCC. In this study, we found that the expression of CHIP was downregulated and significantly correlated with pT status (P = 0.022) and TNM stage (P = 0.022) in 304 RCC and 35 normal renal tissues using tissue microarray. Moreover, low expression of CHIP is a strong and independent negative prognostic value for RCC. In vitro, CHIP negatively regulated   RCC cell   migration, invasion and angiogenesis. In addition, ELISA tests showed that restoration of CHIP inhibited, while knockdown promoted, the secreted level of VEGF. Furthermore, western blot indicated that the VEGFR2 protein level was reduced after CHIP overexpression. Our findings demonstrate for the first time that CHIP may be involved in RCC angiogenesis through regulating VEGF secretion and expression of VEGFR2. CHIP may serve as promising prognostic biomarker of angiogenesis and may constitute a potential therapeutic target in RCC. 
 Transient Receptor Potential Canonical 1 (TRPC1) Channels as Regulators of Sphingolipid and VEGF Receptor Expression: IMPLICATIONS FOR THYROID CANCER CELL MIGRATION AND PROLIFERATION. The identity of calcium channels in the thyroid is unclear. In human follicular thyroid ML-1 cancer cells, sphingolipid sphingosine 1-phosphate (S1P), through S1P receptors 1 and 3 (S1P1/S1P3), and VEGF receptor 2 (VEGFR2) stimulates   migration  . We show that human thyroid cells express several forms of transient receptor potential canonical (TRPC) channels, including TRPC1. In TRPC1 knockdown (TRPC1-KD) ML-1 cells, the basal and S1P-evoked invasion and   migration   was attenuated. Furthermore, the expression of S1P3 and VEGFR2 was significantly down-regulated. Transfecting wild-type ML-1 cells with a nonconducting TRPC1 mutant decreased S1P3 and VEGFR2 expression. In TRPC1-KD cells, receptor-operated calcium entry was decreased. To investigate whether the decreased receptor expression was due to attenuated calcium entry, cells were incubated with the calcium chelator BAPTA-AM (1,2-bis(o-aminophenoxy)ethane-N,N,N',N'-tetraacetic acid). In these cells, and in cells where calmodulin and calmodulin-dependent kinase were blocked pharmacologically, S1P3 and VEGFR2 expression was decreased. In TRPC1-KD cells, both hypoxia-inducible factor 1α expression and the secretion and activity of MMP2 and MMP9 were attenuated, and proliferation was decreased in TRPC1-KD cells. This was due to a prolonged G1 phase of the cell cycle, a significant increase in the expression of the cyclin-dependent kinase inhibitors p21 and p27, and a decrease in the expression of cyclin D2, cyclin D3, and CDK6. Transfecting TRPC1 to TRPC1-KD cells rescued receptor expression,   migratio  n, and proliferation. Thus, the expression of S1P3 and VEGFR2 is mediated by a calcium-dependent mechanism. TRPC1 has a crucial role in this process. This regulation is important for the invasion,   migratio  n, and proliferation of thyroid cancer cells. 
 Inhibition of vascular endothelial growth factor receptor 2-mediated endothelial cell activation by Axl tyrosine kinase receptor. GAS6, the product of a growth arrest specific (GAS) gene, is the ligand of the tyrosine kinase receptor Axl. GAS6 and Axl are both expressed in endothelial cells, where they are involved in many processes such as leukocyte trans  migration   through capillaries and neointima formation in injured vessels. Here, we show that Axl stimulation by GAS6 results in inhibition of the ligand-dependent activation of vascular endothelial growth factor (VEGF) receptor 2 and the consequent activation of an angiogenic program in vascular endothelial cells. GAS6 inhibits chemotaxis of endothelial cells stimulated by VEGF-A isoforms, but not that triggered by fibroblast growth factor-2 or hepatocyte growth factor. Furthermore, it inhibits endothelial cell morphogenesis on Matrigel and VEGF-A-dependent vascularization of chick chorion allantoid membrane. GAS6 activates the tyrosine phosphatase SHP-2 (SH2 domain-containing tyrosine phosphatase 2), which is instrumental in the negative feedback exerted by Axl on VEGF-A activities. A dominant-negative SHP-2 mutant, in which Cys 459 is substituted by Ser, reverted the effect of GAS6 on stimulation of VEGF receptor 2 and endothelial chemotaxis triggered by VEGF-A. These studies provide the first demonstration of a cross talk between Axl and VEGF receptor 2 and add new information on the regulation of VEGF-A activities during tissue vascularization. 
 Tubular network formation by adriamycin-resistant MCF-7 breast cancer cells is closely linked to MMP-9 and VEGFR-2/VEGFR-3 over-expressions. We have previously demonstrated that matrix metalloproteinase-9 (MMP-9) is critical for breast cancer cell   migration   and is necessary but not sufficient for tubular network formation. Given the important angiogenic activity of vascular endothelial growth factor (VEGF), we investigate here its possible contribution in tubular network formation and its link with MMP-9. Exposure of resistant epithelial breast cancer cells (rMCF-7) to Avastin, a VEGF neutralising antibody, suppresses tubular network formation but not cell   migration  . However, their exposure to MMP-9 inhibitor markedly decreases both parameters. Besides, the addition of exogenous VEGF or MMP-9 alone or in combination to sensitive parental cells (sMCF-7) or rMCF-7 cells enhances tubular network formation by rMCF-7 cells but not by sMCF-7 cells. The evaluation of the expression levels of VEGF receptor (VEGFR) subtypes shows that sMCF-7 cells express only small quantities of VEGFR-2 and VEGFR-3 compared with rMCF-7 cells that express strong quantities. However, treatment of sMCF-7 cells by phorbol 12-myristate 13-acetate (PMA), a PKC activator, induces both tubular network formation and VEGFR-2/VEGFR-3 over-expressions. Interestingly, exposure of rMCF-7 cells or PMA-treated sMCF-7 cells to the specific inhibitors of VEGFR-2 and VEGFR-3 reduces markedly the tubular network formation. Together, our results demonstrate that the proteolytic enzyme MMP-9 promotes rMCF-7 cell   migration   and, consequently, tubular network formation through VEGFR-2/ VEGFR-3 activation. Understanding of mechanisms involved in vasculogenic mimicry and cell   migration   related to MMP-9 and VEGF may open new opportunities to improve cancer therapy. 
 Increased VEGFR2 expression during human late endothelial progenitor cells expansion enhances in vitro angiogenesis with up-regulation of integrin alpha(6). In vitro expansion of late endothelial progenitor cells (EPCs) might yield a cell therapy product useful for myocardial and leg ischaemia, but the influence of EPC expansion on the angiogenic properties of these cells is unknown. In the present study, we investigated the effect of in vitro EPC expansion on vascular endothelial growth factor (VEGF) receptor expression. EPCs were obtained from CD34(+) cord blood cells and expanded for up to 5 weeks. Real-time quantitative reverse-transcription polymerase chain reaction (RT-PCR) showed that VEGFR2 expression, contrary to VEGFR1 and VEGFR3 expression, was significantly higher on expanded EPCs than on freshly isolated CD34(+) cells or on human umbilical vein endothelial cells (HUVECs). Quantitative flow cytometry confirmed that VEGFR2 density on EPCs increased during the expansion process and was significantly higher than on HUVECs. The impact of VEGFR2 increase was studied on the three theoretical steps of angiogenesis, i.e., EPC proliferation,   migration   and differentiation. VEGFR2 up-regulation had no effect on VEGF-induced cell proliferation, but significantly enhanced EPC   migration   and pseudotubes formation dependent on integrin alpha(6) subunit overexpression. In vitro expansion of late EPCs increases the expression of VEGFR2, the main VEGF receptor, with possible implications for EPC-based angiogenic therapy. 
 S1P1 and VEGFR-2 form a signaling complex with extracellularly regulated kinase 1/2 and protein kinase C-alpha regulating ML-1 thyroid carcinoma cell   migration  . Sphingosine 1-phosphate (S1P) and vascular endothelial growth factor receptor 2 (VEGFR-2) signaling have been shown to integrate in many biological processes. The follicular thyroid carcinoma cell line ML-1 expresses VEGFR-2 and secretes substantial amounts of both vascular endothelial growth factor (VEGF)-A and VEGF-C. ML-1 cells also express S1P-receptors (S1P(1-3,5)). S1P is able to phosphorylate VEGFR-2, and inhibiting VEGFR-2 attenuates S1P-induced   migration   and down-regulates S1P(1) expression in ML-1 cells. In the present study, we focused on the interactions between S1P(1) and VEGFR-2. We show that S1P receptors form complexes with VEGFR-2 and that the S1P(1)/VEGFR-2 complex associates with protein kinase C (PKC)-alpha and ERK1/2. Furthermore, the complex evokes bidirectional signaling since the S1P-induced ERK1/2 phosphorylation is sensitive to VEGFR-2 kinase inhibition and VEGF-A-induced ERK1/2 phosphorylation is sensitive to pertussis toxin treatment as well as S1P(1) small interfering RNA (siRNA) treatment. Both S1P- and VEGF-A-induced haptotaxis is sensitive to pertussis toxin treatment and S1P(1) siRNA treatment. Phosphorylation of ERK1/2 evoked by both VEGF-A and the S1P(1) agonist SEW-2871 is inhibited by PKC-alpha and PKC-betaI siRNA. We hypothesize that VEGFR-2 forms a signaling complex with S1P(1), evoking bidirectional signaling regulating both ERK1/2 phosphorylation and haptotaxis of ML-1 cells. 
 Neuropilin-1 expression promotes invasiveness of melanoma cells through vascular endothelial growth factor receptor-2-dependent and -independent mechanisms. The majority of human melanoma cell lines secretes vascular endothelial growth factor-A (VEGF-A) and expresses its receptors VEGFR-1, VEGFR-2 and neuropilin-1 (NRP‑1), a co-receptor for VEGF-A that amplifies the signalling through VEGFR-2. Since it is known that the VEGF-A/VEGFR-2 autocrine loop promotes melanoma cell invasiveness, the aim of the present study was to investigate the involvement of NPR-1 in melanoma progression. Syngeneic human melanoma cell lines expressing either VEGFR-2 or NRP-1, both or none of them, were analyzed for their in vitro ability to migrate, invade the extracellular matrix (ECM) and secrete active metalloproteinase-2 (MMP-2). The results indicate that NRP-1 cooperates with VEGFR-2 in melanoma cel  l migrati  on induced by VEGF-A. Moreover, NRP-1 expression is sufficient to promote MMP-2 secretion and melanoma cell invasiveness, as demonstrated by the ability of cells expressing solely NRP-1 to spontaneously invade the ECM. This ability is specifically downregulated by anti-NRP-1 antibodies or by interfering with NRP-1 expression using an shRNA construct. Investigation of the signal transduction pathways triggered by NRP-1 in melanoma cells, indicated that NRP-1-dependent promotion of cell invasiveness involves Akt activation through its phosphorylation on T308. Overall, the results demonstrate that NRP-1 is involved in melanoma progression through VEGFR-2-dependent and -independent mechanisms and suggest NRP-1 as a target for the treatment of the metastatic disease. 
 Lipocortin V may function as a signaling protein for vascular endothelial growth factor receptor-2/Flk-1. Binding of vascular endothelial growth factor (VEGF) to its receptor, VEGFR-2 (Flk-1/KDR), induces dimerization and activation of the tyrosine kinase domain of the receptor, resulting in autophosphorylation of cytoplasmic tyrosine residues used as docking sites for signaling proteins that relay the signals for cell proliferation,   migration  , and permeability enhancement. We explored the VEGF/receptor signaling pathway by performing a two-hybrid screen of a rat lung cDNA library in yeast using the intracellular domain of rat VEGFR-2 as bait. Two clones encoding lipocortin V were isolated. Subsequent studies with the yeast two-hybrid assay showed that the complete intracellular domain of VEGFR-2 was required for the interaction. Co-immunoprecipitation of translated proteins confirmed the interaction between the VEGF receptor and lipocortin V. VEGF induced a rapid tyrosine phosphorylation of lipocortin V in human umbilical vein endothelial cells (HUVEC). Pretreatment of HUVEC with antisense oligodeoxyribonucleotide (ODN) for lipocortin V significantly inhibited VEGF-induced cell proliferation, which was accompanied by a decrease in protein synthesis and tyrosine phosphorylation of lipocortin V. Our results indicate that lipocortin V may function as a signaling protein for VEGFR-2 by directly interacting with the intracellular domain of the receptor and appears to be involved in regulation of vascular endothelial cell proliferation mediated by VEGFR-2. 
 The adaptor protein shb binds to tyrosine 1175 in vascular endothelial growth factor (VEGF) receptor-2 and regulates VEGF-dependent cellular   migration  . Previous studies have shown that the adaptor protein Shb is involved in receptor tyrosine kinase signaling. In this study, we demonstrate that Shb is phosphorylated in an Src-dependent manner upon vascular endothelial growth factor (VEGF) stimulation using porcine aortic endothelial cells expressing the human VEGF receptor 2 (VEGFR-2) (KDR). In co-immunoprecipitation experiments, we could detect an interaction between Shb and the VEGFR-2 in human telomerase-immortalized microvascular endothelial cells. Furthermore, in a glutathione S-transferase pull-down assay, the Src homology 2 domain of Shb was shown to interact with phosphorylated tyrosine 1175 in the C-terminal tail of VEGFR-2. VEGF-induced Shb phosphorylation was lost in porcine aortic endothelial cells expressing a chimeric murine VEGFR-2 (Flk-1) with a mutation at the corresponding position. Shb expression was specifically decreased by 80%, in a transient manner, by using the short interfering RNA technique. Reduced Shb expression led to a loss of stimulation of phosphatidylinositol 3-kinase, phosphorylation of focal adhesion kinase at tyrosine 576, the generation of focal adhesions, and stress fiber formation in response to VEGF. Furthermore, we show that VEGF-induced   migration   is inhibited in Shb short interfering RNA-treated cells. Our data demonstrate that Shb is important for VEGF signaling in endothelial cells. This is achieved by Shb binding to tyrosine 1175 in the VEGFR-2, which regulates VEGF-induced formation of focal adhesions and cell   migration  , of which the latter occurs in a phosphatidylinositol 3-kinase-dependent manner. 
 Galectin-1, a novel ligand of neuropilin-1, activates VEGFR-2 signaling and modulates the   migration   of vascular endothelial cells. Galectin-1 (Gal-1), a homodimeric prototype of the galectins with a single carbohydrate-recognition domain, was recently identified as being overexpressed in tumor-associated capillary endothelial cells. The role of Gal-1 in endothelial cellular functions and the mechanism of action of Gal-1 remain unknown. Neuropilin-1 (NRP1) is a neuronal receptor that mediates repulsive growth cone guidance, and NRP1 functions in endothelial cells as a coreceptor (with vascular endothelial growth factor receptors (VEGFRs)) for VEGF(165). In this study, we found that Gal-1 was overexpressed in the tumor-associated endothelial cells of oral squamous cell carcinomas (P<0.001). Gal-1 increased the proliferation and adhesion of endothelial cells, and enhanced cell   migration   in combination with VEGF(165). Surprisingly, Gal-1 selectively bound NRP1 via the carbohydrate-recognition domain, but did not bind VEGFR-1, VEGFR-2 or VEGFR-3. The Gal-1-NRP1 interaction mediated the   migration   and adhesion of endothelial cells. The binding of Gal-1 to NRP1 enhanced VEGFR-2 phosphorylation and stimulated the activation of the mitogen activated protein (MAP) kinases SAPK1/JNK (stress activated protein kinase-1/c-Jun NH2-terminal kinase). These findings show, for the first time, that Gal-1 can directly bind to NRP1 on endothelial cells, and can promote the NRP1/VEGFR-2-mediated signaling pathway as well as NRP1-mediated biological activities. 
 Differential gene expression in Lin-/VEGF-R2+ bone marrow-derived endothelial progenitor cells isolated from diabetic mice. Diabetes is known to impair the number and function of endothelial progenitor cells in the circulation, causing structural and functional alterations in the micro- and macro-vasculature. The aim of this study was to identify early diabetes-related changes in the expression of genes that have been reported to be closely involved in endothelial progenitor cell   migration   and function.Based on review of current literature, this study examined the expression level of 35 genes that are known to be involved in endothelial progenitor cell   migration   and function in magnetically sorted Lin-/VEGF-R2+ endothelial progenitor cells obtained from the bone marrow of Akita mice in the early stages of diabetes (18 weeks) using RT-PCR and Western blotting. We used the Shapiro-Wilk and D'Agostino & Pearson Omnibus tests to assess normality. Differences between groups were evaluated by Student's t-test for normally distributed data (including Welch correction in cases of unequal variances) or Mann-Whitney test for not normally distributed data.We observed a significant increase in the number of Lin-/VEGF-R2+ endothelial progenitor cells within the bone marrow in diabetic mice compared with non-diabetic mice. Two genes, SDF-1 and SELE, were significantly differentially expressed in diabetic Lin-/VEGF-R2+ endothelial progenitor cells and six other genes, CAV1, eNOS, CLDN5, NANOG, OCLN and BDNF, showed very low levels of expression in diabetic Lin-/VEGF-R2+ progenitor cells.Low SDF-1 expression may contribute to the dysfunctional mobilization of bone marrow Lin-/VEGF-R2+ endothelial progenitor cells, which may contribute to microvascular injury in early diabetes. 
 Regulation of vascular endothelial growth factor receptor 2-mediated phosphorylation of focal adhesion kinase by heat shock protein 90 and Src kinase activities. Exposure of endothelial cells to vascular endothelial growth factor (VEGF) induced tyrosine phosphorylation of focal adhesion kinase (FAK) on site Tyr(407), an effect that required the association of VEGF receptor 2 (VEGFR2) with HSP90. The association of VEGFR2 with HSP90 involved the last 130 amino acids of VEGFR2 and was blocked by geldanamycin, a specific inhibitor of HSP90. Moreover, geldanamycin inhibited the VEGF-induced activation of the small GTPase RhoA, which resulted in an inhibition of phosphorylation of FAK on site Tyr(407). In this context, the inhibition of RhoA kinase (ROCK) with Y27632 or by expression of dominant negative forms of RhoA or ROCK impaired the VEGF-induced phosphorylation of Tyr(407) within FAK. In contrast to phosphorylation of Tyr(861), the phosphorylation of site Tyr(407) was insensitive to Src kinase inhibition by 4-amino-5-(4-chlorophenyl)-7-(t-butyl) pyrazolo[3,4-d] pyrimidine (PP2). We also found that the recruitment of paxillin to FAK was inhibited by geldanamycin but not by PP2, whereas both geldanamycin and PP2 inhibited the recruitment of vinculin to FAK. In accordance, the recruitment of paxillin and vinculin to FAK was inhibited in cells that express the mutant FAK-Y407F, whereas the expression of the mutant Y861F inhibited the recruitment of paxillin but not of vinculin. Importantly, cell   migration   was abolished in cells in which the signal from the VEGFR2-HSP90 pathway was blocked by the expression of Delta130VEGFR2, a deletant of VEGFR2 that does not associate with HSP90. Our findings underscore for the first time the key role played by the VEGFR2-HSP90-RhoA-ROCK-FAK/Tyr(407) pathway in transducing the VEGF signal that leads to the assembly of focal adhesions and endothelial cell   migration  . 
 Phosphorylation of tyrosine 1214 on VEGFR2 is required for VEGF-induced activation of Cdc42 upstream of SAPK2/p38. Activation of the tyrosine kinase receptor vascular endothelial growth factor receptor 2 (VEGFR2) by VEGF leads to the activation of stress-activated protein kinase (SAPK)2/p38 and then to actin polymerization and reorganization into stress fibers in endothelial cells. In turn, this triggers endothelial cell   migration  . Yet, nothing is known about the molecular mechanisms that couple VEGFR2 to SAPK2/p38. Here, we found that VEGF increased by twofold the activity of the small GTPase Cdc42 and that the expression of two different constitutively active forms of Cdc42 (Cdc42 V12 and Cdc42 L61) led to a marked increase in the formation of stress fibers that was sensitive to SAPK2/p38 inhibition by SB203580. Moreover, the expression of a dominant-negative form of Cdc42 (Cdc42 N17) inhibited the activation of SAPK2/p38 and of its direct target MAP kinase-activated protein kinase 2. These results indicate that Cdc42 is upstream of SAPK2/p38 in response to the activation of VEGFR2 by VEGF. In contrast, we found that neither RhoA nor Rac was involved in the SAPK2/p38-mediated actin reorganization induced by VEGF. Using a site-specific mutant of the major autophosphorylation site Y1214 on VEGFR2, we found that the mutant Y1214F inhibited the activation of both Cdc42 and SAPK2/p38 in response to VEGF. We conclude that phosphorylation of Y1214 on VEGFR2 is required to trigger the sequential activation of Cdc42 and SAPK2/p38 and to drive the SAPK2/p38-mediated actin remodeling in stress fibers in endothelial cells exposed to VEGF. 
 VEGF internalization is not required for VEGFR-2 phosphorylation in bioengineered surfaces with covalently linked VEGF. Vascular endothelial growth factor (VEGF) is known to activate proliferation,   migration  , and survival pathways in endothelial cells through phosphorylation of VEGF receptor-2 (VEGFR-2). VEGF has been incorporated into biomaterials through encapsulation, electrostatic sequestration, and covalent attachment, but the effect of these immobilization strategies on VEGF signaling has not been thoroughly investigated. Further, although growth factor internalization along with the receptor generally occurs in a physiological setting, whether this internalization is needed for receptor phosphorylation is not entirely clear. Here we show that VEGF covalently bound through a modified heparin molecule elicits an extended response of pVEGFR-2 in human umbilical vein endothelial cells (HUVECs) and that the covalent linkage reduces internalization of the growth factor during receptor endocytosis. Optical tweezer measurements show that the rupture force required to disrupt the heparin-VEGF-VEGFR-2 interaction increases from 3-8 pN to 6-12 pN when a covalent bond is introduced between VEGF and heparin. Importantly, by covalently binding VEGF to a heparin substrate, the stability (half-life) of VEGF is extended over three-fold. Here, mathematical models support the biological conclusions, further suggesting that VEGF internalization is significantly reduced when covalently bound, and indicating that VEGF is available for repeated phosphorylation events. 
 Potent neutralization of VEGF biological activities with a fully human antibody Fab fragment directed against VEGF receptor 2. Compelling evidence suggest that vascular endothelial growth factor (VEGF) and its receptors, especially receptor 2 (VEGFR2, or kinase insert domain-containing receptor, KDR), play a critical role in angiogenesis under both physiological and pathological conditions, including cancer and angiogenic retinopathies such as age-related macular degeneration (AMD). To this end, inhibition of angiogenesis with antagonists to either VEGF or KDR has yielded significant therapeutic efficacy both in preclinical studies in animal models and in clinical trials in patients with cancer and AMD. We previously reported the identification of a high affinity, fully human anti-KDR antibody fragment, 1121B Fab, through a highly stringent affinity maturation process with a Fab originally isolated from a naïve human antibody phage display library. In this study, we demonstrate that 1121B Fab is able to strongly block KDR/VEGF interaction, resulting in potent inhibition of an array of biological activities of VEGF, including activation of the receptor and its signaling pathway, intracellular calcium mobilization, and   migratio  n and proliferation of endothelial cells. Taken together, our data lend strong support to the further development of 1121B Fab fragment as an anti-angiogenesis agent in both cancer and angiogenic retinopathies. 
 Functional significance of VEGFR-2 on ovarian cancer cells. Vascular endothelial growth factor receptor (VEGFR) has recently been discovered on ovarian cancer cells, but its functional significance is unknown and is the focus of this study. By protein analysis, A2780-par and HeyA8 ovarian cancer cell lines expressed VEGFR-1 and HeyA8 A2774, and SKOV3ip1 expressed VEGFR-2. By in situ hybridization (ISH), 85% of human ovarian cancer specimens showed moderate to high VEGFR-2 expression, whereas only 15% showed moderate to high VEGFR-1 expression. By immunofluorescence, little or no VEGFR-2 was detected in normal ovarian surface epithelial cells, whereas expression was detected in 75% of invasive ovarian cancer specimens. To differentiate between the effects of tumor versus host expression of VEGFR, nude mice were injected with SKOV3ip1 cells and treated with either human VEGFR-2 specific antibody (1121B), murine VEGFR-2 specific antibody (DC101) or the combination. Treatment with 1121B reduced SKOV3ip1 cell   migration   by 68% (p < 0.01) and invasion by 72% (p < 0.01), but exposure to VEGFR-1 antibody had no effect. Treatment with 1121B effectively blocked VEGF-induced phosphorylation of p130Cas. In vivo treatment with either DC101 or 1121B significantly reduced tumor growth alone and in combination in the SKOV3ip1 and A2774 models. Decreased tumor burden after treatment with DC101 or 1121B correlated with increased tumor cell apoptosis, decreased proliferative index, and decreased microvessel density. These effects were significantly greater in the combination group (p < 0.001). We show functionally active VEGFR-2 is present on most ovarian cancer cells. The observed anti-tumor activity of VEGF-targeted therapies may be mediated by both anti-angiogenic and direct anti-tumor effects. 
 Internal and external autocrine VEGF/KDR loops regulate survival of subsets of acute leukemia through distinct signaling pathways. Besides being expressed on endothelial cells, vascular endothelial growth factor receptors (VEGFRs) are also functional on subsets of leukemias, resulting in autocrine loops that sustain leukemia   migration   and proliferation. While recent evidence suggests that VEGF supports hematopoietic stem cell survival via an internal loop, the molecular mechanisms whereby autocrine stimulation of VEGFR-2 (KDR) promotes leukemia growth are not well understood. Here we show on acute myeloid primary leukemias and cell lines that VEGF/KDR autocrine loops operate both internally and externally. First, we demonstrate that KDR is constitutively phosphorylated and located at the nucleus of VEGF-producing leukemias. Treatment with anti-VEGF antibody, which acts externally, blocked KDR nuclear translocation and inhibited nuclear factor kappa B (NF-kappaB; p65 and c-rel) activation. In contrast, a KDR-specific intracellular inhibitor failed to block KDR nuclear translocation, but inhibited the constitutive activation of mitogen activated protein kinase (MAPK)/Erk and the phosphatidylinositol 3-kinase/AKT pathways. Notably, treatment with the anti-VEGF antibody alone had little effect on cell survival, while the internal inhibitor induced leukemia apoptosis, and the 2 drugs produced synergistic effects, together and with chemotherapy, reducing cell survival to a larger extent than either agent alone. Our results demonstrate that internal and external VEGF/KDR autocrine loops regulate leukemia survival via different mechanisms, and suggest that blocking both may have therapeutic potential. 
 VEGF-induced neoangiogenesis is mediated by NAADP and two-pore channel-2-dependent Ca2+ signaling. Vascular endothelial growth factor (VEGF) and its receptors VEGFR1/VEGFR2 play major roles in controlling angiogenesis, including vascularization of solid tumors. Here we describe a specific Ca(2+) signaling pathway linked to the VEGFR2 receptor subtype, controlling the critical angiogenic responses of endothelial cells (ECs) to VEGF. Key steps of this pathway are the involvement of the potent Ca(2+) mobilizing messenger, nicotinic acid adenine-dinucleotide phosphate (NAADP), and the specific engagement of the two-pore channel TPC2 subtype on acidic intracellular Ca(2+) stores, resulting in Ca(2+) release and angiogenic responses. Targeting this intracellular pathway pharmacologically using the NAADP antagonist Ned-19 or genetically using Tpcn2(-/-) mice was found to inhibit angiogenic responses to VEGF in vitro and in vivo. In human umbilical vein endothelial cells (HUVECs) Ned-19 abolished VEGF-induced Ca(2+) release, impairing phosphorylation of ERK1/2, Akt, eNOS, JNK, cell proliferation, cell   migration  , and capillary-like tube formation. Interestingly, Tpcn2 shRNA treatment abolished VEGF-induced Ca(2+) release and capillary-like tube formation. Importantly, in vivo VEGF-induced vessel formation in matrigel plugs in mice was abolished by Ned-19 and, most notably, failed to occur in Tpcn2(-/-) mice, but was unaffected in Tpcn1(-/-) animals. These results demonstrate that a VEGFR2/NAADP/TPC2/Ca(2+) signaling pathway is critical for VEGF-induced angiogenesis in vitro and in vivo. Given that VEGF can elicit both pro- and antiangiogenic responses depending upon the balance of signal transduction pathways activated, targeting specific VEGFR2 downstream signaling pathways could modify this balance, potentially leading to more finely tailored therapeutic strategies. 
 Liver X receptor activation reduces angiogenesis by impairing lipid raft localization and signaling of vascular endothelial growth factor receptor-2. Liver X receptors (LXRα, LXRβ) are master regulators of cholesterol homeostasis. In the endothelium, perturbations of cell cholesterol have an impact on fundamental processes. We, therefore, assessed the effects of LXR activation on endothelial functions related to angiogenesis in vitro and in vivo.LXR agonists (T0901317, GW3965) blunte  d migrati  on, tubulogenesis, and proliferation of human umbilical vein endothelial cells. By affecting endothelial cholesterol homeostasis, LXR activation impaired the compartmentation of vascular endothelial growth factor receptor-2 in lipid rafts/caveolae and led to defective phosphorylation and downstream signaling of vascular endothelial growth factor receptor-2 upon vascular endothelial growth factor-A stimulation. Consistently, the antiangiogenic actions of LXR agonists could be prevented by coadministration of exogenous cholesterol. LXR agonists reduced endothelial sprouting from wild-type but not from LXRα(-/-)/LXRβ(-/-) knockout aortas and blunted the vascularization of implanted angioreactors in vivo. Furthermore, T0901317 reduced the growth of Lewis lung carcinoma grafts in mice by impairing angiogenesis.Pharmacological activation of endothelial LXRs reduces angiogenesis by restraining cholesterol-dependent vascular endothelial growth factor receptor-2 compartmentation and signaling. Thus, administration of LXR agonists could exert therapeutic effects in pathological conditions characterized by uncontrolled angiogenesis. 
 Genetic Variations of Kinase Inserts Domain Receptor (KDR) Gene Are Associated with the Risk of Astrocytomas. Astrocytomas is one of the most common central nervous system (CNS) tumors with high mortality rate. Kinase insert domain receptor (KDR) is involved in the regulation of tumor angiogenesis,   migration  , and vascular permeability. The aim of the study was to explore the relationship between KDR polymorphisms and risk of astrocytomas. Blood samples were collected from 157 astrocytomas patients and 160 healthy controls. Three tag-SNPs (rs2071559C/T, rs2305948T/C, and rs1870377A/T) were identified from the International HapMap Project Databases and genotyped using the method of polymerase chain reaction-restriction fragment length polymorphism (PCR-RFLP). We evaluated the astrocytomas risk caused by individual SNPs and haplotype using odds ratios (ORs) and their 95 % confidence intervals (CIs). In the overall individual SNP analysis, the C allele of rs2071559 was correlated with an increased risk of astrocytomas. However, individuals with mutant allele A and genotype TA + AA of rs1870377 showed a protective effect against astrocytomas. Subgroup analysis based on WHO tumor grade revealed that the C allele of rs2071559 had more influence with the risk of astrocytomas in the grade III-IV (OR = 1.91) subgroup than the grade I-II (OR = 1.47) group. Genotype TT of rs2305948 was found to be significantly associated with susceptibility of astrocytomas only in the grade III-IV subgroup. The protective effect of rs1870377 did not reveal significant difference between the grade III-IV and grade I-II subgroups. Meanwhile, stratified analysis demonstrated that mutation of rs2071559 and rs2305948 could elevate the risk of astrocytomas more significantly in the subgroup of smokers than the nonsmokers. Interestingly, the protective effect of rs1870377 was more obvious in the nonsmokers than the smokers. Additionally, haplotype-specific analysis showed that haplotype CCT and CTT were related with an increased risk of astrocytomas. We found that individual with variants of rs2071559*C and rs2305948*T might significantly elevate the risk of astrocytomas, while mutants of rs1870377*A was associated with the decreased risk of astrocytomas. Further studies about ethnically diverse populations with larger sample size should be performed to confirm the correlation between KDR gene polymorphisms and risk of astrocytomas. 
 The myosin motor Myo1c is required for VEGFR2 delivery to the cell surface and for angiogenic signaling. Vascular endothelial growth factor receptor-2 (VEGFR2) is a receptor tyrosine kinase that is expressed in endothelial cells and regulates angiogenic signal transduction under both physiological and pathological conditions. VEGFR2 turnover at the plasma membrane (PM) is regulated by its transport through endocytic and secretory transport pathways. Short-range cargo trafficking along actin filaments is commonly regulated by motor proteins of myosin superfamily. In the current study, performed in primary human endothelial cells, we demonstrate that unconventional myosin 1c (Myo1c; class I family member) regulates the localization of VEGFR2 at the PM. We further demonstrate that the recruitment of VEGFR2 to the PM and its colocalization with Myo1c and caveolin-1 occur in response to VEGF-A (VEGF) stimulation. In addition, VEGF-induced delivery of VEGFR2 to the cell surface requires Myo1c; surface VEGFR2 levels are reduced in the absence of Myo1c and, more importantly, are restored by the overexpression of wild-type but not mutant Myo1c. Subcellular density gradient fractionation revealed that partitioning of VEGFR2 into caveolin-1- and Myo1c-enriched membrane fractions is dependent on VEGF stimulation. Myo1c depletion resulted in increased VEGF-induced VEGFR2 transport to the lysosomes for degradation and was rescued by applying either brefeldin A, which blocks trafficking between the endoplasmic reticulum and the Golgi complex, or dynasore, an inhibitor of dynamin-mediated endocytosis. Myo1c depletion also reduced VEGF-induced VEGFR2 phosphorylation at Y1175 and phosphorylation-dependent activation of ERK1/2 and c-Src kinase, leading to reduced cell proliferation and cell   migration  . This is the first report demonstrating that Myo1c is an important mediator of VEGF-induced VEGFR2 delivery to the cell surface and plays a role in angiogenic signaling. 
 Norcantharidin inhibits tumor angiogenesis via blocking VEGFR2/MEK/ERK signaling pathways. Norcantharidin (NCTD), the demethylated form of Cantharidin, a reagent isolated from blister beetles, has been shown to be an anti-tumor agent capable of inhibiting proliferation as well as inducing apoptosis in many cancer cell lines. However, little is known about the effect of NCTD in tumor angiogenesis. In this study, we demonstrated that NCTD inhibited vascular endothelial growth factor (VEGF)-induced cell proliferation,   migration  , invasion, and capillary tube formation of primary human umbilical vein endothelial cells (HUVECs) in a dose-dependent manner. Furthermore, we showed NCTD inhibited tumor growth and angiogenesis of colon cancer cells (LOVO) in vivo. We then mechanistically described that NCTD specifically abrogated the phosphorylation/activation of vascular endothelial growth factor receptor-2 (VEGFR2)/MEK/ERK pathway kinases, with little effect on the phosphorylation of p38 MAPK and Akt, and on Cox-2 expression. In summary, our results indicate that NCTD is a potential inhibitor of tumor angiogenesis by blocking VEGFR2/MEK/ERK signaling. 
 Tumor-induced activation of lymphatic endothelial cells via vascular endothelial growth factor receptor-2 is critical for prostate cancer lymphatic metastasis. Prostate cancer disseminates initially and primarily to regional lymph nodes. However, the nature of interactions between tumor cells and lymphatic endothelial cells (LEC) is poorly understood. In the current study, we have isolated prostate LECs and developed a series of two-dimensional and three-dimensional in vitro coculture systems and in vivo orthotopic prostate cancer models to investigate the interactions of prostate cancer cells with prostate LECs. In vitro, highly lymph node metastatic prostate cancer cell lines (PC-3 and LNCaP) and their conditioned medium enhanced prostate LEC tube formation and   migration  , whereas poorly lymph node metastatic prostate cancer cells (DU145) or normal prostate epithelial cells (RWPE-1) or their conditioned medium had no effect. In vivo, the occurrence of lymphatic invasion and lymph node metastasis was observed in PC-3 and LNCaP xenografts but not in DU145 xenografts. Furthermore, vascular endothelial growth factor (VEGF) receptor (VEGFR)-2 is expressed by prostate LECs, and its ligands VEGF-A, VEGF-C, and VEGF-D are up-regulated in highly lymph node metastatic prostate cancer cells. Recombinant VEGF-A and VEGF-C, but not VEGF-C156S, potently promoted prostate LEC tube formation,   migration  , and proliferation in vitro, indicating that signaling via VEGFR-2 rather than VEGFR-3 is involved in these responses. Consistent with this, blockade of VEGFR-2 significantly reduced tumor-induced activation of LECs. These results show that the interaction of prostate tumor cells with LECs via VEGFR-2 modulates LEC behavior and is related to the ability of tumor cells to form lymph node metastases. 
 A cell penetrating peptide derived from azurin inhibits angiogenesis and tumor growth by inhibiting phosphorylation of VEGFR-2, FAK and Akt. Amino acids 50-77 (p28) of azurin, a 128 aa cupredoxin isolated from Pseudomonas aeruginosa, is essentially responsible for azurin's preferential penetration of cancer cells. We now report that p28 also preferentially penetrates human umbilical vein endothelial cells (HUVEC), co-localized with caveolin-1 and VEGFR-2, and inhibits VEGF- and bFGF-induced   migration  , capillary tube formation and neoangiogenesis in multiple xenograft models. The antiangiogenic effect of p28 in HUVEC is associated with a dose-related non-competitive inhibition of VEGFR-2 kinase activity. However, unlike other antiangiogenic agents that inhibit the VEGFR-2 kinase, p28 decreased the downstream phosphorylation of FAK and Akt that normally precedes cellular repositioning of the cytoskeletal (F-actin), focal adhesion (FAK and paxillin), and cell to cell junction protein PECAM-1, inhibiting HUVEC motility and   migration  . The decrease in pFAK and pAkt levels suggests that p28 induces a pFAK-mediated loss of HUVEC motility and   migration   and a parallel Akt-associated reduction in cell matrix attachment and survival. This novel, direct antiangiogenic effect of p28 on endothelial cells may enhance the cell cycle inhibitory and apoptotic properties of this prototype peptide on tumor cell proliferation as it enters a Phase II clinical trial. 
 Dioscin inhibits colon tumor growth and tumor angiogenesis through regulating VEGFR2 and AKT/MAPK signaling pathways. Dioscin has shown cytotoxicity against cancer cells, but its in vivo effects and the mechanisms have not elucidated yet. The purpose of the current study was to assess the antitumor effects and the molecular mechanisms of dioscin. We showed that dioscin could inhibit tumor growth in vivo and has no toxicity at the test condition. The growth suppression was accompanied by obvious blood vessel decrease within solid tumors. We also found dioscin treatment inhibited the proliferation of cancer and endothelial cell lines, and most sensitive to primary cultured human umbilical vein endothelial cells (HUVECs). What's more, analysis of HUVECs   migration  , invasion, and tube formation exhibited that dioscin has significantly inhibitive effects to these actions. Further analysis of blood vessel formation in the matrigel plugs indicated that dioscin could inhibit VEGF-induced blood vessel formation in vivo. We also identified that dioscin could suppress the downstream protein kinases of VEGFR2, including Src, FAK, AKT and Erk1/2, accompanied by the increase of phosphorylated P38MAPK. The results potently suggest that dioscin may be a potential anticancer drug, which efficiently inhibits angiogenesis induced by VEGFR2 signaling pathway as well as AKT/MAPK pathways. 
 Nck and Crk mediate distinct VEGF-induced signaling pathways that serve overlapping functions in focal adhesion turnover and integrin activation. We have asked whether the Nck and Crk adaptor proteins play important roles in the vascular endothelial growth factor (VEGF)-induced signaling pathways that lead to an enhancement in cell   migration  . The introduction into human umbilical vein endothelial cells of a dominant-negative inhibitor for either Nck or Crk blocked the recruitment of both endogenous proteins to the KDR VEGF receptor subtype indicating that both proteins are recruited to the same docking site. The Nck and Crk dominant-negatives led to the formation of abnormally large focal adhesion, blocked VEGF-induced integrin activation, and blocked VEGF-induced actin dynamics. The dominant-negatives had no effects on these properties in cells expressing constitutively active Rac1 or RhoA. Since a DN to either Nck or Crk blocks the cellular responses mediated by both proteins, we performed experiments directed at clarifying signaling pathways specifically mediated by each protein. Inhibition of the interaction between Nck with its downstream effector PAK led to abnormally large focal adhesions, but had no effect on integrin activation or cell adhesiveness. Evidence is presented that Crk complexes with C3G in control cells, and VEGF treatment leads to the recruitment of the complex to the cell surface. Inhibition of the C3G downstream effector Rap1 leads to enlarged focal adhesions and blocks VEGF-induced integrin activation. We conclude that Nck and Crk mediate distinct VEGF-induced signaling pathways that serve overlapping functions in cell   migration  . 
 Dual inhibition using cabozantinib overcomes HGF/MET signaling mediated resistance to pan-VEGFR inhibition in orthotopic and metastatic neuroblastoma tumors. MET is expressed on neuroblastoma cells and may trigger tumor growth, neoangiogenesis and metastasis. MET upregulation further represents an escape mechanism to various anticancer treatments including VEGF signaling inhibitors. We developed in vitro a resistance model to pan-VEGFR inhibition and explored the simultaneous inhibition of VEGFR and MET in neuroblastoma models in vitro and in vivo using cabozantinib, an inhibitor of the tyrosine kinases including VEGFR2, MET, AXL and RET. Resistance in IGR-N91-Luc neuroblastoma cells under continuous in vitro exposure pressure to VEGFR1-3 inhibition using axitinib was associated with HGF and p-ERK overexpression. Cabozantinib exhibited anti-proliferative effects in neuroblastoma cells and reduced ce  ll migrat  ion in vitro as measured by phase-contrast with IncuCyte system. In vivo, an enhanced number of animals with IGR-N91-Luc metastases was noted following axitinib treatment as compared to control animals. Orally administered cabozantinib per gavage at 30 and 60 mg/kg/day significantly inhibited tumor growth of orthotopic adrenal IGR-N91-Luc and metastatic IMR-32-Luc xenografts. Antitumor activity was associated with decreased vascularization, inhibition of p-SRC and induction of apoptotic cell death. Activation of the HGF-mediated MET pathway is involved in escape to selective VEGFR inhibition in neuroblastoma suggesting combined inhibition of MET and VEGFR signaling to reduce secondary resistance and enhanced invasiveness. 
 RNAi-mediated silencing of VEGF-C inhibits non-small cell lung cancer progression by simultaneously down-regulating the CXCR4, CCR7, VEGFR-2 and VEGFR-3-dependent axes-induced ERK, p38 and AKT signalling pathways. Vascular endothelial growth factor C (VEGF-C) expression is associated with the malignant tumour phenotype making it an attractive therapeutic target. We investigated the biological roles of VEGF-C in tumour growth,   migration  , invasion and explored the possibility of VEGF-C as a potential therapeutic target for the treatment of non-small cell lung cancer (NSCLC). A lentivirus-mediated RNA interference (RNAi) technology was used to specifically knockdown the expression of VEGF-C in A549 cells. Quantitative reverse transcriptase-polymerase chain reaction, flow cytometry, Western blot, immunohistochemistry, cellular growth,   migration  , invasion and ELISA assays were used to characterise VEGF-C expression in vitro. A lung cancer xenograft model in nude mice was established to investigate whether knockdown of VEGF-C reduced tumour growth in vivo. Silencing of VEGF-C suppressed tumour cell growth,   migration   and invasion in vitro; suppressed tumour growth, angiogenesis and lymphangiogenesis by tail vein injection of lentivirus encoded shRNA against VEGF-C in vivo. More importantly, silencing of VEGF-C also trapped the VEGFR-2, VEGFR-3, CXCR4, CCR7-dependent axes, and down-regulated the AKT, ERK and p38 signalling pathways. These results suggest that VEGF-C has a multifaceted role in NSCLC growth,   migration   and invasion; that VEGF-C-mediated autocrine loops with their cognate receptors and chemokine receptors are significant factors affecting tumour progression; and that RNAi-mediated silencing of VEGF-C represents a powerful therapeutic approach for controlling NSCLC growth and metastasis. 
 Vascular endothelial growth factor and its high-affinity receptor (VEGFR-2) are highly expressed in the human forebrain and cerebellum during development. Vascular endothelial growth factor (VEGF) is an angiogenic and neurotrophic factor in both adult and neonatal animals, but its expression and role have been incompletely studied in the developing human brain. We analyzed the distribution of VEGF and its high-affinity receptor VEGFR-2 in the human forebrain and cerebellum at developmental stages from 14 weeks' gestation (WG) to the13th postnatal month. Tissue samples free of detectable neuropathologic abnormalities were assessed by immunohistochemistry and confocal microscopy using anti-human VEGF and VEGFR-2 antibodies. The VEGFR-2 was first expressed in the whole cerebral mantle and in migrating cells in the intermediate zone, whereas VEGFwas found in superficial layers of the cortical plate, in radial glia, and in the cerebellar external germinal cell layer. From 23 WG, temporospatial VEGFR-2 expression was superimposable on that ofVEGF in the cortical plate, intermediate zone, basal ganglia, limbicstructures, and external germinal cell layer. The VEGF/VEGFR-2-positive astrocytes were observed during their generation and   migration   from 23 WG to the first postnatal month. The VEGF-positive mature oligodendrocytes were observed in myelinating structures in the forebrain from birth and in the cerebellum from 24WG. These data suggest that VEGF and VEGFR-2 are likely involved in several aspects of human brain development. 
 Chronic hypoxia attenuates VEGF signaling and angiogenic responses by downregulation of KDR in human endothelial cells. Coronary artery disease results in progressive vascular stenosis associated with chronic myocardial ischemia. Vascular endothelial growth factor (VEGF) stimulates endothelial cell angiogenic responses to revascularize ischemic tissues; however, the effect of chronic hypoxia on the responsiveness of endothelial cells to VEGF remains unclear. We, therefore, investigated whether hypoxia alters VEGF-stimulated signaling and angiogenic responses in primary human coronary artery endothelial (HCAE) cells. Exposure of HCAE cells to hypoxia (1% O(2)) for 24 h decreased VEGF-stimulated endothelial cell   migration   ( approximately 82%), proliferation ( approximately 30%), and tube formation. Hypoxia attenuated VEGF-stimulated activation of endothelial nitric oxide (NO) synthase (eNOS) ( approximately 72%) and reduced NO production in VEGF-stimulated cells from 237 +/- 38.8 to 61.3 +/- 28.4 nmol/l. Moreover, hypoxia also decreased the ratio of phosphorylated eNOS to total eNOS in VEGF-stimulated cells by approximately 50%. This effect was not observed in thrombin-stimulated cells, suggesting that hypoxia specifically inhibited VEGF signaling upstream of eNOS phosphorylation. VEGF-induced activation of Akt, ERK1/2, p38, p70S6 kinases, and S6 ribosomal protein was also attenuated in hypoxic cells. Moreover, VEGF-stimulated phosphorylation of VEGF receptor-2 (KDR) at Y996 and Y1175 was decreased by hypoxia. This decrease correlated with a 70 +/- 12% decrease in KDR protein expression. Analysis of mRNA from these cells showed that hypoxia reduced steady-state levels of KDR mRNA by 52 +/- 16% and decreased mRNA stability relative to normoxic cells. Our findings demonstrate that chronic hypoxia attenuates VEGF-stimulated signaling in HCAE cells by specific downregulation of KDR expression. These data provide a novel explanation for the impaired angiogenic responses to VEGF in endothelial cells exposed to chronic hypoxia. 
 Myoferlin regulates vascular endothelial growth factor receptor-2 stability and function. Myoferlin and dysferlin are members of the ferlin family of membrane proteins. Recent studies have shown that mutation or genetic disruption of myoferlin or dysferlin promotes muscular dystrophy-related phenotypes in mice, which are the result of impaired plasma membrane integrity. However, no biological functions have been ascribed to myoferlin in non-muscle tissues. Herein, using a proteomic analysis of endothelial cell (EC) caveolae/lipid raft microdomains we identified myoferlin in these domains and show that myoferlin is highly expressed in ECs and vascular tissues. The loss of myoferlin results in lack of proliferation,   migration  , and nitric oxide (NO) release in response to vascular endothelial growth factor (VEGF). Western blotting and surface biotinylation experiments show that loss of myoferlin reduces the expression level and autophosphorylation of VEGF receptor-2 (VEGFR-2) in native ECs. In a reconstituted cell system, transfection of myoferlin increases VEGFR-2 membrane expression and autophosphorylation in response to VEGF. In vivo, VEGFR-2 levels and VEGF-induced permeability are impaired in myoferlin-deficient mice. Mechanistically, myoferlin forms a complex with dynamin-2 and VEGFR-2, which prevents CBL-dependent VEGFR-2 polyubiquitination and proteasomal degradation. These data are the first to report novel biological activities for myoferlin and reveal the role of membrane integrity to VEGF signaling. 
 Endothelial cell-specific chemotaxis receptor (ECSCR) enhances vascular endothelial growth factor (VEGF) receptor-2/kinase insert domain receptor (KDR) activation and promotes proteolysis of internalized KDR. The endothelial cell-specific chemotaxis receptor (ECSCR) is a cell-surface protein selectively expressed by endothelial cells (ECs), with roles in EC   migration  , apoptosis and proliferation. Our previous study (Verma, A., Bhattacharya, R., Remadevi, I., Li, K., Pramanik, K., Samant, G. V., Horswill, M., Chun, C. Z., Zhao, B., Wang, E., Miao, R. Q., Mukhopadhyay, D., Ramchandran, R., and Wilkinson, G. A. (2010) Blood 115, 4614-4622) showed that loss of ECSCR in primary ECs reduced tyrosine phosphorylation of vascular endothelial growth factor (VEGF) receptor 2/kinase insert domain receptor (KDR) but not VEGF receptor 1/FLT1. Here, we show that ECSCR biochemically associates with KDR but not FLT1 and that the predicted ECSCR cytoplasmic and transmembrane regions can each confer association with KDR. Stimulation with VEGF165 rapidly and transiently increases ECSCR-KDR complex formation, a process blocked by the KDR tyrosine kinase inhibitor compound SU5416 or inhibitors of endosomal acidification. Triple labeling experiments show VEGF-stimulated KDR(+)/ECSCR(+) intracellular co-localization. Silencing of ECSCR disrupts VEGF-induced KDR activation and AKT and ERK phosphorylation and impairs VEGF-stimulated KDR degradation. In zebrafish, ecscr interacts with kdrl during intersomitic vessel sprouting. Human placenta and infantile hemangioma samples highly express ECSCR protein, suggesting a role for ECSCR-KDR interaction in these tissues. 
 Vascular endothelial growth factor receptor 2 as a marker for malignant vascular tumors and mesothelioma: an immunohistochemical study of 262 vascular endothelial and 1640 nonvascular tumors. Vascular endothelial growth factor receptor 2 (VEGFR2) is a primary responder to vascular endothelial growth factor signal and thereby regulates endothelial   migration   and proliferation. This receptor is expressed in endothelial cells and in some vascular tumors, but many reports also detail its expression in carcinomas and lymphomas. VEGFR2 is a potential cell-type marker, and data on VEGFR2 expression may also have therapeutic significance in view of recent availability of VEGFR2 inhibitors. In this study, we immunohistochemically examined 262 vascular endothelial and 1640 nonvascular tumors and selected non-neoplastic tissues with a VEGFR2-specific rabbit monoclonal antibody 55B11. In early human embryo, VEFGR2 was expressed in endothelia of developing capillaries and in the thoracic duct, great vessels, hepatic sinusoids, epidermis, and mesothelia. In late first trimester fetus peripheral soft tissues, VEGFR2 was restricted to capillary endothelia, chondrocytes, and superficial portion of the epidermis. In normal adult tissues, it was restricted to endothelia and mesothelia. VEGFR2 was consistently expressed in angiosarcomas, Kaposi sarcomas, and retiform hemangioendotheliomas. It was detected in only half of epithelioid hemangioendotheliomas (15/27), usually focally. VEGFR2 was strongly expressed in most capillary hemangiomas and weakly or focally in cavernous, venous, and spindle cell hemangiomas and in lymphangiomas. Malignant epithelial mesothelioma was found to be a unique epithelial neoplasm with a strong and nearly consistent VEGFR2 expression, including membrane staining (35/38). Approximately 10% of squamous cell carcinomas and 23% of pulmonary adenocarcinomas contained focal positivity. The only nonendothelial mesenchymal tumors found to be VEGFR2 positive were biphasic synovial sarcoma (focal epithelial expression) and chordoma. All melanomas and lymphomas were negative. VEGFR2 is a promising marker for malignant vascular tumors and malignant epithelioid mesothelioma. Expression in biphasic synovial sarcoma epithelium, chordoma, and some carcinomas has to be considered in differential diagnosis. Information on VEGFR2 tissue expression may be useful in development of targeted oncologic therapy through VEGFR2-specific tyrosine kinase inhibitors. 
 Curcumin inhibits angiogenesis and improves defective hematopoiesis induced by tumor-derived VEGF in tumor model through modulating VEGF-VEGFR2 signaling pathway. Curcumin, a natural polyphenol compound from the perennial herb Curcuma longa, has been proved to be beneficial for tumor-bearing animals through inhibiting tumor neovasculature formation, but the underlying mechanisms are unclear. Here, we aim to test whether curcumin affects VEGF-VEGFR2 signaling pathway and attenuates defective hematopoiesis induced by VEGF in tumor model. We demonstrated that curcumin inhibited proliferation,   migration   of HUVEC under VEGF stimulation and caused HUVEC apoptosis, and blocked VEGFR2 activation and its downstream signaling pathways in vitro. Furthermore, in VEGF over-expressing tumor model, curcumin significantly inhibited the tumor growth accelerated by VEGF in a dose-dependent manner and improved anemia and extramedullary hematopoiesis in livers and spleens of tumor-bearing mice induced by tumor-derived VEGF. Immunohistochemical analysis showed that curcumin normalized vasculature structures of livers and reduced tumor microvessel density. ELISA revealed that curcumin suppressed VEGF secretion from tumor cells both in vitro and in vivo. Survival analysis showed that curcumin significantly improved survival ability of VEGF tumor-bearing mice. Taken together, these findings establish curcumin as a modulator of VEGF and VEGF-VEGFR2 signaling pathway, with potential implication for improving the quality of life of cancer patients. 
 HCPTPA, a protein tyrosine phosphatase that regulates vascular endothelial growth factor receptor-mediated signal transduction and biological activity. Angiogenesis is a tightly controlled process in which signaling by the receptors for vascular endothelial growth factor (VEGF) plays a key role. In order to define signaling pathways downstream of VEGF receptors (VEGFR), the kinase domain of VEGFR2 (Flk-1) was used as a bait to screen a human fetal heart library in the yeast two-hybrid system. One of the signaling molecules identified in this effort was HCPTPA, a low molecular weight, cytoplasmic protein tyrosine phosphatase. Although HCPTPA possesses no identifiable phosphotyrosine binding domains (i.e. SH2 or phosphotyrosine binding domains), it bound specifically to active, autophosphorylated VEGFR2 but not to a mutated, kinase-inactive VEGFR2. Recombinant VEGFR2 and endogenous VEGFR2 were substrates for recombinant HCPTPA, and HCPTPA was co-expressed with VEGFR2 in endothelial cell lines, suggesting that HCPTPA may be a negative regulator of VEGFR2 signal transduction. To pursue this possibility, an adenovirus directing the expression of HCPTPA was constructed. When used to infect cultured endothelial cells, this adenovirus directed high level expression of HCPTPA that resulted in impairment of VEGF-mediated VEGFR2 autophosphorylation and mitogen-activated protein kinase activation. Adenovirus-mediated overexpression of HCPTPA also inhibited VEGF-induced cellular responses (endothelial cell   migration   and proliferation) and inhibited angiogenesis in the rat aortic ring assay. Taken together, these findings indicate that HCPTPA may be an important regulator of VEGF-mediated signaling and biological activity. Potential interactions with other signaling pathways and possible therapeutic implications are discussed. 
 β₂-glycoprotein I inhibits VEGF-induced endothelial cell growth a  nd migrat  ion via suppressing phosphorylation of VEGFR2, ERK1/2, and Akt. β(2)-glycoprotein I (β(2)-GPI) is a plasma glycoprotein with diverse functions, but the impact and molecular effects of β(2)-GPI on vascular biology are as yet unclear. Based on the limited information available on the contribution of β(2)-GPI to endothelial cells, we investigated the effect of β(2)-GPI on cell gro  wth and m  igration in human aortic endothelial cells (HAECs). The regulation of β(2)-GPI as part of intracellular signaling in HAECs was also examined. Vascular endothelial growth factor (VEGF) is a pro-angiogenic factor that may regulate endothelial functions. We found that β(2)-GPI dose-dependently inhibited VEGF-induced endothelial cell growth using the 3-(4,5-dimethylthiazol-2-yl)-2,5-dipenyl tetrazolium bromide assay and cell counts. Using wound healing and Boyden chamber assays, β(2)-GPI remarkably reduced VEGF-incr  eased cel  l migration at the physiological concentration. Furthermore, β(2)-GPI suppressed VEGF-induced phosphorylation of VEGF receptor 2 (VEGFR2), extracellular signal-regulated kinase 1/2 (ERK1/2), and Akt. These results suggest that β(2)-GPI plays an essential role in the down-regulation of VEGF-induced endothelial responses and may be a useful component for anti-angiogenic therapy. 
 Carbon monoxide inhibits sprouting angiogenesis and vascular endothelial growth factor receptor-2 phosphorylation. Carbon monoxide (CO) is a gaseous autacoid known to positively regulate vascular tone; however, its role in angiogenesis is unknown. The aim of this study was to investigate the effect of CO on angiogenesis and vascular endothelial growth factor (VEGF) receptor-2 phosphorylation. Human umbilical vein endothelial cells (HUVECs) were cultured on growth factor-reduced Matrigel and treated with a CO-releasing molecule (CORM-2) or exposed to CO gas (250 ppm). Here, we report the surprising finding that exposure to CO inhibits vascular endothelial growth factor (VEGF)-induced endothelial cell actin reorganisation, cell proliferation,   migration   and capillary-like tube formation. Similarly, CO suppressed VEGF-mediated phosphorylation of VEGFR-2 at tyrosine residue 1175 and 1214 and basic fibroblast growth factor- (FGF-2) and VEGF-mediated Akt phosphorylation. Consistent with these data, mice exposed to 250 ppm CO (1h/day for 14 days) exhibited a marked decrease in FGF-2-induced Matrigel plug angiogenesis (p<0.05). These data establish a new biological function for CO in angiogenesis and point to a potential therapeutic use for CO as an anti-angiogenic agent in tumour suppression. 
 Mechanisms of integrin-vascular endothelial growth factor receptor cross-activation in angiogenesis. The functional responses of endothelial cells are dependent on signaling from peptide growth factors and the cellular adhesion receptors, integrins. These include cell adhesion,   migration  , and proliferation, which, in turn, are essential for more complex processes such as formation of the endothelial tube network during angiogenesis. This study identifies the molecular requirements for the cross-activation between beta3 integrin and tyrosine kinase receptor 2 for vascular endothelial growth factor (VEGF) receptor (VEGFR-2) on endothelium. The relationship between VEGFR-2 and beta3 integrin appears to be synergistic, because VEGFR-2 activation induces beta3 integrin tyrosine phosphorylation, which, in turn, is crucial for VEGF-induced tyrosine phosphorylation of VEGFR-2. We demonstrate here that adhesion- and growth factor-induced beta3 integrin tyrosine phosphorylation are directly mediated by c-Src. VEGF-stimulated recruitment and activation of c-Src and subsequent beta3 integrin tyrosine phosphorylation are critical for interaction between VEGFR-2 and beta3 integrin. Moreover, c-Src mediates growth factor-induced beta3 integrin activation, ligand binding, beta3 integrin-dependent cell adhesion, directional   migration   of endothelial cells, and initiation of angiogenic programming in endothelial cells. Thus, the present study determines the molecular mechanisms and consequences of the synergism between 2 cell surface receptor systems, growth factor receptor and integrins, and opens new avenues for the development of pro- and antiangiogenic strategies. 
 SCF(β-TRCP) suppresses angiogenesis and thyroid cancer cell   migratio  n by promoting ubiquitination and destruction of VEGF receptor 2. The incidence of human papillary thyroid cancer (PTC) is increasing and an aggressive subtype of this disease is resistant to treatment with vascular endothelial growth factor receptor 2 (VEGFR2) inhibitor. VEGFR2 promotes angiogenesis by triggering endothelial cell proliferation and   migratio  n. However, the molecular mechanisms governing VEGFR2 stability in vivo remain unknown. Additionally, whether VEGFR2 influences PTC cell   migratio  n is not clear. We show that the ubiquitin E3 ligase SCF(β-TRCP) promotes ubiquitination and destruction of VEGFR2 in a casein kinase I (CKI)-dependent manner. β-TRCP knockdown or CKI inhibition causes accumulation of VEGFR2, resulting in increased activity of signaling pathways downstream of VEGFR2. β-TRCP-depleted endothelial cells exhibit enhan  ced migra  tion and angiogenesis in vitro. Furthermore, β-TRCP knockdown increased angiogenesis and vessel branching in zebrafish. Importantly, we found an inverse correlation between β-TRCP protein levels and angiogenesis in PTC. We also show that β-TRCP inhibit  s cell mi  gration and decreases sensitivity to the VEGFR2 inhibitor sorafenib in poorly differentiated PTC cells. These results provide a new biomarker that may aid a rational use of tyrosine kinase inhibitors to treat refractory PTC. 
 A heat-shock protein axis regulates VEGFR2 proteolysis, blood vessel development and repair. Vascular endothelial growth factor A (VEGF-A) binds to the VEGFR2 receptor tyrosine kinase, regulating endothelial function, vascular physiology and angiogenesis. However, the mechanism underlying VEGFR2 turnover and degradation in this response is unclear. Here, we tested a role for heat-shock proteins in regulating the presentation of VEGFR2 to a degradative pathway. Pharmacological inhibition of HSP90 stimulated VEGFR2 degradation in primary endothelial cells and blocked VEGF-A-stimulated intracellular signaling via VEGFR2. HSP90 inhibition stimulated the formation of a VEGFR2-HSP70 complex. Clathrin-mediated VEGFR2 endocytosis is required for this HSP-linked degradative pathway for targeting VEGFR2 to the endosome-lysosome system. HSP90 perturbation selectively inhibited VEGF-A-stimulated human endothelial cell   migration   in vitro. A mouse femoral artery model showed that HSP90 inhibition also blocked blood vessel repair in vivo consistent with decreased endothelial regeneration. Depletion of either HSP70 or HSP90 caused defects in blood vessel formation in a transgenic zebrafish model. We conclude that perturbation of the HSP70-HSP90 heat-shock protein axis stimulates degradation of endothelial VEGFR2 and modulates VEGF-A-stimulated intracellular signaling, endothelial cell   migration  , blood vessel development and repair. 
 Regulation of vascular endothelial growth factor receptor 2 trafficking and angiogenesis by Golgi localized t-SNARE syntaxin 6. Vascular endothelial growth factor receptor 2 (VEGFR2) plays a key role in physiologic and pathologic angiogenesis. Plasma membrane (PM) levels of VEGFR2 are regulated by endocytosis and secretory transport through the Golgi apparatus. To date, the mechanism whereby the VEGFR2 traffics through the Golgi apparatus remains incompletely characterized. We show in human endothelial cells that binding of VEGF to the cell surface localized VEGFR2 stimulates exit of intracellular VEGFR2 from the Golgi apparatus. Brefeldin A treatment reduced the level of surface VEGFR2, confirming that VEGFR2 traffics through the Golgi apparatus en route to the PM. Mechanistically, we show that inhibition of syntaxin 6, a Golgi-localized target membrane-soluble N-ethylmaleimide attachment protein receptor (t-SNARE) protein, interferes with VEGFR2 trafficking to the PM and facilitates lysosomal degradation of the VEGFR2. In cell culture, inhibition of syntaxin 6 also reduced VEGF-induced cell proliferation, cell   migration  , and vascular tube formation. Furthermore, in a mouse ear model of angiogenesis, an inhibitory form of syntaxin 6 reduced VEGF-induced neovascularization and permeability. Our data demonstrate the importance of syntaxin 6 in the maintenance of cellular VEGFR2 levels, and suggest that the inhibitory form of syntaxin 6 has good potential as an antiangiogenic agent. 
 PTP1b is a physiologic regulator of vascular endothelial growth factor signaling in endothelial cells. Regulation of vascular endothelial growth factor receptor-2 (VEGFR2) signaling is a control point that determines the extent of vascular tree formation. Recent studies demonstrated an important role played by VEGFR2 endothelial trafficking in control of its activity and suggested the involvement of a phosphotyrosine phosphatase 1b (PTP1b) in this process. This study was designed to define the role of PTP1b in endothelial VEGFR2 signaling and its role in regulation of angiogenesis and arteriogenesis.We generated mice carrying an endothelial-specific deletion of PTP1b and examined the effect of this knockout on VEGF signaling, angiogenesis, and arteriogenesis in vitro and in vivo. PTP1b knockout endothelial cells had increased VEGF-dependent activation of extracellular signal-regulated kinase signaling, sprouting,   migration  , and proliferation compared with controls. Endothelial PTP1b null mice had increased retinal and Matrigel implant angiogenesis and accelerated wound healing, pointing to enhanced angiogenesis. Increased arteriogenesis was demonstrated by observations of faster recovery of arterial blood flow and large numbers of newly formed arterioles in the hindlimb ischemia mouse model. PTP1b endothelial knockout also rescued impaired blood flow recovery after common femoral artery ligation in synectin null mice.PTP1b is a key regulator of endothelial VEGFR2 signaling and plays an important role in regulation of the extent of vascular tree formation. 
 The role of VEGF and a functional link between VEGF and p27Kip1 in acute myeloid leukemia. Alterations in the expression and signalling pathways of vascular endothelial growth factor (VEGF) have been linked to the clinical features and pathogenesis of hematologic malignancies. In this study, we showed that VEGF protein expression was statistically significantly higher in the leukemic blasts than in the normal hematopoietic counterparts. A statistically significant correlation between expression of VEGF and p27(Kip1) was observed in bone marrows from 42 patients with acute myeloid leukemia (P<0.001). We further demonstrated that forced VEGF overexpression or autocrine VEGF stimulation of VEGFR-2 triggers proliferation and   migration  /invasion of U-937 leukemic cells, thereby inducing a more invasive tumor phenotype. U-937 cells overexpressing VEGF were resistant to all-trans-retinoic acid-(ATRA) or camptothecin-induced apoptosis. Finally, we showed that increased p27(Kip1) expression enhanced the ability of VEGF and VEGFR-2 to promote the   migration   of U-937 cells. Taken together, our results suggest that elevated level of VEGF may contribute to the adverse patient outcome by promoting cell growth, survival and   migration   of leukemic cells and by reducing the sensitivity of leukemic cells to therapeutic agents-induced apoptosis. 
 VEGFR2-PLCgamma1 axis is essential for endothelial specification of VEGFR2+ vascular progenitor cells. Vascular endothelial growth factor receptor 2 (VEGFR2) plays crucial roles in vasculogenesis, a process involving cell proliferation,   migration   and differentiation. However, the molecular mechanism by which VEGFR2 signaling directs vascular endothelial differentiation of VEGFR2(+) mesodermal progenitors is not well understood. In this study, we examined the signal transduction pathway downstream of VEGFR2 for endothelial differentiation using an in vitro differentiation system of mouse embryonic stem-cell-derived VEGFR2(+) cells. Using chimeric receptors composed of VEGFR2 and VEGFR3, the third member of the VEGFR family, we found that signaling through tyrosine 1175 (Y1175, corresponding to mouse Y1173) of VEGFR2 is crucial for two processes of endothelial differentiation: endothelial specification of VEGFR2(+) progenitors, and subsequent survival of endothelial cells (ECs). Furthermore, we found that phospholipase Cgamma1 (PLCgamma1), which interacts with VEGFR2 through phosphorylated Y1175, is an inducer of endothelial specification. In contrast to VEGFR2, VEGFR3 does not transmit a signal for endothelial differentiation of VEGFR2(+) cells. We found that VEGFR3 does not activate PLCgamma1, although VEGFR3 has the ability to support endothelial cell survival. Taken together, these findings indicate that VEGFR2-PLCgamma1 signal relay gives rise to the unique function of VEGFR2, thus enabling endothelial differentiation from vascular progenitors. 
 CD146 is a coreceptor for VEGFR-2 in tumor angiogenesis. CD146 is a novel endothelial biomarker and plays an essential role in angiogenesis; however, its role in the molecular mechanism underlying angiogenesis remains poorly understood. In the present study, we show that CD146 interacts directly with VEGFR-2 on endothelial cells and at the molecular level and identify the structural basis of CD146 binding to VEGFR-2. In addition, we show that CD146 is required in VEGF-induced VEGFR-2 phosphorylation, AKT/p38 MAPKs/NF-κB activation, and thus promotion of endothelial cell   migratio  n and microvascular formation. Furthermore, we show that anti-CD146 AA98 or CD146 siRNA abrogates all VEGFR-2 activation induced by VEGF. An in vivo angiogenesis assay showed that VEGF-promoted microvascular formation was impaired in the endothelial conditional knockout of CD146 (CD146(EC-KO)). Our animal experiments demonstrated that anti-CD146 (AA98) and anti-VEGF (bevacizumab) have an additive inhibitory effect on xenografted human pancreatic and melanoma tumors. The results of the present study suggest that CD146 is a new coreceptor for VEGFR-2 and is therefore a promising target for blocking tumor-related angiogenesis. 
 Dentin matrix protein 1 induces membrane expression of VE-cadherin on endothelial cells and inhibits VEGF-induced angiogenesis by blocking VEGFR-2 phosphorylation. Dentin matrix protein 1 (DMP1) is a member of the small integrin-binding ligand N-linked glycoprotein (SIBLING) family, a group of proteins initially described as mineralized extracellular matrices components. More recently, SIBLINGs have been implicated in several key steps of cancer progression, including angiogenesis. Although proangiogenic activities have been demonstrated for 2 SIBLINGs, the role of DMP1 in angiogenesis has not yet been addressed. We demonstrate that this extracellular matrix protein induced the expression of vascular endothelial cadherin (VE-cadherin), a key regulator of intercellular junctions and contact inhibition of growth of endothelial cells that is also known to modulate vascular endothelial growth factor receptor 2 (VEGFR-2) activity, the major high-affinity receptor for VEGF. DMP1 induced VE-cadherin and p27(Kip1) expression followed by cell-cycle arrest in human umbilical vein endothelial cells (HUVECs) in a CD44-dependent manner. VEGF-induced proliferation,   migration  , and tubulogenesis responses were specifically blocked on DMP1 pretreatment of HUVECs. Indeed, after VE-cadherin induction, DMP1 inhibited VEGFR-2 phosphorylation and Src-mediated signaling. However, DMP1 did not interfere with basic fibroblast growth factor-induced angiogenesis. In vivo, DMP1 significantly reduced laser-induced choroidal neovascularization lesions and tumor-associated angiogenesis. These data enable us to put DMP1 on the angiogenic chessboard for the first time and to identify this protein as a new specific inhibitor of VEGF-induced angiogenesis. 
 VEGFR2 inhibition by RNA interference affects cell proliferation,   migration  , invasion, and response to radiation in Calu-1 cells. To investigate the role of the vascular endothelial growth factor receptor 2 (VEGFR2) in the proliferation,   migration  , invasion, and radiation-induced apoptosis of the non-small cell lung cancer (NSCLC) cell line Calu-1.VEGFR2 gene was silenced by RNA interference in Calu-1 cells, and the expression of VEGFR2 was measured by qRT-PCR and Western blot analysis. The cells were divided into control, VEGF-treated, VEGFR2 knockdown, and VEGFR2 knockdown and VEGF-treated groups. A CCK8 assay and Transwell assay were performed to assess cell proliferation,   migration  , and invasion, respectively, after VEGFR2 knockdown. Western blot assays were used to detect signaling proteins downstream of VEGFR2. Cells in the groups listed above were also subjected to radiation treatment, followed by apoptosis analysis.(1) RNA interference of VEGFR2 in Calu-1 cells reduced VEGFR2 mRNA (P < 0.01) and protein levels (P < 0.01). (2) VEGFR2 knockdown inhibited proliferation (P < 0.05),   migration   (P < 0.05), and invasion (P < 0.05) in Calu-1 cells. (3) VEGFR2 knockdown blocked the phosphorylation of protein kinase B (Akt, also known as PKB), extracellular regulated kinase (ERK) 1/2, and p38 mitogen-activated protein kinase (p38 MAPK) to various extent (P < 0.05), but did not change their total protein expression. (4) Knockdown of VEGFR2 suppressed HIF-1α protein synthesis (P < 0.05), and exacerbated apoptosis induced by radiation (P < 0.05).VEGFR2 gene knockdown significantly suppressed a number of cellular activities in Calu-1 cells and increased radiation-induced cell death. 
 VEGF binding to NRP1 is essential for VEGF stimulation of endothelial cell   migration  , complex formation between NRP1 and VEGFR2, and signaling via FAK Tyr407 phosphorylation. In endothelial cells, neuropilin-1 (NRP1) binds vascular endothelial growth factor (VEGF)-A and is thought to act as a coreceptor for kinase insert domain-containing receptor (KDR) by associating with KDR and enhancing VEGF signaling. Here we report mutations in the NRP1 b1 domain (Y297A and D320A), which result in complete loss of VEGF binding. Overexpression of Y297A and D320A NRP1 in human umbilical vein endothelial cells reduced high-affinity VEGF binding and   migration   toward a VEGF gradient, and markedly inhibited VEGF-induced angiogenesis in a coculture cell model. The Y297A NRP1 mutant also disrupted complexation between NRP1 and KDR and decreased VEGF-dependent phosphorylation of focal adhesion kinase at Tyr407, but had little effect on other signaling pathways. Y297A NRP1, however, heterodimerized with wild-type NRP1 and NRP2 indicating that nonbinding NRP1 mutants can act in a dominant-negative manner through formation of NRP1 dimers with reduced binding affinity for VEGF. These findings indicate that VEGF binding to NRP1 has specific effects on endothelial cell signaling and is important for endothelial cell   migration   and angiogenesis mediated via complex formation between NRP1 and KDR and increased signaling to focal adhesions. Identification of key residues essential for VEGF binding and biological functions provides the basis for a rational design of antagonists of VEGF binding to NRP1. 
 Vascular endothelial growth factor overexpression by soft tissue sarcoma cells: implications for tumor growth, metastasis, and chemoresistance. To better elucidate the role of vascular endothelial growth factor (VEGF)(165) in soft tissue sarcoma (STS) growth, metastasis, and chemoresistance, we generated stably transfected human STS cell lines with VEGF(165) to study the effect of VEGF(165) on STS cells in vitro and the effect of culture medium from these cells on human umbilical vascular endothelial cells. Severe combined immunodeficient mice bearing xenografts of transfected cell lines were used to assess the effect of VEGF overexpression and the effect of VEGF receptor (VEGFR) 2 inhibition on STS growth, metastasis, and response to doxorubicin. VEGF(165)-transfected xenografts formed highly vascular tumors with shorter latency, accelerated growth, enhanced chemoresistance, and increased incidence of pulmonary metastases. Blockade of VEGFR2 signaling using DC101 anti-VEGFR2 monoclonal antibody enhanced doxorubicin chemoresponse; this combined biochemotherapy inhibited tumor growth and decreased pulmonary metastases without overt toxicity. Combined therapy reduced microvessel counts while increasing vessel maturation index. VEGF overexpression did not affect on the sarcoma cells per se; however, conditioned medium from VEGF transfectants caused increased endothelial cell proliferation,   migration  , and chemoresistance. Addition of DC101 induced endothelial cell sensitivity to doxorubicin and suppressed the activity of matrix metalloproteinases secreted by endothelial cells. We therefore conclude that VEGF is a critical determinant of STS growth and metastasis and that STS chemoresistance, in our model, is a process induced by the interplay between STS cells and tumor-associated endothelial cells. STS growth and metastasis can be interrupted by combined low-dose doxorubicin and anti-VEGFR2, a strategy that attacks STS-associated endothelial cells. In the future, such therapeutic approaches may be useful in treating STS before the development of clinically apparent metastases. 
 Signaling transduction mechanisms mediating biological actions of the vascular endothelial growth factor family. The central role of vascular endothelial growth factor (VEGF) in angiogenesis in health and disease makes it attractive both as a therapeutic target for anti-angiogenic drugs and as a pro-angiogenic cytokine for the treatment of ischaemic heart disease. While VEGF binds to two receptor protein tyrosine kinases, VEGFR1 (Flt-1) and VEGFR2 (KDR), most biological functions of VEGF are mediated via VEGFR2, and the role of VEGFR1 is currently unknown. Neuropilin-1, a non-tyrosine kinase transmembrane molecule, may function as a co-receptor for VEGFR2. Considerable progress has recently been made towards delineating the signal transduction pathways distal to activation of VEGFR2. Activation of the mitogen-activated protein kinase, protein kinase C and Akt pathways are all strongly implicated in mediating diverse cellular biological functions of VEGF, including cell survival, proliferation, the generation of nitric oxide and prostacyclin and angiogenesis. Upregulation of metalloproteinases, activation of focal adhesion kinase and interactions between VEGF receptors and integrins are strongly implicated in VEGF-induced endothelial cell   migration  . Recent findings suggest important roles for the vasodilators nitric oxide and prostacyclin, in linking post-receptor signaling networks to downstream biological effects and in mediating some in vivo endothelial functions of VEGF. 
 The interaction of uPAR with VEGFR2 promotes VEGF-induced angiogenesis. In endothelial cells, binding of vascular endothelial growth factor (VEGF) to the receptor VEGFR2 activates multiple signaling pathways that trigger processes such as proliferation, survival, and   migration   that are necessary for angiogenesis. VEGF-bound VEGFR2 becomes internalized, which is a key step in the proangiogenic signal. We showed that the urokinase plasminogen activator receptor (uPAR) interacted with VEGFR2 and described the mechanism by which this interaction mediated VEGF signaling and promoted angiogenesis. Knockdown of uPAR in human umbilical vein endothelial cells (HUVECs) impaired VEGFR2 signaling, and uPAR deficiency in mice prevented VEGF-induced angiogenesis. Upon exposure of HUVECs to VEGF, uPAR recruited the low-density lipoprotein receptor-related protein 1 (LRP-1) to VEGFR2, which induced VEGFR2 internalization. Thus, the uPAR-VEGFR2 interaction is crucial for VEGF signaling in endothelial cells. 
 CCL23 up-regulates expression of KDR/Flk-1 and potentiates VEGF-induced proliferation and   migration   of human endothelial cells. CCL23 is a CC chemokine and exerts its biological activities on endothelial cells as well as on immune cells through CCR1. We investigated the potential effect of CCL23 on expression of KDR/Flk-1 receptor in endothelial cells. PCR, confocal microscope and Western blot analysis revealed that CCL23 up-regulated KDR/Flk-1 mRNA and protein levels in endothelial cells. A reporter assay indicated that CCL23-induced KDR/Flk-1 expression primarily occurred at the transcriptional level. In addition, CCL23 stimulated phosphorylation of SAPK/JNK, and an inhibitor of SAPK/JNK blocks the CCL23-induced KDR/Flk-1 expression. Furthermore, VEGF-induced ERK phosphorylation was stimulated by CCL23. Finally, CCL23 promoted VEGF-induced endothelial proliferation and   migration  , which were correlated with the maximal stimulation of KDR/Flk-1 expression by CCL23. Taken together, these findings suggest that CCL23 results in up-regulation of KDR/flk-1 receptor gene transcription and protein expression and that KDR/Flk-1 up-regulation induced by CCL23 may contribute to potentiation of VEGF action in angiogenesis. 
 EMMPRIN promotes melanoma cells malignant properties through a HIF-2alpha mediated up-regulation of VEGF-receptor-2. EMMPRIN's expression in melanoma tissue was reported to be predictive of poor prognosis. Here we demonstrate that EMMPRIN up-regulated VEGF receptor-2 (VEGFR-2) in two different primary melanoma cell lines and consequently increased   migration   and proliferation of these cells while inhibiting their apoptosis. SiRNA inhibition of VEGFR-2 expression abrogated these EMMPRIN effects. EMMPRIN regulation of VEGFR-2 was mediated through the over-expression of HIF-2alpha and its translocation to the nucleus where it forms heterodimers with HIF-1beta. These results were supported by an in vivo correlation between the expression of EMMPRIN with that of VEGFR-2 in human melanoma tissues as well as with the extent of HIF-2alpha localization in the nucleus. They demonstrate a novel mechanism by which EMMPRIN promotes tumor progression through HIF-2alpha/VEGFR-2 mediated mechanism, with an autocrine role in melanoma cell malignancy. The inhibition of EMMPRIN in cancer may thus simultaneously target both the VEGFR-2/VEGF system and the matrix degrading proteases to block tumor cell growth and invasion. 
 Complex formation between tissue transglutaminase II (tTG) and vascular endothelial growth factor receptor 2 (VEGFR-2): proposed mechanism for modulation of endothelial cell response to VEGF. We have recently demonstrated that thrombin-activated FXIII (FXIIIA-subunit), a plasma transglutaminase, activates VEGFR-2 by crosslinking it with the alpha(v)beta(3) integrin on the surface of endothelial cells (EC), thereby stimulating angiogenesis. Tissue transglutaminase (tTG), which is functionally and structurally related to FXIIIA, is expressed by numerous cell types, among them EC. However, its role in EC function has not been fully characterized. In the present study, we investigated the potential involvement of tTG in angiogenesis. Using co-immunoprecipitation and immunofluorescent staining experiments, we observed that tTG forms a complex with VEGFR-2 on the cell surface and within the cytoplasm of EC. Stimulation of EC with VEGF resulted in translocation of the tTG-VEGFR-2 complex from the cytoplasm to the nucleus. In VEGF-treated cells, tTG-VEGFR-2 interaction resulted in incorporation of VEGFR-2 into high molecular weight crosslinked complex (es), as revealed by an antibody against gamma-glutamyl-epsilon-lysine isopeptide bond. tTG -VEGFR-2 association was inhibited by a specific VEGFR-2 protein tyrosine kinase inhibitor (PTKI ), as well as by cystamine, inhibitor of the transglutaminase activity of tTG, but not by bacitracin which inhibits the protein-disulfide isomerase (PDI) activity of tTG. Furthermore, cystamine completely abolished the VEGF-induced nuclear translocation of the tTG-VEGFR-2 complex. Blockade of the crosslinking activity of tTG by cystamine enhanced VEGF-induced   migration   of EC in Boyden chamber by 31% (P < 0.02), and prolonged VEGF-induced signaling response, as demonstrated by sustained activation of the MAP kinase ERK. Taken together, our findings suggest that endothelial cell tTG might be involved in modulation of the cellular response to VEGF by forming an intracellular complex with VEGFR-2, and mediating its translocation into the nucleus upon VEGF stimulation. 
 The angiogenesis induced by HIV-1 tat protein is mediated by the Flk-1/KDR receptor on vascular endothelial cells. The HIV-1 Tat protein transactivates HIV, viral and some host cell genes. Tat can be released by infected cells and acts extracellularly in the microenvironment, regulating functions of immunocompetent and mesenchymal cells. One of the most striking effects of Tat is the induction of a functional program in vascular cells related to angiogenesis and inflammation (  migration  , proliferation and expression of plasminogen activator inhibitor-1 and E selectin). Tat induces growth of Kaposi's sarcoma (KS) spindle cells and is angiogenic in vivo and in transgenic mice10-12. We previously reported that Tat is a direct angiogenic factor and noted the Tat arginine- and lysine-rich sequence is similar to that of other potent angiogenic growth factors, such as vascular endothelial growth factor-A (VEGF-A). It is possible that Tat mimics one of these factors by interacting with its growth factor tyrosine kinase receptor. Here we demonstrate that Tat specifically binds and activates the Flk-1/kinase insert domain receptor (Flk-1/KDR), a VEGF-A tyrosine kinase receptor (for review see ref. 13), and that Tat-induced angiogenesis is blocked by agents blocking the Flk-1/KDR receptor. Endothelial cell stimulation by Tat occurs in the absence of activation of FLT-1, another VEGF-A tyrosine kinase receptor. 
 Corosolic Acid Inhibits Hepatocellular Carcinoma Cell   Migration   by Targeting the VEGFR2/Src/FAK Pathway. Inhibition of VEGFR2 activity has been proposed as an important strategy for the clinical treatment of hepatocellular carcinoma (HCC). In this study, we identified corosolic acid (CA), which exists in the root of Actinidia chinensis, as having a significant anti-cancer effect on HCC cells. We found that CA inhibits VEGFR2 kinase activity by directly interacting with the ATP binding pocket. CA down-regulates the VEGFR2/Src/FAK/cdc42 axis, subsequently decreasing F-actin formation and migratory activity in vitro. In an in vivo model, CA exhibited an effective dose (5 mg/kg/day) on tumor growth. We further demonstrate that CA has a synergistic effect with sorafenib within a wide range of concentrations. In conclusion, this research elucidates the effects and molecular mechanism for CA on HCC cells and suggests that CA could be a therapeutic or adjuvant strategy for patients with aggressive HCC. 
 VEGF receptor-2 Y951 signaling and a role for the adapter molecule TSAd in tumor angiogenesis. Vascular endothelial growth factor receptor-2 (VEGFR-2) activation by VEGF-A is essential in vasculogenesis and angiogenesis. We have generated a pan-phosphorylation site map of VEGFR-2 and identified one major tyrosine phosphorylation site in the kinase insert (Y951), in addition to two major sites in the C-terminal tail (Y1175 and Y1214). In developing vessels, phosphorylation of Y1175 and Y1214 was detected in all VEGFR-2-expressing endothelial cells, whereas phosphorylation of Y951 was identified in a subset of vessels. Phosphorylated Y951 bound the T-cell-specific adapter (TSAd), which was expressed in tumor vessels. Mutation of Y951 to F and introduction of phosphorylated Y951 peptide or TSAd siRNA into endothelial cells blocked VEGF-A-induced actin stress fibers and   migration  , but not mitogenesis. Tumor vascularization and growth was reduced in TSAd-deficient mice, indicating a critical role of Y951-TSAd signaling in pathological angiogenesis. 
 Increased VEGFR-2 gene copy is associated with chemoresistance and shorter survival in patients with non-small-cell lung carcinoma who receive adjuvant chemotherapy. VEGF receptor-2 (VEGFR-2 or kinase insert domain receptor; KDR) is a known endothelial target also expressed in NSCLC tumor cells. We investigated the association between alterations in the KDR gene and clinical outcome in patients with resected non-small-cell lung carcinoma (NSCLC; n = 248). KDR copy number gains (CNG), measured by quantitative PCR and fluorescence in situ hybridization, were detected in 32% of tumors and associated with significantly higher KDR protein and higher microvessel density than tumors without CNGs. KDR CNGs were also associated with significantly increased risk of death (HR = 5.16; P = 0.003) in patients receiving adjuvant platinum-based chemotherapy, but no differences were observed in patients not receiving adjuvant therapy. To investigate potential mechanisms for these associations, we assessed NSCLC cell lines and found that KDR CNGs were significantly associated with in vitro resistance to platinum chemotherapy as well as increased levels of nuclear hypoxia inducible factor-1α (HIF-1α) in both NSCLC tumor specimens and cell lines. Furthermore, KDR knockdown experiments using small interfering RNA reduced platinum resistance, cel  l migrati  on, and HIF-1α levels in cells bearing KDR CNGs, providing evidence for direct involvement of KDR. No KDR mutations were detected in exons 7, 11, and 21 by PCR-based sequencing; however, two variant single nucleotide polymorphism genotypes were associated with favorable overall survival in adenocarcinoma patients. Our findings suggest that tumor cell KDR CNGs may promote a more malignant phenotype including increased chemoresistance, angiogenesis, and HIF-1α levels, and that KDR CNGs may be a useful biomarker for identifying patients at high risk for recurrence after adjuvant therapy, a group that may benefit from VEGFR-2 blockade. 
 Lycopene inhibits angiogenesis both in vitro and in vivo by inhibiting MMP-2/uPA system through VEGFR2-mediated PI3K-Akt and ERK/p38 signaling pathways. Limited in vitro data show that lycopene may be anti-angiogenic but with unclear mechanisms. Here, we employed ex vivo and in vivo assays to substantiate the anti-angiogenic action of lycopene and determined its molecular mechanisms in human umbilical vein endothelial cells (HUVECs).The anti-angiogenic activity of lycopene was confirmed by ex vivo rat aortic ring and in vivo chorioallantoic membrane assays. Furthermore, the in vivo matrigel plug assay in mice demonstrated that lycopene implanted s.c. at the highest dose used (400 μg/plug) completely inhibited the formation of vascular endothelial cells induced by vascular endothelial growth factor (VEGF). As expected, lycopene inhibited tube formation, invasion, and   migratio  n in HUVECs, and such actions were accompanied by reduced activities of matrix metalloproteinase-2, urokinase-type plasminogen activator, and protein expression of Rac1, and by enhancing protein expression of tissue inhibitors of metalloproteinase-2 and plasminogen activator inhibitor-1. Moreover, lycopene attenuated VEGF receptor-2 (VEGFR2)-mediated phosphorylation of extracellular signal-regulated kinase (ERK), p38, and Akt as well as protein expression of PI3K.Our data demonstrate the anti-angiogenic effect of lycopene both in vitro and in vivo. The anti-angiogenic activity of lycopene may involve inhibition of MMP-2/uPA system through VEGFR2-mediated PI3K-Akt and ERK/p38 signaling pathways. 
 VEGF165 modulates proliferation, adhesion,   migration   and differentiation of cultured human outer root sheath cells from central hair follicle epithelium through VEGFR-2 activation in vitro. The functional state of vasculature is tightly controlled by vascular endothelial growth factor receptor-2 (VEGFR-2). Recent studies revealed that VEGFR-2 is expressed on hair follicle keratinocytes.We proposed to investigate its effect on proliferation, adhesion and   migration   of cultured human outer root sheath cells from central hair follicle epithelium.These studies were undertaken in vitro using human outer root sheath cells from central hair follicle epithelium, immunohistochemistry analysis, immunofluorescence microscopy, western blot analysis, MTT, trans well analysis, and RT-PCR.Our results show that VEGFR-2 is expressed in these cells in vivo and in vitro. Furthermore, proliferation and   migration   of cultured human outer root sheath cells from central hair follicle epithelium is increased by VEGF165, while homotypic adhesion is decreased but heterotypic adhesion is increased. VEGF165 upregulates integrin β1 but dowregulates lgr6 expression. In addition, phosphorylation of VEGFR-2, Erk1/2, c-Jun and p38, are increased following VEGF165 treatment and these effects are reversed by a VEGFR-2 neutralizing antibody.Our results suggest a role of VEGF/VEGFR-2 beyond angiogenesis in hair follicle regulation. 
 HIV-Tat dependent chemotaxis and invasion, key aspects of tat mediated pathogenesis. Extracellular Tat acts as a pleiotropic molecule inducing several biological effects on different target cells. Tat stimulates the chemotaxis of numerous cell types and it appears to have oncogenic activities, including acting as a co-factor for Kaposi's sarcoma. The Tat protein has been shown to bind integrins through an RGD amino acid motif. Tat is an angiogenic factor able to induce the   migration   and invasion of endothelial and KS cells through the interaction of its basic domain with the VEGF receptor VEGFR2 (Flk-1/KDR). We have also found that Tat is able to mimic chemokines, activating monocyte   migration   through the chemokine like' cysteine-core domain. Tat is a chemoattractant for dendritic cells, and both the RGD and basic domains appear to be involved in this response. In a recent study we demonstrated that Tat is chemotactic for PMN and induces Ca2+ mobilization in vitro. Experiments using synthetic peptides showed that Tat activities on PMN are mediated by the chemokine like' region. Finally Tat is also able to induce B cell chemotaxis, while its activity on helper T cells has not yet been clarified. Here we review data on Tat-dependent chemotaxis and discuss the possible implications in Tat mediated pathogenesis. 
 Heterotrimeric G alpha q/G alpha 11 proteins function upstream of vascular endothelial growth factor (VEGF) receptor-2 (KDR) phosphorylation in vascular permeability factor/VEGF signaling. Vascular permeability factor/vascular endothelial growth factor (VPF/VEGF) functions by activating two receptor-tyrosine kinases, Flt-1 (VEGF receptor (VEGFR)-1) and KDR (VEGFR-2), both of which are selectively expressed on primary vascular endothelium. KDR is responsible for VPF/VEGF-stimulated endothelial cell proliferation and   migration  , whereas Flt-1 down-modulates KDR-mediated endothelial cell proliferation. Our most recent works show that pertussis toxin-sensitive G proteins and Gbetagamma subunits are required for Flt-1-mediated down-regulation of human umbilical vein endothelial cell (HUVEC) proliferation and that Gq/11 proteins are required for KDR-mediated RhoA activation and HUVEC   migration  . In this study, we demonstrate that Gq/11 proteins are also required for VPF/VEGF-stimulated HUVEC proliferation. Our results further indicate that Gq/11 proteins specifically mediate KDR signaling such as intracellular Ca2+ mobilization rather than Flt-1-induced CDC42 activation and that a Gq/11 antisense oligonucleotide completely inhibits MAPK phosphorylation induced by KDR but has no effect on Flt-1-induced MAPK activation. More importantly, we demonstrate that Gq/11 proteins interact with KDR in vivo, and the interaction of Gq/11 proteins with KDR does not require KDR tyrosine phosphorylation. Surprisingly, the Gq/11 antisense oligonucleotide completely inhibits VPF/VEGF-stimulated KDR phosphorylation. Expression of a constitutively active mutant of G11 but not Gq can cause phosphorylation of KDR and MAPK. In addition, a Gbetagamma minigene, hbetaARK1(495), inhibits VPF/VEGF-stimulated HUVEC proliferation, MAPK phosphorylation, and intracellular Ca2+ mobilization but has no effect on KDR phosphorylation. Taken together, this study demonstrates that Gq/11 proteins mediate KDR tyrosine phosphorylation and KDR-mediated HUVEC proliferation through interaction with KDR. 
 Fully human VEGFR2 monoclonal antibody BC001 attenuates tumor angiogenesis and inhibits tumor growth. The critical role of VEGFR2 in tumor neovascularization and progression has allowed the design of clinically beneficial therapies based on it. Here we show that BC001, a new fully human anti-VEGFR2 monoclonal antibody, inhibits VEGF-stimulated endothelial cell   migration  , tube formation, and effectively suppressed the transdifferentiation of cancer stem cells into endothelial cells in vitro. Since BC001 exhibited no activity against the mouse VEGFR2 and mouse based study was required to confirm its efficacy in vivo, BC101, the mouse analogue of BC001, was developed. BC101 significantly attenuated angiogenesis according to Matrigel plug assay and resulted in ~80% growth inhibition of mouse B16F10 homograft tumors relative to vehicle control. Similarly, human analogue BC001 suppressed the growth of human xenograft tumors HCT116 and BGC823. Furthermore, immunohistochemical results showed reduced expression of CD31, VEGFR2 and Ki-67, as well as increased expression of Caspase 3 in BC001-treated tumor, which indicated BC001 was able to significantly decrease microvessel density, suppress proliferation and promote apoptosis. These results demonstrate the fully human VEGFR2 monoclonal antibody BC001 can work as an effective inhibitor of tumor angiogenesis and tumor growth both in vitro and in vivo. 
 Expression of VEGF-C and activation of its receptors VEGFR-2 and VEGFR-3 in trophoblast. Placental villous development requires the co-ordinated action of angiogenic factors on both endothelial and trophoblast cells. Like vascular endothelial growth factor (VEGF), VEGF-C increases vascular permeability, stimulates endothelial cell proliferation and   migration  . In the present study, we investigated the expression of VEGF-C and its receptors VEGFR-3 and VEGFR-2 in normal and intrauterine growth-restricted (IUGR) placenta. Immunolocalisation studies showed that like VEGF and VEGFR-1, VEGF-C, VEGFR-3 and VEGFR-2 co-localised to the syncytiotrophoblast, to cells in the maternal decidua, as well as to the endothelium of the large placental blood vessels. Western blot analysis demonstrated a significant decrease in placental VEGF-C and VEGFR-3 protein expression in severe IUGR as compared to gestationally-matched third trimester pregnancies. Conditioned medium from VEGF-C producing pancreatic carcinoma (Suit-2) and endometrial epithelial (Hec-1B) cell lines caused an increased association of the phosphorylated extracellular signal regulated kinase (ERK) in VEGFR-3 immunoprecipitates from spontaneously transformed first trimester trophoblast cells. VEGF121 caused dose-dependant phosphorylation of VEGFR-2 in trophoblast cells as well as stimulating DNA synthesis. In addition, premixing VEGF165 with heparin sulphate proteoglycan potentiated trophoblast proliferation and the association of phospho-ERK with the VEGFR-2 receptor. VEGF165-mediated DNA synthesis was inhibited by anti-VEGFR-2 neutralising antibody. The results demonstrate functional VEGFR-2 and VEGFR-3 receptors on trophoblast and suggest that the decreased expression of VEGF-C and VEGFR-3 may contribute to the abnormal villous development observed in IUGR placenta. 
 The protein tyrosine phosphatase TCPTP controls VEGFR2 signalling. Vascular endothelial growth factor (VEGF) is a major angiogenic factor that triggers formation of new vessels under physiological and pathological conditions. However, the mechanisms that limit the VEGF responses in target cells and hence prevent excessive and harmful angiogenesis are not well understood. Here, our objective was to study whether T-cell protein tyrosine phosphatase (TCPTP, also known as PTN2), which we found to be expressed in human endothelial cells, could alter VEGF signalling by controlling phosphorylation of VEGFR2. We show that a TCPTP substrate-trapping mutant interacts with VEGFR2. Moreover, TCPTP dephosphorylates VEGFR2 in a phosphosite-specific manner, inhibits its kinase activity and prevents its internalization from the cell surface. We found that TCPTP activity is induced upon integrin-mediated binding of endothelial cells to collagen matrix. TCPTP activation was also induced by using cell-permeable peptides from the cytoplasmic tail of the collagen-binding integrin alpha1. Controlled activation of TCPTP results in inhibition of VEGF-triggered endothelial cell proliferation, angiogenic sprouting, chemokinesis and chemotaxis. We conclude that matrix-controlled TCPTP phosphatase activity can inhibit VEGFR2 signalling, and the growth,   migration   and differentiation of human endothelial cells. 
 VEGFR-2 expression in carcinoid cancer cells and its role in tumor growth and metastasis. Carcinoid tumors are slow growing and highly vascular neuroendocrine neoplasms that are increasing in incidence. Previously, we showed that carcinoid tumors express vascular endothelial growth factor receptor 2 (VEGFR-2) in the epithelial compartment of carcinoid tumor sections; yet, its role is not completely understood. The purpose of our study was to: (i) assess the expression of VEGFR-2 in the novel human carcinoid cell line BON, (ii) to determine the role of PI3K/Akt signaling on VEGFR-2 expression and (iii) to assess the effect of VEGFR-2 on BON cell invasion,   migration   and proliferation. We found that, although VEGFR-2 is expressed in BON cells, reduction in VEGFR-2 expression actually enhanced proliferation, invasion, and   migration   of the BON cell line. Also, expression of VEGFR-2 was inversely related to PI3K signaling. Carcinoid liver metastases in mice demonstrated decreased VEGFR-2 expression. Furthermore, the expression of a truncated, soluble form of VEGFR-2 (sVEGFR-2), a protein demonstrated to inhibit cell growth, was detected in BON cells. The presence of VEGFR-2 in the epithelial component of carcinoid tumors and in the BON cell line suggests an alternate role for VEGFR-2, in addition to its well-defined role in angiogenesis. The expression of sVEGFR-2 may explain the inverse relationship between VEGFR-2 expression and PI3K/Akt signaling and the inhibitory effect VEGFR-2 has on BON cell proliferation,   migration   and invasion. 
 Cigarette smoke-mediated oxidative stress, shear stress, and endothelial dysfunction: role of VEGFR2. Vascular endothelial growth factor (VEGF) receptor 2 (VEGFR2), a tyrosine kinase receptor, is activated by VEGF and fluid shear stress (FSS), and its downstream signaling is important in regulation of endothelial functions, such as cell   migration  , endothelium-dependent relaxation, and angiogenesis. Inhibition of VEGFR2 augments cigarette smoke (CS)-induced oxidative stress and inflammatory responses leading to endothelial dysfunction. CS-derived reactive oxygen/nitrogen species interact with VEGFR2, causing posttranslational modifications that render VEGFR2 inactive for downstream signaling, resulting in endothelial dysfunction. CS-mediated oxidants/carbonyl stress decreases SIRT1 levels and causes eNOS acetylation, which has ramifications in endothelial dysfunction. CS also affects endothelial cell survival pathway by disrupting VEGF- and FSS-mediated VEGFR2/PI3-kinase signaling, leading to decreased Akt phosphorylation and eNOS acetylation. We describe here the mechanisms whereby CS alters VEGF- and FSS-mediated VEGFR2-eNOS signaling, which may have implications for understanding the pathogenesis of pulmonary and cardiovascular diseases. 
 c-Jun N-terminal kinase mediated VEGFR2 sustained phosphorylation is critical for VEGFA-induced angiogenesis in vitro and in vivo. c-Jun N-terminal kinase/stress-activated protein kinase (JNK/SAPK) is involved in the regulation of various cellular functions including cell cycle, proliferation, apoptosis. However, whether JNK/SAPK directly regulates the angiogenesis of human umbilical vein endothelial cells (HUVECs) induced by vascular endothelial growth factor A (VEGFA) has not yet been fully elucidated. Our present study firstly demonstrated VEGFA-induced angiogenic responses including the increase of cell viability,   migration  , and tube formation with a concentration-dependent manner in HUVECs. Further results showed that VEGFA induced the activation of JNK/SAPK, p38 kinase and extracellular signal-regulated kinases 1 and 2 (ERK1/2), while JNK/SAPK inhibitor SP600125 and specific siRNA both blocked all those angiogenic effects induced by VEGFA. Furthermore, VEGFA induced the phosphorylation of ASK1, SEK1/MKK4, MKK7, and c-Jun, which are upstream or downstream signals of JNK/SAPK. In addition, in vivo matrigel plug assay further showed that SP600125 inhibited VEGFA-induced angiogenesis. Further results showed that SP600125 and JNK/SAPK siRNA decreased VEGFA-induced VEGFR2 (Flk-1/KDR) sustained phosphorylation in HUVECs. Taken together, all these results demonstrate that JNK/SAPK regulates VEGFA-induced VEGFR2 sustained phosphorylation, which plays important roles in VEGFA-induced angiogenesis in HUVECs. 
 Neuropilin-1 binds to VEGF121 and regulates endothelial cell   migration   and sprouting. Neuropilin-1 (NRP1) was first described as a receptor for the axon guidance molecule, Semaphorin3A, regulating the development of the nervous system. It was later shown that NRP1 is an isoform-specific receptor for vascular endothelial growth factor (VEGF), specifically VEGF(165). Much interest has been placed on the role of the various VEGF isoforms in vascular biology. Here we report that blocking NRP1 function, using a recently described antibody that inhibits VEGF(165) binding to NRP1, surprisingly reduces VEGF(121)-induced   migration   and sprout formation of endothelial cells. Intrigued by this observation, direct binding studies of NRP1 to various VEGF isoforms were performed. We show that VEGF(121) binds directly to NRP1; however, unlike VEGF(165), VEGF(121) is not sufficient to bridge the NRP1.VEGFR2 complex. Additionally, we show that VEGFR2 enhances VEGF(165), but not VEGF(121) binding to NRP1. We propose a new model for NRP1 interactions with various VEGF isoforms. 
 Effective suppression of vascular network formation by combination of antibodies blocking VEGFR ligand binding and receptor dimerization. Antibodies that block vascular endothelial growth factor (VEGF) have become an integral part of antiangiogenic tumor therapy, and antibodies targeting other VEGFs and receptors (VEGFRs) are in clinical trials. Typically receptor-blocking antibodies are targeted to the VEGFR ligand-binding site. Here we describe a monoclonal antibody that inhibits VEGFR-3 homodimer and VEGFR-3/VEGFR-2 heterodimer formation, signal transduction, as well as ligand-induced   migration   and sprouting of microvascular endothelial cells. Importantly, we show that combined use of antibodies blocking ligand binding and receptor dimerization improves VEGFR inhibition and results in stronger inhibition of endothelial sprouting and vascular network formation in vivo. These results suggest that receptor dimerization inhibitors could be used to enhance antiangiogenic activity of antibodies blocking ligand binding in tumor therapy. 
 PDGF-induced   migration   of vascular smooth muscle cells is inhibited by heme oxygenase-1 via VEGFR2 upregulation and subsequent assembly of inactive VEGFR2/PDGFRβ heterodimers. In cardiovascular regulation, heme oxygenase-1 (HO-1) activity has been shown to inhibit vascular smooth muscle cell (VSMC) proliferation by promoting cell cycle arrest at the G1/S phase. However, the effect of HO-1 on VSMC   migratio  n remains unclear. We aim to elucidate the mechanism by which HO-1 regulates PDGFBB-induced VSMC   migratio  n.Transduction of HO-1 cDNA adenoviral vector severely impeded human VSMC   migratio  n in a scratch, transmembrane, and directional   migratio  n assay in response to PDGFBB stimulation. Similarly, HO-1 overexpression in the remodeling process during murine retinal vasculature development attenuated VSMC coverage over the major arterial branches as compared with sham vector-transduced eyes. HO-1 expression in VSMCs significantly upregulated VEGFA and VEGFR2 expression, which subsequently promoted the formation of inactive PDGFRβ/VEGFR2 complexes. This compromised PDGFRβ phosphorylation and impeded the downstream cascade of FAK-p38 signaling. siRNA-mediated silencing of VEGFA or VEGFR2 could reverse the inhibitory effect of HO-1 on VS  MC migrat  ion.These findings identify a potent antimigratory function of HO-1 in VSMCs, a mechanism that involves VEGFA and VEGFR2 upregulation, followed by assembly of inactive VEGFR2/PDGFRβ complexes that attenuates effective PDGFRβ signaling. 
 MULTIMERIN2 impairs tumor angiogenesis and growth by interfering with VEGF-A/VEGFR2 pathway. MULTIMERIN2 (MMRN2), also known as Endoglyx-1, is an extracellular matrix glycoprotein whose function has so far remained elusive. Given its specific localization in tight association with the endothelium we hypothesized that this protein could modulate neo-angiogenesis. By multiple assays we showed that MMRN2 significantly impaired endothelial cell (EC)   migration   and organization of a functional vessel network. The interaction of ECs with MMRN2 induced a striking impairment of VEGFR1 and VEGFR2 activation. We focused our attention on VEGFR2, a chief regulator of angiogenesis, and clarified that MMRN2 interfered with the VEGF/VEGFR2 axis through a direct binding with VEGF-A. This novel interaction was assessed in several assays and the affinity was estimated (Kd ∼50  nM). We next questioned whether the anti-angiogenic properties of MMRN2 could impair tumor growth. Although overexpression of MMRN2 by HT1080 cells did not affect their growth and apoptotic rate in vitro, it remarkably affected their growth in vivo. In fact, MMRN2-positive cells failed to efficiently grow and form well-vascularized tumors; a similar outcome was observed following treatment of established tumors with a MMRN2 adenoviral construct. Tumor-section immunostaining revealed a strong co-localization of VEGF-A with the ectopically expressed MMRN2. These novel findings suggest that VEGF may be sequestered by MMRN2 and be less available for the engagement to the receptors. Taken together these results highlight MMRN2 as a crucial player in the regulation of EC function, neo-angiogenesis and hence tumor growth. We hypothesize that secreted and deposited MMRN2 may function as a homeostatic barrier halting the sprouting of novel vessels, and suggest that these studies may embody the potential for the development of novel tools for cancer treatment. 
 Heparin affin regulatory peptide/pleiotrophin negatively affects diverse biological activities in C6 glioma cells. Heparin affin regulatory peptide (HARP) or pleiotrophin seems to be involved in the progression of several tumors of diverse origin. In this study, we tried to determine the role of HARP in rat C6 glioma cells by using an antisense strategy for inhibition of HARP expression. Decrease of the expression of endogenous HARP in C6 cells (AS-C6 cells) significantly increased proliferation,   migration  , and anchorage-independent growth of cells. Implantation of AS-C6 cells onto chicken embryo chorioallantoic membranes resulted in a significant increase of tumor-induced angiogenesis compared with that induced by non-transfected or C6 cells transfected with the plasmid alone (PC-C6 cells). In the same line, conditioned medium from AS-C6 cells significantly increased endothelial cell proliferation,   migration  , and tube formation in vitro compared with the effect of conditioned medium from C6 or PC-C6 cells. Interestingly, vascular endothelial growth factor (VEGF) induced C6 cell proliferation and   migration  , and SU1496, a selective inhibitor of VEGF receptor 2 (VEGFR2), blocked increased glioma cell growth,   migration  , and angiogenicity observed in AS-C6 cell cultures. The above results seem to be due to a direct interaction between HARP and VEGF in the culture medium of C6 and PC-C6 cells, while AS-C6 cells secreted comparable amounts of VEGF that do not interact with HARP. Collectively, these data suggest that HARP negatively affects diverse biological activities in C6 glioma cells, mainly due to binding of HARP to VEGF, which may sequester secreted VEGF from signalling through VEGFR2. 
 A CD44v6 peptide reveals a role of CD44 in VEGFR-2 signaling and angiogenesis. A specific splice variant of the CD44 cell- surface protein family, CD44v6, has been shown to act as a coreceptor for the receptor tyrosine kinase c-Met on epithelial cells. Here we show that also on endothelial cells (ECs), the activity of c-Met is dependent on CD44v6. Furthermore, another receptor tyrosine kinase, VEGFR-2, is also regulated by CD44v6. The CD44v6 ectodomain and a small peptide mimicking a specific extracellular motif of CD44v6 or a CD44v6-specific antibody prevent CD44v6-mediated receptor activation. This indicates that the extracellular part of CD44v6 is required for interaction with c-Met or VEGFR-2. In the cytoplasm, signaling by activated c-Met and VEGFR-2 requires association of the CD44 carboxy-terminus with ezrin that couples CD44v6 to the cytoskeleton. CD44v6 controls EC   migration  , sprouting, and tubule formation induced by hepatocyte growth factor (HGF) or VEGF-A. In vivo the development of blood vessels from grafted EC spheroids and angiogenesis in tumors is impaired by CD44v6 blocking reagents, suggesting that the coreceptor function of CD44v6 for c-Met and VEGFR-2 is a promising target to block angiogenesis in pathologic conditions. 
 Decorin is a novel VEGFR-2-binding antagonist for the human extravillous trophoblast. Extravillous trophoblasts (EVT) of the human placenta invade the uterine decidua and its arteries to ensure successful placentation. We previously identified two decidua-derived molecules, TGF-β and a TGF-β-binding proteoglycan decorin (DCN), as negative regulators of EVT proliferation  , migrati  on, and invasiveness and reported that DCN acts via multiple tyrosine kinase receptors [epidermal growth factor-receptor (EGF-R), IGF receptor-1 (IGFR1), and vascular endothelial growth factor 2 receptor (VEGFR-2)]. Because binding of DCN to VEGFR-2 has never been reported earlier, present study explored this binding, the approximate location of VEGFR-2-binding site in DCN, and its functional role in our human first trimester EVT cell line HTR-8/SVneo. Based on far-Western blotting and coimmunoprecipitation studies, we report that DCN binds both native (EVT expressed) and recombinant VEGFR-2 and that this binding is abrogated with a VEGFR-2 blocking antibody, indicating an overlap between the ligand-binding and the DCN-binding domains of VEGFR-2. We determined that (125)I-labeled VEGF-E (a VEGFR-2 specific ligand) binds EVT with a dissociation constant (K(d)) of 566 pM, and DCN displaced this binding with an inhibition constant (K(i)) of 3.93-5.78 nM, indicating a 7- to 10-fold lower affinity of DCN for VEGFR-2. DCN peptide fragments derived from the leucine rich repeat 5 domain that blocked DCN-VEGFR-2 interactions or VEGF-E binding in EVT cells also blocked VEGF-A- and VEGF-E-induced EVT cell proliferation an  d migrati  on, indicative of functional VEGFR-2-binding sites of DCN. Finally, DCN inhibited VEGF-E-induced EV  T migrati  on by interfering with ERK1/2 activation. Our findings reveal a novel role of DCN as an antagonistic ligand for VEGFR-2, having implications for pathophysiology of preeclampsia, a trophoblast hypoinvasive disorder in pregnancy, and explain its antiangiogenic function. 
 GPR126 protein regulates developmental and pathological angiogenesis through modulation of VEGFR2 receptor signaling. Angiogenesis, the formation of new blood vessels from pre-existing ones, is essential for development, wound healing, and tumor progression. The VEGF pathway plays irreplaceable roles during angiogenesis, but how other signals cross-talk with and modulate VEGF cascades is not clearly elucidated. Here, we identified that Gpr126, an endothelial cell-enriched gene, plays an important role in angiogenesis by regulating endothelial cell proliferation,   migration  , and tube formation. Knockdown of Gpr126 in the mouse retina resulted in the inhibition of hypoxia-induced angiogenesis. Interference of Gpr126 expression in zebrafish embryos led to defects in intersegmental vessel formation. Finally, we identified that GPR126 regulated the expression of VEGFR2 by targeting STAT5 and GATA2 through the cAMP-PKA-cAMP-response element-binding protein signaling pathway during angiogenesis. Our findings illustrate that GPR126 modulates both physiological and pathological angiogenesis through VEGF signaling, providing a potential target for the treatment of angiogenesis-related diseases. 
 Regulation of vascular endothelial growth factor receptor-2 activity by caveolin-1 and plasma membrane cholesterol. The stimulation of vascular endothelial growth factor receptor-2 (VEGFR-2) by tumor-derived VEGF represents a key event in the initiation of angiogenesis. In this work, we report that VEGFR-2 is localized in endothelial caveolae, associated with caveolin-1, and that this complex is rapidly dissociated upon stimulation with VEGF. The kinetics of caveolin-1 dissociation correlated with those of VEGF-dependent VEGFR-2 tyrosine phosphorylation, suggesting that caveolin-1 acts as a negative regulator of VEGF R-2 activity. Interestingly, we observed that in an overexpression system in which VEGFR-2 is constitutively active, caveolin-1 overexpression inhibits VEGFR-2 activity but allows VEGFR-2 to undergo VEGF-dependent activation, suggesting that caveolin-1 can confer ligand dependency to a receptor system. Removal of caveolin and VEGFR-2 from caveolae by cholesterol depletion resulted in an increase in both basal and VEGF-induced phosphorylation of VEGFR-2, but led to the inhibition of VEGF-induced ERK activation and endothelial cell   migration  , suggesting that localization of VEGFR-2 to these domains is crucial for VEGF-mediated signaling. Dissociation of the VEGFR-2/caveolin-1 complex by VEGF or cyclodextrin led to a PP2-sensitive phosphorylation of caveolin-1 on tyrosine 14, suggesting the participation of Src family kinases in this process. Overall, these results suggest that caveolin-1 plays multiple roles in the VEGF-induced signaling cascade. 
 Programmed cell death 6 (PDCD6) inhibits angiogenesis through PI3K/mTOR/p70S6K pathway by interacting of VEGFR-2. Programmed cell death 6 (PDCD6) was originally found as a pro-apoptotic protein, but its molecular mechanism is not well understood. In this study, we have attempted to investigate the effects of PDCD6 on the inhibition of angiogenesis-mediated cell growth as a novel anti-angiogenic protein. Purified recombinant human PDCD6 inhibited cell   migration   in a concentration-time-dependent manner. We also found that overexpressed PDCD6 suppressed vascular endothelial growth factor (VEGF)-induced proliferation, invasion, and capillary-like structure tube formation in vitro. PDCD6 suppressed phosphorylation of signaling regulators downstream from PI3K, including Akt, mammalian target of rapamycin (mTOR), glycogen synthase kinase-3β(GSK-3β), ribosomal protein S6 kinase (p70S6K), and also decreased cyclin D1 expression. We found binding PDCD6 to VEGFR-2, a key player in the PI3K/mTOR/P70S6K signaling pathway. Taken together, these data suggest that PDCD6 plays a significant role in modulating cellular angiogenesis. 
 Decursin inhibited proliferation and angiogenesis of endothelial cells to suppress diabetic retinopathy via VEGFR2. Diabetes induces pathologic proliferation and angiogenesis in the retina that leads to catastrophic loss of vision. Decursin is a novel therapeutic that targets the vascular endothelial growth factor (VEGF) receptor (VEGFR) with putative anti-proliferative and anti-angiogenic activities. Thereby we utilized human retinal microvascular endothelial cells (HRMEC) and human umbilical vein endothelial cells (HUVEC) under conditions of excess glucose to explore dose-dependent responses of decursin on markers of   migration  , angiogenesis, and proliferation. Decursin dose-dependently inhibited tube formation, VEGFR-2 expression, along with relative metabolic activity and 5-bromo-2'-deoxy-uridine (BrdU) activity in both cell lines. We then correlated our findings to the streptozotocin-induced rat model of diabetes. Following three months of decursin treatment VEGFR-2 expression was significantly inhibited. Our data would suggest that decursin may be a potent anti-angiogenic and anti-proliferative agent targeting the VEGFR-2 signaling pathway, which significantly inhibits diabetic retinal neovascularization. 
 Effect of vascular endothelial growth factor and its receptor KDR on the transendothelial   migration   and local trafficking of human T cells in vitro and in vivo. In these studies, we find that the vascular endothelial growth factor (VEGF) receptor KDR is expressed on subsets of mitogen-activated CD4(+) and CD8(+) T cells in vitro. We also found that KDR colocalizes with CD3 on mitogen-activated T cells in vitro and on infiltrates within rejecting human allografts in vivo. To evaluate whether VEGF and KDR mediate lymphocyte   migration   across endothelial cells (ECs), we used an in vitro live-time trans  migration   model and observed that both anti-VEGF and anti-KDR antibodies inhibit the trans  migration   of both CD4(+) and CD8(+) T cells across tumor necrosis factor α (TNFα)-activated, but not unactivated ECs. In addition, we found that interactions among CD4(+) or CD8(+) T cells and TNFα-activated ECs result in the induction of KDR on each T cell subset, and that KDR-expressing lymphocytes preferentially transmigrate across TNFα-activated ECs. Finally, using a humanized severe combined immunodeficient mouse model of lymphocyte trafficking, we found that KDR-expressing lymphocytes migrate into human skin in vivo, and t  hat migra  tion is reduced in mice treated with a blocking anti-VEGF antibody. These observations demonstrate that induced expression of KDR on subsets of T cells, and locally expressed VEGF, facilitate EC-dependent lymphocyte chemotaxis, and thus, the localization of T cells at sites of inflammation. 
 MicroRNA-15b contributes to ginsenoside-Rg1-induced angiogenesis through increased expression of VEGFR-2. Ginsenoside-Rg1 (Rg1) has been identified as potent proangiogenic agent, which plays an important role in wound healing promotion or treatment of ischemic injury. We previously reported that miR-214/eNOS pathway was involved in Rg1-induced angiogenesis. Following the same microRNA microarray profiling data, we proposed miR-15b would be another microRNA candidate involved in Rg1-induced angiogenesis. Using human umbilical vein endothelial cells (HUVECs), it was showed that Rg1 could reduce miR-15b expression rapidly and steadily, leading to a temporal induction of vascular endothelial growth factor receptor-2 (VEGFR-2). The in vitro motility and tubulogenesis via VEGFR-2 in Rg1-treated HUVECs were also demonstrated. Besides, the reduction of VEGFR-2 3'-UTR reporter activity by miR-15b in the luciferase reporter gene assay clearly indicated that miR-15b could affect the VEGFR-2 transcript through targeting its 3'-UTR region. Diminishing expression of endogenous miR-15b could increase VEGFR-2 expression and HUVECs   migration   and tubulogenesis; while over-expression of miR-15b was found to associate with the reduction of VEGFR-2 expression as well as cellular   migration   and tubulogenesis. In vivo, artificial increment of miR-15b by injecting Pre-miR-15b precursor into zebrafish embryos was also found to significantly suppress the subintestinal vessels formation. In conclusion, our results further demonstrated the involvement of microRNAs in Rg1-induced angiogenesis. 
 ARNO regulates VEGF-dependent tissue responses by stabilizing endothelial VEGFR-2 surface expression. The vascular endothelial growth factor (VEGF) stimulates angiogenesis by induction of vessel permeability, proliferation, and   migration   of endothelial cells, an important process in ischaemic diseases. ADP-ribosylation factor (ARF) nucleotide-binding site opener (ARNO) (cytohesin-2) is a guanine exchange factor important for cellular signalling through ARF GTPases. However, a role for ARNO in VEGF-dependent endothelial processes has so far not been documented. Therefore, we investigated whether ARNO has a role in VEGF-dependent activation of endothelial cells and thus vessel permeability.ARNO expression was observed in endothelial cells in vitro by RT-PCR, western blotting, and immunofluorescence as well as ex vivo by immunohistochemical staining of mouse aorta. Treatment with the cytohesin inhibitor SecinH3 or with an ARNO siRNA prevented VEGF-dependent Akt activation, assessed by detection of phosphorylated Akt, and proliferation of endothelial cells in vitro, measured by methylthiazoletetrazolium (MTT) reduction. In addition, ARNO suppression reduced VEGF-induced permeability in vessels of the mouse (C57BL/6) cremaster muscle in vivo, as measured by extravasation of fluorescein isothiocyanate (FITC)-dextran. Moreover, ARNO knock-down accelerated ligand-induced reduction in vascular endothelial growth factor receptor-2 (VEGFR-2) surface expression, internalization, and degradation, as assessed by flow cytometry and western blotting, respectively.Our findings indicate an important and novel role for endothelial ARNO in VEGF-dependent initiation of angiogenesis by regulation of VEGFR-2 internalization in endothelial cells, resulting in the activation of the Akt pathway, vessel permeability, and ultimately endothelial proliferation. Thus, ARNO may be a new essential player in endothelial signalling and angiogenesis. 
 FAS1 domain protein inhibits VEGF165-induced angiogenesis by targeting the interaction between VEGFR-2 and αvβ3 integrin. It is known that VEGF receptors (VEGFR) and integrins interact with each other to regulate angiogenesis. We reported previously that the fasciclin 1 (FAS1) domain-containing protein, TGFBIp/βig-h3 (TGF-β-induced protein) is an angiogenesis regulator that inhibits both endothelial c  ell migra  tion and growth via αvβ3 integrin. In an attempt to target the interaction between VEGFR-2 and αvβ3 integrin, we determined whether the FAS1 domain region of TGFBIp/βig-h3 (FAS1 domain protein) can block the interaction between the two receptors, leading to the suppression of angiogenesis. In this study, we showed that FAS1 domain protein inhibits VEGF165-induced endothelial cell prolifera  tion and   migration via αvβ3 integrin, resulting in the inhibition of VEGF165-induced angiogenesis. We also defined a molecular mechanism by which FAS1 domain protein blocks the association between αvβ3 integrin and VEGFR-2, showing that it binds to αvβ3 integrin but not to VEGFR-2. Blocking the association of these major angiogenic receptors with FAS1 domain protein inhibits signaling pathways downstream of VEGFR-2. Collectively, our results indicate that FAS1 domain protein, in addition to its inhibitory effect on αvβ3 integrin-mediated angiogenesis, also inhibits VEGF165-induced angiogenesis. Thus, FAS1 domain protein can be further developed into a potent anticancer drug that targets two principal angiogenic pathways. 
 p38 MAPK inhibition is critically involved in VEGFR-2-mediated endothelial cell survival. Vascular endothelial growth factor (VEGF) promotes vasculogenesis, arteriogenesis, and angiogenesis by stimulating proliferation,   migration  , and cell survival of endothelial cells. VEGF mediates its actions through activation of two receptor tyrosine kinases, VEGFR-1 and VEGFR-2. Serum starvation led to apoptosis of human umbilical vein endothelial cells (HUVEC), which was accompanied by activation of p38 MAPK and caspase-3. Stimulation of both VEGF-receptors resulted in a considerable decrease of apoptosis, which was associated with the inhibition of p38 MAPK and caspase-3 activity. Selective stimulation of VEGFR-2 showed similar results, whereas the isolated activation of VEGFR-1 was without effect. Incubation of HUVEC with SB203580, a p38 MAPK inhibitor, resulted in similar effects as VEGF-stimulation: p38 MAPK and caspase-3 enzyme activity were reduced and apoptosis was prevented. These data indicate that activation of VEGFR-2 prevents endothelial cell apoptosis by inhibiting p38 MAPK phosphorylation and thus, reducing caspase-3 activity. 
 Expression of VEGFR-2 on HaCaT cells is regulated by VEGF and plays an active role in mediating VEGF induced effects. Vascular endothelial growth factor (VEGF) and its receptor VEGFR-2 play important roles in mitogenesis and chemotaxis of endothelial cells. In normal human skin, VEGF is expressed and secreted by epidermal keratinocytes. Emerging data suggest that keratinocyte-derived VEGF targets other cell types besides the dermal endothelial cells. We have recently showed that keratinocytes from human normal skin expressed all five known VEGF receptors and co-receptors (neuropilin 1 and 2). To define the functional significance of VEGFR-2 in epidermis, we examined its role in a keratinocyte cell line, HaCaT cells, in response to VEGF treatment. Expression of VEGFR-2 on HaCaT cells was confirmed at both RNA and protein levels and was regulated by VEGF165 treatment. Treatment of HaCaT cells with VEGF165 induced tyrosine-autophosphorylation of VEGFR-2 and phosphorylation of PLC-gamma and p44/42 MAPK in a time-dependent manner. Preincubation with a neutralizing antibody for VEGFR-2 (MAB3571) completely abrogated these phosphorylation effects. Furthermore, VEGF165 stimulated proliferation and   migration   of HaCaT cells, and this effect was significantly blocked by a pretreatment with MAB3571. Neutralizing VEGFR-2 in HaCaT cells increased cell adhesion during culture. Our results suggest that VEGFR-2 expressed on HaCaT cells plays a crucial role in VEGF-mediated regulation of cell activity. 
 Evidence for G-quadruplex in the promoter of vegfr-2 and its targeting to inhibit tumor angiogenesis. Tumor angiogenesis is mainly mediated by vascular endothelial growth factor (VEGF), a pro-angiogenic factor produced by cancer cells and active on the endothelium through the VEGF receptor 2 (VEGFR-2). Here we identify a G-rich sequence within the proximal promoter region of vegfr-2, able to form an antiparallel G-quadruplex (G4) structure. This G4 structure can be efficiently stabilized by small molecules with the consequent inhibition of vegfr-2 expression. Functionally, the G4-mediated reduction of VEGFR-2 protein causes a switching off of signaling components that, converging on actin cytoskeleton, regulate the cellular events leading to endothelial cell proliferation,   migration   and differentiation. As a result of endothelial cell function impairment, angiogenic process is strongly inhibited by G4 ligands both in vitro and in vivo. Interestingly, the G4-mediated antiangiogenic effect seems to recapitulate that observed by using a specific interference RNA against vegfr-2, and it is strongly antagonized by overexpressing the vegfr-2 gene. In conclusion, we describe the evidence for the existence of G4 in the promoter of vegfr-2, whose expression and function can be markedly inhibited by G4 ligands, thereby revealing a new, and so far undescribed, way to block VEGFR-2 as target for anticancer therapy. 
 Phosphorylation of Tyr1214 within VEGFR-2 triggers the recruitment of Nck and activation of Fyn leading to SAPK2/p38 activation and endothelial cell   migration   in response to VEGF. VEGFR-2 is the major receptor that regulates the different functions of VEGF in adults. We have previously reported that following VEGF treatment of endothelial cells, VEGFR-2 is phosphorylated on Tyr1214 upstream of the Cdc42-SAPK2/p38-MAPKAP K2 pathway. However, little is known of the earliest molecular events that compose the SAPK2/p38 pathway following VEGFR-2 activation. In this study, we address this question using HA-tagged constructs of either wild-type VEGFR-2 or Y1214F VEGFR-2 mutant in immunoprecipitation assays. We show that the Src family kinase member Fyn, but not c-Src itself, is recruited to VEGFR-2 and is activated in a p-Tyr1214-dependent manner. We also report that the SH2 domain-containing adapter molecule Nck, but not Grb2, is recruited to VEGFR-2 in a p-Tyr1214-dependent manner and that it associates with Fyn. Moreover, PAK-2 is phosphorylated in a Fyn-dependent manner. Using chemical and genetic inhibitors, we show that Fyn activity is required for SAPK2/p38 but not for FAK activation in response to VEGF. In contrast, c-Src permits activation of FAK, but not that of SAPK2/p38. In addition, Fyn is required for stress fiber formation and endothelial cell   migration  . We propose a model in which Fyn forms a molecular complex with Nck and PAK-2 and suggest that this complex assembles in a p-Tyr1214-dependent manner within VEGFR-2 following VEGF treatment. In turn, this triggers the activation of the SAPK2/p38 MAP kinase module, and promotes stress fiber formation and endothelial cell   migration  . 
 Interleukin-6 triggers human cerebral endothelial cells proliferation and   migration  : the role for KDR and MMP-9. Interleukin-6 (IL-6) is involved in angiogenesis. However, the underlying mechanisms are unknown. Using human cerebral endothelial cell (HCEC), we report for the first time that IL-6 triggers HCEC proliferation and   migration   in a dose-dependent manner, specifically associated with enhancement of VEGF expression, up-regulated and phosphorylated VEGF receptor-2 (KDR), and stimulated MMP-9 secretion. We investigated the signal pathway of IL-6/IL-6R responsible for KDR's regulation. Pharmacological inhibitor of PI3K failed to inhibit IL-6-mediated VEGF overexpression, while blocking ERK1/2 with PD98059 could abolish IL-6-induced KDR overexpression. Further, neutralizing endogenous VEGF attenuated KDR expression and phosphorylation, suggesting that IL-6-induced KDR activation is independent of VEGF stimulation. MMP-9 inhibitor GM6001 significantly decreases HCEC proliferation and   migration   (p<0.05), indicating the crucial function of MMP-9 in promoting angiogenic changes in HCECs. We conclude that IL-6 triggers VEGF-induced angiogenic activity through increasing VEGF release, up-regulates KDR expression and phosphorylation through activating ERK1/2 signaling, and stimulates MMP-9 overexpression. 
 Interactions between sphingosine-1-phosphate and vascular endothelial growth factor signalling in ML-1 follicular thyroid carcinoma cells. Sphingosine-1-phosphate (S1P) induces   migration   of human ML-1 thyroid follicular cancer cells and inhibits   migration   of human FRO anaplastic thyroid cancer cells. As tumour cells often secrete vascular endothelial growth factor (VEGF), we investigated a possible interaction between S1P and VEGF signalling in the regulation of thyroid tumour cell   migration  . We found that both ML-1 and FRO cells secreted VEGF-A ( approximately 3.6 and <0.1 ng/10(6) cells/day respectively) and VEGF-C ( approximately 3.0 and 0.14 ng/10(6) cells/day respectively). S1P stimulated VEGF-A secretion in both cell lines, and blocking S1P receptors 1, 2 and 3 attenuated the S1P-evoked secretion of VEGF-A. Neither TSH nor insulin affected the amount of secreted VEGF-A or -C in ML-1 cells, while simultaneous stimulation with insulin and S1P increased VEGF-C secretion in FRO cells. Both cell lines expressed VEGF receptor 2 (VEGFR-2) mRNA and proteins. Serum-evoked   migration   of both ML-1 and FRO cells was attenuated when VEGFR-2 was inhibited. Moreover, inhibiting VEGFR-2 in ML-1 cells resulted in a rapid downregulation of S1P1 mRNA expression and S1P1 protein levels, suppression of S1P-induced   migration   and a decrease in S1P-induced Akt phosphorylation. A VEGF-neutralizing antibody also reduced S1P-induced   migration  . In ML-1 cells, S1P phosphorylated VEGFR-2. In addition, VEGFR-2 inhibition resulted in the upregulation of S1P3 mRNA within 24 h, but a significant increase in S1P3 protein levels was not observed. VEGFR-2 inhibition, but not a VEGF-neutralizing antibody, reduced ML-1 cell proliferation independently of S1P stimulation. The results indicate a complex interaction between S1P and VEGFR-2 in ML-1 cells, particularly in regulating migratory responses. 
 IQGAP1, a novel vascular endothelial growth factor receptor binding protein, is involved in reactive oxygen species--dependent endothelial   migration   and proliferation. Endothelial cell (EC) proliferation and   migration   are important for reendothelialization and angiogenesis. We have demonstrated that reactive oxygen species (ROS) derived from the small GTPase Rac1-dependent NAD(P)H oxidase are involved in vascular endothelial growth factor (VEGF)-mediated endothelial responses mainly through the VEGF type2 receptor (VEGFR2). Little is known about the underlying molecular mechanisms. IQGAP1 is a scaffolding protein that controls cellular motility and morphogenesis by interacting directly with cytoskeletal, cell adhesion, and small G proteins, including Rac1. In this study, we show that IQGAP1 is robustly expressed in ECs and binds to the VEGFR2. A pulldown assay using purified proteins demonstrates that IQGAP1 directly interacts with active VEGFR2. In cultured ECs, VEGF stimulation rapidly promotes recruitment of Rac1 to IQGAP1, which inducibly binds to VEGFR2 and which, in turn, is associated with tyrosine phosphorylation of IQGAP1. Endogenous IQGAP1 knockdown by siRNA shows that IQGAP1 is involved in VEGF-stimulated ROS production, Akt phosphorylation, endothelial   migration  , and proliferation. Wound assays reveal that IQGAP1 and phosphorylated VEGFR2 accumulate and colocalize at the leading edge in actively migrating ECs. Moreover, we found that IQGAP1 expression is dramatically increased in the VEGFR2-positive regenerating EC layer in balloon-injured rat carotid artery. These results suggest that IQGAP1 functions as a VEGFR2-associated scaffold protein to organize ROS-dependent VEGF signaling, thereby promoting EC   migration   and proliferation, which may contribute to repair and maintenance of the functional integrity of established blood vessels. 
 Endorepellin laminin-like globular 1/2 domains bind Ig3-5 of vascular endothelial growth factor (VEGF) receptor 2 and block pro-angiogenic signaling by VEGFA in endothelial cells. Endorepellin, a processed fragment of perlecan protein core, possesses anti-angiogenic activity by antagonizing endothelial cells. Endorepellin contains three laminin G-like (LG) domains and binds simultaneously to vascular endothelial growth factor receptor 2 (VEGFR2) and α2β1 integrin, resulting in dual receptor antagonism. Treatment of endothelial cells with endorepellin inhibits transcription of VEGFA, the natural ligand for VEGFR2, attenuating the pro-survival and migratory activities of VEGFA/VEGFR2 signaling cascade. Here, we investigated the specific binding site of endorepellin within the ectodomain of VEGFR2. Full-length endorepellin was not capable of displacing VEGFA binding from VEGFR2 and LG3 domain alone did not bind VEGFR2. This suggested different binding mechanisms of the extracellular Ig domains of VEGFR2. Therefore, we hypothesized that endorepellin would bind through its proximal LG1/2 domains to VEGFR2 in a different region than VEGFA. Indeed, we found that LG1/2 did not bind Ig1-3, but did bind with high affinity to Ig3-5, distal to the known VEGFA binding site, i.e. Ig2-3. These results support a role for endorepellin as an allosteric inhibitor of VEGFR2. Moreover, we found that LG1/2 blocked the rapid VEGFA activation of VEGFR2 at Tyr1175 in endothelial cells. In contrast, LG1/2 did not result in actin cytoskeletal disassembly in endothelial cells whereas LG3 alone did induce cytoskeletal collapse. However, LG1/2 did inhibit VEGFA-dependent endothelia  l migrati  on through fibrillar collagen I. These studies provide a mechanistic understanding of how the different LG domains of endorepellin signal in endothelial cells while serving as a template for protein design of receptor tyrosine kinase antagonists. 
 The interaction between KDR and interleukin-3 receptor (IL-3R) beta common modulates tumor neovascularization. As vascular endothelial growth factor (VEGF), interleukin-3 (IL-3), released into the tumor microenvironment stimulates motogenic and mitogenic activity of normal and transformed cells. In the present study, we investigate the effects of IL-3 and VEGF on neoplastic vascular growth. Engagement of IL-3 receptor beta common (IL-3R beta c) contributes to both IL-3- and VEGF-induced Rac1 activation, cell   migration   and in vitro tube-like structure formation as shown by the expression of the dominant-negative IL-3R beta c construct (Delta455). In normal and transformed endothelial cells (EC) as well as in HEK 293 cells expressing KDR and IL-3R, VEGF and IL-3 treatment induces the formation of a KDR/IL-3R beta c complex. Moreover, as shown by the IL-3R Delta455 mutant or by the kinase dead KDR, functional receptors are required for this interaction. Consistent with the contribution of IL-3R beta c in both IL-3- and VEGF-mediated angiogenic signal, a reduced number of vessels inside tumors are found in mice injected with cells expressing the IL-3R Delta455 mutant. Thus, these findings provide a novel mechanism through which IL-3 and VEGF support cell survival and tumor neovascularization. 
  GeneRIFs  
 The expression levels of two target genes, Myb and VEGFR2, were affected significantly by miR-16, while glucose administration inhibited miR-16 expression and enhanced tumor cell proliferation and   migration  . 
 blocking the expression of VEGFR2 inhibited proliferation,   migration  , and invasion in the non-small cell lung cancer cell line Calu-1 
 VEGFR family members are expressed in leiomyosarcomas in vivo and in vitro. Upon receptor stimulation, PTK787 is able to inhibit subsequent phosphorylation events and influences cell survival but not metabolic activity and   migration   
 ERK3 regulates endothelial cell   migration  , proliferation and tube formation by upregulating SRC-3/SP-1-mediated VEGFR2 expression. 
 Endorepellin binds through its proximal LG1/2 domains to VEGFR2 and inhibit VEGFA-dependent endothelial   migration  . 
 results demonstrate that the proteolytic enzyme MMP-9 promotes resistant epithelial breast cancer cells   migration   and tubular network formation through VEGFR-2/ VEGFR-3 activation 
 VEGF binding to NRP1 is essential for VEGF stimulation of endothelial cell   migration  , complex formation between NRP1 and VEGFR2, and signaling via FAK Tyr407 phosphorylation. 
 provide evidence that soluble VEGF receptors promote mural cell   migration   through a paracrine mechanism involving interplay in ECs between VEGF/VEGFR-2 and S1P/S1P1 pathways that leads to endothelial nitric oxide synthase activation 
 VEGF might be involved in several aspects of neural cell development, including   migration  , differentiation, synaptogenesis, and myelination, and VEGFR-2 might be one of the main receptors that mediate these processes. 
 Rab GTPase regulation of VEGFR2 trafficking and signaling in endothelial cells; Endothelial cell   migration   was increased by Rab5a depletion but decreased by Rab7a depletion 
 matrix-controlled TCPTP phosphatase activity can inhibit VEGFR2 signalling, and the growth,   migration   and differentiation of human endothelial cells 
 VEGFR2 up-regulation had no effect on VEGF-induced cell proliferation, but significantly enhanced endothelial progenitor cells   migration   and pseudotubes formation dependent on integrin alpha(6) subunit overexpression. 
 Signaling via VEGFR-2 is involved in prostate lymphatic endothelial cell tube formation,   migration  , & proliferation in vitro. Blockade of VEGFR-2 significantly reduced tumor-induced activation of LECs. 
 Shb binds to tyrosine 1175 in the VEGFR-2, which regulates VEGF-induced formation of focal adhesions and cell   migration  , of which the latter occurs in a phosphatidylinositol 3-kinase-dependent manner 
  Summary  
 Vascular endothelial growth factor (VEGF) is a major growth factor for endothelial cells. This gene encodes one of the two receptors of the VEGF. This receptor, known as kinase insert domain receptor, is a type III receptor tyrosine kinase. It functions as the main mediator of VEGF-induced endothelial proliferation, survival,   migration  , tubular morphogenesis and sprouting. The signalling and trafficking of this receptor are regulated by multiple factors, including Rab GTPase, P2Y purine nucleotide receptor, integrin alphaVbeta3, T-cell protein tyrosine phosphatase, etc.. Mutations of this gene are implicated in infantile capillary hemangiomas. [provided by RefSeq, May 2009] 
  ECM  PubMed  
 Expression and regulation of vascular endothelial growth factor ligands and receptors during menstruation and post-menstrual repair of human endometrium. Regeneration and growth of the human endometrium after shedding of the functional layer during menstruation depends on an adequate angiogenic response. We analysed the mRNA expression levels of all known vascular endothelial growth factor (VEGF) ligands and receptors in human endometrium collected in the menstrual and proliferative phases of the menstrual cycle. In addition, we evaluated the expression of VEGF-A, VEGF-R2 and NRP-1 at the protein level. Two periods of elevated mRNA expression of ligands and receptors were observed, separated by a distinct drop at cycle days (CDs) 9 and 10. Immunohistochemical staining showed that VEGF and VEGF-R2 were expressed in epithelial, stromal and endothelial cells. NRP-1 was mainly confined to stroma and blood vessels; only in late-proliferative endometrium, epithelial staining was also observed. Except for endothelial VEGF-R2 expression in CDs 6-8, there were no significant differences in the expression of VEGF, VEGF-R2 or NRP-1 in any of the cell compartments. In contrast, VEGF release by cultured human endometrium explants decreased during the proliferative phase. This output was significantly reduced in menstrual and early-proliferative endometrium by estradiol (E2) treatment. Western blot analysis indicated that part of the VEGF-A was trapped in the extracellular matrix (  ECM  ). Changes in VEGF ligands and receptors were associated with elevated expression of the hypoxia markers HIF1alpha and CA-IX in the menstrual and early proliferative phases. HIF1alpha was also detected in late-proliferative phase endometrium. Our findings indicate that VEGF-A exerts its actions mostly during the first half of the proliferative phase. Furthermore, VEGF-A production appears to be triggered by hypoxia in the menstrual phase and subsequently suppressed by estrogen during the late proliferative phase. 
 Neuropilin-1 expression promotes invasiveness of melanoma cells through vascular endothelial growth factor receptor-2-dependent and -independent mechanisms. The majority of human melanoma cell lines secretes vascular endothelial growth factor-A (VEGF-A) and expresses its receptors VEGFR-1, VEGFR-2 and neuropilin-1 (NRP‑1), a co-receptor for VEGF-A that amplifies the signalling through VEGFR-2. Since it is known that the VEGF-A/VEGFR-2 autocrine loop promotes melanoma cell invasiveness, the aim of the present study was to investigate the involvement of NPR-1 in melanoma progression. Syngeneic human melanoma cell lines expressing either VEGFR-2 or NRP-1, both or none of them, were analyzed for their in vitro ability to migrate, invade the extracellular matrix   (E  CM) and secrete active metalloproteinase-2 (MMP-2). The results indicate that NRP-1 cooperates with VEGFR-2 in melanoma cell migration induced by VEGF-A. Moreover, NRP-1 expression is sufficient to promote MMP-2 secretion and melanoma cell invasiveness, as demonstrated by the ability of cells expressing solely NRP-1 to spontaneously invade th  e E  CM. This ability is specifically downregulated by anti-NRP-1 antibodies or by interfering with NRP-1 expression using an shRNA construct. Investigation of the signal transduction pathways triggered by NRP-1 in melanoma cells, indicated that NRP-1-dependent promotion of cell invasiveness involves Akt activation through its phosphorylation on T308. Overall, the results demonstrate that NRP-1 is involved in melanoma progression through VEGFR-2-dependent and -independent mechanisms and suggest NRP-1 as a target for the treatment of the metastatic disease. 
 Hypoxia differentially regulates arterial and venous smooth muscle cell proliferation via PDGFR-β and VEGFR-2 expression. Despite intensive research studies, theories have yet to focus on the contribution of hypoxia to patency differences observed clinically between arterial vs. venous grafts. This study investigates the differential hypoxic response of smooth muscle cells (SMC) to hypoxia-derived endothelial cell (EC) growth factors. Initiation of SMC proliferation under hypoxia (<5% O(2)) occurred only after incubation with hypoxic endothelial cell-conditioned media (H  -EC  M). After the investigation of several possible growth factors in the H  -EC  M that may be responsible for SMC proliferation, the greatest difference was observed in vascular endothelial growth factor (VEGF-A) and platelet-derived growth factor homodimer B (PDGF-BB) expression. VEGF-A increased (2-fold) significantly (P < 0.05) in arterial-derived smooth muscle cells (ASMC) under hypoxia compared with venous-derived smooth muscle cells (VSMC), which showed no significant change. VSMC showed significant (P < 0.05) increase in VEGFR-2 expression under hypoxia compared with ASMC. Incubation with VEGFR-2-neutralizing antibody/PDGFR antagonist in VSMC before addition of H  -EC  M resulted in decreased proliferation. ASMC proliferation under hypoxia did not decrease during incubation with VEGFR-2-neutralizing antibody but did decrease upon PDGFR antagonist incubation. Current therapies focusing on treating intimal hyperplasia have negated the fact that combinational therapy might be required to combat induction of SMC proliferation. Clinically, therapy with PDGFR antagonists plus anti-VEGFR-2 may prove to be efficacious in managing SMC proliferation in venous-derived grafts. 
  [Ee]xtracellular [Mm]atrix  Process  
   extracellular matrix   organization 
  PubMed  
 Matrix metalloproteinase-1-mediated up-regulation of vascular endothelial growth factor-2 in endothelial cells. Matrix metalloproteinase-1 (MMP-1) is a collagenase that is highly active in   extracellular matrix   and vascular remodeling, angiogenesis, and tumor progression. Vascular endothelial growth factor receptor-2 (VEGFR2), the main receptor for VEGF-A, is expressed on endothelial cells and promotes cell survival, proliferation, and other functions. Although MMP-1 and VEGFR2 co-exist in many normal and pathophysiological conditions, the effect of MMP-1 on cellular VEGFR2 that can promote the above processes is unknown. In this study we test the hypothesis that stimulation of endothelial cells with MMP-1 increases their levels of VEGFR2. The increased VEGFR2 is then available to bind VEGF-A, resulting in increased response. Indeed we found that endothelial cells incubated with active MMP-1 had higher mRNA and protein levels of VEGFR2. Furthermore, VEGF-A-dependent phosphorylation of intracellular signaling molecules and endothelial proliferation were elevated after MMP-1 treatment. MMP-1 caused activation of the nuclear factor-κB (NF-κB) pathway (p65/RelA) in endothelial cells, and this response was dependent upon activation of protease activated receptor-1 (PAR-1). Chromatin immunoprecipitation was used to confirm NF-κB-mediated active transcription of the VEGFR2 (KDR) gene. Elevation in VEGFR2 after MMP-1 stimulation was inhibited by PAR-1 knockdown and NF-κB specific inhibition. We conclude that MMP-1 promotes VEGFR2 expression and proliferation of endothelial cells through stimulation of PAR-1 and activation of NF-κB. These results suggest a mechanism by which MMP-1 may prime or sensitize endothelial cell functions. 
 Urokinase-type Plasminogen Activator (uPA) Promotes Angiogenesis by Attenuating Proline-rich Homeodomain Protein (PRH) Transcription Factor Activity and De-repressing Vascular Endothelial Growth Factor (VEGF) Receptor Expression. Urokinase-type plasminogen activator (uPA) regulates angiogenesis and vascular permeability through proteolytic degradation of   extracellular matrix   and intracellular signaling initiated upon its binding to uPAR/CD87 and other cell surface receptors. Here, we describe an additional mechanism by which uPA regulates angiogenesis. Ex vivo VEGF-induced vascular sprouting from Matrigel-embedded aortic rings isolated from uPA knock-out (uPA(-/-)) mice was impaired compared with vessels emanating from wild-type mice. Endothelial cells isolated from uPA(-/-) mice show less proliferation and migration in response to VEGF than their wild type counterparts or uPA(-/-) endothelial cells in which expression of wild type uPA had been restored. We reported previously that uPA is transported from cell surface receptors to nuclei through a mechanism that requires its kringle domain. Intranuclear uPA modulates gene transcription by binding to a subset of transcription factors. Here we report that wild type single-chain uPA, but not uPA variants incapable of nuclear transport, increases the expression of cell surface VEGF receptor 1 (VEGFR1) and VEGF receptor 2 (VEGFR2) by translocating to the nuclei of ECs. Intranuclear single-chain uPA binds directly to and interferes with the function of the transcription factor hematopoietically expressed homeodomain protein or proline-rich homeodomain protein (HHEX/PRH), which thereby lose their physiologic capacity to repress the activity of vehgr1 and vegfr2 gene promoters. These studies identify uPA-dependent de-repression of vegfr1 and vegfr2 gene transcription through binding to HHEX/PRH as a novel mechanism by which uPA mediates the pro-angiogenic effects of VEGF and identifies a potential new target for control of pathologic angiogenesis. 
 Integrin activation is required for VEGF and FGF receptor protein presence on human microvascular endothelial cells. Endothelial cell proliferation and migration is initiated by growth factors including FGF and VEGF that bind to specific transmembrane receptor tyrosine kinases. Mechanisms that regulate in vivo expression of fibroblast growth factor receptors (FGFR) and vascular endothelial growth factor receptors (VEGFR) are not well understood. Since it is well known that different matrices influence the proliferation and migration of endothelial cells in culture, we hypothesized that changes in the   extracellular matrix   environment can regulate growth factor receptors on endothelial cells. We cultured human microvascular endothelial cells on different matrices (vitronectin, laminin, fibronectin, fibrin, and collagen IV) and examined for the presence of growth factor receptors (FGFR-1, FGFR-2, VEGFR-1, and VEGFR-2). We show that vitronectin increased the presence of all four growth factor receptors and most notably, VEGFR-1. In contrast, fibrin decreased all four receptors, especially FGFR-1 and FGFR-2. Inhibiting phosphotyrosine signaling abolished immunostaining for all four receptors, regardless of the matrix, but was not dependent on activating the Fyn-Shc pathway. Cells plated on vitronectin in the presence of blocking antibodies to integrins alphavbeta3 and alphavbeta5 similarly decreased presence of these growth factor receptors. Our data suggests a possible mechanism of how matrix-integrin interactions regulate endothelial cell responsiveness to growth factors and anchorage-dependent cell growth. 
 Urokinase-type Plasminogen Activator (uPA) Promotes Angiogenesis by Attenuating Proline-rich Homeodomain Protein (PRH) Transcription Factor Activity and De-repressing Vascular Endothelial Growth Factor (VEGF) Receptor Expression. Urokinase-type plasminogen activator (uPA) regulates angiogenesis and vascular permeability through proteolytic degradation of   extracellular matrix   and intracellular signaling initiated upon its binding to uPAR/CD87 and other cell surface receptors. Here, we describe an additional mechanism by which uPA regulates angiogenesis. Ex vivo VEGF-induced vascular sprouting from Matrigel-embedded aortic rings isolated from uPA knock-out (uPA(-/-)) mice was impaired compared with vessels emanating from wild-type mice. Endothelial cells isolated from uPA(-/-) mice show less proliferation and migration in response to VEGF than their wild type counterparts or uPA(-/-) endothelial cells in which expression of wild type uPA had been restored. We reported previously that uPA is transported from cell surface receptors to nuclei through a mechanism that requires its kringle domain. Intranuclear uPA modulates gene transcription by binding to a subset of transcription factors. Here we report that wild type single-chain uPA, but not uPA variants incapable of nuclear transport, increases the expression of cell surface VEGF receptor 1 (VEGFR1) and VEGF receptor 2 (VEGFR2) by translocating to the nuclei of ECs. Intranuclear single-chain uPA binds directly to and interferes with the function of the transcription factor hematopoietically expressed homeodomain protein or proline-rich homeodomain protein (HHEX/PRH), which thereby lose their physiologic capacity to repress the activity of vehgr1 and vegfr2 gene promoters. These studies identify uPA-dependent de-repression of vegfr1 and vegfr2 gene transcription through binding to HHEX/PRH as a novel mechanism by which uPA mediates the pro-angiogenic effects of VEGF and identifies a potential new target for control of pathologic angiogenesis. 
 Matrix metalloproteinase-1-mediated up-regulation of vascular endothelial growth factor-2 in endothelial cells. Matrix metalloproteinase-1 (MMP-1) is a collagenase that is highly active in   extracellular matrix   and vascular remodeling, angiogenesis, and tumor progression. Vascular endothelial growth factor receptor-2 (VEGFR2), the main receptor for VEGF-A, is expressed on endothelial cells and promotes cell survival, proliferation, and other functions. Although MMP-1 and VEGFR2 co-exist in many normal and pathophysiological conditions, the effect of MMP-1 on cellular VEGFR2 that can promote the above processes is unknown. In this study we test the hypothesis that stimulation of endothelial cells with MMP-1 increases their levels of VEGFR2. The increased VEGFR2 is then available to bind VEGF-A, resulting in increased response. Indeed we found that endothelial cells incubated with active MMP-1 had higher mRNA and protein levels of VEGFR2. Furthermore, VEGF-A-dependent phosphorylation of intracellular signaling molecules and endothelial proliferation were elevated after MMP-1 treatment. MMP-1 caused activation of the nuclear factor-κB (NF-κB) pathway (p65/RelA) in endothelial cells, and this response was dependent upon activation of protease activated receptor-1 (PAR-1). Chromatin immunoprecipitation was used to confirm NF-κB-mediated active transcription of the VEGFR2 (KDR) gene. Elevation in VEGFR2 after MMP-1 stimulation was inhibited by PAR-1 knockdown and NF-κB specific inhibition. We conclude that MMP-1 promotes VEGFR2 expression and proliferation of endothelial cells through stimulation of PAR-1 and activation of NF-κB. These results suggest a mechanism by which MMP-1 may prime or sensitize endothelial cell functions. 
 Integrin activation is required for VEGF and FGF receptor protein presence on human microvascular endothelial cells. Endothelial cell proliferation and migration is initiated by growth factors including FGF and VEGF that bind to specific transmembrane receptor tyrosine kinases. Mechanisms that regulate in vivo expression of fibroblast growth factor receptors (FGFR) and vascular endothelial growth factor receptors (VEGFR) are not well understood. Since it is well known that different matrices influence the proliferation and migration of endothelial cells in culture, we hypothesized that changes in the   extracellular matrix   environment can regulate growth factor receptors on endothelial cells. We cultured human microvascular endothelial cells on different matrices (vitronectin, laminin, fibronectin, fibrin, and collagen IV) and examined for the presence of growth factor receptors (FGFR-1, FGFR-2, VEGFR-1, and VEGFR-2). We show that vitronectin increased the presence of all four growth factor receptors and most notably, VEGFR-1. In contrast, fibrin decreased all four receptors, especially FGFR-1 and FGFR-2. Inhibiting phosphotyrosine signaling abolished immunostaining for all four receptors, regardless of the matrix, but was not dependent on activating the Fyn-Shc pathway. Cells plated on vitronectin in the presence of blocking antibodies to integrins alphavbeta3 and alphavbeta5 similarly decreased presence of these growth factor receptors. Our data suggests a possible mechanism of how matrix-integrin interactions regulate endothelial cell responsiveness to growth factors and anchorage-dependent cell growth. 
 Role of alphavbeta3 integrin in the activation of vascular endothelial growth factor receptor-2. Interaction between integrin alphavbeta3 and   extracellular matrix   is crucial for endothelial cells sprouting from capillaries and for angiogenesis. Furthermore, integrin-mediated outside-in signals co-operate with growth factor receptors to promote cell proliferation and motility. To determine a potential regulation of angiogenic inducer receptors by the integrin system, we investigated the interaction between alphavbeta3 integrin and tyrosine kinase vascular endothelial growth factor receptor-2 (VEGFR-2) in human endothelial cells. We report that tyrosine-phosphorylated VEGFR-2 co-immunoprecipitated with beta3 integrin subunit, but not with beta1 or beta5, from cells stimulated with VEGF-A165. VEGFR-2 phosphorylation and mitogenicity induced by VEGF-A165 were enhanced in cells plated on the alphavbeta3 ligand, vitronectin, compared with cells plated on the alpha5beta1 ligand, fibronectin or the alpha2beta1 ligand, collagen. BV4 anti-beta3 integrin mAb, which does not interfere with endothelial cell adhesion to vitronectin, reduced (i) the tyrosine phosphorylation of VEGFR-2; (ii) the activation of downstream transductor phosphoinositide 3-OH kinase; and (iii) biological effects triggered by VEGF-A165. These results indicate a new role for alphavbeta3 integrin in the activation of an in vitro angiogenic program in endothelial cells. Besides being the most important survival system for nascent vessels by regulating cell adhesion to matrix, alphavbeta3 integrin participates in the full activation of VEGFR-2 triggered by VEGF-A, which is an important angiogenic inducer in tumors, inflammation and tissue regeneration. 
 Expression and regulation of vascular endothelial growth factor ligands and receptors during menstruation and post-menstrual repair of human endometrium. Regeneration and growth of the human endometrium after shedding of the functional layer during menstruation depends on an adequate angiogenic response. We analysed the mRNA expression levels of all known vascular endothelial growth factor (VEGF) ligands and receptors in human endometrium collected in the menstrual and proliferative phases of the menstrual cycle. In addition, we evaluated the expression of VEGF-A, VEGF-R2 and NRP-1 at the protein level. Two periods of elevated mRNA expression of ligands and receptors were observed, separated by a distinct drop at cycle days (CDs) 9 and 10. Immunohistochemical staining showed that VEGF and VEGF-R2 were expressed in epithelial, stromal and endothelial cells. NRP-1 was mainly confined to stroma and blood vessels; only in late-proliferative endometrium, epithelial staining was also observed. Except for endothelial VEGF-R2 expression in CDs 6-8, there were no significant differences in the expression of VEGF, VEGF-R2 or NRP-1 in any of the cell compartments. In contrast, VEGF release by cultured human endometrium explants decreased during the proliferative phase. This output was significantly reduced in menstrual and early-proliferative endometrium by estradiol (E2) treatment. Western blot analysis indicated that part of the VEGF-A was trapped in the   extracellular matrix   (ECM). Changes in VEGF ligands and receptors were associated with elevated expression of the hypoxia markers HIF1alpha and CA-IX in the menstrual and early proliferative phases. HIF1alpha was also detected in late-proliferative phase endometrium. Our findings indicate that VEGF-A exerts its actions mostly during the first half of the proliferative phase. Furthermore, VEGF-A production appears to be triggered by hypoxia in the menstrual phase and subsequently suppressed by estrogen during the late proliferative phase. 
 Type I collagen limits VEGFR-2 signaling by a SHP2 protein-tyrosine phosphatase-dependent mechanism 1. During angiogenesis, a combined action between newly secreted   extracellular matrix   proteins and the repertoire of integrins expressed by endothelial cells contributes in the regulation of their biological functions.   Extracellular matrix  -engaged integrins influence tyrosine kinase receptors, thus promoting a regulatory cross-talk between adhesive and soluble stimuli. For instance, vitronectin has been reported to positively regulate VEGFR-2. Here, we show that collagen I downregulates VEGF-A-mediated VEGFR-2 activation. This activity requires the tyrosine phosphatase SHP2, which is recruited to the activated VEGFR-2 when cells are plated on collagen I, but not on vitronectin. Constitutive expression of SHP2(C459S) mutant inhibits the negative role of collagen I on VEGFR-2 phosphorylation. VEGFR-2 undergoes internalisation, which is associated with dynamin II phosphorylation. Expression of SHP2(C459S) impairs receptor internalisation suggesting that SHP2-dependent dephosphorylation regulates this process. These findings demonstrate that collagen I in provisional   extracellular matrix   surrounding nascent capillaries triggers a signaling pathway that negatively regulates angiogenesis. 
 Soluble perlecan domain I enhances vascular endothelial growth factor-165 activity and receptor phosphorylation in human bone marrow endothelial cells. Immobilized recombinant perlecan domain I (PlnDI) binds and modulates the activity of heparin-binding growth factors, in vitro. However, activities for PlnDI, in solution, have not been reported. In this study, we assessed the ability of soluble forms to modulate vascular endothelial growth factor-165 (VEGF165) enhanced capillary tube-like formation, and VEGF receptor-2 phosphorylation of human bone marrow endothelial cells, in vitro.In solution, PlnDI binds VEGF165 in a heparan sulfate and pH dependent manner. Capillary tube-like formation is enhanced by exogenous PlnDI; however, PlnDI/VEGF165 mixtures combine to enhance formation beyond that stimulated by either PlnDI or VEGF165 alone. PlnDI also stimulates VEGF receptor-2 phosphorylation, and mixtures of PlnDI/VEGF165 reduce the time required for peak VEGF receptor-2 phosphorylation (Tyr-951), and increase Akt phosphorylation. PlnDI binds both immobilized neuropilin-1 and VEGF receptor-2, but has a greater affinity for neuropilin-1. PlnDI binding to neuropilin-1, but not to VEGF receptor-2 is dependent upon the heparan sulfate chains adorning PlnDI. Interestingly, the presence of VEGF165 but not VEGF121 significantly enhances PlnDI binding to Neuropilin-1 and VEGF receptor-2.Our observations suggest soluble forms of PlnDI are biologically active. Moreover, PlnDI heparan sulfate chains alone or together with VEGF165 can enhance VEGFR-2 signaling and angiogenic events, in vitro. We propose PlnDI liberated during basement membrane or   extracellular matrix   turnover may have similar activities, in vivo. 
 Neuropilin-1 regulates attachment in human endothelial cells independently of vascular endothelial growth factor receptor-2. Neuropilin-1 (NRP-1) is a type 1 membrane protein that binds the axon guidance factors belonging to the class-3 semaforin family. In endothelial cells, NRP-1 serves as a co-receptor for vascular endothelial growth factor (VEGF) and regulates VEGF receptor 2 (VEGFR-2)-dependent angiogenesis. Although gene-targeting studies documenting embryonic lethality in NRP-1 null mice have demonstrated a critical role for NRP-1 in vascular development, the activities of NRP-1 in mature endothelial cells have been incompletely defined. Using RNA interference-mediated silencing of NRP-1 or VEGFR-2 in primary human endothelial cells, we confirm that NRP-1 modulates VEGFR-2 signaling-dependent mitogenic functions of VEGF. Importantly, we now show that NRP-1 regulates endothelial cell adhesion to   extracellular matrix   proteins independently of VEGFR-2. Based on its dual role as an enhancer of VEGF activity and a mediator of endothelial cell adhesiveness described here, NRP-1 emerges as a promising molecular target for the development of antiangiogenic drugs. 
 Neuropilin-1 expression promotes invasiveness of melanoma cells through vascular endothelial growth factor receptor-2-dependent and -independent mechanisms. The majority of human melanoma cell lines secretes vascular endothelial growth factor-A (VEGF-A) and expresses its receptors VEGFR-1, VEGFR-2 and neuropilin-1 (NRP‑1), a co-receptor for VEGF-A that amplifies the signalling through VEGFR-2. Since it is known that the VEGF-A/VEGFR-2 autocrine loop promotes melanoma cell invasiveness, the aim of the present study was to investigate the involvement of NPR-1 in melanoma progression. Syngeneic human melanoma cell lines expressing either VEGFR-2 or NRP-1, both or none of them, were analyzed for their in vitro ability to migrate, invade th  e extracellular matr  ix (ECM) and secrete active metalloproteinase-2 (MMP-2). The results indicate that NRP-1 cooperates with VEGFR-2 in melanoma cell migration induced by VEGF-A. Moreover, NRP-1 expression is sufficient to promote MMP-2 secretion and melanoma cell invasiveness, as demonstrated by the ability of cells expressing solely NRP-1 to spontaneously invade the ECM. This ability is specifically downregulated by anti-NRP-1 antibodies or by interfering with NRP-1 expression using an shRNA construct. Investigation of the signal transduction pathways triggered by NRP-1 in melanoma cells, indicated that NRP-1-dependent promotion of cell invasiveness involves Akt activation through its phosphorylation on T308. Overall, the results demonstrate that NRP-1 is involved in melanoma progression through VEGFR-2-dependent and -independent mechanisms and suggest NRP-1 as a target for the treatment of the metastatic disease. 
 Plasminogen activator inhibitor-1 inhibits angiogenic signaling by uncoupling vascular endothelial growth factor receptor-2-αVβ3 integrin cross talk. Plasminogen activator inhibitor-1 (PAI-1) regulates angiogenesis via effects o  n extracellular matr  ix proteolysis and cell adhesion. However, no previous study has implicated PAI-1 in controlling vascular endothelial growth factor (VEGF) signaling. We tested the hypothesis that PAI-1 downregulates VEGF receptor-2 (VEGFR-2) activation by inhibiting a vitronectin-dependent cooperative binding interaction between VEGFR-2 and αVβ3.We studied effects of PAI-1 on VEGF signaling in human umbilical vein endothelial cells. PAI-1 inhibited VEGF-induced phosphorylation of VEGFR-2 in human umbilical vein endothelial cells grown on vitronectin, but not on fibronectin or collagen. PAI-1 inhibited the binding of VEGFR-2 to β3 integrin, VEGFR-2 endocytosis, and intracellular signaling pathways downstream of VEGFR-2. The anti-VEGF effect of PAI-1 was mediated by 2 distinct pathways, one requiring binding to vitronectin and another requiring binding to very low-density lipoprotein receptor. PAI-1 inhibited VEGF-induced angiogenesis in vitro and in vivo, and pharmacological inhibition of PAI-1 promoted collateral arteriole development and recovery of hindlimb perfusion after femoral artery interruption.PAI-1 inhibits activation of VEGFR-2 by VEGF by disrupting a vitronectin-dependent proangiogenic binding interaction involving αVβ3 and VEGFR-2. These results broaden our understanding of the roles of PAI-1, vitronectin, and endocytic receptors in regulating VEGFR-2 activation and suggest novel therapeutic strategies for regulating VEGF signaling. 
 Dentin matrix protein 1 induces membrane expression of VE-cadherin on endothelial cells and inhibits VEGF-induced angiogenesis by blocking VEGFR-2 phosphorylation. Dentin matrix protein 1 (DMP1) is a member of the small integrin-binding ligand N-linked glycoprotein (SIBLING) family, a group of proteins initially described as mineralized extracellular matrices components. More recently, SIBLINGs have been implicated in several key steps of cancer progression, including angiogenesis. Although proangiogenic activities have been demonstrated for 2 SIBLINGs, the role of DMP1 in angiogenesis has not yet been addressed. We demonstrate that this   extracellular matrix   protein induced the expression of vascular endothelial cadherin (VE-cadherin), a key regulator of intercellular junctions and contact inhibition of growth of endothelial cells that is also known to modulate vascular endothelial growth factor receptor 2 (VEGFR-2) activity, the major high-affinity receptor for VEGF. DMP1 induced VE-cadherin and p27(Kip1) expression followed by cell-cycle arrest in human umbilical vein endothelial cells (HUVECs) in a CD44-dependent manner. VEGF-induced proliferation, migration, and tubulogenesis responses were specifically blocked on DMP1 pretreatment of HUVECs. Indeed, after VE-cadherin induction, DMP1 inhibited VEGFR-2 phosphorylation and Src-mediated signaling. However, DMP1 did not interfere with basic fibroblast growth factor-induced angiogenesis. In vivo, DMP1 significantly reduced laser-induced choroidal neovascularization lesions and tumor-associated angiogenesis. These data enable us to put DMP1 on the angiogenic chessboard for the first time and to identify this protein as a new specific inhibitor of VEGF-induced angiogenesis. 
 The association of genes involved in the angiogenesis-associated signaling pathway with risk of anterior cruciate ligament rupture. Angiogenesis-associated signaling is a fundamental component in the remodeling of the   extracellular matrix   in response to loading. Genes encoding protein components within this signaling cascade are therefore suitable candidates for investigation into ACL injury susceptibility: namely, vascular endothelial growth factor A isoform (VEGFA), kinase insert-domain receptor (KDR), nerve growth factor (NGF), and hypoxia inducible factor-1α (HIF1A). A case-control genetic association study was conducted on 227 asymptomatic control participants and 227 participants with surgically diagnosed ACL ruptures of which 126 participants reported a non-contact mechanism of rupture. All participants were genotyped for seven polymorphisms within the four genes. The VEGFA rs699947 CC genotype (p=0.010, OR: 1.92, 95% CI: 1.17-3.17) was significantly over-represented within participants with non-contact ACL ruptures. The VEGFA rs1570360 GA genotype was significantly over-represented in the CON group (p=0.007, OR: 1.70, 95% CI: 1.16-2.50). Furthermore, the KDR rs2071559 GA genotype was significantly over-represented in the female controls (p=0.023, OR: 2.16, 95% CI: 1.11-4.22). Inferred haplotype analyses also implicated genomic regions spanning the VEGFA and KDR genes. These novel findings suggest that regions within VEGFA and KDR may be implicated in the pathophysiology of ACL ruptures; highlighting the potential biological significance of angiogenesis-associated signaling in the aetiology of ACL ruptures. 
 MULTIMERIN2 impairs tumor angiogenesis and growth by interfering with VEGF-A/VEGFR2 pathway. MULTIMERIN2 (MMRN2), also known as Endoglyx-1, is an   extracellular matrix   glycoprotein whose function has so far remained elusive. Given its specific localization in tight association with the endothelium we hypothesized that this protein could modulate neo-angiogenesis. By multiple assays we showed that MMRN2 significantly impaired endothelial cell (EC) migration and organization of a functional vessel network. The interaction of ECs with MMRN2 induced a striking impairment of VEGFR1 and VEGFR2 activation. We focused our attention on VEGFR2, a chief regulator of angiogenesis, and clarified that MMRN2 interfered with the VEGF/VEGFR2 axis through a direct binding with VEGF-A. This novel interaction was assessed in several assays and the affinity was estimated (Kd ∼50  nM). We next questioned whether the anti-angiogenic properties of MMRN2 could impair tumor growth. Although overexpression of MMRN2 by HT1080 cells did not affect their growth and apoptotic rate in vitro, it remarkably affected their growth in vivo. In fact, MMRN2-positive cells failed to efficiently grow and form well-vascularized tumors; a similar outcome was observed following treatment of established tumors with a MMRN2 adenoviral construct. Tumor-section immunostaining revealed a strong co-localization of VEGF-A with the ectopically expressed MMRN2. These novel findings suggest that VEGF may be sequestered by MMRN2 and be less available for the engagement to the receptors. Taken together these results highlight MMRN2 as a crucial player in the regulation of EC function, neo-angiogenesis and hence tumor growth. We hypothesize that secreted and deposited MMRN2 may function as a homeostatic barrier halting the sprouting of novel vessels, and suggest that these studies may embody the potential for the development of novel tools for cancer treatment. 
 EMMPRIN promotes angiogenesis through hypoxia-inducible factor-2alpha-mediated regulation of soluble VEGF isoforms and their receptor VEGFR-2.   Extracellular matrix   metalloproteinase inducer (EMMPRIN/CD147) is thought to promote tumor angiogenesis mostly through its protease-inducing function and more recently by its ability to increase tumor cell expression of vascular endothelial growth factor (VEGF). In this study, we present evidence that EMMPRIN can promote angiogenesis by a direct effect on endothelial cells through a paracrine regulation of the VEGF/VEGF-receptor (VEGFR) system. Using human microvascular endothelial cell line-1 endothelial cells, we show that EMMPRIN selectively increased the soluble VEGF isoforms (121 and 165), but not the matrix-bound VEGF 189 form. In addition, EMMPRIN up-regulated the expression of VEGFR-2 without an effect on VEGFR-1. This increase in VEGFR-2 was responsible for the observed EMMPRIN stimulation of the migratory and tube formation capacity of endothelial cells. EMMPRIN's effects, which were matrix metalloproteinase and urokinase-type plasminogen activator independent, were mediated primarily through hypoxia-inducible factor-2alpha expression, also up-regulated by EMMPRIN. VEGFR-2 increase was also observed in vivo in a mouse model of xenograph tumors overexpressing EMMPRIN. These results suggest that in addition to increasing protease production, EMMPRIN may contribute to the formation of a reactive stroma also through the up-regulation of hypoxia-inducible factor-2alpha, VEGFR-2, and the soluble forms of VEGF in endothelial cells, thus directly regulating the angiogenic process. 
  GeneRIFs  
 NRP-1 modulates VEGFR-2 signaling-dependent mitogenic functions of VEGF and regulates endothelial cell adhesion to   extracellular matrix   proteins independently of VEGFR-2. 
  [Ii]nvasive  PubMed  
 Role of the VEGF ligand to receptor ratio in the progression of mismatch repair-proficient colorectal cancer. The VEGF family of ligands and receptors are intimately involved in tumor angiogenesis, lymphangiogenesis and metastasis. The evaluation of VEGF ligand/receptor ratios may provide a more profound understanding of the involvement of these proteins in colorectal tumour progression. The aim of this study was to elucidate the role of the VEGF ligand/receptor ratios on tumour progression and metastasis in patients with mismatch repair-proficient colorectal cancer.Immunohistochemistry for VEGF-A, VEGF-B, VEGF-C, VEGF-D, VEGFR1, VEGFR2 and VEGF3 was carried out on 387 mismatch repair-proficient colorectal cancers using a tissue microarray. Evaluation of immunoreactivity was performed semi-quantitatively and the ligand/receptor expression ratio was obtained.An increased VEGF-A/VEGFR1 ratio, VEGF-A and VEGFR1 was linked to the presence of peritumoral lymphocytic inflammation at the   invasive   front (p = 0.032; p = 0.005; p = 0.032, respectively). VEGFR1 expression was related to poorer outcome in multivariable analysis with pT stage, pN stage, vascular invasion, and post-operative therapy. A higher ratio of VEGF-A/VEGFR2 was linked to advanced TNM stage (p = 0.005) while VEGF-A and VEGFR2 were elevated in tumours with an infiltrating tumour growth pattern (p = 0.006; p = 0.014; p = 0.006). No effect of VEGF-A/VEGFR2, VEGF-A or VEGFR2 on survival time was noted.Our findings highlight an involvement of VEGF-A, VEGR1 and VEGFR2 in events occurring at the   invasive   tumour front and a potential prognostic role of VEGFR1 expression in mismatch repair-proficient colorectal cancers. The VEGF-A ligand to VEGFR1 or VEGFR2 ratio may represent an alternative evaluation system for identifying patients with poorer clinical outcome. 
 Association of polymorphisms of angiogenesis genes with breast cancer. Few studies have systematically explored a pathway approach: to test the association of multiple polymorphisms from multiple genes important to angiogenesis simultaneously with risk of breast cancer. We report our preliminary data evaluating the association of polymorphisms from seven genes known to influence angiogenesis with the likelihood of having breast cancer.We recruited 715 controls and 520 subjects with breast cancer. Subjects provided a blood specimen and completed a questionnaire that included common breast cancer risk factors and breast cancer status. We evaluated candidate polymorphisms in the following genes: Hypoxia Inducible Factor-1 alpha (HIF1alpha), Vascular Endothelial Growth Factor (VEGF), VEGF Receptor 1 (VEGFR-1), VEGFR-2, endothelial Nitric Oxide Synthase (eNOS), Neuropilin-1 (NRP-1) and Neuropilin-2 (NRP-2). Testing for associations between each polymorphism and the presence or absence of breast cancer was performed.VEGF-2578 AA and -1498 CC genotypes were more common in cancer cases than controls (P = 0.06 and P = 0.04, respectively). These two genotypes remained significant predictors of breast cancer status after adjusting for non-genetic risk factors estimated by the Gail model (P = 0.03 and P = 0.03, respectively). When comparing women with   invasive   versus pre-  invasive   breast cancer, the eNOS-786 TT and eNOS 894 GG genotypes were associated with a greater likelihood of   invasive   disease and the eNOS 894 GG genotype was associated with a greater likelihood of having metastatic disease.There is an association of the VEGF-2578A and -1498C alleles with increased breast cancer risk. This association remains significant when adjusted for Gail score-related risk factors. 
 Metastatic properties of prostate cancer cells are controlled by VEGF. Mechanisms of metastasis, the major complication of prostate cancer, are poorly understood. In this study, we define molecular mechanisms that may contribute to the highly   invasive   potential of prostate cancer cells. Vascular endothelial growth factor (VEGF), its receptors (VEGFRs), and alpha5beta1 integrin were expressed by prostate cancer cells in vitro and by prostate tumors in vivo, and their expression was elevated at sites of bone metastasis compared to original prostate tumor. VEGF, through interaction with its receptors, regulated adhesive and migratory properties of the cancer cells. Specifically, the highly metastatic prostate cancer cell subline LNCaP-C4-2 showed a decreased adhesive but an enhanced migratory response to fibronectin, a ligand for alpha5beta1 integrin, compared to its nonmetastatic counterpart. A similar pattern was also observed when bone sialoprotein was used as a ligand in migration assays. Increased migration of metastatic prostate cancer cells to fibronectin and bone sialoprotein was regulated by VEGF via VEGFR-2. Tumor suppressor PTEN was involved in control of VEGF/VEGFR-2 stimulated prostate cancer cell adhesion as well as proliferation. 
 Expression of VEGF and its receptors VEGFR1/VEGFR2 is associated with   invasive  ness of bladder cancer. Vascular endothelial growth factor (VEGF) signaling is frequently altered in   invasive   tumor cells and is associated with patient outcome. In the present study, we examined VEGF, VEGFR1, and VEGFR2 expression in non-muscle   invasive   bladder cancer (NMIBC) and muscle   invasive   bladder cancer (MIBC), and evaluated the association between VEGF and its receptors with disease characteristics and bladder cancer recurrence.Tissue microarrays containing bladder cancer specimens (n=212) and adjacent normal bladder mucosa (n=131) were immunostained using antibodies against VEGF, VEGFR1, and VEGFR2. The association between the expression of these proteins and clinical parameters including stage, lymph node metastasis, and recurrence-free survival were statistically evaluated. VEGF mRNA expression data were extracted from the public Oncomine database.VEGF and VEGFR1 mRNA levels were significantly higher in bladder cancer specimens than that of normal mucosa (for VEGF, p<0.001; for VEGFR1, p=0.02). Analysis of their expression at protein levels showed that levels of VEGF and VEGFR1 were significantly higher in NMIBC than in MIBC (p<0.001), while that of VEGFR2 was significantly higher in all cancer specimens compared to benign urothelial mucosa (p=0.001). Further-more, the expression of VEGFR2 was significantly higher in MIBC, as compared to NMIBC (p<0.001). Patients with higher levels of VEGF, VEGFR1, and VEGFR2 tended to have poorer recurrence-free survival than those with lower levels, but this was not statistically significant.Our results suggest that alterations in the expression of VEGF and VEGF receptors are associated with disease stage and recurrence. 
 Expression of vascular endothelial growth factor and vascular endothelial growth factor receptors 1 and 2 in   invasive   breast carcinoma: prognostic significance and relationship with markers for aggressiveness. Vascular endothelial growth factor (VEGF), VEGF receptor 1 (VEGFR-1) and VEGF receptor 2 (VEGFR-2) play a role in breast cancer growth and angiogenesis. We examined the expression and relationship with clinical outcome and other prognostic factors.Tumour sections from 468 breast cancer patients were immunostained for VEGF, VEGFR-1, and VEGFR-2, and their relationships with tumour vascularity, disseminated tumour cells (DTCs) in bone marrow and other clinicopathological parameters were evaluated. VEGF, VEGFR-1 and VEGFR-2 immunoreactivities were observed in   invasive   breast carcinoma cells. VEGF expression was significantly associated with VEGFR-1 and VEGFR-2 expression (P < 0.001). High-level cytoplasmic expression of VEGFR-1 was associated with significantly reduced distant disease-free survival (DDFS) (P = 0.017, log-rank) and breast cancer-specific survival (BCSS) (P = 0.005, log-rank) for all patients, and for node-negative patients without systemic treatment (DDFS, P = 0.03, log-rank; BCSS, P = 0.009, log-rank). VEGFR-1 expression was significantly associated with histopathological markers of aggressiveness (P < 0.05). Significantly reduced survival was observed in DTC-positive patients as compared with DTC-negative patients in the combined moderate/high VEGFR-1 group (P < 0.001 for DDFS and BCSS), and the same was true for DDFS in the moderate VEGFR-2 group (P = 0.006).High-level expression of VEGFR-1 indicates reduced survival. Higher-level expression of VEGFR-1 or VEGFR-2 in primary breast carcinomas combined with the presence of DTC selects a prognostically unfavourable patient group. 
 GEP100-Arf6-AMAP1-cortactin pathway frequently used in cancer invasion is activated by VEGFR2 to promote angiogenesis. Angiogenesis and cancer   invasive  ness greatly contribute to cancer malignancy.Arf6 and its effector, AMAP1, are frequently overexpressed in breast cancer, and constitute a central pathway to induce the invasion and metastasis. In this pathway, Arf6 is activated by EGFR via GEP100. Arf6 is highly expressed also in human umbilical vein endothelial cells (HUVECs) and is implicated in angiogenesis. Here, we found that HUVECs also highly express AMAP1, and that vascular endothelial growth factor receptor-2 (VEGFR2) recruits GEP100 to activate Arf6. AMAP1 functions by binding to cortactin in cancer invasion and metastasis. We demonstrate that the same GEP100-Arf6-AMAP1-cortactin pathway is essential for angiogenesis activities, including cell migration and tubular formation, as well as for the enhancement of cell permeability and VE-cadherin endocytosis of VEGF-stimulated HUVECs. Components of this pathway are highly expressed in pathologic angiogenesis, and blocking of this pathway effectively inhibits VEGF- or tumor-induced angiogenesis and choroidal neovascularization. The GEP100-Arf6-AMAP1-cortactin pathway, activated by receptor tyrosine kinases, appears to be common in angiogenesis and cancer invasion and metastasis, and provides their new therapeutic targets. 
 Expression of VEGF and its receptors VEGFR1/VEGFR2 is associated with   invasive  ness of bladder cancer. Vascular endothelial growth factor (VEGF) signaling is frequently altered in   invasive   tumor cells and is associated with patient outcome. In the present study, we examined VEGF, VEGFR1, and VEGFR2 expression in non-muscle   invasive   bladder cancer (NMIBC) and muscle   invasive   bladder cancer (MIBC), and evaluated the association between VEGF and its receptors with disease characteristics and bladder cancer recurrence.Tissue microarrays containing bladder cancer specimens (n=212) and adjacent normal bladder mucosa (n=131) were immunostained using antibodies against VEGF, VEGFR1, and VEGFR2. The association between the expression of these proteins and clinical parameters including stage, lymph node metastasis, and recurrence-free survival were statistically evaluated. VEGF mRNA expression data were extracted from the public Oncomine database.VEGF and VEGFR1 mRNA levels were significantly higher in bladder cancer specimens than that of normal mucosa (for VEGF, p<0.001; for VEGFR1, p=0.02). Analysis of their expression at protein levels showed that levels of VEGF and VEGFR1 were significantly higher in NMIBC than in MIBC (p<0.001), while that of VEGFR2 was significantly higher in all cancer specimens compared to benign urothelial mucosa (p=0.001). Further-more, the expression of VEGFR2 was significantly higher in MIBC, as compared to NMIBC (p<0.001). Patients with higher levels of VEGF, VEGFR1, and VEGFR2 tended to have poorer recurrence-free survival than those with lower levels, but this was not statistically significant.Our results suggest that alterations in the expression of VEGF and VEGF receptors are associated with disease stage and recurrence. 
 Expression of vascular endothelial growth factor and vascular endothelial growth factor receptors 1 and 2 in   invasive   breast carcinoma: prognostic significance and relationship with markers for aggressiveness. Vascular endothelial growth factor (VEGF), VEGF receptor 1 (VEGFR-1) and VEGF receptor 2 (VEGFR-2) play a role in breast cancer growth and angiogenesis. We examined the expression and relationship with clinical outcome and other prognostic factors.Tumour sections from 468 breast cancer patients were immunostained for VEGF, VEGFR-1, and VEGFR-2, and their relationships with tumour vascularity, disseminated tumour cells (DTCs) in bone marrow and other clinicopathological parameters were evaluated. VEGF, VEGFR-1 and VEGFR-2 immunoreactivities were observed in   invasive   breast carcinoma cells. VEGF expression was significantly associated with VEGFR-1 and VEGFR-2 expression (P < 0.001). High-level cytoplasmic expression of VEGFR-1 was associated with significantly reduced distant disease-free survival (DDFS) (P = 0.017, log-rank) and breast cancer-specific survival (BCSS) (P = 0.005, log-rank) for all patients, and for node-negative patients without systemic treatment (DDFS, P = 0.03, log-rank; BCSS, P = 0.009, log-rank). VEGFR-1 expression was significantly associated with histopathological markers of aggressiveness (P < 0.05). Significantly reduced survival was observed in DTC-positive patients as compared with DTC-negative patients in the combined moderate/high VEGFR-1 group (P < 0.001 for DDFS and BCSS), and the same was true for DDFS in the moderate VEGFR-2 group (P = 0.006).High-level expression of VEGFR-1 indicates reduced survival. Higher-level expression of VEGFR-1 or VEGFR-2 in primary breast carcinomas combined with the presence of DTC selects a prognostically unfavourable patient group. 
 GEP100-Arf6-AMAP1-cortactin pathway frequently used in cancer invasion is activated by VEGFR2 to promote angiogenesis. Angiogenesis and cancer   invasive  ness greatly contribute to cancer malignancy.Arf6 and its effector, AMAP1, are frequently overexpressed in breast cancer, and constitute a central pathway to induce the invasion and metastasis. In this pathway, Arf6 is activated by EGFR via GEP100. Arf6 is highly expressed also in human umbilical vein endothelial cells (HUVECs) and is implicated in angiogenesis. Here, we found that HUVECs also highly express AMAP1, and that vascular endothelial growth factor receptor-2 (VEGFR2) recruits GEP100 to activate Arf6. AMAP1 functions by binding to cortactin in cancer invasion and metastasis. We demonstrate that the same GEP100-Arf6-AMAP1-cortactin pathway is essential for angiogenesis activities, including cell migration and tubular formation, as well as for the enhancement of cell permeability and VE-cadherin endocytosis of VEGF-stimulated HUVECs. Components of this pathway are highly expressed in pathologic angiogenesis, and blocking of this pathway effectively inhibits VEGF- or tumor-induced angiogenesis and choroidal neovascularization. The GEP100-Arf6-AMAP1-cortactin pathway, activated by receptor tyrosine kinases, appears to be common in angiogenesis and cancer invasion and metastasis, and provides their new therapeutic targets. 
 Role of the VEGF ligand to receptor ratio in the progression of mismatch repair-proficient colorectal cancer. The VEGF family of ligands and receptors are intimately involved in tumor angiogenesis, lymphangiogenesis and metastasis. The evaluation of VEGF ligand/receptor ratios may provide a more profound understanding of the involvement of these proteins in colorectal tumour progression. The aim of this study was to elucidate the role of the VEGF ligand/receptor ratios on tumour progression and metastasis in patients with mismatch repair-proficient colorectal cancer.Immunohistochemistry for VEGF-A, VEGF-B, VEGF-C, VEGF-D, VEGFR1, VEGFR2 and VEGF3 was carried out on 387 mismatch repair-proficient colorectal cancers using a tissue microarray. Evaluation of immunoreactivity was performed semi-quantitatively and the ligand/receptor expression ratio was obtained.An increased VEGF-A/VEGFR1 ratio, VEGF-A and VEGFR1 was linked to the presence of peritumoral lymphocytic inflammation at the   invasive   front (p = 0.032; p = 0.005; p = 0.032, respectively). VEGFR1 expression was related to poorer outcome in multivariable analysis with pT stage, pN stage, vascular invasion, and post-operative therapy. A higher ratio of VEGF-A/VEGFR2 was linked to advanced TNM stage (p = 0.005) while VEGF-A and VEGFR2 were elevated in tumours with an infiltrating tumour growth pattern (p = 0.006; p = 0.014; p = 0.006). No effect of VEGF-A/VEGFR2, VEGF-A or VEGFR2 on survival time was noted.Our findings highlight an involvement of VEGF-A, VEGR1 and VEGFR2 in events occurring at the   invasive   tumour front and a potential prognostic role of VEGFR1 expression in mismatch repair-proficient colorectal cancers. The VEGF-A ligand to VEGFR1 or VEGFR2 ratio may represent an alternative evaluation system for identifying patients with poorer clinical outcome. 
 Association of polymorphisms of angiogenesis genes with breast cancer. Few studies have systematically explored a pathway approach: to test the association of multiple polymorphisms from multiple genes important to angiogenesis simultaneously with risk of breast cancer. We report our preliminary data evaluating the association of polymorphisms from seven genes known to influence angiogenesis with the likelihood of having breast cancer.We recruited 715 controls and 520 subjects with breast cancer. Subjects provided a blood specimen and completed a questionnaire that included common breast cancer risk factors and breast cancer status. We evaluated candidate polymorphisms in the following genes: Hypoxia Inducible Factor-1 alpha (HIF1alpha), Vascular Endothelial Growth Factor (VEGF), VEGF Receptor 1 (VEGFR-1), VEGFR-2, endothelial Nitric Oxide Synthase (eNOS), Neuropilin-1 (NRP-1) and Neuropilin-2 (NRP-2). Testing for associations between each polymorphism and the presence or absence of breast cancer was performed.VEGF-2578 AA and -1498 CC genotypes were more common in cancer cases than controls (P = 0.06 and P = 0.04, respectively). These two genotypes remained significant predictors of breast cancer status after adjusting for non-genetic risk factors estimated by the Gail model (P = 0.03 and P = 0.03, respectively). When comparing women with   invasive   versus pre-  invasive   breast cancer, the eNOS-786 TT and eNOS 894 GG genotypes were associated with a greater likelihood of   invasive   disease and the eNOS 894 GG genotype was associated with a greater likelihood of having metastatic disease.There is an association of the VEGF-2578A and -1498C alleles with increased breast cancer risk. This association remains significant when adjusted for Gail score-related risk factors. 
 Metastatic properties of prostate cancer cells are controlled by VEGF. Mechanisms of metastasis, the major complication of prostate cancer, are poorly understood. In this study, we define molecular mechanisms that may contribute to the highly   invasive   potential of prostate cancer cells. Vascular endothelial growth factor (VEGF), its receptors (VEGFRs), and alpha5beta1 integrin were expressed by prostate cancer cells in vitro and by prostate tumors in vivo, and their expression was elevated at sites of bone metastasis compared to original prostate tumor. VEGF, through interaction with its receptors, regulated adhesive and migratory properties of the cancer cells. Specifically, the highly metastatic prostate cancer cell subline LNCaP-C4-2 showed a decreased adhesive but an enhanced migratory response to fibronectin, a ligand for alpha5beta1 integrin, compared to its nonmetastatic counterpart. A similar pattern was also observed when bone sialoprotein was used as a ligand in migration assays. Increased migration of metastatic prostate cancer cells to fibronectin and bone sialoprotein was regulated by VEGF via VEGFR-2. Tumor suppressor PTEN was involved in control of VEGF/VEGFR-2 stimulated prostate cancer cell adhesion as well as proliferation. 
 Peroxiredoxin 2 is involved in vasculogenic mimicry formation by targeting VEGFR2 activation in colorectal cancer. The mammalian peroxiredoxin 2 (Prdx2) is a member of thiol-dependent antioxidant proteins and plays an important role in the progression of colorectal cancer (CRC). The aim of this study was to confirm the role of Prdx2 in formation of VM and progression of CRC. Immunohistochemistry and CD34/periodic acid Schiff double staining were performed to explore the expression of Prdx2 and VM formation in 70 CRC tissues, and there was a positive correlation between Prdx2 expression and VM formation by the Pearson correlation coefficient (r = 0.282, p < 0.05). Prdx2 was suppressed in poorly differentiated HCT116 cells by Prdx2-siRNA-LV transduction. The expression of Prdx2 at both mRNA and protein levels in HCT116 cells transfected with the Prdx2 siRNA was significantly lower than that of negative control siRNA as confirmed by quantitative real-time PCR and Western blotting analysis, respectively (p < 0.05). The well-established in vitro 3D culture model was chosen to investigate the VM formation of HCT116 cells. The numbers of the tubular structures were significantly fewer in Prdx2 siRNA explants than those of negative control siRNA explants after VEGF induction (p < 0.05). Although VEGFR2 expressions had no change after VEGF induction, we found that VEGFR2 phosphorylation levels were markedly reduced in cells of siPrdx2 over time compared with those of negative control siRNA by Western blotting analysis (p < 0.05, p < 0.01). The effects of Prdx2 s  iRNA on   the invasive capabilities of HCT116 cells with VEGF induction were examined by using Matrigel invasion chamber   assay.   The invasive capabilities of HCT116 cells were significantly declined in Prdx2 siRNA explants than those of negative control siRNA explants (p < 0.05). The effects of Prdx2 siRNA on pathological tumor growth were examined by using a tumor xenograft model in vivo. After implant of HCT116 cells that transduced with Prdx2 siRNA and negative control siRNA as xenografts into nude mice, the growth of xenograft tumors with Prdx2 siRNA was much slower than that of negative control siRNA, and the volumes of tumor xenografts with Prdx2 siRNA were smaller than those of negative control siRNA after 5 weeks (p < 0.05). Further conclusion showed that Prdx2 regulates VM formation by targeting VEGFR2 activation, which now represents as a therapeutic target for RC. 
 Expression of vascular endothelial growth factor, receptor KDR and p53 protein in transitional cell carcinoma of the bladder. To evaluate the expression profile of vascular endothelial growth factor (VEGF), kinase-insert-domain-containing receptor (KDR) and p53 in patients from Northeastern China with transitional cell carcinoma (TCC) of bladder.One hundred and twelve patients with bladder transitional cell carcinoma were involved in this study. The expression of VEGF, KDR and p53 were evaluated by immunohistochemical staining on paraffin-embedded slides. The correlations between the biomarkers with clinical pathological characteristics were assessed; the co-expression of VEGF and KDR in tumor cells as well as the correlation between p53 and VEGF/KDR expression were analyzed.Expression of VEGF, KDR and p53 was observed in 69.6, 55.4 and 48.2% of TCC patients, respectively. VEGF, KDR and p53 expressions were found to be higher in the   invasive   bladder tumors than in the superficial tumors, and KDR and p53 expression were significantly associated with higher tumor grade. Co-expression of VEGF and KDR was found in 51 patients, and VEGF expression was associated with KDR expression. Positive correlations were found between p53 protein and VEGF/KDR expression.Our results suggest that VEGF, KDR and p53 might be valuable biomarkers in prognosis of TCC patients. Co-expression of VEGF and KDR implied an autocrine signaling of VEGF in tumor cells, and the results of immunohistochemical staining supported the role of p53 gene in the process of angiogenesis. 
 The relevance of cell type- and tumor zone-specific VEGFR-2 activation in locally advanced colon cancer. For the successful therapeutic use of inhibitors of the vascular endothelial growth factor receptor (VEGFR) pathway detailed knowledge of the mechanisms leading to tumor progression is indispensable. The main goal of this study was to determine the relevance of the VEGFR-2 activating pathway for colon carcinoma (CC) metastasis. The initial event is ligand-induced receptor activation through tyrosine autophosphorylation.VEGFR-2, its ligands VEGF-C and VEGF-D and the phosphorylated (activated) receptor forms pVEGFR-2(Tyr1175) and pVEGFR-2(Tyr1214) were investigated immunohistochemically in different tumor compartments (intratumoral (zone 1) -   invasive   front (zone 2) - extratumoral soft tissue (zone 3)) and various cell types (tumor cells, inflammatory cells, macro- and microvasculature) in 84 non-metastatic, lymphogenous-metastatic and haematogenous-metastatic CC.VEGF-D produced by tumor cells has an autocrine affinity for its receptor VEGFR-2. In tumor budding regions VEGF-D-induced receptor activation by autophosphorylation at Tyr1214 seems to be a possible initial event of the VEGFR-2-mediated signaling pathway, but without effect on metastatic behaviour. In inflammatory cells of almost all CC VEGFR-2 phosphorylation at Tyr 1175 and Tyr 1214 was detectable without accompanying receptor expression, suggesting receptor activation without cell surface expression. Peritumoral inflammatory cells also expressed paracrine acting VEGF-C. The autocrine VEGF-D/VEGFR-2 signaling axis and receptor autophosphorylation at Tyr1214 appear to be main events for capillaries in all three tumor zones and for small vessels in zone 1 and 2. Independent of the metastatic status a large number of cases with capillary immunopositivity in the angiogenically active   invasive   front was documented, especially for VEGF-D, VEGFR-2 and pVEGFR-2(Tyr1214). VEGFR-2 positive extratumoral capillaries were significantly more common in distant metastatic CC. In all tumor compartments the investigated biomolecules were also detected in different frequencies in the macrovasculature, which is responsible for sufficient tumor vascularization. In addition, vascular paracrine-acting VEGF-C production was widely detected, but without zone and vessel-type dependence.The VEGFR-2 activating pathway is closely involved in tumor cell-associated, vessel-mediated and immuno-inflammatory processes in colon carcinoma and appears to contribute to tumor survival and growth as well as maintenance of the infiltrative phenotype rather than to promote metastasis. 
 VEGF inhibits tumor cell invasion and mesenchymal transition through a MET/VEGFR2 complex. Inhibition of VEGF signaling leads to a pro  invasive   phenotype in mouse models of glioblastoma multiforme (GBM) and in a subset of GBM patients treated with bevacizumab. Here, we demonstrate that vascular endothelial growth factor (VEGF) directly and negatively regulates tumor cell invasion through enhanced recruitment of the protein tyrosine phosphatase 1B (PTP1B) to a MET/VEGFR2 heterocomplex, thereby suppressing HGF-dependent MET phosphorylation and tumor cell migration. Consequently, VEGF blockade restores and increases MET activity in GBM cells in a hypoxia-independent manner, while inducing a program reminiscent of epithelial-to-mesenchymal transition highlighted by a T-cadherin to N-cadherin switch and enhanced mesenchymal features. Inhibition of MET in GBM mouse models blocks mesenchymal transition and invasion provoked by VEGF ablation, resulting in substantial survival benefit. 
 Neuropilin-1 expression promotes   invasive  ness of melanoma cells through vascular endothelial growth factor receptor-2-dependent and -independent mechanisms. The majority of human melanoma cell lines secretes vascular endothelial growth factor-A (VEGF-A) and expresses its receptors VEGFR-1, VEGFR-2 and neuropilin-1 (NRP‑1), a co-receptor for VEGF-A that amplifies the signalling through VEGFR-2. Since it is known that the VEGF-A/VEGFR-2 autocrine loop promotes melanoma cel  l invasi  veness, the aim of the present study was to investigate the involvement of NPR-1 in melanoma progression. Syngeneic human melanoma cell lines expressing either VEGFR-2 or NRP-1, both or none of them, were analyzed for their in vitro ability to migrate, invade the extracellular matrix (ECM) and secrete active metalloproteinase-2 (MMP-2). The results indicate that NRP-1 cooperates with VEGFR-2 in melanoma cell migration induced by VEGF-A. Moreover, NRP-1 expression is sufficient to promote MMP-2 secretion and melanoma cel  l invasi  veness, as demonstrated by the ability of cells expressing solely NRP-1 to spontaneously invade the ECM. This ability is specifically downregulated by anti-NRP-1 antibodies or by interfering with NRP-1 expression using an shRNA construct. Investigation of the signal transduction pathways triggered by NRP-1 in melanoma cells, indicated that NRP-1-dependent promotion of cel  l invasi  veness involves Akt activation through its phosphorylation on T308. Overall, the results demonstrate that NRP-1 is involved in melanoma progression through VEGFR-2-dependent and -independent mechanisms and suggest NRP-1 as a target for the treatment of the metastatic disease. 
 Functional significance of VEGFR-2 on ovarian cancer cells. Vascular endothelial growth factor receptor (VEGFR) has recently been discovered on ovarian cancer cells, but its functional significance is unknown and is the focus of this study. By protein analysis, A2780-par and HeyA8 ovarian cancer cell lines expressed VEGFR-1 and HeyA8 A2774, and SKOV3ip1 expressed VEGFR-2. By in situ hybridization (ISH), 85% of human ovarian cancer specimens showed moderate to high VEGFR-2 expression, whereas only 15% showed moderate to high VEGFR-1 expression. By immunofluorescence, little or no VEGFR-2 was detected in normal ovarian surface epithelial cells, whereas expression was detected in 75% of   invasive   ovarian cancer specimens. To differentiate between the effects of tumor versus host expression of VEGFR, nude mice were injected with SKOV3ip1 cells and treated with either human VEGFR-2 specific antibody (1121B), murine VEGFR-2 specific antibody (DC101) or the combination. Treatment with 1121B reduced SKOV3ip1 cell migration by 68% (p < 0.01) and invasion by 72% (p < 0.01), but exposure to VEGFR-1 antibody had no effect. Treatment with 1121B effectively blocked VEGF-induced phosphorylation of p130Cas. In vivo treatment with either DC101 or 1121B significantly reduced tumor growth alone and in combination in the SKOV3ip1 and A2774 models. Decreased tumor burden after treatment with DC101 or 1121B correlated with increased tumor cell apoptosis, decreased proliferative index, and decreased microvessel density. These effects were significantly greater in the combination group (p < 0.001). We show functionally active VEGFR-2 is present on most ovarian cancer cells. The observed anti-tumor activity of VEGF-targeted therapies may be mediated by both anti-angiogenic and direct anti-tumor effects. 
 Glioma cell VEGFR-2 confers resistance to chemotherapeutic and antiangiogenic treatments in PTEN-deficient glioblastoma. Loss of the tumor suppressor phosphatase and tensin homolog deleted on chromosome 10 (PTEN) is a prerequisite for tumor cell-specific expression of vascular endothelial growth factor receptor (VEGFR)-2 in glioblastoma defining a subgroup prone to develop evasive resistance towards antiangiogenic treatments. Immunohistochemical analysis of human tumor tissues showed VEGFR-2 expression in glioma cells in 19% of specimens examined, mainly in the infiltration zone. Glioma cell VEGFR-2 positivity was restricted to PTEN-deficient tumor specimens. PTEN overexpression reduced VEGFR-2 expression in vitro, as well as knock-down of raptor or rictor. Genetic interference with VEGFR-2 revealed proproliferative, anti  invasive   and chemoprotective functions for VEGFR-2 in glioma cells. VEGFR-2-dependent cellular effects were concomitant with activation of 'kappa-light-chain-enhancer' of activated B-cells, protein kinase B, and N-myc downstream regulated gene 1. Two-photon in vivo microscopy revealed that expression of VEGFR-2 in glioma cells hampers antiangiogenesis. Bevacizumab induces a pro  invasive   response in VEGFR-2-positive glioma cells. Patients with PTEN-negative glioblastomas had a shorter survival after initiation of bevacizumab therapy compared with PTEN-positive glioblastomas. Conclusively, expression of VEGFR-2 in glioma cells indicates an aggressive glioblastoma subgroup developing early resistance to temozolomide or bevacizumab. Loss of PTEN may serve as a biomarker identifying those tumors upfront by routine neuropathological methods. 
 Dual inhibition using cabozantinib overcomes HGF/MET signaling mediated resistance to pan-VEGFR inhibition in orthotopic and metastatic neuroblastoma tumors. MET is expressed on neuroblastoma cells and may trigger tumor growth, neoangiogenesis and metastasis. MET upregulation further represents an escape mechanism to various anticancer treatments including VEGF signaling inhibitors. We developed in vitro a resistance model to pan-VEGFR inhibition and explored the simultaneous inhibition of VEGFR and MET in neuroblastoma models in vitro and in vivo using cabozantinib, an inhibitor of the tyrosine kinases including VEGFR2, MET, AXL and RET. Resistance in IGR-N91-Luc neuroblastoma cells under continuous in vitro exposure pressure to VEGFR1-3 inhibition using axitinib was associated with HGF and p-ERK overexpression. Cabozantinib exhibited anti-proliferative effects in neuroblastoma cells and reduced cell migration in vitro as measured by phase-contrast with IncuCyte system. In vivo, an enhanced number of animals with IGR-N91-Luc metastases was noted following axitinib treatment as compared to control animals. Orally administered cabozantinib per gavage at 30 and 60 mg/kg/day significantly inhibited tumor growth of orthotopic adrenal IGR-N91-Luc and metastatic IMR-32-Luc xenografts. Antitumor activity was associated with decreased vascularization, inhibition of p-SRC and induction of apoptotic cell death. Activation of the HGF-mediated MET pathway is involved in escape to selective VEGFR inhibition in neuroblastoma suggesting combined inhibition of MET and VEGFR signaling to reduce secondary resistance and enh  anced in  vasiveness. 
 In situ validation of VEGFR-2 and α v ß 3 integrin as targets for breast lesion characterization. Vascular endothelial growth factor receptor 2 (VEGFR-2) and α v ß 3 integrin are the most frequently addressed targets in molecular imaging of tumor angiogenesis. In preclinical studies, molecular imaging of angiogenesis has shown potential to detect and differentiate benign and malignant lesions of the breast. Thus, in this retrospective clinical study employing patient tissues, the diagnostic value of VEGFR-2, α v ß 3 integrin and vascular area fraction for the diagnosis and differentiation of breast neoplasia was evaluated. To this end, tissue sections of breast cancer (n = 4  0), pre-  invasive ductal carcinoma in situ (DCIS; n = 8), fibroadenoma (n = 40), radial scar (n = 6) and normal breast tissue (n = 40) were used to quantify (1) endothelial VEGFR-2, (2) endothelial α v ß 3 integrin and (3) total α v ß 3 integrin expression, as well as (4) the vascular area fraction. Sensitivity and specificity to differentiate benign from malignant lesions were calculated for each marker by receiver operating characteristics (ROC) analyses. Whereas vessel density, as commonly used, did not significantly differ between benign and malignant lesions (AUROC: 0.54), VEGFR-2 and α v ß 3 integrin levels were gradually up-regulated in carcinoma versus fibroadenoma versus healthy tissue. The highest diagnostic accuracy for differentiating carcinoma from fibroadenoma was found for total α v ß 3 integrin expression (AUROC: 0.76), followed by VEGFR-2 (AUROC: 0.71) and endothelial α v ß 3 integrin expression (AUROC: 0.68). In conclusion, total α v ß 3 integrin expression is the best discriminator between breast cancer, fibroadenoma and normal breast tissue. With respect to vascular targeting and molecular imaging of angiogenesis, endothelial VEGFR-2 appeared to be slightly superior to endothelial α v ß 3 for differentiating benign from cancerous lesions. 
 VEGFR2 expression and relationship between tumor neovascularization and histologic characteristics in oral squamous cell carcinoma. Neovascularization in the stroma of a tumor plays an important role in tumor growth and the establishment of metastases. The present study examined the immunohistochemical expression of vascular endothelial growth factor receptor 2 (VEGFR2) in 34 cases of oral squamous cell carcinoma (OSCC). Moreover, the relationships between parameters of tumor neovascularization (count of VEGFR2-positive [+] cells and total size of vessel lumen [TSVL]) and those of histology (differentiation type and mode of invasion) were analyzed statistically. Immunohistochemical expression of VEGFR2 was localized in stromal cells at the tumor   invasive   front. The VEGFR2+ cell count around poorly differentiated tumors was significantly higher than that around well differentiated tumors (P = 0.032, one-way ANOVA). The TSVL around the well differentiated type was found to be significantly larger than that around the poorly or moderately differentiated type (P < 0.001, respectively; one-way ANOVA). With regard to the mode of invasion, the TSVL was significantly larger for lower-grade (Grades 1+2) than for higher-grade (Grades 3+4) tumors (P < 0.001, unpaired t-test). On the basis of our results, we suggest that vascular development at the   invasive   front of OSCC is governed by the following factors: the tumor cells themselves may induce hemangiogenesis in the adjoining stromal tissue; hemangiogenic activity is higher when parenchymal intercellular adhesion is looser and when the parenchymal area exposed to the stroma is greater; and the rate of blood flow is higher when parenchymal intercellular adhesion is tighter and parenchymal nests are larger. 
 Comparison of tyrosine kinase receptors HER2, EGFR, and VEGFR expression in micropapillary urothelial carcinoma with   invasive   urothelial carcinoma.   Invasive   micropapillary urothelial carcinomas (MPUC) emerge at higher stages and follow a more aggressive course than conventional   invasive   urothelial carcinomas (UC). Little is known about the target therapies using tyrosine kinase inhibitors in MPUC. This study is to investigate potential effectiveness of tyrosine kinase receptor inhibitors by determining expression of epidermal growth factor receptor (EGFR), human epidermal receptor 2 (HER2), and vascular endothelial growth factor receptor 2 (VEGFR) proteins in MPUC and UC. 16 cases of MPUC and 16 stage-matched UC were identified. Immunohistochemistry for EGFR, HER2, and VEGFR2 and HER2 gene amplification by fluorescence in situ hybridization (FISH) were performed. HER2 and EGFR proteins were expressed in MPUC and UC, with significantly higher HER2 expression in MPUC (ratio 1.82, p < 0.01). HER2 gene amplification was identified in 4 of 16 MPUC (25 %). Amplification was limited to cases with 3+ HER2 expression (100% concordance). EGFR expression in MPUC was slightly higher than UC but not statistically significant (ratio 1.57, p = 0.19). EGFR and HER2 coexpression was noted in 75% of MPUC and 37.5% of UC. No VEGFR expression was identified in the urothelium. Strong VEGFR expression was noted in stromal vessels in both MPUC and UC. In conclusion, EGFR and HER2 are potential targets for neoadjuvant chemotherapy in MPUC and UC. There is no direct anti-tumor effect expected for VEGFR inhibitors. 
 The role of VEGF and a functional link between VEGF and p27Kip1 in acute myeloid leukemia. Alterations in the expression and signalling pathways of vascular endothelial growth factor (VEGF) have been linked to the clinical features and pathogenesis of hematologic malignancies. In this study, we showed that VEGF protein expression was statistically significantly higher in the leukemic blasts than in the normal hematopoietic counterparts. A statistically significant correlation between expression of VEGF and p27(Kip1) was observed in bone marrows from 42 patients with acute myeloid leukemia (P<0.001). We further demonstrated that forced VEGF overexpression or autocrine VEGF stimulation of VEGFR-2 triggers proliferation and migration/invasion of U-937 leukemic cells, thereby inducing a more   invasive   tumor phenotype. U-937 cells overexpressing VEGF were resistant to all-trans-retinoic acid-(ATRA) or camptothecin-induced apoptosis. Finally, we showed that increased p27(Kip1) expression enhanced the ability of VEGF and VEGFR-2 to promote the migration of U-937 cells. Taken together, our results suggest that elevated level of VEGF may contribute to the adverse patient outcome by promoting cell growth, survival and migration of leukemic cells and by reducing the sensitivity of leukemic cells to therapeutic agents-induced apoptosis. 
 VEGF and VEGFR-2 immunohistochemistry in human melanocytic naevi and cutaneous melanomas. Vascular endothelial growth factor (VEGF) and vascular endothelial growth factor receptor-2 (VEGFR-2) play a key role in vasculogenesis and angiogenic sprouting, which are crucial for tumour development and metastasis. In order to determine their possible role in the acquisition of metastatic potential throughout melanocytic tumour progression, VEGF and VEGFR-2 immunohistochemical expression were evaluated in 36 human melanocytic tumours of the skin (24 malignant melanomas and 12 common naevi). Different VEGFR-2 immunostaining patterns were detected in the vast majority of melanomas (21/24; 88%). A nuclear membrane-like pattern was mainly associated with in situ and micro  invasive   melanomas, whereas a combined cytoplasmic membrane and nuclear membrane-like pattern was seen in   invasive   melanomas. A nuclear membrane-like pattern was also observed in 83% (10/12) of common naevi. Cytoplasmic immunostaining for VEGF was observed in 72% (8/11) of in situ/micro  invasive   melanomas, 84% (11/13) of   invasive   melanomas and 91% (11/12) of naevi. CD31 was also investigated as an immunohistochemical marker for microvessel density (MVD) evaluation. No associations were found between MVD and VEGF/VEGFR-2 expression. Taken together, these data indicate that VEGF production is a common event in benign melanocytic tumours, whereas VEGFR-2 expression, co-localized in the cytoplasmic and nuclear membrane, is associated with progression towards   invasive   melanoma. The role exerted by VEGF/VEGFR-2, however, seems to be independent of the development of a tumour-related capillary network. 
 Immunohistochemical study of the angiogenetic network of VEGF, HIF1α, VEGFR-2 and endothelial nitric oxide synthase (eNOS) in human breast cancer. The role of Nitric Oxide (NO) in angiogenesis has not been fully clarified yet. A dual role for NO, either inductive or inhibitory, has been proposed on the basis of different effects that high or low concentrations of NO may exert on the angiogenic process. Additionally, it has been referred that NO may induce VEGF production, while VEGF may induce NO production via up-regulation of the endothelial nitric oxide synthase (eNOS), the two pathways being reverse. The aim of the current study was to investigate the expression of key molecules involved in these opposite pathways in primary breast cancer.Representative tumor samples from 242 patients with early-stage breast cancer   (invasiv  e ductal breast carcinomas) were investigated for the expression of VEGF, VEGFR-2, HIF1α, iNOS, and eNOS using immunohistochemistry.Endothelial NOS was found in 159 cases, VEGF in 131 cases, HIF-1α in 139 cases, VEGFR2 in 185 cases and inducible NOS (iNOS) in 22 cases. There was a significant correlation between the expression of VEGF and VEGFR-2, eNOS and VEGF, eNOS and VEGFR-2, eNOS and HIF1α. No statistically significant correlation was found between iNOS and the rest of the studied molecules.In breast cancer cases, the major molecules regulating NO and VEGF production can be co-expressed in the individual carcinomas implying a possibility for the relevant pathways to be active; however appropriate functional experiments remain to be conducted to prove such a hypothesis. 
 Knockdown of the AKT3 (PKBγ), PI3KCA, and VEGFR2 genes by RNA interference suppresses glioblastoma multiforme T98G cells   invasiv  eness in vitro. Glioblastoma multiforme (GBM) is the most common primary brain malignancy, having a very poor prognosis and is characterized by extensive brain invasion as well as resistance to the therapy. The phosphoinositide 3-kinase (PI3K)/Akt/PTEN signaling pathway is deregulated in GBM. Besides, florid vascularization and aberrantly elevated vascular endothelial growth factor (VEGF) occur very often. The present study was designed to examine the inhibitory effect of AKT3, PI3KCA, and VEGFR2 small interfering RNAs (siRNAs) on GBM cell   invasiv  eness. T98G cells were transfected with AKT3, PI3KCA, and/or VEGFR2 siRNAs. VEGFR2 protein-positive cells were identified by flow cytometry using specific monoclonal anti-VEGFR2 antibodies. Alterations in messenger RNA (mRNA) expression of VEGF, VEGFR2, matrix metalloproteinases (MMPs) (MMP-2, MMP-9, MMP-13, MMP-14), tissue inhibitors of metalloproteinases (TIMPs) (TIMP-1, TIMP-3), c-Fos, c-Jun, hypoxia-inducible factor-1α (HIF-1α), ObRa, and cathepsin D genes were analyzed by qRT-PCR. Cells treated with specific siRNA were also analyzed for invasion using the Matrigel invasion assay. We have found significantly lower mRNA levels of MMPs, cathepsin D, VEGF, VEGFR2, HIF-1α, and c-Fos/c-Jun ratio, as well as significantly higher mRNA level of TIMPs in AKT3 and PI3KCA siRNA transfected cells compared to untransfected cells, while significantly lower mRNA levels of MMPs (MMP-2, MMP-9, MMP-14) and TIMP-1, as well as significantly higher mRNA level of TIMP-3, were shown only in cells transfected with VEGFR2 siRNA. The positive correlation between MMP-13 and ObRa mRNA copy number has been found. Summarizing, transfection of T98G cells with AKT3, PI3KCA, or VEGFR2 siRNAs leads to a significant reduction in c  ell inva  siveness. The siRNA-induced AKT3, PI3KCA, and VEGFR2 mRNA knockdown may offer a novel therapeutic strategy to reduce   the inva  siveness of GBM cells. 
 Decorin is a novel VEGFR-2-binding antagonist for the human extravillous trophoblast. Extravillous trophoblasts (EVT) of the human placenta invade the uterine decidua and its arteries to ensure successful placentation. We previously identified two decidua-derived molecules, TGF-β and a TGF-β-binding proteoglycan decorin (DCN), as negative regulators of EVT proliferation, migration, an  d invasi  veness and reported that DCN acts via multiple tyrosine kinase receptors [epidermal growth factor-receptor (EGF-R), IGF receptor-1 (IGFR1), and vascular endothelial growth factor 2 receptor (VEGFR-2)]. Because binding of DCN to VEGFR-2 has never been reported earlier, present study explored this binding, the approximate location of VEGFR-2-binding site in DCN, and its functional role in our human first trimester EVT cell line HTR-8/SVneo. Based on far-Western blotting and coimmunoprecipitation studies, we report that DCN binds both native (EVT expressed) and recombinant VEGFR-2 and that this binding is abrogated with a VEGFR-2 blocking antibody, indicating an overlap between the ligand-binding and the DCN-binding domains of VEGFR-2. We determined that (125)I-labeled VEGF-E (a VEGFR-2 specific ligand) binds EVT with a dissociation constant (K(d)) of 566 pM, and DCN displaced this binding with an inhibition constant (K(i)) of 3.93-5.78 nM, indicating a 7- to 10-fold lower affinity of DCN for VEGFR-2. DCN peptide fragments derived from the leucine rich repeat 5 domain that blocked DCN-VEGFR-2 interactions or VEGF-E binding in EVT cells also blocked VEGF-A- and VEGF-E-induced EVT cell proliferation and migration, indicative of functional VEGFR-2-binding sites of DCN. Finally, DCN inhibited VEGF-E-induced EVT migration by interfering with ERK1/2 activation. Our findings reveal a novel role of DCN as an antagonistic ligand for VEGFR-2, having implications for pathophysiology of preeclampsia, a trophoblast hy  poinvasi  ve disorder in pregnancy, and explain its antiangiogenic function. 
 Antitumor activity of BIBF 1120, a triple angiokinase inhibitor, and use of VEGFR2+pTyr+ peripheral blood leukocytes as a pharmacodynamic biomarker in vivo. BIBF 1120 is a potent, orally available triple angiokinase inhibitor that inhibits VEGF receptors (VEGFR) 1, 2, and 3, fibroblast growth factor receptors, and platelet-derived growth factor receptors. This study examined the antitumor effects of BIBF 1120 on hepatocellular carcinoma (HCC) and attempted to identify a pharmacodynamic biomarker for use in early clinical trials.We evaluated the antitumor and antiangiogenic effects of BIBF 1120 against HCC cell line both in vitro and in vivo. For the pharmacodynamic study, the phosphorylation levels of VEGFR2 in VEGF-stimulated peripheral blood leukocytes (PBL) were evaluated in mice inoculated with HCC cells and treated with BIBF 1120.BIBF 1120 (0.01 μmol/L) clearly inhibited the VEGFR2 signaling in vitro. The direct growth inhibitory effects of BIBF 1120 on four HCC cell lines were relatively mild in vitro (IC(50) values: 2-5 μmol/L); however, the oral administration of BIBF 1120 (50 or 100 mg/kg/d) significantly inhibited the tumor growth and angiogenesis in a HepG2 xenograft model. A flow cytometric analysis revealed that BIBF 1120 significantly decreased the phosphotyrosine (pTyr) levels of VEGFR2(+)CD45(dim) PBLs and the percentage of VEGFR2(+)pTyr(+) PBLs in vivo; the latter parameter seemed to be a more feasible pharmacodynamic biomarker.We found that BIBF 1120 exhibited potent antitumor and antiangiogenic activity against HCC and identified VEGFR2(+)pTyr(+) PBLs as a feasible and n  oninvasi  ve pharmacodynamic biomarker in vivo. 
 Expression and prognostic significance of VEGFR-2 in breast cancer. Breast cancer is one of the most common cancers among women in the world. Vascular endothelial growth factor receptor 2 (VEGFR-2) was not only found to play a key role in the development of tumor angiogenesis, but has also been located in tumor cells of a variety of tumors. This study investigated the expression pattern of VEGFR-2 in breast cancer tissue specimens in order to evaluate the role of VEGFR-2 in the prognosis of breast cancer. Expression and localization of VEGFR-2 in tumor cells of breast cancer specimens from 98   invasive   breast cancer patients were determined by immunohistochemistry. The relationships between VEGFR-2 expression and clinicopathological features were also analyzed. The results showed that VEGFR-2 expression correlated positively with lymph node (LN) metastasis of breast cancer. Patients with high expression of VEGFR-2 had a significantly worse OS. It was also observed that the expression of epithelial-mesenchymal transition (EMT) marker, including Twist1 and Vimentin, was higher in the tumors with higher VEGFR-2 expression, while the E-cadherin expression was lower in the same tumors, suggesting that VEGFR-2 may serve as a possible mediator of EMT in breast cancer. 
 The VEGF-C/Flt-4 axis promotes invasion and metastasis of cancer cells. Flt-4, a VEGF receptor, is activated by its specific ligand, VEGF-C. The resultant signaling pathway promotes angiogenesis and/or lymphangiogenesis. This report provides evidence that the VEGF-C/Flt-4 axis enhances cancer cell mobility and   invasive  ness and contributes to the promotion of cancer cell metastasis. VEGF-C/Flt-4-mediated invasion and metastasis of cancer cells were found to require upregulation of the neural cell adhesion molecule contactin-1 through activation of the Src-p38 MAPK-C/EBP-dependent pathway. Examination of tumor tissues from various types of cancers revealed high levels of Flt-4 and VEGF-C expression that correlated closely with clinical metastasis and patient survival. The VEGF-C/Flt-4 axis, through upregulation of contactin-1, may regulate the   invasive   capacity in different types of cancer cells. 
  GeneRIFs  
 Activation of the HGF-mediated MET pathway is involved in escape to selective VEGFR inhibition in neuroblastoma suggesting combined inhibition of MET and VEGFR signaling to reduce secondary resistance and enhanced   invasive  ness. 
 there is HER2 and EGFR, but not VEGFR expression in micropapillary urothelial carcinoma with   invasive   urothelial carcinoma 
 AKT3, PI3KCA, and VEGFR2 silencing reduces the   invasive  ness of glioblastoma multiforme T98G cells. 
 VEGFR2 expression was significantly higher in muscle   invasive   bladder cancer than that non-muscle   invasive   bladder cancer which suggests VEGFR2 expression increases with tumor invasion; results suggest that alterations in expression of VEGF and VEGF receptors are associated with disease stage and recurrence 
 VEGF-A, VEGFR1 and VEGFR2 have roles in events occurring at the   invasive   tumour front 
 VEGFR-2 expression, co-localized in the cytoplasmic and nuclear membrane, is associated with progression towards   invasive   melanoma 
  [Mm]etastasize  PubMed  
 Molecular mediators of tumor angiogenesis: enhanced expression and activation of vascular endothelial growth factor receptor KDR in primary breast cancer. The progression of breast cancer growth and its ability to   metastasize   are associated with the process of angiogenesis. In this study, we examined the protein expression of vascular endothelial growth factor (VEGF) and its specific and functional receptor KDR in human breast tissue. We investigated a total of 13 mammary carcinomas, 3 fibroadenomas, 5 specimens with fibrocystic breast disease as well as normal (adjacent to malignant) breast tissue using immunohistochemistry and Western blot analysis. In all carcinomas examined, functional KDR protein was present independent of tumor type, tumor stage and histological grade as demonstrated by tyrosine phosphorylation analysis of KDR. When malignant tissues were compared with their neighboring non-neoplastic regions, activated KDR was found to be expressed to a much higher extent within the malignant tissue samples. In fibroadenomas, KDR was barely detectable, whereas in fibrocystic breast disease KDR expression was variable. Immunostaining of KDR was localized to endothelium and epithelium of mammary ducts in malignant and benign breast tissue, while VEGF immunoreactivity was primarily found in the endothelium and also in tumor cells and macrophages. Our data demonstrate that KDR activation is enhanced in breast cancer in vivo and emphasize the functional role of VEGF and KDR in the development of malignant breast disease. 
  [Mm]etastasis  PubMed  
 Increased expression of vascular endothelial growth factor and its receptors, VEGFR-1 and VEGFR-2, in medullary thyroid carcinoma. Vascular endothelial growth factor (VEGF-A) expression is upregulated in the majority of human tumors, where it stimulates proliferation, migration, and survival of endothelial cells. Studies have suggested that VEGF inhibitors can be used as an alternative therapy in medullary thyroid carcinoma (MTC), but data about expression of VEGF-A and its receptor in this tumor are scarce. The aims of this study were to evaluate VEGF-A, VEGF receptor (VEGFR)-1, VEGFR-2, and microvessel density (MVD) expression in MTC samples and correlate it with clinical parameters.Paraffin-embedded samples from 38 MTC patients were evaluated for VEGF-A, VEGFR-1, VEGFR-2, and MVD expression by immunohistochemistry. Clinical data were retrospectively reviewed in medical records.Thirty-eight patients aged 31.8 +/- 17.1 years were enrolled. Twenty-seven patients had hereditary disease (71.1%). Twenty-five of them were found to have multiple endocrine neoplasia (MEN) 2A and two were found to have MEN 2B. VEGF-A immunohistochemical staining was detected in 95% (36/38), VEGFR-1 in 96% (36/37), and VEGFR-2 in 91% (31/34) of MTC samples. Age at surgery was positively correlated with VEGFR-2 (p = 0.003). There was no correlation between VEGF-A, VEGFR-2, and tumor stage (tumor node   metastasis  ). Nevertheless, VEGFR-1 was found to be inversely correlated with tumor node   metastasis   (p = 0.034). We also observed a trend toward an association between VEGFR-1 signal intensity and cure of disease, although this did not reach statistical significance (p = 0.054). Neither VEGF-A nor VEGFR-2 was associated with disease outcome after a median follow-up period of 5 years (p = 0.882 and p = 0.236, respectively). As expected, MVD was correlated with age at surgery (p = 0.005) and tumor size (p = 0.03). Patients with the hereditary form of the disease had a stronger intensity for VEGFR-1 (p = 0.039), whereas patients with sporadic disease displayed higher MVD counts (44 [27-63] vs. 21 [9-49], p = 0.018).The VEGF-A, VEGFR-1, and VEGFR-2 immunoreactive proteins are overexpressed in MTC lesions and might be implicated in tumor progression. It is not clear, however, if expression of these molecules provides prognostic information regarding the spread or outcome of MTC. 
 [Role of Ang-2, Tie-2 and VEGFR-2 in angiogenesis in colorectal carcinoma and their prognostic value]. To study the expression of angiotensin-2 (Ang-2), Tie-2 and vascular endothelial growth factor receptor-2 (VEGFR-2) in colorectal cancer and analyze their relationship with the occurrence, recurrence,   metastasis  , angiogenesis and prognosis of colorectal cancer.Immunohistochemistry with SP method was used to detect the expressions of Ang-2, Tie-2 and VEGFR-2 in 118 colorectal cancer, 40 adjacent normal tissue and 40 benign colorectal lesion specimens.The positivity rates of Ang-2, Tie-2 and VEGFR-2 in colorectal cancer tissue were 74.58%, 69.49%, and 61.02%, respectively, significantly higher than those in the adjacent normal tissues (25.00%, 17.50%, and 17.50%, P<0.05) and benign colorectal lesion tissues (35.00%, 32.50%, and 32.50%, P<0.05). The rates of two or three coexpression were significantly higher than that of a single expression in the cancer tissues (61.02% vs 15.25%). The microvascular density (MVD) of colorectal cancer tissues was 31.43∓10.50, significantly higher than that of the adjacent normal tissues (10.61∓3.76) and benign colorectal lesions (16.89∓3.83) (P<0.05). The expressions of Ang-2, Tie-2, and VEGFR-2 were positively correlated with carcinoembryonic antigen (CEA) and MVD (P<0.05). The expression of Ang-2, but not Tie-2 and VEGFR-2, was positively correlated with CA199. Ang-2, Tie-2, and VEGFR-2 expressions showed significant differences between cases with tumor recur  rence/meta  stasis and those without 5 years after radical mastectomy, and were all positively correlated with the 5-year survival rates (P<0.05).Ang-2, Tie-2 and VEGFR-2 are involved in the development, inva  sion, meta  stasis, and prognosis of colorectal cancer, and play important roles in the angiogenesis of the tumors. 
 Glucose-regulated protein 78 silencing down-regulates vascular endothelial growth factor/vascular endothelial growth factor receptor 2 pathway to suppress human colon cancer tumor growth. Up to 20% of colorectal cancer (CRC) is diagnosed with distant   metastasis  . The combination of chemotherapy with anti-vascular endothelial growth factor (VEGF) antibody can improve patient survival. Glucose-regulated protein 78 (GRP78) has an important role in cancer progression, but little is known about its role in VEGF production in CRC. The aim of this study was to explore the mechanism of GRP78 in two human colon cancer cell lines.We first checked the expression of GRP78 in human normal and colon cancer tissues and two colon cancer cell lines. Glucose-regulated protein 78 was knocked down using GRP78 small interfering RNA (siRNA) in HT29 and DLD-1 cells. We examined knockdown cells by the cell growth kinetics in vitro and tumor growth rate in vivo, respectively. We also investigated the effect of GRP78 siRNA on the expression of hypoxia inducible factor (HIF-1α), VEGF, and VEGF receptor 2 (VEGFR2).Compared with their adjacent normal tissue, we detected high expression levels of GRP78 of surgically removed colon cancer tissues. Using GRP78 siRNA, we reduced the expression of GRP78 in HT29 and DLD-1 cells. The GRP78 knockdown cells had a lower proliferation rate with fewer colony-forming units in vitro and produced smaller tumors in vivo. In dissecting the mechanism underlying the reduced cell growth, we found that the down-regulation of GRP78 decreased the production of HIF-1α, VEGF, and VEGFR2 and suppressed angiogenesis.Silencing GRP78 not only inhibits tumor, but also decreases the expression of VEGF and VEGFR2. Collectively, therapy targeting for GRP78 may inhibit the formation of colon cancer tumors via the HIF-1α/VEGF/VEGFR2 pathway. 
 Role of the VEGF ligand to receptor ratio in the progression of mismatch repair-proficient colorectal cancer. The VEGF family of ligands and receptors are intimately involved in tumor angiogenesis, lymphangiogenesis and   metastasis  . The evaluation of VEGF ligand/receptor ratios may provide a more profound understanding of the involvement of these proteins in colorectal tumour progression. The aim of this study was to elucidate the role of the VEGF ligand/receptor ratios on tumour progression and   metastasis   in patients with mismatch repair-proficient colorectal cancer.Immunohistochemistry for VEGF-A, VEGF-B, VEGF-C, VEGF-D, VEGFR1, VEGFR2 and VEGF3 was carried out on 387 mismatch repair-proficient colorectal cancers using a tissue microarray. Evaluation of immunoreactivity was performed semi-quantitatively and the ligand/receptor expression ratio was obtained.An increased VEGF-A/VEGFR1 ratio, VEGF-A and VEGFR1 was linked to the presence of peritumoral lymphocytic inflammation at the invasive front (p = 0.032; p = 0.005; p = 0.032, respectively). VEGFR1 expression was related to poorer outcome in multivariable analysis with pT stage, pN stage, vascular invasion, and post-operative therapy. A higher ratio of VEGF-A/VEGFR2 was linked to advanced TNM stage (p = 0.005) while VEGF-A and VEGFR2 were elevated in tumours with an infiltrating tumour growth pattern (p = 0.006; p = 0.014; p = 0.006). No effect of VEGF-A/VEGFR2, VEGF-A or VEGFR2 on survival time was noted.Our findings highlight an involvement of VEGF-A, VEGR1 and VEGFR2 in events occurring at the invasive tumour front and a potential prognostic role of VEGFR1 expression in mismatch repair-proficient colorectal cancers. The VEGF-A ligand to VEGFR1 or VEGFR2 ratio may represent an alternative evaluation system for identifying patients with poorer clinical outcome. 
 The soluble VEGF receptor 1 and 2 expression in cerebral spinal fluid as an indicator for leukemia central nervous system   metastasis  . Over-expression of vascular endothelial growth factor A (VEGF-A) is correlated with leukemia   metastasis  . VEGF-A acts by binding to its membrane receptors R1 and R2 present in soluble forms (sVEGFR1, sVEGFR2) with different functions. sVEGFR could inhibit VEGF-A bioactivities, associated with favorable prognosis in solid tumors. However, its role is obscure in central nervous system leukemia (CNSL). The aim of this study was to investigate sVEGFR1, R2 as biomarkers in CNSL. Paired cerebrospinal fluid (CSF) and serum samples were collected from 35 leukemia cases with or without CNS   metastasis  . Levels of sVEGFR1 and sVEGFR2 in both CSF (sVEGFR1CSF, sVEGFR2CSF) and serum (sVEGFR1Serum, sVEGFR2Serum) were detected by ELISA. Other risk factors related to CNSL prognosis were also analyzed. sVEGFRSerum levels were 2.54-fold (sVEGFR1) and 25.6-fold (sVEGFR2) higher than sVEGFRCSF in both leukemic groups. sVEGFR1CSF in CNSL were 33 % higher than in the non-CNSL, and the levels of sVEGFR2CSF and sVEGFR2Serum had the same trend. Elevated sVEGFR1CSF and sVEGFR2CSF is closely correlated with blood-brain barrier (BBB) values and WBCCSF that is an indicator of CNSL disease burden. Cox regression analysis showed that the sVEGFR2CSF had a positive effect on event-free survival. Our data suggest that sVEGFR2CSF may be more potent than sVEGFR1CSF in predicting the outcome of leukemia patients, the balance between sVEGFR2CSF and VEGF-ACSF levels might be crucial for the progression of CNSL. 
 Immunolocalizations of VEGF, its receptors flt-1, KDR and TGF-beta's in epithelial ovarian tumors. Angiogenesis is an essential factor for growth, differentiation, invasion and   metastasis   of tumors. In this study, we aimed to evaluate the immunolocalizations of vascular endothelial growth factor (VEGF), its receptors flt-1, KDR/flk-1, and transforming growth factor-beta's (TGF-beta) in epithelial ovarian tumors, utilizing indirect immunohistochemistry to understand the role of the angiogenic events in ovarian neoplasia.Tissue blocks from 40 patients who had ovarian pathology (borderline serous-mucinous tumor and malignant serous-mucinous adenocarcinoma of the ovary) were included in this study. All formalin-fixed, paraffin-embedded tissue sections were stained with hematoxylin-eosin or primary antibodies against VEGF, flt-1, KDR/flk-1, TGF-beta1, TGF-beta2 and TGF-beta3 using the avidin-biotin-peroxidase method. H-SCORE, a semi-quantitative grading system, was used to compare immunohistochemical staining intensities.Positive VEGF immunoreactivity was concentrated in the epithelial and stromal parts of all the ovarian samples and the endothelial cells in the stroma were also stained. Increased immunoreactivity of VEGF was observed in malignant ovarian adenocarcinomas compared to the borderline tumors of the ovary. VEGF receptors, flt-1 and KDR/flk-1 immunoreactivities were detected not only in vascular endothelial cells, but also in tumor cells at malignant sites. Immunoreactivities of VEGF and its receptors were coexpressed in tumor cells of the ovarian carcinoma. While immunoreactivities of TGF-beta1 and TGF-beta2 were both overexpressed in malignant ovarian carcinomas, immunoreactivity of TGF-beta3 was still mild.Our results suggest that overexpression of VEGF, its receptors flt-1, KDR/flk-1 and TGF-beta interaction may play an important role in the ovarian cancer biology, with potential effects on tumor growth and angiogenesis. New therapeutic strategies using VEGF and TGF-beta antagonists could obtain an additional approach to the treatment ovarian carcinoma by inhibiting angiogenesis. 
 Klotho endows hepatoma cells with resistance to anoikis via VEGFR2/PAK1 activation in hepatocellular carcinoma. Klotho was originally characterized as an aging suppressor gene that predisposed Klotho-deficient mice to premature aging-like syndrome. Although Klotho was recently reported to exhibit tumor suppressive properties during various malignant transformations, the functional role and molecular mechanism of Klotho in hepatocarcinogenesis remains poorly understood. In our present study, immunohistochemical Klotho staining levels in a clinical follow-up of 52 hepatoma patients were significantly associated with liver cirrhosis, tumor multiplicity and venous invasion. The overall survival rate of hepatoma patients with high Klotho expression was significantly lower than those patients with low Klotho expression. Moreover, Klotho overexpression increased cellular migration, anchorage-independent growth, and anoikis resistance in hepatoma cells. Klotho overexpression elevated p21-activated kinase 1 (PAK1) expression and shRNA-mediated PAK1 knockdown and kinase activity inhibition with kinase dead mutant PAK1 K299R coexpression or allosteric inhibitor IPA3 treatment reversed anoikis resistance in Klotho-overexpressed hepatoma cells. More importantly, the pivotal significance of upregulated VEGFR2 protein levels mediated by Klotho expression was confirmed by VEGFR2 inhibitor Axitinib and blocking antibody treatment in hepatoma cells. Axitinib treatment sensitized anoikis was reversed by constitutive active mutant PAK1 T423E coexpression in Klotho-overexpressed hepatoma cells. Conversely, knockdown of Klotho reduced VEGFR2/PAK1 dependent anoikis resistance, which could be reversed by PAK1 T423E. These results revealed a novel oncogenic function of Klotho in promoting anoikis resistance via activating VEGFR2/PAK1 signaling, thus facilitating tumor migration and invasion during hepatoma progression, which could provide a putative molecular mechanism for tumor   metastasis  . 
 Insulin-like growth factor receptor I (IGF-IR) and vascular endothelial growth factor receptor 2 (VEGFR-2) are expressed on the circulating epithelial tumor cells of breast cancer patients. Circulating epithelial tumor cell (CETC) analysis is a promising diagnostic field for estimating the risk for metastatic relapse and progression in patients with malignant disease. CETCs characterization can be used as a liquid biopsy for prognostic and predictive purposes in breast and other cancers. IGF-IR and VEGFR-2 play an important role in tumor growth and the progression of cancer disease. The purpose of the current study was therefore to investigate their expression on CETCs.CETCs were determined from the blood of 50 patients suffering from breast cancer. The number of vital CETCs and the expression of IGF-IR and VEGFR-2 were investigated using the maintrac® method.IGF-IR and VEGFR-2 expression on the surface of CETCs were detected in 84% of patients. A statistically high correlation was found between IGF-IR and VEGFR-2 (r = 0.745 and p<0.001) on the CETCs. The co-expression of both receptors was confirmed in some experiments and ranged between 70% and 100%. Statistically significant correlations were observed between the number of CETCs and IGF-IR (r = 0.315 and p<0.05) and VEGFR-2 (r = 0.310 and p<0.05) expression. The presence of CETCs and the level of IGF-IR and VEGFR-2 expression were not associated with tumor stage, hormone receptor status or n  odal/dista  nt metastasis.In this study, a parallel and co-expression of IGF-IR and VEGFR-2 was examined on the surface of CETCs in breast cancer patients for the first time. Characterization of CETCs may be a promising approach for the rational design of targeted anticancer therapies. 
 [Expression of vascular endothelial growth factor and its receptors in laryngeal carcinoma cell and its significance]. To study the expression of vascular endothelial growth factor (VEGF) and both the Flt-1 and KDR high affinity VEGF receptors in human laryngeal carcinoma cells and its significance.In this study, we investigated the expression of VEGF mRNA and both the Fit-1 mRNA and KDR mRNA high affinity VEGF receptors in human laryngeal carcinoma cells using techniques of semi-quantify RT-PCR.It was showed that the expression level of VEGF mRNA was significantly increased in human laryngeal carcinoma cells as compared with the normal cells, Hacat cells (P <0.05), and there was the high expression level of Flt-1 mRNA in the Hep-2 cells, but not KDR mRNA.These data raise the possibility that VEGF and its receptors many play key roles in the growth, invasion and   metastasis   of human laryngeal carcinoma cells through either a paracrine or an autocrine mechanism. 
 Expression of vascular endothelial growth factor and its receptors KDR and Flt-1 in gastric cancer cells. The expression of vascular endothelial growth factor (VEGF) and its receptors KDR and Flt-1 by gastric carcinoma tissues and different gastric carcinoma cell lines was detected to elucidate the molecular mechanism of this growth factor in promoting tumor growth.The expression of VEGF, Flt-1 and KDR was determined by reverse transcription-polymerase chain reaction (RT-PCR) in gastric cancer cell lines RF-1, RF-48, AGS-1, NCI-N87, NCI-SNU-1, NCI-SNU-5, NCI-SNU-16 and KATO-III. The expression of Flt-1 and KDR in paraffin-embedded specimens of gastric cancer was determined by immunohistochemistry. The 3-(4,5-dimethylthiazol-2-yl)-2, 5-diphenyltetrazolium bromide (MTT) assay was used to assess the role of VEGF in tumor cell proliferation.All 8 gastric cancer cell lines analyzed expressed VEGF(121) and VEGF(165) and six of them expressed both Flt-1 and KDR, while cell line NCI-SNU-5 expressed Flt-1 only and cell line KATOIII expressed neither Flt-1 nor KDR. The gastric carcinoma tissues expressed Flt-1 and KDR widely, with the positive rate of expression of Flt-1 and KDR being 84.6 % and 70 % respectively. The exogenous VEGF stimulated the growth of KDR-positive cell lines NCI-N87 and AGS-1 in a dose-dependent manner but exhibited no effect on the growth of KDR-negative cell line NCI-N87.VEGF and its receptors KDR and Flt-1 were expressed widely in gastric carcinoma cells and the VEGF stimulated KDR-positive tumor cell growth directly. These results suggest that VEGF may play a role in promoting tumor growth and   metastasis   by participating in both paracrine and autocrine pathways. 
 Gab1 regulates proliferation and migration through the PI3K/Akt signaling pathway in intrahepatic cholangiocarcinoma. Intrahepatic cholangiocarcinoma is the second most common primary malignant tumor of the liver, and it originates from the intrahepatic biliary duct epithelium. Prognosis is poor due to lack of effective comprehensive treatments. In this study, we assessed the expression of Gab1, VEGFR-2, and MMP-9 in intrahepatic cholangiocarcinoma solid tumors by immunohistochemistry and determined whether their expression was associated with clinical and pathological features. We found that expression of Gab1, VEGFR-2, and MMP-9 was highly and positively correlated with each other and with lymph node   metastasis   and TNM stage in intrahepatic cholangiocarcinoma tissues. Interference of Gab1 and VEGFR-2 expression via siRNA in the intrahepatic cholangiocarcinoma cell line RBE resulted in decreased PI3K/Akt pathway activity. Inhibition of Gab1 and VEGFR-2 expression also caused decreased cell proliferation, cell cycle arrested in G1 phase, increased apoptosis, and decreased invasion in RBE cells. These results suggest that Gab1, VEGFR-2, and MMP-9 contribute significantly to the highly malignant behavior of intrahepatic cholangiocarcinoma. The regulation of growth, apoptosis, and invasion by Gab1 through the VEGFR-2/Gab1/PI3K/Akt signaling pathway may represent potential targets for improving the treatment of intrahepatic cholangiocarcinoma. 
 Inhibition of prostate cancer osteoblastic progression with VEGF121/rGel, a single agent targeting osteoblasts, osteoclasts, and tumor neovasculature. A hallmark of prostate cancer (PCa) progression is the development of osteoblastic bone metastases, which respond poorly to available therapies. We previously reported that VEGF(121)/rGel targets osteoclast precursors and tumor neovasculature. Here we tested the hypothesis that targeting nontumor cells expressing these receptors can inhibit tumor progression in a clinically relevant model of osteoblastic PCa.Cells from MDA PCa 118b, a PCa xenograft obtained from a bone   metastasis   in a patient with castrate-resistant PCa, were injected into the femurs of mice. Osteoblastic progression was monitored following systemic administration of VEGF(121)/rGel.VEGF(121)/rGel was cytotoxic in vitro to osteoblast precursor cells. This cytotoxicity was specific as VEGF(121)/rGel internalization into osteoblasts was VEGF(121) receptor driven. Furthermore, VEGF(121)/rGel significantly inhibited PCa-induced bone formation in a mouse calvaria culture assay. In vivo, VEGF(121)/rGel significantly inhibited the osteoblastic progression of PCa cells in the femurs of nude mice. Microcomputed tomographic analysis revealed that VEGF(121)/rGel restored the bone volume fraction of tumor-bearing femurs to values similar to those of the contralateral (non-tumor-bearing) femurs. VEGF(121)/rGel significantly reduced the number of tumor-associated osteoclasts but did not change the numbers of peritumoral osteoblasts. Importantly, VEGF(121)/rGel-treated mice had significantly less tumor burden than control mice. Our results thus indicate that VEGF(121)/rGel inhibits osteoblastic tumor progression by targeting angiogenesis, osteoclastogenesis, and bone formation.Targeting VEGF receptor (VEGFR)-1- or VEGFR-2-expressing cells is effective in controlling the osteoblastic progression of PCa in bone. These findings provide the basis for an effective multitargeted approach for metastatic PCa. 
 Overproduction of VEGF concomitantly expressed with its receptors promotes growth and survival of melanoma cells through MAPK and PI3K signaling. Vascular endothelial growth factor (VEGF) is an important mediator of tumor-associated angiogenesis, and consequently it has been associated with   metastasis  . We report here that the overexpression of VEGF(165) in melanoma xenografts promotes an acceleration of tumor growth and an increase in angiogenesis as well as the spontaneous   metastasis   formation. In addition, VEGF receptors (VEGFR)1, VEGFR2 and neurophilin-1 are expressed in A375 melanoma cells. Forced overexpression of VEGF in these cells induces cell growth and triggers survival activity in serum-starved cultures, by a mechanism dependent on the mitogen-activating protein kinase signaling pathway. Furthermore, these effects are dependent MEK 1/2 activity. Kinase domain region-specific tyrosine kinase inhibitors dramatically reduced DNA synthesis to 20% with respect to the controls, although they did not completely suppress either the p44 or p42-phosphorylated forms of extracellular signal-regulated protein kinase. These inhibitors also provoked a decrease in Akt phosphorylation. We observed a dramatic reduction in survival after treatment with phosphatidylinositol 3'-kinase (PI3K)-specific inhibitor in the presence of specific tyrosinase inhibitors. We suggest that the overproduction of VEGF(165) concomitantly expressed with its receptors favors cell growth and survival of melanoma cells through MAPK and PI3K signaling pathways. These data support the involvement in melanoma growth and survival of a VEGF-dependent internal autocrine loop mechanism, at least in vitro. 
 Circulating serum levels of angiogenic factors and vascular endothelial growth factor receptors 1 and 2 in melanoma patients. Angiogenesis is essential for tumor progression and   metastasis  ; however, the angiogenesis regulators that are biologically relevant for melanoma are still unknown. In this study, we analyzed the circulating serum levels of potent angiogenic factors, including vascular endothelial growth factor (VEGF), angiogenin, transforming growth factor-beta1 and VEGF receptors, VEGFR1 and VEGFR2, in human melanoma patients. One hundred and fourteen patients with histopathologically verified cutaneous melanoma at different stages and 30 healthy controls were investigated. Serum levels of angiogenic factors and VEGF receptors were quantitatively analyzed by solid-phase enzyme-linked immunosorbent assay. The age of the patients (61 men and 53 women) ranged from 18 to 80 years; median age was 51 years. Serum transforming growth factor-beta1 (P < 0.001), VEGF (P = 0.006) and VEGFR1 (P = 0.007) levels were significantly higher in patients with melanoma than in the control group. No significant differences, however, exist in the serum angiogenin and VEGFR2 levels between melanoma patients and the controls. The positive correlations of elevated serum levels of transforming growth factor-beta1, VEGF and VEGFR1 with advanced stages of disease were found. Significant relationship was found only between serum levels of VEGF and VEGFR2. Elevated serum transforming growth factor-beta1 (P < 0.001) and VEGF levels (P = 0.0012) were found to be poor prognostic factors. Serum level of angiogenin and VEGF receptors, however, had no effect on survival. Our data suggest that the angiogenic serum factors, including VEGF, transforming growth factor-beta1 and VEGFR1, but not angiogenin and VEGFR2 were increased in melanoma patients, especially associated with advanced disease stages. The mechanism of VEGF regulation of angiogenesis may in part be due to enhanced proliferation of VEGFRs, especially VEGFR1. 
 Measurement of circulating levels of VEGF-A, -C, and -D and their receptors, VEGFR-1 and -2 in gastric adenocarcinoma. To analyze the serum levels and prognostic significance of vascular endothelial growth factor (VEGF) -A, -C, and -D, and their receptors, VEGFR-1 and -2 in gastric adenocarcinomas.The serum levels of VEGF family members were measured in 76 control subjects and 76 patients with gastric adenocarcinoma using an enzyme-linked immunosorbent assay (ELISA). These measurements were correlated with clinco-pathological features and survival rates.The serum levels of VEGF-A and its receptor, VEGFR-1, were significantly higher in patients with gastric cancer than in healthy donors (t = 2.3, P = 0.02 and t = 4.2, P < 0.0001, respectively). In contrast, the serum levels of VEGF-D were significantly higher in control subjects than in patients (t = 2.9, P = 0.004). There was no significant difference in serum levels of VEGF-C and VEGFR-2 between patients and controls. VEGF-C was associated with advanced tumor stage and presence of   metastasis  . VEGFR-1 was associated with   metastasis  , advanced overall stage, tumor differentiation and survival. VEGFR-2 levels were associated with poor tumor differentiation. There was no significant prognostic value for any of the VEGF family members or their receptors except for VEGFR-1 where high levels were associated with a poor overall survival.Serum VEGF levels vary significantly in the same cohort of patients with variable clinico-pathological features and prognostic values. The simultaneous measurement of VEGF receptors levels in sera may overcome the limitations of a single biomarker assay. 
 Metastatic properties of prostate cancer cells are controlled by VEGF. Mechanisms of   metastasis  , the major complication of prostate cancer, are poorly understood. In this study, we define molecular mechanisms that may contribute to the highly invasive potential of prostate cancer cells. Vascular endothelial growth factor (VEGF), its receptors (VEGFRs), and alpha5beta1 integrin were expressed by prostate cancer cells in vitro and by prostate tumors in vivo, and their expression was elevated at sites of bone   metastasis   compared to original prostate tumor. VEGF, through interaction with its receptors, regulated adhesive and migratory properties of the cancer cells. Specifically, the highly metastatic prostate cancer cell subline LNCaP-C4-2 showed a decreased adhesive but an enhanced migratory response to fibronectin, a ligand for alpha5beta1 integrin, compared to its nonmetastatic counterpart. A similar pattern was also observed when bone sialoprotein was used as a ligand in migration assays. Increased migration of metastatic prostate cancer cells to fibronectin and bone sialoprotein was regulated by VEGF via VEGFR-2. Tumor suppressor PTEN was involved in control of VEGF/VEGFR-2 stimulated prostate cancer cell adhesion as well as proliferation. 
 Expression of VEGF and its receptors VEGFR1/VEGFR2 is associated with invasiveness of bladder cancer. Vascular endothelial growth factor (VEGF) signaling is frequently altered in invasive tumor cells and is associated with patient outcome. In the present study, we examined VEGF, VEGFR1, and VEGFR2 expression in non-muscle invasive bladder cancer (NMIBC) and muscle invasive bladder cancer (MIBC), and evaluated the association between VEGF and its receptors with disease characteristics and bladder cancer recurrence.Tissue microarrays containing bladder cancer specimens (n=212) and adjacent normal bladder mucosa (n=131) were immunostained using antibodies against VEGF, VEGFR1, and VEGFR2. The association between the expression of these proteins and clinical parameters including stage, lymph node   metastasis  , and recurrence-free survival were statistically evaluated. VEGF mRNA expression data were extracted from the public Oncomine database.VEGF and VEGFR1 mRNA levels were significantly higher in bladder cancer specimens than that of normal mucosa (for VEGF, p<0.001; for VEGFR1, p=0.02). Analysis of their expression at protein levels showed that levels of VEGF and VEGFR1 were significantly higher in NMIBC than in MIBC (p<0.001), while that of VEGFR2 was significantly higher in all cancer specimens compared to benign urothelial mucosa (p=0.001). Further-more, the expression of VEGFR2 was significantly higher in MIBC, as compared to NMIBC (p<0.001). Patients with higher levels of VEGF, VEGFR1, and VEGFR2 tended to have poorer recurrence-free survival than those with lower levels, but this was not statistically significant.Our results suggest that alterations in the expression of VEGF and VEGF receptors are associated with disease stage and recurrence. 
 GEP100-Arf6-AMAP1-cortactin pathway frequently used in cancer invasion is activated by VEGFR2 to promote angiogenesis. Angiogenesis and cancer invasiveness greatly contribute to cancer malignancy.Arf6 and its effector, AMAP1, are frequently overexpressed in breast cancer, and constitute a central pathway to induce the invasion and   metastasis  . In this pathway, Arf6 is activated by EGFR via GEP100. Arf6 is highly expressed also in human umbilical vein endothelial cells (HUVECs) and is implicated in angiogenesis. Here, we found that HUVECs also highly express AMAP1, and that vascular endothelial growth factor receptor-2 (VEGFR2) recruits GEP100 to activate Arf6. AMAP1 functions by binding to cortactin in cancer invasion and   metastasis  . We demonstrate that the same GEP100-Arf6-AMAP1-cortactin pathway is essential for angiogenesis activities, including cell migration and tubular formation, as well as for the enhancement of cell permeability and VE-cadherin endocytosis of VEGF-stimulated HUVECs. Components of this pathway are highly expressed in pathologic angiogenesis, and blocking of this pathway effectively inhibits VEGF- or tumor-induced angiogenesis and choroidal neovascularization. The GEP100-Arf6-AMAP1-cortactin pathway, activated by receptor tyrosine kinases, appears to be common in angiogenesis and cancer invasion and   metastasis  , and provides their new therapeutic targets. 
 Differential expression of VEGF ligands and receptors in prostate cancer. Prostate cancer disseminates to regional lymph nodes, however the molecular mechanisms responsible for lymph node   metastasis   are poorly understood. The vascular endothelial growth factor (VEGF) ligand and receptor family have been implicated in the growth and spread of prostate cancer via activation of the blood vasculature and lymphatic systems. The purpose of this study was to comprehensively examine the expression pattern of VEGF ligands and receptors in the glandular epithelium, stroma, lymphatic vasculature and blood vessels in prostate cancer.The localization of VEGF-A, VEGF-C, VEGF-D, VEGF receptor (VEGFR)-1, VEGFR-2, and VEGFR-3 was examined in cancerous and adjacent benign prostate tissue from 52 subjects representing various grades of prostate cancer.Except for VEGFR-2, extensive staining was observed for all ligands and receptors in the prostate specimens. In epithelial cells, VEGF-A and VEGFR-1 expression was higher in tumor tissue compared to benign tissue. VEGF-D and VEGFR-3 expression was significantly higher in benign tissue compared to tumor in the stroma and the endothelium of lymphatic and blood vessels. In addition, the frequency of lymphatic vessels, but not blood vessels, was lower in tumor tissue compared with benign tissue.These results suggest that activation of VEGFR-1 by VEGF-A within the carcinoma, and activation of lymphatic endothelial cell VEGFR-3 by VEGF-D within the adjacent benign stroma may be important signaling mechanisms involved in the progression and subsequent metastatic spread of prostate cancer. Thus inhibition of these pathways may contribute to therapeutic strategies for the management of prostate cancer. 
 Expression of VEGF, VEGFR, EGFR, COX-2 and MVD in cervical carcinoma, in relation with the response to radio-chemotherapy. Despite the improvement in the treatment results due to modern irradiation techniques and to the association of chemo-radiotherapy, cervical cancer remains an unsolved problem of oncology both due to the increased rate of local failures and of the distant   metastasis  . Efforts to implement new therapeutic strategies in order to obtain better results in patients with cervical cancer appear justified. Neovascularization is an important step in the tumor progression and the therapeutic targeting of the tumor blood vessels appears to be a good strategy to follow in the anti-cancer treatment. Thus, even in an incipient phase of the clinical research process, the combination between the anti-angiogenic aimed therapies and the current radio-chemotherapy seems to represent a new, feasible and promising approach. The aim of the present study was to determine the prognostic and/or predictive value of some biological markers of tumor angiogenesis and of their implication in increasing the efficacy of current treatments for this cancer.So far, 54 women were included in a prospective trial: 44 having an advanced cervical carcinoma and 10 healthy women, as controls. A tumor biopsy and a blood sample were obtained from each patient before the start of therapy. The density of microvascularization was assessed using CD34 monoclonal antibody (hot spot technique), the expression of angiogenic factors VEGFR, EGFR and COX-2 were determined in tumor biopsies by specific immunohistochemistry techniques, using primary antibodies anti-EGFR, anti-VEGF and anti-COX-2 respectively. The quantitative polymerase chain reaction (Real Time PCR) was employed for assessing the expression level of the genes involved. Serum VEGF was determined by quantitative ELISA technique.Among the studied clinical and molecular factors, we found to be predictive for the type of response the following factors: tumor size at diagnosis (p=0.01), VEGFR2 expression (p=0.02) and a tendency to significance for patients' age (p=0.06). From the large panel of studied markers it was observed correlation between MVD expression with stromal COX-2 (p=0.01) and a tendency with epithelial COX-2 (p=0.06). Stromal COX-2 has higher correlation with VEGFR2 (p=0.01) and MVD (p=0.01) and also has a lower correlation with tumor size (p=0.08).Univariate analysis demonstrates that the response to radio-chemotherapy in cervical cancer is related to a set of clinical and molecular factors as: the tumor size, the expression of VEGFR2 as mRNA level and the patients' age. Unfortunately, the multivariate analysis by logistic model selects only VEGFR2 expression for prediction of tumor response. The interrelations between the different biomarkers demonstrate the complexity of the tumor progression process and the necessity of further studies to identify new therapeutic targets. 
 Gab1 regulates proliferation and migration through the PI3K/Akt signaling pathway in intrahepatic cholangiocarcinoma. Intrahepatic cholangiocarcinoma is the second most common primary malignant tumor of the liver, and it originates from the intrahepatic biliary duct epithelium. Prognosis is poor due to lack of effective comprehensive treatments. In this study, we assessed the expression of Gab1, VEGFR-2, and MMP-9 in intrahepatic cholangiocarcinoma solid tumors by immunohistochemistry and determined whether their expression was associated with clinical and pathological features. We found that expression of Gab1, VEGFR-2, and MMP-9 was highly and positively correlated with each other and with lymph node   metastasis   and TNM stage in intrahepatic cholangiocarcinoma tissues. Interference of Gab1 and VEGFR-2 expression via siRNA in the intrahepatic cholangiocarcinoma cell line RBE resulted in decreased PI3K/Akt pathway activity. Inhibition of Gab1 and VEGFR-2 expression also caused decreased cell proliferation, cell cycle arrested in G1 phase, increased apoptosis, and decreased invasion in RBE cells. These results suggest that Gab1, VEGFR-2, and MMP-9 contribute significantly to the highly malignant behavior of intrahepatic cholangiocarcinoma. The regulation of growth, apoptosis, and invasion by Gab1 through the VEGFR-2/Gab1/PI3K/Akt signaling pathway may represent potential targets for improving the treatment of intrahepatic cholangiocarcinoma. 
 Glucose-regulated protein 78 silencing down-regulates vascular endothelial growth factor/vascular endothelial growth factor receptor 2 pathway to suppress human colon cancer tumor growth. Up to 20% of colorectal cancer (CRC) is diagnosed with distant   metastasis  . The combination of chemotherapy with anti-vascular endothelial growth factor (VEGF) antibody can improve patient survival. Glucose-regulated protein 78 (GRP78) has an important role in cancer progression, but little is known about its role in VEGF production in CRC. The aim of this study was to explore the mechanism of GRP78 in two human colon cancer cell lines.We first checked the expression of GRP78 in human normal and colon cancer tissues and two colon cancer cell lines. Glucose-regulated protein 78 was knocked down using GRP78 small interfering RNA (siRNA) in HT29 and DLD-1 cells. We examined knockdown cells by the cell growth kinetics in vitro and tumor growth rate in vivo, respectively. We also investigated the effect of GRP78 siRNA on the expression of hypoxia inducible factor (HIF-1α), VEGF, and VEGF receptor 2 (VEGFR2).Compared with their adjacent normal tissue, we detected high expression levels of GRP78 of surgically removed colon cancer tissues. Using GRP78 siRNA, we reduced the expression of GRP78 in HT29 and DLD-1 cells. The GRP78 knockdown cells had a lower proliferation rate with fewer colony-forming units in vitro and produced smaller tumors in vivo. In dissecting the mechanism underlying the reduced cell growth, we found that the down-regulation of GRP78 decreased the production of HIF-1α, VEGF, and VEGFR2 and suppressed angiogenesis.Silencing GRP78 not only inhibits tumor, but also decreases the expression of VEGF and VEGFR2. Collectively, therapy targeting for GRP78 may inhibit the formation of colon cancer tumors via the HIF-1α/VEGF/VEGFR2 pathway. 
 The soluble VEGF receptor 1 and 2 expression in cerebral spinal fluid as an indicator for leukemia central nervous system   metastasis  . Over-expression of vascular endothelial growth factor A (VEGF-A) is correlated with leukemia   metastasis  . VEGF-A acts by binding to its membrane receptors R1 and R2 present in soluble forms (sVEGFR1, sVEGFR2) with different functions. sVEGFR could inhibit VEGF-A bioactivities, associated with favorable prognosis in solid tumors. However, its role is obscure in central nervous system leukemia (CNSL). The aim of this study was to investigate sVEGFR1, R2 as biomarkers in CNSL. Paired cerebrospinal fluid (CSF) and serum samples were collected from 35 leukemia cases with or without CNS   metastasis  . Levels of sVEGFR1 and sVEGFR2 in both CSF (sVEGFR1CSF, sVEGFR2CSF) and serum (sVEGFR1Serum, sVEGFR2Serum) were detected by ELISA. Other risk factors related to CNSL prognosis were also analyzed. sVEGFRSerum levels were 2.54-fold (sVEGFR1) and 25.6-fold (sVEGFR2) higher than sVEGFRCSF in both leukemic groups. sVEGFR1CSF in CNSL were 33 % higher than in the non-CNSL, and the levels of sVEGFR2CSF and sVEGFR2Serum had the same trend. Elevated sVEGFR1CSF and sVEGFR2CSF is closely correlated with blood-brain barrier (BBB) values and WBCCSF that is an indicator of CNSL disease burden. Cox regression analysis showed that the sVEGFR2CSF had a positive effect on event-free survival. Our data suggest that sVEGFR2CSF may be more potent than sVEGFR1CSF in predicting the outcome of leukemia patients, the balance between sVEGFR2CSF and VEGF-ACSF levels might be crucial for the progression of CNSL. 
 Klotho endows hepatoma cells with resistance to anoikis via VEGFR2/PAK1 activation in hepatocellular carcinoma. Klotho was originally characterized as an aging suppressor gene that predisposed Klotho-deficient mice to premature aging-like syndrome. Although Klotho was recently reported to exhibit tumor suppressive properties during various malignant transformations, the functional role and molecular mechanism of Klotho in hepatocarcinogenesis remains poorly understood. In our present study, immunohistochemical Klotho staining levels in a clinical follow-up of 52 hepatoma patients were significantly associated with liver cirrhosis, tumor multiplicity and venous invasion. The overall survival rate of hepatoma patients with high Klotho expression was significantly lower than those patients with low Klotho expression. Moreover, Klotho overexpression increased cellular migration, anchorage-independent growth, and anoikis resistance in hepatoma cells. Klotho overexpression elevated p21-activated kinase 1 (PAK1) expression and shRNA-mediated PAK1 knockdown and kinase activity inhibition with kinase dead mutant PAK1 K299R coexpression or allosteric inhibitor IPA3 treatment reversed anoikis resistance in Klotho-overexpressed hepatoma cells. More importantly, the pivotal significance of upregulated VEGFR2 protein levels mediated by Klotho expression was confirmed by VEGFR2 inhibitor Axitinib and blocking antibody treatment in hepatoma cells. Axitinib treatment sensitized anoikis was reversed by constitutive active mutant PAK1 T423E coexpression in Klotho-overexpressed hepatoma cells. Conversely, knockdown of Klotho reduced VEGFR2/PAK1 dependent anoikis resistance, which could be reversed by PAK1 T423E. These results revealed a novel oncogenic function of Klotho in promoting anoikis resistance via activating VEGFR2/PAK1 signaling, thus facilitating tumor migration and invasion during hepatoma progression, which could provide a putative molecular mechanism for tumor   metastasis  . 
 Insulin-like growth factor receptor I (IGF-IR) and vascular endothelial growth factor receptor 2 (VEGFR-2) are expressed on the circulating epithelial tumor cells of breast cancer patients. Circulating epithelial tumor cell (CETC) analysis is a promising diagnostic field for estimating the risk for metastatic relapse and progression in patients with malignant disease. CETCs characterization can be used as a liquid biopsy for prognostic and predictive purposes in breast and other cancers. IGF-IR and VEGFR-2 play an important role in tumor growth and the progression of cancer disease. The purpose of the current study was therefore to investigate their expression on CETCs.CETCs were determined from the blood of 50 patients suffering from breast cancer. The number of vital CETCs and the expression of IGF-IR and VEGFR-2 were investigated using the maintrac® method.IGF-IR and VEGFR-2 expression on the surface of CETCs were detected in 84% of patients. A statistically high correlation was found between IGF-IR and VEGFR-2 (r = 0.745 and p<0.001) on the CETCs. The co-expression of both receptors was confirmed in some experiments and ranged between 70% and 100%. Statistically significant correlations were observed between the number of CETCs and IGF-IR (r = 0.315 and p<0.05) and VEGFR-2 (r = 0.310 and p<0.05) expression. The presence of CETCs and the level of IGF-IR and VEGFR-2 expression were not associated with tumor stage, hormone receptor status or n  odal/dista  nt metastasis.In this study, a parallel and co-expression of IGF-IR and VEGFR-2 was examined on the surface of CETCs in breast cancer patients for the first time. Characterization of CETCs may be a promising approach for the rational design of targeted anticancer therapies. 
 [Role of Ang-2, Tie-2 and VEGFR-2 in angiogenesis in colorectal carcinoma and their prognostic value]. To study the expression of angiotensin-2 (Ang-2), Tie-2 and vascular endothelial growth factor receptor-2 (VEGFR-2) in colorectal cancer and analyze their relationship with the occurrence, recurrence,   metastasis  , angiogenesis and prognosis of colorectal cancer.Immunohistochemistry with SP method was used to detect the expressions of Ang-2, Tie-2 and VEGFR-2 in 118 colorectal cancer, 40 adjacent normal tissue and 40 benign colorectal lesion specimens.The positivity rates of Ang-2, Tie-2 and VEGFR-2 in colorectal cancer tissue were 74.58%, 69.49%, and 61.02%, respectively, significantly higher than those in the adjacent normal tissues (25.00%, 17.50%, and 17.50%, P<0.05) and benign colorectal lesion tissues (35.00%, 32.50%, and 32.50%, P<0.05). The rates of two or three coexpression were significantly higher than that of a single expression in the cancer tissues (61.02% vs 15.25%). The microvascular density (MVD) of colorectal cancer tissues was 31.43∓10.50, significantly higher than that of the adjacent normal tissues (10.61∓3.76) and benign colorectal lesions (16.89∓3.83) (P<0.05). The expressions of Ang-2, Tie-2, and VEGFR-2 were positively correlated with carcinoembryonic antigen (CEA) and MVD (P<0.05). The expression of Ang-2, but not Tie-2 and VEGFR-2, was positively correlated with CA199. Ang-2, Tie-2, and VEGFR-2 expressions showed significant differences between cases with tumor recur  rence/meta  stasis and those without 5 years after radical mastectomy, and were all positively correlated with the 5-year survival rates (P<0.05).Ang-2, Tie-2 and VEGFR-2 are involved in the development, inva  sion, meta  stasis, and prognosis of colorectal cancer, and play important roles in the angiogenesis of the tumors. 
 Expression of VEGF and its receptors VEGFR1/VEGFR2 is associated with invasiveness of bladder cancer. Vascular endothelial growth factor (VEGF) signaling is frequently altered in invasive tumor cells and is associated with patient outcome. In the present study, we examined VEGF, VEGFR1, and VEGFR2 expression in non-muscle invasive bladder cancer (NMIBC) and muscle invasive bladder cancer (MIBC), and evaluated the association between VEGF and its receptors with disease characteristics and bladder cancer recurrence.Tissue microarrays containing bladder cancer specimens (n=212) and adjacent normal bladder mucosa (n=131) were immunostained using antibodies against VEGF, VEGFR1, and VEGFR2. The association between the expression of these proteins and clinical parameters including stage, lymph node   metastasis  , and recurrence-free survival were statistically evaluated. VEGF mRNA expression data were extracted from the public Oncomine database.VEGF and VEGFR1 mRNA levels were significantly higher in bladder cancer specimens than that of normal mucosa (for VEGF, p<0.001; for VEGFR1, p=0.02). Analysis of their expression at protein levels showed that levels of VEGF and VEGFR1 were significantly higher in NMIBC than in MIBC (p<0.001), while that of VEGFR2 was significantly higher in all cancer specimens compared to benign urothelial mucosa (p=0.001). Further-more, the expression of VEGFR2 was significantly higher in MIBC, as compared to NMIBC (p<0.001). Patients with higher levels of VEGF, VEGFR1, and VEGFR2 tended to have poorer recurrence-free survival than those with lower levels, but this was not statistically significant.Our results suggest that alterations in the expression of VEGF and VEGF receptors are associated with disease stage and recurrence. 
 Differential expression of VEGF ligands and receptors in prostate cancer. Prostate cancer disseminates to regional lymph nodes, however the molecular mechanisms responsible for lymph node   metastasis   are poorly understood. The vascular endothelial growth factor (VEGF) ligand and receptor family have been implicated in the growth and spread of prostate cancer via activation of the blood vasculature and lymphatic systems. The purpose of this study was to comprehensively examine the expression pattern of VEGF ligands and receptors in the glandular epithelium, stroma, lymphatic vasculature and blood vessels in prostate cancer.The localization of VEGF-A, VEGF-C, VEGF-D, VEGF receptor (VEGFR)-1, VEGFR-2, and VEGFR-3 was examined in cancerous and adjacent benign prostate tissue from 52 subjects representing various grades of prostate cancer.Except for VEGFR-2, extensive staining was observed for all ligands and receptors in the prostate specimens. In epithelial cells, VEGF-A and VEGFR-1 expression was higher in tumor tissue compared to benign tissue. VEGF-D and VEGFR-3 expression was significantly higher in benign tissue compared to tumor in the stroma and the endothelium of lymphatic and blood vessels. In addition, the frequency of lymphatic vessels, but not blood vessels, was lower in tumor tissue compared with benign tissue.These results suggest that activation of VEGFR-1 by VEGF-A within the carcinoma, and activation of lymphatic endothelial cell VEGFR-3 by VEGF-D within the adjacent benign stroma may be important signaling mechanisms involved in the progression and subsequent metastatic spread of prostate cancer. Thus inhibition of these pathways may contribute to therapeutic strategies for the management of prostate cancer. 
 GEP100-Arf6-AMAP1-cortactin pathway frequently used in cancer invasion is activated by VEGFR2 to promote angiogenesis. Angiogenesis and cancer invasiveness greatly contribute to cancer malignancy.Arf6 and its effector, AMAP1, are frequently overexpressed in breast cancer, and constitute a central pathway to induce the invasion and   metastasis  . In this pathway, Arf6 is activated by EGFR via GEP100. Arf6 is highly expressed also in human umbilical vein endothelial cells (HUVECs) and is implicated in angiogenesis. Here, we found that HUVECs also highly express AMAP1, and that vascular endothelial growth factor receptor-2 (VEGFR2) recruits GEP100 to activate Arf6. AMAP1 functions by binding to cortactin in cancer invasion and   metastasis  . We demonstrate that the same GEP100-Arf6-AMAP1-cortactin pathway is essential for angiogenesis activities, including cell migration and tubular formation, as well as for the enhancement of cell permeability and VE-cadherin endocytosis of VEGF-stimulated HUVECs. Components of this pathway are highly expressed in pathologic angiogenesis, and blocking of this pathway effectively inhibits VEGF- or tumor-induced angiogenesis and choroidal neovascularization. The GEP100-Arf6-AMAP1-cortactin pathway, activated by receptor tyrosine kinases, appears to be common in angiogenesis and cancer invasion and   metastasis  , and provides their new therapeutic targets. 
 Inhibition of prostate cancer osteoblastic progression with VEGF121/rGel, a single agent targeting osteoblasts, osteoclasts, and tumor neovasculature. A hallmark of prostate cancer (PCa) progression is the development of osteoblastic bone metastases, which respond poorly to available therapies. We previously reported that VEGF(121)/rGel targets osteoclast precursors and tumor neovasculature. Here we tested the hypothesis that targeting nontumor cells expressing these receptors can inhibit tumor progression in a clinically relevant model of osteoblastic PCa.Cells from MDA PCa 118b, a PCa xenograft obtained from a bone   metastasis   in a patient with castrate-resistant PCa, were injected into the femurs of mice. Osteoblastic progression was monitored following systemic administration of VEGF(121)/rGel.VEGF(121)/rGel was cytotoxic in vitro to osteoblast precursor cells. This cytotoxicity was specific as VEGF(121)/rGel internalization into osteoblasts was VEGF(121) receptor driven. Furthermore, VEGF(121)/rGel significantly inhibited PCa-induced bone formation in a mouse calvaria culture assay. In vivo, VEGF(121)/rGel significantly inhibited the osteoblastic progression of PCa cells in the femurs of nude mice. Microcomputed tomographic analysis revealed that VEGF(121)/rGel restored the bone volume fraction of tumor-bearing femurs to values similar to those of the contralateral (non-tumor-bearing) femurs. VEGF(121)/rGel significantly reduced the number of tumor-associated osteoclasts but did not change the numbers of peritumoral osteoblasts. Importantly, VEGF(121)/rGel-treated mice had significantly less tumor burden than control mice. Our results thus indicate that VEGF(121)/rGel inhibits osteoblastic tumor progression by targeting angiogenesis, osteoclastogenesis, and bone formation.Targeting VEGF receptor (VEGFR)-1- or VEGFR-2-expressing cells is effective in controlling the osteoblastic progression of PCa in bone. These findings provide the basis for an effective multitargeted approach for metastatic PCa. 
 Expression of VEGF, VEGFR, EGFR, COX-2 and MVD in cervical carcinoma, in relation with the response to radio-chemotherapy. Despite the improvement in the treatment results due to modern irradiation techniques and to the association of chemo-radiotherapy, cervical cancer remains an unsolved problem of oncology both due to the increased rate of local failures and of the distant   metastasis  . Efforts to implement new therapeutic strategies in order to obtain better results in patients with cervical cancer appear justified. Neovascularization is an important step in the tumor progression and the therapeutic targeting of the tumor blood vessels appears to be a good strategy to follow in the anti-cancer treatment. Thus, even in an incipient phase of the clinical research process, the combination between the anti-angiogenic aimed therapies and the current radio-chemotherapy seems to represent a new, feasible and promising approach. The aim of the present study was to determine the prognostic and/or predictive value of some biological markers of tumor angiogenesis and of their implication in increasing the efficacy of current treatments for this cancer.So far, 54 women were included in a prospective trial: 44 having an advanced cervical carcinoma and 10 healthy women, as controls. A tumor biopsy and a blood sample were obtained from each patient before the start of therapy. The density of microvascularization was assessed using CD34 monoclonal antibody (hot spot technique), the expression of angiogenic factors VEGFR, EGFR and COX-2 were determined in tumor biopsies by specific immunohistochemistry techniques, using primary antibodies anti-EGFR, anti-VEGF and anti-COX-2 respectively. The quantitative polymerase chain reaction (Real Time PCR) was employed for assessing the expression level of the genes involved. Serum VEGF was determined by quantitative ELISA technique.Among the studied clinical and molecular factors, we found to be predictive for the type of response the following factors: tumor size at diagnosis (p=0.01), VEGFR2 expression (p=0.02) and a tendency to significance for patients' age (p=0.06). From the large panel of studied markers it was observed correlation between MVD expression with stromal COX-2 (p=0.01) and a tendency with epithelial COX-2 (p=0.06). Stromal COX-2 has higher correlation with VEGFR2 (p=0.01) and MVD (p=0.01) and also has a lower correlation with tumor size (p=0.08).Univariate analysis demonstrates that the response to radio-chemotherapy in cervical cancer is related to a set of clinical and molecular factors as: the tumor size, the expression of VEGFR2 as mRNA level and the patients' age. Unfortunately, the multivariate analysis by logistic model selects only VEGFR2 expression for prediction of tumor response. The interrelations between the different biomarkers demonstrate the complexity of the tumor progression process and the necessity of further studies to identify new therapeutic targets. 
 [Expression of vascular endothelial growth factor and its receptors in laryngeal carcinoma cell and its significance]. To study the expression of vascular endothelial growth factor (VEGF) and both the Flt-1 and KDR high affinity VEGF receptors in human laryngeal carcinoma cells and its significance.In this study, we investigated the expression of VEGF mRNA and both the Fit-1 mRNA and KDR mRNA high affinity VEGF receptors in human laryngeal carcinoma cells using techniques of semi-quantify RT-PCR.It was showed that the expression level of VEGF mRNA was significantly increased in human laryngeal carcinoma cells as compared with the normal cells, Hacat cells (P <0.05), and there was the high expression level of Flt-1 mRNA in the Hep-2 cells, but not KDR mRNA.These data raise the possibility that VEGF and its receptors many play key roles in the growth, invasion and   metastasis   of human laryngeal carcinoma cells through either a paracrine or an autocrine mechanism. 
 Role of the VEGF ligand to receptor ratio in the progression of mismatch repair-proficient colorectal cancer. The VEGF family of ligands and receptors are intimately involved in tumor angiogenesis, lymphangiogenesis and   metastasis  . The evaluation of VEGF ligand/receptor ratios may provide a more profound understanding of the involvement of these proteins in colorectal tumour progression. The aim of this study was to elucidate the role of the VEGF ligand/receptor ratios on tumour progression and   metastasis   in patients with mismatch repair-proficient colorectal cancer.Immunohistochemistry for VEGF-A, VEGF-B, VEGF-C, VEGF-D, VEGFR1, VEGFR2 and VEGF3 was carried out on 387 mismatch repair-proficient colorectal cancers using a tissue microarray. Evaluation of immunoreactivity was performed semi-quantitatively and the ligand/receptor expression ratio was obtained.An increased VEGF-A/VEGFR1 ratio, VEGF-A and VEGFR1 was linked to the presence of peritumoral lymphocytic inflammation at the invasive front (p = 0.032; p = 0.005; p = 0.032, respectively). VEGFR1 expression was related to poorer outcome in multivariable analysis with pT stage, pN stage, vascular invasion, and post-operative therapy. A higher ratio of VEGF-A/VEGFR2 was linked to advanced TNM stage (p = 0.005) while VEGF-A and VEGFR2 were elevated in tumours with an infiltrating tumour growth pattern (p = 0.006; p = 0.014; p = 0.006). No effect of VEGF-A/VEGFR2, VEGF-A or VEGFR2 on survival time was noted.Our findings highlight an involvement of VEGF-A, VEGR1 and VEGFR2 in events occurring at the invasive tumour front and a potential prognostic role of VEGFR1 expression in mismatch repair-proficient colorectal cancers. The VEGF-A ligand to VEGFR1 or VEGFR2 ratio may represent an alternative evaluation system for identifying patients with poorer clinical outcome. 
 Increased expression of vascular endothelial growth factor and its receptors, VEGFR-1 and VEGFR-2, in medullary thyroid carcinoma. Vascular endothelial growth factor (VEGF-A) expression is upregulated in the majority of human tumors, where it stimulates proliferation, migration, and survival of endothelial cells. Studies have suggested that VEGF inhibitors can be used as an alternative therapy in medullary thyroid carcinoma (MTC), but data about expression of VEGF-A and its receptor in this tumor are scarce. The aims of this study were to evaluate VEGF-A, VEGF receptor (VEGFR)-1, VEGFR-2, and microvessel density (MVD) expression in MTC samples and correlate it with clinical parameters.Paraffin-embedded samples from 38 MTC patients were evaluated for VEGF-A, VEGFR-1, VEGFR-2, and MVD expression by immunohistochemistry. Clinical data were retrospectively reviewed in medical records.Thirty-eight patients aged 31.8 +/- 17.1 years were enrolled. Twenty-seven patients had hereditary disease (71.1%). Twenty-five of them were found to have multiple endocrine neoplasia (MEN) 2A and two were found to have MEN 2B. VEGF-A immunohistochemical staining was detected in 95% (36/38), VEGFR-1 in 96% (36/37), and VEGFR-2 in 91% (31/34) of MTC samples. Age at surgery was positively correlated with VEGFR-2 (p = 0.003). There was no correlation between VEGF-A, VEGFR-2, and tumor stage (tumor node   metastasis  ). Nevertheless, VEGFR-1 was found to be inversely correlated with tumor node   metastasis   (p = 0.034). We also observed a trend toward an association between VEGFR-1 signal intensity and cure of disease, although this did not reach statistical significance (p = 0.054). Neither VEGF-A nor VEGFR-2 was associated with disease outcome after a median follow-up period of 5 years (p = 0.882 and p = 0.236, respectively). As expected, MVD was correlated with age at surgery (p = 0.005) and tumor size (p = 0.03). Patients with the hereditary form of the disease had a stronger intensity for VEGFR-1 (p = 0.039), whereas patients with sporadic disease displayed higher MVD counts (44 [27-63] vs. 21 [9-49], p = 0.018).The VEGF-A, VEGFR-1, and VEGFR-2 immunoreactive proteins are overexpressed in MTC lesions and might be implicated in tumor progression. It is not clear, however, if expression of these molecules provides prognostic information regarding the spread or outcome of MTC. 
 Measurement of circulating levels of VEGF-A, -C, and -D and their receptors, VEGFR-1 and -2 in gastric adenocarcinoma. To analyze the serum levels and prognostic significance of vascular endothelial growth factor (VEGF) -A, -C, and -D, and their receptors, VEGFR-1 and -2 in gastric adenocarcinomas.The serum levels of VEGF family members were measured in 76 control subjects and 76 patients with gastric adenocarcinoma using an enzyme-linked immunosorbent assay (ELISA). These measurements were correlated with clinco-pathological features and survival rates.The serum levels of VEGF-A and its receptor, VEGFR-1, were significantly higher in patients with gastric cancer than in healthy donors (t = 2.3, P = 0.02 and t = 4.2, P < 0.0001, respectively). In contrast, the serum levels of VEGF-D were significantly higher in control subjects than in patients (t = 2.9, P = 0.004). There was no significant difference in serum levels of VEGF-C and VEGFR-2 between patients and controls. VEGF-C was associated with advanced tumor stage and presence of   metastasis  . VEGFR-1 was associated with   metastasis  , advanced overall stage, tumor differentiation and survival. VEGFR-2 levels were associated with poor tumor differentiation. There was no significant prognostic value for any of the VEGF family members or their receptors except for VEGFR-1 where high levels were associated with a poor overall survival.Serum VEGF levels vary significantly in the same cohort of patients with variable clinico-pathological features and prognostic values. The simultaneous measurement of VEGF receptors levels in sera may overcome the limitations of a single biomarker assay. 
 Circulating serum levels of angiogenic factors and vascular endothelial growth factor receptors 1 and 2 in melanoma patients. Angiogenesis is essential for tumor progression and   metastasis  ; however, the angiogenesis regulators that are biologically relevant for melanoma are still unknown. In this study, we analyzed the circulating serum levels of potent angiogenic factors, including vascular endothelial growth factor (VEGF), angiogenin, transforming growth factor-beta1 and VEGF receptors, VEGFR1 and VEGFR2, in human melanoma patients. One hundred and fourteen patients with histopathologically verified cutaneous melanoma at different stages and 30 healthy controls were investigated. Serum levels of angiogenic factors and VEGF receptors were quantitatively analyzed by solid-phase enzyme-linked immunosorbent assay. The age of the patients (61 men and 53 women) ranged from 18 to 80 years; median age was 51 years. Serum transforming growth factor-beta1 (P < 0.001), VEGF (P = 0.006) and VEGFR1 (P = 0.007) levels were significantly higher in patients with melanoma than in the control group. No significant differences, however, exist in the serum angiogenin and VEGFR2 levels between melanoma patients and the controls. The positive correlations of elevated serum levels of transforming growth factor-beta1, VEGF and VEGFR1 with advanced stages of disease were found. Significant relationship was found only between serum levels of VEGF and VEGFR2. Elevated serum transforming growth factor-beta1 (P < 0.001) and VEGF levels (P = 0.0012) were found to be poor prognostic factors. Serum level of angiogenin and VEGF receptors, however, had no effect on survival. Our data suggest that the angiogenic serum factors, including VEGF, transforming growth factor-beta1 and VEGFR1, but not angiogenin and VEGFR2 were increased in melanoma patients, especially associated with advanced disease stages. The mechanism of VEGF regulation of angiogenesis may in part be due to enhanced proliferation of VEGFRs, especially VEGFR1. 
 Immunolocalizations of VEGF, its receptors flt-1, KDR and TGF-beta's in epithelial ovarian tumors. Angiogenesis is an essential factor for growth, differentiation, invasion and   metastasis   of tumors. In this study, we aimed to evaluate the immunolocalizations of vascular endothelial growth factor (VEGF), its receptors flt-1, KDR/flk-1, and transforming growth factor-beta's (TGF-beta) in epithelial ovarian tumors, utilizing indirect immunohistochemistry to understand the role of the angiogenic events in ovarian neoplasia.Tissue blocks from 40 patients who had ovarian pathology (borderline serous-mucinous tumor and malignant serous-mucinous adenocarcinoma of the ovary) were included in this study. All formalin-fixed, paraffin-embedded tissue sections were stained with hematoxylin-eosin or primary antibodies against VEGF, flt-1, KDR/flk-1, TGF-beta1, TGF-beta2 and TGF-beta3 using the avidin-biotin-peroxidase method. H-SCORE, a semi-quantitative grading system, was used to compare immunohistochemical staining intensities.Positive VEGF immunoreactivity was concentrated in the epithelial and stromal parts of all the ovarian samples and the endothelial cells in the stroma were also stained. Increased immunoreactivity of VEGF was observed in malignant ovarian adenocarcinomas compared to the borderline tumors of the ovary. VEGF receptors, flt-1 and KDR/flk-1 immunoreactivities were detected not only in vascular endothelial cells, but also in tumor cells at malignant sites. Immunoreactivities of VEGF and its receptors were coexpressed in tumor cells of the ovarian carcinoma. While immunoreactivities of TGF-beta1 and TGF-beta2 were both overexpressed in malignant ovarian carcinomas, immunoreactivity of TGF-beta3 was still mild.Our results suggest that overexpression of VEGF, its receptors flt-1, KDR/flk-1 and TGF-beta interaction may play an important role in the ovarian cancer biology, with potential effects on tumor growth and angiogenesis. New therapeutic strategies using VEGF and TGF-beta antagonists could obtain an additional approach to the treatment ovarian carcinoma by inhibiting angiogenesis. 
 Metastatic properties of prostate cancer cells are controlled by VEGF. Mechanisms of   metastasis  , the major complication of prostate cancer, are poorly understood. In this study, we define molecular mechanisms that may contribute to the highly invasive potential of prostate cancer cells. Vascular endothelial growth factor (VEGF), its receptors (VEGFRs), and alpha5beta1 integrin were expressed by prostate cancer cells in vitro and by prostate tumors in vivo, and their expression was elevated at sites of bone   metastasis   compared to original prostate tumor. VEGF, through interaction with its receptors, regulated adhesive and migratory properties of the cancer cells. Specifically, the highly metastatic prostate cancer cell subline LNCaP-C4-2 showed a decreased adhesive but an enhanced migratory response to fibronectin, a ligand for alpha5beta1 integrin, compared to its nonmetastatic counterpart. A similar pattern was also observed when bone sialoprotein was used as a ligand in migration assays. Increased migration of metastatic prostate cancer cells to fibronectin and bone sialoprotein was regulated by VEGF via VEGFR-2. Tumor suppressor PTEN was involved in control of VEGF/VEGFR-2 stimulated prostate cancer cell adhesion as well as proliferation. 
 Overproduction of VEGF concomitantly expressed with its receptors promotes growth and survival of melanoma cells through MAPK and PI3K signaling. Vascular endothelial growth factor (VEGF) is an important mediator of tumor-associated angiogenesis, and consequently it has been associated with   metastasis  . We report here that the overexpression of VEGF(165) in melanoma xenografts promotes an acceleration of tumor growth and an increase in angiogenesis as well as the spontaneous   metastasis   formation. In addition, VEGF receptors (VEGFR)1, VEGFR2 and neurophilin-1 are expressed in A375 melanoma cells. Forced overexpression of VEGF in these cells induces cell growth and triggers survival activity in serum-starved cultures, by a mechanism dependent on the mitogen-activating protein kinase signaling pathway. Furthermore, these effects are dependent MEK 1/2 activity. Kinase domain region-specific tyrosine kinase inhibitors dramatically reduced DNA synthesis to 20% with respect to the controls, although they did not completely suppress either the p44 or p42-phosphorylated forms of extracellular signal-regulated protein kinase. These inhibitors also provoked a decrease in Akt phosphorylation. We observed a dramatic reduction in survival after treatment with phosphatidylinositol 3'-kinase (PI3K)-specific inhibitor in the presence of specific tyrosinase inhibitors. We suggest that the overproduction of VEGF(165) concomitantly expressed with its receptors favors cell growth and survival of melanoma cells through MAPK and PI3K signaling pathways. These data support the involvement in melanoma growth and survival of a VEGF-dependent internal autocrine loop mechanism, at least in vitro. 
 Expression of vascular endothelial growth factor and its receptors KDR and Flt-1 in gastric cancer cells. The expression of vascular endothelial growth factor (VEGF) and its receptors KDR and Flt-1 by gastric carcinoma tissues and different gastric carcinoma cell lines was detected to elucidate the molecular mechanism of this growth factor in promoting tumor growth.The expression of VEGF, Flt-1 and KDR was determined by reverse transcription-polymerase chain reaction (RT-PCR) in gastric cancer cell lines RF-1, RF-48, AGS-1, NCI-N87, NCI-SNU-1, NCI-SNU-5, NCI-SNU-16 and KATO-III. The expression of Flt-1 and KDR in paraffin-embedded specimens of gastric cancer was determined by immunohistochemistry. The 3-(4,5-dimethylthiazol-2-yl)-2, 5-diphenyltetrazolium bromide (MTT) assay was used to assess the role of VEGF in tumor cell proliferation.All 8 gastric cancer cell lines analyzed expressed VEGF(121) and VEGF(165) and six of them expressed both Flt-1 and KDR, while cell line NCI-SNU-5 expressed Flt-1 only and cell line KATOIII expressed neither Flt-1 nor KDR. The gastric carcinoma tissues expressed Flt-1 and KDR widely, with the positive rate of expression of Flt-1 and KDR being 84.6 % and 70 % respectively. The exogenous VEGF stimulated the growth of KDR-positive cell lines NCI-N87 and AGS-1 in a dose-dependent manner but exhibited no effect on the growth of KDR-negative cell line NCI-N87.VEGF and its receptors KDR and Flt-1 were expressed widely in gastric carcinoma cells and the VEGF stimulated KDR-positive tumor cell growth directly. These results suggest that VEGF may play a role in promoting tumor growth and   metastasis   by participating in both paracrine and autocrine pathways. 
 Correlation between VEGFR-2 receptor kinase domain-containing receptor (KDR) mRNA and angiotensin II receptor type 1 (AT1-R) mRNA in endometrial cancer. Angiogenesis, a multistep process that results in new blood vessel formation from preexisting vasculature is essential for both the growth of solid tumour and for   metastasis  . Stimulation of vascular endothelial growth factor receptor (VEGFR), a transmembrane glycoprotein, results in mitogenesis. Within this family of receptors, VEGFR 2/kinase-insert-domain containing receptor appears to be principally upregulated during tumorigenesis. The aim of this study was to determine the expression of VEGFR-2/kinase-insert-domain containing receptor (KDR) and its correlation with angiotensin receptor type 1 (AT1-R) and clinical factors in endometrial carcinoma.The expression of KDR and AT1-R was studied in endometrial carcinoma and normal endometrium by Real-time RT-PCR and Western blot analysis in 136 samples. The expression profile was correlated with the clinicopathological characteristics of endometrial adenocarcinoma.We noted a significant correlation between the expression of KDR and AT1-R in tumour grade G1, G2 and G3 (R(s)=0.50; p=0.002, R(s)=0.69; p=0.0001, R(s)=0.52; p=0.005, respectively). In stage I and stage II carcinoma, a significant correlation was also found between the expression of KDR and AT1-R (R(s)=0.70, p=0.0001, R(s)=0.67; p=0.001, respectively). Moreover significant correlation was observed between both KDR and AT1-R in tissue with different myometrial invasion (R(s)=0.54, p=0.0001, R(s)=0.68; p=0.0001; respectively for tumours with invasion into the inner half and invasion into the outer half).Basing on received correlation between AT1-R and KDR expression and previous results we speculate that angiotensin through AT1-R modulates KDR expression and thus have influence on local VEGF level. However, further studies are required to clarify the biological interaction between KDR, AT1-R and other hormonal regulators in endometrial carcinoma. 
 [Expression of VEGFR-2 and VEGFR-3 in papillary renal cell carcinoma and their relationship with prognosis]. To detect the expression of VEGF receptors in papillary renal cell carcinoma and to explore the correlation between their expression and clinical prognosis.Expression of VEGF receptors and PCNA (proliferating cell nuclear antigen) were evaluated in 82 patients with papillary renal cell carcinoma using tissue microarray and SP immunohistochemical staining.The expression of VEGFR-1 in papillary renal cell carcinoma was 82.93%, VEGFR-2 63.41%, VEGFR-3 34.15% and PCNA 67.07%, respectively. Increased VEGFR-2 expression was significantly correlated with tumor size (P = 0.016), histological grade (P = 0.034) and distant   metastasis   (P = 0.002). VEGFR-3 expression was correlated with histological grade (P = 0.028), lymph node status (P = 0.010) and distant   metastasis   (P = 0.018), but not correlated with gender, age, location, tumor size and TNM staging. VEGFR-1 expression had no correlation with any clinic and pathologic factors. PCNA expression was correlated with histological grade (P = 0.011), but not correlated with other factors. The expression of VEGFR-2 and VEGFR-3 in death cases were higher than that in surviving patients.Both VEGFR-2 and VEGFR-3 can serve as markers for prognosis of papillary renal cell carcinoma. Differently, VEGFR-3 is a predictor of lymph node   metastasis  , increased VEGFR-2 expression could be used to predict a potential blood dissemination. 
 Identification of PDCL3 as a novel chaperone protein involved in the generation of functional VEGF receptor 2. Angiogenesis, a hallmark step in tumor   metastasis   and ocular neovascularization, is driven primarily by the function of VEGF ligand on one of its receptors, VEGF receptor 2 (VEGFR-2). Central to the proliferation and ensuing angiogenesis of endothelial cells, the abundance of VEGFR-2 on the surface of endothelial cells is essential for VEGF to recognize and activate VEGFR-2. We have identified phosducin-like 3 (PDCL3, also known as PhLP2A), through a yeast two-hybrid system, as a novel protein involved in the stabilization of VEGFR-2 by serving as a chaperone. PDCL3 binds to the juxtamembrane domain of VEGFR-2 and controls the abundance of VEGFR-2 by inhibiting its ubiquitination and degradation. PDCL3 increases VEGF-induced tyrosine phosphorylation and is required for VEGFR-2-dependent endothelial capillary tube formation and proliferation. Taken together, our data provide strong evidence for the role of PDCL3 in angiogenesis and establishes the molecular mechanism by which it regulates VEGFR-2 expression and function. 
 The relevance of cell type- and tumor zone-specific VEGFR-2 activation in locally advanced colon cancer. For the successful therapeutic use of inhibitors of the vascular endothelial growth factor receptor (VEGFR) pathway detailed knowledge of the mechanisms leading to tumor progression is indispensable. The main goal of this study was to determine the relevance of the VEGFR-2 activating pathway for colon carcinoma (CC)   metastasis  . The initial event is ligand-induced receptor activation through tyrosine autophosphorylation.VEGFR-2, its ligands VEGF-C and VEGF-D and the phosphorylated (activated) receptor forms pVEGFR-2(Tyr1175) and pVEGFR-2(Tyr1214) were investigated immunohistochemically in different tumor compartments (intratumoral (zone 1) - invasive front (zone 2) - extratumoral soft tissue (zone 3)) and various cell types (tumor cells, inflammatory cells, macro- and microvasculature) in 84 non-metastatic, lymphogenous-metastatic and haematogenous-metastatic CC.VEGF-D produced by tumor cells has an autocrine affinity for its receptor VEGFR-2. In tumor budding regions VEGF-D-induced receptor activation by autophosphorylation at Tyr1214 seems to be a possible initial event of the VEGFR-2-mediated signaling pathway, but without effect on metastatic behaviour. In inflammatory cells of almost all CC VEGFR-2 phosphorylation at Tyr 1175 and Tyr 1214 was detectable without accompanying receptor expression, suggesting receptor activation without cell surface expression. Peritumoral inflammatory cells also expressed paracrine acting VEGF-C. The autocrine VEGF-D/VEGFR-2 signaling axis and receptor autophosphorylation at Tyr1214 appear to be main events for capillaries in all three tumor zones and for small vessels in zone 1 and 2. Independent of the metastatic status a large number of cases with capillary immunopositivity in the angiogenically active invasive front was documented, especially for VEGF-D, VEGFR-2 and pVEGFR-2(Tyr1214). VEGFR-2 positive extratumoral capillaries were significantly more common in distant metastatic CC. In all tumor compartments the investigated biomolecules were also detected in different frequencies in the macrovasculature, which is responsible for sufficient tumor vascularization. In addition, vascular paracrine-acting VEGF-C production was widely detected, but without zone and vessel-type dependence.The VEGFR-2 activating pathway is closely involved in tumor cell-associated, vessel-mediated and immuno-inflammatory processes in colon carcinoma and appears to contribute to tumor survival and growth as well as maintenance of the infiltrative phenotype rather than to promote   metastasis  . 
 The effects of VEGF and VEGFR-2 on survival in patients with gastric cancer. Prognostic criteria of the patients with gastric cancer are of critical importance in their management and follow-up. Angiogenesis is essential for the growth and   metastasis   of solid tumors. Tumor angiogenesis is a multi-step interactive process, and vascular endothelial growth factor (VEGF) and its receptors have a major role in tumor angiogenesis. Thus, we investigated the effects of VEGF and VEGF receptor-2 (VEGFR-2, KDR) on survival in patients with gastric cancer. We analyzed 51 patients who had undergone total or subtotal gastric resection. The patients were divided into two subgroups according to their VEGF and VEGFR-2 (KDR) expression in resected specimens. There was no significant difference between sex, surgical method, lymph node   metastasis  , serosal invasion, hematogenous   metastasis  , chemotherapy status of the two subgroups. Mean follow-up time was 24.22 +/- 15.38 months. We found the survival rates of the patients with VEGF positive tumors to be significantly shorter than those of the patients with VEGF negative tumors. There was no significant difference between the survival rates of VEGFR-2 (KDR) positive and negative patients. It was established that the presence of VEGF expression was significantly associated with the short survival rates in patients with gastric cancer. Analysis of VEGF expression in resected specimens may provide additional guidance in determining the prognosis of such patients. If more extensive studies confirm the significance of VEGF and its receptors in gastric cancer, new therapeutic approaches targeting VEGF and its receptors may be considered in gastric cancer management. 
 Anticancer activity of TTAC-0001, a fully human anti-vascular endothelial growth factor receptor 2 (VEGFR-2/KDR) monoclonal antibody, is associated with inhibition of tumor angiogenesis. Vascular endothelial growth factor (VEGF) and its receptors are considered the primary cause of tumor-induced angiogenesis. Specifically, VEGFR-2/kinase insert domain receptor (KDR) is part of the major signaling pathway that plays a significant role in tumor angiogenesis, which is associated with the development of various types of tumor and   metastasis  . In particular, KDR is involved in tumor angiogenesis as well as cancer cell growth and survival. In this study, we evaluated the therapeutic potential of TTAC-0001, a fully human antibody against VEGFR-2/KDR. To assess the efficacy of the antibody and pharmacokinetic (PK) relationship in vivo, we tested the potency of TTAC-0001 in glioblastoma and colorectal cancer xenograft models. Antitumor activity of TTAC-0001 in preclinical models correlated with tumor growth arrest, induction of tumor cell apoptosis, and inhibition of angiogenesis. We also evaluated the combination effect of TTAC-0001 with a chemotherapeutic agent in xenograft models. We were able to determine the relationship between PK and the efficacy of TTAC-0001 through in vivo single-dose PK study. Taken together, our data suggest that targeting VEGFR-2 with TTAC-0001 could be a promising approach for cancer treatment. 
 The -271 G>A polymorphism of kinase insert domain-containing receptor gene regulates its transcription level in patients with non-small cell lung cancer. Kinase insert domain-containing receptor (KDR) plays a critical role in the   metastasis   of cancer and is used as a molecular target in cancer therapy. We investigated the characteristics of the -271 G>A polymorphism of the KDR gene to gain information that may benefit the development of individualized therapies for patients with non-small cell lung cancer (NSCLC).The -271 G>A polymorphism of the KDR gene in 106 lung cancer patients and 203 healthy control individuals was analyzed by polymerase chain reaction (PCR) and DNA sequencing methods. Real-time quantitative PCR and immunohistochemical methods were used to evaluate KDR mRNA and protein expression levels, respectively, in frozen tumor specimens.The -271 G>A polymorphism was associated with the mRNA expression level of the KDR gene in tumor tissues (t = 2.178, P = 0.032, independent samples t-test). Compared with the AG/GG genotype, the AA genotype was associated with higher KDR mRNA expression in tumor tissues. We found no relationship between the genotype and the KDR protein expression level and no significant difference in the distribution of the KDR gene polymorphism genotypes between lung cancer patients and the control group (chi2 = 1.269, P = 0.264, Fisher's exact test).This study is the first to show that the -271 G>A polymorphism of the KDR gene may be a functional polymorphism related to the regulation of gene transcription. These findings may have important implications for therapies targeting KDR in patients with NSCLC. 
 Tumor-induced activation of lymphatic endothelial cells via vascular endothelial growth factor receptor-2 is critical for prostate cancer lymphatic   metastasis  . Prostate cancer disseminates initially and primarily to regional lymph nodes. However, the nature of interactions between tumor cells and lymphatic endothelial cells (LEC) is poorly understood. In the current study, we have isolated prostate LECs and developed a series of two-dimensional and three-dimensional in vitro coculture systems and in vivo orthotopic prostate cancer models to investigate the interactions of prostate cancer cells with prostate LECs. In vitro, highly lymph node metastatic prostate cancer cell lines (PC-3 and LNCaP) and their conditioned medium enhanced prostate LEC tube formation and migration, whereas poorly lymph node metastatic prostate cancer cells (DU145) or normal prostate epithelial cells (RWPE-1) or their conditioned medium had no effect. In vivo, the occurrence of lymphatic invasion and lymph node   metastasis   was observed in PC-3 and LNCaP xenografts but not in DU145 xenografts. Furthermore, vascular endothelial growth factor (VEGF) receptor (VEGFR)-2 is expressed by prostate LECs, and its ligands VEGF-A, VEGF-C, and VEGF-D are up-regulated in highly lymph node metastatic prostate cancer cells. Recombinant VEGF-A and VEGF-C, but not VEGF-C156S, potently promoted prostate LEC tube formation, migration, and proliferation in vitro, indicating that signaling via VEGFR-2 rather than VEGFR-3 is involved in these responses. Consistent with this, blockade of VEGFR-2 significantly reduced tumor-induced activation of LECs. These results show that the interaction of prostate tumor cells with LECs via VEGFR-2 modulates LEC behavior and is related to the ability of tumor cells to form lymph node metastases. 
 Dual inhibition using cabozantinib overcomes HGF/MET signaling mediated resistance to pan-VEGFR inhibition in orthotopic and metastatic neuroblastoma tumors. MET is expressed on neuroblastoma cells and may trigger tumor growth, neoangiogenesis and   metastasis  . MET upregulation further represents an escape mechanism to various anticancer treatments including VEGF signaling inhibitors. We developed in vitro a resistance model to pan-VEGFR inhibition and explored the simultaneous inhibition of VEGFR and MET in neuroblastoma models in vitro and in vivo using cabozantinib, an inhibitor of the tyrosine kinases including VEGFR2, MET, AXL and RET. Resistance in IGR-N91-Luc neuroblastoma cells under continuous in vitro exposure pressure to VEGFR1-3 inhibition using axitinib was associated with HGF and p-ERK overexpression. Cabozantinib exhibited anti-proliferative effects in neuroblastoma cells and reduced cell migration in vitro as measured by phase-contrast with IncuCyte system. In vivo, an enhanced number of animals with IGR-N91-Luc metastases was noted following axitinib treatment as compared to control animals. Orally administered cabozantinib per gavage at 30 and 60 mg/kg/day significantly inhibited tumor growth of orthotopic adrenal IGR-N91-Luc and metastatic IMR-32-Luc xenografts. Antitumor activity was associated with decreased vascularization, inhibition of p-SRC and induction of apoptotic cell death. Activation of the HGF-mediated MET pathway is involved in escape to selective VEGFR inhibition in neuroblastoma suggesting combined inhibition of MET and VEGFR signaling to reduce secondary resistance and enhanced invasiveness. 
 RNAi-mediated silencing of VEGF-C inhibits non-small cell lung cancer progression by simultaneously down-regulating the CXCR4, CCR7, VEGFR-2 and VEGFR-3-dependent axes-induced ERK, p38 and AKT signalling pathways. Vascular endothelial growth factor C (VEGF-C) expression is associated with the malignant tumour phenotype making it an attractive therapeutic target. We investigated the biological roles of VEGF-C in tumour growth, migration, invasion and explored the possibility of VEGF-C as a potential therapeutic target for the treatment of non-small cell lung cancer (NSCLC). A lentivirus-mediated RNA interference (RNAi) technology was used to specifically knockdown the expression of VEGF-C in A549 cells. Quantitative reverse transcriptase-polymerase chain reaction, flow cytometry, Western blot, immunohistochemistry, cellular growth, migration, invasion and ELISA assays were used to characterise VEGF-C expression in vitro. A lung cancer xenograft model in nude mice was established to investigate whether knockdown of VEGF-C reduced tumour growth in vivo. Silencing of VEGF-C suppressed tumour cell growth, migration and invasion in vitro; suppressed tumour growth, angiogenesis and lymphangiogenesis by tail vein injection of lentivirus encoded shRNA against VEGF-C in vivo. More importantly, silencing of VEGF-C also trapped the VEGFR-2, VEGFR-3, CXCR4, CCR7-dependent axes, and down-regulated the AKT, ERK and p38 signalling pathways. These results suggest that VEGF-C has a multifaceted role in NSCLC growth, migration and invasion; that VEGF-C-mediated autocrine loops with their cognate receptors and chemokine receptors are significant factors affecting tumour progression; and that RNAi-mediated silencing of VEGF-C represents a powerful therapeutic approach for controlling NSCLC growth and   metastasis  . 
 VEGFR signaling during lymphatic vascular development: From progenitor cells to functional vessels. Lymphatic vessels are an integral component of the cardiovascular system, serving important roles in fluid homeostasis, lipid absorption, and immune cell trafficking. Defining the mechanisms by which the lymphatic vasculature is constructed and remodeled into a functional vascular network not only provides answers to fascinating biological questions, but is fundamental to understanding how lymphatic vessel growth and development goes awry in human pathologies. While long recognized as dysfunctional in lymphedema and exploited as a route of tumor   metastasis  , recent work has highlighted important roles for lymphatic vessels in modulating immune responses, regulating salt-sensitive hypertension and important for lung inflation at birth. Substantial progress in our understanding of the signaling pathways important for development and morphogenesis of the lymphatic vasculature has been made in recent years. Here, we review advances in our knowledge of the best characterized of these signaling pathways, that involving the vascular endothelial growth factor (VEGF) family members VEGF-C and VEGF-D, together with their receptors VEGFR2 and VEGFR3. Recent work has defined multiple levels at which signal transduction by means of this key axis is regulated; these include control of ligand processing and bioavailability, modulation of receptor activation by interacting proteins, and regulation of receptor endocytosis and trafficking. 
 Generation and characterization of a functional Nanobody against the vascular endothelial growth factor receptor-2; angiogenesis cell receptor. Vascular endothelial growth factor receptor-2 (VEGFR2) is an important tumor-associated receptor and blockade of the VEGF receptor signaling can lead to the inhibition of neovascularization and tumor   metastasis  . Nanobodies are the smallest intact antigen binding fragments derived from heavy chain-only antibodies occurring in camelids. Here, we describe the identification of a VEGFR2-specific Nanobody, named 3VGR19, from dromedaries immunized with a cell line expressing high levels of VEGFR2. We demonstrate by FACS, that 3VGR19 Nanobody specifically binds VEGFR2 on the surface of 293KDR and HUVECs cells. Furthermore, the 3VGR19 Nanobody potently inhibits formation of capillary-like structures. These data show the potential of Nanobodies for the blockade of VEGFR2 signaling and provide a basis for the development of novel cancer therapeutics. 
 Vascular endothelial growth factor receptor 2 gene polymorphisms as predictors for tumor recurrence and overall survival in non-small-cell lung cancer. VEGFR-2 gene displays several functional germline polymorphisms with impact on VEGFR-2 mediated angiogenesis. Our purpose was to evaluate VEGFR-2 polymorphisms as prognostic markers for tumor recurrence and overall survival (OS) in non-small-cell lung cancer (NSCLC).In total, 209 Caucasian patients who had been surgically treated for NSCLC between 1996 and 2010 were included in this study. Genotyping of peripheral blood cells was performed by TaqMan® genotyping assays or polymerase chain reaction for five VEGFR-2 polymorphisms. Chi- square test, Kaplan-Meier estimator, and Cox regression hazard model were used to assess the prognostic value of VEGFR-2 polymorphisms.VEGFR-2+4422 (AC)10-14 polymorphism was identified as a positive prognostic marker for time to   metastasi  s (11/12 vs. 11/11 (AC) repeats: hazard ratio (HR), 0.28; 95% confidence interval (CI), 0.11-0.75; p=0.012) and OS (11/12 vs. 11/11 (AC) repeats: HR, 0.41; 95% CI, 0.21-0.82; p=0.012) in squamous cell carcinoma. For adenocarcinoma, VEGFR-2-906 C>T (C/T vs. CC: HR, 0.19; 95% CI, 0.43-0.82; p=0.027) and VEGFR-2-271 G>A (G/A vs. G/G: HR, 0.25; 95% CI, 0.07-0.86; p=0.027) predicted longer time to local recurrence and VEGFR-2-906 C>T was a predictor for better OS (T/T vs. C/C: HR, 0.28; 95% CI, 0.09-0.84; p=0.024).VEGFR2 germline polymorphisms predict tumor recurrence and OS in NSCLC. 
 Overexpression and activation of EGFR and VEGFR2 in medullary thyroid carcinomas is related to   metastasis  . Therapeutic options for patients with metastatic medullary thyroid carcinoma (MTC) are limited due to lack of effective treatments. Thus, there is a need to thoroughly characterize the pathways of molecular pathogenesis and to identify potential targets for therapy in MTC. Since epidermal growth factor receptor (EGFR) seems to play a crucial role for RET activation, a key feature of MTCs, and several promising EGFR/vascular endothelial growth factor receptor 2 (VEGFR2)-targeted drugs have been developed, the present study was designed to investigate whether these proteins are altered in MTCs. We used a well-characterized series of 153 MTCs to evaluate EGFR activation by sequencing and FISH analysis, and to perform EGFR and VEGFR2 immunohistochemistry. EGFR tyrosine kinase domain mutations were not a feature of MTCs; however, EGFR polysomy and a strong EGFR expression were detected in 15 and 13% of the tumors respectively. Interestingly, EGFR was significantly overexpressed in metastases compared with primary tumors (35 vs 9%, P=0.002). We also studied whether specific RET mutations were associated with EGFR status, and found a decrease in EGFR polysomies (P=0.006) and a tendency towards lower EGFR expression for the most aggressive RET mutations (918, 883). Concerning VEGFR2,   metastasis   showed a higher expression than primary tumors (P=2.8 x 10(-8)). In this first study investigating the relationship between EGFR, RET, and VEGFR2 in a large MTC series, we found an activation of EGFR and VEGFR2 in   metastasis  , using both independent and matched primary/  metastasis   samples. This suggests that some MTC patients may benefit from existing anti-EGFR/VEFGR2 therapies, although additional preclinical and clinical evidence is needed. 
 Inverse correlation of phospho-KDR/Flk-1 expression and stage of colorectal cancer: implication of the significance of neoangiogenesis in activated VEGFR-2 expressing early stage colorectal adenocarcinomas. The activation of vascular endothelial cell growth factor receptors (VEGFRs) plays an essential role in cancer progression. In this study, we investigated the expression of phosphorylated VEGFR-2 (or phospho-KDR/Flk-1), the activated form of VEGFR-2, in human colorectal adenomas and colorectal adenocarcinomas. Phospho-KDR/Flk-1 showed weak expression in the normal colorectal tissue. Phospho-KDR/Flk-1 was mainly stained in the cytoplasm of colorectal adenomas, and was stained in both the cytoplasm and nuclei colorectal adenocarcinomas. There was no indication of increased phospho-KDR/Flk-1 expression in the colorectal adenocarcinomas, as compared to that of colorectal adenomas. Furthermore, there was an inverse relationship of phospho-KDR/Flk-1 expression with cancer stage (p < 0.0001), lymph node   metastasis   (p = 0.011), and distant   metastasis   (p = 0.021) of the colorectal adenocarcinomas. Our results indicate that early stage colorectal adenocarcinomas with highly activated (phosphorylated) VEGFR-2 expression may indicate the significance of neoangiogenesis of the tumors. 
 Genetic variations in the VEGF pathway as prognostic factors in metastatic colorectal cancer patients treated with oxaliplatin-based chemotherapy. Angiogenesis is a significant biological mechanism in the progression and   metastasis   of solid tumors. Vascular endothelial growth factor (VEGF), its receptors and signaling effectors have a central role in tumor-induced angiogenesis. Genetic variation in the VEGF pathway may impact on tumor angiogenesis and, hence, on clinical cancer outcomes. This study evaluates the influence of common genetic variations within the VEGF pathway in the clinical outcomes of 172 metastatic colorectal cancer (mCRC) patients treated with first-line oxaliplatin/5-fluorouracil chemotherapy. A total of 27 single-nucleotide polymorphisms (SNPs) in 16 genes in the VEGF-dependent angionenesis process were genotyped using a dynamic array on the BioMark™ system. After assessing the KRAS mutational status, we found that four SNPs located in three genes (KISS1, KRAS and VEGFR2) were associated with progression-free survival. Five SNPs in three genes (ITGAV, KRAS and VEGFR2) correlated with overall survival. The gene-gene interactions identified in the survival tree analysis support the importance of VEGFR2 rs2071559 and KISS1 rs71745629 in modulating these outcomes. This study provides evidence that functional germline polymorphisms in the VEGF pathway may help to predict outcome in mCRC patients who undergo oxaliplatin/5-fluorouracil chemotherapy. 
 VEGF and VEGFR-2 immunohistochemistry in human melanocytic naevi and cutaneous melanomas. Vascular endothelial growth factor (VEGF) and vascular endothelial growth factor receptor-2 (VEGFR-2) play a key role in vasculogenesis and angiogenic sprouting, which are crucial for tumour development and   metastasis  . In order to determine their possible role in the acquisition of metastatic potential throughout melanocytic tumour progression, VEGF and VEGFR-2 immunohistochemical expression were evaluated in 36 human melanocytic tumours of the skin (24 malignant melanomas and 12 common naevi). Different VEGFR-2 immunostaining patterns were detected in the vast majority of melanomas (21/24; 88%). A nuclear membrane-like pattern was mainly associated with in situ and microinvasive melanomas, whereas a combined cytoplasmic membrane and nuclear membrane-like pattern was seen in invasive melanomas. A nuclear membrane-like pattern was also observed in 83% (10/12) of common naevi. Cytoplasmic immunostaining for VEGF was observed in 72% (8/11) of in situ/microinvasive melanomas, 84% (11/13) of invasive melanomas and 91% (11/12) of naevi. CD31 was also investigated as an immunohistochemical marker for microvessel density (MVD) evaluation. No associations were found between MVD and VEGF/VEGFR-2 expression. Taken together, these data indicate that VEGF production is a common event in benign melanocytic tumours, whereas VEGFR-2 expression, co-localized in the cytoplasmic and nuclear membrane, is associated with progression towards invasive melanoma. The role exerted by VEGF/VEGFR-2, however, seems to be independent of the development of a tumour-related capillary network. 
 Proprotein convertases promote processing of VEGF-D, a critical step for binding the angiogenic receptor VEGFR-2. Vascular endothelial growth factor (VEGF)-D is a secreted glycoprotein that induces angiogenesis and lymphangiogenesis. It consists of a central domain, containing binding sites for VEGF receptor-2 (VEGFR-2) and VEGFR-3, and N- and C-terminal propeptides. It is secreted from the cell as homodimers of the full-length form that can be proteolytically processed to remove the propeptides. It was recently shown, using adenoviral gene delivery, that fully processed VEGF-D induces angiogenesis in vivo, whereas full-length VEGF-D does not. To better understand these observations, we monitored the effect of VEGF-D processing on receptor binding using a full-length VEGF-D mutant that cannot be processed. This mutant binds VEGFR-2, the receptor signaling for angiogenesis, with approximately 17,000-fold lower affinity than mature VEGF-D, indicating the importance of processing for interaction with this receptor. Further, we show that members of the proprotein convertase (PC) family of proteases promote VEGF-D processing, which facilitates the VEGF-D/VEGFR-2 interaction. The PCs furin and PC5 promote cleavage of both propeptides, whereas PC7 promotes cleavage of the C-terminal propeptide only. The finding that PCs promote activation of VEGF-D and other proteins with roles in cancer such as matrix metalloproteinases, emphasizes the importance of these enzymes as potential regulators of tumor progression and   metastasis  . 
 Vascular endothelial growth factor overexpression by soft tissue sarcoma cells: implications for tumor growth,   metastasis  , and chemoresistance. To better elucidate the role of vascular endothelial growth factor (VEGF)(165) in soft tissue sarcoma (STS) growth,   metastasis  , and chemoresistance, we generated stably transfected human STS cell lines with VEGF(165) to study the effect of VEGF(165) on STS cells in vitro and the effect of culture medium from these cells on human umbilical vascular endothelial cells. Severe combined immunodeficient mice bearing xenografts of transfected cell lines were used to assess the effect of VEGF overexpression and the effect of VEGF receptor (VEGFR) 2 inhibition on STS growth,   metastasis  , and response to doxorubicin. VEGF(165)-transfected xenografts formed highly vascular tumors with shorter latency, accelerated growth, enhanced chemoresistance, and increased incidence of pulmonary metastases. Blockade of VEGFR2 signaling using DC101 anti-VEGFR2 monoclonal antibody enhanced doxorubicin chemoresponse; this combined biochemotherapy inhibited tumor growth and decreased pulmonary metastases without overt toxicity. Combined therapy reduced microvessel counts while increasing vessel maturation index. VEGF overexpression did not affect on the sarcoma cells per se; however, conditioned medium from VEGF transfectants caused increased endothelial cell proliferation, migration, and chemoresistance. Addition of DC101 induced endothelial cell sensitivity to doxorubicin and suppressed the activity of matrix metalloproteinases secreted by endothelial cells. We therefore conclude that VEGF is a critical determinant of STS growth and   metastasis   and that STS chemoresistance, in our model, is a process induced by the interplay between STS cells and tumor-associated endothelial cells. STS growth and   metastasis   can be interrupted by combined low-dose doxorubicin and anti-VEGFR2, a strategy that attacks STS-associated endothelial cells. In the future, such therapeutic approaches may be useful in treating STS before the development of clinically apparent metastases. 
 Prognostic significance of epidermal growth factor receptor and vascular endothelial growth factor receptor in colorectal adenocarcinoma. The purpose of this study was to evaluate the association between the expression of growth factors and the clinicopathological variables of colorectal adenocarcinoma. Immunohistochemistry and fluorescence in situ hybridization (FISH) were used to evaluate the amplification and expression of epidermal growth factor receptor (EGFR), vascular endothelial growth factor (VEGF), VEGF-D, VEGF receptor (VEGFR)-2, VEGFR-3, transforming growth factor (TGF)-β1, and insulin-like growth factor-1 receptor (IGF-1R) in a tissue microarray of 292 colorectal adenocarcinomas. The expression of EGFR, VEGF, VEGF-D, VEGFR-2 and VEGFR-3 was detected in 5.1%, 10.0%, 6.8%, 5.2%, and 57.2%. EGFR expression was associated with angioinvasion (p < 0.05) and lymph node   metastasi  s (p < 0.005). VEGFR-3 expression was higher in the rectum than in the colon (p < 0.05). VEGF expression correlated with VEGF-D (p < 0.05) and VEGFR-3 (p < 0.005) expression, while VEGF-D expression showed no significant association with VEGFR-2 or VEGFR-3. EGFR amplification was present in 10.6% and was not associated with EGFR protein expression. VEGFR-2 and VEGFR-3 expression levels were related to poor patient survival. Stage, perineural invasion, and lymph node   metastasi  s were independent prognostic factors based on a Cox analysis. VEGFR-2 and VEGFR-3 expression are markers of a poor prognosis in patients with surgically resected colorectal adenocarcinoma, whereas EGFR has a minor influence. 
 VEGFR-2 expression in carcinoid cancer cells and its role in tumor growth and   metastasis  . Carcinoid tumors are slow growing and highly vascular neuroendocrine neoplasms that are increasing in incidence. Previously, we showed that carcinoid tumors express vascular endothelial growth factor receptor 2 (VEGFR-2) in the epithelial compartment of carcinoid tumor sections; yet, its role is not completely understood. The purpose of our study was to: (i) assess the expression of VEGFR-2 in the novel human carcinoid cell line BON, (ii) to determine the role of PI3K/Akt signaling on VEGFR-2 expression and (iii) to assess the effect of VEGFR-2 on BON cell invasion, migration and proliferation. We found that, although VEGFR-2 is expressed in BON cells, reduction in VEGFR-2 expression actually enhanced proliferation, invasion, and migration of the BON cell line. Also, expression of VEGFR-2 was inversely related to PI3K signaling. Carcinoid liver metastases in mice demonstrated decreased VEGFR-2 expression. Furthermore, the expression of a truncated, soluble form of VEGFR-2 (sVEGFR-2), a protein demonstrated to inhibit cell growth, was detected in BON cells. The presence of VEGFR-2 in the epithelial component of carcinoid tumors and in the BON cell line suggests an alternate role for VEGFR-2, in addition to its well-defined role in angiogenesis. The expression of sVEGFR-2 may explain the inverse relationship between VEGFR-2 expression and PI3K/Akt signaling and the inhibitory effect VEGFR-2 has on BON cell proliferation, migration and invasion. 
 Receptors for advanced glycation end products (RAGE) is associated with microvessel density and is a prognostic biomarker for clear cell renal cell carcinoma. The receptor for advanced glycation end products (RAGE) is involved in a variety of biological processes, including tumorigenisis. Previous studies have demonstrated that RAGE regulates the neo-angiogenesis related downstream molecule - vascular endothelial growth factor receptor 2 (VEGFR-2). Here, we investigated the potential relationship between RAGE, VEGFR-2 and angiogenesis in 80 renal cell carcinoma (RCC) patients. Real-time quantitative PCR and ELISA analysis were used to explore the RAGE and VEGFR-2 gene expression levels and the protein of VEGFR-2 expression. Meanwhile, angiogenesis was detected by the semi-quantification of endothelial cell marker CD34 combined with caldesmon, which was detected by microvessel density (MVD) technique and immunohistochemistry. Tumors were classified as low or high RAGE-expressing using the median as the cut-off. Immunofluorescence staining for RAGE protein was performed as well. Additionally, the median gene expression levels of VEGFR-2 in the tumors were significantly lower expressing low levels of RAGE expression, 0.34 (95% CI, 0.28-0.39) compared to the expressing high levels of RAGE expression, 0.45 (95% CI, 0.29-0.61), (P=0.03). The median MVD was significantly lower in the tumors expressing low levels of RAGE, 6.5 (95% CI, 6.21-7.43), compared to the expressing high levels, 7.9 (95% CI, 6.25-8.93), (P<0.01). Further, a positive association was certified with VEGFR-2 protein levels, P=0.07. Besides, RCC with high levels of RAGE expression are associated with high VEGFR-2 mRNA/protein levels and a higher density of microvessels; conversely, Kaplan-Meier survival analysis suggests that a significant correlation of elevated RAGE expression with decreased overall survival an  d metastas  is-free survival. Our results establish that RAGE was identified as a potential prognostic biomarker for disease prognosis of RCC. 
 Downregulation of KDR expression induces apoptosis in breast cancer cells. Angiogenesis plays a crucial role in the growth, invasion and   metastasis   of breast cancer. Vascular endothelial growth factors (VEGFs) and their receptors (VEGFRs) are the key regulators of tumor angiogenesis. VEGFR-2, known as the kinase insert domain receptor (KDR), is a key receptor involved in malignant angiogenesis. We previously showed that knocking down KDR with short interference RNA (KDR-siRNA) markedly decreased KDR expression and suppressed tumor growth in a xenograft model. However, the mechanisms underlying the anti-cancer effects of KDR-siRNA are not clearly understood. This study aimed to elucidate the molecular mechanisms that induce apoptosis in human breast cancer MCF-7 cells after transfection with KDR-siRNA. We studied the effects of KDR-siRNA on proliferation, apoptosis, antiapoptotic and pro-apoptotic proteins, mitochondrial membrane permeability, cytochrome c release and caspase-3 activity. The results indicated that KDR-siRNA treatment significantly inhibited the proliferation and induced the apoptosis of MCF-7 cells, reduced the levels of the anti-apoptotic proteins, Bcl-2 and Bcl-xl, and increased the level of the pro-apoptotic protein Bax, resulting in a decreased Bcl-2/Bax ratio. KDR-siRNA also enhanced the mitochondrial membrane permeability, induced cytochrome c release from the mitochondria, upregulated apoptotic protease-activating factor-1 (Apaf-1), cleaved caspase-3, and increased caspase-3 activity in MCF-7 cells. Furthermore, KDR-siRNA-induced apoptosis in MCF-7 cells was blocked by the caspase inhibitor Z-VAD-FMK, suggesting a role of caspase activation in the induction of apoptosis. These results indicate that the Bcl-2 family proteins and caspase-related mitochondrial pathways are primarily involved in KDR-siRNAinduced apoptosis in MCF-7 cells and that KDR might be a potential therapeutic target for human breast cancer treatments. 
 Jiedu Xiaozheng Yin, a Chinese herbal formula, inhibits tumor angiogenesis via downregulation of VEGF-A and VEGFR-2 expression in vivo and in vitro. Angiogenesis is crucial for cancer growth and   metastasis   and inhibition of angiogenesis has been recognized to be a promising strategy for the treatment of cancer. Traditional Chinese medicine (TCM) has been used for thousands of years to treat cancer. Jiedu Xiaozheng Yin (JXY), a polyherbal formula of TCM, has been used to treat various tumors in China. However, the mechanism of its anticancer activity has yet to be fully elucidated. Using human umbilical vein endothelial cells (HUVECs), chick chorioallantoic membrane (CAM) and a hepatoma mouse xenograft model, we investigated the underlying molecular mechanisms of ethanol extract of Jiedu Xiaozheng Yin (EE-JXY). EE-JXY treatment significantly inhibited tumor cell growth both in vitro and in the mouse xenograft model (P<0.05). Moreover, EE-JXY reduced tube formation of HUVECs and angiogenesis in the CAM (P<0.01) and microvessel density (MVD) of tumor in vivo (P<0.05). Further studies showed that EE-JXY was able to suppress the expression of vascular endothelial growth factor A (VEGF-A) and vascular endothelial growth factor receptor 2 (VEGFR-2) in both HepG2 cells and HUVECs (P<0.01) and in tumor (P<0.01). Thus, JXY suppressed tumor growth at least by inhibiting angiogenesis. 
 PDGFRα/β and VEGFR2 polymorphisms in colorectal cancer: incidence and implications in clinical outcome. Angiogenesis plays an essential role in tumor growth an  d metastas  is, and is a major target in cancer therapy. VEGFR and PDGFR are key players involved in this process. The purpose of this study was to assess the incidence of genetic variants in these receptors and its potential clinical implications in colorectal cancer (CRC).VEGFR2, PDGFRα and PDGFRβ mutations were evaluated by sequencing their tyrosine kinase domains in 8 CRC cell lines and in 92 samples of patients with CRC. Correlations with clinicopathological features and survival were analyzed.Four SNPs were identified, three in PDGFRα [exon 12 (A12): c.1701A>G; exon 13 (A13): c.1809G>A; and exon 17 (A17): c.2439+58C>A] and one in PDGFRβ [exon 19 (B19): c.2601A>G]. SNP B19, identified in 58% of tumor samples and in 4 cell lines (LS174T, LS180, SW48, COLO205), was associated with higher PDGFR and pPDGFR protein levels. Consistent with this observation, 5-year survival was greater for patients with PDGFR B19 wild type tumors (AA) than for those harboring the G-allele genotype (GA or GG) (51% vs 17%; p=0.073). Multivariate analysis confirmed SNP B19 (p=0.029) was a significant prognostic factor for survival, independent of age (p=0.060) or TNM stage (p<0.001).PDGFRβ exon 19 c.2601A>G SNP is commonly encountered in CRC patients and is associated with increased pathway activation and poorer survival. Implications regarding its potential influence in response to PDGFR-targeted agents remain to be elucidated. 
 VEGF₁₂₁-conjugated mesoporous silica nanoparticle: a tumor targeted drug delivery system. The vascular endothelial growth factor (VEGF)/VEGF receptor (VEGFR) signaling cascade plays a critical role in tumor angiogenesi  s and meta  stasis and has been correlated with several poorly prognostic cancers such as malignant gliomas. Although a number of anti-VEGFR therapies have been conceived, inefficient drug administration still limits their therapeutic efficacy and raises concerns of potential side effects. In the present work, we propose the use of uniform mesoporous silica nanoparticles (MSNs) for VEGFR targeted positron emission tomography imaging and delivery of the anti-VEGFR drug (i.e., sunitinib) in human glioblastoma (U87MG) bearing murine models. MSNs were synthesized, characterized and modified with polyethylene glycol, anti-VEGFR ligand VEGF121 and radioisotope (64)Cu, followed by extensive in vitro, in vivo and ex vivo studies. Our results demonstrated that a significantly higher amount of sunitinib could be delivered to the U87MG tumor by targeting VEGFR when compared with the non-targeted counterparts. The as-developed VEGF121-conjugated MSN could become another attractive nanoplatform for the design of future theranostic nanomedicine. 
 Expression and prognostic significance of VEGFR-2 in breast cancer. Breast cancer is one of the most common cancers among women in the world. Vascular endothelial growth factor receptor 2 (VEGFR-2) was not only found to play a key role in the development of tumor angiogenesis, but has also been located in tumor cells of a variety of tumors. This study investigated the expression pattern of VEGFR-2 in breast cancer tissue specimens in order to evaluate the role of VEGFR-2 in the prognosis of breast cancer. Expression and localization of VEGFR-2 in tumor cells of breast cancer specimens from 98 invasive breast cancer patients were determined by immunohistochemistry. The relationships between VEGFR-2 expression and clinicopathological features were also analyzed. The results showed that VEGFR-2 expression correlated positively with lymph node (LN)   metastasis   of breast cancer. Patients with high expression of VEGFR-2 had a significantly worse OS. It was also observed that the expression of epithelial-mesenchymal transition (EMT) marker, including Twist1 and Vimentin, was higher in the tumors with higher VEGFR-2 expression, while the E-cadherin expression was lower in the same tumors, suggesting that VEGFR-2 may serve as a possible mediator of EMT in breast cancer. 
 Vascular endothelial growth factor receptor isoforms: are they present in oral squamous cell carcinoma? Although the clinical importance of vascular endothelial growth factor (VEGF) overexpression in oral squamous cell carcinoma (OSCC) has been investigated, there are limited data about the overexpression of VEGF receptors (VEGF-Rs) and their clinical importance. VEGF-R isoforms have proven influence on proliferation rates,   metastasis  , and survival in different neoplasms. This study was conducted to investigate VEGF-R expression levels in OSCC samples and to identify any clinical relevance.A retrospective cohort study design (n = 50) was used. Clinical data were gathered from patient charts. Validated immunohistochemical methods were applied to determine VEGF-R isoform expression by tumor cells. Descriptive and inferential statistics with respect to the variable scale were computed. The significance level was set at a P value less than or equal to .05.This study found overexpression of different VEGF-R isoforms in 88% of examined specimens. Statistically important associations were detected between overexpression of specific VEGF-Rs and tumor size, neck node   metastasis  , and tumor-associated death. Furthermore, a history of common OSCC risk factors (smoking and alcohol consumption) were found considerably more often in patients whose OSCC specimens displayed VEGF-R overexpression.These findings show that VEGF-R overexpression occurs frequently in OSCC and could have clinical implications. 
 Overexpression of miRNA-497 inhibits tumor angiogenesis by targeting VEGFR2. Recent studies reported miR-497 exhibited inhibitory effects in various cancers. However, whether miR-497 is involved in inhibiting angiogenesis, which is critical for tumor growth and   metastasis  , is still unknown. The purpose of this study was to investigate the potential role of miR-497 in tumor angiogenesis. In this work, cell proliferation and apoptosis analyses were conducted to explore the potential function of miR-497 in HUVECs by using MTT and TUNEL assays. Western blotting (WB) was employed to validate the downstream targets of miR-497. Furthermore, in order to disclose the role of miR-497 on angiogenesis, VEGFR2-luc transgenic mice were treated with miR-497 mimic and applied to monitor tumor angiogenesis and growth by in vivo bioluminescent imaging (BLI). The results demonstrated that overexpression of miR-497 showed inhibitory effects on VEGFR2 activation and downstream Raf/MEK/ERK signal pathways in vitro and in vivo. Moreover, overexpression of miR-497 effectively induced HUVECs apoptosis by targeting VEGFR2 and downstream PI3K/AKT signaling pathway. Furthermore, miR-497 exhibited anti-angiogenesis and anti-tumor effects in the VEGFR2-luc breast tumor model proven by BLI, WB and immunohistochemistry analysis. In summary, miR-497 inhibits tumor angiogenesis and growth via targeting VEGFR2, indicating miR-497 can be explored as a potential drug candidate for cancer therapy. 
 Molecular characterization of EGFR, PDGFRA and VEGFR2 in cervical adenosquamous carcinoma. Adenosquamous carcinoma of the uterine cervix is an infrequent but aggressive subtype of cervical cancer. A better understanding of its biological behaviour is warranted to define more accurate prognosis and therapeutic targets. Currently, the blockage of receptor tyrosine kinase (RTKs) activity is an efficient therapeutic strategy for many different cancers. The objective of this study was to investigate EGFR, PDGFRA and VEGFR2 RTKs overexpression and activating gene mutations in a cohort of 30 adenosquamous carcinomas of the uterine cervix.EGFR, PDGFRA and VEGFR2 immunohistochemistry was performed in all samples, followed by DNA isolation from the gross macroscopically dissection of the neoplastic area. Screening for EGFR (exons 18-21) and PDGFRA (exons 12, 14 and 18) mutations was done by PCR--single-strand conformational polymorphism (PCR-SSCP).Despite the presence of EGFR immunohistochemical positive reactions in 43% (13/30) of the samples, no EGFR activating mutations in the hotspot region (exons 18-21) were identified. A silent base substitution (CAG>CAA) in EGFR exon 20 at codon 787 (Q787Q) was found in 17 cases (56%). All PDGFRA immunohistochemical reactions were positive and consistently observed in the stromal component, staining fibroblasts and endothelial cells, as well as in the cytoplasm of malignant cells. No activating PDGFRA mutations were found, yet, several silent mutations were observed, such as a base substitution in exon 12 (CCA>CCG) at codon 567 (P567P) in 9 cases and in exon 18 (GTC>GTT) at codon 824 (V824V) in 4 cases. We also observed the presence of base substitutions in intron 14 (IVS14+3G>A and IVS14+49G>A) in two different cases, and in intron 18 (IVS18-50insA) in 4 cases. VEGFR2 positivity was observed in 22 of 30 cases (73.3%), and was significantly associated with lack of   metastasis   (p=0.038).This is the most extensive analysis of EGFR, PDGFRA and VEGFR2 in cervical adenosquamous carcinomas. Despite the absence of EGFR and PDGFRA activating mutations, the presence of overexpression of these three important therapeutic targets in a subset of cases may be important in predicting the sensitivity of adenosquamous carcinoma to specific anti-RTKs drugs. 
 The VEGF-C/Flt-4 axis promotes invasion and   metastasis   of cancer cells. Flt-4, a VEGF receptor, is activated by its specific ligand, VEGF-C. The resultant signaling pathway promotes angiogenesis and/or lymphangiogenesis. This report provides evidence that the VEGF-C/Flt-4 axis enhances cancer cell mobility and invasiveness and contributes to the promotion of cancer cell   metastasis  . VEGF-C/Flt-4-mediated invasion and   metastasis   of cancer cells were found to require upregulation of the neural cell adhesion molecule contactin-1 through activation of the Src-p38 MAPK-C/EBP-dependent pathway. Examination of tumor tissues from various types of cancers revealed high levels of Flt-4 and VEGF-C expression that correlated closely with clinical   metastasis   and patient survival. The VEGF-C/Flt-4 axis, through upregulation of contactin-1, may regulate the invasive capacity in different types of cancer cells. 
  GeneRIFs  
 VEGFR-2 expression correlated positively with lymph node (LN)   metastasis   of breast cancer 
 We found that expression of Gab1, VEGFR-2, and MMP-9 was highly and positively correlated with each other and with lymph node   metastasis   and TNM stage in intrahepatic cholangiocarcinoma tissues 
 Ang-2, Tie-2 and VEGFR-2 are involved in the development, invasion,   metastasis  , and prognosis of colorectal cancer, and play important roles in angiogenesis. 
 Data suggest that GEP100-Arf6-AMAP1-cortactin pathway, activated by VEGFR2, appears to be common in angiogenesis and cancer invasion and   metastasis  , and provides their new therapeutic targets. 
 VEGFR-2 expression was significantly correlated with tumor size, histological grade and distant   metastasis   of papillary renal cell carcinoma. 
 VEGF and its receptors many play key roles in the growth, invasion and   metastasis   of human laryngeal carcinoma cells. 
 Results show a significant positive correlation between the concentrations of tissue VEGF and its receptor VEGFR2 in primary breast neoplasms during neoadjuvant therapy, and suggest that VEGFR2 levels may indicate the level of lymphatic   metastasis  . 
  [Cc]ell [Ii]nvasion  PubMed  
 VEGF inhibits tumor   cell invasion   and mesenchymal transition through a MET/VEGFR2 complex. Inhibition of VEGF signaling leads to a proinvasive phenotype in mouse models of glioblastoma multiforme (GBM) and in a subset of GBM patients treated with bevacizumab. Here, we demonstrate that vascular endothelial growth factor (VEGF) directly and negatively regulates tumor   cell invasion   through enhanced recruitment of the protein tyrosine phosphatase 1B (PTP1B) to a MET/VEGFR2 heterocomplex, thereby suppressing HGF-dependent MET phosphorylation and tumor cell migration. Consequently, VEGF blockade restores and increases MET activity in GBM cells in a hypoxia-independent manner, while inducing a program reminiscent of epithelial-to-mesenchymal transition highlighted by a T-cadherin to N-cadherin switch and enhanced mesenchymal features. Inhibition of MET in GBM mouse models blocks mesenchymal transition and invasion provoked by VEGF ablation, resulting in substantial survival benefit. 
 VEGFR-2 expression in carcinoid cancer cells and its role in tumor growth and metastasis. Carcinoid tumors are slow growing and highly vascular neuroendocrine neoplasms that are increasing in incidence. Previously, we showed that carcinoid tumors express vascular endothelial growth factor receptor 2 (VEGFR-2) in the epithelial compartment of carcinoid tumor sections; yet, its role is not completely understood. The purpose of our study was to: (i) assess the expression of VEGFR-2 in the novel human carcinoid cell line BON, (ii) to determine the role of PI3K/Akt signaling on VEGFR-2 expression and (iii) to assess the effect of VEGFR-2 on BON   cell invasion  , migration and proliferation. We found that, although VEGFR-2 is expressed in BON cells, reduction in VEGFR-2 expression actually enhanced proliferation, invasion, and migration of the BON cell line. Also, expression of VEGFR-2 was inversely related to PI3K signaling. Carcinoid liver metastases in mice demonstrated decreased VEGFR-2 expression. Furthermore, the expression of a truncated, soluble form of VEGFR-2 (sVEGFR-2), a protein demonstrated to inhibit cell growth, was detected in BON cells. The presence of VEGFR-2 in the epithelial component of carcinoid tumors and in the BON cell line suggests an alternate role for VEGFR-2, in addition to its well-defined role in angiogenesis. The expression of sVEGFR-2 may explain the inverse relationship between VEGFR-2 expression and PI3K/Akt signaling and the inhibitory effect VEGFR-2 has on BON cell proliferation, migration and invasion. 
  GeneRIFs  
 demonstrate that vascular endothelial growth factor (VEGF) directly and negatively regulates tumor   cell invasion   through enhanced recruitment of the protein tyrosine phosphatase 1B (PTP1B) to a MET/VEGFR2 heterocomplex 
   
  
 RET 
 
  NCBI Gene  
  GeneRIF  
  Pubmed  
 
 
  Search Term  Rank  Fields  
  [Mm]igration  6  Process(3); PubMed(27)  
  [Ii]nvasive  10  PubMed(10); GeneRIFs(1)  
  [Mm]etastasize  11  PubMed(1)  
  [Mm]etastasis  12  PubMed(33); GeneRIFs(4)  
  [Cc]ell [Ii]nvasion  13  PubMed(1)  
 
 
 hide/show details for RET 
  [Mm]igration  Process  
 lymphocyte   migration   into lymphoid organs 
 neural crest cell   migration   
 positive regulation of cell   migration   
  PubMed  
 Differential membrane compartmentalization of Ret by PTB-adaptor engagement. Glial cell line-derived neurotrophic factor family ligands act through the receptor tyrosine kinase Ret, which plays important roles during embryonic development for cell differentiation, survival, and   migration  . Ret signaling is markedly affected by compartmentalization of receptor complexes into membrane subdomains. Ret can propagate biochemical signaling from within concentrates in cholesterol-rich membrane microdomains or lipid rafts, or outside such regions, but the mechanisms for, and consequences of, Ret translocation between these membrane compartments remain largely unclear. Here we investigate the interaction of Shc and Frs2 phosphotyrosine-binding domain-containing adaptor molecules with Ret and their function in redistributing Ret to specialized membrane compartments. We found that engagement of Ret with the Frs2 adaptor results in an enrichment of Ret in lipid rafts and that signal transduction pathways and chemotaxis responses depend on the integrity of such rafts. The competing Shc adaptor did not promote Ret translocation to equivalent domains, and Shc-mediated effects were less affected by disruption of lipid rafts. However, by expressing a chimeric Shc protein that localizes to lipid rafts, we showed that biochemical signaling downstream of Ret resembled that of Ret signaling via Frs2. We have identified a previously unknown mechanism in which phosphotyrosine-binding domain-containing adaptors, by means of relocating Ret receptor complexes to lipid rafts, segregate diverse signaling and cellular functions mediated by Ret. These results reveal the existence of a novel mechanism that could, by subcellular relocation of Ret, work to amplify ligand gradients during chemotaxis. 
 GFRα1 released by nerves enhances cancer cell perineural invasion through GDNF-RET signaling. The ability of cancer cells to invade along nerves is associated with aggressive disease and diminished patient survival rates. Perineural invasion (PNI) may be mediated by nerve secretion of glial cell line-derived neurotrophic factor (GDNF) attracting cancer cell   migratio  n through activation of cell surface Ret proto-oncogene (RET) receptors. GDNF family receptor (GFR)α1 acts as coreceptor with RET, with both required for response to GDNF. We demonstrate that GFRα1 released by nerves enhances PNI, even in the absence of cancer cell GFRα1 expression. Cancer c  ell migra  tion toward GDNF, RET phosphorylation, and MAPK pathway activity are increased with exposure to soluble GFRα1 in a dose-dependent fashion. Dorsal root ganglia (DRG) release soluble GFRα1, which potentiates RET activation and cancer   cell mig  ration. In vitro DRG coculture assays of PNI show diminished PNI with DRG from GFRα1(+/-) mice compared with GFRα1(+/+) mice. An in vivo murine model of PNI demonstrates that cancer cells lacking GFRα1 maintain an ability to invade nerves and impair nerve function, whereas those lacking RET lose this ability. A tissue microarray of human pancreatic ductal adenocarcinomas demonstrates wide variance of cancer cell GFRα1 expression, suggesting an alternate source of GFRα1 in PNI. These findings collectively demonstrate that GFRα1 released by nerves enhances PNI through GDNF-RET signaling and that GFRα1 expression by cancer cells enhances but is not required for PNI. These results advance a mechanistic understanding of PNI and implicate the nerve itself as a key facilitator of this adverse cancer cell behavior. 
 Cell   migration   by a FRS2-adaptor dependent membrane relocation of ret receptors. During development neural progenitor cells migrate with extraordinary precision to inhabit tissues and organs far from their initial position. Little is known about the cellular basis for directional guidance by tyrosine kinase receptors (RTKs). RET is a RTK with important functions in guiding the   migration   of neuronal cells, and RET dysregulation leads to clinical disease such as agangliosis of the colon. We show here that RET   migration   in neuroepitheliomal and non-neuronal cells is elicited by the activation of specific signaling pathways initiated by the competitive recruitment of the FRS2 adaptor molecule to tyrosine 1062 (Y1062) in RET. FRS2 selectively recruited RET to focal complexes and led to activation of SRC family kinases and focal adhesion kinase (FAK). Activation of SRC depended on its direct interaction with RET at a different intracellular tyrosine (Y981) and activation of molecular signaling from these two separate sites in concert regulated   migration  . Our data suggest that an important function for FRS2 is to concentrate RET in membrane foci, leading to an engagement of specific signaling complexes localized in these membrane domains. 
 Subcellular receptor redistribution and enhanced microspike formation by a Ret receptor preferentially recruiting Dok. Ret is a receptor tyrosine kinase for the GDNF family of ligands and plays important roles during nervous system development for cell proliferation, cell   migration   and neurite growth. Signaling initiated from intracellular tyrosine 1062, by recruitment of several different phosphotyrosine binding (PTB) proteins (i.e. Shc, Frs2 and Dok), is important for these biological effects. By a single amino acid substitution in the PTB domain binding sequence of Ret, we have rewired the receptor such that it preferentially recruits Dok (Ret(Dok+)) with little or no remaining interactions with Shc and Frs2. Ret(Dok+) displays a sustained MAP kinase activation and a loss of Akt signaling compared to Ret(WT). We show that early events after ligand stimulation of Ret(Dok+) include massive formation of fine microspikes that are believed to be priming structures for neurite growth from the cell soma. The Ret(Dok+) receptors relocated in the membrane compartment into focal clusters at the tip of the microspikes, which was associated with Cdc42 activation. These results suggest that engagement of different adaptor proteins by Ret results in very different downstream signaling and functions within neurons and that Dok recruitment leads to a rapid receptor relocation and formation of microspikes. 
 Diminished Ret expression compromises neuronal survival in the colon and causes intestinal aganglionosis in mice. Mutations in the RET gene are the primary cause of Hirschsprung disease (HSCR), or congenital intestinal aganglionosis. However, how RET malfunction leads to HSCR is not known. It has recently been shown that glial cell line-derived neurotrophic factor (GDNF) family receptor alpha1 (GFRalpha1), which binds to GDNF and activates RET, is essential for the survival of enteric neurons. In this study, we investigated Ret regulation of enteric neuron survival and its potential involvement in HSCR. Conditional ablation of Ret in postmigratory enteric neurons caused widespread neuronal death in the colon, which led to colonic aganglionosis. To further examine this finding, we generated a mouse model for HSCR by reducing Ret expression levels. These mice recapitulated the genetic and phenotypic features of HSCR and developed colonic aganglionosis due to impaired   migration   and successive death of enteric neural crest-derived cells. Death of enteric neurons was also induced in the colon, where reduction of Ret expression was induced after the period of enteric neural crest cell   migration  , indicating that diminished Ret expression directly affected the survival of colonic neurons. Thus, enteric neuron survival is sensitive to RET dosage, and cell death is potentially involved in the etiology of HSCR. 
 SLIT2/ROBO1-miR-218-1-RET/PLAG1: a new disease pathway involved in Hirschsprung's disease. Hirschsprung's disease (HSCR) is a rare congenital disease caused by impaired proliferation and   migration   of neural crest cells. We investigated changes in expression of microRNAs (miRNAs) and the genes they regulate in tissues of patients with HSCR. Quantitative real-time PCR and immunoblot analyses were used to measure levels of miRNA, mRNAs, and proteins in colon tissues from 69 patients with HSCR and 49 individuals without HSCR (controls). Direct interactions between miRNAs and specific mRNAs were indentified in vitro, while the function role of miR-218-1 was investigated by using miR-218 transgenic mice. An increased level of miR-218-1 correlated with increased levels of SLIT2 and decreased levels of RET and PLAG1 mRNA and protein. The reductions in RET and PLAG1 by miR-218-1 reduced proliferation and   migration   of SH-SY5Y cells. Overexpression of the secreted form of SLIT2 inhibited cell   migration   via binding to its receptor ROBO1. Bowel tissues from miR-218-1 transgenic mice had nerve fibre hyperplasia and reduced numbers of gangliocytes, compared with wild-type mice. Altered miR-218-1 regulation of SLIT2, RET and PLAG1 might be involved in the pathogenesis of HSCR. 
 miRNA Profiling Reveals Dysregulation of RET and RET-Regulating Pathways in Hirschsprung's Disease. Hirschsprung's disease (HSCR), the most common congenital malformation of the gut, is regulated by multiple signal transduction pathways. Several components of these pathways are important targets for microRNAs (miRNAs). Multiple miRNAs have been associated with the pathophysiology of HSCR, and serum miRNAs profiles of HSCR patients have been reported, but miRNA expression in HSCR colon tissue is almost completely unexplored. Using microarray technology, we screened colon tissue to detect miRNAs whose expression profiles were altered in HSCR and identify targets of differentially expressed miRNAs. Following filtering of low-intensity signals, data normalization, and volcano plot filtering, we identified 168 differentially expressed miRNAs (104 up-regulated and 64 down-regulated). Fifty of these mRNAs represent major targets of dysegulated miRNAs and may thus important roles in the pathophysiology of HSCR. Pathway analysis revealed that 7 of the miRNA targets encode proteins involved in regulation of cell proliferation and   migration   via RET and related signaling pathways (MAPK and PI3K/AKT). Our results identify miRNAs that play key roles in the pathophysiology of the complex multi-factorial disease HSCR. 
 RET modulates cell adhesion via its cleavage by caspase in sympathetic neurons. RET is a tyrosine kinase receptor involved in numerous cellular mechanisms including proliferation, neuronal navigation,   migration  , and differentiation upon binding with glial cell derived neurotrophic factor family ligands. RET is an atypical tyrosine kinase receptor containing four cadherin domains in its extracellular part. Furthermore, it has been shown to act as a dependence receptor. Such a receptor is active in the absence of ligand, triggering apoptosis through a mechanism that requires receptor intracellular caspase cleavage. However, different data suggest that RET is not always associated with the cell death/survival balance but rather provides positional information. We demonstrate here that caspase cleavage of RET is involved in the regulation of adhesion in sympathetic neurons. The cleavage of RET generates an N-terminal truncated fragment that functions as a cadherin accessory protein, modifying cadherin environment and potentiating cadherin-mediated cell aggregation. Thus, the caspase cleavage of RET generates two RET fragments: one intracellular domain that can trigger cell death in apoptotic permissive settings, and one membrane-anchored ectodomain with cadherin accessory activity. We propose that this latter function may notably be important for the adequate development of the superior cervical ganglion. 
 Contribution of RET, NTRK3 and EDN3 to the expression of Hirschsprung disease in a multiplex family. Hirschsprung disease (HSCR) is a developmental disorder caused by a defect in the neural crest neuroblast   migration   process. It is considered to be a paradigm of complex disorders, with many loci contributing to manifestation of the disease. Although HSCR commonly appears as a sporadic trait, approximately 20% of HSCR cases are familial, with complex patterns of inheritance.A multiplex HSCR family with an additive model of inheritance, in which the contribution of three genes (RET, NTRK3, EDN3) leads to the HSCR phenotype is reported.The findings suggest that both RET and NTRK3 mutations acting together are necessary and sufficient for the appearance of the disease, and that the EDN3 mutation is acting as a phenotype-modifier factor in the context of this family, as two different HSCR phenotypes are seen among the affected members: a short segment form, and a total colonic aganglionosis. The results therefore support the complex additive model of inheritance previously proposed for Hirschsprung disease. 
 Functional haplotypes of the RET proto-oncogene promoter are associated with Hirschsprung disease (HSCR). The activation of the RET signaling pathway during embryogenesis is a crucial prerequisite for a directional   migration   of enteric nervous system progenitor cells. Loss-of-function germline mutations of the RET proto-oncogene are reported in familial and sporadic cases of Hirschsprung disease (HSCR) with a variable frequency. Furthermore, variants of several RET polymorphisms are over- or under-represented in HSCR populations. Specifically, the c.135A RET variant has been previously shown to be strongly associated with the HSCR phenotype. We have reported an HSCR-phenotype modifying effect of the RET c.135G>A polymorphism due to a within-gene interaction in patients harboring RET germline mutations, yet the function of the c.135G>A variant is unknown. The basic RET promoter region was investigated by DNA sequencing approach in 80 HSCR patients. Identified polymorphisms were genotyped in the HSCR and in a control population and haplotypes were reconstructed. The dual-luciferase assay was used to evaluate the activity of different RET promoter haplotypes. We demonstrate that variants of two RET promoter polymorphisms -5G>A and -1C>A from the transcription start site are associated with HSCR. Furthermore, the -5G>A polymorphism is in strong linkage disequilibrium with the c.135G>A polymorphism. The promoter haplotype -5/-1AC associated with HSCR has a significantly lower activity in an in vitro dual-luciferase expression assay compared with those haplotypes identified in the majority of normal controls. These data suggest a role for RET haplotypes containing the -5A promoter variant in the etiology of HSCR. 
 Multiple functional effects of RET kinase domain sequence variants in Hirschsprung disease. The REarranged during Transfection (RET) gene encodes a receptor tyrosine kinase required for maturation of the enteric nervous system. RET sequence variants occur in the congenital abnormality Hirschsprung disease (HSCR), characterized by absence of ganglia in the intestinal tract. Although HSCR-RET variants are predicted to inactivate RET, the molecular mechanisms of these events are not well characterized. Using structure-based models of RET, we predicted the molecular consequences of 23 HSCR-associated missense variants and how they lead to receptor dysfunction. We validated our predictions in biochemical and cell-based assays to explore mutational effects on RET protein functions. We found a minority of HSCR-RET variants abrogated RET kinase function, while the remaining mutants were phosphorylated and transduced intracellular signals. HSCR-RET sequence variants also impacted on maturation, stability, and degradation of RET proteins. We showed that each variant conferred a unique combination of effects that together impaired RET protein activity. However, all tested variants impaired RET-mediated cellular functions, including cell transformation and   migration  . Our data indicate that the molecular mechanisms of impaired RET function in HSCR are highly variable. Although a subset of variants cause loss of RET kinase activity and downstream signaling, enzymatic inactivation is not the sole mechanism at play in HSCR. 
 Multiple endocrine neoplasia syndromes, children, Hirschsprung's disease and RET. Multiple endocrine neoplasia (MEN) type 2 syndromes are autosomal dominant clinical associations characterized by a common clinical feature, medullary thyroid carcinoma (MTC). The ability to accurately predict the risk by genetic RET proto-oncogene analysis has resulted in the active follow-up of children at risk for developing early metastatic tumours and which can be prevented by prophylactic thyroidectomy. The C634 and M918T mutations (associated with MEN2A and MEN2B, respectively) are particularly associated with early aggressive behavior and distant metastatic spread requiring early intervention. RET is known to be involved in cellular signalling processes during development and controls the survival, proliferation, differentiation and   migration   of the enteric nervous system (ENS) progenitor cells, as well as the survival and regeneration of sympathetic neural and kidney cells. The centrality of RET in the etiology of both MEN2 and HSCR is now well established with fairly consistent associations existing between RET genotype and phenotype in MEN2. The relationship between Hirschsprung's disease (HSCR) MEN2 syndromes appears to be a highly significant one, sharing a common etiological factor in the RET proto-oncogene. It is now well accepted that most HSCR arises from loss of function, RET mutations, RET haploinsufficiency or RET polymorphisms and haplotypes of the RET promotor region. MEN2 syndromes result from gene up regulation due to germline activating mutations in the RET proto-oncogene (1:500,000). MTC is mostly associated with variations in the 5 cysteine RET radicals and codon-risk management protocols are of considerable value but not infallible. Oncogenic RET mutations may, however, vary between specific population groups. RET analysis in MEN has revolutionized the management of children of MEN2 and allowed surgical prediction and prophylaxis to take place. We discuss the role of genetic testing and possible guidelines for the management of patients from MTC families. The future appears full of promise and the current evaluation of RET-targeting tyrosine kinase and other inhibitors are of considerable interest in the management of these conditions. 
 The modifier role of RET-G691S polymorphism in hereditary medullary thyroid carcinoma: functional characterization and expression/penetrance studies. Hereditary medullary thyroid carcinoma (MTC) is caused by germ-line gain of function mutations in the RET proto-oncogene, and a phenotypic variability among carriers of the same mutation has been reported. We recently observed this phenomenon in a large familial MTC (FMTC) family carrying the RET-S891A mutation. Among genetic modifiers affecting RET-driven MTC, a role has been hypothesized for RET-G691S non-synonymous polymorphism, though the issue remains controversial. Aim of this study was to define the in vitro contribution of RET-G691S to the oncogenic potential of the RET-S891A, previously shown to harbour low transforming activity.The RET-S891A and RET-G691S/S891A mutants were generated by site-directed mutagenesis, transiently transfected in HEK293T cells and stably expressed in NIH3T3 cells. Their oncogenic potential was defined by assessing the   migration   ability by wound healing assay and the anchorage-independent growth by soft agar assay in NIH3T3 cells stably expressing either the single or the double mutants. Two RET-S891A families were characterised for the presence of RET-G691S.The functional studies demonstrated that RET-G691S/S891A double mutant displays a higher oncogenic potential than RET-S891A single mutant, assessed by focus formation and   migration   ability. Moreover, among the 25 RET-S891A carriers, a trend towards an earlier age of diagnosis was found in the MTC patients harboring RET-S891A in association with RET-G691S.We demonstrate that the RET-G691S non-synonymous polymorphism enhances in vitro the oncogenic activity of RET-S891A. Moreover, an effect on the phenotype was observed in the RET-G691S/S891A patients, thus suggesting that the analysis of this polymorphism could contribute to the decision on the more appropriate clinical and follow-up management. 
 Expression of Ret finger protein correlates with outcomes in endometrial cancer. Ret finger protein (RFP) is a nuclear protein with transcriptional repressive activity that is highly expressed in a variety of human and rodent tumor cell lines. We examined the expression of RFP in human endometrial cancer and assessed its clinical significance. Formalin-fixed, paraffin-embedded sections from endometrial cancer tissues were immunostained with the RFP antibody, and the staining intensity was evaluated. The clinicopathological factors examined were age, International Federation of Gynecology and Obstetrics stage, tumor grade, myometrial invasion, and pelvic lymph node metastasis. Overall survival (OS) and progression-free survival (PFS) were evaluated using the Kaplan-Meier method, and multivariate analysis was performed using the Cox proportional hazard analysis. Of the 119 cancer tissues, 57 (47.9%) cases were positive for RFP immunoreactivity. RFP expression was not associated with any of the clinicopathological parameters examined. However, positive RFP expression significantly predicted poorer OS and PFS compared with negative expression (OS, P = 0.0011; PFS, P < 0.0001). In the multivariate analyses, positive RFP expression was an independent prognostic factor for survival in this study. RFP knockdown significantly impaired cancer cell   migration   and invasion in vitro with concomitant decreases of integrins beta1 and alpha2. Positive RFP expression is a predictive marker for an unfavorable clinical outcome in patients with endometrial cancer. 
 RET-mediated cell adhesion and   migration   require multiple integrin subunits. The RET receptor tyrosine kinase is an important mediator of several human diseases, most notably of neuroendocrine cancers. These diseases are characterized by aberrant cell   migration  , a process tightly regulated by integrins.Our goals were to investigate the role of integrins in RET-mediated   migration   in two neoplastic cell models: the neural-derived cell line SH-SY5Y, and the papillary thyroid carcinoma cell line TPC-1. We also evaluated whether multiple integrin subunits have a role in RET-mediated cell   migration  .We evaluated the expression and activation of integrins in response to RET activation using standard cell adhesion and   migration   (wound-healing) assays. We examined focal adhesion formation, using integrin-paxillin coimmunoprecipitations and immunofluorescence, as an indicator of integrin activity.Our data indicate that β1 integrin (ITGB1) is expressed in both SH-SY5Y and TPC-1 cell lines and that these cells adhere strongly to matrices preferentially associated with ITGB1. We showed that RET can activate ITGB1, and that RET-induced cell adhesion and   migratio  n require ITGB1. Furthermore, we showed that β3 integrin (ITGB3) also plays a role in RET-mediated cell adhesion an  d migrati  on in vitro and ITGB3 expression correlates with RET-mediated invasion in a mouse tumor xenograft model, suggesting that RET mediates the activity of multiple integrin subunits.Our data are the first to show that multiple integrin subunits contribute to cell adhesion an  d migrati  on downstream of RET, suggesting that coordinated signaling through these pathways is important for cell interactions with the microenvironment during tumor invasion and progression. 
 Ret inhibition decreases growth and metastatic potential of estrogen receptor positive breast cancer cells. We show that elevated levels of Ret receptor are found in different sub-types of human breast cancers and that high Ret correlates with decreased metastasis-free survival. The role of Ret in ER+ breast cancer models was explored combining in vitro and in vivo approaches. Our analyses revealed that ligand-induced Ret activation: (i) stimulates   migration   of breast cancer cells; (ii) rescues cells from anti-proliferative effects of endocrine treatment and (iii) stimulates expression of cytokines in the presence of endocrine agents. Indeed, we uncovered a positive feed-forward loop between the inflammatory cytokine IL6 and Ret that links them at the expression and the functional level. In vivo inhibition of Ret in a metastatic breast cancer model inhibits tumour outgrowth and metastatic potential. Ret inhibition blocks the feed-forward loop by down-regulating Ret levels, as well as decreasing activity of Fak, an integrator of IL6-Ret signalling. Our results suggest that Ret kinase should be considered as a novel therapeutic target in subsets of breast cancer. 
 Endoneurial macrophages induce perineural invasion of pancreatic cancer cells by secretion of GDNF and activation of RET tyrosine kinase receptor. Perineural invasion of cancer cells (CPNI) is found in most patients with pancreatic adenocarcinomas (PDA), prostate, or head and neck cancers. These patients undergo palliative rather than curative treatment due to dissemination of cancer along nerves, well beyond the extent of any local invasion. Although CPNI is a common source of distant tumor spread and a cause of significant morbidity, its exact mechanism is undefined. Immunohistochemical analysis of specimens excised from patients with PDAs showed a significant increase in the number of endoneurial macrophages (EMΦ) that lie around nerves invaded by cancer compared with normal nerves. Video microscopy and time-lapse analysis revealed that EMΦs are recruited by the tumor cells in response to colony-stimulated factor-1 secreted by invading cancer cells. Conditioned medium (CM) of tumor-activated EMΦs (tEMΦ) induced a 5-fold increase   in migra  tion of PDA cells compared with controls. Compared with resting EMΦs, tEMΦs secreted higher levels of glial-derived neurotrophic factor (GDNF), inducing phosphorylation of RET and downstream activation of extracellular signal-regulated kinases (ERK) in PDA cells. Genetic and pharmacologic inhibition of the GDNF receptors GFRA1 and RET abrogated the migratory effect of EMΦ-CM and reduced ERK phosphorylation. In an in vivo CPNI model, CCR2-deficient mice that have reduced macrophage recruitment and activation showed minimal nerve invasion, whereas wild-type mice developed complete sciatic nerve paralysis due to massive CPNI. Taken together, our results identify a paracrine response between EMΦs and PDA cells that orchestrates the formation of cancer nerve invasion. 
 Glial cell-derived neurotrophic factor (GDNF)-induced   migration   and signal transduction in corneal epithelial cells. To identify signal-transduction pathways induced by glial cell-derived neurotrophic factor (GDNF) in corneal epithelial cells and to characterize its effect on cell   migration  .Expression of GDNF receptor (GFR) alpha-1 in human corneal epithelium was detected by RT-PCR and Western blot analysis. Expression and phosphorylation of Ret, activation of focal adhesion kinase (FAK) and mitogen-associated protein kinase (MAPK) signaling pathways, and phosphorylation of paxillin by GDNF were investigated by immunoprecipitation and Western blot analysis in primary human corneal epithelial cells and a corneal epithelial cell line. The tyrosine kinase inhibitor herbimycin A and Ras farnesyltransferase inhibitor manumycin were used to specifically inhibit GDNF-induced signaling pathways. In vitro wound-healing assays and modified Boyden chamber analysis were performed to investigate the effect of GDNF on epithelial cell   migration  .Expression of GFRalpha-1 was detected in normal and transformed human corneal epithelium. GDNF induced tyrosine phosphorylation of Ret. Furthermore, tyrosine phosphorylation of FAK and phosphotyrosine kinase (Pyk) 2; serine phosphorylation of c-Raf, MEK1, and Elk 1; and tyrosine-threonine phosphorylation of Erk-1 and -2 were time-dependently activated in the presence of GDNF. Tyrosine phosphorylation of paxillin was also induced by GDNF.   Migration   of corneal epithelial cells was significantly stimulated by GDNF. Herbimycin A strongly inhibited the activation of Ret, FAK, c-Raf, and Erk-1 and -2; the phosphorylation of paxillin; and corneal epithelial cell   migration  . More specifically, the Ras inhibitor manumycin inhibited phosphorylation of c-Raf, MEK 1, Erk-1 and -2, and Elk 1, but not that of FAK.Corneal epithelial cells express receptors specific for GDNF that are used by GDNF to induce intracellular signaling. FAK and MAPK pathways seem to be activated by GDNF to modulate gene transcription and cell   migration  . FAK seems to be an upstream regulator of the MAPK cascade for GDNF signal transduction. As an inducer of FAK-dependent corneal epithelial   migration  , GDNF may play an important role in corneal regeneration and wound healing. 
 Hirschsprung's disease and variants in genes that regulate enteric neural crest cell proliferation,   migration   and differentiation. Hirschsprung's disease (HSCR) results from failed colonization of the embryonic gut by enteric neural crest cells (ENCCs); colonization requires RET proto-oncogene (RET) signaling. We sequenced RET to identify coding and splice-site variants in a population-based case group and we tested for associations between HSCR and common variants in RET and candidate genes (ASCL1, homeobox B5 (HOXB5), L1 cell adhesion molecule (L1CAM), paired-like homeobox 2b (PHOX2B), PROK1 and PROKR1) chosen because they are involved in ENCC proliferation,   migration   and differentiation in animal models. We conducted a nested case-control study of 304 HSCR cases and 1215 controls. Among 38 (12.5%) cases with 34 RET coding and splice-site variants, 18 variants were previously unreported. We confirmed associations with common variants in HOXB5 and PHOX2B but the associations with variants in ASCL1, L1CAM and PROK1 were not significant after multiple comparisons adjustment. RET variants were strongly associated with HSCR (P-values between 10(-3) and 10(-31)) but this differed by race/ethnicity: associations were absent in African-Americans. Our population-based study not only identified novel RET variants in HSCR cases, it showed that common RET variants may not contribute to HSCR in all race/ethnic groups. The findings for HOXB5 and PHOX2B provide supportive evidence that genes regulating ENCC proliferation,   migration   and differentiation could be risk factors for HSCR. 
 The tyrosine kinase receptor RET interacts in vivo with aryl hydrocarbon receptor-interacting protein to alter survivin availability. RET is a tyrosine kinase transmembrane receptor expressed in two main alternative isoforms: RET9 and RET51. RET transduces a positive signal leading to survival, differentiation, or   migration   in the presence of its ligand glial cell line-derived neurotrophic factor, whereas in its absence a proapoptotic fragment that initiates a negative signaling for apoptosis is generated. The signal transduction mechanisms leading to apoptosis are still unclear.To shed light on the mechanisms of RET-induced apoptosis, we searched for novel interactors of RET51.The "split ubiquitin yeast two-hybrid system" was used with RET51 as bait against a human brain expression library.We identified aryl hydrocarbon receptor-interacting protein (AIP), a cochaperone recently found mutated in pituitary adenoma patients, as a novel interactor of RET. We showed that RET interacts specifically with AIP both in mammalian cell lines and in vivo in the pituitary gland, regardless of the presence of pituitary adenoma-specific mutations. AIP and RET genes were sequenced in 28 pituitary adenoma, but no relevant mutations were found. In addition, we identified the proapoptotic domain of RET as responsible for the interaction with AIP. Finally, we demonstrated that the AIP-RET interaction does not require RET kinase activity or kinase-dependent signal transduction and that it prevents the formation of the AIP-survivin complex.The identification of the AIP-RET complex represents a starting point to study key cellular processes involved in RET-induced apoptosis. 
 Germline RET sequence variation I852M and occult medullary thyroid cancer: harmless polymorphism or causative mutation? Rearranged during transfection (RET) gene analysis, widely used to identify carriers at risk of medullary thyroid cancer (MTC), occasionally uncovers novel sequence 'variants of unknown clinical significance' including RET I852M. This study aimed to clarify whether RET I852M represents a harmless polymorphism or a pathogenic mutation.Clinical investigation supported by functional characterization of I852M mutant cells in vitro.Genotype-phenotype correlation including five kindreds from a three-generational Caucasian I852M RET family.A node-negative occult MTC was found in the 64-year-old index patient who had increased basal and stimulated peak calcitonin levels of 190 and 13 307 ng/l, respectively. Her 4-year-old grandson had no histopathological evidence of C-cell disease although his serum calcitonin levels had increased within 5 months from 3·2 to 6·3 ng/l basally and from 17·2 to 24·5 ng/l after pentagastrin stimulation. His mother and two 11- and 1·5-year-old siblings, also carrying the gene, had normal basal and stimulated calcitonin levels and hence did not undergo surgery. Functional characterization of transfected NIH3T3 cells in vitro (cell proliferation rate; cell viability; anchorage-independent cell growth;   cell migr  ation; and invasion) indicated that I852M mutant cells have transforming and migratory activities similar to American Thyroid Association (ATA) class A V804M mutants. I852M mutants demonstrated a weaker proliferative potential than fast-proliferating ATA class C C634R mutants and revealed a weaker migratory activity compared with aggressively growing ATA class D A883F mutants.I852M sequence variations represent genuine RET mutations, falling into ATA class A of weakly activating RET germline mutations. 
 Functional RET G691S polymorphism in cutaneous malignant melanoma. RET proto-oncogene encodes a receptor tyrosine kinase whose ligand is glial cell line-derived neurotrophic factor (GDNF), and its polymorphism at G691S juxtamembrane region (RETp) is a germline polymorphism. Cutaneous melanomas, particularly the desmoplastic subtype, are highly neurotropic; thus we sought to determine the frequency of RETp in cutaneous melanoma and its functional responsiveness to GDNF. RETp was assessed in 71 non-desmoplastic cutaneous melanomas (non-DMs) and 70 desmoplastic melanomas (DMs). Melanoma cell lines with RETp, RET wild type (RETwt), BRAF V600E mutation (BRAFmt) or BRAF wild type (BRAFwt) were assessed for functional activity. RETp frequency was significantly higher in DMs (61%) than in non-DMs (31%, P<0.001). BRAFmt was detected in only 11% of DMs. GDNF stimulation significantly amplified cell proliferation,   migration   and invasion in RETp, but not in RETwt melanoma cells. GDNF stimulation of RETp cell lines enhanced phosphorylation of extracellular signal-regulated kinase (ERK) and Akt of the RET-RAS-RAF-ERK and RET-phosphatidylinositol 3-kinase (PI3K)-Akt pathways, respectively. GDNF response of RETp cells in signal transduction and other functional studies were not affected by BRAFmt. The study demonstrates that RETp is frequently found in cutaneous melanoma, particularly desmoplastic subtypes, and responds to GDNF inducing events favorable for tumor progression. 
 MiR-129-5p is down-regulated and involved in the growth, apoptosis and   migration   of medullary thyroid carcinoma cells through targeting RET. Dysregulation of the REarranged during Transfection proto-oncogene (RET) pathway and microRNA (miRNAs) are crucial for the development of medullary thyroid carcinomas (MTC). Here we demonstrate that miR-129-5p is down-regulated in MTC tissues and cell lines and inhibits RET expression by directly binding its 3' untranslated regions. Ectopic expression of miR-129-5p significantly decreases cell growth, induces apoptosis and suppresses   migration   ability in MTC cells through decreasing the phosphorylated AKT, thus functioning as a tumor suppressor. These findings give new clues for understanding MTC carcinogenesis and may help in developing a therapeutic approach for the treatment of RET-activated MTC. 
 Current concepts in RET-related genetics, signaling and therapeutics. The receptor tyrosine kinase RET is expressed in cell lineages derived from the neural crest and has a key role in regulating cell proliferation,   migration  , differentiation and survival during embryogenesis. Germline and somatic mutations in RET that produce constitutively activated receptors cause the cancer syndrome multiple endocrine neoplasia type 2 and several endocrine and neural-crest-derived tumors, whereas mutations resulting in nonfunctional RET or lower expression of RET are found in individuals affected with Hirschsprung disease. This review focuses on the genetics and molecular mechanisms underlying the different inherited human neural-crest-related disorders in which RET dysfunction has a crucial role and discusses RET as a potential therapeutic target. 
 The RET receptor is linked to stress response pathways. RET is a transmembrane receptor required for the development of neuroendocrine and urogenital cell types. Activation of RET has roles in cell growth,   migration  , or differentiation, yet little is known about the gene expression patterns through which these processes are mediated. We have generated cell lines stably expressing either the RET9 or RET51 protein isoforms and have used these to investigate RET-mediated gene expression patterns by cDNA microarray analyses. As seen for many oncogenes, we identified altered expression of genes associated generally with cell-cell or cell-substrate interactions and up-regulation of tumor-specific transcripts. We also saw increased expression of transcripts normally associated with neural crest or other RET-expressing cell types, suggesting these genes may lie downstream of RET activation in development. The most striking pattern of expression was up-regulation of stress response genes. We showed that RET expression significantly up-regulated the genes for heat shock protein (HSP) 70 family members, HSPA1A, HSPA1B, and HSPA1L. Other members of several HSP families and HSP70-interacting molecules that were associated with stress response protein complexes involved in protein maturation were also specifically up-regulated by RET, whereas those associated with the roles of HSP70 in protein degradation were down-regulated or unaffected. The major mechanism of stress response induction is activation of the heat shock transcription factor HSF1. We showed that RET expression leads to increased HSF1 activation, which correlates with increased expression of stress response genes. Together, our data suggest that RET may be directly responsible for expression of stress response proteins and the initiation of stress response. 
 RET proto-oncogene in Sardinia: V804M is the most frequent mutation and may be associated with FMTC/MEN-2A phenotype. Genetic screening of RET proto-oncogene is a powerful tool for the early identification of familial cases of medullary thyroid carcinoma (MTC), comprising isolated familial thyroid medullary carcinoma (FMTC) and multiple endocrine neoplasia syndromes 2A (MEN-2A) and 2B (MEN-2B). We report the results obtained by RET mutation analysis of subjects living in Sardinia, an Italian island whose inhabitants display a peculiar genetic background due to geographic isolation and low im  migration   rate for several centuries.Retrospective study reporting data on 67 patients referred during the last 5 years for RET analysis because affected by MTC or first degree relatives of MTC patients.Only three mutations were identified affecting codons 620 (exon 10), 634 (exon 11), and 804 (exon 14); surprisingly, the most prevalent mutation found was V804M (overall prevalence: 59%). This finding is quite different from previous studies carried out in other Caucasian and non-Caucasian populations, in which the frequency of the V804M mutation is consistently reported less than 5%. The phenotype associated to V804M mutation was mostly FMTC (16/17 cases = 94.1%), but in one case (5.9%) primary hyperparathyroidism was found, suggesting a MEN-2A.These results underline the importance of the genetic background in the distribution of RET mutations and should be taken into consideration when performing genetic evaluation of MTC patients. 
 Identification and characterization of two novel germline RET variants associated with medullary thyroid carcinoma. Activating germline mutations in the RET proto-oncogene are responsible for about 98 % of the familial forms of medullary thyroid carcinoma (MTC), which represent 25 % of all MTC cases. The search for germline mutations in this gene is important for the recognition of hereditary forms of MTC and further identification of at-risk relatives who may benefit from early clinical intervention. Genotype-phenotype correlations are well established for most disease-causing RET mutations, allowing risk stratification. The association of a new RET variant with the MTC phenotype and familial predisposition requires the assessment of its functional and clinical significance. The aim of this study was to evaluate the oncogenic potential of two newly identified RET germline variants associated with late-onset MTC. In vitro functional assays were designed to address the transforming potential of novel RET variants, through their expression in non-transformed cells, and comparing their effect with wild-type RET. The new variants were identified in codons 515 (p.C515W) and 636 (p.T636M) located, respectively, in exons 8 and 11, thus resulting in amino acid substitutions in the extracellular region of the tyrosine kinase receptor RET. Through functional assays, we observed increased cell growth and proliferation, loss of contact inhibition, and a stimulation of cell   migration  , suggesting that these new RET variants hold some relevant transforming potential. The transforming potential of these novel RET variants was of low-grade, when compared to that of RET MEN2A-causing mutation p.C634R, probably explaining the mild phenotype characterized by late onset and low clinical aggressiveness. 
  [Ii]nvasive  PubMed  
 Genetic susceptibility to distinct bladder cancer subphenotypes. Clinical, pathologic, and molecular evidence indicate that bladder cancer is heterogeneous with pathologic/molecular features that define distinct subphenotypes with different prognoses. It is conceivable that specific patterns of genetic susceptibility are associated with particular subphenotypes.To examine evidence for the contribution of germline genetic variation to bladder cancer heterogeneity.The Spanish Bladder Cancer/EPICURO Study is a case-control study based in 18 hospitals located in five areas in Spain. Cases were patients with a newly diagnosed, histologically confirmed, urothelial cell carcinoma of the bladder from 1998 to 2001. Case diagnoses were reviewed and uniformly classified by pathologists following the World Health Organisation/International Society of Urological Pathology 1999 criteria. Controls were hospital-matched patients (n=1149).A total of 1526 candidate variants in 423 candidate genes were analysed. Three distinct subphenotypes were defined according to stage and grade: low-grade nonmuscle   invasive   (n=586), high-grade nonmuscle   invasive   (n=219), and muscle   invasive   (n=246). The association between each variant and subphenotype was assessed by polytomous risk models adjusting for potential confounders. Heterogeneity in genetic susceptibility among subphenotypes was also tested.Two established bladder cancer susceptibility genotypes, NAT2 slow-acetylation and GSTM1-null, exhibited similar associations among the subphenotypes, as did VEGF-rs25648, which was previously identified in our study. Other variants conferred risks for specific tumour subphenotypes such as PMS2-rs6463524 and CD4-rs3213427 (respective heterogeneity p values of 0.006 and 0.004), which were associated with muscle-  invasive   tumours (per-allele odds ratios [95% confidence interval] of 0.56 [0.41-0.77] and 0.71 [0.57-0.88], respectively) but not with non-muscle-  invasive   tumours. Heterogeneity p values were not robust in multiple testing according to their false-discovery rate.These exploratory analyses suggest that genetic susceptibility loci might be related to the molecular/pathologic diversity of bladder cancer. Validation through large-scale replication studies and the study of additional genes and single nucleotide polymorphisms are required. 
 Genetic susceptibility to distinct bladder cancer subphenotypes. Clinical, pathologic, and molecular evidence indicate that bladder cancer is heterogeneous with pathologic/molecular features that define distinct subphenotypes with different prognoses. It is conceivable that specific patterns of genetic susceptibility are associated with particular subphenotypes.To examine evidence for the contribution of germline genetic variation to bladder cancer heterogeneity.The Spanish Bladder Cancer/EPICURO Study is a case-control study based in 18 hospitals located in five areas in Spain. Cases were patients with a newly diagnosed, histologically confirmed, urothelial cell carcinoma of the bladder from 1998 to 2001. Case diagnoses were reviewed and uniformly classified by pathologists following the World Health Organisation/International Society of Urological Pathology 1999 criteria. Controls were hospital-matched patients (n=1149).A total of 1526 candidate variants in 423 candidate genes were analysed. Three distinct subphenotypes were defined according to stage and grade: low-grade nonmuscle   invasive   (n=586), high-grade nonmuscle   invasive   (n=219), and muscle   invasive   (n=246). The association between each variant and subphenotype was assessed by polytomous risk models adjusting for potential confounders. Heterogeneity in genetic susceptibility among subphenotypes was also tested.Two established bladder cancer susceptibility genotypes, NAT2 slow-acetylation and GSTM1-null, exhibited similar associations among the subphenotypes, as did VEGF-rs25648, which was previously identified in our study. Other variants conferred risks for specific tumour subphenotypes such as PMS2-rs6463524 and CD4-rs3213427 (respective heterogeneity p values of 0.006 and 0.004), which were associated with muscle-  invasive   tumours (per-allele odds ratios [95% confidence interval] of 0.56 [0.41-0.77] and 0.71 [0.57-0.88], respectively) but not with non-muscle-  invasive   tumours. Heterogeneity p values were not robust in multiple testing according to their false-discovery rate.These exploratory analyses suggest that genetic susceptibility loci might be related to the molecular/pathologic diversity of bladder cancer. Validation through large-scale replication studies and the study of additional genes and single nucleotide polymorphisms are required. 
 Assessment of RET/PTC oncogene activation and clonality in thyroid nodules with incomplete morphological evidence of papillary carcinoma: a search for the early precursors of papillary cancer. Non  invasive   thyroid nodules that exhibit borderline morphological signs of papillary cancer are difficult to diagnose and we do not know if they represent papillary carcinoma precursor lesions. Forty-six such nodules were analyzed for RET activation by immunohistochemistry and, in selected cases, by reverse transcriptase-polymerase chain reaction performed on RNA extracted after laser capture microdissection (LCM) of the tumor foci with and without papillary carcinoma features and positive RET immunoreactivity. RET immunoreactivity was identified, at least focally, in 30 of 46 (65.2%) of the nodules where it closely paralleled the morphological changes. Enough RNA was obtained after LCM in seven samples. RET/PTC1 or RET/PTC3 were detected in microscopic foci with papillary carcinoma features in most of the thyroid nodules (five of seven cases). No RET/PTC1 or RET/PTC3 rearrangements were detected in areas of the same tumors that lacked the cytological alterations. Analysis of clonality in the same nodules selected for LCM demonstrated that two were monoclonal and six were polyclonal. We conclude that RET activation closely parallels the morphological changes, that it is restricted to those areas of the tumor with the cytological alterations and that it is detectable in both mono- and polyclonal tumors. Although the finding of microscopic foci indicative of papillary carcinoma in a hyperplastic or adenomatous nodule does not justify the interpretation of the entire lesion as papillary carcinoma, it is possible that such foci may precede the development of   invasive   papillary cancer. 
 A rare missense variant in RET exon 8 in a Portuguese family with atypical multiple endocrine neoplasia type 2A. Multiple Endocrine Neoplasia type 2 (MEN2) is a rare genetic disorder characterized by medullary thyroid carcinoma (MTC), pheochromocytoma and primary hyperparathyroidism. MEN2 is an autosomal dominant syndrome caused by mutations in the RET proto-oncogene. In the vast majority of patients, the mutations are localized in exons 10, 11 and 13-15 of the RET gene. Rare variants located in exon 8 were recently identified but their clinical significance remains unclear.We studied two sisters presenting with pheochromocytoma as the first tumor. One of the sisters was diagnosed with a right pheochromocytoma at the age of 44 and at age 53 she developed an   invasive   left pheochromocytoma with no other endocrine neoplasia. The other sister was diagnosed with a left pheochromocytoma at age 50 and at age 64 she had a right phemochromocytoma and MTC. Neither of the two sisters presented evidence of primary hyperparathyroidism. Mutations of the RET proto-oncogene were investigated by DNA sequencing.We detected a germline missense variant in RET exon 8 (p.Cys531Arg) in both sisters. The p.Cys531Arg variant was not present in a third 50-year-old sister who has remained to date clinically unaffected.This is the first case showing the p.Cys531Arg variant in RET exon 8 co-segregating with family members affected by a syndrome reminiscent of MEN2A. Our results suggest that this variant has a specific genotype-phenotype correlation as it is associated with the development of pheochromocytoma before the onset of MTC. 
 CXCR2 and RET single nucleotide polymorphisms in pancreatic cancer. Enhanced angiogenesis and perineural invasion are markers of poor prognosis in patients with pancreatic cancer. Systemic therapies for pancreatic cancer have been largely ineffective, and thus improved, targeted therapies are needed. Single nucleotide polymorphisms (SNP) are DNA sequence variations that result in vast diversity of disease susceptibility and response to disease. CXCR2 is an important mediator of CXC chemokine-induced angiogenesis and is upregulated in pancreatic cancer. In a preclinical corneal micropocket assay, treatment of pancreatic cancer cell lines that express CXCR2 with anti-CXCR2 antibody inhibited angiogenesis. To date, there have not been any CXCR2 SNP associated with pancreatic cancer, but CXCR2 SNP has been postulated to be associated with angiogenesis in systemic sclerosis. The receptor tyrosine kinase encoded by the RET gene and its ligand glial derived neurotrophic factor (GDNF) are upregulated in pancreatic cancer. In vitro treatment of pancreatic cancer cell lines that express RET with anti-RET antibody or RET siRNA-inhibited GDNF-induced   invasive  ness. G691S RET SNP has been previously shown to be associated with enhanced pancreatic cancer   invasive  ness. We suggest that molecular profiling of each patient's tumor for G691S RET SNP, potentially CXCR2 SNP, and also other yet-to-be identified SNP associated with pancreatic cancer will allow for both improved understanding of individual prognosis and allow for utilization of more personalized, targeted adjuvant therapies. 
 Autocrine stimulation by osteopontin plays a pivotal role in the expression of the mitogenic and   invasive   phenotype of RET/PTC-transformed thyroid cells. Papillary thyroid carcinomas are characterized by rearrangements of the RET receptor tyrosine kinase generating RET/PTC oncogenes. Here we show that osteopontin (OPN), a secreted glycoprotein, is a major RET/PTC-induced transcriptional target in PC Cl 3 thyroid follicular cells. OPN upregulation depended on the integrity of the RET/PTC kinase and tyrosines Y1015 and Y1062, two major RET/PTC autophosphorylation sites. RET/PTC also induced a strong overexpression of CD44, a cell surface signalling receptor for OPN. Upregulation of CD44 was dependent on RET/PTC Y1062, as well. Constitutive OPN overexpression or treatment with exogenous recombinant OPN sharply increased proliferation, Matrigel invasion and spreading in collagen gels of RET/PTC-transformed PC Cl 3 cells. These effects were impaired by the treatment of PC Cl 3-RET/PTC cells with OPN- and CD44-locking antibodies. Thus, RET/PTC signalling triggers an autocrine loop involving OPN and CD44 that sustains proliferation and invasion of transfomed PC Cl 3 thyrocytes. 
 Molecular genotyping of papillary thyroid carcinoma follicular variant according to its histological subtypes (encapsulated vs infiltrative) reveals distinct BRAF and RAS mutation patterns. The follicular variant of papillary thyroid carcinoma usually presents as an encapsulated tumor and less commonly as a partially/non-encapsulated infiltrative neoplasm. The encapsulated form rarely metastasizes to lymph node, whereas infiltrative tumor often harbors nodal metastases. The molecular profile of the follicular variant was shown to be close to the follicular adenoma/carcinoma group of tumors with a high RAS and very low BRAF mutation rates. A comprehensive survey of oncogenic mutations in the follicular variant of papillary thyroid carcinoma according to its encapsulated and infiltrative forms has not been performed. Paraffin tissue from 28 patients with encapsulated and 19 with infiltrative follicular variant were subjected to mass spectrometry genotyping encompassing the most significant oncogenes in thyroid carcinomas: 111 mutations in RET, BRAF, NRAS, HRAS, KRAS, PIK3CA, AKT1 and other related genes. There was no difference in age, gender, tumor size and angioinvasion between encapsulated or infiltrative tumors. Infiltrative carcinomas had a much higher frequency of extrathyroid extension, positive margins and nodal metastases than encapsulated tumors (P<0.05). The BRAF 1799T>A mutation was found in 5 of 19 (26%) of the infiltrative tumor and in none of the encapsulated carcinomas (P=0.007). In contrast, RAS mutations were observed in 10 of 28 (36%) of the encapsulated group (5 NRAS_Q61R, 3 HRAS_Q61, 1 HRAS_G13C and 1 KRAS_Q61R) and in only 2 of 19 (10%) of infiltrative tumors (P=0.09). One encapsulated carcinoma showed a PAX8/PPARgamma rearrangement, whereas two infiltrative tumors harbored RET/PTC fusions. Encapsulated follicular variant of papillary thyroid carcinomas have a molecular profile very close to follicular adenomas/carcinomas (high rate of RAS and absence of BRAF mutations). Infiltrative follicular variant has an opposite molecular profile closer to classical papillary thyroid carcinoma than to follicular adenoma/carcinoma (BRAF>RAS mutations). The molecular profile of encapsulated and infiltrative follicular variant parallels their biological behavior (ie, metastatic nodal and   invasive   patterns). 
 RET/PTC1-driven neoplastic transformation and pro  invasive   phenotype of human thyrocytes involve Met induction and beta-catenin nuclear translocation. Activation of the RET gene by chromosomal rearrangements generating RET/PTC oncogenes is a frequent, early, and causative event in papillary thyroid carcinoma (PTC). We have previously shown that, in human primary thyrocytes, RET/PTC1 induces a transcriptional program including the MET proto-oncogene. In PTCs, beta-catenin is frequently mislocated to the cytoplasm nucleus. We investigated the interplay between Ret/ptc1 signaling and Met in regulating the pro  invasive   phenotype and beta-catenin localization in cellular models of human PTC. Here, we show that Met protein is expressed and is constitutively active in human thyrocytes exogenously expressing RET/PTC1 as well as a mutant (Y451F) devoid of the main Ret/ptc1 multidocking site. Both in transformed thyrocytes and in the human PTC cell line TPC-1, Ret/ptc1-Y451-dependent signaling and Met cooperated to promote a pro  invasive   phenotype. Accordingly, gene/functional silencing of either RET/PTC1 or MET abrogated early branching morphogenesis in TPC-1 cells. The same effect was obtained by blocking the common downstream effector Akt. Y451 of Ret/ptc1 was required to promote proliferation and nuclear translocation of beta-catenin, suggesting that these oncogene-driven effects are Met-independent. Pharmacologic inhibition of Ret/ptc1 and Met tyrosine kinases by the multitarget small molecule RPI-1 blocked cell proliferation and   invasive   ability and dislocated beta-catenin from the nucleus. Altogether, these results support that Ret/ptc1 cross talks with Met at transcriptional and signaling levels and promotes beta-catenin transcriptional activity to drive thyrocyte neoplastic transformation. Such molecular network, promoting disease initiation and acquisition of a pro  invasive   phenotype, highlights new options to design multitarget therapeutic strategies for PTCs. 
 Targeting the receptor tyrosine kinase RET sensitizes breast cancer cells to tamoxifen treatment and reveals a role for RET in endocrine resistance. Endocrine therapy is the main therapeutic option for patients with estrogen receptor (ERalpha)-positive breast cancer. Resistance to this treatment is often associated with estrogen-independent activation of ERalpha. In this study, we show that in ERalpha-positive breast cancer cells, activation of the receptor tyrosine kinase RET (REarranged during Transfection) by its ligand GDNF results in increased ERalpha phosphorylation on Ser118 and Ser167 and estrogen-independent activation of ERalpha transcriptional activity. Further, we identify mTOR as a key component in this downstream signaling pathway. In tamoxifen response experiments, RET downregulation resulted in 6.2-fold increase in sensitivity of MCF7 cells to antiproliferative effects of tamoxifen, whereas GDNF stimulation had a protective effect against the drug. In tamoxifen-resistant (TAM(R)-1) MCF7 cells, targeting RET restored tamoxifen sensitivity. Finally, examination of two independent tissue microarrays of primary human breast cancers revealed that expression of RET protein was significantly associated with ERalpha-positive tumors and that in primary tumors from patients who subsequently developed   invasive   recurrence after adjuvant tamoxifen treatment, there was a twofold increase in the number of RET-positive tumors. Together these findings identify RET as a potentially important therapeutic target in ERalpha-positive breast cancers and in particular in tamoxifen-resistant tumors. 
 RET gene abnormalities and thyroid disease: who should be screened and when. Mutations in the RET proto-oncogene have been implicated in the pathogenesis of several forms of medullary thyroid cancer (MTC). Multiple endocrine neoplasia type 2 (MEN-2) is an autosomal dominant syndrome caused by germline activating mutations of the RET proto-oncogene and has been categorized into three distinct clinical forms. MEN-2A is associated with MTC, bilateral pheochromocytoma, and primary hyperparathyroidism. MEN-2B is associated with MTC, bilateral pheochromocytoma, and mucosal neuromas. The rarest clinical form of MEN-2 is familial MTC (FMTC), which is also associated with MTC, but other endocrinopathies are characteristically not present. Each clinical form of MEN-2 results from a specific RET gene mutation, with a strong correlation of phenotype expression with regard to the onset and course of MTC and the presence of other endocrine tumors and a corresponding genotype. Recommendations for screening of RET mutations are necessary as their presence or absence will influence interventional strategies such as the timing of a prophylactic thyroidectomy and extent of surgery. Timing of screenings and development of interventional strategies are extremely important in caring for patients with certain RET mutations as evidence of metastatic MTC has been documented as early as 6 years of age. Interventional strategies should consider the risks of complications of these interventions based on certain characteristics of each individual case such as age of the patient, course of disease in affected family members, and the   invasive  ness of any proposed surgical procedure. 
  GeneRIFs  
 Expression of the mitogenic and   invasive   phenotype of RET/PTC-transformed thyroid cells is stimulated by osteopontin. 
  [Mm]etastasize  PubMed  
 Molecular genotyping of papillary thyroid carcinoma follicular variant according to its histological subtypes (encapsulated vs infiltrative) reveals distinct BRAF and RAS mutation patterns. The follicular variant of papillary thyroid carcinoma usually presents as an encapsulated tumor and less commonly as a partially/non-encapsulated infiltrative neoplasm. The encapsulated form rarely   metastasize  s to lymph node, whereas infiltrative tumor often harbors nodal metastases. The molecular profile of the follicular variant was shown to be close to the follicular adenoma/carcinoma group of tumors with a high RAS and very low BRAF mutation rates. A comprehensive survey of oncogenic mutations in the follicular variant of papillary thyroid carcinoma according to its encapsulated and infiltrative forms has not been performed. Paraffin tissue from 28 patients with encapsulated and 19 with infiltrative follicular variant were subjected to mass spectrometry genotyping encompassing the most significant oncogenes in thyroid carcinomas: 111 mutations in RET, BRAF, NRAS, HRAS, KRAS, PIK3CA, AKT1 and other related genes. There was no difference in age, gender, tumor size and angioinvasion between encapsulated or infiltrative tumors. Infiltrative carcinomas had a much higher frequency of extrathyroid extension, positive margins and nodal metastases than encapsulated tumors (P<0.05). The BRAF 1799T>A mutation was found in 5 of 19 (26%) of the infiltrative tumor and in none of the encapsulated carcinomas (P=0.007). In contrast, RAS mutations were observed in 10 of 28 (36%) of the encapsulated group (5 NRAS_Q61R, 3 HRAS_Q61, 1 HRAS_G13C and 1 KRAS_Q61R) and in only 2 of 19 (10%) of infiltrative tumors (P=0.09). One encapsulated carcinoma showed a PAX8/PPARgamma rearrangement, whereas two infiltrative tumors harbored RET/PTC fusions. Encapsulated follicular variant of papillary thyroid carcinomas have a molecular profile very close to follicular adenomas/carcinomas (high rate of RAS and absence of BRAF mutations). Infiltrative follicular variant has an opposite molecular profile closer to classical papillary thyroid carcinoma than to follicular adenoma/carcinoma (BRAF>RAS mutations). The molecular profile of encapsulated and infiltrative follicular variant parallels their biological behavior (ie, metastatic nodal and invasive patterns). 
  [Mm]etastasis  PubMed  
 The RET p.G533C mutation confers predisposition to multiple endocrine neoplasia type 2A in a Brazilian kindred and is able to induce a malignant phenotype in vitro and in vivo. We have previously described a p.G533C substitution in the rearranged during transfection (RET) oncogene in a large family with medullary thyroid carcinoma. Here, we explore the functional transforming potential of RET p.G533C mutation.Plasmids expressing RET mutants (p.G533C and p.C634Y) and RET wild type were stable transfected into a rat thyroid cell line (PCCL3). Biological and biochemical effects of RET p.G533C were investigated both in vitro and in vivo. Moreover, we report the first case of pheochromocytoma among the RET p.G533C-carriers in this Brazilian family and explore the RET mutational status in DNA isolated from pheochromocytoma.Ectopic expression of RET p.G533C and p.C634Y activates RET/MAPK/ERK pathway at similar levels and significantly increased cell proliferation, compared with RET wild type. We additionally show that p.G533C increased cell viability, anchorage-independent growth, and micronuclei formation while reducing apoptosis, hallmarks of the malignant phenotype. RET p.G533C down-regulates the expression of thyroid specific genes in PCCL3. Moreover, RET p.G533C-expressing cells were able to induce liver   metastasis   in nude mice. Finally, we described two novel RET variants (G548V and S556T) in the DNA isolated from pheochromocytoma while they were absent in the DNA isolated from blood.Our in vitro and in vivo analysis indicates that this mutation confers a malignant phenotype to PCCL3 cells. These findings, in association with the report of first case of pheochromocytoma in the Brazilian kindred, suggest that this noncysteine mutation may be more aggressive than was initially considered. 
 RET protein promotes non-adherent growth of NB-39-nu neuroblastoma cell line. The receptor tyrosine kinase RET is expressed in a number of neuroblastoma tissues and cell lines, but its role in neuroblastoma remains to be determined. In this study, we examined the roles of RET protein in neuroblastoma by the RNA interference technique using the NB-39-nu neuroblastoma cell line. NB-39-nu neuroblastoma cells show high expression and elevated tyrosine phosphorylation of RET, although short interfering RNA against RET (RET siRNA) did not significantly inhibit cell proliferation or suppression of basal levels of phosphorylation of extracellular regulated kinase (ERK)1/2 or protein kinase B (AKT). By the addition of glial cell line-derived neurotrophic factor (GDNF), both the expression and phosphorylation of RET and the phosphorylation of ERK1/2 and AKT were further increased, whereas cell proliferation was not stimulated under normal culture conditions. However, proliferation of cells cultured under non-adherent conditions was significantly increased by GDNF. The increased proliferation was suppressed by RET siRNA, which also caused inhibition of the phosphorylation of ERK1/2 and AKT. These results suggest that RET signaling plays an important role in GDNF-induced enhancement of non-adherent proliferation of NB-39-nu cells, which might contribute to the   metastasis   of neuroblastoma. 
 The RET polymorphic allele S836S is associated with early metastatic disease in patients with hereditary or sporadic medullary thyroid carcinoma. The possible role of RET variants in modifying the natural course of medullary thyroid carcinoma (MTC) is still a matter of debate. Here, we investigate whether the RET variants L769L, S836S, and G691S/S904S influence disease presentation in hereditary or sporadic MTC patients. One hundred and two patients with hereditary MTC and 81 patients with sporadic MTC attending our institution were evaluated. The frequencies of RET polymorphisms in hereditary MTC were as follows: L769L, 17.3%; S836S, 7.95%; and S904S/G691S, 18.2%. No associations were observed between these polymorphisms and pheochromocytoma, hyperparathyroidism, lymph node, or distant   metastasis  . However, patients harboring the S836S variant were younger than those without this allele (17±8.2 vs 28.6±14.4 years, P=0.01), suggesting that these patients had metastases at a young age. Accordingly, the cumulative frequency of local and/or distant metastases as estimated by Kaplan-Meier curves showed that lymph node and distant metastases occurred earlier in patients harboring the S836S variant (P=0.003 and P=0.026 respectively). The S836S allele frequency was higher in sporadic MTC patients than in controls (10.5 vs 3.1%, P=0.01). Individuals harboring the S836S variant were younger (38.6±13.3 vs 48.5±16.7 years, P=0.02) and showed a higher percentage of lymph node and distant metastases (P=0.02 and P=0.04 respectively). Kaplan-Meier estimates of lymph node and distant metastases yielded distinct curves for patients with or without the S836S allele (P=0.002 and P=0.001 respectively). Additional analyses using a COX regression model showed that the S836S variant was independently associated with metastatic disease (hazard ratio 2.82 (95% confidence interval 1.51-5.26), P=0.001). In conclusion, the RET S836S variant is associated with early onset and increased risk for metastatic disease in patients with hereditary or sporadic MTC. 
 Association of RET genetic polymorphisms and haplotypes with papillary thyroid carcinoma in the Portuguese population: a case-control study. Thyroid cancer has a multifactorial aetiology resulting from the interaction of genetic and environmental factors. Several low penetrance susceptibility genes have been identified but their effects often vary between different populations. Somatic point mutations and translocations of the REarranged during Transfection (RET) proto-oncogene are frequently found in thyroid cancer. The aim of this case-control study was to determine the effect of four well known RET single nucleotide polymorphisms (SNPs) on the risk for differentiated thyroid carcinoma. A total of 545 Portuguese patients and 543 controls were genotyped by PCR and restriction enzyme analysis, for the following SNPs: G691S (exon 11, rs1799939 G/A), L769L (exon 13, rs1800861 T/G), S836S (exon 14, rs1800862 C/T), and S904S (exon 15, rs1800863 C/G). The minor allele of S836S was overrepresented in patients with papillary thyroid carcinoma (PTC) when compared to controls (OR 1.57; 95% CI 1.05-2.35; p = 0.026). The GGTC haplotype was also overrepresented in PTC (OR 2.51; 95% CI 1.07-5.91; p = 0.029). No associations were found in follicular thyroid carcinoma (FTC). Multivariate logistic regression analysis showed no differences regarding gender, age at diagnosis, lymph node or   distant me  tastasis. However, a near significant overrepresentation of the minor alleles of G691S and S904S was found in patients with tumours greater than 10 mm of diameter at diagnosis. These data suggest that the RET S836S polymorphism in exon 14 and the GGTC haplotype are risk factors for PTC, but not FTC, and that the G691S/S904S polymorphisms might be associated with tumour behaviour. 
 Effect of 3'UTR RET Variants on RET mRNA Secondary Structure and Disease Presentation in Medullary Thyroid Carcinoma. The RET S836S variant has been associated with early onset and increased risk for metastatic disease in medullary thyroid carcinoma (MTC). However, the mechanism by which this variant modulates MTC pathogenesis is still open to discuss. Of interest, strong linkage disequilibrium (LD) between RET S836S and 3'UTR variants has been reported in Hirschsprung's disease patients.To evaluate the frequency of the RET 3'UTR variants (rs76759170 and rs3026785) in MTC patients and to determine whether these variants are in LD with S836S polymorphism.Our sample comprised 152 patients with sporadic MTC. The RET S836S and 3'UTR (rs76759170 and rs3026785) variants were genotyped using Custom TaqMan Genotyping Assays. Haplotypes were inferred using the phase 2.1 program. RET mRNA structure was assessed by Vienna Package.The mean age of MTC diagnosis was 48.5±15.5 years and 57.9% were women. The minor allele frequencies of RET polymorphisms were as follows: S836S, 5.6%; rs76759170, 5.6%; rs3026785, 6.2%. We observed a strong LD among S836S and 3'UTR variants (|D'| = -1, r2 = 1 and |D'| = -1, r2 = 0,967). Patients harboring the S836S/3'UTR variants presented a higher percentage of lymph node and distant   metastasi  s (P = 0.013 and P<0.001, respectively). Accordingly, RNA folding analyses demonstrated different RNA secondary structure predictions for WT(TCCGT), S836S(TTCGT) or 3'UTR(GTCAC) haplotypes. The S836S/3'UTR haplotype presented a greater number of double helices sections and lower levels of minimal free energy when compared to the wild-type haplotype, suggesting that these variants provides the most thermodynamically stable mRNA structure, which may have functional consequences on the rate of mRNA degradation.The RET S836S polymorphism is in LD with 3'UTR variants. In silico analysis indicate that the 3'UTR variants may affect the secondary structure of RET mRNA, suggesting that these variants might play a role in posttranscriptional control of the RET transcripts. 
 Prognostic and Predictive Values of Subcellular Localisation of RET in Renal Clear-Cell Carcinoma. Metastatic renal cell carcinoma (RCC) presents a poor prognosis and an unpredictable course. To date, no validated biomarkers can predict the outcome of RCC. Ongoing efforts are conducted to identify the molecular markers of RCC progression, as well as the targets for novel therapeutic approaches. RET is a tyrosine kinase receptor which has been investigated as a possible target in other cancers because it is involved in oncogenic activation. To evaluate the predictive and prognostic functions of RET in ccRCC, a tissue microarray study was conducted on 273 ccRCC patients. Results showed that both RET cytoplasmic and nuclear expression were independently associated with PFS and OS, and the combined RET cytoplasmic and nuclear statuses demonstrated that the ratio of high nuclear RET and cytoplasmic RET was the strongest predictor of both PFS and OS. The high cytoplasmic RET expression retained its independent poor prognostic value in targeted drug treated patients. The RET nuclear expression was associated with distant   metastasis  . Moreover, the RET nuclear expression was an independent predictor of ccRCC postoperative   metastasis  . In conclusion, RET may be useful in prognostication and can be used at initial diagnosis to identify patients with high potential to develop   metastasis  . 
 The relationship between overexpression of glial cell-derived neurotrophic factor and its RET receptor with progression and prognosis of human pancreatic cancer. We used immunohistochemical staining to assess protein over-expression of glial cell-derived neurotrophic factor (GDNF) and its RET receptor tyrosine kinase in patients with pancreatic cancer and benign pancreatic neoplasm, and assessed correlations with clinicopathological features and prognosis. Surgically resected pancreatic cancer patients (40/58, 68.9%) showed positive GDNF immunostaining, a significantly higher frequency than in patients with benign pancreatic tumour (3/11, 27.3%). Intrapancreatic neural invasion by cancer cells was significantly related to over-expression of GDNF. Strongly positive expression of GDNF was significantly more frequent than lesser grades of expression in patients with severe back pain before and 12 months after surgery. Expression of RET was significantly related to lymphatic invasion, survival rate after tumour resection and degree of tumour cell differentiation. We conclude that GDNF may be important in pancreatic cancer proliferation and   metastasis  , especially in patients with perineural invasion. Strongly positive expression of GDNF may be an indication for early intensified radiotherapy. RET expression in pancreatic cancer tissues may be a useful prognostic marker. 
 Clinical relevance of RET variants G691S, L769L, S836S and S904S to sporadic medullary thyroid cancer. Based on reports of higher frequencies among patients with sporadic medullary thyroid cancer (MTC) relative to external controls, the RET (REarranged during Transfection) variants G691S, L767L, S836S and S904S have been considered disease modifiers, suggesting greater lifetime risks of MTC. Other studies, employing different external controls, failed to confirm this association. Using a complementary approach, this study aimed at exploring differences in clinico-pathological characteristics among patients with sporadic MTC carrying no (wildtype), one (heterozygotes) or both (homozygotes) homologue RET variants in the germline, with wildtype cases acting as internal controls.Included in this investigation were 150 patients with complete genetic information on G691S, L769L, S836S and S904S RET alleles operated on for sporadic MTC at a tertiary referral centre.Not one statistically significant dose-response relationship was identified between any RET variant (wildtype vs RET heterozygotes vs homologue RET homozygotes) and patient age at MTC diagnosis, gender, primary tumour size, extrathyroidal extension, numbers of involved and removed lymph nodes, or distant   metastasis  . L769L and S836S homozygotes, unlike G691S and S904S homozygotes, were either rare or absent, limiting the analyses to comparisons of heterozygosity versus wildtype. On time-to-event analysis, G691S, L769L, S836S or S904S carriers and noncarriers developed MTC at similar rates.In carriers and noncarriers of the RET variants G691S, L767L, S836S and S904S, sporadic MTC appeared clinically and pathologically indistinguishable. This observation, along with the inconclusive evidence of previous association studies, calls for larger longitudinal association studies with age- and sex-matched external controls and additional functional studies of RET biology. 
 Hereditary paraganglioma-pheochromocytoma syndromes associated with SDHD and RET mutations. Hereditary paraganglioma-pheochromocytoma syndromes (PGL/PCC) are rare tumors arising from neuroendocrine cells.The proband, a 59-year-old white man and his 42-year-old elder son had a medical history of bilateral carotid body PGL and both presented for treatment of abdominal PGLs. His 36-year-old daughter had excision of recurrent malignant carotid body PGL and vertebral   metastasis  . His 33-year-old youngest son presented for excision of a unilateral carotid body PGL. All 4 members had succinate dehydrogenase subunit D (SDHD) mutations, whereas the proband and youngest son also had concurrent rearranged during transfection (RET) mutation.This is the first report of PGL/PCC with SDHD and RET mutations. The role of the RET gene as a modifier remains speculative. Additionally, the family pedigree suggests maternal inheritance of disease from the probands' paternal grandmother. Clinicians should refer PGL/PCC families for mutation analysis as well as being alert to changes in the classification of mutations. 
 Comparative genomic hybridization, BRAF, RAS, RET, and oligo-array analysis in aneuploid papillary thyroid carcinomas. Aneuploidy in papillary thyroid carcinomas (PTCs) is considered a marker of worse prognosis. Multiple genetic surveys have been performed in PTCs, however, we are not aware of any such studies in aneuploid PTCs. In order to contribute to a better comprehension of the genetic basis of this neoplasm's more aggressive behaviour in 17 aneuploid PTCs we performed a comparative genomic hybridization (CGH) analysis, studied the BRAF and RAS mutational status, searched for RET/PTC1 and RET/PTC3 rearrangements and determined their expression profile. Array results were validated by TaqMan and immunohistochemistry. CGH revealed multiple non-random chromosomal abnormalities. BRAFV600E and RAS mutations were found in 41.2% and 33% of the carcinomas respectively. None of the studied cases presented RET/PTC1 or RET/PTC3 rearrangement. When comparing array data with the chromosomal, mutational and clinical data we found that: a) loss of control of cellular transcription was of major relevance in this group of neoplasms, HMGA2 being one of the most overexpressed genes; b) gene expression correlated with the mutational status of PTCs, as in BRAF+ cases cMET and FN1 were concomitantly overexpressed; and c) death from disease and distant   metastasis   was associated to the overexpression of DDR2 and to the down-regulation of genes involved in immune, inflammatory response, signal transduction and cell adhesion processes. In conclusion we have identified in aneuploid PTCs a group of significantly altered molecules that may represent preferential targets for the development of new more efficient therapies in this type of cancer. 
 A family of multiple endocrine neoplasia type 2A (MEN 2A) with Cys630Tyr RET germline mutation: report of a case. Since the majority of multiple endocrine neoplasia type 2A (MEN 2A) patients have missense mutations at codon 634 and those with the Cys630 RET genotype mutations are extremely rare, limited clinical information is available about this rare type. We report here three members of one Japanese MEN 2A family with the Cys630Tyr genotype. A 67-year-old woman presented a firm thyroid nodule, and preoperative examination revealed medullary thyroid carcinoma with primary hyperparthyoidism and no pheochromocytoma. At surgery, bilateral medullary thyroid carcinomas and parathyroid adenoma were found. No lymph node   metastasis   was identified. Computed tomography scans and laboratory examination of blood have shown no evidence of tumor recurrence and no abnormality of parathyroid function during the 4 years after surgery. A 40-year-old man, the proband's son, was shown to have the same RET mutation, underwent total thyroidectomy prophylactically, and only microscopic foci of medullary thyroid carcinoma were found. A 10-year-old boy, the proband's grandson also having the same RET mutation, showed normal basal serum calcitonin level and has been followed up conservatively. To our knowledge, 18 patients of 6 families with the Cys630 mutations have been reported so far. This is only the second reported case with primary hyperparathyroidism. RET 630 mutations might be associated with lower penetrance of primary hyperparthyoidism and pheochromocytoma. 
 Modification of multiple endocrine neoplasia 2A phenotype by cell membrane proximity of RET mutations in exon 10. Rearranged during transfection (RET) germ-line mutations in exon 10 are peculiar because they produce both gain-of-function multiple endocrine neoplasia 2A and loss-of-function Hirschsprung's disease phenotypes. Drawing on 38 medullary thyroid cancer patients harboring germ-line mutations in codon 620 (n=8), 618 (n=19), 611 (n=10), and 609 (n=1), this study aimed to test the hypothesis that closer proximity of RET germ-line mutations in exon 10 to the cell membrane may translate into earlier or more advanced disease. The closer mutations in codon 620, 618, and 611 were located to the transmembrane domain (codons 657-636) of the RET receptor, the greater were mean primary tumor diameters (23.5, 18.7, and 7.5 mm, P=0.020), the frequency of lymph node   metastasis   (75, 68, and 30%, P=0.11) and pheochromocytoma (38, 16, and 0%, P=0.11). Periods of observation were broadly comparable for these groups (mean age 33.4-39.3 years; P=0.71). When mutations in adjoining codons were collapsed (codons 620/618 vs 611/609), the differences in mean primary tumor diameter (20.1 vs 7.4 mm, P=0.005) and lymph node   metastasis   (70 vs 36%; P=0.07) were accentuated. Compared with 80 carriers of exon 11 mutations (codon 634, n=78; codon 630, n=2), the 38 carriers of exon 10 mutations, which are rarer and confer a weaker transforming activity in vitro than exon 11 mutations, required significantly more time to develop fewer tumors. Although limited in numbers, these data suggested that membrane proximity is an important determinant of tumor development in carriers of RET mutations in exon 10. 
 Central role of the threonine residue within the p+1 loop of receptor tyrosine kinase in STAT3 constitutive phosphorylation in metastatic cancer cells. The receptor tyrosine kinases (RTKs) RET, MET, and RON all carry the Met(p+1loop)-->Thr point mutation (i.e., 2B mutation), leading to the formation of tumors with high metastatic potential. Utilizing a novel antibody array, we identified constitutive phosphorylation of STAT3 in cells expressing the 2B mutation but not wild-type RET. MET or RON with the 2B mutation also constitutively phosphorylated STAT3. Members of the EPH, the only group of wild-type RTK that carry Thr(p+1loop) residue, are often expressed unexpectedly in different types of cancers. Ectopic expression of wild-type but not Thr(p+1loop)-->Met substituted EPH family members constitutively phosphorylated STAT3. In both RTK(Metp+1loop) with 2B mutation and wild-type EPH members the Thr(p+1loop) residue is required for constitutive kinase autophosphorylation and STAT3 recruitment. In multiple endocrine neoplasia 2B (MEN-2B) patients expressing RET(M918T), nuclear enrichment of STAT3 and elevated expression of CXCR4 was detected in metastatic thyroid C-cell carcinoma in the liver. In breast adenocarcinoma cell lines expressing multiple EPH members, STAT3 constitutively bound to the promoters of MUC1, MUC4, and MUC5B genes. Inhibiting STAT3 expression resulted in reduced expression of these   metastasis  -related genes and inhibited mobility. These findings provide insight into Thr(p+1loop) residue in RTK autophosphorylation and constitutive activation of STAT3 in metastatic cancer cells. 
 A novel RET kinase-beta-catenin signaling pathway contributes to tumorigenesis in thyroid carcinoma. The RET receptor tyrosine kinase has essential roles in cell survival, differentiation, and proliferation. Oncogenic activation of RET causes the cancer syndrome multiple endocrine neoplasia type 2 (MEN 2) and is a frequent event in sporadic thyroid carcinomas. However, the molecular mechanisms underlying RET's potent transforming and mitogenic signals are still not clear. Here, we show that nuclear localization of beta-catenin is frequent in both thyroid tumors and their metastases from MEN 2 patients, suggesting a novel mechanism of RET-mediated function through the beta-catenin signaling pathway. We show that RET binds to, and tyrosine phosphorylates, beta-catenin and show that the interaction between RET and beta-catenin can be direct and independent of cytoplasmic kinases, such as SRC. As a result of RET-mediated tyrosine phosphorylation, beta-catenin escapes cytosolic down-regulation by the adenomatous polyposis coli/Axin/glycogen synthase kinase-3 complex and accumulates in the nucleus, where it can stimulate beta-catenin-specific transcriptional programs in a RET-dependent fashion. We show that down-regulation of beta-catenin activity decreases RET-mediated cell proliferation, colony formation, and tumor growth in nude mice. Together, our data show that a beta-catenin-RET kinase pathway is a critical contributor to the development and   metastasis   of human thyroid carcinoma. 
 Oncogenic alterations in papillary thyroid cancers of young patients. Papillary thyroid carcinoma (PTC) in young people usually has an aggressive initial presentation, though a good general prognosis despite recurrences in 10%-20% of patients. A number of genetic alterations that activate the mitogen-activated protein kinase (MAPK) pathway have been found in PTC. Some of these alterations have been identified as prognostic factors of PTC in adults. The objective of the current study was to comprehensively characterize all known oncogenic alterations of the MAPK pathway in young people.One hundred three PTCs removed from 9 children, 19 adolescents, and 75 young adults were submitted to molecular analyses.Altogether, 57 alterations were found in 56 PTCs (55%) corresponding to V600E BRAF in 20.3%, RAS mutations in 12.6%, RET/PTC 1 in 11.6%, RET/PTC 3 in 8.7%, and rearrangement of NTRK in 1.9%. The prevalence of all alterations increased with age (22.2% in children; 52.6% in adolescents, 51.4% in adults 20-25 years, and 55.1% in adults 25-35 years). Prevalence increased from 39.2% earlier to 61.3% after 20 years mainly due to BRAF mutations. Classic-type PTC was associated with a larger prevalence of alterations, predominantly BRAF and RET/PTC, whereas the follicular variant was chiefly associated with RAS. RET/PTC (1 and 3) was significantly associated with extrathyroid extension (ET) and lymph node   metastasis   (es) (LNM). This association was found in the adult group. There were no associations of BRAF or RAS mutations with ET or LNM. A 3-year median follow up was available for 90 patients. RET/PTC 1 and 3 was associated with short-term disease dissemination (cervical lymph node recurrences and distant metastases) in young adults (p=0.001). Persistent illness was more prevalent in patients with (15%) than in patients without (7%) genetic alterations.PTCs in young patients display a low prevalence of the already identified oncogenic alterations. The increasing prevalence with age is mainly due to V600E BRAF mutation. There is no relation between tumor aggressiveness and BRAF mutation. There is a relation between the presence of RET/PTC (1 and 3) and the histological and clinical short-term aggressiveness of PTC in the population of young adults. Such a relation is not found in children and adolescents. 
 CD44 and OTP are strong prognostic markers for pulmonary carcinoids. Pulmonary carcinoids are well-differentiated neuroendocrine tumors showing usually a favorable prognosis. However, there is a risk for late recurrence and/or distant   metastasis  . Because histologic classification in typical and atypical carcinoids is difficult and its reliability to predict disease outcome varies, we evaluated three genes as potential prognostic markers, that is, orthopedia homeobox (OTP), CD44, and rearranged during transfection (RET).These genes were analyzed in 56 frozen carcinoids by quantitative real-time PCR (qRT-PCR). RET was further studied by methylation and mutation analysis. Immunohistochemistry for CD44 and OTP protein expression was conducted on 292 carcinoids.Low mRNA expression levels of CD44 (P = 1.8e(-5)) and OTP (P = 0.00054), and high levels of RET (P = 0.025), were strongly associated with a low 20-year survival of carcinoid patients. High RET expression was not related to promoter hypomethylation or gene mutations. A direct link between gene expression and protein levels was confirmed for CD44 and OTP but not for RET. Within all carcinoids as well as atypical carcinoids, absence of CD44 protein was significantly associated with low 20-year survival (P = 0.00014 and 0.00013, respectively). The absence of nuclear OTP followed by complete loss of expression was also significantly associated with unfavorable disease outcome in all carcinoids (P = 5.2(-6)). Multivariate analyses revealed that age at diagnosis, histopathology, stage, and cytoplasmic OTP immunoreactivity were independent predictors of prognosis.Our study indicates that CD44 and OTP are strong indicators of poor outcome. We therefore argue for implementation of these markers in routine diagnostics in addition to histopathology to improve subclassification of pulmonary carcinoids into prognostically relevant categories. 
 Alternative surgical strategies and favorable outcomes in patients with medullary thyroid carcinoma in Japan: experience of a single institution. Medullary thyroid carcinoma (MTC) accounts only for 1.4% of all thyroid malignancies in Japan. Since 1996, we have performed hemithyroidectomy, instead of total thyroidectomy, for sporadic nonhereditary MTC when the primary lesion is located in only one lobe. Regarding lymph node dissection, modified radical neck dissection (MND) at least ipsilateral to the tumor has been routinely performed, even if there is no clinically apparent   metastasis  . We investigated the clinical outcomes of MTC patients in our department.A series of 118 patients with MTC who underwent initial surgery between 1975 and 2005 were enrolled in this study. The RET gene mutations were analyzed for all patients and 46 had germline RET gene mutations. Of those 46 patients, 26 were diagnosed as MEN 2A and 2 were diagnosed as MEN 2B. Postoperative follow-up periods averaged 141 months.Of 115 patients who did not have distant   metastasis   at surgery and who underwent locally curative surgery, 78 (67.8%) were biochemically cured. All patients without pathological lymph node   metastasis   were biochemically cured, and 44.8% of patients with node   metastasis   were also biochemically cured. The 10-year and 20-year disease-free survival rates were 89.0% and 82.5%, respectively. None of the patients who did not show lymph node   metastasis   and only 2 (2.6%) of 78 patients who were biochemically cured showed clinically apparent carcinoma recurrence. The 10-year and 20-year cause-specific survival rates were 96.6% and 91.7%, respectively. Lymph node   metastasis  , tumor size>4 cm, extrathyroid and extranodal tumor extensions significantly affected cause-specific survival of patients.Clinical outcomes of MTC patients in our series were better than those in Western countries, a result that might have resulted in part because of our routine MND regardless of whether clinically apparent node   metastasis   was detected. 
 Case report: a p.C618S RET proto-oncogene germline mutation in a large Chinese pedigree with familial medullary thyroid carcinoma. We report a Chinese pedigree with familial medullary thyroid carcinoma. Direct sequencing of the entire coding sequences of Rearranged during Transfection (RET) identified a recurrent c.T1852A (p.C618S) mutation in 13 of 23 members. The polymorphisms c.A135G (p.A45A), c.A1296G (p.A432A), c.T2307G (p.L769L) and IVS19 + 15T > C were also found in 13 carriers, and c.G2073A (p.G691S) was found in 1 carrier. Of the 13 carriers, seven (mean age: 42.6 years, range: 27-64) presented MTC as the isolated clinical phenotype, with elevated basal serum calcitonin (average: 1077.9 ng/L, range: 504-2,652) and a mean diameter of thyroid nodules of 2.97 cm (range: 1.6-4.3); they underwent a total thyroidectomy with modified bilateral/unilateral neck dissection and/or level VI lymph node dissection. The other 6 carriers did not accept surgery (4 rejected, 2 awaited). These were 2 older patients (63 and 32 years) with elevated calcitonin (1359 and 41.4 ng/L) and multi-centric hypoechoic nodules (1.5 and 0.6 cm) with calcifications in both/left thyroid lobes; and Doppler ultrasound showed normal bilateral thyroids in 4 younger carriers (median age: 8.3 years, range: 4-12) but with increased calcitonin (average: 9.7 ng/L, range: 7.87-12.2) in 3 of them. The phenotype here is consistent with the clinical symptoms reported worldwide. We recommend that screening of hotspot regions of RET should be preferentially carried out, while whole-exon sequencing should be performed when clinical signs fail to reveal hotspot mutations or different phenotype discrepancies. Moreover, we strongly suggest prophylactic thyroidectomy should be performed before age 5 in carriers with p.C618S to prevent the occurrence and   metastasis   of MTC. 
 Tumor genotype determines phenotype and disease-related outcomes in thyroid cancer: a study of 1510 patients. To correlate thyroid cancer genotype with histology and outcomes.The prognostic significance of molecular signature in thyroid cancer (TC) is undefined but can potentially change surgical management.We reviewed a consecutive series of 1510 patients who had initial thyroidectomy for TC with routine testing for BRAF, RAS, RET/PTC, and PAX8/PPARG alterations. Histologic metastatic or recurrent TC was tracked for 6 or more months after oncologic thyroidectomy.Papillary thyroid cancer (PTC) was diagnosed in 97% of patients and poorly differentiated/anaplastic TC in 1.1%. Genetic alterations were detected in 1039 (70%); the most common mutations were BRAFV600E (644/1039, 62%), and RAS isoforms (323/1039, 31%). BRAFV600E-positive PTC was often conventional or tall cell variant (58%), with frequent extrathyroidal extension (51%) and lymph node   metastasis   (46%). Conversely, RAS-positive PTC was commonly follicular variant (87%), with infrequent extrathyroidal extension (4.6%) and lymph node   metastasis   (5.6%). BRAFV600E and RET/PTC-positive PTCs were histologically similar. Analogously, RAS and PAX8/PPARG-positive PTCs were histologically similar. Compared with RAS or PAX8/PPARG-positive TCs, BRAFV600E or RET/PTC-positive TCs were more often associated with stage III/IV disease (40% vs 15%, P < 0.001) and recurrence (10% vs 0.7%, P < 0.001; mean follow-up 33 ± 21 mo). Distant   metastasi  s was highest in patients with RET/PTC-positive TC (10.8%, P = 0.02).In this large study of prospective mutation testing in unselected patients with TC, molecular signature was associated with distinctive phenotypes including cancers, with higher risks of both distant   metastasi  s and early recurrence. Preoperative genotype provides valuable prognostic data to appropriately inform surgery. 
 Genome-wide copy number analysis in a family with p.G533C RET mutation and medullary thyroid carcinoma identified regions potentially associated with a higher predisposition to lymph node   metastasis  . Our group described a p.G533C RET gene mutation in a large family with multiple endocrine neoplasia type 2 syndrome. Clinical heterogeneity, primarily associated with the presence of lymph node metastases, was observed among the p.G533C carriers.The aim of this study was to use single-nucleotide polymorphism-array technology to identify copy number variations (CNVs), which are present in the constitutional DNA and associated with the established clinical and pathological features of aggressive medullary thyroid carcinoma (MTC), primarily the presence of lymph node   metastasis  .Fifteen p.G533C carriers with MTC were chosen for the initial screening. The subjects were divided into two groups according the presence (n = 8) or absence (n = 7) of lymph node   metastasis  . Peripheral blood DNA was independently hybridized using a genome-wide single-nucleotide polymorphism Array 6.0 platform. The results were analyzed using both Genotyping Console and PennCNV software. To identify the possible candidate regions associated with the presence of lymph node   metastasis  , cases (metastatic MTC) were compared with controls (nonmetastatic MTC). The identified CNVs were validated by quantitative PCR in an extended cohort (n = 32).Using two different algorithms, we identified nine CNV regions that may contribute to susceptibility to lymph node   metastasis  . The validation step confirmed that a CNV loss impacting the FMN2 gene was potentially associated with a greater predisposition to lymph node   metastasis   in this family (P = .0179). Finally, we sought to investigate whether the development of lymph node   metastasis   might not depend on a single CNV but rather a combination of various CNVs. These analyses defined a CNV pattern related to a more aggressive phenotype in this family, with CNV deletions being enriched in the metastatic group (P = .0057).Although hereditable specific RET mutations are important to determine cancer risk, germline CNVs in disease-affected individuals may predispose them to MTC aggressiveness. 
 C-Cell Neoplasia in Asymptomatic Carriers of RET Mutation in Extracellular Cysteine-Rich and Intracellular Tyrosine Kinase Domain. Germline mutations in RET proto-oncogene associated with multiple endocrine neoplasia type 2 (MEN2) may affect codons for the extracellular cysteine-rich (ECR) or the intracellular tyrosine kinase (ITK) domain of the transmembrane receptor tyrosine kinase protein. We compared C-cell pathology in asymptomatic carriers of RET mutation affecting the 2 domains. Twenty-two asymptomatic carriers (median age, 9.5 years), 10 with mutations in the ECR (codons 634, 611, 618, and 620) and 12 with mutations in the ITK domain (codons 804, 790, 891, and 918), underwent total thyroidectomy. C-cell hyperplasia was identified in 16 (73%), was multifocal and/or bilateral in 11, and was associated with medullary thyroid carcinoma (MTC) in 10 thyroids. When comparing the ECR and ITK groups in 21 carriers from MEN2A/familial MTC families, C-cell hyperplasia was more frequent in the former (90% versus 55%), as was multifocality (70% versus 27%) and MTC (60% versus 27%), despite the significantly younger median age in the former group (5 versus 23 years, P = .04). One asymptomatic carrier had de novo codon 918 mutation (MEN2B) and showed bilateral microcarcinoma with lymph node   metastasis   at presentation and progressive disease on follow-up. In conclusion, asymptomatic carriers of high-risk RET mutations affecting the ECR were significantly younger and frequently showed C-cell neoplasia, multifocality, and MTC when compared with mutations affecting the ITK domain in the MEN2A/familial MTC families. The presence of C-cell disease, its severity, and aggressiveness correlated with the mutated codon and with increasing age. 
 Characterization of V804M-mutated RET proto-oncogene associated with familial medullary thyroid cancer, report of the largest Turkish family. Analysis of the RET proto-oncogen is very important for diagnosis and prognosis of medullary thyroid cancer (MTC). Genotype-phenotype correlation is also well known. Here we report features of the largest known family in Turkey with the V804M-mutated RET proto-oncogene.Thirty members from three generations were evaluated. A RET proto-oncogen mutation, calcitonin (Ct) measurement and thyroid ultrasound were performed on all individuals. Seventeen members had V804M mutation. Fourteen of these patients underwent total thyroidectomy and additional central lymph node dissection for five subjects.The mean age of patients with MTC was 46.5 (30-61) years. The mean calcitonin level of RET positive members was 13.27 pg/mL (1-49.8 pg/mL). Three had a basal Ct level above normal limits. Seven of the 14 patients were diagnosed with MTC, and two were diagnosed with papillary thyroid cancer without MTC. One patient had central neck   metastasis  . Hyperparathyroidism or pheochromocytoma was not detected in any case. Patients who were RET negative, had normal Ct levels and no suspected nodule on ultrasound examination.Our study revealed a relatively good prognosis in patients with V804M mutation. Despite the surgery was performed in older age no advance disease was observed. 
 Multiple genetic alterations in papillary thyroid cancer are associated with younger age at presentation. There is a significant gender and age disparity in thyroid cancer incidence and outcome. The molecular basis for these divergent clinical presentations and outcome are essentially unknown.The primary tumor genotype in 217 patients with papillary thyroid cancer was determined for six common somatic genetic alterations (RET/PTC1, RET/PTC3, and NTRK1 rearrangements, and BRAF V600E, KRAS, and NRAS hotspot mutations) by PCR and direct sequencing, and nested PCR. Univariate and multivariate analyses were performed to determine the association of genetic changes and age, gender, and other clinicopathologic factors.One hundred twenty-one of the 190 conventional papillary thyroid carcinoma samples (63.7%) had at least one genetic alteration, and 27 of the samples (14.2%) had more than one alteration. In the follicular variant of papillary thyroid carcinomas, 13 of the 27 samples (48.1%) had at least one genetic alteration and three of the 27 samples (11.1%) had more than one. The presence of multiple genetic alterations was associated with younger age at diagnosis (P=0.034), mean difference of 8 y earlier. We found no significant association with the number or type of genetic alterations present by gender, tumor size, extent of tumor differentiation, multicentricity, lymph node   metastasis  , distant metastases, TNM stage, and the AMES risk group. The association of multiple genetic alterations and younger age were independent of tumor size, lymph node or distant   metastasis  , TNM stage, or AMES risk group.Multiple genetic alterations are more common in younger patients with papillary thyroid cancer, but there is no difference in the type or number of genetic alterations by gender. Our findings suggest that multiple genetic alterations in thyroid cancer may be associated with earlier disease initiation and or progression. 
 Medullary thyroid carcinoma. Medullary thyroid carcinoma (MTC) accounts for 5-8% of all thyroid cancers. MTC is mainly sporadic in nature, but an hereditary pattern [multiple endocrine neoplasia type 2 (MEN 2)] is present in 20-30% of cases, transmitted as an autosomal-dominant trait due to germline mutations of the RET proto-oncogene. About 98% of patients with MEN 2 have germline mutations in exons 5, 8, 10, 11, 13, 14, 15 or 16 of the RET gene. The primary treatment of both hereditary and sporadic forms of MTC is total thyroidectomy and removal of all neoplastic tissue present in the neck. The therapeutic option for lymph node surgery should be dictated by the results of presurgical evaluation. After total thyroidectomy, measurements of serum calcitonin (CT) and carcinoembryonic antigen are of paramount importance in the postsurgical follow-up of patients with MTC as they reflect the presence of persistent or recurrent disease. Complete remission is demonstrated by undetectable and stimulated serum CT measurement. On the contrary, if serum CT is detectable under basal conditions or becomes detectable after stimulation, the patient is probably not cured, but imaging techniques will not demonstrate any disease until serum CT approaches levels >150 pg/ml. The tumour   metastasis  es early to both paratracheal and lateral cervical lymph nodes. Metastases outside the neck may occur in the liver, lungs, bones and, less frequently, brain and skin. Surgery is the main treatment for local and distant metastases whenever feasible. Systemic chemotherapy with dacarbazine, 5-fluorouracil and doxorubicin (alone or in combination) has shown very limited efficacy, achieving only partial responses in the range of 10-20% and of short duration. Several kinase inhibitors are currently under evaluation and preliminary results are promising. Familial cases must be identified by searching for RET proto-oncogene mutations in the proband and in family members. Carriers of the RET gene are candidates for prophylactic thyroidectomy at different ages depending on the risk associated with the specific RET mutations. 
 Expression of Ret finger protein correlates with outcomes in endometrial cancer. Ret finger protein (RFP) is a nuclear protein with transcriptional repressive activity that is highly expressed in a variety of human and rodent tumor cell lines. We examined the expression of RFP in human endometrial cancer and assessed its clinical significance. Formalin-fixed, paraffin-embedded sections from endometrial cancer tissues were immunostained with the RFP antibody, and the staining intensity was evaluated. The clinicopathological factors examined were age, International Federation of Gynecology and Obstetrics stage, tumor grade, myometrial invasion, and pelvic lymph node   metastasis  . Overall survival (OS) and progression-free survival (PFS) were evaluated using the Kaplan-Meier method, and multivariate analysis was performed using the Cox proportional hazard analysis. Of the 119 cancer tissues, 57 (47.9%) cases were positive for RFP immunoreactivity. RFP expression was not associated with any of the clinicopathological parameters examined. However, positive RFP expression significantly predicted poorer OS and PFS compared with negative expression (OS, P = 0.0011; PFS, P < 0.0001). In the multivariate analyses, positive RFP expression was an independent prognostic factor for survival in this study. RFP knockdown significantly impaired cancer cell migration and invasion in vitro with concomitant decreases of integrins beta1 and alpha2. Positive RFP expression is a predictive marker for an unfavorable clinical outcome in patients with endometrial cancer. 
 Evaluation of RET polymorphisms in a six-generation family with G533C RET mutation: specific RET variants may modulate age at onset and clinical presentation. We previously described a six-generation family with G533C RET mutation and medullary thyroid carcinoma, in the largest family reported do date. Of particular interest, phenotype variability regarding the age of onset and clinical presentation of the disease, was observed.We evaluate whether single SNPs within RET oncogene or haplotype comprising the RET variants (defined by Haploview) could predispose to early development of MTC in this family and influence the clinical manifestation.Eight SNPs were selected based on their previous association with the clinical course of hereditary or sporadic MTC, in particular promoting an early onset of disease. The variants were initially tested in 77 G533C-carriers and 100 controls using either PCR-direct sequencing or PCR-RFLP. Association between a SNP or haplotype and age at diagnosis or presence of lymph node   metastasis   was tested in 34 G533C-carries with MTC. Different bioinformatic tools were used to evaluate the potential effects on RNA splicing.An association was found between IVS1-126G > T and age at diagnosis. The variant [IVS8 +82A > G; 85-86 insC] was associated with the presence of lymph node metastases at diagnosis. In silico analysis suggested that this variant may induce abnormal splicing. This in silico analysis predicted that the [IVS8 +82A > G; 85-86 insC] could alter the splicing by disrupting and/or creating exonic splicing enhancer motifs.We here identified two RET variants that were associated with phenotype variability in G533C-carriers, which highlights the fact that the modifier effect of a variant might depend on the type of mutation. 
 New mutations in the RET protooncogene-L881V - associated with medullary thyroid carcinoma and -R770Q - in a patient with mixed medullar/follicular thyroid tumour. Clinical studies are needed to classify rare and novel RET mutations associated with hereditary medullary thyroid carcinoma (MTC) into one of the clinical risk groups. Here we describe two new RET mutations/variants, R770Q and L881V, in patients with MTC and analyzed genotype-phenotype correlations associated with these RET mutations in the gene carriers. FAMILY 1: Calcitonin screening in a 42-year-old female patient with multinodular goiter showed elevated levels. RET mutation analysis revealed a new variant in exon 13 R770Q (CGA>CAA) in the patient. A thyroidectomy with central and lateral node dissection was done. Histology showed MTC in a mixed variance with follicular cancer of 2 cm diameter (T2N0M0). Postoperatively there was no increase of calcitonin after pentagastrin stimulation. The patient is biochemically cured concerning MTC and FTC after radioiodine therapy. In the sister of the index patient surprisingly another, previously not described amino-acid substitution Y791N (TAT><) in the RET protooncogene was found. In the parents the R770Q variant was detected in the mother, the Y791N mutation in the father. Another sister carries the R770Q variant. In all other gene carriers (aged 44-70 years), calcitonin levels were in the normal range, therefore, thyroidectomy had not yet been performed. FAMILY 2: In a 46-year-old female patient with nodular goiter thyroidectomy, central and left lateral lymph node dissection was done because of elevated calcitonin levels. Histology revealed a microcarcinoma with one lymph nod  e metastas  is (T1N1(1/8)Mx). RET analysis revealed a new mutation in exon 15 L881V (CTG>GTG). The L881V mutation was detected in five other family members. In the first generation stimulated calcitonin levels were in the normal range, therefore thyroidectomy had not yet been performed. In the sons of the index case thyroidectomy revealed CCH in the older one, no MTC in both. In a cousin thyroidectomy is intended because of elevated basal and stimulated calcitonin.Our clinical findings indicate that the L881V mutation may be associated with late-onset nonaggressive disease. If the germline RET R770Q variant has a causative role in the pathogenesis of the mixed medullar/follicular derived histology of the thyroid tumour in the index patient of family 1 has to be proven. The recommendations for prophylactic thyroidectomy in these mutations should be individualized depending on basal and stimulated calcitonin levels until more data are available. 
 Coexistent familial nonmultiple endocrine neoplasia medullary thyroid carcinoma and papillary thyroid carcinoma associated with RET polymorphism. Familial nonmultiple endocrine neoplasia medullary thyroid cancer accounts for 10% to 15% of hereditary medullary thyroid carcinoma and is characterized by lack of accompanying endocrine or nonendocrine diseases. Simultaneous occurrence of medullary and papillary thyroid carcinoma in the same patient is rare and known as collision tumor. Here, the authors present familial nonmultiple endocrine neoplasia medullary thyroid cancer in 4 sisters, all having RET proto-oncogene polymorphism in exon 15 at codon 904 and 2 having additional polymorphism in exon 13 at codon 769. The index case had concomitant medullary and papillary thyroid carcinomas, which are suggested to be completely different tumors in terms of incidence, cell origin, histopathologic features and prognosis. Histopathologically, she also had Hashimoto thyroiditis in the remaining thyroid tissue and medullary thyroid carcinoma   metastasis   in 3 cervical lymph nodes. This case is the first in the literature to report coexistent familial nonmultiple endocrine neoplasia medullary thyroid cancer and papillary thyroid carcinoma related with a RET polymorphism (S904S in exon 15). 
 Ret inhibition decreases growth and metastatic potential of estrogen receptor positive breast cancer cells. We show that elevated levels of Ret receptor are found in different sub-types of human breast cancers and that high Ret correlates with decreased   metastasis  -free survival. The role of Ret in ER+ breast cancer models was explored combining in vitro and in vivo approaches. Our analyses revealed that ligand-induced Ret activation: (i) stimulates migration of breast cancer cells; (ii) rescues cells from anti-proliferative effects of endocrine treatment and (iii) stimulates expression of cytokines in the presence of endocrine agents. Indeed, we uncovered a positive feed-forward loop between the inflammatory cytokine IL6 and Ret that links them at the expression and the functional level. In vivo inhibition of Ret in a metastatic breast cancer model inhibits tumour outgrowth and metastatic potential. Ret inhibition blocks the feed-forward loop by down-regulating Ret levels, as well as decreasing activity of Fak, an integrator of IL6-Ret signalling. Our results suggest that Ret kinase should be considered as a novel therapeutic target in subsets of breast cancer. 
 A novel activating mutation in the RET tyrosine kinase domain mediates neoplastic transformation. We report the finding of a novel missense mutation at codon 833 in the tyrosine kinase of the RET proto-oncogene in a patient with a carcinoma of the thyroid. In vitro experiments demonstrate that the R833C mutation induces transformed foci only when present in the long 3' splice isoform and, in keeping with a model in which the receptor has to dimerize to be completely activated, glial cell line-derived neurotrophic factor stimulation leads the RET(R833C) receptor to a higher level of activation. Tyrosine kinase assays show that the RET(R833C) long isoform has weak intrinsic kinase activity and phosphorylation of an exogenous substrate is not elevated even in the presence of glial cell line-derived neurotrophic factor. Furthermore, the R833C mutation is capable of sustaining the transformed phenotype in vivo but does not confer upon the transformed cells the ability to degrade the basement membrane in a manner analogous to   metastasis  . Our functional characterization of the R833C substitution suggests that, like the V804M and S891A mutations, this tyrosine kinase mutation confers a weak activating potential upon RET. This is the first report demonstrating that the introduction of an intracellular cysteine can activate RET. However, this does not occur via dimerization in a manner analogous to the extracellular cysteine mutants. 
 A Korean family of familial medullary thyroid cancer with Cys618Ser RET germline mutation. Familial medullary thyroid carcinoma (FMTC) is caused by autosomal dominant gain-of-function mutations in the RET proto-oncogene. An identifiable RET mutation can be detected in about 85% of FMTC families. The majority of germline mutations in FMTC have been found in exons 10 and 11 of the RET proto-oncogene, specifically within the cysteine codons 609, 611, 618, 620, and 634. We screened members of a large Korean family that had a history of FMTC by genetic analyses, and propose a therapeutic approach for managing the disorder. We report a RET mutation in exon10, codon 618 that causes substitution of a cysteine by a serine in the cysteine-rich domain of the RET receptor in a three-generation FMTC family composed of 30 members with 11 carriers. Nine of the gene carriers were clinically affected. The FMTC with cysteine RET mutations found in the Korean population is consistent with the clinical pattern reported worldwide; to date there have been no ethnic differences identified for FMTC. Our results suggest that this genetic profile might be associated with usually aggressive clinical course with regional lymph node   metastasis   but late onset of MTC. 
 A novel mutation (D631del) of the RET gene was associated with MEN2A in a Chinese pedigree. Germline mutations in the RET proto-oncogene (RET gene) are well documented as the genetic causes of multiple endocrine neoplasia type 2A (MEN2A). We performed genetic analysis by direct RET gene mutation analysis in a Chinese MEN2A family and compared these results with biochemical screening tests and pathological examinations. Twenty-one exons and flanking introns of the RET gene were amplified using polymerase chain reaction (PCR). The PCR products were subjected to sequencing directly, or cloned into pGEM-T plasmids and sequenced. Restriction fragment length polymorphism (RFLP) was employed to confirm the mutation on the RET sequence. A novel heterozygous mutation of a 3-bp (GAC) deletion at codon 631 (D631del) of exon 11, resulting in the deletion of an aspartic acid at the locus, was identified in four MEN2A patients and one phenotypically normal family member. The average clinical onset-age of four MEN2A patients was 33.7 years, no cervical lymph node   metastasis   was found in MEN2A patients with medullary thyroid carcinoma in the family. The study indicated that the novel heterozygous deletion mutation at D631 of RET gene was co-segregated with MEN2A phenotype and promoted the development of MEN2A. This report is the first description of the D631del mutation in the family with MEN2A. 
 Malignant progression from C-cell hyperplasia to medullary thyroid carcinoma in 167 carriers of RET germline mutations. Hereditary medullary thyroid carcinoma (MTC) is the most common and potentially life-shortening phenotypic manifestation of RET (rearranged during transfection) germline mutations. If a distinct time lag between the successive stages of malignant progression were identifiable, the information could be used to individualize prophylactic surgery. The study objective was to investigate the impact of RET genotype on disease progression from C-cell hyperplasia (CCH) to MTC.An institutional series of 167 (67 index, 100 nonindex) consecutive carriers of RET gene point mutations in exons 10, 11, 13, 14, or 16 who underwent total thyroidectomy between November 1994 and November 2002.Regarding codons 618, 620, 634, 768, 790, and 804, patient age at diagnosis differed significantly depending on the type of pathology encountered (CCH, MTC without and with nodal   metastasis  ). The variability in age, which may reflect the number of necessary somatic mutations, explained the pathological strata in 38% (codon 634) to 77% (codon 768) of patients. Conversely, 62% (codon 634) to 23% (codon 768) of variability in age at different pathological strata may have been determined by the RET genotype.The pace of malignant progression of the RET genotype should be taken into account when considering prophylactic thyroidectomy in RET gene carriers. 
  GeneRIFs  
 Study shows that the RET nuclear expression was an independent predictor of renal clear cell carcinoma postoperative   metastasis  . 
 Compared with RAS or PAX8/PPARG-positive TCs, BRAFV600E or RET/PTC-positive Thyroid cancers were more often associated with stage III/IV disease and recurrence. Distant   metastasis   was highest in patients positive for RET/PTC. 
 Authors show that elevated levels of Ret receptor are found in different sub-types of human breast cancers and that high Ret correlates with decreased   metastasis  -free survival. 
 a beta-catenin-RET kinase pathway is a critical contributor to the development and   metastasis   of thyroid carcinoma 
  [Cc]ell [Ii]nvasion  PubMed  
 The G691S RET polymorphism increases glial cell line-derived neurotrophic factor-induced pancreatic cancer   cell invasion   by amplifying mitogen-activated protein kinase signaling. Mutations of the RET proto-oncogene are responsible for several inherited human diseases and may function as genetic modifiers of the disease. However, the role of RET mutations in pancreatic cancer has not been studied. Expression of the glial cell line-derived neurotrophic factor (GDNF) receptors RET and GDNF family receptor alpha1 (GFRalpha1) in human pancreatic cancer cells was determined by Western blot, immunofluorescence, and flow cytometry. The effect of GDNF on cell proliferation and invasion was assessed. Small interfering RNA and antibodies were used to evaluate the involvement of RET. The G691S RET polymorphism was analyzed by sequencing and restriction analysis. The modifying effect of G691S RET on GDNF-induced invasion and mitogen-activated protein kinase (MAPK) signaling was evaluated. Transfection studies with wild-type and mutated RET determined the functional role of the G691S polymorphism. Pancreatic cancer specimens and matched tissues were analyzed for the presence of the G691S RET polymorphism. GDNF receptors were found on all cell lines. GDNF increased pancreatic cancer cell proliferation and invasion, which was mediated by RET. The effect of GDNF was more profound in cells with the G691S RET polymorphism (P < 0.01). G691S RET correlated with an enhanced activation of the downstream extracellular signal-regulated kinase pathway. Overexpression of G691S RET increased pancreatic cancer   cell invasion  . The G691S RET polymorphism was also detected in human pancreatic tumors and represented a somatic mutation in some patients. These findings indicate that the G691S RET single nucleotide polymorphism may directly correlate with the aggressive growth of pancreatic cancers and may function as a genetic modifier or even low-penetrance gene. 
   
  
 PDGFRL 
 
  NCBI Gene  
  GeneRIF  
  Pubmed  
 
 
  Search Term  Rank  Fields  
  ECM  7  PubMed(1)  
  [Ee]xtracellular [Mm]atrix  8  PubMed(1)  
  [Mm]etastasis  12  PubMed(1)  
 
 
 hide/show details for PDGFRL 
  ECM  PubMed  
 PDGF/PDGFR signaling and targeting in cancer growth and progression: Focus on tumor microenvironment and cancer-associated fibroblasts. Traditionally, the studies on cancer growth and progression have been focused on the transformed, malignant cells. However, it is now well recognized that the tumour stroma represents a crucial parameter in tumour development, growth and progression. Indeed, several cancers are characterized by a prominent stromal compartment and it is the interactions between cancer cells, stromal cells and extracellular matrix (  ECM  ) components that control the overall tumour growth. Among stromal cells, fibroblasts represent the most important type. They are responsible for deposition and remodeling of   ECM   components, as well as for the release of cytokines and growth factors, including platelet-derived growth factor (PDGF), acting in a paracrine manner on cancer cells. In this review we elucidate the role of tumor stroma interactions, the roles of PDGF receptor signaling in cancer-associated fibroblasts via alteration of stromal matrix composition and the mitogenic effects of cancer-derived PDGFs. Focus on the targeting of tumor microenvironment at the level of PDGF/PDGF receptor (PDGFR) is also presented as to stimulate further studies for designing and development of novel pharmaceutical agents and combined pharmaceutical interventions. Conclusively, PDGF/PDGFR axis is of paramount importance in the tumour microenvironment context and the inhibition of PDGF receptors' activation represents a major target for future anticancer therapies. 
  [Ee]xtracellular [Mm]atrix  PubMed  
 PDGF/PDGFR signaling and targeting in cancer growth and progression: Focus on tumor microenvironment and cancer-associated fibroblasts. Traditionally, the studies on cancer growth and progression have been focused on the transformed, malignant cells. However, it is now well recognized that the tumour stroma represents a crucial parameter in tumour development, growth and progression. Indeed, several cancers are characterized by a prominent stromal compartment and it is the interactions between cancer cells, stromal cells and   extracellular matrix   (ECM) components that control the overall tumour growth. Among stromal cells, fibroblasts represent the most important type. They are responsible for deposition and remodeling of ECM components, as well as for the release of cytokines and growth factors, including platelet-derived growth factor (PDGF), acting in a paracrine manner on cancer cells. In this review we elucidate the role of tumor stroma interactions, the roles of PDGF receptor signaling in cancer-associated fibroblasts via alteration of stromal matrix composition and the mitogenic effects of cancer-derived PDGFs. Focus on the targeting of tumor microenvironment at the level of PDGF/PDGF receptor (PDGFR) is also presented as to stimulate further studies for designing and development of novel pharmaceutical agents and combined pharmaceutical interventions. Conclusively, PDGF/PDGFR axis is of paramount importance in the tumour microenvironment context and the inhibition of PDGF receptors' activation represents a major target for future anticancer therapies. 
  [Mm]etastasis  PubMed  
 The gene expression profiles of primary and metastatic melanoma yields a transition point of tumor progression and   metastasis  . The process of malignant transformation, progression and   metastasis   of melanoma is poorly understood. Gene expression profiling of human cancer has allowed for a unique insight into the genes that are involved in these processes. Thus, we have attempted to utilize this approach through the analysis of a series of primary, non-metastatic cutaneous tumors and metastatic melanoma samples.We have utilized gene microarray analysis and a variety of molecular techniques to compare 40 metastatic melanoma (MM) samples, composed of 22 bulky, macroscopic (replaced) lymph node metastases, 16 subcutaneous and 2 distant metastases (adrenal and brain), to 42 primary cutaneous cancers, comprised of 16 melanoma, 11 squamous cell, 15 basal cell skin cancers. A Human Genome U133 Plus 2.0 array from Affymetrix, Inc. was utilized for each sample. A variety of statistical software, including the Affymetrix MAS 5.0 analysis software, was utilized to compare primary cancers to metastatic melanomas. Separate analyses were performed to directly compare only primary melanoma to metastatic melanoma samples. The expression levels of putative oncogenes and tumor suppressor genes were analyzed by semi- and real-time quantitative RT-PCR (qPCR) and Western blot analysis was performed on select genes.We find that primary basal cell carcinomas, squamous cell carcinomas and thin melanomas express dramatically higher levels of many genes, including SPRR1A/B, KRT16/17, CD24, LOR, GATA3, MUC15, and TMPRSS4, than metastatic melanoma. In contrast, the metastatic melanomas express higher levels of genes such as MAGE, GPR19, BCL2A1, MMP14, SOX5, BUB1, RGS20, and more. The transition from non-metastatic expression levels to metastatic expression levels occurs as melanoma tumors thicken. We further evaluated primary melanomas of varying Breslow's tumor thickness to determine that the transition in expression occurs at different thicknesses for different genes suggesting that the "transition zone" represents a critical time for the emergence of the metastatic phenotype. Several putative tumor oncogenes (SPP-1, MITF, CITED-1, GDF-15, c-Met, HOX loci) and suppressor genes (PITX-1, CST-6, PDGFRL, DSC-3, POU2F3, CLCA2, ST7L), were identified and validated by quantitative PCR as changing expression during this transition period. These are strong candidates for genes involved in the progression or suppression of the metastatic phenotype.The gene expression profiling of primary, non-metastatic cutaneous tumors and metastatic melanoma has resulted in the identification of several genes that may be centrally involved in the progression and metastatic potential of melanoma. This has very important implications as we continue to develop an improved understanding of the metastatic process, allowing us to identify specific genes for prognostic markers and possibly for targeted therapeutic approaches. 
   
  
 PDGFRA 
 
  NCBI Gene  
  GeneRIF  
  Pubmed  
 
 
  Search Term  Rank  Fields  
  [Ff]ilopodia  1  PubMed(1)  
  [Ii]nvadopodia  3  PubMed(1); GeneRIFs(1)  
  [Mm]igration  6  Process(2); PubMed(22); GeneRIFs(1)  
  ECM  7  PubMed(1)  
  [Ee]xtracellular [Mm]atrix  8  PubMed(4)  
  [Ii]nvasive  10  PubMed(9)  
  [Mm]etastasize  11  PubMed(1)  
  [Mm]etastasis  12  PubMed(30); GeneRIFs(6)  
  [Cc]ell [Ii]nvasion  13  PubMed(2)  
 
 
 hide/show details for PDGFRA 
  [Ff]ilopodia  PubMed  
 Platelet-derived growth factor-mediated signaling through the Shb adaptor protein: effects on cytoskeletal organization. The Src homology (SH) 2 domain adaptor protein Shb has previously been shown to interact with the platelet-derived growth factor (PDGF)-beta receptor. In this study we show an association between Shb and the PDGF-alpha receptor which is mediated by the SH2 domain of Shb and involves tyrosine residue 720 in the kinase insert domain of the receptor. To assess the role of Shb in PDGF-mediated signaling, we have overexpressed wild-type Shb or Shb carrying a mutation (R522K) which renders the SH2 domain inactive, in Patch mouse (PhB) fibroblasts expressing both PDGF receptors (PhB/Ralpha). Overexpression of wild-type Shb, but not the R522K Shb mutant, affected PDGF-mediated reorganization of the cytoskeleton by decreasing membrane ruffle formation and stimulating the generation of   filopodia   relative the parental control cells. In addition, the PDGF-induced receptor-associated phosphatidylinositol 3'-kinase activity and phosphorylation of Akt was similar in both PhB/Ralpha/Shb and PhB/Ralpha/ShbR522K cells compared with the parental control, whereas the activation of Rac in response to PDGF-BB was diminished only in the PhB/Ralpha/Shb cells. We conclude that Shb plays a role in PDGF-dependent regulation of certain cytoskeletal changes by modulating the ability of PDGF to activate Rac. 
  [Ii]nvadopodia  PubMed  
 Twist1-induced   invadopodia   formation promotes tumor metastasis. The Twist1 transcription factor is known to promote tumor metastasis and induce Epithelial-Mesenchymal Transition (EMT). Here, we report that Twist1 is capable of promoting the formation of   invadopodia  , specialized membrane protrusions for extracellular matrix degradation. Twist1 induces PDGFRα expression, which in turn activates Src, to promote   invadopodi  a formation. We show that Twist1 and PDGFRα are central mediators o  f invadopod  ia formation in response to various EMT-inducing signals. Induction of PDGFRα a  nd invadopo  dia is essential for Twist1 to promote tumor metastasis. Consistent with PDGFRα being a direct transcriptional target of Twist1, coexpression of Twist1 and PDGFRα predicts poor survival in breast tumor patients. Theref  ore, invado  podia-mediated matrix degradation is a key function of Twist1 in promoting tumor metastasis. 
  GeneRIFs  
 Data show that induction of PDGFRalpha and   invadopodia   is essential for Twist1 to promote tumor metastasis. 
  [Mm]igration  Process  
 positive regulation of cell   migration   
 positive regulation of cell   migration   
  PubMed  
 Activation of Rac1 by Src-dependent phosphorylation of Dock180(Y1811) mediates PDGFRα-stimulated glioma tumorigenesis in mice and humans. Two hallmarks of glioblastoma multiforme, the most common malignant brain cancer in humans, are aggressive growth and the ability of single glioma cells to disperse throughout the brain. These characteristics render tumors resistant to current therapies and account for the poor prognosis of patients. Although it is known that oncogenic signaling caused by overexpression of genes such as PDGFRA is responsible for robust glioma growth and cell infiltration, the mechanisms underlying glioblastoma malignancy remain largely elusive. Here, we report that PDGFRα signaling in glioblastomas leads to Src-dependent phosphorylation of the guanine nucleotide exchange factor Dock180 at tyrosine 1811 (Dock180(Y1811)) that results in activation of the GTPase Rac1 and subsequent cell growth and invasion. In human glioma cells, knockdown of Dock180 and reversion with an RNAi-resistant Dock180(Y1811F) abrogated, whereas an RNAi-resistant Dock180(WT) rescued, PDGFRα-promoted glioma growth, survival, and invasion. Phosphorylation of Dock180(Y1811) enhanced its association with CrkII and p130(Cas), causing activation of Rac1 and consequent cell motility. Dock180 also associated with PDGFRα to promote c  ell migra  tion. Finally, phosphorylated Dock180(Y1811) was detected in clinical samples of gliomas and various types of human cancers, and coexpression of phosphorylated Dock180(Y1811), phosphorylated Src(Y418), and PDGFRα was predictive of extremely poor prognosis of patients with gliomas. Taken together, our findings provide insight into PDGFRα-stimulated gliomagenesis and suggest that phosphorylated Dock180(Y1811) contributes to activation of Rac1 in human cancers with PDGFRA amplification. 
 Activation of Rac1 by Src-dependent phosphorylation of Dock180(Y1811) mediates PDGFRα-stimulated glioma tumorigenesis in mice and humans. Two hallmarks of glioblastoma multiforme, the most common malignant brain cancer in humans, are aggressive growth and the ability of single glioma cells to disperse throughout the brain. These characteristics render tumors resistant to current therapies and account for the poor prognosis of patients. Although it is known that oncogenic signaling caused by overexpression of genes such as PDGFRA is responsible for robust glioma growth and cell infiltration, the mechanisms underlying glioblastoma malignancy remain largely elusive. Here, we report that PDGFRα signaling in glioblastomas leads to Src-dependent phosphorylation of the guanine nucleotide exchange factor Dock180 at tyrosine 1811 (Dock180(Y1811)) that results in activation of the GTPase Rac1 and subsequent cell growth and invasion. In human glioma cells, knockdown of Dock180 and reversion with an RNAi-resistant Dock180(Y1811F) abrogated, whereas an RNAi-resistant Dock180(WT) rescued, PDGFRα-promoted glioma growth, survival, and invasion. Phosphorylation of Dock180(Y1811) enhanced its association with CrkII and p130(Cas), causing activation of Rac1 and consequent cell motility. Dock180 also associated with PDGFRα to promote c  ell migra  tion. Finally, phosphorylated Dock180(Y1811) was detected in clinical samples of gliomas and various types of human cancers, and coexpression of phosphorylated Dock180(Y1811), phosphorylated Src(Y418), and PDGFRα was predictive of extremely poor prognosis of patients with gliomas. Taken together, our findings provide insight into PDGFRα-stimulated gliomagenesis and suggest that phosphorylated Dock180(Y1811) contributes to activation of Rac1 in human cancers with PDGFRA amplification. 
 Signal transduction via platelet-derived growth factor receptors. Platelet-derived growth factor (PDGF) exerts its stimulatory effects on cell growth and motility by binding to two related protein tyrosine kinase receptors. Ligand binding induces receptor dimerization and autophosphorylation, allowing binding and activation of cytoplasmic SH2-domain containing signal transduction molecules. Thereby, a number of different signaling pathways are initiated leading to cell growth, actin reorganization   migration   and differentiation. Recent observations suggest that extensive cross-talk occurs between different signaling pathways, and that stimulatory signals are modulated by inhibitory signals arising in parallel. 
 Structural determinants in the platelet-derived growth factor alpha-receptor implicated in modulation of chemotaxis. Activation of the platelet-derived growth factor (PDGF) beta-receptor leads to cell growth and chemotaxis. The PDGF alpha-receptor also mediates a mitogenic signal, but fails to induce cell   migration   in certain cell types. To examine this difference in signal transduction, a series of point-mutated PDGF alpha-receptors were analyzed. Porcine aortic endothelial cells expressing mutant PDGF alpha-receptors, in which tyrosine residues 768, 993, or 1018 were changed to phenylalanine residues migrated toward PDGF, whereas wild-type alpha-receptors and mutant alpha-receptors changed at tyrosine residues 720, 944, or 988 failed to migrate. All mutant receptors were mitogenically active and their capacity to activate phosphatidylinositol 3'-kinase and phospholipase C-gamma was not different from that of the wild-type receptor. Tyr-768 was found to be phosphorylated in PDGF-stimulated cells; in the Y768F mutant, there was a considerable increase in phosphorylation of Ser-767. Tyr-993 was not phosphorylated, but mutation of this tyrosine residue to a phenylalanine residue resulted in increased efficiency of phosphorylation on Tyr-988. Tyr-1018 is known to be an autophosphorylation site. Phosphorylated Tyr-768 and Tyr-1018 may bind signal transduction molecules involved in negative modulation of the chemotactic signaling capacity, whereas phosphorylated Tyr-988 may mediate increased chemotaxis. Thus our data indicate that the PDGF alpha-receptor has an intrinsic ability to transduce a chemotactic signal, and that this signal is counteracted by overriding negative signals. 
 Exome sequencing of bilateral testicular germ cell tumors suggests independent development lineages. Intratubular germ cell neoplasia, the precursor of testicular germ cell tumors (TGCTs), is hypothesized to arise during embryogenesis from developmentally arrested primordial germ cells (PGCs) or gonocytes. In early embryonal life, the PGCs migrate from the yolk sac to the dorsal body wall where the cell population separates before colonizing the genital ridges. However, whether the malignant transformation takes place before or after this separation is controversial. We have explored the somatic exome-wide mutational spectra of bilateral TGCT to provide novel insight into the in utero critical time frame of malignant transformation and TGCT pathogenesis. Exome sequencing was performed in five patients with bilateral TGCT (eight tumors), of these three patients in whom both tumors were available (six tumors) and two patients each with only one available tumor (two tumors). Selected loci were explored by Sanger sequencing in 71 patients with bilateral TGCT. From the exome-wide mutational spectra, no identical mutations in any of the three bilateral tumor pairs were identified. Exome sequencing of all eight tumors revealed 87 somatic non-synonymous mutations (median 10 per tumor; range 5-21), some in already known cancer genes such as CIITA, NEB, platelet-derived growth factor receptor α (PDGFRA), and WHSC1. SUPT6H was found recurrently mutated in two tumors. We suggest independent development lineages of bilateral TGCT. Thus, malignant transformation into intratubular germ cell neoplasia is likely to occur after the   migratio  n of PGCs. We reveal possible drivers of TGCT pathogenesis, such as mutated PDGFRA, potentially with therapeutic implications for TGCT patients. 
 STC1 expression by cancer-associated fibroblasts drives metastasis of colorectal cancer. Platelet-derived growth factor (PDGF) receptor signaling is a major functional determinant of cancer-associated fibroblasts (CAF). Elevated expression of PDGF receptors on stromal CAFs is associated with metastasis and poor prognosis, but mechanism(s) that underlie these connections are not understood. Here, we report the identification of the secreted glycoprotein stanniocalcin-1 (STC1) as a mediator of metastasis by PDGF receptor function in the setting of colorectal cancer. PDGF-stimulated fibroblasts increased   migration   and invasion of cocultured colorectal cancer cells in an STC1-dependent manner. Analyses of human colorectal cancers revealed significant associations between stromal PDGF receptor and STC1 expression. In an orthotopic mouse model of colorectal cancer, tumors formed in the presence of STC1-deficient fibroblasts displayed reduced intravasation of tumor cells along with fewer and smaller distant metastases formed. Our results reveal a mechanistic basis for understanding the contribution of PDGF-activated CAFs to cancer metastasis. 
 CD140a identifies a population of highly myelinogenic,   migration  -competent and efficiently engrafting human oligodendrocyte progenitor cells. Experimental animals with myelin disorders can be treated by transplanting oligodendrocyte progenitor cells (OPCs) into the affected brain or spinal cord. OPCs have been isolated by their expression of gangliosides recognized by mAb A2B5, but this marker also identifies lineage-restricted astrocytes and immature neurons. To establish a more efficient means of isolating myelinogenic OPCs, we sorted fetal human forebrain cells for CD140a, an epitope of platelet derived growth factor receptor (PDGFR)α, which is differentially expressed by OPCs. CD140a(+) cells were isolated as mitotic bipotential progenitors that initially expressed neither mature neuronal nor astrocytic phenotypic markers, yet could be instructed to either oligodendrocyte or astrocyte fate in vitro. Transplanted CD140a(+) cells were highly migratory and robustly myelinated the hypomyelinated shiverer mouse brain more rapidly and efficiently than did A2B5(+)cells. Microarray analysis of CD140a(+) cells revealed overexpression of the oligodendroglial marker CD9, suggesting that CD9(+)/CD140a(+) cells may constitute an even more highly enriched population of myelinogenic progenitor cells. 
 Transcriptional activation of the Axl and PDGFR-α by c-Met through a ras- and Src-independent mechanism in human bladder cancer. A cross-talk between different receptor tyrosine kinases (RTKs) plays an important role in the pathogenesis of human cancers.Both NIH-Met5 and T24-Met3 cell lines harboring an inducible human c-Met gene were established. C-Met-related RTKs were screened by RTK microarray analysis. The cross-talk of RTKs was demonstrated by Western blotting and confirmed by small interfering RNA (siRNA) silencing, followed by elucidation of the underlying mechanism. The impact of this cross-talk on biological function was demonstrated by Trans-well   migratio  n assay. Finally, the potential clinical importance was examined in a cohort of 65 cases of locally advanced and metastatic bladder cancer patients.A positive association of Axl or platelet-derived growth factor receptor-alpha (PDGFR-α) with c-Met expression was demonstrated at translational level, and confirmed by specific siRNA knock-down. The transactivation of c-Met on Axl or PDGFR-α in vitro was through a ras- and Src-independent activation of mitogen-activated protein kinase/extracellular signal-regulated kinase (MEK/ERK) pathway. In human bladder cancer, co-expression of these RTKs was associated with poor patient survival (p < 0.05), and overexpression of c-Met/Axl/PDGFR-α or c-Met alone showed the most significant correlation with poor survival (p < 0.01).In addition to c-Met, the cross-talk with Axl and/or PDGFR-α also contributes to the progression of human bladder cancer. Evaluation of Axl and PDGFR-α expression status may identify a subset of c-Met-positive bladder cancer patients who may require co-targeting therapy. 
 MicroRNA-34A inhibits the growth, invasion and metastasis of gastric cancer by targeting PDGFR and MET expression. Within the family of RTKs (receptor tyrosine kinases), PDGFR (platelet-derived growth factor receptor) has been implicated in carcinogenesis and tumour development. miRNAs (microRNAs), which can target the mRNAs (messenger RNAs) of cancer-associated genes, are abnormally expressed in various cancers. In this study, our aim was to identify the miRNAs that target PDGFR-α/β and to study the functions of these miRNAs. miR-34a was predicted to target PDGFR, and luciferase reporter assays showed that miR-34a could directly target PDGFR. Meanwhile, we found that miR-34a was down-regulated in gastric cancer tissues and was associated with metastasis. Our findings showed that miR-34a could inhibit gastric cancer cel  l migrati  on, invasion and proliferation, but these tumourigenic properties were only partially restored when PDGFR-α/β was overexpressed. In subsequent experiments, we found that the overexpression of both PDGFR and MET could completely restore the gastric cancer tumourigenic properties. Moreover, the cancer-associated cell signalling pathway was studied, and we found that miR-34a could inhibit Akt [PKB (protein kinase B)] phosphorylation, which was restored by the overexpression of both PDGFR and MET. In conclusion, miR-34a may act as a potential tumour suppressor in gastric cancer and is associated with the mechanisms of gastric cancer metastasis; miR-34a can inhibit gastric cancer tumourigenesis by targeting PDGFR and MET through the PI3K (phosphoinositide 3-kinase)/Akt pathway. 
 Oncostatin M is a FIP1L1/PDGFRA-dependent mediator of cytokine production in chronic eosinophilic leukemia. Chronic eosinophilic leukemia (CEL) is a myeloproliferative neoplasm characterized by expansion of neoplastic eosinophils, tissue infiltration, and organ damage. In a subset of these patients, the FIP1L1/PDGFRA (F/P) oncoprotein is detectable. F/P exhibits constitutive tyrosine kinase activity and activates a number of signaling pathways. So far, however, little is known about the role of F/P-dependent proteins in the pathogenesis of CEL.A screen for F/P-dependent cytokines was performed in growth factor-dependent human cell lines lentivirally transduced with F/P. Signal transduction pathways were characterized in Ba/F3 cells with doxycycline-inducible expression of F/P and in EOL-1 cells. Cytokine expression was confirmed in patients' material by immunohistochemistry, immunofluorescence, and confocal microscopy. Gene expression analysis, proliferation assays, and chemotaxis assays were used to elucidate paracrine interactions between neoplastic eosinophils and stromal cells.We show that F/P upregulates expression of oncostatin M (OSM) in various cell line models in a STAT5-dependent manner. Correspondingly, neoplastic eosinophils in the bone marrow were found to overexpress OSM. OSM derived from F/P + cells stimulated proliferation of stromal cells. Moreover, OSM-containing supernatants from F/P + cells were found to upregulate production of stromal cell-derived factor-1 (SDF-1)/CXCL12 in human fibroblasts. SDF-1, in turn, induced   migration   of EOL-1 cells in a dose-dependent manner.We have identified a F/P-driven paracrine interaction between neoplastic eosinophils and stromal cells that may contribute to tissue fibrosis and accumulation of neoplastic eosinophils in CEL. 
 IFNgamma regulates PDGF-receptor alpha expression in macrophages, THP-1 cells, and arterial smooth muscle cells. The recruitment of monocyte-derived macrophages (MDMs) and arterial smooth muscle cells (ASMCs) contributes to inflammation and development of intimal hyperplasia during atherosclerosis. Platelet-derived growth factor (PDGF) is a potent mitogen for SMC, signalling through PDGF-receptor subunits alpha (Ralpha) and beta (Rbeta). We have previously found that interferon gamma (IFNgamma) upregulates PDGF-Ralpha mRNA expression in human MDM (hMDM) which causes an increased   migration   towards PDGF. In the present study, we found that IFNgamma mediated an upregulation of PDGF-Ralpha mRNA also in THP-1 cells. The induction of PDGF-Ralpha in both hMDM and THP-1 cells was caused by STAT1 binding to the PDGF-Ralpha promoter. In human ASMCs, IFNgamma again stimulated a transient STAT1-binding to the PDGF-Ralpha promoter. However, this was not followed by an upregulation of PDGF-Ralpha mRNA. IFNgamma-stimulation resulted in augmented expression of PDGF-Ralpha protein in differentiated hMDM. Early hMDM only expressed an immature and not fully glycosylated form of the PDGF-Ralpha protein. In contrast, THP-1 cells did not synthesize PDGF-Ralpha protein, implying further posttranscriptional inhibition. Our results contribute to a better understanding of the complex regulation of PDGF-Ralpha expression and how proinflammatory factors may contribute to PDGF-related hyperplasia in vascular diseases. 
 A new look at platelet-derived growth factor in renal disease. The PDGF system, comprising four isoforms (PDGF-A, -B, -C, and -D) and two receptor chains (PDGFR-alpha and -beta), plays important roles in wound healing, atherosclerosis, fibrosis, and malignancy. Components of the system are expressed constitutively or inducibly in most renal cells. They regulate a multitude of pathophysiologic events, ranging from cell proliferation and   migration   to extracellular matrix accumulation, production of pro- and anti-inflammatory mediators, tissue permeability, and regulation of hemodynamics. Genetic deletion of PDGF-B or PDGFR-beta results in an absent glomerular mesangium, whereas PDGF-C and PDGFR-alpha contribute to the formation of the renal cortical interstitium. Almost all experimental and human renal diseases are characterized by altered expression of components of the PDGF system. Infusion or systemic overexpression of PDGF-B or -D induces prominent mesangioproliferative changes and renal fibrosis. Intervention studies identified PDGF-C as a mediator of renal interstitial fibrosis and PDGF-B and -D as key factors involved in mesangioproliferative disease and renal interstitial fibrosis. These data establish PDGF as one of the best characterized growth factors in renal disease and the most potent stimulus of mesangial cell proliferation currently identified. Accordingly, targeted intervention against the various PDGF isoforms offers a promising novel therapeutic approach to renal disease. 
 Overexpression of platelet-derived growth factor receptor alpha promotes tumor progression and indicates poor prognosis in hepatocellular carcinoma. Dysregulation of platelet-derived growth factor receptor alpha (PDGFRα) has been documented in various cancers. However, its role in hepatocellular carcinoma (HCC) remains unknown. We and others have examined that upregulation of PDGFRα might be involved in hepatocarcinogenesis. Here, we report that PDGFRα plays a critical role in HCC progression and prognosis. The expression of PDGFRα was markedly higher in human HCC compared to adjacent liver tissues. Although PDGFRA mRNA was decreased in HCC, PDGF-A mRNA was dramatically increased in HCC. Overexpression of PDGFRα was strongly correlated with microvessel density (MVD) of HCC (p<0.05), as well as macroscopic vascular invasion of the tumors (p<0.05). HCC patients with high PDGFRα expression displayed a shorter overall survival and a higher recurrence rate than those with low PDGFRα expression (p<0.05, respectively). Additionally, stable overexpression of PDGFRα in hepatoma cells promoted cell prolife  ration, m  igration, invasion and epithelial-mesenchymal transition in vitro. Similarly, an in vivo assay showed that PDGFRα overexpression in hepatoma cells exhibited remarkably tumorigenic potential in tumor size and weight in vivo, which displayed markedly elevated MVD than controls. Thus, our study provided the evidence that PDGFRα may serve as a candidate prognostic marker and a novel therapeutic target for HCC. 
 Peroxiredoxin I, platelet-derived growth factor A, and platelet-derived growth factor receptor alpha are overexpressed in carcinoma ex pleomorphic adenoma: association with malignant transformation. Carcinoma ex pleomorphic adenoma is a rare salivary gland malignancy. It constitutes an important model for the study of carcinogenesis, as it can display the tumor in different stages of progression, from benign pleomorphic adenoma to frankly invasive carcinoma. Growth signaling pathways undergo continuous activation in human tumors, commonly as a consequence of the overexpression of ligands and receptors such as platelet-derived growth factor and platelet-derived growth factor receptor. Hydrogen peroxide is produced after platelet-derived growth factor receptor activation, and it is essential for the sequential phosphorylation cascade that drives cell proliferation and   migration  . By their ability to degrade hydrogen peroxide, peroxiredoxins are involved in growth factor signaling regulation and in the oxidative stress response. To verify the potential association of peroxiredoxin I, platelet-derived growth factor-A, and platelet-derived growth factor receptor-alpha with carcinoma ex pleomorphic adenoma progression, we investigated the expression of these molecules in carcinoma ex pleomorphic adenoma showing different degrees of invasion. The peroxiredoxin I, platelet-derived growth factor-A, and platelet-derived growth factor receptor-alpha proteins were present in remnant pleomorphic adenoma to only a small extent, but, collectively, they were highly expressed as soon as the malignant phenotype was achieved and remained at elevated concentrations during progression to the advanced stages of carcinoma ex pleomorphic adenoma. In addition, their locations overlapped significantly, strengthening their connection to this growth-signaling pathway. Our results indicate that carcinoma ex pleomorphic adenoma cells acquire at least 2 significant advantages relative to their normal counterparts: resistance to oxidative stress-induced apoptosis, conferred by high peroxiredoxin I concentrations, and sustained growth, reflecting platelet-derived growth factor-A and platelet-derived growth factor receptor-alpha overexpression. 
 Human cytomegalovirus downregulates expression of receptors for platelet-derived growth factor by smooth muscle cells. Infection by human cytomegalovirus (HCMV) is associated with the development of vascular diseases and may cause severe brain damage in infected fetuses. Platelet-derived growth factor receptors alpha and beta (PDGFR-alpha and -beta) control important cellular processes associated with atherosclerosis and fetal development. In the present investigation, our goal was to determine whether infection by HCMV can influence the expression of PDGFR-alpha and -beta in human smooth muscle cells (SMCs). In connection with HCMV infection in vitro the levels of PDGFR-alpha and -beta at the cell surface and in the total cellular protein of SMCs were reduced in parallel with decreases in the levels of the corresponding mRNAs. These effects were dependent on immediate-early (IE) or early (E) HCMV gene products, since inhibition of late genes did not prevent HCMV from affecting the expression of PDGFR-alpha and -beta. The downregulation of PDGFR caused by HCMV was dose dependent. Furthermore, confocal microscopy revealed that the localization of PDGFR-beta was altered in HCMV-infected cells, in which this protein colocalized with proteins associated with endosomes (Rab4 and -5) and lysosomes (Lamp1 and -2), indicating entrance into pathways for protein degradation. Altogether these observations indicate that an IE and/or E HCMV protein(s) downregulates the expression of PDGFR-alpha and -beta in SMCs. This phenomenon may disrupt cellular processes of importance in connection with cellular differentiation,   migration  , and/or proliferation. These observations may explain why congenital infection with HCMV can cause fetal brain damage. 
 Combined anti-PDGFRalpha and PDGFRbeta targeting in non-small cell lung cancer. Activation of the platelet-derived growth factor (PDGF)-receptors is critically involved into various stromal cell functions including recruitment of stromal cells and vascular endothelial growth factor (VEGF) induction in tumor and perivascular cells. To evaluate the effects of combined PDGFRalpha and -beta inhibition in a non-small cell lung cancer model, we stably transfected A549 lung cancer cells with the PDGF-A mutant PDGF-0. PDGF-0 has been generated by substituting amino acids in the binding region of PDGF-A with the corresponding VEGF-A region, leading to a decreased receptor-binding affinity and activation. Compared with control vector transfected cells, transfection with PDGF-0 had no impact on monolayer growth and apoptosis in vitro, but significantly impaired the number of colony formation in soft agar. After subcutaneous injections, all mice developed tumors within 5 days. While control vector transfected A549 cells were characterized by constant tumor growth, PDGF-0 transfected A549 revealed a reduced tumor mass (p < 0.001) with no further growth beyond 14 days (2 months observation time) and complete regressions in 7 of 13 cases. Immunohistochemical analyses revealed that PDGF-0 transfected tumors demonstrated decreased recruitment of periendothelial cells, while the tumor invasion zone was similar to control vector transfectants. Similarly, conditioned medium from PDGF-0 transfected cells induced significantly less   migration   of smooth muscle cells and fibroblasts in vitro. Interestingly, in PDGF-0 transfectants, neither total vessel count nor VEGF expression were significantly altered. These studies demonstrate that combined inhibition of PDGFRalpha and -beta results in markedly decreased tumor growth in vivo because of impaired recruitment of periendothelial cells. 
 Platelet-derived growth factor receptor signaling is not involved in osteogenic differentiation of human mesenchymal stem cells. Platelet-derived growth factor (PDGF) receptor signaling plays an important role in the regulation of proliferation and   migration   of skeletal cells such as osteoblasts or mesenchymal stem cells (MSCs). However, involvement of these receptors in the process of osteoblastic differentiation of MSCs is still a matter of debate. The aim of our study was to examine the role of PDGF receptor signaling in osteogenic differentiation of human MSCs. For this purpose, we performed PDGF receptor stimulation as well as inhibition experiments. Inhibition experiments were carried out with Tyrphostin AG1296, a potent and specific inhibitor of PDGF receptor activity. As expected, Tyrphostin AG1296 treatment caused a concentration-dependent decrease in fetal calf serum and PDGF-BB-induced proliferation of MSCs and effectively inhibited PDGF-BB-induced phosphorylation of extracellular-regulated kinase 1/2. However, PDGF receptor inhibition had no significant effect on osteoblastic differentiation of MSCs, as evaluated histochemically by von Kossa, Alizarin-Red, and osteocalcin stainings. Moreover, mineralized matrix production, as assayed by quantitative Ca(2+)-measurements, was also not modulated by Tyrphostin AG1296 treatment. These results were noticeable irrespective of whether MSCs were grown under nonosteogenic or osteogenic differentiation conditions. Similarly, PDGF-BB treatment of MSCs in receptor stimulation experiments also failed to modulate mineralization. However, expression of alkaline phosphatase was suppressed by Tyrphostin AG1296 treatment at later stages of osteogenesis but not in the early stages, as assessed by enzyme activity and mRNA expression assays. Expression of other osteogenic marker genes such as osteocalcin, runt-related transcription factor 2, osteopontin, collagen type I, and bone sialoprotein was almost unaffected in our perturbation studies. From these experiments, we conclude that PDGF receptor signaling sustains proliferation without affecting osteogenic differentiation of MSCs. 
 Vascular endothelial growth factor can signal through platelet-derived growth factor receptors. Vascular endothelial growth factor (VEGF-A) is a crucial stimulator of vascular cell   migration   and proliferation. Using bone marrow-derived human adult mesenchymal stem cells (MSCs) that did not express VEGF receptors, we provide evidence that VEGF-A can stimulate platelet-derived growth factor receptors (PDGFRs), thereby regulating MSC   migration   and proliferation. VEGF-A binds to both PDGFRalpha and PDGFRbeta and induces tyrosine phosphorylation that, when inhibited, results in attenuation of VEGF-A-induced MSC   migration   and proliferation. This mechanism was also shown to mediate human dermal fibroblast (HDF)   migration  . VEGF-A/PDGFR signaling has the potential to regulate vascular cell recruitment and proliferation during tissue regeneration and disease. 
 Identification of JAK2 as a mediator of FIP1L1-PDGFRA-induced eosinophil growth and function in CEL. The Fip1-like1 (FIP1L1)-platelet-derived growth factor receptor alpha fusion gene (F/P) arising in the pluripotent hematopoietic stem cell (HSC),causes 14% to 60% of patients with hypereosinophilia syndrome (HES). These patients, classified as having F/P (+) chronic eosinophilic leukemia (CEL), present with clonal eosinophilia and display a more aggressive disease phenotype than patients with F/P (-) HES patients. The mechanisms underlying predominant eosinophil lineage targeting and the cytotoxicity of eosinophils in this leukemia remain unclear. Given that the Janus tyrosine kinase (JAK)/signal transducers and activators of transcription (Stat) signaling pathway is key to cytokine receptor-mediated eosinophil development and activated Stat3 and Stat5 regulate the expression of genes involved in F/P malignant transformation, we investigated whether and how JAK proteins were involved in the pathogenesis of F/P-induced CEL. F/P activation of JAK2, Stat3 and Stat5, were confirmed in all the 11 F/P (+) CEL patients examined. In vitro inhibition of JAK2 in EOL-1, primary F/P(+) CEL cells (PC) and T674I F/P Imatinib resistant cells(IR) by either JAK2-specific short interfering RNA (siRNA) or the tryphostin derivative AG490(AG490), significantly reduced cellular proliferation and induced cellular apoptosis. The F/P can enhance the IL-5-induced JAK2 activation, and further results indicated that JAK2 inhibition blocked IL-5-induced cellular   migration   and activation of the EOL-1 and PC cells in vitro. F/P-stimulation of the JAK2 suppressed cells led to a significantly reduction in Stat3 activation, but relatively normal induction of Stat5 activation. Interestingly, JAK2 inhibition also reduced PI3K, Akt and NF-κB activity in a dose-dependent manner, and suppressed expression levels of c-Myc and Survivin. These results strongly suggest that JAK2 is activated by F/P and is required for F/P stimulation of cellular proliferation and infiltration, possibly through induction of c-Myc and Survivin expression via activation of multiple signaling pathways, including NF-κB, Stat3, and PI3K/Akt. 
 PDGFs and their receptors. The platelet-derived growth factor (PDGF)/PDGFR receptor (PDGFR) family is essential for a vast array of physiological processes such as   migration   and proliferation of percityes that contribute to the formation and proper function of blood vessels. While ligand-dependent de-repression of the PDGFR's kinase activity is the major mode by which the PDGFR is activated, there are additional mechanisms to activate PDGFRs. Deregulated PDGFR activity contributes to various pathological conditions, and hence the PDGF/PDGFR family members are viable therapeutic targets. An increased appreciation of which PDGFR contributes to pathology, biomarkers that indicate the amplitude and mode of activation, and receptor-specific antagonists are necessary for the development of next-generation therapies that target the PDGF/PDGFR family. 
 Chromosomal location, exon structure, and vascular expression patterns of the human PDGFC and PDGFD genes. Platelet-derived growth factor (PDGF), which is a major mitogen for vascular smooth muscle cells and has been implicated in the pathogenesis of arteriosclerosis, is composed of dimers of PDGF-A and PDGF-B polypeptide chains, encoded by different genes. Here, we have analyzed the chromosomal localization, structure, and expression of 2 newly identified human genes of the PDGF family, called PDGFC and PDGFD.We used fluorescence in situ hybridization to locate PDGFC and PDGFD in chromosomes 4q32 and 11q22.3 to 23.2, respectively. Exon structures of PDGFC and PDGFD were determined by sequencing from genomic DNA clones. The coding region of PDGFC consists of 6 and PDGFD of 7 exons, of which the last 2 encode the C-terminal PDGF cystine knot growth factor homology domain. An N-terminal CUB domain is encoded by exons 2 and 3 of both genes, and a region of proteolytic cleavage involved in releasing and activating the growth factor domain is located in exon 4 in PDGFC and exon 5 in PDGFD. PDGF-C was expressed predominantly in smooth muscle cells and PDGF-D in fibroblastic adventitial cells, and both genes were active in cultured endothelial cells and in a variety of tumor cell lines. Both PDGF-C and PDGF-D also stimulated human coronary artery smooth muscle cells.PDGFC and PDGFD have similar genomic structures, which resemble those of the PDGFA and PDGFB genes. Their expression in the arterial wall and cultured vascular cells suggests that they can transduce proliferation/  migration   signals to pericytes and smooth muscle cells. 
 PDGF receptor-α promotes TGF-β signaling in hepatic stellate cells via transcriptional and posttranscriptional regulation of TGF-β receptors. Platelet-derived growth factor (PDGF) and transforming growth factor-β (TGF-β) signaling are required for hepatic stellate cell (HSC) activation under pathological conditions such as liver metastatic tumor growth. These two signaling pathways are functionally divergent; PDGF signaling promotes proliferation   and migr  ation of HSCs, and TGF-β induces transdifferentiation of quiescent HSCs into myofibroblasts. Although PDGF signaling is implicated in TGF-β-mediated epithelial mesenchymal transition of tumor cells, the role of PDGF receptors in TGF-β activation of HSCs has not been investigated. Here we report that PDGF receptor-α (PDGFR-α) is required for TGF-β signaling of cultured human HSCs although HSCs express both PDGF-α and -β receptors. PDGFR-α knockdown inhibits TGF-β-induced phosphorylation and nuclear accumulation of SMAD2 with no influence on AKT or ERK phosphorylation associated with noncanonical TGF-β signaling. PDGFR-α knockdown suppresses TGF-β receptor I (TβRI) but increases TβRII gene transcription. At the protein level, PDGFR-α is recruited to TβRI/TβRII complexes by TGF-β stimulation. PDGFR-α knockdown blocks TGF-β-mediated internalization of TβRII and induces accumulation of TβRII at the plasma membrane, thereby inhibiting TGF-β phosphorylation of SMAD2. Functionally, knockdown of PDGFR-α reduces paracrine effects of HSCs on colorectal   cancer ce  ll proliferation and migration in vitro. In mice and patients, colorectal cancer cell invasion of the liver induces upregulation of PDGFR-α of HSCs. In summary, our finding that PDGFR-α knockdown inhibits SMAD-dependent TGF-β signaling by repressing TβRI transcriptionally and blocking endocytosis of TGF-β receptors highlights a convergence of PDGF and TGF-β signaling for HSC activation and PDGFR-α as a therapeutic target for liver metastasis and other settings of HSC activation. 
  GeneRIFs  
 Thus, malignant transformation into intratubular germ cell neoplasia is likely to occur after the   migration   of PGCs. We reveal possible drivers of TGCT pathogenesis, such as mutated PDGFRA, potentially with therapeutic implications for TGCT patients. 
  ECM  PubMed  
 The oligodendrocyte precursor mitogen PDGF stimulates proliferation by activation of alpha(v)beta3 integrins. Central nervous system development requires precise and localized regulation of neural precursor behaviour. Here we show how the interaction between growth factor and integrin signalling pathways provides a mechanism for such precision in oligodendrocyte progenitor (OP) proliferation. While physiological concentrations of platelet-derived growth factor (PDGF) were not in themselves sufficient to promote OP proliferation, they did so on extracellular matrix (  ECM  ) substrates that bind alpha(v)beta3 integrin. Upon PDGF-AA exposure and alpha(v)beta3 engagement, a physical co-association between both receptors was demonstrated, confirming the interaction between these signalling pathways. Furthermore, we found that PDGFalphaR stimulated a protein kinase C-dependent activation of integrin alpha(v)beta3, which in turn induced OP proliferation via a phosphatidylinositol 3-kinase-dependent signalling pathway. These studies establish a mechanism by which OP proliferation is dependent on the availability of both an   ECM   ligand and a mitogenic growth factor. Growth factor- mediated integrin activation is the critical integrative step in proliferation signalling, and ensures that the response of neural precursor cells to long-range cues can be regulated by their cellular neighbours, allowing precise control of cell behaviour during development. 
  [Ee]xtracellular [Mm]atrix  PubMed  
 Twist1-induced invadopodia formation promotes tumor metastasis. The Twist1 transcription factor is known to promote tumor metastasis and induce Epithelial-Mesenchymal Transition (EMT). Here, we report that Twist1 is capable of promoting the formation of invadopodia, specialized membrane protrusions for   extracellular matrix   degradation. Twist1 induces PDGFRα expression, which in turn activates Src, to promote invadopodia formation. We show that Twist1 and PDGFRα are central mediators of invadopodia formation in response to various EMT-inducing signals. Induction of PDGFRα and invadopodia is essential for Twist1 to promote tumor metastasis. Consistent with PDGFRα being a direct transcriptional target of Twist1, coexpression of Twist1 and PDGFRα predicts poor survival in breast tumor patients. Therefore, invadopodia-mediated matrix degradation is a key function of Twist1 in promoting tumor metastasis. 
 A new look at platelet-derived growth factor in renal disease. The PDGF system, comprising four isoforms (PDGF-A, -B, -C, and -D) and two receptor chains (PDGFR-alpha and -beta), plays important roles in wound healing, atherosclerosis, fibrosis, and malignancy. Components of the system are expressed constitutively or inducibly in most renal cells. They regulate a multitude of pathophysiologic events, ranging from cell proliferation and migration to   extracellular matrix   accumulation, production of pro- and anti-inflammatory mediators, tissue permeability, and regulation of hemodynamics. Genetic deletion of PDGF-B or PDGFR-beta results in an absent glomerular mesangium, whereas PDGF-C and PDGFR-alpha contribute to the formation of the renal cortical interstitium. Almost all experimental and human renal diseases are characterized by altered expression of components of the PDGF system. Infusion or systemic overexpression of PDGF-B or -D induces prominent mesangioproliferative changes and renal fibrosis. Intervention studies identified PDGF-C as a mediator of renal interstitial fibrosis and PDGF-B and -D as key factors involved in mesangioproliferative disease and renal interstitial fibrosis. These data establish PDGF as one of the best characterized growth factors in renal disease and the most potent stimulus of mesangial cell proliferation currently identified. Accordingly, targeted intervention against the various PDGF isoforms offers a promising novel therapeutic approach to renal disease. 
 The oligodendrocyte precursor mitogen PDGF stimulates proliferation by activation of alpha(v)beta3 integrins. Central nervous system development requires precise and localized regulation of neural precursor behaviour. Here we show how the interaction between growth factor and integrin signalling pathways provides a mechanism for such precision in oligodendrocyte progenitor (OP) proliferation. While physiological concentrations of platelet-derived growth factor (PDGF) were not in themselves sufficient to promote OP proliferation, they did so on   extracellular matrix   (ECM) substrates that bind alpha(v)beta3 integrin. Upon PDGF-AA exposure and alpha(v)beta3 engagement, a physical co-association between both receptors was demonstrated, confirming the interaction between these signalling pathways. Furthermore, we found that PDGFalphaR stimulated a protein kinase C-dependent activation of integrin alpha(v)beta3, which in turn induced OP proliferation via a phosphatidylinositol 3-kinase-dependent signalling pathway. These studies establish a mechanism by which OP proliferation is dependent on the availability of both an ECM ligand and a mitogenic growth factor. Growth factor- mediated integrin activation is the critical integrative step in proliferation signalling, and ensures that the response of neural precursor cells to long-range cues can be regulated by their cellular neighbours, allowing precise control of cell behaviour during development. 
 Platelet derived growth factor-evoked Ca2+ wave and matrix gene expression through phospholipase C in human pulmonary fibroblast. The primary role of fibroblasts is production and degradation of   extracellular matrix  , and thus it helps in the structural framework of tissues. The close relation between fibroblast malfunction and many diseases such as chronic obstructive pulmonary disease, asthma, and fibrosis is widely accepted. Fibroblasts are known to respond to different growth factors and cytokines including platelet-derived growth factors (PDGF). However, the intracellular signaling mechanisms are not entirely clear. In addition to complex phosphorylation-driven signaling pathways, PDGF is also known to work through Ca(2+) signaling. We hypothesize that in human pulmonary fibroblasts, Ca(2+) waves play an important role in PDGF-mediated changes. To test this hypothesis, we treated human pulmonary fibroblasts, obtained from the lungs of ten donors, with PDGF acutely or overnight plus/minus a variety of blockers under various conditions. Ca(2+) waves were monitored by confocal [Ca(2+)]i fluorimetry, while gene expression of   extracellular matrix   genes was assessed via RT-PCR method. We found that both acute and overnight PDGF treatment evoked Ca(2+) waves. Removal of external Ca(2+) or depletion of internal Ca(2+) store using Cyclopiazonic acid (CPA) completely occluded PDGF-evoked Ca(2+) waves. Ryanodine, which blocks ryanodine receptor channels, had no effect on PDGF-evoked Ca(2+) wave, whereas the phospholipase C inhibitor U73122 and Xestospongin C, a potent IP3 receptor blocker, reduced the rapid PDGF-response to a relatively slowly-developing rise in [Ca(2+)]i. We also found that PDGF dramatically increased the expression of fibronectin1 and collagen A1 genes, which was reversed by the use of CPA or U73122. Our study indicates that, in human pulmonary fibroblasts, PDGF acts through IP3-induced Ca(2+)-release to trigger Ca(2+) waves, which in turn modulate gene expression of several matrix proteins. 
  [Ii]nvasive  PubMed  
 Platelet-derived growth factor receptor-α promotes lymphatic metastases in papillary thyroid cancer. Lymph node metastases are common in papillary thyroid cancer (PTC) and can be resistant to surgical extirpation or radioiodine ablation. We examined the role of platelet-derived growth factor receptor (PDGFR) in mediating lymph node metastases in PTC. Clinical specimens of PTC (n = 137) were surveyed in a tissue array and by western blots to examine the relationship between expression of the α and β subunits of PDGFR and lymph node metastases. PDGFR-α was found at high levels in primary tumours with known lymphatic metastases but not in those tumours lacking nodal involvement (p < 0.0001). However, PDGFR-β expression was not linked to metastatic disease (p = 0.78) as it was found in virtually all PTC specimens. A matching analysis in fresh PTC specimens (n = 13) confirmed that PDGFR-α expression was strongly linked to metastatic spread (p = 0.0047). PDGFR-α and -β were not found in normal thyroid tissue (p < 0.0001). PTC cell lines selectively expressing PDGFR-α or -β were ass  essed fo  r invasive potential and activation of downstream signal transduction pathways. PTC cell lines expressing PDGFR-α responded to PDGF-BB stimulation with   increas  ed invasive potential and this process can be blocked by the tyrosine kinase receptor inhibitor sunitinib (p < 0.009). Cell lines with only PDGFR-β, or no PDGFR, did not show significant   changes   in invasive potential. Activation of PDGFR-α led to downstream up-regulation of both the MAPK/ERK and PI3K/Akt pathways and disruption of either pathway is sufficient to block PDGFR-mediated   increase  s in invasive potential. Thus, PDGFR-α is associated with lymph node metastases in papillary thyroid carcinoma and PDGFR-α prom  otes inc  reased invasive potential in PTC cell lines. PDGFR-α is a strong candidate for a diagnostic biomarker to identify patients at risk of nodal metastases. Our results also strengthen the rationale for selection of tyrosine kinase receptor inhibitors that target PDGFR in the treatment of progressive, metastatic PTC. 
 Overexpression of platelet-derived growth factor receptor alpha in breast cancer is associated with tumour progression. Receptor tyrosine kinases have been extensively studied owing to their frequently abnormal activation in the development and progression of human cancers. Platelet-derived growth factor receptors (PDGFRs) are receptors with intrinsic tyrosine kinase activity that regulate several functions in normal cells and are widely expressed in a variety of malignancies. After the demonstration that gastrointestinal stromal tumours without c-Kit mutations harbour PDGFR-alpha-activating mutations and that PDGFR-alpha is also a therapeutic target for imatinib mesylate, the interest for this receptor has increased considerably. Because breast cancer is one of the most frequent neoplasias in women worldwide, and only one study has reported PDGFR-alpha expression in breast carcinomas, the aim of this work was to investigate the potential significance of PDGFR-alpha expression in   invasive   mammary carcinomas.We used immunohistochemistry to detect PDGFR-alpha overexpression on a series of 181 formalin-fixed paraffin-embedded   invasive   ductal breast carcinomas and in two breast cancer cell lines: MCF-7 and HS578T. We associated its expression with known prognostic factors and we also performed polymerase chain reaction-single-stranded conformational polymorphism and direct sequencing to screen for PDGFR-alpha mutations.PDGFR-alpha expression was observed in 39.2% of the breast carcinomas and showed an association with lymph node metastasis (P = 0.0079), HER-2 expression (P = 0.0265) and Bcl2 expression (P = 0.0121). A correlation was also found with the expression of platelet-derived growth factor A (PDGF-A; P = 0.0194). The two cell lines tested did not express PDGFR-alpha. Screening for mutations revealed alterations in the PDGFR-alpha gene at the following locations: 2500A-->G, 2529T-->A and 2472C-->T in exon 18 and 1701G-->A in exon 12. We also found an intronic insertion IVS17-50insA at exon 18 in all sequenced cases. None of these genetic alterations was correlated with PDGFR-alpha expression. The cell lines did not reveal any alterations in the PDGFR-alpha gene sequence.PDGFR-alpha is expressed in   invasive   breast carcinomas and is associated with biological aggressiveness. The genetic alterations described were not correlated with protein expression, but other mechanisms such as gene amplification or constitutive activation of a signalling pathway inducing this receptor could still sustain PDGFR-alpha as a potential therapeutic target. 
 Expression and mutational status of PDGFR in thymic tumours. There is an ongoing search for new therapeutic targets in   invasive   non-resectable thymic tumours because of the low response rates in current chemotherapeutic treatment modalities. In this study, the possibility that platelet-derived growth factor receptor A (PDGFRA) and/ or PDGFRB may represent potential therapeutic targets in epithelial tumours of the thymus was investigated.Tissue samples were obtained by thymectomy from 36 different patients with epithelial tumours of the thymus (26 thymomas types A, AB, B1-3 and 10 thymic carcinomas). Normal thymi from three young children were used as controls. The PDGFRA and PDGFRB protein expressions as well as the mutational statuses of exons 12, 14 and 18 of the PDGFRA gene were analyzed.All the subtypes of thymomas and the thymic carcinomas showed staining for PDGFRA, but no mutations in the known mutational hotspots were identified. Only about one third of the tumours stained for PDGFRB. PDGFRA and PDGFRB protein staining were slightly positively correlated.PDGFRA may represent a potential therapeutic target in thymic tumours. 
 Urinary bladder urothelial carcinoma with expression of KIT and PDGFRA and showing diverse differentiations into plasmacytoid, clear cell, acantholytic, nested, and spindle variants, and into adenocarcinoma, signet-ring cell carcinoma, small cell carcinoma, large cell carcinoma, and pleomorphic carcinoma. Various tumors can arise in the urinary bladder (UB); most common is urothelial carcinoma (UC). UC of the UB have many variants. Other types of carcinomas such as adenocarcinoma (AC) and small cell carcinoma (SmCC) can occur in UB carcinomas. Expression of KIT and PDGFRA has not been reported. A 66-year-old man admitted to our hospital because of hematuria. Cystoscopy revealed papillary   invasive   tumor and a transurethral bladder tumorectomy (TUR-BT) was performed. The TUR-BT showed UC, AC, SmCC, large cell carcinoma (LCC), and pleomorphic carcinoma (PC). The UC component showed plasmacytoid, spindle, nested, clear cell, acantholytic variants. The AC element showed tubular adenocarcinoma and signet-ring cell carcinoma (Sig). Immunohistochemically, all of these subtypes were positive for cytokeratin (CK) AE1/3, CK CAM5.2, CK34BE12, CK5, CK6, CK7, CK8, CK18, CK19, CK20, EMA, CEA, p63, CA19-9, p53 (positive 45%), MUC1, NSE, NCAM, KIT, PDGFRA, and Ki-67 (87%). They were negative for vimentin, chromogranin, synaptophysin, S100 protein, CD34, CD14, α-smooth muscle actin, CD31, caldesmon, CD138, CD45, κ-chain, λ-chain, MUC2, MUC5AC and MUC6. Mucin histochemistry revealed mucins in AC element including Sig. A molecular genetic analysis using PCR-direct sequencing method identified no mutations of KIT (exons 9, 11, 13, and 17) and PDGFRA (exons 12 and 18) genes. The carcinoma was highly aggressive and invaded into muscular layer. The nuclear grade was very high, and there were numerous lymphovascular permeations were seen. The surface showed carcinoma in situ involving von-Brunn's nests. This case shows that carcinoma of UB can show diverse differentiations into numerous histological types and variants, and can express KIT and PDGFRA. The both genes showed no mutations in the present case. 
 PDGF receptor alpha inhibition induces apoptosis in glioblastoma cancer stem cells refractory to anti-Notch and anti-EGFR treatment. Cancer stem cells (CSC) represent a rare fraction of cancer cells characterized by resistance to chemotherapy and radiation, therefore nowadays there is great need to develop new targeted therapies for brain tumors and our study aim to target pivotal transmembrane receptors such as Notch, EGFR and PDGFR, which are already under investigation in clinical trials setting for the treatment of Glioblastoma Multiforme (GBM).MTS assay was performed to evaluate cells response to pharmacological treatments. Quantitative RT-PCR and Western blots were performed to state the expression of Notch1, EGFR and PDGFRα/β and the biological effects exerted by either single or combined targeted therapy in GBM CSC. GBM CS  C invasi  ve ability was tested in vitro in absence or presence of Notch and/or EGFR signaling inhibitors.In this study, we investigated gene expression and function of Notch1, EGFR and PDGFR to determine their role among GBM tumor core- (c-CSC) vs. peritumor tissue-derived cancer stem cells (p-CSC) of six cases of GBM. Notch inhibition significantly impaired cell growth of c-CSC compared to p-CSC pools, with no effects observed in cell cycle distribution, apoptosis and cell invasion assays. Instead, anti-EGFR therapy induced cell cycle arrest, sometimes associated with apoptosis and reduction of cel  l invasi  veness in GBM CSC. In two cases, c-CSC pools were more sensitive to simultaneous anti-Notch and anti-EGFR treatment than either therapy alone compared to p-CSC, which were mostly resistant to treatment. We reported the overexpression of PDGFRα and its up-regulation following anti-EGFR therapy in GBM p-CSC compared to c-CSC. RNA interference of PDGFRα significantly reduced cell proliferation rate of p-CSC, while its pharmacological inhibition with Crenolanib impaired survival of both CSC pools, whose effects in combination with EGFR inhibition were maximized.We have used different drugs combination to identify the more effective therapeutic targets for GBM CSC, particularly against GBM peritumor tissue-derived CSC, which are mostly resistant to treatments. Overall, our results provide the rationale for simultaneous targeting of EGFR and PDGFR, which would be beneficial in the treatment of GBM. 
 Low incidence of KIT gene mutations and no PDGFRA gene mutations in primary cutaneous melanoma: an immunohistochemical and molecular genetic study of Japanese cases. Although several studies have reported the incidence of KIT and PDGFRA gene mutations in cutaneous melanomas of Caucasians, only one report is available for Mongoloids, including Japanese. In Japan, melanoma is an uncommon neoplasm compared with countries with Caucasian populations.In this study, protein expression and gene mutations of these two genes were investigated by immunohistochemistry and PCR-direct sequencing in 12 primary cutaneous   invasive   melanomas of Japanese patients. Acral melanoma was detected in 2 cases, chronically sun-damaged skin melanoma in 3 cases, and non-chronically sun-damaged skin melanoma in 7 cases. Genetic analysis was performed in exons 9, 11, 13, and 17 of the KIT gene and in exons 12 and 18 of the PDGFRA gene.Point mutations of the KIT gene were recognized in only one (8%) case, codon 559 of exon 11 (GTT → GCT). This is a melanoma of the sole (acral melanoma). No mutations of the PDGFRA gene were recognized. At the protein level, 11 case (92%) showed membranous expression of KIT; the expression did not correlate with melanoma depth. KIT expression was stronger in the peripheral in situ or mini  mal inva  sive areas than the cent  ral inva  sive areas. Membranous PDGFRA expression was found in all cases (100%).In this study, only 8% of melanomas had the KIT mutation in this Japanese population. The finding of a mutation in one of two acral melanomas suggests that KIT inhibitors, for example imatinib, which are already used in clinics for treatment of GIST, can also be used for melanoma. 
 PDGFRα Regulates Follicular Cell Differentiation Driving Treatment Resistance and Disease Recurrence in Papillary Thyroid Cancer. Dedifferentiation of follicular cells is a central event in resistance to radioactive iodine and patient mortality in papillary thyroid carcinoma (PTC). We reveal that platelet derived growth factor receptor alpha (PDGFRα) specifically drives dedifferentiation in PTC by disrupting the transcriptional activity of thyroid transcription factor-1 (TTF1). PDGFRα activation dephosphorylates TTF1 consequently shifting the localization of this transcription factor from the nucleus to the cytoplasm. TTF1 is required for follicular cell development and disrupting its function abrogates thyroglobulin production and sodium iodide transport. PDGFRα also promotes a m  ore inva  sive and migratory cell phenotype with a dramatic increase in xenograft tumor formation. In patient tumors we confirm that nuclear TTF1 expression is inversely proportional to PDGFRα levels. Patients exhibiting PDGFRα at time of diagnosis are three times more likely to exhibit nodal metastases and are 18 times more likely to recur within 5years than those patients lacking PDGFRα expression. Moreover, high levels of PDGFRα and low levels of nuclear TTF1 predict resistance to radioactive iodine therapy. We demonstrate in SCID xenografts that focused PDGFRα blockade restores iodide transport and decreases tumor burden by >50%. Focused PDGFRα inhibitors, combined with radioactive iodine, represent an additional avenue for treating patients with aggressive variants of PTC. 
 Peroxiredoxin I, platelet-derived growth factor A, and platelet-derived growth factor receptor alpha are overexpressed in carcinoma ex pleomorphic adenoma: association with malignant transformation. Carcinoma ex pleomorphic adenoma is a rare salivary gland malignancy. It constitutes an important model for the study of carcinogenesis, as it can display the tumor in different stages of progression, from benign pleomorphic adenoma to frankly   invasive   carcinoma. Growth signaling pathways undergo continuous activation in human tumors, commonly as a consequence of the overexpression of ligands and receptors such as platelet-derived growth factor and platelet-derived growth factor receptor. Hydrogen peroxide is produced after platelet-derived growth factor receptor activation, and it is essential for the sequential phosphorylation cascade that drives cell proliferation and migration. By their ability to degrade hydrogen peroxide, peroxiredoxins are involved in growth factor signaling regulation and in the oxidative stress response. To verify the potential association of peroxiredoxin I, platelet-derived growth factor-A, and platelet-derived growth factor receptor-alpha with carcinoma ex pleomorphic adenoma progression, we investigated the expression of these molecules in carcinoma ex pleomorphic adenoma showing different degrees of invasion. The peroxiredoxin I, platelet-derived growth factor-A, and platelet-derived growth factor receptor-alpha proteins were present in remnant pleomorphic adenoma to only a small extent, but, collectively, they were highly expressed as soon as the malignant phenotype was achieved and remained at elevated concentrations during progression to the advanced stages of carcinoma ex pleomorphic adenoma. In addition, their locations overlapped significantly, strengthening their connection to this growth-signaling pathway. Our results indicate that carcinoma ex pleomorphic adenoma cells acquire at least 2 significant advantages relative to their normal counterparts: resistance to oxidative stress-induced apoptosis, conferred by high peroxiredoxin I concentrations, and sustained growth, reflecting platelet-derived growth factor-A and platelet-derived growth factor receptor-alpha overexpression. 
 Biologic effects of platelet-derived growth factor receptor α blockade in uterine cancer. Platelet-derived growth factor receptor α (PDGFRα) expression is frequently observed in many kinds of cancer and is a candidate for therapeutic targeting. This preclinical study evaluated the biologic significance of PDGFRα and PDGFRα blockade (using a fully humanized monoclonal antibody, 3G3) in uterine cancer.Expression of PDGFRα was examined in uterine cancer clinical samples and cell lines, and biologic effects of PDGFRα inhibition were evaluated using in vitro (cell viability, apoptosis, and invasion) and in vivo (orthotopic) models of uterine cancer.PDGFRα was highly expressed and activated in uterine cancer samples and cell lines. Treatment with 3G3 resulted in substantial inhibition of PDGFRα phosphorylation and of downstream signaling molecules AKT and mitogen-activated protein kinase (MAPK). Cell viabi  lity and   invasive potential of uterine cancer cells were also inhibited by 3G3 treatment. In orthotopic mouse models of uterine cancer, 3G3 monotherapy had significant antitumor effects in the PDGFRα-positive models (Hec-1A, Ishikawa, Spec-2) but not in the PDGFRα-negative model (OVCA432). Greater therapeutic effects were observed for 3G3 in combination with chemotherapy than for either drug alone in the PDGFRα-positive models. The antitumor effects of therapy were related to increased apoptosis and decreased proliferation and angiogenesis.These findings identify PDGFRα as an attractive target for therapeutic development in uterine cancer. 
  [Mm]etastasize  PubMed  
 Growth factors in tumor microenvironment. Tumor microenvironment plays a critical role in tumor initiation and progression. Components in the microenvironment can modulate the growth of tumor cells, their ability to progress and   metastasize  . A major venue of communication between tumor cells and their microenvironment is through polypeptide growth factors and receptors for these growth factors. This article discusses three major classes of growth-stimulatory polypeptide growth factors and receptors for these growth factors. It also discusses how deregulation of these growth factors or their receptors can drive malignant transformation and progression. 
  [Mm]etastasis  PubMed  
 Expression of CAF-Related Proteins Is Associated with Histologic Grade of Breast Phyllodes Tumor. Purpose. The purpose of this study was to investigate the expression of cancer-associated fibroblast- (CAF-) related proteins and the implications in breast phyllodes tumor (PT). Methods. Tissue microarrays of 194 PT cases (151 benign PT, 27 borderline PT, and 16 malignant PT) were constructed. We performed immunohistochemical staining for CAF-related proteins (podoplanin, prolyl 4-hydroxylase, FAPα, S100A4, PDGFR α/β, and NG2) and analyzed the results according to clinicopathologic parameters. Results. Expression of PDGFRα and PDGFRβ in the stromal component increased with increasing histologic grade of PT (p = 0.003 and p = 0.034, resp.). Among clinicopathologic parameters, only expression of FAPα in stroma was associated with di  stant meta  stasis (p = 0.002). In univariate analysis, stromal expression of PDGFRα was associated with shorter overall survival (p = 0.002). In Cox multivariate analysis, stromal overgrowth and PDGFRα stromal positivity were associated with shorter overall survival (p = 0.006 and p = 0.050, resp.). Furthermore, expression of PDGFRβ in stroma was associated with shorter overall survival in patients with malignant PT (p = 0.041). Conclusion. Stromal expression of PDGFRα and PDGFRβ increased with increasing histologic grade of PT. In addition, PDGFR stromal positivity was associated with shorter overall survival. These results suggest that CAFs are associated with breast PT progression. 
 PDGFRα/β and VEGFR2 polymorphisms in colorectal cancer: incidence and implications in clinical outcome. Angiogenesis plays an essential role in tumor growth an  d metastas  is, and is a major target in cancer therapy. VEGFR and PDGFR are key players involved in this process. The purpose of this study was to assess the incidence of genetic variants in these receptors and its potential clinical implications in colorectal cancer (CRC).VEGFR2, PDGFRα and PDGFRβ mutations were evaluated by sequencing their tyrosine kinase domains in 8 CRC cell lines and in 92 samples of patients with CRC. Correlations with clinicopathological features and survival were analyzed.Four SNPs were identified, three in PDGFRα [exon 12 (A12): c.1701A>G; exon 13 (A13): c.1809G>A; and exon 17 (A17): c.2439+58C>A] and one in PDGFRβ [exon 19 (B19): c.2601A>G]. SNP B19, identified in 58% of tumor samples and in 4 cell lines (LS174T, LS180, SW48, COLO205), was associated with higher PDGFR and pPDGFR protein levels. Consistent with this observation, 5-year survival was greater for patients with PDGFR B19 wild type tumors (AA) than for those harboring the G-allele genotype (GA or GG) (51% vs 17%; p=0.073). Multivariate analysis confirmed SNP B19 (p=0.029) was a significant prognostic factor for survival, independent of age (p=0.060) or TNM stage (p<0.001).PDGFRβ exon 19 c.2601A>G SNP is commonly encountered in CRC patients and is associated with increased pathway activation and poorer survival. Implications regarding its potential influence in response to PDGFR-targeted agents remain to be elucidated. 
 Molecular characterization of EGFR, PDGFRA and VEGFR2 in cervical adenosquamous carcinoma. Adenosquamous carcinoma of the uterine cervix is an infrequent but aggressive subtype of cervical cancer. A better understanding of its biological behaviour is warranted to define more accurate prognosis and therapeutic targets. Currently, the blockage of receptor tyrosine kinase (RTKs) activity is an efficient therapeutic strategy for many different cancers. The objective of this study was to investigate EGFR, PDGFRA and VEGFR2 RTKs overexpression and activating gene mutations in a cohort of 30 adenosquamous carcinomas of the uterine cervix.EGFR, PDGFRA and VEGFR2 immunohistochemistry was performed in all samples, followed by DNA isolation from the gross macroscopically dissection of the neoplastic area. Screening for EGFR (exons 18-21) and PDGFRA (exons 12, 14 and 18) mutations was done by PCR--single-strand conformational polymorphism (PCR-SSCP).Despite the presence of EGFR immunohistochemical positive reactions in 43% (13/30) of the samples, no EGFR activating mutations in the hotspot region (exons 18-21) were identified. A silent base substitution (CAG>CAA) in EGFR exon 20 at codon 787 (Q787Q) was found in 17 cases (56%). All PDGFRA immunohistochemical reactions were positive and consistently observed in the stromal component, staining fibroblasts and endothelial cells, as well as in the cytoplasm of malignant cells. No activating PDGFRA mutations were found, yet, several silent mutations were observed, such as a base substitution in exon 12 (CCA>CCG) at codon 567 (P567P) in 9 cases and in exon 18 (GTC>GTT) at codon 824 (V824V) in 4 cases. We also observed the presence of base substitutions in intron 14 (IVS14+3G>A and IVS14+49G>A) in two different cases, and in intron 18 (IVS18-50insA) in 4 cases. VEGFR2 positivity was observed in 22 of 30 cases (73.3%), and was significantly associated with lack of   metastasis   (p=0.038).This is the most extensive analysis of EGFR, PDGFRA and VEGFR2 in cervical adenosquamous carcinomas. Despite the absence of EGFR and PDGFRA activating mutations, the presence of overexpression of these three important therapeutic targets in a subset of cases may be important in predicting the sensitivity of adenosquamous carcinoma to specific anti-RTKs drugs. 
 Expression of CAF-Related Proteins Is Associated with Histologic Grade of Breast Phyllodes Tumor. Purpose. The purpose of this study was to investigate the expression of cancer-associated fibroblast- (CAF-) related proteins and the implications in breast phyllodes tumor (PT). Methods. Tissue microarrays of 194 PT cases (151 benign PT, 27 borderline PT, and 16 malignant PT) were constructed. We performed immunohistochemical staining for CAF-related proteins (podoplanin, prolyl 4-hydroxylase, FAPα, S100A4, PDGFR α/β, and NG2) and analyzed the results according to clinicopathologic parameters. Results. Expression of PDGFRα and PDGFRβ in the stromal component increased with increasing histologic grade of PT (p = 0.003 and p = 0.034, resp.). Among clinicopathologic parameters, only expression of FAPα in stroma was associated with di  stant meta  stasis (p = 0.002). In univariate analysis, stromal expression of PDGFRα was associated with shorter overall survival (p = 0.002). In Cox multivariate analysis, stromal overgrowth and PDGFRα stromal positivity were associated with shorter overall survival (p = 0.006 and p = 0.050, resp.). Furthermore, expression of PDGFRβ in stroma was associated with shorter overall survival in patients with malignant PT (p = 0.041). Conclusion. Stromal expression of PDGFRα and PDGFRβ increased with increasing histologic grade of PT. In addition, PDGFR stromal positivity was associated with shorter overall survival. These results suggest that CAFs are associated with breast PT progression. 
 PDGFRα/β and VEGFR2 polymorphisms in colorectal cancer: incidence and implications in clinical outcome. Angiogenesis plays an essential role in tumor growth an  d metastas  is, and is a major target in cancer therapy. VEGFR and PDGFR are key players involved in this process. The purpose of this study was to assess the incidence of genetic variants in these receptors and its potential clinical implications in colorectal cancer (CRC).VEGFR2, PDGFRα and PDGFRβ mutations were evaluated by sequencing their tyrosine kinase domains in 8 CRC cell lines and in 92 samples of patients with CRC. Correlations with clinicopathological features and survival were analyzed.Four SNPs were identified, three in PDGFRα [exon 12 (A12): c.1701A>G; exon 13 (A13): c.1809G>A; and exon 17 (A17): c.2439+58C>A] and one in PDGFRβ [exon 19 (B19): c.2601A>G]. SNP B19, identified in 58% of tumor samples and in 4 cell lines (LS174T, LS180, SW48, COLO205), was associated with higher PDGFR and pPDGFR protein levels. Consistent with this observation, 5-year survival was greater for patients with PDGFR B19 wild type tumors (AA) than for those harboring the G-allele genotype (GA or GG) (51% vs 17%; p=0.073). Multivariate analysis confirmed SNP B19 (p=0.029) was a significant prognostic factor for survival, independent of age (p=0.060) or TNM stage (p<0.001).PDGFRβ exon 19 c.2601A>G SNP is commonly encountered in CRC patients and is associated with increased pathway activation and poorer survival. Implications regarding its potential influence in response to PDGFR-targeted agents remain to be elucidated. 
 Molecular characterization of EGFR, PDGFRA and VEGFR2 in cervical adenosquamous carcinoma. Adenosquamous carcinoma of the uterine cervix is an infrequent but aggressive subtype of cervical cancer. A better understanding of its biological behaviour is warranted to define more accurate prognosis and therapeutic targets. Currently, the blockage of receptor tyrosine kinase (RTKs) activity is an efficient therapeutic strategy for many different cancers. The objective of this study was to investigate EGFR, PDGFRA and VEGFR2 RTKs overexpression and activating gene mutations in a cohort of 30 adenosquamous carcinomas of the uterine cervix.EGFR, PDGFRA and VEGFR2 immunohistochemistry was performed in all samples, followed by DNA isolation from the gross macroscopically dissection of the neoplastic area. Screening for EGFR (exons 18-21) and PDGFRA (exons 12, 14 and 18) mutations was done by PCR--single-strand conformational polymorphism (PCR-SSCP).Despite the presence of EGFR immunohistochemical positive reactions in 43% (13/30) of the samples, no EGFR activating mutations in the hotspot region (exons 18-21) were identified. A silent base substitution (CAG>CAA) in EGFR exon 20 at codon 787 (Q787Q) was found in 17 cases (56%). All PDGFRA immunohistochemical reactions were positive and consistently observed in the stromal component, staining fibroblasts and endothelial cells, as well as in the cytoplasm of malignant cells. No activating PDGFRA mutations were found, yet, several silent mutations were observed, such as a base substitution in exon 12 (CCA>CCG) at codon 567 (P567P) in 9 cases and in exon 18 (GTC>GTT) at codon 824 (V824V) in 4 cases. We also observed the presence of base substitutions in intron 14 (IVS14+3G>A and IVS14+49G>A) in two different cases, and in intron 18 (IVS18-50insA) in 4 cases. VEGFR2 positivity was observed in 22 of 30 cases (73.3%), and was significantly associated with lack of   metastasis   (p=0.038).This is the most extensive analysis of EGFR, PDGFRA and VEGFR2 in cervical adenosquamous carcinomas. Despite the absence of EGFR and PDGFRA activating mutations, the presence of overexpression of these three important therapeutic targets in a subset of cases may be important in predicting the sensitivity of adenosquamous carcinoma to specific anti-RTKs drugs. 
 Molecular characterization of EGFR, PDGFRA and VEGFR2 in cervical adenosquamous carcinoma. Adenosquamous carcinoma of the uterine cervix is an infrequent but aggressive subtype of cervical cancer. A better understanding of its biological behaviour is warranted to define more accurate prognosis and therapeutic targets. Currently, the blockage of receptor tyrosine kinase (RTKs) activity is an efficient therapeutic strategy for many different cancers. The objective of this study was to investigate EGFR, PDGFRA and VEGFR2 RTKs overexpression and activating gene mutations in a cohort of 30 adenosquamous carcinomas of the uterine cervix.EGFR, PDGFRA and VEGFR2 immunohistochemistry was performed in all samples, followed by DNA isolation from the gross macroscopically dissection of the neoplastic area. Screening for EGFR (exons 18-21) and PDGFRA (exons 12, 14 and 18) mutations was done by PCR--single-strand conformational polymorphism (PCR-SSCP).Despite the presence of EGFR immunohistochemical positive reactions in 43% (13/30) of the samples, no EGFR activating mutations in the hotspot region (exons 18-21) were identified. A silent base substitution (CAG>CAA) in EGFR exon 20 at codon 787 (Q787Q) was found in 17 cases (56%). All PDGFRA immunohistochemical reactions were positive and consistently observed in the stromal component, staining fibroblasts and endothelial cells, as well as in the cytoplasm of malignant cells. No activating PDGFRA mutations were found, yet, several silent mutations were observed, such as a base substitution in exon 12 (CCA>CCG) at codon 567 (P567P) in 9 cases and in exon 18 (GTC>GTT) at codon 824 (V824V) in 4 cases. We also observed the presence of base substitutions in intron 14 (IVS14+3G>A and IVS14+49G>A) in two different cases, and in intron 18 (IVS18-50insA) in 4 cases. VEGFR2 positivity was observed in 22 of 30 cases (73.3%), and was significantly associated with lack of   metastasis   (p=0.038).This is the most extensive analysis of EGFR, PDGFRA and VEGFR2 in cervical adenosquamous carcinomas. Despite the absence of EGFR and PDGFRA activating mutations, the presence of overexpression of these three important therapeutic targets in a subset of cases may be important in predicting the sensitivity of adenosquamous carcinoma to specific anti-RTKs drugs. 
 Deletion of the KIT gene is associated with liver   metastasis   and poor prognosis in patients with gastrointestinal stromal tumor in the stomach. The goal of this study was to investigate the association of mutations in the KIT gene and the platelet-derived growth factor receptor alpha (PDGFRA) gene with clinicopathological features of patients with gastrointestinal stromal tumor (GIST) localized in the stomach. We evaluated 56 gastric GISTs for KIT and PDGFRA mutations. DNA was extracted from paraffin-embedded tumor specimens, and exons 9, 11, 13 and 17 of the KIT gene and exons 12 and 18 of the PDGFRA gene were amplified by polymerase chain reaction and sequenced. The genetic features were then compared with the clinicopathological features. Immunohistochemistry was performed for KIT, CD34, Ki-67 (as a marker of cell proliferation) and CD31 (as a marker of microvessel density), and apoptosis was assessed by in situ DNA nick-end labeling. Thirty-four (61%) of the 56 GISTs had a mutation in exon 11 of KIT, and 2 (4%) had a mutation in exon 13 of KIT. Deletions in exon 11 of KIT were the most common mutation encountered in the present study. No mutations were found in exon 9 or 17 of KIT. Six of the 20 GISTs lacking KIT mutations had a mutation in exon 18 of PDGFRA, and 1 had a mutation in exon 12 of PDGFRA. The KIT mutation-positive GISTs showed more frequent liver metastases and higher mortality than KIT mutation-negative GISTs. Our data indicate that KIT mutations, especially deletions in exon 11, are markers of poor prognosis for gastric GISTs. 
 PDGFRalpha: a new therapeutic target in the treatment of hepatocellular carcinoma? Hepatocellular carcinoma (HCC) develops most often in a background of chronic inflammatory liver injury from viral infection or alcohol use. Most HCCs are diagnosed at a stage at which surgical resection is not feasible. Even in patients receiving surgery rates of recurrence and   metastasis   remain high. There are few effective HCC therapies and hence a need for novel, rational approaches to treatment. Platelet derived growth factor receptor-alpha (PDGFR-alpha) is involved in tumor angiogenesis and maintenance of the tumor microenvironment and has been implicated in development and   metastasis   of HCC.To examine PDGFR-alpha as a target for therapy of HCC and explore opportunities and strategies for PDGFR-alpha inhibition.A review of relevant literature.Targeted inhibition of PDGFR-alpha is a rational strategy for prevention and therapy of HCC. 
 Deletions affecting codons 557-558 of the c-KIT gene indicate a poor prognosis in patients with completely resected gastrointestinal stromal tumors: a study by the Spanish Group for Sarcoma Research (GEIS). To explore the prognostic value of mutations in c-KIT and PDGFR-alpha genes with respect to relapse-free survival (RFS) in patients with gastrointestinal stromal tumors (GIST). We have investigated the prognostic relevance of the type and position of the mutations, in addition to other clinicopathologic factors, in a large series of patients with GIST.For this study, 162 patients were selected according to the following criteria: completely resected tumors with negative margins attended between 1994 and 2001; no   metastasis   at diagnosis; tumor larger than 2 cm, c-KIT-positive immunostaining; and no other primary tumors.The median follow-up was 42 months for patients free of recurrence. Mutations were detected in 96 tumors (60%): 82 cases involving c-KIT and 14 cases involving PDFGR-alpha. Univariate analysis demonstrated the following as poor prognostic factors for RFS: tumors larger than 10 cm (P < .0001); mitotic count higher than 10 mitoses per 50 high-power fields (P < .0001); high risk index (P < .0001); intestinal GIST location (P = .0041); high cellularity (P < .0001); tumor necrosis (P < .0001); deletions affecting exon 11 (P = .0007); and deletions affecting codons 557 to 558 (P < .0001). After the multivariate analysis, only the high risk index (relative risk [RR], 12.36), high cellularity (RR, 3.97), and deletions affecting codons 557 to 558 of c-KIT (RR, 2.57) corresponded to independent prognostic factors for RFS in GIST patients.Deletions affecting codons 557 to 558 are relevant for the prognosis of RFS in GIST patients. This critical genetic alteration should be considered to be a new prognostic stratification variable for randomized trials exploring imatinib mesylate in the adjuvant setting in GIST patients. 
 PDGFR-A is a therapeutic target in alveolar rhabdomyosarcoma. Alveolar rhabdomyosarcoma is an aggressive skeletal muscle cancer of childhood. Our initial studies of rhabdomyosarcoma gene expression for patients enrolled in a national clinical trial suggested that platelet-derived growth factor receptor A (PDGFR-A) may be a mediator of disease progression and   metastasis  . Using our conditional mouse tumor models that authentically recapitulate the primary mutations and metastatic progression of alveolar rhabdomyosarcomas in humans, we found by immunoblotting and immunokinase assays that PDGFR-A and its downstream effectors, mitogen-activated protein kinase and Akt, were highly activated in both primary and metastatic tumors. Inhibition of PDGFR-A by RNA interference, small molecule inhibitor or neutralizing antibody had a dramatic effect on tumor cell growth both in vitro and in vivo, although resistance evolved in one-third of tumors. These results establish proof-of-principal for PDGFR-A as a therapeutic target in alveolar rhabdomyosarcoma. 
 Prognostic prediction in patients with metastatic renal cell carcinoma treated with sorafenib based on expression levels of potential molecular markers in radical nephrectomy specimens. To investigate the expression levels of multiple molecular markers in radical nephrectomy specimens from patients with metastatic renal cell carcinoma (RCC) treated with sorafenib in order to identify factors predicting susceptibility to this agent.This study included 45 consecutive patients undergoing radical nephrectomy for clear cell RCC who were diagnosed as having metastatic diseases refractory to cytokine therapy and subsequently treated with sorafenib. Expression levels of 19 molecular markers involved in the regulation of apoptosis, cell cycle, signal transduction, and angiogenesis in primary RCC specimens were measured by immunohistochemical staining.There was no molecular marker having significant impact on the prediction of response to sorafenib. However, progression-free survival (PFS) was significantly associated with the expression levels of Bcl-xL and platelet-derived growth factor receptor (PDGFR)-α in addition to the presence of bone   metastasi  s and C-reactive protein level on univariate analysis. Of these significant factors, PDGFR-α expression and the presence of bon  e metastas  is appeared to be independently related to PFS by multivariate analysis. Furthermore, there were significant differences in PFS according to positive numbers of these 2 independent risk factors; that is, disease progression occurred in 2 of 7 patients who were negative for risk factor, 19 of 34 positive for a single risk factor, and 6 of 6 positive for both risk factors.Collectively, these findings suggest that it would be useful to consider expression levels of potential molecular markers, particularly PDGFR-α, as well as clinical parameters to select metastatic RCC patients likely to benefit from treatment with sorafenib. 
 Overexpression of platelet-derived growth factor receptor alpha in breast cancer is associated with tumour progression. Receptor tyrosine kinases have been extensively studied owing to their frequently abnormal activation in the development and progression of human cancers. Platelet-derived growth factor receptors (PDGFRs) are receptors with intrinsic tyrosine kinase activity that regulate several functions in normal cells and are widely expressed in a variety of malignancies. After the demonstration that gastrointestinal stromal tumours without c-Kit mutations harbour PDGFR-alpha-activating mutations and that PDGFR-alpha is also a therapeutic target for imatinib mesylate, the interest for this receptor has increased considerably. Because breast cancer is one of the most frequent neoplasias in women worldwide, and only one study has reported PDGFR-alpha expression in breast carcinomas, the aim of this work was to investigate the potential significance of PDGFR-alpha expression in invasive mammary carcinomas.We used immunohistochemistry to detect PDGFR-alpha overexpression on a series of 181 formalin-fixed paraffin-embedded invasive ductal breast carcinomas and in two breast cancer cell lines: MCF-7 and HS578T. We associated its expression with known prognostic factors and we also performed polymerase chain reaction-single-stranded conformational polymorphism and direct sequencing to screen for PDGFR-alpha mutations.PDGFR-alpha expression was observed in 39.2% of the breast carcinomas and showed an association with lymph node   metastasis   (P = 0.0079), HER-2 expression (P = 0.0265) and Bcl2 expression (P = 0.0121). A correlation was also found with the expression of platelet-derived growth factor A (PDGF-A; P = 0.0194). The two cell lines tested did not express PDGFR-alpha. Screening for mutations revealed alterations in the PDGFR-alpha gene at the following locations: 2500A-->G, 2529T-->A and 2472C-->T in exon 18 and 1701G-->A in exon 12. We also found an intronic insertion IVS17-50insA at exon 18 in all sequenced cases. None of these genetic alterations was correlated with PDGFR-alpha expression. The cell lines did not reveal any alterations in the PDGFR-alpha gene sequence.PDGFR-alpha is expressed in invasive breast carcinomas and is associated with biological aggressiveness. The genetic alterations described were not correlated with protein expression, but other mechanisms such as gene amplification or constitutive activation of a signalling pathway inducing this receptor could still sustain PDGFR-alpha as a potential therapeutic target. 
 PDGFRalpha/beta expression correlates with the metastatic behavior of human colorectal cancer: a possible rationale for a molecular targeting strategy. As new multi-target tyrosine kinase inhibitors are emerging in the therapy of various malignancies, our aim was to define the co-expression pattern of receptor-tyrosine-kinase platelet-derived growth factor receptors alpha and beta (PDGFRalpha/beta) in human colorectal cancer. The co-expression pattern of PDGFRalpha/beta was analyzed by RT-PCR in 99 histologically confirmed human colorectal carcinomas and five colorectal cancer cell lines. In addition, immunohistochemical (IHC) staining was applied for confirmation of expression and analysis of receptor tyrosine kinase (RTK) localisation. The colorectal cancer cell lines that were analysed revealed varying expression intensities of PDGFRalpha and PDGFRbeta. The majority of human colorectal cancer specimens revealed a PDGFRalpha (83%) or PDGFRbeta (60%) expression. While PDGFRalpha showed a predominantly cytoplasmic staining in tumor cells as well as in stromal pericytes, PDGFRbeta was restricted to stromal pericytes only. Furthermore, PDGFRalpha expression significantly correlated with lymph node   metastasis   (P=0.0082) and advanced UICC stages III/IV (P=0.018) in older patients (P=0.043). PDGFRbeta expression only revealed a trend towards lymphatic dissemination (P=0.099). Co-expression of PDGFRalpha/beta occurred in 57% of the colorectal cancer samples, whereas another 29% of the samples depicted mono-expression of PDGFRalpha or PDGFRbeta. Notably, PDGFRalpha/beta expression significantly correlated with lymphatic   metastasis   (P=0.007) and advanced UICC stages III/IV (P=0.017) in older patients (P=0.03). In summary, our results revealed that PDGFRalpha/beta expression significantly correlates with lymphatic dissemination and therefore encourages application of PDGFRalpha/beta RTK-inhibitors within a combination therapy. 
 STC1 expression by cancer-associated fibroblasts drives   metastasis   of colorectal cancer. Platelet-derived growth factor (PDGF) receptor signaling is a major functional determinant of cancer-associated fibroblasts (CAF). Elevated expression of PDGF receptors on stromal CAFs is associated with   metastasis   and poor prognosis, but mechanism(s) that underlie these connections are not understood. Here, we report the identification of the secreted glycoprotein stanniocalcin-1 (STC1) as a mediator of   metastasis   by PDGF receptor function in the setting of colorectal cancer. PDGF-stimulated fibroblasts increased migration and invasion of cocultured colorectal cancer cells in an STC1-dependent manner. Analyses of human colorectal cancers revealed significant associations between stromal PDGF receptor and STC1 expression. In an orthotopic mouse model of colorectal cancer, tumors formed in the presence of STC1-deficient fibroblasts displayed reduced intravasation of tumor cells along with fewer and smaller distant metastases formed. Our results reveal a mechanistic basis for understanding the contribution of PDGF-activated CAFs to cancer   metastasis  . 
 Detection of mutations in the BRAF gene in patients with KIT and PDGFRA wild-type gastrointestinal stromal tumors. Gastrointestinal stromal tumors (GISTs) are characterized by mutations in exons 9, 11, 13, and 17 of KIT or exons 12, 14, and 18 of PDGFRA gene. However, approximately 10 to 15 % of GISTs lack the mutations in KIT and PDGFRA, and these are referred to as wild-type GISTs which are less sensitive to tyrosine-kinase inhibitors. The aim of this study was to detect BRAF mutations in patients with wild-type GISTs. We applied a sensitive allele-specific PCR, which was optimized using the V600E mutation-harboring cell line RKO, followed by verification of the results by dideoxy sequencing. We selected 149 GIST patients without detectable mutations in KIT and PDGFRA genes from the Slovak national GIST register and analyzed biopsy specimens for the presence of BRAF mutations in exon 15. We identified nine patients with the V600E mutation. The BRAF-driven GISTs were primary gastric (n = 3), small intestinal (n = 3), colon (n = 1), and of uncertain origin (n = 1). We also included   a liver m  etastasis of a patient with a simultaneous KIT exon 11-mutated intra-a  bdominal m  etastasis. We conclude that genome analysis of wild-type GISTs for mutations should include the BRAF gene, as its mutation status contributes to understanding of pathogenesis and might be important for decisions on therapy. 
 Twist1-induced invadopodia formation promotes tumor   metastasis  . The Twist1 transcription factor is known to promote tumor   metastasis   and induce Epithelial-Mesenchymal Transition (EMT). Here, we report that Twist1 is capable of promoting the formation of invadopodia, specialized membrane protrusions for extracellular matrix degradation. Twist1 induces PDGFRα expression, which in turn activates Src, to promote invadopodia formation. We show that Twist1 and PDGFRα are central mediators of invadopodia formation in response to various EMT-inducing signals. Induction of PDGFRα and invadopodia is essential for Twist1 to promote tum  or metasta  sis. Consistent with PDGFRα being a direct transcriptional target of Twist1, coexpression of Twist1 and PDGFRα predicts poor survival in breast tumor patients. Therefore, invadopodia-mediated matrix degradation is a key function of Twist1 in promoting t  umor metas  tasis. 
 Potential role of imatinib mesylate (Gleevec, STI-571) in the treatment of vestibular schwannoma. To determine the expression of the tyrosine kinases platelet-derived growth factor receptor (PDGFR) and c-Kit in vestibular schwannoma (VS) and to determine the potential role of imatinib mesylate (Gleevec) in regulating the growth and cell death of this tumor.Protein tyrosine kinases are transmembrane tyrosine kinase receptors that transduce signals from inside and outside the cell and function as relay points for signaling pathways. They have a key role in numerous processes that affect cell proliferation, tumorigenesis, cancer invasion,   metastasis  , and modulation of apoptosis. A few of these kinases have been demonstrated to be overexpressed and dysregulated in many carcinomas, sarcomas, and benign tumors.Immunohistochemical staining was used to investigate the expression of PDGFR and c-Kit in archived acoustic neuroma tissue. Clinical data including size of tumors, age, sex, and symptoms were correlated with kinase expression, whereas Western blot analysis and immunofluorescence were performed to demonstrate the expression and localization of PDGFR and c-Kit in HEI193, an immortalized VS cell line. Clonogenic survival assays were performed to assess proliferation inhibition by Gleevec. Gleevec's effect on the cell cycle profile also was investigated via flow cytometry analysis.Expression of PDGFR in the formalin-fixed VS tumor tissue was observed in 23 (67.5%) of the 34 samples. C-kit was expressed in 18 (52.9%) of the 34 samples. Western blot analysis demonstrates positive expression of c-Kit and PDGFR-Q in HEI193 and a primary VS culture. Western blot analysis showed downregulation of phospho-c-kit and phospho-PDGFR-Q with 5 and 10 uM Gleevec. Immunofluorescent staining of this cell line also reveals that PDGFR-β is localized primarily in the cytoplasm, whereas c-Kit is both nuclear and cytoplasmic. Cell cycle analysis of HEI193 96 hours after incubation with Gleevec indicates a dose-dependent increase in G1 from 61.6% to 70.7% and 74% at 5 and 10 uM of Gleevec, respectively. Colony formation assays demonstrate dose-dependent growth inhibition by Gleevec, in the HEI193 cell line as well as in a VS cell culture derived from a fresh tumor.The expression of PDGFR-Q and c-Kit in VS tissue may indicate novel molecular targets involved in the development of this tumor. Direct inhibition of these molecules by Gleevec may have relevant therapeutic applications. 
 Prognostic value of KIT/PDGFRA mutations in gastrointestinal stromal tumors: a meta-analysis. The postulated relationship between KIT/PDGFRA mutations and their prognostic value in gastrointestinal stromal tumors (GISTs) has generated intense attention during the past decade, despite the fact that a great deal of studies have been conducted on this subject. To provide a strong quantitative estimate of this postulated relationship, we carried out a meta-analysis which combined, compared, and summarized the results of existing relevant studies.Studies were identified by searching databases and reviewing citations in relevant articles. Of 48 potentially relevant studies, we combined individual patient data from 18 studies which involved 1,487 patients with GISTs, by which we made a comparison between the positive KIT mutation subgroup and the negative KIT mutation subgroup (PDGFRA mutation and wild type). We tabulated and analyzed the patient characteristics from each study, including general information such as age and gender, histopathological parameters, and clinical follow-up outcomes.KIT mutations, compared with PDGFRA mutations and wild type, showed a marked increased risk not only for tumor size (>5 cm) but also for higher mitotic activity (>5), suggesting that KIT mutations significantly correlated with the National Comprehensive Cancer Network (NCCN) high risk or National Institutes of Health (NIH) high risk (1.74 (95% CI, 1.20 to 2.53) and 2.00 (95% CI, 1.08 to 3.68), respectively). Moreover, higher recurrence and   metastasis   was observed in GISTs with KIT mutations, revealing its closer correlation with clinical malignant risk (P<0.001 for each, with odds ratio (OR) of 2.06 (95%, 1.37 to 3.11) and 2.77 (95%, 1.64 to 4.67), respectively). High risk or malignant GISTs with KIT mutations had a significantly poorer prognosis, as measured by 3-year overall survival, compared to those with PDGFRA mutations and wild type (0.47 (95% CI, 0.25 to 0.90)).KIT mutations, compared with PDGFRA mutations and wild type, represent a poorer prognostic marker in high risk or malignant GISTs. 
 Cervical squamous cancer mRNA profiles reveal the key genes of   metastasis   and invasion. To investigate mRNA expression profiles associated with cervical squamous carcinoma progression and to identify key genes involved in invasion and   metastasis   of cervical squamous cancer.The authors extracted the mRNA expression profile of eight normal cervical tissues by human whole genome microarray. The main functions of differentially expressed genes were identified by gene ontology (GO) analysis. Gene-networks were established based on bioinformatic approaches. Microarray data of the expressions level of key genes verified by qRT-PCR.The authors identified 2036 differentially expressed genes between two groups including 1,282 down-regulated genes and 754 up-regulated genes (p < 0.05, FDR < 0.05). Gene-network revealed that PDGFRA, CAV1, and GJA-1 were critical for cervical cancer invasion and   metastasis  .PDGFRA, CAV1, and GJA-1 were revealed as key node genes for cervical cancer invasion and   metastasis  . The results may provide new evidences and ideas for early diagnosis and prognosis assessment of cervical cancer. 
 MicroRNA-34A inhibits the growth, invasion and   metastasis   of gastric cancer by targeting PDGFR and MET expression. Within the family of RTKs (receptor tyrosine kinases), PDGFR (platelet-derived growth factor receptor) has been implicated in carcinogenesis and tumour development. miRNAs (microRNAs), which can target the mRNAs (messenger RNAs) of cancer-associated genes, are abnormally expressed in various cancers. In this study, our aim was to identify the miRNAs that target PDGFR-α/β and to study the functions of these miRNAs. miR-34a was predicted to target PDGFR, and luciferase reporter assays showed that miR-34a could directly target PDGFR. Meanwhile, we found that miR-34a was down-regulated in gastric cancer tissues and was associated wit  h metastas  is. Our findings showed that miR-34a could inhibit gastric cancer cell migration, invasion and proliferation, but these tumourigenic properties were only partially restored when PDGFR-α/β was overexpressed. In subsequent experiments, we found that the overexpression of both PDGFR and MET could completely restore the gastric cancer tumourigenic properties. Moreover, the cancer-associated cell signalling pathway was studied, and we found that miR-34a could inhibit Akt [PKB (protein kinase B)] phosphorylation, which was restored by the overexpression of both PDGFR and MET. In conclusion, miR-34a may act as a potential tumour suppressor in gastric cancer and is associated with the mechanisms of gastric can  cer metast  asis; miR-34a can inhibit gastric cancer tumourigenesis by targeting PDGFR and MET through the PI3K (phosphoinositide 3-kinase)/Akt pathway. 
 PDGF: the nuts and bolts of signalling toolbox. PDGF is a growth factor and is extensively involved in multi-dimensional cellular dynamics. It switches on a plethora of molecules other than its classical pathway. It is engaged in various transitions of development; however, if the unleashed potentials lead astray, it brings forth tumourigenesis. Conventionally, it has been assumed that the components of this signalling pathway show fidelity and act with a high degree of autonomy. However, as illustrated by the PDGF signal transduction, reinterpretation of recent data suggests that machinery is often shared between multiple pathways, and other components crosstalk to each other through multiple mechanisms. It is important to note that metastatic cascade is an intricate process that we have only begun to understand in recent years. Many of the early steps of this PDGF cascade are not readily targetable in the clinic. In this review, we will unravel the paradoxes with reference to mitrons and cellular plasticity and discuss how disruption of signalling cascade triggers cellular proliferation phase transition and   metastasis  . We will also focus on the therapeutic interventions to counteract resultant molecular disorders. 
 Expression of platelet-derived growth factor-alpha receptor and c-kit in giant cell tumours of bone. Platelet-derived growth factor-alpha receptor (PDGFRA) and c-kit are tyrosine kinase receptors expressed in many neoplasms. We investigated protein expression in 34 giant cell tumours of bone and two specimens of lung metastases.The expression of PDGFRA and c-kit was analysed by immunohistochemistry. Additionally, capillary sequencing of genomic DNA was performed to search for mutations in therapeutically relevant exons 12 and 18 of the PDGFRA gene and exons 9 and 11 of the c-kit gene.PDGFRA expression was detected in all specimens and all cellular components such as reactive osteoclast-like giant cells and neoplastic stromal cells. C-kit expression was found in six cases of giant cell tumour and one specimen of lung   metastasis   in all cellular components ranging from 5 to 50% (mean 4.8%, SD 12.4). A further 13 cases showed labelling of osteoclast-like giant cells. DNA sequence of exons 12 and 18 of the PDGFRA gene and exons 9 and 11 of the c-kit gene were not altered in these tumours.PDGFRA and c-kit play a functional role in the growth of giant cell tumours of bone but genetic changes of therapeutically relevant exons of the PDGFRA gene and the c-kit gene are not seen in these tumours. 
 The analysis of status and clinical implication of KIT and PDGFRA mutations in gastrointestinal stromal tumor (GIST). To analyze the frequency and spectrum of KIT and platelet-derived growth factor receptor-alpha (PDGFRA) gene in a large series of study and to explore the clinical implication of mutations in the gastrointestinal stromal tumors (GISTs) in China.A total of 141 GISTs were subject to mutation analysis of KIT (exons 9, 11, 13, and 17) and PDGFRA (exons 12 and 18) using PCR amplification and direct sequencing. Clinicopathologic characteristics were correlated to gene mutations.Of the 141 tumors studied, approximately 76.6% had KIT gene mutations, 2.8% had PDGFRA gene mutations and 20.6% had a wild-type gene of KIT and PDGFRA. Among those with KIT gene mutations, 70.2% occurred in exon 11, 5.7% in exon 9, 0.7% in exon 13, and no mutation was detected in exons 17. The most frequent sites of mutation were in exon 11 and the mutations clustered in the classic "hot spot" at the 5'end of the exon mostly heterogeneous and the second "hot spot" were internal tandem duplications (ITD) at the 3'end of the exon. The overall mutation rate was significantly lower in GISTs originated from colorectum or extra-gastrointestinal tract (chi(2) = 6.728, P = 0.009; chi(2) = 4.059, P = 0.044), however, mutation rate on exon 11 was significantly increased in gastric stromal tumor (chi(2) = 5.713, P = 0.017; chi(2) = 4.341, P = 0.037). There were no significant differences in terms of age, gender, tumor size, mitotic counts, grade of malignant potential and liver   metastasis   in patients with or without gene mutations.KIT and PDGFRA gene were frequently found in patients with GISTs. Gene mutation rate varies in originated organ. 
 KIT and PDGFRA mutations and PDGFRA immunostaining in gastrointestinal stromal tumors. In the present study, we investigated the association of PDGFRA and KIT mutations as well as PDGFRA immunohistochemical expression with clinicopathologic features and prognosis in a series of gastrointestinal stromal tumors (GISTs). Tumor DNA from 40 GISTs was sequenced for the presence of mutations in KIT exons 9, 11, 13 and 17, and in PDGFRA exons 12 and 18. Tissue sections were stained with polyclonal anti-PDGFRA antibody. KIT mutations occurred in 26 cases. There were 13 deletions, 6 substitutions, 3 deletion-substitutions, 3 duplications and 1 insertion. Tumors with KIT deletions/insertion were large with a high mitotic index (MI), and were associated with a high rate of symptoms at diagnosis, invasion into adjacent organs, distant   metastasis  , relapse and a short disease-free survival (DFS). PDGFRA mutations occurred in 6 gastric GISTs. There were 4 deletions and 2 substitutions. Tumors with PDGFRA mutations were small, with a low MI and Ki67 score, and were associated with a very low rate of symptoms at diagnosis, invasion into adjacent organs and distant   metastasis  . PDGFRA immunopositivity was found in 23 cases: a peculiar 'dotlike' staining was found in 5 out of 6 PDGFRA mutated cases. Patients with positive PDGFRA immunostaining had a longer DFS than those with negative staining. Our data confirm that the type of KIT mutation is associated with various clinicopathologic features of GISTs, and indicate that PDGFRA mutations are associated with rather indolent tumors. PDGFRA immunopositivity reflects PDGFRA mutational status and is associated with a favorable outcome. 
 Amelanotic malignant melanoma of the esophagus: report of two cases with immunohistochemical and molecular genetic study of KIT and PDGFRA. The author reports herein two cases of amelanotic malignant melanoma of the esophagus. Case 1 is an 87-year-old woman who was admitted to our hospital because of nausea and vomiting. Endoscopic examination revealed an ulcerated tumor of the distal esophagus, and a biopsy was taken. The biopsy showed malignant polygonal and spindle cells. No melanin pigment was recognized. Immunohistochemically, the tumor cells were positive for melanosome (HMB45), S100 protein, KIT and Platelet derived growth factor receptor-alpha (PDGFRA). The patient was treated by chemotherapy and radiation, but died of systemic   metastasis   12 mo after the presentation. Case 2 is a 56-year-old man presenting with dysphagia. Endoscopic examination revealed a polypoid tumor in the middle esophagus, and a biopsy was obtained. The biopsy showed malignant spindle cells without melanin pigment. Immunohistochemically, the tumor cells were positively labeled for melanosome, S100 protein, KIT and PDGFRA. The patient refused operation, and was treated by palliative chemotherapy and radiation. He died of   metastasis   7 mo after the admission. In both cases, molecular genetic analyses of KIT gene (exons 9, 11, 13 and 17) and PDGFRA gene (exons 12 and 18) were performed by the PCR direct sequencing method, which showed no mutations of KIT and PDGFRA genes. This is the first report of esophageal malignant melanoma with an examination of the expression of KIT and PDGFRA and the mutational status of KIT and PDGFRA genes. 
 Fluorescence in situ hybridization analysis and immunophenotyping of c-Kit/PDGFRA and Bcl-2 expression in gastrointestinal stromal tumors. To investigate c-Kit/PDGFRA genomic alterations, KIT-PDGFRA coexpression in gastrointestinal stromal tumors (GISTs) and the role of Bcl-2.We analyzed 70 primary tumors, 6 recurrences, 4 metastases and 1 recurrence plus   metastasis  , all molecularly characterized. Alterations in gene copy number were detected by fluorescence in situ hybridization (FISH) and expression of KIT, PDGFRA and Bcl-2 by immunohistochemistry.c-Kit/PDGFRA gene alterations affected 38% of all cases and 39% of primary tumors, with major changes accounting for 15% in both all the cases and primary tumors. Cytoplasmic KIT/PDGFRA coexpression was present in 96.5% of the c-Kit-mutated cases, 100% of the wt c-Kit/PDGFRA cases and 66.6% of the PDGFRA-mutated cases. Bcl-2 immunoreactivity was present in 70% of cases, with expression levels of +++ in 29%, ++ in 38% and + in 33%.FISH confirmed cytogenetic alterations in about 40% of primary GISTs at the onset. The high rate of c-Kit/PDGFRA coexpression suggests that both receptors are involved in oncogenicity and may affect imatinib efficacy. The assumption that Bcl-2 expression is supported by the KIT pathway and that its imatinib-mediated down-regulation contributes to autophagic cell death, although attractive, needs to be further confirmed. 
 PDGF receptor-α promotes TGF-β signaling in hepatic stellate cells via transcriptional and posttranscriptional regulation of TGF-β receptors. Platelet-derived growth factor (PDGF) and transforming growth factor-β (TGF-β) signaling are required for hepatic stellate cell (HSC) activation under pathological conditions such as liver metastatic tumor growth. These two signaling pathways are functionally divergent; PDGF signaling promotes proliferation and migration of HSCs, and TGF-β induces transdifferentiation of quiescent HSCs into myofibroblasts. Although PDGF signaling is implicated in TGF-β-mediated epithelial mesenchymal transition of tumor cells, the role of PDGF receptors in TGF-β activation of HSCs has not been investigated. Here we report that PDGF receptor-α (PDGFR-α) is required for TGF-β signaling of cultured human HSCs although HSCs express both PDGF-α and -β receptors. PDGFR-α knockdown inhibits TGF-β-induced phosphorylation and nuclear accumulation of SMAD2 with no influence on AKT or ERK phosphorylation associated with noncanonical TGF-β signaling. PDGFR-α knockdown suppresses TGF-β receptor I (TβRI) but increases TβRII gene transcription. At the protein level, PDGFR-α is recruited to TβRI/TβRII complexes by TGF-β stimulation. PDGFR-α knockdown blocks TGF-β-mediated internalization of TβRII and induces accumulation of TβRII at the plasma membrane, thereby inhibiting TGF-β phosphorylation of SMAD2. Functionally, knockdown of PDGFR-α reduces paracrine effects of HSCs on colorectal cancer cell proliferation and migration in vitro. In mice and patients, colorectal cancer cell invasion of the liver induces upregulation of PDGFR-α of HSCs. In summary, our finding that PDGFR-α knockdown inhibits SMAD-dependent TGF-β signaling by repressing TβRI transcriptionally and blocking endocytosis of TGF-β receptors highlights a convergence of PDGF and TGF-β signaling for HSC activation and PDGFR-  α as a th  erapeutic target for liver metastasis and other settings of HSC activation. 
 Platelet-derived growth factor and platelet-derived growth factor receptor-α expression in the normal human thymus and thymoma. Platelet-derived growth factor (PDGF) and its receptors (PDGFRs) are strongly involved in the normal development of several organs, tumour angiogenesis and malignant progression and   metastasi  s. Few studies concerning their expression, distribution and role in normal and pathological human thymus are available in the literature. The aim of this study has been to analyse the immunohistochemical expression of PDGF and PDGFR-α in prenatal and postnatal normal human thymus and thymomal biopsy specimens. The results demonstrated immunoreactivity to both PDGF and PDGFR-α in all specimens, but the intensity, distribution and number of positive cells were different in normal thymus and thymomas, and also among different tumour types. PDGF and PDGFR-α were weakly expressed in foetal and postnatal humans with a different distribution between cortex and medulla in both blood vessels and epithelial cells, whereas they were overexpressed in thymoma, especially in type B2 and B3, in the tumour epithelial cells. Overall, these data suggest that PDGF and PDGFR-α may be involved in the pathophysiology of the human thymus. 
 PDGFRα and β play critical roles in mediating Foxq1-driven breast cancer stemness and chemoresistance. Many epithelial-mesenchymal transition (EMT)-promoting transcription factors have been implicated in tumorigenesis an  d metastas  is as well as chemoresistance of cancer. However, the underlying mechanisms mediating these processes are unclear. Here, we report that Foxq1, a forkhead box-containing transcription factor and EMT-inducing gene, promotes stemness traits and chemoresistance in mammary epithelial cells. Using an expression profiling assay, we identified Twist1, Zeb2, and PDGFRα and β as Foxq1 downstream targets. We further show that PDGFRα and β can be directly regulated by Foxq1 or indirectly regulated through the Foxq1/Twist1 axis. Knockdown of both PDGFRα and β results in more significant effects on reversing Foxq1-promoted oncogenesis in vitro and in vivo than knockdown of either PDGFRα or β alone. In addition, PDGFRβ is a more potent mediator of Foxq1-promoted stemness traits than PDGFRα. Finally, pharmacologic inhibition or gene silencing of PDGFRs sensitizes mammary epithelial cells to chemotherapeutic agents in vitro and in vivo. These findings collectively implicate PDGFRs as critical mediators of breast cancer oncogenesis and chemoresistance driven by Foxq1, with potential implications for developing novel therapeutic combinations to treat breast cancer. 
  GeneRIFs  
 Microarray data of the expression level of key genes reveals that PDGFRA is critical for cervical cancer invasion and   metastasis  . 
 PDGFRA mutations are associated with recurrence and   metastasis   in gastrointestinal stromal tumors. 
 Data indicate that the secreted glycoprotein stanniocalcin-1 (STC1) as a mediator of   metastasis   by PDGF receptor function in the setting of colorectal cancer. 
 Data show that induction of PDGFRalpha and invadopodia is essential for Twist1 to promote tumor   metastasis  . 
 PDGFRA is involved in tumor angiogenesis & maintenance of the tumor microenvironment & is implicated in development &   metastasis   of HCC, as well as playing a role in normal angiogenesis and embryo development. Review. 
 PDGFRalpha expression in colorectal cancer significantly correlated with lymph node   metastasis  . 
  [Cc]ell [Ii]nvasion  PubMed  
 PDGF receptor alpha inhibition induces apoptosis in glioblastoma cancer stem cells refractory to anti-Notch and anti-EGFR treatment. Cancer stem cells (CSC) represent a rare fraction of cancer cells characterized by resistance to chemotherapy and radiation, therefore nowadays there is great need to develop new targeted therapies for brain tumors and our study aim to target pivotal transmembrane receptors such as Notch, EGFR and PDGFR, which are already under investigation in clinical trials setting for the treatment of Glioblastoma Multiforme (GBM).MTS assay was performed to evaluate cells response to pharmacological treatments. Quantitative RT-PCR and Western blots were performed to state the expression of Notch1, EGFR and PDGFRα/β and the biological effects exerted by either single or combined targeted therapy in GBM CSC. GBM CSC invasive ability was tested in vitro in absence or presence of Notch and/or EGFR signaling inhibitors.In this study, we investigated gene expression and function of Notch1, EGFR and PDGFR to determine their role among GBM tumor core- (c-CSC) vs. peritumor tissue-derived cancer stem cells (p-CSC) of six cases of GBM. Notch inhibition significantly impaired cell growth of c-CSC compared to p-CSC pools, with no effects observed in cell cycle distribution, apoptosis an  d cell invasi  on assays. Instead, anti-EGFR therapy induced cell cycle arrest, sometimes associated with apoptosis and reduction of cell invasiveness in GBM CSC. In two cases, c-CSC pools were more sensitive to simultaneous anti-Notch and anti-EGFR treatment than either therapy alone compared to p-CSC, which were mostly resistant to treatment. We reported the overexpression of PDGFRα and its up-regulation following anti-EGFR therapy in GBM p-CSC compared to c-CSC. RNA interference of PDGFRα significantly reduced cell proliferation rate of p-CSC, while its pharmacological inhibition with Crenolanib impaired survival of both CSC pools, whose effects in combination with EGFR inhibition were maximized.We have used different drugs combination to identify the more effective therapeutic targets for GBM CSC, particularly against GBM peritumor tissue-derived CSC, which are mostly resistant to treatments. Overall, our results provide the rationale for simultaneous targeting of EGFR and PDGFR, which would be beneficial in the treatment of GBM. 
 PDGF receptor-α promotes TGF-β signaling in hepatic stellate cells via transcriptional and posttranscriptional regulation of TGF-β receptors. Platelet-derived growth factor (PDGF) and transforming growth factor-β (TGF-β) signaling are required for hepatic stellate cell (HSC) activation under pathological conditions such as liver metastatic tumor growth. These two signaling pathways are functionally divergent; PDGF signaling promotes proliferation and migration of HSCs, and TGF-β induces transdifferentiation of quiescent HSCs into myofibroblasts. Although PDGF signaling is implicated in TGF-β-mediated epithelial mesenchymal transition of tumor cells, the role of PDGF receptors in TGF-β activation of HSCs has not been investigated. Here we report that PDGF receptor-α (PDGFR-α) is required for TGF-β signaling of cultured human HSCs although HSCs express both PDGF-α and -β receptors. PDGFR-α knockdown inhibits TGF-β-induced phosphorylation and nuclear accumulation of SMAD2 with no influence on AKT or ERK phosphorylation associated with noncanonical TGF-β signaling. PDGFR-α knockdown suppresses TGF-β receptor I (TβRI) but increases TβRII gene transcription. At the protein level, PDGFR-α is recruited to TβRI/TβRII complexes by TGF-β stimulation. PDGFR-α knockdown blocks TGF-β-mediated internalization of TβRII and induces accumulation of TβRII at the plasma membrane, thereby inhibiting TGF-β phosphorylation of SMAD2. Functionally, knockdown of PDGFR-α reduces paracrine effects of HSCs on colorectal cancer cell proliferation and migration in vitro. In mice an  d patients, c  olorectal cancer cell invasion of the liver induces upregulation of PDGFR-α of HSCs. In summary, our finding that PDGFR-α knockdown inhibits SMAD-dependent TGF-β signaling by repressing TβRI transcriptionally and blocking endocytosis of TGF-β receptors highlights a convergence of PDGF and TGF-β signaling for HSC activation and PDGFR-α as a therapeutic target for liver metastasis and other settings of HSC activation. 
   
  
 FLT3 
 
  NCBI Gene  
  GeneRIF  
  Pubmed  
 
 
  Search Term  Rank  Fields  
  [Mm]igration  6  PubMed(9)  
  [Ii]nvasive  10  PubMed(2)  
  [Mm]etastasis  12  PubMed(2)  
 
 
 hide/show details for FLT3 
  [Mm]igration  PubMed  
 Internal tandem duplication mutations in FLT3 gene augment chemotaxis to Cxcl12 protein by blocking the down-regulation of the Rho-associated kinase via the Cxcl12/Cxcr4 signaling axis. Internal tandem duplication mutations in the Flt3 gene (ITD-FLT3) enhance cell   migration   toward the chemokine Cxcl12, which is highly expressed in the therapy-protective bone marrow niche, providing a potential mechanism underlying the poor prognosis of ITD-FLT3(+) acute myeloid leukemia. We aimed to investigate the mechanisms linking ITD-FLT3 to increased cell   migration   toward Cxcl12. Classification of the expression of Cxcl12-regulated genes in ITD-FLT3(+) cells demonstrated that the enhanced   migration   of ITD-FLT3(+) cells toward Cxcl12 was associated with the differential expression of genes downstream of Cxcl12/Cxcr4, which are functionally distinct from those expressed in ITD-FLT3(-) cells but are independent of the Cxcr4 expression levels. Among these differentially regulated genes, the expression of Rock1 in the ITD-FLT3(+) cells that migrated toward Cxcl12 was significantly higher than in ITD-FLT3(-) cells that migrated toward Cxcl12. In ITD-FLT3(-) cells, Rock1 expression and Mypt1 phosphorylation were transiently up-regulated but were subsequently down-regulated by Cxcl12. In contrast, the presence of ITD-FLT3 blocked the Cxcl12-induced down-regulation of Rock1 and early Mypt1 dephosphorylation. Likewise, the FLT3 ligand counteracted the Cxcl12-induced down-regulation of Rock1 in ITD-FLT3(-) cells, which coincided with enhanced cell   migration   toward Cxcl12. Rock1 antagonists or Rock1 shRNA abolished the enhanced   migration   of ITD-FLT3(+) cells toward Cxcl12. Our findings demonstrate that ITD-FLT3 increases cell   migration   toward Cxcl12 by antagonizing the down-regulation of Rock1 expression. These findings suggest that the aberrant modulation of Rock1 expression and activity induced by ITD-FLT3 may enhance acute myeloid leukemia cell chemotaxis to the therapy-protective bone marrow niche, where Cxcl12 is abundantly expressed. 
 Association of the EGF-TM7 receptor CD97 expression with FLT3-ITD in acute myeloid leukemia. Internal tandem duplications within the juxtamembrane region of the FMS-like tyrosine kinase receptor FLT3 (FLT3-ITD) represents one of the most common mutations in patients with acute myeloid leukemia (AML) which results in constitutive aberrant activation, increased proliferation of leukemic progenitors and is associated with an aggressive clinical phenotype. The expression of CD97, an EGF-TM7 receptor, has been linked to invasive behavior in thyroid and colorectal cancer. Here, we have investigated the association of CD97 with FLT3-ITD and its functional consequences in AML.Higher CD97 expression levels have been detected in 208 out of 385 primary AML samples. This was accompanied by a significantly increased bone marrow blast count as well as by mutations in the FLT3 gene. FLT3-ITD expressing cell lines as MV4-11 and MOLM-13 revealed significantly higher CD97 levels than FLT3 wildtype EOL-1, OCI-AML3 and HL-60 cells which were clearly decreased by the tyrosine kinase inhibitors PKC412 and SU5614. CD97 knock down by short hairpin RNA in MV4-11 cells resulted in inhibited trans-well   migration   towards fetal calf serum (FCS) and lysophosphatidic acid (LPA) being at least in part Rho-A dependent. Moreover, knock down of CD97 led to an altered mechanical phenotype, reduced adhesion to a stromal layer and lower wildtype FLT3 expression.Our results, thus, constitute the first evidence for the functional relevance of CD97 expression in FLT3-ITD AML cells rendering it a potential new theragnostic target. 
 FLT3-driven redox-modulation of Ezrin regulates leukaemic cell   migration  . The concept of reactive oxygen species (ROS) being produced via the activation of specific oncogenes provides a basis for generating genomic instability and pro-survival signalling in tumour cells. The purpose of this study was to identify downstream targets of NADPH oxidase (Nox)-derived ROS signalling in acute myeloid leukaemia cells, by performing a proteomic analysis utilizing two-dimensional phosphotyrosine immunoblotting. The majority of the targets identified were cytoskeletal-associated proteins including Ezrin, a known regulator of the cytoskeleton, which was examined further. The study demonstrated that inhibition of Nox enzymes, using diphenyleneiodonium chloride in the acute myeloid leukaemia cell line MOLM-13, resulted in a decrease in Ezrin tyrosine phosphorylation and also triggered a shift in Ezrin sub-cellular localization as detected by immunofluorescence. The change in Ezrin localization coincided with altered cell morphology, observed using scanning electron microscopy and a decreased ability to migrate through a polycarbonate transwell membrane. Similar effects were observed upon inhibition of the oncogenic receptor tyrosine kinase FLT3 using the staurosporine derivate PKC412, implicating a role for FLT3 as an upstream regulator of Ezrin. Our results indicate that FLT3 drives production of ROS by Nox, which stimulates changes in Ezrin tyrosine phosphorylation and localization via redox regulation of Src. Furthermore, inhibition of FLT3 signalling leads to alterations in MOLM-13 cell morphology and has a significant influence on cell motility. 
 Impact of CXCR4 inhibition on FLT3-ITD-positive human AML blasts. Internal tandem duplication (ITD) mutations of the FLT3 receptor are associated with a high incidence of relapse in acute myeloid leukemia (AML). Expression of the CXCR4 receptor in FLT3-ITD-positive AML is correlated with poor outcome, and inhibition of CXCR4 was shown to sensitize AML blasts toward chemotherapy. The aim of this study was to evaluate the impact of FLT3-ITD on cell proliferation and CXCR4-dependent   migration   in human hematopoietic progenitor cells and to investigate their response to CXCR4 inhibition.We used primary blasts from patients with FLT3-ITD or FLT3 wild-type AML. In addition, human CD34(+) hematopoietic progenitor cells were transduced to >70% with retroviral vectors containing human FLT3-ITD.We found that FLT3-ITD transgene overexpressing human hematopoietic progenitor cells show strongly reduced   migration   toward stromal-derived factor-1 in vitro and display significantly reduced bone marrow homing in nonobese diabetic severe combined immunodeficient mice. Cocultivation of FLT3-ITD-positive AML blasts or hematopoietic progenitor cells on bone marrow stromal cells resulted in a strong proliferation advantage and increased early cobblestone area-forming cells compared to FLT3-wild-type AML blasts. Addition of the CXCR4 inhibitor AMD3100 to the coculture significantly reduced both cobblestone area-forming cells and proliferation of FLT3-ITD-positive cells, but did not affect FLT3-wild-type cells-highlighting the critical interaction between CXCR4 and FLT3-ITD.CXCR4 inhibition to decrease cell proliferation and to control the leukemic burden may provide a novel therapeutic strategy in patients with advanced FLT3-ITD-positive AML. 
 Y654 of β-catenin is essential for FLT3/ITD-related tyrosine phosphorylation and nuclear localization of β-catenin. β-Catenin plays a dual role as a key effecter in the regulation of adherens junctions as well as a transcriptional co-activator. Tyrosine phosphorylation of β-catenin affects the cell adhesi  on, migra  tion, and gene transcription in many types of human cancer cells, including acute myeloid leukemia cells with FLT3 internal tandem duplication (FLT3/ITD-AML). Here, we investigated the relationship between three tyrosine residues (Y86, Y142, and Y654) in β-catenin and oncogenic FLT3/ITD kinase. In the experiments using COS-7 cells expressing FLT3/ITD and Wt or mutant β-catenin, FLT3/ITD phosphorylated Y654, and this residue was essential for β-catenin's nuclear localization by FLT3/ITD. Promoter-reporter assays demonstrated that Y654 phosphorylation of β-catenin was closely related to TCF transcriptional activity. In vitro kinase assays, using recombinant FLT3 and biotinylated β-catenin peptide including Y654 showed that FLT3 directly phosphorylated Y654 of β-catenin. These results explain how FLT3/ITD affects the tyrosine phosphorylation, nuclear localization, and transcriptional activity of β-catenin. Targeting Y654 phosphorylation may lead to the development of novel approaches to therapy for FLT3/ITD-AML. 
 Targeting BTK for the treatment of FLT3-ITD mutated acute myeloid leukemia. Approximately 20% of patients with acute myeloid leukaemia (AML) have a mutation in FMS-like-tyrosine-kinase-3 (FLT3). FLT3 is a trans-membrane receptor with a tyrosine kinase domain which, when activated, initiates a cascade of phosphorylated proteins including the SRC family of kinases. Recently our group and others have shown that pharmacologic inhibition and genetic knockdown of Bruton's tyrosine kinase (BTK) blocks AML blast proliferation, leukaemic cell adhesion to bone marrow stromal cells as well as   migration   of AML blasts. The anti-proliferative effects of BTK inhibition in human AML are mediated via inhibition of downstream NF-κB pro-survival signalling however the upstream drivers of BTK activation in human AML have yet to be fully characterised. Here we place the FLT3-ITD upstream of BTK in AML and show that the BTK inhibitor ibrutinib inhibits the survival and proliferation of FLT3-ITD primary AML blasts and AML cell lines. Furthermore ibrutinib inhibits the activation of downstream kinases including MAPK, AKT and STAT5. In addition we show that BTK RNAi inhibits proliferation of FLT3-ITD AML cells. Finally we report that ibrutinib reverses the cyto-protective role of BMSC on FLT3-ITD AML survival. These results argue for the evaluation of ibrutinib in patients with FLT3-ITD mutated AML. 
 Autotaxin is expressed in FLT3-ITD positive acute myeloid leukemia and hematopoietic stem cells and promotes cell   migration   and proliferation. Autotaxin (ATX) has been reported to act as a motility and growth factor in a variety of cancer cells. The ATX protein acts as a secreted lysophospholipase D by converting lysophosphatidylcholine (LPC) to lysophosphatidic acid (LPA), which signals via G-protein-coupled receptors and has important functions in cell   migration   and proliferation. This study demonstrates that ATX expression is specifically upregulated and functionally active in acute myeloid leukemia (AML) harboring an internal tandem duplication (ITD) mutation of the FLT3 receptor gene. Moreover, ATX expression was also found in normal human CD34+ progenitor cells and selected myeloid and lymphoid subpopulations. Enforced expression of mutant FLT3-ITD by retroviral vector transduction increased ATX mRNA in selected cell lines, whereas inhibition of FLT3-ITD signaling by sublethal doses of PKC412 or SU5614 led to a significant downregulation of ATX mRNA and protein levels. In the presence of LPC, ATX expression significantly increased proliferation. LPA induced proliferation, regardless of ATX expression, and induced chemotaxis in all tested human leukemic cell lines and human CD34(+) progenitors. LPC increased chemotaxis only in cells with high expression of endogenous ATX by at least 80%, demonstrating the autocrine action of ATX. Inhibition of ATX using a small molecule inhibitor selectively induced killing of ATX-expressing cell lines and reduced motility in these cells. Our data suggest that the production of bioactive LPA through ATX is involved in controlling proliferation and   migration   during hematopoiesis and that deregulation of ATX contributes to the pathogenesis of AML. 
 FLT3-mediated p38-MAPK activation participates in the control of megakaryopoiesis in primary myelofibrosis. Primary myelofibrosis (PMF) is characterized by increased number of hematopoietic progenitors and a dysmegakaryopoiesis which supports the stromal reaction defining this disease. We showed that increased ligand (FL) levels in plasma, hematopoietic progenitors, and stromal cells from PMF patients were associated with upregulation of the cognate Flt3 receptor on megakaryocytic (MK) cells. This connection prompted us to study a functional role for the FL/Flt3 couple in PMF dysmegakaryopoiesis, as a route to reveal insights into pathobiology and therapy in this disease. Analysis of PMF CD34(+) and MK cell transcriptomes revealed deregulation of the mitogen-activated protein kinase (MAPK) pathway along with Flt3 expression. In PMF patients, a higher proportion of circulating Flt3(+)CD34(+)CD41(+) cells exhibited an increased MAPK effector phosphorylation independently of Jak2(V617F) mutation. Activation of FL/Flt3 axis in PMF MK cell cultures, in response to FL, induced activation of the p38-MAPK cascade, which is known to be involved in inflammation, also increasing expression of its target genes (NFATC4, p53, AP-1, IL-8). Inhibiting Flt3 or MAPK or especially p38 by chemical, antibody, or silencing strategies restored megakaryopoiesis and reduced phosphorylation of Flt3 and p38 pathway effectors, confirming the involvement of Flt3 in PMF dysmegakaryopoiesis via p38 activation. In addition, in contrast to healthy donors, MK cells derived from PMF CD34(+) cells exhibited an FL-induced   migration   that could be reversed by p38 inhibition. Taken together, our results implicate the FL/Flt3 ligand-receptor complex in PMF dysmegakaryopoiesis through persistent p38-MAPK activation, with implications for therapeutic prospects to correct altered megakaryopoiesis in an inflammatory context. 
 Ligand-independent FLT3 activation does not cooperate with MLL-AF4 to immortalize/transform cord blood CD34+ cells. MLL-AF4 fusion is hallmark in high-risk infant pro-B-acute lymphoblastic leukemia (pro-B-ALL). Our limited understanding of MLL-AF4-mediated transformation reflects the absence of human models reproducing this leukemia. Hematopoietic stem/progenitor cells (HSPCs) constitute likely targets for transformation. We previously reported that MLL-AF4 enhanced hematopoietic engraftment and clonogenic potential in cord blood (CB)-derived CD34+ HSPCs but was not sufficient for leukemogenesis, suggesting that additional oncogenic lesions are required for MLL-AF4-mediated transformation. MLL-AF4+ pro-B-ALL display enormous levels of FLT3, and occasionally FLT3-activating mutations, thus representing a candidate cooperating event in MLL-AF4+ pro-B-ALL. We have explored whether FLT3.TKD (tyrosine kinase domain) mutation or increased expression of FLT3.WT (wild type) cooperates with MLL-AF4 to immortalize/transform CB-CD34+ HSPCs. In vivo, FLT3.TKD/FLT3.WT alone, or in combination with MLL-AF4, enhances hematopoietic repopulating function of CB-CD34+ HSPCs without impairing   migration   or hematopoietic differentiation. None of the animals transplanted with MLL-AF4+FLT3.TKD/WT-CD34+ HSPCs showed any sign of disease after 16 weeks. In vitro, enforced expression of FLT3.TKD/FLT3.WT conveys a transient overexpansion of MLL-AF4-expressing CD34+ HSPCs associated to higher proportion of cycling cells coupled to lower apoptotic levels, but does not augment clonogenic potential nor confer stable replating. Together, FLT3 activation does not suffice to immortalize/transform MLL-AF4-expressing CB-CD34+ HSPCs, suggesting the need of alternative (epi)-genetic cooperating oncogenic lesions. 
  [Ii]nvasive  PubMed  
 Association of the EGF-TM7 receptor CD97 expression with FLT3-ITD in acute myeloid leukemia. Internal tandem duplications within the juxtamembrane region of the FMS-like tyrosine kinase receptor FLT3 (FLT3-ITD) represents one of the most common mutations in patients with acute myeloid leukemia (AML) which results in constitutive aberrant activation, increased proliferation of leukemic progenitors and is associated with an aggressive clinical phenotype. The expression of CD97, an EGF-TM7 receptor, has been linked to   invasive   behavior in thyroid and colorectal cancer. Here, we have investigated the association of CD97 with FLT3-ITD and its functional consequences in AML.Higher CD97 expression levels have been detected in 208 out of 385 primary AML samples. This was accompanied by a significantly increased bone marrow blast count as well as by mutations in the FLT3 gene. FLT3-ITD expressing cell lines as MV4-11 and MOLM-13 revealed significantly higher CD97 levels than FLT3 wildtype EOL-1, OCI-AML3 and HL-60 cells which were clearly decreased by the tyrosine kinase inhibitors PKC412 and SU5614. CD97 knock down by short hairpin RNA in MV4-11 cells resulted in inhibited trans-well migration towards fetal calf serum (FCS) and lysophosphatidic acid (LPA) being at least in part Rho-A dependent. Moreover, knock down of CD97 led to an altered mechanical phenotype, reduced adhesion to a stromal layer and lower wildtype FLT3 expression.Our results, thus, constitute the first evidence for the functional relevance of CD97 expression in FLT3-ITD AML cells rendering it a potential new theragnostic target. 
 MLL-AF9 and FLT3 cooperation in acute myelogenous leukemia: development of a model for rapid therapeutic assessment. Human leukemias harboring chromosomal translocations involving the mixed lineage leukemia (MLL, HRX, ALL-1) gene possess high-level expression, and frequent activating mutations of the receptor tyrosine kinase FLT3. We used a murine bone marrow transplant model to assess cooperation between MLL translocation and FLT3 activation. We demonstrate that MLL-AF9 expression induces acute myelogenous leukemia (AML) in approximately 70 days, whereas the combination of MLL-AF9 and FLT3-ITD does so in less than 30 days. Secondary transplantation of splenic cells from diseased mice established that leukemia stem cells are present at a very high frequency of approximately 1:100 in both diseases. Importantly, prospectively isolated granulocyte macrophage progenitors (GMPs) coinfected with MLL-AF9 and FLT3-ITD give rise to a similar AML, with shorter latency than from GMP transduced with MLL-AF9 alone. Cooperation between MLL-AF9 and FLT3-ITD was further verified by real-time assessment of leukemogenesis using non  invasive   bioluminescence imaging. We used this model to demonstrate that MLL-AF9/FLT3-ITD-induced leukemias are sensitive to FLT3 inhibition in a 2-3 week in vivo assay. These data show that activated FLT3 cooperates with MLL-AF9 to accelerate onset of an AML from whole bone marrow as well as a committed hematopoietic progenitor, and provide a new genetically defined model system that should prove useful for rapid assessment of potential therapeutics in vivo. 
  [Mm]etastasis  PubMed  
 Oncogenic roles of PRL-3 in FLT3-ITD induced acute myeloid leukaemia. FLT3-ITD mutations are prevalent mutations in acute myeloid leukaemia (AML). PRL-3, a   metastasis  -associated phosphatase, is a downstream target of FLT3-ITD. This study investigates the regulation and function of PRL-3 in leukaemia cell lines and AML patients associated with FLT3-ITD mutations. PRL-3 expression is upregulated by the FLT3-STAT5 signalling pathway in leukaemia cells, leading an activation of AP-1 transcription factors via ERK and JNK pathways. PRL-3-depleted AML cells showed a significant decrease in cell growth. Clinically, high PRL-3 mRNA expression was associated with FLT3-ITD mutations in four independent AML datasets with 1158 patients. Multivariable Cox-regression analysis on our Cohort 1 with 221 patients identified PRL-3 as a novel prognostic marker independent of other clinical parameters. Kaplan-Meier analysis showed high PRL-3 mRNA expression was significantly associated with poorer survival among 491 patients with normal karyotype. Targeting PRL-3 reversed the oncogenic effects in FLT3-ITD AML models in vitro and in vivo. Herein, we suggest that PRL-3 could serve as a prognostic marker to predict poorer survival and as a promising novel therapeutic target for AML patients. 
 PRL-3, a   metastasis   associated tyrosine phosphatase, is involved in FLT3-ITD signaling and implicated in anti-AML therapy. Combination with other small molecule drugs represents a promising strategy to improve therapeutic efficacy of FLT3 inhibitors in the clinic. We demonstrated that combining ABT-869, a FLT3 inhibitor, with SAHA, a HDAC inhibitor, led to synergistic killing of the AML cells with FLT3 mutations and suppression of colony formation. We identified a core gene signature that is uniquely induced by the combination treatment in 2 different leukemia cell lines. Among these, we showed that downregulation of PTP4A3 (PRL-3) played a role in this synergism. PRL-3 is downstream of FLT3 signaling and ectopic expression of PRL-3 conferred therapeutic resistance through upregulation of STAT (signal transducers and activators of transcription) pathway activity and anti-apoptotic Mcl-1 protein. PRL-3 interacts with HDAC4 and SAHA downregulates PRL-3 via a proteasome dependent pathway. In addition, PRL-3 protein was identified in 47% of AML cases, but was absent in myeloid cells in normal bone marrows. Our results suggest such combination therapies may significantly improve the therapeutic efficacy of FLT3 inhibitors. PRL-3 plays a potential pathological role in AML and it might be a useful therapeutic target in AML, and warrant clinical investigation. 
   
  
 FGFR1 
 
  NCBI Gene  
  GeneRIF  
  Pubmed  
 
 
  Search Term  Rank  Fields  
  [Ff]ilopodia  1  PubMed(1)  
  [Mm]igration  6  Process(2); PubMed(40); GeneRIFs(4)  
  [Ee]xtracellular [Mm]atrix  8  PubMed(14)  
  [Ii]nvasive  10  PubMed(15); GeneRIFs(1)  
  [Mm]etastasize  11  PubMed(3)  
  [Mm]etastasis  12  PubMed(23); GeneRIFs(8)  
  [Cc]ell [Ii]nvasion  13  PubMed(2); GeneRIFs(1)  
 
 
 hide/show details for FGFR1 
  [Ff]ilopodia  PubMed  
 The F-BAR protein NOSTRIN participates in FGF signal transduction and vascular development. F-BAR proteins are multivalent adaptors that link plasma membrane and cytoskeleton and coordinate cellular processes such as membrane protrusion and migration. Yet, little is known about the function of F-BAR proteins in vivo. Here we report, that the F-BAR protein NOSTRIN is necessary for proper vascular development in zebrafish and postnatal retinal angiogenesis in mice. The loss of NOSTRIN impacts on the migration of endothelial tip cells and leads to a reduction of tip cell   filopodia   number and length. NOSTRIN forms a complex with the GTPase Rac1 and its exchange factor Sos1 and overexpression of NOSTRIN in cells induces Rac1 activation. Furthermore, NOSTRIN is required for fibroblast growth factor 2 dependent activation of Rac1 in primary endothelial cells and the angiogenic response to fibroblast growth factor 2 in the in vivo matrigel plug assay. We propose a novel regulatory circuit, in which NOSTRIN assembles a signalling complex containing FGFR1, Rac1 and Sos1 thereby facilitating the activation of Rac1 in endothelial cells during developmental angiogenesis. 
  [Mm]igration  Process  
 cell   migration   
 neuron   migration   
  PubMed  
 MicroRNA-16 and microRNA-424 regulate cell-autonomous angiogenic functions in endothelial cells via targeting vascular endothelial growth factor receptor-2 and fibroblast growth factor receptor-1. MicroRNAs play key roles in modulating a variety of cellular processes by posttranscriptional regulation of their target genes. Vascular endothelial growth factor (VEGF), VEGF receptor-2 (VEGFR2), and fibroblast growth factor receptor-1 (FGFR1) were identified by bioinformatic approaches and subsequently validated as targets of microRNA (miR)-16 and miR-424 in endothelial cells (ECs).Mimetics of these microRNAs reduced VEGF, VEGFR2, and FGFR1 expression, whereas specific antagonists enhanced their expression. Expression of mature miR-16 and miR-424 was upregulated on VEGF or basic fibroblast growth factor (bFGF) treatment. This upregulation was accompanied by a parallel increase in primary transcript (pri-miR)-16-1 and pri-miR-16-2 but not in pri-miR-424 levels, indicating a VEGF/bFGF-dependent transcriptional and posttranscriptional regulation of miR-16 and miR-424, respectively. Reduced expression of VEGFR2 and FGFR1 by miR-16 or miR-424 overexpression regulated VEGF and bFGF signaling through these receptors, thereby affecting the activity of downstream components of the pathways. Functionally, miR-16 or miR-424 overexpression reduced proliferation,   migration  , and cord formation of ECs in vitro, and lentiviral overexpression of miR-16 reduced the ability of ECs to form blood vessels in vivo.We conclude that these miRNAs fine-tune the expression of selected endothelial angiogenic mediators in response to these growth factors. Altogether, these findings suggest that miR-16 and miR-424 play important roles in regulating cell-intrinsic angiogenic activity of ECs. 
 Integrin activation is required for VEGF and FGF receptor protein presence on human microvascular endothelial cells. Endothelial cell proliferation and   migration   is initiated by growth factors including FGF and VEGF that bind to specific transmembrane receptor tyrosine kinases. Mechanisms that regulate in vivo expression of fibroblast growth factor receptors (FGFR) and vascular endothelial growth factor receptors (VEGFR) are not well understood. Since it is well known that different matrices influence the proliferation and   migration   of endothelial cells in culture, we hypothesized that changes in the extracellular matrix environment can regulate growth factor receptors on endothelial cells. We cultured human microvascular endothelial cells on different matrices (vitronectin, laminin, fibronectin, fibrin, and collagen IV) and examined for the presence of growth factor receptors (FGFR-1, FGFR-2, VEGFR-1, and VEGFR-2). We show that vitronectin increased the presence of all four growth factor receptors and most notably, VEGFR-1. In contrast, fibrin decreased all four receptors, especially FGFR-1 and FGFR-2. Inhibiting phosphotyrosine signaling abolished immunostaining for all four receptors, regardless of the matrix, but was not dependent on activating the Fyn-Shc pathway. Cells plated on vitronectin in the presence of blocking antibodies to integrins alphavbeta3 and alphavbeta5 similarly decreased presence of these growth factor receptors. Our data suggests a possible mechanism of how matrix-integrin interactions regulate endothelial cell responsiveness to growth factors and anchorage-dependent cell growth. 
 Fibronectin induces endothelial cell   migration   through β1 integrin and Src-dependent phosphorylation of fibroblast growth factor receptor-1 at tyrosines 653/654 and 766. The extracellular matrix microenvironment regulates cell phenotype and function. One mechanism by which this is achieved is the transactivation of receptor tyrosine kinases by specific matrix molecules. Here, we demonstrate that the provisional matrix protein, fibronectin (FN), activates fibroblast growth factor (FGF) receptor-1 (FGFR1) independent of FGF ligand in liver endothelial cells. FN activation of FGFR1 requires β1 integrin, as evidenced by neutralizing antibody and siRNA-based studies. Complementary genetic and pharmacologic approaches identify that the non-receptor tyrosine kinase Src is required for FN transactivation of FGFR1. Whereas FGF ligand-induced phosphorylation of FGFR1 preferentially activates ERK, FN-induced phosphorylation of FGFR1 preferentially activates AKT, indicating differential downstream signaling of FGFR1 in response to alternate stimuli. Mutation analysis of known tyrosine residues of FGFR1 reveals that tyrosine 653/654 and 766 residues are required for FN-FGFR1 activation of AKT and chemotaxis. Thus, our study mechanistically dissects a new signaling pathway by which FN achieves endothelial cell chemotaxis, demonstrates how differential phosphorylation profiles of FGFR1 can achieve alternate downstream signals, and, more broadly, highlights the diversity of mechanisms by which the extracellular matrix microenvironment regulates cell behavior through transactivation of receptor tyrosine kinases. 
 Fibronectin induces endothelial cell   migration   through β1 integrin and Src-dependent phosphorylation of fibroblast growth factor receptor-1 at tyrosines 653/654 and 766. The extracellular matrix microenvironment regulates cell phenotype and function. One mechanism by which this is achieved is the transactivation of receptor tyrosine kinases by specific matrix molecules. Here, we demonstrate that the provisional matrix protein, fibronectin (FN), activates fibroblast growth factor (FGF) receptor-1 (FGFR1) independent of FGF ligand in liver endothelial cells. FN activation of FGFR1 requires β1 integrin, as evidenced by neutralizing antibody and siRNA-based studies. Complementary genetic and pharmacologic approaches identify that the non-receptor tyrosine kinase Src is required for FN transactivation of FGFR1. Whereas FGF ligand-induced phosphorylation of FGFR1 preferentially activates ERK, FN-induced phosphorylation of FGFR1 preferentially activates AKT, indicating differential downstream signaling of FGFR1 in response to alternate stimuli. Mutation analysis of known tyrosine residues of FGFR1 reveals that tyrosine 653/654 and 766 residues are required for FN-FGFR1 activation of AKT and chemotaxis. Thus, our study mechanistically dissects a new signaling pathway by which FN achieves endothelial cell chemotaxis, demonstrates how differential phosphorylation profiles of FGFR1 can achieve alternate downstream signals, and, more broadly, highlights the diversity of mechanisms by which the extracellular matrix microenvironment regulates cell behavior through transactivation of receptor tyrosine kinases. 
 MicroRNA-16 and microRNA-424 regulate cell-autonomous angiogenic functions in endothelial cells via targeting vascular endothelial growth factor receptor-2 and fibroblast growth factor receptor-1. MicroRNAs play key roles in modulating a variety of cellular processes by posttranscriptional regulation of their target genes. Vascular endothelial growth factor (VEGF), VEGF receptor-2 (VEGFR2), and fibroblast growth factor receptor-1 (FGFR1) were identified by bioinformatic approaches and subsequently validated as targets of microRNA (miR)-16 and miR-424 in endothelial cells (ECs).Mimetics of these microRNAs reduced VEGF, VEGFR2, and FGFR1 expression, whereas specific antagonists enhanced their expression. Expression of mature miR-16 and miR-424 was upregulated on VEGF or basic fibroblast growth factor (bFGF) treatment. This upregulation was accompanied by a parallel increase in primary transcript (pri-miR)-16-1 and pri-miR-16-2 but not in pri-miR-424 levels, indicating a VEGF/bFGF-dependent transcriptional and posttranscriptional regulation of miR-16 and miR-424, respectively. Reduced expression of VEGFR2 and FGFR1 by miR-16 or miR-424 overexpression regulated VEGF and bFGF signaling through these receptors, thereby affecting the activity of downstream components of the pathways. Functionally, miR-16 or miR-424 overexpression reduced proliferation,   migration  , and cord formation of ECs in vitro, and lentiviral overexpression of miR-16 reduced the ability of ECs to form blood vessels in vivo.We conclude that these miRNAs fine-tune the expression of selected endothelial angiogenic mediators in response to these growth factors. Altogether, these findings suggest that miR-16 and miR-424 play important roles in regulating cell-intrinsic angiogenic activity of ECs. 
 Integrin activation is required for VEGF and FGF receptor protein presence on human microvascular endothelial cells. Endothelial cell proliferation and   migration   is initiated by growth factors including FGF and VEGF that bind to specific transmembrane receptor tyrosine kinases. Mechanisms that regulate in vivo expression of fibroblast growth factor receptors (FGFR) and vascular endothelial growth factor receptors (VEGFR) are not well understood. Since it is well known that different matrices influence the proliferation and   migration   of endothelial cells in culture, we hypothesized that changes in the extracellular matrix environment can regulate growth factor receptors on endothelial cells. We cultured human microvascular endothelial cells on different matrices (vitronectin, laminin, fibronectin, fibrin, and collagen IV) and examined for the presence of growth factor receptors (FGFR-1, FGFR-2, VEGFR-1, and VEGFR-2). We show that vitronectin increased the presence of all four growth factor receptors and most notably, VEGFR-1. In contrast, fibrin decreased all four receptors, especially FGFR-1 and FGFR-2. Inhibiting phosphotyrosine signaling abolished immunostaining for all four receptors, regardless of the matrix, but was not dependent on activating the Fyn-Shc pathway. Cells plated on vitronectin in the presence of blocking antibodies to integrins alphavbeta3 and alphavbeta5 similarly decreased presence of these growth factor receptors. Our data suggests a possible mechanism of how matrix-integrin interactions regulate endothelial cell responsiveness to growth factors and anchorage-dependent cell growth. 
 Fibroblast growth factor receptor-1-mediated endothelial cell proliferation is dependent on the Src homology (SH) 2/SH3 domain-containing adaptor protein Crk. Stimulation of fibroblast growth factor receptor-1 (FGFR-1) expressed on endothelial cells leads to cellular   migration   and proliferation. We have examined the role of the Src homology (SH) 2/SH3 domain-containing adaptor protein Crk in these processes. Transient tyrosine phosphorylation of Crk in fibroblast growth factor-2-stimulated endothelial cells was dependent on the juxtamembrane tyrosine residue 463 in FGFR-1, and a Crk SH2 domain precipitated FGFR-1 via phosphorylated Tyr-463, indicating direct complex formation between Crk and FGFR-1. Furthermore, Crk SH2 and SH3 domains formed ligand-independent complexes with Shc, C3G, and the Crk-associated substrate (Cas). Tyrosine phosphorylation of C3G and Cas increased as a consequence of growth factor treatment. We examined the role of Crk in FGFR-1-mediated cellular responses by use of cells expressing chimeric platelet-derived growth factor receptor-alpha/FGFR-1 (alphaR/FR) wild type and mutant Y463F receptors. The kinase activity of alphaR/FR Y463F was intact, but both Crk and the adaptor FRS-2 were no longer tyrosine-phosphorylated in the mutant cells. Both wild type and mutant receptor cells migrated efficiently, whereas cells expressing the mutant alphaR/FR Y463F failed to proliferate and Erk2 and Jun kinase activities were suppressed in these cells. In wild type alphaR/FR cells transiently expressing an SH2 domain mutant of Crk, Erk and Jun kinase activities as well as DNA synthesis were attenuated. Our data indicate that Crk participates in signaling complexes downstream of FGFR-1, which propagate mitogenic signals. 
 Mutations in fibroblast growth factor receptor 1 cause Kallmann syndrome with a wide spectrum of reproductive phenotypes. Kallmann's syndrome (KS) is a clinically and genetically heterogeneous disorder consisting of idiopathic hypogonadotropic hypogonadism (IHH) and anosmia. Mutations in KAL1 causing the X-linked form of KS have been identified in 10% of all KS patients and consistently result in a severe reproductive phenotype. KAL1 gene encodes for anosmin-1, a key protein involved in olfactory and GnRH neuronal   migration   through a putative interaction with FGFR1. Heterozygous mutations in the FGFR1 gene accompanied by a high frequency of cleft palate and other facial dysmorphisms were recently identified in 8% of a large KS cohort, yet the reproductive phenotype of KS patients harboring FGFR1 mutations has not been described.One hundred and fifty probands with KS (130 males and 20 females) were studied to determine the frequency and distribution of FGFR1 mutations and their detailed reproductive phenotypes. Fifteen heterozygous mutations in unrelated probands were identified. Twelve missense mutations (p.R78C, p.V102I, p.D224H, p.G237D, p.R254Q, p.V273M, p.E274G, p.Y339C, p.S346C, p.I538V, p.G703S and p.G703R) were distributed among the first, second and third immunoglobulin-like domains (D1-D3), as well as the tyrosine kinase domain (TKD). The mutations Y339C and S346C are located in exon 8B and code for the isoform FGFR1c. Additionally, two nonsense mutations (p.T585X and p.R622X) were documented in the TKD of the protein. A wide spectrum of reproductive function was observed among KS probands including: (1) a severe phenotype demonstrated by microphallus, cryptorchidism, no pubertal development, undetectable serum gonadotropins and low serum testosterone (T) and inhibin B; (2) partial pubertal development; (3) the fertile eunuch variant of IHH with normal testicular size and active spermatogenesis with a reversal of HH after T therapy. In addition, we found an even wider spectrum of reproductive function within pedigrees carrying an FGFR1 mutation ranging from IHH to delayed puberty to normal reproductive function (anosmia only or asymptomatic carriers). These observations strongly suggest a role for other genes that modify the phenotype of FGFR1 mutations.KS patients and family members carrying an FGFR1 mutation present a broad spectrum of pubertal development in contrast to the almost uniform severe clinical phenotype described in KS subjects with a KAL1 mutation. Additionally, this report implicates the isoform FGFR1c in the pathogenesis of KS. 
 Inhibition of endogenous SPARC enhances pancreatic cancer cell growth: modulation by FGFR1-III isoform expression. Secreted protein acidic and rich in cysteine (SPARC) is a multi-faceted protein-modulating cell-cell and cell-matrix interactions. In cancer, SPARC can be not only associated with a highly aggressive phenotype, but also acts as a tumour suppressor. The aim of this study was to characterise the function of SPARC and its modulation by fibroblast growth factor receptor (FGFR) 1 isoforms in pancreatic ductal adenocarcinoma (PDAC).Exogenous SPARC inhibited growth, movement, and   migration  . ShRNA inhibition of endogenous SPARC in ASPC-1 and PANC-1 cells resulted in increased anchorage-dependent and -independent growth, transwell   migration  , and xenograft growth as well as increased mitogenic efficacy of fibroblast growth factor (FGF) 1 and FGF2. Endogenous SPARC expression in PANC-1 cells was increased in FGFR1-IIIb over-expressing cells, but decreased in FGFR1-IIIc over-expressing cells. The up-regulation of endogenous SPARC was abrogated by the p38-mitogen-activated protein kinase inhibitor SB203580. SPARC was detectable in conditioned medium of pancreatic stellate cells (PSCs), but not PDAC cells. Conditioned medium of PDAC cells reduced endogenous SPARC expression of PSCs.Endogenous SPARC inhibits the malignant phenotype of PDAC cells and may, therefore, act as a tumour suppressor in PDAC. Endogenous SPARC expression can be modulated by FGFR1-III isoform expression. In addition, PDAC cells may inhibit endogenous SPARC expression in surrounding PSCs by paracrine actions. 
 FGF1-FGFR1 axis promotes tongue squamous cell carcinoma (TSCC) metastasis through epithelial-mesenchymal transition (EMT). Increasing evidences suggest a close association between tumor metastasis and the inflammatory factors secreted by tumor microenvironment. It has been reported that epithelial mesenchymal-transition (EMT) plays a significant role during multiple types of tumor metastasis and progression induced by inflammatory factor from tumor microenvironment. Previous researches implied that fibroblast growth factor 1 (FGF1) can promote tumor progression and cause poor prognosis in several types of malignant tumors via interacting with its receptor fibroblast growth factor receptor 1 (FGFR1). However, the effects of FGF1-FGFR1 on tongue squamous cell carcinoma (TSCC) are not yet completely understood. In the present study, we evaluated the effects and function of FGF1-FGFR1 axis on TSCC metastasis. In addition, we investigated whether the EMT pathway is involved in these effects, thus modulating the TSCC progression. The expression of FGFR1 was measured both in tongue cancer cell lines and tissues by qRT-PCR and western blot. We found that FGFR1 was up-regulated in TSCC tissues compared to non-neoplastic tongue tissues. Additionally, overexpression of FGFR1 is positively associated with poor differentiation and metastasis potential. Furthermore, the function of FGF1-FGFR1 was examined in TSCC cell line. The results implied that FGF1 can obviously promote Cal27 cells   migration   and invasion abilities through FGFR1, while the motile and invasive capabilities can be severely attenuated when knockdown the expression of FGFR1 by specific siRNAs. Further investigation results show that FGF1-FGFR1 axis promotes TSCC metastasis by modulating EMT pathway. However, this effect can be inhibited by blocking the FGF1-FGFR1 axis using FGFR1 specific siRNAs. In conclusion, our findings of the present study provide the evidences that FGF1-FGFR1 axis promotes the TSCC metastasis through the EMT pathway. 
 FGFR1 cleavage and nuclear translocation regulates breast cancer cell behavior. FGF-10 and its receptors, FGFR1 and FGFR2, have been implicated in breast cancer susceptibility and progression, suggesting that fibroblast growth factor (FGF) signaling may be co-opted by breast cancer cells. We identify a novel pathway downstream of FGFR1 activation, whereby the receptor is cleaved and traffics to the nucleus, where it can regulate specific target genes. We confirm Granzyme B (GrB) as the protease responsible for cleavage and show that blocking GrB activity stopped FGFR1 trafficking to the nucleus and abrogates the promigratory effect of FGF stimulation. We confirm the in vivo relevance of our findings, showing that FGFR1 localized to the nucleus specifically in invading cells in both clinical material and a three-dimensional model of breast cancer. We identify target genes for FGFR1, which exert significant effects on cell   migration   and may represent an invasive signature. Our experiments identify a novel mechanism by which FGF signaling can regulate cancer cell behavior and provide a novel therapeutic target for treatment of invasive breast cancer. 
 Activation of the FGFR1 signalling pathway by the Epstein-Barr virus-encoded LMP1 promotes aerobic glycolysis and transformation of human nasopharyngeal epithelial cells. Non-keratinizing nasopharyngeal carcinoma (NPC) is closely associated with Epstein-Barr virus (EBV) infection. The EBV-encoded latent membrane protein 1 (LMP1) is believed to play an important role in NPC pathogenesis by virtue of its ability to activate multiple cell signalling pathways which collectively promote cell proliferation, transformation, angiogenesis, and invasiveness, as well as modulation of energy metabolism. In this study, we report that LMP1 increases cellular uptake of glucose and glutamine, enhances LDHA activity and lactate production, but reduces pyruvate kinase activity and pyruvate concentrations. LMP1 also increases the phosphorylation of PKM2, LDHA, and FGFR1, as well as the expression of PDHK1, FGFR1, c-Myc, and HIF-1α, regardless of oxygen availability. Collectively, these findings suggest that LMP1 promotes aerobic glycolysis. With respect to FGFR1 signalling, LMP1 not only increases FGFR1 expression, but also up-regulates FGF2, leading to constitutive activation of the FGFR1 signalling pathway. Furthermore, two inhibitors of FGFR1 (PD161570 and SU5402) attenuate LMP1-mediated aerobic glycolysis, cellular transformation (proliferation and anchorage-independent growth), cell   migratio  n, and invasion in nasopharyngeal epithelial cells, identifying FGFR1 signalling as a key pathway in LMP1-mediated growth transformation. Immunohistochemical staining revealed that high levels of phosphorylated FGFR1 are common in primary NPC specimens and that this correlated with the expression of LMP1. In addition, FGFR1 inhibitors suppress cell proliferation and anchorage-independent growth of NPC cells. Our current findings demonstrate that LMP1-mediated FGFR1 activation contributes to aerobic glycolysis and transformation of epithelial cells, thereby implicating FGF2/FGFR1 signalling activation in the EBV-driven pathogenesis of NPC. 
 Analysis of non-canonical fibroblast growth factor receptor 1 (FGFR1) interaction reveals regulatory and activating domains of neurofascin. Fibroblast growth factor receptors (FGFRs) are important for many different mechanisms, including cell   migration  , proliferation, differentiation, and survival. Here, we show a new link between FGFR1 and the cell adhesion molecule neurofascin, which is important for neurite outgrowth. After overexpression in HEK293 cells, embryonal neurofascin isoform NF166 was able to associate with FGFR1, whereas the adult isoform NF186, differing from NF166 in additional extracellular sequences, was deficient. Pharmacological inhibitors and overexpression of dominant negative components of the FGFR signaling pathway pointed to the activation of FGFR1 after association with neurofascin in neurite outgrowth assays in chick tectal neurons and rat PC12-E2 cells. Both extra- and intracellular domains of embryonal neurofascin isoform NF166 were able to form complexes with FGFR1 independently. However, the cytosolic domain was both necessary and sufficient for the activation of FGFR1. Cytosolic serine residues 56 and 100 were shown to be essential for the neurite outgrowth-promoting activity of neurofascin, whereas both amino acid residues were dispensable for FGFR1 association. In conclusion, the data suggest a neurofascin intracellular domain, which activates FGFR1 for neurite outgrowth, whereas the extracellular domain functions as an additional, regulatory FGFR1 interaction domain in the course of development. 
 Direct binding of integrin alphavbeta3 to FGF1 plays a role in FGF1 signaling. Integrins play a role in fibroblast growth factor (FGF) signaling through cross-talk with FGF receptors (FGFRs), but the mechanism underlying the cross-talk is unknown. We discovered that FGF1 directly bound to soluble and cell-surface integrin alphavbeta3 (K(D) about 1 microm). Antagonists to alphavbeta3 (monoclonal antibody 7E3 and cyclic RGDfV) blocked this interaction. alphavbeta3 was the predominant, if not the only, integrin that bound to FGF1, because FGF1 bound only weakly to several beta1 integrins tested. We presented evidence that the CYDMKTTC sequence (the specificity loop) within the ligand-binding site of beta3 plays a role in FGF1 binding. We found that the integrin-binding site of FGF1 overlaps with the heparin-binding site but is distinct from the FGFR-binding site using docking simulation and mutagenesis. We identified an FGF1 mutant (R50E) that was defective in integrin binding but still bound to heparin and FGFR. R50E was defective in inducing DNA synthesis, cell proliferation, cell   migration  , and chemotaxis, suggesting that the direct integrin binding to FGF1 is critical for FGF signaling. Nevertheless, R50E induced phosphorylation of FGFR1 and FRS2alpha and activation of AKT and ERK1/2. These results suggest that the defect in R50E in FGF signaling is not in the initial activation of FGF signaling pathway components, but in the later steps in FGF signaling. We propose that R50E is a useful tool to identify the role of integrins in FGF signaling. 
 Inhibition of FGFR signaling by PD173074 improves antitumor immunity and impairs breast cancer metastasis. Aberrant fibroblast growth factor (FGF) and FGF receptor (FGFR) system have been associated with breast cancer. The objectives of our study were to investigate the effects and mechanisms of FGFR inhibition on tumor growth and metastasis on breast cancer. Our studies showed that the FGFR inhibitor PD173074 decreased the viability of several human breast cancer cells, as well as 4T1 murine mammary tumor cells. Therefore, we chose 4T1 cells to study PD173074's antitumor mechanism. Flow cytometry showed that PD173074 induced 4T1 cell apoptosis in a concentration-dependent manner. Western blot demonstrated that PD173074-induced apoptosis was correlated with the inhibition of Mcl-1 and survivin. Moreover, PD173074 also significantly increased the ratio of Bax/Bcl-2. PD173074 could also block 4T1 cell   migration   and invasion in vitro. In 4T1 tumor-bearing mice, PD173074 significantly inhibited tumor growth without obvious side effects. Meanwhile, PD173074 functionally reduced microvessel density and proliferation index and induced tumor apoptosis. Importantly, we found that FGFR inhibition by PD173074 reduced myeloid-derived suppressor cells (MDSCs) in the blood, spleens and tumors, accompanied by the increased infiltration of CD4(+) and CD8(+) T cells in the spleens and tumors. Furthermore, PD173074 significantly inhibited breast tumor metastasis to the lung of inoculated 4T1 breast cancer cells, which was accompanied by a reduction in MDSCs. Our findings suggested that FGFR inhibition could delay breast tumor progression, impair lung metastasis and break immunosuppression by effecting on tumor microenvironment, which may provide a promising therapeutic approach for breast cancer patient. 
 Enhanced Expression of Integrin αvβ3 Induced by TGF-β Is Required for the Enhancing Effect of Fibroblast Growth Factor 1 (FGF1) in TGF-β-Induced Epithelial-Mesenchymal Transition (EMT) in Mammary Epithelial Cells. Epithelial-to-mesenchymal transition (EMT) plays a critical role in cancer metastasis, and is regulated by growth factors such as transforming growth factor β (TGF-β) and fibroblast growth factors (FGF) secreted from the stromal and tumor cells. However, the role of growth factors in EMT has not been fully established. Several integrins are upregulated by TGF-β1 during EMT. Integrins are involved in growth factor signaling through integrin-growth factor receptor crosstalk. We previously reported that FGF1 directly binds to integrin αvβ3 and the interaction was required for FGF1 functions such as cell prolifera  tion and   migration. We studied the role of αvβ3 induced by TGF-β on TGF-β-induced EMT. Here, we describe that FGF1 augmented EMT induced by TGF-β1 in MCF10A and MCF12A mammary epithelial cells. TGF-β1 markedly amplified integrin αvβ3 and FGFR1 (but not FGFR2). We studied if the enhancing effect of FGF1 on TGF-β1-induced EMT requires enhanced levels of both integrin αvβ3 expression and FGFR1. Knockdown of β3 suppressed the enhancement by FGF1 of TGF-β1-induced EMT in MCF10A cells. Antagonists to FGFR suppressed the enhancing effect of FGF1 on EMT. Integrin-binding defective FGF1 mutant did not augment TGF-β1-induced EMT in MCF10A cells. These findings suggest that enhanced integrin αvβ3 expression in addition to enhanced FGFR1 expression is critical for FGF1 to augment TGF-β1-induced EMT in mammary epithelial cells. 
 The evaluation of basic fibroblast growth factor and fibroblastic growth factor receptor 1 levels in saliva and serum of patients with salivary gland tumor. Basic fibroblast growth factor (FGF2) is a well-known endothelial mitogen that regulates endothelial cell proliferation,   migration  , differentiation, and survival. In the present study, we investigated the levels of FGF2 and fibroblastic growth factor receptor 1 (FGFR1) in saliva and serum of patients with salivary gland tumors. Saliva and serum samples were collected from 43 patients with salivary gland tumors and 40 healthy volunteers. The FGF2 and FGFR1 concentrations in saliva and serum samples were measured by enzyme-linked immunosorbent assay. We found that the levels of FGF2 and FGFR1 in saliva and serum from patients with salivary gland tumors were significantly higher than those from healthy control subjects. These results suggest that salivary FGF2 and FGFR1 can be used as potential biomarkers in the diagnosis of salivary gland tumors. 
 Novel mechanisms of fibroblast growth factor receptor 1 regulation by extracellular matrix protein anosmin-1. Activation of fibroblast growth factor (FGF) signaling is initiated by a multiprotein complex formation between FGF, FGF receptor (FGFR), and heparan sulfate proteoglycan on the cell membrane. Cross-talk with other factors could affect this complex assembly and modulate the biological response of cells to FGF. We have previously demonstrated that anosmin-1, a glycosylated extracellular matrix protein, interacts with the FGFR1 signaling complex and enhances its activity in an IIIc isoform-specific and HS-dependent manner. The molecular mechanism of anosmin-1 action on FGFR1 signaling, however, remains unknown. Here, we show that anosmin-1 directly binds to FGFR1 with high affinity. This interaction involves domains in the N terminus of anosmin-1 (cysteine-rich region, whey acidic protein-like domain and the first fibronectin type III domain) and the D2-D3 extracellular domains of FGFR1. In contrast, anosmin-1 binds to FGFR2IIIc with much lower affinity and displays negligible binding to FGFR3IIIc. We also show that FGFR1-bound anosmin-1, although capable of binding to FGF2 alone, cannot bind to a FGF2.heparin complex, thus preventing FGFR1.FGF2.heparin complex formation. By contrast, heparin-bound anosmin-1 binds to pre-formed FGF2.FGFR1 complex, generating an anosmin-1.FGFR1.FGF2.heparin complex. Furthermore, a functional interaction between anosmin-1 and the FGFR1 signaling complex is demonstrated by immunofluorescence co-localization and Transwell   migration   assays where anosmin-1 was shown to induce opposing effects during chemotaxis of human neuronal cells. Our study provides molecular and cellular evidence for a modulatory action of anosmin-1 on FGFR1 signaling, whereby binding of anosmin-1 to FGFR1 and heparin can play a dual role in assembly and activity of the ternary FGFR1.FGF2.heparin complex. 
 FGFR1-induced epithelial to mesenchymal transition through MAPK/PLCγ/COX-2-mediated mechanisms. Tumour invasion and metastasis is the most common cause of death from cancer. For epithelial cells to invade surrounding tissues and metastasise, an epithelial-mesenchymal transition (EMT) is required. We have demonstrated that FGFR1 expression is increased in bladder cancer and that activation of FGFR1 induces an EMT in urothelial carcinoma (UC) cell lines. Here, we created an in vitro FGFR1-inducible model of EMT, and used this model to identify regulators of urothelial EMT. FGFR1 activation promoted EMT over a period of 72 hours. Initially a rapid increase in actin stress fibres occurred, followed by an increase in cell size, altered morphology and increased   migratio  n and invasion. By using site-directed mutagenesis and small molecule inhibitors we demonstrated that combined activation of the mitogen activated protein kinase (MAPK) and phospholipase C gamma (PLCγ) pathways regulated this EMT. Actin stress fibre formation was regulated by PLCγ activation, and was also important for the increase in cell siz  e, migrat  ion and altered morphology. MAPK activation regulat  ed migrat  ion and E-cadherin expression, indicating that combined activation of PLCγ and MAPK is required for a full EMT. We used expression microarrays to assess changes in gene expression downstream of these signalling cascades. COX-2 was transcriptionally upregulated by FGFR1 and caused increased intracellular prostaglandin E(2) levels, which promo  ted migra  tion. In conclusion, we have demonstrated that FGFR1 activation in UC cells lines promotes EMT via coordinated activation of multiple signalling pathways and by promoting activation of prostaglandin synthesis. 
 Immunolocalization of fibroblast growth factor-1 (FGF-1), its receptor (FGFR-1), and fibroblast-specific protein-1 (FSP-1) in inflammatory renal disease. The fibroblast growth factor (FGF) family has functions in development, cell proliferation,   migration  , and differentiation. While FGF-2 induces fibrosis, the role of FGF-1 in inflammation and fibrosis is less defined. We examined the expression of FGF-1 and FGF receptor (FGFR-1) to determine if renal diseases with varying etiologies of inflammation, including lupus nephritis (LN), acute interstitial nephritis (AIN) and acute rejection superimposed on chronic allograft nephropathy (CAN), showed varying patterns of expression. We also examined the expression of fibroblast-specific protein-1 (FSP-1), which has been linked to epithelial-mesenchymal transition (EMT) and fibrosis, to determine whether it was linked to potential profibrotic and inflammatory FGF-1 mechanisms.Proliferative LN (PLN) (N= 12), nonproliferative lupus nephritis (NPLN) (N= 5), AIN (N= 6), CAN (N= 4), and normal kidneys (N= 3) were studied. FGF, FGFR-1, and FSP-1 were localized by immunohistochemistry, and intensity scored on a 0 to 3+ scale. Double staining with CD68 and separate immunohistochemical staining for CD4 and CD8 with serial sections analysis were done to identify if T lymphocytes or macrophages showed staining for FGF-1 and FGFR-1 or FSP-1.In normal kidneys, FGF-1 was expressed in mesangial cells (0.67 +/- 0.58), glomerular endothelial (0.67 +/- 0.58), visceral, and parietal epithelial cells (1.67 +/- 0.58). FGFR-1 showed a similar pattern of staining but also was expressed in tubular epithelium, and arterial endothelium and smooth muscle. Expression of FGF-1 was increased over normal in glomerular parenchymal cells only in CAN in podocytes (2.30 +/- 0.58 vs. 3.00 +/- 0.00) (P < 0.05) and parietal epithelial cells (1.67 +/- 0.58 vs. 2.25 +/- 0.50) (P < 0.05). Infiltrating glomerular and interstitial inflammatory cells in diseased glomeruli also expressed FGF-1 and FGFR-1. Tubular cells expressed slightly increased FGFR-1 in renal diseases vs. normal, whereas tubules remained negative for FGF-1 in diseased kidneys. FSP-1 expression was prominent in the interstitium in all kidneys with interstitial inflammation, and most prominent in CAN. Interstitial FSP-1+ cells were consistent with a myofibroblast-type morphology, and did not stain with CD-68. FSP-1 expression was closely associated with inflammatory cells expressing FGF-1 and FGFR-1. FSP-1 also showed positivity within crescents and occasional podocytes in PLN.The expression of FGF-1 and FGFR-1 in infiltrating lymphocytes and macrophages, and of FGFR-1 in tubules, is supportive, but does not prove causality, of the possibility that FGF-1 might have both autocrine and paracrine functions in renal inflammation. However, the initial stimulus for renal inflammation, whether immune complex, hypersensitivity or rejection, did not alter expression patterns of FGF-1 or its receptor. The colocalization of inflammatory infiltrates with interstitial fibrosis supports the possibility of a contribution of FGF-1 for chemotaxis and associated fibrosis, further supported by interstitial FSP-1 expression closely associated with these inflammatory cells expressing FGF-1 and FGFR-1. 
 Sef inhibits fibroblast growth factor signaling by inhibiting FGFR1 tyrosine phosphorylation and subsequent ERK activation. Signaling through fibroblast growth factor receptors (FGFRs) is essential for many cellular processes including proliferation and   migration   as well as differentiation events such as angiogenesis, osteogenesis, and chondrogenesis. Recently, genetic screens in Drosophila and gene expression screens in zebrafish have resulted in the identification of several feedback inhibitors of FGF signaling. One of these, Sef (similar expression to fgf genes), encodes a transmembrane protein that belongs to the FGF synexpression group. Here we show that like zebrafish Sef (zSef), mouse Sef (mSef) interacts with FGFR1 and that the cytoplasmic domain of mSef mediates this interaction. Overexpression of mSef in NIH3T3 cells results in a decrease in FGF-induced cell proliferation associated with a decrease in Tyr phosphorylation of FGFR1 and FRS2. As a consequence, there is a reduction in the phosphorylation of Raf-1 at Ser(338), MEK1/2 at Ser(217) and Ser(221), and ERK1/2 at Thr(202) and Tyr(204). Furthermore, mSef inhibits ERK activation mediated by a constitutively activated FGFR1 but not by a constitutively active Ras and decreases FGF but not PDGF-mediated activation of Akt. These results indicate that Sef exerts its inhibitory effects at the level of FGFR and upstream of Ras providing an additional level of negative regulation of FGF signaling. 
 Targeting FGFR Pathway in Human Hepatocellular Carcinoma: Expressing pFGFR and pMET for Antitumor Activity. The MET receptor tyrosine kinase, the receptor for hepatocyte growth factor (HGF), has been implicated in cancer growth, invasion,   migration  , angiogenesis, and metastasis in a broad variety of human cancers, including human hepatocellular carcinoma (HCC). Recently, MET was suggested to be a potential target for the personalized treatment of HCC with an active HGF-MET signaling pathway. However, the mechanisms of resistance to MET inhibitors need to be elucidated to provide effective treatment. Here, we show that HCC cells exhibit different sensitivities to the MET inhibitor PHA665752, depending on the phosphorylation status of FGFR. Treatment of cells expressing both phospho-FGFR and phospho-MET with the inhibitor PHA665752 did not cause growth inhibition and cell death, whereas treatment with AZD4547, a pan-FGFR inhibitor, resulted in decreased colony formation and cleavage of caspase-3. Moreover, silencing of endogenous FGFR1 and FGFR2 by RNAi of HCC cells expressing phospho-FGFR, phospho-FGFR2, and phospho-MET overcame the resistance to PHA665752 treatment. Treatment of primary cancer cells from patients with HCC expressing both phospho-FGFR and phospho-MET with PHA665752 did not induce cell death, whereas AZD4547 treatment induced cell death through the cleavage of caspase-3. In addition, treatment of cells resistant to PHA665752 with AZD4547 abrogated the activation of downstream effectors of cell growth, proliferation, and survival. On the basis of these results, we conclude that the FGFR pathway is critical for HCC survival, and that targeting this pathway with AZD4547 may be beneficial for the treatment of patients with HCC-expressing phospho-FGFR and phospho-MET. 
 Cellular signaling by fibroblast growth factor receptors. The 22 members of the fibroblast growth factor (FGF) family of growth factors mediate their cellular responses by binding to and activating the different isoforms encoded by the four receptor tyrosine kinases (RTKs) designated FGFR1, FGFR2, FGFR3 and FGFR4. Unlike other growth factors, FGFs act in concert with heparin or heparan sulfate proteoglycan (HSPG) to activate FGFRs and to induce the pleiotropic responses that lead to the variety of cellular responses induced by this large family of growth factors. A variety of human skeletal dysplasias have been linked to specific point mutations in FGFR1, FGFR2 and FGFR3 leading to severe impairment in cranial, digital and skeletal development. Gain of function mutations in FGFRs were also identified in a variety of human cancers such as myeloproliferative syndromes, lymphomas, prostate and breast cancers as well as other malignant diseases. The binding of FGF and HSPG to the extracellular ligand domain of FGFR induces receptor dimerization, activation and autophosphorylation of multiple tyrosine residues in the cytoplasmic domain of the receptor molecule. A variety of signaling proteins are phosphorylated in response to FGF stimulation including Shc, phospholipase-Cgamma, STAT1, Gab1 and FRS2alpha leading to stimulation of intracellular signaling pathways that control cell proliferation, cell differentiation, cell   migration  , cell survival and cell shape. The docking proteins FRS2alpha and FRS2beta are major mediators of the Ras/MAPK and PI-3 kinase/Akt signaling pathways as well as negative feedback mechanisms that fine-tune the signal that is initiated at the cell surface following FGFR stimulation. 
 Alternative splicing generates at least five different isoforms of the human basic-FGF receptor. Fibroblast growth factors (FGFs) are polypeptide mitogens that induce the proliferation of a wide variety of cell types. Of the seven family members, the best characterized are basic and acidic FGF. In addition to their mitogenic effects, they participate in angiogenesis, differentiation and maintenance of survival of neurons, cell   migration   and embryonal development. Of all family members, keratinocyte growth factor (KGF) is unique in that it is a specific mitogen for epithelial cells and does not interact with the FGF receptor of fibroblasts. To study the interactions between KGF and its receptor, we isolated KGF and FGF receptors from keratinocytes and fibroblasts, respectively. In the course of this study, we isolated five different variants of the FGF receptor from human fibroblasts and showed that all were derived from a single genetic locus. Four of these variants encode transmembrane receptors and can be divided into two subgroups that differ from one another with respect to the number (two or three) of immunoglobulin (Ig)-like domains. Within each subgroup, one receptor differed from the other by the presence of a two-codon insertion. Thus, all the variations among the four isoforms are localized to their ligand binding domains. The fifth isoform encodes a molecule truncated just 3' to the first Ig-like domain and thus could be secreted from the cell. The transcripts encoding the long and short isoforms were found to be expressed in many cell types, but their relative levels of expression varied greatly depending on the cell type. These findings indicate that alternative splicing generates diverse FGF receptor isoforms in human cells. 
 EphA4 promotes cell proliferation and   migration   through a novel EphA4-FGFR1 signaling pathway in the human glioma U251 cell line. The Eph receptor tyrosine kinases and their ephrin ligands form a unique cell-cell contact-mediated bidirectional signaling mechanism for regulating cell localization and organization. High expression of Eph receptors in a wide variety of human tumors indicates some roles in tumor progression, which makes these proteins potential targets for anticancer therapy. For this purpose, we did gene expression profiling for 47 surgical specimens of brain tumors including 32 high-grade glioma using a microarray technique. The analysis, focused on the receptor tyrosine kinases, showed that EphA4 mRNA in the tumors was 4-fold higher than in normal brain tissue. To investigate the biological significance of EphA4 overexpression in these tumors, we analyzed EphA4-induced phenotypic changes and the signaling mechanisms using human glioma U251 cells. EphA4 promoted fibroblast growth factor 2-mediated cell proliferation and   migration   accompanied with enhancement of fibroblast growth factor 2-triggered mitogen-activated protein kinase and Akt phosphorylation. In addition, active forms of Rac1 and Cdc42 increased in the EphA4-overexpressing cells. Furthermore, we found that EphA4 formed a heteroreceptor complex with fibroblast growth factor receptor 1 (FGFR1) in the cells and that the EphA4-FGFR1 complex potentiated FGFR-mediated downstream signaling. Thus, our results indicate that EphA4 plays an important role in malignant phenotypes of glioblastoma by enhancing cell proliferation and   migration   through accelerating a canonical FGFR signaling pathway. 
 FGFR1 mediates recombinant thrombomodulin domain-induced angiogenesis. The recombinant epidermal growth factor-like domain plus the serine/threonine-rich domain of thrombomodulin (rTMD23) promotes angiogenesis and accelerates the generation of activated protein C (APC), which facilitates angiogenesis. The aim of this study was to elucidate the molecular mechanisms underlying the angiogenic activity of rTMD23.We prepared rTMD23 and its mutants that did not possess the ability to promote APC generation and investigated their angiogenic activities in vitro and in vivo. rTMD23 mutants promoted proliferation,   migration  , and tube formation of human umbilical vein endothelial cells in vitro and induced neovascularization in vivo; these effects were similar to those exerted by wild-type rTMD23. To investigate its interaction with rTMD23, Type I fibroblast growth factor receptor (FGFR1) was precipitated along with syndecan-4 by rTMD23-conjugated Sepharose in human umbilical vein endothelial cells and FGFR1-expressing human embryonic kidney 293 cells. Additionally, the kinetics of the interaction between rTMD23 and FGFR1 were analysed using surface plasmon resonance. rTMD23-induced FGFR1 activation and tube formation were inhibited by an FGFR1-specific tyrosine kinase inhibitor, PD173074, or by knockdown of FGFR1 using siRNA technology. We observed an improvement in rat hindlimb recovery in an ischaemic model following rTMD23 treatment, and this was associated with increased neovascularization and FGFR1 phosphorylation.rTMD23 induced angiogenesis via FGFR1, a process that is independent of the APC pathway. 
 Reversible kallmann syndrome, delayed puberty, and isolated anosmia occurring in a single family with a mutation in the fibroblast growth factor receptor 1 gene. Kallmann syndrome (KS) is a clinically and genetically heterogeneous disorder. Recently, loss-of-function mutations in the fibroblast growth factor receptor 1 (FGFR1) gene have been shown to cause autosomal dominant KS. To date, the detailed reproductive phenotype of KS associated with mutations in the FGFR1 has yet to be described. We report a kindred comprising a male proband with KS and spontaneous reversibility, whose mother had delayed puberty and whose maternal grandfather isolated anosmia. The proband presented at age 18 yr with KS and was subsequently treated with testosterone (T) therapy. Upon discontinuation of T therapy, he recovered from his hypogonadotropic hypogonadism, as evidenced by a normal LH secretion pattern, sustained normal serum T levels, and active spermatogenesis. The three members of this single family harbor the same FGFR1 mutation (Arg(622)X) in the tyrosine kinase domain. This report demonstrates 1) the first genetic cause of the rare variant of reversible KS, 2) the reversal of hypogonadotropic hypogonadism in a proband carrying an FGFR1 mutation suggests a role of FGFR1 beyond embryonic GnRH neuron   migration  , and 3) a loss of function mutation in the FGFR1 gene causing delayed puberty. 
 Fibroblast growth factor receptor 1 activation in mammary tumor cells promotes macrophage recruitment in a CX3CL1-dependent manner. Tumor formation is an extensive process requiring complex interactions that involve both tumor cell-intrinsic pathways and soluble mediators within the microenvironment. Tumor cells exploit the intrinsic functions of many soluble molecules, including chemokines and their receptors, to regulate pro-tumorigenic phenotypes that are required for growth and progression of the primary tumor. Previous studies have shown that activation of inducible FGFR1 (iFGFR1) in mammary epithelial cells resulted in increased proliferation,   migration  , and invasion in vitro and tumor formation in vivo. These studies also demonstrated that iFGFR1 activation stimulated recruitment of macrophages to the epithelium where macrophages contributed to iFGFR1-mediated epithelial cell proliferation and angiogenesis. The studies presented here further utilize this model to identify the mechanisms that regulate FGFR1-induced macrophage recruitment. Results from this study elucidate a novel role for the inflammatory chemokine CX3CL1 in FGFR1-induced macrophage   migration  . Specifically, we illustrate that activation of both the inducible FGFR1 construct in mouse mammary epithelial cells and endogenous FGFR in the triple negative breast cancer cell line, HS578T, leads to expression of the chemokine CX3CL1. Furthermore, we demonstrate that FGFR-induced CX3CL1 is sufficient to recruit CX3CR1-expressing macrophages in vitro. Finally, blocking CX3CR1 in vivo leads to decreased iFGFR1-induced macrophage recruitment, which correlates with decreased angiogenesis. While CX3CL1 is a known target of FGF signaling in the wound healing environment, these studies demonstrate that FGFR activation also leads to induction of CX3CL1 in a tumor setting. Furthermore, these results define a novel role for CX3CL1 in promoting macrophage recruitment during mammary tumor formation, suggesting that the CX3CL1/CX3CR1 axis may represent a potential therapeutic approach for targeting breast cancers associated with high levels of tumor-associated macrophages. 
 FGFR1 mutations in Kallmann syndrome. Kallmann syndrome (KS) is a heterogeneous genetic disorder that associates variable gonadotropin-releasing hormone (GnRH) deficiency with anosmia and, sometimes, other non-reproductive clinical features. X-linked recessive, as well as autosomal recessive and dominant modes of transmission have been described. The first KS-related gene to be described (KAL1) was in the X-linked form. The second gene (KAL2) was initially unexpected, as it was known to encode the fibroblast growth receptor 1 (FGFR1). Its consideration as a candidate gene arose as a result of cases with contiguous gene syndrome and, initially, mutations were mostly described in familial cases with autosomal dominant transmission of KS. Since its initial discovery, numerous mutations of FGFR1 have been described in several functional domains of the receptor. Genotype-phenotype correlations have shown that some clinical features associated with KS, such as loss of nasal cartilage, hearing impairment, and anomalies of the limbs seem to be mainly associated with KAL2 mutations. The role of FGFR1 in the normal development of the olfactory bulb explains the association of anosmia with GnRH deficiency in FGFR1-mutated patients. Phenotype analysis indicates that FGFR1 is involved in normal   migration   of GnRH fetal neurons, but this is clearly not the whole story as a substantial proportion of KAL2-mutated individuals have normosmic GnRH deficiency. Finally, recent additional observations, notably using animal models, but also the description of human mutations in a specific ligand of FGFR1, FGF8, are beginning to shed light on the pathogenesis of GnRH deficiency in general, not just KS. 
 RSK2 regulates endocytosis of FGF receptor 1 by phosphorylation on serine 789. FGFR1 (fibroblast growth factor receptor 1) regulates many key cellular responses including proliferation,   migration   and differentiation through activation of signaling pathways. Irregularities in FGFR1 signaling have been implicated in several pathological conditions, including human cancer. In order to discover novel regulators of FGFR1 signaling, we performed yeast two-hybrid screens and identified RSK2 (p90 ribosomal S6 kinase 2) as a potential FGFR1 interaction partner. RSK2 belongs to the family of serine/threonine kinases that are activated through the Ras-MAPK signal transduction pathway. Both in vitro and in vivo experiments confirmed the interaction and we show that phosphorylated RSK2 binds to and phosphorylates serine 789 in the C-terminal tail of FGFR1. Inhibition of RSK2 activity led to prolonged tyrosine transphosphorylation of FGFR1. Furthermore, prevention of FGFR1 phosphorylation by inhibition of RSK2 activity or mutation of serine 789 to alanine reduced FGFR1 endocytosis and ubiquitination explaining mechanistically the prolonged signaling activity. We propose a novel regulatory mechanism whereby activated RSK2 directly interacts with and phosphorylates FGFR1, thereby modulating receptor signaling through regulation of endocytosis. 
 The F-BAR protein NOSTRIN participates in FGF signal transduction and vascular development. F-BAR proteins are multivalent adaptors that link plasma membrane and cytoskeleton and coordinate cellular processes such as membrane protrusion and   migration  . Yet, little is known about the function of F-BAR proteins in vivo. Here we report, that the F-BAR protein NOSTRIN is necessary for proper vascular development in zebrafish and postnatal retinal angiogenesis in mice. The loss of NOSTRIN impacts on the   migration   of endothelial tip cells and leads to a reduction of tip cell filopodia number and length. NOSTRIN forms a complex with the GTPase Rac1 and its exchange factor Sos1 and overexpression of NOSTRIN in cells induces Rac1 activation. Furthermore, NOSTRIN is required for fibroblast growth factor 2 dependent activation of Rac1 in primary endothelial cells and the angiogenic response to fibroblast growth factor 2 in the in vivo matrigel plug assay. We propose a novel regulatory circuit, in which NOSTRIN assembles a signalling complex containing FGFR1, Rac1 and Sos1 thereby facilitating the activation of Rac1 in endothelial cells during developmental angiogenesis. 
 Syndecan 4 regulates FGFR1 signaling in endothelial cells by directing macropinocytosis. Fibroblast growth factor 2 (FGF2) induces endothelial cell   migration   and angiogenesis through two classes of receptors: receptor tyrosine kinases, such as FGF receptor 1 (FGFR1), and heparan sulfate proteoglycans, such as syndecan 4 (S4). We examined the distinct contributions of FGFR1 and S4 in shaping the endothelial response to FGF2. S4 determined the kinetics and magnitude of FGF2-induced mitogen-activated protein kinase (MAPK) signaling by promoting the macropinocytosis of the FGFR1-S4-FGF2 signaling complex. Internalization of the S4 receptor complex was independent of clathrin and dynamin, proceeded from lipid raft-enriched membranes, and required activation of the guanosine triphosphatases RhoG and Rab5. Genetic knockout of S4, disruption of S4 function, or inhibition of Rab5 led to increased endocytosis and MAPK signaling. These data define the mechanism by which FGFR1 and S4 coordinate downstream signaling upon FGF2 stimulation: FGFR1 initiates MAPK signaling, whereas S4-dependent FGFR1 macropinocytosis modulates the kinetics of MAPK activation. Our studies identify S4 as a regulator of MAPK signaling and address the question of how distinct classes of FGFRs individually contribute to signal transduction in endothelial cells. 
 Anosmin-1 modulates the FGF-2-dependent   migration   of oligodendrocyte precursors in the developing optic nerve. Oligodendrocyte precursors (OPCs) originate at specific domains within the neural tube before migrating to colonize the entire CNS. Once in their target areas, these cells differentiate into oligodendrocytes, the myelin-forming cells in the CNS. Using the embryonic mouse optic nerve as an experimental model, we have analyzed the influence of FGF-2 on OPC development. FGF-2 exerts a dose-dependent motogenic effect on the   migration   of plp-dm20+ and it also acts as a chemoattractant on these cells. These effects produced by FGF-2 are principally mediated by the FGFR1 receptor, which is expressed by OPCs. Anosmin-1 is the protein that is defective in the X-linked form of human Kallmann syndrome. This protein is expressed by retinal axons and it also interacts with FGFR1, thereby impairing the   migration   of OPCs. Because both Anosmin-1 and FGF-2 are present in the optic nerve in vivo, we propose a model whereby the relative concentration of these two proteins modulates the   migration   of OPCs during development through their interaction with FGFR1. This FGF-2/FGFR1/Anosmin-1 system may be relevant in the context of demyelinating diseases. 
 Activation of FGFR1beta signaling pathway promotes survival,   migration   and resistance to chemotherapy in acute myeloid leukemia cells. Fibroblast growth factors (FGFs) are important regulators of hematopoiesis and have been implicated in the tumorigenesis of solid tumors. Recent evidence suggests that FGF signaling through FGF receptors (FGFRs) may play a role in the proliferation of subsets of acute myeloid leukemias (AMLs). However, the precise mechanism and specific FGF receptors that support leukemic cell growth are not known. We show that FGF-2, through activation of FGFR1beta signaling, promotes survival, proliferation and   migration   of AML cells. Stimulation of FGFR1beta results in phosphoinositide 3-kinase (PI3-K)/Akt activation and inhibits chemotherapy-induced apoptosis of leukemic cells. Neutralizing FGFR1-specific antibody abrogates the physiologic and chemoprotective effects of FGF-2/FGFR1beta signaling and inhibits tumor growth in mice xenotransplanted with human AML. These data suggest that activation of FGF-2/FGFR1beta supports progression and chemoresistance in subsets of AML. Therefore, FGFR1 targeting may be of therapeutic benefit in subsets of AML. 
 Fibroblast growth factor receptor inhibition is active against mesothelioma and synergizes with radio- and chemotherapy. Malignant pleural mesothelioma is an aggressive malignancy characterized by frequent resistance to chemo- and radiotherapy, poor outcome, and limited therapeutic options. Fibroblast growth factors (FGFs) and their receptors are potential targets for cancer therapy, but their significance in mesothelioma has remained largely undefined.To investigate the antimesothelioma potential of FGF receptor 1 (FGFR1) inhibition.Expression of FGFs and their receptors was analyzed in mesothelioma cell lines and tissue specimens. Several cell models were used to investigate FGFR1 inhibition in vitro and in combination with cisplatin and irradiation. Mouse intraperitoneal xenotransplant models were used for in vivo validation.FGFR1, FGF2, and FGF18 were overexpressed in mesothelioma. Stimulation with FGF2 led to increased cell proliferation,   migration  , and transition to a more sarcomatoid phenotype in subsets of mesothelioma cell lines. In contrast, inhibition of FGFR1 by a specific kinase inhibitor or a dominant-negative FGFR1 construct led to significantly decreased proliferation, clonogenicity,   migration  , spheroid formation, and G1 cell cycle arrest in several mesothelioma cell lines, accompanied by apoptosis induction and decreased mitogen-activated protein kinase pathway activity. Reduced tumor growth, proliferation, mitogenic signaling, and apoptosis induction were observed in vivo. Inhibition of FGFR1 synergistically enhanced the cytotoxic effects of ionizing radiation and cisplatin.Our data suggest that the malignant phenotype of mesothelioma cells depends on intact FGF signals, which should be considered as therapeutic targets with a promising chemo- and radiosensitizing potential. 
 The adhesion molecule NCAM promotes ovarian cancer progression via FGFR signalling. Epithelial ovarian carcinoma (EOC) is an aggressive neoplasm, which mainly disseminates to organs of the peritoneal cavity, an event mediated by molecular mechanisms that remain elusive. Here, we investigated the expression and functional role of neural cell adhesion molecule (NCAM), a cell surface glycoprotein involved in brain development and plasticity, in EOC. NCAM is absent from normal ovarian epithelium but becomes highly expressed in a subset of human EOC, in which NCAM expression is associated with high tumour grade, suggesting a causal role in cancer aggressiveness. We demonstrate that NCAM stimulates EOC cell   migration   and invasion in vitro and promotes metastatic dissemination in mice. This pro-malignant function of NCAM is mediated by its interaction with fibroblast growth factor receptor (FGFR). Indeed, not only FGFR signalling is required for NCAM-induced EOC cell motility, but targeting the NCAM/FGFR interplay with a monoclonal antibody abolishes the metastatic dissemination of EOC in mice. Our results point to NCAM-mediated stimulation of FGFR as a novel mechanism underlying EOC malignancy and indicate that this interplay may represent a valuable therapeutic target. 
 Expression of FGFR1 is an independent prognostic factor in triple-negative breast cancer. Triple-negative breast cancers (TNBCs) are clinically aggressive tumors with limited treatment options. We examined the clinicopathological associations and prognostic implications of FGFR1 and FGFR2 expression in TNBCs. Tissue microarrays constructed from TNBCs were immunostained with FGFR1 and FGFR2, and scored by intensity and percentage of tumor cells stained per intensity for each subcellular compartment, which were correlated with clinicopathological parameters and survival. Cell   migration   following siRNA-mediated silencing of the FGFR1 gene in TNBC cell lines was also performed. 714 cases were informative for FGFR1 and FGFR2 immunostaining. Thresholds were defined as at least 1 % of cells stained and H-score of 100 or more. Proportions positive by each threshold were, respectively, 89.9 %, 7.1 % for FGFR1 (cytoplasm); 36.8 %, 7.8 % for FGFR2 (cytoplasm); and 33.5 %, 5.2 % for FGFR2 (membrane). Significant associations included FGFR1 and FGFR2 immunostaining for lobular subtype, FGFR2 immunostaining with lower grade, and more basal-like cancers with H-scores of 100 or more FGFR1 immunostaining. Multivariate Cox regression analysis showed FGFR1 expression in TNBCs to be independently prognostic for overall survival (OS) at both thresholds. Cases completely negative (less than 1 % staining) for FGFR1 immunostaining showed improved OS, while those with H-score of 100 or more immunostaining had the worst OS. Cell line studies revealed up-regulation of the FGFR1 gene in the MDA-MB-231 and Hs578T TNBC cells, and specific knockdown of FGFR1 expression significantly reduc  ed cell m  igration in MDA-MB-231 cell line. In conclusion, FGFR1 expression in TNBCs is independently prognostic of OS, and H-score of 100 or more FGFR1 immunostaining may define tumors that have treatment potential via FGFR signaling inhibition. 
 Prostaglandin E2 regulates angiogenesis via activation of fibroblast growth factor receptor-1. Prostaglandin E(2) (PGE(2)) behaves as a mitogen in epithelial tumor cells as well as in many other cell types. We investigated the actions of PGE(2) on microvascular endothelial cells (capillary venular endothelial cells) with the purpose of delineating the signaling pathway leading to the acquisition of the angiogenic phenotype and to new vessel formation. PGE(2) (100 nM) produced activation of the fibroblast growth factor receptor 1 (FGFR-1), as measured by its phosphorylation, but not of vascular endothelial growth factor receptor 2. PGE(2) stimulated the EP3 subtype receptor, as deduced by abrogation of EP3 Galpha(i) subunit activity through pertussis toxin. Consistent with this result, in human umbilical venular endothelial cells missing the EP3 receptor, PGE(2) did not phosphorylate FGFR-1. Upon binding to its receptor, PGE(2) initiated an autocrine/paracrine signaling cascade involving the intracellular activation of c-Src, activation of matrix metalloproteinase (predominantly MMP2), which in turn caused the mobilization of membrane-anchored fibroblast growth factor-2 (FGF-2). In fact, in cells unable to release FGF-2 the transfection with both FGFR-1 and EP3 did not result in FGFR-1 phosphorylation in response to PGE(2). Relevance for the FGF2-FGFR-1 system was highlighted by confocal analysis, showing receptor internalization after cell exposure to the prostanoid. ERK1/2 appeared to be the distal signal involved, its phosphorylation being sensitive to either cSrc inhibitor or FGFR-1 blocker. Finally, PGE(2) stimulated cell   migration   and capillary formation in aortic rings, which were severely reduced by inhibitors of signaling molecules or by receptor antagonist. In conclusion, this study provides evidence for the involvement of FGFR-1 through FGF2 in eliciting PGE(2) angiogenic responses. This signaling pattern is similar to the autocrine-paracrine mechanism which operates in endothelial cells to support neovascular growth. 
 Identification of microRNA-214 as a negative regulator of colorectal cancer liver metastasis by way of regulation of fibroblast growth factor receptor 1 expression. The purpose of this study was to identify microRNAs (miRNAs) involved in the pathology of colorectal cancer (CRC) liver metastasis and investigate their underlying mechanisms. A total of 39 miRNAs were identified to be differentially expressed between 16 primary CRC tissues with liver metastases and 16 CRC tissues without liver metastases from 32 patients by Affymetric miRNA microarrays. A panel of eight miRNAs were confirmed to be significantly and differentially expressed between CRC tissues with and without liver metastases through quantitative reverse-transcription polymerase chain reaction (RT-PCR) analysis in the 32 patients. In a validated cohort of 99 CRC patients (44 with and 55 without liver metastases), only miR-214 was validated to be significantly down-regulated in CRC with liver metastases, which was associated with an unfavorable prognosis. Ectopic expression of miR-214 suppressed proliferation,   migration  , and invasion in vitro, tumor growth and liver metastasis in an in vivo xenograft mouse model, whereas miR-214 knockdown promoted proliferation,   migration  , and invasion in CRC cell lines. Further studies indicated that fibroblast growth factor receptor 1 (FGFR1) was a potential target of miR-214. Restoring miR-214 expression in CRC cells decreased endogenous FGFR1 messenger RNA (mRNA) and protein levels. FGFR1 knockdown mimicked the tumor suppressive effect of miR-214 on CRC cells, while reintroduction of FGFR1 abolished the tumor suppressive effect of miR-214 on CRC cells. Moreover, miR-214 expression levels were inversely correlated with FGFR1 in CRC patients.Down-regulation of miR-214 expression was correlated with increased FGFR1 expression levels, which may contribute to increased CRC liver metastasis. miR-214 may serve as a potential marker to predict survival, and the miR-214-FGFR1 axis may be a therapeutic target in CRC patients. 
 Diversity in fibroblast growth factor receptor 1 regulation: learning from the investigation of Kallmann syndrome. The unravelling of the genetic basis of the hypogonadotrophic hypogonadal disorders, including Kallmann syndrome (KS), has led to renewed interest into the developmental biology of gonadotrophin-releasing hormone (GnRH) neurones and, more generally, into the molecular mechanisms of reproduction. KS is characterised by the association of GnRH deficiency with diminished olfaction. Until recently, only two KS-associated genes were known: KAL1 and KAL2. KAL1 encodes the cell membrane and extracellular matrix-associated secreted protein anosmin-1 which is implicated in the X-linked form of KS. Anosmin-1 shows high affinity binding to heparan sulphate (HS) and its function remains the focus of ongoing investigation, although a role in axonal guidance and neuronal   migration  , which are processes essential for normal GnRH ontogeny and olfactory bulb histogenesis, has been suggested. KAL2, identified as the fibroblast growth factor receptor 1 (FGFR1) gene, has now been recognised to be the underlying genetic defect for an autosomal dominant form of KS. The diverse signalling pathways initiated upon FGFR activation can elicit pleiotropic cellular responses depending on the cellular context. Signalling through FGFR requires HS for receptor dimerisation and ligand binding. Current evidence supports a HS-dependent interaction between anosmin-1 and FGFR1, where anosmin-1 serves as a co-ligand activator enhancing the signal activity, the finer details of whose mechanism remain the subject of intense investigation. Recently, mutations in the genes encoding prokineticin 2 (PK2) and prokineticin receptor 2 (PKR2) were reported in a cohort of KS patients, further reinforcing the view of KS as a multigenic trait involving divergent pathways. Here, we review the historical and current understandings of KS and discuss the latest findings from the molecular and cellular studies of the KS-associated proteins, and describe the evidence that suggests convergence of several of these pathways during normal GnRH and olfactory neuronal ontogeny. 
  GeneRIFs  
 Fibronectin induces endothelial cell   migration   through beta1 integrin and Src-dependent phosphorylation of fibroblast growth factor receptor-1 at tyrosines 653/654 and 766. 
 These results indicate that EphA4 plays an important role in malignant phenotypes of glioblastoma by enhancing cell proliferation and   migration   through accelerating a canonical FGFR signaling pathway. 
 Data suggest that the relative concentrations of Anosmin-1 and FGF-2 modulate the   migration   of oligodendrocyte precursors during development through their interaction with FGFR1. 
 the reversal of hypogonadotropic hypogonadism in a proband carrying an FGFR1 mutation suggests a role of FGFR1 beyond embryonic GnRH neuron   migration  , and a loss of function mutation in the FGFR1 gene causing delayed puberty. 
  [Ee]xtracellular [Mm]atrix  PubMed  
 Integrin activation is required for VEGF and FGF receptor protein presence on human microvascular endothelial cells. Endothelial cell proliferation and migration is initiated by growth factors including FGF and VEGF that bind to specific transmembrane receptor tyrosine kinases. Mechanisms that regulate in vivo expression of fibroblast growth factor receptors (FGFR) and vascular endothelial growth factor receptors (VEGFR) are not well understood. Since it is well known that different matrices influence the proliferation and migration of endothelial cells in culture, we hypothesized that changes in the   extracellular matrix   environment can regulate growth factor receptors on endothelial cells. We cultured human microvascular endothelial cells on different matrices (vitronectin, laminin, fibronectin, fibrin, and collagen IV) and examined for the presence of growth factor receptors (FGFR-1, FGFR-2, VEGFR-1, and VEGFR-2). We show that vitronectin increased the presence of all four growth factor receptors and most notably, VEGFR-1. In contrast, fibrin decreased all four receptors, especially FGFR-1 and FGFR-2. Inhibiting phosphotyrosine signaling abolished immunostaining for all four receptors, regardless of the matrix, but was not dependent on activating the Fyn-Shc pathway. Cells plated on vitronectin in the presence of blocking antibodies to integrins alphavbeta3 and alphavbeta5 similarly decreased presence of these growth factor receptors. Our data suggests a possible mechanism of how matrix-integrin interactions regulate endothelial cell responsiveness to growth factors and anchorage-dependent cell growth. 
 Fibronectin induces endothelial cell migration through β1 integrin and Src-dependent phosphorylation of fibroblast growth factor receptor-1 at tyrosines 653/654 and 766. The   extracellular matri  x microenvironment regulates cell phenotype and function. One mechanism by which this is achieved is the transactivation of receptor tyrosine kinases by specific matrix molecules. Here, we demonstrate that the provisional matrix protein, fibronectin (FN), activates fibroblast growth factor (FGF) receptor-1 (FGFR1) independent of FGF ligand in liver endothelial cells. FN activation of FGFR1 requires β1 integrin, as evidenced by neutralizing antibody and siRNA-based studies. Complementary genetic and pharmacologic approaches identify that the non-receptor tyrosine kinase Src is required for FN transactivation of FGFR1. Whereas FGF ligand-induced phosphorylation of FGFR1 preferentially activates ERK, FN-induced phosphorylation of FGFR1 preferentially activates AKT, indicating differential downstream signaling of FGFR1 in response to alternate stimuli. Mutation analysis of known tyrosine residues of FGFR1 reveals that tyrosine 653/654 and 766 residues are required for FN-FGFR1 activation of AKT and chemotaxis. Thus, our study mechanistically dissects a new signaling pathway by which FN achieves endothelial cell chemotaxis, demonstrates how differential phosphorylation profiles of FGFR1 can achieve alternate downstream signals, and, more broadly, highlights the diversity of mechanisms by which th  e extracellular matr  ix microenvironment regulates cell behavior through transactivation of receptor tyrosine kinases. 
 Fibronectin induces endothelial cell migration through β1 integrin and Src-dependent phosphorylation of fibroblast growth factor receptor-1 at tyrosines 653/654 and 766. The   extracellular matri  x microenvironment regulates cell phenotype and function. One mechanism by which this is achieved is the transactivation of receptor tyrosine kinases by specific matrix molecules. Here, we demonstrate that the provisional matrix protein, fibronectin (FN), activates fibroblast growth factor (FGF) receptor-1 (FGFR1) independent of FGF ligand in liver endothelial cells. FN activation of FGFR1 requires β1 integrin, as evidenced by neutralizing antibody and siRNA-based studies. Complementary genetic and pharmacologic approaches identify that the non-receptor tyrosine kinase Src is required for FN transactivation of FGFR1. Whereas FGF ligand-induced phosphorylation of FGFR1 preferentially activates ERK, FN-induced phosphorylation of FGFR1 preferentially activates AKT, indicating differential downstream signaling of FGFR1 in response to alternate stimuli. Mutation analysis of known tyrosine residues of FGFR1 reveals that tyrosine 653/654 and 766 residues are required for FN-FGFR1 activation of AKT and chemotaxis. Thus, our study mechanistically dissects a new signaling pathway by which FN achieves endothelial cell chemotaxis, demonstrates how differential phosphorylation profiles of FGFR1 can achieve alternate downstream signals, and, more broadly, highlights the diversity of mechanisms by which th  e extracellular matr  ix microenvironment regulates cell behavior through transactivation of receptor tyrosine kinases. 
 Integrin activation is required for VEGF and FGF receptor protein presence on human microvascular endothelial cells. Endothelial cell proliferation and migration is initiated by growth factors including FGF and VEGF that bind to specific transmembrane receptor tyrosine kinases. Mechanisms that regulate in vivo expression of fibroblast growth factor receptors (FGFR) and vascular endothelial growth factor receptors (VEGFR) are not well understood. Since it is well known that different matrices influence the proliferation and migration of endothelial cells in culture, we hypothesized that changes in the   extracellular matrix   environment can regulate growth factor receptors on endothelial cells. We cultured human microvascular endothelial cells on different matrices (vitronectin, laminin, fibronectin, fibrin, and collagen IV) and examined for the presence of growth factor receptors (FGFR-1, FGFR-2, VEGFR-1, and VEGFR-2). We show that vitronectin increased the presence of all four growth factor receptors and most notably, VEGFR-1. In contrast, fibrin decreased all four receptors, especially FGFR-1 and FGFR-2. Inhibiting phosphotyrosine signaling abolished immunostaining for all four receptors, regardless of the matrix, but was not dependent on activating the Fyn-Shc pathway. Cells plated on vitronectin in the presence of blocking antibodies to integrins alphavbeta3 and alphavbeta5 similarly decreased presence of these growth factor receptors. Our data suggests a possible mechanism of how matrix-integrin interactions regulate endothelial cell responsiveness to growth factors and anchorage-dependent cell growth. 
 Novel mechanisms of fibroblast growth factor receptor 1 regulation by   extracellular matrix   protein anosmin-1. Activation of fibroblast growth factor (FGF) signaling is initiated by a multiprotein complex formation between FGF, FGF receptor (FGFR), and heparan sulfate proteoglycan on the cell membrane. Cross-talk with other factors could affect this complex assembly and modulate the biological response of cells to FGF. We have previously demonstrated that anosmin-1, a glycosylated   extracellular matrix   protein, interacts with the FGFR1 signaling complex and enhances its activity in an IIIc isoform-specific and HS-dependent manner. The molecular mechanism of anosmin-1 action on FGFR1 signaling, however, remains unknown. Here, we show that anosmin-1 directly binds to FGFR1 with high affinity. This interaction involves domains in the N terminus of anosmin-1 (cysteine-rich region, whey acidic protein-like domain and the first fibronectin type III domain) and the D2-D3 extracellular domains of FGFR1. In contrast, anosmin-1 binds to FGFR2IIIc with much lower affinity and displays negligible binding to FGFR3IIIc. We also show that FGFR1-bound anosmin-1, although capable of binding to FGF2 alone, cannot bind to a FGF2.heparin complex, thus preventing FGFR1.FGF2.heparin complex formation. By contrast, heparin-bound anosmin-1 binds to pre-formed FGF2.FGFR1 complex, generating an anosmin-1.FGFR1.FGF2.heparin complex. Furthermore, a functional interaction between anosmin-1 and the FGFR1 signaling complex is demonstrated by immunofluorescence co-localization and Transwell migration assays where anosmin-1 was shown to induce opposing effects during chemotaxis of human neuronal cells. Our study provides molecular and cellular evidence for a modulatory action of anosmin-1 on FGFR1 signaling, whereby binding of anosmin-1 to FGFR1 and heparin can play a dual role in assembly and activity of the ternary FGFR1.FGF2.heparin complex. 
 Distribution of fibroblast growth factor (FGF)-2 and FGF receptor-1 messenger RNA expression and protein presence in the mid-trimester human fetus. Fibroblast growth factors (FGF) are known to have key roles in embryonic growth and morphogenesis, but their presence and contributions to fetal development are unclear. In particular, little information exists as to the relevance of FGF and their specific receptors to human fetal development. We studied the anatomical distribution of messenger RNA encoding FGF-2 and one of its high affinity receptors, FGFR1, using in situ hybridization in a variety of human fetal tissues in early second trimester. Corresponding protein distributions were determined by immunohistochemistry. Both FGF-2 and FGFR1 mRNA and proteins were found to be present in every organ and tissue examined, but with defined cellular localizations. In skeletal muscle, both FGF-2 and FGFR1 mRNA and peptides were present in differentiated fibers, and both co-localized to proliferating chondrocytes of the epiphyseal growth plate. FGF-2 and FGFR1 mRNA and peptides were also present within cardiac or gastrointestinal smooth muscle. Within the gastrointestinal tract FGF-2 mRNA and peptide were located in the submucosal tissue, whereas FGFR1 was expressed within the overlying mucosa. Similarly, in skin, FGF-2 was expressed within the dermis whereas FGFR1 mRNA and peptide were most apparent in the stratum germinativum of the epidermis. In kidney and lung, FGFR1 mRNA was located in the tubular and alveolar epithelia respectively, whereas FGF-2 was expressed in both epithelial and mesenchymal cell populations. Both growth factor and receptor were widespread in both neuroblasts and glioblasts in the cerebral cortex of the brain. Immunoreactivity for FGF-2 and FGFR1 was seen in all vascular endothelial cells of major vessels and capillaries. Within the skin, kidney, lung, and intestine FGF-2 immunoreactivity was found in basement membranes underlying epithelia, and was associated with the   extracellular matrix   and plasma membranes of many cell types. The results show that FGF-2 and one of its receptors are widely expressed anatomically in the mid-trimester human fetus. 
 Loss-of-function mutations in FGFR1 cause autosomal dominant Kallmann syndrome. We took advantage of overlapping interstitial deletions at chromosome 8p11-p12 in two individuals with contiguous gene syndromes and defined an interval of roughly 540 kb associated with a dominant form of Kallmann syndrome, KAL2. We establish here that loss-of-function mutations in FGFR1 underlie KAL2 whereas a gain-of-function mutation in FGFR1 has been shown to cause a form of craniosynostosis. Moreover, we suggest that the KAL1 gene product, the   extracellular matrix   protein anosmin-1, is involved in FGF signaling and propose that the gender difference in anosmin-1 dosage (because KAL1 partially escapes X inactivation) explains the higher prevalence of the disease in males. 
 Expression of a fibroblast growth factor-binding protein during the development of adenocarcinoma of the pancreas and colon. The activity of growth factors is crucial for tumor progression. We previously characterized a secreted fibroblast growth factor-binding protein (FGF-BP1) as a chaperone molecule, which enhances the biological functions of FGFs by releasing FGFs from the   extracellular matrix  . Here, we characterize the frequency and pattern of FGF-BP1 expression during the malignant progression of pancreas and colorectal carcinoma. For this, we generated monoclonal antibodies that detect FGF-BP1 protein in formalin-fixed, paraffin-embedded tissues and applied in situ hybridization to detect FGF-BP1 mRNA in adjacent tissue sections. FGF-BP1 protein and mRNA were found up-regulated (>70% positive) in parallel (r = 0.70, P < 0.0001) in colon adenoma (n = 9) as well as primary (n = 46) and metastatic (n = 71) colorectal cancers relative to normal colon epithelia (all P < 0.0001, versus normal). Similarly, pancreatitis (n = 17), pancreatic intraepithelial neoplasia (n = 80), and pancreatic adenocarcinoma (n = 67) showed a significant up-regulation of FGF-BP1 compared with normal pancreas (n = 42; all P < 0.0001, relative to normal). Furthermore, the biological activity of FGF-BP1 is neutralized by one of the antibodies, suggesting the potential for antibody-based therapeutic targeting. We propose that the up-regulation of the secreted FGF-BP1 protein during initiation of pancreas and colon neoplasia could make this protein a possible serum marker indicating the presence of high-risk premalignant lesions. 
 K-sam-related gene, N-sam, encodes fibroblast growth factor receptor and is expressed in T-lymphocytic tumors. We recently reported the isolation of the K-sam complementary DNA (cDNA), which was amplified preferentially in poorly differentiated types of stomach cancer and codes for one of the heparin-binding growth factor or fibroblast growth factor (FGF) receptor families. The K-sam-related gene, N-sam (NCC-IT-cell-derived sam), was isolated by screening of the cDNA libraries of human immature teratoma cells, NCC-IT. Sequence analysis of the N-sam cDNAs showed that N-sam encodes a human FGF receptor, the FLG protein. N-sam was expressed in lymphocytic leukemia/lymphoma cells, predominantly in the thymic T-cell phenotype. In a T-cell leukemia line, MOLT3, N-sam mRNA expression was markedly enhanced by 12-O-tetradecanoylphorbol-13-acetate treatment and was also up-regulated by basic FGF exposure. These results indicate that N-sam expression is regulated during T-cell ontogeny and modulated by its putative ligand exposure. The results also suggested that interaction between immature T-cell and marrow or thymic interstitial cells might be mediated by N-sam and basic FGF stored in the   extracellular matrix   of stromal cells. 
 Structure of a heparin-linked biologically active dimer of fibroblast growth factor. The fibroblast growth factors (FGFs) form a large family of structurally related, multifunctional proteins that regulate various biological responses. They mediate cellular functions by binding to transmembrane FGF receptors, which are protein tyrosine kinases. FGF receptors are activated by oligomerization, and both this activation and FGF-stimulated biological responses require heparin-like molecules as well as FGF. Heparins are linear anionic polysaccharide chains; they are typically heterogeneously sulphated on alternating L-iduronic and D-glucosamino sugars, and are nearly ubiquitous in animal tissues as heparan sulphate proteoglycans on cell surfaces and in the   extracellular matrix  . Although several crystal structures have been described for FGF molecules in complexes with heparin-like sugars, the nature of a biologically active complex has been unknown until now. Here we describe the X-ray crystal structure, at 2.9 A resolution, of a biologically active dimer of human acidic FGF in a complex with a fully sulphated, homogeneous heparin decassacharide. The dimerization of heparin-linked acidic FGF observed here is an elegant mechanism for the modulation of signalling through combinatorial homodimerization and heterodimerization of the 12 known members of the FGF family. 
 Fibroblast growth factor receptor 1 is a key inhibitor of TGFβ signaling in the endothelium. Abnormal vascular homeostasis can lead to increased proliferation of smooth muscle cells and deposition of   extracellular matri  x, resulting in neointima formation, which contributes to vascular lumen narrowing, a pathology that underlies diseases including transplant vasculopathy, the recurrence of stenosis, and atherosclerosis. Growth of neointima is in part due to endothelial-to-mesenchymal transition (EndMT), a transforming growth factor-β (TGFβ)-driven process, which leads to increased numbers of smooth muscle cells and fibroblasts and deposition   of extracellular mat  rix. We reported that endothelial cell-specific knockout of fibroblast growth factor receptor 1 (FGFR1) led to activation of TGFβ signaling and development of EndMT in vitro and in vivo. Furthermore, EndMT in human diseased vasculature correlated with decreased abundance of FGFR1. These findings identify FGFR1 as the key regulator of TGFβ signaling and EndMT development. 
 Costimulation of T cell receptor-triggered IL-2 production by Jurkat T cells via fibroblast growth factor receptor 1 upon its engagement by CD56. Recent studies have demonstrated that neural cell adhesion molecule (NCAM) is involved in multiple adhesive interactions with several different classes of ligands on the cell surface and in the   extracellular matrix  . One of these ligands is fibroblast growth factor receptor (FGFR) that is expressed on neural cells. While it is known that CD56 is a molecular isoform of NCAM expressed on human NK cells and a subset of T cells, it remains poorly characterized, with its ligand unidentified. Therefore, we were prompted to examine if CD56 molecules on NK cells interact with FGFR expressed on T cells. We demonstrate that ligation of FGFR1 beta on J.C2-14 Jurkat T cells by CD56 on fixed NK-92 cells costimulates TCR/CD3-triggered IL-2 production. CD56-binding mAbs inhibited the costimulatory effect of NK-92 cells in 50-75%. Flow cytometric analysis and cell adhesion assays showed that FGFR1 beta/Fc and FGFR2 beta/Fc chimeric proteins bind to NK-92 cells. The binding of FGFR1 beta/Fc protein to CD56 molecules was verified by immunoprecipitation of CD56 with anti-CD56 mAb followed by Western blotting with FGFR1 beta/Fc. These findings suggest that ligation of FGFR1 by CD56 may contribute to the interaction between NK cells and T cells that we have postulated in our previous studies. 
 Diversity in fibroblast growth factor receptor 1 regulation: learning from the investigation of Kallmann syndrome. The unravelling of the genetic basis of the hypogonadotrophic hypogonadal disorders, including Kallmann syndrome (KS), has led to renewed interest into the developmental biology of gonadotrophin-releasing hormone (GnRH) neurones and, more generally, into the molecular mechanisms of reproduction. KS is characterised by the association of GnRH deficiency with diminished olfaction. Until recently, only two KS-associated genes were known: KAL1 and KAL2. KAL1 encodes the cell membrane and   extracellular matrix  -associated secreted protein anosmin-1 which is implicated in the X-linked form of KS. Anosmin-1 shows high affinity binding to heparan sulphate (HS) and its function remains the focus of ongoing investigation, although a role in axonal guidance and neuronal migration, which are processes essential for normal GnRH ontogeny and olfactory bulb histogenesis, has been suggested. KAL2, identified as the fibroblast growth factor receptor 1 (FGFR1) gene, has now been recognised to be the underlying genetic defect for an autosomal dominant form of KS. The diverse signalling pathways initiated upon FGFR activation can elicit pleiotropic cellular responses depending on the cellular context. Signalling through FGFR requires HS for receptor dimerisation and ligand binding. Current evidence supports a HS-dependent interaction between anosmin-1 and FGFR1, where anosmin-1 serves as a co-ligand activator enhancing the signal activity, the finer details of whose mechanism remain the subject of intense investigation. Recently, mutations in the genes encoding prokineticin 2 (PK2) and prokineticin receptor 2 (PKR2) were reported in a cohort of KS patients, further reinforcing the view of KS as a multigenic trait involving divergent pathways. Here, we review the historical and current understandings of KS and discuss the latest findings from the molecular and cellular studies of the KS-associated proteins, and describe the evidence that suggests convergence of several of these pathways during normal GnRH and olfactory neuronal ontogeny. 
 Effect of FGF-binding protein 3 on vascular permeability. Fibroblast growth factor-binding protein 1 (FGF-BP1 is BP1) is involved in the regulation of embryonic development, tumor growth, and angiogenesis by mobilizing endogenous FGFs from their   extracellular matrix   storage. Here we describe a new member of the FGF-BP family, human BP3. We show that the hBP3 protein is secreted from cells, binds to FGF2 in vitro and in intact cells, and inhibits FGF2 binding to heparin. To determine the function of hBP3 in vivo, hBP3 was transiently expressed in chicken embryos and resulted in > 50% lethality within 24 h because of vascular leakage. The onset of vascular permeability was monitored by recording the extravasation kinetics of FITC-labeled 40-kDa dextran microperfused into the vitelline vein of 3-day-old embryos. Vascular permeability increased as early as 8 h after expression of hBP3. The increased vascular permeability caused by hBP3 was prevented by treatment of embryos with PD173074, a selective FGFR kinase inhibitor. Interestingly, a C-terminal 66-amino acid fragment (C66) of hBP3, which contains the predicted FGF binding domain, still inhibited binding of FGF2 to heparin similar to full-length hBP3. However, expression of the C66 fragment did not increase vascular permeability on its own, but required the administration of exogenous FGF2 protein. We conclude that the FGF binding domain and the heparin binding domain are necessary for the hBP3 interaction with endogenous FGF and the activation of FGFR signaling in vivo. 
  [Ii]nvasive  PubMed  
 Basic fibroblast growth factor in the bone microenvironment enhances cell motility and invasion of Ewing's sarcoma family of tumours by activating the FGFR1-PI3K-Rac1 pathway. Ewing's sarcoma family of tumours (ESFT) is a malignant small round-cell tumour of the bone and soft tissues. It is characterised by a strong tendency to invade and form metastases. The microenvironment of the bone marrow is a large repository for many growth factors, including the basic fibroblast growth factor (bFGF). However, the role of bFGF in the   invasive   and metastatic phenotype of ESFT has not been investigated.The motility and invasion of ESFT cells were assessed by a wound-healing assay, chemotaxis assay, and invasion assay. The expression and activation of FGF receptors (FGFRs) in ESFT cell lines and clinical samples were detected by RT-PCR, western blotting, and immunohistochemistry. The morphology of ESFT cells was investigated by phase-contrast microscopy and fluorescence staining for actin. Activation of Rac1 was analysed by a pull-down assay.bFGF strongly induced the motility and invasion of ESFT cells. Furthermore, FGFR1 was found to be expressed and activated in clinical samples of ESFT. Basic FGF-induced cell motility was mediated through the FGFR1-phosphatidylinositol 3-kinase (PI3K)-Rac1 pathway. Conditioned medium from bone marrow stromal cells induced the motility of ESFT cells by activating bFGF/FGFR1 signalling.The bFGF-FGFR1-PI3K-Rac1 pathway in the bone microenvironment may have a significant role in the invasion and metastasis of ESFT. 
 Basic fibroblast growth factor in the bone microenvironment enhances cell motility and invasion of Ewing's sarcoma family of tumours by activating the FGFR1-PI3K-Rac1 pathway. Ewing's sarcoma family of tumours (ESFT) is a malignant small round-cell tumour of the bone and soft tissues. It is characterised by a strong tendency to invade and form metastases. The microenvironment of the bone marrow is a large repository for many growth factors, including the basic fibroblast growth factor (bFGF). However, the role of bFGF in the   invasive   and metastatic phenotype of ESFT has not been investigated.The motility and invasion of ESFT cells were assessed by a wound-healing assay, chemotaxis assay, and invasion assay. The expression and activation of FGF receptors (FGFRs) in ESFT cell lines and clinical samples were detected by RT-PCR, western blotting, and immunohistochemistry. The morphology of ESFT cells was investigated by phase-contrast microscopy and fluorescence staining for actin. Activation of Rac1 was analysed by a pull-down assay.bFGF strongly induced the motility and invasion of ESFT cells. Furthermore, FGFR1 was found to be expressed and activated in clinical samples of ESFT. Basic FGF-induced cell motility was mediated through the FGFR1-phosphatidylinositol 3-kinase (PI3K)-Rac1 pathway. Conditioned medium from bone marrow stromal cells induced the motility of ESFT cells by activating bFGF/FGFR1 signalling.The bFGF-FGFR1-PI3K-Rac1 pathway in the bone microenvironment may have a significant role in the invasion and metastasis of ESFT. 
 Non  invasive   dynamic fluorescence imaging of human melanomas reveals that targeted inhibition of bFGF or FGFR-1 in melanoma cells blocks tumor growth by apoptosis. Two prominent biological features of the advanced stages of human melanoma are their high degree of vascularity and high-level expression of basic fibroblast growth factor (bFGF) and fibroblast growth factor receptor-1 (FGFR-1). Given these characteristics, human melanoma serves as an ideal model to address an important question regarding the efficacy of angiogenesis-based cancer therapy. To induce tumor growth arrest and regression, does it suffice to block expression of bFGF and/or FGFR-1 in only the melanoma cells, or is it essential to inhibit expression of bFGF and/or FGFR-1 in both the melanoma cells and the melanoma cell-interspersing vasculature?Primary and metastatic human melanomas, grown as subcutaneous tumors in nude mice, were injected twice a week with vector constructs containing the human tyrosinase promoter and antisense- oriented human bFGF or FGFR-1 cDNA. On alternating days, the bFGF and FGFR-1 antisense-targeted tumors received injections of cyanine fluorochrome-conjugated antibodies to a human melanoma and mouse blood vessel marker. Non  invasive  , dynamic fluorescence imaging was used to document the cellular events that took place inside the tumors as the result of blocking expression of bFGF or FGFR-1 in the melanoma cells.In vivo, ex vivo, and in vitro fluorescence imaging of the bFGF and FGFR-1 antisense-targeted tumors demonstrated that inhibiting bFGF and FGFR-1 signaling in only the melanoma cells suffices to inhibit tumor growth due to massive induction of melanoma cell apoptosis.The investigations presented in this study document that inhibiting expression of bFGF or FGFR-1 in only the melanoma cells is as effective in blocking tumor growth as simultaneously inhibiting bFGF or FGFR-1 synthesis in the melanoma cells and the melanoma cell-interspersing vasculature. Furthermore, blocking expression of bFGF or FGFR-1 in the melanoma cells did not lead to activation or increased production of another angiogenic molecule, suggesting the absence of a "salvage pathway" that can circumvent or rescue the blockage of bFGF/FGFR-1 in the melanoma cells. 
 FGF1-FGFR1 axis promotes tongue squamous cell carcinoma (TSCC) metastasis through epithelial-mesenchymal transition (EMT). Increasing evidences suggest a close association between tumor metastasis and the inflammatory factors secreted by tumor microenvironment. It has been reported that epithelial mesenchymal-transition (EMT) plays a significant role during multiple types of tumor metastasis and progression induced by inflammatory factor from tumor microenvironment. Previous researches implied that fibroblast growth factor 1 (FGF1) can promote tumor progression and cause poor prognosis in several types of malignant tumors via interacting with its receptor fibroblast growth factor receptor 1 (FGFR1). However, the effects of FGF1-FGFR1 on tongue squamous cell carcinoma (TSCC) are not yet completely understood. In the present study, we evaluated the effects and function of FGF1-FGFR1 axis on TSCC metastasis. In addition, we investigated whether the EMT pathway is involved in these effects, thus modulating the TSCC progression. The expression of FGFR1 was measured both in tongue cancer cell lines and tissues by qRT-PCR and western blot. We found that FGFR1 was up-regulated in TSCC tissues compared to non-neoplastic tongue tissues. Additionally, overexpression of FGFR1 is positively associated with poor differentiation and metastasis potential. Furthermore, the function of FGF1-FGFR1 was examined in TSCC cell line. The results implied that FGF1 can obviously promote Cal27 cells migration and invasion abilities through FGFR1, while the motile and   invasive   capabilities can be severely attenuated when knockdown the expression of FGFR1 by specific siRNAs. Further investigation results show that FGF1-FGFR1 axis promotes TSCC metastasis by modulating EMT pathway. However, this effect can be inhibited by blocking the FGF1-FGFR1 axis using FGFR1 specific siRNAs. In conclusion, our findings of the present study provide the evidences that FGF1-FGFR1 axis promotes the TSCC metastasis through the EMT pathway. 
 FGFR1 cleavage and nuclear translocation regulates breast cancer cell behavior. FGF-10 and its receptors, FGFR1 and FGFR2, have been implicated in breast cancer susceptibility and progression, suggesting that fibroblast growth factor (FGF) signaling may be co-opted by breast cancer cells. We identify a novel pathway downstream of FGFR1 activation, whereby the receptor is cleaved and traffics to the nucleus, where it can regulate specific target genes. We confirm Granzyme B (GrB) as the protease responsible for cleavage and show that blocking GrB activity stopped FGFR1 trafficking to the nucleus and abrogates the promigratory effect of FGF stimulation. We confirm the in vivo relevance of our findings, showing that FGFR1 localized to the nucleus specifically in invading cells in both clinical material and a three-dimensional model of breast cancer. We identify target genes for FGFR1, which exert significant effects on cell migration and may represent an   invasive   signature. Our experiments identify a novel mechanism by which FGF signaling can regulate cancer cell behavior and provide a novel therapeutic target for treatment of   invasive   breast cancer. 
 Activation of the FGFR1 signalling pathway by the Epstein-Barr virus-encoded LMP1 promotes aerobic glycolysis and transformation of human nasopharyngeal epithelial cells. Non-keratinizing nasopharyngeal carcinoma (NPC) is closely associated with Epstein-Barr virus (EBV) infection. The EBV-encoded latent membrane protein 1 (LMP1) is believed to play an important role in NPC pathogenesis by virtue of its ability to activate multiple cell signalling pathways which collectively promote cell proliferation, transformation, angiogenesis, and   invasive  ness, as well as modulation of energy metabolism. In this study, we report that LMP1 increases cellular uptake of glucose and glutamine, enhances LDHA activity and lactate production, but reduces pyruvate kinase activity and pyruvate concentrations. LMP1 also increases the phosphorylation of PKM2, LDHA, and FGFR1, as well as the expression of PDHK1, FGFR1, c-Myc, and HIF-1α, regardless of oxygen availability. Collectively, these findings suggest that LMP1 promotes aerobic glycolysis. With respect to FGFR1 signalling, LMP1 not only increases FGFR1 expression, but also up-regulates FGF2, leading to constitutive activation of the FGFR1 signalling pathway. Furthermore, two inhibitors of FGFR1 (PD161570 and SU5402) attenuate LMP1-mediated aerobic glycolysis, cellular transformation (proliferation and anchorage-independent growth), cell migration, and invasion in nasopharyngeal epithelial cells, identifying FGFR1 signalling as a key pathway in LMP1-mediated growth transformation. Immunohistochemical staining revealed that high levels of phosphorylated FGFR1 are common in primary NPC specimens and that this correlated with the expression of LMP1. In addition, FGFR1 inhibitors suppress cell proliferation and anchorage-independent growth of NPC cells. Our current findings demonstrate that LMP1-mediated FGFR1 activation contributes to aerobic glycolysis and transformation of epithelial cells, thereby implicating FGF2/FGFR1 signalling activation in the EBV-driven pathogenesis of NPC. 
 Fibroblast growth factor receptor-1 protein expression is associated with prognosis in estrogen receptor-positive/human epidermal growth factor receptor-2-negative primary breast cancer. Recently, research into the development of new targeted therapies has focused on specific genetic alterations to create advanced, more personalized treatment. One of the target genes, fibroblast growth factor receptor-1 (FGFR1), has been reported to be amplified in estrogen receptor (ER)-positive subtype breast cancer, and is considered one possible mechanism of endocrine resistance through cross-talk between ER and growth factor receptor signaling. We performed a comprehensive analysis of FGFR1 at the levels of gene copy number, transcript and protein expression, and examined the relationships between FGFR1 status and clinicopathological parameters, including prognosis in 307 ER-positive/HER2-negative primary breast cancer patients treated with standard care at our institute. Most notably, a high level of FGFR1 protein expression was observed in 85 patients (27.7%), and was positively associated with   invasive   tumor size (P = 0.039). Furthermore, univariate analysis revealed that high FGFR1 protein expression was significantly correlated with poor relapse-free survival rate (P = 0.0019, HR: 2.63, 95% confidence interval: 1.17-5.98), and showed a tendency towards an increase in recurrent events if the observation period extended beyond the 5 years of the standard endocrine treatment term. FGFR1 gain/amplification was found in 43 (14.0%) patients, which was only associated with higher nuclear grade (P = 0.010). No correlation was found between FGFR1 mRNA expression levels and any clinicopathological factors. Overall, the level of FGFR1 protein expression may be a biomarker of ER-positive/HER2-negative primary breast cancer with possible resistance to standard treatment, and may be a useful tool to identify more specific patients who would benefit from FGFR-1 targeted therapy. 
 Activation of FGF receptor signaling promotes invasion of non-small-cell lung cancer. The molecular regulation of metastasis of non-small-cell lung cancer (NSCLC) remains not completely defined. Here we showed significant higher MMP26 in the resected NSCLC than adjacent healthy tissue from the patients. Moreover, a strong correlation between MMP26 and the phosphorylated fibroblast growth factor receptor 1 (FGFR1) was detected. To examine the causal relationship between activated FGFR signaling and MMP26, we studied a human NSCLC cell line, A549. We found that FGF1-induced FGFR1 phosphorylation in A549 cells activated MMP26, resulting in an increase in cancer   invasive  ness. Inhibition of FGFR1 phosphorylation abolished FGF1-stimulated MMP26 activation, suggesting that activation of FGFR signaling pathway in NSCLC promotes cancer metastasis through MMP26. To define the signal transduction cascades downstream of FGFR1 activation for MMP26 activation, we used specific inhibitors for PI3K, ERK/MAPK, and JNK, respectively, to the FGF1-stimulated A549 cells. We found that only inhibition of JNK significantly decreased the activation of MMP26 in response to FGF1 stimulation, suggesting that activation of FGFR1 signaling may activate JNK to activate MMP26 in NSCLC. Our study thus highlights FGFR signaling pathway and MMP26 as novel therapeutic targets for NSCLC therapy. 
 Fibroblast growth factor receptor (FGFR) gene amplifications are rare events in bladder cancer. Activating point mutations and protein overexpression of fibroblast growth factor receptors (FGFRs), especially FGFR3, are frequent events in bladder cancer. Little is known about gene amplifications, therefore we characterized amplification of FGFR1-3 by fluorescence in-situ hybridization (FISH).Tumours of 153 patients (n = 65 pTa low-grade, n = 15 pTa high-grade, n = 37 pT1, n = 20 pT2, n = 10 pT3, n = 6 pT4) were analysed by FISH for FGFR1-3 copy numbers and screened for FGFR3 mutations and immunohistochemical protein expression. Amplifications of FGFR1 were found in 1.6% (two of 122), FGFR2 in 0.8% (one of 121) and FGFR3 in 3.4% (five of 145). All amplifications were high-level amplifications, not overlapping with polysomy. Amplifications were found in papillar  y/papill  ary-invasive tumour parts, and predominantly in tumours with enhanced Ki67 index (>10%), aberrant CK20 expression, and low p53 expression. All FGFR3-amplified samples showed concomitant FGFR3 mutations and FGFR3 protein overexpression. FGFR amplifications were not associated significantly with gender, age, grade or stage in statistical analyses.FGFR amplifications are rare events in bladder cancer, with FGFR3 amplification being the most prevalent (3.4% of cases). Concomitant FGFR3 mutations and protein overexpression indicate that FGFR3-mediated signalling in these tumours would probably be highly active. This patient subgroup may be particularly suited to FGFR-targeted pharmacotherapy. 
 Fibroblast growth factor receptor 1 and cytokeratin 20 expressions and their relation to prognostic variables in bladder cancer. Tumor grade and stage are currently the most important prognostic variables in bladder cancer but establishing additional criteria is still needed for effective treatment.The aim of the study was to assess the expression of fibroblast growth factor receptor 1 (FGFR1) and cytokeratin 20 (CK20) in cancer bladder (CB) and to evaluate their association with the clinicopathological features of the disease.The study included 80 patients diagnosed as bladder cancer of different stages and grades and 80 patients with nonmalignant urothelial diseases of matched age and sex to the malignant group. The expressions of FGFR1 and CK20 in tissue samples were determined by RT-PCR and immunohistochemistry.The expression levels of FGFR1 and CK20 were increased in the malignant group when compared to the control group (P<0.001 for each). Analysis of their expression showed that levels of FGFR1 and CK20 were significantly higher in   invasive   tumor stages (pT2-pT4) than in non-  invasive   stages (pTis, pTa, pT1) (P<0.001). Interestingly, the sensitivity and specificity of combined detection with CK20 and FGFR1 for the differentiation between   invasive   and non-  invasive   stages of bladder cancer reached 97.5% and 92.5%, respectively.Our results determined overexpression of both FGFR1 and CK20 in CB specimens. The alterations in the expression of FGFR1 and CK20 were associated with disease stage and grade. Lastly, combined detection of FGFR1 and CK20 had a high predictive prognostic value in differentiating   invasive   from non-  invasive   carcinoma. 
 Time-resolved FRET reports FGFR1 dimerization and formation of a complex with its effector PLCγ1. In vitro and in vivo imaging of protein tyrosine kinase activity requires minimal  ly invas  ive, molecularly precise optical probes to provide spatiotemporal mechanistic information of dimerization and complex formation with downstream effectors. We present here a construct with genetically encoded, site-specifically incorporated, bioorthogonal reporter that can be selectively labelled with exogenous fluorogenic probes to monitor the structure and function of fibroblast growth factor receptor (FGFR). GyrB.FGFR1KD.TC contains a coumermycin-induced artificial dimerizer (GyrB), FGFR1 kinase domain (KD) and a tetracysteine (TC) motif that enables fluorescent labelling with biarsenical dyes FlAsH-EDT2 and ReAsH-EDT2. We generated bimolecular system for time-resolved FRET (TR-FRET) studies, which pairs FlAsH-tagged GyrB.FGFR1KD.TC and N-terminal Src homology 2 (nSH2) domain of phospholipase Cγ (PLCγ), a downstream effector of FGFR1, fused to mTurquoise fluorescent protein (mTFP). We demonstrated phosphorylation-dependent TR-FRET readout of complex formation between mTFP.nSH2 and GyrB.FGFR1KD.TC. By further application of TR-FRET, we also demonstrated formation of the GyrB.FGFR1KD.TC homodimer by coumermycin-induced dimerization. Herein, we present a spectroscopic FRET approach to facilitate and propagate studies that would provide structural and functional insights for FGFR and other tyrosine kinases. 
 Nuclear translocation of FGFR1 and FGF2 in pancreatic stellate cells facilitates pancreatic cancer cell invasion. Pancreatic cancer is characterised by desmoplasia, driven by activated pancreatic stellate cells (PSCs). Over-expression of FGFs and their receptors is a feature of pancreatic cancer and correlates with poor prognosis, but whether their expression impacts on PSCs is unclear. At the   invasive   front of human pancreatic cancer, FGF2 and FGFR1 localise to the nucleus in activated PSCs but not cancer cells. In vitro, inhibiting FGFR1 and FGF2 in PSCs, using RNAi or chemical inhibition, resulted in significantly reduced cell proliferation, which was not seen in cancer cells. In physiomimetic organotypic co-cultures, FGFR inhibition prevented PSC as well as cancer cell invasion. FGFR inhibition resulted in cytoplasmic localisation of FGFR1 and FGF2, in contrast to vehicle-treated conditions where PSCs with nuclear FGFR1 and FGF2 led cancer cells to invade the underlying extra-cellular matrix. Strikingly, abrogation of nuclear FGFR1 and FGF2 in PSCs abolished cancer cell invasion. These findings suggest a novel therapeutic approach, where preventing nuclear FGF/FGFR mediated proliferation and invasion in PSCs leads to disruption of the tumour microenvironment, preventing pancreatic cancer cell invasion. 
 FGFR1 is amplified during the progression of in situ to   invasive   breast carcinoma. Gene amplification is an important mechanism for activating oncogenes in malignant tumors. Although amplification of HER2, C-MYC, CCND1 and FGFR1 has been reported in breast cancers, their role in the progression of in situ to   invasive   breast carcinoma is unclear. To investigate this question we compared the amplification frequencies of these genes in pure ductal carcinoma in situ (DCIS), DCIS associated with   invasive   carcinoma, and   invasive   carcinoma.We performed fluorescence in situ hybridization of the selected genes on tissue microarrays composed of 179 pure DCIS and 438   invasive   carcinomas. Two hundred and sixteen of the latter had DCIS components, and in those cases we compared gene amplification in the intraductal and   invasive   components of each carcinoma.The rate of amplification of FGFR1 was higher in   invasive   carcinomas than in the pure DCIS, but the opposite was true for HER2 amplification. These findings applied consistently to high-grade tumors, but not to low/intermediate-grade tumors. The amplification status of HER2, C-MYC, CCND1 and FGFR1 was generally similar in the matched   invasive   and DCIS components of the same tumors. However, FGFR1 amplification was more common in the   invasive   components than in the DCIS components. In survival analyses, FGFR1 amplification was found to be an independent prognostic factor for poor disease-free survival for all patients with   invasive   carcinoma and for the hormone receptor-positive subgroup.Amplification of HER2, C-MYC and CCND1 seems to play a role in the early development of breast cancer, but not in its progression. However, the increased frequency of FGFR1 amplification in   invasive   carcinomas compared with pure DCIS and in the   invasive   components of individual tumors, and its association with decreased disease-free survival, suggests a role for FGFR1 amplification in the progression of breast cancer including in situ-to-  invasive   transition, as well as initiation. 
 Expression of basic fibroblast growth factor, its receptors and syndecans in bladder cancer. Basic fibroblast growth factor (bFGF) is a heparin-binding cationic protein involved in a variety of pathological conditions including angiogenesis and solid tumour growth. The basic fibroblast growth factor receptor (FGFR) family comprises at least 4 high affinity tyrosine kinase receptors that require syndecans for their function. Mounting evidence indicates that syndecans, that bind both bFGF and their FGFRs, will act as stimulators, whereas syndecans that only bind bFGF will act as inhibitors of signaling by sequestering the growth factor. Recent findings have highlighted the importance of syndecans in urological cancers. The aim of this study is to investigate the expression of bFGF, its receptors (R1 and R2) and syndecans (1-4) in   invasive   urothelial carcinoma and normal-looking urothelium by Western blotting, RT-PCR, and immunohistochemistry analyses. Interestingly, bFGF, FGFR1 and FGFR2 protein levels statistically increased in bladder cancer tissues. mRNA of FGFR1 and syndecans (1-4), showed a statistically significant increase while an mRNA increase in the other molecules analysed was not significant. bFGF, its receptors and syndecan immunostaining were mainly present in the urothelium both in normal-looking tissues and urothelial neoplastic cells. In conclusion, our data report that the bFGF, FGFR and syndecan expressions are altered in bladder tumours. 
 Differential expression of receptor tyrosine kinases (RTKs) and IGF-I pathway activation in human uterine leiomyomas. Uterine leiomyomas (fibroids) are benign tumors that are prevalent in women of reproductive age. Research suggests that activated receptor tyrosine kinases (RTKs) play an important role in the enhanced proliferation observed in fibroids. In this study, a phospho-RTK array technique was used to detect RTK activity in leiomyomas compared with myometrial tissue. We found that fifteen out of seventeen RTKs evaluated in this study were highly expressed (P < 0.02-0.03) in the leiomyomas, and included the IGF-I/IGF-IR, EGF/EGFR, FGF/FGF-R, HGF/HGF-R, and PDGF/PDGF-R gene families. Due to the higher protein levels of IGF-IR observed in leiomyomas by us in earlier studies, we decided to focus on the activation of the IGF-IR, its downstream effectors, and MAPKp44/42 to confirm our earlier findings; and validate the significance of the increased IGF-IR phosphorylation observed by RTK array analysis in this study. We used immunolocalization, western blot, or immunoprecipitation studies and confirmed that leiomyomas overexpressed IGF-IRbeta and phosphorylated IGF-IRbeta. Additionally, we showed that the downstream effectors, Shc, Grb2, and MAPKp44/42 (P < 0.02-0.001) were also overexpressed and involved in IGF-IR signaling in these tumors, while IRS-I, PI3K, and AKT were not. In vitro studies showed that IGF-I (100 ng/mL) increased the proliferation of uterine leiomyoma cells (UtLM) (P < 0.0001), and that phosphorylated IGF-IRbeta, Shc, and MAPKp44/42 were also overexpressed in IGF-I-treated UtLM cells (P < 0.05), similar to the tissue findings. A neutralizing antibody against the IGF-IRbeta blocked these effects. These data indicate that overexpression of RTKs and, in particular, activation of the IGF-IR signaling pathway through Shc/Grb2/MAPK are important in mediating uterine leiomyoma growth. These data may provide new anti-tumor targets for non  invasive   treatment of fibroids. 
  GeneRIFs  
 FGFR1 is amplified during the progression of in situ to   invasive   breast carcinoma. 
  [Mm]etastasize  PubMed  
 Growth factors in tumor microenvironment. Tumor microenvironment plays a critical role in tumor initiation and progression. Components in the microenvironment can modulate the growth of tumor cells, their ability to progress and   metastasize  . A major venue of communication between tumor cells and their microenvironment is through polypeptide growth factors and receptors for these growth factors. This article discusses three major classes of growth-stimulatory polypeptide growth factors and receptors for these growth factors. It also discusses how deregulation of these growth factors or their receptors can drive malignant transformation and progression. 
 Growth factors in tumor microenvironment. Tumor microenvironment plays a critical role in tumor initiation and progression. Components in the microenvironment can modulate the growth of tumor cells, their ability to progress and   metastasize  . A major venue of communication between tumor cells and their microenvironment is through polypeptide growth factors and receptors for these growth factors. This article discusses three major classes of growth-stimulatory polypeptide growth factors and receptors for these growth factors. It also discusses how deregulation of these growth factors or their receptors can drive malignant transformation and progression. 
 Fibroblast growth factor receptor 1 gene amplification in gastric adenocarcinoma. Gastric adenocarcinomas are associated with a poor prognosis due to the fact that the tumor has often   metastasize  d by the time of diagnosis. Thus, identification of novel therapeutic targets is highly desirable. Here, we examined gene copy number of fibroblast growth factor receptor 1 (FGFR1), a potential target for tyrosine kinase inhibitors, and clinicopathologic parameters in a large cohort of gastric adenocarcinomas. We performed fluorescence in situ hybridization analysis of 293 gastric adenocarcinomas using tissue microarrays. Amplification of the FGFR1 gene is a rare but noticeable event that can be found in 2% (6/293) of cases and was associated with poor 10-year survival (median 15.3 months in FGFR1-amplified cases versus 36 months in nonamplified cases, P = .047) and a higher rate of distant metastasis (P = .025). FGFR1 appears to represent a potential new therapeutic target in a subset of patients with gastric carcinoma. Identification of gastric cancers harboring FGFR1 amplification may be important in preselecting patients and/or interpreting clinical studies using tyrosine kinase inhibitors. 
  [Mm]etastasis  PubMed  
 Basic fibroblast growth factor in the bone microenvironment enhances cell motility and invasion of Ewing's sarcoma family of tumours by activating the FGFR1-PI3K-Rac1 pathway. Ewing's sarcoma family of tumours (ESFT) is a malignant small round-cell tumour of the bone and soft tissues. It is characterised by a strong tendency to invade and form metastases. The microenvironment of the bone marrow is a large repository for many growth factors, including the basic fibroblast growth factor (bFGF). However, the role of bFGF in the invasive and metastatic phenotype of ESFT has not been investigated.The motility and invasion of ESFT cells were assessed by a wound-healing assay, chemotaxis assay, and invasion assay. The expression and activation of FGF receptors (FGFRs) in ESFT cell lines and clinical samples were detected by RT-PCR, western blotting, and immunohistochemistry. The morphology of ESFT cells was investigated by phase-contrast microscopy and fluorescence staining for actin. Activation of Rac1 was analysed by a pull-down assay.bFGF strongly induced the motility and invasion of ESFT cells. Furthermore, FGFR1 was found to be expressed and activated in clinical samples of ESFT. Basic FGF-induced cell motility was mediated through the FGFR1-phosphatidylinositol 3-kinase (PI3K)-Rac1 pathway. Conditioned medium from bone marrow stromal cells induced the motility of ESFT cells by activating bFGF/FGFR1 signalling.The bFGF-FGFR1-PI3K-Rac1 pathway in the bone microenvironment may have a significant role in the invasion and   metastasis   of ESFT. 
 Basic fibroblast growth factor in the bone microenvironment enhances cell motility and invasion of Ewing's sarcoma family of tumours by activating the FGFR1-PI3K-Rac1 pathway. Ewing's sarcoma family of tumours (ESFT) is a malignant small round-cell tumour of the bone and soft tissues. It is characterised by a strong tendency to invade and form metastases. The microenvironment of the bone marrow is a large repository for many growth factors, including the basic fibroblast growth factor (bFGF). However, the role of bFGF in the invasive and metastatic phenotype of ESFT has not been investigated.The motility and invasion of ESFT cells were assessed by a wound-healing assay, chemotaxis assay, and invasion assay. The expression and activation of FGF receptors (FGFRs) in ESFT cell lines and clinical samples were detected by RT-PCR, western blotting, and immunohistochemistry. The morphology of ESFT cells was investigated by phase-contrast microscopy and fluorescence staining for actin. Activation of Rac1 was analysed by a pull-down assay.bFGF strongly induced the motility and invasion of ESFT cells. Furthermore, FGFR1 was found to be expressed and activated in clinical samples of ESFT. Basic FGF-induced cell motility was mediated through the FGFR1-phosphatidylinositol 3-kinase (PI3K)-Rac1 pathway. Conditioned medium from bone marrow stromal cells induced the motility of ESFT cells by activating bFGF/FGFR1 signalling.The bFGF-FGFR1-PI3K-Rac1 pathway in the bone microenvironment may have a significant role in the invasion and   metastasis   of ESFT. 
 Fibroblast growth factor receptor splice variants are stable markers of oncogenic transforming growth factor β1 signaling in metastatic breast cancers. Epithelial-mesenchymal transition (EMT) and mesenchymal-epithelial transition (MET) facilitate breast cancer (BC)   metastasi  s; however, stable molecular changes that result as a consequence of these processes remain poorly defined. Therefore, with the hope of targeting unique aspects of metastatic tumor outgrowth, we sought to identify molecular markers that could identify tumor cells that had completed the EMT:MET cycle.An in vivo reporter system for epithelial cadherin (E-cad) expression was used to quantify its regulation in metastatic BC cells during primary and metastatic tumor growth. Exogenous addition of transforming growth factor β1 (TGF-β1) was used to induce EMT in an in situ model of BC. Microarray analysis was employed to examine gene expression changes in cells chronically treated with and withdrawn from TGF-β1, thus completing one full EMT:MET cycle. Changes in fibroblast growth factor receptor type 1 (FGFR1) isoform expression were validated using PCR analyses of patient-derived tumor tissues versus matched normal tissues. FGFR1 gene expression was manipulated using short hairpin RNA depletion and cDNA rescue. Preclinical pharmacological inhibition of FGFR kinase was employed using the orally available compound BGJ-398.Metastatic BC cells undergo spontaneous downregulation of E-cad during primary tumor growth, and its expression subsequently returns following initiation of metastatic outgrowth. Exogenous exposure to TGF-β1 was sufficient to drive   the metas  tasis of an otherwise in situ model of BC and was similarly associated with a depletion and return of E-cad expression during metastatic progression. BC cells treated and withdrawn from TGF-β stably upregulate a truncated FGFR1-β splice variant that lacks the outermost extracellular immunoglobulin domain. Identification of this FGFR1 splice variant was verified in metastatic human BC cell lines and patient-derived tumor samples. Expression of FGFR1-β was also dominant in a model of metastatic outgrowth where depletion of FGFR1 and pharmacologic inhibition of FGFR kinase activity both inhibited pulmonary tumor outgrowth. Highlighting the dichotomous nature of FGFR splice variants and recombinant expression of full-length FGFR1-α also blocked pulmonary tumor outgrowth.The results of our study strongly suggest that FGFR1-β is required for the pulmonary outgrowth of metastatic BC. Moreover, FGFR1 isoform expression can be used as a predictive biomarker for therapeutic application of its kinase inhibitors. 
 Long-Pentraxin 3 Derivative as a Small-Molecule FGF Trap for Cancer Therapy. The fibroblast growth factor (FGF)/FGF receptor (FGFR) system plays a crucial role in cancer by affecting tumor growth, angiogenesis, drug resistance, and escape from anti-angiogenic anti-vascular endothelial growth factor therapy. The soluble pattern recognition receptor long-pentraxin 3 (PTX3) acts as a multi-FGF antagonist. Here we demonstrate that human PTX3 overexpression in transgenic mice driven by the Tie2 promoter inhibits tumor growth, angiogenesis, and   metastasis   in heterotopic, orthotopic, and autochthonous FGF-dependent tumor models. Using pharmacophore modeling of the interaction of a minimal PTX3-derived FGF-binding pentapeptide with FGF2, we identified a small-molecule chemical (NSC12) that acts as an extracellular FGF trap with significant implications in cancer therapy. 
 FGF1-FGFR1 axis promotes tongue squamous cell carcinoma (TSCC)   metastasis   through epithelial-mesenchymal transition (EMT). Increasing evidences suggest a close association between tumor   metastasis   and the inflammatory factors secreted by tumor microenvironment. It has been reported that epithelial mesenchymal-transition (EMT) plays a significant role during multiple types of tumor   metastasis   and progression induced by inflammatory factor from tumor microenvironment. Previous researches implied that fibroblast growth factor 1 (FGF1) can promote tumor progression and cause poor prognosis in several types of malignant tumors via interacting with its receptor fibroblast growth factor receptor 1 (FGFR1). However, the effects of FGF1-FGFR1 on tongue squamous cell carcinoma (TSCC) are not yet completely understood. In the present study, we evaluated the effects and function of FGF1-FGFR1 axis on TSCC   metastasis  . In addition, we investigated whether the EMT pathway is involved in these effects, thus modulating the TSCC progression. The expression of FGFR1 was measured both in tongue cancer cell lines and tissues by qRT-PCR and western blot. We found that FGFR1 was up-regulated in TSCC tissues compared to non-neoplastic tongue tissues. Additionally, overexpression of FGFR1 is positively associated with poor differentiation and   metastasis   potential. Furthermore, the function of FGF1-FGFR1 was examined in TSCC cell line. The results implied that FGF1 can obviously promote Cal27 cells migration and invasion abilities through FGFR1, while the motile and invasive capabilities can be severely attenuated when knockdown the expression of FGFR1 by specific siRNAs. Further investigation results show that FGF1-FGFR1 axis promotes TSCC   metastasis   by modulating EMT pathway. However, this effect can be inhibited by blocking the FGF1-FGFR1 axis using FGFR1 specific siRNAs. In conclusion, our findings of the present study provide the evidences that FGF1-FGFR1 axis promotes the TSCC   metastasis   through the EMT pathway. 
 FGFR1 amplification in lung squamous cell carcinoma: a systematic review with meta-analysis. Current targeted therapy proves no effective outcomes in lung squamous cell carcinoma (SQCC). Recent studies suggested that FGFR1 would be promising. This systematic review elaborated FGFR1 amplification in lung SQCC.An electronic search was conducted on PubMed, EMBASE, Web of SCI, Google Scholar and Cochrane Library. Eligible studies regarding incidence of FGFR1 amplification in lung SQCC and correlation between FGFR1 amplification and clinicopathological features or survival were extracted and analyzed.We identified 13 eligible studies with a total of 1798 patients. The results showed about 19% of FGFR1 amplification (95% CI: 0.15-0.24; I(2)=84.5%; p=0.000). Using the same test method: FISH, definition and ethnicity, the rates were 17% (95% CI: 0.14-0.20; I(2)=53.1%; p=0.037), 21% (95% CI: 0.18-0.24; I(2)=0; p=0.615), and 16% (95% CI: 0.13-0.19; I(2)=72.1%; p=0.028), respectively. Pearson's correlation analysis suggested that smoking status was highly correlated with FGFR1 amplification (coefficient=0.961, p<0.001). FGFR1 amplification was significantly correlated with lymph node   metastasis   (OR: 2.27; 95% CI: 1.62-3.20; p=0.000), but not correlated with gender (OR: 1.12; 95% CI: 0.90-1.38; p=0.91), differentiation (OR: 1.02; 95% CI: 0.76-1.38; p=0.959) and stage (OR: 0.93; 95% CI: 0.73-1.19; p=0.877) in lung SQCC patients. With respect to survival, FGFR1 amplification had no influence on PFS (HR: 1.57; 95% CI: 0.85-2.30; p=0.259) and OS (HR: 1.40; 95% CI: 0.90-1.89; p=0.416) for SQCC patients.FGFR1 amplification is about 19%. Gender, stage, differentiation, ethnicities and test methods have no influence on FGFR1 amplification. FGFR1 amplification trends to correlate with lymph node   metastasis   and smoking. Whether FGFR1 amplification has effect on survival remains controversial. 
 Inhibition of FGFR signaling by PD173074 improves antitumor immunity and impairs breast cancer   metastasis  . Aberrant fibroblast growth factor (FGF) and FGF receptor (FGFR) system have been associated with breast cancer. The objectives of our study were to investigate the effects and mechanisms of FGFR inhibition on tumor growth and   metastasis   on breast cancer. Our studies showed that the FGFR inhibitor PD173074 decreased the viability of several human breast cancer cells, as well as 4T1 murine mammary tumor cells. Therefore, we chose 4T1 cells to study PD173074's antitumor mechanism. Flow cytometry showed that PD173074 induced 4T1 cell apoptosis in a concentration-dependent manner. Western blot demonstrated that PD173074-induced apoptosis was correlated with the inhibition of Mcl-1 and survivin. Moreover, PD173074 also significantly increased the ratio of Bax/Bcl-2. PD173074 could also block 4T1 cell migration and invasion in vitro. In 4T1 tumor-bearing mice, PD173074 significantly inhibited tumor growth without obvious side effects. Meanwhile, PD173074 functionally reduced microvessel density and proliferation index and induced tumor apoptosis. Importantly, we found that FGFR inhibition by PD173074 reduced myeloid-derived suppressor cells (MDSCs) in the blood, spleens and tumors, accompanied by the increased infiltration of CD4(+) and CD8(+) T cells in the spleens and tumors. Furthermore, PD173074 significantly inhibited breast tumor   metastasis   to the lung of inoculated 4T1 breast cancer cells, which was accompanied by a reduction in MDSCs. Our findings suggested that FGFR inhibition could delay breast tumor progression, impair lung   metastasis   and break immunosuppression by effecting on tumor microenvironment, which may provide a promising therapeutic approach for breast cancer patient. 
 Enhanced Expression of Integrin αvβ3 Induced by TGF-β Is Required for the Enhancing Effect of Fibroblast Growth Factor 1 (FGF1) in TGF-β-Induced Epithelial-Mesenchymal Transition (EMT) in Mammary Epithelial Cells. Epithelial-to-mesenchymal transition (EMT) plays a critical role in can  cer metast  asis, and is regulated by growth factors such as transforming growth factor β (TGF-β) and fibroblast growth factors (FGF) secreted from the stromal and tumor cells. However, the role of growth factors in EMT has not been fully established. Several integrins are upregulated by TGF-β1 during EMT. Integrins are involved in growth factor signaling through integrin-growth factor receptor crosstalk. We previously reported that FGF1 directly binds to integrin αvβ3 and the interaction was required for FGF1 functions such as cell proliferation and migration. We studied the role of αvβ3 induced by TGF-β on TGF-β-induced EMT. Here, we describe that FGF1 augmented EMT induced by TGF-β1 in MCF10A and MCF12A mammary epithelial cells. TGF-β1 markedly amplified integrin αvβ3 and FGFR1 (but not FGFR2). We studied if the enhancing effect of FGF1 on TGF-β1-induced EMT requires enhanced levels of both integrin αvβ3 expression and FGFR1. Knockdown of β3 suppressed the enhancement by FGF1 of TGF-β1-induced EMT in MCF10A cells. Antagonists to FGFR suppressed the enhancing effect of FGF1 on EMT. Integrin-binding defective FGF1 mutant did not augment TGF-β1-induced EMT in MCF10A cells. These findings suggest that enhanced integrin αvβ3 expression in addition to enhanced FGFR1 expression is critical for FGF1 to augment TGF-β1-induced EMT in mammary epithelial cells. 
 Activation of FGF receptor signaling promotes invasion of non-small-cell lung cancer. The molecular regulation of   metastasis   of non-small-cell lung cancer (NSCLC) remains not completely defined. Here we showed significant higher MMP26 in the resected NSCLC than adjacent healthy tissue from the patients. Moreover, a strong correlation between MMP26 and the phosphorylated fibroblast growth factor receptor 1 (FGFR1) was detected. To examine the causal relationship between activated FGFR signaling and MMP26, we studied a human NSCLC cell line, A549. We found that FGF1-induced FGFR1 phosphorylation in A549 cells activated MMP26, resulting in an increase in cancer invasiveness. Inhibition of FGFR1 phosphorylation abolished FGF1-stimulated MMP26 activation, suggesting that activation of FGFR signaling pathway in NSCLC promotes cancer   metastasis   through MMP26. To define the signal transduction cascades downstream of FGFR1 activation for MMP26 activation, we used specific inhibitors for PI3K, ERK/MAPK, and JNK, respectively, to the FGF1-stimulated A549 cells. We found that only inhibition of JNK significantly decreased the activation of MMP26 in response to FGF1 stimulation, suggesting that activation of FGFR1 signaling may activate JNK to activate MMP26 in NSCLC. Our study thus highlights FGFR signaling pathway and MMP26 as novel therapeutic targets for NSCLC therapy. 
 Overexpression of the fibroblast growth factor receptor-1 gene correlates with liver   metastasis   in colorectal cancer. Expression of the fibroblast growth factor (FGF)-1, FGF-2, fibroblast growth factor receptor (FGFR)-1, and FGFR-2 genes has been reported in various cancers and is associated with poor outcomes in patients with solid tumors. This study examined the relations between the relative expression of the FGF genes and clinicopathological factors, especially invasion and   metastasis  , in patients with colorectal cancer. We studied surgical specimens of cancer tissue and adjacent normal mucosa obtained from 202 patients with untreated colorectal carcinoma. The relative expression levels of FGF-1, FGF-2, FGFR-1, and FGFR-2 mRNA in cancer and in normal adjacent mucosa were measured by quantitative real-time, reverse-transcription polymerase chain reaction. The relative expression level of the FGFR-2 gene was higher in normal adjacent mucosa than in cancer, whereas the relative expression levels of the FGF-1, FGF-2, and FGFR-1 genes were similar. FGFR-1 gene expression levels were higher in the presence than in the absence of liver   metastasis  . An analysis of the relation between clinicopathological features and gene expression showed that overexpression of FGFR-1 correlated with liver   metastasis  . Our results suggested that overexpression of the FGFR-1 gene might lead to liver   metastasis   in colorectal cancer. Overexpression of the FGFR-1 gene may thus be a useful predictor of liver   metastasis   in patients with colorectal cancer. 
 FGFR1-WNT-TGF-β signaling in prostate cancer mouse models recapitulates human reactive stroma. The reactive stroma surrounding tumor lesions performs critical roles ranging from supporting tumor cell proliferation to inducing tumorigenesis and   metastasi  s. Therefore, it is critical to understand the cellular components and signaling control mechanisms that underlie the etiology of reactive stroma. Previous studies have individually implicated fibroblast growth factor receptor 1 (FGFR1) and canonical WNT/β-catenin signaling in prostate cancer progression and the initiation and maintenance of a reactive stroma; however, both pathways are frequently found to be coactivated in cancer tissue. Using autochthonous transgenic mouse models for inducible FGFR1 (JOCK1) and prostate-specific and ubiquitously expressed inducible β-catenin (Pro-Cat and Ubi-Cat, respectively) and bigenic crosses between these lines (Pro-Cat × JOCK1 and Ubi-Cat × JOCK1), we describe WNT-induced synergistic acceleration of FGFR1-driven adenocarcinoma, associated with a pronounced fibroblastic reactive stroma activation surrounding prostatic intraepithelial neoplasia (mPIN) lesions found both in in situ and reconstitution assays. Both mouse and human reactive stroma exhibited increased transforming growth factor-β (TGF-β) signaling adjacent to pathologic lesions likely contributing to invasion. Furthermore, elevated stromal TGF-β signaling was associated with higher Gleason scores in archived human biopsies, mirroring murine patterns. Our findings establish the importance of the FGFR1-WNT-TGF-β signaling axes as driving forces behind reactive stroma in aggressive prostate adenocarcinomas, deepening their relevance as therapeutic targets. 
 FGFR1-induced epithelial to mesenchymal transition through MAPK/PLCγ/COX-2-mediated mechanisms. Tumour invasion and   metastasi  s is the most common cause of death from cancer. For epithelial cells to invade surrounding tissues and   metastasi  se, an epithelial-mesenchymal transition (EMT) is required. We have demonstrated that FGFR1 expression is increased in bladder cancer and that activation of FGFR1 induces an EMT in urothelial carcinoma (UC) cell lines. Here, we created an in vitro FGFR1-inducible model of EMT, and used this model to identify regulators of urothelial EMT. FGFR1 activation promoted EMT over a period of 72 hours. Initially a rapid increase in actin stress fibres occurred, followed by an increase in cell size, altered morphology and increased migration and invasion. By using site-directed mutagenesis and small molecule inhibitors we demonstrated that combined activation of the mitogen activated protein kinase (MAPK) and phospholipase C gamma (PLCγ) pathways regulated this EMT. Actin stress fibre formation was regulated by PLCγ activation, and was also important for the increase in cell size, migration and altered morphology. MAPK activation regulated migration and E-cadherin expression, indicating that combined activation of PLCγ and MAPK is required for a full EMT. We used expression microarrays to assess changes in gene expression downstream of these signalling cascades. COX-2 was transcriptionally upregulated by FGFR1 and caused increased intracellular prostaglandin E(2) levels, which promoted migration. In conclusion, we have demonstrated that FGFR1 activation in UC cells lines promotes EMT via coordinated activation of multiple signalling pathways and by promoting activation of prostaglandin synthesis. 
 High rate of FGFR1 amplifications in brain metastases of squamous and non-squamous lung cancer. FGFR1 amplifications are common in squamous cell carcinoma and rare in adenocarcinoma of the lung, but have not been investigated in brain metastases of non-small cell lung cancer (NSCLC).We performed fluorescent in situ hybridization (FISH) for FGFR1 and immunohistochemistry for pAKT, PI3K, HIF1a and Ki67 in 175 NSCLC brain metastases and 11 matched primary tumors. ALK gene rearrangement status was available from a previous study. We performed statistical correlations of clinical, histopathological and molecular data.FGFR1 amplifications were found in a total of 30/175 (17%) brain metastases: 4/21 (19%) squamous cell carcinomas, 20/130 (15.3%) adenocarcinomas, 2/12 (16.6%) adenosquamous carcinomas, 4/9 (44.4%) large cell carcinomas and 0/3 neuroendocrine large cell carcinoma. FGFR1 gene status was identical between primary tumors and brain metastases in 9/11 evaluable cases. In 2/11 cases (1 adenosquamous and 1 large cell carcinoma), FGFR1 amplifications were present only in the brain   metastasis   and not in the primary tumor. Furthermore, we found a significant positive correlation of ALK and FGFR1 gene amplification status in brain metastases (p<0.001, Chi square test). Patients with high-level FGFR1 amplifications had significantly higher number of visceral metastases (p<0.001, Chi square test).Our findings argue for an enrichment of FGFR1 amplifications in brain metastases of adenocarcinomas (where they were 5-fold more frequent than reported for primary tumors) and possibly also of other non-squamous carcinomas, but not in squamous cell carcinomas of the lung. These results may be relevant for targeted therapy and prophylaxis of NSCLC brain metastases. 
 Targeting FGFR Pathway in Human Hepatocellular Carcinoma: Expressing pFGFR and pMET for Antitumor Activity. The MET receptor tyrosine kinase, the receptor for hepatocyte growth factor (HGF), has been implicated in cancer growth, invasion, migration, angiogenesis, and   metastasis   in a broad variety of human cancers, including human hepatocellular carcinoma (HCC). Recently, MET was suggested to be a potential target for the personalized treatment of HCC with an active HGF-MET signaling pathway. However, the mechanisms of resistance to MET inhibitors need to be elucidated to provide effective treatment. Here, we show that HCC cells exhibit different sensitivities to the MET inhibitor PHA665752, depending on the phosphorylation status of FGFR. Treatment of cells expressing both phospho-FGFR and phospho-MET with the inhibitor PHA665752 did not cause growth inhibition and cell death, whereas treatment with AZD4547, a pan-FGFR inhibitor, resulted in decreased colony formation and cleavage of caspase-3. Moreover, silencing of endogenous FGFR1 and FGFR2 by RNAi of HCC cells expressing phospho-FGFR, phospho-FGFR2, and phospho-MET overcame the resistance to PHA665752 treatment. Treatment of primary cancer cells from patients with HCC expressing both phospho-FGFR and phospho-MET with PHA665752 did not induce cell death, whereas AZD4547 treatment induced cell death through the cleavage of caspase-3. In addition, treatment of cells resistant to PHA665752 with AZD4547 abrogated the activation of downstream effectors of cell growth, proliferation, and survival. On the basis of these results, we conclude that the FGFR pathway is critical for HCC survival, and that targeting this pathway with AZD4547 may be beneficial for the treatment of patients with HCC-expressing phospho-FGFR and phospho-MET. 
 Rationale for treatment of metastatic squamous cell carcinoma of the lung using fibroblast growth factor receptor inhibitors. We previously identified amplification of the fibroblast growth factor receptor 1 gene (FGFR1) as a potential therapeutic target for small-molecule inhibitor therapy in squamous cell lung cancer (L-SCC). Currently, clinical phase I trials are underway to examine whether patients with FGFR1-amplified L-SCC benefit from a targeted therapy approach using small-molecule inhibitors. Because most patients with lung cancer present with metastatic disease, we investigated whether lymph node metastases in L-SCC share the FGFR1 amplification status of their corresponding primary tumor.The study cohort consisted of 72 patients with L-SCC, 39 with regional lymph node metastases. Tissue microarrays were constructed from formalin-fixed, paraffin-embedded tissue of the primary tumors and, where present, of the corresponding lymph node   metastasis  . A biotin-labeled target probe spanning the FGFR1 locus (8p11.22-23) was used to determine the FGFR1 amplification status by fluorescence in situ hybridization.FGFR1 amplification was detected in 16% (12 of 72) of all primary L-SCCs. In metastatic tumors, 18% (seven of 39) of the lymph node metastases displayed FGFR1 amplification with an exact correlation of FGFR1 amplification status between tumor and metastatic tissue.FGFR1 amplification is a common genetic event occurring at a frequency of 16% in L-SCCs. Moreover, lymph node metastases derived from FGFR1-amplified L-SCCs also exhibit FGFR1 amplification. Therefore, we suggest that the FGFR1 amplification is a clonal event in tumor progression. Beyond this biologically relevant observation, the findings carry potential therapeutic implications in that small-molecule inhibitors may be applicable to the treatment of a subset of patients with metastatic L-SCC. 
 Downregulation of microRNA-214 and overexpression of FGFR-1 contribute to hepatocellular carcinoma   metastasis  . miR-214 is one of the most significantly downregulated microRNAs (miRNAs) in hepatocellular carcinoma (HCC). Fibroblast growth factor receptor 1 (FGFR-1) is a miR-214 target gene implicated in the progression of HCC. However, the roles of miR-214 and FGFR-1 in HCC are not fully understood. Here, we analyzed the expression of miR-214 and FGFR-1 in 65 cases of HCC and paired non-neoplastic tissue specimens using real-time PCR and Western blot (WB), respectively. Our data indicated that miR-214 was downregulated and FGFR-1 was overexpressed in HCC compared to the paired non-neoplastic tissues. The low miR-214 expression was correlated with portal vein invasion (p=0.016) and early recurrence (p=0.045) in HCC patients. Moreover, the low miR-214 expression was correlated with high positive rate of FGFR-1 in HCC cases (p=0.020). Our data further demonstrated that miR-214 overexpression in SK-HEP1 and HepG2 cells downregulated FGFR-1 expression and inhibited liver cancer cell invasion. The Luciferase assay results further demonstrated the targeted regulation of FGFR-1 by miR-214. In conclusion, our data indicate that the downregulation of miR-214 in HCC and the upregulation of its target gene FGFR-1 is associated with HCC progression. Therefore, miR-214 and FGFR-1 are potential prognostic markers and therapeutic targets in HCC. 
 MUC4 potentiates invasion and   metastasis   of pancreatic cancer cells through stabilization of fibroblast growth factor receptor 1. MUC4 is a type-1 transmembrane mucin differentially expressed in multiple cancers and has previously been shown to potentiate progression and   metastasis   of pancreatic cancer. In this study, we investigated the molecular mechanisms associated with the MUC4-induced invasion and   metastasis   in pancreatic cancer. Stable silencing of MUC4 in multiple pancreatic cancer cells resulted in the downregulation of N-cadherin and its interacting partner fibroblast growth factor receptor 1 (FGFR1) through downregulation of partly by pFAK, pMKK7, pJNK and pc-Jun pathway and partly through PI-3K/Akt pathway. The downregulation of FGFR1 in turn led to downregulation of pAkt, pERK1/2, pNF-κB, pIkBα, uPA, MMP-9, vimentin, N-cadherin, Twist, Slug and Zeb1 and upregulation of E-cadherin, Occludin, Cytokeratin-18 and Caspase-9 in MUC4 knockdown BXPC3 and Capan1 cells compared with scramble vector transfected cells. Further, downregulation of FGFR1 was associated with a significant change in morphology and reorganization of the actin-cytoskeleton, leading to a significant decrease in motility (P < 0.00001) and invasion (P < 0.0001) in vitro and decreased tumorigenicity and incidence o  f metastas  is in vivo upon orthotopic implantation in the athymic mice. Taken together, the results of the present study suggest that MUC4 promotes invasion an  d metastas  is by FGFR1 stabilization through the N-cadherin upregulation. 
 Combinatorial targeting of FGF and ErbB receptors blocks growth and metastatic spread of breast cancer models. Targeting receptor tyrosine kinases (RTKs) with kinase inhibitors is a clinically validated anti-cancer approach. However, blocking one signaling pathway is often not sufficient to cause tumor regression and the effectiveness of individual inhibitors is often short-lived. As alterations in fibroblast growth factor receptor (FGFR) activity have been implicated in breast cancer, we examined in breast cancer models with autocrine FGFR activity the impact of targeting FGFRs in vivo with a selective kinase inhibitor in combination with an inhibitor of PI3K/mTOR or with a pan-ErbB inhibitor.Using 4T1 or 67NR models of basal-like breast cancer, tumor growth was measured in mice treated with an FGFR inhibitor (dovitinib/TKI258), a PI3K/mTOR inhibitor (NVP-BEZ235) or a pan-ErbB inhibitor (AEE788) individually or in combination. To uncover mechanisms underlying inhibitor action, signaling pathway activity was examined in tumor lysates and transcriptome analysis carried out to identify pathways upregulated by FGFR inhibition. Anti-phosphotyrosine receptor antibody arrays (P-Tyr RTK) were also used to screen 4T1 tumors.The combination of dovitinib + NVP-BEZ235 causes tumor stasis and strong down-regulation of the FRS2/Erk and PI3K/Akt/mTOR signaling pathways. P-Tyr RTK arrays identified high levels of P-EGFR and P-ErbB2 in 4T1 tumors. Testing AEE788 in the tumor models revealed that the combination of dovitinib + AEE788 resulted in blockade of the PI3K/Akt/mTOR pathway, prolonged tumor stasis and in the 4T1 model, a significant decrease in lung   metastasis  . The results show that in vivo these breast cancer models become dependent upon co-activation of FGFR and ErbB receptors for PI3K pathway activity.The work presented here shows that in the breast cancer models examined, the combination of dovitinib + NVP-BEZ235 or dovitinib + AEE788 results in strong inhibition of tumor growth and a block in metastatic spread. Only these combinations strongly down-regulate the FGFR/FRS2/Erk and PI3K/Akt/mTOR signaling pathways. The resultant decrease in mitosis and increase in apoptosis was consistently stronger in the dovitinib + AEE788 treatment-group, suggesting that targeting ErbB receptors has broader downstream effects compared to targeting only PI3K/mTOR. Considering that sub-classes of human breast tumors co-express ErbB receptors and FGFRs, these results have implications for targeted therapy. 
 Fibroblast Growth Factor Receptor 1 Gene Copy Number and mRNA Expression in Primary Colorectal Cancer and Its Clinicopathologic Correlation. Fibroblast growth factor receptor 1 (FGFR1) has been reported to be overexpressed in colorectal cancer (CRC) and suggested to be a therapeutic target. In this study, we investigated FGFR1 expression and amplification in CRC and its correlation with clinicopathologic parameters.FGFR1 dual-color fluorescence in situ hybridization and mRNA in situ hybridization were performed on tissue array blocks composed of 291 consecutive primary CRCs.Of the 291 CRC cases, FGFR1 gene amplification was found in 11 (3.8%) cases, high FGFR1 polysomy in 4 (1.4%) cases, and FGFR1 gene copy number (GCN) gain (GCN >2) in 77 (26.5%) cases. FGFR1 GCN gain was significantly associated with left-sided location, lymph node   metastasis  , distant   metastasis  , and higher TNM stage (p < 0.05). FGFR1 GCN gain also correlated with poor patient survival (p = 0.015). FGFR1 mRNA overexpression (score 3-4) was present in 11.7% (34/291) of the patients and was significantly associated with FGFR1 GCN alteration (Pearson correlation coefficient, r = 0.463; p < 0.001).FGFR1 GCN gain was more frequently found (26.5%) than gene amplification (3.8%) and correlated with aggressive clinical behavior in consecutive CRC patients. FGFR1 GCN alteration was associated with a high FGFR1 mRNA level. 
 EGFR, HER2 and HER3 expression in esophageal primary tumours and corresponding metastases. The expression of EGFR, HER2 and HER3 receptors were analyzed in immunohistochemical preparations from primary esophageal tumours and corresponding lymph node metastases. The goal was to evaluate whether any of these receptors are suitable as targets for radionuclide based imaging and therapy. The receptor expressions were evaluated in parallel samples, primary tumour and   metastasis  , from each patient (n=51). The majority of the cases were esophageal squamous cell carcinomas, ESCC (n=40). The HercepTest scoring was used for the analysis of both HER2 and EGFR expression (0, 1+, 2+ or 3+). HER3 was only evaluated as negative, weak or strong staining. EGFR overexpression (2+/3+) was found in 67.5% (27/40) of both the ESCC primary tumours and the corresponding lymph node metastases. There were only a few changes in these EGFR-scores: two cases from 2+/3+ to 0/1+ when the primary tumours were compared to the corresponding metastases and 2 changes the other way around. HER2 overexpression (2+/3+) was found in only 3 of the primary ESCC tumours and 2 of the lymph node metastases. EGFR and HER2 stainings were found mainly in the cell membranes. The HER3 staining (weak or strong) was mainly cytoplasmic and granular and was observed in about half (20/39) of the cases, for both the ESCC primary tumours and the corresponding lymph node metastases. It was concluded that ESCC lymph node metastases generally have a strong expression of EGFR in their cell membranes and to the same extent as in the primary tumours. The stability in EGFR expression is encouraging for efforts to develop radionuclide based EGFR imaging agents. It is also possible that EGFR targeting agents (e.g. Iressa, Tarceva, Erbitux or radiolabelled antibodies) can be applied for therapy of ESCC. 
 Fibroblast growth factor receptors-1 and -3 play distinct roles in the regulation of bladder cancer growth and   metastasis  : implications for therapeutic targeting. Fibroblast growth factor receptors (FGFRs) are activated by mutation and overexpressed in bladder cancers (BCs), and FGFR inhibitors are currently being evaluated in clinical trials in BC patients. However, BC cells display marked heterogeneity in their responses to FGFR inhibitors, and the biological mechanisms underlying this heterogeneity are not well defined. Here we used a novel inhibitor of FGFRs 1-3 and RNAi to determine the effects of inhibiting FGFR1 or FGFR3 in a panel of human BC cell lines. We observed that FGFR1 was expressed in BC cells that also expressed the "mesenchymal" markers ZEB1 and vimentin, whereas FGFR3 expression was restricted to the E-cadherin- and p63-positive "epithelial" subset. Sensitivity to the growth-inhibitory effects of BGJ-398 was also restricted to the "epithelial" BC cells and it correlated directly with FGFR3 mRNA levels but not with the presence of activating FGFR3 mutations. In contrast, BGJ-398 did not strongly inhibit proliferation but did block invasion in the "mesenchymal" BC cells in vitro. Similarly, BGJ-398 did not inhibit primary tumor growth but blocked the production of circulating tumor cells (CTCs) and the formation of lymph node and distant metastases in mice bearing orthotopically implanted "mesenchymal" UM-UC3 cells. Together, our data demonstrate that FGFR1 and FGFR3 have largely non-overlapping roles in regulating invasion/  metastasis   and proliferation in distinct "mesenchymal" and "epithelial" subsets of human BC cells. The results suggest that the tumor EMT phenotype will be an important determinant of the biological effects of FGFR inhibitors in patients. 
 Fibroblast growth factor receptor 1 gene amplification in gastric adenocarcinoma. Gastric adenocarcinomas are associated with a poor prognosis due to the fact that the tumor has often metastasized by the time of diagnosis. Thus, identification of novel therapeutic targets is highly desirable. Here, we examined gene copy number of fibroblast growth factor receptor 1 (FGFR1), a potential target for tyrosine kinase inhibitors, and clinicopathologic parameters in a large cohort of gastric adenocarcinomas. We performed fluorescence in situ hybridization analysis of 293 gastric adenocarcinomas using tissue microarrays. Amplification of the FGFR1 gene is a rare but noticeable event that can be found in 2% (6/293) of cases and was associated with poor 10-year survival (median 15.3 months in FGFR1-amplified cases versus 36 months in nonamplified cases, P = .047) and a higher rate of distant   metastasis   (P = .025). FGFR1 appears to represent a potential new therapeutic target in a subset of patients with gastric carcinoma. Identification of gastric cancers harboring FGFR1 amplification may be important in preselecting patients and/or interpreting clinical studies using tyrosine kinase inhibitors. 
 Identification of microRNA-214 as a negative regulator of colorectal cancer liver   metastasis   by way of regulation of fibroblast growth factor receptor 1 expression. The purpose of this study was to identify microRNAs (miRNAs) involved in the pathology of colorectal cancer (CRC) liver   metastasis   and investigate their underlying mechanisms. A total of 39 miRNAs were identified to be differentially expressed between 16 primary CRC tissues with liver metastases and 16 CRC tissues without liver metastases from 32 patients by Affymetric miRNA microarrays. A panel of eight miRNAs were confirmed to be significantly and differentially expressed between CRC tissues with and without liver metastases through quantitative reverse-transcription polymerase chain reaction (RT-PCR) analysis in the 32 patients. In a validated cohort of 99 CRC patients (44 with and 55 without liver metastases), only miR-214 was validated to be significantly down-regulated in CRC with liver metastases, which was associated with an unfavorable prognosis. Ectopic expression of miR-214 suppressed proliferation, migration, and invasion in vitro, tumor growth and liver   metastasis   in an in vivo xenograft mouse model, whereas miR-214 knockdown promoted proliferation, migration, and invasion in CRC cell lines. Further studies indicated that fibroblast growth factor receptor 1 (FGFR1) was a potential target of miR-214. Restoring miR-214 expression in CRC cells decreased endogenous FGFR1 messenger RNA (mRNA) and protein levels. FGFR1 knockdown mimicked the tumor suppressive effect of miR-214 on CRC cells, while reintroduction of FGFR1 abolished the tumor suppressive effect of miR-214 on CRC cells. Moreover, miR-214 expression levels were inversely correlated with FGFR1 in CRC patients.Down-regulation of miR-214 expression was correlated with increased FGFR1 expression levels, which may contribute to increased CRC liver   metastasis  . miR-214 may serve as a potential marker to predict survival, and the miR-214-FGFR1 axis may be a therapeutic target in CRC patients. 
  GeneRIFs  
 FGF1-FGFR1 axis promotes tongue squamous cell carcinoma   metastasis   through the epithelial-mesenchymal transition pathway 
 Gender, stage, differentiation, ethnicities and test methods have no influence on FGFR1 amplification. FGFR1 amplification trends to correlate with lymph node   metastasis   and smoking. Whether FGFR1 amplification has effect on survival remains controversial 
 Down-regulation of miR-214 expression was correlated with increased FGFR1 expression levels, which may contribute to increased colorectal liver   metastasis  . 
 Our findings suggested that FGFR inhibition could delay breast tumor progression, impair lung   metastasis   and break immunosuppression by effecting on tumor microenvironment, which may provide a promising therapeutic approach for breast cancer patient. 
 data demonstrate that FGFR1 and FGFR3 have largely non-overlapping roles in regulating invasion/  metastasis   and proliferation in distinct "mesenchymal" and "epithelial" subsets of human BC cells 
 results of the present study suggest that MUC4 promotes invasion and   metastasis   by FGFR1 stabilization through the N-cadherin upregulation. 
 The bFGF-FGFR1-PI3K-Rac1 pathway in the bone microenvironment may have a significant role in the invasion and   metastasis   of Ewing sarcoma. 
 Overexpression of the FGFR-1 gene may thus be a useful predictor of liver   metastasis   in patients with colorectal cancer. 
  [Cc]ell [Ii]nvasion  PubMed  
 Nuclear translocation of FGFR1 and FGF2 in pancreatic stellate cells facilitates pancreatic cancer   cell invasion  . Pancreatic cancer is characterised by desmoplasia, driven by activated pancreatic stellate cells (PSCs). Over-expression of FGFs and their receptors is a feature of pancreatic cancer and correlates with poor prognosis, but whether their expression impacts on PSCs is unclear. At the invasive front of human pancreatic cancer, FGF2 and FGFR1 localise to the nucleus in activated PSCs but not cancer cells. In vitro, inhibiting FGFR1 and FGF2 in PSCs, using RNAi or chemical inhibition, resulted in significantly reduced cell proliferation, which was not seen in cancer cells. In physiomimetic organotypic co-cultures, FGFR inhibition prevented PSC as well as cancer   cell invasion  . FGFR inhibition resulted in cytoplasmic localisation of FGFR1 and FGF2, in contrast to vehicle-treated conditions where PSCs with nuclear FGFR1 and FGF2 led cancer cells to invade the underlying extra-cellular matrix. Strikingly, abrogation of nuclear FGFR1 and FGF2 in PSCs abolished cancer   cell invasion  . These findings suggest a novel therapeutic approach, where preventing nuclear FGF/FGFR mediated proliferation and invasion in PSCs leads to disruption of the tumour microenvironment, preventing pancreatic cancer   cell invasion  . 
 Downregulation of microRNA-214 and overexpression of FGFR-1 contribute to hepatocellular carcinoma metastasis. miR-214 is one of the most significantly downregulated microRNAs (miRNAs) in hepatocellular carcinoma (HCC). Fibroblast growth factor receptor 1 (FGFR-1) is a miR-214 target gene implicated in the progression of HCC. However, the roles of miR-214 and FGFR-1 in HCC are not fully understood. Here, we analyzed the expression of miR-214 and FGFR-1 in 65 cases of HCC and paired non-neoplastic tissue specimens using real-time PCR and Western blot (WB), respectively. Our data indicated that miR-214 was downregulated and FGFR-1 was overexpressed in HCC compared to the paired non-neoplastic tissues. The low miR-214 expression was correlated with portal vein invasion (p=0.016) and early recurrence (p=0.045) in HCC patients. Moreover, the low miR-214 expression was correlated with high positive rate of FGFR-1 in HCC cases (p=0.020). Our data further demonstrated that miR-214 overexpression in SK-HEP1 and HepG2 cells downregulated FGFR-1 expression and inhibited liver cancer   cell invasion  . The Luciferase assay results further demonstrated the targeted regulation of FGFR-1 by miR-214. In conclusion, our data indicate that the downregulation of miR-214 in HCC and the upregulation of its target gene FGFR-1 is associated with HCC progression. Therefore, miR-214 and FGFR-1 are potential prognostic markers and therapeutic targets in HCC. 
  GeneRIFs  
 Nuclear targeting of FGFR1 and FGF2 in pancreatic stellate cells facilitates pancreatic cancer   cell invasion  . 
   
  
 FGFR3 
 
  NCBI Gene  
  GeneRIF  
  Pubmed  
 
 
  Search Term  Rank  Fields  
  [Mm]igration  6  PubMed(9); GeneRIFs(2)  
  ECM  7  PubMed(1)  
  [Ee]xtracellular [Mm]atrix  8  PubMed(4)  
  [Ii]nvasive  10  PubMed(62); GeneRIFs(11)  
  [Mm]etastasize  11  PubMed(1)  
  [Mm]etastasis  12  PubMed(7); GeneRIFs(2)  
  [Cc]ell [Ii]nvasion  13  PubMed(2)  
 
 
 hide/show details for FGFR3 
  [Mm]igration  PubMed  
 The physical basis of FGFR3 response to fgf1 and fgf2. Fibroblast growth factors (fgfs) play important roles in embryonic development and in adult life by controlling cell proliferation, differentiation, and   migration  . There are 18 known fgfs which activate four fibroblast growth factor receptors (FGFRs), with different isoforms due to alternative splicing. The physical basis behind the specificity of the biological responses mediated by different fgf-FGFR pairs is currently unknown. To gain insight into the specificity of FGFR3c, a membrane receptor which is critical for bone development, we studied, analyzed, and compared the activation of FGFR3c over a wide range of fgf1 and fgf2 concentrations. We found that while the strength of fgf2 binding to FGFR3c is lower than the strength of fgf1 binding, the fgf2-bound dimers exhibit higher phosphorylation of the critical tyrosines in the activation loop. As a result, fgf1 and fgf2 elicit a similar FGFR3c response at low, but not at high, concentrations. The results demonstrate the versatility of FGFR3c response to fgf1 and fgf2 and highlight the complexity in fgf signaling. 
 [R248C FGFR3 mutation. Effect on cell growth, apoptosis and attachment in HaCaT keratinocytes]. Activating FGFR3 mutations have been identified in a variety of benign skin lesions (seborrheic keratosis, epidermal nevus, solar lentigo). However, the functional consequences of these mutations in the human epidermis are unknown. We therefore analyzed functional effects of the common R248C mutation in HaCaT keratinocytes. The cells were stably transduced with the R248C FGFR3 mutation or FGFR3-IIIb wildtype sequence using a retroviral system. The R248C mutant keratinocytes revealed significantly enhanced cell growth compared with wildtype cells after reaching confluence. Likewise, apoptosis and attachment to fibronectin were significantly reduced in mutant cells. In contrast, there was no difference regarding   migration   and oncogene-induced senescence. Gene expression analysis revealed only a few differentially expressed genes between mutant and wildtype HaCaT keratinocytes. ERK1/2 appear to be involved in the FGFR3-dependent signalling of R248C mutant keratinocytes. Our results indicate that an increased cell number at confluence along with reduced apoptosis may contribute to the growth of benign acanthotic tumors in the human epidermis. 
 Fibroblast growth factor receptor 3-IIIc mediates colorectal cancer growth and   migration  . Deregulation of fibroblast growth factor receptor 3 (FGFR3) is involved in several malignancies. Its role in colorectal cancer has not been assessed before.Expression of FGFR3 in human colorectal tumour specimens was analysed using splice variant-specific real-time reverse transcriptase PCR assays. To analyse the impact of FGFR3-IIIc expression on tumour cell biology, colon cancer cell models overexpressing wild-type (WT-3b and WT3c) or dominant-negative FGFR3 variants (KD3c and KD3b) were generated by either plasmid transfection or adenoviral transduction.Although FGFR3 mRNA expression is downregulated in colorectal cancer, alterations mainly affected the FGFR3-IIIb splice variant, resulting in an increased IIIc/IIIb ratio predominantly in a subgroup of advanced tumours. Overexpression of WT3c increased proliferation, survival and colony formation in all colon cancer cell models tested, whereas WT3b had little activity. In addition, it conferred sensitivity to autocrine FGF18-mediated growth and   migration   signals in SW480 cells with low endogenous FGFR3-IIIc expression. Disruption of FGFR3-IIIc-dependent signalling by dominant-negative FGFR3-IIIc or small interfering RNA-mediated FGFR3-IIIc knockdown resulted in inhibition of cell growth and induction of apoptosis, which could not be observed when FGFR3-IIIb was blocked. In addition, KD3c expression blocked colony formation and   migration   and distinctly attenuated tumour growth in SCID mouse xenograft models.Our data show that FGFR3-IIIc exerts oncogenic functions by mediating FGF18 effects in colorectal cancer and may constitute a promising new target for therapeutic interventions. 
 Interaction of fibroblast growth factor receptor 3 and the adapter protein SH2-B. A role in STAT5 activation. Fibroblast growth factor receptor 3 (FGFR3) influences a diverse array of biological processes, including cell growth, differentiation, and   migration  . Activating mutations in FGFR3 are associated with multiple myeloma, cervical carcinoma, and bladder cancer. To identify proteins that interact with FGFR3 and which may mediate FGFR3-dependent signaling, a yeast two-hybrid screen was employed using the cytoplasmic kinase domain of FGFR3 as bait. We identified the adapter protein SH2-B as an FGFR3-interacting protein. Coimmunoprecipitation experiments demonstrate binding of the SH2-B beta isoform to FGFR3 in 293T cells. Tyrosine phosphorylation of SH2-B beta was observed when coexpressed with activated FGFR3 mutants such as the weakly activated mutant N540K or the strongly activated mutant K650E, both associated with human developmental syndromes. The extent of tyrosine phosphorylation of SH2-B beta correlates with receptor activation, suggesting that FGFR3 activation mediates tyrosine phosphorylation of SH2-B beta. Furthermore, two tyrosine phosphorylation sites of FGFR3, Tyr-724 and Tyr-760, are required for optimal binding of the Src homology-2 (SH2) domain of SH2-B beta. We also demonstrate the phosphorylation and nuclear translocation of Stat5 by activated FGFR3, which increases in response to overexpression of SH2-B beta. Taken together, our results identify SH2-B beta as a novel FGFR3 binding partner that mediates signal transduction. 
 [Hereditary skeletal dysplasias and FGFR3 and PTHR1 signaling pathways]. Skeletal development is a highly sophisticated process involving, as a first step,   migration   and condensation of mesenchymal cells into osteoprogenitor cells. These cells further differentiate into chondrocytes and osteoblasts through multiple differentiation stages requiring a set of specific transcriptional factors. Defective endochondral ossification in human is associated with a large number of inherited skeletal dysplasias caused by mutations in genes encoding extracellular matrix components, growth factors and their receptors, signaling molecules and transcription factors. This review summarizes some of the recent findings on a series of chondrodysplasias caused by mutations in FGFR3 and PTHR1, two receptors expressed in the cartilage growth plate and mediating two main signaling pathways. Data from human diseases and relevant animal models provide new clues for understanding how signaling molecules and their interaction with key transcription factors control and regulate the development and growth of long bones. 
 FGFR3 promotes angiogenesis-dependent metastasis of hepatocellular carcinoma via facilitating MCP-1-mediated vascular formation. The biological role of fibroblast growth factor receptor 3 (FGFR3) in tumor angiogenesis of hepatocellular carcinoma (HCC) has not been discussed before. Our previous work had indicated FGFR3 was overexpressed in HCC, and silencing FGFR3 in Hu7 cells could regulate tumorigenesis via down-regulating the phosphorylation level of key members of classic signaling pathways including ERK and AKT. In the present work, we explored the role of FGFR3 in angiogenesis-dependent metastasis by using SMMC-7721 and QGY-7703 stable cell lines. Our results indicated FGFR3 could regulate in vitro cell   migration   ability and in vivo lung metastasis ability of HCC, which was in accordance with increased angiogenesis ability in vitro and in vivo. Using the supernatant from SMMC-7721/FGFR3 cells, we conducted a human angiogenesis protein microarray including 43 angiogenesis factors and found that FGFR3 modulated angiogenesis and metastasis of HCC mainly by promoting the protein level of monocyte chemotactic protein 1 (MCP-1). Silencing FGFR3 by short hairpin RNA (shRNA) could reduce MCP-1 level in lysates and supernatant of QGY-7703 cells and SMMC-7721 cells. Silencing MCP-1 in QGY-7703 or SMMC-7721 cells could induce similar phenotypes compared with silencing FGFR3. Our results suggested FGFR3 promoted metastasis potential of HCC, at least partially if not all, via facilitating MCP-1-mediated angiogenesis, in addition to previously found cell growth and metastasis. MCP-1, a key medium between HCC cells and HUVECs, might be a novel anti-vascular target in HCC. 
 FGFR3 mutation affects cell growth, apoptosis and attachment in keratinocytes. FGFR3 mutations have recently been identified in several benign epidermal skin lesions such as seborrheic keratosis, epidermal nevus and solar lentigo. The functional consequences of these mutations in human skin are as yet unknown. In this study we analyzed the functional effects of the most common FGFR3 mutation in benign skin tumors, the R248C FGFR3 hotspot mutation, in human HaCaT keratinocytes. The cells were stably transduced with either the R248C or wildtype FGFR3 IIIb cDNA using a retroviral vector system. FGFR3 mutant and wildtype cells showed similar growth rates at subconfluence. However, at confluence FGFR3 mutant keratinocytes revealed a significantly higher cell number than wildtype cells. Furthermore, FGFR3 mutant cells showed significantly lower levels of apoptosis and decreased attachment to fibronectin compared with FGFR3 wildtype cells. Expression of mutant FGFR3 did not alter   migration   and senescence. Microarray analysis revealed only a few differentially expressed genes between FGFR3 mutant and wildtype keratinocytes. Enhanced phosphorylation of ERK1/2 was observed in confluent R248C mutant HaCaT cells compared with wildtype keratinocytes. Our results suggest that an increased cell number at confluence along with a decreased apoptosis may contribute to the development of acanthotic tumors in FGFR3 mutant skin in vivo. 
 Loss of heterozygosity at 4p16.3 and mutation of FGFR3 in transitional cell carcinoma. 4p16.3 has previously been identified as a region of non-random LOH in transitional cell carcinoma, suggesting the presence of a tumour suppressor gene. One candidate within this region is fibroblast growth factor receptor 3 (FGFR3). Germline mutations in FGFR3 are known to cause several autosomal dominant skeletal dysplasias, the severity of which depends on the position and nature of the mutation in the protein. We investigated the frequency and nature of FGFR3 mutations in a panel of transitional cell carcinomas and cell lines and studied the possible link between mutation and loss of heterozygosity (LOH) on 4p16.3. FGFR3 coding sequence from 63 transitional cell carcinomas (TCC) of various stages and grades, and 18 cell lines was analysed by fluorescent SSCP. Samples with abnormal   migration   patterns were sequenced to identify the mutation or polymorphism. Thirty-one of the 63 tumours had previously been assessed to have LOH at 4p16.3. Twenty-six of the 63 tumours (41%) and 4/18 (22%) of the cell lines had missense mutations in FGFR3. All mutations detected in our panel have been reported in the germline where all apart from one cause lethal conditions. One tumour contained K652Q which has recently been identified in less severe cases of skeletal dysplasia. Tumours with and without LOH at 4p16.3 had mutations in FGFR3 suggesting that these two events are not causally linked. The frequency of FGFR3 mutation indicates that this protein plays an important role in TCC. 
 Colorectal cancer cells activate adjacent fibroblasts resulting in FGF1/FGFR3 signaling and increased invasion. Cancer-associated fibroblasts expressing fibroblast activation protein (FAP) have been implicated in the invasive behavior of colorectal cancer. In this study, we use FAP expression as a marker of fibroblast activation and analyze the effect of activated fibroblasts on colorectal cancer   migration   and invasion in experimental cell studies. We also investigated the expression pattern of FAP in cancer-associated fibroblasts during transformation from benign to malignant colorectal tumors. In immunohistochemical analyses, FAP was expressed in fibroblasts in all colorectal cancer samples examined, whereas all normal colon, hyperplastic polyps, or adenoma samples were negative. In in vitro studies, conditioned medium from colon cancer cells, but not adenoma cells, activated fibroblasts by inducing FAP expression. These activated fibroblasts increased the   migration   and invasion of colon cancer cells in Boyden chamber experiments and in a three-dimensional cell culture model. We identify fibroblast growth factor 1/fibroblast growth factor receptor 3 (FGF1/FGFR-3) signaling as mediators leading to the increased   migration   and invasion. Activated fibroblasts increase their expression of FGF1, and by adding a fibroblast growth factor receptor inhibitor, as well as an FGF1-neutralizing antibody, we reduced the   migration   of colon cancer cells. Our findings provide evidence of a possible molecular mechanism involved in the cross talk between cancer cells and fibroblasts leading to cancer cell invasion. 
  GeneRIFs  
 Fibroblast growth factor 1/fibroblast growth factor receptor 3 (FGF1/FGFR-3) signaling are mediators leading to the increased   migration   and invasion. 
 mediates growth and   migration   in colorectal cancer 
  ECM  PubMed  
 New insight on FGFR3-related chondrodysplasias molecular physiopathology revealed by human chondrocyte gene expression profiling. Endochondral ossification is the process by which the appendicular skeleton, facial bones, vertebrae and medial clavicles are formed and relies on the tight control of chondrocyte maturation. Fibroblast growth factor receptor (FGFR)3 plays a role in bone development and maintenance and belongs to a family of proteins which differ in their ligand affinities and tissue distribution. Activating mutations of the FGFR3 gene lead to craniosynostosis and multiple types of skeletal dysplasia with varying degrees of severity: thanatophoric dysplasia (TD), achondroplasia and hypochondroplasia. Despite progress in the characterization of FGFR3-mediated regulation of cartilage development, many aspects remain unclear. The aim and the novelty of our study was to examine whole gene expression differences occurring in primary human chondrocytes isolated from normal cartilage or pathological cartilage from TD-affected fetuses, using Affymetrix technology. The phenotype of the primary cells was confirmed by the high expression of chondrocytic markers. Altered expression of genes associated with many cellular processes was observed, including cell growth and proliferation, cell cycle, cell adhesion, cell motility, metabolic pathways, signal transduction, cell cycle process and cell signaling. Most of the cell cycle process genes were down-regulated and consisted of genes involved in cell cycle progression, DNA biosynthesis, spindle dynamics and cytokinesis. About eight percent of all modulated genes were found to impact extracellular matrix (  ECM  ) structure and turnover, especially glycosaminoglycan (GAG) and proteoglycan biosynthesis and sulfation. Altogether, the gene expression analyses provide new insight into the consequences of FGFR3 mutations in cell cycle regulation, onset of pre-hypertrophic differentiation and concomitant metabolism changes. Moreover, impaired motility and   ECM   properties may also provide clues about growth plate disorganization. These results also suggest that many signaling pathways may be directly or indirectly altered by FGFR3 and confirm the crucial role of FGFR3 in the control of growth plate development. 
  [Ee]xtracellular [Mm]atrix  PubMed  
 Alteration of cell-cell and cell-matrix adhesion in urothelial cells: an oncogenic mechanism for mutant FGFR3. Activating mutations of FGFR3 are a common and early event in bladder cancer. Ectopic expression of mutant FGFR3 in normal urothelial cells has both pro-proliferative and antiapoptotic effects at confluence, suggesting that mutant cells are insensitive to cell-cell contact inhibition. Herein, detailed analysis revealed that these cells have reduced cell-cell adhesion, with large intercellular spaces observable at confluence, and diminished cell-substrate adhesion to collagen IV, collagen I, and fibronectin. These phenotypic alterations are accompanied by changes in the expression of genes involved in cell adhesion and   extracellular matrix   remodeling. Silencing of endogenous mutant FGFR3 in bladder cancer cells induced converse changes in transcript levels of CDH16, PLAU, MMP10, EPCAM, TNC, and HAS3, confirming them as downstream gene targets of mutant FGFR3. Overexpression of EPCAM, HAS3, and MMP10 transcripts was found in a large fraction of primary bladder tumors analyzed, supporting their key role in bladder tumorigenesis in vivo. However, no correlation was found between their protein and/or mRNA expression and FGFR3 mutation status in tumor specimens, indicating that these genes may be targeted by several converging oncogenic pathways. Overall, these results indicate that mutant FGFR3 favors the development and progression of premalignant bladder lesions by altering key genes regulating the cell-cell and cell-matrix adhesive properties of urothelial cells.The ability of mutant FGFR3 to drive transcriptional expression profiles involved in tumor cell adhesion suggests a mechanism for expansion of premalignant urothelial lesions. 
 Effect of PPADS on achondroplasic chondrocytes: inhibition of FGF receptor type 3 over-activity. Achondroplasia, results from a mutation in the FGF receptor type 3, leading to receptor hyperactivation and subsequent amplification of FGF receptor type 3 signals. We have tested the ability of pyridoxal-5'-phosphate-6-azophenyl-2', 4'-disulfonate (PPADS) to decrease the overactivation and signalling of FGF receptor type 3 in achondroplasic chondrocytes. PPADS reduced the tyrosine phosphorylation of FGF receptor type 3 triggered by fibroblast growth factor 9 (FGF9) (50% reduction), as well as the activation of extracellular signal-regulated kinases 1 and 2 (ERK1/2) pathway. As a consequence of this inhibitory effect on ERK1/2 activity the loss of   extracellular matrix   was also reversed by PPADS. The action of PPADS seems to be due to a mechanism independent of P2 receptor antagonism. 
 [Hereditary skeletal dysplasias and FGFR3 and PTHR1 signaling pathways]. Skeletal development is a highly sophisticated process involving, as a first step, migration and condensation of mesenchymal cells into osteoprogenitor cells. These cells further differentiate into chondrocytes and osteoblasts through multiple differentiation stages requiring a set of specific transcriptional factors. Defective endochondral ossification in human is associated with a large number of inherited skeletal dysplasias caused by mutations in genes encoding   extracellular matrix   components, growth factors and their receptors, signaling molecules and transcription factors. This review summarizes some of the recent findings on a series of chondrodysplasias caused by mutations in FGFR3 and PTHR1, two receptors expressed in the cartilage growth plate and mediating two main signaling pathways. Data from human diseases and relevant animal models provide new clues for understanding how signaling molecules and their interaction with key transcription factors control and regulate the development and growth of long bones. 
 New insight on FGFR3-related chondrodysplasias molecular physiopathology revealed by human chondrocyte gene expression profiling. Endochondral ossification is the process by which the appendicular skeleton, facial bones, vertebrae and medial clavicles are formed and relies on the tight control of chondrocyte maturation. Fibroblast growth factor receptor (FGFR)3 plays a role in bone development and maintenance and belongs to a family of proteins which differ in their ligand affinities and tissue distribution. Activating mutations of the FGFR3 gene lead to craniosynostosis and multiple types of skeletal dysplasia with varying degrees of severity: thanatophoric dysplasia (TD), achondroplasia and hypochondroplasia. Despite progress in the characterization of FGFR3-mediated regulation of cartilage development, many aspects remain unclear. The aim and the novelty of our study was to examine whole gene expression differences occurring in primary human chondrocytes isolated from normal cartilage or pathological cartilage from TD-affected fetuses, using Affymetrix technology. The phenotype of the primary cells was confirmed by the high expression of chondrocytic markers. Altered expression of genes associated with many cellular processes was observed, including cell growth and proliferation, cell cycle, cell adhesion, cell motility, metabolic pathways, signal transduction, cell cycle process and cell signaling. Most of the cell cycle process genes were down-regulated and consisted of genes involved in cell cycle progression, DNA biosynthesis, spindle dynamics and cytokinesis. About eight percent of all modulated genes were found to impact   extracellular matrix   (ECM) structure and turnover, especially glycosaminoglycan (GAG) and proteoglycan biosynthesis and sulfation. Altogether, the gene expression analyses provide new insight into the consequences of FGFR3 mutations in cell cycle regulation, onset of pre-hypertrophic differentiation and concomitant metabolism changes. Moreover, impaired motility and ECM properties may also provide clues about growth plate disorganization. These results also suggest that many signaling pathways may be directly or indirectly altered by FGFR3 and confirm the crucial role of FGFR3 in the control of growth plate development. 
  [Ii]nvasive  PubMed  
 Focal amplifications are associated with high grade and recurrences in stage Ta bladder carcinoma. Urinary bladder cancer is a heterogeneous disease with tumors ranging from papillary non  invasive   (stage Ta) to solid muscle infiltrating tumors (stage T2+). The risk of progression and death for the most frequent diagnosed type, Ta, is low, but the high incidence of recurrences has a significant effect on the patients' quality of life and poses substantial costs for health care systems. Consequently, the purpose of this study was to search for predictive factors of recurrence on the basis of genetic profiling. A clinically well characterized cohort of Ta bladder carcinomas, selected by the presence or absence of recurrences, was evaluated by an integrated analysis of DNA copy number changes and gene expression (clone-based 32K, respectively, U133Plus2.0 arrays). Only a few chromosomal aberrations have previously been defined in superficial bladder cancer. Surprisingly, the profiling of Ta tumors with a high-resolution array showed that DNA copy alterations are relatively common in this tumor type. Furthermore, we observed an overrepresentation of focal amplifications within high-grade and recurrent cases. Known (FGFR3, CCND1, MYC, MDM2) and novel candidate genes were identified within the loci. For example, MYBL2, a nuclear transcription factor involved in cell-cycle progression; YWHAB, an antiapoptotic protein; and SDC4, an important component of focal adhesions represent interesting candidates detected within two amplicons on chromosome 20, for which DNA amplification correlated with transcript up-regulation. The observed overrepresentation of amplicons within high-grade and recurrent cases may be clinically useful for the identification of patients who will benefit from a more aggressive therapy. 
 Fibroblast growth factor receptor (FGFR) gene amplifications are rare events in bladder cancer. Activating point mutations and protein overexpression of fibroblast growth factor receptors (FGFRs), especially FGFR3, are frequent events in bladder cancer. Little is known about gene amplifications, therefore we characterized amplification of FGFR1-3 by fluorescence in-situ hybridization (FISH).Tumours of 153 patients (n = 65 pTa low-grade, n = 15 pTa high-grade, n = 37 pT1, n = 20 pT2, n = 10 pT3, n = 6 pT4) were analysed by FISH for FGFR1-3 copy numbers and screened for FGFR3 mutations and immunohistochemical protein expression. Amplifications of FGFR1 were found in 1.6% (two of 122), FGFR2 in 0.8% (one of 121) and FGFR3 in 3.4% (five of 145). All amplifications were high-level amplifications, not overlapping with polysomy. Amplifications were found in papillar  y/papill  ary-invasive tumour parts, and predominantly in tumours with enhanced Ki67 index (>10%), aberrant CK20 expression, and low p53 expression. All FGFR3-amplified samples showed concomitant FGFR3 mutations and FGFR3 protein overexpression. FGFR amplifications were not associated significantly with gender, age, grade or stage in statistical analyses.FGFR amplifications are rare events in bladder cancer, with FGFR3 amplification being the most prevalent (3.4% of cases). Concomitant FGFR3 mutations and protein overexpression indicate that FGFR3-mediated signalling in these tumours would probably be highly active. This patient subgroup may be particularly suited to FGFR-targeted pharmacotherapy. 
 A sequence variant at 4p16.3 confers susceptibility to urinary bladder cancer. Previously, we reported germline DNA variants associated with risk of urinary bladder cancer (UBC) in Dutch and Icelandic subjects. Here we expanded the Icelandic sample set and tested the top 20 markers from the combined analysis in several European case-control sample sets, with a total of 4,739 cases and 45,549 controls. The T allele of rs798766 on 4p16.3 was found to associate with UBC (odds ratio = 1.24, P = 9.9 x 10(-12)). rs798766 is located in an intron of TACC3, 70 kb from FGFR3, which often harbors activating somatic mutations in low-grade, non  invasive   UBC. Notably, rs798766[T] shows stronger association with low-grade and low-stage UBC than with more aggressive forms of the disease and is associated with higher risk of recurrence in low-grade stage Ta tumors. The frequency of rs798766[T] is higher in Ta tumors that carry an activating mutation in FGFR3 than in Ta tumors with wild-type FGFR3. Our results show a link between germline variants, somatic mutations of FGFR3 and risk of UBC. 
 Differential expression of receptor tyrosine kinases (RTKs) and IGF-I pathway activation in human uterine leiomyomas. Uterine leiomyomas (fibroids) are benign tumors that are prevalent in women of reproductive age. Research suggests that activated receptor tyrosine kinases (RTKs) play an important role in the enhanced proliferation observed in fibroids. In this study, a phospho-RTK array technique was used to detect RTK activity in leiomyomas compared with myometrial tissue. We found that fifteen out of seventeen RTKs evaluated in this study were highly expressed (P < 0.02-0.03) in the leiomyomas, and included the IGF-I/IGF-IR, EGF/EGFR, FGF/FGF-R, HGF/HGF-R, and PDGF/PDGF-R gene families. Due to the higher protein levels of IGF-IR observed in leiomyomas by us in earlier studies, we decided to focus on the activation of the IGF-IR, its downstream effectors, and MAPKp44/42 to confirm our earlier findings; and validate the significance of the increased IGF-IR phosphorylation observed by RTK array analysis in this study. We used immunolocalization, western blot, or immunoprecipitation studies and confirmed that leiomyomas overexpressed IGF-IRbeta and phosphorylated IGF-IRbeta. Additionally, we showed that the downstream effectors, Shc, Grb2, and MAPKp44/42 (P < 0.02-0.001) were also overexpressed and involved in IGF-IR signaling in these tumors, while IRS-I, PI3K, and AKT were not. In vitro studies showed that IGF-I (100 ng/mL) increased the proliferation of uterine leiomyoma cells (UtLM) (P < 0.0001), and that phosphorylated IGF-IRbeta, Shc, and MAPKp44/42 were also overexpressed in IGF-I-treated UtLM cells (P < 0.05), similar to the tissue findings. A neutralizing antibody against the IGF-IRbeta blocked these effects. These data indicate that overexpression of RTKs and, in particular, activation of the IGF-IR signaling pathway through Shc/Grb2/MAPK are important in mediating uterine leiomyoma growth. These data may provide new anti-tumor targets for non  invasive   treatment of fibroids. 
 Fibroblast growth factor receptor (FGFR) gene amplifications are rare events in bladder cancer. Activating point mutations and protein overexpression of fibroblast growth factor receptors (FGFRs), especially FGFR3, are frequent events in bladder cancer. Little is known about gene amplifications, therefore we characterized amplification of FGFR1-3 by fluorescence in-situ hybridization (FISH).Tumours of 153 patients (n = 65 pTa low-grade, n = 15 pTa high-grade, n = 37 pT1, n = 20 pT2, n = 10 pT3, n = 6 pT4) were analysed by FISH for FGFR1-3 copy numbers and screened for FGFR3 mutations and immunohistochemical protein expression. Amplifications of FGFR1 were found in 1.6% (two of 122), FGFR2 in 0.8% (one of 121) and FGFR3 in 3.4% (five of 145). All amplifications were high-level amplifications, not overlapping with polysomy. Amplifications were found in papillar  y/papill  ary-invasive tumour parts, and predominantly in tumours with enhanced Ki67 index (>10%), aberrant CK20 expression, and low p53 expression. All FGFR3-amplified samples showed concomitant FGFR3 mutations and FGFR3 protein overexpression. FGFR amplifications were not associated significantly with gender, age, grade or stage in statistical analyses.FGFR amplifications are rare events in bladder cancer, with FGFR3 amplification being the most prevalent (3.4% of cases). Concomitant FGFR3 mutations and protein overexpression indicate that FGFR3-mediated signalling in these tumours would probably be highly active. This patient subgroup may be particularly suited to FGFR-targeted pharmacotherapy. 
 Focal amplifications are associated with high grade and recurrences in stage Ta bladder carcinoma. Urinary bladder cancer is a heterogeneous disease with tumors ranging from papillary non  invasive   (stage Ta) to solid muscle infiltrating tumors (stage T2+). The risk of progression and death for the most frequent diagnosed type, Ta, is low, but the high incidence of recurrences has a significant effect on the patients' quality of life and poses substantial costs for health care systems. Consequently, the purpose of this study was to search for predictive factors of recurrence on the basis of genetic profiling. A clinically well characterized cohort of Ta bladder carcinomas, selected by the presence or absence of recurrences, was evaluated by an integrated analysis of DNA copy number changes and gene expression (clone-based 32K, respectively, U133Plus2.0 arrays). Only a few chromosomal aberrations have previously been defined in superficial bladder cancer. Surprisingly, the profiling of Ta tumors with a high-resolution array showed that DNA copy alterations are relatively common in this tumor type. Furthermore, we observed an overrepresentation of focal amplifications within high-grade and recurrent cases. Known (FGFR3, CCND1, MYC, MDM2) and novel candidate genes were identified within the loci. For example, MYBL2, a nuclear transcription factor involved in cell-cycle progression; YWHAB, an antiapoptotic protein; and SDC4, an important component of focal adhesions represent interesting candidates detected within two amplicons on chromosome 20, for which DNA amplification correlated with transcript up-regulation. The observed overrepresentation of amplicons within high-grade and recurrent cases may be clinically useful for the identification of patients who will benefit from a more aggressive therapy. 
 Differential expression of receptor tyrosine kinases (RTKs) and IGF-I pathway activation in human uterine leiomyomas. Uterine leiomyomas (fibroids) are benign tumors that are prevalent in women of reproductive age. Research suggests that activated receptor tyrosine kinases (RTKs) play an important role in the enhanced proliferation observed in fibroids. In this study, a phospho-RTK array technique was used to detect RTK activity in leiomyomas compared with myometrial tissue. We found that fifteen out of seventeen RTKs evaluated in this study were highly expressed (P < 0.02-0.03) in the leiomyomas, and included the IGF-I/IGF-IR, EGF/EGFR, FGF/FGF-R, HGF/HGF-R, and PDGF/PDGF-R gene families. Due to the higher protein levels of IGF-IR observed in leiomyomas by us in earlier studies, we decided to focus on the activation of the IGF-IR, its downstream effectors, and MAPKp44/42 to confirm our earlier findings; and validate the significance of the increased IGF-IR phosphorylation observed by RTK array analysis in this study. We used immunolocalization, western blot, or immunoprecipitation studies and confirmed that leiomyomas overexpressed IGF-IRbeta and phosphorylated IGF-IRbeta. Additionally, we showed that the downstream effectors, Shc, Grb2, and MAPKp44/42 (P < 0.02-0.001) were also overexpressed and involved in IGF-IR signaling in these tumors, while IRS-I, PI3K, and AKT were not. In vitro studies showed that IGF-I (100 ng/mL) increased the proliferation of uterine leiomyoma cells (UtLM) (P < 0.0001), and that phosphorylated IGF-IRbeta, Shc, and MAPKp44/42 were also overexpressed in IGF-I-treated UtLM cells (P < 0.05), similar to the tissue findings. A neutralizing antibody against the IGF-IRbeta blocked these effects. These data indicate that overexpression of RTKs and, in particular, activation of the IGF-IR signaling pathway through Shc/Grb2/MAPK are important in mediating uterine leiomyoma growth. These data may provide new anti-tumor targets for non  invasive   treatment of fibroids. 
 [Preliminary study of p53 and FGFR3 gene mutations in the urine for bladder tumors]. Two major pathways are described in bladder carcinogenesis: one for   invasive   or high grade tumors characterized by alteration of the p53 tumor suppressor gene and the other for non-  invasive   tumors or low grade involving mutations FGFR3. The objective of our study was to validate the research in the urine of mutations in these two genes in patients with a bladder tumor.In our preliminary study, we investigated 36 patients the FGFR3 and p53 mutations in tumors and urine collected during endoscopic resection. The p53 mutations were sought in FASAY, which allows a functional analysis of the protein P53. The FGFR3 mutations were sought in SNaPshot that searches the eight most frequent mutation points of this gene.For 24 patients (66% of cases), we found at least one of the two mutations in the tumor. This mutation was present in the urine in 15 patients (sensitivity=62.5%). In only one patient, we found a mutation in the urinary sediment that did not exist in the tumor (specificity=91.7%).The search for mutations of p53 and FGFR3 in the urine was a simple and non-  invasive   assay, which seems superior to urinary cytology for the detection of bladder tumors, raising hopes of an interest in this bio-assay for surveillance of bladder tumors and screening risk populations. 
 [Update on FGFR3 mutation and multiple regional epigenetic silencing (MRES) phenotype in urothelial carcinogenesis]. FGFR3 mutation leads to a constitutive activation of the receptor 3 to Fibroblast Growth Factor. This mutation is early in urothelial carcinogenesis and is strongly associated to low grade papillary tumors. Multiple regional epigenetic silencing (MRES) phenotype corresponds to the transcriptional inactivation of chromosomal regions in muscle   invasive   bladder cancer, and is strongly associated to the molecular signature of carcinoma in situ. These alterations could be targeted by new specific therapies. 
 Integrated genetic and epigenetic analysis of bladder cancer reveals an additive diagnostic value of FGFR3 mutations and hypermethylation events. The bladder cancer genome harbors numerous oncogenic mutations and aberrantly methylated gene promoters. The aim of our study was to generate a profile of these alterations and investigate their use as biomarkers in urine sediments for non  invasive   detection of bladder cancer. We systematically screened FGFR3, PIK3CA, TP53, HRAS, NRAS and KRAS for mutations and quantitatively assessed the methylation status of APC, ARF, DBC1, INK4A, RARB, RASSF1A, SFRP1, SFRP2, SFRP4, SFRP5 and WIF1 in a prospective series of tumor biopsies (N = 105) and urine samples (N = 113) from 118 bladder tumor patients. We also analyzed urine samples from 33 patients with noncancerous urinary lesions. A total of 95 oncogenic mutations and 189 hypermethylation events were detected in the 105 tumor biopsies. The total panel of markers provided a sensitivity of 93%, whereas mutation and methylation markers alone provided sensitivities of 72% and 70%, respectively. In urine samples, the sensitivity was 70% for all markers, 50% for mutation markers and 52% for methylation markers. FGFR3 mutations occurred more frequently in tumors with no methylation events than in tumors with one or more methylation events (78% vs. 33%; p < 0.0001). FGFR3 mutation in combination with three methylation markers (APC, RASSF1A and SFRP2) provided a sensitivity of 90% in tumors and 62% in urine with 100% specificity. These results suggest an inverse correlation between FGFR3 mutations and hypermethylation events, which may be used to improve non  invasive  , DNA-based detection of bladder cancer. 
 The role of mutations and overexpression of the fibroblast growth factor receptor-3 in bladder cancer. Bladder cancer (BC) is the seventh most common cancer worldwide. Throughout the last decade, several studies demonstrated that the fibroblast growth factor (FGF) signalling is altered in a significant proportion of patients with BC. FGF receptor (FGFR) 3 may thus serve as a promising biomarker for BC. Mutations of this gene are prevalent in BC (e.g., found in 74% of non-  invasive   papillary tumours), suggesting that FGFR3 status is an important event in BC. The aim of this review was to overview the outcomes of different mutations in FGFR3 receptor in the context of BC. We first described FGFR3 receptor and continue with mutations of FGFR3 gene, including activating mutations and overexpression of this gene. Finally, we addressed the clinical relevance of mutated FGFR3 gene. 
 FGFR3 and TP53 gene mutations define two distinct pathways in urothelial cell carcinoma of the bladder. FGFR3 and TP53 mutations are frequent in superficial papillary and   invasive   disease, respectively. We used denaturing high-performance liquid chromatography and sequencing to screen for FGFR3 and TP53 mutations in 81 newly diagnosed urothelial cell carcinomas. Tumors were classified as follows: 31 pTa, 1 carcinoma in situ, 30 pT1, and 19 pT2-T4. Tumor grades were as follows: 10 G1, 29 G2, and 42 G3. FGFR3 mutations were associated with low-stage (P < 0.0001), low-grade (P < 0.008) tumors, whereas TP53 mutations were associated with high-stage (P < 0.003), high-grade (P < 0.02) tumors. Mutations in these two genes were almost mutually exclusive. Our results suggest that FGFR3 and TP53 mutations define separate pathways at initial diagnosis of urothelial cell carcinoma. 
 Fibroblast growth factor receptor-3 in urothelial tumorigenesis. Fibroblast growth factor receptor-3 (FGFR3) is a receptor tyrosine kinase implicated in the tumorigenesis of multiple malignancies, including bladder and other urothelial cancers, multiple myeloma, and cervical cancer. In urothelial carcinoma (UC), constitutive receptor activation occurs most commonly through substitution of a wild-type residue with cysteine in the extracellular domain of FGFR3, thereby resulting in dimerization (through disulfide bridge formation) and subsequent stimulation of tyrosine kinase activity. Activating mutations of FGFR3 have been observed in up to 70% of non-muscle-  invasive   bladder tumors, while overexpression of a wild-type receptor, found in approximately 40% of tumors, has been correlated with more   invasive   disease. The identification of FGFR3 mutations in UC has sparked substantial interest in the therapeutic exploitation of these aberrations, and in vitro studies have provided evidence that such alterations may represent driver oncogenic lesions. In this review, we discuss the biologic and prognostic impact of FGFR3 mutations in UC as well as FGFR3 as a potential target for novel therapeutics. 
 Prediction of progression of non-muscle-  invasive   bladder cancer by WHO 1973 and 2004 grading and by FGFR3 mutation status: a prospective study. The clinical management of non-muscle-  invasive   urothelial cell carcinoma of the bladder (UCC) is challenging, as it has a marked tendency to recur and to progress. Aim of this study was to investigate the prognostic value of the WHO 1973 and 2004 grading systems and biomarkers FGFR3, CK20 and Ki-67.In a prospective study, tumours from 221 patients were studied for the expression of CK20 and Ki-67 by immunohistochemistry, and FGFR3 status by SNaPshot mutation detection. Staging and grading were performed according to the WHO classification systems of 1973 and 2004.: Median follow-up was 35 mo. Recurrence occurred in 72 of 221 patients. None of the parameters was able to predict disease recurrence. CK20, Ki-67, FGFR3 mutation, molecular grade using FGFR3 mutation analysis and Ki-67, and histological grading and staging were significantly associated with disease progression in stage. In multivariable analyses, WHO 1973 and 2004 grading systems remained statistically significant and independent predictors of progression, with p=0.005 for WHO 1973 and p=0.004 for 2004. FGFR3 status was able to discriminate progressors from nonprogressors in a subset of patients with high-grade UCC (p=0.009).This is the first prospective study comparing the WHO 1973 and 2004 grading systems. We show that both grading systems contribute valuable independent information. Therefore, it should be considered whether a better grading system could be developed that incorporates essential elements from both. The combination of WHO 2004 grading with FGFR3 status allows a better risk stratification for patients with high-grade non-muscle-  invasive   UCC. 
 FGFR3 mutation analysis in voided urine samples to decrease cystoscopies and cost in nonmuscle   invasive   bladder cancer surveillance: a comparison of 3 strategies. We determined whether FGFR3 mutation analysis of voided urine samples would be cost-effective to partly replace cystoscopy in the surveillance of patients treated for nonmuscle   invasive   urothelial carcinoma.In this decision analytical study we analyzed data on 70 Dutch patients with FGFR3 positive primary tumors and a median followup of 8.8 years. Surveillance strategies were compared in a Markov model. Modified surveillance consisted of FGFR3 mutation analysis of voided urine samples every 3 months, and cystoscopy at 3, 12 and 24 months. Standard surveillance was defined as cystoscopy every 3 months and minimal surveillance was defined as cystoscopy at 3, 12 and 24 months. Analysis was stratified for 3 risk profiles, including surveillance after 1) the primary tumor, 2) the first to third recurrence and 3) the fourth recurrence or more. Sensitivity analysis was performed to evaluate the impact of variations in cost, sensitivity and specificity.The probability of no recurrence after 2 years of surveillance after a primary tumor was higher for modified surveillance than for standard and minimal surveillance, eg after primary tumors (95.7% vs 95.0% and 93.9%, respectively). The total cost of surveillance after the primary tumor was lower for minimal and modified surveillance (€2,254 and €2,558, respectively) than for standard surveillance (€5,861). Results were robust to changing inputs over plausible ranges and consistent for each of the 3 risk profiles.Surveillance in which cystoscopy is partly replaced by FGFR3 mutation analysis of urine seems a safe, effective and cost-effective surveillance strategy. Further validation in larger cohorts is required. 
 FGFR3, HRAS, KRAS, NRAS and PIK3CA mutations in bladder cancer and their potential as biomarkers for surveillance and therapy. Fifty percent of patients with muscle-  invasive   bladder cancer (MI-BC) die from their disease and current chemotherapy treatment only marginally increases survival. Novel therapies targeting receptor tyrosine kinases or activated oncogenes may improve outcome. Hence, it is necessary to stratify patients based on mutations in relevant oncogenes. Patients with non-muscle-  invasive   bladder cancer (NMI-BC) have excellent survival, however two-thirds develop recurrences. Tumor specific mutations can be used to detect recurrences in urine assays, presenting a more patient-friendly diagnostic procedure than cystoscopy.To address these issues, we developed a mutation assay for the simultaneous detection of 19 possible mutations in the HRAS, KRAS, and NRAS genes. With this assay and mutation assays for the FGFR3 and PIK3CA oncogenes, we screened primary bladder tumors of 257 patients and 184 recurrences from 54 patients. Additionally, in primary tumors p53 expression was obtained by immunohistochemistry. Of primary tumors 64% were mutant for FGFR3, 11% for RAS, 24% for PIK3CA, and 26% for p53. FGFR3 mutations were mutually exclusive with RAS mutations (p = 0.001) and co-occurred with PIK3CA mutations (p = 0.016). P53 overexpression was mutually exclusive with PIK3CA and FGFR3 mutations (p≤0.029). Mutations in the RAS and PIK3CA genes were not predictors for recurrence-free, progression-free and disease-specific survival. In patients presenting with NMI-BC grade 3 and MI-BC, 33 and 36% of the primary tumors were mutant. In patients with low-grade NMI-BC, 88% of the primary tumors carried a mutation and 88% of the recurrences were mutant.The mutation assays present a companion diagnostic to define patients for targeted therapies. In addition, the assays are a potential biomarker to detect recurrences during surveillance. We showed that 88% of patients presenting with low-grade NMI-BC are eligible for such a follow-up. This may contribute to a reduction in the number of cystoscopical examinations. 
 No mutations of FGFR3 in normal urothelium in the vicinity of urothelial carcinoma of the bladder harbouring activating FGFR3 mutations in patients with bladder cancer. Mutations in the fibroblast growth factor receptor 3 (FGFR3) gene causing constitutive oncogenic protein activation have been shown to be frequent in papillary non  invasive   bladder tumours and are associated with a low risk of progression and a favourable outcome. FGFR3 alterations have also been found in benign urothelial papilloma and flat urothelial hyperplasia suggesting FGFR3 alterations as an early event in bladder tumorigenesis. To date there is no data available on FGFR3 mutations in normal urothelium from patients with bladder cancer. We therefore analysed 64 samples of histopathological unsuspicious normal urothelium from 38 patients with FGFR3 mutated bladder tumours and 15 samples of urothelium from patients (n = 15) without any urothelial malignancy as a control group. Urothelial cells were microdissected from formalin-fixed, paraffin-embedded material. After DNA isolation whole genome amplification was done by I-PEP-PCR. FGFR3 mutations were detected using SNaPshot analysis. All samples could successfully be investigated. FGFR3 analyses did not reveal any mutation in the urothelium from neither the control group nor the bladder cancer group. All urothelial samples showed a wildtype sequence for FGFR3. These data suggest that mutations in the FGFR3 gene are not the earliest genetic alterations in bladder carcinogenesis and are associated with a hyperproliferative (hyperplastic) phenotype in the urothelium. Chromosomal alterations like deletions on chromosome 9q or aberrant promoter hypermethylation could play more important roles in early urothelial transformation than mutational FGFR3 activation. 
 Immunohistochemical detection of fibroblast growth factor receptor 3 in human breast cancer: correlation with clinicopathological/molecular parameters and prognosis. Reportedly, fibroblast growth factor receptor 3 (FGFR3) that regulates embryonic growth and development may function as an oncoprotein in certain malignancies. We aimed to investigate the biological significance of FGFR3 expression in   invasive   breast cancer.FGFR3 expression was investigated in 50   invasive   breast cancer specimens by immunohistochemistry. The association between FGFR3 expression and clinicopathological/molecular parameters or prognosis was evaluated.Weak FGFR3 expression was observed in myoepithelial cells, but not in duct epithelial cells, of the normal mammary ducts and lobules. FGFR3 expression in breast cancer cells was observed in 19 of 50 (38.0%) cases (9 weak positive and 10 strong positive). Besides the cytoplasm and cell membrane, nuclear staining was observed in 3 of 10 strong-positive cases. FGFR3 was further detected in non-neoplastic duct epithelial cells or duct papillomatosis in 5 strong-positive cases. No significant correlation was observed between FGFR3 expression and specific clinicopathological/molecular parameters. In contrast, FGFR3 expression was found to be significantly associated with overall survival in our cohort.FGFR3 expression in   invasive   breast cancer was not found to be significantly associated with specific clinicopathological/molecular parameters, but might be used as a candidate marker for a poor prognosis. 
 Prospective study of FGFR3 mutations as a prognostic factor in nonmuscle   invasive   urothelial bladder carcinomas. To determine the frequency and the prognostic value of fibroblast growth factor receptor 3 (FGFR3) mutations in patients with nonmuscle   invasive   bladder tumors according to tumor stage and grade.Seven hundred seventy-two patients with newly diagnosed bladder tumors were recruited. Tumors were reviewed by expert pathologists. Patients were prospectively followed-up (median, 62.6 months for disease-free patients) through review of hospital records and telephone interviews. The sequence of exons 7 and 10 of FGFR3 was analyzed by polymerase chain reaction and direct sequencing. We assessed the association of mutations with stage and grade. The predictive value of mutations for recurrence, progression, and mortality were assessed using Kaplan-Meier and Cox multivariable models.Mutations were more common among low malignant potential neoplasms (LMPN; 77%) and TaG1/TaG2 tumors (61%/58%) than among TaG3 tumors (34%) and T1G3 tumors (17%). The S249C, Y375C, S248C, and G372C mutations accounted for 91.5% of all sequence changes. The A393E substitution was associated with LMPN (P < .001). The F386L polymorphism was more frequent among patients with low-grade tumors (odds ratio, 6.97; 95%CI, 1.40 to 47.06; P = .009). In the multivariable analysis of all superficial tumors, mutations were associated with increased risk of recurrence. However, in the stratified analyses only patients with TaG1 tumors had a significantly higher risk of recurrence (hazard ratio, 2.12; 95%CI, 1.28 to 3.53; P = .004).The findings of this large study strongly support the notion that FGFR3 mutations characterize a subgroup of bladder cancers with good prognosis; patients with mutant TaG1 tumors have a higher risk of recurrence; and the F386L variant is selectively associated with low-grade tumors. 
 Fibroblast growth factor receptor 3 mutations in bladder tumors correlate with low frequency of chromosome alterations. The aim of this study was to analyze the distribution of FGFR3 mutations in bladder tumors of different grade and stage and determine the relation of mutations to chromosomal alterations detected by comparative genomic hybridization (CGH). One hundred bladder cancer samples served as templates for manual microdissection. DNA was isolated from dissected samples containing at least 80% tumor cells. Mutations in FGFR3 were analyzed by SNaPshot analysis. CGH was carried out according to standard protocols. FGFR3 mutations were detected in 45 of 92 samples (48.9%). Concerning T-category, the following mutation frequencies occurred: pTa, 69%; pT1, 38%; and pT2-3, 0%. The mutation frequency was significantly associated with tumor grade: G1, 72%; G2, 56%; and G3, 4%. In pTaG1 tumors, mutations were found in 74%. A significantly lower number of genetic alterations per tumor detected by CGH was associated with FGFR3 mutations (2 vs 8). This association was also seen in pTaG1 tumors: 2.5 (with mutation) vs 7.5 (without mutation). FGFR3 mutations characterize non  invasive   low-risk tumors of low malignancy. The low malignant potential of these tumors is underlined by a low number of genetic alterations per tumor. Therefore, FGFR3 represents a valuable prognostic marker of tumors with low malignant potential and can be used as surrogate marker for the detection of genetically stable bladder tumors. 
 Clinical and biological characteristics of cervical neoplasias with FGFR3 mutation. We have previously reported activating mutations of the gene coding for the fibroblast growth factor receptor 3 (FGFR3) in   invasive   cervical carcinoma. To further analyze the role of FGFR3 in cervical tumor progression, we extended our study to screen a total of 75   invasive   tumors and 80 cervical intraepithelial neoplasias (40 low-grade and 40 high-grade lesions).Using single strand conformation polymorphism (SSCP) followed by DNA sequencing, we found FGFR3 mutation (S249C in all cases) in 5% of   invasive   cervical carcinomas and no mutation in intraepithelial lesions. These results suggest that, unlike in bladder carcinoma, FGFR3 mutation does not or rarely occur in non   invasive   lesions. Compared to patients with wildtype FGFR3 tumor, patients with S249C FGFR3 mutated tumors were older (mean age 64 vs. 49.4 years, P = 0.02), and were more likely to be associated with a non-16/18 HPV type in their tumor. Gene expression analysis demonstrated that FGFR3 mutated tumors were associated with higher FGFR3b mRNA expression levels compared to wildtype FGFR3 tumors. Supervised analysis of Affymetrix expression data identified a significant number of genes specifically differentially expressed in tumors with respect to FGFR3 mutation status.This study suggest that tumors with FGFR3 mutation appear to have distinctive clinical and biological characteristics that may help in defining a population of patients for FGFR3 mutation screening. 
 FGFR3 protein expression and its relationship to mutation status and prognostic variables in bladder cancer. FGFR3 is frequently activated by mutation in urothelial carcinoma (UC) and represents a potential target for therapy. In multiple myeloma, both over-expression and mutation of FGFR3 contribute to tumour development. To define the population of UC patients who may benefit from FGFR-targeted therapy, we assessed both mutation and receptor over-expression in primary UCs from a population of new patients. Manual or laser capture microdissection was used to isolate pure tumour cell populations. Where present, non-  invasive   and   invasive   components in the same section were microdissected. A screen of the region of the highest tumour stage in each sample yielded a mutation frequency of 42%. Mutations comprised 61 single and five double mutations, all in hotspot codons previously identified in UC. There was a significant association of mutation with low tumour grade and stage. Subsequently, non-  invasive   areas from the 43 tumours with both non-  invasive   and   invasive   components were analysed separately; 18 of these had mutation in at least one region, including nine with mutation in all regions examined, eight with mutation in only the non-  invasive   component and one with different mutations in different regions. Of the eight with mutation in only the non-  invasive   component, six were predicted to represent a single tumour and two showed morphological dissimilarity of fragments within the block, indicating the possible presence of distinct tumour clones. Immunohistochemistry showed over-expression of FGFR3 protein in many tumours compared to normal bladder and ureteric controls. Increased expression was associated with mutation (85% of mutant tumours showed high-level expression). Overall, 42% of tumours with no detectable mutation showed over-expression, including many muscle-  invasive   tumours. This may represent a non-mutant subset of tumours in which FGFR3 signalling contributes to the transformed phenotype and which may benefit from FGFR-targeted therapies. 
 PIK3CA mutations are an early genetic alteration associated with FGFR3 mutations in superficial papillary bladder tumors. Bladder tumors constitute a very heterogeneous disease. Superficial tumors are characterized by a high prevalence of FGFR3 mutations and chromosome 9 alterations. High-grade and muscle-  invasive   tumors are characterized by Tp53 mutations and aneuploidy. We have analyzed the sequence of exons 9 and 20 of PIK3CA in a panel of bladder tumors covering the whole spectrum of the disease. DNA from formalin-fixed, paraffin-embedded tumor sections was amplified by PCR and products were sequenced. In an unselected panel of tumors representative of the disease, the PIK3CA mutation prevalence was 13% (11 of 87). Mutations occurred mainly at the previously identified hotspots (codons 542, 545, 1007, and 1047). The distribution according to stage was as follows: papillary urothelial neoplasms of uncertain malignant potential (PUNLMP; 11 of 43, 25.6%), T(a) (9 of 57, 16%), T(1) (2 of 10, 20%), and muscle-  invasive   tumors (0 of 20, 0%; P = 0.019). Mutations were associated with low-grade tumors: grade 1 (6 of 27, 22.2%), grade 2 (3 of 23, 13%), and grade 3 (2 of 37, 5.4%; P = 0.047). Overall, PIK3CA mutations were strongly associated with FGFR3 mutations: 18 of 69 (26%) FGFR3(mut) tumors were PIK3CA(mut), versus 4 of 58 (6.9%) FGFR3(wt) tumors (P = 0.005). Our findings indicate that PIK3CA mutations are a common event that can occur early in bladder carcinogenesis and support the notion that papillary and muscle-  invasive   tumors arise through different molecular pathways. PIK3CA may constitute a novel diagnostic and prognostic tool, as well as a therapeutic target, in bladder cancer. 
 FGFR3 mutational status and protein expression in patients with bladder cancer in a Jordanian population. Bladder cancer accounts for nearly 5% of all newly diagnosed cancers in Jordan, with a much higher frequency in males. Recent studies have shown that activating mutations in FGFR3 are the most common findings in non-  invasive   low grade bladder tumors. In this study, we, retrospectively, investigated a cohort of 121 bladder cancer patients with various grades and stages of the tumor for molecular changes in FGFR3. Overexpression of FGFR3 was observed in 49%, 34%, 15%, and 2% of pTa, pT1, pT2, and pT3 cases, respectively. Further, FGFR3 expression was positive in 45%, 26%, and 30% of G1, G2 and G3 cases, respectively. Mutational analysis of exons 7, 10 and 15 of FGFR3 identified four previously reported mutations, namely R248C (n=4; 10%), S249C (n=23; 59%), Y375C (n=7; 18%), G382R (n=4; 10%), and one novel mutation, G382E (n=1; 3%). Our results indicate that both mutations and overexpression of FGFR3 are correlated together, and are more prevalent in early stage (pTa and pT1) and low grade (G1 and G2) bladder tumors. Survival analysis showed no contribution of changes in FGFR3 on the patient's survival. Multivariate Cox proportional hazards model analysis of overall survival for the following variables: age, gender, stage and grade of tumor, and FGFR3 (expression and mutation) revealed that age, stage and grade of tumor are independent predictors of overall survival in patients with bladder cancer. Our work is the first to address the molecular status of FGFR3 in Jordanian patients with bladder cancer, and provides further support for FGFR3 as a key player in the initiation of bladder tumors. 
 Activation of extracellular regulated kinases (ERK1/2) predicts poor prognosis in urothelial bladder carcinoma and is not associated with B-Raf gene mutations. The analysis of the presence of B-Raf gene mutations in relation to ERK1/2 activation in bladder urothelial carcinoma (UC), in order to determine their potential role in tumour aggressiveness and patients' survival.Polymerase chain reaction-single strand confirmation polymorphism (PCR-SSCP) and sequencing analysis were used for B-Raf gene mutation detection. pERK1/2 and FGFR3 expression were examined by immunohistochemistry in 152 and 116 primary UCs, respectively.None of the cases displayed mutations in exon 15 of B-Raf gene. Nuclear or cytoplasmic pERK immunoreactivity was displayed in 99.3% and 96.7% of cases, respectively. pERK nuclear expression increased with histological grade and with T-category. Nuclear and cytoplasmic pERK expression was unrelated to FGFR3 expression. In univariate survival analysis of muscle-  invasive   carcinomas, advanced T-category and higher pERK nuclear expression (p = 0.018) adversely affected survival. However, multivariate analysis in non-  invasive   as well as in muscle-  invasive   carcinomas selected only T-category as a significant prognosticator.Our findings suggest that elevated pERK expression occurs in UCs in the absence of B-Raf mutations and is not correlated with FGFR3 over-expression. Moreover, it implicates ERK activation in the acquisition of a more aggressive phenotype. However, the assessment of pERK1/2 expression does not seem to add to the prognostic information provided by classical prognosticators. 
 Fibroblast growth factor receptor 3 mutation in voided urine is a useful diagnostic marker and significant indicator of tumor recurrence in non-muscle   invasive   bladder cancer. The fibroblast growth factor receptor (FGFR)-3 gene encodes a receptor tyrosine kinase that is frequently mutated in non-muscle   invasive   bladder cancer (NMIBC). A sensitive and quantitative assay using peptide nucleic acid-mediated real-time PCR was developed for detecting FGFR3 mutations in the urine samples and evaluated as a molecular marker for detecting intravesical recurrence of NMIBC in patients undergoing transurethral resection of bladder tumor. FGFR3 mutation was examined in tumor tissues and serially taken pre- and postoperative urine sediments in 45 NMIBC patients with a median follow up of 32 months. FGFR3 mutations were detected in 53.3% (24/45) of primary tumor tissues, among which intravesical recurrence developed in 37.5% (9/24) of cases. FGFR3 mutation in the primary tumor was not a significant prognostic indicator for recurrence, while the proportion of FGFR3 mutation (i.e. tumor cellularity was >or=11%) in the preoperative urine sediments was a significant indicator for recurrence in patients with FGFR3 mutations in the primary tumors. FGFR3 mutations were detected in 78% (7/9) of postoperative urine samples from recurrent cases with FGFR3 mutations in the tumor, while no mutations were detected in the urine of 15 non-recurrent cases. Urine cytology was negative in all cases with FGFR3 mutations in the primary tumors, while the sensitivity of cytological examination was as high as 56% (5/9) in cases showing wild-type FGFR3 in the primary tumors. Urine FGFR3 mutation assay and cytological examination may be available in the future as complementary diagnostic modalities in postoperative management of NMIBC. 
 Prenatal DNA diagnosis of a single-gene disorder from maternal plasma. Achondroplasia is a short-limb disorder caused by a point mutation in a single gene. To diagnose such a disorder prenatally requires the use of   invasive   procedures such as amniocentesis. However, using PCR and restriction fragment length polymorphism analysis, we were able to detect the mutation in the plasma of a woman carrying a fetus suspected of having achondroplasia. The detection of a fetus-derived mutant gene from maternal plasma may therefore permit non-  invasive   prenatal diagnosis of single-gene disorders. 
 CDKN2A homozygous deletion is associated with muscle invasion in FGFR3-mutated urothelial bladder carcinoma. The gene cyclin-dependent kinase inhibitor 2A (CDKN2A) is frequently inactivated by deletion in bladder carcinoma. However, its role in bladder tumourigenesis remains unclear. We investigated the role of CDKN2A deletion in urothelial carcinogenesis, as a function of FGFR3 mutation status, a marker for one of the two pathways of bladder tumour progression, the Ta pathway. We studied 288 bladder carcinomas: 177 non-muscle-  invasive   (123 Ta, 54 T1) and 111 muscle-  invasive   (T2-4) tumours. CDKN2A copy number was determined by multiplex ligation-dependent probe amplification, and FGFR3 mutations by SNaPshot analysis. FGFR3 mutation was detected in 124 tumours (43.1%) and CDKN2A homozygous deletion in 56 tumours (19.4%). CDKN2A homozygous deletion was significantly more frequent in FGFR3-mutated tumours than in wild-type FGFR3 tumours (p = 0.0015). This event was associated with muscle-  invasive   tumours within the FGFR3-mutated subgroup (p < 0.0001) but not in wild-type FGFR3 tumours. Similar findings were obtained for an independent series of 101 bladder carcinomas. The impact of CDKN2A deletions on recurrence-free and progression-free survival was then analysed in 89 patients with non-muscle-  invasive   FGFR3-mutated tumours. Kaplan-Meier survival analysis showed that CDKN2A losses (hemizygous and homozygous) were associated with progression (p = 0.0002), but not with recurrence, in these tumours. Multivariate Cox regression analysis identified CDKN2A loss as a predictor of progression independent of stage and grade. These findings highlight the crucial role of CDKN2A loss in the progression of non-muscle-  invasive   FGFR3-mutated bladder carcinomas and provide a potentially useful clinical marker for adapting the treatment of such tumours, which account for about 50% of cases at initial clinical presentation. 
 A 3-plex methylation assay combined with the FGFR3 mutation assay sensitively detects recurrent bladder cancer in voided urine. DNA methylation is associated with bladder cancer and these modifications could serve as useful biomarkers. FGFR3 mutations are present in 60% to 70% of non-muscle   invasive   bladder cancer (NMIBC). Low-grade bladder cancer recurs in more than 50% of patients. The aim of this study is to determine the sensitivity and specificity of a urine assay for the diagnosis of recurrences in patients with a previous primary NMIBC G1/G2 by using cystoscopy as the reference standard.We selected eight CpG islands (CGI) methylated in bladder cancer from our earlier genome-wide study. Sensitivity of the CGIs for recurrences detection was investigated on a test set of 101 preTUR urines. Specificity was determined on 70 urines from healthy males aged more than 50 years. A 3-plex assay for the best combination was developed and validated on an independent set of 95 preTUR, recurrence free, and nonmalignant urines (n=130).The 3-plex assay identified recurrent bladder cancer in voided urine with a sensitivity of 74% in the validation set. In combination with the FGFR3 mutation assay, a sensitivity of 79% was reached (specificity of 77%). Sensitivity of FGFR3 and cytology was 52% and 57%, respectively.The combination of methylation and FGFR3 assays efficiently detects recurrent bladder cancer without the need for stratification of patients regarding methylation/mutation status of the primary tumor. We conclude that the sensitivity of this combination is in the same range as cystoscopy and paves the way for a subsequent study that investigates a modified surveillance protocol consisting of the urine test followed by cystoscopy only when the urine test is positive. 
 Prognostic value of molecular markers, sub-stage and European Organisation for the Research and Treatment of Cancer risk scores in primary T1 bladder cancer. What's known on the subject? and What does the study add? The stakes are high when making treatment decisions in T1 bladder cancer (BC). Conservative management may lead to progression and possibly death from BC. Conversely, radical cystectomy could be over-treatment of non-progressive disease. The problem for clinicians is that reliable prognostic indices are lacking. We performed a head-to-head comparison of two substaging systems, European Organisation for the Research and Treatment of Cancer (EORTC) risk scores and four molecular markers in T1 carcinomas of the bladder treated conservatively with BCG. T1 sub-stage according to a new system (micro-  invasive   [T1m] and extensive-  invasive   [T1e]) was the most important clinical variable for predicting progression to carcinoma invading bladder muscle. The performance of the EORTC risk scores was disappointing for this T1 sub-group. Molecular markers were not significant in multivariable analysis for predicting progression. Future studies may lead to the incorporation of sub-stage (T1m/T1e) in the TNM classification system for urinary BC to guide clinical decision-making in T1 BC.To evaluate the prognostic significance of four molecular markers, sub-stage and European Organisation for the Research and Treatment of Cancer (EORTC) risk scores in primary T1 bladder cancer (BC) treated with adjuvant bacille Calmette-Guérin.The slides of 129 carcinomas of the bladder from two university hospitals were reviewed and the T1 diagnosis was confirmed. T1 sub-staging was done in two separate rounds, using a new system that identifies micro  -invasiv  e (T1m) and extensive  -invasiv  e (T1e) T1BC, and then according to invasion of the muscularis mucosae (T1a/T1b/T1c). The EORTC risk scores for recurrence and progression were calculated. Uni- and multivariable analyses for recurrence and progression were performed using clinicopathological variables, T1 sub-stage, EORTC risk scores and molecular markers (fibroblast growth factor receptor 3 gene mutation and Ki-67, P53, P27 expression).The median follow-up was 6.5 years. Forty-two patients remained recurrence-free (33%). Progression to T2 or metastasis was observed in 38 (30%) patients. In multivariable analysis for recurrence, multiplicity was significant. In multivariable analysis for progression, female gender, sub-stage (T1m/T1e) and carcinoma in situ (CIS) were significant. Molecular markers were significant in univariable and in multivariable analyses for recurrence. EORTC risk scores were not significant.CIS, female gender and sub-stage (T1m/T1e) were the most important variables for progression. The additional value of molecular markers was modest. Sub-stage (T1m/T1e) could potentially be incorporated in future tumour-node-metastasis classifications. 
 FGFR3 mutations, but not FGFR3 expression and FGFR3 copy-number variations, are associated with favourable non-muscle   invasive   bladder cancer. The fibroblast growth factor receptor 3 (FGFR3) is a tyrosine kinase receptor frequently activated by point mutations in bladder cancer (BC). These mutations are associated with genetically stable, Ta and low-grade BC, representing the favourable BC pathway. Conversely, FGFR3 over-expression was recently found in 40 % of muscle   invasive   BC. We examined FGFR3 mutation status and protein expression in patients originally diagnosed as T1. We also investigated copy-number variations in FGFR3 as a possible alternative mechanism to activate FGFR3. We included 84 patients with T1 BC as their initial diagnosis. A uropathologist reviewed the slides for grade and (sub)stage. The FGFR3 mutation status was examined by PCR-SNaPshot and FGFR3 protein expression by standard immuno-histochemistry (FGFR3-B9). Copy-number status was determined in 69/84 cases with nine probes covering nine exons of the FGFR3 gene (MLPA). Of 27 BC with FGFR3 mutations, 26 (96 %) showed FGFR3 over-expression. Of the 57 wild-type BC, 27 (47 %) BC showed over-expression. Pathological parameters significantly differed (p < 0.01) between mutant and wild-type tumours with the FGFR3 mutation pointing to more favourable BC. However, if the BC exhibited wild-type FGFR3, FGFR3 protein status had no influence on grade and (sub)stage. We found six tumours with more than or equal to three copies of FGFR3. Only 1 of 22 wild-type tumours with over-expression of FGFR3 had more than or equal to three gene copies. In initially diagnosed T1 BC, only the FGFR3 mutation was significantly associated with favourable BC disease characteristics. In addition to almost all FGFR3 mutant BC, 47 % of wild-type BC displayed FGFR3 over-expression, suggesting an alternative mechanism to activate FGFR3. Increased FGFR3 copy number was a rare event and did not account for this mechanism. Nevertheless, FGFR3 wild-type tumours with over-expression of the protein may still represent a subset that might potentially benefit from FGFR3-targeted therapy. 
 Molecular basis of urinary bladder cancer. Bladder cancer is a relatively common and potentially life-threatening neoplasm. The diagnosis of urothelial carcinoma usually entails a lifelong surveillance to detect recurrent disease. In recent years, significant progress has been made in understanding the molecular mechanisms of carcinogenesis in urinary bladder. An early step in the process of carcinoma development is establishment of a premalignant abnormal urothelial patch that may give rise to various types of urothelial carcinoma and may provide a fertile ground for development of multifocal synchronous and metachronous tumors. Two distinct molecular pathways are involved. Low-grade papillary carcinoma is associated with mutation in the FGFR3 or in some cases mutations in RAS genes. High-grade in situ/muscle-  invasive   carcinoma on the other hand is characterized by alteration of p53 and pRB. Loss of function of these key genes, which play a crucial role in the control of cell cycle, leads to accumulation of additional mutations and deletions of genes resulting in an aggressive phenotype. It is hoped that a thorough understanding of the molecular basis of urothelial cancer will facilitate early diagnosis and will lead to development of new modalities for the management and treatment of these carcinomas. 
 FGFR3 expression in primary and metastatic urothelial carcinoma of the bladder. While fibroblast growth factor receptor 3 (FGFR3) is frequently mutated or overexpressed in nonmuscle-  invasive   urothelial carcinoma (UC), the prevalence of FGFR3 protein expression and mutation remains unknown in muscle-  invasive   disease. FGFR3 protein and mRNA expression, mutational status, and copy number variation were retrospectively analyzed in 231 patients with formalin-fixed paraffin-embedded primary UCs, 33 metastases, and 14 paired primary and metastatic tumors using the following methods: immunohistochemistry, NanoString nCounterTM, OncoMap or Affymetrix OncoScanTM array, and Gain and Loss of Analysis of DNA and Genomic Identification of Significant Targets in Cancer software. FGFR3 immunohistochemistry staining was present in 29% of primary UCs and 49% of metastases and did not impact overall survival (P = 0.89, primary tumors; P = 0.78, metastases). FGFR3 mutations were observed in 2% of primary tumors and 9% of metastases. Mutant tumors expressed higher levels of FGFR3 mRNA than wild-type tumors (P < 0.001). FGFR3 copy number gain and loss were rare events in primary and metastatic tumors (0.8% each; 3.0% and 12.3%, respectively). FGFR3 immunohistochemistry staining is present in one third of primary muscle-  invasive   UCs and half of metastases, while FGFR3 mutations and copy number changes are relatively uncommon. 
 FGFR3 mutations and a normal CK20 staining pattern define low-grade non  invasive   urothelial bladder tumours. Molecular markers superior to conventional clinicopathologic parameters are needed to predict disease courses in bladder cancer patients. In this study, we investigated four markers (Ki-67, TP53, CK20, FGFR3) in primary urothelial bladder tumours and compared them with traditional pathologic features.Tissue microarrays were used to analyse CK20, TP53, and Ki-67 expression immunohistochemically in 255 unselected patients. FGFR3 mutations were detected by SNaPshot analysis.Abnormal CK20 expression was strongly associated with higher tumour grades and stages (p < 0.001); however, 65% of pTa tumours revealed an abnormal CK20 pattern. In the group of pTaG1 tumours, 59% presented with an abnormal CK20 pattern, whereas 82% carried the FGFR3 mutation. In the group of bladder tumours with normal CK20 pattern, the FGFR3 gene was mutated in 89%, whereas a mutated FGFR3 gene was found in only 37% of cases with abnormal CK20 expression (p < 0.001). All markers proved to be strong predictors of disease-specific survival in univariate studies. However, in multivariate analyses they were not independent from classical pathologic parameters. None of the molecular markers was significantly associated with tumour recurrence.Dysregulation of CK20 expression is an early event in the carcinogenesis of papillary non  invasive   bladder cancer, but occurs later than FGFR3 mutations. The group of low-grade non  invasive   papillary tumours is defined by the presence of an FGFR3 mutation and a normal CK20 expression pattern. 
 Urothelial carcinoma following augmentation cystoplasty: an aggressive variant with distinct clinicopathological characteristics and molecular genetic alterations. Patients who have undergone intestinal augmentation cystoplasty are at risk for developing latent vesicle malignancy. The aim was to evaluate the histological and immunohistochemical characteristics and molecular genetic alterations in these neoplasms.Four patients developing urothelial neoplasms after augmentation cystoplasty were included in the current study. The mean age of the patients, including two men and two women, was 37 years. The latency from bladder augmentation to developing malignancy ranged from 17 to 21 years (mean 19 years). All patients died of cancer shortly after diagnosis (mean 5 months). In the morphological evaluation, all tumours were high-grade (grade 3)   invasive   urothelial carcinoma comprising various architectural patterns with brisk mitoses and tumour necrosis. Three harboured glandular differentiation and the remaining one showed squamous differentiation. All cases revealed abnormal decreasing beta-catenin expression. Two tumours showed nuclear expression of CDX2. On UroVysion fluorescence in situ hybridization (FISH) analysis, all tumours displayed characteristic chromosomal abnormalities. Point mutations of both FGFR3 and p53 genes were identified in one case.Urothelial carcinomas developed after augmentation cystoplasty are extremely aggressive and exhibit distinct morphological, immunohistochemical and genetic characteristics. UroVysion FISH analysis may offer a surveillance strategy in patients who undergo augmentation cystoplasty. 
 ARID1A alterations are associated with FGFR3-wild type, poor-prognosis, urothelial bladder tumors. Urothelial bladder cancer (UBC) is heterogeneous at the clinical, pathological, genetic, and epigenetic levels. Exome sequencing has identified ARID1A as a novel tumor suppressor gene coding for a chromatin remodeling protein that is mutated in UBC. Here, we assess ARID1A alterations in two series of patients with UBC. In the first tumor series, we analyze exons 2-20 in 52 primary UBC and find that all mutant tumors belong to the aggressive UBC phenotype (high grade non-muscle   invasive   and muscle   invasive   tumors) (P = 0.05). In a second series (n = 84), we assess ARID1A expression using immunohistochemistry, a surrogate for mutation analysis, and find that loss of expression increases with higher stage/grade, it is inversely associated with FGFR3 overexpression (P = 0.03) but it is not correlated with p53 overexpression (P = 0.30). We also analyzed the expression of cytokeratins in the same set of tumor and find, using unsupervised clustering, that tumors with ARID1A loss of expression are generally KRT5/6-low. In this patient series, loss of ARID1A expression is also associated with worse prognosis, likely reflecting the higher prevalence of losses found in tumors of higher stage and grade. The independent findings in these two sets of patients strongly support the notion that ARID1A inactivation is a key player in bladder carcinogenesis occurring predominantly in FGFR3 wild type tumors. 
 FGFR3 and Cyclin D3 as urine biomarkers of bladder cancer recurrence. To assess the diagnostic performance of FGFR3 and Cyclin D3 urinary protein levels in detecting bladder cancer recurrence.Urine of 321 patients in follow-up for bladder cancer and 150 non-neoplastic urine controls was included. Cytology, cystoscopy and FGFR3 and Cyclin D3 expression by western blot were performed.One hundred ten (34.3%) patients had evidence of tumor recurrence. The sensitivity and specificity of cytology/cystoscopy was 80 and 84%, and for FGFR3/Cyclin D3 was of 73 and 90%.Combined urinary FGFR3/Cyclin D3 expression shows improved detection rates for bladder cancer recurrence with high specificity and sensitivity, and within the same range of detection shown by cystoscopy, therefore supporting its potential use as non  invasive   diagnostic biomarker for bladder cancer recurrence. 
 Molecular grade (FGFR3/MIB-1) and EORTC risk scores are predictive in primary non-muscle-  invasive   bladder cancer. The European Organization for Research and Treatment of Cancer (EORTC) risk scores are not validated in an independent patient population. Molecular grade (mG) based on fibroblast growth factor receptor 3 (FGFR3) gene mutation status and MIB-1 expression was proposed as an alternative to pathologic grade in bladder cancer (BCa) [1].To validate the EORTC risk score and to determine its relation to mG in a series with long-term follow-up as well as to determine reproducibility of pathologic grade and mG.In this multicenter study, we included 230 patients with primary non-muscle-  invasive   BCa (NMIBC).Four uropathologists reviewed the slides. FGFR3 mutation status was examined by two assays. MIB-1 was assessed by immunohistochemistry. The EORTC risk scores for recurrence and progression were determined. Multivariable analyses were used to find prognostic factors.Median follow-up was 8.62 yr (interquartile range: 6.6-11.8). FGFR3 mutations were significantly related to favorable disease parameters, whereas altered MIB-1 was frequently seen with pT1, high grade, and high EORTC risk scores. EORTC risk scores were significant in multivariable analyses for recurrence and progression. In multivariable analyses for progression and disease-specific survival, the mG had independent significance. The addition of mG to the multivariable model for progression increased the predictive accuracy from 74.9% to 81.7% (p<0.001; Mantel-Haenszel test). The mG (89%) was more reproducible than the pathologic grade (41-74%).We validated the EORTC risk scores for primary NMIBC in a clinical and biomarker setting. Next to EORTC risk score, mG proved highly reproducible and predictive. Our long-term results justify an independent prospective analysis of mG and EORTC risk scores. 
 FGFR3 overexpression is prognostic of adverse outcome for muscle-  invasive   bladder carcinoma treated with adjuvant chemotherapy. Alterations in fibroblast growth factor receptor 3 (FGFR3) have been implicated in the pathogenesis of urothelial carcinoma. However, its clinicopathological significance has not been clearly established, especially in muscle-  invasive   bladder cancer (MIBC).The aim of our study was to investigate the mutation and overexpression of FGFR3 in MIBC cases from radical cystectomy and to analyze the prognostic and predictive significance in the groups with or without adjuvant chemotherapy.Study cohorts included 72 cases of MIBC including 42 patients who were treated with adjuvant chemotherapy. The mutation status of FGFR3 exons 7, 10, and 15 was investigated and protein expression was evaluated. The findings were analyzed for the association with relevant clinicopathological findings.FGFR3 mutations were found in 7 patients (9.7%) and were correlated with a pattern of papillary growth, moderate histologic grade (G2 vs. G3), and moderately advanced TNM stage (II-III vs. IV). FGFR3 protein overexpression was detected in 33 cases (45.8%) but was not associated with the relevant clinicopathological parameters. In patients treated with adjuvant chemotherapy, FGFR3 overexpression was correlated with shorter disease-free survival (P = 0.067, 95% confidence interval: 14.8-29.6, marginal significance) and overall survival (P = 0.035), remaining as a significant independent prognostic factor for disease-free survival and overall survival in multivariate analysis using Cox proportional hazards model. In patients without adjuvant chemotherapy, FGFR3 mutation or overexpression did not have prognostic significance in multivariate analysis.We report the FGFR3 alterations in MIBCs, and discuss their biological implication in subsets of patients. FGFR3 overexpression was predictive of adverse outcome in patients with adjuvant cisplatin-based chemotherapy after radical cystectomy. The utility of FGFR3 as a therapeutic target is suggested. 
 Fibroblast growth factor receptor 3 is overexpressed in urinary tract carcinomas and modulates the neoplastic cell growth. Fibroblast growth factor receptor 3 (FGFR3) mutations have been associated with achondroplastic syndromes and urinary bladder carcinomas. Here we describe changes in FGFR3 mRNA and protein expression in transitional carcinomas and determine the effect of monoclonal antibodies against FGFR3 in RT-112 cell line proliferation.We used microarray tools to evaluate FGFR3 mRNA expression in 22 urinary bladder carcinomas at different stages (non  invasive   pTa, lamina propria   invasive   pT1, and muscular   invasive   pT2) and 7 nonneoplastic tissue controls. FGFR3 protein expression was evaluated by Western blotting in 15 different carcinomas and 3 nonneoplastic controls. Two hundred thirty-seven urinary bladder and renal pelvis carcinomas and 21 negative controls were tested on tissue microarrays by immunohistochemistry. The effect on cell proliferation in the RT-112 bladder cancer cell line of monoclonal antibodies against FGFR3 was also evaluated.Overexpression of FGFR3 mRNA was found in pTa and pT1 stage carcinomas (fold change >8) and in pT2 carcinomas (fold change >4). Nonneoplastic urinary bladder samples do not express FGFR3 protein. However, 83% of pTa, 100% of pT1, and 50% of pT2 carcinomas expressed FGFR3 as determined by Western blotting. By immunohistochemistry, FGFR3 was positive in 71.4% of pTa, 72% of pT1, and 49.2% of pT2 cases as well as 61.5% of upper urinary tract carcinomas. Proliferation of the RT-112 cell line was inhibited with monoclonal antibodies against FGFR3.FGFR3 seems to play an important role in transitional cell carcinoma development. Our results suggest that FGFR3 antagonists could be developed as possible therapeutics for treatment of urinary tract carcinoma. 
 FGFR3 mutations indicate better survival in   invasive   upper urinary tract and bladder tumours. Promoter hypermethylation and microsatellite instability are frequent in tumours of the upper urinary tract (UTT) and infrequent in bladder tumours. FGFR3 mutations are common findings in bladder tumours and are associated with a good prognosis.To investigate the occurrence of FGFR3 mutations in UTT and determine the prognostic effect of these genetic changes.Tissue from the initial tumour was obtained from 280 patients (117 bladder tumours and 163 UTT). Patients were selected from pathologic archives to represent the disease spectrum of UCC throughout the urinary tract. Following UCC excision, patients underwent surveillance for a median of 56 mo (range 1-216 mo) or until death.FGFR3 mutation analysis was successfully performed on 252 of the 280 primary tumours using the SNaPshot method. Two-tailed statistical analyses were done using the chi(2), Fisher exact tests, and log rank tests. Cox proportional hazard ratios were estimated to obtain risks of recurrence, progression, and death, and to find independent prognostic factors in a multivariate model.FGFR3 mutations occurred with the same frequency in bladder and upper tract tumours. Mutations were associated with low-stage tumours and a milder disease course in bladder, ureter, and renal pelvis tumours. Strikingly, our data suggest that these mutations indicate a better survival in patients with   invasive   tumours from the bladder and upper urinary tract.FGFR3 mutation status might be used to select patients with   invasive   UCC who have a lower risk of death. 
 Hypoxia regulates FGFR3 expression via HIF-1α and miR-100 and contributes to cell survival in non-muscle   invasiv  e bladder cancer. Non-muscle   invasiv  e (NMI) bladder cancer is characterised by increased expression and activating mutations of FGFR3. We have previously investigated the role of microRNAs in bladder cancer and have shown that FGFR3 is a target of miR-100. In this study, we investigated the effects of hypoxia on miR-100 and FGFR3 expression, and the link between miR-100 and FGFR3 in hypoxia.Bladder cancer cell lines were exposed to normoxic or hypoxic conditions and examined for the expression of FGFR3 by quantitative PCR (qPCR) and western blotting, and miR-100 by qPCR. The effect of FGFR3 and miR-100 on cell viability in two-dimensional (2-D) and three-dimensional (3-D) was examined by transfecting siRNA or mimic-100, respectively.In NMI bladder cancer cell lines, FGFR3 expression was induced by hypoxia in a transcriptional and HIF-1α-dependent manner. Increased FGFR3 was also in part dependent on miR-100 levels, which decreased in hypoxia. Knockdown of FGFR3 led to a decrease in phosphorylation of the downstream kinases mitogen-activated protein kinase (MAPK) and protein kinase B (PKB), which was more pronounced under hypoxic conditions. Furthermore, transfection of mimic-100 also decreased phosphorylation of MAPK and PKB. Finally, knocking down FGFR3 profoundly decreased 2-D and 3-D cell growth, whereas introduction of mimic-100 decreased 3-D growth of cells.Hypoxia, in part via suppression of miR-100, induces FGFR3 expression in bladder cancer, both of which have an important role in maintaining cell viability under conditions of stress. 
 Strong immunohistochemical expression of fibroblast growth factor receptor 3, superficial staining pattern of cytokeratin 20, and low proliferative activity define those papillary urothelial neoplasms of low malignant potential that do not recur. Papillary urothelial neoplasm of low malignant potential (PUNLMP) is a clinically significant lesion because recurrence occurs in approximately 35% of patients. To date, it is not possible to identify those cases that will recur based on conventional histopathologic assessment. The objective of the current study was to evaluate immunohistochemically tissue expression of fibroblast growth factor receptor 3 (FGFR3), cytokeratin 20 (CK20), and MIB-1 in nonrecurrent and recurrent PUNLMP.FGFR3, CK20, and MIB-1 were investigated by immunohistochemistry (IHC) in 80 PUNLMP cases (41 nonrecurrent and 39 recurrent), in 4 cases of normal urothelium (NU), and in 5 cases of muscle   invasive   pT2 urothelial carcinoma (UC). Statistics included discriminant analysis.NU demonstrated a weak to moderate FRFG3 staining intensity, a superficial pattern of CK20 staining, and low proliferative activity. UC was found to have FGFR3 staining similar to NU, an abnormal CK20 expression, and high proliferative activity. The nonrecurrent PUNLMP group demonstrated strong FGFR3 intensity in 80.5% of the cases (vs 56.4% of the recurrent cases), a superficial CK20 staining pattern in 53.7% of the cases (vs 28.2% of the recurrent cases), and a percentage of MIB-1-positive nuclei below the median value of all the PUNLMP cases in 61% of the cases (35.9% in the recurrent cases). The differences were statistically significant. Discriminant analysis based on these 3 features demonstrated that 67.5% of cross-validated grouped PUNLMP cases were correctly allocated, with 73.2% of the nonrecurrent and 61.5% of the nonrecurrent cases being correctly classified. The specificity and sensitivity were 73.1% and 61.5%, respectively.Strong immunohistochemical expression of FGFR3, a superficial staining pattern of CK20, and a low proliferative activity define those papillary urothelial neoplasms of low malignant potential that do not recur. 
 Optimization of nonmuscle   invasive   bladder cancer recurrence detection using a urine based FGFR3 mutation assay. FGFR3 mutations occur in 70% of nonmuscle   invasive   bladder tumors. Although urine based FGFR3 mutation analysis can detect recurrence, its sensitivity may be limited if samples have few or no tumor cells. We determined whether test sensitivity depends on tumor size and the time point of urine collection, and how to increase sensitivity.A total of 440 urine samples from 18 patients with a suspicious bladder lesion at cystoscopy were collected during 6 days before surgery. Eight patients (300 samples) had an FGFR3 mutant tumor, including 4 each with a tumor greater than 3 and less than 1.5 cm. Polymerase chain reaction based FGFR3 analysis was done on all tumors and urine samples.FGFR3 mutations were detected in 257 of the 300 urine samples (86%) from patients with an FGFR3 mutant tumor. Assay sensitivity was 100% for tumors greater than 3 cm and 75% for tumors less than 1.5 cm. It increased to 100% in patients with a less than 1.5 cm tumor when samples were pooled during 24 hours. Sensitivity was not influenced by the time of urine collection. All urine samples from patients with an FGFR3 wild-type tumor were negative for FGFR3 mutation.The sensitivity of tumor detection increased with tumor size. FGFR3 assay sensitivity depends on the number of shed tumor cells and improves by increasing urine volume. These findings suggest that there is an upper limit to the sensitivity of the FGFR3 assay when 1 urine sample is analyzed. This may also apply to other DNA or RNA based assays. 
 Expression of the endothelin axis in non  invasive   and superficially   invasive   bladder cancer: relation to clinicopathologic and molecular prognostic parameters. The endothelin (ET) axis plays a role in cancer biology and plays a potential role as a target for molecular therapy in urogenital tumours. Alterations of several proteins of the ET axis were detected in   invasive   bladder cancer.To examine the potential role of the expression of ET axis proteins compared to other prognostic parameters (kinase inhibitor 67 [Ki-67], tumour protein 53 [TP53], and fibroblast growth factor receptor 3 gene [FGFR3] mutations) in non  invasive   and   invasive   bladder cancer.Tissue microarrays from 154 consecutive patients with pTa-pT2 urothelial bladder cancer were immunohistochemically stained for endothelin 1 (ET-1), endothelin A and B receptors (ET(A)R, ET(B)R), TP53, and Ki-67. FGFR3 mutations were detected by SNaPshot analysis.The results were correlated with clinicopathologic parameters and disease-specific survival, overall survival, and recurrence-free survival.Proteins of the ET axis were frequently expressed in bladder cancer (ET-1 in 62% of tumours, ET(A)R in 93% of tumours, and ET(B)R in 84% of tumours). ET-1 expression was strongly correlated with tumour stage (p=0.015), histologic grade (p=0.008), and low proliferation status (p=0.003). ET(A)R immunostaining was only associated with low proliferation status (p=0.015). Kaplan-Meier survival analysis showed a significantly longer overall survival for patients with ET-1-expressing tumours (p=0.007). A significantly longer disease-free survival was found in patients with ET(A)R-expressing tumours (p=0.040), whereas ET(B)R expression was significantly correlated to a longer disease-free survival only in subgroups of patients with multifocal tumours (p=0.031), low proliferation index (Ki-67 ≤10; p=0.050), low TP53 expression (≤10; p=0.018), and tumours with an FGFR3 mutation (p=0.026). In the global model for recurrence-free survival, only high-grade (p=0.048) and negative ET(A)R immunoreactivity (p=0.048) were correlated with poor prognosis.In addition to other factors, particularly age at diagnosis and growth pattern, lack of ET-1 expression may be an independent negative prognostic factor for the overall-survival probability of bladder cancer patients. Lack of ET(A)R expression may be an independent negative marker for recurrence-free survival. 
 TP53 and FGFR3 Gene Mutation Assessment in Urine: Pilot Study for Bladder Cancer Diagnosis. To assess, in a prospective clinical research study, a new non-  invasive   and reliable test to accurately detect tumor protein 53 (TP53) and fibroblast growth factor receptor-3 (FGFR3) mutations in cells in urine.TP53 mutations were analyzed using the functional analysis of separated allele in yeast (FASAY) method, which allows functional analysis of the P53 protein, and FGFR3 mutations were assessed with the SNaPshot system, detecting the eight most frequent point-mutations of this gene. Chi-square test or Fisher's exact test were used to compare TP53 and FGFR3 mutations in the tumors according to tumor stage and grade.TP53 and FGFR3 mutations in bladder tumors increased and decreased respectively with increasing tumor stage and cellular grade (p<0.05 and p<0.001, respectively). A total of 103 tumor/urinary sediment couples were analyzed. TP53 or FGFR3 mutations were observed in 76 tumors. The sensitivity for the detection of this type of mutation in urine was 46%, the specificity was 81%, the positive predictive value was 94% and the negative predictive value was 37%.Our original data confirmed the feasibility of TP53 and FGFR3 mutation detection in urine sediment. These measurements, together with urine cytology, may increase tumor detection. The sensitivity of the TP53/FGFR3 phenotype test in the urine was less than 50% and was not able to replace standard cystoscopy in the diagnosis of bladder tumors. 
 Somatic mutation of fibroblast growth factor receptor-3 (FGFR3) defines a distinct morphological subtype of high-grade urothelial carcinoma. FGFR3 mutations are common in low-grade urothelial carcinoma and represent a potential therapeutic target in this disease. Their incidence and functional role in high-grade urothelial carcinoma (HGUC), which displays an increased propensity for recurrence and muscularis propria invasion, is less well defined. We developed a mass spectrometry-based genotyping assay to define the incidence of FGFR3 mutations in a large clinically annotated set of urothelial carcinomas. FGFR3 mutations were found in 17% of HGUC versus 84% of low-grade lesions. Retrospective pathological review of the class of FGFR3 mutant HGUC revealed unique histological features, characterized by a bulky, exophytic component with branching papillary architecture as well as irregular nuclei with a koilocytoid appearance. The predictive value of this histological appearance was confirmed using a prospective set of 49 additional HGUCs. Prospective histological review was able to correctly predict for the presence of an FGFR3 mutation in 13/24 HGUC specimens that exhibited the distinct morphology (54%). All 25 specimens lacking the defined histological features were FGFR3 wild-type for a negative predictive value of 100%. Macrodissection of individual tumours confirmed the presence of the FGFR3 mutant allele in non-  invasive   and   invasive  , low and high-grade regions of individual tumours and in the lymph node metastases of patients whose tumours possessed the characteristic morphological signature, suggesting that FGFR3 mutations are not restricted to the more clinically indolent regions of HGUCs. These data suggest that histological screening of HGUCs followed by confirmatory genotyping can be used to enrich for the population of HGUCs most likely to harbour activating mutations in the FGFR-3 receptor tyrosine kinase. Histological review could thus aid in the development of targeted inhibitors of FGFR-3 by facilitating the identification of the subset of patients most likely to harbour activating mutations in the FGFR3 gene. 
 The Diagnostic and Prognostic Performance of Urinary FGFR3 Mutation Analysis in Bladder Cancer Surveillance: A Prospective Multicenter Study. To assess the diagnostic and prognostic performance of a non  invasive   FGFR3 mutation analysis. After transurethral resection (TUR) of non  invasive   bladder transitional cell carcinoma (B-TCC), recurrence occurs in 70% of patients, thus justifying cystoscopic surveillance.A prospective multicenter study was carried out with a 2-year follow-up of patients with superficial B-TCC. Urine samples were collected before TUR and then before each cystoscopy during follow-up. Screening for the most prevalent FGFR3 mutations was done using urinary cells. The prognostic significance of an FGFR3 mutation at the time of the initial diagnosis was determined. The performance of the test in diagnosing and/or predicting recurrence during follow-up was assessed by calculating sensitivity and specificity.Of 191 patients studied, 74 (39%) had a positive analysis before TUR (FGFR3 mutation group). The presence of an FGFR3 mutation at the time of diagnosis was associated with a shorter time to recurrence (P = .02). During follow-up, 68 patients from the FGFR3 mutation group were evaluated. FGFR3 mutation analysis showed a sensitivity of 0.73 and a specificity of 0.87 when compared with the results of cystoscopy. A positive urine test was predictive of recurrence either at the time of the positive result or later during the 2-year follow-up, with a sensitivity of 0.70 and a specificity of 0.87.Among patients with an FGFR3 mutation in the initial tumor, a n  oninvasi  ve urine test during follow-up can be valuable in diagnosing or predicting subsequent recurrence. 
 A simple and fast method for the simultaneous detection of nine fibroblast growth factor receptor 3 mutations in bladder cancer and voided urine. Mutations in the fibroblast growth factor receptor 3 (FGFR3) occur in 50% of primary bladder tumors. An FGFR3 mutation is associated with good prognosis, illustrated by significantly lower percentage of patients with progression and disease-specific mortality. FGFR3 mutations are especially prevalent in low grade/stage tumors, with pTa tumors harboring mutations in 85% of the cases. These tumors recur in 70% of patients. Efficient FGFR3 mutation detection for prognostic purposes and for detection of recurrences in urine is an important clinical issue. In this paper, we describe a simple assay for the simultaneous detection of nine different FGFR3 mutations.The assay consists of one multiplex PCR, followed by extension of primers for each mutation with a labeled dideoxynucleotide. The extended primers are separated by capillary electrophoresis, and the identity of the incorporated nucleotide indicates the presence or absence of a mutation.The assay was found to be more sensitive than single-strand conformation polymorphism analysis. Mutations could still be detected with an input of only 1 ng of genomic DNA and in a 20-fold excess of wild-type DNA. Moreover, in urine samples from patients with a mutant tumor, the sensitivity of mutation detection was 62%.We have developed a fast, easy to use assay for the simultaneous detection of FGFR3 mutations, which can be of assistance in clinical decision-making and as an alternative for the follow-up of patients by   invasive   cystoscopy for the detection of recurrences in urine. 
 Sensitive detection of FGFR3 mutations in bladder cancer and urine sediments by peptide nucleic acid-mediated real-time PCR clamping. Somatic mutations of the fibroblast growth factor receptor 3 (FGFR3) gene were detected by peptide nucleic acid (PNA)-mediated real-time PCR clamping. Mutation was detected in negative control containing only wild-type DNA due to a misincorporation of dNTPs to PNA binding sites when the amount of template DNA was decreased to 1 ng. Thus, the amount of template DNA was critical determinant of the assay sensitivity in PNA-mediated PCR clamping. Assay conditions were optimized to detect FGFR3 mutations in exons 7, 10, and 15, at a concentration of more than 1% mutated DNA using 50 ng of genomic DNA as the template. Mutations were detected in 12 of 13 (92.3%) tumor tissues and 11 of 13 (84.6%) urine samples from patients with superficial bladder cancer, while no mutations were detected in tissues and/or urine samples from patients with muscle-  invasive   bladder cancer or chronic cystitis. 
 Combined analysis of smoking, TP53, and FGFR3 mutations in Tunisian patients with   invasive   and superficial high-grade bladder tumors. In our cohort, FGFR3 mutations were detected in 31.1% of bladder tumors and are associated with lower stage and grade. Concerning TP53, 62 mutations were found in tumors from 44 cases (48.88%) and are associated with advanced forms. The combined analysis of FGFR3 and TP53 mutations in our cohort showed an independent distribution. In addition, we have reported that FGFR3 mutations spectrum depends on the intensity of tobacco use (pack years: PY). Finally, we have found that the FGFR3wt/TP53mut genotype, which was associated with advanced bladder tumors; was overrepresented in light smokers (PY < 40) compared to nonsmoker patients (p =.01). 
 Fibroblast growth factor receptor 3 mutation analysis on voided urine for surveillance of patients with low-grade non-muscle-  invasive   bladder cancer. Mutations in the fibroblast growth factor receptor 3 (FGFR3) have been found in 70% of the low-grade non-muscle-  invasive   bladder cancer (NMI-BC) tumors. We aim to determine the potential of FGFR3 mutation analysis on voided urine to detect recurrences during surveillance of patients with low-grade NMI-BC.FGFR3 mutation status of the study inclusion tumor was determined from 200 low-grade NMI-BC patients. Patients with an FGFR3-mutant inclusion tumor were selected for analysis and monitored by cystoscopy, and voided urine samples were collected. FGFR3 mutation analysis was done on 463 prospectively collected urines. Sensitivity and predictive value of the assay were determined for detection of concomitant recurrences. Longitudinal and Cox time-to-event analyses were done to determine the predictive value for detection of future recurrences.Median follow-up was 3.5 years. The sensitivity of the assay for detection of concomitant recurrences was 26 of 45 (58%). Of the 105 positive urine samples, 85 (81%) were associated with a concomitant or a future recurrence. An FGFR3-positive urine was associated with a 3.8-fold (P < 0.0001) higher risk of having a recurrence in the Cox analysis. In contrast, only 41 of 358 (11%) FGFR3-negative urine samples were associated with a recurrence. Positive predictive value increased from 25% to 90% in patients having consecutive FGFR3-positive urine tests.FGFR3 mutation analysis on voided urine is a simple and non  invasive   diagnostic method for detection of recurrences during surveillance of patients presenting with a low-grade FGFR3-mutant NMI-BC tumor. 
 Chromosome 9 deletions are more frequent than FGFR3 mutations in flat urothelial hyperplasias of the bladder. Flat urothelial hyperplasias (FUHs) in patients with papillary bladder tumours frequently show deletions of chromosome 9, suggesting that FUH could be the first neoplastic step in the development of papillary bladder cancer. FGFR3 mutations are frequent in non-  invasive   papillary tumours with low risk of progression. Our aim was to investigate the frequency of FGFR3 mutations and deletions of chromosomes 9p/q and 8p/q in FUH. Thirty FUH and 9 simultaneous or consecutive tumours were detected by 5-ALA-based photodynamic cystoscopy. DNA was isolated from frozen sections and whole genome amplification was done by I-PEP-PCR, followed by LOH analysis on chromosomes 8p/q and 9p/q. FGFR3 mutations were detected by SNaPshot analysis. LOH analysis on FUH revealed deletions at 9p/q (11/30, 37%) and 8p/q (3/30, 10%). FGFR3 mutations were found in 7/30 FUH (23%). Only 2 FUH showed an FGFR3 mutation without deletions of chromosome 9. In contrast, 6 FUH revealed chromosome 9 deletions but wild type FGFR3 (p = 0.03). These results suggest that chromosome 9 deletions are the earliest genetic alterations in bladder cancer. The detection of FGFR3 mutations in FUH further supports the role of this lesion as precursor of papillary bladder cancer. 
 Mutations of fibroblast growth factor receptor 3 gene (FGFR3) in transitional cell carcinoma of urinary bladder in Thai patients [Revision-2a]. Determine the incidence of FGFR3 mutations in Thai patients with bladder transitional cell carcinoma (TCC), and evaluate their correlation with pathological characteristics.One hundred twenty two frozen tissue samples from TCC patients were analyzed for mutations in exons 7, 10, and 15 of FGFR3 by polymerase chain reaction and direct DNA sequencing.FGFR3 mutations were detected in 22 of 122 cases (18%) studied, all of which were found within previously identified hotspots, including S249C (13 cases; 59%) and R248C (4 cases; 18%) in exon 7, and Y375C (5 cases; 23%) in exon 10, but no mutations in exon 15. Sixty-five patients (53%) were categorized as non-muscle-  invasive   TCC (pTa-pT1). The incidence of mutations is significantly higher in non-muscle-  invasive   tumors (28%) compared to the muscle-invading group (7%) (p < 0.01). Patients with grade (G) 1 TCC have significantly higher mutation frequency (40%) compared to other grades (4%) (p < 0.01). When T stage and grade were considered together, mutations were most commonly found in Ta-T1/G1 TCC (18/45 cases, 40%). Mean follow-up period was 45.1 months. Two-year and four-year overall survival (OS) was 70% and 56% respectively. Three-year OS in non-muscle-  invasive   TCC (80%) is significantly higher than that of muscle invading TCC (41%) (p < 0.01). However three-year OS in cases with an FGFR3 mutation (73%) is not significantly different from cases without a mutation (61%). In 16 cases with an FGFR3 mutation and recurrent disease, no mutations were detected in metachronous disease.The overall incidence of FGFR3 mutations in Thai patients with TCC was lower than similar reports from other ethnic groups. In the presented cases, although FGFR3 mutations were frequently detected in low-grade, non-muscle-  invasive   TCC, identical mutation was not conserved in metachronous disease, thereby precluding the use of this marker in detection of tumor recurrence. 
 Colorectal cancer cells activate adjacent fibroblasts resulting in FGF1/FGFR3 signaling and increased invasion. Cancer-associated fibroblasts expressing fibroblast activation protein (FAP) have been implicated in the   invasive   behavior of colorectal cancer. In this study, we use FAP expression as a marker of fibroblast activation and analyze the effect of activated fibroblasts on colorectal cancer migration and invasion in experimental cell studies. We also investigated the expression pattern of FAP in cancer-associated fibroblasts during transformation from benign to malignant colorectal tumors. In immunohistochemical analyses, FAP was expressed in fibroblasts in all colorectal cancer samples examined, whereas all normal colon, hyperplastic polyps, or adenoma samples were negative. In in vitro studies, conditioned medium from colon cancer cells, but not adenoma cells, activated fibroblasts by inducing FAP expression. These activated fibroblasts increased the migration and invasion of colon cancer cells in Boyden chamber experiments and in a three-dimensional cell culture model. We identify fibroblast growth factor 1/fibroblast growth factor receptor 3 (FGF1/FGFR-3) signaling as mediators leading to the increased migration and invasion. Activated fibroblasts increase their expression of FGF1, and by adding a fibroblast growth factor receptor inhibitor, as well as an FGF1-neutralizing antibody, we reduced the migration of colon cancer cells. Our findings provide evidence of a possible molecular mechanism involved in the cross talk between cancer cells and fibroblasts leading to cancer cell invasion. 
 [Correlation of FGFR3 mutations and chromosomal alterations in bladder cancer]. It has been suggested that mutation of FGFR3 is associated with non-  invasive   tumors of low malignant potential and low risk of recurrence and progression. The aim of this study was to analyze the distribution of FGFR3 mutations in bladder tumors of different grade and stage and to determine the relation of FGFR3 mutations to chromosomal alterations detected by CGH. Frozen sections of 100 bladder cancer samples served as templates for manual microdissection. DNA was isolated from dissected samples containing at least 80% tumor cells. Mutations in FGFR3 were analyzed by SNaPshot analysis. CGH was carried out according to standard protocols. FGFR3 mutations were detected in 45 out of 92 samples (48.9 %). Concerning T-category, the following mutation frequencies occurred: pTa - 69 %, pT1 - 38 %, pT2/3 - 0 %. The mutation frequency was significantly associated with tumor grade: G1 - 72%, G2 - 56%, G3 - 4%. In pTaG1 tumors, mutations were found in 74 %. A significant lower number of genetic alterations per tumor detected by CGH was associated with FGFR3 mutations (2 vs. 8). This association was also seen in pTaG1 tumors: 2.5 (with mutation) vs. 7.5 (without mutation). Our results confirm that FGFR3 mutations characterize non-  invasive   low-risk tumors of low malignancy. The low malignant potential of these tumors is underlined by a low number of chromosomal alterations per tumor. Therefore, FGFR3 could represent a prognostic marker of chromosomally stable tumors with low malignant potential. 
 The FGFR3 mutation is related to favorable pT1 bladder cancer. Stage pT1 bladder cancer comprises a heterogeneous group of tumors for which different management options are advocated. FGFR3 mutations are linked to favorable (low grade/stage) pTa bladder cancer while altered P53 is common in cases of high grade, muscle   invasive   (pT2 or greater) bladder cancer. We determined the frequency of FGFR3 mutations and P53 alterations in patients with pT1 bladder cancer and correlated these data to histopathological variables and clinical outcomes.We included 132 patients with primary pT1 bladder cancer from a total of 2 academic centers. A uropathologist reviewed the slides for grade and confirmed the pT1 diagnosis. FGFR3 mutation status was examined by SNaPshot® analysis and P53 expression was determined by standard immunohistochemistry. Kaplan-Meier and multivariate analyses were used to assess progression.FGFR3 mutations were detected in 37 of 132 pT1 bladder cancer cases (28%) and altered P53 was seen in 71 (54%). Only 8% of patients had the 2 molecular alterations (p = 0.001). FGFR3 mutation correlated with lower grade and altered P53 correlated with high grade pT1 bladder cancer. Median followup was 6.5 years. FGFR3 mutation status and carcinoma in situ were significant for predicting progression on univariate and multivariate analyses but P53 status was not.FGFR3 mutations selectively identify patients with pT1 bladder cancer who have favorable disease characteristics. Further study may confirm that FGFR3 identifies those who would benefit from a conservative approach to the disease. 
 A meta-analysis of the relationship between FGFR3 and TP53 mutations in bladder cancer. TP53 and FGFR3 mutations are the most common mutations in bladder cancers. FGFR3 mutations are most frequent in low-grade low-stage tumours, whereas TP53 mutations are most frequent in high-grade high-stage tumours. Several studies have reported FGFR3 and TP53 mutations to be mutually exclusive events, whereas others have reported them to be independent. We carried out a meta-analysis of published findings for FGFR3 and TP53 mutations in bladder cancer (535 tumours, 6 publications) and additional unpublished data for 382 tumours. TP53 and FGFR3 mutations were not independent events for all tumours considered together (OR = 0.25 [0.18-0.37], p = 0.0001) or for pT1 tumours alone (OR = 0.47 [0.28-0.79], p = 0.0009). However, if the analysis was restricted to pTa tumou  rs or to   muscle-invasive tumours alone, FGFR3 and TP53 mutations were independent events (OR = 0.56 [0.23-1.36] (p = 0.12) and OR = 0.99 [0.37-2.7] (p = 0.35), respectively). After stratification of the tumours by stage and grade, no dependence was detected in the five tumour groups considered (pTaG1 and pTaG2 together, pTaG3, pT1G2, pT1G3, pT2-4). These differences in findings can be attributed to the putative existence of two different pathways of tumour progression in bladder cancer: the CIS pathway, in which FGFR3 mutations are rare, and the Ta pathway, in which FGFR3 mutations are frequent. TP53 mutations occur at the earliest stage of the CIS pathway, whereas they occur would much later in the Ta   pathway  , at the T1G3 or muscle-invasive stage. 
 FGFR3 expression in primary   invasive   bladder cancers and matched lymph node metastases. FGFR3 is considered a good therapeutic target for bladder cancer. However, to our knowledge it is unknown whether the FGFR3 status of primary tumors is a surrogate for related metastases, which must be targeted by FGFR targeted systemic therapies. We assessed FGFR3 protein expression in primary bladder tumors and matched nodal metastases.We examined matched primary tumor and nodal metastases from 150 patients with bladder cancer clinically staged as N0M0. Four samples per patient were incorporated into a tissue microarray and FGFR3 expression was assessed by immunohistochemistry. FGFR3 expression was tested for an association with categorical clinical data using the Fisher exact test, and with overall and recurrence-free survival by Kaplan-Meier analysis.Duplicate spots from primary tumors and lymph node metastases were highly concordant (OR 8.6 and 16.7, respectively, each p <0.001). Overall FGFR protein expression levels did not differ between primary and metastatic lesions (p = 0.78). Up-regulated expression was recorded in 53 of 106 evaluable primary tumor spots and 56 matched metastases. Concordance of FGFR3 expression levels in 79 matched primary tumor and metastasis specimens was high (OR 8.45, p <0.001). In 15 and 12 patients expression was up-regulated in only metastasis and in only the primary tumor, respectively. Overall and recurrence-free survival was not related to FGFR3 expression.FGFR3 expression in matched primary and metastasized bladder cancer specimens showed good but not absolute concordance. Thus, in most patients primary tumor FGFR3 status can guide the selection of FGFR targeted therapy. 
 The development of multiple bladder tumour recurrences in relation to the FGFR3 mutation status of the primary tumour. Non-muscle   invasive   bladder cancers (NMI-BCs) represent 75% of bladder cancers upon presentation. After removal of the primary tumour by transurethral resection, multiple recurrences continue to develop in 70% of patients. Consequently, prolonged and costly surveillance by cystoscopy is required. Mutations in the FGFR3 oncogene are common in NMI-BCs and are associated with a lower chance of progression to muscle-  invasive   disease. Here we analysed the consistency of FGFR3 mutations in primary and recurrent tumours. This knowledge is of crucial importance if FGFR3 mutation analysis on urinary cells is to be used as an alternative for cystoscopical surveillance. To this end, we monitored the disease process and FGFR3 mutation status of primary and recurrent tumours in 118 patients with NMI-BC. During median follow-up of 8.8 years, these patients underwent 2133 cystoscopies and 80 patients developed 414 recurrences. FGFR3 mutations were equally prevalent in primary and recurrent tumours (63%). Patients can have different types of FGFR3 mutations in different tumours. Recurrence risk was not significantly different for patients with a mutant or wild-type primary tumour. Recurrence rates varied widely between patients but were constant for a patient and were unrelated to FGFR3 status. In the mutant patient group, in contrast to the wild-type group, recurrences continued to develop after 10 years. In 81% of the recurrences of patients with a mutant primary tumour, a mutation was found. Moreover, recurrences in this patient group were of lower stage and grade than those of patients with a wild-type primary tumour (p < 0.001). These results suggest that surveillance by FGFR3 mutation analysis on voided urine in combination with a reduced cystoscopy frequency of patients presenting with an FGFR3 mutant tumour is worth investigating. 
 Increased angiogenesis and FGFR protein expression indicate a favourable prognosis in bladder cancer. Compared to other members of the fibroblast growth factor receptor (FGFR) family, only few studies investigate FGFR3 in tumour angiogenesis. We investigated the connection between angiogenesis and FGF/FGFR expression including FGFR3 mutation status in urothelial carcinomas. Immunohistochemistry was performed in   invasive   and non-  invasive   urothelial cancers of 61 patients. Protein expression of CD31, factor VIII (FVIII), FGF-1/2, FGFR1, FGFR3 and FGFR4 and FGFR3 mutation status were evaluated. Morphometric assessment of angiogenesis including microvessel count (MVC) and vascular surface area (VSA) was analysed. Correlation and survival analyses (overall survival (OS) and disease-free survival (DFS)) with univariate and multivariate analyses were performed. CD31 values (MVC and VSA) significantly correlated with OS and DFS. OS and DFS were significantly better in patients with FGFR3 overexpression. Multivariate analysis revealed FGFR3 protein expression and tumour grading (WHO classification 2004) as independent prognostic factors of OS and VSA of CD31 and FGFR3 protein expression of DFS. FGFR3 mutation status was correlated with VSA measured by FVIII. FGFR3 may be able to induce a pro-angiogenic phenotype in urothelial carcinomas and significantly influence prognosis. Consequently, FGFR3 is a potential therapeutic target also from the angiogenesis perspective. 
 Knockdown by shRNA identifies S249C mutant FGFR3 as a potential therapeutic target in bladder cancer. More than 60% of low-grade non-  invasive   papillary urothelial cell carcinomas contain activating point mutations of fibroblast growth factor receptor 3 (FGFR3). The phenotypic consequences of constitutive activation of FGFR3 in bladder cancer have not been elucidated and further studies are required to confirm the consequences of inhibiting receptor activity in urothelial cells. We measured FGFR3 transcript levels and demonstrated that transcript levels were significantly more abundant in low-stage and grade tumours. We identified a tumour cell line, 97-7, expressing the most common FGFR3 mutation (S249C) at similar FGFR3 transcript levels to low-stage and grade tumours. In these cells, S249C FGFR3 protein formed stable homodimers and was constitutively phosphorylated. We used retrovirus-mediated delivery of shRNA to knockdown S249C FGFR3. This induced cell flattening, decreased cell proliferation and reduced clonogenicity on plastic and in soft agar. However, no effects of knockdown of wild-type FGFR3 were observed in telomerase immortalized normal human urothelial cells, indicating possible dependence of the tumour cell line on mutant FGFR3. Re-expression of S249C FGFR3 in shRNA-expressing 97-7 cells resulted in a reversal of phenotypic changes, confirming the specificity of the shRNA. These results indicate that targeted inhibition of S249C FGFR3 may represent a useful therapeutic approach in superficial bladder cancer. 
  GeneRIFs  
 Among patients with an FGFR3 mutation in the initial tumor, a non  invasive   urine test during follow-up can be valuable in diagnosing or predicting subsequent recurrence. 
 FGFR3 mutations, but not FGFR3 expression and FGFR3 copy-number variations, are associated with favourable non-muscle   invasive   bladder cancer. 
 Data indicat that fibroblast growth factor receptor 3 (FGFR3) overexpression was predictive of adverse outcome in muscle-  invasive   bladder carcinoma with adjuvant cisplatin-based chemotherapy after radical cystectomy. 
 Highlight the crucial role of CDKN2A loss in the progression of non-muscle-  invasive   FGFR3-mutated bladder carcinomas and provide a potentially useful clinical marker for adapting the treatment of such tumours. 
 results suggest an inverse correlation between FGFR3 mutations and hypermethylation events, which may be used to improve non  invasive  , DNA-based detection of bladder cancer 
 FGFR3 expression in   invasive   breast cancer was not found to be significantly associated with specific clinicopathological/molecular parameters, but might be used as a candidate marker for a poor prognosis. 
 Urine FGFR3 mutation assay and cytological examination may be available in the future as complementary diagnostic modalities in postoperative management ofnon-muscle   invasive   bladder cancer. 
 Combined analysis of smoking, TP53, and FGFR3 mutations in Tunisian patients with   invasive   and superficial high-grade bladder tumors. 
 FGFR3 mutation status might be used to select patients with   invasive   UCC who have a lower risk of death 
 FGFR3 status allows a better risk stratification for patients with high-grade non-muscle-  invasive   UCC 
 The group of low-grade non  invasive   papillary tumours is defined by the presence of an FGFR3 mutation 
  [Mm]etastasize  PubMed  
 FGFR3 expression in primary invasive bladder cancers and matched lymph node metastases. FGFR3 is considered a good therapeutic target for bladder cancer. However, to our knowledge it is unknown whether the FGFR3 status of primary tumors is a surrogate for related metastases, which must be targeted by FGFR targeted systemic therapies. We assessed FGFR3 protein expression in primary bladder tumors and matched nodal metastases.We examined matched primary tumor and nodal metastases from 150 patients with bladder cancer clinically staged as N0M0. Four samples per patient were incorporated into a tissue microarray and FGFR3 expression was assessed by immunohistochemistry. FGFR3 expression was tested for an association with categorical clinical data using the Fisher exact test, and with overall and recurrence-free survival by Kaplan-Meier analysis.Duplicate spots from primary tumors and lymph node metastases were highly concordant (OR 8.6 and 16.7, respectively, each p <0.001). Overall FGFR protein expression levels did not differ between primary and metastatic lesions (p = 0.78). Up-regulated expression was recorded in 53 of 106 evaluable primary tumor spots and 56 matched metastases. Concordance of FGFR3 expression levels in 79 matched primary tumor and metastasis specimens was high (OR 8.45, p <0.001). In 15 and 12 patients expression was up-regulated in only metastasis and in only the primary tumor, respectively. Overall and recurrence-free survival was not related to FGFR3 expression.FGFR3 expression in matched primary and   metastasize  d bladder cancer specimens showed good but not absolute concordance. Thus, in most patients primary tumor FGFR3 status can guide the selection of FGFR targeted therapy. 
  [Mm]etastasis  PubMed  
 Fibroblast growth factor receptors-1 and -3 play distinct roles in the regulation of bladder cancer growth and   metastasis  : implications for therapeutic targeting. Fibroblast growth factor receptors (FGFRs) are activated by mutation and overexpressed in bladder cancers (BCs), and FGFR inhibitors are currently being evaluated in clinical trials in BC patients. However, BC cells display marked heterogeneity in their responses to FGFR inhibitors, and the biological mechanisms underlying this heterogeneity are not well defined. Here we used a novel inhibitor of FGFRs 1-3 and RNAi to determine the effects of inhibiting FGFR1 or FGFR3 in a panel of human BC cell lines. We observed that FGFR1 was expressed in BC cells that also expressed the "mesenchymal" markers ZEB1 and vimentin, whereas FGFR3 expression was restricted to the E-cadherin- and p63-positive "epithelial" subset. Sensitivity to the growth-inhibitory effects of BGJ-398 was also restricted to the "epithelial" BC cells and it correlated directly with FGFR3 mRNA levels but not with the presence of activating FGFR3 mutations. In contrast, BGJ-398 did not strongly inhibit proliferation but did block invasion in the "mesenchymal" BC cells in vitro. Similarly, BGJ-398 did not inhibit primary tumor growth but blocked the production of circulating tumor cells (CTCs) and the formation of lymph node and distant metastases in mice bearing orthotopically implanted "mesenchymal" UM-UC3 cells. Together, our data demonstrate that FGFR1 and FGFR3 have largely non-overlapping roles in regulating invasion/  metastasis   and proliferation in distinct "mesenchymal" and "epithelial" subsets of human BC cells. The results suggest that the tumor EMT phenotype will be an important determinant of the biological effects of FGFR inhibitors in patients. 
 Fibroblast growth factor receptors-1 and -3 play distinct roles in the regulation of bladder cancer growth and   metastasis  : implications for therapeutic targeting. Fibroblast growth factor receptors (FGFRs) are activated by mutation and overexpressed in bladder cancers (BCs), and FGFR inhibitors are currently being evaluated in clinical trials in BC patients. However, BC cells display marked heterogeneity in their responses to FGFR inhibitors, and the biological mechanisms underlying this heterogeneity are not well defined. Here we used a novel inhibitor of FGFRs 1-3 and RNAi to determine the effects of inhibiting FGFR1 or FGFR3 in a panel of human BC cell lines. We observed that FGFR1 was expressed in BC cells that also expressed the "mesenchymal" markers ZEB1 and vimentin, whereas FGFR3 expression was restricted to the E-cadherin- and p63-positive "epithelial" subset. Sensitivity to the growth-inhibitory effects of BGJ-398 was also restricted to the "epithelial" BC cells and it correlated directly with FGFR3 mRNA levels but not with the presence of activating FGFR3 mutations. In contrast, BGJ-398 did not strongly inhibit proliferation but did block invasion in the "mesenchymal" BC cells in vitro. Similarly, BGJ-398 did not inhibit primary tumor growth but blocked the production of circulating tumor cells (CTCs) and the formation of lymph node and distant metastases in mice bearing orthotopically implanted "mesenchymal" UM-UC3 cells. Together, our data demonstrate that FGFR1 and FGFR3 have largely non-overlapping roles in regulating invasion/  metastasis   and proliferation in distinct "mesenchymal" and "epithelial" subsets of human BC cells. The results suggest that the tumor EMT phenotype will be an important determinant of the biological effects of FGFR inhibitors in patients. 
 FGFR3 and TP53 mutations in a prospective cohort of Belarusian bladder cancer patients. The aim of this study was to determine the frequencies of FGFR3 and TP53 mutations in a prospective cohort of 150 bladder cancer patients and to assess the relationship between their mutational status and clinicopathological variables.The FGFR3 and TP53 mutations were detected by the SNaPshot method and PCR-single-strand conformational polymorphism analysis followed by DNA sequencing.The activating FGFR3 mutations were found in 71 (47.3%) whereas TP53 mutations were observed in 31 (20.7%) urothelial carcinomas. FGFR3-mutant tumors significantly correlated with lower tumor stage and grade, papillary form of bladder cancer and the absence of metastases while TP53-mutant tumors were strongly associated with higher tumor stage and grade as well as the presence of   metastasis  . We also found significant inverse correlation between FGFR3 mutations and TP53 alterations in urothelial carcinomas (p=0.03). Four possible genotypes were observed in the whole studied cohort, namely FGFR3mut/TP53wt (41.3%), FGFR3wt/TP53wt (38%), FGFR3wt/TP53mut (14.7%), and FGFR3mut/TP53mut (6%). Tumors with FGFR3wt/TP53wt genotype comprised the subgroup, in which all stages and grades were equally distributed.Our findings confirm the alternative role of FGFR3 and TP53 mutations in the development of bladder cancer. Together these two genetic markers are attributed to 62% of the tumors studied. Tumors with both wild type genes included urothelial carcinomas of all stages and grades and may develop through another genetic pathway. To elucidate complete molecular profile of bladder tumors further additional studies are needed. 
 Prognostic value of molecular markers, sub-stage and European Organisation for the Research and Treatment of Cancer risk scores in primary T1 bladder cancer. What's known on the subject? and What does the study add? The stakes are high when making treatment decisions in T1 bladder cancer (BC). Conservative management may lead to progression and possibly death from BC. Conversely, radical cystectomy could be over-treatment of non-progressive disease. The problem for clinicians is that reliable prognostic indices are lacking. We performed a head-to-head comparison of two substaging systems, European Organisation for the Research and Treatment of Cancer (EORTC) risk scores and four molecular markers in T1 carcinomas of the bladder treated conservatively with BCG. T1 sub-stage according to a new system (micro-invasive [T1m] and extensive-invasive [T1e]) was the most important clinical variable for predicting progression to carcinoma invading bladder muscle. The performance of the EORTC risk scores was disappointing for this T1 sub-group. Molecular markers were not significant in multivariable analysis for predicting progression. Future studies may lead to the incorporation of sub-stage (T1m/T1e) in the TNM classification system for urinary BC to guide clinical decision-making in T1 BC.To evaluate the prognostic significance of four molecular markers, sub-stage and European Organisation for the Research and Treatment of Cancer (EORTC) risk scores in primary T1 bladder cancer (BC) treated with adjuvant bacille Calmette-Guérin.The slides of 129 carcinomas of the bladder from two university hospitals were reviewed and the T1 diagnosis was confirmed. T1 sub-staging was done in two separate rounds, using a new system that identifies micro-invasive (T1m) and extensive-invasive (T1e) T1BC, and then according to invasion of the muscularis mucosae (T1a/T1b/T1c). The EORTC risk scores for recurrence and progression were calculated. Uni- and multivariable analyses for recurrence and progression were performed using clinicopathological variables, T1 sub-stage, EORTC risk scores and molecular markers (fibroblast growth factor receptor 3 gene mutation and Ki-67, P53, P27 expression).The median follow-up was 6.5 years. Forty-two patients remained recurrence-free (33%). Progression to T2 or   metastasi  s was observed in 38 (30%) patients. In multivariable analysis for recurrence, multiplicity was significant. In multivariable analysis for progression, female gender, sub-stage (T1m/T1e) and carcinoma in situ (CIS) were significant. Molecular markers were significant in univariable and in multivariable analyses for recurrence. EORTC risk scores were not significant.CIS, female gender and sub-stage (T1m/T1e) were the most important variables for progression. The additional value of molecular markers was modest. Sub-stage (T1m/T1e) could potentially be incorporated in future tumour-node  -metastasi  s classifications. 
 FGFR3 promotes angiogenesis-dependent   metastasis   of hepatocellular carcinoma via facilitating MCP-1-mediated vascular formation. The biological role of fibroblast growth factor receptor 3 (FGFR3) in tumor angiogenesis of hepatocellular carcinoma (HCC) has not been discussed before. Our previous work had indicated FGFR3 was overexpressed in HCC, and silencing FGFR3 in Hu7 cells could regulate tumorigenesis via down-regulating the phosphorylation level of key members of classic signaling pathways including ERK and AKT. In the present work, we explored the role of FGFR3 in angiogenesis-dependent   metastasis   by using SMMC-7721 and QGY-7703 stable cell lines. Our results indicated FGFR3 could regulate in vitro cell migration ability and in vivo lung   metastasis   ability of HCC, which was in accordance with increased angiogenesis ability in vitro and in vivo. Using the supernatant from SMMC-7721/FGFR3 cells, we conducted a human angiogenesis protein microarray including 43 angiogenesis factors and found that FGFR3 modulated angiogenesis and   metastasis   of HCC mainly by promoting the protein level of monocyte chemotactic protein 1 (MCP-1). Silencing FGFR3 by short hairpin RNA (shRNA) could reduce MCP-1 level in lysates and supernatant of QGY-7703 cells and SMMC-7721 cells. Silencing MCP-1 in QGY-7703 or SMMC-7721 cells could induce similar phenotypes compared with silencing FGFR3. Our results suggested FGFR3 promoted   metastasis   potential of HCC, at least partially if not all, via facilitating MCP-1-mediated angiogenesis, in addition to previously found cell growth and   metastasis  . MCP-1, a key medium between HCC cells and HUVECs, might be a novel anti-vascular target in HCC. 
 Long noncoding RNA FGFR3-AS1 promotes osteosarcoma growth through regulating its natural antisense transcript FGFR3. Long noncoding RNAs (lncRNAs), a new class of RNAs with no protein-coding potential, have been reported to have crucial roles in the regulation of a variety of tumors. However, the functions and molecular mechanisms of lncRNAs to osteosarcoma are still largely unknown. The purpose of this study is to examine the expression, functions and molecular mechanisms of a new lncRNA FGFR3 antisense transcript 1 (FGFR3-AS1) in osteosarcoma. The expression of FGFR3-AS1 was examined by real-time quantitative PCR. The regulation of FGFR3 by FGFR3-AS1 was examined by RNase protection assay, real-time quantitative PCR, western blotting, and luciferase reporter assay. The effects of FGFR3-AS1 on osteosarcoma cell proliferation and cell cycle were determined by Cell Counting Kit-8, Ethynyl deoxyuridine incorporation assay and flow cytometry. FGFR3-AS1 was upregulated in osteosarcoma. Increased FGFR3-AS1 expression correlates with large tumor size, advanced Enneking stage,   metastasis   and poor survival. Through antisense pairing with FGFR3 3'UTR, FGFR3-AS1 increases FGFR3 mRNA stability and upregulates FGFR3 expression. The expression of FGFR3-AS1 and FGFR3 is positively correlated in osteosarcoma tissues. Knockdown of FGFR3-AS1 inhibits the proliferation and cell cycle progression of osteosarcoma cells in vitro. Moreover, knockdown of FGFR3-AS1 inhibits xenograft tumor growth of osteosarcoma cells in vivo. These data demonstrate the mechanisms of how antisense noncoding RNA regulate the expression of sense genes, and show the pivotal functions of FGFR3-AS1 in osteosarcoma. 
 FGFR3 expression in primary invasive bladder cancers and matched lymph node metastases. FGFR3 is considered a good therapeutic target for bladder cancer. However, to our knowledge it is unknown whether the FGFR3 status of primary tumors is a surrogate for related metastases, which must be targeted by FGFR targeted systemic therapies. We assessed FGFR3 protein expression in primary bladder tumors and matched nodal metastases.We examined matched primary tumor and nodal metastases from 150 patients with bladder cancer clinically staged as N0M0. Four samples per patient were incorporated into a tissue microarray and FGFR3 expression was assessed by immunohistochemistry. FGFR3 expression was tested for an association with categorical clinical data using the Fisher exact test, and with overall and recurrence-free survival by Kaplan-Meier analysis.Duplicate spots from primary tumors and lymph node metastases were highly concordant (OR 8.6 and 16.7, respectively, each p <0.001). Overall FGFR protein expression levels did not differ between primary and metastatic lesions (p = 0.78). Up-regulated expression was recorded in 53 of 106 evaluable primary tumor spots and 56 matched metastases. Concordance of FGFR3 expression levels in 79 matched primary tumor and   metastasis   specimens was high (OR 8.45, p <0.001). In 15 and 12 patients expression was up-regulated in only   metastasis   and in only the primary tumor, respectively. Overall and recurrence-free survival was not related to FGFR3 expression.FGFR3 expression in matched primary and metastasized bladder cancer specimens showed good but not absolute concordance. Thus, in most patients primary tumor FGFR3 status can guide the selection of FGFR targeted therapy. 
  GeneRIFs  
 FGFR3 promotes angiogenesis-dependent   metastasis   of hepatocellular carcinoma via facilitating MCP-1-mediated vascular formation. 
 data demonstrate that FGFR1 and FGFR3 have largely non-overlapping roles in regulating invasion/  metastasis   and proliferation in distinct "mesenchymal" and "epithelial" subsets of human BC cells 
  [Cc]ell [Ii]nvasion  PubMed  
 Photofrin based photodynamic therapy and miR-99a transfection inhibited FGFR3 and PI3K/Akt signaling mechanisms to control growth of human glioblastoma In vitro and in vivo. Glioblastoma is the most common malignant brain tumor in humans. We explored the molecular mechanisms how the efficacy of photofrin based photodynamic therapy (PDT) was enhanced by miR-99a transfection in human glioblastoma cells. Our results showed almost similar uptake of photofrin after 24 h in different glioblastoma cells, but p53 wild-type cells were more sensitive to radiation and photofrin doses than p53 mutant cells. Photofrin based PDT induced apoptosis, inhibited   cell invasion  , prevented angiogenic network formation, and promoted DNA fragmentation and laddering in U87MG and U118MG cells harvoring p53 wild-type. Western blotting showed that photofrin based PDT was efficient to block the angiogenesis and cell survival pathways. Further, photofrin based PDT followed by miR-99a transfection dramatically increased miR-99a expression and also increased apoptosis in glioblastoma cell cultures and drastically reduced tumor growth in athymic nude mice, due to down regulation of fibroblast growth factor receptor 3 (FGFR3) and PI3K/Akt signaling mechanisms leading to inhibition of cell proliferation and induction of molecular mechanisms of apoptosis. Therefore, our results indicated that the anti-tumor effects of photofrin based PDT was strongly augmented by miR-99a overexpression and this novel combination therapeutic strategy could be used for controlling growth of human p53 wild-type glioblastomas both in vitro and in vivo. 
 Colorectal cancer cells activate adjacent fibroblasts resulting in FGF1/FGFR3 signaling and increased invasion. Cancer-associated fibroblasts expressing fibroblast activation protein (FAP) have been implicated in the invasive behavior of colorectal cancer. In this study, we use FAP expression as a marker of fibroblast activation and analyze the effect of activated fibroblasts on colorectal cancer migration and invasion in experimental cell studies. We also investigated the expression pattern of FAP in cancer-associated fibroblasts during transformation from benign to malignant colorectal tumors. In immunohistochemical analyses, FAP was expressed in fibroblasts in all colorectal cancer samples examined, whereas all normal colon, hyperplastic polyps, or adenoma samples were negative. In in vitro studies, conditioned medium from colon cancer cells, but not adenoma cells, activated fibroblasts by inducing FAP expression. These activated fibroblasts increased the migration and invasion of colon cancer cells in Boyden chamber experiments and in a three-dimensional cell culture model. We identify fibroblast growth factor 1/fibroblast growth factor receptor 3 (FGF1/FGFR-3) signaling as mediators leading to the increased migration and invasion. Activated fibroblasts increase their expression of FGF1, and by adding a fibroblast growth factor receptor inhibitor, as well as an FGF1-neutralizing antibody, we reduced the migration of colon cancer cells. Our findings provide evidence of a possible molecular mechanism involved in the cross talk between cancer cells and fibroblasts leading to cancer   cell invasion  . 
   
  
 STYK1 
 
  NCBI Gene  
  GeneRIF  
  Pubmed  
 
 
  Search Term  Rank  Fields  
  [Mm]igration  6  Process(1); PubMed(3)  
  [Mm]etastasis  12  PubMed(5); GeneRIFs(2)  
 
 
 hide/show details for STYK1 
  [Mm]igration  Process  
 cell   migration   
  PubMed  
 A novel protein tyrosine kinase NOK that shares homology with platelet- derived growth factor/fibroblast growth factor receptors induces tumorigenesis and metastasis in nude mice. Receptor protein tyrosine kinases (RPTKs) play important roles in the regulation of a variety of cellular processes including cell   migration  , proliferation, and protection from apoptosis. Here, we report the identification and characterization of a novel RPTK-like molecule that has a critical role in induction of tumorigenesis and metastasis and is termed Novel Oncogene with Kinase-domain (NOK). NOK contains a putative single transmembrane domain and a conserved intracellular tyrosine kinase domain that shares homology with members of the platelet-derived growth factor/fibroblast growth factor receptor superfamily. NOK was exclusively located in the cytoplasm. NOK mRNAs were detected in limited human organs and expressed with the highest abundance in the prostate. A variety of tumor cells also expressed the NOK mRNAs. We demonstrated that NIH3T3 and BaF3 cells could be strongly transformed by the expression of the NOK gene as examined by colony formation experiment. In addition, BaF3 cells with the stable expression of NOK induced rapid tumorigenesis in nude mice. Interestingly, these NOK-expressing tumor cells could promptly invade and spread into various distinct organs and form metastatic foci, eventually leading to the rapid death of these animals. Moreover, molecular mechanism studies indicated that NOK could concomitantly activate both MAP kinase and phosphatidylinositol 3'-kinases (PI3K) pathways in stable BaF3 cells. Thus, our results both in vitro and in vivo suggest that NOK is a novel oncogene with the capacity of promoting cell transformation, tumorigenesis, and metastasis. 
 High Novel Oncogene with Kinase-Domain (NOK) Gene Expression is Associated with the Progression of Renal Cell Carcinoma. To study the expression levels of the NOK (novel oncogene with kinase-domain) gene in renal cell carcinoma and its association with the progression of this cancer.In this study, immunohistochemistry (IHC) and Western blot analyses were applied to investigate the NOK expression level in RCC and adjacent normal renal tissue samples. MTT, colony formation, and   migration   assays were also utilized to evaluate the role of NOK in RCC cell lines.Knocked-down expression of NOK in an RCC cell line (786-0) suppressed cellular proliferation and   migration   by restraining the activation of AKT and ERK. We found that the expression level of NOK was significantly higher in RCC tissues than in their adjacent tissues, and more importantly, overexpression of NOK was evidently correlated with the tumor TNM stage and Fuhrman grade (p < 0.001). A high level of NOK was also associated with poor overall survival (p < 0.05) and disease-free survival (p < 0.05) by Kaplan-Meier analysis.NOK expression increased in RCC and was significantly correlated with TNM stage, Fuhrman grade, poor overall survival, poor disease-free survival, metastasis, and proliferation in RCC cells by regulating the activation of AKT and ERK, suggesting that NOK may play important roles as a positive regulator to RCC. 
 Depletion of STYK1 inhibits intrahepatic cholangiocarcinoma development both in vitro and in vivo. Intrahepatic cholangiocarcinoma (ICC) has been reported to be the second most common primary hepatic carcinoma worldwide, and very limited therapies are currently available. Serine threonine tyrosine kinase (STYK1), a member of the receptor tyrosine kinase family, exhibits tumorigenicity in many types of cancers and is a potential therapeutic target for ICC. In this study, STYK1 was knocked down in the ICC cell lines HCCC-9810 and RBE via a lentivirus-mediated system using short hairpin RNA (shRNA). Next, cell proliferation, colony formation, cell cycle progression, tumor formation in nude mice,   migration   and invasion, and the expression levels of cell cycle proteins in Lv-sh STYK1- or Lv-sh Con-infected cells were analyzed by CCK-8 assay, colony formation evaluation, flow cytometry, tumor formation evaluation, wound scratch assay, transwell assay, and western blotting. The results indicated that depletion of STYK1 inhibits ICC development both in vitro and in vivo. 
  [Mm]etastasis  PubMed  
 NOK/STYK1 has a strong tendency towards forming aggregates and colocalises with epidermal growth factor receptor in endosomes. Our previous studies showed that the overexpression of Novel Oncogene with Kinase-domain (NOK)/STYK1 led to cellular transformation, tumorigenesis and   metastasis  . This report characterises the subcellular distribution of NOK in HeLa cells and its localisation in early endosomes. Confocal immunolocalisation studies indicated that NOK had structural subtypes and was distributed into two distinct expression patterns: a dot pattern (DP) and an aggregation pattern (AP). The results of an immunohistochemistry (IHC) analysis of pathological tissues also showed that high expression level of endogenous NOK was expressed in an aggregate-like structure in vivo. Importantly, we found that NOK was localised in endosomes and colocalised with epidermal growth factor receptor (EGFR) in activated endosomal vesicles. However, as the stimulation time increased, NOK and EGFR began to progress through different pathways. EGFR was gradually degraded after treatment with EGF for approximately 20 min, whereas NOK levels were not reduced. This result suggests that NOK mainly plays a role in facilitating the trafficking of EGFR from early endosomes to later endosomes/lysosomes. Taken together, NOK has a strong tendency towards forming aggregates, which may have physiological implications and provide the first evidence that this novel receptor kinase is colocalised with EGFR in endosomes to participate in a post-internalisation step of EGFR. 
 A novel protein tyrosine kinase NOK that shares homology with platelet- derived growth factor/fibroblast growth factor receptors induces tumorigenesis and   metastasis   in nude mice. Receptor protein tyrosine kinases (RPTKs) play important roles in the regulation of a variety of cellular processes including cell migration, proliferation, and protection from apoptosis. Here, we report the identification and characterization of a novel RPTK-like molecule that has a critical role in induction of tumorigenesis and   metastasis   and is termed Novel Oncogene with Kinase-domain (NOK). NOK contains a putative single transmembrane domain and a conserved intracellular tyrosine kinase domain that shares homology with members of the platelet-derived growth factor/fibroblast growth factor receptor superfamily. NOK was exclusively located in the cytoplasm. NOK mRNAs were detected in limited human organs and expressed with the highest abundance in the prostate. A variety of tumor cells also expressed the NOK mRNAs. We demonstrated that NIH3T3 and BaF3 cells could be strongly transformed by the expression of the NOK gene as examined by colony formation experiment. In addition, BaF3 cells with the stable expression of NOK induced rapid tumorigenesis in nude mice. Interestingly, these NOK-expressing tumor cells could promptly invade and spread into various distinct organs and form metastatic foci, eventually leading to the rapid death of these animals. Moreover, molecular mechanism studies indicated that NOK could concomitantly activate both MAP kinase and phosphatidylinositol 3'-kinases (PI3K) pathways in stable BaF3 cells. Thus, our results both in vitro and in vivo suggest that NOK is a novel oncogene with the capacity of promoting cell transformation, tumorigenesis, and   metastasis  . 
 High Novel Oncogene with Kinase-Domain (NOK) Gene Expression is Associated with the Progression of Renal Cell Carcinoma. To study the expression levels of the NOK (novel oncogene with kinase-domain) gene in renal cell carcinoma and its association with the progression of this cancer.In this study, immunohistochemistry (IHC) and Western blot analyses were applied to investigate the NOK expression level in RCC and adjacent normal renal tissue samples. MTT, colony formation, and migration assays were also utilized to evaluate the role of NOK in RCC cell lines.Knocked-down expression of NOK in an RCC cell line (786-0) suppressed cellular proliferation and migration by restraining the activation of AKT and ERK. We found that the expression level of NOK was significantly higher in RCC tissues than in their adjacent tissues, and more importantly, overexpression of NOK was evidently correlated with the tumor TNM stage and Fuhrman grade (p < 0.001). A high level of NOK was also associated with poor overall survival (p < 0.05) and disease-free survival (p < 0.05) by Kaplan-Meier analysis.NOK expression increased in RCC and was significantly correlated with TNM stage, Fuhrman grade, poor overall survival, poor disease-free survival,   metastasis  , and proliferation in RCC cells by regulating the activation of AKT and ERK, suggesting that NOK may play important roles as a positive regulator to RCC. 
 Serine threonine tyrosine kinase 1 is a potential prognostic marker in colorectal cancer. Aberrant expression of serine threonine tyrosine kinase 1 (STYK1) has been reported in several human malignancies including colorectal cancer (CRC). However, the prognostic significance of STYK1 expression in CRC remains unknown.STYK1 protein expression in paraffin-embedded CRC specimens was determined immunohistochemically. The correlation of STYK1 expression with clinicopathologic features was assessed in a cohort containing 353 patients with primary CRC. Kaplan-Meier and Cox proportional regression analyses were used to evaluate the association between STYK1 expression and patients' survival.STYK1 expression was frequently up-regulated in CRC clinical samples at the protein levels and was significantly associated with tumor differentiation grade (p = 0.030), lymph n  ode metast  asis (p = 0.004), TNM stage (p = 0.007) and patient death (p < 0.001). Kaplan-Meier analysis indicated that patients with high intratumoral STYK1 expression had a significantly shorter disease-specific survival (DSS) than those with low expression (p < 0.001). Importantly, high levels of STYK1 protein predicted poor DSS for both stage II (p < 0.001) and stage III (p = 0.004) patients. Furthermore, multivariate analyses revealed that STYK1 protein expression was an independent prognostic indicator for both stage II (hazard ratio [HR], 2.472; p = 0.001) and stage III (HR, 2.001; p = 0.004) patients.Our results suggest that increased STYK1 protein expression corr  elates wit  h disease progression and metastasis and may serve as a predictor of poor survival in CRC. 
 Clinicopathologic features and prognostic implications of NOK/STYK1 protein expression in non-small cell lung cancer. The expression of novel oncogenic kinase (NOK), a member of the protein tyrosine kinase (PTK) family, has been observed in several human malignancies including non-small cell lung cancer (NSCLC). However, the clinic relevance of NOK expression in NSCLC remains unclear.In this study, NOK expression in tumor cells was assessed using immunohistochemical methods in 191 patients with resected NSCLC. The association of NOK expression with clinicopathological parameters, including the Ki-67 labeling index (LI), was also evaluated. Kaplan-Meier survival analysis and Cox proportional hazards models were used to estimate the effect of NOK expression on survival.Data showed that NOK was expressed in 75.4% and 14.1% of cancer lesions and corresponding adjacent non-cancerous tissue, respectively. Out of all the clinicopathological factors analyzed, NOK expression was significantly correlated with the grade of tumor differentiation (P=0.035), pTNM stage (P=0.020), lymphatic   metastasis   (P=0.005) and high Ki-67 LI (P<0.001). NOK positive NSCLC patients had a significantly shorter survival time (P=0.004, Log-rank test) and the prognostic significance of NOK expression was apparent in squamous cell carcinoma patients (P=0.022). Multivariate analysis indicated that NOK expression may be an independent prognostic factor in NSCLC (hazard ratio [HR], 1.731; P=0.043).Our results indicate that NOK expression is of clinical significance and can serve as a prognostic biomarker in NSCLC. 
  GeneRIFs  
 increased STYK1 protein expression correlates with disease progression and   metastasis   and may serve as a predictor of poor survival in CRC. 
 NOK expression increased in renal cell carcinoma and was significantly correlated with TNM stage, Fuhrman grade, poor overall survival, poor disease-free survival,   metastasis  , and proliferation. 
   
  
 FLT4 
 
  NCBI Gene  
  GeneRIF  
  Pubmed  
 
 
  Search Term  Rank  Fields  
  [Ff]ilopodia  1  PubMed(4)  
  [Mm]igration  6  Process(1); PubMed(33); GeneRIFs(5)  
  ECM  7  PubMed(3)  
  [Ee]xtracellular [Mm]atrix  8  PubMed(8); GeneRIFs(1)  
  [Ii]nvasive  10  PubMed(8); GeneRIFs(1)  
  [Mm]etastasize  11  PubMed(2)  
  [Mm]etastasis  12  PubMed(67); GeneRIFs(23)  
  [Cc]ell [Ii]nvasion  13  PubMed(7)  
 
 
 hide/show details for FLT4 
  [Ff]ilopodia  PubMed  
 VEGF receptor 2/-3 heterodimers detected in situ by proximity ligation on angiogenic sprouts. The vascular endothelial growth factors VEGFA and VEGFC are crucial regulators of vascular development. They exert their effects by dimerization and activation of the cognate receptors VEGFR2 and VEGFR3. Here, we have used in situ proximity ligation to detect receptor complexes in intact endothelial cells. We show that both VEGFA and VEGFC potently induce formation of VEGFR2/-3 heterodimers. Receptor heterodimers were found in both developing blood vessels and immature lymphatic structures in embryoid bodies. We present evidence that heterodimers frequently localize to tip cell   filopodia  . Interestingly, in the presence of VEGFC, heterodimers were enriched in the leading tip cells as compared with trailing stalk cells of growing sprouts. Neutralization of VEGFR3 to prevent heterodimer formation in response to VEGFA decreased the extent of angiogenic sprouting. We conclude that VEGFR2/-3 heterodimers on angiogenic sprouts induced by VEGFA or VEGFC may serve to positively regulate angiogenic sprouting. 
 Angiopoietin-1 promotes lymphatic sprouting and hyperplasia. Angiopoietin 1 (Ang1), a ligand for the receptor tyrosine kinase Tie2, regulates the formation and stabilization of the blood vessel network during embryogenesis. In adults, Ang1 is associated with blood vessel stabilization and recruitment of perivascular cells, whereas Ang2 acts to counter these actions. Recent results from gene-targeted mice have shown that Ang2 is also essential for the proper patterning of lymphatic vessels and that Ang1 can be substituted for this function. In order to characterize the effects of the angiopoietins on lymphatic vessels, we employed viral vectors for overexpression of Ang1 in adult mouse tissues. We found that Ang1 activated lymphatic vessel endothelial proliferation, vessel enlargement, and generation of long endothelial cell   filopodia   that eventually fused, leading to new sprouts and vessel development. Cutaneous lymphatic hyperplasia was also detected in transgenic mice expressing Ang1 in the basal epidermal cells. Tie2 was expressed in the lymphatic endothelial cells and Ang1 stimulation of these cells resulted in up-regulation of vascular endothelial growth factor receptor 3 (VEGFR-3). Furthermore, a soluble form of VEGFR-3 inhibited the observed lymphatic sprouting. Our results reinforce the concept that Ang1 therapy may be useful in settings of tissue edema. 
 VEGF receptor 2/-3 heterodimers detected in situ by proximity ligation on angiogenic sprouts. The vascular endothelial growth factors VEGFA and VEGFC are crucial regulators of vascular development. They exert their effects by dimerization and activation of the cognate receptors VEGFR2 and VEGFR3. Here, we have used in situ proximity ligation to detect receptor complexes in intact endothelial cells. We show that both VEGFA and VEGFC potently induce formation of VEGFR2/-3 heterodimers. Receptor heterodimers were found in both developing blood vessels and immature lymphatic structures in embryoid bodies. We present evidence that heterodimers frequently localize to tip cell   filopodia  . Interestingly, in the presence of VEGFC, heterodimers were enriched in the leading tip cells as compared with trailing stalk cells of growing sprouts. Neutralization of VEGFR3 to prevent heterodimer formation in response to VEGFA decreased the extent of angiogenic sprouting. We conclude that VEGFR2/-3 heterodimers on angiogenic sprouts induced by VEGFA or VEGFC may serve to positively regulate angiogenic sprouting. 
 Angiopoietin-1 promotes lymphatic sprouting and hyperplasia. Angiopoietin 1 (Ang1), a ligand for the receptor tyrosine kinase Tie2, regulates the formation and stabilization of the blood vessel network during embryogenesis. In adults, Ang1 is associated with blood vessel stabilization and recruitment of perivascular cells, whereas Ang2 acts to counter these actions. Recent results from gene-targeted mice have shown that Ang2 is also essential for the proper patterning of lymphatic vessels and that Ang1 can be substituted for this function. In order to characterize the effects of the angiopoietins on lymphatic vessels, we employed viral vectors for overexpression of Ang1 in adult mouse tissues. We found that Ang1 activated lymphatic vessel endothelial proliferation, vessel enlargement, and generation of long endothelial cell   filopodia   that eventually fused, leading to new sprouts and vessel development. Cutaneous lymphatic hyperplasia was also detected in transgenic mice expressing Ang1 in the basal epidermal cells. Tie2 was expressed in the lymphatic endothelial cells and Ang1 stimulation of these cells resulted in up-regulation of vascular endothelial growth factor receptor 3 (VEGFR-3). Furthermore, a soluble form of VEGFR-3 inhibited the observed lymphatic sprouting. Our results reinforce the concept that Ang1 therapy may be useful in settings of tissue edema. 
  [Mm]igration  Process  
 positive regulation of endothelial cell   migration   
  PubMed  
 The inhibition of tyrosine kinase receptor signalling in leiomyosarcoma cells using the small molecule kinase inhibitor PTK787/ZK222584 (Vatalanib®). Leiomyosarcomas remain challenging tumors to manage and novel therapy strategies besides radiation and conventional chemotherapy are needed. Targeting angiogenesis by inhibition of vascular endothelial growth factor (VEGF) receptor tyrosine kinases (RTKs) of the tumor vasculature with small molecules is a promising new therapy. It has been shown recently that these receptors are not only expressed on tumor endothelium but also on tumor cells themselves. Thus, we investigated the expression of members of the VEGF receptor (VEGFR) family and corresponding growth factors in leiomyosarcoma tissue specimens and in the leiomyosarcoma cell lines SK-LMS-1 and SK-UT-1. We evaluated the influence of the VEGFR inhibitor PTK787/ZK222584 (PTK787) on cell growth  , migrati  on, apoptosis and phosphorylation of intracellular signalling molecules. In human leiomyosarcoma tissue specimens VEGFR‑1/-2 and platelet-derived growth factor receptor (PDGFR-β) were strongly expressed. Both leiomyosarcoma cell lines expressed VEGFR‑1/-3 and PDGFR-β but VEGFR-2 protein expression was positive only in SK-UT-1. SK-LMS-1 and SK-UT-1 cells secreted high and low amounts of VEGF-A, respectively, whereas PDGF-BB secretion was similar in both cell lines. Application of PTK787 led to partial inhibition of PDGF-BB-activated AKT/p90RSK and ERK1/2 signalling pathways. In contrast, protein phosphorylation was not affected by PTK787 in VEGF-A-treated cells. PTK787 turned out to inhib  it cell m  igration even though no effects were observed upon stimulation with VEGF-A or PDGF-BB. In line, cell growth in leiomyosarcoma cell lines remained unchanged upon PTK787 treatment alone and with subsequent VEGF-A- or PDGF-BB-stimulation. However, VEGF-A, but not PDGF-BB-treated cells showed increased cell death upon PTK787 treatment. VEGFR family members are expressed in leiomyosarcomas in vivo and in vitro. Upon receptor stimulation, PTK787 is able to inhibit subsequent phosphorylation events and influences cell survival but not metabolic activ  ity and m  igration. Thus, the inhibitor is possibly an additional option in the treatment of leiomyosarcomas. 
 Immunolocalization and expression of vascular endothelial growth factor receptors (VEGFRs) and neuropilins (NRPs) on keratinocytes in human epidermis. Vascular endothelial growth factor (VEGF) plays an important role in normal and pathological angiogenesis. VEGF receptors (VEGFRs, including VEGFR-1, VEGFR-2, and VEGFR-3) and neuropilins (NRPs, including NRP-1 and NRP-2) are high-affinity receptors for VEGF and are typically considered to be specific for endothelial cells. Here we showed expression of VEGFRs and NRPs on cultured epidermal keratinocytes at both mRNA and protein levels. We further localized these receptors by immunofluorescence (IF) staining in the epidermis of surgical skin specimens. We found positive staining for VEGFRs and NRPs in all layers of the epidermis except for the stratum corneum. VEGFR-1 and VEGFR-2 are primarily expressed on the cytoplasmic membrane of basal cells and the adjacent spinosum keratinocytes. All layers of the epidermis except for the horny cell layer demonstrated a uniform pattern of VEGFR-3, NRP-1, and NRP-2. Sections staining for NRP-1 and NRP-2 also showed diffuse intense fluorescence and were localized to the cell membrane and cytoplasm of keratinocytes. In another panel of experiments, keratinocytes were treated with different concentrations of VEGF, with or without VEGFR-2 neutralizing antibody in culture. VEGF enhanced the proliferation and   migration   of keratinocytes, and these effects were partially inhibited by pretreatment with VEGFR-2 neutralizing antibody. Adhesion of keratinocytes to type IV collagen-coated culture plates was decreased by VEGF treatment, but this reduction could be completely reversed by pretreatment with VEGFR-2 neutralizing antibody. Taken together, our results suggest that the expression of VEGFRs and NRPs on keratinocytes may constitute important regulators for its activity and may possibly be responsible for the autocrine signaling in the epidermis. 
 RNAi-mediated silencing of VEGF-C inhibits non-small cell lung cancer progression by simultaneously down-regulating the CXCR4, CCR7, VEGFR-2 and VEGFR-3-dependent axes-induced ERK, p38 and AKT signalling pathways. Vascular endothelial growth factor C (VEGF-C) expression is associated with the malignant tumour phenotype making it an attractive therapeutic target. We investigated the biological roles of VEGF-C in tumour growth,   migration  , invasion and explored the possibility of VEGF-C as a potential therapeutic target for the treatment of non-small cell lung cancer (NSCLC). A lentivirus-mediated RNA interference (RNAi) technology was used to specifically knockdown the expression of VEGF-C in A549 cells. Quantitative reverse transcriptase-polymerase chain reaction, flow cytometry, Western blot, immunohistochemistry, cellular growth,   migration  , invasion and ELISA assays were used to characterise VEGF-C expression in vitro. A lung cancer xenograft model in nude mice was established to investigate whether knockdown of VEGF-C reduced tumour growth in vivo. Silencing of VEGF-C suppressed tumour cell growth,   migration   and invasion in vitro; suppressed tumour growth, angiogenesis and lymphangiogenesis by tail vein injection of lentivirus encoded shRNA against VEGF-C in vivo. More importantly, silencing of VEGF-C also trapped the VEGFR-2, VEGFR-3, CXCR4, CCR7-dependent axes, and down-regulated the AKT, ERK and p38 signalling pathways. These results suggest that VEGF-C has a multifaceted role in NSCLC growth,   migration   and invasion; that VEGF-C-mediated autocrine loops with their cognate receptors and chemokine receptors are significant factors affecting tumour progression; and that RNAi-mediated silencing of VEGF-C represents a powerful therapeutic approach for controlling NSCLC growth and metastasis. 
 Kaposi's sarcoma-associated herpesvirus activation of vascular endothelial growth factor receptor 3 alters endothelial function and enhances infection. Kaposi's sarcoma-associated herpesvirus (KSHV; also known as human herpesvirus 8) is the etiologic agent of Kaposi's sarcoma, an endothelial neoplasm. This gamma-herpesvirus encodes for several unique proteins that alter target cell function, including the virion envelope-associated glycoprotein B (gB). Glycoprotein B has an RGD (Arg-Gly-Asp) motif at the extracellular amino terminus region and binds to the alpha3beta1 surface integrin, which enhances virus entry. We now report that gB can activate the vascular endothelial growth factor receptor 3 (VEGFR-3) on the surface of microvascular endothelial cells and trigger receptor signaling, which can modulate endothelial   migration   and proliferation. Furthermore, we observed that VEGFR-3 expression and activation enhance KSHV infection and participate in KSHV-mediated transformation. These functional changes in the endothelium may contribute to the pathogenesis of Kaposi's sarcoma and suggest that interventions that inhibit gB activation of VEGFR-3 could be useful in the treatment of this neoplasm. 
 Stimulation of beta 1 integrin induces tyrosine phosphorylation of vascular endothelial growth factor receptor-3 and modulates cell   migration  . Interactions between integrins and tyrosine kinase receptors can modulate a variety of cell functions. We observed a cooperative interaction between the beta(1) integrin and vascular endothelial growth factor receptor-3 (VEGFR-3 or Flt4) that appeared to be required for cell   migration  . By using VEGFR-3-transfected 293 cells (293/VEGFR-3) or primary dermal microvascular endothelial cells (DMEC), we found that stimulation with either soluble or immobilized extracellular matrix (ECM) proteins, collagen or fibronectin (FN), resulted in the increased tyrosine phosphorylation of VEGFR-3 in the absence of a cognate ligand. This increased tyrosine phosphorylation of VEGFR-3 was diminished by pretreatment with a blocking antibody against the beta(1) integrin. Cross-linking with anti-beta(1) integrin antibody induced a similar degree of tyrosine phosphorylation of VEGFR-3. Stimulation with collagen or FN induced an association between beta(1) integrin and VEGFR-3 in both 293/VEGFR-3 and primary DMEC cells. Collagen or FN-induced tyrosine phosphorylation of VEGFR-3 was inhibited by treatment with cytochalasin D, an inhibitor of actin polymerization. Collagen or FN was able to induce the   migration   of 293/VEGFR-3 or DMEC cells to a limited extent. However,   migration   was dramatically enhanced when a gradient of the cognate ligand, VEGF-D, was added. VEGF-D failed to induce cell   migration   in the absence of ECM proteins. Introducing a mutation at the kinase domain of VEGFR-3 or treatment with blocking antibody against either VEGFR-3 or beta(1) integrin inhibited cell   migration   induced by ECM and VEGF-D, indicating that signals from both beta(1) integrin and VEGFR-3 are required for this cell function. 
 Extracellular matrix regulates endothelial functions through interaction of VEGFR-3 and integrin alpha5beta1. Endothelium extracellular matrix (ECM) interactions can provide distinct spatial and molecular signals which control cellular proliferation,   migration  , and differentiation. Here, we investigated the role of fibronectin (FN), a major ECM protein, on the functions of lymphatic endothelial cells (LEC). We observed that FN, the ligand for integrin alpha5beta1, selectively promoted the growth of LEC as compared with vitronectin (VN) in the presence of the ligand for vascular endothelial growth factor receptor 3 [VEGFR-3 (VEGF-C156S)]. Upon investigating the mechanisms whereby ECM components regulate VEGFR-3 signaling, we found that FN transactivated VEGFR-3 and significantly enhanced the phosphorylation of VEGFR-3 induced by VEGF-C156S as compared to VN. An enhanced association of the integrin subunit alpha5 or beta1 with VEGFR-3, after stimulation with VEGF-C156S, was observed by co-immunoprecipitation. While blockade of integrin alpha5beta1 inhibited the VEGF-C156S-induced phosphorylation of VEGFR-3, no similar effect was obtained by blocking integrin alphavbeta3. FN also protected the endothelial cells from serum deprivation-induced apoptosis. Moreover, while the specific PI3 kinase inhibitor, LY294002, abolished this FN-mediated cell survival, the MAPK kinase inhibitor, PD98059, had no significant effect. Furthermore, a dominant-negative mutant of VEGFR-3 (G857R) reduced VEGF-C156S or FN-mediated cell survival, as well as the activities of PI3 kinase/Akt. Our results indicate that integrin alpha5beta1 participates in the activation of both VEGFR-3 and its downstream PI3 kinase/Akt signaling pathway, which is essential for FN-mediated lymphatic endothelial cell survival and proliferation. 
 Malignant hematopoietic cells induce an increased expression of VEGFR-1 and VEGFR-3 on bone marrow endothelial cells via AKT and mTOR signalling pathways. Angiogenesis plays a significant role in a variety of malignant hematologic diseases, and it is recognized that it has prognostic value. However, the cellular mechanisms by which malignant hematologic cells induce angiogenesis are not well understood. In order to investigate the role of cells from B-cell chronic lymphocytic leukemia (B-CLL) and multiple myeloma (MM) in angiogenesis on human bone marrow endothelial cells (HBMEC), we analyzed the impact of factors secreted by B-CLL cells and by MM cells on HBMEC capillary tube formation on matrigel. It was found that, in addition to the secretion of angiogenic factors VEGF and b-FGF by B-CLL and MM cells, MM cells (but not B-CLL cells) induced a dramatic increase in expression of VEGFR-1 and VEGFR-3 on human bone marrow endothelial cells (HBMEC). It would seem that this increase in VEGFR-3 occurred via the ERK and mTOR pathways, since their respective inhibitors U0126, LY294002 or rapamycin were responsible for a decrease of VEGFR-3. In response to MM cells-increased VEGF receptors on HBMEC, endothelial cell   migration   was enhanced in a wound artificially produced in a semi-confluent HBMEC culture, a phenomenon which was also down-regulated by the same inhibitors that reversed the increase in VEGF receptors. The present study suggests that, in addition to the classic angiogenic pathway, another mechanism related to an increased expression of VEGFRs on HBMEC might exist in malignant hematopoietic angiogenesis. 
 Tubular network formation by adriamycin-resistant MCF-7 breast cancer cells is closely linked to MMP-9 and VEGFR-2/VEGFR-3 over-expressions. We have previously demonstrated that matrix metalloproteinase-9 (MMP-9) is critical for breast cancer cell   migration   and is necessary but not sufficient for tubular network formation. Given the important angiogenic activity of vascular endothelial growth factor (VEGF), we investigate here its possible contribution in tubular network formation and its link with MMP-9. Exposure of resistant epithelial breast cancer cells (rMCF-7) to Avastin, a VEGF neutralising antibody, suppresses tubular network formation but not cell   migration  . However, their exposure to MMP-9 inhibitor markedly decreases both parameters. Besides, the addition of exogenous VEGF or MMP-9 alone or in combination to sensitive parental cells (sMCF-7) or rMCF-7 cells enhances tubular network formation by rMCF-7 cells but not by sMCF-7 cells. The evaluation of the expression levels of VEGF receptor (VEGFR) subtypes shows that sMCF-7 cells express only small quantities of VEGFR-2 and VEGFR-3 compared with rMCF-7 cells that express strong quantities. However, treatment of sMCF-7 cells by phorbol 12-myristate 13-acetate (PMA), a PKC activator, induces both tubular network formation and VEGFR-2/VEGFR-3 over-expressions. Interestingly, exposure of rMCF-7 cells or PMA-treated sMCF-7 cells to the specific inhibitors of VEGFR-2 and VEGFR-3 reduces markedly the tubular network formation. Together, our results demonstrate that the proteolytic enzyme MMP-9 promotes rMCF-7 cell   migration   and, consequently, tubular network formation through VEGFR-2/ VEGFR-3 activation. Understanding of mechanisms involved in vasculogenic mimicry and cell   migration   related to MMP-9 and VEGF may open new opportunities to improve cancer therapy. 
 Inflammation induces lymphangiogenesis through up-regulation of VEGFR-3 mediated by NF-kappaB and Prox1. The concept of inflammation-induced lymphangiogenesis (ie, formation of new lymphatic vessels) has long been recognized, but the molecular mechanisms remained largely unknown. The 2 primary mediators of lymphangiogenesis are vascular endothelial growth factor receptor-3 (VEGFR-3) and Prox1. The key factors that regulate inflammation-induced transcription are members of the nuclear factor-kappaB (NF-kappaB) family; however, the role of NF-kappaB in regulation of lymphatic-specific genes has not been defined. Here, we identified VEGFR-3 and Prox1 as downstream targets of the NF-kappaB pathway. In vivo time-course analysis of inflammation-induced lymphangiogenesis showed activation of NF-kappaB followed by sequential up-regulation of Prox1 and VEGFR-3 that preceded lymphangiogenesis by 4 and 2 days, respectively. Activation of NF-kappaB by inflammatory stimuli also elevated Prox1 and VEGFR-3 expression in cultured lymphatic endothelial cells, resulting in increased proliferation and   migration  . We also show that Prox1 synergizes with the p50 of NF-kappaB to control VEGFR-3 expression. Collectively, our findings suggest that induction of the NF-kappaB pathway by inflammatory stimuli activates Prox1, and both NF-kappaB and Prox1 activate the VEGFR-3 promoter leading to increased receptor expression in lymphatic endothelial cells. This, in turn, enhances the responsiveness of preexisting lymphatic endothelium to VEGFR-3 binding factors, VEGF-C and VEGF-D, ultimately resulting in robust lymphangiogenesis. 
 Early endocardial morphogenesis requires Scl/Tal1. The primitive heart tube is composed of an outer myocardial and an inner endocardial layer that will give rise to the cardiac valves and septa. Specification and differentiation of these two cell layers are among the earliest events in heart development, but the embryonic origins and genetic regulation of early endocardial development remain largely undefined. We have analyzed early endocardial development in the zebrafish using time-lapse confocal microscopy and show that the endocardium seems to originate from a region in the lateral plate mesoderm that will give rise to hematopoietic cells of the primitive myeloid lineage. Endocardial precursors appear to rapidly migrate to the site of heart tube formation, where they arrive prior to the bilateral myocardial primordia. Analysis of a newly discovered zebrafish Scl/Tal1 mutant showed an additional and previously undescribed role of this transcription factor during the development of the endocardium. In Scl/Tal1 mutant embryos, endocardial precursors are specified, but   migration   is severely defective and endocardial cells aggregate at the ventricular pole of the heart. We further show that the initial fusion of the bilateral myocardial precursor populations occurs independently of the endocardium and tal1 function. Our results suggest early separation of the two components of the primitive heart tube and imply Scl/Tal1 as an indispensable component of the molecular hierarchy that controls endocardium morphogenesis. 
 Effective suppression of vascular network formation by combination of antibodies blocking VEGFR ligand binding and receptor dimerization. Antibodies that block vascular endothelial growth factor (VEGF) have become an integral part of antiangiogenic tumor therapy, and antibodies targeting other VEGFs and receptors (VEGFRs) are in clinical trials. Typically receptor-blocking antibodies are targeted to the VEGFR ligand-binding site. Here we describe a monoclonal antibody that inhibits VEGFR-3 homodimer and VEGFR-3/VEGFR-2 heterodimer formation, signal transduction, as well as ligand-induced   migration   and sprouting of microvascular endothelial cells. Importantly, we show that combined use of antibodies blocking ligand binding and receptor dimerization improves VEGFR inhibition and results in stronger inhibition of endothelial sprouting and vascular network formation in vivo. These results suggest that receptor dimerization inhibitors could be used to enhance antiangiogenic activity of antibodies blocking ligand binding in tumor therapy. 
 The inhibition of tyrosine kinase receptor signalling in leiomyosarcoma cells using the small molecule kinase inhibitor PTK787/ZK222584 (Vatalanib®). Leiomyosarcomas remain challenging tumors to manage and novel therapy strategies besides radiation and conventional chemotherapy are needed. Targeting angiogenesis by inhibition of vascular endothelial growth factor (VEGF) receptor tyrosine kinases (RTKs) of the tumor vasculature with small molecules is a promising new therapy. It has been shown recently that these receptors are not only expressed on tumor endothelium but also on tumor cells themselves. Thus, we investigated the expression of members of the VEGF receptor (VEGFR) family and corresponding growth factors in leiomyosarcoma tissue specimens and in the leiomyosarcoma cell lines SK-LMS-1 and SK-UT-1. We evaluated the influence of the VEGFR inhibitor PTK787/ZK222584 (PTK787) on cell growth  , migrati  on, apoptosis and phosphorylation of intracellular signalling molecules. In human leiomyosarcoma tissue specimens VEGFR‑1/-2 and platelet-derived growth factor receptor (PDGFR-β) were strongly expressed. Both leiomyosarcoma cell lines expressed VEGFR‑1/-3 and PDGFR-β but VEGFR-2 protein expression was positive only in SK-UT-1. SK-LMS-1 and SK-UT-1 cells secreted high and low amounts of VEGF-A, respectively, whereas PDGF-BB secretion was similar in both cell lines. Application of PTK787 led to partial inhibition of PDGF-BB-activated AKT/p90RSK and ERK1/2 signalling pathways. In contrast, protein phosphorylation was not affected by PTK787 in VEGF-A-treated cells. PTK787 turned out to inhib  it cell m  igration even though no effects were observed upon stimulation with VEGF-A or PDGF-BB. In line, cell growth in leiomyosarcoma cell lines remained unchanged upon PTK787 treatment alone and with subsequent VEGF-A- or PDGF-BB-stimulation. However, VEGF-A, but not PDGF-BB-treated cells showed increased cell death upon PTK787 treatment. VEGFR family members are expressed in leiomyosarcomas in vivo and in vitro. Upon receptor stimulation, PTK787 is able to inhibit subsequent phosphorylation events and influences cell survival but not metabolic activ  ity and m  igration. Thus, the inhibitor is possibly an additional option in the treatment of leiomyosarcomas. 
 Tubular network formation by adriamycin-resistant MCF-7 breast cancer cells is closely linked to MMP-9 and VEGFR-2/VEGFR-3 over-expressions. We have previously demonstrated that matrix metalloproteinase-9 (MMP-9) is critical for breast cancer cell   migration   and is necessary but not sufficient for tubular network formation. Given the important angiogenic activity of vascular endothelial growth factor (VEGF), we investigate here its possible contribution in tubular network formation and its link with MMP-9. Exposure of resistant epithelial breast cancer cells (rMCF-7) to Avastin, a VEGF neutralising antibody, suppresses tubular network formation but not cell   migration  . However, their exposure to MMP-9 inhibitor markedly decreases both parameters. Besides, the addition of exogenous VEGF or MMP-9 alone or in combination to sensitive parental cells (sMCF-7) or rMCF-7 cells enhances tubular network formation by rMCF-7 cells but not by sMCF-7 cells. The evaluation of the expression levels of VEGF receptor (VEGFR) subtypes shows that sMCF-7 cells express only small quantities of VEGFR-2 and VEGFR-3 compared with rMCF-7 cells that express strong quantities. However, treatment of sMCF-7 cells by phorbol 12-myristate 13-acetate (PMA), a PKC activator, induces both tubular network formation and VEGFR-2/VEGFR-3 over-expressions. Interestingly, exposure of rMCF-7 cells or PMA-treated sMCF-7 cells to the specific inhibitors of VEGFR-2 and VEGFR-3 reduces markedly the tubular network formation. Together, our results demonstrate that the proteolytic enzyme MMP-9 promotes rMCF-7 cell   migration   and, consequently, tubular network formation through VEGFR-2/ VEGFR-3 activation. Understanding of mechanisms involved in vasculogenic mimicry and cell   migration   related to MMP-9 and VEGF may open new opportunities to improve cancer therapy. 
 RNAi-mediated silencing of VEGF-C inhibits non-small cell lung cancer progression by simultaneously down-regulating the CXCR4, CCR7, VEGFR-2 and VEGFR-3-dependent axes-induced ERK, p38 and AKT signalling pathways. Vascular endothelial growth factor C (VEGF-C) expression is associated with the malignant tumour phenotype making it an attractive therapeutic target. We investigated the biological roles of VEGF-C in tumour growth,   migration  , invasion and explored the possibility of VEGF-C as a potential therapeutic target for the treatment of non-small cell lung cancer (NSCLC). A lentivirus-mediated RNA interference (RNAi) technology was used to specifically knockdown the expression of VEGF-C in A549 cells. Quantitative reverse transcriptase-polymerase chain reaction, flow cytometry, Western blot, immunohistochemistry, cellular growth,   migration  , invasion and ELISA assays were used to characterise VEGF-C expression in vitro. A lung cancer xenograft model in nude mice was established to investigate whether knockdown of VEGF-C reduced tumour growth in vivo. Silencing of VEGF-C suppressed tumour cell growth,   migration   and invasion in vitro; suppressed tumour growth, angiogenesis and lymphangiogenesis by tail vein injection of lentivirus encoded shRNA against VEGF-C in vivo. More importantly, silencing of VEGF-C also trapped the VEGFR-2, VEGFR-3, CXCR4, CCR7-dependent axes, and down-regulated the AKT, ERK and p38 signalling pathways. These results suggest that VEGF-C has a multifaceted role in NSCLC growth,   migration   and invasion; that VEGF-C-mediated autocrine loops with their cognate receptors and chemokine receptors are significant factors affecting tumour progression; and that RNAi-mediated silencing of VEGF-C represents a powerful therapeutic approach for controlling NSCLC growth and metastasis. 
 Inflammation induces lymphangiogenesis through up-regulation of VEGFR-3 mediated by NF-kappaB and Prox1. The concept of inflammation-induced lymphangiogenesis (ie, formation of new lymphatic vessels) has long been recognized, but the molecular mechanisms remained largely unknown. The 2 primary mediators of lymphangiogenesis are vascular endothelial growth factor receptor-3 (VEGFR-3) and Prox1. The key factors that regulate inflammation-induced transcription are members of the nuclear factor-kappaB (NF-kappaB) family; however, the role of NF-kappaB in regulation of lymphatic-specific genes has not been defined. Here, we identified VEGFR-3 and Prox1 as downstream targets of the NF-kappaB pathway. In vivo time-course analysis of inflammation-induced lymphangiogenesis showed activation of NF-kappaB followed by sequential up-regulation of Prox1 and VEGFR-3 that preceded lymphangiogenesis by 4 and 2 days, respectively. Activation of NF-kappaB by inflammatory stimuli also elevated Prox1 and VEGFR-3 expression in cultured lymphatic endothelial cells, resulting in increased proliferation and   migration  . We also show that Prox1 synergizes with the p50 of NF-kappaB to control VEGFR-3 expression. Collectively, our findings suggest that induction of the NF-kappaB pathway by inflammatory stimuli activates Prox1, and both NF-kappaB and Prox1 activate the VEGFR-3 promoter leading to increased receptor expression in lymphatic endothelial cells. This, in turn, enhances the responsiveness of preexisting lymphatic endothelium to VEGFR-3 binding factors, VEGF-C and VEGF-D, ultimately resulting in robust lymphangiogenesis. 
 Extracellular matrix regulates endothelial functions through interaction of VEGFR-3 and integrin alpha5beta1. Endothelium extracellular matrix (ECM) interactions can provide distinct spatial and molecular signals which control cellular proliferation,   migration  , and differentiation. Here, we investigated the role of fibronectin (FN), a major ECM protein, on the functions of lymphatic endothelial cells (LEC). We observed that FN, the ligand for integrin alpha5beta1, selectively promoted the growth of LEC as compared with vitronectin (VN) in the presence of the ligand for vascular endothelial growth factor receptor 3 [VEGFR-3 (VEGF-C156S)]. Upon investigating the mechanisms whereby ECM components regulate VEGFR-3 signaling, we found that FN transactivated VEGFR-3 and significantly enhanced the phosphorylation of VEGFR-3 induced by VEGF-C156S as compared to VN. An enhanced association of the integrin subunit alpha5 or beta1 with VEGFR-3, after stimulation with VEGF-C156S, was observed by co-immunoprecipitation. While blockade of integrin alpha5beta1 inhibited the VEGF-C156S-induced phosphorylation of VEGFR-3, no similar effect was obtained by blocking integrin alphavbeta3. FN also protected the endothelial cells from serum deprivation-induced apoptosis. Moreover, while the specific PI3 kinase inhibitor, LY294002, abolished this FN-mediated cell survival, the MAPK kinase inhibitor, PD98059, had no significant effect. Furthermore, a dominant-negative mutant of VEGFR-3 (G857R) reduced VEGF-C156S or FN-mediated cell survival, as well as the activities of PI3 kinase/Akt. Our results indicate that integrin alpha5beta1 participates in the activation of both VEGFR-3 and its downstream PI3 kinase/Akt signaling pathway, which is essential for FN-mediated lymphatic endothelial cell survival and proliferation. 
 Malignant hematopoietic cells induce an increased expression of VEGFR-1 and VEGFR-3 on bone marrow endothelial cells via AKT and mTOR signalling pathways. Angiogenesis plays a significant role in a variety of malignant hematologic diseases, and it is recognized that it has prognostic value. However, the cellular mechanisms by which malignant hematologic cells induce angiogenesis are not well understood. In order to investigate the role of cells from B-cell chronic lymphocytic leukemia (B-CLL) and multiple myeloma (MM) in angiogenesis on human bone marrow endothelial cells (HBMEC), we analyzed the impact of factors secreted by B-CLL cells and by MM cells on HBMEC capillary tube formation on matrigel. It was found that, in addition to the secretion of angiogenic factors VEGF and b-FGF by B-CLL and MM cells, MM cells (but not B-CLL cells) induced a dramatic increase in expression of VEGFR-1 and VEGFR-3 on human bone marrow endothelial cells (HBMEC). It would seem that this increase in VEGFR-3 occurred via the ERK and mTOR pathways, since their respective inhibitors U0126, LY294002 or rapamycin were responsible for a decrease of VEGFR-3. In response to MM cells-increased VEGF receptors on HBMEC, endothelial cell   migration   was enhanced in a wound artificially produced in a semi-confluent HBMEC culture, a phenomenon which was also down-regulated by the same inhibitors that reversed the increase in VEGF receptors. The present study suggests that, in addition to the classic angiogenic pathway, another mechanism related to an increased expression of VEGFRs on HBMEC might exist in malignant hematopoietic angiogenesis. 
 Immunolocalization and expression of vascular endothelial growth factor receptors (VEGFRs) and neuropilins (NRPs) on keratinocytes in human epidermis. Vascular endothelial growth factor (VEGF) plays an important role in normal and pathological angiogenesis. VEGF receptors (VEGFRs, including VEGFR-1, VEGFR-2, and VEGFR-3) and neuropilins (NRPs, including NRP-1 and NRP-2) are high-affinity receptors for VEGF and are typically considered to be specific for endothelial cells. Here we showed expression of VEGFRs and NRPs on cultured epidermal keratinocytes at both mRNA and protein levels. We further localized these receptors by immunofluorescence (IF) staining in the epidermis of surgical skin specimens. We found positive staining for VEGFRs and NRPs in all layers of the epidermis except for the stratum corneum. VEGFR-1 and VEGFR-2 are primarily expressed on the cytoplasmic membrane of basal cells and the adjacent spinosum keratinocytes. All layers of the epidermis except for the horny cell layer demonstrated a uniform pattern of VEGFR-3, NRP-1, and NRP-2. Sections staining for NRP-1 and NRP-2 also showed diffuse intense fluorescence and were localized to the cell membrane and cytoplasm of keratinocytes. In another panel of experiments, keratinocytes were treated with different concentrations of VEGF, with or without VEGFR-2 neutralizing antibody in culture. VEGF enhanced the proliferation and   migration   of keratinocytes, and these effects were partially inhibited by pretreatment with VEGFR-2 neutralizing antibody. Adhesion of keratinocytes to type IV collagen-coated culture plates was decreased by VEGF treatment, but this reduction could be completely reversed by pretreatment with VEGFR-2 neutralizing antibody. Taken together, our results suggest that the expression of VEGFRs and NRPs on keratinocytes may constitute important regulators for its activity and may possibly be responsible for the autocrine signaling in the epidermis. 
 Kaposi's sarcoma-associated herpesvirus activation of vascular endothelial growth factor receptor 3 alters endothelial function and enhances infection. Kaposi's sarcoma-associated herpesvirus (KSHV; also known as human herpesvirus 8) is the etiologic agent of Kaposi's sarcoma, an endothelial neoplasm. This gamma-herpesvirus encodes for several unique proteins that alter target cell function, including the virion envelope-associated glycoprotein B (gB). Glycoprotein B has an RGD (Arg-Gly-Asp) motif at the extracellular amino terminus region and binds to the alpha3beta1 surface integrin, which enhances virus entry. We now report that gB can activate the vascular endothelial growth factor receptor 3 (VEGFR-3) on the surface of microvascular endothelial cells and trigger receptor signaling, which can modulate endothelial   migration   and proliferation. Furthermore, we observed that VEGFR-3 expression and activation enhance KSHV infection and participate in KSHV-mediated transformation. These functional changes in the endothelium may contribute to the pathogenesis of Kaposi's sarcoma and suggest that interventions that inhibit gB activation of VEGFR-3 could be useful in the treatment of this neoplasm. 
 Role of the VEGFR3/VEGFD receptor axis in TGFbeta1 activation of primary prostate cell lines. Reports indicate that vascular endothelial growth factor receptor type 3 (VEGFR3) regulates cellular functions such as invasion, proliferation, and chemo-resistance. However, the exact function of the VEGFR3 signaling axis in prostate epithelial cells is poorly characterized.The goal of this study was to evaluate whether TGFbeta1 in combination with VEGFD can promote pre-malignant invasive activities of intermediate basal cells (IBC-10a) isolated from human prostate cancer (Gleason score 6).hTERT immortalized IBC-10a cells normally grew as confluent "cobblestoned" monolayers, but treatment with TGFbeta1 (10 ng/ml for 2-6 hr) dissociated the cell-cell junctions and induced VEGFR3 translocation to the cell surface. This event was not inhibited by 10 microM cycloheximide or puromycin, indicating transcription and protein synthesis were not required. We further discovered that TGFbeta1 in combination with VEGFD induced a significant increase in the invasive activity of IBC-10a cells (>26% and 53% after 24 and 48 hr, respectively) in modified Boyden Chamber assays. TGFbetaRII receptor antibodies specifically blocked TGFbeta1 induction of VEGFR3 translocation to the cell surface and blocked VEGFD-induced invasion. Zymograms revealed that TGFbeta1 (and not VEGFR3) stimulated the secretion of MMP-2 and MMP-9, presumably to promote cell invasion. The cell invasion assays confirmed that antibodies specific for TGFbetaII receptor, MMP-2 and MMP-9 and VEGFR3, independently blocked TGFbeta1-induced invasion.For the first time, we have demonstrated the mechanism by which TGFbeta1 stimulates VEGFD/VEGFR3 receptor axis activation leading to increased cell   migration   and invasion by primary intermediate basal cell cultures. 
 Endothelial cell adhesion to the extracellular matrix induces c-Src-dependent VEGFR-3 phosphorylation without the activation of the receptor intrinsic kinase activity. Integrins cooperate with growth factor receptors to promote downstream signaling for cell proliferation and   migration  . However, the mechanism of receptor activation is still unknown.To analyze the mechanism of phosphorylation of the vascular endothelial growth factor receptor (VEGFR)-3 by cell adhesion.We show that VEGFR-3 phosphorylation, induced by cell attachment to the extracellular matrix, is independent from the intrinsic kinase activity of the receptor, as evidenced from phosphorylation cell adhesion experiments with a mutant kinase dead receptor or in the presence of the specific kinase inhibitor MAZ 51. Cell adhesion experiments in the presence of the c-Src inhibitor PP2 or in fibroblast triple knockout for c-Src, Yes, and Fyn (SYF) demonstrate that VEGFR-3 phosphorylation, induced by extracellular matrix, is mediated by c-Src. Kinase assays in vitro with recombinant c-Src show that VEGFR-3 is a direct c-Src target and mass spectrometry analysis identified the sites phosphorylated by c-Src as tyrosine 830, 833, 853, 1063, 1333, and 1337, demonstrating that integrin-mediated receptor phosphorylation induces a phosphorylation pattern that is distinct from that induced by growth factors. Furthermore, pull-down assays show that integrin-mediated VEGFR-3 phosphorylation activates the recruitment to the receptor of the adaptor proteins CRKI/II and SHC inducing activation of JNK.These data suggest that cell adhesion to extracellular matrix induces a downstream signaling using the tyrosine kinase receptor VEGFR-3 as scaffold. 
 Pathway-related molecules of VEGFC/D-VEGFR3/NRP2 axis in tumor lymphangiogenesis and lymphatic metastasis. Precondition for tumor lymphatic metastasis is that tumor cells induce formation of original and newborn lymphatic vessels and invade surrounding lymphatic vessels in tumor stroma, while some pathway-related molecules play an important role in mechanisms associated with proliferation and   migration   of lymphatic endothelial cells (LECs) and tumor cells. In lymphangiogenesis and lymphatic metastasis, the pathway-related molecules of VEGFC/D-VEGFR3/NRP2 axis, such as Furin-like enzyme, CNTN1, Prox1, LYVE-1, Podoplanin, SOX18, SDF1 and CXCR4, are direct constitutors as a portion of VEGFC/D-VEGFR3/NRP2 axis, and their biological activities rely on this ligand-receptor system. These axis-related signal molecules could gradually produce waterfall-like cascading effects, mediate differentiation and maturation of LECs, remodel original and neonatal lymphatic vessels, as well as ultimately promote tumor cell chemotaxis,   migration  , invasion and metastasis to lymphoid tracts. This review summarizes the structure and function features of pathway-related molecules of VEGFC/D-VEGFR3/NRP2 axis, the expression changes of these molecules in different anatomic organs or histopathologic types or development stages of various tumors, the characteristics of transduction, implementation, integration of signal networks, the interactive effects on biological behaviors between tumor cells and lymphatic endothelial cells, and their molecular mechanisms and significances in tumor lymphangiogenesis and lymphatic metastasis. 
 Mirtron microRNA-1236 inhibits VEGFR-3 signaling during inflammatory lymphangiogenesis. Vascular endothelial growth factor receptor(VEGFR)-3 is a critical regulator of developmental and adult vasculogenesis and lymphangiogenesis through its interactions with select members of the VEGF family. The goal of this study was to investigate how VEGFR-3 expression is regulated during inflammatory lymphangiogenesis.In this study, we present for the first time evidence that VEGFR-3 can be negatively regulated by a mirtron, hsa-miR-1236 (miR-1236), which is expressed in primary human lymphatic endothelial cells. In human lymphatic endothelial cells, miR-1236 is upregulated in response to IL-1β, a negative regulator of VEGFR-3. miR-1236 binds the 3' untranslated region of Vegfr3, resulting in translational inhibition. Overexpression of miR-1236 significantly decreased expression of VEGFR-3, but not VEGFR-2, in human lymphatic endothelial cells. Compared to a control miR, overexpression of miR-1236 also led to decreased VEGFR-3 signaling. However, VEGFR-2-specific signaling was not affected. miR-1236 can attenuate human lymphatic endothelial cell   migratio  n and tube formation, as well as in vivo lymphangiogenesis.Our data suggest that miR-1236 may function as a negative regulator of VEGFR-3 signaling during inflammatory lymphangiogenesis. 
 Direct recruitment of CRK and GRB2 to VEGFR-3 induces proliferation,   migration  , and survival of endothelial cells through the activation of ERK, AKT, and JNK pathways. Vascular endothelial growth factor receptor-3 (VEGFR-3) plays a key role for the remodeling of the primary capillary plexus in the embryo and contributes to angiogenesis and lymphangiogenesis in the adult. However, VEGFR-3 signal transduction pathways remain to be elucidated. Here we investigated VEGFR-3 signaling in primary human umbilical vein endothelial cells (HUVECs) by the systematic mutation of the tyrosine residues potentially involved in VEGFR-3 signaling and identified the tyrosines critical for its function. Y1068 was shown to be essential for the kinase activity of the receptor. Y1063 signals the receptor-mediated survival by recruiting CRKI/II to the activated receptor, inducing a signaling cascade that, via mitogen-activated protein kinase kinase-4 (MKK4), activates c-Jun N-terminal kinase-1/2 (JNK1/2). Inhibition of JNK1/2 function either by specific peptide inhibitor JNKI1 or by RNA interference (RNAi) demonstrated that activation of JNK1/2 is required for a VEGFR-3-dependent prosurvival signaling. Y1230/Y1231 contributes, together with Y1337, to proliferation,   migration  , and survival of endothelial cells. Phospho-Y1230/Y1231 directly recruits growth factor receptor-bonus protein (GRB2) to the receptor, inducing the activation of both AKT and extracellular signal-related kinase 1/2 (ERK1/2) signaling. Finally, we observed that Y1063 and Y1230/Y1231 signaling converge to induce c-JUN expression, and RNAi experiments demonstrated that c-JUN is required for growth factor-induced prosurvival signaling in primary endothelial cells. 
 Vascular endothelial growth factor receptor-3 directly interacts with phosphatidylinositol 3-kinase to regulate lymphangiogenesis. Dysfunctional lymphatic vessel formation has been implicated in a number of pathological conditions including cancer metastasis, lymphedema, and impaired wound healing. The vascular endothelial growth factor (VEGF) family is a major regulator of lymphatic endothelial cell (LEC) function and lymphangiogenesis. Indeed, dissemination of malignant cells into the regional lymph nodes, a common occurrence in many cancers, is stimulated by VEGF family members. This effect is generally considered to be mediated via VEGFR-2 and VEGFR-3. However, the role of specific receptors and their downstream signaling pathways is not well understood.Here we delineate the VEGF-C/VEGF receptor (VEGFR)-3 signaling pathway in LECs and show that VEGF-C induces activation of PI3K/Akt and MEK/Erk. Furthermore, activation of PI3K/Akt by VEGF-C/VEGFR-3 resulted in phosphorylation of P70S6K, eNOS, PLCγ1, and Erk1/2. Importantly, a direct interaction between PI3K and VEGFR-3 in LECs was demonstrated both in vitro and in clinical cancer specimens. This interaction was strongly associated with the presence of lymph node metastases in primary small cell carcinoma of the lung in clinical specimens. Blocking PI3K activity abolished VEGF-C-stimulated LEC tube formation and   migratio  n.Our findings demonstrate that specific VEGFR-3 signaling pathways are activated in LECs by VEGF-C. The importance of PI3K in VEGF-C/VEGFR-3-mediated lymphangiogenesis provides a potential therapeutic target for the inhibition of lymphatic metastasis. 
 Genistein suppresses FLT4 and inhibits human colorectal cancer metastasis. Dietary consumption of genistein, found in soy, has been associated with a potentially protective role in colorectal cancer (CRC) development and progression. Herein we demonstrate that genistein will inhibit human CRC cell invasion and   migration  , that it does so at non-cytotoxic concentrations and we demonstrate this in multiple human CRC cell lines. After orthotopic implantation of human CRC tumors into mice, oral genistein did not inhibit tumor growth, but did inhibit distant metastasis formation, and was non-toxic to mice. Using a qPCR array, we screened for genistein-induced changes in gene expression, followed by Western blot confirmation, demonstrating that genistein downregulated matrix metalloproteinase 2 and Fms-Related Tyrosine Kinase 4 (FLT4; vascular endothelial growth factor receptor 3). After demonstrating that genistein suppressed neo-angiogenesis in mouse tumors, we examined FLT4 expression in primary CRC and adjacent normal colonic tissue from 60 human subjects, demonstrating that increased FLT4 significantly correlates with increased stage and decreased survival. In summary, we demonstrate for the first time that genistein inhibits human CRC metastasis at dietary, non-toxic, doses. FLT4 is identified as a marker of metastatic disease, and as a response marker for small molecule therapeutics that inhibit CRC metastasis. 
 Vascular endothelial growth factor (VEGF)-C signaling through FLT-4 (VEGFR-3) mediates leukemic cell proliferation, survival, and resistance to chemotherapy. Similar to solid tumors, growth of leukemias may also be angiogenesis dependent. Furthermore, tyrosine kinase receptors specific to endothelial cells are expressed on certain subsets of leukemias. We have previously demonstrated the existence of a VEGF/VEGFR-2 autocrine loop on leukemic cells that supports their growth and   migration  . Here, we demonstrate that in response to leukemia-derived proangiogenic and proinflammatory cytokines such as basic fibroblast growth factor and IL-1, endothelial cells release increasing amounts of another vascular endothelial growth factor (VEGF) family member, VEGF-C. In turn, interaction of VEGF-C with its receptor VEGFR-3 (FLT-4) promotes leukemia survival and proliferation. We demonstrate in 2 cell lines and 5 FLT-4(+) leukemias that VEGF-C and a mutant form of the molecule that lacks the KDR-binding motif induce receptor phosphorylation, leukemia proliferation, and increased survival, as determined by increased Bcl-2/Bax ratios. Moreover, VEGF-C protected leukemic cells from the apoptotic effects of 3 chemotherapeutic agents. Because most leukemic cells release proangiogenic as well as proinflammatory cytokines, our data suggest that the generation of a novel paracrine angiogenic loop involving VEGF-C and FLT-4 may promote the survival of a subset of leukemias and protect them from chemotherapy-induced apoptosis. These results identify the VEGF-C/FLT-4 pathway as a novel therapeutic target for the treatment of subsets of acute leukemia. 
 Vascular endothelial growth factor c promotes ovarian carcinoma progression through paracrine and autocrine mechanisms. Vascular endothelial growth factor C (VEGFC) has been reported to promote tumor progression in several tumor types, mainly through the stimulation of lymphangiogenesis and lymphatic metastasis. However, the expression and biological significance of the VEGFC/VEGF receptor (VEGFR)-3 pathway in ovarian cancer growth and dissemination are unclear, and have been investigated in this study. Soluble VEGFC was detected in the plasma and ascites of patients with ovarian carcinoma, and VEGFR3 expression was found in their tumor tissues. In human ovarian carcinoma xenograft models, high levels of soluble VEGFC in ascites and serum were detected, in association with disease progression, tumor burden, and volume of ascites. Peak VEGFC expression preceded para-aortic lymph node infiltration by HOC8 neoplastic cells. Histological detection of tumor cells in blood and lymphatic vessels indicated both hematogenous and lymphatic dissemination. Overexpression of VEGFC in the VEGFR3-positive and luciferase-expressing IGROV1 cells promoted carcinoma dissemination after orthotopic transplantation in the ovary of immunodeficient mice. In vitro, VEGFC released by the tumor cells stimulated tumor cell   migration   in an autocrine manner. Cediranib, an inhibitor of VEGFR1-3 and c-kit, inhibited in vivo metastasis of VEGFC-overexpressing IGROV1 and in vitro autocrine effects. These findings suggest that the VEGFC/VEGFR3 pathway acts as an enhancer of ovarian cancer progression through autocrine and paracrine mechanisms, hence offering a potential target for therapy. 
 Hypoxia preconditioning of mesenchymal stromal cells enhances PC3 cell lymphatic metastasis accompanied by VEGFR-3/CCR7 activation. Mesenchymal stromal cells (MSCs) in bone marrow may enhance tumor metastases through the secretion of chemokines. MSCs have been reported to home toward the hypoxic tumor microenvironment in vivo. In this study, we investigated prostate cancer PC3 cell behavior under the influence of hypoxia preconditioned MSCs and explored the related mechanism of prostate cancer lymphatic metastases in mice. Transwell assays revealed that VEGF-C receptor, VEGFR-3, as well as chemokine CCL21 receptor, CC chemokine receptor 7 (CCR7), were responsible for the   migration   of PC3 cells toward hypoxia preconditioned MSCs. Knock-in Ccr7 in PC3 cells also improved cell   migration   in vitro. Furthermore, when PC3 cells were labeled using the hrGfp-lentiviral vector, and were combined with hypoxia preconditioned MSCs for xenografting, it resulted in an enhancement of lymph node metastases accompanied by up-regulation of VEGFR-3 and CCR7 in primary tumors. Both PI3K/Akt/IκBα and JAK2/STAT3 signaling pathways were activated in xenografts in the presence of hypoxia-preconditioned MSCs. Unexpectedly, the p-VEGFR-2/VEGFR-2 ratio was attenuated accompanied by decreased JAK1 expression, indicating a switching-off of potential vascular signal within xenografts in the presence of hypoxia-preconditioned MSCs. Unlike results from other studies, VEGF-C maintained a stable expression in both conditions, which indicated that hypoxia preconditioning of MSCs did not influence VEGF-C secretion. Our results provide the new insights into the functional molecular events and signalings influencing prostate tumor metastases, suggesting a hopeful diagnosis and treatment in new approaches. 
 Neuropilin-2 mediates lymphangiogenesis of colorectal carcinoma via a VEGFC/VEGFR3 independent signaling. Lymphangiogenesis critically contributes to the lymphatic metastasis of colorectal carcinomas (CRCs), but the underlying mechanism of CRC lymphangiogenesis remains largely elusive. We have previously demonstrated that Semaphorin-3F (SEMA3F) is critically involved in CRC metastasis, and the receptor of SEMA3F, neuropilin-2 (NRP2), originally described as an axon guiding chemorepulsant implicated in nerve development, has been suggested in promoting lymphangiogenesis via acting as an obligate co-receptor of VEGFR3 cooperatively enhancing the activity of VEGF-C. Our present study revealed that in colorectal carcinomas, NRP2 expression levels of tumor-associated lymphatic endothelial cells (LECs) are significantly correlated with the density of tumor lymphatic vessels. In vitro, activation of NRP2 in LECs substantially facilitates their   migration  , sprouting, and tubulogenesis capacity via regulating the rearrangement of cytoskeleton polarity. In vivo model further showed that in the xenografts generated from SEMA3F knockdown CRC cells, NRP2 is substantially activated in tumor-associated LECs, resulting in a significantly increased tumor lymphangiogenesis. Further evidence demonstrated that CRC cell induces the activation of NRP2 in LECs to promote tumor lymphangiogenesis via integrinα9β1/FAK/Erk pathway independent VEGF-C/VEGFR3 signaling. Our study for the first time revealed the novel molecular mechanism of NRP2-mediated-lymphangiogenesis in CRCs, suggesting NRP2 as a potential therapeutic target in preventing lymphatic metastasis of CRCs. 
 Endothelin-1 induces the transactivation of vascular endothelial growth factor receptor-3 and modulates cell   migration   and vasculogenic mimicry in melanoma cells. Endothelin receptor B (ET(B)R) is a G-protein-coupled receptor overexpressed in melanoma, blood, and lymphatic endothelial cells. Given that aberrant signal transduction can be mediated through cross talk between receptors, here, we explore the functional relationship between ET(B)R and the vascular endothelial growth factor receptor (VEGFR)-3 system and how this cross talk might influence the aggressive behavior of melanoma cells. The expression of VEGFR-3 and its ligands, VEGF-C and VEGF-D, significantly increased after activating ET(B)R by ET-1 in primary and metastatic melanoma cell lines. These effects, similarly to those induced by hypoxia, were mediated by hypoxia-inducible factor (HIF)-1α and HIF-2α. ET-1 caused the phosphorylation of VEGFR-3, which was accompanied by the activation of the downstream signaling molecules, such as MAPK and AKT. Inhibition of c-Src activity or silencing of the scaffold protein β-arrestin-1 reduced ET-1-induced VEGFR-3 phosphorylation, demonstrating that, upon ET-1 stimulus, β-arrestin-1 is involved with c-Src in the ET(B)R-mediated VEGFR-3 transactivation. Moreover, ET-1 in combination with VEGF-C further increased VEGFR-3, MAPK, and AKT phosphorylation and markedly promoted c  ell migra  tion and vasculogenic mimicry. Dual inhibition of ET(B)R and VEGFR-3 was required for the effective inhibition of these effects, as well as for VEGFR-3 phosphorylation, demonstrating that ET(B)R cross talk with VEGFR-3 enhances cell plasticity and motility. Finally, in melanoma xenografts, ET(B)R antagonist inhibited tumor growth and the activation of the VEGF-C/VEGFR-3 axis, indicating that targeting ET(B)R may improve melanoma treatment acting directly or indirectly by impairing ET(B)R cross talk with VEGFR-3. 
 Isolated lymphatic endothelial cells transduce growth, survival and migratory signals via the VEGF-C/D receptor VEGFR-3. Vascular endothelial growth factor receptor-3 (VEGFR-3/Flt4) binds two known members of the VEGF ligand family, VEGF-C and VEGF-D, and has a critical function in the remodelling of the primary capillary vasculature of midgestation embryos. Later during development, VEGFR-3 regulates the growth and maintenance of the lymphatic vessels. In the present study, we have isolated and cultured stable lineages of blood vascular and lymphatic endothelial cells from human primary microvascular endothelium by using antibodies against the extracellular domain of VEGFR-3. We show that VEGFR-3 stimulation alone protects the lymphatic endothelial cells from serum deprivation-induced apoptosis and induces their growth and   migration  . At least some of these signals are transduced via a protein kinase C-dependent activation of the p42/p44 MAPK signalling cascade and via a wortmannin-sensitive induction of Akt phosphorylation. These results define the critical role of VEGF-C/VEGFR-3 signalling in the growth and survival of lymphatic endothelial cells. The culture of isolated lymphatic endothelial cells should now allow further studies of the molecular properties of these cells. 
 Molecular controls of lymphatic VEGFR3 signaling. Vascular endothelial growth factor receptor 3 (VEGFR3) plays important roles both in lymphangiogenesis and angiogenesis. On stimulation by its ligand VEGF-C, VEGFR3 is able to form both homodimers as well as heterodimers with VEGFR2 and activates several downstream signal pathways, including extracellular signal-regulated kinases (ERK)1/2 and protein kinase B (AKT). Despite certain similarities with VEGFR2, molecular features of VEGFR3 signaling are still largely unknown.Human dermal lymphatic endothelial cells were used to examine VEGF-C-driven activation of signaling. Compared with VEGF-A activation of VEGFR2, VEGF-C-induced VEGFR3 activation led to a more extensive AKT activation, whereas activation of ERK1/2 displayed a distinctly different kinetics. Furthermore, VEGF-C, but not VEGF-A, induced formation of VEGFR3/VEGFR2 complexes. Silencing VEGFR2 or its partner neuropilin 1 specifically abolished VEGF-C-induced AKT but not ERK activation, whereas silencing of neuropilin 2 had little effect on either signaling pathway. Finally, suppression of vascular endothelial phosphotyrosine phosphatase but not other phosphotyrosine phosphatases enhanced VEGF-C-induced activation of both ERK and AKT pathways. Functionally, both ERK and AKT pathways are important for lymphatic endothelial cells   migration  .VEGF-C activates AKT signaling via formation of VEGFR3/VEGFR2 complex, whereas ERK is activated by VEGFR3 homodimer. Neuropilin 1 and vascular endothelial phosphotyrosine phosphatase are involved in regulation of VEGFR3 signaling. 
  GeneRIFs  
 VEGFR family members are expressed in leiomyosarcomas in vivo and in vitro. Upon receptor stimulation, PTK787 is able to inhibit subsequent phosphorylation events and influences cell survival but not metabolic activity and   migration   
 Transwell assays revealed that VEGF-C receptor, VEGFR-3, as well as chemokine CCL21 receptor, CC chemokine receptor 7 (CCR7), were responsible for the   migration   of PC3 cells toward hypoxia preconditioned MSCs 
 results demonstrate that the proteolytic enzyme MMP-9 promotes resistant epithelial breast cancer cells   migration   and tubular network formation through VEGFR-2/ VEGFR-3 activation 
 TGFbeta1 stimulates VEGFD/VEGFR3 receptor axis activation leading to increased cell   migration   and invasion by primary intermediate prostate cancer basal cell cultures 
 KSHV gB can activate VEGFR-3 on the microvascular endothelium and modulate endothelial cell   migration   and proliferation via an interaction between the alpha3beta1 integrin and the VEGFR-3 receptor 
  ECM  PubMed  
 Stimulation of beta 1 integrin induces tyrosine phosphorylation of vascular endothelial growth factor receptor-3 and modulates cell migration. Interactions between integrins and tyrosine kinase receptors can modulate a variety of cell functions. We observed a cooperative interaction between the beta(1) integrin and vascular endothelial growth factor receptor-3 (VEGFR-3 or Flt4) that appeared to be required for cell migration. By using VEGFR-3-transfected 293 cells (293/VEGFR-3) or primary dermal microvascular endothelial cells (DMEC), we found that stimulation with either soluble or immobilized extracellular matrix (  ECM  ) proteins, collagen or fibronectin (FN), resulted in the increased tyrosine phosphorylation of VEGFR-3 in the absence of a cognate ligand. This increased tyrosine phosphorylation of VEGFR-3 was diminished by pretreatment with a blocking antibody against the beta(1) integrin. Cross-linking with anti-beta(1) integrin antibody induced a similar degree of tyrosine phosphorylation of VEGFR-3. Stimulation with collagen or FN induced an association between beta(1) integrin and VEGFR-3 in both 293/VEGFR-3 and primary DMEC cells. Collagen or FN-induced tyrosine phosphorylation of VEGFR-3 was inhibited by treatment with cytochalasin D, an inhibitor of actin polymerization. Collagen or FN was able to induce the migration of 293/VEGFR-3 or DMEC cells to a limited extent. However, migration was dramatically enhanced when a gradient of the cognate ligand, VEGF-D, was added. VEGF-D failed to induce cell migration in the absence of   ECM   proteins. Introducing a mutation at the kinase domain of VEGFR-3 or treatment with blocking antibody against either VEGFR-3 or beta(1) integrin inhibited cell migration induced by   ECM   and VEGF-D, indicating that signals from both beta(1) integrin and VEGFR-3 are required for this cell function. 
 Extracellular matrix regulates endothelial functions through interaction of VEGFR-3 and integrin alpha5beta1. Endothelium extracellular matrix (  ECM  ) interactions can provide distinct spatial and molecular signals which control cellular proliferation, migration, and differentiation. Here, we investigated the role of fibronectin (FN), a major   ECM   protein, on the functions of lymphatic endothelial cells (LEC). We observed that FN, the ligand for integrin alpha5beta1, selectively promoted the growth of LEC as compared with vitronectin (VN) in the presence of the ligand for vascular endothelial growth factor receptor 3 [VEGFR-3 (VEGF-C156S)]. Upon investigating the mechanisms whereby   ECM   components regulate VEGFR-3 signaling, we found that FN transactivated VEGFR-3 and significantly enhanced the phosphorylation of VEGFR-3 induced by VEGF-C156S as compared to VN. An enhanced association of the integrin subunit alpha5 or beta1 with VEGFR-3, after stimulation with VEGF-C156S, was observed by co-immunoprecipitation. While blockade of integrin alpha5beta1 inhibited the VEGF-C156S-induced phosphorylation of VEGFR-3, no similar effect was obtained by blocking integrin alphavbeta3. FN also protected the endothelial cells from serum deprivation-induced apoptosis. Moreover, while the specific PI3 kinase inhibitor, LY294002, abolished this FN-mediated cell survival, the MAPK kinase inhibitor, PD98059, had no significant effect. Furthermore, a dominant-negative mutant of VEGFR-3 (G857R) reduced VEGF-C156S or FN-mediated cell survival, as well as the activities of PI3 kinase/Akt. Our results indicate that integrin alpha5beta1 participates in the activation of both VEGFR-3 and its downstream PI3 kinase/Akt signaling pathway, which is essential for FN-mediated lymphatic endothelial cell survival and proliferation. 
 Extracellular matrix regulates endothelial functions through interaction of VEGFR-3 and integrin alpha5beta1. Endothelium extracellular matrix (  ECM  ) interactions can provide distinct spatial and molecular signals which control cellular proliferation, migration, and differentiation. Here, we investigated the role of fibronectin (FN), a major   ECM   protein, on the functions of lymphatic endothelial cells (LEC). We observed that FN, the ligand for integrin alpha5beta1, selectively promoted the growth of LEC as compared with vitronectin (VN) in the presence of the ligand for vascular endothelial growth factor receptor 3 [VEGFR-3 (VEGF-C156S)]. Upon investigating the mechanisms whereby   ECM   components regulate VEGFR-3 signaling, we found that FN transactivated VEGFR-3 and significantly enhanced the phosphorylation of VEGFR-3 induced by VEGF-C156S as compared to VN. An enhanced association of the integrin subunit alpha5 or beta1 with VEGFR-3, after stimulation with VEGF-C156S, was observed by co-immunoprecipitation. While blockade of integrin alpha5beta1 inhibited the VEGF-C156S-induced phosphorylation of VEGFR-3, no similar effect was obtained by blocking integrin alphavbeta3. FN also protected the endothelial cells from serum deprivation-induced apoptosis. Moreover, while the specific PI3 kinase inhibitor, LY294002, abolished this FN-mediated cell survival, the MAPK kinase inhibitor, PD98059, had no significant effect. Furthermore, a dominant-negative mutant of VEGFR-3 (G857R) reduced VEGF-C156S or FN-mediated cell survival, as well as the activities of PI3 kinase/Akt. Our results indicate that integrin alpha5beta1 participates in the activation of both VEGFR-3 and its downstream PI3 kinase/Akt signaling pathway, which is essential for FN-mediated lymphatic endothelial cell survival and proliferation. 
  [Ee]xtracellular [Mm]atrix  PubMed  
 Identification of fetal and maternal single nucleotide polymorphisms in candidate genes that predispose to spontaneous preterm labor with intact membranes. The purpose of this study was to determine whether maternal/fetal single nucleotide polymorphisms (SNPs) in candidate genes are associated with spontaneous preterm labor/delivery.A genetic association study was conducted in 223 mothers and 179 fetuses (preterm labor with intact membranes who delivered <37 weeks of gestation [preterm birth (PTB)]), and 599 mothers and 628 fetuses (normal pregnancy); 190 candidate genes and 775 SNPs were studied. Single locus/haplotype association analyses were performed; the false discovery rate was used to correct for multiple testing.The strongest single locus associations with PTB were interleukin-6 receptor 1 (fetus; P=.000148) and tissue inhibitor of metalloproteinase 2 (mother; P=.000197), which remained significant after correction for multiple comparisons. Global haplotype analysis indicated an association between a fetal DNA variant in insulin-like growth factor F2 and maternal alpha 3 type IV collagen isoform 1 (global, P=.004 and .007, respectively).An SNP involved in controlling fetal inflammation (interleukin-6 receptor 1) and DNA variants in maternal genes encoding for proteins involved in   extracellular matrix   metabolism approximately doubled the risk of PTB. 
 Stimulation of beta 1 integrin induces tyrosine phosphorylation of vascular endothelial growth factor receptor-3 and modulates cell migration. Interactions between integrins and tyrosine kinase receptors can modulate a variety of cell functions. We observed a cooperative interaction between the beta(1) integrin and vascular endothelial growth factor receptor-3 (VEGFR-3 or Flt4) that appeared to be required for cell migration. By using VEGFR-3-transfected 293 cells (293/VEGFR-3) or primary dermal microvascular endothelial cells (DMEC), we found that stimulation with either soluble or immobilized   extracellular matrix   (ECM) proteins, collagen or fibronectin (FN), resulted in the increased tyrosine phosphorylation of VEGFR-3 in the absence of a cognate ligand. This increased tyrosine phosphorylation of VEGFR-3 was diminished by pretreatment with a blocking antibody against the beta(1) integrin. Cross-linking with anti-beta(1) integrin antibody induced a similar degree of tyrosine phosphorylation of VEGFR-3. Stimulation with collagen or FN induced an association between beta(1) integrin and VEGFR-3 in both 293/VEGFR-3 and primary DMEC cells. Collagen or FN-induced tyrosine phosphorylation of VEGFR-3 was inhibited by treatment with cytochalasin D, an inhibitor of actin polymerization. Collagen or FN was able to induce the migration of 293/VEGFR-3 or DMEC cells to a limited extent. However, migration was dramatically enhanced when a gradient of the cognate ligand, VEGF-D, was added. VEGF-D failed to induce cell migration in the absence of ECM proteins. Introducing a mutation at the kinase domain of VEGFR-3 or treatment with blocking antibody against either VEGFR-3 or beta(1) integrin inhibited cell migration induced by ECM and VEGF-D, indicating that signals from both beta(1) integrin and VEGFR-3 are required for this cell function. 
   Extracellular matrix   regulates endothelial functions through interaction of VEGFR-3 and integrin alpha5beta1. Endothelium   extracellular matrix   (ECM) interactions can provide distinct spatial and molecular signals which control cellular proliferation, migration, and differentiation. Here, we investigated the role of fibronectin (FN), a major ECM protein, on the functions of lymphatic endothelial cells (LEC). We observed that FN, the ligand for integrin alpha5beta1, selectively promoted the growth of LEC as compared with vitronectin (VN) in the presence of the ligand for vascular endothelial growth factor receptor 3 [VEGFR-3 (VEGF-C156S)]. Upon investigating the mechanisms whereby ECM components regulate VEGFR-3 signaling, we found that FN transactivated VEGFR-3 and significantly enhanced the phosphorylation of VEGFR-3 induced by VEGF-C156S as compared to VN. An enhanced association of the integrin subunit alpha5 or beta1 with VEGFR-3, after stimulation with VEGF-C156S, was observed by co-immunoprecipitation. While blockade of integrin alpha5beta1 inhibited the VEGF-C156S-induced phosphorylation of VEGFR-3, no similar effect was obtained by blocking integrin alphavbeta3. FN also protected the endothelial cells from serum deprivation-induced apoptosis. Moreover, while the specific PI3 kinase inhibitor, LY294002, abolished this FN-mediated cell survival, the MAPK kinase inhibitor, PD98059, had no significant effect. Furthermore, a dominant-negative mutant of VEGFR-3 (G857R) reduced VEGF-C156S or FN-mediated cell survival, as well as the activities of PI3 kinase/Akt. Our results indicate that integrin alpha5beta1 participates in the activation of both VEGFR-3 and its downstream PI3 kinase/Akt signaling pathway, which is essential for FN-mediated lymphatic endothelial cell survival and proliferation. 
 A genetic association study of maternal and fetal candidate genes that predispose to preterm prelabor rupture of membranes (PROM). We sought to determine whether maternal/fetal single-nucleotide polymorphisms (SNPs) in candidate genes are associated with preterm prelabor rupture of membranes (pPROM).A case-control study was conducted in patients with pPROM (225 mothers and 155 fetuses) and 599 mothers and 628 fetuses with a normal pregnancy; 190 candidate genes and 775 SNPs were studied. Single locus/haplotype association analyses were performed; false discovery rate was used to correct for multiple testing (q* = 0.15).First, a SNP in tissue inhibitor of metalloproteinase 2 in mothers was significantly associated with pPROM (odds ratio, 2.12; 95% confidence interval, 1.47-3.07; P = .000068), and this association remained significant after correction for multiple comparisons. Second, haplotypes for Alpha 3 type IV collagen isoform precursor in the mother were associated with pPROM (global P = .003). Third, multilocus analysis identified a 3-locus model, which included maternal SNPs in collagen type I alpha 2, defensin alpha 5 gene, and endothelin 1.DNA variants in a maternal gene involved in   extracellular matrix   metabolism doubled the risk of pPROM. 
 Identification of fetal and maternal single nucleotide polymorphisms in candidate genes that predispose to spontaneous preterm labor with intact membranes. The purpose of this study was to determine whether maternal/fetal single nucleotide polymorphisms (SNPs) in candidate genes are associated with spontaneous preterm labor/delivery.A genetic association study was conducted in 223 mothers and 179 fetuses (preterm labor with intact membranes who delivered <37 weeks of gestation [preterm birth (PTB)]), and 599 mothers and 628 fetuses (normal pregnancy); 190 candidate genes and 775 SNPs were studied. Single locus/haplotype association analyses were performed; the false discovery rate was used to correct for multiple testing.The strongest single locus associations with PTB were interleukin-6 receptor 1 (fetus; P=.000148) and tissue inhibitor of metalloproteinase 2 (mother; P=.000197), which remained significant after correction for multiple comparisons. Global haplotype analysis indicated an association between a fetal DNA variant in insulin-like growth factor F2 and maternal alpha 3 type IV collagen isoform 1 (global, P=.004 and .007, respectively).An SNP involved in controlling fetal inflammation (interleukin-6 receptor 1) and DNA variants in maternal genes encoding for proteins involved in   extracellular matrix   metabolism approximately doubled the risk of PTB. 
 A genetic association study of maternal and fetal candidate genes that predispose to preterm prelabor rupture of membranes (PROM). We sought to determine whether maternal/fetal single-nucleotide polymorphisms (SNPs) in candidate genes are associated with preterm prelabor rupture of membranes (pPROM).A case-control study was conducted in patients with pPROM (225 mothers and 155 fetuses) and 599 mothers and 628 fetuses with a normal pregnancy; 190 candidate genes and 775 SNPs were studied. Single locus/haplotype association analyses were performed; false discovery rate was used to correct for multiple testing (q* = 0.15).First, a SNP in tissue inhibitor of metalloproteinase 2 in mothers was significantly associated with pPROM (odds ratio, 2.12; 95% confidence interval, 1.47-3.07; P = .000068), and this association remained significant after correction for multiple comparisons. Second, haplotypes for Alpha 3 type IV collagen isoform precursor in the mother were associated with pPROM (global P = .003). Third, multilocus analysis identified a 3-locus model, which included maternal SNPs in collagen type I alpha 2, defensin alpha 5 gene, and endothelin 1.DNA variants in a maternal gene involved in   extracellular matrix   metabolism doubled the risk of pPROM. 
   Extracellular matrix   regulates endothelial functions through interaction of VEGFR-3 and integrin alpha5beta1. Endothelium   extracellular matrix   (ECM) interactions can provide distinct spatial and molecular signals which control cellular proliferation, migration, and differentiation. Here, we investigated the role of fibronectin (FN), a major ECM protein, on the functions of lymphatic endothelial cells (LEC). We observed that FN, the ligand for integrin alpha5beta1, selectively promoted the growth of LEC as compared with vitronectin (VN) in the presence of the ligand for vascular endothelial growth factor receptor 3 [VEGFR-3 (VEGF-C156S)]. Upon investigating the mechanisms whereby ECM components regulate VEGFR-3 signaling, we found that FN transactivated VEGFR-3 and significantly enhanced the phosphorylation of VEGFR-3 induced by VEGF-C156S as compared to VN. An enhanced association of the integrin subunit alpha5 or beta1 with VEGFR-3, after stimulation with VEGF-C156S, was observed by co-immunoprecipitation. While blockade of integrin alpha5beta1 inhibited the VEGF-C156S-induced phosphorylation of VEGFR-3, no similar effect was obtained by blocking integrin alphavbeta3. FN also protected the endothelial cells from serum deprivation-induced apoptosis. Moreover, while the specific PI3 kinase inhibitor, LY294002, abolished this FN-mediated cell survival, the MAPK kinase inhibitor, PD98059, had no significant effect. Furthermore, a dominant-negative mutant of VEGFR-3 (G857R) reduced VEGF-C156S or FN-mediated cell survival, as well as the activities of PI3 kinase/Akt. Our results indicate that integrin alpha5beta1 participates in the activation of both VEGFR-3 and its downstream PI3 kinase/Akt signaling pathway, which is essential for FN-mediated lymphatic endothelial cell survival and proliferation. 
 Endothelial cell adhesion to the   extracellular matrix   induces c-Src-dependent VEGFR-3 phosphorylation without the activation of the receptor intrinsic kinase activity. Integrins cooperate with growth factor receptors to promote downstream signaling for cell proliferation and migration. However, the mechanism of receptor activation is still unknown.To analyze the mechanism of phosphorylation of the vascular endothelial growth factor receptor (VEGFR)-3 by cell adhesion.We show that VEGFR-3 phosphorylation, induced by cell attachment to the   extracellular matrix  , is independent from the intrinsic kinase activity of the receptor, as evidenced from phosphorylation cell adhesion experiments with a mutant kinase dead receptor or in the presence of the specific kinase inhibitor MAZ 51. Cell adhesion experiments in the presence of the c-Src inhibitor PP2 or in fibroblast triple knockout for c-Src, Yes, and Fyn (SYF) demonstrate that VEGFR-3 phosphorylation, induced by   extracellular matrix  , is mediated by c-Src. Kinase assays in vitro with recombinant c-Src show that VEGFR-3 is a direct c-Src target and mass spectrometry analysis identified the sites phosphorylated by c-Src as tyrosine 830, 833, 853, 1063, 1333, and 1337, demonstrating that integrin-mediated receptor phosphorylation induces a phosphorylation pattern that is distinct from that induced by growth factors. Furthermore, pull-down assays show that integrin-mediated VEGFR-3 phosphorylation activates the recruitment to the receptor of the adaptor proteins CRKI/II and SHC inducing activation of JNK.These data suggest that cell adhesion to   extracellular matrix   induces a downstream signaling using the tyrosine kinase receptor VEGFR-3 as scaffold. 
  GeneRIFs  
 Cell adhesion to   extracellular matrix   induces a downstream signaling using the tyrosine kinase receptor VEGFR-3 as scaffold. 
  [Ii]nvasive  PubMed  
 Vascular endothelial growth factor C enhances cervical cancer cell   invasive  ness via upregulation of galectin-3 protein. Vascular endothelial growth factor C (VEGF-C) promotes cervical cancer metastasis, while the detailed mechanism remains obscure. Recent evidence shows that galectin-3 (Gal-3), a glycan binding protein, interacts with the VEGF receptors and reinforces their signal transduction. In this study, we investigated the role of Gal-3 in VEGF-C-induced cervical cancer cell invasion. On cervical carcinoma cell line SiHa cells, silencing of Gal-3 expression with specific siRNA largely impaired VEGF-C-enhanced cell invasion. Treatment with VEGF-C for 12-48 h enhanced Gal-3 protein expression, which was inhibited by the addition of NF-κB inhibitor pyrrolidine dithiocarbamate (PDTC). Moreover, the silencing of NF-κB subunit p65 expression with specific siRNA attenuated VEGF-C-enhanced Gal-3 expression, suggesting that NF-κB is the key intermediate. Under VEGF-C stimulation, an enhanced interaction between VEGF receptor-3 (VEGF-R3) and Gal-3 was found, which may possibly lead to VEGF-R3 activation since exogenous Gal-3 induced VEGF-R3 phosphorylation in a dose- and time-dependent manner. In conclusion, our findings implied that VEGF-C enhanced cervical ca  ncer inv  asiveness via upregulation of Gal-3 protein through NF-κB pathway, which may shed light on potential therapeutic strategies for cervical cancer therapy. 
 The VEGF-C/Flt-4 axis promotes invasion and metastasis of cancer cells. Flt-4, a VEGF receptor, is activated by its specific ligand, VEGF-C. The resultant signaling pathway promotes angiogenesis and/or lymphangiogenesis. This report provides evidence that the VEGF-C/Flt-4 axis enhances cancer cell mobility and   invasive  ness and contributes to the promotion of cancer cell metastasis. VEGF-C/Flt-4-mediated invasion and metastasis of cancer cells were found to require upregulation of the neural cell adhesion molecule contactin-1 through activation of the Src-p38 MAPK-C/EBP-dependent pathway. Examination of tumor tissues from various types of cancers revealed high levels of Flt-4 and VEGF-C expression that correlated closely with clinical metastasis and patient survival. The VEGF-C/Flt-4 axis, through upregulation of contactin-1, may regulate the   invasive   capacity in different types of cancer cells. 
 Vascular endothelial growth factor C enhances cervical cancer cell   invasive  ness via upregulation of galectin-3 protein. Vascular endothelial growth factor C (VEGF-C) promotes cervical cancer metastasis, while the detailed mechanism remains obscure. Recent evidence shows that galectin-3 (Gal-3), a glycan binding protein, interacts with the VEGF receptors and reinforces their signal transduction. In this study, we investigated the role of Gal-3 in VEGF-C-induced cervical cancer cell invasion. On cervical carcinoma cell line SiHa cells, silencing of Gal-3 expression with specific siRNA largely impaired VEGF-C-enhanced cell invasion. Treatment with VEGF-C for 12-48 h enhanced Gal-3 protein expression, which was inhibited by the addition of NF-κB inhibitor pyrrolidine dithiocarbamate (PDTC). Moreover, the silencing of NF-κB subunit p65 expression with specific siRNA attenuated VEGF-C-enhanced Gal-3 expression, suggesting that NF-κB is the key intermediate. Under VEGF-C stimulation, an enhanced interaction between VEGF receptor-3 (VEGF-R3) and Gal-3 was found, which may possibly lead to VEGF-R3 activation since exogenous Gal-3 induced VEGF-R3 phosphorylation in a dose- and time-dependent manner. In conclusion, our findings implied that VEGF-C enhanced cervical ca  ncer inv  asiveness via upregulation of Gal-3 protein through NF-κB pathway, which may shed light on potential therapeutic strategies for cervical cancer therapy. 
 Role of the VEGFR3/VEGFD receptor axis in TGFbeta1 activation of primary prostate cell lines. Reports indicate that vascular endothelial growth factor receptor type 3 (VEGFR3) regulates cellular functions such as invasion, proliferation, and chemo-resistance. However, the exact function of the VEGFR3 signaling axis in prostate epithelial cells is poorly characterized.The goal of this study was to evaluate whether TGFbeta1 in combination with VEGFD can promote pre-malignant   invasive   activities of intermediate basal cells (IBC-10a) isolated from human prostate cancer (Gleason score 6).hTERT immortalized IBC-10a cells normally grew as confluent "cobblestoned" monolayers, but treatment with TGFbeta1 (10 ng/ml for 2-6 hr) dissociated the cell-cell junctions and induced VEGFR3 translocation to the cell surface. This event was not inhibited by 10 microM cycloheximide or puromycin, indicating transcription and protein synthesis were not required. We further discovered that TGFbeta1 in combination with VEGFD induced a significant increase in the   invasive   activity of IBC-10a cells (>26% and 53% after 24 and 48 hr, respectively) in modified Boyden Chamber assays. TGFbetaRII receptor antibodies specifically blocked TGFbeta1 induction of VEGFR3 translocation to the cell surface and blocked VEGFD-induced invasion. Zymograms revealed that TGFbeta1 (and not VEGFR3) stimulated the secretion of MMP-2 and MMP-9, presumably to promote cell invasion. The cell invasion assays confirmed that antibodies specific for TGFbetaII receptor, MMP-2 and MMP-9 and VEGFR3, independently blocked TGFbeta1-induced invasion.For the first time, we have demonstrated the mechanism by which TGFbeta1 stimulates VEGFD/VEGFR3 receptor axis activation leading to increased cell migration and invasion by primary intermediate basal cell cultures. 
 Expression of vascular endothelial growth factor (VEGF)-C and VEGF-D, and their receptor VEGFR-3, during different stages of cervical carcinogenesis. Cervical carcinogenesis has well-defined stages of disease progression including three grades of pre-  invasive   lesions--cervical intraepithelial neoplasia grades 1-3 (CIN 1-3)--and   invasive   cervical cancer. However, the biological properties of CIN lesions prone to develop   invasive   disease are not well defined. Recent observations suggest that early   invasive   disease spreads to regional lymph nodes in several tumour types and that growth factors (VEGF-C and VEGF-D) involved in new lymphatic vessel formation may play a crucial role in this process. The present study has assessed the expression of VEGF-C and VEGF-D, and their receptor VEGFR-3, in 152 cervical lesions (33 CIN 1, 33 CIN 2, 37 CIN 3, and 49 squamous cell carcinomas) to determine whether expression of lymphangiogenic factors occurs prior to invasion. The presence of lymphatic vessels was determined using LYVE-1 and podoplanin staining, as well as double immunostaining for LYVE-1/CD34 and podoplanin/CD34. In situ hybridization was performed to determine VEGFR-3 mRNA expression. A significant positive correlation was found between VEGF-C, VEGF-D, and VEGFR-3 expression through the different stages of cervical carcinogenesis. Significant differences in protein expression for VEGF-C, VEGF-D, and VEGFR-3 were found between CIN 1-2 and CIN 3 (p<0.001 for all), but not between CIN 3 and cervical cancer. More than 50% of the CIN 3 lesions showed moderate to strong staining for VEGF-C and VEGF-D, whereas most of the early pre-cancerous lesions (CIN 1 and 2) were negative. In cervical cancer, similar observations to those in CIN 3 were found. VEGFR-3 mRNA expression was found in the cytoplasm of epithelial neoplastic cells and VEGFR3 protein expression was found in more than 50% of CIN 3 lesions and cervical cancers, compared with 15% in CIN 1 and 2. These findings suggest an autocrine growth stimulation pattern via VEGFR-3. Adjacent CIN 3 was present in nine cervical cancers and displayed strong expression for VEGF-C, VEGF-D, and VEGFR-3. These results suggest that in cervical carcinogenesis a switch to the lymphangiogenic phenotype may occur at the stage of CIN 3. 
 Notch alters VEGF responsiveness in human and murine endothelial cells by direct regulation of VEGFR-3 expression. The Notch family of cell surface receptors and its ligands are highly conserved proteins that regulate cell fate determination, including those involved in mammalian vascular development. We report that Notch induces VEGFR-3 expression in vitro in human endothelial cells and in vivo in mice. In vitro, Notch in complex with the DNA-binding protein CBF-1/suppressor of hairless/Lag1 (CSL) bound the VEGFR-3 promoter and transactivated VEGFR-3 specifically in endothelial cells. Through induction of VEGFR-3, Notch increased endothelial cell responsiveness to VEGF-C, promoting endothelial cell survival and morphological changes. In vivo, VEGFR-3 was upregulated in endothelial cells with active Notch signaling. Mice heterozygous for null alleles of both Notch1 and VEGFR-3 had significantly reduced viability and displayed midgestational vascular patterning defects analogous to Notch1 nullizygous embryos. We found that Notch1 and Notch4 were expressed in normal and tumor lymphatic endothelial cells and that Notch1 was activated in lymphatic endothelium of   invasive   mammary micropapillary carcinomas. These results demonstrate that Notch1 and VEGFR-3 interact genetically, that Notch directly induces VEGFR-3 in blood endothelial cells to regulate vascular development, and that Notch may function in tumor lymphangiogenesis. 
 Prox 1, VEGF-C and VEGFR3 expression during cervical neoplasia progression as evidence of an early lymphangiogenic switch. Prox1 is a key regulator of lymphatic endothelial cell commitment during embryonic development. No correlations between Prox1 and VEGF-C/VEGFR3 expression in cervical cancer has been done until now. The aim of the present study was to evaluate the peculiarities of Prox1, VEGF-C and VEGFR3 expression during uterine cervix neoplasia progression. Material and methods. One hundred and four specimens taken from women with macroscopically detectable lesions were classified by histopathology and analyzed by immunohistochemistry for Prox1, VEGFR3 and VEGF-C expression. Results. The presence of Prox1 nuclear expression was detected starting from CIN2 and CIN3 lesions to micro  invasive   carcinoma, in the nuclei of lymphatic and venous endothelial cells and scattered stromal cells. All Prox1 positive lymphatic vessels were positive for VEGFR3. A significant correlation was found between expression of VEGF-C in tumor cells and nuclear density of Prox1 positive lymphatic cells (p=0.044). Conclusion. The commitment of Prox1 positive cells through a lymphatic lineage is an early event for cervical neoplastic progression, being present starting with intraepithelial cervical lesions, and is strongly associated with VEGFR3 and VEGF-C expression. These findings suggest an early active lymphangiogenesis during cervical neoplasia progression and explain, in part, the early presence of lymph node metastasis in cervical cancer. By the detection of Prox1 expression in lymphatic and venous endothelial cells, and also in stromal cells, it has been suggested that there are at least three different mechanism of lymph vessel development during cervical neoplasia progression. 
 Lymphatic microvessels density, VEGF-C, and VEGFR-3 expression in 25 cases of breast   invasive   lobular carcinoma.   Invasive   lobular carcinoma (ILC) is the second most common type of   invasive   breast cancer, having distinct morphologically but also prognostic and therapeutic features. This type of breast cancer shows a higher rate of multiple metastases with a more frequent axillary-lymph-node involvement. Related to these dissemination and metastatic features, we aimed to study the immunohistochemical expression of D2-40, VEGF-C and VEGFR-3 in 25 cases of ILCs stratified according to the histopathological and molecular classification. Regardless of histopathological or molecular subtype, the statistical tests proved that for ILC, the highest D2-40 lymphatic microvessels density (LMVD) was in the peritumoral areas. In classical subtype, the LMVD values were positively correlated with the degree of tumor differentiation and pTNM clinical stages and when these cases were classified based on the molecular criteria the highest recorded values were found in the luminal B subtype. In addition, regardless of the histopathological and molecular subtypes, the D2-40 LMVD varied in the same direction for both VEGF-C and VEGFR-3 categories, with the highest LMVD values recorded in those cases with the highest VEGF-C and VEGFR-3 reactivity, especially in the peritumoral areas. Considering only the molecular luminal A and B subtypes, we have noted that VEGF-C and VEGFR-3 expression was significantly higher in luminal A subtype compared to luminal B. This immunoprofile suggests the existence of a tumor type-specific lymphangiogenesis that may have certain prognostic and therapeutic implications. 
  GeneRIFs  
 Data suggest that VEGFC (vascular endothelial growth factor C) enhances cervical cancer   invasive  ness via up-regulation of galectin-3 via stimulation of NFkappaB/RELA pathway; galectin-3 interacts/activates VEGFR3. 
  [Mm]etastasize  PubMed  
 Retiform hemangioendotheliomas usually do not express D2-40 and VEGFR-3. Retiform hemangioendothelioma (RH) is a rare vascular neoplasm most often occurring in the limbs of middle-aged females. This entity is characterized by infiltrative vascular spaces arranged in a pattern similar to the rete testis. RH differs from angiosarcoma by lacking cytologic atypia and high mitotic rates. This neoplasm frequently recurs but rarely   metastasize  s. RH tumor cells react with vascular endothelial markers CD31, CD34, and factor VIII-related antigen. A review of the English literature provides only one attempt at staining RH with D2-40, a marker of endothelium of lymphatic vessels, which was negative, and one reported staining of RH with lymphatic endothelial marker VEGFR-3, which was positive. The etiology of RH is unknown. RH has previously been considered closely related to Dabska tumors, which are positive for lymphatic endothelial marker D2-40. We stained 4 RHs with mouse monoclonal antibodies against D2-40 and CD31 and 3 of the 4 RHs with vascular endothelial growth factor receptor 3 (VEGFR-3), to further evaluate whether RH had lymphatic differentiation, in addition to vascular differentiation. Three of the 4 RH biopsies failed to demonstrate D2-40, none expressed VEGFR-3, whereas CD31 was strongly positive, suggesting that RH is a vascular entity which usually does not have lymphatic differentiation, but may rarely express D2-40. 
 Lymphangiogenic VEGF-C and VEGFR-3 expression in genetically characterised gastrointestinal stromal tumours. This study aimed to assess the distribution of VEGF-C and VEGFR-3 expression in gastrointestinal stromal tumours (GISTs), and to analyse the value of lymphatic vessel density (LVD) in a tumour that is believed to preferentially   metastasize   through blood vessel conduits. A panel of immunohistochemical antibodies was used to evaluate 51 cases of genetically characterised GISTs: VEGF-C, VEGFR-3, D2-40 (for LVD assessment) and CD31 (for blood vessel density--BDV--assessment). The results were correlated with the clinical-pathological data. The large majority of cases (86.2%; 44/51) showed a mutation of the KIT gene, most of them (72.5%; 37/51) revealing mutations in exon 11. VEGFR-3 was predominantly expressed in KIT mutated GISTs (p=0.019). High LVD was correlated with the absence of metastasis (p=0.010) and high BVD showed a positive correlation with the occurrence of metastasis (p=0.049). The strong expression of VEGF-C and VEGFR-3 in GIST's cells was not correlated with the clinical parameters of aggressiveness, nor with high LVD. 
  [Mm]etastasis  PubMed  
 Vascular endothelial growth factor C enhances cervical cancer cell invasiveness via upregulation of galectin-3 protein. Vascular endothelial growth factor C (VEGF-C) promotes cervical cancer   metastasis  , while the detailed mechanism remains obscure. Recent evidence shows that galectin-3 (Gal-3), a glycan binding protein, interacts with the VEGF receptors and reinforces their signal transduction. In this study, we investigated the role of Gal-3 in VEGF-C-induced cervical cancer cell invasion. On cervical carcinoma cell line SiHa cells, silencing of Gal-3 expression with specific siRNA largely impaired VEGF-C-enhanced cell invasion. Treatment with VEGF-C for 12-48 h enhanced Gal-3 protein expression, which was inhibited by the addition of NF-κB inhibitor pyrrolidine dithiocarbamate (PDTC). Moreover, the silencing of NF-κB subunit p65 expression with specific siRNA attenuated VEGF-C-enhanced Gal-3 expression, suggesting that NF-κB is the key intermediate. Under VEGF-C stimulation, an enhanced interaction between VEGF receptor-3 (VEGF-R3) and Gal-3 was found, which may possibly lead to VEGF-R3 activation since exogenous Gal-3 induced VEGF-R3 phosphorylation in a dose- and time-dependent manner. In conclusion, our findings implied that VEGF-C enhanced cervical cancer invasiveness via upregulation of Gal-3 protein through NF-κB pathway, which may shed light on potential therapeutic strategies for cervical cancer therapy. 
 [Expression of VEGFR-2 and VEGFR-3 in papillary renal cell carcinoma and their relationship with prognosis]. To detect the expression of VEGF receptors in papillary renal cell carcinoma and to explore the correlation between their expression and clinical prognosis.Expression of VEGF receptors and PCNA (proliferating cell nuclear antigen) were evaluated in 82 patients with papillary renal cell carcinoma using tissue microarray and SP immunohistochemical staining.The expression of VEGFR-1 in papillary renal cell carcinoma was 82.93%, VEGFR-2 63.41%, VEGFR-3 34.15% and PCNA 67.07%, respectively. Increased VEGFR-2 expression was significantly correlated with tumor size (P = 0.016), histological grade (P = 0.034) and distant   metastasis   (P = 0.002). VEGFR-3 expression was correlated with histological grade (P = 0.028), lymph node status (P = 0.010) and distant   metastasis   (P = 0.018), but not correlated with gender, age, location, tumor size and TNM staging. VEGFR-1 expression had no correlation with any clinic and pathologic factors. PCNA expression was correlated with histological grade (P = 0.011), but not correlated with other factors. The expression of VEGFR-2 and VEGFR-3 in death cases were higher than that in surviving patients.Both VEGFR-2 and VEGFR-3 can serve as markers for prognosis of papillary renal cell carcinoma. Differently, VEGFR-3 is a predictor of lymph node   metastasis  , increased VEGFR-2 expression could be used to predict a potential blood dissemination. 
 Prognostic roles of cross-talk between peritumoral hepatocytes and stromal cells in hepatocellular carcinoma involving peritumoral VEGF-C, VEGFR-1 and VEGFR-3. Peritumoral liver tissue could play a potential role in hepatocellular carcinoma (HCC) progression and patient survival via angiogenesis- and lymphangiogensis-related factors. The prognostic role of these factors in hepatocytes and stromal cells in HCC patients after curative resection remains to be explored.Tumor tissue and surrounding peritumoral tissue were obtained from 145 resected HCC patients without lymph node   metastasis   (LNM) and 37 resected HCC patients with LNM. Tissue microarrays were constructed from duplicate cores of tumor tissue and surrounding peritumoral tissue from each resected specimen. Immunohistochemistry and real-time polymerase chain reaction were used to evaluate the expression of vascular endothelial growth factor-A (VEGF-A), VEGF-C, VEGF receptor-1(VEGFR-1), VEGFR-2, and VEGFR-3. Macrophage infiltration was determined by CD68 staining. Correlations between the expression of these factors and overall survival (OS) and time to recurrence (TTR) were studied.The peritumoral expression of VEGF-A, VEGF-C, VEGFR-1, VEGFR-2, and VEGFR-3 were significantly higher than expression of these factors in tumors. VEGFR-1 was mostly located in peritumoral macrophages, while VEGF-C and VEGFR-3 were mostly located in peritumoral hepatocytes. HCC with high peritumoral co-expression of VEGF-C, VEGFR-1, and VEGFR-3 was associated with higher peritumoral distribution of macrophages (0.87%±0.26% versus 0.45%±0.20%), LNM (32.4% versus 12.0%), shorter TTR (10.2 months versus 34.5 months), and poor prognosis (19.4 months versus 49.3 months).Expression of VEGF-C, VEGFR-1, and VEGFR-3 in peritumoral liver tissue is associated with a unique type of HCC that has a poorer outcome after hepatectomy. 
 Angiogenesis-related protein expression in bevacizumab-treated metastatic colorectal cancer: NOTCH1 detrimental to overall survival. The development of targeted therapies has undoubtedly broadened therapeutic options for patients with colorectal cancer (CRC). The use of bevacizumab to reduce angiogenesis has been associated with improved clinical outcomes. However, an urgent need for prognostic/predictive biomarkers for anti-angiogenic therapies still exists.Clinical data of 105 CRC patients treated with bevacizumab in conjunction with chemotherapy were analyzed. The expression of vascular endothelial growth factor (VEGF) receptors, NOTCH1 receptor and its ligand DLL4 were determined by immunohistochemistry. Tumor samples were arranged on a tissue microarray. The association between protein expression and clinicopathological characteristics and outcomes was determined.Bevacizumab was administered as a first-line of treatment in 70.5 % of our cases. The median progression-free survival (PFS) was 10.2 months. The median overall survival (OS) of the total cohort was 24.4 months. Bevacizumab, as the first-line of treatment, and the presence of liver   metastasis   were independently associated with objective response rate. Membrane VEGFR1 and VEGFR3 expressions were associated with the presence of lung   metastasis  ; interestingly, VEGFR3 was associated with less liver   metastasis  . NOTCH1 expression was associated with lymph node   metastasis  . There was a trend toward association between improved PFS and lower NOTCH1 expression (p = 0.06). Improved OS was significantly associated with lower NOTCH1 expression (p = 0.01). In a multivariate analysis, ECOG (Eastern Cooperative Oncology Group) performance status  , liver me  tastasis, histological grade, and NOTCH1 expression were independently associated with OS.Our findings illustrated the expression profile of angiogenesis-related proteins and their association with clinicopathological characteristics and outcomes. NOTCH1 expression is a detrimental prognostic factor in metastatic CRC patients treated with chemotherapy plus bevacizumab. 
 Vascular endothelial growth factor receptors 1,3 and caveolin-1 are implicated in colorectal cancer aggressiveness and prognosis--correlations with epidermal growth factor receptor, CD44v6, focal adhesion kinase, and c-Met. Vascular endothelial growth factor receptor-1 (VEGFR-1) and caveolin-1 have been shown to act both as tumor-promoting and tumor-suppressing proteins in various malignancies as well as in colorectal cancer (CRC), while VEGFR-3's lymphagiogenic involvement and connection to tumor parameters has yielded heterogenic results. This study was designed to investigate the expression of these molecules in 183 human CRC tissue specimens and explore their effect in both clinicopathological parameters and disease prognosis. We also utilize our previous results regarding epidermal growth factor receptor (EGFR), c-Met, CD44v6, and focal adhesion kinase, in an attempt to further clarify their distinct role in tumor prognosis and their crosstalk. Caveolin-1 was more freely distributed in the neoplasms of the right colon and restricted towards the left and the rectal cancer samples (p = 0.022); VEGFR-3 was associated with higher nodal   metastasis  ' status (p = 0.001) and staging (p = 0.006), and loss of VEGFR-1 predicted distant   metastasis   (p = 0.026) and advanced stage (p = 0.049). Prompted by previous reports, we performed all analyses also in the patient group of early (I and II) tumor stage where it was evident that VEGFR-1 was more frequently expressed in patients under 60 years old (p = 0.014) and VEGFR-3 was significantly elevated in left colon cancers (p = 0.039) and female patients (p = 0.038). Within the advanced stage (III and IV), the absence of VEGFR-1 exhibited a tendency for higher M status (p = 0.067) and lack of caveolin-1 signified worse AJCC classification (p = 0.053). Additionally, patient survival was influenced from VEGFR-3 (p = 0.019) for the whole sample, whereas subgroup analyses provided a correlation between caveolin-1 expression and improved survival in the early detection group of patients (p = 0.022). Using Cox regression for all available markers, FAK, CD44v6, and Caveolin-1 [corrected] emerged in this study as potential surrogate markers, the latter having positive prognostic significance. We further explored the multiple receptor correlations that were identified. 
 RNAi-mediated silencing of VEGF-C inhibits non-small cell lung cancer progression by simultaneously down-regulating the CXCR4, CCR7, VEGFR-2 and VEGFR-3-dependent axes-induced ERK, p38 and AKT signalling pathways. Vascular endothelial growth factor C (VEGF-C) expression is associated with the malignant tumour phenotype making it an attractive therapeutic target. We investigated the biological roles of VEGF-C in tumour growth, migration, invasion and explored the possibility of VEGF-C as a potential therapeutic target for the treatment of non-small cell lung cancer (NSCLC). A lentivirus-mediated RNA interference (RNAi) technology was used to specifically knockdown the expression of VEGF-C in A549 cells. Quantitative reverse transcriptase-polymerase chain reaction, flow cytometry, Western blot, immunohistochemistry, cellular growth, migration, invasion and ELISA assays were used to characterise VEGF-C expression in vitro. A lung cancer xenograft model in nude mice was established to investigate whether knockdown of VEGF-C reduced tumour growth in vivo. Silencing of VEGF-C suppressed tumour cell growth, migration and invasion in vitro; suppressed tumour growth, angiogenesis and lymphangiogenesis by tail vein injection of lentivirus encoded shRNA against VEGF-C in vivo. More importantly, silencing of VEGF-C also trapped the VEGFR-2, VEGFR-3, CXCR4, CCR7-dependent axes, and down-regulated the AKT, ERK and p38 signalling pathways. These results suggest that VEGF-C has a multifaceted role in NSCLC growth, migration and invasion; that VEGF-C-mediated autocrine loops with their cognate receptors and chemokine receptors are significant factors affecting tumour progression; and that RNAi-mediated silencing of VEGF-C represents a powerful therapeutic approach for controlling NSCLC growth and   metastasis  . 
 Differential expression of VEGF ligands and receptors in prostate cancer. Prostate cancer disseminates to regional lymph nodes, however the molecular mechanisms responsible for lymph node   metastasis   are poorly understood. The vascular endothelial growth factor (VEGF) ligand and receptor family have been implicated in the growth and spread of prostate cancer via activation of the blood vasculature and lymphatic systems. The purpose of this study was to comprehensively examine the expression pattern of VEGF ligands and receptors in the glandular epithelium, stroma, lymphatic vasculature and blood vessels in prostate cancer.The localization of VEGF-A, VEGF-C, VEGF-D, VEGF receptor (VEGFR)-1, VEGFR-2, and VEGFR-3 was examined in cancerous and adjacent benign prostate tissue from 52 subjects representing various grades of prostate cancer.Except for VEGFR-2, extensive staining was observed for all ligands and receptors in the prostate specimens. In epithelial cells, VEGF-A and VEGFR-1 expression was higher in tumor tissue compared to benign tissue. VEGF-D and VEGFR-3 expression was significantly higher in benign tissue compared to tumor in the stroma and the endothelium of lymphatic and blood vessels. In addition, the frequency of lymphatic vessels, but not blood vessels, was lower in tumor tissue compared with benign tissue.These results suggest that activation of VEGFR-1 by VEGF-A within the carcinoma, and activation of lymphatic endothelial cell VEGFR-3 by VEGF-D within the adjacent benign stroma may be important signaling mechanisms involved in the progression and subsequent metastatic spread of prostate cancer. Thus inhibition of these pathways may contribute to therapeutic strategies for the management of prostate cancer. 
 Prognostic significance of epidermal growth factor receptor and vascular endothelial growth factor receptor in colorectal adenocarcinoma. The purpose of this study was to evaluate the association between the expression of growth factors and the clinicopathological variables of colorectal adenocarcinoma. Immunohistochemistry and fluorescence in situ hybridization (FISH) were used to evaluate the amplification and expression of epidermal growth factor receptor (EGFR), vascular endothelial growth factor (VEGF), VEGF-D, VEGF receptor (VEGFR)-2, VEGFR-3, transforming growth factor (TGF)-β1, and insulin-like growth factor-1 receptor (IGF-1R) in a tissue microarray of 292 colorectal adenocarcinomas. The expression of EGFR, VEGF, VEGF-D, VEGFR-2 and VEGFR-3 was detected in 5.1%, 10.0%, 6.8%, 5.2%, and 57.2%. EGFR expression was associated with angioinvasion (p < 0.05) and lymph node   metastasi  s (p < 0.005). VEGFR-3 expression was higher in the rectum than in the colon (p < 0.05). VEGF expression correlated with VEGF-D (p < 0.05) and VEGFR-3 (p < 0.005) expression, while VEGF-D expression showed no significant association with VEGFR-2 or VEGFR-3. EGFR amplification was present in 10.6% and was not associated with EGFR protein expression. VEGFR-2 and VEGFR-3 expression levels were related to poor patient survival. Stage, perineural invasion, and lymph node   metastasi  s were independent prognostic factors based on a Cox analysis. VEGFR-2 and VEGFR-3 expression are markers of a poor prognosis in patients with surgically resected colorectal adenocarcinoma, whereas EGFR has a minor influence. 
 The VEGF-C/Flt-4 axis promotes invasion and   metastasis   of cancer cells. Flt-4, a VEGF receptor, is activated by its specific ligand, VEGF-C. The resultant signaling pathway promotes angiogenesis and/or lymphangiogenesis. This report provides evidence that the VEGF-C/Flt-4 axis enhances cancer cell mobility and invasiveness and contributes to the promotion of cancer cell   metastasis  . VEGF-C/Flt-4-mediated invasion and   metastasis   of cancer cells were found to require upregulation of the neural cell adhesion molecule contactin-1 through activation of the Src-p38 MAPK-C/EBP-dependent pathway. Examination of tumor tissues from various types of cancers revealed high levels of Flt-4 and VEGF-C expression that correlated closely with clinical   metastasis   and patient survival. The VEGF-C/Flt-4 axis, through upregulation of contactin-1, may regulate the invasive capacity in different types of cancer cells. 
 VEGFR signaling during lymphatic vascular development: From progenitor cells to functional vessels. Lymphatic vessels are an integral component of the cardiovascular system, serving important roles in fluid homeostasis, lipid absorption, and immune cell trafficking. Defining the mechanisms by which the lymphatic vasculature is constructed and remodeled into a functional vascular network not only provides answers to fascinating biological questions, but is fundamental to understanding how lymphatic vessel growth and development goes awry in human pathologies. While long recognized as dysfunctional in lymphedema and exploited as a route of tumor   metastasis  , recent work has highlighted important roles for lymphatic vessels in modulating immune responses, regulating salt-sensitive hypertension and important for lung inflation at birth. Substantial progress in our understanding of the signaling pathways important for development and morphogenesis of the lymphatic vasculature has been made in recent years. Here, we review advances in our knowledge of the best characterized of these signaling pathways, that involving the vascular endothelial growth factor (VEGF) family members VEGF-C and VEGF-D, together with their receptors VEGFR2 and VEGFR3. Recent work has defined multiple levels at which signal transduction by means of this key axis is regulated; these include control of ligand processing and bioavailability, modulation of receptor activation by interacting proteins, and regulation of receptor endocytosis and trafficking. 
 Angiogenesis-related protein expression in bevacizumab-treated metastatic colorectal cancer: NOTCH1 detrimental to overall survival. The development of targeted therapies has undoubtedly broadened therapeutic options for patients with colorectal cancer (CRC). The use of bevacizumab to reduce angiogenesis has been associated with improved clinical outcomes. However, an urgent need for prognostic/predictive biomarkers for anti-angiogenic therapies still exists.Clinical data of 105 CRC patients treated with bevacizumab in conjunction with chemotherapy were analyzed. The expression of vascular endothelial growth factor (VEGF) receptors, NOTCH1 receptor and its ligand DLL4 were determined by immunohistochemistry. Tumor samples were arranged on a tissue microarray. The association between protein expression and clinicopathological characteristics and outcomes was determined.Bevacizumab was administered as a first-line of treatment in 70.5 % of our cases. The median progression-free survival (PFS) was 10.2 months. The median overall survival (OS) of the total cohort was 24.4 months. Bevacizumab, as the first-line of treatment, and the presence of liver   metastasis   were independently associated with objective response rate. Membrane VEGFR1 and VEGFR3 expressions were associated with the presence of lung   metastasis  ; interestingly, VEGFR3 was associated with less liver   metastasis  . NOTCH1 expression was associated with lymph node   metastasis  . There was a trend toward association between improved PFS and lower NOTCH1 expression (p = 0.06). Improved OS was significantly associated with lower NOTCH1 expression (p = 0.01). In a multivariate analysis, ECOG (Eastern Cooperative Oncology Group) performance status  , liver me  tastasis, histological grade, and NOTCH1 expression were independently associated with OS.Our findings illustrated the expression profile of angiogenesis-related proteins and their association with clinicopathological characteristics and outcomes. NOTCH1 expression is a detrimental prognostic factor in metastatic CRC patients treated with chemotherapy plus bevacizumab. 
 VEGFR signaling during lymphatic vascular development: From progenitor cells to functional vessels. Lymphatic vessels are an integral component of the cardiovascular system, serving important roles in fluid homeostasis, lipid absorption, and immune cell trafficking. Defining the mechanisms by which the lymphatic vasculature is constructed and remodeled into a functional vascular network not only provides answers to fascinating biological questions, but is fundamental to understanding how lymphatic vessel growth and development goes awry in human pathologies. While long recognized as dysfunctional in lymphedema and exploited as a route of tumor   metastasis  , recent work has highlighted important roles for lymphatic vessels in modulating immune responses, regulating salt-sensitive hypertension and important for lung inflation at birth. Substantial progress in our understanding of the signaling pathways important for development and morphogenesis of the lymphatic vasculature has been made in recent years. Here, we review advances in our knowledge of the best characterized of these signaling pathways, that involving the vascular endothelial growth factor (VEGF) family members VEGF-C and VEGF-D, together with their receptors VEGFR2 and VEGFR3. Recent work has defined multiple levels at which signal transduction by means of this key axis is regulated; these include control of ligand processing and bioavailability, modulation of receptor activation by interacting proteins, and regulation of receptor endocytosis and trafficking. 
 Vascular endothelial growth factor C enhances cervical cancer cell invasiveness via upregulation of galectin-3 protein. Vascular endothelial growth factor C (VEGF-C) promotes cervical cancer   metastasis  , while the detailed mechanism remains obscure. Recent evidence shows that galectin-3 (Gal-3), a glycan binding protein, interacts with the VEGF receptors and reinforces their signal transduction. In this study, we investigated the role of Gal-3 in VEGF-C-induced cervical cancer cell invasion. On cervical carcinoma cell line SiHa cells, silencing of Gal-3 expression with specific siRNA largely impaired VEGF-C-enhanced cell invasion. Treatment with VEGF-C for 12-48 h enhanced Gal-3 protein expression, which was inhibited by the addition of NF-κB inhibitor pyrrolidine dithiocarbamate (PDTC). Moreover, the silencing of NF-κB subunit p65 expression with specific siRNA attenuated VEGF-C-enhanced Gal-3 expression, suggesting that NF-κB is the key intermediate. Under VEGF-C stimulation, an enhanced interaction between VEGF receptor-3 (VEGF-R3) and Gal-3 was found, which may possibly lead to VEGF-R3 activation since exogenous Gal-3 induced VEGF-R3 phosphorylation in a dose- and time-dependent manner. In conclusion, our findings implied that VEGF-C enhanced cervical cancer invasiveness via upregulation of Gal-3 protein through NF-κB pathway, which may shed light on potential therapeutic strategies for cervical cancer therapy. 
 Prognostic roles of cross-talk between peritumoral hepatocytes and stromal cells in hepatocellular carcinoma involving peritumoral VEGF-C, VEGFR-1 and VEGFR-3. Peritumoral liver tissue could play a potential role in hepatocellular carcinoma (HCC) progression and patient survival via angiogenesis- and lymphangiogensis-related factors. The prognostic role of these factors in hepatocytes and stromal cells in HCC patients after curative resection remains to be explored.Tumor tissue and surrounding peritumoral tissue were obtained from 145 resected HCC patients without lymph node   metastasis   (LNM) and 37 resected HCC patients with LNM. Tissue microarrays were constructed from duplicate cores of tumor tissue and surrounding peritumoral tissue from each resected specimen. Immunohistochemistry and real-time polymerase chain reaction were used to evaluate the expression of vascular endothelial growth factor-A (VEGF-A), VEGF-C, VEGF receptor-1(VEGFR-1), VEGFR-2, and VEGFR-3. Macrophage infiltration was determined by CD68 staining. Correlations between the expression of these factors and overall survival (OS) and time to recurrence (TTR) were studied.The peritumoral expression of VEGF-A, VEGF-C, VEGFR-1, VEGFR-2, and VEGFR-3 were significantly higher than expression of these factors in tumors. VEGFR-1 was mostly located in peritumoral macrophages, while VEGF-C and VEGFR-3 were mostly located in peritumoral hepatocytes. HCC with high peritumoral co-expression of VEGF-C, VEGFR-1, and VEGFR-3 was associated with higher peritumoral distribution of macrophages (0.87%±0.26% versus 0.45%±0.20%), LNM (32.4% versus 12.0%), shorter TTR (10.2 months versus 34.5 months), and poor prognosis (19.4 months versus 49.3 months).Expression of VEGF-C, VEGFR-1, and VEGFR-3 in peritumoral liver tissue is associated with a unique type of HCC that has a poorer outcome after hepatectomy. 
 Vascular endothelial growth factor receptors 1,3 and caveolin-1 are implicated in colorectal cancer aggressiveness and prognosis--correlations with epidermal growth factor receptor, CD44v6, focal adhesion kinase, and c-Met. Vascular endothelial growth factor receptor-1 (VEGFR-1) and caveolin-1 have been shown to act both as tumor-promoting and tumor-suppressing proteins in various malignancies as well as in colorectal cancer (CRC), while VEGFR-3's lymphagiogenic involvement and connection to tumor parameters has yielded heterogenic results. This study was designed to investigate the expression of these molecules in 183 human CRC tissue specimens and explore their effect in both clinicopathological parameters and disease prognosis. We also utilize our previous results regarding epidermal growth factor receptor (EGFR), c-Met, CD44v6, and focal adhesion kinase, in an attempt to further clarify their distinct role in tumor prognosis and their crosstalk. Caveolin-1 was more freely distributed in the neoplasms of the right colon and restricted towards the left and the rectal cancer samples (p = 0.022); VEGFR-3 was associated with higher nodal   metastasis  ' status (p = 0.001) and staging (p = 0.006), and loss of VEGFR-1 predicted distant   metastasis   (p = 0.026) and advanced stage (p = 0.049). Prompted by previous reports, we performed all analyses also in the patient group of early (I and II) tumor stage where it was evident that VEGFR-1 was more frequently expressed in patients under 60 years old (p = 0.014) and VEGFR-3 was significantly elevated in left colon cancers (p = 0.039) and female patients (p = 0.038). Within the advanced stage (III and IV), the absence of VEGFR-1 exhibited a tendency for higher M status (p = 0.067) and lack of caveolin-1 signified worse AJCC classification (p = 0.053). Additionally, patient survival was influenced from VEGFR-3 (p = 0.019) for the whole sample, whereas subgroup analyses provided a correlation between caveolin-1 expression and improved survival in the early detection group of patients (p = 0.022). Using Cox regression for all available markers, FAK, CD44v6, and Caveolin-1 [corrected] emerged in this study as potential surrogate markers, the latter having positive prognostic significance. We further explored the multiple receptor correlations that were identified. 
 Differential expression of VEGF ligands and receptors in prostate cancer. Prostate cancer disseminates to regional lymph nodes, however the molecular mechanisms responsible for lymph node   metastasis   are poorly understood. The vascular endothelial growth factor (VEGF) ligand and receptor family have been implicated in the growth and spread of prostate cancer via activation of the blood vasculature and lymphatic systems. The purpose of this study was to comprehensively examine the expression pattern of VEGF ligands and receptors in the glandular epithelium, stroma, lymphatic vasculature and blood vessels in prostate cancer.The localization of VEGF-A, VEGF-C, VEGF-D, VEGF receptor (VEGFR)-1, VEGFR-2, and VEGFR-3 was examined in cancerous and adjacent benign prostate tissue from 52 subjects representing various grades of prostate cancer.Except for VEGFR-2, extensive staining was observed for all ligands and receptors in the prostate specimens. In epithelial cells, VEGF-A and VEGFR-1 expression was higher in tumor tissue compared to benign tissue. VEGF-D and VEGFR-3 expression was significantly higher in benign tissue compared to tumor in the stroma and the endothelium of lymphatic and blood vessels. In addition, the frequency of lymphatic vessels, but not blood vessels, was lower in tumor tissue compared with benign tissue.These results suggest that activation of VEGFR-1 by VEGF-A within the carcinoma, and activation of lymphatic endothelial cell VEGFR-3 by VEGF-D within the adjacent benign stroma may be important signaling mechanisms involved in the progression and subsequent metastatic spread of prostate cancer. Thus inhibition of these pathways may contribute to therapeutic strategies for the management of prostate cancer. 
 [Expression of VEGFR-2 and VEGFR-3 in papillary renal cell carcinoma and their relationship with prognosis]. To detect the expression of VEGF receptors in papillary renal cell carcinoma and to explore the correlation between their expression and clinical prognosis.Expression of VEGF receptors and PCNA (proliferating cell nuclear antigen) were evaluated in 82 patients with papillary renal cell carcinoma using tissue microarray and SP immunohistochemical staining.The expression of VEGFR-1 in papillary renal cell carcinoma was 82.93%, VEGFR-2 63.41%, VEGFR-3 34.15% and PCNA 67.07%, respectively. Increased VEGFR-2 expression was significantly correlated with tumor size (P = 0.016), histological grade (P = 0.034) and distant   metastasis   (P = 0.002). VEGFR-3 expression was correlated with histological grade (P = 0.028), lymph node status (P = 0.010) and distant   metastasis   (P = 0.018), but not correlated with gender, age, location, tumor size and TNM staging. VEGFR-1 expression had no correlation with any clinic and pathologic factors. PCNA expression was correlated with histological grade (P = 0.011), but not correlated with other factors. The expression of VEGFR-2 and VEGFR-3 in death cases were higher than that in surviving patients.Both VEGFR-2 and VEGFR-3 can serve as markers for prognosis of papillary renal cell carcinoma. Differently, VEGFR-3 is a predictor of lymph node   metastasis  , increased VEGFR-2 expression could be used to predict a potential blood dissemination. 
 RNAi-mediated silencing of VEGF-C inhibits non-small cell lung cancer progression by simultaneously down-regulating the CXCR4, CCR7, VEGFR-2 and VEGFR-3-dependent axes-induced ERK, p38 and AKT signalling pathways. Vascular endothelial growth factor C (VEGF-C) expression is associated with the malignant tumour phenotype making it an attractive therapeutic target. We investigated the biological roles of VEGF-C in tumour growth, migration, invasion and explored the possibility of VEGF-C as a potential therapeutic target for the treatment of non-small cell lung cancer (NSCLC). A lentivirus-mediated RNA interference (RNAi) technology was used to specifically knockdown the expression of VEGF-C in A549 cells. Quantitative reverse transcriptase-polymerase chain reaction, flow cytometry, Western blot, immunohistochemistry, cellular growth, migration, invasion and ELISA assays were used to characterise VEGF-C expression in vitro. A lung cancer xenograft model in nude mice was established to investigate whether knockdown of VEGF-C reduced tumour growth in vivo. Silencing of VEGF-C suppressed tumour cell growth, migration and invasion in vitro; suppressed tumour growth, angiogenesis and lymphangiogenesis by tail vein injection of lentivirus encoded shRNA against VEGF-C in vivo. More importantly, silencing of VEGF-C also trapped the VEGFR-2, VEGFR-3, CXCR4, CCR7-dependent axes, and down-regulated the AKT, ERK and p38 signalling pathways. These results suggest that VEGF-C has a multifaceted role in NSCLC growth, migration and invasion; that VEGF-C-mediated autocrine loops with their cognate receptors and chemokine receptors are significant factors affecting tumour progression; and that RNAi-mediated silencing of VEGF-C represents a powerful therapeutic approach for controlling NSCLC growth and   metastasis  . 
 Prognostic significance of epidermal growth factor receptor and vascular endothelial growth factor receptor in colorectal adenocarcinoma. The purpose of this study was to evaluate the association between the expression of growth factors and the clinicopathological variables of colorectal adenocarcinoma. Immunohistochemistry and fluorescence in situ hybridization (FISH) were used to evaluate the amplification and expression of epidermal growth factor receptor (EGFR), vascular endothelial growth factor (VEGF), VEGF-D, VEGF receptor (VEGFR)-2, VEGFR-3, transforming growth factor (TGF)-β1, and insulin-like growth factor-1 receptor (IGF-1R) in a tissue microarray of 292 colorectal adenocarcinomas. The expression of EGFR, VEGF, VEGF-D, VEGFR-2 and VEGFR-3 was detected in 5.1%, 10.0%, 6.8%, 5.2%, and 57.2%. EGFR expression was associated with angioinvasion (p < 0.05) and lymph node   metastasi  s (p < 0.005). VEGFR-3 expression was higher in the rectum than in the colon (p < 0.05). VEGF expression correlated with VEGF-D (p < 0.05) and VEGFR-3 (p < 0.005) expression, while VEGF-D expression showed no significant association with VEGFR-2 or VEGFR-3. EGFR amplification was present in 10.6% and was not associated with EGFR protein expression. VEGFR-2 and VEGFR-3 expression levels were related to poor patient survival. Stage, perineural invasion, and lymph node   metastasi  s were independent prognostic factors based on a Cox analysis. VEGFR-2 and VEGFR-3 expression are markers of a poor prognosis in patients with surgically resected colorectal adenocarcinoma, whereas EGFR has a minor influence. 
 MTA1 Is Up-regulated in Colorectal Cancer and Is Inversely Correlated with Lymphatic   Metastasis  .   Metastasis  -associated protein 1 (MTA1) plays an important role in tumourigenesis and progression of certain cancer types. In the current study, we analyzed the relationship between MTA1 expression and disease progression of colorectal cancer (CRC).CRC tissues (n=93) and adjacent normal colorectal tissues (n=70) were analyzed by quantitative real-time polymerase chain reaction. MTA1 knockdown was established in RKO and HT115 cells using MTA1 siRNA.The expression of MTA1 was significantly increased in CRC tissues compared to paired normal colorectal tissues, but decreased expression of MTA1 was correlated with poor prognosis (higher lymph node involvement stage, TNM stage, local invasion and recurrence) that was associated with increased expression of VEGFC and -D and the receptor VEGFR3.MTA1 is up-regulated in CRC. MTA1 expression is inversely associated with lymphatic metastases and the expression of VEGFC, VEGFD and VEGFR3. 
 Expression of GLI1 correlates with expression of lymphangiogenesis proteins, vascular endothelial growth factor C and vascular endothelial growth factor receptor 3, in colorectal cancer. Aberrant activation of the hedgehog (Hh) signaling pathway is associated with tumorigenesis in various tissues. In colorectal cancer (CRC), evidence for Hh activation is inconsistent, and the relationship between the Hh signaling pathway and lymphangiogenesis has not been studied. The aim of this study was to determine the relationship between Hh signaling and lymphangiogenesis and the association of this relationship with lymph node   metastasis   in CRC. We investigated 189 patients who underwent curative surgical resection for CRC between 2002 and 2004 at Korea University Guro Hospital. Paraffin-embedded specimens of colorectal adenocarcinoma and adjacent normal mucosa were evaluated. Immunohistochemical staining for Sonic hedgehog (Shh), Gli1, vascular endothelial growth factor C (VEGFC), and VEGF receptor 3 (VEGFR3) was performed for each specimen. Tumor specimen showed significantly strong staining of Shh, Gli1, VEGFC, and VEGFR3 compared with a normal specimen. Shh expression was not associated with Gli1 expression. Gli1 expression correlated positively with VEGFC and VEGFR3 expression (P < 0.05 in both) but not with lymph node   metastasis  . Activation of the Hh signaling pathway associated with Gli1 promotes expression of lymphangiogenesis proteins, VEGFC and VEGFR3, in CRC. Further studies are necessary to determine the association of this relationship with lymph node   metastasis   in CRC. 
 Anti-VEGF-A therapy reduces lymphatic vessel density and expression of VEGFR-3 in an orthotopic breast tumor model. Because   metastasis   contributes significantly to cancer mortality, understanding its mechanisms is crucial to developing effective therapy.   Metastasis   is facilitated by lymphangiogenesis, the growth of new intratumoral or peritumoral lymphatic vessels from pre-existing vessels. Vascular endothelial growth factor A (VEGF-A) is a well-known angiogenic factor. Increasing evidence implicates VEGF-A in lymphangiogenesis, although the mechanism of its pro-lymphangiogenic effect is poorly understood. We examined the effect of the anti-VEGF-A neutralizing antibody 2C3 on tumor lymphangiogenesis and   metastasis   in an orthotopic breast carcinoma model using MDA-MB-231 cells and its luciferase-tagged derivative, 231-Luc(+) cells. Anti-VEGF-A antibody therapy reduced blood and lymphatic vessel densities by 70% and 80%, respectively, compared with the control antibody. Treatment with 2C3 antibody also decreased incidence of lymphatic and pulmonary metastases by 3.2- and 4.5-fold, respectively. Macrophage infiltration was reduced in 2C3-treated tumors by 32%, but VEGF-C expression was unchanged. In contrast, neoplastic cells and blood vessels in tumors from 2C3-treated mice expressed significantly less angiopoietin-2 (Ang-2) than tumors from control mice. The reduction in Ang-2 was associated with inhibition of VEGFR-3 expression in intratumoral lymphatic endothelial cells. Both VEGF-A and Ang-2 upregulated the expression of VEGFR-3 in cultured lymphatic endothelial cells. VEGF-A induced proliferation of lymphatic endothelial cells was reduced by 50% by soluble Tie-2, suggesting that Ang-2 is an intermediary of the pro-lymphangiogenic VEGF-A effect. These results suggest a novel mechanism by which anti-VEGF-A therapy may suppress tumor lymphangiogenesis and subsequent   metastasis   supporting the use of anti-VEGF-A therapy to control   metastasis   clinically. 
 Expression and significance of VEGF-C and FLT-4 in gastric cancer. To investigate the expression of pathological factors of VEGF-C and its receptor FLT-4 in primary gastric cancer and adjacent normal tissues.The expression of VEGF-C and FLT-4 was studied in 80 primary gastric cancers and adjacent normal tissues from the same patients by semi-quantitative reverse transcriptase-polymerase chain reaction (RT-PCR) and immumohistochemistry.Both primary gastric cancer and adjacent normal tissue could express VEGF-C and FLT-4, and FLT-4 expression was also detected in endothelial cells of stromal blood vessels and lymphatic vessels. There was a significant difference in expression of VEGF-C between primary tumor and adjacent normal tissue samples (P=0.01), and a statistical correlation between VEGF-C and FLT-4 expression in tumors (P=0.00886). With regard to VEGF-C expression, there was a significant difference between moderate-poor differential type and high differential type (P=0.032), and a significant difference between positive and negative lymph node metastases (P=0.024). However, there was no significant difference between positive and negative serosal invasions (P=0.219).VEGF-C and its receptor FLT-4 play a role in the development of gastric cancer, and the tumors with expression of VEGF-C and FLT-4 are more likely to have lymph node   metastasis  . 
 Co-expression of PDGF-B and VEGFR-3 strongly correlates with lymph node   metastasis   and poor survival in non-small-cell lung cancer. Platelet-derived growth factors (PDGFs) and vascular endothelial growth factors and their receptors [platelet-derived growth factor receptors (PDGFRs) and vascular endothelial growth factor receptors (VEGFRs)] are related to both angiogenesis and lymphangiogenesis and are important targets in new cancer treatment strategies. We aimed to study the PDGFs/PDGFRs and correlations with lymph node   metastasis   (LNM) and investigate the prognostic impact of the co-expression of PDGF-B and VEGFR-3 and its correlation with LNM.Tumor tissue samples from 335 resected patients with stage I-IIIA non-small-cell lung cancer (NSCLC) were obtained and tissue microarrays were constructed from duplicate cores of tumor cells and tumor-related stroma from each specimen. Immunohistochemistry was used to evaluate the expression of the molecular markers PDGF-A, PDGF-B, PDGF-C, PDGF-D, PDGFR-alpha, PDGFR-beta, VEGFR-3 and D2-40.There were 232 N0 and 103 N+ patients (76 N1 and 27 N2). In multivariate analyses, high tumor cell PDGF-A expression (P = 0.017) correlated with LNM. Tumor cell co-expression of VEGFR-3 and PDGF-B correlated with nodal   metastasis   and was an independent indicator of poor prognosis (hazard ratio 4.8, confidence interval 95% 2.80-8.31, P < 0.001).Tumor cell PDGF-A expression correlates with LNM, and the co-expression of PDGF-B and VEGFR-3 is strongly associated with poor survival in NSCLC patients. 
 Interleukin-8: A potent promoter of human lymphatic endothelial cell growth in gastric cancer. Lymphatic   metastasis   is a major progression route of gastric cancer. Interleukin-8 (IL-8), as an inflammatory cytokine, is induced by Helicobacter pylori infection and is strongly associated with gastric cancer development and   metastasis  . The blood and lymphatic systems are similar in their function and gene expression profiles. It has been proposed that IL-8 activates angiogenesis. However, the direct role of IL-8 in lymphangiogenesis in gastric cancer remains unclear. We investigated the effect of IL-8 on the growth of human lymphatic endothelial cells (LECs). In addition, protein and mRNA expression of selected lymphangiogenesis markers was assessed in these cells. LECs were co-cultured with gastric cancer SGC7901 cells and exposed to various concentrations of IL-8 (0, 0.2, 0.5, 0.8 and 1.0 ng/ml). The Cell Counting Kit-8 was used to evaluate LEC proliferation (cultured for 1-6 days). Then, protein (immunofluorescence and western blotting) and mRNA [quantitative transcription-polymerase chain reaction (qPCR)] levels were measured in samples obtained from the 24-h cultured cells, for lymphatic vessel endothelial hyaluronic acid receptor-1 (LYVE-1), vascular endothelial growth factor (VEGF)-C, VEGF-D and vascular endothelial growth factor receptor-3 (VEGFR-3). The data presented herein demonstrated that IL-8 promotes the proliferation of LECs and enhances the protein and mRNA expression of LYVE-1. Notably, IL-8 inhibited VEGF-C, VEGF-D and VEGFR-3 protein expression as well as VEGF-D and VEGFR-3 mRNA expression. These findings suggest that IL-8 may be a potent inducer of LECs, although this effect does not appear to involve the VEGF-C/VEGF-D and VEGFR-3 signaling pathway. 
 Expression of angiopoietin-2 and vascular endothelial growth factor receptor-3 correlates with lymphangiogenesis and angiogenesis and affects survival of oral squamous cell carcinoma. Both Ang-2 and VEGFR-3 are major regulators of angiogenesis and lymphangiogenesis, respectively, and thus may affect prognosis of OSCC. We sought to determine the associations between Ang-2 and VEGFR-3 expression and survival of OSCC.Ang-2 and VEGFR-3 expression was determined immunohistochemically in tumor tissues from 112 patients with OSCC; OSCC-adjacent noncancerous oral tissue from 85 OSCC patients; and normal oral mucosa from 37 cancer-free individuals. A log-rank test and Cox proportional hazard models were used to compare survival among different groups with expression of Ang-2 and VEGFR-3.Ang-2 and VEGFR-3 expression was upregulated in OSCC compared to nontumor tissue (all P<0.05). High Ang-2 expression positively correlated with microvessel density (MVD) (P<0.01), and high VEGFR-3 expression positively correlated with lymph node   metastasis   (P<0.01) and lymphatic vessel density (LVD) (P<0.01). The patients with high expression of Ang-2 alone or in combination with VEGFR-3 had a significantly worse survival than in patients with low expression of Ang-2 or any other co-expression status (all P<0.05), respectively. Furthermore, multivariable analysis showed that patients with high expression of Ang-2 alone or in combination with VEGFR-3 had a significantly increased risk of death compared with those with low expression of Ang-2 or any other co-expression status (HR, 2.7, 95% CI, 1.1-6.2 and 5.0, 1.3-15.4, respectively).These results suggest that increased expression in tumors of Ang-2 may individually, or in combination with VEGFR-3, predict poor prognosis of OSCC. 
 Associations of nm23H1, VEGF-C, and VEGF-3 receptor in human prostate cancer. We studied the expression of the non-metastatic clone 23 type 1 (nm23H1) gene, vascular endothelial growth factor (VEGF)-C, and its receptor VEGFR-3 using an in situ hybridization technique and immunohistochemical analyses with prostate cancer tissues and adjacent benign tissues of 52 human archival cases. The association between VEGF-C expression, microlymphatic count (MLC), and staining intensity for nm23H1 and VEGFR-3 was used to evaluate tumor   metastasis   and survival rate. MLC values were significantly higher in tumorous tissue than in non-cancerous tissue. VEGF-C mRNA, VEGFR-3, and nm23H1 were highly expressed in tumorous tissue. VEGFR-3 expression was greater in VEGF-C mRNA-positive tumors than in VEGF-C mRNA-negative tumors. The association of VEGFR-3 expression with VEGF-C mRNA and MLC suggested that the poor prognosis and tumor   metastasis   associated with VEGFR-3 expression may be due, in part, to its role in promoting angiogenesis. VEGF-C expression was significantly associated with tumor lymphangiogenesis, angiogenesis, and immune response as a potent multifunctional stimulating factor in prostate cancer. Expression of nm23H1 was significantly inversely correlated with lymph node   metastasis  . Furthermore, there was a strong negative correlation between the expression of nm23H1, VEGF-C mRNA, and MLC. These findings provide important information for prophylactic, diagnostic, and therapeutic strategies for prostate cancer. 
 Induction of lymphangiogenesis through vascular endothelial growth factor-C/vascular endothelial growth factor receptor 3 axis and its correlation with lymph node   metastasis   in nasopharyngeal carcinoma. The contribution of the lymphatic system to tumor   metastasis   is being increasingly appreciated through studies of human cancers. As the biological behavior of nasopharyngeal carcinoma (NPC) depends on its nodal status, patients with advanced nodal status show a higher tendency toward a poor outcome. Here, we examined the role of lymphangiogenesis on lymphatic spread of NPC. We also evaluated the involvement of vascular endothelial growth factor (VEGF)-C/VEGF receptor 3 (VEGFR3) signaling pathway on lymphangiogenesis in NPC. Furthermore, we tested whether Epstein-Barr virus (EBV)-latent membrane protein (LMP) 1 induces VEGF-C. Forty-one patients with NPC were evaluated for expressions of VEGF-C and its receptor, VEGFR3, and LMP1 proteins and lymphatic vessel counts (LVC) highlighted by anti-podoplanin employing immunohistochemistry. The VEGF-C induction by LMP1 was then tested with Western blotting and enzyme-linked immunosorbent assay in vitro. The LVC and VEGF-C expression were significantly higher in cases with advanced regional lymph node   metastasis   (N2,3) than those with no or limited lymph node involvement (N0,1) (p=0.0380 and p=0.0109, respectively). In VEGF-C/VEGFR3-positive cases, the LVC were significantly increased compared with VEGF-C/VEGFR3-negative cases (p=0.0007). However, LMP1 expression did not show significant associations with LVC and VEGF-C-expression scores (p=0.1210 and p=0.1324, respectively). Induction of VEGF-C protein by LMP1 was not detected in vitro. These results suggest the involvement of the VEGF-C/VEGFR3 axis in the induction of lymphangiogenesis which results in lymphatic spread of NPC. However, EBV-LMP1 was not associated with the mechanism. 
 IGFBP7 functions as a potential lymphangiogenesis inducer in non-small cell lung carcinoma. Lymphangiogenesis is not only involved in the processes of embryonic development, tissue repair and chronic inflammation, but also in tumor lymphatic   metastasis  . Metastatic tumor cells spreading through lymphatic vessels occur in non-small cell lung carcinoma (NSCLC), with regional lymph node   metastasis   often being the most important prognostic factor for carcinoma patients. Recent research has identified a range of lymphangiogenic growth factors that could conceivably play a great role in promoting tumor lymphangiogenesis and lymphatic   metastasis  . The most extensively accepted signaling pathways promoting lymphangiogenesis in tumors include the secreted lymphangiogenic proteins: vascular endothelial growth factor-C (VEGF-C) and VEGF-D, and their cognate receptor on lymphatic endothelium VEGF receptor-3 (VEGFR-3). Targeting VEGF pathway strategy sometimes failed to decrease tumor   metastasis   in vivo experiments and clinical trials. It is unclear whether the tumor cells induced the lymphangiogenesis process, while VEGF pathway could not completely illustrate the mechanism of tumor cell lymphatic   metastasis  . To explore the novel tumor lymphangiogenesis targets, we screened 181 candidate genes between high lymphatic vascular density (LVD) and low LVD in lung adenocarcinomas using Human Genome U133 Plus 2.0 Microarray. Insulin-like growth factor binding protein 7 (IGFBP7) was proven to be associated with metastatic clinicopathological features and high LVD. Furthermore, by assessing the capability of lymphatic endothelial cell forming lymphatic vessel-like structures in vitro, it appears to enhance lymphangiogenesis. 
 Expression of the vascular endothelial growth factor receptor-3 (VEGFR-3) and its ligand VEGF-C in human colorectal adenocarcinoma. Vascular endothelial growth factors (VEGF) are secreted by many tumor types, and are believed to affect tumor growth by promoting angiogenesis through binding to their receptors present on vascular endothelium. Recently, mRNA for VEGF-C the ligand for VEGFR-3, was found to be up-regulated in colorectal adenocarcinoma (CRC). The aim of this work was to determine: 1) the distribution of VEGF-C and VEGFR-3 in CRC, and 2) the biological significance of such expression. Sections of formalin-fixed and paraffin-embedded tissues from 56 CRC were immunohistochemically stained for VEGF-C and VEGFR-3. The type and percent of positive cells was recorded. Survival analysis was performed using the Kaplan-Meier method. All CRC were positive for VEGF-C which was present in the cancer cells themselves, as well as in stromal cells. Normal colon epithelium was usually negative. Only ten (17%) of the 56 CRC completely lacked VEGFR-3 expression. VEGFR-3 immunoreactivity was detected in <25% of the cancer cells in 22 cases and in >25% of the cells in 34 cases. Expression of VEGFR-3 in >25% of the cancer cells was associated with significantly poorer overall survival (p<0.05), but not with lymph node   metastasis   or depth of tumor invasion. Our results suggest that VEGFs promote cancer growth not only by stimulating angiogenesis, but also by acting on receptors present on the cancer cells themselves. 
 [Expression of vascular endothelial growth factor, vascular endothelial growth factor receptor-3 and nm23-H1 in oral squamous cell carcinoma]. To explore the role of vascular endothelial growth factor C (VEGF-C), VEGFR-3 and nm23-H(1) in lymphatic   metastasis   of oral squamous cell carcinoma (OSCC).The expression of VEGF-C, VEGFR-3 and nm23-H(1) were examined by immunohistochemical SP method. The lymphatic vessels density (LVD) in tumors was counted and analyzed with clinicopathologic parameters.VEGF-C, LVD and nm23-H(1) expressions were significantly different between OSCC and benign and precancerous lesions (P < 0.05). VEGF-C was correlated with lymph node   metastasis   and LVD (P < 0.01). nm23-H(1) was related to clinical stage, histological grade and lymphatic   metastasis   (P < 0.05).The high expressions of VEGF-C/VEGFR-3 and inactivation of nm23-H(1) may play an important role in lymphatic   metastasis   in OSCC. 
 Comparison of (18)F-fluorodeoxyglucose PET/CT findings with vascular endothelial growth factors and receptors in colorectal cancer. The purpose of this study was to evaluate the association of (18)F-fluorodeoxyglucose (FDG)-PET/CT findings with the vascular endothelial growth factor (VEGF) family and its receptor (VEGFR) levels in metastatic and nonmetastatic colorectal cancer (CRC). Fluorine-18 FDG-PET/CT scans were performed for initial staging and restaging of patients with CRC. FDG-PET/CT findings of tumor (such as the presence of a primary tumor, the lymphatic or distance metastases, and the maximum standardized uptake value (SUVmax) of the primary tumor), serum VEGF A-C-D-E levels, and serum VEGF receptor 1-2-3 levels were analyzed. A total of 63 patients were included into the study (35 males, mean age 61.3 ± 11.9 years). Patients were divided into two groups, based on positive and negative PET/CT findings. Patients were also categorized according to the presen  ce of meta  stasis. All evaluated parameters were significantly higher in the PET/CT-positive group than the PET/CT-negative group (p < 0.001). All those parameters were also positively correlated with each other. The highest correlation for SUVmax of primary tumor was found with VEGFR-3 (p < 0.001, r = 0.665). Patients with metastases had high levels of VEGF-D, VEGF-A, VEGF-C, VEGF-E, and VEGFR-3 than those without metastases. These parameters had better specificity and sensitivity values than the SUVmax of the primary tumor for detection of metastases. However, VEGF-D was the   best indic  ator of metastasis in all of those parameters (VEGF-D vs SUVmax; sensitivity 100 vs 100 %; specificity 76 vs 76 %; AUC 0.903 vs 0.835; p < 0.001, respectively). Vascular endothelial growth factor family and its receptors were significantly higher in metastatic CRC patients. VEGF-D wa  s the best   indicator of metastasis than all VEGF family, VEGFR-3, and primary tumor SUVmax. VEGF family (A-C-D-E) and VEGFR-3 may help to determine the prognosis and management of CRC. 
 Signaling for lymphangiogenesis via VEGFR-3 is required for the early events of   metastasis  .   Metastasis   to regional lymph nodes is an important and early event in many tumors. Vascular endothelial growth factor-C (VEGF-C), VEGF-D and their receptor VEGFR-3, play a role in tumor spread via the lymphatics, although the timing of their involvement is not understood. In contrast, VEGFR-2, activated by VEGF-A, VEGF-C and VEGF-D, is a mediator of angiogenesis and drives primary tumor growth. We demonstrate the critical role for VEGFR-3, but not VEGFR-2, in the early events of   metastasis  . In a tumor model exhibiting both VEGF-D-dependent angiogenesis and lymphangiogenesis, an antibody to VEGFR-2 (DC101) was capable of inhibiting angiogenesis (79 % reduction in PECAM + blood vessels) and growth (93 % reduction in tumor volume). However, unlike an anti-VEGFR-3 Mab (mF4-31C1), DC101 was not capable of eliminating either tumor lymphangiogenesis or lymphogenous   metastasis   (60 % reduction of lymph node   metastasis   by DC101 vs 95 % by mF4-31C1). Early excision of the primary tumors demonstrated that VEGF-D-mediated tumor spread precedes angiogenesis-induced growth. Small but highly metastatic primary human breast cancers had significantly higher lymphatic vessel density (23.1 vessels/mm(2)) than size-matched (11.7) or larger non-metastatic tumors (12.4) thus supporting the importance of lymphatic vessels, as opposed to angiogenesis-mediated primary tumor growth, for nodal   metastasis  . These results suggest that lymphangiogenesis via VEGF-D is more critical than angiogenesis for nodal   metastasis  . 
 Pathway-related molecules of VEGFC/D-VEGFR3/NRP2 axis in tumor lymphangiogenesis and lymphatic   metastasis  . Precondition for tumor lymphatic   metastasis   is that tumor cells induce formation of original and newborn lymphatic vessels and invade surrounding lymphatic vessels in tumor stroma, while some pathway-related molecules play an important role in mechanisms associated with proliferation and migration of lymphatic endothelial cells (LECs) and tumor cells. In lymphangiogenesis and lymphatic   metastasis  , the pathway-related molecules of VEGFC/D-VEGFR3/NRP2 axis, such as Furin-like enzyme, CNTN1, Prox1, LYVE-1, Podoplanin, SOX18, SDF1 and CXCR4, are direct constitutors as a portion of VEGFC/D-VEGFR3/NRP2 axis, and their biological activities rely on this ligand-receptor system. These axis-related signal molecules could gradually produce waterfall-like cascading effects, mediate differentiation and maturation of LECs, remodel original and neonatal lymphatic vessels, as well as ultimately promote tumor cell chemotaxis, migration, invasion and   metastasis   to lymphoid tracts. This review summarizes the structure and function features of pathway-related molecules of VEGFC/D-VEGFR3/NRP2 axis, the expression changes of these molecules in different anatomic organs or histopathologic types or development stages of various tumors, the characteristics of transduction, implementation, integration of signal networks, the interactive effects on biological behaviors between tumor cells and lymphatic endothelial cells, and their molecular mechanisms and significances in tumor lymphangiogenesis and lymphatic   metastasis  . 
 Expression of vascular endothelial growth factor-C and its receptor in osteosarcomas. Vascular endothelial growth factor-C (VEGF-C) and its receptor, vascular endothelial growth factor receptor-3 (VEGFR-3), have been implicated as important factors in the formation of lymphatic vessels, but its role in osteosarcomas has not yet been fully investigated. This study aims to define the expression of VEGF-C and VEGFR-3 in primary and metastatic osteosarcomas and their relationship to various clinicopathologic parameters. Thirty-three primary osteosarcomas and two pulmonary metastatic samples were immunostained for VEGF-C and VEGFR-3. In addition, VEGF-C and vascular endothelial growth factor-D (VEGF-D) mRNA expression levels in three different human osteosarcoma cell lines and control fibroblasts were evaluated by real-time quantitative polymerase chain reaction (PCR). Both VEGF-C and VEGFR-3 were expressed mainly in the cytoplasm of the tumor cells. Of the 35 patients with osteosarcoma, 16 patients (45.7%) showed strong positive reaction with VEGF-C. Four cases (11.4%) were negative, and 15 cases (42.9%) showed weak immunoreactivity. For VEGFR-3, 12 patients (34.3%) showed strong positive reaction. Fifteen cases (42.9%) were negative, and eight cases (22.8%) showed weak immunoreactivity. A significant, positive correlation (Rs=0.42, p=0.01) was found between the expression of VEGF-C and VEGFR-3 in osteosarcomas. The expression of VEGF-C was significantly associated with the osteoblastic subtype and high histologic grade in osteosarcomas. However, the expression of VEGF-C showed no significant correlation with the presence of   metastasis  . Expression of VEGFR-3 was not related to any clinicopathologic features analyzed. Two of the three osteosarcoma cell lines tested showed amplification of VEGF-C mRNA compared with control cells. No amplification of VEGF-D was noted in these cell lines. Our data suggest that VEGF-C and its receptor are expressed in osteosarcomas. Although the level of VEGF-C was high, it does not seem to have a direct influence on tumor   metastasis   in osteosarcomas. 
 Structural and mechanistic insights into VEGF receptor 3 ligand binding and activation. Vascular endothelial growth factors (VEGFs) and their receptors (VEGFRs) are key drivers of blood and lymph vessel formation in development, but also in several pathological processes. VEGF-C signaling through VEGFR-3 promotes lymphangiogenesis, which is a clinically relevant target for treating lymphatic insufficiency and for blocking tumor angiogenesis and   metastasis  . The extracellular domain of VEGFRs consists of seven Ig homology domains; domains 1-3 (D1-3) are responsible for ligand binding, and the membrane-proximal domains 4-7 (D4-7) are involved in structural rearrangements essential for receptor dimerization and activation. Here we analyzed the crystal structures of VEGF-C in complex with VEGFR-3 domains D1-2 and of the VEGFR-3 D4-5 homodimer. The structures revealed a conserved ligand-binding interface in D2 and a unique mechanism for VEGFR dimerization and activation, with homotypic interactions in D5. Mutation of the conserved residues mediating the D5 interaction (Thr446 and Lys516) and the D7 interaction (Arg737) compromised VEGF-C induced VEGFR-3 activation. A thermodynamic analysis of VEGFR-3 deletion mutants showed that D3, D4-5, and D6-7 all contribute to ligand binding. A structural model of the VEGF-C/VEGFR-3 D1-7 complex derived from small-angle X-ray scattering data is consistent with the homotypic interactions in D5 and D7. Taken together, our data show that ligand-dependent homotypic interactions in D5 and D7 are essential for VEGFR activation, opening promising possibilities for the design of VEGFR-specific drugs. 
 Expression of a flt-4 (VEGFR3) splicing variant in primary human prostate tumors. VEGF D and flt-4t(Delta773-1081) overexpression is diagnostic for sentinel lymph node   metastasis  . Utilizing a cDNA expression library established from human prostate PC-3ML tumor cells, we have cloned a truncated flt-4 gene, termed flt-4t(Delta773-1081). We have then utilized RNase protection and ELISA to measure the relative levels of VEGF B, C, D and flt-1, KDR, flt-4 and flt-4t(Delta773-1081) expression in freshly isolated benign prostatic hyperplasia or BPH tissue (n=21), primary prostate cancers (n=82) and matching sentinel lymph node metastases from stage T2a-T2b/T3 tumors (n=52). Comparisons of the primary tumors with BPH showed that there was a significant upregulation of VEGF-B (P=0.003), VEGF D (P=0.005), flt-1 (P=0.003), KDR (P=0.002), flt-4 (P=0.007), and flt-4t(Delta773-1081) (P=0.001), but not VEGF-C (P=0.543). There was no correlation between VEGF-B and its receptor flt-1 (P=0.545), or VEGF-C and flt-4 (P=0.16) and KDR (P=0.23) receptor expression in tumor specimens. Conversely, there was no significant relationship between VEGF-D and the flt-4t(Delta773-1081) receptor (P=0.516) expression. Statistical analysis further showed that there was no significant correlation between VEGF-B, VEGF-C, VEGF-D, flt-1, KDR, flt-4 and flt-4t(Delta773-1081) with patient age (P>0.10), stage (P>0.10), PSA value (P>0.15) or tumor size (P>0.15). Likewise, there was no significant correlation between VEGF-B, VEGF-C, flt-1, KDR, and flt-4 with Gleason score (P>0.15). In comparison, flt-4t(Delta773-1081) levels clearly increased significantly in Gleason score 7 and Gleason score 8-10 tumors as well as in stage T2a-T2b/T3 tumors. The studies were extended to compare gene expression profiles in T2a-T2b and T3 tumors with (n=26) and without (n=26) matching sentinel lymph node metastases. The data showed that VEGF D and flt-4t(Delta773-1081) expression levels were significantly elevated in primary tumors with sentinel lymph node involvement compared to those lacking lymph node involvement (P>0.0022 and 0.006, respectively). These data suggest that targeting VEGF D and flt-4t(Delta773-1081) receptors may be particularly effective in the prevention of lymph node metastases. 
 High interstitial fluid pressure promotes tumor progression through inducing lymphatic   metastasis  -related protein expressions in oral squamous cell carcinoma. The present study was carried out to analyze the interstitial fluid pressure (IFP) values in patients with oral squamous cell carcinoma and identify the relationship between the IFP and tumor prognosis.We investigated tumor lymphatic   metastasis  -related protein (VEGFC, VEGFD and VEGFR3) expressions change on SCC-4 and SCC-9 cells subjected to increased extracellular pressure in vitro and analyzed the relationship between these proteins and IFP, and prognosis of patients with oral squamous cell carcinoma.The results showed that the elevated extracellular pressure significantly resulted in a dramatic increase of VEGFC, VEGFD and VEGFR3 protein expressions in OSCC. More important, the activation of VEGFC, VEGFD and VEGFR3 proteins through IFP elevation contributed to poor prognosis for patients with OSCC.These results suggest an important potential clinical application of measuring IFP, which can be used as a generic marker of prognosis evaluation and response to therapy. Furthermore, VEGFC, VEGFD and VEGFR3 may be useful targets in developing novel and specific therapeutic tool for OSCC patients with high IFP. 
 Relationship between LYVE-1, VEGFR-3 and CD44 gene expressions and lymphatic   metastasis   in gastric cancer. To investigate the expression levels of lymphatic vessel endothelial hyaluronan receptor-1 (LYVE-1), vascular endothelial growth factor receptor-3 (VEGFR-3) and CD44 genes and the relationship between their levels and clinicopathological parameters in gastric cancer.Tissue samples were obtained from 33 patients (8 females) with gastric cancer. mRNA levels of LYVE-1, VEGFR-3 and CD44 in normal and tumor tissues were quantitatively measured using real time polymerase chain reaction. The results were correlated with lymph node   metastasis  , histological type and differentiation of the tumor, T-stage, and presence of vascular, perineural and lymphatic invasions. The distribution of molecules in the tissue was evaluated using immunohistochemistry.LYVE-1, CD44 and VEGFR-3 gene expression levels were significantly higher in gastric cancer than in normal tissue. While there was no correlation between gene expressions and clinicopathologic features such as histologic type, differentiation and stage, gene expression levels were found to be increased in conjunction with positive lymph node/total lymph node ratio and the presence of perineural invasion. A significant correlation was also found between LYVE-1 and CD44 over-expressions and perineural invasion and lymph node positivity in gastric cancers. When the distribution of LYVE-1 antibody-stained lymphatic vessels in tissue was evaluated, lymphatic vessels were located intra-tumorally in 13% and peri-tumorally in 27% of the patients. Moreover, lymph node metastases were also positive in all patients with LYVE-1-staining.LYVE-1, VEGFR-3 and CD44 all play an important role in lymphangiogenesis, invasion and   metastasis  . LYVE-1 is a perfectly reliable lymphatic vessel marker and useful for immunohistochemistry. 
 Expression of VEGF-C/VEGFR-3 in human laryngeal squamous cell carcinomas and its significance for lymphatic   metastasis  . Expression of vascular endothelial growth factor C (VEGF-C)and vascular endothelial growth factor receptor-3 (VEGFR-3) in laryngeal squamous carcinoma and its relationship to lymph node   metastasis   were investigated.VEGF-C and VEGFR-3 gene expression in 30 cases of normal laryngeal mucosa tissue (NLM), primary laryngeal carcinoma cell carcinomas (PLC) and cervical lymph nodes (CLN) was examined by reverse transcription polymerase chain reaction (RT-PCR). Protein levels of VEGF-C expression were determined by immunohistochemical staining in 60 cases of PLC.Expression of VEGF-C and VEGFR-3 different among NLM, PLC and CLN in the same patient. In PLC, expression was significantly higher in lymph node positive group than in the lymph node negative group and associated with histological grade of differentiation; Expression of VEGF-C and VEGFR-3 was not linked with age, sex, site or T stage.A close correlation was found between VEGF-C/VEGFR-3 expression and lymph node   metastasis   in PLC, suggesting a role in   metastasis   of laryngeal carcinomas. 
 Protein tyrosine phosphatase PTPN14 is a regulator of lymphatic function and choanal development in humans. The lymphatic vasculature is essential for the recirculation of extracellular fluid, fat absorption, and immune function and as a route of tumor   metastasis  . The dissection of molecular mechanisms underlying lymphangiogenesis has been accelerated by the identification of tissue-specific lymphatic endothelial markers and the study of congenital lymphedema syndromes. We report the results of genetic analyses of a kindred inheriting a unique autosomal-recessive lymphedema-choanal atresia syndrome. These studies establish linkage of the trait to chromosome 1q32-q41 and identify a loss-of-function mutation in PTPN14, which encodes a nonreceptor tyrosine phosphatase. The causal role of PTPN14 deficiency was confirmed by the generation of a murine Ptpn14 gene trap model that manifested lymphatic hyperplasia with lymphedema. Biochemical studies revealed a potential interaction between PTPN14 and the vascular endothelial growth factor receptor 3 (VEGFR3), a receptor tyrosine kinase essential for lymphangiogenesis. These results suggest a unique and conserved role for PTPN14 in the regulation of lymphatic development in mammals and a nonconserved role in choanal development in humans. 
 Interaction of tumor cells and lymphatic vessels in cancer progression. Metastatic spread of cancer through the lymphatic system affects hundreds of thousands of patients yearly. Growth of new lymphatic vessels, lymphangiogenesis, is activated in cancer and inflammation, but is largely inactive in normal physiology, and therefore offers therapeutic potential. Key mediators of lymphangiogenesis have been identified in developmental studies. During embryonic development, lymphatic endothelial cells derive from the blood vascular endothelium and differentiate under the guidance of lymphatic-specific regulators, such as the prospero homeobox 1 transcription factor. Vascular endothelial growth factor-C (VEGF-C) and VEGF receptor 3 signaling are essential for the further development of lymphatic vessels and therefore they provide a promising target for inhibition of tumor lymphangiogenesis. Lymphangiogenesis is important for the progression of solid tumors as shown for melanoma and breast cancer. Tumor cells may use chemokine gradients as guidance cues and enter lymphatic vessels through intercellular openings between endothelial cell junctions or, possibly, by inducing larger discontinuities in the endothelial cell layer. Tumor-draining sentinel lymph nodes show enhanced lymphangiogenesis even before cancer   metastasis   and they may function as a permissive 'lymphovascular niche' for the survival of metastatic cells. Although our current knowledge indicates that the development of anti-lymphangiogenic therapies may be beneficial for the treatment of cancer patients, several open questions remain with regard to the frequency, mechanisms and biological importance of lymphatic metastases. 
 Vascular endothelial growth factor receptor-3 directly interacts with phosphatidylinositol 3-kinase to regulate lymphangiogenesis. Dysfunctional lymphatic vessel formation has been implicated in a number of pathological conditions including cancer   metastasis  , lymphedema, and impaired wound healing. The vascular endothelial growth factor (VEGF) family is a major regulator of lymphatic endothelial cell (LEC) function and lymphangiogenesis. Indeed, dissemination of malignant cells into the regional lymph nodes, a common occurrence in many cancers, is stimulated by VEGF family members. This effect is generally considered to be mediated via VEGFR-2 and VEGFR-3. However, the role of specific receptors and their downstream signaling pathways is not well understood.Here we delineate the VEGF-C/VEGF receptor (VEGFR)-3 signaling pathway in LECs and show that VEGF-C induces activation of PI3K/Akt and MEK/Erk. Furthermore, activation of PI3K/Akt by VEGF-C/VEGFR-3 resulted in phosphorylation of P70S6K, eNOS, PLCγ1, and Erk1/2. Importantly, a direct interaction between PI3K and VEGFR-3 in LECs was demonstrated both in vitro and in clinical cancer specimens. This interaction was strongly associated with the presence of lymph node metastases in primary small cell carcinoma of the lung in clinical specimens. Blocking PI3K activity abolished VEGF-C-stimulated LEC tube formation and migration.Our findings demonstrate that specific VEGFR-3 signaling pathways are activated in LECs by VEGF-C. The importance of PI3K in VEGF-C/VEGFR-3-mediated lymphangiogenesis provides a potential therapeutic target for the inhibition of lymphatic   metastasi  s. 
 Expression of vascular endothelial growth factor receptor-3 by lymphatic endothelial cells is associated with lymph node   metastasis   in prostate cancer. The molecular mechanisms underlying lymph node   metastasis   are poorly understood, despite the well-established clinical importance of lymph node status in many human cancers. Recently, vascular endothelial growth factor (VEGF)-C and VEGF-D have been implicated in the regulation of tumor lymphangiogenesis and enhancement of lymphatic invasion via activation of VEGF receptor-3. The purpose of this study was to determine the expression pattern of the VEGF-C/VEGF-D/VEGF receptor-3 axis in prostate cancer and its relationship with lymph node   metastasis  .The expression pattern of VEGF-C, VEGF-D, and VEGF receptor-3 in localized prostate cancer specimens (n = 37) was determined using immunohistochemistry.Widespread, heterogeneous staining for VEGF-C and VEGF-D was observed in all cancer specimens. Intensity of VEGF-C staining was lower in benign prostate epithelium than in adjacent carcinoma, whereas no difference between benign epithelium and carcinoma was observed for VEGF-D staining. VEGF receptor-3 immunostaining was detected in endothelial cells of lymphatic vessels in 18 of 37 tissue samples. The presence of VEGF receptor-3-positive vessels was associated with lymph node   metastasis   (P = 0.0002), Gleason grade (P < 0.0001), extracapsular extension (P = 0.0382), and surgical margin status (P = 0.0069). In addition, VEGF receptor-3 staining highlighted lymphatic invasion by VEGF-C-positive/VEGF-D-positive carcinoma cells.Together, these results suggest that paracrine activation of lymphatic endothelial cell VEGF receptor-3 by VEGF-C and/or VEGF-D may be involved in lymphatic   metastasis  . Thus the VEGF-C/VEGF-D/VEGF receptor-3 signaling pathway may provide a target for antilymphangiogenic therapy in prostate cancer. 
 Lymphangiogenesis and its relationship with lymphatic   metastasis   and prognosis in malignant melanoma. Regional lymph node   metastasis   is one of the important indicators of cutaneous malignant melanoma. Newly formed lymphatic vessels are considered to provide a route whereby tumor cells can migrate to the lymph nodes. Both vascular endothelial growth factors (VEGF) -C and -D have been confirmed to participate in tumor lymphangiogenesis, but the prognostic significance of VEGF-C, VEGF-D, and lymphangiogenesis in cutaneous malignant melanoma remains controversial. To clarify the effects of these factors and to evaluate the relationships between lymphangiogenesis, lymph node   metastasis  , and prognosis in patients with malignant melanoma, the expressions of VEGF-C, VEGF-D, and their receptor (VEGFR) -3 were detected by immunohistochemistry and reverse transcriptase-polymerase chain reaction. The expressions of both VEGF-C and VEGF-D proteins were concomitantly detected in the cytoplasm of the malignant cells. VEGF-C and VEGF-D expressions were associated with VEGFR-3 expression and were significantly correlated with both peritumoral lymphangiogenesis and lymph node   metastasis  . The incidence of peritumoral lymphatic vessels was significantly higher in lymph node metastatic melanomas than that in nonmetastatic melanomas. Univariate and multivariate analyses indicated that VEGF-C and VEGF-D were independent prognostic factors for overall survival and disease-free survival in patients with malignant melanoma. This study suggests that both VEGF-C and VEGF-D are involved in peritumoral lymphangiogenesis and lymphatic   metastasis  . VEGF-C and VEGF-D expression may be clinically useful indicators for prognostic evaluation in patients with cutaneous malignant melanoma. 
 Genistein suppresses FLT4 and inhibits human colorectal cancer   metastasis  . Dietary consumption of genistein, found in soy, has been associated with a potentially protective role in colorectal cancer (CRC) development and progression. Herein we demonstrate that genistein will inhibit human CRC cell invasion and migration, that it does so at non-cytotoxic concentrations and we demonstrate this in multiple human CRC cell lines. After orthotopic implantation of human CRC tumors into mice, oral genistein did not inhibit tumor growth, but did inhibit distant   metastasis   formation, and was non-toxic to mice. Using a qPCR array, we screened for genistein-induced changes in gene expression, followed by Western blot confirmation, demonstrating that genistein downregulated matrix metalloproteinase 2 and Fms-Related Tyrosine Kinase 4 (FLT4; vascular endothelial growth factor receptor 3). After demonstrating that genistein suppressed neo-angiogenesis in mouse tumors, we examined FLT4 expression in primary CRC and adjacent normal colonic tissue from 60 human subjects, demonstrating that increased FLT4 significantly correlates with increased stage and decreased survival. In summary, we demonstrate for the first time that genistein inhibits human CRC   metastasis   at dietary, non-toxic, doses. FLT4 is identified as a marker of metastatic disease, and as a response marker for small molecule therapeutics that inhibit CRC   metastasis  . 
 Suppression of lymph node and lung metastases of endometrial cancer by muscle-mediated expression of soluble vascular endothelial growth factor receptor-3. Lymph node   metastasis   is the most important prognostic factor of endometrial cancer. However, effective therapy has not been established against lymph node   metastasis  . In this study, we explored the efficacy of gene therapy targeting lymph node   metastasis   of endometrial cancer by suppressing the action of vascular endothelial growth factor (VEGF)-C through soluble VEGF receptor-3 (sVEGFR-3) expression. For this purpose, we first conducted a model experiment by introducing sVEGFR-3 cDNA into an endometrial cancer cell line HEC1A and established HEC1A/sVEGFR-3 cell line with high sVEGFR-3 expression. The conditioned medium of HEC1A/sVEGFR-3 cells inhibited lymphatic endothelial cell growth in vitro, and sVEGFR-3 expression in HEC1A cells suppressed in vivo lymph node and lung metastases without inhibiting the growth of a subcutaneously inoculated tumor. To validate the therapeutic efficacy, adeno-associated virus vectors encoding sVEGFR-3 were injected into the skeletal muscle of mice with lymph node   metastasis  . Lymph node and lung metastases of HEC1A cells were completely suppressed by the muscle-mediated expression of sVEGFR-3 using adeno-associated virus vectors. These results suggest the possibility of gene therapy against lymph node and lung metastases of endometrial cancer by using muscle-mediated expression of sVEGFR-3. 
 Suppression of tumor lymphangiogenesis and lymph node   metastasis   by blocking vascular endothelial growth factor receptor 3 signaling. Vascular endothelial growth factor C (VEGF-C) stimulates tumor lymphangiogenesis (i.e., formation of lymphatic vessels) and   metastasis   to regional lymph nodes by interacting with VEGF receptor 3 (VEGFR-3). We sought to determine whether inhibiting VEGFR-3 signaling, and thus tumor lymphangiogenesis, would inhibit tumor   metastasis  .We used the highly metastatic human lung cancer cell line NCI-H460-LNM35 (LNM35) and its parental line NCI-H460-N15 (N15) with low metastatic capacity. We inserted genes by transfection and established a stable N15 cell line secreting VEGF-C and a LNM35 cell line secreting the soluble fusion protein VEGF receptor 3-immunoglobulin (VEGFR-3-Ig, which binds VEGF-C and inhibits VEGFR-3 signaling). Control lines were transfected with mock vectors. Tumor cells were implanted subcutaneously into severe combined immunodeficient mice (n = 6 in each group), and tumors and metastases were examined 6 weeks later. In another approach, recombinant adenoviruses expressing VEGFR-3-Ig (AdR3-Ig) or beta-galactosidase (AdLacZ) were injected intravenously into LNM35 tumor-bearing mice (n = 14 and 7, respectively).LNM35 cells expressed higher levels of VEGF-C RNA and protein than did N15 cells. Xenograft mock vector-transfected LNM35 tumors showed more intratumoral lymphatic vessels (15.3 vessels per grid; 95% confidence interval [CI] = 13.3 to 17.4) and more metastases in draining lymph nodes (12 of 12) than VEGFR-3-Ig-transfected LNM35 tumors (4.1 vessels per grid; 95% CI = 3.4 to 4.7; P<.001, two-sided t test; and four lymph nodes with metastases of 12 lymph nodes examined). Lymph node   metastasis   was also inhibited in AdR3-Ig-treated mice (AdR3-Ig = 0 of 28 lymph nodes; AdLacZ = 11 of 14 lymph nodes). However,   metastasis   to the lungs occurred in all mice, suggesting that LNM35 cells can also spread via other mechanisms. N15 tumors overexpressing VEGF-C contained more lymphatic vessels than vector-transfected tumors but did not have increased metastatic ability.Lymph node   metastasis   appears to be regulated by additional factors besides VEGF-C. Inhibition of VEGFR-3 signaling can suppress tumor lymphangiogenesis and   metastasis   to regional lymph nodes but not to lungs. 
 Endothelial VEGFR-3 expression in colorectal carcinomas is associated with hematogenous   metastasis  . Vascular endothelial growth factor receptor 3 (VEGFR-3) is a major inducer of lymphangiogenic signalling and seems to be involved also in angiogenesis. Since both processes are closely linked with tumor   metastasis   this study investigated the expression of VEGFR-3 in tumor-associated vessels in colorectal carcinomas and evaluated its relevance for lymphogenous and hematogenous   metastasis  . In a comparative study between microvascular endothelial cells isolated from the tumor (HCTEC) and the corresponding non-neoplastic tissue (HCMEC) from five patients with colorectal cancer VEGFR-3 expression was measured using a specific ELISA. The expression pattern was individually different, with cases showing reduced, elevated and unchanged protein levels. Under hypoxic culture conditions (3% O2 for 24 h), which are more realistic for the tumor situation, the levels remained unchanged. In contrast, hypoxia exposure of macrovascular human umbilical vein endothelial cells (HUVEC) led to a consistent downregulation of VEGFR-3 protein. These data indicate a 'hypoxia-resistant' behaviour of VEGFR-3 in colonic microvasculature. Using immunohistochemistry the endothelial expression pattern of VEGFR-3 in 74 non-metastatic, lymphogenously-metastatic and hematogenously-metastatic colorectal carcinoma specimens was assessed. Positive VEGFR-3 expression was highly significantly associated with those cases showing distant   metastasis   (p=0.0003). In contrast, significant differences in the expression of VEGFR-3 between non-metastatic tumors and carcinomas with lymph node   metastasis   were not found. The majority of the detectable intratumoral VEGFR-3-positive vessels were of blood vascular origin (CD31 positive, D2-40 negative). Whereas intratumoral lymphatic vessels were collapsed, VEGFR-3 positive peritumoral lymphatic vessels had mostly open lumina. These morphological observations provide evidence for a predominant significance of VEGFR-3-positive, possibly angiogenesis-mediated, tumor-associated blood vessels in hematogenous   metastasis   of colorectal cancer. In addition, due to their patency VEGFR-3-positive peritumoral, but not intratumoral lymphatics could be the vascular substrate functionally mediating lymphogenous   metastasis  . 
 Vascular endothelial growth factor c promotes ovarian carcinoma progression through paracrine and autocrine mechanisms. Vascular endothelial growth factor C (VEGFC) has been reported to promote tumor progression in several tumor types, mainly through the stimulation of lymphangiogenesis and lymphatic   metastasis  . However, the expression and biological significance of the VEGFC/VEGF receptor (VEGFR)-3 pathway in ovarian cancer growth and dissemination are unclear, and have been investigated in this study. Soluble VEGFC was detected in the plasma and ascites of patients with ovarian carcinoma, and VEGFR3 expression was found in their tumor tissues. In human ovarian carcinoma xenograft models, high levels of soluble VEGFC in ascites and serum were detected, in association with disease progression, tumor burden, and volume of ascites. Peak VEGFC expression preceded para-aortic lymph node infiltration by HOC8 neoplastic cells. Histological detection of tumor cells in blood and lymphatic vessels indicated both hematogenous and lymphatic dissemination. Overexpression of VEGFC in the VEGFR3-positive and luciferase-expressing IGROV1 cells promoted carcinoma dissemination after orthotopic transplantation in the ovary of immunodeficient mice. In vitro, VEGFC released by the tumor cells stimulated tumor cell migration in an autocrine manner. Cediranib, an inhibitor of VEGFR1-3 and c-kit, inhibited in vivo   metastasis   of VEGFC-overexpressing IGROV1 and in vitro autocrine effects. These findings suggest that the VEGFC/VEGFR3 pathway acts as an enhancer of ovarian cancer progression through autocrine and paracrine mechanisms, hence offering a potential target for therapy. 
 Suppression of vascular endothelial growth factor receptor 3 (VEGFR3) and vascular endothelial growth factor C (VEGFC) inhibits hypoxia-induced lymph node metastases in cervix cancer. We have examined the role of VEGFC/VEGFR3 signaling in lymph node   metastasis   and growth of orthotopic human ME180 and SiHa cervical xenograft models following exposure to hypoxia. Our previous studies showed that growth of these tumors under conditions of cyclic hypoxia increased nodal   metastasis  .Mice bearing orthotopic tumors were subjected to cyclic hypoxia at 7% O(2)/air 10min cycles 4h/day/2weeks. Knockdown of vegfc was carried out by shRNA and inhibition of VEGFR3 was conducted by blocking antibodies for the mouse and human proteins. VEGFR3 protein expression was detected by Western blotting. Immunohistochemical staining was used to assess CA9, CD31, LYVE1 and Ki67 labeling. Gene expression was determined by real time PCR.Knock down of vegfc or inhibition of VEGFR3 with blocking antibody reduced metastases under normoxic and cyclic hypoxia conditions. A reduction in lymphatics and blood vessel formation and a decrease in tumor cell proliferation was observed following vegfc knockdown and VEGFR3 inhibition. VEGFR3 expression was upregulated at the mRNA and protein levels following hypoxia.Collectively, our results indicate that anti-VEGFR3 antibody inhibits vegfc-induced tumor lymphangiogenesis and   metastasis   and that vegfc knockdown in the tumor cells causes a similar inhibitory effect on lymph node   metastasis  . These results suggest that the effects of vegfc/VEGFR3 on the progression of tumor cells to form lymph node metastases occur primarily under an hypoxic tumor microenvironment. 
 Hypoxia preconditioning of mesenchymal stromal cells enhances PC3 cell lymphatic   metastasis   accompanied by VEGFR-3/CCR7 activation. Mesenchymal stromal cells (MSCs) in bone marrow may enhance tumor metastases through the secretion of chemokines. MSCs have been reported to home toward the hypoxic tumor microenvironment in vivo. In this study, we investigated prostate cancer PC3 cell behavior under the influence of hypoxia preconditioned MSCs and explored the related mechanism of prostate cancer lymphatic metastases in mice. Transwell assays revealed that VEGF-C receptor, VEGFR-3, as well as chemokine CCL21 receptor, CC chemokine receptor 7 (CCR7), were responsible for the migration of PC3 cells toward hypoxia preconditioned MSCs. Knock-in Ccr7 in PC3 cells also improved cell migration in vitro. Furthermore, when PC3 cells were labeled using the hrGfp-lentiviral vector, and were combined with hypoxia preconditioned MSCs for xenografting, it resulted in an enhancement of lymph node metastases accompanied by up-regulation of VEGFR-3 and CCR7 in primary tumors. Both PI3K/Akt/IκBα and JAK2/STAT3 signaling pathways were activated in xenografts in the presence of hypoxia-preconditioned MSCs. Unexpectedly, the p-VEGFR-2/VEGFR-2 ratio was attenuated accompanied by decreased JAK1 expression, indicating a switching-off of potential vascular signal within xenografts in the presence of hypoxia-preconditioned MSCs. Unlike results from other studies, VEGF-C maintained a stable expression in both conditions, which indicated that hypoxia preconditioning of MSCs did not influence VEGF-C secretion. Our results provide the new insights into the functional molecular events and signalings influencing prostate tumor metastases, suggesting a hopeful diagnosis and treatment in new approaches. 
 Characterization of the interaction between endostatin short peptide and VEGF receptor 3. Corneal angiogenesis and lymphangiogenesis are induced by vascular endothelial growth factors (VEGFs) signaling through its receptors VEGFR-1, -2, and -3. Endostatin is a peptide antagonist of these receptors that causes inhibition of bFGF-induced corneal angiogenesis and lymphangiogenesis. Here we show that binding of VEGF-C and endostatin to recombinant VEGFR-3 is competitive. Alignments of the primary amino acid sequences of VEGF-C and the C-terminal endostatin peptide (mEP: LEQKAASCHNSYIVLCIENSFMTSFSK) identified two conserved cysteine residues separated by seven amino acids. Peptides of VEGF-C and mEP containing these conserved residues bound to VEGFR-3. However, substitution of alanine for either of the cysteines in the mEP peptide perturbed the secondary structure, and this mutated peptide was unable to bind to VEGFR-3. Analysis by surface plasmon resonance demonstrated that the binding of the mEP peptide for recombinant VEGFR-3 had a Ka of 1.41 x 10⁷ M⁻¹ s⁻¹, Kd of 0.6718 s⁻¹, and a KD of 4.78 x 10⁻⁸ M. Characterization of the mechanism of endostatin binding to VEGFR-3 may lead to the development of novel therapies for lymphangiogenesis-related disorders, such as transplant rejection, lymphede  ma, and ca  ncer metastasis. 
 [Roles of VEGF-C and its receptor Flt-4 in proliferation and   metastasis   of primary breast cancer]. Vascular endothelial growth factor-C(VEGF-C) is a member of VEGF family and the only factor that can combine receptor VEGFR-3 (fms-like tyrosine kinase, Flt-4) located at endothelium of lymphatic vessel and modulates the physiological function of lymphatic vessel. Previous study showed that VEGF-C/Flt-4 system play a modulating role in   metastasis   of many kinds of tumors, but there were few reports about its function for primary breast cancer at home and abroad. The objective of this study was to identify the function of VEGF-C/Flt-4 system in proliferation and   metastasis   of primary breast cancer and its significance.A series of 101 primary breast cancer specimens were detected for the expression of VEGF-C, Flt-4,and PCNA by Immunohistochemical methods.Among 101 cases of breast cancer, the positive rate of VEGF-C was 93.1% (94/101), the positive rate of flt-4 was 86.1% (87/101). With the increase of the expression of VEGF-C, the positive index of flt-4 increased (r=0.816,P< 0.001). The positive rate of PCNA was 88.8% (89/101). With the increase of the expression of VEGF-C, the proliferation activity of PCNA was stronger (r=0.673,P< 0.001). The positive index of VEGF-C in lymph node metastases group (61.89+/-17.79) was significantly higher than that of no lymph node   metastasis   group (44.28+/-17.87)(P< 0.05). With the increase of VEGF-C protein level, the number of flt-4 positive vessels increased and significant differences among these groups were observed (P< 0.001). The number of flt-4 positive vessels in lymph node metastases group (15.55+/-3.63)was significantly higher than that of no lymph node   metastasis   group (10.71+/-2.90 ) (P< 0.05).VEGF-C and Flt-4 are overexpressed in primary breast cancer and related to lymph node   metastasis  . VEGF-C can promote proliferation of breast cancer cell. VEGF-C/flt-4 system can promote vasculogenesis in stroma of breast cancer. The number of Flt-4 positive vessels is closely related to lymph node   metastasis  . 
 Neuropilin-2 mediates lymphangiogenesis of colorectal carcinoma via a VEGFC/VEGFR3 independent signaling. Lymphangiogenesis critically contributes to the lymphatic   metastasis   of colorectal carcinomas (CRCs), but the underlying mechanism of CRC lymphangiogenesis remains largely elusive. We have previously demonstrated that Semaphorin-3F (SEMA3F) is critically involved in CRC   metastasis  , and the receptor of SEMA3F, neuropilin-2 (NRP2), originally described as an axon guiding chemorepulsant implicated in nerve development, has been suggested in promoting lymphangiogenesis via acting as an obligate co-receptor of VEGFR3 cooperatively enhancing the activity of VEGF-C. Our present study revealed that in colorectal carcinomas, NRP2 expression levels of tumor-associated lymphatic endothelial cells (LECs) are significantly correlated with the density of tumor lymphatic vessels. In vitro, activation of NRP2 in LECs substantially facilitates their migration, sprouting, and tubulogenesis capacity via regulating the rearrangement of cytoskeleton polarity. In vivo model further showed that in the xenografts generated from SEMA3F knockdown CRC cells, NRP2 is substantially activated in tumor-associated LECs, resulting in a significantly increased tumor lymphangiogenesis. Further evidence demonstrated that CRC cell induces the activation of NRP2 in LECs to promote tumor lymphangiogenesis via integrinα9β1/FAK/Erk pathway independent VEGF-C/VEGFR3 signaling. Our study for the first time revealed the novel molecular mechanism of NRP2-mediated-lymphangiogenesis in CRCs, suggesting NRP2 as a potential therapeutic target in preventing lymphati  c metastas  is of CRCs. 
 [Effect of arsenic trioxide on vascular endothelial growth factor-C and its receptor (VEGFR-3) in nude mice with gastric cancer]. This study was aimed to investigate the effect of arsenic trioxide (As2O3) on expression of vascular endothelial growth factor-C (VEGF-C) and its receptor VEGFR-3 in gastric cancer in order to clarify the role of As2O3 in lymphangiogenesis and   metastasis   of tumor. The gastric cancer model was established in nude mice by using gastric cancer cell line SGC-7901. As2O3 was injected to the two treatment groups (2.5 mg/kg and 5 mg/kg) and the same volume of saline solution was injected to the control group. Expression of VEGF-C and VEGFR-3 were detected by immunohistochemistry and were analyzed with QWin550cW image Acquiring & Analysis System. The results showed that the expression of VEGF-C and VEGFR-3 in cancer cells significantly reduced in the arsenic -treated groups. The expression of VEGF-C and VEGFR-3 in 5 mg/kg group was significantly less than that in 2.5 mg/kg group. The gray ratio analysis confirmed that there were significant difference between control group and two treated group, as well as between 2.5 mg/kg-treated group and 5 mg/kg-treated group. It is concluded that As2O3 can inhibit expression of VEGF-C and VEGFR-3 of human gastric cancer xenografts in nude mice, which suggests that As2O3 may inhibit the lymphangiogenesis by suppressing the expression of VEGF-C and VEGFR-3. 
 Lymphangiogenic VEGF-C and VEGFR-3 expression in genetically characterised gastrointestinal stromal tumours. This study aimed to assess the distribution of VEGF-C and VEGFR-3 expression in gastrointestinal stromal tumours (GISTs), and to analyse the value of lymphatic vessel density (LVD) in a tumour that is believed to preferentially metastasize through blood vessel conduits. A panel of immunohistochemical antibodies was used to evaluate 51 cases of genetically characterised GISTs: VEGF-C, VEGFR-3, D2-40 (for LVD assessment) and CD31 (for blood vessel density--BDV--assessment). The results were correlated with the clinical-pathological data. The large majority of cases (86.2%; 44/51) showed a mutation of the KIT gene, most of them (72.5%; 37/51) revealing mutations in exon 11. VEGFR-3 was predominantly expressed in KIT mutated GISTs (p=0.019). High LVD was correlated with the absence of   metastasis   (p=0.010) and high BVD showed a positive correlation with the occurrence of   metastasis   (p=0.049). The strong expression of VEGF-C and VEGFR-3 in GIST's cells was not correlated with the clinical parameters of aggressiveness, nor with high LVD. 
 [Correlation between the expression of VEGF-C mRNA, VEGFR-3, CD31 and tumor metastases in Chinese with prostate cancer]. The expressions of VEGF-C mRNA, VEGFR-3 and CD31 were studied in order to investigate the correlation between them and neoangiogenesis, hyperplasia of micro-lymphatics and tumor metastases. 34 cases of prostate cancer tissue and 12 cases of adjacent nontumorous tissue specimens were tested. They were marked by VEGFR-3 and CD31 with immunohistochemistic staining and analyzed with image, the micro-lymphatics count (MLC) and microvessel density (MVD) were counted using Weidner's highest vessel density count method; the expression of VEGF-C mRNA was inspected in situ hybridization. The expression of VEGF-C mRNA was 44.12% positive in 34 cases of prostate cancer, the MLC (8.26 +/- 2.73)mm2 and MVD (74.82 +/- 11.76)mm2 in prostate cancer were significantly higher than those in adjacent nontumorous tissue (MLC, 4.82 +/- 3.48/mm2; MVD, 32.86 +/- 5.41/mm2). In addition, there was a correlation between the expression of VEGF-C mRNA and micro-lymphatics metastases and there was a positive correlation between the expression of VEGFR-3 and CD31. The expressions of VEGF-C mRNA , MLC, MVD in stage III and IV and those who have lymph   metastasis   were higher than those in stage I and II and those who have no lymph   metastasis  ; the expressions of VEGFR-3 and CD31 in VEGF-C mRNA positive groups were significantly higher than those in negative groups. The difference of histopathologic grading in prostate cancer had no statistical significance. VEGF-C could accelerate the hyperplasia of micro-lymphatics and neoangiogenesis induced by tumor and play an important role in tumor lymph metastases. There was a close correlation between the expressions of VEGFR-3, CD31 and tumor metastases. The increase of MLC and MVD on prostate cancer indicated the hyperplasia of new micro-lymphatics and neoangiogenesis in the tumor tissue, which could also be a signal to determine the tumor metastases in clinic. 
 Use of NRP1, a novel biomarker, along with VEGF-C, VEGFR-3, CCR7 and SEMA3E, to predict lymph node   metastasis   in squamous cell carcinoma of the tongue. Lymph node (LN)   metastasi  s has been suggested as a major prognostic factor for oral cancer. Knockdown of the growth factors and receptors involved in these metastatic mechanisms could significantly reduce LN   metastasi  s and improve the survival of oral cancer patients after treatment. The present study, therefore, aimed to evaluate the expression levels of the following growth factors and receptors in squamous cell carcinoma (SCC) of the tongue: the vascular endothelial growth factor (VEGF)‑C and VEGF‑D, which bind to the cell surface tyrosine kinase receptor VEGF receptor‑3 (VEGFR‑3); C‑C motif chemokine receptor 7 (CCR7); neuropilin (NRP)1 and NRP2; and semaphorin 3E (SEMA3E). Furthermore, we assessed microvessel density (MVD) and lymphatic vessel density (LVD) to demonstrate the correlation between these fact  ors and re  gional LN metastasis, with respect to the clinicopathological features. Finally, we analyzed the correlation between these proteins and overall or disease‑free survival, in order to demonstrate their prognostic value. Univariate analysis revealed a significant a  ssociation   between LN metastasis and the expression levels of VEGF‑C, VEGFR‑3, CCR7, NRP1, and SEMA3E, as well as LVD, in SCC cells. In contrast, multivariate analysis identif  ied associ  ations between LN metastasis and NRP1 expres  sion, as   well as between LN metastasis and LVD; however, no cor  relation w  as found between LN metastasis and the expression levels of the other proteins. The expression levels of VEGF‑C, VEGFR‑3, NRP1, and SEMA3E, as well as LVD, were correlated with disease‑free survival   time. The  se results indicate that LN metastasis is associated with poor survival in SCC. This study suggests that NRP1 expression and LVD are independent factors that   are likel  y to predict the risk of LN metastasis in SCC of the tongue, whereas the expression of VEGF‑C, VEGFR‑3, CCR7, and SEMA3E are non‑independent predictive factors. 
 Prox 1, VEGF-C and VEGFR3 expression during cervical neoplasia progression as evidence of an early lymphangiogenic switch. Prox1 is a key regulator of lymphatic endothelial cell commitment during embryonic development. No correlations between Prox1 and VEGF-C/VEGFR3 expression in cervical cancer has been done until now. The aim of the present study was to evaluate the peculiarities of Prox1, VEGF-C and VEGFR3 expression during uterine cervix neoplasia progression. Material and methods. One hundred and four specimens taken from women with macroscopically detectable lesions were classified by histopathology and analyzed by immunohistochemistry for Prox1, VEGFR3 and VEGF-C expression. Results. The presence of Prox1 nuclear expression was detected starting from CIN2 and CIN3 lesions to microinvasive carcinoma, in the nuclei of lymphatic and venous endothelial cells and scattered stromal cells. All Prox1 positive lymphatic vessels were positive for VEGFR3. A significant correlation was found between expression of VEGF-C in tumor cells and nuclear density of Prox1 positive lymphatic cells (p=0.044). Conclusion. The commitment of Prox1 positive cells through a lymphatic lineage is an early event for cervical neoplastic progression, being present starting with intraepithelial cervical lesions, and is strongly associated with VEGFR3 and VEGF-C expression. These findings suggest an early active lymphangiogenesis during cervical neoplasia progression and explain, in part, the early presence of lymph node   metastasis   in cervical cancer. By the detection of Prox1 expression in lymphatic and venous endothelial cells, and also in stromal cells, it has been suggested that there are at least three different mechanism of lymph vessel development during cervical neoplasia progression. 
 [The expression and role of VEGF-C and its receptor FLT-4 in laryngeal and hypopharyngeal squamous cell carcinoma   metastasis  ]. To investigate the expression of VEGF-C and its receptor FLT-4 in laryngeal and hypopharyngeal squamous cell carcinoma, and to analyse its role in lymphatic   metastasis   and micro  metastasis  .Forty nine cases postoperative specimens verified by histopathology were studied. The expression of VEGF-C, FLT-4 were detected with immunohistochemical SP method, image analysis method. The clinicopathological parameters of tumor were compared with the immunohistochemical results of VEGF-C and FLT-4, followed by the further analysis on the relationship between VEGF-C/FLT-4 expression and lymph nodal   metastasis   or micro  metastasis  .There was a statistical correlation between the expression of VEGF-C and grades of pathological differentiation in laryngeal and hypopharyngeal squamous cell carcinoma. Moreover there was also a statistical correlation between the expression of VEGF-C and lymph node metastases. But no correlation was observed between the expression of VEGF-C and clinical T grades. The statistical correlation also existed between VEGF-C and FLT-4 expression in laryngeal or hypopharyngeal tumors. There was a significant difference of expression of FLT-4 in laryngeal and hypopharyngeal tumours with positive and negative lymph node metastases. No correlation was observed between FLT-4 and grades of pathological differentiation or clinical T grades. There was no statistical correlation between the expression of VEGF-C/FLT-4 and nodal micro  metastasis   (P > 0.05).VEGF-C and its receptor FLT-4 play an important role in the lymphatic   metastasis   of laryngeal and hypopharyngeal squamous cell carcinoma. VEGF-C/FLT-4 may be a useful biological marker to foresee the cervical lymph node   metastasis   and prognosis. 
 High VEGFR-3-positive circulating lymphatic/vascular endothelial progenitor cell level is associated with poor prognosis in human small cell lung cancer. The newly identified bone marrow-derived cell population, called lymphatic/vascular endothelial progenitor cells (LVEPC), has been shown to contribute to lymph capillary growth in experimental tumor systems. The clinical significance of these cells has not yet been investigated in a human malignancy. Our aim was to study whether peripheral blood circulating LVEPCs participate in the progression of human small cell lung cancer (SCLC).A total of 88 patients with limited-stage SCLC and 32 tumor-free control subjects were included. Peripheral blood circulating LVEPC labeled with CD34 and vascular endothelial growth factor receptor-3 (VEGFR3) antibodies and the serum levels of the key lymphangiogenic molecule VEGF-C were measured by flow cytometry and ELISA, respectively.CD34-positive/VEGFR3-positive LVEPC levels were significantly increased in patients (versus controls; P<0.01), and there was also a significant relationship between LVEPC counts and lymph node   metastasis   (P<0.01). High pretreatment circulating LVEPC numbers correlated with poor overall survival (P<0.01). Although we observed significantly elevated VEGF-C concentrations in patients (versus controls; P<0.01), there was no significant correlation between VEGF-C and LVEPC levels. Moreover, no significant differences in peripheral blood VEGF-C levels were seen between patients subgrouped by clinicopathologic variables including tumor and lymph node stages and survival.Peripheral blood levels of bone marrow-derived LVEPCs are significantly increased in patients with SCLC and correlate with lymphatic involvement and prognosis. This is the first study that shows evidence of increased numbers of circulating LVEPC in patients with a malignant tumor. 
 The balance of VEGF-C and VEGFR-3 mRNA is a predictor of lymph node   metastasis   in non-small cell lung cancer. A positive association between vascular endothelial growth factor-C (VEGF-C) expression and lymph node   metastasis   has been reported in several cancers. However, the relationship of VEGF-C and lymph node   metastasis   in some cancers, including non-small cell lung cancer (NSCLC), is controversial. We evaluated the VEGF-C and vascular endothelial growth factor receptor-3 (VEGFR-3) expression in NSCLC samples from patients who had undergone surgery between 1998 and 2002 using real-time quantitative RT-PCR and immunohistochemical staining. We failed to find a positive association between VEGF-C and VEGFR-3 mRNA expression and lymph node   metastasis   in NSCLC. An immunohistological study demonstrated that VEGF-C was expressed not only in cancer cells, but also in macrophages in NSCLC, and that VEGFR-3 was expressed in cancer cells, macrophages, type II pneumocytes and lymph vessels. The VEGF-C/VEGFR-3 ratio of the node-positive group was significantly higher than that of the node-negative group. Immunohistochemical staining showed that VEGFR-3 was mainly expressed in cancer cells. The immunoreactivity of VEGF-C and VEGFR-3 was roughly correlated to the mRNA levels of VEGF-C and VEGFR-3 in real-time PCR. VEGF-C mRNA alone has no positive association with lymph node   metastasis   in NSCLC. The VEGF-C/VEGFR-3 ratio was positively associated with lymph node   metastasis   in NSCLC. This suggests that VEGF-C promotes lymph node   metastasis   while being influenced by the strength of the VEGF-C autocrine loop, and the VEGF-C/VEGFR-3 ratio can be a useful predictor of lymph node   metastasis   in NSCLC. 
 High levels of circulating CD34+/VEGFR3+ lymphatic/vascular endothelial progenitor cells is correlated with lymph node   metastasis   in patients with epithelial ovarian cancer. Lymph node   metastasis   is one of the predictive factors associated with poor prognosis of epithelial ovarian cancer. To clarify the role of CD34 and vascular endothelial growth factor receptor-3-positive (CD34+/VEGFR3+) lymphatic/vascular endothelial progenitor cells (LVEPC) in patients with lymph node   metastasis   and epithelial ovarian cancer progression, the levels of circulating CD34+/VEGFR3+ LVEPC in epithelial ovarian cancer patients were detected. We also tested the plasma protein levels of VEGF and stromal cell-derived factor to find out their possible relationships with lymph node   metastasis   in our epithelial ovarian cancer cohort.Peripheral blood samples were collected from 54 patients diagnosed as epithelial ovarian cancer, and 31 normal samples as control. The circulating levels of LVEPC were carried out by flow cytometry, and blood protein levels of biomarkers were measured by enzyme-linked immunosorbent assay (ELISA).The level of circulating LVEPC was significantly higher in patients with ovarian cancer compared with that of healthy controls. There was also a statistically significant correlation between LVEPC levels and surgical staging of epithelial ovarian cancer (P < 0.01).The circulating levels of bone marrow-derived LVEPC are significantly increased in epithelial ovarian cancer patients and these levels correlate with lymph node   metastasis   too. 
 TNFR1 mediates TNF-α-induced tumour lymphangiogenesis and   metastasi  s by modulating VEGF-C-VEGFR3 signalling. Inflammation and lymphangiogenesis are two cohesively coupled processes that promote tumour growth and invasion. Here we report that TNF-α markedly promotes tumour lymphangiogenesis and lymphati  c metastas  is. The TNF-α-TNFR1 signalling pathway directly stimulates lymphatic endothelial cell activity through a VEGFR3-independent mechanism. However, VEGFR3-induced lymphatic endothelial cell tips are a prerequisite for lymphatic vessel growth in vivo, and a VEGFR3 blockade completely ablates TNF-α-induced lymphangiogenesis. Moreover, TNF-α-TNFR1-activated inflammatory macrophages produce high levels of VEGF-C to coordinately activate VEGFR3. Genetic deletion of TNFR1 (Tnfr1(-/-)) in mice or depletion of tumour-associated macrophages (TAMs) virtually eliminates TNF-α-induced lymphangiogenesis and lymp  hatic meta  stasis. Gain-of-function experiments show that reconstitution of Tnfr1(+/+) macrophages in Tnfr1(-/-) mice largely restores tumour lymphangiogenesis and lymp  hatic meta  stasis. These findings shed mechanistic light on the intimate interplay between inflammation and lymphangiogenesis in c  ancer meta  stasis, and propose therapeutic intervention of lymp  hatic meta  stasis by targeting the TNF-α-TNFR1 pathway. 
 Vascular endothelial growth factor-C and its receptor VEGFR-3 in non-small-cell lung cancer: concurrent expression in cancer cells from primary tumour and metastatic lymph node. Investigation of the role of vascular endothelial growth factor-C (VEGF-C) and VEGF receptor-3 (VEGFR-3) in non-small-cell lung cancer (NSCLC) has mainly focused on lymph node (LN)   metastasis   related to lymphangiogenesis. However, the coexpression of VEGF-C/VEGFR-3 by tumour cells can independently play an important role. The present study was therefore designed to evaluate VEGF-C/VEGFR-3 coexpression in tumour cells from the primary tumour and corresponding LN metastases.VEGF-C and VEGFR-3 expression in cancer cells were evaluated by immunohistochemistry in 92 NSCLC samples and 45 metastatic LNs. Ki67 expression and mitotic index (MI) in tumours and clinicopathological data were analysed concurrently.VEGFR-3 and VEGF-C expression were observed in 42% and 74% of tumours, respectively. Concurrent expression of VEGF-C and VEGFR-3, observed in 39% of tumours, was significantly associated with a higher proliferation rate and a higher incidence of LN metastases. VEGF-C expression in tumour cells was observed in 100% of metastatic LN and VEGF-C/VEGFR-3 coexpression was observed in 71% of metastatic LN. Finally, concurrent expression of VEGF-C/VEGFR-3 in the primary tumour was associated with poor disease-free survival on univariate analysis.In NSCLC cancer cells, VEGF-C/VEGFR-3 coexpression suggests an autocrine/paracrine loop responsible for a high proliferation rate in tumour cells. As VEGF-C/VEGFR-3 coexpression is very frequent in metastatic LN tumour cells, it can be hypothesised that this coexpression participates in the growth of LN   metastasis  . 
 Expression of vascular endothelial growth factor C (VEGF-C) and VEGF receptor-3 in human prostate cancer is associated with regional lymph node   metastasis  . Vascular endothelial growth factor C (VEGF-C) and its receptor, VEGFR-3, have been implicated as important factors in the formation of lymphatic vessels and recent evidence suggests that tumor lymphangiogenesis promotes lymphatic   metastasis  .The expression of VEGF-C and VEGFR-3 was examined in 22 human prostate cancer specimens with immunohistochemistry. A semi-quantitative scoring system was used for evaluation of staining.Expression of VEGF-C was stronger in prostate cancer areas in comparison to adjacent benign glands. In addition, patients with lymph node metastases had a significantly higher expression of VEGF-C than patients without lymph node metastases. Interestingly, VEGFR-3 was expressed in malignant prostate epithelial cells and its expression was significantly higher in the lymph node positive group compared to the lymph node negative group.The results of the present study indicate that increased expression of VEGF-C and VEGFR-3 play a role in prostate cancer progression and in   metastasis   to regional lymph nodes. 
  GeneRIFs  
 This study suggests that NRP1 expression and LVD are independent factors that are likely to predict the risk of LN   metastasis   in squamous cell carcinoma (SCC)of the tongue, whereas the expression of VEGFC, VEGFR3, CCR7, and SEMA3E are nonindependent predictive factors 
 Data indicate that vascular endothelial growth factor D (VEGF-D) was the best indicator of   metastasis   and vascular endothelial growth factors and receptor-3 (VEGFR-3) may help to determine the prognosis and management of colorectal cancer (CRC). 
 The summarizes the structure and function features of pathway-related molecules of VEGFC/D-VEGFR3/NRP2 axis, stages of various tumors and their molecular mechanisms and significances in tuthe expression changes of these molecules in different anatomic organs or histopathologic types or development lymphatic   metastasis  . 
 High expression levels of VEGFR1 and VEGFR3 were associated with a higher rate of lung   metastasis  . 
 Genistein suppresses FLT4 and inhibits human colorectal cancer   metastasis  . 
 TNFR1 has a role in mediating TNF-alpha-induced tumour lymphangiogenesis and   metastasis   by modulating VEGF-C-VEGFR3 signalling 
 Expression of VEGFR-3 was highly correlated with tumor   metastasis   in prostate cancer patients. 
 unlike an anti-VEGFR-3 Mab (mF4-31C1), DC101 was not capable of eliminating either tumor lymphangiogenesis or lymphogenous   metastasis   (60 % reduction of lymph node   metastasis   by DC101 vs 95 % by mF4-31C1). 
 Data suggest that circulating VEGFR3/CD34 are biomarkers for epithelial ovarian cancer (EOC); circulating bone marrow-derived lymphatic/vascular endothelial progenitor cells are significantly increased in EOC and correlate with lymph node   metastasis  . 
 Shh, Gli1, VEGFC, and VEGFR3 expression was higher in colorectal adenocarcinoma than in normal mucosa. Gli1 expression correlated with VEGFC and VEGFR3 expression but not lymphatic   metastasis  . 
 High VEGFR-3 gene expression is associated with lymphatic   metastasis   in laryngeal squamous cell carcinomas. 
 LYVE-1, VEGFR-3 and CD44 all play an important role in lymphangiogenesis, invasion and   metastasis   
 VEGFR-3 expression was correlated with histological grade, lymph node status and distant   metastasis  , but not correlated with gender, age, location, tumor size and TNM staging of papillary renal cell carcinoma. 
 VEGF-C and its receptor FLT-4 play an important role in the lymphatic   metastasis   of laryngeal and hypopharyngeal squamous cell carcinoma. 
 The high expressions of VEGF-C/VEGFR-3 and inactivation of nm23-H(1) may play an important role in lymphatic   metastasis   in oral squamous cell carcinoma. 
 VEGF-C/flt-4 system can promote vasculogenesis in stroma of breast cancer. The number of Flt-4 positive vessels is closely related to lymph node   metastasis  . 
 High endothelial VEGFR-3 expression in colorectal carcinomas is associated with hematogenous   metastasis  . 
 VEGF-C and VEGF-D expressions were associated with VEGFR-3 expression and were significantly correlated with both peritumoral lymphangiogenesis and lymph node   metastasis  . 
 Suppression of tumor lymphangiogenesis and lymph node   metastasis   by blocking vascular endothelial growth factor receptor 3 signaling. 
 vascular endothelial growth factor receptor-3 may have a role in lymph node   metastasis   in prostate cancer 
 increased expression of VEGF-C and VEGFR-3 play a role in prostate cancer progression and in   metastasis   to regional lymph nodes 
 VEGF-C and its receptor FLT-4 play a role in the development of gastric cancer, and the tumors with expression of VEGF-C and FLT-4 are more likely to have lymph node   metastasis  . 
 The VEGF-3/VEGFR-c ratio was positively associated with lymph node   metastasis   in non-small cell lung cancer . 
  [Cc]ell [Ii]nvasion  PubMed  
 Vascular endothelial growth factor C enhances cervical cancer cell invasiveness via upregulation of galectin-3 protein. Vascular endothelial growth factor C (VEGF-C) promotes cervical cancer metastasis, while the detailed mechanism remains obscure. Recent evidence shows that galectin-3 (Gal-3), a glycan binding protein, interacts with the VEGF receptors and reinforces their signal transduction. In this study, we investigated the role of Gal-3 in VEGF-C-induced cervical cancer   cell invasion  . On cervical carcinoma cell line SiHa cells, silencing of Gal-3 expression with specific siRNA largely impaired VEGF-C-enhanced   cell invasion  . Treatment with VEGF-C for 12-48 h enhanced Gal-3 protein expression, which was inhibited by the addition of NF-κB inhibitor pyrrolidine dithiocarbamate (PDTC). Moreover, the silencing of NF-κB subunit p65 expression with specific siRNA attenuated VEGF-C-enhanced Gal-3 expression, suggesting that NF-κB is the key intermediate. Under VEGF-C stimulation, an enhanced interaction between VEGF receptor-3 (VEGF-R3) and Gal-3 was found, which may possibly lead to VEGF-R3 activation since exogenous Gal-3 induced VEGF-R3 phosphorylation in a dose- and time-dependent manner. In conclusion, our findings implied that VEGF-C enhanced cervical cancer invasiveness via upregulation of Gal-3 protein through NF-κB pathway, which may shed light on potential therapeutic strategies for cervical cancer therapy. 
 Effect of lentivirus-mediated shRNA targeting VEGFR-3 on proliferation, apoptosis and invasion of gastric cancer cells. It has been reported that vascular endothelial growth factor receptor 3 (VEGFR-3) is highly expressed in most tumor tissues, including gastric cancer. However, the effects of VEGFR-3 knockdown on the proliferation, apoptosis and invasion of gastric cancer cells and downstream signaling molecules have not yet been well established. In the present study, four short hairpin RNA (shRNA) sequences targeting the VEGFR-3 gene (NM_002020) were designed and cloned into a lentiviral vector, pRNAT-U6.2/Lenti, to construct four recombinant lentiviral vectors. The vectors with the two highest interfering efficiencies were selected to be co-transfected with packaging vectors in HEK293T cells to assemble lentivirus particles. Results from Western blot analysis showed that the VEGFR-3 shRNA-4 lentivirus-infected group (sh#4) had the highest efficiency of gene silencing in the MKN45 cell line compared with the parental and control group. The sh#4 group significantly slowed cell proliferation, decreased the mean percentage of S-phase cells and increased the mean percentage of G1 phase cells, promoted cell apoptosis, and also significantly inhibited   cell invasion   of MKN45 compared with the other two groups. Furthermore, the expression of the anti-apoptotic factor Bcl-2 was significantly decreased in the sh#4 group compared to that of the other two groups. Moreover, results from qRT-PCR revealed that knockdown of VEGFR-3 with the shRNA lentiviral vector resulted in down-regulation of the downstream neural cell adhesion molecule contactin-1 (CNTN-1). In conclusion, the recombinant lentivirus particles were able to remarkably suppress VEGFR-3 expression, regulate the cell cycle, inhibit proliferation and induce apoptosis in the MKN45 cell lines. 
 Vascular endothelial growth factor C enhances cervical cancer cell invasiveness via upregulation of galectin-3 protein. Vascular endothelial growth factor C (VEGF-C) promotes cervical cancer metastasis, while the detailed mechanism remains obscure. Recent evidence shows that galectin-3 (Gal-3), a glycan binding protein, interacts with the VEGF receptors and reinforces their signal transduction. In this study, we investigated the role of Gal-3 in VEGF-C-induced cervical cancer   cell invasion  . On cervical carcinoma cell line SiHa cells, silencing of Gal-3 expression with specific siRNA largely impaired VEGF-C-enhanced   cell invasion  . Treatment with VEGF-C for 12-48 h enhanced Gal-3 protein expression, which was inhibited by the addition of NF-κB inhibitor pyrrolidine dithiocarbamate (PDTC). Moreover, the silencing of NF-κB subunit p65 expression with specific siRNA attenuated VEGF-C-enhanced Gal-3 expression, suggesting that NF-κB is the key intermediate. Under VEGF-C stimulation, an enhanced interaction between VEGF receptor-3 (VEGF-R3) and Gal-3 was found, which may possibly lead to VEGF-R3 activation since exogenous Gal-3 induced VEGF-R3 phosphorylation in a dose- and time-dependent manner. In conclusion, our findings implied that VEGF-C enhanced cervical cancer invasiveness via upregulation of Gal-3 protein through NF-κB pathway, which may shed light on potential therapeutic strategies for cervical cancer therapy. 
 Effect of lentivirus-mediated shRNA targeting VEGFR-3 on proliferation, apoptosis and invasion of gastric cancer cells. It has been reported that vascular endothelial growth factor receptor 3 (VEGFR-3) is highly expressed in most tumor tissues, including gastric cancer. However, the effects of VEGFR-3 knockdown on the proliferation, apoptosis and invasion of gastric cancer cells and downstream signaling molecules have not yet been well established. In the present study, four short hairpin RNA (shRNA) sequences targeting the VEGFR-3 gene (NM_002020) were designed and cloned into a lentiviral vector, pRNAT-U6.2/Lenti, to construct four recombinant lentiviral vectors. The vectors with the two highest interfering efficiencies were selected to be co-transfected with packaging vectors in HEK293T cells to assemble lentivirus particles. Results from Western blot analysis showed that the VEGFR-3 shRNA-4 lentivirus-infected group (sh#4) had the highest efficiency of gene silencing in the MKN45 cell line compared with the parental and control group. The sh#4 group significantly slowed cell proliferation, decreased the mean percentage of S-phase cells and increased the mean percentage of G1 phase cells, promoted cell apoptosis, and also significantly inhibited   cell invasion   of MKN45 compared with the other two groups. Furthermore, the expression of the anti-apoptotic factor Bcl-2 was significantly decreased in the sh#4 group compared to that of the other two groups. Moreover, results from qRT-PCR revealed that knockdown of VEGFR-3 with the shRNA lentiviral vector resulted in down-regulation of the downstream neural cell adhesion molecule contactin-1 (CNTN-1). In conclusion, the recombinant lentivirus particles were able to remarkably suppress VEGFR-3 expression, regulate the cell cycle, inhibit proliferation and induce apoptosis in the MKN45 cell lines. 
 Role of the VEGFR3/VEGFD receptor axis in TGFbeta1 activation of primary prostate cell lines. Reports indicate that vascular endothelial growth factor receptor type 3 (VEGFR3) regulates cellular functions such as invasion, proliferation, and chemo-resistance. However, the exact function of the VEGFR3 signaling axis in prostate epithelial cells is poorly characterized.The goal of this study was to evaluate whether TGFbeta1 in combination with VEGFD can promote pre-malignant invasive activities of intermediate basal cells (IBC-10a) isolated from human prostate cancer (Gleason score 6).hTERT immortalized IBC-10a cells normally grew as confluent "cobblestoned" monolayers, but treatment with TGFbeta1 (10 ng/ml for 2-6 hr) dissociated the cell-cell junctions and induced VEGFR3 translocation to the cell surface. This event was not inhibited by 10 microM cycloheximide or puromycin, indicating transcription and protein synthesis were not required. We further discovered that TGFbeta1 in combination with VEGFD induced a significant increase in the invasive activity of IBC-10a cells (>26% and 53% after 24 and 48 hr, respectively) in modified Boyden Chamber assays. TGFbetaRII receptor antibodies specifically blocked TGFbeta1 induction of VEGFR3 translocation to the cell surface and blocked VEGFD-induced invasion. Zymograms revealed that TGFbeta1 (and not VEGFR3) stimulated the secretion of MMP-2 and MMP-9, presumably to promote   cell invasion  . The   cell invasion   assays confirmed that antibodies specific for TGFbetaII receptor, MMP-2 and MMP-9 and VEGFR3, independently blocked TGFbeta1-induced invasion.For the first time, we have demonstrated the mechanism by which TGFbeta1 stimulates VEGFD/VEGFR3 receptor axis activation leading to increased cell migration and invasion by primary intermediate basal cell cultures. 
 Genistein suppresses FLT4 and inhibits human colorectal cancer metastasis. Dietary consumption of genistein, found in soy, has been associated with a potentially protective role in colorectal cancer (CRC) development and progression. Herein we demonstrate that genistein will inhibit human CRC   cell invasion   and migration, that it does so at non-cytotoxic concentrations and we demonstrate this in multiple human CRC cell lines. After orthotopic implantation of human CRC tumors into mice, oral genistein did not inhibit tumor growth, but did inhibit distant metastasis formation, and was non-toxic to mice. Using a qPCR array, we screened for genistein-induced changes in gene expression, followed by Western blot confirmation, demonstrating that genistein downregulated matrix metalloproteinase 2 and Fms-Related Tyrosine Kinase 4 (FLT4; vascular endothelial growth factor receptor 3). After demonstrating that genistein suppressed neo-angiogenesis in mouse tumors, we examined FLT4 expression in primary CRC and adjacent normal colonic tissue from 60 human subjects, demonstrating that increased FLT4 significantly correlates with increased stage and decreased survival. In summary, we demonstrate for the first time that genistein inhibits human CRC metastasis at dietary, non-toxic, doses. FLT4 is identified as a marker of metastatic disease, and as a response marker for small molecule therapeutics that inhibit CRC metastasis. 
 Vascular endothelial growth factor receptor-3 promotes breast cancer cell proliferation, motility and survival in vitro and tumor formation in vivo. Vascular endothelial growth factor receptor-3 is a receptor tyrosine kinase that is overexpressed in some human carcinomas, but its role in tumorigenesis has not been fully elucidated. We examined VEGFR-3 expression in normal, nonneoplastic and early stage malignant breast tissues and have shown that VEGFR-3 upregulation in breast cancer preceded tumor   cell invasion  , suggesting that VEGFR-3 may function as a survival signal. We characterized the biological effects of VEGFR-3 over-expression in human breast cancer cells based on two approaches: gain of function by overexpressing VEGFR-3 in MCF-7 breast cancer cells and loss of function by RNAi-mediated silencing of VEGFR-3 in MCF-7-VEGFR-3 and BT474 cells. VEGFR-3 overexpression increased cellular proliferation by 40% when MCF7-VEGFR-3 cells were compared to parental MCF7 cells, and proliferation was reduced by more than 40% when endogenous VEGFR-3 was downregulated in BT474 cells. VEGFR-3 overexpression promoted a three-fold increase in motility and invasion and both motility and invasion were inhibited by downregulation of VEGFR-3. Furthermore, VEGFR-3 overexpression promoted cellular survival under stress conditions induced by staurosporine treatment and led to anchorage-independent growth. VEGFR-3 overexpression dramatically increased tumor formation in both hormone-dependent and independent xenograft models. With estrogen stimulation, MCF7-VEGFR-3 xenografts were ten times larger than control xenografts. Finally, downregulation of VEGFR-3 expression in both xenograft model cell lines led to a significant reduction of tumor growth. For the first time, we have demonstrated that VEGFR-3 overexpression promotes breast cancer cell proliferation, motility, survival, anchorage-independent growth and tumorogenicity in the absence of ligand expression. 
   
  
 FGFR2 
 
  NCBI Gene  
  GeneRIF  
  Pubmed  
 
 
  Search Term  Rank  Fields  
  [Mm]igration  6  PubMed(25); GeneRIFs(1)  
  ECM  7  PubMed(2)  
  [Ee]xtracellular [Mm]atrix  8  PubMed(7); Component(1)  
  [Ii]nvasive  10  PubMed(39); GeneRIFs(4)  
  [Mm]etastasis  12  PubMed(25); GeneRIFs(4)  
  [Cc]ell [Ii]nvasion  13  GeneRIFs(1)  
 
 
 hide/show details for FGFR2 
  [Mm]igration  PubMed  
 Integrin activation is required for VEGF and FGF receptor protein presence on human microvascular endothelial cells. Endothelial cell proliferation and   migration   is initiated by growth factors including FGF and VEGF that bind to specific transmembrane receptor tyrosine kinases. Mechanisms that regulate in vivo expression of fibroblast growth factor receptors (FGFR) and vascular endothelial growth factor receptors (VEGFR) are not well understood. Since it is well known that different matrices influence the proliferation and   migration   of endothelial cells in culture, we hypothesized that changes in the extracellular matrix environment can regulate growth factor receptors on endothelial cells. We cultured human microvascular endothelial cells on different matrices (vitronectin, laminin, fibronectin, fibrin, and collagen IV) and examined for the presence of growth factor receptors (FGFR-1, FGFR-2, VEGFR-1, and VEGFR-2). We show that vitronectin increased the presence of all four growth factor receptors and most notably, VEGFR-1. In contrast, fibrin decreased all four receptors, especially FGFR-1 and FGFR-2. Inhibiting phosphotyrosine signaling abolished immunostaining for all four receptors, regardless of the matrix, but was not dependent on activating the Fyn-Shc pathway. Cells plated on vitronectin in the presence of blocking antibodies to integrins alphavbeta3 and alphavbeta5 similarly decreased presence of these growth factor receptors. Our data suggests a possible mechanism of how matrix-integrin interactions regulate endothelial cell responsiveness to growth factors and anchorage-dependent cell growth. 
 Targeting FGFR Pathway in Human Hepatocellular Carcinoma: Expressing pFGFR and pMET for Antitumor Activity. The MET receptor tyrosine kinase, the receptor for hepatocyte growth factor (HGF), has been implicated in cancer growth, invasion,   migration  , angiogenesis, and metastasis in a broad variety of human cancers, including human hepatocellular carcinoma (HCC). Recently, MET was suggested to be a potential target for the personalized treatment of HCC with an active HGF-MET signaling pathway. However, the mechanisms of resistance to MET inhibitors need to be elucidated to provide effective treatment. Here, we show that HCC cells exhibit different sensitivities to the MET inhibitor PHA665752, depending on the phosphorylation status of FGFR. Treatment of cells expressing both phospho-FGFR and phospho-MET with the inhibitor PHA665752 did not cause growth inhibition and cell death, whereas treatment with AZD4547, a pan-FGFR inhibitor, resulted in decreased colony formation and cleavage of caspase-3. Moreover, silencing of endogenous FGFR1 and FGFR2 by RNAi of HCC cells expressing phospho-FGFR, phospho-FGFR2, and phospho-MET overcame the resistance to PHA665752 treatment. Treatment of primary cancer cells from patients with HCC expressing both phospho-FGFR and phospho-MET with PHA665752 did not induce cell death, whereas AZD4547 treatment induced cell death through the cleavage of caspase-3. In addition, treatment of cells resistant to PHA665752 with AZD4547 abrogated the activation of downstream effectors of cell growth, proliferation, and survival. On the basis of these results, we conclude that the FGFR pathway is critical for HCC survival, and that targeting this pathway with AZD4547 may be beneficial for the treatment of patients with HCC-expressing phospho-FGFR and phospho-MET. 
 Indirect recruitment of the signalling adaptor Shc to the fibroblast growth factor receptor 2 (FGFR2). The adaptor protein Shc (Src homology and collagen-containing protein) plays an important role in the activation of signalling pathways downstream of RTKs (receptor tyrosine kinases) regulating diverse cellular functions, such as differentiation, adhesion,   migration   and mitogenesis. Despite being phosphorylated downstream of members of the FGFR (fibroblast growth factor receptor) family, a direct interaction of Shc with this receptor family has not been described to date. Various studies have suggested potential binding sites for the Shc PTB domain (phosphotyrosine-binding domain) and/or the SH2 (Src homology 2) domain on FGFR1, but no interaction of full-length Shc with these sites has been reported in vivo. In the present study, we investigated the importance of the SH2 domain and the PTB domain in recruitment of Shc to FGFR2(IIIc) to characterize the interaction of these two proteins. Confocal microscopy revealed extensive co-localization of Shc with FGFR2. The PTB domain was identified as the critical component of Shc which mediates membrane localization. Results from FLIM (fluorescence lifetime imaging microscopy) revealed that the interaction between Shc and FGFR2 is indirect, suggesting that the adaptor protein forms part of a signalling complex containing the receptor. We identified the non-RTK Src as a protein which potentially mediates the formation of such a ternary complex. Although an interaction between Src and Shc has been described previously, in the present study we implicate the Shc SH2 domain as a novel mediator of this association. The recruitment of Shc to FGFR2 via an indirect mechanism provides new insight into the regulation of protein assembly and activation of various signalling pathways downstream of this RTK. 
 Targeting FGFR Pathway in Human Hepatocellular Carcinoma: Expressing pFGFR and pMET for Antitumor Activity. The MET receptor tyrosine kinase, the receptor for hepatocyte growth factor (HGF), has been implicated in cancer growth, invasion,   migration  , angiogenesis, and metastasis in a broad variety of human cancers, including human hepatocellular carcinoma (HCC). Recently, MET was suggested to be a potential target for the personalized treatment of HCC with an active HGF-MET signaling pathway. However, the mechanisms of resistance to MET inhibitors need to be elucidated to provide effective treatment. Here, we show that HCC cells exhibit different sensitivities to the MET inhibitor PHA665752, depending on the phosphorylation status of FGFR. Treatment of cells expressing both phospho-FGFR and phospho-MET with the inhibitor PHA665752 did not cause growth inhibition and cell death, whereas treatment with AZD4547, a pan-FGFR inhibitor, resulted in decreased colony formation and cleavage of caspase-3. Moreover, silencing of endogenous FGFR1 and FGFR2 by RNAi of HCC cells expressing phospho-FGFR, phospho-FGFR2, and phospho-MET overcame the resistance to PHA665752 treatment. Treatment of primary cancer cells from patients with HCC expressing both phospho-FGFR and phospho-MET with PHA665752 did not induce cell death, whereas AZD4547 treatment induced cell death through the cleavage of caspase-3. In addition, treatment of cells resistant to PHA665752 with AZD4547 abrogated the activation of downstream effectors of cell growth, proliferation, and survival. On the basis of these results, we conclude that the FGFR pathway is critical for HCC survival, and that targeting this pathway with AZD4547 may be beneficial for the treatment of patients with HCC-expressing phospho-FGFR and phospho-MET. 
 Indirect recruitment of the signalling adaptor Shc to the fibroblast growth factor receptor 2 (FGFR2). The adaptor protein Shc (Src homology and collagen-containing protein) plays an important role in the activation of signalling pathways downstream of RTKs (receptor tyrosine kinases) regulating diverse cellular functions, such as differentiation, adhesion,   migration   and mitogenesis. Despite being phosphorylated downstream of members of the FGFR (fibroblast growth factor receptor) family, a direct interaction of Shc with this receptor family has not been described to date. Various studies have suggested potential binding sites for the Shc PTB domain (phosphotyrosine-binding domain) and/or the SH2 (Src homology 2) domain on FGFR1, but no interaction of full-length Shc with these sites has been reported in vivo. In the present study, we investigated the importance of the SH2 domain and the PTB domain in recruitment of Shc to FGFR2(IIIc) to characterize the interaction of these two proteins. Confocal microscopy revealed extensive co-localization of Shc with FGFR2. The PTB domain was identified as the critical component of Shc which mediates membrane localization. Results from FLIM (fluorescence lifetime imaging microscopy) revealed that the interaction between Shc and FGFR2 is indirect, suggesting that the adaptor protein forms part of a signalling complex containing the receptor. We identified the non-RTK Src as a protein which potentially mediates the formation of such a ternary complex. Although an interaction between Src and Shc has been described previously, in the present study we implicate the Shc SH2 domain as a novel mediator of this association. The recruitment of Shc to FGFR2 via an indirect mechanism provides new insight into the regulation of protein assembly and activation of various signalling pathways downstream of this RTK. 
 Integrin activation is required for VEGF and FGF receptor protein presence on human microvascular endothelial cells. Endothelial cell proliferation and   migration   is initiated by growth factors including FGF and VEGF that bind to specific transmembrane receptor tyrosine kinases. Mechanisms that regulate in vivo expression of fibroblast growth factor receptors (FGFR) and vascular endothelial growth factor receptors (VEGFR) are not well understood. Since it is well known that different matrices influence the proliferation and   migration   of endothelial cells in culture, we hypothesized that changes in the extracellular matrix environment can regulate growth factor receptors on endothelial cells. We cultured human microvascular endothelial cells on different matrices (vitronectin, laminin, fibronectin, fibrin, and collagen IV) and examined for the presence of growth factor receptors (FGFR-1, FGFR-2, VEGFR-1, and VEGFR-2). We show that vitronectin increased the presence of all four growth factor receptors and most notably, VEGFR-1. In contrast, fibrin decreased all four receptors, especially FGFR-1 and FGFR-2. Inhibiting phosphotyrosine signaling abolished immunostaining for all four receptors, regardless of the matrix, but was not dependent on activating the Fyn-Shc pathway. Cells plated on vitronectin in the presence of blocking antibodies to integrins alphavbeta3 and alphavbeta5 similarly decreased presence of these growth factor receptors. Our data suggests a possible mechanism of how matrix-integrin interactions regulate endothelial cell responsiveness to growth factors and anchorage-dependent cell growth. 
 Epithelial splicing regulatory protein 1 is a favorable prognostic factor in pancreatic cancer that attenuates pancreatic metastases. Epithelial splicing regulatory protein 1 (ESRP1) binds the FGFR-2 auxiliary cis-element ISE/ISS-3, located in the intron between exon IIIb and IIIc, and primarily promotes FGFR-2 IIIb expression. Here we assessed the role of ESRP1 in pancreatic ductal adenocarcinoma (PDAC). Immunohistochemical analysis was performed using anti-ESRP1, FGFR-2 IIIb and FGFR-2 IIIc antibodies in 123 PDAC cases. ESRP1 expression vector and small interference RNA (siRNA) targeting ESRP1 were transfected into human PDAC cells, and cell growth,   migration   and invasion were analyzed. In vivo heterotopic and orthotopic implantations using ESRP1 overexpression clones were performed and effects on pancreatic tumor volumes and hepatic and pulmonary metastases determined. ESRP1 immunoreactivity was strong in the nuclei of cancer cells in well-to-moderately differentiated PDACs but weak in poorly differentiated cancers. Well-to-moderately differentiated cancers also exhibited high FGFR-2 IIIb and low FGFR-2 IIIc expression, whereas this ratio was reversed in the poorly differentiated cancers. Increased ESRP1 expression was associated with longer survival in comparison with low ESRP1 expression, and PANC-1 cells engineered to express ESRP1 exhibited increased FGFR-2 IIIb expression and decreased   migration   and invasion in vitro, whereas ESRP1 siRNA-transfected KLM-1 cells exhibited increased FGFR-2 IIIc expression and increased cell growth,   migration   and invasion. In vivo, ESRP1-overexpressing clones formed significantly fewer liver metastases as compared with control clones. ESRP1 regulates the expression pattern of FGFR-2 isoforms, attenuates cell growth,   migration  , invasion and metastasis, and is a favorable prognostic factor in PDAC. Therefore, devising mechanisms to upregulate ESRP1 may exert a beneficial therapeutic effect in PDAC. 
 Inhibition of fibroblast growth factor receptor 2 attenuates proliferation and invasion of pancreatic cancer. The alternative splicing of the extracellular domain of fibroblast growth factor receptor (FGFR)-2 generates the IIIb and IIIc isoforms. Expression of FGFR-2 IIIb correlates with vascular endothelial growth factor-A (VEGF-A) expression and venous invasion of pancreatic ductal adenocarcinoma (PDAC). By contrast, FGFR-2 IIIc expression correlates with faster development of liver metastasis after surgery, and increased proliferation rates and invasion of the cancer. In this study, we analyzed the expression and roles of total FGFR-2 (both isoforms) to determine the effectiveness of FGFR-2-targeting therapy for PDAC. Immunohistochemically, FGFR-2 was highly expressed in 25/48 (52.1%) PDAC cases, and correlated with advanced stage cancer. In FISH analysis, FGFR2 was amplified in 3/7 PDAC cell lines. We stably transfected an FGFR-2 shRNA targeting the IIIb and IIIc isoforms into FGFR2-amplified PDAC cells. The proliferation rates,   migration  , and invasion of FGFR-2-shRNA-transfected cells were lower than those of control cells in vitro. In response to FGF-2, FGFR-2-shRNA-transfected cells showed decreased phosphorylation of ERK compared with control cells. The FGFR-2-shRNA-transfected cells also expressed lower levels of vascular endothelial growth factor-A than control cells, and formed smaller s.c. tumors in nude mice. These findings suggest that FGFR-2 is a therapeutic target for inhibition in PDAC. 
 Interactions between FGFR2 and RSK2-implications for breast cancer prognosis. We have previously demonstrated that fibroblast growth factor receptor 2 (FGFR2) activates ribosomal s6 kinase 2 (RSK2) in mammary epithelial cells and that this pathway promotes in vitro cell growth and   migration  . Potential clinical significance of FGFR2 and RSK2 association has never been investigated. Herein, we have undertaken an evaluation of a possible relationship between FGFR2/RSK2 interdependence and disease outcome in breast cancer (BCa) patients. The clinical analysis was complemented by an in vitro investigation of an involvement of RSK2 in the regulation of FGFR2 function. Primary tumour samples from 152 stage I-III BCa patients were examined for FGFR2 and RSK2 gene and protein expression. FGFR2 showed a positive correlation with RSK2 at both protein (p = 0.003) and messenger RNA (mRNA) (p = 0.001) levels. Lack of both FGFR2 and activated RSK (RSK-P) significantly correlated with better disease-free survival (DFS) (p = 0.01). Patients with tumours displaying immunoreactivity for either or both FGFR2 and RSK-P had 4.89-fold higher risk of recurrence when compared to the FGFR2/RSK-P-negative subgroup. FGFR2-RSK2 interactions were verified by co-immunoprecipitation and internalization assays in HB2 mammary epithelial cell line (characterized by high endogenous FGFR2 and RSK2 expression). In vitro analyses revealed that FGFR2 and RSK2 formed an indirect complex and that activated RSK exerted a significant impact on fibroblast growth factor 2 (FGF2)-triggered internalization of FGFR2. Our results suggest that the FGFR2-RSK2 signalling pathway is involved in pathophysiology of BCa and evaluation of FGFR2/RSK-P expression may be useful in disease prognostication. 
 [Expression and action of keratinocyte growth factor, keratinocyte growth factor receptor on CaSki cell]. To investigate the expressions of keratinocyte growth factor (KGF), keratinocyte growth factor receptor (KGFR) in CaSki cell and action of keratinocyte growth factor on proliferation,   migration   of the CaSki cell.ELISA and Western blot methods were used to determine the protein expressions of the KGF and KGFR in CaSki cell respectively: 3H-Thymidine incorporation method was used to determine the effect of recombinant human KGF and anti-KGF on CaSki cell proliferation; Millicell-PCF was used to determine the effect of recombinant human KGF, anti-KGF on CaSki cell   migration  .Both KGF and KGFR were expressing in the CaSki cell; Recombinant human KGF resulted in a increase in the proliferation and   migration   of CaSki cells; The proliferation and   migration   of CaSki cell became weak due to autocrine KGF neutralized by KGF antibodies (P < 0.05).Current results demonstrate that KGF and KGFR express in CaSki cell; Both autocrine and recombinant human KGF have the effect on CaSki cell proliferation and   migration  . 
 Fibroblast growth factor receptor 2 IIIc as a therapeutic target for colorectal cancer cells. A high percentage of colorectal carcinomas overexpress a lot of growth factors and their receptors, including fibroblast growth factor (FGF) and FGF receptor (FGFR). We previously reported that FGFR2 overexpression was associated with distant metastasis and that FGFR2 inhibition suppressed cell growth,   migration  , and invasion. The FGFR2 splicing isoform FGFR2IIIb is associated with well-differentiated histologic type, tumor angiogenesis, and adhesion to extracellular matrices. Another isoform, FGFR2IIIc, correlates with the aggressiveness of various types of cancer. In the present study, we examined the expression and roles of FGFR2IIIc in colorectal carcinoma to determine the effectiveness of FGFR2IIIc-targeting therapy. In normal colorectal tissues, FGFR2IIIc expression was weakly detected in superficial colorectal epithelial cells and was not detected in proliferative zone cells. FGFR2IIIc-positive cells were detected by immunohistochemistry in the following lesions, listed in the order of increasing percentage: hyperplastic polyps < low-grade adenomas < high-grade adenomas < carcinomas. FGFR2IIIc immunoreactivity was expressed in 27% of colorectal carcinoma cases, and this expression correlated with distant metastasis and poor prognosis. FGFR2IIIc-transfected colorectal carcinoma cells showed increased cell growth, soft agar colony formation,   migration  , and invasion, as well as decreased adhesion to extracellular matrices. Furthermore, FGFR2IIIc-transfected colorectal carcinoma cells formed larger tumors in subcutaneous tissues and the cecum of nude mice. Fully human anti-FGFR2IIIc monoclonal antibody inhibited the growth and   migration   of colorectal carcinoma cells through alterations in cell   migration  , cell death, and development-related genes. In conclusion, FGFR2IIIc plays an important role in colorectal carcinogenesis and tumor progression. Monoclonal antibody against FGFR2IIIc has promising potential in colorectal carcinoma therapy. 
 Formononetin, a novel FGFR2 inhibitor, potently inhibits angiogenesis and tumor growth in preclinical models. Most anti-angiogenic therapies currently being evaluated in clinical trials target vascular endothelial growth factor (VEGF) pathway, however, the tumor vasculature can acquire resistance to VEGF-targeted therapy by shifting to other angiogenesis mechanisms. Therefore, other potential therapeutic agents that block non-VEGF angiogenic pathways need to be evaluated. Here we identified formononetin as a novel agent with potential anti-angiogenic and anti-cancer activities. Formononetin demonstrated inhibition of endothelial cell proliferation,   migration  , and tube formation in response to basic fibroblast growth factor 2 (FGF2). In ex vivo and in vivo angiogenesis assays, formononetin suppressed FGF2-induced microvessel sprouting of rat aortic rings and angiogenesis. To understand the underlying molecular basis, we examined the effects of formononetin on different molecular components in treated endothelial cell, and found that formononetin suppressed FGF2-triggered activation of FGFR2 and protein kinase B (Akt) signaling. Moreover, formononetin directly inhibited proliferation and blocked the oncogenic signaling pathways in breast cancer cell. In vivo, using xenograft models of breast cancer, formononetin showed growth-inhibitory activity associated with inhibition of tumor angiogenesis. Moreover, formononetin enhanced the effect of VEGFR2 inhibitor sunitinib on tumor growth inhibition. Taken together, our results indicate that formononetin targets the FGFR2-mediated Akt signaling pathway, leading to the suppression of tumor growth and angiogenesis. 
 [Expression and significance of FGFR2IIIc in chemoresistant bladder cancer cells]. To investigate the role of fibroblast growth factor receptor-2 (FGFR2) splice variant FGFR2IIIc in the regulation of mesenchymal-epithelial transition (MET) in doxorubicin-resistant bladder cancer cells.A doxorubicin-resistant human bladder cell line (253J/DOX) was generated from the bladder cancer cell line 253J by being continuously exposed to gradually increasing doses of doxorubicin. Chemosensitivity to doxorubicin was determined by MTT assay. The expressions of P-glycoprotein and FGFR2IIIc were evaluated by Western blotting and real-time RT-PCR, respectively. Changes in E-cadherin and vimentin were detected by Western blot analysis.   Migration   ability of 253J and 253J/DOX cells was analyzed by in vitro wound healing assay.The resistant cells, 253J/DOX, were more resistant to doxorubicin than the parent cells. Western blotting and RT-PCR analysis indicated the higher levels of P-glycoprotein and FGFR2IIIc in 253J/DOX cells (P<0.05). Additionally, compared with the 253J cells, 253J/DOX cells presented the upregulation of E-cadherin, the downregulation of vimentin and the inhibition of   migration   ability.FGFR2IIIc-induced MET in chemoresistant bladder cancer cells may play an important role in the formation of metastatic lesions. 
 C1GALT1 overexpression promotes the invasive behavior of colon cancer cells through modifying O-glycosylation of FGFR2. Core 1 β1,3-galactosyltransferase (C1GALT1) transfers galactose (Gal) to N-acetylgalactosamine (GalNAc) to form Galβ1,3GalNAc (T antigen). Aberrant O-glycans, such as T antigen, are commonly found in colorectal cancer. However, the role of C1GALT1 in colorectal cancer remains unclear. Here we showed that C1GALT1 was frequently overexpressed in colorectal tumors and is associated with poor survival. C1GALT1 overexpression promoted cell survival  , migrati  on, invasion, and sphere formation as well as tumor growth and metastasis of colon cancer cells. Conversely, knockdown of C1GALT1 with small interference (si) RNA was sufficient to suppress these malignant phenotypes in vitro and in vivo. Moreover, we are the first to show that fibroblast growth factor receptor (FGFR) 2 carried O-glycans in colon cancer cells. Mechanistic investigations showed that C1GALT1 modified the O-glycans on FGFR2 and enhanced bFGF-triggered activation of FGFR2 as well as increased bFGF-mediated malignant phenotypes. In addition, BGJ398, a selective inhibitor of FGFR, blocked the effects of C1GALT1. These findings suggest that C1GALT1 overexpression modifies O-glycans on FGFR2 and enhances its phosphorylation to promote the invasive behavior and cancer stem-like property in colon cancer cells, indicating a critical role of O-glycosylation in the pathogenesis of colorectal cancer. 
 Predicting the impact of deleterious mutations in the protein kinase domain of FGFR2 in the context of function, structure, and pathogenesis--a bioinformatics approach. Fibroblast growth factor receptor 2 (FGFR2) controls a wide range of biological functions by regulating the cellular proliferation, survival,   migration   and differentiation. A growing body of preclinical data demonstrated that deregulation of the FGFR signalling through genetic modification was observed in various types of cancers. However, the extent to which genetic modifications interfere with gene regulation and their involvement in cancer susceptibility remains largely unknown. In this work, we performed in silico profiling of harmful non-synonymous single nucleotide polymorphisms (SNPs) in the protein kinase domain of FGFR2. Tolerance index, position-specific independent count score, change in free energy score (ΔΔG), Eris and FoldX indicated that seven mutations were found to be deleterious and may alter the protein function and structure. Furthermore, based on physico-chemical properties, two mutations K659N and R747H were found to be most deleterious in protein kinase domain and taken for further structural analysis. Docking study showed a complete loss of binding affinity followed by interference in hydrogen bonding and surrounding residues due to K659N and R747H mutations. In order to elucidate the mechanism behind the impact of mutation that can generate a ripple effect throughout the protein structure and ultimately affect the function, in-depth molecular dynamics simulation and principal component analysis were performed. The obtained results indicate that K659N and R747H mutations have a distinct effect on the dynamic behaviour of FGFR2 protein. Our strategy may be helpful for understanding SNP effects on proteins with function and their role in human genetic diseases and for the development of novel pharmacological strategies. 
 Antitumor effects and molecular mechanisms of ponatinib on endometrial cancer cells harboring activating FGFR2 mutations. Aberrant mutational activation of FGFR2 is associated with endometrial cancers (ECs). AP24534 (ponatinib) currently undergoing clinical trials has been known to be an orally available multi-targeted tyrosine kinase inhibitor. Our biochemical kinase assay showed that AP24534 is potent against wild-type FGFR1-4 and 5 mutant FGFRs (V561M-FGFR1, N549H-FGFR2, K650E-FGFR3, G697C-FGFR3, N535K-FGFR4) and possesses the strongest kinase-inhibitory activity on N549H-FGFR2 (IC50 of 0.5 nM) among all FGFRs tested. We therefore investigated the effects of AP24534 on endometrial cancer cells harboring activating FGFR2 mutations and explored the underlying molecular mechanisms. AP24534 significantly inhibited the proliferation of endometrial cancer cells bearing activating FGFR2 mutations (N549K, K310R/N549K, S252W) and mainly induced G1/S cell cycle arrest leading to apoptosis. AP24534 also diminished the kinase activity of immunoprecipitated FGFR2 derived from MFE-296 and MFE-280 cells and reduced the phosphorylation of FGFR2 and FRS2 on MFE-296 and AN3CA cells. AP24534 caused substantial reductions in ERK phosphorylation, PLCγ signaling and STAT5 signal transduction on ECs bearing FGFR2 activating mutations. Akt signaling pathway was also deactivated by AP24534. AP24534 causes the chemotherapeutic effect through mainly the blockade of ERK, PLCγ and STAT5 signal transduction on ECs. Moreover, AP24534 inhibite  d migrati  on and invasion of endometrial cancer cells with FGFR2 mutations. In addition, AP24534 significantly blocked anchorage-independent growth of endometrial cancer cells. We, for the first time, report the molecular mechanisms by which AP24534 exerts antitumor effects on ECs with FGFR2 activating mutations, which would provide mechanistic insight into ongoing clinical investigations of AP24534 for ECs. 
 FGFR2 is overexpressed in myxoid liposarcoma and inhibition of FGFR signaling impairs tumor growth in vitro. Myxoid liposarcomas account for more than one third of liposarcomas and about 10% of all adult soft tissue sarcomas. The tumors are characterized by specific chromosomal translocations leading to the chimeric oncogenes FUS-DDIT3 or EWS1R-DDIT3. The encoded fusion proteins act as aberrant transcription factors. Therefore, we implemented comparative expression analyses using whole-genome microarrays in tumor and fat tissue samples. We aimed at identifying differentially expressed genes which may serve as diagnostic or prognostic biomarkers or as therapeutic targets. Microarray analyses revealed overexpression of FGFR2 and other members of the FGF/FGFR family. Overexpression of FGFR2 was validated by qPCR, immunohistochemistry and western blot analysis in primary tumor samples. Treatment of the myxoid liposarcoma cell lines MLS 402 and MLS 1765 with the FGFR inhibitors PD173074, TKI258 (dovitinib) and BGJ398 as well as specific siRNAs reduced cell proliferation, induced apoptosis and delayed cell   migration  . Combination of FGFR inhibitors with trabectedin further increased the effect. Our study demonstrates overexpression of FGFR2 and a functional role of FGFR signaling in myxoid liposarcoma. As FGFR inhibition showed effects on proliferation and cell   migration   and induced apoptosis in vitro, our data indicate the potential use of FGFR inhibitors as a targeted therapy for these tumors. 
 Overexpressed fibroblast growth factor receptor 2 in the invasive front of colorectal cancer: a potential therapeutic target in colorectal cancer. Fibroblast growth factor receptor 2 (FGFR2) is considered a novel therapeutic target for various cancer. We used a silencing strategy to clarify the effect of reduced FGFR2 expression in human colorectal cancer (CRC) cells. The invasive front of cancer cells exhibited stronger FGFR2 expression than the surface area of the cancers. FGFR2 shRNA-transfected LoVo cells inhibited cell   migration  , invasion and tumor growth in vitro and in vivo. Thus, FGFR2 plays important roles in CRC progression in association with tumor cell   migration  , invasion and growth, and FGFR2 might be a novel therapeutic target for CRC. 
 Enhanced expression of fibroblast growth factor receptor 2 IIIc promotes human pancreatic cancer cell proliferation. In pancreatic ductal adenocarcinoma (PDAC), the fibroblast growth factor receptor 1 (FGFR-1) IIIb isoform correlates with the inhibition of cancer cell proliferation,   migration  , and invasion, whereas FGFR-1 IIIc enhances cancer cell proliferation. The FGFR-2 IIIb isoform is expressed in PDAC, and its expression correlates with increased venous invasion. We examined the role of FGFR-2 IIIc in PDAC. FGFR-2 IIIc was expressed in all six pancreatic cancer cell lines examined and was highest in PANC-1 cells. FGFR-2 IIIc was abundant in the cancer cells from 83 of 117 PDAC cases, which correlated with decreased duration to development of liver metastasis after surgery. FGFR-2 IIIc-transfected cells exhibited increased proliferation in vitro and formed larger subcutaneous and orthotopic tumors, the latter producing more liver metastases. Moreover, FGF-2 exerted a more rapid stimulatory effect on the levels of phosphorylated extracellular signal-regulated kinase (p-ERK) in FGFR-2 IIIc stably transfected PANC-1 cells, compared with control cells. FGFR-2 IIIc-transfected cells also formed more spheres and contained more side population cells. Suppression of FGFR-2 IIIc expression inhibited the proliferation of PANC-1 cells, whereas an anti-FGFR-2 IIIc antibody inhibited the proliferation and   migration   of PANC-1 cells. Thus, high FGFR-2 IIIc levels in PDAC contribute to disease aggressiveness and confer to pancreatic cancer cells features suggestive of cancer stem cells, indicating that FGFR-2 IIIc may be a novel and important therapeutic target in PDAC. 
 Fibroblast growth factor receptor 2-positive fibroblasts provide a suitable microenvironment for tumor development and progression in esophageal carcinoma. Tumor fibroblasts (TF) have been suggested to play an essential role in the complex process of tumor-stroma interactions and tumorigenesis. The aim of the present study was to investigate the specific role of TF in the esophageal cancer microenvironment.An Affymetrix expression microarray was used to compare gene expression profiles between six pairs of TFs and normal fibroblasts from esophageal squamous cell carcinoma (ESCC). Differentially expressed genes were identified, and a subset was evaluated by quantitative real-time PCR and immunohistochemistry.About 43% (126 of 292) of known deregulated genes in TFs were associated with cell proliferation, extracellular matrix remodeling, and immune response. Up-regulation of fibroblast growth factor receptor 2 (FGFR2), which showed the most significant change, was detected in all six tested TFs compared with their paired normal fibroblasts. A further study found that FGFR2-positive fibroblasts were only observed inside the tumor tissues and not in tumor-surrounding stromal tissues, suggesting that FGFR2 could be used as a TF-specific marker in ESCC. Moreover, the conditioned medium from TFs was found to be able to promote ESCC tumor cell growth,   migration  , and invasion in vitro.Our study provides new candidate genes for the esophageal cancer microenvironment. Based on our results, we hypothesize that FGFR2(+)-TFs might provide cancer cells with a suitable microenvironment via secretion of proteins that could promote cancer development and progression through stimulation of cancer cell proliferation, induction of angiogenesis, inhibition of cell adhesion, enhancement of cell mobility, and promotion of the epithelial-mesenchymal transition. 
 FGFR2 mutation confers a less drastic gain of function in mesenchymal stem cells than in fibroblasts. Gain-of-function mutations in FGFR2 cause Apert syndrome (AS), a disease characterized by craniosynostosis and limb bone defects both due to abnormalities in bone differentiation and remodeling. Although the periosteum is an important cell source for bone remodeling, its role in craniosynostosis remains poorly characterized. We hypothesized that periosteal mesenchymal stem cells (MSCs) and fibroblasts from AS patients have abnormal cell phenotypes that contribute to the recurrent fusion of the coronal sutures. MSCs and fibroblasts were obtained from the periostea of 3 AS patients (S252W) and 3 control individuals (WT). We evaluated the proliferation,   migration  , and osteogenic differentiation of these cells. Interestingly, S252W mutation had opposite effects on different cell types: S252W MSCs proliferated less than WT MSCs, while S252W fibroblasts proliferated more than WT fibroblasts. Under restrictive media conditions, only S252W fibroblasts showed enhanced   migration  . The presence of S252W mutation increased in vitro and in vivo osteogenic differentiation in both studied cell types, though the difference compared to WT cells was more pronounced in S252W fibroblasts. This osteogenic differentiation was reversed through inhibition of JNK. We demonstrated that S252W fibroblasts can induce osteogenic differentiation in periosteal MSCs but not in MSCs from another tissue. MSCs and fibroblasts responded differently to the pathogenic effects of the FGFR2(S252W) mutation. We propose that cells from the periosteum have a more important role in the premature fusion of cranial sutures than previously thought and that molecules in JNK pathway are strong candidates for the treatment of AS patients. 
 An in vitro analysis of mechanical wounding-induced ligand-independent KGFR activation. KGFR (keratinocyte growth factor receptor), exclusively expressed in epithelial cells, plays an important role in wound healing. However, mechanisms of KGFR activation and signaling in wound healing are not clearly understood.We utilized an in vitro mechanical wounding model to examine ligand-independent KGFR activation, its regulation by reactive oxygen species (ROS) and the functional significance of this activation mechanism.Confluent HaCaT cell line cultures were mechanically wounded and KGFR internalization and phosphorylation were examined using immunostaining with confocal microscopy and immunoprecipitation with Western blotting. Wounding-induced generation of reactive oxygen species and ligand-independent activation of KGFR were examined. In addition, phosphorylation of its associated molecules FRS2 and c-Src were examined in the presence and absence of the ROS and pathway specific inhibitors. The importance of this activation process on cell   migration   was also examined in the presence and absence of these inhibitors.Mechanical wounding induced ligand-independent KGFR activation and internalization. KGFR internalization and phosphorylation was associated with ROS generation along the wound edge and scavenging of ROS with NAC inhibited KGFR phosphorylation. Intracellularly, c-Src was phosphorylated by wounding but its inhibitor, PP1, significantly inhibited KGFR activation and associated FRS2 phosphorylation. Mechanical wounding induced wound edge   migration  , which was significantly reduced by the selective receptor and pathway inhibitors PP1 (82.7%), KGFR inhibitor SU5402 (70%) and MAPK inhibitor PD98059 (57%).Mechanical wounding induces significant ROS generation at the wound edge which, in turn, induced ligand-independent KGFR and FRS2 activation via c-Src kinase signaling. Functionally, downstream MAPK signaling induced wound edge cell   migration  . 
